# Supplementary material for: Tigerfish designs oligonucleotide-based in situ hybridization probes targeting intervals of highly repetitive DNA at the scale of genomes
Source: Nat Commun. 2024 Feb 3;15:1027. doi: 10.1038/s41467-024-45385-x (PMC10838309; doi:10.1038/s41467-024-45385-x)
Supplement: Supplementary file 8 — Supplementary Software [file 41467_2024_45385_MOESM8_ESM.zip › TigerFISH-master/example_run/probe_candidate_binding_test/expected_output/04_supplementary_output/06_generate_chromomap/chrX_chromomap.html]

chromoMap
