# Supplementary figures and images for: Tigerfish designs oligonucleotide-based in situ hybridization probes targeting intervals of highly repetitive DNA at the scale of genomes
Source: Nat Commun. 2024 Feb 3;15:1027. doi: 10.1038/s41467-024-45385-x (PMC10838309; doi:10.1038/s41467-024-45385-x)

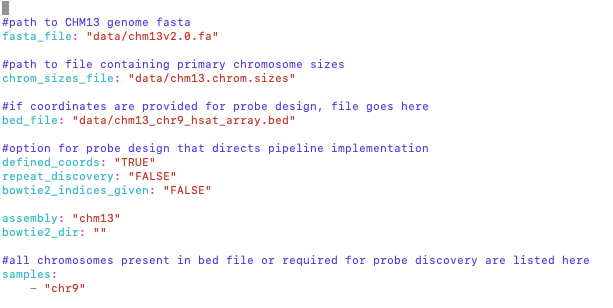

Supplement: Supplementary file 8 — Supplementary Software [file 41467_2024_45385_MOESM8_ESM.zip › TigerFISH-master/docs/source/imgs/chm13_yaml.png]

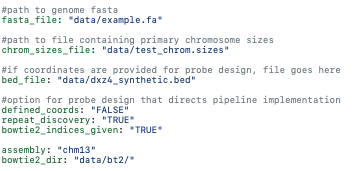

Supplement: Supplementary file 8 — Supplementary Software [file 41467_2024_45385_MOESM8_ESM.zip › TigerFISH-master/docs/source/imgs/rd_params.png]

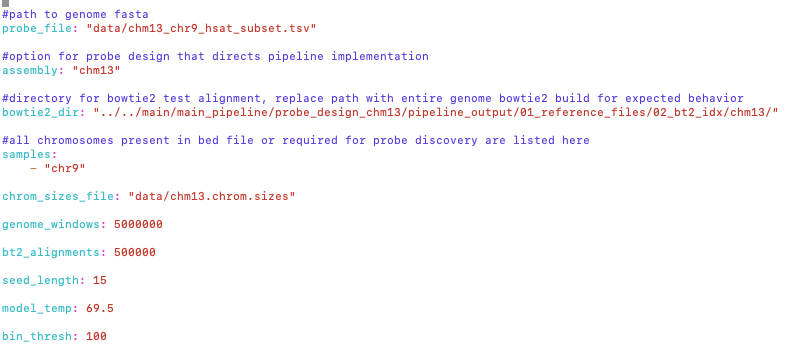

Supplement: Supplementary file 8 — Supplementary Software [file 41467_2024_45385_MOESM8_ESM.zip › TigerFISH-master/docs/source/imgs/chm13_chr9_yaml.png]

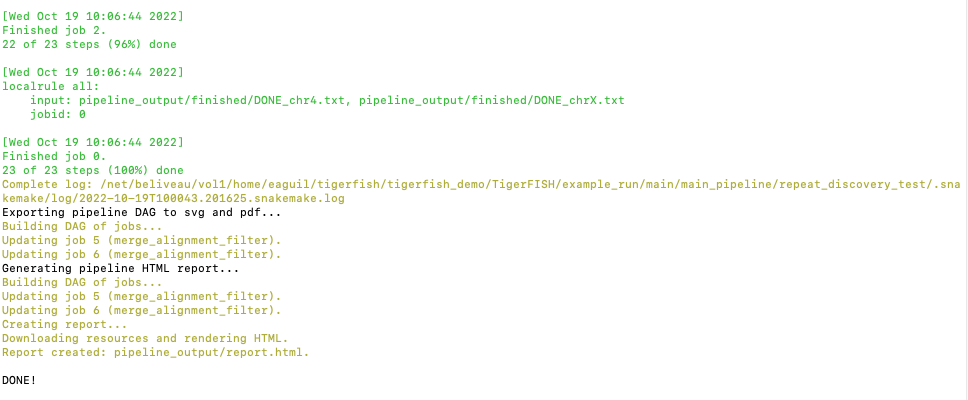

Supplement: Supplementary file 8 — Supplementary Software [file 41467_2024_45385_MOESM8_ESM.zip › TigerFISH-master/docs/source/imgs/step_6_repeat_disc.png]

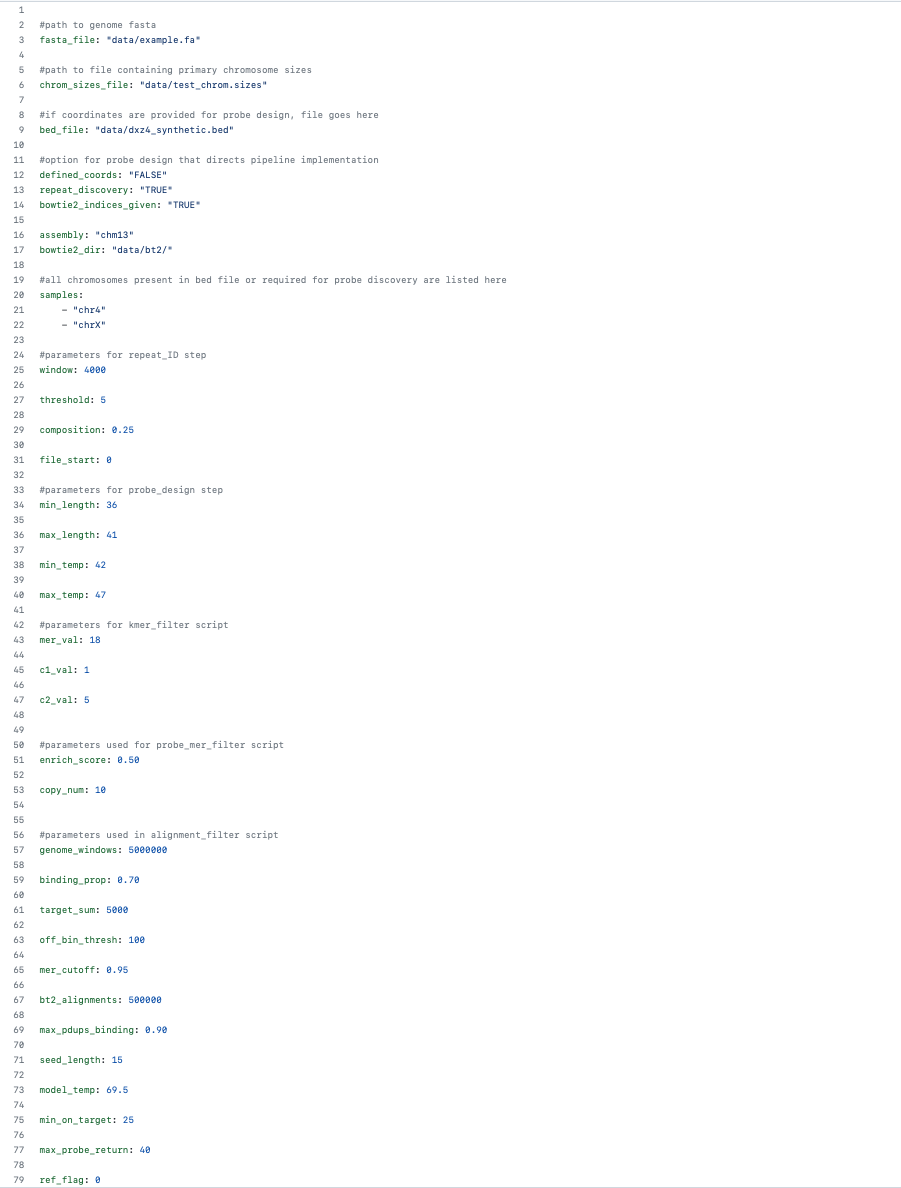

Supplement: Supplementary file 8 — Supplementary Software [file 41467_2024_45385_MOESM8_ESM.zip › TigerFISH-master/docs/source/imgs/repeat_discovery_config.png]

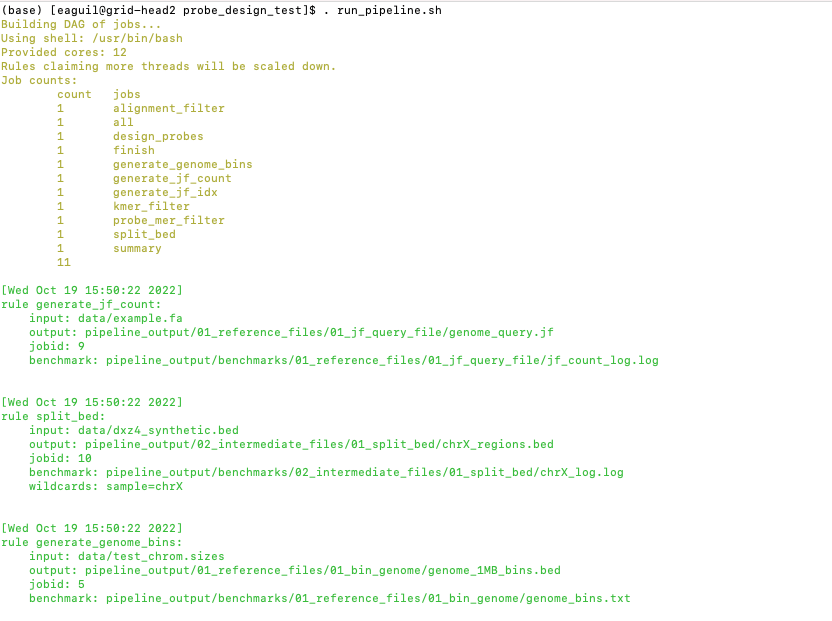

Supplement: Supplementary file 8 — Supplementary Software [file 41467_2024_45385_MOESM8_ESM.zip › TigerFISH-master/docs/source/imgs/step_2_probe_design.png]

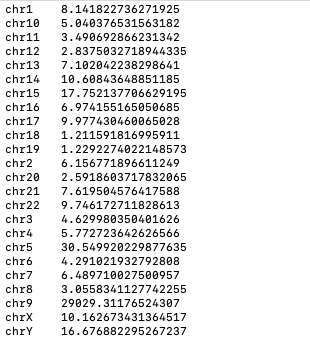

Supplement: Supplementary file 8 — Supplementary Software [file 41467_2024_45385_MOESM8_ESM.zip › TigerFISH-master/docs/source/imgs/genome_wide_binding_chr9.png]

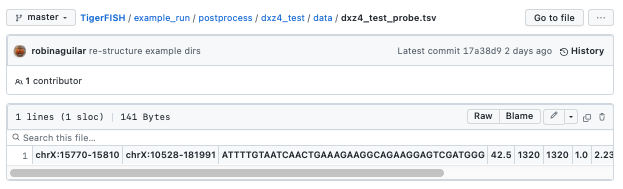

Supplement: Supplementary file 8 — Supplementary Software [file 41467_2024_45385_MOESM8_ESM.zip › TigerFISH-master/docs/source/imgs/sample_out_probe.png]

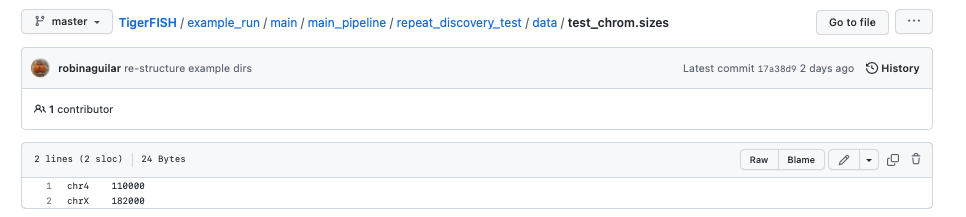

Supplement: Supplementary file 8 — Supplementary Software [file 41467_2024_45385_MOESM8_ESM.zip › TigerFISH-master/docs/source/imgs/chrom_sizes_repeat_disc.png]

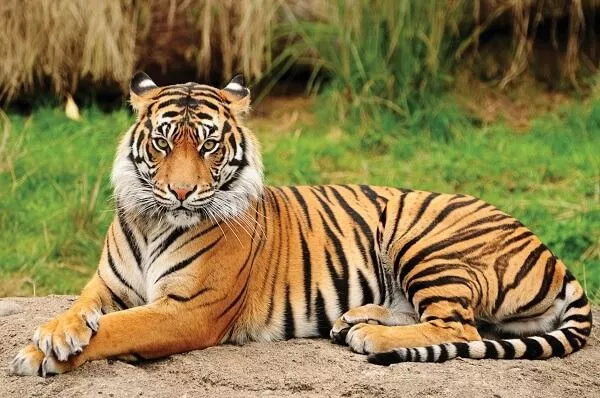

Supplement: Supplementary file 8 — Supplementary Software [file 41467_2024_45385_MOESM8_ESM.zip › TigerFISH-master/docs/source/imgs/tiger.jpg]

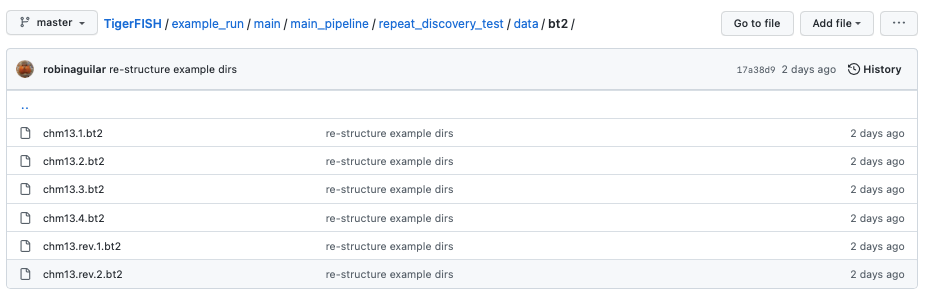

Supplement: Supplementary file 8 — Supplementary Software [file 41467_2024_45385_MOESM8_ESM.zip › TigerFISH-master/docs/source/imgs/bt2_repeat_disc.png]

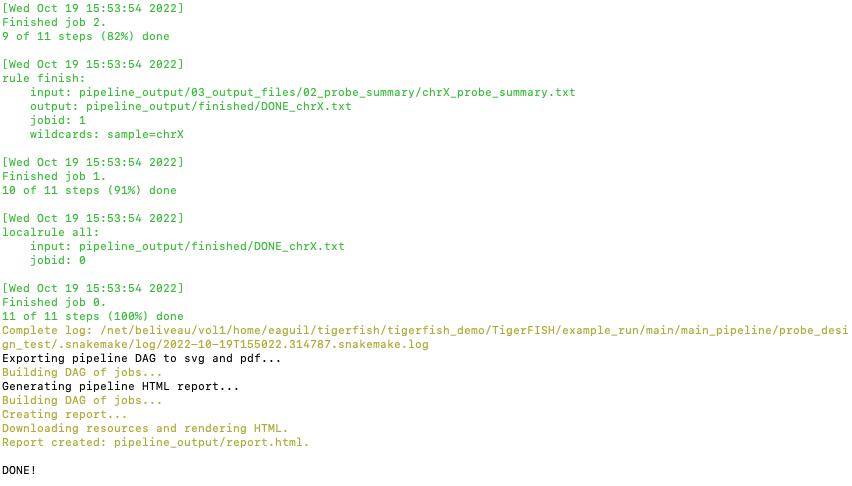

Supplement: Supplementary file 8 — Supplementary Software [file 41467_2024_45385_MOESM8_ESM.zip › TigerFISH-master/docs/source/imgs/step_3_probe_design.png]

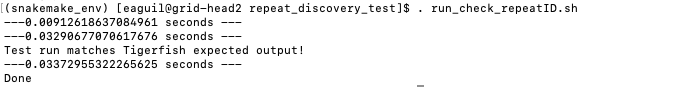

Supplement: Supplementary file 8 — Supplementary Software [file 41467_2024_45385_MOESM8_ESM.zip › TigerFISH-master/docs/source/imgs/step_7_repeat_disc.png]

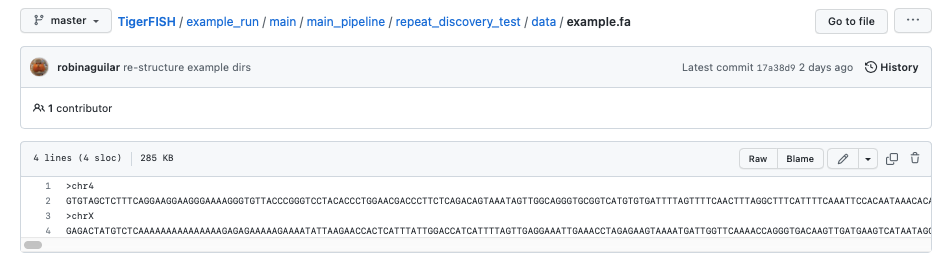

Supplement: Supplementary file 8 — Supplementary Software [file 41467_2024_45385_MOESM8_ESM.zip › TigerFISH-master/docs/source/imgs/repeat_disc_fasta.png]

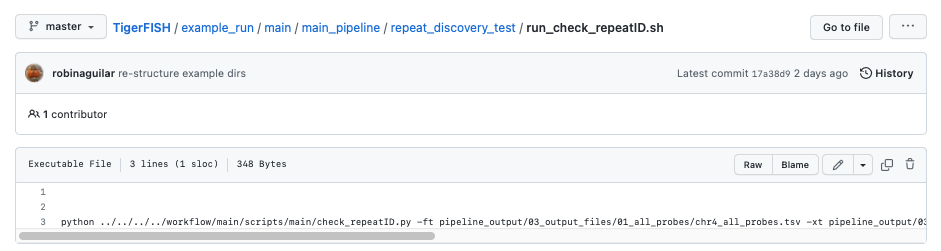

Supplement: Supplementary file 8 — Supplementary Software [file 41467_2024_45385_MOESM8_ESM.zip › TigerFISH-master/docs/source/imgs/check_repeat_disc.png]

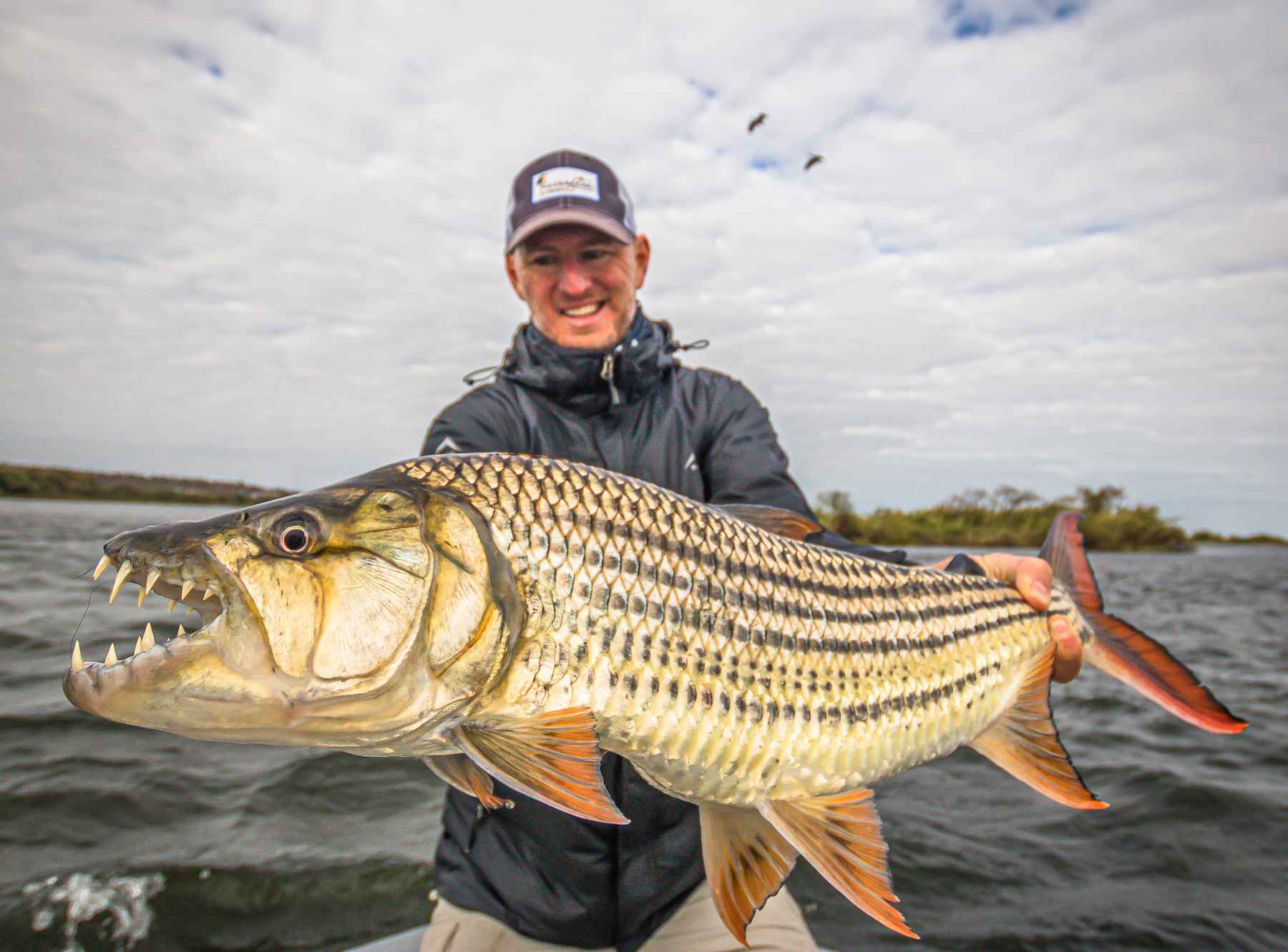

Supplement: Supplementary file 8 — Supplementary Software [file 41467_2024_45385_MOESM8_ESM.zip › TigerFISH-master/docs/source/imgs/tigerfish_fish.jpg]

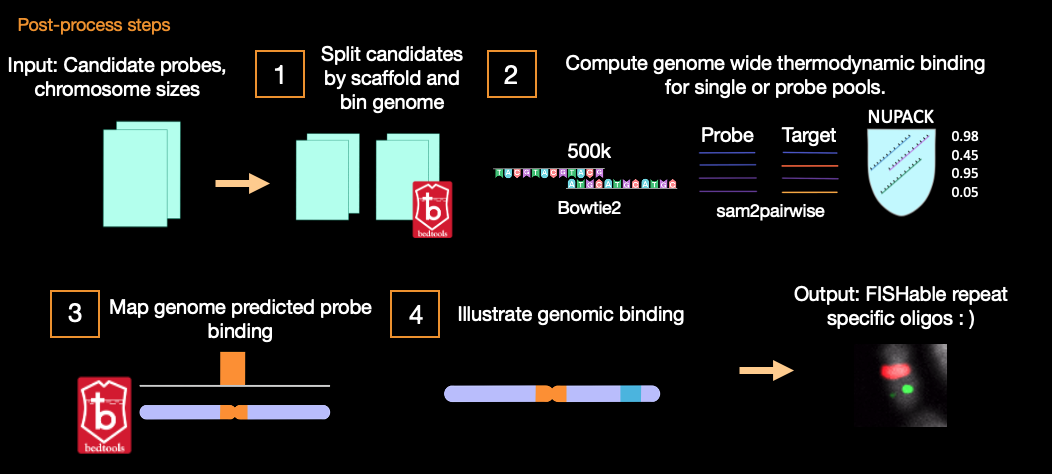

Supplement: Supplementary file 8 — Supplementary Software [file 41467_2024_45385_MOESM8_ESM.zip › TigerFISH-master/docs/source/imgs/tigerfish_postprocess_overview.png]

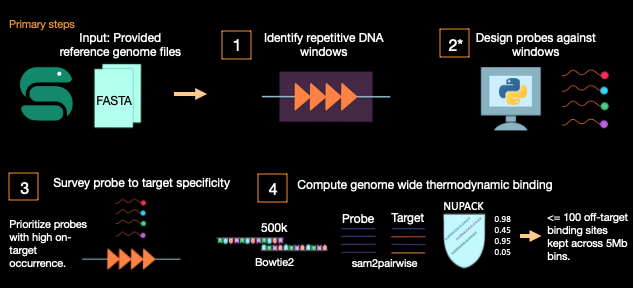

Supplement: Supplementary file 8 — Supplementary Software [file 41467_2024_45385_MOESM8_ESM.zip › TigerFISH-master/docs/source/imgs/tigerfish_main_overview.png]

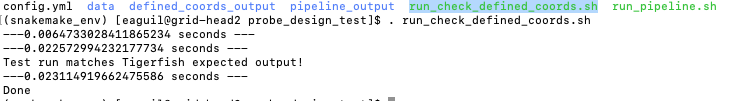

Supplement: Supplementary file 8 — Supplementary Software [file 41467_2024_45385_MOESM8_ESM.zip › TigerFISH-master/docs/source/imgs/step_4_probe_design.png]

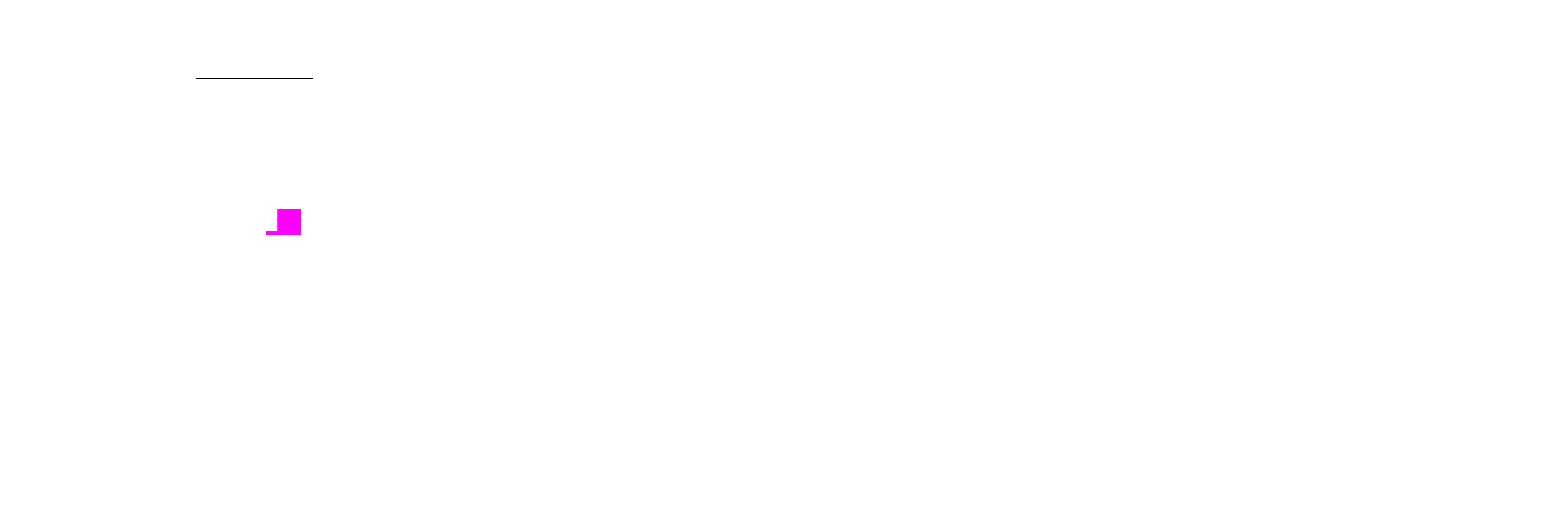

Supplement: Supplementary file 8 — Supplementary Software [file 41467_2024_45385_MOESM8_ESM.zip › TigerFISH-master/docs/source/imgs/chr9_genome_view.png]

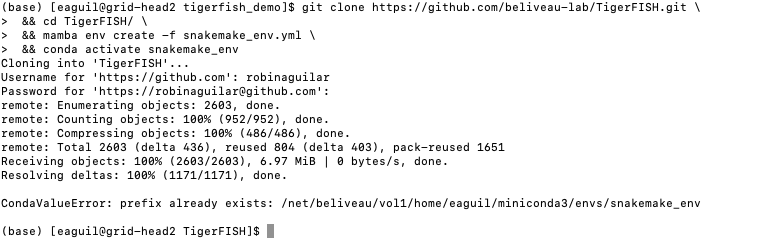

Supplement: Supplementary file 8 — Supplementary Software [file 41467_2024_45385_MOESM8_ESM.zip › TigerFISH-master/docs/source/imgs/step_2_repeat_disc.png]

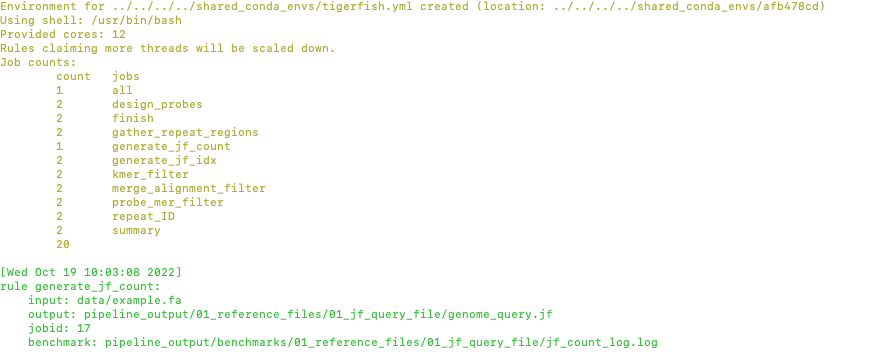

Supplement: Supplementary file 8 — Supplementary Software [file 41467_2024_45385_MOESM8_ESM.zip › TigerFISH-master/docs/source/imgs/step_5_repeat_disc.png]

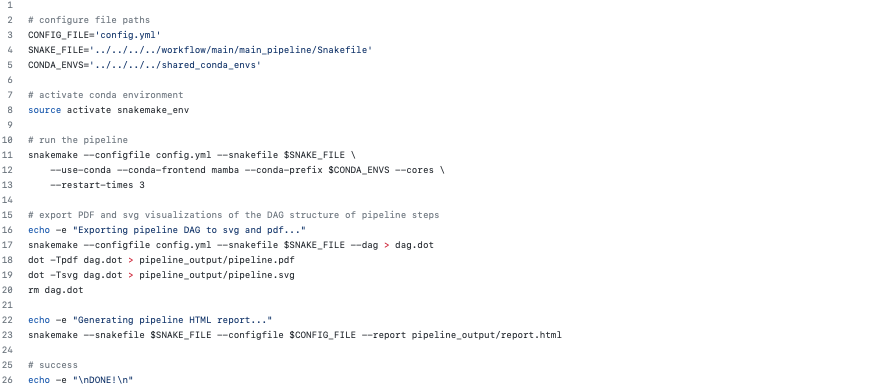

Supplement: Supplementary file 8 — Supplementary Software [file 41467_2024_45385_MOESM8_ESM.zip › TigerFISH-master/docs/source/imgs/run_pipeline_repeat_disc.png]

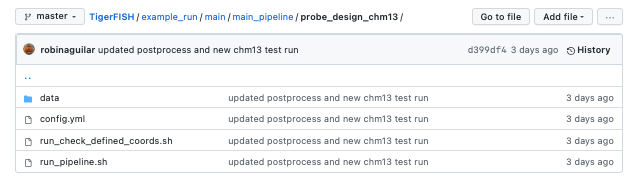

Supplement: Supplementary file 8 — Supplementary Software [file 41467_2024_45385_MOESM8_ESM.zip › TigerFISH-master/docs/source/imgs/chm13_probe_design_dir.png]

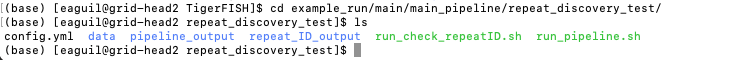

Supplement: Supplementary file 8 — Supplementary Software [file 41467_2024_45385_MOESM8_ESM.zip › TigerFISH-master/docs/source/imgs/step_4_repeat_disc.png]

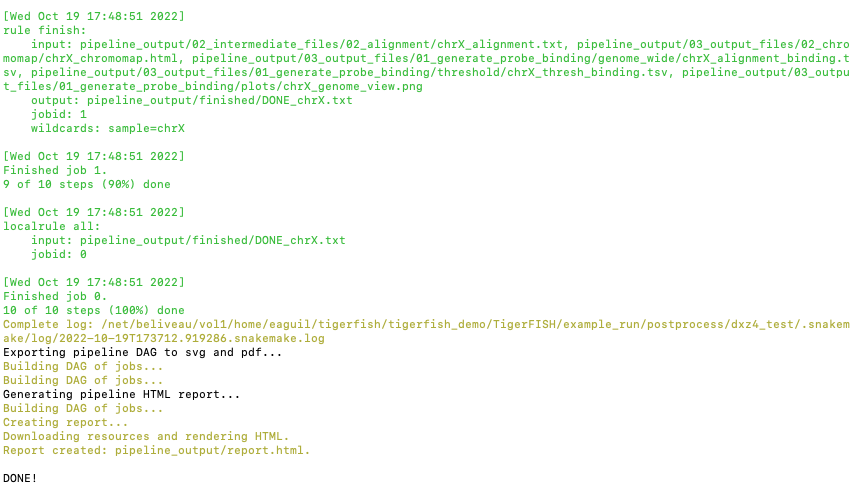

Supplement: Supplementary file 8 — Supplementary Software [file 41467_2024_45385_MOESM8_ESM.zip › TigerFISH-master/docs/source/imgs/postprocess_step_3.png]

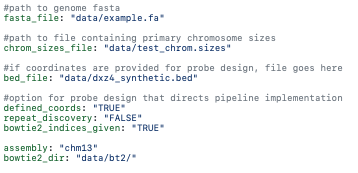

Supplement: Supplementary file 8 — Supplementary Software [file 41467_2024_45385_MOESM8_ESM.zip › TigerFISH-master/docs/source/imgs/pd_params.png]

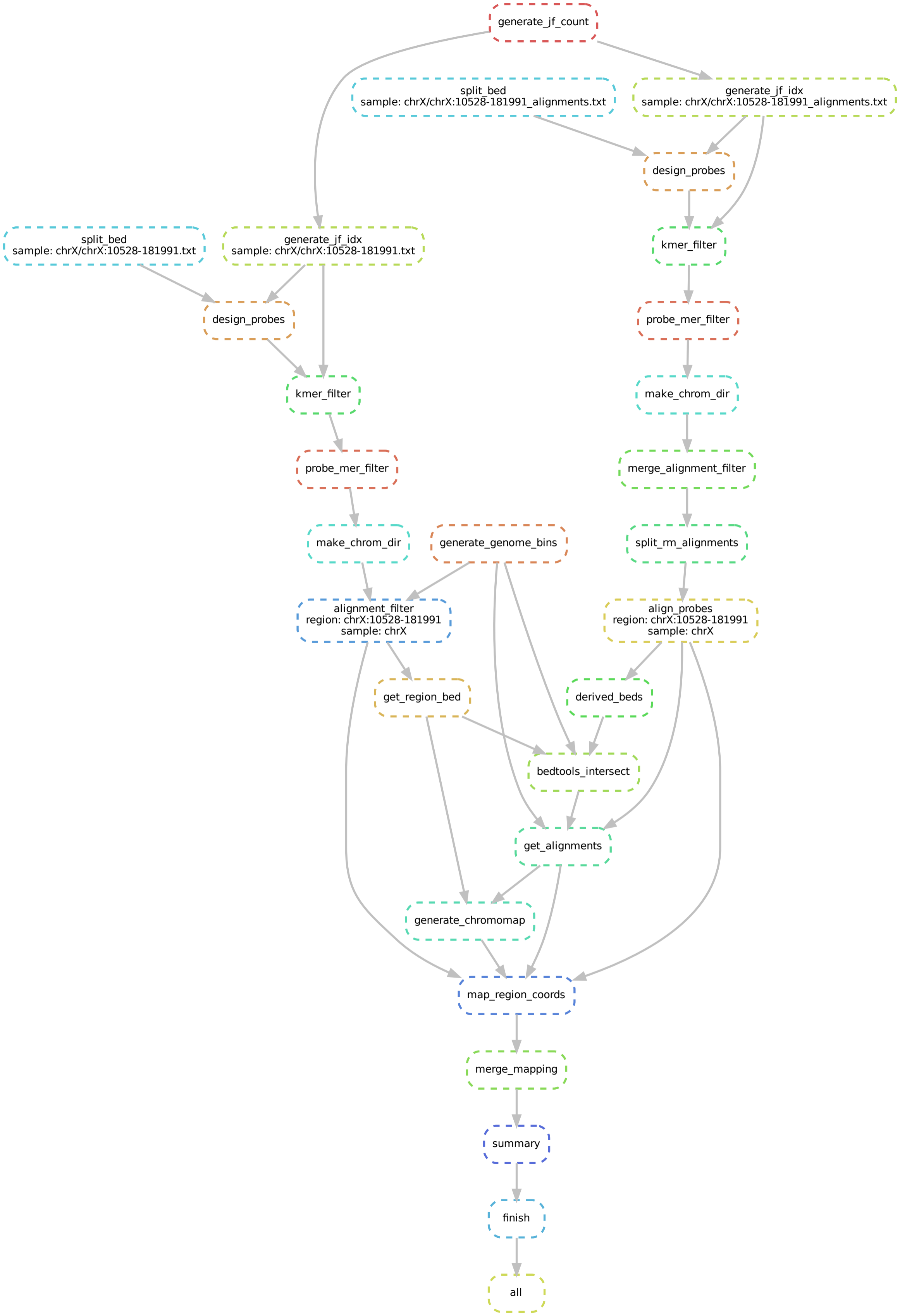

Supplement: Supplementary file 8 — Supplementary Software [file 41467_2024_45385_MOESM8_ESM.zip › TigerFISH-master/docs/source/imgs/DAG.pdf]

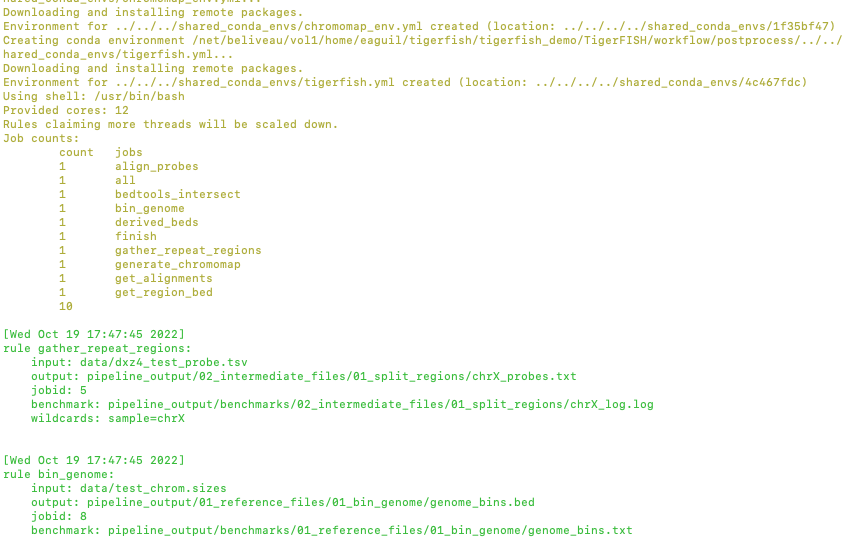

Supplement: Supplementary file 8 — Supplementary Software [file 41467_2024_45385_MOESM8_ESM.zip › TigerFISH-master/docs/source/imgs/postprocess_step_2.png]

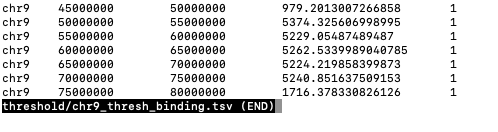

Supplement: Supplementary file 8 — Supplementary Software [file 41467_2024_45385_MOESM8_ESM.zip › TigerFISH-master/docs/source/imgs/genome_threshold_chr9.png]

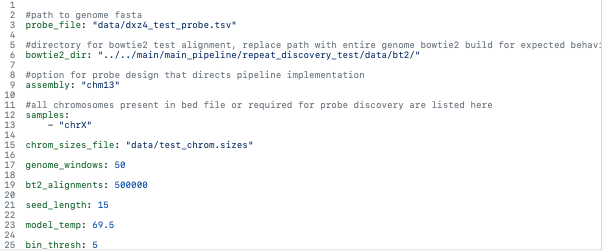

Supplement: Supplementary file 8 — Supplementary Software [file 41467_2024_45385_MOESM8_ESM.zip › TigerFISH-master/docs/source/imgs/postprocess_config.png]

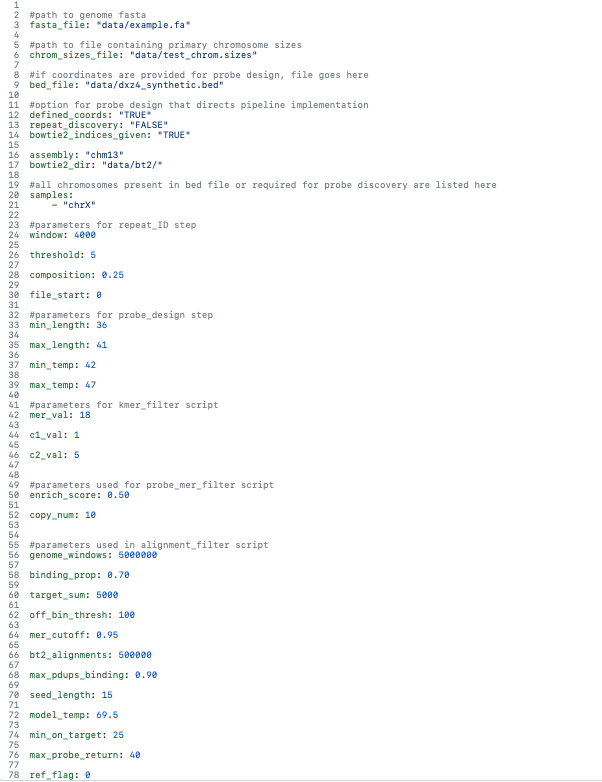

Supplement: Supplementary file 8 — Supplementary Software [file 41467_2024_45385_MOESM8_ESM.zip › TigerFISH-master/docs/source/imgs/probe_design_config.png]

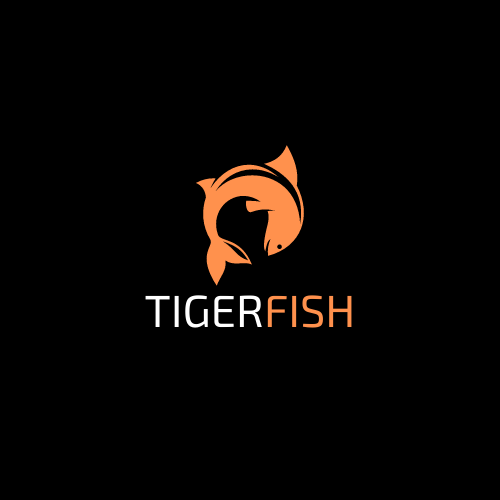

Supplement: Supplementary file 8 — Supplementary Software [file 41467_2024_45385_MOESM8_ESM.zip › TigerFISH-master/docs/source/imgs/tigerfish_logo.png]

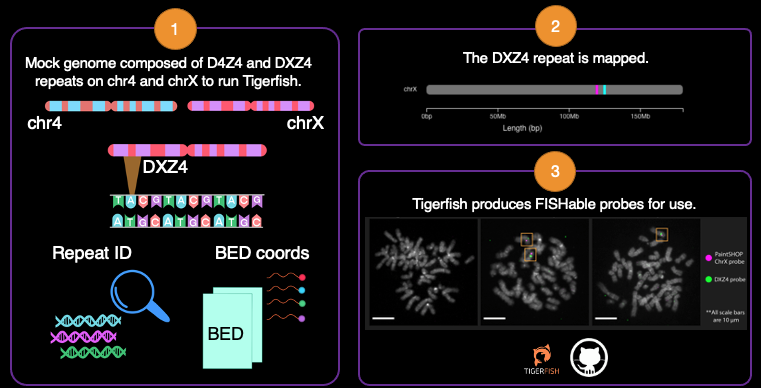

Supplement: Supplementary file 8 — Supplementary Software [file 41467_2024_45385_MOESM8_ESM.zip › TigerFISH-master/docs/source/imgs/tutorials_summary.png]

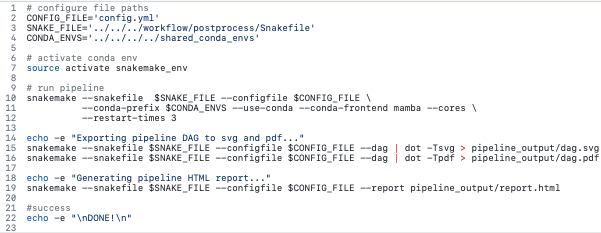

Supplement: Supplementary file 8 — Supplementary Software [file 41467_2024_45385_MOESM8_ESM.zip › TigerFISH-master/docs/source/imgs/postprocess_run.png]

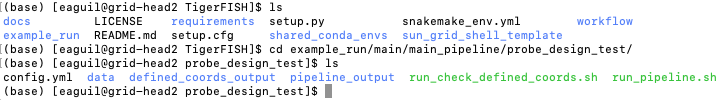

Supplement: Supplementary file 8 — Supplementary Software [file 41467_2024_45385_MOESM8_ESM.zip › TigerFISH-master/docs/source/imgs/step_1_probe_design.png]

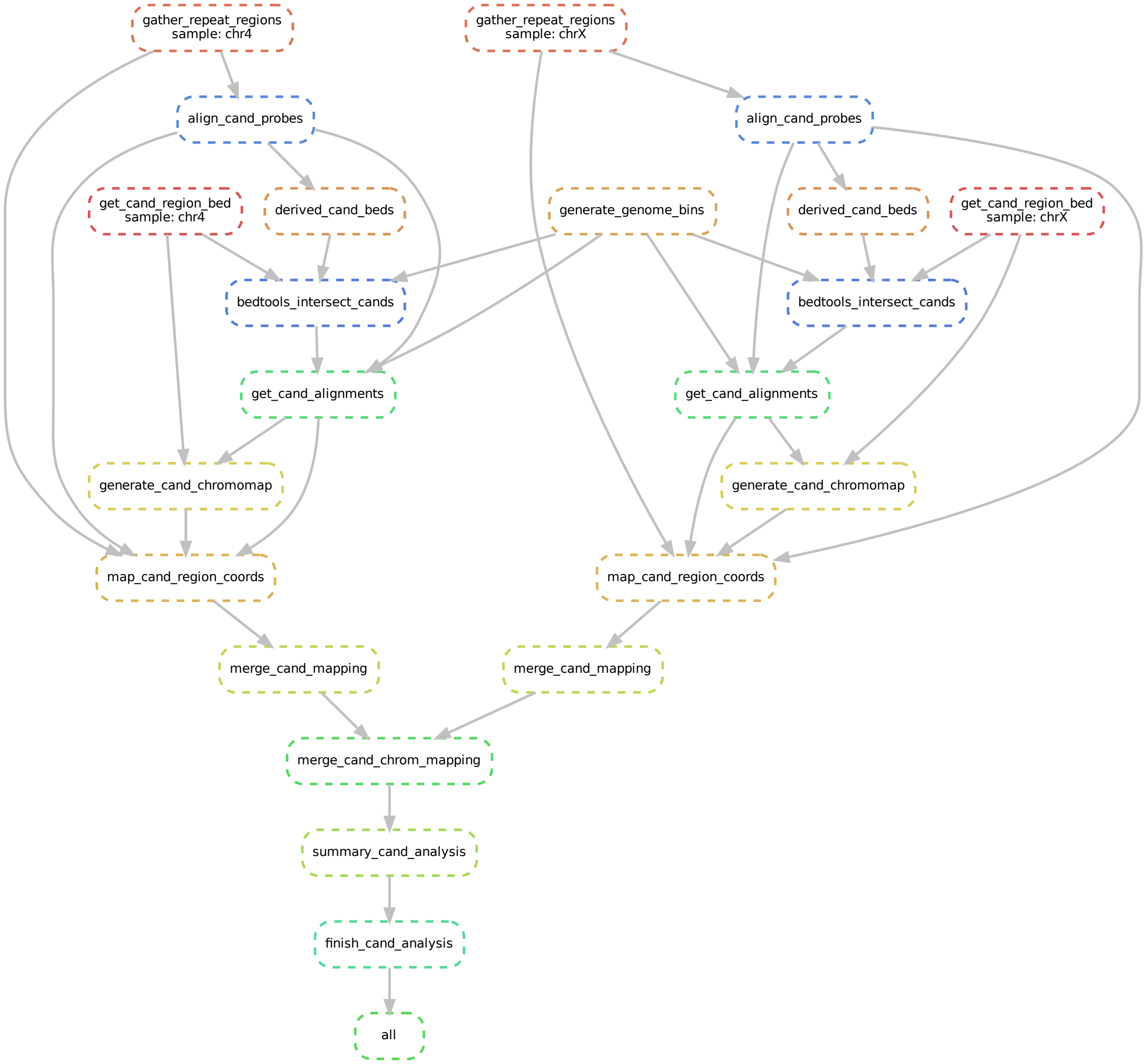

Supplement: Supplementary file 8 — Supplementary Software [file 41467_2024_45385_MOESM8_ESM.zip › TigerFISH-master/example_run/probe_candidate_binding_test/expected_output/pipeline.pdf]

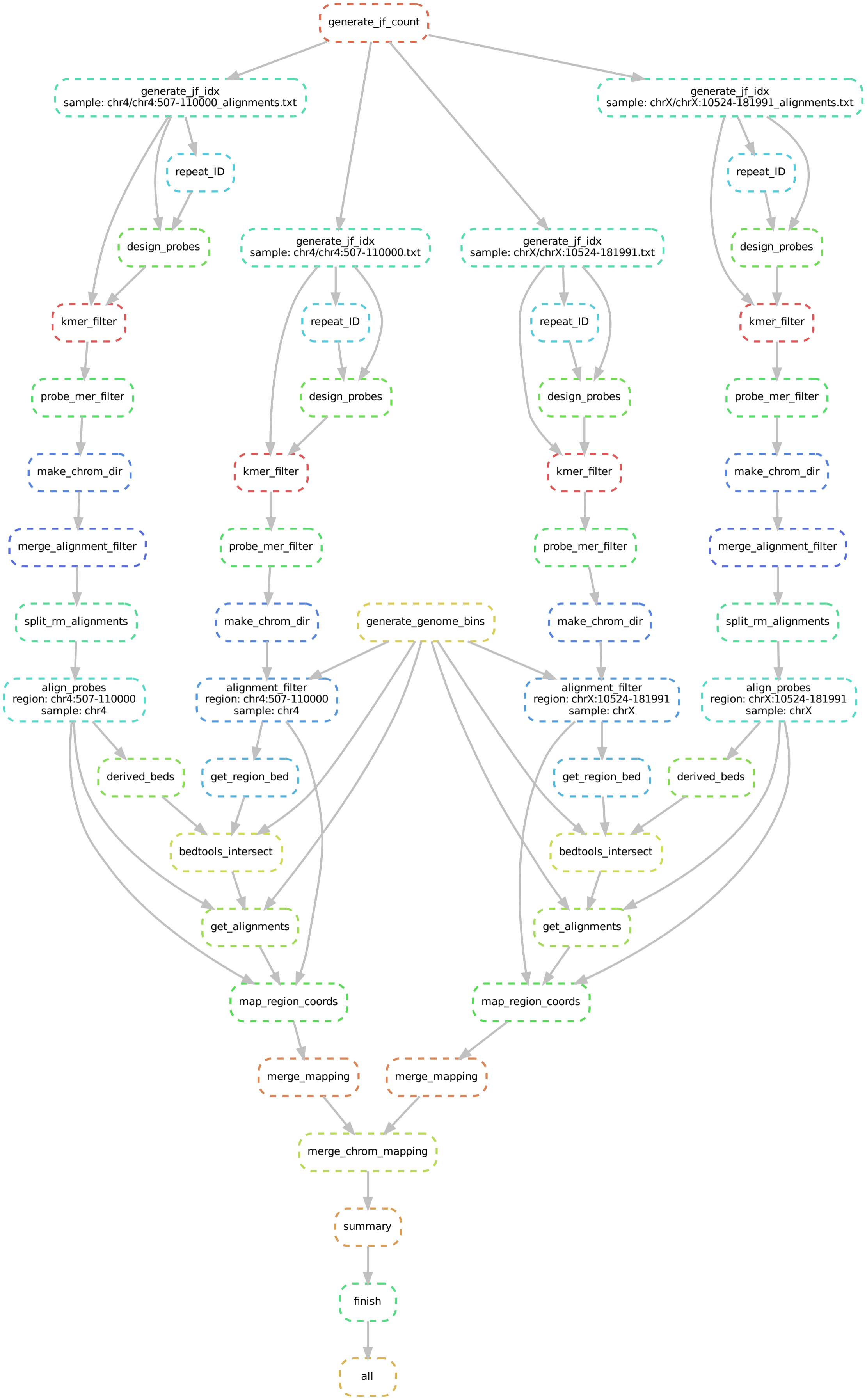

Supplement: Supplementary file 8 — Supplementary Software [file 41467_2024_45385_MOESM8_ESM.zip › TigerFISH-master/example_run/repeat_discovery_test/expected_output/pipeline.pdf]

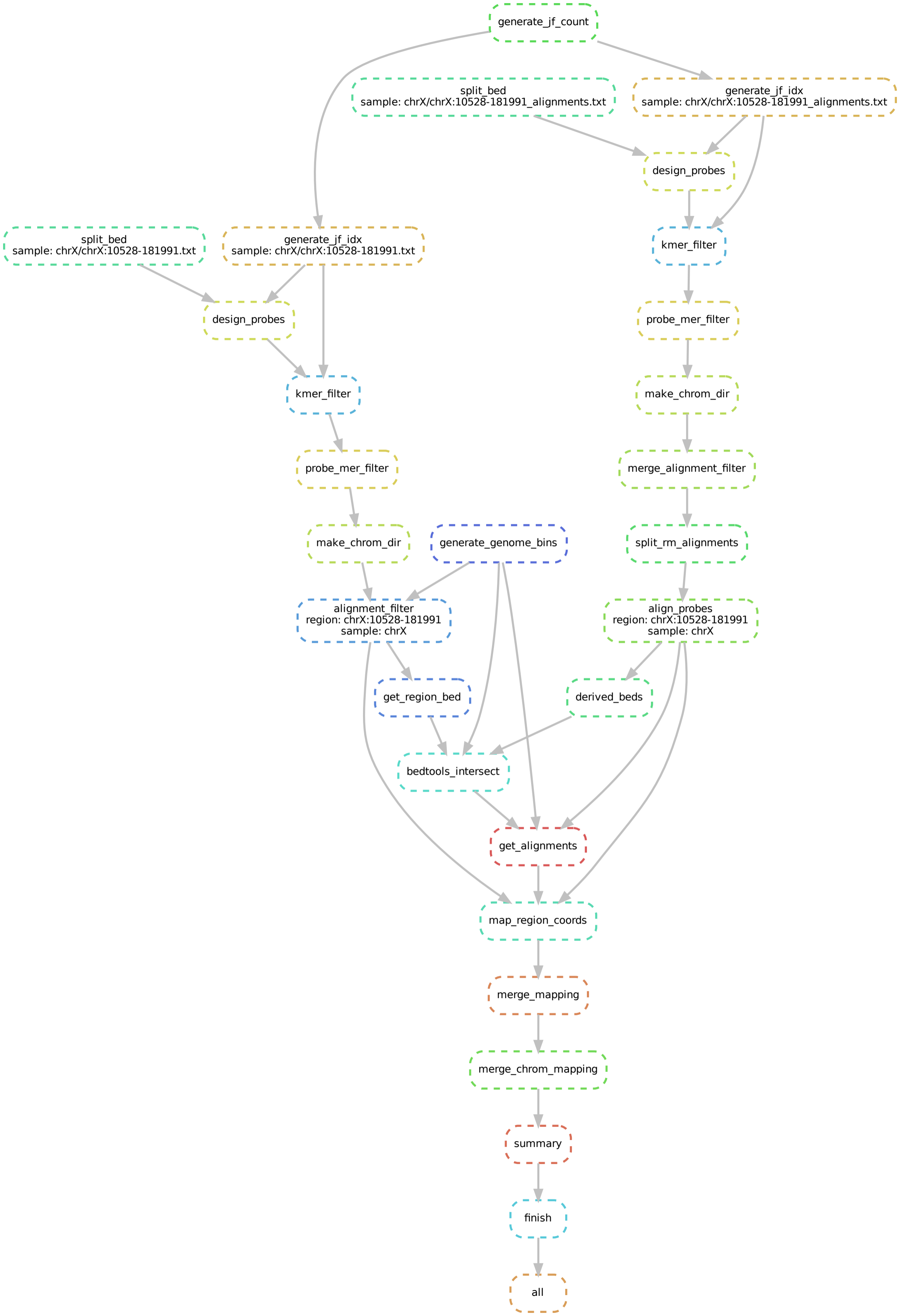

Supplement: Supplementary file 8 — Supplementary Software [file 41467_2024_45385_MOESM8_ESM.zip › TigerFISH-master/example_run/probe_design_test/expected_output/pipeline.pdf]

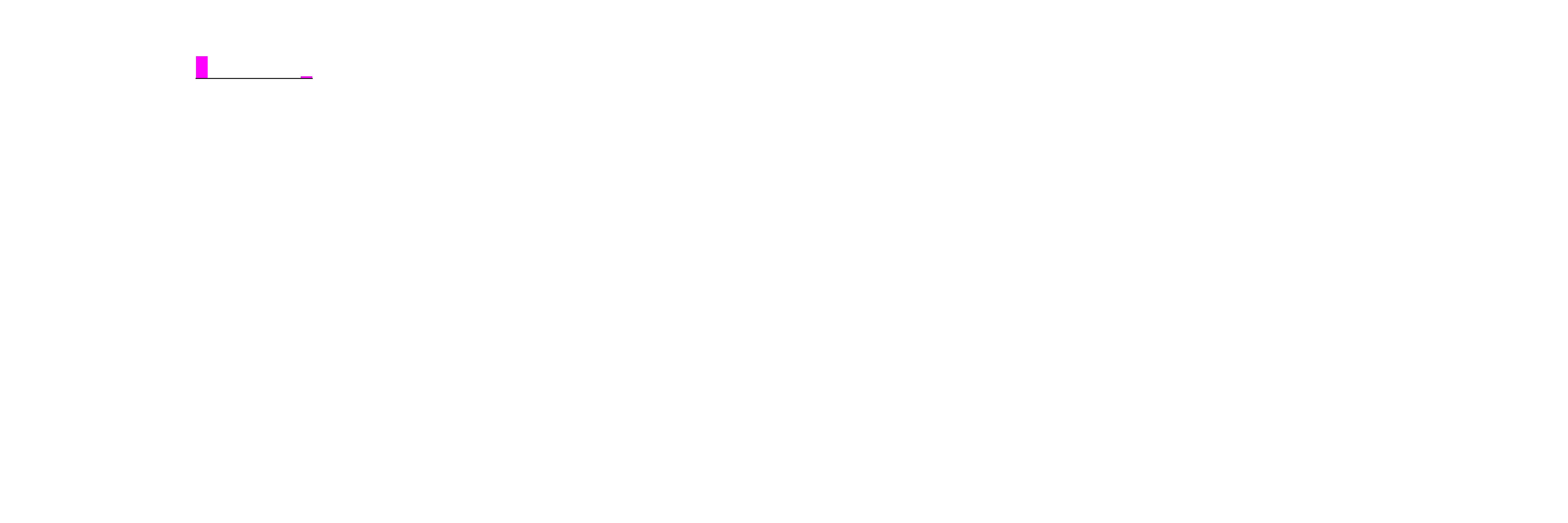

Supplement: Supplementary file 8 — Supplementary Software [file 41467_2024_45385_MOESM8_ESM.zip › TigerFISH-master/example_run/probe_candidate_binding_test/expected_output/04_supplementary_output/03_genome_wide_binding_plots/chr4_genome_view.png]

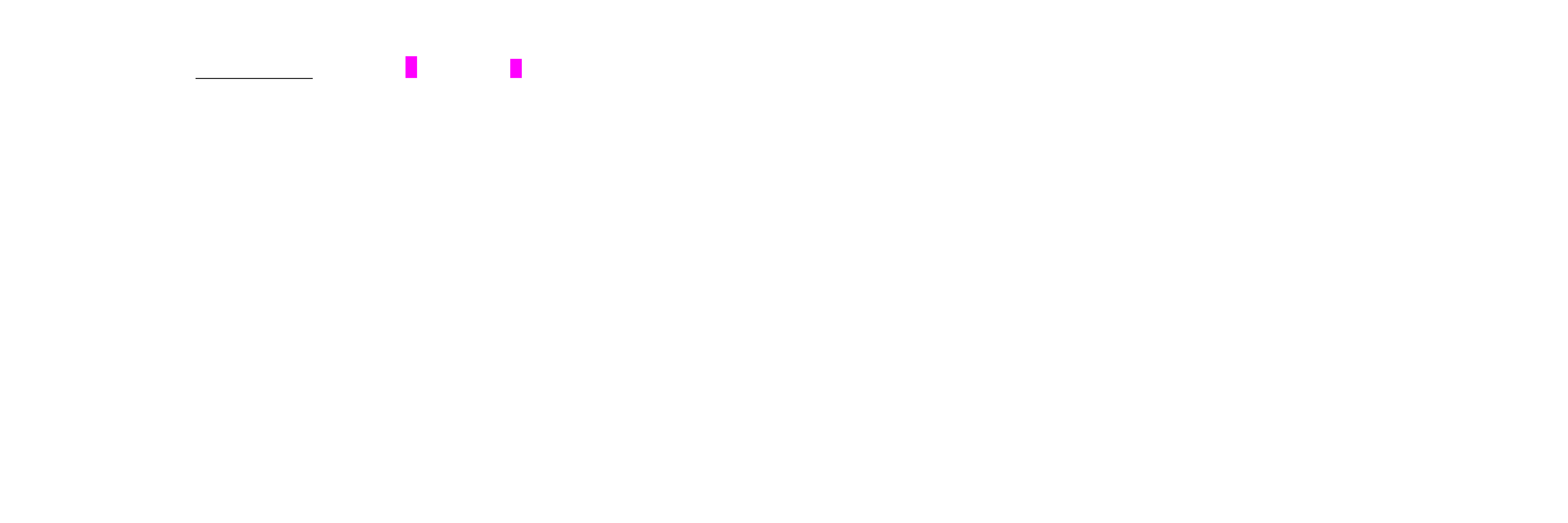

Supplement: Supplementary file 8 — Supplementary Software [file 41467_2024_45385_MOESM8_ESM.zip › TigerFISH-master/example_run/probe_candidate_binding_test/expected_output/04_supplementary_output/03_genome_wide_binding_plots/chrX_genome_view.png]
