# Supplementary material for: Tigerfish designs oligonucleotide-based in situ hybridization probes targeting intervals of highly repetitive DNA at the scale of genomes
Source: Nat Commun. 2024 Feb 3;15:1027. doi: 10.1038/s41467-024-45385-x (PMC10838309; doi:10.1038/s41467-024-45385-x)
Supplement: Supplementary file 9 — Reporting Summary [file 41467_2024_45385_MOESM9_ESM.pdf]

nature portfolio

Corresponding author(s): William S.Noble
Brian J. Beliveau

Last updated by author(s): Jan 8, 2024

Reporting Summary

Nature Portfolio wishes to improve the reproducibility of the work that we publish. This form provides structure for consistency and transparency in reporting. For further information on Nature Portfolio policies, see our Editorial Policies and the Editorial Policy Checklist.

Statistics

For all statistical analyses, confirm that the following items are present in the figure legend, table legend, main text, or Methods section.

n/a	Confirmed	
☐	☒	The exact sample size (*n*) for each experimental group/condition, given as a discrete number and unit of measurement
☐	☒	A statement on whether measurements were taken from distinct samples or whether the same sample was measured repeatedly
☐	☒	The statistical test(s) used AND whether they are one- or two-sided<br>*Only common tests should be described solely by name; describe more complex techniques in the Methods section.*
☒	☐	A description of all covariates tested
☒	☐	A description of any assumptions or corrections, such as tests of normality and adjustment for multiple comparisons
☐	☒	A full description of the statistical parameters including central tendency (e.g. means) or other basic estimates (e.g. regression coefficient) AND variation (e.g. standard deviation) or associated estimates of uncertainty (e.g. confidence intervals)
☐	☒	For null hypothesis testing, the test statistic (e.g. *F*, *t*, *r*) with confidence intervals, effect sizes, degrees of freedom and *P* value noted<br>*Give P values as exact values whenever suitable.*
☒	☐	For Bayesian analysis, information on the choice of priors and Markov chain Monte Carlo settings
☒	☐	For hierarchical and complex designs, identification of the appropriate level for tests and full reporting of outcomes
☒	☐	Estimates of effect sizes (e.g. Cohen's *d*, Pearson's *r*), indicating how they were calculated

Our web collection on statistics for biologists contains articles on many of the points above.

Software and code

Policy information about availability of computer code

Data collection	Nikon Elements AR 5.20.00 was used to acquire microscopy images om the Nikon Ti-2 system.
Data analysis	Tigerfish probe design was performed with open source code hosted here: https://github.com/beliveau-lab/TigerFISH.<br>Tigerfish is written in Python 3.7.8 with dependencies that include Biopython 1.77, Bowtie 2.3.5.1, NUPACK 4.0, BEDtools 2.29.2, Numpy 1.18.5, Pandas 1.0.5, pip 20.1.1, pybedtools 0.8.1, sam2pairwise 1.0.0, samtools 1.9, scikit-learn 0.23.1, scipy 1.5.0, zip 3.0, matplotlib 3.3.4, seaborn 0.11.1, pytest 6.2, and Jellyfish 2.2.10. All Tigerfish probe collections were generated using a pipeline implemented with Snakemake 7.19.

For manuscripts utilizing custom algorithms or software that are central to the research but not yet described in published literature, software must be made available to editors and reviewers. We strongly encourage code deposition in a community repository (e.g. GitHub). See the Nature Portfolio guidelines for submitting code & software for further information.

Data

Policy information about availability of data

All manuscripts must include a data availability statement. This statement should provide the following information, where applicable:

- Accession codes, unique identifiers, or web links for publicly available datasets
- A description of any restrictions on data availability
- For clinical datasets or third party data, please ensure that the statement adheres to our policy

The primary bioinformatic data can be accessed via the Source Data file that accompanies this manuscript. Primary microscopy data will be made available upon request. The CHM13 genome assembly versions 1.0, 1.1, and 2 (+ HG002 chrY) can be downloaded without repeat masking from the T2T consortium at https://github.com/marbl/CHM13.

Research involving human participants, their data, or biological material

Policy information about studies with human participants or human data. See also policy information about sex, gender (identity/presentation), and sexual orientation and race, ethnicity and racism.

Reporting on sex and gender	N/A
Reporting on race, ethnicity, or other socially relevant groupings	N/A
Population characteristics	N/A
Recruitment	N/A
Ethics oversight	N/A

Note that full information on the approval of the study protocol must also be provided in the manuscript.

Field-specific reporting

Please select the one below that is the best fit for your research. If you are not sure, read the appropriate sections before making your selection.

☒ Life sciences ☐ Behavioural & social sciences ☐ Ecological, evolutionary & environmental sciences

For a reference copy of the document with all sections, see nature.com/documents/nr-reporting-summary-flat.pdf

Life sciences study design

All studies must disclose on these points even when the disclosure is negative.

Sample size	>10 fields of view were manually examined in each imaging experiment to ensure the observed staining patterns were present in different fields of view. This yielded >30 nuclei per sample, which is a field standard for collecting information about staining pattern variation and is in line with previous studies we have published validating software tools for computational probe design.
Data exclusions	No data were excluded from analyses.
Replication	All experiments were repeated at least three times to verify the observed staining patterns were consistent and reproducible.
Randomization	Randomization was not part of the experimental design as no experimental work sought to investigate biological differences between different cell populations.
Blinding	Blinding was not possible as experimental conditions were immediately apparent from the imaging data.

Reporting for specific materials, systems and methods

We require information from authors about some types of materials, experimental systems and methods used in many studies. Here, indicate whether each material, system or method listed is relevant to your study. If you are not sure if a list item applies to your research, read the appropriate section before selecting a response.

Materials & experimental systems

n/a	Involved in the study
☒	☐ Antibodies
☐	☒ Eukaryotic cell lines
☒	☐ Palaeontology and archaeology
☒	☐ Animals and other organisms
☒	☐ Clinical data
☒	☐ Dual use research of concern
☒	☐ Plants

Methods

n/a	Involved in the study
☒	☐ ChIP-seq
☒	☐ Flow cytometry
☒	☐ MRI-based neuroimaging

Eukaryotic cell lines

Policy information about cell lines and Sex and Gender in Research

Cell line source(s)	Human metaphase chromosome spread slides (which contain both chromosome spreads and intact primary lymphoblast nuclei were obtained from Applied Genetic La
Authentication	We authenticated the metaphase chromosome spread slides by performing FISH against non-repetitive intervals using validated probe sets designed by PaintSHOP.
Mycoplasma contamination	None of the cell lines used were tested for Mycoplasma contamination.
Commonly misidentified lines (See ICLAC register)	No commonly misidentified cell lines were used in this study.

Plants

Seed stocks	N/A
Novel plant genotypes	N/A
Authentication	N/A
