# Supplementary material for: Tigerfish designs oligonucleotide-based in situ hybridization probes targeting intervals of highly repetitive DNA at the scale of genomes
Source: Nat Commun. 2024 Feb 3;15:1027. doi: 10.1038/s41467-024-45385-x (PMC10838309; doi:10.1038/s41467-024-45385-x)
Supplement: Supplementary file 10 — Source Data [file 41467_2024_45385_MOESM10_ESM.gz › Tigerfish_full_data/Figure_S1_HOR_TRF_Analysis/TRF/trf_output/chr2_8_21_Y_hor.fa.s3.2.5.7.80.10.50.2000.1.txt.html]

chr2\_8\_21\_Y\_hor.fa.s3.2.5.7.80.10.50.2000.txt.html

```
Tandem Repeats Finder Program written by:

                 Gary Benson
      Program in Bioinformatics
          Boston University

Version 4.09

Sequence: chr21:10962853-11311467

Parameters: 2 5 7 80 10 50 2000

Pmatch=0.80,Pindel=0.10
tuple sizes 0,4,5,7
tuple distances 0, 29, 159, 2000

Length: 348614
ACGTcount: A:0.31, C:0.22, G:0.17, T:0.30


Found at i:788 original size:171 final size:171

Alignment explanation  
  

    Indices: 18--348425  Score: 316476
    Period size: 171  Copynumber: 2050.0  Consensus size: 171

          8 AATTCCGTGC

                   *     *       *                *       *          *       
         18 TTCCAACTAAATCCTCAAAGCAATCCAAATATCCACTTGCAGATTCCACAAAAAGAGGGTTTCAA
          1 TTCCAACGAAATCATCAAAGCGATCCAAATATCCACTTCCAGATTCTACAAAAAGAGTGTTTCAA

             *            *  *  *            *   **  *     *     *  *        
         83 ATCTGCTCTATCAATAGCAAGGTTCAACTCTGTTAGTATAGGACACATATCACCAACAAGTTTCT
         66 AACTGCTCTATCAAAAGAAATGTTCAACTCTGTGAGTTGAGTACACACATCACAAAGAAGTTTCT

                             *   *   **  *      *    
        148 GAGAATGCTTCTGTCTACTTTCTATGGGAGGATATTTCCTT
        131 GAGAATGCTTCTGTCTAGTTTTTATATGAAGATATTCCCTT

              *  ** * ** **          *  * *          *  *          *         
        189 TTTCACTGTAGGCGACAAAGCGATCAAATTGTCCA-TTCCATATACTACAAAAAGTGTGTTTCAA
          1 TTCCAACGAAATCATCAAAGCGATCCAAATATCCACTTCCAGATTCTACAAAAAGAGTGTTTCAA

             *     *   *   * *   *    *    *  *     * ** * *    *      *  *  
        253 ACCTGCTTTATGAAAGGTAATATTCATCTCTATGTGTTGAATGGAAATATCAGAAAGAAATTGCT
         66 AACTGCTCTATCAAAAGAAATGTTCAACTCTGTGAGTTGAGTACACACATCACAAAGAAGTTTCT

                  *  *       **                    **
        318 GAGAATTCTGCTGTCTACATTTTATAT---GA-ATTCCCGC
        131 GAGAATGCTTCTGTCTAGTTTTTATATGAAGATATTCCCTT

                         *     * *                *       *                  
        355 TTCCAACGAAATCCTCAAATCAATCCAAATATCCACTTGCAGATTCCACAAAAAGAGTGTTTCAA
          1 TTCCAACGAAATCATCAAAGCGATCCAAATATCCACTTCCAGATTCTACAAAAAGAGTGTTTCAA

                          *     *          * *       *              *        
        420 AACTGCTCTATCAATAGAAAGGTTCAACTCTTTTAGTTGAGAACACACATCACAAACAAGTTTCT
         66 AACTGCTCTATCAAAAGAAATGTTCAACTCTGTGAGTTGAGTACACACATCACAAAGAAGTTTCT

                        *   * *      **     **  *    
        485 GAGAATGCTTCTCTCTCGCTTTTATGGGAAGACGTTTCC-T
        131 GAGAATGCTTCTGTCTAGTTTTTATATGAAGATATTCCCTT

                 * *  **        * *       *                  *               
        525 TTCCACCAAAGGCATCAAAGTGTTCCAAATGTCCACTTCCAGATTCTACCAAAAGAGTGTTTCAA
          1 TTCCAACGAAATCATCAAAGCGATCCAAATATCCACTTCCAGATTCTACAAAAAGAGTGTTTCAA

             **         **  * *                 *    * *  * *     *   *      
        590 ACGTGCTC-ATAGTAAGGGAATGTTCAACTCTGTGACTTGAATGCAGATATCACCAAGTAGTTTC
         66 AACTGCTCTAT-CAAAAGAAATGTTCAACTCTGTGAGTTGAGTACACACATCACAAAGAAGTTTC

             * * *             *     *    *         * 
        654 TAATAGTGCTTCTGTCTAGATTTTAGATGATGATATTCCCGT
        130 TGAGAATGCTTCTGTCTAGTTTTTATATGAAGATATTCCCTT

                         * * *   *        *    *  *   *      *     *       * 
        696 TTCCAACGAAATCGTTAGAGCTATCCAAATGTCCAGTTACAGTTTCTACCAAAAGGGTGTTTCCA
          1 TTCCAACGAAATCATCAAAGCGATCCAAATATCCACTTCCAGATTCTACAAAAAGAGTGTTTCAA

              *    **           *            *       *                     * 
        761 AATTGCTGCATCAAAAGAAAGGTTCAACTCTGTTAGTTGAGGACACACATCACAAAGAAGTTTGT
         66 AACTGCTCTATCAAAAGAAATGTTCAACTCTGTGAGTTGAGTACACACATCACAAAGAAGTTTCT

                              *    *     *           
        826 GAGAATGCTTCTGTCTAGATTTTGTATGACGATATTCCCTT
        131 GAGAATGCTTCTGTCTAGTTTTTATATGAAGATATTCCCTT

                     *   * *     *   *       * *  *    *  *       *  *       
        867 TTCCAACGATATCGTTAAAGCAATCTAAATATCAATTTGCAGAATCCACAAAAATAGAGTTTCAA
          1 TTCCAACGAAATCATCAAAGCGATCCAAATATCCACTTCCAGATTCTACAAAAAGAGTGTTTCAA

             *       * *        *    *       *  *                   ***      
        932 AGCTGCTCTGTAAAAAGAAAGGTTCCACTCTGTTAGCTGAGTACACACATCACAAACTTGTTTCT
         66 AACTGCTCTATCAAAAGAAATGTTCAACTCTGTGAGTTGAGTACACACATCACAAAGAAGTTTCT

                  *         *        **         **   
        997 GAGAATCCTTCTGTCTCGTTTTTATGGGAAGATATTTACTT
        131 GAGAATGCTTCTGTCTAGTTTTTATATGAAGATATTCCCTT

                 *  * **          *       *     *       *  *  *              
       1038 TTCCACCGTAGGCATCAAAGCGCTCCAAATGTCCACATCCAGATACTCCAGAAAGAGTGTTTCAA
          1 TTCCAACGAAATCATCAAAGCGATCCAAATATCCACTTCCAGATTCTACAAAAAGAGTGTTTCAA

             *         *   * *   *         *        * *  *      *      *     
       1103 ACCTGCTCTATGAAAGGGAATCTTCAACTCTATGAGTTGAATGCAGACATCAGAAAGAAATTTCT
         66 AACTGCTCTATCAAAAGAAATGTTCAACTCTGTGAGTTGAGTACACACATCACAAAGAAGTTTCT

                     *       **          *         **
       1168 GAGAATGCTGCTGTCTACCTTTTAT-T--TGA-ATTCCCGC
        131 GAGAATGCTTCTGTCTAGTTTTTATATGAAGATATTCCCTT

                         *  *    *              * *  **   *    *             
       1205 TTCCAACGAAATCCTCCAAGCTATCCAAATATCCACCTGCATTTTCCACAACAAGAGTGTTTCAA
          1 TTCCAACGAAATCATCAAAGCGATCCAAATATCCACTTCCAGATTCTACAAAAAGAGTGTTTCAA

                          *                 *    *  *                *       
       1270 AACTGCTCTATCAATAGAAATGTTCAACTCCTTTG-GCTGGGTACACACATCACAAACAAGTTTC
         66 AACTGCTCTATCAAAAGAAATGTTCAACT-CTGTGAGTTGAGTACACACATCACAAAGAAGTTTC

                                      **     *        
       1334 TGAGAATGCTTCTGTCTAGTTTTTATGGGAAGACATTCCCTT
        130 TGAGAATGCTTCTGTCTAGTTTTTATATGAAGATATTCCCTT

              *  * *  **          *       *            **                    
       1376 TTTCACCAAAGGCATCAAAGCGCTCCAAATGTCCACTTCCAGACACTACAAAAAGAGTGTTTCAA
          1 TTCCAACGAAATCATCAAAGCGATCCAAATATCCACTTCCAGATTCTACAAAAAGAGTGTTTCAA

             **       **    **                 *    * *  * *      *  *       
       1441 ACGTGCTCTAAGAAAACGAATGTTCAACTCTGTGACTTGAATGCAGATATCACAGAGTAGTTTCT
         66 AACTGCTCTATCAAAAGAAATGTTCAACTCTGTGAGTTGAGTACACACATCACAAAGAAGTTTCT

                **            *     *    *         * 
       1506 GAGAGGGCTTCTGTCTAGATTTTAGATGATGATATTCCCGT
        131 GAGAATGCTTCTGTCTAGTTTTTATATGAAGATATTCCCTT

                           * *   *                *   *                    * 
       1547 TTCCAACGAAATCATTAGAGCTATCCAAATATCCACTTACAGTTTCTACAAAAAGAGTGTTTCCA
          1 TTCCAACGAAATCATCAAAGCGATCCAAATATCCACTTCCAGATTCTACAAAAAGAGTGTTTCAA

                   **         * *    *       *  *                   ***      
       1612 AACTGCTGCATCAAAAGAGAGGTTCCACTCTGTTAGCTGAGTACACACATCACAAACTTGTTTCT
         66 AACTGCTCTATCAAAAGAAATGTTCAACTCTGTGAGTTGAGTACACACATCACAAAGAAGTTTCT

            *     *         *        **         **   
       1677 CAGAATCCTTCTGTCTCGTTTTTATGGGAAGATATTTACTT
        131 GAGAATGCTTCTGTCTAGTTTTTATATGAAGATATTCCCTT

              *  *  * ***         *       *     *       **    *       *      
       1718 TTTCACCGTAGGTATCAAAGCGCTCCAAATGTCCACATCCAGATAGTACAGAAAGAGTTTTTCAA
          1 TTCCAACGAAATCATCAAAGCGATCCAAATATCCACTTCCAGATTCTACAAAAAGAGTGTTTCAA

             *         *   * *   *         *        * *  *      *      *     
       1783 ACCTGCTCTATGAAAGGGAATCTTCAACTCTATGAGTTGAATGCAGACATCAGAAAGAAATTTCT
         66 AACTGCTCTATCAAAAGAAATGTTCAACTCTGTGAGTTGAGTACACACATCACAAAGAAGTTTCT

                     *       **          *         **
       1848 GAGAATGCTGCTGTCTACCTTTTAT-T--TGA-ATTCCCGC
        131 GAGAATGCTTCTGTCTAGTTTTTATATGAAGATATTCCCTT

                         *  *    *                *       *                  
       1885 TTCCAACGAAATCCTCCAAGCTATCCAAATATCCACTTGCAGATTCCACAAAAAGAGTGTTTCAA
          1 TTCCAACGAAATCATCAAAGCGATCCAAATATCCACTTCCAGATTCTACAAAAAGAGTGTTTCAA

                            ** *  *            *       *     *     *  *      
       1950 AACTGCTCTCTATCAATGGCAAAGTTCAACTCTGTTAGTTGAGGACACATATCACCAACAAGTTT
         66 AACTG--CTCTATCAAAAGAAATGTTCAACTCTGTGAGTTGAGTACACACATCACAAAGAAGTTT

                               *       **         *    
       2015 CTGAGAATGCTTCTGTCTATTTTTTATGGGAAGATATTTCCTT
        129 CTGAGAATGCTTCTGTCTAGTTTTTATATGAAGATATTCCCTT

              *  *  * ** *    *      *    *          * **                    
       2058 TTTCACCGTAGGCGTCAAGGCGATCGAAATGTCCACTTCCACAAACTACAAAAAGAGTGTTTCAA
          1 TTCCAACGAAATCATCAAAGCGATCCAAATATCCACTTCCAGATTCTACAAAAAGAGTGTTTCAA

             *         *   * **       *    *        * ** * *   **      *     
       2123 ACCTGCTCTATGAAAGGCCATGTTCATCTCTATGAGTTGAATGGAAATATCCGAAAGAAATTTCT
         66 AACTGCTCTATCAAAAGAAATGTTCAACTCTGTGAGTTGAGTACACACATCACAAAGAAGTTTCT

             *       *          *        *         **
       2188 GGGAATGCTGCTGTCTAG-TGTT-TAT-ACGA-ATTCCCGC
        131 GAGAATGCTTCTGTCTAGTTTTTATATGAAGATATTCCCTT

                         *       *                *    *  *                  
       2225 TTCCAACGAAATCCTCAAAGCAATCCAAATATCCACTTGCAGAATCCACAAAAAGAGTGTTTCAA
          1 TTCCAACGAAATCATCAAAGCGATCCAAATATCCACTTCCAGATTCTACAAAAAGAGTGTTTCAA

                          *     *          * *                   *  *        
       2290 AACTGCTCTATCAATAGAAAGGTTCAACTCTTTTAGTTGAGTACACACATCACGAACAAGTTTCT
         66 AACTGCTCTATCAAAAGAAATGTTCAACTCTGTGAGTTGAGTACACACATCACAAAGAAGTTTCT

                            * *      **     **  *    
       2355 GAGAATGCTTCTGTCTGGCTTTTATTGGAAGACGTTTCCTT
        131 GAGAATGCTTCTGTCTAGTTTTTATATGAAGATATTCCCTT

                 * *  **        * *       *                  *               
       2396 TT-CACCAAAGGCATCAAAGTGCTCCAAATGTCCACTTCCAGATTCTACCAAAAGAGTGTTTCAA
          1 TTCCAACGAAATCATCAAAGCGATCCAAATATCCACTTCCAGATTCTACAAAAAGAGTGTTTCAA

             **         **  * *                 *    * *  * *     *   *      
       2460 ACGTGCTC-ATAGTAAGGGAATGTTCAACTCTGTGACTTGAATGCAGATATCACCAAGTAGTTTC
         66 AACTGCTCTAT-CAAAAGAAATGTTCAACTCTGTGAGTTGAGTACACACATCACAAAGAAGTTTC

             * * *             *     *    *         * 
       2524 TAATAGTGCTTCTGTCTAGATTTTAGATGATGATATTCCCGT
        130 TGAGAATGCTTCTGTCTAGTTTTTATATGAAGATATTCCCTT

                         *** *   *             *  *   *      *     *       * 
       2566 TTCCAACGAAATCGCTAGAGCTATCCAAATATCCAGTTACAGTTTCTACCAAAAGGGTGTTTCCA
          1 TTCCAACGAAATCATCAAAGCGATCCAAATATCCACTTCCAGATTCTACAAAAAGAGTGTTTCAA

              *    **           *            *       *      *              * 
       2631 AATTGCTGCATCAAAAGAAAGGTTCAACTCTGTTAGTTGAGGACACACGTCACAAAGAAGTTTGT
         66 AACTGCTCTATCAAAAGAAATGTTCAACTCTGTGAGTTGAGTACACACATCACAAAGAAGTTTCT

                              *    *     *           
       2696 GAGAATGCTTCTGTCTAGATTTTGTATGACGATATTCCCTT
        131 GAGAATGCTTCTGTCTAGTTTTTATATGAAGATATTCCCTT

                     *   * *     *   *       * *  *    *  *       *  *       
       2737 TTCCAACGATATCGTTAAAGCAATCTAAATATCAATTTGCAGAATCCACAAAAATAGAGTTTCAA
          1 TTCCAACGAAATCATCAAAGCGATCCAAATATCCACTTCCAGATTCTACAAAAAGAGTGTTTCAA

             *       * *        *    *       *  *                   ***      
       2802 AGCTGCTCTGTAAAAAGAAAGGTTCCACTCTGTTAGCTGAGTACACACATCACAAACTTGTTTCT
         66 AACTGCTCTATCAAAAGAAATGTTCAACTCTGTGAGTTGAGTACACACATCACAAAGAAGTTTCT

                  *         *        **         **   
       2867 GAGAATCCTTCTGTCTCGTTTTTATGGGAAGATATTTACTT
        131 GAGAATGCTTCTGTCTAGTTTTTATATGAAGATATTCCCTT

              *  *  * **          *       *     *       *  *  *              
       2908 TTTCACCGTAGGCATCAAAGCGCTCCAAATGTCCACATCCAGATACTCCAGAAAGAGTGTTTCAA
          1 TTCCAACGAAATCATCAAAGCGATCCAAATATCCACTTCCAGATTCTACAAAAAGAGTGTTTCAA

             *         *   * *   *         *        * *  *      *      *     
       2973 ACCTGCTCTATGAAAGGCAATCTTCAACTCTATGAGTTGAATGCAGACATCAGAAAGAAATTTCT
         66 AACTGCTCTATCAAAAGAAATGTTCAACTCTGTGAGTTGAGTACACACATCACAAAGAAGTTTCT

                     *       **          *         **
       3038 GAGAATGCTGCTGTCTACCTTTTAT-T--TGA-ATTCCCGC
        131 GAGAATGCTTCTGTCTAGTTTTTATATGAAGATATTCCCTT

                         *  *    *              * *  **   *    *             
       3075 TTCCAACGAAATCCTCCAAGCTATCCAAATATCCACCTGCATTTTCCACAACAAGAGTGTTTCAA
          1 TTCCAACGAAATCATCAAAGCGATCCAAATATCCACTTCCAGATTCTACAAAAAGAGTGTTTCAA

                          *                 *    *  *                *       
       3140 AACTGCTCTATCAATAGAAATGTTCAACTCCTTTG-GCTGGGTACACACATCACAAACAAGTTTC
         66 AACTGCTCTATCAAAAGAAATGTTCAACT-CTGTGAGTTGAGTACACACATCACAAAGAAGTTTC

                                      **     **       
       3204 TGAGAATGCTTCTGTCTAGTTTTTATGGGAAGACGTTCCCTT
        130 TGAGAATGCTTCTGTCTAGTTTTTATATGAAGATATTCCCTT

              *  * *  **          *       *            **                  * 
       3246 TTTCACCAAAGGCATCAAAGCGCTCCAAATGTCCACTTCCAGACACTACAAAAAGAGTGTTTCCA
          1 TTCCAACGAAATCATCAAAGCGATCCAAATATCCACTTCCAGATTCTACAAAAAGAGTGTTTCAA

             **   *   **   ***                 *    * *  * *         *       
       3311 ACGTGCCCTAAGAAAGCGAATGTTCAACTCTGTGACTTGAATGCAGATATCACAAAGTAGTTTCT
         66 AACTGCTCTATCAAAAGAAATGTTCAACTCTGTGAGTTGAGTACACACATCACAAAGAAGTTTCT

                **            *     *    *         * 
       3376 GAGAGGGCTTCTGTCTAGATTTTAGATGATGATATTCCCGT
        131 GAGAATGCTTCTGTCTAGTTTTTATATGAAGATATTCCCTT

                           * *   *                *   *                    * 
       3417 TTCCAACGAAATCATTAGAGCTATCCAAATATCCACTTACAGTTTCTACAAAAAGAGTGTTTCCA
          1 TTCCAACGAAATCATCAAAGCGATCCAAATATCCACTTCCAGATTCTACAAAAAGAGTGTTTCAA

                   **         * *    *       *  *                   ***      
       3482 AACTGCTGCATCAAAAGAGAGGTTCCACTCTGTTAGCTGAGTACACACATCACAAACTTGTTTCT
         66 AACTGCTCTATCAAAAGAAATGTTCAACTCTGTGAGTTGAGTACACACATCACAAAGAAGTTTCT

            *     *         *        **         **   
       3547 CAGAATCCTTCTGTCTCGTTTTTATGGGAAGATATTTACTT
        131 GAGAATGCTTCTGTCTAGTTTTTATATGAAGATATTCCCTT

              *  *  * **          *       *     *       *  *  *              
       3588 TTTCACCGTAGGCATCAAAGCGCTCCAAATGTCCACATCCAGATACTCCAGAAAGAGTGTTTCAA
          1 TTCCAACGAAATCATCAAAGCGATCCAAATATCCACTTCCAGATTCTACAAAAAGAGTGTTTCAA

             *         *   * *   *         *        * *  *      *      *     
       3653 ACCTGCTCTATGAAAGGGAATCTTCAACTCTATGAGTTGAATGCAGACATCAGAAAGAAATTTCT
         66 AACTGCTCTATCAAAAGAAATGTTCAACTCTGTGAGTTGAGTACACACATCACAAAGAAGTTTCT

                     *       **          *         **
       3718 GAGAATGCTGCTGTCTACCTTTTAT-T--TGA-ATTCCCGC
        131 GAGAATGCTTCTGTCTAGTTTTTATATGAAGATATTCCCTT

                         *  *    *                *       *                  
       3755 TTCCAACGAAATCCTCCAAGCTATCCAAATATCCACTTGCAGATTCCACAAAAAGAGTGTTTCAA
          1 TTCCAACGAAATCATCAAAGCGATCCAAATATCCACTTCCAGATTCTACAAAAAGAGTGTTTCAA

                            ** *  *            *       *     *     *  *      
       3820 AACTGCTCTCTATCAATGGCAAAGTTCAACTCTGTTAGTTGAGGACACATATCACCAACAAGTTT
         66 AACTG--CTCTATCAAAAGAAATGTTCAACTCTGTGAGTTGAGTACACACATCACAAAGAAGTTT

                               *       **         *    
       3885 CTGAGAATGCTTCTGTCTATTTTTTATGGGAAGATATTTCCTT
        129 CTGAGAATGCTTCTGTCTAGTTTTTATATGAAGATATTCCCTT

              *  *  * ** *    *      *    *          * **                    
       3928 TTTCACCGTAGGCGTCAAGGCGATCGAAATGTCCACTTCCACAAACTACAAAAAGAGTGTTTCAA
          1 TTCCAACGAAATCATCAAAGCGATCCAAATATCCACTTCCAGATTCTACAAAAAGAGTGTTTCAA

             *         *   * **       *    *        * ** * *   **      *     
       3993 ACCTGCTCTATGAAAGGCCATGTTCATCTCTATGAGTTGAATGGAAATATCCGAAAGAAATTTCT
         66 AACTGCTCTATCAAAAGAAATGTTCAACTCTGTGAGTTGAGTACACACATCACAAAGAAGTTTCT

             *       *          *        *         **
       4058 GGGAATGCTGCTGTCTAG-TGTT-TAT-ACGA-ATTCCCGC
        131 GAGAATGCTTCTGTCTAGTTTTTATATGAAGATATTCCCTT

                         *       *                *    *  *                  
       4095 TTCCAACGAAATCCTCAAAGCAATCCAAATATCCACTTGCAGAATCCACAAAAAGAGTGTTTCAA
          1 TTCCAACGAAATCATCAAAGCGATCCAAATATCCACTTCCAGATTCTACAAAAAGAGTGTTTCAA

                          *     *          * *                   *  *        
       4160 AACTGCTCTATCAATAGAAAGGTTCAACTCTTTTAGTTGAGTACACACATCACGAACAAGTTTCT
         66 AACTGCTCTATCAAAAGAAATGTTCAACTCTGTGAGTTGAGTACACACATCACAAAGAAGTTTCT

                            * *      **     **  *    
       4225 GAGAATGCTTCTGTCTGGCTTTTATTGGAAGACGTTTCCTT
        131 GAGAATGCTTCTGTCTAGTTTTTATATGAAGATATTCCCTT

                 * *  **          *       *                * *               
       4266 TT-CACCAAAGGCATCAAAGCGCTCCAAATGTCCACTTCCAGATTCTTCCAAAAGAGTGTTTCAA
          1 TTCCAACGAAATCATCAAAGCGATCCAAATATCCACTTCCAGATTCTACAAAAAGAGTGTTTCAA

             **     * ***  * *                 *    * *  * *     *   *       
       4330 ACGTGCTCAAAGTAAGGGAATGTTCAACTCTGTGACTTGAATGCAGATATCACCAAGTAGTTTCT
         66 AACTGCTCTATCAAAAGAAATGTTCAACTCTGTGAGTTGAGTACACACATCACAAAGAAGTTTCT

            * * *             *   * *    *         * 
       4395 AATAGTGCTTCTGTCTAGATTTCAGATGATGATATTCCCGT
        131 GAGAATGCTTCTGTCTAGTTTTTATATGAAGATATTCCCTT

                         * * *   *                *   *                    * 
       4436 TTCCAACGAAATCGTTAGAGCTATCCAAATATCCACTTACAGTTTCTACAAAAAGAGTGTTTCCA
          1 TTCCAACGAAATCATCAAAGCGATCCAAATATCCACTTCCAGATTCTACAAAAAGAGTGTTTCAA

                   **           *            *       *                     * 
       4501 AACTGCTGCATCAAAAGAAAGGTTCAACTCTGTTAGTTGAGGACACACATCACAAAGAAGTTTGT
         66 AACTGCTCTATCAAAAGAAATGTTCAACTCTGTGAGTTGAGTACACACATCACAAAGAAGTTTCT

                              *    *     *           
       4566 GAGAATGCTTCTGTCTAGATTTTGTATGACGATATTCCCTT
        131 GAGAATGCTTCTGTCTAGTTTTTATATGAAGATATTCCCTT

                     *   * *     *   *       * *  *    *  *       *  *       
       4607 TTCCAACGATATCGTTAAAGCAATCTAAATATCAATTTGCAGAATCCACAAAAATAGAGTTTCAA
          1 TTCCAACGAAATCATCAAAGCGATCCAAATATCCACTTCCAGATTCTACAAAAAGAGTGTTTCAA

             *       * *    *   *    *       *  *                   ***      
       4672 AGCTGCTCTGTAAAAATAAAGGTTCCACTCTGTTAGCTGAGTACACACATCACAAACTTGTTTCT
         66 AACTGCTCTATCAAAAGAAATGTTCAACTCTGTGAGTTGAGTACACACATCACAAAGAAGTTTCT

                  *         *        **         **   
       4737 GAGAATCCTTCTGTCTCGTTTTTATGGGAAGATATTTACTT
        131 GAGAATGCTTCTGTCTAGTTTTTATATGAAGATATTCCCTT

              *  *  * **          *       *     *       *  *  *     *        
       4778 TTTCACCGTAGGCATCAAAGCGCTCCAAATGTCCACATCCAGATACTCCAGAAAGACTGTTTCAA
          1 TTCCAACGAAATCATCAAAGCGATCCAAATATCCACTTCCAGATTCTACAAAAAGAGTGTTTCAA

             *         *   * *   *         *        * *  *      *      *     
       4843 ACCTGCTCTATGAAAGGGAATCTTCAACTCTATGAGTTGAATGCAGACATCAGAAAGAAATTTCT
         66 AACTGCTCTATCAAAAGAAATGTTCAACTCTGTGAGTTGAGTACACACATCACAAAGAAGTTTCT

                     *       **          *         **
       4908 GAGAATGCTGCTGTCTACCTTTTAT-T--TGA-ATTCCCGC
        131 GAGAATGCTTCTGTCTAGTTTTTATATGAAGATATTCCCTT

                         *  *    *                *  **   *                  
       4945 TTCCAACGAAATCCTCCAAGCTATCCAAATATCCACTTGCATTTTCCACAAAAAGAGTGTTTCAA
          1 TTCCAACGAAATCATCAAAGCGATCCAAATATCCACTTCCAGATTCTACAAAAAGAGTGTTTCAA

                          *               ** *  *  *                *        
       5010 AACTGCTCTATCAATAGAAATGTTCAACTCCTTTAGCTGGGTACACACATCACAAACAAGTTTCT
         66 AACTGCTCTATCAAAAGAAATGTTCAACTCTGTGAGTTGAGTACACACATCACAAAGAAGTTTCT

                                     **     **       
       5075 GAGAATGCTTCTGTCTAGTTTTTATGGGAAGACGTTCCCTT
        131 GAGAATGCTTCTGTCTAGTTTTTATATGAAGATATTCCCTT

              *  * *  **          *       *            **                    
       5116 TTTCACCAAAGGCATCAAAGCGCTCCAAATGTCCACTTCCAGACACTACAAAAAGAGTGTTTCAA
          1 TTCCAACGAAATCATCAAAGCGATCCAAATATCCACTTCCAGATTCTACAAAAAGAGTGTTTCAA

             **       **   ***                 *    * *  * *         *       
       5181 ACGTGCTCTAAGAAAGCGAATGTTCAACTCTGTGACTTGAATGCAGATATCACAAAGTAGTTTCT
         66 AACTGCTCTATCAAAAGAAATGTTCAACTCTGTGAGTTGAGTACACACATCACAAAGAAGTTTCT

                **            *     *    *         * 
       5246 GAGAGGGCTTCTGTCTAGATTTTAGATGATGATATTCCCGT
        131 GAGAATGCTTCTGTCTAGTTTTTATATGAAGATATTCCCTT

                           * *   *                *   *                    * 
       5287 TTCCAACGAAATCATTAGAGCTATCCAAATATCCACTTACAGTTTCTACAAAAAGAGTGTTTCCA
          1 TTCCAACGAAATCATCAAAGCGATCCAAATATCCACTTCCAGATTCTACAAAAAGAGTGTTTCAA

                   **         * *    *       *  *                   ***      
       5352 AACTGCTGCATCAAAAGAGAGGTTCCACTCTGTTAGCTGAGTACACACATCACAAACTTGTTTCT
         66 AACTGCTCTATCAAAAGAAATGTTCAACTCTGTGAGTTGAGTACACACATCACAAAGAAGTTTCT

            *     *         *        **         **   
       5417 CAGAATCCTTCTGTCTCGTTTTTATGGGAAGATATTTACTT
        131 GAGAATGCTTCTGTCTAGTTTTTATATGAAGATATTCCCTT

              *  *  * **          *       *     *       *  *  *              
       5458 TTTCACCGTAGGCATCAAAGCGCTCCAAATGTCCACATCCAGATACTCCAGAAAGAGTGTTTCAA
          1 TTCCAACGAAATCATCAAAGCGATCCAAATATCCACTTCCAGATTCTACAAAAAGAGTGTTTCAA

             *         *   * *   *         *        * *  *      *      *     
       5523 ACCTGCTCTATGAAAGGGAATCTTCAACTCTATGAGTTGAATGCAGACATCAGAAAGAAATTTCT
         66 AACTGCTCTATCAAAAGAAATGTTCAACTCTGTGAGTTGAGTACACACATCACAAAGAAGTTTCT

                     *       **          *         **
       5588 GAGAATGCTGCTGTCTACCTTTTAT-T--TGA-ATTCCCGC
        131 GAGAATGCTTCTGTCTAGTTTTTATATGAAGATATTCCCTT

                         *  *    *                *       *                  
       5625 TTCCAACGAAATCCTCCAAGCTATCCAAATATCCACTTGCAGATTCCACAAAAAGAGTGTTTCAA
          1 TTCCAACGAAATCATCAAAGCGATCCAAATATCCACTTCCAGATTCTACAAAAAGAGTGTTTCAA

                            ** *  *            *       *     *     *  *      
       5690 AACTGCTCTCTATCAATGGCAAAGTTCAACTCTGTTAGTTGAGGACACATATCACCAACAAGTTT
         66 AACTG--CTCTATCAAAAGAAATGTTCAACTCTGTGAGTTGAGTACACACATCACAAAGAAGTTT

                               *       **         *    
       5755 CTGAGAATGCTTCTGTCTATTTTTTATGGGAAGATATTTCCTT
        129 CTGAGAATGCTTCTGTCTAGTTTTTATATGAAGATATTCCCTT

              *  *  * ** *    *      *    *          * **                    
       5798 TTTCACCGTAGGCGTCAAGGCGATCGAAATGTCCACTTCCACAAACTACAAAAAGAGTGTTTCAA
          1 TTCCAACGAAATCATCAAAGCGATCCAAATATCCACTTCCAGATTCTACAAAAAGAGTGTTTCAA

             *         *   * **       *    *        * ** * *   **      *     
       5863 ACCTGCTCTATGAAAGGCGATGTTCATCTCTATGAGTTGAATGGAAATATCCGAAAGAAATTTCT
         66 AACTGCTCTATCAAAAGAAATGTTCAACTCTGTGAGTTGAGTACACACATCACAAAGAAGTTTCT

             *       *                             **
       5928 GGGAATGCTGCTGTCTAGTTTTTATAT---GA-ATTCCCGC
        131 GAGAATGCTTCTGTCTAGTTTTTATATGAAGATATTCCCTT

                         *       *                *    *  *         *        
       5965 TTCCAACGAAATCCTCAAAGCAATCCAAATATCCACTTGCAGAATCCACAAAAAGATTGTTTCAA
          1 TTCCAACGAAATCATCAAAGCGATCCAAATATCCACTTCCAGATTCTACAAAAAGAGTGTTTCAA

                          *     *          * *                      *        
       6030 AACTGCTCTATCAATAGAAAGGTTCAACTCTTTTAGTTGAGTACACACATCACAAACAAGTTTCT
         66 AACTGCTCTATCAAAAGAAATGTTCAACTCTGTGAGTTGAGTACACACATCACAAAGAAGTTTCT

                            * *      **     **  *    
       6095 GAGAATGCTTCTGTCTGGCTTTTATTGGAAGACGTTTCCTT
        131 GAGAATGCTTCTGTCTAGTTTTTATATGAAGATATTCCCTT

                 * *  **        * *       *                * *               
       6136 TT-CACCAAAGGCATCAAAGTGCTCCAAATGTCCACTTCCAGATTCTTCCAAAAGAGTGTTTCAA
          1 TTCCAACGAAATCATCAAAGCGATCCAAATATCCACTTCCAGATTCTACAAAAAGAGTGTTTCAA

             **  *  * ***  * *                 *    * *  * *     *   *       
       6200 ACGTGGTCGAAGTAAGGGAATGTTCAACTCTGTGACTTGAATGCAGATATCACCAAGTAGTTTCT
         66 AACTGCTCTATCAAAAGAAATGTTCAACTCTGTGAGTTGAGTACACACATCACAAAGAAGTTTCT

            * * *            **     *    *         * 
       6265 AATAGTGCTTCTGTCTACATTTTAGATGATGATATTCCCGT
        131 GAGAATGCTTCTGTCTAGTTTTTATATGAAGATATTCCCTT

                         * * *   *             *  *   *      *     *       * 
       6306 TTCCAACGAAATCGTTAGAGCTATCCAAATATCCAGTTACAGTTTCTACCAAAAGGGTGTTTCCA
          1 TTCCAACGAAATCATCAAAGCGATCCAAATATCCACTTCCAGATTCTACAAAAAGAGTGTTTCAA

              *    **           *            *       *                     * 
       6371 AATTGCTGCATCAAAAGAAAGGTTCAACTCTGTTAGTTGAGGACACACATCACAAAGAAGTTTGT
         66 AACTGCTCTATCAAAAGAAATGTTCAACTCTGTGAGTTGAGTACACACATCACAAAGAAGTTTCT

                              *    *     *           
       6436 GAGAATGCTTCTGTCTAGATTTTGTATGACGATATTCCCTT
        131 GAGAATGCTTCTGTCTAGTTTTTATATGAAGATATTCCCTT

                     *   * *     *   *       * *  *    *  *       *  *       
       6477 TTCCAACGATATCGTTAAAGCAATCTAAATATCAATTTGCAGAATCCACAAAAATAGAGTTTCAA
          1 TTCCAACGAAATCATCAAAGCGATCCAAATATCCACTTCCAGATTCTACAAAAAGAGTGTTTCAA

             *       * *        *    *       *  *                   ***      
       6542 AGCTGCTCTGTAAAAAGAAAGGTTCCACTCTGTTAGCTGAGTACACACATCACAAACTTGTTTCT
         66 AACTGCTCTATCAAAAGAAATGTTCAACTCTGTGAGTTGAGTACACACATCACAAAGAAGTTTCT

                  *         *        **         **   
       6607 GAGAATCCTTCTGTCTCGTTTTTATGGGAAGATATTTACTT
        131 GAGAATGCTTCTGTCTAGTTTTTATATGAAGATATTCCCTT

                 *  * **          *       *     *       *  *  *  *           
       6648 TTCCACCGTAGGCATCAAAGCGCTCCAAATGTCCACATCCAGATACTCCAGAACGAGTGTTTCAA
          1 TTCCAACGAAATCATCAAAGCGATCCAAATATCCACTTCCAGATTCTACAAAAAGAGTGTTTCAA

             *         *   * *   *         *        * *  *      *      *     
       6713 ACCTGCTCTATGAAAGGGAATCTTCAACTCTATGAGTTGAATGCAGACATCAGAAAGAAATTTCT
         66 AACTGCTCTATCAAAAGAAATGTTCAACTCTGTGAGTTGAGTACACACATCACAAAGAAGTTTCT

                     *       **          *         **
       6778 GAGAATGCTGCTGTCTACCTTTTAT-T--TGA-ATTCCCGC
        131 GAGAATGCTTCTGTCTAGTTTTTATATGAAGATATTCCCTT

                         *  *    *              * *  **   *    *             
       6815 TTCCAACGAAATCCTCCAAGCTATCCAAATATCCACCTGCATTTTCCACAACAAGAGTGTTTCAA
          1 TTCCAACGAAATCATCAAAGCGATCCAAATATCCACTTCCAGATTCTACAAAAAGAGTGTTTCAA

                          *                 *    *  *                *       
       6880 AACTGCTCTATCAATAGAAATGTTCAACTCCTTTG-GCTGGGTACACACATCACAAACAAGTTTC
         66 AACTGCTCTATCAAAAGAAATGTTCAACT-CTGTGAGTTGAGTACACACATCACAAAGAAGTTTC

                                      **     **       
       6944 TGAGAATGCTTCTGTCTAGTTTTTATGGGAAGACGTTCCCTT
        130 TGAGAATGCTTCTGTCTAGTTTTTATATGAAGATATTCCCTT

              *  * *  **          *       *            **                  * 
       6986 TTTCACCAAAGGCATCAAAGCGCTCCAAATGTCCACTTCCAGACACTACAAAAAGAGTGTTTCCA
          1 TTCCAACGAAATCATCAAAGCGATCCAAATATCCACTTCCAGATTCTACAAAAAGAGTGTTTCAA

             **   *   **   ***                 *    * *  * *         *       
       7051 ACGTGCCCTAAGAAAGCGAATGTTCAACTCTGTGACTTGAATGCAGATATCACAAAGTAGTTTCT
         66 AACTGCTCTATCAAAAGAAATGTTCAACTCTGTGAGTTGAGTACACACATCACAAAGAAGTTTCT

                **            *     *    *         * 
       7116 GAGAGGGCTTCTGTCTAGATTTTAGATGATGATATTCCCGT
        131 GAGAATGCTTCTGTCTAGTTTTTATATGAAGATATTCCCTT

                           * *   *                *   *                    * 
       7157 TTCCAACGAAATCATTAGAGCTATCCAAATATCCACTTACAGTTTCTACAAAAAGAGTGTTTCCA
          1 TTCCAACGAAATCATCAAAGCGATCCAAATATCCACTTCCAGATTCTACAAAAAGAGTGTTTCAA

                   **         * *    *       *  *                   ***      
       7222 AACTGCTGCATCAAAAGAGAGGTTCCACTCTGTTAGCTGAGTACACACATCACAAACTTGTTTCT
         66 AACTGCTCTATCAAAAGAAATGTTCAACTCTGTGAGTTGAGTACACACATCACAAAGAAGTTTCT

            *     *         *        **         **   
       7287 CAGAATCCTTCTGTCTCGTTTTTATGGGAAGATATTTACTT
        131 GAGAATGCTTCTGTCTAGTTTTTATATGAAGATATTCCCTT

              *  *  * **          *       *     *       **            *      
       7328 TTTCACCGTAGGCATCAAAGCGCTCCAAATGTCCACATCCAGATAGTAC----AGAGTTTTTCAA
          1 TTCCAACGAAATCATCAAAGCGATCCAAATATCCACTTCCAGATTCTACAAAAAGAGTGTTTCAA

             *         *   * *   *         *        * *  *      *      *     
       7389 ACCTGCTCTATGAAAGGGAATCTTCAACTCTATGAGTTGAATGCAGACATCAGAAAGAAATTTCT
         66 AACTGCTCTATCAAAAGAAATGTTCAACTCTGTGAGTTGAGTACACACATCACAAAGAAGTTTCT

                     *       **          *         **
       7454 GAGAATGCTGCTGTCTACCTTTTAT-T--TGA-ATTCCCGC
        131 GAGAATGCTTCTGTCTAGTTTTTATATGAAGATATTCCCTT

                         *  *    *                *       *                  
       7491 TTCCAACGAAATCCTCCAAGCTATCCAAATATCCACTTGCAGATTCCACAAAAAGAGTGTTTCAA
          1 TTCCAACGAAATCATCAAAGCGATCCAAATATCCACTTCCAGATTCTACAAAAAGAGTGTTTCAA

                            ** *  *            *       *     *     *  *      
       7556 AACTGCTCTCTATCAATGGCAAAGTTCAACTCTGTTAGTTGAGGACACATATCACCAACAAGTTT
         66 AACTG--CTCTATCAAAAGAAATGTTCAACTCTGTGAGTTGAGTACACACATCACAAAGAAGTTT

                               *       **         *    
       7621 CTGAGAATGCTTCTGTCTATTTTTTATGGGAAGATATTTCCTT
        129 CTGAGAATGCTTCTGTCTAGTTTTTATATGAAGATATTCCCTT

              *  *  * ** *    *      *    *          * **                    
       7664 TTTCACCGTAGGCGTCAAGGCGATCGAAATGTCCACTTCCACAAACTACAAAAAGAGTGTTTCAA
          1 TTCCAACGAAATCATCAAAGCGATCCAAATATCCACTTCCAGATTCTACAAAAAGAGTGTTTCAA

             *         *   * **       *    *        * ** * *   **      *     
       7729 ACCTGCTCTATGAAAGGCCATGTTCATCTCTATGAGTTGAATGGAAATATCCGAAAGAAATTTCT
         66 AACTGCTCTATCAAAAGAAATGTTCAACTCTGTGAGTTGAGTACACACATCACAAAGAAGTTTCT

             *       *          *        *         **
       7794 GGGAATGCTGCTGTCTAG-TGTT-TAT-ACGA-ATTCCCGC
        131 GAGAATGCTTCTGTCTAGTTTTTATATGAAGATATTCCCTT

                         *       *                *    *  *                  
       7831 TTCCAACGAAATCCTCAAAGCAATCCAAATATCCACTTGCAGAATCCACAAAAAGAGTGTTTCAA
          1 TTCCAACGAAATCATCAAAGCGATCCAAATATCCACTTCCAGATTCTACAAAAAGAGTGTTTCAA

                          *     *          * *                   *  *        
       7896 AACTGCTCTATCAATAGAAAGGTTCAACTCTTTTAGTTGAGTACACACATCACGAACAAGTTTCT
         66 AACTGCTCTATCAAAAGAAATGTTCAACTCTGTGAGTTGAGTACACACATCACAAAGAAGTTTCT

                            * *      **     **  *    
       7961 GAGAATGCTTCTGTCTGGCTTTTATTGGAAGACGTTTCCTT
        131 GAGAATGCTTCTGTCTAGTTTTTATATGAAGATATTCCCTT

                 * *  **        * *       *                  *               
       8002 TT-CACCAAAGGCATCAAAGTGCTCCAAATGTCCACTTCCAGATTCTACCAAAAGAGTGTTTCAA
          1 TTCCAACGAAATCATCAAAGCGATCCAAATATCCACTTCCAGATTCTACAAAAAGAGTGTTTCAA

             **         **  * *                 *    * *  * *     *   *      
       8066 ACGTGCTC-ATAGTAAGGGAATGTTCAACTCTGTGACTTGAATGCAGATATCACCAAGTAGTTTC
         66 AACTGCTCTAT-CAAAAGAAATGTTCAACTCTGTGAGTTGAGTACACACATCACAAAGAAGTTTC

             * * *             *     *    *         * 
       8130 TAATAGTGCTTCTGTCTAGATTTTAGATGATGATATTCCCGT
        130 TGAGAATGCTTCTGTCTAGTTTTTATATGAAGATATTCCCTT

                         *** *   *             *  *   *      *     *       * 
       8172 TTCCAACGAAATCGCTAGAGCTATCCAAATATCCAGTTACAGTTTCTACCAAAAGGGTGTTTCCA
          1 TTCCAACGAAATCATCAAAGCGATCCAAATATCCACTTCCAGATTCTACAAAAAGAGTGTTTCAA

              *    **           *            *       *      *              * 
       8237 AATTGCTGCATCAAAAGAAAGGTTCAACTCTGTTAGTTGAGGACACACGTCACAAAGAAGTTTGT
         66 AACTGCTCTATCAAAAGAAATGTTCAACTCTGTGAGTTGAGTACACACATCACAAAGAAGTTTCT

                              *    *     *           
       8302 GAGAATGCTTCTGTCTAGATTTTGTATGACGATATTCCCTT
        131 GAGAATGCTTCTGTCTAGTTTTTATATGAAGATATTCCCTT

                     *   * *     *   *       * *  *    *  *       *  *       
       8343 TTCCAACGATATCGTTAAAGCAATCTAAATATCAATTTGCAGAATCCACAAAAATAGAGTTTCAA
          1 TTCCAACGAAATCATCAAAGCGATCCAAATATCCACTTCCAGATTCTACAAAAAGAGTGTTTCAA

             *       * *        *    *       *  *                   ***      
       8408 AGCTGCTCTGTAAAAAGAAAGGTTCCACTCTGTTAGCTGAGTACACACATCACAAACTTGTTTCT
         66 AACTGCTCTATCAAAAGAAATGTTCAACTCTGTGAGTTGAGTACACACATCACAAAGAAGTTTCT

                  *         *        **         **   
       8473 GAGAATCCTTCTGTCTCGTTTTTATGGGAAGATATTTACTT
        131 GAGAATGCTTCTGTCTAGTTTTTATATGAAGATATTCCCTT

                 *  * **          *       *     *       *  *  *  *           
       8514 TTCCACCGTAGGCATCAAAGCGCTCCAAATGTCCACATCCAGATACTCCAGAACGAGTGTTTCAA
          1 TTCCAACGAAATCATCAAAGCGATCCAAATATCCACTTCCAGATTCTACAAAAAGAGTGTTTCAA

             *         *   * *   *         *        * *  *      *      *     
       8579 ACCTGCTCTATGAAAGGGAATCTTCAACTCTATGAGTTGAATGCAGACATCAGAAAGAAATTTCT
         66 AACTGCTCTATCAAAAGAAATGTTCAACTCTGTGAGTTGAGTACACACATCACAAAGAAGTTTCT

                     *       **          *         **
       8644 GAGAATGCTGCTGTCTACCTTTTAT-T--TGA-ATTCCCGC
        131 GAGAATGCTTCTGTCTAGTTTTTATATGAAGATATTCCCTT

                         *  *    *              * *  **   *    *             
       8681 TTCCAACGAAATCCTCCAAGCTATCCAAATATCCACCTGCATTTTCCACAACAAGAGTGTTTCAA
          1 TTCCAACGAAATCATCAAAGCGATCCAAATATCCACTTCCAGATTCTACAAAAAGAGTGTTTCAA

                          *                 *    *  *                *       
       8746 AACTGCTCTATCAATAGAAATGTTCAACTCCTTTG-GCTGGGTACACACATCACAAACAAGTTTC
         66 AACTGCTCTATCAAAAGAAATGTTCAACT-CTGTGAGTTGAGTACACACATCACAAAGAAGTTTC

                                      **     **       
       8810 TGAGAATGCTTCTGTCTAGTTTTTATGGGAAGACGTTCCCTT
        130 TGAGAATGCTTCTGTCTAGTTTTTATATGAAGATATTCCCTT

              *  * *  **          *       *            **                  * 
       8852 TTTCACCAAAGGCATCAAAGCGCTCCAAATGTCCACTTCCAGACACTACAAAAAGAGTGTTTCCA
          1 TTCCAACGAAATCATCAAAGCGATCCAAATATCCACTTCCAGATTCTACAAAAAGAGTGTTTCAA

             **   *   **   ***                 *    * *  * *         *       
       8917 ACGTGCCCTAAGAAAGCGAATGTTCAACTCTGTGACTTGAATGCAGATATCACAAAGTAGTTTCT
         66 AACTGCTCTATCAAAAGAAATGTTCAACTCTGTGAGTTGAGTACACACATCACAAAGAAGTTTCT

                **            *     *    *         * 
       8982 GAGAGGGCTTCTGTCTAGATTTTAGATGATGATATTCCCGT
        131 GAGAATGCTTCTGTCTAGTTTTTATATGAAGATATTCCCTT

                           * *   *                *   *                    * 
       9023 TTCCAACGAAATCATTAGAGCTATCCAAATATCCACTTACAGTTTCTACAAAAAGAGTGTTTCCA
          1 TTCCAACGAAATCATCAAAGCGATCCAAATATCCACTTCCAGATTCTACAAAAAGAGTGTTTCAA

                   **         * *    *       *  *                   ***      
       9088 AACTGCTGCATCAAAAGAGAGGTTCCACTCTGTTAGCTGAGTACACACATCACAAACTTGTTTCT
         66 AACTGCTCTATCAAAAGAAATGTTCAACTCTGTGAGTTGAGTACACACATCACAAAGAAGTTTCT

            *     *         *        **         **   
       9153 CAGAATCCTTCTGTCTCGTTTTTATGGGAAGATATTTACTT
        131 GAGAATGCTTCTGTCTAGTTTTTATATGAAGATATTCCCTT

              *  *  * **          *       *     *       *  *  *              
       9194 TTTCACCGTAGGCATCAAAGCGCTCCAAATGTCCACATCCAGATACTCCAGAAAGAGTGTTTCAA
          1 TTCCAACGAAATCATCAAAGCGATCCAAATATCCACTTCCAGATTCTACAAAAAGAGTGTTTCAA

             *         *   * *   *         *        * *  *      *      *     
       9259 ACCTGCTCTATGAAAGGGAATCTTCAACTCTATGAGTTGAATGCAGACATCAGAAAGAAATTTCT
         66 AACTGCTCTATCAAAAGAAATGTTCAACTCTGTGAGTTGAGTACACACATCACAAAGAAGTTTCT

                     *       **          *         **
       9324 GAGAATGCTGCTGTCTACCTTTTAT-T--TGA-ATTCCCGC
        131 GAGAATGCTTCTGTCTAGTTTTTATATGAAGATATTCCCTT

                         *  *    *                *       *                  
       9361 TTCCAACGAAATCCTCCAAGCTATCCAAATATCCACTTGCAGATTCCACAAAAAGAGTGTTTCAA
          1 TTCCAACGAAATCATCAAAGCGATCCAAATATCCACTTCCAGATTCTACAAAAAGAGTGTTTCAA

                            ** *  *            *       *     *     *  *      
       9426 AACTGCTCTCTATCAATGGCAAAGTTCAACTCTGTTAGTTGAGGACACATATCACCAACAAGTTT
         66 AACTG--CTCTATCAAAAGAAATGTTCAACTCTGTGAGTTGAGTACACACATCACAAAGAAGTTT

                               *       **         *    
       9491 CTGAGAATGCTTCTGTCTATTTTTTATGGGAAGATATTTCCTT
        129 CTGAGAATGCTTCTGTCTAGTTTTTATATGAAGATATTCCCTT

              *  *  * ** *    *      *    *          * **          *         
       9534 TTTCACCGTAGGCGTCAAGGCGATCGAAATGTCCACTTCCACAAACTACAAAAAGTGTGTTTCAA
          1 TTCCAACGAAATCATCAAAGCGATCCAAATATCCACTTCCAGATTCTACAAAAAGAGTGTTTCAA

             *         *   * **       *    *        * ** * *   **      *     
       9599 ACCTGCTCTATGAAAGGCCATGTTCATCTCTATGAGTTGAATGGAAATATCCGAAAGAAATTTCT
         66 AACTGCTCTATCAAAAGAAATGTTCAACTCTGTGAGTTGAGTACACACATCACAAAGAAGTTTCT

             *       *          *        *         **
       9664 GGGAATGCTGCTGTCTAG-TGTT-TAT-ACGA-ATTCCCGC
        131 GAGAATGCTTCTGTCTAGTTTTTATATGAAGATATTCCCTT

                         *       *                *    *  *                  
       9701 TTCCAACGAAATCCTCAAAGCAATCCAAATATCCACTTGCAGAATCCACAAAAAGAGTGTTTCAA
          1 TTCCAACGAAATCATCAAAGCGATCCAAATATCCACTTCCAGATTCTACAAAAAGAGTGTTTCAA

                          *     *          * *                   *  *        
       9766 AACTGCTCTATCAATAGAAAGGTTCAACTCTTTTAGTTGAGTACACACATCACGAACAAGTTTCT
         66 AACTGCTCTATCAAAAGAAATGTTCAACTCTGTGAGTTGAGTACACACATCACAAAGAAGTTTCT

                            * *      **     **  *    
       9831 GAGAATGCTTCTGTCTGGCTTTTATTGGAAGACGTTTCCTT
        131 GAGAATGCTTCTGTCTAGTTTTTATATGAAGATATTCCCTT

                 * *  **          *       *                * *               
       9872 TT-CACCAAAGGCATCAAAGCGCTCCAAATGTCCACTTCCAGATTCTTCCAAAAGAGTGTTTCAA
          1 TTCCAACGAAATCATCAAAGCGATCCAAATATCCACTTCCAGATTCTACAAAAAGAGTGTTTCAA

             **     * ***  * *                 *    * *  * *     *   *       
       9936 ACGTGCTCAAAGTAAGGGAATGTTCAACTCTGTGACTTGAATGCAGATATCACCAAGTAGTTTCT
         66 AACTGCTCTATCAAAAGAAATGTTCAACTCTGTGAGTTGAGTACACACATCACAAAGAAGTTTCT

            * * *             *   * *    *         * 
      10001 AATAGTGCTTCTGTCTAGATTTCAGATGATGATATTCCCGT
        131 GAGAATGCTTCTGTCTAGTTTTTATATGAAGATATTCCCTT

                         * * *   * **          *  *   *      *     *       * 
      10042 TTCCAACGAAATCGTTAGAGCTAAGCAAATATCCAGTTACAGTTTCTACCAAAAGGGTGTTTCCA
          1 TTCCAACGAAATCATCAAAGCGATCCAAATATCCACTTCCAGATTCTACAAAAAGAGTGTTTCAA

              *    **           *            *       *                     * 
      10107 AATTGCTGCATCAAAAGAAAGGTTCAACTCTGTTAGTTGAGGACACACATCACAAAGAAGTTTGT
         66 AACTGCTCTATCAAAAGAAATGTTCAACTCTGTGAGTTGAGTACACACATCACAAAGAAGTTTCT

                              *    *     *           
      10172 GAGAATGCTTCTGTCTAGATTTTGTATGACGATATTCCCTT
        131 GAGAATGCTTCTGTCTAGTTTTTATATGAAGATATTCCCTT

                     *   * *     *   *       * *  *    *  *       *  *       
      10213 TTCCAACGATATCGTTAAAGCAATCTAAATATCAATTTGCAGAATCCACAAAAATAGAGTTTCAA
          1 TTCCAACGAAATCATCAAAGCGATCCAAATATCCACTTCCAGATTCTACAAAAAGAGTGTTTCAA

             *       * *        *    *       *  *                   ***      
      10278 AGCTGCTCTGTAAAAAGAAAGGTTCCACTCTGTTAGCTGAGTACACACATCACAAACTTGTTTCT
         66 AACTGCTCTATCAAAAGAAATGTTCAACTCTGTGAGTTGAGTACACACATCACAAAGAAGTTTCT

                  *         *        **         **   
      10343 GAGAATCCTTCTGTCTCGTTTTTATGGGAAGATATTTACTT
        131 GAGAATGCTTCTGTCTAGTTTTTATATGAAGATATTCCCTT

                 *  * **          *       *     *       *  *  *  *           
      10384 TTCCACCGTAGGCATCAAAGCGCTCCAAATGTCCACATCCAGATACTCCAGAACGAGTGTTTCAA
          1 TTCCAACGAAATCATCAAAGCGATCCAAATATCCACTTCCAGATTCTACAAAAAGAGTGTTTCAA

             *         *   * *   *         *        * *  *      *      *     
      10449 ACCTGCTCTATGAAAGGGAATCTTCAACTCTATGAGTTGAATGCAGACATCAGAAAGAAATTTCT
         66 AACTGCTCTATCAAAAGAAATGTTCAACTCTGTGAGTTGAGTACACACATCACAAAGAAGTTTCT

                     *       **          *         **
      10514 GAGAATGCTGCTGTCTACCTTTTAT-T--TGA-ATTCCCGC
        131 GAGAATGCTTCTGTCTAGTTTTTATATGAAGATATTCCCTT

                         *  *    *              * *  **   *    *             
      10551 TTCCAACGAAATCCTCCAAGCTATCCAAATATCCACCTGCATTTTCCACAACAAGAGTGTTTCAA
          1 TTCCAACGAAATCATCAAAGCGATCCAAATATCCACTTCCAGATTCTACAAAAAGAGTGTTTCAA

                          *                 *    *  *                *       
      10616 AACTGCTCTATCAATAGAAATGTTCAACTCCTTTG-GCTGGGTACACACATCACAAACAAGTTTC
         66 AACTGCTCTATCAAAAGAAATGTTCAACT-CTGTGAGTTGAGTACACACATCACAAAGAAGTTTC

                                      **     **       
      10680 TGAGAATGCTTCTGTCTAGTTTTTATGGGAAGACGTTCCCTT
        130 TGAGAATGCTTCTGTCTAGTTTTTATATGAAGATATTCCCTT

              *  * *  **          *       *            **                  * 
      10722 TTTCACCAAAGGCATCAAAGCGCTCCAAATGTCCACTTCCAGACACTACAAAAAGAGTGTTTCCA
          1 TTCCAACGAAATCATCAAAGCGATCCAAATATCCACTTCCAGATTCTACAAAAAGAGTGTTTCAA

             **   *   **   ***                 *    * *  * *         *       
      10787 ACGTGCCCTAAGAAAGCGAATGTTCAACTCTGTGACTTGAATGCAGATATCACAAAGTAGTTTCT
         66 AACTGCTCTATCAAAAGAAATGTTCAACTCTGTGAGTTGAGTACACACATCACAAAGAAGTTTCT

                **            *     *    *         * 
      10852 GAGAGGGCTTCTGTCTAGATTTTAGATGATGATATTCCCGT
        131 GAGAATGCTTCTGTCTAGTTTTTATATGAAGATATTCCCTT

                           * *   *                *   *                    * 
      10893 TTCCAACGAAATCATTAGAGCTATCCAAATATCCACTTACAGTTTCTACAAAAAGAGTGTTTCCA
          1 TTCCAACGAAATCATCAAAGCGATCCAAATATCCACTTCCAGATTCTACAAAAAGAGTGTTTCAA

                   **         * *    *       *  *                   ***      
      10958 AACTGCTGCATCAAAAGAGAGGTTCCACTCTGTTAGCTGAGTACACACATCACAAACTTGTTTCT
         66 AACTGCTCTATCAAAAGAAATGTTCAACTCTGTGAGTTGAGTACACACATCACAAAGAAGTTTCT

            *     *         *        **         **   
      11023 CAGAATCCTTCTGTCTCGTTTTTATGGGAAGATATTTACTT
        131 GAGAATGCTTCTGTCTAGTTTTTATATGAAGATATTCCCTT

              *  *  * **          *       *     *       *  *  *              
      11064 TTTCACCGTAGGCATCAAAGCGCTCCAAATGTCCACATCCAGATACTCCAGAAAGAGTGTTTCAA
          1 TTCCAACGAAATCATCAAAGCGATCCAAATATCCACTTCCAGATTCTACAAAAAGAGTGTTTCAA

             *         *   * *   *         *        * *  *      *      *     
      11129 ACCTGCTCTATGAAAGGGAATCTTCAACTCTATGAGTTGAATGCAGACATCAGAAAGAAATTTCT
         66 AACTGCTCTATCAAAAGAAATGTTCAACTCTGTGAGTTGAGTACACACATCACAAAGAAGTTTCT

                     *       **          *         **
      11194 GAGAATGCTGCTGTCTACCTTTTAT-T--TGA-ATTCCCGC
        131 GAGAATGCTTCTGTCTAGTTTTTATATGAAGATATTCCCTT

                         *  *    *                *       *                  
      11231 TTCCAACGAAATCCTCCAAGCTATCCAAATATCCACTTGCAGATTCCACAAAAAGAGTGTTTCAA
          1 TTCCAACGAAATCATCAAAGCGATCCAAATATCCACTTCCAGATTCTACAAAAAGAGTGTTTCAA

                            ** *  *            *       *     *     *  *      
      11296 AACTGCTCTCTATCAATGGCAAAGTTCAACTCTGTTAGTTGAGGACACATATCACCAACAAGTTT
         66 AACTG--CTCTATCAAAAGAAATGTTCAACTCTGTGAGTTGAGTACACACATCACAAAGAAGTTT

                               *       **         *    
      11361 CTGAGAATGCTTCTGTCTATTTTTTATGGGAAGATATTTCCTT
        129 CTGAGAATGCTTCTGTCTAGTTTTTATATGAAGATATTCCCTT

              *  *  * ** *    *      *    *          * **                    
      11404 TTTCACCGTAGGCGTCAAGGCGATCGAAATGTCCACTTCCACAAACTACAAAAAGAGTGTTTCAA
          1 TTCCAACGAAATCATCAAAGCGATCCAAATATCCACTTCCAGATTCTACAAAAAGAGTGTTTCAA

             *         *   * **       *    *        * ** * *   **      *     
      11469 ACCTGCTCTATGAAAGGCCATGTTCATCTCTATGAGTTGAATGGAAATATCCGAAAGAAATTTCT
         66 AACTGCTCTATCAAAAGAAATGTTCAACTCTGTGAGTTGAGTACACACATCACAAAGAAGTTTCT

             *       *                             **
      11534 GGGAATGCTGCTGTCTAGTTTTTATAT---GA-ATTCCCGC
        131 GAGAATGCTTCTGTCTAGTTTTTATATGAAGATATTCCCTT

                         *       *                *    *  *                  
      11571 TTCCAACGAAATCCTCAAAGCAATCCAAATATCCACTTGCAGAATCCACAAAAAGAGTGTTTCAA
          1 TTCCAACGAAATCATCAAAGCGATCCAAATATCCACTTCCAGATTCTACAAAAAGAGTGTTTCAA

                          *     *          * *                      *        
      11636 AACTGCTCTATCAATAGAAAGGTTCAACTCTTTTAGTTGAGTACACACATCACAAACAAGTTTCT
         66 AACTGCTCTATCAAAAGAAATGTTCAACTCTGTGAGTTGAGTACACACATCACAAAGAAGTTTCT

                            * *      **     **  *    
      11701 GAGAATGCTTCTGTCTGGCTTTTATTGGAAGACGTTTCCTT
        131 GAGAATGCTTCTGTCTAGTTTTTATATGAAGATATTCCCTT

                 * *  **          *       *                * *               
      11742 TT-CACCAAAGGCATCAAAGCGCTCCAAATGTCCACTTCCAGATTCTTCCAAAAGAGTGTTTCAA
          1 TTCCAACGAAATCATCAAAGCGATCCAAATATCCACTTCCAGATTCTACAAAAAGAGTGTTTCAA

             **  *  * ***  * *                 *    * *  * *     *   *       
      11806 ACGTGGTCGAAGTAAGGGAATGTTCAACTCTGTGACTTGAATGCAGATATCACCAAGTAGTTTCT
         66 AACTGCTCTATCAAAAGAAATGTTCAACTCTGTGAGTTGAGTACACACATCACAAAGAAGTTTCT

            * * *            **     *    *         * 
      11871 AATAGTGCTTCTGTCTACATTTTAGATGATGATATTCCCGT
        131 GAGAATGCTTCTGTCTAGTTTTTATATGAAGATATTCCCTT

                         * * *   * **          *  *   *      *     *       * 
      11912 TTCCAACGAAATCGTTAGAGCTAAGCAAATATCCAGTTACAGTTTCTACCAAAAGGGTGTTTCCA
          1 TTCCAACGAAATCATCAAAGCGATCCAAATATCCACTTCCAGATTCTACAAAAAGAGTGTTTCAA

              *    **           *            *       *                     * 
      11977 AATTGCTGCATCAAAAGAAAGGTTCAACTCTGTTAGTTGAGGACACACATCACAAAGAAGTTTGT
         66 AACTGCTCTATCAAAAGAAATGTTCAACTCTGTGAGTTGAGTACACACATCACAAAGAAGTTTCT

                              *    *     *           
      12042 GAGAATGCTTCTGTCTAGATTTTGTATGACGATATTCCCTT
        131 GAGAATGCTTCTGTCTAGTTTTTATATGAAGATATTCCCTT

                     *   * *     *   *       * *  *    *  *       *  *       
      12083 TTCCAACGATATCGTTAAAGCAATCTAAATATCAATTTGCAGAATCCACAAAAATAGAGTTTCAA
          1 TTCCAACGAAATCATCAAAGCGATCCAAATATCCACTTCCAGATTCTACAAAAAGAGTGTTTCAA

             *       * *        *    *       *  *                   ***      
      12148 AGCTGCTCTGTAAAAAGAAAGGTTCCACTCTGTTAGCTGAGTACACACATCACAAACTTGTTTCT
         66 AACTGCTCTATCAAAAGAAATGTTCAACTCTGTGAGTTGAGTACACACATCACAAAGAAGTTTCT

                  *         *        **         **   
      12213 GAGAATCCTTCTGTCTCGTTTTTATGGGAAGATATTTACTT
        131 GAGAATGCTTCTGTCTAGTTTTTATATGAAGATATTCCCTT

                 *  * **          *       *     *       *  *  *              
      12254 TTCCACCGTAGGCATCAAAGCGCTCCAAATGTCCACATCCAGATACTCCAGAAAGAGTGTTTCAA
          1 TTCCAACGAAATCATCAAAGCGATCCAAATATCCACTTCCAGATTCTACAAAAAGAGTGTTTCAA

             *         *   * *   *         *        * *  *      *      *     
      12319 ACCTGCTCTATGAAAGGGAATCTTCAACTCTATGAGTTGAATGCAGACATCAGAAAGAAATTTCT
         66 AACTGCTCTATCAAAAGAAATGTTCAACTCTGTGAGTTGAGTACACACATCACAAAGAAGTTTCT

                     *       **          *         **
      12384 GAGAATGCTGCTGTCTACCTTTTAT-T--TGA-ATTCCCGC
        131 GAGAATGCTTCTGTCTAGTTTTTATATGAAGATATTCCCTT

                         *  *    *              * *  **   *                  
      12421 TTCCAACGAAATCCTCCAAGCTATCCAAATATCCACCTGCATTTTCCACAAAAAGAGTGTTTCAA
          1 TTCCAACGAAATCATCAAAGCGATCCAAATATCCACTTCCAGATTCTACAAAAAGAGTGTTTCAA

                          *                 *    *  *                *       
      12486 AACTGCTCTATCAATAGAAATGTTCAACTCCTTTG-GCTGGGTACACACATCACAAACAAGTTTC
         66 AACTGCTCTATCAAAAGAAATGTTCAACT-CTGTGAGTTGAGTACACACATCACAAAGAAGTTTC

                                      **     **       
      12550 TGAGAATGCTTCTGTCTAGTTTTTATGGGAAGACGTTCCCTT
        130 TGAGAATGCTTCTGTCTAGTTTTTATATGAAGATATTCCCTT

              *  * *  **          *       *            **                    
      12592 TTTCACCAAAGGCATCAAAGCGCTCCAAATGTCCACTTCCAGACACTACAAAAAGAGTGTTTCAA
          1 TTCCAACGAAATCATCAAAGCGATCCAAATATCCACTTCCAGATTCTACAAAAAGAGTGTTTCAA

             **       **   ***                 *    * *  * *         *       
      12657 ACGTGCTCTAAGAAAGCGAATGTTCAACTCTGTGACTTGAATGCAGATATCACAAAGTAGTTTCT
         66 AACTGCTCTATCAAAAGAAATGTTCAACTCTGTGAGTTGAGTACACACATCACAAAGAAGTTTCT

                **            *     *    *         * 
      12722 GAGAGGGCTTCTGTCTAGATTTTAGATGATGATATTCCCGT
        131 GAGAATGCTTCTGTCTAGTTTTTATATGAAGATATTCCCTT

                           * *   *                *   *                    * 
      12763 TTCCAACGAAATCATTAGAGCTATCCAAATATCCACTTACAGTTTCTACAAAAAGAGTGTTTCCA
          1 TTCCAACGAAATCATCAAAGCGATCCAAATATCCACTTCCAGATTCTACAAAAAGAGTGTTTCAA

                   **         * *    *       *  *                   ***      
      12828 AACTGCTGCATCAAAAGAGAGGTTCCACTCTGTTAGCTGAGTACACACATCACAAACTTGTTTCT
         66 AACTGCTCTATCAAAAGAAATGTTCAACTCTGTGAGTTGAGTACACACATCACAAAGAAGTTTCT

            *     *         *        **         **   
      12893 CAGAATCCTTCTGTCTCGTTTTTATGGGAAGATATTTACTT
        131 GAGAATGCTTCTGTCTAGTTTTTATATGAAGATATTCCCTT

              *  *  * **          *       *     *       **    *       *      
      12934 TTTCACCGTAGGCATCAAAGCGCTCCAAATGTCCACATCCAGATAGTACAGAAAGAGTTTTTCAA
          1 TTCCAACGAAATCATCAAAGCGATCCAAATATCCACTTCCAGATTCTACAAAAAGAGTGTTTCAA

             *         *   * *   *         *        * *  *      *      *     
      12999 ACCTGCTCTATGAAAGGGAATCTTCAACTCTATGAGTTGAATGCAGACATCAGAAAGAAATTTCT
         66 AACTGCTCTATCAAAAGAAATGTTCAACTCTGTGAGTTGAGTACACACATCACAAAGAAGTTTCT

                     *       **          *         **
      13064 GAGAATGCTGCTGTCTACCTTTTAT-T--TGA-ATTCCCGC
        131 GAGAATGCTTCTGTCTAGTTTTTATATGAAGATATTCCCTT

                         *  *    *                *       *                  
      13101 TTCCAACGAAATCCTCCAAGCTATCCAAATATCCACTTGCAGATTCCACAAAAAGAGTGTTTCAA
          1 TTCCAACGAAATCATCAAAGCGATCCAAATATCCACTTCCAGATTCTACAAAAAGAGTGTTTCAA

                            ** *  *            *       *     *     *  *      
      13166 AACTGCTCTCTATCAATGGCAAAGTTCAACTCTGTTAGTTGAGGACACATATCACCAACAAGTTT
         66 AACTG--CTCTATCAAAAGAAATGTTCAACTCTGTGAGTTGAGTACACACATCACAAAGAAGTTT

                               *       **         *    
      13231 CTGAGAATGCTTCTGTCTATTTTTTATGGGAAGATATTTCCTT
        129 CTGAGAATGCTTCTGTCTAGTTTTTATATGAAGATATTCCCTT

              *  *  * ** *    *      *    *          * **                    
      13274 TTTCACCGTAGGCGTCAAGGCGATCGAAATGTCCACTTCCACAAACTACAAAAAGAGTGTTTCAA
          1 TTCCAACGAAATCATCAAAGCGATCCAAATATCCACTTCCAGATTCTACAAAAAGAGTGTTTCAA

             *         *   * **       *    *        * ** * *   **      *     
      13339 ACCTGCTCTATGAAAGGCCATGTTCATCTCTATGAGTTGAATGGAAATATCCGAAAGAAATTTCT
         66 AACTGCTCTATCAAAAGAAATGTTCAACTCTGTGAGTTGAGTACACACATCACAAAGAAGTTTCT

             *       *          *        *         **
      13404 GGGAATGCTGCTGTCTAG-TGTT-TAT-ACGA-ATTCCCGC
        131 GAGAATGCTTCTGTCTAGTTTTTATATGAAGATATTCCCTT

                         *       *                *    *  *                  
      13441 TTCCAACGAAATCCTCAAAGCAATCCAAATATCCACTTGCAGAATCCACAAAAAGAGTGTTTCAA
          1 TTCCAACGAAATCATCAAAGCGATCCAAATATCCACTTCCAGATTCTACAAAAAGAGTGTTTCAA

                          *     *          * *                   *  *        
      13506 AACTGCTCTATCAATAGAAAGGTTCAACTCTTTTAGTTGAGTACACACATCACGAACAAGTTTCT
         66 AACTGCTCTATCAAAAGAAATGTTCAACTCTGTGAGTTGAGTACACACATCACAAAGAAGTTTCT

                            * *      **     **  *    
      13571 GAGAATGCTTCTGTCTGGCTTTTATTGGAAGACGTTTCCTT
        131 GAGAATGCTTCTGTCTAGTTTTTATATGAAGATATTCCCTT

                   *  **          *       *                * *               
      13612 TT-CAACAAAGGCATCAAAGCGCTCCAAATGTCCACTTCCAGATTCTTCCAAAAGAGTGTTTCAA
          1 TTCCAACGAAATCATCAAAGCGATCCAAATATCCACTTCCAGATTCTACAAAAAGAGTGTTTCAA

             **     * ***  * *                 *    * *  * *     *   *       
      13676 ACGTGCTCGAAGTAAGGGAATGTTCAACTCTGTGACTTGAATGCAGATATCACCAAGTAGTTTCT
         66 AACTGCTCTATCAAAAGAAATGTTCAACTCTGTGAGTTGAGTACACACATCACAAAGAAGTTTCT

            * * *            **     *    *         * 
      13741 AATAGTGCTTCTGTCTACATTTTAGATGATGATATTCCCGT
        131 GAGAATGCTTCTGTCTAGTTTTTATATGAAGATATTCCCTT

                         * * *   *             *  *   *      *     *       * 
      13782 TTCCAACGAAATCGTTAGAGCTATCCAAATATCCAGTTACAGTTTCTACCAAAAGGGTGTTTCCA
          1 TTCCAACGAAATCATCAAAGCGATCCAAATATCCACTTCCAGATTCTACAAAAAGAGTGTTTCAA

              *    **           *            *       *                     * 
      13847 AATTGCTGCATCAAAAGAAAGGTTCAACTCTGTTAGTTGAGGACACACATCACAAAGAAGTTTGT
         66 AACTGCTCTATCAAAAGAAATGTTCAACTCTGTGAGTTGAGTACACACATCACAAAGAAGTTTCT

                              *    *     *           
      13912 GAGAATGCTTCTGTCTAGATTTTGTATGACGATATTCCCTT
        131 GAGAATGCTTCTGTCTAGTTTTTATATGAAGATATTCCCTT

                     *   * *     *   *       * *  *    *  *       *  *       
      13953 TTCCAACGATATCGTTAAAGCAATCTAAATATCAATTTGCAGAATCCACAAAAATAGAGTTTCAA
          1 TTCCAACGAAATCATCAAAGCGATCCAAATATCCACTTCCAGATTCTACAAAAAGAGTGTTTCAA

             *       * *        *    *       *  *                   ***      
      14018 AGCTGCTCTGTAAAAAGAAAGGTTCCACTCTGTTAGCTGAGTACACACATCACAAACTTGTTTCT
         66 AACTGCTCTATCAAAAGAAATGTTCAACTCTGTGAGTTGAGTACACACATCACAAAGAAGTTTCT

                  *         *        **         **   
      14083 GAGAATCCTTCTGTCTCGTTTTTATGGGAAGATATTTACTT
        131 GAGAATGCTTCTGTCTAGTTTTTATATGAAGATATTCCCTT

              *  *  * **          *       *     *       *  *  *  *           
      14124 TTTCACCGTAGGCATCAAAGCGCTCCAAATGTCCACATCCAGATACTCCAGAACGAGTGTTTCAA
          1 TTCCAACGAAATCATCAAAGCGATCCAAATATCCACTTCCAGATTCTACAAAAAGAGTGTTTCAA

             *         *   * *   *         *        * *  *      *      *     
      14189 ACCTGCTCTATGAAAGGGAATCTTCAACTCTATGAGTTGAATGCAGACATCAGAAAGAAATTTCT
         66 AACTGCTCTATCAAAAGAAATGTTCAACTCTGTGAGTTGAGTACACACATCACAAAGAAGTTTCT

                     *       **          *         **
      14254 GAGAATGCTGCTGTCTACCTTTTAT-T--TGA-ATTCCCGC
        131 GAGAATGCTTCTGTCTAGTTTTTATATGAAGATATTCCCTT

                         *  *    *              * *  **   *    *             
      14291 TTCCAACGAAATCCTCCAAGCTATCCAAATATCCACCTGCATTTTCCACAACAAGAGTGTTTCAA
          1 TTCCAACGAAATCATCAAAGCGATCCAAATATCCACTTCCAGATTCTACAAAAAGAGTGTTTCAA

                          *                 *    *  *                *       
      14356 AACTGCTCTATCAATAGAAATGTTCAACTCCTTTG-GCTGGGTACACACATCACAAACAAGTTTC
         66 AACTGCTCTATCAAAAGAAATGTTCAACT-CTGTGAGTTGAGTACACACATCACAAAGAAGTTTC

                                      **     **       
      14420 TGAGAATGCTTCTGTCTAGTTTTTATGGGAAGACGTTCCCTT
        130 TGAGAATGCTTCTGTCTAGTTTTTATATGAAGATATTCCCTT

              *  * *  **          *       *            **                  * 
      14462 TTTCACCAAAGGCATCAAAGCGCTCCAAATGTCCACTTCCAGACACTACAAAAAGAGTGTTTCCA
          1 TTCCAACGAAATCATCAAAGCGATCCAAATATCCACTTCCAGATTCTACAAAAAGAGTGTTTCAA

             **   *   **   ***                 *    * *  * *         *       
      14527 ACGTGCCCTAAGAAAGCGAATGTTCAACTCTGTGACTTGAATGCAGATATCACAAAGTAGTTTCT
         66 AACTGCTCTATCAAAAGAAATGTTCAACTCTGTGAGTTGAGTACACACATCACAAAGAAGTTTCT

                **            *     *    *         * 
      14592 GAGAGGGCTTCTGTCTAGATTTTAGATGATGATATTCCCGT
        131 GAGAATGCTTCTGTCTAGTTTTTATATGAAGATATTCCCTT

                           * *   *                *   *                    * 
      14633 TTCCAACGAAATCATTAGAGCTATCCAAATATCCACTTACAGTTTCTACAAAAAGAGTGTTTCCA
          1 TTCCAACGAAATCATCAAAGCGATCCAAATATCCACTTCCAGATTCTACAAAAAGAGTGTTTCAA

                   **         * *    *       *  *                   ***      
      14698 AACTGCTGCATCAAAAGAGAGGTTCCACTCTGTTAGCTGAGTACACACATCACAAACTTGTTTCT
         66 AACTGCTCTATCAAAAGAAATGTTCAACTCTGTGAGTTGAGTACACACATCACAAAGAAGTTTCT

            *     *         *        **         **   
      14763 CAGAATCCTTCTGTCTCGTTTTTATGGGAAGATATTTACTT
        131 GAGAATGCTTCTGTCTAGTTTTTATATGAAGATATTCCCTT

              *  *  * **          *       *     *       *  *  *              
      14804 TTTCACCGTAGGCATCAAAGCGCTCCAAATGTCCACATCCAGATACTCCAGAAAGAGTGTTTCAA
          1 TTCCAACGAAATCATCAAAGCGATCCAAATATCCACTTCCAGATTCTACAAAAAGAGTGTTTCAA

             *         *   * *   *         *        * *  *      *      *     
      14869 ACCTGCTCTATGAAAGGGAATCTTCAACTCTATGAGTTGAATGCAGACATCAGAAAGAAATTTCT
         66 AACTGCTCTATCAAAAGAAATGTTCAACTCTGTGAGTTGAGTACACACATCACAAAGAAGTTTCT

                     *       **          *         **
      14934 GAGAATGCTGCTGTCTACCTTTTAT-T--TGA-ATTCCCGC
        131 GAGAATGCTTCTGTCTAGTTTTTATATGAAGATATTCCCTT

                         *  *    *                *       *                  
      14971 TTCCAACGAAATCCTCCAAGCTATCCAAATATCCACTTGCAGATTCCACAAAAAGAGTGTTTCAA
          1 TTCCAACGAAATCATCAAAGCGATCCAAATATCCACTTCCAGATTCTACAAAAAGAGTGTTTCAA

                            ** *  *            *       *     *     *  *      
      15036 AACTGCTCTCTATCAATGGCAAAGTTCAACTCTGTTAGTTGAGGACACATATCACCAACAAGTTT
         66 AACTG--CTCTATCAAAAGAAATGTTCAACTCTGTGAGTTGAGTACACACATCACAAAGAAGTTT

                               *       **         *    
      15101 CTGAGAATGCTTCTGTCTATTTTTTATGGGAAGATATTTCCTT
        129 CTGAGAATGCTTCTGTCTAGTTTTTATATGAAGATATTCCCTT

              *  *  * ** *    *      *    *          * **                    
      15144 TTTCACCGTAGGCGTCAAGGCGATCGAAATGTCCACTTCCACAAACTACAAAAAGAGTGTTTCAA
          1 TTCCAACGAAATCATCAAAGCGATCCAAATATCCACTTCCAGATTCTACAAAAAGAGTGTTTCAA

             *         *   * **       *    *        * ** * *   **      *     
      15209 ACCTGCTCTATGAAAGGCCATGTTCATCTCTATGAGTTGAATGGAAATATCCGAAAGAAATTTCT
         66 AACTGCTCTATCAAAAGAAATGTTCAACTCTGTGAGTTGAGTACACACATCACAAAGAAGTTTCT

             *       *                             **
      15274 GGGAATGCTGCTGTCTAGTTTTTATAT---GA-ATTCCCGC
        131 GAGAATGCTTCTGTCTAGTTTTTATATGAAGATATTCCCTT

                         *       *                *    *  *                  
      15311 TTCCAACGAAATCCTCAAAGCAATCCAAATATCCACTTGCAGAATCCACAAAAAGAGTGTTTCAA
          1 TTCCAACGAAATCATCAAAGCGATCCAAATATCCACTTCCAGATTCTACAAAAAGAGTGTTTCAA

                          *     *          * *                      *        
      15376 AACTGCTCTATCAATAGAAAGGTTCAACTCTTTTAGTTGAGTACACACATCACAAACAAGTTTCT
         66 AACTGCTCTATCAAAAGAAATGTTCAACTCTGTGAGTTGAGTACACACATCACAAAGAAGTTTCT

                            * *      **     **  *    
      15441 GAGAATGCTTCTGTCTGGCTTTTATTGGAAGACGTTTCCTT
        131 GAGAATGCTTCTGTCTAGTTTTTATATGAAGATATTCCCTT

                 * *  **          *       *                * *               
      15482 TT-CACCAAAGGCATCAAAGCGCTCCAAATGTCCACTTCCAGATTCTTCCAAAAGAGTGTTTCAA
          1 TTCCAACGAAATCATCAAAGCGATCCAAATATCCACTTCCAGATTCTACAAAAAGAGTGTTTCAA

             **  *  * ***  * *                 *    * *  * *     *   *       
      15546 ACGTGGTCGAAGTAAGGGAATGTTCAACTCTGTGACTTGAATGCAGATATCACCAAGTAGTTTCT
         66 AACTGCTCTATCAAAAGAAATGTTCAACTCTGTGAGTTGAGTACACACATCACAAAGAAGTTTCT

            * * *             *     *    *         * 
      15611 AATAGTGCTTCTGTCTAGATTTTAGATGATGATATTCCCGT
        131 GAGAATGCTTCTGTCTAGTTTTTATATGAAGATATTCCCTT

                         * * *   *             *  *   *      *     *       * 
      15652 TTCCAACGAAATCGTTAGAGCTATCCAAATATCCAGTTACAGTTTCTACCAAAAGGGTGTTTCCA
          1 TTCCAACGAAATCATCAAAGCGATCCAAATATCCACTTCCAGATTCTACAAAAAGAGTGTTTCAA

              *    **           *            *       *                     * 
      15717 AATTGCTGCATCAAAAGAAAGGTTCAACTCTGTTAGTTGAGGACACACATCACAAAGAAGTTTGT
         66 AACTGCTCTATCAAAAGAAATGTTCAACTCTGTGAGTTGAGTACACACATCACAAAGAAGTTTCT

                              *    *     *           
      15782 GAGAATGCTTCTGTCTAGATTTTGTATGACGATATTCCCTT
        131 GAGAATGCTTCTGTCTAGTTTTTATATGAAGATATTCCCTT

                     *   * *     *   *       * *  *    *  *       *  *       
      15823 TTCCAACGATATCGTTAAAGCAATCTAAATATCAATTTGCAGAATCCACAAAAATAGAGTTTCAA
          1 TTCCAACGAAATCATCAAAGCGATCCAAATATCCACTTCCAGATTCTACAAAAAGAGTGTTTCAA

             *       * *        *    *       *  *                   ***      
      15888 AGCTGCTCTGTAAAAAGAAAGGTTCCACTCTGTTAGCTGAGTACACACATCACAAACTTGTTTCT
         66 AACTGCTCTATCAAAAGAAATGTTCAACTCTGTGAGTTGAGTACACACATCACAAAGAAGTTTCT

                  *         *        **         **   
      15953 GAGAATCCTTCTGTCTCGTTTTTATGGGAAGATATTTACTT
        131 GAGAATGCTTCTGTCTAGTTTTTATATGAAGATATTCCCTT

                 *  * **        * *       *     *       *  *  *              
      15994 TTCCACCGTAGGCATCAAAGTGCTCCAAATGTCCACATCCAGATACTCCAGAAAGAGTGTTTCAA
          1 TTCCAACGAAATCATCAAAGCGATCCAAATATCCACTTCCAGATTCTACAAAAAGAGTGTTTCAA

             *         *   * *   *         *        * *  *      *      *     
      16059 ACCTGCTCTATGAAAGGGAATCTTCAACTCTATGAGTTGAATGCAGACATCAGAAAGAAATTTCT
         66 AACTGCTCTATCAAAAGAAATGTTCAACTCTGTGAGTTGAGTACACACATCACAAAGAAGTTTCT

                     *       **          *         **
      16124 GAGAATGCTGCTGTCTACCTTTTAT-T--TGA-ATTCCCGC
        131 GAGAATGCTTCTGTCTAGTTTTTATATGAAGATATTCCCTT

                         *  *    *              * *  **   *    *             
      16161 TTCCAACGAAATCCTCCAAGCTATCCAAATATCCACCTGCATTTTCCACAACAAGAGTGTTTCAA
          1 TTCCAACGAAATCATCAAAGCGATCCAAATATCCACTTCCAGATTCTACAAAAAGAGTGTTTCAA

                          *                 *    *  *                *       
      16226 AACTGCTCTATCAATAGAAATGTTCAACTCCTTTG-GCTGGGTACACACATCACAAACAAGTTTC
         66 AACTGCTCTATCAAAAGAAATGTTCAACT-CTGTGAGTTGAGTACACACATCACAAAGAAGTTTC

                                      **     **       
      16290 TGAGAATGCTTCTGTCTAGTTTTTATGGGAAGACGTTCCCTT
        130 TGAGAATGCTTCTGTCTAGTTTTTATATGAAGATATTCCCTT

              *  * *  **          *       *            **                    
      16332 TTTCACCAAAGGCATCAAAGCGCTCCAAATGTCCACTTCCAGACACTACAAAAAGAGTGTTTCAA
          1 TTCCAACGAAATCATCAAAGCGATCCAAATATCCACTTCCAGATTCTACAAAAAGAGTGTTTCAA

             **       **   ***                 *    * *  * *         *       
      16397 ACGTGCTCTAAGAAAGCGAATGTTCAACTCTGTGACTTGAATGCAGATATCACAAAGTAGTTTCT
         66 AACTGCTCTATCAAAAGAAATGTTCAACTCTGTGAGTTGAGTACACACATCACAAAGAAGTTTCT

                *             *     * *  *         * 
      16462 GAGAGTGCTTCTGTCTAGATTTTAGACGATGATATTCCCGT
        131 GAGAATGCTTCTGTCTAGTTTTTATATGAAGATATTCCCTT

                           * *   *                *   *                    * 
      16503 TTCCAACGAAATCATTAGAGCTATCCAAATATCCACTTACAGTTTCTACAAAAAGAGTGTTTCCA
          1 TTCCAACGAAATCATCAAAGCGATCCAAATATCCACTTCCAGATTCTACAAAAAGAGTGTTTCAA

                   **         * *    *       *  *                   ***      
      16568 AACTGCTGCATCAAAAGAGAGGTTCCACTCTGTTAGCTGAGTACACACATCACAAACTTGTTTCT
         66 AACTGCTCTATCAAAAGAAATGTTCAACTCTGTGAGTTGAGTACACACATCACAAAGAAGTTTCT

            *     *         *        **         **   
      16633 CAGAATCCTTCTGTCTCGTTTTTATGGGAAGATATTTACTT
        131 GAGAATGCTTCTGTCTAGTTTTTATATGAAGATATTCCCTT

              *  *  * **          *       *     *       **    *       *      
      16674 TTTCACCGTAGGCATCAAAGCGCTCCAAATGTCCACATCCAGATAGTACAGAAAGAGTTTTTCAA
          1 TTCCAACGAAATCATCAAAGCGATCCAAATATCCACTTCCAGATTCTACAAAAAGAGTGTTTCAA

             *         *   * *   *         *        * *  *      *      *     
      16739 ACCTGCTCTATGAAAGGGAATCTTCAACTCTATGAGTTGAATGCAGACATCAGAAAGAAATTTCT
         66 AACTGCTCTATCAAAAGAAATGTTCAACTCTGTGAGTTGAGTACACACATCACAAAGAAGTTTCT

                     *       **          *         **
      16804 GAGAATGCTGCTGTCTACCTTTTAT-T--TGA-ATTCCCGC
        131 GAGAATGCTTCTGTCTAGTTTTTATATGAAGATATTCCCTT

                         *  *    *                *       *                  
      16841 TTCCAACGAAATCCTCCAAGCTATCCAAATATCCACTTGCAGATTCCACAAAAAGAGTGTTTCAA
          1 TTCCAACGAAATCATCAAAGCGATCCAAATATCCACTTCCAGATTCTACAAAAAGAGTGTTTCAA

                            ** *  *            *       *     *     *  *      
      16906 AACTGCTCTCTATCAATGGCAAAGTTCAACTCTGTTAGTTGAGGACACATATCACCAACAAGTTT
         66 AACTG--CTCTATCAAAAGAAATGTTCAACTCTGTGAGTTGAGTACACACATCACAAAGAAGTTT

                               *       **         *    
      16971 CTGAGAATGCTTCTGTCTATTTTTTATGGGAAGATATTTCCTT
        129 CTGAGAATGCTTCTGTCTAGTTTTTATATGAAGATATTCCCTT

              *  *  * ** *    *      *    *          * **                    
      17014 TTTCACCGTAGGCGTCAAGGCGATCGAAATGTCCACTTCCACAAACTACAAAAAGAGTGTTTCAA
          1 TTCCAACGAAATCATCAAAGCGATCCAAATATCCACTTCCAGATTCTACAAAAAGAGTGTTTCAA

             *         *   * **       *    *        * ** * *   **      *     
      17079 ACCTGCTCTATGAAAGGCCATGTTCATCTCTATGAGTTGAATGGAAATATCCGAAAGAAATTTCT
         66 AACTGCTCTATCAAAAGAAATGTTCAACTCTGTGAGTTGAGTACACACATCACAAAGAAGTTTCT

             *       *          *        *         **
      17144 GGGAATGCTGCTGTCTAG-TGTT-TAT-ACGA-ATTCCCGC
        131 GAGAATGCTTCTGTCTAGTTTTTATATGAAGATATTCCCTT

                         *       *                *    *  *                  
      17181 TTCCAACGAAATCCTCAAAGCAATCCAAATATCCACTTGCAGAATCCACAAAAAGAGTGTTTCAA
          1 TTCCAACGAAATCATCAAAGCGATCCAAATATCCACTTCCAGATTCTACAAAAAGAGTGTTTCAA

                          *     *          * *                   *  *        
      17246 AACTGCTCTATCAATAGAAAGGTTCAACTCTTTTAGTTGAGTACACACATCACGAACAAGTTTCT
         66 AACTGCTCTATCAAAAGAAATGTTCAACTCTGTGAGTTGAGTACACACATCACAAAGAAGTTTCT

                            * *      **     **  *    
      17311 GAGAATGCTTCTGTCTGGCTTTTATTGGAAGACGTTTCCTT
        131 GAGAATGCTTCTGTCTAGTTTTTATATGAAGATATTCCCTT

                   *  **          *       *                * *               
      17352 TT-CAACAAAGGCATCAAAGCGCTCCAAATGTCCACTTCCAGATTCTTCCAAAAGAGTGTTTCAA
          1 TTCCAACGAAATCATCAAAGCGATCCAAATATCCACTTCCAGATTCTACAAAAAGAGTGTTTCAA

             **     * ***  * *                 *    * *  * *     *   *       
      17416 ACGTGCTCGAAGTAAGGGAATGTTCAACTCTGTGACTTGAATGCAGATATCACCAAGTAGTTTCT
         66 AACTGCTCTATCAAAAGAAATGTTCAACTCTGTGAGTTGAGTACACACATCACAAAGAAGTTTCT

            * * *            **     *    *         * 
      17481 AATAGTGCTTCTGTCTACATTTTAGATGATGATATTCCCGT
        131 GAGAATGCTTCTGTCTAGTTTTTATATGAAGATATTCCCTT

                         * * *   *             *  *   *      *     *       * 
      17522 TTCCAACGAAATCGTTAGAGCTATCCAAATATCCAGTTACAGTTTCTACCAAAAGGGTGTTTCCA
          1 TTCCAACGAAATCATCAAAGCGATCCAAATATCCACTTCCAGATTCTACAAAAAGAGTGTTTCAA

              *    **           *            *       *                     * 
      17587 AATTGCTGCATCAAAAGAAAGGTTCAACTCTGTTAGTTGAGGACACACATCACAAAGAAGTTTGT
         66 AACTGCTCTATCAAAAGAAATGTTCAACTCTGTGAGTTGAGTACACACATCACAAAGAAGTTTCT

                              *    *     *           
      17652 GAGAATGCTTCTGTCTAGATTTTGTATGACGATATTCCCTT
        131 GAGAATGCTTCTGTCTAGTTTTTATATGAAGATATTCCCTT

                     *   * *     *   *       * *  *    *  *       *  *       
      17693 TTCCAACGATATCGTTAAAGCAATCTAAATATCAATTTGCAGAATCCACAAAAATAGAGTTTCAA
          1 TTCCAACGAAATCATCAAAGCGATCCAAATATCCACTTCCAGATTCTACAAAAAGAGTGTTTCAA

             *       * *        *    *       *  *                   ***      
      17758 AGCTGCTCTGTAAAAAGAAAGGTTCCACTCTGTTAGCTGAGTACACACATCACAAACTTGTTTCT
         66 AACTGCTCTATCAAAAGAAATGTTCAACTCTGTGAGTTGAGTACACACATCACAAAGAAGTTTCT

                  *         *        **         **   
      17823 GAGAATCCTTCTGTCTCGTTTTTATGGGAAGATATTTACTT
        131 GAGAATGCTTCTGTCTAGTTTTTATATGAAGATATTCCCTT

                 *  * **          *       *     *       *  *  *  *           
      17864 TTCCACCGTAGGCATCAAAGCGCTCCAAATGTCCACATCCAGATACTCCAGAACGAGTGTTTCAA
          1 TTCCAACGAAATCATCAAAGCGATCCAAATATCCACTTCCAGATTCTACAAAAAGAGTGTTTCAA

             *         *   * *   *         *        * *  *      *      *     
      17929 ACCTGCTCTATGAAAGGGAATCTTCAACTCTATGAGTTGAATGCAGACATCAGAAAGAAATTTCT
         66 AACTGCTCTATCAAAAGAAATGTTCAACTCTGTGAGTTGAGTACACACATCACAAAGAAGTTTCT

                     *       **          *         **
      17994 GAGAATGCTGCTGTCTACCTTTTAT-T--TGA-ATTCCCGC
        131 GAGAATGCTTCTGTCTAGTTTTTATATGAAGATATTCCCTT

                         *  *    *              * *  **   *    *             
      18031 TTCCAACGAAATCCTCCAAGCTATCCAAATATCCACCTGCATTTTCCACAACAAGAGTGTTTCAA
          1 TTCCAACGAAATCATCAAAGCGATCCAAATATCCACTTCCAGATTCTACAAAAAGAGTGTTTCAA

                          *                 *    *  *                *       
      18096 AACTGCTCTATCAATAGAAATGTTCAACTCCTTTG-GCTGGGTACACACATCACAAACAAGTTTC
         66 AACTGCTCTATCAAAAGAAATGTTCAACT-CTGTGAGTTGAGTACACACATCACAAAGAAGTTTC

                                      **     **       
      18160 TGAGAATGCTTCTGTCTAGTTTTTATGGGAAGACGTTCCCTT
        130 TGAGAATGCTTCTGTCTAGTTTTTATATGAAGATATTCCCTT

              *  * *  **          *       *            **                  * 
      18202 TTTCACCAAAGGCATCAAAGCGCTCCAAATGTCCACTTCCAGACACTACAAAAAGAGTGTTTCCA
          1 TTCCAACGAAATCATCAAAGCGATCCAAATATCCACTTCCAGATTCTACAAAAAGAGTGTTTCAA

             **       **    **                 *    * *  * *      *  *       
      18267 ACGTGCTCTAAGAAAACGAATGTTCAACTCTGTGACTTGAATGCAGATATCACAGAGTAGTTTCT
         66 AACTGCTCTATCAAAAGAAATGTTCAACTCTGTGAGTTGAGTACACACATCACAAAGAAGTTTCT

                **            *     *    *         * 
      18332 GAGAGGGCTTCTGTCTAGATTTTAGATGATGATATTCCCGT
        131 GAGAATGCTTCTGTCTAGTTTTTATATGAAGATATTCCCTT

                           * *   *                *   *                    * 
      18373 TTCCAACGAAATCATTAGAGCTATCCAAATATCCACTTACAGTTTCTACAAAAAGAGTGTTTCCA
          1 TTCCAACGAAATCATCAAAGCGATCCAAATATCCACTTCCAGATTCTACAAAAAGAGTGTTTCAA

                   **         * *    *       *  *                *  ***      
      18438 AACTGCTGCATCAAAAGAGAGGTTCCACTCTGTTAGCTGAGTACACACATCACGAACTTGTTTCT
         66 AACTGCTCTATCAAAAGAAATGTTCAACTCTGTGAGTTGAGTACACACATCACAAAGAAGTTTCT

            *     *         *        **         **   
      18503 CAGAATCCTTCTGTCTCGTTTTTATGGGAAGATATTTACTT
        131 GAGAATGCTTCTGTCTAGTTTTTATATGAAGATATTCCCTT

              *  *  * **          *       *     *       *  *  *              
      18544 TTTCACCGTAGGCATCAAAGCGCTCCAAATGTCCACATCCAGATACTCCAGAAAGAGTGTTTCAA
          1 TTCCAACGAAATCATCAAAGCGATCCAAATATCCACTTCCAGATTCTACAAAAAGAGTGTTTCAA

             *         *   * *   *         *        * *  *      *      *     
      18609 ACCTGCTCTATGAAAGGGAATCTTCAACTCTATGAGTTGAATGCAGACATCAGAAAGAAATTTCT
         66 AACTGCTCTATCAAAAGAAATGTTCAACTCTGTGAGTTGAGTACACACATCACAAAGAAGTTTCT

                     *       **          *         **
      18674 GAGAATGCTGCTGTCTACCTTTTAT-T--TGA-ATTCCCGC
        131 GAGAATGCTTCTGTCTAGTTTTTATATGAAGATATTCCCTT

                         *  *    *                *       *                  
      18711 TTCCAACGAAATCCTCCAAGCTATCCAAATATCCACTTGCAGATTCCACAAAAAGAGTGTTTCAA
          1 TTCCAACGAAATCATCAAAGCGATCCAAATATCCACTTCCAGATTCTACAAAAAGAGTGTTTCAA

                            ** *  *            *       *     *     *  *      
      18776 AACTGCTCTCTATCAATGGCAAAGTTCAACTCTGTTAGTTGAGGACACATATCACCAACAAGTTT
         66 AACTG--CTCTATCAAAAGAAATGTTCAACTCTGTGAGTTGAGTACACACATCACAAAGAAGTTT

                               *       **         *    
      18841 CTGAGAATGCTTCTGTCTATTTTTTATGGGAAGATATTTCCTT
        129 CTGAGAATGCTTCTGTCTAGTTTTTATATGAAGATATTCCCTT

              *  *  * ** *    *      *    *          * **                    
      18884 TTTCACCGTAGGCGTCAAGGCGATCGAAATGTCCACTTCCACAAACTACAAAAAGAGTGTTTCAA
          1 TTCCAACGAAATCATCAAAGCGATCCAAATATCCACTTCCAGATTCTACAAAAAGAGTGTTTCAA

             *         *   * **       *    *        * ** * *   **      *     
      18949 ACCTGCTCTATGAAAGGCGATGTTCATCTCTATGAGTTGAATGGAAATATCCGAAAGAAATTTCT
         66 AACTGCTCTATCAAAAGAAATGTTCAACTCTGTGAGTTGAGTACACACATCACAAAGAAGTTTCT

             *       *          *        *         **
      19014 GGGAATGCTGCTGTCTAG-TGTT-TAT-ACGA-ATTCCCGC
        131 GAGAATGCTTCTGTCTAGTTTTTATATGAAGATATTCCCTT

                         *       *                *    *  *                  
      19051 TTCCAACGAAATCCTCAAAGCAATCCAAATATCCACTTGCAGAATCCACAAAAAGAGTGTTTCAA
          1 TTCCAACGAAATCATCAAAGCGATCCAAATATCCACTTCCAGATTCTACAAAAAGAGTGTTTCAA

                          *     *          * *                   *  *        
      19116 AACTGCTCTATCAATAGAAAGGTTCAACTCTTTTAGTTGAGTACACACATCACGAACAAGTTTCT
         66 AACTGCTCTATCAAAAGAAATGTTCAACTCTGTGAGTTGAGTACACACATCACAAAGAAGTTTCT

                            * *      **     **  *    
      19181 GAGAATGCTTCTGTCTGGCTTTTATTGGAAGACGTTTCCTT
        131 GAGAATGCTTCTGTCTAGTTTTTATATGAAGATATTCCCTT

                 * *  **          *       *                * *               
      19222 TT-CACCAAAGGCATCAAAGCGCTCCAAATGTCCACTTCCAGATTCTTCCAAAAGAGTGTTTCAA
          1 TTCCAACGAAATCATCAAAGCGATCCAAATATCCACTTCCAGATTCTACAAAAAGAGTGTTTCAA

             **     * ***  * *                 *    * *  * *     *   *       
      19286 ACGTGCTCAAAGTAAGGGAATGTTCAACTCTGTGACTTGAATGCAGATATCACCAAGTAGTTTCT
         66 AACTGCTCTATCAAAAGAAATGTTCAACTCTGTGAGTTGAGTACACACATCACAAAGAAGTTTCT

            * * *             *     *    *         * 
      19351 AATAGTGCTTCTGTCTAGATTTTAGATGATGATATTCCCGT
        131 GAGAATGCTTCTGTCTAGTTTTTATATGAAGATATTCCCTT

                         * * *   *             *  *   *                    * 
      19392 TTCCAACGAAATCGTTAGAGCTATCCAAATATCCAGTTACAGTTTCTACAAAAAGAGTGTTTCCA
          1 TTCCAACGAAATCATCAAAGCGATCCAAATATCCACTTCCAGATTCTACAAAAAGAGTGTTTCAA

                   **           *            *       *                     * 
      19457 AACTGCTGCATCAAAAGAAAGGTTCAACTCTGTTAGTTGAGGACACACATCACAAAGAAGTTTGT
         66 AACTGCTCTATCAAAAGAAATGTTCAACTCTGTGAGTTGAGTACACACATCACAAAGAAGTTTCT

                              *    *     *           
      19522 GAGAATGCTTCTGTCTAGATTTTGTATGACGATATTCCCTT
        131 GAGAATGCTTCTGTCTAGTTTTTATATGAAGATATTCCCTT

                     *   * *     *   *       * *  *    *  *       *  *       
      19563 TTCCAACGATATCGTTAAAGCAATCTAAATATCAATTTGCAGAATCCACAAAAATAGAGTTTCAA
          1 TTCCAACGAAATCATCAAAGCGATCCAAATATCCACTTCCAGATTCTACAAAAAGAGTGTTTCAA

             *       * *    *   *    *       *  *                   ***      
      19628 AGCTGCTCTGTAAAAATAAAAGTTCCACTCTGTTAGCTGAGTACACACATCACAAACTTGTTTCT
         66 AACTGCTCTATCAAAAGAAATGTTCAACTCTGTGAGTTGAGTACACACATCACAAAGAAGTTTCT

                  *         *        **         **   
      19693 GAGAATCCTTCTGTCTCGTTTTTATGGGAAGATATTTACTT
        131 GAGAATGCTTCTGTCTAGTTTTTATATGAAGATATTCCCTT

              *  *  * **          *       *     *       *  *  *     *        
      19734 TTTCACCGTAGGCATCAAAGCGCTCCAAATGTCCACATCCAGATACTCCAGAAAGACTGTTTCAA
          1 TTCCAACGAAATCATCAAAGCGATCCAAATATCCACTTCCAGATTCTACAAAAAGAGTGTTTCAA

             *         *   * *   *         *        * *  *      *      *     
      19799 ACCTGCTCTATGAAAGGGAATCTTCAACTCTATGAGTTGAATGCAGACATCAGAAAGAAATTTCT
         66 AACTGCTCTATCAAAAGAAATGTTCAACTCTGTGAGTTGAGTACACACATCACAAAGAAGTTTCT

                     *       **          *         **
      19864 GAGAATGCTGCTGTCTACCTTTTAT-T--TGA-ATTCCCGC
        131 GAGAATGCTTCTGTCTAGTTTTTATATGAAGATATTCCCTT

                         *  *    *                *  **   *                  
      19901 TTCCAACGAAATCCTCCAAGCTATCCAAATATCCACTTGCATTTTCCACAAAAAGAGTGTTTCAA
          1 TTCCAACGAAATCATCAAAGCGATCCAAATATCCACTTCCAGATTCTACAAAAAGAGTGTTTCAA

                          *               ** *  *  *                *        
      19966 AACTGCTCTATCAATAGAAATGTTCAACTCCTTTAGCTGGGTACACACATCACAAACAAGTTTCT
         66 AACTGCTCTATCAAAAGAAATGTTCAACTCTGTGAGTTGAGTACACACATCACAAAGAAGTTTCT

                                     **     **       
      20031 GAGAATGCTTCTGTCTAGTTTTTATGGGAAGACGTTCCCTT
        131 GAGAATGCTTCTGTCTAGTTTTTATATGAAGATATTCCCTT

              *  * *  **          *       *            **                    
      20072 TTTCACCAAAGGCATCAAAGCGCTCCAAATGTCCACTTCCAGACACTACAAAAAGAGTGTTTCAA
          1 TTCCAACGAAATCATCAAAGCGATCCAAATATCCACTTCCAGATTCTACAAAAAGAGTGTTTCAA

             **       **   ***                 *    * *  * *         *       
      20137 ACGTGCTCTAAGAAAGCGAATGTTCAACTCTGTGACTTGAATGCAGATATCACAAAGTAGTTTCT
         66 AACTGCTCTATCAAAAGAAATGTTCAACTCTGTGAGTTGAGTACACACATCACAAAGAAGTTTCT

                **            *     *    *         * 
      20202 GAGAGGGCTTCTGTCTAGATTTTAGATGATGATATTCCCGT
        131 GAGAATGCTTCTGTCTAGTTTTTATATGAAGATATTCCCTT

                           * *   *                *   *                    * 
      20243 TTCCAACGAAATCATTAGAGCTATCCAAATATCCACTTACAGTTTCTACAAAAAGAGTGTTTCCA
          1 TTCCAACGAAATCATCAAAGCGATCCAAATATCCACTTCCAGATTCTACAAAAAGAGTGTTTCAA

                   **         * *    *       *  **                  ***      
      20308 AACTGCTGCATCAAAAGAGAGGTTCCACTCTGTTAGCCGAGTACACACATCACAAACTTGTTTCT
         66 AACTGCTCTATCAAAAGAAATGTTCAACTCTGTGAGTTGAGTACACACATCACAAAGAAGTTTCT

            *     *         *        **         **   
      20373 CAGAATCCTTCTGTCTCGTTTTTATGGGAAGATATTTACTT
        131 GAGAATGCTTCTGTCTAGTTTTTATATGAAGATATTCCCTT

              *  *  * **          *       *     *       *  *  *              
      20414 TTTCACCGTAGGCATCAAAGCGCTCCAAATGTCCACATCCAGATACTCCAGAAAGAGTGTTTCAA
          1 TTCCAACGAAATCATCAAAGCGATCCAAATATCCACTTCCAGATTCTACAAAAAGAGTGTTTCAA

             *         *   * *   *         *        * *  *      *      *     
      20479 ACCTGCTCTATGAAAGGGAATCTTCAACTCTATGAGTTGAATGCAGACATCAGAAAGAAATTTCT
         66 AACTGCTCTATCAAAAGAAATGTTCAACTCTGTGAGTTGAGTACACACATCACAAAGAAGTTTCT

                     *       **          *         **
      20544 GAGAATGCTGCTGTCTACCTTTTAT-T--TGA-ATTCCCGC
        131 GAGAATGCTTCTGTCTAGTTTTTATATGAAGATATTCCCTT

                         *  *    *                *       *                  
      20581 TTCCAACGAAATCCTCCAAGCTATCCAAATATCCACTTGCAGATTCCACAAAAAGAGTGTTTCAA
          1 TTCCAACGAAATCATCAAAGCGATCCAAATATCCACTTCCAGATTCTACAAAAAGAGTGTTTCAA

                            ** *  *            *       *     *     *  *      
      20646 AACTGCTCTCTATCAATGGCAAAGTTCAACTCTGTTAGTTGAGGACACATATCACCAACAAGTTT
         66 AACTG--CTCTATCAAAAGAAATGTTCAACTCTGTGAGTTGAGTACACACATCACAAAGAAGTTT

                               *       **         *    
      20711 CTGAGAATGCTTCTGTCTATTTTTTATGGGAAGATATTTCCTT
        129 CTGAGAATGCTTCTGTCTAGTTTTTATATGAAGATATTCCCTT

              *  *  * ** *    *      *    *          * **                    
      20754 TTTCACCGTAGGCGTCAAGGCGATCGAAATGTCCACTTCCACAAACTACAAAAAGAGTGTTTCAA
          1 TTCCAACGAAATCATCAAAGCGATCCAAATATCCACTTCCAGATTCTACAAAAAGAGTGTTTCAA

             *         *   * **       *    *        * ** * *   **      *     
      20819 ACCTGCTCTATGAAAGGCCATGTTCATCTCTATGAGTTGAATGGAAATATCCGAAAGAAATTTCT
         66 AACTGCTCTATCAAAAGAAATGTTCAACTCTGTGAGTTGAGTACACACATCACAAAGAAGTTTCT

             *       *          *        *         **
      20884 GGGAATGCTGCTGTCTAG-TGTT-TAT-ACGA-ATTCCCGC
        131 GAGAATGCTTCTGTCTAGTTTTTATATGAAGATATTCCCTT

                         *       *                *    *  *                  
      20921 TTCCAACGAAATCCTCAAAGCAATCCAAATATCCACTTGCAGAATCCACAAAAAGAGTGTTTCAA
          1 TTCCAACGAAATCATCAAAGCGATCCAAATATCCACTTCCAGATTCTACAAAAAGAGTGTTTCAA

                          *     *          * *                   *  *        
      20986 AACTGCTCTATCAATAGAAAGGTTCAACTCTTTTAGTTGAGTACACACATCACGAACAAGTTTCT
         66 AACTGCTCTATCAAAAGAAATGTTCAACTCTGTGAGTTGAGTACACACATCACAAAGAAGTTTCT

                            * *      **     **  *    
      21051 GAGAATGCTTCTGTCTGGCTTTTATTGGAAGACGTTTCCTT
        131 GAGAATGCTTCTGTCTAGTTTTTATATGAAGATATTCCCTT

                 * *  **          *       *                * *               
      21092 TT-CACCAAAGGCATCAAAGCGCTCCAAATGTCCACTTCCAGATTCTTCCAAAAGAGTGTTTCAA
          1 TTCCAACGAAATCATCAAAGCGATCCAAATATCCACTTCCAGATTCTACAAAAAGAGTGTTTCAA

             **     * ***  * *                 *    * *  * *     *   *       
      21156 ACGTGCTCAAAGTAAGGGAATGTTCAACTCTGTGACTTGAATGCAGATATCACCAAGTAGTTTCT
         66 AACTGCTCTATCAAAAGAAATGTTCAACTCTGTGAGTTGAGTACACACATCACAAAGAAGTTTCT

            * * *             *   * *    *         * 
      21221 AATAGTGCTTCTGTCTAGATTTCAGATGATGATATTCCCGT
        131 GAGAATGCTTCTGTCTAGTTTTTATATGAAGATATTCCCTT

                         * * *   * **          *  *   *      *     *       * 
      21262 TTCCAACGAAATCGTTAGAGCTAAGCAAATATCCAGTTACAGTTTCTACCAAAAGGGTGTTTCCA
          1 TTCCAACGAAATCATCAAAGCGATCCAAATATCCACTTCCAGATTCTACAAAAAGAGTGTTTCAA

              *    **           *            *       *                     * 
      21327 AATTGCTGCATCAAAAGAAAGGTTCAACTCTGTTAGTTGAGGACACACATCACAAAGAAGTTTGT
         66 AACTGCTCTATCAAAAGAAATGTTCAACTCTGTGAGTTGAGTACACACATCACAAAGAAGTTTCT

                              *    *     *           
      21392 GAGAATGCTTCTGTCTAGATTTTGTATGACGATATTCCCTT
        131 GAGAATGCTTCTGTCTAGTTTTTATATGAAGATATTCCCTT

                     *   * *     *   *       * *  *    *  *       *  *       
      21433 TTCCAACGATATCGTTAAAGCAATCTAAATATCAATTTGCAGAATCCACAAAAATAGAGTTTCAA
          1 TTCCAACGAAATCATCAAAGCGATCCAAATATCCACTTCCAGATTCTACAAAAAGAGTGTTTCAA

             *       * *        *    *       *  *                   ***      
      21498 AGCTGCTCTGTAAAAAGAAAGGTTCCACTCTGTTAGCTGAGTACACACATCACAAACTTGTTTCT
         66 AACTGCTCTATCAAAAGAAATGTTCAACTCTGTGAGTTGAGTACACACATCACAAAGAAGTTTCT

                  *         *        **         **   
      21563 GAGAATCCTTCTGTCTCGTTTTTATGGGAAGATATTTACTT
        131 GAGAATGCTTCTGTCTAGTTTTTATATGAAGATATTCCCTT

                 *  * **          *       *     *       *  *  *              
      21604 TTCCACCGTAGGCATCAAAGCGCTCCAAATGTCCACATCCAGATACTCCAGAAAGAGTGTTTCAA
          1 TTCCAACGAAATCATCAAAGCGATCCAAATATCCACTTCCAGATTCTACAAAAAGAGTGTTTCAA

             *         *   * *   *         *        * *  *      *      *     
      21669 ACCTGCTCTATGAAAGGCAATCTTCAACTCTATGAGTTGAATGCAGACATCAGAAAGAAATTTCT
         66 AACTGCTCTATCAAAAGAAATGTTCAACTCTGTGAGTTGAGTACACACATCACAAAGAAGTTTCT

                     *       **          *         **
      21734 GAGAATGCTGCTGTCTACCTTTTAT-T--TGA-ATTCCCGC
        131 GAGAATGCTTCTGTCTAGTTTTTATATGAAGATATTCCCTT

                         *  *    *              * *  **   *                  
      21771 TTCCAACGAAATCCTCCAAGCTATCCAAATATCCACCTGCATTTTCCACAAAAAGAGTGTTTCAA
          1 TTCCAACGAAATCATCAAAGCGATCCAAATATCCACTTCCAGATTCTACAAAAAGAGTGTTTCAA

                          *                 *    *  *                *       
      21836 AACTGCTCTATCAATAGAAATGTTCAACTCCTTTG-GCTGGGTACACACATCACAAACAAGTTTC
         66 AACTGCTCTATCAAAAGAAATGTTCAACT-CTGTGAGTTGAGTACACACATCACAAAGAAGTTTC

                *                     **     *        
      21900 TGAGCATGCTTCTGTCTAGTTTTTATGGGAAGACATTCCCTT
        130 TGAGAATGCTTCTGTCTAGTTTTTATATGAAGATATTCCCTT

              *  * *  **          *       *            **                    
      21942 TTTCACCAAAGGCATCAAAGCGCTCCAAATGTCCACTTCCAGACACTACAAAAAGAGTGTTTCAA
          1 TTCCAACGAAATCATCAAAGCGATCCAAATATCCACTTCCAGATTCTACAAAAAGAGTGTTTCAA

             **       **    **                 *    * *  * *      *  *       
      22007 ACGTGCTCTAAGAAAACGAATGTTCAACTCTGTGACTTGAATGCAGATATCACAGAGTAGTTTCT
         66 AACTGCTCTATCAAAAGAAATGTTCAACTCTGTGAGTTGAGTACACACATCACAAAGAAGTTTCT

                **            *     *    *         * 
      22072 GAGAGGGCTTCTGTCTAGATTTTAGATGATGATATTCCCGT
        131 GAGAATGCTTCTGTCTAGTTTTTATATGAAGATATTCCCTT

                           * *   *                *   *                    * 
      22113 TTCCAACGAAATCATTAGAGCTATCCAAATATCCACTTACAGTTTCTACAAAAAGAGTGTTTCCA
          1 TTCCAACGAAATCATCAAAGCGATCCAAATATCCACTTCCAGATTCTACAAAAAGAGTGTTTCAA

                   **         * *    *       *  *                   ***      
      22178 AACTGCTGCATCAAAAGAGAGGTTCCACTCTGTTAGCTGAGTACACACATCACAAACTTGTTTCT
         66 AACTGCTCTATCAAAAGAAATGTTCAACTCTGTGAGTTGAGTACACACATCACAAAGAAGTTTCT

            *     *         *        **         **   
      22243 CAGAATCCTTCTGTCTCGTTTTTATGGGAAGATATTTACTT
        131 GAGAATGCTTCTGTCTAGTTTTTATATGAAGATATTCCCTT

              *  *  * **          *       *     *       *  *  *              
      22284 TTTCACCGTAGGCATCAAAGCGCTCCAAATGTCCACATCCAGATACTCCAGAAAGAGTGTTTCAA
          1 TTCCAACGAAATCATCAAAGCGATCCAAATATCCACTTCCAGATTCTACAAAAAGAGTGTTTCAA

             *         *   * *   *         *        * *  *      *      *     
      22349 ACCTGCTCTATGAAAGGGAATCTTCAACTCTATGAGTTGAATGCAGACATCAGAAAGAAATTTCT
         66 AACTGCTCTATCAAAAGAAATGTTCAACTCTGTGAGTTGAGTACACACATCACAAAGAAGTTTCT

                     *       **          *         **
      22414 GAGAATGCTGCTGTCTACCTTTTAT-T--TGA-ATTCCCGC
        131 GAGAATGCTTCTGTCTAGTTTTTATATGAAGATATTCCCTT

                         *  *    *                *       *                  
      22451 TTCCAACGAAATCCTCCAAGCTATCCAAATATCCACTTGCAGATTCCACAAAAAGAGTGTTTCAA
          1 TTCCAACGAAATCATCAAAGCGATCCAAATATCCACTTCCAGATTCTACAAAAAGAGTGTTTCAA

                            ** *  *            *       *     *     *  *      
      22516 AACTGCTCTCTATCAATGGCAAAGTTCAACTCTGTTAGTTGAGGACACATATCACCAACAAGTTT
         66 AACTG--CTCTATCAAAAGAAATGTTCAACTCTGTGAGTTGAGTACACACATCACAAAGAAGTTT

                               *       **         *    
      22581 CTGAGAATGCTTCTGTCTATTTTTTATGGGAAGATATTTCCTT
        129 CTGAGAATGCTTCTGTCTAGTTTTTATATGAAGATATTCCCTT

              *  *  * ** *    *      *    *          * **                    
      22624 TTTCACCGTAGGCGTCAAGGCGATCGAAATGTCCACTTCCACAAACTACAAAAAGAGTGTTTCAA
          1 TTCCAACGAAATCATCAAAGCGATCCAAATATCCACTTCCAGATTCTACAAAAAGAGTGTTTCAA

             *         *   * **       *    *        * ** * *   **      *     
      22689 ACCTGCTCTATGAAAGGCCATGTTCATCTCTATGAGTTGAATGGAAATATCCGAAAGAAATTTCT
         66 AACTGCTCTATCAAAAGAAATGTTCAACTCTGTGAGTTGAGTACACACATCACAAAGAAGTTTCT

             *       *          *        *         **
      22754 GGGAATGCTGCTGTCTAG-TGTT-TAT-ACGA-ATTCCCGC
        131 GAGAATGCTTCTGTCTAGTTTTTATATGAAGATATTCCCTT

                         *       *                *    *  *                  
      22791 TTCCAACGAAATCCTCAAAGCAATCCAAATATCCACTTGCAGAATCCACAAAAAGAGTGTTTCAA
          1 TTCCAACGAAATCATCAAAGCGATCCAAATATCCACTTCCAGATTCTACAAAAAGAGTGTTTCAA

                          *     *          * *                   *  *        
      22856 AACTGCTCTATCAATAGAAAGGTTCAACTCTTTTAGTTGAGTACACACATCACGAACAAGTTTCT
         66 AACTGCTCTATCAAAAGAAATGTTCAACTCTGTGAGTTGAGTACACACATCACAAAGAAGTTTCT

                            * *      **     **  *    
      22921 GAGAATGCTTCTGTCTGGCTTTTATTGGAAGACGTTTCCTT
        131 GAGAATGCTTCTGTCTAGTTTTTATATGAAGATATTCCCTT

                 * *  **          *       *                * *               
      22962 TT-CACCAAAGGCATCAAAGCGCTCCAAATGTCCACTTCCAGATTCTTCCAAAAGAGTGTTTCAA
          1 TTCCAACGAAATCATCAAAGCGATCCAAATATCCACTTCCAGATTCTACAAAAAGAGTGTTTCAA

             **     * ***  * *                 *    * *  * *     *   *       
      23026 ACGTGCTCAAAGTAAGGGAATGTTCAACTCTGTGACTTGAATGCAGATATCACCAAGTAGTTTCT
         66 AACTGCTCTATCAAAAGAAATGTTCAACTCTGTGAGTTGAGTACACACATCACAAAGAAGTTTCT

            * * *             *     *    *         * 
      23091 AATAGTGCTTCTGTCTAGATTTTAGATGATGATATTCCCGT
        131 GAGAATGCTTCTGTCTAGTTTTTATATGAAGATATTCCCTT

                         * * *   *             *  *   *                    * 
      23132 TTCCAACGAAATCGTTAGAGCTATCCAAATATCCAGTTACAGTTTCTACAAAAAGAGTGTTTCCA
          1 TTCCAACGAAATCATCAAAGCGATCCAAATATCCACTTCCAGATTCTACAAAAAGAGTGTTTCAA

                   **           *            *       *                     * 
      23197 AACTGCTGCATCAAAAGAAAGGTTCAACTCTGTTAGTTGAGGACACACATCACAAAGAAGTTTGT
         66 AACTGCTCTATCAAAAGAAATGTTCAACTCTGTGAGTTGAGTACACACATCACAAAGAAGTTTCT

                              *    *     *           
      23262 GAGAATGCTTCTGTCTAGATTTTGTATGACGATATTCCCTT
        131 GAGAATGCTTCTGTCTAGTTTTTATATGAAGATATTCCCTT

                     *   * *     *   *       * *  *    *  *       *  *       
      23303 TTCCAACGATATCGTTAAAGCAATCTAAATATCAATTTGCAGAATCCACAAAAATAGAGTTTCAA
          1 TTCCAACGAAATCATCAAAGCGATCCAAATATCCACTTCCAGATTCTACAAAAAGAGTGTTTCAA

             *       * *    *   *    *       *  *                   ***      
      23368 AGCTGCTCTGTAAAAATAAAAGTTCCACTCTGTTAGCTGAGTACACACATCACAAACTTGTTTCT
         66 AACTGCTCTATCAAAAGAAATGTTCAACTCTGTGAGTTGAGTACACACATCACAAAGAAGTTTCT

                  *         *        **         **   
      23433 GAGAATCCTTCTGTCTCGTTTTTATGGGAAGATATTTACTT
        131 GAGAATGCTTCTGTCTAGTTTTTATATGAAGATATTCCCTT

              *  *  * **          *       *     *       *  *  *     *        
      23474 TTTCACCGTAGGCATCAAAGCGCTCCAAATGTCCACATCCAGATACTCCAGAAAGACTGTTTCAA
          1 TTCCAACGAAATCATCAAAGCGATCCAAATATCCACTTCCAGATTCTACAAAAAGAGTGTTTCAA

             *         *   * *   *         *        * *  *      *      *     
      23539 ACCTGCTCTATGAAAGGGAATCTTCAACTCTATGAGTTGAATGCAGACATCAGAAAGAAATTTCT
         66 AACTGCTCTATCAAAAGAAATGTTCAACTCTGTGAGTTGAGTACACACATCACAAAGAAGTTTCT

                     *       **          *         **
      23604 GAGAATGCTGCTGTCTACCTTTTAT-T--TGA-ATTCCCGC
        131 GAGAATGCTTCTGTCTAGTTTTTATATGAAGATATTCCCTT

                         *  *    *                *  **   *                  
      23641 TTCCAACGAAATCCTCCAAGCTATCCAAATATCCACTTGCATTTTCCACAAAAAGAGTGTTTCAA
          1 TTCCAACGAAATCATCAAAGCGATCCAAATATCCACTTCCAGATTCTACAAAAAGAGTGTTTCAA

                          *               ** *  *  *                *        
      23706 AACTGCTCTATCAATAGAAATGTTCAACTCCTTTAGCTGGGTACACACATCACAAACAAGTTTCT
         66 AACTGCTCTATCAAAAGAAATGTTCAACTCTGTGAGTTGAGTACACACATCACAAAGAAGTTTCT

                                     **     **       
      23771 GAGAATGCTTCTGTCTAGTTTTTATGGGAAGACGTTCCCTT
        131 GAGAATGCTTCTGTCTAGTTTTTATATGAAGATATTCCCTT

              *  * *  **          *       *            **                    
      23812 TTTCACCAAAGGCATCAAAGCGCTCCAAATGTCCACTTCCAGACACTACAAAAAGAGTGTTTCAA
          1 TTCCAACGAAATCATCAAAGCGATCCAAATATCCACTTCCAGATTCTACAAAAAGAGTGTTTCAA

             **       **   ***                 *    * *  * *         *       
      23877 ACGTGCTCTAAGAAAGCGAATGTTCAACTCTGTGACTTGAATGCAGATATCACAAAGTAGTTTCT
         66 AACTGCTCTATCAAAAGAAATGTTCAACTCTGTGAGTTGAGTACACACATCACAAAGAAGTTTCT

                **            *     *    *         * 
      23942 GAGAGGGCTTCTGTCTAGATTTTAGATGATGATATTCCCGT
        131 GAGAATGCTTCTGTCTAGTTTTTATATGAAGATATTCCCTT

                           * *   *                *   *                    * 
      23983 TTCCAACGAAATCATTAGAGCTATCCAAATATCCACTTACAGTTTCTACAAAAAGAGTGTTTCCA
          1 TTCCAACGAAATCATCAAAGCGATCCAAATATCCACTTCCAGATTCTACAAAAAGAGTGTTTCAA

                   **         * *    *       *  **                  ***      
      24048 AACTGCTGCATCAAAAGAGAGGTTCCACTCTGTTAGCCGAGTACACACATCACAAACTTGTTTCT
         66 AACTGCTCTATCAAAAGAAATGTTCAACTCTGTGAGTTGAGTACACACATCACAAAGAAGTTTCT

            *     *         *        **         **   
      24113 CAGAATCCTTCTGTCTCGTTTTTATGGGAAGATATTTACTT
        131 GAGAATGCTTCTGTCTAGTTTTTATATGAAGATATTCCCTT

              *  *  * **          *       *     *       *  *  *              
      24154 TTTCACCGTAGGCATCAAAGCGCTCCAAATGTCCACATCCAGATACTCCAGAAAGAGTGTTTCAA
          1 TTCCAACGAAATCATCAAAGCGATCCAAATATCCACTTCCAGATTCTACAAAAAGAGTGTTTCAA

             *         *   * *   *         *        * *  *      *      *     
      24219 ACCTGCTCTATGAAAGGGAATCTTCAACTCTATGAGTTGAATGCAGACATCAGAAAGAAATTTCT
         66 AACTGCTCTATCAAAAGAAATGTTCAACTCTGTGAGTTGAGTACACACATCACAAAGAAGTTTCT

                     *       **          *         **
      24284 GAGAATGCTGCTGTCTACCTTTTAT-T--TGA-ATTCCCGC
        131 GAGAATGCTTCTGTCTAGTTTTTATATGAAGATATTCCCTT

                         *  *    *                *       *                  
      24321 TTCCAACGAAATCCTCCAAGCTATCCAAATATCCACTTGCAGATTCCACAAAAAGAGTGTTTCAA
          1 TTCCAACGAAATCATCAAAGCGATCCAAATATCCACTTCCAGATTCTACAAAAAGAGTGTTTCAA

                            ** *  *            *       *     *     *  *      
      24386 AACTGCTCTCTATCAATGGCAAAGTTCAACTCTGTTAGTTGAGGACACATATCACCAACAAGTTT
         66 AACTG--CTCTATCAAAAGAAATGTTCAACTCTGTGAGTTGAGTACACACATCACAAAGAAGTTT

                               *       **         *    
      24451 CTGAGAATGCTTCTGTCTATTTTTTATGGGAAGATATTTCCTT
        129 CTGAGAATGCTTCTGTCTAGTTTTTATATGAAGATATTCCCTT

              *  *  * ** *    *   *  *    *          * **                    
      24494 TTTCACCGTAGGCGTCAACGCGTTCGAAATGTCCACTTCCACAAACTACAAAAAGAGTGTTTCAA
          1 TTCCAACGAAATCATCAAAGCGATCCAAATATCCACTTCCAGATTCTACAAAAAGAGTGTTTCAA

             *         *   * **       *    *        * ** * *   **      *     
      24559 ACCTGCTCTATGAAAGGCCATGTTCATCTCTATGAGTTGAATGGAAATATCCGAAAGAAATTTCT
         66 AACTGCTCTATCAAAAGAAATGTTCAACTCTGTGAGTTGAGTACACACATCACAAAGAAGTTTCT

             *       *          *        *         **
      24624 GGGAATGCTGCTGTCTAG-TGTT-TAT-ACGA-ATTCCCGC
        131 GAGAATGCTTCTGTCTAGTTTTTATATGAAGATATTCCCTT

                         *       *                *    *  *                  
      24661 TTCCAACGAAATCCTCAAAGCAATCCAAATATCCACTTGCAGAATCCACAAAAAGAGTGTTTCAA
          1 TTCCAACGAAATCATCAAAGCGATCCAAATATCCACTTCCAGATTCTACAAAAAGAGTGTTTCAA

                          *     *          * *                   *  *        
      24726 AACTGCTCTATCAATAGAAAGGTTCAACTCTTTTAGTTGAGTACACACATCACGAACAAGTTTCT
         66 AACTGCTCTATCAAAAGAAATGTTCAACTCTGTGAGTTGAGTACACACATCACAAAGAAGTTTCT

                            * *      **     **  *    
      24791 GAGAATGCTTCTGTCTGGCTTTTATTGGAAGACGTTTCCTT
        131 GAGAATGCTTCTGTCTAGTTTTTATATGAAGATATTCCCTT

                 * *  **          *       *                * *               
      24832 TT-CACCAAAGGCATCAAAGCGCTCCAAATGTCCACTTCCAGATTCTTCCAAAAGAGTGTTTCAA
          1 TTCCAACGAAATCATCAAAGCGATCCAAATATCCACTTCCAGATTCTACAAAAAGAGTGTTTCAA

             **     * ***  * *                 *    * *  * *     *   *       
      24896 ACGTGCTCAAAGTAAGGGAATGTTCAACTCTGTGACTTGAATGCAGATATCACCAAGTAGTTTCT
         66 AACTGCTCTATCAAAAGAAATGTTCAACTCTGTGAGTTGAGTACACACATCACAAAGAAGTTTCT

            * * *             *   * *    *         * 
      24961 AATAGTGCTTCTGTCTAGATTTCAGATGATGATATTCCCGT
        131 GAGAATGCTTCTGTCTAGTTTTTATATGAAGATATTCCCTT

                         * * *   * **          *  *   *      *     *       * 
      25002 TTCCAACGAAATCGTTAGAGCTAAGCAAATATCCAGTTACAGTTTCTACCAAAAGGGTGTTTCCA
          1 TTCCAACGAAATCATCAAAGCGATCCAAATATCCACTTCCAGATTCTACAAAAAGAGTGTTTCAA

              *    **           *            *       *                     * 
      25067 AATTGCTGCATCAAAAGAAAGGTTCAACTCTGTTAGTTGAGGACACACATCACAAAGAAGTTTGT
         66 AACTGCTCTATCAAAAGAAATGTTCAACTCTGTGAGTTGAGTACACACATCACAAAGAAGTTTCT

                              *    *     *           
      25132 GAGAATGCTTCTGTCTAGATTTTGTATGACGATATTCCCTT
        131 GAGAATGCTTCTGTCTAGTTTTTATATGAAGATATTCCCTT

                     *   * *     *   *       * *  *    *  *       *  *       
      25173 TTCCAACGATATCGTTAAAGCAATCTAAATATCAATTTGCAGAATCCACAAAAATAGAGTTTCAA
          1 TTCCAACGAAATCATCAAAGCGATCCAAATATCCACTTCCAGATTCTACAAAAAGAGTGTTTCAA

             *       * *        *    *       *  *                   ***      
      25238 AGCTGCTCTGTAAAAAGAAAGGTTCCACTCTGTTAGCTGAGTACACACATCACAAACTTGTTTCT
         66 AACTGCTCTATCAAAAGAAATGTTCAACTCTGTGAGTTGAGTACACACATCACAAAGAAGTTTCT

                  *         *        **         **   
      25303 GAGAATCCTTCTGTCTCGTTTTTATGGGAAGATATTTACTT
        131 GAGAATGCTTCTGTCTAGTTTTTATATGAAGATATTCCCTT

                 *  * **          *       *     *       *  *  *              
      25344 TTCCACCGTAGGCATCAAAGCGCTCCAAATGTCCACATCCAGATACTCCAGAAAGAGTGTTTCAA
          1 TTCCAACGAAATCATCAAAGCGATCCAAATATCCACTTCCAGATTCTACAAAAAGAGTGTTTCAA

             *         *   * *   *         *        * *  *      *      *     
      25409 ACCTGCTCTATGAAAGGCAATCTTCAACTCTATGAGTTGAATGCAGACATCAGAAAGAAATTTCT
         66 AACTGCTCTATCAAAAGAAATGTTCAACTCTGTGAGTTGAGTACACACATCACAAAGAAGTTTCT

                     *       **          *         **
      25474 GAGAATGCTGCTGTCTACCTTTTAT-T--TGA-ATTCCCGC
        131 GAGAATGCTTCTGTCTAGTTTTTATATGAAGATATTCCCTT

                         *  *    *              * *  **   *                  
      25511 TTCCAACGAAATCCTCCAAGCTATCCAAATATCCACCTGCATTTTCCACAAAAAGAGTGTTTCAA
          1 TTCCAACGAAATCATCAAAGCGATCCAAATATCCACTTCCAGATTCTACAAAAAGAGTGTTTCAA

                          *                 *    *  *                *       
      25576 AACTGCTCTATCAATAGAAATGTTCAACTCCTTTG-GCTGGGTACACACATCACAAACAAGTTTC
         66 AACTGCTCTATCAAAAGAAATGTTCAACT-CTGTGAGTTGAGTACACACATCACAAAGAAGTTTC

                *                     **     *        
      25640 TGAGCATGCTTCTGTCTAGTTTTTATGGGAAGACATTCCCTT
        130 TGAGAATGCTTCTGTCTAGTTTTTATATGAAGATATTCCCTT

              *  * *  **          *       *            **                    
      25682 TTTCACCAAAGGCATCAAAGCGCTCCAAATGTCCACTTCCAGACACTACAAAAAGAGTGTTTCAA
          1 TTCCAACGAAATCATCAAAGCGATCCAAATATCCACTTCCAGATTCTACAAAAAGAGTGTTTCAA

             **       **    **                 *    * *  * *      *  *       
      25747 ACGTGCTCTAAGAAAACGAATGTTCAACTCTGTGACTTGAATGCAGATATCACAGAGTAGTTTCT
         66 AACTGCTCTATCAAAAGAAATGTTCAACTCTGTGAGTTGAGTACACACATCACAAAGAAGTTTCT

                **            *     *    *         * 
      25812 GAGAGGGCTTCTGTCTAGATTTTAGATGATGATATTCCCGT
        131 GAGAATGCTTCTGTCTAGTTTTTATATGAAGATATTCCCTT

                           * *   *                *   *                    * 
      25853 TTCCAACGAAATCATTAGAGCTATCCAAATATCCACTTACAGTTTCTACAAAAAGAGTGTTTCCA
          1 TTCCAACGAAATCATCAAAGCGATCCAAATATCCACTTCCAGATTCTACAAAAAGAGTGTTTCAA

                   **         * *    *       *  *                   ***      
      25918 AACTGCTGCATCAAAAGAGAGGTTCCACTCTGTTAGCTGAGTACACACATCACAAACTTGTTTCT
         66 AACTGCTCTATCAAAAGAAATGTTCAACTCTGTGAGTTGAGTACACACATCACAAAGAAGTTTCT

            *     *         *        **         **   
      25983 CAGAATCCTTCTGTCTCGTTTTTATGGGAAGATATTTACTT
        131 GAGAATGCTTCTGTCTAGTTTTTATATGAAGATATTCCCTT

              *  *  * **          *       *     *       *  *  *              
      26024 TTTCACCGTAGGCATCAAAGCGCTCCAAATGTCCACATCCAGATACTCCAGAAAGAGTGTTTCAA
          1 TTCCAACGAAATCATCAAAGCGATCCAAATATCCACTTCCAGATTCTACAAAAAGAGTGTTTCAA

             *         *   * *   *         *        * *  *      *      *     
      26089 ACCTGCTCTATGAAAGGGAATCTTCAACTCTATGAGTTGAATGCAGACATCAGAAAGAAATTTCT
         66 AACTGCTCTATCAAAAGAAATGTTCAACTCTGTGAGTTGAGTACACACATCACAAAGAAGTTTCT

                     *       **          *         **
      26154 GAGAATGCTGCTGTCTACCTTTTAT-T--TGA-ATTCCCGC
        131 GAGAATGCTTCTGTCTAGTTTTTATATGAAGATATTCCCTT

                         *  *    *                *       *                  
      26191 TTCCAACGAAATCCTCCAAGCTATCCAAATATCCACTTGCAGATTCCACAAAAAGAGTGTTTCAA
          1 TTCCAACGAAATCATCAAAGCGATCCAAATATCCACTTCCAGATTCTACAAAAAGAGTGTTTCAA

                            ** *  *            *       *     *     *  *      
      26256 AACTGCTCTCTATCAATGGCAAAGTTCAACTCTGTTAGTTGAGGACACATATCACCAACAAGTTT
         66 AACTG--CTCTATCAAAAGAAATGTTCAACTCTGTGAGTTGAGTACACACATCACAAAGAAGTTT

                               *       **         *    
      26321 CTGAGAATGCTTCTGTCTATTTTTTATGGGAAGATATTTCCTT
        129 CTGAGAATGCTTCTGTCTAGTTTTTATATGAAGATATTCCCTT

              *  *  * ** *    *      *    *          * **                    
      26364 TTTCACCGTAGGCGTCAAGGCGATCGAAATGTCCACTTCCACAAACTACAAAAAGAGTGTTTCAA
          1 TTCCAACGAAATCATCAAAGCGATCCAAATATCCACTTCCAGATTCTACAAAAAGAGTGTTTCAA

             *         *   * **       *    *        * ** * *   **      *     
      26429 ACCTGCTCTATGAAAGGCCATGTTCATCTCTATGAGTTGAATGGAAATATCCGAAAGAAATTTCT
         66 AACTGCTCTATCAAAAGAAATGTTCAACTCTGTGAGTTGAGTACACACATCACAAAGAAGTTTCT

             *       *          *        *         **
      26494 GGGAATGCTGCTGTCTAG-TGTT-TAT-ACGA-ATTCCCGC
        131 GAGAATGCTTCTGTCTAGTTTTTATATGAAGATATTCCCTT

                         *       *                *    *  *                  
      26531 TTCCAACGAAATCCTCAAAGCAATCCAAATATCCACTTGCAGAATCCACAAAAAGAGTGTTTCAA
          1 TTCCAACGAAATCATCAAAGCGATCCAAATATCCACTTCCAGATTCTACAAAAAGAGTGTTTCAA

                          *     *          * *                   *  *        
      26596 AACTGCTCTATCAATAGAAAGGTTCAACTCTTTTAGTTGAGTACACACATCACGAACAAGTTTCT
         66 AACTGCTCTATCAAAAGAAATGTTCAACTCTGTGAGTTGAGTACACACATCACAAAGAAGTTTCT

                            * *      **     **  *    
      26661 GAGAATGCTTCTGTCTGGCTTTTATTGGAAGACGTTTCCTT
        131 GAGAATGCTTCTGTCTAGTTTTTATATGAAGATATTCCCTT

                 * *  **          *       *                * *               
      26702 TT-CACCAAAGGCATCAAAGCGCTCCAAATGTCCACTTCCAGATTCTTCCAAAAGAGTGTTTCAA
          1 TTCCAACGAAATCATCAAAGCGATCCAAATATCCACTTCCAGATTCTACAAAAAGAGTGTTTCAA

             **     * ***  * *                 *    * *  * *     *   *       
      26766 ACGTGCTCAAAGTAAGGGAATGTTCAACTCTGTGACTTGAATGCAGATATCACCAAGTAGTTTCT
         66 AACTGCTCTATCAAAAGAAATGTTCAACTCTGTGAGTTGAGTACACACATCACAAAGAAGTTTCT

            * * *             *     *    *         * 
      26831 AATAGTGCTTCTGTCTAGATTTTAGATGATGATATTCCCGT
        131 GAGAATGCTTCTGTCTAGTTTTTATATGAAGATATTCCCTT

                         * * *   *             *  *   *                    * 
      26872 TTCCAACGAAATCGTTAGAGCTATCCAAATATCCAGTTACAGTTTCTACAAAAAGAGTGTTTCCA
          1 TTCCAACGAAATCATCAAAGCGATCCAAATATCCACTTCCAGATTCTACAAAAAGAGTGTTTCAA

                   **           *            *       *                     * 
      26937 AACTGCTGCATCAAAAGAAAGGTTCAACTCTGTTAGTTGAGGACACACATCACAAAGAAGTTTGT
         66 AACTGCTCTATCAAAAGAAATGTTCAACTCTGTGAGTTGAGTACACACATCACAAAGAAGTTTCT

                              *    *     *           
      27002 GAGAATGCTTCTGTCTAGATTTTGTATGACGATATTCCCTT
        131 GAGAATGCTTCTGTCTAGTTTTTATATGAAGATATTCCCTT

                     *   * *     *   *       * *  *    *  *       *  *       
      27043 TTCCAACGATATCGTTAAAGCAATCTAAATATCAATTTGCAGAATCCACAAAAATAGAGTTTCAA
          1 TTCCAACGAAATCATCAAAGCGATCCAAATATCCACTTCCAGATTCTACAAAAAGAGTGTTTCAA

             *       * *    *   *    *       *  *                   ***      
      27108 AGCTGCTCTGTAAAAATAAAAGTTCCACTCTGTTAGCTGAGTACACACATCACAAACTTGTTTCT
         66 AACTGCTCTATCAAAAGAAATGTTCAACTCTGTGAGTTGAGTACACACATCACAAAGAAGTTTCT

                  *         *        **         **   
      27173 GAGAATCCTTCTGTCTCGTTTTTATGGGAAGATATTTACTT
        131 GAGAATGCTTCTGTCTAGTTTTTATATGAAGATATTCCCTT

              *  *  * **          *       *     *       *  *  *     *        
      27214 TTTCACCGTAGGCATCAAAGCGCTCCAAATGTCCACATCCAGATACTCCAGAAAGACTGTTTCAA
          1 TTCCAACGAAATCATCAAAGCGATCCAAATATCCACTTCCAGATTCTACAAAAAGAGTGTTTCAA

             *         *   * *   *         *        * *  *      *      *     
      27279 ACCTGCTCTATGAAAGGGAATCTTCAACTCTATGAGTTGAATGCAGACATCAGAAAGAAATTTCT
         66 AACTGCTCTATCAAAAGAAATGTTCAACTCTGTGAGTTGAGTACACACATCACAAAGAAGTTTCT

                     *       **          *         **
      27344 GAGAATGCTGCTGTCTACCTTTTAT-T--TGA-ATTCCCGC
        131 GAGAATGCTTCTGTCTAGTTTTTATATGAAGATATTCCCTT

                         *  *    *                *  **   *                  
      27381 TTCCAACGAAATCCTCCAAGCTATCCAAATATCCACTTGCATTTTCCACAAAAAGAGTGTTTCAA
          1 TTCCAACGAAATCATCAAAGCGATCCAAATATCCACTTCCAGATTCTACAAAAAGAGTGTTTCAA

                          *               ** *  *  *                *        
      27446 AACTGCTCTATCAATAGAAATGTTCAACTCCTTTAGCTGGGTACACACATCACAAACAAGTTTCT
         66 AACTGCTCTATCAAAAGAAATGTTCAACTCTGTGAGTTGAGTACACACATCACAAAGAAGTTTCT

                                     **     **       
      27511 GAGAATGCTTCTGTCTAGTTTTTATGGGAAGACGTTCCCTT
        131 GAGAATGCTTCTGTCTAGTTTTTATATGAAGATATTCCCTT

              *  * *  **          *       *            **                  * 
      27552 TTTCACCAAAGGCATCAAAGCGCTCCAAATGTCCACTTCCAGACACTACAAAAAGAGTGTTTCCA
          1 TTCCAACGAAATCATCAAAGCGATCCAAATATCCACTTCCAGATTCTACAAAAAGAGTGTTTCAA

             **       **   ***                 *    * *  * *         *       
      27617 ACGTGCTCTAAGAAAGCGAATGTTCAACTCTGTGACTTGAATGCAGATATCACAAAGTAGTTTCT
         66 AACTGCTCTATCAAAAGAAATGTTCAACTCTGTGAGTTGAGTACACACATCACAAAGAAGTTTCT

                **            *     *    *         * 
      27682 GAGAGGGCTTCTGTCTAGATTTTAGATGATGATATTCCCGT
        131 GAGAATGCTTCTGTCTAGTTTTTATATGAAGATATTCCCTT

                           * *   *                *   *                    * 
      27723 TTCCAACGAAATCATTAGAGCTATCCAAATATCCACTTACAGTTTCTACAAAAAGAGTGTTTCCA
          1 TTCCAACGAAATCATCAAAGCGATCCAAATATCCACTTCCAGATTCTACAAAAAGAGTGTTTCAA

                   **         * *    *       *  **                  ***      
      27788 AACTGCTGCATCAAAAGAGAGGTTCCACTCTGTTAGCCGAGTACACACATCACAAACTTGTTTCT
         66 AACTGCTCTATCAAAAGAAATGTTCAACTCTGTGAGTTGAGTACACACATCACAAAGAAGTTTCT

            *     *         *        **         **   
      27853 CAGAATCCTTCTGTCTCGTTTTTATGGGAAGATATTTACTT
        131 GAGAATGCTTCTGTCTAGTTTTTATATGAAGATATTCCCTT

              *  *  * **          *       *     *       **    *              
      27894 TTTCACCGTAGGCATCAAAGCGCTCCAAATGTCCACATCCAGATAGTACAGAAAGAGTGTTTCAA
          1 TTCCAACGAAATCATCAAAGCGATCCAAATATCCACTTCCAGATTCTACAAAAAGAGTGTTTCAA

             *         *   * *   *         *        * *  *      *      *     
      27959 ACCTGCTCTATGAAAGGGAATCTTCAACTCTATGAGTTGAATGCAGACATCAGAAAGAAATTTCT
         66 AACTGCTCTATCAAAAGAAATGTTCAACTCTGTGAGTTGAGTACACACATCACAAAGAAGTTTCT

                     *       **          *         **
      28024 GAGAATGCTGCTGTCTACCTTTTAT-T--TGA-ATTCCCGC
        131 GAGAATGCTTCTGTCTAGTTTTTATATGAAGATATTCCCTT

                         *  *    *                *       *                  
      28061 TTCCAACGAAATCCTCCAAGCTATCCAAATATCCACTTGCAGATTCCACAAAAAGAGTGTTTCAA
          1 TTCCAACGAAATCATCAAAGCGATCCAAATATCCACTTCCAGATTCTACAAAAAGAGTGTTTCAA

                            ** *  *            *       *     *     *  *      
      28126 AACTGCTCTCTATCAATGGCAAAGTTCAACTCTGTTAGTTGAGGACACATATCACCAACAAGTTT
         66 AACTG--CTCTATCAAAAGAAATGTTCAACTCTGTGAGTTGAGTACACACATCACAAAGAAGTTT

                               *       **         *    
      28191 CTGAGAATGCTTCTGTCTATTTTTTATGGGAAGATATTTCCTT
        129 CTGAGAATGCTTCTGTCTAGTTTTTATATGAAGATATTCCCTT

              *  *  * ** *    *      *    *          * **                    
      28234 TTTCACCGTAGGCGTCAAGGCGATCGAAATGTCCACTTCCACAAACTACAAAAAGAGTGTTTCAA
          1 TTCCAACGAAATCATCAAAGCGATCCAAATATCCACTTCCAGATTCTACAAAAAGAGTGTTTCAA

             *         *   * **       *    *        * ** * *   **      *     
      28299 ACCTGCTCTATGAAAGGCCATGTTCATCTCTATGAGTTGAATGGAAATATCCGAAAGAAATTTCT
         66 AACTGCTCTATCAAAAGAAATGTTCAACTCTGTGAGTTGAGTACACACATCACAAAGAAGTTTCT

             *       *          *        *         **
      28364 GGGAATGCTGCTGTCTAG-TGTT-TAT-ACGA-ATTCCCGC
        131 GAGAATGCTTCTGTCTAGTTTTTATATGAAGATATTCCCTT

                         *       *                *    *  *                  
      28401 TTCCAACGAAATCCTCAAAGCAATCCAAATATCCACTTGCAGAATCCACAAAAAGAGTGTTTCAA
          1 TTCCAACGAAATCATCAAAGCGATCCAAATATCCACTTCCAGATTCTACAAAAAGAGTGTTTCAA

                          *     *          * *                   *  *        
      28466 AACTGCTCTATCAATAGAAAGGTTCAACTCTTTTAGTTGAGTACACACATCACGAACAAGTTTCT
         66 AACTGCTCTATCAAAAGAAATGTTCAACTCTGTGAGTTGAGTACACACATCACAAAGAAGTTTCT

                            * *      **     **  *    
      28531 GAGAATGCTTCTGTCTGGCTTTTATTGGAAGACGTTTCCTT
        131 GAGAATGCTTCTGTCTAGTTTTTATATGAAGATATTCCCTT

                 * *  **          *       *                * *               
      28572 TT-CACCAAAGGCATCAAAGCGCTCCAAATGTCCACTTCCAGATTCTTCCAAAAGAGTGTTTCAA
          1 TTCCAACGAAATCATCAAAGCGATCCAAATATCCACTTCCAGATTCTACAAAAAGAGTGTTTCAA

             **     * ***  * *                 *    * *  * *     *   *       
      28636 ACGTGCTCAAAGTAAGGGAATGTTCAACTCTGTGACTTGAATGCAGATATCACCAAGTAGTTTCT
         66 AACTGCTCTATCAAAAGAAATGTTCAACTCTGTGAGTTGAGTACACACATCACAAAGAAGTTTCT

            * * *             *   * *    *         * 
      28701 AATAGTGCTTCTGTCTAGATTTCAGATGATGATATTCCCGT
        131 GAGAATGCTTCTGTCTAGTTTTTATATGAAGATATTCCCTT

                         * * *   * **          *  *   *      *     *       * 
      28742 TTCCAACGAAATCGTTAGAGCTAAGCAAATATCCAGTTACAGTTTCTACCAAAAGGGTGTTTCCA
          1 TTCCAACGAAATCATCAAAGCGATCCAAATATCCACTTCCAGATTCTACAAAAAGAGTGTTTCAA

              *    **           *            *       *                     * 
      28807 AATTGCTGCATCAAAAGAAAGGTTCAACTCTGTTAGTTGAGGACACACATCACAAAGAAGTTTGT
         66 AACTGCTCTATCAAAAGAAATGTTCAACTCTGTGAGTTGAGTACACACATCACAAAGAAGTTTCT

                              *    *     *           
      28872 GAGAATGCTTCTGTCTAGATTTTGTATGACGATATTCCCTT
        131 GAGAATGCTTCTGTCTAGTTTTTATATGAAGATATTCCCTT

                     *   * *     *   *       * *  *    *  *       *  *       
      28913 TTCCAACGATATCGTTAAAGCAATCTAAATATCAATTTGCAGAATCCACAAAAATAGAGTTTCAA
          1 TTCCAACGAAATCATCAAAGCGATCCAAATATCCACTTCCAGATTCTACAAAAAGAGTGTTTCAA

             *       * *        *    *       *  *                   ***      
      28978 AGCTGCTCTGTAAAAAGAAAGGTTCCACTCTGTTAGCTGAGTACACACATCACAAACTTGTTTCT
         66 AACTGCTCTATCAAAAGAAATGTTCAACTCTGTGAGTTGAGTACACACATCACAAAGAAGTTTCT

                  *         *        **         **   
      29043 GAGAATCCTTCTGTCTCGTTTTTATGGGAAGATATTTACTT
        131 GAGAATGCTTCTGTCTAGTTTTTATATGAAGATATTCCCTT

                 *  * **          *       *     *       *  *  *              
      29084 TTCCACCGTAGGCATCAAAGCGCTCCAAATGTCCACATCCAGATACTCCAGAAAGAGTGTTTCAA
          1 TTCCAACGAAATCATCAAAGCGATCCAAATATCCACTTCCAGATTCTACAAAAAGAGTGTTTCAA

             *         *   * *   *         *        * *  *      *      *     
      29149 ACCTGCTCTATGAAAGGCAATCTTCAACTCTATGAGTTGAATGCAGACATCAGAAAGAAATTTCT
         66 AACTGCTCTATCAAAAGAAATGTTCAACTCTGTGAGTTGAGTACACACATCACAAAGAAGTTTCT

                     *       **          *         **
      29214 GAGAATGCTGCTGTCTACCTTTTAT-T--TGA-ATTCCCGC
        131 GAGAATGCTTCTGTCTAGTTTTTATATGAAGATATTCCCTT

                         *  *    *              * *  **   *                  
      29251 TTCCAACGAAATCCTCCAAGCTATCCAAATATCCACCTGCATTTTCCACAAAAAGAGTGTTTCAA
          1 TTCCAACGAAATCATCAAAGCGATCCAAATATCCACTTCCAGATTCTACAAAAAGAGTGTTTCAA

                          *                 *    *  *                *       
      29316 AACTGCTCTATCAATAGAAATGTTCAACTCCTTTG-GCTGGGTACACACATCACAAACAAGTTTC
         66 AACTGCTCTATCAAAAGAAATGTTCAACT-CTGTGAGTTGAGTACACACATCACAAAGAAGTTTC

                *                     **     *        
      29380 TGAGCATGCTTCTGTCTAGTTTTTATGGGAAGACATTCCCTT
        130 TGAGAATGCTTCTGTCTAGTTTTTATATGAAGATATTCCCTT

              *  * *  **          *       *            **                  * 
      29422 TTTCACCAAAGGCATCAAAGCGCTCCAAATGTCCACTTCCAGACACTACAAAAAGAGTGTTTCCA
          1 TTCCAACGAAATCATCAAAGCGATCCAAATATCCACTTCCAGATTCTACAAAAAGAGTGTTTCAA

             **       **    **                 *    * *  * *      *  *       
      29487 ACGTGCTCTAAGAAAACGAATGTTCAACTCTGTGACTTGAATGCAGATATCACAGAGTAGTTTCT
         66 AACTGCTCTATCAAAAGAAATGTTCAACTCTGTGAGTTGAGTACACACATCACAAAGAAGTTTCT

                **            *     *    *         * 
      29552 GAGAGGGCTTCTGTCTAGATTTTAGATGATGATATTCCCGT
        131 GAGAATGCTTCTGTCTAGTTTTTATATGAAGATATTCCCTT

                           * *   *                *   *                    * 
      29593 TTCCAACGAAATCATTAGAGCTATCCAAATATCCACTTACAGTTTCTACAAAAAGAGTGTTTCCA
          1 TTCCAACGAAATCATCAAAGCGATCCAAATATCCACTTCCAGATTCTACAAAAAGAGTGTTTCAA

                   **         * *    *       *  *                   ***      
      29658 AACTGCTGCATCAAAAGAGAGGTTCCACTCTGTTAGCTGAGTACACACATCACAAACTTGTTTCT
         66 AACTGCTCTATCAAAAGAAATGTTCAACTCTGTGAGTTGAGTACACACATCACAAAGAAGTTTCT

            *     *         *        **         **   
      29723 CAGAATCCTTCTGTCTCGTTTTTATGGGAAGATATTTACTT
        131 GAGAATGCTTCTGTCTAGTTTTTATATGAAGATATTCCCTT

              *  *  * **          *       *     *       *  *  *              
      29764 TTTCACCGTAGGCATCAAAGCGCTCCAAATGTCCACATCCAGATACTCCAGAAAGAGTGTTTCAA
          1 TTCCAACGAAATCATCAAAGCGATCCAAATATCCACTTCCAGATTCTACAAAAAGAGTGTTTCAA

             *         *   * *   *         *        * *  *      *      *     
      29829 ACCTGCTCTATGAAAGGGAATCTTCAACTCTATGAGTTGAATGCAGACATCAGAAAGAAATTTCT
         66 AACTGCTCTATCAAAAGAAATGTTCAACTCTGTGAGTTGAGTACACACATCACAAAGAAGTTTCT

                     *       **          *         **
      29894 GAGAATGCTGCTGTCTACCTTTTAT-T--TGA-ATTCCCGC
        131 GAGAATGCTTCTGTCTAGTTTTTATATGAAGATATTCCCTT

                         *  *    *                *       *                  
      29931 TTCCAACGAAATCCTCCAAGCTATCCAAATATCCACTTGCAGATTCCACAAAAAGAGTGTTTCAA
          1 TTCCAACGAAATCATCAAAGCGATCCAAATATCCACTTCCAGATTCTACAAAAAGAGTGTTTCAA

                            ** *  *            *       *     *     *  *      
      29996 AACTGCTCTCTATCAATGGCAAAGTTCAACTCTGTTAGTTGAGGACACATATCACCAACAAGTTT
         66 AACTG--CTCTATCAAAAGAAATGTTCAACTCTGTGAGTTGAGTACACACATCACAAAGAAGTTT

                               *       **         *    
      30061 CTGAGAATGCTTCTGTCTATTTTTTATGGGAAGATATTTCCTT
        129 CTGAGAATGCTTCTGTCTAGTTTTTATATGAAGATATTCCCTT

              *  *  * ** *    *      *    *          * **                    
      30104 TTTCACCGTAGGCGTCAAGGCGATCGAAATGTCCACTTCCACAAACTACAAAAAGAGTGTTTCAA
          1 TTCCAACGAAATCATCAAAGCGATCCAAATATCCACTTCCAGATTCTACAAAAAGAGTGTTTCAA

             *         *   * **       *    *        * ** * *   **      *     
      30169 ACCTGCTCTATGAAAGGCGATGTTCATCTCTATGAGTTGAATGGAAATATCCGAAAGAAATTTCT
         66 AACTGCTCTATCAAAAGAAATGTTCAACTCTGTGAGTTGAGTACACACATCACAAAGAAGTTTCT

             *       *          *        *         **
      30234 GGGAATGCTGCTGTCTAG-TGTT-TAT-ACGA-ATTCCCGC
        131 GAGAATGCTTCTGTCTAGTTTTTATATGAAGATATTCCCTT

                         *       *                *    *  *                  
      30271 TTCCAACGAAATCCTCAAAGCAATCCAAATATCCACTTGCAGAATCCACAAAAAGAGTGTTTCAA
          1 TTCCAACGAAATCATCAAAGCGATCCAAATATCCACTTCCAGATTCTACAAAAAGAGTGTTTCAA

                          *     *          * *                   *  *        
      30336 AACTGCTCTATCAATAGAAAGGTTCAACTCTTTTAGTTGAGTACACACATCACGAACAAGTTTCT
         66 AACTGCTCTATCAAAAGAAATGTTCAACTCTGTGAGTTGAGTACACACATCACAAAGAAGTTTCT

                            * *      **     **  *    
      30401 GAGAATGCTTCTGTCTGGCTTTTATTGGAAGACGTTTCCTT
        131 GAGAATGCTTCTGTCTAGTTTTTATATGAAGATATTCCCTT

                 * *  **          *       *                * *               
      30442 TT-CACCAAAGGCATCAAAGCGCTCCAAATGTCCACTTCCAGATTCTTCCAAAAGAGTGTTTCAA
          1 TTCCAACGAAATCATCAAAGCGATCCAAATATCCACTTCCAGATTCTACAAAAAGAGTGTTTCAA

             **     * ***  * *                 *    * *  * *     *   *       
      30506 ACGTGCTCAAAGTAAGGGAATGTTCAACTCTGTGACTTGAATGCAGATATCACCAAGTAGTTTCT
         66 AACTGCTCTATCAAAAGAAATGTTCAACTCTGTGAGTTGAGTACACACATCACAAAGAAGTTTCT

            * * *             *     *    *         * 
      30571 AATAGTGCTTCTGTCTAGATTTTAGATGATGATATTCCCGT
        131 GAGAATGCTTCTGTCTAGTTTTTATATGAAGATATTCCCTT

                         * * *   *             *  *   *                    * 
      30612 TTCCAACGAAATCGTTAGAGCTATCCAAATATCCAGTTACAGTTTCTACAAAAAGAGTGTTTCCA
          1 TTCCAACGAAATCATCAAAGCGATCCAAATATCCACTTCCAGATTCTACAAAAAGAGTGTTTCAA

                   **           *            *       *                     * 
      30677 AACTGCTGCATCAAAAGAAAGGTTCAACTCTGTTAGTTGAGGACACACATCACAAAGAAGTTTGT
         66 AACTGCTCTATCAAAAGAAATGTTCAACTCTGTGAGTTGAGTACACACATCACAAAGAAGTTTCT

                              *    *     *           
      30742 GAGAATGCTTCTGTCTAGATTTTGTATGACGATATTCCCTT
        131 GAGAATGCTTCTGTCTAGTTTTTATATGAAGATATTCCCTT

                     *   * *     *   *       * *  *    *  *       *  *       
      30783 TTCCAACGATATCGTTAAAGCAATCTAAATATCAATTTGCAGAATCCACAAAAATAGAGTTTCAA
          1 TTCCAACGAAATCATCAAAGCGATCCAAATATCCACTTCCAGATTCTACAAAAAGAGTGTTTCAA

             *       * *    *   *    *       *  *                   ***      
      30848 AGCTGCTCTGTAAAAATAAAAGTTCCACTCTGTTAGCTGAGTACACACATCACAAACTTGTTTCT
         66 AACTGCTCTATCAAAAGAAATGTTCAACTCTGTGAGTTGAGTACACACATCACAAAGAAGTTTCT

                  *         *        **         **   
      30913 GAGAATCCTTCTGTCTCGTTTTTATGGGAAGATATTTACTT
        131 GAGAATGCTTCTGTCTAGTTTTTATATGAAGATATTCCCTT

              *  *  * **          *       *     *       *  *  *     *        
      30954 TTTCACCGTAGGCATCAAAGCGCTCCAAATGTCCACATCCAGATACTCCAGAAAGACTGTTTCAA
          1 TTCCAACGAAATCATCAAAGCGATCCAAATATCCACTTCCAGATTCTACAAAAAGAGTGTTTCAA

             *         *   * *   *         *        * *  *      *      *     
      31019 ACCTGCTCTATGAAAGGGAATCTTCAACTCTATGAGTTGAATGCAGACATCAGAAAGAAATTTCT
         66 AACTGCTCTATCAAAAGAAATGTTCAACTCTGTGAGTTGAGTACACACATCACAAAGAAGTTTCT

                     *       **          *         **
      31084 GAGAATGCTGCTGTCTACCTTTTAT-T--TGA-ATTCCCGC
        131 GAGAATGCTTCTGTCTAGTTTTTATATGAAGATATTCCCTT

                         *  *    *                *  **   *                  
      31121 TTCCAACGAAATCCTCCAAGCTATCCAAATATCCACTTGCATTTTCCACAAAAAGAGTGTTTCAA
          1 TTCCAACGAAATCATCAAAGCGATCCAAATATCCACTTCCAGATTCTACAAAAAGAGTGTTTCAA

                          *               ** *  *  *                *        
      31186 AACTGCTCTATCAATAGAAATGTTCAACTCCTTTAGCTGGGTACACACATCACAAACAAGTTTCT
         66 AACTGCTCTATCAAAAGAAATGTTCAACTCTGTGAGTTGAGTACACACATCACAAAGAAGTTTCT

                                     **     **       
      31251 GAGAATGCTTCTGTCTAGTTTTTATGGGAAGACGTTCCCTT
        131 GAGAATGCTTCTGTCTAGTTTTTATATGAAGATATTCCCTT

              *  * *  **          *       *            **                    
      31292 TTTCACCAAAGGCATCAAAGCGCTCCAAATGTCCACTTCCAGACACTACAAAAAGAGTGTTTCAA
          1 TTCCAACGAAATCATCAAAGCGATCCAAATATCCACTTCCAGATTCTACAAAAAGAGTGTTTCAA

             **       **   ***                 *    * *  * *         *       
      31357 ACGTGCTCTAAGAAAGCGAATGTTCAACTCTGTGACTTGAATGCAGATATCACAAAGTAGTTTCT
         66 AACTGCTCTATCAAAAGAAATGTTCAACTCTGTGAGTTGAGTACACACATCACAAAGAAGTTTCT

                **            *     *    *         * 
      31422 GAGAGGGCTTCTGTCTAGATTTTAGATGATGATATTCCCGT
        131 GAGAATGCTTCTGTCTAGTTTTTATATGAAGATATTCCCTT

                           * *   *                *   *                    * 
      31463 TTCCAACGAAATCATTAGAGCTATCCAAATATCCACTTACAGTTTCTACAAAAAGAGTGTTTCCA
          1 TTCCAACGAAATCATCAAAGCGATCCAAATATCCACTTCCAGATTCTACAAAAAGAGTGTTTCAA

                   **         * *    *       *  **                  ***      
      31528 AACTGCTGCATCAAAAGAGAGGTTCCACTCTGTTAGCCGAGTACACACATCACAAACTTGTTTCT
         66 AACTGCTCTATCAAAAGAAATGTTCAACTCTGTGAGTTGAGTACACACATCACAAAGAAGTTTCT

            *     *         *        **         **   
      31593 CAGAATCCTTCTGTCTCGTTTTTATGGGAAGATATTTACTT
        131 GAGAATGCTTCTGTCTAGTTTTTATATGAAGATATTCCCTT

              *  *  * **          *       *     *       *  *  *              
      31634 TTTCACCGTAGGCATCAAAGCGCTCCAAATGTCCACATCCAGATACTCCAGAAAGAGTGTTTCAA
          1 TTCCAACGAAATCATCAAAGCGATCCAAATATCCACTTCCAGATTCTACAAAAAGAGTGTTTCAA

             *         *   * *   *         *        * *  *      *      *     
      31699 ACCTGCTCTATGAAAGGGAATCTTCAACTCTATGAGTTGAATGCAGACATCAGAAAGAAATTTCT
         66 AACTGCTCTATCAAAAGAAATGTTCAACTCTGTGAGTTGAGTACACACATCACAAAGAAGTTTCT

                     *       **          *         **
      31764 GAGAATGCTGCTGTCTACCTTTTAT-T--TGA-ATTCCCGC
        131 GAGAATGCTTCTGTCTAGTTTTTATATGAAGATATTCCCTT

                         *  *    *                *       *                  
      31801 TTCCAACGAAATCCTCCAAGCTATCCAAATATCCACTTGCAGATTCCACAAAAAGAGTGTTTCAA
          1 TTCCAACGAAATCATCAAAGCGATCCAAATATCCACTTCCAGATTCTACAAAAAGAGTGTTTCAA

                            ** *  *            *       *     *     *  *      
      31866 AACTGCTCTCTATCAATGGCAAAGTTCAACTCTGTTAGTTGAGGACACATATCACCAACAAGTTT
         66 AACTG--CTCTATCAAAAGAAATGTTCAACTCTGTGAGTTGAGTACACACATCACAAAGAAGTTT

                               *       **         *    
      31931 CTGAGAATGCTTCTGTCTATTTTTTATGGGAAGATATTTCCTT
        129 CTGAGAATGCTTCTGTCTAGTTTTTATATGAAGATATTCCCTT

              *  *  * ** *    *   *  *    *          * **                    
      31974 TTTCACCGTAGGCGTCAACGCGTTCGAAATGTCCACTTCCACAAACTACAAAAAGAGTGTTTCAA
          1 TTCCAACGAAATCATCAAAGCGATCCAAATATCCACTTCCAGATTCTACAAAAAGAGTGTTTCAA

             *         *   * **       *    *        * ** * *   **      *     
      32039 ACCTGCTCTATGAAAGGCCATGTTCATCTCTATGAGTTGAATGGAAATATCCGAAAGAAATTTCT
         66 AACTGCTCTATCAAAAGAAATGTTCAACTCTGTGAGTTGAGTACACACATCACAAAGAAGTTTCT

             *       *          *        *         **
      32104 GGGAATGCTGCTGTCTAG-TGTT-TAT-ACGA-ATTCCCGC
        131 GAGAATGCTTCTGTCTAGTTTTTATATGAAGATATTCCCTT

                         *       *                *    *  *                  
      32141 TTCCAACGAAATCCTCAAAGCAATCCAAATATCCACTTGCAGAATCCACAAAAAGAGTGTTTCAA
          1 TTCCAACGAAATCATCAAAGCGATCCAAATATCCACTTCCAGATTCTACAAAAAGAGTGTTTCAA

                          *     *          * *                   *  *        
      32206 AACTGCTCTATCAATAGAAAGGTTCAACTCTTTTAGTTGAGTACACACATCACGAACAAGTTTCT
         66 AACTGCTCTATCAAAAGAAATGTTCAACTCTGTGAGTTGAGTACACACATCACAAAGAAGTTTCT

                            * *      **     **  *    
      32271 GAGAATGCTTCTGTCTGGCTTTTATTGGAAGACGTTTCCTT
        131 GAGAATGCTTCTGTCTAGTTTTTATATGAAGATATTCCCTT

                 * *  **          *       *                * *               
      32312 TT-CACCAAAGGCATCAAAGCGCTCCAAATGTCCACTTCCAGATTCTTCCAAAAGAGTGTTTCAA
          1 TTCCAACGAAATCATCAAAGCGATCCAAATATCCACTTCCAGATTCTACAAAAAGAGTGTTTCAA

             **     * ***  * *                 *    * *  * *     *   *       
      32376 ACGTGCTCAAAGTAAGGGAATGTTCAACTCTGTGACTTGAATGCAGATATCACCAAGTAGTTTCT
         66 AACTGCTCTATCAAAAGAAATGTTCAACTCTGTGAGTTGAGTACACACATCACAAAGAAGTTTCT

            * * *             *   * *    *         * 
      32441 AATAGTGCTTCTGTCTAGATTTCAGATGATGATATTCCCGT
        131 GAGAATGCTTCTGTCTAGTTTTTATATGAAGATATTCCCTT

                         * * *   * **          *  *   *      *     *       * 
      32482 TTCCAACGAAATCGTTAGAGCTAAGCAAATATCCAGTTACAGTTTCTACCAAAAGGGTGTTTCCA
          1 TTCCAACGAAATCATCAAAGCGATCCAAATATCCACTTCCAGATTCTACAAAAAGAGTGTTTCAA

              *    **           *            *       *                     * 
      32547 AATTGCTGCATCAAAAGAAAGGTTCAACTCTGTTAGTTGAGGACACACATCACAAAGAAGTTTGT
         66 AACTGCTCTATCAAAAGAAATGTTCAACTCTGTGAGTTGAGTACACACATCACAAAGAAGTTTCT

                              *    *     *           
      32612 GAGAATGCTTCTGTCTAGATTTTGTATGACGATATTCCCTT
        131 GAGAATGCTTCTGTCTAGTTTTTATATGAAGATATTCCCTT

                     *   * *     *   *       * *  *    *  *       *  *       
      32653 TTCCAACGATATCGTTAAAGCAATCTAAATATCAATTTGCAGAATCCACAAAAATAGAGTTTCAA
          1 TTCCAACGAAATCATCAAAGCGATCCAAATATCCACTTCCAGATTCTACAAAAAGAGTGTTTCAA

             *       * *        *    *       *  *                   ***      
      32718 AGCTGCTCTGTAAAAAGAAAGGTTCCACTCTGTTAGCTGAGTACACACATCACAAACTTGTTTCT
         66 AACTGCTCTATCAAAAGAAATGTTCAACTCTGTGAGTTGAGTACACACATCACAAAGAAGTTTCT

                  *         *        **         **   
      32783 GAGAATCCTTCTGTCTCGTTTTTATGGGAAGATATTTACTT
        131 GAGAATGCTTCTGTCTAGTTTTTATATGAAGATATTCCCTT

                 *  * **          *       *     *       *  *  *              
      32824 TTCCACCGTAGGCATCAAAGCGCTCCAAATGTCCACATCCAGATACTCCAGAAAGAGTGTTTCAA
          1 TTCCAACGAAATCATCAAAGCGATCCAAATATCCACTTCCAGATTCTACAAAAAGAGTGTTTCAA

             *         *   * *   *         *        * *  *      *      *     
      32889 ACCTGCTCTATGAAAGGCAATCTTCAACTCTATGAGTTGAATGCAGACATCAGAAAGAAATTTCT
         66 AACTGCTCTATCAAAAGAAATGTTCAACTCTGTGAGTTGAGTACACACATCACAAAGAAGTTTCT

                     *       **          *         **
      32954 GAGAATGCTGCTGTCTACCTTTTAT-T--TGA-ATTCCCGC
        131 GAGAATGCTTCTGTCTAGTTTTTATATGAAGATATTCCCTT

                         *  *    *              * *  **   *                  
      32991 TTCCAACGAAATCCTCCAAGCTATCCAAATATCCACCTGCATTTTCCACAAAAAGAGTGTTTCAA
          1 TTCCAACGAAATCATCAAAGCGATCCAAATATCCACTTCCAGATTCTACAAAAAGAGTGTTTCAA

                          *                 *    *  *                *       
      33056 AACTGCTCTATCAATAGAAATGTTCAACTCCTTTG-GCTGGGTACACACATCACAAACAAGTTTC
         66 AACTGCTCTATCAAAAGAAATGTTCAACT-CTGTGAGTTGAGTACACACATCACAAAGAAGTTTC

                *                     **     *        
      33120 TGAGCATGCTTCTGTCTAGTTTTTATGGGAAGACATTCCCTT
        130 TGAGAATGCTTCTGTCTAGTTTTTATATGAAGATATTCCCTT

              *  * *  **          *       *            **                    
      33162 TTTCACCAAAGGCATCAAAGCGCTCCAAATGTCCACTTCCAGACACTACAAAAAGAGTGTTTCAA
          1 TTCCAACGAAATCATCAAAGCGATCCAAATATCCACTTCCAGATTCTACAAAAAGAGTGTTTCAA

             **       **    **                 *    * *  * *      *  *       
      33227 ACGTGCTCTAAGAAAACGAATGTTCAACTCTGTGACTTGAATGCAGATATCACAGAGTAGTTTCT
         66 AACTGCTCTATCAAAAGAAATGTTCAACTCTGTGAGTTGAGTACACACATCACAAAGAAGTTTCT

                **            *     *    *         * 
      33292 GAGAGGGCTTCTGTCTAGATTTTAGATGATGATATTCCCGT
        131 GAGAATGCTTCTGTCTAGTTTTTATATGAAGATATTCCCTT

                           * *   *                *   *                    * 
      33333 TTCCAACGAAATCATTAGAGCTATCCAAATATCCACTTACAGTTTCTACAAAAAGAGTGTTTCCA
          1 TTCCAACGAAATCATCAAAGCGATCCAAATATCCACTTCCAGATTCTACAAAAAGAGTGTTTCAA

                   **         * *    *       *  *                   ***      
      33398 AACTGCTGCATCAAAAGAGAGGTTCCACTCTGTTAGCTGAGTACACACATCACAAACTTGTTTCT
         66 AACTGCTCTATCAAAAGAAATGTTCAACTCTGTGAGTTGAGTACACACATCACAAAGAAGTTTCT

            *     *         *        **         **   
      33463 CAGAATCCTTCTGTCTCGTTTTTATGGGAAGATATTTACTT
        131 GAGAATGCTTCTGTCTAGTTTTTATATGAAGATATTCCCTT

              *  *  * **          *       *     *       *  *  *              
      33504 TTTCACCGTAGGCATCAAAGCGCTCCAAATGTCCACATCCAGATACTCCAGAAAGAGTGTTTCAA
          1 TTCCAACGAAATCATCAAAGCGATCCAAATATCCACTTCCAGATTCTACAAAAAGAGTGTTTCAA

             *         *   * *   *         *        * *  *      *      *     
      33569 ACCTGCTCTATGAAAGGGAATCTTCAACTCTATGAGTTGAATGCAGACATCAGAAAGAAATTTCT
         66 AACTGCTCTATCAAAAGAAATGTTCAACTCTGTGAGTTGAGTACACACATCACAAAGAAGTTTCT

                     *       **          *         **
      33634 GAGAATGCTGCTGTCTACCTTTTAT-T--TGA-ATTCCCGC
        131 GAGAATGCTTCTGTCTAGTTTTTATATGAAGATATTCCCTT

                         *  *    *                *       *                  
      33671 TTCCAACGAAATCCTCCAAGCTATCCAAATATCCACTTGCAGATTCCACAAAAAGAGTGTTTCAA
          1 TTCCAACGAAATCATCAAAGCGATCCAAATATCCACTTCCAGATTCTACAAAAAGAGTGTTTCAA

                            ** *  *            *       *     *     *  *      
      33736 AACTGCTCTCTATCAATGGCAAAGTTCAACTCTGTTAGTTGAGGACACATATCACCAACAAGTTT
         66 AACTG--CTCTATCAAAAGAAATGTTCAACTCTGTGAGTTGAGTACACACATCACAAAGAAGTTT

                               *       **         *    
      33801 CTGAGAATGCTTCTGTCTATTTTTTATGGGAAGATATTTCCTT
        129 CTGAGAATGCTTCTGTCTAGTTTTTATATGAAGATATTCCCTT

              *  *  * ** *    *      *    *          * **                    
      33844 TTTCACCGTAGGCGTCAAGGCGATCGAAATGTCCACTTCCACAAACTACAAAAAGAGTGTTTCAA
          1 TTCCAACGAAATCATCAAAGCGATCCAAATATCCACTTCCAGATTCTACAAAAAGAGTGTTTCAA

             *         *   * **       *    *        * ** * *   **      *     
      33909 ACCTGCTCTATGAAAGGCCATGTTCATCTCTATGAGTTGAATGGAAATATCCGAAAGAAATTTCT
         66 AACTGCTCTATCAAAAGAAATGTTCAACTCTGTGAGTTGAGTACACACATCACAAAGAAGTTTCT

             *       *          *        *         **
      33974 GGGAATGCTGCTGTCTAG-TGTT-TAT-ACGA-ATTCCCGC
        131 GAGAATGCTTCTGTCTAGTTTTTATATGAAGATATTCCCTT

                         *       *                *    *  *                  
      34011 TTCCAACGAAATCCTCAAAGCAATCCAAATATCCACTTGCAGAATCCACAAAAAGAGTGTTTCAA
          1 TTCCAACGAAATCATCAAAGCGATCCAAATATCCACTTCCAGATTCTACAAAAAGAGTGTTTCAA

                          *     *          * *                   *  *        
      34076 AACTGCTCTATCAATAGAAAGGTTCAACTCTTTTAGTTGAGTACACACATCACGAACAAGTTTCT
         66 AACTGCTCTATCAAAAGAAATGTTCAACTCTGTGAGTTGAGTACACACATCACAAAGAAGTTTCT

                            * *      **     **  *    
      34141 GAGAATGCTTCTGTCTGGCTTTTATTGGAAGACGTTTCCTT
        131 GAGAATGCTTCTGTCTAGTTTTTATATGAAGATATTCCCTT

                 * *  **          *       *                * *               
      34182 TT-CACCAAAGGCATCAAAGCGCTCCAAATGTCCACTTCCAGATTCTTCCAAAAGAGTGTTTCAA
          1 TTCCAACGAAATCATCAAAGCGATCCAAATATCCACTTCCAGATTCTACAAAAAGAGTGTTTCAA

             **     * ***  * *                 *    * *  * *     *   *       
      34246 ACGTGCTCAAAGTAAGGGAATGTTCAACTCTGTGACTTGAATGCAGATATCACCAAGTAGTTTCT
         66 AACTGCTCTATCAAAAGAAATGTTCAACTCTGTGAGTTGAGTACACACATCACAAAGAAGTTTCT

            * * *             *     *    *         * 
      34311 AATAGTGCTTCTGTCTAGATTTTAGATGATGATATTCCCGT
        131 GAGAATGCTTCTGTCTAGTTTTTATATGAAGATATTCCCTT

                         * * *   *             *  *   *                    * 
      34352 TTCCAACGAAATCGTTAGAGCTATCCAAATATCCAGTTACAGTTTCTACAAAAAGAGTGTTTCCA
          1 TTCCAACGAAATCATCAAAGCGATCCAAATATCCACTTCCAGATTCTACAAAAAGAGTGTTTCAA

                   **           *            *       *                     * 
      34417 AACTGCTGCATCAAAAGAAAGGTTCAACTCTGTTAGTTGAGGACACACATCACAAAGAAGTTTGT
         66 AACTGCTCTATCAAAAGAAATGTTCAACTCTGTGAGTTGAGTACACACATCACAAAGAAGTTTCT

                              *    *     *           
      34482 GAGAATGCTTCTGTCTAGATTTTGTATGACGATATTCCCTT
        131 GAGAATGCTTCTGTCTAGTTTTTATATGAAGATATTCCCTT

                     *   * *     *   *       * *  *    *  *       *  *       
      34523 TTCCAACGATATCGTTAAAGCAATCTAAATATCAATTTGCAGAATCCACAAAAATAGAGTTTCAA
          1 TTCCAACGAAATCATCAAAGCGATCCAAATATCCACTTCCAGATTCTACAAAAAGAGTGTTTCAA

             *       * *    *   *    *       *  *                   ***      
      34588 AGCTGCTCTGTAAAAATAAAAGTTCCACTCTGTTAGCTGAGTACACACATCACAAACTTGTTTCT
         66 AACTGCTCTATCAAAAGAAATGTTCAACTCTGTGAGTTGAGTACACACATCACAAAGAAGTTTCT

                  *         *        **         **   
      34653 GAGAATCCTTCTGTCTCGTTTTTATGGGAAGATATTTACTT
        131 GAGAATGCTTCTGTCTAGTTTTTATATGAAGATATTCCCTT

              *  *  * **          *       *     *       *  *  *     *        
      34694 TTTCACCGTAGGCATCAAAGCGCTCCAAATGTCCACATCCAGATACTCCAGAAAGACTGTTTCAA
          1 TTCCAACGAAATCATCAAAGCGATCCAAATATCCACTTCCAGATTCTACAAAAAGAGTGTTTCAA

             *         *   * *   *         *        * *  *      *      *     
      34759 ACCTGCTCTATGAAAGGGAATCTTCAACTCTATGAGTTGAATGCAGACATCAGAAAGAAATTTCT
         66 AACTGCTCTATCAAAAGAAATGTTCAACTCTGTGAGTTGAGTACACACATCACAAAGAAGTTTCT

                     *       **          *         **
      34824 GAGAATGCTGCTGTCTACCTTTTAT-T--TGA-ATTCCCGC
        131 GAGAATGCTTCTGTCTAGTTTTTATATGAAGATATTCCCTT

                         *  *    *                *  **   *                  
      34861 TTCCAACGAAATCCTCCAAGCTATCCAAATATCCACTTGCATTTTCCACAAAAAGAGTGTTTCAA
          1 TTCCAACGAAATCATCAAAGCGATCCAAATATCCACTTCCAGATTCTACAAAAAGAGTGTTTCAA

                          *               ** *  *  *                *        
      34926 AACTGCTCTATCAATAGAAATGTTCAACTCCTTTAGCTGGGTACACACATCACAAACAAGTTTCT
         66 AACTGCTCTATCAAAAGAAATGTTCAACTCTGTGAGTTGAGTACACACATCACAAAGAAGTTTCT

                                     **     **       
      34991 GAGAATGCTTCTGTCTAGTTTTTATGGGAAGACGTTCCCTT
        131 GAGAATGCTTCTGTCTAGTTTTTATATGAAGATATTCCCTT

              *  * *  **          *       *            **                    
      35032 TTTCACCAAAGGCATCAAAGCGCTCCAAATGTCCACTTCCAGACACTACAAAAAGAGTGTTTCAA
          1 TTCCAACGAAATCATCAAAGCGATCCAAATATCCACTTCCAGATTCTACAAAAAGAGTGTTTCAA

             **       **   ***                 *    * *  * *         *       
      35097 ACGTGCTCTAAGAAAGCGAATGTTCAACTCTGTGACTTGAATGCAGATATCACAAAGTAGTTTCT
         66 AACTGCTCTATCAAAAGAAATGTTCAACTCTGTGAGTTGAGTACACACATCACAAAGAAGTTTCT

                **            *     *    *         * 
      35162 GAGAGGGCTTCTGTCTAGATTTTAGATGATGATATTCCCGT
        131 GAGAATGCTTCTGTCTAGTTTTTATATGAAGATATTCCCTT

                           * *   *                *   *                    * 
      35203 TTCCAACGAAATCATTAGAGCTATCCAAATATCCACTTACAGTTTCTACAAAAAGAGTGTTTCCA
          1 TTCCAACGAAATCATCAAAGCGATCCAAATATCCACTTCCAGATTCTACAAAAAGAGTGTTTCAA

                   **         * *    *       *  **                  ***      
      35268 AACTGCTGCATCAAAAGAGAGGTTCCACTCTGTTAGCCGAGTACACACATCACAAACTTGTTTCT
         66 AACTGCTCTATCAAAAGAAATGTTCAACTCTGTGAGTTGAGTACACACATCACAAAGAAGTTTCT

            *     *         *        **         **   
      35333 CAGAATCCTTCTGTCTCGTTTTTATGGGAAGATATTTACTT
        131 GAGAATGCTTCTGTCTAGTTTTTATATGAAGATATTCCCTT

              *  *  * **          *       *     *       **    *              
      35374 TTTCACCGTAGGCATCAAAGCGCTCCAAATGTCCACATCCAGATAGTACAGAAAGAGTGTTTCAA
          1 TTCCAACGAAATCATCAAAGCGATCCAAATATCCACTTCCAGATTCTACAAAAAGAGTGTTTCAA

             *         *   * *   *         *        * *  *      *      *     
      35439 ACCTGCTCTATGAAAGGGAATCTTCAACTCTATGAGTTGAATGCAGACATCAGAAAGAAATTTCT
         66 AACTGCTCTATCAAAAGAAATGTTCAACTCTGTGAGTTGAGTACACACATCACAAAGAAGTTTCT

                     *       **          *         **
      35504 GAGAATGCTGCTGTCTACCTTTTAT-T--TGA-ATTCCCGC
        131 GAGAATGCTTCTGTCTAGTTTTTATATGAAGATATTCCCTT

                         *  *    *                *       *                  
      35541 TTCCAACGAAATCCTCCAAGCTATCCAAATATCCACTTGCAGATTCCACAAAAAGAGTGTTTCAA
          1 TTCCAACGAAATCATCAAAGCGATCCAAATATCCACTTCCAGATTCTACAAAAAGAGTGTTTCAA

                            ** *  *            *       *     *     *  *      
      35606 AACTGCTCTCTATCAATGGCAAAGTTCAACTCTGTTAGTTGAGGACACATATCACCAACAAGTTT
         66 AACTG--CTCTATCAAAAGAAATGTTCAACTCTGTGAGTTGAGTACACACATCACAAAGAAGTTT

                               *       **         *    
      35671 CTGAGAATGCTTCTGTCTATTTTTTATGGGAAGATATTTCCTT
        129 CTGAGAATGCTTCTGTCTAGTTTTTATATGAAGATATTCCCTT

              *  *  * ** *    *      *    *          * **                    
      35714 TTTCACCGTAGGCGTCAAGGCGATCGAAATGTCCACTTCCACAAACTACAAAAAGAGTGTTTCAA
          1 TTCCAACGAAATCATCAAAGCGATCCAAATATCCACTTCCAGATTCTACAAAAAGAGTGTTTCAA

             *         *   * **       *    *        * ** * *   **      *     
      35779 ACCTGCTCTATGAAAGGCCATGTTCATCTCTATGAGTTGAATGGAAATATCCGAAAGAAATTTCT
         66 AACTGCTCTATCAAAAGAAATGTTCAACTCTGTGAGTTGAGTACACACATCACAAAGAAGTTTCT

             *       *          *        *         **
      35844 GGGAATGCTGCTGTCTAG-TGTT-TAT-ACGA-ATTCCCGC
        131 GAGAATGCTTCTGTCTAGTTTTTATATGAAGATATTCCCTT

                         *       *                *    *  *                  
      35881 TTCCAACGAAATCCTCAAAGCAATCCAAATATCCACTTGCAGAATCCACAAAAAGAGTGTTTCAA
          1 TTCCAACGAAATCATCAAAGCGATCCAAATATCCACTTCCAGATTCTACAAAAAGAGTGTTTCAA

                          *     *          * *                   *  *        
      35946 AACTGCTCTATCAATAGAAAGGTTCAACTCTTTTAGTTGAGTACACACATCACGAACAAGTTTCT
         66 AACTGCTCTATCAAAAGAAATGTTCAACTCTGTGAGTTGAGTACACACATCACAAAGAAGTTTCT

                            * *      **     **  *    
      36011 GAGAATGCTTCTGTCTGGCTTTTATTGGAAGACGTTTCCTT
        131 GAGAATGCTTCTGTCTAGTTTTTATATGAAGATATTCCCTT

                 * *  **          *       *                * *               
      36052 TT-CACCAAAGGCATCAAAGCGCTCCAAATGTCCACTTCCAGATTCTTCCAAAAGAGTGTTTCAA
          1 TTCCAACGAAATCATCAAAGCGATCCAAATATCCACTTCCAGATTCTACAAAAAGAGTGTTTCAA

             **     * ***  * *                 *    * *  * *     *   *       
      36116 ACGTGCTCAAAGTAAGGGAATGTTCAACTCTGTGACTTGAATGCAGATATCACCAAGTAGTTTCT
         66 AACTGCTCTATCAAAAGAAATGTTCAACTCTGTGAGTTGAGTACACACATCACAAAGAAGTTTCT

            * * *             *   * *    *         * 
      36181 AATAGTGCTTCTGTCTAGATTTCAGATGATGATATTCCCGT
        131 GAGAATGCTTCTGTCTAGTTTTTATATGAAGATATTCCCTT

                         * * *   * **          *  *   *      *     *       * 
      36222 TTCCAACGAAATCGTTAGAGCTAAGCAAATATCCAGTTACAGTTTCTACCAAAAGGGTGTTTCCA
          1 TTCCAACGAAATCATCAAAGCGATCCAAATATCCACTTCCAGATTCTACAAAAAGAGTGTTTCAA

              *    **           *            *       *                     * 
      36287 AATTGCTGCATCAAAAGAAAGGTTCAACTCTGTTAGTTGAGGACACACATCACAAAGAAGTTTGT
         66 AACTGCTCTATCAAAAGAAATGTTCAACTCTGTGAGTTGAGTACACACATCACAAAGAAGTTTCT

                              *    *     *           
      36352 GAGAATGCTTCTGTCTAGATTTTGTATGACGATATTCCCTT
        131 GAGAATGCTTCTGTCTAGTTTTTATATGAAGATATTCCCTT

                     *   * *     *   *       * *  *    *  *       *  *       
      36393 TTCCAACGATATCGTTAAAGCAATCTAAATATCAATTTGCAGAATCCACAAAAATAGAGTTTCAA
          1 TTCCAACGAAATCATCAAAGCGATCCAAATATCCACTTCCAGATTCTACAAAAAGAGTGTTTCAA

             *       * *        *    *       *  *                   ***      
      36458 AGCTGCTCTGTAAAAAGAAAGGTTCCACTCTGTTAGCTGAGTACACACATCACAAACTTGTTTCT
         66 AACTGCTCTATCAAAAGAAATGTTCAACTCTGTGAGTTGAGTACACACATCACAAAGAAGTTTCT

                  *         *        **         **   
      36523 GAGAATCCTTCTGTCTCGTTTTTATGGGAAGATATTTACTT
        131 GAGAATGCTTCTGTCTAGTTTTTATATGAAGATATTCCCTT

                 *  * **          *       *     *       *  *  *              
      36564 TTCCACCGTAGGCATCAAAGCGCTCCAAATGTCCACATCCAGATACTCCAGAAAGAGTGTTTCAA
          1 TTCCAACGAAATCATCAAAGCGATCCAAATATCCACTTCCAGATTCTACAAAAAGAGTGTTTCAA

             *         *   * *   *         *        * *  *      *      *     
      36629 ACCTGCTCTATGAAAGGCAATCTTCAACTCTATGAGTTGAATGCAGACATCAGAAAGAAATTTCT
         66 AACTGCTCTATCAAAAGAAATGTTCAACTCTGTGAGTTGAGTACACACATCACAAAGAAGTTTCT

                     *       **          *         **
      36694 GAGAATGCTGCTGTCTACCTTTTAT-T--TGA-ATTCCCGC
        131 GAGAATGCTTCTGTCTAGTTTTTATATGAAGATATTCCCTT

                         *  *    *              * *  **   *                  
      36731 TTCCAACGAAATCCTCCAAGCTATCCAAATATCCACCTGCATTTTCCACAAAAAGAGTGTTTCAA
          1 TTCCAACGAAATCATCAAAGCGATCCAAATATCCACTTCCAGATTCTACAAAAAGAGTGTTTCAA

                          *                 *    *  *                *       
      36796 AACTGCTCTATCAATAGAAATGTTCAACTCCTTTG-GCTGGGTACACACATCACAAACAAGTTTC
         66 AACTGCTCTATCAAAAGAAATGTTCAACT-CTGTGAGTTGAGTACACACATCACAAAGAAGTTTC

                *                     **     *        
      36860 TGAGCATGCTTCTGTCTAGTTTTTATGGGAAGACATTCCCTT
        130 TGAGAATGCTTCTGTCTAGTTTTTATATGAAGATATTCCCTT

              *  * *  **          *       *            **                  * 
      36902 TTTCACCAAAGGCATCAAAGCGCTCCAAATGTCCACTTCCAGACACTACAAAAAGAGTGTTTCCA
          1 TTCCAACGAAATCATCAAAGCGATCCAAATATCCACTTCCAGATTCTACAAAAAGAGTGTTTCAA

             **       **    **                 *    * *  * *      *  *       
      36967 ACGTGCTCTAAGAAAACGAATGTTCAACTCTGTGACTTGAATGCAGATATCACAGAGTAGTTTCT
         66 AACTGCTCTATCAAAAGAAATGTTCAACTCTGTGAGTTGAGTACACACATCACAAAGAAGTTTCT

                **            *     *    *         * 
      37032 GAGAGGGCTTCTGTCTAGATTTTAGATGATGATATTCCCGT
        131 GAGAATGCTTCTGTCTAGTTTTTATATGAAGATATTCCCTT

                           * *   *                *   *                    * 
      37073 TTCCAACGAAATCATTAGAGCTATCCAAATATCCACTTACAGTTTCTACAAAAAGAGTGTTTCCA
          1 TTCCAACGAAATCATCAAAGCGATCCAAATATCCACTTCCAGATTCTACAAAAAGAGTGTTTCAA

                   **         * *    *       *  *                   ***      
      37138 AACTGCTGCATCAAAAGAGAGGTTCCACTCTGTTAGCTGAGTACACACATCACAAACTTGTTTCT
         66 AACTGCTCTATCAAAAGAAATGTTCAACTCTGTGAGTTGAGTACACACATCACAAAGAAGTTTCT

            *     *         *        **         **   
      37203 CAGAATCCTTCTGTCTCGTTTTTATGGGAAGATATTTACTT
        131 GAGAATGCTTCTGTCTAGTTTTTATATGAAGATATTCCCTT

              *  *  * **          *       *     *       *  *  *              
      37244 TTTCACCGTAGGCATCAAAGCGCTCCAAATGTCCACATCCAGATACTCCAGAAAGAGTGTTTCAA
          1 TTCCAACGAAATCATCAAAGCGATCCAAATATCCACTTCCAGATTCTACAAAAAGAGTGTTTCAA

             *         *   * *   *         *        * *  *      *      *     
      37309 ACCTGCTCTATGAAAGGGAATCTTCAACTCTATGAGTTGAATGCAGACATCAGAAAGAAATTTCT
         66 AACTGCTCTATCAAAAGAAATGTTCAACTCTGTGAGTTGAGTACACACATCACAAAGAAGTTTCT

                     *       **          *         **
      37374 GAGAATGCTGCTGTCTACCTTTTAT-T--TGA-ATTCCCGC
        131 GAGAATGCTTCTGTCTAGTTTTTATATGAAGATATTCCCTT

                         *  *    *                *       *                  
      37411 TTCCAACGAAATCCTCCAAGCTATCCAAATATCCACTTGCAGATTCCACAAAAAGAGTGTTTCAA
          1 TTCCAACGAAATCATCAAAGCGATCCAAATATCCACTTCCAGATTCTACAAAAAGAGTGTTTCAA

                            ** *  *            *       *     *     *  *      
      37476 AACTGCTCTCTATCAATGGCAAAGTTCAACTCTGTTAGTTGAGGACACATATCACCAACAAGTTT
         66 AACTG--CTCTATCAAAAGAAATGTTCAACTCTGTGAGTTGAGTACACACATCACAAAGAAGTTT

                               *       **         *    
      37541 CTGAGAATGCTTCTGTCTATTTTTTATGGGAAGATATTTCCTT
        129 CTGAGAATGCTTCTGTCTAGTTTTTATATGAAGATATTCCCTT

              *  *  * ** *    *      *    *          * **                    
      37584 TTTCACCGTAGGCGTCAAGGCGATCGAAATGTCCACTTCCACAAACTACAAAAAGAGTGTTTCAA
          1 TTCCAACGAAATCATCAAAGCGATCCAAATATCCACTTCCAGATTCTACAAAAAGAGTGTTTCAA

             *         *   * **       *    *        * ** * *   **      *     
      37649 ACCTGCTCTATGAAAGGCGATGTTCATCTCTATGAGTTGAATGGAAATATCCGAAAGAAATTTCT
         66 AACTGCTCTATCAAAAGAAATGTTCAACTCTGTGAGTTGAGTACACACATCACAAAGAAGTTTCT

             *       *          *        *         **
      37714 GGGAATGCTGCTGTCTAG-TGTT-TAT-ACGA-ATTCCCGC
        131 GAGAATGCTTCTGTCTAGTTTTTATATGAAGATATTCCCTT

                         *       *                *    *  *                  
      37751 TTCCAACGAAATCCTCAAAGCAATCCAAATATCCACTTGCAGAATCCACAAAAAGAGTGTTTCAA
          1 TTCCAACGAAATCATCAAAGCGATCCAAATATCCACTTCCAGATTCTACAAAAAGAGTGTTTCAA

                          *     *          * *                   *  *        
      37816 AACTGCTCTATCAATAGAAAGGTTCAACTCTTTTAGTTGAGTACACACATCACGAACAAGTTTCT
         66 AACTGCTCTATCAAAAGAAATGTTCAACTCTGTGAGTTGAGTACACACATCACAAAGAAGTTTCT

                            * *      **     **  *    
      37881 GAGAATGCTTCTGTCTGGCTTTTATTGGAAGACGTTTCCTT
        131 GAGAATGCTTCTGTCTAGTTTTTATATGAAGATATTCCCTT

                 * *  **          *       *                * *               
      37922 TT-CACCAAAGGCATCAAAGCGCTCCAAATGTCCACTTCCAGATTCTTCCAAAAGAGTGTTTCAA
          1 TTCCAACGAAATCATCAAAGCGATCCAAATATCCACTTCCAGATTCTACAAAAAGAGTGTTTCAA

             **     * ***  * *                 *    * *  * *     *   *       
      37986 ACGTGCTCAAAGTAAGGGAATGTTCAACTCTGTGACTTGAATGCAGATATCACCAAGTAGTTTCT
         66 AACTGCTCTATCAAAAGAAATGTTCAACTCTGTGAGTTGAGTACACACATCACAAAGAAGTTTCT

            * * *             *     *    *         * 
      38051 AATAGTGCTTCTGTCTAGATTTTAGATGATGATATTCCCGT
        131 GAGAATGCTTCTGTCTAGTTTTTATATGAAGATATTCCCTT

                         * * *   *             *  *   *                    * 
      38092 TTCCAACGAAATCGTTAGAGCTATCCAAATATCCAGTTACAGTTTCTACAAAAAGAGTGTTTCCA
          1 TTCCAACGAAATCATCAAAGCGATCCAAATATCCACTTCCAGATTCTACAAAAAGAGTGTTTCAA

                   **           *            *       *                     * 
      38157 AACTGCTGCATCAAAAGAAAGGTTCAACTCTGTTAGTTGAGGACACACATCACAAAGAAGTTTGT
         66 AACTGCTCTATCAAAAGAAATGTTCAACTCTGTGAGTTGAGTACACACATCACAAAGAAGTTTCT

                              *    *     *           
      38222 GAGAATGCTTCTGTCTAGATTTTGTATGACGATATTCCCTT
        131 GAGAATGCTTCTGTCTAGTTTTTATATGAAGATATTCCCTT

                     *   * *     *   *       * *  *    *  *       *  *       
      38263 TTCCAACGATATCGTTAAAGCAATCTAAATATCAATTTGCAGAATCCACAAAAATAGAGTTTCAA
          1 TTCCAACGAAATCATCAAAGCGATCCAAATATCCACTTCCAGATTCTACAAAAAGAGTGTTTCAA

             *       * *    *   *    *       *  *                   ***      
      38328 AGCTGCTCTGTAAAAATAAAAGTTCCACTCTGTTAGCTGAGTACACACATCACAAACTTGTTTCT
         66 AACTGCTCTATCAAAAGAAATGTTCAACTCTGTGAGTTGAGTACACACATCACAAAGAAGTTTCT

                  *         *        **         **   
      38393 GAGAATCCTTCTGTCTCGTTTTTATGGGAAGATATTTACTT
        131 GAGAATGCTTCTGTCTAGTTTTTATATGAAGATATTCCCTT

              *  *  * **          *       *     *       *  *  *     *        
      38434 TTTCACCGTAGGCATCAAAGCGCTCCAAATGTCCACATCCAGATACTCCAGAAAGACTGTTTCAA
          1 TTCCAACGAAATCATCAAAGCGATCCAAATATCCACTTCCAGATTCTACAAAAAGAGTGTTTCAA

             *         *   * *   *         *        * *  *      *      *     
      38499 ACCTGCTCTATGAAAGGGAATCTTCAACTCTATGAGTTGAATGCAGACATCAGAAAGAAATTTCT
         66 AACTGCTCTATCAAAAGAAATGTTCAACTCTGTGAGTTGAGTACACACATCACAAAGAAGTTTCT

                     *       **          *         **
      38564 GAGAATGCTGCTGTCTACCTTTTAT-T--TGA-ATTCCCGC
        131 GAGAATGCTTCTGTCTAGTTTTTATATGAAGATATTCCCTT

                         *  *    *                *  **   *                  
      38601 TTCCAACGAAATCCTCCAAGCTATCCAAATATCCACTTGCATTTTCCACAAAAAGAGTGTTTCAA
          1 TTCCAACGAAATCATCAAAGCGATCCAAATATCCACTTCCAGATTCTACAAAAAGAGTGTTTCAA

                          *               ** *  *  *                *        
      38666 AACTGCTCTATCAATAGAAATGTTCAACTCCTTTAGCTGGGTACACACATCACAAACAAGTTTCT
         66 AACTGCTCTATCAAAAGAAATGTTCAACTCTGTGAGTTGAGTACACACATCACAAAGAAGTTTCT

                                     **     **       
      38731 GAGAATGCTTCTGTCTAGTTTTTATGGGAAGACGTTCCCTT
        131 GAGAATGCTTCTGTCTAGTTTTTATATGAAGATATTCCCTT

              *  * *  **          *       *            **                    
      38772 TTTCACCAAAGGCATCAAAGCGCTCCAAATGTCCACTTCCAGACACTACAAAAAGAGTGTTTCAA
          1 TTCCAACGAAATCATCAAAGCGATCCAAATATCCACTTCCAGATTCTACAAAAAGAGTGTTTCAA

             **       **   ***                 *    * *  * *         *       
      38837 ACGTGCTCTAAGAAAGCGAATGTTCAACTCTGTGACTTGAATGCAGATATCACAAAGTAGTTTCT
         66 AACTGCTCTATCAAAAGAAATGTTCAACTCTGTGAGTTGAGTACACACATCACAAAGAAGTTTCT

                **            *     *    *         * 
      38902 GAGAGGGCTTCTGTCTAGATTTTAGATGATGATATTCCCGT
        131 GAGAATGCTTCTGTCTAGTTTTTATATGAAGATATTCCCTT

                           * *   *                *   *                    * 
      38943 TTCCAACGAAATCATTAGAGCTATCCAAATATCCACTTACAGTTTCTACAAAAAGAGTGTTTCCA
          1 TTCCAACGAAATCATCAAAGCGATCCAAATATCCACTTCCAGATTCTACAAAAAGAGTGTTTCAA

                   **         * *    *       *  **                  ***      
      39008 AACTGCTGCATCAAAAGAGAGGTTCCACTCTGTTAGCCGAGTACACACATCACAAACTTGTTTCT
         66 AACTGCTCTATCAAAAGAAATGTTCAACTCTGTGAGTTGAGTACACACATCACAAAGAAGTTTCT

            *     *         *        **         **   
      39073 CAGAATCCTTCTGTCTCGTTTTTATGGGAAGATATTTACTT
        131 GAGAATGCTTCTGTCTAGTTTTTATATGAAGATATTCCCTT

              *  *  * **          *       *     *       *  *  *              
      39114 TTTCACCGTAGGCATCAAAGCGCTCCAAATGTCCACATCCAGATACTCCAGAAAGAGTGTTTCAA
          1 TTCCAACGAAATCATCAAAGCGATCCAAATATCCACTTCCAGATTCTACAAAAAGAGTGTTTCAA

             *         *   * *   *         *        * *  *      *      *     
      39179 ACCTGCTCTATGAAAGGGAATCTTCAACTCTATGAGTTGAATGCAGACATCAGAAAGAAATTTCT
         66 AACTGCTCTATCAAAAGAAATGTTCAACTCTGTGAGTTGAGTACACACATCACAAAGAAGTTTCT

                     *       **          *         **
      39244 GAGAATGCTGCTGTCTACCTTTTAT-T--TGA-ATTCCCGC
        131 GAGAATGCTTCTGTCTAGTTTTTATATGAAGATATTCCCTT

                         *  *    *                *       *                  
      39281 TTCCAACGAAATCCTCCAAGCTATCCAAATATCCACTTGCAGATTCCACAAAAAGAGTGTTTCAA
          1 TTCCAACGAAATCATCAAAGCGATCCAAATATCCACTTCCAGATTCTACAAAAAGAGTGTTTCAA

                            ** *  *            *       *     *     *  *      
      39346 AACTGCTCTCTATCAATGGCAAAGTTCAACTCTGTTAGTTGAGGACACATATCACCAACAAGTTT
         66 AACTG--CTCTATCAAAAGAAATGTTCAACTCTGTGAGTTGAGTACACACATCACAAAGAAGTTT

                               *       **         *    
      39411 CTGAGAATGCTTCTGTCTATTTTTTATGGGAAGATATTTCCTT
        129 CTGAGAATGCTTCTGTCTAGTTTTTATATGAAGATATTCCCTT

              *  *  * ** *    *   *  *    *          * **                    
      39454 TTTCACCGTAGGCGTCAACGCGTTCGAAATGTCCACTTCCACAAACTACAAAAAGAGTGTTTCAA
          1 TTCCAACGAAATCATCAAAGCGATCCAAATATCCACTTCCAGATTCTACAAAAAGAGTGTTTCAA

             *         *   * **       *    *        * ** * *   **      *     
      39519 ACCTGCTCTATGAAAGGCCATGTTCATCTCTATGAGTTGAATGGAAATATCCGAAAGAAATTTCT
         66 AACTGCTCTATCAAAAGAAATGTTCAACTCTGTGAGTTGAGTACACACATCACAAAGAAGTTTCT

             *       *          *        *         **
      39584 GGGAATGCTGCTGTCTAG-TGTT-TAT-ACGA-ATTCCCGC
        131 GAGAATGCTTCTGTCTAGTTTTTATATGAAGATATTCCCTT

                         *       *                *    *  *                  
      39621 TTCCAACGAAATCCTCAAAGCAATCCAAATATCCACTTGCAGAATCCACAAAAAGAGTGTTTCAA
          1 TTCCAACGAAATCATCAAAGCGATCCAAATATCCACTTCCAGATTCTACAAAAAGAGTGTTTCAA

                          *     *          * *                   *  *        
      39686 AACTGCTCTATCAATAGAAAGGTTCAACTCTTTTAGTTGAGTACACACATCACGAACAAGTTTCT
         66 AACTGCTCTATCAAAAGAAATGTTCAACTCTGTGAGTTGAGTACACACATCACAAAGAAGTTTCT

                            * *      **     **  *    
      39751 GAGAATGCTTCTGTCTGGCTTTTATTGGAAGACGTTTCCTT
        131 GAGAATGCTTCTGTCTAGTTTTTATATGAAGATATTCCCTT

                 * *  **          *       *                * *               
      39792 TT-CACCAAAGGCATCAAAGCGCTCCAAATGTCCACTTCCAGATTCTTCCAAAAGAGTGTTTCAA
          1 TTCCAACGAAATCATCAAAGCGATCCAAATATCCACTTCCAGATTCTACAAAAAGAGTGTTTCAA

             **     * ***  * *                 *    * *  * *     *   *       
      39856 ACGTGCTCAAAGTAAGGGAATGTTCAACTCTGTGACTTGAATGCAGATATCACCAAGTAGTTTCT
         66 AACTGCTCTATCAAAAGAAATGTTCAACTCTGTGAGTTGAGTACACACATCACAAAGAAGTTTCT

            * * *             *   * *    *         * 
      39921 AATAGTGCTTCTGTCTAGATTTCAGATGATGATATTCCCGT
        131 GAGAATGCTTCTGTCTAGTTTTTATATGAAGATATTCCCTT

                         * * *   * **          *  *   *      *     *       * 
      39962 TTCCAACGAAATCGTTAGAGCTAAGCAAATATCCAGTTACAGTTTCTACCAAAAGGGTGTTTCCA
          1 TTCCAACGAAATCATCAAAGCGATCCAAATATCCACTTCCAGATTCTACAAAAAGAGTGTTTCAA

              *    **           *            *       *                     * 
      40027 AATTGCTGCATCAAAAGAAAGGTTCAACTCTGTTAGTTGAGGACACACATCACAAAGAAGTTTGT
         66 AACTGCTCTATCAAAAGAAATGTTCAACTCTGTGAGTTGAGTACACACATCACAAAGAAGTTTCT

                              *    *     *           
      40092 GAGAATGCTTCTGTCTAGATTTTGTATGACGATATTCCCTT
        131 GAGAATGCTTCTGTCTAGTTTTTATATGAAGATATTCCCTT

                     *   * *     *   *       * *  *    *  *       *  *       
      40133 TTCCAACGATATCGTTAAAGCAATCTAAATATCAATTTGCAGAATCCACAAAAATAGAGTTTCAA
          1 TTCCAACGAAATCATCAAAGCGATCCAAATATCCACTTCCAGATTCTACAAAAAGAGTGTTTCAA

             *       * *        *    *       *  *                   ***      
      40198 AGCTGCTCTGTAAAAAGAAAGGTTCCACTCTGTTAGCTGAGTACACACATCACAAACTTGTTTCT
         66 AACTGCTCTATCAAAAGAAATGTTCAACTCTGTGAGTTGAGTACACACATCACAAAGAAGTTTCT

                  *         *        **         **   
      40263 GAGAATCCTTCTGTCTCGTTTTTATGGGAAGATATTTACTT
        131 GAGAATGCTTCTGTCTAGTTTTTATATGAAGATATTCCCTT

                 *  * **          *       *     *       *  *  *              
      40304 TTCCACCGTAGGCATCAAAGCGCTCCAAATGTCCACATCCAGATACTCCAGAAAGAGTGTTTCAA
          1 TTCCAACGAAATCATCAAAGCGATCCAAATATCCACTTCCAGATTCTACAAAAAGAGTGTTTCAA

             *         *   * *   *         *        * *  *      *      *     
      40369 ACCTGCTCTATGAAAGGCAATCTTCAACTCTATGAGTTGAATGCAGACATCAGAAAGAAATTTCT
         66 AACTGCTCTATCAAAAGAAATGTTCAACTCTGTGAGTTGAGTACACACATCACAAAGAAGTTTCT

                     *       **          *         **
      40434 GAGAATGCTGCTGTCTACCTTTTAT-T--TGA-ATTCCCGC
        131 GAGAATGCTTCTGTCTAGTTTTTATATGAAGATATTCCCTT

                         *  *    *              * *  **   *                  
      40471 TTCCAACGAAATCCTCCAAGCTATCCAAATATCCACCTGCATTTTCCACAAAAAGAGTGTTTCAA
          1 TTCCAACGAAATCATCAAAGCGATCCAAATATCCACTTCCAGATTCTACAAAAAGAGTGTTTCAA

                          *                 *    *  *                *       
      40536 AACTGCTCTATCAATAGAAATGTTCAACTCCTTTG-GCTGGGTACACACATCACAAACAAGTTTC
         66 AACTGCTCTATCAAAAGAAATGTTCAACT-CTGTGAGTTGAGTACACACATCACAAAGAAGTTTC

                *                     **     *        
      40600 TGAGCATGCTTCTGTCTAGTTTTTATGGGAAGACATTCCCTT
        130 TGAGAATGCTTCTGTCTAGTTTTTATATGAAGATATTCCCTT

              *  * *  **          *       *            **                    
      40642 TTTCACCAAAGGCATCAAAGCGCTCCAAATGTCCACTTCCAGACACTACAAAAAGAGTGTTTCAA
          1 TTCCAACGAAATCATCAAAGCGATCCAAATATCCACTTCCAGATTCTACAAAAAGAGTGTTTCAA

             **       **    **                 *    * *  * *      *  *       
      40707 ACGTGCTCTAAGAAAACGAATGTTCAACTCTGTGACTTGAATGCAGATATCACAGAGTAGTTTCT
         66 AACTGCTCTATCAAAAGAAATGTTCAACTCTGTGAGTTGAGTACACACATCACAAAGAAGTTTCT

                **            *     *    *         * 
      40772 GAGAGGGCTTCTGTCTAGATTTTAGATGATGATATTCCCGT
        131 GAGAATGCTTCTGTCTAGTTTTTATATGAAGATATTCCCTT

                           * *   *                *   *                    * 
      40813 TTCCAACGAAATCATTAGAGCTATCCAAATATCCACTTACAGTTTCTACAAAAAGAGTGTTTCCA
          1 TTCCAACGAAATCATCAAAGCGATCCAAATATCCACTTCCAGATTCTACAAAAAGAGTGTTTCAA

                   **         * *    *       *  *                   ***      
      40878 AACTGCTGCATCAAAAGAGAGGTTCCACTCTGTTAGCTGAGTACACACATCACAAACTTGTTTCT
         66 AACTGCTCTATCAAAAGAAATGTTCAACTCTGTGAGTTGAGTACACACATCACAAAGAAGTTTCT

            *     *         *        **         **   
      40943 CAGAATCCTTCTGTCTCGTTTTTATGGGAAGATATTTACTT
        131 GAGAATGCTTCTGTCTAGTTTTTATATGAAGATATTCCCTT

              *  *  * **          *       *     *       *  *  *              
      40984 TTTCACCGTAGGCATCAAAGCGCTCCAAATGTCCACATCCAGATACTCCAGAAAGAGTGTTTCAA
          1 TTCCAACGAAATCATCAAAGCGATCCAAATATCCACTTCCAGATTCTACAAAAAGAGTGTTTCAA

             *         *   * *   *         *        * *  *      *      *     
      41049 ACCTGCTCTATGAAAGGGAATCTTCAACTCTATGAGTTGAATGCAGACATCAGAAAGAAATTTCT
         66 AACTGCTCTATCAAAAGAAATGTTCAACTCTGTGAGTTGAGTACACACATCACAAAGAAGTTTCT

                     *       **          *         **
      41114 GAGAATGCTGCTGTCTACCTTTTAT-T--TGA-ATTCCCGC
        131 GAGAATGCTTCTGTCTAGTTTTTATATGAAGATATTCCCTT

                         *  *    *                *       *                  
      41151 TTCCAACGAAATCCTCCAAGCTATCCAAATATCCACTTGCAGATTCCACAAAAAGAGTGTTTCAA
          1 TTCCAACGAAATCATCAAAGCGATCCAAATATCCACTTCCAGATTCTACAAAAAGAGTGTTTCAA

                            ** *  *            *       *     *     *  *      
      41216 AACTGCTCTCTATCAATGGCAAAGTTCAACTCTGTTAGTTGAGGACACATATCACCAACAAGTTT
         66 AACTG--CTCTATCAAAAGAAATGTTCAACTCTGTGAGTTGAGTACACACATCACAAAGAAGTTT

                               *       **         *    
      41281 CTGAGAATGCTTCTGTCTATTTTTTATGGGAAGATATTTCCTT
        129 CTGAGAATGCTTCTGTCTAGTTTTTATATGAAGATATTCCCTT

              *  *  * ** *    *      *    *          * **                    
      41324 TTTCACCGTAGGCGTCAAGGCGATCGAAATGTCCACTTCCACAAACTACAAAAAGAGTGTTTCAA
          1 TTCCAACGAAATCATCAAAGCGATCCAAATATCCACTTCCAGATTCTACAAAAAGAGTGTTTCAA

             *         *   * **       *    *        * ** * *   **      *     
      41389 ACCTGCTCTATGAAAGGCCATGTTCATCTCTATGAGTTGAATGGAAATATCCGAAAGAAATTTCT
         66 AACTGCTCTATCAAAAGAAATGTTCAACTCTGTGAGTTGAGTACACACATCACAAAGAAGTTTCT

             *       *          *        *         **
      41454 GGGAATGCTGCTGTCTAG-TGTT-TAT-ACGA-ATTCCCGC
        131 GAGAATGCTTCTGTCTAGTTTTTATATGAAGATATTCCCTT

                         *       *                *    *  *                  
      41491 TTCCAACGAAATCCTCAAAGCAATCCAAATATCCACTTGCAGAATCCACAAAAAGAGTGTTTCAA
          1 TTCCAACGAAATCATCAAAGCGATCCAAATATCCACTTCCAGATTCTACAAAAAGAGTGTTTCAA

                          *     *          * *                   *  *        
      41556 AACTGCTCTATCAATAGAAAGGTTCAACTCTTTTAGTTGAGTACACACATCACGAACAAGTTTCT
         66 AACTGCTCTATCAAAAGAAATGTTCAACTCTGTGAGTTGAGTACACACATCACAAAGAAGTTTCT

                            * *      **     **  *    
      41621 GAGAATGCTTCTGTCTGGCTTTTATTGGAAGACGTTTCCTT
        131 GAGAATGCTTCTGTCTAGTTTTTATATGAAGATATTCCCTT

                   *  **          *       *                * *               
      41662 TT-CAACAAAGGCATCAAAGCGCTCCAAATGTCCACTTCCAGATTCTTCCAAAAGAGTGTTTCAA
          1 TTCCAACGAAATCATCAAAGCGATCCAAATATCCACTTCCAGATTCTACAAAAAGAGTGTTTCAA

             **     * ***  * *                 *    * *  * *    **   *       
      41726 ACGTGCTCGAAGTAAGGGAATGTTCAACTCTGTGACTTGAATGCAGATATCATCAAGTAGTTTCT
         66 AACTGCTCTATCAAAAGAAATGTTCAACTCTGTGAGTTGAGTACACACATCACAAAGAAGTTTCT

            * * *            **     *    *         * 
      41791 AATAGTGCTTCTGTCTACATTTTAGATGATGATATTCCCGT
        131 GAGAATGCTTCTGTCTAGTTTTTATATGAAGATATTCCCTT

                         * * *   *             *  *   *      *     *       * 
      41832 TTCCAACGAAATCGTTAGAGCTATCCAAATATCCAGTTACAGTTTCTACCAAAAGGGTGTTTCCA
          1 TTCCAACGAAATCATCAAAGCGATCCAAATATCCACTTCCAGATTCTACAAAAAGAGTGTTTCAA

              *    **           *            *       *                     * 
      41897 AATTGCTGCATCAAAAGAAAGGTTCAACTCTGTTAGTTGAGGACACACATCACAAAGAAGTTTGT
         66 AACTGCTCTATCAAAAGAAATGTTCAACTCTGTGAGTTGAGTACACACATCACAAAGAAGTTTCT

                              *    *     *           
      41962 GAGAATGCTTCTGTCTAGATTTTGTATGACGATATTCCCTT
        131 GAGAATGCTTCTGTCTAGTTTTTATATGAAGATATTCCCTT

                     *   * *     *   *       * *  *    *  *       *  *       
      42003 TTCCAACGATATCGTTAAAGCAATCTAAATATCAATTTGCAGAATCCACAAAAATAGAGTTTCAA
          1 TTCCAACGAAATCATCAAAGCGATCCAAATATCCACTTCCAGATTCTACAAAAAGAGTGTTTCAA

             *       * *        *    *       *  *                   ***      
      42068 AGCTGCTCTGTAAAAAGAAAGGTTCCACTCTGTTAGCTGAGTACACACATCACAAACTTGTTTCT
         66 AACTGCTCTATCAAAAGAAATGTTCAACTCTGTGAGTTGAGTACACACATCACAAAGAAGTTTCT

                  *         *        **         **   
      42133 GAGAATCCTTCTGTCTCGTTTTTATGGGAAGATATTTACTT
        131 GAGAATGCTTCTGTCTAGTTTTTATATGAAGATATTCCCTT

                 *  * **          *       *     *       *  *  *  *           
      42174 TTCCACCGTAGGCATCAAAGCGCTCCAAATGTCCACATCCAGATACTCCAGAACGAGTGTTTCAA
          1 TTCCAACGAAATCATCAAAGCGATCCAAATATCCACTTCCAGATTCTACAAAAAGAGTGTTTCAA

             *         *   * *   *         *        * *  *      *      *     
      42239 ACCTGCTCTATGAAAGGGAATCTTCAACTCTATGAGTTGAATGCAGACATCAGAAAGAAATTTCT
         66 AACTGCTCTATCAAAAGAAATGTTCAACTCTGTGAGTTGAGTACACACATCACAAAGAAGTTTCT

                     *       **          *         **
      42304 GAGAATGCTGCTGTCTACCTTTTAT-T--TGA-ATTCCCGC
        131 GAGAATGCTTCTGTCTAGTTTTTATATGAAGATATTCCCTT

                         *  *    *              * *  **   *    *             
      42341 TTCCAACGAAATCCTCCAAGCTATCCAAATATCCACCTGCATTTTCCACAACAAGAGTGTTTCAA
          1 TTCCAACGAAATCATCAAAGCGATCCAAATATCCACTTCCAGATTCTACAAAAAGAGTGTTTCAA

                          *                 *    *  *                *       
      42406 AACTGCTCTATCAATAGAAATGTTCAACTCCTTTG-GCTGGGTACACACATCACAAACAAGTTTC
         66 AACTGCTCTATCAAAAGAAATGTTCAACT-CTGTGAGTTGAGTACACACATCACAAAGAAGTTTC

                *                     **     *        
      42470 TGAGCATGCTTCTGTCTAGTTTTTATGGGAAGACATTCCCTT
        130 TGAGAATGCTTCTGTCTAGTTTTTATATGAAGATATTCCCTT

              *  * *  **          *       *            **                    
      42512 TTTCACCAAAGGCATCAAAGCGCTCCAAATGTCCACTTCCAGACACTACAAAAAGAGTGTTTCAA
          1 TTCCAACGAAATCATCAAAGCGATCCAAATATCCACTTCCAGATTCTACAAAAAGAGTGTTTCAA

             **       **    **                 *    * *  * *      *  *       
      42577 ACGTGCTCTAAGAAAACGAATGTTCAACTCTGTGACTTGAATGCAGATATCACAGAGTAGTTTCT
         66 AACTGCTCTATCAAAAGAAATGTTCAACTCTGTGAGTTGAGTACACACATCACAAAGAAGTTTCT

                **            *     *    *         * 
      42642 GAGAGGGCTTCTGTCTAGATTTTAGATGATGATATTCCCGT
        131 GAGAATGCTTCTGTCTAGTTTTTATATGAAGATATTCCCTT

                           * *   *                *   *                    * 
      42683 TTCCAACGAAATCATTAGAGCTATCCAAATATCCACTTACAGTTTCTACAAAAAGAGTGTTTCCA
          1 TTCCAACGAAATCATCAAAGCGATCCAAATATCCACTTCCAGATTCTACAAAAAGAGTGTTTCAA

                   **         * *    *       *  *                   ***      
      42748 AACTGCTGCATCAAAAGAGAGGTTCCACTCTGTTAGCTGAGTACACACATCACAAACTTGTTTCT
         66 AACTGCTCTATCAAAAGAAATGTTCAACTCTGTGAGTTGAGTACACACATCACAAAGAAGTTTCT

            *     *         *        **         **   
      42813 CAGAATCCTTCTGTCTCGTTTTTATGGGAAGATATTTACTT
        131 GAGAATGCTTCTGTCTAGTTTTTATATGAAGATATTCCCTT

              *  *  * **          *       *     *       *  *  *              
      42854 TTTCACCGTAGGCATCAAAGCGCTCCAAATGTCCACATCCAGATACTCCAGAAAGAGTGTTTCAA
          1 TTCCAACGAAATCATCAAAGCGATCCAAATATCCACTTCCAGATTCTACAAAAAGAGTGTTTCAA

             *         *   * *   *         *        * *  *      *      *     
      42919 ACCTGCTCTATGAAAGGGAATCTTCAACTCTATGAGTTGAATGCAGACATCAGAAAGAAATTTCT
         66 AACTGCTCTATCAAAAGAAATGTTCAACTCTGTGAGTTGAGTACACACATCACAAAGAAGTTTCT

                     *       **          *         **
      42984 GAGAATGCTGCTGTCTACCTTTTAT-T--TGA-ATTCCCGC
        131 GAGAATGCTTCTGTCTAGTTTTTATATGAAGATATTCCCTT

                         *  *    *                *       *                  
      43021 TTCCAACGAAATCCTCCAAGCTATCCAAATATCCACTTGCAGATTCCACAAAAAGAGTGTTTCAA
          1 TTCCAACGAAATCATCAAAGCGATCCAAATATCCACTTCCAGATTCTACAAAAAGAGTGTTTCAA

                            ** *  *            *       *     *     *  *      
      43086 AACTGCTCTCTATCAATGGCAAAGTTCAACTCTGTTAGTTGAGGACACATATCACCAACAAGTTT
         66 AACTG--CTCTATCAAAAGAAATGTTCAACTCTGTGAGTTGAGTACACACATCACAAAGAAGTTT

                               *       **         *    
      43151 CTGAGAATGCTTCTGTCTATTTTTTATGGGAAGATATTTCCTT
        129 CTGAGAATGCTTCTGTCTAGTTTTTATATGAAGATATTCCCTT

              *  *  * ** *    *      *    *          * **                    
      43194 TTTCACCGTAGGCGTCAAGGCGATCGAAATGTCCACTTCCACAAACTACAAAAAGAGTGTTTCAA
          1 TTCCAACGAAATCATCAAAGCGATCCAAATATCCACTTCCAGATTCTACAAAAAGAGTGTTTCAA

             *         *   * **       *    *        * ** * *   **      *     
      43259 ACCTGCTCTATGAAAGGCGATGTTCATCTCTATGAGTTGAATGGAAATATCCGAAAGAAATTTCT
         66 AACTGCTCTATCAAAAGAAATGTTCAACTCTGTGAGTTGAGTACACACATCACAAAGAAGTTTCT

             *       *          *        *         **
      43324 GGGAATGCTGCTGTCTAG-TGTT-TAT-ACGA-ATTCCCGC
        131 GAGAATGCTTCTGTCTAGTTTTTATATGAAGATATTCCCTT

                         *       *                *    *  *                  
      43361 TTCCAACGAAATCCTCAAAGCAATCCAAATATCCACTTGCAGAATCCACAAAAAGAGTGTTTCAA
          1 TTCCAACGAAATCATCAAAGCGATCCAAATATCCACTTCCAGATTCTACAAAAAGAGTGTTTCAA

                          *     *          * *                   *  *        
      43426 AACTGCTCTATCAATAGAAAGGTTCAACTCTTTTAGTTGAGTACACACATCACGAACAAGTTTCT
         66 AACTGCTCTATCAAAAGAAATGTTCAACTCTGTGAGTTGAGTACACACATCACAAAGAAGTTTCT

                            * *      **     **  *    
      43491 GAGAATGCTTCTGTCTGGCTTTTATTGGAAGACGTTTCCTT
        131 GAGAATGCTTCTGTCTAGTTTTTATATGAAGATATTCCCTT

                 * *  **          *       *                * *               
      43532 TT-CACCAAAGGCATCAAAGCGCTCCAAATGTCCACTTCCAGATTCTTCCAAAAGAGTGTTTCAA
          1 TTCCAACGAAATCATCAAAGCGATCCAAATATCCACTTCCAGATTCTACAAAAAGAGTGTTTCAA

             **     * ***  * *                 *    * *  * *     *   *       
      43596 ACGTGCTCAAAGTAAGGGAATGTTCAACTCTGTGACTTGAATGCAGATATCACCAAGTAGTTTCT
         66 AACTGCTCTATCAAAAGAAATGTTCAACTCTGTGAGTTGAGTACACACATCACAAAGAAGTTTCT

            * * *             *     *    *         * 
      43661 AATAGTGCTTCTGTCTAGATTTTAGATGATGATATTCCCGT
        131 GAGAATGCTTCTGTCTAGTTTTTATATGAAGATATTCCCTT

                         * * *   *                *   *                    * 
      43702 TTCCAACGAAATCGTTAGAGCTATCCAAATATCCACTTACAGTTTCTACAAAAAGAGTGTTTCCA
          1 TTCCAACGAAATCATCAAAGCGATCCAAATATCCACTTCCAGATTCTACAAAAAGAGTGTTTCAA

                   **           *            *       *                     * 
      43767 AACTGCTGCATCAAAAGAAAGGTTCAACTCTGTTAGTTGAGGACACACATCACAAAGAAGTTTGT
         66 AACTGCTCTATCAAAAGAAATGTTCAACTCTGTGAGTTGAGTACACACATCACAAAGAAGTTTCT

                              *    *     *           
      43832 GAGAATGCTTCTGTCTAGATTTTGTATGACGATATTCCCTT
        131 GAGAATGCTTCTGTCTAGTTTTTATATGAAGATATTCCCTT

                     *   * *     *   *       * *  *    *  *       *  *       
      43873 TTCCAACGATATCGTTAAAGCAATCTAAATATCAATTTGCAGAATCCACAAAAATAGAGTTTCAA
          1 TTCCAACGAAATCATCAAAGCGATCCAAATATCCACTTCCAGATTCTACAAAAAGAGTGTTTCAA

             *       * *    *   *    *       *  *                   ***      
      43938 AGCTGCTCTGTAAAAATAAAAGTTCCACTCTGTTAGCTGAGTACACACATCACAAACTTGTTTCT
         66 AACTGCTCTATCAAAAGAAATGTTCAACTCTGTGAGTTGAGTACACACATCACAAAGAAGTTTCT

                  *         *        **         **   
      44003 GAGAATCCTTCTGTCTCGTTTTTATGGGAAGATATTTACTT
        131 GAGAATGCTTCTGTCTAGTTTTTATATGAAGATATTCCCTT

              *  *  * **          *       *     *       *  *  *     *        
      44044 TTTCACCGTAGGCATCAAAGCGCTCCAAATGTCCACATCCAGATACTCCAGAAAGACTGTTTCAA
          1 TTCCAACGAAATCATCAAAGCGATCCAAATATCCACTTCCAGATTCTACAAAAAGAGTGTTTCAA

             *         *   * *   *         *        * *  *      *      *     
      44109 ACCTGCTCTATGAAAGGGAATCTTCAACTCTATGAGTTGAATGCAGACATCAGAAAGAAATTTCT
         66 AACTGCTCTATCAAAAGAAATGTTCAACTCTGTGAGTTGAGTACACACATCACAAAGAAGTTTCT

                     *       **          *         **
      44174 GAGAATGCTGCTGTCTACCTTTTAT-T--TGA-ATTCCCGC
        131 GAGAATGCTTCTGTCTAGTTTTTATATGAAGATATTCCCTT

                         *  *    *              * *  **   *                  
      44211 TTCCAACGAAATCCTCCAAGCTATCCAAATATCCACCTGCATTTTCCACAAAAAGAGTGTTTCAA
          1 TTCCAACGAAATCATCAAAGCGATCCAAATATCCACTTCCAGATTCTACAAAAAGAGTGTTTCAA

                          *                 *    *  *                *       
      44276 AACTGCTCTATCAATAGAAATGTTCAACTCCTTTG-GCTGGGTACACACATCACAAACAAGTTTC
         66 AACTGCTCTATCAAAAGAAATGTTCAACT-CTGTGAGTTGAGTACACACATCACAAAGAAGTTTC

                                      **     **       
      44340 TGAGAATGCTTCTGTCTAGTTTTTATGGGAAGACGTTCCCTT
        130 TGAGAATGCTTCTGTCTAGTTTTTATATGAAGATATTCCCTT

              *  * *  **          *       *            **                    
      44382 TTTCACCAAAGGCATCAAAGCGCTCCAAATGTCCACTTCCAGACACTACAAAAAGAGTGTTTCAA
          1 TTCCAACGAAATCATCAAAGCGATCCAAATATCCACTTCCAGATTCTACAAAAAGAGTGTTTCAA

             **       **   ***                 *    * *  * *         *       
      44447 ACGTGCTCTAAGAAAGCGAATGTTCAACTCTGTGACTTGAATGCAGATATCACAAAGTAGTTTCT
         66 AACTGCTCTATCAAAAGAAATGTTCAACTCTGTGAGTTGAGTACACACATCACAAAGAAGTTTCT

                *             *     * *  *         * 
      44512 GAGAGTGCTTCTGTCTAGATTTTAGACGATGATATTCCCGT
        131 GAGAATGCTTCTGTCTAGTTTTTATATGAAGATATTCCCTT

                           * *   *                *   *                    * 
      44553 TTCCAACGAAATCATTAGAGCTATCCAAATATCCACTTACAGTTTCTACAAAAAGAGTGTTTCCA
          1 TTCCAACGAAATCATCAAAGCGATCCAAATATCCACTTCCAGATTCTACAAAAAGAGTGTTTCAA

                   **         * *    *       *  *                   ***      
      44618 AACTGCTGCATCAAAAGAGAGGTTCCACTCTGTTAGCTGAGTACACACATCACAAACTTGTTTCT
         66 AACTGCTCTATCAAAAGAAATGTTCAACTCTGTGAGTTGAGTACACACATCACAAAGAAGTTTCT

            *     *         *        **         **   
      44683 CAGAATCCTTCTGTCTCGTTTTTATGGGAAGATATTTACTT
        131 GAGAATGCTTCTGTCTAGTTTTTATATGAAGATATTCCCTT

              *  *  * **          *       *     *       **    *       *      
      44724 TTTCACCGTAGGCATCAAAGCGCTCCAAATGTCCACATCCAGATAGTACAGAAAGAGTTTTTCAA
          1 TTCCAACGAAATCATCAAAGCGATCCAAATATCCACTTCCAGATTCTACAAAAAGAGTGTTTCAA

             *         *   * *   *         *        * *  *      *      *     
      44789 ACCTGCTCTATGAAAGGGAATCTTCAACTCTATGAGTTGAATGCAGACATCAGAAAGAAATTTCT
         66 AACTGCTCTATCAAAAGAAATGTTCAACTCTGTGAGTTGAGTACACACATCACAAAGAAGTTTCT

                     *       **          *         **
      44854 GAGAATGCTGCTGTCTACCTTTTAT-T--TGA-ATTCCCGC
        131 GAGAATGCTTCTGTCTAGTTTTTATATGAAGATATTCCCTT

                         *  *    *                *       *                  
      44891 TTCCAACGAAATCCTCCAAGCTATCCAAATATCCACTTGCAGATTCCACAAAAAGAGTGTTTCAA
          1 TTCCAACGAAATCATCAAAGCGATCCAAATATCCACTTCCAGATTCTACAAAAAGAGTGTTTCAA

                            ** *  *            *       *     *     *  *      
      44956 AACTGCTCTCTATCAATGGCAAAGTTCAACTCTGTTAGTTGAGGACACATATCACCAACAAGTTT
         66 AACTG--CTCTATCAAAAGAAATGTTCAACTCTGTGAGTTGAGTACACACATCACAAAGAAGTTT

                               *       **         *    
      45021 CTGAGAATGCTTCTGTCTATTTTTTATGGGAAGATATTTCCTT
        129 CTGAGAATGCTTCTGTCTAGTTTTTATATGAAGATATTCCCTT

              *  *  * ** *    *      *    *          * **                    
      45064 TTTCACCGTAGGCGTCAAGGCGATCGAAATGTCCACTTCCACAAACTACAAAAAGAGTGTTTCAA
          1 TTCCAACGAAATCATCAAAGCGATCCAAATATCCACTTCCAGATTCTACAAAAAGAGTGTTTCAA

             *         *   * **       *    *        * ** * *   **      *     
      45129 ACCTGCTCTATGAAAGGCGATGTTCATCTCTATGAGTTGAATGGAAATATCCGAAAGAAATTTCT
         66 AACTGCTCTATCAAAAGAAATGTTCAACTCTGTGAGTTGAGTACACACATCACAAAGAAGTTTCT

             *       *          *        *         **
      45194 GGGAATGCTGCTGTCTAG-TGTT-TAT-ACGA-ATTCCCGC
        131 GAGAATGCTTCTGTCTAGTTTTTATATGAAGATATTCCCTT

                         *       *                *    *  *                  
      45231 TTCCAACGAAATCCTCAAAGCAATCCAAATATCCACTTGCAGAATCCACAAAAAGAGTGTTTCAA
          1 TTCCAACGAAATCATCAAAGCGATCCAAATATCCACTTCCAGATTCTACAAAAAGAGTGTTTCAA

                          *     *          * *                   *  *        
      45296 AACTGCTCTATCAATAGAAAGGTTCAACTCTTTTAGTTGAGTACACACATCACGAACAAGTTTCT
         66 AACTGCTCTATCAAAAGAAATGTTCAACTCTGTGAGTTGAGTACACACATCACAAAGAAGTTTCT

                            * *      **     **  *    
      45361 GAGAATGCTTCTGTCTGGCTTTTATTGGAAGACGTTTCCTT
        131 GAGAATGCTTCTGTCTAGTTTTTATATGAAGATATTCCCTT

                 * *  **          *       *                * *               
      45402 TT-CACCAAAGGCATCAAAGCGCTCCAAATGTCCACTTCCAGATTCTTCCAAAAGAGTGTTTCAA
          1 TTCCAACGAAATCATCAAAGCGATCCAAATATCCACTTCCAGATTCTACAAAAAGAGTGTTTCAA

             **     * ***  * *                 *    * *  * *     *   *       
      45466 ACGTGCTCAAAGTAAGGGAATGTTCAACTCTGTGACTTGAATGCAGATATCACCAAGTAGTTTCT
         66 AACTGCTCTATCAAAAGAAATGTTCAACTCTGTGAGTTGAGTACACACATCACAAAGAAGTTTCT

            * * *             *     *    *         * 
      45531 AATAGTGCTTCTGTCTAGATTTTAGATGATGATATTCCCGT
        131 GAGAATGCTTCTGTCTAGTTTTTATATGAAGATATTCCCTT

                         * * *   *                *   *                    * 
      45572 TTCCAACGAAATCGTTAGAGCTATCCAAATATCCACTTACAGTTTCTACAAAAAGAGTGTTTCCA
          1 TTCCAACGAAATCATCAAAGCGATCCAAATATCCACTTCCAGATTCTACAAAAAGAGTGTTTCAA

                   **           *            *       *                     * 
      45637 AACTGCTGCATCAAAAGAAAGGTTCAACTCTGTTAGTTGAGGACACACATCACAAAGAAGTTTGT
         66 AACTGCTCTATCAAAAGAAATGTTCAACTCTGTGAGTTGAGTACACACATCACAAAGAAGTTTCT

                              *    *     *           
      45702 GAGAATGCTTCTGTCTAGATTTTGTATGACGATATTCCCTT
        131 GAGAATGCTTCTGTCTAGTTTTTATATGAAGATATTCCCTT

                     *   * *     *   *       * *  *    *  *       *  *       
      45743 TTCCAACGATATCGTTAAAGCAATCTAAATATCAATTTGCAGAATCCACAAAAATAGAGTTTCAA
          1 TTCCAACGAAATCATCAAAGCGATCCAAATATCCACTTCCAGATTCTACAAAAAGAGTGTTTCAA

             *       * *    *   *    *       *  *                   ***      
      45808 AGCTGCTCTGTAAAAATAAAAGTTCCACTCTGTTAGCTGAGTACACACATCACAAACTTGTTTCT
         66 AACTGCTCTATCAAAAGAAATGTTCAACTCTGTGAGTTGAGTACACACATCACAAAGAAGTTTCT

                  *         *        **         **   
      45873 GAGAATCCTTCTGTCTCGTTTTTATGGGAAGATATTTACTT
        131 GAGAATGCTTCTGTCTAGTTTTTATATGAAGATATTCCCTT

              *  *  * **          *       *     *       *  *  *     *        
      45914 TTTCACCGTAGGCATCAAAGCGCTCCAAATGTCCACATCCAGATACTCCAGAAAGACTGTTTCAA
          1 TTCCAACGAAATCATCAAAGCGATCCAAATATCCACTTCCAGATTCTACAAAAAGAGTGTTTCAA

             *         *   * *   *         *        * *  *      *      *     
      45979 ACCTGCTCTATGAAAGGGAATCTTCAACTCTATGAGTTGAATGCAGACATCAGAAAGAAATTTCT
         66 AACTGCTCTATCAAAAGAAATGTTCAACTCTGTGAGTTGAGTACACACATCACAAAGAAGTTTCT

                     *       **          *         **
      46044 GAGAATGCTGCTGTCTACCTTTTAT-T--TGA-ATTCCCGC
        131 GAGAATGCTTCTGTCTAGTTTTTATATGAAGATATTCCCTT

                         *  *    *              * *  **   *                  
      46081 TTCCAACGAAATCCTCCAAGCTATCCAAATATCCACCTGCATTTTCCACAAAAAGAGTGTTTCAA
          1 TTCCAACGAAATCATCAAAGCGATCCAAATATCCACTTCCAGATTCTACAAAAAGAGTGTTTCAA

                          *                 *    *  *                *       
      46146 AACTGCTCTATCAATAGAAATGTTCAACTCCTTTG-GCTGGGTACACACATCACAAACAAGTTTC
         66 AACTGCTCTATCAAAAGAAATGTTCAACT-CTGTGAGTTGAGTACACACATCACAAAGAAGTTTC

                                      **     **       
      46210 TGAGAATGCTTCTGTCTAGTTTTTATGGGAAGACGTTCCCTT
        130 TGAGAATGCTTCTGTCTAGTTTTTATATGAAGATATTCCCTT

              *  * *  **          *       *            **                    
      46252 TTTCACCAAAGGCATCAAAGCGCTCCAAATGTCCACTTCCAGACACTACAAAAAGAGTGTTTCAA
          1 TTCCAACGAAATCATCAAAGCGATCCAAATATCCACTTCCAGATTCTACAAAAAGAGTGTTTCAA

             **       **   ***                 *    * *  * *         *       
      46317 ACGTGCTCTAAGAAAGCGAATGTTCAACTCTGTGACTTGAATGCAGATATCACAAAGTAGTTTCT
         66 AACTGCTCTATCAAAAGAAATGTTCAACTCTGTGAGTTGAGTACACACATCACAAAGAAGTTTCT

                *             *     * *  *         * 
      46382 GAGAGTGCTTCTGTCTAGATTTTAGACGATGATATTCCCGT
        131 GAGAATGCTTCTGTCTAGTTTTTATATGAAGATATTCCCTT

                           * *   *                *   *                    * 
      46423 TTCCAACGAAATCATTAGAGCTATCCAAATATCCACTTACAGTTTCTACAAAAAGAGTGTTTCCA
          1 TTCCAACGAAATCATCAAAGCGATCCAAATATCCACTTCCAGATTCTACAAAAAGAGTGTTTCAA

                   **         * *    *       *  *                   ***      
      46488 AACTGCTGCATCAAAAGAGAGGTTCCACTCTGTTAGCTGAGTACACACATCACAAACTTGTTTCT
         66 AACTGCTCTATCAAAAGAAATGTTCAACTCTGTGAGTTGAGTACACACATCACAAAGAAGTTTCT

            *     *         *        **         **   
      46553 CAGAATCCTTCTGTCTCGTTTTTATGGGAAGATATTTACTT
        131 GAGAATGCTTCTGTCTAGTTTTTATATGAAGATATTCCCTT

              *  *  * **          *       *     *       **    *       *      
      46594 TTTCACCGTAGGCATCAAAGCGCTCCAAATGTCCACATCCAGATAGTACAGAAAGAGTTTTTCAA
          1 TTCCAACGAAATCATCAAAGCGATCCAAATATCCACTTCCAGATTCTACAAAAAGAGTGTTTCAA

             *         *   * *   *         *        * *  *      *      *     
      46659 ACCTGCTCTATGAAAGGGAATCTTCAACTCTATGAGTTGAATGCAGACATCAGAAAGAAATTTCT
         66 AACTGCTCTATCAAAAGAAATGTTCAACTCTGTGAGTTGAGTACACACATCACAAAGAAGTTTCT

                     *       **          *         **
      46724 GAGAATGCTGCTGTCTACCTTTTAT-T--TGA-ATTCCCGC
        131 GAGAATGCTTCTGTCTAGTTTTTATATGAAGATATTCCCTT

                         *  *    *                *       *                  
      46761 TTCCAACGAAATCCTCCAAGCTATCCAAATATCCACTTGCAGATTCCACAAAAAGAGTGTTTCAA
          1 TTCCAACGAAATCATCAAAGCGATCCAAATATCCACTTCCAGATTCTACAAAAAGAGTGTTTCAA

                            ** *  *            *       *     *     *  *      
      46826 AACTGCTCTCTATCAATGGCAAAGTTCAACTCTGTTAGTTGAGGACACATATCACCAACAAGTTT
         66 AACTG--CTCTATCAAAAGAAATGTTCAACTCTGTGAGTTGAGTACACACATCACAAAGAAGTTT

                               *       **         *    
      46891 CTGAGAATGCTTCTGTCTATTTTTTATGGGAAGATATTTCCTT
        129 CTGAGAATGCTTCTGTCTAGTTTTTATATGAAGATATTCCCTT

              *  *  * ** *    *      *    *          * **                    
      46934 TTTCACCGTAGGCGTCAAGGCGATCGAAATGTCCACTTCCACAAACTACAAAAAGAGTGTTTCAA
          1 TTCCAACGAAATCATCAAAGCGATCCAAATATCCACTTCCAGATTCTACAAAAAGAGTGTTTCAA

             *         *   * **       *    *        * ** * *   **      *     
      46999 ACCTGCTCTATGAAAGGCCATGTTCATCTCTATGAGTTGAATGGAAATATCCGAAAGAAATTTCT
         66 AACTGCTCTATCAAAAGAAATGTTCAACTCTGTGAGTTGAGTACACACATCACAAAGAAGTTTCT

             *       *          *        *         **
      47064 GGGAATGCTGCTGTCTAG-TGTT-TAT-ACGA-ATTCCCGC
        131 GAGAATGCTTCTGTCTAGTTTTTATATGAAGATATTCCCTT

                         *       *                *    *  *                  
      47101 TTCCAACGAAATCCTCAAAGCAATCCAAATATCCACTTGCAGAATCCACAAAAAGAGTGTTTCAA
          1 TTCCAACGAAATCATCAAAGCGATCCAAATATCCACTTCCAGATTCTACAAAAAGAGTGTTTCAA

                          *     *          * *                   *  *        
      47166 AACTGCTCTATCAATAGAAAGGTTCAACTCTTTTAGTTGAGTACACACATCACGAACAAGTTTCT
         66 AACTGCTCTATCAAAAGAAATGTTCAACTCTGTGAGTTGAGTACACACATCACAAAGAAGTTTCT

                            * *      **     **  *    
      47231 GAGAATGCTTCTGTCTGGCTTTTATTGGAAGACGTTTCCTT
        131 GAGAATGCTTCTGTCTAGTTTTTATATGAAGATATTCCCTT

                 * *  **          *       *                * *               
      47272 TT-CACCAAAGGCATCAAAGCGCTCCAAATGTCCACTTCCAGATTCTTCCAAAAGAGTGTTTCAA
          1 TTCCAACGAAATCATCAAAGCGATCCAAATATCCACTTCCAGATTCTACAAAAAGAGTGTTTCAA

             **     * ***  * *                 *    * *  * *     *   *       
      47336 ACGTGCTCAAAGTAAGGGAATGTTCAACTCTGTGACTTGAATGCAGATATCACCAAGTAGTTTCT
         66 AACTGCTCTATCAAAAGAAATGTTCAACTCTGTGAGTTGAGTACACACATCACAAAGAAGTTTCT

            * * *             *     *    *         * 
      47401 AATAGTGCTTCTGTCTAGATTTTAGATGATGATATTCCCGT
        131 GAGAATGCTTCTGTCTAGTTTTTATATGAAGATATTCCCTT

                         * * *   *                *   *                    * 
      47442 TTCCAACGAAATCGTTAGAGCTATCCAAATATCCACTTACAGTTTCTACAAAAAGAGTGTTTCCA
          1 TTCCAACGAAATCATCAAAGCGATCCAAATATCCACTTCCAGATTCTACAAAAAGAGTGTTTCAA

                   **           *            *       *                     * 
      47507 AACTGCTGCATCAAAAGAAAGGTTCAACTCTGTTAGTTGAGGACACACATCACAAAGAAGTTTGT
         66 AACTGCTCTATCAAAAGAAATGTTCAACTCTGTGAGTTGAGTACACACATCACAAAGAAGTTTCT

                              *    *     *           
      47572 GAGAATGCTTCTGTCTAGATTTTGTATGACGATATTCCCTT
        131 GAGAATGCTTCTGTCTAGTTTTTATATGAAGATATTCCCTT

                     *   * *     *   *       * *  *    *  *       *  *       
      47613 TTCCAACGATATCGTTAAAGCAATCTAAATATCAATTTGCAGAATCCACAAAAATAGAGTTTCAA
          1 TTCCAACGAAATCATCAAAGCGATCCAAATATCCACTTCCAGATTCTACAAAAAGAGTGTTTCAA

             *       * *    *   *    *       *  *                   ***      
      47678 AGCTGCTCTGTAAAAATAAAAGTTCCACTCTGTTAGCTGAGTACACACATCACAAACTTGTTTCT
         66 AACTGCTCTATCAAAAGAAATGTTCAACTCTGTGAGTTGAGTACACACATCACAAAGAAGTTTCT

                  *         *        **         **   
      47743 GAGAATCCTTCTGTCTCGTTTTTATGGGAAGATATTTACTT
        131 GAGAATGCTTCTGTCTAGTTTTTATATGAAGATATTCCCTT

              *  *  * **          *       *     *       *  *  *     *        
      47784 TTTCACCGTAGGCATCAAAGCGCTCCAAATGTCCACATCCAGATACTCCAGAAAGACTGTTTCAA
          1 TTCCAACGAAATCATCAAAGCGATCCAAATATCCACTTCCAGATTCTACAAAAAGAGTGTTTCAA

             *         *   * *   *         *        * *  *      *      *     
      47849 ACCTGCTCTATGAAAGGGAATCTTCAACTCTATGAGTTGAATGCAGACATCAGAAAGAAATTTCT
         66 AACTGCTCTATCAAAAGAAATGTTCAACTCTGTGAGTTGAGTACACACATCACAAAGAAGTTTCT

                     *       **          *         **
      47914 GAGAATGCTGCTGTCTACCTTTTAT-T--TGA-ATTCCCGC
        131 GAGAATGCTTCTGTCTAGTTTTTATATGAAGATATTCCCTT

                         *  *    *              * *  **   *                  
      47951 TTCCAACGAAATCCTCCAAGCTATCCAAATATCCACCTGCATTTTCCACAAAAAGAGTGTTTCAA
          1 TTCCAACGAAATCATCAAAGCGATCCAAATATCCACTTCCAGATTCTACAAAAAGAGTGTTTCAA

                          *                 *    *  *                *       
      48016 AACTGCTCTATCAATAGAAATGTTCAACTCCTTTG-GCTGGGTACACACATCACAAACAAGTTTC
         66 AACTGCTCTATCAAAAGAAATGTTCAACT-CTGTGAGTTGAGTACACACATCACAAAGAAGTTTC

                                      **     **       
      48080 TGAGAATGCTTCTGTCTAGTTTTTATGGGAAGACGTTCCCTT
        130 TGAGAATGCTTCTGTCTAGTTTTTATATGAAGATATTCCCTT

              *  * *  **          *       *            **                    
      48122 TTTCACCAAAGGCATCAAAGCGCTCCAAATGTCCACTTCCAGACACTACAAAAAGAGTGTTTCAA
          1 TTCCAACGAAATCATCAAAGCGATCCAAATATCCACTTCCAGATTCTACAAAAAGAGTGTTTCAA

             **       **   ***                 *    * *  * *         *       
      48187 ACGTGCTCTAAGAAAGCGAATGTTCAACTCTGTGACTTGAATGCAGATATCACAAAGTAGTTTCT
         66 AACTGCTCTATCAAAAGAAATGTTCAACTCTGTGAGTTGAGTACACACATCACAAAGAAGTTTCT

                *             *     * *  *         * 
      48252 GAGAGTGCTTCTGTCTAGATTTTAGACGATGATATTCCCGT
        131 GAGAATGCTTCTGTCTAGTTTTTATATGAAGATATTCCCTT

                           * *   *                *   *                    * 
      48293 TTCCAACGAAATCATTAGAGCTATCCAAATATCCACTTACAGTTTCTACAAAAAGAGTGTTTCCA
          1 TTCCAACGAAATCATCAAAGCGATCCAAATATCCACTTCCAGATTCTACAAAAAGAGTGTTTCAA

                   **         * *    *       *  *                   ***      
      48358 AACTGCTGCATCAAAAGAGAGGTTCCACTCTGTTAGCTGAGTACACACATCACAAACTTGTTTCT
         66 AACTGCTCTATCAAAAGAAATGTTCAACTCTGTGAGTTGAGTACACACATCACAAAGAAGTTTCT

            *     *         *        **         **   
      48423 CAGAATCCTTCTGTCTCGTTTTTATGGGAAGATATTTACTT
        131 GAGAATGCTTCTGTCTAGTTTTTATATGAAGATATTCCCTT

              *  *  * **          *       *     *       **    *       *      
      48464 TTTCACCGTAGGCATCAAAGCGCTCCAAATGTCCACATCCAGATAGTACAGAAAGAGTTTTTCAA
          1 TTCCAACGAAATCATCAAAGCGATCCAAATATCCACTTCCAGATTCTACAAAAAGAGTGTTTCAA

             *         *   * *   *         *        * *  *      *      *     
      48529 ACCTGCTCTATGAAAGGGAATCTTCAACTCTATGAGTTGAATGCAGACATCAGAAAGAAATTTCT
         66 AACTGCTCTATCAAAAGAAATGTTCAACTCTGTGAGTTGAGTACACACATCACAAAGAAGTTTCT

                     *       **          *         **
      48594 GAGAATGCTGCTGTCTACCTTTTAT-T--TGA-ATTCCCGC
        131 GAGAATGCTTCTGTCTAGTTTTTATATGAAGATATTCCCTT

                         *  *    *                *       *                  
      48631 TTCCAACGAAATCCTCCAAGCTATCCAAATATCCACTTGCAGATTCCACAAAAAGAGTGTTTCAA
          1 TTCCAACGAAATCATCAAAGCGATCCAAATATCCACTTCCAGATTCTACAAAAAGAGTGTTTCAA

                            ** *  *            *       *     *     *  *      
      48696 AACTGCTCTCTATCAATGGCAAAGTTCAACTCTGTTAGTTGAGGACACATATCACCAACAAGTTT
         66 AACTG--CTCTATCAAAAGAAATGTTCAACTCTGTGAGTTGAGTACACACATCACAAAGAAGTTT

                               *       **         *    
      48761 CTGAGAATGCTTCTGTCTATTTTTTATGGGAAGATATTTCCTT
        129 CTGAGAATGCTTCTGTCTAGTTTTTATATGAAGATATTCCCTT

              *  *  * ** *    *      *    *          * **                    
      48804 TTTCACCGTAGGCGTCAAGGCGATCGAAATGTCCACTTCCACAAACTACAAAAAGAGTGTTTCAA
          1 TTCCAACGAAATCATCAAAGCGATCCAAATATCCACTTCCAGATTCTACAAAAAGAGTGTTTCAA

             *         *   * **       *    *        * ** * *   **      *     
      48869 ACCTGCTCTATGAAAGGCGATGTTCATCTCTATGAGTTGAATGGAAATATCCGAAAGAAATTTCT
         66 AACTGCTCTATCAAAAGAAATGTTCAACTCTGTGAGTTGAGTACACACATCACAAAGAAGTTTCT

             *       *          *        *         **
      48934 GGGAATGCTGCTGTCTAG-TGTT-TAT-ACGA-ATTCCCGC
        131 GAGAATGCTTCTGTCTAGTTTTTATATGAAGATATTCCCTT

                         *       *                *    *  *                  
      48971 TTCCAACGAAATCCTCAAAGCAATCCAAATATCCACTTGCAGAATCCACAAAAAGAGTGTTTCAA
          1 TTCCAACGAAATCATCAAAGCGATCCAAATATCCACTTCCAGATTCTACAAAAAGAGTGTTTCAA

                          *     *          * *                   *  *        
      49036 AACTGCTCTATCAATAGAAAGGTTCAACTCTTTTAGTTGAGTACACACATCACGAACAAGTTTCT
         66 AACTGCTCTATCAAAAGAAATGTTCAACTCTGTGAGTTGAGTACACACATCACAAAGAAGTTTCT

                            * *      **     **  *    
      49101 GAGAATGCTTCTGTCTGGCTTTTATTGGAAGACGTTTCCTT
        131 GAGAATGCTTCTGTCTAGTTTTTATATGAAGATATTCCCTT

                 * *  **          *       *                * *               
      49142 TT-CACCAAAGGCATCAAAGCGCTCCAAATGTCCACTTCCAGATTCTTCCAAAAGAGTGTTTCAA
          1 TTCCAACGAAATCATCAAAGCGATCCAAATATCCACTTCCAGATTCTACAAAAAGAGTGTTTCAA

             **     * ***  * *                 *    * *  * *     *   *       
      49206 ACGTGCTCAAAGTAAGGGAATGTTCAACTCTGTGACTTGAATGCAGATATCACCAAGTAGTTTCT
         66 AACTGCTCTATCAAAAGAAATGTTCAACTCTGTGAGTTGAGTACACACATCACAAAGAAGTTTCT

            * * *             *     *    *         * 
      49271 AATAGTGCTTCTGTCTAGATTTTAGATGATGATATTCCCGT
        131 GAGAATGCTTCTGTCTAGTTTTTATATGAAGATATTCCCTT

                         * * *   *                *   *                    * 
      49312 TTCCAACGAAATCGTTAGAGCTATCCAAATATCCACTTACAGTTTCTACAAAAAGAGTGTTTCCA
          1 TTCCAACGAAATCATCAAAGCGATCCAAATATCCACTTCCAGATTCTACAAAAAGAGTGTTTCAA

                   **           *            *       *                     * 
      49377 AACTGCTGCATCAAAAGAAAGGTTCAACTCTGTTAGTTGAGGACACACATCACAAAGAAGTTTGT
         66 AACTGCTCTATCAAAAGAAATGTTCAACTCTGTGAGTTGAGTACACACATCACAAAGAAGTTTCT

                              *    *     *           
      49442 GAGAATGCTTCTGTCTAGATTTTGTATGACGATATTCCCTT
        131 GAGAATGCTTCTGTCTAGTTTTTATATGAAGATATTCCCTT

                     *   * *     *   *       * *  *    *  *       *  *       
      49483 TTCCAACGATATCGTTAAAGCAATCTAAATATCAATTTGCAGAATCCACAAAAATAGAGTTTCAA
          1 TTCCAACGAAATCATCAAAGCGATCCAAATATCCACTTCCAGATTCTACAAAAAGAGTGTTTCAA

             *       * *    *   *    *       *  *                   ***      
      49548 AGCTGCTCTGTAAAAATAAAAGTTCCACTCTGTTAGCTGAGTACACACATCACAAACTTGTTTCT
         66 AACTGCTCTATCAAAAGAAATGTTCAACTCTGTGAGTTGAGTACACACATCACAAAGAAGTTTCT

                  *         *        **         **   
      49613 GAGAATCCTTCTGTCTCGTTTTTATGGGAAGATATTTACTT
        131 GAGAATGCTTCTGTCTAGTTTTTATATGAAGATATTCCCTT

              *  *  * **          *       *     *       *  *  *     *        
      49654 TTTCACCGTAGGCATCAAAGCGCTCCAAATGTCCACATCCAGATACTCCAGAAAGACTGTTTCAA
          1 TTCCAACGAAATCATCAAAGCGATCCAAATATCCACTTCCAGATTCTACAAAAAGAGTGTTTCAA

             *         *   * *   *         *        * *  *      *      *     
      49719 ACCTGCTCTATGAAAGGGAATCTTCAACTCTATGAGTTGAATGCAGACATCAGAAAGAAATTTCT
         66 AACTGCTCTATCAAAAGAAATGTTCAACTCTGTGAGTTGAGTACACACATCACAAAGAAGTTTCT

                     *       **          *         **
      49784 GAGAATGCTGCTGTCTACCTTTTAT-T--TGA-ATTCCCGC
        131 GAGAATGCTTCTGTCTAGTTTTTATATGAAGATATTCCCTT

                         *  *    *              * *  **   *                  
      49821 TTCCAACGAAATCCTCCAAGCTATCCAAATATCCACCTGCATTTTCCACAAAAAGAGTGTTTCAA
          1 TTCCAACGAAATCATCAAAGCGATCCAAATATCCACTTCCAGATTCTACAAAAAGAGTGTTTCAA

                          *                 *    *  *                *       
      49886 AACTGCTCTATCAATAGAAATGTTCAACTCCTTTG-GCTGGGTACACACATCACAAACAAGTTTC
         66 AACTGCTCTATCAAAAGAAATGTTCAACT-CTGTGAGTTGAGTACACACATCACAAAGAAGTTTC

                                      **     **       
      49950 TGAGAATGCTTCTGTCTAGTTTTTATGGGAAGACGTTCCCTT
        130 TGAGAATGCTTCTGTCTAGTTTTTATATGAAGATATTCCCTT

              *  * *  **          *       *            **                    
      49992 TTTCACCAAAGGCATCAAAGCGCTCCAAATGTCCACTTCCAGACACTACAAAAAGAGTGTTTCAA
          1 TTCCAACGAAATCATCAAAGCGATCCAAATATCCACTTCCAGATTCTACAAAAAGAGTGTTTCAA

             **       **   ***                 *    * *  * *         *       
      50057 ACGTGCTCTAAGAAAGCGAATGTTCAACTCTGTGACTTGAATGCAGATATCACAAAGTAGTTTCT
         66 AACTGCTCTATCAAAAGAAATGTTCAACTCTGTGAGTTGAGTACACACATCACAAAGAAGTTTCT

                *             *     * *  *         * 
      50122 GAGAGTGCTTCTGTCTAGATTTTAGACGATGATATTCCCGT
        131 GAGAATGCTTCTGTCTAGTTTTTATATGAAGATATTCCCTT

                           * *   *                *   *                    * 
      50163 TTCCAACGAAATCATTAGAGCTATCCAAATATCCACTTACAGTTTCTACAAAAAGAGTGTTTCCA
          1 TTCCAACGAAATCATCAAAGCGATCCAAATATCCACTTCCAGATTCTACAAAAAGAGTGTTTCAA

                   **         * *    *       *  *                   ***      
      50228 AACTGCTGCATCAAAAGAGAGGTTCCACTCTGTTAGCTGAGTACACACATCACAAACTTGTTTCT
         66 AACTGCTCTATCAAAAGAAATGTTCAACTCTGTGAGTTGAGTACACACATCACAAAGAAGTTTCT

            *     *         *        **         **   
      50293 CAGAATCCTTCTGTCTCGTTTTTATGGGAAGATATTTACTT
        131 GAGAATGCTTCTGTCTAGTTTTTATATGAAGATATTCCCTT

              *  *  * **          *       *     *       **    *       *      
      50334 TTTCACCGTAGGCATCAAAGCGCTCCAAATGTCCACATCCAGATAGTACAGAAAGAGTTTTTCAA
          1 TTCCAACGAAATCATCAAAGCGATCCAAATATCCACTTCCAGATTCTACAAAAAGAGTGTTTCAA

             *         *   * *   *         *        * *  *      *      *     
      50399 ACCTGCTCTATGAAAGGGAATCTTCAACTCTATGAGTTGAATGCAGACATCAGAAAGAAATTTCT
         66 AACTGCTCTATCAAAAGAAATGTTCAACTCTGTGAGTTGAGTACACACATCACAAAGAAGTTTCT

                     *       **          *         **
      50464 GAGAATGCTGCTGTCTACCTTTTAT-T--TGA-ATTCCCGC
        131 GAGAATGCTTCTGTCTAGTTTTTATATGAAGATATTCCCTT

                         *  *    *                *       *                  
      50501 TTCCAACGAAATCCTCCAAGCTATCCAAATATCCACTTGCAGATTCCACAAAAAGAGTGTTTCAA
          1 TTCCAACGAAATCATCAAAGCGATCCAAATATCCACTTCCAGATTCTACAAAAAGAGTGTTTCAA

                            ** *  *            *       *     *     *  *      
      50566 AACTGCTCTCTATCAATGGCAAAGTTCAACTCTGTTAGTTGAGGACACATATCACCAACAAGTTT
         66 AACTG--CTCTATCAAAAGAAATGTTCAACTCTGTGAGTTGAGTACACACATCACAAAGAAGTTT

                               *       **         *    
      50631 CTGAGAATGCTTCTGTCTATTTTTTATGGGAAGATATTTCCTT
        129 CTGAGAATGCTTCTGTCTAGTTTTTATATGAAGATATTCCCTT

              *  *  * ** *    *      *    *          * **                    
      50674 TTTCACCGTAGGCGTCAAGGCGATCGAAATGTCCACTTCCACAAACTACAAAAAGAGTGTTTCAA
          1 TTCCAACGAAATCATCAAAGCGATCCAAATATCCACTTCCAGATTCTACAAAAAGAGTGTTTCAA

             *         *   * **       *    *        * ** * *   **      *     
      50739 ACCTGCTCTATGAAAGGCGATGTTCATCTCTATGAGTTGAATGGAAATATCCGAAAGAAATTTCT
         66 AACTGCTCTATCAAAAGAAATGTTCAACTCTGTGAGTTGAGTACACACATCACAAAGAAGTTTCT

             *       *          *        *         **
      50804 GGGAATGCTGCTGTCTAG-TGTT-TAT-ACGA-ATTCCCGC
        131 GAGAATGCTTCTGTCTAGTTTTTATATGAAGATATTCCCTT

                         *       *                *    *  *                  
      50841 TTCCAACGAAATCCTCAAAGCAATCCAAATATCCACTTGCAGAATCCACAAAAAGAGTGTTTCAA
          1 TTCCAACGAAATCATCAAAGCGATCCAAATATCCACTTCCAGATTCTACAAAAAGAGTGTTTCAA

                          *     *          * *                   *  *        
      50906 AACTGCTCTATCAATAGAAAGGTTCAACTCTTTTAGTTGAGTACACACATCACGAACAAGTTTCT
         66 AACTGCTCTATCAAAAGAAATGTTCAACTCTGTGAGTTGAGTACACACATCACAAAGAAGTTTCT

                            * *      **     **  *    
      50971 GAGAATGCTTCTGTCTGGCTTTTATTGGAAGACGTTTCCTT
        131 GAGAATGCTTCTGTCTAGTTTTTATATGAAGATATTCCCTT

                 * *  **          *       *                * *               
      51012 TT-CACCAAAGGCATCAAAGCGCTCCAAATGTCCACTTCCAGATTCTTCCAAAAGAGTGTTTCAA
          1 TTCCAACGAAATCATCAAAGCGATCCAAATATCCACTTCCAGATTCTACAAAAAGAGTGTTTCAA

             **     * ***  * *                 *    * *  * *     *   *       
      51076 ACGTGCTCAAAGTAAGGGAATGTTCAACTCTGTGACTTGAATGCAGATATCACCAAGTAGTTTCT
         66 AACTGCTCTATCAAAAGAAATGTTCAACTCTGTGAGTTGAGTACACACATCACAAAGAAGTTTCT

            * * *             *     *    *         * 
      51141 AATAGTGCTTCTGTCTAGATTTTAGATGATGATATTCCCGT
        131 GAGAATGCTTCTGTCTAGTTTTTATATGAAGATATTCCCTT

                         * * *   *                *   *                    * 
      51182 TTCCAACGAAATCGTTAGAGCTATCCAAATATCCACTTACAGTTTCTACAAAAAGAGTGTTTCCA
          1 TTCCAACGAAATCATCAAAGCGATCCAAATATCCACTTCCAGATTCTACAAAAAGAGTGTTTCAA

                   **           *            *       *                     * 
      51247 AACTGCTGCATCAAAAGAAAGGTTCAACTCTGTTAGTTGAGGACACACATCACAAAGAAGTTTGT
         66 AACTGCTCTATCAAAAGAAATGTTCAACTCTGTGAGTTGAGTACACACATCACAAAGAAGTTTCT

                              *    *     *           
      51312 GAGAATGCTTCTGTCTAGATTTTGTATGACGATATTCCCTT
        131 GAGAATGCTTCTGTCTAGTTTTTATATGAAGATATTCCCTT

                     *   * *     *   *       * *  *    *  *       *  *       
      51353 TTCCAACGATATCGTTAAAGCAATCTAAATATCAATTTGCAGAATCCACAAAAATAGAGTTTCAA
          1 TTCCAACGAAATCATCAAAGCGATCCAAATATCCACTTCCAGATTCTACAAAAAGAGTGTTTCAA

             *       * *    *   *    *       *  *                   ***      
      51418 AGCTGCTCTGTAAAAATAAAAGTTCCACTCTGTTAGCTGAGTACACACATCACAAACTTGTTTCT
         66 AACTGCTCTATCAAAAGAAATGTTCAACTCTGTGAGTTGAGTACACACATCACAAAGAAGTTTCT

                  *         *        **         **   
      51483 GAGAATCCTTCTGTCTCGTTTTTATGGGAAGATATTTACTT
        131 GAGAATGCTTCTGTCTAGTTTTTATATGAAGATATTCCCTT

              *  *  * **          *       *     *       *  *  *     *        
      51524 TTTCACCGTAGGCATCAAAGCGCTCCAAATGTCCACATCCAGATACTCCAGAAAGACTGTTTCAA
          1 TTCCAACGAAATCATCAAAGCGATCCAAATATCCACTTCCAGATTCTACAAAAAGAGTGTTTCAA

             *         *   * *   *         *        * *  *      *      *     
      51589 ACCTGCTCTATGAAAGGGAATCTTCAACTCTATGAGTTGAATGCAGACATCAGAAAGAAATTTCT
         66 AACTGCTCTATCAAAAGAAATGTTCAACTCTGTGAGTTGAGTACACACATCACAAAGAAGTTTCT

                     *       **          *         **
      51654 GAGAATGCTGCTGTCTACCTTTTAT-T--TGA-ATTCCCGC
        131 GAGAATGCTTCTGTCTAGTTTTTATATGAAGATATTCCCTT

                         *  *    *              * *  **   *                  
      51691 TTCCAACGAAATCCTCCAAGCTATCCAAATATCCACCTGCATTTTCCACAAAAAGAGTGTTTCAA
          1 TTCCAACGAAATCATCAAAGCGATCCAAATATCCACTTCCAGATTCTACAAAAAGAGTGTTTCAA

                          *                 *    *  *                *       
      51756 AACTGCTCTATCAATAGAAATGTTCAACTCCTTTG-GCTGGGTACACACATCACAAACAAGTTTC
         66 AACTGCTCTATCAAAAGAAATGTTCAACT-CTGTGAGTTGAGTACACACATCACAAAGAAGTTTC

                                      **     **       
      51820 TGAGAATGCTTCTGTCTAGTTTTTATGGGAAGACGTTCCCTT
        130 TGAGAATGCTTCTGTCTAGTTTTTATATGAAGATATTCCCTT

              *  * *  **          *       *            **                    
      51862 TTTCACCAAAGGCATCAAAGCGCTCCAAATGTCCACTTCCAGACACTACAAAAAGAGTGTTTCAA
          1 TTCCAACGAAATCATCAAAGCGATCCAAATATCCACTTCCAGATTCTACAAAAAGAGTGTTTCAA

             **       **   ***                 *    * *  * *         *       
      51927 ACGTGCTCTAAGAAAGCGAATGTTCAACTCTGTGACTTGAATGCAGATATCACAAAGTAGTTTCT
         66 AACTGCTCTATCAAAAGAAATGTTCAACTCTGTGAGTTGAGTACACACATCACAAAGAAGTTTCT

                *             *     * *  *         * 
      51992 GAGAGTGCTTCTGTCTAGATTTTAGACGATGATATTCCCGT
        131 GAGAATGCTTCTGTCTAGTTTTTATATGAAGATATTCCCTT

                           * *   *                *   *                    * 
      52033 TTCCAACGAAATCATTAGAGCTATCCAAATATCCACTTACAGTTTCTACAAAAAGAGTGTTTCCA
          1 TTCCAACGAAATCATCAAAGCGATCCAAATATCCACTTCCAGATTCTACAAAAAGAGTGTTTCAA

                   **         * *    *       *  *                   ***      
      52098 AACTGCTGCATCAAAAGAGAGGTTCCACTCTGTTAGCTGAGTACACACATCACAAACTTGTTTCT
         66 AACTGCTCTATCAAAAGAAATGTTCAACTCTGTGAGTTGAGTACACACATCACAAAGAAGTTTCT

            *     *         *        **         **   
      52163 CAGAATCCTTCTGTCTCGTTTTTATGGGAAGATATTTACTT
        131 GAGAATGCTTCTGTCTAGTTTTTATATGAAGATATTCCCTT

              *  *  * **          *       *     *       **    *       *      
      52204 TTTCACCGTAGGCATCAAAGCGCTCCAAATGTCCACATCCAGATAGTACAGAAAGAGTTTTTCAA
          1 TTCCAACGAAATCATCAAAGCGATCCAAATATCCACTTCCAGATTCTACAAAAAGAGTGTTTCAA

             *         *   * *   *         *        * *  *      *      *     
      52269 ACCTGCTCTATGAAAGGGAATCTTCAACTCTATGAGTTGAATGCAGACATCAGAAAGAAATTTCT
         66 AACTGCTCTATCAAAAGAAATGTTCAACTCTGTGAGTTGAGTACACACATCACAAAGAAGTTTCT

                     *       **          *         **
      52334 GAGAATGCTGCTGTCTACCTTTTAT-T--TGA-ATTCCCGC
        131 GAGAATGCTTCTGTCTAGTTTTTATATGAAGATATTCCCTT

                         *  *    *                *       *                  
      52371 TTCCAACGAAATCCTCCAAGCTATCCAAATATCCACTTGCAGATTCCACAAAAAGAGTGTTTCAA
          1 TTCCAACGAAATCATCAAAGCGATCCAAATATCCACTTCCAGATTCTACAAAAAGAGTGTTTCAA

                            ** *  *            *       *     *     *  *      
      52436 AACTGCTCTCTATCAATGGCAAAGTTCAACTCTGTTAGTTGAGGACACATATCACCAACAAGTTT
         66 AACTG--CTCTATCAAAAGAAATGTTCAACTCTGTGAGTTGAGTACACACATCACAAAGAAGTTT

                               *       **         *    
      52501 CTGAGAATGCTTCTGTCTATTTTTTATGGGAAGATATTTCCTT
        129 CTGAGAATGCTTCTGTCTAGTTTTTATATGAAGATATTCCCTT

              *  *  * ** *    *      *    *          * **                    
      52544 TTTCACCGTAGGCGTCAAGGCGATCGAAATGTCCACTTCCACAAACTACAAAAAGAGTGTTTCAA
          1 TTCCAACGAAATCATCAAAGCGATCCAAATATCCACTTCCAGATTCTACAAAAAGAGTGTTTCAA

             *         *   * **       *    *        * ** * *   **      *     
      52609 ACCTGCTCTATGAAAGGCCATGTTCATCTCTATGAGTTGAATGGAAATATCCGAAAGAAATTTCT
         66 AACTGCTCTATCAAAAGAAATGTTCAACTCTGTGAGTTGAGTACACACATCACAAAGAAGTTTCT

             *       *          *        *         **
      52674 GGGAATGCTGCTGTCTAG-TGTT-TAT-ACGA-ATTCCCGC
        131 GAGAATGCTTCTGTCTAGTTTTTATATGAAGATATTCCCTT

                         *       *                *    *  *                  
      52711 TTCCAACGAAATCCTCAAAGCAATCCAAATATCCACTTGCAGAATCCACAAAAAGAGTGTTTCAA
          1 TTCCAACGAAATCATCAAAGCGATCCAAATATCCACTTCCAGATTCTACAAAAAGAGTGTTTCAA

                          *     *          * *                   *  *        
      52776 AACTGCTCTATCAATAGAAAGGTTCAACTCTTTTAGTTGAGTACACACATCACGAACAAGTTTCT
         66 AACTGCTCTATCAAAAGAAATGTTCAACTCTGTGAGTTGAGTACACACATCACAAAGAAGTTTCT

                            * *      **     **  *    
      52841 GAGAATGCTTCTGTCTGGCTTTTATTGGAAGACGTTTCCTT
        131 GAGAATGCTTCTGTCTAGTTTTTATATGAAGATATTCCCTT

                   *  **          *       *                * *               
      52882 TT-CAACAAAGGCATCAAAGCGCTCCAAATGTCCACTTCCAGATTCTTCCAAAAGAGTGTTTCAA
          1 TTCCAACGAAATCATCAAAGCGATCCAAATATCCACTTCCAGATTCTACAAAAAGAGTGTTTCAA

             **     * ***  * *                 *    * *  * *     *   *       
      52946 ACGTGCTCGAAGTAAGGGAATGTTCAACTCTGTGACTTGAATGCAGATATCACCAAGTAGTTTCT
         66 AACTGCTCTATCAAAAGAAATGTTCAACTCTGTGAGTTGAGTACACACATCACAAAGAAGTTTCT

            * * *            **     *    *         * 
      53011 AATAGTGCTTCTGTCTACATTTTAGATGATGATATTCCCGT
        131 GAGAATGCTTCTGTCTAGTTTTTATATGAAGATATTCCCTT

                         * * *   *             *  *   *      *     *       * 
      53052 TTCCAACGAAATCGTTAGAGCTATCCAAATATCCAGTTACAGTTTCTACCAAAAGGGTGTTTCCA
          1 TTCCAACGAAATCATCAAAGCGATCCAAATATCCACTTCCAGATTCTACAAAAAGAGTGTTTCAA

              *    **           *            *       *                     * 
      53117 AATTGCTGCATCAAAAGAAAGGTTCAACTCTGTTAGTTGAGGACACACATCACAAAGAAGTTTGT
         66 AACTGCTCTATCAAAAGAAATGTTCAACTCTGTGAGTTGAGTACACACATCACAAAGAAGTTTCT

                              *    *     *           
      53182 GAGAATGCTTCTGTCTAGATTTTGTATGACGATATTCCCTT
        131 GAGAATGCTTCTGTCTAGTTTTTATATGAAGATATTCCCTT

                     *   * *     *   *       * *  *    *  *       *  *       
      53223 TTCCAACGATATCGTTAAAGCAATCTAAATATCAATTTGCAGAATCCACAAAAATAGAGTTTCAA
          1 TTCCAACGAAATCATCAAAGCGATCCAAATATCCACTTCCAGATTCTACAAAAAGAGTGTTTCAA

             *       * *        *    *       *  *                   ***      
      53288 AGCTGCTCTGTAAAAAGAAAGGTTCCACTCTGTTAGCTGAGTACACACATCACAAACTTGTTTCT
         66 AACTGCTCTATCAAAAGAAATGTTCAACTCTGTGAGTTGAGTACACACATCACAAAGAAGTTTCT

                  *         *        **         **   
      53353 GAGAATCCTTCTGTCTCGTTTTTATGGGAAGATATTTACTT
        131 GAGAATGCTTCTGTCTAGTTTTTATATGAAGATATTCCCTT

                 *  * **          *       *     *       *  *  *  *           
      53394 TTCCACCGTAGGCATCAAAGCGCTCCAAATGTCCACATCCAGATACTCCAGAACGAGTGTTTCAA
          1 TTCCAACGAAATCATCAAAGCGATCCAAATATCCACTTCCAGATTCTACAAAAAGAGTGTTTCAA

             *         *   * *   *         *        * *  *      *      *     
      53459 ACCTGCTCTATGAAAGGGAATCTTCAACTCTATGAGTTGAATGCAGACATCAGAAAGAAATTTCT
         66 AACTGCTCTATCAAAAGAAATGTTCAACTCTGTGAGTTGAGTACACACATCACAAAGAAGTTTCT

                     *       **          *         **
      53524 GAGAATGCTGCTGTCTACCTTTTAT-T--TGA-ATTCCCGC
        131 GAGAATGCTTCTGTCTAGTTTTTATATGAAGATATTCCCTT

                         *  *    *              * *  **   *    *             
      53561 TTCCAACGAAATCCTCCAAGCTATCCAAATATCCACCTGCATTTTCCACAACAAGAGTGTTTCAA
          1 TTCCAACGAAATCATCAAAGCGATCCAAATATCCACTTCCAGATTCTACAAAAAGAGTGTTTCAA

                          *                 *    *  *                *       
      53626 AACTGCTCTATCAATAGAAATGTTCAACTCCTTTG-GCTGGGTACACACATCACAAACAAGTTTC
         66 AACTGCTCTATCAAAAGAAATGTTCAACT-CTGTGAGTTGAGTACACACATCACAAAGAAGTTTC

                *                     **     *        
      53690 TGAGCATGCTTCTGTCTAGTTTTTATGGGAAGACATTCCCTT
        130 TGAGAATGCTTCTGTCTAGTTTTTATATGAAGATATTCCCTT

              *  * *  **          *       *            **                    
      53732 TTTCACCAAAGGCATCAAAGCGCTCCAAATGTCCACTTCCAGACACTACAAAAAGAGTGTTTCAA
          1 TTCCAACGAAATCATCAAAGCGATCCAAATATCCACTTCCAGATTCTACAAAAAGAGTGTTTCAA

             **       **    **                 *    * *  * *      *  *       
      53797 ACGTGCTCTAAGAAAACGAATGTTCAACTCTGTGACTTGAATGCAGATATCACAGAGTAGTTTCT
         66 AACTGCTCTATCAAAAGAAATGTTCAACTCTGTGAGTTGAGTACACACATCACAAAGAAGTTTCT

                **            *     *    *         * 
      53862 GAGAGGGCTTCTGTCTAGATTTTAGATGATGATATTCCCGT
        131 GAGAATGCTTCTGTCTAGTTTTTATATGAAGATATTCCCTT

                           * *   *                *   *                    * 
      53903 TTCCAACGAAATCATTAGAGCTATCCAAATATCCACTTACAGTTTCTACAAAAAGAGTGTTTCCA
          1 TTCCAACGAAATCATCAAAGCGATCCAAATATCCACTTCCAGATTCTACAAAAAGAGTGTTTCAA

                   **         * *    *       *  *                   ***      
      53968 AACTGCTGCATCAAAAGAGAGGTTCCACTCTGTTAGCTGAGTACACACATCACAAACTTGTTTCT
         66 AACTGCTCTATCAAAAGAAATGTTCAACTCTGTGAGTTGAGTACACACATCACAAAGAAGTTTCT

            *     *         *        **         **   
      54033 CAGAATCCTTCTGTCTCGTTTTTATGGGAAGATATTTACTT
        131 GAGAATGCTTCTGTCTAGTTTTTATATGAAGATATTCCCTT

              *  *  * **          *       *     *       *  *  *              
      54074 TTTCACCGTAGGCATCAAAGCGCTCCAAATGTCCACATCCAGATACTCCAGAAAGAGTGTTTCAA
          1 TTCCAACGAAATCATCAAAGCGATCCAAATATCCACTTCCAGATTCTACAAAAAGAGTGTTTCAA

             *         *   * *   *         *        * *  *      *      *     
      54139 ACCTGCTCTATGAAAGGGAATCTTCAACTCTATGAGTTGAATGCAGACATCAGAAAGAAATTTCT
         66 AACTGCTCTATCAAAAGAAATGTTCAACTCTGTGAGTTGAGTACACACATCACAAAGAAGTTTCT

                     *       **          *         **
      54204 GAGAATGCTGCTGTCTACCTTTTAT-T--TGA-ATTCCCGC
        131 GAGAATGCTTCTGTCTAGTTTTTATATGAAGATATTCCCTT

                         *  *    *                *       *                  
      54241 TTCCAACGAAATCCTCCAAGCTATCCAAATATCCACTTGCAGATTCCACAAAAAGAGTGTTTCAA
          1 TTCCAACGAAATCATCAAAGCGATCCAAATATCCACTTCCAGATTCTACAAAAAGAGTGTTTCAA

                            ** *  *            *       *     *     *  *      
      54306 AACTGCTCTCTATCAATGGCAAAGTTCAACTCTGTTAGTTGAGGACACATATCACCAACAAGTTT
         66 AACTG--CTCTATCAAAAGAAATGTTCAACTCTGTGAGTTGAGTACACACATCACAAAGAAGTTT

                               *       **         *    
      54371 CTGAGAATGCTTCTGTCTATTTTTTATGGGAAGATATTTCCTT
        129 CTGAGAATGCTTCTGTCTAGTTTTTATATGAAGATATTCCCTT

              *  *  * ** *    *      *    *          * **                    
      54414 TTTCACCGTAGGCGTCAAGGCGATCGAAATGTCCACTTCCACAAACTACAAAAAGAGTGTTTCAA
          1 TTCCAACGAAATCATCAAAGCGATCCAAATATCCACTTCCAGATTCTACAAAAAGAGTGTTTCAA

             *         *   * **       *    *        * ** * *   **      *     
      54479 ACCTGCTCTATGAAAGGCGATGTTCATCTCTATGAGTTGAATGGAAATATCCGAAAGAAATTTCT
         66 AACTGCTCTATCAAAAGAAATGTTCAACTCTGTGAGTTGAGTACACACATCACAAAGAAGTTTCT

             *       *          *        *         **
      54544 GGGAATGCTGCTGTCTAG-TGTT-TAT-ACGA-ATTCCCGC
        131 GAGAATGCTTCTGTCTAGTTTTTATATGAAGATATTCCCTT

                         *       *                *    *  *                  
      54581 TTCCAACGAAATCCTCAAAGCAATCCAAATATCCACTTGCAGAATCCACAAAAAGAGTGTTTCAA
          1 TTCCAACGAAATCATCAAAGCGATCCAAATATCCACTTCCAGATTCTACAAAAAGAGTGTTTCAA

                          *     *          * *                   *  *        
      54646 AACTGCTCTATCAATAGAAAGGTTCAACTCTTTTAGTTGAGTACACACATCACGAACAAGTTTCT
         66 AACTGCTCTATCAAAAGAAATGTTCAACTCTGTGAGTTGAGTACACACATCACAAAGAAGTTTCT

                            * *      **     **  *    
      54711 GAGAATGCTTCTGTCTGGCTTTTATTGGAAGACGTTTCCTT
        131 GAGAATGCTTCTGTCTAGTTTTTATATGAAGATATTCCCTT

                 * *  **          *       *                * *               
      54752 TT-CACCAAAGGCATCAAAGCGCTCCAAATGTCCACTTCCAGATTCTTCCAAAAGAGTGTTTCAA
          1 TTCCAACGAAATCATCAAAGCGATCCAAATATCCACTTCCAGATTCTACAAAAAGAGTGTTTCAA

             **     * ***  * *                 *    * *  * *     *   *       
      54816 ACGTGCTCGAAGTAAGGGAATGTTCAACTCTGTGACTTGAATGCAGATATCACCAAGTAGTTTCT
         66 AACTGCTCTATCAAAAGAAATGTTCAACTCTGTGAGTTGAGTACACACATCACAAAGAAGTTTCT

            * * *             *     *    *         * 
      54881 AATAGTGCTTCTGTCTAGATTTTAGATGATGATATTCCCGT
        131 GAGAATGCTTCTGTCTAGTTTTTATATGAAGATATTCCCTT

                         * * *   *                *   *                    * 
      54922 TTCCAACGAAATCGTTAGAGCTATCCAAATATCCACTTACAGTTTCTACAAAAAGAGTGTTTCCA
          1 TTCCAACGAAATCATCAAAGCGATCCAAATATCCACTTCCAGATTCTACAAAAAGAGTGTTTCAA

                   **           *            *       *                     * 
      54987 AACTGCTGCATCAAAAGAAAGGTTCAACTCTGTTAGTTGAGGACACACATCACAAAGAAGTTTGT
         66 AACTGCTCTATCAAAAGAAATGTTCAACTCTGTGAGTTGAGTACACACATCACAAAGAAGTTTCT

                              *    *     *           
      55052 GAGAATGCTTCTGTCTAGATTTTGTATGACGATATTCCCTT
        131 GAGAATGCTTCTGTCTAGTTTTTATATGAAGATATTCCCTT

                     *   * *     *   *       * *  *    *  *       *  *       
      55093 TTCCAACGATATCGTTAAAGCAATCTAAATATCAATTTGCAGAATCCACAAAAATAGAGTTTCAA
          1 TTCCAACGAAATCATCAAAGCGATCCAAATATCCACTTCCAGATTCTACAAAAAGAGTGTTTCAA

             *       * *    *   *    *       *  *                   ***      
      55158 AGCTGCTCTGTAAAAATAAAAGTTCCACTCTGTTAGCTGAGTACACACATCACAAACTTGTTTCT
         66 AACTGCTCTATCAAAAGAAATGTTCAACTCTGTGAGTTGAGTACACACATCACAAAGAAGTTTCT

                  *         *        **         **   
      55223 GAGAATCCTTCTGTCTCGTTTTTATGGGAAGATATTTACTT
        131 GAGAATGCTTCTGTCTAGTTTTTATATGAAGATATTCCCTT

              *  *  * **          *       *     *       *  *  *     *        
      55264 TTTCACCGTAGGCATCAAAGCGCTCCAAATGTCCACATCCAGATACTCCAGAAAGACTGTTTCAA
          1 TTCCAACGAAATCATCAAAGCGATCCAAATATCCACTTCCAGATTCTACAAAAAGAGTGTTTCAA

             *         *   * *   *         *        * *  *      *      *     
      55329 ACCTGCTCTATGAAAGGGAATCTTCAACTCTATGAGTTGAATGCAGACATCAGAAAGAAATTTCT
         66 AACTGCTCTATCAAAAGAAATGTTCAACTCTGTGAGTTGAGTACACACATCACAAAGAAGTTTCT

                     *       **          *         **
      55394 GAGAATGCTGCTGTCTACCTTTTAT-T--TGA-ATTCCCGC
        131 GAGAATGCTTCTGTCTAGTTTTTATATGAAGATATTCCCTT

                         *  *    *              * *  **   *                  
      55431 TTCCAACGAAATCCTCCAAGCTATCCAAATATCCACCTGCATTTTCCACAAAAAGAGTGTTTCAA
          1 TTCCAACGAAATCATCAAAGCGATCCAAATATCCACTTCCAGATTCTACAAAAAGAGTGTTTCAA

                          *                 *    *  *                *       
      55496 AACTGCTCTATCAATAGAAATGTTCAACTCCTTTG-GCTGGGTACACACATCACAAACAAGTTTC
         66 AACTGCTCTATCAAAAGAAATGTTCAACT-CTGTGAGTTGAGTACACACATCACAAAGAAGTTTC

                                      **     **       
      55560 TGAGAATGCTTCTGTCTAGTTTTTATGGGAAGACGTTCCCTT
        130 TGAGAATGCTTCTGTCTAGTTTTTATATGAAGATATTCCCTT

              *  * *  **          *       *            **                    
      55602 TTTCACCAAAGGCATCAAAGCGCTCCAAATGTCCACTTCCAGACACTACAAAAAGAGTGTTTCAA
          1 TTCCAACGAAATCATCAAAGCGATCCAAATATCCACTTCCAGATTCTACAAAAAGAGTGTTTCAA

             **       **   ***                 *    * *  * *         *       
      55667 ACGTGCTCTAAGAAAGCGAATGTTCAACTCTGTGACTTGAATGCAGATATCACAAAGTAGTTTCT
         66 AACTGCTCTATCAAAAGAAATGTTCAACTCTGTGAGTTGAGTACACACATCACAAAGAAGTTTCT

                *             *     * *  *         * 
      55732 GAGAGTGCTTCTGTCTAGATTTTAGACGATGATATTCCCGT
        131 GAGAATGCTTCTGTCTAGTTTTTATATGAAGATATTCCCTT

                           * *   *                *   *                    * 
      55773 TTCCAACGAAATCATTAGAGCTATCCAAATATCCACTTACAGTTTCTACAAAAAGAGTGTTTCCA
          1 TTCCAACGAAATCATCAAAGCGATCCAAATATCCACTTCCAGATTCTACAAAAAGAGTGTTTCAA

                   **         * *    *       *  *                   ***      
      55838 AACTGCTGCATCAAAAGAGAGGTTCCACTCTGTTAGCTGAGTACACACATCACAAACTTGTTTCT
         66 AACTGCTCTATCAAAAGAAATGTTCAACTCTGTGAGTTGAGTACACACATCACAAAGAAGTTTCT

            *     *         *        **         **   
      55903 CAGAATCCTTCTGTCTCGTTTTTATGGGAAGATATTTACTT
        131 GAGAATGCTTCTGTCTAGTTTTTATATGAAGATATTCCCTT

              *  *  * **          *       *     *       *  *  *              
      55944 TTTCACCGTAGGCATCAAAGCGCTCCAAATGTCCACATCCAGATACTCCAGAAAGAGTGTTTCAA
          1 TTCCAACGAAATCATCAAAGCGATCCAAATATCCACTTCCAGATTCTACAAAAAGAGTGTTTCAA

             *         *   * *   *         *        * *  *      *      *     
      56009 ACCTGCTCTATGAAAGGGAATCTTCAACTCTATGAGTTGAATGCAGACATCAGAAAGAAATTTCT
         66 AACTGCTCTATCAAAAGAAATGTTCAACTCTGTGAGTTGAGTACACACATCACAAAGAAGTTTCT

                     *       **          *         **
      56074 GAGAATGCTGCTGTCTACCTTTTAT-T--TGA-ATTCCCGC
        131 GAGAATGCTTCTGTCTAGTTTTTATATGAAGATATTCCCTT

                         *  *    *                *       *                  
      56111 TTCCAACGAAATCCTCCAAGCTATCCAAATATCCACTTGCAGATTCCACAAAAAGAGTGTTTCAA
          1 TTCCAACGAAATCATCAAAGCGATCCAAATATCCACTTCCAGATTCTACAAAAAGAGTGTTTCAA

                            ** *  *            *       *     *     *  *      
      56176 AACTGCTCTCTATCAATGGCAAAGTTCAACTCTGTTAGTTGAGGACACATATCACCAACAAGTTT
         66 AACTG--CTCTATCAAAAGAAATGTTCAACTCTGTGAGTTGAGTACACACATCACAAAGAAGTTT

                               *       **         *    
      56241 CTGAGAATGCTTCTGTCTATTTTTTATGGGAAGATATTTCCTT
        129 CTGAGAATGCTTCTGTCTAGTTTTTATATGAAGATATTCCCTT

              *  *  * ** *    *      *    *          * **                    
      56284 TTTCACCGTAGGCGTCAAGGCGATCGAAATGTCCACTTCCACAAACTACAAAAAGAGTGTTTCAA
          1 TTCCAACGAAATCATCAAAGCGATCCAAATATCCACTTCCAGATTCTACAAAAAGAGTGTTTCAA

             *         *   * **       *    *        * ** * *   **      *     
      56349 ACCTGCTCTATGAAAGGCCATGTTCATCTCTATGAGTTGAATGGAAATATCCGAAAGAAATTTCT
         66 AACTGCTCTATCAAAAGAAATGTTCAACTCTGTGAGTTGAGTACACACATCACAAAGAAGTTTCT

             *       *          *        *         **
      56414 GGGAATGCTGCTGTCTAG-TGTT-TAT-ACGA-ATTCCCGC
        131 GAGAATGCTTCTGTCTAGTTTTTATATGAAGATATTCCCTT

                         *       *                *    *  *                  
      56451 TTCCAACGAAATCCTCAAAGCAATCCAAATATCCACTTGCAGAATCCACAAAAAGAGTGTTTCAA
          1 TTCCAACGAAATCATCAAAGCGATCCAAATATCCACTTCCAGATTCTACAAAAAGAGTGTTTCAA

                          *     *          * *                   *  *        
      56516 AACTGCTCTATCAATAGAAAGGTTCAACTCTTTTAGTTGAGTACACACATCACGAACAAGTTTCT
         66 AACTGCTCTATCAAAAGAAATGTTCAACTCTGTGAGTTGAGTACACACATCACAAAGAAGTTTCT

                            * *      **     **  *    
      56581 GAGAATGCTTCTGTCTGGCTTTTATTGGAAGACGTTTCCTT
        131 GAGAATGCTTCTGTCTAGTTTTTATATGAAGATATTCCCTT

                   *  **          *       *                * *               
      56622 TT-CAACAAAGGCATCAAAGCGCTCCAAATGTCCACTTCCAGATTCTTCCAAAAGAGTGTTTCAA
          1 TTCCAACGAAATCATCAAAGCGATCCAAATATCCACTTCCAGATTCTACAAAAAGAGTGTTTCAA

             **     * ***  * *                 *    * *  * *     *   *       
      56686 ACGTGCTCGAAGTAAGGGAATGTTCAACTCTGTGACTTGAATGCAGATATCACCAAGTAGTTTCT
         66 AACTGCTCTATCAAAAGAAATGTTCAACTCTGTGAGTTGAGTACACACATCACAAAGAAGTTTCT

            * * *            **     *    *         * 
      56751 AATAGTGCTTCTGTCTACATTTTAGATGATGATATTCCCGT
        131 GAGAATGCTTCTGTCTAGTTTTTATATGAAGATATTCCCTT

                         * * *   *             *  *   *      *     *       * 
      56792 TTCCAACGAAATCGTTAGAGCTATCCAAATATCCAGTTACAGTTTCTACCAAAAGGGTGTTTCCA
          1 TTCCAACGAAATCATCAAAGCGATCCAAATATCCACTTCCAGATTCTACAAAAAGAGTGTTTCAA

              *    **           *            *       *                     * 
      56857 AATTGCTGCATCAAAAGAAAGGTTCAACTCTGTTAGTTGAGGACACACATCACAAAGAAGTTTGT
         66 AACTGCTCTATCAAAAGAAATGTTCAACTCTGTGAGTTGAGTACACACATCACAAAGAAGTTTCT

                              *    *     *           
      56922 GAGAATGCTTCTGTCTAGATTTTGTATGACGATATTCCCTT
        131 GAGAATGCTTCTGTCTAGTTTTTATATGAAGATATTCCCTT

                     *   * *     *   *       * *  *    *  *       *  *       
      56963 TTCCAACGATATCGTTAAAGCAATCTAAATATCAATTTGCAGAATCCACAAAAATAGAGTTTCAA
          1 TTCCAACGAAATCATCAAAGCGATCCAAATATCCACTTCCAGATTCTACAAAAAGAGTGTTTCAA

             *       * *        *    *       *  *                   ***      
      57028 AGCTGCTCTGTAAAAAGAAAGGTTCCACTCTGTTAGCTGAGTACACACATCACAAACTTGTTTCT
         66 AACTGCTCTATCAAAAGAAATGTTCAACTCTGTGAGTTGAGTACACACATCACAAAGAAGTTTCT

                  *         *        **         **   
      57093 GAGAATCCTTCTGTCTCGTTTTTATGGGAAGATATTTACTT
        131 GAGAATGCTTCTGTCTAGTTTTTATATGAAGATATTCCCTT

                 *  * **          *       *     *       *  *  *  *           
      57134 TTCCACCGTAGGCATCAAAGCGCTCCAAATGTCCACATCCAGATACTCCAGAACGAGTGTTTCAA
          1 TTCCAACGAAATCATCAAAGCGATCCAAATATCCACTTCCAGATTCTACAAAAAGAGTGTTTCAA

             *         *   * *   *         *        * *  *      *      *     
      57199 ACCTGCTCTATGAAAGGGAATCTTCAACTCTATGAGTTGAATGCAGACATCAGAAAGAAATTTCT
         66 AACTGCTCTATCAAAAGAAATGTTCAACTCTGTGAGTTGAGTACACACATCACAAAGAAGTTTCT

                     *       **          *         **
      57264 GAGAATGCTGCTGTCTACCTTTTAT-T--TGA-ATTCCCGC
        131 GAGAATGCTTCTGTCTAGTTTTTATATGAAGATATTCCCTT

                         *  *    *              * *  **   *    *             
      57301 TTCCAACGAAATCCTCCAAGCTATCCAAATATCCACCTGCATTTTCCACAACAAGAGTGTTTCAA
          1 TTCCAACGAAATCATCAAAGCGATCCAAATATCCACTTCCAGATTCTACAAAAAGAGTGTTTCAA

                          *                 *    *  *                *       
      57366 AACTGCTCTATCAATAGAAATGTTCAACTCCTTTG-GCTGGGTACACACATCACAAACAAGTTTC
         66 AACTGCTCTATCAAAAGAAATGTTCAACT-CTGTGAGTTGAGTACACACATCACAAAGAAGTTTC

                *                     **     *        
      57430 TGAGCATGCTTCTGTCTAGTTTTTATGGGAAGACATTCCCTT
        130 TGAGAATGCTTCTGTCTAGTTTTTATATGAAGATATTCCCTT

              *  * *  **          *       *            **                    
      57472 TTTCACCAAAGGCATCAAAGCGCTCCAAATGTCCACTTCCAGACACTACAAAAAGAGTGTTTCAA
          1 TTCCAACGAAATCATCAAAGCGATCCAAATATCCACTTCCAGATTCTACAAAAAGAGTGTTTCAA

             **       **    **                 *    * *  * *      *  *       
      57537 ACGTGCTCTAAGAAAACGAATGTTCAACTCTGTGACTTGAATGCAGATATCACAGAGTAGTTTCT
         66 AACTGCTCTATCAAAAGAAATGTTCAACTCTGTGAGTTGAGTACACACATCACAAAGAAGTTTCT

                **            *     *    *         * 
      57602 GAGAGGGCTTCTGTCTAGATTTTAGATGATGATATTCCCGT
        131 GAGAATGCTTCTGTCTAGTTTTTATATGAAGATATTCCCTT

                           * *   *                *   *                    * 
      57643 TTCCAACGAAATCATTAGAGCTATCCAAATATCCACTTACAGTTTCTACAAAAAGAGTGTTTCCA
          1 TTCCAACGAAATCATCAAAGCGATCCAAATATCCACTTCCAGATTCTACAAAAAGAGTGTTTCAA

                   **         * *    *       *  *                   ***      
      57708 AACTGCTGCATCAAAAGAGAGGTTCCACTCTGTTAGCTGAGTACACACATCACAAACTTGTTTCT
         66 AACTGCTCTATCAAAAGAAATGTTCAACTCTGTGAGTTGAGTACACACATCACAAAGAAGTTTCT

            *     *         *        **         **   
      57773 CAGAATCCTTCTGTCTCGTTTTTATGGGAAGATATTTACTT
        131 GAGAATGCTTCTGTCTAGTTTTTATATGAAGATATTCCCTT

              *  *  * **          *       *     *       *  *  *              
      57814 TTTCACCGTAGGCATCAAAGCGCTCCAAATGTCCACATCCAGATACTCCAGAAAGAGTGTTTCAA
          1 TTCCAACGAAATCATCAAAGCGATCCAAATATCCACTTCCAGATTCTACAAAAAGAGTGTTTCAA

             *         *   * *   *         *        * *  *      *      *     
      57879 ACCTGCTCTATGAAAGGGAATCTTCAACTCTATGAGTTGAATGCAGACATCAGAAAGAAATTTCT
         66 AACTGCTCTATCAAAAGAAATGTTCAACTCTGTGAGTTGAGTACACACATCACAAAGAAGTTTCT

                     *       **          *         **
      57944 GAGAATGCTGCTGTCTACCTTTTAT-T--TGA-ATTCCCGC
        131 GAGAATGCTTCTGTCTAGTTTTTATATGAAGATATTCCCTT

                         *  *    *                *       *                  
      57981 TTCCAACGAAATCCTCCAAGCTATCCAAATATCCACTTGCAGATTCCACAAAAAGAGTGTTTCAA
          1 TTCCAACGAAATCATCAAAGCGATCCAAATATCCACTTCCAGATTCTACAAAAAGAGTGTTTCAA

                            ** *  *            *       *     *     *  *      
      58046 AACTGCTCTCTATCAATGGCAAAGTTCAACTCTGTTAGTTGAGGACACATATCACCAACAAGTTT
         66 AACTG--CTCTATCAAAAGAAATGTTCAACTCTGTGAGTTGAGTACACACATCACAAAGAAGTTT

                               *       **         *    
      58111 CTGAGAATGCTTCTGTCTATTTTTTATGGGAAGATATTTCCTT
        129 CTGAGAATGCTTCTGTCTAGTTTTTATATGAAGATATTCCCTT

              *  *  * ** *    *      *    *          * **                    
      58154 TTTCACCGTAGGCGTCAAGGCGATCGAAATGTCCACTTCCACAAACTACAAAAAGAGTGTTTCAA
          1 TTCCAACGAAATCATCAAAGCGATCCAAATATCCACTTCCAGATTCTACAAAAAGAGTGTTTCAA

             *         *   * **       *    *        * ** * *   **      *     
      58219 ACCTGCTCTATGAAAGGCCATGTTCATCTCTATGAGTTGAATGGAAATATCCGAAAGAAATTTCT
         66 AACTGCTCTATCAAAAGAAATGTTCAACTCTGTGAGTTGAGTACACACATCACAAAGAAGTTTCT

             *       *          *        *         **
      58284 GGGAATGCTGCTGTCTAG-TGTT-TAT-ACGA-ATTCCCGC
        131 GAGAATGCTTCTGTCTAGTTTTTATATGAAGATATTCCCTT

                         *       *                *    *  *                  
      58321 TTCCAACGAAATCCTCAAAGCAATCCAAATATCCACTTGCAGAATCCACAAAAAGAGTGTTTCAA
          1 TTCCAACGAAATCATCAAAGCGATCCAAATATCCACTTCCAGATTCTACAAAAAGAGTGTTTCAA

                          *     *          * *                   *  *        
      58386 AACTGCTCTATCAATAGAAAGGTTCAACTCTTTTAGTTGAGTACACACATCACGAACAAGTTTCT
         66 AACTGCTCTATCAAAAGAAATGTTCAACTCTGTGAGTTGAGTACACACATCACAAAGAAGTTTCT

                            * *      **     **  *    
      58451 GAGAATGCTTCTGTCTGGCTTTTATTGGAAGACGTTTCCTT
        131 GAGAATGCTTCTGTCTAGTTTTTATATGAAGATATTCCCTT

                 * *  **          *       *                * *               
      58492 TT-CACCAAAGGCATCAAAGCGCTCCAAATGTCCACTTCCAGATTCTTCCAAAAGAGTGTTTCAA
          1 TTCCAACGAAATCATCAAAGCGATCCAAATATCCACTTCCAGATTCTACAAAAAGAGTGTTTCAA

             **     * ***  * *                 *    * *  * *     *   *       
      58556 ACGTGCTCAAAGTAAGGGAATGTTCAACTCTGTGACTTGAATGCAGATATCACCAAGTAGTTTCT
         66 AACTGCTCTATCAAAAGAAATGTTCAACTCTGTGAGTTGAGTACACACATCACAAAGAAGTTTCT

            * * *             *   * *    *         * 
      58621 AATAGTGCTTCTGTCTAGATTTCAGATGATGATATTCCCGT
        131 GAGAATGCTTCTGTCTAGTTTTTATATGAAGATATTCCCTT

                         * * *   *             *  *   *                    * 
      58662 TTCCAACGAAATCGTTAGAGCTATCCAAATATCCAGTTACAGTTTCTACAAAAAGAGTGTTTCCA
          1 TTCCAACGAAATCATCAAAGCGATCCAAATATCCACTTCCAGATTCTACAAAAAGAGTGTTTCAA

                   **           *            *       *                     * 
      58727 AACTGCTGCATCAAAAGAAAGGTTCAACTCTGTTAGTTGAGGACACACATCACAAAGAAGTTTGT
         66 AACTGCTCTATCAAAAGAAATGTTCAACTCTGTGAGTTGAGTACACACATCACAAAGAAGTTTCT

                              *    *     *           
      58792 GAGAATGCTTCTGTCTAGATTTTGTATGACGATATTCCCTT
        131 GAGAATGCTTCTGTCTAGTTTTTATATGAAGATATTCCCTT

                     *   * *     *   *       * *  *    *  *       *  *       
      58833 TTCCAACGATATCGTTAAAGCAATCTAAATATCAATTTGCAGAATCCACAAAAATAGAGTTTCAA
          1 TTCCAACGAAATCATCAAAGCGATCCAAATATCCACTTCCAGATTCTACAAAAAGAGTGTTTCAA

             *       * *        *    *       *  *                   ***      
      58898 AGCTGCTCTGTAAAAAGAAAGGTTCCACTCTGTTAGCTGAGTACACACATCACAAACTTGTTTCT
         66 AACTGCTCTATCAAAAGAAATGTTCAACTCTGTGAGTTGAGTACACACATCACAAAGAAGTTTCT

                  *         *        **         **   
      58963 GAGAATCCTTCTGTCTCGTTTTTATGGGAAGATATTTACTT
        131 GAGAATGCTTCTGTCTAGTTTTTATATGAAGATATTCCCTT

              *  *  * **          *       *     *       *  *  *     *        
      59004 TTTCACCGTAGGCATCAAAGCGCTCCAAATGTCCACATCCAGATACTCCAGAAAGACTGTTTCAA
          1 TTCCAACGAAATCATCAAAGCGATCCAAATATCCACTTCCAGATTCTACAAAAAGAGTGTTTCAA

             *         *   * *   *         *        * *  *      *      *     
      59069 ACCTGCTCTATGAAAGGGAATCTTCAACTCTATGAGTTGAATGCAGACATCAGAAAGAAATTTCT
         66 AACTGCTCTATCAAAAGAAATGTTCAACTCTGTGAGTTGAGTACACACATCACAAAGAAGTTTCT

                     *       **          *         **
      59134 GAGAATGCTGCTGTCTACCTTTTAT-T--TGA-ATTCCCGC
        131 GAGAATGCTTCTGTCTAGTTTTTATATGAAGATATTCCCTT

                         *  *    *                *  **   *                  
      59171 TTCCAACGAAATCCTCCAAGCTATCCAAATATCCACTTGCATTTTCCACAAAAAGAGTGTTTCAA
          1 TTCCAACGAAATCATCAAAGCGATCCAAATATCCACTTCCAGATTCTACAAAAAGAGTGTTTCAA

                          *               ** *  *  *                *        
      59236 AACTGCTCTATCAATAGAAATGTTCAACTCCTTTAGCTGGGTACACACATCACAAACAAGTTTCT
         66 AACTGCTCTATCAAAAGAAATGTTCAACTCTGTGAGTTGAGTACACACATCACAAAGAAGTTTCT

                                     **     **       
      59301 GAGAATGCTTCTGTCTAGTTTTTATGGGAAGACGTTCCCTT
        131 GAGAATGCTTCTGTCTAGTTTTTATATGAAGATATTCCCTT

              *  * *  **          *       *            **                    
      59342 TTTCACCAAAGGCATCAAAGCGCTCCAAATGTCCACTTCCAGACACTACAAAAAGAGTGTTTCAA
          1 TTCCAACGAAATCATCAAAGCGATCCAAATATCCACTTCCAGATTCTACAAAAAGAGTGTTTCAA

             **       **   ***                 *    * *  * *         *       
      59407 ACGTGCTCTAAGAAAGCGAATGTTCAACTCTGTGACTTGAATGCAGATATCACAAAGTAGTTTCT
         66 AACTGCTCTATCAAAAGAAATGTTCAACTCTGTGAGTTGAGTACACACATCACAAAGAAGTTTCT

                **            *     *    *         * 
      59472 GAGAGGGCTTCTGTCTAGATTTTAGATGATGATATTCCCGT
        131 GAGAATGCTTCTGTCTAGTTTTTATATGAAGATATTCCCTT

                           * *   *                *   *                    * 
      59513 TTCCAACGAAATCATTAGAGCTATCCAAATATCCACTTACAGTTTCTACAAAAAGAGTGTTTCCA
          1 TTCCAACGAAATCATCAAAGCGATCCAAATATCCACTTCCAGATTCTACAAAAAGAGTGTTTCAA

                   **         * *    *       *  **                  ***      
      59578 AACTGCTGCATCAAAAGAGAGGTTCCACTCTGTTAGCCGAGTACACACATCACAAACTTGTTTCT
         66 AACTGCTCTATCAAAAGAAATGTTCAACTCTGTGAGTTGAGTACACACATCACAAAGAAGTTTCT

            *     *         *        **         **   
      59643 CAGAATCCTTCTGTCTCGTTTTTATGGGAAGATATTTACTT
        131 GAGAATGCTTCTGTCTAGTTTTTATATGAAGATATTCCCTT

              *  *  * **          *       *     *       *  *  *              
      59684 TTTCACCGTAGGCATCAAAGCGCTCCAAATGTCCACATCCAGATACTCCAGAAAGAGTGTTTCAA
          1 TTCCAACGAAATCATCAAAGCGATCCAAATATCCACTTCCAGATTCTACAAAAAGAGTGTTTCAA

             *         *   * *   *         *        * *  *      *      *     
      59749 ACCTGCTCTATGAAAGGGAATCTTCAACTCTATGAGTTGAATGCAGACATCAGAAAGAAATTTCT
         66 AACTGCTCTATCAAAAGAAATGTTCAACTCTGTGAGTTGAGTACACACATCACAAAGAAGTTTCT

                     *       **          *         **
      59814 GAGAATGCTGCTGTCTACCTTTTAT-T--TGA-ATTCCCGC
        131 GAGAATGCTTCTGTCTAGTTTTTATATGAAGATATTCCCTT

                         *  *    *                *       *                  
      59851 TTCCAACGAAATCCTCCAAGCTATCCAAATATCCACTTGCAGATTCCACAAAAAGAGTGTTTCAA
          1 TTCCAACGAAATCATCAAAGCGATCCAAATATCCACTTCCAGATTCTACAAAAAGAGTGTTTCAA

                            ** *  *            *       *     *     *  *      
      59916 AACTGCTCTCTATCAATGGCAAAGTTCAACTCTGTTAGTTGAGGACACATATCACCAACAAGTTT
         66 AACTG--CTCTATCAAAAGAAATGTTCAACTCTGTGAGTTGAGTACACACATCACAAAGAAGTTT

                               *       **         *    
      59981 CTGAGAATGCTTCTGTCTATTTTTTATGGGAAGATATTTCCTT
        129 CTGAGAATGCTTCTGTCTAGTTTTTATATGAAGATATTCCCTT

              *  *  * ** *    *      *    *          * **                    
      60024 TTTCACCGTAGGCGTCAAGGCGATCGAAATGTCCACTTCCACAAACTACAAAAAGAGTGTTTCAA
          1 TTCCAACGAAATCATCAAAGCGATCCAAATATCCACTTCCAGATTCTACAAAAAGAGTGTTTCAA

             *         *   * **       *    *        * ** * *   **      *     
      60089 ACCTGCTCTATGAAAGGCCATGTTCATCTCTATGAGTTGAATGGAAATATCCGAAAGAAATTTCT
         66 AACTGCTCTATCAAAAGAAATGTTCAACTCTGTGAGTTGAGTACACACATCACAAAGAAGTTTCT

             *       *          *        *         **
      60154 GGGAATGCTGCTGTCTAG-TGTT-TAT-ACGA-ATTCCCGC
        131 GAGAATGCTTCTGTCTAGTTTTTATATGAAGATATTCCCTT

                         *       *                *    *  *                  
      60191 TTCCAACGAAATCCTCAAAGCAATCCAAATATCCACTTGCAGAATCCACAAAAAGAGTGTTTCAA
          1 TTCCAACGAAATCATCAAAGCGATCCAAATATCCACTTCCAGATTCTACAAAAAGAGTGTTTCAA

                          *     *          * *                   *  *        
      60256 AACTGCTCTATCAATAGAAAGGTTCAACTCTTTTAGTTGAGTACACACATCACGAACAAGTTTCT
         66 AACTGCTCTATCAAAAGAAATGTTCAACTCTGTGAGTTGAGTACACACATCACAAAGAAGTTTCT

                            * *      **     **  *    
      60321 GAGAATGCTTCTGTCTGGCTTTTATTGGAAGACGTTTCCTT
        131 GAGAATGCTTCTGTCTAGTTTTTATATGAAGATATTCCCTT

                   *  **          *       *                * *               
      60362 TT-CAACAAAGGCATCAAAGCGCTCCAAATGTCCACTTCCAGATTCTTCCAAAAGAGTGTTTCAA
          1 TTCCAACGAAATCATCAAAGCGATCCAAATATCCACTTCCAGATTCTACAAAAAGAGTGTTTCAA

             **     * ***  * *                 *    * *  * *     *   *       
      60426 ACGTGCTCGAAGTAAGGGAATGTTCAACTCTGTGACTTGAATGCAGATATCACCAAGTAGTTTCT
         66 AACTGCTCTATCAAAAGAAATGTTCAACTCTGTGAGTTGAGTACACACATCACAAAGAAGTTTCT

            * * *            **     *    *         * 
      60491 AATAGTGCTTCTGTCTACATTTTAGATGATGATATTCCCGT
        131 GAGAATGCTTCTGTCTAGTTTTTATATGAAGATATTCCCTT

                         * * *   * **          *  *   *      *     *       * 
      60532 TTCCAACGAAATCGTTAGAGCTAAGCAAATATCCAGTTACAGTTTCTACCAAAAGGGTGTTTCCA
          1 TTCCAACGAAATCATCAAAGCGATCCAAATATCCACTTCCAGATTCTACAAAAAGAGTGTTTCAA

              *    **           *            *       *                     * 
      60597 AATTGCTGCATCAAAAGAAAGGTTCAACTCTGTTAGTTGAGGACACACATCACAAAGAAGTTTGT
         66 AACTGCTCTATCAAAAGAAATGTTCAACTCTGTGAGTTGAGTACACACATCACAAAGAAGTTTCT

                              *    *     *           
      60662 GAGAATGCTTCTGTCTAGATTTTGTATGACGATATTCCCTT
        131 GAGAATGCTTCTGTCTAGTTTTTATATGAAGATATTCCCTT

                     *   * *     *   *       * *  *    *  *       *  *       
      60703 TTCCAACGATATCGTTAAAGCAATCTAAATATCAATTTGCAGAATCCACAAAAATAGAGTTTCAA
          1 TTCCAACGAAATCATCAAAGCGATCCAAATATCCACTTCCAGATTCTACAAAAAGAGTGTTTCAA

             *       * *        *    *       *  *                   ***      
      60768 AGCTGCTCTGTAAAAAGAAAGGTTCCACTCTGTTAGCTGAGTACACACATCACAAACTTGTTTCT
         66 AACTGCTCTATCAAAAGAAATGTTCAACTCTGTGAGTTGAGTACACACATCACAAAGAAGTTTCT

                  *         *        **         **   
      60833 GAGAATCCTTCTGTCTCGTTTTTATGGGAAGATATTTACTT
        131 GAGAATGCTTCTGTCTAGTTTTTATATGAAGATATTCCCTT

                 *  * **          *       *     *       *  *  *  *           
      60874 TTCCACCGTAGGCATCAAAGCGCTCCAAATGTCCACATCCAGATACTCCAGAACGAGTGTTTCAA
          1 TTCCAACGAAATCATCAAAGCGATCCAAATATCCACTTCCAGATTCTACAAAAAGAGTGTTTCAA

             *         *   * *   *         *        * *  *      *      *     
      60939 ACCTGCTCTATGAAAGGGAATCTTCAACTCTATGAGTTGAATGCAGACATCAGAAAGAAATTTCT
         66 AACTGCTCTATCAAAAGAAATGTTCAACTCTGTGAGTTGAGTACACACATCACAAAGAAGTTTCT

                     *       **          *         **
      61004 GAGAATGCTGCTGTCTACCTTTTAT-T--TGA-ATTCCCGC
        131 GAGAATGCTTCTGTCTAGTTTTTATATGAAGATATTCCCTT

                         *  *    *              * *  **   *    *             
      61041 TTCCAACGAAATCCTCCAAGCTATCCAAATATCCACCTGCATTTTCCACAACAAGAGTGTTTCAA
          1 TTCCAACGAAATCATCAAAGCGATCCAAATATCCACTTCCAGATTCTACAAAAAGAGTGTTTCAA

                          *                 *    *  *                *       
      61106 AACTGCTCTATCAATAGAAATGTTCAACTCCTTTG-GCTGGGTACACACATCACAAACAAGTTTC
         66 AACTGCTCTATCAAAAGAAATGTTCAACT-CTGTGAGTTGAGTACACACATCACAAAGAAGTTTC

                *                     **     *        
      61170 TGAGCATGCTTCTGTCTAGTTTTTATGGGAAGACATTCCCTT
        130 TGAGAATGCTTCTGTCTAGTTTTTATATGAAGATATTCCCTT

              *  * *  **          *       *            **                    
      61212 TTTCACCAAAGGCATCAAAGCGCTCCAAATGTCCACTTCCAGACACTACAAAAAGAGTGTTTCAA
          1 TTCCAACGAAATCATCAAAGCGATCCAAATATCCACTTCCAGATTCTACAAAAAGAGTGTTTCAA

             **       **    **                 *    * *  * *      *  *       
      61277 ACGTGCTCTAAGAAAACGAATGTTCAACTCTGTGACTTGAATGCAGATATCACAGAGTAGTTTCT
         66 AACTGCTCTATCAAAAGAAATGTTCAACTCTGTGAGTTGAGTACACACATCACAAAGAAGTTTCT

                **            *     *    *         * 
      61342 GAGAGGGCTTCTGTCTAGATTTTAGATGATGATATTCCCGT
        131 GAGAATGCTTCTGTCTAGTTTTTATATGAAGATATTCCCTT

                           * *   *                *   *                    * 
      61383 TTCCAACGAAATCATTAGAGCTATCCAAATATCCACTTACAGTTTCTACAAAAAGAGTGTTTCCA
          1 TTCCAACGAAATCATCAAAGCGATCCAAATATCCACTTCCAGATTCTACAAAAAGAGTGTTTCAA

                   **         * *    *       *  *                   ***      
      61448 AACTGCTGCATCAAAAGAGAGGTTCCACTCTGTTAGCTGAGTACACACATCACAAACTTGTTTCT
         66 AACTGCTCTATCAAAAGAAATGTTCAACTCTGTGAGTTGAGTACACACATCACAAAGAAGTTTCT

            *     *         *        **         **   
      61513 CAGAATCCTTCTGTCTCGTTTTTATGGGAAGATATTTACTT
        131 GAGAATGCTTCTGTCTAGTTTTTATATGAAGATATTCCCTT

              *  *  * **          *       *     *       *  *  *              
      61554 TTTCACCGTAGGCATCAAAGCGCTCCAAATGTCCACATCCAGATACTCCAGAAAGAGTGTTTCAA
          1 TTCCAACGAAATCATCAAAGCGATCCAAATATCCACTTCCAGATTCTACAAAAAGAGTGTTTCAA

             *         *   * *   *         *        * *  *      *      *     
      61619 ACCTGCTCTATGAAAGGGAATCTTCAACTCTATGAGTTGAATGCAGACATCAGAAAGAAATTTCT
         66 AACTGCTCTATCAAAAGAAATGTTCAACTCTGTGAGTTGAGTACACACATCACAAAGAAGTTTCT

                     *       **          *         **
      61684 GAGAATGCTGCTGTCTACCTTTTAT-T--TGA-ATTCCCGC
        131 GAGAATGCTTCTGTCTAGTTTTTATATGAAGATATTCCCTT

                         *  *    *                *       *                  
      61721 TTCCAACGAAATCCTCCAAGCTATCCAAATATCCACTTGCAGATTCCACAAAAAGAGTGTTTCAA
          1 TTCCAACGAAATCATCAAAGCGATCCAAATATCCACTTCCAGATTCTACAAAAAGAGTGTTTCAA

                            ** *  *            *       *     *     *  *      
      61786 AACTGCTCTCTATCAATGGCAAAGTTCAACTCTGTTAGTTGAGGACACATATCACCAACAAGTTT
         66 AACTG--CTCTATCAAAAGAAATGTTCAACTCTGTGAGTTGAGTACACACATCACAAAGAAGTTT

                               *       **         *    
      61851 CTGAGAATGCTTCTGTCTATTTTTTATGGGAAGATATTTCCTT
        129 CTGAGAATGCTTCTGTCTAGTTTTTATATGAAGATATTCCCTT

              *  *  * ** *    *      *    *          * **                    
      61894 TTTCACCGTAGGCGTCAAGGCGATCGAAATGTCCACTTCCACAAACTACAAAAAGAGTGTTTCAA
          1 TTCCAACGAAATCATCAAAGCGATCCAAATATCCACTTCCAGATTCTACAAAAAGAGTGTTTCAA

             *         *   * **       *    *        * ** * *   **      *     
      61959 ACCTGCTCTATGAAAGGCCATGTTCATCTCTATGAGTTGAATGGAAATATCCGAAAGAAATTTCT
         66 AACTGCTCTATCAAAAGAAATGTTCAACTCTGTGAGTTGAGTACACACATCACAAAGAAGTTTCT

             *       *          *        *         **
      62024 GGGAATGCTGCTGTCTAG-TGTT-TAT-ACGA-ATTCCCGC
        131 GAGAATGCTTCTGTCTAGTTTTTATATGAAGATATTCCCTT

                         *       *                *    *  *                  
      62061 TTCCAACGAAATCCTCAAAGCAATCCAAATATCCACTTGCAGAATCCACAAAAAGAGTGTTTCAA
          1 TTCCAACGAAATCATCAAAGCGATCCAAATATCCACTTCCAGATTCTACAAAAAGAGTGTTTCAA

                          *     *          * *                   *  *        
      62126 AACTGCTCTATCAATAGAAAGGTTCAACTCTTTTAGTTGAGTACACACATCACGAACAAGTTTCT
         66 AACTGCTCTATCAAAAGAAATGTTCAACTCTGTGAGTTGAGTACACACATCACAAAGAAGTTTCT

                            * *      **     **  *    
      62191 GAGAATGCTTCTGTCTGGCTTTTATTGGAAGACGTTTCCTT
        131 GAGAATGCTTCTGTCTAGTTTTTATATGAAGATATTCCCTT

                 * *  **          *       *                * *               
      62232 TT-CACCAAAGGCATCAAAGCGCTCCAAATGTCCACTTCCAGATTCTTCCAAAAGAGTGTTTCAA
          1 TTCCAACGAAATCATCAAAGCGATCCAAATATCCACTTCCAGATTCTACAAAAAGAGTGTTTCAA

             **     * ***  * *                 *    * *  * *     *   *       
      62296 ACGTGCTCAAAGTAAGGGAATGTTCAACTCTGTGACTTGAATGCAGATATCACCAAGTAGTTTCT
         66 AACTGCTCTATCAAAAGAAATGTTCAACTCTGTGAGTTGAGTACACACATCACAAAGAAGTTTCT

            * * *             *     *    *         * 
      62361 AATAGTGCTTCTGTCTAGATTTTAGATGATGATATTCCCGT
        131 GAGAATGCTTCTGTCTAGTTTTTATATGAAGATATTCCCTT

                         * * *   *                *   *                    * 
      62402 TTCCAACGAAATCGTTAGAGCTATCCAAATATCCACTTACAGTTTCTACAAAAAGAGTGTTTCCA
          1 TTCCAACGAAATCATCAAAGCGATCCAAATATCCACTTCCAGATTCTACAAAAAGAGTGTTTCAA

                   **           *            *       *                     * 
      62467 AACTGCTGCATCAAAAGAAAGGTTCAACTCTGTTAGTTGAGGACACACATCACAAAGAAGTTTGT
         66 AACTGCTCTATCAAAAGAAATGTTCAACTCTGTGAGTTGAGTACACACATCACAAAGAAGTTTCT

                              *    *     *           
      62532 GAGAATGCTTCTGTCTAGATTTTGTATGACGATATTCCCTT
        131 GAGAATGCTTCTGTCTAGTTTTTATATGAAGATATTCCCTT

                     *   * *     *   *       * *  *    *  *       *  *       
      62573 TTCCAACGATATCGTTAAAGCAATCTAAATATCAATTTGCAGAATCCACAAAAATAGAGTTTCAA
          1 TTCCAACGAAATCATCAAAGCGATCCAAATATCCACTTCCAGATTCTACAAAAAGAGTGTTTCAA

             *       * *    *   *    *       *  *                   ***      
      62638 AGCTGCTCTGTAAAAATAAAAGTTCCACTCTGTTAGCTGAGTACACACATCACAAACTTGTTTCT
         66 AACTGCTCTATCAAAAGAAATGTTCAACTCTGTGAGTTGAGTACACACATCACAAAGAAGTTTCT

                  *         *        **         **   
      62703 GAGAATCCTTCTGTCTCGTTTTTATGGGAAGATATTTACTT
        131 GAGAATGCTTCTGTCTAGTTTTTATATGAAGATATTCCCTT

              *  *  * **          *       *     *       *  *  *     *        
      62744 TTTCACCGTAGGCATCAAAGCGCTCCAAATGTCCACATCCAGATACTCCAGAAAGACTGTTTCAA
          1 TTCCAACGAAATCATCAAAGCGATCCAAATATCCACTTCCAGATTCTACAAAAAGAGTGTTTCAA

             *         *   * *   *         *        * *  *      *      *     
      62809 ACCTGCTCTATGAAAGGGAATCTTCAACTCTATGAGTTGAATGCAGACATCAGAAAGAAATTTCT
         66 AACTGCTCTATCAAAAGAAATGTTCAACTCTGTGAGTTGAGTACACACATCACAAAGAAGTTTCT

                     *       **          *         **
      62874 GAGAATGCTGCTGTCTACCTTTTAT-T--TGA-ATTCCCGC
        131 GAGAATGCTTCTGTCTAGTTTTTATATGAAGATATTCCCTT

                         *  *    *              * *  **   *                  
      62911 TTCCAACGAAATCCTCCAAGCTATCCAAATATCCACCTGCATTTTCCACAAAAAGAGTGTTTCAA
          1 TTCCAACGAAATCATCAAAGCGATCCAAATATCCACTTCCAGATTCTACAAAAAGAGTGTTTCAA

                          *                 *    *  *                *       
      62976 AACTGCTCTATCAATAGAAATGTTCAACTCCTTTG-GCTGGGTACACACATCACAAACAAGTTTC
         66 AACTGCTCTATCAAAAGAAATGTTCAACT-CTGTGAGTTGAGTACACACATCACAAAGAAGTTTC

                                      **     **       
      63040 TGAGAATGCTTCTGTCTAGTTTTTATGGGAAGACGTTCCCTT
        130 TGAGAATGCTTCTGTCTAGTTTTTATATGAAGATATTCCCTT

              *  * *  **          *       *            **                    
      63082 TTTCACCAAAGGCATCAAAGCGCTCCAAATGTCCACTTCCAGACACTACAAAAAGAGTGTTTCAA
          1 TTCCAACGAAATCATCAAAGCGATCCAAATATCCACTTCCAGATTCTACAAAAAGAGTGTTTCAA

             **       **   ***                 *    * *  * *         *       
      63147 ACGTGCTCTAAGAAAGCGAATGTTCAACTCTGTGACTTGAATGCAGATATCACAAAGTAGTTTCT
         66 AACTGCTCTATCAAAAGAAATGTTCAACTCTGTGAGTTGAGTACACACATCACAAAGAAGTTTCT

                *             *     * *  *         * 
      63212 GAGAGTGCTTCTGTCTAGATTTTAGACGATGATATTCCCGT
        131 GAGAATGCTTCTGTCTAGTTTTTATATGAAGATATTCCCTT

                           * *   *                *   *                    * 
      63253 TTCCAACGAAATCATTAGAGCTATCCAAATATCCACTTACAGTTTCTACAAAAAGAGTGTTTCCA
          1 TTCCAACGAAATCATCAAAGCGATCCAAATATCCACTTCCAGATTCTACAAAAAGAGTGTTTCAA

                   **         * *    *       *  *                   ***      
      63318 AACTGCTGCATCAAAAGAGAGGTTCCACTCTGTTAGCTGAGTACACACATCACAAACTTGTTTCT
         66 AACTGCTCTATCAAAAGAAATGTTCAACTCTGTGAGTTGAGTACACACATCACAAAGAAGTTTCT

            *     *         *        **         **   
      63383 CAGAATCCTTCTGTCTCGTTTTTATGGGAAGATATTTACTT
        131 GAGAATGCTTCTGTCTAGTTTTTATATGAAGATATTCCCTT

              *  *  * **          *       *     *       **    *       *      
      63424 TTTCACCGTAGGCATCAAAGCGCTCCAAATGTCCACATCCAGATAGTACAGAAAGAGTTTTTCAA
          1 TTCCAACGAAATCATCAAAGCGATCCAAATATCCACTTCCAGATTCTACAAAAAGAGTGTTTCAA

             *         *   * *   *         *        * *  *      *      *     
      63489 ACCTGCTCTATGAAAGGGAATCTTCAACTCTATGAGTTGAATGCAGACATCAGAAAGAAATTTCT
         66 AACTGCTCTATCAAAAGAAATGTTCAACTCTGTGAGTTGAGTACACACATCACAAAGAAGTTTCT

                     *       **          *         **
      63554 GAGAATGCTGCTGTCTACCTTTTAT-T--TGA-ATTCCCGC
        131 GAGAATGCTTCTGTCTAGTTTTTATATGAAGATATTCCCTT

                         *  *    *                *       *                  
      63591 TTCCAACGAAATCCTCCAAGCTATCCAAATATCCACTTGCAGATTCCACAAAAAGAGTGTTTCAA
          1 TTCCAACGAAATCATCAAAGCGATCCAAATATCCACTTCCAGATTCTACAAAAAGAGTGTTTCAA

                            ** *  *            *       *     *     *  *      
      63656 AACTGCTCTCTATCAATGGCAAAGTTCAACTCTGTTAGTTGAGGACACATATCACCAACAAGTTT
         66 AACTG--CTCTATCAAAAGAAATGTTCAACTCTGTGAGTTGAGTACACACATCACAAAGAAGTTT

                               *       **         *    
      63721 CTGAGAATGCTTCTGTCTATTTTTTATGGGAAGATATTTCCTT
        129 CTGAGAATGCTTCTGTCTAGTTTTTATATGAAGATATTCCCTT

              *  *  * ** *    *      *    *          * **                    
      63764 TTTCACCGTAGGCGTCAAGGCGATCGAAATGTCCACTTCCACAAACTACAAAAAGAGTGTTTCAA
          1 TTCCAACGAAATCATCAAAGCGATCCAAATATCCACTTCCAGATTCTACAAAAAGAGTGTTTCAA

             *         *   * **       *    *        * ** * *   **      *     
      63829 ACCTGCTCTATGAAAGGCCATGTTCATCTCTATGAGTTGAATGGAAATATCCGAAAGAAATTTCT
         66 AACTGCTCTATCAAAAGAAATGTTCAACTCTGTGAGTTGAGTACACACATCACAAAGAAGTTTCT

             *       *          *        *         **
      63894 GGGAATGCTGCTGTCTAG-TGTT-TAT-ACGA-ATTCCCGC
        131 GAGAATGCTTCTGTCTAGTTTTTATATGAAGATATTCCCTT

                         *       *                *    *  *                  
      63931 TTCCAACGAAATCCTCAAAGCAATCCAAATATCCACTTGCAGAATCCACAAAAAGAGTGTTTCAA
          1 TTCCAACGAAATCATCAAAGCGATCCAAATATCCACTTCCAGATTCTACAAAAAGAGTGTTTCAA

                          *     *          * *                   *  *        
      63996 AACTGCTCTATCAATAGAAAGGTTCAACTCTTTTAGTTGAGTACACACATCACGAACAAGTTTCT
         66 AACTGCTCTATCAAAAGAAATGTTCAACTCTGTGAGTTGAGTACACACATCACAAAGAAGTTTCT

                            * *      **     **  *    
      64061 GAGAATGCTTCTGTCTGGCTTTTATTGGAAGACGTTTCCTT
        131 GAGAATGCTTCTGTCTAGTTTTTATATGAAGATATTCCCTT

                   *  **          *       *                * *               
      64102 TT-CAACAAAGGCATCAAAGCGCTCCAAATGTCCACTTCCAGATTCTTCCAAAAGAGTGTTTCAA
          1 TTCCAACGAAATCATCAAAGCGATCCAAATATCCACTTCCAGATTCTACAAAAAGAGTGTTTCAA

             **     * ***  * *                 *    * *  * *     *   *       
      64166 ACGTGCTCGAAGTAAGGGAATGTTCAACTCTGTGACTTGAATGCAGATATCACCAAGTAGTTTCT
         66 AACTGCTCTATCAAAAGAAATGTTCAACTCTGTGAGTTGAGTACACACATCACAAAGAAGTTTCT

            * * *            **     *    *         * 
      64231 AATAGTGCTTCTGTCTACATTTTAGATGATGATATTCCCGT
        131 GAGAATGCTTCTGTCTAGTTTTTATATGAAGATATTCCCTT

                         * * *   *             *  *   *      *     *       * 
      64272 TTCCAACGAAATCGTTAGAGCTATCCAAATATCCAGTTACAGTTTCTACCAAAAGGGTGTTTCCA
          1 TTCCAACGAAATCATCAAAGCGATCCAAATATCCACTTCCAGATTCTACAAAAAGAGTGTTTCAA

              *    **           *            *       *                     * 
      64337 AATTGCTGCATCAAAAGAAAGGTTCAACTCTGTTAGTTGAGGACACACATCACAAAGAAGTTTGT
         66 AACTGCTCTATCAAAAGAAATGTTCAACTCTGTGAGTTGAGTACACACATCACAAAGAAGTTTCT

                              *    *     *           
      64402 GAGAATGCTTCTGTCTAGATTTTGTATGACGATATTCCCTT
        131 GAGAATGCTTCTGTCTAGTTTTTATATGAAGATATTCCCTT

                     *   * *     *   *       * *  *    *  *       *  *       
      64443 TTCCAACGATATCGTTAAAGCAATCTAAATATCAATTTGCAGAATCCACAAAAATAGAGTTTCAA
          1 TTCCAACGAAATCATCAAAGCGATCCAAATATCCACTTCCAGATTCTACAAAAAGAGTGTTTCAA

             *       * *        *    *       *  *                   ***      
      64508 AGCTGCTCTGTAAAAAGAAAGGTTCCACTCTGTTAGCTGAGTACACACATCACAAACTTGTTTCT
         66 AACTGCTCTATCAAAAGAAATGTTCAACTCTGTGAGTTGAGTACACACATCACAAAGAAGTTTCT

                  *         *        **         **   
      64573 GAGAATCCTTCTGTCTCGTTTTTATGGGAAGATATTTACTT
        131 GAGAATGCTTCTGTCTAGTTTTTATATGAAGATATTCCCTT

                 *  * **          *       *     *       *  *  *  *           
      64614 TTCCACCGTAGGCATCAAAGCGCTCCAAATGTCCACATCCAGATACTCCAGAACGAGTGTTTCAA
          1 TTCCAACGAAATCATCAAAGCGATCCAAATATCCACTTCCAGATTCTACAAAAAGAGTGTTTCAA

             *         *   * *   *         *        * *  *      *      *     
      64679 ACCTGCTCTATGAAAGGGAATCTTCAACTCTATGAGTTGAATGCAGACATCAGAAAGAAATTTCT
         66 AACTGCTCTATCAAAAGAAATGTTCAACTCTGTGAGTTGAGTACACACATCACAAAGAAGTTTCT

                     *       **          *         **
      64744 GAGAATGCTGCTGTCTACCTTTTAT-T--TGA-ATTCCCGC
        131 GAGAATGCTTCTGTCTAGTTTTTATATGAAGATATTCCCTT

                         *  *    *              * *  **   *    *             
      64781 TTCCAACGAAATCCTCCAAGCTATCCAAATATCCACCTGCATTTTCCACAACAAGAGTGTTTCAA
          1 TTCCAACGAAATCATCAAAGCGATCCAAATATCCACTTCCAGATTCTACAAAAAGAGTGTTTCAA

                          *                 *    *  *                *       
      64846 AACTGCTCTATCAATAGAAATGTTCAACTCCTTTG-GCTGGGTACACACATCACAAACAAGTTTC
         66 AACTGCTCTATCAAAAGAAATGTTCAACT-CTGTGAGTTGAGTACACACATCACAAAGAAGTTTC

                *                     **     *        
      64910 TGAGCATGCTTCTGTCTAGTTTTTATGGGAAGACATTCCCTT
        130 TGAGAATGCTTCTGTCTAGTTTTTATATGAAGATATTCCCTT

              *  * *  **          *       *            **                    
      64952 TTTCACCAAAGGCATCAAAGCGCTCCAAATGTCCACTTCCAGACACTACAAAAAGAGTGTTTCAA
          1 TTCCAACGAAATCATCAAAGCGATCCAAATATCCACTTCCAGATTCTACAAAAAGAGTGTTTCAA

             **       **    **                 *    * *  * *      *  *       
      65017 ACGTGCTCTAAGAAAACGAATGTTCAACTCTGTGACTTGAATGCAGATATCACAGAGTAGTTTCT
         66 AACTGCTCTATCAAAAGAAATGTTCAACTCTGTGAGTTGAGTACACACATCACAAAGAAGTTTCT

                **            *     *    *         * 
      65082 GAGAGGGCTTCTGTCTAGATTTTAGATGATGATATTCCCGT
        131 GAGAATGCTTCTGTCTAGTTTTTATATGAAGATATTCCCTT

                           * *   *                *   *                    * 
      65123 TTCCAACGAAATCATTAGAGCTATCCAAATATCCACTTACAGTTTCTACAAAAAGAGTGTTTCCA
          1 TTCCAACGAAATCATCAAAGCGATCCAAATATCCACTTCCAGATTCTACAAAAAGAGTGTTTCAA

                   **         * *    *       *  *                   ***      
      65188 AACTGCTGCATCAAAAGAGAGGTTCCACTCTGTTAGCTGAGTACACACATCACAAACTTGTTTCT
         66 AACTGCTCTATCAAAAGAAATGTTCAACTCTGTGAGTTGAGTACACACATCACAAAGAAGTTTCT

            *     *         *        **         **   
      65253 CAGAATCCTTCTGTCTCGTTTTTATGGGAAGATATTTACTT
        131 GAGAATGCTTCTGTCTAGTTTTTATATGAAGATATTCCCTT

              *  *  * **          *       *     *       *  *  *              
      65294 TTTCACCGTAGGCATCAAAGCGCTCCAAATGTCCACATCCAGATACTCCAGAAAGAGTGTTTCAA
          1 TTCCAACGAAATCATCAAAGCGATCCAAATATCCACTTCCAGATTCTACAAAAAGAGTGTTTCAA

             *         *   * *   *         *        * *  *      *      *     
      65359 ACCTGCTCTATGAAAGGGAATCTTCAACTCTATGAGTTGAATGCAGACATCAGAAAGAAATTTCT
         66 AACTGCTCTATCAAAAGAAATGTTCAACTCTGTGAGTTGAGTACACACATCACAAAGAAGTTTCT

                     *       **          *         **
      65424 GAGAATGCTGCTGTCTACCTTTTAT-T--TGA-ATTCCCGC
        131 GAGAATGCTTCTGTCTAGTTTTTATATGAAGATATTCCCTT

                         *  *    *                *       *                  
      65461 TTCCAACGAAATCCTCCAAGCTATCCAAATATCCACTTGCAGATTCCACAAAAAGAGTGTTTCAA
          1 TTCCAACGAAATCATCAAAGCGATCCAAATATCCACTTCCAGATTCTACAAAAAGAGTGTTTCAA

                            ** *  *            *       *     *     *  *      
      65526 AACTGCTCTCTATCAATGGCAAAGTTCAACTCTGTTAGTTGAGGACACATATCACCAACAAGTTT
         66 AACTG--CTCTATCAAAAGAAATGTTCAACTCTGTGAGTTGAGTACACACATCACAAAGAAGTTT

                               *       **         *    
      65591 CTGAGAATGCTTCTGTCTATTTTTTATGGGAAGATATTTCCTT
        129 CTGAGAATGCTTCTGTCTAGTTTTTATATGAAGATATTCCCTT

              *  *  * ** *    *      *    *          * **                    
      65634 TTTCACCGTAGGCGTCAAGGCGATCGAAATGTCCACTTCCACAAACTACAAAAAGAGTGTTTCAA
          1 TTCCAACGAAATCATCAAAGCGATCCAAATATCCACTTCCAGATTCTACAAAAAGAGTGTTTCAA

             *         *   * **       *    *        * ** * *   **      *     
      65699 ACCTGCTCTATGAAAGGCCATGTTCATCTCTATGAGTTGAATGGAAATATCCGAAAGAAATTTCT
         66 AACTGCTCTATCAAAAGAAATGTTCAACTCTGTGAGTTGAGTACACACATCACAAAGAAGTTTCT

             *       *          *        *         **
      65764 GGGAATGCTGCTGTCTAG-TGTT-TAT-ACGA-ATTCCCGC
        131 GAGAATGCTTCTGTCTAGTTTTTATATGAAGATATTCCCTT

                         *       *                *    *  *                  
      65801 TTCCAACGAAATCCTCAAAGCAATCCAAATATCCACTTGCAGAATCCACAAAAAGAGTGTTTCAA
          1 TTCCAACGAAATCATCAAAGCGATCCAAATATCCACTTCCAGATTCTACAAAAAGAGTGTTTCAA

                          *     *          * *                   *  *        
      65866 AACTGCTCTATCAATAGAAAGGTTCAACTCTTTTAGTTGAGTACACACATCACGAACAAGTTTCT
         66 AACTGCTCTATCAAAAGAAATGTTCAACTCTGTGAGTTGAGTACACACATCACAAAGAAGTTTCT

                            * *      **     **  *    
      65931 GAGAATGCTTCTGTCTGGCTTTTATTGGAAGACGTTTCCTT
        131 GAGAATGCTTCTGTCTAGTTTTTATATGAAGATATTCCCTT

                 * *  **          *       *                * *               
      65972 TT-CACCAAAGGCATCAAAGCGCTCCAAATGTCCACTTCCAGATTCTTCCAAAAGAGTGTTTCAA
          1 TTCCAACGAAATCATCAAAGCGATCCAAATATCCACTTCCAGATTCTACAAAAAGAGTGTTTCAA

             **     * ***  * *                 *    * *  * *     *   *       
      66036 ACGTGCTCAAAGTAAGGGAATGTTCAACTCTGTGACTTGAATGCAGATATCACCAAGTAGTTTCT
         66 AACTGCTCTATCAAAAGAAATGTTCAACTCTGTGAGTTGAGTACACACATCACAAAGAAGTTTCT

            * * *             *   * *    *         * 
      66101 AATAGTGCTTCTGTCTAGATTTCAGATGATGATATTCCCGT
        131 GAGAATGCTTCTGTCTAGTTTTTATATGAAGATATTCCCTT

                         * * *   *             *  *   *                    * 
      66142 TTCCAACGAAATCGTTAGAGCTATCCAAATATCCAGTTACAGTTTCTACAAAAAGAGTGTTTCCA
          1 TTCCAACGAAATCATCAAAGCGATCCAAATATCCACTTCCAGATTCTACAAAAAGAGTGTTTCAA

                   **           *            *       *                     * 
      66207 AACTGCTGCATCAAAAGAAAGGTTCAACTCTGTTAGTTGAGGACACACATCACAAAGAAGTTTGT
         66 AACTGCTCTATCAAAAGAAATGTTCAACTCTGTGAGTTGAGTACACACATCACAAAGAAGTTTCT

                              *    *     *           
      66272 GAGAATGCTTCTGTCTAGATTTTGTATGACGATATTCCCTT
        131 GAGAATGCTTCTGTCTAGTTTTTATATGAAGATATTCCCTT

                     *   * *     *   *       * *  *    *  *       *  *       
      66313 TTCCAACGATATCGTTAAAGCAATCTAAATATCAATTTGCAGAATCCACAAAAATAGAGTTTCAA
          1 TTCCAACGAAATCATCAAAGCGATCCAAATATCCACTTCCAGATTCTACAAAAAGAGTGTTTCAA

             *       * *        *    *       *  *                   ***      
      66378 AGCTGCTCTGTAAAAAGAAAGGTTCCACTCTGTTAGCTGAGTACACACATCACAAACTTGTTTCT
         66 AACTGCTCTATCAAAAGAAATGTTCAACTCTGTGAGTTGAGTACACACATCACAAAGAAGTTTCT

                  *         *        **         **   
      66443 GAGAATCCTTCTGTCTCGTTTTTATGGGAAGATATTTACTT
        131 GAGAATGCTTCTGTCTAGTTTTTATATGAAGATATTCCCTT

              *  *  * **          *       *     *       *  *  *     *        
      66484 TTTCACCGTAGGCATCAAAGCGCTCCAAATGTCCACATCCAGATACTCCAGAAAGACTGTTTCAA
          1 TTCCAACGAAATCATCAAAGCGATCCAAATATCCACTTCCAGATTCTACAAAAAGAGTGTTTCAA

             *         *   * *   *         *        * *  *      *      *     
      66549 ACCTGCTCTATGAAAGGGAATCTTCAACTCTATGAGTTGAATGCAGACATCAGAAAGAAATTTCT
         66 AACTGCTCTATCAAAAGAAATGTTCAACTCTGTGAGTTGAGTACACACATCACAAAGAAGTTTCT

                     *       **          *         **
      66614 GAGAATGCTGCTGTCTACCTTTTAT-T--TGA-ATTCCCGC
        131 GAGAATGCTTCTGTCTAGTTTTTATATGAAGATATTCCCTT

                         *  *    *                *  **   *                  
      66651 TTCCAACGAAATCCTCCAAGCTATCCAAATATCCACTTGCATTTTCCACAAAAAGAGTGTTTCAA
          1 TTCCAACGAAATCATCAAAGCGATCCAAATATCCACTTCCAGATTCTACAAAAAGAGTGTTTCAA

                          *               ** *  *  *                *        
      66716 AACTGCTCTATCAATAGAAATGTTCAACTCCTTTAGCTGGGTACACACATCACAAACAAGTTTCT
         66 AACTGCTCTATCAAAAGAAATGTTCAACTCTGTGAGTTGAGTACACACATCACAAAGAAGTTTCT

                                     **     **       
      66781 GAGAATGCTTCTGTCTAGTTTTTATGGGAAGACGTTCCCTT
        131 GAGAATGCTTCTGTCTAGTTTTTATATGAAGATATTCCCTT

              *  * *  **          *       *            **                    
      66822 TTTCACCAAAGGCATCAAAGCGCTCCAAATGTCCACTTCCAGACACTACAAAAAGAGTGTTTCAA
          1 TTCCAACGAAATCATCAAAGCGATCCAAATATCCACTTCCAGATTCTACAAAAAGAGTGTTTCAA

             **       **   ***                 *    * *  * *         *       
      66887 ACGTGCTCTAAGAAAGCGAATGTTCAACTCTGTGACTTGAATGCAGATATCACAAAGTAGTTTCT
         66 AACTGCTCTATCAAAAGAAATGTTCAACTCTGTGAGTTGAGTACACACATCACAAAGAAGTTTCT

                **            *     *    *         * 
      66952 GAGAGGGCTTCTGTCTAGATTTTAGATGATGATATTCCCGT
        131 GAGAATGCTTCTGTCTAGTTTTTATATGAAGATATTCCCTT

                           * *   *                *   *                    * 
      66993 TTCCAACGAAATCATTAGAGCTATCCAAATATCCACTTACAGTTTCTACAAAAAGAGTGTTTCCA
          1 TTCCAACGAAATCATCAAAGCGATCCAAATATCCACTTCCAGATTCTACAAAAAGAGTGTTTCAA

                   **         * *    *       *  **                  ***      
      67058 AACTGCTGCATCAAAAGAGAGGTTCCACTCTGTTAGCCGAGTACACACATCACAAACTTGTTTCT
         66 AACTGCTCTATCAAAAGAAATGTTCAACTCTGTGAGTTGAGTACACACATCACAAAGAAGTTTCT

            *     *         *        **         **   
      67123 CAGAATCCTTCTGTCTCGTTTTTATGGGAAGATATTTACTT
        131 GAGAATGCTTCTGTCTAGTTTTTATATGAAGATATTCCCTT

              *  *  * **          *       * *   *       *  *  *              
      67164 TTTCACCGTAGGCATCAAAGCGCTCCAAATGTTCACATCCAGATACTCCAGAAAGAGTGTTTCAA
          1 TTCCAACGAAATCATCAAAGCGATCCAAATATCCACTTCCAGATTCTACAAAAAGAGTGTTTCAA

             *         *   * *   *         *        * *  *      *      *     
      67229 ACCTGCTCTATGAAAGGGAATCTTCAACTCTATGAGTTGAATGCAGACATCAGAAAGAAATTTCT
         66 AACTGCTCTATCAAAAGAAATGTTCAACTCTGTGAGTTGAGTACACACATCACAAAGAAGTTTCT

                     *       **          *         **
      67294 GAGAATGCTGCTGTCTACCTTTTAT-T--TGA-ATTCCCGC
        131 GAGAATGCTTCTGTCTAGTTTTTATATGAAGATATTCCCTT

                         *  *    *                *       *                  
      67331 TTCCAACGAAATCCTCCAAGCTATCCAAATATCCACTTGCAGATTCCACAAAAAGAGTGTTTCAA
          1 TTCCAACGAAATCATCAAAGCGATCCAAATATCCACTTCCAGATTCTACAAAAAGAGTGTTTCAA

                            ** *  *            *       *     *     *  *      
      67396 AACTGCTCTCTATCAATGGCAAAGTTCAACTCTGTTAGTTGAGGACACATATCACCAACAAGTTT
         66 AACTG--CTCTATCAAAAGAAATGTTCAACTCTGTGAGTTGAGTACACACATCACAAAGAAGTTT

                               *       **         *    
      67461 CTGAGAATGCTTCTGTCTATTTTTTATGGGAAGATATTTCCTT
        129 CTGAGAATGCTTCTGTCTAGTTTTTATATGAAGATATTCCCTT

              *  *  * ** *    *   *  *    *          * **                    
      67504 TTTCACCGTAGGCGTCAAGGCGTTCGAAATGTCCACTTCCACAAACTACAAAAAGAGTGTTTCAA
          1 TTCCAACGAAATCATCAAAGCGATCCAAATATCCACTTCCAGATTCTACAAAAAGAGTGTTTCAA

             *         *   * **       *    *        * ** * *   **      *     
      67569 ACCTGCTCTATGAAAGGCCATGTTCATCTCTATGAGTTGAATGGAAATATCCGAAAGAAATTTCT
         66 AACTGCTCTATCAAAAGAAATGTTCAACTCTGTGAGTTGAGTACACACATCACAAAGAAGTTTCT

             *       *          *        *         **
      67634 GGGAATGCTGCTGTCTAG-TGTT-TAT-ACGA-ATTCCCGC
        131 GAGAATGCTTCTGTCTAGTTTTTATATGAAGATATTCCCTT

                         *       *                *    *  *                  
      67671 TTCCAACGAAATCCTCAAAGCAATCCAAATATCCACTTGCAGAATCCACAAAAAGAGTGTTTCAA
          1 TTCCAACGAAATCATCAAAGCGATCCAAATATCCACTTCCAGATTCTACAAAAAGAGTGTTTCAA

                          *     *          * *                   *  *        
      67736 AACTGCTCTATCAATAGAAAGGTTCAACTCTTTTAGTTGAGTACACACATCACGAACAAGTTTCT
         66 AACTGCTCTATCAAAAGAAATGTTCAACTCTGTGAGTTGAGTACACACATCACAAAGAAGTTTCT

                            * *      **     **  *    
      67801 GAGAATGCTTCTGTCTGGCTTTTATTGGAAGACGTTTCCTT
        131 GAGAATGCTTCTGTCTAGTTTTTATATGAAGATATTCCCTT

                 * *  **          *       *                * *               
      67842 TT-CACCAAAGGCATCAAAGCGCTCCAAATGTCCACTTCCAGATTCTTCCAAAAGAGTGTTTCAA
          1 TTCCAACGAAATCATCAAAGCGATCCAAATATCCACTTCCAGATTCTACAAAAAGAGTGTTTCAA

             **     * ***  * *                 *    * *  * *     *   *       
      67906 ACGTGCTCAAAGTAAGGGAATGTTCAACTCTGTGACTTGAATGCAGATATCACCAAGTAGTTTCT
         66 AACTGCTCTATCAAAAGAAATGTTCAACTCTGTGAGTTGAGTACACACATCACAAAGAAGTTTCT

            * * *             *   * *    *         * 
      67971 AATAGTGCTTCTGTCTAGATTTCAGATGATGATATTCCCGT
        131 GAGAATGCTTCTGTCTAGTTTTTATATGAAGATATTCCCTT

                         * * *   * **          *  *   *      *     *       * 
      68012 TTCCAACGAAATCGTTAGAGCTAAGCAAATATCCAGTTACAGTTTCTACCAAAAGGGTGTTTCCA
          1 TTCCAACGAAATCATCAAAGCGATCCAAATATCCACTTCCAGATTCTACAAAAAGAGTGTTTCAA

              *    **           *            *       *                     * 
      68077 AATTGCTGCATCAAAAGAAAGGTTCAACTCTGTTAGTTGAGGACACACATCACAAAGAAGTTTGT
         66 AACTGCTCTATCAAAAGAAATGTTCAACTCTGTGAGTTGAGTACACACATCACAAAGAAGTTTCT

                              *    *     *           
      68142 GAGAATGCTTCTGTCTAGATTTTGTATGACGATATTCCCTT
        131 GAGAATGCTTCTGTCTAGTTTTTATATGAAGATATTCCCTT

                     *   * *     *   *       * *  *    *  *       *  *       
      68183 TTCCAACGATATCGTTAAAGCAATCTAAATATCAATTTGCAGAATCCACAAAAATAGAGTTTCAA
          1 TTCCAACGAAATCATCAAAGCGATCCAAATATCCACTTCCAGATTCTACAAAAAGAGTGTTTCAA

             *       * *        *    *       *  *                   ***      
      68248 AGCTGCTCTGTAAAAAGAAAGGTTCCACTCTGTTAGCTGAGTACACACATCACAAACTTGTTTCT
         66 AACTGCTCTATCAAAAGAAATGTTCAACTCTGTGAGTTGAGTACACACATCACAAAGAAGTTTCT

                  *         *        **         **   
      68313 GAGAATCCTTCTGTCTCGTTTTTATGGGAAGATATTTACTT
        131 GAGAATGCTTCTGTCTAGTTTTTATATGAAGATATTCCCTT

                 *  * **          *       *     *       *  *  *  *           
      68354 TTCCACCGTAGGCATCAAAGCGCTCCAAATGTCCACATCCAGATACTCCAGAACGAGTGTTTCAA
          1 TTCCAACGAAATCATCAAAGCGATCCAAATATCCACTTCCAGATTCTACAAAAAGAGTGTTTCAA

             *         *   * *   *         *        * *  *      *      *     
      68419 ACCTGCTCTATGAAAGGGAATCTTCAACTCTATGAGTTGAATGCAGACATCAGAAAGAAATTTCT
         66 AACTGCTCTATCAAAAGAAATGTTCAACTCTGTGAGTTGAGTACACACATCACAAAGAAGTTTCT

                     *       **          *         **
      68484 GAGAATGCTGCTGTCTACCTTTTAT-T--TGA-ATTCCCGC
        131 GAGAATGCTTCTGTCTAGTTTTTATATGAAGATATTCCCTT

                         *  *    *              * *  **   *    *             
      68521 TTCCAACGAAATCCTCCAAGCTATCCAAATATCCACCTGCATTTTCCACAACAAGAGTGTTTCAA
          1 TTCCAACGAAATCATCAAAGCGATCCAAATATCCACTTCCAGATTCTACAAAAAGAGTGTTTCAA

                          *                 *    *  *                *       
      68586 AACTGCTCTATCAATAGAAATGTTCAACTCCTTTG-GCTGGGTACACACATCACAAACAAGTTTC
         66 AACTGCTCTATCAAAAGAAATGTTCAACT-CTGTGAGTTGAGTACACACATCACAAAGAAGTTTC

                *                     **     *        
      68650 TGAGCATGCTTCTGTCTAGTTTTTATGGGAAGACATTCCCTT
        130 TGAGAATGCTTCTGTCTAGTTTTTATATGAAGATATTCCCTT

              *  * *  **          *       *            **                    
      68692 TTTCACCAAAGGCATCAAAGCGCTCCAAATGTCCACTTCCAGACACTACAAAAAGAGTGTTTCAA
          1 TTCCAACGAAATCATCAAAGCGATCCAAATATCCACTTCCAGATTCTACAAAAAGAGTGTTTCAA

             **       **    **                 *    * *  * *      *  *       
      68757 ACGTGCTCTAAGAAAACGAATGTTCAACTCTGTGACTTGAATGCAGATATCACAGAGTAGTTTCT
         66 AACTGCTCTATCAAAAGAAATGTTCAACTCTGTGAGTTGAGTACACACATCACAAAGAAGTTTCT

                **            *     *    *         * 
      68822 GAGAGGGCTTCTGTCTAGATTTTAGATGATGATATTCCCGT
        131 GAGAATGCTTCTGTCTAGTTTTTATATGAAGATATTCCCTT

                           * *   *                *   *                    * 
      68863 TTCCAACGAAATCATTAGAGCTATCCAAATATCCACTTACAGTTTCTACAAAAAGAGTGTTTCCA
          1 TTCCAACGAAATCATCAAAGCGATCCAAATATCCACTTCCAGATTCTACAAAAAGAGTGTTTCAA

                   **         * *    *       *  *                   ***      
      68928 AACTGCTGCATCAAAAGAGAGGTTCCACTCTGTTAGCTGAGTACACACATCACAAACTTGTTTCT
         66 AACTGCTCTATCAAAAGAAATGTTCAACTCTGTGAGTTGAGTACACACATCACAAAGAAGTTTCT

            *     *         *        **         **   
      68993 CAGAATCCTTCTGTCTCGTTTTTATGGGAAGATATTTACTT
        131 GAGAATGCTTCTGTCTAGTTTTTATATGAAGATATTCCCTT

              *  *  * **          *       *     *       *  *  *              
      69034 TTTCACCGTAGGCATCAAAGCGCTCCAAATGTCCACATCCAGATACTCCAGAAAGAGTGTTTCAA
          1 TTCCAACGAAATCATCAAAGCGATCCAAATATCCACTTCCAGATTCTACAAAAAGAGTGTTTCAA

             *         *   * *   *         *        * *  *      *      *     
      69099 ACCTGCTCTATGAAAGGGAATCTTCAACTCTATGAGTTGAATGCAGACATCAGAAAGAAATTTCT
         66 AACTGCTCTATCAAAAGAAATGTTCAACTCTGTGAGTTGAGTACACACATCACAAAGAAGTTTCT

                     *       **          *         **
      69164 GAGAATGCTGCTGTCTACCTTTTAT-T--TGA-ATTCCCGC
        131 GAGAATGCTTCTGTCTAGTTTTTATATGAAGATATTCCCTT

                         *  *    *                *       *                  
      69201 TTCCAACGAAATCCTCCAAGCTATCCAAATATCCACTTGCAGATTCCACAAAAAGAGTGTTTCAA
          1 TTCCAACGAAATCATCAAAGCGATCCAAATATCCACTTCCAGATTCTACAAAAAGAGTGTTTCAA

                            ** *  *            *       *     *     *  *      
      69266 AACTGCTCTCTATCAATGGCAAAGTTCAACTCTGTTAGTTGAGGACACATATCACCAACAAGTTT
         66 AACTG--CTCTATCAAAAGAAATGTTCAACTCTGTGAGTTGAGTACACACATCACAAAGAAGTTT

                               *       **         *    
      69331 CTGAGAATGCTTCTGTCTATTTTTTATGGGAAGATATTTCCTT
        129 CTGAGAATGCTTCTGTCTAGTTTTTATATGAAGATATTCCCTT

              *  *  * ** *    *      *    *          * **                    
      69374 TTTCACCGTAGGCGTCAAGGCGATCGAAATGTCCACTTCCACAAACTACAAAAAGAGTGTTTCAA
          1 TTCCAACGAAATCATCAAAGCGATCCAAATATCCACTTCCAGATTCTACAAAAAGAGTGTTTCAA

             *         *   * **       *    *        * ** * *   **      *     
      69439 ACCTGCTCTATGAAAGGCCATGTTCATCTCTATGAGTTGAATGGAAATATCCGAAAGAAATTTCT
         66 AACTGCTCTATCAAAAGAAATGTTCAACTCTGTGAGTTGAGTACACACATCACAAAGAAGTTTCT

             *       *          *        *         **
      69504 GGGAATGCTGCTGTCTAG-TGTT-TAT-ACGA-ATTCCCGC
        131 GAGAATGCTTCTGTCTAGTTTTTATATGAAGATATTCCCTT

                         *       *                *    *  *                  
      69541 TTCCAACGAAATCCTCAAAGCAATCCAAATATCCACTTGCAGAATCCACAAAAAGAGTGTTTCAA
          1 TTCCAACGAAATCATCAAAGCGATCCAAATATCCACTTCCAGATTCTACAAAAAGAGTGTTTCAA

                          *     *          * *                   *  *        
      69606 AACTGCTCTATCAATAGAAAGGTTCAACTCTTTTAGTTGAGTACACACATCACGAACAAGTTTCT
         66 AACTGCTCTATCAAAAGAAATGTTCAACTCTGTGAGTTGAGTACACACATCACAAAGAAGTTTCT

                            * *      **     **  *    
      69671 GAGAATGCTTCTGTCTGGCTTTTATTGGAAGACGTTTCCTT
        131 GAGAATGCTTCTGTCTAGTTTTTATATGAAGATATTCCCTT

                 * *  **          *       *                * *               
      69712 TT-CACCAAAGGCATCAAAGCGCTCCAAATGTCCACTTCCAGATTCTTCCAAAAGAGTGTTTCAA
          1 TTCCAACGAAATCATCAAAGCGATCCAAATATCCACTTCCAGATTCTACAAAAAGAGTGTTTCAA

             **     * ***  * *                 *    * *  * *     *   *       
      69776 ACGTGCTCAAAGTAAGGGAATGTTCAACTCTGTGACTTGAATGCAGATATCACCAAGTAGTTTCT
         66 AACTGCTCTATCAAAAGAAATGTTCAACTCTGTGAGTTGAGTACACACATCACAAAGAAGTTTCT

            * * *             *   * *    *         * 
      69841 AATAGTGCTTCTGTCTAGATTTCAGATGATGATATTCCCGT
        131 GAGAATGCTTCTGTCTAGTTTTTATATGAAGATATTCCCTT

                         * * *   *             *  *   *      *     *       * 
      69882 TTCCAACGAAATCGTTAGAGCTATCCAAATATCCAGTTACAGTTTCTACCAAAAGGGTGTTTCCA
          1 TTCCAACGAAATCATCAAAGCGATCCAAATATCCACTTCCAGATTCTACAAAAAGAGTGTTTCAA

              *    **           *            *       *                     * 
      69947 AATTGCTGCATCAAAAGAAAGGTTCAACTCTGTTAGTTGAGGACACACATCACAAAGAAGTTTGT
         66 AACTGCTCTATCAAAAGAAATGTTCAACTCTGTGAGTTGAGTACACACATCACAAAGAAGTTTCT

                              *    *     *           
      70012 GAGAATGCTTCTGTCTAGATTTTGTATGACGATATTCCCTT
        131 GAGAATGCTTCTGTCTAGTTTTTATATGAAGATATTCCCTT

                     *   * *     *   *       * *  *    *  *       *  *       
      70053 TTCCAACGATATCGTTAAAGCAATCTAAATATCAATTTGCAGAATCCACAAAAATAGAGTTTCAA
          1 TTCCAACGAAATCATCAAAGCGATCCAAATATCCACTTCCAGATTCTACAAAAAGAGTGTTTCAA

             *       * *        *    *       *  *                   ***      
      70118 AGCTGCTCTGTAAAAAGAAAGGTTCCACTCTGTTAGCTGAGTACACACATCACAAACTTGTTTCT
         66 AACTGCTCTATCAAAAGAAATGTTCAACTCTGTGAGTTGAGTACACACATCACAAAGAAGTTTCT

                  *         *        **         **   
      70183 GAGAATCCTTCTGTCTCGTTTTTATGGGAAGATATTTACTT
        131 GAGAATGCTTCTGTCTAGTTTTTATATGAAGATATTCCCTT

                 *  * **          *       *     *       *  *  *     *        
      70224 TTCCACCGTAGGCATCAAAGCGCTCCAAATGTCCACATCCAGATACTCCAGAAAGACTGTTTCAA
          1 TTCCAACGAAATCATCAAAGCGATCCAAATATCCACTTCCAGATTCTACAAAAAGAGTGTTTCAA

             *         *   * *   *         *        * *  *      *      *     
      70289 ACCTGCTCTATGAAAGGGAATCTTCAACTCTATGAGTTGAATGCAGACATCAGAAAGAAATTTCT
         66 AACTGCTCTATCAAAAGAAATGTTCAACTCTGTGAGTTGAGTACACACATCACAAAGAAGTTTCT

                     *       **          *         **
      70354 GAGAATGCTGCTGTCTACCTTTTAT-T--TGA-ATTCCCGC
        131 GAGAATGCTTCTGTCTAGTTTTTATATGAAGATATTCCCTT

                         *  *    *              * *  **   *                  
      70391 TTCCAACGAAATCCTCCAAGCTATCCAAATATCCACCTGCATTTTCCACAAAAAGAGTGTTTCAA
          1 TTCCAACGAAATCATCAAAGCGATCCAAATATCCACTTCCAGATTCTACAAAAAGAGTGTTTCAA

                          *                 *    *  *                *       
      70456 AACTGCTCTATCAATAGAAATGTTCAACTCCTTTG-GCTGGGTACACACATCACAAACAAGTTTC
         66 AACTGCTCTATCAAAAGAAATGTTCAACT-CTGTGAGTTGAGTACACACATCACAAAGAAGTTTC

                *                     **     *        
      70520 TGAGCATGCTTCTGTCTAGTTTTTATGGGAAGACATTCCCTT
        130 TGAGAATGCTTCTGTCTAGTTTTTATATGAAGATATTCCCTT

              *  * *  **          *       *            **                    
      70562 TTTCACCAAAGGCATCAAAGCGCTCCAAATGTCCACTTCCAGACACTACAAAAAGAGTGTTTCAA
          1 TTCCAACGAAATCATCAAAGCGATCCAAATATCCACTTCCAGATTCTACAAAAAGAGTGTTTCAA

             **       **    **                 *    * *  * *      *  *       
      70627 ACGTGCTCTAAGAAAACGAATGTTCAACTCTGTGACTTGAATGCAGATATCACAGAGTAGTTTCT
         66 AACTGCTCTATCAAAAGAAATGTTCAACTCTGTGAGTTGAGTACACACATCACAAAGAAGTTTCT

                **            *     *    *         * 
      70692 GAGAGGGCTTCTGTCTAGATTTTAGATGATGATATTCCCGT
        131 GAGAATGCTTCTGTCTAGTTTTTATATGAAGATATTCCCTT

                           * *   *                *   *                    * 
      70733 TTCCAACGAAATCATTAGAGCTATCCAAATATCCACTTACAGTTTCTACAAAAAGAGTGTTTCCA
          1 TTCCAACGAAATCATCAAAGCGATCCAAATATCCACTTCCAGATTCTACAAAAAGAGTGTTTCAA

                   **         * *    *       *  *                   ***      
      70798 AACTGCTGCATCAAAAGAGAGGTTCCACTCTGTTAGCTGAGTACACACATCACAAACTTGTTTCT
         66 AACTGCTCTATCAAAAGAAATGTTCAACTCTGTGAGTTGAGTACACACATCACAAAGAAGTTTCT

            *     *         *        **         **   
      70863 CAGAATCCTTCTGTCTCGTTTTTATGGGAAGATATTTACTT
        131 GAGAATGCTTCTGTCTAGTTTTTATATGAAGATATTCCCTT

              *  *  * **          *       *     *       *  *  *              
      70904 TTTCACCGTAGGCATCAAAGCGCTCCAAATGTCCACATCCAGATACTCCAGAAAGAGTGTTTCAA
          1 TTCCAACGAAATCATCAAAGCGATCCAAATATCCACTTCCAGATTCTACAAAAAGAGTGTTTCAA

             *         *   * *   *         *        * *  *      *      *     
      70969 ACCTGCTCTATGAAAGGGAATCTTCAACTCTATGAGTTGAATGCAGACATCAGAAAGAAATTTCT
         66 AACTGCTCTATCAAAAGAAATGTTCAACTCTGTGAGTTGAGTACACACATCACAAAGAAGTTTCT

                     *       **          *         **
      71034 GAGAATGCTGCTGTCTACCTTTTAT-T--TGA-ATTCCCGC
        131 GAGAATGCTTCTGTCTAGTTTTTATATGAAGATATTCCCTT

                         *  *    *                *       *                  
      71071 TTCCAACGAAATCCTCCAAGCTATCCAAATATCCACTTGCAGATTCCACAAAAAGAGTGTTTCAA
          1 TTCCAACGAAATCATCAAAGCGATCCAAATATCCACTTCCAGATTCTACAAAAAGAGTGTTTCAA

                            ** *  *            *       *     *     *  *      
      71136 AACTGCTCTCTATCAATGGCAAAGTTCAACTCTGTTAGTTGAGGACACATATCACCAACAAGTTT
         66 AACTG--CTCTATCAAAAGAAATGTTCAACTCTGTGAGTTGAGTACACACATCACAAAGAAGTTT

                               *       **         *    
      71201 CTGAGAATGCTTCTGTCTATTTTTTATGGGAAGATATTTCCTT
        129 CTGAGAATGCTTCTGTCTAGTTTTTATATGAAGATATTCCCTT

              *  *  * ** *    *      *    *          * **                    
      71244 TTTCACCGTAGGCGTCAAGGCGATCGAAATGTCCACTTCCACAAACTACAAAAAGAGTGTTTCAA
          1 TTCCAACGAAATCATCAAAGCGATCCAAATATCCACTTCCAGATTCTACAAAAAGAGTGTTTCAA

             *         *   * **       *    *        * ** * *   **      *     
      71309 ACCTGCTCTATGAAAGGCCATGTTCATCTCTATGAGTTGAATGGAAATATCCGAAAGAAATTTCT
         66 AACTGCTCTATCAAAAGAAATGTTCAACTCTGTGAGTTGAGTACACACATCACAAAGAAGTTTCT

             *       *          *        *         **
      71374 GGGAATGCTGCTGTCTAG-TGTT-TAT-ACGA-ATTCCCGC
        131 GAGAATGCTTCTGTCTAGTTTTTATATGAAGATATTCCCTT

                         *       *                *    *  *                  
      71411 TTCCAACGAAATCCTCAAAGCAATCCAAATATCCACTTGCAGAATCCACAAAAAGAGTGTTTCAA
          1 TTCCAACGAAATCATCAAAGCGATCCAAATATCCACTTCCAGATTCTACAAAAAGAGTGTTTCAA

                          *     *          * *                   *  *        
      71476 AACTGCTCTATCAATAGAAAGGTTCAACTCTTTTAGTTGAGTACACACATCACGAACAAGTTTCT
         66 AACTGCTCTATCAAAAGAAATGTTCAACTCTGTGAGTTGAGTACACACATCACAAAGAAGTTTCT

                            * *      **     **  *    
      71541 GAGAATGCTTCTGTCTGGCTTTTATTGGAAGACGTTTCCTT
        131 GAGAATGCTTCTGTCTAGTTTTTATATGAAGATATTCCCTT

                 * *  **          *       *                * *               
      71582 TT-CACCAAAGGCATCAAAGCGCTCCAAATGTCCACTTCCAGATTCTTCCAAAAGAGTGTTTCAA
          1 TTCCAACGAAATCATCAAAGCGATCCAAATATCCACTTCCAGATTCTACAAAAAGAGTGTTTCAA

             **  *  * ***  * *                 *    * *  * *     *   *       
      71646 ACGTGGTCGAAGTAAGGGAATGTTCAACTCTGTGACTTGAATGCAGATATCACCAAGTAGTTTCT
         66 AACTGCTCTATCAAAAGAAATGTTCAACTCTGTGAGTTGAGTACACACATCACAAAGAAGTTTCT

            * * *             *     *    *         * 
      71711 AATAGTGCTTCTGTCTAGATTTTAGATGATGATATTCCCGT
        131 GAGAATGCTTCTGTCTAGTTTTTATATGAAGATATTCCCTT

                         * * *   * **          *  *   *      *     *       * 
      71752 TTCCAACGAAATCGTTAGAGCTAAGCAAATATCCAGTTACAGTTTCTACCAAAAGGGTGTTTCCA
          1 TTCCAACGAAATCATCAAAGCGATCCAAATATCCACTTCCAGATTCTACAAAAAGAGTGTTTCAA

              *    **           *            *       *                     * 
      71817 AATTGCTGCATCAAAAGAAAGGTTCAACTCTGTTAGTTGAGGACACACATCACAAAGAAGTTTGT
         66 AACTGCTCTATCAAAAGAAATGTTCAACTCTGTGAGTTGAGTACACACATCACAAAGAAGTTTCT

                              *    *     *           
      71882 GAGAATGCTTCTGTCTAGATTTTGTATGACGATATTCCCTT
        131 GAGAATGCTTCTGTCTAGTTTTTATATGAAGATATTCCCTT

                     *   * *     *   *       * *  *    *  *       *  *       
      71923 TTCCAACGATATCGTTAAAGCAATCTAAATATCAATTTGCAGAATCCACAAAAATAGCGTTTCAA
          1 TTCCAACGAAATCATCAAAGCGATCCAAATATCCACTTCCAGATTCTACAAAAAGAGTGTTTCAA

             *       * *        *    *       *  *                   ***      
      71988 AGCTGCTCTGTAAAAAGAAAGGTTCCACTCTGTTAGCTGAGTACACACATCACAAACTTGTTTCT
         66 AACTGCTCTATCAAAAGAAATGTTCAACTCTGTGAGTTGAGTACACACATCACAAAGAAGTTTCT

                  *         *        **         **   
      72053 GAGAATCCTTCTGTCTCGTTTTTATGGGAAGATATTTACTT
        131 GAGAATGCTTCTGTCTAGTTTTTATATGAAGATATTCCCTT

                 *  * **          *       *     *       *  *  *  *           
      72094 TTCCACCGTAGGCATCAAAGCGCTCCAAATGTCCACATCCAGATACTCCAGAACGAGTGTTTCAA
          1 TTCCAACGAAATCATCAAAGCGATCCAAATATCCACTTCCAGATTCTACAAAAAGAGTGTTTCAA

             *         *   * *   *         *        * *  *      *      *     
      72159 ACCTGCTCTATGAAAGGGAATCTTCAACTCTATGAGTTGAATGCAGACATCAGAAAGAAATTTCT
         66 AACTGCTCTATCAAAAGAAATGTTCAACTCTGTGAGTTGAGTACACACATCACAAAGAAGTTTCT

                     *       **          *         **
      72224 GAGAATGCTGCTGTCTACCTTTTAT-T--TGA-ATTCCCGC
        131 GAGAATGCTTCTGTCTAGTTTTTATATGAAGATATTCCCTT

                         *  *    *              * *  **   *    *             
      72261 TTCCAACGAAATCCTCCAAGCTATCCAAATATCCACCTGCATTTTCCACAACAAGAGTGTTTCAA
          1 TTCCAACGAAATCATCAAAGCGATCCAAATATCCACTTCCAGATTCTACAAAAAGAGTGTTTCAA

                          *                 *    *  *                *       
      72326 AACTGCTCTATCAATAGAAATGTTCAACTCCTTTG-GCTGGGTACACACATCACAAACAAGTTTC
         66 AACTGCTCTATCAAAAGAAATGTTCAACT-CTGTGAGTTGAGTACACACATCACAAAGAAGTTTC

                                      **     **       
      72390 TGAGAATGCTTCTGTCTAGTTTTTATGGGAAGACGTTCCCTT
        130 TGAGAATGCTTCTGTCTAGTTTTTATATGAAGATATTCCCTT

              *  * *  **          *       *            **                  * 
      72432 TTTCACCAAAGGCATCAAAGCGCTCCAAATGTCCACTTCCAGACACTACAAAAAGAGTGTTTCCA
          1 TTCCAACGAAATCATCAAAGCGATCCAAATATCCACTTCCAGATTCTACAAAAAGAGTGTTTCAA

             **   *   **   ***                 *    * *  * *         *       
      72497 ACGTGCCCTAAGAAAGCGAATGTTCAACTCTGTGACTTGAATGCAGATATCACAAAGTAGTTTCT
         66 AACTGCTCTATCAAAAGAAATGTTCAACTCTGTGAGTTGAGTACACACATCACAAAGAAGTTTCT

                **            *     *    *         * 
      72562 GAGAGGGCTTCTGTCTAGATTTTAGATGATGATATTCCCGT
        131 GAGAATGCTTCTGTCTAGTTTTTATATGAAGATATTCCCTT

                           * *   *                *   *                    * 
      72603 TTCCAACGAAATCATTAGAGCTATCCAAATATCCACTTACAGTTTCTACAAAAAGAGTGTTTCCA
          1 TTCCAACGAAATCATCAAAGCGATCCAAATATCCACTTCCAGATTCTACAAAAAGAGTGTTTCAA

                   **         * *    *       *  *                   ***      
      72668 AACTGCTGCATCAAAAGAGAGGTTCCACTCTGTTAGCTGAGTACACACATCACAAACTTGTTTCT
         66 AACTGCTCTATCAAAAGAAATGTTCAACTCTGTGAGTTGAGTACACACATCACAAAGAAGTTTCT

            *     *         *        **         **   
      72733 CAGAATCCTTCTGTCTCGTTTTTATGGGAAGATATTTACTT
        131 GAGAATGCTTCTGTCTAGTTTTTATATGAAGATATTCCCTT

              *  *  * **          *       *     *       *  *  *              
      72774 TTTCACCGTAGGCATCAAAGCGCTCCAAATGTCCACATCCAGATACTCCAGAAAGAGTGTTTCAA
          1 TTCCAACGAAATCATCAAAGCGATCCAAATATCCACTTCCAGATTCTACAAAAAGAGTGTTTCAA

             *         *   * *   *         *        * *  *      *      *     
      72839 ACCTGCTCTATGAAAGGGAATCTTCAACTCTATGAGTTGAATGCAGACATCAGAAAGAAATTTCT
         66 AACTGCTCTATCAAAAGAAATGTTCAACTCTGTGAGTTGAGTACACACATCACAAAGAAGTTTCT

                     *       **          *         **
      72904 GAGAATGCTGCTGTCTACCTTTTAT-T--TGA-ATTCCCGC
        131 GAGAATGCTTCTGTCTAGTTTTTATATGAAGATATTCCCTT

                         *  *    *                *       *                  
      72941 TTCCAACGAAATCCTCCAAGCTATCCAAATATCCACTTGCAGATTCCACAAAAAGAGTGTTTCAA
          1 TTCCAACGAAATCATCAAAGCGATCCAAATATCCACTTCCAGATTCTACAAAAAGAGTGTTTCAA

                            ** *  *            *       *     *     *  *      
      73006 AACTGCTCTCTATCAATGGCAAAGTTCAACTCTGTTAGTTGAGGACACATATCACCAACAAGTTT
         66 AACTG--CTCTATCAAAAGAAATGTTCAACTCTGTGAGTTGAGTACACACATCACAAAGAAGTTT

                               *       **         *    
      73071 CTGAGAATGCTTCTGTCTATTTTTTATGGGAAGATATTTCCTT
        129 CTGAGAATGCTTCTGTCTAGTTTTTATATGAAGATATTCCCTT

              *  *  * ** *    *      *    *          * **                    
      73114 TTTCACCGTAGGCGTCAAGGCGATCGAAATGTCCACTTCCACAAACTACAAAAAGAGTGTTTCAA
          1 TTCCAACGAAATCATCAAAGCGATCCAAATATCCACTTCCAGATTCTACAAAAAGAGTGTTTCAA

             *         *   * **       *    *        * ** * *   **      *     
      73179 ACCTGCTCTATGAAAGGCGATGTTCATCTCTATGAGTTGAATGGAAATATCCGAAAGAAATTTCT
         66 AACTGCTCTATCAAAAGAAATGTTCAACTCTGTGAGTTGAGTACACACATCACAAAGAAGTTTCT

             *       *          *        *         **
      73244 GGGAATGCTGCTGTCTAG-TGTT-TAT-ACGA-ATTCCCGC
        131 GAGAATGCTTCTGTCTAGTTTTTATATGAAGATATTCCCTT

                         *       *                *    *  *                  
      73281 TTCCAACGAAATCCTCAAAGCAATCCAAATATCCACTTGCAGAATCCACAAAAAGAGTGTTTCAA
          1 TTCCAACGAAATCATCAAAGCGATCCAAATATCCACTTCCAGATTCTACAAAAAGAGTGTTTCAA

                          *     *          * *                   *  *        
      73346 AACTGCTCTATCAATAGAAAGGTTCAACTCTTTTAGTTGAGTACACACATCACGAACAAGTTTCT
         66 AACTGCTCTATCAAAAGAAATGTTCAACTCTGTGAGTTGAGTACACACATCACAAAGAAGTTTCT

                            * *      **     **  *    
      73411 GAGAATGCTTCTGTCTGGCTTTTATTGGAAGACGTTTCCTT
        131 GAGAATGCTTCTGTCTAGTTTTTATATGAAGATATTCCCTT

            *    * *  **          *       *                * *               
      73452 AT-CACCAAAGGCATCAAAGCGCTCCAAATGTCCACTTCCAGATTCTTCCAAAAGAGTGTTTCAA
          1 TTCCAACGAAATCATCAAAGCGATCCAAATATCCACTTCCAGATTCTACAAAAAGAGTGTTTCAA

             **     * ***  * *                 *    * *  * *     *   *       
      73516 ACGTGCTCAAAGTAAGGGAATGTTCAACTCTGTGACTTGAATGCAGATATCACCAAGTAGTTTCT
         66 AACTGCTCTATCAAAAGAAATGTTCAACTCTGTGAGTTGAGTACACACATCACAAAGAAGTTTCT

            * * *             *     *    *         * 
      73581 AATAGTGCTTCTGTCTAGATTTTAGATGATGATATTCCCGT
        131 GAGAATGCTTCTGTCTAGTTTTTATATGAAGATATTCCCTT

                         * * *   *             *  *   *                    * 
      73622 TTCCAACGAAATCGTTAGAGCTATCCAAATATCCAGTTACAGTTTCTACAAAAAGAGTGTTTCCA
          1 TTCCAACGAAATCATCAAAGCGATCCAAATATCCACTTCCAGATTCTACAAAAAGAGTGTTTCAA

                   **           *            *       *                     * 
      73687 AACTGCTGCATCAAAAGAAAGGTTCAACTCTGTTAGTTGAGGACACACATCACAAAGAAGTTTGT
         66 AACTGCTCTATCAAAAGAAATGTTCAACTCTGTGAGTTGAGTACACACATCACAAAGAAGTTTCT

                              *    *     *           
      73752 GAGAATGCTTCTGTCTAGATTTTGTATGACGATATTCCCTT
        131 GAGAATGCTTCTGTCTAGTTTTTATATGAAGATATTCCCTT

                     *   * *     *   *       * *  *    *  *       *  *       
      73793 TTCCAACGATATCGTTAAAGCAATCTAAATATCAATTTGCAGAATCCACAAAAATAGAGTTTCAA
          1 TTCCAACGAAATCATCAAAGCGATCCAAATATCCACTTCCAGATTCTACAAAAAGAGTGTTTCAA

             *       * *    *   *    *       *  *                   ***      
      73858 AGCTGCTCTGTAAAAATAAAAGTTCCACTCTGTTAGCTGAGTACACACATCACAAACTTGTTTCT
         66 AACTGCTCTATCAAAAGAAATGTTCAACTCTGTGAGTTGAGTACACACATCACAAAGAAGTTTCT

                  *         *        **         **   
      73923 GAGAATCCTTCTGTCTCGTTTTTATGGGAAGATATTTACTT
        131 GAGAATGCTTCTGTCTAGTTTTTATATGAAGATATTCCCTT

              *  *  * **          *       *     *       *  *  *     *        
      73964 TTTCACCGTAGGCATCAAAGCGCTCCAAATGTCCACATCCAGATACTCCAGAAAGACTGTTTCAA
          1 TTCCAACGAAATCATCAAAGCGATCCAAATATCCACTTCCAGATTCTACAAAAAGAGTGTTTCAA

             *         *   * *   *         *        * *  *      *      *     
      74029 ACCTGCTCTATGAAAGGGAATCTTCAACTCTATGAGTTGAATGCAGACATCAGAAAGAAATTTCT
         66 AACTGCTCTATCAAAAGAAATGTTCAACTCTGTGAGTTGAGTACACACATCACAAAGAAGTTTCT

                     *       **          *         **
      74094 GAGAATGCTGCTGTCTACCTTTTAT-T--TGA-ATTCCCGC
        131 GAGAATGCTTCTGTCTAGTTTTTATATGAAGATATTCCCTT

                         *  *    *                *  **   *                  
      74131 TTCCAACGAAATCCTCCAAGCTATCCAAATATCCACTTGCATTTTCCACAAAAAGAGTGTTTCAA
          1 TTCCAACGAAATCATCAAAGCGATCCAAATATCCACTTCCAGATTCTACAAAAAGAGTGTTTCAA

                          *               ** *  *  *                *        
      74196 AACTGCTCTATCAATAGAAATGTTCAACTCCTTTAGCTGGGTACACACATCACAAACAAGTTTCT
         66 AACTGCTCTATCAAAAGAAATGTTCAACTCTGTGAGTTGAGTACACACATCACAAAGAAGTTTCT

                                     **     **       
      74261 GAGAATGCTTCTGTCTAGTTTTTATGGGAAGACGTTCCCTT
        131 GAGAATGCTTCTGTCTAGTTTTTATATGAAGATATTCCCTT

              *  * *  **          *       *            **                    
      74302 TTTCACCAAAGGCATCAAAGCGCTCCAAATGTCCACTTCCAGACACTACAAAAAGAGTGTTTCAA
          1 TTCCAACGAAATCATCAAAGCGATCCAAATATCCACTTCCAGATTCTACAAAAAGAGTGTTTCAA

             **       **   ***                 *    * *  * *         *       
      74367 ACGTGCTCTAAGAAAGCGAATGTTCAACTCTGTGACTTGAATGCAGATATCACAAAGTAGTTTCT
         66 AACTGCTCTATCAAAAGAAATGTTCAACTCTGTGAGTTGAGTACACACATCACAAAGAAGTTTCT

                **            *     *    *         * 
      74432 GAGAGGGCTTCTGTCTAGATTTTAGATGATGATATTCCCGT
        131 GAGAATGCTTCTGTCTAGTTTTTATATGAAGATATTCCCTT

                           * *   *                *   *     *              * 
      74473 TTCCAACGAAATCATTAGAGCTATCCAAATATCCACTTACAGTTTCTATAAAAAGAGTGTTTCCA
          1 TTCCAACGAAATCATCAAAGCGATCCAAATATCCACTTCCAGATTCTACAAAAAGAGTGTTTCAA

                   **         * *    *       *  **                  ***      
      74538 AACTGCTGCATCAAAAGAGAGGTTCCACTCTGTTAGCCGAGTACACACATCACAAACTTGTTTCT
         66 AACTGCTCTATCAAAAGAAATGTTCAACTCTGTGAGTTGAGTACACACATCACAAAGAAGTTTCT

            *     *         *        **         **   
      74603 CAGAATCCTTCTGTCTCGTTTTTATGGGAAGATATTTACTT
        131 GAGAATGCTTCTGTCTAGTTTTTATATGAAGATATTCCCTT

                 *  * **          *       *     *       *  *  *              
      74644 TTCCACCGTAGGCATCAAAGCGCTCCAAATGTCCACATCCAGATACTCCAGAAAGAGTGTTTCAA
          1 TTCCAACGAAATCATCAAAGCGATCCAAATATCCACTTCCAGATTCTACAAAAAGAGTGTTTCAA

             *         *   * *   *         *        * *  *      *      *     
      74709 ACCTGCTCTATGAAAGGGAATCTTCAACTCTATGAGTTGAATGCAGACATCAGAAAGAAATTTCT
         66 AACTGCTCTATCAAAAGAAATGTTCAACTCTGTGAGTTGAGTACACACATCACAAAGAAGTTTCT

                     *       **          *         **
      74774 GAGAATGCTGCTGTCTACCTTTTAT-T--TGA-ATTCCCGC
        131 GAGAATGCTTCTGTCTAGTTTTTATATGAAGATATTCCCTT

                         *  *    *              * *  **   *    *             
      74811 TTCCAACGAAATCCTCCAAGCTATCCAAATATCCACCTGCATTTTCCACAACAAGAGTGTTTCAA
          1 TTCCAACGAAATCATCAAAGCGATCCAAATATCCACTTCCAGATTCTACAAAAAGAGTGTTTCAA

                          *                 *    *  *                *       
      74876 AACTGCTCTATCAATAGAAATGTTCAACTCCTTTG-GCTGGGTACACACATCACAAACAAGTTTC
         66 AACTGCTCTATCAAAAGAAATGTTCAACT-CTGTGAGTTGAGTACACACATCACAAAGAAGTTTC

                *                     **     *        
      74940 TGAGCATGCTTCTGTCTAGTTTTTATGGGAAGACATTCCCTT
        130 TGAGAATGCTTCTGTCTAGTTTTTATATGAAGATATTCCCTT

              *  * *  **          *       *            **                    
      74982 TTTCACCAAAGGCATCAAAGCGCTCCAAATGTCCACTTCCAGACACTACAAAAAGAGTGTTTCAA
          1 TTCCAACGAAATCATCAAAGCGATCCAAATATCCACTTCCAGATTCTACAAAAAGAGTGTTTCAA

             **       **    **                 *    * *  * *      *  *       
      75047 ACGTGCTCTAAGAAAACGAATGTTCAACTCTGTGACTTGAATGCAGATATCACAGAGTAGTTTCT
         66 AACTGCTCTATCAAAAGAAATGTTCAACTCTGTGAGTTGAGTACACACATCACAAAGAAGTTTCT

                **            *     *    *         * 
      75112 GAGAGGGCTTCTGTCTAGATTTTAGATGATGATATTCCCGT
        131 GAGAATGCTTCTGTCTAGTTTTTATATGAAGATATTCCCTT

                           * *   *                *   *                    * 
      75153 TTCCAACGAAATCATTAGAGCTATCCAAATATCCACTTACAGTTTCTACAAAAAGAGTGTTTCCA
          1 TTCCAACGAAATCATCAAAGCGATCCAAATATCCACTTCCAGATTCTACAAAAAGAGTGTTTCAA

                   **         * *    *       *  *                   ***      
      75218 AACTGCTGCATCAAAAGAGAGGTTCCACTCTGTTAGCTGAGTACACACATCACAAACTTGTTTCT
         66 AACTGCTCTATCAAAAGAAATGTTCAACTCTGTGAGTTGAGTACACACATCACAAAGAAGTTTCT

            *     *         *        **         **   
      75283 CAGAATCCTTCTGTCTCGTTTTTATGGGAAGATATTTACTT
        131 GAGAATGCTTCTGTCTAGTTTTTATATGAAGATATTCCCTT

              *  *  * **          *       *     *       *  *  *              
      75324 TTTCACCGTAGGCATCAAAGCGCTCCAAATGTCCACATCCAGATACTCCAGAAAGAGTGTTTCAA
          1 TTCCAACGAAATCATCAAAGCGATCCAAATATCCACTTCCAGATTCTACAAAAAGAGTGTTTCAA

             *         *   * *   *         *        * *  *      *      *     
      75389 ACCTGCTCTATGAAAGGGAATCTTCAACTCTATGAGTTGAATGCAGACATCAGAAAGAAATTTCT
         66 AACTGCTCTATCAAAAGAAATGTTCAACTCTGTGAGTTGAGTACACACATCACAAAGAAGTTTCT

                     *       **          *         **
      75454 GAGAATGCTGCTGTCTACCTTTTAT-T--TGA-ATTCCCGC
        131 GAGAATGCTTCTGTCTAGTTTTTATATGAAGATATTCCCTT

                         *  *    *                *       *                  
      75491 TTCCAACGAAATCCTCCAAGCTATCCAAATATCCACTTGCAGATTCCACAAAAAGAGTGTTTCAA
          1 TTCCAACGAAATCATCAAAGCGATCCAAATATCCACTTCCAGATTCTACAAAAAGAGTGTTTCAA

                            ** *  *            *       *     *     *  *      
      75556 AACTGCTCTCTATCAATGGCAAAGTTCAACTCTGTTAGTTGAGGACACATATCACCAACAAGTTT
         66 AACTG--CTCTATCAAAAGAAATGTTCAACTCTGTGAGTTGAGTACACACATCACAAAGAAGTTT

                               *       **         *    
      75621 CTGAGAATGCTTCTGTCTATTTTTTATGGGAAGATATTTCCTT
        129 CTGAGAATGCTTCTGTCTAGTTTTTATATGAAGATATTCCCTT

              *  *  * ** *    *      *    *          * **                    
      75664 TTTCACCGTAGGCGTCAAGGCGATCGAAATGTCCACTTCCACAAACTACAAAAAGAGTGTTTCAA
          1 TTCCAACGAAATCATCAAAGCGATCCAAATATCCACTTCCAGATTCTACAAAAAGAGTGTTTCAA

             *         *   * **       *    *        * ** * *   **      *     
      75729 ACCTGCTCTATGAAAGGCCATGTTCATCTCTATGAGTTGAATGGAAATATCCGAAAGAAATTTCT
         66 AACTGCTCTATCAAAAGAAATGTTCAACTCTGTGAGTTGAGTACACACATCACAAAGAAGTTTCT

             *       *          *        *         **
      75794 GGGAATGCTGCTGTCTAG-TGTT-TAT-ACGA-ATTCCCGC
        131 GAGAATGCTTCTGTCTAGTTTTTATATGAAGATATTCCCTT

                         *       *                *    *  *                  
      75831 TTCCAACGAAATCCTCAAAGCAATCCAAATATCCACTTGCAGAATCCACAAAAAGAGTGTTTCAA
          1 TTCCAACGAAATCATCAAAGCGATCCAAATATCCACTTCCAGATTCTACAAAAAGAGTGTTTCAA

                          *     *          * *                   *  *        
      75896 AACTGCTCTATCAATAGAAAGGTTCAACTCTTTTAGTTGAGTACACACATCACGAACAAGTTTCT
         66 AACTGCTCTATCAAAAGAAATGTTCAACTCTGTGAGTTGAGTACACACATCACAAAGAAGTTTCT

                            * *      **     **  *    
      75961 GAGAATGCTTCTGTCTGGCTTTTATTGGAAGACGTTTCCTT
        131 GAGAATGCTTCTGTCTAGTTTTTATATGAAGATATTCCCTT

                 * *  **          *       *                * *               
      76002 TT-CACCAAAGGCATCAAAGCGCTCCAAATGTCCACTTCCAGATTCTTCCAAAAGAGTGTTTCAA
          1 TTCCAACGAAATCATCAAAGCGATCCAAATATCCACTTCCAGATTCTACAAAAAGAGTGTTTCAA

             **  *  * ***  * *                 *    * *  * *     *   *       
      76066 ACGTGGTCGAAGTAAGGGAATGTTCAACTCTGTGACTTGAATGCAGATATCACCAAGTAGTTTCT
         66 AACTGCTCTATCAAAAGAAATGTTCAACTCTGTGAGTTGAGTACACACATCACAAAGAAGTTTCT

            * * *             *     *    *         * 
      76131 AATAGTGCTTCTGTCTAGATTTTAGATGATGATATTCCCGT
        131 GAGAATGCTTCTGTCTAGTTTTTATATGAAGATATTCCCTT

                         * * *   * **          *  *   *      *     *       * 
      76172 TTCCAACGAAATCGTTAGAGCTAAGCAAATATCCAGTTACAGTTTCTACCAAAAGGGTGTTTCCA
          1 TTCCAACGAAATCATCAAAGCGATCCAAATATCCACTTCCAGATTCTACAAAAAGAGTGTTTCAA

              *    **           *            *       *                     * 
      76237 AATTGCTGCATCAAAAGAAAGGTTCAACTCTGTTAGTTGAGGACACACATCACAAAGAAGTTTGT
         66 AACTGCTCTATCAAAAGAAATGTTCAACTCTGTGAGTTGAGTACACACATCACAAAGAAGTTTCT

                              *    *     *           
      76302 GAGAATGCTTCTGTCTAGATTTTGTATGACGATATTCCCTT
        131 GAGAATGCTTCTGTCTAGTTTTTATATGAAGATATTCCCTT

                     *   * *     *   *       * *  *    *  *       *  *       
      76343 TTCCAACGATATCGTTAAAGCAATCTAAATATCAATTTGCAGAATCCACAAAAATAGCGTTTCAA
          1 TTCCAACGAAATCATCAAAGCGATCCAAATATCCACTTCCAGATTCTACAAAAAGAGTGTTTCAA

             *       * *        *    *       *  *                   ***      
      76408 AGCTGCTCTGTAAAAAGAAAGGTTCCACTCTGTTAGCTGAGTACACACATCACAAACTTGTTTCT
         66 AACTGCTCTATCAAAAGAAATGTTCAACTCTGTGAGTTGAGTACACACATCACAAAGAAGTTTCT

                  *         *        **         **   
      76473 GAGAATCCTTCTGTCTCGTTTTTATGGGAAGATATTTACTT
        131 GAGAATGCTTCTGTCTAGTTTTTATATGAAGATATTCCCTT

                 *  * **          *       *     *       *  *  *  *           
      76514 TTCCACCGTAGGCATCAAAGCGCTCCAAATGTCCACATCCAGATACTCCAGAACGAGTGTTTCAA
          1 TTCCAACGAAATCATCAAAGCGATCCAAATATCCACTTCCAGATTCTACAAAAAGAGTGTTTCAA

             *         *   * *   *         *        * *  *      *      *     
      76579 ACCTGCTCTATGAAAGGGAATCTTCAACTCTATGAGTTGAATGCAGACATCAGAAAGAAATTTCT
         66 AACTGCTCTATCAAAAGAAATGTTCAACTCTGTGAGTTGAGTACACACATCACAAAGAAGTTTCT

                     *       **          *         **
      76644 GAGAATGCTGCTGTCTACCTTTTAT-T--TGA-ATTCCCGC
        131 GAGAATGCTTCTGTCTAGTTTTTATATGAAGATATTCCCTT

                         *  *    *              * *  **   *    *             
      76681 TTCCAACGAAATCCTCCAAGCTATCCAAATATCCACCTGCATTTTCCACAACAAGAGTGTTTCAA
          1 TTCCAACGAAATCATCAAAGCGATCCAAATATCCACTTCCAGATTCTACAAAAAGAGTGTTTCAA

                          *                 *    *  *                *       
      76746 AACTGCTCTATCAATAGAAATGTTCAACTCCTTTG-GCTGGGTACACACATCACAAACAAGTTTC
         66 AACTGCTCTATCAAAAGAAATGTTCAACT-CTGTGAGTTGAGTACACACATCACAAAGAAGTTTC

                                      **     **       
      76810 TGAGAATGCTTCTGTCTAGTTTTTATGGGAAGACGTTCCCTT
        130 TGAGAATGCTTCTGTCTAGTTTTTATATGAAGATATTCCCTT

              *  * *  **          *       *            **                  * 
      76852 TTTCACCAAAGGCATCAAAGCGCTCCAAATGTCCACTTCCAGACACTACAAAAAGAGTGTTTCCA
          1 TTCCAACGAAATCATCAAAGCGATCCAAATATCCACTTCCAGATTCTACAAAAAGAGTGTTTCAA

             **   *   **   ***                 *    * *  * *         *       
      76917 ACGTGCCCTAAGAAAGCGAATGTTCAACTCTGTGACTTGAATGCAGATATCACAAAGTAGTTTCT
         66 AACTGCTCTATCAAAAGAAATGTTCAACTCTGTGAGTTGAGTACACACATCACAAAGAAGTTTCT

                **            *     *    *         * 
      76982 GAGAGGGCTTCTGTCTAGATTTTAGATGATGATATTCCCGT
        131 GAGAATGCTTCTGTCTAGTTTTTATATGAAGATATTCCCTT

                           * *   *                *   *                    * 
      77023 TTCCAACGAAATCATTAGAGCTATCCAAATATCCACTTACAGTTTCTACAAAAAGAGTGTTTCCA
          1 TTCCAACGAAATCATCAAAGCGATCCAAATATCCACTTCCAGATTCTACAAAAAGAGTGTTTCAA

                   **         * *    *       *  *                   ***      
      77088 AACTGCTGCATCAAAAGAGAGGTTCCACTCTGTTAGCTGAGTACACACATCACAAACTTGTTTCT
         66 AACTGCTCTATCAAAAGAAATGTTCAACTCTGTGAGTTGAGTACACACATCACAAAGAAGTTTCT

            *     *         *        **         **   
      77153 CAGAATCCTTCTGTCTCGTTTTTATGGGAAGATATTTACTT
        131 GAGAATGCTTCTGTCTAGTTTTTATATGAAGATATTCCCTT

              *  *  * **          *       *     *       *  *  *              
      77194 TTTCACCGTAGGCATCAAAGCGCTCCAAATGTCCACATCCAGATACTCCAGAAAGAGTGTTTCAA
          1 TTCCAACGAAATCATCAAAGCGATCCAAATATCCACTTCCAGATTCTACAAAAAGAGTGTTTCAA

             *         *   * *   *         *        * *  *      *      *     
      77259 ACCTGCTCTATGAAAGGGAATCTTCAACTCTATGAGTTGAATGCAGACATCAGAAAGAAATTTCT
         66 AACTGCTCTATCAAAAGAAATGTTCAACTCTGTGAGTTGAGTACACACATCACAAAGAAGTTTCT

                     *       **          *         **
      77324 GAGAATGCTGCTGTCTACCTTTTAT-T--TGA-ATTCCCGC
        131 GAGAATGCTTCTGTCTAGTTTTTATATGAAGATATTCCCTT

                         *  *    *                *       *                  
      77361 TTCCAACGAAATCCTCCAAGCTATCCAAATATCCACTTGCAGATTCCACAAAAAGAGTGTTTCAA
          1 TTCCAACGAAATCATCAAAGCGATCCAAATATCCACTTCCAGATTCTACAAAAAGAGTGTTTCAA

                            ** *  *            *       *     *     *  *      
      77426 AACTGCTCTCTATCAATGGCAAAGTTCAACTCTGTTAGTTGAGGACACATATCACCAACAAGTTT
         66 AACTG--CTCTATCAAAAGAAATGTTCAACTCTGTGAGTTGAGTACACACATCACAAAGAAGTTT

                               *       **         *    
      77491 CTGAGAATGCTTCTGTCTATTTTTTATGGGAAGATATTTCCTT
        129 CTGAGAATGCTTCTGTCTAGTTTTTATATGAAGATATTCCCTT

              *  *  * ** *    *      *    *          * **                    
      77534 TTTCACCGTAGGCGTCAAGGCGATCGAAATGTCCACTTCCACAAACTACAAAAAGAGTGTTTCAA
          1 TTCCAACGAAATCATCAAAGCGATCCAAATATCCACTTCCAGATTCTACAAAAAGAGTGTTTCAA

             *         *   * **       *    *        * ** * *   **      *     
      77599 ACCTGCTCTATGAAAGGCGATGTTCATCTCTATGAGTTGAATGGAAATATCCGAAAGAAATTTCT
         66 AACTGCTCTATCAAAAGAAATGTTCAACTCTGTGAGTTGAGTACACACATCACAAAGAAGTTTCT

             *       *          *        *         **
      77664 GGGAATGCTGCTGTCTAG-TGTT-TAT-ACGA-ATTCCCGC
        131 GAGAATGCTTCTGTCTAGTTTTTATATGAAGATATTCCCTT

                         *       *                *    *  *                  
      77701 TTCCAACGAAATCCTCAAAGCAATCCAAATATCCACTTGCAGAATCCACAAAAAGAGTGTTTCAA
          1 TTCCAACGAAATCATCAAAGCGATCCAAATATCCACTTCCAGATTCTACAAAAAGAGTGTTTCAA

                          *     *          * *                   *  *        
      77766 AACTGCTCTATCAATAGAAAGGTTCAACTCTTTTAGTTGAGTACACACATCACGAACAAGTTTCT
         66 AACTGCTCTATCAAAAGAAATGTTCAACTCTGTGAGTTGAGTACACACATCACAAAGAAGTTTCT

                            * *      **     **  *    
      77831 GAGAATGCTTCTGTCTGGCTTTTATTGGAAGACGTTTCCTT
        131 GAGAATGCTTCTGTCTAGTTTTTATATGAAGATATTCCCTT

            *    * *  **          *       *                * *               
      77872 AT-CACCAAAGGCATCAAAGCGCTCCAAATGTCCACTTCCAGATTCTTCCAAAAGAGTGTTTCAA
          1 TTCCAACGAAATCATCAAAGCGATCCAAATATCCACTTCCAGATTCTACAAAAAGAGTGTTTCAA

             **     * ***  * *                 *    * *  * *     *   *       
      77936 ACGTGCTCAAAGTAAGGGAATGTTCAACTCTGTGACTTGAATGCAGATATCACCAAGTAGTTTCT
         66 AACTGCTCTATCAAAAGAAATGTTCAACTCTGTGAGTTGAGTACACACATCACAAAGAAGTTTCT

            * * *             *     *    *         * 
      78001 AATAGTGCTTCTGTCTAGATTTTAGATGATGATATTCCCGT
        131 GAGAATGCTTCTGTCTAGTTTTTATATGAAGATATTCCCTT

                         * * *   *             *  *   *                    * 
      78042 TTCCAACGAAATCGTTAGAGCTATCCAAATATCCAGTTACAGTTTCTACAAAAAGAGTGTTTCCA
          1 TTCCAACGAAATCATCAAAGCGATCCAAATATCCACTTCCAGATTCTACAAAAAGAGTGTTTCAA

                   **           *            *       *                     * 
      78107 AACTGCTGCATCAAAAGAAAGGTTCAACTCTGTTAGTTGAGGACACACATCACAAAGAAGTTTGT
         66 AACTGCTCTATCAAAAGAAATGTTCAACTCTGTGAGTTGAGTACACACATCACAAAGAAGTTTCT

                              *    *     *           
      78172 GAGAATGCTTCTGTCTAGATTTTGTATGACGATATTCCCTT
        131 GAGAATGCTTCTGTCTAGTTTTTATATGAAGATATTCCCTT

                     *   * *     *   *       * *  *    *  *       *  *       
      78213 TTCCAACGATATCGTTAAAGCAATCTAAATATCAATTTGCAGAATCCACAAAAATAGAGTTTCAA
          1 TTCCAACGAAATCATCAAAGCGATCCAAATATCCACTTCCAGATTCTACAAAAAGAGTGTTTCAA

             *       * *    *   *    *       *  *                   ***      
      78278 AGCTGCTCTGTAAAAATAAAAGTTCCACTCTGTTAGCTGAGTACACACATCACAAACTTGTTTCT
         66 AACTGCTCTATCAAAAGAAATGTTCAACTCTGTGAGTTGAGTACACACATCACAAAGAAGTTTCT

                  *         *        **         **   
      78343 GAGAATCCTTCTGTCTCGTTTTTATGGGAAGATATTTACTT
        131 GAGAATGCTTCTGTCTAGTTTTTATATGAAGATATTCCCTT

              *  *  * **          *       *     *       *  *  *     *        
      78384 TTTCACCGTAGGCATCAAAGCGCTCCAAATGTCCACATCCAGATACTCCAGAAAGACTGTTTCAA
          1 TTCCAACGAAATCATCAAAGCGATCCAAATATCCACTTCCAGATTCTACAAAAAGAGTGTTTCAA

             *         *   * *   *         *        * *  *      *      *     
      78449 ACCTGCTCTATGAAAGGGAATCTTCAACTCTATGAGTTGAATGCAGACATCAGAAAGAAATTTCT
         66 AACTGCTCTATCAAAAGAAATGTTCAACTCTGTGAGTTGAGTACACACATCACAAAGAAGTTTCT

                     *       **          *         **
      78514 GAGAATGCTGCTGTCTACCTTTTAT-T--TGA-ATTCCCGC
        131 GAGAATGCTTCTGTCTAGTTTTTATATGAAGATATTCCCTT

                         *  *    *                *  **   *                  
      78551 TTCCAACGAAATCCTCCAAGCTATCCAAATATCCACTTGCATTTTCCACAAAAAGAGTGTTTCAA
          1 TTCCAACGAAATCATCAAAGCGATCCAAATATCCACTTCCAGATTCTACAAAAAGAGTGTTTCAA

                          *               ** *  *  *                *        
      78616 AACTGCTCTATCAATAGAAATGTTCAACTCCTTTAGCTGGGTACACACATCACAAACAAGTTTCT
         66 AACTGCTCTATCAAAAGAAATGTTCAACTCTGTGAGTTGAGTACACACATCACAAAGAAGTTTCT

                                     **     **       
      78681 GAGAATGCTTCTGTCTAGTTTTTATGGGAAGACGTTCCCTT
        131 GAGAATGCTTCTGTCTAGTTTTTATATGAAGATATTCCCTT

              *  * *  **          *       *            **                    
      78722 TTTCACCAAAGGCATCAAAGCGCTCCAAATGTCCACTTCCAGACACTACAAAAAGAGTGTTTCAA
          1 TTCCAACGAAATCATCAAAGCGATCCAAATATCCACTTCCAGATTCTACAAAAAGAGTGTTTCAA

             **       **   ***                 *    * *  * *         *       
      78787 ACGTGCTCTAAGAAAGCGAATGTTCAACTCTGTGACTTGAATGCAGATATCACAAAGTAGTTTCT
         66 AACTGCTCTATCAAAAGAAATGTTCAACTCTGTGAGTTGAGTACACACATCACAAAGAAGTTTCT

                **            *     *    *         * 
      78852 GAGAGGGCTTCTGTCTAGATTTTAGATGATGATATTCCCGT
        131 GAGAATGCTTCTGTCTAGTTTTTATATGAAGATATTCCCTT

                           * *   *                *   *     *              * 
      78893 TTCCAACGAAATCATTAGAGCTATCCAAATATCCACTTACAGTTTCTATAAAAAGAGTGTTTCCA
          1 TTCCAACGAAATCATCAAAGCGATCCAAATATCCACTTCCAGATTCTACAAAAAGAGTGTTTCAA

                   **         * *    *       *  **                  ***      
      78958 AACTGCTGCATCAAAAGAGAGGTTCCACTCTGTTAGCCGAGTACACACATCACAAACTTGTTTCT
         66 AACTGCTCTATCAAAAGAAATGTTCAACTCTGTGAGTTGAGTACACACATCACAAAGAAGTTTCT

            *     *         *        **         **   
      79023 CAGAATCCTTCTGTCTCGTTTTTATGGGAAGATATTTACTT
        131 GAGAATGCTTCTGTCTAGTTTTTATATGAAGATATTCCCTT

                 *  * **          *       *     *       *  *  *              
      79064 TTCCACCGTAGGCATCAAAGCGCTCCAAATGTCCACATCCAGATACTCCAGAAAGAGTGTTTCAA
          1 TTCCAACGAAATCATCAAAGCGATCCAAATATCCACTTCCAGATTCTACAAAAAGAGTGTTTCAA

             *         *   * *   *         *        * *  *      *      *     
      79129 ACCTGCTCTATGAAAGGGAATCTTCAACTCTATGAGTTGAATGCAGACATCAGAAAGAAATTTCT
         66 AACTGCTCTATCAAAAGAAATGTTCAACTCTGTGAGTTGAGTACACACATCACAAAGAAGTTTCT

                     *       **          *         **
      79194 GAGAATGCTGCTGTCTACCTTTTAT-T--TGA-ATTCCCGC
        131 GAGAATGCTTCTGTCTAGTTTTTATATGAAGATATTCCCTT

                         *  *    *              * *  **   *    *             
      79231 TTCCAACGAAATCCTCCAAGCTATCCAAATATCCACCTGCATTTTCCACAACAAGAGTGTTTCAA
          1 TTCCAACGAAATCATCAAAGCGATCCAAATATCCACTTCCAGATTCTACAAAAAGAGTGTTTCAA

                          *                 *    *  *                *       
      79296 AACTGCTCTATCAATAGAAATGTTCAACTCCTTTG-GCTGGGTACACACATCACAAACAAGTTTC
         66 AACTGCTCTATCAAAAGAAATGTTCAACT-CTGTGAGTTGAGTACACACATCACAAAGAAGTTTC

                *                     **     *        
      79360 TGAGCATGCTTCTGTCTAGTTTTTATGGGAAGACATTCCCTT
        130 TGAGAATGCTTCTGTCTAGTTTTTATATGAAGATATTCCCTT

              *  * *  **          *       *            **                    
      79402 TTTCACCAAAGGCATCAAAGCGCTCCAAATGTCCACTTCCAGACACTACAAAAAGAGTGTTTCAA
          1 TTCCAACGAAATCATCAAAGCGATCCAAATATCCACTTCCAGATTCTACAAAAAGAGTGTTTCAA

             **       **    **                 *    * *  * *      *  *       
      79467 ACGTGCTCTAAGAAAACGAATGTTCAACTCTGTGACTTGAATGCAGATATCACAGAGTAGTTTCT
         66 AACTGCTCTATCAAAAGAAATGTTCAACTCTGTGAGTTGAGTACACACATCACAAAGAAGTTTCT

                **            *     *    *         * 
      79532 GAGAGGGCTTCTGTCTAGATTTTAGATGATGATATTCCCGT
        131 GAGAATGCTTCTGTCTAGTTTTTATATGAAGATATTCCCTT

                           * *   *                *   *                    * 
      79573 TTCCAACGAAATCATTAGAGCTATCCAAATATCCACTTACAGTTTCTACAAAAAGAGTGTTTCCA
          1 TTCCAACGAAATCATCAAAGCGATCCAAATATCCACTTCCAGATTCTACAAAAAGAGTGTTTCAA

                   **         * *    *       *  *                   ***      
      79638 AACTGCTGCATCAAAAGAGAGGTTCCACTCTGTTAGCTGAGTACACACATCACAAACTTGTTTCT
         66 AACTGCTCTATCAAAAGAAATGTTCAACTCTGTGAGTTGAGTACACACATCACAAAGAAGTTTCT

            *     *         *        **         **   
      79703 CAGAATCCTTCTGTCTCGTTTTTATGGGAAGATATTTACTT
        131 GAGAATGCTTCTGTCTAGTTTTTATATGAAGATATTCCCTT

              *  *  * **          *       *     *       *  *  *              
      79744 TTTCACCGTAGGCATCAAAGCGCTCCAAATGTCCACATCCAGATACTCCAGAAAGAGTGTTTCAA
          1 TTCCAACGAAATCATCAAAGCGATCCAAATATCCACTTCCAGATTCTACAAAAAGAGTGTTTCAA

             *         *   * *   *         *        * *  *      *      *     
      79809 ACCTGCTCTATGAAAGGGAATCTTCAACTCTATGAGTTGAATGCAGACATCAGAAAGAAATTTCT
         66 AACTGCTCTATCAAAAGAAATGTTCAACTCTGTGAGTTGAGTACACACATCACAAAGAAGTTTCT

                     *       **          *         **
      79874 GAGAATGCTGCTGTCTACCTTTTAT-T--TGA-ATTCCCGC
        131 GAGAATGCTTCTGTCTAGTTTTTATATGAAGATATTCCCTT

                         *  *    *                *       *                  
      79911 TTCCAACGAAATCCTCCAAGCTATCCAAATATCCACTTGCAGATTCCACAAAAAGAGTGTTTCAA
          1 TTCCAACGAAATCATCAAAGCGATCCAAATATCCACTTCCAGATTCTACAAAAAGAGTGTTTCAA

                            ** *  *            *       *     *     *  *      
      79976 AACTGCTCTCTATCAATGGCAAAGTTCAACTCTGTTAGTTGAGGACACATATCACCAACAAGTTT
         66 AACTG--CTCTATCAAAAGAAATGTTCAACTCTGTGAGTTGAGTACACACATCACAAAGAAGTTT

                               *       **         *    
      80041 CTGAGAATGCTTCTGTCTATTTTTTATGGGAAGATATTTCCTT
        129 CTGAGAATGCTTCTGTCTAGTTTTTATATGAAGATATTCCCTT

              *  *  * ** *    *      *    *          * **                    
      80084 TTTCACCGTAGGCGTCAAGGCGATCGAAATGTCCACTTCCACAAACTACAAAAAGAGTGTTTCAA
          1 TTCCAACGAAATCATCAAAGCGATCCAAATATCCACTTCCAGATTCTACAAAAAGAGTGTTTCAA

             *         *   * **       *    *        * ** * *   **      *     
      80149 ACCTGCTCTATGAAAGGCCATGTTCATCTCTATGAGTTGAATGGAAATATCCGAAAGAAATTTCT
         66 AACTGCTCTATCAAAAGAAATGTTCAACTCTGTGAGTTGAGTACACACATCACAAAGAAGTTTCT

             *       *          *        *         **
      80214 GGGAATGCTGCTGTCTAG-TGTT-TAT-ACGA-ATTCCCGC
        131 GAGAATGCTTCTGTCTAGTTTTTATATGAAGATATTCCCTT

                         *       *                *    *  *                  
      80251 TTCCAACGAAATCCTCAAAGCAATCCAAATATCCACTTGCAGAATCCACAAAAAGAGTGTTTCAA
          1 TTCCAACGAAATCATCAAAGCGATCCAAATATCCACTTCCAGATTCTACAAAAAGAGTGTTTCAA

                          *     *          * *                   *  *        
      80316 AACTGCTCTATCAATAGAAAGGTTCAACTCTTTTAGTTGAGTACACACATCACGAACAAGTTTCT
         66 AACTGCTCTATCAAAAGAAATGTTCAACTCTGTGAGTTGAGTACACACATCACAAAGAAGTTTCT

                            * *      **     **  *    
      80381 GAGAATGCTTCTGTCTGGCTTTTATTGGAAGACGTTTCCTT
        131 GAGAATGCTTCTGTCTAGTTTTTATATGAAGATATTCCCTT

                 * *  **          *       *                * *               
      80422 TT-CACCAAAGGCATCAAAGCGCTCCAAATGTCCACTTCCAGATTCTTCCAAAAGAGTGTTTCAA
          1 TTCCAACGAAATCATCAAAGCGATCCAAATATCCACTTCCAGATTCTACAAAAAGAGTGTTTCAA

             **     * ***  * *                 *    * *  * *     *   *       
      80486 ACGTGCTCAAAGTAAGGGAATGTTCAACTCTGTGACTTGAATGCAGATATCACCAAGTAGTTTCT
         66 AACTGCTCTATCAAAAGAAATGTTCAACTCTGTGAGTTGAGTACACACATCACAAAGAAGTTTCT

            * * *             *   * *    *         * 
      80551 AATAGTGCTTCTGTCTAGATTTCAGATGATGATATTCCCGT
        131 GAGAATGCTTCTGTCTAGTTTTTATATGAAGATATTCCCTT

                         * * *   * **          *  *   *      *     *       * 
      80592 TTCCAACGAAATCGTTAGAGCTAAGCAAATATCCAGTTACAGTTTCTACCAAAAGGGTGTTTCCA
          1 TTCCAACGAAATCATCAAAGCGATCCAAATATCCACTTCCAGATTCTACAAAAAGAGTGTTTCAA

              *    **           *            *       *                     * 
      80657 AATTGCTGCATCAAAAGAAAGGTTCAACTCTGTTAGTTGAGGACACACATCACAAAGAAGTTTGT
         66 AACTGCTCTATCAAAAGAAATGTTCAACTCTGTGAGTTGAGTACACACATCACAAAGAAGTTTCT

                              *    *     *           
      80722 GAGAATGCTTCTGTCTAGATTTTGTATGACGATATTCCCTT
        131 GAGAATGCTTCTGTCTAGTTTTTATATGAAGATATTCCCTT

                     *   * *     *   *       * *  *    *  *       *  *       
      80763 TTCCAACGATATCGTTAAAGCAATCTAAATATCAATTTGCAGAATCCACAAAAATAGAGTTTCAA
          1 TTCCAACGAAATCATCAAAGCGATCCAAATATCCACTTCCAGATTCTACAAAAAGAGTGTTTCAA

             *       * *        *    *       *  *                   ***      
      80828 AGCTGCTCTGTAAAAAGAAAGGTTCCACTCTGTTAGCTGAGTACACACATCACAAACTTGTTTCT
         66 AACTGCTCTATCAAAAGAAATGTTCAACTCTGTGAGTTGAGTACACACATCACAAAGAAGTTTCT

                  *         *        **         **   
      80893 GAGAATCCTTCTGTCTCGTTTTTATGGGAAGATATTTACTT
        131 GAGAATGCTTCTGTCTAGTTTTTATATGAAGATATTCCCTT

                 *  * **          *       *     *       *  *  *              
      80934 TTCCACCGTAGGCATCAAAGCGCTCCAAATGTCCACATCCAGATACTCCAGAAAGAGTGTTTCAA
          1 TTCCAACGAAATCATCAAAGCGATCCAAATATCCACTTCCAGATTCTACAAAAAGAGTGTTTCAA

             *         *   * *   *         *        * *  *      *      *     
      80999 ACCTGCTCTATGAAAGGGAATCTTCAACTCTATGAGTTGAATGCAGACATCAGAAAGAAATTTCT
         66 AACTGCTCTATCAAAAGAAATGTTCAACTCTGTGAGTTGAGTACACACATCACAAAGAAGTTTCT

                     *       **          *         **
      81064 GAGAATGCTGCTGTCTACCTTTTAT-T--TGA-ATTCCCGC
        131 GAGAATGCTTCTGTCTAGTTTTTATATGAAGATATTCCCTT

                         *  *    *              * *  **   *                  
      81101 TTCCAACGAAATCCTCCAAGCTATCCAAATATCCACCTGCATTTTCCACAAAAAGAGTGTTTCAA
          1 TTCCAACGAAATCATCAAAGCGATCCAAATATCCACTTCCAGATTCTACAAAAAGAGTGTTTCAA

                          *                 *    *  *                *       
      81166 AACTGCTCTATCAATAGAAATGTTCAACTCCTTTG-GCTGGGTACACACATCACAAACAAGTTTC
         66 AACTGCTCTATCAAAAGAAATGTTCAACT-CTGTGAGTTGAGTACACACATCACAAAGAAGTTTC

                *                     **     *        
      81230 TGAGCATGCTTCTGTCTAGTTTTTATGGGAAGACATTCCCTT
        130 TGAGAATGCTTCTGTCTAGTTTTTATATGAAGATATTCCCTT

              *  * *  **          *       *            **                    
      81272 TTTCACCAAAGGCATCAAAGCGCTCCAAATGTCCACTTCCAGACACTACAAAAAGAGTGTTTCAA
          1 TTCCAACGAAATCATCAAAGCGATCCAAATATCCACTTCCAGATTCTACAAAAAGAGTGTTTCAA

             **       **    **                 *    * *  * *      *  *       
      81337 ACGTGCTCTAAGAAAACGAATGTTCAACTCTGTGACTTGAATGCAGATATCACAGAGTAGTTTCT
         66 AACTGCTCTATCAAAAGAAATGTTCAACTCTGTGAGTTGAGTACACACATCACAAAGAAGTTTCT

                **            *     *    *         * 
      81402 GAGAGGGCTTCTGTCTAGATTTTAGATGATGATATTCCCGT
        131 GAGAATGCTTCTGTCTAGTTTTTATATGAAGATATTCCCTT

                           * *   *                *   *                    * 
      81443 TTCCAACGAAATCATTAGAGCTATCCAAATATCCACTTACAGTTTCTACAAAAAGAGTGTTTCCA
          1 TTCCAACGAAATCATCAAAGCGATCCAAATATCCACTTCCAGATTCTACAAAAAGAGTGTTTCAA

                   **         * *    *       *  *                   ***      
      81508 AACTGCTGCATCAAAAGAGAGGTTCCACTCTGTTAGCTGAGTACACACATCACAAACTTGTTTCT
         66 AACTGCTCTATCAAAAGAAATGTTCAACTCTGTGAGTTGAGTACACACATCACAAAGAAGTTTCT

            *     *         *        **         **   
      81573 CAGAATCCTTCTGTCTCGTTTTTATGGGAAGATATTTACTT
        131 GAGAATGCTTCTGTCTAGTTTTTATATGAAGATATTCCCTT

              *  *  * **          *       *     *       **    *       *      
      81614 TTTCACCGTAGGCATCAAAGCGCTCCAAATGTCCACATCCAGATAGTACAGAAAGAGTTTTTCAA
          1 TTCCAACGAAATCATCAAAGCGATCCAAATATCCACTTCCAGATTCTACAAAAAGAGTGTTTCAA

             *         *   * *   *         *        * *  *      *      *     
      81679 ACCTGCTCTATGAAAGGGAATCTTCAACTCTATGAGTTGAATGCAGACATCAGAAAGAAATTTCT
         66 AACTGCTCTATCAAAAGAAATGTTCAACTCTGTGAGTTGAGTACACACATCACAAAGAAGTTTCT

                     *       **          *         **
      81744 GAGAATGCTGCTGTCTACCTTTTAT-T--TGA-ATTCCCGC
        131 GAGAATGCTTCTGTCTAGTTTTTATATGAAGATATTCCCTT

                         *  *    *                *       *                  
      81781 TTCCAACGAAATCCTCCAAGCTATCCAAATATCCACTTGCAGATTCCACAAAAAGAGTGTTTCAA
          1 TTCCAACGAAATCATCAAAGCGATCCAAATATCCACTTCCAGATTCTACAAAAAGAGTGTTTCAA

                            ** *  *            *       *     *     *  *      
      81846 AACTGCTCTCTATCAATGGCAAAGTTCAACTCTGTTAGTTGAGGACACATATCACCAACAAGTTT
         66 AACTG--CTCTATCAAAAGAAATGTTCAACTCTGTGAGTTGAGTACACACATCACAAAGAAGTTT

                               *       **         *    
      81911 CTGAGAATGCTTCTGTCTATTTTTTATGGGAAGATATTTCCTT
        129 CTGAGAATGCTTCTGTCTAGTTTTTATATGAAGATATTCCCTT

              *  *  * ** *    *      *    *          * **                    
      81954 TTTCACCGTAGGCGTCAAGGCGATCGAAATGTCCACTTCCACAAACTACAAAAAGAGTGTTTCAA
          1 TTCCAACGAAATCATCAAAGCGATCCAAATATCCACTTCCAGATTCTACAAAAAGAGTGTTTCAA

             *         *   * **       *    *        * ** * *   **      *     
      82019 ACCTGCTCTATGAAAGGCCATGTTCATCTCTATGAGTTGAATGGAAATATCCGAAAGAAATTTCT
         66 AACTGCTCTATCAAAAGAAATGTTCAACTCTGTGAGTTGAGTACACACATCACAAAGAAGTTTCT

             *       *          *        *         **
      82084 GGGAATGCTGCTGTCTAG-TGTT-TAT-ACGA-ATTCCCGC
        131 GAGAATGCTTCTGTCTAGTTTTTATATGAAGATATTCCCTT

                         *       *                *    *  *                  
      82121 TTCCAACGAAATCCTCAAAGCAATCCAAATATCCACTTGCAGAATCCACAAAAAGAGTGTTTCAA
          1 TTCCAACGAAATCATCAAAGCGATCCAAATATCCACTTCCAGATTCTACAAAAAGAGTGTTTCAA

                          *     *          * *                   *  *        
      82186 AACTGCTCTATCAATAGAAAGGTTCAACTCTTTTAGTTGAGTACACACATCACGAACAAGTTTCT
         66 AACTGCTCTATCAAAAGAAATGTTCAACTCTGTGAGTTGAGTACACACATCACAAAGAAGTTTCT

                            * *      **     **  *    
      82251 GAGAATGCTTCTGTCTGGCTTTTATTGGAAGACGTTTCCTT
        131 GAGAATGCTTCTGTCTAGTTTTTATATGAAGATATTCCCTT

                 * *  **          *       *                * *               
      82292 TT-CACCAAAGGCATCAAAGCGCTCCAAATGTCCACTTCCAGATTCTTCCAAAAGAGTGTTTCAA
          1 TTCCAACGAAATCATCAAAGCGATCCAAATATCCACTTCCAGATTCTACAAAAAGAGTGTTTCAA

             **     * ***  * *                 *    * *  * *     *   *       
      82356 ACGTGCTCAAAGTAAGGGAATGTTCAACTCTGTGACTTGAATGCAGATATCACCAAGTAGTTTCT
         66 AACTGCTCTATCAAAAGAAATGTTCAACTCTGTGAGTTGAGTACACACATCACAAAGAAGTTTCT

            * * *             *   * *    *         * 
      82421 AATAGTGCTTCTGTCTAGATTTCAGATGATGATATTCCCGT
        131 GAGAATGCTTCTGTCTAGTTTTTATATGAAGATATTCCCTT

                         * * *   * **          *  *   *      *     *       * 
      82462 TTCCAACGAAATCGTTAGAGCTAAGCAAATATCCAGTTACAGTTTCTACCAAAAGGGTGTTTCCA
          1 TTCCAACGAAATCATCAAAGCGATCCAAATATCCACTTCCAGATTCTACAAAAAGAGTGTTTCAA

              *    **           *            *       *                     * 
      82527 AATTGCTGCATCAAAAGAAAGGTTCAACTCTGTTAGTTGAGGACACACATCACAAAGAAGTTTGT
         66 AACTGCTCTATCAAAAGAAATGTTCAACTCTGTGAGTTGAGTACACACATCACAAAGAAGTTTCT

                              *    *     *           
      82592 GAGAATGCTTCTGTCTAGATTTTGTATGACGATATTCCCTT
        131 GAGAATGCTTCTGTCTAGTTTTTATATGAAGATATTCCCTT

                     *   * *     *   *       * *  *    *  *       *  *       
      82633 TTCCAACGATATCGTTAAAGCAATCTAAATATCAATTTGCAGAATCCACAAAAATAGAGTTTCAA
          1 TTCCAACGAAATCATCAAAGCGATCCAAATATCCACTTCCAGATTCTACAAAAAGAGTGTTTCAA

             *       * *        *    *       *  *                   ***      
      82698 AGCTGCTCTGTAAAAAGAAAGGTTCCACTCTGTTAGCTGAGTACACACATCACAAACTTGTTTCT
         66 AACTGCTCTATCAAAAGAAATGTTCAACTCTGTGAGTTGAGTACACACATCACAAAGAAGTTTCT

                  *         *        **         **   
      82763 GAGAATCCTTCTGTCTCGTTTTTATGGGAAGATATTTACTT
        131 GAGAATGCTTCTGTCTAGTTTTTATATGAAGATATTCCCTT

                 *  * **          *       *     *       *  *  *  *           
      82804 TTCCACCGTAGGCATCAAAGCGCTCCAAATGTCCACATCCAGATACTCCAGAACGAGTGTTTCAA
          1 TTCCAACGAAATCATCAAAGCGATCCAAATATCCACTTCCAGATTCTACAAAAAGAGTGTTTCAA

             *         *   * *   *         *        * *  *      *      *     
      82869 ACCTGCTCTATGAAAGGGAATCTTCAACTCTATGAGTTGAATGCAGACATCAGAAAGAAATTTCT
         66 AACTGCTCTATCAAAAGAAATGTTCAACTCTGTGAGTTGAGTACACACATCACAAAGAAGTTTCT

                     *       **          *         **
      82934 GAGAATGCTGCTGTCTACCTTTTAT-T--TGA-ATTCCCGC
        131 GAGAATGCTTCTGTCTAGTTTTTATATGAAGATATTCCCTT

                         *  *    *              * *  **   *    *             
      82971 TTCCAACGAAATCCTCCAAGCTATCCAAATATCCACCTGCATTTTCCACAACAAGAGTGTTTCAA
          1 TTCCAACGAAATCATCAAAGCGATCCAAATATCCACTTCCAGATTCTACAAAAAGAGTGTTTCAA

                          *                 *    *  *                *       
      83036 AACTGCTCTATCAATAGAAATGTTCAACTCCTTTG-GCTGGGTACACACATCACAAACAAGTTTC
         66 AACTGCTCTATCAAAAGAAATGTTCAACT-CTGTGAGTTGAGTACACACATCACAAAGAAGTTTC

                                      **     **       
      83100 TGAGAATGCTTCTGTCTAGTTTTTATGGGAAGACGTTCCCTT
        130 TGAGAATGCTTCTGTCTAGTTTTTATATGAAGATATTCCCTT

              *  * *  **          *       *            **                    
      83142 TTTCACCAAAGGCATCAAAGCGCTCCAAATGTCCACTTCCAGACACTACAAAAAGAGTGTTTCAA
          1 TTCCAACGAAATCATCAAAGCGATCCAAATATCCACTTCCAGATTCTACAAAAAGAGTGTTTCAA

             **       **   ***                 *    * *  * *         *       
      83207 ACGTGCTCTAAGAAAGCGAATGTTCAACTCTGTGACTTGAATGCAGATATCACAAAGTAGTTTCT
         66 AACTGCTCTATCAAAAGAAATGTTCAACTCTGTGAGTTGAGTACACACATCACAAAGAAGTTTCT

                **            *     *    *         * 
      83272 GAGAGGGCTTCTGTCTAGATTTTAGATGATGATATTCCCGT
        131 GAGAATGCTTCTGTCTAGTTTTTATATGAAGATATTCCCTT

                           * *   *                *   *                    * 
      83313 TTCCAACGAAATCATTAGAGCTATCCAAATATCCACTTACAGTTTCTACAAAAAGAGTGTTTCCA
          1 TTCCAACGAAATCATCAAAGCGATCCAAATATCCACTTCCAGATTCTACAAAAAGAGTGTTTCAA

                   **         * *    *       *  *                   ***      
      83378 AACTGCTGCATCAAAAGAGAGGTTCCACTCTGTTAGCTGAGTACACACATCACAAACTTGTTTCT
         66 AACTGCTCTATCAAAAGAAATGTTCAACTCTGTGAGTTGAGTACACACATCACAAAGAAGTTTCT

            *     *         *        **         **   
      83443 CAGAATCCTTCTGTCTCGTTTTTATGGGAAGATATTTACTT
        131 GAGAATGCTTCTGTCTAGTTTTTATATGAAGATATTCCCTT

              *  *  * **          *       *     *       *  *  *              
      83484 TTTCACCGTAGGCATCAAAGCGCTCCAAATGTCCACATCCAGATACTCCAGAAAGAGTGTTTCAA
          1 TTCCAACGAAATCATCAAAGCGATCCAAATATCCACTTCCAGATTCTACAAAAAGAGTGTTTCAA

             *         *   * *   *         *        * *  *      *      *     
      83549 ACCTGCTCTATGAAAGGGAATCTTCAACTCTATGAGTTGAATGCAGACATCAGAAAGAAATTTCT
         66 AACTGCTCTATCAAAAGAAATGTTCAACTCTGTGAGTTGAGTACACACATCACAAAGAAGTTTCT

                     *       **          *         **
      83614 GAGAATGCTGCTGTCTACCTTTTAT-T--TGA-ATTCCCGC
        131 GAGAATGCTTCTGTCTAGTTTTTATATGAAGATATTCCCTT

                         *  *    *                *       *                  
      83651 TTCCAACGAAATCCTCCAAGCTATCCAAATATCCACTTGCAGATTCCACAAAAAGAGTGTTTCAA
          1 TTCCAACGAAATCATCAAAGCGATCCAAATATCCACTTCCAGATTCTACAAAAAGAGTGTTTCAA

                            ** *  *            *       *     *     *  *      
      83716 AACTGCTCTCTATCAATGGCAAAGTTCAACTCTGTTAGTTGAGGACACATATCACCAACAAGTTT
         66 AACTG--CTCTATCAAAAGAAATGTTCAACTCTGTGAGTTGAGTACACACATCACAAAGAAGTTT

                               *       **         *    
      83781 CTGAGAATGCTTCTGTCTATTTTTTATGGGAAGATATTTCCTT
        129 CTGAGAATGCTTCTGTCTAGTTTTTATATGAAGATATTCCCTT

              *  *  * ** *    *      *    *          * **                    
      83824 TTTCACCGTAGGCGTCAAGGCGATCGAAATGTCCACTTCCACAAACTACAAAAAGAGTGTTTCAA
          1 TTCCAACGAAATCATCAAAGCGATCCAAATATCCACTTCCAGATTCTACAAAAAGAGTGTTTCAA

             *         *   * **       *    *        * ** * *   **      *     
      83889 ACCTGCTCTATGAAAGGCCATGTTCATCTCTATGAGTTGAATGGAAATATCCGAAAGAAATTTCT
         66 AACTGCTCTATCAAAAGAAATGTTCAACTCTGTGAGTTGAGTACACACATCACAAAGAAGTTTCT

             *       *          *        *         **
      83954 GGGAATGCTGCTGTCTAG-TGTT-TAT-ACGA-ATTCCCGC
        131 GAGAATGCTTCTGTCTAGTTTTTATATGAAGATATTCCCTT

                         *       *                *    *  *                  
      83991 TTCCAACGAAATCCTCAAAGCAATCCAAATATCCACTTGCAGAATCCACAAAAAGAGTGTTTCAA
          1 TTCCAACGAAATCATCAAAGCGATCCAAATATCCACTTCCAGATTCTACAAAAAGAGTGTTTCAA

                          *     *          * *                   *  *        
      84056 AACTGCTCTATCAATAGAAAGGTTCAACTCTTTTAGTTGAGTACACACATCACGAACAAGTTTCT
         66 AACTGCTCTATCAAAAGAAATGTTCAACTCTGTGAGTTGAGTACACACATCACAAAGAAGTTTCT

                            * *      **     **  *    
      84121 GAGAATGCTTCTGTCTGGCTTTTATTGGAAGACGTTTCCTT
        131 GAGAATGCTTCTGTCTAGTTTTTATATGAAGATATTCCCTT

                 * *  **          *       *                * *               
      84162 TT-CACCAAAGGCATCAAAGCGCTCCAAATGTCCACTTCCAGATTCTTCCAAAAGAGTGTTTCAA
          1 TTCCAACGAAATCATCAAAGCGATCCAAATATCCACTTCCAGATTCTACAAAAAGAGTGTTTCAA

             **     * ***  * *                 *    * *  * *     *   *       
      84226 ACGTGCTCAAAGTAAGGGAATGTTCAACTCTGTGACTTGAATGCAGATATCACCAAGTAGTTTCT
         66 AACTGCTCTATCAAAAGAAATGTTCAACTCTGTGAGTTGAGTACACACATCACAAAGAAGTTTCT

            * * *             *   * *    *         * 
      84291 AATAGTGCTTCTGTCTAGATTTCAGATGATGATATTCCCGT
        131 GAGAATGCTTCTGTCTAGTTTTTATATGAAGATATTCCCTT

                         * * *   * **          *  *   *      *     *       * 
      84332 TTCCAACGAAATCGTTAGAGCTAAGCAAATATCCAGTTACAGTTTCTACCAAAAGGGTGTTTCCA
          1 TTCCAACGAAATCATCAAAGCGATCCAAATATCCACTTCCAGATTCTACAAAAAGAGTGTTTCAA

              *    **           *            *       *                     * 
      84397 AATTGCTGCATCAAAAGAAAGGTTCAACTCTGTTAGTTGAGGACACACATCACAAAGAAGTTTGT
         66 AACTGCTCTATCAAAAGAAATGTTCAACTCTGTGAGTTGAGTACACACATCACAAAGAAGTTTCT

                              *    *     *           
      84462 GAGAATGCTTCTGTCTAGATTTTGTATGACGATATTCCCTT
        131 GAGAATGCTTCTGTCTAGTTTTTATATGAAGATATTCCCTT

                     *   * *     *   *       * *  *    *  *       *  *       
      84503 TTCCAACGATATCGTTAAAGCAATCTAAATATCAATTTGCAGAATCCACAAAAATAGCGTTTCAA
          1 TTCCAACGAAATCATCAAAGCGATCCAAATATCCACTTCCAGATTCTACAAAAAGAGTGTTTCAA

             *       * *        *    *       *  *                   ***      
      84568 AGCTGCTCTGTAAAAAGAAAGGTTCCACTCTGTTAGCTGAGTACACACATCACAAACTTGTTTCT
         66 AACTGCTCTATCAAAAGAAATGTTCAACTCTGTGAGTTGAGTACACACATCACAAAGAAGTTTCT

                  *         *        **         **   
      84633 GAGAATCCTTCTGTCTCGTTTTTATGGGAAGATATTTACTT
        131 GAGAATGCTTCTGTCTAGTTTTTATATGAAGATATTCCCTT

                 *  * **          *       *     *       *  *  *  *           
      84674 TTCCACCGTAGGCATCAAAGCGCTCCAAATGTCCACATCCAGATACTCCAGAACGAGTGTTTCAA
          1 TTCCAACGAAATCATCAAAGCGATCCAAATATCCACTTCCAGATTCTACAAAAAGAGTGTTTCAA

             *         *   * *   *         *        * *  *      *      *     
      84739 ACCTGCTCTATGAAAGGGAATCTTCAACTCTATGAGTTGAATGCAGACATCAGAAAGAAATTTCT
         66 AACTGCTCTATCAAAAGAAATGTTCAACTCTGTGAGTTGAGTACACACATCACAAAGAAGTTTCT

                     *       **          *         **
      84804 GAGAATGCTGCTGTCTACCTTTTAT-T--TGA-ATTCCCGC
        131 GAGAATGCTTCTGTCTAGTTTTTATATGAAGATATTCCCTT

                         *  *    *              * *  **   *    *             
      84841 TTCCAACGAAATCCTCCAAGCTATCCAAATATCCACCTGCATTTTCCACAACAAGAGTGTTTCAA
          1 TTCCAACGAAATCATCAAAGCGATCCAAATATCCACTTCCAGATTCTACAAAAAGAGTGTTTCAA

                          *                 *    *  *                *       
      84906 AACTGCTCTATCAATAGAAATGTTCAACTCCTTTG-GCTGGGTACACACATCACAAACAAGTTTC
         66 AACTGCTCTATCAAAAGAAATGTTCAACT-CTGTGAGTTGAGTACACACATCACAAAGAAGTTTC

                                      **     **       
      84970 TGAGAATGCTTCTGTCTAGTTTTTATGGGAAGACGTTCCCTT
        130 TGAGAATGCTTCTGTCTAGTTTTTATATGAAGATATTCCCTT

              *  * *  **          *       *            **                    
      85012 TTTCACCAAAGGCATCAAAGCGCTCCAAATGTCCACTTCCAGACACTACAAAAAGAGTGTTTCAA
          1 TTCCAACGAAATCATCAAAGCGATCCAAATATCCACTTCCAGATTCTACAAAAAGAGTGTTTCAA

             **       **   ***                 *    * *  * *         *       
      85077 ACGTGCTCTAAGAAAGCGAATGTTCAACTCTGTGACTTGAATGCAGATATCACAAAGTAGTTTCT
         66 AACTGCTCTATCAAAAGAAATGTTCAACTCTGTGAGTTGAGTACACACATCACAAAGAAGTTTCT

                **            *     *    *         * 
      85142 GAGAGGGCTTCTGTCTAGATTTTAGATGATGATATTCCCGT
        131 GAGAATGCTTCTGTCTAGTTTTTATATGAAGATATTCCCTT

                           * *   *                *   *                    * 
      85183 TTCCAACGAAATCATTAGAGCTATCCAAATATCCACTTACAGTTTCTACAAAAAGAGTGTTTCCA
          1 TTCCAACGAAATCATCAAAGCGATCCAAATATCCACTTCCAGATTCTACAAAAAGAGTGTTTCAA

                   **         * *    *       *  **                  ***      
      85248 AACTGCTGCATCAAAAGAGAGGTTCCACTCTGTTAGCCGAGTACACACATCACAAACTTGTTTCT
         66 AACTGCTCTATCAAAAGAAATGTTCAACTCTGTGAGTTGAGTACACACATCACAAAGAAGTTTCT

            *     *         *        **         **   
      85313 CAGAATCCTTCTGTCTCGTTTTTATGGGAAGATATTTACTT
        131 GAGAATGCTTCTGTCTAGTTTTTATATGAAGATATTCCCTT

              *  *  * **          *       *     *       *  *  *              
      85354 TTTCACCGTAGGCATCAAAGCGCTCCAAATGTCCACATCCAGATACTCCAGAAAGAGTGTTTCAA
          1 TTCCAACGAAATCATCAAAGCGATCCAAATATCCACTTCCAGATTCTACAAAAAGAGTGTTTCAA

             *         *   * *   *         *        * *  *      *      *     
      85419 ACCTGCTCTATGAAAGGGAATCTTCAACTCTATGAGTTGAATGCAGACATCAGAAAGAAATTTCT
         66 AACTGCTCTATCAAAAGAAATGTTCAACTCTGTGAGTTGAGTACACACATCACAAAGAAGTTTCT

                     *       **          *         **
      85484 GAGAATGCTGCTGTCTACCTTTTAT-T--TGA-ATTCCCGC
        131 GAGAATGCTTCTGTCTAGTTTTTATATGAAGATATTCCCTT

                         *  *    *                *       *                  
      85521 TTCCAACGAAATCCTCCAAGCTATCCAAATATCCACTTGCAGATTCCACAAAAAGAGTGTTTCAA
          1 TTCCAACGAAATCATCAAAGCGATCCAAATATCCACTTCCAGATTCTACAAAAAGAGTGTTTCAA

                          ** *  *            *       *     *     *  *        
      85586 AACTG--CTATCAATGGCAAAGTTCAACTCTGTTAGTTGAGGACACATATCACCAACAAGTTTCT
         66 AACTGCTCTATCAAAAGAAATGTTCAACTCTGTGAGTTGAGTACACACATCACAAAGAAGTTTCT

                             *  *    **         *    
      85649 GAGAATGCTTCTGTCTATTTCTTATGGGAAGATATTTCCTT
        131 GAGAATGCTTCTGTCTAGTTTTTATATGAAGATATTCCCTT

              *  *  * ** *    *      *    *          * **                    
      85690 TTTCAGCGTAGGCGTCAAGGCGATCGAAATGTCCACTTCCACAAACTACAAAAAGAGTGTTTCAA
          1 TTCCAACGAAATCATCAAAGCGATCCAAATATCCACTTCCAGATTCTACAAAAAGAGTGTTTCAA

             *         *   * **       *    *        * ** * *   **      *     
      85755 ACCTGCTCTATGAAAGGCCATGTTCATCTCTATGAGTTGAATGGAAATATCCGAAAGAAATTTCT
         66 AACTGCTCTATCAAAAGAAATGTTCAACTCTGTGAGTTGAGTACACACATCACAAAGAAGTTTCT

             *       *          *        *         **
      85820 GGGAATGCTGCTGTCTAG-TGTT-TAT-ACGA-ATTCCCGC
        131 GAGAATGCTTCTGTCTAGTTTTTATATGAAGATATTCCCTT

                         *       *                *    *                     
      85857 TTCCAACGAAATCCTCAAAGCAATCCAAATATCCACTTGCAGAATCTACAAAAAGAGTGTTTCAA
          1 TTCCAACGAAATCATCAAAGCGATCCAAATATCCACTTCCAGATTCTACAAAAAGAGTGTTTCAA

                          *     *      *   * *                   *  *        
      85922 AACTGCTCTATCAATAGAAAGGTTCAAATCTTTTAGTTGAGTACACACATCACGAACAAGTTTCT
         66 AACTGCTCTATCAAAAGAAATGTTCAACTCTGTGAGTTGAGTACACACATCACAAAGAAGTTTCT

                            * *      **     **  *    
      85987 GAGAATGCTTCTGTCTGGCTTTTATTGGAAGACGTTTCCTT
        131 GAGAATGCTTCTGTCTAGTTTTTATATGAAGATATTCCCTT

                 * *  **        * *       *                  *               
      86028 TT-CACCAAAGGCATCAAAGTGCTCCAAATGTCCACTTCCAGATTCTACCAAAAGAGTGTTTCAA
          1 TTCCAACGAAATCATCAAAGCGATCCAAATATCCACTTCCAGATTCTACAAAAAGAGTGTTTCAA

             **         **  * *                 *    * *  * *     *   *      
      86092 ACGTGCTC-ATAGTAAGGGAATGTTCAACTCTGTGACTTGAATGCAGATATCACCAAGTAGTTTC
         66 AACTGCTCTAT-CAAAAGAAATGTTCAACTCTGTGAGTTGAGTACACACATCACAAAGAAGTTTC

             * * *             *     *    *         * 
      86156 TAATAGTGCTTCTGTCTAGATTTTAGATGATGATATTCCCGT
        130 TGAGAATGCTTCTGTCTAGTTTTTATATGAAGATATTCCCTT

                         *** *   *             *  *   *      *     *       * 
      86198 TTCCAACGAAATCGCTAGAGCTATCCAAATATCCAGTTACAGTTTCTACCAAAAGGGTGTTTCCA
          1 TTCCAACGAAATCATCAAAGCGATCCAAATATCCACTTCCAGATTCTACAAAAAGAGTGTTTCAA

              *    **           *            *       *                     * 
      86263 AATTGCTGCATCAAAAGAAAGGTTCAACTCTGTTAGTTGAGGACACACATCACAAAGAAGTTTGT
         66 AACTGCTCTATCAAAAGAAATGTTCAACTCTGTGAGTTGAGTACACACATCACAAAGAAGTTTCT

                              *    *     *           
      86328 GAGAATGCTTCTGTCTAGATTTTGTATGACGATATTCCCTT
        131 GAGAATGCTTCTGTCTAGTTTTTATATGAAGATATTCCCTT

                     *   * *     *   *       * *  *    *  *       *  *       
      86369 TTCCAACGATATCGTTAAAGCAATCTAAATATCAATTTGCAGAATCCACAAAAATAGAGTTTCAA
          1 TTCCAACGAAATCATCAAAGCGATCCAAATATCCACTTCCAGATTCTACAAAAAGAGTGTTTCAA

             *       * *        *    *       *  *                   ***      
      86434 AGCTGCTCTGTAAAAAGAAAGGTTCCACTCTGTTAGCTGAGTACACACATCACAAACTTGTTTCT
         66 AACTGCTCTATCAAAAGAAATGTTCAACTCTGTGAGTTGAGTACACACATCACAAAGAAGTTTCT

                  *         *        **         **   
      86499 GAGAATCCTTCTGTCTCGTTTTTATGGGAAGATATTTACTT
        131 GAGAATGCTTCTGTCTAGTTTTTATATGAAGATATTCCCTT

                 *  * **          *       *     *       *  *  *  *           
      86540 TTCCACCGTAGGCATCAAAGCGCTCCAAATGTCCACATCCAGATACTCCAGAACGAGTGTTTCAA
          1 TTCCAACGAAATCATCAAAGCGATCCAAATATCCACTTCCAGATTCTACAAAAAGAGTGTTTCAA

             *         *   * *   *         *        * *  *      *      *     
      86605 ACCTGCTCTATGAAAGGGAATCTTCAACTCTATGAGTTGAATGCAGACATCAGAAAGAAATTTCT
         66 AACTGCTCTATCAAAAGAAATGTTCAACTCTGTGAGTTGAGTACACACATCACAAAGAAGTTTCT

                     *       **          *         **
      86670 GAGAATGCTGCTGTCTACCTTTTAT-T--TGA-ATTCCCGC
        131 GAGAATGCTTCTGTCTAGTTTTTATATGAAGATATTCCCTT

                         *  *    *              * *  **   *    *             
      86707 TTCCAACGAAATCCTCCAAGCTATCCAAATATCCACCTGCATTTTCCACAACAAGAGTGTTTCAA
          1 TTCCAACGAAATCATCAAAGCGATCCAAATATCCACTTCCAGATTCTACAAAAAGAGTGTTTCAA

                          *                 *    *  *                *       
      86772 AACTGCTCTATCAATAGAAATGTTCAACTCCTTTG-GCTGGGTACACACATCACAAACAAGTTTC
         66 AACTGCTCTATCAAAAGAAATGTTCAACT-CTGTGAGTTGAGTACACACATCACAAAGAAGTTTC

                                      **     **       
      86836 TGAGAATGCTTCTGTCTAGTTTTTATGGGAAGACGTTCCCTT
        130 TGAGAATGCTTCTGTCTAGTTTTTATATGAAGATATTCCCTT

              *  * *  **          *       *            **                  * 
      86878 TTTCACCAAAGGCATCAAAGCGCTCCAAATGTCCACTTCCAGACACTACAAAAAGAGTGTTTCCA
          1 TTCCAACGAAATCATCAAAGCGATCCAAATATCCACTTCCAGATTCTACAAAAAGAGTGTTTCAA

             **   *   **   ***                 *    * *  * *         *       
      86943 ACGTGCCCTAAGAAAGCGAATGTTCAACTCTGTGACTTGAATGCAGATATCACAAAGTAGTTTCT
         66 AACTGCTCTATCAAAAGAAATGTTCAACTCTGTGAGTTGAGTACACACATCACAAAGAAGTTTCT

                **            *     *    *         * 
      87008 GAGAGGGCTTCTGTCTAGATTTTAGATGATGATATTCCCGT
        131 GAGAATGCTTCTGTCTAGTTTTTATATGAAGATATTCCCTT

                           * *   *                *   *                    * 
      87049 TTCCAACGAAATCATTAGAGCTATCCAAATATCCACTTACAGTTTCTACAAAAAGAGTGTTTCCA
          1 TTCCAACGAAATCATCAAAGCGATCCAAATATCCACTTCCAGATTCTACAAAAAGAGTGTTTCAA

                   **         * *    *       *  *                   ***      
      87114 AACTGCTGCATCAAAAGAGAGGTTCCACTCTGTTAGCTGAGTACACACATCACAAACTTGTTTCT
         66 AACTGCTCTATCAAAAGAAATGTTCAACTCTGTGAGTTGAGTACACACATCACAAAGAAGTTTCT

            *     *         *        **         **   
      87179 CAGAATCCTTCTGTCTCGTTTTTATGGGAAGATATTTACTT
        131 GAGAATGCTTCTGTCTAGTTTTTATATGAAGATATTCCCTT

              *  *  * **          *       *     *       *  *  *              
      87220 TTTCACCGTAGGCATCAAAGCGCTCCAAATGTCCACATCCAGATACTCCAGAAAGAGTGTTTCAA
          1 TTCCAACGAAATCATCAAAGCGATCCAAATATCCACTTCCAGATTCTACAAAAAGAGTGTTTCAA

             *         *   * *   *         *        * *  *      *      *     
      87285 ACCTGCTCTATGAAAGGGAATCTTCAACTCTATGAGTTGAATGCAGACATCAGAAAGAAATTTCT
         66 AACTGCTCTATCAAAAGAAATGTTCAACTCTGTGAGTTGAGTACACACATCACAAAGAAGTTTCT

                     *       **          *         **
      87350 GAGAATGCTGCTGTCTACCTTTTAT-T--TGA-ATTCCCGC
        131 GAGAATGCTTCTGTCTAGTTTTTATATGAAGATATTCCCTT

                         *  *    *                *       *                  
      87387 TTCCAACGAAATCCTCCAAGCTATCCAAATATCCACTTGCAGATTCCACAAAAAGAGTGTTTCAA
          1 TTCCAACGAAATCATCAAAGCGATCCAAATATCCACTTCCAGATTCTACAAAAAGAGTGTTTCAA

                            ** *  *            *       *     *     *  *      
      87452 AACTGCTCTCTATCAATGGCAAAGTTCAACTCTGTTAGTTGAGGACACATATCACCAACAAGTTT
         66 AACTG--CTCTATCAAAAGAAATGTTCAACTCTGTGAGTTGAGTACACACATCACAAAGAAGTTT

                               *       **         *    
      87517 CTGAGAATGCTTCTGTCTATTTTTTATGGGAAGATATTTCCTT
        129 CTGAGAATGCTTCTGTCTAGTTTTTATATGAAGATATTCCCTT

              *  *  * ** *    *      *    *          * **          *         
      87560 TTTCACCGTAGGCGTCAAGGCGATCGAAATGTCCACTTCCACAAACTACAAAAAGTGTGTTTCAA
          1 TTCCAACGAAATCATCAAAGCGATCCAAATATCCACTTCCAGATTCTACAAAAAGAGTGTTTCAA

             *         *   * **       *    *        * ** * *   **      *     
      87625 ACCTGCTCTATGAAAGGCCATGTTCATCTCTATGAGTTGAATGGAAATATCCGAAAGAAATTTCT
         66 AACTGCTCTATCAAAAGAAATGTTCAACTCTGTGAGTTGAGTACACACATCACAAAGAAGTTTCT

             *       *          *        *         **
      87690 GGGAATGCTGCTGTCTAG-TGTT-TAT-ACGA-ATTCCCGC
        131 GAGAATGCTTCTGTCTAGTTTTTATATGAAGATATTCCCTT

                         *       *                *    *  *                  
      87727 TTCCAACGAAATCCTCAAAGCAATCCAAATATCCACTTGCAGAATCCACAAAAAGAGTGTTTCAA
          1 TTCCAACGAAATCATCAAAGCGATCCAAATATCCACTTCCAGATTCTACAAAAAGAGTGTTTCAA

                          *     *          * *                   *  *        
      87792 AACTGCTCTATCAATAGAAAGGTTCAACTCTTTTAGTTGAGTACACACATCACGAACAAGTTTCT
         66 AACTGCTCTATCAAAAGAAATGTTCAACTCTGTGAGTTGAGTACACACATCACAAAGAAGTTTCT

                            * *      **     **  *    
      87857 GAGAATGCTTCTGTCTGGCTTTTATTGGAAGACGTTTCCTT
        131 GAGAATGCTTCTGTCTAGTTTTTATATGAAGATATTCCCTT

                 * *  **          *       *                * *               
      87898 TT-CACCAAAGGCATCAAAGCGCTCCAAATGTCCACTTCCAGATTCTTCCAAAAGAGTGTTTCAA
          1 TTCCAACGAAATCATCAAAGCGATCCAAATATCCACTTCCAGATTCTACAAAAAGAGTGTTTCAA

             **     * ***  * *                 *    * *  * *     *   *       
      87962 ACGTGCTCAAAGTAAGGGAATGTTCAACTCTGTGACTTGAATGCAGATATCACCAAGTAGTTTCT
         66 AACTGCTCTATCAAAAGAAATGTTCAACTCTGTGAGTTGAGTACACACATCACAAAGAAGTTTCT

            * * *             *     *    *         * 
      88027 AATAGTGCTTCTGTCTAGATTTTAGATGATGATATTCCCGT
        131 GAGAATGCTTCTGTCTAGTTTTTATATGAAGATATTCCCTT

                    *    * * *   *          *  *  *   *      *     *       * 
      88068 TTCCAACGGAATCGTTAGAGCTATCCAAATATACAGTTACAGTTTCTACCAAAAGGGTGTTTCCA
          1 TTCCAACGAAATCATCAAAGCGATCCAAATATCCACTTCCAGATTCTACAAAAAGAGTGTTTCAA

              *    **           *            *       *                     * 
      88133 AATTGCTGCATCAAAAGAAAGGTTCAACTCTGTTAGTTGAGGACACACATCACAAAGAAGTTTGT
         66 AACTGCTCTATCAAAAGAAATGTTCAACTCTGTGAGTTGAGTACACACATCACAAAGAAGTTTCT

                              *    *     *           
      88198 GAGAATGCTTCTGTCTAGATTTTGTATGACGATATTCCCTT
        131 GAGAATGCTTCTGTCTAGTTTTTATATGAAGATATTCCCTT

                     *   * *     *   *       * *  *    *  *       *  *       
      88239 TTCCAACGATATCGTTAAAGCAATCTAAATATCAATTTGCAGAATCCACAAAAATAGAGTTTCAA
          1 TTCCAACGAAATCATCAAAGCGATCCAAATATCCACTTCCAGATTCTACAAAAAGAGTGTTTCAA

             *       * *        *    *       *  *                   *** *    
      88304 AGCTGCTCTGTAAAAAGAAAGGTTCCACTCTGTTAGCTGAGTACACACATCACAAACTTGATTCT
         66 AACTGCTCTATCAAAAGAAATGTTCAACTCTGTGAGTTGAGTACACACATCACAAAGAAGTTTCT

                  *         *        **         **   
      88369 GAGAATCCTTCTGTCTCGTTTTTATGGGAAGATATTTACTT
        131 GAGAATGCTTCTGTCTAGTTTTTATATGAAGATATTCCCTT

                 *  * **          *       *     *       *  *  *  *           
      88410 TTCCACCGTAGGCATCAAAGCGCTCCAAATGTCCACATCCAGATACTCCAGAACGAGTGTTTCAA
          1 TTCCAACGAAATCATCAAAGCGATCCAAATATCCACTTCCAGATTCTACAAAAAGAGTGTTTCAA

             *         *   * *   *         *        * *  *      *      *     
      88475 ACCTGCTCTATGAAAGGGAATCTTCAACTCTATGAGTTGAATGCAGACATCAGAAAGAAATTTCT
         66 AACTGCTCTATCAAAAGAAATGTTCAACTCTGTGAGTTGAGTACACACATCACAAAGAAGTTTCT

                     *       **          *         **
      88540 GAGAATGCTGCTGTCTACCTTTTAT-T--TGA-ATTCCCGC
        131 GAGAATGCTTCTGTCTAGTTTTTATATGAAGATATTCCCTT

                         *  *    *              * *  **   *    *             
      88577 TTCCAACGAAATCCTCCAAGCTATCCAAATATCCACCTGCATTTTCCACAACAAGAGTGTTTCAA
          1 TTCCAACGAAATCATCAAAGCGATCCAAATATCCACTTCCAGATTCTACAAAAAGAGTGTTTCAA

                          *                 *    *  *                *       
      88642 AACTGCTCTATCAATAGAAATGTTCAACTCCTTTG-GCTGGGTACACACATCACAAACAAGTTTC
         66 AACTGCTCTATCAAAAGAAATGTTCAACT-CTGTGAGTTGAGTACACACATCACAAAGAAGTTTC

                                      **     **       
      88706 TGAGAATGCTTCTGTCTAGTTTTTATGGGAAGACGTTCCCTT
        130 TGAGAATGCTTCTGTCTAGTTTTTATATGAAGATATTCCCTT

              *  * *  **          *       *            **                  * 
      88748 TTTCACCAAAGGCATCAAAGCGCTCCAAATGTCCACTTCCAGACACTACAAAAAGAGTGTTTCCA
          1 TTCCAACGAAATCATCAAAGCGATCCAAATATCCACTTCCAGATTCTACAAAAAGAGTGTTTCAA

             **   *   **   ***                 *    * *  * *         *       
      88813 ACGTGCCCTAAGAAAGCGAATGTTCAACTCTGTGACTTGAATGCAGATATCACAAAGTAGTTTCT
         66 AACTGCTCTATCAAAAGAAATGTTCAACTCTGTGAGTTGAGTACACACATCACAAAGAAGTTTCT

                **            *     *    *         * 
      88878 GAGAGGGCTTCTGTCTAGATTTTAGATGATGATATTCCCGT
        131 GAGAATGCTTCTGTCTAGTTTTTATATGAAGATATTCCCTT

                           * *   *                *   *                    * 
      88919 TTCCAACGAAATCATTAGAGCTATCCAAATATCCACTTACAGTTTCTACAAAAAGAGTGTTTCCA
          1 TTCCAACGAAATCATCAAAGCGATCCAAATATCCACTTCCAGATTCTACAAAAAGAGTGTTTCAA

                   **         * *    *       *  *                   ***      
      88984 AACTGCTGCATCAAAAGAGAGGTTCCACTCTGTTAGCTGAGTACACACATCACAAACTTGTTTCT
         66 AACTGCTCTATCAAAAGAAATGTTCAACTCTGTGAGTTGAGTACACACATCACAAAGAAGTTTCT

            *     *         *        **         **   
      89049 CAGAATCCTTCTGTCTCGTTTTTATGGGAAGATATTTACTT
        131 GAGAATGCTTCTGTCTAGTTTTTATATGAAGATATTCCCTT

              *  *  * **          *       *     *       *  *  *              
      89090 TTTCACCGTAGGCATCAAAGCGCTCCAAATGTCCACATCCAGATACTCCAGAAAGAGTGTTTCAA
          1 TTCCAACGAAATCATCAAAGCGATCCAAATATCCACTTCCAGATTCTACAAAAAGAGTGTTTCAA

             *         *   * *   *         *        * *  *      *      *     
      89155 ACCTGCTCTATGAAAGGGAATCTTCAACTCTATGAGTTGAATGCAGACATCAGAAAGAAATTTCT
         66 AACTGCTCTATCAAAAGAAATGTTCAACTCTGTGAGTTGAGTACACACATCACAAAGAAGTTTCT

                     *       **          *         **
      89220 GAGAATGCTGCTGTCTACCTTTTAT-T--TGA-ATTCCCGC
        131 GAGAATGCTTCTGTCTAGTTTTTATATGAAGATATTCCCTT

                         *  *    *                *       *                  
      89257 TTCCAACGAAATCCTCCAAGCTATCCAAATATCCACTTGCAGATTCCACAAAAAGAGTGTTTCAA
          1 TTCCAACGAAATCATCAAAGCGATCCAAATATCCACTTCCAGATTCTACAAAAAGAGTGTTTCAA

                            ** *  *            *       *     *     *  *      
      89322 AACTGCTCTCTATCAATGGCAAAGTTCAACTCTGTTAGTTGAGGACACATATCACCAACAAGTTT
         66 AACTG--CTCTATCAAAAGAAATGTTCAACTCTGTGAGTTGAGTACACACATCACAAAGAAGTTT

                               *       **         *    
      89387 CTGAGAATGCTTCTGTCTATTTTTTATGGGAAGATATTTCCTT
        129 CTGAGAATGCTTCTGTCTAGTTTTTATATGAAGATATTCCCTT

              *  *  * ** *    *      *    *          * **          *         
      89430 TTTCACCGTAGGCGTCAAGGCGATCGAAATGTCCACTTCCACAAACTACAAAAAGTGTGTTTCAA
          1 TTCCAACGAAATCATCAAAGCGATCCAAATATCCACTTCCAGATTCTACAAAAAGAGTGTTTCAA

             *         *   * **       *    *        * ** * *   **      *     
      89495 ACCTGCTCTATGAAAGGCCATGTTCATCTCTATGAGTTGAATGGAAATATCCGAAAGAAATTTCT
         66 AACTGCTCTATCAAAAGAAATGTTCAACTCTGTGAGTTGAGTACACACATCACAAAGAAGTTTCT

             *       *          *        *         **
      89560 GGGAATGCTGCTGTCTAG-TGTT-TAT-ACGA-ATTCCCGC
        131 GAGAATGCTTCTGTCTAGTTTTTATATGAAGATATTCCCTT

                         *       *                *    *  *                  
      89597 TTCCAACGAAATCCTCAAAGCAATCCAAATATCCACTTGCAGAATCCACAAAAAGAGTGTTTCAA
          1 TTCCAACGAAATCATCAAAGCGATCCAAATATCCACTTCCAGATTCTACAAAAAGAGTGTTTCAA

                          *     *          * *                   *  *        
      89662 AACTGCTCTATCAATAGAAAGGTTCAACTCTTTTAGTTGAGTACACACATCACGAACAAGTTTCT
         66 AACTGCTCTATCAAAAGAAATGTTCAACTCTGTGAGTTGAGTACACACATCACAAAGAAGTTTCT

                            * *      **     **  *    
      89727 GAGAATGCTTCTGTCTGGCTTTTATTGGAAGACGTTTCCTT
        131 GAGAATGCTTCTGTCTAGTTTTTATATGAAGATATTCCCTT

                 * *  **          *       *                * *               
      89768 TT-CACCAAAGGCATCAAAGCGCTCCAAATGTCCACTTCCAGATTCTTCCAAAAGAGTGTTTCAA
          1 TTCCAACGAAATCATCAAAGCGATCCAAATATCCACTTCCAGATTCTACAAAAAGAGTGTTTCAA

             **     * ***  * *                 *    * *  * *     *   *       
      89832 ACGTGCTCAAAGTAAGGGAATGTTCAACTCTGTGACTTGAATGCAGATATCACCAAGTAGTTTCT
         66 AACTGCTCTATCAAAAGAAATGTTCAACTCTGTGAGTTGAGTACACACATCACAAAGAAGTTTCT

            * * *             *     *    *         * 
      89897 AATAGTGCTTCTGTCTAGATTTTAGATGATGATATTCCCGT
        131 GAGAATGCTTCTGTCTAGTTTTTATATGAAGATATTCCCTT

                    *    * * *   *          *  *  *   *      *     *       * 
      89938 TTCCAACGGAATCGTTAGAGCTATCCAAATATACAGTTACAGTTTCTACCAAAAGGGTGTTTCCA
          1 TTCCAACGAAATCATCAAAGCGATCCAAATATCCACTTCCAGATTCTACAAAAAGAGTGTTTCAA

              *    **           *            *       *                     * 
      90003 AATTGCTGCATCAAAAGAAAGGTTCAACTCTGTTAGTTGAGGACACACATCACAAAGAAGTTTGT
         66 AACTGCTCTATCAAAAGAAATGTTCAACTCTGTGAGTTGAGTACACACATCACAAAGAAGTTTCT

                             **    *     *           
      90068 GAGAATGCTTCTGTCTACATTTTGTATGACGATATTCCCTT
        131 GAGAATGCTTCTGTCTAGTTTTTATATGAAGATATTCCCTT

                     *   * *     *   *       * *  *    *  *       *  *       
      90109 TTCCAACGATATCGTTAAAGCAATCTAAATATCAATTTGCAGAATCCACAAAAATAGAGTTTCAA
          1 TTCCAACGAAATCATCAAAGCGATCCAAATATCCACTTCCAGATTCTACAAAAAGAGTGTTTCAA

             *       * *        *    *       *  *                   *** *    
      90174 AGCTGCTCTGTAAAAAGAAAGGTTCCACTCTGTTAGCTGAGTACACACATCACAAACTTGATTCT
         66 AACTGCTCTATCAAAAGAAATGTTCAACTCTGTGAGTTGAGTACACACATCACAAAGAAGTTTCT

                  *         *        **         **   
      90239 GAGAATCCTTCTGTCTCGTTTTTATGGGAAGATATTTACTT
        131 GAGAATGCTTCTGTCTAGTTTTTATATGAAGATATTCCCTT

                 *  * **          *       *     *       *  *  *  *           
      90280 TTCCACCGTAGGCATCAAAGCGCTCCAAATGTCCACATCCAGATACTCCAGAACGAGTGTTTCAA
          1 TTCCAACGAAATCATCAAAGCGATCCAAATATCCACTTCCAGATTCTACAAAAAGAGTGTTTCAA

             *         *   * *   *         *        * *  *      *      *     
      90345 ACCTGCTCTATGAAAGGGAATCTTCAACTCTATGAGTTGAATGCAGACATCAGAAAGAAATTTCT
         66 AACTGCTCTATCAAAAGAAATGTTCAACTCTGTGAGTTGAGTACACACATCACAAAGAAGTTTCT

                     *       **          *         **
      90410 GAGAATGCTGCTGTCTACCTTTTAT-T--TGA-ATTCCCGC
        131 GAGAATGCTTCTGTCTAGTTTTTATATGAAGATATTCCCTT

                         *  *    *              * *  **   *    *             
      90447 TTCCAACGAAATCCTCCAAGCTATCCAAATATCCACCTGCATTTTCCACAACAAGAGTGTTTCAA
          1 TTCCAACGAAATCATCAAAGCGATCCAAATATCCACTTCCAGATTCTACAAAAAGAGTGTTTCAA

                          *                 *    *  *                *       
      90512 AACTGCTCTATCAATAGAAATGTTCAACTCCTTTG-GCTGGGTACACACATCACAAACAAGTTTC
         66 AACTGCTCTATCAAAAGAAATGTTCAACT-CTGTGAGTTGAGTACACACATCACAAAGAAGTTTC

                                      **     **       
      90576 TGAGAATGCTTCTGTCTAGTTTTTATGGGAAGACGTTCCCTT
        130 TGAGAATGCTTCTGTCTAGTTTTTATATGAAGATATTCCCTT

              *  * *  **          *       *            **                  * 
      90618 TTTCACCAAAGGCATCAAAGCGCTCCAAATGTCCACTTCCAGACACTACAAAAAGAGTGTTTCCA
          1 TTCCAACGAAATCATCAAAGCGATCCAAATATCCACTTCCAGATTCTACAAAAAGAGTGTTTCAA

             **   *   **   ***                 *    * *  * *         *       
      90683 ACGTGCCCTAAGAAAGCGAATGTTCAACTCTGTGACTTGAATGCAGATATCACAAAGTAGTTTCT
         66 AACTGCTCTATCAAAAGAAATGTTCAACTCTGTGAGTTGAGTACACACATCACAAAGAAGTTTCT

                **            *     *    *         * 
      90748 GAGAGGGCTTCTGTCTAGATTTTAGATGATGATATTCCCGT
        131 GAGAATGCTTCTGTCTAGTTTTTATATGAAGATATTCCCTT

                           * *   *                *   *                    * 
      90789 TTCCAACGAAATCATTAGAGCTATCCAAATATCCACTTACAGTTTCTACAAAAAGAGTGTTTCCA
          1 TTCCAACGAAATCATCAAAGCGATCCAAATATCCACTTCCAGATTCTACAAAAAGAGTGTTTCAA

                   **         * *    *       *  *                   ***      
      90854 AACTGCTGCATCAAAAGAGAGGTTCCACTCTGTTAGCTGAGTACACACATCACAAACTTGTTTCT
         66 AACTGCTCTATCAAAAGAAATGTTCAACTCTGTGAGTTGAGTACACACATCACAAAGAAGTTTCT

            *     *         *        **         **   
      90919 CAGAATCCTTCTGTCTCGTTTTTATGGGAAGATATTTACTT
        131 GAGAATGCTTCTGTCTAGTTTTTATATGAAGATATTCCCTT

              *  *  * **          *       *     *       *  *  *              
      90960 TTTCACCGTAGGCATCAAAGCGCTCCAAATGTCCACATCCAGATACTCCAGAAAGAGTGTTTCAA
          1 TTCCAACGAAATCATCAAAGCGATCCAAATATCCACTTCCAGATTCTACAAAAAGAGTGTTTCAA

             *         *   * *   *         *        * *  *      *      *     
      91025 ACCTGCTCTATGAAAGGGAATCTTCAACTCTATGAGTTGAATGCAGACATCAGAAAGAAATTTCT
         66 AACTGCTCTATCAAAAGAAATGTTCAACTCTGTGAGTTGAGTACACACATCACAAAGAAGTTTCT

                     *       **          *         **
      91090 GAGAATGCTGCTGTCTACCTTTTAT-T--TGA-ATTCCCGC
        131 GAGAATGCTTCTGTCTAGTTTTTATATGAAGATATTCCCTT

                         *  *    *                *       *                  
      91127 TTCCAACGAAATCCTCCAAGCTATCCAAATATCCACTTGCAGATTCCACAAAAAGAGTGTTTCAA
          1 TTCCAACGAAATCATCAAAGCGATCCAAATATCCACTTCCAGATTCTACAAAAAGAGTGTTTCAA

                            ** *  *            *       *     *     *  *      
      91192 AACTGCTCTCTATCAATGGCAAAGTTCAACTCTGTTAGTTGAGGACACATATCACCAACAAGTTT
         66 AACTG--CTCTATCAAAAGAAATGTTCAACTCTGTGAGTTGAGTACACACATCACAAAGAAGTTT

                    *         *        **         **   
      91257 CTGAGAATCCTTCTGTCTCGTTTTTATGGGAAGATATTTACTT
        129 CTGAGAATGCTTCTGTCTAGTTTTTATATGAAGATATTCCCTT

                 *  * **          *       *     *       *  *  *  *           
      91300 TTCCACCGTAGGCATCAAAGCGCTCCAAATGTCCACATCCAGATACTCCAGAACGAGTGTTTCAA
          1 TTCCAACGAAATCATCAAAGCGATCCAAATATCCACTTCCAGATTCTACAAAAAGAGTGTTTCAA

             *         *   * **       *    *        * ** * *   **      *     
      91365 ACCTGCTCTATGAAAGGCCATGTTCATCTCTATGAGTTGAATGGAAATATCCGAAAGAAATTTCT
         66 AACTGCTCTATCAAAAGAAATGTTCAACTCTGTGAGTTGAGTACACACATCACAAAGAAGTTTCT

             *       *          *        *         **
      91430 GGGAATGCTGCTGTCTAG-TGTT-TAT-ACGA-ATTCCCGC
        131 GAGAATGCTTCTGTCTAGTTTTTATATGAAGATATTCCCTT

                         *       *                *    *  *                  
      91467 TTCCAACGAAATCCTCAAAGCAATCCAAATATCCACTTGCAGAATCCACAAAAAGAGTGTTTCAA
          1 TTCCAACGAAATCATCAAAGCGATCCAAATATCCACTTCCAGATTCTACAAAAAGAGTGTTTCAA

               *          *     *          * *                      *        
      91532 AACCGCTCTATCAATAGAAAGGTTCAACTCTTTTAGTTGAGTACACACATCACAAACAAGTTTCT
         66 AACTGCTCTATCAAAAGAAATGTTCAACTCTGTGAGTTGAGTACACACATCACAAAGAAGTTTCT

                            * *      **     **  *    
      91597 GAGAATGCTTCTGTCTGGCTTTTATTGGAAGACGTTTCCTT
        131 GAGAATGCTTCTGTCTAGTTTTTATATGAAGATATTCCCTT

                 * *  **          *       *                * *               
      91638 TT-CACCAAAGGCATCAAAGCGCTCCAAATGTCCACTTCCAGATTCTTCCAAAAGAGTGTTTCAA
          1 TTCCAACGAAATCATCAAAGCGATCCAAATATCCACTTCCAGATTCTACAAAAAGAGTGTTTCAA

             **  *  * ***  * *                 *    * *  * *     *   *       
      91702 ACGTGGTCGAAGTAAGGGAATGTTCAACTCTGTGACTTGAATGCAGATATCACCAAGTAGTTTCT
         66 AACTGCTCTATCAAAAGAAATGTTCAACTCTGTGAGTTGAGTACACACATCACAAAGAAGTTTCT

            * * *             *     *    *         * 
      91767 AATAGTGCTTCTGTCTAGATTTTAGATGATGATATTCCCGT
        131 GAGAATGCTTCTGTCTAGTTTTTATATGAAGATATTCCCTT

                         * * *   *             *  *   *      *     *       * 
      91808 TTCCAACGAAATCGTTAGAGCTATCCAAATATCCAGTTACAGTTTCTACCAAAAGGGTGTTTCCA
          1 TTCCAACGAAATCATCAAAGCGATCCAAATATCCACTTCCAGATTCTACAAAAAGAGTGTTTCAA

              *    **           *            *       *                     * 
      91873 AATTGCTGCATCAAAAGAAAGGTTCAACTCTGTTAGTTGAGGACACACATCACAAAGAAGTTTGT
         66 AACTGCTCTATCAAAAGAAATGTTCAACTCTGTGAGTTGAGTACACACATCACAAAGAAGTTTCT

                              *    *     *           
      91938 GAGAATGCTTCTGTCTAGATTTTGTATGACGATATTCCCTT
        131 GAGAATGCTTCTGTCTAGTTTTTATATGAAGATATTCCCTT

                     *   * *     *   *       * *  *    *  *       *  *       
      91979 TTCCAACGATATCGTTAAAGCAATCTAAATATCAATTTGCAGAATCCACAAAAATAGAGTTTCAA
          1 TTCCAACGAAATCATCAAAGCGATCCAAATATCCACTTCCAGATTCTACAAAAAGAGTGTTTCAA

             *       * *        *    *       *  *                   ***      
      92044 AGCTGCTCTGTAAAAAGAAAGGTTCCACTCTGTTAGCTGAGTACACACATCACAAACTTGTTTCT
         66 AACTGCTCTATCAAAAGAAATGTTCAACTCTGTGAGTTGAGTACACACATCACAAAGAAGTTTCT

                 **         *        **         **   
      92109 GAGAAACCTTCTGTCTCGTTTTTATGGGAAGATATTTACTT
        131 GAGAATGCTTCTGTCTAGTTTTTATATGAAGATATTCCCTT

                 ** * **          *       *     *       *  *  *  *           
      92150 TTCCACTGTAGGCATCAAAGCGCTCCAAATGTCCACATCCAGATACTCCAGAACGAGTGTTTCAA
          1 TTCCAACGAAATCATCAAAGCGATCCAAATATCCACTTCCAGATTCTACAAAAAGAGTGTTTCAA

             *         *   * *   *         *        * *  *      *      *     
      92215 ACCTGCTCTATGAAAGGGAATCTTCAACTCTATGAGTTGAATGCAGA-ATCAGAAAGAAATTTCT
         66 AACTGCTCTATCAAAAGAAATGTTCAACTCTGTGAGTTGAGTACACACATCACAAAGAAGTTTCT

                     *       **          *         **
      92279 GAGAATGCTGCTGTCTACCTTTTAT-T--TGA-ATTCCCGC
        131 GAGAATGCTTCTGTCTAGTTTTTATATGAAGATATTCCCTT

                         *  *    *              * *  **   *    *             
      92316 TTCCAACGAAATCCTCCAAGCTATCCAAATATCCACCTGCATTTTCCACAACAAGAGTGTTTCAA
          1 TTCCAACGAAATCATCAAAGCGATCCAAATATCCACTTCCAGATTCTACAAAAAGAGTGTTTCAA

                          *                 *    *  *                *       
      92381 AACTGCTCTATCAATAGAAATGTTCAACTCCTTTG-GCTGGGTACACACATCACAAACAAGTTTC
         66 AACTGCTCTATCAAAAGAAATGTTCAACT-CTGTGAGTTGAGTACACACATCACAAAGAAGTTTC

                                      **     **       
      92445 TGAGAATGCTTCTGTCTAGTTTTTATGGGAAGACGTTCCCTT
        130 TGAGAATGCTTCTGTCTAGTTTTTATATGAAGATATTCCCTT

              *  * *  **          *       *            **                    
      92487 TTTCACCAAAGGCATCAAAGCGCTCCAAATGTCCACTTCCAGACACTACAAAAAGAGTGTTTCAA
          1 TTCCAACGAAATCATCAAAGCGATCCAAATATCCACTTCCAGATTCTACAAAAAGAGTGTTTCAA

             **       **    **                 *    * *  * *         *       
      92552 ACGTGCTCTAAGAAAACGAATGTTCAACTCTGTGACTTGAATGCAGATATCACAAAGTAGTTTCT
         66 AACTGCTCTATCAAAAGAAATGTTCAACTCTGTGAGTTGAGTACACACATCACAAAGAAGTTTCT

                **            *     *    *         * 
      92617 GAGAGGGCTTCTGTCTAGATTTTAGATGATGATATTCCCGT
        131 GAGAATGCTTCTGTCTAGTTTTTATATGAAGATATTCCCTT

                           * *   *                *   *                    * 
      92658 TTCCAACGAAATCATTAGAGCTATCCAAATATCCACTTACAGTTTCTACAAAAAGAGTGTTTCCA
          1 TTCCAACGAAATCATCAAAGCGATCCAAATATCCACTTCCAGATTCTACAAAAAGAGTGTTTCAA

                   **         * *    *       *  **                  ***      
      92723 AACTGCTGCATCAAAAGAGAGGTTCCACTCTGTTAGCCGAGTACACACATCACAAACTTGTTTCT
         66 AACTGCTCTATCAAAAGAAATGTTCAACTCTGTGAGTTGAGTACACACATCACAAAGAAGTTTCT

            *     *         *        **         **   
      92788 CAGAATCCTTCTGTCTCGTTTTTATGGGAAGATATTTACTT
        131 GAGAATGCTTCTGTCTAGTTTTTATATGAAGATATTCCCTT

              *  *  * **          *       *     *       *  *  *              
      92829 TTTCACCGTAGGCATCAAAGCGCTCCAAATGTCCACATCCAGATACTCCAGAAAGAGTGTTTCAA
          1 TTCCAACGAAATCATCAAAGCGATCCAAATATCCACTTCCAGATTCTACAAAAAGAGTGTTTCAA

             *         *   * *   *         *        * *  *      *      *     
      92894 ACCTGCTCTATGAAAGGGAATCTTCAACTCTATGAGTTGAATGCAGACATCAGAAAGAAATTTCT
         66 AACTGCTCTATCAAAAGAAATGTTCAACTCTGTGAGTTGAGTACACACATCACAAAGAAGTTTCT

                     *       **          *         **
      92959 GAGAATGCTGCTGTCTACCTTTTAT-T--TGA-ATTCCCGC
        131 GAGAATGCTTCTGTCTAGTTTTTATATGAAGATATTCCCTT

                         *  *    *                *       *                  
      92996 TTCCAACGAAATCCTCCAAGCTATCCAAATATCCACTTGCAGATTCCACAAAAAGAGTGTTTCAA
          1 TTCCAACGAAATCATCAAAGCGATCCAAATATCCACTTCCAGATTCTACAAAAAGAGTGTTTCAA

                            ** *  *            *       *     *     *  *      
      93061 AACTGCTCTCTATCAATGGCAAAGTTCAACTCTGTTAGTTGAGGACACATATCACCAACAAGTTT
         66 AACTG--CTCTATCAAAAGAAATGTTCAACTCTGTGAGTTGAGTACACACATCACAAAGAAGTTT

                               *       **         *    
      93126 CTGAGAATGCTTCTGTCTATTTTTTATGGGAAGATATTTCCTT
        129 CTGAGAATGCTTCTGTCTAGTTTTTATATGAAGATATTCCCTT

              *  *  * ** *    *      *    *          * **                    
      93169 TTTCACCGTAGGCGTCAAGGCGATCGAAATGTCCACTTCCACAAACTACAAAAAGAGTGTTTCAA
          1 TTCCAACGAAATCATCAAAGCGATCCAAATATCCACTTCCAGATTCTACAAAAAGAGTGTTTCAA

             *         *   * **       *    *        * ** * *   **      *     
      93234 ACCTGCTCTATGAAAGGCCATGTTCATCTCTATGAGTTGAATGGAAATATCCGAAAGAAATTTCT
         66 AACTGCTCTATCAAAAGAAATGTTCAACTCTGTGAGTTGAGTACACACATCACAAAGAAGTTTCT

             *       *                             **
      93299 GGGAATGCTGCTGTCTAGTTTTTATAT---GA-ATTCCCGC
        131 GAGAATGCTTCTGTCTAGTTTTTATATGAAGATATTCCCTT

                         *       *                *    *  *                  
      93336 TTCCAACGAAATCCTCAAAGCAATCCAAATATCCACTTGCAGAATCCACAAAAAGAGTGTTTCAA
          1 TTCCAACGAAATCATCAAAGCGATCCAAATATCCACTTCCAGATTCTACAAAAAGAGTGTTTCAA

                          *     *          * *                      *        
      93401 AACTGCTCTATCAATAGAAAGGTTCAACTCTTTTAGTTGAGTACACACATCACAAACAAGTTTCT
         66 AACTGCTCTATCAAAAGAAATGTTCAACTCTGTGAGTTGAGTACACACATCACAAAGAAGTTTCT

                            * *      **     **  *    
      93466 GAGAATGCTTCTGTCTGGCTTTTATTGGAAGACGTTTCCTT
        131 GAGAATGCTTCTGTCTAGTTTTTATATGAAGATATTCCCTT

                 * *  **          *      **                * *               
      93507 TT-CACCAAAGGCATCAAAGCGCTCCAAAGGTCCACTTCCAGATTCTTCCAAAAGAGTGTTTCAA
          1 TTCCAACGAAATCATCAAAGCGATCCAAATATCCACTTCCAGATTCTACAAAAAGAGTGTTTCAA

             **          *  * *                 *    * *  * *     *   *      
      93571 ACGTGCTC-ATACTAAGGGAATGTTCAACTCTGTGACTTGAATGCAGATATCACCAAGTAGTTTC
         66 AACTGCTCTAT-CAAAAGAAATGTTCAACTCTGTGAGTTGAGTACACACATCACAAAGAAGTTTC

             * * *             *   * *    *         * 
      93635 TAATAGTGCTTCTGTCTAGATTTCAGATGATGATATTCCCGT
        130 TGAGAATGCTTCTGTCTAGTTTTTATATGAAGATATTCCCTT

                         *** *   *             *  *   *      *     *       * 
      93677 TTCCAACGAAATCGCTAGAGCTATCCAAATATCCAGTTACAGTTTCTACCAAAAGGGTGTTTCCA
          1 TTCCAACGAAATCATCAAAGCGATCCAAATATCCACTTCCAGATTCTACAAAAAGAGTGTTTCAA

              *    **           *            *       *                     * 
      93742 AATTGCTGCATCAAAAGAAAGGTTCAACTCTGTTAGTTGAGGACACACATCACAAAGAAGTTTGT
         66 AACTGCTCTATCAAAAGAAATGTTCAACTCTGTGAGTTGAGTACACACATCACAAAGAAGTTTCT

                             **    *     *           
      93807 GAGAATGCTTCTGTCTACATTTTGTATGACGATATTCCCTT
        131 GAGAATGCTTCTGTCTAGTTTTTATATGAAGATATTCCCTT

                     *   * *     *   *       * *  *    *  *       *  *       
      93848 TTCCAACGATATCGTTAAAGCAATCTAAATATCAATTTGCAGAATCCACAAAAATAGAGTTTCAA
          1 TTCCAACGAAATCATCAAAGCGATCCAAATATCCACTTCCAGATTCTACAAAAAGAGTGTTTCAA

             *       * *        *    *       *  *                   ***      
      93913 AGCTGCTCTGTAAAAAGAAAGGTTCCACTCTGTTAGCTGAGTACACACATCACAAACTTGTTTCT
         66 AACTGCTCTATCAAAAGAAATGTTCAACTCTGTGAGTTGAGTACACACATCACAAAGAAGTTTCT

                  *         *        **         **   
      93978 GAGAATCCTTCTGTCTCGTTTTTATGGGAAGATATTTACTT
        131 GAGAATGCTTCTGTCTAGTTTTTATATGAAGATATTCCCTT

                 *  * **          *       *                * *               
      94019 TTCCACCGTAGGCATCAAAGCGCTCCAAATGTCCACTTCCAGATTCTTCCAAAAGAGTGTTTCAA
          1 TTCCAACGAAATCATCAAAGCGATCCAAATATCCACTTCCAGATTCTACAAAAAGAGTGTTTCAA

             **  *  * ***  * *                 *    * *  * *     *   *       
      94084 ACGTGGTCGAAGTAAGGGAATGTTCAACTCTGTGACTTGAATGCAGATATCACCAAGTAGTTTCT
         66 AACTGCTCTATCAAAAGAAATGTTCAACTCTGTGAGTTGAGTACACACATCACAAAGAAGTTTCT

            * * *             *     *    *         * 
      94149 AATAGTGCTTCTGTCTAGATTTTAGATGATGATATTCCCGT
        131 GAGAATGCTTCTGTCTAGTTTTTATATGAAGATATTCCCTT

                         * * *   *             *  *   *      *     *       * 
      94190 TTCCAACGAAATCGTTAGAGCTATCCAAATATCCAGTTACAGTTTCTACCAAAAGGGTGTTTCCA
          1 TTCCAACGAAATCATCAAAGCGATCCAAATATCCACTTCCAGATTCTACAAAAAGAGTGTTTCAA

              *    **           *            *       *                     * 
      94255 AATTGCTGCATCAAAAGAAAGGTTCAACTCTGTTAGTTGAGGACACACATCACAAAGAAGTTTGT
         66 AACTGCTCTATCAAAAGAAATGTTCAACTCTGTGAGTTGAGTACACACATCACAAAGAAGTTTCT

                              *    *     *           
      94320 GAGAATGCTTCTGTCTAGATTTTGTATGACGATATTCCCTT
        131 GAGAATGCTTCTGTCTAGTTTTTATATGAAGATATTCCCTT

                     *   * *     *   *       * *  *    *  *       *  *       
      94361 TTCCAACGATATCGTTAAAGCAATCTAAATATCAATTTGCAGAATCCACAAAAATAGAGTTTCAA
          1 TTCCAACGAAATCATCAAAGCGATCCAAATATCCACTTCCAGATTCTACAAAAAGAGTGTTTCAA

             *       * *        *    *       *  *                   ***      
      94426 AGCTGCTCTGTAAAAAGAAAGGTTCCACTCTGTTAGCTGAGTACACACATCACAAACTTGTTTCT
         66 AACTGCTCTATCAAAAGAAATGTTCAACTCTGTGAGTTGAGTACACACATCACAAAGAAGTTTCT

                 **         *        **         **   
      94491 GAGAAACCTTCTGTCTCGTTTTTATGGGAAGATATTTACTT
        131 GAGAATGCTTCTGTCTAGTTTTTATATGAAGATATTCCCTT

                 ** * **          *       *     *       *  *  *  *           
      94532 TTCCACTGTAGGCATCAAAGCGCTCCAAATGTCCACATCCAGATACTCCAGAACGAGTGTTTCAA
          1 TTCCAACGAAATCATCAAAGCGATCCAAATATCCACTTCCAGATTCTACAAAAAGAGTGTTTCAA

             *         *   * *   *         *        * *  *      *      *     
      94597 ACCTGCTCTATGAAAGGGAATCTTCAACTCTATGAGTTGAATGCAGA-ATCAGAAAGAAATTTCT
         66 AACTGCTCTATCAAAAGAAATGTTCAACTCTGTGAGTTGAGTACACACATCACAAAGAAGTTTCT

                     *       **          *         **
      94661 GAGAATGCTGCTGTCTACCTTTTAT-T--TGA-ATTCCCGC
        131 GAGAATGCTTCTGTCTAGTTTTTATATGAAGATATTCCCTT

                         *  *    *              * *  **   *    *             
      94698 TTCCAACGAAATCCTCCAAGCTATCCAAATATCCACCTGCATTTTCCACAACAAGAGTGTTTCAA
          1 TTCCAACGAAATCATCAAAGCGATCCAAATATCCACTTCCAGATTCTACAAAAAGAGTGTTTCAA

                          *                 *    *  *                *       
      94763 AACTGCTCTATCAATAGAAATGTTCAACTCCTTTG-GCTGGGTACACACATCACAAACAAGTTTC
         66 AACTGCTCTATCAAAAGAAATGTTCAACT-CTGTGAGTTGAGTACACACATCACAAAGAAGTTTC

                                      **     **       
      94827 TGAGAATGCTTCTGTCTAGTTTTTATGGGAAGACGTTCCCTT
        130 TGAGAATGCTTCTGTCTAGTTTTTATATGAAGATATTCCCTT

              *  * *  **          *       *            **                    
      94869 TTTCACCAAAGGCATCAAAGCGCTCCAAATGTCCACTTCCAGACACTACAAAAAGAGTGTTTCAA
          1 TTCCAACGAAATCATCAAAGCGATCCAAATATCCACTTCCAGATTCTACAAAAAGAGTGTTTCAA

             **       **    **                 *    * *  * *         *       
      94934 ACGTGCTCTAAGAAAACGAATGTTCAACTCTGTGACTTGAATGCAGATATCACAAAGTAGTTTCT
         66 AACTGCTCTATCAAAAGAAATGTTCAACTCTGTGAGTTGAGTACACACATCACAAAGAAGTTTCT

                **            *     *    *         * 
      94999 GAGAGGGCTTCTGTCTAGATTTTAGATGATGATATTCCCGT
        131 GAGAATGCTTCTGTCTAGTTTTTATATGAAGATATTCCCTT

                           * *   *                *   *                    * 
      95040 TTCCAACGAAATCATTAGAGCTATCCAAATATCCACTTACAGTTTCTACAAAAAGAGTGTTTCCA
          1 TTCCAACGAAATCATCAAAGCGATCCAAATATCCACTTCCAGATTCTACAAAAAGAGTGTTTCAA

                   **         * *    *       *  **                  ***      
      95105 AACTGCTGCATCAAAAGAGAGGTTCCACTCTGTTAGCCGAGTACACACATCACAAACTTGTTTCT
         66 AACTGCTCTATCAAAAGAAATGTTCAACTCTGTGAGTTGAGTACACACATCACAAAGAAGTTTCT

            *     *         *        **         **   
      95170 CAGAATCCTTCTGTCTCGTTTTTATGGGAAGATATTTACTT
        131 GAGAATGCTTCTGTCTAGTTTTTATATGAAGATATTCCCTT

              *  *  * **          *       *     *       *  *  *              
      95211 TTTCACCGTAGGCATCAAAGCGCTCCAAATGTCCACATCCAGATACTCCAGAAAGAGTGTTTCAA
          1 TTCCAACGAAATCATCAAAGCGATCCAAATATCCACTTCCAGATTCTACAAAAAGAGTGTTTCAA

             *         *   * *   *         *        * *  *      *      *     
      95276 ACCTGCTCTATGAAAGGGAATCTTCAACTCTATGAGTTGAATGCAGACATCAGAAAGAAATTTCT
         66 AACTGCTCTATCAAAAGAAATGTTCAACTCTGTGAGTTGAGTACACACATCACAAAGAAGTTTCT

                     *       **          *         **
      95341 GAGAATGCTGCTGTCTACCTTTTAT-T--TGA-ATTCCCGC
        131 GAGAATGCTTCTGTCTAGTTTTTATATGAAGATATTCCCTT

                         *  *    *                *       *                  
      95378 TTCCAACGAAATCCTCCAAGCTATCCAAATATCCACTTGCAGATTCCACAAAAAGAGTGTTTCAA
          1 TTCCAACGAAATCATCAAAGCGATCCAAATATCCACTTCCAGATTCTACAAAAAGAGTGTTTCAA

                            ** *  *            *       *     *     *  *      
      95443 AACTGCTCTCTATCAATGGCAAAGTTCAACTCTGTTAGTTGAGGACACATATCACCAACAAGTTT
         66 AACTG--CTCTATCAAAAGAAATGTTCAACTCTGTGAGTTGAGTACACACATCACAAAGAAGTTT

                               *       **         *    
      95508 CTGAGAATGCTTCTGTCTATTTTTTATGGGAAGATATTTCCTT
        129 CTGAGAATGCTTCTGTCTAGTTTTTATATGAAGATATTCCCTT

              *  *  * ** *    * *    *    *          * **                    
      95551 TTTCACCGTAGGCGTCAAGGTGATCGAAATGTCCACTTCCACAAACTACAAAAAGAGTGTTTCAA
          1 TTCCAACGAAATCATCAAAGCGATCCAAATATCCACTTCCAGATTCTACAAAAAGAGTGTTTCAA

             *         *   * **       *    *        * ** * *   **      *     
      95616 ACCTGCTCTATGAAAGGCCATGTTCATCTCTATGAGTTGAATGGAAATATCCGAAAGAAATTTCT
         66 AACTGCTCTATCAAAAGAAATGTTCAACTCTGTGAGTTGAGTACACACATCACAAAGAAGTTTCT

             *       *                             **
      95681 GGGAATGCTGCTGTCTAGTTTTTATAT---GA-ATTCCCGC
        131 GAGAATGCTTCTGTCTAGTTTTTATATGAAGATATTCCCTT

                         *       *                *    *  *                  
      95718 TTCCAACGAAATCCTCAAAGCAATCCAAATATCCACTTGCAGAATCCACAAAAAGAGTGTTTCAA
          1 TTCCAACGAAATCATCAAAGCGATCCAAATATCCACTTCCAGATTCTACAAAAAGAGTGTTTCAA

                          *     *          * *                      *        
      95783 AACTGCTCTATCAATAGAAAGGTTCAACTCTTTTAGTTGAGTACACACATCACAAACAAGTTTCT
         66 AACTGCTCTATCAAAAGAAATGTTCAACTCTGTGAGTTGAGTACACACATCACAAAGAAGTTTCT

                            * *      **     **  *    
      95848 GAGAATGCTTCTGTCTGGCTTTTATTGGAAGACGTTTCCTT
        131 GAGAATGCTTCTGTCTAGTTTTTATATGAAGATATTCCCTT

                 * *  **          *      **                * *               
      95889 TT-CACCAAAGGCATCAAAGCGCTCCAAAGGTCCACTTCCAGATTCTTCCAAAAGAGTGTTTCAA
          1 TTCCAACGAAATCATCAAAGCGATCCAAATATCCACTTCCAGATTCTACAAAAAGAGTGTTTCAA

             **         **  * *                 *    * *  * *     *   *      
      95953 ACGTGCTC-ATAGTAAGGGAATGTTCAACTCTGTGACTTGAATGCAGATATCACCAAGTAGTTTC
         66 AACTGCTCTAT-CAAAAGAAATGTTCAACTCTGTGAGTTGAGTACACACATCACAAAGAAGTTTC

             * * *             *     *    *         * 
      96017 TAATAGTGCTTCTGTCTAGATTTTAGATGATGATATTCCCGT
        130 TGAGAATGCTTCTGTCTAGTTTTTATATGAAGATATTCCCTT

                         *** *   *             *  *   *      *     *       * 
      96059 TTCCAACGAAATCGCTAGAGCTATCCAAATATCCAGTTACAGTTTCTACCAAAAGGGTGTTTCCA
          1 TTCCAACGAAATCATCAAAGCGATCCAAATATCCACTTCCAGATTCTACAAAAAGAGTGTTTCAA

              *    **           *            *       *                     * 
      96124 AATTGCTGCATCAAAAGAAAGGTTCAACTCTGTTAGTTGAGGACACACATCACAAAGAAGTTTGT
         66 AACTGCTCTATCAAAAGAAATGTTCAACTCTGTGAGTTGAGTACACACATCACAAAGAAGTTTCT

                             **    *     *           
      96189 GAGAATGCTTCTGTCTACATTTTGTATGACGATATTCCCTT
        131 GAGAATGCTTCTGTCTAGTTTTTATATGAAGATATTCCCTT

                     *   * *     *   *       * *  *    *  *       *  *       
      96230 TTCCAACGATATCGTTAAAGCAATCTAAATATCAATTTGCAGAATCCACAAAAATAGAGTTTCAA
          1 TTCCAACGAAATCATCAAAGCGATCCAAATATCCACTTCCAGATTCTACAAAAAGAGTGTTTCAA

             *       * *        *    *       *  *                   ***      
      96295 AGCTGCTCTGTAAAAAGAAAGGTTCCACTCTGTTAGCTGAGTACACACATCACAAACTTGTTTCT
         66 AACTGCTCTATCAAAAGAAATGTTCAACTCTGTGAGTTGAGTACACACATCACAAAGAAGTTTCT

                  *         *        **         **   
      96360 GAGAATCCTTCTGTCTCGTTTTTATGGGAAGATATTTACTT
        131 GAGAATGCTTCTGTCTAGTTTTTATATGAAGATATTCCCTT

                 *  * **          *       *     *       *  *  *  *           
      96401 TTCCACCGTAGGCATCAAAGCGCTCCAAATGTCCACATCCAGATACTCCAGAACGAGTGTTTCAA
          1 TTCCAACGAAATCATCAAAGCGATCCAAATATCCACTTCCAGATTCTACAAAAAGAGTGTTTCAA

             *         *   * *   *         *        * *  *      *      *     
      96466 ACCTGCTCTATGAAAGGGAATCTTCAACTCTATGAGTTGAATGCAGACATCAGAAAGAAATTTCT
         66 AACTGCTCTATCAAAAGAAATGTTCAACTCTGTGAGTTGAGTACACACATCACAAAGAAGTTTCT

                     *       **          *         **
      96531 GAGAATGCTGCTGTCTACCTTTTAT-T--TGA-ATTCCCGC
        131 GAGAATGCTTCTGTCTAGTTTTTATATGAAGATATTCCCTT

                         *  *    *              * *  **   *    *             
      96568 TTCCAACGAAATCCTCCAAGCTATCCAAATATCCACCTGCATTTTCCACAACAAGAGTGTTTCAA
          1 TTCCAACGAAATCATCAAAGCGATCCAAATATCCACTTCCAGATTCTACAAAAAGAGTGTTTCAA

                          *                 *    *  *                *       
      96633 AACTGCTCTATCAATAGAAATGTTCAACTCCTTTG-GCTGGGTACACACATCACAAACAAGTTTC
         66 AACTGCTCTATCAAAAGAAATGTTCAACT-CTGTGAGTTGAGTACACACATCACAAAGAAGTTTC

                                      **     **       
      96697 TGAGAATGCTTCTGTCTAGTTTTTATGGGAAGACGTTCCCTT
        130 TGAGAATGCTTCTGTCTAGTTTTTATATGAAGATATTCCCTT

              *  * *  **          *       *            **                    
      96739 TTTCACCAAAGGCATCAAAGCGCTCCAAATGTCCACTTCCAGACACTACAAAAAGAGTGTTTCAA
          1 TTCCAACGAAATCATCAAAGCGATCCAAATATCCACTTCCAGATTCTACAAAAAGAGTGTTTCAA

             **       **   ***                 *    * *  * *         *       
      96804 ACGTGCTCTAAGAAAGCGAATGTTCAACTCTGTGACTTGAATGCAGATATCACAAAGTAGTTTCT
         66 AACTGCTCTATCAAAAGAAATGTTCAACTCTGTGAGTTGAGTACACACATCACAAAGAAGTTTCT

                **            *     *    *         * 
      96869 GAGAGGGCTTCTGTCTAGATTTTAGATGATGATATTCCCGT
        131 GAGAATGCTTCTGTCTAGTTTTTATATGAAGATATTCCCTT

                           * *   *                *   *                    * 
      96910 TTCCAACGAAATCATTAGAGCTATCCAAATATCCACTTACAGTTTCTACAAAAAGAGTGTTTCCA
          1 TTCCAACGAAATCATCAAAGCGATCCAAATATCCACTTCCAGATTCTACAAAAAGAGTGTTTCAA

                   **         * *    *       *  *                   ***      
      96975 AACTGCTGCATCAAAAGAGAGGTTCCACTCTGTTAGCTGAGTACACACATCACAAACTTGTTTCT
         66 AACTGCTCTATCAAAAGAAATGTTCAACTCTGTGAGTTGAGTACACACATCACAAAGAAGTTTCT

            *     *         *        **         **   
      97040 CAGAATCCTTCTGTCTCGTTTTTATGGGAAGATATTTACTT
        131 GAGAATGCTTCTGTCTAGTTTTTATATGAAGATATTCCCTT

              *  *  * **          *       *     *       *  *  *              
      97081 TTTCACCGTAGGCATCAAAGCGCTCCAAATGTCCACATCCAGATACTCCAGAAAGAGTGTTTCAA
          1 TTCCAACGAAATCATCAAAGCGATCCAAATATCCACTTCCAGATTCTACAAAAAGAGTGTTTCAA

             *         *   * *   *         *        * *  *      *      *     
      97146 ACCTGCTCTATGAAAGGGAATCTTCAACTCTATGAGTTGAATGCAGACATCAGAAAGAAATTTCT
         66 AACTGCTCTATCAAAAGAAATGTTCAACTCTGTGAGTTGAGTACACACATCACAAAGAAGTTTCT

                     *       **          *         **
      97211 GAGAATGCTGCTGTCTACCTTTTAT-T--TGA-ATTCCCGC
        131 GAGAATGCTTCTGTCTAGTTTTTATATGAAGATATTCCCTT

                         *  *    *                *       *                  
      97248 TTCCAACGAAATCCTCCAAGCTATCCAAATATCCACTTGCAGATTCCACAAAAAGAGTGTTTCAA
          1 TTCCAACGAAATCATCAAAGCGATCCAAATATCCACTTCCAGATTCTACAAAAAGAGTGTTTCAA

                            ** *  *            *       *     *     *  *      
      97313 AACTGCTCTCTATCAATGGCAAAGTTCAACTCTGTTAGTTGAGGACACATATCACCAACAAGTTT
         66 AACTG--CTCTATCAAAAGAAATGTTCAACTCTGTGAGTTGAGTACACACATCACAAAGAAGTTT

                               *       **         *    
      97378 CTGAGAATGCTTCTGTCTATTTTTTATGGGAAGATATTTCCTT
        129 CTGAGAATGCTTCTGTCTAGTTTTTATATGAAGATATTCCCTT

              *  *  * ** *    *      *    *          * **                    
      97421 TTTCACCGTAGGCGTCAAGGCGATCGAAATGTCCACTTCCACAAACTACAAAAAGAGTGTTTCAA
          1 TTCCAACGAAATCATCAAAGCGATCCAAATATCCACTTCCAGATTCTACAAAAAGAGTGTTTCAA

             *         *   * **       *    *        * ** * *   **      *     
      97486 ACCTGCTCTATGAAAGGCCATGTTCATCTCTATGAGTTGAATGGAAATATCCGAAAGAAATTTCT
         66 AACTGCTCTATCAAAAGAAATGTTCAACTCTGTGAGTTGAGTACACACATCACAAAGAAGTTTCT

             *       *          *        *         **
      97551 GGGAATGCTGCTGTCTAG-TGTT-TAT-ACGA-ATTCCCGC
        131 GAGAATGCTTCTGTCTAGTTTTTATATGAAGATATTCCCTT

                         *       *                *    *  *                  
      97588 TTCCAACGAAATCCTCAAAGCAATCCAAATATCCACTTGCAGAATCCACAAAAAGAGTGTTTCAA
          1 TTCCAACGAAATCATCAAAGCGATCCAAATATCCACTTCCAGATTCTACAAAAAGAGTGTTTCAA

               *          *     *          * *                      *        
      97653 AACCGCTCTATCAATAGAAAGGTTCAACTCTTTTAGTTGAGTACACACATCACAAACAAGTTTCT
         66 AACTGCTCTATCAAAAGAAATGTTCAACTCTGTGAGTTGAGTACACACATCACAAAGAAGTTTCT

                            * *      **     **  *    
      97718 GAGAATGCTTCTGTCTGGCTTTTATTGGAAGACGTTTCCTT
        131 GAGAATGCTTCTGTCTAGTTTTTATATGAAGATATTCCCTT

                 * *  **          *       *                * *               
      97759 TT-CACCAAAGGCATCAAAGCGCTCCAAATGTCCACTTCCAGATTCTTCCAAAAGAGTGTTTCAA
          1 TTCCAACGAAATCATCAAAGCGATCCAAATATCCACTTCCAGATTCTACAAAAAGAGTGTTTCAA

             **  *  * ***  * *                 *    * *  * *     *   *       
      97823 ACGTGGTCGAAGTAAGGGAATGTTCAACTCTGTGACTTGAATGCAGATATCACCAAGTAGTTTCT
         66 AACTGCTCTATCAAAAGAAATGTTCAACTCTGTGAGTTGAGTACACACATCACAAAGAAGTTTCT

            * * *             *     *    *         * 
      97888 AATAGTGCTTCTGTCTAGATTTTAGATGATGATATTCCCGT
        131 GAGAATGCTTCTGTCTAGTTTTTATATGAAGATATTCCCTT

                         * * *   *             *  *   *      *     *       * 
      97929 TTCCAACGAAATCGTTAGAGCTATCCAAATATCCAGTTACAGTTTCTACCAAAAGGGTGTTTCCA
          1 TTCCAACGAAATCATCAAAGCGATCCAAATATCCACTTCCAGATTCTACAAAAAGAGTGTTTCAA

              *    **           *            *       *                     * 
      97994 AATTGCTGCATCAAAAGAAAGGTTCAACTCTGTTAGTTGAGGACACACATCACAAAGAAGTTTGT
         66 AACTGCTCTATCAAAAGAAATGTTCAACTCTGTGAGTTGAGTACACACATCACAAAGAAGTTTCT

                              *    *     *           
      98059 GAGAATGCTTCTGTCTAGATTTTGTATGACGATATTCCCTT
        131 GAGAATGCTTCTGTCTAGTTTTTATATGAAGATATTCCCTT

                     *   * *     *   *       * *  *    *  *       *  *       
      98100 TTCCAACGATATCGTTAAAGCAATCTAAATATCAATTTGCAGAATCCACAAAAATAGAGTTTCAA
          1 TTCCAACGAAATCATCAAAGCGATCCAAATATCCACTTCCAGATTCTACAAAAAGAGTGTTTCAA

             *       * *        *    *       *  *                   ***      
      98165 AGCTGCTCTGTAAAAAGAAAGGTTCCACTCTGTTAGCTGAGTACACACATCACAAACTTGTTTCT
         66 AACTGCTCTATCAAAAGAAATGTTCAACTCTGTGAGTTGAGTACACACATCACAAAGAAGTTTCT

                 **         *        **         **   
      98230 GAGAAACCTTCTGTCTCGTTTTTATGGGAAGATATTTACTT
        131 GAGAATGCTTCTGTCTAGTTTTTATATGAAGATATTCCCTT

                 ** * **          *       *     *       *  *  *  *           
      98271 TTCCACTGTAGGCATCAAAGCGCTCCAAATGTCCACATCCAGATACTCCAGAACGAGTGTTTCAA
          1 TTCCAACGAAATCATCAAAGCGATCCAAATATCCACTTCCAGATTCTACAAAAAGAGTGTTTCAA

             *         *   * *   *         *        * *  *      *      *     
      98336 ACCTGCTCTATGAAAGGGAATCTTCAACTCTATGAGTTGAATGCAGA-ATCAGAAAGAAATTTCT
         66 AACTGCTCTATCAAAAGAAATGTTCAACTCTGTGAGTTGAGTACACACATCACAAAGAAGTTTCT

                     *       **          *         **
      98400 GAGAATGCTGCTGTCTACCTTTTAT-T--TGA-ATTCCCGC
        131 GAGAATGCTTCTGTCTAGTTTTTATATGAAGATATTCCCTT

                         *  *    *              * *  **   *    *             
      98437 TTCCAACGAAATCCTCCAAGCTATCCAAATATCCACCTGCATTTTCCACAACAAGAGTGTTTCAA
          1 TTCCAACGAAATCATCAAAGCGATCCAAATATCCACTTCCAGATTCTACAAAAAGAGTGTTTCAA

                          *                 *    *  *                *       
      98502 AACTGCTCTATCAATAGAAATGTTCAACTCCTTTG-GCTGGGTACACACATCACAAACAAGTTTC
         66 AACTGCTCTATCAAAAGAAATGTTCAACT-CTGTGAGTTGAGTACACACATCACAAAGAAGTTTC

                                      **     **       
      98566 TGAGAATGCTTCTGTCTAGTTTTTATGGGAAGACGTTCCCTT
        130 TGAGAATGCTTCTGTCTAGTTTTTATATGAAGATATTCCCTT

              *  * *  **          *       *            **                    
      98608 TTTCACCAAAGGCATCAAAGCGCTCCAAATGTCCACTTCCAGACACTACAAAAAGAGTGTTTCAA
          1 TTCCAACGAAATCATCAAAGCGATCCAAATATCCACTTCCAGATTCTACAAAAAGAGTGTTTCAA

             **       **    **                 *    * *  * *         *       
      98673 ACGTGCTCTAAGAAAACGAATGTTCAACTCTGTGACTTGAATGCAGATATCACAAAGTAGTTTCT
         66 AACTGCTCTATCAAAAGAAATGTTCAACTCTGTGAGTTGAGTACACACATCACAAAGAAGTTTCT

                **            *     *    *         * 
      98738 GAGAGGGCTTCTGTCTAGATTTTAGATGATGATATTCCCGT
        131 GAGAATGCTTCTGTCTAGTTTTTATATGAAGATATTCCCTT

                           * *   *                *   *     *              * 
      98779 TTCCAACGAAATCATTAGAGCTATCCAAATATCCACTTACAGTTTCTATAAAAAGAGTGTTTCCA
          1 TTCCAACGAAATCATCAAAGCGATCCAAATATCCACTTCCAGATTCTACAAAAAGAGTGTTTCAA

                   **         * *    *       *  **                  ***      
      98844 AACTGCTGCATCAAAAGAGAGGTTCCACTCTGTTAGCCGAGTACACACATCACAAACTTGTTTCT
         66 AACTGCTCTATCAAAAGAAATGTTCAACTCTGTGAGTTGAGTACACACATCACAAAGAAGTTTCT

            *     *         *        **         **   
      98909 CAGAATCCTTCTGTCTCGTTTTTATGGGAAGATATTTACTT
        131 GAGAATGCTTCTGTCTAGTTTTTATATGAAGATATTCCCTT

              *  *  * **          *       *     *       *  *  *              
      98950 TTTCACCGTAGGCATCAAAGCGCTCCAAATGTCCACATCCAGATACTCCAGAAAGAGTGTTTCAA
          1 TTCCAACGAAATCATCAAAGCGATCCAAATATCCACTTCCAGATTCTACAAAAAGAGTGTTTCAA

             *         *   * *   *         *        * *  *      *      *     
      99015 ACCTGCTCTATGAAAGGGAATCTTCAACTCTATGAGTTGAATGCAGACATCAGAAAGAAATTTCT
         66 AACTGCTCTATCAAAAGAAATGTTCAACTCTGTGAGTTGAGTACACACATCACAAAGAAGTTTCT

                     *       **          *         **
      99080 GAGAATGCTGCTGTCTACCTTTTAT-T--TGA-ATTCCCGC
        131 GAGAATGCTTCTGTCTAGTTTTTATATGAAGATATTCCCTT

                         *  *    *                *       *                  
      99117 TTCCAACGAAATCCTCCAAGCTATCCAAATATCCACTTGCAGATTCCACAAAAAGAGTGTTTCAA
          1 TTCCAACGAAATCATCAAAGCGATCCAAATATCCACTTCCAGATTCTACAAAAAGAGTGTTTCAA

                            ** *  *            *       *     *     *  *      
      99182 AACTGCTCTCTATCAATGGCAAAGTTCAACTCTGTTAGTTGAGGACACATATCACCAACAAGTTT
         66 AACTG--CTCTATCAAAAGAAATGTTCAACTCTGTGAGTTGAGTACACACATCACAAAGAAGTTT

                               *       **         *    
      99247 CTGAGAATGCTTCTGTCTATTTTTTATGGGAAGATATTTCCTT
        129 CTGAGAATGCTTCTGTCTAGTTTTTATATGAAGATATTCCCTT

              *  *  * ** *    *      *    *          * **                    
      99290 TTTCACCGTAGGCGTCAAGGCGATCGAAATGTCCACTTCCACAAACTACAAAAAGAGTGTTTCAA
          1 TTCCAACGAAATCATCAAAGCGATCCAAATATCCACTTCCAGATTCTACAAAAAGAGTGTTTCAA

             *         *   * **       *    *        * ** * *   **      *     
      99355 ACCTGCTCTATGAAAGGCCATGTTCATCTCTATGAGTTGAATGGAAATATCCGAAAGAAATTTCT
         66 AACTGCTCTATCAAAAGAAATGTTCAACTCTGTGAGTTGAGTACACACATCACAAAGAAGTTTCT

             *       *                             **
      99420 GGGAATGCTGCTGTCTAGTTTTTATAT---GA-ATTCCCGC
        131 GAGAATGCTTCTGTCTAGTTTTTATATGAAGATATTCCCTT

                         *       *                *    *  *                  
      99457 TTCCAACGAAATCCTCAAAGCAATCCAAATATCCACTTGCAGAATCCACAAAAAGAGTGTTTCAA
          1 TTCCAACGAAATCATCAAAGCGATCCAAATATCCACTTCCAGATTCTACAAAAAGAGTGTTTCAA

                          *     *          * *                      *        
      99522 AACTGCTCTATCAATAGAAAGGTTCAACTCTTTTAGTTGAGTACACACATCACAAACAAGTTTCT
         66 AACTGCTCTATCAAAAGAAATGTTCAACTCTGTGAGTTGAGTACACACATCACAAAGAAGTTTCT

                            * *      **     **  *    
      99587 GAGAATGCTTCTGTCTGGCTTTTATTGGAAGACGTTTCCTT
        131 GAGAATGCTTCTGTCTAGTTTTTATATGAAGATATTCCCTT

                 * *  **          *      **                * *               
      99628 TT-CACCAAAGGCATCAAAGCGCTCCAAAGGTCCACTTCCAGATTCTTCCAAAAGAGTGTTTCAA
          1 TTCCAACGAAATCATCAAAGCGATCCAAATATCCACTTCCAGATTCTACAAAAAGAGTGTTTCAA

             **         **  * *                 *    * *  * *     *   *      
      99692 ACGTGCTC-ATAGTAAGGGAATGTTCAACTCTGTGACTTGAATGCAGATATCACCAAGTAGTTTC
         66 AACTGCTCTAT-CAAAAGAAATGTTCAACTCTGTGAGTTGAGTACACACATCACAAAGAAGTTTC

             * * *             *     *    *         * 
      99756 TAATAGTGCTTCTGTCTAGATTTTAGATGATGATATTCCCGT
        130 TGAGAATGCTTCTGTCTAGTTTTTATATGAAGATATTCCCTT

                         *** *   *             *  *   *      *     *       * 
      99798 TTCCAACGAAATCGCTAGAGCTATCCAAATATCCAGTTACAGTTTCTACCAAAAGGGTGTTTCCA
          1 TTCCAACGAAATCATCAAAGCGATCCAAATATCCACTTCCAGATTCTACAAAAAGAGTGTTTCAA

              *    **           *            *       *                     * 
      99863 AATTGCTGCATCAAAAGAAAGGTTCAACTCTGTTAGTTGAGGACACACATCACAAAGAAGTTTGT
         66 AACTGCTCTATCAAAAGAAATGTTCAACTCTGTGAGTTGAGTACACACATCACAAAGAAGTTTCT

                             **    *     *           
      99928 GAGAATGCTTCTGTCTACATTTTGTATGACGATATTCCCTT
        131 GAGAATGCTTCTGTCTAGTTTTTATATGAAGATATTCCCTT

                     *   * *     *   *       * *  *    *  *       *  *       
      99969 TTCCAACGATATCGTTAAAGCAATCTAAATATCAATTTGCAGAATCCACAAAAATAGAGTTTCAA
          1 TTCCAACGAAATCATCAAAGCGATCCAAATATCCACTTCCAGATTCTACAAAAAGAGTGTTTCAA

             *       * *        *    *       *  *                   ***      
     100034 AGCTGCTCTGTAAAAAGAAAGGTTCCACTCTGTTAGCTGAGTACACACATCACAAACTTGTTTCT
         66 AACTGCTCTATCAAAAGAAATGTTCAACTCTGTGAGTTGAGTACACACATCACAAAGAAGTTTCT

                  *         *        **         **   
     100099 GAGAATCCTTCTGTCTCGTTTTTATGGGAAGATATTTACTT
        131 GAGAATGCTTCTGTCTAGTTTTTATATGAAGATATTCCCTT

                 *  * **          *       *     *       *  *  *  *           
     100140 TTCCACCGTAGGCATCAAAGCGCTCCAAATGTCCACATCCAGATACTCCAGAACGAGTGTTTCAA
          1 TTCCAACGAAATCATCAAAGCGATCCAAATATCCACTTCCAGATTCTACAAAAAGAGTGTTTCAA

             *         *   * *   *       * *        * *  *      *      *     
     100205 ACCTGCTCTATGAAAGGGAATCTTCAACTGTATGAGTTGAATGCAGACATCAGAAAGAAATTTCT
         66 AACTGCTCTATCAAAAGAAATGTTCAACTCTGTGAGTTGAGTACACACATCACAAAGAAGTTTCT

                     *       **          *         **
     100270 GAGAATGCTGCTGTCTACCTTTTAT-T--TGA-ATTCCCGC
        131 GAGAATGCTTCTGTCTAGTTTTTATATGAAGATATTCCCTT

                         *  *    *              * *  **   *    *             
     100307 TTCCAACGAAATCCTCCAAGCTATCCAAATATCCACCTGCATTTTCCACAACAAGAGTGTTTCAA
          1 TTCCAACGAAATCATCAAAGCGATCCAAATATCCACTTCCAGATTCTACAAAAAGAGTGTTTCAA

                          *                 *    *  *                *       
     100372 AACTGCTCTATCAATAGAAATGTTCAACTCCTTTG-GCTGGGTACACACATCACAAACAAGTTTC
         66 AACTGCTCTATCAAAAGAAATGTTCAACT-CTGTGAGTTGAGTACACACATCACAAAGAAGTTTC

                                      **     **       
     100436 TGAGAATGCTTCTGTCTAGTTTTTATGGGAAGACGTTCCCTT
        130 TGAGAATGCTTCTGTCTAGTTTTTATATGAAGATATTCCCTT

              *  * *  **          *       *            **                    
     100478 TTTCACCAAAGGCATCAAAGCGCTCCAAATGTCCACTTCCAGACACTACAAAAAGAGTGTTTCAA
          1 TTCCAACGAAATCATCAAAGCGATCCAAATATCCACTTCCAGATTCTACAAAAAGAGTGTTTCAA

             **       **   ***                 *    * *  * *         *       
     100543 ACGTGCTCTAAGAAAGCGAATGTTCAACTCTGTGACTTGAATGCAGATATCACAAAGTAGTTTCT
         66 AACTGCTCTATCAAAAGAAATGTTCAACTCTGTGAGTTGAGTACACACATCACAAAGAAGTTTCT

                **            *     *    *         * 
     100608 GAGAGGGCTTCTGTCTAGATTTTAGATGATGATATTCCCGT
        131 GAGAATGCTTCTGTCTAGTTTTTATATGAAGATATTCCCTT

                           * *   *                *   *                    * 
     100649 TTCCAACGAAATCATTAGAGCTATCCAAATATCCACTTACAGTTTCTACAAAAAGAGTGTTTCCA
          1 TTCCAACGAAATCATCAAAGCGATCCAAATATCCACTTCCAGATTCTACAAAAAGAGTGTTTCAA

                   **         * *    *       *  *                   ***      
     100714 AACTGCTGCATCAAAAGAGAGGTTCCACTCTGTTAGCTGAGTACACACATCACAAACTTGTTTCT
         66 AACTGCTCTATCAAAAGAAATGTTCAACTCTGTGAGTTGAGTACACACATCACAAAGAAGTTTCT

            *     *         *        **         **   
     100779 CAGAATCCTTCTGTCTCGTTTTTATGGGAAGATATTTACTT
        131 GAGAATGCTTCTGTCTAGTTTTTATATGAAGATATTCCCTT

              *  *  * **          *       *     *       *  *  *              
     100820 TTTCACCGTAGGCATCAAAGCGCTCCAAATGTCCACATCCAGATACTCCAGAAAGAGTGTTTCAA
          1 TTCCAACGAAATCATCAAAGCGATCCAAATATCCACTTCCAGATTCTACAAAAAGAGTGTTTCAA

             *         *   * *   *         *        * *  *      *      *     
     100885 ACCTGCTCTATGAAAGGGAATCTTCAACTCTATGAGTTGAATGCAGACATCAGAAAGAAATTTCT
         66 AACTGCTCTATCAAAAGAAATGTTCAACTCTGTGAGTTGAGTACACACATCACAAAGAAGTTTCT

                     *       **          *         **
     100950 GAGAATGCTGCTGTCTACCTTTTAT-T--TGA-ATTCCCGC
        131 GAGAATGCTTCTGTCTAGTTTTTATATGAAGATATTCCCTT

                         *  *    *                *       *                  
     100987 TTCCAACGAAATCCTCCAAGCTATCCAAATATCCACTTGCAGATTCCACAAAAAGAGTGTTTCAA
          1 TTCCAACGAAATCATCAAAGCGATCCAAATATCCACTTCCAGATTCTACAAAAAGAGTGTTTCAA

                            ** *  *            *       *     *     *  *      
     101052 AACTGCTCTCTATCAATGGCAAAGTTCAACTCTGTTAGTTGAGGACACATATCACCAACAAGTTT
         66 AACTG--CTCTATCAAAAGAAATGTTCAACTCTGTGAGTTGAGTACACACATCACAAAGAAGTTT

                               *       **         *    
     101117 CTGAGAATGCTTCTGTCTATTTTTTATGGGAAGATATTTCCTT
        129 CTGAGAATGCTTCTGTCTAGTTTTTATATGAAGATATTCCCTT

              *  *  * ** *    *      *    *          * **                    
     101160 TTTCACCGTAGGCGTCAAGGCGATCGAAATGTCCACTTCCACAAACTACAAAAAGAGTGTTTCAA
          1 TTCCAACGAAATCATCAAAGCGATCCAAATATCCACTTCCAGATTCTACAAAAAGAGTGTTTCAA

             *         *   * **       *    *        * ** * *   **      *     
     101225 ACCTGCTCTATGAAAGGCCATGTTCATCTCTATGAGTTGAATGGAAATATCCGAAAGAAATTTCT
         66 AACTGCTCTATCAAAAGAAATGTTCAACTCTGTGAGTTGAGTACACACATCACAAAGAAGTTTCT

             *       *          *        *         **
     101290 GGGAATGCTGCTGTCTAG-TGTT-TAT-ACGA-ATTCCCGC
        131 GAGAATGCTTCTGTCTAGTTTTTATATGAAGATATTCCCTT

                         *       *                *    *  *                  
     101327 TTCCAACGAAATCCTCAAAGCAATCCAAATATCCACTTGCAGAATCCACAAAAAGAGTGTTTCAA
          1 TTCCAACGAAATCATCAAAGCGATCCAAATATCCACTTCCAGATTCTACAAAAAGAGTGTTTCAA

                          *     *          * *                      *        
     101392 AACTGCTCTATCAATAGAAAGGTTCAACTCTTTTAGTTGAGTACACACATCACAAACAAGTTTCT
         66 AACTGCTCTATCAAAAGAAATGTTCAACTCTGTGAGTTGAGTACACACATCACAAAGAAGTTTCT

                            * *      **     **  *    
     101457 GAGAATGCTTCTGTCTGGCTTTTATTGGAAGACGTTTCCTT
        131 GAGAATGCTTCTGTCTAGTTTTTATATGAAGATATTCCCTT

                 * *  **          *       *                * *               
     101498 TT-CACCAAAGGCATCAAAGCGCTCCAAATGTCCACTTCCAGATTCTTCCAAAAGAGTGTTTCAA
          1 TTCCAACGAAATCATCAAAGCGATCCAAATATCCACTTCCAGATTCTACAAAAAGAGTGTTTCAA

             **  *  * ***  * *                 *    * *  * *     *   *       
     101562 ACGTGGTCGAAGTAAGGGAATGTTCAACTCTGTGACTTGAATGCAGATATCACCAAGTAGTTTCT
         66 AACTGCTCTATCAAAAGAAATGTTCAACTCTGTGAGTTGAGTACACACATCACAAAGAAGTTTCT

            * * *             *     *    *         * 
     101627 AATAGTGCTTCTGTCTAGATTTTAGATGATGATATTCCCGT
        131 GAGAATGCTTCTGTCTAGTTTTTATATGAAGATATTCCCTT

                         * * *   *             *  *   *      *     *       * 
     101668 TTCCAACGAAATCGTTAGAGCTATCCAAATATCCAGTTACAGTTTCTACCAAAAGGGTGTTTCCA
          1 TTCCAACGAAATCATCAAAGCGATCCAAATATCCACTTCCAGATTCTACAAAAAGAGTGTTTCAA

              *    **           *            *       *                     * 
     101733 AATTGCTGCATCAAAAGAAAGGTTCAACTCTGTTAGTTGAGGACACACATCACAAAGAAGTTTGT
         66 AACTGCTCTATCAAAAGAAATGTTCAACTCTGTGAGTTGAGTACACACATCACAAAGAAGTTTCT

                              *    *     *           
     101798 GAGAATGCTTCTGTCTAGATTTTGTATGACGATATTCCCTT
        131 GAGAATGCTTCTGTCTAGTTTTTATATGAAGATATTCCCTT

                     *   * *     *   *       * *  *    *  *       *  *       
     101839 TTCCAACGATATCGTTAAAGCAATCTAAATATCAATTTGCAGAATCCACAAAAATAGAGTTTCAA
          1 TTCCAACGAAATCATCAAAGCGATCCAAATATCCACTTCCAGATTCTACAAAAAGAGTGTTTCAA

             *       * *        *    *       *  *                   ***      
     101904 AGCTGCTCTGTAAAAAGAAAGGTTCCACTCTGTTAGCTGAGTACACACATCACAAACTTGTTTCT
         66 AACTGCTCTATCAAAAGAAATGTTCAACTCTGTGAGTTGAGTACACACATCACAAAGAAGTTTCT

                 **         *        **         **   
     101969 GAGAAACCTTCTGTCTCGTTTTTATGGGAAGATATTTACTT
        131 GAGAATGCTTCTGTCTAGTTTTTATATGAAGATATTCCCTT

                 *  * **          *       *     *       *  *  *  *           
     102010 TTCCACCGTAGGCATCAAAGCGCTCCAAATGTCCACATCCAGATACTCCAGAACGAGTGTTTCAA
          1 TTCCAACGAAATCATCAAAGCGATCCAAATATCCACTTCCAGATTCTACAAAAAGAGTGTTTCAA

             *         *   * *   *         *        * *  *      *      *     
     102075 ACCTGCTCTATGAAAGGGAATCTTCAACTCTATGAGTTGAATGCAGA-ATCAGAAAGAAATTTCT
         66 AACTGCTCTATCAAAAGAAATGTTCAACTCTGTGAGTTGAGTACACACATCACAAAGAAGTTTCT

                     *       **          *         **
     102139 GAGAATGCTGCTGTCTACCTTTTAT-T--TGA-ATTCCCGC
        131 GAGAATGCTTCTGTCTAGTTTTTATATGAAGATATTCCCTT

                         *  *    *              * *  **   *    *             
     102176 TTCCAACGAAATCCTCCAAGCTATCCAAATATCCACCTGCATTTTCCACAACAAGAGTGTTTCAA
          1 TTCCAACGAAATCATCAAAGCGATCCAAATATCCACTTCCAGATTCTACAAAAAGAGTGTTTCAA

                          *                 *    *  *                *       
     102241 AACTGCTCTATCAATAGAAATGTTCAACTCCTTTG-GCTGGGTACACACATCACAAACAAGTTTC
         66 AACTGCTCTATCAAAAGAAATGTTCAACT-CTGTGAGTTGAGTACACACATCACAAAGAAGTTTC

                                      **     **       
     102305 TGAGAATGCTTCTGTCTAGTTTTTATGGGAAGACGTTCCCTT
        130 TGAGAATGCTTCTGTCTAGTTTTTATATGAAGATATTCCCTT

              *  * *  **          *       *            **                    
     102347 TTTCACCAAAGGCATCAAAGCGCTCCAAATGTCCACTTCCAGACACTACAAAAAGAGTGTTTCAA
          1 TTCCAACGAAATCATCAAAGCGATCCAAATATCCACTTCCAGATTCTACAAAAAGAGTGTTTCAA

             **       **    **                 *    * *  * *         *       
     102412 ACGTGCTCTAAGAAAACGAATGTTCAACTCTGTGACTTGAATGCAGATATCACAAAGTAGTTTCT
         66 AACTGCTCTATCAAAAGAAATGTTCAACTCTGTGAGTTGAGTACACACATCACAAAGAAGTTTCT

                **            *     *    *         * 
     102477 GAGAGGGCTTCTGTCTAGATTTTAGATGATGATATTCCCGT
        131 GAGAATGCTTCTGTCTAGTTTTTATATGAAGATATTCCCTT

                           * *   *                *   *     *              * 
     102518 TTCCAACGAAATCATTAGAGCTATCCAAATATCCACTTACAGTTTCTATAAAAAGAGTGTTTCCA
          1 TTCCAACGAAATCATCAAAGCGATCCAAATATCCACTTCCAGATTCTACAAAAAGAGTGTTTCAA

                   **         * *    *       *  **                  ***      
     102583 AACTGCTGCATCAAAAGAGAGGTTCCACTCTGTTAGCCGAGTACACACATCACAAACTTGTTTCT
         66 AACTGCTCTATCAAAAGAAATGTTCAACTCTGTGAGTTGAGTACACACATCACAAAGAAGTTTCT

            *     *         *        **         **   
     102648 CAGAATCCTTCTGTCTCGTTTTTATGGGAAGATATTTACTT
        131 GAGAATGCTTCTGTCTAGTTTTTATATGAAGATATTCCCTT

              *  *  * **          *       *     *       *  *  *              
     102689 TTTCACCGTAGGCATCAAAGCGCTCCAAATGTCCACATCCAGATACTCCAGAAAGAGTGTTTCAA
          1 TTCCAACGAAATCATCAAAGCGATCCAAATATCCACTTCCAGATTCTACAAAAAGAGTGTTTCAA

             *         *   * *   *         *        * *  *      *      *     
     102754 ACCTGCTCTATGAAAGGGAATCTTCAACTCTATGAGTTGAATGCAGACATCAGAAAGAAATTTCT
         66 AACTGCTCTATCAAAAGAAATGTTCAACTCTGTGAGTTGAGTACACACATCACAAAGAAGTTTCT

                     *  *    **          *         **
     102819 GAGAATGCTGCTCTCTACCTTTTAT-T--TGA-ATTCCCGC
        131 GAGAATGCTTCTGTCTAGTTTTTATATGAAGATATTCCCTT

                         *  *    *                *       *                  
     102856 TTCCAACGAAATCCTCCAAGCTATCCAAATATCCACTTGCAGATTCCACAAAAAGAGTGTTTCAA
          1 TTCCAACGAAATCATCAAAGCGATCCAAATATCCACTTCCAGATTCTACAAAAAGAGTGTTTCAA

                            ** *  *            *       *     *     *  *      
     102921 AACTGCTCTCTATCAATGGCAAAGTTCAACTCTGTTAGTTGAGGACACATATCACCAACAAGTTT
         66 AACTG--CTCTATCAAAAGAAATGTTCAACTCTGTGAGTTGAGTACACACATCACAAAGAAGTTT

                               *       **         *    
     102986 CTGAGAATGCTTCTGTCTATTTTTTATGGGAAGATATTTCCTT
        129 CTGAGAATGCTTCTGTCTAGTTTTTATATGAAGATATTCCCTT

              *  *  * ** *    *      *    *          * **                    
     103029 TTTCACCGTAGGCGTCAAGGCGATCGAAATGTCCACTTCCACAAACTACAAAAAGAGTGTTTCAA
          1 TTCCAACGAAATCATCAAAGCGATCCAAATATCCACTTCCAGATTCTACAAAAAGAGTGTTTCAA

             *         *   * **       *    *        * ** * *   **      *     
     103094 ACCTGCTCTATGAAAGGCCATGTTCATCTCTATGAGTTGAATGGAAATATCCGAAAGAAATTTCT
         66 AACTGCTCTATCAAAAGAAATGTTCAACTCTGTGAGTTGAGTACACACATCACAAAGAAGTTTCT

             *       *                             **
     103159 GGGAATGCTGCTGTCTAGTTTTTATAT---GA-ATTCCCGC
        131 GAGAATGCTTCTGTCTAGTTTTTATATGAAGATATTCCCTT

                         *       *                *    *  *                  
     103196 TTCCAACGAAATCCTCAAAGCAATCCAAATATCCACTTGCAGAATCCACAAAAAGAGTGTTTCAA
          1 TTCCAACGAAATCATCAAAGCGATCCAAATATCCACTTCCAGATTCTACAAAAAGAGTGTTTCAA

                          *     *          * *                      *        
     103261 AACTGCTCTATCAATAGAAAGGTTCAACTCTTTTAGTTGAGTACACACATCACAAACAAGTTTCT
         66 AACTGCTCTATCAAAAGAAATGTTCAACTCTGTGAGTTGAGTACACACATCACAAAGAAGTTTCT

                            * *      **     **  *    
     103326 GAGAATGCTTCTGTCTGGCTTTTATTGGAAGACGTTTCCTT
        131 GAGAATGCTTCTGTCTAGTTTTTATATGAAGATATTCCCTT

                 * *  **          *      **                * *               
     103367 TT-CACCAAAGGCATCAAAGCGCTCCAAAGGTCCACTTCCAGATTCTTCCAAAAGAGTGTTTCAA
          1 TTCCAACGAAATCATCAAAGCGATCCAAATATCCACTTCCAGATTCTACAAAAAGAGTGTTTCAA

             **         **  * *                 *    * *  * *     *   *      
     103431 ACGTGCTC-ATAGTAAGGGAATGTTCAACTCTGTGACTTGAATGCAGATATCACCAAGTAGTTTC
         66 AACTGCTCTAT-CAAAAGAAATGTTCAACTCTGTGAGTTGAGTACACACATCACAAAGAAGTTTC

             * * *             *     *    *         * 
     103495 TAATAGTGCTTCTGTCTAGATTTTAGATGATGATATTCCCGT
        130 TGAGAATGCTTCTGTCTAGTTTTTATATGAAGATATTCCCTT

                         *** *   *             *  *   *      *     *       * 
     103537 TTCCAACGAAATCGCTAGAGCTATCCAAATATCCAGTTACAGTTTCTACCAAAAGGGTGTTTCCA
          1 TTCCAACGAAATCATCAAAGCGATCCAAATATCCACTTCCAGATTCTACAAAAAGAGTGTTTCAA

              *    **           *            *       *                     * 
     103602 AATTGCTGCATCAAAAGAAAGGTTCAACTCTGTTAGTTGAGGACACACATCACAAAGAAGTTTGT
         66 AACTGCTCTATCAAAAGAAATGTTCAACTCTGTGAGTTGAGTACACACATCACAAAGAAGTTTCT

                             **    *     *           
     103667 GAGAATGCTTCTGTCTACATTTTGTATGACGATATTCCCTT
        131 GAGAATGCTTCTGTCTAGTTTTTATATGAAGATATTCCCTT

                     *   * *     *   *       * *  *    *  *       *  *       
     103708 TTCCAACGATATCGTTAAAGCAATCTAAATATCAATTTGCAGAATCCACAAAAATAGAGTTTCAA
          1 TTCCAACGAAATCATCAAAGCGATCCAAATATCCACTTCCAGATTCTACAAAAAGAGTGTTTCAA

             *       * *        *    *       *  *                   ***      
     103773 AGCTGCTCTGTAAAAAGAAAGGTTCCACTCTGTTAGCTGAGTACACACATCACAAACTTGTTTCT
         66 AACTGCTCTATCAAAAGAAATGTTCAACTCTGTGAGTTGAGTACACACATCACAAAGAAGTTTCT

                  *         *        **         **   
     103838 GAGAATCCTTCTGTCTCGTTTTTATGGGAAGATATTTACTT
        131 GAGAATGCTTCTGTCTAGTTTTTATATGAAGATATTCCCTT

                 *  * **          *       *     *       *  *  *  *           
     103879 TTCCACCGTAGGCATCAAAGCGCTCCAAATGTCCACATCCAGATACTCCAGAACGAGTGTTTCAA
          1 TTCCAACGAAATCATCAAAGCGATCCAAATATCCACTTCCAGATTCTACAAAAAGAGTGTTTCAA

             *         *   * *   *         *        * *  *      *      *     
     103944 ACCTGCTCTATGAAAGGGAATCTTCAACTCTATGAGTTGAATGCAGACATCAGAAAGAAATTTCT
         66 AACTGCTCTATCAAAAGAAATGTTCAACTCTGTGAGTTGAGTACACACATCACAAAGAAGTTTCT

                     *       **          *         **
     104009 GAGAATGCTGCTGTCTACCTTTTAT-T--TGA-ATTCCCGC
        131 GAGAATGCTTCTGTCTAGTTTTTATATGAAGATATTCCCTT

                         *  *    *              * *  **   *    *             
     104046 TTCCAACGAAATCCTCCAAGCTATCCAAATATCCACCTGCATTTTCCACAACAAGAGTGTTTCAA
          1 TTCCAACGAAATCATCAAAGCGATCCAAATATCCACTTCCAGATTCTACAAAAAGAGTGTTTCAA

                          *                 *    *  *                *       
     104111 AACTGCTCTATCAATAGAAATGTTCAACTCCTTTG-GCTGGGTACACACATCACAAACAAGTTTC
         66 AACTGCTCTATCAAAAGAAATGTTCAACT-CTGTGAGTTGAGTACACACATCACAAAGAAGTTTC

                                      **     **       
     104175 TGAGAATGCTTCTGTCTAGTTTTTATGGGAAGACGTTCCCTT
        130 TGAGAATGCTTCTGTCTAGTTTTTATATGAAGATATTCCCTT

              *  * *  **          *       *            **                    
     104217 TTTCACCAAAGGCATCAAAGCGCTCCAAATGTCCACTTCCAGACACTACAAAAAGAGTGTTTCAA
          1 TTCCAACGAAATCATCAAAGCGATCCAAATATCCACTTCCAGATTCTACAAAAAGAGTGTTTCAA

             **       **   ***                 *    * *  * *         *       
     104282 ACGTGCTCTAAGAAAGCGAATGTTCAACTCTGTGACTTGAATGCAGATATCACAAAGTAGTTTCT
         66 AACTGCTCTATCAAAAGAAATGTTCAACTCTGTGAGTTGAGTACACACATCACAAAGAAGTTTCT

                **            *     *    *         * 
     104347 GAGAGGGCTTCTGTCTAGATTTTAGATGATGATATTCCCGT
        131 GAGAATGCTTCTGTCTAGTTTTTATATGAAGATATTCCCTT

                           * *   *                *   *                    * 
     104388 TTCCAACGAAATCATTAGAGCTATCCAAATATCCACTTACAGTTTCTACAAAAAGAGTGTTTCCA
          1 TTCCAACGAAATCATCAAAGCGATCCAAATATCCACTTCCAGATTCTACAAAAAGAGTGTTTCAA

                   **         * *    *       *  *                   ***      
     104453 AACTGCTGCATCAAAAGAGAGGTTCCACTCTGTTAGCTGAGTACACACATCACAAACTTGTTTCT
         66 AACTGCTCTATCAAAAGAAATGTTCAACTCTGTGAGTTGAGTACACACATCACAAAGAAGTTTCT

            *     *         *        **         **   
     104518 CAGAATCCTTCTGTCTCGTTTTTATGGGAAGATATTTACTT
        131 GAGAATGCTTCTGTCTAGTTTTTATATGAAGATATTCCCTT

              *  *  * **          *       *     *       *  *  *              
     104559 TTTCACCGTAGGCATCAAAGCGCTCCAAATGTCCACATCCAGATACTCCAGAAAGAGTGTTTCAA
          1 TTCCAACGAAATCATCAAAGCGATCCAAATATCCACTTCCAGATTCTACAAAAAGAGTGTTTCAA

             *         *   * *   *         *        * *  *      *      *     
     104624 ACCTGCTCTATGAAAGGGAATCTTCAACTCTATGAGTTGAATGCAGACATCAGAAAGAAATTTCT
         66 AACTGCTCTATCAAAAGAAATGTTCAACTCTGTGAGTTGAGTACACACATCACAAAGAAGTTTCT

                     *       **          *         **
     104689 GAGAATGCTGCTGTCTACCTTTTAT-T--TGA-ATTCCCGC
        131 GAGAATGCTTCTGTCTAGTTTTTATATGAAGATATTCCCTT

                         *  *    *                *       *                  
     104726 TTCCAACGAAATCCTCCAAGCTATCCAAATATCCACTTGCAGATTCCACAAAAAGAGTGTTTCAA
          1 TTCCAACGAAATCATCAAAGCGATCCAAATATCCACTTCCAGATTCTACAAAAAGAGTGTTTCAA

                            ** *  *            *       *     *     *  *      
     104791 AACTGCTCTCTATCAATGGCAAAGTTCAACTCTGTTAGTTGAGGACACATATCACCAACAAGTTT
         66 AACTG--CTCTATCAAAAGAAATGTTCAACTCTGTGAGTTGAGTACACACATCACAAAGAAGTTT

                               *       **         *    
     104856 CTGAGAATGCTTCTGTCTATTTTTTATGGGAAGATATTTCCTT
        129 CTGAGAATGCTTCTGTCTAGTTTTTATATGAAGATATTCCCTT

              *  *  * ** *    *      *    *          * **                    
     104899 TTTCACCGTAGGCGTCAAGGCGATCGAAATGTCCACTTCCACAAACTACAAAAAGAGTGTTTCAA
          1 TTCCAACGAAATCATCAAAGCGATCCAAATATCCACTTCCAGATTCTACAAAAAGAGTGTTTCAA

             *         *   * **       *    *        * ** * *   **      *     
     104964 ACCTGCTCTATGAAAGGCCATGTTCATCTCTATGAGTTGAATGGAAATATCCGAAAGAAATTTCT
         66 AACTGCTCTATCAAAAGAAATGTTCAACTCTGTGAGTTGAGTACACACATCACAAAGAAGTTTCT

             *       *          *        *         **
     105029 GGGAATGCTGCTGTCTAG-TGTT-TAT-ACGA-ATTCCCGC
        131 GAGAATGCTTCTGTCTAGTTTTTATATGAAGATATTCCCTT

                         *       *                *    *  *                  
     105066 TTCCAACGAAATCCTCAAAGCAATCCAAATATCCACTTGCAGAATCCACAAAAAGAGTGTTTCAA
          1 TTCCAACGAAATCATCAAAGCGATCCAAATATCCACTTCCAGATTCTACAAAAAGAGTGTTTCAA

               *          *     *          * *                      *        
     105131 AACCGCTCTATCAATAGAAAGGTTCAACTCTTTTAGTTGAGTACACACATCACAAACAAGTTTCT
         66 AACTGCTCTATCAAAAGAAATGTTCAACTCTGTGAGTTGAGTACACACATCACAAAGAAGTTTCT

                            * *      **     **  *    
     105196 GAGAATGCTTCTGTCTGGCTTTTATTGGAAGACGTTTCCTT
        131 GAGAATGCTTCTGTCTAGTTTTTATATGAAGATATTCCCTT

                 * *  **          *       *                * *               
     105237 TT-CACCAAAGGCATCAAAGCGCTCCAAATGTCCACTTCCAGATTCTTCCAAAAGAGTGTTTCAA
          1 TTCCAACGAAATCATCAAAGCGATCCAAATATCCACTTCCAGATTCTACAAAAAGAGTGTTTCAA

             **  *  * ***  * *                 *    * *  * *     *   *       
     105301 ACGTGGTCGAAGTAAGGGAATGTTCAACTCTGTGACTTGAATGCAGATATCACCAAGTAGTTTCT
         66 AACTGCTCTATCAAAAGAAATGTTCAACTCTGTGAGTTGAGTACACACATCACAAAGAAGTTTCT

            * * *             *     *    *         * 
     105366 AATAGTGCTTCTGTCTAGATTTTAGATGATGATATTCCCGT
        131 GAGAATGCTTCTGTCTAGTTTTTATATGAAGATATTCCCTT

                         * * *   *             *  *   *      *     *       * 
     105407 TTCCAACGAAATCGTTAGAGCTATCCAAATATCCAGTTACAGTTTCTACCAAAAGGGTGTTTCCA
          1 TTCCAACGAAATCATCAAAGCGATCCAAATATCCACTTCCAGATTCTACAAAAAGAGTGTTTCAA

              *    **           *            *       *                     * 
     105472 AATTGCTGCATCAAAAGAAAGGTTCAACTCTGTTAGTTGAGGACACACATCACAAAGAAGTTTGT
         66 AACTGCTCTATCAAAAGAAATGTTCAACTCTGTGAGTTGAGTACACACATCACAAAGAAGTTTCT

                              *    *     *           
     105537 GAGAATGCTTCTGTCTAGATTTTGTATGACGATATTCCCTT
        131 GAGAATGCTTCTGTCTAGTTTTTATATGAAGATATTCCCTT

                     *   * *     *   *       * *  *    *  *       *  *       
     105578 TTCCAACGATATCGTTAAAGCAATCTAAATATCAATTTGCAGAATCCACAAAAATAGAGTTTCAA
          1 TTCCAACGAAATCATCAAAGCGATCCAAATATCCACTTCCAGATTCTACAAAAAGAGTGTTTCAA

             *       * *        *    *       *  *                   ***      
     105643 AGCTGCTCTGTAAAAAGAAAGGTTCCACTCTGTTAGCTGAGTACACACATCACAAACTTGTTTCT
         66 AACTGCTCTATCAAAAGAAATGTTCAACTCTGTGAGTTGAGTACACACATCACAAAGAAGTTTCT

                 **         *        **         **   
     105708 GAGAAACCTTCTGTCTCGTTTTTATGGGAAGATATTTACTT
        131 GAGAATGCTTCTGTCTAGTTTTTATATGAAGATATTCCCTT

                 ** * **          *       *     *       *  *  *  *           
     105749 TTCCACTGTAGGCATCAAAGCGCTCCAAATGTCCACATCCAGATACTCCAGAACGAGTGTTTCAA
          1 TTCCAACGAAATCATCAAAGCGATCCAAATATCCACTTCCAGATTCTACAAAAAGAGTGTTTCAA

             *         *   * *   *         *        * *  *      *      *     
     105814 ACCTGCTCTATGAAAGGGAATCTTCAACTCTATGAGTTGAATGCAGA-ATCAGAAAGAAATTTCT
         66 AACTGCTCTATCAAAAGAAATGTTCAACTCTGTGAGTTGAGTACACACATCACAAAGAAGTTTCT

                     *       **          *         **
     105878 GAGAATGCTGCTGTCTACCTTTTAT-T--TGA-ATTCCCGC
        131 GAGAATGCTTCTGTCTAGTTTTTATATGAAGATATTCCCTT

                         *  *    *              * *  **   *    *             
     105915 TTCCAACGAAATCCTCCAAGCTATCCAAATATCCACCTGCATTTTCCACAACAAGAGTGTTTCAA
          1 TTCCAACGAAATCATCAAAGCGATCCAAATATCCACTTCCAGATTCTACAAAAAGAGTGTTTCAA

                          *                 *    *  *                *       
     105980 AACTGCTCTATCAATAGAAATGTTCAACTCCTTTG-GCTGGGTACACACATCACAAACAAGTTTC
         66 AACTGCTCTATCAAAAGAAATGTTCAACT-CTGTGAGTTGAGTACACACATCACAAAGAAGTTTC

                                      **     **       
     106044 TGAGAATGCTTCTGTCTAGTTTTTATGGGAAGACGTTCCCTT
        130 TGAGAATGCTTCTGTCTAGTTTTTATATGAAGATATTCCCTT

              *  * *  **          *       *            **                    
     106086 TTTCACCAAAGGCATCAAAGCGCTCCAAATGTCCACTTCCAGACACTACAAAAAGAGTGTTTCAA
          1 TTCCAACGAAATCATCAAAGCGATCCAAATATCCACTTCCAGATTCTACAAAAAGAGTGTTTCAA

             **       **    **                 *    * *  * *         *       
     106151 ACGTGCTCTAAGAAAACGAATGTTCAACTCTGTGACTTGAATGCAGATATCACAAAGTAGTTTCT
         66 AACTGCTCTATCAAAAGAAATGTTCAACTCTGTGAGTTGAGTACACACATCACAAAGAAGTTTCT

                **            *     *    *         * 
     106216 GAGAGGGCTTCTGTCTAGATTTTAGATGATGATATTCCCGT
        131 GAGAATGCTTCTGTCTAGTTTTTATATGAAGATATTCCCTT

                           * *   *                *   *                    * 
     106257 TTCCAACGAAATCATTAGAGCTATCCAAATATCCACTTACAGTTTCTACAAAAAGAGTGTTTCCA
          1 TTCCAACGAAATCATCAAAGCGATCCAAATATCCACTTCCAGATTCTACAAAAAGAGTGTTTCAA

                   **         * *    *       *  **                  ***      
     106322 AACTGCTGCATCAAAAGAGAGGTTCCACTCTGTTAGCCGAGTACACACATCACAAACTTGTTTCT
         66 AACTGCTCTATCAAAAGAAATGTTCAACTCTGTGAGTTGAGTACACACATCACAAAGAAGTTTCT

            *     *         *        **         **   
     106387 CAGAATCCTTCTGTCTCGTTTTTATGGGAAGATATTTACTT
        131 GAGAATGCTTCTGTCTAGTTTTTATATGAAGATATTCCCTT

              *  *  * **          *       *     *       *  *  *              
     106428 TTTCACCGTAGGCATCAAAGCGCTCCAAATGTCCACATCCAGATACTCCAGAAAGAGTGTTTCAA
          1 TTCCAACGAAATCATCAAAGCGATCCAAATATCCACTTCCAGATTCTACAAAAAGAGTGTTTCAA

             *         *   * *   *         *        * *  *      *      *     
     106493 ACCTGCTCTATGAAAGGGAATCTTCAACTCTATGAGTTGAATGCAGACATCAGAAAGAAATTTCT
         66 AACTGCTCTATCAAAAGAAATGTTCAACTCTGTGAGTTGAGTACACACATCACAAAGAAGTTTCT

                     *       **          *         **
     106558 GAGAATGCTGCTGTCTACCTTTTAT-T--TGA-ATTCCCGC
        131 GAGAATGCTTCTGTCTAGTTTTTATATGAAGATATTCCCTT

                         *  *    *                *       *                  
     106595 TTCCAACGAAATCCTCCAAGCTATCCAAATATCCACTTGCAGATTCCACAAAAAGAGTGTTTCAA
          1 TTCCAACGAAATCATCAAAGCGATCCAAATATCCACTTCCAGATTCTACAAAAAGAGTGTTTCAA

                            ** *  *            *       *     *     *  *      
     106660 AACTGCTCTCTATCAATGGCAAAGTTCAACTCTGTTAGTTGAGGACACATATCACCAACAAGTTT
         66 AACTG--CTCTATCAAAAGAAATGTTCAACTCTGTGAGTTGAGTACACACATCACAAAGAAGTTT

                               *       **         *    
     106725 CTGAGAATGCTTCTGTCTATTTTTTATGGGAAGATATTTCCTT
        129 CTGAGAATGCTTCTGTCTAGTTTTTATATGAAGATATTCCCTT

              *  *  * ** *    *      *    *          * **                    
     106768 TTTCACCGTAGGCGTCAAGGCGATCGAAATGTCCACTTCCACAAACTACAAAAAGAGTGTTTCAA
          1 TTCCAACGAAATCATCAAAGCGATCCAAATATCCACTTCCAGATTCTACAAAAAGAGTGTTTCAA

             *         *   * **       *    *        * ** * *   **      *     
     106833 ACCTGCTCTATGAAAGGCCATGTTCATCTCTATGAGTTGAATGGAAATATCCGAAAGAAATTTCT
         66 AACTGCTCTATCAAAAGAAATGTTCAACTCTGTGAGTTGAGTACACACATCACAAAGAAGTTTCT

             *       *                             **
     106898 GGGAATGCTGCTGTCTAGTTTTTATAT---GA-ATTCCCGC
        131 GAGAATGCTTCTGTCTAGTTTTTATATGAAGATATTCCCTT

                         *       *                *    *  *                  
     106935 TTCCAACGAAATCCTCAAAGCAATCCAAATATCCACTTGCAGAATCCACAAAAAGAGTGTTTCAA
          1 TTCCAACGAAATCATCAAAGCGATCCAAATATCCACTTCCAGATTCTACAAAAAGAGTGTTTCAA

                          *     *          * *                      *        
     107000 AACTGCTCTATCAATAGAAAGGTTCAACTCTTTTAGTTGAGTACACACATCACAAACAAGTTTCT
         66 AACTGCTCTATCAAAAGAAATGTTCAACTCTGTGAGTTGAGTACACACATCACAAAGAAGTTTCT

                            * *      **     **  *    
     107065 GAGAATGCTTCTGTCTGGCTTTTATTGGAAGACGTTTCCTT
        131 GAGAATGCTTCTGTCTAGTTTTTATATGAAGATATTCCCTT

                 * *  **          *      **                * *               
     107106 TT-CACCAAAGGCATCAAAGCGCTCCAAAGGTCCACTTCCAGATTCTTCCAAAAGAGTGTTTCAA
          1 TTCCAACGAAATCATCAAAGCGATCCAAATATCCACTTCCAGATTCTACAAAAAGAGTGTTTCAA

             **          *  * *                 *    * *  * *     *   *      
     107170 ACGTGCTC-ATACTAAGGGAATGTTCAACTCTGTGACTTGAATGCAGATATCACCAAGTAGTTTC
         66 AACTGCTCTAT-CAAAAGAAATGTTCAACTCTGTGAGTTGAGTACACACATCACAAAGAAGTTTC

             * * *            **     *    *         * 
     107234 TAATAGTGCTTCTGTCTATATTTTAGATGATGATATTCCCGT
        130 TGAGAATGCTTCTGTCTAGTTTTTATATGAAGATATTCCCTT

                         *** *   *             *  *   *      *     *       * 
     107276 TTCCAACGAAATCGCTAGAGCTATCCAAATATCCAGTTACAGTTTCTACCAAAAGGGTGTTTCCA
          1 TTCCAACGAAATCATCAAAGCGATCCAAATATCCACTTCCAGATTCTACAAAAAGAGTGTTTCAA

              *    **           *            *       *                     * 
     107341 AATTGCTGCATCAAAAGAAAGGTTCAACTCTGTTAGTTGAGGACACACATCACAAAGAAGTTTGT
         66 AACTGCTCTATCAAAAGAAATGTTCAACTCTGTGAGTTGAGTACACACATCACAAAGAAGTTTCT

                              *    *     *           
     107406 GAGAATGCTTCTGTCTAGATTTTGTATGACGATATTCCCTT
        131 GAGAATGCTTCTGTCTAGTTTTTATATGAAGATATTCCCTT

                     *   * *     *   *       * *  *    *  *       *  *       
     107447 TTCCAACGATATCGTTAAAGCAATCTAAATATCAATTTGCAGAATCCACAAAAATAGAGTTTCAA
          1 TTCCAACGAAATCATCAAAGCGATCCAAATATCCACTTCCAGATTCTACAAAAAGAGTGTTTCAA

             *       * *        *    *       *  *                   ***      
     107512 AGCTGCTCTGTAAAAAGAAAGGTTCCACTCTGTTAGCTGAGTACACACATCACAAACTTGTTTCT
         66 AACTGCTCTATCAAAAGAAATGTTCAACTCTGTGAGTTGAGTACACACATCACAAAGAAGTTTCT

                  *         *        **         **   
     107577 GAGAATCCTTCTGTCTCGTTTTTATGGGAAGATATTTACTT
        131 GAGAATGCTTCTGTCTAGTTTTTATATGAAGATATTCCCTT

                 *  * **          *       *     *       *  *  *  *           
     107618 TTCCACCGTAGGCATCAAAGCGCTCCAAATGTCCACATCCAGATACTCCAGAACGAGTGTTTCAA
          1 TTCCAACGAAATCATCAAAGCGATCCAAATATCCACTTCCAGATTCTACAAAAAGAGTGTTTCAA

             *         *   * *   *         *        * *  *      *      *     
     107683 ACCTGCTCTATGAAAGGGAATCTTCAACTCTATGAGTTGAATGCAGACATCAGAAAGAAATTTCT
         66 AACTGCTCTATCAAAAGAAATGTTCAACTCTGTGAGTTGAGTACACACATCACAAAGAAGTTTCT

                     *       **          *         **
     107748 GAGAATGCTGCTGTCTACCTTTTAT-T--TGA-ATTCCCGC
        131 GAGAATGCTTCTGTCTAGTTTTTATATGAAGATATTCCCTT

                         *  *    *              * *  **   *    *             
     107785 TTCCAACGAAATCCTCCAAGCTATCCAAATATCCACCTGCATTTTCCACAACAAGAGTGTTTCAA
          1 TTCCAACGAAATCATCAAAGCGATCCAAATATCCACTTCCAGATTCTACAAAAAGAGTGTTTCAA

                          *                 *    *  *                *       
     107850 AACTGCTCTATCAATAGAAATGTTCAACTCCTTTG-GCTGGGTACACACATCACAAACAAGTTTC
         66 AACTGCTCTATCAAAAGAAATGTTCAACT-CTGTGAGTTGAGTACACACATCACAAAGAAGTTTC

                                      **     **       
     107914 TGAGAATGCTTCTGTCTAGTTTTTATGGGAAGACGTTCCCTT
        130 TGAGAATGCTTCTGTCTAGTTTTTATATGAAGATATTCCCTT

              *  * *  **          *       *            **                    
     107956 TTTCACCAAAGGCATCAAAGCGCTCCAAATGTCCACTTCCAGACACTACAAAAAGAGTGTTTCAA
          1 TTCCAACGAAATCATCAAAGCGATCCAAATATCCACTTCCAGATTCTACAAAAAGAGTGTTTCAA

             **       **   ***                 *    * *  * *         *       
     108021 ACGTGCTCTAAGAAAGCGAATGTTCAACTCTGTGACTTGAATGCAGATATCACAAAGTAGTTTCT
         66 AACTGCTCTATCAAAAGAAATGTTCAACTCTGTGAGTTGAGTACACACATCACAAAGAAGTTTCT

                **            *     *    *         * 
     108086 GAGAGGGCTTCTGTCTAGATTTTAGATGATGATATTCCCGT
        131 GAGAATGCTTCTGTCTAGTTTTTATATGAAGATATTCCCTT

                           * *   *                *   *                    * 
     108127 TTCCAACGAAATCATTAGAGCTATCCAAATATCCACTTACAGTTTCTACAAAAAGAGTGTTTCCA
          1 TTCCAACGAAATCATCAAAGCGATCCAAATATCCACTTCCAGATTCTACAAAAAGAGTGTTTCAA

                   **         * *    *       *  *                   ***      
     108192 AACTGCTGCATCAAAAGAGAGGTTCCACTCTGTTAGCTGAGTACACACATCACAAACTTGTTTCT
         66 AACTGCTCTATCAAAAGAAATGTTCAACTCTGTGAGTTGAGTACACACATCACAAAGAAGTTTCT

            *     *         *        **         **   
     108257 CAGAATCCTTCTGTCTCGTTTTTATGGGAAGATATTTACTT
        131 GAGAATGCTTCTGTCTAGTTTTTATATGAAGATATTCCCTT

              *  *  * **          *       *     *       *  *  *              
     108298 TTTCACCGTAGGCATCAAAGCGCTCCAAATGTCCACATCCAGATACTCCAGAAAGAGTGTTTCAA
          1 TTCCAACGAAATCATCAAAGCGATCCAAATATCCACTTCCAGATTCTACAAAAAGAGTGTTTCAA

             *         *   * *   *         *        * *  *      *      *     
     108363 ACCTGCTCTATGAAAGGGAATCTTCAACTCTATGAGTTGAATGCAGACATCAGAAAGAAATTTCT
         66 AACTGCTCTATCAAAAGAAATGTTCAACTCTGTGAGTTGAGTACACACATCACAAAGAAGTTTCT

                     *       **          *         **
     108428 GAGAATGCTGCTGTCTACCTTTTAT-T--TGA-ATTCCCGC
        131 GAGAATGCTTCTGTCTAGTTTTTATATGAAGATATTCCCTT

                         *  *    *                *       *                  
     108465 TTCCAACGAAATCCTCCAAGCTATCCAAATATCCACTTGCAGATTCCACAAAAAGAGTGTTTCAA
          1 TTCCAACGAAATCATCAAAGCGATCCAAATATCCACTTCCAGATTCTACAAAAAGAGTGTTTCAA

                            ** *  *            *       *     *     *  *      
     108530 AACTGCTCTCTATCAATGGCAAAGTTCAACTCTGTTAGTTGAGGACACATATCACCAACAAGTTT
         66 AACTG--CTCTATCAAAAGAAATGTTCAACTCTGTGAGTTGAGTACACACATCACAAAGAAGTTT

                               *       **         *    
     108595 CTGAGAATGCTTCTGTCTATTTTTTATGGGAAGATATTTCCTT
        129 CTGAGAATGCTTCTGTCTAGTTTTTATATGAAGATATTCCCTT

              *  *  * ** *    *      *    *          * **                    
     108638 TTTCACCGTAGGCGTCAAGGCGATCGAAATGTCCACTTCCACAAACTACAAAAAGAGTGTTTCAA
          1 TTCCAACGAAATCATCAAAGCGATCCAAATATCCACTTCCAGATTCTACAAAAAGAGTGTTTCAA

             *         *   * **       *    *        * ** * *   **      *     
     108703 ACCTGCTCTATGAAAGGCCATGTTCATCTCTATGAGTTGAATGGAAATATCCGAAAGAAATTTCT
         66 AACTGCTCTATCAAAAGAAATGTTCAACTCTGTGAGTTGAGTACACACATCACAAAGAAGTTTCT

             *       *          *        *         **
     108768 GGGAATGCTGCTGTCTAG-TGTT-TAT-ACGA-ATTCCCGC
        131 GAGAATGCTTCTGTCTAGTTTTTATATGAAGATATTCCCTT

                         *       *                *    *  *                  
     108805 TTCCAACGAAATCCTCAAAGCAATCCAAATATCCACTTGCAGAATCCACAAAAAGAGTGTTTCAA
          1 TTCCAACGAAATCATCAAAGCGATCCAAATATCCACTTCCAGATTCTACAAAAAGAGTGTTTCAA

               *          *     *          * *                      *        
     108870 AACCGCTCTATCAATAGAAAGGTTCAACTCTTTTAGTTGAGTACACACATCACAAACAAGTTTCT
         66 AACTGCTCTATCAAAAGAAATGTTCAACTCTGTGAGTTGAGTACACACATCACAAAGAAGTTTCT

                            * *      **     **  *    
     108935 GAGAATGCTTCTGTCTGGCTTTTATTGGAAGACGTTTCCTT
        131 GAGAATGCTTCTGTCTAGTTTTTATATGAAGATATTCCCTT

                 * *  **          *       *                * *               
     108976 TT-CACCAAAGGCATCAAAGCGCTCCAAATGTCCACTTCCAGATTCTTCCAAAAGAGTGTTTCAA
          1 TTCCAACGAAATCATCAAAGCGATCCAAATATCCACTTCCAGATTCTACAAAAAGAGTGTTTCAA

             **  *  * ***  * *                 *    * *  * *     *   *       
     109040 ACGTGGTCGAAGTAAGGGAATGTTCAACTCTGTGACTTGAATGCAGATATCACCAAGTAGTTTCT
         66 AACTGCTCTATCAAAAGAAATGTTCAACTCTGTGAGTTGAGTACACACATCACAAAGAAGTTTCT

            * * *             *     *    *         * 
     109105 AATAGTGCTTCTGTCTAGATTTTAGATGATGATATTCCCGT
        131 GAGAATGCTTCTGTCTAGTTTTTATATGAAGATATTCCCTT

                         * * *   *             *  *   *      *     *       * 
     109146 TTCCAACGAAATCGTTAGAGCTATCCAAATATCCAGTTACAGTTTCTACCAAAAGGGTGTTTCCA
          1 TTCCAACGAAATCATCAAAGCGATCCAAATATCCACTTCCAGATTCTACAAAAAGAGTGTTTCAA

              *    **           *            *       *                     * 
     109211 AATTGCTGCATCAAAAGAAAGGTTCAACTCTGTTAGTTGAGGACACACATCACAAAGAAGTTTGT
         66 AACTGCTCTATCAAAAGAAATGTTCAACTCTGTGAGTTGAGTACACACATCACAAAGAAGTTTCT

                              *    *     *           
     109276 GAGAATGCTTCTGTCTAGATTTTGTATGACGATATTCCCTT
        131 GAGAATGCTTCTGTCTAGTTTTTATATGAAGATATTCCCTT

                     *   * *     *   *       * *  *    *  *       *  *       
     109317 TTCCAACGATATCGTTAAAGCAATCTAAATATCAATTTGCAGAATCCACAAAAATAGAGTTTCAA
          1 TTCCAACGAAATCATCAAAGCGATCCAAATATCCACTTCCAGATTCTACAAAAAGAGTGTTTCAA

             *       * *        *    *       *  *                   ***      
     109382 AGCTGCTCTGTAAAAAGAAAGGTTCCACTCTGTTAGCTGAGTACACACATCACAAACTTGTTTCT
         66 AACTGCTCTATCAAAAGAAATGTTCAACTCTGTGAGTTGAGTACACACATCACAAAGAAGTTTCT

                 **         *        **         **   
     109447 GAGAAACCTTCTGTCTCGTTTTTATGGGAAGATATTTACTT
        131 GAGAATGCTTCTGTCTAGTTTTTATATGAAGATATTCCCTT

                 ** * **          *       *     *       *  *  *  *           
     109488 TTCCACTGTAGGCATCAAAGCGCTCCAAATGTCCACATCCAGATACTCCAGAACGAGTGTTTCAA
          1 TTCCAACGAAATCATCAAAGCGATCCAAATATCCACTTCCAGATTCTACAAAAAGAGTGTTTCAA

             *         *   * *   *         *        * *  *      *      *     
     109553 ACCTGCTCTATGAAAGGGAATCTTCAACTCTATGAGTTGAATGCAGA-ATCAGAAAGAAATTTCT
         66 AACTGCTCTATCAAAAGAAATGTTCAACTCTGTGAGTTGAGTACACACATCACAAAGAAGTTTCT

                     *       **          *         **
     109617 GAGAATGCTGCTGTCTACCTTTTAT-T--TGA-ATTCCCGC
        131 GAGAATGCTTCTGTCTAGTTTTTATATGAAGATATTCCCTT

                         *  *    *              * *  **   *    *             
     109654 TTCCAACGAAATCCTCCAAGCTATCCAAATATCCACCTGCATTTTCCACAACAAGAGTGTTTCAA
          1 TTCCAACGAAATCATCAAAGCGATCCAAATATCCACTTCCAGATTCTACAAAAAGAGTGTTTCAA

                          *                 *    *  *                *       
     109719 AACTGCTCTATCAATAGAAATGTTCAACTCCTTTG-GCTGGGTACACACATCACAAACAAGTTTC
         66 AACTGCTCTATCAAAAGAAATGTTCAACT-CTGTGAGTTGAGTACACACATCACAAAGAAGTTTC

                                      **     **       
     109783 TGAGAATGCTTCTGTCTAGTTTTTATGGGAAGACGTTCCCTT
        130 TGAGAATGCTTCTGTCTAGTTTTTATATGAAGATATTCCCTT

              *  * *  **          *       *            **                    
     109825 TTTCACCAAAGGCATCAAAGCGCTCCAAATGTCCACTTCCAGACACTACAAAAAGAGTGTTTCAA
          1 TTCCAACGAAATCATCAAAGCGATCCAAATATCCACTTCCAGATTCTACAAAAAGAGTGTTTCAA

             **       **    **                 *    * *  * *         *       
     109890 ACGTGCTCTAAGAAAACGAATGTTCAACTCTGTGACTTGAATGCAGATATCACAAAGTAGTTTCT
         66 AACTGCTCTATCAAAAGAAATGTTCAACTCTGTGAGTTGAGTACACACATCACAAAGAAGTTTCT

                **            *     *    *         * 
     109955 GAGAGGGCTTCTGTCTAGATTTTAGATGATGATATTCCCGT
        131 GAGAATGCTTCTGTCTAGTTTTTATATGAAGATATTCCCTT

                           * *   *                *   *                    * 
     109996 TTCCAACGAAATCATTAGAGCTATCCAAATATCCACTTACAGTTTCTACAAAAAGAGTGTTTCCA
          1 TTCCAACGAAATCATCAAAGCGATCCAAATATCCACTTCCAGATTCTACAAAAAGAGTGTTTCAA

                   **         * *    *       *  **                  ***      
     110061 AACTGCTGCATCAAAAGAGAGGTTCCACTCTGTTAGCCGAGTACACACATCACAAACTTGTTTCT
         66 AACTGCTCTATCAAAAGAAATGTTCAACTCTGTGAGTTGAGTACACACATCACAAAGAAGTTTCT

            *     *         *        **         **   
     110126 CAGAATCCTTCTGTCTCGTTTTTATGGGAAGATATTTACTT
        131 GAGAATGCTTCTGTCTAGTTTTTATATGAAGATATTCCCTT

              *  *  * **          *       *     *       *  *  *              
     110167 TTTCACCGTAGGCATCAAAGCGCTCCAAATGTCCACATCCAGATACTCCAGAAAGAGTGTTTCAA
          1 TTCCAACGAAATCATCAAAGCGATCCAAATATCCACTTCCAGATTCTACAAAAAGAGTGTTTCAA

             *         *   * *   *         *        * *  *      *      *     
     110232 ACCTGCTCTATGAAAGGGAATCTTCAACTCTATGAGTTGAATGCAGACATCAGAAAGAAATTTCT
         66 AACTGCTCTATCAAAAGAAATGTTCAACTCTGTGAGTTGAGTACACACATCACAAAGAAGTTTCT

                     *       **          *         **
     110297 GAGAATGCTGCTGTCTACCTTTTAT-T--TGA-ATTCCCGC
        131 GAGAATGCTTCTGTCTAGTTTTTATATGAAGATATTCCCTT

                         *  *    *                *       *                  
     110334 TTCCAACGAAATCCTCCAAGCTATCCAAATATCCACTTGCAGATTCCACAAAAAGAGTGTTTCAA
          1 TTCCAACGAAATCATCAAAGCGATCCAAATATCCACTTCCAGATTCTACAAAAAGAGTGTTTCAA

                            ** *  *            *       *     *     *  *      
     110399 AACTGCTCTCTATCAATGGCAAAGTTCAACTCTGTTAGTTGAGGACACATATCACCAACAAGTTT
         66 AACTG--CTCTATCAAAAGAAATGTTCAACTCTGTGAGTTGAGTACACACATCACAAAGAAGTTT

                               *       **         *    
     110464 CTGAGAATGCTTCTGTCTATTTTTTATGGGAAGATATTTCCTT
        129 CTGAGAATGCTTCTGTCTAGTTTTTATATGAAGATATTCCCTT

              *  *  * ** *    *      *    *          * **                    
     110507 TTTCACCGTAGGCGTCAAGGCGATCGAAATGTCCACTTCCACAAACTACAAAAAGAGTGTTTCAA
          1 TTCCAACGAAATCATCAAAGCGATCCAAATATCCACTTCCAGATTCTACAAAAAGAGTGTTTCAA

             *         *   * **       *    *        * ** * *   **      *     
     110572 ACCTGCTCTATGAAAGGCCATGTTCATCTCTATGAGTTGAATGGAAATATCCGAAAGAAATTTCT
         66 AACTGCTCTATCAAAAGAAATGTTCAACTCTGTGAGTTGAGTACACACATCACAAAGAAGTTTCT

             *       *                             **
     110637 GGGAATGCTGCTGTCTAGTTTTTATAT---GA-ATTCCCGC
        131 GAGAATGCTTCTGTCTAGTTTTTATATGAAGATATTCCCTT

                         *       *                *    *  *                  
     110674 TTCCAACGAAATCCTCAAAGCAATCCAAATATCCACTTGCAGAATCCACAAAAAGAGTGTTTCAA
          1 TTCCAACGAAATCATCAAAGCGATCCAAATATCCACTTCCAGATTCTACAAAAAGAGTGTTTCAA

                          *     *          * *                      *        
     110739 AACTGCTCTATCAATAGAAAGGTTCAACTCTTTTAGTTGAGTACACACATCACAAACAAGTTTCT
         66 AACTGCTCTATCAAAAGAAATGTTCAACTCTGTGAGTTGAGTACACACATCACAAAGAAGTTTCT

                            * *      **     **  *    
     110804 GAGAATGCTTCTGTCTGGCTTTTATTGGAAGACGTTTCCTT
        131 GAGAATGCTTCTGTCTAGTTTTTATATGAAGATATTCCCTT

                 * *  **          *      **                * *               
     110845 TT-CACCAAAGGCATCAAAGCGCTCCAAAGGTCCACTTCCAGATTCTTCCAAAAGAGTGTTTCAA
          1 TTCCAACGAAATCATCAAAGCGATCCAAATATCCACTTCCAGATTCTACAAAAAGAGTGTTTCAA

             **         **  * *                 *    * *  * *     *   *      
     110909 ACGTGCTC-ATAGTAAGGGAATGTTCAACTCTGTGACTTGAATGCAGATATCACCAAGTAGTTTC
         66 AACTGCTCTAT-CAAAAGAAATGTTCAACTCTGTGAGTTGAGTACACACATCACAAAGAAGTTTC

             * * *             *     *    *         * 
     110973 TAATAGTGCTTCTGTCTAGATTTTAGATGATGATATTCCCGT
        130 TGAGAATGCTTCTGTCTAGTTTTTATATGAAGATATTCCCTT

                         *** *   *             *  *   *      *     *       * 
     111015 TTCCAACGAAATCGCTAGAGCTATCCAAATATCCAGTTACAGTTTCTACCAAAAGGGTGTTTCCA
          1 TTCCAACGAAATCATCAAAGCGATCCAAATATCCACTTCCAGATTCTACAAAAAGAGTGTTTCAA

              *    **           *            *       *                     * 
     111080 AATTGCTGCATCAAAAGAAAGGTTCAACTCTGTTAGTTGAGGACACACATCACAAAGAAGTTTGT
         66 AACTGCTCTATCAAAAGAAATGTTCAACTCTGTGAGTTGAGTACACACATCACAAAGAAGTTTCT

                             **    *     *           
     111145 GAGAATGCTTCTGTCTACATTTTGTATGACGATATTCCCTT
        131 GAGAATGCTTCTGTCTAGTTTTTATATGAAGATATTCCCTT

                     *   * *     *   *       * *  *    *  *       *  *       
     111186 TTCCAACGATATCGTTAAAGCAATCTAAATATCAATTTGCAGAATCCACAAAAATAGAGTTTCAA
          1 TTCCAACGAAATCATCAAAGCGATCCAAATATCCACTTCCAGATTCTACAAAAAGAGTGTTTCAA

             *       * *        *    *       *  *                   ***      
     111251 AGCTGCTCTGTAAAAAGAAAGGTTCCACTCTGTTAGCTGAGTACACACATCACAAACTTGTTTCT
         66 AACTGCTCTATCAAAAGAAATGTTCAACTCTGTGAGTTGAGTACACACATCACAAAGAAGTTTCT

                  *         *        **         **   
     111316 GAGAATCCTTCTGTCTCGTTTTTATGGGAAGATATTTACTT
        131 GAGAATGCTTCTGTCTAGTTTTTATATGAAGATATTCCCTT

                 *  * **          *       *     *       *  *  *  *           
     111357 TTCCACCGTAGGCATCAAAGCGCTCCAAATGTCCACATCCAGATACTCCAGAACGAGTGTTTCAA
          1 TTCCAACGAAATCATCAAAGCGATCCAAATATCCACTTCCAGATTCTACAAAAAGAGTGTTTCAA

             *         *   * *   *         *        * *  *      *      *     
     111422 ACCTGCTCTATGAAAGGGAATCTTCAACTCTATGAGTTGAATGCAGACATCAGAAAGAAATTTCT
         66 AACTGCTCTATCAAAAGAAATGTTCAACTCTGTGAGTTGAGTACACACATCACAAAGAAGTTTCT

                     *       **          *         **
     111487 GAGAATGCTGCTGTCTACCTTTTAT-T--TGA-ATTCCCGC
        131 GAGAATGCTTCTGTCTAGTTTTTATATGAAGATATTCCCTT

                         *  *    *              * *  **   *    *             
     111524 TTCCAACGAAATCCTCCAAGCTATCCAAATATCCACCTGCATTTTCCACAACAAGAGTGTTTCAA
          1 TTCCAACGAAATCATCAAAGCGATCCAAATATCCACTTCCAGATTCTACAAAAAGAGTGTTTCAA

                          *                 *    *  *                *       
     111589 AACTGCTCTATCAATAGAAATGTTCAACTCCTTTG-GCTGGGTACACACATCACAAACAAGTTTC
         66 AACTGCTCTATCAAAAGAAATGTTCAACT-CTGTGAGTTGAGTACACACATCACAAAGAAGTTTC

                                      **     **       
     111653 TGAGAATGCTTCTGTCTAGTTTTTATGGGAAGACGTTCCCTT
        130 TGAGAATGCTTCTGTCTAGTTTTTATATGAAGATATTCCCTT

              *  * *  **          *       *            **                  * 
     111695 TTTCACCAAAGGCATCAAAGCGCTCCAAATGTCCACTTCCAGACACTACAAAAAGAGTGTTTCCA
          1 TTCCAACGAAATCATCAAAGCGATCCAAATATCCACTTCCAGATTCTACAAAAAGAGTGTTTCAA

             **   *   **   ***                 *    * *  * *         *       
     111760 ACGTGCCCTAAGAAAGCGAATGTTCAACTCTGTGACTTGAATGCAGATATCACAAAGTAGTTTCT
         66 AACTGCTCTATCAAAAGAAATGTTCAACTCTGTGAGTTGAGTACACACATCACAAAGAAGTTTCT

                **            *     *    *         * 
     111825 GAGAGGGCTTCTGTCTAGATTTTAGATGATGATATTCCCGT
        131 GAGAATGCTTCTGTCTAGTTTTTATATGAAGATATTCCCTT

                           * *   *                *   *                    * 
     111866 TTCCAACGAAATCATTAGAGCTATCCAAATATCCACTTACAGTTTCTACAAAAAGAGTGTTTCCA
          1 TTCCAACGAAATCATCAAAGCGATCCAAATATCCACTTCCAGATTCTACAAAAAGAGTGTTTCAA

                   **         * *    *       *  *                   ***      
     111931 AACTGCTGCATCAAAAGAGAGGTTCCACTCTGTTAGCTGAGTACACACATCACAAACTTGTTTCT
         66 AACTGCTCTATCAAAAGAAATGTTCAACTCTGTGAGTTGAGTACACACATCACAAAGAAGTTTCT

            *     *         *        **         **   
     111996 CAGAATCCTTCTGTCTCGTTTTTATGGGAAGATATTTACTT
        131 GAGAATGCTTCTGTCTAGTTTTTATATGAAGATATTCCCTT

              *  *  * **          *       *     *       *  *  *              
     112037 TTTCACCGTAGGCATCAAAGCGCTCCAAATGTCCACATCCAGATACTCCAGAAAGAGTGTTTCAA
          1 TTCCAACGAAATCATCAAAGCGATCCAAATATCCACTTCCAGATTCTACAAAAAGAGTGTTTCAA

             *         *   * *   *         *        * *  *      *      *     
     112102 ACCTGCTCTATGAAAGGGAATCTTCAACTCTATGAGTTGAATGCAGACATCAGAAAGAAATTTCT
         66 AACTGCTCTATCAAAAGAAATGTTCAACTCTGTGAGTTGAGTACACACATCACAAAGAAGTTTCT

                     *       **          *         **
     112167 GAGAATGCTGCTGTCTACCTTTTAT-T--TGA-ATTCCCGC
        131 GAGAATGCTTCTGTCTAGTTTTTATATGAAGATATTCCCTT

                         *  *    *                *       *                  
     112204 TTCCAACGAAATCCTCCAAGCTATCCAAATATCCACTTGCAGATTCCACAAAAAGAGTGTTTCAA
          1 TTCCAACGAAATCATCAAAGCGATCCAAATATCCACTTCCAGATTCTACAAAAAGAGTGTTTCAA

                            ** *  *            *       *     *     *  *      
     112269 AACTGCTCTCTATCAATGGCAAAGTTCAACTCTGTTAGTTGAGGACACATATCACCAACAAGTTT
         66 AACTG--CTCTATCAAAAGAAATGTTCAACTCTGTGAGTTGAGTACACACATCACAAAGAAGTTT

                               *       **         *    
     112334 CTGAGAATGCTTCTGTCTATTTTTTATGGGAAGATATTTCCTT
        129 CTGAGAATGCTTCTGTCTAGTTTTTATATGAAGATATTCCCTT

              *  *  * ** *    *      *    *          * **          *         
     112377 TTTCACCGTAGGCGTCAAGGCGATCGAAATGTCCACTTCCACAAACTACAAAAAGTGTGTTTCAA
          1 TTCCAACGAAATCATCAAAGCGATCCAAATATCCACTTCCAGATTCTACAAAAAGAGTGTTTCAA

             *         *   * **       *    *        * ** * *   **      *     
     112442 ACCTGCTCTATGAAAGGCCATGTTCATCTCTATGAGTTGAATGGAAATATCCGAAAGAAATTTCT
         66 AACTGCTCTATCAAAAGAAATGTTCAACTCTGTGAGTTGAGTACACACATCACAAAGAAGTTTCT

             *       *          *        *         **
     112507 GGGAATGCTGCTGTCTAG-TGTT-TAT-ACGA-ATTCCCGC
        131 GAGAATGCTTCTGTCTAGTTTTTATATGAAGATATTCCCTT

                         *       *                *    *  *                  
     112544 TTCCAACGAAATCCTCAAAGCAATCCAAATATCCACTTGCAGAATCCACAAAAAGAGTGTTTCAA
          1 TTCCAACGAAATCATCAAAGCGATCCAAATATCCACTTCCAGATTCTACAAAAAGAGTGTTTCAA

                          *     *          * *                   *  *        
     112609 AACTGCTCTATCAATAGAAAGGTTCAACTCTTTTAGTTGAGTACACACATCACGAACAAGTTTCT
         66 AACTGCTCTATCAAAAGAAATGTTCAACTCTGTGAGTTGAGTACACACATCACAAAGAAGTTTCT

                            * *      **     **  *    
     112674 GAGAATGCTTCTGTCTGGCTTTTATTGGAAGACGTTTCCTT
        131 GAGAATGCTTCTGTCTAGTTTTTATATGAAGATATTCCCTT

                 * *  **          *       *                * *               
     112715 TT-CACCAAAGGCATCAAAGCGCTCCAAATGTCCACTTCCAGATTCTTCCAAAAGAGTGTTTCAA
          1 TTCCAACGAAATCATCAAAGCGATCCAAATATCCACTTCCAGATTCTACAAAAAGAGTGTTTCAA

             **     * ***  * *                 *    * *  * *     *   *       
     112779 ACGTGCTCAAAGTAAGGGAATGTTCAACTCTGTGACTTGAATGCAGATATCACCAAGTAGTTTCT
         66 AACTGCTCTATCAAAAGAAATGTTCAACTCTGTGAGTTGAGTACACACATCACAAAGAAGTTTCT

            * * *             *     *    *         * 
     112844 AATAGTGCTTCTGTCTAGATTTTAGATGATGATATTCCCGT
        131 GAGAATGCTTCTGTCTAGTTTTTATATGAAGATATTCCCTT

                         * * *   * **          *  *   *      *     *       * 
     112885 TTCCAACGAAATCGTTAGAGCTAAGCAAATATCCAGTTACAGTTTCTACCAAAAGGGTGTTTCCA
          1 TTCCAACGAAATCATCAAAGCGATCCAAATATCCACTTCCAGATTCTACAAAAAGAGTGTTTCAA

              *    **           *            *       *                     * 
     112950 AATTGCTGCATCAAAAGAAAGGTTCAACTCTGTTAGTTGAGGACACACATCACAAAGAAGTTTGT
         66 AACTGCTCTATCAAAAGAAATGTTCAACTCTGTGAGTTGAGTACACACATCACAAAGAAGTTTCT

                              *    *     *           
     113015 GAGAATGCTTCTGTCTAGATTTTGTATGACGATATTCCCTT
        131 GAGAATGCTTCTGTCTAGTTTTTATATGAAGATATTCCCTT

                     *   * *     *   *       * *  *    *  *       *  *       
     113056 TTCCAACGATATCGTTAAAGCAATCTAAATATCAATTTGCAGAATCCACAAAAATAGAGTTTCAA
          1 TTCCAACGAAATCATCAAAGCGATCCAAATATCCACTTCCAGATTCTACAAAAAGAGTGTTTCAA

             *       * *        *    *       *  *                   *** *    
     113121 AGCTGCTCTGTAAAAAGAAAGGTTCCACTCTGTTAGCTGAGTACACACATCACAAACTTGATTCT
         66 AACTGCTCTATCAAAAGAAATGTTCAACTCTGTGAGTTGAGTACACACATCACAAAGAAGTTTCT

                  *         *        **         **   
     113186 GAGAATCCTTCTGTCTCGTTTTTATGGGAAGATATTTACTT
        131 GAGAATGCTTCTGTCTAGTTTTTATATGAAGATATTCCCTT

                 *  * **          *       *     *       *  *  *  *           
     113227 TTCCACCGTAGGCATCAAAGCGCTCCAAATGTCCACATCCAGATACTCCAGAACGAGTGTTTCAA
          1 TTCCAACGAAATCATCAAAGCGATCCAAATATCCACTTCCAGATTCTACAAAAAGAGTGTTTCAA

             *         *   * *   *         *        * *  *      *      *     
     113292 ACCTGCTCTATGAAAGGGAATCTTCAACTCTATGAGTTGAATGCAGACATCAGAAAGAAATTTCT
         66 AACTGCTCTATCAAAAGAAATGTTCAACTCTGTGAGTTGAGTACACACATCACAAAGAAGTTTCT

                     *       **          *         **
     113357 GAGAATGCTGCTGTCTACCTTTTAT-T--TGA-ATTCCCGC
        131 GAGAATGCTTCTGTCTAGTTTTTATATGAAGATATTCCCTT

                         *  *    *              * *  **   *    *             
     113394 TTCCAACGAAATCCTCCAAGCTATCCAAATATCCACCTGCATTTTCCACAACAAGAGTGTTTCAA
          1 TTCCAACGAAATCATCAAAGCGATCCAAATATCCACTTCCAGATTCTACAAAAAGAGTGTTTCAA

                          *                 *    *  *                *       
     113459 AACTGCTCTATCAATAGAAATGTTCAACTCCTTTG-GCTGGGTACACACATCACAAACAAGTTTC
         66 AACTGCTCTATCAAAAGAAATGTTCAACT-CTGTGAGTTGAGTACACACATCACAAAGAAGTTTC

                                      **     **       
     113523 TGAGAATGCTTCTGTCTAGTTTTTATGGGAAGACGTTCCCTT
        130 TGAGAATGCTTCTGTCTAGTTTTTATATGAAGATATTCCCTT

              *  * *  **          *       *            **                    
     113565 TTTCACCAAAGGCATCAAAGCGCTCCAAATGTCCACTTCCAGACACTACAAAAAGAGTGTTTCAA
          1 TTCCAACGAAATCATCAAAGCGATCCAAATATCCACTTCCAGATTCTACAAAAAGAGTGTTTCAA

             **       **   ***                 *    * *  * *         *       
     113630 ACGTGCTCTAAGAAAGCGAATGTTCAACTCTGTGACTTGAATGCAGATATCACAAAGTAGTTTCT
         66 AACTGCTCTATCAAAAGAAATGTTCAACTCTGTGAGTTGAGTACACACATCACAAAGAAGTTTCT

                **            *     *    *         * 
     113695 GAGAGGGCTTCTGTCTAGATTTTAGATGATGATATTCCCGT
        131 GAGAATGCTTCTGTCTAGTTTTTATATGAAGATATTCCCTT

                           * *   *                *   *                    * 
     113736 TTCCAACGAAATCATTAGAGCTATCCAAATATCCACTTACAGTTTCTACAAAAAGAGTGTTTCCA
          1 TTCCAACGAAATCATCAAAGCGATCCAAATATCCACTTCCAGATTCTACAAAAAGAGTGTTTCAA

                   **         * *    *       *  **                  ***      
     113801 AACTGCTGCATCAAAAGAGAGGTTCCACTCTGTTAGCCGAGTACACACATCACAAACTTGTTTCT
         66 AACTGCTCTATCAAAAGAAATGTTCAACTCTGTGAGTTGAGTACACACATCACAAAGAAGTTTCT

            *     *      *  *        **         **   
     113866 CAGAATCCTTCTGACTCGTTTTTATGGGAAGATATTTACTT
        131 GAGAATGCTTCTGTCTAGTTTTTATATGAAGATATTCCCTT

              *  *  * **          *       *     *       *  *  *              
     113907 TTTCACCGTAGGCATCAAAGCGCTCCAAATGTCCACATCCAGATACTCCAGAAAGAGTGTTTCAA
          1 TTCCAACGAAATCATCAAAGCGATCCAAATATCCACTTCCAGATTCTACAAAAAGAGTGTTTCAA

             *         *   * *   *         *        * *  *      *      *     
     113972 ACCTGCTCTATGAAAGGGAATCTTCAACTCTATGAGTTGAATGCAGACATCAGAAAGAAATTTCT
         66 AACTGCTCTATCAAAAGAAATGTTCAACTCTGTGAGTTGAGTACACACATCACAAAGAAGTTTCT

                     *       **          *         **
     114037 GAGAATGCTGCTGTCTACCTTTTAT-T--TGA-ATTCCCGC
        131 GAGAATGCTTCTGTCTAGTTTTTATATGAAGATATTCCCTT

                         *  *    *                *       *                  
     114074 TTCCAACGAAATCCTCCAAGCTATCCAAATATCCACTTGCAGATTCCACAAAAAGAGTGTTTCAA
          1 TTCCAACGAAATCATCAAAGCGATCCAAATATCCACTTCCAGATTCTACAAAAAGAGTGTTTCAA

                            ** *  *            *       *     *     *  *      
     114139 AACTGCTCTCTATCAATGGCAAAGTTCAACTCTGTTAGTTGAGGACACATATCACCAACAAGTTT
         66 AACTG--CTCTATCAAAAGAAATGTTCAACTCTGTGAGTTGAGTACACACATCACAAAGAAGTTT

                     *         *       **         *    
     114204 CTGAGAATGTTTCTGTCTATTTTTTATGGGAAGATATTTCCTT
        129 CTGAGAATGCTTCTGTCTAGTTTTTATATGAAGATATTCCCTT

              *  *  * ** *    *      *    *          * **                    
     114247 TTTCACCGTAGGCGTCAAGGCGATCGAAATGTCCACTTCCACAAACTACAAAAAGAGTGTTTCAA
          1 TTCCAACGAAATCATCAAAGCGATCCAAATATCCACTTCCAGATTCTACAAAAAGAGTGTTTCAA

             *         *   * **       *    *        * ** * *   **      *     
     114312 ACCTGCTCTATGAAAGGCCATGTTCATCTCTATGAGTTGAATGGAAATATCCGAAAGAAATTTCT
         66 AACTGCTCTATCAAAAGAAATGTTCAACTCTGTGAGTTGAGTACACACATCACAAAGAAGTTTCT

             *       *          *        *         **
     114377 GGGAATGCTGCTGTCTAG-TGTT-TAT-ACGA-ATTCCCGC
        131 GAGAATGCTTCTGTCTAGTTTTTATATGAAGATATTCCCTT

                         *       *                *    *  *                  
     114414 TTCCAACGAAATCCTCAAAGCAATCCAAATATCCACTTGCAGAATCCACAAAAAGAGTGTTTCAA
          1 TTCCAACGAAATCATCAAAGCGATCCAAATATCCACTTCCAGATTCTACAAAAAGAGTGTTTCAA

                          *     *          * *                      *        
     114479 AACTGCTCTATCAATAGAAAGGTTCAACTCTTTTAGTTGAGTACACACATCACAAACAAGTTTCT
         66 AACTGCTCTATCAAAAGAAATGTTCAACTCTGTGAGTTGAGTACACACATCACAAAGAAGTTTCT

                            * *      **     **  *    
     114544 GAGAATGCTTCTGTCTGGCTTTTATTGGAAGACGTTTCCTT
        131 GAGAATGCTTCTGTCTAGTTTTTATATGAAGATATTCCCTT

                   *  **          *       *                * *               
     114585 TT-CAACAAAGGCATCAAAGCGCTCCAAATGTCCACTTCCAGATTCTTCCAAAAGAGTGTTTCAA
          1 TTCCAACGAAATCATCAAAGCGATCCAAATATCCACTTCCAGATTCTACAAAAAGAGTGTTTCAA

             **  *  * ***  * *                 *    * *  * *     *   *       
     114649 ACGTGGTCGAAGTAAGGGAATGTTCAACTCTGTGACTTGAATGCAGATATCACCAAGTAGTTTCT
         66 AACTGCTCTATCAAAAGAAATGTTCAACTCTGTGAGTTGAGTACACACATCACAAAGAAGTTTCT

            * * *            **     *    *         * 
     114714 AATAGTGCTTCTGTCTACATTTTAGATGATGATATTCCCGT
        131 GAGAATGCTTCTGTCTAGTTTTTATATGAAGATATTCCCTT

                         * * *   *             *  *   *      *     *       * 
     114755 TTCCAACGAAATCGTTAGAGCTATCCAAATATCCAGTTACAGTTTCTACCAAAAGGGTGTTTCCA
          1 TTCCAACGAAATCATCAAAGCGATCCAAATATCCACTTCCAGATTCTACAAAAAGAGTGTTTCAA

              *    **           *            *       *                     * 
     114820 AATTGCTGCATCAAAAGAAAGGTTCAACTCTGTTAGTTGAGGACACACATCACAAAGAAGTTTGT
         66 AACTGCTCTATCAAAAGAAATGTTCAACTCTGTGAGTTGAGTACACACATCACAAAGAAGTTTCT

                              *    *     *           
     114885 GAGAATGCTTCTGTCTAGATTTTGTATGACGATATTCCCTT
        131 GAGAATGCTTCTGTCTAGTTTTTATATGAAGATATTCCCTT

                     *   * *     *   *       * *  *    *  *       *  *       
     114926 TTCCAACGATATCGTTAAAGCAATCTAAATATCAATTTGCAGAATCCACAAAAATAGAGTTTCAA
          1 TTCCAACGAAATCATCAAAGCGATCCAAATATCCACTTCCAGATTCTACAAAAAGAGTGTTTCAA

             *       * *        *    *       *  *                   ***      
     114991 AGCTGCTCTGTAAAAAGAAAGGTTCCACTCTGTTAGCTGAGTACACACATCACAAACTTGTTTCT
         66 AACTGCTCTATCAAAAGAAATGTTCAACTCTGTGAGTTGAGTACACACATCACAAAGAAGTTTCT

                 **         *        **         **   
     115056 GAGAAACCTTCTGTCTCGTTTTTATGGGAAGATATTTACTT
        131 GAGAATGCTTCTGTCTAGTTTTTATATGAAGATATTCCCTT

                 ** * **          *       *     *       *  *  *  *           
     115097 TTCCACTGTAGGCATCAAAGCGCTCCAAATGTCCACATCCAGATACTCCAGAACGAGTGTTTCAA
          1 TTCCAACGAAATCATCAAAGCGATCCAAATATCCACTTCCAGATTCTACAAAAAGAGTGTTTCAA

             *         *   * *   *         *        * *  *      *      *     
     115162 ACCTGCTCTATGAAAGGGAATCTTCAACTCTATGAGTTGAATGCAGA-ATCAGAAAGAAATTTCT
         66 AACTGCTCTATCAAAAGAAATGTTCAACTCTGTGAGTTGAGTACACACATCACAAAGAAGTTTCT

                     *       **          *         **
     115226 GAGAATGCTGCTGTCTACCTTTTAT-T--TGA-ATTCCCGC
        131 GAGAATGCTTCTGTCTAGTTTTTATATGAAGATATTCCCTT

                         *  *    *              * *  **   *                  
     115263 TTCCAACGAAATCCTCCAAGCTATCCAAATATCCACCTGCATTTTCCACAAAAAGAGTGTTTCAA
          1 TTCCAACGAAATCATCAAAGCGATCCAAATATCCACTTCCAGATTCTACAAAAAGAGTGTTTCAA

                          *                 *    *  *                *       
     115328 AACTGCTCTATCAATAGAAATGTTCAACTCCTTTG-GCTGGGTACACACATCACAAACAAGTTTC
         66 AACTGCTCTATCAAAAGAAATGTTCAACT-CTGTGAGTTGAGTACACACATCACAAAGAAGTTTC

                                      **     **       
     115392 TGAGAATGCTTCTGTCTAGTTTTTATGGGAAGACGTTCCCTT
        130 TGAGAATGCTTCTGTCTAGTTTTTATATGAAGATATTCCCTT

              *  * *  **          *       *            **                    
     115434 TTTCACCAAAGGCATCAAAGCGCTCCAAATGTCCACTTCCAGACACTACAAAAAGAGTGTTTCAA
          1 TTCCAACGAAATCATCAAAGCGATCCAAATATCCACTTCCAGATTCTACAAAAAGAGTGTTTCAA

             **       **   ***                 *    * *  * *         *       
     115499 ACGTGCTCTAAGAAAGCGAATGTTCAACTCTGTGACTTGAATGCAGATATCACAAAGTAGTTTCT
         66 AACTGCTCTATCAAAAGAAATGTTCAACTCTGTGAGTTGAGTACACACATCACAAAGAAGTTTCT

                **            *     *    *         * 
     115564 GAGAGGGCTTCTGTCTAGATTTTAGATGATGATATTCCCGT
        131 GAGAATGCTTCTGTCTAGTTTTTATATGAAGATATTCCCTT

                           * *   *                *   *                    * 
     115605 TTCCAACGAAATCATTAGAGCTATCCAAATATCCACTTACAGTTTCTACAAAAAGAGTGTTTCCA
          1 TTCCAACGAAATCATCAAAGCGATCCAAATATCCACTTCCAGATTCTACAAAAAGAGTGTTTCAA

                   **         * *    *       *  **                  ***      
     115670 AACTGCTGCATCAAAAGAGAGGTTCCACTCTGTTAGCCGAGTACACACATCACAAACTTGTTTCT
         66 AACTGCTCTATCAAAAGAAATGTTCAACTCTGTGAGTTGAGTACACACATCACAAAGAAGTTTCT

            *     *         *        **         **   
     115735 CAGAATCCTTCTGTCTCGTTTTTATGGGAAGATATTTACTT
        131 GAGAATGCTTCTGTCTAGTTTTTATATGAAGATATTCCCTT

              *  *  * **          *       *     *       *  *  *              
     115776 TTTCACCGTAGGCATCAAAGCGCTCCAAATGTCCACATCCAGATACTCCAGAAAGAGTGTTTCAA
          1 TTCCAACGAAATCATCAAAGCGATCCAAATATCCACTTCCAGATTCTACAAAAAGAGTGTTTCAA

             *         *   * *   *         *        * *  *      *      *     
     115841 ACCTGCTCTATGAAAGGGAATCTTCAACTCTATGAGTTGAATGCAGACATCAGAAAGAAATTTCT
         66 AACTGCTCTATCAAAAGAAATGTTCAACTCTGTGAGTTGAGTACACACATCACAAAGAAGTTTCT

                     *       **          *         **
     115906 GAGAATGCTGCTGTCTACCTTTTAT-T--TGA-ATTCCCGC
        131 GAGAATGCTTCTGTCTAGTTTTTATATGAAGATATTCCCTT

                         *  *    *                *       *                  
     115943 TTCCAACGAAATCCTCCAAGCTATCCAAATATCCACTTGCAGATTCCACAAAAAGAGTGTTTCAA
          1 TTCCAACGAAATCATCAAAGCGATCCAAATATCCACTTCCAGATTCTACAAAAAGAGTGTTTCAA

                            ** *  *            *       *     *     *  *      
     116008 AACTGCTCTCTATCAATGGCAAAGTTCAACTCTGTTAGTTGAGGACACATATCACCAACAAGTTT
         66 AACTG--CTCTATCAAAAGAAATGTTCAACTCTGTGAGTTGAGTACACACATCACAAAGAAGTTT

                               *       **         *    
     116073 CTGAGAATGCTTCTGTCTATTTTTTATGGGAAGATATTTCCTT
        129 CTGAGAATGCTTCTGTCTAGTTTTTATATGAAGATATTCCCTT

              *  *  * ** *    *      *    *          * **                    
     116116 TTTCACCGTAGGCGTCAAGGCGATCGAAATGTCCACTTCCACAAACTACAAAAAGAGTGTTTCAA
          1 TTCCAACGAAATCATCAAAGCGATCCAAATATCCACTTCCAGATTCTACAAAAAGAGTGTTTCAA

             *         *   * **       *    *        * ** * *   **      *     
     116181 ACCTGCTCTATGAAAGGCCATGTTCATCTCTATGAGTTGAATGGAAATATCCGAAAGAAATTTCT
         66 AACTGCTCTATCAAAAGAAATGTTCAACTCTGTGAGTTGAGTACACACATCACAAAGAAGTTTCT

             *       *          *        *         **
     116246 GGGAATGCTGCTGTCTAG-TGTT-TAT-ACGA-ATTCCCGC
        131 GAGAATGCTTCTGTCTAGTTTTTATATGAAGATATTCCCTT

                         *       *                *    *  *                  
     116283 TTCCAACGAAATCCTCAAAGCAATCCAAATATCCACTTGCAGAATCCACAAAAAGAGTGTTTCAA
          1 TTCCAACGAAATCATCAAAGCGATCCAAATATCCACTTCCAGATTCTACAAAAAGAGTGTTTCAA

               *          *     *          * *                      *        
     116348 AACCGCTCTATCAATAGAAAGGTTCAACTCTTTTAGTTGAGTACACACATCACAAACAAGTTTCT
         66 AACTGCTCTATCAAAAGAAATGTTCAACTCTGTGAGTTGAGTACACACATCACAAAGAAGTTTCT

                            * *      **     **  *    
     116413 GAGAATGCTTCTGTCTGGCTTTTATTGGAAGACGTTTCCTT
        131 GAGAATGCTTCTGTCTAGTTTTTATATGAAGATATTCCCTT

                 * *  **          *       *                * *               
     116454 TT-CACCAAAGGCATCAAAGCGCTCCAAATGTCCACTTCCAGATTCTTCCAAAAGAGTGTTTCAA
          1 TTCCAACGAAATCATCAAAGCGATCCAAATATCCACTTCCAGATTCTACAAAAAGAGTGTTTCAA

             **  *  * ***  * *                 *    * *  * *     *   *       
     116518 ACGTGGTCGAAGTAAGGGAATGTTCAACTCTGTGACTTGAATGCAGATATCACCAAGTAGTTTCT
         66 AACTGCTCTATCAAAAGAAATGTTCAACTCTGTGAGTTGAGTACACACATCACAAAGAAGTTTCT

            * * *             *     *    *         * 
     116583 AATAGTGCTTCTGTCTAGATTTTAGATGATGATATTCCCGT
        131 GAGAATGCTTCTGTCTAGTTTTTATATGAAGATATTCCCTT

                         * * *   *             *  *   *      *     *       * 
     116624 TTCCAACGAAATCGTTAGAGCTATCCAAATATCCAGTTACAGTTTCTACCAAAAGGGTGTTTCCA
          1 TTCCAACGAAATCATCAAAGCGATCCAAATATCCACTTCCAGATTCTACAAAAAGAGTGTTTCAA

              *    **           *            *       *                     * 
     116689 AATTGCTGCATCAAAAGAAAGGTTCAACTCTGTTAGTTGAGGACACACATCACAAAGAAGTTTGT
         66 AACTGCTCTATCAAAAGAAATGTTCAACTCTGTGAGTTGAGTACACACATCACAAAGAAGTTTCT

                              *    *     *           
     116754 GAGAATGCTTCTGTCTAGATTTTGTATGACGATATTCCCTT
        131 GAGAATGCTTCTGTCTAGTTTTTATATGAAGATATTCCCTT

                     *   * *     *   *       * *  *    *  *       *  *       
     116795 TTCCAACGATATCGTTAAAGCAATCTAAATATCAATTTGCAGAATCCACAAAAATAGAGTTTCAA
          1 TTCCAACGAAATCATCAAAGCGATCCAAATATCCACTTCCAGATTCTACAAAAAGAGTGTTTCAA

             *       * *        *    *       *  *                   ***      
     116860 AGCTGCTCTGTAAAAAGAAAGGTTCCACTCTGTTAGCTGAGTACACACATCACAAACTTGTTTCT
         66 AACTGCTCTATCAAAAGAAATGTTCAACTCTGTGAGTTGAGTACACACATCACAAAGAAGTTTCT

                 **         *        **         **   
     116925 GAGAAACCTTCTGTCTCGTTTTTATGGGAAGATATTTACTT
        131 GAGAATGCTTCTGTCTAGTTTTTATATGAAGATATTCCCTT

                 ** * **          *       *     *       *  *  *  *           
     116966 TTCCACTGTAGGCATCAAAGCGCTCCAAATGTCCACATCCAGATACTCCAGAACGAGTGTTTCAA
          1 TTCCAACGAAATCATCAAAGCGATCCAAATATCCACTTCCAGATTCTACAAAAAGAGTGTTTCAA

             *         *   * *   *         *        * *  *      *      *     
     117031 ACCTGCTCTATGAAAGGGAATCTTCAACTCTATGAGTTGAATGCAGA-ATCAGAAAGAAATTTCT
         66 AACTGCTCTATCAAAAGAAATGTTCAACTCTGTGAGTTGAGTACACACATCACAAAGAAGTTTCT

                     *       **          *         **
     117095 GAGAATGCTGCTGTCTACCTTTTAT-T--TGA-ATTCCCGC
        131 GAGAATGCTTCTGTCTAGTTTTTATATGAAGATATTCCCTT

                         *  *    *              * *  **   *    *             
     117132 TTCCAACGAAATCCTCCAAGCTATCCAAATATCCACCTGCATTTTCCACAACAAGAGTGTTTCAA
          1 TTCCAACGAAATCATCAAAGCGATCCAAATATCCACTTCCAGATTCTACAAAAAGAGTGTTTCAA

                          *                 *    *  *                *       
     117197 AACTGCTCTATCAATAGAAATGTTCAACTCCTTTG-GCTGGGTACACACATCACAAACAAGTTTC
         66 AACTGCTCTATCAAAAGAAATGTTCAACT-CTGTGAGTTGAGTACACACATCACAAAGAAGTTTC

                                      **     **       
     117261 TGAGAATGCTTCTGTCTAGTTTTTATGGGAAGACGTTCCCTT
        130 TGAGAATGCTTCTGTCTAGTTTTTATATGAAGATATTCCCTT

              *  * *  **          *       *            **                    
     117303 TTTCACCAAAGGCATCAAAGCGCTCCAAATGTCCACTTCCAGACACTACAAAAAGAGTGTTTCAA
          1 TTCCAACGAAATCATCAAAGCGATCCAAATATCCACTTCCAGATTCTACAAAAAGAGTGTTTCAA

             **       **    **                 *    * *  * *         *       
     117368 ACGTGCTCTAAGAAAACGAATGTTCAACTCTGTGACTTGAATGCAGATATCACAAAGTAGTTTCT
         66 AACTGCTCTATCAAAAGAAATGTTCAACTCTGTGAGTTGAGTACACACATCACAAAGAAGTTTCT

                **            *     *    *         * 
     117433 GAGAGGGCTTCTGTCTAGATTTTAGATGATGATATTCCCGT
        131 GAGAATGCTTCTGTCTAGTTTTTATATGAAGATATTCCCTT

                           * *   *                *   *                    * 
     117474 TTCCAACGAAATCATTAGAGCTATCCAAATATCCACTTACAGTTTCTACAAAAAGAGTGTTTCCA
          1 TTCCAACGAAATCATCAAAGCGATCCAAATATCCACTTCCAGATTCTACAAAAAGAGTGTTTCAA

                   **         * *    *       *  **                  ***      
     117539 AACTGCTGCATCAAAAGAGAGGTTCCACTCTGTTAGCCGAGTACACACATCACAAACTTGTTTCT
         66 AACTGCTCTATCAAAAGAAATGTTCAACTCTGTGAGTTGAGTACACACATCACAAAGAAGTTTCT

            *     *         *        **         **   
     117604 CAGAATCCTTCTGTCTCGTTTTTATGGGAAGATATTTACTT
        131 GAGAATGCTTCTGTCTAGTTTTTATATGAAGATATTCCCTT

              *  *  * **          *       *     *       *  *  *              
     117645 TTTCACCGTAGGCATCAAAGCGCTCCAAATGTCCACATCCAGATACTCCAGAAAGAGTGTTTCAA
          1 TTCCAACGAAATCATCAAAGCGATCCAAATATCCACTTCCAGATTCTACAAAAAGAGTGTTTCAA

             *         *   * *   *         *        * *  *      *      *     
     117710 ACCTGCTCTATGAAAGGGAATCTTCAACTCTATGAGTTGAATGCAGACATCAGAAAGAAATTTCT
         66 AACTGCTCTATCAAAAGAAATGTTCAACTCTGTGAGTTGAGTACACACATCACAAAGAAGTTTCT

                     *       **          *         **
     117775 GAGAATGCTGCTGTCTACCTTTTAT-T--TGA-ATTCCCGC
        131 GAGAATGCTTCTGTCTAGTTTTTATATGAAGATATTCCCTT

                         *  *    *                *       *                  
     117812 TTCCAACGAAATCCTCCAAGCTATCCAAATATCCACTTGCAGATTCCACAAAAAGAGTGTTTCAA
          1 TTCCAACGAAATCATCAAAGCGATCCAAATATCCACTTCCAGATTCTACAAAAAGAGTGTTTCAA

                            ** *  *            *       *     *     *  *      
     117877 AACTGCTCTCTATCAATGGCAAAGTTCAACTCTGTTAGTTGAGGACACATATCACCAACAAGTTT
         66 AACTG--CTCTATCAAAAGAAATGTTCAACTCTGTGAGTTGAGTACACACATCACAAAGAAGTTT

                               *       **         *    
     117942 CTGAGAATGCTTCTGTCTATTTTTTATGGGAAGATATTTCCTT
        129 CTGAGAATGCTTCTGTCTAGTTTTTATATGAAGATATTCCCTT

              *  *  * ** *    *      *    *          * **                    
     117985 TTTCACCGTAGGCGTCAAGGCGATCGAAATGTCCACTTCCACAAACTACAAAAAGAGTGTTTCAA
          1 TTCCAACGAAATCATCAAAGCGATCCAAATATCCACTTCCAGATTCTACAAAAAGAGTGTTTCAA

             *         *   * **       *    *        * ** * *   **      *     
     118050 ACCTGCTCTATGAAAGGCCATGTTCATCTCTATGAGTTGAATGGAAATATCCGAAAGAAATTTCT
         66 AACTGCTCTATCAAAAGAAATGTTCAACTCTGTGAGTTGAGTACACACATCACAAAGAAGTTTCT

             *       *                             **
     118115 GGGAATGCTGCTGTCTAGTTTTTATAT---GA-ATTCCCGC
        131 GAGAATGCTTCTGTCTAGTTTTTATATGAAGATATTCCCTT

                         *       *                *    *  *                  
     118152 TTCCAACGAAATCCTCAAAGCAATCCAAATATCCACTTGCAGAATCCACAAAAAGAGTGTTTCAA
          1 TTCCAACGAAATCATCAAAGCGATCCAAATATCCACTTCCAGATTCTACAAAAAGAGTGTTTCAA

                          *     *          * *                      *        
     118217 AACTGCTCTATCAATAGAAAGGTTCAACTCTTTTAGTTGAGTACACACATCACAAACAAGTTTCT
         66 AACTGCTCTATCAAAAGAAATGTTCAACTCTGTGAGTTGAGTACACACATCACAAAGAAGTTTCT

                            * *      **     **  *    
     118282 GAGAATGCTTCTGTCTGGCTTTTATTGGAAGACGTTTCCTT
        131 GAGAATGCTTCTGTCTAGTTTTTATATGAAGATATTCCCTT

                 * *  **          *       *                * *               
     118323 TT-CACCAAAGGCATCAAAGCGCTCCAAATGTCCACTTCCAGATTCTTCCAAAAGAGTGTTTCAA
          1 TTCCAACGAAATCATCAAAGCGATCCAAATATCCACTTCCAGATTCTACAAAAAGAGTGTTTCAA

             **         **  * *                 *    * *  * *     *   *      
     118387 ACGTGCTC-ATAGTAAGGGAATGTTCAACTCTGTGACTTGAATGCAGATATCACCAAGTAGTTTC
         66 AACTGCTCTAT-CAAAAGAAATGTTCAACTCTGTGAGTTGAGTACACACATCACAAAGAAGTTTC

             * * *             *     *    *         * 
     118451 TAATAGTGCTTCTGTCTAGATTTTAGATGATGATATTCCCGT
        130 TGAGAATGCTTCTGTCTAGTTTTTATATGAAGATATTCCCTT

                         *** *   *             *  *   *      *     *       * 
     118493 TTCCAACGAAATCGCTAGAGCTATCCAAATATCCAGTTACAGTTTCTACCAAAAGGGTGTTTCCA
          1 TTCCAACGAAATCATCAAAGCGATCCAAATATCCACTTCCAGATTCTACAAAAAGAGTGTTTCAA

              *    **           *            *       *                     * 
     118558 AATTGCTGCATCAAAAGAAAGGTTCAACTCTGTTAGTTGAGGACACACATCACAAAGAAGTTTGT
         66 AACTGCTCTATCAAAAGAAATGTTCAACTCTGTGAGTTGAGTACACACATCACAAAGAAGTTTCT

                              *    *     *           
     118623 GAGAATGCTTCTGTCTAGATTTTGTATGACGATATTCCCTT
        131 GAGAATGCTTCTGTCTAGTTTTTATATGAAGATATTCCCTT

                     *   * *     *   *       * *  *    *  *       *  *       
     118664 TTCCAACGATATCGTTAAAGCAATCTAAATATCAATTTGCAGAATCCACAAAAATAGAGTTTCAA
          1 TTCCAACGAAATCATCAAAGCGATCCAAATATCCACTTCCAGATTCTACAAAAAGAGTGTTTCAA

             *       * *        *    *       *  *                   ***      
     118729 AGCTGCTCTGTAAAAAGAAAGGTTCCACTCTGTTAGCTGAGTACACACATCACAAACTTGTTTCT
         66 AACTGCTCTATCAAAAGAAATGTTCAACTCTGTGAGTTGAGTACACACATCACAAAGAAGTTTCT

                  *         *        **         **   
     118794 GAGAATCCTTCTGTCTCGTTTTTATGGGAAGATATTTACTT
        131 GAGAATGCTTCTGTCTAGTTTTTATATGAAGATATTCCCTT

                 *  * **          *       *     *       *  *  *  *           
     118835 TTCCACCGTAGGCATCAAAGCGCTCCAAATGTCCACATCCAGATACTCCAGAACGAGTGTTTCAA
          1 TTCCAACGAAATCATCAAAGCGATCCAAATATCCACTTCCAGATTCTACAAAAAGAGTGTTTCAA

             *         *   * *   *         *        * *  *      *      *     
     118900 ACCTGCTCTATGAAAGGGAATCTTCAACTCTATGAGTTGAATGCAGACATCAGAAAGAAATTTCT
         66 AACTGCTCTATCAAAAGAAATGTTCAACTCTGTGAGTTGAGTACACACATCACAAAGAAGTTTCT

                     *       **          *         **
     118965 GAGAATGCTGCTGTCTACCTTTTAT-T--TGA-ATTCCCGC
        131 GAGAATGCTTCTGTCTAGTTTTTATATGAAGATATTCCCTT

                         *  *    *              * *  **   *    *             
     119002 TTCCAACGAAATCCTCCAAGCTATCCAAATATCCACCTGCATTTTCCACAACAAGAGTGTTTCAA
          1 TTCCAACGAAATCATCAAAGCGATCCAAATATCCACTTCCAGATTCTACAAAAAGAGTGTTTCAA

                          *                 *    *  *                *       
     119067 AACTGCTCTATCAATAGAAATGTTCAACTCCTTTG-GCTGGGTACACACATCACAAACAAGTTTC
         66 AACTGCTCTATCAAAAGAAATGTTCAACT-CTGTGAGTTGAGTACACACATCACAAAGAAGTTTC

                                      **     **       
     119131 TGAGAATGCTTCTGTCTAGTTTTTATGGGAAGACGTTCCCTT
        130 TGAGAATGCTTCTGTCTAGTTTTTATATGAAGATATTCCCTT

              *  * *  **          *       *            **                  * 
     119173 TTTCACCAAAGGCATCAAAGCGCTCCAAATGTCCACTTCCAGACACTACAAAAAGAGTGTTTCCA
          1 TTCCAACGAAATCATCAAAGCGATCCAAATATCCACTTCCAGATTCTACAAAAAGAGTGTTTCAA

             **   *   **   ***                 *    * *  * *         *       
     119238 ACGTGCCCTAAGAAAGCGAATGTTCAACTCTGTGACTTGAATGCAGATATCACAAAGTAGTTTCT
         66 AACTGCTCTATCAAAAGAAATGTTCAACTCTGTGAGTTGAGTACACACATCACAAAGAAGTTTCT

                **            *     *    *         * 
     119303 GAGAGGGCTTCTGTCTAGATTTTAGATGATGATATTCCCGT
        131 GAGAATGCTTCTGTCTAGTTTTTATATGAAGATATTCCCTT

                           * *   *             *  *   *                    * 
     119344 TTCCAACGAAATCATTAGAGCTATCCAAATATCCAATTACAGTTTCTACAAAAAGAGTGTTTCCA
          1 TTCCAACGAAATCATCAAAGCGATCCAAATATCCACTTCCAGATTCTACAAAAAGAGTGTTTCAA

                   **         * *    *       *  *                   ***      
     119409 AACTGCTGCATCAAAAGAGAGGTTCCACTCTGTTAGCTGAGTACACACATCACAAACTTGTTTCT
         66 AACTGCTCTATCAAAAGAAATGTTCAACTCTGTGAGTTGAGTACACACATCACAAAGAAGTTTCT

            *     *         *        **         **   
     119474 CAGAATCCTTCTGTCTCGTTTTTATGGGAAGATATTTACTT
        131 GAGAATGCTTCTGTCTAGTTTTTATATGAAGATATTCCCTT

              *  *  * **          *       *     *       *  *  *              
     119515 TTTCACCGTAGGCATCAAAGCGCTCCAAATGTCCACATCCAGATACTCCAGAAAGAGTGTTTCAA
          1 TTCCAACGAAATCATCAAAGCGATCCAAATATCCACTTCCAGATTCTACAAAAAGAGTGTTTCAA

             *         *   * *   *         *        * *  *      *      *     
     119580 ACCTGCTCTATGAAAGGGAATCTTCAACTCTATGAGTTGAATGCAGACATCAGAAAGAAATTTCT
         66 AACTGCTCTATCAAAAGAAATGTTCAACTCTGTGAGTTGAGTACACACATCACAAAGAAGTTTCT

                     *       **          *         **
     119645 GAGAATGCTGCTGTCTACCTTTTAT-T--TGA-ATTCCCGC
        131 GAGAATGCTTCTGTCTAGTTTTTATATGAAGATATTCCCTT

                         *  *    *                *       *                  
     119682 TTCCAACGAAATCCTCCAAGCTATCCAAATATCCACTTGCAGATTCCACAAAAAGAGTGTTTCAA
          1 TTCCAACGAAATCATCAAAGCGATCCAAATATCCACTTCCAGATTCTACAAAAAGAGTGTTTCAA

                            ** *  *            *       *     *     *  *      
     119747 AACTGCTCTCTATCAATGGCAAAGTTCAACTCTGTTAGTTGAGGACACATATCACCAACAAGTTT
         66 AACTG--CTCTATCAAAAGAAATGTTCAACTCTGTGAGTTGAGTACACACATCACAAAGAAGTTT

                               *       **         *    
     119812 CTGAGAATGCTTCTGTCTATTTTTTATGGGAAGATATTTCCTT
        129 CTGAGAATGCTTCTGTCTAGTTTTTATATGAAGATATTCCCTT

              *  *  * ** *    *      *    *          * **                    
     119855 TTTCACCGTAGGCGTCAAGGCGATCGAAATGTCCACTTCCACAAACTACAAAAAGAGTGTTTCAA
          1 TTCCAACGAAATCATCAAAGCGATCCAAATATCCACTTCCAGATTCTACAAAAAGAGTGTTTCAA

             *         *   * **       *    *        * ** * *   **      *     
     119920 ACCTGCTCTATGAAAGGCCATGTTCATCTCTATGAGTTGAATGGAAATATCCGAAAGAAATTTCT
         66 AACTGCTCTATCAAAAGAAATGTTCAACTCTGTGAGTTGAGTACACACATCACAAAGAAGTTTCT

             *       *                             **
     119985 GGGAATGCTGCTGTCTAGTTTTTATAT---GA-ATTCCCGC
        131 GAGAATGCTTCTGTCTAGTTTTTATATGAAGATATTCCCTT

                         *       *                *    *  *                  
     120022 TTCCAACGAAATCCTCAAAGCAATCCAAATATCCACTTGCAGAATCCACAAAAAGAGTGTTTCAA
          1 TTCCAACGAAATCATCAAAGCGATCCAAATATCCACTTCCAGATTCTACAAAAAGAGTGTTTCAA

                          *     *          * *                      *        
     120087 AACTGCTCTATCAATAGAAAGGTTCAACTCTTTTAGTTGAGTACACACATCACAAACAAGTTTCT
         66 AACTGCTCTATCAAAAGAAATGTTCAACTCTGTGAGTTGAGTACACACATCACAAAGAAGTTTCT

                            * *      **     **  *    
     120152 GAGAATGCTTCTGTCTGGCTTTTATTGGAAGACGTTTCCTT
        131 GAGAATGCTTCTGTCTAGTTTTTATATGAAGATATTCCCTT

                 * *  **          *      **                * *               
     120193 TT-CACCAAAGGCATCAAAGCGCTCCAAAGGTCCACTTCCAGATTCTTCCAAAAGAGTGTTTCAA
          1 TTCCAACGAAATCATCAAAGCGATCCAAATATCCACTTCCAGATTCTACAAAAAGAGTGTTTCAA

             **         **  * *                 *    * *  * *     *   *      
     120257 ACGTGCTC-ATAGTAAGGGAATGTTCAACTCTGTGACTTGAATGCAGATATCACCAAGTAGTTTC
         66 AACTGCTCTAT-CAAAAGAAATGTTCAACTCTGTGAGTTGAGTACACACATCACAAAGAAGTTTC

             * * *             *     *    *         * 
     120321 TAATAGTGCTTCTGTCTAGATTTTAGATGATGATATTCCCGT
        130 TGAGAATGCTTCTGTCTAGTTTTTATATGAAGATATTCCCTT

                         *** *   *             *  *   *      *     *       * 
     120363 TTCCAACGAAATCGCTAGAGCTATCCAAATATCCAGTTACAGTTTCTACCAAAAGGGTGTTTCCA
          1 TTCCAACGAAATCATCAAAGCGATCCAAATATCCACTTCCAGATTCTACAAAAAGAGTGTTTCAA

              *    **           *            *       *                     * 
     120428 AATTGCTGCATCAAAAGAAAGGTTCAACTCTGTTAGTTGAGGACACACATCACAAAGAAGTTTGT
         66 AACTGCTCTATCAAAAGAAATGTTCAACTCTGTGAGTTGAGTACACACATCACAAAGAAGTTTCT

                             **    *     *           
     120493 GAGAATGCTTCTGTCTACATTTTGTATGACGATATTCCCTT
        131 GAGAATGCTTCTGTCTAGTTTTTATATGAAGATATTCCCTT

                     *   * *     *   *       * *  *    *  *       *  *       
     120534 TTCCAACGATATCGTTAAAGCAATCTAAATATCAATTTGCAGAATCCACAAAAATAGAGTTTCAA
          1 TTCCAACGAAATCATCAAAGCGATCCAAATATCCACTTCCAGATTCTACAAAAAGAGTGTTTCAA

             *       * *        *    *       *  *                   ***      
     120599 AGCTGCTCTGTAAAAAGAAAGGTTCCACTCTGTTAGCTGAGTACACACATCACAAACTTGTTTCT
         66 AACTGCTCTATCAAAAGAAATGTTCAACTCTGTGAGTTGAGTACACACATCACAAAGAAGTTTCT

                  *         *        **         **   
     120664 GAGAATCCTTCTGTCTCGTTTTTATGGGAAGATATTTACTT
        131 GAGAATGCTTCTGTCTAGTTTTTATATGAAGATATTCCCTT

                 *  * **          *       *     *       *  *  *  *           
     120705 TTCCACCGTAGGCATCAAAGCGCTCCAAATGTCCACATCCAGATACTCCAGAACGAGTGTTTCAA
          1 TTCCAACGAAATCATCAAAGCGATCCAAATATCCACTTCCAGATTCTACAAAAAGAGTGTTTCAA

             *         *   * *   *         *        * *  *      *      *     
     120770 ACCTGCTCTATGAAAGGGAATCTTCAACTCTATGAGTTGAATGCAGACATCAGAAAGAAATTTCT
         66 AACTGCTCTATCAAAAGAAATGTTCAACTCTGTGAGTTGAGTACACACATCACAAAGAAGTTTCT

                     *       **          *         **
     120835 GAGAATGCTGCTGTCTACCTTTTAT-T--TGA-ATTCCCGC
        131 GAGAATGCTTCTGTCTAGTTTTTATATGAAGATATTCCCTT

                         *  *    *              * *  **   *    *             
     120872 TTCCAACGAAATCCTCCAAGCTATCCAAATATCCACCTGCATTTTCCACAACAAGAGTGTTTCAA
          1 TTCCAACGAAATCATCAAAGCGATCCAAATATCCACTTCCAGATTCTACAAAAAGAGTGTTTCAA

                          *                 *    *  *                *       
     120937 AACTGCTCTATCAATAGAAATGTTCAACTCCTTTG-GCTGGGTACACACATCACAAACAAGTTTC
         66 AACTGCTCTATCAAAAGAAATGTTCAACT-CTGTGAGTTGAGTACACACATCACAAAGAAGTTTC

                                      **     **       
     121001 TGAGAATGCTTCTGTCTAGTTTTTATGGGAAGACGTTCCCTT
        130 TGAGAATGCTTCTGTCTAGTTTTTATATGAAGATATTCCCTT

              *  * *  **          *       *            **                  * 
     121043 TTTCACCAAAGGCATCAAAGCGCTCCAAATGTCCACTTCCAGACACTACAAAAAGAGTGTTTCCA
          1 TTCCAACGAAATCATCAAAGCGATCCAAATATCCACTTCCAGATTCTACAAAAAGAGTGTTTCAA

             **   *   **   ***                 *    * *  * *         *       
     121108 ACGTGCCCTAAGAAAGCGAATGTTCAACTCTGTGACTTGAATGCAGATATCACAAAGTAGTTTCT
         66 AACTGCTCTATCAAAAGAAATGTTCAACTCTGTGAGTTGAGTACACACATCACAAAGAAGTTTCT

                **            *     *    *         * 
     121173 GAGAGGGCTTCTGTCTAGATTTTAGATGATGATATTCCCGT
        131 GAGAATGCTTCTGTCTAGTTTTTATATGAAGATATTCCCTT

                           * *   *                *   *                    * 
     121214 TTCCAACGAAATCATTAGAGCTATCCAAATATCCACTTACAGTTTCTACAAAAAGAGTGTTTCCA
          1 TTCCAACGAAATCATCAAAGCGATCCAAATATCCACTTCCAGATTCTACAAAAAGAGTGTTTCAA

                   **         * *    *       *  *                   ***      
     121279 AACTGCTGCATCAAAAGAGAGGTTCCACTCTGTTAGCTGAGTACACACATCACAAACTTGTTTCT
         66 AACTGCTCTATCAAAAGAAATGTTCAACTCTGTGAGTTGAGTACACACATCACAAAGAAGTTTCT

            *     *         *        **         **   
     121344 CAGAATCCTTCTGTCTCGTTTTTATGGGAAGATATTTACTT
        131 GAGAATGCTTCTGTCTAGTTTTTATATGAAGATATTCCCTT

              *  *  * **          *       *     *       *  *  *              
     121385 TTTCACCGTAGGCATCAAAGCGCTCCAAATGTCCACATCCAGATACTCCAGAAAGAGTGTTTCAA
          1 TTCCAACGAAATCATCAAAGCGATCCAAATATCCACTTCCAGATTCTACAAAAAGAGTGTTTCAA

             *         *   * *   *         *        * *  *      *      *     
     121450 ACCTGCTCTATGAAAGGGAATCTTCAACTCTATGAGTTGAATGCAGACATCAGAAAGAAATTTCT
         66 AACTGCTCTATCAAAAGAAATGTTCAACTCTGTGAGTTGAGTACACACATCACAAAGAAGTTTCT

                     *       **          *         **
     121515 GAGAATGCTGCTGTCTACCTTTTAT-T--TGA-ATTCCCGC
        131 GAGAATGCTTCTGTCTAGTTTTTATATGAAGATATTCCCTT

                         *  *    *                *       *                  
     121552 TTCCAACGAAATCCTCCAAGCTATCCAAATATCCACTTGCAGATTCCACAAAAAGAGTGTTTCAA
          1 TTCCAACGAAATCATCAAAGCGATCCAAATATCCACTTCCAGATTCTACAAAAAGAGTGTTTCAA

                            ** *  *            *       *     *     *  *      
     121617 AACTGCTCTCTATCAATGGCAAAGTTCAACTCTGTTAGTTGAGGACACATATCACCAACAAGTTT
         66 AACTG--CTCTATCAAAAGAAATGTTCAACTCTGTGAGTTGAGTACACACATCACAAAGAAGTTT

                               *       **         *    
     121682 CTGAGAATGCTTCTGTCTATTTTTTATGGGAAGATATTTCCTT
        129 CTGAGAATGCTTCTGTCTAGTTTTTATATGAAGATATTCCCTT

              *  *  * ** *    *      *    *          * **          *         
     121725 TTTCACCGTAGGCGTCAAGGCGATCGAAATGTCCACTTCCACAAACTACAAAAAGTGTGTTTCAA
          1 TTCCAACGAAATCATCAAAGCGATCCAAATATCCACTTCCAGATTCTACAAAAAGAGTGTTTCAA

             *         *   * **       *    *        * ** * *   **      *     
     121790 ACCTGCTCTATGAAAGGCCATGTTCATCTCTATGAGTTGAATGGAAATATCCGAAAGAAATTTCT
         66 AACTGCTCTATCAAAAGAAATGTTCAACTCTGTGAGTTGAGTACACACATCACAAAGAAGTTTCT

             *       *          *        *         **
     121855 GGGAATGCTGCTGTCTAG-TGTT-TAT-ACGA-ATTCCCGC
        131 GAGAATGCTTCTGTCTAGTTTTTATATGAAGATATTCCCTT

                         *       *                *    *  *                  
     121892 TTCCAACGAAATCCTCAAAGCAATCCAAATATCCACTTGCAGAATCCACAAAAAGAGTGTTTCAA
          1 TTCCAACGAAATCATCAAAGCGATCCAAATATCCACTTCCAGATTCTACAAAAAGAGTGTTTCAA

                          *     *          * *                   *  *        
     121957 AACTGCTCTATCAATAGAAAGGTTCAACTCTTTTAGTTGAGTACACACATCACGAACAAGTTTCT
         66 AACTGCTCTATCAAAAGAAATGTTCAACTCTGTGAGTTGAGTACACACATCACAAAGAAGTTTCT

                            * *      **     **  *    
     122022 GAGAATGCTTCTGTCTGGCTTTTATTGGAAGACGTTTCCTT
        131 GAGAATGCTTCTGTCTAGTTTTTATATGAAGATATTCCCTT

                 * *  **          *       *                * *               
     122063 TT-CACCAAAGGCATCAAAGCGCTCCAAATGTCCACTTCCAGATTCTTCCAAAAGAGTGTTTCAA
          1 TTCCAACGAAATCATCAAAGCGATCCAAATATCCACTTCCAGATTCTACAAAAAGAGTGTTTCAA

             **     * ***  * *                 *    * *  * *     *   *       
     122127 ACGTGCTCAAAGTAAGGGAATGTTCAACTCTGTGACTTGAATGCAGATATCACCAAGTAGTTTCT
         66 AACTGCTCTATCAAAAGAAATGTTCAACTCTGTGAGTTGAGTACACACATCACAAAGAAGTTTCT

            * * *             *     *    *         * 
     122192 AATAGTGCTTCTGTCTAGATTTTAGATGATGATATTCCCGT
        131 GAGAATGCTTCTGTCTAGTTTTTATATGAAGATATTCCCTT

                         * * *   * **          *  *   *      *     *       * 
     122233 TTCCAACGAAATCGTTAGAGCTAAGCAAATATCCAGTTACAGTTTCTACCAAAAGGGTGTTTCCA
          1 TTCCAACGAAATCATCAAAGCGATCCAAATATCCACTTCCAGATTCTACAAAAAGAGTGTTTCAA

              *    **           *            *       *                     * 
     122298 AATTGCTGCATCAAAAGAAAGGTTCAACTCTGTTAGTTGAGGACACACATCACAAAGAAGTTTGT
         66 AACTGCTCTATCAAAAGAAATGTTCAACTCTGTGAGTTGAGTACACACATCACAAAGAAGTTTCT

                              *    *     *           
     122363 GAGAATGCTTCTGTCTAGATTTTGTATGACGATATTCCCTT
        131 GAGAATGCTTCTGTCTAGTTTTTATATGAAGATATTCCCTT

                     *   * *     *   *       * *  *    *  *       *  *       
     122404 TTCCAACGATATCGTTAAAGCAATCTAAATATCAATTTGCAGAATCCACAAAAATAGAGTTTCAA
          1 TTCCAACGAAATCATCAAAGCGATCCAAATATCCACTTCCAGATTCTACAAAAAGAGTGTTTCAA

             *       * *        *    *       *  *                   *** *    
     122469 AGCTGCTCTGTAAAAAGAAAGGTTCCACTCTGTTAGCTGAGTACACACATCACAAACTTGATTCT
         66 AACTGCTCTATCAAAAGAAATGTTCAACTCTGTGAGTTGAGTACACACATCACAAAGAAGTTTCT

                  *         *        **         **   
     122534 GAGAATCCTTCTGTCTCGTTTTTATGGGAAGATATTTACTT
        131 GAGAATGCTTCTGTCTAGTTTTTATATGAAGATATTCCCTT

                 *  * **          *       *     *       *  *  *  *           
     122575 TTCCACCGTAGGCATCAAAGCGCTCCAAATGTCCACATCCAGATACTCCAGAACGAGTGTTTCAA
          1 TTCCAACGAAATCATCAAAGCGATCCAAATATCCACTTCCAGATTCTACAAAAAGAGTGTTTCAA

             *         *   * *   *         *        * *  *      *      *     
     122640 ACCTGCTCTATGAAAGGGAATCTTCAACTCTATGAGTTGAATGCAGACATCAGAAAGAAATTTCT
         66 AACTGCTCTATCAAAAGAAATGTTCAACTCTGTGAGTTGAGTACACACATCACAAAGAAGTTTCT

                     *       **          *         **
     122705 GAGAATGCTGCTGTCTACCTTTTAT-T--TGA-ATTCCCGC
        131 GAGAATGCTTCTGTCTAGTTTTTATATGAAGATATTCCCTT

                         *  *    *              * *  **   *    *             
     122742 TTCCAACGAAATCCTCCAAGCTATCCAAATATCCACCTGCATTTTCCACAACAAGAGTGTTTCAA
          1 TTCCAACGAAATCATCAAAGCGATCCAAATATCCACTTCCAGATTCTACAAAAAGAGTGTTTCAA

                          *                 *    *  *                *       
     122807 AACTGCTCTATCAATAGAAATGTTCAACTCCTTTG-GCTGGGTACACACATCACAAACAAGTTTC
         66 AACTGCTCTATCAAAAGAAATGTTCAACT-CTGTGAGTTGAGTACACACATCACAAAGAAGTTTC

                                      **     **       
     122871 TGAGAATGCTTCTGTCTAGTTTTTATGGGAAGACGTTCCCTT
        130 TGAGAATGCTTCTGTCTAGTTTTTATATGAAGATATTCCCTT

              *  * *  **          *       *            **                    
     122913 TTTCACCAAAGGCATCAAAGCGCTCCAAATGTCCACTTCCAGACACTACAAAAAGAGTGTTTCAA
          1 TTCCAACGAAATCATCAAAGCGATCCAAATATCCACTTCCAGATTCTACAAAAAGAGTGTTTCAA

             **       **   ***                 *    * *  * *         *       
     122978 ACGTGCTCTAAGAAAGCGAATGTTCAACTCTGTGACTTGAATGCAGATATCACAAAGTAGTTTCT
         66 AACTGCTCTATCAAAAGAAATGTTCAACTCTGTGAGTTGAGTACACACATCACAAAGAAGTTTCT

                **            *     *    *         * 
     123043 GAGAGGGCTTCTGTCTAGATTTTAGATGATGATATTCCCGT
        131 GAGAATGCTTCTGTCTAGTTTTTATATGAAGATATTCCCTT

                           * *   *                *   *                    * 
     123084 TTCCAACGAAATCATTAGAGCTATCCAAATATCCACTTACAGTTTCTACAAAAAGAGTGTTTCCA
          1 TTCCAACGAAATCATCAAAGCGATCCAAATATCCACTTCCAGATTCTACAAAAAGAGTGTTTCAA

                   **         * *    *       *  **                  ***      
     123149 AACTGCTGCATCAAAAGAGAGGTTCCACTCTGTTAGCCGAGTACACACATCACAAACTTGTTTCT
         66 AACTGCTCTATCAAAAGAAATGTTCAACTCTGTGAGTTGAGTACACACATCACAAAGAAGTTTCT

            *     *      *  *        **         **   
     123214 CAGAATCCTTCTGACTCGTTTTTATGGGAAGATATTTACTT
        131 GAGAATGCTTCTGTCTAGTTTTTATATGAAGATATTCCCTT

              *  *  * **          *       *     *       *  *  *              
     123255 TTTCACCGTAGGCATCAAAGCGCTCCAAATGTCCACATCCAGATACTCCAGAAAGAGTGTTTCAA
          1 TTCCAACGAAATCATCAAAGCGATCCAAATATCCACTTCCAGATTCTACAAAAAGAGTGTTTCAA

             *         *   * *   *         *        * *  *      *      *     
     123320 ACCTGCTCTATGAAAGGGAATCTTCAACTCTATGAGTTGAATGCAGACATCAGAAAGAAATTTCT
         66 AACTGCTCTATCAAAAGAAATGTTCAACTCTGTGAGTTGAGTACACACATCACAAAGAAGTTTCT

                     *       **          *         **
     123385 GAGAATGCTGCTGTCTACCTTTTAT-T--TGA-ATTCCCGC
        131 GAGAATGCTTCTGTCTAGTTTTTATATGAAGATATTCCCTT

                         *  *    *                *       *                  
     123422 TTCCAACGAAATCCTCCAAGCTATCCAAATATCCACTTGCAGATTCCACAAAAAGAGTGTTTCAA
          1 TTCCAACGAAATCATCAAAGCGATCCAAATATCCACTTCCAGATTCTACAAAAAGAGTGTTTCAA

                            ** *  *            *       *     *     *  *      
     123487 AACTGCTCTCTATCAATGGCAAAGTTCAACTCTGTTAGTTGAGGACACATATCACCAACAAGTTT
         66 AACTG--CTCTATCAAAAGAAATGTTCAACTCTGTGAGTTGAGTACACACATCACAAAGAAGTTT

                               *       **         *    
     123552 CTGAGAATGCTTCTGTCTATTTTTTATGGGAAGATATTTCCTT
        129 CTGAGAATGCTTCTGTCTAGTTTTTATATGAAGATATTCCCTT

              *  *  * ** *    *      *    *          * **                    
     123595 TTTCACCGTAGGCGTCAAGGCGATCGAAATGTCCACTTCCACAAACTACAAAAAGAGTGTTTCAA
          1 TTCCAACGAAATCATCAAAGCGATCCAAATATCCACTTCCAGATTCTACAAAAAGAGTGTTTCAA

             *         *   * **       *    *        * ** * *   **      *     
     123660 ACCTGCTCTATGAAAGGCCATGTTCATCTCTATGAGTTGAATGGAAATATCCGAAAGAAATTTCT
         66 AACTGCTCTATCAAAAGAAATGTTCAACTCTGTGAGTTGAGTACACACATCACAAAGAAGTTTCT

             *       *          *        *         **
     123725 GGGAATGCTGCTGTCTAG-TGTT-TAT-ACGA-ATTCCCGC
        131 GAGAATGCTTCTGTCTAGTTTTTATATGAAGATATTCCCTT

                         *       *                *    *  *                  
     123762 TTCCAACGAAATCCTCAAAGCAATCCAAATATCCACTTGCAGAATCCACAAAAAGAGTGTTTCAA
          1 TTCCAACGAAATCATCAAAGCGATCCAAATATCCACTTCCAGATTCTACAAAAAGAGTGTTTCAA

                          *     *          * *                      *        
     123827 AACTGCTCTATCAATAGAAAGGTTCAACTCTTTTAGTTGAGTACACACATCACAAACAAGTTTCT
         66 AACTGCTCTATCAAAAGAAATGTTCAACTCTGTGAGTTGAGTACACACATCACAAAGAAGTTTCT

                            * *      **     **  *    
     123892 GAGAATGCTTCTGTCTGGCTTTTATTGGAAGACGTTTCCTT
        131 GAGAATGCTTCTGTCTAGTTTTTATATGAAGATATTCCCTT

                   *  **          *       *                * *               
     123933 TT-CAACAAAGGCATCAAAGCGCTCCAAATGTCCACTTCCAGATTCTTCCAAAAGAGTGTTTCAA
          1 TTCCAACGAAATCATCAAAGCGATCCAAATATCCACTTCCAGATTCTACAAAAAGAGTGTTTCAA

             **  *  * ***  * *                 *    * *  * *     *   *       
     123997 ACGTGGTCGAAGTAAGGGAATGTTCAACTCTGTGACTTGAATGCAGATATCACCAAGTAGTTTCT
         66 AACTGCTCTATCAAAAGAAATGTTCAACTCTGTGAGTTGAGTACACACATCACAAAGAAGTTTCT

            * * *            **     *    *         * 
     124062 AATAGTGCTTCTGTCTACATTTTAGATGATGATATTCCCGT
        131 GAGAATGCTTCTGTCTAGTTTTTATATGAAGATATTCCCTT

                         * * *   *             *  *   *      *     *       * 
     124103 TTCCAACGAAATCGTTAGAGCTATCCAAATATCCAGTTACAGTTTCTACCAAAAGGGTGTTTCCA
          1 TTCCAACGAAATCATCAAAGCGATCCAAATATCCACTTCCAGATTCTACAAAAAGAGTGTTTCAA

              *    **           *            *       *                     * 
     124168 AATTGCTGCATCAAAAGAAAGGTTCAACTCTGTTAGTTGAGGACACACATCACAAAGAAGTTTGT
         66 AACTGCTCTATCAAAAGAAATGTTCAACTCTGTGAGTTGAGTACACACATCACAAAGAAGTTTCT

                              *    *     *           
     124233 GAGAATGCTTCTGTCTAGATTTTGTATGACGATATTCCCTT
        131 GAGAATGCTTCTGTCTAGTTTTTATATGAAGATATTCCCTT

                     *   * *     *   *       * *  *    *  *       *  *       
     124274 TTCCAACGATATCGTTAAAGCAATCTAAATATCAATTTGCAGAATCCACAAAAATAGAGTTTCAA
          1 TTCCAACGAAATCATCAAAGCGATCCAAATATCCACTTCCAGATTCTACAAAAAGAGTGTTTCAA

             *       * *        *    *       *  *                   ***      
     124339 AGCTGCTCTGTAAAAAGAAAGGTTCCACTCTGTTAGCTGAGTACACACATCACAAACTTGTTTCT
         66 AACTGCTCTATCAAAAGAAATGTTCAACTCTGTGAGTTGAGTACACACATCACAAAGAAGTTTCT

                 **         *        **         **   
     124404 GAGAAACCTTCTGTCTCGTTTTTATGGGAAGATATTTACTT
        131 GAGAATGCTTCTGTCTAGTTTTTATATGAAGATATTCCCTT

                 ** * **          *       *     *       *  *  *  *           
     124445 TTCCACTGTAGGCATCAAAGCGCTCCAAATGTCCACATCCAGATACTCCAGAACGAGTGTTTCAA
          1 TTCCAACGAAATCATCAAAGCGATCCAAATATCCACTTCCAGATTCTACAAAAAGAGTGTTTCAA

             *         *   * *   *         *        * *  *      *      *     
     124510 ACCTGCTCTATGAAAGGGAATCTTCAACTCTATGAGTTGAATGCAGA-ATCAGAAAGAAATTTCT
         66 AACTGCTCTATCAAAAGAAATGTTCAACTCTGTGAGTTGAGTACACACATCACAAAGAAGTTTCT

                     *       **          *         **
     124574 GAGAATGCTGCTGTCTACCTTTTAT-T--TGA-ATTCCCGC
        131 GAGAATGCTTCTGTCTAGTTTTTATATGAAGATATTCCCTT

                         *  *    *              * *  **   *                  
     124611 TTCCAACGAAATCCTCCAAGCTATCCAAATATCCACCTGCATTTTCCACAAAAAGAGTGTTTCAA
          1 TTCCAACGAAATCATCAAAGCGATCCAAATATCCACTTCCAGATTCTACAAAAAGAGTGTTTCAA

                          *                 *    *  *                *       
     124676 AACTGCTCTATCAATAGAAATGTTCAACTCCTTTG-GCTGGGTACACACATCACAAACAAGTTTC
         66 AACTGCTCTATCAAAAGAAATGTTCAACT-CTGTGAGTTGAGTACACACATCACAAAGAAGTTTC

                                      **     **       
     124740 TGAGAATGCTTCTGTCTAGTTTTTATGGGAAGACGTTCCCTT
        130 TGAGAATGCTTCTGTCTAGTTTTTATATGAAGATATTCCCTT

              *  * *  **          *       *            **                  * 
     124782 TTTCACCAAAGGCATCAAAGCGCTCCAAATGTCCACTTCCAGACACTACAAAAAGAGTGTTTCCA
          1 TTCCAACGAAATCATCAAAGCGATCCAAATATCCACTTCCAGATTCTACAAAAAGAGTGTTTCAA

             **       **   ***                 *    * *  * *         *       
     124847 ACGTGCTCTAAGAAAGCGAATGTTCAACTCTGTGACTTGAATGCAGATATCACAAAGTAGTTTCT
         66 AACTGCTCTATCAAAAGAAATGTTCAACTCTGTGAGTTGAGTACACACATCACAAAGAAGTTTCT

                **            *     *    *         * 
     124912 GAGAGGGCTTCTGTCTAGATTTTAGATGATGATATTCCCGT
        131 GAGAATGCTTCTGTCTAGTTTTTATATGAAGATATTCCCTT

                           * *   *                *   *                    * 
     124953 TTCCAACGAAATCATTAGAGCTATCCAAATATCCACTTACAGTTTCTACAAAAAGAGTGTTTCCA
          1 TTCCAACGAAATCATCAAAGCGATCCAAATATCCACTTCCAGATTCTACAAAAAGAGTGTTTCAA

                   **         * *    *       *  **                  ***      
     125018 AACTGCTGCATCAAAAGAGAGGTTCCACTCTGTTAGCCGAGTACACACATCACAAACTTGTTTCT
         66 AACTGCTCTATCAAAAGAAATGTTCAACTCTGTGAGTTGAGTACACACATCACAAAGAAGTTTCT

            *     *         *        **         **   
     125083 CAGAATCCTTCTGTCTCGTTTTTATGGGAAGATATTTACTT
        131 GAGAATGCTTCTGTCTAGTTTTTATATGAAGATATTCCCTT

              *  *  * **          *       *     *       *  *  *              
     125124 TTTCACCGTAGGCATCAAAGCGCTCCAAATGTCCACATCCAGATACTCCAGAAAGAGTGTTTCAA
          1 TTCCAACGAAATCATCAAAGCGATCCAAATATCCACTTCCAGATTCTACAAAAAGAGTGTTTCAA

             *         *   * *   *         *        * *  *      *      *     
     125189 ACCTGCTCTATGAAAGGGAATCTTCAACTCTATGAGTTGAATGCAGACATCAGAAAGAAATTTCT
         66 AACTGCTCTATCAAAAGAAATGTTCAACTCTGTGAGTTGAGTACACACATCACAAAGAAGTTTCT

                     *       **          *         **
     125254 GAGAATGCTGCTGTCTACCTTTTAT-T--TGA-ATTCCCGC
        131 GAGAATGCTTCTGTCTAGTTTTTATATGAAGATATTCCCTT

                         *  *    *                *       *                  
     125291 TTCCAACGAAATCCTCCAAGCTATCCAAATATCCACTTGCAGATTCCACAAAAAGAGTGTTTCAA
          1 TTCCAACGAAATCATCAAAGCGATCCAAATATCCACTTCCAGATTCTACAAAAAGAGTGTTTCAA

                            ** *  *            *       *     *     *  *      
     125356 AACTGCTCTCTATCAATGGCAAAGTTCAACTCTGTTAGTTGAGGACACATATCACCAACAAGTTT
         66 AACTG--CTCTATCAAAAGAAATGTTCAACTCTGTGAGTTGAGTACACACATCACAAAGAAGTTT

                               *       **         *    
     125421 CTGAGAATGCTTCTGTCTATTTTTTATGGGAAGATATTTCCTT
        129 CTGAGAATGCTTCTGTCTAGTTTTTATATGAAGATATTCCCTT

              *  *  * ** *    *      *    *          * **                    
     125464 TTTCACCGTAGGCGTCAAGGCGATCGAAATGTCCACTTCCACAAACTACAAAAAGAGTGTTTCAA
          1 TTCCAACGAAATCATCAAAGCGATCCAAATATCCACTTCCAGATTCTACAAAAAGAGTGTTTCAA

             *         *   * **       *    *        * ** * *   **      *     
     125529 ACCTGCTCTATGAAAGGCCATGTTCATCTCTATGAGTTGAATGGAAATATCCGAAAGAAATTTCT
         66 AACTGCTCTATCAAAAGAAATGTTCAACTCTGTGAGTTGAGTACACACATCACAAAGAAGTTTCT

             *       *          *        *         **
     125594 GGGAATGCTGCTGTCTAG-TGTT-TAT-ACGA-ATTCCCGC
        131 GAGAATGCTTCTGTCTAGTTTTTATATGAAGATATTCCCTT

                         *       *                *    *  *                  
     125631 TTCCAACGAAATCCTCAAAGCAATCCAAATATCCACTTGCAGAATCCACAAAAAGAGTGTTTCAA
          1 TTCCAACGAAATCATCAAAGCGATCCAAATATCCACTTCCAGATTCTACAAAAAGAGTGTTTCAA

               *          *     *          * *                      *        
     125696 AACCGCTCTATCAATAGAAAGGTTCAACTCTTTTAGTTGAGTACACACATCACAAACAAGTTTCT
         66 AACTGCTCTATCAAAAGAAATGTTCAACTCTGTGAGTTGAGTACACACATCACAAAGAAGTTTCT

                            * *      **     **  *    
     125761 GAGAATGCTTCTGTCTGGCTTTTATTGGAAGACGTTTCCTT
        131 GAGAATGCTTCTGTCTAGTTTTTATATGAAGATATTCCCTT

                 * *  **          *       *                * *               
     125802 TT-CACCAAAGGCATCAAAGCGCTCCAAATGTCCACTTCCAGATTCTTCCAAAAGAGTGTTTCAA
          1 TTCCAACGAAATCATCAAAGCGATCCAAATATCCACTTCCAGATTCTACAAAAAGAGTGTTTCAA

             **  *  * ***  * *                 *    * *  * *     *   *       
     125866 ACGTGGTCGAAGTAAGGGAATGTTCAACTCTGTGACTTGAATGCAGATATCACCAAGTAGTTTCT
         66 AACTGCTCTATCAAAAGAAATGTTCAACTCTGTGAGTTGAGTACACACATCACAAAGAAGTTTCT

            * * *             *     *    *         * 
     125931 AATAGTGCTTCTGTCTAGATTTTAGATGATGATATTCCCGT
        131 GAGAATGCTTCTGTCTAGTTTTTATATGAAGATATTCCCTT

                         * * *   *             *  *   *      *     *       * 
     125972 TTCCAACGAAATCGTTAGAGCTATCCAAATATCCAGTTACAGTTTCTACCAAAAGGGTGTTTCCA
          1 TTCCAACGAAATCATCAAAGCGATCCAAATATCCACTTCCAGATTCTACAAAAAGAGTGTTTCAA

              *    **           *            *       *                     * 
     126037 AATTGCTGCATCAAAAGAAAGGTTCAACTCTGTTAGTTGAGGACACACATCACAAAGAAGTTTGT
         66 AACTGCTCTATCAAAAGAAATGTTCAACTCTGTGAGTTGAGTACACACATCACAAAGAAGTTTCT

                              *    *     *           
     126102 GAGAATGCTTCTGTCTAGATTTTGTATGACGATATTCCCTT
        131 GAGAATGCTTCTGTCTAGTTTTTATATGAAGATATTCCCTT

                     *   * *     *   *       * *  *    *  *       *  *       
     126143 TTCCAACGATATCGTTAAAGCAATCTAAATATCAATTTGCAGAATCCACAAAAATAGAGTTTCAA
          1 TTCCAACGAAATCATCAAAGCGATCCAAATATCCACTTCCAGATTCTACAAAAAGAGTGTTTCAA

             *       * *        *    *       *  *                   ***      
     126208 AGCTGCTCTGTAAAAAGAAAGGTTCCACTCTGTTAGCTGAGTACACACATCACAAACTTGTTTCT
         66 AACTGCTCTATCAAAAGAAATGTTCAACTCTGTGAGTTGAGTACACACATCACAAAGAAGTTTCT

                 **         *        **         **   
     126273 GAGAAACCTTCTGTCTCGTTTTTATGGGAAGATATTTACTT
        131 GAGAATGCTTCTGTCTAGTTTTTATATGAAGATATTCCCTT

                 ** * **          *       *     *       *  *  *  *           
     126314 TTCCACTGTAGGCATCAAAGCGCTCCAAATGTCCACATCCAGATACTCCAGAACGAGTGTTTCAA
          1 TTCCAACGAAATCATCAAAGCGATCCAAATATCCACTTCCAGATTCTACAAAAAGAGTGTTTCAA

             *         *   * *   *         *        * *  *      *      *     
     126379 ACCTGCTCTATGAAAGGGAATCTTCAACTCTATGAGTTGAATGCAGA-ATCAGAAAGAAATTTCT
         66 AACTGCTCTATCAAAAGAAATGTTCAACTCTGTGAGTTGAGTACACACATCACAAAGAAGTTTCT

                     *       **          *         **
     126443 GAGAATGCTGCTGTCTACCTTTTAT-T--TGA-ATTCCCGC
        131 GAGAATGCTTCTGTCTAGTTTTTATATGAAGATATTCCCTT

                         *  *    *              * *  **   *    *             
     126480 TTCCAACGAAATCCTCCAAGCTATCCAAATATCCACCTGCATTTTCCACAACAAGAGTGTTTCAA
          1 TTCCAACGAAATCATCAAAGCGATCCAAATATCCACTTCCAGATTCTACAAAAAGAGTGTTTCAA

                          *                 *    *  *                *       
     126545 AACTGCTCTATCAATAGAAATGTTCAACTCCTTTG-GCTGGGTACACACATCACAAACAAGTTTC
         66 AACTGCTCTATCAAAAGAAATGTTCAACT-CTGTGAGTTGAGTACACACATCACAAAGAAGTTTC

                                      **     **       
     126609 TGAGAATGCTTCTGTCTAGTTTTTATGGGAAGACGTTCCCTT
        130 TGAGAATGCTTCTGTCTAGTTTTTATATGAAGATATTCCCTT

              *  * *  **          *       *            **                    
     126651 TTTCACCAAAGGCATCAAAGCGCTCCAAATGTCCACTTCCAGACACTACAAAAAGAGTGTTTCAA
          1 TTCCAACGAAATCATCAAAGCGATCCAAATATCCACTTCCAGATTCTACAAAAAGAGTGTTTCAA

             **       **    **                 *    * *  * *         *       
     126716 ACGTGCTCTAAGAAAACGAATGTTCAACTCTGTGACTTGAATGCAGATATCACAAAGTAGTTTCT
         66 AACTGCTCTATCAAAAGAAATGTTCAACTCTGTGAGTTGAGTACACACATCACAAAGAAGTTTCT

                **            *     *    *         * 
     126781 GAGAGGGCTTCTGTCTAGATTTTAGATGATGATATTCCCGT
        131 GAGAATGCTTCTGTCTAGTTTTTATATGAAGATATTCCCTT

                           * *   *                *   *                    * 
     126822 TTCCAACGAAATCATTAGAGCTATCCAAATATCCACTTACAGTTTCTACAAAAAGAGTGTTTCCA
          1 TTCCAACGAAATCATCAAAGCGATCCAAATATCCACTTCCAGATTCTACAAAAAGAGTGTTTCAA

                   **         * *    *       *  **                  ***      
     126887 AACTGCTGCATCAAAAGAGAGGTTCCACTCTGTTAGCCGAGTACACACATCACAAACTTGTTTCT
         66 AACTGCTCTATCAAAAGAAATGTTCAACTCTGTGAGTTGAGTACACACATCACAAAGAAGTTTCT

            *     *         *        **         **   
     126952 CAGAATCCTTCTGTCTCGTTTTTATGGGAAGATATTTACTT
        131 GAGAATGCTTCTGTCTAGTTTTTATATGAAGATATTCCCTT

              *  *  * **          *       *     *       *  *  *              
     126993 TTTCACCGTAGGCATCAAAGCGCTCCAAATGTCCACATCCAGATACTCCAGAAAGAGTGTTTCAA
          1 TTCCAACGAAATCATCAAAGCGATCCAAATATCCACTTCCAGATTCTACAAAAAGAGTGTTTCAA

             *         *   * *   *         *        * *  *      *      *     
     127058 ACCTGCTCTATGAAAGGGAATCTTCAACTCTATGAGTTGAATGCAGACATCAGAAAGAAATTTCT
         66 AACTGCTCTATCAAAAGAAATGTTCAACTCTGTGAGTTGAGTACACACATCACAAAGAAGTTTCT

                     *       **          *         **
     127123 GAGAATGCTGCTGTCTACCTTTTAT-T--TGA-ATTCCCGC
        131 GAGAATGCTTCTGTCTAGTTTTTATATGAAGATATTCCCTT

                         *  *    *                *       *                  
     127160 TTCCAACGAAATCCTCCAAGCTATCCAAATATCCACTTGCAGATTCCACAAAAAGAGTGTTTCAA
          1 TTCCAACGAAATCATCAAAGCGATCCAAATATCCACTTCCAGATTCTACAAAAAGAGTGTTTCAA

                            ** *  *            *       *     *     *  *      
     127225 AACTGCTCTCTATCAATGGCAAAGTTCAACTCTGTTAGTTGAGGACACATATCACCAACAAGTTT
         66 AACTG--CTCTATCAAAAGAAATGTTCAACTCTGTGAGTTGAGTACACACATCACAAAGAAGTTT

                               *       **         *    
     127290 CTGAGAATGCTTCTGTCTATTTTTTATGGGAAGATATTTCCTT
        129 CTGAGAATGCTTCTGTCTAGTTTTTATATGAAGATATTCCCTT

              *  *  * ** *    *      *    *          * **                    
     127333 TTTCACCGTAGGCGTCAAGGCGATCGAAATGTCCACTTCCACAAACTACAAAAAGAGTGTTTCAA
          1 TTCCAACGAAATCATCAAAGCGATCCAAATATCCACTTCCAGATTCTACAAAAAGAGTGTTTCAA

             *         *   * **       *    *        * ** * *   **      *     
     127398 ACCTGCTCTATGAAAGGCCATGTTCATCTCTATGAGTTGAATGGAAATATCCGAAAGAAATTTCT
         66 AACTGCTCTATCAAAAGAAATGTTCAACTCTGTGAGTTGAGTACACACATCACAAAGAAGTTTCT

             *       *                             **
     127463 GGGAATGCTGCTGTCTAGTTTTTATAT---GA-ATTCCCGC
        131 GAGAATGCTTCTGTCTAGTTTTTATATGAAGATATTCCCTT

                         *       *                *    *  *                  
     127500 TTCCAACGAAATCCTCAAAGCAATCCAAATATCCACTTGCAGAATCCACAAAAAGAGTGTTTCAA
          1 TTCCAACGAAATCATCAAAGCGATCCAAATATCCACTTCCAGATTCTACAAAAAGAGTGTTTCAA

                          *     *          * *                      *        
     127565 AACTGCTCTATCAATAGAAAGGTTCAACTCTTTTAGTTGAGTACACACATCACAAACAAGTTTCT
         66 AACTGCTCTATCAAAAGAAATGTTCAACTCTGTGAGTTGAGTACACACATCACAAAGAAGTTTCT

                            * *      **     **  *    
     127630 GAGAATGCTTCTGTCTGGCTTTTATTGGAAGACGTTTCCTT
        131 GAGAATGCTTCTGTCTAGTTTTTATATGAAGATATTCCCTT

                 * *  **          *      **                * *               
     127671 TT-CACCAAAGGCATCAAAGCGCTCCAAAGGTCCACTTCCAGATTCTTCCAAAAGAGTGTTTCAA
          1 TTCCAACGAAATCATCAAAGCGATCCAAATATCCACTTCCAGATTCTACAAAAAGAGTGTTTCAA

             **         **  * *                 *    * *  * *     *   *      
     127735 ACGTGCTC-ATAGTAAGGGAATGTTCAACTCTGTGACTTGAATGCAGATATCACCAAGTAGTTTC
         66 AACTGCTCTAT-CAAAAGAAATGTTCAACTCTGTGAGTTGAGTACACACATCACAAAGAAGTTTC

             * * *             *     *    *         * 
     127799 TAATAGTGCTTCTGTCTAGATTTTAGATGATGATATTCCCGT
        130 TGAGAATGCTTCTGTCTAGTTTTTATATGAAGATATTCCCTT

                         *** *   *             *  *   *      *     *       * 
     127841 TTCCAACGAAATCGCTAGAGCTATCCAAATATCCAGTTACAGTTTCTACCAAAAGGGTGTTTCCA
          1 TTCCAACGAAATCATCAAAGCGATCCAAATATCCACTTCCAGATTCTACAAAAAGAGTGTTTCAA

              *    **           *            *       *                     * 
     127906 AATTGCTGCATCAAAAGAAAGGTTCAACTCTGTTAGTTGAGGACACACATCACAAAGAAGTTTGT
         66 AACTGCTCTATCAAAAGAAATGTTCAACTCTGTGAGTTGAGTACACACATCACAAAGAAGTTTCT

                              *    *     *           
     127971 GAGAATGCTTCTGTCTAGATTTTGTATGACGATATTCCCTT
        131 GAGAATGCTTCTGTCTAGTTTTTATATGAAGATATTCCCTT

                     *   * *     *   *       * *  *    *  *       *  *       
     128012 TTCCAACGATATCGTTAAAGCAATCTAAATATCAATTTGCAGAATCCACAAAAATAGAGTTTCAA
          1 TTCCAACGAAATCATCAAAGCGATCCAAATATCCACTTCCAGATTCTACAAAAAGAGTGTTTCAA

             *       * *        *    *       *  *                   ***      
     128077 AGCTGCTCTGTAAAAAGAAAGGTTCCACTCTGTTAGCTGAGTACACACATCACAAACTTGTTTCT
         66 AACTGCTCTATCAAAAGAAATGTTCAACTCTGTGAGTTGAGTACACACATCACAAAGAAGTTTCT

                  *         *        **         **   
     128142 GAGAATCCTTCTGTCTCGTTTTTATGGGAAGATATTTACTT
        131 GAGAATGCTTCTGTCTAGTTTTTATATGAAGATATTCCCTT

                 *  * **          *       *     *       *  *  *  *           
     128183 TTCCACCGTAGGCATCAAAGCGCTCCAAATGTCCACATCCAGATACTCCAGAACGAGTGTTTCAA
          1 TTCCAACGAAATCATCAAAGCGATCCAAATATCCACTTCCAGATTCTACAAAAAGAGTGTTTCAA

             *         *   * *   *         *        * *  *      *      *     
     128248 ACCTGCTCTATGAAAGGGAATCTTCAACTCTATGAGTTGAATGCAGACATCAGAAAGAAATTTCT
         66 AACTGCTCTATCAAAAGAAATGTTCAACTCTGTGAGTTGAGTACACACATCACAAAGAAGTTTCT

                     *       **          *         **
     128313 GAGAATGCTGCTGTCTACCTTTTAT-T--TGA-ATTCCCGC
        131 GAGAATGCTTCTGTCTAGTTTTTATATGAAGATATTCCCTT

                         *  *    *              * *  **   *    *             
     128350 TTCCAACGAAATCCTCCAAGCTATCCAAATATCCACCTGCATTTTCCACAACAAGAGTGTTTCAA
          1 TTCCAACGAAATCATCAAAGCGATCCAAATATCCACTTCCAGATTCTACAAAAAGAGTGTTTCAA

                          *                 *    *  *                *       
     128415 AACTGCTCTATCAATAGAAATGTTCAACTCCTTTG-GCTGGGTACACACATCACAAACAAGTTTC
         66 AACTGCTCTATCAAAAGAAATGTTCAACT-CTGTGAGTTGAGTACACACATCACAAAGAAGTTTC

                                      **     **       
     128479 TGAGAATGCTTCTGTCTAGTTTTTATGGGAAGACGTTCCCTT
        130 TGAGAATGCTTCTGTCTAGTTTTTATATGAAGATATTCCCTT

              *  * *  **          *       *            **                  * 
     128521 TTTCACCAAAGGCATCAAAGCGCTCCAAATGTCCACTTCCAGACACTACAAAAAGAGTGTTTCCA
          1 TTCCAACGAAATCATCAAAGCGATCCAAATATCCACTTCCAGATTCTACAAAAAGAGTGTTTCAA

             **   *   **   ***                 *    * *  * *         *       
     128586 ACGTGCCCTAAGAAAGCGAATGTTCAACTCTGTGACTTGAATGCAGATATCACAAAGTAGTTTCT
         66 AACTGCTCTATCAAAAGAAATGTTCAACTCTGTGAGTTGAGTACACACATCACAAAGAAGTTTCT

                **            *     *    *         * 
     128651 GAGAGGGCTTCTGTCTAGATTTTAGATGATGATATTCCCGT
        131 GAGAATGCTTCTGTCTAGTTTTTATATGAAGATATTCCCTT

                           * *   *             *  *   *                    * 
     128692 TTCCAACGAAATCATTAGAGCTATCCAAATATCCAATTACAGTTTCTACAAAAAGAGTGTTTCCA
          1 TTCCAACGAAATCATCAAAGCGATCCAAATATCCACTTCCAGATTCTACAAAAAGAGTGTTTCAA

                   **         * *    *       *  *                   ***      
     128757 AACTGCTGCATCAAAAGAGAGGTTCCACTCTGTTAGCTGAGTACACACATCACAAACTTGTTTCT
         66 AACTGCTCTATCAAAAGAAATGTTCAACTCTGTGAGTTGAGTACACACATCACAAAGAAGTTTCT

            *     *         *        **         **   
     128822 CAGAATCCTTCTGTCTCGTTTTTATGGGAAGATATTTACTT
        131 GAGAATGCTTCTGTCTAGTTTTTATATGAAGATATTCCCTT

              *  *  * **          *       *     *       *  *  *              
     128863 TTTCACCGTAGGCATCAAAGCGCTCCAAATGTCCACATCCAGATACTCCAGAAAGAGTGTTTCAA
          1 TTCCAACGAAATCATCAAAGCGATCCAAATATCCACTTCCAGATTCTACAAAAAGAGTGTTTCAA

             *         *   * *   *         *        * *  *      *      *     
     128928 ACCTGCTCTATGAAAGGGAATCTTCAACTCTATGAGTTGAATGCAGACATCAGAAAGAAATTTCT
         66 AACTGCTCTATCAAAAGAAATGTTCAACTCTGTGAGTTGAGTACACACATCACAAAGAAGTTTCT

                     *       **          *         **
     128993 GAGAATGCTGCTGTCTACCTTTTAT-T--TGA-ATTCCCGC
        131 GAGAATGCTTCTGTCTAGTTTTTATATGAAGATATTCCCTT

                         *  *    *                *       *                  
     129030 TTCCAACGAAATCCTCCAAGCTATCCAAATATCCACTTGCAGATTCCACAAAAAGAGTGTTTCAA
          1 TTCCAACGAAATCATCAAAGCGATCCAAATATCCACTTCCAGATTCTACAAAAAGAGTGTTTCAA

                            ** *  *            *       *     *     *  *      
     129095 AACTGCTCTCTATCAATGGCAAAGTTCAACTCTGTTAGTTGAGGACACATATCACCAACAAGTTT
         66 AACTG--CTCTATCAAAAGAAATGTTCAACTCTGTGAGTTGAGTACACACATCACAAAGAAGTTT

                               *       **         *    
     129160 CTGAGAATGCTTCTGTCTATTTTTTATGGGAAGATATTTCCTT
        129 CTGAGAATGCTTCTGTCTAGTTTTTATATGAAGATATTCCCTT

              *  *  * ** *    *      *    *          * **          *         
     129203 TTTCACCGTAGGCGTCAAGGCGATCGAAATGTCCACTTCCACAAACTACAAAAAGTGTGTTTCAA
          1 TTCCAACGAAATCATCAAAGCGATCCAAATATCCACTTCCAGATTCTACAAAAAGAGTGTTTCAA

             *         *   * **       *    *        * ** * *   **      *     
     129268 ACCTGCTCTATGAAAGGCCATGTTCATCTCTATGAGTTGAATGGAAATATCCGAAAGAAATTTCT
         66 AACTGCTCTATCAAAAGAAATGTTCAACTCTGTGAGTTGAGTACACACATCACAAAGAAGTTTCT

             *       *          *        *         **
     129333 GGGAATGCTGCTGTCTAG-TGTT-TAT-ACGA-ATTCCCGC
        131 GAGAATGCTTCTGTCTAGTTTTTATATGAAGATATTCCCTT

                         *       *                *    *  *                  
     129370 TTCCAACGAAATCCTCAAAGCAATCCAAATATCCACTTGCAGAATCCACAAAAAGAGTGTTTCAA
          1 TTCCAACGAAATCATCAAAGCGATCCAAATATCCACTTCCAGATTCTACAAAAAGAGTGTTTCAA

                          *     *          * *                   *  *        
     129435 AACTGCTCTATCAATAGAAAGGTTCAACTCTTTTAGTTGAGTACACACATCACGAACAAGTTTCT
         66 AACTGCTCTATCAAAAGAAATGTTCAACTCTGTGAGTTGAGTACACACATCACAAAGAAGTTTCT

                            * *      **     **  *    
     129500 GAGAATGCTTCTGTCTGGCTTTTATTGGAAGACGTTTCCTT
        131 GAGAATGCTTCTGTCTAGTTTTTATATGAAGATATTCCCTT

                 * *  **          *       *                * *               
     129541 TT-CACCAAAGGCATCAAAGCGCTCCAAATGTCCACTTCCAGATTCTTCCAAAAGAGTGTTTCAA
          1 TTCCAACGAAATCATCAAAGCGATCCAAATATCCACTTCCAGATTCTACAAAAAGAGTGTTTCAA

             **     * ***  * *                 *    * *  * *     *   *       
     129605 ACGTGCTCAAAGTAAGGGAATGTTCAACTCTGTGACTTGAATGCAGATATCACCAAGTAGTTTCT
         66 AACTGCTCTATCAAAAGAAATGTTCAACTCTGTGAGTTGAGTACACACATCACAAAGAAGTTTCT

            * * *             *     *    *         * 
     129670 AATAGTGCTTCTGTCTAGATTTTAGATGATGATATTCCCGT
        131 GAGAATGCTTCTGTCTAGTTTTTATATGAAGATATTCCCTT

                         * * *   * **          *  *   *      *     *       * 
     129711 TTCCAACGAAATCGTTAGAGCTAAGCAAATATCCAGTTACAGTTTCTACCAAAAGGGTGTTTCCA
          1 TTCCAACGAAATCATCAAAGCGATCCAAATATCCACTTCCAGATTCTACAAAAAGAGTGTTTCAA

              *    **           *            *       *                     * 
     129776 AATTGCTGCATCAAAAGAAAGGTTCAACTCTGTTAGTTGAGGACACACATCACAAAGAAGTTTGT
         66 AACTGCTCTATCAAAAGAAATGTTCAACTCTGTGAGTTGAGTACACACATCACAAAGAAGTTTCT

                              *    *     *           
     129841 GAGAATGCTTCTGTCTAGATTTTGTATGACGATATTCCCTT
        131 GAGAATGCTTCTGTCTAGTTTTTATATGAAGATATTCCCTT

                     *   * *     *   *       * *  *    *  *       *  *       
     129882 TTCCAACGATATCGTTAAAGCAATCTAAATATCAATTTGCAGAATCCACAAAAATAGAGTTTCAA
          1 TTCCAACGAAATCATCAAAGCGATCCAAATATCCACTTCCAGATTCTACAAAAAGAGTGTTTCAA

             *       * *        *    *       *  *                   *** *    
     129947 AGCTGCTCTGTAAAAAGAAAGGTTCCACTCTGTTAGCTGAGTACACACATCACAAACTTGATTCT
         66 AACTGCTCTATCAAAAGAAATGTTCAACTCTGTGAGTTGAGTACACACATCACAAAGAAGTTTCT

                  *         *        **         **   
     130012 GAGAATCCTTCTGTCTCGTTTTTATGGGAAGATATTTACTT
        131 GAGAATGCTTCTGTCTAGTTTTTATATGAAGATATTCCCTT

                 *  * **          *       *     *       *  *  *  *           
     130053 TTCCACCGTAGGCATCAAAGCGCTCCAAATGTCCACATCCAGATACTCCAGAACGAGTGTTTCAA
          1 TTCCAACGAAATCATCAAAGCGATCCAAATATCCACTTCCAGATTCTACAAAAAGAGTGTTTCAA

             *         *   * *   *         *        * *  *      *      *     
     130118 ACCTGCTCTATGAAAGGGAATCTTCAACTCTATGAGTTGAATGCAGACATCAGAAAGAAATTTCT
         66 AACTGCTCTATCAAAAGAAATGTTCAACTCTGTGAGTTGAGTACACACATCACAAAGAAGTTTCT

                     *       **          *         **
     130183 GAGAATGCTGCTGTCTACCTTTTAT-T--TGA-ATTCCCGC
        131 GAGAATGCTTCTGTCTAGTTTTTATATGAAGATATTCCCTT

                         *  *    *              * *  **   *    *             
     130220 TTCCAACGAAATCCTCCAAGCTATCCAAATATCCACCTGCATTTTCCACAACAAGAGTGTTTCAA
          1 TTCCAACGAAATCATCAAAGCGATCCAAATATCCACTTCCAGATTCTACAAAAAGAGTGTTTCAA

                          *                 *    *  *                *       
     130285 AACTGCTCTATCAATAGAAATGTTCAACTCCTTTG-GCTGGGTACACACATCACAAACAAGTTTC
         66 AACTGCTCTATCAAAAGAAATGTTCAACT-CTGTGAGTTGAGTACACACATCACAAAGAAGTTTC

                                      **     **       
     130349 TGAGAATGCTTCTGTCTAGTTTTTATGGGAAGACGTTCCCTT
        130 TGAGAATGCTTCTGTCTAGTTTTTATATGAAGATATTCCCTT

              *  * *  **          *       *            **                    
     130391 TTTCACCAAAGGCATCAAAGCGCTCCAAATGTCCACTTCCAGACACTACAAAAAGAGTGTTTCAA
          1 TTCCAACGAAATCATCAAAGCGATCCAAATATCCACTTCCAGATTCTACAAAAAGAGTGTTTCAA

             **       **   ***                 *    * *  * *         *       
     130456 ACGTGCTCTAAGAAAGCGAATGTTCAACTCTGTGACTTGAATGCAGATATCACAAAGTAGTTTCT
         66 AACTGCTCTATCAAAAGAAATGTTCAACTCTGTGAGTTGAGTACACACATCACAAAGAAGTTTCT

                **            *     *    *         * 
     130521 GAGAGGGCTTCTGTCTAGATTTTAGATGATGATATTCCCGT
        131 GAGAATGCTTCTGTCTAGTTTTTATATGAAGATATTCCCTT

                           * *   *                *   *                    * 
     130562 TTCCAACGAAATCATTAGAGCTATCCAAATATCCACTTACAGTTTCTACAAAAAGAGTGTTTCCA
          1 TTCCAACGAAATCATCAAAGCGATCCAAATATCCACTTCCAGATTCTACAAAAAGAGTGTTTCAA

                   **         * *    *       *  **                  ***      
     130627 AACTGCTGCATCAAAAGAGAGGTTCCACTCTGTTAGCCGAGTACACACATCACAAACTTGTTTCT
         66 AACTGCTCTATCAAAAGAAATGTTCAACTCTGTGAGTTGAGTACACACATCACAAAGAAGTTTCT

            *     *      *  *        **         **   
     130692 CAGAATCCTTCTGACTCGTTTTTATGGGAAGATATTTACTT
        131 GAGAATGCTTCTGTCTAGTTTTTATATGAAGATATTCCCTT

              *  *  * **          *       *     *       *  *  *              
     130733 TTTCACCGTAGGCATCAAAGCGCTCCAAATGTCCACATCCAGATACTCCAGAAAGAGTGTTTCAA
          1 TTCCAACGAAATCATCAAAGCGATCCAAATATCCACTTCCAGATTCTACAAAAAGAGTGTTTCAA

             *         *   * *   *         *        * *  *      *      *     
     130798 ACCTGCTCTATGAAAGGGAATCTTCAACTCTATGAGTTGAATGCAGACATCAGAAAGAAATTTCT
         66 AACTGCTCTATCAAAAGAAATGTTCAACTCTGTGAGTTGAGTACACACATCACAAAGAAGTTTCT

                     *       **          *         **
     130863 GAGAATGCTGCTGTCTACCTTTTAT-T--TGA-ATTCCCGC
        131 GAGAATGCTTCTGTCTAGTTTTTATATGAAGATATTCCCTT

                         *  *    *                *       *             *    
     130900 TTCCAACGAAATCCTCCAAGCTATCCAAATATCCACTTGCAGATTCCACAAAAAGAGTGTATCAA
          1 TTCCAACGAAATCATCAAAGCGATCCAAATATCCACTTCCAGATTCTACAAAAAGAGTGTTTCAA

                            ** *  *            *       *     *     *  *      
     130965 AACTGCTCTCTATCAATGGCAAAGTTCAACTCTGTTAGTTGAGGACACATATCACCAACAAGTTT
         66 AACTG--CTCTATCAAAAGAAATGTTCAACTCTGTGAGTTGAGTACACACATCACAAAGAAGTTT

                               *       **         *    
     131030 CTGAGAATGCTTCTGTCTATTTTTTATGGGAAGATATTTCCTT
        129 CTGAGAATGCTTCTGTCTAGTTTTTATATGAAGATATTCCCTT

              *  *  * ** *    *      *    *          * **                    
     131073 TTTCACCGTAGGCGTCAAGGCGATCGAAATGTCCACTTCCACAAACTACAAAAAGAGTGTTTCAA
          1 TTCCAACGAAATCATCAAAGCGATCCAAATATCCACTTCCAGATTCTACAAAAAGAGTGTTTCAA

             *         *   * **       *    *        * ** * *   **      *     
     131138 ACCTGCTCTATGAAAGGCCATGTTCATCTCTATGAGTTGAATGGAAATATCCGAAAGAAATTTCT
         66 AACTGCTCTATCAAAAGAAATGTTCAACTCTGTGAGTTGAGTACACACATCACAAAGAAGTTTCT

             *       *          *        *         **
     131203 GGGAATGCTGCTGTCTAG-TGTT-TAT-ACGA-ATTCCCGC
        131 GAGAATGCTTCTGTCTAGTTTTTATATGAAGATATTCCCTT

                         *       *                *    *  *                  
     131240 TTCCAACGAAATCCTCAAAGCAATCCAAATATCCACTTGCAGAATCCACAAAAAGAGTGTTTCAA
          1 TTCCAACGAAATCATCAAAGCGATCCAAATATCCACTTCCAGATTCTACAAAAAGAGTGTTTCAA

                          *     *          * *                   *  *        
     131305 AACTGCTCTATCAATAGAAAGGTTCAACTCTTTTAGTTGAGTACACACATCACGAACAAGTTTCT
         66 AACTGCTCTATCAAAAGAAATGTTCAACTCTGTGAGTTGAGTACACACATCACAAAGAAGTTTCT

                            * *      **     **  *    
     131370 GAGAATGCTTCTGTCTGGCTTTTATTGGAAGACGTTTCCTT
        131 GAGAATGCTTCTGTCTAGTTTTTATATGAAGATATTCCCTT

                 * *  **          *       *                * *               
     131411 TT-CACCAAAGGCATCAAAGCGCTCCAAATGTCCACTTCCAGATTCTTCCAAAAGAGTGTTTCAA
          1 TTCCAACGAAATCATCAAAGCGATCCAAATATCCACTTCCAGATTCTACAAAAAGAGTGTTTCAA

             **     * ***  * *                 *    * *  * *     *   *       
     131475 ACGTGCTCAAAGTAAGGGAATGTTCAACTCTGTGACTTGAATGCAGATATCACCAAGTAGTTTCT
         66 AACTGCTCTATCAAAAGAAATGTTCAACTCTGTGAGTTGAGTACACACATCACAAAGAAGTTTCT

            * * *             *     *    *         * 
     131540 AATAGTGCTTCTGTCTAGATTTTAGATGATGATATTCCCGT
        131 GAGAATGCTTCTGTCTAGTTTTTATATGAAGATATTCCCTT

                         *** *   *             *  *   *      *     *       * 
     131581 TTCCAACGAAATCGCTAGAGCTATCCAAATATCCAGTTACAGTTTCTACCAAAAGGGTGTTTCCA
          1 TTCCAACGAAATCATCAAAGCGATCCAAATATCCACTTCCAGATTCTACAAAAAGAGTGTTTCAA

              *    **           *            *       *                     * 
     131646 AATTGCTGCATCAAAAGAAAGGTTCAACTCTGTTAGTTGAGGACACACATCACAAAGAAGTTTGT
         66 AACTGCTCTATCAAAAGAAATGTTCAACTCTGTGAGTTGAGTACACACATCACAAAGAAGTTTCT

                              *    *     *           
     131711 GAGAATGCTTCTGTCTAGATTTTGTATGACGATATTCCCTT
        131 GAGAATGCTTCTGTCTAGTTTTTATATGAAGATATTCCCTT

                     *   * *     *   *       * *  *    *  *       *  *       
     131752 TTCCAACGATATCGTTAAAGCAATCTAAATATCAATTTGCAGAATCCACAAAAATAGAGTTTCAA
          1 TTCCAACGAAATCATCAAAGCGATCCAAATATCCACTTCCAGATTCTACAAAAAGAGTGTTTCAA

             *       * *        *    *       *  *                   ***      
     131817 AGCTGCTCTGTAAAAAGAAAGGTTCCACTCTGTTAGCTGAGTACACACATCACAAACTTGTTTCT
         66 AACTGCTCTATCAAAAGAAATGTTCAACTCTGTGAGTTGAGTACACACATCACAAAGAAGTTTCT

                 **         *        **         **   
     131882 GAGAAACCTTCTGTCTCGTTTTTATGGGAAGATATTTACTT
        131 GAGAATGCTTCTGTCTAGTTTTTATATGAAGATATTCCCTT

                 ** * **          *       *     *       *  *  *  *           
     131923 TTCCACTGTAGGCATCAAAGCGCTCCAAATGTCCACATCCAGATACTCCAGAACGAGTGTTTCAA
          1 TTCCAACGAAATCATCAAAGCGATCCAAATATCCACTTCCAGATTCTACAAAAAGAGTGTTTCAA

             *         *   * *   *         *        * *  *      *      *     
     131988 ACCTGCTCTATGAAAGGGAATCTTCAACTCTATGAGTTGAATGCAGA-ATCAGAAAGAAATTTCT
         66 AACTGCTCTATCAAAAGAAATGTTCAACTCTGTGAGTTGAGTACACACATCACAAAGAAGTTTCT

                     *       **          *         **
     132052 GAGAATGCTGCTGTCTACCTTTTAT-T--TGA-ATTCCCGC
        131 GAGAATGCTTCTGTCTAGTTTTTATATGAAGATATTCCCTT

                         *  *    *              * *  **   *                  
     132089 TTCCAACGAAATCCTCCAAGCTATCCAAATATCCACCTGCATTTTCCACAAAAAGAGTGTTTCAA
          1 TTCCAACGAAATCATCAAAGCGATCCAAATATCCACTTCCAGATTCTACAAAAAGAGTGTTTCAA

                          *                 *    *  *                *       
     132154 AACTGCTCTATCAATAGAAATGTTCAACTCCTTTG-GCTGGGTACACACATCACAAACAAGTTTC
         66 AACTGCTCTATCAAAAGAAATGTTCAACT-CTGTGAGTTGAGTACACACATCACAAAGAAGTTTC

                                      **     **       
     132218 TGAGAATGCTTCTGTCTAGTTTTTATGGGAAGACGTTCCCTT
        130 TGAGAATGCTTCTGTCTAGTTTTTATATGAAGATATTCCCTT

              *  * *  **          *       *            **                    
     132260 TTTCACCAAAGGCATCAAAGCGCTCCAAATGTCCACTTCCAGACACTACAAAAAGAGTGTTTCAA
          1 TTCCAACGAAATCATCAAAGCGATCCAAATATCCACTTCCAGATTCTACAAAAAGAGTGTTTCAA

             **       **   ***                 *    * *  * *         *       
     132325 ACGTGCTCTAAGAAAGCGAATGTTCAACTCTGTGACTTGAATGCAGATATCACAAAGTAGTTTCT
         66 AACTGCTCTATCAAAAGAAATGTTCAACTCTGTGAGTTGAGTACACACATCACAAAGAAGTTTCT

                **            *     *    *         * 
     132390 GAGAGGGCTTCTGTCTAGATTTTAGATGATGATATTCCCGT
        131 GAGAATGCTTCTGTCTAGTTTTTATATGAAGATATTCCCTT

                           * *   *                *   *                    * 
     132431 TTCCAACGAAATCATTAGAGCTATCCAAATATCCACTTACAGTTTCTACAAAAAGAGTGTTTCCA
          1 TTCCAACGAAATCATCAAAGCGATCCAAATATCCACTTCCAGATTCTACAAAAAGAGTGTTTCAA

                   **         * *    *       *  **                  ***      
     132496 AACTGCTGCATCAAAAGAGAGGTTCCACTCTGTTAGCCGAGTACACACATCACAAACTTGTTTCT
         66 AACTGCTCTATCAAAAGAAATGTTCAACTCTGTGAGTTGAGTACACACATCACAAAGAAGTTTCT

            *     *         *        **         **   
     132561 CAGAATCCTTCTGTCTCGTTTTTATGGGAAGATATTTACTT
        131 GAGAATGCTTCTGTCTAGTTTTTATATGAAGATATTCCCTT

              *  *  * **          *       *     *       *  *  *              
     132602 TTTCACCGTAGGCATCAAAGCGCTCCAAATGTCCACATCCAGATACTCCAGAAAGAGTGTTTCAA
          1 TTCCAACGAAATCATCAAAGCGATCCAAATATCCACTTCCAGATTCTACAAAAAGAGTGTTTCAA

             *         *   * *   *         *        * *  *      *      *     
     132667 ACCTGCTCTATGAAAGGGAATCTTCAACTCTATGAGTTGAATGCAGACATCAGAAAGAAATTTCT
         66 AACTGCTCTATCAAAAGAAATGTTCAACTCTGTGAGTTGAGTACACACATCACAAAGAAGTTTCT

                     *       **          *         **
     132732 GAGAATGCTGCTGTCTACCTTTTAT-T--TGA-ATTCCCGC
        131 GAGAATGCTTCTGTCTAGTTTTTATATGAAGATATTCCCTT

                         *  *    *                *       *                  
     132769 TTCCAACGAAATCCTCCAAGCTATCCAAATATCCACTTGCAGATTCCACAAAAAGAGTGTTTCAA
          1 TTCCAACGAAATCATCAAAGCGATCCAAATATCCACTTCCAGATTCTACAAAAAGAGTGTTTCAA

                            ** *  *            *       *     *     *  *      
     132834 AACTGCTCTCTATCAATGGCAAAGTTCAACTCTGTTAGTTGAGGACACATATCACCAACAAGTTT
         66 AACTG--CTCTATCAAAAGAAATGTTCAACTCTGTGAGTTGAGTACACACATCACAAAGAAGTTT

                               *       **         *    
     132899 CTGAGAATGCTTCTGTCTATTTTTTATGGGAAGATATTTCCTT
        129 CTGAGAATGCTTCTGTCTAGTTTTTATATGAAGATATTCCCTT

              *  *  * ** *    *      *    *          * **                    
     132942 TTTCACCGTAGGCGTCAAGGCGATCGAAATGTCCACTTCCACAAACTACAAAAAGAGTGTTTCAA
          1 TTCCAACGAAATCATCAAAGCGATCCAAATATCCACTTCCAGATTCTACAAAAAGAGTGTTTCAA

             *         *   * **       *    *        * ** * *   **      *     
     133007 ACCTGCTCTATGAAAGGCCATGTTCATCTCTATGAGTTGAATGGAAATATCCGAAAGAAATTTCT
         66 AACTGCTCTATCAAAAGAAATGTTCAACTCTGTGAGTTGAGTACACACATCACAAAGAAGTTTCT

             *       *          *        *         **
     133072 GGGAATGCTGCTGTCTAG-TGTT-TAT-ACGA-ATTCCCGC
        131 GAGAATGCTTCTGTCTAGTTTTTATATGAAGATATTCCCTT

                         *       *                *    *  *                  
     133109 TTCCAACGAAATCCTCAAAGCAATCCAAATATCCACTTGCAGAATCCACAAAAAGAGTGTTTCAA
          1 TTCCAACGAAATCATCAAAGCGATCCAAATATCCACTTCCAGATTCTACAAAAAGAGTGTTTCAA

                          *     *          * *                      *        
     133174 AACTGCTCTATCAATAGAAAGGTTCAACTCTTTTAGTTGAGTACACACATCACAAACAAGTTTCT
         66 AACTGCTCTATCAAAAGAAATGTTCAACTCTGTGAGTTGAGTACACACATCACAAAGAAGTTTCT

                            * *      **     **  *    
     133239 GAGAATGCTTCTGTCTGGCTTTTATTGGAAGACGTTTCCTT
        131 GAGAATGCTTCTGTCTAGTTTTTATATGAAGATATTCCCTT

                 * *  **          *       *                * *               
     133280 TT-CACCAAAGGCATCAAAGCGCTCCAAATGTCCACTTCCAGATTCTTCCAAAAGAGTGTTTCAA
          1 TTCCAACGAAATCATCAAAGCGATCCAAATATCCACTTCCAGATTCTACAAAAAGAGTGTTTCAA

             **  *  * ***  * *                 *    * *  * *     *   *       
     133344 ACGTGGTCGAAGTAAGGGAATGTTCAACTCTGTGACTTGAATGCAGATATCACCAAGTAGTTTCT
         66 AACTGCTCTATCAAAAGAAATGTTCAACTCTGTGAGTTGAGTACACACATCACAAAGAAGTTTCT

            * * *            **     *    *         * 
     133409 AATAGTGCTTCTGTCTACATTTTAGATGATGATATTCCCGT
        131 GAGAATGCTTCTGTCTAGTTTTTATATGAAGATATTCCCTT

                         * * *   *             *  *   *      *     *       * 
     133450 TTCCAACGAAATCGTTAGAGCTATCCAAATATCCAGTTACAGTTTCTACCAAAAGGGTGTTTCCA
          1 TTCCAACGAAATCATCAAAGCGATCCAAATATCCACTTCCAGATTCTACAAAAAGAGTGTTTCAA

              *    **           *            *       *                     * 
     133515 AATTGCTGCATCAAAAGAAAGGTTCAACTCTGTTAGTTGAGGACACACATCACAAAGAAGTTTGT
         66 AACTGCTCTATCAAAAGAAATGTTCAACTCTGTGAGTTGAGTACACACATCACAAAGAAGTTTCT

                              *    *     *           
     133580 GAGAATGCTTCTGTCTAGATTTTGTATGACGATATTCCCTT
        131 GAGAATGCTTCTGTCTAGTTTTTATATGAAGATATTCCCTT

                     *   * *     *   *       * *  *    *  *       *  *       
     133621 TTCCAACGATATCGTTAAAGCAATCTAAATATCAATTTGCAGAATCCACAAAAATAGAGTTTCAA
          1 TTCCAACGAAATCATCAAAGCGATCCAAATATCCACTTCCAGATTCTACAAAAAGAGTGTTTCAA

             *       * *        *    *       *  *                   ***      
     133686 AGCTGCTCTGTAAAAAGAAAGGTTCCACTCTGTTAGCTGAGTACACACATCACAAACTTGTTTCT
         66 AACTGCTCTATCAAAAGAAATGTTCAACTCTGTGAGTTGAGTACACACATCACAAAGAAGTTTCT

                 **         *        **         **   
     133751 GAGAAACCTTCTGTCTCGTTTTTATGGGAAGATATTTACTT
        131 GAGAATGCTTCTGTCTAGTTTTTATATGAAGATATTCCCTT

                 ** * **          *       *     *       *  *  *  *           
     133792 TTCCACTGTAGGCATCAAAGCGCTCCAAATGTCCACATCCAGATACTCCAGAACGAGTGTTTCAA
          1 TTCCAACGAAATCATCAAAGCGATCCAAATATCCACTTCCAGATTCTACAAAAAGAGTGTTTCAA

             *         *   * *   *         *        * *  *      *      *     
     133857 ACCTGCTCTATGAAAGGGAATCTTCAACTCTATGAGTTGAATGCAGA-ATCAGAAAGAAATTTCT
         66 AACTGCTCTATCAAAAGAAATGTTCAACTCTGTGAGTTGAGTACACACATCACAAAGAAGTTTCT

                     *       **          *         **
     133921 GAGAATGCTGCTGTCTACCTTTTAT-T--TGA-ATTCCCGC
        131 GAGAATGCTTCTGTCTAGTTTTTATATGAAGATATTCCCTT

                         *  *    *              * *  **   *                  
     133958 TTCCAACGAAATCCTCCAAGCTATCCAAATATCCACCTGCATTTTCCACAAAAAGAGTGTTTCAA
          1 TTCCAACGAAATCATCAAAGCGATCCAAATATCCACTTCCAGATTCTACAAAAAGAGTGTTTCAA

                          *                 *    *  *                *       
     134023 AACTGCTCTATCAATAGAAATGTTCAACTCCTTTG-GCTGGGTACACACATCACAAACAAGTTTC
         66 AACTGCTCTATCAAAAGAAATGTTCAACT-CTGTGAGTTGAGTACACACATCACAAAGAAGTTTC

                                      **     **       
     134087 TGAGAATGCTTCTGTCTAGTTTTTATGGGAAGACGTTCCCTT
        130 TGAGAATGCTTCTGTCTAGTTTTTATATGAAGATATTCCCTT

              *  * *  **          *       *            **                    
     134129 TTTCACCAAAGGCATCAAAGCGCTCCAAATGTCCACTTCCAGACACTACAAAAAGAGTGTTTCAA
          1 TTCCAACGAAATCATCAAAGCGATCCAAATATCCACTTCCAGATTCTACAAAAAGAGTGTTTCAA

             **       **   ***                 *    * *  * *         *       
     134194 ACGTGCTCTAAGAAAGCGAATGTTCAACTCTGTGACTTGAATGCAGATATCACAAAGTAGTTTCT
         66 AACTGCTCTATCAAAAGAAATGTTCAACTCTGTGAGTTGAGTACACACATCACAAAGAAGTTTCT

                **            *     *    *         * 
     134259 GAGAGGGCTTCTGTCTAGATTTTAGATGATGATATTCCCGT
        131 GAGAATGCTTCTGTCTAGTTTTTATATGAAGATATTCCCTT

                           * *   *                *   *                    * 
     134300 TTCCAACGAAATCATTAGAGCTATCCAAATATCCACTTACAGTTTCTACAAAAAGAGTGTTTCCA
          1 TTCCAACGAAATCATCAAAGCGATCCAAATATCCACTTCCAGATTCTACAAAAAGAGTGTTTCAA

                   **         * *    *       *  **                  ***      
     134365 AACTGCTGCATCAAAAGAGAGGTTCCACTCTGTTAGCCGAGTACACACATCACAAACTTGTTTCT
         66 AACTGCTCTATCAAAAGAAATGTTCAACTCTGTGAGTTGAGTACACACATCACAAAGAAGTTTCT

            *     *         *        **         **   
     134430 CAGAATCCTTCTGTCTCGTTTTTATGGGAAGATATTTACTT
        131 GAGAATGCTTCTGTCTAGTTTTTATATGAAGATATTCCCTT

              *  *  * **          *       *     *       *  *  *              
     134471 TTTCACCGTAGGCATCAAAGCGCTCCAAATGTCCACATCCAGATACTCCAGAAAGAGTGTTTCAA
          1 TTCCAACGAAATCATCAAAGCGATCCAAATATCCACTTCCAGATTCTACAAAAAGAGTGTTTCAA

             *         *   * *   *         *        * *  *      *      *     
     134536 ACCTGCTCTATGAAAGGGAATCTTCAACTCTATGAGTTGAATGCAGACATCAGAAAGAAATTTCT
         66 AACTGCTCTATCAAAAGAAATGTTCAACTCTGTGAGTTGAGTACACACATCACAAAGAAGTTTCT

                     *       **          *         **
     134601 GAGAATGCTGCTGTCTACCTTTTAT-T--TGA-ATTCCCGC
        131 GAGAATGCTTCTGTCTAGTTTTTATATGAAGATATTCCCTT

                         *  *    *                *       *                  
     134638 TTCCAACGAAATCCTCCAAGCTATCCAAATATCCACTTGCAGATTCCACAAAAAGAGTGTTTCAA
          1 TTCCAACGAAATCATCAAAGCGATCCAAATATCCACTTCCAGATTCTACAAAAAGAGTGTTTCAA

                            ** *  *            *       *     *     *  *      
     134703 AACTGCTCTCTATCAATGGCAAAGTTCAACTCTGTTAGTTGAGGACACATATCACCAACAAGTTT
         66 AACTG--CTCTATCAAAAGAAATGTTCAACTCTGTGAGTTGAGTACACACATCACAAAGAAGTTT

                               *       **         *    
     134768 CTGAGAATGCTTCTGTCTATTTTTTATGGGAAGATATTTCCTT
        129 CTGAGAATGCTTCTGTCTAGTTTTTATATGAAGATATTCCCTT

              *  *  * ** *    *      *    *          * **                    
     134811 TTTCACCGTAGGCGTCAAGGCGATCGAAATGTCCACTTCCACAAACTACAAAAAGAGTGTTTCAA
          1 TTCCAACGAAATCATCAAAGCGATCCAAATATCCACTTCCAGATTCTACAAAAAGAGTGTTTCAA

             *         *   * **       *    *        * ** * *   **      *     
     134876 ACCTGCTCTATGAAAGGCCATGTTCATCTCTATGAGTTGAATGGAAATATCCGAAAGAAATTTCT
         66 AACTGCTCTATCAAAAGAAATGTTCAACTCTGTGAGTTGAGTACACACATCACAAAGAAGTTTCT

             *       *                            ***
     134941 GGGAATGCTGCTGTCTAGTTTTTATAT---GA-ATTCCGGC
        131 GAGAATGCTTCTGTCTAGTTTTTATATGAAGATATTCCCTT

                         *       *                *    *  *                  
     134978 TTCCAACGAAATCCTCAAAGCAATCCAAATATCCACTTGCAGAATCCACAAAAAGAGTGTTTCAA
          1 TTCCAACGAAATCATCAAAGCGATCCAAATATCCACTTCCAGATTCTACAAAAAGAGTGTTTCAA

                          *     *          * *                      *        
     135043 AACTGCTCTATCAATAGAAAGGTTCAACTCTTTTAGTTGAGTACACACATCACAAACAAGTTTCT
         66 AACTGCTCTATCAAAAGAAATGTTCAACTCTGTGAGTTGAGTACACACATCACAAAGAAGTTTCT

                            * *      **     **  *    
     135108 GAGAATGCTTCTGTCTGGCTTTTATTGGAAGACGTTTCCTT
        131 GAGAATGCTTCTGTCTAGTTTTTATATGAAGATATTCCCTT

                 * *  **          *       *                * *               
     135149 TT-CACCAAAGGCATCAAAGCGCTCCAAATGTCCACTTCCAGATTCTTCCAAAAGAGTGTTTCAA
          1 TTCCAACGAAATCATCAAAGCGATCCAAATATCCACTTCCAGATTCTACAAAAAGAGTGTTTCAA

             **     * ***  * *       *         *    * *  * *     *   *       
     135213 ACGTGCTCGAAGTAAGGGAATGTTCTACTCTGTGACTTGAATGCAGATATCACCAAGTAGTTTCT
         66 AACTGCTCTATCAAAAGAAATGTTCAACTCTGTGAGTTGAGTACACACATCACAAAGAAGTTTCT

            * * *             *     *    *         * 
     135278 AATAGTGCTTCTGTCTAGATTTTAGATGATGATATTCCCGT
        131 GAGAATGCTTCTGTCTAGTTTTTATATGAAGATATTCCCTT

                         *** *   *             *  *   *      *     *       * 
     135319 TTCCAACGAAATCGCTAGAGCTATCCAAATATCCAGTTACAGTTTCTACCAAAAGGGTGTTTCCA
          1 TTCCAACGAAATCATCAAAGCGATCCAAATATCCACTTCCAGATTCTACAAAAAGAGTGTTTCAA

              *    **           *            *       *                     * 
     135384 AATTGCTGCATCAAAAGAAAGGTTCAACTCTGTTAGTTGAGGACACACATCACAAAGAAGTTTGT
         66 AACTGCTCTATCAAAAGAAATGTTCAACTCTGTGAGTTGAGTACACACATCACAAAGAAGTTTCT

                              *    *     *           
     135449 GAGAATGCTTCTGTCTAGATTTTGTATGACGATATTCCCTT
        131 GAGAATGCTTCTGTCTAGTTTTTATATGAAGATATTCCCTT

                     *   * *     *   *       * *  *    *  *       *  *       
     135490 TTCCAACGATATCGTTAAAGCAATCTAAATATCAATTTGCAGAATCCACAAAAATAGAGTTTCAA
          1 TTCCAACGAAATCATCAAAGCGATCCAAATATCCACTTCCAGATTCTACAAAAAGAGTGTTTCAA

             *       * *        *    *       *  *                   ***      
     135555 AGCTGCTCTGTAAAAAGAAAGGTTCCACTCTGTTAGCTGAGTACACACATCACAAACTTGTTTCT
         66 AACTGCTCTATCAAAAGAAATGTTCAACTCTGTGAGTTGAGTACACACATCACAAAGAAGTTTCT

            *     *         *        **       * **   
     135620 CAGAATCCTTCTGTCTCGTTTTTATGGGAAGATACTTACTT
        131 GAGAATGCTTCTGTCTAGTTTTTATATGAAGATATTCCCTT

                 *  * **          *       *     *       *  *  *  *           
     135661 TTCCACCGTAGGCATCAAAGCGCTCCAAATGTCCACATCCAGATACTCCAGAACGAGTGTTTCAA
          1 TTCCAACGAAATCATCAAAGCGATCCAAATATCCACTTCCAGATTCTACAAAAAGAGTGTTTCAA

             *         *   * *   *         *        * *  *      *      *     
     135726 ACCTGCTCTATGAAAGGGAATCTTCAACTCTATGAGTTGAATGCAGACATCAGAAAGAAATTTCT
         66 AACTGCTCTATCAAAAGAAATGTTCAACTCTGTGAGTTGAGTACACACATCACAAAGAAGTTTCT

                     *       **          *         **
     135791 GAGAATGCTGCTGTCTACCTTTTAT-T--TGA-ATTCCCGC
        131 GAGAATGCTTCTGTCTAGTTTTTATATGAAGATATTCCCTT

                         *  *    *              * *  **   *    *             
     135828 TTCCAACGAAATCCTCCAAGCTATCCAAATATCCACCTGCATTTTCCACAACAAGAGTGTTTCAA
          1 TTCCAACGAAATCATCAAAGCGATCCAAATATCCACTTCCAGATTCTACAAAAAGAGTGTTTCAA

                          *                 *    *  *                *       
     135893 AACTGCTCTATCAATAGAAATGTTCAACTCCTTTG-GCTGGGTACACACATCACAAACAAGTTTC
         66 AACTGCTCTATCAAAAGAAATGTTCAACT-CTGTGAGTTGAGTACACACATCACAAAGAAGTTTC

                                      **     **       
     135957 TGAGAATGCTTCTGTCTAGTTTTTATGGGAAGACGTTCCCTT
        130 TGAGAATGCTTCTGTCTAGTTTTTATATGAAGATATTCCCTT

              *  * *  **          *       *            **                  * 
     135999 TTTCACCAAAGGCATCAAAGCGCTCCAAATGTCCACTTCCAGACACTACAAAAAGAGTGTTTCCA
          1 TTCCAACGAAATCATCAAAGCGATCCAAATATCCACTTCCAGATTCTACAAAAAGAGTGTTTCAA

             **       **   ***                 *    * *  * *         *       
     136064 ACGTGCTCTAAGAAAGCGAATGTTCAACTCTGTGACTTGAATGCAGATATCACAAAGTAGTTTCT
         66 AACTGCTCTATCAAAAGAAATGTTCAACTCTGTGAGTTGAGTACACACATCACAAAGAAGTTTCT

                **            *     *    *         * 
     136129 GAGAGGGCTTCTGTCTAGATTTTAGATGATGATATTCCCGT
        131 GAGAATGCTTCTGTCTAGTTTTTATATGAAGATATTCCCTT

                   *       * *    *               *   *                    * 
     136170 TTCCAACAAAATCATTAGAGC-TTCCAAATATCCACTTACAGTTTCTACAAAAAGAGTGTTTCCA
          1 TTCCAACGAAATCATCAAAGCGATCCAAATATCCACTTCCAGATTCTACAAAAAGAGTGTTTCAA

                   **         * *    *       *  **                  ***      
     136234 AACTGCTGCATCAAAAGAGAGGTTCCACTCTGTTAGCCGAGTACACACATCACAAACTTGTTTCT
         66 AACTGCTCTATCAAAAGAAATGTTCAACTCTGTGAGTTGAGTACACACATCACAAAGAAGTTTCT

            *     *         *        **         **   
     136299 CAGAATCCTTCTGTCTCGTTTTTATGGGAAGATATTTACTT
        131 GAGAATGCTTCTGTCTAGTTTTTATATGAAGATATTCCCTT

              *  *  * **          *       *     *       *  *  *              
     136340 TTTCACCGTAGGCATCAAAGCGCTCCAAATGTCCACATCCAGATACTCCAGAAAGAGTGTTTCAA
          1 TTCCAACGAAATCATCAAAGCGATCCAAATATCCACTTCCAGATTCTACAAAAAGAGTGTTTCAA

             *         *   * *   *         *        * *  *      *      *     
     136405 ACCTGCTCTATGAAAGGGAATCTTCAACTCTATGAGTTGAATGCAGACATCAGAAAGAAATTTCT
         66 AACTGCTCTATCAAAAGAAATGTTCAACTCTGTGAGTTGAGTACACACATCACAAAGAAGTTTCT

                     *       **          *         **
     136470 GAGAATGCTGCTGTCTACCTTTTAT-T--TGA-ATTCCCGC
        131 GAGAATGCTTCTGTCTAGTTTTTATATGAAGATATTCCCTT

                         *  *    *                *       *                  
     136507 TTCCAACGAAATCCTCCAAGCTATCCAAATATCCACTTGCAGATTCCACAAAAAGAGTGTTTCAA
          1 TTCCAACGAAATCATCAAAGCGATCCAAATATCCACTTCCAGATTCTACAAAAAGAGTGTTTCAA

                            ** *  *            *       *     *     *  *      
     136572 AACTGCTCTCTATCAATGGCAAAGTTCAACTCTGTTAGTTGAGGACACATATCACCAACAAGTTT
         66 AACTG--CTCTATCAAAAGAAATGTTCAACTCTGTGAGTTGAGTACACACATCACAAAGAAGTTT

                               *       **         *    
     136637 CTGAGAATGCTTCTGTCTATTTTTTATGGGAAGATATTTCCTT
        129 CTGAGAATGCTTCTGTCTAGTTTTTATATGAAGATATTCCCTT

              *  *  * ** *    *      *    *          * **                    
     136680 TTTCACCGTAGGCGTCAAGGCGATCGAAATGTCCACTTCCACAAACTACAAAAAGAGTGTTTCAA
          1 TTCCAACGAAATCATCAAAGCGATCCAAATATCCACTTCCAGATTCTACAAAAAGAGTGTTTCAA

             *         *   * **       *    *        * ** * *   **      *     
     136745 ACCTGCTCTATGAAAGGCCATGTTCATCTCTATGAGTTGAATGGAAATATCCGAAAGAAATTTCT
         66 AACTGCTCTATCAAAAGAAATGTTCAACTCTGTGAGTTGAGTACACACATCACAAAGAAGTTTCT

             *       *                             **
     136810 GGGAATGCTGCTGTCTAGTTTTTATAT---GA-ATTCCCGC
        131 GAGAATGCTTCTGTCTAGTTTTTATATGAAGATATTCCCTT

                         *       *                *    *  *                  
     136847 TTCCAACGAAATCCTCAAAGCAATCCAAATATCCACTTGCAGAATCCACAAAAAGAGTGTTTCAA
          1 TTCCAACGAAATCATCAAAGCGATCCAAATATCCACTTCCAGATTCTACAAAAAGAGTGTTTCAA

                          *     *          * *                      *        
     136912 AACTGCTCTATCAATAGAAAGGTTCAACTCTTTTAGTTGAGTACACACATCACAAACAAGTTTCT
         66 AACTGCTCTATCAAAAGAAATGTTCAACTCTGTGAGTTGAGTACACACATCACAAAGAAGTTTCT

                            * *      **     **  *    
     136977 GAGAATGCTTCTGTCTGGCTTTTATTGGAAGACGTTTCCTT
        131 GAGAATGCTTCTGTCTAGTTTTTATATGAAGATATTCCCTT

                 * *  **          *      **                * *               
     137018 TT-CACCAAAGGCATCAAAGCGCTCCAAAGGTCCACTTCCAGATTCTTCCAAAAGAGTGTTTCAA
          1 TTCCAACGAAATCATCAAAGCGATCCAAATATCCACTTCCAGATTCTACAAAAAGAGTGTTTCAA

             **         **  * *                 *    * *  * *     *   *      
     137082 ACGTGCTC-ATAGTAAGGGAATGTTCAACTCTGTGACTTGAATGCAGATATCACCAAGTAGTTTC
         66 AACTGCTCTAT-CAAAAGAAATGTTCAACTCTGTGAGTTGAGTACACACATCACAAAGAAGTTTC

             * * *             *     *    *         * 
     137146 TAATAGTGCTTCTGTCTAGATTTTAGATGATGATATTCCCGT
        130 TGAGAATGCTTCTGTCTAGTTTTTATATGAAGATATTCCCTT

                         *** *   *             *  *   *      *     *       * 
     137188 TTCCAACGAAATCGCTAGAGCTATCCAAATATCCAGTTACAGTTTCTACCAAAAGGGTGTTTCCA
          1 TTCCAACGAAATCATCAAAGCGATCCAAATATCCACTTCCAGATTCTACAAAAAGAGTGTTTCAA

              *    **           *            *       *                     * 
     137253 AATTGCTGCATCAAAAGAAAGGTTCAACTCTGTTAGTTGAGGACACACATCACAAAGAAGTTTGT
         66 AACTGCTCTATCAAAAGAAATGTTCAACTCTGTGAGTTGAGTACACACATCACAAAGAAGTTTCT

                              *    *     *           
     137318 GAGAATGCTTCTGTCTAGATTTTGTATGACGATATTCCCTT
        131 GAGAATGCTTCTGTCTAGTTTTTATATGAAGATATTCCCTT

                     *   * *     *   *       * *  *    *  *       *  *       
     137359 TTCCAACGATATCGTTAAAGCAATCTAAATATCAATTTGCAGAATCCACAAAAATAGAGTTTCAA
          1 TTCCAACGAAATCATCAAAGCGATCCAAATATCCACTTCCAGATTCTACAAAAAGAGTGTTTCAA

             *       * *        *    *       *  *                   ***      
     137424 AGCTGCTCTGTAAAAAGAAAGGTTCCACTCTGTTAGCTGAGTACACACATCACAAACTTGTTTCT
         66 AACTGCTCTATCAAAAGAAATGTTCAACTCTGTGAGTTGAGTACACACATCACAAAGAAGTTTCT

                  *         *        **         **   
     137489 GAGAATCCTTCTGTCTCGTTTTTATGGGAAGATATTTACTT
        131 GAGAATGCTTCTGTCTAGTTTTTATATGAAGATATTCCCTT

                 *  * **          *       *     *       *  *  *  *           
     137530 TTCCACCGTAGGCATCAAAGCGCTCCAAATGTCCACATCCAGATACTCCAGAACGAGTGTTTCAA
          1 TTCCAACGAAATCATCAAAGCGATCCAAATATCCACTTCCAGATTCTACAAAAAGAGTGTTTCAA

             *         *   * *   *         *        * *  *      *      *     
     137595 ACCTGCTCTATGAAAGGGAATCTTCAACTCTATGAGTTGAATGCAGACATCAGAAAGAAATTTCT
         66 AACTGCTCTATCAAAAGAAATGTTCAACTCTGTGAGTTGAGTACACACATCACAAAGAAGTTTCT

                     *       **          *         **
     137660 GAGAATGCTGCTGTCTACCTTTTAT-T--TGA-ATTCCCGC
        131 GAGAATGCTTCTGTCTAGTTTTTATATGAAGATATTCCCTT

                         *  *    *              * *  **   *    *             
     137697 TTCCAACGAAATCCTCCAAGCTATCCAAATATCCACCTGCATTTTCCACAACAAGAGTGTTTCAA
          1 TTCCAACGAAATCATCAAAGCGATCCAAATATCCACTTCCAGATTCTACAAAAAGAGTGTTTCAA

                          *                 *    *  *                *       
     137762 AACTGCTCTATCAATAGAAATGTTCAACTCCTTTG-GCTGGGTACACACATCACAAACAAGTTTC
         66 AACTGCTCTATCAAAAGAAATGTTCAACT-CTGTGAGTTGAGTACACACATCACAAAGAAGTTTC

                                      **     **       
     137826 TGAGAATGCTTCTGTCTAGTTTTTATGGGAAGACGTTCCCTT
        130 TGAGAATGCTTCTGTCTAGTTTTTATATGAAGATATTCCCTT

              *  * *  **          *       *            **                  * 
     137868 TTTCACCAAAGGCATCAAAGCGCTCCAAATGTCCACTTCCAGACACTACAAAAAGAGTGTTTCCA
          1 TTCCAACGAAATCATCAAAGCGATCCAAATATCCACTTCCAGATTCTACAAAAAGAGTGTTTCAA

             **   *   **   ***                 *    * *  * *         *       
     137933 ACGTGCCCTAAGAAAGCGAATGTTCAACTCTGTGACTTGAATGCAGATATCACAAAGTAGTTTCT
         66 AACTGCTCTATCAAAAGAAATGTTCAACTCTGTGAGTTGAGTACACACATCACAAAGAAGTTTCT

                **            *     *    *         * 
     137998 GAGAGGGCTTCTGTCTAGATTTTAGATGATGATATTCCCGT
        131 GAGAATGCTTCTGTCTAGTTTTTATATGAAGATATTCCCTT

                           * *   *             *  *   *                    * 
     138039 TTCCAACGAAATCATTAGAGCTATCCAAATATCCAATTACAGTTTCTACAAAAAGAGTGTTTCCA
          1 TTCCAACGAAATCATCAAAGCGATCCAAATATCCACTTCCAGATTCTACAAAAAGAGTGTTTCAA

                   **         * *    *       *  *                   ***      
     138104 AACTGCTGCATCAAAAGAGAGGTTCCACTCTGTTAGCTGAGTACACACATCACAAACTTGTTTCT
         66 AACTGCTCTATCAAAAGAAATGTTCAACTCTGTGAGTTGAGTACACACATCACAAAGAAGTTTCT

            *     *         *        **         **   
     138169 CAGAATCCTTCTGTCTCGTTTTTATGGGAAGATATTTACTT
        131 GAGAATGCTTCTGTCTAGTTTTTATATGAAGATATTCCCTT

              *  *  * **          *       *     *       *  *  *              
     138210 TTTCACCGTAGGCATCAAAGCGCTCCAAATGTCCACATCCAGATACTCCAGAAAGAGTGTTTCAA
          1 TTCCAACGAAATCATCAAAGCGATCCAAATATCCACTTCCAGATTCTACAAAAAGAGTGTTTCAA

             *         *   * *   *         *        * *  *      *      *     
     138275 ACCTGCTCTATGAAAGGGAATCTTCAACTCTATGAGTTGAATGCAGACATCAGAAAGAAATTTCT
         66 AACTGCTCTATCAAAAGAAATGTTCAACTCTGTGAGTTGAGTACACACATCACAAAGAAGTTTCT

                     *       **          *         **
     138340 GAGAATGCTGCTGTCTACCTTTTAT-T--TGA-ATTCCCGC
        131 GAGAATGCTTCTGTCTAGTTTTTATATGAAGATATTCCCTT

                         *  *    *                *       *                  
     138377 TTCCAACGAAATCCTCCAAGCTATCCAAATATCCACTTGCAGATTCCACAAAAAGAGTGTTTCAA
          1 TTCCAACGAAATCATCAAAGCGATCCAAATATCCACTTCCAGATTCTACAAAAAGAGTGTTTCAA

                            ** *  *            *       *     *     *  *      
     138442 AACTGCTCTCTATCAATGGCAAAGTTCAACTCTGTTAGTTGAGGACACATATCACCAACAAGTTT
         66 AACTG--CTCTATCAAAAGAAATGTTCAACTCTGTGAGTTGAGTACACACATCACAAAGAAGTTT

                               *       **         *    
     138507 CTGAGAATGCTTCTGTCTATTTTTTATGGGAAGATATTTCCTT
        129 CTGAGAATGCTTCTGTCTAGTTTTTATATGAAGATATTCCCTT

              *  *  * ** *    *      *    *          * **                    
     138550 TTTCACCGTAGGCGTCAAGGCGATCGAAATGTCCACTTCCACAAACTACAAAAAGAGTGTTTCAA
          1 TTCCAACGAAATCATCAAAGCGATCCAAATATCCACTTCCAGATTCTACAAAAAGAGTGTTTCAA

             *         *   * **       *    *        * ** * *   **      *     
     138615 ACCTGCTCTATGAAAGGCCATGTTCATCTCTATGAGTTGAATGGAAATATCCGAAAGAAATTTCT
         66 AACTGCTCTATCAAAAGAAATGTTCAACTCTGTGAGTTGAGTACACACATCACAAAGAAGTTTCT

             *       *          *        *         **
     138680 GGGAATGCTGCTGTCTAG-TGTT-TAT-ACGA-ATTCCCGC
        131 GAGAATGCTTCTGTCTAGTTTTTATATGAAGATATTCCCTT

                         *       *                *    *  *                  
     138717 TTCCAACGAAATCCTCAAAGCAATCCAAATATCCACTTGCAGAATCCACAAAAAGAGTGTTTCAA
          1 TTCCAACGAAATCATCAAAGCGATCCAAATATCCACTTCCAGATTCTACAAAAAGAGTGTTTCAA

                          *     *          * *                   *  *        
     138782 AACTGCTCTATCAATAGAAAGGTTCAACTCTTTTAGTTGAGTACACACATCACGAACAAGTTTCT
         66 AACTGCTCTATCAAAAGAAATGTTCAACTCTGTGAGTTGAGTACACACATCACAAAGAAGTTTCT

                            * *      **     **  *    
     138847 GAGAATGCTTCTGTCTGGCTTTTATTGGAAGACGTTTCCTT
        131 GAGAATGCTTCTGTCTAGTTTTTATATGAAGATATTCCCTT

                 * *  **          *       *                * *               
     138888 TT-CACCAAAGGCATCAAAGCGCTCCAAATGTCCACTTCCAGATTCTTCCAAAAGAGTGTTTCAA
          1 TTCCAACGAAATCATCAAAGCGATCCAAATATCCACTTCCAGATTCTACAAAAAGAGTGTTTCAA

             **     * ***  * *                 *    * *  * *     *   *       
     138952 ACGTGCTCAAAGTAAGGGAATGTTCAACTCTGTGACTTGAATGCAGATATCACCAAGTAGTTTCT
         66 AACTGCTCTATCAAAAGAAATGTTCAACTCTGTGAGTTGAGTACACACATCACAAAGAAGTTTCT

            * * *             *     *    *         * 
     139017 AATAGTGCTTCTGTCTAGATTTTAGATGATGATATTCCCGT
        131 GAGAATGCTTCTGTCTAGTTTTTATATGAAGATATTCCCTT

                         * * *   * **          *  *   *      *     *       * 
     139058 TTCCAACGAAATCGTTAGAGCTAAGCAAATATCCAGTTACAGTTTCTACCAAAAGGGTGTTTCCA
          1 TTCCAACGAAATCATCAAAGCGATCCAAATATCCACTTCCAGATTCTACAAAAAGAGTGTTTCAA

              *    **           *            *       *                     * 
     139123 AATTGCTGCATCAAAAGAAAGGTTCAACTCTGTTAGTTGAGGACACACATCACAAAGAAGTTTGT
         66 AACTGCTCTATCAAAAGAAATGTTCAACTCTGTGAGTTGAGTACACACATCACAAAGAAGTTTCT

                              *    *     *           
     139188 GAGAATGCTTCTGTCTAGATTTTGTATGACGATATTCCCTT
        131 GAGAATGCTTCTGTCTAGTTTTTATATGAAGATATTCCCTT

                     *   * *     *   *       * *  *    *  *       *  *       
     139229 TTCCAACGATATCGTTAAAGCAATCTAAATATCAATTTGCAGAATCCACAAAAATAGAGTTTCAA
          1 TTCCAACGAAATCATCAAAGCGATCCAAATATCCACTTCCAGATTCTACAAAAAGAGTGTTTCAA

             *       * *        *    *       *  *                   *** *    
     139294 AGCTGCTCTGTAAAAAGAAAGGTTCCACTCTGTTAGCTGAGTACACACATCACAAACTTGATTCT
         66 AACTGCTCTATCAAAAGAAATGTTCAACTCTGTGAGTTGAGTACACACATCACAAAGAAGTTTCT

                  *         *        **         **   
     139359 GAGAATCCTTCTGTCTCGTTTTTATGGGAAGATATTTACTT
        131 GAGAATGCTTCTGTCTAGTTTTTATATGAAGATATTCCCTT

                 *  * **          *       *     *       *  *  *  *           
     139400 TTCCACCGTAGGCATCAAAGCGCTCCAAATGTCCACATCCAGATACTCCAGAACGAGTGTTTCAA
          1 TTCCAACGAAATCATCAAAGCGATCCAAATATCCACTTCCAGATTCTACAAAAAGAGTGTTTCAA

             *         *   * *   *         *        * *  *      *      *     
     139465 ACCTGCTCTATGAAAGGGAATCTTCAACTCTATGAGTTGAATGCAGACATCAGAAAGAAATTTCT
         66 AACTGCTCTATCAAAAGAAATGTTCAACTCTGTGAGTTGAGTACACACATCACAAAGAAGTTTCT

                     *       **          *         **
     139530 GAGAATGCTGCTGTCTACCTTTTAT-T--TGA-ATTCCCGC
        131 GAGAATGCTTCTGTCTAGTTTTTATATGAAGATATTCCCTT

                         *  *    *              * *  **   *    *             
     139567 TTCCAACGAAATCCTCCAAGCTATCCAAATATCCACCTGCATTTTCCACAACAAGAGTGTTTCAA
          1 TTCCAACGAAATCATCAAAGCGATCCAAATATCCACTTCCAGATTCTACAAAAAGAGTGTTTCAA

                          *                 *    *  *                *       
     139632 AACTGCTCTATCAATAGAAATGTTCAACTCCTTTG-GCTGGGTACACACATCACAAACAAGTTTC
         66 AACTGCTCTATCAAAAGAAATGTTCAACT-CTGTGAGTTGAGTACACACATCACAAAGAAGTTTC

                                      **     **       
     139696 TGAGAATGCTTCTGTCTAGTTTTTATGGGAAGACGTTCCCTT
        130 TGAGAATGCTTCTGTCTAGTTTTTATATGAAGATATTCCCTT

              *  * *  **          *       *            **                    
     139738 TTTCACCAAAGGCATCAAAGCGCTCCAAATGTCCACTTCCAGACACTACAAAAAGAGTGTTTCAA
          1 TTCCAACGAAATCATCAAAGCGATCCAAATATCCACTTCCAGATTCTACAAAAAGAGTGTTTCAA

             **       **   ***                 *    * *  * *         *       
     139803 ACGTGCTCTAAGAAAGCGAATGTTCAACTCTGTGACTTGAATGCAGATATCACAAAGTAGTTTCT
         66 AACTGCTCTATCAAAAGAAATGTTCAACTCTGTGAGTTGAGTACACACATCACAAAGAAGTTTCT

                **            *     *    *         * 
     139868 GAGAGGGCTTCTGTCTAGATTTTAGATGATGATATTCCCGT
        131 GAGAATGCTTCTGTCTAGTTTTTATATGAAGATATTCCCTT

                           * *   *                *   *                    * 
     139909 TTCCAACGAAATCATTAGAGCTATCCAAATATCCACTTACAGTTTCTACAAAAAGAGTGTTTCCA
          1 TTCCAACGAAATCATCAAAGCGATCCAAATATCCACTTCCAGATTCTACAAAAAGAGTGTTTCAA

                   **         * *    *       *  **                  ***      
     139974 AACTGCTGCATCAAAAGAGAGGTTCCACTCTGTTAGCCGAGTACACACATCACAAACTTGTTTCT
         66 AACTGCTCTATCAAAAGAAATGTTCAACTCTGTGAGTTGAGTACACACATCACAAAGAAGTTTCT

            *     *      *  *        **         **   
     140039 CAGAATCCTTCTGACTCGTTTTTATGGGAAGATATTTACTT
        131 GAGAATGCTTCTGTCTAGTTTTTATATGAAGATATTCCCTT

              *  *  * **          *       *     *       *  *  *              
     140080 TTTCACCGTAGGCATCAAAGCGCTCCAAATGTCCACATCCAGATACTCCAGAAAGAGTGTTTCAA
          1 TTCCAACGAAATCATCAAAGCGATCCAAATATCCACTTCCAGATTCTACAAAAAGAGTGTTTCAA

             *         *   * *   *         *        * *  *      *      *     
     140145 ACCTGCTCTATGAAAGGGAATCTTCAACTCTATGAGTTGAATGCAGACATCAGAAAGAAATTTCT
         66 AACTGCTCTATCAAAAGAAATGTTCAACTCTGTGAGTTGAGTACACACATCACAAAGAAGTTTCT

                     *       **          *         **
     140210 GAGAATGCTGCTGTCTACCTTTTAT-T--TGA-ATTCCCGC
        131 GAGAATGCTTCTGTCTAGTTTTTATATGAAGATATTCCCTT

                         *  *    *                *       *             *    
     140247 TTCCAACGAAATCCTCCAAGCTATCCAAATATCCACTTGCAGATTCCACAAAAAGAGTGTATCAA
          1 TTCCAACGAAATCATCAAAGCGATCCAAATATCCACTTCCAGATTCTACAAAAAGAGTGTTTCAA

                            ** *  *            *       *     *     *  *      
     140312 AACTGCTCTCTATCAATGGCAAAGTTCAACTCTGTTAGTTGAGGACACATATCACCAACAAGTTT
         66 AACTG--CTCTATCAAAAGAAATGTTCAACTCTGTGAGTTGAGTACACACATCACAAAGAAGTTT

                               *       **         *    
     140377 CTGAGAATGCTTCTGTCTATTTTTTATGGGAAGATATTTCCTT
        129 CTGAGAATGCTTCTGTCTAGTTTTTATATGAAGATATTCCCTT

              *  *  * ** *    *      *    *          * **                    
     140420 TTTCACCGTAGGCGTCAAGGCGATCGAAATGTCCACTTCCACAAACTACAAAAAGAGTGTTTCAA
          1 TTCCAACGAAATCATCAAAGCGATCCAAATATCCACTTCCAGATTCTACAAAAAGAGTGTTTCAA

             *         *   * **       *    *        * ** * *   **      *     
     140485 ACCTGCTCTATGAAAGGCCATGTTCATCTCTATGAGTTGAATGGAAATATCCGAAAGAAATTTCT
         66 AACTGCTCTATCAAAAGAAATGTTCAACTCTGTGAGTTGAGTACACACATCACAAAGAAGTTTCT

             *       *          *        *         **
     140550 GGGAATGCTGCTGTCTAG-TGTT-TAT-ACGA-ATTCCCGC
        131 GAGAATGCTTCTGTCTAGTTTTTATATGAAGATATTCCCTT

                         *       *                *    *  *                  
     140587 TTCCAACGAAATCCTCAAAGCAATCCAAATATCCACTTGCAGAATCCACAAAAAGAGTGTTTCAA
          1 TTCCAACGAAATCATCAAAGCGATCCAAATATCCACTTCCAGATTCTACAAAAAGAGTGTTTCAA

                          *     *          * *                   *  *        
     140652 AACTGCTCTATCAATAGAAAGGTTCAACTCTTTTAGTTGAGTACACACATCACGAACAAGTTTCT
         66 AACTGCTCTATCAAAAGAAATGTTCAACTCTGTGAGTTGAGTACACACATCACAAAGAAGTTTCT

                            * *      **     **  *    
     140717 GAGAATGCTTCTGTCTGGCTTTTATTGGAAGACGTTTCCTT
        131 GAGAATGCTTCTGTCTAGTTTTTATATGAAGATATTCCCTT

                 * *  **          *       *                * *               
     140758 TT-CACCAAAGGCATCAAAGCGCTCCAAATGTCCACTTCCAGATTCTTCCAAAAGAGTGTTTCAA
          1 TTCCAACGAAATCATCAAAGCGATCCAAATATCCACTTCCAGATTCTACAAAAAGAGTGTTTCAA

             **     * ***  * *                 *    * *  * *     *   *       
     140822 ACGTGCTCAAAGTAAGGGAATGTTCAACTCTGTGACTTGAATGCAGATATCACCAAGTAGTTTCT
         66 AACTGCTCTATCAAAAGAAATGTTCAACTCTGTGAGTTGAGTACACACATCACAAAGAAGTTTCT

            * * *             *     *    *         * 
     140887 AATAGTGCTTCTGTCTAGATTTTAGATGATGATATTCCCGT
        131 GAGAATGCTTCTGTCTAGTTTTTATATGAAGATATTCCCTT

                         *** *   *             *  *   *      *     *       * 
     140928 TTCCAACGAAATCGCTAGAGCTATCCAAATATCCAGTTACAGTTTCTACCAAAAGGGTGTTTCCA
          1 TTCCAACGAAATCATCAAAGCGATCCAAATATCCACTTCCAGATTCTACAAAAAGAGTGTTTCAA

              *    **           *            *       *                     * 
     140993 AATTGCTGCATCAAAAGAAAGGTTCAACTCTGTTAGTTGAGGACACACATCACAAAGAAGTTTGT
         66 AACTGCTCTATCAAAAGAAATGTTCAACTCTGTGAGTTGAGTACACACATCACAAAGAAGTTTCT

                              *    *     *           
     141058 GAGAATGCTTCTGTCTAGATTTTGTATGACGATATTCCCTT
        131 GAGAATGCTTCTGTCTAGTTTTTATATGAAGATATTCCCTT

                     *   * *     *   *       * *  *    *  *       *  *       
     141099 TTCCAACGATATCGTTAAAGCAATCTAAATATCAATTTGCAGAATCCACAAAAATAGAGTTTCAA
          1 TTCCAACGAAATCATCAAAGCGATCCAAATATCCACTTCCAGATTCTACAAAAAGAGTGTTTCAA

             *       * *        *    *       *  *                   ***      
     141164 AGCTGCTCTGTAAAAAGAAAGGTTCCACTCTGTTAGCTGAGTACACACATCACAAACTTGTTTCT
         66 AACTGCTCTATCAAAAGAAATGTTCAACTCTGTGAGTTGAGTACACACATCACAAAGAAGTTTCT

                 **         *        **         **   
     141229 GAGAAACCTTCTGTCTCGTTTTTATGGGAAGATATTTACTT
        131 GAGAATGCTTCTGTCTAGTTTTTATATGAAGATATTCCCTT

                 ** * **          *       *     *       *  *  *  *           
     141270 TTCCACTGTAGGCATCAAAGCGCTCCAAATGTCCACATCCAGATACTCCAGAACGAGTGTTTCAA
          1 TTCCAACGAAATCATCAAAGCGATCCAAATATCCACTTCCAGATTCTACAAAAAGAGTGTTTCAA

             *         *   * *   *         *        * *  *      *      *     
     141335 ACCTGCTCTATGAAAGGGAATCTTCAACTCTATGAGTTGAATGCAGA-ATCAGAAAGAAATTTCT
         66 AACTGCTCTATCAAAAGAAATGTTCAACTCTGTGAGTTGAGTACACACATCACAAAGAAGTTTCT

                     *       **          *         **
     141399 GAGAATGCTGCTGTCTACCTTTTAT-T--TGA-ATTCCCGC
        131 GAGAATGCTTCTGTCTAGTTTTTATATGAAGATATTCCCTT

                         *  *    *              * *  **   *                  
     141436 TTCCAACGAAATCCTCCAAGCTATCCAAATATCCACCTGCATTTTCCACAAAAAGAGTGTTTCAA
          1 TTCCAACGAAATCATCAAAGCGATCCAAATATCCACTTCCAGATTCTACAAAAAGAGTGTTTCAA

                          *                 *    *  *                *       
     141501 AACTGCTCTATCAATAGAAATGTTCAACTCCTTTG-GCTGGGTACACACATCACAAACAAGTTTC
         66 AACTGCTCTATCAAAAGAAATGTTCAACT-CTGTGAGTTGAGTACACACATCACAAAGAAGTTTC

                                      **     **       
     141565 TGAGAATGCTTCTGTCTAGTTTTTATGGGAAGACGTTCCCTT
        130 TGAGAATGCTTCTGTCTAGTTTTTATATGAAGATATTCCCTT

              *  * *  **          *       *            **                    
     141607 TTTCACCAAAGGCATCAAAGCGCTCCAAATGTCCACTTCCAGACACTACAAAAAGAGTGTTTCAA
          1 TTCCAACGAAATCATCAAAGCGATCCAAATATCCACTTCCAGATTCTACAAAAAGAGTGTTTCAA

             **       **   ***                 *    * *  * *         *       
     141672 ACGTGCTCTAAGAAAGCGAATGTTCAACTCTGTGACTTGAATGCAGATATCACAAAGTAGTTTCT
         66 AACTGCTCTATCAAAAGAAATGTTCAACTCTGTGAGTTGAGTACACACATCACAAAGAAGTTTCT

                **            *     *    *         * 
     141737 GAGAGGGCTTCTGTCTAGATTTTAGATGATGATATTCCCGT
        131 GAGAATGCTTCTGTCTAGTTTTTATATGAAGATATTCCCTT

                           * *   *                *   *                    * 
     141778 TTCCAACGAAATCATTAGAGCTATCCAAATATCCACTTACAGTTTCTACAAAAAGAGTGTTTCCA
          1 TTCCAACGAAATCATCAAAGCGATCCAAATATCCACTTCCAGATTCTACAAAAAGAGTGTTTCAA

                   **         * *    *       *  **                  ***      
     141843 AACTGCTGCATCAAAAGAGAGGTTCCACTCTGTTAGCCGAGTACACACATCACAAACTTGTTTCT
         66 AACTGCTCTATCAAAAGAAATGTTCAACTCTGTGAGTTGAGTACACACATCACAAAGAAGTTTCT

            *     *         *        **         **   
     141908 CAGAATCCTTCTGTCTCGTTTTTATGGGAAGATATTTACTT
        131 GAGAATGCTTCTGTCTAGTTTTTATATGAAGATATTCCCTT

              *  *  * **          *       *     *       *  *  *              
     141949 TTTCACCGTAGGCATCAAAGCGCTCCAAATGTCCACATCCAGATACTCCAGAAAGAGTGTTTCAA
          1 TTCCAACGAAATCATCAAAGCGATCCAAATATCCACTTCCAGATTCTACAAAAAGAGTGTTTCAA

             *         *   * *   *         *        * *  *      *      *     
     142014 ACCTGCTCTATGAAAGGGAATCTTCAACTCTATGAGTTGAATGCAGACATCAGAAAGAAATTTCT
         66 AACTGCTCTATCAAAAGAAATGTTCAACTCTGTGAGTTGAGTACACACATCACAAAGAAGTTTCT

                     *       **          *         **
     142079 GAGAATGCTGCTGTCTACCTTTTAT-T--TGA-ATTCCCGC
        131 GAGAATGCTTCTGTCTAGTTTTTATATGAAGATATTCCCTT

                         *  *    *                *       *                  
     142116 TTCCAACGAAATCCTCCAAGCTATCCAAATATCCACTTGCAGATTCCACAAAAAGAGTGTTTCAA
          1 TTCCAACGAAATCATCAAAGCGATCCAAATATCCACTTCCAGATTCTACAAAAAGAGTGTTTCAA

                            ** *  *            *       *     *     *  *      
     142181 AACTGCTCTCTATCAATGGCAAAGTTCAACTCTGTTAGTTGAGGACACATATCACCAACAAGTTT
         66 AACTG--CTCTATCAAAAGAAATGTTCAACTCTGTGAGTTGAGTACACACATCACAAAGAAGTTT

                               *       **         *    
     142246 CTGAGAATGCTTCTGTCTATTTTTTATGGGAAGATATTTCCTT
        129 CTGAGAATGCTTCTGTCTAGTTTTTATATGAAGATATTCCCTT

              *  *  * ** *    *      *    *          * **                    
     142289 TTTCACCGTAGGCGTCAAGGCGATCGAAATGTCCACTTCCACAAACTACAAAAAGAGTGTTTCAA
          1 TTCCAACGAAATCATCAAAGCGATCCAAATATCCACTTCCAGATTCTACAAAAAGAGTGTTTCAA

             *         *   * **       *    *        * ** * *   **      *     
     142354 ACCTGCTCTATGAAAGGCCATGTTCATCTCTATGAGTTGAATGGAAATATCCGAAAGAAATTTCT
         66 AACTGCTCTATCAAAAGAAATGTTCAACTCTGTGAGTTGAGTACACACATCACAAAGAAGTTTCT

             *       *          *        *         **
     142419 GGGAATGCTGCTGTCTAG-TGTT-TAT-ACGA-ATTCCCGC
        131 GAGAATGCTTCTGTCTAGTTTTTATATGAAGATATTCCCTT

                         *       *                *    *  *                  
     142456 TTCCAACGAAATCCTCAAAGCAATCCAAATATCCACTTGCAGAATCCACAAAAAGAGTGTTTCAA
          1 TTCCAACGAAATCATCAAAGCGATCCAAATATCCACTTCCAGATTCTACAAAAAGAGTGTTTCAA

                          *     *          * *                      *        
     142521 AACTGCTCTATCAATAGAAAGGTTCAACTCTTTTAGTTGAGTACACACATCACAAACAAGTTTCT
         66 AACTGCTCTATCAAAAGAAATGTTCAACTCTGTGAGTTGAGTACACACATCACAAAGAAGTTTCT

                            * *      **     **  *    
     142586 GAGAATGCTTCTGTCTGGCTTTTATTGGAAGACGTTTCCTT
        131 GAGAATGCTTCTGTCTAGTTTTTATATGAAGATATTCCCTT

                 * *  **          *       *                * *               
     142627 TT-CACCAAAGGCATCAAAGCGCTCCAAATGTCCACTTCCAGATTCTTCCAAAAGAGTGTTTCAA
          1 TTCCAACGAAATCATCAAAGCGATCCAAATATCCACTTCCAGATTCTACAAAAAGAGTGTTTCAA

             **  *  * ***  * *                 *    * *  * *     *   *       
     142691 ACGTGGTCGAAGTAAGGGAATGTTCAACTCTGTGACTTGAATGCAGATATCACCAAGTAGTTTCT
         66 AACTGCTCTATCAAAAGAAATGTTCAACTCTGTGAGTTGAGTACACACATCACAAAGAAGTTTCT

            * * *            **     *    *         * 
     142756 AATAGTGCTTCTGTCTACATTTTAGATGATGATATTCCCGT
        131 GAGAATGCTTCTGTCTAGTTTTTATATGAAGATATTCCCTT

                         * * *   *             *  *   *      *     *       * 
     142797 TTCCAACGAAATCGTTAGAGCTATCCAAATATCCAGTTACAGTTTCTACCAAAAGGGTGTTTCCA
          1 TTCCAACGAAATCATCAAAGCGATCCAAATATCCACTTCCAGATTCTACAAAAAGAGTGTTTCAA

              *    **           *            *       *                     * 
     142862 AATTGCTGCATCAAAAGAAAGGTTCAACTCTGTTAGTTGAGGACACACATCACAAAGAAGTTTGT
         66 AACTGCTCTATCAAAAGAAATGTTCAACTCTGTGAGTTGAGTACACACATCACAAAGAAGTTTCT

                              *    *     *           
     142927 GAGAATGCTTCTGTCTAGATTTTGTATGACGATATTCCCTT
        131 GAGAATGCTTCTGTCTAGTTTTTATATGAAGATATTCCCTT

                     *   * *     *   *       * *  *    *  *       *  *       
     142968 TTCCAACGATATCGTTAAAGCAATCTAAATATCAATTTGCAGAATCCACAAAAATAGAGTTTCAA
          1 TTCCAACGAAATCATCAAAGCGATCCAAATATCCACTTCCAGATTCTACAAAAAGAGTGTTTCAA

             *       * *        *    *       *  *                   ***      
     143033 AGCTGCTCTGTAAAAAGAAAGGTTCCACTCTGTTAGCTGAGTACACACATCACAAACTTGTTTCT
         66 AACTGCTCTATCAAAAGAAATGTTCAACTCTGTGAGTTGAGTACACACATCACAAAGAAGTTTCT

                 **         *        **         **   
     143098 GAGAAACCTTCTGTCTCGTTTTTATGGGAAGATATTTACTT
        131 GAGAATGCTTCTGTCTAGTTTTTATATGAAGATATTCCCTT

                 ** * **          *       *     *       *  *  *  *           
     143139 TTCCACTGTAGGCATCAAAGCGCTCCAAATGTCCACATCCAGATACTCCAGAACGAGTGTTTCAA
          1 TTCCAACGAAATCATCAAAGCGATCCAAATATCCACTTCCAGATTCTACAAAAAGAGTGTTTCAA

             *         *   * *   *         *        * *  *      *      *     
     143204 ACCTGCTCTATGAAAGGGAATCTTCAACTCTATGAGTTGAATGCAGA-ATCAGAAAGAAATTTCT
         66 AACTGCTCTATCAAAAGAAATGTTCAACTCTGTGAGTTGAGTACACACATCACAAAGAAGTTTCT

                     *       **          *         **
     143268 GAGAATGCTGCTGTCTACCTTTTAT-T--TGA-ATTCCCGC
        131 GAGAATGCTTCTGTCTAGTTTTTATATGAAGATATTCCCTT

                         *  *    *              * *  **   *                  
     143305 TTCCAACGAAATCCTCCAAGCTATCCAAATATCCACCTGCATTTTCCACAAAAAGAGTGTTTCAA
          1 TTCCAACGAAATCATCAAAGCGATCCAAATATCCACTTCCAGATTCTACAAAAAGAGTGTTTCAA

                          *                 *    *  *                *       
     143370 AACTGCTCTATCAATAGAAATGTTCAACTCCTTTG-GCTGGGTACACACATCACAAACAAGTTTC
         66 AACTGCTCTATCAAAAGAAATGTTCAACT-CTGTGAGTTGAGTACACACATCACAAAGAAGTTTC

                                      **     **       
     143434 TGAGAATGCTTCTGTCTAGTTTTTATGGGAAGACGTTCCCTT
        130 TGAGAATGCTTCTGTCTAGTTTTTATATGAAGATATTCCCTT

              *  * *  **          *       *            **                    
     143476 TTTCACCAAAGGCATCAAAGCGCTCCAAATGTCCACTTCCAGACACTACAAAAAGAGTGTTTCAA
          1 TTCCAACGAAATCATCAAAGCGATCCAAATATCCACTTCCAGATTCTACAAAAAGAGTGTTTCAA

             **       **   ***                 *    * *  * *         *       
     143541 ACGTGCTCTAAGAAAGCGAATGTTCAACTCTGTGACTTGAATGCAGATATCACAAAGTAGTTTCT
         66 AACTGCTCTATCAAAAGAAATGTTCAACTCTGTGAGTTGAGTACACACATCACAAAGAAGTTTCT

                **            *     *    *         * 
     143606 GAGAGGGCTTCTGTCTAGATTTTAGATGATGATATTCCCGT
        131 GAGAATGCTTCTGTCTAGTTTTTATATGAAGATATTCCCTT

                           * *   *                *   *                    * 
     143647 TTCCAACGAAATCATTAGAGCTATCCAAATATCCACTTACAGTTTCTACAAAAAGAGTGTTTCCA
          1 TTCCAACGAAATCATCAAAGCGATCCAAATATCCACTTCCAGATTCTACAAAAAGAGTGTTTCAA

                   **         * *    *       *  **                  ***      
     143712 AACTGCTGCATCAAAAGAGAGGTTCCACTCTGTTAGCCGAGTACACACATCACAAACTTGTTTCT
         66 AACTGCTCTATCAAAAGAAATGTTCAACTCTGTGAGTTGAGTACACACATCACAAAGAAGTTTCT

            *     *         *        **         **   
     143777 CAGAATCCTTCTGTCTCGTTTTTATGGGAAGATATTTACTT
        131 GAGAATGCTTCTGTCTAGTTTTTATATGAAGATATTCCCTT

              *  *  * **          *       *     *       *  *  *              
     143818 TTTCACCGTAGGCATCAAAGCGCTCCAAATGTCCACATCCAGATACTCCAGAAAGAGTGTTTCAA
          1 TTCCAACGAAATCATCAAAGCGATCCAAATATCCACTTCCAGATTCTACAAAAAGAGTGTTTCAA

             *         *   * *   *         *        * *  *      *      *     
     143883 ACCTGCTCTATGAAAGGGAATCTTCAACTCTATGAGTTGAATGCAGACATCAGAAAGAAATTTCT
         66 AACTGCTCTATCAAAAGAAATGTTCAACTCTGTGAGTTGAGTACACACATCACAAAGAAGTTTCT

                     *       **          *         **
     143948 GAGAATGCTGCTGTCTACCTTTTAT-T--TGA-ATTCCCGC
        131 GAGAATGCTTCTGTCTAGTTTTTATATGAAGATATTCCCTT

                         *  *    *                *       *                  
     143985 TTCCAACGAAATCCTCCAAGCTATCCAAATATCCACTTGCAGATTCCACAAAAAGAGTGTTTCAA
          1 TTCCAACGAAATCATCAAAGCGATCCAAATATCCACTTCCAGATTCTACAAAAAGAGTGTTTCAA

                            ** *  *            *       *     *     *  *      
     144050 AACTGCTCTCTATCAATGGCAAAGTTCAACTCTGTTAGTTGAGGACACATATCACCAACAAGTTT
         66 AACTG--CTCTATCAAAAGAAATGTTCAACTCTGTGAGTTGAGTACACACATCACAAAGAAGTTT

                               *       **         *    
     144115 CTGAGAATGCTTCTGTCTATTTTTTATGGGAAGATATTTCCTT
        129 CTGAGAATGCTTCTGTCTAGTTTTTATATGAAGATATTCCCTT

              *  *  * ** *    *      *    *          * **                    
     144158 TTTCACCGTAGGCGTCAAGGCGATCGAAATGTCCACTTCCACAAACTACAAAAAGAGTGTTTCAA
          1 TTCCAACGAAATCATCAAAGCGATCCAAATATCCACTTCCAGATTCTACAAAAAGAGTGTTTCAA

             *         *   * **       *    *        * ** * *   **      *     
     144223 ACCTGCTCTATGAAAGGCCATGTTCATCTCTATGAGTTGAATGGAAATATCCGAAAGAAATTTCT
         66 AACTGCTCTATCAAAAGAAATGTTCAACTCTGTGAGTTGAGTACACACATCACAAAGAAGTTTCT

             *       *                            ***
     144288 GGGAATGCTGCTGTCTAGTTTTTATAT---GA-ATTCCGGC
        131 GAGAATGCTTCTGTCTAGTTTTTATATGAAGATATTCCCTT

                         *       *                *    *  *                  
     144325 TTCCAACGAAATCCTCAAAGCAATCCAAATATCCACTTGCAGAATCCACAAAAAGAGTGTTTCAA
          1 TTCCAACGAAATCATCAAAGCGATCCAAATATCCACTTCCAGATTCTACAAAAAGAGTGTTTCAA

                          *     *          * *                      *        
     144390 AACTGCTCTATCAATAGAAAGGTTCAACTCTTTTAGTTGAGTACACACATCACAAACAAGTTTCT
         66 AACTGCTCTATCAAAAGAAATGTTCAACTCTGTGAGTTGAGTACACACATCACAAAGAAGTTTCT

                            * *      **     **  *    
     144455 GAGAATGCTTCTGTCTGGCTTTTATTGGAAGACGTTTCCTT
        131 GAGAATGCTTCTGTCTAGTTTTTATATGAAGATATTCCCTT

                 * *  **          *       *                * *               
     144496 TT-CACCAAAGGCATCAAAGCGCTCCAAATGTCCACTTCCAGATTCTTCCAAAAGAGTGTTTCAA
          1 TTCCAACGAAATCATCAAAGCGATCCAAATATCCACTTCCAGATTCTACAAAAAGAGTGTTTCAA

             **     * ***  * *       *         *    * *  * *     *   *       
     144560 ACGTGCTCGAAGTAAGGGAATGTTCTACTCTGTGACTTGAATGCAGATATCACCAAGTAGTTTCT
         66 AACTGCTCTATCAAAAGAAATGTTCAACTCTGTGAGTTGAGTACACACATCACAAAGAAGTTTCT

            * * *             *     *    *         * 
     144625 AATAGTGCTTCTGTCTAGATTTTAGATGATGATATTCCCGT
        131 GAGAATGCTTCTGTCTAGTTTTTATATGAAGATATTCCCTT

                         *** *   *             *  *   *      *     *       * 
     144666 TTCCAACGAAATCGCTAGAGCTATCCAAATATCCAGTTACAGTTTCTACCAAAAGGGTGTTTCCA
          1 TTCCAACGAAATCATCAAAGCGATCCAAATATCCACTTCCAGATTCTACAAAAAGAGTGTTTCAA

              *    **           *            *       *                     * 
     144731 AATTGCTGCATCAAAAGAAAGGTTCAACTCTGTTAGTTGAGGACACACATCACAAAGAAGTTTGT
         66 AACTGCTCTATCAAAAGAAATGTTCAACTCTGTGAGTTGAGTACACACATCACAAAGAAGTTTCT

                              *    *     *           
     144796 GAGAATGCTTCTGTCTAGATTTTGTATGACGATATTCCCTT
        131 GAGAATGCTTCTGTCTAGTTTTTATATGAAGATATTCCCTT

                     *   * *     *   *       * *  *    *  *       *  *       
     144837 TTCCAACGATATCGTTAAAGCAATCTAAATATCAATTTGCAGAATCCACAAAAATAGAGTTTCAA
          1 TTCCAACGAAATCATCAAAGCGATCCAAATATCCACTTCCAGATTCTACAAAAAGAGTGTTTCAA

             *       * *        *    *       *  *                   ***      
     144902 AGCTGCTCTGTAAAAAGAAAGGTTCCACTCTGTTAGCTGAGTACACACATCACAAACTTGTTTCT
         66 AACTGCTCTATCAAAAGAAATGTTCAACTCTGTGAGTTGAGTACACACATCACAAAGAAGTTTCT

            *     *         *        **       * **   
     144967 CAGAATCCTTCTGTCTCGTTTTTATGGGAAGATACTTACTT
        131 GAGAATGCTTCTGTCTAGTTTTTATATGAAGATATTCCCTT

                 *  * **          *       *     *       *  *  *  *           
     145008 TTCCACCGTAGGCATCAAAGCGCTCCAAATGTCCACATCCAGATACTCCAGAACGAGTGTTTCAA
          1 TTCCAACGAAATCATCAAAGCGATCCAAATATCCACTTCCAGATTCTACAAAAAGAGTGTTTCAA

             *         *   * *   *         *        * *  *      *      *     
     145073 ACCTGCTCTATGAAAGGGAATCTTCAACTCTATGAGTTGAATGCAGACATCAGAAAGAAATTTCT
         66 AACTGCTCTATCAAAAGAAATGTTCAACTCTGTGAGTTGAGTACACACATCACAAAGAAGTTTCT

                     *       **          *         **
     145138 GAGAATGCTGCTGTCTACCTTTTAT-T--TGA-ATTCCCGC
        131 GAGAATGCTTCTGTCTAGTTTTTATATGAAGATATTCCCTT

                         *  *    *              * *  **   *    *             
     145175 TTCCAACGAAATCCTCCAAGCTATCCAAATATCCACCTGCATTTTCCACAACAAGAGTGTTTCAA
          1 TTCCAACGAAATCATCAAAGCGATCCAAATATCCACTTCCAGATTCTACAAAAAGAGTGTTTCAA

                          *                 *    *  *                *       
     145240 AACTGCTCTATCAATAGAAATGTTCAACTCCTTTG-GCTGGGTACACACATCACAAACAAGTTTC
         66 AACTGCTCTATCAAAAGAAATGTTCAACT-CTGTGAGTTGAGTACACACATCACAAAGAAGTTTC

                                      **     **       
     145304 TGAGAATGCTTCTGTCTAGTTTTTATGGGAAGACGTTCCCTT
        130 TGAGAATGCTTCTGTCTAGTTTTTATATGAAGATATTCCCTT

              *  * *  **          *       *            **                  * 
     145346 TTTCACCAAAGGCATCAAAGCGCTCCAAATGTCCACTTCCAGACACTACAAAAAGAGTGTTTCCA
          1 TTCCAACGAAATCATCAAAGCGATCCAAATATCCACTTCCAGATTCTACAAAAAGAGTGTTTCAA

             **       **   ***                 *    * *  * *         *       
     145411 ACGTGCTCTAAGAAAGCGAATGTTCAACTCTGTGACTTGAATGCAGATATCACAAAGTAGTTTCT
         66 AACTGCTCTATCAAAAGAAATGTTCAACTCTGTGAGTTGAGTACACACATCACAAAGAAGTTTCT

                **            *     *    *         * 
     145476 GAGAGGGCTTCTGTCTAGATTTTAGATGATGATATTCCCGT
        131 GAGAATGCTTCTGTCTAGTTTTTATATGAAGATATTCCCTT

                   *       * *    *               *   *                    * 
     145517 TTCCAACAAAATCATTAGAGC-TTCCAAATATCCACTTACAGTTTCTACAAAAAGAGTGTTTCCA
          1 TTCCAACGAAATCATCAAAGCGATCCAAATATCCACTTCCAGATTCTACAAAAAGAGTGTTTCAA

                   **         * *    *       *  *                   ***      
     145581 AACTGCTGCATCAAAAGAGAGGTTCCACTCTGTTAGCTGAGTACACACATCACAAACTTGTTTCT
         66 AACTGCTCTATCAAAAGAAATGTTCAACTCTGTGAGTTGAGTACACACATCACAAAGAAGTTTCT

            *     *         *        **         **   
     145646 CAGAATCCTTCTGTCTCGTTTTTATGGGAAGATATTTACTT
        131 GAGAATGCTTCTGTCTAGTTTTTATATGAAGATATTCCCTT

              *  *  * **          *       *     *       *  *  *              
     145687 TTTCACCGTAGGCATCAAAGCGCTCCAAATGTCCACATCCAGATACTCCAGAAAGAGTGTTTCAA
          1 TTCCAACGAAATCATCAAAGCGATCCAAATATCCACTTCCAGATTCTACAAAAAGAGTGTTTCAA

             *         *   * *   *         *        * *  *      *      *     
     145752 ACCTGCTCTATGAAAGGGAATCTTCAACTCTATGAGTTGAATGCAGACATCAGAAAGAAATTTCT
         66 AACTGCTCTATCAAAAGAAATGTTCAACTCTGTGAGTTGAGTACACACATCACAAAGAAGTTTCT

                     *       **          *         **
     145817 GAGAATGCTGCTGTCTACCTTTTAT-T--TGA-ATTCCCGC
        131 GAGAATGCTTCTGTCTAGTTTTTATATGAAGATATTCCCTT

                         *  *    *                *       *                  
     145854 TTCCAACGAAATCCTCCAAGCTATCCAAATATCCACTTGCAGATTCCACAAAAAGAGTGTTTCAA
          1 TTCCAACGAAATCATCAAAGCGATCCAAATATCCACTTCCAGATTCTACAAAAAGAGTGTTTCAA

                            ** *  *            *       *     *     *  *      
     145919 AACTGCTCTCTATCAATGGCAAAGTTCAACTCTGTTAGTTGAGGACACATATCACCAACAAGTTT
         66 AACTG--CTCTATCAAAAGAAATGTTCAACTCTGTGAGTTGAGTACACACATCACAAAGAAGTTT

                               *       **         *    
     145984 CTGAGAATGCTTCTGTCTATTTTTTATGGGAAGATATTTCCTT
        129 CTGAGAATGCTTCTGTCTAGTTTTTATATGAAGATATTCCCTT

              *  *  * ** *    *      *    *          * **                    
     146027 TTTCACCGTAGGCGTCAAGGCGATCGAAATGTCCACTTCCACAAACTACAAAAAGAGTGTTTCAA
          1 TTCCAACGAAATCATCAAAGCGATCCAAATATCCACTTCCAGATTCTACAAAAAGAGTGTTTCAA

             *         *   * **       *    *        * ** * *   **      *     
     146092 ACCTGCTCTATGAAAGGCCATGTTCATCTCTATGAGTTGAATGGAAATATCCGAAAGAAATTTCT
         66 AACTGCTCTATCAAAAGAAATGTTCAACTCTGTGAGTTGAGTACACACATCACAAAGAAGTTTCT

             *       *          *        *         **
     146157 GGGAATGCTGCTGTCTAG-TGTT-TAT-ACGA-ATTCCCGC
        131 GAGAATGCTTCTGTCTAGTTTTTATATGAAGATATTCCCTT

                         *       *                *    *  *                  
     146194 TTCCAACGAAATCCTCAAAGCAATCCAAATATCCACTTGCAGAATCCACAAAAAGAGTGTTTCAA
          1 TTCCAACGAAATCATCAAAGCGATCCAAATATCCACTTCCAGATTCTACAAAAAGAGTGTTTCAA

                          *     *          * *                   *  *        
     146259 AACTGCTCTATCAATAGAAAGGTTCAACTCTTTTAGTTGAGTACACACATCACGAACAAGTTTCT
         66 AACTGCTCTATCAAAAGAAATGTTCAACTCTGTGAGTTGAGTACACACATCACAAAGAAGTTTCT

                            * *      **     **  *    
     146324 GAGAATGCTTCTGTCTGGCTTTTATTGGAAGACGTTTCCTT
        131 GAGAATGCTTCTGTCTAGTTTTTATATGAAGATATTCCCTT

                 * *  **          *       *                * *               
     146365 TT-CACCAAAGGCATCAAAGCGCTCCAAATGTCCACTTCCAGATTCTTCCAAAAGAGTGTTTCAA
          1 TTCCAACGAAATCATCAAAGCGATCCAAATATCCACTTCCAGATTCTACAAAAAGAGTGTTTCAA

             **     * ***  * *                 *    * *  * *     *   *       
     146429 ACGTGCTCAAAGTAAGGGAATGTTCAACTCTGTGACTTGAATGCAGATATCACCAAGTAGTTTCT
         66 AACTGCTCTATCAAAAGAAATGTTCAACTCTGTGAGTTGAGTACACACATCACAAAGAAGTTTCT

            * * *             *     *    *         * 
     146494 AATAGTGCTTCTGTCTAGATTTTAGATGATGATATTCCCGT
        131 GAGAATGCTTCTGTCTAGTTTTTATATGAAGATATTCCCTT

                         * * *   *             *  *   *      *     *       * 
     146535 TTCCAACGAAATCGTTAGAGCTATCCAAATATCCAGTTACAGTTTCTACCAAAAGGGTGTTTCCA
          1 TTCCAACGAAATCATCAAAGCGATCCAAATATCCACTTCCAGATTCTACAAAAAGAGTGTTTCAA

              *    **           *            *       *                     * 
     146600 AATTGCTGCATCAAAAGAAAGGTTCAACTCTGTTAGTTGAGGACACACATCACAAAGAAGTTTGT
         66 AACTGCTCTATCAAAAGAAATGTTCAACTCTGTGAGTTGAGTACACACATCACAAAGAAGTTTCT

                              *    *     *           
     146665 GAGAATGCTTCTGTCTAGATTTTGTATGACGATATTCCCTT
        131 GAGAATGCTTCTGTCTAGTTTTTATATGAAGATATTCCCTT

                     *   * *     *   *       * *  *    *  *       *  *       
     146706 TTCCAACGATATCGTTAAAGCAATCTAAATATCAATTTGCAGAATCCACAAAAATAGAGTTTCAA
          1 TTCCAACGAAATCATCAAAGCGATCCAAATATCCACTTCCAGATTCTACAAAAAGAGTGTTTCAA

             *       * *        *    *       *  *                   ***      
     146771 AGCTGCTCTGTAAAAAGAAAGGTTCCACTCTGTTAGCTGAGTACACACATCACAAACTTGTTTCT
         66 AACTGCTCTATCAAAAGAAATGTTCAACTCTGTGAGTTGAGTACACACATCACAAAGAAGTTTCT

                  *         *        **         **   
     146836 GAGAATCCTTCTGTCTCGTTTTTATGGGAAGATATTTACTT
        131 GAGAATGCTTCTGTCTAGTTTTTATATGAAGATATTCCCTT

                 *  * **          *       *     *       *  *  *  *           
     146877 TTCCACCGTAGGCATCAAAGCGCTCCAAATGTCCACATCCAGATACTCCAGAACGAGTGTTTCAA
          1 TTCCAACGAAATCATCAAAGCGATCCAAATATCCACTTCCAGATTCTACAAAAAGAGTGTTTCAA

             *         *   * *   *         *        * *  *      *      *     
     146942 ACCTGCTCTATGAAAGGGAATCTTCAACTCTATGAGTTGAATGCAGACATCAGAAAGAAATTTCT
         66 AACTGCTCTATCAAAAGAAATGTTCAACTCTGTGAGTTGAGTACACACATCACAAAGAAGTTTCT

                     *       **          *         **
     147007 GAGAATGCTGCTGTCTACCTTTTAT-T--TGA-ATTCCCGC
        131 GAGAATGCTTCTGTCTAGTTTTTATATGAAGATATTCCCTT

                         *  *    *                *  **   *    *             
     147044 TTCCAACGAAATCCTCCAAGCTATCCAAATATCCACTTGCATTTTCCACAACAAGAGTGTTTCAA
          1 TTCCAACGAAATCATCAAAGCGATCCAAATATCCACTTCCAGATTCTACAAAAAGAGTGTTTCAA

                          *                 *    *  *                *       
     147109 AACTGCTCTATCAATAGAAATGTTCAACTCCTTTG-GCTGGGTACACACATCACAAACAAGTTTC
         66 AACTGCTCTATCAAAAGAAATGTTCAACT-CTGTGAGTTGAGTACACACATCACAAAGAAGTTTC

                                      **     *        
     147173 TGAGAATGCTTCTGTCTAGTTTTTATGGGAAGACATTCCCTT
        130 TGAGAATGCTTCTGTCTAGTTTTTATATGAAGATATTCCCTT

              *  * *  **          *       *            **                    
     147215 TTTCACCAAAGGCATCAAAGCGCTCCAAATGTCCACTTCCAGACACTACAAAAAGAGTGTTTCAA
          1 TTCCAACGAAATCATCAAAGCGATCCAAATATCCACTTCCAGATTCTACAAAAAGAGTGTTTCAA

             **       **   ***                 *    * *  * *         *       
     147280 ACGTGCTCTAAGAAAGCGAATGTTCAACTCTGTGACTTGAATGCAGATATCACAAAGTAGTTTCT
         66 AACTGCTCTATCAAAAGAAATGTTCAACTCTGTGAGTTGAGTACACACATCACAAAGAAGTTTCT

                *             *     *    *         * 
     147345 GAGAGTGCTTCTGTCTAGATTTTAGATGATGATATTCCCGT
        131 GAGAATGCTTCTGTCTAGTTTTTATATGAAGATATTCCCTT

                  *        * *   *                *   *                    * 
     147386 TTCCAAAGAAATCATTAGAGCTATCCAAATATCCACTTACAGTTTCTACAAAAAGAGTGTTTCCA
          1 TTCCAACGAAATCATCAAAGCGATCCAAATATCCACTTCCAGATTCTACAAAAAGAGTGTTTCAA

                   **         * *    *       *  *                   ***      
     147451 AACTGCTGCATCAAAAGAGAGGTTCCACTCTGTTAGCTGAGTACACACATCACAAACTTGTTTCT
         66 AACTGCTCTATCAAAAGAAATGTTCAACTCTGTGAGTTGAGTACACACATCACAAAGAAGTTTCT

            *     *         *        **         **   
     147516 CAGAATCCTTCTGTCTCGTTTTTATGGGAAGATATTTACTT
        131 GAGAATGCTTCTGTCTAGTTTTTATATGAAGATATTCCCTT

              *  *  * **          *       *     *       *  *  *              
     147557 TTTCACCGTAGGCATCAAAGCGCTCCAAATGTCCACATCCAGATACTCCAGAAAGAGTGTTTCAA
          1 TTCCAACGAAATCATCAAAGCGATCCAAATATCCACTTCCAGATTCTACAAAAAGAGTGTTTCAA

             *         *   * *   *         *        * *  *      *      *     
     147622 ACCTGCTCTATGAAAGGGAATCTTCAACTCTATGAGTTGAATGCAGACATCAGAAAGAAATTTCT
         66 AACTGCTCTATCAAAAGAAATGTTCAACTCTGTGAGTTGAGTACACACATCACAAAGAAGTTTCT

                     *       **          *         **
     147687 GAGAATGCTGCTGTCTACCTTTTAT-T--TGA-ATTCCCGC
        131 GAGAATGCTTCTGTCTAGTTTTTATATGAAGATATTCCCTT

                         *  *    *                *       *                  
     147724 TTCCAACGAAATCCTCCAAGCTATCCAAATATCCACTTGCAGATTCCACAAAAAGAGTGTTTCAA
          1 TTCCAACGAAATCATCAAAGCGATCCAAATATCCACTTCCAGATTCTACAAAAAGAGTGTTTCAA

                            ** *  *            *       *     *     *  *      
     147789 AACTGCTCTCTATCAATGGCAAAGTTCAACTCTGTTAGTTGAGGACACATATCACCAACAAGTTT
         66 AACTG--CTCTATCAAAAGAAATGTTCAACTCTGTGAGTTGAGTACACACATCACAAAGAAGTTT

                               *       **         *    
     147854 CTGAGAATGCTTCTGTCTATTTTTTATGGGAAGATATTTCCTT
        129 CTGAGAATGCTTCTGTCTAGTTTTTATATGAAGATATTCCCTT

              *  *  * ** *    *  *   *    *         ** **                    
     147897 TTTCACCGTAGGCGTCAAGGCAATCGAAATGTCCACTTCCGCAAACTACAAAAAGAGTGTTTCAA
          1 TTCCAACGAAATCATCAAAGCGATCCAAATATCCACTTCCAGATTCTACAAAAAGAGTGTTTCAA

             *         *   * **       *    *        * ** * *   **      *     
     147962 ACCTGCTCTATGAAAGGCCATGTTCATCTCTATGAGTTGAATGGAAATATCCGAAAGAAATTTCT
         66 AACTGCTCTATCAAAAGAAATGTTCAACTCTGTGAGTTGAGTACACACATCACAAAGAAGTTTCT

             *       *                             **
     148027 GGGAATGCTGCTGTCTAGTTTTTATAT---GA-ATTCCCGC
        131 GAGAATGCTTCTGTCTAGTTTTTATATGAAGATATTCCCTT

                         *       *                *    *  *                  
     148064 TTCCAACGAAATCCTCAAAGCAATCCAAATATCCACTTGCAGAATCCACAAAAAGAGTGTTTCAA
          1 TTCCAACGAAATCATCAAAGCGATCCAAATATCCACTTCCAGATTCTACAAAAAGAGTGTTTCAA

                          *     *          * *                      *        
     148129 AACTGCTCTATCAATAGAAAGGTTCAACTCTTTTAGTTGAGTACACACATCACAAACAAGTTTCT
         66 AACTGCTCTATCAAAAGAAATGTTCAACTCTGTGAGTTGAGTACACACATCACAAAGAAGTTTCT

                            * *      **     **  *    
     148194 GAGAATGCTTCTGTCTGGCTTTTATTGGAAGACGTTTCCTT
        131 GAGAATGCTTCTGTCTAGTTTTTATATGAAGATATTCCCTT

                 * *  **          *       *                * *               
     148235 TT-CACCAAAGGCATCAAAGCGCTCCAAATGTCCACTTCCAGATTCTTCCAAAAGAGTGTTTCAA
          1 TTCCAACGAAATCATCAAAGCGATCCAAATATCCACTTCCAGATTCTACAAAAAGAGTGTTTCAA

             **  *  * ***  * *                 *    * *  * *     *   *       
     148299 ACGTGGTCGAAGTAAGGGAATGTTCAACTCTGTGACTTGAATGCAGATATCACCAAGTAGTTTCT
         66 AACTGCTCTATCAAAAGAAATGTTCAACTCTGTGAGTTGAGTACACACATCACAAAGAAGTTTCT

            * * *             *     *    *         * 
     148364 AATAGTGCTTCTGTCTAGATTTTAGATGATGATATTCCCGT
        131 GAGAATGCTTCTGTCTAGTTTTTATATGAAGATATTCCCTT

                         * * *   *             *  *   *      *     *       * 
     148405 TTCCAACGAAATCGTTAGAGCTATCCAAATATCCAGTTACAGTTTCTACCAAAAGGGTGTTTCCA
          1 TTCCAACGAAATCATCAAAGCGATCCAAATATCCACTTCCAGATTCTACAAAAAGAGTGTTTCAA

              *    **           *            *       *                     * 
     148470 AATTGCTGCATCAAAAGAAAGGTTCAACTCTGTTAGTTGAGGACACACATCACAAAGAAGTTTGT
         66 AACTGCTCTATCAAAAGAAATGTTCAACTCTGTGAGTTGAGTACACACATCACAAAGAAGTTTCT

                              *    *     *           
     148535 GAGAATGCTTCTGTCTAGATTTTGTATGACGATATTCCCTT
        131 GAGAATGCTTCTGTCTAGTTTTTATATGAAGATATTCCCTT

                     *   * *     *   *       * *  *    *  *       *  *       
     148576 TTCCAACGATATCGTTAAAGCAATCTAAATATCAATTTGCAGAATCCACAAAAATAGAGTTTCAA
          1 TTCCAACGAAATCATCAAAGCGATCCAAATATCCACTTCCAGATTCTACAAAAAGAGTGTTTCAA

             *       * *        *    *       *  *                   ***      
     148641 AGCTGCTCTGTAAAAAGAAAGGTTCCACTCTGTTAGCTGAGTACACACATCACAAACTTGTTTCT
         66 AACTGCTCTATCAAAAGAAATGTTCAACTCTGTGAGTTGAGTACACACATCACAAAGAAGTTTCT

                 **         *        **         **   
     148706 GAGAAACCTTCTGTCTCGTTTTTATGGGAAGATATTTACTT
        131 GAGAATGCTTCTGTCTAGTTTTTATATGAAGATATTCCCTT

                 ** * **          *       *     *       *  *  *  *           
     148747 TTCCACTGTAGGCATCAAAGCGCTCCAAATGTCCACATCCAGATACTCCAGAACGAGTGTTTCAA
          1 TTCCAACGAAATCATCAAAGCGATCCAAATATCCACTTCCAGATTCTACAAAAAGAGTGTTTCAA

             *         *   * *   *         *        * *  *      *      *     
     148812 ACCTGCTCTATGAAAGGGAATCTTCAACTCTATGAGTTGAATGCAGA-ATCAGAAAGAAATTTCT
         66 AACTGCTCTATCAAAAGAAATGTTCAACTCTGTGAGTTGAGTACACACATCACAAAGAAGTTTCT

                     *       **          *         **
     148876 GAGAATGCTGCTGTCTACCTTTTAT-T--TGA-ATTCCCGC
        131 GAGAATGCTTCTGTCTAGTTTTTATATGAAGATATTCCCTT

                         *  *    *              * *  **   *    *             
     148913 TTCCAACGAAATCCTCCAAGCTATCCAAATATCCACCTGCATTTTCCACAACAAGAGTGTTTCAA
          1 TTCCAACGAAATCATCAAAGCGATCCAAATATCCACTTCCAGATTCTACAAAAAGAGTGTTTCAA

                          *                 *    *  *                *       
     148978 AACTGCTCTATCAATAGAAATGTTCAACTCCTTTG-GCTGGGTACACACATCACAAACAAGTTTC
         66 AACTGCTCTATCAAAAGAAATGTTCAACT-CTGTGAGTTGAGTACACACATCACAAAGAAGTTTC

                                      **     **       
     149042 TGAGAATGCTTCTGTCTAGTTTTTATGGGAAGACGTTCCCTT
        130 TGAGAATGCTTCTGTCTAGTTTTTATATGAAGATATTCCCTT

              *  * *  **          *       *            **                    
     149084 TTTCACCAAAGGCATCAAAGCGCTCCAAATGTCCACTTCCAGACACTACAAAAAGAGTGTTTCAA
          1 TTCCAACGAAATCATCAAAGCGATCCAAATATCCACTTCCAGATTCTACAAAAAGAGTGTTTCAA

             **       **   ***                 *    * *    *         *       
     149149 ACGTGCTCTAAGAAAGCGAATGTTCAACTCTGTGACTTGAATGCACATATCACAAAGTAGTTTCT
         66 AACTGCTCTATCAAAAGAAATGTTCAACTCTGTGAGTTGAGTACACACATCACAAAGAAGTTTCT

                **            *     *    *         * 
     149214 GAGAGGGCTTCTGTCTAGATTTTAGATGATGATATTCCCGT
        131 GAGAATGCTTCTGTCTAGTTTTTATATGAAGATATTCCCTT

                   *       * *    *               *   *                    * 
     149255 TTCCAACAAAATCATTAGAGC-TTCCAAATATCCACTTACAGTTTCTACAAAAAGAGTGTTTCCA
          1 TTCCAACGAAATCATCAAAGCGATCCAAATATCCACTTCCAGATTCTACAAAAAGAGTGTTTCAA

                   **         * *    *       *  *                   ***      
     149319 AACTGCTGCATCAAAAGAGAGGTTCCACTCTGTTAGCTGAGTACACACATCACAAACTTGTTTCT
         66 AACTGCTCTATCAAAAGAAATGTTCAACTCTGTGAGTTGAGTACACACATCACAAAGAAGTTTCT

            *     *         *        **        ***   
     149384 CAGAATCCTTCTGTCTCGTTTTTATGGGAAGAT-TATACTT
        131 GAGAATGCTTCTGTCTAGTTTTTATATGAAGATATTCCCTT

              *  *  * **          *       *     *       *  *  *              
     149424 TTTCACCGTAGGCATCAAAGCGCTCCAAATGTCCACATCCAGATACTCCAGAAAGAGTGTTTCAA
          1 TTCCAACGAAATCATCAAAGCGATCCAAATATCCACTTCCAGATTCTACAAAAAGAGTGTTTCAA

             *         *   * *   *         *        * *  *      *      *     
     149489 ACCTGCTCTATGAAAGGGAATCTTCAACTCTATGAGTTGAATGCAGACATCAGAAAGAAATTTCT
         66 AACTGCTCTATCAAAAGAAATGTTCAACTCTGTGAGTTGAGTACACACATCACAAAGAAGTTTCT

                     *       **          *         **
     149554 GAGAATGCTGCTGTCTACCTTTTAT-T--TGA-ATTCCCGC
        131 GAGAATGCTTCTGTCTAGTTTTTATATGAAGATATTCCCTT

                         *  *    *                *       *                  
     149591 TTCCAACGAAATCCTCCAAGCTATCCAAATATCCACTTGCAGATTCCACAAAAAGAGTGTTTCAA
          1 TTCCAACGAAATCATCAAAGCGATCCAAATATCCACTTCCAGATTCTACAAAAAGAGTGTTTCAA

                            ** *  *            *       *     *     *  *      
     149656 AACTGCTCTCTATCAATGGCAAAGTTCAACTCTGTTAGTTGAGGACACATATCACCAACAAGTTT
         66 AACTG--CTCTATCAAAAGAAATGTTCAACTCTGTGAGTTGAGTACACACATCACAAAGAAGTTT

                               *       **         *    
     149721 CTGAGAATGCTTCTGTCTATTTTTTATGGGAAGATATTTCCTT
        129 CTGAGAATGCTTCTGTCTAGTTTTTATATGAAGATATTCCCTT

              *  *  * ** *    *      *    *          * **                    
     149764 TTTCACCGTAGGCGTCAAGGCGATCGAAATGTCCACTTCCACAAACTACAAAAAGAGTGTTTCAA
          1 TTCCAACGAAATCATCAAAGCGATCCAAATATCCACTTCCAGATTCTACAAAAAGAGTGTTTCAA

             *         *   * **       *    *        * ** * *   **      *     
     149829 ACCTGCTCTATGAAAGGCCATGTTCATCTCTATGAGTTGAATGGAAATATCCGAAAGAAATTTCT
         66 AACTGCTCTATCAAAAGAAATGTTCAACTCTGTGAGTTGAGTACACACATCACAAAGAAGTTTCT

             *       *          *        *         **
     149894 GGGAATGCTGCTGTCTAG-TGTT-TAT-ACGA-ATTCCCGC
        131 GAGAATGCTTCTGTCTAGTTTTTATATGAAGATATTCCCTT

                         *       *                *    *  *                  
     149931 TTCCAACGAAATCCTCAAAGCAATCCAAATATCCACTTGCAGAATCCACAAAAAGAGTGTTTCAA
          1 TTCCAACGAAATCATCAAAGCGATCCAAATATCCACTTCCAGATTCTACAAAAAGAGTGTTTCAA

                          *     *          * *                   *  *        
     149996 AACTGCTCTATCAATAGAAAGGTTCAACTCTTTTAGTTGAGTACACACATCACGAACAAGTTTCT
         66 AACTGCTCTATCAAAAGAAATGTTCAACTCTGTGAGTTGAGTACACACATCACAAAGAAGTTTCT

                            * *      **     **  *    
     150061 GAGAATGCTTCTGTCTGGCTTTTATTGGAAGACGTTTCCTT
        131 GAGAATGCTTCTGTCTAGTTTTTATATGAAGATATTCCCTT

                 * *  **          *       *                * *               
     150102 TT-CACCAAAGGCATCAAAGCGCTCCAAATGTCCACTTCCAGATTCTTCCAAAAGAGTGTTTCAA
          1 TTCCAACGAAATCATCAAAGCGATCCAAATATCCACTTCCAGATTCTACAAAAAGAGTGTTTCAA

             **     * ***  * *                 *    * *  * *     *   *       
     150166 ACGTGCTCAAAGTAAGGGAATGTTCAACTCTGTGACTTGAATGCAGATATCACCAAGTAGTTTCT
         66 AACTGCTCTATCAAAAGAAATGTTCAACTCTGTGAGTTGAGTACACACATCACAAAGAAGTTTCT

            * * *            **     *    *         * 
     150231 AATAGTGCTTCTGTCTACATTTTAGATGATGATATTCCCGT
        131 GAGAATGCTTCTGTCTAGTTTTTATATGAAGATATTCCCTT

                         * * *   *             *  *   *      *     *       * 
     150272 TTCCAACGAAATCGTTAGAGCTATCCAAATATCCAGTTACAGTTTCTACCAAAAGGGTGTTTCCA
          1 TTCCAACGAAATCATCAAAGCGATCCAAATATCCACTTCCAGATTCTACAAAAAGAGTGTTTCAA

              *    **           *            *       *                     * 
     150337 AATTGCTGCATCAAAAGAAAGGTTCAACTCTGTTAGTTGAGGACACACATCACAAAGAAGTTTGT
         66 AACTGCTCTATCAAAAGAAATGTTCAACTCTGTGAGTTGAGTACACACATCACAAAGAAGTTTCT

                              *    *     *           
     150402 GAGAATGCTTCTGTCTAGATTTTGTATGACGATATTCCCTT
        131 GAGAATGCTTCTGTCTAGTTTTTATATGAAGATATTCCCTT

                     *   * *     *   *       * *  *    *  *       *  *       
     150443 TTCCAACGATATCGTTAAAGCAATCTAAATATCAATTTGCAGAATCCACAAAAATAGAGTTTCAA
          1 TTCCAACGAAATCATCAAAGCGATCCAAATATCCACTTCCAGATTCTACAAAAAGAGTGTTTCAA

             *       * *        *    *       *  *                   ***      
     150508 AGCTGCTCTGTAAAAAGAAAGGTTCCACTCTGTTAGCTGAGTACACACATCACAAACTTGTTTCT
         66 AACTGCTCTATCAAAAGAAATGTTCAACTCTGTGAGTTGAGTACACACATCACAAAGAAGTTTCT

                  *         *        **         **   
     150573 GAGAATCCTTCTGTCTCGTTTTTATGGGAAGATATTTACTT
        131 GAGAATGCTTCTGTCTAGTTTTTATATGAAGATATTCCCTT

                 *  * **          *       *     *       *  *  *  *           
     150614 TTCCACCGTAGGCATCAAAGCGCTCCAAATGTCCACATCCAGATACTCCAGAACGAGTGTTTCAA
          1 TTCCAACGAAATCATCAAAGCGATCCAAATATCCACTTCCAGATTCTACAAAAAGAGTGTTTCAA

             *         *   * *   *         *        * *  *      *      *     
     150679 ACCTGCTCTATGAAAGGGAATCTTCAACTCTATGAGTTGAATGCAGACATCAGAAAGAAATTTCT
         66 AACTGCTCTATCAAAAGAAATGTTCAACTCTGTGAGTTGAGTACACACATCACAAAGAAGTTTCT

                     *       **          *         **
     150744 GAGAATGCTGCTGTCTACCTTTTAT-T--TGA-ATTCCCGC
        131 GAGAATGCTTCTGTCTAGTTTTTATATGAAGATATTCCCTT

                         *  *    *              * *  **   *    *             
     150781 TTCCAACGAAATCCTCCAAGCTATCCAAATATCCACCTGCATTTTCCACAACAAGAGTGTTTCAA
          1 TTCCAACGAAATCATCAAAGCGATCCAAATATCCACTTCCAGATTCTACAAAAAGAGTGTTTCAA

             *            *                 *    *  *                *       
     150846 ACCTGCTCTATCAATAGAAATGTTCAACTCCTTTG-GCTGGGTACACACATCACAAACAAGTTTC
         66 AACTGCTCTATCAAAAGAAATGTTCAACT-CTGTGAGTTGAGTACACACATCACAAAGAAGTTTC

                                      **     **       
     150910 TGAGAATGCTTCTGTCTAGTTTTTATGGGAAGACGTTCCCTT
        130 TGAGAATGCTTCTGTCTAGTTTTTATATGAAGATATTCCCTT

              *  * *  **          *       *            **                    
     150952 TTTCACCAAAGGCATCAAAGCGCTCCAAATGTCCACTTCCAGACACTACAAAAAGAGTGTTTCAA
          1 TTCCAACGAAATCATCAAAGCGATCCAAATATCCACTTCCAGATTCTACAAAAAGAGTGTTTCAA

             **       **   ***                 *    * *  * *         *       
     151017 ACGTGCTCTAAGAAAGCGAATGTTCAACTCTGTGACTTGAATGCAGATATCACAAAGTAGTTTCT
         66 AACTGCTCTATCAAAAGAAATGTTCAACTCTGTGAGTTGAGTACACACATCACAAAGAAGTTTCT

                **            *     *    *         * 
     151082 GAGAGGGCTTCTGTCTAGATTTTAGATGATGATATTCCCGT
        131 GAGAATGCTTCTGTCTAGTTTTTATATGAAGATATTCCCTT

                           * *   *                *   *                    * 
     151123 TTCCAACGAAATCATTAGAGCTATCCAAATATCCACTTACAGTTTCTACAAAAAGAGTGTTTCCA
          1 TTCCAACGAAATCATCAAAGCGATCCAAATATCCACTTCCAGATTCTACAAAAAGAGTGTTTCAA

                   **         * *    *       *  *                   ***      
     151188 AACTGCTGCATCAAAAGAGAGGTTCCACTCTGTTAGCTGAGTACACACATCACAAACTTGTTTCT
         66 AACTGCTCTATCAAAAGAAATGTTCAACTCTGTGAGTTGAGTACACACATCACAAAGAAGTTTCT

            *     *         *        **         **   
     151253 CAGAATCCTTCTGTCTCGTTTTTATGGGAAGATATTTACTT
        131 GAGAATGCTTCTGTCTAGTTTTTATATGAAGATATTCCCTT

              *  *  * **          *       *     *       *  *  *              
     151294 TTTCACCGTAGGCATCAAAGCGCTCCAAATGTCCACATCCAGATACTCCAGAAAGAGTGTTTCAA
          1 TTCCAACGAAATCATCAAAGCGATCCAAATATCCACTTCCAGATTCTACAAAAAGAGTGTTTCAA

             *         *   * *   *         *        * *  *      *      *     
     151359 ACCTGCTCTATGAAAGGGAATCTTCAACTCTATGAGTTGAATGCAGACATCAGAAAGAAATTTCT
         66 AACTGCTCTATCAAAAGAAATGTTCAACTCTGTGAGTTGAGTACACACATCACAAAGAAGTTTCT

                     *       **          *         **
     151424 GAGAATGCTGCTGTCTACCTTTTAT-T--TGA-ATTCCCGC
        131 GAGAATGCTTCTGTCTAGTTTTTATATGAAGATATTCCCTT

                         *  *    *                *       *                  
     151461 TTCCAACGAAATCCTCCAAGCTATCCAAATATCCACTTGCAGATTCCACAAAAAGAGTGTTTCAA
          1 TTCCAACGAAATCATCAAAGCGATCCAAATATCCACTTCCAGATTCTACAAAAAGAGTGTTTCAA

                            ** *  *            *       *     *     *  *      
     151526 AACTGCTCTCTATCAATGGCAAAGTTCAACTCTGTTAGTTGAGGACACATATCACCAACAAGTTT
         66 AACTG--CTCTATCAAAAGAAATGTTCAACTCTGTGAGTTGAGTACACACATCACAAAGAAGTTT

                               *       **         *    
     151591 CTGAGAATGCTTCTGTCTATTTTTTATGGGAAGATATTTCCTT
        129 CTGAGAATGCTTCTGTCTAGTTTTTATATGAAGATATTCCCTT

              *  *  * ** *    *      *    *          * **                    
     151634 TTTCACCGTAGGCGTCAAGGCGATCGAAATGTCCACTTCCACAAACTACAAAAAGAGTGTTTCAA
          1 TTCCAACGAAATCATCAAAGCGATCCAAATATCCACTTCCAGATTCTACAAAAAGAGTGTTTCAA

             *         *   * **       *    *        * ** * *   **      *     
     151699 ACCTGCTCTATGAAAGGCCATGTTCATCTCTATGAGTTGAATGGAAATATCCGAAAGAAATTTCT
         66 AACTGCTCTATCAAAAGAAATGTTCAACTCTGTGAGTTGAGTACACACATCACAAAGAAGTTTCT

             *       *          *        *         **
     151764 GGGAATGCTGCTGTCTAG-TGTT-TAT-ACGA-ATTCCCGC
        131 GAGAATGCTTCTGTCTAGTTTTTATATGAAGATATTCCCTT

                         *       *                *    *  *                  
     151801 TTCCAACGAAATCCTCAAAGCAATCCAAATATCCACTTGCAGAATCCACAAAAAGAGTGTTTCAA
          1 TTCCAACGAAATCATCAAAGCGATCCAAATATCCACTTCCAGATTCTACAAAAAGAGTGTTTCAA

                          *     *          * *                   *  *        
     151866 AACTGCTCTATCAATAGAAAGGTTCAACTCTTTTAGTTGAGTACACACATCACGAACAAGTTTCT
         66 AACTGCTCTATCAAAAGAAATGTTCAACTCTGTGAGTTGAGTACACACATCACAAAGAAGTTTCT

                            * *      **     **  *    
     151931 GAGAATGCTTCTGTCTGGCTTTTATTGGAAGACGTTTCCTT
        131 GAGAATGCTTCTGTCTAGTTTTTATATGAAGATATTCCCTT

                 * *  **          *       *                * *               
     151972 TT-CACCAAAGGCATCAAAGCGCTCCAAATGTCCACTTCCAGATTCTTCCAAAAGAGTGTTTCAA
          1 TTCCAACGAAATCATCAAAGCGATCCAAATATCCACTTCCAGATTCTACAAAAAGAGTGTTTCAA

             **     * ***  * *                 *    * *  * *     *   *       
     152036 ACGTGCTCAAAGTAAGGGAATGTTCAACTCTGTGACTTGAATGCAGATATCACCAAGTAGTTTCT
         66 AACTGCTCTATCAAAAGAAATGTTCAACTCTGTGAGTTGAGTACACACATCACAAAGAAGTTTCT

            * * *            **     *    *         * 
     152101 AATAGTGCTTCTGTCTACATTTTAGATGATGATATTCCCGT
        131 GAGAATGCTTCTGTCTAGTTTTTATATGAAGATATTCCCTT

                         * * *   *             *  *   *      *     *       * 
     152142 TTCCAACGAAATCGTTAGAGCTATCCAAATATCCAGTTACAGTTTCTACCAAAAGGGTGTTTCCA
          1 TTCCAACGAAATCATCAAAGCGATCCAAATATCCACTTCCAGATTCTACAAAAAGAGTGTTTCAA

              *    **           *            *       *                     * 
     152207 AATTGCTGCATCAAAAGAAAGGTTCAACTCTGTTAGTTGAGGACACACATCACAAAGAAGTTTGT
         66 AACTGCTCTATCAAAAGAAATGTTCAACTCTGTGAGTTGAGTACACACATCACAAAGAAGTTTCT

                              *    *     *           
     152272 GAGAATGCTTCTGTCTAGATTTTGTATGACGATATTCCCTT
        131 GAGAATGCTTCTGTCTAGTTTTTATATGAAGATATTCCCTT

                     *   * *     *   *       * *  *    *  *       *  *       
     152313 TTCCAACGATATCGTTAAAGCAATCTAAATATCAATTTGCAGAATCCACAAAAATAGAGTTTCAA
          1 TTCCAACGAAATCATCAAAGCGATCCAAATATCCACTTCCAGATTCTACAAAAAGAGTGTTTCAA

             *       * *        *    *       *  *                   ***      
     152378 AGCTGCTCTGTAAAAAGAAAGGTTCCACTCTGTTAGCTGAGTACACACATCACAAACTTGTTTCT
         66 AACTGCTCTATCAAAAGAAATGTTCAACTCTGTGAGTTGAGTACACACATCACAAAGAAGTTTCT

                  *         *        **         **   
     152443 GAGAATCCTTCTGTCTCGTTTTTATGGGAAGATATTTACTT
        131 GAGAATGCTTCTGTCTAGTTTTTATATGAAGATATTCCCTT

                 *  * **          *       *     *       *  *  *  *           
     152484 TTCCACCGTAGGCATCAAAGCGCTCCAAATGTCCACATCCAGATACTCCAGAACGAGTGTTTCAA
          1 TTCCAACGAAATCATCAAAGCGATCCAAATATCCACTTCCAGATTCTACAAAAAGAGTGTTTCAA

             *         *   * *   *         *        * *  *      *      *     
     152549 ACCTGCTCTATGAAAGGGAATCTTCAACTCTATGAGTTGAATGCAGACATCAGAAAGAAATTTCT
         66 AACTGCTCTATCAAAAGAAATGTTCAACTCTGTGAGTTGAGTACACACATCACAAAGAAGTTTCT

                     *       **          *         **
     152614 GAGAATGCTGCTGTCTACCTTTTAT-T--TGA-ATTCCCGC
        131 GAGAATGCTTCTGTCTAGTTTTTATATGAAGATATTCCCTT

                         *  *    *              * *  **   *    *             
     152651 TTCCAACGAAATCCTCCAAGCTATCCAAATATCCACCTGCATTTTCCACAACAAGAGTGTTTCAA
          1 TTCCAACGAAATCATCAAAGCGATCCAAATATCCACTTCCAGATTCTACAAAAAGAGTGTTTCAA

                          *                 *    *  *                *       
     152716 AACTGCTCTATCAATAGAAATGTTCAACTCCTTTG-GCTGGGTACACACATCACAAACAAGTTTC
         66 AACTGCTCTATCAAAAGAAATGTTCAACT-CTGTGAGTTGAGTACACACATCACAAAGAAGTTTC

                                      **     **       
     152780 TGAGAATGCTTCTGTCTAGTTTTTATGGGAAGACGTTCCCTT
        130 TGAGAATGCTTCTGTCTAGTTTTTATATGAAGATATTCCCTT

              *  * *  **          *       *            **                    
     152822 TTTCACCAAAGGCATCAAAGCGCTCCAAATGTCCACTTCCAGACACTACAAAAAGAGTGTTTCAA
          1 TTCCAACGAAATCATCAAAGCGATCCAAATATCCACTTCCAGATTCTACAAAAAGAGTGTTTCAA

             **       **   ***                 *    * *  * *         *       
     152887 ACGTGCTCTAAGAAAGCGAATGTTCAACTCTGTGACTTGAATGCAGATATCACAAAGTAGTTTCT
         66 AACTGCTCTATCAAAAGAAATGTTCAACTCTGTGAGTTGAGTACACACATCACAAAGAAGTTTCT

                **            *     *    *         * 
     152952 GAGAGGGCTTCTGTCTAGATTTTAGATGATGATATTCCCGT
        131 GAGAATGCTTCTGTCTAGTTTTTATATGAAGATATTCCCTT

                           * *   *                *   *                    * 
     152993 TTCCAACGAAATCATTAGAGCTATCCAAATATCCACTTACAGTTTCTACAAAAAGAGTGTTTCCA
          1 TTCCAACGAAATCATCAAAGCGATCCAAATATCCACTTCCAGATTCTACAAAAAGAGTGTTTCAA

                   **         * *    *       *  *                   ***      
     153058 AACTGCTGCATCAAAAGAGAGGTTCCACTCTGTTAGCTGAGTACACACATCACAAACTTGTTTCT
         66 AACTGCTCTATCAAAAGAAATGTTCAACTCTGTGAGTTGAGTACACACATCACAAAGAAGTTTCT

            *     *         *        **   *     **   
     153123 CAGAATCCTTCTGTCTCGTTTTTATGGGAAAATATTTACTT
        131 GAGAATGCTTCTGTCTAGTTTTTATATGAAGATATTCCCTT

              *  *  * **          *       *     *       *  *  *              
     153164 TTTCACCGTAGGCATCAAAGCGCTCCAAATGTCCACATCCAGATACTCCAGAAAGAGTGTTTCAA
          1 TTCCAACGAAATCATCAAAGCGATCCAAATATCCACTTCCAGATTCTACAAAAAGAGTGTTTCAA

             *         *   * *   *         *        * *  *      *      *     
     153229 ACCTGCTCTATGAAAGGGAATCTTCAACTCTATGAGTTGAATGCAGACATCAGAAAGAAATTTCT
         66 AACTGCTCTATCAAAAGAAATGTTCAACTCTGTGAGTTGAGTACACACATCACAAAGAAGTTTCT

                     *       **          *         **
     153294 GAGAATGCTGCTGTCTACCTTTTAT-T--TGA-ATTCCCGC
        131 GAGAATGCTTCTGTCTAGTTTTTATATGAAGATATTCCCTT

                         *  *    *                *       *                  
     153331 TTCCAACGAAATCCTCCAAGCTATCCAAATATCCACTTGCAGATTCCACAAAAAGAGTGTTTCAA
          1 TTCCAACGAAATCATCAAAGCGATCCAAATATCCACTTCCAGATTCTACAAAAAGAGTGTTTCAA

                            ** *  *            *       *     *     *  *      
     153396 AACTGCTCTCTATCAATGGCAAAGTTCAACTCTGTTAGTTGAGGACACATATCACCAACAAGTTT
         66 AACTG--CTCTATCAAAAGAAATGTTCAACTCTGTGAGTTGAGTACACACATCACAAAGAAGTTT

                               *       **         *    
     153461 CTGAGAATGCTTCTGTCTATTTTTTATGGGAAGATATTTCCTT
        129 CTGAGAATGCTTCTGTCTAGTTTTTATATGAAGATATTCCCTT

              *  *  * ** *    *      *    *          * **                    
     153504 TTTCACCGTAGGCGTCAAGGCGATCGAAATGTCCACTTCCACAAACTACAAAAAGAGTGTTTCAA
          1 TTCCAACGAAATCATCAAAGCGATCCAAATATCCACTTCCAGATTCTACAAAAAGAGTGTTTCAA

             *         *   * **       *    *        * ** * *   **      *     
     153569 ACCTGCTCTATGAAAGGCCATGTTCATCTCTATGAGTTGAATGGAAATATCCGAAAGAAATTTCT
         66 AACTGCTCTATCAAAAGAAATGTTCAACTCTGTGAGTTGAGTACACACATCACAAAGAAGTTTCT

             *       *          *        *         **
     153634 GGGAATGCTGCTGTCTAG-TGTT-TAT-ACGA-ATTCCCGC
        131 GAGAATGCTTCTGTCTAGTTTTTATATGAAGATATTCCCTT

                         *       *                *    *  *                  
     153671 TTCCAACGAAATCCTCAAAGCAATCCAAATATCCACTTGCAGAATCCACAAAAAGAGTGTTTCAA
          1 TTCCAACGAAATCATCAAAGCGATCCAAATATCCACTTCCAGATTCTACAAAAAGAGTGTTTCAA

                          *     *          * *                      *        
     153736 AACTGCTCTATCAATAGAAAGGTTCAACTCTTTTAGTTGAGTACACACATCACAAACAAGTTTCT
         66 AACTGCTCTATCAAAAGAAATGTTCAACTCTGTGAGTTGAGTACACACATCACAAAGAAGTTTCT

                            * *      **     **  *    
     153801 GAGAATGCTTCTGTCTGGCTTTTATTGGAAGACGTTTCCTT
        131 GAGAATGCTTCTGTCTAGTTTTTATATGAAGATATTCCCTT

                 * *  **          *       *                * *               
     153842 TT-CACCAAAGGCATCAAAGCGCTCCAAATGTCCACTTCCAGATTCTTCCAAAAGAGTGTTTCAA
          1 TTCCAACGAAATCATCAAAGCGATCCAAATATCCACTTCCAGATTCTACAAAAAGAGTGTTTCAA

             **     * ***  * *                 *    * *  * *     *   *       
     153906 ACGTGCTCAAAGTAAGGGAATGTTCAACTCTGTGACTTGAATGCAGATATCACCAAGTAGTTTCT
         66 AACTGCTCTATCAAAAGAAATGTTCAACTCTGTGAGTTGAGTACACACATCACAAAGAAGTTTCT

            * * *             *     *    *         * 
     153971 AATAGTGCTTCTGTCTAGATTTTAGATGATGATATTCCCGT
        131 GAGAATGCTTCTGTCTAGTTTTTATATGAAGATATTCCCTT

                         * * *   *             *  *   *                    * 
     154012 TTCCAACGAAATCGTTAGAGCTATCCAAATATCCAGTTACAGTTTCTACAAAAAGAGTGTTTCCA
          1 TTCCAACGAAATCATCAAAGCGATCCAAATATCCACTTCCAGATTCTACAAAAAGAGTGTTTCAA

                   **           *            *       *                     * 
     154077 AACTGCTGCATCAAAAGAAAGGTTCAACTCTGTTAGTTGAGGACACACATCACAAAGAAGTTTGT
         66 AACTGCTCTATCAAAAGAAATGTTCAACTCTGTGAGTTGAGTACACACATCACAAAGAAGTTTCT

                              *    *     *           
     154142 GAGAATGCTTCTGTCTAGATTTTGTATGACGATATTCCCTT
        131 GAGAATGCTTCTGTCTAGTTTTTATATGAAGATATTCCCTT

                     *   * *     *   *       * *  *    *  *       *  *       
     154183 TTCCAACGATATCGTTAAAGCAATCTAAATATCAATTTGCAGAATCCACAAAAATAGAGTTTCAA
          1 TTCCAACGAAATCATCAAAGCGATCCAAATATCCACTTCCAGATTCTACAAAAAGAGTGTTTCAA

             *       * *    *   *    *       *  *                   ***      
     154248 AGCTGCTCTGTAAAAATAAAAGTTCCACTCTGTTAGCTGAGTACACACATCACAAACTTGTTTCT
         66 AACTGCTCTATCAAAAGAAATGTTCAACTCTGTGAGTTGAGTACACACATCACAAAGAAGTTTCT

                  *         *        **         **   
     154313 GAGAATCCTTCTGTCTCGTTTTTATGGGAAGATATTTACTT
        131 GAGAATGCTTCTGTCTAGTTTTTATATGAAGATATTCCCTT

              *  *  * **          *       *     *       *  *  *     *        
     154354 TTTCACCGTAGGCATCAAAGCGCTCCAAATGTCCACATCCAGATACTCCAGAAAGACTGTTTCAA
          1 TTCCAACGAAATCATCAAAGCGATCCAAATATCCACTTCCAGATTCTACAAAAAGAGTGTTTCAA

             *         *   * *   *         *        * *  *      *      *     
     154419 ACCTGCTCTATGAAAGGGAATCTTCAACTCTATGAGTTGAATGCAGACATCAGAAAGAAATTTCT
         66 AACTGCTCTATCAAAAGAAATGTTCAACTCTGTGAGTTGAGTACACACATCACAAAGAAGTTTCT

                     *       **          *         **
     154484 GAGAATGCTGCTGTCTACCTTTTAT-T--TGA-ATTCCCGC
        131 GAGAATGCTTCTGTCTAGTTTTTATATGAAGATATTCCCTT

                         *  *    *                *  **   *    *             
     154521 TTCCAACGAAATCCTCCAAGCTATCCAAATATCCACTTGCATTTTCCACAACAAGAGTGTTTCAA
          1 TTCCAACGAAATCATCAAAGCGATCCAAATATCCACTTCCAGATTCTACAAAAAGAGTGTTTCAA

                          *               ** *  *  *                *        
     154586 AACTGCTCTATCAATAGAAATGTTCAACTCCTTTAGCTGGGTACACACATCACAAACAAGTTTCT
         66 AACTGCTCTATCAAAAGAAATGTTCAACTCTGTGAGTTGAGTACACACATCACAAAGAAGTTTCT

                                     **     **       
     154651 GAGAATGCTTCTGTCTAGTTTTTATGGGAAGACGTTCCCTT
        131 GAGAATGCTTCTGTCTAGTTTTTATATGAAGATATTCCCTT

              *  * *  **          *       *            **                    
     154692 TTTCACCAAAGGCATCAAAGCGCTCCAAATGTCCACTTCCAGACACTACAAAAAGAGTGTTTCAA
          1 TTCCAACGAAATCATCAAAGCGATCCAAATATCCACTTCCAGATTCTACAAAAAGAGTGTTTCAA

             **       **   ***                 *    * *  * *         *    *  
     154757 ACGTGCTCTAAGAAAGCGAATGTTCAACTCTGTGACTTGAATGCAGATATCACAAAGTAGTTACT
         66 AACTGCTCTATCAAAAGAAATGTTCAACTCTGTGAGTTGAGTACACACATCACAAAGAAGTTTCT

                **            *     *    *         * 
     154822 GAGAGGGCTTCTGTCTAGATTTTAGATGATGATATTCCCGT
        131 GAGAATGCTTCTGTCTAGTTTTTATATGAAGATATTCCCTT

                           * *   *                *   *                    * 
     154863 TTCCAACGAAATCATTAGAGCTATCCAAATATCCACTTACAGTTTCTACAAAAAGAGTGTTTCCA
          1 TTCCAACGAAATCATCAAAGCGATCCAAATATCCACTTCCAGATTCTACAAAAAGAGTGTTTCAA

                   **         * *    *       *  **                  ***      
     154928 AACTGCTGCATCAAAAGAGAGGTTCCACTCTGTTAGCCGAGTACACACATCACAAACTTGTTTCT
         66 AACTGCTCTATCAAAAGAAATGTTCAACTCTGTGAGTTGAGTACACACATCACAAAGAAGTTTCT

            *     *         *        **         **   
     154993 CAGAATCCTTCTGTCTCGTTTTTATGGGAAGATATTTACTT
        131 GAGAATGCTTCTGTCTAGTTTTTATATGAAGATATTCCCTT

              *  *  * **          *       *     *       *  *  *              
     155034 TTTCACCGTAGGCATCAAAGCGCTCCAAATGTCCACATCCAGATACTCCAGAAAGAGTGTTTCAA
          1 TTCCAACGAAATCATCAAAGCGATCCAAATATCCACTTCCAGATTCTACAAAAAGAGTGTTTCAA

             *         *   * *   *         *        * *  *      *      *     
     155099 ACCTGCTCTATGAAAGGGAATCTTCAACTCTATGAGTTGAATGCAGACATCAGAAAGAAATTTCT
         66 AACTGCTCTATCAAAAGAAATGTTCAACTCTGTGAGTTGAGTACACACATCACAAAGAAGTTTCT

                     *       **          *         **
     155164 GAGAATGCTGCTGTCTACCTTTTAT-T--TGA-ATTCCCGC
        131 GAGAATGCTTCTGTCTAGTTTTTATATGAAGATATTCCCTT

                         *  *    *                *       *                  
     155201 TTCCAACGAAATCCTCCAAGCTATCCAAATATCCACTTGCAGATTCCACAAAAAGAGTGTTTCAA
          1 TTCCAACGAAATCATCAAAGCGATCCAAATATCCACTTCCAGATTCTACAAAAAGAGTGTTTCAA

                            ** *  *            *       *     *     *  *      
     155266 AACTGCTCTCTATCAATGGCAAAGTTCAACTCTGTTAGTTGAGGACACATATCACCAACAAGTTT
         66 AACTG--CTCTATCAAAAGAAATGTTCAACTCTGTGAGTTGAGTACACACATCACAAAGAAGTTT

                               *       **         *    
     155331 CTGAGAATGCTTCTGTCTATTTTTTATGGGAAGATATTTCCTT
        129 CTGAGAATGCTTCTGTCTAGTTTTTATATGAAGATATTCCCTT

              *  *  * ** *    *      *    *          * **                    
     155374 TTTCACCGTAGGCGTCAAGGCGATCGAAATGTCCACTTCCACAAACTACAAAAAGAGTGTTTCAA
          1 TTCCAACGAAATCATCAAAGCGATCCAAATATCCACTTCCAGATTCTACAAAAAGAGTGTTTCAA

             *         *   * **       *    *        * ** * *   **      *     
     155439 ACCTGCTCTATGAAAGGCCATGTTCATCTCTATGAGTTGAATGGAAATATCCGAAAGAAATTTCT
         66 AACTGCTCTATCAAAAGAAATGTTCAACTCTGTGAGTTGAGTACACACATCACAAAGAAGTTTCT

             *       *          *        *         **
     155504 GGGAATGCTGCTGTCTAG-TGTT-TAT-ACGA-ATTCCCGC
        131 GAGAATGCTTCTGTCTAGTTTTTATATGAAGATATTCCCTT

                         *       *                *    *  *                  
     155541 TTCCAACGAAATCCTCAAAGCAATCCAAATATCCACTTGCAGAATCCACAAAAAGAGTGTTTCAA
          1 TTCCAACGAAATCATCAAAGCGATCCAAATATCCACTTCCAGATTCTACAAAAAGAGTGTTTCAA

                          *     *          * *                   *  *        
     155606 AACTGCTCTATCAATAGAAAGGTTCAACTCTTTTAGTTGAGTACACACATCACGAACAAGTTTCT
         66 AACTGCTCTATCAAAAGAAATGTTCAACTCTGTGAGTTGAGTACACACATCACAAAGAAGTTTCT

                            * *      **     **  *    
     155671 GAGAATGCTTCTGTCTGGCTTTTATTGGAAGACGTTTCCTT
        131 GAGAATGCTTCTGTCTAGTTTTTATATGAAGATATTCCCTT

                 * *  **          *       *                * *               
     155712 TT-CACCAAAGGCATCAAAGCGCTCCAAATGTCCACTTCCAGATTCTTCCAAAAGAGTGTTTCAA
          1 TTCCAACGAAATCATCAAAGCGATCCAAATATCCACTTCCAGATTCTACAAAAAGAGTGTTTCAA

             **     * ***  * *                 *    * *  * *     *   *       
     155776 ACGTGCTCAAAGTAAGGGAATGTTCAACTCTGTGACTTGAATGCAGATATCACCAAGTAGTTTCT
         66 AACTGCTCTATCAAAAGAAATGTTCAACTCTGTGAGTTGAGTACACACATCACAAAGAAGTTTCT

            * * *            **     *    *           
     155841 AATAGTGCTTCTGTCTACATTTTAGATGATGATATTCCCTT
        131 GAGAATGCTTCTGTCTAGTTTTTATATGAAGATATTCCCTT

                         * * *   *             *  *   *      *     *       * 
     155882 TTCCAACGAAATCGTTAGAGCTATCCAAATATCCAGTTACAGTTTCTACCAAAAGGGTGTTTCCA
          1 TTCCAACGAAATCATCAAAGCGATCCAAATATCCACTTCCAGATTCTACAAAAAGAGTGTTTCAA

              *    **           *            *       *                     * 
     155947 AATTGCTGCATCAAAAGAAAGGTTCAACTCTGTTAGTTGAGGACACACATCACAAAGAAGTTTGT
         66 AACTGCTCTATCAAAAGAAATGTTCAACTCTGTGAGTTGAGTACACACATCACAAAGAAGTTTCT

                              *    *     *           
     156012 GAGAATGCTTCTGTCTAGATTTTGTATGACGATATTCCCTT
        131 GAGAATGCTTCTGTCTAGTTTTTATATGAAGATATTCCCTT

                     *   * *     *   *       * *  *    *  *       *  *       
     156053 TTCCAACGATATCGTTAAAGCAATCTAAATATCAATTTGCAGAATCCACAAAAATAGAGTTTCAA
          1 TTCCAACGAAATCATCAAAGCGATCCAAATATCCACTTCCAGATTCTACAAAAAGAGTGTTTCAA

             *       * *        *    *       *  *                   ***      
     156118 AGCTGCTCTGTAAAAAGAAAGGTTCCACTCTGTTAGCTGAGTACACACATCACAAACTTGTTTCT
         66 AACTGCTCTATCAAAAGAAATGTTCAACTCTGTGAGTTGAGTACACACATCACAAAGAAGTTTCT

                  *         *        **         **   
     156183 GAGAATCCTTCTGTCTCGTTTTTATGGGAAGATATTTACTT
        131 GAGAATGCTTCTGTCTAGTTTTTATATGAAGATATTCCCTT

              *  *  * **          *       *     *       *  *  *  *           
     156224 TTTCACCGTAGGCATCAAAGCGCTCCAAATGTCCACATCCAGATACTCCAGAACGAGTGTTTCAA
          1 TTCCAACGAAATCATCAAAGCGATCCAAATATCCACTTCCAGATTCTACAAAAAGAGTGTTTCAA

             *         *   * *   *         *        * *  *      *      *     
     156289 ACCTGCTCTATGAAAGGGAATCTTCAACTCTATGAGTTGAATGCAGACATCAGAAAGAAATTTCT
         66 AACTGCTCTATCAAAAGAAATGTTCAACTCTGTGAGTTGAGTACACACATCACAAAGAAGTTTCT

                     *       **          *         **
     156354 GAGAATGCTGCTGTCTACCTTTTAT-T--TGA-ATTCCCGC
        131 GAGAATGCTTCTGTCTAGTTTTTATATGAAGATATTCCCTT

                         *  *    *                *       *                  
     156391 TTCCAACGAAATCCTCCAAGCTATCCAAATATCCACTTGCAGATTCCACAAAAAGAGTGTTTCAA
          1 TTCCAACGAAATCATCAAAGCGATCCAAATATCCACTTCCAGATTCTACAAAAAGAGTGTTTCAA

                            ** *  *            *       *     *     *  *      
     156456 AACTGCTCTCTATCAATGGCAAAGTTCAACTCTGTTAGTTGAGGACACATATCACCAACAAGTTT
         66 AACTG--CTCTATCAAAAGAAATGTTCAACTCTGTGAGTTGAGTACACACATCACAAAGAAGTTT

                               *       **         *    
     156521 CTGAGAATGCTTCTGTCTATTTTTTATGGGAAGATATTTCCTT
        129 CTGAGAATGCTTCTGTCTAGTTTTTATATGAAGATATTCCCTT

              *  *  * ** *    *      *    *          * **                    
     156564 TTTCAGCGTAGGCGTCAAGGCGATCGAAATGTCCACTTCCACAAACTACAAAAAGAGTGTTTCAA
          1 TTCCAACGAAATCATCAAAGCGATCCAAATATCCACTTCCAGATTCTACAAAAAGAGTGTTTCAA

             *         *   * **       *    *        * ** * *   **      *     
     156629 ACCTGCTCTATGAAAGGCCATGTTCATCTCTATGAGTTGAATGGAAATATCCGAAAGAAATTTCT
         66 AACTGCTCTATCAAAAGAAATGTTCAACTCTGTGAGTTGAGTACACACATCACAAAGAAGTTTCT

             *       *          *        *         **
     156694 GGGAATGCTGCTGTCTAG-TGTT-TAT-ACGA-ATTCCCGC
        131 GAGAATGCTTCTGTCTAGTTTTTATATGAAGATATTCCCTT

                         *       *                *    *  *                  
     156731 TTCCAACGAAATCCTCAAAGCAATCCAAATATCCACTTGCAGAATCCACAAAAAGAGTGTTTCAA
          1 TTCCAACGAAATCATCAAAGCGATCCAAATATCCACTTCCAGATTCTACAAAAAGAGTGTTTCAA

                          *     *          * *                   *  *        
     156796 AACTGCTCTATCAATAGAAAGGTTCAACTCTTTTAGTTGAGTACACACATCACGAACAAGTTTCT
         66 AACTGCTCTATCAAAAGAAATGTTCAACTCTGTGAGTTGAGTACACACATCACAAAGAAGTTTCT

                            * *      **     **  *    
     156861 GAGAATGCTTCTGTCTGGCTTTTATTGGAAGACGTTTCCTT
        131 GAGAATGCTTCTGTCTAGTTTTTATATGAAGATATTCCCTT

                 * *  **          *       *                * *               
     156902 TT-CACCAAAGGCATCAAAGCGCTCCAAATGTCCACTTCCAGATTCTTCCAAAAGAGTGTTTCAA
          1 TTCCAACGAAATCATCAAAGCGATCCAAATATCCACTTCCAGATTCTACAAAAAGAGTGTTTCAA

             **     * ***  * *                 *    * *  * *     *   *       
     156966 ACGTGCTCAAAGTAAGGGAATGTTCAACTCTGTGACTTGAATGCAGATATCACCAAGTAGTTTCT
         66 AACTGCTCTATCAAAAGAAATGTTCAACTCTGTGAGTTGAGTACACACATCACAAAGAAGTTTCT

            * * *            **     *    *         * 
     157031 AATAGTGCTTCTGTCTACATTTTAGATGATGATATTCCCGT
        131 GAGAATGCTTCTGTCTAGTTTTTATATGAAGATATTCCCTT

                         * * *   *             *  *   *      *     *       * 
     157072 TTCCAACGAAATCGTTAGAGCTATCCAAATATCCAGTTACAGTTTCTACCAAAAGGGTGTTTCCA
          1 TTCCAACGAAATCATCAAAGCGATCCAAATATCCACTTCCAGATTCTACAAAAAGAGTGTTTCAA

              *    **           *            *       *                     * 
     157137 AATTGCTGCATCAAAAGAAAGGTTCAACTCTGTTAGTTGAGGACACACATCACAAAGAAGTTTGT
         66 AACTGCTCTATCAAAAGAAATGTTCAACTCTGTGAGTTGAGTACACACATCACAAAGAAGTTTCT

                              *    *     *           
     157202 GAGAATGCTTCTGTCTAGATTTTGTATGACGATATTCCCTT
        131 GAGAATGCTTCTGTCTAGTTTTTATATGAAGATATTCCCTT

                     *   * *     *   *       * *  *    *  *       *  *       
     157243 TTCCAACGATATCGTTAAAGCAATCTAAATATCAATTTGCAGAATCCACAAAAATAGAGTTTCAA
          1 TTCCAACGAAATCATCAAAGCGATCCAAATATCCACTTCCAGATTCTACAAAAAGAGTGTTTCAA

             *       * *        *    *       *  *                   ***      
     157308 AGCTGCTCTGTAAAAAGAAAGGTTCCACTCTGTTAGCTGAGTACACACATCACAAACTTGTTTCT
         66 AACTGCTCTATCAAAAGAAATGTTCAACTCTGTGAGTTGAGTACACACATCACAAAGAAGTTTCT

                  *         *        **         **   
     157373 GAGAATCCTTCTGTCTCGTTTTTATGGGAAGATATTTACTT
        131 GAGAATGCTTCTGTCTAGTTTTTATATGAAGATATTCCCTT

                 *  * **          *       *     *       *  *  *  *           
     157414 TTCCACCGTAGGCATCAAAGCGCTCCAAATGTCCACATCCAGATACTCCAGAACGAGTGTTTCAA
          1 TTCCAACGAAATCATCAAAGCGATCCAAATATCCACTTCCAGATTCTACAAAAAGAGTGTTTCAA

             *         *   * *   *         *        * *  *      *      *     
     157479 ACCTGCTCTATGAAAGGGAATCTTCAACTCTATGAGTTGAATGCAGACATCAGAAAGAAATTTCT
         66 AACTGCTCTATCAAAAGAAATGTTCAACTCTGTGAGTTGAGTACACACATCACAAAGAAGTTTCT

                     *       **          *         **
     157544 GAGAATGCTGCTGTCTACCTTTTAT-T--TGA-ATTCCCGC
        131 GAGAATGCTTCTGTCTAGTTTTTATATGAAGATATTCCCTT

                         *  *    *              * *  **   *    *             
     157581 TTCCAACGAAATCCTCCAAGCTATCCAAATATCCACCTGCATTTTCCACAACAAGAGTGTTTCAA
          1 TTCCAACGAAATCATCAAAGCGATCCAAATATCCACTTCCAGATTCTACAAAAAGAGTGTTTCAA

                          *                 *    *  *                *       
     157646 AACTGCTCTATCAATAGAAATGTTCAACTCCTTTG-GCTGGGTACACACATCACAAACAAGTTTC
         66 AACTGCTCTATCAAAAGAAATGTTCAACT-CTGTGAGTTGAGTACACACATCACAAAGAAGTTTC

                                      **     **       
     157710 TGAGAATGCTTCTGTCTAGTTTTTATGGGAAGACGTTCCCTT
        130 TGAGAATGCTTCTGTCTAGTTTTTATATGAAGATATTCCCTT

              *  * *  **          *       *            **                    
     157752 TTTCACCAAAGGCATCAAAGCGCTCCAAATGTCCACTTCCAGACACTACAAAAAGAGTGTTTCAA
          1 TTCCAACGAAATCATCAAAGCGATCCAAATATCCACTTCCAGATTCTACAAAAAGAGTGTTTCAA

             **       **   ***                 *    * *  * *         *       
     157817 ACGTGCTCTAAGAAAGCGAATGTTCAACTCTGTGACTTGAATGCAGATATCACAAAGTAGTTTCT
         66 AACTGCTCTATCAAAAGAAATGTTCAACTCTGTGAGTTGAGTACACACATCACAAAGAAGTTTCT

                **            *     *    *         * 
     157882 GAGAGGGCTTCTGTCTAGATTTTAGATGATGATATTCCCGT
        131 GAGAATGCTTCTGTCTAGTTTTTATATGAAGATATTCCCTT

                           * *   *                *   *                    * 
     157923 TTCCAACGAAATCATTAGAGCTATCCAAATATCCACTTACAGTTTCTACAAAAAGAGTGTTTCCA
          1 TTCCAACGAAATCATCAAAGCGATCCAAATATCCACTTCCAGATTCTACAAAAAGAGTGTTTCAA

                   **         * *    *       *  *                   ***      
     157988 AACTGCTGCATCAAAAGAGAGGTTCCACTCTGTTAGCTGAGTACACACATCACAAACTTGTTTCT
         66 AACTGCTCTATCAAAAGAAATGTTCAACTCTGTGAGTTGAGTACACACATCACAAAGAAGTTTCT

            *     *         *        **         **   
     158053 CAGAATCCTTCTGTCTCGTTTTTATGGGAAGATATTTACTT
        131 GAGAATGCTTCTGTCTAGTTTTTATATGAAGATATTCCCTT

              *  *  * **          *       *     *       *  *  *              
     158094 TTTCACCGTAGGCATCAAAGCGCTCCAAATGTCCACATCCAGATACTCCAGAAAGAGTGTTTCAA
          1 TTCCAACGAAATCATCAAAGCGATCCAAATATCCACTTCCAGATTCTACAAAAAGAGTGTTTCAA

             *         *   * *   *         *        * *  *      *      *     
     158159 ACCTGCTCTATGAAAGGGAATCTTCAACTCTATGAGTTGAATGCAGACATCAGAAAGAAATTTCT
         66 AACTGCTCTATCAAAAGAAATGTTCAACTCTGTGAGTTGAGTACACACATCACAAAGAAGTTTCT

                     *       **          *         **
     158224 GAGAATGCTGCTGTCTACCTTTTAT-T--TGA-ATTCCCGC
        131 GAGAATGCTTCTGTCTAGTTTTTATATGAAGATATTCCCTT

                         *  *    *                *       *                  
     158261 TTCCAACGAAATCCTCCAAGCTATCCAAATATCCACTTGCAGATTCCACAAAAAGAGTGTTTCAA
          1 TTCCAACGAAATCATCAAAGCGATCCAAATATCCACTTCCAGATTCTACAAAAAGAGTGTTTCAA

                            ** *  *            *       *     *     *  *      
     158326 AACTGCTCTCTATCAATGGCAAAGTTCAACTCTGTTAGTTGAGGACACATATCACCAACAAGTTT
         66 AACTG--CTCTATCAAAAGAAATGTTCAACTCTGTGAGTTGAGTACACACATCACAAAGAAGTTT

                               *       **         *    
     158391 CTGAGAATGCTTCTGTCTATTTTTTATGGGAAGATATTTCCTT
        129 CTGAGAATGCTTCTGTCTAGTTTTTATATGAAGATATTCCCTT

              *  *  * ** *    *      *    *          * **                    
     158434 TTTCACCGTAGGCGTCAAGGCGATCGAAATGTCCACTTCCACAAACTACAAAAAGAGTGTTTCAA
          1 TTCCAACGAAATCATCAAAGCGATCCAAATATCCACTTCCAGATTCTACAAAAAGAGTGTTTCAA

             *         *   * **       *    *        * ** * *   **      *     
     158499 ACCTGCTCTATGAAAGGCCATGTTCATCTCTATGAGTTGAATGGAAATATCCGAAAGAAATTTCT
         66 AACTGCTCTATCAAAAGAAATGTTCAACTCTGTGAGTTGAGTACACACATCACAAAGAAGTTTCT

             *       *          *        *         **
     158564 GGGAATGCTGCTGTCTAG-TGTT-TAT-ACGA-ATTCCCGC
        131 GAGAATGCTTCTGTCTAGTTTTTATATGAAGATATTCCCTT

                    *    *       *                *    *  *                  
     158601 TTCCAACGTAATCCTCAAAGCAATCCAAATATCCACTTGCAGAATCCACAAAAAGAGTGTTTCAA
          1 TTCCAACGAAATCATCAAAGCGATCCAAATATCCACTTCCAGATTCTACAAAAAGAGTGTTTCAA

                          *     *          * *                   *  *        
     158666 AACTGCTCTATCAATAGAAAGGTTCAACTCTTTTAGTTGAGTACACACATCACGAACAAGTTTCT
         66 AACTGCTCTATCAAAAGAAATGTTCAACTCTGTGAGTTGAGTACACACATCACAAAGAAGTTTCT

                            * *      **     **  *    
     158731 GAGAATGCTTCTGTCTGGCTTTTATTGGAAGACGTTTCCTT
        131 GAGAATGCTTCTGTCTAGTTTTTATATGAAGATATTCCCTT

                 * *  **          *       *                * *               
     158772 TT-CACCAAAGGCATCAAAGCGCTCCAAATGTCCACTTCCAGATTCTTCCAAAAGAGTGTTTCAA
          1 TTCCAACGAAATCATCAAAGCGATCCAAATATCCACTTCCAGATTCTACAAAAAGAGTGTTTCAA

             **     * ***  * *                 *    * *  * *     *   *       
     158836 ACGTGCTCAAAGTAAGGGAATGTTCAACTCTGTGACTTGAATGCAGATATCACCAAGTAGTTTCT
         66 AACTGCTCTATCAAAAGAAATGTTCAACTCTGTGAGTTGAGTACACACATCACAAAGAAGTTTCT

            * * *            **     *    *         * 
     158901 AATAGTGCTTCTGTCTACATTTTAGATGATGATATTCCCGT
        131 GAGAATGCTTCTGTCTAGTTTTTATATGAAGATATTCCCTT

                         *** *   *             *  *   *      *     *       * 
     158942 TTCCAACGAAATCGCTAGAGCTATCCAAATATCCAGTTACAGTTTCTACCAAAAGGGTGTTTCCA
          1 TTCCAACGAAATCATCAAAGCGATCCAAATATCCACTTCCAGATTCTACAAAAAGAGTGTTTCAA

              *    **           *            *       *                     * 
     159007 AATTGCTGCATCAAAAGAAAGGTTCAACTCTGTTAGTTGAGGACACACATCACAAAGAAGTTTGT
         66 AACTGCTCTATCAAAAGAAATGTTCAACTCTGTGAGTTGAGTACACACATCACAAAGAAGTTTCT

                              *    *     *           
     159072 GAGAATGCTTCTGTCTAGGTTTTGTATGACGATATTCCCTT
        131 GAGAATGCTTCTGTCTAGTTTTTATATGAAGATATTCCCTT

                     *   * *     *   *       * *  *    *  *       *  *       
     159113 TTCCAACGATATCGTTAAAGCAATCTAAATATCAATTTGCAGAATCCACAAAAATAGAGTTTCAA
          1 TTCCAACGAAATCATCAAAGCGATCCAAATATCCACTTCCAGATTCTACAAAAAGAGTGTTTCAA

             *       * *        *    *       *  *                   ***      
     159178 AGCTGCTCTGTAAAAAGAAAGGTTCCACTCTGTTAGCTGAGTACACACATCACAAACTTGTTTCT
         66 AACTGCTCTATCAAAAGAAATGTTCAACTCTGTGAGTTGAGTACACACATCACAAAGAAGTTTCT

            *     *         *        **       * **   
     159243 CAGAATCCTTCTGTCTCGTTTTTATGGGAAGATACTTACTT
        131 GAGAATGCTTCTGTCTAGTTTTTATATGAAGATATTCCCTT

                 *  * **          *       *     *       *  *  *  *           
     159284 TTCCACCGTAGGCATCAAAGCGCTCCAAATGTCCACATCCAGATACTCCAGAACGAGTGTTTCAA
          1 TTCCAACGAAATCATCAAAGCGATCCAAATATCCACTTCCAGATTCTACAAAAAGAGTGTTTCAA

             *         *   * *   *         *        * *  *      *      *     
     159349 ACCTGCTCTATGAAAGGGAATCTTCAACTCTATGAGTTGAATGCAGACATCAGAAAGAAATTTCT
         66 AACTGCTCTATCAAAAGAAATGTTCAACTCTGTGAGTTGAGTACACACATCACAAAGAAGTTTCT

                     *       **          *         **
     159414 GAGAATGCTGCTGTCTACCTTTTAT-T--TGA-ATTCCCGC
        131 GAGAATGCTTCTGTCTAGTTTTTATATGAAGATATTCCCTT

                         *  *    *              * *  **   *    *             
     159451 TTCCAACGAAATCCTCCAAGCTATCCAAATATCCACCTGCATTTTCCACAACAAGAGTGTTTCAA
          1 TTCCAACGAAATCATCAAAGCGATCCAAATATCCACTTCCAGATTCTACAAAAAGAGTGTTTCAA

                          *                 *    *  *                *       
     159516 AACTGCTCTATCAATAGAAATGTTCAACTCCTTTG-GCTGGGTACACACATCACAAACAAGTTTC
         66 AACTGCTCTATCAAAAGAAATGTTCAACT-CTGTGAGTTGAGTACACACATCACAAAGAAGTTTC

                                      **     **       
     159580 TGAGAATGCTTCTGTCTAGTTTTTATGGGAAGACGTTCCCTT
        130 TGAGAATGCTTCTGTCTAGTTTTTATATGAAGATATTCCCTT

              *  * *  **          *       *            **                    
     159622 TTTCACCAAAGGCATCAAAGCGCTCCAAATGTCCACTTCCAGACACTACAAAAAGAGTGTTTCAA
          1 TTCCAACGAAATCATCAAAGCGATCCAAATATCCACTTCCAGATTCTACAAAAAGAGTGTTTCAA

             **       **   ***                 *    * *    *         *       
     159687 ACGTGCTCTAAGAAAGCGAATGTTCAACTCTGTGACTTGAATGCACATATCACAAAGTAGTTTCT
         66 AACTGCTCTATCAAAAGAAATGTTCAACTCTGTGAGTTGAGTACACACATCACAAAGAAGTTTCT

                **            *     *    *         * 
     159752 GAGAGGGCTTCTGTCTAGATTTTAGATGATGATATTCCCGT
        131 GAGAATGCTTCTGTCTAGTTTTTATATGAAGATATTCCCTT

                   *       * *    *               *   *                    * 
     159793 TTCCAACAAAATCATTAGAGC-TTCCAAATATCCACTTACAGTTTCTACAAAAAGAGTGTTTCCA
          1 TTCCAACGAAATCATCAAAGCGATCCAAATATCCACTTCCAGATTCTACAAAAAGAGTGTTTCAA

                   **         * *    *       *  *                   ***      
     159857 AACTGCTGCATCAAAAGAGAGGTTCCACTCTGTTAGCTGAGTACACACATCACAAACTTGTTTCT
         66 AACTGCTCTATCAAAAGAAATGTTCAACTCTGTGAGTTGAGTACACACATCACAAAGAAGTTTCT

            *     *         *        **         **   
     159922 CAGAATCCTTCTGTCTCGTTTTTATGGGAAGATATTTACTT
        131 GAGAATGCTTCTGTCTAGTTTTTATATGAAGATATTCCCTT

              *  *  * **          *       *     *       *  *  *              
     159963 TTTCACCGTAGGCATCAAAGCGCTCCAAATGTCCACATCCAGATACTCCAGAAAGAGTGTTTCAA
          1 TTCCAACGAAATCATCAAAGCGATCCAAATATCCACTTCCAGATTCTACAAAAAGAGTGTTTCAA

             *    *    *   * *   *         *        * *  *      *      *     
     160028 ACCTGCCCTATGAAAGGGAATCTTCAACTCTATGAGTTGAATGCAGACATCAGAAAGAAATTTCT
         66 AACTGCTCTATCAAAAGAAATGTTCAACTCTGTGAGTTGAGTACACACATCACAAAGAAGTTTCT

                     *       **          *         **
     160093 GAGAATGCTGCTGTCTACCTTTTAT-T--TGA-ATTCCCGC
        131 GAGAATGCTTCTGTCTAGTTTTTATATGAAGATATTCCCTT

                         *  *    *                *       *                  
     160130 TTCCAACGAAATCCTCCAAGCTATCCAAATATCCACTTGCAGATTCCACAAAAAGAGTGTTTCAA
          1 TTCCAACGAAATCATCAAAGCGATCCAAATATCCACTTCCAGATTCTACAAAAAGAGTGTTTCAA

                            ** *  *            *       *     *     *  *      
     160195 AACTGCTCTCTATCAATGGCAAAGTTCAACTCTGTTAGTTGAGGACACATATCACCAACAAGTTT
         66 AACTG--CTCTATCAAAAGAAATGTTCAACTCTGTGAGTTGAGTACACACATCACAAAGAAGTTT

                               *       **         *    
     160260 CTGAGAATGCTTCTGTCTATTTTTTATGGGAAGATATTTCCTT
        129 CTGAGAATGCTTCTGTCTAGTTTTTATATGAAGATATTCCCTT

              *  *  * ** *    *      *    *          * **                    
     160303 TTTCACCGTAGGCGTCAAGGCGATCGAAATGTCCACTTCCACAAACTACAAAAAGAGTGTTTCAA
          1 TTCCAACGAAATCATCAAAGCGATCCAAATATCCACTTCCAGATTCTACAAAAAGAGTGTTTCAA

             *         *   * **       *    *        * ** * *   **      *     
     160368 ACCTGCTCTATGAAAGGCCATGTTCATCTCTATGAGTTGAATGGAAATATCCGAAAGAAATTTCT
         66 AACTGCTCTATCAAAAGAAATGTTCAACTCTGTGAGTTGAGTACACACATCACAAAGAAGTTTCT

             *       *          *        *         **
     160433 GGGAATGCTGCTGTCTAG-TGTT-TAT-ACGA-ATTCCCGC
        131 GAGAATGCTTCTGTCTAGTTTTTATATGAAGATATTCCCTT

                         *       *                *    *  *                  
     160470 TTCCAACGAAATCCTCAAAGCAATCCAAATATCCACTTGCAGAATCCACAAAAAGAGTGTTTCAA
          1 TTCCAACGAAATCATCAAAGCGATCCAAATATCCACTTCCAGATTCTACAAAAAGAGTGTTTCAA

                          *     *          * *                   *  *        
     160535 AACTGCTCTATCAATAGAAAGGTTCAACTCTTTTAGTTGAGTACACACATCACGAACAAGTTTCT
         66 AACTGCTCTATCAAAAGAAATGTTCAACTCTGTGAGTTGAGTACACACATCACAAAGAAGTTTCT

                            * *      **     **  *    
     160600 GAGAATGCTTCTGTCTGGCTTTTATTGGAAGACGTTTCCTT
        131 GAGAATGCTTCTGTCTAGTTTTTATATGAAGATATTCCCTT

                   *  **          *       *                * *               
     160641 TT-CAACAAAGGCATCAAAGCGCTCCAAATGTCCACTTCCAGATTCTTCCAAAAGAGTGTTTCAA
          1 TTCCAACGAAATCATCAAAGCGATCCAAATATCCACTTCCAGATTCTACAAAAAGAGTGTTTCAA

             **     * ***    *                 *    * *  * *     *   *       
     160705 ACGTGCTCAAAGTAAAGGAATGTTCAACTCTGTGACTTGAATGCAGATATCACCAAGTAGTTTCT
         66 AACTGCTCTATCAAAAGAAATGTTCAACTCTGTGAGTTGAGTACACACATCACAAAGAAGTTTCT

            * * *        *   **     *    *           
     160770 AATAGTGCTTCTGCCTACATTTTAGATGATGATATTCCCTT
        131 GAGAATGCTTCTGTCTAGTTTTTATATGAAGATATTCCCTT

                         * * *   *             *  *   *      *     *       * 
     160811 TTCCAACGAAATCGTTAGAGCTATCCAAATATCCAGTTACAGTTTCTACCAAAAGGGTGTTTCCA
          1 TTCCAACGAAATCATCAAAGCGATCCAAATATCCACTTCCAGATTCTACAAAAAGAGTGTTTCAA

              *    **           *            *       *                     * 
     160876 AATTGCTGCATCAAAAGAAAGGTTCAACTCTGTTAGTTGAGGACACACATCACAAAGAAGTTTGT
         66 AACTGCTCTATCAAAAGAAATGTTCAACTCTGTGAGTTGAGTACACACATCACAAAGAAGTTTCT

                              *    *     *           
     160941 GAGAATGCTTCTGTCTAGATTTTGTATGACGATATTCCCTT
        131 GAGAATGCTTCTGTCTAGTTTTTATATGAAGATATTCCCTT

                     *   * *     *   *       * *  *    *  *       *  *       
     160982 TTCCAACGATATCGTTAAAGCAATCTAAATATCAATTTGCAGAATCCACAAAAATAGAGTTTCAA
          1 TTCCAACGAAATCATCAAAGCGATCCAAATATCCACTTCCAGATTCTACAAAAAGAGTGTTTCAA

             *       * *        *    *       *  *                   ***      
     161047 AGCTGCTCTGTAAAAAGAAAGGTTCCACTCTGTTAGCTGAGTACACACATCACAAACTTGTTTCT
         66 AACTGCTCTATCAAAAGAAATGTTCAACTCTGTGAGTTGAGTACACACATCACAAAGAAGTTTCT

                  *         *        **         **   
     161112 GAGAATCCTTCTGTCTCGTTTTTATGGGAAGATATTTACTT
        131 GAGAATGCTTCTGTCTAGTTTTTATATGAAGATATTCCCTT

                 *  * **          *       *     *       *  *  *  *           
     161153 TTCCACCGTAGGCATCAAAGCGCTCCAAATGTCCACATCCAGATACTCCAGAACGAGTGTTTCAA
          1 TTCCAACGAAATCATCAAAGCGATCCAAATATCCACTTCCAGATTCTACAAAAAGAGTGTTTCAA

             *         *   * *   *         *        * *  *      *      *     
     161218 ACCTGCTCTATGAAAGGGAATCTTCAACTCTATGAGTTGAATGCAGACATCAGAAAGAAATTTCT
         66 AACTGCTCTATCAAAAGAAATGTTCAACTCTGTGAGTTGAGTACACACATCACAAAGAAGTTTCT

                     *       **          *         **
     161283 GAGAATGCTGCTGTCTACCTTTTAT-T--TGA-ATTCCCGC
        131 GAGAATGCTTCTGTCTAGTTTTTATATGAAGATATTCCCTT

                         *  *    *              * *  **   *                  
     161320 TTCCAACGAAATCCTCCAAGCTATCCAAATATCCACCTGCATTTTCCACAAAAAGAGTGTTTCAA
          1 TTCCAACGAAATCATCAAAGCGATCCAAATATCCACTTCCAGATTCTACAAAAAGAGTGTTTCAA

                          *                 *    *  *                *       
     161385 AACTGCTCTATCAATAGAAATGTTCAACTCCTTTG-GCTGGGTACACACATCACAAACAAGTTTC
         66 AACTGCTCTATCAAAAGAAATGTTCAACT-CTGTGAGTTGAGTACACACATCACAAAGAAGTTTC

                                      **     *        
     161449 TGAGAATGCTTCTGTCTAGTTTTTATGGGAAGACATTCCCTT
        130 TGAGAATGCTTCTGTCTAGTTTTTATATGAAGATATTCCCTT

              *  * *  **          *       *            **                    
     161491 TTTCACCAAAGACATCAAAGCGCTCCAAATGTCCACTTCCAGACACTACAAAAAGAGTGTTTCAA
          1 TTCCAACGAAATCATCAAAGCGATCCAAATATCCACTTCCAGATTCTACAAAAAGAGTGTTTCAA

             **       **   ***           *     *    * *  * *         *       
     161556 ACGTGCTCTAAGAAAGCGAATGTTCAACTTTGTGACTTGAATGCAGATATCACAAAGTAGTTTCT
         66 AACTGCTCTATCAAAAGAAATGTTCAACTCTGTGAGTTGAGTACACACATCACAAAGAAGTTTCT

                **            *     *    *         * 
     161621 GAGAGGGCTTCTGTCTAGATTTTAGATGATGATATTCCCGT
        131 GAGAATGCTTCTGTCTAGTTTTTATATGAAGATATTCCCTT

                           * *   *                *   *                    * 
     161662 TTCCAACGAAATCATTAGAGCTATCCAAATATCCACTTACAGTTTCTACAAAAAGAGTGTTTCCA
          1 TTCCAACGAAATCATCAAAGCGATCCAAATATCCACTTCCAGATTCTACAAAAAGAGTGTTTCAA

                   **         * *    *       *  *                   ***      
     161727 AACTGCTGCATCAAAAGAGAGGTTCCACTCTGTTAGCTGAGTACACACATCACAAACTTGTTTCT
         66 AACTGCTCTATCAAAAGAAATGTTCAACTCTGTGAGTTGAGTACACACATCACAAAGAAGTTTCT

            *     *         *        **         **   
     161792 CAGAATCCTTCTGTCTCGTTTTTATGGGAAGATATTTACTT
        131 GAGAATGCTTCTGTCTAGTTTTTATATGAAGATATTCCCTT

              *  *  * **          *       *     *       *  *  *              
     161833 TTTCACCGTAGGCATCAAAGCGCTCCAAATGTCCACATCCAGATACTCCAGAAAGAGTGTTTCAA
          1 TTCCAACGAAATCATCAAAGCGATCCAAATATCCACTTCCAGATTCTACAAAAAGAGTGTTTCAA

             *         *   * *   *         *        * *  *      *      *     
     161898 ACCTGCTCTATGAAAGGGAATCTTCAACTCTATGAGTTGAATGCAGACATCAGAAAGAAATTTCT
         66 AACTGCTCTATCAAAAGAAATGTTCAACTCTGTGAGTTGAGTACACACATCACAAAGAAGTTTCT

                     *       **          *         **
     161963 GAGAATGCTGCTGTCTACCTTTTAT-T--TGA-ATTCCCGC
        131 GAGAATGCTTCTGTCTAGTTTTTATATGAAGATATTCCCTT

                         *  *    *                *       *                  
     162000 TTCCAACGAAATCCTCCAAGCTATCCAAATATCCACTTGCAGATTCCACAAAAAGAGTGTTTCAA
          1 TTCCAACGAAATCATCAAAGCGATCCAAATATCCACTTCCAGATTCTACAAAAAGAGTGTTTCAA

                            ** *  *            *       *     *     *  *      
     162065 AACTGCTCTCTATCAATGGCAAAGTTCAACTCTGTTAGTTGAGGACACATATCACCAACAAGTTT
         66 AACTG--CTCTATCAAAAGAAATGTTCAACTCTGTGAGTTGAGTACACACATCACAAAGAAGTTT

                               *       **         *    
     162130 CTGAGAATGCTTCTGTCTATTTTTTATGGGAAGATATTTCCTT
        129 CTGAGAATGCTTCTGTCTAGTTTTTATATGAAGATATTCCCTT

              *  *  * ** *    *      *    *          * **                    
     162173 TTTCACCGTAGGCGTCAAGGCGATCGAAATGTCCACTTCCACAAACTACAAAAAGAGTGTTTCAA
          1 TTCCAACGAAATCATCAAAGCGATCCAAATATCCACTTCCAGATTCTACAAAAAGAGTGTTTCAA

             *         *   * **       *    *        * ** * *   **      *     
     162238 ACCTGCTCTATGAAAGGCCATGTTCATCTCTATGAGTTGAATGGAAATATCCGAAAGAAATTTCT
         66 AACTGCTCTATCAAAAGAAATGTTCAACTCTGTGAGTTGAGTACACACATCACAAAGAAGTTTCT

             *       *          *        *         **
     162303 GGGAATGCTGCTGTCTAG-TGTT-TAT-ACGA-ATTCCCGC
        131 GAGAATGCTTCTGTCTAGTTTTTATATGAAGATATTCCCTT

                    *    *       *                *    *  *                  
     162340 TTCCAACGTAATCCTCAAAGCAATCCAAATATCCACTTGCAGAATCCACAAAAAGAGTGTTTCAA
          1 TTCCAACGAAATCATCAAAGCGATCCAAATATCCACTTCCAGATTCTACAAAAAGAGTGTTTCAA

                          *     *          * *                   *  *        
     162405 AACTGCTCTATCAATAGAAAGGTTCAACTCTTTTAGTTGAGTACACACATCACGAACAAGTTTCT
         66 AACTGCTCTATCAAAAGAAATGTTCAACTCTGTGAGTTGAGTACACACATCACAAAGAAGTTTCT

                            * *      **     **  *    
     162470 GAGAATGCTTCTGTCTGGCTTTTATTGGAAGACGTTTCCTT
        131 GAGAATGCTTCTGTCTAGTTTTTATATGAAGATATTCCCTT

                 * *  **          *       *                * *               
     162511 TT-CACCAAAGGCATCAAAGCGCTCCAAATGTCCACTTCCAGATTCTTCCAAAAGAGTGTTTCAA
          1 TTCCAACGAAATCATCAAAGCGATCCAAATATCCACTTCCAGATTCTACAAAAAGAGTGTTTCAA

             **     * ***  * *                 *    * *  * *     *   *       
     162575 ACGTGCTCAAAGTAAGGGAATGTTCAACTCTGTGACTTGAATGCAGATATCACCAAGTAGTTTCT
         66 AACTGCTCTATCAAAAGAAATGTTCAACTCTGTGAGTTGAGTACACACATCACAAAGAAGTTTCT

            * * *            **     *    *         * 
     162640 AATAGTGCTTCTGTCTACATTTTAGATGATGATATTCCCGT
        131 GAGAATGCTTCTGTCTAGTTTTTATATGAAGATATTCCCTT

                         *** *   *             *  *   *      *     *       * 
     162681 TTCCAACGAAATCGCTAGAGCTATCCAAATATCCAGTTACAGTTTCTACCAAAAGGGTGTTTCCA
          1 TTCCAACGAAATCATCAAAGCGATCCAAATATCCACTTCCAGATTCTACAAAAAGAGTGTTTCAA

              *    **           *            *       *                     * 
     162746 AATTGCTGCATCAAAAGAAAGGTTCAACTCTGTTAGTTGAGGACACACATCACAAAGAAGTTTGT
         66 AACTGCTCTATCAAAAGAAATGTTCAACTCTGTGAGTTGAGTACACACATCACAAAGAAGTTTCT

                              *    *     *           
     162811 GAGAATGCTTCTGTCTAGGTTTTGTATGACGATATTCCCTT
        131 GAGAATGCTTCTGTCTAGTTTTTATATGAAGATATTCCCTT

                     *   * *     *   *       * *  *    *  *       *  *       
     162852 TTCCAACGATATCGTTAAAGCAATCTAAATATCAATTTGCAGAATCCACAAAAATAGAGTTTCAA
          1 TTCCAACGAAATCATCAAAGCGATCCAAATATCCACTTCCAGATTCTACAAAAAGAGTGTTTCAA

             *       * *        *    *       *  *                   ***      
     162917 AGCTGCTCTGTAAAAAGAAAGGTTCCACTCTGTTAGCTGAGTACACACATCACAAACTTGTTTCT
         66 AACTGCTCTATCAAAAGAAATGTTCAACTCTGTGAGTTGAGTACACACATCACAAAGAAGTTTCT

            *     *         *        **       * **   
     162982 CAGAATCCTTCTGTCTCGTTTTTATGGGAAGATACTTACTT
        131 GAGAATGCTTCTGTCTAGTTTTTATATGAAGATATTCCCTT

                 *  * **          *       *     *       *  *  *  *           
     163023 TTCCACCGTAGGCATCAAAGCGCTCCAAATGTCCACATCCAGATACTCCAGAACGAGTGTTTCAA
          1 TTCCAACGAAATCATCAAAGCGATCCAAATATCCACTTCCAGATTCTACAAAAAGAGTGTTTCAA

             *         *   * *   *         *        * *  *      *      *     
     163088 ACCTGCTCTATGAAAGGGAATCTTCAACTCTATGAGTTGAATGCAGACATCAGAAAGAAATTTCT
         66 AACTGCTCTATCAAAAGAAATGTTCAACTCTGTGAGTTGAGTACACACATCACAAAGAAGTTTCT

                     *       **          *         **
     163153 GAGAATGCTGCTGTCTACCTTTTAT-T--TGA-ATTCCCGC
        131 GAGAATGCTTCTGTCTAGTTTTTATATGAAGATATTCCCTT

                         *  *    *              * *  **   *    *             
     163190 TTCCAACGAAATCCTCCAAGCTATCCAAATATCCACCTGCATTTTCCACAACAAGAGTGTTTCAA
          1 TTCCAACGAAATCATCAAAGCGATCCAAATATCCACTTCCAGATTCTACAAAAAGAGTGTTTCAA

                          *                 *    *  *                *       
     163255 AACTGCTCTATCAATAGAAATGTTCAACTCCTTTG-GCTGGGTACACACATCACAAACAAGTTTC
         66 AACTGCTCTATCAAAAGAAATGTTCAACT-CTGTGAGTTGAGTACACACATCACAAAGAAGTTTC

                                      **     **       
     163319 TGAGAATGCTTCTGTCTAGTTTTTATGGGAAGACGTTCCCTT
        130 TGAGAATGCTTCTGTCTAGTTTTTATATGAAGATATTCCCTT

              *  * *  **          *       *            **                    
     163361 TTTCACCAAAGGCATCAAAGCGCTCCAAATGTCCACTTCCAGACACTACAAAAAGAGTGTTTCAA
          1 TTCCAACGAAATCATCAAAGCGATCCAAATATCCACTTCCAGATTCTACAAAAAGAGTGTTTCAA

             **       **   ***                 *    * *    *         *       
     163426 ACGTGCTCTAAGAAAGCGAATGTTCAACTCTGTGACTTGAATGCACATATCACAAAGTAGTTTCT
         66 AACTGCTCTATCAAAAGAAATGTTCAACTCTGTGAGTTGAGTACACACATCACAAAGAAGTTTCT

                **            *     *    *         * 
     163491 GAGAGGGCTTCTGTCTAGATTTTAGATGATGATATTCCCGT
        131 GAGAATGCTTCTGTCTAGTTTTTATATGAAGATATTCCCTT

                   *       * *    *               *   *                    * 
     163532 TTCCAACAAAATCATTAGAGC-TTCCAAATATCCACTTACAGTTTCTACAAAAAGAGTGTTTCCA
          1 TTCCAACGAAATCATCAAAGCGATCCAAATATCCACTTCCAGATTCTACAAAAAGAGTGTTTCAA

                   **         * *    *       *  *                   ***      
     163596 AACTGCTGCATCAAAAGAGAGGTTCCACTCTGTTAGCTGAGTACACACATCACAAACTTGTTTCT
         66 AACTGCTCTATCAAAAGAAATGTTCAACTCTGTGAGTTGAGTACACACATCACAAAGAAGTTTCT

            *     *         *        **         **   
     163661 CAGAATCCTTCTGTCTCGTTTTTATGGGAAGATATTTACTT
        131 GAGAATGCTTCTGTCTAGTTTTTATATGAAGATATTCCCTT

              *  *  * **          *       *     *       *  *  *              
     163702 TTTCACCGTAGGCATCAAAGCGCTCCAAATGTCCACATCCAGATACTCCAGAAAGAGTGTTTCAA
          1 TTCCAACGAAATCATCAAAGCGATCCAAATATCCACTTCCAGATTCTACAAAAAGAGTGTTTCAA

             *    *    *   * *   *         *        * *  *      *      *     
     163767 ACCTGCCCTATGAAAGGGAATCTTCAACTCTATGAGTTGAATGCAGACATCAGAAAGAAATTTCT
         66 AACTGCTCTATCAAAAGAAATGTTCAACTCTGTGAGTTGAGTACACACATCACAAAGAAGTTTCT

                     *       **          *         **
     163832 GAGAATGCTGCTGTCTACCTTTTAT-T--TGA-ATTCCCGC
        131 GAGAATGCTTCTGTCTAGTTTTTATATGAAGATATTCCCTT

                         *  *    *                *       *                  
     163869 TTCCAACGAAATCCTCCAAGCTATCCAAATATCCACTTGCAGATTCCACAAAAAGAGTGTTTCAA
          1 TTCCAACGAAATCATCAAAGCGATCCAAATATCCACTTCCAGATTCTACAAAAAGAGTGTTTCAA

                            ** *  *            *       *     *     *  *      
     163934 AACTGCTCTCTATCAATGGCAAAGTTCAACTCTGTTAGTTGAGGACACATATCACCAACAAGTTT
         66 AACTG--CTCTATCAAAAGAAATGTTCAACTCTGTGAGTTGAGTACACACATCACAAAGAAGTTT

                               *       **         *    
     163999 CTGAGAATGCTTCTGTCTATTTTTTATGGGAAGATATTTCCTT
        129 CTGAGAATGCTTCTGTCTAGTTTTTATATGAAGATATTCCCTT

              *  *  * ** *    *      *    *          * **                    
     164042 TTTCACCGTAGGCGTCAAGGCGATCGAAATGTCCACTTCCACAAACTACAAAAAGAGTGTTTCAA
          1 TTCCAACGAAATCATCAAAGCGATCCAAATATCCACTTCCAGATTCTACAAAAAGAGTGTTTCAA

             *         *   * **       *    *        * ** * *   **      *     
     164107 ACCTGCTCTATGAAAGGCCATGTTCATCTCTATGAGTTGAATGGAAATATCCGAAAGAAATTTCT
         66 AACTGCTCTATCAAAAGAAATGTTCAACTCTGTGAGTTGAGTACACACATCACAAAGAAGTTTCT

             *       *          *        *         **
     164172 GGGAATGCTGCTGTCTAG-TGTT-TAT-ACGA-ATTCCCGC
        131 GAGAATGCTTCTGTCTAGTTTTTATATGAAGATATTCCCTT

                         *       *                *    *  *                  
     164209 TTCCAACGAAATCCTCAAAGCAATCCAAATATCCACTTGCAGAATCCACAAAAAGAGTGTTTCAA
          1 TTCCAACGAAATCATCAAAGCGATCCAAATATCCACTTCCAGATTCTACAAAAAGAGTGTTTCAA

                          *     *          * *                   *  *        
     164274 AACTGCTCTATCAATAGAAAGGTTCAACTCTTTTAGTTGAGTACACACATCACGAACAAGTTTCT
         66 AACTGCTCTATCAAAAGAAATGTTCAACTCTGTGAGTTGAGTACACACATCACAAAGAAGTTTCT

                            * *      **     **  *    
     164339 GAGAATGCTTCTGTCTGGCTTTTATTGGAAGACGTTTCCTT
        131 GAGAATGCTTCTGTCTAGTTTTTATATGAAGATATTCCCTT

                   *  **          *       *                * *               
     164380 TT-CAACAAAGGCATCAAAGCGCTCCAAATGTCCACTTCCAGATTCTTCCAAAAGAGTGTTTCAA
          1 TTCCAACGAAATCATCAAAGCGATCCAAATATCCACTTCCAGATTCTACAAAAAGAGTGTTTCAA

             **     * ***    *                 *    * *  * *     *   *       
     164444 ACGTGCTCAAAGTAAAGGAATGTTCAACTCTGTGACTTGAATGCAGATATCACCAAGTAGTTTCT
         66 AACTGCTCTATCAAAAGAAATGTTCAACTCTGTGAGTTGAGTACACACATCACAAAGAAGTTTCT

            * * *        *   **     *    *           
     164509 AATAGTGCTTCTGCCTACATTTTAGATGATGATATTCCCTT
        131 GAGAATGCTTCTGTCTAGTTTTTATATGAAGATATTCCCTT

                         * * *   *             *  *   *      *     *       * 
     164550 TTCCAACGAAATCGTTAGAGCTATCCAAATATCCAGTTACAGTTTCTACCAAAAGGGTGTTTCCA
          1 TTCCAACGAAATCATCAAAGCGATCCAAATATCCACTTCCAGATTCTACAAAAAGAGTGTTTCAA

              *    **           *            *       *                     * 
     164615 AATTGCTGCATCAAAAGAAAGGTTCAACTCTGTTAGTTGAGGACACACATCACAAAGAAGTTTGT
         66 AACTGCTCTATCAAAAGAAATGTTCAACTCTGTGAGTTGAGTACACACATCACAAAGAAGTTTCT

                              *    *     *           
     164680 GAGAATGCTTCTGTCTAGATTTTGTATGACGATATTCCCTT
        131 GAGAATGCTTCTGTCTAGTTTTTATATGAAGATATTCCCTT

                     *   * *     *   *       * *  *    *  *       *  *       
     164721 TTCCAACGATATCGTTAAAGCAATCTAAATATCAATTTGCAGAATCCACAAAAATAGAGTTTCAA
          1 TTCCAACGAAATCATCAAAGCGATCCAAATATCCACTTCCAGATTCTACAAAAAGAGTGTTTCAA

             *       * *        *    *       *  *                   ***      
     164786 AGCTGCTCTGTAAAAAGAAAGGTTCCACTCTGTTAGCTGAGTACACACATCACAAACTTGTTTCT
         66 AACTGCTCTATCAAAAGAAATGTTCAACTCTGTGAGTTGAGTACACACATCACAAAGAAGTTTCT

                  *         *        **         **   
     164851 GAGAATCCTTCTGTCTCGTTTTTATGGGAAGATATTTACTT
        131 GAGAATGCTTCTGTCTAGTTTTTATATGAAGATATTCCCTT

                 *  * **          *       *     *       *  *  *  *           
     164892 TTCCACCGTAGGCATCAAAGCGCTCCAAATGTCCACATCCAGATACTCCAGAACGAGTGTTTCAA
          1 TTCCAACGAAATCATCAAAGCGATCCAAATATCCACTTCCAGATTCTACAAAAAGAGTGTTTCAA

             *         *   * *   *         *        * *  *      *      *     
     164957 ACCTGCTCTATGAAAGGGAATCTTCAACTCTATGAGTTGAATGCAGACATCAGAAAGAAATTTCT
         66 AACTGCTCTATCAAAAGAAATGTTCAACTCTGTGAGTTGAGTACACACATCACAAAGAAGTTTCT

                     *       **          *         **
     165022 GAGAATGCTGCTGTCTACCTTTTAT-T--TGA-ATTCCCGC
        131 GAGAATGCTTCTGTCTAGTTTTTATATGAAGATATTCCCTT

                         *  *    *              * *  **   *                  
     165059 TTCCAACGAAATCCTCCAAGCTATCCAAATATCCACCTGCATTTTCCACAAAAAGAGTGTTTCAA
          1 TTCCAACGAAATCATCAAAGCGATCCAAATATCCACTTCCAGATTCTACAAAAAGAGTGTTTCAA

                          *                 *    *  *                *       
     165124 AACTGCTCTATCAATAGAAATGTTCAACTCCTTTG-GCTGGGTACACACATCACAAACAAGTTTC
         66 AACTGCTCTATCAAAAGAAATGTTCAACT-CTGTGAGTTGAGTACACACATCACAAAGAAGTTTC

                                      **     *        
     165188 TGAGAATGCTTCTGTCTAGTTTTTATGGGAAGACATTCCCTT
        130 TGAGAATGCTTCTGTCTAGTTTTTATATGAAGATATTCCCTT

              *  * *  **          *       *            **                    
     165230 TTTCACCAAAGACATCAAAGCGCTCCAAATGTCCACTTCCAGACACTACAAAAAGAGTGTTTCAA
          1 TTCCAACGAAATCATCAAAGCGATCCAAATATCCACTTCCAGATTCTACAAAAAGAGTGTTTCAA

             **       **   ***           *     *    * *  * *         *       
     165295 ACGTGCTCTAAGAAAGCGAATGTTCAACTTTGTGACTTGAATGCAGATATCACAAAGTAGTTTCT
         66 AACTGCTCTATCAAAAGAAATGTTCAACTCTGTGAGTTGAGTACACACATCACAAAGAAGTTTCT

                **            *     *    *         * 
     165360 GAGAGGGCTTCTGTCTAGATTTTAGATGATGATATTCCCGT
        131 GAGAATGCTTCTGTCTAGTTTTTATATGAAGATATTCCCTT

                           * *   *                *   *                    * 
     165401 TTCCAACGAAATCATTAGAGCTATCCAAATATCCACTTACAGTTTCTACAAAAAGAGTGTTTCCA
          1 TTCCAACGAAATCATCAAAGCGATCCAAATATCCACTTCCAGATTCTACAAAAAGAGTGTTTCAA

                   **         * *    *       *  *                   ***      
     165466 AACTGCTGCATCAAAAGAGAGGTTCCACTCTGTTAGCTGAGTACACACATCACAAACTTGTTTCT
         66 AACTGCTCTATCAAAAGAAATGTTCAACTCTGTGAGTTGAGTACACACATCACAAAGAAGTTTCT

            *     *         *        **         **   
     165531 CAGAATCCTTCTGTCTCGTTTTTATGGGAAGATATTTACTT
        131 GAGAATGCTTCTGTCTAGTTTTTATATGAAGATATTCCCTT

              *  *  * **          *       *     *       *  *  *              
     165572 TTTCACCGTAGGCATCAAAGCGCTCCAAATGTCCACATCCAGATACTCCAGAAAGAGTGTTTCAA
          1 TTCCAACGAAATCATCAAAGCGATCCAAATATCCACTTCCAGATTCTACAAAAAGAGTGTTTCAA

             *         *   * *   *         *        * *  *      *      *     
     165637 ACCTGCTCTATGAAAGGGAATCTTCAACTCTATGAGTTGAATGCAGACATCAGAAAGAAATTTCT
         66 AACTGCTCTATCAAAAGAAATGTTCAACTCTGTGAGTTGAGTACACACATCACAAAGAAGTTTCT

                     *       **          *         **
     165702 GAGAATGCTGCTGTCTACCTTTTAT-T--TGA-ATTCCCGC
        131 GAGAATGCTTCTGTCTAGTTTTTATATGAAGATATTCCCTT

                         *  *    *                *       *                  
     165739 TTCCAACGAAATCCTCCAAGCTATCCAAATATCCACTTGCAGATTCCACAAAAAGAGTGTTTCAA
          1 TTCCAACGAAATCATCAAAGCGATCCAAATATCCACTTCCAGATTCTACAAAAAGAGTGTTTCAA

                            ** *  *            *       *     *     *  *      
     165804 AACTGCTCTCTATCAATGGCAAAGTTCAACTCTGTTAGTTGAGGACACATATCACCAACAAGTTT
         66 AACTG--CTCTATCAAAAGAAATGTTCAACTCTGTGAGTTGAGTACACACATCACAAAGAAGTTT

                               *       **         *    
     165869 CTGAGAATGCTTCTGTCTATTTTTTATGGGAAGATATTTCCTT
        129 CTGAGAATGCTTCTGTCTAGTTTTTATATGAAGATATTCCCTT

              *  *  * ** *    *      *    *          * **                    
     165912 TTTCACCGTAGGCGTCAAGGCGATCGAAATGTCCACTTCCACAAACTACAAAAAGAGTGTTTCAA
          1 TTCCAACGAAATCATCAAAGCGATCCAAATATCCACTTCCAGATTCTACAAAAAGAGTGTTTCAA

             *         *   * **       *    *        * ** * *   **      *     
     165977 ACCTGCTCTATGAAAGGCCATGTTCATCTCTATGAGTTGAATGGAAATATCCGAAAGAAATTTCT
         66 AACTGCTCTATCAAAAGAAATGTTCAACTCTGTGAGTTGAGTACACACATCACAAAGAAGTTTCT

             *       *          *        *         **
     166042 GGGAATGCTGCTGTCTAG-TGTT-TAT-ACGA-ATTCCCGC
        131 GAGAATGCTTCTGTCTAGTTTTTATATGAAGATATTCCCTT

                    *    *       *                *    *  *                  
     166079 TTCCAACGTAATCCTCAAAGCAATCCAAATATCCACTTGCAGAATCCACAAAAAGAGTGTTTCAA
          1 TTCCAACGAAATCATCAAAGCGATCCAAATATCCACTTCCAGATTCTACAAAAAGAGTGTTTCAA

                          *     *          * *                   *  *        
     166144 AACTGCTCTATCAATAGAAAGGTTCAACTCTTTTAGTTGAGTACACACATCACGAACAAGTTTCT
         66 AACTGCTCTATCAAAAGAAATGTTCAACTCTGTGAGTTGAGTACACACATCACAAAGAAGTTTCT

                            * *      **     **  *    
     166209 GAGAATGCTTCTGTCTGGCTTTTATTGGAAGACGTTTCCTT
        131 GAGAATGCTTCTGTCTAGTTTTTATATGAAGATATTCCCTT

                 * *  **          *       *                * *               
     166250 TT-CACCAAAGGCATCAAAGCGCTCCAAATGTCCACTTCCAGATTCTTCCAAAAGAGTGTTTCAA
          1 TTCCAACGAAATCATCAAAGCGATCCAAATATCCACTTCCAGATTCTACAAAAAGAGTGTTTCAA

             **     * ***  * *                 *    * *  * *     *   *       
     166314 ACGTGCTCAAAGTAAGGGAATGTTCAACTCTGTGACTTGAATGCAGATATCACCAAGTAGTTTCT
         66 AACTGCTCTATCAAAAGAAATGTTCAACTCTGTGAGTTGAGTACACACATCACAAAGAAGTTTCT

            * * *            **     *    *         * 
     166379 AATAGTGCTTCTGTCTACATTTTAGATGATGATATTCCCGT
        131 GAGAATGCTTCTGTCTAGTTTTTATATGAAGATATTCCCTT

                         *** *   *             *  *   *      *     *       * 
     166420 TTCCAACGAAATCGCTAGAGCTATCCAAATATCCAGTTACAGTTTCTACCAAAAGGGTGTTTCCA
          1 TTCCAACGAAATCATCAAAGCGATCCAAATATCCACTTCCAGATTCTACAAAAAGAGTGTTTCAA

              *    **           *            *       *                     * 
     166485 AATTGCTGCATCAAAAGAAAGGTTCAACTCTGTTAGTTGAGGACACACATCACAAAGAAGTTTGT
         66 AACTGCTCTATCAAAAGAAATGTTCAACTCTGTGAGTTGAGTACACACATCACAAAGAAGTTTCT

                              *    *     *           
     166550 GAGAATGCTTCTGTCTAGGTTTTGTATGACGATATTCCCTT
        131 GAGAATGCTTCTGTCTAGTTTTTATATGAAGATATTCCCTT

                     *   * *     *   *       * *  *    *  *       *  *       
     166591 TTCCAACGATATCGTTAAAGCAATCTAAATATCAATTTGCAGAATCCACAAAAATAGAGTTTCAA
          1 TTCCAACGAAATCATCAAAGCGATCCAAATATCCACTTCCAGATTCTACAAAAAGAGTGTTTCAA

             *       * *        *    *       *  *                   ***      
     166656 AGCTGCTCTGTAAAAAGAAAGGTTCCACTCTGTTAGCTGAGTACACACATCACAAACTTGTTTCT
         66 AACTGCTCTATCAAAAGAAATGTTCAACTCTGTGAGTTGAGTACACACATCACAAAGAAGTTTCT

            *     *         *        **       * **   
     166721 CAGAATCCTTCTGTCTCGTTTTTATGGGAAGATACTTACTT
        131 GAGAATGCTTCTGTCTAGTTTTTATATGAAGATATTCCCTT

                 *  * **          *       *     *       *  *  *  *           
     166762 TTCCACCGTAGGCATCAAAGCGCTCCAAATGTCCACATCCAGATACTCCAGAACGAGTGTTTCAA
          1 TTCCAACGAAATCATCAAAGCGATCCAAATATCCACTTCCAGATTCTACAAAAAGAGTGTTTCAA

             *         *   * *   *         *        * *  *      *      *     
     166827 ACCTGCTCTATGAAAGGGAATCTTCAACTCTATGAGTTGAATGCAGACATCAGAAAGAAATTTCT
         66 AACTGCTCTATCAAAAGAAATGTTCAACTCTGTGAGTTGAGTACACACATCACAAAGAAGTTTCT

                     *       **          *         **
     166892 GAGAATGCTGCTGTCTACCTTTTAT-T--TGA-ATTCCCGC
        131 GAGAATGCTTCTGTCTAGTTTTTATATGAAGATATTCCCTT

                         *  *    *              * *  **   *    *             
     166929 TTCCAACGAAATCCTCCAAGCTATCCAAATATCCACCTGCATTTTCCACAACAAGAGTGTTTCAA
          1 TTCCAACGAAATCATCAAAGCGATCCAAATATCCACTTCCAGATTCTACAAAAAGAGTGTTTCAA

                          *                 *    *  *                *       
     166994 AACTGCTCTATCAATAGAAATGTTCAACTCCTTTG-GCTGGGTACACACATCACAAACAAGTTTC
         66 AACTGCTCTATCAAAAGAAATGTTCAACT-CTGTGAGTTGAGTACACACATCACAAAGAAGTTTC

                                      **     **       
     167058 TGAGAATGCTTCTGTCTAGTTTTTATGGGAAGACGTTCCCTT
        130 TGAGAATGCTTCTGTCTAGTTTTTATATGAAGATATTCCCTT

              *  * *  **          *       *            **                    
     167100 TTTCACCAAAGGCATCAAAGCGCTCCAAATGTCCACTTCCAGACACTACAAAAAGAGTGTTTCAA
          1 TTCCAACGAAATCATCAAAGCGATCCAAATATCCACTTCCAGATTCTACAAAAAGAGTGTTTCAA

             **       **   ***                 *    * *    *         *       
     167165 ACGTGCTCTAAGAAAGCGAATGTTCAACTCTGTGACTTGAATGCACATATCACAAAGTAGTTTCT
         66 AACTGCTCTATCAAAAGAAATGTTCAACTCTGTGAGTTGAGTACACACATCACAAAGAAGTTTCT

                **            *     *    *         * 
     167230 GAGAGGGCTTCTGTCTAGATTTTAGATGATGATATTCCCGT
        131 GAGAATGCTTCTGTCTAGTTTTTATATGAAGATATTCCCTT

                   *       * *    *               *   *                    * 
     167271 TTCCAACAAAATCATTAGAGC-TTCCAAATATCCACTTACAGTTTCTACAAAAAGAGTGTTTCCA
          1 TTCCAACGAAATCATCAAAGCGATCCAAATATCCACTTCCAGATTCTACAAAAAGAGTGTTTCAA

                   **         * *    *       *  *                   ***      
     167335 AACTGCTGCATCAAAAGAGAGGTTCCACTCTGTTAGCTGAGTACACACATCACAAACTTGTTTCT
         66 AACTGCTCTATCAAAAGAAATGTTCAACTCTGTGAGTTGAGTACACACATCACAAAGAAGTTTCT

            *     *         *        **         **   
     167400 CAGAATCCTTCTGTCTCGTTTTTATGGGAAGATATTTACTT
        131 GAGAATGCTTCTGTCTAGTTTTTATATGAAGATATTCCCTT

              *  *  * **          *       *     *       *  *  *              
     167441 TTTCACCGTAGGCATCAAAGCGCTCCAAATGTCCACATCCAGATACTCCAGAAAGAGTGTTTCAA
          1 TTCCAACGAAATCATCAAAGCGATCCAAATATCCACTTCCAGATTCTACAAAAAGAGTGTTTCAA

             *    *    *   * *   *         *        * *  *      *      *     
     167506 ACCTGCCCTATGAAAGGGAATCTTCAACTCTATGAGTTGAATGCAGACATCAGAAAGAAATTTCT
         66 AACTGCTCTATCAAAAGAAATGTTCAACTCTGTGAGTTGAGTACACACATCACAAAGAAGTTTCT

                     *       **          *         **
     167571 GAGAATGCTGCTGTCTACCTTTTAT-T--TGA-ATTCCCGC
        131 GAGAATGCTTCTGTCTAGTTTTTATATGAAGATATTCCCTT

                         *  *    *                *       *                  
     167608 TTCCAACGAAATCCTCCAAGCTATCCAAATATCCACTTGCAGATTCCACAAAAAGAGTGTTTCAA
          1 TTCCAACGAAATCATCAAAGCGATCCAAATATCCACTTCCAGATTCTACAAAAAGAGTGTTTCAA

                            ** *  *            *       *     *     *  *      
     167673 AACTGCTCTCTATCAATGGCAAAGTTCAACTCTGTTAGTTGAGGACACATATCACCAACAAGTTT
         66 AACTG--CTCTATCAAAAGAAATGTTCAACTCTGTGAGTTGAGTACACACATCACAAAGAAGTTT

                               *       **         *    
     167738 CTGAGAATGCTTCTGTCTATTTTTTATGGGAAGATATTTCCTT
        129 CTGAGAATGCTTCTGTCTAGTTTTTATATGAAGATATTCCCTT

              *  *  * ** *    *      *    *          * **                    
     167781 TTTCACCGTAGGCGTCAAGGCGATCGAAATGTCCACTTCCACAAACTACAAAAAGAGTGTTTCAA
          1 TTCCAACGAAATCATCAAAGCGATCCAAATATCCACTTCCAGATTCTACAAAAAGAGTGTTTCAA

             *         *   * **       *    *        * ** * *   **      *     
     167846 ACCTGCTCTATGAAAGGCCATGTTCATCTCTATGAGTTGAATGGAAATATCCGAAAGAAATTTCT
         66 AACTGCTCTATCAAAAGAAATGTTCAACTCTGTGAGTTGAGTACACACATCACAAAGAAGTTTCT

             *       *          *        *         **
     167911 GGGAATGCTGCTGTCTAG-TGTT-TAT-ACGA-ATTCCCGC
        131 GAGAATGCTTCTGTCTAGTTTTTATATGAAGATATTCCCTT

                         *       *                *    *  *                  
     167948 TTCCAACGAAATCCTCAAAGCAATCCAAATATCCACTTGCAGAATCCACAAAAAGAGTGTTTCAA
          1 TTCCAACGAAATCATCAAAGCGATCCAAATATCCACTTCCAGATTCTACAAAAAGAGTGTTTCAA

                          *     *          * *                   *  *        
     168013 AACTGCTCTATCAATAGAAAGGTTCAACTCTTTTAGTTGAGTACACACATCACGAACAAGTTTCT
         66 AACTGCTCTATCAAAAGAAATGTTCAACTCTGTGAGTTGAGTACACACATCACAAAGAAGTTTCT

                            * *      **     **  *    
     168078 GAGAATGCTTCTGTCTGGCTTTTATTGGAAGACGTTTCCTT
        131 GAGAATGCTTCTGTCTAGTTTTTATATGAAGATATTCCCTT

                   *  **          *       *                * *               
     168119 TT-CAACAAAGGCATCAAAGCGCTCCAAATGTCCACTTCCAGATTCTTCCAAAAGAGTGTTTCAA
          1 TTCCAACGAAATCATCAAAGCGATCCAAATATCCACTTCCAGATTCTACAAAAAGAGTGTTTCAA

             **     * ***  * *                 *    * *  * *     *   *       
     168183 ACGTGCTCAAAGTAAGGGAATGTTCAACTCTGTGACTTGAATGCAGATATCACCAAGTAGTTTCT
         66 AACTGCTCTATCAAAAGAAATGTTCAACTCTGTGAGTTGAGTACACACATCACAAAGAAGTTTCT

            * * *        *   **     *    *           
     168248 AATAGTGCTTCTGCCTACATTTTAGATGATGATATTCCCTT
        131 GAGAATGCTTCTGTCTAGTTTTTATATGAAGATATTCCCTT

                         * * *   *             *  *   *      *     *       * 
     168289 TTCCAACGAAATCGTTAGAGCTATCCAAATATCCAGTTACAGTTTCTACCAAAAGGGTGTTTCCA
          1 TTCCAACGAAATCATCAAAGCGATCCAAATATCCACTTCCAGATTCTACAAAAAGAGTGTTTCAA

              *    **           *            *       *                     * 
     168354 AATTGCTGCATCAAAAGAAAGGTTCAACTCTGTTAGTTGAGGACACACATCACAAAGAAGTTTGT
         66 AACTGCTCTATCAAAAGAAATGTTCAACTCTGTGAGTTGAGTACACACATCACAAAGAAGTTTCT

                              *    *     *           
     168419 GAGAATGCTTCTGTCTAGATTTTGTATGACGATATTCCCTT
        131 GAGAATGCTTCTGTCTAGTTTTTATATGAAGATATTCCCTT

                     *   * *     *   *       * *  *    *  *       *  *       
     168460 TTCCAACGATATCGTTAAAGCAATCTAAATATCAATTTGCAGAATCCACAAAAATAGAGTTTCAA
          1 TTCCAACGAAATCATCAAAGCGATCCAAATATCCACTTCCAGATTCTACAAAAAGAGTGTTTCAA

             *       * *        *    *       *  *                   ***      
     168525 AGCTGCTCTGTAAAAAGAAAGGTTCCACTCTGTTAGCTGAGTACACACATCACAAACTTGTTTCT
         66 AACTGCTCTATCAAAAGAAATGTTCAACTCTGTGAGTTGAGTACACACATCACAAAGAAGTTTCT

                  *         *        **         **   
     168590 GAGAATCCTTCTGTCTCGTTTTTATGGGAAGATATTTACTT
        131 GAGAATGCTTCTGTCTAGTTTTTATATGAAGATATTCCCTT

                 *  * **          *       *     *       *  *  *  *           
     168631 TTCCACCGTAGGCATCAAAGCGCTCCAAATGTCCACATCCAGATACTCCAGAACGAGTGTTTCAA
          1 TTCCAACGAAATCATCAAAGCGATCCAAATATCCACTTCCAGATTCTACAAAAAGAGTGTTTCAA

             *         *   * *   *         *        * *  *      *      *     
     168696 ACCTGCTCTATGAAAGGGAATCTTCAACTCTATGAGTTGAATGCAGACATCAGAAAGAAATTTCT
         66 AACTGCTCTATCAAAAGAAATGTTCAACTCTGTGAGTTGAGTACACACATCACAAAGAAGTTTCT

                     *       **          *         **
     168761 GAGAATGCTGCTGTCTACCTTTTAT-T--TGA-ATTCCCGC
        131 GAGAATGCTTCTGTCTAGTTTTTATATGAAGATATTCCCTT

                         *  *    *              * *  **   *                  
     168798 TTCCAACGAAATCCTCCAAGCTATCCAAATATCCACCTGCATTTTCCACAAAAAGAGTGTTTCAA
          1 TTCCAACGAAATCATCAAAGCGATCCAAATATCCACTTCCAGATTCTACAAAAAGAGTGTTTCAA

                          *                 *    *  *                *       
     168863 AACTGCTCTATCAATAGAAATGTTCAACTCCTTTG-GCTGGGTACACACATCACAAACAAGTTTC
         66 AACTGCTCTATCAAAAGAAATGTTCAACT-CTGTGAGTTGAGTACACACATCACAAAGAAGTTTC

                                      **     *        
     168927 TGAGAATGCTTCTGTCTAGTTTTTATGGGAAGACATTCCCTT
        130 TGAGAATGCTTCTGTCTAGTTTTTATATGAAGATATTCCCTT

              *  * *  **          *       *            **                    
     168969 TTTCACCAAAGACATCAAAGCGCTCCAAATGTCCACTTCCAGACACTACAAAAAGAGTGTTTCAA
          1 TTCCAACGAAATCATCAAAGCGATCCAAATATCCACTTCCAGATTCTACAAAAAGAGTGTTTCAA

             **       **   ***                 *    * *  * *         *       
     169034 ACGTGCTCTAAGAAAGCGAATGTTCAACTCTGTGACTTGAATGCAGATATCACAAAGTAGTTTCT
         66 AACTGCTCTATCAAAAGAAATGTTCAACTCTGTGAGTTGAGTACACACATCACAAAGAAGTTTCT

                **            *     *    *         * 
     169099 GAGAGGGCTTCTGTCTAGATTTTAGATGATGATATTCCCGT
        131 GAGAATGCTTCTGTCTAGTTTTTATATGAAGATATTCCCTT

                           * *   *                *   *                    * 
     169140 TTCCAACGAAATCATTAGAGCTATCCAAATATCCACTTACAGTTTCTACAAAAAGAGTGTTTCCA
          1 TTCCAACGAAATCATCAAAGCGATCCAAATATCCACTTCCAGATTCTACAAAAAGAGTGTTTCAA

                   **         * *    *       *  *                   ***      
     169205 AACTGCTGCATCAAAAGAGAGGTTCCACTCTGTTAGCTGAGTACACACATCACAAACTTGTTTCT
         66 AACTGCTCTATCAAAAGAAATGTTCAACTCTGTGAGTTGAGTACACACATCACAAAGAAGTTTCT

            *     *         *        **         **   
     169270 CAGAATCCTTCTGTCTCGTTTTTATGGGAAGATATTTACTT
        131 GAGAATGCTTCTGTCTAGTTTTTATATGAAGATATTCCCTT

              *  *  * **          *       *     *       *  *  *              
     169311 TTTCACCGTAGGCATCAAAGCGCTCCAAATGTCCACATCCAGATACTCCAGAAAGAGTGTTTCAA
          1 TTCCAACGAAATCATCAAAGCGATCCAAATATCCACTTCCAGATTCTACAAAAAGAGTGTTTCAA

             *         *   * *   *         *        * *  *      *      *     
     169376 ACCTGCTCTATGAAAGGGAATCTTCAACTCTATGAGTTGAATGCAGACATCAGAAAGAAATTTCT
         66 AACTGCTCTATCAAAAGAAATGTTCAACTCTGTGAGTTGAGTACACACATCACAAAGAAGTTTCT

                     *       **          *         **
     169441 GAGAATGCTGCTGTCTACCTTTTAT-T--TGA-ATTCCCGC
        131 GAGAATGCTTCTGTCTAGTTTTTATATGAAGATATTCCCTT

                         *  *    *                *       *                  
     169478 TTCCAACGAAATCCTCCAAGCTATCCAAATATCCACTTGCAGATTCCACAAAAAGAGTGTTTCAA
          1 TTCCAACGAAATCATCAAAGCGATCCAAATATCCACTTCCAGATTCTACAAAAAGAGTGTTTCAA

                            ** *  *            *       *     *     *  *      
     169543 AACTGCTCTCTATCAATGGCAAAGTTCAACTCTGTTAGTTGAGGACACATATCACCAACAAGTTT
         66 AACTG--CTCTATCAAAAGAAATGTTCAACTCTGTGAGTTGAGTACACACATCACAAAGAAGTTT

                               *       **         *    
     169608 CTGAGAATGCTTCTGTCTATTTTTTATGGGAAGATATTTCCTT
        129 CTGAGAATGCTTCTGTCTAGTTTTTATATGAAGATATTCCCTT

              *  *  * ** *    *      *    *          * **                    
     169651 TTTCACCGTAGGCGTCAAGGCGATCGAAATGTCCACTTCCACAAACTACAAAAAGAGTGTTTCAA
          1 TTCCAACGAAATCATCAAAGCGATCCAAATATCCACTTCCAGATTCTACAAAAAGAGTGTTTCAA

             *         *   * **       *    *        * ** * *   **      *     
     169716 ACCTGCTCTATGAAAGGCCATGTTCATCTCTATGAGTTGAATGGAAATATCCGAAAGAAATTTCT
         66 AACTGCTCTATCAAAAGAAATGTTCAACTCTGTGAGTTGAGTACACACATCACAAAGAAGTTTCT

             *       *          *        *         **
     169781 GGGAATGCTGCTGTCTAG-TGTT-TAT-ACGA-ATTCCCGC
        131 GAGAATGCTTCTGTCTAGTTTTTATATGAAGATATTCCCTT

                    *    *       *                *    *  *                  
     169818 TTCCAACGTAATCCTCAAAGCAATCCAAATATCCACTTGCAGAATCCACAAAAAGAGTGTTTCAA
          1 TTCCAACGAAATCATCAAAGCGATCCAAATATCCACTTCCAGATTCTACAAAAAGAGTGTTTCAA

                          *     *          * *                   *  *        
     169883 AACTGCTCTATCAATAGAAAGGTTCAACTCTTTTAGTTGAGTACACACATCACGAACAAGTTTCT
         66 AACTGCTCTATCAAAAGAAATGTTCAACTCTGTGAGTTGAGTACACACATCACAAAGAAGTTTCT

                            * *      **     **  *    
     169948 GAGAATGCTTCTGTCTGGCTTTTATTGGAAGACGTTTCCTT
        131 GAGAATGCTTCTGTCTAGTTTTTATATGAAGATATTCCCTT

                 * *  **          *       *                * *               
     169989 TT-CACCAAAGGCATCAAAGCGCTCCAAATGTCCACTTCCAGATTCTTCCAAAAGAGTGTTTCAA
          1 TTCCAACGAAATCATCAAAGCGATCCAAATATCCACTTCCAGATTCTACAAAAAGAGTGTTTCAA

             **     * ***  * *                 *    * *  * *     *   *       
     170053 ACGTGCTCAAAGTAAGGGAATGTTCAACTCTGTGACTTGAATGCAGATATCACCAAGTAGTTTCT
         66 AACTGCTCTATCAAAAGAAATGTTCAACTCTGTGAGTTGAGTACACACATCACAAAGAAGTTTCT

            * * *            **     *    *         * 
     170118 AATAGTGCTTCTGTCTACATTTTAGATGATGATATTCCCGT
        131 GAGAATGCTTCTGTCTAGTTTTTATATGAAGATATTCCCTT

                         *** *   *             *  *   *      *     *       * 
     170159 TTCCAACGAAATCGCTAGAGCTATCCAAATATCCAGTTACAGTTTCTACCAAAAGGGTGTTTCCA
          1 TTCCAACGAAATCATCAAAGCGATCCAAATATCCACTTCCAGATTCTACAAAAAGAGTGTTTCAA

              *    **           *            *       *                     * 
     170224 AATTGCTGCATCAAAAGAAAGGTTCAACTCTGTTAGTTGAGGACACACATCACAAAGAAGTTTGT
         66 AACTGCTCTATCAAAAGAAATGTTCAACTCTGTGAGTTGAGTACACACATCACAAAGAAGTTTCT

                              *    *     *           
     170289 GAGAATGCTTCTGTCTAGATTTTGTATGACGATATTCCCTT
        131 GAGAATGCTTCTGTCTAGTTTTTATATGAAGATATTCCCTT

                     *   * *     *   *       * *  *    *  *       *  *       
     170330 TTCCAACGATATCGTTAAAGCAATCTAAATATCAATTTGCAGAATCCACAAAAATAGAGTTTCAA
          1 TTCCAACGAAATCATCAAAGCGATCCAAATATCCACTTCCAGATTCTACAAAAAGAGTGTTTCAA

             *       * *        *    *       *  *                   ***      
     170395 AGCTGCTCTGTAAAAAGAAAGGTTCCACTCTGTTAGCTGAGTACACACATCACAAACTTGTTTCT
         66 AACTGCTCTATCAAAAGAAATGTTCAACTCTGTGAGTTGAGTACACACATCACAAAGAAGTTTCT

            *     *         *        **       * **   
     170460 CAGAATCCTTCTGTCTCGTTTTTATGGGAAGATACTTACTT
        131 GAGAATGCTTCTGTCTAGTTTTTATATGAAGATATTCCCTT

                 *  * **          *       *     *       *  *  *  *           
     170501 TTCCACCGTAGGCATCAAAGCGCTCCAAATGTCCACATCCAGATACTCCAGAACGAGTGTTTCAA
          1 TTCCAACGAAATCATCAAAGCGATCCAAATATCCACTTCCAGATTCTACAAAAAGAGTGTTTCAA

             *         *   * *   *         *        * *  *      *      *     
     170566 ACCTGCTCTATGAAAGGGAATCTTCAACTCTATGAGTTGAATGCAGACATCAGAAAGAAATTTCT
         66 AACTGCTCTATCAAAAGAAATGTTCAACTCTGTGAGTTGAGTACACACATCACAAAGAAGTTTCT

                     *       **          *         **
     170631 GAGAATGCTGCTGTCTACCTTTTAT-T--TGA-ATTCCCGC
        131 GAGAATGCTTCTGTCTAGTTTTTATATGAAGATATTCCCTT

                         *  *    *              * *  **   *    *             
     170668 TTCCAACGAAATCCTCCAAGCTATCCAAATATCCACCTGCATTTTCCACAACAAGAGTGTTTCAA
          1 TTCCAACGAAATCATCAAAGCGATCCAAATATCCACTTCCAGATTCTACAAAAAGAGTGTTTCAA

                          *                 *    *  *                *       
     170733 AACTGCTCTATCAATAGAAATGTTCAACTCCTTTG-GCTGGGTACACACATCACAAACAAGTTTC
         66 AACTGCTCTATCAAAAGAAATGTTCAACT-CTGTGAGTTGAGTACACACATCACAAAGAAGTTTC

                                      **     **       
     170797 TGAGAATGCTTCTGTCTAGTTTTTATGGGAAGACGTTCCCTT
        130 TGAGAATGCTTCTGTCTAGTTTTTATATGAAGATATTCCCTT

              *  * *  **    *     *       *     *       *  *  *              
     170839 TTTCACCAAAGGCATCCAAGCGCTCCAAATGTCCACATCCAGATACTCCAGAAAGAGTGTTTCAA
          1 TTCCAACGAAATCATCAAAGCGATCCAAATATCCACTTCCAGATTCTACAAAAAGAGTGTTTCAA

             *         *   * *   *         *        * *  *      *      *     
     170904 ACCTGCTCTATGAAAGGGAATCTTCAACTCTATGAGTTGAATGCAGACATCAGAAAGAAATTTCT
         66 AACTGCTCTATCAAAAGAAATGTTCAACTCTGTGAGTTGAGTACACACATCACAAAGAAGTTTCT

                     *       **          *         **
     170969 GAGAATGCTGCTGTCTACCTTTTAT-T--TGA-ATTCCCGC
        131 GAGAATGCTTCTGTCTAGTTTTTATATGAAGATATTCCCTT

                         *  *    *                *       *                  
     171006 TTCCAACGAAATCCTCCAAGCTATCCAAATATCCACTTGCAGATTCCACAAAAAGAGTGTTTCAA
          1 TTCCAACGAAATCATCAAAGCGATCCAAATATCCACTTCCAGATTCTACAAAAAGAGTGTTTCAA

                            ** *  *            *       *     *     *  *      
     171071 AACTGCTCTCTATCAATGGCAAAGTTCAACTCTGTTAGTTGAGGACACATATCACCAACAAGTTT
         66 AACTG--CTCTATCAAAAGAAATGTTCAACTCTGTGAGTTGAGTACACACATCACAAAGAAGTTT

                               *       **         *    
     171136 CTGAGAATGCTTCTGTCTATTTTTTATGGGAAGATATTTCCTT
        129 CTGAGAATGCTTCTGTCTAGTTTTTATATGAAGATATTCCCTT

              *  * ** ** *    *      *    *          * **                    
     171179 TTTCACCATAGGCGTCAAGGCGATCGAAATGTCCACTTCCACAAACTACAAAAAGAGTGTTTCAA
          1 TTCCAACGAAATCATCAAAGCGATCCAAATATCCACTTCCAGATTCTACAAAAAGAGTGTTTCAA

             *         *   * **       *    *        * ** * *   **      *     
     171244 ACCTGCTCTATGAAAGGCCATGTTCATCTCTATGAGTTGAATGGAAATATCCGAAAGAAATTTCT
         66 AACTGCTCTATCAAAAGAAATGTTCAACTCTGTGAGTTGAGTACACACATCACAAAGAAGTTTCT

             *       *          *        *         **
     171309 GGGAATGCTGCTGTCTAG-TGTT-TAT-ACGA-ATTCCCGC
        131 GAGAATGCTTCTGTCTAGTTTTTATATGAAGATATTCCCTT

                         *       *                *    *  *                  
     171346 TTCCAACGAAATCCTCAAAGCAATCCAAATATCCACTTGCAGAATCCACAAAAAGAGTGTTTCAA
          1 TTCCAACGAAATCATCAAAGCGATCCAAATATCCACTTCCAGATTCTACAAAAAGAGTGTTTCAA

                          *     *          * *                   *  *        
     171411 AACTGCTCTATCAATAGAAAGGTTCAACTCTTTTAGTTGAGTACACACATCACGAACAAGTTTCT
         66 AACTGCTCTATCAAAAGAAATGTTCAACTCTGTGAGTTGAGTACACACATCACAAAGAAGTTTCT

                            * *      **     **  *    
     171476 GAGAATGCTTCTGTCTGGCTTTTATTGGAAGACGTTTCCTT
        131 GAGAATGCTTCTGTCTAGTTTTTATATGAAGATATTCCCTT

                   *  **          *       *                * *               
     171517 TT-CAACAAAGGCATCAAAGCGCTCCAAATGTCCACTTCCAGATTCTTCCAAAAGAGTGTTTCAA
          1 TTCCAACGAAATCATCAAAGCGATCCAAATATCCACTTCCAGATTCTACAAAAAGAGTGTTTCAA

             **     * ***  * *                 *    * *  * *     *   *       
     171581 ACGTGCTCAAAGTAAGGGAATGTTCAACTCTGTGACTTGAATGCAGATATCACCAAGTAGTTTCT
         66 AACTGCTCTATCAAAAGAAATGTTCAACTCTGTGAGTTGAGTACACACATCACAAAGAAGTTTCT

            * * *        *   **     *    *           
     171646 AATAGTGCTTCTGCCTACATTTTAGATGATGATATTCCCTT
        131 GAGAATGCTTCTGTCTAGTTTTTATATGAAGATATTCCCTT

                         * * *   *             *  *   *      *     *       * 
     171687 TTCCAACGAAATCGTTAGAGCTATCCAAATATCCAGTTACAGTTTCTACCAAAAGGGTGTTTCCA
          1 TTCCAACGAAATCATCAAAGCGATCCAAATATCCACTTCCAGATTCTACAAAAAGAGTGTTTCAA

              *    **           *            *       *                     * 
     171752 AATTGCTGCATCAAAAGAAAGGTTCAACTCTGTTAGTTGAGGACACACATCACAAAGAAGTTTGT
         66 AACTGCTCTATCAAAAGAAATGTTCAACTCTGTGAGTTGAGTACACACATCACAAAGAAGTTTCT

                              *    *     *           
     171817 GAGAATGCTTCTGTCTAGATTTTGTATGACGATATTCCCTT
        131 GAGAATGCTTCTGTCTAGTTTTTATATGAAGATATTCCCTT

                     *   * *     *   *       * *  *    *  *       *  *       
     171858 TTCCAACGATATCGTTAAAGCAATCTAAATATCAATTTGCAGAATCCACAAAAATAGAGTTTCAA
          1 TTCCAACGAAATCATCAAAGCGATCCAAATATCCACTTCCAGATTCTACAAAAAGAGTGTTTCAA

             *       * *        *    *       *  *                   ***      
     171923 AGCTGCTCTGTAAAAAGAAAGGTTCCACTCTGTTAGCTGAGTACACACATCACAAACTTGTTTCT
         66 AACTGCTCTATCAAAAGAAATGTTCAACTCTGTGAGTTGAGTACACACATCACAAAGAAGTTTCT

                  *         *        **         **   
     171988 GAGAATCCTTCTGTCTCGTTTTTATGGGAAGATATTTACTT
        131 GAGAATGCTTCTGTCTAGTTTTTATATGAAGATATTCCCTT

                 *  * **          *       *     *       *  *  *  *           
     172029 TTCCACCGTAGGCATCAAAGCGCTCCAAATGTCCACATCCAGATACTCCAGAACGAGTGTTTCAA
          1 TTCCAACGAAATCATCAAAGCGATCCAAATATCCACTTCCAGATTCTACAAAAAGAGTGTTTCAA

             *         *   * *   *         *        * *  *      *      *     
     172094 ACCTGCTCTATGAAAGGGAATCTTCAACTCTATGAGTTGAATGCAGACATCAGAAAGAAATTTCT
         66 AACTGCTCTATCAAAAGAAATGTTCAACTCTGTGAGTTGAGTACACACATCACAAAGAAGTTTCT

                     *       **          *         **
     172159 GAGAATGCTGCTGTCTACCTTTTAT-T--TGA-ATTCCCGC
        131 GAGAATGCTTCTGTCTAGTTTTTATATGAAGATATTCCCTT

                         *  *    *              * *  **   *                  
     172196 TTCCAACGAAATCCTCCAAGCTATCCAAATATCCACCTGCATTTTCCACAAAAAGAGTGTTTCAA
          1 TTCCAACGAAATCATCAAAGCGATCCAAATATCCACTTCCAGATTCTACAAAAAGAGTGTTTCAA

                          *                 *    *  *                *       
     172261 AACTGCTCTATCAATAGAAATGTTCAACTCCTTTG-GCTGGGTACACACATCACAAACAAGTTTC
         66 AACTGCTCTATCAAAAGAAATGTTCAACT-CTGTGAGTTGAGTACACACATCACAAAGAAGTTTC

                                      **     *        
     172325 TGAGAATGCTTCTGTCTAGTTTTTATGGGAAGACATTCCCTT
        130 TGAGAATGCTTCTGTCTAGTTTTTATATGAAGATATTCCCTT

              *  * *  **          *       *            **                    
     172367 TTTCACCAAAGACATCAAAGCGCTCCAAATGTCCACTTCCAGACACTACAAAAAGAGTGTTTCAA
          1 TTCCAACGAAATCATCAAAGCGATCCAAATATCCACTTCCAGATTCTACAAAAAGAGTGTTTCAA

             **       **   ***                 *    * *  * *         *       
     172432 ACGTGCTCTAAGAAAGCGAATGTTCAACTCTGTGACTTGAATGCAGATATCACAAAGTAGTTTCT
         66 AACTGCTCTATCAAAAGAAATGTTCAACTCTGTGAGTTGAGTACACACATCACAAAGAAGTTTCT

                **            *     *    *         * 
     172497 GAGAGGGCTTCTGTCTAGATTTTAGATGATGATATTCCCGT
        131 GAGAATGCTTCTGTCTAGTTTTTATATGAAGATATTCCCTT

                           * *   *                *   *                    * 
     172538 TTCCAACGAAATCATTAGAGCTATCCAAATATCCACTTACAGTTTCTACAAAAAGAGTGTTTCCA
          1 TTCCAACGAAATCATCAAAGCGATCCAAATATCCACTTCCAGATTCTACAAAAAGAGTGTTTCAA

                   **         * *    *       *  *                   ***      
     172603 AACTGCTGCATCAAAAGAGAGGTTCCACTCTGTTAGCTGAGTACACACATCACAAACTTGTTTCT
         66 AACTGCTCTATCAAAAGAAATGTTCAACTCTGTGAGTTGAGTACACACATCACAAAGAAGTTTCT

            *     *         *        **         **   
     172668 CAGAATCCTTCTGTCTCGTTTTTATGGGAAGATATTTACTT
        131 GAGAATGCTTCTGTCTAGTTTTTATATGAAGATATTCCCTT

              *  *  * **          *       *     *       *  *  *              
     172709 TTTCACCGTAGGCATCAAAGCGCTCCAAATGTCCACATCCAGATACTCCAGAAAGAGTGTTTCAA
          1 TTCCAACGAAATCATCAAAGCGATCCAAATATCCACTTCCAGATTCTACAAAAAGAGTGTTTCAA

             *         *   * *   *         *        * *  *      *      *     
     172774 ACCTGCTCTATGAAAGGGAATCTTCAACTCTATGAGTTGAATGCAGACATCAGAAAGAAATTTCT
         66 AACTGCTCTATCAAAAGAAATGTTCAACTCTGTGAGTTGAGTACACACATCACAAAGAAGTTTCT

                     *       **          *         **
     172839 GAGAATGCTGCTGTCTACCTTTTAT-T--TGA-ATTCCCGC
        131 GAGAATGCTTCTGTCTAGTTTTTATATGAAGATATTCCCTT

                         *  *    *                *       *                  
     172876 TTCCAACGAAATCCTCCAAGCTATCCAAATATCCACTTGCAGATTCCACAAAAAGAGTGTTTCAA
          1 TTCCAACGAAATCATCAAAGCGATCCAAATATCCACTTCCAGATTCTACAAAAAGAGTGTTTCAA

                            ** *  *            *       *     *     *  *      
     172941 AACTGCTCTCTATCAATGGCAAAGTTCAACTCTGTTAGTTGAGGACACATATCACCAACAAGTTT
         66 AACTG--CTCTATCAAAAGAAATGTTCAACTCTGTGAGTTGAGTACACACATCACAAAGAAGTTT

                               *       **         *    
     173006 CTGAGAATGCTTCTGTCTATTTTTTATGGGAAGATATTTCCTT
        129 CTGAGAATGCTTCTGTCTAGTTTTTATATGAAGATATTCCCTT

              *  *  * ** *    *      *    *          * **                    
     173049 TTTCACCGTAGGCGTCAAGGCGATCGAAATGTCCACTTCCACAAACTACAAAAAGAGTGTTTCAA
          1 TTCCAACGAAATCATCAAAGCGATCCAAATATCCACTTCCAGATTCTACAAAAAGAGTGTTTCAA

             *         *   * **       *    *        * ** * *   **      *     
     173114 ACCTGCTCTATGAAAGGCCATGTTCATCTCTATGAGTTGAATGGAAATATCCGAAAGAAATTTCT
         66 AACTGCTCTATCAAAAGAAATGTTCAACTCTGTGAGTTGAGTACACACATCACAAAGAAGTTTCT

             *       *          *        *         **
     173179 GGGAATGCTGCTGTCTAG-TGTT-TAT-ACGA-ATTCCCGC
        131 GAGAATGCTTCTGTCTAGTTTTTATATGAAGATATTCCCTT

                    *    *       *                *    *  *                  
     173216 TTCCAACGTAATCCTCAAAGCAATCCAAATATCCACTTGCAGAATCCACAAAAAGAGTGTTTCAA
          1 TTCCAACGAAATCATCAAAGCGATCCAAATATCCACTTCCAGATTCTACAAAAAGAGTGTTTCAA

                          *     *          * *                   *  *        
     173281 AACTGCTCTATCAATAGAAAGGTTCAACTCTTTTAGTTGAGTACACACATCACGAACAAGTTTCT
         66 AACTGCTCTATCAAAAGAAATGTTCAACTCTGTGAGTTGAGTACACACATCACAAAGAAGTTTCT

                            * *      **     **  *    
     173346 GAGAATGCTTCTGTCTGGCTTTTATTGGAAGACGTTTCCTT
        131 GAGAATGCTTCTGTCTAGTTTTTATATGAAGATATTCCCTT

                 * *  **          *       *                * *               
     173387 TT-CACCAAAGGCATCAAAGCGCTCCAAATGTCCACTTCCAGATTCTTCCAAAAGAGTGTTTCAA
          1 TTCCAACGAAATCATCAAAGCGATCCAAATATCCACTTCCAGATTCTACAAAAAGAGTGTTTCAA

             **     * ***  * *                 *    * *  * *     *   *       
     173451 ACGTGCTCAAAGTAAGGGAATGTTCAACTCTGTGACTTGAATGCAGATATCACCAAGTAGTTTCT
         66 AACTGCTCTATCAAAAGAAATGTTCAACTCTGTGAGTTGAGTACACACATCACAAAGAAGTTTCT

            * * *            **     *    *         * 
     173516 AATAGTGCTTCTGTCTACATTTTAGATGATGATATTCCCGT
        131 GAGAATGCTTCTGTCTAGTTTTTATATGAAGATATTCCCTT

                         *** *   *             *  *   *      *     *       * 
     173557 TTCCAACGAAATCGCTAGAGCTATCCAAATATCCAGTTACAGTTTCTACCAAAAGGGTGTTTCCA
          1 TTCCAACGAAATCATCAAAGCGATCCAAATATCCACTTCCAGATTCTACAAAAAGAGTGTTTCAA

              *    **           *            *       *                     * 
     173622 AATTGCTGCATCAAAAGAAAGGTTCAACTCTGTTAGTTGAGGACACACATCACAAAGAAGTTTGT
         66 AACTGCTCTATCAAAAGAAATGTTCAACTCTGTGAGTTGAGTACACACATCACAAAGAAGTTTCT

                              *    *     *           
     173687 GAGAATGCTTCTGTCTAGGTTTTGTATGACGATATTCCCTT
        131 GAGAATGCTTCTGTCTAGTTTTTATATGAAGATATTCCCTT

                     *   * *     *   *       * *  *    *  *       *  *       
     173728 TTCCAACGATATCGTTAAAGCAATCTAAATATCAATTTGCAGAATCCACAAAAATAGAGTTTCAA
          1 TTCCAACGAAATCATCAAAGCGATCCAAATATCCACTTCCAGATTCTACAAAAAGAGTGTTTCAA

             *       * *        *    *       *  *                   ***      
     173793 AGCTGCTCTGTAAAAAGAAAGGTTCCACTCTGTTAGCTGAGTACACACATCACAAACTTGTTTCT
         66 AACTGCTCTATCAAAAGAAATGTTCAACTCTGTGAGTTGAGTACACACATCACAAAGAAGTTTCT

            *     *         *        **       * **   
     173858 CAGAATCCTTCTGTCTCGTTTTTATGGGAAGATACTTACTT
        131 GAGAATGCTTCTGTCTAGTTTTTATATGAAGATATTCCCTT

                 *  * **          *       *     *       *  *  *  *           
     173899 TTCCACCGTAGGCATCAAAGCGCTCCAAATGTCCACATCCAGATACTCCAGAACGAGTGTTTCAA
          1 TTCCAACGAAATCATCAAAGCGATCCAAATATCCACTTCCAGATTCTACAAAAAGAGTGTTTCAA

             *         *   * *   *         *        * *  *      *      *     
     173964 ACCTGCTCTATGAAAGGGAATCTTCAACTCTATGAGTTGAATGCAGACATCAGAAAGAAATTTCT
         66 AACTGCTCTATCAAAAGAAATGTTCAACTCTGTGAGTTGAGTACACACATCACAAAGAAGTTTCT

                     *       **          *         **
     174029 GAGAATGCTGCTGTCTACCTTTTAT-T--TGA-ATTCCCGC
        131 GAGAATGCTTCTGTCTAGTTTTTATATGAAGATATTCCCTT

                         *  *    *              * *  **   *    *             
     174066 TTCCAACGAAATCCTCCAAGCTATCCAAATATCCACCTGCATTTTCCACAACAAGAGTGTTTCAA
          1 TTCCAACGAAATCATCAAAGCGATCCAAATATCCACTTCCAGATTCTACAAAAAGAGTGTTTCAA

                          *                 *    *  *                *       
     174131 AACTGCTCTATCAATAGAAATGTTCAACTCCTTTG-GCTGGGTACACACATCACAAACAAGTTTC
         66 AACTGCTCTATCAAAAGAAATGTTCAACT-CTGTGAGTTGAGTACACACATCACAAAGAAGTTTC

                                      **     **       
     174195 TGAGAATGCTTCTGTCTAGTTTTTATGGGAAGACGTTCCCTT
        130 TGAGAATGCTTCTGTCTAGTTTTTATATGAAGATATTCCCTT

              *  * *  **          *       *            **                    
     174237 TTTCACCAAAGGCATCAAAGCGCTCCAAATGTCCACTTCCAGACACTACAAAAAGAGTGTTTCAA
          1 TTCCAACGAAATCATCAAAGCGATCCAAATATCCACTTCCAGATTCTACAAAAAGAGTGTTTCAA

             **       **   ***                 *    * *    *         *       
     174302 ACGTGCTCTAAGAAAGCGAATGTTCAACTCTGTGACTTGAATGCACATATCACAAAGTAGTTTCT
         66 AACTGCTCTATCAAAAGAAATGTTCAACTCTGTGAGTTGAGTACACACATCACAAAGAAGTTTCT

                **            *     *    *         * 
     174367 GAGAGGGCTTCTGTCTAGATTTTAGATGATGATATTCCCGT
        131 GAGAATGCTTCTGTCTAGTTTTTATATGAAGATATTCCCTT

                   *       * *    *               *   *                    * 
     174408 TTCCAACAAAATCATTAGAGC-TTCCAAATATCCACTTACAGTTTCTACAAAAAGAGTGTTTCCA
          1 TTCCAACGAAATCATCAAAGCGATCCAAATATCCACTTCCAGATTCTACAAAAAGAGTGTTTCAA

                   **         * *    *       *  *                   ***      
     174472 AACTGCTGCATCAAAAGAGAGGTTCCACTCTGTTAGCTGAGTACACACATCACAAACTTGTTTCT
         66 AACTGCTCTATCAAAAGAAATGTTCAACTCTGTGAGTTGAGTACACACATCACAAAGAAGTTTCT

            *     *         *        **        ***   
     174537 CAGAATCCTTCTGTCTCGTTTTTATGGGAAGAT-TATACTT
        131 GAGAATGCTTCTGTCTAGTTTTTATATGAAGATATTCCCTT

              *  *  * **          *       *     *       *  *  *              
     174577 TTTCACCGTAGGCATCAAAGCGCTCCAAATGTCCACATCCAGATACTCCAGAAAGAGTGTTTCAA
          1 TTCCAACGAAATCATCAAAGCGATCCAAATATCCACTTCCAGATTCTACAAAAAGAGTGTTTCAA

             *    *    *   * *   *         *        * *  *      *      *     
     174642 ACCTGCCCTATGAAAGGGAATCTTCAACTCTATGAGTTGAATGCAGACATCAGAAAGAAATTTCT
         66 AACTGCTCTATCAAAAGAAATGTTCAACTCTGTGAGTTGAGTACACACATCACAAAGAAGTTTCT

                     *       **          *         **
     174707 GAGAATGCTGCTGTCTACCTTTTAT-T--TGA-ATTCCCGC
        131 GAGAATGCTTCTGTCTAGTTTTTATATGAAGATATTCCCTT

                         *  *    *                *       *                  
     174744 TTCCAACGAAATCCTCCAAGCTATCCAAATATCCACTTGCAGATTCCACAAAAAGAGTGTTTCAA
          1 TTCCAACGAAATCATCAAAGCGATCCAAATATCCACTTCCAGATTCTACAAAAAGAGTGTTTCAA

                            ** *  *            *       *     *     *  *      
     174809 AACTGCTCTCTATCAATGGCAAAGTTCAACTCTGTTAGTTGAGGACACATATCACCAACAAGTTT
         66 AACTG--CTCTATCAAAAGAAATGTTCAACTCTGTGAGTTGAGTACACACATCACAAAGAAGTTT

                               *       **         *    
     174874 CTGAGAATGCTTCTGTCTATTTTTTATGGGAAGATATTTCCTT
        129 CTGAGAATGCTTCTGTCTAGTTTTTATATGAAGATATTCCCTT

              *  *  * ** *    *      *    *          * **                    
     174917 TTTCACCGTAGGCGTCAAGGCGATCGAAATGTCCACTTCCACAAACTACAAAAAGAGTGTTTCAA
          1 TTCCAACGAAATCATCAAAGCGATCCAAATATCCACTTCCAGATTCTACAAAAAGAGTGTTTCAA

             *         *   * **       *    *        * ** * *   **      *     
     174982 ACCTGCTCTATGAAAGGCCATGTTCATCTCTATGAGTTGAATGGAAATATCCGAAAGAAATTTCT
         66 AACTGCTCTATCAAAAGAAATGTTCAACTCTGTGAGTTGAGTACACACATCACAAAGAAGTTTCT

             *       *          *        *         **
     175047 GGGAATGCTGCTGTCTAG-TGTT-TAT-ACGA-ATTCCCGC
        131 GAGAATGCTTCTGTCTAGTTTTTATATGAAGATATTCCCTT

                         *       *                *    *  *                  
     175084 TTCCAACGAAATCCTCAAAGCAATCCAAATATCCACTTGCAGAATCCACAAAAAGAGTGTTTCAA
          1 TTCCAACGAAATCATCAAAGCGATCCAAATATCCACTTCCAGATTCTACAAAAAGAGTGTTTCAA

                          *     *          * *                   *  *        
     175149 AACTGCTCTATCAATAGAAAGGTTCAACTCTTTTAGTTGAGTACACACATCACGAACAAGTTTCT
         66 AACTGCTCTATCAAAAGAAATGTTCAACTCTGTGAGTTGAGTACACACATCACAAAGAAGTTTCT

                            * *      **     **  *    
     175214 GAGAATGCTTCTGTCTGGCTTTTATTGGAAGACGTTTCCTT
        131 GAGAATGCTTCTGTCTAGTTTTTATATGAAGATATTCCCTT

                   *  **          *       *                * *               
     175255 TT-CAACAAAGGCATCAAAGCGCTCCAAATGTCCACTTCCAGATTCTTCCAAAAGAGTGTTTCAA
          1 TTCCAACGAAATCATCAAAGCGATCCAAATATCCACTTCCAGATTCTACAAAAAGAGTGTTTCAA

             **     * ***  * *                 *    * *  * *     *   *       
     175319 ACGTGCTCAAAGTAAGGGAATGTTCAACTCTGTGACTTGAATGCAGATATCACCAAGTAGTTTCT
         66 AACTGCTCTATCAAAAGAAATGTTCAACTCTGTGAGTTGAGTACACACATCACAAAGAAGTTTCT

            * * *        *   **     *    *           
     175384 AATAGTGCTTCTGCCTACATTTTAGATGATGATATTCCCTT
        131 GAGAATGCTTCTGTCTAGTTTTTATATGAAGATATTCCCTT

                         * * *   *             *  *   *      *     *       * 
     175425 TTCCAACGAAATCGTTAGAGCTATCCAAATATCCAGTTACAGTTTCTACCAAAAGGGTGTTTCCA
          1 TTCCAACGAAATCATCAAAGCGATCCAAATATCCACTTCCAGATTCTACAAAAAGAGTGTTTCAA

              *    **           *            *       *                     * 
     175490 AATTGCTGCATCAAAAGAAAGGTTCAACTCTGTTAGTTGAGGACACACATCACAAAGAAGTTTGT
         66 AACTGCTCTATCAAAAGAAATGTTCAACTCTGTGAGTTGAGTACACACATCACAAAGAAGTTTCT

                              *    *     *           
     175555 GAGAATGCTTCTGTCTAGATTTTGTATGACGATATTCCCTT
        131 GAGAATGCTTCTGTCTAGTTTTTATATGAAGATATTCCCTT

                     *   * *     *   *       * *  *    *  *       *  *       
     175596 TTCCAACGATATCGTTAAAGCAATCTAAATATCAATTTGCAGAATCCACAAAAATAGAGTTTCAA
          1 TTCCAACGAAATCATCAAAGCGATCCAAATATCCACTTCCAGATTCTACAAAAAGAGTGTTTCAA

             *       * *        *    *       *  *                   ***      
     175661 AGCTGCTCTGTAAAAAGAAAGGTTCCACTCTGTTAGCTGAGTACACACATCACAAACTTGTTTCT
         66 AACTGCTCTATCAAAAGAAATGTTCAACTCTGTGAGTTGAGTACACACATCACAAAGAAGTTTCT

                  *         *        **         **   
     175726 GAGAATCCTTCTGTCTCGTTTTTATGGGAAGATATTTACTT
        131 GAGAATGCTTCTGTCTAGTTTTTATATGAAGATATTCCCTT

                 *  * **          *       *     *       *  *  *  *           
     175767 TTCCACCGTAGGCATCAAAGCGCTCCAAATGTCCACATCCAGATACTCCAGAACGAGTGTTTCAA
          1 TTCCAACGAAATCATCAAAGCGATCCAAATATCCACTTCCAGATTCTACAAAAAGAGTGTTTCAA

             *         *   * *   *         *        * *  *      *      *     
     175832 ACCTGCTCTATGAAAGGGAATCTTCAACTCTATGAGTTGAATGCAGACATCAGAAAGAAATTTCT
         66 AACTGCTCTATCAAAAGAAATGTTCAACTCTGTGAGTTGAGTACACACATCACAAAGAAGTTTCT

                     *       **          *         **
     175897 GAGAATGCTGCTGTCTACCTTTTAT-T--TGA-ATTCCCGC
        131 GAGAATGCTTCTGTCTAGTTTTTATATGAAGATATTCCCTT

                         *  *    *              * *  **   *                  
     175934 TTCCAACGAAATCCTCCAAGCTATCCAAATATCCACCTGCATTTTCCACAAAAAGAGTGTTTCAA
          1 TTCCAACGAAATCATCAAAGCGATCCAAATATCCACTTCCAGATTCTACAAAAAGAGTGTTTCAA

                          *                 *    *  *                *       
     175999 AACTGCTCTATCAATAGAAATGTTCAACTCCTTTG-GCTGGGTACACACATCACAAACAAGTTTC
         66 AACTGCTCTATCAAAAGAAATGTTCAACT-CTGTGAGTTGAGTACACACATCACAAAGAAGTTTC

                                      **     *        
     176063 TGAGAATGCTTCTGTCTAGTTTTTATGGGAAGACATTCCCTT
        130 TGAGAATGCTTCTGTCTAGTTTTTATATGAAGATATTCCCTT

              *  * *  **          *       *            **                    
     176105 TTTCACCAAAGACATCAAAGCGCTCCAAATGTCCACTTCCAGACACTACAAAAAGAGTGTTTCAA
          1 TTCCAACGAAATCATCAAAGCGATCCAAATATCCACTTCCAGATTCTACAAAAAGAGTGTTTCAA

             **       **   ***                 *    * *  * *         *       
     176170 ACGTGCTCTAAGAAAGCGAATGTTCAACTCTGTGACTTGAATGCAGATATCACAAAGTAGTTTCT
         66 AACTGCTCTATCAAAAGAAATGTTCAACTCTGTGAGTTGAGTACACACATCACAAAGAAGTTTCT

                **            *     *    *         * 
     176235 GAGAGGGCTTCTGTCTAGATTTTAGATGATGATATTCCCGT
        131 GAGAATGCTTCTGTCTAGTTTTTATATGAAGATATTCCCTT

                           * *   *                *   *                    * 
     176276 TTCCAACGAAATCATTAGAGCTATCCAAATATCCACTTACAGTTTCTACAAAAAGAGTGTTTCCA
          1 TTCCAACGAAATCATCAAAGCGATCCAAATATCCACTTCCAGATTCTACAAAAAGAGTGTTTCAA

                   **         * *    *       *  **                  ***      
     176341 AACTGCTGCATCAAAAGAGAGGTTCCACTCTGTTAGCCGAGTACACACATCACAAACTTGTTTCT
         66 AACTGCTCTATCAAAAGAAATGTTCAACTCTGTGAGTTGAGTACACACATCACAAAGAAGTTTCT

            *     *         *        **         **   
     176406 CAGAATCCTTCTGTCTCGTTTTTATGGGAAGATATTTACTT
        131 GAGAATGCTTCTGTCTAGTTTTTATATGAAGATATTCCCTT

              *  *  * **          *       *     *       *  *  *              
     176447 TTTCACCGTAGGCATCAAAGCGCTCCAAATGTCCACATCCAGATACTCCAGAAAGAGTGTTTCAA
          1 TTCCAACGAAATCATCAAAGCGATCCAAATATCCACTTCCAGATTCTACAAAAAGAGTGTTTCAA

             *         *   * *   *         *        * *  *      *      *     
     176512 ACCTGCTCTATGAAAGGGAATCTTCAACTCTATGAGTTGAATGCAGACATCAGAAAGAAATTTCT
         66 AACTGCTCTATCAAAAGAAATGTTCAACTCTGTGAGTTGAGTACACACATCACAAAGAAGTTTCT

                     *       **          *         **
     176577 GAGAATGCTGCTGTCTACCTTTTAT-T--TGA-ATTCCCGC
        131 GAGAATGCTTCTGTCTAGTTTTTATATGAAGATATTCCCTT

                         *  *    *                *       *                  
     176614 TTCCAACGAAATCCTCCAAGCTATCCAAATATCCACTTGCAGATTCCACAAAAAGAGTGTTTCAA
          1 TTCCAACGAAATCATCAAAGCGATCCAAATATCCACTTCCAGATTCTACAAAAAGAGTGTTTCAA

                            ** *  *            *       *     *     *  *      
     176679 AACTGCTCTCTATCAATGGCAAAGTTCAACTCTGTTAGTTGAGGACACATATCACCAACAAGTTT
         66 AACTG--CTCTATCAAAAGAAATGTTCAACTCTGTGAGTTGAGTACACACATCACAAAGAAGTTT

                               *       **         *    
     176744 CTGAGAATGCTTCTGTCTATTTTTTATGGGAAGATATTTCCTT
        129 CTGAGAATGCTTCTGTCTAGTTTTTATATGAAGATATTCCCTT

              *  *  * ** *    *      *    *          * **                    
     176787 TTTCACCGTAGGCGTCAAGGCGATCGAAATGTCCACTTCCACAAACTACAAAAAGAGTGTTTCAA
          1 TTCCAACGAAATCATCAAAGCGATCCAAATATCCACTTCCAGATTCTACAAAAAGAGTGTTTCAA

             *         *   * **       *    *        * ** * *   **      *     
     176852 ACCTGCTCTATGAAAGGCCATGTTCATCTCTATGAGTTGAATGGAAATATCCGAAAGAAATTTCT
         66 AACTGCTCTATCAAAAGAAATGTTCAACTCTGTGAGTTGAGTACACACATCACAAAGAAGTTTCT

             *       *          *        *         **
     176917 GGGAATGCTGCTGTCTAG-TGTT-TAT-ACGA-ATTCCCGC
        131 GAGAATGCTTCTGTCTAGTTTTTATATGAAGATATTCCCTT

                         *       *                *    *  *                  
     176954 TTCCAACGAAATCCTCAAAGCAATCCAAATATCCACTTGCAGAATCCACAAAAAGAGTGTTTCAA
          1 TTCCAACGAAATCATCAAAGCGATCCAAATATCCACTTCCAGATTCTACAAAAAGAGTGTTTCAA

                          *     *          * *                   *  *        
     177019 AACTGCTCTATCAATAGAAAGGTTCAACTCTTTTAGTTGAGTACACACATCACGAACAAGTTTCT
         66 AACTGCTCTATCAAAAGAAATGTTCAACTCTGTGAGTTGAGTACACACATCACAAAGAAGTTTCT

                            * *      **     **  *    
     177084 GAGAATGCTTCTGTCTGGCTTTTATTGGAAGACGTTTCCTT
        131 GAGAATGCTTCTGTCTAGTTTTTATATGAAGATATTCCCTT

                 * *  **          *       *                * *               
     177125 TT-CACCAAAGGCATCAAAGCGCTCCAAATGTCCACTTCCAGATTCTTCCAAAAGAGTGTTTCAA
          1 TTCCAACGAAATCATCAAAGCGATCCAAATATCCACTTCCAGATTCTACAAAAAGAGTGTTTCAA

             **     * ***  * *                 *    * *  * *     *   *       
     177189 ACGTGCTCAAAGTAAGGGAATGTTCAACTCTGTGACTTGAATGCAGATATCACCAAGTAGTTTCT
         66 AACTGCTCTATCAAAAGAAATGTTCAACTCTGTGAGTTGAGTACACACATCACAAAGAAGTTTCT

            * * *            **     *    *         * 
     177254 AATAGTGCTTCTGTCTACATTTTAGATGATGATATTCCCGT
        131 GAGAATGCTTCTGTCTAGTTTTTATATGAAGATATTCCCTT

                         *** *   *             *  *   *      *     *       * 
     177295 TTCCAACGAAATCGCTAGAGCTATCCAAATATCCAGTTACAGTTTCTACCAAAAGGGTGTTTCCA
          1 TTCCAACGAAATCATCAAAGCGATCCAAATATCCACTTCCAGATTCTACAAAAAGAGTGTTTCAA

              *    **           *            *       *                     * 
     177360 AATTGCTGCATCAAAAGAAAGGTTCAACTCTGTTAGTTGAGGACACACATCACAAAGAAGTTTGT
         66 AACTGCTCTATCAAAAGAAATGTTCAACTCTGTGAGTTGAGTACACACATCACAAAGAAGTTTCT

                              *    *     *           
     177425 GAGAATGCTTCTGTCTAGATTTTGTATGACGATATTCCCTT
        131 GAGAATGCTTCTGTCTAGTTTTTATATGAAGATATTCCCTT

                     *   * *     *   *       * *  *    *  *       *  *       
     177466 TTCCAACGATATCGTTAAAGCAATCTAAATATCAATTTGCAGAATCCACAAAAATAGAGTTTCAA
          1 TTCCAACGAAATCATCAAAGCGATCCAAATATCCACTTCCAGATTCTACAAAAAGAGTGTTTCAA

             *       * *        *    *       *  *                   ***      
     177531 AGCTGCTCTGTAAAAAGAAAGGTTCCACTCTGTTAGCTGAGTACACACATCACAAACTTGTTTCT
         66 AACTGCTCTATCAAAAGAAATGTTCAACTCTGTGAGTTGAGTACACACATCACAAAGAAGTTTCT

            *     *         *        **       * **   
     177596 CAGAATCCTTCTGTCTCGTTTTTATGGGAAGATACTTACTT
        131 GAGAATGCTTCTGTCTAGTTTTTATATGAAGATATTCCCTT

                 *  * **          *       *     *       *  *  *  *           
     177637 TTCCACCGTAGGCATCAAAGCGCTCCAAATGTCCACATCCAGATACTCCAGAACGAGTGTTTCAA
          1 TTCCAACGAAATCATCAAAGCGATCCAAATATCCACTTCCAGATTCTACAAAAAGAGTGTTTCAA

             *         *   * *   *         *        * *  *      *      *     
     177702 ACCTGCTCTATGAAAGGGAATCTTCAACTCTATGAGTTGAATGCAGACATCAGAAAGAAATTTCT
         66 AACTGCTCTATCAAAAGAAATGTTCAACTCTGTGAGTTGAGTACACACATCACAAAGAAGTTTCT

                     *       **          *         **
     177767 GAGAATGCTGCTGTCTACCTTTTAT-T--TGA-ATTCCCGC
        131 GAGAATGCTTCTGTCTAGTTTTTATATGAAGATATTCCCTT

                         *  *    *              * *  **   *    *             
     177804 TTCCAACGAAATCCTCCAAGCTATCCAAATATCCACCTGCATTTTCCACAACAAGAGTGTTTCAA
          1 TTCCAACGAAATCATCAAAGCGATCCAAATATCCACTTCCAGATTCTACAAAAAGAGTGTTTCAA

                          *                 *    *  *                *       
     177869 AACTGCTCTATCAATAGAAATGTTCAACTCCTTTG-GCTGGGTACACACATCACAAACAAGTTTC
         66 AACTGCTCTATCAAAAGAAATGTTCAACT-CTGTGAGTTGAGTACACACATCACAAAGAAGTTTC

                                      **     **       
     177933 TGAGAATGCTTCTGTCTAGTTTTTATGGGAAGACGTTCCCTT
        130 TGAGAATGCTTCTGTCTAGTTTTTATATGAAGATATTCCCTT

              *  * *  **    *     *       *     *       *  *  *              
     177975 TTTCACCAAAGGCATCCAAGCGCTCCAAATGTCCACATCCAGATACTCCAGAAAGAGTGTTTCAA
          1 TTCCAACGAAATCATCAAAGCGATCCAAATATCCACTTCCAGATTCTACAAAAAGAGTGTTTCAA

             *         *   * *   *         *        * *  *      *      *     
     178040 ACCTGCTCTATGAAAGGGAATCTTCAACTCTATGAGTTGAATGCAGACATCAGAAAGAAATTTCT
         66 AACTGCTCTATCAAAAGAAATGTTCAACTCTGTGAGTTGAGTACACACATCACAAAGAAGTTTCT

                     *       **          *         **
     178105 GAGAATGCTGCTGTCTACCTTTTAT-T--TGA-ATTCCCGC
        131 GAGAATGCTTCTGTCTAGTTTTTATATGAAGATATTCCCTT

                         *  *    *                *       *                  
     178142 TTCCAACGAAATCCTCCAAGCTATCCAAATATCCACTTGCAGATTCCACAAAAAGAGTGTTTCAA
          1 TTCCAACGAAATCATCAAAGCGATCCAAATATCCACTTCCAGATTCTACAAAAAGAGTGTTTCAA

                            ** *  *            *       *     *     *  *      
     178207 AACTGCTCTCTATCAATGGCAAAGTTCAACTCTGTTAGTTGAGGACACATATCACCAACAAGTTT
         66 AACTG--CTCTATCAAAAGAAATGTTCAACTCTGTGAGTTGAGTACACACATCACAAAGAAGTTT

                               *       **         *    
     178272 CTGAGAATGCTTCTGTCTATTTTTTATGGGAAGATATTTCCTT
        129 CTGAGAATGCTTCTGTCTAGTTTTTATATGAAGATATTCCCTT

              *  * ** ** *    *      *    *          * **                    
     178315 TTTCACCATAGGCGTCAAGGCGATCGAAATGTCCACTTCCACAAACTACAAAAAGAGTGTTTCAA
          1 TTCCAACGAAATCATCAAAGCGATCCAAATATCCACTTCCAGATTCTACAAAAAGAGTGTTTCAA

             *         *   * **       *    *        * ** * *   **      *     
     178380 ACCTGCTCTATGAAAGGCCATGTTCATCTCTATGAGTTGAATGGAAATATCCGAAAGAAATTTCT
         66 AACTGCTCTATCAAAAGAAATGTTCAACTCTGTGAGTTGAGTACACACATCACAAAGAAGTTTCT

             *       *          *        *         **
     178445 GGGAATGCTGCTGTCTAG-TGTT-TAT-ACGA-ATTCCCGC
        131 GAGAATGCTTCTGTCTAGTTTTTATATGAAGATATTCCCTT

                         *       *                *    *  *                  
     178482 TTCCAACGAAATCCTCAAAGCAATCCAAATATCCACTTGCAGAATCCACAAAAAGAGTGTTTCAA
          1 TTCCAACGAAATCATCAAAGCGATCCAAATATCCACTTCCAGATTCTACAAAAAGAGTGTTTCAA

                          *     *          * *                   *  *        
     178547 AACTGCTCTATCAATAGAAAGGTTCAACTCTTTTAGTTGAGTACACACATCACGAACAAGTTTCT
         66 AACTGCTCTATCAAAAGAAATGTTCAACTCTGTGAGTTGAGTACACACATCACAAAGAAGTTTCT

                            * *      **     **  *    
     178612 GAGAATGCTTCTGTCTGGCTTTTATTGGAAGACGTTTCCTT
        131 GAGAATGCTTCTGTCTAGTTTTTATATGAAGATATTCCCTT

                   *  **          *       *                * *               
     178653 TT-CAACAAAGGCATCAAAGCGCTCCAAATGTCCACTTCCAGATTCTTCCAAAAGAGTGTTTCAA
          1 TTCCAACGAAATCATCAAAGCGATCCAAATATCCACTTCCAGATTCTACAAAAAGAGTGTTTCAA

             **     * ***  * *                 *    * *  * *     *   *       
     178717 ACGTGCTCAAAGTAAGGGAATGTTCAACTCTGTGACTTGAATGCAGATATCACCAAGTAGTTTCT
         66 AACTGCTCTATCAAAAGAAATGTTCAACTCTGTGAGTTGAGTACACACATCACAAAGAAGTTTCT

            * * *        *   **     *    *           
     178782 AATAGTGCTTCTGCCTACATTTTAGATGATGATATTCCCTT
        131 GAGAATGCTTCTGTCTAGTTTTTATATGAAGATATTCCCTT

                         * * *   *             *  *   *      *     *       * 
     178823 TTCCAACGAAATCGTTAGAGCTATCCAAATATCCAGTTACAGTTTCTACCAAAAGGGTGTTTCCA
          1 TTCCAACGAAATCATCAAAGCGATCCAAATATCCACTTCCAGATTCTACAAAAAGAGTGTTTCAA

              *    **           *            *       *                     * 
     178888 AATTGCTGCATCAAAAGAAAGGTTCAACTCTGTTAGTTGAGGACACACATCACAAAGAAGTTTGT
         66 AACTGCTCTATCAAAAGAAATGTTCAACTCTGTGAGTTGAGTACACACATCACAAAGAAGTTTCT

                              *    *     *           
     178953 GAGAATGCTTCTGTCTAGATTTTGTATGACGATATTCCCTT
        131 GAGAATGCTTCTGTCTAGTTTTTATATGAAGATATTCCCTT

                     *   * *     *   *       * *  *    *  *       *  *       
     178994 TTCCAACGATATCGTTAAAGCAATCTAAATATCAATTTGCAGAATCCACAAAAATAGAGTTTCAA
          1 TTCCAACGAAATCATCAAAGCGATCCAAATATCCACTTCCAGATTCTACAAAAAGAGTGTTTCAA

             *       * *        *    *       *  *                   ***      
     179059 AGCTGCTCTGTAAAAAGAAAGGTTCCACTCTGTTAGCTGAGTACACACATCACAAACTTGTTTCT
         66 AACTGCTCTATCAAAAGAAATGTTCAACTCTGTGAGTTGAGTACACACATCACAAAGAAGTTTCT

                  *         *        **         **   
     179124 GAGAATCCTTCTGTCTCGTTTTTATGGGAAGATATTTACTT
        131 GAGAATGCTTCTGTCTAGTTTTTATATGAAGATATTCCCTT

                 *  * **          *       *     *       *  *  *  *           
     179165 TTCCACCGTAGGCATCAAAGCGCTCCAAATGTCCACATCCAGATACTCCAGAACGAGTGTTTCAA
          1 TTCCAACGAAATCATCAAAGCGATCCAAATATCCACTTCCAGATTCTACAAAAAGAGTGTTTCAA

             *         *   * *   *         *        * *  *      *      *     
     179230 ACCTGCTCTATGAAAGGGAATCTTCAACTCTATGAGTTGAATGCAGACATCAGAAAGAAATTTCT
         66 AACTGCTCTATCAAAAGAAATGTTCAACTCTGTGAGTTGAGTACACACATCACAAAGAAGTTTCT

                     *       **          *         **
     179295 GAGAATGCTGCTGTCTACCTTTTAT-T--TGA-ATTCCCGC
        131 GAGAATGCTTCTGTCTAGTTTTTATATGAAGATATTCCCTT

                         *  *    *              * *  **   *                  
     179332 TTCCAACGAAATCCTCCAAGCTATCCAAATATCCACCTGCATTTTCCACAAAAAGAGTGTTTCAA
          1 TTCCAACGAAATCATCAAAGCGATCCAAATATCCACTTCCAGATTCTACAAAAAGAGTGTTTCAA

                          *                 *    *  *                *       
     179397 AACTGCTCTATCAATAGAAATGTTCAACTCCTTTG-GCTGGGTACACACATCACAAACAAGTTTC
         66 AACTGCTCTATCAAAAGAAATGTTCAACT-CTGTGAGTTGAGTACACACATCACAAAGAAGTTTC

                                      **     *        
     179461 TGAGAATGCTTCTGTCTAGTTTTTATGGGAAGACATTCCCTT
        130 TGAGAATGCTTCTGTCTAGTTTTTATATGAAGATATTCCCTT

              *  * *  **          *       *            **                    
     179503 TTTCACCAAAGACATCAAAGCGCTCCAAATGTCCACTTCCAGACACTACAAAAAGAGTGTTTCAA
          1 TTCCAACGAAATCATCAAAGCGATCCAAATATCCACTTCCAGATTCTACAAAAAGAGTGTTTCAA

             **       **   ***                 *    * *  * *         *       
     179568 ACGTGCTCTAAGAAAGCGAATGTTCAACTCTGTGACTTGAATGCAGATATCACAAAGTAGTTTCT
         66 AACTGCTCTATCAAAAGAAATGTTCAACTCTGTGAGTTGAGTACACACATCACAAAGAAGTTTCT

                **            *     *    *         * 
     179633 GAGAGGGCTTCTGTCTAGATTTTAGATGATGATATTCCCGT
        131 GAGAATGCTTCTGTCTAGTTTTTATATGAAGATATTCCCTT

                           * *   *                *   *                    * 
     179674 TTCCAACGAAATCATTAGAGCTATCCAAATATCCACTTACAGTTTCTACAAAAAGAGTGTTTCCA
          1 TTCCAACGAAATCATCAAAGCGATCCAAATATCCACTTCCAGATTCTACAAAAAGAGTGTTTCAA

                   **         * *    *       *  *                   ***      
     179739 AACTGCTGCATCAAAAGAGAGGTTCCACTCTGTTAGCTGAGTACACACATCACAAACTTGTTTCT
         66 AACTGCTCTATCAAAAGAAATGTTCAACTCTGTGAGTTGAGTACACACATCACAAAGAAGTTTCT

            *     *         *        **         **   
     179804 CAGAATCCTTCTGTCTCGTTTTTATGGGAAGATATTTACTT
        131 GAGAATGCTTCTGTCTAGTTTTTATATGAAGATATTCCCTT

              *  *  * **          *       *     *       *  *  *              
     179845 TTTCACCGTAGGCATCAAAGCGCTCCAAATGTCCACATCCAGATACTCCAGAAAGAGTGTTTCAA
          1 TTCCAACGAAATCATCAAAGCGATCCAAATATCCACTTCCAGATTCTACAAAAAGAGTGTTTCAA

             *         *   * *   *         *        * *  *      *      *     
     179910 ACCTGCTCTATGAAAGGGAATCTTCAACTCTATGAGTTGAATGCAGACATCAGAAAGAAATTTCT
         66 AACTGCTCTATCAAAAGAAATGTTCAACTCTGTGAGTTGAGTACACACATCACAAAGAAGTTTCT

                     *       **          *         **
     179975 GAGAATGCTGCTGTCTACCTTTTAT-T--TGA-ATTCCCGC
        131 GAGAATGCTTCTGTCTAGTTTTTATATGAAGATATTCCCTT

                         *  *    *                *       *                  
     180012 TTCCAACGAAATCCTCCAAGCTATCCAAATATCCACTTGCAGATTCCACAAAAAGAGTGTTTCAA
          1 TTCCAACGAAATCATCAAAGCGATCCAAATATCCACTTCCAGATTCTACAAAAAGAGTGTTTCAA

                            ** *  *            *       *     *     *  *      
     180077 AACTGCTCTCTATCAATGGCAAAGTTCAACTCTGTTAGTTGAGGACACATATCACCAACAAGTTT
         66 AACTG--CTCTATCAAAAGAAATGTTCAACTCTGTGAGTTGAGTACACACATCACAAAGAAGTTT

                               *       **         *    
     180142 CTGAGAATGCTTCTGTCTATTTTTTATGGGAAGATATTTCCTT
        129 CTGAGAATGCTTCTGTCTAGTTTTTATATGAAGATATTCCCTT

              *  *  * ** *    *      *    *          * **                    
     180185 TTTCACCGTAGGCGTCAAGGCGATCGAAATGTCCACTTCCACAAACTACAAAAAGAGTGTTTCAA
          1 TTCCAACGAAATCATCAAAGCGATCCAAATATCCACTTCCAGATTCTACAAAAAGAGTGTTTCAA

             *         *   * **       *    *        * ** * *   **      *     
     180250 ACCTGCTCTATGAAAGGCCATGTTCATCTCTATGAGTTGAATGGAAATATCCGAAAGAAATTTCT
         66 AACTGCTCTATCAAAAGAAATGTTCAACTCTGTGAGTTGAGTACACACATCACAAAGAAGTTTCT

             *       *          *        *         **
     180315 GGGAATGCTGCTGTCTAG-TGTT-TAT-ACGA-ATTCCCGC
        131 GAGAATGCTTCTGTCTAGTTTTTATATGAAGATATTCCCTT

                    *    *       *                *    *  *                  
     180352 TTCCAACGTAATCCTCAAAGCAATCCAAATATCCACTTGCAGAATCCACAAAAAGAGTGTTTCAA
          1 TTCCAACGAAATCATCAAAGCGATCCAAATATCCACTTCCAGATTCTACAAAAAGAGTGTTTCAA

                          *     *          * *                   *  *        
     180417 AACTGCTCTATCAATAGAAAGGTTCAACTCTTTTAGTTGAGTACACACATCACGAACAAGTTTCT
         66 AACTGCTCTATCAAAAGAAATGTTCAACTCTGTGAGTTGAGTACACACATCACAAAGAAGTTTCT

                            * *      **     **  *    
     180482 GAGAATGCTTCTGTCTGGCTTTTATTGGAAGACGTTTCCTT
        131 GAGAATGCTTCTGTCTAGTTTTTATATGAAGATATTCCCTT

                 * *  **          *       *                * *               
     180523 TT-CACCAAAGGCATCAAAGCGCTCCAAATGTCCACTTCCAGATTCTTCCAAAAGAGTGTTTCAA
          1 TTCCAACGAAATCATCAAAGCGATCCAAATATCCACTTCCAGATTCTACAAAAAGAGTGTTTCAA

             **     * ***  * *                 *    * *  * *     *   *       
     180587 ACGTGCTCAAAGTAAGGGAATGTTCAACTCTGTGACTTGAATGCAGATATCACCAAGTAGTTTCT
         66 AACTGCTCTATCAAAAGAAATGTTCAACTCTGTGAGTTGAGTACACACATCACAAAGAAGTTTCT

            * * *            **     *    *         * 
     180652 AATAGTGCTTCTGTCTACATTTTAGATGATGATATTCCCGT
        131 GAGAATGCTTCTGTCTAGTTTTTATATGAAGATATTCCCTT

                         *** *   *             *  *   *      *     *       * 
     180693 TTCCAACGAAATCGCTAGAGCTATCCAAATATCCAGTTACAGTTTCTACCAAAAGGGTGTTTCCA
          1 TTCCAACGAAATCATCAAAGCGATCCAAATATCCACTTCCAGATTCTACAAAAAGAGTGTTTCAA

              *    **           *            *       *                     * 
     180758 AATTGCTGCATCAAAAGAAAGGTTCAACTCTGTTAGTTGAGGACACACATCACAAAGAAGTTTGT
         66 AACTGCTCTATCAAAAGAAATGTTCAACTCTGTGAGTTGAGTACACACATCACAAAGAAGTTTCT

                              *    *     *           
     180823 GAGAATGCTTCTGTCTAGGTTTTGTATGACGATATTCCCTT
        131 GAGAATGCTTCTGTCTAGTTTTTATATGAAGATATTCCCTT

                     *   * *     *   *       * *  *    *  *       *  *       
     180864 TTCCAACGATATCGTTAAAGCAATCTAAATATCAATTTGCAGAATCCACAAAAATAGAGTTTCAA
          1 TTCCAACGAAATCATCAAAGCGATCCAAATATCCACTTCCAGATTCTACAAAAAGAGTGTTTCAA

             *       * *        *    *       *  *                   ***      
     180929 AGCTGCTCTGTAAAAAGAAAGGTTCCACTCTGTTAGCTGAGTACACACATCACAAACTTGTTTCT
         66 AACTGCTCTATCAAAAGAAATGTTCAACTCTGTGAGTTGAGTACACACATCACAAAGAAGTTTCT

                  *         *        **       * **   
     180994 GAGAATCCTTCTGTCTCGTTTTTATGGGAAGATACTTACTT
        131 GAGAATGCTTCTGTCTAGTTTTTATATGAAGATATTCCCTT

                 *  * **          *       *     *       *  *  *  *           
     181035 TTCCACCGTAGGCATCAAAGCGCTCCAAATGTCCACATCCAGATACTCCAGAACGAGTGTTTCAA
          1 TTCCAACGAAATCATCAAAGCGATCCAAATATCCACTTCCAGATTCTACAAAAAGAGTGTTTCAA

             *         *   * *   *         *        * *  *      *      *     
     181100 ACCTGCTCTATGAAAGGGAATCTTCAACTCTATGAGTTGAATGCAGACATCAGAAAGAAATTTCT
         66 AACTGCTCTATCAAAAGAAATGTTCAACTCTGTGAGTTGAGTACACACATCACAAAGAAGTTTCT

                     *       **          *         **
     181165 GAGAATGCTGCTGTCTACCTTTTAT-T--TGA-ATTCCCGC
        131 GAGAATGCTTCTGTCTAGTTTTTATATGAAGATATTCCCTT

                         *  *    *              * *  **   *    *             
     181202 TTCCAACGAAATCCTCCAAGCTATCCAAATATCCACCTGCATTTTCCACAACAAGAGTGTTTCAA
          1 TTCCAACGAAATCATCAAAGCGATCCAAATATCCACTTCCAGATTCTACAAAAAGAGTGTTTCAA

                          *                 *    *  *                *       
     181267 AACTGCTCTATCAATAGAAATGTTCAACTCCTTTG-GCTGGGTACACACATCACAAACAAGTTTC
         66 AACTGCTCTATCAAAAGAAATGTTCAACT-CTGTGAGTTGAGTACACACATCACAAAGAAGTTTC

                                      **     **       
     181331 TGAGAATGCTTCTGTCTAGTTTTTATGGGAAGACGTTCCCTT
        130 TGAGAATGCTTCTGTCTAGTTTTTATATGAAGATATTCCCTT

              *  * *  **          *       *            **                    
     181373 TTTCACCAAAGGCATCAAAGCGCTCCAAATGTCCACTTCCAGACACTACAAAAAGAGTGTTTCAA
          1 TTCCAACGAAATCATCAAAGCGATCCAAATATCCACTTCCAGATTCTACAAAAAGAGTGTTTCAA

             **       **   ***                 *    * *    *         *    *  
     181438 ACGTGCTCTAAGAAAGCGAATGTTCAACTCTGTGACTTGAATGCACATATCACAAAGTAGTTCCT
         66 AACTGCTCTATCAAAAGAAATGTTCAACTCTGTGAGTTGAGTACACACATCACAAAGAAGTTTCT

                **            *     *    *         * 
     181503 GAGAGGGCTTCTGTCTAGATTTTAGATGATGATATTCCCGT
        131 GAGAATGCTTCTGTCTAGTTTTTATATGAAGATATTCCCTT

                   *       * *    *               *   *                    * 
     181544 TTCCAACAAAATCATTAGAGC-TTCCAAATATCCACTTACAGTTTCTACAAAAAGAGTGTTTCCA
          1 TTCCAACGAAATCATCAAAGCGATCCAAATATCCACTTCCAGATTCTACAAAAAGAGTGTTTCAA

                   **         * *    *       *  *                   ***      
     181608 AACTGCTGCATCAAAAGAGAGGTTCCACTCTGTTAGCTGAGTACACACATCACAAACTTGTTTCT
         66 AACTGCTCTATCAAAAGAAATGTTCAACTCTGTGAGTTGAGTACACACATCACAAAGAAGTTTCT

            *     *         *        **        ***   
     181673 CAGAATCCTTCTGTCTCGTTTTTATGGGAAGAT-TATACTT
        131 GAGAATGCTTCTGTCTAGTTTTTATATGAAGATATTCCCTT

              *  *  * **          *       *     *       *  *  *              
     181713 TTTCACCGTAGGCATCAAAGCGCTCCAAATGTCCACATCCAGATACTCCAGAAAGAGTGTTTCAA
          1 TTCCAACGAAATCATCAAAGCGATCCAAATATCCACTTCCAGATTCTACAAAAAGAGTGTTTCAA

             *    *    *   * *   *         *        * *  *      *      *     
     181778 ACCTGCCCTATGAAAGGGAATCTTCAACTCTATGAGTTGAATGCAGACATCAGAAAGAAATTTCT
         66 AACTGCTCTATCAAAAGAAATGTTCAACTCTGTGAGTTGAGTACACACATCACAAAGAAGTTTCT

                     *       **          *         **
     181843 GAGAATGCTGCTGTCTACCTTTTAT-T--TGA-ATTCCCGC
        131 GAGAATGCTTCTGTCTAGTTTTTATATGAAGATATTCCCTT

                         *  *    *                *       *                  
     181880 TTCCAACGAAATCCTCCAAGCTATCCAAATATCCACTTGCAGATTCCACAAAAAGAGTGTTTCAA
          1 TTCCAACGAAATCATCAAAGCGATCCAAATATCCACTTCCAGATTCTACAAAAAGAGTGTTTCAA

                            ** *  *            *       *     *     *  *      
     181945 AACTGCTCTCTATCAATGGCAAAGTTCAACTCTGTTAGTTGAGGACACATATCACCAACAAGTTT
         66 AACTG--CTCTATCAAAAGAAATGTTCAACTCTGTGAGTTGAGTACACACATCACAAAGAAGTTT

                               *       **         *    
     182010 CTGAGAATGCTTCTGTCTATTTTTTATGGGAAGATATTTCCTT
        129 CTGAGAATGCTTCTGTCTAGTTTTTATATGAAGATATTCCCTT

              *  *  * ** *    *      *    *          * **                    
     182053 TTTCACCGTAGGCGTCAAGGCGATCGAAATGTCCACTTCCACAAACTACAAAAAGAGTGTTTCAA
          1 TTCCAACGAAATCATCAAAGCGATCCAAATATCCACTTCCAGATTCTACAAAAAGAGTGTTTCAA

             *         *   * **       *    *        * ** * *   **      *     
     182118 ACCTGCTCTATGAAAGGCCATGTTCATCTCTATGAGTTGAATGGAAATATCCGAAAGAAATTTCT
         66 AACTGCTCTATCAAAAGAAATGTTCAACTCTGTGAGTTGAGTACACACATCACAAAGAAGTTTCT

             *       *          *        *         **
     182183 GGGAATGCTGCTGTCTAG-TGTT-TAT-ACGA-ATTCCCGC
        131 GAGAATGCTTCTGTCTAGTTTTTATATGAAGATATTCCCTT

                         *       *                *    *  *                  
     182220 TTCCAACGAAATCCTCAAAGCAATCCAAATATCCACTTGCAGAATCCACAAAAAGAGTGTTTCAA
          1 TTCCAACGAAATCATCAAAGCGATCCAAATATCCACTTCCAGATTCTACAAAAAGAGTGTTTCAA

                          *     *          * *                   *  *        
     182285 AACTGCTCTATCAATAGAAAGGTTCAACTCTTTTAGTTGAGTACACACATCACGAACAAGTTTCT
         66 AACTGCTCTATCAAAAGAAATGTTCAACTCTGTGAGTTGAGTACACACATCACAAAGAAGTTTCT

                            * *      **     **  *    
     182350 GAGAATGCTTCTGTCTGGCTTTTATTGGAAGACGTTTCCTT
        131 GAGAATGCTTCTGTCTAGTTTTTATATGAAGATATTCCCTT

                 * *  **          *       *                * *               
     182391 TT-CACCAAAGGCATCAAAGCGCTCCAAATGTCCACTTCCAGATTCTTCCAAAAGAGTGTTTCAA
          1 TTCCAACGAAATCATCAAAGCGATCCAAATATCCACTTCCAGATTCTACAAAAAGAGTGTTTCAA

             **     * ***  * *                 *    * *  * *     *   *       
     182455 ACGTGCTCAAAGTAAGGGAATGTTCAACTCTGTGACTTGAATGCAGATATCACCAAGTAGTTTCT
         66 AACTGCTCTATCAAAAGAAATGTTCAACTCTGTGAGTTGAGTACACACATCACAAAGAAGTTTCT

            * * *        *   **     *    *           
     182520 AATAGTGCTTCTGCCTACATTTTAGATGATGATATTCCCTT
        131 GAGAATGCTTCTGTCTAGTTTTTATATGAAGATATTCCCTT

                         * * *   *             *  *   *      *     *       * 
     182561 TTCCAACGAAATCGTTAGAGCTATCCAAATATCCAGTTACAGTTTCTACCAAAAGGGTGTTTCCA
          1 TTCCAACGAAATCATCAAAGCGATCCAAATATCCACTTCCAGATTCTACAAAAAGAGTGTTTCAA

              *    **           *            *       *                     * 
     182626 AATTGCTGCATCAAAAGAAAGGTTCAACTCTGTTAGTTGAGGACACACATCACAAAGAAGTTTGT
         66 AACTGCTCTATCAAAAGAAATGTTCAACTCTGTGAGTTGAGTACACACATCACAAAGAAGTTTCT

                              *    *     *           
     182691 GAGAATGCTTCTGTCTAGATTTTGTATGACGATATTCCCTT
        131 GAGAATGCTTCTGTCTAGTTTTTATATGAAGATATTCCCTT

                     *   * *     *   *       * *  *    *  *       *  *       
     182732 TTCCAACGATATCGTTAAAGCAATCTAAATATCAATTTGCAGAATCCACAAAAATAGAGTTTCAA
          1 TTCCAACGAAATCATCAAAGCGATCCAAATATCCACTTCCAGATTCTACAAAAAGAGTGTTTCAA

             *       * *        *    *       *  *                   ***      
     182797 AGCTGCTCTGTAAAAAGAAAGGTTCCACTCTGTTAGCTGAGTACACACATCACAAACTTGTTTCT
         66 AACTGCTCTATCAAAAGAAATGTTCAACTCTGTGAGTTGAGTACACACATCACAAAGAAGTTTCT

                  *         *        **         **   
     182862 GAGAATCCTTCTGTCTCGTTTTTATGGGAAGATATTTACTT
        131 GAGAATGCTTCTGTCTAGTTTTTATATGAAGATATTCCCTT

                 *  * **          *       *     *       *  *  *  *           
     182903 TTCCACCGTAGGCATCAAAGCGCTCCAAATGTCCACATCCAGATACTCCAGAACGAGTGTTTCAA
          1 TTCCAACGAAATCATCAAAGCGATCCAAATATCCACTTCCAGATTCTACAAAAAGAGTGTTTCAA

             *         *   * *   *         *        * *  *      *      *     
     182968 ACCTGCTCTATGAAAGGGAATCTTCAACTCTATGAGTTGAATGCAGACATCAGAAAGAAATTTCT
         66 AACTGCTCTATCAAAAGAAATGTTCAACTCTGTGAGTTGAGTACACACATCACAAAGAAGTTTCT

                     *       **          *         **
     183033 GAGAATGCTGCTGTCTACCTTTTAT-T--TGA-ATTCCCGC
        131 GAGAATGCTTCTGTCTAGTTTTTATATGAAGATATTCCCTT

                         *  *    *              * *  **   *                  
     183070 TTCCAACGAAATCCTCCAAGCTATCCAAATATCCACCTGCATTTTCCACAAAAAGAGTGTTTCAA
          1 TTCCAACGAAATCATCAAAGCGATCCAAATATCCACTTCCAGATTCTACAAAAAGAGTGTTTCAA

                          *                 *    *  *                *       
     183135 AACTGCTCTATCAATAGAAATGTTCAACTCCTTTG-GCTGGGTACACACATCACAAACAAGTTTC
         66 AACTGCTCTATCAAAAGAAATGTTCAACT-CTGTGAGTTGAGTACACACATCACAAAGAAGTTTC

                                      **     *        
     183199 TGAGAATGCTTCTGTCTAGTTTTTATGGGAAGACATTCCCTT
        130 TGAGAATGCTTCTGTCTAGTTTTTATATGAAGATATTCCCTT

              *  * *  **          *       *            **                    
     183241 TTTCACCAAAGACATCAAAGCGCTCCAAATGTCCACTTCCAGACACTACAAAAAGAGTGTTTCAA
          1 TTCCAACGAAATCATCAAAGCGATCCAAATATCCACTTCCAGATTCTACAAAAAGAGTGTTTCAA

             **       **   ***                 *    * *  * *         *       
     183306 ACGTGCTCTAAGAAAGCGAATGTTCAACTCTGTGACTTGAATGCAGATATCACAAAGTAGTTTCT
         66 AACTGCTCTATCAAAAGAAATGTTCAACTCTGTGAGTTGAGTACACACATCACAAAGAAGTTTCT

                **            *     *    *         * 
     183371 GAGAGGGCTTCTGTCTAGATTTTAGATGATGATATTCCCGT
        131 GAGAATGCTTCTGTCTAGTTTTTATATGAAGATATTCCCTT

                           * *   *                *   *                    * 
     183412 TTCCAACGAAATCATTAGAGCTATCCAAATATCCACTTACAGTTTCTACAAAAAGAGTGTTTCCA
          1 TTCCAACGAAATCATCAAAGCGATCCAAATATCCACTTCCAGATTCTACAAAAAGAGTGTTTCAA

                   **         * *    *       *  **                  ***      
     183477 AACTGCTGCATCAAAAGAGAGGTTCCACTCTGTTAGCCGAGTACACACATCACAAACTTGTTTCT
         66 AACTGCTCTATCAAAAGAAATGTTCAACTCTGTGAGTTGAGTACACACATCACAAAGAAGTTTCT

            *     *         *        **         **   
     183542 CAGAATCCTTCTGTCTCGTTTTTATGGGAAGATATTTACTT
        131 GAGAATGCTTCTGTCTAGTTTTTATATGAAGATATTCCCTT

              *  *  * **          *       *     *       *  *  *              
     183583 TTTCACCGTAGGCATCAAAGCGCTCCAAATGTCCACATCCAGATACTCCAGAAAGAGTGTTTCAA
          1 TTCCAACGAAATCATCAAAGCGATCCAAATATCCACTTCCAGATTCTACAAAAAGAGTGTTTCAA

             *         *   * *   *         *        * *  *      *      *     
     183648 ACCTGCTCTATGAAAGGGAATCTTCAACTCTATGAGTTGAATGCAGACATCAGAAAGAAATTTCT
         66 AACTGCTCTATCAAAAGAAATGTTCAACTCTGTGAGTTGAGTACACACATCACAAAGAAGTTTCT

                     *       **          *         **
     183713 GAGAATGCTGCTGTCTACCTTTTAT-T--TGA-ATTCCCGC
        131 GAGAATGCTTCTGTCTAGTTTTTATATGAAGATATTCCCTT

                         *  *    *                *       *                  
     183750 TTCCAACGAAATCCTCCAAGCTATCCAAATATCCACTTGCAGATTCCACAAAAAGAGTGTTTCAA
          1 TTCCAACGAAATCATCAAAGCGATCCAAATATCCACTTCCAGATTCTACAAAAAGAGTGTTTCAA

                            ** *  *            *       *     *     *  *      
     183815 AACTGCTCTCTATCAATGGCAAAGTTCAACTCTGTTAGTTGAGGACACATATCACCAACAAGTTT
         66 AACTG--CTCTATCAAAAGAAATGTTCAACTCTGTGAGTTGAGTACACACATCACAAAGAAGTTT

                               *       **         *    
     183880 CTGAGAATGCTTCTGTCTATTTTTTATGGGAAGATATTTCCTT
        129 CTGAGAATGCTTCTGTCTAGTTTTTATATGAAGATATTCCCTT

              *  *  * ** *    *      *    *          * **                    
     183923 TTTCACCGTAGGCGTCAAGGCGATCGAAATGTCCACTTCCACAAACTACAAAAAGAGTGTTTCAA
          1 TTCCAACGAAATCATCAAAGCGATCCAAATATCCACTTCCAGATTCTACAAAAAGAGTGTTTCAA

             *         *   * **       *    *        * ** * *   **      *     
     183988 ACCTGCTCTATGAAAGGCCATGTTCATCTCTATGAGTTGAATGGAAATATCCGAAAGAAATTTCT
         66 AACTGCTCTATCAAAAGAAATGTTCAACTCTGTGAGTTGAGTACACACATCACAAAGAAGTTTCT

             *       *          *        *         **
     184053 GGGAATGCTGCTGTCTAG-TGTT-TAT-ACGA-ATTCCCGC
        131 GAGAATGCTTCTGTCTAGTTTTTATATGAAGATATTCCCTT

                    *    *       *                *    *  *                  
     184090 TTCCAACGTAATCCTCAAAGCAATCCAAATATCCACTTGCAGAATCCACAAAAAGAGTGTTTCAA
          1 TTCCAACGAAATCATCAAAGCGATCCAAATATCCACTTCCAGATTCTACAAAAAGAGTGTTTCAA

                          *     *          * *                   *  *        
     184155 AACTGCTCTATCAATAGAAAGGTTCAACTCTTTTAGTTGAGTACACACATCACGAACAAGTTTCT
         66 AACTGCTCTATCAAAAGAAATGTTCAACTCTGTGAGTTGAGTACACACATCACAAAGAAGTTTCT

                            * *      **     **  *    
     184220 GAGAATGCTTCTGTCTGGCTTTTATTGGAAGACGTTTCCTT
        131 GAGAATGCTTCTGTCTAGTTTTTATATGAAGATATTCCCTT

                 * *  **          *       *                * *               
     184261 TT-CACCAAAGGCATCAAAGCGCTCCAAATGTCCACTTCCAGATTCTTCCAAAAGAGTGTTTCAA
          1 TTCCAACGAAATCATCAAAGCGATCCAAATATCCACTTCCAGATTCTACAAAAAGAGTGTTTCAA

             **     * ***  * *                 *    * *  * *     *   *       
     184325 ACGTGCTCAAAGTAAGGGAATGTTCAACTCTGTGACTTGAATGCAGATATCACCAAGTAGTTTCT
         66 AACTGCTCTATCAAAAGAAATGTTCAACTCTGTGAGTTGAGTACACACATCACAAAGAAGTTTCT

            * * *            **     *    *         * 
     184390 AATAGTGCTTCTGTCTACATTTTAGATGATGATATTCCCGT
        131 GAGAATGCTTCTGTCTAGTTTTTATATGAAGATATTCCCTT

                         *** *   *             *  *   *      *     *       * 
     184431 TTCCAACGAAATCGCTAGAGCTATCCAAATATCCAGTTACAGTTTCTACCAAAAGGGTGTTTCCA
          1 TTCCAACGAAATCATCAAAGCGATCCAAATATCCACTTCCAGATTCTACAAAAAGAGTGTTTCAA

              *    **           *            *       *                     * 
     184496 AATTGCTGCATCAAAAGAAAGGTTCAACTCTGTTAGTTGAGGACACACATCACAAAGAAGTTTGT
         66 AACTGCTCTATCAAAAGAAATGTTCAACTCTGTGAGTTGAGTACACACATCACAAAGAAGTTTCT

                              *    *     *           
     184561 GAGAATGCTTCTGTCTAGATTTTGTATGACGATATTCCCTT
        131 GAGAATGCTTCTGTCTAGTTTTTATATGAAGATATTCCCTT

                     *   * *     *   *       * *  *    *  *       *  *       
     184602 TTCCAACGATATCGTTAAAGCAATCTAAATATCAATTTGCAGAATCCACAAAAATAGAGTTTCAA
          1 TTCCAACGAAATCATCAAAGCGATCCAAATATCCACTTCCAGATTCTACAAAAAGAGTGTTTCAA

             *       * *        *    *       *  *                   ***      
     184667 AGCTGCTCTGTAAAAAGAAAGGTTCCACTCTGTTAGCTGAGTACACACATCACAAACTTGTTTCT
         66 AACTGCTCTATCAAAAGAAATGTTCAACTCTGTGAGTTGAGTACACACATCACAAAGAAGTTTCT

            *     *         *        **       * **   
     184732 CAGAATCCTTCTGTCTCGTTTTTATGGGAAGATACTTACTT
        131 GAGAATGCTTCTGTCTAGTTTTTATATGAAGATATTCCCTT

                 *  * **          *       *     *       *  *  *  *           
     184773 TTCCACCGTAGGCATCAAAGCGCTCCAAATGTCCACATCCAGATACTCCAGAACGAGTGTTTCAA
          1 TTCCAACGAAATCATCAAAGCGATCCAAATATCCACTTCCAGATTCTACAAAAAGAGTGTTTCAA

             *         *   * *   *         *        * *  *      *      *     
     184838 ACCTGCTCTATGAAAGGGAATCTTCAACTCTATGAGTTGAATGCAGACATCAGAAAGAAATTTCT
         66 AACTGCTCTATCAAAAGAAATGTTCAACTCTGTGAGTTGAGTACACACATCACAAAGAAGTTTCT

                     *       **          *         **
     184903 GAGAATGCTGCTGTCTACCTTTTAT-T--TGA-ATTCCCGC
        131 GAGAATGCTTCTGTCTAGTTTTTATATGAAGATATTCCCTT

                         *  *    *              * *  **   *    *             
     184940 TTCCAACGAAATCCTCCAAGCTATCCAAATATCCACCTGCATTTTCCACAACAAGAGTGTTTCAA
          1 TTCCAACGAAATCATCAAAGCGATCCAAATATCCACTTCCAGATTCTACAAAAAGAGTGTTTCAA

                          *                 *    *  *                *       
     185005 AACTGCTCTATCAATAGAAATGTTCAACTCCTTTG-GCTGGGTACACACATCACAAACAAGTTTC
         66 AACTGCTCTATCAAAAGAAATGTTCAACT-CTGTGAGTTGAGTACACACATCACAAAGAAGTTTC

                                      **     **       
     185069 TGAGAATGCTTCTGTCTAGTTTTTATGGGAAGACGTTCCCTT
        130 TGAGAATGCTTCTGTCTAGTTTTTATATGAAGATATTCCCTT

              *  * *  **    *     *       *     *       *  *  *              
     185111 TTTCACCAAAGGCATCCAAGCGCTCCAAATGTCCACATCCAGATACTCCAGAAAGAGTGTTTCAA
          1 TTCCAACGAAATCATCAAAGCGATCCAAATATCCACTTCCAGATTCTACAAAAAGAGTGTTTCAA

             *         *   * *   *         *        * *  *      *      *     
     185176 ACCTGCTCTATGAAAGGGAATCTTCAACTCTATGAGTTGAATGCAGACATCAGAAAGAAATTTCT
         66 AACTGCTCTATCAAAAGAAATGTTCAACTCTGTGAGTTGAGTACACACATCACAAAGAAGTTTCT

                     *       **          *         **
     185241 GAGAATGCTGCTGTCTACCTTTTAT-T--TGA-ATTCCCGC
        131 GAGAATGCTTCTGTCTAGTTTTTATATGAAGATATTCCCTT

                         *  *    *                *       *                  
     185278 TTCCAACGAAATCCTCCAAGCTATCCAAATATCCACTTGCAGATTCCACAAAAAGAGTGTTTCAA
          1 TTCCAACGAAATCATCAAAGCGATCCAAATATCCACTTCCAGATTCTACAAAAAGAGTGTTTCAA

                            ** *  *            *       *     *     *  *      
     185343 AACTGCTCTCTATCAATGGCAAAGTTCAACTCTGTTAGTTGAGGACACATATCACCAACAAGTTT
         66 AACTG--CTCTATCAAAAGAAATGTTCAACTCTGTGAGTTGAGTACACACATCACAAAGAAGTTT

                               *       **         *    
     185408 CTGAGAATGCTTCTGTCTATTTTTTATGGGAAGATATTTCCTT
        129 CTGAGAATGCTTCTGTCTAGTTTTTATATGAAGATATTCCCTT

              *  * ** ** *    *      *    *          * **                    
     185451 TTTCACCATAGGCGTCAAGGCGATCGAAATGTCCACTTCCACAAACTACAAAAAGAGTGTTTCAA
          1 TTCCAACGAAATCATCAAAGCGATCCAAATATCCACTTCCAGATTCTACAAAAAGAGTGTTTCAA

             *         *   * **       *    *        * ** * *   **      *     
     185516 ACCTGCTCTATGAAAGGCCATGTTCATCTCTATGAGTTGAATGGAAATATCCGAAAGAAATTTCT
         66 AACTGCTCTATCAAAAGAAATGTTCAACTCTGTGAGTTGAGTACACACATCACAAAGAAGTTTCT

             *       *          *        *         **
     185581 GGGAATGCTGCTGTCTAG-TGTT-TAT-ACGA-ATTCCCGC
        131 GAGAATGCTTCTGTCTAGTTTTTATATGAAGATATTCCCTT

                         *       *                *    *  *                  
     185618 TTCCAACGAAATCCTCAAAGCAATCCAAATATCCACTTGCAGAATCCACAAAAAGAGTGTTTCAA
          1 TTCCAACGAAATCATCAAAGCGATCCAAATATCCACTTCCAGATTCTACAAAAAGAGTGTTTCAA

                          *     *          * *                   *  *        
     185683 AACTGCTCTATCAATAGAAAGGTTCAACTCTTTTAGTTGAGTACACACATCACGAACAAGTTTCT
         66 AACTGCTCTATCAAAAGAAATGTTCAACTCTGTGAGTTGAGTACACACATCACAAAGAAGTTTCT

                            * *      **     **  *    
     185748 GAGAATGCTTCTGTCTGGCTTTTATTGGAAGACGTTTCCTT
        131 GAGAATGCTTCTGTCTAGTTTTTATATGAAGATATTCCCTT

                   *  **          *       *                * *               
     185789 TT-CAACAAAGGCATCAAAGCGCTCCAAATGTCCACTTCCAGATTCTTCCAAAAGAGTGTTTCAA
          1 TTCCAACGAAATCATCAAAGCGATCCAAATATCCACTTCCAGATTCTACAAAAAGAGTGTTTCAA

             **     * ***  * *                 *    * *  * *     *   *       
     185853 ACGTGCTCAAAGTAAGGGAATGTTCAACTCTGTGACTTGAATGCAGATATCACCAAGTAGTTTCT
         66 AACTGCTCTATCAAAAGAAATGTTCAACTCTGTGAGTTGAGTACACACATCACAAAGAAGTTTCT

            * * *        *   **     *    *           
     185918 AATAGTGCTTCTGCCTACATTTTAGATGATGATATTCCCTT
        131 GAGAATGCTTCTGTCTAGTTTTTATATGAAGATATTCCCTT

                         * * *   *             *  *   *      *     *       * 
     185959 TTCCAACGAAATCGTTAGAGCTATCCAAATATCCAGTTACAGTTTCTACCAAAAGGGTGTTTCCA
          1 TTCCAACGAAATCATCAAAGCGATCCAAATATCCACTTCCAGATTCTACAAAAAGAGTGTTTCAA

              *    **           *            *       *                     * 
     186024 AATTGCTGCATCAAAAGAAAGGTTCAACTCTGTTAGTTGAGGACACACATCACAAAGAAGTTTGT
         66 AACTGCTCTATCAAAAGAAATGTTCAACTCTGTGAGTTGAGTACACACATCACAAAGAAGTTTCT

                              *    *     *           
     186089 GAGAATGCTTCTGTCTAGATTTTGTATGACGATATTCCCTT
        131 GAGAATGCTTCTGTCTAGTTTTTATATGAAGATATTCCCTT

                     *   * *     *   *       * *  *    *  *       *  *       
     186130 TTCCAACGATATCGTTAAAGCAATCTAAATATCAATTTGCAGAATCCACAAAAATAGAGTTTCAA
          1 TTCCAACGAAATCATCAAAGCGATCCAAATATCCACTTCCAGATTCTACAAAAAGAGTGTTTCAA

             *       * *        *    *       *  *                   ***      
     186195 AGCTGCTCTGTAAAAAGAAAGGTTCCACTCTGTTAGCTGAGTACACACATCACAAACTTGTTTCT
         66 AACTGCTCTATCAAAAGAAATGTTCAACTCTGTGAGTTGAGTACACACATCACAAAGAAGTTTCT

                  *         *        **         **   
     186260 GAGAATCCTTCTGTCTCGTTTTTATGGGAAGATATTTACTT
        131 GAGAATGCTTCTGTCTAGTTTTTATATGAAGATATTCCCTT

                 *  * **          *       *     *       *  *  *  *           
     186301 TTCCACCGTAGGCATCAAAGCGCTCCAAATGTCCACATCCAGATACTCCAGAACGAGTGTTTCAA
          1 TTCCAACGAAATCATCAAAGCGATCCAAATATCCACTTCCAGATTCTACAAAAAGAGTGTTTCAA

             *         *   * *   *         *        * *  *      *      *     
     186366 ACCTGCTCTATGAAAGGGAATCTTCAACTCTATGAGTTGAATGCAGACATCAGAAAGAAATTTCT
         66 AACTGCTCTATCAAAAGAAATGTTCAACTCTGTGAGTTGAGTACACACATCACAAAGAAGTTTCT

                     *       **          *         **
     186431 GAGAATGCTGCTGTCTACCTTTTAT-T--TGA-ATTCCCGC
        131 GAGAATGCTTCTGTCTAGTTTTTATATGAAGATATTCCCTT

                         *  *    *              * *  **   *                  
     186468 TTCCAACGAAATCCTCCAAGCTATCCAAATATCCACCTGCATTTTCCACAAAAAGAGTGTTTCAA
          1 TTCCAACGAAATCATCAAAGCGATCCAAATATCCACTTCCAGATTCTACAAAAAGAGTGTTTCAA

                          *                 *    *  *                *       
     186533 AACTGCTCTATCAATAGAAATGTTCAACTCCTTTG-GCTGGGTACACACATCACAAACAAGTTTC
         66 AACTGCTCTATCAAAAGAAATGTTCAACT-CTGTGAGTTGAGTACACACATCACAAAGAAGTTTC

                                      **     *        
     186597 TGAGAATGCTTCTGTCTAGTTTTTATGGGAAGACATTCCCTT
        130 TGAGAATGCTTCTGTCTAGTTTTTATATGAAGATATTCCCTT

              *  * *  **          *       *            **                    
     186639 TTTCACCAAAGACATCAAAGCGCTCCAAATGTCCACTTCCAGACACTACAAAAAGAGTGTTTCAA
          1 TTCCAACGAAATCATCAAAGCGATCCAAATATCCACTTCCAGATTCTACAAAAAGAGTGTTTCAA

             **       **   ***                 *    * *    *         *       
     186704 ACGTGCTCTAAGAAAGCGAATGTTCAACTCTGTGACTTGAATGCACATATCACAAAGTAGTTTCT
         66 AACTGCTCTATCAAAAGAAATGTTCAACTCTGTGAGTTGAGTACACACATCACAAAGAAGTTTCT

                **            *     *    *         * 
     186769 GAGAGGGCTTCTGTCTAGATTTTAGATGATGATATTCCCGT
        131 GAGAATGCTTCTGTCTAGTTTTTATATGAAGATATTCCCTT

                   *       * *    *               *   *                    * 
     186810 TTCCAACAAAATCATTAGAGC-TTCCAAATATCCACTTACAGTTTCTACAAAAAGAGTGTTTCCA
          1 TTCCAACGAAATCATCAAAGCGATCCAAATATCCACTTCCAGATTCTACAAAAAGAGTGTTTCAA

                   **         * *    *       *  *                   ***      
     186874 AACTGCTGCATCAAAAGAGAGGTTCCACTCTGTTAGCTGAGTACACACATCACAAACTTGTTTCT
         66 AACTGCTCTATCAAAAGAAATGTTCAACTCTGTGAGTTGAGTACACACATCACAAAGAAGTTTCT

            *     *         *        **        ***   
     186939 CAGAATCCTTCTGTCTCGTTTTTATGGGAAGAT-TATACTT
        131 GAGAATGCTTCTGTCTAGTTTTTATATGAAGATATTCCCTT

              *  *  * **          *       *     *       *  *  *              
     186979 TTTCACCGTAGGCATCAAAGCGCTCCAAATGTCCACATCCAGATACTCCAGAAAGAGTGTTTCAA
          1 TTCCAACGAAATCATCAAAGCGATCCAAATATCCACTTCCAGATTCTACAAAAAGAGTGTTTCAA

             *    *    *   * *   *         *        * *  *      *      *     
     187044 ACCTGCCCTATGAAAGGGAATCTTCAACTCTATGAGTTGAATGCAGACATCAGAAAGAAATTTCT
         66 AACTGCTCTATCAAAAGAAATGTTCAACTCTGTGAGTTGAGTACACACATCACAAAGAAGTTTCT

                     *       **          *         **
     187109 GAGAATGCTGCTGTCTACCTTTTAT-T--TGA-ATTCCCGC
        131 GAGAATGCTTCTGTCTAGTTTTTATATGAAGATATTCCCTT

                         *  *    *                *       *                  
     187146 TTCCAACGAAATCCTCCAAGCTATCCAAATATCCACTTGCAGATTCCACAAAAAGAGTGTTTCAA
          1 TTCCAACGAAATCATCAAAGCGATCCAAATATCCACTTCCAGATTCTACAAAAAGAGTGTTTCAA

                            ** *  *            *       *     *     *  *      
     187211 AACTGCTCTCTATCAATGGCAAAGTTCAACTCTGTTAGTTGAGGACACATATCACCAACAAGTTT
         66 AACTG--CTCTATCAAAAGAAATGTTCAACTCTGTGAGTTGAGTACACACATCACAAAGAAGTTT

                               *       **         *    
     187276 CTGAGAATGCTTCTGTCTATTTTTTATGGGAAGATATTTCCTT
        129 CTGAGAATGCTTCTGTCTAGTTTTTATATGAAGATATTCCCTT

              *  *  * ** *    *      *    *          * **                    
     187319 TTTCACCGTAGGCGTCAAGGCGATCGAAATGTCCACTTCCACAAACTACAAAAAGAGTGTTTCAA
          1 TTCCAACGAAATCATCAAAGCGATCCAAATATCCACTTCCAGATTCTACAAAAAGAGTGTTTCAA

             *         *   * **       *    *        * ** * *   **      *     
     187384 ACCTGCTCTATGAAAGGCCATGTTCATCTCTATGAGTTGAATGGAAATATCCGAAAGAAATTTCT
         66 AACTGCTCTATCAAAAGAAATGTTCAACTCTGTGAGTTGAGTACACACATCACAAAGAAGTTTCT

             *       *          *        *         **
     187449 GGGAATGCTGCTGTCTAG-TGTT-TAT-ACGA-ATTCCCGC
        131 GAGAATGCTTCTGTCTAGTTTTTATATGAAGATATTCCCTT

                         *       *                *    *  *                  
     187486 TTCCAACGAAATCCTCAAAGCAATCCAAATATCCACTTGCAGAATCCACAAAAAGAGTGTTTCAA
          1 TTCCAACGAAATCATCAAAGCGATCCAAATATCCACTTCCAGATTCTACAAAAAGAGTGTTTCAA

                          *     *          * *                   *  *        
     187551 AACTGCTCTATCAATAGAAAGGTTCAACTCTTTTAGTTGAGTACACACATCACGAACAAGTTTCT
         66 AACTGCTCTATCAAAAGAAATGTTCAACTCTGTGAGTTGAGTACACACATCACAAAGAAGTTTCT

                            * *      **     **  *    
     187616 GAGAATGCTTCTGTCTGGCTTTTATTGGAAGACGTTTCCTT
        131 GAGAATGCTTCTGTCTAGTTTTTATATGAAGATATTCCCTT

                   *  **          *       *                * *               
     187657 TT-CAACAAAGGCATCAAAGCGCTCCAAATGTCCACTTCCAGATTCTTCCAAAAGAGTGTTTCAA
          1 TTCCAACGAAATCATCAAAGCGATCCAAATATCCACTTCCAGATTCTACAAAAAGAGTGTTTCAA

             **     * ***  * *                 *    * *  * *     *   *       
     187721 ACGTGCTCAAAGTAAGGGAATGTTCAACTCTGTGACTTGAATGCAGATATCACCAAGTAGTTTCT
         66 AACTGCTCTATCAAAAGAAATGTTCAACTCTGTGAGTTGAGTACACACATCACAAAGAAGTTTCT

            * * *        *   **     *    *           
     187786 AATAGTGCTTCTGCCTACATTTTAGATGATGATATTCCCTT
        131 GAGAATGCTTCTGTCTAGTTTTTATATGAAGATATTCCCTT

                         * * *   *             *  *   *      *     *       * 
     187827 TTCCAACGAAATCGTTAGAGCTATCCAAATATCCAGTTACAGTTTCTACCAAAAGGGTGTTTCCA
          1 TTCCAACGAAATCATCAAAGCGATCCAAATATCCACTTCCAGATTCTACAAAAAGAGTGTTTCAA

              *    **           *            *       *                     * 
     187892 AATTGCTGCATCAAAAGAAAGGTTCAACTCTGTTAGTTGAGGACACACATCACAAAGAAGTTTGT
         66 AACTGCTCTATCAAAAGAAATGTTCAACTCTGTGAGTTGAGTACACACATCACAAAGAAGTTTCT

                              *    *     *           
     187957 GAGAATGCTTCTGTCTAGATTTTGTATGACGATATTCCCTT
        131 GAGAATGCTTCTGTCTAGTTTTTATATGAAGATATTCCCTT

                     *   * *     *   *       * *  *    *  *       *  *       
     187998 TTCCAACGATATCGTTAAAGCAATCTAAATATCAATTTGCAGAATCCACAAAAATAGAGTTTCAA
          1 TTCCAACGAAATCATCAAAGCGATCCAAATATCCACTTCCAGATTCTACAAAAAGAGTGTTTCAA

             *       * *        *    *       *  *                   ***      
     188063 AGCTGCTCTGTAAAAAGAAAGGTTCCACTCTGTTAGCTGAGTACACACATCACAAACTTGTTTCT
         66 AACTGCTCTATCAAAAGAAATGTTCAACTCTGTGAGTTGAGTACACACATCACAAAGAAGTTTCT

                  *         *        **         **   
     188128 GAGAATCCTTCTGTCTCGTTTTTATGGGAAGATATTTACTT
        131 GAGAATGCTTCTGTCTAGTTTTTATATGAAGATATTCCCTT

                 *  * **          *       *     *       *  *  *  *           
     188169 TTCCACCGTAGGCATCAAAGCGCTCCAAATGTCCACATCCAGATACTCCAGAACGAGTGTTTCAA
          1 TTCCAACGAAATCATCAAAGCGATCCAAATATCCACTTCCAGATTCTACAAAAAGAGTGTTTCAA

             *         *   * *   *         *        * *  *      *      *     
     188234 ACCTGCTCTATGAAAGGGAATCTTCAACTCTATGAGTTGAATGCAGACATCAGAAAGAAATTTCT
         66 AACTGCTCTATCAAAAGAAATGTTCAACTCTGTGAGTTGAGTACACACATCACAAAGAAGTTTCT

                     *       **          *         **
     188299 GAGAATGCTGCTGTCTACCTTTTAT-T--TGA-ATTCCCGC
        131 GAGAATGCTTCTGTCTAGTTTTTATATGAAGATATTCCCTT

                         *  *    *              * *  **   *                  
     188336 TTCCAACGAAATCCTCCAAGCTATCCAAATATCCACCTGCATTTTCCACAAAAAGAGTGTTTCAA
          1 TTCCAACGAAATCATCAAAGCGATCCAAATATCCACTTCCAGATTCTACAAAAAGAGTGTTTCAA

                          *                 *    *  *                *       
     188401 AACTGCTCTATCAATAGAAATGTTCAACTCCTTTG-GCTGGGTACACACATCACAAACAAGTTTC
         66 AACTGCTCTATCAAAAGAAATGTTCAACT-CTGTGAGTTGAGTACACACATCACAAAGAAGTTTC

                                      **     *        
     188465 TGAGAATGCTTCTGTCTAGTTTTTATGGGAAGACATTCCCTT
        130 TGAGAATGCTTCTGTCTAGTTTTTATATGAAGATATTCCCTT

              *  * *  **          *       *            **                    
     188507 TTTCACCAAAGACATCAAAGCGCTCCAAATGTCCACTTCCAGACACTACAAAAAGAGTGTTTCAA
          1 TTCCAACGAAATCATCAAAGCGATCCAAATATCCACTTCCAGATTCTACAAAAAGAGTGTTTCAA

             **       **   ***                 *    * *  * *         *       
     188572 ACGTGCTCTAAGAAAGCGAATGTTCAACTCTGTGACTTGAATGCAGATATCACAAAGTAGTTTCT
         66 AACTGCTCTATCAAAAGAAATGTTCAACTCTGTGAGTTGAGTACACACATCACAAAGAAGTTTCT

                **            *     *    *         * 
     188637 GAGAGGGCTTCTGTCTAGATTTTAGATGATGATATTCCCGT
        131 GAGAATGCTTCTGTCTAGTTTTTATATGAAGATATTCCCTT

                           * *   *                *   *                    * 
     188678 TTCCAACGAAATCATTAGAGCTATCCAAATATCCACTTACAGTTTCTACAAAAAGAGTGTTTCCA
          1 TTCCAACGAAATCATCAAAGCGATCCAAATATCCACTTCCAGATTCTACAAAAAGAGTGTTTCAA

                   **         * *    *       *  **                  ***      
     188743 AACTGCTGCATCAAAAGAGAGGTTCCACTCTGTTAGCCGAGTACACACATCACAAACTTGTTTCT
         66 AACTGCTCTATCAAAAGAAATGTTCAACTCTGTGAGTTGAGTACACACATCACAAAGAAGTTTCT

            *     *         *        **         **   
     188808 CAGAATCCTTCTGTCTCGTTTTTATGGGAAGATATTTACTT
        131 GAGAATGCTTCTGTCTAGTTTTTATATGAAGATATTCCCTT

              *  *  * **          *       *     *       *  *  *              
     188849 TTTCACCGTAGGCATCAAAGCGCTCCAAATGTCCACATCCAGATACTCCAGAAAGAGTGTTTCAA
          1 TTCCAACGAAATCATCAAAGCGATCCAAATATCCACTTCCAGATTCTACAAAAAGAGTGTTTCAA

             *         *   * *   *         *        * *  *      *      *     
     188914 ACCTGCTCTATGAAAGGGAATCTTCAACTCTATGAGTTGAATGCAGACATCAGAAAGAAATTTCT
         66 AACTGCTCTATCAAAAGAAATGTTCAACTCTGTGAGTTGAGTACACACATCACAAAGAAGTTTCT

                     *       **          *         **
     188979 GAGAATGCTGCTGTCTACCTTTTAT-T--TGA-ATTCCCGC
        131 GAGAATGCTTCTGTCTAGTTTTTATATGAAGATATTCCCTT

                         *  *    *                *       *                  
     189016 TTCCAACGAAATCCTCCAAGCTATCCAAATATCCACTTGCAGATTCCACAAAAAGAGTGTTTCAA
          1 TTCCAACGAAATCATCAAAGCGATCCAAATATCCACTTCCAGATTCTACAAAAAGAGTGTTTCAA

                            ** *  *            *       *     *     *  *      
     189081 AACTGCTCTCTATCAATGGCAAAGTTCAACTCTGTTAGTTGAGGACACATATCACCAACAAGTTT
         66 AACTG--CTCTATCAAAAGAAATGTTCAACTCTGTGAGTTGAGTACACACATCACAAAGAAGTTT

                               *       **         *    
     189146 CTGAGAATGCTTCTGTCTATTTTTTATGGGAAGATATTTCCTT
        129 CTGAGAATGCTTCTGTCTAGTTTTTATATGAAGATATTCCCTT

              *  *  * ** *    *      *    *          * **                    
     189189 TTTCACCGTAGGCGTCAAGGCGATCGAAATGTCCACTTCCACAAACTACAAAAAGAGTGTTTCAA
          1 TTCCAACGAAATCATCAAAGCGATCCAAATATCCACTTCCAGATTCTACAAAAAGAGTGTTTCAA

             *         *   * **       *    *        * ** * *   **      *     
     189254 ACCTGCTCTATGAAAGGCCATGTTCATCTCTATGAGTTGAATGGAAATATCCGAAAGAAATTTCT
         66 AACTGCTCTATCAAAAGAAATGTTCAACTCTGTGAGTTGAGTACACACATCACAAAGAAGTTTCT

             *       *          *        *         **
     189319 GGGAATGCTGCTGTCTAG-TGTT-TAT-ACGA-ATTCCCGC
        131 GAGAATGCTTCTGTCTAGTTTTTATATGAAGATATTCCCTT

                         *       *                *    *  *                  
     189356 TTCCAACGAAATCCTCAAAGCAATCCAAATATCCACTTGCAGAATCCACAAAAAGAGTGTTTCAA
          1 TTCCAACGAAATCATCAAAGCGATCCAAATATCCACTTCCAGATTCTACAAAAAGAGTGTTTCAA

                          *     *          * *                   *  *        
     189421 AACTGCTCTATCAATAGAAAGGTTCAACTCTTTTAGTTGAGTACACACATCACGAACAAGTTTCT
         66 AACTGCTCTATCAAAAGAAATGTTCAACTCTGTGAGTTGAGTACACACATCACAAAGAAGTTTCT

                            * *      **     **  *    
     189486 GAGAATGCTTCTGTCTGGCTTTTATTGGAAGACGTTTCCTT
        131 GAGAATGCTTCTGTCTAGTTTTTATATGAAGATATTCCCTT

                 * *  **          *       *                * *               
     189527 TT-CACCAAAGGCATCAAAGCGCTCCAAATGTCCACTTCCAGATTCTTCCAAAAGAGTGTTTCAA
          1 TTCCAACGAAATCATCAAAGCGATCCAAATATCCACTTCCAGATTCTACAAAAAGAGTGTTTCAA

             **     * ***  * *                 *    * *  * *     *   *       
     189591 ACGTGCTCAAAGTAAGGGAATGTTCAACTCTGTGACTTGAATGCAGATATCACCAAGTAGTTTCT
         66 AACTGCTCTATCAAAAGAAATGTTCAACTCTGTGAGTTGAGTACACACATCACAAAGAAGTTTCT

            * * *            **     *    *         * 
     189656 AATAGTGCTTCTGTCTACATTTTAGATGATGATATTCCCGT
        131 GAGAATGCTTCTGTCTAGTTTTTATATGAAGATATTCCCTT

                         *** *   *             *  *   *      *     *       * 
     189697 TTCCAACGAAATCGCTAGAGCTATCCAAATATCCAGTTACAGTTTCTACCAAAAGGGTGTTTCCA
          1 TTCCAACGAAATCATCAAAGCGATCCAAATATCCACTTCCAGATTCTACAAAAAGAGTGTTTCAA

              *    **           *            *       *                     * 
     189762 AATTGCTGCATCAAAAGAAAGGTTCAACTCTGTTAGTTGAGGACACACATCACAAAGAAGTTTGT
         66 AACTGCTCTATCAAAAGAAATGTTCAACTCTGTGAGTTGAGTACACACATCACAAAGAAGTTTCT

                              *    *     *           
     189827 GAGAATGCTTCTGTCTAGATTTTGTATGACGATATTCCCTT
        131 GAGAATGCTTCTGTCTAGTTTTTATATGAAGATATTCCCTT

                     *   * *     *   *       * *  *    *  *       *  *       
     189868 TTCCAACGATATCGTTAAAGCAATCTAAATATCAATTTGCAGAATCCACAAAAATAGAGTTTCAA
          1 TTCCAACGAAATCATCAAAGCGATCCAAATATCCACTTCCAGATTCTACAAAAAGAGTGTTTCAA

             *       * *        *    *       *  *                   ***      
     189933 AGCTGCTCTGTAAAAAGAAAGGTTCCACTCTGTTAGCTGAGTACACACATCACAAACTTGTTTCT
         66 AACTGCTCTATCAAAAGAAATGTTCAACTCTGTGAGTTGAGTACACACATCACAAAGAAGTTTCT

            *     *         *        **       * **   
     189998 CAGAATCCTTCTGTCTCGTTTTTATGGGAAGATACTTACTT
        131 GAGAATGCTTCTGTCTAGTTTTTATATGAAGATATTCCCTT

                 *  * **          *       *     *       *  *  *  *           
     190039 TTCCACCGTAGGCATCAAAGCGCTCCAAATGTCCACATCCAGATACTCCAGAACGAGTGTTTCAA
          1 TTCCAACGAAATCATCAAAGCGATCCAAATATCCACTTCCAGATTCTACAAAAAGAGTGTTTCAA

             *         *   * *   *         *        * *  *      *      *     
     190104 ACCTGCTCTATGAAAGGGAATCTTCAACTCTATGAGTTGAATGCAGACATCAGAAAGAAATTTCT
         66 AACTGCTCTATCAAAAGAAATGTTCAACTCTGTGAGTTGAGTACACACATCACAAAGAAGTTTCT

                     *       **          *         **
     190169 GAGAATGCTGCTGTCTACCTTTTAT-T--TGA-ATTCCCGC
        131 GAGAATGCTTCTGTCTAGTTTTTATATGAAGATATTCCCTT

                         *  *    *              * *  **   *    *             
     190206 TTCCAACGAAATCCTCCAAGCTATCCAAATATCCACCTGCATTTTCCACAACAAGAGTGTTTCAA
          1 TTCCAACGAAATCATCAAAGCGATCCAAATATCCACTTCCAGATTCTACAAAAAGAGTGTTTCAA

                          *                 *    *  *                *       
     190271 AACTGCTCTATCAATAGAAATGTTCAACTCCTTTG-GCTGGGTACACACATCACAAACAAGTTTC
         66 AACTGCTCTATCAAAAGAAATGTTCAACT-CTGTGAGTTGAGTACACACATCACAAAGAAGTTTC

                                      **     **       
     190335 TGAGAATGCTTCTGTCTAGTTTTTATGGGAAGACGTTCCCTT
        130 TGAGAATGCTTCTGTCTAGTTTTTATATGAAGATATTCCCTT

              *  * *  **    *     *       *     *       *  *  *              
     190377 TTTCACCAAAGGCATCCAAGCGCTCCAAATGTCCACATCCAGATACTCCAGAAAGAGTGTTTCAA
          1 TTCCAACGAAATCATCAAAGCGATCCAAATATCCACTTCCAGATTCTACAAAAAGAGTGTTTCAA

             *         *   * *   *         *        * *  *      *      *     
     190442 ACCTGCTCTATGAAAGGGAATCTTCAACTCTATGAGTTGAATGCAGACATCAGAAAGAAATTTCT
         66 AACTGCTCTATCAAAAGAAATGTTCAACTCTGTGAGTTGAGTACACACATCACAAAGAAGTTTCT

                     *       **          *         **
     190507 GAGAATGCTGCTGTCTACCTTTTAT-T--TGA-ATTCCCGC
        131 GAGAATGCTTCTGTCTAGTTTTTATATGAAGATATTCCCTT

                         *  *    *                *       *                  
     190544 TTCCAACGAAATCCTCCAAGCTATCCAAATATCCACTTGCAGATTCCACAAAAAGAGTGTTTCAA
          1 TTCCAACGAAATCATCAAAGCGATCCAAATATCCACTTCCAGATTCTACAAAAAGAGTGTTTCAA

                            ** *  *            *       *     *     *  *      
     190609 AACTGCTCTCTATCAATGGCAAAGTTCAACTCTGTTAGTTGAGGACACATATCACCAACAAGTTT
         66 AACTG--CTCTATCAAAAGAAATGTTCAACTCTGTGAGTTGAGTACACACATCACAAAGAAGTTT

                               *       **         *    
     190674 CTGAGAATGCTTCTGTCTATTTTTTATGGGAAGATATTTCCTT
        129 CTGAGAATGCTTCTGTCTAGTTTTTATATGAAGATATTCCCTT

              *  * ** ** *    *      *    *          * **                    
     190717 TTTCACCATAGGCGTCAAGGCGATCGAAATGTCCACTTCCACAAACTACAAAAAGAGTGTTTCAA
          1 TTCCAACGAAATCATCAAAGCGATCCAAATATCCACTTCCAGATTCTACAAAAAGAGTGTTTCAA

             *         *   * **       *    *        * ** * *   **      *     
     190782 ACCTGCTCTATGAAAGGCCATGTTCATCTCTATGAGTTGAATGGAAATATCCGAAAGAAATTTCT
         66 AACTGCTCTATCAAAAGAAATGTTCAACTCTGTGAGTTGAGTACACACATCACAAAGAAGTTTCT

             *       *          *        *         **
     190847 GGGAATGCTGCTGTCTAG-TGTT-TAT-ACGA-ATTCCCGC
        131 GAGAATGCTTCTGTCTAGTTTTTATATGAAGATATTCCCTT

                         *       *                *    *  *                  
     190884 TTCCAACGAAATCCTCAAAGCAATCCAAATATCCACTTGCAGAATCCACAAAAAGAGTGTTTCAA
          1 TTCCAACGAAATCATCAAAGCGATCCAAATATCCACTTCCAGATTCTACAAAAAGAGTGTTTCAA

                          *     *          * *                   *  *        
     190949 AACTGCTCTATCAATAGAAAGGTTCAACTCTTTTAGTTGAGTACACACATCACGAACAAGTTTCT
         66 AACTGCTCTATCAAAAGAAATGTTCAACTCTGTGAGTTGAGTACACACATCACAAAGAAGTTTCT

                            * *      **     **  *    
     191014 GAGAATGCTTCTGTCTGGCTTTTATTGGAAGACGTTTCCTT
        131 GAGAATGCTTCTGTCTAGTTTTTATATGAAGATATTCCCTT

                   *  **          *       *                * *               
     191055 TT-CAACAAAGGCATCAAAGCGCTCCAAATGTCCACTTCCAGATTCTTCCAAAAGAGTGTTTCAA
          1 TTCCAACGAAATCATCAAAGCGATCCAAATATCCACTTCCAGATTCTACAAAAAGAGTGTTTCAA

             **     * ***  *                   *    * *  * *     *   *       
     191119 ACGTGCTCAAAGTAAGGAAATGTTCAACTCTGTGACTTGAATGCAGATATCACCAAGTAGTTTCT
         66 AACTGCTCTATCAAAAGAAATGTTCAACTCTGTGAGTTGAGTACACACATCACAAAGAAGTTTCT

            * * *        *   **     *    *           
     191184 AATAGTGCTTCTGCCTACATTTTAGATGATGATATTCCCTT
        131 GAGAATGCTTCTGTCTAGTTTTTATATGAAGATATTCCCTT

                         * * *   *             *  *   *      *     *       * 
     191225 TTCCAACGAAATCGTTAGAGCTATCCAAATATCCAGTTACAGTTTCTACCAAAAGGGTGTTTCCA
          1 TTCCAACGAAATCATCAAAGCGATCCAAATATCCACTTCCAGATTCTACAAAAAGAGTGTTTCAA

              *    **           *            *       *                     * 
     191290 AATTGCTGCATCAAAAGAAAGGTTCAACTCTGTTAGTTGAGGACACACATCACAAAGAAGTTTGT
         66 AACTGCTCTATCAAAAGAAATGTTCAACTCTGTGAGTTGAGTACACACATCACAAAGAAGTTTCT

                              *    *     *           
     191355 GAGAATGCTTCTGTCTAGATTTTGTATGACGATATTCCCTT
        131 GAGAATGCTTCTGTCTAGTTTTTATATGAAGATATTCCCTT

                     *   * *     *   *       * *  *    *  *       *  *       
     191396 TTCCAACGATATCGTTAAAGCAATCTAAATATCAATTTGCAGAATCCACAAAAATAGAGTTTCAA
          1 TTCCAACGAAATCATCAAAGCGATCCAAATATCCACTTCCAGATTCTACAAAAAGAGTGTTTCAA

             *       * *        *    *       *  *                   ***      
     191461 AGCTGCTCTGTAAAAAGAAAGGTTCCACTCTGTTAGCTGAGTACACACATCACAAACTTGTTTCT
         66 AACTGCTCTATCAAAAGAAATGTTCAACTCTGTGAGTTGAGTACACACATCACAAAGAAGTTTCT

                  *         *        **         **   
     191526 GAGAATCCTTCTGTCTCGTTTTTATGGGAAGATATTTACTT
        131 GAGAATGCTTCTGTCTAGTTTTTATATGAAGATATTCCCTT

                 *  * **          *       *     *       *  *  *  *           
     191567 TTCCACCGTAGGCATCAAAGCGCTCCAAATGTCCACATCCAGATACTCCAGAACGAGTGTTTCAA
          1 TTCCAACGAAATCATCAAAGCGATCCAAATATCCACTTCCAGATTCTACAAAAAGAGTGTTTCAA

             *         *   * *   *         *        * *  *      *      *     
     191632 ACCTGCTCTATGAAAGGGAATCTTCAACTCTATGAGTTGAATGCAGACATCAGAAAGAAATTTCT
         66 AACTGCTCTATCAAAAGAAATGTTCAACTCTGTGAGTTGAGTACACACATCACAAAGAAGTTTCT

                     *       **          *         **
     191697 GAGAATGCTGCTGTCTACCTTTTAT-T--TGA-ATTCCCGC
        131 GAGAATGCTTCTGTCTAGTTTTTATATGAAGATATTCCCTT

                         *  *    *              * *  **   *                  
     191734 TTCCAACGAAATCCTCCAAGCTATCCAAATATCCACCTGCATTTTCCACAAAAAGAGTGTTTCAA
          1 TTCCAACGAAATCATCAAAGCGATCCAAATATCCACTTCCAGATTCTACAAAAAGAGTGTTTCAA

                          *                 *    *  *                *       
     191799 AACTGCTCTATCAATAGAAATGTTCAACTCCTTTG-GCTGGGTACACACATCACAAACAAGTTTC
         66 AACTGCTCTATCAAAAGAAATGTTCAACT-CTGTGAGTTGAGTACACACATCACAAAGAAGTTTC

                                      **     *        
     191863 TGAGAATGCTTCTGTCTAGTTTTTATGGGAAGACATTCCCTT
        130 TGAGAATGCTTCTGTCTAGTTTTTATATGAAGATATTCCCTT

              *  * *  **          *       *            **                    
     191905 TTTCACCAAAGACATCAAAGCGCTCCAAATGTCCACTTCCAGACACTACAAAAAGAGTGTTTCAA
          1 TTCCAACGAAATCATCAAAGCGATCCAAATATCCACTTCCAGATTCTACAAAAAGAGTGTTTCAA

             **       **   ***                 *    * *  * *         *       
     191970 ACGTGCTCTAAGAAAGCGAATGTTCAACTCTGTGACTTGAATGCAGATATCACAAAGTAGTTTCT
         66 AACTGCTCTATCAAAAGAAATGTTCAACTCTGTGAGTTGAGTACACACATCACAAAGAAGTTTCT

                **            *     *    *         * 
     192035 GAGAGGGCTTCTGTCTAGATTTTAGATGATGATATTCCCGT
        131 GAGAATGCTTCTGTCTAGTTTTTATATGAAGATATTCCCTT

                           * *   *                *   *                    * 
     192076 TTCCAACGAAATCATTAGAGCTATCCAAATATCCACTTACAGTTTCTACAAAAAGAGTGTTTCCA
          1 TTCCAACGAAATCATCAAAGCGATCCAAATATCCACTTCCAGATTCTACAAAAAGAGTGTTTCAA

                   **         * *    *       *  *                   ***      
     192141 AACTGCTGCATCAAAAGAGAGGTTCCACTCTGTTAGCTGAGTACACACATCACAAACTTGTTTCT
         66 AACTGCTCTATCAAAAGAAATGTTCAACTCTGTGAGTTGAGTACACACATCACAAAGAAGTTTCT

            *     *         *        **         **   
     192206 CAGAATCCTTCTGTCTCGTTTTTATGGGAAGATATTTACTT
        131 GAGAATGCTTCTGTCTAGTTTTTATATGAAGATATTCCCTT

              *  *  * **          *       *     *       *  *  *              
     192247 TTTCACCGTAGGCATCAAAGCGCTCCAAATGTCCACATCCAGATACTCCAGAAAGAGTGTTTCAA
          1 TTCCAACGAAATCATCAAAGCGATCCAAATATCCACTTCCAGATTCTACAAAAAGAGTGTTTCAA

             *         *   * *   *         *        * *  *      *      *     
     192312 ACCTGCTCTATGAAAGGGAATCTTCAACTCTATGAGTTGAATGCAGACATCAGAAAGAAATTTCT
         66 AACTGCTCTATCAAAAGAAATGTTCAACTCTGTGAGTTGAGTACACACATCACAAAGAAGTTTCT

                     *       **          *         **
     192377 GAGAATGCTGCTGTCTACCTTTTAT-T--TGA-ATTCCCGC
        131 GAGAATGCTTCTGTCTAGTTTTTATATGAAGATATTCCCTT

                         *  *    *                *       *                  
     192414 TTCCAACGAAATCCTCCAAGCTATCCAAATATCCACTTGCAGATTCCACAAAAAGAGTGTTTCAA
          1 TTCCAACGAAATCATCAAAGCGATCCAAATATCCACTTCCAGATTCTACAAAAAGAGTGTTTCAA

                            ** *  *            *       *     *     *  *      
     192479 AACTGCTCTCTATCAATGGCAAAGTTCAACTCTGTTAGTTGAGGACACATATCACCAACAAGTTT
         66 AACTG--CTCTATCAAAAGAAATGTTCAACTCTGTGAGTTGAGTACACACATCACAAAGAAGTTT

                               *       **         *    
     192544 CTGAGAATGCTTCTGTCTATTTTTTATGGGAAGATATTTCCTT
        129 CTGAGAATGCTTCTGTCTAGTTTTTATATGAAGATATTCCCTT

              *  *  * ** *    *      *    *          * **                    
     192587 TTTCACCGTAGGCGTCAAGGCGATCGAAATGTCCACTTCCACAAACTACAAAAAGAGTGTTTCAA
          1 TTCCAACGAAATCATCAAAGCGATCCAAATATCCACTTCCAGATTCTACAAAAAGAGTGTTTCAA

             *         *   * **       *    *        * ** * *   **      *     
     192652 ACCTGCTCTATGAAAGGCCATGTTCATCTCTATGAGTTGAATGGAAATATCCGAAAGAAATTTCT
         66 AACTGCTCTATCAAAAGAAATGTTCAACTCTGTGAGTTGAGTACACACATCACAAAGAAGTTTCT

             *       *          *        *         **
     192717 GGGAATGCTGCTGTCTAG-TGTT-TAT-ACGA-ATTCCCGC
        131 GAGAATGCTTCTGTCTAGTTTTTATATGAAGATATTCCCTT

                    *    *       *                *    *  *                  
     192754 TTCCAACGTAATCCTCAAAGCAATCCAAATATCCACTTGCAGAATCCACAAAAAGAGTGTTTCAA
          1 TTCCAACGAAATCATCAAAGCGATCCAAATATCCACTTCCAGATTCTACAAAAAGAGTGTTTCAA

                          *     *          * *                   *  *        
     192819 AACTGCTCTATCAATAGAAAGGTTCAACTCTTTTAGTTGAGTACACACATCACGAACAAGTTTCT
         66 AACTGCTCTATCAAAAGAAATGTTCAACTCTGTGAGTTGAGTACACACATCACAAAGAAGTTTCT

                            * *      **     **  *    
     192884 GAGAATGCTTCTGTCTGGCTTTTATTGGAAGACGTTTCCTT
        131 GAGAATGCTTCTGTCTAGTTTTTATATGAAGATATTCCCTT

                 * *  **          *       *                * *               
     192925 TT-CACCAAAGGCATCAAAGCGCTCCAAATGTCCACTTCCAGATTCTTCCAAAAGAGTGTTTCAA
          1 TTCCAACGAAATCATCAAAGCGATCCAAATATCCACTTCCAGATTCTACAAAAAGAGTGTTTCAA

             **     * ***  * *                 *    * *  * *     *   *       
     192989 ACGTGCTCAAAGTAAGGGAATGTTCAACTCTGTGACTTGAATGCAGATATCACCAAGTAGTTTCT
         66 AACTGCTCTATCAAAAGAAATGTTCAACTCTGTGAGTTGAGTACACACATCACAAAGAAGTTTCT

            * * *            **     *    *         * 
     193054 AATAGTGCTTCTGTCTACATTTTAGATGATGATATTCCCGT
        131 GAGAATGCTTCTGTCTAGTTTTTATATGAAGATATTCCCTT

                         *** *   *             *  *   *      *     *       * 
     193095 TTCCAACGAAATCGCTAGAGCTATCCAAATATCCAGTTACAGTTTCTACCAAAAGGGTGTTTCCA
          1 TTCCAACGAAATCATCAAAGCGATCCAAATATCCACTTCCAGATTCTACAAAAAGAGTGTTTCAA

              *    **           *            *       *                     * 
     193160 AATTGCTGCATCAAAAGAAAGGTTCAACTCTGTTAGTTGAGGACACACATCACAAAGAAGTTTGT
         66 AACTGCTCTATCAAAAGAAATGTTCAACTCTGTGAGTTGAGTACACACATCACAAAGAAGTTTCT

                              *    *     *           
     193225 GAGAATGCTTCTGTCTAGGTTTTGTATGACGATATTCCCTT
        131 GAGAATGCTTCTGTCTAGTTTTTATATGAAGATATTCCCTT

                     *   * *     *   *       * *  *    *  *       *  *       
     193266 TTCCAACGATATCGTTAAAGCAATCTAAATATCAATTTGCAGAATCCACAAAAATAGAGTTTCAA
          1 TTCCAACGAAATCATCAAAGCGATCCAAATATCCACTTCCAGATTCTACAAAAAGAGTGTTTCAA

             *       * *        *    *       *  *                   ***      
     193331 AGCTGCTCTGTAAAAAGAAAGGTTCCACTCTGTTAGCTGAGTACACACATCACAAATTTGTTTCT
         66 AACTGCTCTATCAAAAGAAATGTTCAACTCTGTGAGTTGAGTACACACATCACAAAGAAGTTTCT

                  *         *        **       * **   
     193396 GAGAATCCTTCTGTCTCGTTTTTATGGGAAGATACTTACTT
        131 GAGAATGCTTCTGTCTAGTTTTTATATGAAGATATTCCCTT

                 *  * **          *       *     *       *  *  *  *           
     193437 TTCCACCGTAGGCATCAAAGCGCTCCAAATGTCCACATCCAGATACTCCAGAACGAGTGTTTCAA
          1 TTCCAACGAAATCATCAAAGCGATCCAAATATCCACTTCCAGATTCTACAAAAAGAGTGTTTCAA

             *         *   * *   *         *        * *  *      *      *     
     193502 ACCTGCTCTATGAAAGGGAATCTTCAACTCTATGAGTTGAATGCAGACATCAGAAAGAAATTTCT
         66 AACTGCTCTATCAAAAGAAATGTTCAACTCTGTGAGTTGAGTACACACATCACAAAGAAGTTTCT

                     *       **          *         **
     193567 GAGAATGCTGCTGTCTACCTTTTAT-T--TGA-ATTCCCGC
        131 GAGAATGCTTCTGTCTAGTTTTTATATGAAGATATTCCCTT

                         *  *    *              * *  **   *    *             
     193604 TTCCAACGAAATCCTCCAAGCTATCCAAATATCCACCTGCATTTTCCACAACAAGAGTGTTTCAA
          1 TTCCAACGAAATCATCAAAGCGATCCAAATATCCACTTCCAGATTCTACAAAAAGAGTGTTTCAA

                          *                 *    *  *                *       
     193669 AACTGCTCTATCAATAGAAATGTTCAACTCCTTTG-GCTGGGTACACACATCACAAACAAGTTTC
         66 AACTGCTCTATCAAAAGAAATGTTCAACT-CTGTGAGTTGAGTACACACATCACAAAGAAGTTTC

                                      **     **       
     193733 TGAGAATGCTTCTGTCTAGTTTTTATGGGAAGACGTTCCCTT
        130 TGAGAATGCTTCTGTCTAGTTTTTATATGAAGATATTCCCTT

              *  * *  **          *       *            **                    
     193775 TTTCACCAAAGGCATCAAAGCGCTCCAAATGTCCACTTCCAGACACTACAAAAAGAGTGTTTCAA
          1 TTCCAACGAAATCATCAAAGCGATCCAAATATCCACTTCCAGATTCTACAAAAAGAGTGTTTCAA

             **       **   ***                 *    * *    *         *    *  
     193840 ACGTGCTCTAAGAAAGCGAATGTTCAACTCTGTGACTTGAATGCACATATCACAAAGTAGTTCCT
         66 AACTGCTCTATCAAAAGAAATGTTCAACTCTGTGAGTTGAGTACACACATCACAAAGAAGTTTCT

                **            *     *    *         * 
     193905 GAGAGGGCTTCTGTCTAGATTTTAGATGATGATATTCCCGT
        131 GAGAATGCTTCTGTCTAGTTTTTATATGAAGATATTCCCTT

                   *       * *    *               *   *                    * 
     193946 TTCCAACAAAATCATTAGAGC-TTCCAAATATCCACTTACAGTTTCTACAAAAAGAGTGTTTCCA
          1 TTCCAACGAAATCATCAAAGCGATCCAAATATCCACTTCCAGATTCTACAAAAAGAGTGTTTCAA

                   **         * *    *       *  *                   ***      
     194010 AACTGCTGCATCAAAAGAGAGGTTCCACTCTGTTAGCTGAGTACACACATCACAAACTTGTTTCT
         66 AACTGCTCTATCAAAAGAAATGTTCAACTCTGTGAGTTGAGTACACACATCACAAAGAAGTTTCT

            *     *         *        **        ***   
     194075 CAGAATCCTTCTGTCTCGTTTTTATGGGAAGAT-TATACTT
        131 GAGAATGCTTCTGTCTAGTTTTTATATGAAGATATTCCCTT

              *  *  * **          *       *     *       *  *  *              
     194115 TTTCACCGTAGGCATCAAAGCGCTCCAAATGTCCACATCCAGATACTCCAGAAAGAGTGTTTCAA
          1 TTCCAACGAAATCATCAAAGCGATCCAAATATCCACTTCCAGATTCTACAAAAAGAGTGTTTCAA

             *    *    *   * *   *         *        * *  *      *      *     
     194180 ACCTGCCCTATGAAAGGGAATCTTCAACTCTATGAGTTGAATGCAGACATCAGAAAGAAATTTCT
         66 AACTGCTCTATCAAAAGAAATGTTCAACTCTGTGAGTTGAGTACACACATCACAAAGAAGTTTCT

                     *       **          *         **
     194245 GAGAATGCTGCTGTCTACCTTTTAT-T--TGA-ATTCCCGC
        131 GAGAATGCTTCTGTCTAGTTTTTATATGAAGATATTCCCTT

                         *  *    *                *       *                  
     194282 TTCCAACGAAATCCTCCAAGCTATCCAAATATCCACTTGCAGATTCCACAAAAAGAGTGTTTCAA
          1 TTCCAACGAAATCATCAAAGCGATCCAAATATCCACTTCCAGATTCTACAAAAAGAGTGTTTCAA

                            ** *  *            *       *     *     *  *      
     194347 AACTGCTCTCTATCAATGGCAAAGTTCAACTCTGTTAGTTGAGGACACATATCACCAACAAGTTT
         66 AACTG--CTCTATCAAAAGAAATGTTCAACTCTGTGAGTTGAGTACACACATCACAAAGAAGTTT

                               *       **         *    
     194412 CTGAGAATGCTTCTGTCTATTTTTTATGGGAAGATATTTCCTT
        129 CTGAGAATGCTTCTGTCTAGTTTTTATATGAAGATATTCCCTT

              *  *  * ** *    *      *    *          * **                    
     194455 TTTCACCGTAGGCGTCAAGGCGATCGAAATGTCCACTTCCACAAACTACAAAAAGAGTGTTTCAA
          1 TTCCAACGAAATCATCAAAGCGATCCAAATATCCACTTCCAGATTCTACAAAAAGAGTGTTTCAA

             *         *   * **       *    *        * ** * *   **      *     
     194520 ACCTGCTCTATGAAAGGCCATGTTCATCTCTATGAGTTGAATGGAAATATCCGAAAGAAATTTCT
         66 AACTGCTCTATCAAAAGAAATGTTCAACTCTGTGAGTTGAGTACACACATCACAAAGAAGTTTCT

             *       *          *        *         **
     194585 GGGAATGCTGCTGTCTAG-TGTT-TAT-ACGA-ATTCCCGC
        131 GAGAATGCTTCTGTCTAGTTTTTATATGAAGATATTCCCTT

                         *       *                *    *  *                  
     194622 TTCCAACGAAATCCTCAAAGCAATCCAAATATCCACTTGCAGAATCCACAAAAAGAGTGTTTCAA
          1 TTCCAACGAAATCATCAAAGCGATCCAAATATCCACTTCCAGATTCTACAAAAAGAGTGTTTCAA

                          *     *          * *                   *  *        
     194687 AACTGCTCTATCAATAGAAAGGTTCAACTCTTTTAGTTGAGTACACACATCACGAACAAGTTTCT
         66 AACTGCTCTATCAAAAGAAATGTTCAACTCTGTGAGTTGAGTACACACATCACAAAGAAGTTTCT

                            * *      **     **  *    
     194752 GAGAATGCTTCTGTCTGGCTTTTATTGGAAGACGTTTCCTT
        131 GAGAATGCTTCTGTCTAGTTTTTATATGAAGATATTCCCTT

                 * *  **          *       *                * *               
     194793 TT-CACCAAAGGCATCAAAGCGCTCCAAATGTCCACTTCCAGATTCTTCCAAAAGAGTGTTTCAA
          1 TTCCAACGAAATCATCAAAGCGATCCAAATATCCACTTCCAGATTCTACAAAAAGAGTGTTTCAA

             **     * ***  * *                 *    * *  * *     *   *       
     194857 ACGTGCTCAAAGTAAGGGAATGTTCAACTCTGTGACTTGAATGCAGATATCACCAAGTAGTTTCT
         66 AACTGCTCTATCAAAAGAAATGTTCAACTCTGTGAGTTGAGTACACACATCACAAAGAAGTTTCT

            * * *        *   **     *    *           
     194922 AATAGTGCTTCTGCCTACATTTTAGATGATGATATTCCCTT
        131 GAGAATGCTTCTGTCTAGTTTTTATATGAAGATATTCCCTT

                         * * *   *             *  *   *      *     *       * 
     194963 TTCCAACGAAATCGTTAGAGCTATCCAAATATCCAGTTACAGTTTCTACCAAAAGGGTGTTTCCA
          1 TTCCAACGAAATCATCAAAGCGATCCAAATATCCACTTCCAGATTCTACAAAAAGAGTGTTTCAA

              *    **           *            *       *                     * 
     195028 AATTGCTGCATCAAAAGAAAGGTTCAACTCTGTTAGTTGAGGACACACATCACAAAGAAGTTTGT
         66 AACTGCTCTATCAAAAGAAATGTTCAACTCTGTGAGTTGAGTACACACATCACAAAGAAGTTTCT

                              *    *     *           
     195093 GAGAATGCTTCTGTCTAGATTTTGTATGACGATATTCCCTT
        131 GAGAATGCTTCTGTCTAGTTTTTATATGAAGATATTCCCTT

                     *   * *     *   *       * *  *    *  *       *  *       
     195134 TTCCAACGATATCGTTAAAGCAATCTAAATATCAATTTGCAGAATCCACAAAAATAGAGTTTCAA
          1 TTCCAACGAAATCATCAAAGCGATCCAAATATCCACTTCCAGATTCTACAAAAAGAGTGTTTCAA

             *       * *        *    *       *  *                   ***      
     195199 AGCTGCTCTGTAAAAAGAAAGGTTCCACTCTGTTAGCTGAGTACACACATCACAAACTTGTTTCT
         66 AACTGCTCTATCAAAAGAAATGTTCAACTCTGTGAGTTGAGTACACACATCACAAAGAAGTTTCT

                  *         *        **         **   
     195264 GAGAATCCTTCTGTCTCGTTTTTATGGGAAGATATTTACTT
        131 GAGAATGCTTCTGTCTAGTTTTTATATGAAGATATTCCCTT

                 *  * **          *       *     *       *  *  *  *           
     195305 TTCCACCGTAGGCATCAAAGCGCTCCAAATGTCCACATCCAGATACTCCAGAACGAGTGTTTCAA
          1 TTCCAACGAAATCATCAAAGCGATCCAAATATCCACTTCCAGATTCTACAAAAAGAGTGTTTCAA

             *         *   * *   *         *        * *  *      *      *     
     195370 ACCTGCTCTATGAAAGGGAATCTTCAACTCTATGAGTTGAATGCAGACATCAGAAAGAAATTTCT
         66 AACTGCTCTATCAAAAGAAATGTTCAACTCTGTGAGTTGAGTACACACATCACAAAGAAGTTTCT

                     *       **          *         **
     195435 GAGAATGCTGCTGTCTACCTTTTAT-T--TGA-ATTCCCGC
        131 GAGAATGCTTCTGTCTAGTTTTTATATGAAGATATTCCCTT

                         *  *    *              * *  **   *                  
     195472 TTCCAACGAAATCCTCCAAGCTATCCAAATATCCACCTGCATTTTCCACAAAAAGAGTGTTTCAA
          1 TTCCAACGAAATCATCAAAGCGATCCAAATATCCACTTCCAGATTCTACAAAAAGAGTGTTTCAA

                          *                 *    *  *                *       
     195537 AACTGCTCTATCAATAGAAATGTTCAACTCCTTTG-GCTGGGTACACACATCACAAACAAGTTTC
         66 AACTGCTCTATCAAAAGAAATGTTCAACT-CTGTGAGTTGAGTACACACATCACAAAGAAGTTTC

                                      **     *        
     195601 TGAGAATGCTTCTGTCTAGTTTTTATGGGAAGACATTCCCTT
        130 TGAGAATGCTTCTGTCTAGTTTTTATATGAAGATATTCCCTT

              *  * *  **          *       *            **                    
     195643 TTTCACCAAAGACATCAAAGCGCTCCAAATGTCCACTTCCAGACACTACAAAAAGAGTGTTTCAA
          1 TTCCAACGAAATCATCAAAGCGATCCAAATATCCACTTCCAGATTCTACAAAAAGAGTGTTTCAA

             **       **   ***                 *    * *  * *         *       
     195708 ACGTGCTCTAAGAAAGCGAATGTTCAACTCTGTGACTTGAATGCAGATATCACAAAGTAGTTTCT
         66 AACTGCTCTATCAAAAGAAATGTTCAACTCTGTGAGTTGAGTACACACATCACAAAGAAGTTTCT

                **            *     *    *         * 
     195773 GAGAGGGCTTCTGTCTAGATTTTAGATGATGATATTCCCGT
        131 GAGAATGCTTCTGTCTAGTTTTTATATGAAGATATTCCCTT

                           * *   *                *   *                    * 
     195814 TTCCAACGAAATCATTAGAGCTATCCAAATATCCACTTACAGTTTCTACAAAAAGAGTGTTTCCA
          1 TTCCAACGAAATCATCAAAGCGATCCAAATATCCACTTCCAGATTCTACAAAAAGAGTGTTTCAA

                   **         * *    *       *  **                  ***      
     195879 AACTGCTGCATCAAAAGAGAGGTTCCACTCTGTTAGCCGAGTACACACATCACAAACTTGTTTCT
         66 AACTGCTCTATCAAAAGAAATGTTCAACTCTGTGAGTTGAGTACACACATCACAAAGAAGTTTCT

            *     *         *        **         **   
     195944 CAGAATCCTTCTGTCTCGTTTTTATGGGAAGATATTTACTT
        131 GAGAATGCTTCTGTCTAGTTTTTATATGAAGATATTCCCTT

              *  *  * **          *       *     *       *  *  *              
     195985 TTTCACCGTAGGCATCAAAGCGCTCCAAATGTCCACATCCAGATACTCCAGAAAGAGTGTTTCAA
          1 TTCCAACGAAATCATCAAAGCGATCCAAATATCCACTTCCAGATTCTACAAAAAGAGTGTTTCAA

             *         *   * *   *         *        * *  *      *      *     
     196050 ACCTGCTCTATGAAAGGGAATCTTCAACTCTATGAGTTGAATGCAGACATCAGAAAGAAATTTCT
         66 AACTGCTCTATCAAAAGAAATGTTCAACTCTGTGAGTTGAGTACACACATCACAAAGAAGTTTCT

                     *       **          *         **
     196115 GAGAATGCTGCTGTCTACCTTTTAT-T--TGA-ATTCCCGC
        131 GAGAATGCTTCTGTCTAGTTTTTATATGAAGATATTCCCTT

                         *  *    *                *       *                  
     196152 TTCCAACGAAATCCTCCAAGCTATCCAAATATCCACTTGCAGATTCCACAAAAAGAGTGTTTCAA
          1 TTCCAACGAAATCATCAAAGCGATCCAAATATCCACTTCCAGATTCTACAAAAAGAGTGTTTCAA

                            ** *  *            *       *     *     *  *      
     196217 AACTGCTCTCTATCAATGGCAAAGTTCAACTCTGTTAGTTGAGGACACATATCACCAACAAGTTT
         66 AACTG--CTCTATCAAAAGAAATGTTCAACTCTGTGAGTTGAGTACACACATCACAAAGAAGTTT

                               *       **         *    
     196282 CTGAGAATGCTTCTGTCTATTTTTTATGGGAAGATATTTCCTT
        129 CTGAGAATGCTTCTGTCTAGTTTTTATATGAAGATATTCCCTT

              *  *  * ** *    *      *    *          * **                    
     196325 TTTCACCGTAGGCGTCAAGGCGATCGAAATGTCCACTTCCACAAACTACAAAAAGAGTGTTTCAA
          1 TTCCAACGAAATCATCAAAGCGATCCAAATATCCACTTCCAGATTCTACAAAAAGAGTGTTTCAA

             *         *   * **       *    *        * ** * *   **      *     
     196390 ACCTGCTCTATGAAAGGCCATGTTCATCTCTATGAGTTGAATGGAAATATCCGAAAGAAATTTCT
         66 AACTGCTCTATCAAAAGAAATGTTCAACTCTGTGAGTTGAGTACACACATCACAAAGAAGTTTCT

             *       *          *        *         **
     196455 GGGAATGCTGCTGTCTAG-TGTT-TAT-ACGA-ATTCCCGC
        131 GAGAATGCTTCTGTCTAGTTTTTATATGAAGATATTCCCTT

                    *    *       *                *    *  *                  
     196492 TTCCAACGTAATCCTCAAAGCAATCCAAATATCCACTTGCAGAATCCACAAAAAGAGTGTTTCAA
          1 TTCCAACGAAATCATCAAAGCGATCCAAATATCCACTTCCAGATTCTACAAAAAGAGTGTTTCAA

                          *     *          * *                   *  *        
     196557 AACTGCTCTATCAATAGAAAGGTTCAACTCTTTTAGTTGAGTACACACATCACGAACAAGTTTCT
         66 AACTGCTCTATCAAAAGAAATGTTCAACTCTGTGAGTTGAGTACACACATCACAAAGAAGTTTCT

                            * *      **     **  *    
     196622 GAGAATGCTTCTGTCTGGCTTTTATTGGAAGACGTTTCCTT
        131 GAGAATGCTTCTGTCTAGTTTTTATATGAAGATATTCCCTT

                 * *  **          *       *                * *               
     196663 TT-CACCAAAGGCATCAAAGCGCTCCAAATGTCCACTTCCAGATTCTTCCAAAAGAGTGTTTCAA
          1 TTCCAACGAAATCATCAAAGCGATCCAAATATCCACTTCCAGATTCTACAAAAAGAGTGTTTCAA

             **     * ***  * *                 *    * *  * *     *   *       
     196727 ACGTGCTCAAAGTAAGGGAATGTTCAACTCTGTGACTTGAATGCAGATATCACCAAGTAGTTTCT
         66 AACTGCTCTATCAAAAGAAATGTTCAACTCTGTGAGTTGAGTACACACATCACAAAGAAGTTTCT

            * * *            **     *    *         * 
     196792 AATAGTGCTTCTGTCTACATTTTAGATGATGATATTCCCGT
        131 GAGAATGCTTCTGTCTAGTTTTTATATGAAGATATTCCCTT

                         *** *   *             *  *   *      *     *       * 
     196833 TTCCAACGAAATCGCTAGAGCTATCCAAATATCCAGTTACAGTTTCTACCAAAAGGGTGTTTCCA
          1 TTCCAACGAAATCATCAAAGCGATCCAAATATCCACTTCCAGATTCTACAAAAAGAGTGTTTCAA

              *    **           *            *       *                     * 
     196898 AATTGCTGCATCAAAAGAAAGGTTCAACTCTGTTAGTTGAGGACACACATCACAAAGAAGTTTGT
         66 AACTGCTCTATCAAAAGAAATGTTCAACTCTGTGAGTTGAGTACACACATCACAAAGAAGTTTCT

                              *    *     *           
     196963 GAGAATGCTTCTGTCTAGATTTTGTATGACGATATTCCCTT
        131 GAGAATGCTTCTGTCTAGTTTTTATATGAAGATATTCCCTT

                     *   * *     *   *       * *  *    *  *       *  *       
     197004 TTCCAACGATATCGTTAAAGCAATCTAAATATCAATTTGCAGAATCCACAAAAATAGAGTTTCAA
          1 TTCCAACGAAATCATCAAAGCGATCCAAATATCCACTTCCAGATTCTACAAAAAGAGTGTTTCAA

             *       * *        *    *       *  *                   ***      
     197069 AGCTGCTCTGTAAAAAGAAAGGTTCCACTCTGTTAGCTGAGTACACACATCACAAACTTGTTTCT
         66 AACTGCTCTATCAAAAGAAATGTTCAACTCTGTGAGTTGAGTACACACATCACAAAGAAGTTTCT

            *     *         *        **       * **   
     197134 CAGAATCCTTCTGTCTCGTTTTTATGGGAAGATACTTACTT
        131 GAGAATGCTTCTGTCTAGTTTTTATATGAAGATATTCCCTT

                 *  * **          *       *     *       *  *  *  *           
     197175 TTCCACCGTAGGCATCAAAGCGCTCCAAATGTCCACATCCAGATACTCCAGAACGAGTGTTTCAA
          1 TTCCAACGAAATCATCAAAGCGATCCAAATATCCACTTCCAGATTCTACAAAAAGAGTGTTTCAA

             *         *   * *   *         *        * *  *      *      *     
     197240 ACCTGCTCTATGAAAGGGAATCTTCAACTCTATGAGTTGAATGCAGACATCAGAAAGAAATTTCT
         66 AACTGCTCTATCAAAAGAAATGTTCAACTCTGTGAGTTGAGTACACACATCACAAAGAAGTTTCT

                     *       **          *         **
     197305 GAGAATGCTGCTGTCTACCTTTTAT-T--TGA-ATTCCCGC
        131 GAGAATGCTTCTGTCTAGTTTTTATATGAAGATATTCCCTT

                         *  *    *              * *  **   *    *             
     197342 TTCCAACGAAATCCTCCAAGCTATCCAAATATCCACCTGCATTTTCCACAACAAGAGTGTTTCAA
          1 TTCCAACGAAATCATCAAAGCGATCCAAATATCCACTTCCAGATTCTACAAAAAGAGTGTTTCAA

                          *                 *    *  *                *       
     197407 AACTGCTCTATCAATAGAAATGTTCAACTCCTTTG-GCTGGGTACACACATCACAAACAAGTTTC
         66 AACTGCTCTATCAAAAGAAATGTTCAACT-CTGTGAGTTGAGTACACACATCACAAAGAAGTTTC

                                      **     **       
     197471 TGAGAATGCTTCTGTCTAGTTTTTATGGGAAGACGTTCCCTT
        130 TGAGAATGCTTCTGTCTAGTTTTTATATGAAGATATTCCCTT

              *  * *  **    *     *       *     *       *  *  *              
     197513 TTTCACCAAAGGCATCCAAGCGCTCCAAATGTCCACATCCAGATACTCCAGAAAGAGTGTTTCAA
          1 TTCCAACGAAATCATCAAAGCGATCCAAATATCCACTTCCAGATTCTACAAAAAGAGTGTTTCAA

             *         *   * *   *         *        * *  *      *      *     
     197578 ACCTGCTCTATGAAAGGGAATCTTCAACTCTATGAGTTGAATGCAGACATCAGAAAGAAATTTCT
         66 AACTGCTCTATCAAAAGAAATGTTCAACTCTGTGAGTTGAGTACACACATCACAAAGAAGTTTCT

                     *       **          *         **
     197643 GAGAATGCTGCTGTCTACCTTTTAT-T--TGA-ATTCCCGC
        131 GAGAATGCTTCTGTCTAGTTTTTATATGAAGATATTCCCTT

                         *  *    *                *       *                  
     197680 TTCCAACGAAATCCTCCAAGCTATCCAAATATCCACTTGCAGATTCCACAAAAAGAGTGTTTCAA
          1 TTCCAACGAAATCATCAAAGCGATCCAAATATCCACTTCCAGATTCTACAAAAAGAGTGTTTCAA

                            ** *  *            *       *     *     *  *      
     197745 AACTGCTCTCTATCAATGGCAAAGTTCAACTCTGTTAGTTGAGGACACATATCACCAACAAGTTT
         66 AACTG--CTCTATCAAAAGAAATGTTCAACTCTGTGAGTTGAGTACACACATCACAAAGAAGTTT

                               *       **         *    
     197810 CTGAGAATGCTTCTGTCTATTTTTTATGGGAAGATATTTCCTT
        129 CTGAGAATGCTTCTGTCTAGTTTTTATATGAAGATATTCCCTT

              *  * ** ** *    *      *    *          * **                    
     197853 TTTCACCATAGGCGTCAAGGCGATCGAAATGTCCACTTCCACAAACTACAAAAAGAGTGTTTCAA
          1 TTCCAACGAAATCATCAAAGCGATCCAAATATCCACTTCCAGATTCTACAAAAAGAGTGTTTCAA

             *         *   * **       *    *        * ** * *   **      *     
     197918 ACCTGCTCTATGAAAGGCCATGTTCATCTCTATGAGTTGAATGGAAATATCCGAAAGAAATTTCT
         66 AACTGCTCTATCAAAAGAAATGTTCAACTCTGTGAGTTGAGTACACACATCACAAAGAAGTTTCT

             *       *          *        *         **
     197983 GGGAATGCTGCTGTCTAG-TGTT-TAT-ACGA-ATTCCCGC
        131 GAGAATGCTTCTGTCTAGTTTTTATATGAAGATATTCCCTT

                         *       *                *    *  *                  
     198020 TTCCAACGAAATCCTCAAAGCAATCCAAATATCCACTTGCAGAATCCACAAAAAGAGTGTTTCAA
          1 TTCCAACGAAATCATCAAAGCGATCCAAATATCCACTTCCAGATTCTACAAAAAGAGTGTTTCAA

                          *     *          * *                   *  *        
     198085 AACTGCTCTATCAATAGAAAGGTTCAACTCTTTTAGTTGAGTACACACATCACGAACAAGTTTCT
         66 AACTGCTCTATCAAAAGAAATGTTCAACTCTGTGAGTTGAGTACACACATCACAAAGAAGTTTCT

                            * *      **     **  *    
     198150 GAGAATGCTTCTGTCTGGCTTTTATTGGAAGACGTTTCCTT
        131 GAGAATGCTTCTGTCTAGTTTTTATATGAAGATATTCCCTT

                   *  **          *       *                * *               
     198191 TT-CAACAAAGGCATCAAAGCGCTCCAAATGTCCACTTCCAGATTCTTCCAAAAGAGTGTTTCAA
          1 TTCCAACGAAATCATCAAAGCGATCCAAATATCCACTTCCAGATTCTACAAAAAGAGTGTTTCAA

             **     * ***  * *                 *    * *  * *     *   *       
     198255 ACGTGCTCAAAGTAAGGGAATGTTCAACTCTGTGACTTGAATGCAGATATCACCAAGTAGTTTCT
         66 AACTGCTCTATCAAAAGAAATGTTCAACTCTGTGAGTTGAGTACACACATCACAAAGAAGTTTCT

            * * *        *   **     *    *           
     198320 AATAGTGCTTCTGCCTACATTTTAGATGATGATATTCCCTT
        131 GAGAATGCTTCTGTCTAGTTTTTATATGAAGATATTCCCTT

                         * * *   *             *  *   *      *     *       * 
     198361 TTCCAACGAAATCGTTAGAGCTATCCAAATATCCAGTTACAGTTTCTACCAAAAGGGTGTTTCCA
          1 TTCCAACGAAATCATCAAAGCGATCCAAATATCCACTTCCAGATTCTACAAAAAGAGTGTTTCAA

              *    **           *            *       *                     * 
     198426 AATTGCTGCATCAAAAGAAAGGTTCAACTCTGTTAGTTGAGGACACACATCACAAAGAAGTTTGT
         66 AACTGCTCTATCAAAAGAAATGTTCAACTCTGTGAGTTGAGTACACACATCACAAAGAAGTTTCT

                              *    *     *           
     198491 GAGAATGCTTCTGTCTAGATTTTGTATGACGATATTCCCTT
        131 GAGAATGCTTCTGTCTAGTTTTTATATGAAGATATTCCCTT

                     *   * *     *   *       * *  *    *  *       *  *       
     198532 TTCCAACGATATCGTTAAAGCAATCTAAATATCAATTTGCAGAATCCACAAAAATAGAGTTTCAA
          1 TTCCAACGAAATCATCAAAGCGATCCAAATATCCACTTCCAGATTCTACAAAAAGAGTGTTTCAA

             *       * *        *    *       *  *                   ***      
     198597 AGCTGCTCTGTAAAAAGAAAGGTTCCACTCTGTTAGCTGAGTACACACATCACAAACTTGTTTCT
         66 AACTGCTCTATCAAAAGAAATGTTCAACTCTGTGAGTTGAGTACACACATCACAAAGAAGTTTCT

                  *         *        **         **   
     198662 GAGAATCCTTCTGTCTCGTTTTTATGGGAAGATATTTACTT
        131 GAGAATGCTTCTGTCTAGTTTTTATATGAAGATATTCCCTT

                 *  * **          *       *     *       *  *  *  *           
     198703 TTCCACCGTAGGCATCAAAGCGCTCCAAATGTCCACATCCAGATACTCCAGAACGAGTGTTTCAA
          1 TTCCAACGAAATCATCAAAGCGATCCAAATATCCACTTCCAGATTCTACAAAAAGAGTGTTTCAA

             *         *   * *   *         *        * *  *      *      *     
     198768 ACCTGCTCTATGAAAGGGAATCTTCAACTCTATGAGTTGAATGCAGACATCAGAAAGAAATTTCT
         66 AACTGCTCTATCAAAAGAAATGTTCAACTCTGTGAGTTGAGTACACACATCACAAAGAAGTTTCT

                     *       **          *         **
     198833 GAGAATGCTGCTGTCTACCTTTTAT-T--TGA-ATTCCCGC
        131 GAGAATGCTTCTGTCTAGTTTTTATATGAAGATATTCCCTT

                         *  *    *              * *  **   *                  
     198870 TTCCAACGAAATCCTCCAAGCTATCCAAATATCCACCTGCATTTTCCACAAAAAGAGTGTTTCAA
          1 TTCCAACGAAATCATCAAAGCGATCCAAATATCCACTTCCAGATTCTACAAAAAGAGTGTTTCAA

                          *                 *    *  *                *       
     198935 AACTGCTCTATCAATAGAAATGTTCAACTCCTTTG-GCTGGGTACACACATCACAAACAAGTTTC
         66 AACTGCTCTATCAAAAGAAATGTTCAACT-CTGTGAGTTGAGTACACACATCACAAAGAAGTTTC

                                      **     *        
     198999 TGAGAATGCTTCTGTCTAGTTTTTATGGGAAGACATTCCCTT
        130 TGAGAATGCTTCTGTCTAGTTTTTATATGAAGATATTCCCTT

              *  * *  **          *       *            **                    
     199041 TTTCACCAAAGACATCAAAGCGCTCCAAATGTCCACTTCCAGACACTACAAAAAGAGTGTTTCAA
          1 TTCCAACGAAATCATCAAAGCGATCCAAATATCCACTTCCAGATTCTACAAAAAGAGTGTTTCAA

             **       **   ***                 *    * *  * *         *       
     199106 ACGTGCTCTAAGAAAGCGAATGTTCAACTCTGTGACTTGAATGCAGATATCACAAAGTAGTTTCT
         66 AACTGCTCTATCAAAAGAAATGTTCAACTCTGTGAGTTGAGTACACACATCACAAAGAAGTTTCT

                **            *     *    *         * 
     199171 GAGAGGGCTTCTGTCTAGATTTTAGATGATGATATTCCCGT
        131 GAGAATGCTTCTGTCTAGTTTTTATATGAAGATATTCCCTT

                           * *   *                *   *                    * 
     199212 TTCCAACGAAATCATTAGAGCTATCCAAATATCCACTTACAGTTTCTACAAAAAGAGTGTTTCCA
          1 TTCCAACGAAATCATCAAAGCGATCCAAATATCCACTTCCAGATTCTACAAAAAGAGTGTTTCAA

                   **         * *    *       *  *                   ***      
     199277 AACTGCTGCATCAAAAGAGAGGTTCCACTCTGTTAGCTGAGTACACACATCACAAACTTGTTTCT
         66 AACTGCTCTATCAAAAGAAATGTTCAACTCTGTGAGTTGAGTACACACATCACAAAGAAGTTTCT

            *     *         *        **         **   
     199342 CAGAATCCTTCTGTCTCGTTTTTATGGGAAGATATTTACTT
        131 GAGAATGCTTCTGTCTAGTTTTTATATGAAGATATTCCCTT

              *  *  * **          *       *     *       *  *  *              
     199383 TTTCACCGTAGGCATCAAAGCGCTCCAAATGTCCACATCCAGATACTCCAGAAAGAGTGTTTCAA
          1 TTCCAACGAAATCATCAAAGCGATCCAAATATCCACTTCCAGATTCTACAAAAAGAGTGTTTCAA

             *         *   * *   *         *        * *  *      *      *     
     199448 ACCTGCTCTATGAAAGGGAATCTTCAACTCTATGAGTTGAATGCAGACATCAGAAAGAAATTTCT
         66 AACTGCTCTATCAAAAGAAATGTTCAACTCTGTGAGTTGAGTACACACATCACAAAGAAGTTTCT

                     *       **          *         **
     199513 GAGAATGCTGCTGTCTACCTTTTAT-T--TGA-ATTCCCGC
        131 GAGAATGCTTCTGTCTAGTTTTTATATGAAGATATTCCCTT

                         *  *    *                *       *                  
     199550 TTCCAACGAAATCCTCCAAGCTATCCAAATATCCACTTGCAGATTCCACAAAAAGAGTGTTTCAA
          1 TTCCAACGAAATCATCAAAGCGATCCAAATATCCACTTCCAGATTCTACAAAAAGAGTGTTTCAA

                            ** *  *            *       *     *     *  *      
     199615 AACTGCTCTCTATCAATGGCAAAGTTCAACTCTGTTAGTTGAGGACACATATCACCAACAAGTTT
         66 AACTG--CTCTATCAAAAGAAATGTTCAACTCTGTGAGTTGAGTACACACATCACAAAGAAGTTT

                               *       **         *    
     199680 CTGAGAATGCTTCTGTCTATTTTTTATGGGAAGATATTTCCTT
        129 CTGAGAATGCTTCTGTCTAGTTTTTATATGAAGATATTCCCTT

              *  *  * ** *    *      *    *          * **                    
     199723 TTTCACCGTAGGCGTCAAGGCGATCGAAATGTCCACTTCCACAAACTACAAAAAGAGTGTTTCAA
          1 TTCCAACGAAATCATCAAAGCGATCCAAATATCCACTTCCAGATTCTACAAAAAGAGTGTTTCAA

             *         *   * **       *    *        * ** * *   **      *     
     199788 ACCTGCTCTATGAAAGGCCATGTTCATCTCTATGAGTTGAATGGAAATATCCGAAAGAAATTTCT
         66 AACTGCTCTATCAAAAGAAATGTTCAACTCTGTGAGTTGAGTACACACATCACAAAGAAGTTTCT

             *       *          *        *         **
     199853 GGGAATGCTGCTGTCTAG-TGTT-TAT-ACGA-ATTCCCGC
        131 GAGAATGCTTCTGTCTAGTTTTTATATGAAGATATTCCCTT

                         *       *                *    *  *                  
     199890 TTCCAACGAAATCCTCAAAGCAATCCAAATATCCACTTGCAGAATCCACAAAAAGAGTGTTTCAA
          1 TTCCAACGAAATCATCAAAGCGATCCAAATATCCACTTCCAGATTCTACAAAAAGAGTGTTTCAA

                          *     *          * *                   *  *        
     199955 AACTGCTCTATCAATAGAAAGGTTCAACTCTTTTAGTTGAGTACACACATCACGAACAAGTTTCT
         66 AACTGCTCTATCAAAAGAAATGTTCAACTCTGTGAGTTGAGTACACACATCACAAAGAAGTTTCT

                            * *      **     **  *    
     200020 GAGAATGCTTCTGTCTGGCTTTTATTGGAAGACGTTTCCTT
        131 GAGAATGCTTCTGTCTAGTTTTTATATGAAGATATTCCCTT

                   *  **          *       *                * *               
     200061 TT-CAACAAAGGCATCAAAGCGCTCCAAATGTCCACTTCCAGATTCTTCCAAAAGAGTGTTTCAA
          1 TTCCAACGAAATCATCAAAGCGATCCAAATATCCACTTCCAGATTCTACAAAAAGAGTGTTTCAA

             **     * ***  * *                 *    * *  * *     *   *       
     200125 ACGTGCTCAAAGTAAGGGAATGTTCAACTCTGTGACTTGAATGCAGATATCACCAAGTAGTTTCT
         66 AACTGCTCTATCAAAAGAAATGTTCAACTCTGTGAGTTGAGTACACACATCACAAAGAAGTTTCT

            * * *        *   **     *    *           
     200190 AATAGTGCTTCTGCCTACATTTTAGATGATGATATTCCCTT
        131 GAGAATGCTTCTGTCTAGTTTTTATATGAAGATATTCCCTT

                         * * *   *             *  *   *      *     *       * 
     200231 TTCCAACGAAATCGTTAGAGCTATCCAAATATCCAGTTACAGTTTCTACCAAAAGGGTGTTTCCA
          1 TTCCAACGAAATCATCAAAGCGATCCAAATATCCACTTCCAGATTCTACAAAAAGAGTGTTTCAA

              *    **           *            *       *                     * 
     200296 AATTGCTGCATCAAAAGAAAGGTTCAACTCTGTTAGTTGAGGACACACATCACAAAGAAGTTTGT
         66 AACTGCTCTATCAAAAGAAATGTTCAACTCTGTGAGTTGAGTACACACATCACAAAGAAGTTTCT

                              *    *     *           
     200361 GAGAATGCTTCTGTCTAGATTTTGTATGACGATATTCCCTT
        131 GAGAATGCTTCTGTCTAGTTTTTATATGAAGATATTCCCTT

                     *   * *     *   *       * *  *    *  *       *  *       
     200402 TTCCAACGATATCGTTAAAGCAATCTAAATATCAATTTGCAGAATCCACAAAAATAGAGTTTCAA
          1 TTCCAACGAAATCATCAAAGCGATCCAAATATCCACTTCCAGATTCTACAAAAAGAGTGTTTCAA

             *       * *        *    *       *  *                   ***      
     200467 AGCTGCTCTGTAAAAAGAAAGGTTCCACTCTGTTAGCTGAGTACACACATCACAAACTTGTTTCT
         66 AACTGCTCTATCAAAAGAAATGTTCAACTCTGTGAGTTGAGTACACACATCACAAAGAAGTTTCT

                  *         *        **         **   
     200532 GAGAATCCTTCTGTCTCGTTTTTATGGGAAGATATTTACTT
        131 GAGAATGCTTCTGTCTAGTTTTTATATGAAGATATTCCCTT

                 *  * **          *       *     *       *  *  *  *           
     200573 TTCCACCGTAGGCATCAAAGCGCTCCAAATGTCCACATCCAGATACTCCAGAACGAGTGTTTCAA
          1 TTCCAACGAAATCATCAAAGCGATCCAAATATCCACTTCCAGATTCTACAAAAAGAGTGTTTCAA

             *         *   * *   *         *        * *  *      *      *     
     200638 ACCTGCTCTATGAAAGGGAATCTTCAACTCTATGAGTTGAATGCAGACATCAGAAAGAAATTTCT
         66 AACTGCTCTATCAAAAGAAATGTTCAACTCTGTGAGTTGAGTACACACATCACAAAGAAGTTTCT

                     *       **          *         **
     200703 GAGAATGCTGCTGTCTACCTTTTAT-T--TGA-ATTCCCGC
        131 GAGAATGCTTCTGTCTAGTTTTTATATGAAGATATTCCCTT

                         *  *    *              * *  **   *                  
     200740 TTCCAACGAAATCCTCCAAGCTATCCAAATATCCACCTGCATTTTCCACAAAAAGAGTGTTTCAA
          1 TTCCAACGAAATCATCAAAGCGATCCAAATATCCACTTCCAGATTCTACAAAAAGAGTGTTTCAA

                          *                 *    *  *                *       
     200805 AACTGCTCTATCAATAGAAATGTTCAACTCCTTTG-GCTGGGTACACACATCACAAACAAGTTTC
         66 AACTGCTCTATCAAAAGAAATGTTCAACT-CTGTGAGTTGAGTACACACATCACAAAGAAGTTTC

                                      **     *        
     200869 TGAGAATGCTTCTGTCTAGTTTTTATGGGAAGACATTCCCTT
        130 TGAGAATGCTTCTGTCTAGTTTTTATATGAAGATATTCCCTT

              *  * *  **          *       *            **                    
     200911 TTTCACCAAAGACATCAAAGCGCTCCAAATGTCCACTTCCAGACACTACAAAAAGAGTGTTTCAA
          1 TTCCAACGAAATCATCAAAGCGATCCAAATATCCACTTCCAGATTCTACAAAAAGAGTGTTTCAA

             **       **   ***                 *    * *  * *         *       
     200976 ACGTGCTCTAAGAAAGCGAATGTTCAACTCTGTGACTTGAATGCAGATATCACAAAGTAGTTTCT
         66 AACTGCTCTATCAAAAGAAATGTTCAACTCTGTGAGTTGAGTACACACATCACAAAGAAGTTTCT

                **            *     *    *         * 
     201041 GAGAGGGCTTCTGTCTAGATTTTAGATGATGATATTCCCGT
        131 GAGAATGCTTCTGTCTAGTTTTTATATGAAGATATTCCCTT

                           * *   *                *   *                    * 
     201082 TTCCAACGAAATCATTAGAGCTATCCAAATATCCACTTACAGTTTCTACAAAAAGAGTGTTTCCA
          1 TTCCAACGAAATCATCAAAGCGATCCAAATATCCACTTCCAGATTCTACAAAAAGAGTGTTTCAA

                   **         * *    *       *  *                   ***      
     201147 AACTGCTGCATCAAAAGAGAGGTTCCACTCTGTTAGCTGAGTACACACATCACAAACTTGTTTCT
         66 AACTGCTCTATCAAAAGAAATGTTCAACTCTGTGAGTTGAGTACACACATCACAAAGAAGTTTCT

            *     *         *        **         **   
     201212 CAGAATCCTTCTGTCTCGTTTTTATGGGAAGATATTTACTT
        131 GAGAATGCTTCTGTCTAGTTTTTATATGAAGATATTCCCTT

              *  *  * **          *       *     *       *  *  *              
     201253 TTTCACCGTAGGCATCAAAGCGCTCCAAATGTCCACATCCAGATACTCCAGAAAGAGTGTTTCAA
          1 TTCCAACGAAATCATCAAAGCGATCCAAATATCCACTTCCAGATTCTACAAAAAGAGTGTTTCAA

             *         *   * *   *         *        * *  *      *      *     
     201318 ACCTGCTCTATGAAAGGGAATCTTCAACTCTATGAGTTGAATGCAGACATCAGAAAGAAATTTCT
         66 AACTGCTCTATCAAAAGAAATGTTCAACTCTGTGAGTTGAGTACACACATCACAAAGAAGTTTCT

                     *       **          *         **
     201383 GAGAATGCTGCTGTCTACCTTTTAT-T--TGA-ATTCCCGC
        131 GAGAATGCTTCTGTCTAGTTTTTATATGAAGATATTCCCTT

                         *  *    *                *       *                  
     201420 TTCCAACGAAATCCTCCAAGCTATCCAAATATCCACTTGCAGATTCCACAAAAAGAGTGTTTCAA
          1 TTCCAACGAAATCATCAAAGCGATCCAAATATCCACTTCCAGATTCTACAAAAAGAGTGTTTCAA

                            ** *  *            *       *     *     *  *      
     201485 AACTGCTCTCTATCAATGGCAAAGTTCAACTCTGTTAGTTGAGGACACATATCACCAACAAGTTT
         66 AACTG--CTCTATCAAAAGAAATGTTCAACTCTGTGAGTTGAGTACACACATCACAAAGAAGTTT

                               *       **         *    
     201550 CTGAGAATGCTTCTGTCTATTTTTTATGGGAAGATATTTCCTT
        129 CTGAGAATGCTTCTGTCTAGTTTTTATATGAAGATATTCCCTT

              *  *  * ** *    *      *    *          * **                    
     201593 TTTCACCGTAGGCGTCAAGGCGATCGAAATGTCCACTTCCACAAACTACAAAAAGAGTGTTTCAA
          1 TTCCAACGAAATCATCAAAGCGATCCAAATATCCACTTCCAGATTCTACAAAAAGAGTGTTTCAA

             *         *   * **       *    *        * ** * *   **      *     
     201658 ACCTGCTCTATGAAAGGCCATGTTCATCTCTATGAGTTGAATGGAAATATCCGAAAGAAATTTCT
         66 AACTGCTCTATCAAAAGAAATGTTCAACTCTGTGAGTTGAGTACACACATCACAAAGAAGTTTCT

             *       *          *        *         **
     201723 GGGAATGCTGCTGTCTAG-TGTT-TAT-ACGA-ATTCCCGC
        131 GAGAATGCTTCTGTCTAGTTTTTATATGAAGATATTCCCTT

                    *    *       *                *    *  *                  
     201760 TTCCAACGTAATCCTCAAAGCAATCCAAATATCCACTTGCAGAATCCACAAAAAGAGTGTTTCAA
          1 TTCCAACGAAATCATCAAAGCGATCCAAATATCCACTTCCAGATTCTACAAAAAGAGTGTTTCAA

                          *     *          * *                   *  *        
     201825 AACTGCTCTATCAATAGAAAGGTTCAACTCTTTTAGTTGAGTACACACATCACGAACAAGTTTCT
         66 AACTGCTCTATCAAAAGAAATGTTCAACTCTGTGAGTTGAGTACACACATCACAAAGAAGTTTCT

                            * *      **     **  *    
     201890 GAGAATGCTTCTGTCTGGCTTTTATTGGAAGACGTTTCCTT
        131 GAGAATGCTTCTGTCTAGTTTTTATATGAAGATATTCCCTT

                 * *  **          *       *                * *               
     201931 TT-CACCAAAGGCATCAAAGCGCTCCAAATGTCCACTTCCAGATTCTTCCAAAAGAGTGTTTCAA
          1 TTCCAACGAAATCATCAAAGCGATCCAAATATCCACTTCCAGATTCTACAAAAAGAGTGTTTCAA

             **     * ***  * *                 *    * *  * *     *   *       
     201995 ACGTGCTCAAAGTAAGGGAATGTTCAACTCTGTGACTTGAATGCAGATATCACCAAGTAGTTTCT
         66 AACTGCTCTATCAAAAGAAATGTTCAACTCTGTGAGTTGAGTACACACATCACAAAGAAGTTTCT

            * * *        *   **     *    *           
     202060 AATAGTGCTTCTGCCTACATTTTAGATGATGATATTCCCTT
        131 GAGAATGCTTCTGTCTAGTTTTTATATGAAGATATTCCCTT

                         * * *   *             *  *   *      *     *       * 
     202101 TTCCAACGAAATCGTTAGAGCTATCCAAATATCCAGTTACAGTTTCTACCAAAAGGGTGTTTCCA
          1 TTCCAACGAAATCATCAAAGCGATCCAAATATCCACTTCCAGATTCTACAAAAAGAGTGTTTCAA

              *    **           *            *       *                     * 
     202166 AATTGCTGCATCAAAAGAAAGGTTCAACTCTGTTAGTTGAGGACACACATCACAAAGAAGTTTGT
         66 AACTGCTCTATCAAAAGAAATGTTCAACTCTGTGAGTTGAGTACACACATCACAAAGAAGTTTCT

                              *    *     *           
     202231 GAGAATGCTTCTGTCTAGATTTTGTATGACGATATTCCCTT
        131 GAGAATGCTTCTGTCTAGTTTTTATATGAAGATATTCCCTT

                     *   * *     *   *       * *  *    *  *       *  *       
     202272 TTCCAACGATATCGTTAAAGCAATCTAAATATCAATTTGCAGAATCCACAAAAATAGAGTTTCAA
          1 TTCCAACGAAATCATCAAAGCGATCCAAATATCCACTTCCAGATTCTACAAAAAGAGTGTTTCAA

             *       * *        *    *       *  *                   ***      
     202337 AGCTGCTCTGTAAAAAGAAAGGTTCCACTCTGTTAGCTGAGTACACACATCACAAACTTGTTTCT
         66 AACTGCTCTATCAAAAGAAATGTTCAACTCTGTGAGTTGAGTACACACATCACAAAGAAGTTTCT

                  *         *        **         **   
     202402 GAGAATCCTTCTGTCTCGTTTTTATGGGAAGATATTTACTT
        131 GAGAATGCTTCTGTCTAGTTTTTATATGAAGATATTCCCTT

                 *  * **          *       *     *       *  *  *  *           
     202443 TTCCACCGTAGGCATCAAAGCGCTCCAAATGTCCACATCCAGATACTCCAGAACGAGTGTTTCAA
          1 TTCCAACGAAATCATCAAAGCGATCCAAATATCCACTTCCAGATTCTACAAAAAGAGTGTTTCAA

             *         *   * *   *         *        * *  *      *      *     
     202508 ACCTGCTCTATGAAAGGGAATCTTCAACTCTATGAGTTGAATGCAGACATCAGAAAGAAATTTCT
         66 AACTGCTCTATCAAAAGAAATGTTCAACTCTGTGAGTTGAGTACACACATCACAAAGAAGTTTCT

                     *       **          *         **
     202573 GAGAATGCTGCTGTCTACCTTTTAT-T--TGA-ATTCCCGC
        131 GAGAATGCTTCTGTCTAGTTTTTATATGAAGATATTCCCTT

                         *  *    *              * *  **   *                  
     202610 TTCCAACGAAATCCTCCAAGCTATCCAAATATCCACCTGCATTTTCCACAAAAAGAGTGTTTCAA
          1 TTCCAACGAAATCATCAAAGCGATCCAAATATCCACTTCCAGATTCTACAAAAAGAGTGTTTCAA

                          *                 *    *  *                *       
     202675 AACTGCTCTATCAATAGAAATGTTCAACTCCTTTG-GCTGGGTACACACATCACAAACAAGTTTC
         66 AACTGCTCTATCAAAAGAAATGTTCAACT-CTGTGAGTTGAGTACACACATCACAAAGAAGTTTC

                                      **     *        
     202739 TGAGAATGCTTCTGTCTAGTTTTTATGGGAAGACATTCCCTT
        130 TGAGAATGCTTCTGTCTAGTTTTTATATGAAGATATTCCCTT

              *  * *  **          *       *            **                    
     202781 TTTCACCAAAGACATCAAAGCGCTCCAAATGTCCACTTCCAGACACTACAAAAAGAGTGTTTCAA
          1 TTCCAACGAAATCATCAAAGCGATCCAAATATCCACTTCCAGATTCTACAAAAAGAGTGTTTCAA

             **       **   ***                 *    * *  * *         *       
     202846 ACGTGCTCTAAGAAAGCGAATGTTCAACTCTGTGACTTGAATGCAGATATCACAAAGTAGTTTCT
         66 AACTGCTCTATCAAAAGAAATGTTCAACTCTGTGAGTTGAGTACACACATCACAAAGAAGTTTCT

                **            *     *    *         * 
     202911 GAGAGGGCTTCTGTCTAGATTTTAGATGATGATATTCCCGT
        131 GAGAATGCTTCTGTCTAGTTTTTATATGAAGATATTCCCTT

                           * *   *                *   *                    * 
     202952 TTCCAACGAAATCATTAGAGCTATCCAAATATCCACTTACAGTTTCTACAAAAAGAGTGTTTCCA
          1 TTCCAACGAAATCATCAAAGCGATCCAAATATCCACTTCCAGATTCTACAAAAAGAGTGTTTCAA

                   **         * *    *       *  **                  ***      
     203017 AACTGCTGCATCAAAAGAGAGGTTCCACTCTGTTAGCCGAGTACACACATCACAAACTTGTTTCT
         66 AACTGCTCTATCAAAAGAAATGTTCAACTCTGTGAGTTGAGTACACACATCACAAAGAAGTTTCT

            *     *         *        **         **   
     203082 CAGAATCCTTCTGTCTCGTTTTTATGGGAAGATATTTACTT
        131 GAGAATGCTTCTGTCTAGTTTTTATATGAAGATATTCCCTT

              *  *  * **          *       *     *       *  *  *              
     203123 TTTCACCGTAGGCATCAAAGCGCTCCAAATGTCCACATCCAGATACTCCAGAAAGAGTGTTTCAA
          1 TTCCAACGAAATCATCAAAGCGATCCAAATATCCACTTCCAGATTCTACAAAAAGAGTGTTTCAA

             *         *   * *   *         *        * *  *      *      *     
     203188 ACCTGCTCTATGAAAGGGAATCTTCAACTCTATGAGTTGAATGCAGACATCAGAAAGAAATTTCT
         66 AACTGCTCTATCAAAAGAAATGTTCAACTCTGTGAGTTGAGTACACACATCACAAAGAAGTTTCT

                     *       **          *         **
     203253 GAGAATGCTGCTGTCTACCTTTTAT-T--TGA-ATTCCCGC
        131 GAGAATGCTTCTGTCTAGTTTTTATATGAAGATATTCCCTT

                         *  *    *                *       *                  
     203290 TTCCAACGAAATCCTCCAAGCTATCCAAATATCCACTTGCAGATTCCACAAAAAGAGTGTTTCAA
          1 TTCCAACGAAATCATCAAAGCGATCCAAATATCCACTTCCAGATTCTACAAAAAGAGTGTTTCAA

                            ** *  *            *       *     *     *  *      
     203355 AACTGCTCTCTATCAATGGCAAAGTTCAACTCTGTTAGTTGAGGACACATATCACCAACAAGTTT
         66 AACTG--CTCTATCAAAAGAAATGTTCAACTCTGTGAGTTGAGTACACACATCACAAAGAAGTTT

                               *       **         *    
     203420 CTGAGAATGCTTCTGTCTATTTTTTATGGGAAGATATTTCCTT
        129 CTGAGAATGCTTCTGTCTAGTTTTTATATGAAGATATTCCCTT

              *  *  * ** *    *      *    *          * **                    
     203463 TTTCACCGTAGGCGTCAAGGCGATCGAAATGTCCACTTCCACAAACTACAAAAAGAGTGTTTCAA
          1 TTCCAACGAAATCATCAAAGCGATCCAAATATCCACTTCCAGATTCTACAAAAAGAGTGTTTCAA

             *         *   * **       *    *        * ** * *   **      *     
     203528 ACCTGCTCTATGAAAGGCCATGTTCATCTCTATGAGTTGAATGGAAATATCCGAAAGAAATTTCT
         66 AACTGCTCTATCAAAAGAAATGTTCAACTCTGTGAGTTGAGTACACACATCACAAAGAAGTTTCT

             *       *          *        *         **
     203593 GGGAATGCTGCTGTCTAG-TGTT-TAT-ACGA-ATTCCCGC
        131 GAGAATGCTTCTGTCTAGTTTTTATATGAAGATATTCCCTT

                    *    *       *                *    *  *                  
     203630 TTCCAACGTAATCCTCAAAGCAATCCAAATATCCACTTGCAGAATCCACAAAAAGAGTGTTTCAA
          1 TTCCAACGAAATCATCAAAGCGATCCAAATATCCACTTCCAGATTCTACAAAAAGAGTGTTTCAA

                          *     *          * *                   *  *        
     203695 AACTGCTCTATCAATAGAAAGGTTCAACTCTTTTAGTTGAGTACACACATCACGAACAAGTTTCT
         66 AACTGCTCTATCAAAAGAAATGTTCAACTCTGTGAGTTGAGTACACACATCACAAAGAAGTTTCT

                            * *      **     **  *    
     203760 GAGAATGCTTCTGTCTGGCTTTTATTGGAAGACGTTTCCTT
        131 GAGAATGCTTCTGTCTAGTTTTTATATGAAGATATTCCCTT

                 * *  **          *       *                * *               
     203801 TT-CACCAAAGGCATCAAAGCGCTCCAAATGTCCACTTCCAGATTCTTCCAAAAGAGTGTTTCAA
          1 TTCCAACGAAATCATCAAAGCGATCCAAATATCCACTTCCAGATTCTACAAAAAGAGTGTTTCAA

             **     * ***  * *                 *    * *  * *     *   *       
     203865 ACGTGCTCAAAGTAAGGGAATGTTCAACTCTGTGACTTGAATGCAGATATCACCAAGTAGTTTCT
         66 AACTGCTCTATCAAAAGAAATGTTCAACTCTGTGAGTTGAGTACACACATCACAAAGAAGTTTCT

            * * *            **     *    *         * 
     203930 AATAGTGCTTCTGTCTACATTTTAGATGATGATATTCCCGT
        131 GAGAATGCTTCTGTCTAGTTTTTATATGAAGATATTCCCTT

                         *** *   *             *  *   *      *     *       * 
     203971 TTCCAACGAAATCGCTAGAGCTATCCAAATATCCAGTTACAGTTTCTACCAAAAGGGTGTTTCCA
          1 TTCCAACGAAATCATCAAAGCGATCCAAATATCCACTTCCAGATTCTACAAAAAGAGTGTTTCAA

              *    **           *            *       *                     * 
     204036 AATTGCTGCATCAAAAGAAAGGTTCAACTCTGTTAGTTGAGGACACACATCACAAAGAAGTTTGT
         66 AACTGCTCTATCAAAAGAAATGTTCAACTCTGTGAGTTGAGTACACACATCACAAAGAAGTTTCT

                              *    *     *           
     204101 GAGAATGCTTCTGTCTAGATTTTGTATGACGATATTCCCTT
        131 GAGAATGCTTCTGTCTAGTTTTTATATGAAGATATTCCCTT

                     *   * *     *   *       * *  *    *  *       *  *       
     204142 TTCCAACGATATCGTTAAAGCAATCTAAATATCAATTTGCAGAATCCACAAAAATAGAGTTTCAA
          1 TTCCAACGAAATCATCAAAGCGATCCAAATATCCACTTCCAGATTCTACAAAAAGAGTGTTTCAA

             *       * *        *    *       *  *                   ***      
     204207 AGCTGCTCTGTAAAAAGAAAGGTTCCACTCTGTTAGCTGAGTACACACATCACAAACTTGTTTCT
         66 AACTGCTCTATCAAAAGAAATGTTCAACTCTGTGAGTTGAGTACACACATCACAAAGAAGTTTCT

            *     *         *        **       * **   
     204272 CAGAATCCTTCTGTCTCGTTTTTATGGGAAGATACTTACTT
        131 GAGAATGCTTCTGTCTAGTTTTTATATGAAGATATTCCCTT

                 *  * **          *       *     *       *  *  *  *           
     204313 TTCCACCGTAGGCATCAAAGCGCTCCAAATGTCCACATCCAGATACTCCAGAACGAGTGTTTCAA
          1 TTCCAACGAAATCATCAAAGCGATCCAAATATCCACTTCCAGATTCTACAAAAAGAGTGTTTCAA

             *         *   * *   *         *        * *  *      *      *     
     204378 ACCTGCTCTATGAAAGGGAATCTTCAACTCTATGAGTTGAATGCAGACATCAGAAAGAAATTTCT
         66 AACTGCTCTATCAAAAGAAATGTTCAACTCTGTGAGTTGAGTACACACATCACAAAGAAGTTTCT

                     *       **          *         **
     204443 GAGAATGCTGCTGTCTACCTTTTAT-T--TGA-ATTCCCGC
        131 GAGAATGCTTCTGTCTAGTTTTTATATGAAGATATTCCCTT

                         *  *    *              * *  **   *    *             
     204480 TTCCAACGAAATCCTCCAAGCTATCCAAATATCCACCTGCATTTTCCACAACAAGAGTGTTTCAA
          1 TTCCAACGAAATCATCAAAGCGATCCAAATATCCACTTCCAGATTCTACAAAAAGAGTGTTTCAA

                          *                 *    *  *                *       
     204545 AACTGCTCTATCAATAGAAATGTTCAACTCCTTTG-GCTGGGTACACACATCACAAACAAGTTTC
         66 AACTGCTCTATCAAAAGAAATGTTCAACT-CTGTGAGTTGAGTACACACATCACAAAGAAGTTTC

                                      **     **       
     204609 TGAGAATGCTTCTGTCTAGTTTTTATGGGAAGACGTTCCCTT
        130 TGAGAATGCTTCTGTCTAGTTTTTATATGAAGATATTCCCTT

              *  * *  **    *     *       *     *       *  *  *              
     204651 TTTCACCAAAGGCATCCAAGCGCTCCAAATGTCCACATCCAGATACTCCAGAAAGAGTGTTTCAA
          1 TTCCAACGAAATCATCAAAGCGATCCAAATATCCACTTCCAGATTCTACAAAAAGAGTGTTTCAA

             *         *   * *   *         *        * *  *      *      *     
     204716 ACCTGCTCTATGAAAGGGAATCTTCAACTCTATGAGTTGAATGCAGACATCAGAAAGAAATTTCT
         66 AACTGCTCTATCAAAAGAAATGTTCAACTCTGTGAGTTGAGTACACACATCACAAAGAAGTTTCT

                     *       **          *         **
     204781 GAGAATGCTGCTGTCTACCTTTTAT-T--TGA-ATTCCCGC
        131 GAGAATGCTTCTGTCTAGTTTTTATATGAAGATATTCCCTT

                         *  *    *                *       *                  
     204818 TTCCAACGAAATCCTCCAAGCTATCCAAATATCCACTTGCAGATTCCACAAAAAGAGTGTTTCAA
          1 TTCCAACGAAATCATCAAAGCGATCCAAATATCCACTTCCAGATTCTACAAAAAGAGTGTTTCAA

                            ** *  *            *       *     *     *  *      
     204883 AACTGCTCTCTATCAATGGCAAAGTTCAACTCTGTTAGTTGAGGACACATATCACCAACAAGTTT
         66 AACTG--CTCTATCAAAAGAAATGTTCAACTCTGTGAGTTGAGTACACACATCACAAAGAAGTTT

                               *       **         *    
     204948 CTGAGAATGCTTCTGTCTATTTTTTATGGGAAGATATTTCCTT
        129 CTGAGAATGCTTCTGTCTAGTTTTTATATGAAGATATTCCCTT

              *  * ** ** *    *      *    *          * **                    
     204991 TTTCACCATAGGCGTCAAGGCGATCGAAATGTCCACTTCCACAAACTACAAAAAGAGTGTTTCAA
          1 TTCCAACGAAATCATCAAAGCGATCCAAATATCCACTTCCAGATTCTACAAAAAGAGTGTTTCAA

             *         *   * **       *    *        * ** * *   **      *     
     205056 ACCTGCTCTATGAAAGGCCATGTTCATCTCTATGAGTTGAATGGAAATATCCGAAAGAAATTTCT
         66 AACTGCTCTATCAAAAGAAATGTTCAACTCTGTGAGTTGAGTACACACATCACAAAGAAGTTTCT

             *       *          *        *         **
     205121 GGGAATGCTGCTGTCTAG-TGTT-TAT-ACGA-ATTCCCGC
        131 GAGAATGCTTCTGTCTAGTTTTTATATGAAGATATTCCCTT

                         *       *                *    *  *                  
     205158 TTCCAACGAAATCCTCAAAGCAATCCAAATATCCACTTGCAGAATCCACAAAAAGAGTGTTTCAA
          1 TTCCAACGAAATCATCAAAGCGATCCAAATATCCACTTCCAGATTCTACAAAAAGAGTGTTTCAA

                          *     *          * *                   *  *        
     205223 AACTGCTCTATCAATAGAAAGGTTCAACTCTTTTAGTTGAGTACACACATCACGAACAAGTTTCT
         66 AACTGCTCTATCAAAAGAAATGTTCAACTCTGTGAGTTGAGTACACACATCACAAAGAAGTTTCT

                            * *      **     **  *    
     205288 GAGAATGCTTCTGTCTGGCTTTTATTGGAAGACGTTTCCTT
        131 GAGAATGCTTCTGTCTAGTTTTTATATGAAGATATTCCCTT

                   *  **          *       *                * *               
     205329 TT-CAACAAAGGCATCAAAGCGCTCCAAATGTCCACTTCCAGATTCTTCCAAAAGAGTGTTTCAA
          1 TTCCAACGAAATCATCAAAGCGATCCAAATATCCACTTCCAGATTCTACAAAAAGAGTGTTTCAA

             **     * ***  * *                 *    * *  * *     *   *       
     205393 ACGTGCTCAAAGTAAGGGAATGTTCAACTCTGTGACTTGAATGCAGATATCACCAAGTAGTTTCT
         66 AACTGCTCTATCAAAAGAAATGTTCAACTCTGTGAGTTGAGTACACACATCACAAAGAAGTTTCT

            * * *        *   **     *    *           
     205458 AATAGTGCTTCTGCCTACATTTTAGATGATGATATTCCCTT
        131 GAGAATGCTTCTGTCTAGTTTTTATATGAAGATATTCCCTT

                         * * *   *             *  *   *      *     *       * 
     205499 TTCCAACGAAATCGTTAGAGCTATCCAAATATCCAGTTACAGTTTCTACCAAAAGGGTGTTTCCA
          1 TTCCAACGAAATCATCAAAGCGATCCAAATATCCACTTCCAGATTCTACAAAAAGAGTGTTTCAA

              *    **           *            *       *                     * 
     205564 AATTGCTGCATCAAAAGAAAGGTTCAACTCTGTTAGTTGAGGACACACATCACAAAGAAGTTTGT
         66 AACTGCTCTATCAAAAGAAATGTTCAACTCTGTGAGTTGAGTACACACATCACAAAGAAGTTTCT

                              *    *     *           
     205629 GAGAATGCTTCTGTCTAGATTTTGTATGACGATATTCCCTT
        131 GAGAATGCTTCTGTCTAGTTTTTATATGAAGATATTCCCTT

                     *   * *     *   *       * *  *    *  *       *  *       
     205670 TTCCAACGATATCGTTAAAGCAATCTAAATATCAATTTGCAGAATCCACAAAAATAGAGTTTCAA
          1 TTCCAACGAAATCATCAAAGCGATCCAAATATCCACTTCCAGATTCTACAAAAAGAGTGTTTCAA

             *       * *        *    *       *  *                   ***      
     205735 AGCTGCTCTGTAAAAAGAAAGGTTCCACTCTGTTAGCTGAGTACACACATCACAAACTTGTTTCT
         66 AACTGCTCTATCAAAAGAAATGTTCAACTCTGTGAGTTGAGTACACACATCACAAAGAAGTTTCT

                  *         *        **         **   
     205800 GAGAATCCTTCTGTCTCGTTTTTATGGGAAGATATTTACTT
        131 GAGAATGCTTCTGTCTAGTTTTTATATGAAGATATTCCCTT

                 *  * **          *       *     *       *  *  *  *           
     205841 TTCCACCGTAGGCATCAAAGCGCTCCAAATGTCCACATCCAGATACTCCAGAACGAGTGTTTCAA
          1 TTCCAACGAAATCATCAAAGCGATCCAAATATCCACTTCCAGATTCTACAAAAAGAGTGTTTCAA

             *         *   * *   *         *        * *  *      *      *     
     205906 ACCTGCTCTATGAAAGGGAATCTTCAACTCTATGAGTTGAATGCAGACATCAGAAAGAAATTTCT
         66 AACTGCTCTATCAAAAGAAATGTTCAACTCTGTGAGTTGAGTACACACATCACAAAGAAGTTTCT

                     *       **          *         **
     205971 GAGAATGCTGCTGTCTACCTTTTAT-T--TGA-ATTCCCGC
        131 GAGAATGCTTCTGTCTAGTTTTTATATGAAGATATTCCCTT

                         *  *    *              * *  **   *                  
     206008 TTCCAACGAAATCCTCCAAGCTATCCAAATATCCACCTGCATTTTCCACAAAAAGAGTGTTTCAA
          1 TTCCAACGAAATCATCAAAGCGATCCAAATATCCACTTCCAGATTCTACAAAAAGAGTGTTTCAA

                          *                 *    *  *                *       
     206073 AACTGCTCTATCAATAGAAATGTTCAACTCCTTTG-GCTGGGTACACACATCACAAACAAGTTTC
         66 AACTGCTCTATCAAAAGAAATGTTCAACT-CTGTGAGTTGAGTACACACATCACAAAGAAGTTTC

                                      **     *        
     206137 TGAGAATGCTTCTGTCTAGTTTTTATGGGAAGACATTCCCTT
        130 TGAGAATGCTTCTGTCTAGTTTTTATATGAAGATATTCCCTT

              *  * *  **          *       *            **                    
     206179 TTTCACCAAAGACATCAAAGCGCTCCAAATGTCCACTTCCAGACACTACAAAAAGAGTGTTTCAA
          1 TTCCAACGAAATCATCAAAGCGATCCAAATATCCACTTCCAGATTCTACAAAAAGAGTGTTTCAA

             **       **   ***                 *    * *  * *         *       
     206244 ACGTGCTCTAAGAAAGCGAATGTTCAACTCTGTGACTTGAATGCAGATATCACAAAGTAGTTTCT
         66 AACTGCTCTATCAAAAGAAATGTTCAACTCTGTGAGTTGAGTACACACATCACAAAGAAGTTTCT

                **            *     *    *         * 
     206309 GAGAGGGCTTCTGTCTAGATTTTAGATGATGATATTCCCGT
        131 GAGAATGCTTCTGTCTAGTTTTTATATGAAGATATTCCCTT

                           * *   *                *   *                    * 
     206350 TTCCAACGAAATCATTAGAGCTATCCAAATATCCACTTACAGTTTCTACAAAAAGAGTGTTTCCA
          1 TTCCAACGAAATCATCAAAGCGATCCAAATATCCACTTCCAGATTCTACAAAAAGAGTGTTTCAA

                   **         * *    *       *  *                   ***      
     206415 AACTGCTGCATCAAAAGAGAGGTTCCACTCTGTTAGCTGAGTACACACATCACAAACTTGTTTCT
         66 AACTGCTCTATCAAAAGAAATGTTCAACTCTGTGAGTTGAGTACACACATCACAAAGAAGTTTCT

            *     *         *        **         **   
     206480 CAGAATCCTTCTGTCTCGTTTTTATGGGAAGATATTTACTT
        131 GAGAATGCTTCTGTCTAGTTTTTATATGAAGATATTCCCTT

              *  *  * **          *       *     *       *  *  *              
     206521 TTTCACCGTAGGCATCAAAGCGCTCCAAATGTCCACATCCAGATACTCCAGAAAGAGTGTTTCAA
          1 TTCCAACGAAATCATCAAAGCGATCCAAATATCCACTTCCAGATTCTACAAAAAGAGTGTTTCAA

             *         *   * *   *         *        * *  *      *      *     
     206586 ACCTGCTCTATGAAAGGGAATCTTCAACTCTATGAGTTGAATGCAGACATCAGAAAGAAATTTCT
         66 AACTGCTCTATCAAAAGAAATGTTCAACTCTGTGAGTTGAGTACACACATCACAAAGAAGTTTCT

                     *       **          *         **
     206651 GAGAATGCTGCTGTCTACCTTTTAT-T--TGA-ATTCCCGC
        131 GAGAATGCTTCTGTCTAGTTTTTATATGAAGATATTCCCTT

                         *  *    *                *       *                  
     206688 TTCCAACGAAATCCTCCAAGCTATCCAAATATCCACTTGCAGATTCCACAAAAAGAGTGTTTCAA
          1 TTCCAACGAAATCATCAAAGCGATCCAAATATCCACTTCCAGATTCTACAAAAAGAGTGTTTCAA

                            ** *  *            *       *     *     *  *      
     206753 AACTGCTCTCTATCAATGGCAAAGTTCAACTCTGTTAGTTGAGGACACATATCACCAACAAGTTT
         66 AACTG--CTCTATCAAAAGAAATGTTCAACTCTGTGAGTTGAGTACACACATCACAAAGAAGTTT

                               *       **         *    
     206818 CTGAGAATGCTTCTGTCTATTTTTTATGGGAAGATATTTCCTT
        129 CTGAGAATGCTTCTGTCTAGTTTTTATATGAAGATATTCCCTT

              *  *  * ** *    *      *    *          * **                    
     206861 TTTCACCGTAGGCGTCAAGGCGATCGAAATGTCCACTTCCACAAACTACAAAAAGAGTGTTTCAA
          1 TTCCAACGAAATCATCAAAGCGATCCAAATATCCACTTCCAGATTCTACAAAAAGAGTGTTTCAA

             *         *   * **       *    *        * ** * *   **      *     
     206926 ACCTGCTCTATGAAAGGCCATGTTCATCTCTATGAGTTGAATGGAAATATCCGAAAGAAATTTCT
         66 AACTGCTCTATCAAAAGAAATGTTCAACTCTGTGAGTTGAGTACACACATCACAAAGAAGTTTCT

             *       *          *        *         **
     206991 GGGAATGCTGCTGTCTAG-TGTT-TAT-ACGA-ATTCCCGC
        131 GAGAATGCTTCTGTCTAGTTTTTATATGAAGATATTCCCTT

                    *    *       *                *    *  *                  
     207028 TTCCAACGTAATCCTCAAAGCAATCCAAATATCCACTTGCAGAATCCACAAAAAGAGTGTTTCAA
          1 TTCCAACGAAATCATCAAAGCGATCCAAATATCCACTTCCAGATTCTACAAAAAGAGTGTTTCAA

                          *     *          * *                   *  *        
     207093 AACTGCTCTATCAATAGAAAGGTTCAACTCTTTTAGTTGAGTACACACATCACGAACAAGTTTCT
         66 AACTGCTCTATCAAAAGAAATGTTCAACTCTGTGAGTTGAGTACACACATCACAAAGAAGTTTCT

                            * *      **     **  *    
     207158 GAGAATGCTTCTGTCTGGCTTTTATTGGAAGACGTTTCCTT
        131 GAGAATGCTTCTGTCTAGTTTTTATATGAAGATATTCCCTT

                 * *  **          *       *                * *               
     207199 TT-CACCAAAGGCATCAAAGCGCTCCAAATGTCCACTTCCAGATTCTTCCAAAAGAGTGTTTCAA
          1 TTCCAACGAAATCATCAAAGCGATCCAAATATCCACTTCCAGATTCTACAAAAAGAGTGTTTCAA

             **     * ***  * *                 *    * *  * *     *   *       
     207263 ACGTGCTCAAAGTAAGGGAATGTTCAACTCTGTGACTTGAATGCAGATATCACCAAGTAGTTTCT
         66 AACTGCTCTATCAAAAGAAATGTTCAACTCTGTGAGTTGAGTACACACATCACAAAGAAGTTTCT

            * * *            **     *    *         * 
     207328 AATAGTGCTTCTGTCTACATTTTAGATGATGATATTCCCGT
        131 GAGAATGCTTCTGTCTAGTTTTTATATGAAGATATTCCCTT

                         *** *   *             *  *   *      *     *       * 
     207369 TTCCAACGAAATCGCTAGAGCTATCCAAATATCCAGTTACAGTTTCTACCAAAAGGGTGTTTCCA
          1 TTCCAACGAAATCATCAAAGCGATCCAAATATCCACTTCCAGATTCTACAAAAAGAGTGTTTCAA

              *    **           *            *       *                     * 
     207434 AATTGCTGCATCAAAAGAAAGGTTCAACTCTGTTAGTTGAGGACACACATCACAAAGAAGTTTGT
         66 AACTGCTCTATCAAAAGAAATGTTCAACTCTGTGAGTTGAGTACACACATCACAAAGAAGTTTCT

                              *    *     *           
     207499 GAGAATGCTTCTGTCTAGGTTTTGTATGACGATATTCCCTT
        131 GAGAATGCTTCTGTCTAGTTTTTATATGAAGATATTCCCTT

                     *   * *     *   *       * *  *    *  *       *  *       
     207540 TTCCAACGATATCGTTAAAGCAATCTAAATATCAATTTGCAGAATCCACAAAAATAGAGTTTCAA
          1 TTCCAACGAAATCATCAAAGCGATCCAAATATCCACTTCCAGATTCTACAAAAAGAGTGTTTCAA

             *       * *        *    *       *  *                   ***      
     207605 AGCTGCTCTGTAAAAAGAAAGGTTCCACTCTGTTAGCTGAGTACACACATCACAAACTTGTTTCT
         66 AACTGCTCTATCAAAAGAAATGTTCAACTCTGTGAGTTGAGTACACACATCACAAAGAAGTTTCT

            *     *         *        **       * **   
     207670 CAGAATCCTTCTGTCTCGTTTTTATGGGAAGATACTTACTT
        131 GAGAATGCTTCTGTCTAGTTTTTATATGAAGATATTCCCTT

                 *  * **          *       *     *       *  *  *  *           
     207711 TTCCACCGTAGGCATCAAAGCGCTCCAAATGTCCACATCCAGATACTCCAGAACGAGTGTTTCAA
          1 TTCCAACGAAATCATCAAAGCGATCCAAATATCCACTTCCAGATTCTACAAAAAGAGTGTTTCAA

             *         *   * *   *         *        * *  *      *      *     
     207776 ACCTGCTCTATGAAAGGGAATCTTCAACTCTATGAGTTGAATGCAGACATCAGAAAGAAATTTCT
         66 AACTGCTCTATCAAAAGAAATGTTCAACTCTGTGAGTTGAGTACACACATCACAAAGAAGTTTCT

                     *       **          *         **
     207841 GAGAATGCTGCTGTCTACCTTTTAT-T--TGA-ATTCCCGC
        131 GAGAATGCTTCTGTCTAGTTTTTATATGAAGATATTCCCTT

                         *  *    *              * *  **   *    *             
     207878 TTCCAACGAAATCCTCCAAGCTATCCAAATATCCACCTGCATTTTCCACAACAAGAGTGTTTCAA
          1 TTCCAACGAAATCATCAAAGCGATCCAAATATCCACTTCCAGATTCTACAAAAAGAGTGTTTCAA

                          *                 *    *  *                *       
     207943 AACTGCTCTATCAATAGAAATGTTCAACTCCTTTG-GCTGGGTACACACATCACAAACAAGTTTC
         66 AACTGCTCTATCAAAAGAAATGTTCAACT-CTGTGAGTTGAGTACACACATCACAAAGAAGTTTC

                                      **     **       
     208007 TGAGAATGCTTCTGTCTAGTTTTTATGGGAAGACGTTCCCTT
        130 TGAGAATGCTTCTGTCTAGTTTTTATATGAAGATATTCCCTT

              *  * *  **          *       *            **                    
     208049 TTTCACCAAAGGCATCAAAGCGCTCCAAATGTCCACTTCCAGACACTACAAAAAGAGTGTTTCAA
          1 TTCCAACGAAATCATCAAAGCGATCCAAATATCCACTTCCAGATTCTACAAAAAGAGTGTTTCAA

             **       **   ***                 *    * *    *         *       
     208114 ACGTGCTCTAAGAAAGCGAATGTTCAACTCTGTGACTTGAATGCACATATCACAAAGTAGTTTCT
         66 AACTGCTCTATCAAAAGAAATGTTCAACTCTGTGAGTTGAGTACACACATCACAAAGAAGTTTCT

                **            *     *    *         * 
     208179 GAGAGGGCTTCTGTCTAGATTTTAGATGATGATATTCCCGT
        131 GAGAATGCTTCTGTCTAGTTTTTATATGAAGATATTCCCTT

                   *       * *    *               *   *                    * 
     208220 TTCCAACAAAATCATTAGAGC-TTCCAAATATCCACTTACAGTTTCTACAAAAAGAGTGTTTCCA
          1 TTCCAACGAAATCATCAAAGCGATCCAAATATCCACTTCCAGATTCTACAAAAAGAGTGTTTCAA

                   **         * *    *       *  *                   ***      
     208284 AACTGCTGCATCAAAAGAGAGGTTCCACTCTGTTAGCTGAGTACACACATCACAAACTTGTTTCT
         66 AACTGCTCTATCAAAAGAAATGTTCAACTCTGTGAGTTGAGTACACACATCACAAAGAAGTTTCT

            *     *         *        **        ***   
     208349 CAGAATCCTTCTGTCTCGTTTTTATGGGAAGAT-TATACTT
        131 GAGAATGCTTCTGTCTAGTTTTTATATGAAGATATTCCCTT

              *  *  * **          *       *     *       *  *  *              
     208389 TTTCACCGTAGGCATCAAAGCGCTCCAAATGTCCACATCCAGATACTCCAGAAAGAGTGTTTCAA
          1 TTCCAACGAAATCATCAAAGCGATCCAAATATCCACTTCCAGATTCTACAAAAAGAGTGTTTCAA

             *    *    *   * *   *         *        * *  *      *      *     
     208454 ACCTGCCCTATGAAAGGGAATCTTCAACTCTATGAGTTGAATGCAGACATCAGAAAGAAATTTCT
         66 AACTGCTCTATCAAAAGAAATGTTCAACTCTGTGAGTTGAGTACACACATCACAAAGAAGTTTCT

                     *       **          *         **
     208519 GAGAATGCTGCTGTCTACCTTTTAT-T--TGA-ATTCCCGC
        131 GAGAATGCTTCTGTCTAGTTTTTATATGAAGATATTCCCTT

                         *  *    *                *       *                  
     208556 TTCCAACGAAATCCTCCAAGCTATCCAAATATCCACTTGCAGATTCCACAAAAAGAGTGTTTCAA
          1 TTCCAACGAAATCATCAAAGCGATCCAAATATCCACTTCCAGATTCTACAAAAAGAGTGTTTCAA

                            ** *  *            *       *     *     *  *      
     208621 AACTGCTCTCTATCAATGGCAAAGTTCAACTCTGTTAGTTGAGGACACATATCACCAACAAGTTT
         66 AACTG--CTCTATCAAAAGAAATGTTCAACTCTGTGAGTTGAGTACACACATCACAAAGAAGTTT

                               *       **         *    
     208686 CTGAGAATGCTTCTGTCTATTTTTTATGGGAAGATATTTCCTT
        129 CTGAGAATGCTTCTGTCTAGTTTTTATATGAAGATATTCCCTT

              *  *  * ** *    *      *    *          * **                    
     208729 TTTCACCGTAGGCGTCAAGGCGATCGAAATGTCCACTTCCACAAACTACAAAAAGAGTGTTTCAA
          1 TTCCAACGAAATCATCAAAGCGATCCAAATATCCACTTCCAGATTCTACAAAAAGAGTGTTTCAA

             *         *   * **       *    *        * ** * *   **      *     
     208794 ACCTGCTCTATGAAAGGCCATGTTCATCTCTATGAGTTGAATGGAAATATCCGAAAGAAATTTCT
         66 AACTGCTCTATCAAAAGAAATGTTCAACTCTGTGAGTTGAGTACACACATCACAAAGAAGTTTCT

             *       *          *        *         **
     208859 GGGAATGCTGCTGTCTAG-TGTT-TAT-ACGA-ATTCCCGC
        131 GAGAATGCTTCTGTCTAGTTTTTATATGAAGATATTCCCTT

                         *       *                *    *  *                  
     208896 TTCCAACGAAATCCTCAAAGCAATCCAAATATCCACTTGCAGAATCCACAAAAAGAGTGTTTCAA
          1 TTCCAACGAAATCATCAAAGCGATCCAAATATCCACTTCCAGATTCTACAAAAAGAGTGTTTCAA

                          *     *          * *                   *  *        
     208961 AACTGCTCTATCAATAGAAAGGTTCAACTCTTTTAGTTGAGTACACACATCACGAACAAGTTTCT
         66 AACTGCTCTATCAAAAGAAATGTTCAACTCTGTGAGTTGAGTACACACATCACAAAGAAGTTTCT

                            * *      **     **  *    
     209026 GAGAATGCTTCTGTCTGGCTTTTATTGGAAGACGTTTCCTT
        131 GAGAATGCTTCTGTCTAGTTTTTATATGAAGATATTCCCTT

                 * *  **          *       *                * *               
     209067 TT-CACCAAAGGCATCAAAGCGCTCCAAATGTCCACTTCCAGATTCTTCCAAAAGAGTGTTTCAA
          1 TTCCAACGAAATCATCAAAGCGATCCAAATATCCACTTCCAGATTCTACAAAAAGAGTGTTTCAA

             **     * ***  * *                 *    * *  * *     *   *       
     209131 ACGTGCTCAAAGTAAGGGAATGTTCAACTCTGTGACTTGAATGCAGATATCACCAAGTAGTTTCT
         66 AACTGCTCTATCAAAAGAAATGTTCAACTCTGTGAGTTGAGTACACACATCACAAAGAAGTTTCT

            * * *        *   **     *    *           
     209196 AATAGTGCTTCTGCCTACATTTTAGATGATGATATTCCCTT
        131 GAGAATGCTTCTGTCTAGTTTTTATATGAAGATATTCCCTT

                         * * *   *             *  *   *      *     *       * 
     209237 TTCCAACGAAATCGTTAGAGCTATCCAAATATCCAGTTACAGTTTCTACCAAAAGGGTGTTTCCA
          1 TTCCAACGAAATCATCAAAGCGATCCAAATATCCACTTCCAGATTCTACAAAAAGAGTGTTTCAA

              *    **           *            *       *                     * 
     209302 AATTGCTGCATCAAAAGAAAGGTTCAACTCTGTTAGTTGAGGACACACATCACAAAGAAGTTTGT
         66 AACTGCTCTATCAAAAGAAATGTTCAACTCTGTGAGTTGAGTACACACATCACAAAGAAGTTTCT

                              *    *     *           
     209367 GAGAATGCTTCTGTCTAGATTTTGTATGACGATATTCCCTT
        131 GAGAATGCTTCTGTCTAGTTTTTATATGAAGATATTCCCTT

                     *   * *     *   *       * *  *    *  *       *  *       
     209408 TTCCAACGATATCGTTAAAGCAATCTAAATATCAATTTGCAGAATCCACAAAAATAGAGTTTCAA
          1 TTCCAACGAAATCATCAAAGCGATCCAAATATCCACTTCCAGATTCTACAAAAAGAGTGTTTCAA

             *       * *        *    *       *  *                   ***      
     209473 AGCTGCTCTGTAAAAAGAAAGGTTCCACTCTGTTAGCTGAGTACACACATCACAAACTTGTTTCT
         66 AACTGCTCTATCAAAAGAAATGTTCAACTCTGTGAGTTGAGTACACACATCACAAAGAAGTTTCT

                  *         *        **         **   
     209538 GAGAATCCTTCTGTCTCGTTTTTATGGGAAGATATTTACTT
        131 GAGAATGCTTCTGTCTAGTTTTTATATGAAGATATTCCCTT

                 *  * **          *       *     *       *  *  *  *           
     209579 TTCCACCGTAGGCATCAAAGCGCTCCAAATGTCCACATCCAGATACTCCAGAACGAGTGTTTCAA
          1 TTCCAACGAAATCATCAAAGCGATCCAAATATCCACTTCCAGATTCTACAAAAAGAGTGTTTCAA

             *         *   * *   *         *        * *  *      *      *     
     209644 ACCTGCTCTATGAAAGGGAATCTTCAACTCTATGAGTTGAATGCAGACATCAGAAAGAAATTTCT
         66 AACTGCTCTATCAAAAGAAATGTTCAACTCTGTGAGTTGAGTACACACATCACAAAGAAGTTTCT

                     *       **          *         **
     209709 GAGAATGCTGCTGTCTACCTTTTAT-T--TGA-ATTCCCGC
        131 GAGAATGCTTCTGTCTAGTTTTTATATGAAGATATTCCCTT

                         *  *    *              * *  **   *                  
     209746 TTCCAACGAAATCCTCCAAGCTATCCAAATATCCACCTGCATTTTCCACAAAAAGAGTGTTTCAA
          1 TTCCAACGAAATCATCAAAGCGATCCAAATATCCACTTCCAGATTCTACAAAAAGAGTGTTTCAA

                          *                 *    *  *                *       
     209811 AACTGCTCTATCAATAGAAATGTTCAACTCCTTTG-GCTGGGTACACACATCACAAACAAGTTTC
         66 AACTGCTCTATCAAAAGAAATGTTCAACT-CTGTGAGTTGAGTACACACATCACAAAGAAGTTTC

                                      **     *        
     209875 TGAGAATGCTTCTGTCTAGTTTTTATGGGAAGACATTCCCTT
        130 TGAGAATGCTTCTGTCTAGTTTTTATATGAAGATATTCCCTT

              *  * *  **          *       *            **                    
     209917 TTTCACCAAAGACATCAAAGCGCTCCAAATGTCCACTTCCAGACACTACAAAAAGAGTGTTTCAA
          1 TTCCAACGAAATCATCAAAGCGATCCAAATATCCACTTCCAGATTCTACAAAAAGAGTGTTTCAA

             **       **   ***                 *    * *  * *         *       
     209982 ACGTGCTCTAAGAAAGCGAATGTTCAACTCTGTGACTTGAATGCAGATATCACAAAGTAGTTTCT
         66 AACTGCTCTATCAAAAGAAATGTTCAACTCTGTGAGTTGAGTACACACATCACAAAGAAGTTTCT

                **            *     *    *         * 
     210047 GAGAGGGCTTCTGTCTAGATTTTAGATGATGATATTCCCGT
        131 GAGAATGCTTCTGTCTAGTTTTTATATGAAGATATTCCCTT

                           * *   *                *   *                    * 
     210088 TTCCAACGAAATCATTAGAGCTATCCAAATATCCACTTACAGTTTCTACAAAAAGAGTGTTTCCA
          1 TTCCAACGAAATCATCAAAGCGATCCAAATATCCACTTCCAGATTCTACAAAAAGAGTGTTTCAA

                   **         * *    *       *  **                  ***      
     210153 AACTGCTGCATCAAAAGAGAGGTTCCACTCTGTTAGCCGAGTACACACATCACAAACTTGTTTCT
         66 AACTGCTCTATCAAAAGAAATGTTCAACTCTGTGAGTTGAGTACACACATCACAAAGAAGTTTCT

            *     *         *        **         **   
     210218 CAGAATCCTTCTGTCTCGTTTTTATGGGAAGATATTTACTT
        131 GAGAATGCTTCTGTCTAGTTTTTATATGAAGATATTCCCTT

              *  *  * **          *       *     *       *  *  *              
     210259 TTTCACCGTAGGCATCAAAGCGCTCCAAATGTCCACATCCAGATACTCCAGAAAGAGTGTTTCAA
          1 TTCCAACGAAATCATCAAAGCGATCCAAATATCCACTTCCAGATTCTACAAAAAGAGTGTTTCAA

             *         *   * *   *         *        * *  *      *      *     
     210324 ACCTGCTCTATGAAAGGGAATCTTCAACTCTATGAGTTGAATGCAGACATCAGAAAGAAATTTCT
         66 AACTGCTCTATCAAAAGAAATGTTCAACTCTGTGAGTTGAGTACACACATCACAAAGAAGTTTCT

                     *       **          *         **
     210389 GAGAATGCTGCTGTCTACCTTTTAT-T--TGA-ATTCCCGC
        131 GAGAATGCTTCTGTCTAGTTTTTATATGAAGATATTCCCTT

                         *  *    *                *       *                  
     210426 TTCCAACGAAATCCTCCAAGCTATCCAAATATCCACTTGCAGATTCCACAAAAAGAGTGTTTCAA
          1 TTCCAACGAAATCATCAAAGCGATCCAAATATCCACTTCCAGATTCTACAAAAAGAGTGTTTCAA

                            ** *  *            *       *     *     *  *      
     210491 AACTGCTCTCTATCAATGGCAAAGTTCAACTCTGTTAGTTGAGGACACATATCACCAACAAGTTT
         66 AACTG--CTCTATCAAAAGAAATGTTCAACTCTGTGAGTTGAGTACACACATCACAAAGAAGTTT

                               *       **         *    
     210556 CTGAGAATGCTTCTGTCTATTTTTTATGGGAAGATATTTCCTT
        129 CTGAGAATGCTTCTGTCTAGTTTTTATATGAAGATATTCCCTT

              *  *  * ** *    *      *    *          * **                    
     210599 TTTCACCGTAGGCGTCAAGGCGATCGAAATGTCCACTTCCACAAACTACAAAAAGAGTGTTTCAA
          1 TTCCAACGAAATCATCAAAGCGATCCAAATATCCACTTCCAGATTCTACAAAAAGAGTGTTTCAA

             *         *   * **       *    *        * ** * *   **      *     
     210664 ACCTGCTCTATGAAAGGCCATGTTCATCTCTATGAGTTGAATGGAAATATCCGAAAGAAATTTCT
         66 AACTGCTCTATCAAAAGAAATGTTCAACTCTGTGAGTTGAGTACACACATCACAAAGAAGTTTCT

             *       *          *        *         **
     210729 GGGAATGCTGCTGTCTAG-TGTT-TAT-ACGA-ATTCCCGC
        131 GAGAATGCTTCTGTCTAGTTTTTATATGAAGATATTCCCTT

                    *    *       *                *    *  *                  
     210766 TTCCAACGTAATCCTCAAAGCAATCCAAATATCCACTTGCAGAATCCACAAAAAGAGTGTTTCAA
          1 TTCCAACGAAATCATCAAAGCGATCCAAATATCCACTTCCAGATTCTACAAAAAGAGTGTTTCAA

                          *     *          * *                   *  *        
     210831 AACTGCTCTATCAATAGAAAGGTTCAACTCTTTTAGTTGAGTACACACATCACGAACAAGTTTCT
         66 AACTGCTCTATCAAAAGAAATGTTCAACTCTGTGAGTTGAGTACACACATCACAAAGAAGTTTCT

                            * *      **     **  *    
     210896 GAGAATGCTTCTGTCTGGCTTTTATTGGAAGACGTTTCCTT
        131 GAGAATGCTTCTGTCTAGTTTTTATATGAAGATATTCCCTT

                 * *  **          *       *                * *               
     210937 TT-CACCAAAGGCATCAAAGCGCTCCAAATGTCCACTTCCAGATTCTTCCAAAAGAGTGTTTCAA
          1 TTCCAACGAAATCATCAAAGCGATCCAAATATCCACTTCCAGATTCTACAAAAAGAGTGTTTCAA

             **     * ***  * *                 *    * *  * *     *   *       
     211001 ACGTGCTCAAAGTAAGGGAATGTTCAACTCTGTGACTTGAATGCAGATATCACCAAGTAGTTTCT
         66 AACTGCTCTATCAAAAGAAATGTTCAACTCTGTGAGTTGAGTACACACATCACAAAGAAGTTTCT

            * * *            **     *    *         * 
     211066 AATAGTGCTTCTGTCTACATTTTAGATGATGATATTCCCGT
        131 GAGAATGCTTCTGTCTAGTTTTTATATGAAGATATTCCCTT

               *         *** *   *             *  *   *      *     *       * 
     211107 TTCAAACGAAATCGCTAGAGCTATCCAAATATCCAGTTACAGTTTCTACCAAAAGGGTGTTTCCA
          1 TTCCAACGAAATCATCAAAGCGATCCAAATATCCACTTCCAGATTCTACAAAAAGAGTGTTTCAA

              *    **           *            *       *                     * 
     211172 AATTGCTGCATCAAAAGAAAGGTTCAACTCTGTTAGTTGAGGACACACATCACAAAGAAGTTTGT
         66 AACTGCTCTATCAAAAGAAATGTTCAACTCTGTGAGTTGAGTACACACATCACAAAGAAGTTTCT

                              *    *     *           
     211237 GAGAATGCTTCTGTCTAGATTTTGTATGACGATATTCCCTT
        131 GAGAATGCTTCTGTCTAGTTTTTATATGAAGATATTCCCTT

                     *   * *     *   *       * *  *    *  *       *  *       
     211278 TTCCAACGATATCGTTAAAGCAATCTAAATATCAATTTGCAGAATCCACAAAAATAGAGTTTCAA
          1 TTCCAACGAAATCATCAAAGCGATCCAAATATCCACTTCCAGATTCTACAAAAAGAGTGTTTCAA

             *       * *        *    *       *  *                   ***      
     211343 AGCTGCTCTGTAAAAAGAAAGGTTCCACTCTGTTAGCTGAGTACACACATCACAAACTTGTTTCT
         66 AACTGCTCTATCAAAAGAAATGTTCAACTCTGTGAGTTGAGTACACACATCACAAAGAAGTTTCT

            *     *         *        **       * **   
     211408 CAGAATCCTTCTGTCTCGTTTTTATGGGAAGATACTTACTT
        131 GAGAATGCTTCTGTCTAGTTTTTATATGAAGATATTCCCTT

                 *  * **          *       *     *       *  *  *  *           
     211449 TTCCACCGTAGGCATCAAAGCGCTCCAAATGTCCACATCCAGATACTCCAGAACGAGTGTTTCAA
          1 TTCCAACGAAATCATCAAAGCGATCCAAATATCCACTTCCAGATTCTACAAAAAGAGTGTTTCAA

             *         *   * *   *         *        * *  *      *      *     
     211514 ACCTGCTCTATGAAAGGGAATCTTCAACTCTATGAGTTGAATGCAGACATCAGAAAGAAATTTCT
         66 AACTGCTCTATCAAAAGAAATGTTCAACTCTGTGAGTTGAGTACACACATCACAAAGAAGTTTCT

                     *       **          *         **
     211579 GAGAATGCTGCTGTCTACCTTTTAT-T--TGA-ATTCCCGC
        131 GAGAATGCTTCTGTCTAGTTTTTATATGAAGATATTCCCTT

                         *  *    *              * *  **   *    *             
     211616 TTCCAACGAAATCCTCCAAGCTATCCAAATATCCACCTGCATTTTCCACAACAAGAGTGTTTCAA
          1 TTCCAACGAAATCATCAAAGCGATCCAAATATCCACTTCCAGATTCTACAAAAAGAGTGTTTCAA

                          *                 *    *  *                *       
     211681 AACTGCTCTATCAATAGAAATGTTCAACTCCTTTG-GCTGGGTACACACATCACAAACAAGTTTC
         66 AACTGCTCTATCAAAAGAAATGTTCAACT-CTGTGAGTTGAGTACACACATCACAAAGAAGTTTC

                                      **     **       
     211745 TGAGAATGCTTCTGTCTAGTTTTTATGGGAAGACGTTCCCTT
        130 TGAGAATGCTTCTGTCTAGTTTTTATATGAAGATATTCCCTT

              *  * *  **    *     *       *     *       *  *  *              
     211787 TTTCACCAAAGGCATCCAAGCGCTCCAAATGTCCACATCCAGATACTCCAGAAAGAGTGTTTCAA
          1 TTCCAACGAAATCATCAAAGCGATCCAAATATCCACTTCCAGATTCTACAAAAAGAGTGTTTCAA

             *         *   * *   *         *        * *  *      *      *     
     211852 ACCTGCTCTATGAAAGGGAATCTTCAACTCTATGAGTTGAATGCAGACATCAGAAAGAAATTTCT
         66 AACTGCTCTATCAAAAGAAATGTTCAACTCTGTGAGTTGAGTACACACATCACAAAGAAGTTTCT

                     *       **          *         **
     211917 GAGAATGCTGCTGTCTACCTTTTAT-T--TGA-ATTCCCGC
        131 GAGAATGCTTCTGTCTAGTTTTTATATGAAGATATTCCCTT

                         *  *    *                *       *                  
     211954 TTCCAACGAAATCCTCCAAGCTATCCAAATATCCACTTGCAGATTCCACAAAAAGAGTGTTTCAA
          1 TTCCAACGAAATCATCAAAGCGATCCAAATATCCACTTCCAGATTCTACAAAAAGAGTGTTTCAA

                            ** *  *            *       *     *     *  *      
     212019 AACTGCTCTCTATCAATGGCAAAGTTCAACTCTGTTAGTTGAGGACACATATCACCAACAAGTTT
         66 AACTG--CTCTATCAAAAGAAATGTTCAACTCTGTGAGTTGAGTACACACATCACAAAGAAGTTT

                               *       **         *    
     212084 CTGAGAATGCTTCTGTCTATTTTTTATGGGAAGATATTTCCTT
        129 CTGAGAATGCTTCTGTCTAGTTTTTATATGAAGATATTCCCTT

              *  *  * ** *    *      *    *          * **                    
     212127 TTTCACCGTAGGCGTCAAGGCGATCGAAATGTCCACTTCCACAAACTACAAAAAGAGTGTTTCAA
          1 TTCCAACGAAATCATCAAAGCGATCCAAATATCCACTTCCAGATTCTACAAAAAGAGTGTTTCAA

             *         *   * **       *    *        * ** * *   **      *     
     212192 ACCTGCTCTATGAAAGGCCATGTTCATCTCTATGAGTTGAATGGAAATATCCGAAAGAAATTTCT
         66 AACTGCTCTATCAAAAGAAATGTTCAACTCTGTGAGTTGAGTACACACATCACAAAGAAGTTTCT

             *       *          *        *         **
     212257 GGGAATGCTGCTGTCTAG-TGTT-TAT-ACGA-ATTCCCGC
        131 GAGAATGCTTCTGTCTAGTTTTTATATGAAGATATTCCCTT

                         *       *                *    *  *                  
     212294 TTCCAACGAAATCCTCAAAGCAATCCAAATATCCACTTGCAGAATCCACAAAAAGAGTGTTTCAA
          1 TTCCAACGAAATCATCAAAGCGATCCAAATATCCACTTCCAGATTCTACAAAAAGAGTGTTTCAA

                          *     *          * *                   *  *        
     212359 AACTGCTCTATCAATAGAAAGGTTCAACTCTTTTAGTTGAGTACACACATCACGAACAAGTTTCT
         66 AACTGCTCTATCAAAAGAAATGTTCAACTCTGTGAGTTGAGTACACACATCACAAAGAAGTTTCT

                            * *      **     **  *    
     212424 GAGAATGCTTCTGTCTGGCTTTTATTGGAAGACGTTTCCTT
        131 GAGAATGCTTCTGTCTAGTTTTTATATGAAGATATTCCCTT

                   *  **          *       *                * *               
     212465 TT-CAACAAAGGCATCAAAGCGCTCCAAATGTCCACTTCCAGATTCTTCCAAAAGAGTGTTTCAA
          1 TTCCAACGAAATCATCAAAGCGATCCAAATATCCACTTCCAGATTCTACAAAAAGAGTGTTTCAA

             **     * ***  * *                 *    * *  * *     *   *       
     212529 ACGTGCTCAAAGTAAGGGAATGTTCAACTCTGTGACTTGAATGCAGATATCACCAAGTAGTTTCT
         66 AACTGCTCTATCAAAAGAAATGTTCAACTCTGTGAGTTGAGTACACACATCACAAAGAAGTTTCT

            * * *            **     *    *           
     212594 AATAGTGCTTCTGTCTACATTTTAGATGATGATATTCCCTT
        131 GAGAATGCTTCTGTCTAGTTTTTATATGAAGATATTCCCTT

                         * * *   *             *  *   *      *     *       * 
     212635 TTCCAACGAAATCGTTAGAGCTATCCAAATATCCAGTTACAGTTTCTACCAAAAGGGTGTTTCCA
          1 TTCCAACGAAATCATCAAAGCGATCCAAATATCCACTTCCAGATTCTACAAAAAGAGTGTTTCAA

              *    **           *            *       *                     * 
     212700 AATTGCTGCATCAAAAGAAAGGTTCAACTCTGTTAGTTGAGGACACACATCACAAAGAAGTTTGT
         66 AACTGCTCTATCAAAAGAAATGTTCAACTCTGTGAGTTGAGTACACACATCACAAAGAAGTTTCT

                              *    *     *           
     212765 GAGAATGCTTCTGTCTAGATTTTGTATGACGATATTCCCTT
        131 GAGAATGCTTCTGTCTAGTTTTTATATGAAGATATTCCCTT

                     *   * *     *   *       * *  *    *  *       *  *       
     212806 TTCCAACGATATCGTTAAAGCAATCTAAATATCAATTTGCAGAATCCACAAAAATAGAGTTTCAA
          1 TTCCAACGAAATCATCAAAGCGATCCAAATATCCACTTCCAGATTCTACAAAAAGAGTGTTTCAA

             *       * *        *    *       *  *                   ***      
     212871 AGCTGCTCTGTAAAAAGAAAGGTTCCACTCTGTTAGCTGAGTACACACATCACAAACTTGTTTCT
         66 AACTGCTCTATCAAAAGAAATGTTCAACTCTGTGAGTTGAGTACACACATCACAAAGAAGTTTCT

                  *         *        **         **   
     212936 GAGAATCCTTCTGTCTCGTTTTTATGGGAAGATATTTACTT
        131 GAGAATGCTTCTGTCTAGTTTTTATATGAAGATATTCCCTT

                 *  * **          *       *     *       *  *  *  *           
     212977 TTCCACCGTAGGCATCAAAGCGCTCCAAATGTCCACATCCAGATACTCCAGAACGAGTGTTTCAA
          1 TTCCAACGAAATCATCAAAGCGATCCAAATATCCACTTCCAGATTCTACAAAAAGAGTGTTTCAA

             *         *   * *   *         *        * *  *      *      *     
     213042 ACCTGCTCTATGAAAGGGAATCTTCAACTCTATGAGTTGAATGCAGACATCAGAAAGAAATTTCT
         66 AACTGCTCTATCAAAAGAAATGTTCAACTCTGTGAGTTGAGTACACACATCACAAAGAAGTTTCT

                     *       **          *         **
     213107 GAGAATGCTGCTGTCTACCTTTTAT-T--TGA-ATTCCCGC
        131 GAGAATGCTTCTGTCTAGTTTTTATATGAAGATATTCCCTT

                         *  *    *              * *  **   *                  
     213144 TTCCAACGAAATCCTCCAAGCTATCCAAATATCCACCTGCATTTTCCACAAAAAGAGTGTTTCAA
          1 TTCCAACGAAATCATCAAAGCGATCCAAATATCCACTTCCAGATTCTACAAAAAGAGTGTTTCAA

                          *                 *    *  *                *       
     213209 AACTGCTCTATCAATAGAAATGTTCAACTCCTTTG-GCTGGGTACACACATCACAAACAAGTTTC
         66 AACTGCTCTATCAAAAGAAATGTTCAACT-CTGTGAGTTGAGTACACACATCACAAAGAAGTTTC

                                      **     *        
     213273 TGAGAATGCTTCTGTCTAGTTTTTATGGGAAGACATTCCCTT
        130 TGAGAATGCTTCTGTCTAGTTTTTATATGAAGATATTCCCTT

              *  * *  **          *       *            **                    
     213315 TTTCACCAAAGACATCAAAGCGCTCCAAATGTCCACTTCCAGACACTACAAAAAGAGTGTTTCAA
          1 TTCCAACGAAATCATCAAAGCGATCCAAATATCCACTTCCAGATTCTACAAAAAGAGTGTTTCAA

             **       **   ***                 *    * *  * *         *       
     213380 ACGTGCTCTAAGAAAGCGAATGTTCAACTCTGTGACTTGAATGCAGATATCACAAAGTAGTTTCT
         66 AACTGCTCTATCAAAAGAAATGTTCAACTCTGTGAGTTGAGTACACACATCACAAAGAAGTTTCT

                **            *     *    *         * 
     213445 GAGAGGGCTTCTGTCTAGATTTTAGATGATGATATTCCCGT
        131 GAGAATGCTTCTGTCTAGTTTTTATATGAAGATATTCCCTT

                           * *   *                *   *                    * 
     213486 TTCCAACGAAATCATTAGAGCTATCCAAATATCCACTTACAGTTTCTACAAAAAGAGTGTTTCCA
          1 TTCCAACGAAATCATCAAAGCGATCCAAATATCCACTTCCAGATTCTACAAAAAGAGTGTTTCAA

                   **         * *    *       *  **                  ***      
     213551 AACTGCTGCATCAAAAGAGAGGTTCCACTCTGTTAGCCGAGTACACACATCACAAACTTGTTTCT
         66 AACTGCTCTATCAAAAGAAATGTTCAACTCTGTGAGTTGAGTACACACATCACAAAGAAGTTTCT

            *     *         *        **         **   
     213616 CAGAATCCTTCTGTCTCGTTTTTATGGGAAGATATTTACTT
        131 GAGAATGCTTCTGTCTAGTTTTTATATGAAGATATTCCCTT

              *  *  * **          *       *     *       *  *  *              
     213657 TTTCACCGTAGGCATCAAAGCGCTCCAAATGTCCACATCCAGATACTCCAGAAAGAGTGTTTCAA
          1 TTCCAACGAAATCATCAAAGCGATCCAAATATCCACTTCCAGATTCTACAAAAAGAGTGTTTCAA

             *         *   * *   *         *        * *  *      *      *     
     213722 ACCTGCTCTATGAAAGGGAATCTTCAACTCTATGAGTTGAATGCAGACATCAGAAAGAAATTTCT
         66 AACTGCTCTATCAAAAGAAATGTTCAACTCTGTGAGTTGAGTACACACATCACAAAGAAGTTTCT

                     *       **          *         **
     213787 GAGAATGCTGCTGTCTACCTTTTAT-T--TGA-ATTCCCGC
        131 GAGAATGCTTCTGTCTAGTTTTTATATGAAGATATTCCCTT

                         *  *    *                *       *                  
     213824 TTCCAACGAAATCCTCCAAGCTATCCAAATATCCACTTGCAGATTCCACAAAAAGAGTGTTTCAA
          1 TTCCAACGAAATCATCAAAGCGATCCAAATATCCACTTCCAGATTCTACAAAAAGAGTGTTTCAA

                            ** *  *            *       *     *     *  *      
     213889 AACTGCTCTCTATCAATGGCAAAGTTCAACTCTGTTAGTTGAGGACACATATCACCAACAAGTTT
         66 AACTG--CTCTATCAAAAGAAATGTTCAACTCTGTGAGTTGAGTACACACATCACAAAGAAGTTT

                               *       **         *    
     213954 CTGAGAATGCTTCTGTCTATTTTTTATGGGAAGATATTTCCTT
        129 CTGAGAATGCTTCTGTCTAGTTTTTATATGAAGATATTCCCTT

              *  *  * ** *    *      *    *          * **                    
     213997 TTTCACCGTAGGCGTCAAGGCGATCGAAATGTCCACTTCCACAAACTACAAAAAGAGTGTTTCAA
          1 TTCCAACGAAATCATCAAAGCGATCCAAATATCCACTTCCAGATTCTACAAAAAGAGTGTTTCAA

             *         *   * **       *    *        * ** * *   **      *     
     214062 ACCTGCTCTATGAAAGGCCATGTTCATCTCTATGAGTTGAATGGAAATATCCGAAAGAAATTTCT
         66 AACTGCTCTATCAAAAGAAATGTTCAACTCTGTGAGTTGAGTACACACATCACAAAGAAGTTTCT

             *       *          *        *         **
     214127 GGGAATGCTGCTGTCTAG-TGTT-TAT-ACGA-ATTCCCGC
        131 GAGAATGCTTCTGTCTAGTTTTTATATGAAGATATTCCCTT

                    *    *       *                *    *  *                  
     214164 TTCCAACGTAATCCTCAAAGCAATCCAAATATCCACTTGCAGAATCCACAAAAAGAGTGTTTCAA
          1 TTCCAACGAAATCATCAAAGCGATCCAAATATCCACTTCCAGATTCTACAAAAAGAGTGTTTCAA

                          *     *          * *                   *  *        
     214229 AACTGCTCTATCAATAGAAAGGTTCAACTCTTTTAGTTGAGTACACACATCACGAACAAGTTTCT
         66 AACTGCTCTATCAAAAGAAATGTTCAACTCTGTGAGTTGAGTACACACATCACAAAGAAGTTTCT

                            * *      **     **  *    
     214294 GAGAATGCTTCTGTCTGGCTTTTATTGGAAGACGTTTCCTT
        131 GAGAATGCTTCTGTCTAGTTTTTATATGAAGATATTCCCTT

                 * *  **          *       *                * *               
     214335 TT-CACCAAAGGCATCAAAGCGCTCCAAATGTCCACTTCCAGATTCTTCCAAAAGAGTGTTTCAA
          1 TTCCAACGAAATCATCAAAGCGATCCAAATATCCACTTCCAGATTCTACAAAAAGAGTGTTTCAA

             **     * ***  * *                 *    * *  * *     *   *       
     214399 ACGTGCTCAAAGTAAGGGAATGTTCAACTCTGTGACTTGAATGCAGATATCACCAAGTAGTTTCT
         66 AACTGCTCTATCAAAAGAAATGTTCAACTCTGTGAGTTGAGTACACACATCACAAAGAAGTTTCT

            * * *            **     *    *         * 
     214464 AATAGTGCTTCTGTCTACATTTTAGATGATGATATTCCCGT
        131 GAGAATGCTTCTGTCTAGTTTTTATATGAAGATATTCCCTT

                         *** *   *             *  *   *      *     *       * 
     214505 TTCCAACGAAATCGCTAGAGCTATCCAAATATCCAGTTACAGTTTCTACCAAAAGGGTGTTTCCA
          1 TTCCAACGAAATCATCAAAGCGATCCAAATATCCACTTCCAGATTCTACAAAAAGAGTGTTTCAA

              *    **           *            *       *                     * 
     214570 AATTGCTGCATCAAAAGAAAGGTTCAACTCTGTTAGTTGAGGACACACATCACAAAGAAGTTTGT
         66 AACTGCTCTATCAAAAGAAATGTTCAACTCTGTGAGTTGAGTACACACATCACAAAGAAGTTTCT

                              *    *     *           
     214635 GAGAATGCTTCTGTCTAGATTTTGTATGACGATATTCCCTT
        131 GAGAATGCTTCTGTCTAGTTTTTATATGAAGATATTCCCTT

                     *   * *     *   *       * *  *    *  *       *  *       
     214676 TTCCAACGATATCGTTAAAGCAATCTAAATATCAATTTGCAGAATCCACAAAAATAGAGTTTCAA
          1 TTCCAACGAAATCATCAAAGCGATCCAAATATCCACTTCCAGATTCTACAAAAAGAGTGTTTCAA

             *       * *        *    *       *  *                   ***      
     214741 AGCTGCTCTGTAAAAAGAAAGGTTCCACTCTGTTAGCTGAGTACACACATCACAAACTTGTTTCT
         66 AACTGCTCTATCAAAAGAAATGTTCAACTCTGTGAGTTGAGTACACACATCACAAAGAAGTTTCT

            *     *         *        **       * **   
     214806 CAGAATCCTTCTGTCTCGTTTTTATGGGAAGATACTTACTT
        131 GAGAATGCTTCTGTCTAGTTTTTATATGAAGATATTCCCTT

                 *  * **          *       *     *       *  *  *  *           
     214847 TTCCACCGTAGGCATCAAAGCGCTCCAAATGTCCACATCCAGATACTCCAGAACGAGTGTTTCAA
          1 TTCCAACGAAATCATCAAAGCGATCCAAATATCCACTTCCAGATTCTACAAAAAGAGTGTTTCAA

             *         *   * *   *         *        * *  *      *      *     
     214912 ACCTGCTCTATGAAAGGGAATCTTCAACTCTATGAGTTGAATGCAGACATCAGAAAGAAATTTCT
         66 AACTGCTCTATCAAAAGAAATGTTCAACTCTGTGAGTTGAGTACACACATCACAAAGAAGTTTCT

                     *       **          *         **
     214977 GAGAATGCTGCTGTCTACCTTTTAT-T--TGA-ATTCCCGC
        131 GAGAATGCTTCTGTCTAGTTTTTATATGAAGATATTCCCTT

                         *  *    *              * *  **   *    *             
     215014 TTCCAACGAAATCCTCCAAGCTATCCAAATATCCACCTGCATTTTCCACAACAAGAGTGTTTCAA
          1 TTCCAACGAAATCATCAAAGCGATCCAAATATCCACTTCCAGATTCTACAAAAAGAGTGTTTCAA

                          *                 *    *  *                *       
     215079 AACTGCTCTATCAATAGAAATGTTCAACTCCTTTG-GCTGGGTACACACATCACAAACAAGTTTC
         66 AACTGCTCTATCAAAAGAAATGTTCAACT-CTGTGAGTTGAGTACACACATCACAAAGAAGTTTC

                                      **     **       
     215143 TGAGAATGCTTCTGTCTAGTTTTTATGGGAAGACGTTCCCTT
        130 TGAGAATGCTTCTGTCTAGTTTTTATATGAAGATATTCCCTT

              *  * *  **    *     *       *     *       *  *  *              
     215185 TTTCACCAAAGGCATCCAAGCGCTCCAAATGTCCACATCCAGATACTCCAGAAAGAGTGTTTCAA
          1 TTCCAACGAAATCATCAAAGCGATCCAAATATCCACTTCCAGATTCTACAAAAAGAGTGTTTCAA

             *         *   * *   *         *        * *  *      *      *     
     215250 ACCTGCTCTATGAAAGGGAATCTTCAACTCTATGAGTTGAATGCAGACATCAGAAAGAAATTTCT
         66 AACTGCTCTATCAAAAGAAATGTTCAACTCTGTGAGTTGAGTACACACATCACAAAGAAGTTTCT

                     *       **          *         **
     215315 GAGAATGCTGCTGTCTACCTTTTAT-T--TGA-ATTCCCGC
        131 GAGAATGCTTCTGTCTAGTTTTTATATGAAGATATTCCCTT

                         *  *    *                *       *                  
     215352 TTCCAACGAAATCCTCCAAGCTATCCAAATATCCACTTGCAGATTCCACAAAAAGAGTGTTTCAA
          1 TTCCAACGAAATCATCAAAGCGATCCAAATATCCACTTCCAGATTCTACAAAAAGAGTGTTTCAA

                            ** *  *            *       *     *     *  *      
     215417 AACTGCTCTCTATCAATGGCAAAGTTCAACTCTGTTAGTTGAGGACACATATCACCAACAAGTTT
         66 AACTG--CTCTATCAAAAGAAATGTTCAACTCTGTGAGTTGAGTACACACATCACAAAGAAGTTT

                               *       **         *    
     215482 CTGAGAATGCTTCTGTCTATTTTTTATGGGAAGATATTTCCTT
        129 CTGAGAATGCTTCTGTCTAGTTTTTATATGAAGATATTCCCTT

              *  *  * ** *    *      *    *          * **                    
     215525 TTTCACCGTAGGCGTCAAGGCGATCGAAATGTCCACTTCCACAAACTACAAAAAGAGTGTTTCAA
          1 TTCCAACGAAATCATCAAAGCGATCCAAATATCCACTTCCAGATTCTACAAAAAGAGTGTTTCAA

             *         *   * **       *    *        * ** * *   **      *     
     215590 ACCTGCTCTATGAAAGGCCATGTTCATCTCTATGAGTTGAATGGAAATATCCGAAAGAAATTTCT
         66 AACTGCTCTATCAAAAGAAATGTTCAACTCTGTGAGTTGAGTACACACATCACAAAGAAGTTTCT

             *       *          *        *         **
     215655 GGGAATGCTGCTGTCTAG-TGTT-TAT-ACGA-ATTCCCGC
        131 GAGAATGCTTCTGTCTAGTTTTTATATGAAGATATTCCCTT

                         *       *                *    *  *                  
     215692 TTCCAACGAAATCCTCAAAGCAATCCAAATATCCACTTGCAGAATCCACAAAAAGAGTGTTTCAA
          1 TTCCAACGAAATCATCAAAGCGATCCAAATATCCACTTCCAGATTCTACAAAAAGAGTGTTTCAA

                          *     *          * *                   *  *        
     215757 AACTGCTCTATCAATAGAAAGGTTCAACTCTTTTAGTTGAGTACACACATCACGAACAAGTTTCT
         66 AACTGCTCTATCAAAAGAAATGTTCAACTCTGTGAGTTGAGTACACACATCACAAAGAAGTTTCT

                            * *      **     **  *    
     215822 GAGAATGCTTCTGTCTGGCTTTTATTGGAAGACGTTTCCTT
        131 GAGAATGCTTCTGTCTAGTTTTTATATGAAGATATTCCCTT

                   *  **          *       *                * *               
     215863 TT-CAACAAAGGCATCAAAGCGCTCCAAATGTCCACTTCCAGATTCTTCCAAAAGAGTGTTTCAA
          1 TTCCAACGAAATCATCAAAGCGATCCAAATATCCACTTCCAGATTCTACAAAAAGAGTGTTTCAA

             **     * ***  * *                 *    * *  * *     *   *       
     215927 ACGTGCTCAAAGTAAGGGAATGTTCAACTCTGTGACTTGAATGCAGATATCACCAAGTAGTTTCT
         66 AACTGCTCTATCAAAAGAAATGTTCAACTCTGTGAGTTGAGTACACACATCACAAAGAAGTTTCT

            * * *        *   **     *    *           
     215992 AATAGTGCTTCTGCCTACATTTTAGATGATGATATTCCCTT
        131 GAGAATGCTTCTGTCTAGTTTTTATATGAAGATATTCCCTT

                         * * *   *             *  *   *      *     *       * 
     216033 TTCCAACGAAATCGTTAGAGCTATCCAAATATCCAGTTACAGTTTCTACCAAAAGGGTGTTTCCA
          1 TTCCAACGAAATCATCAAAGCGATCCAAATATCCACTTCCAGATTCTACAAAAAGAGTGTTTCAA

              *    **           *            *       *                     * 
     216098 AATTGCTGCATCAAAAGAAAGGTTCAACTCTGTTAGTTGAGGACACACATCACAAAGAAGTTTGT
         66 AACTGCTCTATCAAAAGAAATGTTCAACTCTGTGAGTTGAGTACACACATCACAAAGAAGTTTCT

                              *    *     *           
     216163 GAGAATGCTTCTGTCTAGATTTTGTATGACGATATTCCCTT
        131 GAGAATGCTTCTGTCTAGTTTTTATATGAAGATATTCCCTT

                     *   * *     *   *       * *  *    *  *       *  *       
     216204 TTCCAACGATATCGTTAAAGCAATCTAAATATCAATTTGCAGAATCCACAAAAATAGAGTTTCAA
          1 TTCCAACGAAATCATCAAAGCGATCCAAATATCCACTTCCAGATTCTACAAAAAGAGTGTTTCAA

             *       * *        *    *       *  *                   ***      
     216269 AGCTGCTCTGTAAAAAGAAAGGTTCCACTCTGTTAGCTGAGTACACACATCACAAACTTGTTTCT
         66 AACTGCTCTATCAAAAGAAATGTTCAACTCTGTGAGTTGAGTACACACATCACAAAGAAGTTTCT

                  *         *        **         **   
     216334 GAGAATCCTTCTGTCTCGTTTTTATGGGAAGATATTTACTT
        131 GAGAATGCTTCTGTCTAGTTTTTATATGAAGATATTCCCTT

                 *  * **          *       *     *       *  *  *  *           
     216375 TTCCACCGTAGGCATCAAAGCGCTCCAAATGTCCACATCCAGATACTCCAGAACGAGTGTTTCAA
          1 TTCCAACGAAATCATCAAAGCGATCCAAATATCCACTTCCAGATTCTACAAAAAGAGTGTTTCAA

             *         *   * *   *         *        * *  *      *      *     
     216440 ACCTGCTCTATGAAAGGGAATCTTCAACTCTATGAGTTGAATGCAGACATCAGAAAGAAATTTCT
         66 AACTGCTCTATCAAAAGAAATGTTCAACTCTGTGAGTTGAGTACACACATCACAAAGAAGTTTCT

                     *       **          *         **
     216505 GAGAATGCTGCTGTCTACCTTTTAT-T--TGA-ATTCCCGC
        131 GAGAATGCTTCTGTCTAGTTTTTATATGAAGATATTCCCTT

                         *  *    *              * *  **   *                  
     216542 TTCCAACGAAATCCTCCAAGCTATCCAAATATCCACCTGCATTTTCCACAAAAAGAGTGTTTCAA
          1 TTCCAACGAAATCATCAAAGCGATCCAAATATCCACTTCCAGATTCTACAAAAAGAGTGTTTCAA

                          *                 *    *  *                *       
     216607 AACTGCTCTATCAATAGAAATGTTCAACTCCTTTG-GCTGGGTACACACATCACAAACAAGTTTC
         66 AACTGCTCTATCAAAAGAAATGTTCAACT-CTGTGAGTTGAGTACACACATCACAAAGAAGTTTC

                                      **     *        
     216671 TGAGAATGCTTCTGTCTAGTTTTTATGGGAAGACATTCCCTT
        130 TGAGAATGCTTCTGTCTAGTTTTTATATGAAGATATTCCCTT

              *  * *  **          *       *            **                    
     216713 TTTCACCAAAGACATCAAAGCGCTCCAAATGTCCACTTCCAGACACTACAAAAAGAGTGTTTCAA
          1 TTCCAACGAAATCATCAAAGCGATCCAAATATCCACTTCCAGATTCTACAAAAAGAGTGTTTCAA

             **       **   ***                 *    * *  * *         *       
     216778 ACGTGCTCTAAGAAAGCGAATGTTCAACTCTGTGACTTGAATGCAGATATCACAAAGTAGTTTCT
         66 AACTGCTCTATCAAAAGAAATGTTCAACTCTGTGAGTTGAGTACACACATCACAAAGAAGTTTCT

                **            *     *    *         * 
     216843 GAGAGGGCTTCTGTCTAGATTTTAGATGATGATATTCCCGT
        131 GAGAATGCTTCTGTCTAGTTTTTATATGAAGATATTCCCTT

                           * *   *                *   *                    * 
     216884 TTCCAACGAAATCATTAGAGCTATCCAAATATCCACTTACAGTTTCTACAAAAAGAGTGTTTCCA
          1 TTCCAACGAAATCATCAAAGCGATCCAAATATCCACTTCCAGATTCTACAAAAAGAGTGTTTCAA

                   **         * *    *       *  *                   ***      
     216949 AACTGCTGCATCAAAAGAGAGGTTCCACTCTGTTAGCTGAGTACACACATCACAAACTTGTTTCT
         66 AACTGCTCTATCAAAAGAAATGTTCAACTCTGTGAGTTGAGTACACACATCACAAAGAAGTTTCT

            *     *         *        **         **   
     217014 CAGAATCCTTCTGTCTCGTTTTTATGGGAAGATATTTACTT
        131 GAGAATGCTTCTGTCTAGTTTTTATATGAAGATATTCCCTT

              *  *  * **          *       *     *       *  *  *              
     217055 TTTCACCGTAGGCATCAAAGCGCTCCAAATGTCCACATCCAGATACTCCAGAAAGAGTGTTTCAA
          1 TTCCAACGAAATCATCAAAGCGATCCAAATATCCACTTCCAGATTCTACAAAAAGAGTGTTTCAA

             *         *   * *   *         *        * *  *      *      *     
     217120 ACCTGCTCTATGAAAGGGAATCTTCAACTCTATGAGTTGAATGCAGACATCAGAAAGAAATTTCT
         66 AACTGCTCTATCAAAAGAAATGTTCAACTCTGTGAGTTGAGTACACACATCACAAAGAAGTTTCT

                     *       **          *         **
     217185 GAGAATGCTGCTGTCTACCTTTTAT-T--TGA-ATTCCCGC
        131 GAGAATGCTTCTGTCTAGTTTTTATATGAAGATATTCCCTT

                         *  *    *                *       *                  
     217222 TTCCAACGAAATCCTCCAAGCTATCCAAATATCCACTTGCAGATTCCACAAAAAGAGTGTTTCAA
          1 TTCCAACGAAATCATCAAAGCGATCCAAATATCCACTTCCAGATTCTACAAAAAGAGTGTTTCAA

                            ** *  *            *       *     *     *  *      
     217287 AACTGCTCTCTATCAATGGCAAAGTTCAACTCTGTTAGTTGAGGACACATATCACCAACAAGTTT
         66 AACTG--CTCTATCAAAAGAAATGTTCAACTCTGTGAGTTGAGTACACACATCACAAAGAAGTTT

                               *       **         *    
     217352 CTGAGAATGCTTCTGTCTATTTTTTATGGGAAGATATTTCCTT
        129 CTGAGAATGCTTCTGTCTAGTTTTTATATGAAGATATTCCCTT

              *  *  * ** *    *      *    *          * **                    
     217395 TTTCACCGTAGGCGTCAAGGCGATCGAAATGTCCACTTCCACAAACTACAAAAAGAGTGTTTCAA
          1 TTCCAACGAAATCATCAAAGCGATCCAAATATCCACTTCCAGATTCTACAAAAAGAGTGTTTCAA

             *         *   * **       *    *        * ** * *   **      *     
     217460 ACCTGCTCTATGAAAGGCCATGTTCATCTCTATGAGTTGAATGGAAATATCCGAAAGAAATTTCT
         66 AACTGCTCTATCAAAAGAAATGTTCAACTCTGTGAGTTGAGTACACACATCACAAAGAAGTTTCT

             *       *          *        *         **
     217525 GGGAATGCTGCTGTCTAG-TGTT-TAT-ACGA-ATTCCCGC
        131 GAGAATGCTTCTGTCTAGTTTTTATATGAAGATATTCCCTT

                         *       *                *    *  *                  
     217562 TTCCAACGAAATCCTCAAAGCAATCCAAATATCCACTTGCAGAATCCACAAAAAGAGTGTTTCAA
          1 TTCCAACGAAATCATCAAAGCGATCCAAATATCCACTTCCAGATTCTACAAAAAGAGTGTTTCAA

                          *     *          * *                   *  *        
     217627 AACTGCTCTATCAATAGAAAGGTTCAACTCTTTTAGTTGAGTACACACATCACGAACAAGTTTCT
         66 AACTGCTCTATCAAAAGAAATGTTCAACTCTGTGAGTTGAGTACACACATCACAAAGAAGTTTCT

                            * *      **     **  *    
     217692 GAGAATGCTTCTGTCTGGCTTTTATTGGAAGACGTTTCCTT
        131 GAGAATGCTTCTGTCTAGTTTTTATATGAAGATATTCCCTT

                   *  **          *       *                * *               
     217733 TT-CAACAAAGGCATCAAAGCGCTCCAAATGTCCACTTCCAGATTCTTCCAAAAGAGTGTTTCAA
          1 TTCCAACGAAATCATCAAAGCGATCCAAATATCCACTTCCAGATTCTACAAAAAGAGTGTTTCAA

             **     * ***  * *                 *    * *  * *     *   *       
     217797 ACGTGCTCAAAGTAAGGGAATGTTCAACTCTGTGACTTGAATGCAGATATCACCAAGTAGTTTCT
         66 AACTGCTCTATCAAAAGAAATGTTCAACTCTGTGAGTTGAGTACACACATCACAAAGAAGTTTCT

            * * *        *   **     *    *           
     217862 AATAGTGCTTCTGCCTACATTTTAGATGATGATATTCCCTT
        131 GAGAATGCTTCTGTCTAGTTTTTATATGAAGATATTCCCTT

                         * * *   *             *  *   *      *     *       * 
     217903 TTCCAACGAAATCGTTAGAGCTATCCAAATATCCAGTTACAGTTTCTACCAAAAGGGTGTTTCCA
          1 TTCCAACGAAATCATCAAAGCGATCCAAATATCCACTTCCAGATTCTACAAAAAGAGTGTTTCAA

              *    **           *            *       *                     * 
     217968 AATTGCTGCATCAAAAGAAAGGTTCAACTCTGTTAGTTGAGGACACACATCACAAAGAAGTTTGT
         66 AACTGCTCTATCAAAAGAAATGTTCAACTCTGTGAGTTGAGTACACACATCACAAAGAAGTTTCT

                              *    *     *           
     218033 GAGAATGCTTCTGTCTAGATTTTGTATGACGATATTCCCTT
        131 GAGAATGCTTCTGTCTAGTTTTTATATGAAGATATTCCCTT

                     *   * *     *   *       * *  *    *  *       *  *       
     218074 TTCCAACGATATCGTTAAAGCAATCTAAATATCAATTTGCAGAATCCACAAAAATAGAGTTTCAA
          1 TTCCAACGAAATCATCAAAGCGATCCAAATATCCACTTCCAGATTCTACAAAAAGAGTGTTTCAA

             *       * *        *    *       *  *                   ***      
     218139 AGCTGCTCTGTAAAAAGAAAGGTTCCACTCTGTTAGCTGAGTACACACATCACAAACTTGTTTCT
         66 AACTGCTCTATCAAAAGAAATGTTCAACTCTGTGAGTTGAGTACACACATCACAAAGAAGTTTCT

                  *         *        **         **   
     218204 GAGAATCCTTCTGTCTCGTTTTTATGGGAAGATATTTACTT
        131 GAGAATGCTTCTGTCTAGTTTTTATATGAAGATATTCCCTT

                 *  * **          *       *     *       *  *  *  *           
     218245 TTCCACCGTAGGCATCAAAGCGCTCCAAATGTCCACATCCAGATACTCCAGAACGAGTGTTTCAA
          1 TTCCAACGAAATCATCAAAGCGATCCAAATATCCACTTCCAGATTCTACAAAAAGAGTGTTTCAA

             *         *   * *   *         *        * *  *      *      *     
     218310 ACCTGCTCTATGAAAGGGAATCTTCAACTCTATGAGTTGAATGCAGACATCAGAAAGAAATTTCT
         66 AACTGCTCTATCAAAAGAAATGTTCAACTCTGTGAGTTGAGTACACACATCACAAAGAAGTTTCT

                     *       **          *         **
     218375 GAGAATGCTGCTGTCTACCTTTTAT-T--TGA-ATTCCCGC
        131 GAGAATGCTTCTGTCTAGTTTTTATATGAAGATATTCCCTT

                         *  *    *              * *  **   *                  
     218412 TTCCAACGAAATCCTCCAAGCTATCCAAATATCCACCTGCATTTTCCACAAAAAGAGTGTTTCAA
          1 TTCCAACGAAATCATCAAAGCGATCCAAATATCCACTTCCAGATTCTACAAAAAGAGTGTTTCAA

                          *                 *    *  *                *       
     218477 AACTGCTCTATCAATAGAAATGTTCAACTCCTTTG-GCTGGGTACACACATCACAAACAAGTTTC
         66 AACTGCTCTATCAAAAGAAATGTTCAACT-CTGTGAGTTGAGTACACACATCACAAAGAAGTTTC

                                      **     *        
     218541 TGAGAATGCTTCTGTCTAGTTTTTATGGGAAGACATTCCCTT
        130 TGAGAATGCTTCTGTCTAGTTTTTATATGAAGATATTCCCTT

              *  * *  **          *       *            **                    
     218583 TTTCACCAAAGACATCAAAGCGCTCCAAATGTCCACTTCCAGACACTACAAAAAGAGTGTTTCAA
          1 TTCCAACGAAATCATCAAAGCGATCCAAATATCCACTTCCAGATTCTACAAAAAGAGTGTTTCAA

             **       **   ***                 *    * *  * *         *       
     218648 ACGTGCTCTAAGAAAGCGAATGTTCAACTCTGTGACTTGAATGCAGATATCACAAAGTAGTTTCT
         66 AACTGCTCTATCAAAAGAAATGTTCAACTCTGTGAGTTGAGTACACACATCACAAAGAAGTTTCT

                **            *     *    *         * 
     218713 GAGAGGGCTTCTGTCTAGATTTTAGATGATGATATTCCCGT
        131 GAGAATGCTTCTGTCTAGTTTTTATATGAAGATATTCCCTT

                           * *   *                *   *                    * 
     218754 TTCCAACGAAATCATTAGAGCTATCCAAATATCCACTTACAGTTTCTACAAAAAGAGTGTTTCCA
          1 TTCCAACGAAATCATCAAAGCGATCCAAATATCCACTTCCAGATTCTACAAAAAGAGTGTTTCAA

                   **         * *    *       *  *                   ***      
     218819 AACTGCTGCATCAAAAGAGAGGTTCCACTCTGTTAGCTGAGTACACACATCACAAACTTGTTTCT
         66 AACTGCTCTATCAAAAGAAATGTTCAACTCTGTGAGTTGAGTACACACATCACAAAGAAGTTTCT

            *     *         *        **         **   
     218884 CAGAATCCTTCTGTCTCGTTTTTATGGGAAGATATTTACTT
        131 GAGAATGCTTCTGTCTAGTTTTTATATGAAGATATTCCCTT

              *  *  * **          *       *     *       *  *  *              
     218925 TTTCACCGTAGGCATCAAAGCGCTCCAAATGTCCACATCCAGATACTCCAGAAAGAGTGTTTCAA
          1 TTCCAACGAAATCATCAAAGCGATCCAAATATCCACTTCCAGATTCTACAAAAAGAGTGTTTCAA

             *         *   * *   *         *        * *  *      *      *     
     218990 ACCTGCTCTATGAAAGGGAATCTTCAACTCTATGAGTTGAATGCAGACATCAGAAAGAAATTTCT
         66 AACTGCTCTATCAAAAGAAATGTTCAACTCTGTGAGTTGAGTACACACATCACAAAGAAGTTTCT

                     *       **          *         **
     219055 GAGAATGCTGCTGTCTACCTTTTAT-T--TGA-ATTCCCGC
        131 GAGAATGCTTCTGTCTAGTTTTTATATGAAGATATTCCCTT

                         *  *    *                *       *                  
     219092 TTCCAACGAAATCCTCCAAGCTATCCAAATATCCACTTGCAGATTCCACAAAAAGAGTGTTTCAA
          1 TTCCAACGAAATCATCAAAGCGATCCAAATATCCACTTCCAGATTCTACAAAAAGAGTGTTTCAA

                            ** *  *            *       *     *     *  *      
     219157 AACTGCTCTCTATCAATGGCAAAGTTCAACTCTGTTAGTTGAGGACACATATCACCAACAAGTTT
         66 AACTG--CTCTATCAAAAGAAATGTTCAACTCTGTGAGTTGAGTACACACATCACAAAGAAGTTT

                               *       **         *    
     219222 CTGAGAATGCTTCTGTCTATTTTTTATGGGAAGATATTTCCTT
        129 CTGAGAATGCTTCTGTCTAGTTTTTATATGAAGATATTCCCTT

              *  *  * ** *    *      *    *          * **                    
     219265 TTTCACCGTAGGCGTCAAGGCGATCGAAATGTCCACTTCCACAAACTACAAAAAGAGTGTTTCAA
          1 TTCCAACGAAATCATCAAAGCGATCCAAATATCCACTTCCAGATTCTACAAAAAGAGTGTTTCAA

             *         *   * **       *    *        * ** * *   **      *     
     219330 ACCTGCTCTATGAAAGGCCATGTTCATCTCTATGAGTTGAATGGAAATATCCGAAAGAAATTTCT
         66 AACTGCTCTATCAAAAGAAATGTTCAACTCTGTGAGTTGAGTACACACATCACAAAGAAGTTTCT

             *       *          *        *         **
     219395 GGGAATGCTGCTGTCTAG-TGTT-TAT-ACGA-ATTCCCGC
        131 GAGAATGCTTCTGTCTAGTTTTTATATGAAGATATTCCCTT

                    *    *       *                *    *  *                  
     219432 TTCCAACGTAATCCTCAAAGCAATCCAAATATCCACTTGCAGAATCCACAAAAAGAGTGTTTCAA
          1 TTCCAACGAAATCATCAAAGCGATCCAAATATCCACTTCCAGATTCTACAAAAAGAGTGTTTCAA

                          *     *          * *                   *  *        
     219497 AACTGCTCTATCAATAGAAAGGTTCAACTCTTTTAGTTGAGTACACACATCACGAACAAGTTTCT
         66 AACTGCTCTATCAAAAGAAATGTTCAACTCTGTGAGTTGAGTACACACATCACAAAGAAGTTTCT

                            * *      **     **  *    
     219562 GAGAATGCTTCTGTCTGGCTTTTATTGGAAGACGTTTCCTT
        131 GAGAATGCTTCTGTCTAGTTTTTATATGAAGATATTCCCTT

                 * *  **          *       *                * *               
     219603 TT-CACCAAAGGCATCAAAGCGCTCCAAATGTCCACTTCCAGATTCTTCCAAAAGAGTGTTTCAA
          1 TTCCAACGAAATCATCAAAGCGATCCAAATATCCACTTCCAGATTCTACAAAAAGAGTGTTTCAA

             **     * ***  * *                 *    * *  * *     *   *       
     219667 ACGTGCTCAAAGTAAGGGAATGTTCAACTCTGTGACTTGAATGCAGATATCACCAAGTAGTTTCT
         66 AACTGCTCTATCAAAAGAAATGTTCAACTCTGTGAGTTGAGTACACACATCACAAAGAAGTTTCT

            * * *            **     *    *         * 
     219732 AATAGTGCTTCTGTCTACATTTTAGATGATGATATTCCCGT
        131 GAGAATGCTTCTGTCTAGTTTTTATATGAAGATATTCCCTT

                         *** *   *             *  *   *      *     *       * 
     219773 TTCCAACGAAATCGCTAGAGCTATCCAAATATCCAGTTACAGTTTCTACCAAAAGGGTGTTTCCA
          1 TTCCAACGAAATCATCAAAGCGATCCAAATATCCACTTCCAGATTCTACAAAAAGAGTGTTTCAA

              *    **           *            *       *                     * 
     219838 AATTGCTGCATCAAAAGAAAGGTTCAACTCTGTTAGTTGAGGACACACATCACAAAGAAGTTTGT
         66 AACTGCTCTATCAAAAGAAATGTTCAACTCTGTGAGTTGAGTACACACATCACAAAGAAGTTTCT

                              *    *     *           
     219903 GAGAATGCTTCTGTCTAGATTTTGTATGACGATATTCCCTT
        131 GAGAATGCTTCTGTCTAGTTTTTATATGAAGATATTCCCTT

                     *   * *     *   *       * *  *    *  *       *  *       
     219944 TTCCAACGATATCGTTAAAGCAATCTAAATATCAATTTGCAGAATCCACAAAAATAGAGTTTCAA
          1 TTCCAACGAAATCATCAAAGCGATCCAAATATCCACTTCCAGATTCTACAAAAAGAGTGTTTCAA

             *       * *        *    *       *  *                   ***      
     220009 AGCTGCTCTGTAAAAAGAAAGGTTCCACTCTGTTAGCTGAGTACACACATCACAAACTTGTTTCT
         66 AACTGCTCTATCAAAAGAAATGTTCAACTCTGTGAGTTGAGTACACACATCACAAAGAAGTTTCT

            *     *         *        **       * **   
     220074 CAGAATCCTTCTGTCTCGTTTTTATGGGAAGATACTTACTT
        131 GAGAATGCTTCTGTCTAGTTTTTATATGAAGATATTCCCTT

                 *  * **          *       *     *       *  *  *  *           
     220115 TTCCACCGTAGGCATCAAAGCGCTCCAAATGTCCACATCCAGATACTCCAGAACGAGTGTTTCAA
          1 TTCCAACGAAATCATCAAAGCGATCCAAATATCCACTTCCAGATTCTACAAAAAGAGTGTTTCAA

             *         *   * *   *         *        * *  *      *      *     
     220180 ACCTGCTCTATGAAAGGGAATCTTCAACTCTATGAGTTGAATGCAGACATCAGAAAGAAATTTCT
         66 AACTGCTCTATCAAAAGAAATGTTCAACTCTGTGAGTTGAGTACACACATCACAAAGAAGTTTCT

                     *       **          *         **
     220245 GAGAATGCTGCTGTCTACCTTTTAT-T--TGA-ATTCCCGC
        131 GAGAATGCTTCTGTCTAGTTTTTATATGAAGATATTCCCTT

                         *  *    *              * *  **   *    *             
     220282 TTCCAACGAAATCCTCCAAGCTATCCAAATATCCACCTGCATTTTCCACAACAAGAGTGTTTCAA
          1 TTCCAACGAAATCATCAAAGCGATCCAAATATCCACTTCCAGATTCTACAAAAAGAGTGTTTCAA

                          *                 *    *  *                *       
     220347 AACTGCTCTATCAATAGAAATGTTCAACTCCTTTG-GCTGGGTACACACATCACAAACAAGTTTC
         66 AACTGCTCTATCAAAAGAAATGTTCAACT-CTGTGAGTTGAGTACACACATCACAAAGAAGTTTC

                                      **     **       
     220411 TGAGAATGCTTCTGTCTAGTTTTTATGGGAAGACGTTCCCTT
        130 TGAGAATGCTTCTGTCTAGTTTTTATATGAAGATATTCCCTT

              *  * *  **    *     *       *     *       *  *  *              
     220453 TTTCACCAAAGGCATCCAAGCGCTCCAAATGTCCACATCCAGATACTCCAGAAAGAGTGTTTCAA
          1 TTCCAACGAAATCATCAAAGCGATCCAAATATCCACTTCCAGATTCTACAAAAAGAGTGTTTCAA

             *         *   * *   *         *        * *  *      *      *     
     220518 ACCTGCTCTATGAAAGGGAATCTTCAACTCTATGAGTTGAATGCAGACATCAGAAAGAAATTTCT
         66 AACTGCTCTATCAAAAGAAATGTTCAACTCTGTGAGTTGAGTACACACATCACAAAGAAGTTTCT

                     *       **          *         **
     220583 GAGAATGCTGCTGTCTACCTTTTAT-T--TGA-ATTCCCGC
        131 GAGAATGCTTCTGTCTAGTTTTTATATGAAGATATTCCCTT

                         *  *    *                *       *                  
     220620 TTCCAACGAAATCCTCCAAGCTATCCAAATATCCACTTGCAGATTCCACAAAAAGAGTGTTTCAA
          1 TTCCAACGAAATCATCAAAGCGATCCAAATATCCACTTCCAGATTCTACAAAAAGAGTGTTTCAA

                            ** *  *            *       *     *     *  *      
     220685 AACTGCTCTCTATCAATGGCAAAGTTCAACTCTGTTAGTTGAGGACACATATCACCAACAAGTTT
         66 AACTG--CTCTATCAAAAGAAATGTTCAACTCTGTGAGTTGAGTACACACATCACAAAGAAGTTT

                               *       **         *    
     220750 CTGAGAATGCTTCTGTCTATTTTTTATGGGAAGATATTTCCTT
        129 CTGAGAATGCTTCTGTCTAGTTTTTATATGAAGATATTCCCTT

              *  *  * ** *    *      *    *          * **                    
     220793 TTTCACCGTAGGCGTCAAGGCGATCGAAATGTCCACTTCCACAAACTACAAAAAGAGTGTTTCAA
          1 TTCCAACGAAATCATCAAAGCGATCCAAATATCCACTTCCAGATTCTACAAAAAGAGTGTTTCAA

             *         *   * **       *    *        * ** * *   **      *     
     220858 ACCTGCTCTATGAAAGGCCATGTTCATCTCTATGAGTTGAATGGAAATATCCGAAAGAAATTTCT
         66 AACTGCTCTATCAAAAGAAATGTTCAACTCTGTGAGTTGAGTACACACATCACAAAGAAGTTTCT

             *       *          *        *         **
     220923 GGGAATGCTGCTGTCTAG-TGTT-TAT-ACGA-ATTCCCGC
        131 GAGAATGCTTCTGTCTAGTTTTTATATGAAGATATTCCCTT

                         *       *                *    *  *                  
     220960 TTCCAACGAAATCCTCAAAGCAATCCAAATATCCACTTGCAGAATCCACAAAAAGAGTGTTTCAA
          1 TTCCAACGAAATCATCAAAGCGATCCAAATATCCACTTCCAGATTCTACAAAAAGAGTGTTTCAA

                          *     *          * *                   *  *        
     221025 AACTGCTCTATCAATAGAAAGGTTCAACTCTTTTAGTTGAGTACACACATCACGAACAAGTTTCT
         66 AACTGCTCTATCAAAAGAAATGTTCAACTCTGTGAGTTGAGTACACACATCACAAAGAAGTTTCT

                            * *      **     **  *    
     221090 GAGAATGCTTCTGTCTGGCTTTTATTGGAAGACGTTTCCTT
        131 GAGAATGCTTCTGTCTAGTTTTTATATGAAGATATTCCCTT

                   *  **          *       *                * *               
     221131 TT-CAACAAAGGCATCAAAGCGCTCCAAATGTCCACTTCCAGATTCTTCCAAAAGAGTGTTTCAA
          1 TTCCAACGAAATCATCAAAGCGATCCAAATATCCACTTCCAGATTCTACAAAAAGAGTGTTTCAA

             **     * ***  * *                 *    * *  * *     *   *       
     221195 ACGTGCTCAAAGTAAGGGAATGTTCAACTCTGTGACTTGAATGCAGATATCACCAAGTAGTTTCT
         66 AACTGCTCTATCAAAAGAAATGTTCAACTCTGTGAGTTGAGTACACACATCACAAAGAAGTTTCT

            * * *        *   **     *    *           
     221260 AATAGTGCTTCTGCCTACATTTTAGATGATGATATTCCCTT
        131 GAGAATGCTTCTGTCTAGTTTTTATATGAAGATATTCCCTT

                         * * *   *             *  *   *      *     *       * 
     221301 TTCCAACGAAATCGTTAGAGCTATCCAAATATCCAGTTACAGTTTCTACCAAAAGGGTGTTTCCA
          1 TTCCAACGAAATCATCAAAGCGATCCAAATATCCACTTCCAGATTCTACAAAAAGAGTGTTTCAA

              *    **           *            *       *                     * 
     221366 AATTGCTGCATCAAAAGAAAGGTTCAACTCTGTTAGTTGAGGACACACATCACAAAGAAGTTTGT
         66 AACTGCTCTATCAAAAGAAATGTTCAACTCTGTGAGTTGAGTACACACATCACAAAGAAGTTTCT

                              *    *     *           
     221431 GAGAATGCTTCTGTCTAGATTTTGTATGACGATATTCCCTT
        131 GAGAATGCTTCTGTCTAGTTTTTATATGAAGATATTCCCTT

                     *   * *     *   *       * *  *    *  *       *  *       
     221472 TTCCAACGATATCGTTAAAGCAATCTAAATATCAATTTGCAGAATCCACAAAAATAGAGTTTCAA
          1 TTCCAACGAAATCATCAAAGCGATCCAAATATCCACTTCCAGATTCTACAAAAAGAGTGTTTCAA

             *       * *        *    *       *  *                   ***      
     221537 AGCTGCTCTGTAAAAAGAAAGGTTCCACTCTGTTAGCTGAGTACACACATCACAAACTTGTTTCT
         66 AACTGCTCTATCAAAAGAAATGTTCAACTCTGTGAGTTGAGTACACACATCACAAAGAAGTTTCT

                  *         *        **         **   
     221602 GAGAATCCTTCTGTCTCGTTTTTATGGGAAGATATTTACTT
        131 GAGAATGCTTCTGTCTAGTTTTTATATGAAGATATTCCCTT

                 *  * **          *       *     *       *  *  *  *           
     221643 TTCCACCGTAGGCATCAAAGCGCTCCAAATGTCCACATCCAGATACTCCAGAACGAGTGTTTCAA
          1 TTCCAACGAAATCATCAAAGCGATCCAAATATCCACTTCCAGATTCTACAAAAAGAGTGTTTCAA

             *         *   * *   *         *        * *  *      *      *     
     221708 ACCTGCTCTATGAAAGGGAATCTTCAACTCTATGAGTTGAATGCAGACATCAGAAAGAAATTTCT
         66 AACTGCTCTATCAAAAGAAATGTTCAACTCTGTGAGTTGAGTACACACATCACAAAGAAGTTTCT

                     *       **          *         **
     221773 GAGAATGCTGCTGTCTACCTTTTAT-T--TGA-ATTCCCGC
        131 GAGAATGCTTCTGTCTAGTTTTTATATGAAGATATTCCCTT

                         *  *    *              * *  **   *                  
     221810 TTCCAACGAAATCCTCCAAGCTATCCAAATATCCACCTGCATTTTCCACAAAAAGAGTGTTTCAA
          1 TTCCAACGAAATCATCAAAGCGATCCAAATATCCACTTCCAGATTCTACAAAAAGAGTGTTTCAA

                          *                 *    *  *                *       
     221875 AACTGCTCTATCAATAGAAATGTTCAACTCCTTTG-GCTGGGTACACACATCACAAACAAGTTTC
         66 AACTGCTCTATCAAAAGAAATGTTCAACT-CTGTGAGTTGAGTACACACATCACAAAGAAGTTTC

                                      **     *        
     221939 TGAGAATGCTTCTGTCTAGTTTTTATGGGAAGACATTCCCTT
        130 TGAGAATGCTTCTGTCTAGTTTTTATATGAAGATATTCCCTT

              *  * *  **          *       *            **                    
     221981 TTTCACCAAAGACATCAAAGCGCTCCAAATGTCCACTTCCAGACACTACAAAAAGAGTGTTTCAA
          1 TTCCAACGAAATCATCAAAGCGATCCAAATATCCACTTCCAGATTCTACAAAAAGAGTGTTTCAA

             **       **   ***                 *    * *  * *         *       
     222046 ACGTGCTCTAAGAAAGCGAATGTTCAACTCTGTGACTTGAATGCAGATATCACAAAGTAGTTTCT
         66 AACTGCTCTATCAAAAGAAATGTTCAACTCTGTGAGTTGAGTACACACATCACAAAGAAGTTTCT

                **            *     *    *         * 
     222111 GAGAGGGCTTCTGTCTAGATTTTAGATGATGATATTCCCGT
        131 GAGAATGCTTCTGTCTAGTTTTTATATGAAGATATTCCCTT

                           * *   *                *   *                    * 
     222152 TTCCAACGAAATCATTAGAGCTATCCAAATATCCACTTACAGTTTCTACAAAAAGAGTGTTTCCA
          1 TTCCAACGAAATCATCAAAGCGATCCAAATATCCACTTCCAGATTCTACAAAAAGAGTGTTTCAA

                   **         * *    *       *  **                  ***      
     222217 AACTGCTGCATCAAAAGAGAGGTTCCACTCTGTTAGCCGAGTACACACATCACAAACTTGTTTCT
         66 AACTGCTCTATCAAAAGAAATGTTCAACTCTGTGAGTTGAGTACACACATCACAAAGAAGTTTCT

            *     *         *        **         **   
     222282 CAGAATCCTTCTGTCTCGTTTTTATGGGAAGATATTTACTT
        131 GAGAATGCTTCTGTCTAGTTTTTATATGAAGATATTCCCTT

              *  *  * **          *       *     *       *  *  *              
     222323 TTTCACCGTAGGCATCAAAGCGCTCCAAATGTCCACATCCAGATACTCCAGAAAGAGTGTTTCAA
          1 TTCCAACGAAATCATCAAAGCGATCCAAATATCCACTTCCAGATTCTACAAAAAGAGTGTTTCAA

             *         *   * *   *         *        * *  *      *      *     
     222388 ACCTGCTCTATGAAAGGGAATCTTCAACTCTATGAGTTGAATGCAGACATCAGAAAGAAATTTCT
         66 AACTGCTCTATCAAAAGAAATGTTCAACTCTGTGAGTTGAGTACACACATCACAAAGAAGTTTCT

                     *       **          *         **
     222453 GAGAATGCTGCTGTCTACCTTTTAT-T--TGA-ATTCCCGC
        131 GAGAATGCTTCTGTCTAGTTTTTATATGAAGATATTCCCTT

                         *  *    *                *       *                  
     222490 TTCCAACGAAATCCTCCAAGCTATCCAAATATCCACTTGCAGATTCCACAAAAAGAGTGTTTCAA
          1 TTCCAACGAAATCATCAAAGCGATCCAAATATCCACTTCCAGATTCTACAAAAAGAGTGTTTCAA

                            ** *  *            *       *     *     *  *      
     222555 AACTGCTCTCTATCAATGGCAAAGTTCAACTCTGTTAGTTGAGGACACATATCACCAACAAGTTT
         66 AACTG--CTCTATCAAAAGAAATGTTCAACTCTGTGAGTTGAGTACACACATCACAAAGAAGTTT

                               *       **         *    
     222620 CTGAGAATGCTTCTGTCTATTTTTTATGGGAAGATATTTCCTT
        129 CTGAGAATGCTTCTGTCTAGTTTTTATATGAAGATATTCCCTT

              *  *  * ** *    *      *    *          * **                    
     222663 TTTCACCGTAGGCGTCAAGGCGATCGAAATGTCCACTTCCACAAACTACAAAAAGAGTGTTTCAA
          1 TTCCAACGAAATCATCAAAGCGATCCAAATATCCACTTCCAGATTCTACAAAAAGAGTGTTTCAA

             *         *   * **       *    *        * ** * *   **      *     
     222728 ACCTGCTCTATGAAAGGCCATGTTCATCTCTATGAGTTGAATGGAAATATCCGAAAGAAATTTCT
         66 AACTGCTCTATCAAAAGAAATGTTCAACTCTGTGAGTTGAGTACACACATCACAAAGAAGTTTCT

             *       *          *        *         **
     222793 GGGAATGCTGCTGTCTAG-TGTT-TAT-ACGA-ATTCCCGC
        131 GAGAATGCTTCTGTCTAGTTTTTATATGAAGATATTCCCTT

                    *    *       *                *    *  *                  
     222830 TTCCAACGTAATCCTCAAAGCAATCCAAATATCCACTTGCAGAATCCACAAAAAGAGTGTTTCAA
          1 TTCCAACGAAATCATCAAAGCGATCCAAATATCCACTTCCAGATTCTACAAAAAGAGTGTTTCAA

                          *     *          * *                   *  *        
     222895 AACTGCTCTATCAATAGAAAGGTTCAACTCTTTTAGTTGAGTACACACATCACGAACAAGTTTCT
         66 AACTGCTCTATCAAAAGAAATGTTCAACTCTGTGAGTTGAGTACACACATCACAAAGAAGTTTCT

                            * *      **     **  *    
     222960 GAGAATGCTTCTGTCTGGCTTTTATTGGAAGACGTTTCCTT
        131 GAGAATGCTTCTGTCTAGTTTTTATATGAAGATATTCCCTT

                 * *  **          *       *                * *               
     223001 TT-CACCAAAGGCATCAAAGCGCTCCAAATGTCCACTTCCAGATTCTTCCAAAAGAGTGTTTCAA
          1 TTCCAACGAAATCATCAAAGCGATCCAAATATCCACTTCCAGATTCTACAAAAAGAGTGTTTCAA

             **     * ***  * *                 *    * *  * *     *   *       
     223065 ACGTGCTCAAAGTAAGGGAATGTTCAACTCTGTGACTTGAATGCAGATATCACCAAGTAGTTTCT
         66 AACTGCTCTATCAAAAGAAATGTTCAACTCTGTGAGTTGAGTACACACATCACAAAGAAGTTTCT

            * * *            **     *    *         * 
     223130 AATAGTGCTTCTGTCTACATTTTAGATGATGATATTCCCGT
        131 GAGAATGCTTCTGTCTAGTTTTTATATGAAGATATTCCCTT

                         *** *   *             *  *   *      *     *       * 
     223171 TTCCAACGAAATCGCTAGAGCTATCCAAATATCCAGTTACAGTTTCTACCAAAAGGGTGTTTCCA
          1 TTCCAACGAAATCATCAAAGCGATCCAAATATCCACTTCCAGATTCTACAAAAAGAGTGTTTCAA

              *    **           *            *       *                     * 
     223236 AATTGCTGCATCAAAAGAAAGGTTCAACTCTGTTAGTTGAGGACACACATCACAAAGAAGTTTGT
         66 AACTGCTCTATCAAAAGAAATGTTCAACTCTGTGAGTTGAGTACACACATCACAAAGAAGTTTCT

                              *    *     *           
     223301 GAGAATGCTTCTGTCTAGATTTTGTATGACGATATTCCCTT
        131 GAGAATGCTTCTGTCTAGTTTTTATATGAAGATATTCCCTT

                     *   * *     *   *       * *  *    *  *       *  *       
     223342 TTCCAACGATATCGTTAAAGCAATCTAAATATCAATTTGCAGAATCCACAAAAATAGAGTTTCAA
          1 TTCCAACGAAATCATCAAAGCGATCCAAATATCCACTTCCAGATTCTACAAAAAGAGTGTTTCAA

             *       * *        *    *       *  *                   ***      
     223407 AGCTGCTCTGTAAAAAGAAAGGTTCCACTCTGTTAGCTGAGTACACACATCACAAACTTGTTTCT
         66 AACTGCTCTATCAAAAGAAATGTTCAACTCTGTGAGTTGAGTACACACATCACAAAGAAGTTTCT

            *     *         *        **       * **   
     223472 CAGAATCCTTCTGTCTCGTTTTTATGGGAAGATACTTACTT
        131 GAGAATGCTTCTGTCTAGTTTTTATATGAAGATATTCCCTT

                 *  * **          *       *     *       *  *  *  *           
     223513 TTCCACCGTAGGCATCAAAGCGCTCCAAATGTCCACATCCAGATACTCCAGAACGAGTGTTTCAA
          1 TTCCAACGAAATCATCAAAGCGATCCAAATATCCACTTCCAGATTCTACAAAAAGAGTGTTTCAA

             *         *   * *   *         *        * *  *      *      *     
     223578 ACCTGCTCTATGAAAGGGAATCTTCAACTCTATGAGTTGAATGCAGACATCAGAAAGAAATTTCT
         66 AACTGCTCTATCAAAAGAAATGTTCAACTCTGTGAGTTGAGTACACACATCACAAAGAAGTTTCT

                     *       **          *         **
     223643 GAGAATGCTGCTGTCTACCTTTTAT-T--TGA-ATTCCCGC
        131 GAGAATGCTTCTGTCTAGTTTTTATATGAAGATATTCCCTT

                         *  *    *              * *  **   *    *             
     223680 TTCCAACGAAATCCTCCAAGCTATCCAAATATCCACCTGCATTTTCCACAACAAGAGTGTTTCAA
          1 TTCCAACGAAATCATCAAAGCGATCCAAATATCCACTTCCAGATTCTACAAAAAGAGTGTTTCAA

                          *                 *    *  *                *       
     223745 AACTGCTCTATCAATAGAAATGTTCAACTCCTTTG-GCTGGGTACACACATCACAAACAAGTTTC
         66 AACTGCTCTATCAAAAGAAATGTTCAACT-CTGTGAGTTGAGTACACACATCACAAAGAAGTTTC

                                      **     **       
     223809 TGAGAATGCTTCTGTCTAGTTTTTATGGGAAGACGTTCCCTT
        130 TGAGAATGCTTCTGTCTAGTTTTTATATGAAGATATTCCCTT

              *  * *  **    *     *       *     *       *  *  *              
     223851 TTTCACCAAAGGCATCCAAGCGCTCCAAATGTCCACATCCAGATACTCCAGAAAGAGTGTTTCAA
          1 TTCCAACGAAATCATCAAAGCGATCCAAATATCCACTTCCAGATTCTACAAAAAGAGTGTTTCAA

             *         *   * *   *         *        * *  *      *      *     
     223916 ACCTGCTCTATGAAAGGGAATCTTCAACTCTATGAGTTGAATGCAGACATCAGAAAGAAATTTCT
         66 AACTGCTCTATCAAAAGAAATGTTCAACTCTGTGAGTTGAGTACACACATCACAAAGAAGTTTCT

                     *       **          *         **
     223981 GAGAATGCTGCTGTCTACCTTTTAT-T--TGA-ATTCCCGC
        131 GAGAATGCTTCTGTCTAGTTTTTATATGAAGATATTCCCTT

                         *  *    *                *       *                  
     224018 TTCCAACGAAATCCTCCAAGCTATCCAAATATCCACTTGCAGATTCCACAAAAAGAGTGTTTCAA
          1 TTCCAACGAAATCATCAAAGCGATCCAAATATCCACTTCCAGATTCTACAAAAAGAGTGTTTCAA

                            ** *  *            *       *     *     *  *      
     224083 AACTGCTCTCTATCAATGGCAAAGTTCAACTCTGTTAGTTGAGGACACATATCACCAACAAGTTT
         66 AACTG--CTCTATCAAAAGAAATGTTCAACTCTGTGAGTTGAGTACACACATCACAAAGAAGTTT

                               *       **         *    
     224148 CTGAGAATGCTTCTGTCTATTTTTTATGGGAAGATATTTCCTT
        129 CTGAGAATGCTTCTGTCTAGTTTTTATATGAAGATATTCCCTT

              *  *  * ** *    *      *    *          * **                    
     224191 TTTCACCGTAGGCGTCAAGGCGATCGAAATGTCCACTTCCACAAACTACAAAAAGAGTGTTTCAA
          1 TTCCAACGAAATCATCAAAGCGATCCAAATATCCACTTCCAGATTCTACAAAAAGAGTGTTTCAA

             *         *   * **       *    *        * ** * *   **      *     
     224256 ACCTGCTCTATGAAAGGCCATGTTCATCTCTATGAGTTGAATGGAAATATCCGAAAGAAATTTCT
         66 AACTGCTCTATCAAAAGAAATGTTCAACTCTGTGAGTTGAGTACACACATCACAAAGAAGTTTCT

             *       *          *        *         **
     224321 GGGAATGCTGCTGTCTAG-TGTT-TAT-ACGA-ATTCCCGC
        131 GAGAATGCTTCTGTCTAGTTTTTATATGAAGATATTCCCTT

                         *       *                *    *  *                  
     224358 TTCCAACGAAATCCTCAAAGCAATCCAAATATCCACTTGCAGAATCCACAAAAAGAGTGTTTCAA
          1 TTCCAACGAAATCATCAAAGCGATCCAAATATCCACTTCCAGATTCTACAAAAAGAGTGTTTCAA

                          *     *          * *                   *  *        
     224423 AACTGCTCTATCAATAGAAAGGTTCAACTCTTTTAGTTGAGTACACACATCACGAACAAGTTTCT
         66 AACTGCTCTATCAAAAGAAATGTTCAACTCTGTGAGTTGAGTACACACATCACAAAGAAGTTTCT

                            * *      **     **  *    
     224488 GAGAATGCTTCTGTCTGGCTTTTATTGGAAGACGTTTCCTT
        131 GAGAATGCTTCTGTCTAGTTTTTATATGAAGATATTCCCTT

                   *  **          *       *                * *               
     224529 TT-CAACAAAGGCATCAAAGCGCTCCAAATGTCCACTTCCAGATTCTTCCAAAAGAGTGTTTCAA
          1 TTCCAACGAAATCATCAAAGCGATCCAAATATCCACTTCCAGATTCTACAAAAAGAGTGTTTCAA

             **     * ***  * *                 *    * *  * *     *   *       
     224593 ACGTGCTCAAAGTAAGGGAATGTTCAACTCTGTGACTTGAATGCAGATATCACCAAGTAGTTTCT
         66 AACTGCTCTATCAAAAGAAATGTTCAACTCTGTGAGTTGAGTACACACATCACAAAGAAGTTTCT

            * * *        *   **     *    *           
     224658 AATAGTGCTTCTGCCTACATTTTAGATGATGATATTCCCTT
        131 GAGAATGCTTCTGTCTAGTTTTTATATGAAGATATTCCCTT

                         * * *   *             *  *   *      *     *       * 
     224699 TTCCAACGAAATCGTTAGAGCTATCCAAATATCCAGTTACAGTTTCTACCAAAAGGGTGTTTCCA
          1 TTCCAACGAAATCATCAAAGCGATCCAAATATCCACTTCCAGATTCTACAAAAAGAGTGTTTCAA

              *    **           *            *       *                     * 
     224764 AATTGCTGCATCAAAAGAAAGGTTCAACTCTGTTAGTTGAGGACACACATCACAAAGAAGTTTGT
         66 AACTGCTCTATCAAAAGAAATGTTCAACTCTGTGAGTTGAGTACACACATCACAAAGAAGTTTCT

                              *    *     *           
     224829 GAGAATGCTTCTGTCTAGATTTTGTATGACGATATTCCCTT
        131 GAGAATGCTTCTGTCTAGTTTTTATATGAAGATATTCCCTT

                     *   * *     *   *       * *  *    *  *       *  *       
     224870 TTCCAACGATATCGTTAAAGCAATCTAAATATCAATTTGCAGAATCCACAAAAATAGAGTTTCAA
          1 TTCCAACGAAATCATCAAAGCGATCCAAATATCCACTTCCAGATTCTACAAAAAGAGTGTTTCAA

             *       * *        *    *       *  *                   ***      
     224935 AGCTGCTCTGTAAAAAGAAAGGTTCCACTCTGTTAGCTGAGTACACACATCACAAACTTGTTTCT
         66 AACTGCTCTATCAAAAGAAATGTTCAACTCTGTGAGTTGAGTACACACATCACAAAGAAGTTTCT

                  *         *        **     *   **   
     225000 GAGAATCCTTCTGTCTCGTTTTTATGGGAAGACATTTACTT
        131 GAGAATGCTTCTGTCTAGTTTTTATATGAAGATATTCCCTT

                 *  * **          *       *     *       *  *  *  *           
     225041 TTCCACCGTAGGCATCAAAGCGCTCCAAATGTCCACATCCAGATACTCCAGAACGAGTGTTTCAA
          1 TTCCAACGAAATCATCAAAGCGATCCAAATATCCACTTCCAGATTCTACAAAAAGAGTGTTTCAA

             *         *   * *   *         *        * *  *      *      *     
     225106 ACCTGCTCTATGAAAGGGAATCTTCAACTCTATGAGTTGAATGCAGACATCAGAAAGAAATTTCT
         66 AACTGCTCTATCAAAAGAAATGTTCAACTCTGTGAGTTGAGTACACACATCACAAAGAAGTTTCT

                     *       **          *         **
     225171 GAGAATGCTGCTGTCTACCTTTTAT-T--TGA-ATTCCCGC
        131 GAGAATGCTTCTGTCTAGTTTTTATATGAAGATATTCCCTT

                         *  *    *              * *  **   *                  
     225208 TTCCAACGAAATCCTCCAAGCTATCCAAATATCCACCTGCATTTTCCACAAAAAGAGTGTTTCAA
          1 TTCCAACGAAATCATCAAAGCGATCCAAATATCCACTTCCAGATTCTACAAAAAGAGTGTTTCAA

                          *                 *    *  *                *       
     225273 AACTGCTCTATCAATAGAAATGTTCAACTCCTTTG-GCTGGGTACACACATCACAAACAAGTTTC
         66 AACTGCTCTATCAAAAGAAATGTTCAACT-CTGTGAGTTGAGTACACACATCACAAAGAAGTTTC

                                      **     *        
     225337 TGAGAATGCTTCTGTCTAGTTTTTATGGGAAGACATTCCCTT
        130 TGAGAATGCTTCTGTCTAGTTTTTATATGAAGATATTCCCTT

              *  * *  **          *       *            **                    
     225379 TTTCACCAAAGACATCAAAGCGCTCCAAATGTCCACTTCCAGACACTACAAAAAGAGTGTTTCAA
          1 TTCCAACGAAATCATCAAAGCGATCCAAATATCCACTTCCAGATTCTACAAAAAGAGTGTTTCAA

             **       **   ***                 *    * *  * *         *       
     225444 ACGTGCTCTAAGAAAGCGAATGTTCAACTCTGTGACTTGAATGCAGATATCACAAAGTAGTTTCT
         66 AACTGCTCTATCAAAAGAAATGTTCAACTCTGTGAGTTGAGTACACACATCACAAAGAAGTTTCT

                **            *     *    *         * 
     225509 GAGAGGGCTTCTGTCTAGATTTTAGATGATGATATTCCCGT
        131 GAGAATGCTTCTGTCTAGTTTTTATATGAAGATATTCCCTT

                           * *   *                *   *                    * 
     225550 TTCCAACGAAATCATTAGAGCTATCCAAATATCCACTTACAGTTTCTACAAAAAGAGTGTTTCCA
          1 TTCCAACGAAATCATCAAAGCGATCCAAATATCCACTTCCAGATTCTACAAAAAGAGTGTTTCAA

                   **         * *    *       *  **                  ***      
     225615 AACTGCTGCATCAAAAGAGAGGTTCCACTCTGTTAGCCGAGTACACACATCACAAACTTGTTTCT
         66 AACTGCTCTATCAAAAGAAATGTTCAACTCTGTGAGTTGAGTACACACATCACAAAGAAGTTTCT

            *     *         *        **         **   
     225680 CAGAATCCTTCTGTCTCGTTTTTATGGGAAGATATTTACTT
        131 GAGAATGCTTCTGTCTAGTTTTTATATGAAGATATTCCCTT

              *  *  * **          *       *     *       *  *  *              
     225721 TTTCACCGTAGGCATCAAAGCGCTCCAAATGTCCACATCCAGATACTCCAGAAAGAGTGTTTCAA
          1 TTCCAACGAAATCATCAAAGCGATCCAAATATCCACTTCCAGATTCTACAAAAAGAGTGTTTCAA

             *         *   * *   *         *        * *  *      *      *     
     225786 ACCTGCTCTATGAAAGGGAATCTTCAACTCTATGAGTTGAATGCAGACATCAGAAAGAAATTTCT
         66 AACTGCTCTATCAAAAGAAATGTTCAACTCTGTGAGTTGAGTACACACATCACAAAGAAGTTTCT

                     *       **          *         **
     225851 GAGAATGCTGCTGTCTACCTTTTAT-T--TGA-ATTCCCGC
        131 GAGAATGCTTCTGTCTAGTTTTTATATGAAGATATTCCCTT

                         *  *    *                *       *                  
     225888 TTCCAACGAAATCCTCCAAGCTATCCAAATATCCACTTGCAGATTCCACAAAAAGAGTGTTTCAA
          1 TTCCAACGAAATCATCAAAGCGATCCAAATATCCACTTCCAGATTCTACAAAAAGAGTGTTTCAA

                            ** *  *            *       *     *     *  *      
     225953 AACTGCTCTCTATCAATGGCAAAGTTCAACTCTGTTAGTTGAGGACACATATCACCAACAAGTTT
         66 AACTG--CTCTATCAAAAGAAATGTTCAACTCTGTGAGTTGAGTACACACATCACAAAGAAGTTT

                               *       **         *    
     226018 CTGAGAATGCTTCTGTCTATTTTTTATGGGAAGATATTTCCTT
        129 CTGAGAATGCTTCTGTCTAGTTTTTATATGAAGATATTCCCTT

              *  *  * ** *    *      *    *          * **                    
     226061 TTTCACCGTAGGCGTCAAGGCGATCGAAATGTCCACTTCCACAAACTACAAAAAGAGTGTTTCAA
          1 TTCCAACGAAATCATCAAAGCGATCCAAATATCCACTTCCAGATTCTACAAAAAGAGTGTTTCAA

             *         *   * **       *    *        * ** * *   **      *     
     226126 ACCTGCTCTATGAAAGGCCATGTTCATCTCTATGAGTTGAATGGAAATATCCGAAAGAAATTTCT
         66 AACTGCTCTATCAAAAGAAATGTTCAACTCTGTGAGTTGAGTACACACATCACAAAGAAGTTTCT

             *       *          *        *         **
     226191 GGGAATGCTGCTGTCTAG-TGTT-TAT-ACGA-ATTCCCGC
        131 GAGAATGCTTCTGTCTAGTTTTTATATGAAGATATTCCCTT

                    *    *       *                *    *  *                  
     226228 TTCCAACGTAATCCTCAAAGCAATCCAAATATCCACTTGCAGAATCCACAAAAAGAGTGTTTCAA
          1 TTCCAACGAAATCATCAAAGCGATCCAAATATCCACTTCCAGATTCTACAAAAAGAGTGTTTCAA

                          *     *          * *                   *  *        
     226293 AACTGCTCTATCAATAGAAAGGTTCAACTCTTTTAGTTGAGTACACACATCACGAACAAGTTTCT
         66 AACTGCTCTATCAAAAGAAATGTTCAACTCTGTGAGTTGAGTACACACATCACAAAGAAGTTTCT

                            * *      **     **  *    
     226358 GAGAATGCTTCTGTCTGGCTTTTATTGGAAGACGTTTCCTT
        131 GAGAATGCTTCTGTCTAGTTTTTATATGAAGATATTCCCTT

                 * *  **          *       *                * *               
     226399 TT-CACCAAAGGCATCAAAGCGCTCCAAATGTCCACTTCCAGATTCTTCCAAAAGAGTGTTTCAA
          1 TTCCAACGAAATCATCAAAGCGATCCAAATATCCACTTCCAGATTCTACAAAAAGAGTGTTTCAA

             **     * ***  * *                 *    * *  * *     *   *       
     226463 ACGTGCTCAAAGTAAGGGAATGTTCAACTCTGTGACTTGAATGCAGATATCACCAAGTAGTTTCT
         66 AACTGCTCTATCAAAAGAAATGTTCAACTCTGTGAGTTGAGTACACACATCACAAAGAAGTTTCT

            * * *            **     *    *         * 
     226528 AATAGTGCTTCTGTCTACATTTTAGATGATGATATTCCCGT
        131 GAGAATGCTTCTGTCTAGTTTTTATATGAAGATATTCCCTT

                         *** *   *             *  *   *      *     *       * 
     226569 TTCCAACGAAATCGCTAGAGCTATCCAAATATCCAGTTACAGTTTCTACCAAAAGGGTGTTTCCA
          1 TTCCAACGAAATCATCAAAGCGATCCAAATATCCACTTCCAGATTCTACAAAAAGAGTGTTTCAA

              *    **           *            *       *                     * 
     226634 AATTGCTGCATCAAAAGAAAGGTTCAACTCTGTTAGTTGAGGACACACATCACAAAGAAGTTTGT
         66 AACTGCTCTATCAAAAGAAATGTTCAACTCTGTGAGTTGAGTACACACATCACAAAGAAGTTTCT

                              *    *     *           
     226699 GAGAATGCTTCTGTCTAGATTTTGTATGACGATATTCCCTT
        131 GAGAATGCTTCTGTCTAGTTTTTATATGAAGATATTCCCTT

                     *   * *     *   *       * *  *    *  *       *  *       
     226740 TTCCAACGATATCGTTAAAGCAATCTAAATATCAATTTGCAGAATCCACAAAAATAGAGTTTCAA
          1 TTCCAACGAAATCATCAAAGCGATCCAAATATCCACTTCCAGATTCTACAAAAAGAGTGTTTCAA

             *       * *        *    *       *  *                   ***      
     226805 AGCTGCTCTGTAAAAAGAAAGGTTCCACTCTGTTAGCTGAGTACACACATCACAAACTTGTTTCT
         66 AACTGCTCTATCAAAAGAAATGTTCAACTCTGTGAGTTGAGTACACACATCACAAAGAAGTTTCT

            *     *         *        **       * **   
     226870 CAGAATCCTTCTGTCTCGTTTTTATGGGAAGATACTTACTT
        131 GAGAATGCTTCTGTCTAGTTTTTATATGAAGATATTCCCTT

                 *  * **          *       *     *       *  *  *  *           
     226911 TTCCACCGTAGGCATCAAAGCGCTCCAAATGTCCACATCCAGATACTCCAGAACGAGTGTTTCAA
          1 TTCCAACGAAATCATCAAAGCGATCCAAATATCCACTTCCAGATTCTACAAAAAGAGTGTTTCAA

             *         *   * *   *         *        * *  *      *      *     
     226976 ACCTGCTCTATGAAAGGGAATCTTCAACTCTATGAGTTGAATGCAGACATCAGAAAGAAATTTCT
         66 AACTGCTCTATCAAAAGAAATGTTCAACTCTGTGAGTTGAGTACACACATCACAAAGAAGTTTCT

                     *       **          *         **
     227041 GAGAATGCTGCTGTCTACCTTTTAT-T--TGA-ATTCCCGC
        131 GAGAATGCTTCTGTCTAGTTTTTATATGAAGATATTCCCTT

                         *  *    *              * *  **   *    *             
     227078 TTCCAACGAAATCCTCCAAGCTATCCAAATATCCACCTGCATTTTCCACAACAAGAGTGTTTCAA
          1 TTCCAACGAAATCATCAAAGCGATCCAAATATCCACTTCCAGATTCTACAAAAAGAGTGTTTCAA

                          *                 *    *  *                *       
     227143 AACTGCTCTATCAATAGAAATGTTCAACTCCTTTG-GCTGGGTACACACATCACAAACAAGTTTC
         66 AACTGCTCTATCAAAAGAAATGTTCAACT-CTGTGAGTTGAGTACACACATCACAAAGAAGTTTC

                                      **     **       
     227207 TGAGAATGCTTCTGTCTAGTTTTTATGGGAAGACGTTCCCTT
        130 TGAGAATGCTTCTGTCTAGTTTTTATATGAAGATATTCCCTT

              *  * *  **    *     *       *     *       *  *  *              
     227249 TTTCACCAAAGGCATCCAAGCGCTCCAAATGTCCACATCCAGATACTCCAGAAAGAGTGTTTCAA
          1 TTCCAACGAAATCATCAAAGCGATCCAAATATCCACTTCCAGATTCTACAAAAAGAGTGTTTCAA

             *         *   * *   *         *        * *  *      *      *     
     227314 ACCTGCTCTATGAAAGGGAATCTTCAACTCTATGAGTTGAATGCAGACATCAGAAAGAAATTTCT
         66 AACTGCTCTATCAAAAGAAATGTTCAACTCTGTGAGTTGAGTACACACATCACAAAGAAGTTTCT

                     *       **          *         **
     227379 GAGAATGCTGCTGTCTACCTTTTAT-T--TGA-ATTCCCGC
        131 GAGAATGCTTCTGTCTAGTTTTTATATGAAGATATTCCCTT

                         *  *    *                *       *                  
     227416 TTCCAACGAAATCCTCCAAGCTATCCAAATATCCACTTGCAGATTCCACAAAAAGAGTGTTTCAA
          1 TTCCAACGAAATCATCAAAGCGATCCAAATATCCACTTCCAGATTCTACAAAAAGAGTGTTTCAA

                            ** *  *            *       *     *     *  *      
     227481 AACTGCTCTCTATCAATGGCAAAGTTCAACTCTGTTAGTTGAGGACACATATCACCAACAAGTTT
         66 AACTG--CTCTATCAAAAGAAATGTTCAACTCTGTGAGTTGAGTACACACATCACAAAGAAGTTT

                               *       **         *    
     227546 CTGAGAATGCTTCTGTCTATTTTTTATGGGAAGATATTTCCTT
        129 CTGAGAATGCTTCTGTCTAGTTTTTATATGAAGATATTCCCTT

              *  *  * ** *    *      *    *          * **                    
     227589 TTTCACCGTAGGCGTCAAGGCGATCGAAATGTCCACTTCCACAAACTACAAAAAGAGTGTTTCAA
          1 TTCCAACGAAATCATCAAAGCGATCCAAATATCCACTTCCAGATTCTACAAAAAGAGTGTTTCAA

             *         *   * **       *    *        * ** * *   **      *     
     227654 ACCTGCTCTATGAAAGGCCATGTTCATCTCTATGAGTTGAATGGAAATATCCGAAAGAAATTTCT
         66 AACTGCTCTATCAAAAGAAATGTTCAACTCTGTGAGTTGAGTACACACATCACAAAGAAGTTTCT

             *       *          *        *         **
     227719 GGGAATGCTGCTGTCTAG-TGTT-TAT-ACGA-ATTCCCGC
        131 GAGAATGCTTCTGTCTAGTTTTTATATGAAGATATTCCCTT

                         *       *                *    *  *                  
     227756 TTCCAACGAAATCCTCAAAGCAATCCAAATATCCACTTGCAGAATCCACAAAAAGAGTGTTTCAA
          1 TTCCAACGAAATCATCAAAGCGATCCAAATATCCACTTCCAGATTCTACAAAAAGAGTGTTTCAA

                          *     *          * *                   *  *        
     227821 AACTGCTCTATCAATAGAAAGGTTCAACTCTTTTAGTTGAGTACACACATCACGAACAAGTTTCT
         66 AACTGCTCTATCAAAAGAAATGTTCAACTCTGTGAGTTGAGTACACACATCACAAAGAAGTTTCT

                            * *      **     **  *    
     227886 GAGAATGCTTCTGTCTGGCTTTTATTGGAAGACGTTTCCTT
        131 GAGAATGCTTCTGTCTAGTTTTTATATGAAGATATTCCCTT

                   *  **          *       *                * *               
     227927 TT-CAACAAAGGCATCAAAGCGCTCCAAATGTCCACTTCCAGATTCTTCCAAAAGAGTGTTTCAA
          1 TTCCAACGAAATCATCAAAGCGATCCAAATATCCACTTCCAGATTCTACAAAAAGAGTGTTTCAA

             **     * ***  * *                 *    * *  * *     *   *       
     227991 ACGTGCTCAAAGTAAGGGAATGTTCAACTCTGTGACTTGAATGCAGATATCACCAAGTAGTTTCT
         66 AACTGCTCTATCAAAAGAAATGTTCAACTCTGTGAGTTGAGTACACACATCACAAAGAAGTTTCT

            * * *        *   **     *    *           
     228056 AATAGTGCTTCTGCCTACATTTTAGATGATGATATTCCCTT
        131 GAGAATGCTTCTGTCTAGTTTTTATATGAAGATATTCCCTT

                         * * *   *             *  *   *      *     *       * 
     228097 TTCCAACGAAATCGTTAGAGCTATCCAAATATCCAGTTACAGTTTCTACCAAAAGGGTGTTTCCA
          1 TTCCAACGAAATCATCAAAGCGATCCAAATATCCACTTCCAGATTCTACAAAAAGAGTGTTTCAA

              *    **           *            *       *                     * 
     228162 AATTGCTGCATCAAAAGAAAGGTTCAACTCTGTTAGTTGAGGACACACATCACAAAGAAGTTTGT
         66 AACTGCTCTATCAAAAGAAATGTTCAACTCTGTGAGTTGAGTACACACATCACAAAGAAGTTTCT

                              *    *     *           
     228227 GAGAATGCTTCTGTCTAGATTTTGTATGACGATATTCCCTT
        131 GAGAATGCTTCTGTCTAGTTTTTATATGAAGATATTCCCTT

                     *   * *     *   *       * *  *    *  *       *  *       
     228268 TTCCAACGATATCGTTAAAGCAATCTAAATATCAATTTGCAGAATCCACAAAAATAGAGTTTCAA
          1 TTCCAACGAAATCATCAAAGCGATCCAAATATCCACTTCCAGATTCTACAAAAAGAGTGTTTCAA

             *       * *        *    *       *  *                   ***      
     228333 AGCTGCTCTGTAAAAAGAAAGGTTCCACTCTGTTAGCTGAGTACACACATCACAAACTTGTTTCT
         66 AACTGCTCTATCAAAAGAAATGTTCAACTCTGTGAGTTGAGTACACACATCACAAAGAAGTTTCT

                  *         *        **     *   **   
     228398 GAGAATCCTTCTGTCTCGTTTTTATGGGAAGACATTTACTT
        131 GAGAATGCTTCTGTCTAGTTTTTATATGAAGATATTCCCTT

                 *  * **          *       *     *       *  *  *  *           
     228439 TTCCACCGTAGGCATCAAAGCGCTCCAAATGTCCACATCCAGATACTCCAGAACGAGTGTTTCAA
          1 TTCCAACGAAATCATCAAAGCGATCCAAATATCCACTTCCAGATTCTACAAAAAGAGTGTTTCAA

             *         *   * *   *         *        * *  *      *      *     
     228504 ACCTGCTCTATGAAAGGGAATCTTCAACTCTATGAGTTGAATGCAGACATCAGAAAGAAATTTCT
         66 AACTGCTCTATCAAAAGAAATGTTCAACTCTGTGAGTTGAGTACACACATCACAAAGAAGTTTCT

                     *       **          *         **
     228569 GAGAATGCTGCTGTCTACCTTTTAT-T--TGA-ATTCCCGC
        131 GAGAATGCTTCTGTCTAGTTTTTATATGAAGATATTCCCTT

                         *  *    *                *  **   *                  
     228606 TTCCAACGAAATCCTCCAAGCTATCCAAATATCCACTTGCATTTTCCACAAAAAGAGTGTTTCAA
          1 TTCCAACGAAATCATCAAAGCGATCCAAATATCCACTTCCAGATTCTACAAAAAGAGTGTTTCAA

                          *                 *    *  *                *       
     228671 AACTGCTCTATCAATAGAAATGTTCAACTCCTTTG-GCTGGGTACACACATCACAAACAAGTTTC
         66 AACTGCTCTATCAAAAGAAATGTTCAACT-CTGTGAGTTGAGTACACACATCACAAAGAAGTTTC

                                      **     **       
     228735 TGAGAATGCTTCTGTCTAGTTTTTATGGGAAGACGTTCCCTT
        130 TGAGAATGCTTCTGTCTAGTTTTTATATGAAGATATTCCCTT

              *  * *  **          *       *            **                  * 
     228777 TTTCACCAAAGGCATCAAAGCGCTCCAAATGTCCACTTCCAGACACTACAAAAAGAGTGTTTCCA
          1 TTCCAACGAAATCATCAAAGCGATCCAAATATCCACTTCCAGATTCTACAAAAAGAGTGTTTCAA

             **       **   ***                 *    * *  * *         *       
     228842 ACGTGCTCTAAGAAAGCGAATGTTCAACTCTGTGACTTGAATGCAGATATCACAAAGTAGTTTCT
         66 AACTGCTCTATCAAAAGAAATGTTCAACTCTGTGAGTTGAGTACACACATCACAAAGAAGTTTCT

                **            *     *    *         * 
     228907 GAGAGGGCTTCTGTCTAGATTTTAGATGATGATATTCCCGT
        131 GAGAATGCTTCTGTCTAGTTTTTATATGAAGATATTCCCTT

                           * *   *                *   *                    * 
     228948 TTCCAACGAAATCATTAGAGCTATCCAAATATCCACTTACAGTTTCTACAAAAAGAGTGTTTCCA
          1 TTCCAACGAAATCATCAAAGCGATCCAAATATCCACTTCCAGATTCTACAAAAAGAGTGTTTCAA

                   **         * *    *       *  *                   ***      
     229013 AACTGCTGCATCAAAAGAGAGGTTCCACTCTGTTAGCTGAGTACACACATCACAAACTTGTTTCT
         66 AACTGCTCTATCAAAAGAAATGTTCAACTCTGTGAGTTGAGTACACACATCACAAAGAAGTTTCT

                  *         *        **         **   
     229078 GAGAATCCTTCTGTCTCGTTTTTATGGGAAGATATTTACTT
        131 GAGAATGCTTCTGTCTAGTTTTTATATGAAGATATTCCCTT

              *  *  * **          *       *   * *       *  *  *              
     229119 TTTCACCGTAGGCATCAAAGCGCTCCAAATGTCCTCATCCAGATACTCCAGAAAGAGTGTTTCAA
          1 TTCCAACGAAATCATCAAAGCGATCCAAATATCCACTTCCAGATTCTACAAAAAGAGTGTTTCAA

             *         *   * *   *         *        * *  *      *      *     
     229184 ACCTGCTCTATGAAAGGGAATCTTCAACTCTATGAGTTGAATGCAGACATCAGAAAGAAATTTCT
         66 AACTGCTCTATCAAAAGAAATGTTCAACTCTGTGAGTTGAGTACACACATCACAAAGAAGTTTCT

                     *       **          *         **
     229249 GAGAATGCTGCTGTCTACCTTTTAT-T--TGA-ATTCCCGC
        131 GAGAATGCTTCTGTCTAGTTTTTATATGAAGATATTCCCTT

                         *  *    *                *       *                  
     229286 TTCCAACGAAATCCTCCAAGCTATCCAAATATCCACTTGCAGATTCCACAAAAAGAGTGTTTCAA
          1 TTCCAACGAAATCATCAAAGCGATCCAAATATCCACTTCCAGATTCTACAAAAAGAGTGTTTCAA

                            *     *            *       *     *     *  *      
     229351 AACTGCTCTCTATCAATAGAAAGGTTCAACTCTGTTAGTTGAGGACACATATCACCAACAAGTTT
         66 AACTG--CTCTATCAAAAGAAATGTTCAACTCTGTGAGTTGAGTACACACATCACAAAGAAGTTT

                               *       **         *    
     229416 CTGAGAATGCTTCTGTCTATTTTTTATGGGAAGATATTTCCTT
        129 CTGAGAATGCTTCTGTCTAGTTTTTATATGAAGATATTCCCTT

              *  *  * ** *    *      *    *          * **                    
     229459 TTTCACCGTAGGCGTCAAGGCGATCGAAATGTCCACTTCCACAAACTACAAAAAGAGTGTTTCAA
          1 TTCCAACGAAATCATCAAAGCGATCCAAATATCCACTTCCAGATTCTACAAAAAGAGTGTTTCAA

             *         *   * **       *    *        * ** * *   **      *     
     229524 ACCTGCTCTATGAAAGGCCATGTTCATCTCTATGAGTTGAATGGATATATCCGAAAGAAATTTCT
         66 AACTGCTCTATCAAAAGAAATGTTCAACTCTGTGAGTTGAGTACACACATCACAAAGAAGTTTCT

             *       *                   *         **
     229589 GGGAATGCTGCTGTCTAG-TTTT-TAT-ACGA-ATTCCCGC
        131 GAGAATGCTTCTGTCTAGTTTTTATATGAAGATATTCCCTT

                         *  *    *                *       *                  
     229626 TTCCAACGAAATCCTCCAAGCTATCCAAATATCCACTTGCAGATTCCACAAAAAGAGTGTTTCAA
          1 TTCCAACGAAATCATCAAAGCGATCCAAATATCCACTTCCAGATTCTACAAAAAGAGTGTTTCAA

                            *     *            *       *     *     *  *      
     229691 AACTGCTCTCTATCAATAGAAAGGTTCAACTCTGTTAGTTGAGGACACATATCACCAACAAGTTT
         66 AACTG--CTCTATCAAAAGAAATGTTCAACTCTGTGAGTTGAGTACACACATCACAAAGAAGTTT

                               *       **         *    
     229756 CTGAGAATGCTTCTGTCTATTTTTTATGGGAAGATATTTCCTT
        129 CTGAGAATGCTTCTGTCTAGTTTTTATATGAAGATATTCCCTT

              *  *  * ** *    *      *    *          * **                    
     229799 TTTCACCGTAGGCGTCAAGGCGATCGAAATGTCCACTTCCACAAACTACAAAAAGAGTGTTTCAA
          1 TTCCAACGAAATCATCAAAGCGATCCAAATATCCACTTCCAGATTCTACAAAAAGAGTGTTTCAA

             *         *   * **       *    *        * ** * *   **      *     
     229864 ACCTGCTCTATGAAAGGCCATGTTCATCTCTATGAGTTGAATGGAAATATCCGAAAGAAATTTCT
         66 AACTGCTCTATCAAAAGAAATGTTCAACTCTGTGAGTTGAGTACACACATCACAAAGAAGTTTCT

             *       *          *        *         **
     229929 GGGAATGCTGCTGTCTAG-TGTT-TAT-ACGA-ATTCCCGC
        131 GAGAATGCTTCTGTCTAGTTTTTATATGAAGATATTCCCTT

                         *       *                *    *  *                  
     229966 TTCCAACGAAATCCTCAAAGCAATCCAAATATCCACTTGCAGAATCCACAAAAAGAGTGTTTCAA
          1 TTCCAACGAAATCATCAAAGCGATCCAAATATCCACTTCCAGATTCTACAAAAAGAGTGTTTCAA

                          *     *          * *                   *  *        
     230031 AACTGCTCTATCAATAGAAAGGTTCAACTCTTTTAGTTGAGTACACACATCACGAACAAGTTTCT
         66 AACTGCTCTATCAAAAGAAATGTTCAACTCTGTGAGTTGAGTACACACATCACAAAGAAGTTTCT

                            * *      **     **  *    
     230096 GAGAATGCTTCTGTCTGGCTTTTATTGGAAGACGTTTCCTT
        131 GAGAATGCTTCTGTCTAGTTTTTATATGAAGATATTCCCTT

                 * *  **          *       *                * *               
     230137 TT-CACCAAAGGCATCAAAGCGCTCCAAATGTCCACTTCCAGATTCTTCCAAAAGAGTGTTTCAA
          1 TTCCAACGAAATCATCAAAGCGATCCAAATATCCACTTCCAGATTCTACAAAAAGAGTGTTTCAA

             **     * ***  * *                 *    * *  * *     *   *       
     230201 ACGTGCTCAAAGTAAGGGAATGTTCAACTCTGTGACTTGAATGCAGATATCACCAAGTAGTTTCT
         66 AACTGCTCTATCAAAAGAAATGTTCAACTCTGTGAGTTGAGTACACACATCACAAAGAAGTTTCT

            * * *            **     *    *         * 
     230266 AATAGTGCTTCTGTCTACATTTTAGATGATGATATTCCCGT
        131 GAGAATGCTTCTGTCTAGTTTTTATATGAAGATATTCCCTT

                         * * *   *             *  *   *      *     *       * 
     230307 TTCCAACGAAATCGTTAGAGCTATCCAAATATCCAGTTACAGTTTCTACCAAAAGGGTGTTTCCA
          1 TTCCAACGAAATCATCAAAGCGATCCAAATATCCACTTCCAGATTCTACAAAAAGAGTGTTTCAA

              *    **           *            *  *    *                     * 
     230372 AATTGCTGCATCAAAAGAAAGGTTCAACTCTGTTAGCTGAGGACACACATCACAAAGAAGTTTGT
         66 AACTGCTCTATCAAAAGAAATGTTCAACTCTGTGAGTTGAGTACACACATCACAAAGAAGTTTCT

                              *    *     *           
     230437 GAGAATGCTTCTGTCTAGATTTTGTATGACGATATTCCCTT
        131 GAGAATGCTTCTGTCTAGTTTTTATATGAAGATATTCCCTT

                     *   * *     *   *       * *  *    *  *       *  *       
     230478 TTCCAACGATATCGTTAAAGCAATCTAAATATCAATTTGCAGAATCCACAAAAATAGAGTTTCAA
          1 TTCCAACGAAATCATCAAAGCGATCCAAATATCCACTTCCAGATTCTACAAAAAGAGTGTTTCAA

             *       * *        *    *       *  *                   ***      
     230543 AGCTGCTCTGTAAAAAGAAAGGTTCCACTCTGTTAGCTGAGTACACACATCACAAACTTGTTTCT
         66 AACTGCTCTATCAAAAGAAATGTTCAACTCTGTGAGTTGAGTACACACATCACAAAGAAGTTTCT

                  *         *        **         **   
     230608 GAGAATCCTTCTGTCTCGTTTTTATGGGAAGATATTTACTT
        131 GAGAATGCTTCTGTCTAGTTTTTATATGAAGATATTCCCTT

                 *  * **          *       *     *       *  *  *              
     230649 TTCCACCGTAGGCATCAAAGCGCTCCAAATGTCCACATCCAGATACTCCAGAAAGAGTGTTTCAA
          1 TTCCAACGAAATCATCAAAGCGATCCAAATATCCACTTCCAGATTCTACAAAAAGAGTGTTTCAA

             *         *   * *   *         *        * *  *      *      *     
     230714 ACCTGCTCTATGAAAGGGAATCTTCAACTCTATGAGTTGAATGCAGACATCAGAAAGAAATTTCT
         66 AACTGCTCTATCAAAAGAAATGTTCAACTCTGTGAGTTGAGTACACACATCACAAAGAAGTTTCT

                     *       **          *         **
     230779 GAGAATGCTGCTGTCTACCTTTTAT-T--TGA-ATTCCCGC
        131 GAGAATGCTTCTGTCTAGTTTTTATATGAAGATATTCCCTT

                     *   *  *    *                *  **   *                  
     230816 TTCCAACGATATCCTCCAAGCTATCCAAATATCCACTTGCATTTTCCACAAAAAGAGTGTTTCAA
          1 TTCCAACGAAATCATCAAAGCGATCCAAATATCCACTTCCAGATTCTACAAAAAGAGTGTTTCAA

                          *                 *    *  *                *       
     230881 AACTGCTCTATCAATAGAAATGTTCAACTCCTTTG-GCTGGGTACACACATCACAAACAAGTTTC
         66 AACTGCTCTATCAAAAGAAATGTTCAACT-CTGTGAGTTGAGTACACACATCACAAAGAAGTTTC

                                      **     **       
     230945 TGAGAATGCTTCTGTCTAGTTTTTATGGGAAGACGTTCCCTT
        130 TGAGAATGCTTCTGTCTAGTTTTTATATGAAGATATTCCCTT

              *  * *  **          *       *            **                  * 
     230987 TTTCACCAAAGGCATCAAAGCGCTCCAAATGTCCACTTCCAGACACTACAAAAAGAGTGTTTCCA
          1 TTCCAACGAAATCATCAAAGCGATCCAAATATCCACTTCCAGATTCTACAAAAAGAGTGTTTCAA

             **       **   ***                 *    * *  * *         *       
     231052 ACGTGCTCTAAGAAAGCGAATGTTCAACTCTGTGACTTGAATGCAGATATCACAAAGTAGTTTCT
         66 AACTGCTCTATCAAAAGAAATGTTCAACTCTGTGAGTTGAGTACACACATCACAAAGAAGTTTCT

                **            *     *    *         * 
     231117 GAGAGGGCTTCTGTCTAGATTTTAGATGATGATATTCCCGT
        131 GAGAATGCTTCTGTCTAGTTTTTATATGAAGATATTCCCTT

                           * *   *                *   *                    * 
     231158 TTCCAACGAAATCATTAGAGCTATCCAAATATCCACTTACAGTTTCTACAAAAAGAGTGTTTCCA
          1 TTCCAACGAAATCATCAAAGCGATCCAAATATCCACTTCCAGATTCTACAAAAAGAGTGTTTCAA

                   **         * *    *       *  *                   ***      
     231223 AACTGCTGCATCAAAAGAGAGGTTCCACTCTGTTAGCTGAGTACACACATCACAAACTTGTTTCT
         66 AACTGCTCTATCAAAAGAAATGTTCAACTCTGTGAGTTGAGTACACACATCACAAAGAAGTTTCT

            *     * *       *        **         **   
     231288 CAGAATCCGTCTGTCTCGTTTTTATGGGAAGATATTTACTT
        131 GAGAATGCTTCTGTCTAGTTTTTATATGAAGATATTCCCTT

              *  *  * **          *       *     *       *     *              
     231329 TTTCACCGTAGGCATCAAAGCGCTCCAAATGTCCACATCCAGATACTACAGAAAGAGTGTTTCAA
          1 TTCCAACGAAATCATCAAAGCGATCCAAATATCCACTTCCAGATTCTACAAAAAGAGTGTTTCAA

             *         *   * *   *         *        * *  *      *      *     
     231394 ACCTGCTCTATGAAAGGGAATCTTCAACTCTATGAGTTGAATGCAGACATCAGAAAGAAATTTCT
         66 AACTGCTCTATCAAAAGAAATGTTCAACTCTGTGAGTTGAGTACACACATCACAAAGAAGTTTCT

                     *       **          *         **
     231459 GAGAATGCTGCTGTCTACCTTTTAT-T--TGA-ATTCCCGC
        131 GAGAATGCTTCTGTCTAGTTTTTATATGAAGATATTCCCTT

                         *  *    *                *       *                * 
     231496 TTCCAACGAAATCCTCCAAGCTATCCAAATATCCACTTGCAGATTCCACAAAAAGAGTGTTTCTA
          1 TTCCAACGAAATCATCAAAGCGATCCAAATATCCACTTCCAGATTCTACAAAAAGAGTGTTTCAA

                            ** *  *            *       *     *     *  *      
     231561 AACTGCTCTCTATCAATGGCAAAGTTCAACTCTGTTAGTTGAGGACACATATCACCAACAAGTTT
         66 AACTG--CTCTATCAAAAGAAATGTTCAACTCTGTGAGTTGAGTACACACATCACAAAGAAGTTT

                               *       **         *    
     231626 CTGAGAATGCTTCTGTCTATTTTTTATGGGAAGATATTTCCTT
        129 CTGAGAATGCTTCTGTCTAGTTTTTATATGAAGATATTCCCTT

              *  *  * ** *    *      *    *          * **                    
     231669 TTTCACCGTAGGCGTCAAGGCGATCGAAATGTCCACTTCCACAAACTACAAAAAGAGTGTTTCAA
          1 TTCCAACGAAATCATCAAAGCGATCCAAATATCCACTTCCAGATTCTACAAAAAGAGTGTTTCAA

             *         *   * **       *    *        * ** * *   **      *     
     231734 ACCTGCTCTATGAAAGGCCATGTTCATCTCTATGAGTTGAATGGAAATATCCGAAAGAAATTTCT
         66 AACTGCTCTATCAAAAGAAATGTTCAACTCTGTGAGTTGAGTACACACATCACAAAGAAGTTTCT

             *       *          *        *         **
     231799 GGGAATGCTGCTGTCTAG-TGTT-TAT-ACGA-ATTCCCGC
        131 GAGAATGCTTCTGTCTAGTTTTTATATGAAGATATTCCCTT

                         *       *                *    *  *                  
     231836 TTCCAACGAAATCCTCAAAGCAATCCAAATATCCACTTGCAGAATCCACAAAAAGAGTGTTTCAA
          1 TTCCAACGAAATCATCAAAGCGATCCAAATATCCACTTCCAGATTCTACAAAAAGAGTGTTTCAA

                          *     *     *    * *                   *  *        
     231901 AACTGCTCTATCAATAGAAAGGTTCATCTCTTTTAGTTGAGTACACACATCACGAACAAGTTTCT
         66 AACTGCTCTATCAAAAGAAATGTTCAACTCTGTGAGTTGAGTACACACATCACAAAGAAGTTTCT

                            * *      **      *  *    
     231966 GAGAATGCTTCTGTCTGGCTTTTATTGGAAGATGTTTCCTT
        131 GAGAATGCTTCTGTCTAGTTTTTATATGAAGATATTCCCTT

                 * *  **          *       *                * *               
     232007 TT-CACCAAAGGCATCAAAGCGCTCCAAATGTCCACTTCCAGATTCTTCCAAAAGAGTGTTTCAA
          1 TTCCAACGAAATCATCAAAGCGATCCAAATATCCACTTCCAGATTCTACAAAAAGAGTGTTTCAA

             **     * ***  * *                 *    * *  * *     *   *       
     232071 ACGTGCTCAAAGTAAGGGAATGTTCAACTCTGTGACTTGAATGCAGATATCACCAAGTAGTTTCT
         66 AACTGCTCTATCAAAAGAAATGTTCAACTCTGTGAGTTGAGTACACACATCACAAAGAAGTTTCT

            * * *            **     *    *         * 
     232136 AATAGTGCTTCTGTCTACATTTT-GATGATGATATTCCCGT
        131 GAGAATGCTTCTGTCTAGTTTTTATATGAAGATATTCCCTT

                         * * *   *          *  *  *   *                    * 
     232176 TTCCAACGAAATCGTTAGAGCTATCCAAATATGCAGTTACAGTTTCTACAAAAAGAGTGTTTCCA
          1 TTCCAACGAAATCATCAAAGCGATCCAAATATCCACTTCCAGATTCTACAAAAAGAGTGTTTCAA

                   **           *            *       *                     * 
     232241 AACTGCTGCATCAAAAGAAAGGTTCAACTCTGTTAGTTGAGGACACACATCACAAAGAAGTTTGT
         66 AACTGCTCTATCAAAAGAAATGTTCAACTCTGTGAGTTGAGTACACACATCACAAAGAAGTTTCT

                              *    *     *           
     232306 GAGAATGCTTCTGTCTAGATTTTGTATGACGATATTCCCTT
        131 GAGAATGCTTCTGTCTAGTTTTTATATGAAGATATTCCCTT

                     *   * *     *   *       * *  *    *  *       *  *       
     232347 TTCCAACGATATCGTTAAAGCAATCTAAATATCAATTTGCAGAATCCACAAAAATAGAGTTTCAA
          1 TTCCAACGAAATCATCAAAGCGATCCAAATATCCACTTCCAGATTCTACAAAAAGAGTGTTTCAA

             *       * *        **   *       *  *                   ***      
     232412 AGCTGCTCTGTAAAAAGAAAGTTTCCACTCTGTTAGCTGAGTACACACATCACAAACTTGTTTCT
         66 AACTGCTCTATCAAAAGAAATGTTCAACTCTGTGAGTTGAGTACACACATCACAAAGAAGTTTCT

                  *         *        **         **   
     232477 GAGAATCCTTCTGTCTCGTTTTTATGGGAAGATATTTACTT
        131 GAGAATGCTTCTGTCTAGTTTTTATATGAAGATATTCCCTT

              *  *  * **          *       *     *       *  *  *     *        
     232518 TTTCACCGTAGGCATCAAAGCGCTCCAAATGTCCACATCCAGATACTCCAGAAAGACTGTTTCAA
          1 TTCCAACGAAATCATCAAAGCGATCCAAATATCCACTTCCAGATTCTACAAAAAGAGTGTTTCAA

             *         *   * *   *         *        * *  *      *      *     
     232583 ACCTGCTCTATGAAAGGGAATCTTCAACTCTATGAGTTGAATGCAGACATCAGAAAGAAATTTCT
         66 AACTGCTCTATCAAAAGAAATGTTCAACTCTGTGAGTTGAGTACACACATCACAAAGAAGTTTCT

                     *       **          *         **
     232648 GAGAATGCTGCTGTCTACCTTTTAT-T--TGA-ATTCCCGC
        131 GAGAATGCTTCTGTCTAGTTTTTATATGAAGATATTCCCTT

                         *  *    *                *  **   *                  
     232685 TTCCAACGAAATCCTCCAAGCTATCCAAATATCCACTTGCATTTTCCACAAAAAGAGTGTTTCAA
          1 TTCCAACGAAATCATCAAAGCGATCCAAATATCCACTTCCAGATTCTACAAAAAGAGTGTTTCAA

                          *               ** *  *  *                *        
     232750 AACTGCTCTATCAATAGAAATGTTCAACTCCTTTAGCTGGGTACACACATCACAAACAAGTTTCT
         66 AACTGCTCTATCAAAAGAAATGTTCAACTCTGTGAGTTGAGTACACACATCACAAAGAAGTTTCT

                                     **     *        
     232815 GAGAATGCTTCTGTCTAGTTTTTATGGGAAGACATTCCCTT
        131 GAGAATGCTTCTGTCTAGTTTTTATATGAAGATATTCCCTT

              *  * *  **          *       *            **                    
     232856 TTTCACCAAAGACATCAAAGCGCTCCAAATGTCCACTTCCAGACACTACAAAAAGAGTGTTTCAA
          1 TTCCAACGAAATCATCAAAGCGATCCAAATATCCACTTCCAGATTCTACAAAAAGAGTGTTTCAA

             **       **   ***                 *    * *  * *         *       
     232921 ACGTGCTCTAAGAAAGCGAATGTTCAACTCTGTGACTTGAATGCAGATATCACAAAGTAGTTTCT
         66 AACTGCTCTATCAAAAGAAATGTTCAACTCTGTGAGTTGAGTACACACATCACAAAGAAGTTTCT

                **            *     *    *         * 
     232986 GAGAGGGCTTCTGTCTAGATTTTAGATGATGATATTCCCGT
        131 GAGAATGCTTCTGTCTAGTTTTTATATGAAGATATTCCCTT

                           * *   *                *   *                    * 
     233027 TTCCAACGAAATCATTAGAGCTATCCAAATATCCACTTACAGTTTCTACAAAAAGAGTGTTTCCA
          1 TTCCAACGAAATCATCAAAGCGATCCAAATATCCACTTCCAGATTCTACAAAAAGAGTGTTTCAA

                   **         * *    *       *  *                   ***      
     233092 AACTGCTGCATCAAAAGAGAGGTTCCACTCTGTTAGCTGAGTACACACATCACAAACTTGTTTCT
         66 AACTGCTCTATCAAAAGAAATGTTCAACTCTGTGAGTTGAGTACACACATCACAAAGAAGTTTCT

            *     *         *        **         **   
     233157 CAGAATCCTTCTGTCTCGTTTTTATGGGAAGATATTTACTT
        131 GAGAATGCTTCTGTCTAGTTTTTATATGAAGATATTCCCTT

              *  *  * **          *       *     *       ** *  *              
     233198 TTTCACCGTAGGCATCAAAGCGCTCCAAATGTCCACATCCAGATAATCCAGAAAGAGTGTTTCAA
          1 TTCCAACGAAATCATCAAAGCGATCCAAATATCCACTTCCAGATTCTACAAAAAGAGTGTTTCAA

             *         *   * *   *         *        * *  *      *      *     
     233263 ACCTGCTCTATGAAAGGGAATCTTCAACTCTATGAGTTGAATGCAGACATCAGAAAGAAATTTCT
         66 AACTGCTCTATCAAAAGAAATGTTCAACTCTGTGAGTTGAGTACACACATCACAAAGAAGTTTCT

                     *       **          *         **
     233328 GAGAATGCTGCTGTCTACCTTTTAT-T--TGA-ATTCCCGC
        131 GAGAATGCTTCTGTCTAGTTTTTATATGAAGATATTCCCTT

                         *  *    *                *       *                  
     233365 TTCCAACGAAATCCTCCAAGCTATCCAAATATCCACTTGCAGATTCCACAAAAAGAGTGTTTCAA
          1 TTCCAACGAAATCATCAAAGCGATCCAAATATCCACTTCCAGATTCTACAAAAAGAGTGTTTCAA

                            ** *  *            *       *     *     *  *      
     233430 AACTGCTCTCTATCAATGGCAAAGTTCAACTCTGTTAGTTGAGGACACATATCACCAACAAGTTT
         66 AACTG--CTCTATCAAAAGAAATGTTCAACTCTGTGAGTTGAGTACACACATCACAAAGAAGTTT

                               *       **         *    
     233495 CTGAGAATGCTTCTGTCTATTTTTTATGGGAAGATATTTCCTT
        129 CTGAGAATGCTTCTGTCTAGTTTTTATATGAAGATATTCCCTT

              *  *  * ** *    *      *    *          * **        *           
     233538 TTTCACCGTAGGCGTCAAGGCGATCGAAATGTCCACTTCCACAAACTACAAAATGAGTGTTTCAA
          1 TTCCAACGAAATCATCAAAGCGATCCAAATATCCACTTCCAGATTCTACAAAAAGAGTGTTTCAA

             *         *   * **       *    *        * ** * *   **      *     
     233603 ACCTGCTCTATGAAAGGCCATGTTCATCTCTATGAGTTGAATGGAAATATCCGAAAGAAATTTCT
         66 AACTGCTCTATCAAAAGAAATGTTCAACTCTGTGAGTTGAGTACACACATCACAAAGAAGTTTCT

             *       *          *        *         **
     233668 GGGAATGCTGCTGTCTAG-TGTT-TAT-ACGA-ATTCCCGC
        131 GAGAATGCTTCTGTCTAGTTTTTATATGAAGATATTCCCTT

                         *       *                *    *  *                  
     233705 TTCCAACGAAATCCTCAAAGCAATCCAAATATCCACTTGCAGAATCCACAAAAAGAGTGTTTCAA
          1 TTCCAACGAAATCATCAAAGCGATCCAAATATCCACTTCCAGATTCTACAAAAAGAGTGTTTCAA

                          *     *          * *                      *        
     233770 AACTGCTCTATCAATAGAAAGGTTCAACTCTTTTAGTTGAGTACACACATCACAAACAAGTTTCT
         66 AACTGCTCTATCAAAAGAAATGTTCAACTCTGTGAGTTGAGTACACACATCACAAAGAAGTTTCT

                            * *      **     **  *    
     233835 GAGAATGCTTCTGTCTGGCTTTTATTGGAAGACGTTTCCTT
        131 GAGAATGCTTCTGTCTAGTTTTTATATGAAGATATTCCCTT

                 * *  **          *       *            **                    
     233876 TT-CACCAAAGGCATCAAAGCGCTCCAAATGTCCACTTCCAGACACTACAAAAAGAGTGTTTCAA
          1 TTCCAACGAAATCATCAAAGCGATCCAAATATCCACTTCCAGATTCTACAAAAAGAGTGTTTCAA

             **       **   **       *          *    * *  * *         *       
     233940 ACGTGCTCTAAGAAAGCAAATGTTGAACTCTGTGACTTGAATGCAGATATCACAAAGTAGTTTCT
         66 AACTGCTCTATCAAAAGAAATGTTCAACTCTGTGAGTTGAGTACACACATCACAAAGAAGTTTCT

                *             *     *    *           
     234005 GAGAGTGCTTCTGTCTAGATTTTAGATGATGATATTCCCTT
        131 GAGAATGCTTCTGTCTAGTTTTTATATGAAGATATTCCCTT

                           * *   *                *   *                    * 
     234046 TTCCAACGAAATCATTAGAGCTATCCAAATATCCACTTACAGTTTCTACAAAAAGAGTGTTTCCA
          1 TTCCAACGAAATCATCAAAGCGATCCAAATATCCACTTCCAGATTCTACAAAAAGAGTGTTTCAA

                   **         * *    *       *  *                   ***      
     234111 AACTGCTGCATCAAAAGAGAGGTTCCACTCTGTTAGCTGAGTACACACATCACAAACTTGTTTCT
         66 AACTGCTCTATCAAAAGAAATGTTCAACTCTGTGAGTTGAGTACACACATCACAAAGAAGTTTCT

            *     *         *        **         *    
     234176 CAGAATCCTTCTGTCTCGTTTTTATGGGAAGATATTTCCTT
        131 GAGAATGCTTCTGTCTAGTTTTTATATGAAGATATTCCCTT

              *  *  * ** *    *      *    *          * **                    
     234217 TTTCACCGTAGGCGTCAAGGCGATCGAAATGTCCACTTCCACAAACTACAAAAAGAGTGTTTCAA
          1 TTCCAACGAAATCATCAAAGCGATCCAAATATCCACTTCCAGATTCTACAAAAAGAGTGTTTCAA

             *         *   * *   *         *        * *  *      *      *     
     234282 ACCTGCTCTATGAAAGGGAATCTTCAACTCTATGAGTTGAATGCAGACATCAGAAAGAAATTTCT
         66 AACTGCTCTATCAAAAGAAATGTTCAACTCTGTGAGTTGAGTACACACATCACAAAGAAGTTTCT

                     *       **          *         **
     234347 GAGAATGCTGCTGTCTACCTTTTAT-T--TGA-ATTCCCGC
        131 GAGAATGCTTCTGTCTAGTTTTTATATGAAGATATTCCCTT

                         *  *   **              * *  **   *           *      
     234384 TTCCAACGAAATCCTCCAAGGTATCCAAATATCCACCTGCATTTTCCACAAAAAGAGTATTTCAA
          1 TTCCAACGAAATCATCAAAGCGATCCAAATATCCACTTCCAGATTCTACAAAAAGAGTGTTTCAA

                 *        *                 *    *  *                *       
     234449 AACTGTTCTATCAATAGAAATGTTCAACTCCTTTG-GCTGGGTACACACATCACAAACAAGTTTC
         66 AACTGCTCTATCAAAAGAAATGTTCAACT-CTGTGAGTTGAGTACACACATCACAAAGAAGTTTC

                                      **     *        
     234513 TGAGAATGCTTCTGTCTAGTTTTTATGGGAAGACATTCCCTT
        130 TGAGAATGCTTCTGTCTAGTTTTTATATGAAGATATTCCCTT

              *  * *  **          *       *            **                  * 
     234555 TTTCACCAAAGGCATCAAAGCGCTCCAAATGTCCACTTCCAGACACTACAAAAAGAGTGTTTCCA
          1 TTCCAACGAAATCATCAAAGCGATCCAAATATCCACTTCCAGATTCTACAAAAAGAGTGTTTCAA

             **       **   ***                 *    * *  * *         *       
     234620 ACGTGCTCTAAGAAAGCGAATGTTCAACTCTGTGACTTGAATGCAGATATCACAAAGTAGTTTCT
         66 AACTGCTCTATCAAAAGAAATGTTCAACTCTGTGAGTTGAGTACACACATCACAAAGAAGTTTCT

                *             *     *    *         * 
     234685 GAGAGTGCTTCTGTCTAGATTTTAGATGATGATATTCCCGT
        131 GAGAATGCTTCTGTCTAGTTTTTATATGAAGATATTCCCTT

                           * *   *                *   *                    * 
     234726 TTCCAACGAAATCATTAGAGCTATCCAAATATCCACTTACAGTTTCTACAAAAAGAGTGTTTCCA
          1 TTCCAACGAAATCATCAAAGCGATCCAAATATCCACTTCCAGATTCTACAAAAAGAGTGTTTCAA

                   **         * *    *       *  *                   ***      
     234791 AACTGCTGCATCAAAAGAGAGGTTCCACTCTGTTAGCTGAGTACACACATCACAAACTTGTTTCT
         66 AACTGCTCTATCAAAAGAAATGTTCAACTCTGTGAGTTGAGTACACACATCACAAAGAAGTTTCT

            *     *         *        **         **   
     234856 CAGAATCCTTCTGTCTCGTTTTTATGGGAAGATATTTACTT
        131 GAGAATGCTTCTGTCTAGTTTTTATATGAAGATATTCCCTT

              *  *  * **          *       *     *       *  *  *              
     234897 TTTCACCGTAGGCATCAAAGCGCTCCAAATGTCCACATCCAGATACTCCAGAAAGAGTGTTTCAA
          1 TTCCAACGAAATCATCAAAGCGATCCAAATATCCACTTCCAGATTCTACAAAAAGAGTGTTTCAA

             *         *   * *         *   *        * *  *      *      *     
     234962 ACCTGCTCTATGAAAGGGAATGTTCAATTCTATGAGTTGAATGCAGACATCAGAAAGAAATTTCT
         66 AACTGCTCTATCAAAAGAAATGTTCAACTCTGTGAGTTGAGTACACACATCACAAAGAAGTTTCT

                     *       **          *         **
     235027 GAGAATGCTGCTGTCTACCTTTTAT-T--TGA-ATTCCCGC
        131 GAGAATGCTTCTGTCTAGTTTTTATATGAAGATATTCCCTT

                  *      *  *    *                *       *                  
     235064 TTCCAATGAAATCCTCCAAGCTATCCAAATATCCACTTGCAGATTCCACAAAAAGAGTGTTTCAA
          1 TTCCAACGAAATCATCAAAGCGATCCAAATATCCACTTCCAGATTCTACAAAAAGAGTGTTTCAA

                            ** *  *            *       *     *     *  *      
     235129 AACTGCTCTCTATCAATGGCAAAGTTCAACTCTGTTAGTTGAGGACACATATCACCAACAAGTTT
         66 AACTG--CTCTATCAAAAGAAATGTTCAACTCTGTGAGTTGAGTACACACATCACAAAGAAGTTT

                               *       **         *    
     235194 CTGAGAATGCTTCTGTCTATTTTTTATGGGAAGATATTTCCTT
        129 CTGAGAATGCTTCTGTCTAGTTTTTATATGAAGATATTCCCTT

              *  *  * ** *    *      *    *          * **                    
     235237 TTTCACCGTAGGCGTCAAGGCGATCGAAATGTCCACTTCCACAAACTACAAAAAGAGTGTTTCAA
          1 TTCCAACGAAATCATCAAAGCGATCCAAATATCCACTTCCAGATTCTACAAAAAGAGTGTTTCAA

             *         *   * **       *    *        * ** * *   **      *     
     235302 ACCTGCTCTATGAAAGGCCATGTTCATCTCTATGAGTTGAATGGAAATATCCGAAAGAAATTTCT
         66 AACTGCTCTATCAAAAGAAATGTTCAACTCTGTGAGTTGAGTACACACATCACAAAGAAGTTTCT

             *       *          *        *         **
     235367 GGGAATGCTGCTGTCTAG-TGTT-TAT-ACGA-ATTCCCGC
        131 GAGAATGCTTCTGTCTAGTTTTTATATGAAGATATTCCCTT

                         *       *                *    *  *                  
     235404 TTCCAACGAAATCCTCAAAGCAATCCAAATATCCACTTGCAGAATCCACAAAAAGAGTGTTTCAA
          1 TTCCAACGAAATCATCAAAGCGATCCAAATATCCACTTCCAGATTCTACAAAAAGAGTGTTTCAA

                          *     *          * *                   *  *        
     235469 AACTGCTCTATCAATAGAAAGGTTCAACTCTTTTAGTTGAGTACACACATCACGAACAAGTTTCT
         66 AACTGCTCTATCAAAAGAAATGTTCAACTCTGTGAGTTGAGTACACACATCACAAAGAAGTTTCT

                            * *      **     **  *    
     235534 GAGAATGCTTCTGTCTGGCTTTTATTGGAAGACGTTTCCTT
        131 GAGAATGCTTCTGTCTAGTTTTTATATGAAGATATTCCCTT

                   *  **          *       *                * *               
     235575 TT-CAACAAAGGCATCAAAGCGCTCCAAATGTCCACTTCCAGATTCTTCCAAAAGAGTGTTTCAA
          1 TTCCAACGAAATCATCAAAGCGATCCAAATATCCACTTCCAGATTCTACAAAAAGAGTGTTTCAA

             **     * ***  * *                 *    * *  * *     *   *       
     235639 ACGTGCTCAAAGTAAGGGAATGTTCAACTCTGTGACTTGAATGCAGATATCACCAAGTAGTTTCT
         66 AACTGCTCTATCAAAAGAAATGTTCAACTCTGTGAGTTGAGTACACACATCACAAAGAAGTTTCT

            * * *             *     *    *         * 
     235704 AATAGTGCTTCTGTCTAGATTTTAGATGATGATATTCCCGT
        131 GAGAATGCTTCTGTCTAGTTTTTATATGAAGATATTCCCTT

                         *** *   *             *  *   *      *     *       * 
     235745 TTCCAACGAAATCGCTAGAGCTATCCAAATATCCAGTTACAGTTTCTACCAAAAGGGTGTTTCCA
          1 TTCCAACGAAATCATCAAAGCGATCCAAATATCCACTTCCAGATTCTACAAAAAGAGTGTTTCAA

              *    **           *            *       *                     * 
     235810 AATTGCTGCATCAAAAGAAAGGTTCAACTCTGTTAGTTGAGGACACACATCACAAAGAAGTTTGT
         66 AACTGCTCTATCAAAAGAAATGTTCAACTCTGTGAGTTGAGTACACACATCACAAAGAAGTTTCT

                              *    *     *           
     235875 GAGAATGCTTCTGTCTAGATTTTGTATGACGATATTCCCTT
        131 GAGAATGCTTCTGTCTAGTTTTTATATGAAGATATTCCCTT

                     *   * *     *   *       * *  *    *  *       *  *       
     235916 TTCCAACGATATCGTTAAAGCAATCTAAATATCAATTTGCAGAATCCACAAAAATAGAGTTTCAA
          1 TTCCAACGAAATCATCAAAGCGATCCAAATATCCACTTCCAGATTCTACAAAAAGAGTGTTTCAA

             *       * *        *    *       *  *                   ***      
     235981 AGCTGCTCTGTAAAAAGAAAGGTTCCACTCTGTTAGCTGAGTACACACATCACAAACTTGTTTCT
         66 AACTGCTCTATCAAAAGAAATGTTCAACTCTGTGAGTTGAGTACACACATCACAAAGAAGTTTCT

                  *         *        **        ***   
     236046 GAGAATCCTTCTGTCTCGTTTTTATGGGAAGATATATACTT
        131 GAGAATGCTTCTGTCTAGTTTTTATATGAAGATATTCCCTT

                 *  * **          *       *     *       *  *  *  *           
     236087 TTCCACCGTAGGCATCAAAGCGCTCCAAATGTCCACATCCAGATACTCCAGAACGAGTGTTTCAA
          1 TTCCAACGAAATCATCAAAGCGATCCAAATATCCACTTCCAGATTCTACAAAAAGAGTGTTTCAA

             *         *   * *   *         *        * *  *      *      *     
     236152 ACCTGCTCTATGAAAGGGAATCTTCAACTCTATGAGTTGAATGCAGACATCAGAAAGAAATTTCT
         66 AACTGCTCTATCAAAAGAAATGTTCAACTCTGTGAGTTGAGTACACACATCACAAAGAAGTTTCT

                     *       **          *         **
     236217 GAGAATGCTGCTGTCTACCTTTTAT-T--TGA-ATTCCCGC
        131 GAGAATGCTTCTGTCTAGTTTTTATATGAAGATATTCCCTT

                         *  *    *              * *  **   *    *             
     236254 TTCCAACGAAATCCTCCAAGCTATCCAAATATCCACCTGCATTTTCCACAACAAGAGTGTTTCAA
          1 TTCCAACGAAATCATCAAAGCGATCCAAATATCCACTTCCAGATTCTACAAAAAGAGTGTTTCAA

                          *                 *    *  *                *       
     236319 AACTGCTCTATCAATAGAAATGTTCAACTCCTTTG-GCTGGGTACACACATCACAAACAAGTTTC
         66 AACTGCTCTATCAAAAGAAATGTTCAACT-CTGTGAGTTGAGTACACACATCACAAAGAAGTTTC

                                      **     **       
     236383 TGAGAATGCTTCTGTCTAGTTTTTATGGGAAGACGTTCCCTT
        130 TGAGAATGCTTCTGTCTAGTTTTTATATGAAGATATTCCCTT

              *  * *  **          *       *            **                  * 
     236425 TTTCACCAAAGGCATCAAAGCGCTCCAAATGTCCACTTCCAGACACTACAAAAAGAGTGTTTCCA
          1 TTCCAACGAAATCATCAAAGCGATCCAAATATCCACTTCCAGATTCTACAAAAAGAGTGTTTCAA

             **       **   ***                 *    * *  * *         *       
     236490 ACGTGCTCTAAGAAAGCGAATGTTCAACTCTGTGACTTGAATGCAGATATCACAAAGTAGTTTCT
         66 AACTGCTCTATCAAAAGAAATGTTCAACTCTGTGAGTTGAGTACACACATCACAAAGAAGTTTCT

                **            *     *    *         * 
     236555 GAGAGGGCTTCTGTCTAGATTTTAGATGATGATATTCCCGT
        131 GAGAATGCTTCTGTCTAGTTTTTATATGAAGATATTCCCTT

                           * *   *                *   *                    * 
     236596 TTCCAACGAAATCATTAGAGCTATCCAAATATCCACTTACAGTTTCTACAAAAAGAGTGTTTCCA
          1 TTCCAACGAAATCATCAAAGCGATCCAAATATCCACTTCCAGATTCTACAAAAAGAGTGTTTCAA

                   **         * *    *       *  *                   ***      
     236661 AACTGCTGCATCAAAAGAGAGGTTCCACTCTGTTAGCTGAGTACACACATCACAAACTTGTTTCT
         66 AACTGCTCTATCAAAAGAAATGTTCAACTCTGTGAGTTGAGTACACACATCACAAAGAAGTTTCT

            *     *         *        **         **   
     236726 CAGAATCCTTCTGTCTCGTTTTTATGGGAAGATATTTACTT
        131 GAGAATGCTTCTGTCTAGTTTTTATATGAAGATATTCCCTT

              *  *  * **          *       *     *       *  *  *              
     236767 TTTCACCGTAGGCATCAAAGCGCTCCAAATGTCCACATCCAGATACTCCAGAAAGAGTGTTTCAA
          1 TTCCAACGAAATCATCAAAGCGATCCAAATATCCACTTCCAGATTCTACAAAAAGAGTGTTTCAA

             *         *   * *             *        * *  *      *      *     
     236832 ACCTGCTCTATGAAAGGGAATGTTCAACTCTATGAGTTGAATGCAGACATCAGAAAGAAATTTCT
         66 AACTGCTCTATCAAAAGAAATGTTCAACTCTGTGAGTTGAGTACACACATCACAAAGAAGTTTCT

                     *       **          *         **
     236897 GAGAATGCTGCTGTCTACCTTTTAT-T--TGA-ATTCCCGC
        131 GAGAATGCTTCTGTCTAGTTTTTATATGAAGATATTCCCTT

                         *  *    *                *       *                  
     236934 TTCCAACGAAATCCTCCAAGCTATCCAAATATCCACTTGCAGATTCCACAAAAAGAGTGTTTCAA
          1 TTCCAACGAAATCATCAAAGCGATCCAAATATCCACTTCCAGATTCTACAAAAAGAGTGTTTCAA

                            ** *  *            *       *     *     *  *      
     236999 AACTGCTCTCTATCAATGGCAAAGTTCAACTCTGTTAGTTGAGGACACATATCACCAACAAGTTT
         66 AACTG--CTCTATCAAAAGAAATGTTCAACTCTGTGAGTTGAGTACACACATCACAAAGAAGTTT

                               *       **         *    
     237064 CTGAGAATGCTTCTGTCTATTTTTTATGGGAAGATATTTCCTT
        129 CTGAGAATGCTTCTGTCTAGTTTTTATATGAAGATATTCCCTT

              *  *  * ** *    *      *    *          * **                    
     237107 TTTCACCGTAGGCGTCAAGGCGATCGAAATGTCCACTTCCACAAACTACAAAAAGAGTGTTTCAA
          1 TTCCAACGAAATCATCAAAGCGATCCAAATATCCACTTCCAGATTCTACAAAAAGAGTGTTTCAA

             *         *   * **       *    *        * ** * *   **      *     
     237172 ACCTGCTCTATGAAAGGCCATGTTCATCTCTATGAGTTGAATGGATATATCGGAAAGAAATTTCT
         66 AACTGCTCTATCAAAAGAAATGTTCAACTCTGTGAGTTGAGTACACACATCACAAAGAAGTTTCT

             *       *                   *         **
     237237 GGGAATGCTGCTGTCTAG-TTTT-TAT-ACGA-ATTCCCGC
        131 GAGAATGCTTCTGTCTAGTTTTTATATGAAGATATTCCCTT

                         *       *                *    *  *                  
     237274 TTCCAACGAAATCCTCAAAGCAATCCAAATATCCACTTGCAGAATCCACAAAAAGAGTGTTTCAA
          1 TTCCAACGAAATCATCAAAGCGATCCAAATATCCACTTCCAGATTCTACAAAAAGAGTGTTTCAA

                          *     *          * *                   *  *        
     237339 AACTGCTCTATCAATAGAAAGGTTCAACTCTTTTAGTTGAGTACACACATCACGAACAAGTTTCT
         66 AACTGCTCTATCAAAAGAAATGTTCAACTCTGTGAGTTGAGTACACACATCACAAAGAAGTTTCT

                            * *      **     **  *    
     237404 GAGAATGCTTCTGTCTGGCTTTTATTGGAAGACGTTTCCTT
        131 GAGAATGCTTCTGTCTAGTTTTTATATGAAGATATTCCCTT

                 * *  **          *       *                * *               
     237445 TT-CACCAAAGGCATCAAAGCGCTCCAAATGTCCACTTCCAGATTCTTCCAAAAGAGTGTTTCAA
          1 TTCCAACGAAATCATCAAAGCGATCCAAATATCCACTTCCAGATTCTACAAAAAGAGTGTTTCAA

             **     * ***  * *                 *    * *  * *     *   *       
     237509 ACGTGCTCAAAGTAAGGGAATGTTCAACTCTGTGACTTGAATGCAGATATCACCAAGTAGTTTCT
         66 AACTGCTCTATCAAAAGAAATGTTCAACTCTGTGAGTTGAGTACACACATCACAAAGAAGTTTCT

            * * *            **     *    *         * 
     237574 AATAGTGCTTCTGTCTACATTTTAGATGATGATATTCCCGT
        131 GAGAATGCTTCTGTCTAGTTTTTATATGAAGATATTCCCTT

                         * * *   *             *  *   *      *     *       * 
     237615 TTCCAACGAAATCGTTAGAGCTATCCAAATATCCAGTTACAGTTTCTACCAAAAGGGTGTTTCCA
          1 TTCCAACGAAATCATCAAAGCGATCCAAATATCCACTTCCAGATTCTACAAAAAGAGTGTTTCAA

              *    **           *            *       *                     * 
     237680 AATTGCTGCATCAAAAGAAAGGTTCAACTCTGTTAGTTGAGGACACACATCACAAAGAAGTTTGT
         66 AACTGCTCTATCAAAAGAAATGTTCAACTCTGTGAGTTGAGTACACACATCACAAAGAAGTTTCT

                              *    *     *           
     237745 GAGAATGCTTCTGTCTAGATTTTGTATGACGATATTCCCTT
        131 GAGAATGCTTCTGTCTAGTTTTTATATGAAGATATTCCCTT

                     *   * *     *   *       * *  *    *  *       *  *       
     237786 TTCCAACGATATCGTTAAAGCAATCTAAATATCAATTTGCAGAATCCACAAAAATAGAGTTTCAA
          1 TTCCAACGAAATCATCAAAGCGATCCAAATATCCACTTCCAGATTCTACAAAAAGAGTGTTTCAA

             *       * *        *    *       *  *                   ***      
     237851 AGCTGCTCTGTAAAAAGAAAGGTTCCACTCTGTTAGCTGAGTACACACATCACAAACTTGTTTCT
         66 AACTGCTCTATCAAAAGAAATGTTCAACTCTGTGAGTTGAGTACACACATCACAAAGAAGTTTCT

                  *         *        **         **   
     237916 GAGAATCCTTCTGTCTCGTTTTTATGGGAAGATATTTACTT
        131 GAGAATGCTTCTGTCTAGTTTTTATATGAAGATATTCCCTT

                 *  * **          *       *     *       *  *  *              
     237957 TTCCACCGTAGGCATCAAAGCGCTCCAAATGTCCACATCCAGATACTCCAGAAAGAGTGTTTCAA
          1 TTCCAACGAAATCATCAAAGCGATCCAAATATCCACTTCCAGATTCTACAAAAAGAGTGTTTCAA

             *         *   * *   *         *        * *  *      *      *     
     238022 ACCTGCTCTATGAAAGGGAATCTTCAACTCTATGAGTTGAATGCAGACATCAGAAAGAAATTTCT
         66 AACTGCTCTATCAAAAGAAATGTTCAACTCTGTGAGTTGAGTACACACATCACAAAGAAGTTTCT

                     *       **          *         **
     238087 GAGAATGCTGCTGTCTACCTTTTAT-T--TGA-ATTCCCGC
        131 GAGAATGCTTCTGTCTAGTTTTTATATGAAGATATTCCCTT

                         *  *    *              * *  **   *                  
     238124 TTCCAACGAAATCCTCCAAGCTATCCAAATATCCACCTGCATTTTCCACAAAAAGAGTGTTTCAA
          1 TTCCAACGAAATCATCAAAGCGATCCAAATATCCACTTCCAGATTCTACAAAAAGAGTGTTTCAA

                          *                 *    *  *                *       
     238189 AACTGCTCTATCAATAGAAATGTTCAACTCCTTTG-GCTGGGTACACACATCACAAACAAGTTTC
         66 AACTGCTCTATCAAAAGAAATGTTCAACT-CTGTGAGTTGAGTACACACATCACAAAGAAGTTTC

                                      **     *        
     238253 TGAGAATGCTTCTGTCTAGTTTTTATGGGAAGACATTCCCTT
        130 TGAGAATGCTTCTGTCTAGTTTTTATATGAAGATATTCCCTT

              *  * *  **          *       *            **                  * 
     238295 TTTCACCAAAGGCATCAAAGCGCTCCAAATGTCCACTTCCAGACACTACAAAAAGAGTGTTTCCA
          1 TTCCAACGAAATCATCAAAGCGATCCAAATATCCACTTCCAGATTCTACAAAAAGAGTGTTTCAA

             **       **   ***                 *    * *  * *         *       
     238360 ACGTGCTCTAAGAAAGCGAATGTTCAACTCTGTGACTTGAATGCAGATATCACAAAGTAGTTTCT
         66 AACTGCTCTATCAAAAGAAATGTTCAACTCTGTGAGTTGAGTACACACATCACAAAGAAGTTTCT

                *             *     *    *         * 
     238425 GAGAGTGCTTCTGTCTAGATTTTAGATGATGATATTCCCGT
        131 GAGAATGCTTCTGTCTAGTTTTTATATGAAGATATTCCCTT

                           * *   *                *   *                    * 
     238466 TTCCAACGAAATCATTAGAGCTATCCAAATATCCACTTACAGTTTCTACAAAAAGAGTGTTTCCA
          1 TTCCAACGAAATCATCAAAGCGATCCAAATATCCACTTCCAGATTCTACAAAAAGAGTGTTTCAA

                   **         * *    *       *  *                   ***      
     238531 AACTGCTGCATCAAAAGAGAGGTTCCACTCTGTTAGCTGAGTACACACATCACAAACTTGTTTCT
         66 AACTGCTCTATCAAAAGAAATGTTCAACTCTGTGAGTTGAGTACACACATCACAAAGAAGTTTCT

            *     *         *        **         **   
     238596 CAGAATCCTTCTGTCTCGTTTTTATGGGAAGATATTTACTT
        131 GAGAATGCTTCTGTCTAGTTTTTATATGAAGATATTCCCTT

              *  *  * **          *       *     *       *  *  *              
     238637 TTTCACCGTAGGCATCAAAGCGCTCCAAATGTCCACATCCAGATACTCCAGAAAGAGTGTTTCAA
          1 TTCCAACGAAATCATCAAAGCGATCCAAATATCCACTTCCAGATTCTACAAAAAGAGTGTTTCAA

             *         *   * *   *         *        * *  *      *      *     
     238702 ACCTGCTCTATGAAAGGGAATCTTCAACTCTATGAGTTGAATGCAGACATCAGAAAGAAATTTCT
         66 AACTGCTCTATCAAAAGAAATGTTCAACTCTGTGAGTTGAGTACACACATCACAAAGAAGTTTCT

                     *       **          *         **
     238767 GAGAATGCTGCTGTCTACCTTTTAT-T--TGA-ATTCCCGC
        131 GAGAATGCTTCTGTCTAGTTTTTATATGAAGATATTCCCTT

                         *  *    *                *       *                  
     238804 TTCCAACGAAATCCTCCAAGCTATCCAAATATCCACTTGCAGATTCCACAAAAAGAGTGTTTCAA
          1 TTCCAACGAAATCATCAAAGCGATCCAAATATCCACTTCCAGATTCTACAAAAAGAGTGTTTCAA

                            ** *  *            *       *     *     *  *      
     238869 AACTGCTCTCTATCAATGGCAAAGTTCAACTCTGTTAGTTGAGGACACATATCACCAACAAGTTT
         66 AACTG--CTCTATCAAAAGAAATGTTCAACTCTGTGAGTTGAGTACACACATCACAAAGAAGTTT

                               *       **         *    
     238934 CTGAGAATGCTTCTGTCTATTTTTTATGGGAAGATATTTCCTT
        129 CTGAGAATGCTTCTGTCTAGTTTTTATATGAAGATATTCCCTT

              *  *  * ** *    *      *    *          * **                    
     238977 TTTCACCGTAGGCGTCAAGGCGATCGAAATGTCCACTTCCACAAACTACAAAAAGAGTGTTTCAA
          1 TTCCAACGAAATCATCAAAGCGATCCAAATATCCACTTCCAGATTCTACAAAAAGAGTGTTTCAA

             *         *   * **       *    *        * ** * *   **      *     
     239042 ACCTGCTCTATGAAAGGCCATGTTCATCTCTATGAGTTGAATGGAAATATCCGAAAGAAATTTCT
         66 AACTGCTCTATCAAAAGAAATGTTCAACTCTGTGAGTTGAGTACACACATCACAAAGAAGTTTCT

             * *     *          *        *         **
     239107 GGGCATGCTGCTGTCTAG-TGTT-TAT-ACGA-ATTCCCGC
        131 GAGAATGCTTCTGTCTAGTTTTTATATGAAGATATTCCCTT

                         *       *                *    *  *                  
     239144 TTCCAACGAAATCCTCAAAGCAATCCAAATATCCACTTGCAGAATCCACAAAAAGAGTGTTTCAA
          1 TTCCAACGAAATCATCAAAGCGATCCAAATATCCACTTCCAGATTCTACAAAAAGAGTGTTTCAA

                          *     *          * *                   *  *        
     239209 AACTGCTCTATCAATAGAAAGGTTCAACTCTTTTAGTTGAGTACACACATCACGAACAAGTTTCT
         66 AACTGCTCTATCAAAAGAAATGTTCAACTCTGTGAGTTGAGTACACACATCACAAAGAAGTTTCT

                            * *      **     **  *    
     239274 GAGAATGCTTCTGTCTGGCTTTTATTGGAAGACGTTTCCTT
        131 GAGAATGCTTCTGTCTAGTTTTTATATGAAGATATTCCCTT

                 * *  **          *       *                * *               
     239315 TT-CACCAAAGGCATCAAAGCGCTCCAAATGTCCACTTCCAGATTCTTCCAAAAGAGTGTTTCAA
          1 TTCCAACGAAATCATCAAAGCGATCCAAATATCCACTTCCAGATTCTACAAAAAGAGTGTTTCAA

             **     * ***  * *                 *    * *  * *     *   *       
     239379 ACGTGCTCAAAGTAAGGGAATGTTCAACTCTGTGACTTGAATGCAGATATCACCAAGTAGTTTCT
         66 AACTGCTCTATCAAAAGAAATGTTCAACTCTGTGAGTTGAGTACACACATCACAAAGAAGTTTCT

            * * *             *     *    *         * 
     239444 AATAGTGCTTCTGTCTAGATTTTAGATGATGATATTCCCGT
        131 GAGAATGCTTCTGTCTAGTTTTTATATGAAGATATTCCCTT

                         *** *   *             *  *   *      *     *       * 
     239485 TTCCAACGAAATCGCTAGAGCTATCCAAATATCCAGTTACAGTTTCTACTAAAAGGGTGTTTCCA
          1 TTCCAACGAAATCATCAAAGCGATCCAAATATCCACTTCCAGATTCTACAAAAAGAGTGTTTCAA

              *    **           *            *       *                     * 
     239550 AATTGCTGCATCAAAAGAAAGGTTCAACTCTGTTAGTTGAGGACACACATCACAAAGAAGTTTGT
         66 AACTGCTCTATCAAAAGAAATGTTCAACTCTGTGAGTTGAGTACACACATCACAAAGAAGTTTCT

                              *    *     *           
     239615 GAGAATGCTTCTGTCTAGATTTTGTATGACGATATTCCCTT
        131 GAGAATGCTTCTGTCTAGTTTTTATATGAAGATATTCCCTT

                     *   * *     *   *       * *  *    *  *       *  *       
     239656 TTCCAACGATATCGTTAAAGCAATCTAAATATCAATTTGCAGAATCCACAAAAATAGAGTTTCAA
          1 TTCCAACGAAATCATCAAAGCGATCCAAATATCCACTTCCAGATTCTACAAAAAGAGTGTTTCAA

             *       * *        *    *       *  *                   ***      
     239721 AGCTGCTCTGTAAAAAGAAAGGTTCCACTCTGTTAGCTGAGTACACACATCACAAACTTGTTTCT
         66 AACTGCTCTATCAAAAGAAATGTTCAACTCTGTGAGTTGAGTACACACATCACAAAGAAGTTTCT

                  *         *        **         **   
     239786 GAGAATCCTTCTGTCTCGTTTTTATGGGAAGATATTTACTT
        131 GAGAATGCTTCTGTCTAGTTTTTATATGAAGATATTCCCTT

                 *  * **          *       *     *       *  *  *  *           
     239827 TTCCACCGTAGGCATCAAAGCGCTCCAAATGTCCACATCCAGATACTCCAGAACGAGTGTTTCAA
          1 TTCCAACGAAATCATCAAAGCGATCCAAATATCCACTTCCAGATTCTACAAAAAGAGTGTTTCAA

             *         *   * *   *         *        * *  *      *      *     
     239892 ACCTGCTCTATGAAAGGGAATCTTCAACTCTATGAGTTGAATGCAGACATCAGAAAGAAATTTCT
         66 AACTGCTCTATCAAAAGAAATGTTCAACTCTGTGAGTTGAGTACACACATCACAAAGAAGTTTCT

                     *       **          *         **
     239957 GAGAATGCTGCTGTCTACCTTTTAT-T--TGA-ATTCCCGC
        131 GAGAATGCTTCTGTCTAGTTTTTATATGAAGATATTCCCTT

                         *  *    *              * *  **   *    *             
     239994 TTCCAACGAAATCCTCCAAGCTATCCAAATATCCACCTGCATTTTCCACAACAAGAGTGTTTCAA
          1 TTCCAACGAAATCATCAAAGCGATCCAAATATCCACTTCCAGATTCTACAAAAAGAGTGTTTCAA

                          *                 *    *  *                *       
     240059 AACTGCTCTATCAATAGAAATGTTCAACTCCTTTG-GCTGGGTACACACATCACAAACAAGTTTC
         66 AACTGCTCTATCAAAAGAAATGTTCAACT-CTGTGAGTTGAGTACACACATCACAAAGAAGTTTC

                                      **     **       
     240123 TGAGAATGCTTCTGTCTAGTTTTTATGGGAAGACGTTCCCTT
        130 TGAGAATGCTTCTGTCTAGTTTTTATATGAAGATATTCCCTT

              *  * *  **          *       *            **                  * 
     240165 TTTCACCAAAGGCATCAAAGCGCTCCAAATGTCCACTTCCAGACACTACAAAAAGAGTGTTTCCA
          1 TTCCAACGAAATCATCAAAGCGATCCAAATATCCACTTCCAGATTCTACAAAAAGAGTGTTTCAA

             **       **   ***                 *    * *  * *         *       
     240230 ACGTGCTCTAAGAAAGCGAATGTTCAACTCTGTGACTTGAATGCAGATATCACAAAGTAGTTTCT
         66 AACTGCTCTATCAAAAGAAATGTTCAACTCTGTGAGTTGAGTACACACATCACAAAGAAGTTTCT

                **            *     *    *         * 
     240295 GAGAGGGCTTCTGTCTAGATTTTAGATGATGATATTCCCGT
        131 GAGAATGCTTCTGTCTAGTTTTTATATGAAGATATTCCCTT

                           * *   *                *   *                    * 
     240336 TTCCAACGAAATCATTAGAGCTATCCAAATATCCACTTACAGTTTCTACAAAAAGAGTGTTTCCA
          1 TTCCAACGAAATCATCAAAGCGATCCAAATATCCACTTCCAGATTCTACAAAAAGAGTGTTTCAA

                   **         * *    *       *  *                   ***      
     240401 AACTGCTGCATCAAAAGAGAGGTTCCACTCTGTTAGCTGAGTACACACATCACAAACTTGTTTCT
         66 AACTGCTCTATCAAAAGAAATGTTCAACTCTGTGAGTTGAGTACACACATCACAAAGAAGTTTCT

            *     *         *        **         **   
     240466 CAGAATCCTTCTGTCTCGTTTTTATGGGAAGATATTTACTT
        131 GAGAATGCTTCTGTCTAGTTTTTATATGAAGATATTCCCTT

              *  *  * **          *       *     *       *  *  *              
     240507 TTTCACCGTAGGCATCAAAGCGCTCCAAATGTCCACATCCAGATACTCCAGAAAGAGTGTTTCAA
          1 TTCCAACGAAATCATCAAAGCGATCCAAATATCCACTTCCAGATTCTACAAAAAGAGTGTTTCAA

             *         *   * *   *  *      *        * *  *      *      *     
     240572 ACCTGCTCTATGAAAGGGAATCTTGAACTCTATGAGTTGAATGCAGACATCAGAAAGAAATTTCT
         66 AACTGCTCTATCAAAAGAAATGTTCAACTCTGTGAGTTGAGTACACACATCACAAAGAAGTTTCT

                     *       **          *         **
     240637 GAGAATGCTGCTGTCTACCTTTTAT-T--TGA-ATTCCCGC
        131 GAGAATGCTTCTGTCTAGTTTTTATATGAAGATATTCCCTT

                         *  *    *                *       *                  
     240674 TTCCAACGAAATCCTCCAAGCTATCCAAATATCCACTTGCAGATTCCACAAAAAGAGTGTTTCAA
          1 TTCCAACGAAATCATCAAAGCGATCCAAATATCCACTTCCAGATTCTACAAAAAGAGTGTTTCAA

                            ** *  *            *       *     *     *  *      
     240739 AACTGCTCTCTATCAATGGCAAAGTTCAACTCTGTTAGTTGAGGACACATATCACCAACAAGTTT
         66 AACTG--CTCTATCAAAAGAAATGTTCAACTCTGTGAGTTGAGTACACACATCACAAAGAAGTTT

                               *       **         *    
     240804 CTGAGAATGCTTCTGTCTATTTTTTATGGGAAGATATTTCCTT
        129 CTGAGAATGCTTCTGTCTAGTTTTTATATGAAGATATTCCCTT

              *  *  * ** *    *      *    *          * **                    
     240847 TTTCACCGTAGGCGTCAAGGCGATCGAAATGTCCACTTCCACAAACTACAAAAAGAGTGTTTCAA
          1 TTCCAACGAAATCATCAAAGCGATCCAAATATCCACTTCCAGATTCTACAAAAAGAGTGTTTCAA

             *         *   * **       *    *        * ** * *   **      *     
     240912 ACCTGCTCTATGAAAGGCCATGTTCATCTCTATGAGTTGAATGGATATATCCGAAAGAAATTTCT
         66 AACTGCTCTATCAAAAGAAATGTTCAACTCTGTGAGTTGAGTACACACATCACAAAGAAGTTTCT

             *       *                   *         **
     240977 GGGAATGCTGCTGTCTAG-TTTT-TAT-ACGA-ATTCCCGA
        131 GAGAATGCTTCTGTCTAGTTTTTATATGAAGATATTCCCTT

                         *       *                *    *  *                  
     241014 TTCCAACGAAATCCTCAAAGCAATCCAAATATCCACTTGCAGAATCCACAAAAAGAGTGTTTCAA
          1 TTCCAACGAAATCATCAAAGCGATCCAAATATCCACTTCCAGATTCTACAAAAAGAGTGTTTCAA

                          *     *          * *                   *  *        
     241079 AACTGCTCTATCAATAGAAAGGTTCAACTCTTTTAGTTGAGTACACACATCACGAACAAGTTTCT
         66 AACTGCTCTATCAAAAGAAATGTTCAACTCTGTGAGTTGAGTACACACATCACAAAGAAGTTTCT

                            * *      **     **  *    
     241144 GAGAATGCTTCTGTCTGGCTTTTATTGGAAGACGTTTCCTT
        131 GAGAATGCTTCTGTCTAGTTTTTATATGAAGATATTCCCTT

                 * *  **          *       *                * *               
     241185 TT-CACCAAAGGCATCAAAGCGCTCCAAATGTCCACTTCCAGATTCTTCCAAAAGAGTGTTTCAA
          1 TTCCAACGAAATCATCAAAGCGATCCAAATATCCACTTCCAGATTCTACAAAAAGAGTGTTTCAA

             **     * ***  * *                 *    * *  * *     *   *       
     241249 ACGTGCTCAAAGTAAGGGAATGTTCAACTCTGTGACTTGAATGCAGATATCACCAAGTAGTTTCT
         66 AACTGCTCTATCAAAAGAAATGTTCAACTCTGTGAGTTGAGTACACACATCACAAAGAAGTTTCT

            * * *             *     *    *         * 
     241314 AATAGTGCTTCTGTCTAGATTTTAGATGATGATATTCCCGT
        131 GAGAATGCTTCTGTCTAGTTTTTATATGAAGATATTCCCTT

                         *** *   *             *  *   *      *     *       * 
     241355 TTCCAACGAAATCGCTAGAGCTATCCAAATATCCAGTTACAGTTTCTACTAAAAGGGTGTTTCCA
          1 TTCCAACGAAATCATCAAAGCGATCCAAATATCCACTTCCAGATTCTACAAAAAGAGTGTTTCAA

              *    **           *            *       *                     * 
     241420 AATTGCTGCATCAAAAGAAAGGTTCAACTCTGTTAGTTGAGGACACACATCACAAAGAAGTTTGT
         66 AACTGCTCTATCAAAAGAAATGTTCAACTCTGTGAGTTGAGTACACACATCACAAAGAAGTTTCT

                              *    *     *           
     241485 GAGAATGCTTCTGTCTAGATTTTGTATGACGATATTCCCTT
        131 GAGAATGCTTCTGTCTAGTTTTTATATGAAGATATTCCCTT

                     *   * *     *   *       * *  *    *  *       *  *       
     241526 TTCCAACGATATCGTTAAAGCAATCTAAATATCAATTTGCAGAATCCACAAAAATAGAGTTTCAA
          1 TTCCAACGAAATCATCAAAGCGATCCAAATATCCACTTCCAGATTCTACAAAAAGAGTGTTTCAA

             *       * *        *    *       *  *                   ***      
     241591 AGCTGCTCTGTAAAAAGAAAGGTTCCACTCTGTTAGCTGAGTACACACATCACAAACTTGTTTCT
         66 AACTGCTCTATCAAAAGAAATGTTCAACTCTGTGAGTTGAGTACACACATCACAAAGAAGTTTCT

                  *         *   *    **        ***   
     241656 GAGAATCCTTCTGTCTCGTTCTTATGGGAAGATATATACTT
        131 GAGAATGCTTCTGTCTAGTTTTTATATGAAGATATTCCCTT

                 *    **          *       *     *       *  *  *  *           
     241697 TTCCACCGAAGGCATCAAAGCGCTCCAAATGTCCACATCCAGATACTCCAGAACGAGTGTTTCAA
          1 TTCCAACGAAATCATCAAAGCGATCCAAATATCCACTTCCAGATTCTACAAAAAGAGTGTTTCAA

             *         *   * *   *         *        * *  *      *      *     
     241762 ACCTGCTCTATGAAAGGGAATCTTCAACTCTATGAGTTGAATGCAGACATCAGAAAGAAATTTCT
         66 AACTGCTCTATCAAAAGAAATGTTCAACTCTGTGAGTTGAGTACACACATCACAAAGAAGTTTCT

                     *       **          *         **
     241827 GAGAATGCTGCTGTCTACCTTTTAT-T--TGA-ATTCCCGC
        131 GAGAATGCTTCTGTCTAGTTTTTATATGAAGATATTCCCTT

                         *  *    *              * *  **   *    *             
     241864 TTCCAACGAAATCCTCCAAGCTATCCAAATATCCACCTGCATTTTCCACAACAAGAGTGTTTCAA
          1 TTCCAACGAAATCATCAAAGCGATCCAAATATCCACTTCCAGATTCTACAAAAAGAGTGTTTCAA

                          *                 *    *  *                *       
     241929 AACTGCTCTATCAATAGAAATGTTCAACTCCTTTG-GCTGGGTACACACATCACAAACAAGTTTC
         66 AACTGCTCTATCAAAAGAAATGTTCAACT-CTGTGAGTTGAGTACACACATCACAAAGAAGTTTC

                                      **     **       
     241993 TGAGAATGCTTCTGTCTAGTTTTTATGGGAAGACGTTCCCTT
        130 TGAGAATGCTTCTGTCTAGTTTTTATATGAAGATATTCCCTT

              *  * *  **          *       *            **                  * 
     242035 TTTCACCAAAGGCATCAAAGCGCTCCAAATGTCCACTTCCAGACACTACAAAAAGAGTGTTTCCA
          1 TTCCAACGAAATCATCAAAGCGATCCAAATATCCACTTCCAGATTCTACAAAAAGAGTGTTTCAA

             **       **   ***                 *    * *  * *         *       
     242100 ACGTGCTCTAAGAAAGCGAATGTTCAACTCTGTGACTTGAATGCAGATATCACAAAGTAGTTTCT
         66 AACTGCTCTATCAAAAGAAATGTTCAACTCTGTGAGTTGAGTACACACATCACAAAGAAGTTTCT

                **            *     *    *         * 
     242165 GAGAGGGCTTCTGTCTAGATTTTAGATGATGATATTCCCGT
        131 GAGAATGCTTCTGTCTAGTTTTTATATGAAGATATTCCCTT

                           * *   *                *   *                    * 
     242206 TTCCAACGAAATCATTAGAGCTATCCAAATATCCACTTACAGTTTCTACAAAAAGAGTGTTTCCA
          1 TTCCAACGAAATCATCAAAGCGATCCAAATATCCACTTCCAGATTCTACAAAAAGAGTGTTTCAA

                   **         * *    *       *  *                   ***      
     242271 AACTGCTGCATCAAAAGAGAGGTTCCACTCTGTTAGCTGAGTACACACATCACAAACTTGTTTCT
         66 AACTGCTCTATCAAAAGAAATGTTCAACTCTGTGAGTTGAGTACACACATCACAAAGAAGTTTCT

            *     *         *        **         **   
     242336 CAGAATCCTTCTGTCTCGTTTTTATGGGAAGATATTTACTT
        131 GAGAATGCTTCTGTCTAGTTTTTATATGAAGATATTCCCTT

              *  *  * **          *       *     *       *  *  *              
     242377 TTTCACCGTAGGCATCAAAGCGCTCCAAATGTCCACATCCAGATACTCCAGAAAGAGTGTTTCAA
          1 TTCCAACGAAATCATCAAAGCGATCCAAATATCCACTTCCAGATTCTACAAAAAGAGTGTTTCAA

             *         *   * *   *         *        * *  *      *      *     
     242442 ACCTGCTCTATGAAAGGGAATCTTCAACTCTATGAGTTGAATGCAGACATCAGAAAGAAATTTCT
         66 AACTGCTCTATCAAAAGAAATGTTCAACTCTGTGAGTTGAGTACACACATCACAAAGAAGTTTCT

                     *       **          *         **
     242507 GAGAATGCTGCTGTCTACCTTTTAT-T--TGA-ATTCCCGC
        131 GAGAATGCTTCTGTCTAGTTTTTATATGAAGATATTCCCTT

                         *  *    *                *       *                  
     242544 TTCCAACGAAATCCTCCAAGCTATCCAAATATCCACTTGCAGATTCCACAAAAAGAGTGTTTCAA
          1 TTCCAACGAAATCATCAAAGCGATCCAAATATCCACTTCCAGATTCTACAAAAAGAGTGTTTCAA

                            ** *  *            *       *     *     *  *      
     242609 AACTGCTCTCTATCAATGGCAAAGTTCAACTCTGTTAGTTGAGGACACATATCACCAACAAGTTT
         66 AACTG--CTCTATCAAAAGAAATGTTCAACTCTGTGAGTTGAGTACACACATCACAAAGAAGTTT

                               *       **         *    
     242674 CTGAGAATGCTTCTGTCTATTTTTTATGGGAAGATATTTCCTT
        129 CTGAGAATGCTTCTGTCTAGTTTTTATATGAAGATATTCCCTT

              *  *  * ** *    *      *    *          * **                    
     242717 TTTCACCGTAGGCGTCAAGGCGATCGAAATGTCCACTTCCACAAACTACAAAAAGAGTGTTTCAA
          1 TTCCAACGAAATCATCAAAGCGATCCAAATATCCACTTCCAGATTCTACAAAAAGAGTGTTTCAA

             *         *   * **       *    *        * ** * *   **      *     
     242782 ACCTGCTCTATGAAAGGCCATGTTCATCTCTATGAGTTGAATGGATATATCCGAAAGAAATTTCT
         66 AACTGCTCTATCAAAAGAAATGTTCAACTCTGTGAGTTGAGTACACACATCACAAAGAAGTTTCT

             *       *                   *         **
     242847 GGGAATGCTGCTGTCTAG-TTTT-TAT-ACGA-ATTCCCGC
        131 GAGAATGCTTCTGTCTAGTTTTTATATGAAGATATTCCCTT

                         *       *                *    *  *                  
     242884 TTCCAACGAAATCCTCAAAGCAATCCAAATATCCACTTGCAGAATCCACAAAAAGAGTGTTTCAA
          1 TTCCAACGAAATCATCAAAGCGATCCAAATATCCACTTCCAGATTCTACAAAAAGAGTGTTTCAA

                          *     *          * *                   *  *        
     242949 AACTGCTCTATCAATAGAAAGGTTCAACTCTTTTAGTTGAGTACACACATCACGAACAAGTTTCT
         66 AACTGCTCTATCAAAAGAAATGTTCAACTCTGTGAGTTGAGTACACACATCACAAAGAAGTTTCT

                            * *      **     **  *    
     243014 GAGAATGCTTCTGTCTGGCTTTTATTGGAAGACGTTTCCTT
        131 GAGAATGCTTCTGTCTAGTTTTTATATGAAGATATTCCCTT

                 * *  **          *       *                * *               
     243055 TT-CACCAAAGGCATCAAAGCGCTCCAAATGTCCACTTCCAGATTCTTCCAAAAGAGTGTTTCAA
          1 TTCCAACGAAATCATCAAAGCGATCCAAATATCCACTTCCAGATTCTACAAAAAGAGTGTTTCAA

             **     * ***  * *                 *    * *  * *     *   *       
     243119 ACGTGCTCAAAGTAAGGGAATGTTCAACTCTGTGACTTGAATGCAGATATCACCAAGTAGTTTCT
         66 AACTGCTCTATCAAAAGAAATGTTCAACTCTGTGAGTTGAGTACACACATCACAAAGAAGTTTCT

            * * *            **     *    *         * 
     243184 AATAGTGCTTCTGTCTACATTTTAGATGATGATATTCCCGT
        131 GAGAATGCTTCTGTCTAGTTTTTATATGAAGATATTCCCTT

                         * * *   *             *  *   *      *     *       * 
     243225 TTCCAACGAAATCGTTAGAGCTATCCAAATATCCAGTTACAGTTTCTACCAAAAGGGTGTTTCCA
          1 TTCCAACGAAATCATCAAAGCGATCCAAATATCCACTTCCAGATTCTACAAAAAGAGTGTTTCAA

              *    **           *            *       *                     * 
     243290 AATTGCTGCATCAAAAGAAAGGTTCAACTCTGTTAGTTGAGGACACACATCACAAAGAAGTTTGT
         66 AACTGCTCTATCAAAAGAAATGTTCAACTCTGTGAGTTGAGTACACACATCACAAAGAAGTTTCT

                              *    *     *           
     243355 GAGAATGCTTCTGTCTAGATTTTGTATGACGATATTCCCTT
        131 GAGAATGCTTCTGTCTAGTTTTTATATGAAGATATTCCCTT

                     *   * *     *   *       * *  *    *          *  *       
     243396 TTCCAACGATATCGTTAAAGCAATCTAAATATCAATTTGCAGAATCTACAAAAATAGAGTTTCAA
          1 TTCCAACGAAATCATCAAAGCGATCCAAATATCCACTTCCAGATTCTACAAAAAGAGTGTTTCAA

             *       * *        *    *       *  *                   ***      
     243461 AGCTGCTCTGTAAAAAGAAAGGTTCCACTCTGTTAGCTGAGTACACACATCACAAACTTGTTTCT
         66 AACTGCTCTATCAAAAGAAATGTTCAACTCTGTGAGTTGAGTACACACATCACAAAGAAGTTTCT

                  *         *        **         **   
     243526 GAGAATCCTTCTGTCTCGTTTTTATGGGAAGATATTTACTT
        131 GAGAATGCTTCTGTCTAGTTTTTATATGAAGATATTCCCTT

                 *  * **          *       *     *       *  *  *  *           
     243567 TTCCACCGTAGGCATCAAAGCGCTCCAAATGTCCACATCCAGATACTCCAGAACGAGTGTTTCAA
          1 TTCCAACGAAATCATCAAAGCGATCCAAATATCCACTTCCAGATTCTACAAAAAGAGTGTTTCAA

             *         *   * *   *         *        * *  *      *      *     
     243632 ACCTGCTCTATGAAAGGGAATCTTCAACTCTATGAGTTGAATGCAGACATCAGAAAGAAATTTCT
         66 AACTGCTCTATCAAAAGAAATGTTCAACTCTGTGAGTTGAGTACACACATCACAAAGAAGTTTCT

                     *       **          *         **
     243697 GAGAATGCTGCTGTCTACCTTTTAT-T--TGA-ATTCCCGC
        131 GAGAATGCTTCTGTCTAGTTTTTATATGAAGATATTCCCTT

                         *  *    *              * *  **  **                  
     243734 TTCCAACGAAATCCTCCAAGCTATCCAAATATCCACCTGCATTTTACACAAAAAGAGTGTTTCAA
          1 TTCCAACGAAATCATCAAAGCGATCCAAATATCCACTTCCAGATTCTACAAAAAGAGTGTTTCAA

                            ** *  *            *       *     *     *  *      
     243799 AACTGCTCTCTATCAATGGCAAAGTTCAACTCTGTTAGTTGAGGACACATATCACCAACAAGTTT
         66 AACTG--CTCTATCAAAAGAAATGTTCAACTCTGTGAGTTGAGTACACACATCACAAAGAAGTTT

                               *       **         *    
     243864 CTGAGAATGCTTCTGTCTATTTTTTATGGGAAGATATTTCCTT
        129 CTGAGAATGCTTCTGTCTAGTTTTTATATGAAGATATTCCCTT

              *  *  * ** *    *      *    *          * **                    
     243907 TTTCACCGTAGGCGTCAAGGCGATCGAAATGTCCACTTCCACAAACTACAAAAAGAGTGTTTCAA
          1 TTCCAACGAAATCATCAAAGCGATCCAAATATCCACTTCCAGATTCTACAAAAAGAGTGTTTCAA

             *         *   * **       *    *        * ** * **  **      *     
     243972 ACCTGCTCTATGAAAGGCCATGTTCATCTCTATGAGTTGAATGGAAATTTCCGAAAGAAATTTCT
         66 AACTGCTCTATCAAAAGAAATGTTCAACTCTGTGAGTTGAGTACACACATCACAAAGAAGTTTCT

             *       *          *        *         **
     244037 GGGAATGCTGCTGTCTAG-TGTT-TAT-ACGA-ATTCCCGC
        131 GAGAATGCTTCTGTCTAGTTTTTATATGAAGATATTCCCTT

                         *       *                *    *  *                  
     244074 TTCCAACGAAATCCTCAAAGCAATCCAAATATCCACTTGCAGAATCCACAAAAAGAGTGTTTCAA
          1 TTCCAACGAAATCATCAAAGCGATCCAAATATCCACTTCCAGATTCTACAAAAAGAGTGTTTCAA

                          *     *          * *                   *  *        
     244139 AACTGCTCTATCAATAGAAAGGTTCAACTCTTTTAGTTGAGTACACACATCACGAACAAGTTTCT
         66 AACTGCTCTATCAAAAGAAATGTTCAACTCTGTGAGTTGAGTACACACATCACAAAGAAGTTTCT

                            * *      **     **  *    
     244204 GAGAATGCTTCTGTCTGGCTTTTATTGGAAGACGTTTCCTT
        131 GAGAATGCTTCTGTCTAGTTTTTATATGAAGATATTCCCTT

                 * *  **          *       *                * *               
     244245 TT-CAGCAAAGGCATCAAAGCGCTCCAAATGTCCACTTCCAGATTCTTCCAAAAGAGTGTTTCAA
          1 TTCCAACGAAATCATCAAAGCGATCCAAATATCCACTTCCAGATTCTACAAAAAGAGTGTTTCAA

             **     * ***  * *                 *    * *  * *     *   *       
     244309 ACGTGCTCAAAGTAAGGGAATGTTCAACTCTGTGACTTGAATGCAGATATCACCAAGTAGTTTCT
         66 AACTGCTCTATCAAAAGAAATGTTCAACTCTGTGAGTTGAGTACACACATCACAAAGAAGTTTCT

            * * *            **     *    *         * 
     244374 AATAGTGCTTCTGTCTACATTTTAGATGATGATATTCCCGT
        131 GAGAATGCTTCTGTCTAGTTTTTATATGAAGATATTCCCTT

                         * * *   *             *  *   *      *     *       * 
     244415 TTCCAACGAAATCGTTAGAGCTATCCAAATATCCAGTTACAGTTTCTACCAAAAGGGTGTTTCCA
          1 TTCCAACGAAATCATCAAAGCGATCCAAATATCCACTTCCAGATTCTACAAAAAGAGTGTTTCAA

              *    **           *            *       *                     * 
     244480 AATTGCTGCATCAAAAGAAAGGTTCAACTCTGTTAGTTGAGGACACACATCACAAAGAAGTTTGT
         66 AACTGCTCTATCAAAAGAAATGTTCAACTCTGTGAGTTGAGTACACACATCACAAAGAAGTTTCT

                              *    *     *           
     244545 GAGAATGCTTCTGTCTAGATTTTGTATGACGATATTCCCTT
        131 GAGAATGCTTCTGTCTAGTTTTTATATGAAGATATTCCCTT

                     *   * *     *   *       * *  *    *  *       *  *       
     244586 TTCCAACGATATCGTTAAAGCAATCTAAATATCAATTTGCAGAATCCACAAAAATAGAGTTTCAA
          1 TTCCAACGAAATCATCAAAGCGATCCAAATATCCACTTCCAGATTCTACAAAAAGAGTGTTTCAA

             *       * *        *    *       *  *                   ***      
     244651 AGCTGCTCTGTAAAAAGAAAGGTTCCACTCTGTTAGCTGAGTACACACATCACAAACTTGTTTCT
         66 AACTGCTCTATCAAAAGAAATGTTCAACTCTGTGAGTTGAGTACACACATCACAAAGAAGTTTCT

                  *         *        **         **   
     244716 GAGAATCCTTCTGTCTCGTTTTTATGGGAAGATATTTACTT
        131 GAGAATGCTTCTGTCTAGTTTTTATATGAAGATATTCCCTT

                 *  * **          *       *     *       *  *  *              
     244757 TTCCACCGTAGGCATCAAAGCGCTCCAAATGTCCACATCCAGATACTCCAGAAAGAGTGTTTCAA
          1 TTCCAACGAAATCATCAAAGCGATCCAAATATCCACTTCCAGATTCTACAAAAAGAGTGTTTCAA

             *         *   * *   *         *        * *  *      *      *     
     244822 ACCTGCTCTATGAAAGGGAATCTTCAACTCTATGAGTTGAATGCAGACATCAGAAAGAAATTTCT
         66 AACTGCTCTATCAAAAGAAATGTTCAACTCTGTGAGTTGAGTACACACATCACAAAGAAGTTTCT

                     *       **          *         **
     244887 GAGAATGCTGCTGTCTACCTTTTAT-T--TGA-ATTCCCGC
        131 GAGAATGCTTCTGTCTAGTTTTTATATGAAGATATTCCCTT

                         *  *    *                *  **   *                  
     244924 TTCCAACGAAATCCTCCAAGCTATCCAAATATCCACTTGCATTTTCCACAAAAAGAGTGTTTCAA
          1 TTCCAACGAAATCATCAAAGCGATCCAAATATCCACTTCCAGATTCTACAAAAAGAGTGTTTCAA

                          *                 *    *  *                *       
     244989 AACTGCTCTATCAATAGAAATGTTCAACTCCTTTG-GCTGGGTACACACATCACAAACAAGTTTC
         66 AACTGCTCTATCAAAAGAAATGTTCAACT-CTGTGAGTTGAGTACACACATCACAAAGAAGTTTC

                                      **     **       
     245053 TGAGAATGCTTCTGTCTAGTTTTTATGGGAAGACGTTCCCTT
        130 TGAGAATGCTTCTGTCTAGTTTTTATATGAAGATATTCCCTT

              *  * *  **          *       *            **                  * 
     245095 TTTCACCAAAGGCATCAAAGCGCTCCAAATGTCCACTTCCAGACACTACAAAAAGAGTGTTTCCA
          1 TTCCAACGAAATCATCAAAGCGATCCAAATATCCACTTCCAGATTCTACAAAAAGAGTGTTTCAA

             **       **   ***                 *    * *  * *         *       
     245160 ACGTGCTCTAAGAAAGCGAATGTTCAACTCTGTGACTTGAATGCAGATATCACAAAGTAGTTTCT
         66 AACTGCTCTATCAAAAGAAATGTTCAACTCTGTGAGTTGAGTACACACATCACAAAGAAGTTTCT

                **            *     *    *         * 
     245225 GAGAGGGCTTCTGTCTAGATTTTAGATGATGATATTCCCGT
        131 GAGAATGCTTCTGTCTAGTTTTTATATGAAGATATTCCCTT

                           * *   *                *   *                    * 
     245266 TTCCAACGAAATCATTAGAGCTATCCAAATATCCACTTACAGTTTCTACAAAAAGAGTGTTTCCA
          1 TTCCAACGAAATCATCAAAGCGATCCAAATATCCACTTCCAGATTCTACAAAAAGAGTGTTTCAA

                   **         * *    *       *  *                   ***      
     245331 AACTGCTGCATCAAAAGAGAGGTTCCACTCTGTTAGCTGAGTACACACATCACAAACTTGTTTCT
         66 AACTGCTCTATCAAAAGAAATGTTCAACTCTGTGAGTTGAGTACACACATCACAAAGAAGTTTCT

            *     *         *        **         **   
     245396 CAGAATCCTTCTGTCTCGTTTTTATGGGAAGATATTTACTT
        131 GAGAATGCTTCTGTCTAGTTTTTATATGAAGATATTCCCTT

                 *  * **          *       *     *       *  *  *              
     245437 TTCCACCGTAGGCATCAAAGCGCTCCAAATGTCCACATCCAGATACTCCAGAAAGAGTGTTTCAA
          1 TTCCAACGAAATCATCAAAGCGATCCAAATATCCACTTCCAGATTCTACAAAAAGAGTGTTTCAA

             *         *   * *             *        * *  *      *      *     
     245502 ACCTGCTCTATGAAAGGGAATGTTCAACTCTATGAGTTGAATGCAGACATCAGAAAGAAATTTCT
         66 AACTGCTCTATCAAAAGAAATGTTCAACTCTGTGAGTTGAGTACACACATCACAAAGAAGTTTCT

                     *       **          *         **
     245567 GAGAATGCTGCTGTCTACCTTTTAT-T--TGA-ATTCCCGC
        131 GAGAATGCTTCTGTCTAGTTTTTATATGAAGATATTCCCTT

                         *  *    *                *       *                  
     245604 TTCCAACGAAATCCTCCAAGCTATCCAAATATCCACTTGCAGATTCCACAAAAAGAGTGTTTCAA
          1 TTCCAACGAAATCATCAAAGCGATCCAAATATCCACTTCCAGATTCTACAAAAAGAGTGTTTCAA

                            ** *  *            *       *     *     *  *      
     245669 AACTGCTCTCTATCAATGGCAAAGTTCAACTCTGTTAGTTGAGGACACATATCACCAACAAGTTT
         66 AACTG--CTCTATCAAAAGAAATGTTCAACTCTGTGAGTTGAGTACACACATCACAAAGAAGTTT

                               *       **         *    
     245734 CTGAGAATGCTTCTGTCTATTTTTTATGGGAAGATATTTCCTT
        129 CTGAGAATGCTTCTGTCTAGTTTTTATATGAAGATATTCCCTT

              *  *  * ** *    *      *    *          * **                    
     245777 TTTCACCGTAGGCGTCAAGGCGATCGAAATGTCCACTTCCACAAACTACAAAAAGAGTGTTTCAA
          1 TTCCAACGAAATCATCAAAGCGATCCAAATATCCACTTCCAGATTCTACAAAAAGAGTGTTTCAA

             *         *   * **       *    *        * ** * *   **      *     
     245842 ACCTGCTCTATGAAAGGCCATGTTCATCTCTATGAGTTGAATGGAAATATCCGAAAGAAATTTCT
         66 AACTGCTCTATCAAAAGAAATGTTCAACTCTGTGAGTTGAGTACACACATCACAAAGAAGTTTCT

             *       *          *        *         **
     245907 GGGAATGCTGCTGTCTAG-TGTT-TAT-ACGA-ATTCCCGC
        131 GAGAATGCTTCTGTCTAGTTTTTATATGAAGATATTCCCTT

                         *       *                *    *  *                  
     245944 TTCCAACGAAATCCTCAAAGCAATCCAAATATCCACTTGCAGAATCCACAAAAAGAGTGTTTCAA
          1 TTCCAACGAAATCATCAAAGCGATCCAAATATCCACTTCCAGATTCTACAAAAAGAGTGTTTCAA

                          *     *          * *                   *  *        
     246009 AACTGCTCTATCAATAGAAAGGTTCAACTCTTTTAGTTGAGTACACACATCACGAACAAGTTTCT
         66 AACTGCTCTATCAAAAGAAATGTTCAACTCTGTGAGTTGAGTACACACATCACAAAGAAGTTTCT

                            * *      **     **  *    
     246074 GAGAATGCTTCTGTCTGGCTTTTATTGGAAGACGTTTCCTT
        131 GAGAATGCTTCTGTCTAGTTTTTATATGAAGATATTCCCTT

                 * *  **          *       *                * *               
     246115 TT-CACCAAAGGCATCAAAGCGCTCCAAATGTCCACTTCCAGATTCTTCCAAAAGAGTGTTTCAA
          1 TTCCAACGAAATCATCAAAGCGATCCAAATATCCACTTCCAGATTCTACAAAAAGAGTGTTTCAA

             **     * ***  * *                 *    * *  * *     *   *       
     246179 ACGTGCTCAAAGTAAGGGAATGTTCAACTCTGTGACTTGAATGCAGATATCACCAAGTAGTTTCT
         66 AACTGCTCTATCAAAAGAAATGTTCAACTCTGTGAGTTGAGTACACACATCACAAAGAAGTTTCT

            * * *            **     *    *         * 
     246244 AATAGTGCTTCTGTCTACATTTTAGATGATGATATTCCCGT
        131 GAGAATGCTTCTGTCTAGTTTTTATATGAAGATATTCCCTT

                         * * *   *             *  *   *      *     *       * 
     246285 TTCCAACGAAATCGTTAGAGCTATCCAAATATCCAGTTACAGTTTCTACCAAAAGGGTGTTTCCA
          1 TTCCAACGAAATCATCAAAGCGATCCAAATATCCACTTCCAGATTCTACAAAAAGAGTGTTTCAA

              *    **           *            *       *                     * 
     246350 AATTGCTGCATCAAAAGAAAGGTTCAACTCTGTTAGTTGAGGACACACATCACAAAGAAGTTTGT
         66 AACTGCTCTATCAAAAGAAATGTTCAACTCTGTGAGTTGAGTACACACATCACAAAGAAGTTTCT

                              *    *     *           
     246415 GAGAATGCTTCTGTCTAGATTTTGTATGACGATATTCCCTT
        131 GAGAATGCTTCTGTCTAGTTTTTATATGAAGATATTCCCTT

                     *   * *     *   *       * *  *    *  *       *  *       
     246456 TTCCAACGATATCGTTAAAGCAATCTAAATATCAATTTGCAGAATCCACAAAAATAGAGTTTCAA
          1 TTCCAACGAAATCATCAAAGCGATCCAAATATCCACTTCCAGATTCTACAAAAAGAGTGTTTCAA

             *       * *        *    *     * *  *                   ***      
     246521 AGCTGCTCTGTAAAAAGAAAGGTTCCACTCTTTTAGCTGAGTACACACATCACAAACTTGTTTCT
         66 AACTGCTCTATCAAAAGAAATGTTCAACTCTGTGAGTTGAGTACACACATCACAAAGAAGTTTCT

                  *         *        **         **   
     246586 GAGAATCCTTCTGTCTCGTTTTTATGGGAAGATATTTACTT
        131 GAGAATGCTTCTGTCTAGTTTTTATATGAAGATATTCCCTT

                 *  * **  *       *       *     *       *  *  *              
     246627 TTCCACCGTAGGCACCAAAGCGCTCCAAATGTCCACATCCAGATACTCCAGAAAGAGTGTTTCAA
          1 TTCCAACGAAATCATCAAAGCGATCCAAATATCCACTTCCAGATTCTACAAAAAGAGTGTTTCAA

             *         *   * *   *         *        * *  *      *      *     
     246692 ACCTGCTCTATGAAAGGGAATCTTCAACTCTATGAGTTGAATGCAGACATCAGAAAGAAATTTCT
         66 AACTGCTCTATCAAAAGAAATGTTCAACTCTGTGAGTTGAGTACACACATCACAAAGAAGTTTCT

                     *       **   *      *         **
     246757 GAGAATGCTGCTGTCTACCTTTCAT-T--TGA-ATTCCCGC
        131 GAGAATGCTTCTGTCTAGTTTTTATATGAAGATATTCCCTT

                         *  *    *              * *  **   *    *             
     246794 TTCCAACGAAATCCTCCAAGCTATCCAAATATCCACCTGCATTTTCCACAACAAGAGTGTTTCAA
          1 TTCCAACGAAATCATCAAAGCGATCCAAATATCCACTTCCAGATTCTACAAAAAGAGTGTTTCAA

                          *                 *    *  *                *       
     246859 AACTGCTCTATCAATAGAAATGTTCAACTCCTTTG-GCTGGGTACACACATCACAAACAAGTTTC
         66 AACTGCTCTATCAAAAGAAATGTTCAACT-CTGTGAGTTGAGTACACACATCACAAAGAAGTTTC

                                      **     **       
     246923 TGAGAATGCTTCTGTCTAGTTTTTATGGGAAGACGTTCCCTT
        130 TGAGAATGCTTCTGTCTAGTTTTTATATGAAGATATTCCCTT

              *  * *  **          *       *            **                  * 
     246965 TTTCACCAAAGGCATCAAAGCGCTCCAAATGTCCACTTCCAGACACTACAAAAAGAGTGTTTCCA
          1 TTCCAACGAAATCATCAAAGCGATCCAAATATCCACTTCCAGATTCTACAAAAAGAGTGTTTCAA

             **       **   ***                 *    * *  * *         *       
     247030 ACGTGCTCTAAGAAAGCGAATGTTCAACTCTGTGACTTGAATGCAGATATCACAAAGTAGTTTCT
         66 AACTGCTCTATCAAAAGAAATGTTCAACTCTGTGAGTTGAGTACACACATCACAAAGAAGTTTCT

                **            *     *    *         * 
     247095 GAGAGGGCTTCTGTCTAGATTTTAGATGATGATATTCCCGT
        131 GAGAATGCTTCTGTCTAGTTTTTATATGAAGATATTCCCTT

                           * *   *                *   *                    * 
     247136 TTCCAACGAAATCATTAGAGCTATCCAAATATCCACTTACAGTTTCTACAAAAAGAGTGTTTCCA
          1 TTCCAACGAAATCATCAAAGCGATCCAAATATCCACTTCCAGATTCTACAAAAAGAGTGTTTCAA

                   **         * *    *       *  *                   ***      
     247201 AACTGCTGCATCAAAAGAGAGGTTCCACTCTGTTAGCTGAGTACACACATCACAAACTTGTTTCT
         66 AACTGCTCTATCAAAAGAAATGTTCAACTCTGTGAGTTGAGTACACACATCACAAAGAAGTTTCT

            *     *         *        **         **   
     247266 CAGAATCCTTCTGTCTCGTTTTTATGGGAAGATATTTACTT
        131 GAGAATGCTTCTGTCTAGTTTTTATATGAAGATATTCCCTT

              *  *  * **          *       *     *       *  *  *              
     247307 TTTCACCGTAGGCATCAAAGCGCTCCAAATGTCCACATCCAGATACTCCAGAAAGAGTGTTTCAA
          1 TTCCAACGAAATCATCAAAGCGATCCAAATATCCACTTCCAGATTCTACAAAAAGAGTGTTTCAA

             *         *   * *             *        * *  *      *      *     
     247372 ACCTGCTCTATGAAAGGGAATGTTCAACTCTATGAGTTGAATGCAGACATCAGAAAGAAATTTCT
         66 AACTGCTCTATCAAAAGAAATGTTCAACTCTGTGAGTTGAGTACACACATCACAAAGAAGTTTCT

                     *       **          *         **
     247437 GAGAATGCTGCTGTCTACCTTTTAT-T--TGA-ATTCCCGC
        131 GAGAATGCTTCTGTCTAGTTTTTATATGAAGATATTCCCTT

                         *  *    *                *       *                  
     247474 TTCCAACGAAATCCTCCAAGCTATCCAAATATCCACTTGCAGATTCCACAAAAAGAGTGTTTCAA
          1 TTCCAACGAAATCATCAAAGCGATCCAAATATCCACTTCCAGATTCTACAAAAAGAGTGTTTCAA

                            ** *  *            *       *     *     *  *      
     247539 AACTGCTCTCTATCAATGGCAAAGTTCAACTCTGTTAGTTGAGGACACATATCACCAACAAGTTT
         66 AACTG--CTCTATCAAAAGAAATGTTCAACTCTGTGAGTTGAGTACACACATCACAAAGAAGTTT

                               *      ***              
     247604 CTGAGAATGCTTCTGTCTATTTTTTAAGGGAAGATATTCCCTT
        129 CTGAGAATGCTTCTGTCTAGTTTTTATATGAAGATATTCCCTT

              *  *  * ** *    *      *    *       *  * **                    
     247647 TTTCACCGTAGGCGTCAAGGCGATCGAAATGTCCACTTTCACAAACTACAAAAAGAGTGTTTCAA
          1 TTCCAACGAAATCATCAAAGCGATCCAAATATCCACTTCCAGATTCTACAAAAAGAGTGTTTCAA

             *         *   * **       *    *        * ** * *   **      *     
     247712 ACCTGCTCTATGAAAGGCCATGTTCATCTCTATGAGTTGAATGGATATATCCGAAAGAAATTTCT
         66 AACTGCTCTATCAAAAGAAATGTTCAACTCTGTGAGTTGAGTACACACATCACAAAGAAGTTTCT

             *       *          *        *         **
     247777 GGGAATGCTGCTGTCTAG-TGTT-TAT-ACGA-ATTCCCGA
        131 GAGAATGCTTCTGTCTAGTTTTTATATGAAGATATTCCCTT

                         *       *                *    *  *                  
     247814 TTCCAACGAAATCCTCAAAGCAATCCAAATATCCACTTGCAGAATCCACAAAAAGAGTGTTTCAA
          1 TTCCAACGAAATCATCAAAGCGATCCAAATATCCACTTCCAGATTCTACAAAAAGAGTGTTTCAA

                          *                * *                   *  *        
     247879 AACTGCTCTATCAATAGAAATGTTCAACTCTTTTAGTTGAGTACACACATCACGAACAAGTTTCT
         66 AACTGCTCTATCAAAAGAAATGTTCAACTCTGTGAGTTGAGTACACACATCACAAAGAAGTTTCT

                            * *      **     **  *    
     247944 GAGAATGCTTCTGTCTGGCTTTTATTGGAAGACGTTTCCTT
        131 GAGAATGCTTCTGTCTAGTTTTTATATGAAGATATTCCCTT

                 * *  **          *    *  *                * *      *        
     247985 TT-CACCAAAGGCATCAAAGCGCTCCATATGTCCACTTCCAGATTCTTCCAAAAGACTGTTTCAA
          1 TTCCAACGAAATCATCAAAGCGATCCAAATATCCACTTCCAGATTCTACAAAAAGAGTGTTTCAA

             **     * ***  * *                 *    * *  * *     *   *       
     248049 ACGTGCTCAAAGTAAGGGAATGTTCAACTCTGTGACTTGAATGCAGATATCACCAAGTAGTTTCT
         66 AACTGCTCTATCAAAAGAAATGTTCAACTCTGTGAGTTGAGTACACACATCACAAAGAAGTTTCT

            * * *             *     *    *         * 
     248114 AATAGTGCTTCTGTCTAGATTTTAGATGATGATATTCCCGT
        131 GAGAATGCTTCTGTCTAGTTTTTATATGAAGATATTCCCTT

                         * * *   *             *  *   *      *     *       * 
     248155 TTCCAACGAAATCGTTAGAGCTATCCAAATATCCAGTTACAGTTTCTACCAAAAGGGTGTTTCCA
          1 TTCCAACGAAATCATCAAAGCGATCCAAATATCCACTTCCAGATTCTACAAAAAGAGTGTTTCAA

              *    **           *            *       *                     * 
     248220 AATTGCTGCATCAAAAGAAAGGTTCAACTCTGTTAGTTGAGGACACACATCACAAAGAAGTTTGT
         66 AACTGCTCTATCAAAAGAAATGTTCAACTCTGTGAGTTGAGTACACACATCACAAAGAAGTTTCT

                              *    *     *           
     248285 GAGAATGCTTCTGTCTAGATTTTGTATGACGATATTCCCTT
        131 GAGAATGCTTCTGTCTAGTTTTTATATGAAGATATTCCCTT

                     *   * *     *   *       * *  *    *  *       *  *       
     248326 TTCCAACGATATCGTTAAAGCAATCTAAATATCAATTTGCAGAATCCACAAAAATAGAGTTTCAA
          1 TTCCAACGAAATCATCAAAGCGATCCAAATATCCACTTCCAGATTCTACAAAAAGAGTGTTTCAA

             *       * *        *    *       *  *                   ***      
     248391 AGCTGCTCTGTAAAAAGAAAGGTTCCACTCTGTTAGCTGAGTACACACATCACAAACTTGTTTCT
         66 AACTGCTCTATCAAAAGAAATGTTCAACTCTGTGAGTTGAGTACACACATCACAAAGAAGTTTCT

                  *         *        **         **   
     248456 GAGAATCCTTCTGTCTCGTTTTTATGGGAAGATATTTACTT
        131 GAGAATGCTTCTGTCTAGTTTTTATATGAAGATATTCCCTT

                 *  * **          *       *     *       *  *  *              
     248497 TTCCACCGTAGGCATCAAAGCGCTCCAAATGTCCACATCCAGATACTCCAGAAAGAGTGTTTCAA
          1 TTCCAACGAAATCATCAAAGCGATCCAAATATCCACTTCCAGATTCTACAAAAAGAGTGTTTCAA

             *         *   * *   *         *        * *  *      *      *     
     248562 ACCTGCTCTATGAAAGGGAATCTTCAACTCTATGAGTTGAATGCAGACATCAGAAAGAAATTTCT
         66 AACTGCTCTATCAAAAGAAATGTTCAACTCTGTGAGTTGAGTACACACATCACAAAGAAGTTTCT

                     *       **          *         **
     248627 GAGAATGCTGCTGTCTACCTTTTAT-T--TGA-ATTCCCGC
        131 GAGAATGCTTCTGTCTAGTTTTTATATGAAGATATTCCCTT

                         *  *    *              * *  **   *                  
     248664 TTCCAACGAAATCCTCCAAGCTATCCAAATATCCACCTGCATTTTCCACAAAAAGAGTGTTTCAA
          1 TTCCAACGAAATCATCAAAGCGATCCAAATATCCACTTCCAGATTCTACAAAAAGAGTGTTTCAA

                          *                 *    *  *                *       
     248729 AACTGCTCTATCAATAGAAATGTTCAACTCCTTTG-GCTGGGTACACACATCACAAACAAGTTTC
         66 AACTGCTCTATCAAAAGAAATGTTCAACT-CTGTGAGTTGAGTACACACATCACAAAGAAGTTTC

                                      **     **       
     248793 TGAGAATGCTTCTGTCTAGTTTTTATGGGAAGACGTTCCCTT
        130 TGAGAATGCTTCTGTCTAGTTTTTATATGAAGATATTCCCTT

              *  * *  **          *       *            **                    
     248835 TTTCACCAAAGGCATCAAAGCGCTCCAAATGTCCACTTCCAGACACTACAAAAAGAGTGTTTCAA
          1 TTCCAACGAAATCATCAAAGCGATCCAAATATCCACTTCCAGATTCTACAAAAAGAGTGTTTCAA

             **       **   ***                 *    * *  * *         *       
     248900 ACGTGCTCTAAGAAAGCGAATGTTCAACTCTGTGACTTGAATGCAGATATCACAAAGTAGTTTCT
         66 AACTGCTCTATCAAAAGAAATGTTCAACTCTGTGAGTTGAGTACACACATCACAAAGAAGTTTCT

                **            *     *    *         * 
     248965 GAGAGGGCTTCTGTCTAGATTTTAGATGATGATATTCCCGT
        131 GAGAATGCTTCTGTCTAGTTTTTATATGAAGATATTCCCTT

                           * *   *                *   *                    * 
     249006 TTCCAACGAAATCATTAGAGCTATCCAAATATCCACTTACAGTTTCTACAAAAAGAGTGTTTCCA
          1 TTCCAACGAAATCATCAAAGCGATCCAAATATCCACTTCCAGATTCTACAAAAAGAGTGTTTCAA

                   **         * *    *       *  *                   ***      
     249071 AACTGCTGCATCAAAAGAGAGGTTCCACTCTGTTAGCTGAGTACACACATCACAAACTTGTTTCT
         66 AACTGCTCTATCAAAAGAAATGTTCAACTCTGTGAGTTGAGTACACACATCACAAAGAAGTTTCT

            *     * *       *        **         **   
     249136 CAGAATCCGTCTGTCTCGTTTTTATGGGAAGATATTTACTT
        131 GAGAATGCTTCTGTCTAGTTTTTATATGAAGATATTCCCTT

              *  *  * **          *       *     *       *  *  *              
     249177 TTTCACCGTAGGCATCAAAGCGCTCCAAATGTCCACATCCAGATACTCCAGAAAGAGTGTTTCAA
          1 TTCCAACGAAATCATCAAAGCGATCCAAATATCCACTTCCAGATTCTACAAAAAGAGTGTTTCAA

             *         *   * *             *        * *  *      *      *     
     249242 ACCTGCTCTATGAAAGGGAATGTTCAACTCTATGAGTTGAATGCAGACATCAGAAAGAAATTTCT
         66 AACTGCTCTATCAAAAGAAATGTTCAACTCTGTGAGTTGAGTACACACATCACAAAGAAGTTTCT

                     *       **          *         **
     249307 GAGAATGCTGCTGTCTACCTTTTAT-T--TGA-ATTCCCGC
        131 GAGAATGCTTCTGTCTAGTTTTTATATGAAGATATTCCCTT

                         *  *    *                *       *                  
     249344 TTCCAACGAAATCCTCCAAGCTATCCAAATATCCACTTGCAGATTCCACAAAAAGAGTGTTTCAA
          1 TTCCAACGAAATCATCAAAGCGATCCAAATATCCACTTCCAGATTCTACAAAAAGAGTGTTTCAA

                            ** *  *            *       *     *     *  *      
     249409 AACTGCTCTCTATCAATGGCAAAGTTCAACTCTGTTAGTTGAGGACACATATCACCAACAAGTTT
         66 AACTG--CTCTATCAAAAGAAATGTTCAACTCTGTGAGTTGAGTACACACATCACAAAGAAGTTT

                               *       **         *    
     249474 CTGAGAATGCTTCTGTCTATTTTTTATGGGAAGATATTTCCTT
        129 CTGAGAATGCTTCTGTCTAGTTTTTATATGAAGATATTCCCTT

              *  *  * ** *    *      *    *          * **                    
     249517 TTTCACCGTAGGCGTCAAGGCGATCGAAATGTCCACTTCCACAAACTACAAAAAGAGTGTTTCAA
          1 TTCCAACGAAATCATCAAAGCGATCCAAATATCCACTTCCAGATTCTACAAAAAGAGTGTTTCAA

             *         *   * **       *    *        * ** * *   **      *     
     249582 ACCTGCTCTATGAAAGGCCATGTTCATCTCTATGAGTTGAATGGAAATATCCGAAAGAAATTTCT
         66 AACTGCTCTATCAAAAGAAATGTTCAACTCTGTGAGTTGAGTACACACATCACAAAGAAGTTTCT

             *       *                             **
     249647 GGGAATGCTGCTGTCTAGTTTTTATAT---GA-ATTCCCGC
        131 GAGAATGCTTCTGTCTAGTTTTTATATGAAGATATTCCCTT

                         *       *                *    *  *                  
     249684 TTCCAACGAAATCCTCAAAGCAATCCAAATATCCACTTGCAGAATCCACAAAAAGAGTGTTTCAA
          1 TTCCAACGAAATCATCAAAGCGATCCAAATATCCACTTCCAGATTCTACAAAAAGAGTGTTTCAA

                          *     *          * *                      *        
     249749 AACTGCTCTATCAATAGAAAGGTTCAACTCTTTTAGTTGAGTACACACATCACAAACAAGTTTCT
         66 AACTGCTCTATCAAAAGAAATGTTCAACTCTGTGAGTTGAGTACACACATCACAAAGAAGTTTCT

                            * *      **     **  *    
     249814 GAGAATGCTTCTGTCTGGCTTTTATTGGAAGACGTTTCCTT
        131 GAGAATGCTTCTGTCTAGTTTTTATATGAAGATATTCCCTT

                 * *  **          *       *                * *               
     249855 TT-CACCAAAGGCATCAAAGCGCTCCAAATGTCCACTTCCAGATTCTTCCAAAAGAGTGTTTCAA
          1 TTCCAACGAAATCATCAAAGCGATCCAAATATCCACTTCCAGATTCTACAAAAAGAGTGTTTCAA

             **     * ***  * *                 *    * *  * *     *   *  *    
     249919 ACGTGCTCAAAGTAAGGGAATGTTCAACTCTGTGACTTGAATGCAGATATCACCAAGTAGCTTCT
         66 AACTGCTCTATCAAAAGAAATGTTCAACTCTGTGAGTTGAGTACACACATCACAAAGAAGTTTCT

            * * *             *     *    *         * 
     249984 AATAGTGCTTCTGTCTAGATTTTAGATGATGATATTCCCGT
        131 GAGAATGCTTCTGTCTAGTTTTTATATGAAGATATTCCCTT

                         * * *   *             *  *   *      *     *       * 
     250025 TTCCAACGAAATCGTTAGAGCTATCCAAATATCCAGTTACAGTTTCTACCAAAAGGGTGTTTCCA
          1 TTCCAACGAAATCATCAAAGCGATCCAAATATCCACTTCCAGATTCTACAAAAAGAGTGTTTCAA

              *    **           *            *       *                     * 
     250090 AATTGCTGCATCAAAAGAAAGGTTCAACTCTGTTAGTTGAGGACACACATCACAAAGAAGTTTGT
         66 AACTGCTCTATCAAAAGAAATGTTCAACTCTGTGAGTTGAGTACACACATCACAAAGAAGTTTCT

                              *    *     *           
     250155 GAGAATGCTTCTGTCTAGATTTTGTATGACGATATTCCCTT
        131 GAGAATGCTTCTGTCTAGTTTTTATATGAAGATATTCCCTT

                     *   * *     *   *       * *  *    *  *       *  *       
     250196 TTCCAACGATATCGTTAAAGCAATCTAAATATCAATTTGCAGAATCCACAAAAATAGAGTTTCAA
          1 TTCCAACGAAATCATCAAAGCGATCCAAATATCCACTTCCAGATTCTACAAAAAGAGTGTTTCAA

             *       * *        *    *       *  *                   ***      
     250261 AGCTGCTCTGTAAAAAGAAAGGTTCCACTCTGTTAGCTGAGTACACACATCACAAACTTGTTTCT
         66 AACTGCTCTATCAAAAGAAATGTTCAACTCTGTGAGTTGAGTACACACATCACAAAGAAGTTTCT

                  *         *        **         **   
     250326 GAGAATCCTTCTGTCTCGTTTTTATGGGAAGATATTTACTT
        131 GAGAATGCTTCTGTCTAGTTTTTATATGAAGATATTCCCTT

                 *  * **          *       *     *       *  *  *  *           
     250367 TTCCACCGTAGGCATCAAAGCGCTCCAAATGTCCACATCCAGATACTCCAGAACGAGTGTTTCAA
          1 TTCCAACGAAATCATCAAAGCGATCCAAATATCCACTTCCAGATTCTACAAAAAGAGTGTTTCAA

             *         *   * *   *         *        * *  *      *      *     
     250432 ACCTGCTCTATGAAAGGGAATCTTCAACTCTATGAGTTGAATGCAGACATCAGAAAGAAATTTCT
         66 AACTGCTCTATCAAAAGAAATGTTCAACTCTGTGAGTTGAGTACACACATCACAAAGAAGTTTCT

                     *       **          *         **
     250497 GAGAATGCTGCTGTCTACCTTTTAT-T--TGA-ATTCCCGC
        131 GAGAATGCTTCTGTCTAGTTTTTATATGAAGATATTCCCTT

                         *  *    *              * *  **   *    *             
     250534 TTCCAACGAAATCCTCCAAGCTATCCAAATATCCACCTGCATTTTCCACAACAAGAGTGTTTCAA
          1 TTCCAACGAAATCATCAAAGCGATCCAAATATCCACTTCCAGATTCTACAAAAAGAGTGTTTCAA

                          *                 *    *  *                *       
     250599 AACTGCTCTATCAATAGAAATGTTCAACTCCTTTG-GCTGGGTACACACATCACAAACAAGTTTC
         66 AACTGCTCTATCAAAAGAAATGTTCAACT-CTGTGAGTTGAGTACACACATCACAAAGAAGTTTC

                                      **     **       
     250663 TGAGAATGCTTCTGTCTAGTTTTTATGGGAAGACGTTCCCTT
        130 TGAGAATGCTTCTGTCTAGTTTTTATATGAAGATATTCCCTT

              *  * *  **          *       *            **                  * 
     250705 TTTCACCAAAGGCATCAAAGCGCTCCAAATGTCCACTTCCAGACACTACAAAAAGAGTGTTTCCA
          1 TTCCAACGAAATCATCAAAGCGATCCAAATATCCACTTCCAGATTCTACAAAAAGAGTGTTTCAA

             **       **   ***                 *    * *  * *         *       
     250770 ACGTGCTCTAAGAAAGCGAATGTTCAACTCTGTGACTTGAATGCAGATATCACAAAGTAGTTTCT
         66 AACTGCTCTATCAAAAGAAATGTTCAACTCTGTGAGTTGAGTACACACATCACAAAGAAGTTTCT

                **            *     *    *         * 
     250835 GAGAGGGCTTCTGTCTAGATTTTAGATGATGATATTCCCGT
        131 GAGAATGCTTCTGTCTAGTTTTTATATGAAGATATTCCCTT

                           * *   *                *   *                    * 
     250876 TTCCAACGAAATCATTAGAGCTATCCAAATATCCACTTACAGTTTCTACAAAAAGAGTGTTTCCA
          1 TTCCAACGAAATCATCAAAGCGATCCAAATATCCACTTCCAGATTCTACAAAAAGAGTGTTTCAA

                   **         * *    *       *  *                   ***      
     250941 AACTGCTGCATCAAAAGAGAGGTTCCACTCTGTTAGCTGAGTACACACATCACAAACTTGTTTCT
         66 AACTGCTCTATCAAAAGAAATGTTCAACTCTGTGAGTTGAGTACACACATCACAAAGAAGTTTCT

            *     *         *        **         **   
     251006 CAGAATCCTTCTGTCTCGTTTTTATGGGAAGATATTTACTT
        131 GAGAATGCTTCTGTCTAGTTTTTATATGAAGATATTCCCTT

              *  *  * **          *       *     *       *  *  *              
     251047 TTTCACCGTAGGCATCAAAGCGCTCCAAATGTCCACATCCAGATACTCCAGAAAGAGTGTTTCAA
          1 TTCCAACGAAATCATCAAAGCGATCCAAATATCCACTTCCAGATTCTACAAAAAGAGTGTTTCAA

             *         *   * *   *  *      *        * *  *      *      *     
     251112 ACCTGCTCTATGAAAGGGAATCTTGAACTCTATGAGTTGAATGCAGACATCAGAAAGAAATTTCT
         66 AACTGCTCTATCAAAAGAAATGTTCAACTCTGTGAGTTGAGTACACACATCACAAAGAAGTTTCT

                     *       **          *         **
     251177 GAGAATGCTGCTGTCTACCTTTTAT-T--TGA-ATTCCCGC
        131 GAGAATGCTTCTGTCTAGTTTTTATATGAAGATATTCCCTT

                         *  *    *                *       *                  
     251214 TTCCAACGAAATCCTCCAAGCTATCCAAATATCCACTTGCAGATTCCACAAAAAGAGTGTTTCAA
          1 TTCCAACGAAATCATCAAAGCGATCCAAATATCCACTTCCAGATTCTACAAAAAGAGTGTTTCAA

                            ** *  *            *       *     *     *  *      
     251279 AACTGCTCTCTATCAATGGCAAAGTTCAACTCTGTTAGTTGAGGACACATATCACCAACAAGTTT
         66 AACTG--CTCTATCAAAAGAAATGTTCAACTCTGTGAGTTGAGTACACACATCACAAAGAAGTTT

                               *       **         *    
     251344 CTGAGAATGCTTCTGTCTATTTTTTATGGGAAGATATTTCCTT
        129 CTGAGAATGCTTCTGTCTAGTTTTTATATGAAGATATTCCCTT

              *  *  * ** *    *      *    *          * **                    
     251387 TTTCACCGTAGGCGTCAAGGCGATCGAAATGTCCACTTCCACAAACTACAAAAAGAGTGTTTCAA
          1 TTCCAACGAAATCATCAAAGCGATCCAAATATCCACTTCCAGATTCTACAAAAAGAGTGTTTCAA

             *         *   * **       *    *        * ** * *   **      *     
     251452 ACCTGCTCTATGAAAGGCCATGTTCATCTCTATGAGTTGAATGGATATATCCGAAAGAAATTTCT
         66 AACTGCTCTATCAAAAGAAATGTTCAACTCTGTGAGTTGAGTACACACATCACAAAGAAGTTTCT

             *       *                   *         **
     251517 GGGAATGCTGCTGTCTAG-TTTT-TAT-ACGA-ATTCCCGA
        131 GAGAATGCTTCTGTCTAGTTTTTATATGAAGATATTCCCTT

                         *       *                *    *  *                  
     251554 TTCCAACGAAATCCTCAAAGCAATCCAAATATCCACTTGCAGAATCCACAAAAAGAGTGTTTCAA
          1 TTCCAACGAAATCATCAAAGCGATCCAAATATCCACTTCCAGATTCTACAAAAAGAGTGTTTCAA

                          *     *          * *                   *  *        
     251619 AACTGCTCTATCAATAGAAAGGTTCAACTCTTTTAGTTGAGTACACACATCACGAACAAGTTTCT
         66 AACTGCTCTATCAAAAGAAATGTTCAACTCTGTGAGTTGAGTACACACATCACAAAGAAGTTTCT

                            * *      **     **  *    
     251684 GAGAATGCTTCTGTCTGGCTTTTATTGGAAGACGTTTCCTT
        131 GAGAATGCTTCTGTCTAGTTTTTATATGAAGATATTCCCTT

                 * *  **          *       *                * *               
     251725 TT-CACCAAAGGCATCAAAGCGCTCCAAATGTCCACTTCCAGATTCTTCCAAAAGAGTGTTTCAA
          1 TTCCAACGAAATCATCAAAGCGATCCAAATATCCACTTCCAGATTCTACAAAAAGAGTGTTTCAA

             **     * ***  * *                 *    * *  * *     *   *       
     251789 ACGTGCTCAAAGTAAGGGAATGTTCAACTCTGTGACTTGAATGCAGATATCACCAAGTAGTTTCT
         66 AACTGCTCTATCAAAAGAAATGTTCAACTCTGTGAGTTGAGTACACACATCACAAAGAAGTTTCT

            * * *             *     *    *         * 
     251854 AATAGTGCTTCTGTCTAGATTTTAGATGATGATATTCCCGT
        131 GAGAATGCTTCTGTCTAGTTTTTATATGAAGATATTCCCTT

                         *** *   *             *  *   *      *     *       * 
     251895 TTCCAACGAAATCGCTAGAGCTATCCAAATATCCAGTTACAGTTTCTACTAAAAGGGTGTTTCCA
          1 TTCCAACGAAATCATCAAAGCGATCCAAATATCCACTTCCAGATTCTACAAAAAGAGTGTTTCAA

              *    **           *            *       *                     * 
     251960 AATTGCTGCATCAAAAGAAAGGTTCAACTCTGTTAGTTGAGGACACACATCACAAAGAAGTTTGT
         66 AACTGCTCTATCAAAAGAAATGTTCAACTCTGTGAGTTGAGTACACACATCACAAAGAAGTTTCT

                              *    *     *           
     252025 GAGAATGCTTCTGTCTAGATTTTGTATGACGATATTCCCTT
        131 GAGAATGCTTCTGTCTAGTTTTTATATGAAGATATTCCCTT

                     *   * *     *   *       * *  *    *  *       *  *       
     252066 TTCCAACGATATCGTTAAAGCAATCTAAATATCAATTTGCAGAATCCACAAAAATAGAGTTTCAA
          1 TTCCAACGAAATCATCAAAGCGATCCAAATATCCACTTCCAGATTCTACAAAAAGAGTGTTTCAA

             *       * *        *    *       *  *                   ***      
     252131 AGCTGCTCTGTAAAAAGAAAGGTTCCACTCTGTTAGCTGAGTACACACATCACAAACTTGTTTCT
         66 AACTGCTCTATCAAAAGAAATGTTCAACTCTGTGAGTTGAGTACACACATCACAAAGAAGTTTCT

                  *         *   *    **        ***   
     252196 GAGAATCCTTCTGTCTCGTTCTTATGGGAAGATATATACTT
        131 GAGAATGCTTCTGTCTAGTTTTTATATGAAGATATTCCCTT

                 *    **          *       *     *       *  *  *  *           
     252237 TTCCACCGAAGGCATCAAAGCGCTCCAAATGTCCACATCCAGATACTCCAGAACGAGTGTTTCAA
          1 TTCCAACGAAATCATCAAAGCGATCCAAATATCCACTTCCAGATTCTACAAAAAGAGTGTTTCAA

             *         *   * *   *         *        * *  *      *      *     
     252302 ACCTGCTCTATGAAAGGGAATCTTCAACTCTATGAGTTGAATGCAGACATCAGAAAGAAATTTCT
         66 AACTGCTCTATCAAAAGAAATGTTCAACTCTGTGAGTTGAGTACACACATCACAAAGAAGTTTCT

                     *       **          *         **
     252367 GAGAATGCTGCTGTCTACCTTTTAT-T--TGA-ATTCCCGC
        131 GAGAATGCTTCTGTCTAGTTTTTATATGAAGATATTCCCTT

                         *  *    *              * *  **   *    *             
     252404 TTCCAACGAAATCCTCCAAGCTATCCAAATATCCACCTGCATTTTCCACAACAAGAGTGTTTCAA
          1 TTCCAACGAAATCATCAAAGCGATCCAAATATCCACTTCCAGATTCTACAAAAAGAGTGTTTCAA

                          *                 *    *  *                *       
     252469 AACTGCTCTATCAATAGAAATGTTCAACTCCTTTG-GCTGGGTACACACATCACAAACAAGTTTC
         66 AACTGCTCTATCAAAAGAAATGTTCAACT-CTGTGAGTTGAGTACACACATCACAAAGAAGTTTC

                                      **     **       
     252533 TGAGAATGCTTCTGTCTAGTTTTTATGGGAAGACGTTCCCTT
        130 TGAGAATGCTTCTGTCTAGTTTTTATATGAAGATATTCCCTT

              *  * *  **          *       *            **                  * 
     252575 TTTCACCAAAGGCATCAAAGCGCTCCAAATGTCCACTTCCAGACACTACAAAAAGAGTGTTTCCA
          1 TTCCAACGAAATCATCAAAGCGATCCAAATATCCACTTCCAGATTCTACAAAAAGAGTGTTTCAA

             **       **   ***                 *    * *  * *         *       
     252640 ACGTGCTCTAAGAAAGCGAATGTTCAACTCTGTGACTTGAATGCAGATATCACAAAGTAGTTTCT
         66 AACTGCTCTATCAAAAGAAATGTTCAACTCTGTGAGTTGAGTACACACATCACAAAGAAGTTTCT

                **            *     *    *         * 
     252705 GAGAGGGCTTCTGTCTAGATTTTAGATGATGATATTCCCGT
        131 GAGAATGCTTCTGTCTAGTTTTTATATGAAGATATTCCCTT

                           * *   *                *   *                    * 
     252746 TTCCAACGAAATCATTAGAGCTATCCAAATATCCACTTACAGTTTCTACAAAAAGAGTGTTTCCA
          1 TTCCAACGAAATCATCAAAGCGATCCAAATATCCACTTCCAGATTCTACAAAAAGAGTGTTTCAA

                   **         * *    *       *  *                   ***      
     252811 AACTGCTGCATCAAAAGAGAGGTTCCACTCTGTTAGCTGAGTACACACATCACAAACTTGTTTCT
         66 AACTGCTCTATCAAAAGAAATGTTCAACTCTGTGAGTTGAGTACACACATCACAAAGAAGTTTCT

            *     *         *        **         **   
     252876 CAGAATCCTTCTGTCTCGTTTTTATGGGAAGATATTTACTT
        131 GAGAATGCTTCTGTCTAGTTTTTATATGAAGATATTCCCTT

              *  *  * **          *       *     *       *  *  *              
     252917 TTTCACCGTAGGCATCAAAGCGCTCCAAATGTCCACATCCAGATACTCCAGAAAGAGTGTTTCAA
          1 TTCCAACGAAATCATCAAAGCGATCCAAATATCCACTTCCAGATTCTACAAAAAGAGTGTTTCAA

             *         *   * *   *         *        * *  *      *      *     
     252982 ACCTGCTCTATGAAAGGGAATCTTCAACTCTATGAGTTGAATGCAGACATCAGAAAGAAATTTCT
         66 AACTGCTCTATCAAAAGAAATGTTCAACTCTGTGAGTTGAGTACACACATCACAAAGAAGTTTCT

                     *       **          *         **
     253047 GAGAATGCTGCTGTCTACCTTTTAT-T--TGA-ATTCCCGC
        131 GAGAATGCTTCTGTCTAGTTTTTATATGAAGATATTCCCTT

                         *  *    *                *       *                  
     253084 TTCCAACGAAATCCTCCAAGCTATCCAAATATCCACTTGCAGATTCCACAAAAAGAGTGTTTCAA
          1 TTCCAACGAAATCATCAAAGCGATCCAAATATCCACTTCCAGATTCTACAAAAAGAGTGTTTCAA

                            ** *  *            *       *     *     *  *      
     253149 AACTGCTCTCTATCAATGGCAAAGTTCAACTCTGTTAGTTGAGGACACATATCACCAACAAGTTT
         66 AACTG--CTCTATCAAAAGAAATGTTCAACTCTGTGAGTTGAGTACACACATCACAAAGAAGTTT

                               *       **         *    
     253214 CTGAGAATGCTTCTGTCTATTTTTTATGGGAAGATATTTCCTT
        129 CTGAGAATGCTTCTGTCTAGTTTTTATATGAAGATATTCCCTT

              *  *  * ** *    *      *    *          * **                    
     253257 TTTCACCGTAGGCGTCAAGGCGATCGAAATGTCCACTTCCACAAACTACAAAAAGAGTGTTTCAA
          1 TTCCAACGAAATCATCAAAGCGATCCAAATATCCACTTCCAGATTCTACAAAAAGAGTGTTTCAA

             *         *   * **       *    *        * ** * *   **      *     
     253322 ACCTGCTCTATGAAAGGCCATGTTCATCTCTATGAGTTGAATGGATATATCCGAAAGAAATTTCT
         66 AACTGCTCTATCAAAAGAAATGTTCAACTCTGTGAGTTGAGTACACACATCACAAAGAAGTTTCT

             *       *                   *         **
     253387 GGGAATGCTGCTGTCTAG-TTTT-TAT-ACGA-ATTCCCGC
        131 GAGAATGCTTCTGTCTAGTTTTTATATGAAGATATTCCCTT

                         *       *                *    *  *                  
     253424 TTCCAACGAAATCCTCAAAGCAATCCAAATATCCACTTGCAGAATCCACAAAAAGAGTGTTTCAA
          1 TTCCAACGAAATCATCAAAGCGATCCAAATATCCACTTCCAGATTCTACAAAAAGAGTGTTTCAA

                          *     *          * *                   *  *        
     253489 AACTGCTCTATCAATAGAAAGGTTCAACTCTTTTAGTTGAGTACACACATCACGAACAAGTTTCT
         66 AACTGCTCTATCAAAAGAAATGTTCAACTCTGTGAGTTGAGTACACACATCACAAAGAAGTTTCT

                            * *      **     **  *    
     253554 GAGAATGCTTCTGTCTGGCTTTTATTGGAAGACGTTTCCTT
        131 GAGAATGCTTCTGTCTAGTTTTTATATGAAGATATTCCCTT

                 * *  **          *       *                * *               
     253595 TT-CACCAAAGGCATCAAAGCGCTCCAAATGTCCACTTCCAGATTCTTCCAAAAGAGTGTTTCAA
          1 TTCCAACGAAATCATCAAAGCGATCCAAATATCCACTTCCAGATTCTACAAAAAGAGTGTTTCAA

             **     * ***  * *                 *    * *  * *     *   *       
     253659 ACGTGCTCAAAGTAAGGGAATGTTCAACTCTGTGACTTGAATGCAGATATCACCAAGTAGTTTCT
         66 AACTGCTCTATCAAAAGAAATGTTCAACTCTGTGAGTTGAGTACACACATCACAAAGAAGTTTCT

            * * *            **     *    *         * 
     253724 AATAGTGCTTCTGTCTACATTTTAGATGATGATATTCCCGT
        131 GAGAATGCTTCTGTCTAGTTTTTATATGAAGATATTCCCTT

                         * * *   *             *  *   *      *     *       * 
     253765 TTCCAACGAAATCGTTAGAGCTATCCAAATATCCAGTTACAGTTTCTACCAAAAGGGTGTTTCCA
          1 TTCCAACGAAATCATCAAAGCGATCCAAATATCCACTTCCAGATTCTACAAAAAGAGTGTTTCAA

              *    **           *            *       *                     * 
     253830 AATTGCTGCATCAAAAGAAAGGTTCAACTCTGTTAGTTGAGGACACACATCACAAAGAAGTTTGT
         66 AACTGCTCTATCAAAAGAAATGTTCAACTCTGTGAGTTGAGTACACACATCACAAAGAAGTTTCT

                              *    *     *           
     253895 GAGAATGCTTCTGTCTAGATTTTGTATGACGATATTCCCTT
        131 GAGAATGCTTCTGTCTAGTTTTTATATGAAGATATTCCCTT

                     *   * *     *   *       * *  *    *          *  *       
     253936 TTCCAACGATATCGTTAAAGCAATCTAAATATCAATTTGCAGAATCTACAAAAATAGAGTTTCAA
          1 TTCCAACGAAATCATCAAAGCGATCCAAATATCCACTTCCAGATTCTACAAAAAGAGTGTTTCAA

             *       * *        *    *       *  *                   ***      
     254001 AGCTGCTCTGTAAAAAGAAAGGTTCCACTCTGTTAGCTGAGTACACACATCACAAACTTGTTTCT
         66 AACTGCTCTATCAAAAGAAATGTTCAACTCTGTGAGTTGAGTACACACATCACAAAGAAGTTTCT

                  *         *        **         **   
     254066 GAGAATCCTTCTGTCTCGTTTTTATGGGAAGATATTTACTT
        131 GAGAATGCTTCTGTCTAGTTTTTATATGAAGATATTCCCTT

                 *  * **          *       *     *       *  *  *  *           
     254107 TTCCACCGTAGGCATCAAAGCGCTCCAAATGTCCACATCCAGATACTCCAGAACGAGTGTTTCAA
          1 TTCCAACGAAATCATCAAAGCGATCCAAATATCCACTTCCAGATTCTACAAAAAGAGTGTTTCAA

             *         *   * *   *         *        * *  *      *      *     
     254172 ACCTGCTCTATGAAAGGGAATCTTCAACTCTATGAGTTGAATGCAGACATCAGAAAGAAATTTCT
         66 AACTGCTCTATCAAAAGAAATGTTCAACTCTGTGAGTTGAGTACACACATCACAAAGAAGTTTCT

                     *       **          *         **
     254237 GAGAATGCTGCTGTCTACCTTTTAT-T--TGA-ATTCCCGC
        131 GAGAATGCTTCTGTCTAGTTTTTATATGAAGATATTCCCTT

                         *  *    *              * *  **   *                  
     254274 TTCCAACGAAATCCTCCAAGCTATCCAAATATCCACCTGCATTTTCCACAAAAAGAGTGTTTCAA
          1 TTCCAACGAAATCATCAAAGCGATCCAAATATCCACTTCCAGATTCTACAAAAAGAGTGTTTCAA

                            ** *  *            *       *     *     *  *      
     254339 AACTGCTCTCTATCAATGGCAAAGTTCAACTCTGTTAGTTGAGGACACATATCACCAACAAGTTT
         66 AACTG--CTCTATCAAAAGAAATGTTCAACTCTGTGAGTTGAGTACACACATCACAAAGAAGTTT

                               *       **         *    
     254404 CTGAGAATGCTTCTGTCTATTTTTTATGGGAAGATATTTCCTT
        129 CTGAGAATGCTTCTGTCTAGTTTTTATATGAAGATATTCCCTT

              *  *  * ** *    *      *    *          * **                    
     254447 TTTCACCGTAGGCGTCAAGGCGATCGAAATGTCCACTTCCACAAACTACAAAAAGAGTGTTTCAA
          1 TTCCAACGAAATCATCAAAGCGATCCAAATATCCACTTCCAGATTCTACAAAAAGAGTGTTTCAA

             *         *   * **       *    *        * ** * **  **      *     
     254512 ACCTGCTCTATGAAAGGCCATGTTCATCTCTATGAGTTGAATGGAAATTTCCGAAAGAAATTTCT
         66 AACTGCTCTATCAAAAGAAATGTTCAACTCTGTGAGTTGAGTACACACATCACAAAGAAGTTTCT

             *       *          *        *         **
     254577 GGGAATGCTGCTGTCTAG-TGTT-TAT-ACGA-ATTCCCGC
        131 GAGAATGCTTCTGTCTAGTTTTTATATGAAGATATTCCCTT

                         *       *                *    *  *                  
     254614 TTCCAACGAAATCCTCAAAGCAATCCAAATATCCACTTGCAGAATCCACAAAAAGAGTGTTTCAA
          1 TTCCAACGAAATCATCAAAGCGATCCAAATATCCACTTCCAGATTCTACAAAAAGAGTGTTTCAA

                          *     *          * *                   *  *        
     254679 AACTGCTCTATCAATAGAAAGGTTCAACTCTTTTAGTTGAGTACACACATCACGAACAAGTTTCT
         66 AACTGCTCTATCAAAAGAAATGTTCAACTCTGTGAGTTGAGTACACACATCACAAAGAAGTTTCT

                            * *      **     **  *    
     254744 GAGAATGCTTCTGTCTGGCTTTTATTGGAAGACGTTTCCTT
        131 GAGAATGCTTCTGTCTAGTTTTTATATGAAGATATTCCCTT

                 * *  **          *       *                * *               
     254785 TT-CAGCAAAGGCATCAAAGCGCTCCAAATGTCCACTTCCAGATTCTTCCAAAAGAGTGTTTCAA
          1 TTCCAACGAAATCATCAAAGCGATCCAAATATCCACTTCCAGATTCTACAAAAAGAGTGTTTCAA

             **     * ***  * *                 *    * *  * *     *   *       
     254849 ACGTGCTCAAAGTAAGGGAATGTTCAACTCTGTGACTTGAATGCAGATATCACCAAGTAGTTTCT
         66 AACTGCTCTATCAAAAGAAATGTTCAACTCTGTGAGTTGAGTACACACATCACAAAGAAGTTTCT

            * * *            **     *    *         * 
     254914 AATAGTGCTTCTGTCTACATTTTAGATGATGATATTCCCGT
        131 GAGAATGCTTCTGTCTAGTTTTTATATGAAGATATTCCCTT

                         * * *   *             *  *   *      *     *       * 
     254955 TTCCAACGAAATCGTTAGAGCTATCCAAATATCCAGTTACAGTTTCTACCAAAAGGGTGTTTCCA
          1 TTCCAACGAAATCATCAAAGCGATCCAAATATCCACTTCCAGATTCTACAAAAAGAGTGTTTCAA

              *    **           *            *       *                     * 
     255020 AATTGCTGCATCAAAAGAAAGGTTCAACTCTGTTAGTTGAGGACACACATCACAAAGAAGTTTGT
         66 AACTGCTCTATCAAAAGAAATGTTCAACTCTGTGAGTTGAGTACACACATCACAAAGAAGTTTCT

                              *    *     *           
     255085 GAGAATGCTTCTGTCTAGATTTTGTATGACGATATTCCCTT
        131 GAGAATGCTTCTGTCTAGTTTTTATATGAAGATATTCCCTT

                     *   * *     *   *       * *  *    *  *       *  *       
     255126 TTCCAACGATATCGTTAAAGCAATCTAAATATCAATTTGCAGAATCCACAAAAATAGAGTTTCAA
          1 TTCCAACGAAATCATCAAAGCGATCCAAATATCCACTTCCAGATTCTACAAAAAGAGTGTTTCAA

             *       * *        *    *       *  *                   ***      
     255191 AGCTGCTCTGTAAAAAGAAAGGTTCCACTCTGTTAGCTGAGTACACACATCACAAACTTGTTTCT
         66 AACTGCTCTATCAAAAGAAATGTTCAACTCTGTGAGTTGAGTACACACATCACAAAGAAGTTTCT

                  *         *        **         **   
     255256 GAGAATCCTTCTGTCTCGTTTTTATGGGAAGATATTTACTT
        131 GAGAATGCTTCTGTCTAGTTTTTATATGAAGATATTCCCTT

                 *  * **          *       *     *       *  *  *              
     255297 TTCCACCGTAGGCATCAAAGCGCTCCAAATGTCCACATCCAGATACTCCAGAAAGAGTGTTTCAA
          1 TTCCAACGAAATCATCAAAGCGATCCAAATATCCACTTCCAGATTCTACAAAAAGAGTGTTTCAA

             *         *   * *   *         *        * *  *      *      *     
     255362 ACCTGCTCTATGAAAGGGAATCTTCAACTCTATGAGTTGAATGCAGACATCAGAAAGAAATTTCT
         66 AACTGCTCTATCAAAAGAAATGTTCAACTCTGTGAGTTGAGTACACACATCACAAAGAAGTTTCT

                     *       **          *         **
     255427 GAGAATGCTGCTGTCTACCTTTTAT-T--TGA-ATTCCCGC
        131 GAGAATGCTTCTGTCTAGTTTTTATATGAAGATATTCCCTT

                         *  *    *                *  **   *                  
     255464 TTCCAACGAAATCCTCCAAGCTATCCAAATATCCACTTGCATTTTCCACAAAAAGAGTGTTTCAA
          1 TTCCAACGAAATCATCAAAGCGATCCAAATATCCACTTCCAGATTCTACAAAAAGAGTGTTTCAA

                          *                 *    *  *                *       
     255529 AACTGCTCTATCAATAGAAATGTTCAACTCCTTTG-GCTGGGTACACACATCACAAACAAGTTTC
         66 AACTGCTCTATCAAAAGAAATGTTCAACT-CTGTGAGTTGAGTACACACATCACAAAGAAGTTTC

                                      **     **       
     255593 TGAGAATGCTTCTGTCTAGTTTTTATGGGAAGACGTTCCCTT
        130 TGAGAATGCTTCTGTCTAGTTTTTATATGAAGATATTCCCTT

              *  * *  **          *       *            **                  * 
     255635 TTTCACCAAAGGCATCAAAGCGCTCCAAATGTCCACTTCCAGACACTACAAAAAGAGTGTTTCCA
          1 TTCCAACGAAATCATCAAAGCGATCCAAATATCCACTTCCAGATTCTACAAAAAGAGTGTTTCAA

             **       **   ***                 *    * *  * *         *       
     255700 ACGTGCTCTAAGAAAGCGAATGTTCAACTCTGTGACTTGAATGCAGATATCACAAAGTAGTTTCT
         66 AACTGCTCTATCAAAAGAAATGTTCAACTCTGTGAGTTGAGTACACACATCACAAAGAAGTTTCT

                **            *     *    *         * 
     255765 GAGAGGGCTTCTGTCTAGATTTTAGATGATGATATTCCCGT
        131 GAGAATGCTTCTGTCTAGTTTTTATATGAAGATATTCCCTT

                           * *   *                *   *                    * 
     255806 TTCCAACGAAATCATTAGAGCTATCCAAATATCCACTTACAGTTTCTACAAAAAGAGTGTTTCCA
          1 TTCCAACGAAATCATCAAAGCGATCCAAATATCCACTTCCAGATTCTACAAAAAGAGTGTTTCAA

                   **         * *    *       *  *                   ***      
     255871 AACTGCTGCATCAAAAGAGAGGTTCCACTCTGTTAGCTGAGTACACACATCACAAACTTGTTTCT
         66 AACTGCTCTATCAAAAGAAATGTTCAACTCTGTGAGTTGAGTACACACATCACAAAGAAGTTTCT

            *     *         *        **         **   
     255936 CAGAATCCTTCTGTCTCGTTTTTATGGGAAGATATTTACTT
        131 GAGAATGCTTCTGTCTAGTTTTTATATGAAGATATTCCCTT

                 *  * **          *       *     *       *  *  *              
     255977 TTCCACCGTAGGCATCAAAGCGCTCCAAATGTCCACATCCAGATACTCCAGAAAGAGTGTTTCAA
          1 TTCCAACGAAATCATCAAAGCGATCCAAATATCCACTTCCAGATTCTACAAAAAGAGTGTTTCAA

             *         *   * *             *        * *  *      *      *     
     256042 ACCTGCTCTATGAAAGGGAATGTTCAACTCTATGAGTTGAATGCAGACATCAGAAAGAAATTTCT
         66 AACTGCTCTATCAAAAGAAATGTTCAACTCTGTGAGTTGAGTACACACATCACAAAGAAGTTTCT

                     *       **          *         **
     256107 GAGAATGCTGCTGTCTACCTTTTAT-T--TGA-ATTCCCGC
        131 GAGAATGCTTCTGTCTAGTTTTTATATGAAGATATTCCCTT

                         *  *    *                *       *                  
     256144 TTCCAACGAAATCCTCCAAGCTATCCAAATATCCACTTGCAGATTCCACAAAAAGAGTGTTTCAA
          1 TTCCAACGAAATCATCAAAGCGATCCAAATATCCACTTCCAGATTCTACAAAAAGAGTGTTTCAA

                            ** *  *            *       *     *     *  *      
     256209 AACTGCTCTCTATCAATGGCAAAGTTCAACTCTGTTAGTTGAGGACACATATCACCAACAAGTTT
         66 AACTG--CTCTATCAAAAGAAATGTTCAACTCTGTGAGTTGAGTACACACATCACAAAGAAGTTT

                               *       **         *    
     256274 CTGAGAATGCTTCTGTCTATTTTTTATGGGAAGATATTTCCTT
        129 CTGAGAATGCTTCTGTCTAGTTTTTATATGAAGATATTCCCTT

              *  *  * ** *    *      *    *          * **                    
     256317 TTTCACCGTAGGCGTCAAGGCGATCGAAATGTCCACTTCCACAAACTACAAAAAGAGTGTTTCAA
          1 TTCCAACGAAATCATCAAAGCGATCCAAATATCCACTTCCAGATTCTACAAAAAGAGTGTTTCAA

             *         *   * **       *    *        * ** * *   **      *     
     256382 ACCTGCTCTATGAAAGGCCATGTTCATCTCTATGAGTTGAATGGAAATATCCGAAAGAAATTTCT
         66 AACTGCTCTATCAAAAGAAATGTTCAACTCTGTGAGTTGAGTACACACATCACAAAGAAGTTTCT

             *       *          *        *         **
     256447 GGGAATGCTGCTGTCTAG-TGTT-TAT-ACGA-ATTCCCGC
        131 GAGAATGCTTCTGTCTAGTTTTTATATGAAGATATTCCCTT

                         *       *                *    *  *                  
     256484 TTCCAACGAAATCCTCAAAGCAATCCAAATATCCACTTGCAGAATCCACAAAAAGAGTGTTTCAA
          1 TTCCAACGAAATCATCAAAGCGATCCAAATATCCACTTCCAGATTCTACAAAAAGAGTGTTTCAA

                          *     *          * *                   *  *        
     256549 AACTGCTCTATCAATAGAAAGGTTCAACTCTTTTAGTTGAGTACACACATCACGAACAAGTTTCT
         66 AACTGCTCTATCAAAAGAAATGTTCAACTCTGTGAGTTGAGTACACACATCACAAAGAAGTTTCT

                            * *      **     **  *    
     256614 GAGAATGCTTCTGTCTGGCTTTTATTGGAAGACGTTTCCTT
        131 GAGAATGCTTCTGTCTAGTTTTTATATGAAGATATTCCCTT

                 * *  **          *       *                * *               
     256655 TT-CACCAAAGGCATCAAAGCGCTCCAAATGTCCACTTCCAGATTCTTCCAAAAGAGTGTTTCAA
          1 TTCCAACGAAATCATCAAAGCGATCCAAATATCCACTTCCAGATTCTACAAAAAGAGTGTTTCAA

             **     * ***  * *                 *    * *  * *     *   *       
     256719 ACGTGCTCAAAGTAAGGGAATGTTCAACTCTGTGACTTGAATGCAGATATCACCAAGTAGTTTCT
         66 AACTGCTCTATCAAAAGAAATGTTCAACTCTGTGAGTTGAGTACACACATCACAAAGAAGTTTCT

            * * *            **     *    *         * 
     256784 AATAGTGCTTCTGTCTACATTTTAGATGATGATATTCCCGT
        131 GAGAATGCTTCTGTCTAGTTTTTATATGAAGATATTCCCTT

                         * * *   *             *  *   *      *     *       * 
     256825 TTCCAACGAAATCGTTAGAGCTATCCAAATATCCAGTTACAGTTTCTACCAAAAGGGTGTTTCCA
          1 TTCCAACGAAATCATCAAAGCGATCCAAATATCCACTTCCAGATTCTACAAAAAGAGTGTTTCAA

              *    **           *            *       *                     * 
     256890 AATTGCTGCATCAAAAGAAAGGTTCAACTCTGTTAGTTGAGGACACACATCACAAAGAAGTTTGT
         66 AACTGCTCTATCAAAAGAAATGTTCAACTCTGTGAGTTGAGTACACACATCACAAAGAAGTTTCT

                              *    *     *           
     256955 GAGAATGCTTCTGTCTAGATTTTGTATGACGATATTCCCTT
        131 GAGAATGCTTCTGTCTAGTTTTTATATGAAGATATTCCCTT

                     *   * *     *   *       * *  *    *  *       *  *       
     256996 TTCCAACGATATCGTTAAAGCAATCTAAATATCAATTTGCAGAATCCACAAAAATAGAGTTTCAA
          1 TTCCAACGAAATCATCAAAGCGATCCAAATATCCACTTCCAGATTCTACAAAAAGAGTGTTTCAA

             *       * *        *    *     * *  *                   ***      
     257061 AGCTGCTCTGTAAAAAGAAAGGTTCCACTCTTTTAGCTGAGTACACACATCACAAACTTGTTTCT
         66 AACTGCTCTATCAAAAGAAATGTTCAACTCTGTGAGTTGAGTACACACATCACAAAGAAGTTTCT

                  *         *        **         **   
     257126 GAGAATCCTTCTGTCTCGTTTTTATGGGAAGATATTTACTT
        131 GAGAATGCTTCTGTCTAGTTTTTATATGAAGATATTCCCTT

                 *  * **  *       *       *     *       *  *  *              
     257167 TTCCACCGTAGGCACCAAAGCGCTCCAAATGTCCACATCCAGATACTCCAGAAAGAGTGTTTCAA
          1 TTCCAACGAAATCATCAAAGCGATCCAAATATCCACTTCCAGATTCTACAAAAAGAGTGTTTCAA

             *         *   * *   *         *        * *  *      *      *     
     257232 ACCTGCTCTATGAAAGGGAATCTTCAACTCTATGAGTTGAATGCAGACATCAGAAAGAAATTTCT
         66 AACTGCTCTATCAAAAGAAATGTTCAACTCTGTGAGTTGAGTACACACATCACAAAGAAGTTTCT

                     *       **          *         **
     257297 GAGAATGCTGCTGTCTACCTTTTAT-T--TGA-ATTCCCGC
        131 GAGAATGCTTCTGTCTAGTTTTTATATGAAGATATTCCCTT

                         *  *   **                *  **   *    *             
     257334 TTCCAACGAAATCCTCCAAGGTATCCAAATATCCACTTGCATTTTCCACAACAAGAGTGTTTCAA
          1 TTCCAACGAAATCATCAAAGCGATCCAAATATCCACTTCCAGATTCTACAAAAAGAGTGTTTCAA

                          *                 *    *  *                *       
     257399 AACTGCTCTATCAATAGAAATGTTCAACTCCTTTG-GCTGGGTACACACATCACAAACAAGTTTC
         66 AACTGCTCTATCAAAAGAAATGTTCAACT-CTGTGAGTTGAGTACACACATCACAAAGAAGTTTC

                                      **     *        
     257463 TGAGAATGCTTCTGTCTAGTTTTTATGGGAAGACATTCCCTT
        130 TGAGAATGCTTCTGTCTAGTTTTTATATGAAGATATTCCCTT

              *  * *  **          *       *            **                  * 
     257505 TTTCACCAAAGGCATCAAAGCGCTCCAAATGTCCACTTCCAGACACTACAAAAAGAGTGTTTCCA
          1 TTCCAACGAAATCATCAAAGCGATCCAAATATCCACTTCCAGATTCTACAAAAAGAGTGTTTCAA

             **       **   ***                 *    * *  * *         *       
     257570 ACGTGCTCTAAGAAAGCGAATGTTCAACTCTGTGACTTGAATGCAGATATCACAAAGTAGTTTCT
         66 AACTGCTCTATCAAAAGAAATGTTCAACTCTGTGAGTTGAGTACACACATCACAAAGAAGTTTCT

              * *             *     *    *         * 
     257635 GAAAGTGCTTCTGTCTAGATTTTAGATGATGATATTCCCGT
        131 GAGAATGCTTCTGTCTAGTTTTTATATGAAGATATTCCCTT

                           * *   *                *   *                    * 
     257676 TTCCAACGAAATCATTAGAGCTATCCAAATATCCACTTACAGTTTCTACAAAAAGAGTGTTTCCA
          1 TTCCAACGAAATCATCAAAGCGATCCAAATATCCACTTCCAGATTCTACAAAAAGAGTGTTTCAA

                   **         * *    *       *  *                   ***      
     257741 AACTGCTGCATCAAAAGAGAGGTTCCACTCTGTTAGCTGAGTACACACATCACAAACTTGTTTCT
         66 AACTGCTCTATCAAAAGAAATGTTCAACTCTGTGAGTTGAGTACACACATCACAAAGAAGTTTCT

            *     *         *        **         **   
     257806 CAGAATCCTTCTGTCTCGTTTTTATGGGAAGATATTTACTT
        131 GAGAATGCTTCTGTCTAGTTTTTATATGAAGATATTCCCTT

              *  *  * **          *       *     *       *  *  *              
     257847 TTTCACCGTAGGCATCAAAGCGCTCCAAATGTCCACATCCAGATACTCCAGAAAGAGTGTTTCAA
          1 TTCCAACGAAATCATCAAAGCGATCCAAATATCCACTTCCAGATTCTACAAAAAGAGTGTTTCAA

             *         *   * *   *         *        * *  *      *      *     
     257912 ACCTGCTCTATGAAAGGGAATCTTCAACTCTATGAGTTGAATGCAGACATCAGAAAGAAATTTCT
         66 AACTGCTCTATCAAAAGAAATGTTCAACTCTGTGAGTTGAGTACACACATCACAAAGAAGTTTCT

                     *       **          *         **
     257977 GAGAATGCTGCTGTCTACCTTTTAT-T--TGA-ATTCCCGC
        131 GAGAATGCTTCTGTCTAGTTTTTATATGAAGATATTCCCTT

                         *  *    *                *       *                  
     258014 TTCCAACGAAATCCTCCAAGCTATCCAAATATCCACTTGCAGATTCCACAAAAAGAGTGTTTCAA
          1 TTCCAACGAAATCATCAAAGCGATCCAAATATCCACTTCCAGATTCTACAAAAAGAGTGTTTCAA

                            ** *  *            *       *     *     *  *      
     258079 AACTGCTCTCTATCAATGGCAAAGTTCAACTCTGTTAGTTGAGGACACATATCACCAACAAGTTT
         66 AACTG--CTCTATCAAAAGAAATGTTCAACTCTGTGAGTTGAGTACACACATCACAAAGAAGTTT

                               *       **         *    
     258144 CTGAGAATGCTTCTGTCTATTTTTTATGGGAAGATATTTCCTT
        129 CTGAGAATGCTTCTGTCTAGTTTTTATATGAAGATATTCCCTT

              *  *  * ** *    *      *    *          * **                    
     258187 TTTCACCGTAGGCGTCAAGGCGATCGAAATGTCCACTTCCACAAACTACAAAAAGAGTGTTTCAA
          1 TTCCAACGAAATCATCAAAGCGATCCAAATATCCACTTCCAGATTCTACAAAAAGAGTGTTTCAA

             *         *   * **       *    *        * ** * *   **      *     
     258252 ACCTGCTCTATGAAAGGCCATGTTCATCTCTATGAGTTGAATGGAAATATCCGAAAGAAATTTCT
         66 AACTGCTCTATCAAAAGAAATGTTCAACTCTGTGAGTTGAGTACACACATCACAAAGAAGTTTCT

             *       *          *        *         **
     258317 GGGAATGCTGCTGTCTAG-TGTT-TAT-ACGA-ATTCCCGC
        131 GAGAATGCTTCTGTCTAGTTTTTATATGAAGATATTCCCTT

                         *       *                *    *  *                  
     258354 TTCCAACGAAATCCTCAAAGCAATCCAAATATCCACTTGCAGAATCCACAAAAAGAGTGTTTCAA
          1 TTCCAACGAAATCATCAAAGCGATCCAAATATCCACTTCCAGATTCTACAAAAAGAGTGTTTCAA

                          *     *          * *                   *  *        
     258419 AACTGCTCTATCAATAGAAAGGTTCAACTCTTTTAGTTGAGTACACACATCACGAACAAGTTTCT
         66 AACTGCTCTATCAAAAGAAATGTTCAACTCTGTGAGTTGAGTACACACATCACAAAGAAGTTTCT

                            * *      **     **  *    
     258484 GAGAATGCTTCTGTCTGGCTTTTATTGGAAGACGTTTCCTT
        131 GAGAATGCTTCTGTCTAGTTTTTATATGAAGATATTCCCTT

                 * *  **          *       *                * *               
     258525 TT-CACCAAAGGCATCAAAGCGCTCCAAATGTCCACTTCCAGATTCTTCCAAAAGAGTGTTTCAA
          1 TTCCAACGAAATCATCAAAGCGATCCAAATATCCACTTCCAGATTCTACAAAAAGAGTGTTTCAA

             **     * ***  * *                 *    * *  * *     *   *       
     258589 ACGTGCTCAAAGTAAGGGAATGTTCAACTCTGTGACTTGAATGCAGATATCACCAAGTAGTTTCT
         66 AACTGCTCTATCAAAAGAAATGTTCAACTCTGTGAGTTGAGTACACACATCACAAAGAAGTTTCT

            * * *             *     *    *         * 
     258654 AATAGTGCTTCTGTCTAGATTTTAGATGATGATATTCCCGT
        131 GAGAATGCTTCTGTCTAGTTTTTATATGAAGATATTCCCTT

                         *** *   *             *  *   *      *     *       * 
     258695 TTCCAACGAAATCGCTAGAGCTATCCAAATATCCAGTTACAGTTTCTACCAAAAGGGTGTTTCCA
          1 TTCCAACGAAATCATCAAAGCGATCCAAATATCCACTTCCAGATTCTACAAAAAGAGTGTTTCAA

              *    **           *            *       *                     * 
     258760 AATTGCTGCATCAAAAGAAAGGTTCAACTCTGTTAGTTGAGGACACACATCACAAAGAAGTTTGT
         66 AACTGCTCTATCAAAAGAAATGTTCAACTCTGTGAGTTGAGTACACACATCACAAAGAAGTTTCT

                              *    *     *           
     258825 GAGAATGCTTCTGTCTAGATTTTGTATGACGATATTCCCTT
        131 GAGAATGCTTCTGTCTAGTTTTTATATGAAGATATTCCCTT

                     *   * *     *   *       * *  *    *  *       *  *       
     258866 TTCCAACGATATCGTTAAAGCAATCTAAATATCAATTTGCAGAATCCACAAAAATAGAGTTTCAA
          1 TTCCAACGAAATCATCAAAGCGATCCAAATATCCACTTCCAGATTCTACAAAAAGAGTGTTTCAA

             *       * *        *    *       *  *                   ***      
     258931 AGCTGCTCTGTAAAAAGAAAGGTTCCACTCTGTTAGCTGAGTACACACATCACAAACTTGTTTCT
         66 AACTGCTCTATCAAAAGAAATGTTCAACTCTGTGAGTTGAGTACACACATCACAAAGAAGTTTCT

                  *         *        **         **   
     258996 GAGAATCCTTCTGTCTCGTTTTTATGGGAAGATATTTACTT
        131 GAGAATGCTTCTGTCTAGTTTTTATATGAAGATATTCCCTT

                 *  * **          *       *     *       *  *  *              
     259037 TTCCACCGTAGGCATCAAAGCGCTCCAAATGTCCACATCCAGATACTCCAGAAAGAGTGTTTCAA
          1 TTCCAACGAAATCATCAAAGCGATCCAAATATCCACTTCCAGATTCTACAAAAAGAGTGTTTCAA

             *         *   * *   *         *        * *  *      *      *     
     259102 ACCTGCTCTATGAAAGGGAATCTTCAACTCTATGAGTTGAATGCAGACATCAGAAAGAAATTTCT
         66 AACTGCTCTATCAAAAGAAATGTTCAACTCTGTGAGTTGAGTACACACATCACAAAGAAGTTTCT

                     *       **   *      *         **
     259167 GAGAATGCTGCTGTCTACCTTTCAT-T--TGA-ATTCCCGC
        131 GAGAATGCTTCTGTCTAGTTTTTATATGAAGATATTCCCTT

                         *  *    *              * *  **   *    *             
     259204 TTCCAACGAAATCCTCCAAGCTATCCAAATATCCACCTGCATTTTCCACAACAAGAGTGTTTCAA
          1 TTCCAACGAAATCATCAAAGCGATCCAAATATCCACTTCCAGATTCTACAAAAAGAGTGTTTCAA

                          *                 *    *  *                *       
     259269 AACTGCTCTATCAATAGAAATGTTCAACTCCTTTG-GCTGGGTACACACATCACAAACAAGTTTC
         66 AACTGCTCTATCAAAAGAAATGTTCAACT-CTGTGAGTTGAGTACACACATCACAAAGAAGTTTC

                                      **     **       
     259333 TGAGAATGCTTCTGTCTAGTTTTTATGGGAAGACGTTCCCTT
        130 TGAGAATGCTTCTGTCTAGTTTTTATATGAAGATATTCCCTT

              *  * *  **          *       *            **                  * 
     259375 TTTCACCAAAGGCATCAAAGCGCTCCAAATGTCCACTTCCAGACACTACAAAAAGAGTGTTTCCA
          1 TTCCAACGAAATCATCAAAGCGATCCAAATATCCACTTCCAGATTCTACAAAAAGAGTGTTTCAA

             **       **   ***                 *    * *  * *         *       
     259440 ACGTGCTCTAAGAAAGCGAATGTTCAACTCTGTGACTTGAATGCAGATATCACAAAGTAGTTTCT
         66 AACTGCTCTATCAAAAGAAATGTTCAACTCTGTGAGTTGAGTACACACATCACAAAGAAGTTTCT

                **            *     *    *         * 
     259505 GAGAGGGCTTCTGTCTAGATTTTAGATGATGATATTCCCGT
        131 GAGAATGCTTCTGTCTAGTTTTTATATGAAGATATTCCCTT

                           * *   *                *   *                    * 
     259546 TTCCAACGAAATCATTAGAGCTATCCAAATATCCACTTACAGTTTCTACAAAAAGAGTGTTTCCA
          1 TTCCAACGAAATCATCAAAGCGATCCAAATATCCACTTCCAGATTCTACAAAAAGAGTGTTTCAA

                   **         * *    *       *  *                   ***      
     259611 AACTGCTGCATCAAAAGAGAGGTTCCACTCTGTTAGCTGAGTACACACATCACAAACTTGTTTCT
         66 AACTGCTCTATCAAAAGAAATGTTCAACTCTGTGAGTTGAGTACACACATCACAAAGAAGTTTCT

            *     *         *        **         **   
     259676 CAGAATCCTTCTGTCTCGTTTTTATGGGAAGATATTTACTT
        131 GAGAATGCTTCTGTCTAGTTTTTATATGAAGATATTCCCTT

              *  *  * **          *       *     *       *  *  *              
     259717 TTTCACCGTAGGCATCAAAGCGCTCCAAATGTCCACATCCAGATACTCCAGAAAGAGTGTTTCAA
          1 TTCCAACGAAATCATCAAAGCGATCCAAATATCCACTTCCAGATTCTACAAAAAGAGTGTTTCAA

             *         *   * *             *        * *  *      *      *     
     259782 ACCTGCTCTATGAAAGGGAATGTTCAACTCTATGAGTTGAATGCAGACATCAGAAAGAAATTTCT
         66 AACTGCTCTATCAAAAGAAATGTTCAACTCTGTGAGTTGAGTACACACATCACAAAGAAGTTTCT

                     *       **          *         **
     259847 GAGAATGCTGCTGTCTACCTTTTAT-T--TGA-ATTCCCGC
        131 GAGAATGCTTCTGTCTAGTTTTTATATGAAGATATTCCCTT

                         *  *    *                *       *                  
     259884 TTCCAACGAAATCCTCCAAGCTATCCAAATATCCACTTGCAGATTCCACAAAAAGAGTGTTTCAA
          1 TTCCAACGAAATCATCAAAGCGATCCAAATATCCACTTCCAGATTCTACAAAAAGAGTGTTTCAA

                            ** *  *            *       *     *     *  *      
     259949 AACTGCTCTCTATCAATGGCAAAGTTCAACTCTGTTAGTTGAGGACACATATCACCAACAAGTTT
         66 AACTG--CTCTATCAAAAGAAATGTTCAACTCTGTGAGTTGAGTACACACATCACAAAGAAGTTT

                               *      ***              
     260014 CTGAGAATGCTTCTGTCTATTTTTTAAGGGAAGATATTCCCTT
        129 CTGAGAATGCTTCTGTCTAGTTTTTATATGAAGATATTCCCTT

              *  *  * ** *    *      *    *       *  * **                    
     260057 TTTCACCGTAGGCGTCAAGGCGATCGAAATGTCCACTTTCACAAACTACAAAAAGAGTGTTTCAA
          1 TTCCAACGAAATCATCAAAGCGATCCAAATATCCACTTCCAGATTCTACAAAAAGAGTGTTTCAA

             *         *   * **       *    *        * ** * *   **      *     
     260122 ACCTGCTCTATGAAAGGCCATGTTCATCTCTATGAGTTGAATGGATATATCCGAAAGAAATTTCT
         66 AACTGCTCTATCAAAAGAAATGTTCAACTCTGTGAGTTGAGTACACACATCACAAAGAAGTTTCT

             *       *          *        *         **
     260187 GGGAATGCTGCTGTCTAG-TGTT-TAT-ACGA-ATTCCCGA
        131 GAGAATGCTTCTGTCTAGTTTTTATATGAAGATATTCCCTT

                         *       *                *    *  *                  
     260224 TTCCAACGAAATCCTCAAAGCAATCCAAATATCCACTTGCAGAATCCACAAAAAGAGTGTTTCAA
          1 TTCCAACGAAATCATCAAAGCGATCCAAATATCCACTTCCAGATTCTACAAAAAGAGTGTTTCAA

                          *                * *                   *  *        
     260289 AACTGCTCTATCAATAGAAATGTTCAACTCTTTTAGTTGAGTACACACATCACGAACAAGTTTCT
         66 AACTGCTCTATCAAAAGAAATGTTCAACTCTGTGAGTTGAGTACACACATCACAAAGAAGTTTCT

                            * *      **     **  *    
     260354 GAGAATGCTTCTGTCTGGCTTTTATTGGAAGACGTTTCCTT
        131 GAGAATGCTTCTGTCTAGTTTTTATATGAAGATATTCCCTT

                 * *  **          *    *  *                * *      *        
     260395 TT-CACCAAAGGCATCAAAGCGCTCCATATGTCCACTTCCAGATTCTTCCAAAAGACTGTTTCAA
          1 TTCCAACGAAATCATCAAAGCGATCCAAATATCCACTTCCAGATTCTACAAAAAGAGTGTTTCAA

             **     * ***  * *                 *    * *  * *     *   *       
     260459 ACGTGCTCAAAGTAAGGGAATGTTCAACTCTGTGACTTGAATGCAGATATCACCAAGTAGTTTCT
         66 AACTGCTCTATCAAAAGAAATGTTCAACTCTGTGAGTTGAGTACACACATCACAAAGAAGTTTCT

            * * *             *     *    *         * 
     260524 AATAGTGCTTCTGTCTAGATTTTAGATGATGATATTCCCGT
        131 GAGAATGCTTCTGTCTAGTTTTTATATGAAGATATTCCCTT

                         * * *   *             *  *   *      *     *       * 
     260565 TTCCAACGAAATCGTTAGAGCTATCCAAATATCCAGTTACAGTTTCTACCAAAAGGGTGTTTCCA
          1 TTCCAACGAAATCATCAAAGCGATCCAAATATCCACTTCCAGATTCTACAAAAAGAGTGTTTCAA

              *    **           *            *       *                     * 
     260630 AATTGCTGCATCAAAAGAAAGGTTCAACTCTGTTAGTTGAGGACACACATCACAAAGAAGTTTGT
         66 AACTGCTCTATCAAAAGAAATGTTCAACTCTGTGAGTTGAGTACACACATCACAAAGAAGTTTCT

                              *    *     *           
     260695 GAGAATGCTTCTGTCTAGATTTTGTATGACGATATTCCCTT
        131 GAGAATGCTTCTGTCTAGTTTTTATATGAAGATATTCCCTT

                     *   * *     *   *       * *  *    *  *       *  *       
     260736 TTCCAACGATATCGTTAAAGCAATCTAAATATCAATTTGCAGAATCCACAAAAATAGAGTTTCAA
          1 TTCCAACGAAATCATCAAAGCGATCCAAATATCCACTTCCAGATTCTACAAAAAGAGTGTTTCAA

             *       * *        *    *       *  *                   ***      
     260801 AGCTGCTCTGTAAAAAGAAAGGTTCCACTCTGTTAGCTGAGTACACACATCACAAACTTGTTTCT
         66 AACTGCTCTATCAAAAGAAATGTTCAACTCTGTGAGTTGAGTACACACATCACAAAGAAGTTTCT

                  *         *        **         **   
     260866 GAGAATCCTTCTGTCTCGTTTTTATGGGAAGATATTTACTT
        131 GAGAATGCTTCTGTCTAGTTTTTATATGAAGATATTCCCTT

                 *  * **          *       *     *       *  *  *              
     260907 TTCCACCGTAGGCATCAAAGCGCTCCAAATGTCCACATCCAGATACTCCAGAAAGAGTGTTTCAA
          1 TTCCAACGAAATCATCAAAGCGATCCAAATATCCACTTCCAGATTCTACAAAAAGAGTGTTTCAA

             *         *   * *   *         *        * *  *      *      *     
     260972 ACCTGCTCTATGAAAGGGAATCTTCAACTCTATGAGTTGAATGCAGACATCAGAAAGAAATTTCT
         66 AACTGCTCTATCAAAAGAAATGTTCAACTCTGTGAGTTGAGTACACACATCACAAAGAAGTTTCT

                     *       **          *         **
     261037 GAGAATGCTGCTGTCTACCTTTTAT-T--TGA-ATTCCCGC
        131 GAGAATGCTTCTGTCTAGTTTTTATATGAAGATATTCCCTT

                         *  *    *              * *  **   *                  
     261074 TTCCAACGAAATCCTCCAAGCTATCCAAATATCCACCTGCATTTTCCACAAAAAGAGTGTTTCAA
          1 TTCCAACGAAATCATCAAAGCGATCCAAATATCCACTTCCAGATTCTACAAAAAGAGTGTTTCAA

                          *                 *    *  *                *       
     261139 AACTGCTCTATCAATAGAAATGTTCAACTCCTTTG-GCTGGGTACACACATCACAAACAAGTTTC
         66 AACTGCTCTATCAAAAGAAATGTTCAACT-CTGTGAGTTGAGTACACACATCACAAAGAAGTTTC

                                      **     **       
     261203 TGAGAATGCTTCTGTCTAGTTTTTATGGGAAGACGTTCCCTT
        130 TGAGAATGCTTCTGTCTAGTTTTTATATGAAGATATTCCCTT

              *  * *  **          *       *            **                    
     261245 TTTCACCAAAGGCATCAAAGCGCTCCAAATGTCCACTTCCAGACACTACAAAAAGAGTGTTTCAA
          1 TTCCAACGAAATCATCAAAGCGATCCAAATATCCACTTCCAGATTCTACAAAAAGAGTGTTTCAA

             **       **   ***                 *    * *  * *         *       
     261310 ACGTGCTCTAAGAAAGCGAATGTTCAACTCTGTGACTTGAATGCAGATATCACAAAGTAGTTTCT
         66 AACTGCTCTATCAAAAGAAATGTTCAACTCTGTGAGTTGAGTACACACATCACAAAGAAGTTTCT

                **            *     *    *         * 
     261375 GAGAGGGCTTCTGTCTAGATTTTAGATGATGATATTCCCGT
        131 GAGAATGCTTCTGTCTAGTTTTTATATGAAGATATTCCCTT

                           * *   *                *   *                    * 
     261416 TTCCAACGAAATCATTAGAGCTATCCAAATATCCACTTACAGTTTCTACAAAAAGAGTGTTTCCA
          1 TTCCAACGAAATCATCAAAGCGATCCAAATATCCACTTCCAGATTCTACAAAAAGAGTGTTTCAA

                   **         * *    *       *  *                   ***      
     261481 AACTGCTGCATCAAAAGAGAGGTTCCACTCTGTTAGCTGAGTACACACATCACAAACTTGTTTCT
         66 AACTGCTCTATCAAAAGAAATGTTCAACTCTGTGAGTTGAGTACACACATCACAAAGAAGTTTCT

            *     * *       *        **         **   
     261546 CAGAATCCGTCTGTCTCGTTTTTATGGGAAGATATTTACTT
        131 GAGAATGCTTCTGTCTAGTTTTTATATGAAGATATTCCCTT

              *  *  * **          *       *     *       *  *  *              
     261587 TTTCACCGTAGGCATCAAAGCGCTCCAAATGTCCACATCCAGATACTCCAGAAAGAGTGTTTCAA
          1 TTCCAACGAAATCATCAAAGCGATCCAAATATCCACTTCCAGATTCTACAAAAAGAGTGTTTCAA

             *         *   * *             *        * *  *      *      *     
     261652 ACCTGCTCTATGAAAGGGAATGTTCAACTCTATGAGTTGAATGCAGACATCAGAAAGAAATTTCT
         66 AACTGCTCTATCAAAAGAAATGTTCAACTCTGTGAGTTGAGTACACACATCACAAAGAAGTTTCT

                     *       **          *         **
     261717 GAGAATGCTGCTGTCTACCTTTTAT-T--TGA-ATTCCCGC
        131 GAGAATGCTTCTGTCTAGTTTTTATATGAAGATATTCCCTT

                         *  *    *                *       *                  
     261754 TTCCAACGAAATCCTCCAAGCTATCCAAATATCCACTTGCAGATTCCACAAAAAGAGTGTTTCAA
          1 TTCCAACGAAATCATCAAAGCGATCCAAATATCCACTTCCAGATTCTACAAAAAGAGTGTTTCAA

                            ** *  *            *       *     *     *  *      
     261819 AACTGCTCTCTATCAATGGCAAAGTTCAACTCTGTTAGTTGAGGACACATATCACCAACAAGTTT
         66 AACTG--CTCTATCAAAAGAAATGTTCAACTCTGTGAGTTGAGTACACACATCACAAAGAAGTTT

                               *       **         *    
     261884 CTGAGAATGCTTCTGTCTATTTTTTATGGGAAGATATTTCCTT
        129 CTGAGAATGCTTCTGTCTAGTTTTTATATGAAGATATTCCCTT

              *  *  * ** *    *      *    *          * **                    
     261927 TTTCACCGTAGGCGTCAAGGCGATCGAAATGTCCACTTCCACAAACTACAAAAAGAGTGTTTCAA
          1 TTCCAACGAAATCATCAAAGCGATCCAAATATCCACTTCCAGATTCTACAAAAAGAGTGTTTCAA

             *         *   * **       *    *        * ** * *   **      *     
     261992 ACCTGCTCTATGAAAGGCCATGTTCATCTCTATGAGTTGAATGGAAATATCCGAAAGAAATTTCT
         66 AACTGCTCTATCAAAAGAAATGTTCAACTCTGTGAGTTGAGTACACACATCACAAAGAAGTTTCT

             *       *                             **
     262057 GGGAATGCTGCTGTCTAGTTTTTATAT---GA-ATTCCCGC
        131 GAGAATGCTTCTGTCTAGTTTTTATATGAAGATATTCCCTT

                         *       *                *    *  *                  
     262094 TTCCAACGAAATCCTCAAAGCAATCCAAATATCCACTTGCAGAATCCACAAAAAGAGTGTTTCAA
          1 TTCCAACGAAATCATCAAAGCGATCCAAATATCCACTTCCAGATTCTACAAAAAGAGTGTTTCAA

                          *     *          * *                      *        
     262159 AACTGCTCTATCAATAGAAAGGTTCAACTCTTTTAGTTGAGTACACACATCACAAACAAGTTTCT
         66 AACTGCTCTATCAAAAGAAATGTTCAACTCTGTGAGTTGAGTACACACATCACAAAGAAGTTTCT

                            * *      **     **  *    
     262224 GAGAATGCTTCTGTCTGGCTTTTATTGGAAGACGTTTCCTT
        131 GAGAATGCTTCTGTCTAGTTTTTATATGAAGATATTCCCTT

                 * *  **          *       *                * *               
     262265 TT-CACCAAAGGCATCAAAGCGCTCCAAATGTCCACTTCCAGATTCTTCCAAAAGAGTGTTTCAA
          1 TTCCAACGAAATCATCAAAGCGATCCAAATATCCACTTCCAGATTCTACAAAAAGAGTGTTTCAA

             **     * ***  * *                 *    * *  * *     *   *  *    
     262329 ACGTGCTCAAAGTAAGGGAATGTTCAACTCTGTGACTTGAATGCAGATATCACCAAGTAGCTTCT
         66 AACTGCTCTATCAAAAGAAATGTTCAACTCTGTGAGTTGAGTACACACATCACAAAGAAGTTTCT

            * * *             *     *    *         * 
     262394 AATAGTGCTTCTGTCTAGATTTTAGATGATGATATTCCCGT
        131 GAGAATGCTTCTGTCTAGTTTTTATATGAAGATATTCCCTT

                         * * *   *             *  *   *      *     *       * 
     262435 TTCCAACGAAATCGTTAGAGCTATCCAAATATCCAGTTACAGTTTCTACCAAAAGGGTGTTTCCA
          1 TTCCAACGAAATCATCAAAGCGATCCAAATATCCACTTCCAGATTCTACAAAAAGAGTGTTTCAA

              *    **           *            *       *                     * 
     262500 AATTGCTGCATCAAAAGAAAGGTTCAACTCTGTTAGTTGAGGACACACATCACAAAGAAGTTTGT
         66 AACTGCTCTATCAAAAGAAATGTTCAACTCTGTGAGTTGAGTACACACATCACAAAGAAGTTTCT

                              *    *     *           
     262565 GAGAATGCTTCTGTCTAGATTTTGTATGACGATATTCCCTT
        131 GAGAATGCTTCTGTCTAGTTTTTATATGAAGATATTCCCTT

                     *   * *     *   *       * *  *    *  *       *  *       
     262606 TTCCAACGATATCGTTAAAGCAATCTAAATATCAATTTGCAGAATCCACAAAAATAGAGTTTCAA
          1 TTCCAACGAAATCATCAAAGCGATCCAAATATCCACTTCCAGATTCTACAAAAAGAGTGTTTCAA

             *       * *        *    *       *  *                   ***      
     262671 AGCTGCTCTGTAAAAAGAAAGGTTCCACTCTGTTAGCTGAGTACACACATCACAAACTTGTTTCT
         66 AACTGCTCTATCAAAAGAAATGTTCAACTCTGTGAGTTGAGTACACACATCACAAAGAAGTTTCT

                  *         *        **         **   
     262736 GAGAATCCTTCTGTCTCGTTTTTATGGGAAGATATTTACTT
        131 GAGAATGCTTCTGTCTAGTTTTTATATGAAGATATTCCCTT

                 *  * **          *       *     *       *  *  *  *           
     262777 TTCCACCGTAGGCATCAAAGCGCTCCAAATGTCCACATCCAGATACTCCAGAACGAGTGTTTCAA
          1 TTCCAACGAAATCATCAAAGCGATCCAAATATCCACTTCCAGATTCTACAAAAAGAGTGTTTCAA

             *         *   * *   *         *        * *  *      *      *     
     262842 ACCTGCTCTATGAAAGGGAATCTTCAACTCTATGAGTTGAATGCAGACATCAGAAAGAAATTTCT
         66 AACTGCTCTATCAAAAGAAATGTTCAACTCTGTGAGTTGAGTACACACATCACAAAGAAGTTTCT

                     *       **          *         **
     262907 GAGAATGCTGCTGTCTACCTTTTAT-T--TGA-ATTCCCGC
        131 GAGAATGCTTCTGTCTAGTTTTTATATGAAGATATTCCCTT

                         *  *    *              * *  **   *                  
     262944 TTCCAACGAAATCCTCCAAGCTATCCAAATATCCACCTGCATTTTCCACAAAAAGAGTGTTTCAA
          1 TTCCAACGAAATCATCAAAGCGATCCAAATATCCACTTCCAGATTCTACAAAAAGAGTGTTTCAA

                          *                 *    *  *                *       
     263009 AACTGCTCTATCAATAGAAATGTTCAACTCCTTTG-GCTGGGTACACACATCACAAACAAGTTTC
         66 AACTGCTCTATCAAAAGAAATGTTCAACT-CTGTGAGTTGAGTACACACATCACAAAGAAGTTTC

                                      **     **       
     263073 TGAGAATGCTTCTGTCTAGTTTTTATGGGAAGACGTTCCCTT
        130 TGAGAATGCTTCTGTCTAGTTTTTATATGAAGATATTCCCTT

              *  * *  **          *       *            **                    
     263115 TTTCACCAAAGGCATCAAAGCGCTCCAAATGTCCACTTCCAGACACTACAAAAAGAGTGTTTCAA
          1 TTCCAACGAAATCATCAAAGCGATCCAAATATCCACTTCCAGATTCTACAAAAAGAGTGTTTCAA

             **       **   ***            *    *    * *  * *         *       
     263180 ACGTGCTCTAAGAAAGCGAATGTTCAACTCGGTGACTTGAATGCAGATATCACAAAGTAGTTTCT
         66 AACTGCTCTATCAAAAGAAATGTTCAACTCTGTGAGTTGAGTACACACATCACAAAGAAGTTTCT

                **            *     *    *         * 
     263245 GAGAGGGCTTCTGTCTAGATTTTAGATGATGATATTCCCGT
        131 GAGAATGCTTCTGTCTAGTTTTTATATGAAGATATTCCCTT

                           * *   *                *   *                    * 
     263286 TTCCAACGAAATCATTAGAGCTATCCAAATATCCACTTACAGTTTCTACAAAAAGAGTGTTTCCA
          1 TTCCAACGAAATCATCAAAGCGATCCAAATATCCACTTCCAGATTCTACAAAAAGAGTGTTTCAA

                   **         * *    *       *  *                   ***      
     263351 AACTGCTGCATCAAAAGAGAGGTTCCACTCTGTTAGCTGAGTACACACATCACAAACTTGTTTCT
         66 AACTGCTCTATCAAAAGAAATGTTCAACTCTGTGAGTTGAGTACACACATCACAAAGAAGTTTCT

            *   * *         *        **         **   
     263416 CAGATTCCTTCTGTCTCGTTTTTATGGGAAGATATTTACTT
        131 GAGAATGCTTCTGTCTAGTTTTTATATGAAGATATTCCCTT

              *  *  * **          *       *     *      **  *  *              
     263457 TTTCACCGTAGGCATCAAAGCGCTCCAAATGTCCACATCCAGAAACTCCAGAAAGAGTGTTTCAA
          1 TTCCAACGAAATCATCAAAGCGATCCAAATATCCACTTCCAGATTCTACAAAAAGAGTGTTTCAA

             *         *   * *             *        * *  *      *      *     
     263522 ACCTGCTCTATGAAAGGGAATGTTCAACTCTATGAGTTGAATGCAGACATCAGAAAGAAATTTCT
         66 AACTGCTCTATCAAAAGAAATGTTCAACTCTGTGAGTTGAGTACACACATCACAAAGAAGTTTCT

                     *       **          *         **
     263587 GAGAATGCTGCTGTCTACCTTTTAT-T--TGA-ATTCCCAC
        131 GAGAATGCTTCTGTCTAGTTTTTATATGAAGATATTCCCTT

                         *  *    *                *       *                  
     263624 TTCCAACGAAATCCTCCAAGCTATCCAAATATCCACTTGCAGATTCCACAAAAAGAGTGTTTCAA
          1 TTCCAACGAAATCATCAAAGCGATCCAAATATCCACTTCCAGATTCTACAAAAAGAGTGTTTCAA

                            ** *  *            *       *     *     *  *      
     263689 AACTGCTCTCTATCAATGGCAAAGTTCAACTCTGTTAGTTGAGGACACATATCACCAACAAGTTT
         66 AACTG--CTCTATCAAAAGAAATGTTCAACTCTGTGAGTTGAGTACACACATCACAAAGAAGTTT

                               *       **         *    
     263754 CTGAGAATGCTTCTGTCTATTTTTTATGGGAAGATATTTCCTT
        129 CTGAGAATGCTTCTGTCTAGTTTTTATATGAAGATATTCCCTT

              *  *  * ** *    *      *    *          * **    *               
     263797 TTTCAGCGTAGGCGTCAAGGCGATCAAAATGTCCACTTCCACAAACTACCAAAAGAGTGTTTCAA
          1 TTCCAACGAAATCATCAAAGCGATCCAAATATCCACTTCCAGATTCTACAAAAAGAGTGTTTCAA

             *         *   * **       *    *        * ** * *   **      *     
     263862 ACCTGCTCTATGAAAGGCCATGTTCATCTCTATGAGTTGAATGGAAATATCCGAAAGAAATTTCT
         66 AACTGCTCTATCAAAAGAAATGTTCAACTCTGTGAGTTGAGTACACACATCACAAAGAAGTTTCT

             *       *                             **
     263927 GGGAATGCTGCTGTCTAGTTTTTATAT---GA-ATTCCCGC
        131 GAGAATGCTTCTGTCTAGTTTTTATATGAAGATATTCCCTT

                 *       *       *                *    *  *                  
     263964 TTCCACCGAAATCCTCAAAGCAATCCAAATATCCACTTGCAGAATCCACAAAAAGAGTGTTTCAA
          1 TTCCAACGAAATCATCAAAGCGATCCAAATATCCACTTCCAGATTCTACAAAAAGAGTGTTTCAA

                  *       *     *          * *                      *        
     264029 AACTGCGCTATCAATAGAAAGGTTCAACTCTTTTAGTTGAGTACACACATCACAAACAAGTTTCT
         66 AACTGCTCTATCAAAAGAAATGTTCAACTCTGTGAGTTGAGTACACACATCACAAAGAAGTTTCT

                            * *      **     **  *    
     264094 GAGAATGCTTCTGTCTGGCTTTTATTGGAAGACGTTTCCTT
        131 GAGAATGCTTCTGTCTAGTTTTTATATGAAGATATTCCCTT

                 * *  **          *       *                * *               
     264135 TT-CACCAAAGGCATCAAAGCGCTCCAAATGTCCACTTCCAGATTCTTCCAAAAGAGTGTTTCAA
          1 TTCCAACGAAATCATCAAAGCGATCCAAATATCCACTTCCAGATTCTACAAAAAGAGTGTTTCAA

             **     * *** ** *                 *    * *  * *     *   *       
     264199 ACGTGCTCAAAGTACGGGAATGTTCAACTCTGTGACTTGAATGCAGATATCACCAAGTAGTTTCT
         66 AACTGCTCTATCAAAAGAAATGTTCAACTCTGTGAGTTGAGTACACACATCACAAAGAAGTTTCT

            * * *             *     *    *         * 
     264264 AATAGTGCTTCTGTCTAGATTTTAGATGATGATATTCCCGT
        131 GAGAATGCTTCTGTCTAGTTTTTATATGAAGATATTCCCTT

                         * * *   *             *  *   *      *     *       * 
     264305 TTCCAACGAAATCGTTAGAGCTATCCAAATATCCAGTTACAGTTTCTACCAAAAGGGTGTTTCCA
          1 TTCCAACGAAATCATCAAAGCGATCCAAATATCCACTTCCAGATTCTACAAAAAGAGTGTTTCAA

              *    **           *            *       *                     * 
     264370 AATTGCTGCATCAAAAGAAAGGTTCAACTCTGTTAGTTGAGGACACACATCACAAAGAAGTTTGT
         66 AACTGCTCTATCAAAAGAAATGTTCAACTCTGTGAGTTGAGTACACACATCACAAAGAAGTTTCT

                              *    *     *           
     264435 GAGAATGCTTCTGTCTAGATTTTGTATGACGATATTCCCTT
        131 GAGAATGCTTCTGTCTAGTTTTTATATGAAGATATTCCCTT

                     *   * *     *   *       * *  *    *  *       *  *       
     264476 TTCCAACGATATCGTTAAAGCAATCTAAATATCAATTTGCAGAATCCACAAAAATAGAGTTTCAA
          1 TTCCAACGAAATCATCAAAGCGATCCAAATATCCACTTCCAGATTCTACAAAAAGAGTGTTTCAA

             *       * *        *    *       *  *                   ***      
     264541 AGCTGCTCTGTAAAAAGAAAGGTTCCACTCTGTTAGCTGAGTACACACATCACAAACTTGTTTCT
         66 AACTGCTCTATCAAAAGAAATGTTCAACTCTGTGAGTTGAGTACACACATCACAAAGAAGTTTCT

                  *         *        **         **   
     264606 GAGAATCCTTCTGTCTCGTTTTTATGGGAAGATATTTACTT
        131 GAGAATGCTTCTGTCTAGTTTTTATATGAAGATATTCCCTT

                 *  * **          *       *     *       *  *  *  *           
     264647 TTCCACCGTAGGCATCAAAGCGCTCCAAATGTCCACATCCAGATACTCCAGAAGGAGTGTTTCAA
          1 TTCCAACGAAATCATCAAAGCGATCCAAATATCCACTTCCAGATTCTACAAAAAGAGTGTTTCAA

             *         *   * *   *         *        * *  *      *      *     
     264712 ACCTGCTCTATGAAAGGGAATCTTCAACTCTATGAGTTGAATGCAGACATCAGAAAGAAATTTCT
         66 AACTGCTCTATCAAAAGAAATGTTCAACTCTGTGAGTTGAGTACACACATCACAAAGAAGTTTCT

                     *       **          *         **
     264777 GAGAATGCTGCTGTCTACCTTTTAT-T--TGA-ATTCCCGC
        131 GAGAATGCTTCTGTCTAGTTTTTATATGAAGATATTCCCTT

                         *  *    *                *  **   *    *             
     264814 TTCCAACGAAATCCTCCAAGCTATCCAAATATCCACTTGCATTTTCCACAACAAGAGTGTTTCAA
          1 TTCCAACGAAATCATCAAAGCGATCCAAATATCCACTTCCAGATTCTACAAAAAGAGTGTTTCAA

                          *                 *    *  *                *       
     264879 AACTGCTCTATCAATAGAAATGTTCAACTCCTTTG-GCTGGGTACACACATCACAAACAAGTTTC
         66 AACTGCTCTATCAAAAGAAATGTTCAACT-CTGTGAGTTGAGTACACACATCACAAAGAAGTTTC

                                      **     *        
     264943 TGAGAATGCTTCTGTCTAGTTTTTATGGGAAGACATTCCCTT
        130 TGAGAATGCTTCTGTCTAGTTTTTATATGAAGATATTCCCTT

              *  * *  **          *       *            **                    
     264985 TTTCACCAAAGGCATCAAAGCGCTCCAAATGTCCACTTCCAGACACTACAAAAAGAGTGTTTCAA
          1 TTCCAACGAAATCATCAAAGCGATCCAAATATCCACTTCCAGATTCTACAAAAAGAGTGTTTCAA

             **       **   ***                 *    * *  * *         *       
     265050 ACGTGCTCTAAGAAAGCGAATGTTCAACTCTGTGACTTGAATGCAGATATCACAAAGTAGTTTCT
         66 AACTGCTCTATCAAAAGAAATGTTCAACTCTGTGAGTTGAGTACACACATCACAAAGAAGTTTCT

                *             *     *    *         * 
     265115 GAGAGTGCTTCTGTCTAGATTTTAGATGATGATATTCCCGT
        131 GAGAATGCTTCTGTCTAGTTTTTATATGAAGATATTCCCTT

                           * *   *                *   *                    * 
     265156 TTCCAACGAAATCATTAGAGCTATCCAAATATCCACTTACAGTTTCTACAAAAAGAGTGTTTCCA
          1 TTCCAACGAAATCATCAAAGCGATCCAAATATCCACTTCCAGATTCTACAAAAAGAGTGTTTCAA

                   **         * *    *       *  *                   ***      
     265221 AACTGCTGCATCAAAAGAGAGGTTCCACTCTGTTAGCTGAGTACACACATCACAAACTTGTTTCT
         66 AACTGCTCTATCAAAAGAAATGTTCAACTCTGTGAGTTGAGTACACACATCACAAAGAAGTTTCT

            *     *         *        **         **   
     265286 CAGAATCCTTCTGTCTCGTTTTTATGGGAAGATATTTACTT
        131 GAGAATGCTTCTGTCTAGTTTTTATATGAAGATATTCCCTT

              *  *  * **          *       *     *       *  *  *              
     265327 TTTCACCGTAGGCATCAAAGCGCTCCAAATGTCCACATCCAGATACTCCAGAAAGAGTGTTTCAA
          1 TTCCAACGAAATCATCAAAGCGATCCAAATATCCACTTCCAGATTCTACAAAAAGAGTGTTTCAA

             *         *   * *   *         *        * *  *      *      *     
     265392 ACCTGCTCTATGAAAGGGAATCTTCAACTCTATGAGTTGAATGCAGACATCAGAAAGAAATTTCT
         66 AACTGCTCTATCAAAAGAAATGTTCAACTCTGTGAGTTGAGTACACACATCACAAAGAAGTTTCT

                     *       **          *         **
     265457 GAGAATGCTGCTGTCTACCTTTTAT-T--TGA-ATTCCCGC
        131 GAGAATGCTTCTGTCTAGTTTTTATATGAAGATATTCCCTT

                         *  *    *                *       *                  
     265494 TTCCAACGAAATCCTCCAAGCTATCCAAATATCCACTTGCAGATTCCACAAAAAGAGTGTTTCAA
          1 TTCCAACGAAATCATCAAAGCGATCCAAATATCCACTTCCAGATTCTACAAAAAGAGTGTTTCAA

                            ** *  *            *       *     *     *  *      
     265559 AACTGCTCTCTATCAATGGCAAAGTTCAACTCTGTTAGTTGAGGACACATATCACCAACAAGTTT
         66 AACTG--CTCTATCAAAAGAAATGTTCAACTCTGTGAGTTGAGTACACACATCACAAAGAAGTTT

                               *       **         *    
     265624 CTGAGAATGCTTCTGTCTATTTTTTATGGGAAGATATTTCCTT
        129 CTGAGAATGCTTCTGTCTAGTTTTTATATGAAGATATTCCCTT

              *  *  * ** *    *      *    *          * **          *         
     265667 TTTCACCGTAGGCGTCAAGGCGATCGAAATGTCCACTTCCACAAACTACAAAAAGCGTGTTTCAA
          1 TTCCAACGAAATCATCAAAGCGATCCAAATATCCACTTCCAGATTCTACAAAAAGAGTGTTTCAA

             *         *   * **       *    *        * ** * *   **      *     
     265732 ACCTGCTCTATGAAAGGCCATGTTCATCTCTATGAGTTGAATGGAAATATCCGAAAGAAATTTCT
         66 AACTGCTCTATCAAAAGAAATGTTCAACTCTGTGAGTTGAGTACACACATCACAAAGAAGTTTCT

             *       *          *        *         **
     265797 GGGAATGCTGCTGTCTAG-TGTT-TAT-ACGA-ATTCCCGC
        131 GAGAATGCTTCTGTCTAGTTTTTATATGAAGATATTCCCTT

                         *       *                *    *  *                  
     265834 TTCCAACGAAATCCTCAAAGCAATCCAAATATCCACTTGCAGAATCCACAAAAAGAGTGTTTCAA
          1 TTCCAACGAAATCATCAAAGCGATCCAAATATCCACTTCCAGATTCTACAAAAAGAGTGTTTCAA

                          *     *          * *                   *  *        
     265899 AACTGCTCTATCAATAGAAAGGTTCAACTCTTTTAGTTGAGTACACACATCACGAACAAGTTTCT
         66 AACTGCTCTATCAAAAGAAATGTTCAACTCTGTGAGTTGAGTACACACATCACAAAGAAGTTTCT

                            * *      **     **  *    
     265964 GAGAATGCTTCTGTCTGGCTTTTATTGGAAGACGTTTCCTT
        131 GAGAATGCTTCTGTCTAGTTTTTATATGAAGATATTCCCTT

                 * *  **          *       *                * *               
     266005 TT-CACCAAAGGCATCAAAGCGCTCCAAATGTCCACTTCCAGATTCTTCCAAAAGAGTGTTTCAA
          1 TTCCAACGAAATCATCAAAGCGATCCAAATATCCACTTCCAGATTCTACAAAAAGAGTGTTTCAA

             **     * ***  * *                 *    * *  * *     *   *       
     266069 ACGTGCTCAAAGTAAGGGAATGTTCAACTCTGTGACTTGAATGCAGATATCACCAAGTAGTTTCT
         66 AACTGCTCTATCAAAAGAAATGTTCAACTCTGTGAGTTGAGTACACACATCACAAAGAAGTTTCT

            * * *             *     *    *         * 
     266134 AATAGTGCTTCTGTCTAGATTTTAGATGATGATATTCCCGT
        131 GAGAATGCTTCTGTCTAGTTTTTATATGAAGATATTCCCTT

                         *** *   *             *  *   *      *     *       * 
     266175 TTCCAACGAAATCGCTAGAGCTATCCAAATATCCAGTTACAGTTTCTACTAAAAGGGTGTTTCCA
          1 TTCCAACGAAATCATCAAAGCGATCCAAATATCCACTTCCAGATTCTACAAAAAGAGTGTTTCAA

              *    **           *            *       *                     * 
     266240 AATTGCTGCATCAAAAGAAAGGTTCAACTCTGTTAGTTGAGGACACACATCACAAAGAAGTTTGT
         66 AACTGCTCTATCAAAAGAAATGTTCAACTCTGTGAGTTGAGTACACACATCACAAAGAAGTTTCT

                              *    *     *           
     266305 GAGAATGCTTCTGTCTAGATTTTGTATGACGATATTCCCTT
        131 GAGAATGCTTCTGTCTAGTTTTTATATGAAGATATTCCCTT

                     *   * *     *   *       * *  *    *  *       *  *       
     266346 TTCCAACGATATCGTTAAAGCAATCTAAATATCAATTTGCAGAATCCACAAAAATAGAGTTTCAA
          1 TTCCAACGAAATCATCAAAGCGATCCAAATATCCACTTCCAGATTCTACAAAAAGAGTGTTTCAA

             *       * *        *    *       *  *                   ***      
     266411 AGCTGCTCTGTAAAAAGAAAGGTTCCACTCTGTTAGCTGAGTACACACATCACAAACTTGTTTCT
         66 AACTGCTCTATCAAAAGAAATGTTCAACTCTGTGAGTTGAGTACACACATCACAAAGAAGTTTCT

                  *         *   *    **        ***   
     266476 GAGAATCCTTCTGTCTCGTTCTTATGGGAAGATATATACTT
        131 GAGAATGCTTCTGTCTAGTTTTTATATGAAGATATTCCCTT

                 *    **          *       *     *       *  *  *  *           
     266517 TTCCACCGAAGGCATCAAAGCGCTCCAAATGTCCACATCCAGATACTCCAGAACGAGTGTTTCAA
          1 TTCCAACGAAATCATCAAAGCGATCCAAATATCCACTTCCAGATTCTACAAAAAGAGTGTTTCAA

             *         *   * *   *         *        * *  *      *      *     
     266582 ACCTGCTCTATGAAAGGGAATCTTCAACTCTATGAGTTGAATGCAGACATCAGAAAGAAATTTCT
         66 AACTGCTCTATCAAAAGAAATGTTCAACTCTGTGAGTTGAGTACACACATCACAAAGAAGTTTCT

                     *       **          *         **
     266647 GAGAATGCTGCTGTCTACCTTTTAT-T--TGA-ATTCCCGC
        131 GAGAATGCTTCTGTCTAGTTTTTATATGAAGATATTCCCTT

                         *  *    *              * *  **   *    *             
     266684 TTCCAACGAAATCCTCCAAGCTATCCAAATATCCACCTGCATTTTCCACAACAAGAGTGTTTCAA
          1 TTCCAACGAAATCATCAAAGCGATCCAAATATCCACTTCCAGATTCTACAAAAAGAGTGTTTCAA

                          *                 *    *  *                *       
     266749 AACTGCTCTATCAATAGAAATGTTCAACTCCTTTG-GCTGGGTACACACATCACAAACAAGTTTC
         66 AACTGCTCTATCAAAAGAAATGTTCAACT-CTGTGAGTTGAGTACACACATCACAAAGAAGTTTC

                                      **     **       
     266813 TGAGAATGCTTCTGTCTAGTTTTTATGGGAAGACGTTCCCTT
        130 TGAGAATGCTTCTGTCTAGTTTTTATATGAAGATATTCCCTT

              *  * *  **          *       *            **                  * 
     266855 TTTCACCAAAGGCATCAAAGCGCTCCAAATGTCCACTTCCAGACACTACAAAAAGAGTGTTTCCA
          1 TTCCAACGAAATCATCAAAGCGATCCAAATATCCACTTCCAGATTCTACAAAAAGAGTGTTTCAA

             **       **   ***                 *    * *  * *         *       
     266920 ACGTGCTCTAAGAAAGCGAATGTTCAACTCTGTGACTTGAATGCAGATATCACAAAGTAGTTTCT
         66 AACTGCTCTATCAAAAGAAATGTTCAACTCTGTGAGTTGAGTACACACATCACAAAGAAGTTTCT

                **            *     *    *         * 
     266985 GAGAGGGCTTCTGTCTAGATTTTAGATGATGATATTCCCGT
        131 GAGAATGCTTCTGTCTAGTTTTTATATGAAGATATTCCCTT

                           * *   *                *   *                    * 
     267026 TTCCAACGAAATCATTAGAGCTATCCAAATATCCACTTACAGTTTCTACAAAAAGAGTGTTTCCA
          1 TTCCAACGAAATCATCAAAGCGATCCAAATATCCACTTCCAGATTCTACAAAAAGAGTGTTTCAA

                   **         * *    *       *  *                   ***      
     267091 AACTGCTGCATCAAAAGAGAGGTTCCACTCTGTTAGCTGAGTACACACATCACAAACTTGTTTCT
         66 AACTGCTCTATCAAAAGAAATGTTCAACTCTGTGAGTTGAGTACACACATCACAAAGAAGTTTCT

            *     *         *        **         **   
     267156 CAGAATCCTTCTGTCTCGTTTTTATGGGAAGATATTTACTT
        131 GAGAATGCTTCTGTCTAGTTTTTATATGAAGATATTCCCTT

              *  *  * **          *       *     *       *  *  *              
     267197 TTTCACCGTAGGCATCAAAGCGCTCCAAATGTCCACATCCAGATACTCCAGAAAGAGTGTTTCAA
          1 TTCCAACGAAATCATCAAAGCGATCCAAATATCCACTTCCAGATTCTACAAAAAGAGTGTTTCAA

             *         *   * *   *         *        * *  *      *      *     
     267262 ACCTGCTCTATGAAAGGGAATCTTCAACTCTATGAGTTGAATGCAGACATCAGAAAGAAATTTCT
         66 AACTGCTCTATCAAAAGAAATGTTCAACTCTGTGAGTTGAGTACACACATCACAAAGAAGTTTCT

                     *       **          *         **
     267327 GAGAATGCTGCTGTCTACCTTTTAT-T--TGA-ATTCCCGC
        131 GAGAATGCTTCTGTCTAGTTTTTATATGAAGATATTCCCTT

                         *  *    *                *       *                  
     267364 TTCCAACGAAATCCTCCAAGCTATCCAAATATCCACTTGCAGATTCCACAAAAAGAGTGTTTCAA
          1 TTCCAACGAAATCATCAAAGCGATCCAAATATCCACTTCCAGATTCTACAAAAAGAGTGTTTCAA

                            ** *  *            *       *     *     *  *      
     267429 AACTGCTCTCTATCAATGGCAAAGTTCAACTCTGTTAGTTGAGGACACATATCACCAACAAGTTT
         66 AACTG--CTCTATCAAAAGAAATGTTCAACTCTGTGAGTTGAGTACACACATCACAAAGAAGTTT

                               *       **         *    
     267494 CTGAGAATGCTTCTGTCTATTTTTTATGGGAAGATATTTCCTT
        129 CTGAGAATGCTTCTGTCTAGTTTTTATATGAAGATATTCCCTT

              *  *  * ** *    *      *    *          * **                    
     267537 TTTCACCGTAGGCGTCAAGGCGATCGAAATGTCCACTTCCACAAACTACAAAAAGAGTGTTTCAA
          1 TTCCAACGAAATCATCAAAGCGATCCAAATATCCACTTCCAGATTCTACAAAAAGAGTGTTTCAA

             *         *   * **       *    *        * ** * *   **      *     
     267602 ACCTGCTCTATGAAAGGCCATGTTCATCTCTATGAGTTGAATGGATATATCCGAAAGAAATTTCT
         66 AACTGCTCTATCAAAAGAAATGTTCAACTCTGTGAGTTGAGTACACACATCACAAAGAAGTTTCT

             *       *                   *         **
     267667 GGGAATGCTGCTGTCTAG-TTTT-TAT-ACGA-ATTCCCGC
        131 GAGAATGCTTCTGTCTAGTTTTTATATGAAGATATTCCCTT

                         *       *                *    *  *                  
     267704 TTCCAACGAAATCCTCAAAGCAATCCAAATATCCACTTGCAGAATCCACAAAAAGAGTGTTTCAA
          1 TTCCAACGAAATCATCAAAGCGATCCAAATATCCACTTCCAGATTCTACAAAAAGAGTGTTTCAA

                          *     *          * *                   *  *        
     267769 AACTGCTCTATCAATAGAAAGGTTCAACTCTTTTAGTTGAGTACACACATCACGAACAAGTTTCT
         66 AACTGCTCTATCAAAAGAAATGTTCAACTCTGTGAGTTGAGTACACACATCACAAAGAAGTTTCT

                            * *      **     **  *    
     267834 GAGAATGCTTCTGTCTGGCTTTTATTGGAAGACGTTTCCTT
        131 GAGAATGCTTCTGTCTAGTTTTTATATGAAGATATTCCCTT

                 * *  **          *       *                * *               
     267875 TT-CACCAAAGGCATCAAAGCGCTCCAAATGTCCACTTCCAGATTCTTCCAAAAGAGTGTTTCAA
          1 TTCCAACGAAATCATCAAAGCGATCCAAATATCCACTTCCAGATTCTACAAAAAGAGTGTTTCAA

             **     * ***  * *                 *    * *  * *     *   *       
     267939 ACGTGCTCAAAGTAAGGGAATGTTCAACTCTGTGACTTGAATGCAGATATCACCAAGTAGTTTCT
         66 AACTGCTCTATCAAAAGAAATGTTCAACTCTGTGAGTTGAGTACACACATCACAAAGAAGTTTCT

            * * *            **     *    *         * 
     268004 AATAGTGCTTCTGTCTACATTTTAGATGATGATATTCCCGT
        131 GAGAATGCTTCTGTCTAGTTTTTATATGAAGATATTCCCTT

                         * * *   *             *  *   *      *     *       * 
     268045 TTCCAACGAAATCGTTAGAGCTATCCAAATATCCAGTTACAGTTTCTACCAAAAGGGTGTTTCCA
          1 TTCCAACGAAATCATCAAAGCGATCCAAATATCCACTTCCAGATTCTACAAAAAGAGTGTTTCAA

              *    **           *            *       *                     * 
     268110 AATTGCTGCATCAAAAGAAAGGTTCAACTCTGTTAGTTGAGGACACACATCACAAAGAAGTTTGT
         66 AACTGCTCTATCAAAAGAAATGTTCAACTCTGTGAGTTGAGTACACACATCACAAAGAAGTTTCT

                              *    *     *           
     268175 GAGAATGCTTCTGTCTAGATTTTGTATGACGATATTCCCTT
        131 GAGAATGCTTCTGTCTAGTTTTTATATGAAGATATTCCCTT

                     *   * *     *   *       * *  *    *  *       *  *       
     268216 TTCCAACGATATCGTTAAAGCAATCTAAATATCAATTTGCAGAATCCACAAAAATAGAGTTTCAA
          1 TTCCAACGAAATCATCAAAGCGATCCAAATATCCACTTCCAGATTCTACAAAAAGAGTGTTTCAA

             *       * *        *    *       *  *                   ***      
     268281 AGCTGCTCTGTAAAAAGAAAGGTTCCACTCTGTTAGCTGAGTACACACATCACAAACTTGTTTCT
         66 AACTGCTCTATCAAAAGAAATGTTCAACTCTGTGAGTTGAGTACACACATCACAAAGAAGTTTCT

                  *         *        **         **   
     268346 GAGAATCCTTCTGTCTCGTTTTTATGGGAAGATATTTACTT
        131 GAGAATGCTTCTGTCTAGTTTTTATATGAAGATATTCCCTT

                 *  * **          *       *     *       *  *  *              
     268387 TTCCACCGTAGGCATCAAAGCGCTCCAAATGTCCACATCCAGATACTCCAGAAAGAGTGTTTCAA
          1 TTCCAACGAAATCATCAAAGCGATCCAAATATCCACTTCCAGATTCTACAAAAAGAGTGTTTCAA

             *         *   * *   *         *        * *  *      *      *     
     268452 ACCTGCTCTATGAAAGGGAATCTTCAACTCTATGAGTTGAATGCAGACATCAGAAAGAAATTTCT
         66 AACTGCTCTATCAAAAGAAATGTTCAACTCTGTGAGTTGAGTACACACATCACAAAGAAGTTTCT

                     *       **          *         **
     268517 GAGAATGCTGCTGTCTACCTTTTAT-T--TGA-ATTCCCGC
        131 GAGAATGCTTCTGTCTAGTTTTTATATGAAGATATTCCCTT

                         *  *    *                *  **   *                  
     268554 TTCCAACGAAATCCTCCAAGCTATCCAAATATCCACTTGCATTTTCCACAAAAAGAGTGTTTCAA
          1 TTCCAACGAAATCATCAAAGCGATCCAAATATCCACTTCCAGATTCTACAAAAAGAGTGTTTCAA

                          *                 *    *  *                *       
     268619 AACTGCTCTATCAATAGAAATGTTCAACTCCTTTG-GCTGGGTACACACATCACAAACAAGTTTC
         66 AACTGCTCTATCAAAAGAAATGTTCAACT-CTGTGAGTTGAGTACACACATCACAAAGAAGTTTC

                                      **     **       
     268683 TGAGAATGCTTCTGTCTAGTTTTTATGGGAAGACGTTCCCTT
        130 TGAGAATGCTTCTGTCTAGTTTTTATATGAAGATATTCCCTT

              *  * *  **          *       *            **                  * 
     268725 TTTCACCAAAGGCATCAAAGCGCTCCAAATGTCCACTTCCAGACACTACAAAAAGAGTGTTTCCA
          1 TTCCAACGAAATCATCAAAGCGATCCAAATATCCACTTCCAGATTCTACAAAAAGAGTGTTTCAA

             **       **   ***                 *    * *  * *         *       
     268790 ACGTGCTCTAAGAAAGCGAATGTTCAACTCTGTGACTTGAATGCAGATATCACAAAGTAGTTTCT
         66 AACTGCTCTATCAAAAGAAATGTTCAACTCTGTGAGTTGAGTACACACATCACAAAGAAGTTTCT

                **            *     *    *         * 
     268855 GAGAGGGCTTCTGTCTAGATTTTAGATGATGATATTCCCGT
        131 GAGAATGCTTCTGTCTAGTTTTTATATGAAGATATTCCCTT

                           * *   *                *   *                    * 
     268896 TTCCAACGAAATCATTAGAGCTATCCAAATATCCACTTACAGTTTCTACAAAAAGAGTGTTTCCA
          1 TTCCAACGAAATCATCAAAGCGATCCAAATATCCACTTCCAGATTCTACAAAAAGAGTGTTTCAA

                   **         * *    *       *  *                   ***      
     268961 AACTGCTGCATCAAAAGAGAGGTTCCACTCTGTTAGCTGAGTACACACATCACAAACTTGTTTCT
         66 AACTGCTCTATCAAAAGAAATGTTCAACTCTGTGAGTTGAGTACACACATCACAAAGAAGTTTCT

            *     *         *        **         **   
     269026 CAGAATCCTTCTGTCTCGTTTTTATGGGAAGATATTTACTT
        131 GAGAATGCTTCTGTCTAGTTTTTATATGAAGATATTCCCTT

                 *  * **          *       *     *       *  *  *              
     269067 TTCCACCGTAGGCATCAAAGCGCTCCAAATGTCCACATCCAGATACTCCAGAAAGAGTGTTTCAA
          1 TTCCAACGAAATCATCAAAGCGATCCAAATATCCACTTCCAGATTCTACAAAAAGAGTGTTTCAA

             *         *   * *             *        * *  *      *      *     
     269132 ACCTGCTCTATGAAAGGGAATGTTCAACTCTATGAGTTGAATGCAGACATCAGAAAGAAATTTCT
         66 AACTGCTCTATCAAAAGAAATGTTCAACTCTGTGAGTTGAGTACACACATCACAAAGAAGTTTCT

                     *       **          *         **
     269197 GAGAATGCTGCTGTCTACCTTTTAT-T--TGA-ATTCCCGC
        131 GAGAATGCTTCTGTCTAGTTTTTATATGAAGATATTCCCTT

                         *  *    *                *       *                  
     269234 TTCCAACGAAATCCTCCAAGCTATCCAAATATCCACTTGCAGATTCCACAAAAAGAGTGTTTCAA
          1 TTCCAACGAAATCATCAAAGCGATCCAAATATCCACTTCCAGATTCTACAAAAAGAGTGTTTCAA

                            ** *  *            *       *     *     *  *      
     269299 AACTGCTCTCTATCAATGGCAAAGTTCAACTCTGTTAGTTGAGGACACATATCACCAACAAGTTT
         66 AACTG--CTCTATCAAAAGAAATGTTCAACTCTGTGAGTTGAGTACACACATCACAAAGAAGTTT

                               *       **         *    
     269364 CTGAGAATGCTTCTGTCTATTTTTTATGGGAAGATATTTCCTT
        129 CTGAGAATGCTTCTGTCTAGTTTTTATATGAAGATATTCCCTT

              *  *  * ** *    *      *    *          * **                    
     269407 TTTCACCGTAGGCGTCAAGGCGATCGAAATGTCCACTTCCACAAACTACAAAAAGAGTGTTTCAA
          1 TTCCAACGAAATCATCAAAGCGATCCAAATATCCACTTCCAGATTCTACAAAAAGAGTGTTTCAA

             *         *   * **       *    *        * ** * *   **      *     
     269472 ACCTGCTCTATGAAAGGCCATGTTCATCTCTATGAGTTGAATGGAAATATCCGAAAGAAATTTCT
         66 AACTGCTCTATCAAAAGAAATGTTCAACTCTGTGAGTTGAGTACACACATCACAAAGAAGTTTCT

             *       *          *        *         **
     269537 GGGAATGCTGCTGTCTAG-TGTT-TAT-ACGA-ATTCCCGC
        131 GAGAATGCTTCTGTCTAGTTTTTATATGAAGATATTCCCTT

                         *       *                *    *  *                  
     269574 TTCCAACGAAATCCTCAAAGCAATCCAAATATCCACTTGCAGAATCCACAAAAAGAGTGTTTCAA
          1 TTCCAACGAAATCATCAAAGCGATCCAAATATCCACTTCCAGATTCTACAAAAAGAGTGTTTCAA

                          *     *          * *                   *  *        
     269639 AACTGCTCTATCAATAGAAAGGTTCAACTCTTTTAGTTGAGTACACACATCACGAACAAGTTTCT
         66 AACTGCTCTATCAAAAGAAATGTTCAACTCTGTGAGTTGAGTACACACATCACAAAGAAGTTTCT

                            * *      **     **  *    
     269704 GAGAATGCTTCTGTCTGGCTTTTATTGGAAGACGTTTCCTT
        131 GAGAATGCTTCTGTCTAGTTTTTATATGAAGATATTCCCTT

                 * *  **          *       *                * *               
     269745 TT-CACCAAAGGCATCAAAGCGCTCCAAATGTCCACTTCCAGATTCTTCCAAAAGAGTGTTTCAA
          1 TTCCAACGAAATCATCAAAGCGATCCAAATATCCACTTCCAGATTCTACAAAAAGAGTGTTTCAA

             **     * ***  * *                 *    * *  * *     *   *       
     269809 ACGTGCTCAAAGTAAGGGAATGTTCAACTCTGTGACTTGAATGCAGATATCACCAAGTAGTTTCT
         66 AACTGCTCTATCAAAAGAAATGTTCAACTCTGTGAGTTGAGTACACACATCACAAAGAAGTTTCT

            * * *            **     *    *         * 
     269874 AATAGTGCTTCTGTCTACATTTTAGATGATGATATTCCCGT
        131 GAGAATGCTTCTGTCTAGTTTTTATATGAAGATATTCCCTT

                         * * *   *             *  *   *      *     *       * 
     269915 TTCCAACGAAATCGTTAGAGCTATCCAAATATCCAGTTACAGTTTCTACCAAAAGGGTGTTTCCA
          1 TTCCAACGAAATCATCAAAGCGATCCAAATATCCACTTCCAGATTCTACAAAAAGAGTGTTTCAA

              *    **           *            *       *                     * 
     269980 AATTGCTGCATCAAAAGAAAGGTTCAACTCTGTTAGTTGAGGACACACATCACAAAGAAGTTTGT
         66 AACTGCTCTATCAAAAGAAATGTTCAACTCTGTGAGTTGAGTACACACATCACAAAGAAGTTTCT

                              *    *     *           
     270045 GAGAATGCTTCTGTCTAGATTTTGTATGACGATATTCCCTT
        131 GAGAATGCTTCTGTCTAGTTTTTATATGAAGATATTCCCTT

                     *   * *     *   *       * *  *    *  *       *  *       
     270086 TTCCAACGATATCGTTAAAGCAATCTAAATATCAATTTGCAGAATCCACAAAAATAGAGTTTCAA
          1 TTCCAACGAAATCATCAAAGCGATCCAAATATCCACTTCCAGATTCTACAAAAAGAGTGTTTCAA

             *       * *        *    *       *  *                   ***      
     270151 AGCTGCTCTGTAAAAAGAAAGGTTCCACTCTGTTAGCTGAGTACACACATCACAAACTTGTTTCT
         66 AACTGCTCTATCAAAAGAAATGTTCAACTCTGTGAGTTGAGTACACACATCACAAAGAAGTTTCT

                  *         *        **         **   
     270216 GAGAATCCTTCTGTCTCGTTTTTATGGGAAGATATTTACTT
        131 GAGAATGCTTCTGTCTAGTTTTTATATGAAGATATTCCCTT

                 *  * **          *       *     *       *  *  *              
     270257 TTCCACCGTAGGCATCAAAGCGCTCCAAATGTCCACATCCAGATACTCCAGAAAGAGTGTTTCAA
          1 TTCCAACGAAATCATCAAAGCGATCCAAATATCCACTTCCAGATTCTACAAAAAGAGTGTTTCAA

             *         *   * *   *         *        * *  *      *      *     
     270322 ACCTGCTCTATGAAAGGGAATCTTCAACTCTATGAGTTGAATGCAGACATCAGAAAGAAATTTCT
         66 AACTGCTCTATCAAAAGAAATGTTCAACTCTGTGAGTTGAGTACACACATCACAAAGAAGTTTCT

                     *       **          *         **
     270387 GAGAATGCTGCTGTCTACCTTTTAT-T--TGA-ATTCCCGC
        131 GAGAATGCTTCTGTCTAGTTTTTATATGAAGATATTCCCTT

                         *  *    *                *  **   *                  
     270424 TTCCAACGAAATCCTCCAAGCTATCCAAATATCCACTTGCATTTTCCACAAAAAGAGTGTTTCAA
          1 TTCCAACGAAATCATCAAAGCGATCCAAATATCCACTTCCAGATTCTACAAAAAGAGTGTTTCAA

                          *                 *    *  *                *       
     270489 AACTGCTCTATCAATAGAAATGTTCAACTCCTTTG-GCTGGGTACACACATCACAAACAAGTTTC
         66 AACTGCTCTATCAAAAGAAATGTTCAACT-CTGTGAGTTGAGTACACACATCACAAAGAAGTTTC

                                      **     **       
     270553 TGAGAATGCTTCTGTCTAGTTTTTATGGGAAGACGTTCCCTT
        130 TGAGAATGCTTCTGTCTAGTTTTTATATGAAGATATTCCCTT

              *  * *  **          *       *            **                  * 
     270595 TTTCACCAAAGGCATCAAAGCGCTCCAAATGTCCACTTCCAGACACTACAAAAAGAGTGTTTCCA
          1 TTCCAACGAAATCATCAAAGCGATCCAAATATCCACTTCCAGATTCTACAAAAAGAGTGTTTCAA

             **       **   ***                 *    * *  * *         *       
     270660 ACGTGCTCTAAGAAAGCGAATGTTCAACTCTGTGACTTGAATGCAGATATCACAAAGTAGTTTCT
         66 AACTGCTCTATCAAAAGAAATGTTCAACTCTGTGAGTTGAGTACACACATCACAAAGAAGTTTCT

                **            *     *    *         * 
     270725 GAGAGGGCTTCTGTCTAGATTTTAGATGATGATATTCCCGT
        131 GAGAATGCTTCTGTCTAGTTTTTATATGAAGATATTCCCTT

                           * *   *                *   *                    * 
     270766 TTCCAACGAAATCATTAGAGCTATCCAAATATCCACTTACAGTTTCTACAAAAAGAGTGTTTCCA
          1 TTCCAACGAAATCATCAAAGCGATCCAAATATCCACTTCCAGATTCTACAAAAAGAGTGTTTCAA

                   **         * *    *       *  *                   ***      
     270831 AACTGCTGCATCAAAAGAGAGGTTCCACTCTGTTAGCTGAGTACACACATCACAAACTTGTTTCT
         66 AACTGCTCTATCAAAAGAAATGTTCAACTCTGTGAGTTGAGTACACACATCACAAAGAAGTTTCT

            *     *         *        **         **   
     270896 CAGAATCCTTCTGTCTCGTTTTTATGGGAAGATATTTACTT
        131 GAGAATGCTTCTGTCTAGTTTTTATATGAAGATATTCCCTT

                 *  * **          *       *     *       *  *  *              
     270937 TTCCACCGTAGGCATCAAAGCGCTCCAAATGTCCACATCCAGATACTCCAGAAAGAGTGTTTCAA
          1 TTCCAACGAAATCATCAAAGCGATCCAAATATCCACTTCCAGATTCTACAAAAAGAGTGTTTCAA

             *         *   * *             *        * *  *      *      *     
     271002 ACCTGCTCTATGAAAGGGAATGTTCAACTCTATGAGTTGAATGCAGACATCAGAAAGAAATTTCT
         66 AACTGCTCTATCAAAAGAAATGTTCAACTCTGTGAGTTGAGTACACACATCACAAAGAAGTTTCT

                     *       **          *         **
     271067 GAGAATGCTGCTGTCTACCTTTTAT-T--TGA-ATTCCCGC
        131 GAGAATGCTTCTGTCTAGTTTTTATATGAAGATATTCCCTT

                         *  *    *                *       *                  
     271104 TTCCAACGAAATCCTCCAAGCTATCCAAATATCCACTTGCAGATTCCACAAAAAGAGTGTTTCAA
          1 TTCCAACGAAATCATCAAAGCGATCCAAATATCCACTTCCAGATTCTACAAAAAGAGTGTTTCAA

                            ** *  *            *       *     *     *  *      
     271169 AACTGCTCTCTATCAATGGCAAAGTTCAACTCTGTTAGTTGAGGACACATATCACCAACAAGTTT
         66 AACTG--CTCTATCAAAAGAAATGTTCAACTCTGTGAGTTGAGTACACACATCACAAAGAAGTTT

                               *       **         *    
     271234 CTGAGAATGCTTCTGTCTATTTTTTATGGGAAGATATTTCCTT
        129 CTGAGAATGCTTCTGTCTAGTTTTTATATGAAGATATTCCCTT

              *  *  * ** *    *      *    *          * **                    
     271277 TTTCACCGTAGGCGTCAAGGCGATCGAAATGTCCACTTCCACAAACTACAAAAAGAGTGTTTCAA
          1 TTCCAACGAAATCATCAAAGCGATCCAAATATCCACTTCCAGATTCTACAAAAAGAGTGTTTCAA

             *         *   * **       *    *        * ** * *   **      *     
     271342 ACCTGCTCTATGAAAGGCCATGTTCATCTCTATGAGTTGAATGGAAATATCCGAAAGAAATTTCT
         66 AACTGCTCTATCAAAAGAAATGTTCAACTCTGTGAGTTGAGTACACACATCACAAAGAAGTTTCT

             *       *          *        *         **
     271407 GGGAATGCTGCTGTCTAG-TGTT-TAT-ACGA-ATTCCCGC
        131 GAGAATGCTTCTGTCTAGTTTTTATATGAAGATATTCCCTT

                         *       *                *    *  *                  
     271444 TTCCAACGAAATCCTCAAAGCAATCCAAATATCCACTTGCAGAATCCACAAAAAGAGTGTTTCAA
          1 TTCCAACGAAATCATCAAAGCGATCCAAATATCCACTTCCAGATTCTACAAAAAGAGTGTTTCAA

                          *     *          * *                   *  *        
     271509 AACTGCTCTATCAATAGAAAGGTTCAACTCTTTTAGTTGAGTACACACATCACGAACAAGTTTCT
         66 AACTGCTCTATCAAAAGAAATGTTCAACTCTGTGAGTTGAGTACACACATCACAAAGAAGTTTCT

                            * *      **     **  *    
     271574 GAGAATGCTTCTGTCTGGCTTTTATTGGAAGACGTTTCCTT
        131 GAGAATGCTTCTGTCTAGTTTTTATATGAAGATATTCCCTT

                 * *  **          *       *                * *               
     271615 TT-CACCAAAGGCATCAAAGCGCTCCAAATGTCCACTTCCAGATTCTTCCAAAAGAGTGTTTCAA
          1 TTCCAACGAAATCATCAAAGCGATCCAAATATCCACTTCCAGATTCTACAAAAAGAGTGTTTCAA

             **     * ***  * *                 *    * *  * *     *   *       
     271679 ACGTGCTCAAAGTAAGGGAATGTTCAACTCTGTGACTTGAATGCAGATATCACCAAGTAGTTTCT
         66 AACTGCTCTATCAAAAGAAATGTTCAACTCTGTGAGTTGAGTACACACATCACAAAGAAGTTTCT

            * * *            **     *    *         * 
     271744 AATAGTGCTTCTGTCTACATTTTAGATGATGATATTCCCGT
        131 GAGAATGCTTCTGTCTAGTTTTTATATGAAGATATTCCCTT

                         * * *   *             *  *   *      *     *       * 
     271785 TTCCAACGAAATCGTTAGAGCTATCCAAATATCCAGTTACAGTTTCTACCAAAAGGGTGTTTCCA
          1 TTCCAACGAAATCATCAAAGCGATCCAAATATCCACTTCCAGATTCTACAAAAAGAGTGTTTCAA

              *    **           *            *       *                     * 
     271850 AATTGCTGCATCAAAAGAAAGGTTCAACTCTGTTAGTTGAGGACACACATCACAAAGAAGTTTGT
         66 AACTGCTCTATCAAAAGAAATGTTCAACTCTGTGAGTTGAGTACACACATCACAAAGAAGTTTCT

                              *    *     *           
     271915 GAGAATGCTTCTGTCTAGATTTTGTATGACGATATTCCCTT
        131 GAGAATGCTTCTGTCTAGTTTTTATATGAAGATATTCCCTT

                     *   * *     *   *       * *  *    *  *       *  *       
     271956 TTCCAACGATATCGTTAAAGCAATCTAAATATCAATTTGCAGAATCCACAAAAATAGAGTTTCAA
          1 TTCCAACGAAATCATCAAAGCGATCCAAATATCCACTTCCAGATTCTACAAAAAGAGTGTTTCAA

             *       * *        *    *       *  *                   ***      
     272021 AGCTGCTCTGTAAAAAGAAAGGTTCCACTCTGTTAGCTGAGTACACACATCACAAACTTGTTTCT
         66 AACTGCTCTATCAAAAGAAATGTTCAACTCTGTGAGTTGAGTACACACATCACAAAGAAGTTTCT

                  *         *        **         **   
     272086 GAGAATCCTTCTGTCTCGTTTTTATGGGAAGATATTTACTT
        131 GAGAATGCTTCTGTCTAGTTTTTATATGAAGATATTCCCTT

                 *  * **          *       *     *       *  *  *              
     272127 TTCCACCGTAGGCATCAAAGCGCTCCAAATGTCCACATCCAGATACTCCAGAAAGAGTGTTTCAA
          1 TTCCAACGAAATCATCAAAGCGATCCAAATATCCACTTCCAGATTCTACAAAAAGAGTGTTTCAA

             *         *   * *   *         *        * *  *      *      *     
     272192 ACCTGCTCTATGAAAGGGAATCTTCAACTCTATGAGTTGAATGCAGACATCAGAAAGAAATTTCT
         66 AACTGCTCTATCAAAAGAAATGTTCAACTCTGTGAGTTGAGTACACACATCACAAAGAAGTTTCT

                     *       **          *         **
     272257 GAGAATGCTGCTGTCTACCTTTTAT-T--TGA-ATTCCCGC
        131 GAGAATGCTTCTGTCTAGTTTTTATATGAAGATATTCCCTT

                         *  *    *                *  **   *                  
     272294 TTCCAACGAAATCCTCCAAGCTATCCAAATATCCACTTGCATTTTCCACAAAAAGAGTGTTTCAA
          1 TTCCAACGAAATCATCAAAGCGATCCAAATATCCACTTCCAGATTCTACAAAAAGAGTGTTTCAA

                          *                 *    *  *                *       
     272359 AACTGCTCTATCAATAGAAATGTTCAACTCCTTTG-GCTGGGTACACACATCACAAACAAGTTTC
         66 AACTGCTCTATCAAAAGAAATGTTCAACT-CTGTGAGTTGAGTACACACATCACAAAGAAGTTTC

                                      **     **       
     272423 TGAGAATGCTTCTGTCTAGTTTTTATGGGAAGACGTTCCCTT
        130 TGAGAATGCTTCTGTCTAGTTTTTATATGAAGATATTCCCTT

              *  * *  **          *       *            **                  * 
     272465 TTTCACCAAAGGCATCAAAGCGCTCCAAATGTCCACTTCCAGACACTACAAAAAGAGTGTTTCCA
          1 TTCCAACGAAATCATCAAAGCGATCCAAATATCCACTTCCAGATTCTACAAAAAGAGTGTTTCAA

             **       **   ***                 *    * *  * *         *       
     272530 ACGTGCTCTAAGAAAGCGAATGTTCAACTCTGTGACTTGAATGCAGATATCACAAAGTAGTTTCT
         66 AACTGCTCTATCAAAAGAAATGTTCAACTCTGTGAGTTGAGTACACACATCACAAAGAAGTTTCT

                **            *     *    *         * 
     272595 GAGAGGGCTTCTGTCTAGATTTTAGATGATGATATTCCCGT
        131 GAGAATGCTTCTGTCTAGTTTTTATATGAAGATATTCCCTT

                           * *   *                *   *                    * 
     272636 TTCCAACGAAATCATTAGAGCTATCCAAATATCCACTTACAGTTTCTACAAAAAGAGTGTTTCCA
          1 TTCCAACGAAATCATCAAAGCGATCCAAATATCCACTTCCAGATTCTACAAAAAGAGTGTTTCAA

                   **         * *    *       *  *                   ***      
     272701 AACTGCTGCATCAAAAGAGAGGTTCCACTCTGTTAGCTGAGTACACACATCACAAACTTGTTTCT
         66 AACTGCTCTATCAAAAGAAATGTTCAACTCTGTGAGTTGAGTACACACATCACAAAGAAGTTTCT

            *     *         *        **         **   
     272766 CAGAATCCTTCTGTCTCGTTTTTATGGGAAGATATTTACTT
        131 GAGAATGCTTCTGTCTAGTTTTTATATGAAGATATTCCCTT

                 *  * **          *       *     *       *  *  *              
     272807 TTCCACCGTAGGCATCAAAGCGCTCCAAATGTCCACATCCAGATACTCCAGAAAGAGTGTTTCAA
          1 TTCCAACGAAATCATCAAAGCGATCCAAATATCCACTTCCAGATTCTACAAAAAGAGTGTTTCAA

             *         *   * *             *        * *  *      *      *     
     272872 ACCTGCTCTATGAAAGGGAATGTTCAACTCTATGAGTTGAATGCAGACATCAGAAAGAAATTTCT
         66 AACTGCTCTATCAAAAGAAATGTTCAACTCTGTGAGTTGAGTACACACATCACAAAGAAGTTTCT

                     *       **          *         **
     272937 GAGAATGCTGCTGTCTACCTTTTAT-T--TGA-ATTCCCGC
        131 GAGAATGCTTCTGTCTAGTTTTTATATGAAGATATTCCCTT

                         *  *    *                *       *                  
     272974 TTCCAACGAAATCCTCCAAGCTATCCAAATATCCACTTGCAGATTCCACAAAAAGAGTGTTTCAA
          1 TTCCAACGAAATCATCAAAGCGATCCAAATATCCACTTCCAGATTCTACAAAAAGAGTGTTTCAA

                            ** *  *            *       *     *     *  *      
     273039 AACTGCTCTCTATCAATGGCAAAGTTCAACTCTGTTAGTTGAGGACACATATCACCAACAAGTTT
         66 AACTG--CTCTATCAAAAGAAATGTTCAACTCTGTGAGTTGAGTACACACATCACAAAGAAGTTT

                               *       **         *    
     273104 CTGAGAATGCTTCTGTCTATTTTTTATGGGAAGATATTTCCTT
        129 CTGAGAATGCTTCTGTCTAGTTTTTATATGAAGATATTCCCTT

              *  *  * ** *    *      *    *          * **                    
     273147 TTTCACCGTAGGCGTCAAGGCGATCGAAATGTCCACTTCCACAAACTACAAAAAGAGTGTTTCAA
          1 TTCCAACGAAATCATCAAAGCGATCCAAATATCCACTTCCAGATTCTACAAAAAGAGTGTTTCAA

             *         *   * **       *    *        * ** * *   **      *     
     273212 ACCTGCTCTATGAAAGGCCATGTTCATCTCTATGAGTTGAATGGAAATATCCGAAAGAAATTTCT
         66 AACTGCTCTATCAAAAGAAATGTTCAACTCTGTGAGTTGAGTACACACATCACAAAGAAGTTTCT

             *       *          *        *         **
     273277 GGGAATGCTGCTGTCTAG-TGTT-TAT-ACGA-ATTCCCGC
        131 GAGAATGCTTCTGTCTAGTTTTTATATGAAGATATTCCCTT

                         *       *                *    *  *                  
     273314 TTCCAACGAAATCCTCAAAGCAATCCAAATATCCACTTGCAGAATCCACAAAAAGAGTGTTTCAA
          1 TTCCAACGAAATCATCAAAGCGATCCAAATATCCACTTCCAGATTCTACAAAAAGAGTGTTTCAA

                          *     *          * *                   *  *        
     273379 AACTGCTCTATCAATAGAAAGGTTCAACTCTTTTAGTTGAGTACACACATCACGAACAAGTTTCT
         66 AACTGCTCTATCAAAAGAAATGTTCAACTCTGTGAGTTGAGTACACACATCACAAAGAAGTTTCT

                            * *      **     **  *    
     273444 GAGAATGCTTCTGTCTGGCTTTTATTGGAAGACGTTTCCTT
        131 GAGAATGCTTCTGTCTAGTTTTTATATGAAGATATTCCCTT

                 * *  **          *       *                * *               
     273485 TT-CACCAAAGGCATCAAAGCGCTCCAAATGTCCACTTCCAGATTCTTCCAAAAGAGTGTTTCAA
          1 TTCCAACGAAATCATCAAAGCGATCCAAATATCCACTTCCAGATTCTACAAAAAGAGTGTTTCAA

             **     * ***  * *                 *    * *  * *     *   *       
     273549 ACGTGCTCAAAGTAAGGGAATGTTCAACTCTGTGACTTGAATGCAGATATCACCAAGTAGTTTCT
         66 AACTGCTCTATCAAAAGAAATGTTCAACTCTGTGAGTTGAGTACACACATCACAAAGAAGTTTCT

            * * *            **     *    *         * 
     273614 AATAGTGCTTCTGTCTACATTTTAGATGATGATATTCCCGT
        131 GAGAATGCTTCTGTCTAGTTTTTATATGAAGATATTCCCTT

                         * * *   *             *  *   *      *     *       * 
     273655 TTCCAACGAAATCGTTAGAGCTATCCAAATATCCAGTTACAGTTTCTACCAAAAGGGTGTTTCCA
          1 TTCCAACGAAATCATCAAAGCGATCCAAATATCCACTTCCAGATTCTACAAAAAGAGTGTTTCAA

              *    **           *            *       *                     * 
     273720 AATTGCTGCATCAAAAGAAAGGTTCAACTCTGTTAGTTGAGGACACACATCACAAAGAAGTTTGT
         66 AACTGCTCTATCAAAAGAAATGTTCAACTCTGTGAGTTGAGTACACACATCACAAAGAAGTTTCT

                              *    *     *           
     273785 GAGAATGCTTCTGTCTAGATTTTGTATGACGATATTCCCTT
        131 GAGAATGCTTCTGTCTAGTTTTTATATGAAGATATTCCCTT

                     *   * *     *   *       * *  *    *  *       *  *       
     273826 TTCCAACGATATCGTTAAAGCAATCTAAATATCAATTTGCAGAATCCACAAAAATAGAGTTTCAA
          1 TTCCAACGAAATCATCAAAGCGATCCAAATATCCACTTCCAGATTCTACAAAAAGAGTGTTTCAA

             *       * *        *    *       *  *                   ***      
     273891 AGCTGCTCTGTAAAAAGAAAGGTTCCACTCTGTTAGCTGAGTACACACATCACAAACTTGTTTCT
         66 AACTGCTCTATCAAAAGAAATGTTCAACTCTGTGAGTTGAGTACACACATCACAAAGAAGTTTCT

                  *         *        **         **   
     273956 GAGAATCCTTCTGTCTCGTTTTTATGGGAAGATATTTACTT
        131 GAGAATGCTTCTGTCTAGTTTTTATATGAAGATATTCCCTT

                 *  * **          *       *     *       *  *  *              
     273997 TTCCACCGTAGGCATCAAAGCGCTCCAAATGTCCACATCCAGATACTCCAGAAAGAGTGTTTCAA
          1 TTCCAACGAAATCATCAAAGCGATCCAAATATCCACTTCCAGATTCTACAAAAAGAGTGTTTCAA

             *         *   * *   *         *        * *  *      *      *     
     274062 ACCTGCTCTATGAAAGGGAATCTTCAACTCTATGAGTTGAATGCAGACATCAGAAAGAAATTTCT
         66 AACTGCTCTATCAAAAGAAATGTTCAACTCTGTGAGTTGAGTACACACATCACAAAGAAGTTTCT

                     *       **          *         **
     274127 GAGAATGCTGCTGTCTACCTTTTAT-T--TGA-ATTCCCGC
        131 GAGAATGCTTCTGTCTAGTTTTTATATGAAGATATTCCCTT

                         *  *    *                *  **   *                  
     274164 TTCCAACGAAATCCTCCAAGCTATCCAAATATCCACTTGCATTTTCCACAAAAAGAGTGTTTCAA
          1 TTCCAACGAAATCATCAAAGCGATCCAAATATCCACTTCCAGATTCTACAAAAAGAGTGTTTCAA

                          *                 *    *  *                *       
     274229 AACTGCTCTATCAATAGAAATGTTCAACTCCTTTG-GCTGGGTACACACATCACAAACAAGTTTC
         66 AACTGCTCTATCAAAAGAAATGTTCAACT-CTGTGAGTTGAGTACACACATCACAAAGAAGTTTC

                                      **     **       
     274293 TGAGAATGCTTCTGTCTAGTTTTTATGGGAAGACGTTCCCTT
        130 TGAGAATGCTTCTGTCTAGTTTTTATATGAAGATATTCCCTT

              *  * *  **          *       *            **                  * 
     274335 TTTCACCAAAGGCATCAAAGCGCTCCAAATGTCCACTTCCAGACACTACAAAAAGAGTGTTTCCA
          1 TTCCAACGAAATCATCAAAGCGATCCAAATATCCACTTCCAGATTCTACAAAAAGAGTGTTTCAA

             **       **   ***                 *    * *  * *         *       
     274400 ACGTGCTCTAAGAAAGCGAATGTTCAACTCTGTGACTTGAATGCAGATATCACAAAGTAGTTTCT
         66 AACTGCTCTATCAAAAGAAATGTTCAACTCTGTGAGTTGAGTACACACATCACAAAGAAGTTTCT

                **            *     *    *         * 
     274465 GAGAGGGCTTCTGTCTAGATTTTAGATGATGATATTCCCGT
        131 GAGAATGCTTCTGTCTAGTTTTTATATGAAGATATTCCCTT

                           * *   *                *   *                    * 
     274506 TTCCAACGAAATCATTAGAGCTATCCAAATATCCACTTACAGTTTCTACAAAAAGAGTGTTTCCA
          1 TTCCAACGAAATCATCAAAGCGATCCAAATATCCACTTCCAGATTCTACAAAAAGAGTGTTTCAA

                   **         * *    *       *  *                   ***      
     274571 AACTGCTGCATCAAAAGAGAGGTTCCACTCTGTTAGCTGAGTACACACATCACAAACTTGTTTCT
         66 AACTGCTCTATCAAAAGAAATGTTCAACTCTGTGAGTTGAGTACACACATCACAAAGAAGTTTCT

            *     *         *        **         **   
     274636 CAGAATCCTTCTGTCTCGTTTTTATGGGAAGATATTTACTT
        131 GAGAATGCTTCTGTCTAGTTTTTATATGAAGATATTCCCTT

                 *  * **          *       *     *       *  *  *              
     274677 TTCCACCGTAGGCATCAAAGCGCTCCAAATGTCCACATCCAGATACTCCAGAAAGAGTGTTTCAA
          1 TTCCAACGAAATCATCAAAGCGATCCAAATATCCACTTCCAGATTCTACAAAAAGAGTGTTTCAA

             *         *   * *             *        * *  *      *      *     
     274742 ACCTGCTCTATGAAAGGGAATGTTCAACTCTATGAGTTGAATGCAGACATCAGAAAGAAATTTCT
         66 AACTGCTCTATCAAAAGAAATGTTCAACTCTGTGAGTTGAGTACACACATCACAAAGAAGTTTCT

                     *       **          *         **
     274807 GAGAATGCTGCTGTCTACCTTTTAT-T--TGA-ATTCCCGC
        131 GAGAATGCTTCTGTCTAGTTTTTATATGAAGATATTCCCTT

                         *  *    *                *       *                  
     274844 TTCCAACGAAATCCTCCAAGCTATCCAAATATCCACTTGCAGATTCCACAAAAAGAGTGTTTCAA
          1 TTCCAACGAAATCATCAAAGCGATCCAAATATCCACTTCCAGATTCTACAAAAAGAGTGTTTCAA

                            ** *  *            *       *     *     *  *      
     274909 AACTGCTCTCTATCAATGGCAAAGTTCAACTCTGTTAGTTGAGGACACATATCACCAACAAGTTT
         66 AACTG--CTCTATCAAAAGAAATGTTCAACTCTGTGAGTTGAGTACACACATCACAAAGAAGTTT

                               *       **         *    
     274974 CTGAGAATGCTTCTGTCTATTTTTTATGGGAAGATATTTCCTT
        129 CTGAGAATGCTTCTGTCTAGTTTTTATATGAAGATATTCCCTT

              *  *  * ** *    *      *    *          * **                    
     275017 TTTCACCGTAGGCGTCAAGGCGATCGAAATGTCCACTTCCACAAACTACAAAAAGAGTGTTTCAA
          1 TTCCAACGAAATCATCAAAGCGATCCAAATATCCACTTCCAGATTCTACAAAAAGAGTGTTTCAA

             *         *   * **       *    *        * ** * *   **      *     
     275082 ACCTGCTCTATGAAAGGCCATGTTCATCTCTATGAGTTGAATGGAAATATCCGAAAGAAATTTCT
         66 AACTGCTCTATCAAAAGAAATGTTCAACTCTGTGAGTTGAGTACACACATCACAAAGAAGTTTCT

             *       *          *        *         **
     275147 GGGAATGCTGCTGTCTAG-TGTT-TAT-ACGA-ATTCCCGC
        131 GAGAATGCTTCTGTCTAGTTTTTATATGAAGATATTCCCTT

                         *       *                *    *  *                  
     275184 TTCCAACGAAATCCTCAAAGCAATCCAAATATCCACTTGCAGAATCCACAAAAAGAGTGTTTCAA
          1 TTCCAACGAAATCATCAAAGCGATCCAAATATCCACTTCCAGATTCTACAAAAAGAGTGTTTCAA

                          *     *          * *                   *  *        
     275249 AACTGCTCTATCAATAGAAAGGTTCAACTCTTTTAGTTGAGTACACACATCACGAACAAGTTTCT
         66 AACTGCTCTATCAAAAGAAATGTTCAACTCTGTGAGTTGAGTACACACATCACAAAGAAGTTTCT

                            * *      **     **  *    
     275314 GAGAATGCTTCTGTCTGGCTTTTATTGGAAGACGTTTCCTT
        131 GAGAATGCTTCTGTCTAGTTTTTATATGAAGATATTCCCTT

                 * *  **          *       *                * *               
     275355 TT-CACCAAAGGCATCAAAGCGCTCCAAATGTCCACTTCCAGATTCTTCCAAAAGAGTGTTTCAA
          1 TTCCAACGAAATCATCAAAGCGATCCAAATATCCACTTCCAGATTCTACAAAAAGAGTGTTTCAA

             **     * ***  * *                 *    * *  * *     *   *       
     275419 ACGTGCTCAAAGTAAGGGAATGTTCAACTCTGTGACTTGAATGCAGATATCACCAAGTAGTTTCT
         66 AACTGCTCTATCAAAAGAAATGTTCAACTCTGTGAGTTGAGTACACACATCACAAAGAAGTTTCT

            * * *            **     *    *         * 
     275484 AATAGTGCTTCTGTCTACATTTTAGATGATGATATTCCCGT
        131 GAGAATGCTTCTGTCTAGTTTTTATATGAAGATATTCCCTT

                         * * *   *             *  *   *      *     *       * 
     275525 TTCCAACGAAATCGTTAGAGCTATCCAAATATCCAGTTACAGTTTCTACCAAAAGGGTGTTTCCA
          1 TTCCAACGAAATCATCAAAGCGATCCAAATATCCACTTCCAGATTCTACAAAAAGAGTGTTTCAA

              *    **           *            *       *                     * 
     275590 AATTGCTGCATCAAAAGAAAGGTTCAACTCTGTTAGTTGAGGACACACATCACAAAGAAGTTTGT
         66 AACTGCTCTATCAAAAGAAATGTTCAACTCTGTGAGTTGAGTACACACATCACAAAGAAGTTTCT

                              *    *     *           
     275655 GAGAATGCTTCTGTCTAGATTTTGTATGACGATATTCCCTT
        131 GAGAATGCTTCTGTCTAGTTTTTATATGAAGATATTCCCTT

                     *   * *     *   *       * *  *    *  *       *  *       
     275696 TTCCAACGATATCGTTAAAGCAATCTAAATATCAATTTGCAGAATCCACAAAAATAGAGTTTCAA
          1 TTCCAACGAAATCATCAAAGCGATCCAAATATCCACTTCCAGATTCTACAAAAAGAGTGTTTCAA

             *       * *        *    *       *  *                   ***      
     275761 AGCTGCTCTGTAAAAAGAAAGGTTCCACTCTGTTAGCTGAGTACACACATCACAAACTTGTTTCT
         66 AACTGCTCTATCAAAAGAAATGTTCAACTCTGTGAGTTGAGTACACACATCACAAAGAAGTTTCT

                  *         *        **         **   
     275826 GAGAATCCTTCTGTCTCGTTTTTATGGGAAGATATTTACTT
        131 GAGAATGCTTCTGTCTAGTTTTTATATGAAGATATTCCCTT

                 *  * **          *       *     *       *  *  *              
     275867 TTCCACCGTAGGCATCAAAGCGCTCCAAATGTCCACATCCAGATACTCCAGAAAGAGTGTTTCAA
          1 TTCCAACGAAATCATCAAAGCGATCCAAATATCCACTTCCAGATTCTACAAAAAGAGTGTTTCAA

             *         *   * *   *         *        * *  *      *      *     
     275932 ACCTGCTCTATGAAAGGGAATCTTCAACTCTATGAGTTGAATGCAGACATCAGAAAGAAATTTCT
         66 AACTGCTCTATCAAAAGAAATGTTCAACTCTGTGAGTTGAGTACACACATCACAAAGAAGTTTCT

                     *       **          *         **
     275997 GAGAATGCTGCTGTCTACCTTTTAT-T--TGA-ATTCCCGC
        131 GAGAATGCTTCTGTCTAGTTTTTATATGAAGATATTCCCTT

                         *  *    *                *  **   *                  
     276034 TTCCAACGAAATCCTCCAAGCTATCCAAATATCCACTTGCATTTTCCACAAAAAGAGTGTTTCAA
          1 TTCCAACGAAATCATCAAAGCGATCCAAATATCCACTTCCAGATTCTACAAAAAGAGTGTTTCAA

                          *                 *    *  *                *       
     276099 AACTGCTCTATCAATAGAAATGTTCAACTCCTTTG-GCTGGGTACACACATCACAAACAAGTTTC
         66 AACTGCTCTATCAAAAGAAATGTTCAACT-CTGTGAGTTGAGTACACACATCACAAAGAAGTTTC

                                      **     **       
     276163 TGAGAATGCTTCTGTCTAGTTTTTATGGGAAGACGTTCCCTT
        130 TGAGAATGCTTCTGTCTAGTTTTTATATGAAGATATTCCCTT

              *  * *  **          *       *            **                  * 
     276205 TTTCACCAAAGGCATCAAAGCGCTCCAAATGTCCACTTCCAGACACTACAAAAAGAGTGTTTCCA
          1 TTCCAACGAAATCATCAAAGCGATCCAAATATCCACTTCCAGATTCTACAAAAAGAGTGTTTCAA

             **       **   ***                 *    * *  * *         *       
     276270 ACGTGCTCTAAGAAAGCGAATGTTCAACTCTGTGACTTGAATGCAGATATCACAAAGTAGTTTCT
         66 AACTGCTCTATCAAAAGAAATGTTCAACTCTGTGAGTTGAGTACACACATCACAAAGAAGTTTCT

                **            *     *    *         * 
     276335 GAGAGGGCTTCTGTCTAGATTTTAGATGATGATATTCCCGT
        131 GAGAATGCTTCTGTCTAGTTTTTATATGAAGATATTCCCTT

                           * *   *                *   *                    * 
     276376 TTCCAACGAAATCATTAGAGCTATCCAAATATCCACTTACAGTTTCTACAAAAAGAGTGTTTCCA
          1 TTCCAACGAAATCATCAAAGCGATCCAAATATCCACTTCCAGATTCTACAAAAAGAGTGTTTCAA

                   **         * *    *       *  *                   ***      
     276441 AACTGCTGCATCAAAAGAGAGGTTCCACTCTGTTAGCTGAGTACACACATCACAAACTTGTTTCT
         66 AACTGCTCTATCAAAAGAAATGTTCAACTCTGTGAGTTGAGTACACACATCACAAAGAAGTTTCT

            *     *         *        **         **   
     276506 CAGAATCCTTCTGTCTCGTTTTTATGGGAAGATATTTACTT
        131 GAGAATGCTTCTGTCTAGTTTTTATATGAAGATATTCCCTT

                 *  * **          *       *     *       *  *  *              
     276547 TTCCACCGTAGGCATCAAAGCGCTCCAAATGTCCACATCCAGATACTCCAGAAAGAGTGTTTCAA
          1 TTCCAACGAAATCATCAAAGCGATCCAAATATCCACTTCCAGATTCTACAAAAAGAGTGTTTCAA

             *         *   * *             *        * *  *      *      *     
     276612 ACCTGCTCTATGAAAGGGAATGTTCAACTCTATGAGTTGAATGCAGACATCAGAAAGAAATTTCT
         66 AACTGCTCTATCAAAAGAAATGTTCAACTCTGTGAGTTGAGTACACACATCACAAAGAAGTTTCT

                     *       **          *         **
     276677 GAGAATGCTGCTGTCTACCTTTTAT-T--TGA-ATTCCCGC
        131 GAGAATGCTTCTGTCTAGTTTTTATATGAAGATATTCCCTT

                         *  *    *                *       *                  
     276714 TTCCAACGAAATCCTCCAAGCTATCCAAATATCCACTTGCAGATTCCACAAAAAGAGTGTTTCAA
          1 TTCCAACGAAATCATCAAAGCGATCCAAATATCCACTTCCAGATTCTACAAAAAGAGTGTTTCAA

                            ** *  *            *       *     *     *  *      
     276779 AACTGCTCTCTATCAATGGCAAAGTTCAACTCTGTTAGTTGAGGACACATATCACCAACAAGTTT
         66 AACTG--CTCTATCAAAAGAAATGTTCAACTCTGTGAGTTGAGTACACACATCACAAAGAAGTTT

                               *       **         *    
     276844 CTGAGAATGCTTCTGTCTATTTTTTATGGGAAGATATTTCCTT
        129 CTGAGAATGCTTCTGTCTAGTTTTTATATGAAGATATTCCCTT

              *  *  * ** *    *      *    *          * **                    
     276887 TTTCACCGTAGGCGTCAAGGCGATCGAAATGTCCACTTCCACAAACTACAAAAAGAGTGTTTCAA
          1 TTCCAACGAAATCATCAAAGCGATCCAAATATCCACTTCCAGATTCTACAAAAAGAGTGTTTCAA

             *         *   * **       *    *        * ** * *   **      *     
     276952 ACCTGCTCTATGAAAGGCCATGTTCATCTCTATGAGTTGAATGGAAATATCCGAAAGAAATTTCT
         66 AACTGCTCTATCAAAAGAAATGTTCAACTCTGTGAGTTGAGTACACACATCACAAAGAAGTTTCT

             *       *          *        *         **
     277017 GGGAATGCTGCTGTCTAG-TGTT-TAT-ACGA-ATTCCCGC
        131 GAGAATGCTTCTGTCTAGTTTTTATATGAAGATATTCCCTT

                         *       *                *    *  *                  
     277054 TTCCAACGAAATCCTCAAAGCAATCCAAATATCCACTTGCAGAATCCACAAAAAGAGTGTTTCAA
          1 TTCCAACGAAATCATCAAAGCGATCCAAATATCCACTTCCAGATTCTACAAAAAGAGTGTTTCAA

                          *     *          * *                   *  *        
     277119 AACTGCTCTATCAATAGAAAGGTTCAACTCTTTTAGTTGAGTACACACATCACGAACAAGTTTCT
         66 AACTGCTCTATCAAAAGAAATGTTCAACTCTGTGAGTTGAGTACACACATCACAAAGAAGTTTCT

                            * *      **     **  *    
     277184 GAGAATGCTTCTGTCTGGCTTTTATTGGAAGACGTTTCCTT
        131 GAGAATGCTTCTGTCTAGTTTTTATATGAAGATATTCCCTT

                 * *  **          *       *                * *               
     277225 TT-CACCAAAGGCATCAAAGCGCTCCAAATGTCCACTTCCAGATTCTTCCAAAAGAGTGTTTCAA
          1 TTCCAACGAAATCATCAAAGCGATCCAAATATCCACTTCCAGATTCTACAAAAAGAGTGTTTCAA

             **     * ***  * *                 *    * *  * *     *   *       
     277289 ACGTGCTCAAAGTAAGGGAATGTTCAACTCTGTGACTTGAATGCAGATATCACCAAGTAGTTTCT
         66 AACTGCTCTATCAAAAGAAATGTTCAACTCTGTGAGTTGAGTACACACATCACAAAGAAGTTTCT

            * * *            **     *    *         * 
     277354 AATAGTGCTTCTGTCTACATTTTAGATGATGATATTCCCGT
        131 GAGAATGCTTCTGTCTAGTTTTTATATGAAGATATTCCCTT

                         * * *   *             *  *   *      *     *       * 
     277395 TTCCAACGAAATCGTTAGAGCTATCCAAATATCCAGTTACAGTTTCTACCAAAAGGGTGTTTCCA
          1 TTCCAACGAAATCATCAAAGCGATCCAAATATCCACTTCCAGATTCTACAAAAAGAGTGTTTCAA

              *    **           *            *       *                     * 
     277460 AATTGCTGCATCAAAAGAAAGGTTCAACTCTGTTAGTTGAGGACACACATCACAAAGAAGTTTGT
         66 AACTGCTCTATCAAAAGAAATGTTCAACTCTGTGAGTTGAGTACACACATCACAAAGAAGTTTCT

                              *    *     *           
     277525 GAGAATGCTTCTGTCTAGATTTTGTATGACGATATTCCCTT
        131 GAGAATGCTTCTGTCTAGTTTTTATATGAAGATATTCCCTT

                     *   * *     *   *       * *  *    *  *       *  *       
     277566 TTCCAACGATATCGTTAAAGCAATCTAAATATCAATTTGCAGAATCCACAAAAATAGAGTTTCAA
          1 TTCCAACGAAATCATCAAAGCGATCCAAATATCCACTTCCAGATTCTACAAAAAGAGTGTTTCAA

             *       * *        *    *       *  *                   ***      
     277631 AGCTGCTCTGTAAAAAGAAAGGTTCCACTCTGTTAGCTGAGTACACACATCACAAACTTGTTTCT
         66 AACTGCTCTATCAAAAGAAATGTTCAACTCTGTGAGTTGAGTACACACATCACAAAGAAGTTTCT

                  *         *        **         **   
     277696 GAGAATCCTTCTGTCTCGTTTTTATGGGAAGATATTTACTT
        131 GAGAATGCTTCTGTCTAGTTTTTATATGAAGATATTCCCTT

                 *  * **          *       *     *       *  *  *              
     277737 TTCCACCGTAGGCATCAAAGCGCTCCAAATGTCCACATCCAGATACTCCAGAAAGAGTGTTTCAA
          1 TTCCAACGAAATCATCAAAGCGATCCAAATATCCACTTCCAGATTCTACAAAAAGAGTGTTTCAA

             *         *   * *   *         *        * *  *      *      *     
     277802 ACCTGCTCTATGAAAGGGAATCTTCAACTCTATGAGTTGAATGCAGACATCAGAAAGAAATTTCT
         66 AACTGCTCTATCAAAAGAAATGTTCAACTCTGTGAGTTGAGTACACACATCACAAAGAAGTTTCT

                     *       **          *         **
     277867 GAGAATGCTGCTGTCTACCTTTTAT-T--TGA-ATTCCCGC
        131 GAGAATGCTTCTGTCTAGTTTTTATATGAAGATATTCCCTT

                         *  *    *                *  **   *                  
     277904 TTCCAACGAAATCCTCCAAGCTATCCAAATATCCACTTGCATTTTCCACAAAAAGAGTGTTTCAA
          1 TTCCAACGAAATCATCAAAGCGATCCAAATATCCACTTCCAGATTCTACAAAAAGAGTGTTTCAA

                          *                 *    *  *                *       
     277969 AACTGCTCTATCAATAGAAATGTTCAACTCCTTTG-GCTGGGTACACACATCACAAACAAGTTTC
         66 AACTGCTCTATCAAAAGAAATGTTCAACT-CTGTGAGTTGAGTACACACATCACAAAGAAGTTTC

                                      **     **       
     278033 TGAGAATGCTTCTGTCTAGTTTTTATGGGAAGACGTTCCCTT
        130 TGAGAATGCTTCTGTCTAGTTTTTATATGAAGATATTCCCTT

              *  * *  **          *       *            **                  * 
     278075 TTTCACCAAAGGCATCAAAGCGCTCCAAATGTCCACTTCCAGACACTACAAAAAGAGTGTTTCCA
          1 TTCCAACGAAATCATCAAAGCGATCCAAATATCCACTTCCAGATTCTACAAAAAGAGTGTTTCAA

             **       **   ***                 *    * *  * *         *       
     278140 ACGTGCTCTAAGAAAGCGAATGTTCAACTCTGTGACTTGAATGCAGATATCACAAAGTAGTTTCT
         66 AACTGCTCTATCAAAAGAAATGTTCAACTCTGTGAGTTGAGTACACACATCACAAAGAAGTTTCT

                **            *     *    *         * 
     278205 GAGAGGGCTTCTGTCTAGATTTTAGATGATGATATTCCCGT
        131 GAGAATGCTTCTGTCTAGTTTTTATATGAAGATATTCCCTT

                           * *   *                *   *                    * 
     278246 TTCCAACGAAATCATTAGAGCTATCCAAATATCCACTTACAGTTTCTACAAAAAGAGTGTTTCCA
          1 TTCCAACGAAATCATCAAAGCGATCCAAATATCCACTTCCAGATTCTACAAAAAGAGTGTTTCAA

                   **         * *    *       *  *                   ***      
     278311 AACTGCTGCATCAAAAGAGAGGTTCCACTCTGTTAGCTGAGTACACACATCACAAACTTGTTTCT
         66 AACTGCTCTATCAAAAGAAATGTTCAACTCTGTGAGTTGAGTACACACATCACAAAGAAGTTTCT

            *     *         *        **         **   
     278376 CAGAATCCTTCTGTCTCGTTTTTATGGGAAGATATTTACTT
        131 GAGAATGCTTCTGTCTAGTTTTTATATGAAGATATTCCCTT

                 *  * **          *       *     *       *  *  *              
     278417 TTCCACCGTAGGCATCAAAGCGCTCCAAATGTCCACATCCAGATACTCCAGAAAGAGTGTTTCAA
          1 TTCCAACGAAATCATCAAAGCGATCCAAATATCCACTTCCAGATTCTACAAAAAGAGTGTTTCAA

             *         *   * *             *        * *  *      *      *     
     278482 ACCTGCTCTATGAAAGGGAATGTTCAACTCTATGAGTTGAATGCAGACATCAGAAAGAAATTTCT
         66 AACTGCTCTATCAAAAGAAATGTTCAACTCTGTGAGTTGAGTACACACATCACAAAGAAGTTTCT

                     *       **          *         **
     278547 GAGAATGCTGCTGTCTACCTTTTAT-T--TGA-ATTCCCGC
        131 GAGAATGCTTCTGTCTAGTTTTTATATGAAGATATTCCCTT

                         *  *    *                *       *                  
     278584 TTCCAACGAAATCCTCCAAGCTATCCAAATATCCACTTGCAGATTCCACAAAAAGAGTGTTTCAA
          1 TTCCAACGAAATCATCAAAGCGATCCAAATATCCACTTCCAGATTCTACAAAAAGAGTGTTTCAA

                            ** *  *            *       *     *     *  *      
     278649 AACTGCTCTCTATCAATGGCAAAGTTCAACTCTGTTAGTTGAGGACACATATCACCAACAAGTTT
         66 AACTG--CTCTATCAAAAGAAATGTTCAACTCTGTGAGTTGAGTACACACATCACAAAGAAGTTT

                               *       **         *    
     278714 CTGAGAATGCTTCTGTCTATTTTTTATGGGAAGATATTTCCTT
        129 CTGAGAATGCTTCTGTCTAGTTTTTATATGAAGATATTCCCTT

              *  *  * ** *    *      *    *          * **                    
     278757 TTTCACCGTAGGCGTCAAGGCGATCGAAATGTCCACTTCCACAAACTACAAAAAGAGTGTTTCAA
          1 TTCCAACGAAATCATCAAAGCGATCCAAATATCCACTTCCAGATTCTACAAAAAGAGTGTTTCAA

             *         *   * **       *    *        * ** * *   **      *     
     278822 ACCTGCTCTATGAAAGGCCATGTTCATCTCTATGAGTTGAATGGAAATATCCGAAAGAAATTTCT
         66 AACTGCTCTATCAAAAGAAATGTTCAACTCTGTGAGTTGAGTACACACATCACAAAGAAGTTTCT

             *       *          *        *         **
     278887 GGGAATGCTGCTGTCTAG-TGTT-TAT-ACGA-ATTCCCGC
        131 GAGAATGCTTCTGTCTAGTTTTTATATGAAGATATTCCCTT

                         *       *                *    *  *                  
     278924 TTCCAACGAAATCCTCAAAGCAATCCAAATATCCACTTGCAGAATCCACAAAAAGAGTGTTTCAA
          1 TTCCAACGAAATCATCAAAGCGATCCAAATATCCACTTCCAGATTCTACAAAAAGAGTGTTTCAA

                          *     *          * *                   *  *        
     278989 AACTGCTCTATCAATAGAAAGGTTCAACTCTTTTAGTTGAGTACACACATCACGAACAAGTTTCT
         66 AACTGCTCTATCAAAAGAAATGTTCAACTCTGTGAGTTGAGTACACACATCACAAAGAAGTTTCT

                            * *      **     **  *    
     279054 GAGAATGCTTCTGTCTGGCTTTTATTGGAAGACGTTTCCTT
        131 GAGAATGCTTCTGTCTAGTTTTTATATGAAGATATTCCCTT

                 * *  **          *       *                * *               
     279095 TT-CACCAAAGGCATCAAAGCGCTCCAAATGTCCACTTCCAGATTCTTCCAAAAGAGTGTTTCAA
          1 TTCCAACGAAATCATCAAAGCGATCCAAATATCCACTTCCAGATTCTACAAAAAGAGTGTTTCAA

             **     * ***  * *                 *    * *  * *     *   *       
     279159 ACGTGCTCAAAGTAAGGGAATGTTCAACTCTGTGACTTGAATGCAGATATCACCAAGTAGTTTCT
         66 AACTGCTCTATCAAAAGAAATGTTCAACTCTGTGAGTTGAGTACACACATCACAAAGAAGTTTCT

            * * *            **     *    *         * 
     279224 AATAGTGCTTCTGTCTACATTTTAGATGATGATATTCCCGT
        131 GAGAATGCTTCTGTCTAGTTTTTATATGAAGATATTCCCTT

                         * * *   *             *  *   *      *     *       * 
     279265 TTCCAACGAAATCGTTAGAGCTATCCAAATATCCAGTTACAGTTTCTACCAAAAGGGTGTTTCCA
          1 TTCCAACGAAATCATCAAAGCGATCCAAATATCCACTTCCAGATTCTACAAAAAGAGTGTTTCAA

              *    **           *            *       *                     * 
     279330 AATTGCTGCATCAAAAGAAAGGTTCAACTCTGTTAGTTGAGGACACACATCACAAAGAAGTTTGT
         66 AACTGCTCTATCAAAAGAAATGTTCAACTCTGTGAGTTGAGTACACACATCACAAAGAAGTTTCT

                              *    *     *           
     279395 GAGAATGCTTCTGTCTAGATTTTGTATGACGATATTCCCTT
        131 GAGAATGCTTCTGTCTAGTTTTTATATGAAGATATTCCCTT

                     *   * *     *   *       * *  *    *  *       *  *       
     279436 TTCCAACGATATCGTTAAAGCAATCTAAATATCAATTTGCAGAATCCACAAAAATAGAGTTTCAA
          1 TTCCAACGAAATCATCAAAGCGATCCAAATATCCACTTCCAGATTCTACAAAAAGAGTGTTTCAA

             *       * *        *    *       *  *                   ***      
     279501 AGCTGCTCTGTAAAAAGAAAGGTTCCACTCTGTTAGCTGAGTACACACATCACAAACTTGTTTCT
         66 AACTGCTCTATCAAAAGAAATGTTCAACTCTGTGAGTTGAGTACACACATCACAAAGAAGTTTCT

                  *         *        **         **   
     279566 GAGAATCCTTCTGTCTCGTTTTTATGGGAAGATATTTACTT
        131 GAGAATGCTTCTGTCTAGTTTTTATATGAAGATATTCCCTT

                 *  * **          *       *     *       *  *  *              
     279607 TTCCACCGTAGGCATCAAAGCGCTCCAAATGTCCACATCCAGATACTCCAGAAAGAGTGTTTCAA
          1 TTCCAACGAAATCATCAAAGCGATCCAAATATCCACTTCCAGATTCTACAAAAAGAGTGTTTCAA

             *         *   * *   *         *        * *  *      *      *     
     279672 ACCTGCTCTATGAAAGGGAATCTTCAACTCTATGAGTTGAATGCAGACATCAGAAAGAAATTTCT
         66 AACTGCTCTATCAAAAGAAATGTTCAACTCTGTGAGTTGAGTACACACATCACAAAGAAGTTTCT

                     *       **          *         **
     279737 GAGAATGCTGCTGTCTACCTTTTAT-T--TGA-ATTCCCGC
        131 GAGAATGCTTCTGTCTAGTTTTTATATGAAGATATTCCCTT

                         *  *    *                *  **   *                  
     279774 TTCCAACGAAATCCTCCAAGCTATCCAAATATCCACTTGCATTTTCCACAAAAAGAGTGTTTCAA
          1 TTCCAACGAAATCATCAAAGCGATCCAAATATCCACTTCCAGATTCTACAAAAAGAGTGTTTCAA

                          *                 *    *  *                *       
     279839 AACTGCTCTATCAATAGAAATGTTCAACTCCTTTG-GCTGGGTACACACATCACAAACAAGTTTC
         66 AACTGCTCTATCAAAAGAAATGTTCAACT-CTGTGAGTTGAGTACACACATCACAAAGAAGTTTC

                                      **     **       
     279903 TGAGAATGCTTCTGTCTAGTTTTTATGGGAAGACGTTCCCTT
        130 TGAGAATGCTTCTGTCTAGTTTTTATATGAAGATATTCCCTT

              *  * *  **          *       *            **                  * 
     279945 TTTCACCAAAGGCATCAAAGCGCTCCAAATGTCCACTTCCAGACACTACAAAAAGAGTGTTTCCA
          1 TTCCAACGAAATCATCAAAGCGATCCAAATATCCACTTCCAGATTCTACAAAAAGAGTGTTTCAA

             **       **   ***                 *    * *  * *         *       
     280010 ACGTGCTCTAAGAAAGCGAATGTTCAACTCTGTGACTTGAATGCAGATATCACAAAGTAGTTTCT
         66 AACTGCTCTATCAAAAGAAATGTTCAACTCTGTGAGTTGAGTACACACATCACAAAGAAGTTTCT

                **            *     *    *         * 
     280075 GAGAGGGCTTCTGTCTAGATTTTAGATGATGATATTCCCGT
        131 GAGAATGCTTCTGTCTAGTTTTTATATGAAGATATTCCCTT

                           * *   *                *   *                    * 
     280116 TTCCAACGAAATCATTAGAGCTATCCAAATATCCACTTACAGTTTCTACAAAAAGAGTGTTTCCA
          1 TTCCAACGAAATCATCAAAGCGATCCAAATATCCACTTCCAGATTCTACAAAAAGAGTGTTTCAA

                   **         * *    *       *  *                   ***      
     280181 AACTGCTGCATCAAAAGAGAGGTTCCACTCTGTTAGCTGAGTACACACATCACAAACTTGTTTCT
         66 AACTGCTCTATCAAAAGAAATGTTCAACTCTGTGAGTTGAGTACACACATCACAAAGAAGTTTCT

            *     *         *        **         **   
     280246 CAGAATCCTTCTGTCTCGTTTTTATGGGAAGATATTTACTT
        131 GAGAATGCTTCTGTCTAGTTTTTATATGAAGATATTCCCTT

                 *  * **          *       *     *       *  *  *              
     280287 TTCCACCGTAGGCATCAAAGCGCTCCAAATGTCCACATCCAGATACTCCAGAAAGAGTGTTTCAA
          1 TTCCAACGAAATCATCAAAGCGATCCAAATATCCACTTCCAGATTCTACAAAAAGAGTGTTTCAA

             *         *   * *             *        * *  *      *      *     
     280352 ACCTGCTCTATGAAAGGGAATGTTCAACTCTATGAGTTGAATGCAGACATCAGAAAGAAATTTCT
         66 AACTGCTCTATCAAAAGAAATGTTCAACTCTGTGAGTTGAGTACACACATCACAAAGAAGTTTCT

                     *       **          *         **
     280417 GAGAATGCTGCTGTCTACCTTTTAT-T--TGA-ATTCCCGC
        131 GAGAATGCTTCTGTCTAGTTTTTATATGAAGATATTCCCTT

                         *  *    *                *       *                  
     280454 TTCCAACGAAATCCTCCAAGCTATCCAAATATCCACTTGCAGATTCCACAAAAAGAGTGTTTCAA
          1 TTCCAACGAAATCATCAAAGCGATCCAAATATCCACTTCCAGATTCTACAAAAAGAGTGTTTCAA

                            ** *  *            *       *     *     *  *      
     280519 AACTGCTCTCTATCAATGGCAAAGTTCAACTCTGTTAGTTGAGGACACATATCACCAACAAGTTT
         66 AACTG--CTCTATCAAAAGAAATGTTCAACTCTGTGAGTTGAGTACACACATCACAAAGAAGTTT

                               *       **         *    
     280584 CTGAGAATGCTTCTGTCTATTTTTTATGGGAAGATATTTCCTT
        129 CTGAGAATGCTTCTGTCTAGTTTTTATATGAAGATATTCCCTT

              *  *  * ** *    *      *    *          * **                    
     280627 TTTCACCGTAGGCGTCAAGGCGATCGAAATGTCCACTTCCACAAACTACAAAAAGAGTGTTTCAA
          1 TTCCAACGAAATCATCAAAGCGATCCAAATATCCACTTCCAGATTCTACAAAAAGAGTGTTTCAA

             *         *   * **       *    *        * ** * *   **      *     
     280692 ACCTGCTCTATGAAAGGCCATGTTCATCTCTATGAGTTGAATGGAAATATCCGAAAGAAATTTCT
         66 AACTGCTCTATCAAAAGAAATGTTCAACTCTGTGAGTTGAGTACACACATCACAAAGAAGTTTCT

             *       *          *        *         **
     280757 GGGAATGCTGCTGTCTAG-TGTT-TAT-ACGA-ATTCCCGC
        131 GAGAATGCTTCTGTCTAGTTTTTATATGAAGATATTCCCTT

                         *       *                *    *  *                  
     280794 TTCCAACGAAATCCTCAAAGCAATCCAAATATCCACTTGCAGAATCCACAAAAAGAGTGTTTCAA
          1 TTCCAACGAAATCATCAAAGCGATCCAAATATCCACTTCCAGATTCTACAAAAAGAGTGTTTCAA

                          *     *          * *                   *  *        
     280859 AACTGCTCTATCAATAGAAAGGTTCAACTCTTTTAGTTGAGTACACACATCACGAACAAGTTTCT
         66 AACTGCTCTATCAAAAGAAATGTTCAACTCTGTGAGTTGAGTACACACATCACAAAGAAGTTTCT

                            * *      **     **  *    
     280924 GAGAATGCTTCTGTCTGGCTTTTATTGGAAGACGTTTCCTT
        131 GAGAATGCTTCTGTCTAGTTTTTATATGAAGATATTCCCTT

                 * *  **          *       *                * *               
     280965 TT-CACCAAAGGCATCAAAGCGCTCCAAATGTCCACTTCCAGATTCTTCCAAAAGAGTGTTTCAA
          1 TTCCAACGAAATCATCAAAGCGATCCAAATATCCACTTCCAGATTCTACAAAAAGAGTGTTTCAA

             **     * ***  * *                 *    * *  * *     *   *       
     281029 ACGTGCTCAAAGTAAGGGAATGTTCAACTCTGTGACTTGAATGCAGATATCACCAAGTAGTTTCT
         66 AACTGCTCTATCAAAAGAAATGTTCAACTCTGTGAGTTGAGTACACACATCACAAAGAAGTTTCT

            * * *            **     *    *         * 
     281094 AATAGTGCTTCTGTCTACATTTTAGATGATGATATTCCCGT
        131 GAGAATGCTTCTGTCTAGTTTTTATATGAAGATATTCCCTT

                         * * *   *             *  *   *      *     *       * 
     281135 TTCCAACGAAATCGTTAGAGCTATCCAAATATCCAGTTACAGTTTCTACCAAAAGGGTGTTTCCA
          1 TTCCAACGAAATCATCAAAGCGATCCAAATATCCACTTCCAGATTCTACAAAAAGAGTGTTTCAA

              *    **           *            *       *                     * 
     281200 AATTGCTGCATCAAAAGAAAGGTTCAACTCTGTTAGTTGAGGACACACATCACAAAGAAGTTTGT
         66 AACTGCTCTATCAAAAGAAATGTTCAACTCTGTGAGTTGAGTACACACATCACAAAGAAGTTTCT

                              *    *     *           
     281265 GAGAATGCTTCTGTCTAGATTTTGTATGACGATATTCCCTT
        131 GAGAATGCTTCTGTCTAGTTTTTATATGAAGATATTCCCTT

                     *   * *     *   *       * *  *    *  *       *  *       
     281306 TTCCAACGATATCGTTAAAGCAATCTAAATATCAATTTGCAGAATCCACAAAAATAGAGTTTCAA
          1 TTCCAACGAAATCATCAAAGCGATCCAAATATCCACTTCCAGATTCTACAAAAAGAGTGTTTCAA

             *       * *        *    *       *  *                   ***      
     281371 AGCTGCTCTGTAAAAAGAAAGGTTCCACTCTGTTAGCTGAGTACACACATCACAAACTTGTTTCT
         66 AACTGCTCTATCAAAAGAAATGTTCAACTCTGTGAGTTGAGTACACACATCACAAAGAAGTTTCT

                  *         *        **         **   
     281436 GAGAATCCTTCTGTCTCGTTTTTATGGGAAGATATTTACTT
        131 GAGAATGCTTCTGTCTAGTTTTTATATGAAGATATTCCCTT

                 *  * **          *       *     *       *  *  *              
     281477 TTCCACCGTAGGCATCAAAGCGCTCCAAATGTCCACATCCAGATACTCCAGAAAGAGTGTTTCAA
          1 TTCCAACGAAATCATCAAAGCGATCCAAATATCCACTTCCAGATTCTACAAAAAGAGTGTTTCAA

             *         *   * *   *         *        * *  *      *      *     
     281542 ACCTGCTCTATGAAAGGGAATCTTCAACTCTATGAGTTGAATGCAGACATCAGAAAGAAATTTCT
         66 AACTGCTCTATCAAAAGAAATGTTCAACTCTGTGAGTTGAGTACACACATCACAAAGAAGTTTCT

                     *       **          *         **
     281607 GAGAATGCTGCTGTCTACCTTTTAT-T--TGA-ATTCCCGC
        131 GAGAATGCTTCTGTCTAGTTTTTATATGAAGATATTCCCTT

                         *  *    *                *  **   *                  
     281644 TTCCAACGAAATCCTCCAAGCTATCCAAATATCCACTTGCATTTTCCACAAAAAGAGTGTTTCAA
          1 TTCCAACGAAATCATCAAAGCGATCCAAATATCCACTTCCAGATTCTACAAAAAGAGTGTTTCAA

                          *                 *    *  *                *       
     281709 AACTGCTCTATCAATAGAAATGTTCAACTCCTTTG-GCTGGGTACACACATCACAAACAAGTTTC
         66 AACTGCTCTATCAAAAGAAATGTTCAACT-CTGTGAGTTGAGTACACACATCACAAAGAAGTTTC

                                      **     **       
     281773 TGAGAATGCTTCTGTCTAGTTTTTATGGGAAGACGTTCCCTT
        130 TGAGAATGCTTCTGTCTAGTTTTTATATGAAGATATTCCCTT

              *  * *  **          *       *            **                  * 
     281815 TTTCACCAAAGGCATCAAAGCGCTCCAAATGTCCACTTCCAGACACTACAAAAAGAGTGTTTCCA
          1 TTCCAACGAAATCATCAAAGCGATCCAAATATCCACTTCCAGATTCTACAAAAAGAGTGTTTCAA

             **       **   ***                 *    * *  * *         *       
     281880 ACGTGCTCTAAGAAAGCGAATGTTCAACTCTGTGACTTGAATGCAGATATCACAAAGTAGTTTCT
         66 AACTGCTCTATCAAAAGAAATGTTCAACTCTGTGAGTTGAGTACACACATCACAAAGAAGTTTCT

                **            *     *    *         * 
     281945 GAGAGGGCTTCTGTCTAGATTTTAGATGATGATATTCCCGT
        131 GAGAATGCTTCTGTCTAGTTTTTATATGAAGATATTCCCTT

                           * *   *                *   *                    * 
     281986 TTCCAACGAAATCATTAGAGCTATCCAAATATCCACTTACAGTTTCTACAAAAAGAGTGTTTCCA
          1 TTCCAACGAAATCATCAAAGCGATCCAAATATCCACTTCCAGATTCTACAAAAAGAGTGTTTCAA

                   **         * *    *       *  *                   ***      
     282051 AACTGCTGCATCAAAAGAGAGGTTCCACTCTGTTAGCTGAGTACACACATCACAAACTTGTTTCT
         66 AACTGCTCTATCAAAAGAAATGTTCAACTCTGTGAGTTGAGTACACACATCACAAAGAAGTTTCT

            *     *         *        **         **   
     282116 CAGAATCCTTCTGTCTCGTTTTTATGGGAAGATATTTACTT
        131 GAGAATGCTTCTGTCTAGTTTTTATATGAAGATATTCCCTT

                 *  * **          *       *     *       *  *  *              
     282157 TTCCACCGTAGGCATCAAAGCGCTCCAAATGTCCACATCCAGATACTCCAGAAAGAGTGTTTCAA
          1 TTCCAACGAAATCATCAAAGCGATCCAAATATCCACTTCCAGATTCTACAAAAAGAGTGTTTCAA

             *         *   * *             *        * *  *      *      *     
     282222 ACCTGCTCTATGAAAGGGAATGTTCAACTCTATGAGTTGAATGCAGACATCAGAAAGAAATTTCT
         66 AACTGCTCTATCAAAAGAAATGTTCAACTCTGTGAGTTGAGTACACACATCACAAAGAAGTTTCT

                     *       **          *         **
     282287 GAGAATGCTGCTGTCTACCTTTTAT-T--TGA-ATTCCCGC
        131 GAGAATGCTTCTGTCTAGTTTTTATATGAAGATATTCCCTT

                         *  *    *                *       *                  
     282324 TTCCAACGAAATCCTCCAAGCTATCCAAATATCCACTTGCAGATTCCACAAAAAGAGTGTTTCAA
          1 TTCCAACGAAATCATCAAAGCGATCCAAATATCCACTTCCAGATTCTACAAAAAGAGTGTTTCAA

                            ** *  *            *       *     *     *  *      
     282389 AACTGCTCTCTATCAATGGCAAAGTTCAACTCTGTTAGTTGAGGACACATATCACCAACAAGTTT
         66 AACTG--CTCTATCAAAAGAAATGTTCAACTCTGTGAGTTGAGTACACACATCACAAAGAAGTTT

                               *       **         *    
     282454 CTGAGAATGCTTCTGTCTATTTTTTATGGGAAGATATTTCCTT
        129 CTGAGAATGCTTCTGTCTAGTTTTTATATGAAGATATTCCCTT

              *  *  * ** *    *      *    *          * **                    
     282497 TTTCACCGTAGGCGTCAAGGCGATCGAAATGTCCACTTCCACAAACTACAAAAAGAGTGTTTCAA
          1 TTCCAACGAAATCATCAAAGCGATCCAAATATCCACTTCCAGATTCTACAAAAAGAGTGTTTCAA

             *         *   * **       *    *        * ** * *   **      *     
     282562 ACCTGCTCTATGAAAGGCCATGTTCATCTCTATGAGTTGAATGGAAATATCCGAAAGAAATTTCT
         66 AACTGCTCTATCAAAAGAAATGTTCAACTCTGTGAGTTGAGTACACACATCACAAAGAAGTTTCT

             *       *          *        *         **
     282627 GGGAATGCTGCTGTCTAG-TGTT-TAT-ACGA-ATTCCCGC
        131 GAGAATGCTTCTGTCTAGTTTTTATATGAAGATATTCCCTT

                         *       *                *    *  *                  
     282664 TTCCAACGAAATCCTCAAAGCAATCCAAATATCCACTTGCAGAATCCACAAAAAGAGTGTTTCAA
          1 TTCCAACGAAATCATCAAAGCGATCCAAATATCCACTTCCAGATTCTACAAAAAGAGTGTTTCAA

                          *     *          * *                   *  *        
     282729 AACTGCTCTATCAATAGAAAGGTTCAACTCTTTTAGTTGAGTACACACATCACGAACAAGTTTCT
         66 AACTGCTCTATCAAAAGAAATGTTCAACTCTGTGAGTTGAGTACACACATCACAAAGAAGTTTCT

                            * *      **     **  *    
     282794 GAGAATGCTTCTGTCTGGCTTTTATTGGAAGACGTTTCCTT
        131 GAGAATGCTTCTGTCTAGTTTTTATATGAAGATATTCCCTT

                 * *  **          *       *                * *               
     282835 TT-CACCAAAGGCATCAAAGCGCTCCAAATGTCCACTTCCAGATTCTTCCAAAAGAGTGTTTCAA
          1 TTCCAACGAAATCATCAAAGCGATCCAAATATCCACTTCCAGATTCTACAAAAAGAGTGTTTCAA

             **     * ***  * *                 *    * *  * *     *   *       
     282899 ACGTGCTCAAAGTAAGGGAATGTTCAACTCTGTGACTTGAATGCAGATATCACCAAGTAGTTTCT
         66 AACTGCTCTATCAAAAGAAATGTTCAACTCTGTGAGTTGAGTACACACATCACAAAGAAGTTTCT

            * * *            **     *    *         * 
     282964 AATAGTGCTTCTGTCTACATTTTAGATGATGATATTCCCGT
        131 GAGAATGCTTCTGTCTAGTTTTTATATGAAGATATTCCCTT

                         * * *   *             *  *   *      *     *       * 
     283005 TTCCAACGAAATCGTTAGAGCTATCCAAATATCCAGTTACAGTTTCTACCAAAAGGGTGTTTCCA
          1 TTCCAACGAAATCATCAAAGCGATCCAAATATCCACTTCCAGATTCTACAAAAAGAGTGTTTCAA

              *    **           *            *       *                     * 
     283070 AATTGCTGCATCAAAAGAAAGGTTCAACTCTGTTAGTTGAGGACACACATCACAAAGAAGTTTGT
         66 AACTGCTCTATCAAAAGAAATGTTCAACTCTGTGAGTTGAGTACACACATCACAAAGAAGTTTCT

                              *    *     *           
     283135 GAGAATGCTTCTGTCTAGATTTTGTATGACGATATTCCCTT
        131 GAGAATGCTTCTGTCTAGTTTTTATATGAAGATATTCCCTT

                     *   * *     *   *       * *  *    *  *       *  *       
     283176 TTCCAACGATATCGTTAAAGCAATCTAAATATCAATTTGCAGAATCCACAAAAATAGAGTTTCAA
          1 TTCCAACGAAATCATCAAAGCGATCCAAATATCCACTTCCAGATTCTACAAAAAGAGTGTTTCAA

             *       * *        *    *     * *  *                   ***      
     283241 AGCTGCTCTGTAAAAAGAAAGGTTCCACTCTTTTAGCTGAGTACACACATCACAAACTTGTTTCT
         66 AACTGCTCTATCAAAAGAAATGTTCAACTCTGTGAGTTGAGTACACACATCACAAAGAAGTTTCT

                  *         *        **         **   
     283306 GAGAATCCTTCTGTCTCGTTTTTATGGGAAGATATTTACTT
        131 GAGAATGCTTCTGTCTAGTTTTTATATGAAGATATTCCCTT

                 *  * **  *       *       *     *       *  *  *              
     283347 TTCCACCGTAGGCACCAAAGCGCTCCAAATGTCCACATCCAGATACTCCAGAAAGAGTGTTTCAA
          1 TTCCAACGAAATCATCAAAGCGATCCAAATATCCACTTCCAGATTCTACAAAAAGAGTGTTTCAA

             *         *   * *   *         *        * *  *      *      *     
     283412 ACCTGCTCTATGAAAGGGAATCTTCAACTCTATGAGTTGAATGCAGACATCAGAAAGAAATTTCT
         66 AACTGCTCTATCAAAAGAAATGTTCAACTCTGTGAGTTGAGTACACACATCACAAAGAAGTTTCT

                     *       **          *         **
     283477 GAGAATGCTGCTGTCTACCTTTTAT-T--TGA-ATTCCCGC
        131 GAGAATGCTTCTGTCTAGTTTTTATATGAAGATATTCCCTT

                         *  *   **                *  **   *    *             
     283514 TTCCAACGAAATCCTCCAAGGTATCCAAATATCCACTTGCATTTTCCACAACAAGAGTGTTTCAA
          1 TTCCAACGAAATCATCAAAGCGATCCAAATATCCACTTCCAGATTCTACAAAAAGAGTGTTTCAA

                          *                 *    *  *                *       
     283579 AACTGCTCTATCAATAGAAATGTTCAACTCCTTTG-GCTGGGTACACACATCACAAACAAGTTTC
         66 AACTGCTCTATCAAAAGAAATGTTCAACT-CTGTGAGTTGAGTACACACATCACAAAGAAGTTTC

                                      **     *        
     283643 TGAGAATGCTTCTGTCTAGTTTTTATGGGAAGACATTCCCTT
        130 TGAGAATGCTTCTGTCTAGTTTTTATATGAAGATATTCCCTT

              *  * *  **          *       *            **                  * 
     283685 TTTCACCAAAGGCATCAAAGCGCTCCAAATGTCCACTTCCAGACACTACAAAAAGAGTGTTTCCA
          1 TTCCAACGAAATCATCAAAGCGATCCAAATATCCACTTCCAGATTCTACAAAAAGAGTGTTTCAA

             **       **   ***                 *    * *  * *         *       
     283750 ACGTGCTCTAAGAAAGCGAATGTTCAACTCTGTGACTTGAATGCAGATATCACAAAGTAGTTTCT
         66 AACTGCTCTATCAAAAGAAATGTTCAACTCTGTGAGTTGAGTACACACATCACAAAGAAGTTTCT

              * *             *     *    *         * 
     283815 GAAAGTGCTTCTGTCTAGATTTTAGATGATGATATTCCCGT
        131 GAGAATGCTTCTGTCTAGTTTTTATATGAAGATATTCCCTT

                           * *   *                *   *                    * 
     283856 TTCCAACGAAATCATTAGAGCTATCCAAATATCCACTTACAGTTTCTACAAAAAGAGTGTTTCCA
          1 TTCCAACGAAATCATCAAAGCGATCCAAATATCCACTTCCAGATTCTACAAAAAGAGTGTTTCAA

                   **         * *    *       *  *                   ***      
     283921 AACTGCTGCATCAAAAGAGAGGTTCCACTCTGTTAGCTGAGTACACACATCACAAACTTGTTTCT
         66 AACTGCTCTATCAAAAGAAATGTTCAACTCTGTGAGTTGAGTACACACATCACAAAGAAGTTTCT

            *     *         *        **         **   
     283986 CAGAATCCTTCTGTCTCGTTTTTATGGGAAGATATTTACTT
        131 GAGAATGCTTCTGTCTAGTTTTTATATGAAGATATTCCCTT

              *  *  * **          *       *     *       *  *  *              
     284027 TTTCACCGTAGGCATCAAAGCGCTCCAAATGTCCACATCCAGATACTCCAGAAAGAGTGTTTCAA
          1 TTCCAACGAAATCATCAAAGCGATCCAAATATCCACTTCCAGATTCTACAAAAAGAGTGTTTCAA

             *         *   * *   *         *        * *  *      *      *     
     284092 ACCTGCTCTATGAAAGGGAATCTTCAACTCTATGAGTTGAATGCAGACATCAGAAAGAAATTTCT
         66 AACTGCTCTATCAAAAGAAATGTTCAACTCTGTGAGTTGAGTACACACATCACAAAGAAGTTTCT

                     *       **          *         **
     284157 GAGAATGCTGCTGTCTACCTTTTAT-T--TGA-ATTCCCGC
        131 GAGAATGCTTCTGTCTAGTTTTTATATGAAGATATTCCCTT

                         *  *    *                *       *                  
     284194 TTCCAACGAAATCCTCCAAGCTATCCAAATATCCACTTGCAGATTCCACAAAAAGAGTGTTTCAA
          1 TTCCAACGAAATCATCAAAGCGATCCAAATATCCACTTCCAGATTCTACAAAAAGAGTGTTTCAA

                            ** *  *            *       *     *     *  *      
     284259 AACTGCTCTCTATCAATGGCAAAGTTCAACTCTGTTAGTTGAGGACACATATCACCAACAAGTTT
         66 AACTG--CTCTATCAAAAGAAATGTTCAACTCTGTGAGTTGAGTACACACATCACAAAGAAGTTT

                               *       **         *    
     284324 CTGAGAATGCTTCTGTCTATTTTTTATGGGAAGATATTTCCTT
        129 CTGAGAATGCTTCTGTCTAGTTTTTATATGAAGATATTCCCTT

              *  *  * ** *    *      *    *          * **                    
     284367 TTTCACCGTAGGCGTCAAGGCGATCGAAATGTCCACTTCCACAAACTACAAAAAGAGTGTTTCAA
          1 TTCCAACGAAATCATCAAAGCGATCCAAATATCCACTTCCAGATTCTACAAAAAGAGTGTTTCAA

             *         *   * **       *    *        * ** * *   **      *     
     284432 ACCTGCTCTATGAAAGGCCATGTTCATCTCTATGAGTTGAATGGAAATATCCGAAAGAAATTTCT
         66 AACTGCTCTATCAAAAGAAATGTTCAACTCTGTGAGTTGAGTACACACATCACAAAGAAGTTTCT

             *       *          *        *         **
     284497 GGGAATGCTGCTGTCTAG-TGTT-TAT-ACGA-ATTCCCGC
        131 GAGAATGCTTCTGTCTAGTTTTTATATGAAGATATTCCCTT

                         *       *                *    *  *                  
     284534 TTCCAACGAAATCCTCAAAGCAATCCAAATATCCACTTGCAGAATCCACAAAAAGAGTGTTTCAA
          1 TTCCAACGAAATCATCAAAGCGATCCAAATATCCACTTCCAGATTCTACAAAAAGAGTGTTTCAA

                          *     *          * *                   *  *        
     284599 AACTGCTCTATCAATAGAAAGGTTCAACTCTTTTAGTTGAGTACACACATCACGAACAAGTTTCT
         66 AACTGCTCTATCAAAAGAAATGTTCAACTCTGTGAGTTGAGTACACACATCACAAAGAAGTTTCT

                            * *      **     **  *    
     284664 GAGAATGCTTCTGTCTGGCTTTTATTGGAAGACGTTTCCTT
        131 GAGAATGCTTCTGTCTAGTTTTTATATGAAGATATTCCCTT

                 * *  **          *       *                * *               
     284705 TT-CACCAAAGGCATCAAAGCGCTCCAAATGTCCACTTCCAGATTCTTCCAAAAGAGTGTTTCAA
          1 TTCCAACGAAATCATCAAAGCGATCCAAATATCCACTTCCAGATTCTACAAAAAGAGTGTTTCAA

             **     * ***  * *                 *    * *  * *     *   *       
     284769 ACGTGCTCAAAGTAAGGGAATGTTCAACTCTGTGACTTGAATGCAGATATCACCAAGTAGTTTCT
         66 AACTGCTCTATCAAAAGAAATGTTCAACTCTGTGAGTTGAGTACACACATCACAAAGAAGTTTCT

            * * *             *     *    *         * 
     284834 AATAGTGCTTCTGTCTAGATTTTAGATGATGATATTCCCGT
        131 GAGAATGCTTCTGTCTAGTTTTTATATGAAGATATTCCCTT

                         *** *   *             *  *   *      *     *       * 
     284875 TTCCAACGAAATCGCTAGAGCTATCCAAATATCCAGTTACAGTTTCTACCAAAAGGGTGTTTCCA
          1 TTCCAACGAAATCATCAAAGCGATCCAAATATCCACTTCCAGATTCTACAAAAAGAGTGTTTCAA

              *    **           *            *       *                     * 
     284940 AATTGCTGCATCAAAAGAAAGGTTCAACTCTGTTAGTTGAGGACACACATCACAAAGAAGTTTGT
         66 AACTGCTCTATCAAAAGAAATGTTCAACTCTGTGAGTTGAGTACACACATCACAAAGAAGTTTCT

                              *    *     *           
     285005 GAGAATGCTTCTGTCTAGATTTTGTATGACGATATTCCCTT
        131 GAGAATGCTTCTGTCTAGTTTTTATATGAAGATATTCCCTT

                     *   * *     *   *       * *  *    *  *       *  *       
     285046 TTCCAACGATATCGTTAAAGCAATCTAAATATCAATTTGCAGAATCCACAAAAATAGAGTTTCAA
          1 TTCCAACGAAATCATCAAAGCGATCCAAATATCCACTTCCAGATTCTACAAAAAGAGTGTTTCAA

             *       * *        *    *       *  *                   ***      
     285111 AGCTGCTCTGTAAAAAGAAAGGTTCCACTCTGTTAGCTGAGTACACACATCACAAACTTGTTTCT
         66 AACTGCTCTATCAAAAGAAATGTTCAACTCTGTGAGTTGAGTACACACATCACAAAGAAGTTTCT

                  *         *        **         **   
     285176 GAGAATCCTTCTGTCTCGTTTTTATGGGAAGATATTTACTT
        131 GAGAATGCTTCTGTCTAGTTTTTATATGAAGATATTCCCTT

                 *  * **          *       *     *       *  *  *              
     285217 TTCCACCGTAGGCATCAAAGCGCTCCAAATGTCCACATCCAGATACTCCAGAAAGAGTGTTTCAA
          1 TTCCAACGAAATCATCAAAGCGATCCAAATATCCACTTCCAGATTCTACAAAAAGAGTGTTTCAA

             *         *   * *   *         *        * *  *      *      *     
     285282 ACCTGCTCTATGAAAGGGAATCTTCAACTCTATGAGTTGAATGCAGACATCAGAAAGAAATTTCT
         66 AACTGCTCTATCAAAAGAAATGTTCAACTCTGTGAGTTGAGTACACACATCACAAAGAAGTTTCT

                     *       **   *      *         **
     285347 GAGAATGCTGCTGTCTACCTTTCAT-T--TGA-ATTCCCGC
        131 GAGAATGCTTCTGTCTAGTTTTTATATGAAGATATTCCCTT

                         *  *    *              * *  **   *    *             
     285384 TTCCAACGAAATCCTCCAAGCTATCCAAATATCCACCTGCATTTTCCACAACAAGAGTGTTTCAA
          1 TTCCAACGAAATCATCAAAGCGATCCAAATATCCACTTCCAGATTCTACAAAAAGAGTGTTTCAA

                          *                 *    *  *                *       
     285449 AACTGCTCTATCAATAGAAATGTTCAACTCCTTTG-GCTGGGTACACACATCACAAACAAGTTTC
         66 AACTGCTCTATCAAAAGAAATGTTCAACT-CTGTGAGTTGAGTACACACATCACAAAGAAGTTTC

                                      **     **       
     285513 TGAGAATGCTTCTGTCTAGTTTTTATGGGAAGACGTTCCCTT
        130 TGAGAATGCTTCTGTCTAGTTTTTATATGAAGATATTCCCTT

              *  * *  **          *       *            **                  * 
     285555 TTTCACCAAAGGCATCAAAGCGCTCCAAATGTCCACTTCCAGACACTACAAAAAGAGTGTTTCCA
          1 TTCCAACGAAATCATCAAAGCGATCCAAATATCCACTTCCAGATTCTACAAAAAGAGTGTTTCAA

             **       **   ***                 *    * *  * *         *       
     285620 ACGTGCTCTAAGAAAGCGAATGTTCAACTCTGTGACTTGAATGCAGATATCACAAAGTAGTTTCT
         66 AACTGCTCTATCAAAAGAAATGTTCAACTCTGTGAGTTGAGTACACACATCACAAAGAAGTTTCT

                **            *     *    *         * 
     285685 GAGAGGGCTTCTGTCTAGATTTTAGATGATGATATTCCCGT
        131 GAGAATGCTTCTGTCTAGTTTTTATATGAAGATATTCCCTT

                           * *   *                *   *                    * 
     285726 TTCCAACGAAATCATTAGAGCTATCCAAATATCCACTTACAGTTTCTACAAAAAGAGTGTTTCCA
          1 TTCCAACGAAATCATCAAAGCGATCCAAATATCCACTTCCAGATTCTACAAAAAGAGTGTTTCAA

                   **         * *    *       *  *                   ***      
     285791 AACTGCTGCATCAAAAGAGAGGTTCCACTCTGTTAGCTGAGTACACACATCACAAACTTGTTTCT
         66 AACTGCTCTATCAAAAGAAATGTTCAACTCTGTGAGTTGAGTACACACATCACAAAGAAGTTTCT

            *     *         *        **         **   
     285856 CAGAATCCTTCTGTCTCGTTTTTATGGGAAGATATTTACTT
        131 GAGAATGCTTCTGTCTAGTTTTTATATGAAGATATTCCCTT

              *  *  * **          *       *     *       *  *  *              
     285897 TTTCACCGTAGGCATCAAAGCGCTCCAAATGTCCACATCCAGATACTCCAGAAAGAGTGTTTCAA
          1 TTCCAACGAAATCATCAAAGCGATCCAAATATCCACTTCCAGATTCTACAAAAAGAGTGTTTCAA

             *         *   * *             *        * *  *      *      *     
     285962 ACCTGCTCTATGAAAGGGAATGTTCAACTCTATGAGTTGAATGCAGACATCAGAAAGAAATTTCT
         66 AACTGCTCTATCAAAAGAAATGTTCAACTCTGTGAGTTGAGTACACACATCACAAAGAAGTTTCT

                     *       **          *         **
     286027 GAGAATGCTGCTGTCTACCTTTTAT-T--TGA-ATTCCCGC
        131 GAGAATGCTTCTGTCTAGTTTTTATATGAAGATATTCCCTT

                         *  *    *                *       *                  
     286064 TTCCAACGAAATCCTCCAAGCTATCCAAATATCCACTTGCAGATTCCACAAAAAGAGTGTTTCAA
          1 TTCCAACGAAATCATCAAAGCGATCCAAATATCCACTTCCAGATTCTACAAAAAGAGTGTTTCAA

                            ** *  *            *       *     *     *  *      
     286129 AACTGCTCTCTATCAATGGCAAAGTTCAACTCTGTTAGTTGAGGACACATATCACCAACAAGTTT
         66 AACTG--CTCTATCAAAAGAAATGTTCAACTCTGTGAGTTGAGTACACACATCACAAAGAAGTTT

                               *      ***              
     286194 CTGAGAATGCTTCTGTCTATTTTTTAAGGGAAGATATTCCCTT
        129 CTGAGAATGCTTCTGTCTAGTTTTTATATGAAGATATTCCCTT

              *  *  * ** *    *      *    *       *  * **                    
     286237 TTTCACCGTAGGCGTCAAGGCGATCGAAATGTCCACTTTCACAAACTACAAAAAGAGTGTTTCAA
          1 TTCCAACGAAATCATCAAAGCGATCCAAATATCCACTTCCAGATTCTACAAAAAGAGTGTTTCAA

             *         *   * **       *    *        * ** * *   **      *     
     286302 ACCTGCTCTATGAAAGGCCATGTTCATCTCTATGAGTTGAATGGATATATCCGAAAGAAATTTCT
         66 AACTGCTCTATCAAAAGAAATGTTCAACTCTGTGAGTTGAGTACACACATCACAAAGAAGTTTCT

             *       *          *        *         **
     286367 GGGAATGCTGCTGTCTAG-TGTT-TAT-ACGA-ATTCCCGA
        131 GAGAATGCTTCTGTCTAGTTTTTATATGAAGATATTCCCTT

                         *       *                *    *  *                  
     286404 TTCCAACGAAATCCTCAAAGCAATCCAAATATCCACTTGCAGAATCCACAAAAAGAGTGTTTCAA
          1 TTCCAACGAAATCATCAAAGCGATCCAAATATCCACTTCCAGATTCTACAAAAAGAGTGTTTCAA

                          *                * *                   *  *        
     286469 AACTGCTCTATCAATAGAAATGTTCAACTCTTTTAGTTGAGTACACACATCACGAACAAGTTTCT
         66 AACTGCTCTATCAAAAGAAATGTTCAACTCTGTGAGTTGAGTACACACATCACAAAGAAGTTTCT

                            * *      **     **  *    
     286534 GAGAATGCTTCTGTCTGGCTTTTATTGGAAGACGTTTCCTT
        131 GAGAATGCTTCTGTCTAGTTTTTATATGAAGATATTCCCTT

                 * *  **          *    *  *                * *      *        
     286575 TT-CACCAAAGGCATCAAAGCGCTCCATATGTCCACTTCCAGATTCTTCCAAAAGACTGTTTCAA
          1 TTCCAACGAAATCATCAAAGCGATCCAAATATCCACTTCCAGATTCTACAAAAAGAGTGTTTCAA

             **     * ***  * *                 *    * *  * *     *   *       
     286639 ACGTGCTCAAAGTAAGGGAATGTTCAACTCTGTGACTTGAATGCAGATATCACCAAGTAGTTTCT
         66 AACTGCTCTATCAAAAGAAATGTTCAACTCTGTGAGTTGAGTACACACATCACAAAGAAGTTTCT

            * * *             *     *    *         * 
     286704 AATAGTGCTTCTGTCTAGATTTTAGATGATGATATTCCCGT
        131 GAGAATGCTTCTGTCTAGTTTTTATATGAAGATATTCCCTT

                         * * *   *             *  *   *      *     *       * 
     286745 TTCCAACGAAATCGTTAGAGCTATCCAAATATCCAGTTACAGTTTCTACCAAAAGGGTGTTTCCA
          1 TTCCAACGAAATCATCAAAGCGATCCAAATATCCACTTCCAGATTCTACAAAAAGAGTGTTTCAA

              *    **           *            *       *                     * 
     286810 AATTGCTGCATCAAAAGAAAGGTTCAACTCTGTTAGTTGAGGACACACATCACAAAGAAGTTTGT
         66 AACTGCTCTATCAAAAGAAATGTTCAACTCTGTGAGTTGAGTACACACATCACAAAGAAGTTTCT

                              *    *     *           
     286875 GAGAATGCTTCTGTCTAGATTTTGTATGACGATATTCCCTT
        131 GAGAATGCTTCTGTCTAGTTTTTATATGAAGATATTCCCTT

                     *   * *     *   *       * *  *    *  *       *  *       
     286916 TTCCAACGATATCGTTAAAGCAATCTAAATATCAATTTGCAGAATCCACAAAAATAGAGTTTCAA
          1 TTCCAACGAAATCATCAAAGCGATCCAAATATCCACTTCCAGATTCTACAAAAAGAGTGTTTCAA

             *       * *        *    *       *  *                   ***      
     286981 AGCTGCTCTGTAAAAAGAAAGGTTCCACTCTGTTAGCTGAGTACACACATCACAAACTTGTTTCT
         66 AACTGCTCTATCAAAAGAAATGTTCAACTCTGTGAGTTGAGTACACACATCACAAAGAAGTTTCT

                  *         *        **         **   
     287046 GAGAATCCTTCTGTCTCGTTTTTATGGGAAGATATTTACTT
        131 GAGAATGCTTCTGTCTAGTTTTTATATGAAGATATTCCCTT

                 *  * **          *       *     *       *  *  *              
     287087 TTCCACCGTAGGCATCAAAGCGCTCCAAATGTCCACATCCAGATACTCCAGAAAGAGTGTTTCAA
          1 TTCCAACGAAATCATCAAAGCGATCCAAATATCCACTTCCAGATTCTACAAAAAGAGTGTTTCAA

             *         *   * *   *         *        * *  *      *      *     
     287152 ACCTGCTCTATGAAAGGGAATCTTCAACTCTATGAGTTGAATGCAGACATCAGAAAGAAATTTCT
         66 AACTGCTCTATCAAAAGAAATGTTCAACTCTGTGAGTTGAGTACACACATCACAAAGAAGTTTCT

                     *       **          *         **
     287217 GAGAATGCTGCTGTCTACCTTTTAT-T--TGA-ATTCCCGC
        131 GAGAATGCTTCTGTCTAGTTTTTATATGAAGATATTCCCTT

                         *  *    *              * *  **   *                  
     287254 TTCCAACGAAATCCTCCAAGCTATCCAAATATCCACCTGCATTTTCCACAAAAAGAGTGTTTCAA
          1 TTCCAACGAAATCATCAAAGCGATCCAAATATCCACTTCCAGATTCTACAAAAAGAGTGTTTCAA

                          *                 *    *  *                *       
     287319 AACTGCTCTATCAATAGAAATGTTCAACTCCTTTG-GCTGGGTACACACATCACAAACAAGTTTC
         66 AACTGCTCTATCAAAAGAAATGTTCAACT-CTGTGAGTTGAGTACACACATCACAAAGAAGTTTC

                                      **     **       
     287383 TGAGAATGCTTCTGTCTAGTTTTTATGGGAAGACGTTCCCTT
        130 TGAGAATGCTTCTGTCTAGTTTTTATATGAAGATATTCCCTT

              *  * *  **          *       *            **                    
     287425 TTTCACCAAAGGCATCAAAGCGCTCCAAATGTCCACTTCCAGACACTACAAAAAGAGTGTTTCAA
          1 TTCCAACGAAATCATCAAAGCGATCCAAATATCCACTTCCAGATTCTACAAAAAGAGTGTTTCAA

             **       **   ***                 *    * *  * *         *       
     287490 ACGTGCTCTAAGAAAGCGAATGTTCAACTCTGTGACTTGAATGCAGATATCACAAAGTAGTTTCT
         66 AACTGCTCTATCAAAAGAAATGTTCAACTCTGTGAGTTGAGTACACACATCACAAAGAAGTTTCT

                **            *     *    *         * 
     287555 GAGAGGGCTTCTGTCTAGATTTTAGATGATGATATTCCCGT
        131 GAGAATGCTTCTGTCTAGTTTTTATATGAAGATATTCCCTT

                           * *   *                *   *                    * 
     287596 TTCCAACGAAATCATTAGAGCTATCCAAATATCCACTTACAGTTTCTACAAAAAGAGTGTTTCCA
          1 TTCCAACGAAATCATCAAAGCGATCCAAATATCCACTTCCAGATTCTACAAAAAGAGTGTTTCAA

                   **         * *    *       *  *                   ***      
     287661 AACTGCTGCATCAAAAGAGAGGTTCCACTCTGTTAGCTGAGTACACACATCACAAACTTGTTTCT
         66 AACTGCTCTATCAAAAGAAATGTTCAACTCTGTGAGTTGAGTACACACATCACAAAGAAGTTTCT

            *     * *       *        **         **   
     287726 CAGAATCCGTCTGTCTCGTTTTTATGGGAAGATATTTACTT
        131 GAGAATGCTTCTGTCTAGTTTTTATATGAAGATATTCCCTT

              *  *  * **          *       *     *       *  *  *              
     287767 TTTCACCGTAGGCATCAAAGCGCTCCAAATGTCCACATCCAGATACTCCAGAAAGAGTGTTTCAA
          1 TTCCAACGAAATCATCAAAGCGATCCAAATATCCACTTCCAGATTCTACAAAAAGAGTGTTTCAA

             *         *   * *             *        * *  *      *      *     
     287832 ACCTGCTCTATGAAAGGGAATGTTCAACTCTATGAGTTGAATGCAGACATCAGAAAGAAATTTCT
         66 AACTGCTCTATCAAAAGAAATGTTCAACTCTGTGAGTTGAGTACACACATCACAAAGAAGTTTCT

                     *       **          *         **
     287897 GAGAATGCTGCTGTCTACCTTTTAT-T--TGA-ATTCCCGC
        131 GAGAATGCTTCTGTCTAGTTTTTATATGAAGATATTCCCTT

                         *  *    *                *       *                  
     287934 TTCCAACGAAATCCTCCAAGCTATCCAAATATCCACTTGCAGATTCCACAAAAAGAGTGTTTCAA
          1 TTCCAACGAAATCATCAAAGCGATCCAAATATCCACTTCCAGATTCTACAAAAAGAGTGTTTCAA

                            ** *  *            *       *     *     *  *      
     287999 AACTGCTCTCTATCAATGGCAAAGTTCAACTCTGTTAGTTGAGGACACATATCACCAACAAGTTT
         66 AACTG--CTCTATCAAAAGAAATGTTCAACTCTGTGAGTTGAGTACACACATCACAAAGAAGTTT

                               *       **         *    
     288064 CTGAGAATGCTTCTGTCTATTTTTTATGGGAAGATATTTCCTT
        129 CTGAGAATGCTTCTGTCTAGTTTTTATATGAAGATATTCCCTT

              *  *  * ** *    *      *    *          * **                    
     288107 TTTCACCGTAGGCGTCAAGGCGATCGAAATGTCCACTTCCACAAACTACAAAAAGAGTGTTTCAA
          1 TTCCAACGAAATCATCAAAGCGATCCAAATATCCACTTCCAGATTCTACAAAAAGAGTGTTTCAA

             *         *   * **       *    *        * ** * *   **      *     
     288172 ACCTGCTCTATGAAAGGCCATGTTCATCTCTATGAGTTGAATGGAAATATCCGAAAGAAATTTCT
         66 AACTGCTCTATCAAAAGAAATGTTCAACTCTGTGAGTTGAGTACACACATCACAAAGAAGTTTCT

             *       *                             **
     288237 GGGAATGCTGCTGTCTAGTTTTTATAT---GA-ATTCCCGC
        131 GAGAATGCTTCTGTCTAGTTTTTATATGAAGATATTCCCTT

                         *       *                *    *  *                  
     288274 TTCCAACGAAATCCTCAAAGCAATCCAAATATCCACTTGCAGAATCCACAAAAAGAGTGTTTCAA
          1 TTCCAACGAAATCATCAAAGCGATCCAAATATCCACTTCCAGATTCTACAAAAAGAGTGTTTCAA

                          *     *          * *                      *        
     288339 AACTGCTCTATCAATAGAAAGGTTCAACTCTTTTAGTTGAGTACACACATCACAAACAAGTTTCT
         66 AACTGCTCTATCAAAAGAAATGTTCAACTCTGTGAGTTGAGTACACACATCACAAAGAAGTTTCT

                            * *      **     **  *    
     288404 GAGAATGCTTCTGTCTGGCTTTTATTGGAAGACGTTTCCTT
        131 GAGAATGCTTCTGTCTAGTTTTTATATGAAGATATTCCCTT

                 * *  **          *       *                * *               
     288445 TT-CACCAAAGGCATCAAAGCGCTCCAAATGTCCACTTCCAGATTCTTCCAAAAGAGTGTTTCAA
          1 TTCCAACGAAATCATCAAAGCGATCCAAATATCCACTTCCAGATTCTACAAAAAGAGTGTTTCAA

             **     * ***  * *                 *    * *  * *     *   *  *    
     288509 ACGTGCTCAAAGTAAGGGAATGTTCAACTCTGTGACTTGAATGCAGATATCACCAAGTAGCTTCT
         66 AACTGCTCTATCAAAAGAAATGTTCAACTCTGTGAGTTGAGTACACACATCACAAAGAAGTTTCT

            * * *             *     *    *         * 
     288574 AATAGTGCTTCTGTCTAGATTTTAGATGATGATATTCCCGT
        131 GAGAATGCTTCTGTCTAGTTTTTATATGAAGATATTCCCTT

                         * * *   *             *  *   *      *     *       * 
     288615 TTCCAACGAAATCGTTAGAGCTATCCAAATATCCAGTTACAGTTTCTACCAAAAGGGTGTTTCCA
          1 TTCCAACGAAATCATCAAAGCGATCCAAATATCCACTTCCAGATTCTACAAAAAGAGTGTTTCAA

              *    **           *            *       *                     * 
     288680 AATTGCTGCATCAAAAGAAAGGTTCAACTCTGTTAGTTGAGGACACACATCACAAAGAAGTTTGT
         66 AACTGCTCTATCAAAAGAAATGTTCAACTCTGTGAGTTGAGTACACACATCACAAAGAAGTTTCT

                              *    *     *           
     288745 GAGAATGCTTCTGTCTAGATTTTGTATGACGATATTCCCTT
        131 GAGAATGCTTCTGTCTAGTTTTTATATGAAGATATTCCCTT

                     *   * *     *   *       * *  *    *  *       *  *       
     288786 TTCCAACGATATCGTTAAAGCAATCTAAATATCAATTTGCAGAATCCACAAAAATAGAGTTTCAA
          1 TTCCAACGAAATCATCAAAGCGATCCAAATATCCACTTCCAGATTCTACAAAAAGAGTGTTTCAA

             *       * *        *    *       *  *                   ***      
     288851 AGCTGCTCTGTAAAAAGAAAGGTTCCACTCTGTTAGCTGAGTACACACATCACAAACTTGTTTCT
         66 AACTGCTCTATCAAAAGAAATGTTCAACTCTGTGAGTTGAGTACACACATCACAAAGAAGTTTCT

                  *         *        **         **   
     288916 GAGAATCCTTCTGTCTCGTTTTTATGGGAAGATATTTACTT
        131 GAGAATGCTTCTGTCTAGTTTTTATATGAAGATATTCCCTT

                 *  * **          *       *     *       *  *  *  *           
     288957 TTCCACCGTAGGCATCAAAGCGCTCCAAATGTCCACATCCAGATACTCCAGAACGAGTGTTTCAA
          1 TTCCAACGAAATCATCAAAGCGATCCAAATATCCACTTCCAGATTCTACAAAAAGAGTGTTTCAA

             *         *   * *   *         *        * *  *      *      *     
     289022 ACCTGCTCTATGAAAGGGAATCTTCAACTCTATGAGTTGAATGCAGACATCAGAAAGAAATTTCT
         66 AACTGCTCTATCAAAAGAAATGTTCAACTCTGTGAGTTGAGTACACACATCACAAAGAAGTTTCT

                     *       **          *         **
     289087 GAGAATGCTGCTGTCTACCTTTTAT-T--TGA-ATTCCCGC
        131 GAGAATGCTTCTGTCTAGTTTTTATATGAAGATATTCCCTT

                         *  *    *              * *  **   *                  
     289124 TTCCAACGAAATCCTCCAAGCTATCCAAATATCCACCTGCATTTTCCACAAAAAGAGTGTTTCAA
          1 TTCCAACGAAATCATCAAAGCGATCCAAATATCCACTTCCAGATTCTACAAAAAGAGTGTTTCAA

                          *                 *    *  *                *       
     289189 AACTGCTCTATCAATAGAAATGTTCAACTCCTTTG-GCTGGGTACACACATCACAAACAAGTTTC
         66 AACTGCTCTATCAAAAGAAATGTTCAACT-CTGTGAGTTGAGTACACACATCACAAAGAAGTTTC

                                      **     **       
     289253 TGAGAATGCTTCTGTCTAGTTTTTATGGGAAGACGTTCCCTT
        130 TGAGAATGCTTCTGTCTAGTTTTTATATGAAGATATTCCCTT

              *  * *  **          *       *            **                    
     289295 TTTCACCAAAGGCATCAAAGCGCTCCAAATGTCCACTTCCAGACACTACAAAAAGAGTGTTTCAA
          1 TTCCAACGAAATCATCAAAGCGATCCAAATATCCACTTCCAGATTCTACAAAAAGAGTGTTTCAA

             **       **   ***            *    *    * *  * *         *       
     289360 ACGTGCTCTAAGAAAGCGAATGTTCAACTCGGTGACTTGAATGCAGATATCACAAAGTAGTTTCT
         66 AACTGCTCTATCAAAAGAAATGTTCAACTCTGTGAGTTGAGTACACACATCACAAAGAAGTTTCT

                **            *     *    *         * 
     289425 GAGAGGGCTTCTGTCTAGATTTTAGATGATGATATTCCCGT
        131 GAGAATGCTTCTGTCTAGTTTTTATATGAAGATATTCCCTT

                             *   *                *   *                    * 
     289466 TTCCAACG-AA--ATCAGAGCTATCCAAATATCCACTTACAGTTTCTACAAAAAGAGTGTTTCCA
          1 TTCCAACGAAATCATCAAAGCGATCCAAATATCCACTTCCAGATTCTACAAAAAGAGTGTTTCAA

                   **         * *    *       *  *                   ***      
     289528 AACTGCTGCATCAAAAGAGAGGTTCCACTCTGTTAGCTGAGTACACACATCACAAACTTGTTTCT
         66 AACTGCTCTATCAAAAGAAATGTTCAACTCTGTGAGTTGAGTACACACATCACAAAGAAGTTTCT

            *   * *         *        **         **   
     289593 CAGATTCCTTCTGTCTCGTTTTTATGGGAAGATATTTACTT
        131 GAGAATGCTTCTGTCTAGTTTTTATATGAAGATATTCCCTT

              *  *  * **          *       *     *      **  *  *              
     289634 TTTCACCGTAGGCATCAAAGCGCTCCAAATGTCCACATCCAGAAACTCCAGAAAGAGTGTTTCAA
          1 TTCCAACGAAATCATCAAAGCGATCCAAATATCCACTTCCAGATTCTACAAAAAGAGTGTTTCAA

             *         *   * *             *        * *  *      *      *     
     289699 ACCTGCTCTATGAAAGGGAATGTTCAACTCTATGAGTTGAATGCAGACATCAGAAAGAAATTTCT
         66 AACTGCTCTATCAAAAGAAATGTTCAACTCTGTGAGTTGAGTACACACATCACAAAGAAGTTTCT

                     *       **          *         **
     289764 GAGAATGCTGCTGTCTACCTTTTAT-T--TGA-ATTCCCAC
        131 GAGAATGCTTCTGTCTAGTTTTTATATGAAGATATTCCCTT

                         *  *    *                *       *                  
     289801 TTCCAACGAAATCCTCCAAGCTATCCAAATATCCACTTGCAGATTCCACAAAAAGAGTGTTTCAA
          1 TTCCAACGAAATCATCAAAGCGATCCAAATATCCACTTCCAGATTCTACAAAAAGAGTGTTTCAA

                            ** *  *            *       *     *     *  *      
     289866 AACTGCTCTCTATCAATGGCAAAGTTCAACTCTGTTAGTTGAGGACACATATCACCAACAAGTTT
         66 AACTG--CTCTATCAAAAGAAATGTTCAACTCTGTGAGTTGAGTACACACATCACAAAGAAGTTT

                               *       **         *    
     289931 CTGAGAATGCTTCTGTCTATTTTTTATGGGAAGATATTTCCTT
        129 CTGAGAATGCTTCTGTCTAGTTTTTATATGAAGATATTCCCTT

              *  *  * ** *    *      *    *          * **    *               
     289974 TTTCAGCGTAGGCGTCAAGGCGATCAAAATGTCCACTTCCACAAACTACCAAAAGAGTGTTTCAA
          1 TTCCAACGAAATCATCAAAGCGATCCAAATATCCACTTCCAGATTCTACAAAAAGAGTGTTTCAA

             *         *   * **       *    *        * ** * *   **      *     
     290039 ACCTGCTCTATGAAAGGCCATGTTCATCTCTATGAGTTGAATGGAAATATCCGAAAGAAATTTCT
         66 AACTGCTCTATCAAAAGAAATGTTCAACTCTGTGAGTTGAGTACACACATCACAAAGAAGTTTCT

             *       *                             **
     290104 GGGAATGCTGCTGTCTAGTTTTTATAT---GA-ATTCCCGC
        131 GAGAATGCTTCTGTCTAGTTTTTATATGAAGATATTCCCTT

                 *       *       *                *    *  *                  
     290141 TTCCACCGAAATCCTCAAAGCAATCCAAATATCCACTTGCAGAATCCACAAAAAGAGTGTTTCAA
          1 TTCCAACGAAATCATCAAAGCGATCCAAATATCCACTTCCAGATTCTACAAAAAGAGTGTTTCAA

                  *       *     *          * *                      *        
     290206 AACTGCGCTATCAATAGAAAGGTTCAACTCTTTTAGTTGAGTACACACATCACAAACAAGTTTCT
         66 AACTGCTCTATCAAAAGAAATGTTCAACTCTGTGAGTTGAGTACACACATCACAAAGAAGTTTCT

                            * *      **     **  *    
     290271 GAGAATGCTTCTGTCTGGCTTTTATTGGAAGACGTTTCCTT
        131 GAGAATGCTTCTGTCTAGTTTTTATATGAAGATATTCCCTT

                 * *  **          *       *                * *               
     290312 TT-CACCAAAGGCATCAAAGCGCTCCAAATGTCCACTTCCAGATTCTTCCAAAAGAGTGTTTCAA
          1 TTCCAACGAAATCATCAAAGCGATCCAAATATCCACTTCCAGATTCTACAAAAAGAGTGTTTCAA

             **     * ***  * *                 *    * *  * *     *   *       
     290376 ACGTGCTCAAAGTAAGGGAATGTTCAACTCTGTGACTTGAATGCAGATATCACCAAGTAGTTTCT
         66 AACTGCTCTATCAAAAGAAATGTTCAACTCTGTGAGTTGAGTACACACATCACAAAGAAGTTTCT

            * * *             *     *    *         * 
     290441 AATAGTGCTTCTGTCTAGATTTTAGATGATGATATTCCCGT
        131 GAGAATGCTTCTGTCTAGTTTTTATATGAAGATATTCCCTT

                         *** *   *             *  *   *      *     *       * 
     290482 TTCCAACGAAATCGCTAGAGCTATCCAAATATCCAGTTACAGTTTCTACCAAAAGGGTGTTTCCA
          1 TTCCAACGAAATCATCAAAGCGATCCAAATATCCACTTCCAGATTCTACAAAAAGAGTGTTTCAA

              *    **           *            *       *                     * 
     290547 AATTGCTGCATCAAAAGAAAGGTTCAACTCTGTTAGTTGAGGACACACATCACAAAGAAGTTTGT
         66 AACTGCTCTATCAAAAGAAATGTTCAACTCTGTGAGTTGAGTACACACATCACAAAGAAGTTTCT

                              *    *     *           
     290612 GAGAATGCTTCTGTCTAGATTTTGTATGACGATATTCCCTT
        131 GAGAATGCTTCTGTCTAGTTTTTATATGAAGATATTCCCTT

                     *   * *     *   *       * *  *    *  *       *  *       
     290653 TTCCAACGATATCGTTAAAGCAATCTAAATATCAATTTGCAGAATCCACAAAAATAGAGTTTCAA
          1 TTCCAACGAAATCATCAAAGCGATCCAAATATCCACTTCCAGATTCTACAAAAAGAGTGTTTCAA

             *       * *        *    *       *  *                   ***      
     290718 AGCTGCTCTGTAAAAAGAAAGGTTCCACTCTGTTAGCTGAGTACACACATCACAAACTTGTTTCT
         66 AACTGCTCTATCAAAAGAAATGTTCAACTCTGTGAGTTGAGTACACACATCACAAAGAAGTTTCT

                  *         *        **         **   
     290783 GAGAATCCTTCTGTCTCGTTTTTATGGGAAGATATTTACTT
        131 GAGAATGCTTCTGTCTAGTTTTTATATGAAGATATTCCCTT

                 *  * **          *       *     *       *  *  *              
     290824 TTCCACCGTAGGCATCAAAGCGCTCCAAATGTCCACATCCAGATACTCCAGAAAGAGTGTTTCAA
          1 TTCCAACGAAATCATCAAAGCGATCCAAATATCCACTTCCAGATTCTACAAAAAGAGTGTTTCAA

             *         *   * *   *         *        * *  *      *      *     
     290889 ACCTGCTCTATGAAAGGGAATCTTCAACTCTATGAGTTGAATGCAGACATCAGAAAGAAATTTCT
         66 AACTGCTCTATCAAAAGAAATGTTCAACTCTGTGAGTTGAGTACACACATCACAAAGAAGTTTCT

                     *       **   *      *         **
     290954 GAGAATGCTGCTGTCTACCTTTCAT-T--TGA-ATTCCCGC
        131 GAGAATGCTTCTGTCTAGTTTTTATATGAAGATATTCCCTT

                         *  *    *              * *  **   *    *             
     290991 TTCCAACGAAATCCTCCAAGCTATCCAAATATCCACCTGCATTTTCCACAACAAGAGTGTTTCAA
          1 TTCCAACGAAATCATCAAAGCGATCCAAATATCCACTTCCAGATTCTACAAAAAGAGTGTTTCAA

                          *                 *    *  *                *       
     291056 AACTGCTCTATCAATAGAAATGTTCAACTCCTTTG-GCTGGGTACACACATCACAAACAAGTTTC
         66 AACTGCTCTATCAAAAGAAATGTTCAACT-CTGTGAGTTGAGTACACACATCACAAAGAAGTTTC

                                      **     **       
     291120 TGAGAATGCTTCTGTCTAGTTTTTATGGGAAGACGTTCCCTT
        130 TGAGAATGCTTCTGTCTAGTTTTTATATGAAGATATTCCCTT

              *  * *  **          *       *            **                  * 
     291162 TTTCACCAAAGGCATCAAAGCGCTCCAAATGTCCACTTCCAGACACTACAAAAAGAGTGTTTCCA
          1 TTCCAACGAAATCATCAAAGCGATCCAAATATCCACTTCCAGATTCTACAAAAAGAGTGTTTCAA

             **       **   ***                 *    * *  * *         *       
     291227 ACGTGCTCTAAGAAAGCGAATGTTCAACTCTGTGACTTGAATGCAGATATCACAAAGTAGTTTCT
         66 AACTGCTCTATCAAAAGAAATGTTCAACTCTGTGAGTTGAGTACACACATCACAAAGAAGTTTCT

                **            *     *    *         * 
     291292 GAGAGGGCTTCTGTCTAGATTTTAGATGATGATATTCCCGT
        131 GAGAATGCTTCTGTCTAGTTTTTATATGAAGATATTCCCTT

                           * *   *                *   *                    * 
     291333 TTCCAACGAAATCATTAGAGCTATCCAAATATCCACTTACAGTTTCTACAAAAAGAGTGTTTCCA
          1 TTCCAACGAAATCATCAAAGCGATCCAAATATCCACTTCCAGATTCTACAAAAAGAGTGTTTCAA

                   **         * *    *       *  *                   ***      
     291398 AACTGCTGCATCAAAAGAGAGGTTCCACTCTGTTAGCTGAGTACACACATCACAAACTTGTTTCT
         66 AACTGCTCTATCAAAAGAAATGTTCAACTCTGTGAGTTGAGTACACACATCACAAAGAAGTTTCT

            *     *         *        **         **   
     291463 CAGAATCCTTCTGTCTCGTTTTTATGGGAAGATATTTACTT
        131 GAGAATGCTTCTGTCTAGTTTTTATATGAAGATATTCCCTT

              *  *  * **          *       *     *       *  *  *              
     291504 TTTCACCGTAGGCATCAAAGCGCTCCAAATGTCCACATCCAGATACTCCAGAAAGAGTGTTTCAA
          1 TTCCAACGAAATCATCAAAGCGATCCAAATATCCACTTCCAGATTCTACAAAAAGAGTGTTTCAA

             *         *   * *             *        * *  *      *      *     
     291569 ACCTGCTCTATGAAAGGGAATGTTCAACTCTATGAGTTGAATGCAGACATCAGAAAGAAATTTCT
         66 AACTGCTCTATCAAAAGAAATGTTCAACTCTGTGAGTTGAGTACACACATCACAAAGAAGTTTCT

                     *       **          *         **
     291634 GAGAATGCTGCTGTCTACCTTTTAT-T--TGA-ATTCCCGC
        131 GAGAATGCTTCTGTCTAGTTTTTATATGAAGATATTCCCTT

                         *  *    *                *       *                  
     291671 TTCCAACGAAATCCTCCAAGCTATCCAAATATCCACTTGCAGATTCCACAAAAAGAGTGTTTCAA
          1 TTCCAACGAAATCATCAAAGCGATCCAAATATCCACTTCCAGATTCTACAAAAAGAGTGTTTCAA

                            ** *  *            *       *     *     *  *      
     291736 AACTGCTCTCTATCAATGGCAAAGTTCAACTCTGTTAGTTGAGGACACATATCACCAACAAGTTT
         66 AACTG--CTCTATCAAAAGAAATGTTCAACTCTGTGAGTTGAGTACACACATCACAAAGAAGTTT

                               *      ***              
     291801 CTGAGAATGCTTCTGTCTATTTTTTAAGGGAAGATATTCCCTT
        129 CTGAGAATGCTTCTGTCTAGTTTTTATATGAAGATATTCCCTT

              *  *  * ** *    *      *    *       *  * **                    
     291844 TTTCACCGTAGGCGTCAAGGCGATCGAAATGTCCACTTTCACAAACTACAAAAAGAGTGTTTCAA
          1 TTCCAACGAAATCATCAAAGCGATCCAAATATCCACTTCCAGATTCTACAAAAAGAGTGTTTCAA

             *         *   * **       *    *        * ** * *   **      *     
     291909 ACCTGCTCTATGAAAGGCCATGTTCATCTCTATGAGTTGAATGGATATATCCGAAAGAAATTTCT
         66 AACTGCTCTATCAAAAGAAATGTTCAACTCTGTGAGTTGAGTACACACATCACAAAGAAGTTTCT

             *       *          *        *         **
     291974 GGGAATGCTGCTGTCTAG-TGTT-TAT-ACGA-ATTCCCGA
        131 GAGAATGCTTCTGTCTAGTTTTTATATGAAGATATTCCCTT

                         *       *                *    *  *                  
     292011 TTCCAACGAAATCCTCAAAGCAATCCAAATATCCACTTGCAGAATCCACAAAAAGAGTGTTTCAA
          1 TTCCAACGAAATCATCAAAGCGATCCAAATATCCACTTCCAGATTCTACAAAAAGAGTGTTTCAA

                          *                * *                   *  *        
     292076 AACTGCTCTATCAATAGAAATGTTCAACTCTTTTAGTTGAGTACACACATCACGAACAAGTTTCT
         66 AACTGCTCTATCAAAAGAAATGTTCAACTCTGTGAGTTGAGTACACACATCACAAAGAAGTTTCT

                            * *      **     **  *    
     292141 GAGAATGCTTCTGTCTGGCTTTTATTGGAAGACGTTTCCTT
        131 GAGAATGCTTCTGTCTAGTTTTTATATGAAGATATTCCCTT

                 * *  **          *    *  *                * *      *        
     292182 TT-CACCAAAGGCATCAAAGCGCTCCATATGTCCACTTCCAGATTCTTCCAAAAGACTGTTTCAA
          1 TTCCAACGAAATCATCAAAGCGATCCAAATATCCACTTCCAGATTCTACAAAAAGAGTGTTTCAA

             **     * ***  * *                 *    * *  * *     *   *       
     292246 ACGTGCTCAAAGTAAGGGAATGTTCAACTCTGTGACTTGAATGCAGATATCACCAAGTAGTTTCT
         66 AACTGCTCTATCAAAAGAAATGTTCAACTCTGTGAGTTGAGTACACACATCACAAAGAAGTTTCT

            * * *             *     *    *         * 
     292311 AATAGTGCTTCTGTCTAGATTTTAGATGATGATATTCCCGT
        131 GAGAATGCTTCTGTCTAGTTTTTATATGAAGATATTCCCTT

                         * * *   *             *  *   *      *     *       * 
     292352 TTCCAACGAAATCGTTAGAGCTATCCAAATATCCAGTTACAGTTTCTACCAAAAGGGTGTTTCCA
          1 TTCCAACGAAATCATCAAAGCGATCCAAATATCCACTTCCAGATTCTACAAAAAGAGTGTTTCAA

              *    **           *            *       *                     * 
     292417 AATTGCTGCATCAAAAGAAAGGTTCAACTCTGTTAGTTGAGGACACACATCACAAAGAAGTTTGT
         66 AACTGCTCTATCAAAAGAAATGTTCAACTCTGTGAGTTGAGTACACACATCACAAAGAAGTTTCT

                              *    *     *           
     292482 GAGAATGCTTCTGTCTAGATTTTGTATGACGATATTCCCTT
        131 GAGAATGCTTCTGTCTAGTTTTTATATGAAGATATTCCCTT

                     *   * *     *   *       * *  *    *  *       *  *       
     292523 TTCCAACGATATCGTTAAAGCAATCTAAATATCAATTTGCAGAATCCACAAAAATAGAGTTTCAA
          1 TTCCAACGAAATCATCAAAGCGATCCAAATATCCACTTCCAGATTCTACAAAAAGAGTGTTTCAA

             *       * *        *    *       *  *                   ***      
     292588 AGCTGCTCTGTAAAAAGAAAGGTTCCACTCTGTTAGCTGAGTACACACATCACAAACTTGTTTCT
         66 AACTGCTCTATCAAAAGAAATGTTCAACTCTGTGAGTTGAGTACACACATCACAAAGAAGTTTCT

                  *         *        **         **   
     292653 GAGAATCCTTCTGTCTCGTTTTTATGGGAAGATATTTACTT
        131 GAGAATGCTTCTGTCTAGTTTTTATATGAAGATATTCCCTT

                 *  * **          *       *     *       *  *  *              
     292694 TTCCACCGTAGGCATCAAAGCGCTCCAAATGTCCACATCCAGATACTCCAGAAAGAGTGTTTCAA
          1 TTCCAACGAAATCATCAAAGCGATCCAAATATCCACTTCCAGATTCTACAAAAAGAGTGTTTCAA

             *         *   * *   *         *        * *  *      *      *     
     292759 ACCTGCTCTATGAAAGGGAATCTTCAACTCTATGAGTTGAATGCAGACATCAGAAAGAAATTTCT
         66 AACTGCTCTATCAAAAGAAATGTTCAACTCTGTGAGTTGAGTACACACATCACAAAGAAGTTTCT

                     *       **          *         **
     292824 GAGAATGCTGCTGTCTACCTTTTAT-T--TGA-ATTCCCGC
        131 GAGAATGCTTCTGTCTAGTTTTTATATGAAGATATTCCCTT

                         *  *    *              * *  **   *                  
     292861 TTCCAACGAAATCCTCCAAGCTATCCAAATATCCACCTGCATTTTCCACAAAAAGAGTGTTTCAA
          1 TTCCAACGAAATCATCAAAGCGATCCAAATATCCACTTCCAGATTCTACAAAAAGAGTGTTTCAA

                          *                 *    *  *                *       
     292926 AACTGCTCTATCAATAGAAATGTTCAACTCCTTTG-GCTGGGTACACACATCACAAACAAGTTTC
         66 AACTGCTCTATCAAAAGAAATGTTCAACT-CTGTGAGTTGAGTACACACATCACAAAGAAGTTTC

                                      **     **       
     292990 TGAGAATGCTTCTGTCTAGTTTTTATGGGAAGACGTTCCCTT
        130 TGAGAATGCTTCTGTCTAGTTTTTATATGAAGATATTCCCTT

              *  * *  **          *       *            **                    
     293032 TTTCACCAAAGGCATCAAAGCGCTCCAAATGTCCACTTCCAGACACTACAAAAAGAGTGTTTCAA
          1 TTCCAACGAAATCATCAAAGCGATCCAAATATCCACTTCCAGATTCTACAAAAAGAGTGTTTCAA

             **       **   ***                 *    * *  * *         *       
     293097 ACGTGCTCTAAGAAAGCGAATGTTCAACTCTGTGACTTGAATGCAGATATCACAAAGTAGTTTCT
         66 AACTGCTCTATCAAAAGAAATGTTCAACTCTGTGAGTTGAGTACACACATCACAAAGAAGTTTCT

                **            *     *    *         * 
     293162 GAGAGGGCTTCTGTCTAGATTTTAGATGATGATATTCCCGT
        131 GAGAATGCTTCTGTCTAGTTTTTATATGAAGATATTCCCTT

                           * *   *                *   *                    * 
     293203 TTCCAACGAAATCATTAGAGCTATCCAAATATCCACTTACAGTTTCTACAAAAAGAGTGTTTCCA
          1 TTCCAACGAAATCATCAAAGCGATCCAAATATCCACTTCCAGATTCTACAAAAAGAGTGTTTCAA

                   **         * *    *       *  *                   ***      
     293268 AACTGCTGCATCAAAAGAGAGGTTCCACTCTGTTAGCTGAGTACACACATCACAAACTTGTTTCT
         66 AACTGCTCTATCAAAAGAAATGTTCAACTCTGTGAGTTGAGTACACACATCACAAAGAAGTTTCT

            *     * *       *        **         **   
     293333 CAGAATCCGTCTGTCTCGTTTTTATGGGAAGATATTTACTT
        131 GAGAATGCTTCTGTCTAGTTTTTATATGAAGATATTCCCTT

              *  *  * **          *       *     *       *  *  *              
     293374 TTTCACCGTAGGCATCAAAGCGCTCCAAATGTCCACATCCAGATACTCCAGAAAGAGTGTTTCAA
          1 TTCCAACGAAATCATCAAAGCGATCCAAATATCCACTTCCAGATTCTACAAAAAGAGTGTTTCAA

             *         *   * *             *        * *  *      *      *     
     293439 ACCTGCTCTATGAAAGGGAATGTTCAACTCTATGAGTTGAATGCAGACATCAGAAAGAAATTTCT
         66 AACTGCTCTATCAAAAGAAATGTTCAACTCTGTGAGTTGAGTACACACATCACAAAGAAGTTTCT

                     *       **          *         **
     293504 GAGAATGCTGCTGTCTACCTTTTAT-T--TGA-ATTCCCGC
        131 GAGAATGCTTCTGTCTAGTTTTTATATGAAGATATTCCCTT

                         *  *    *                *       *                  
     293541 TTCCAACGAAATCCTCCAAGCTATCCAAATATCCACTTGCAGATTCCACAAAAAGAGTGTTTCAA
          1 TTCCAACGAAATCATCAAAGCGATCCAAATATCCACTTCCAGATTCTACAAAAAGAGTGTTTCAA

                            ** *  *            *       *     *     *  *      
     293606 AACTGCTCTCTATCAATGGCAAAGTTCAACTCTGTTAGTTGAGGACACATATCACCAACAAGTTT
         66 AACTG--CTCTATCAAAAGAAATGTTCAACTCTGTGAGTTGAGTACACACATCACAAAGAAGTTT

                               *       **         *    
     293671 CTGAGAATGCTTCTGTCTATTTTTTATGGGAAGATATTTCCTT
        129 CTGAGAATGCTTCTGTCTAGTTTTTATATGAAGATATTCCCTT

              *  *  * ** *    *      *    *          * **                    
     293714 TTTCACCGTAGGCGTCAAGGCGATCGAAATGTCCACTTCCACAAACTACAAAAAGAGTGTTTCAA
          1 TTCCAACGAAATCATCAAAGCGATCCAAATATCCACTTCCAGATTCTACAAAAAGAGTGTTTCAA

             *         *   * **       *    *        * ** * *   **      *     
     293779 ACCTGCTCTATGAAAGGCCATGTTCATCTCTATGAGTTGAATGGAAATATCCGAAAGAAATTTCT
         66 AACTGCTCTATCAAAAGAAATGTTCAACTCTGTGAGTTGAGTACACACATCACAAAGAAGTTTCT

             *       *                             **
     293844 GGGAATGCTGCTGTCTAGTTTTTATAT---GA-ATTCCCGC
        131 GAGAATGCTTCTGTCTAGTTTTTATATGAAGATATTCCCTT

                         *       *                *    *  *                  
     293881 TTCCAACGAAATCCTCAAAGCAATCCAAATATCCACTTGCAGAATCCACAAAAAGAGTGTTTCAA
          1 TTCCAACGAAATCATCAAAGCGATCCAAATATCCACTTCCAGATTCTACAAAAAGAGTGTTTCAA

                          *     *          * *                      *        
     293946 AACTGCTCTATCAATAGAAAGGTTCAACTCTTTTAGTTGAGTACACACATCACAAACAAGTTTCT
         66 AACTGCTCTATCAAAAGAAATGTTCAACTCTGTGAGTTGAGTACACACATCACAAAGAAGTTTCT

                            * *      **     **  *    
     294011 GAGAATGCTTCTGTCTGGCTTTTATTGGAAGACGTTTCCTT
        131 GAGAATGCTTCTGTCTAGTTTTTATATGAAGATATTCCCTT

                 * *  **          *       *                * *               
     294052 TT-CACCAAAGGCATCAAAGCGCTCCAAATGTCCACTTCCAGATTCTTCCAAAAGAGTGTTTCAA
          1 TTCCAACGAAATCATCAAAGCGATCCAAATATCCACTTCCAGATTCTACAAAAAGAGTGTTTCAA

             **     * ***  * *                 *    * *  * *     *   *  *    
     294116 ACGTGCTCAAAGTAAGGGAATGTTCAACTCTGTGACTTGAATGCAGATATCACCAAGTAGCTTCT
         66 AACTGCTCTATCAAAAGAAATGTTCAACTCTGTGAGTTGAGTACACACATCACAAAGAAGTTTCT

            * * *             *     *    *         * 
     294181 AATAGTGCTTCTGTCTAGATTTTAGATGATGATATTCCCGT
        131 GAGAATGCTTCTGTCTAGTTTTTATATGAAGATATTCCCTT

                         * * *   *             *  *   *      *     *       * 
     294222 TTCCAACGAAATCGTTAGAGCTATCCAAATATCCAGTTACAGTTTCTACCAAAAGGGTGTTTCCA
          1 TTCCAACGAAATCATCAAAGCGATCCAAATATCCACTTCCAGATTCTACAAAAAGAGTGTTTCAA

              *    **           *            *       *                     * 
     294287 AATTGCTGCATCAAAAGAAAGGTTCAACTCTGTTAGTTGAGGACACACATCACAAAGAAGTTTGT
         66 AACTGCTCTATCAAAAGAAATGTTCAACTCTGTGAGTTGAGTACACACATCACAAAGAAGTTTCT

                              *    *     *           
     294352 GAGAATGCTTCTGTCTAGATTTTGTATGACGATATTCCCTT
        131 GAGAATGCTTCTGTCTAGTTTTTATATGAAGATATTCCCTT

                     *   * *     *   *       * *  *    *  *       *  *       
     294393 TTCCAACGATATCGTTAAAGCAATCTAAATATCAATTTGCAGAATCCACAAAAATAGAGTTTCAA
          1 TTCCAACGAAATCATCAAAGCGATCCAAATATCCACTTCCAGATTCTACAAAAAGAGTGTTTCAA

             *       * *        *    *       *  *                   ***      
     294458 AGCTGCTCTGTAAAAAGAAAGGTTCCACTCTGTTAGCTGAGTACACACATCACAAACTTGTTTCT
         66 AACTGCTCTATCAAAAGAAATGTTCAACTCTGTGAGTTGAGTACACACATCACAAAGAAGTTTCT

                  *         *        **         **   
     294523 GAGAATCCTTCTGTCTCGTTTTTATGGGAAGATATTTACTT
        131 GAGAATGCTTCTGTCTAGTTTTTATATGAAGATATTCCCTT

                 *  * **          *       *     *       *  *  *  *           
     294564 TTCCACCGTAGGCATCAAAGCGCTCCAAATGTCCACATCCAGATACTCCAGAACGAGTGTTTCAA
          1 TTCCAACGAAATCATCAAAGCGATCCAAATATCCACTTCCAGATTCTACAAAAAGAGTGTTTCAA

             *         *   * *   *         *        * *  *      *      *     
     294629 ACCTGCTCTATGAAAGGGAATCTTCAACTCTATGAGTTGAATGCAGACATCAGAAAGAAATTTCT
         66 AACTGCTCTATCAAAAGAAATGTTCAACTCTGTGAGTTGAGTACACACATCACAAAGAAGTTTCT

                     *       **          *         **
     294694 GAGAATGCTGCTGTCTACCTTTTAT-T--TGA-ATTCCCGC
        131 GAGAATGCTTCTGTCTAGTTTTTATATGAAGATATTCCCTT

                         *  *    *              * *  **   *                  
     294731 TTCCAACGAAATCCTCCAAGCTATCCAAATATCCACCTGCATTTTCCACAAAAAGAGTGTTTCAA
          1 TTCCAACGAAATCATCAAAGCGATCCAAATATCCACTTCCAGATTCTACAAAAAGAGTGTTTCAA

                          *                 *    *  *                *       
     294796 AACTGCTCTATCAATAGAAATGTTCAACTCCTTTG-GCTGGGTACACACATCACAAACAAGTTTC
         66 AACTGCTCTATCAAAAGAAATGTTCAACT-CTGTGAGTTGAGTACACACATCACAAAGAAGTTTC

                                      **     **       
     294860 TGAGAATGCTTCTGTCTAGTTTTTATGGGAAGACGTTCCCTT
        130 TGAGAATGCTTCTGTCTAGTTTTTATATGAAGATATTCCCTT

              *  * *  **          *       *            **                    
     294902 TTTCACCAAAGGCATCAAAGCGCTCCAAATGTCCACTTCCAGACACTACAAAAAGAGTGTTTCAA
          1 TTCCAACGAAATCATCAAAGCGATCCAAATATCCACTTCCAGATTCTACAAAAAGAGTGTTTCAA

             **       **   ***            *    *    * *  * *         *       
     294967 ACGTGCTCTAAGAAAGCGAATGTTCAACTCGGTGACTTGAATGCAGATATCACAAAGTAGTTTCT
         66 AACTGCTCTATCAAAAGAAATGTTCAACTCTGTGAGTTGAGTACACACATCACAAAGAAGTTTCT

                **            *     *    *         * 
     295032 GAGAGGGCTTCTGTCTAGATTTTAGATGATGATATTCCCGT
        131 GAGAATGCTTCTGTCTAGTTTTTATATGAAGATATTCCCTT

                             *   *                *   *                    * 
     295073 TTCCAACG-AA--ATCAGAGCTATCCAAATATCCACTTACAGTTTCTACAAAAAGAGTGTTTCCA
          1 TTCCAACGAAATCATCAAAGCGATCCAAATATCCACTTCCAGATTCTACAAAAAGAGTGTTTCAA

                   **         * *    *       *  *                   ***      
     295135 AACTGCTGCATCAAAAGAGAGGTTCCACTCTGTTAGCTGAGTACACACATCACAAACTTGTTTCT
         66 AACTGCTCTATCAAAAGAAATGTTCAACTCTGTGAGTTGAGTACACACATCACAAAGAAGTTTCT

            *   * *         *        **         **   
     295200 CAGATTCCTTCTGTCTCGTTTTTATGGGAAGATATTTACTT
        131 GAGAATGCTTCTGTCTAGTTTTTATATGAAGATATTCCCTT

              *  *  * **          *       *     *      **  *  *              
     295241 TTTCACCGTAGGCATCAAAGCGCTCCAAATGTCCACATCCAGAAACTCCAGAAAGAGTGTTTCAA
          1 TTCCAACGAAATCATCAAAGCGATCCAAATATCCACTTCCAGATTCTACAAAAAGAGTGTTTCAA

             *         *   * *             *        * *  *      *      *     
     295306 ACCTGCTCTATGAAAGGGAATGTTCAACTCTATGAGTTGAATGCAGACATCAGAAAGAAATTTCT
         66 AACTGCTCTATCAAAAGAAATGTTCAACTCTGTGAGTTGAGTACACACATCACAAAGAAGTTTCT

                     *       **          *         **
     295371 GAGAATGCTGCTGTCTACCTTTTAT-T--TGA-ATTCCCAC
        131 GAGAATGCTTCTGTCTAGTTTTTATATGAAGATATTCCCTT

                         *  *    *                *       *                  
     295408 TTCCAACGAAATCCTCCAAGCTATCCAAATATCCACTTGCAGATTCCACAAAAAGAGTGTTTCAA
          1 TTCCAACGAAATCATCAAAGCGATCCAAATATCCACTTCCAGATTCTACAAAAAGAGTGTTTCAA

                            ** *  *            *       *     *     *  *      
     295473 AACTGCTCTCTATCAATGGCAAAGTTCAACTCTGTTAGTTGAGGACACATATCACCAACAAGTTT
         66 AACTG--CTCTATCAAAAGAAATGTTCAACTCTGTGAGTTGAGTACACACATCACAAAGAAGTTT

                               *       **         *    
     295538 CTGAGAATGCTTCTGTCTATTTTTTATGGGAAGATATTTCCTT
        129 CTGAGAATGCTTCTGTCTAGTTTTTATATGAAGATATTCCCTT

              *  *  * ** *    *      *    *          * **    *               
     295581 TTTCAGCGTAGGCGTCAAGGCGATCAAAATGTCCACTTCCACAAACTACCAAAAGAGTGTTTCAA
          1 TTCCAACGAAATCATCAAAGCGATCCAAATATCCACTTCCAGATTCTACAAAAAGAGTGTTTCAA

             *         *   * **       *    *        * ** * *   **      *     
     295646 ACCTGCTCTATGAAAGGCCATGTTCATCTCTATGAGTTGAATGGAAATATCCGAAAGAAATTTCT
         66 AACTGCTCTATCAAAAGAAATGTTCAACTCTGTGAGTTGAGTACACACATCACAAAGAAGTTTCT

             *       *                             **
     295711 GGGAATGCTGCTGTCTAGTTTTTATAT---GA-ATTCCCGC
        131 GAGAATGCTTCTGTCTAGTTTTTATATGAAGATATTCCCTT

                 *       *       *                *    *  *                  
     295748 TTCCACCGAAATCCTCAAAGCAATCCAAATATCCACTTGCAGAATCCACAAAAAGAGTGTTTCAA
          1 TTCCAACGAAATCATCAAAGCGATCCAAATATCCACTTCCAGATTCTACAAAAAGAGTGTTTCAA

                  *       *     *          * *                      *        
     295813 AACTGCGCTATCAATAGAAAGGTTCAACTCTTTTAGTTGAGTACACACATCACAAACAAGTTTCT
         66 AACTGCTCTATCAAAAGAAATGTTCAACTCTGTGAGTTGAGTACACACATCACAAAGAAGTTTCT

                            * *      **     **  *    
     295878 GAGAATGCTTCTGTCTGGCTTTTATTGGAAGACGTTTCCTT
        131 GAGAATGCTTCTGTCTAGTTTTTATATGAAGATATTCCCTT

                 * *  **          *       *                * *               
     295919 TT-CACCAAAGGCATCAAAGCGCTCCAAATGTCCACTTCCAGATTCTTCCAAAAGAGTGTTTCAA
          1 TTCCAACGAAATCATCAAAGCGATCCAAATATCCACTTCCAGATTCTACAAAAAGAGTGTTTCAA

             **     * *** ** *                 *    * *  * *     *   *       
     295983 ACGTGCTCAAAGTACGGGAATGTTCAACTCTGTGACTTGAATGCAGATATCACCAAGTAGTTTCT
         66 AACTGCTCTATCAAAAGAAATGTTCAACTCTGTGAGTTGAGTACACACATCACAAAGAAGTTTCT

            * * *             *     *    *         * 
     296048 AATAGTGCTTCTGTCTAGATTTTAGATGATGATATTCCCGT
        131 GAGAATGCTTCTGTCTAGTTTTTATATGAAGATATTCCCTT

                         * * *   *             *  *   *      *     *       * 
     296089 TTCCAACGAAATCGTTAGAGCTATCCAAATATCCAGTTACAGTTTCTACCAAAAGGGTGTTTCCA
          1 TTCCAACGAAATCATCAAAGCGATCCAAATATCCACTTCCAGATTCTACAAAAAGAGTGTTTCAA

              *    **           *            *       *                     * 
     296154 AATTGCTGCATCAAAAGAAAGGTTCAACTCTGTTAGTTGAGGACACACATCACAAAGAAGTTTGT
         66 AACTGCTCTATCAAAAGAAATGTTCAACTCTGTGAGTTGAGTACACACATCACAAAGAAGTTTCT

                              *    *     *           
     296219 GAGAATGCTTCTGTCTAGATTTTGTATGACGATATTCCCTT
        131 GAGAATGCTTCTGTCTAGTTTTTATATGAAGATATTCCCTT

                     *   * *     *   *       * *  *    *  *       *  *       
     296260 TTCCAACGATATCGTTAAAGCAATCTAAATATCAATTTGCAGAATCCACAAAAATAGAGTTTCAA
          1 TTCCAACGAAATCATCAAAGCGATCCAAATATCCACTTCCAGATTCTACAAAAAGAGTGTTTCAA

             *       * *        *    *       *  *                   ***      
     296325 AGCTGCTCTGTAAAAAGAAAGGTTCCACTCTGTTAGCTGAGTACACACATCACAAACTTGTTTCT
         66 AACTGCTCTATCAAAAGAAATGTTCAACTCTGTGAGTTGAGTACACACATCACAAAGAAGTTTCT

                  *         *        **         **   
     296390 GAGAATCCTTCTGTCTCGTTTTTATGGGAAGATATTTACTT
        131 GAGAATGCTTCTGTCTAGTTTTTATATGAAGATATTCCCTT

                 *  * **          *       *     *       *  *  *  *           
     296431 TTCCACCGTAGGCATCAAAGCGCTCCAAATGTCCACATCCAGATACTCCAGAAGGAGTGTTTCAA
          1 TTCCAACGAAATCATCAAAGCGATCCAAATATCCACTTCCAGATTCTACAAAAAGAGTGTTTCAA

             *         *   * *   *         *        * *  *      *      *     
     296496 ACCTGCTCTATGAAAGGGAATCTTCAACTCTATGAGTTGAATGCAGACATCAGAAAGAAATTTCT
         66 AACTGCTCTATCAAAAGAAATGTTCAACTCTGTGAGTTGAGTACACACATCACAAAGAAGTTTCT

                     *       **          *         **
     296561 GAGAATGCTGCTGTCTACCTTTTAT-T--TGA-ATTCCCGC
        131 GAGAATGCTTCTGTCTAGTTTTTATATGAAGATATTCCCTT

                         *  *    *                *  **   *    *             
     296598 TTCCAACGAAATCCTCCAAGCTATCCAAATATCCACTTGCATTTTCCACAACAAGAGTGTTTCAA
          1 TTCCAACGAAATCATCAAAGCGATCCAAATATCCACTTCCAGATTCTACAAAAAGAGTGTTTCAA

                          *                 *    *  *                *       
     296663 AACTGCTCTATCAATAGAAATGTTCAACTCCTTTG-GCTGGGTACACACATCACAAACAAGTTTC
         66 AACTGCTCTATCAAAAGAAATGTTCAACT-CTGTGAGTTGAGTACACACATCACAAAGAAGTTTC

                                      **     *        
     296727 TGAGAATGCTTCTGTCTAGTTTTTATGGGAAGACATTCCCTT
        130 TGAGAATGCTTCTGTCTAGTTTTTATATGAAGATATTCCCTT

              *  * *  **          *       *            **                    
     296769 TTTCACCAAAGGCATCAAAGCGCTCCAAATGTCCACTTCCAGACACTACAAAAAGAGTGTTTCAA
          1 TTCCAACGAAATCATCAAAGCGATCCAAATATCCACTTCCAGATTCTACAAAAAGAGTGTTTCAA

             **       **   ***                 *    * *  * *         *       
     296834 ACGTGCTCTAAGAAAGCGAATGTTCAACTCTGTGACTTGAATGCAGATATCACAAAGTAGTTTCT
         66 AACTGCTCTATCAAAAGAAATGTTCAACTCTGTGAGTTGAGTACACACATCACAAAGAAGTTTCT

                *             *     *    *         * 
     296899 GAGAGTGCTTCTGTCTAGATTTTAGATGATGATATTCCCGT
        131 GAGAATGCTTCTGTCTAGTTTTTATATGAAGATATTCCCTT

                           * *   *                *   *                    * 
     296940 TTCCAACGAAATCATTAGAGCTATCCAAATATCCACTTACAGTTTCTACAAAAAGAGTGTTTCCA
          1 TTCCAACGAAATCATCAAAGCGATCCAAATATCCACTTCCAGATTCTACAAAAAGAGTGTTTCAA

                   **         * *    *       *  *                   ***      
     297005 AACTGCTGCATCAAAAGAGAGGTTCCACTCTGTTAGCTGAGTACACACATCACAAACTTGTTTCT
         66 AACTGCTCTATCAAAAGAAATGTTCAACTCTGTGAGTTGAGTACACACATCACAAAGAAGTTTCT

            *     *         *        **         **   
     297070 CAGAATCCTTCTGTCTCGTTTTTATGGGAAGATATTTACTT
        131 GAGAATGCTTCTGTCTAGTTTTTATATGAAGATATTCCCTT

              *  *  * **          *       *     *       *  *  *              
     297111 TTTCACCGTAGGCATCAAAGCGCTCCAAATGTCCACATCCAGATACTCCAGAAAGAGTGTTTCAA
          1 TTCCAACGAAATCATCAAAGCGATCCAAATATCCACTTCCAGATTCTACAAAAAGAGTGTTTCAA

             *         *   * *   *         *        * *  *      *      *     
     297176 ACCTGCTCTATGAAAGGGAATCTTCAACTCTATGAGTTGAATGCAGACATCAGAAAGAAATTTCT
         66 AACTGCTCTATCAAAAGAAATGTTCAACTCTGTGAGTTGAGTACACACATCACAAAGAAGTTTCT

                     *       **          *         **
     297241 GAGAATGCTGCTGTCTACCTTTTAT-T--TGA-ATTCCCGC
        131 GAGAATGCTTCTGTCTAGTTTTTATATGAAGATATTCCCTT

                         *  *    *                *       *                  
     297278 TTCCAACGAAATCCTCCAAGCTATCCAAATATCCACTTGCAGATTCCACAAAAAGAGTGTTTCAA
          1 TTCCAACGAAATCATCAAAGCGATCCAAATATCCACTTCCAGATTCTACAAAAAGAGTGTTTCAA

                            ** *  *            *       *     *     *  *      
     297343 AACTGCTCTCTATCAATGGCAAAGTTCAACTCTGTTAGTTGAGGACACATATCACCAACAAGTTT
         66 AACTG--CTCTATCAAAAGAAATGTTCAACTCTGTGAGTTGAGTACACACATCACAAAGAAGTTT

                               *       **         *    
     297408 CTGAGAATGCTTCTGTCTATTTTTTATGGGAAGATATTTCCTT
        129 CTGAGAATGCTTCTGTCTAGTTTTTATATGAAGATATTCCCTT

              *  *  * ** *    *      *    *          * **          *         
     297451 TTTCACCGTAGGCGTCAAGGCGATCGAAATGTCCACTTCCACAAACTACAAAAAGCGTGTTTCAA
          1 TTCCAACGAAATCATCAAAGCGATCCAAATATCCACTTCCAGATTCTACAAAAAGAGTGTTTCAA

             *         *   * **       *    *        * ** * *   **      *     
     297516 ACCTGCTCTATGAAAGGCCATGTTCATCTCTATGAGTTGAATGGAAATATCCGAAAGAAATTTCT
         66 AACTGCTCTATCAAAAGAAATGTTCAACTCTGTGAGTTGAGTACACACATCACAAAGAAGTTTCT

             *       *          *        *         **
     297581 GGGAATGCTGCTGTCTAG-TGTT-TAT-ACGA-ATTCCCGC
        131 GAGAATGCTTCTGTCTAGTTTTTATATGAAGATATTCCCTT

                         *       *                *    *  *                  
     297618 TTCCAACGAAATCCTCAAAGCAATCCAAATATCCACTTGCAGAATCCACAAAAAGAGTGTTTCAA
          1 TTCCAACGAAATCATCAAAGCGATCCAAATATCCACTTCCAGATTCTACAAAAAGAGTGTTTCAA

                          *     *          * *                   *  *        
     297683 AACTGCTCTATCAATAGAAAGGTTCAACTCTTTTAGTTGAGTACACACATCACGAACAAGTTTCT
         66 AACTGCTCTATCAAAAGAAATGTTCAACTCTGTGAGTTGAGTACACACATCACAAAGAAGTTTCT

                            * *      **     **  *    
     297748 GAGAATGCTTCTGTCTGGCTTTTATTGGAAGACGTTTCCTT
        131 GAGAATGCTTCTGTCTAGTTTTTATATGAAGATATTCCCTT

                 * *  **          *       *                * *               
     297789 TT-CACCAAAGGCATCAAAGCGCTCCAAATGTCCACTTCCAGATTCTTCCAAAAGAGTGTTTCAA
          1 TTCCAACGAAATCATCAAAGCGATCCAAATATCCACTTCCAGATTCTACAAAAAGAGTGTTTCAA

             **  *  * ***  * *                 *    * *  * *     *   *       
     297853 ACGTGGTCGAAGTAAGGGAATGTTCAACTCTGTGACTTGAATGCAGATATCACCAAGTAGTTTCT
         66 AACTGCTCTATCAAAAGAAATGTTCAACTCTGTGAGTTGAGTACACACATCACAAAGAAGTTTCT

            * * *            **     *    *         * 
     297918 AATAGTGCTTCTGTCTACATTTT-GATGATGATATTCCCGT
        131 GAGAATGCTTCTGTCTAGTTTTTATATGAAGATATTCCCTT

                         * * *   *             *      *      *     *       * 
     297958 TTCCAACGAAATCGTTAGAGCTATCCAAATATCCAGTT--AGTTTCTACCAAAAGGGTGTTTCCA
          1 TTCCAACGAAATCATCAAAGCGATCCAAATATCCACTTCCAGATTCTACAAAAAGAGTGTTTCAA

              *    **           *            *       *                     * 
     298021 AATTGCTGCATCAAAAGAAAGGTTCAACTCTGTTAGTTGAGGACACACATCACAAAGAAGTTTGT
         66 AACTGCTCTATCAAAAGAAATGTTCAACTCTGTGAGTTGAGTACACACATCACAAAGAAGTTTCT

                              *    *     *           
     298086 GAGAATGCTTCTGTCTAGATTTTGTATGACGATATTCCCTT
        131 GAGAATGCTTCTGTCTAGTTTTTATATGAAGATATTCCCTT

                  *  *   * *     *   *       * *  *    *  *       *  *       
     298127 TTCCAATGATATCGTTAAAGCAATCTAAATATCAATTTGCAGAATCCACAAAAATAGAGTTTCAA
          1 TTCCAACGAAATCATCAAAGCGATCCAAATATCCACTTCCAGATTCTACAAAAAGAGTGTTTCAA

             *       * *        *    *       *  *                   ***      
     298192 AGCTGCTCTGTAAAAAGAAAGGTTCCACTCTGTTAGCTGAGTACACACATCACAAACTTGTTTCT
         66 AACTGCTCTATCAAAAGAAATGTTCAACTCTGTGAGTTGAGTACACACATCACAAAGAAGTTTCT

                  *         *        **         **   
     298257 GAGAATCCTTCTGTCTCGTTTTTATGGGAAGATATTTACTT
        131 GAGAATGCTTCTGTCTAGTTTTTATATGAAGATATTCCCTT

              *  *  * **          *       *     *       *  *  *  *           
     298298 TTTCACCGTAGGCATCAAAGCGCTCCAAATGTCCACATCCAGATACTCCAGAACGAGTGTTTCAA
          1 TTCCAACGAAATCATCAAAGCGATCCAAATATCCACTTCCAGATTCTACAAAAAGAGTGTTTCAA

             *  *      *   * *   *         *        * *  *      *      *     
     298363 ACCTACTCTATGAAAGGGAATCTTCAACTCTATGAGTTGAATGCAGACATCAGAAAGAAATTTCT
         66 AACTGCTCTATCAAAAGAAATGTTCAACTCTGTGAGTTGAGTACACACATCACAAAGAAGTTTCT

                     *       **          *         **
     298428 GAGAATGCTGCTGTCTACCTTTTAT-T--TGA-ATTCCCGC
        131 GAGAATGCTTCTGTCTAGTTTTTATATGAAGATATTCCCTT

                         *  *    *                *  **   *    *             
     298465 TTCCAACGAAATCCTCCAAGCTATCCAAATATCCACTTGCATTTTCCACAACAAGAGTGTTTCAA
          1 TTCCAACGAAATCATCAAAGCGATCCAAATATCCACTTCCAGATTCTACAAAAAGAGTGTTTCAA

                          *                 *    *  *                *       
     298530 AACTGCTCTATCAATAGAAATGTTCAACTCCTTTG-GCTGGGTACACACATCACAAACAAGTTTC
         66 AACTGCTCTATCAAAAGAAATGTTCAACT-CTGTGAGTTGAGTACACACATCACAAAGAAGTTTC

                                      **     *        
     298594 TGAGAATGCTTCTGTCTAGTTTTTATGGGAAGACATTCCCTT
        130 TGAGAATGCTTCTGTCTAGTTTTTATATGAAGATATTCCCTT

              *  * *  **          *       *            **                    
     298636 TTTCACCAAAGGCATCAAAGCGCTCCAAATGTCCACTTCCAGACACTACAAAAAGAGTGTTTCAA
          1 TTCCAACGAAATCATCAAAGCGATCCAAATATCCACTTCCAGATTCTACAAAAAGAGTGTTTCAA

             **       **   ***                 *    * *  * *         *       
     298701 ACGTGCTCTAAGAAAGCGAATGTTCAACTCTGTGACTTGAATGCAGATATCACAAAGTAGTTTCT
         66 AACTGCTCTATCAAAAGAAATGTTCAACTCTGTGAGTTGAGTACACACATCACAAAGAAGTTTCT

                *             **    *    *         * 
     298766 GAGAGTGCTTCTGTCTAGACTTTAGATGATGATATTCCCGT
        131 GAGAATGCTTCTGTCTAGTTTTTATATGAAGATATTCCCTT

                           * *   *                *   *                    * 
     298807 TTCCAACGAAATCATTAGAGCTATCCAAATATCCACTTACAGTTTCTACAAAAAGAGTGTTTCCA
          1 TTCCAACGAAATCATCAAAGCGATCCAAATATCCACTTCCAGATTCTACAAAAAGAGTGTTTCAA

                   **  *      * *    *       *  *                   ***      
     298872 AACTGCTGCATGAAAAGAGAGGTTCCACTCTGTTAGCTGAGTACACACATCACAAACTTGTTTCT
         66 AACTGCTCTATCAAAAGAAATGTTCAACTCTGTGAGTTGAGTACACACATCACAAAGAAGTTTCT

            *     * *       *        **         **   
     298937 CAGAATCCGTCTGTCTCGTTTTTATGGGAAGATATTTACTT
        131 GAGAATGCTTCTGTCTAGTTTTTATATGAAGATATTCCCTT

              *  *  * **          *       *     *       *  *  *              
     298978 TTTCACCGTAGGCATCAAAGCGCTCCAAATGTCCACATCCAGATACTCCAGAAAGAGTGTTTCAA
          1 TTCCAACGAAATCATCAAAGCGATCCAAATATCCACTTCCAGATTCTACAAAAAGAGTGTTTCAA

             *         *   * *   *         *        * *  *      *      *     
     299043 ACCTGCTCTATGAAAGGGAATCTTCAACTCTATGAGTTGAATGCAGACATCAGAAAGAAATTTCT
         66 AACTGCTCTATCAAAAGAAATGTTCAACTCTGTGAGTTGAGTACACACATCACAAAGAAGTTTCT

                     *       **          *         **
     299108 GAGAATGCTGCTGTCTACCTTTTAT-T--TGA-ATTCCCGC
        131 GAGAATGCTTCTGTCTAGTTTTTATATGAAGATATTCCCTT

                         *  *    *                *       *                  
     299145 TTCCAACGAAATCCTCCAAGCTATCCAAATATCCACTTGCAGATTCCACAAAAAGAGTGTTTCAA
          1 TTCCAACGAAATCATCAAAGCGATCCAAATATCCACTTCCAGATTCTACAAAAAGAGTGTTTCAA

                            ** *  *            *       *     *     *  *      
     299210 AACTGCTCTCTATCAATGGCAAAGTTCAACTCTGTTAGTTGAGGACACATATCACCAACAAGTTT
         66 AACTG--CTCTATCAAAAGAAATGTTCAACTCTGTGAGTTGAGTACACACATCACAAAGAAGTTT

                               *       **         *    
     299275 CTGAGAATGCTTCTGTCTATTTTTTATGGGAAGATATTTCCTT
        129 CTGAGAATGCTTCTGTCTAGTTTTTATATGAAGATATTCCCTT

              *  *  * ** *    *      *    *          * **          *         
     299318 TTTCACCGTAGGCGTCAAGGCGATCGAAATGTCCACTTCCACAAACTACAAAAAGCGTGTTTCAA
          1 TTCCAACGAAATCATCAAAGCGATCCAAATATCCACTTCCAGATTCTACAAAAAGAGTGTTTCAA

             *         *   * **       *    *        * ** * *   **      *     
     299383 ACCTGCTCTATGAAAGGCCATGTTCATCTCTATGAGTTGAATGGAAATATCCGAAAGAAATTTCT
         66 AACTGCTCTATCAAAAGAAATGTTCAACTCTGTGAGTTGAGTACACACATCACAAAGAAGTTTCT

             *       *          *        *         **
     299448 GGGAATGCTGCTGTCTAG-TGTT-TAT-ACGA-ATTCCCGC
        131 GAGAATGCTTCTGTCTAGTTTTTATATGAAGATATTCCCTT

                         *       *                *    *  *                  
     299485 TTCCAACGAAATCCTCAAAGCAATCCAAATATCCACTTGCAGAATCCACAAAAAGAGTGTTTCAA
          1 TTCCAACGAAATCATCAAAGCGATCCAAATATCCACTTCCAGATTCTACAAAAAGAGTGTTTCAA

                          *     *          * *                   *  *        
     299550 AACTGCTCTATCAATAGAAAGGTTCAACTCTTTTAGTTGAGTACACACATCACGAACAAGTTTCT
         66 AACTGCTCTATCAAAAGAAATGTTCAACTCTGTGAGTTGAGTACACACATCACAAAGAAGTTTCT

                            * *      **     **  *    
     299615 GAGAATGCTTCTGTCTGGCTTTTATTGGAAGACGTTTCCTT
        131 GAGAATGCTTCTGTCTAGTTTTTATATGAAGATATTCCCTT

                 * *  **          *       *                * *               
     299656 TT-CACCAAAGGCATCAAAGCGCTCCAAATGTCCACTTCCAGATTCTTCCAAAAGAGTGTTTCAA
          1 TTCCAACGAAATCATCAAAGCGATCCAAATATCCACTTCCAGATTCTACAAAAAGAGTGTTTCAA

             **  *  * ***  * *                 *    * *  * *     *   *       
     299720 ACGTGGTCGAAGTAAGGGAATGTTCAACTCTGTGACTTGAATGCAGATATCACCAAGTAGTTTCT
         66 AACTGCTCTATCAAAAGAAATGTTCAACTCTGTGAGTTGAGTACACACATCACAAAGAAGTTTCT

            * * *            **     *    *         * 
     299785 AATAGTGCTTCTGTCTACATTTT-GATGATGATATTCCCGT
        131 GAGAATGCTTCTGTCTAGTTTTTATATGAAGATATTCCCTT

                         * * *   *             *      *      *     *       * 
     299825 TTCCAACGAAATCGTTAGAGCTATCCAAATATCCAGTT--AGTTTCTACCAAAAGGGTGTTTCCA
          1 TTCCAACGAAATCATCAAAGCGATCCAAATATCCACTTCCAGATTCTACAAAAAGAGTGTTTCAA

              *    **           *            *       *                     * 
     299888 AATTGCTGCATCAAAAGAAAGGTTCAACTCTGTTAGTTGAGGACACACATCACAAAGAAGTTTGT
         66 AACTGCTCTATCAAAAGAAATGTTCAACTCTGTGAGTTGAGTACACACATCACAAAGAAGTTTCT

                              *    *     *           
     299953 GAGAATGCTTCTGTCTAGATTTTGTATGACGATATTCCCTT
        131 GAGAATGCTTCTGTCTAGTTTTTATATGAAGATATTCCCTT

                  *  *   * *     *   *       * *  *    *  *       *  *       
     299994 TTCCAATGATATCGTTAAAGCAATCTAAATATCAATTTGCAGAATCCACAAAAATAGAGTTTCAA
          1 TTCCAACGAAATCATCAAAGCGATCCAAATATCCACTTCCAGATTCTACAAAAAGAGTGTTTCAA

             *       * *        *    *       *  *                   ***      
     300059 AGCTGCTCTGTAAAAAGAAAGGTTCCACTCTGTTAGCTGAGTACACACATCACAAACTTGTTTCT
         66 AACTGCTCTATCAAAAGAAATGTTCAACTCTGTGAGTTGAGTACACACATCACAAAGAAGTTTCT

                  *         *        **         **   
     300124 GAGAATCCTTCTGTCTCGTTTTTATGGGAAGATATTTACTT
        131 GAGAATGCTTCTGTCTAGTTTTTATATGAAGATATTCCCTT

              *  *  * **          *       *     *       *  *  *  *           
     300165 TTTCACCGTAGGCATCAAAGCGCTCCAAATGTCCACATCCAGATACTCCAGAACGAGTGTTTCAA
          1 TTCCAACGAAATCATCAAAGCGATCCAAATATCCACTTCCAGATTCTACAAAAAGAGTGTTTCAA

             *  *      *   * *   *         *        * *  *      *      *     
     300230 ACCTACTCTATGAAAGGGAATCTTCAACTCTATGAGTTGAATGCAGACATCAGAAAGAAATTTCT
         66 AACTGCTCTATCAAAAGAAATGTTCAACTCTGTGAGTTGAGTACACACATCACAAAGAAGTTTCT

                     *       **          *         **
     300295 GAGAATGCTGCTGTCTACCTTTTAT-T--TGA-ATTCCCGC
        131 GAGAATGCTTCTGTCTAGTTTTTATATGAAGATATTCCCTT

                         *  *    *                *  **   *    *             
     300332 TTCCAACGAAATCCTCCAAGCTATCCAAATATCCACTTGCATTTTCCACAACAAGAGTGTTTCAA
          1 TTCCAACGAAATCATCAAAGCGATCCAAATATCCACTTCCAGATTCTACAAAAAGAGTGTTTCAA

                          *                 *    *  *                *       
     300397 AACTGCTCTATCAATAGAAATGTTCAACTCCTTTG-GCTGGGTACACACATCACAAACAAGTTTC
         66 AACTGCTCTATCAAAAGAAATGTTCAACT-CTGTGAGTTGAGTACACACATCACAAAGAAGTTTC

                                      **     *        
     300461 TGAGAATGCTTCTGTCTAGTTTTTATGGGAAGACATTCCCTT
        130 TGAGAATGCTTCTGTCTAGTTTTTATATGAAGATATTCCCTT

              *  * *  **          *       *            **                    
     300503 TTTCACCAAAGGCATCAAAGCGCTCCAAATGTCCACTTCCAGACACTACAAAAAGAGTGTTTCAA
          1 TTCCAACGAAATCATCAAAGCGATCCAAATATCCACTTCCAGATTCTACAAAAAGAGTGTTTCAA

             **       **   ***                 *    * *  * *         *       
     300568 ACGTGCTCTAAGAAAGCGAATGTTCAACTCTGTGACTTGAATGCAGATATCACAAAGTAGTTTCT
         66 AACTGCTCTATCAAAAGAAATGTTCAACTCTGTGAGTTGAGTACACACATCACAAAGAAGTTTCT

                *             **    *    *         * 
     300633 GAGAGTGCTTCTGTCTAGACTTTAGATGATGATATTCCCGT
        131 GAGAATGCTTCTGTCTAGTTTTTATATGAAGATATTCCCTT

                           * *   *                *   *                    * 
     300674 TTCCAACGAAATCATTAGAGCTATCCAAATATCCACTTACAGTTTCTACAAAAAGAGTGTTTCCA
          1 TTCCAACGAAATCATCAAAGCGATCCAAATATCCACTTCCAGATTCTACAAAAAGAGTGTTTCAA

                   **  *      * *    *       *  *                   ***      
     300739 AACTGCTGCATGAAAAGAGAGGTTCCACTCTGTTAGCTGAGTACACACATCACAAACTTGTTTCT
         66 AACTGCTCTATCAAAAGAAATGTTCAACTCTGTGAGTTGAGTACACACATCACAAAGAAGTTTCT

            *     * *       *        **         **   
     300804 CAGAATCCGTCTGTCTCGTTTTTATGGGAAGATATTTACTT
        131 GAGAATGCTTCTGTCTAGTTTTTATATGAAGATATTCCCTT

              *  *  * **          *       *     *       *  *  *              
     300845 TTTCACCGTAGGCATCAAAGCGCTCCAAATGTCCACATCCAGATACTCCAGAAAGAGTGTTTCAA
          1 TTCCAACGAAATCATCAAAGCGATCCAAATATCCACTTCCAGATTCTACAAAAAGAGTGTTTCAA

             *         *   * *   *         *        * *  *      *      *     
     300910 ACCTGCTCTATGAAAGGGAATCTTCAACTCTATGAGTTGAATGCAGACATCAGAAAGAAATTTCT
         66 AACTGCTCTATCAAAAGAAATGTTCAACTCTGTGAGTTGAGTACACACATCACAAAGAAGTTTCT

                     *       **          *         **
     300975 GAGAATGCTGCTGTCTACCTTTTAT-T--TGA-ATTCCCGC
        131 GAGAATGCTTCTGTCTAGTTTTTATATGAAGATATTCCCTT

                         *  *    *                *       *                  
     301012 TTCCAACGAAATCCTCCAAGCTATCCAAATATCCACTTGCAGATTCCACAAAAAGAGTGTTTCAA
          1 TTCCAACGAAATCATCAAAGCGATCCAAATATCCACTTCCAGATTCTACAAAAAGAGTGTTTCAA

                            ** *  *            *       *     *     *  *      
     301077 AACTGCTCTCTATCAATGGCAAAGTTCAACTCTGTTAGTTGAGGACACATATCACCAACAAGTTT
         66 AACTG--CTCTATCAAAAGAAATGTTCAACTCTGTGAGTTGAGTACACACATCACAAAGAAGTTT

                               *       **         *    
     301142 CTGAGAATGCTTCTGTCTATTTTTTATGGGAAGATATTTCCTT
        129 CTGAGAATGCTTCTGTCTAGTTTTTATATGAAGATATTCCCTT

              *  *  * ** *    *      *    *          * **          *         
     301185 TTTCACCGTAGGCGTCAAGGCGATCGAAATGTCCACTTCCACAAACTACAAAAAGCGTGTTTCAA
          1 TTCCAACGAAATCATCAAAGCGATCCAAATATCCACTTCCAGATTCTACAAAAAGAGTGTTTCAA

             *         *   * **       *    *        * ** * *   **      *     
     301250 ACCTGCTCTATGAAAGGCCATGTTCATCTCTATGAGTTGAATGGAAATATCCGAAAGAAATTTCT
         66 AACTGCTCTATCAAAAGAAATGTTCAACTCTGTGAGTTGAGTACACACATCACAAAGAAGTTTCT

             *       *          *        *         **
     301315 GGGAATGCTGCTGTCTAG-TGTT-TAT-ACGA-ATTCCCGC
        131 GAGAATGCTTCTGTCTAGTTTTTATATGAAGATATTCCCTT

                         *       *                *    *  *                  
     301352 TTCCAACGAAATCCTCAAAGCAATCCAAATATCCACTTGCAGAATCCACAAAAAGAGTGTTTCAA
          1 TTCCAACGAAATCATCAAAGCGATCCAAATATCCACTTCCAGATTCTACAAAAAGAGTGTTTCAA

                          *     *          * *                   *  *        
     301417 AACTGCTCTATCAATAGAAAGGTTCAACTCTTTTAGTTGAGTACACACATCACGAACAAGTTTCT
         66 AACTGCTCTATCAAAAGAAATGTTCAACTCTGTGAGTTGAGTACACACATCACAAAGAAGTTTCT

                            * *      **     **  *    
     301482 GAGAATGCTTCTGTCTGGCTTTTATTGGAAGACGTTTCCTT
        131 GAGAATGCTTCTGTCTAGTTTTTATATGAAGATATTCCCTT

                 * *  **          *       *                * *               
     301523 TT-CACCAAAGGCATCAAAGCGCTCCAAATGTCCACTTCCAGATTCTTCCAAAAGAGTGTTTCAA
          1 TTCCAACGAAATCATCAAAGCGATCCAAATATCCACTTCCAGATTCTACAAAAAGAGTGTTTCAA

             **  *  * ***  * *                 *    * *  * *     *   *       
     301587 ACGTGGTCGAAGTAAGGGAATGTTCAACTCTGTGACTTGAATGCAGATATCACCAAGTAGTTTCT
         66 AACTGCTCTATCAAAAGAAATGTTCAACTCTGTGAGTTGAGTACACACATCACAAAGAAGTTTCT

            * * *            **     *    *         * 
     301652 AATAGTGCTTCTGTCTACATTTT-GATGATGATATTCCCGT
        131 GAGAATGCTTCTGTCTAGTTTTTATATGAAGATATTCCCTT

                         * * *   *             *      *      *     *       * 
     301692 TTCCAACGAAATCGTTAGAGCTATCCAAATATCCAGTT--AGTTTCTACCAAAAGGGTGTTTCCA
          1 TTCCAACGAAATCATCAAAGCGATCCAAATATCCACTTCCAGATTCTACAAAAAGAGTGTTTCAA

              *    **           *            *       *                     * 
     301755 AATTGCTGCATCAAAAGAAAGGTTCAACTCTGTTAGTTGAGGACACACATCACAAAGAAGTTTGT
         66 AACTGCTCTATCAAAAGAAATGTTCAACTCTGTGAGTTGAGTACACACATCACAAAGAAGTTTCT

                              *    *     *           
     301820 GAGAATGCTTCTGTCTAGATTTTGTATGACGATATTCCCTT
        131 GAGAATGCTTCTGTCTAGTTTTTATATGAAGATATTCCCTT

                  *  *   * *     *   *       * *  *    *  *       *  *       
     301861 TTCCAATGATATCGTTAAAGCAATCTAAATATCAATTTGCAGAATCCACAAAAATAGAGTTTCAA
          1 TTCCAACGAAATCATCAAAGCGATCCAAATATCCACTTCCAGATTCTACAAAAAGAGTGTTTCAA

             *       * *        *    *       *  *                   ***      
     301926 AGCTGCTCTGTAAAAAGAAAGGTTCCACTCTGTTAGCTGAGTACACACATCACAAACTTGTTTCT
         66 AACTGCTCTATCAAAAGAAATGTTCAACTCTGTGAGTTGAGTACACACATCACAAAGAAGTTTCT

                  *         *        **         **   
     301991 GAGAATCCTTCTGTCTCGTTTTTATGGGAAGATATTTACTT
        131 GAGAATGCTTCTGTCTAGTTTTTATATGAAGATATTCCCTT

              *  *  * **          *       *     *       *  *  *  *           
     302032 TTTCACCGTAGGCATCAAAGCGCTCCAAATGTCCACATCCAGATACTCCAGAACGAGTGTTTCAA
          1 TTCCAACGAAATCATCAAAGCGATCCAAATATCCACTTCCAGATTCTACAAAAAGAGTGTTTCAA

             *  *      *   * *   *         *        * *  *      *      *     
     302097 ACCTACTCTATGAAAGGGAATCTTCAACTCTATGAGTTGAATGCAGACATCAGAAAGAAATTTCT
         66 AACTGCTCTATCAAAAGAAATGTTCAACTCTGTGAGTTGAGTACACACATCACAAAGAAGTTTCT

                     *       **          *         **
     302162 GAGAATGCTGCTGTCTACCTTTTAT-T--TGA-ATTCCCGC
        131 GAGAATGCTTCTGTCTAGTTTTTATATGAAGATATTCCCTT

                         *  *    *                *  **   *    *             
     302199 TTCCAACGAAATCCTCCAAGCTATCCAAATATCCACTTGCATTTTCCACAACAAGAGTGTTTCAA
          1 TTCCAACGAAATCATCAAAGCGATCCAAATATCCACTTCCAGATTCTACAAAAAGAGTGTTTCAA

                          *                 *    *  *                *       
     302264 AACTGCTCTATCAATAGAAATGTTCAACTCCTTTG-GCTGGGTACACACATCACAAACAAGTTTC
         66 AACTGCTCTATCAAAAGAAATGTTCAACT-CTGTGAGTTGAGTACACACATCACAAAGAAGTTTC

                                      **     *        
     302328 TGAGAATGCTTCTGTCTAGTTTTTATGGGAAGACATTCCCTT
        130 TGAGAATGCTTCTGTCTAGTTTTTATATGAAGATATTCCCTT

              *  * *  **          *       *            **                    
     302370 TTTCACCAAAGGCATCAAAGCGCTCCAAATGTCCACTTCCAGACACTACAAAAAGAGTGTTTCAA
          1 TTCCAACGAAATCATCAAAGCGATCCAAATATCCACTTCCAGATTCTACAAAAAGAGTGTTTCAA

             **       **   ***                 *    * *  * *         *       
     302435 ACGTGCTCTAAGAAAGCGAATGTTCAACTCTGTGACTTGAATGCAGATATCACAAAGTAGTTTCT
         66 AACTGCTCTATCAAAAGAAATGTTCAACTCTGTGAGTTGAGTACACACATCACAAAGAAGTTTCT

                *             **    *    *         * 
     302500 GAGAGTGCTTCTGTCTAGACTTTAGATGATGATATTCCCGT
        131 GAGAATGCTTCTGTCTAGTTTTTATATGAAGATATTCCCTT

                           * *   *                *   *                    * 
     302541 TTCCAACGAAATCATTAGAGCTATCCAAATATCCACTTACAGTTTCTACAAAAAGAGTGTTTCCA
          1 TTCCAACGAAATCATCAAAGCGATCCAAATATCCACTTCCAGATTCTACAAAAAGAGTGTTTCAA

                   **  *      * *    *       *  *                   ***      
     302606 AACTGCTGCATGAAAAGAGAGGTTCCACTCTGTTAGCTGAGTACACACATCACAAACTTGTTTCT
         66 AACTGCTCTATCAAAAGAAATGTTCAACTCTGTGAGTTGAGTACACACATCACAAAGAAGTTTCT

            *     * *       *        **         **   
     302671 CAGAATCCGTCTGTCTCGTTTTTATGGGAAGATATTTACTT
        131 GAGAATGCTTCTGTCTAGTTTTTATATGAAGATATTCCCTT

              *  *  * **          *       *     *       *  *  *              
     302712 TTTCACCGTAGGCATCAAAGCGCTCCAAATGTCCACATCCAGATACTCCAGAAAGAGTGTTTCAA
          1 TTCCAACGAAATCATCAAAGCGATCCAAATATCCACTTCCAGATTCTACAAAAAGAGTGTTTCAA

             *         *   * *             *        * *  *      *      *     
     302777 ACCTGCTCTATGAAAGGGAATGTTCAACTCTATGAGTTGAATGCAGACATCAGAAAGAAATTTCT
         66 AACTGCTCTATCAAAAGAAATGTTCAACTCTGTGAGTTGAGTACACACATCACAAAGAAGTTTCT

                     *       **          *         **
     302842 GAGAATGCTGCTGTCTACCTTTTAT-T--TGA-ATTCCCGC
        131 GAGAATGCTTCTGTCTAGTTTTTATATGAAGATATTCCCTT

                         *  *    *                *       *                  
     302879 TTCCAACGAAATCCTCCAAGCTATCCAAATATCCACTTGCAGATTCCACAAAAAGAGTGTTTCAA
          1 TTCCAACGAAATCATCAAAGCGATCCAAATATCCACTTCCAGATTCTACAAAAAGAGTGTTTCAA

                            ** *  *            *       *     *     *  *      
     302944 AACTGCTCTCTATCAATGGCAAAGTTCAACTCTGTTAGTTGAGGACACATATCACCAACAAGTTT
         66 AACTG--CTCTATCAAAAGAAATGTTCAACTCTGTGAGTTGAGTACACACATCACAAAGAAGTTT

                               *       **         *    
     303009 CTGAGAATGCTTCTGTCTATTTTTTATGGGAAGATATTTCCTT
        129 CTGAGAATGCTTCTGTCTAGTTTTTATATGAAGATATTCCCTT

              *  *  * ** *    *      *    *          * **                    
     303052 TTTCACCGTAGGCGTCAAGGCGATCGAAATGTCCACTTCCACAAACTACAAAAAGAGTGTTTCAA
          1 TTCCAACGAAATCATCAAAGCGATCCAAATATCCACTTCCAGATTCTACAAAAAGAGTGTTTCAA

             *         *   * **       *    *        * ** * *   **   *  *     
     303117 ACCTGCTCTATGAAAGGCCATGTTCATCTCTATGAGTTGAATGGAAATATCCGAAAAAAATTTCT
         66 AACTGCTCTATCAAAAGAAATGTTCAACTCTGTGAGTTGAGTACACACATCACAAAGAAGTTTCT

             *       *                             **
     303182 GGGAATGCTGCTGTCTAGTTTTTATAT---GA-ATTCCCGC
        131 GAGAATGCTTCTGTCTAGTTTTTATATGAAGATATTCCCTT

                         *       *                *    *  *                  
     303219 TTCCAACGAAATCCTCAAAGCAATCCAAATATCCACTTGCAGAATCCACAAAAAGAGTGTTTCAA
          1 TTCCAACGAAATCATCAAAGCGATCCAAATATCCACTTCCAGATTCTACAAAAAGAGTGTTTCAA

                          *     *          * *                      *        
     303284 AACTGCTCTATCAATAGAAAGGTTCAACTCTTTTAGTTGAGTACACACATCACAAACAAGTTTCT
         66 AACTGCTCTATCAAAAGAAATGTTCAACTCTGTGAGTTGAGTACACACATCACAAAGAAGTTTCT

                            * *      **     **  *    
     303349 GAGAATGCTTCTGTCTGGCTTTTATTGGAAGACGTTTCCTT
        131 GAGAATGCTTCTGTCTAGTTTTTATATGAAGATATTCCCTT

                 * *  **          *       *                * *               
     303390 TT-CACCAAAGGCATCAAAGCGCTCCAAATGTCCACTTCCAGATTCTTCCAAAAGAGTGTTTCAA
          1 TTCCAACGAAATCATCAAAGCGATCCAAATATCCACTTCCAGATTCTACAAAAAGAGTGTTTCAA

             **  *  * ***  * *       *         *    * *  * *     *   *       
     303454 ACGTGGTCGAAGTAAGGGAATGTTCTACTCTGTGACTTGAATGCAGATATCACCAAGTAGTTTCT
         66 AACTGCTCTATCAAAAGAAATGTTCAACTCTGTGAGTTGAGTACACACATCACAAAGAAGTTTCT

            * * *             *     *    *         * 
     303519 AATAGTGCTTCTGTCTAGATTTTAGATGATGATATTCCCGT
        131 GAGAATGCTTCTGTCTAGTTTTTATATGAAGATATTCCCTT

                         * * *   *             *  *   *      *     *       * 
     303560 TTCCAACGAAATCGTTAGAGCTATCCAAATATCCAGTTACAGTTTCTACCAAAAGGGTGTTTCCA
          1 TTCCAACGAAATCATCAAAGCGATCCAAATATCCACTTCCAGATTCTACAAAAAGAGTGTTTCAA

              *    **           *            *       *                     * 
     303625 AATTGCTGCATCAAAAGAAAGGTTCAACTCTGTTAGTTGAGGACACACATCACAAAGAAGTTTGT
         66 AACTGCTCTATCAAAAGAAATGTTCAACTCTGTGAGTTGAGTACACACATCACAAAGAAGTTTCT

                              *    *     *           
     303690 GAGAATGCTTCTGTCTAGATTTTGTATGACGATATTCCCTT
        131 GAGAATGCTTCTGTCTAGTTTTTATATGAAGATATTCCCTT

                     *   * *     *   *       * *  *    *  *       *  *       
     303731 TTCCAACGATATCGTTAAAGCAATCTAAATATCAATTTGCAGAATCCACAAAAATAGAGTTTCAA
          1 TTCCAACGAAATCATCAAAGCGATCCAAATATCCACTTCCAGATTCTACAAAAAGAGTGTTTCAA

             *       * *        *    *       *  *                   ***      
     303796 AGCTGCTCTGTAAAAAGAAAGGTTCCACTCTGTTAGCTGAGTACACACATCACAAACTTGTTTCT
         66 AACTGCTCTATCAAAAGAAATGTTCAACTCTGTGAGTTGAGTACACACATCACAAAGAAGTTTCT

                  *         *        **         **   
     303861 GAGAATCCTTCTGTCTCGTTTTTATGGGAAGATATTTACTT
        131 GAGAATGCTTCTGTCTAGTTTTTATATGAAGATATTCCCTT

                 *  * **          *       *     *       *  *  *              
     303902 TTCCACCGTAGGCATCAAAGCGCTCCAAATGTCCACATCCAGATACTCCAGAAAGAGTGTTTCAA
          1 TTCCAACGAAATCATCAAAGCGATCCAAATATCCACTTCCAGATTCTACAAAAAGAGTGTTTCAA

             *         *   * *   *         *        * *  *      *      *     
     303967 ACCTGCTCTATGAAAGGGAATCTTCAACTCTATGAGTTGAATGCAGACATCAGAAAGAAATTTCT
         66 AACTGCTCTATCAAAAGAAATGTTCAACTCTGTGAGTTGAGTACACACATCACAAAGAAGTTTCT

                     *       **          *         **
     304032 GAGAATGCTGCTGTCTACCTTTTAT-T--TGA-ATTCCCGC
        131 GAGAATGCTTCTGTCTAGTTTTTATATGAAGATATTCCCTT

                         *  *    *                *  **   *                  
     304069 TTCCAACGAAATCCTCCAAGCTATCCAAATATCCACTTGCATTTTCCACAAAAAGAGTGTTTCAA
          1 TTCCAACGAAATCATCAAAGCGATCCAAATATCCACTTCCAGATTCTACAAAAAGAGTGTTTCAA

                          *                 *    *  *                *       
     304134 AACTGCTCTATCAATAGAAATGTTCAACTCCTTTG-GCTGGGTACACACATCACAAACAAGTTTC
         66 AACTGCTCTATCAAAAGAAATGTTCAACT-CTGTGAGTTGAGTACACACATCACAAAGAAGTTTC

                                      **     *        
     304198 TGAGAATGCTTCTGTCTAGTTTTTATGGGAAGACATTCCCTT
        130 TGAGAATGCTTCTGTCTAGTTTTTATATGAAGATATTCCCTT

              *  * *  **          *       *            **                    
     304240 TTTCACCAAAGGCATCAAAGCGCTCCAAATGTCCACTTCCAGACACTACAAAAAGAGTGTTTCAA
          1 TTCCAACGAAATCATCAAAGCGATCCAAATATCCACTTCCAGATTCTACAAAAAGAGTGTTTCAA

             **       **   ***                 *    * *  * *         *       
     304305 ACGTGCTCTAAGAAAGCGAATGTTCAACTCTGTGACTTGAATGCAGATATCACAAAGTAGTTTCT
         66 AACTGCTCTATCAAAAGAAATGTTCAACTCTGTGAGTTGAGTACACACATCACAAAGAAGTTTCT

                **            *     *    *         * 
     304370 GAGAGGGCTTCTGTCTAGATTTTAGATGATGATATTCCCGT
        131 GAGAATGCTTCTGTCTAGTTTTTATATGAAGATATTCCCTT

                           * *   *  *             * * *                    * 
     304411 TTCCAACGAAATCATTAGAGCTATACAAATATCCACTTACTGTTTCTACAAAAAGAGTGTTTCCA
          1 TTCCAACGAAATCATCAAAGCGATCCAAATATCCACTTCCAGATTCTACAAAAAGAGTGTTTCAA

                   **         * *    *       *  *                   ***      
     304476 AACTGCTGCATCAAAAGAGAGGTTCCACTCTGTTAGCTGAGTACACACATCACAAACTTGTTTCT
         66 AACTGCTCTATCAAAAGAAATGTTCAACTCTGTGAGTTGAGTACACACATCACAAAGAAGTTTCT

            *     *         *        **         **   
     304541 CAGAATCCTTCTGTCTCGTTTTTATGGGAAGATATTTACTT
        131 GAGAATGCTTCTGTCTAGTTTTTATATGAAGATATTCCCTT

              *  *  * **          *       *     *       *  *  *              
     304582 TTTCACCGTAGGCATCAAAGCGCTCCAAATGTCCACATCCAGATACTCCAGAAAGAGTGTTTCAA
          1 TTCCAACGAAATCATCAAAGCGATCCAAATATCCACTTCCAGATTCTACAAAAAGAGTGTTTCAA

             *         *   * *   *         *        * *  *      *      *     
     304647 ACCTGCTCTATGAAAGGGAATCTTCAACTCTATGAGTTGAATGCAGACATCAGAAAGAAATTTCT
         66 AACTGCTCTATCAAAAGAAATGTTCAACTCTGTGAGTTGAGTACACACATCACAAAGAAGTTTCT

                     *       **          *         **
     304712 GAGAATGCTGCTGTCTACCTTTTAT-T--TGA-ATTCCCGC
        131 GAGAATGCTTCTGTCTAGTTTTTATATGAAGATATTCCCTT

                         *  *    *                *       *                  
     304749 TTCCAACGAAATCCTCCAAGCTATCCAAATATCCACTTGCAGATTCCACAAAAAGAGTGTTTCAA
          1 TTCCAACGAAATCATCAAAGCGATCCAAATATCCACTTCCAGATTCTACAAAAAGAGTGTTTCAA

                            ** *  *            *       *     *     *  *      
     304814 AACTGCTCTCTATCAATGGCAAAGTTCAACTCTGTTAGTTGAGGACACATATCACCAACAAGTTT
         66 AACTG--CTCTATCAAAAGAAATGTTCAACTCTGTGAGTTGAGTACACACATCACAAAGAAGTTT

                               *       **         *    
     304879 CTGAGAATGCTTCTGTCTATTTTTTATGGGAAGATATTTCCTT
        129 CTGAGAATGCTTCTGTCTAGTTTTTATATGAAGATATTCCCTT

              *  *  * ** *    *      *    *          * **                    
     304922 TTTCACCGTAGGCGTCAAGGCGATCGAAATGTCCACTTCCACAAACTACAAAAAGAGTGTTTCAA
          1 TTCCAACGAAATCATCAAAGCGATCCAAATATCCACTTCCAGATTCTACAAAAAGAGTGTTTCAA

             *         *   * **       *    *        * ** * *   **   *  *     
     304987 ACCTGCTCTATGAAAGGCCATGTTCATCTCTATGAGTTGAATGGAAATATCCGAAAAAAATTTCT
         66 AACTGCTCTATCAAAAGAAATGTTCAACTCTGTGAGTTGAGTACACACATCACAAAGAAGTTTCT

             *       *                             **
     305052 GGGAATGCTGCTGTCTAGTTTTTATAT---GA-ATTCCCGC
        131 GAGAATGCTTCTGTCTAGTTTTTATATGAAGATATTCCCTT

                         *       *                *    *  *                  
     305089 TTCCAACGAAATCCTCAAAGCAATCCAAATATCCACTTGCAGAATCCACAAAAAGAGTGTTTCAA
          1 TTCCAACGAAATCATCAAAGCGATCCAAATATCCACTTCCAGATTCTACAAAAAGAGTGTTTCAA

                          *     *          * *                   *  *        
     305154 AACTGCTCTATCAATAGAAAGGTTCAACTCTTTTAGTTGAGTACACACATCACGAACAAGTTTCT
         66 AACTGCTCTATCAAAAGAAATGTTCAACTCTGTGAGTTGAGTACACACATCACAAAGAAGTTTCT

                            * *      **     **  *    
     305219 GAGAATGCTTCTGTCTGGCTTTTATTGGAAGACGTTTCCTT
        131 GAGAATGCTTCTGTCTAGTTTTTATATGAAGATATTCCCTT

                 * *  **          *       *                * *               
     305260 TT-CACCAAAGGCATCAAAGCGCTCCAAATGTCCACTTCCAGATTCTTCCAAAAGAGTGTTTCAA
          1 TTCCAACGAAATCATCAAAGCGATCCAAATATCCACTTCCAGATTCTACAAAAAGAGTGTTTCAA

             **     * ***  * *       *         *    * *  * *     *   *       
     305324 ACGTGCTCGAAGTAAGGGAATGTTCTACTCTGTGACTTGAATGCAGATATCACCAAGTAGTTTCT
         66 AACTGCTCTATCAAAAGAAATGTTCAACTCTGTGAGTTGAGTACACACATCACAAAGAAGTTTCT

            * * *             *     *    *         * 
     305389 AATAGTGCTTCTGTCTAGATTTTAGATGATGATATTCCCGT
        131 GAGAATGCTTCTGTCTAGTTTTTATATGAAGATATTCCCTT

                         * * *   *             *  *   *      *     *       * 
     305430 TTCCAACGAAATCGTTAGAGCTATCCAAATATCCAGTTACAGTTTCTACCAAAAGGGTGTTTCCA
          1 TTCCAACGAAATCATCAAAGCGATCCAAATATCCACTTCCAGATTCTACAAAAAGAGTGTTTCAA

              *    **           *            *       *                     * 
     305495 AATTGCTGCATCAAAAGAAAGGTTCAACTCTGTTAGTTGAGGACACACATCACAAAGAAGTTTGT
         66 AACTGCTCTATCAAAAGAAATGTTCAACTCTGTGAGTTGAGTACACACATCACAAAGAAGTTTCT

                              *    *     *           
     305560 GAGAATGCTTCTGTCTAGATTTTGTATGACGATATTCCCTT
        131 GAGAATGCTTCTGTCTAGTTTTTATATGAAGATATTCCCTT

                     *   * *     *   *       * *  *    *  *       *  *       
     305601 TTCCAACGATATCGTTAAAGCAATCTAAATATCAATTTGCAGAATCCACAAAAATAGAGTTTCAA
          1 TTCCAACGAAATCATCAAAGCGATCCAAATATCCACTTCCAGATTCTACAAAAAGAGTGTTTCAA

             *       * *        *    *       *  *                   ***      
     305666 AGCTGCTCTGTAAAAAGAAAGGTTCCACTCTGTTAGCTGAGTACACACATCACAAACTTGTTTCT
         66 AACTGCTCTATCAAAAGAAATGTTCAACTCTGTGAGTTGAGTACACACATCACAAAGAAGTTTCT

                  *         *        **         **   
     305731 GAGAATCCTTCTGTCTCGTTTTTATGGGAAGATATTTACTT
        131 GAGAATGCTTCTGTCTAGTTTTTATATGAAGATATTCCCTT

                 *  * **          *       *     *       *  *  *  *           
     305772 TTCCACCGTAGGCATCAAAGCGCTCCAAATGTCCACATCCAGATACTCCAGAACGAGTGTTTCAA
          1 TTCCAACGAAATCATCAAAGCGATCCAAATATCCACTTCCAGATTCTACAAAAAGAGTGTTTCAA

             *         *   * *   *         *        * *  *      *      *     
     305837 ACCTGCTCTATGAAAGGGAATCTTCAACTCTATGAGTTGAATGCAGACATCAGAAAGAAATTTCT
         66 AACTGCTCTATCAAAAGAAATGTTCAACTCTGTGAGTTGAGTACACACATCACAAAGAAGTTTCT

                     *       **          *         **
     305902 GAGAATGCTGCTGTCTACCTTTTAT-T--TGA-ATTCCCGC
        131 GAGAATGCTTCTGTCTAGTTTTTATATGAAGATATTCCCTT

                         *  *    *                *  **   *                  
     305939 TTCCAACGAAATCCTCCAAGCTATCCAAATATCCACTTGCATTTTCCACAAAAAGAGTGTTTCAA
          1 TTCCAACGAAATCATCAAAGCGATCCAAATATCCACTTCCAGATTCTACAAAAAGAGTGTTTCAA

                          *                 *    *  *                *       
     306004 AACTGCTCTATCAATAGAAATGTTCAACTCCTTTG-GCTGGGTACACACATCACAAACAAGTTTC
         66 AACTGCTCTATCAAAAGAAATGTTCAACT-CTGTGAGTTGAGTACACACATCACAAAGAAGTTTC

                                      **     **       
     306068 TGAGAATGCTTCTGTCTAGTTTTTATGGGAAGACGTTCCCTT
        130 TGAGAATGCTTCTGTCTAGTTTTTATATGAAGATATTCCCTT

              *  * *  **          *       *            **                  * 
     306110 TTTCACCAAAGGCATCAAAGCGCTCCAAATGTCCACTTCCAGACACTACAAAAAGAGTGTTTCCA
          1 TTCCAACGAAATCATCAAAGCGATCCAAATATCCACTTCCAGATTCTACAAAAAGAGTGTTTCAA

             **       **   ***                 *    * *  * *         *       
     306175 ACGTGCTCTAAGAAAGCGAATGTTCAACTCTGTGACTTGAATGCAGATATCACAAAGTAGTTTCT
         66 AACTGCTCTATCAAAAGAAATGTTCAACTCTGTGAGTTGAGTACACACATCACAAAGAAGTTTCT

                **            *     *    *         * 
     306240 GAGAGGGCTTCTGTCTAGATTTTAGATGATGATATTCCCGT
        131 GAGAATGCTTCTGTCTAGTTTTTATATGAAGATATTCCCTT

                           * *   *                *   *                    * 
     306281 TTCCAACGAAATCATTAGAGCTATCCAAATATCCACTTACAGTTTCTACAAAAAGAGTGTTTCCA
          1 TTCCAACGAAATCATCAAAGCGATCCAAATATCCACTTCCAGATTCTACAAAAAGAGTGTTTCAA

                   **         * *    *       *  *                   ***      
     306346 AACTGCTGCATCAAAAGAGAGGTTCCACTCTGTTAGCTGAGTACACACATCACAAACTTGTTTCT
         66 AACTGCTCTATCAAAAGAAATGTTCAACTCTGTGAGTTGAGTACACACATCACAAAGAAGTTTCT

            *     *         *        **         **   
     306411 CAGAATCCTTCTGTCTCGTTTTTATGGGAAGATATTTACTT
        131 GAGAATGCTTCTGTCTAGTTTTTATATGAAGATATTCCCTT

              *  *  * **          *       *     *       *  *  *              
     306452 TTTCACCGTAGGCATCAAAGCGCTCCAAATGTCCACATCCAGATACTCCAGAAAGAGTGTTTCAA
          1 TTCCAACGAAATCATCAAAGCGATCCAAATATCCACTTCCAGATTCTACAAAAAGAGTGTTTCAA

             *         *   * *   *         *        * *  *      *      *     
     306517 ACCTGCTCTATGAAAGGGAATCTTCAACTCTATGAGTTGAATGCAGACATCAGAAAGAAATTTCT
         66 AACTGCTCTATCAAAAGAAATGTTCAACTCTGTGAGTTGAGTACACACATCACAAAGAAGTTTCT

                     *       **          *         **
     306582 GAGAATGCTGCTGTCTACCTTTTAT-T--TGA-ATTCCCGC
        131 GAGAATGCTTCTGTCTAGTTTTTATATGAAGATATTCCCTT

                         *  *    *                *       *                  
     306619 TTCCAACGAAATCCTCCAAGCTATCCAAATATCCACTTGCAGATTCCACAAAAAGAGTGTTTCAA
          1 TTCCAACGAAATCATCAAAGCGATCCAAATATCCACTTCCAGATTCTACAAAAAGAGTGTTTCAA

                            ** *  *            *       *     *     *  *      
     306684 AACTGCTCTCTATCAATGGCAAAGTTCAACTCTGTTAGTTGAGGACACATATCACCAACAAGTTT
         66 AACTG--CTCTATCAAAAGAAATGTTCAACTCTGTGAGTTGAGTACACACATCACAAAGAAGTTT

                               *       **         *    
     306749 CTGAGAATGCTTCTGTCTATTTTTTATGGGAAGATATTTCCTT
        129 CTGAGAATGCTTCTGTCTAGTTTTTATATGAAGATATTCCCTT

             **  *  * ** *    *      *    *          * **                    
     306792 TATCACCGTAGGCGTCAAGGCGATCGAAATGTCCACTTCCACAAACTACAAAAAGAGTGTTTCAA
          1 TTCCAACGAAATCATCAAAGCGATCCAAATATCCACTTCCAGATTCTACAAAAAGAGTGTTTCAA

             *         *   * **       *    *        * ** * *   **      *     
     306857 ACCTGCTCTATGAAAGGCCATGTTCATCTCTATGAGTTGAATGGAAATATCCGAAAGAAATTTCT
         66 AACTGCTCTATCAAAAGAAATGTTCAACTCTGTGAGTTGAGTACACACATCACAAAGAAGTTTCT

             *       *          *        *         **
     306922 GGGAATGCTGCTGTCTAG-TGTT-TAT-ACGA-ATTCCCGC
        131 GAGAATGCTTCTGTCTAGTTTTTATATGAAGATATTCCCTT

                         *       *                *    *  *                  
     306959 TTCCAACGAAATCCTCAAAGCAATCCAAATATCCACTTGCAGAATCCACAAAAAGAGTGTTTCAA
          1 TTCCAACGAAATCATCAAAGCGATCCAAATATCCACTTCCAGATTCTACAAAAAGAGTGTTTCAA

               *          *     *          * *                   *  *        
     307024 AACCGCTCTATCAATAGAAAGGTTCAACTCTTTTAGTTGAGTACACACATCACGAACAAGTTTCT
         66 AACTGCTCTATCAAAAGAAATGTTCAACTCTGTGAGTTGAGTACACACATCACAAAGAAGTTTCT

                            * *      **     **  *    
     307089 GAGAATGCTTCTGTCTGGCTTTTATTGGAAGACGTTTCCTT
        131 GAGAATGCTTCTGTCTAGTTTTTATATGAAGATATTCCCTT

                 * *  **          *       *                * *               
     307130 TT-CACCAAAGGCATCAAAGCGCTCCAAATGTCCACTTCCAGATTCTTCCAAAAGAGTGTTTCAA
          1 TTCCAACGAAATCATCAAAGCGATCCAAATATCCACTTCCAGATTCTACAAAAAGAGTGTTTCAA

             **     * ***  * *        *        *    * *  * *     *   *       
     307194 ACGTGCTCGAAGTAAGGGAATGTTCATCTCTGTGACTTGAATGCAGATATCACCAAGTAGTTTCT
         66 AACTGCTCTATCAAAAGAAATGTTCAACTCTGTGAGTTGAGTACACACATCACAAAGAAGTTTCT

            * * *             *     *    *         * 
     307259 AATAGTGCTTCTGTCTAGATTTTAGATGATGATATTCCCGT
        131 GAGAATGCTTCTGTCTAGTTTTTATATGAAGATATTCCCTT

                         * * * * *             *  *   *      *     *       * 
     307300 TTCCAACGAAATCGTTAGATCTATCCAAATATCCAGTTACAGTTTCTACCAAAAGGGTGTTTCCA
          1 TTCCAACGAAATCATCAAAGCGATCCAAATATCCACTTCCAGATTCTACAAAAAGAGTGTTTCAA

              *    **           *            *       *                     * 
     307365 AATTGCTGCATCAAAAGAAAGGTTCAACTCTGTTAGTTGAGGACACACATCACAAAGAAGTTTGT
         66 AACTGCTCTATCAAAAGAAATGTTCAACTCTGTGAGTTGAGTACACACATCACAAAGAAGTTTCT

                              *    *     *           
     307430 GAGAATGCTTCTGTCTAGATTTTGTATGATGATATTCCCTT
        131 GAGAATGCTTCTGTCTAGTTTTTATATGAAGATATTCCCTT

                     *   * *     *   *       * *  *    *  *       *  *       
     307471 TTCCAACGATATCGTTAAAGCAATCTAAATATCAATTTGCAGAATCCACAAAAATAGAGTTTCAA
          1 TTCCAACGAAATCATCAAAGCGATCCAAATATCCACTTCCAGATTCTACAAAAAGAGTGTTTCAA

             *       * *        *    *       *  *                   ***      
     307536 AGCTGCTCTGTAAAAAGAAAGGTTCCACTCTGTTAGCTGAGTACACACATCACAAACTTGTTTCT
         66 AACTGCTCTATCAAAAGAAATGTTCAACTCTGTGAGTTGAGTACACACATCACAAAGAAGTTTCT

                  *         *        **         **   
     307601 GAGAATCCTTCTGTCTCGTTTTTATGGGAAGATATTTACTT
        131 GAGAATGCTTCTGTCTAGTTTTTATATGAAGATATTCCCTT

                 *  * **          *       *     *       *  *  *  *           
     307642 TTCCACCGTAGGCATCAAAGCGCTCCAAATGTCCACATCCAGATACTCCAGAACGAGTGTTTCAA
          1 TTCCAACGAAATCATCAAAGCGATCCAAATATCCACTTCCAGATTCTACAAAAAGAGTGTTTCAA

             *         *   * *   *         *        * *  *      *      *     
     307707 ACCTGCTCTATGAAAGGGAATCTTCAACTCTATGAGTTGAATGCAGACATCAGAAAGAAATTTCT
         66 AACTGCTCTATCAAAAGAAATGTTCAACTCTGTGAGTTGAGTACACACATCACAAAGAAGTTTCT

                     *       **          *         **
     307772 GAGAATGCTGCTGTCTACCTTTTAT-T--TGA-ATTCCCGC
        131 GAGAATGCTTCTGTCTAGTTTTTATATGAAGATATTCCCTT

                         *  *    *                *  **   *                  
     307809 TTCCAACGAAATCCTCCAAGCTATCCAAATATCCACTTGCATTTTCCACAAAAAGAGTGTTTCAA
          1 TTCCAACGAAATCATCAAAGCGATCCAAATATCCACTTCCAGATTCTACAAAAAGAGTGTTTCAA

                          *                 *    *  *                *       
     307874 AACTGCTCTATCAATAGAAATGTTCAACTCCTTTG-GCTGGGTACACACATCACAAACAAGTTTC
         66 AACTGCTCTATCAAAAGAAATGTTCAACT-CTGTGAGTTGAGTACACACATCACAAAGAAGTTTC

                                      **     **       
     307938 TGAGAATGCTTCTGTCTAGTTTTTATGGGAAGACGTTCCCTT
        130 TGAGAATGCTTCTGTCTAGTTTTTATATGAAGATATTCCCTT

              *  * *  **          *       *            **                  * 
     307980 TTTCACCAAAGGCATCAAAGCGCTCCAAATGTCCACTTCCAGACACTACAAAAAGAGTGTTTCCA
          1 TTCCAACGAAATCATCAAAGCGATCCAAATATCCACTTCCAGATTCTACAAAAAGAGTGTTTCAA

             **       **   ***                 *    * *  * *         *       
     308045 ACGTGCTCTAAGAAAGCGAATGTTCAACTCTGTGACTTGAATGCAGATATCACAAAGTAGTTTCT
         66 AACTGCTCTATCAAAAGAAATGTTCAACTCTGTGAGTTGAGTACACACATCACAAAGAAGTTTCT

                **            *     *    *         * 
     308110 GAGAGGGCTTCTGTCTAGATTTTAGATGATGATATTCCCGT
        131 GAGAATGCTTCTGTCTAGTTTTTATATGAAGATATTCCCTT

                           * *   *                *   *                    * 
     308151 TTCCAACGAAATCATTAGAGCTATCCAAATATCCACTTACAGTTTCTACAAAAAGAGTGTTTCCA
          1 TTCCAACGAAATCATCAAAGCGATCCAAATATCCACTTCCAGATTCTACAAAAAGAGTGTTTCAA

                   **         * *    *       *  *                   ***      
     308216 AACTGCTGCATCAAAAGAGAGGTTCCACTCTGTTAGCTGAGTACACACATCACAAACTTGTTTCT
         66 AACTGCTCTATCAAAAGAAATGTTCAACTCTGTGAGTTGAGTACACACATCACAAAGAAGTTTCT

            *     *         *        **         **   
     308281 CAGAATCCTTCTGTCTCGTTTTTATGGGAAGATATTTACTT
        131 GAGAATGCTTCTGTCTAGTTTTTATATGAAGATATTCCCTT

              *  *  * **          *       *     *       *  *  *              
     308322 TTTCACCGTAGGCATCAAAGCGCTCCAAATGTCCACATCCAGATACTCCAGAAAGAGTGTTTCAA
          1 TTCCAACGAAATCATCAAAGCGATCCAAATATCCACTTCCAGATTCTACAAAAAGAGTGTTTCAA

             *         *   * *   *         *        * *  *      *      *     
     308387 ACCTGCTCTATGAAAGGGAATCTTCAACTCTATGAGTTGAATGCAGACATCAGAAAGAAATTTCT
         66 AACTGCTCTATCAAAAGAAATGTTCAACTCTGTGAGTTGAGTACACACATCACAAAGAAGTTTCT

                     *       **          *         **
     308452 GAGAATGCTGCTGTCTACCTTTTAT-T--TGA-ATTCCCGC
        131 GAGAATGCTTCTGTCTAGTTTTTATATGAAGATATTCCCTT

                         *  *    *                *       *                  
     308489 TTCCAACGAAATCCTCCAAGCTATCCAAATATCCACTTGCAGATTCCACAAAAAGAGTGTTTCAA
          1 TTCCAACGAAATCATCAAAGCGATCCAAATATCCACTTCCAGATTCTACAAAAAGAGTGTTTCAA

                            ** *  *            *       *     *     *  *      
     308554 AACTGCTCTCTATCAATGGCAAAGTTCAACTCTGTTAGTTGAGGACACATATCACCAACAAGTTT
         66 AACTG--CTCTATCAAAAGAAATGTTCAACTCTGTGAGTTGAGTACACACATCACAAAGAAGTTT

                               *       **         *    
     308619 CTGAGAATGCTTCTGTCTATTTTTTATGGGAAGATATTTCCTT
        129 CTGAGAATGCTTCTGTCTAGTTTTTATATGAAGATATTCCCTT

              *  *  * ** *    *      *    *          * **                    
     308662 TTTCACCGTAGGCGTCAAGGCGATCGAAATGTCCACTTCCACAAACTACAAAAAGAGTGTTTCAA
          1 TTCCAACGAAATCATCAAAGCGATCCAAATATCCACTTCCAGATTCTACAAAAAGAGTGTTTCAA

             *         *   * **       *    *        * ** * *   **      *     
     308727 ACCTGCTCTATGAAAGGCCATGTTCATCTCTATGAGTTGAATGGAAATATCCGAAAGAAATTTCT
         66 AACTGCTCTATCAAAAGAAATGTTCAACTCTGTGAGTTGAGTACACACATCACAAAGAAGTTTCT

             *       *         *      *            **
     308792 GGGAATGCTGCTGTCTAGTGTTTATACG-A-AT-TT-CCGC
        131 GAGAATGCTTCTGTCTAGTTTTTATATGAAGATATTCCCTT

                         *       *                *    *  *                  
     308829 TTCCAACGAAATCCTCAAAGCAATCCAAATATCCACTTGCAGAATCCACAAAAAGAGTGTTTCAA
          1 TTCCAACGAAATCATCAAAGCGATCCAAATATCCACTTCCAGATTCTACAAAAAGAGTGTTTCAA

                          *     *          * *                   *  *        
     308894 AACTGCTCTATCAATAGAAAGGTTCAACTCTTTTAGTTGAGTACACACATCACGAACAAGTTTCT
         66 AACTGCTCTATCAAAAGAAATGTTCAACTCTGTGAGTTGAGTACACACATCACAAAGAAGTTTCT

                            * *      **     **  *    
     308959 GAGAATGCTTCTGTCTGGCTTTTATTGGAAGACGTTTCCTT
        131 GAGAATGCTTCTGTCTAGTTTTTATATGAAGATATTCCCTT

                 * *  **          *       *                * *               
     309000 TT-CACCAAAGGCATCAAAGCGCTCCAAATGTCCACTTCCAGATTCTTCCAAAAGAGTGTTTCAA
          1 TTCCAACGAAATCATCAAAGCGATCCAAATATCCACTTCCAGATTCTACAAAAAGAGTGTTTCAA

             **     * ***  * *                 *    * *  * *     *   *       
     309064 ACGTGCTCAAAGTAAGGGAATGTTCAACTCTGTGACTTGAATGCAGATATCACCAAGTAGTTTCT
         66 AACTGCTCTATCAAAAGAAATGTTCAACTCTGTGAGTTGAGTACACACATCACAAAGAAGTTTCT

            * * *            **     *    *         * 
     309129 AATAGTGCTTCTGTCTACATTTTAGATGATGATATTCCCGT
        131 GAGAATGCTTCTGTCTAGTTTTTATATGAAGATATTCCCTT

               *         * * *   *             *  *   *      *     *       * 
     309170 TTCAAACGAAATCGTTAGAGCTATCCAAATATCCAGTTACAGTTTCTACCAAAAGGGTGTTTCCA
          1 TTCCAACGAAATCATCAAAGCGATCCAAATATCCACTTCCAGATTCTACAAAAAGAGTGTTTCAA

              *    **           *            *       *                     * 
     309235 AATTGCTGCATCAAAAGAAAGGTTCAACTCTGTTAGTTGAGGACACACATCACAAAGAAGTTTGT
         66 AACTGCTCTATCAAAAGAAATGTTCAACTCTGTGAGTTGAGTACACACATCACAAAGAAGTTTCT

                              *    *     *           
     309300 GAGAATGCTTCTGTCTAGATTTTGTATGACGATATTCCCTT
        131 GAGAATGCTTCTGTCTAGTTTTTATATGAAGATATTCCCTT

                     *   * *     *   *       * *  *    *  *       *  *       
     309341 TTCCAACGATATCGTTAAAGCAATCTAAATATCAATTTGCAGAATCCACAAAAATAGAGTTTCAA
          1 TTCCAACGAAATCATCAAAGCGATCCAAATATCCACTTCCAGATTCTACAAAAAGAGTGTTTCAA

             *       * *        *    *       *  *                   ***      
     309406 AGCTGCTCTGTAAAAAGAAAGGTTCCACTCTGTTAGCTGAGTACACACATCACAAACTTGTTTCT
         66 AACTGCTCTATCAAAAGAAATGTTCAACTCTGTGAGTTGAGTACACACATCACAAAGAAGTTTCT

            *     *         *        **         **   
     309471 CAGAATCCTTCTGTCTCGTTTTTATGGGAAGATATTTACTT
        131 GAGAATGCTTCTGTCTAGTTTTTATATGAAGATATTCCCTT

                 *  * **          *       *     *       *  *  *              
     309512 TTCCACCGTAGGCATCAAAGCGCTCCAAATGTCCACATCCAGATACTCCAGAAAGAGTGTTTCAA
          1 TTCCAACGAAATCATCAAAGCGATCCAAATATCCACTTCCAGATTCTACAAAAAGAGTGTTTCAA

             *         *   * *             *        * *  *      *      *     
     309577 ACCTGCTCTATGAAAGGGAATGTTCAACTCTATGAGTTGAATGCAGACATCAGAAAGAAATTTCT
         66 AACTGCTCTATCAAAAGAAATGTTCAACTCTGTGAGTTGAGTACACACATCACAAAGAAGTTTCT

                     *       **          *         **
     309642 GAGAATGCTGCTGTCTACCTTTTAT-T--TGA-ATTCCCGC
        131 GAGAATGCTTCTGTCTAGTTTTTATATGAAGATATTCCCTT

                         *  *    *                *  **   *                  
     309679 TTCCAACGAAATCCTCCAAGCTATCCAAATATCCACTTGCATTTTCCACAAAAAGAGTGTTTCAA
          1 TTCCAACGAAATCATCAAAGCGATCCAAATATCCACTTCCAGATTCTACAAAAAGAGTGTTTCAA

                          *               ** *  *  *                *        
     309744 AACTGCTCTATCAATAGAAATGTTCAACTCCTTTAGCTGGGTACACACATCACAAACAAGTTTCT
         66 AACTGCTCTATCAAAAGAAATGTTCAACTCTGTGAGTTGAGTACACACATCACAAAGAAGTTTCT

                                     **     **       
     309809 GAGAATGCTTCTGTCTAGTTTTTATGGGAAGACGTTCCCTT
        131 GAGAATGCTTCTGTCTAGTTTTTATATGAAGATATTCCCTT

              *  * *  **          *       *        *   **                  * 
     309850 TTTCACCAAAGGCATCAAAGCGCTCCAAATGTCCACTTCAAGACACTACAAAAAGAGTGTTTCCA
          1 TTCCAACGAAATCATCAAAGCGATCCAAATATCCACTTCCAGATTCTACAAAAAGAGTGTTTCAA

             **       **   ***                 *    * *  * *         *       
     309915 ACGTGCTCTAAGAAAGCGAATGTTCAACTCTGTGACTTGAATGCAGATATCACAAAGTAGTTTCT
         66 AACTGCTCTATCAAAAGAAATGTTCAACTCTGTGAGTTGAGTACACACATCACAAAGAAGTTTCT

                **            *     *    *         * 
     309980 GAGAGGGCTTCTGTCTAGATTTTAGATGATGATATTCCCGT
        131 GAGAATGCTTCTGTCTAGTTTTTATATGAAGATATTCCCTT

                           * *   *                *   *                    * 
     310021 TTCCAACGAAATCATTAGAGCTATCCAAATATCCACTTACAGTTTCTACAAAAAGAGTGTTTCCA
          1 TTCCAACGAAATCATCAAAGCGATCCAAATATCCACTTCCAGATTCTACAAAAAGAGTGTTTCAA

                   **         * *    *       *  *                   ***      
     310086 AACTGCTGCATCAAAAGAGAGGTTCCACTCTGTTAGCTGAGTACACACATCACAAACTTGTTTCT
         66 AACTGCTCTATCAAAAGAAATGTTCAACTCTGTGAGTTGAGTACACACATCACAAAGAAGTTTCT

            *     *         *        **         **   
     310151 CAGAATCCTTCTGTCTCGTTTTTATGGGAAGATATTTACTT
        131 GAGAATGCTTCTGTCTAGTTTTTATATGAAGATATTCCCTT

              *  *  * **          *       *     *       *  *  *              
     310192 TTTCACCGTAGGCATCAAAGCGCTCCAAATGTCCACATCCAGATACTCCAGAAAGAGTGTTTCAA
          1 TTCCAACGAAATCATCAAAGCGATCCAAATATCCACTTCCAGATTCTACAAAAAGAGTGTTTCAA

             *         *   * *   *         *        * *  *      *      *     
     310257 ACCTGCTCTATGAAAGGGAATCTTCAACTCTATGAGTTGAATGCAGACATCAGAAAGAAATTTCT
         66 AACTGCTCTATCAAAAGAAATGTTCAACTCTGTGAGTTGAGTACACACATCACAAAGAAGTTTCT

                     *       **          *         **
     310322 GAGAATGCTGCTGTCTACCTTTTAT-T--TGA-ATTCCCGC
        131 GAGAATGCTTCTGTCTAGTTTTTATATGAAGATATTCCCTT

                         *  *    *                *       *                  
     310359 TTCCAACGAAATCCTCCAAGCTATCCAAATATCCACTTGCAGATTCCACAAAAAGAGTGTTTCAA
          1 TTCCAACGAAATCATCAAAGCGATCCAAATATCCACTTCCAGATTCTACAAAAAGAGTGTTTCAA

                            ** *  *            *       *     *     *  *      
     310424 AACTGCTCTCTATCAATGGCAAAGTTCAACTCTGTTAGTTGAGGACACATATCACCAACAAGTTT
         66 AACTG--CTCTATCAAAAGAAATGTTCAACTCTGTGAGTTGAGTACACACATCACAAAGAAGTTT

                               *       **         *    
     310489 CTGAGAATGCTTCTGTCTATTTTTTATGGGAAGATATTTCCTT
        129 CTGAGAATGCTTCTGTCTAGTTTTTATATGAAGATATTCCCTT

              *  *  * ** *    *      *    *          * **                    
     310532 TTTCACCGTAGGCGTCAAGGCGATCGAAATGTCCACTTCCACAAACTACAAAAAGAGTGTTTCAA
          1 TTCCAACGAAATCATCAAAGCGATCCAAATATCCACTTCCAGATTCTACAAAAAGAGTGTTTCAA

             *         *   * **       *    *        * ** * *   **      *     
     310597 ACCTGCTCTATGAAAGGCCATGTTCATCTCTATGAGTTGAATGGAAATATCCGAAAGAAATTTCT
         66 AACTGCTCTATCAAAAGAAATGTTCAACTCTGTGAGTTGAGTACACACATCACAAAGAAGTTTCT

             *       *          *        *         **
     310662 GGGAATGCTGCTGTCTAG-TGTT-TAT-ACGA-ATTCCCAC
        131 GAGAATGCTTCTGTCTAGTTTTTATATGAAGATATTCCCTT

                         *       *                *    *  *                  
     310699 TTCCAACGAAATCCTCAAAGCAATCCAAATATCCACTTGCAGAATCCACAAAAAGAGTGTTTCAA
          1 TTCCAACGAAATCATCAAAGCGATCCAAATATCCACTTCCAGATTCTACAAAAAGAGTGTTTCAA

                          *     *          * *                   *  *        
     310764 AACTGCTCTATCAATAGAAAGGTTCAACTCTTTTAGTTGAGTACACACATCACGAACAAGTTTCT
         66 AACTGCTCTATCAAAAGAAATGTTCAACTCTGTGAGTTGAGTACACACATCACAAAGAAGTTTCT

                            * *      **     **  *    
     310829 GAGAATGCTTCTGTCTGGCTTTTATTGGAAGACGTTTCCTT
        131 GAGAATGCTTCTGTCTAGTTTTTATATGAAGATATTCCCTT

                 * *  **          *       *                * *               
     310870 TT-CACCAAAGGCATCAAAGCGCTCCAAATGTCCACTTCCAGATTCTTCCAAAAGAGTGTTTCAA
          1 TTCCAACGAAATCATCAAAGCGATCCAAATATCCACTTCCAGATTCTACAAAAAGAGTGTTTCAA

             **     * ***  * *                 *    * *  * *     *   *       
     310934 ACGTGCTCAAAGTAAGGGAATGTTCAACTCTGTGACTTGAATGCAGATATCACCAAGTAGTTTCT
         66 AACTGCTCTATCAAAAGAAATGTTCAACTCTGTGAGTTGAGTACACACATCACAAAGAAGTTTCT

            * * *             *     *    *         * 
     310999 AATAGTGCTTCTGTCTAGATTTTAGATGATGATATTCCCGT
        131 GAGAATGCTTCTGTCTAGTTTTTATATGAAGATATTCCCTT

                         * * *   *                *   *                    * 
     311040 TTCCAACGAAATCGTTAGAGCTATCCAAATATCCACTTACAGTTTCTACAAAAAGAGTGTTTCCA
          1 TTCCAACGAAATCATCAAAGCGATCCAAATATCCACTTCCAGATTCTACAAAAAGAGTGTTTCAA

                   **           *            *       *                     * 
     311105 AACTGCTGCATCAAAAGAAAGGTTCAACTCTGTTAGTTGAGGACACACATCACAAAGAAGTTTGT
         66 AACTGCTCTATCAAAAGAAATGTTCAACTCTGTGAGTTGAGTACACACATCACAAAGAAGTTTCT

                              *    *     *           
     311170 GAGAATGCTTCTGTCTAGATTTTGTATGACGATATTCCCTT
        131 GAGAATGCTTCTGTCTAGTTTTTATATGAAGATATTCCCTT

                     *   * *     *   *       * *  *    *  *       *  *       
     311211 TTCCAACGATATCGTTAAAGCAATCTAAATATCAATTTGCAGAATCCACAAAAATAGAGTTTCAA
          1 TTCCAACGAAATCATCAAAGCGATCCAAATATCCACTTCCAGATTCTACAAAAAGAGTGTTTCAA

             *       * *        *    *       *  *                   ***      
     311276 AGCTGCTCTGTAAAAAGAAAGGTTCCACTCTGTTAGCTGAGTACACACATCACAAACTTGTTTCT
         66 AACTGCTCTATCAAAAGAAATGTTCAACTCTGTGAGTTGAGTACACACATCACAAAGAAGTTTCT

            *     *         *        **         **   
     311341 CAGAATCCTTCTGTCTCGTTTTTATGGGAAGATATTTACTT
        131 GAGAATGCTTCTGTCTAGTTTTTATATGAAGATATTCCCTT

                 *  * **          *       *     *       *  *  *  *           
     311382 TTCCACCGTAGGCATCAAAGCGCTCCAAATGTCCACATCCAGATACTCCAGAACGAGTGTTTCAA
          1 TTCCAACGAAATCATCAAAGCGATCCAAATATCCACTTCCAGATTCTACAAAAAGAGTGTTTCAA

             *         *   * *   *         *        * *  *      *      *     
     311447 ACCTGCTCTATGAAAGGGAATCTTCAACTCTATGAGTTGAATGCAGACATCAGAAAGAAATTTCT
         66 AACTGCTCTATCAAAAGAAATGTTCAACTCTGTGAGTTGAGTACACACATCACAAAGAAGTTTCT

                     *       **          *         **
     311512 GAGAATGCTGCTGTCTACCTTTTAT-T--TGA-ATTCCCGC
        131 GAGAATGCTTCTGTCTAGTTTTTATATGAAGATATTCCCTT

                         *  *    *                *  **   *                  
     311549 TTCCAACGAAATCCTCCAAGCTATCCAAATATCCACTTGCATTTTCCACAAAAAGAGTGTTTCAA
          1 TTCCAACGAAATCATCAAAGCGATCCAAATATCCACTTCCAGATTCTACAAAAAGAGTGTTTCAA

                          *                 *    *  *                *       
     311614 AACTGCTCTATCAATAGAAATGTTCAACTCCTTTG-GCTGGGTACACACATCACAAACAAGTTTC
         66 AACTGCTCTATCAAAAGAAATGTTCAACT-CTGTGAGTTGAGTACACACATCACAAAGAAGTTTC

                                      **     **       
     311678 TGAGAATGCTTCTGTCTAGTTTTTATGGGAAGACGTTCCCTT
        130 TGAGAATGCTTCTGTCTAGTTTTTATATGAAGATATTCCCTT

              *  * *  **          *       *            **                  * 
     311720 TTTCACCAAAGGCATCAAAGCGCTCCAAATGTCCACTTCCAGACACTACAAAAAGAGTGTTTCCA
          1 TTCCAACGAAATCATCAAAGCGATCCAAATATCCACTTCCAGATTCTACAAAAAGAGTGTTTCAA

             **       **   ***                 *    * *  * *         *       
     311785 ACGTGCTCTAAGAAAGCGAATGTTCAACTCTGTGACTTGAATGCAGATATCACAAAGTAGTTTCT
         66 AACTGCTCTATCAAAAGAAATGTTCAACTCTGTGAGTTGAGTACACACATCACAAAGAAGTTTCT

                **            *     *    *         * 
     311850 GAGAGGGCTTCTGTCTAGATTTTAGATGATGATATTCCCGT
        131 GAGAATGCTTCTGTCTAGTTTTTATATGAAGATATTCCCTT

                           * *   *                *   *                    * 
     311891 TTCCAACGAAATCATTAGAGCTATCCAAATATCCACTTACAGTTTCTACAAAAAGAGTGTTTCCA
          1 TTCCAACGAAATCATCAAAGCGATCCAAATATCCACTTCCAGATTCTACAAAAAGAGTGTTTCAA

                   **         * *    *       *  *                   ***      
     311956 AACTGCTGCATCAAAAGAGAGGTTCCACTCTGTTAGCTGAGTACACACATCACAAACTTGTTTCT
         66 AACTGCTCTATCAAAAGAAATGTTCAACTCTGTGAGTTGAGTACACACATCACAAAGAAGTTTCT

            *     *         *        **         **   
     312021 CAGAATCCTTCTGTCTCGTTTTTATGGGAAGATATTTACTT
        131 GAGAATGCTTCTGTCTAGTTTTTATATGAAGATATTCCCTT

              *  *  * **          *       *     *       *  *  *              
     312062 TTTCACCGTAGGCATCAAAGCGCTCCAAATGTCCACATCCAGATACTCCAGAAAGAGTGTTTCAA
          1 TTCCAACGAAATCATCAAAGCGATCCAAATATCCACTTCCAGATTCTACAAAAAGAGTGTTTCAA

             *         *   * *   *         *        * *  *      *      *     
     312127 ACCTGCTCTATGAAAGGGAATCTTCAACTCTATGAGTTGAATGCAGACATCAGAAAGAAATTTCT
         66 AACTGCTCTATCAAAAGAAATGTTCAACTCTGTGAGTTGAGTACACACATCACAAAGAAGTTTCT

                     *       **          *         **
     312192 GAGAATGCTGCTGTCTACCTTTTAT-T--TGA-ATTCCCGC
        131 GAGAATGCTTCTGTCTAGTTTTTATATGAAGATATTCCCTT

                         *  *    *                *       *                  
     312229 TTCCAACGAAATCCTCCAAGCTATCCAAATATCCACTTGCAGATTCCACAAAAAGAGTGTTTCAA
          1 TTCCAACGAAATCATCAAAGCGATCCAAATATCCACTTCCAGATTCTACAAAAAGAGTGTTTCAA

                            ** *  *            *       *     *     *  *      
     312294 AACTGCTCTCTATCAATGGCAAAGTTCAACTCTGTTAGTTGAGGACACATATCACCAACAAGTTT
         66 AACTG--CTCTATCAAAAGAAATGTTCAACTCTGTGAGTTGAGTACACACATCACAAAGAAGTTT

                               *       **         *    
     312359 CTGAGAATGCTTCTGTCTATTTTTTATGGGAAGATATTTCCTT
        129 CTGAGAATGCTTCTGTCTAGTTTTTATATGAAGATATTCCCTT

              *  *  * ** *    *      *    *          * **                    
     312402 TTTCACCGTAGGCGTCAAGGCGATCGAAATGTCCACTTCCACAAACTACAAAAAGAGTGTTTCAA
          1 TTCCAACGAAATCATCAAAGCGATCCAAATATCCACTTCCAGATTCTACAAAAAGAGTGTTTCAA

             *         *   * **       *    *        * ** * *   **   *  *     
     312467 ACCTGCTCTATGAAAGGCCATGTTCATCTCTATGAGTTGAATGGAAATATCCGAAAAAAATTTCT
         66 AACTGCTCTATCAAAAGAAATGTTCAACTCTGTGAGTTGAGTACACACATCACAAAGAAGTTTCT

             *       *                             **
     312532 GGGAATGCTGCTGTCTAGTTTTTATAT---GA-ATTCCCGC
        131 GAGAATGCTTCTGTCTAGTTTTTATATGAAGATATTCCCTT

                         *       *                *    *  *                  
     312569 TTCCAACGAAATCCTCAAAGCAATCCAAATATCCACTTGCAGAATCCACAAAAAGAGTGTTTCAA
          1 TTCCAACGAAATCATCAAAGCGATCCAAATATCCACTTCCAGATTCTACAAAAAGAGTGTTTCAA

                          *     *          * *                   *  *        
     312634 AACTGCTCTATCAATAGAAAGGTTCAACTCTTTTAGTTGAGTACACACATCACGAACAAGTTTCT
         66 AACTGCTCTATCAAAAGAAATGTTCAACTCTGTGAGTTGAGTACACACATCACAAAGAAGTTTCT

                            * *      **     **  *    
     312699 GAGAATGCTTCTGTCTGGCTTTTATTGGAAGACGTTTCCTT
        131 GAGAATGCTTCTGTCTAGTTTTTATATGAAGATATTCCCTT

                 * *  **          *       *                * *               
     312740 TT-CACCAAAGGCATCAAAGCGCTCCAAATGTCCACTTCCAGATTCTTCCAAAAGAGTGTTTCAA
          1 TTCCAACGAAATCATCAAAGCGATCCAAATATCCACTTCCAGATTCTACAAAAAGAGTGTTTCAA

             **     * ***  * *       *         *    * *  * *     *   *       
     312804 ACGTGCTCGAAGTAAGGGAATGTTCTACTCTGTGACTTGAATGCAGATATCACCAAGTAGTTTCT
         66 AACTGCTCTATCAAAAGAAATGTTCAACTCTGTGAGTTGAGTACACACATCACAAAGAAGTTTCT

            * * *             *     *    *         * 
     312869 AATAGTGCTTCTGTCTAGATTTTAGATGATGATATTCCCGT
        131 GAGAATGCTTCTGTCTAGTTTTTATATGAAGATATTCCCTT

                         * * *   *             *  *   *      *     *       * 
     312910 TTCCAACGAAATCGTTAGAGCTATCCAAATATCCAGTTACAGTTTCTACCAAAAGGGTGTTTCCA
          1 TTCCAACGAAATCATCAAAGCGATCCAAATATCCACTTCCAGATTCTACAAAAAGAGTGTTTCAA

              *    **           *            *       *                     * 
     312975 AATTGCTGCATCAAAAGAAAGGTTCAACTCTGTTAGTTGAGGACACACATCACAAAGAAGTTTGT
         66 AACTGCTCTATCAAAAGAAATGTTCAACTCTGTGAGTTGAGTACACACATCACAAAGAAGTTTCT

                              *    *     *           
     313040 GAGAATGCTTCTGTCTAGATTTTGTATGACGATATTCCCTT
        131 GAGAATGCTTCTGTCTAGTTTTTATATGAAGATATTCCCTT

                     *   * *     *   *       * *  *    *  *       *  *       
     313081 TTCCAACGATATCGTTAAAGCAATCTAAATATCAATTTGCAGAATCCACAAAAATAGAGTTTCAA
          1 TTCCAACGAAATCATCAAAGCGATCCAAATATCCACTTCCAGATTCTACAAAAAGAGTGTTTCAA

             *       * *        *    *       *  *                   ***      
     313146 AGCTGCTCTGTAAAAAGAAAGGTTCCACTCTGTTAGCTGAGTACACACATCACAAACTTGTTTCT
         66 AACTGCTCTATCAAAAGAAATGTTCAACTCTGTGAGTTGAGTACACACATCACAAAGAAGTTTCT

                  *         *        **         **   
     313211 GAGAATCCTTCTGTCTCGTTTTTATGGGAAGATATTTACTT
        131 GAGAATGCTTCTGTCTAGTTTTTATATGAAGATATTCCCTT

                 *  * **          *       *     *       *  *  *  *           
     313252 TTCCACCGTAGGCATCAAAGCGCTCCAAATGTCCACATCCAGATACTCCAGAACGAGTGTTTCAA
          1 TTCCAACGAAATCATCAAAGCGATCCAAATATCCACTTCCAGATTCTACAAAAAGAGTGTTTCAA

             *         *   * *   *         *        * *  *      *      *     
     313317 ACCTGCTCTATGAAAGGGAATCTTCAACTCTATGAGTTGAATGCAGACATCAGAAAGAAATTTCT
         66 AACTGCTCTATCAAAAGAAATGTTCAACTCTGTGAGTTGAGTACACACATCACAAAGAAGTTTCT

                     *       **          *         **
     313382 GAGAATGCTGCTGTCTACCTTTTAT-T--TGA-ATTCCCGC
        131 GAGAATGCTTCTGTCTAGTTTTTATATGAAGATATTCCCTT

                         *  *    *                *  **   *                  
     313419 TTCCAACGAAATCCTCCAAGCTATCCAAATATCCACTTGCATTTTCCACAAAAAGAGTGTTTCAA
          1 TTCCAACGAAATCATCAAAGCGATCCAAATATCCACTTCCAGATTCTACAAAAAGAGTGTTTCAA

                          *                 *    *  *                *       
     313484 AACTGCTCTATCAATAGAAATGTTCAACTCCTTTG-GCTGGGTACACACATCACAAACAAGTTTC
         66 AACTGCTCTATCAAAAGAAATGTTCAACT-CTGTGAGTTGAGTACACACATCACAAAGAAGTTTC

                                      **     **       
     313548 TGAGAATGCTTCTGTCTAGTTTTTATGGGAAGACGTTCCCTT
        130 TGAGAATGCTTCTGTCTAGTTTTTATATGAAGATATTCCCTT

              *  * *  **          *       *            **                  * 
     313590 TTTCACCAAAGGCATCAAAGCGCTCCAAATGTCCACTTCCAGACACTACAAAAAGAGTGTTTCCA
          1 TTCCAACGAAATCATCAAAGCGATCCAAATATCCACTTCCAGATTCTACAAAAAGAGTGTTTCAA

             **       **   ***                 *    * *  * *         *       
     313655 ACGTGCTCTAAGAAAGCGAATGTTCAACTCTGTGACTTGAATGCAGATATCACAAAGTAGTTTCT
         66 AACTGCTCTATCAAAAGAAATGTTCAACTCTGTGAGTTGAGTACACACATCACAAAGAAGTTTCT

                **            *     *    *         * 
     313720 GAGAGGGCTTCTGTCTAGATTTTAGATGATGATATTCCCGT
        131 GAGAATGCTTCTGTCTAGTTTTTATATGAAGATATTCCCTT

                           * *   *                *   *                    * 
     313761 TTCCAACGAAATCATTAGAGCTATCCAAATATCCACTTACAGTTTCTACAAAAAGAGTGTTTCCA
          1 TTCCAACGAAATCATCAAAGCGATCCAAATATCCACTTCCAGATTCTACAAAAAGAGTGTTTCAA

                   **         * *    *       *  *                   ***      
     313826 AACTGCTGCATCAAAAGAGAGGTTCCACTCTGTTAGCTGAGTACACACATCACAAACTTGTTTCT
         66 AACTGCTCTATCAAAAGAAATGTTCAACTCTGTGAGTTGAGTACACACATCACAAAGAAGTTTCT

            *     *         *        **         **   
     313891 CAGAATCCTTCTGTCTCGTTTTTATGGGAAGATATTTACTT
        131 GAGAATGCTTCTGTCTAGTTTTTATATGAAGATATTCCCTT

              *  *  * **          *       *     *       *  *  *              
     313932 TTTCACCGTAGGCATCAAAGCGCTCCAAATGTCCACATCCAGATACTCCAGAAAGAGTGTTTCAA
          1 TTCCAACGAAATCATCAAAGCGATCCAAATATCCACTTCCAGATTCTACAAAAAGAGTGTTTCAA

             *         *   * *   *         *        * *  *      *      *     
     313997 ACCTGCTCTATGAAAGGGAATCTTCAACTCTATGAGTTGAATGCAGACATCAGAAAGAAATTTCT
         66 AACTGCTCTATCAAAAGAAATGTTCAACTCTGTGAGTTGAGTACACACATCACAAAGAAGTTTCT

                     *       **          *         **
     314062 GAGAATGCTGCTGTCTACCTTTTAT-T--TGA-ATTCCCGC
        131 GAGAATGCTTCTGTCTAGTTTTTATATGAAGATATTCCCTT

                         *  *    *                *       *                  
     314099 TTCCAACGAAATCCTCCAAGCTATCCAAATATCCACTTGCAGATTCCACAAAAAGAGTGTTTCAA
          1 TTCCAACGAAATCATCAAAGCGATCCAAATATCCACTTCCAGATTCTACAAAAAGAGTGTTTCAA

                            ** *  *            *       *     *     *  *      
     314164 AACTGCTCTCTATCAATGGCAAAGTTCAACTCTGTTAGTTGAGGACACATATCACCAACAAGTTT
         66 AACTG--CTCTATCAAAAGAAATGTTCAACTCTGTGAGTTGAGTACACACATCACAAAGAAGTTT

                               *       **         *    
     314229 CTGAGAATGCTTCTGTCTATTTTTTATGGGAAGATATTTCCTT
        129 CTGAGAATGCTTCTGTCTAGTTTTTATATGAAGATATTCCCTT

             **  *  * ** *    *      *    *          * **                    
     314272 TATCACCGTAGGCGTCAAGGCGATCGAAATGTCCACTTCCACAAACTACAAAAAGAGTGTTTCAA
          1 TTCCAACGAAATCATCAAAGCGATCCAAATATCCACTTCCAGATTCTACAAAAAGAGTGTTTCAA

             *         *   * **       *    *        * ** * *   **      *     
     314337 ACCTGCTCTATGAAAGGCCATGTTCATCTCTATGAGTTGAATGGAAATATCCGAAAGAAATTTCT
         66 AACTGCTCTATCAAAAGAAATGTTCAACTCTGTGAGTTGAGTACACACATCACAAAGAAGTTTCT

             *       *         *                   **
     314402 GGGAATGCTGCTGTCTAGTGTTTATAT---GA-ATTCCCGC
        131 GAGAATGCTTCTGTCTAGTTTTTATATGAAGATATTCCCTT

                         *       *                *    *  *                  
     314439 TTCCAACGAAATCCTCAAAGCAATCCAAATATCCACTTGCAGAATCCACAAAAAGAGTGTTTCAA
          1 TTCCAACGAAATCATCAAAGCGATCCAAATATCCACTTCCAGATTCTACAAAAAGAGTGTTTCAA

               *          *     *          * *                   *  *        
     314504 AACCGCTCTATCAATAGAAAGGTTCAACTCTTTTAGTTGAGTACACACATCACGAACAAGTTTCT
         66 AACTGCTCTATCAAAAGAAATGTTCAACTCTGTGAGTTGAGTACACACATCACAAAGAAGTTTCT

                            * *      **     **  *    
     314569 GAGAATGCTTCTGTCTGGCTTTTATTGGAAGACGTTTCCTT
        131 GAGAATGCTTCTGTCTAGTTTTTATATGAAGATATTCCCTT

                 * *  **          *       *                * *               
     314610 TT-CACCAAAGGCATCAAAGCGCTCCAAATGTCCACTTCCAGATTCTTCCAAAAGAGTGTTTCAA
          1 TTCCAACGAAATCATCAAAGCGATCCAAATATCCACTTCCAGATTCTACAAAAAGAGTGTTTCAA

             **     * ***  * *        *        *    * *  * *     *   *       
     314674 ACGTGCTCGAAGTAAGGGAATGTTCATCTCTGTGACTTGAATGCAGATATCACCAAGTAGTTTCT
         66 AACTGCTCTATCAAAAGAAATGTTCAACTCTGTGAGTTGAGTACACACATCACAAAGAAGTTTCT

            * * *             *     *    *         * 
     314739 AATAGTGCTTCTGTCTAGATTTTAGATGATGATATTCCCGT
        131 GAGAATGCTTCTGTCTAGTTTTTATATGAAGATATTCCCTT

                         * * * * *             *  *   *      *     *       * 
     314780 TTCCAACGAAATCGTTAGATCTATCCAAATATCCAGTTACAGTTTCTACCAAAAGGGTGTTTCCA
          1 TTCCAACGAAATCATCAAAGCGATCCAAATATCCACTTCCAGATTCTACAAAAAGAGTGTTTCAA

              *    **           *            *       *                     * 
     314845 AATTGCTGCATCAAAAGAAAGGTTCAACTCTGTTAGTTGAGGACACACATCACAAAGAAGTTTGT
         66 AACTGCTCTATCAAAAGAAATGTTCAACTCTGTGAGTTGAGTACACACATCACAAAGAAGTTTCT

                              *    *     *           
     314910 GAGAATGCTTCTGTCTAGATTTTGTATGATGATATTCCCTT
        131 GAGAATGCTTCTGTCTAGTTTTTATATGAAGATATTCCCTT

                     *   * *     *   *       * *  *    *  *       *  *       
     314951 TTCCAACGATATCGTTAAAGCAATCTAAATATCAATTTGCAGAATCCACAAAAATAGAGTTTCAA
          1 TTCCAACGAAATCATCAAAGCGATCCAAATATCCACTTCCAGATTCTACAAAAAGAGTGTTTCAA

             *       * *        *    *       *  *                   ***      
     315016 AGCTGCTCTGTAAAAAGAAAGGTTCCACTCTGTTAGCTGAGTACACACATCACAAACTTGTTTCT
         66 AACTGCTCTATCAAAAGAAATGTTCAACTCTGTGAGTTGAGTACACACATCACAAAGAAGTTTCT

                  *         *        **         **   
     315081 GAGAATCCTTCTGTCTCGTTTTTATGGGAAGATATTTACTT
        131 GAGAATGCTTCTGTCTAGTTTTTATATGAAGATATTCCCTT

                 *  * **          *       *     *       *  *  *              
     315122 TTCCACCGTAGGCATCAAAGCGCTCCAAATGTCCACATCCAGATACTCCAGAAAGAGTGTTTCAA
          1 TTCCAACGAAATCATCAAAGCGATCCAAATATCCACTTCCAGATTCTACAAAAAGAGTGTTTCAA

             *         *   * *   *         *        * *  *      *      *     
     315187 ACCTGCTCTATGAAAGGGAATCTTCAACTCTATGAGTTGAATGCAGACATCAGAAAGAAATTTCT
         66 AACTGCTCTATCAAAAGAAATGTTCAACTCTGTGAGTTGAGTACACACATCACAAAGAAGTTTCT

                     *       **          *         **
     315252 GAGAATGCTGCTGTCTACCTTTTAT-T--TGA-ATTCCCGC
        131 GAGAATGCTTCTGTCTAGTTTTTATATGAAGATATTCCCTT

                         *  *    *                *  **   *                  
     315289 TTCCAACGAAATCCTCCAAGCTATCCAAATATCCACTTGCATTTTCCACAAAAAGAGTGTTTCAA
          1 TTCCAACGAAATCATCAAAGCGATCCAAATATCCACTTCCAGATTCTACAAAAAGAGTGTTTCAA

                          *                 *    *  *                *       
     315354 AACTGCTCTATCAATAGAAATGTTCAACTCCTTTG-GCTGGGTACACACATCACAAACAAGTTTC
         66 AACTGCTCTATCAAAAGAAATGTTCAACT-CTGTGAGTTGAGTACACACATCACAAAGAAGTTTC

                                      **     **       
     315418 TGAGAATGCTTCTGTCTAGTTTTTATGGGAAGACGTTCCCTT
        130 TGAGAATGCTTCTGTCTAGTTTTTATATGAAGATATTCCCTT

              *  * *  **          *       *            **                  * 
     315460 TTTCACCAAAGGCATCAAAGCGCTCCAAATGTCCACTTCCAGACACTACAAAAAGAGTGTTTCCA
          1 TTCCAACGAAATCATCAAAGCGATCCAAATATCCACTTCCAGATTCTACAAAAAGAGTGTTTCAA

             **       **   ***                 *    * *  * *         *       
     315525 ACGTGCTCTAAGAAAGCGAATGTTCAACTCTGTGACTTGAATGCAGATATCACAAAGTAGTTTCT
         66 AACTGCTCTATCAAAAGAAATGTTCAACTCTGTGAGTTGAGTACACACATCACAAAGAAGTTTCT

                **            *     *    *         * 
     315590 GAGAGGGCTTCTGTCTAGATTTTAGATGATGATATTCCCGT
        131 GAGAATGCTTCTGTCTAGTTTTTATATGAAGATATTCCCTT

                           * *   *                *   *                    * 
     315631 TTCCAACGAAATCATTAGAGCTATCCAAATATCCACTTACAGTTTCTACAAAAAGAGTGTTTCCA
          1 TTCCAACGAAATCATCAAAGCGATCCAAATATCCACTTCCAGATTCTACAAAAAGAGTGTTTCAA

                   **         * *    *       *  *                   ***      
     315696 AACTGCTGCATCAAAAGAGAGGTTCCACTCTGTTAGCTGAGTACACACATCACAAACTTGTTTCT
         66 AACTGCTCTATCAAAAGAAATGTTCAACTCTGTGAGTTGAGTACACACATCACAAAGAAGTTTCT

            *     *         *        **         **   
     315761 CAGAATCCTTCTGTCTCGTTTTTATGGGAAGATATTTACTT
        131 GAGAATGCTTCTGTCTAGTTTTTATATGAAGATATTCCCTT

              *  *  * **          *       *     *       *  *  *              
     315802 TTTCACCGTAGGCATCAAAGCGCTCCAAATGTCCACATCCAGATACTCCAGAAAGAGTGTTTCAA
          1 TTCCAACGAAATCATCAAAGCGATCCAAATATCCACTTCCAGATTCTACAAAAAGAGTGTTTCAA

             *         *   * *   *         *        * *  *      *      *     
     315867 ACCTGCTCTATGAAAGGGAATCTTCAACTCTATGAGTTGAATGCAGACATCAGAAAGAAATTTCT
         66 AACTGCTCTATCAAAAGAAATGTTCAACTCTGTGAGTTGAGTACACACATCACAAAGAAGTTTCT

                     *       **          *         **
     315932 GAGAATGCTGCTGTCTACCTTTTAT-T--TGA-ATTCCCGC
        131 GAGAATGCTTCTGTCTAGTTTTTATATGAAGATATTCCCTT

                         *  *    *                *       *                  
     315969 TTCCAACGAAATCCTCCAAGCTATCCAAATATCCACTTGCAGATTCCACAAAAAGAGTGTTTCAA
          1 TTCCAACGAAATCATCAAAGCGATCCAAATATCCACTTCCAGATTCTACAAAAAGAGTGTTTCAA

                            ** *  *            *       *     *     *  *      
     316034 AACTGCTCTCTATCAATGGCAAAGTTCAACTCTGTTAGTTGAGGACACATATCACCAACAAGTTT
         66 AACTG--CTCTATCAAAAGAAATGTTCAACTCTGTGAGTTGAGTACACACATCACAAAGAAGTTT

                               *       **         *    
     316099 CTGAGAATGCTTCTGTCTATTTTTTATGGGAAGATATTTCCTT
        129 CTGAGAATGCTTCTGTCTAGTTTTTATATGAAGATATTCCCTT

              *  *  * ** *    *      *    *          * **                    
     316142 TTTCACCGTAGGCGTCAAGGCGATCGAAATGTCCACTTCCACAAACTACAAAAAGAGTGTTTCAA
          1 TTCCAACGAAATCATCAAAGCGATCCAAATATCCACTTCCAGATTCTACAAAAAGAGTGTTTCAA

             *         *   * **       *    *        * ** * *   **      *     
     316207 ACCTGCTCTATGAAAGGCCATGTTCATCTCTATGAGTTGAATGGAAATATCCGAAAGAAATTTCT
         66 AACTGCTCTATCAAAAGAAATGTTCAACTCTGTGAGTTGAGTACACACATCACAAAGAAGTTTCT

             *       *         *      *            **
     316272 GGGAATGCTGCTGTCTAGTGTTTATACG-A-AT-TT-CCGC
        131 GAGAATGCTTCTGTCTAGTTTTTATATGAAGATATTCCCTT

                         *       *                *    *  *                  
     316309 TTCCAACGAAATCCTCAAAGCAATCCAAATATCCACTTGCAGAATCCACAAAAAGAGTGTTTCAA
          1 TTCCAACGAAATCATCAAAGCGATCCAAATATCCACTTCCAGATTCTACAAAAAGAGTGTTTCAA

                          *     *          * *                   *  *        
     316374 AACTGCTCTATCAATAGAAAGGTTCAACTCTTTTAGTTGAGTACACACATCACGAACAAGTTTCT
         66 AACTGCTCTATCAAAAGAAATGTTCAACTCTGTGAGTTGAGTACACACATCACAAAGAAGTTTCT

                            * *      **     **  *    
     316439 GAGAATGCTTCTGTCTGGCTTTTATTGGAAGACGTTTCCTT
        131 GAGAATGCTTCTGTCTAGTTTTTATATGAAGATATTCCCTT

                 * *  **          *       *                * *               
     316480 TT-CACCAAAGGCATCAAAGCGCTCCAAATGTCCACTTCCAGATTCTTCCAAAAGAGTGTTTCAA
          1 TTCCAACGAAATCATCAAAGCGATCCAAATATCCACTTCCAGATTCTACAAAAAGAGTGTTTCAA

             **     * ***  * *                 *    * *  * *     *   *       
     316544 ACGTGCTCAAAGTAAGGGAATGTTCAACTCTGTGACTTGAATGCAGATATCACCAAGTAGTTTCT
         66 AACTGCTCTATCAAAAGAAATGTTCAACTCTGTGAGTTGAGTACACACATCACAAAGAAGTTTCT

            * * *            **     *    *         * 
     316609 AATAGTGCTTCTGTCTACATTTTAGATGATGATATTCCCGT
        131 GAGAATGCTTCTGTCTAGTTTTTATATGAAGATATTCCCTT

               *         * * *   *             *  *   *      *     *       * 
     316650 TTCAAACGAAATCGTTAGAGCTATCCAAATATCCAGTTACAGTTTCTACCAAAAGGGTGTTTCCA
          1 TTCCAACGAAATCATCAAAGCGATCCAAATATCCACTTCCAGATTCTACAAAAAGAGTGTTTCAA

              *    **           *            *       *                     * 
     316715 AATTGCTGCATCAAAAGAAAGGTTCAACTCTGTTAGTTGAGGACACACATCACAAAGAAGTTTGT
         66 AACTGCTCTATCAAAAGAAATGTTCAACTCTGTGAGTTGAGTACACACATCACAAAGAAGTTTCT

                              *    *     *           
     316780 GAGAATGCTTCTGTCTAGATTTTGTATGACGATATTCCCTT
        131 GAGAATGCTTCTGTCTAGTTTTTATATGAAGATATTCCCTT

                     *   * *     *   *       * *  *    *  *       *  *       
     316821 TTCCAACGATATCGTTAAAGCAATCTAAATATCAATTTGCAGAATCCACAAAAATAGAGTTTCAA
          1 TTCCAACGAAATCATCAAAGCGATCCAAATATCCACTTCCAGATTCTACAAAAAGAGTGTTTCAA

             *       * *        *    *       *  *                   ***      
     316886 AGCTGCTCTGTAAAAAGAAAGGTTCCACTCTGTTAGCTGAGTACACACATCACAAACTTGTTTCT
         66 AACTGCTCTATCAAAAGAAATGTTCAACTCTGTGAGTTGAGTACACACATCACAAAGAAGTTTCT

            *     *         *        **         **   
     316951 CAGAATCCTTCTGTCTCGTTTTTATGGGAAGATATTTACTT
        131 GAGAATGCTTCTGTCTAGTTTTTATATGAAGATATTCCCTT

                 *  * **          *       *     *       *  *  *              
     316992 TTCCACCGTAGGCATCAAAGCGCTCCAAATGTCCACATCCAGATACTCCAGAAAGAGTGTTTCAA
          1 TTCCAACGAAATCATCAAAGCGATCCAAATATCCACTTCCAGATTCTACAAAAAGAGTGTTTCAA

             *         *   * *             *        * *  *      *      *     
     317057 ACCTGCTCTATGAAAGGGAATGTTCAACTCTATGAGTTGAATGCAGACATCAGAAAGAAATTTCT
         66 AACTGCTCTATCAAAAGAAATGTTCAACTCTGTGAGTTGAGTACACACATCACAAAGAAGTTTCT

                     *       **          *         **
     317122 GAGAATGCTGCTGTCTACCTTTTAT-T--TGA-ATTCCCGC
        131 GAGAATGCTTCTGTCTAGTTTTTATATGAAGATATTCCCTT

                         *  *    *                *  **   *                  
     317159 TTCCAACGAAATCCTCCAAGCTATCCAAATATCCACTTGCATTTTCCACAAAAAGAGTGTTTCAA
          1 TTCCAACGAAATCATCAAAGCGATCCAAATATCCACTTCCAGATTCTACAAAAAGAGTGTTTCAA

                          *                 *    *  *                *       
     317224 AACTGCTCTATCAATAGAAATGTTCAACTCCTTTG-GCTGGGTACACACATCACAAACAAGTTTC
         66 AACTGCTCTATCAAAAGAAATGTTCAACT-CTGTGAGTTGAGTACACACATCACAAAGAAGTTTC

                                      **     **       
     317288 TGAGAATGCTTCTGTCTAGTTTTTATGGGAAGACGTTCCCTT
        130 TGAGAATGCTTCTGTCTAGTTTTTATATGAAGATATTCCCTT

              *  * *  **          *       *        *   **                  * 
     317330 TTTCACCAAAGGCATCAAAGCGCTCCAAATGTCCACTTCAAGACACTACAAAAAGAGTGTTTCCA
          1 TTCCAACGAAATCATCAAAGCGATCCAAATATCCACTTCCAGATTCTACAAAAAGAGTGTTTCAA

             **       **   ***                 *    * *  * *         *       
     317395 ACGTGCTCTAAGAAAGCGAATGTTCAACTCTGTGACTTGAATGCAGATATCACAAAGTAGTTTCT
         66 AACTGCTCTATCAAAAGAAATGTTCAACTCTGTGAGTTGAGTACACACATCACAAAGAAGTTTCT

                **            *     *    *         * 
     317460 GAGAGGGCTTCTGTCTAGATTTTAGATGATGATATTCCCGT
        131 GAGAATGCTTCTGTCTAGTTTTTATATGAAGATATTCCCTT

                           * *   *                *   *                    * 
     317501 TTCCAACGAAATCATTAGAGCTATCCAAATATCCACTTACAGTTTCTACAAAAAGAGTGTTTCCA
          1 TTCCAACGAAATCATCAAAGCGATCCAAATATCCACTTCCAGATTCTACAAAAAGAGTGTTTCAA

                   **         * *    *       *  *                   ***      
     317566 AACTGCTGCATCAAAAGAGAGGTTCCACTCTGTTAGCTGAGTACACACATCACAAACTTGTTTCT
         66 AACTGCTCTATCAAAAGAAATGTTCAACTCTGTGAGTTGAGTACACACATCACAAAGAAGTTTCT

            *     *         *        **         **   
     317631 CAGAATCCTTCTGTCTCGTTTTTATGGGAAGATATTTACTT
        131 GAGAATGCTTCTGTCTAGTTTTTATATGAAGATATTCCCTT

              *  *  * **          *       *     *       *  *  *              
     317672 TTTCACCGTAGGCATCAAAGCGCTCCAAATGTCCACATCCAGATACTCCAGAAAGAGTGTTTCAA
          1 TTCCAACGAAATCATCAAAGCGATCCAAATATCCACTTCCAGATTCTACAAAAAGAGTGTTTCAA

             *         *   * *   *         *        * *  *      *      *     
     317737 ACCTGCTCTATGAAAGGGAATCTTCAACTCTATGAGTTGAATGCAGACATCAGAAAGAAATTTCT
         66 AACTGCTCTATCAAAAGAAATGTTCAACTCTGTGAGTTGAGTACACACATCACAAAGAAGTTTCT

                     *       **          *         **
     317802 GAGAATGCTGCTGTCTACCTTTTAT-T--TGA-ATTCCCGC
        131 GAGAATGCTTCTGTCTAGTTTTTATATGAAGATATTCCCTT

                         *  *    *                *       *                  
     317839 TTCCAACGAAATCCTCCAAGCTATCCAAATATCCACTTGCAGATTCCACAAAAAGAGTGTTTCAA
          1 TTCCAACGAAATCATCAAAGCGATCCAAATATCCACTTCCAGATTCTACAAAAAGAGTGTTTCAA

                            ** *  *            *       *     *     *  *      
     317904 AACTGCTCTCTATCAATGGCAAAGTTCAACTCTGTTAGTTGAGGACACATATCACCAACAAGTTT
         66 AACTG--CTCTATCAAAAGAAATGTTCAACTCTGTGAGTTGAGTACACACATCACAAAGAAGTTT

                               *       **         *    
     317969 CTGAGAATGCTTCTGTCTATTTTTTATGGGAAGATATTTCCTT
        129 CTGAGAATGCTTCTGTCTAGTTTTTATATGAAGATATTCCCTT

              *  *  * ** *    *      *    *          * **                    
     318012 TTTCACCGTAGGCGTCAAGGCGATCGAAATGTCCACTTCCACAAACTACAAAAAGAGTGTTTCAA
          1 TTCCAACGAAATCATCAAAGCGATCCAAATATCCACTTCCAGATTCTACAAAAAGAGTGTTTCAA

             *         *   * **       *    *        * ** * *   **      *     
     318077 ACCTGCTCTATGAAAGGCCATGTTCATCTCTATGAGTTGAATGGAAATATCCGAAAGAAATTTCT
         66 AACTGCTCTATCAAAAGAAATGTTCAACTCTGTGAGTTGAGTACACACATCACAAAGAAGTTTCT

             *       *          *        *         **
     318142 GGGAATGCTGCTGTCTAG-TGTT-TAT-ACGA-ATTCCCAC
        131 GAGAATGCTTCTGTCTAGTTTTTATATGAAGATATTCCCTT

                         *       *                *    *  *                  
     318179 TTCCAACGAAATCCTCAAAGCAATCCAAATATCCACTTGCAGAATCCACAAAAAGAGTGTTTCAA
          1 TTCCAACGAAATCATCAAAGCGATCCAAATATCCACTTCCAGATTCTACAAAAAGAGTGTTTCAA

                          *     *          * *                   *  *        
     318244 AACTGCTCTATCAATAGAAAGGTTCAACTCTTTTAGTTGAGTACACACATCACGAACAAGTTTCT
         66 AACTGCTCTATCAAAAGAAATGTTCAACTCTGTGAGTTGAGTACACACATCACAAAGAAGTTTCT

                            * *      **     **  *    
     318309 GAGAATGCTTCTGTCTGGCTTTTATTGGAAGACGTTTCCTT
        131 GAGAATGCTTCTGTCTAGTTTTTATATGAAGATATTCCCTT

                 * *  **          *       *                * *               
     318350 TT-CACCAAAGGCATCAAAGCGCTCCAAATGTCCACTTCCAGATTCTTCCAAAAGAGTGTTTCAA
          1 TTCCAACGAAATCATCAAAGCGATCCAAATATCCACTTCCAGATTCTACAAAAAGAGTGTTTCAA

             **     * ***  * *                 *    * *  * *     *   *       
     318414 ACGTGCTCAAAGTAAGGGAATGTTCAACTCTGTGACTTGAATGCAGATATCACCAAGTAGTTTCT
         66 AACTGCTCTATCAAAAGAAATGTTCAACTCTGTGAGTTGAGTACACACATCACAAAGAAGTTTCT

            * * *             *     *    *         * 
     318479 AATAGTGCTTCTGTCTAGATTTTAGATGATGATATTCCCGT
        131 GAGAATGCTTCTGTCTAGTTTTTATATGAAGATATTCCCTT

                         * * *   *                *   *                    * 
     318520 TTCCAACGAAATCGTTAGAGCTATCCAAATATCCACTTACAGTTTCTACAAAAAGAGTGTTTCCA
          1 TTCCAACGAAATCATCAAAGCGATCCAAATATCCACTTCCAGATTCTACAAAAAGAGTGTTTCAA

                   **           *            *       *                     * 
     318585 AACTGCTGCATCAAAAGAAAGGTTCAACTCTGTTAGTTGAGGACACACATCACAAAGAAGTTTGT
         66 AACTGCTCTATCAAAAGAAATGTTCAACTCTGTGAGTTGAGTACACACATCACAAAGAAGTTTCT

                              *    *     *           
     318650 GAGAATGCTTCTGTCTAGATTTTGTATGACGATATTCCCTT
        131 GAGAATGCTTCTGTCTAGTTTTTATATGAAGATATTCCCTT

                     *   * *     *   *       * *  *    *  *       *          
     318691 TTCCAACGATATCGTTAAAGCAATCTAAATATCAATTTGCAGAATCCACAAAAATA--GTTTCAA
          1 TTCCAACGAAATCATCAAAGCGATCCAAATATCCACTTCCAGATTCTACAAAAAGAGTGTTTCAA

             *       * *        *    *       *  *                   ***      
     318754 AGCTGCTCTGTAAAAAGAAAGGTTCCACTCTGTTAGCTGAGTACACACATCACAAACTTGTTTCT
         66 AACTGCTCTATCAAAAGAAATGTTCAACTCTGTGAGTTGAGTACACACATCACAAAGAAGTTTCT

            *     *         *        **         **   
     318819 CAGAATCCTTCTGTCTCGTTTTTATGGGAAGATATTTACTT
        131 GAGAATGCTTCTGTCTAGTTTTTATATGAAGATATTCCCTT

                 *  * **          *       *     *       *  *  *  *           
     318860 TTCCACCGTAGGCATCAAAGCGCTCCAAATGTCCACATCCAGATACTCCAGAACGAGTGTTTCAA
          1 TTCCAACGAAATCATCAAAGCGATCCAAATATCCACTTCCAGATTCTACAAAAAGAGTGTTTCAA

             *         *   * *   *         *        * *  *      *      *     
     318925 ACCTGCTCTATGAAAGGGAATCTTCAACTCTATGAGTTGAATGCAGACATCAGAAAGAAATTTCT
         66 AACTGCTCTATCAAAAGAAATGTTCAACTCTGTGAGTTGAGTACACACATCACAAAGAAGTTTCT

                     *       **          *         **
     318990 GAGAATGCTGCTGTCTACCTTTTAT-T--TGA-ATTCCCGC
        131 GAGAATGCTTCTGTCTAGTTTTTATATGAAGATATTCCCTT

                         *  *    *                *  **   *                  
     319027 TTCCAACGAAATCCTCCAAGCTATCCAAATATCCACTTGCATTTTCCACAAAAAGAGTGTTTCAA
          1 TTCCAACGAAATCATCAAAGCGATCCAAATATCCACTTCCAGATTCTACAAAAAGAGTGTTTCAA

                          *                 *    *  *                *       
     319092 AACTGCTCTATCAATAGAAATGTTCAACTCCTTTG-GCTGGGTACACACATCACAAACAAGTTTC
         66 AACTGCTCTATCAAAAGAAATGTTCAACT-CTGTGAGTTGAGTACACACATCACAAAGAAGTTTC

                                      **     **       
     319156 TGAGAATGCTTCTGTCTAGTTTTTATGGGAAGACGTTCCCTT
        130 TGAGAATGCTTCTGTCTAGTTTTTATATGAAGATATTCCCTT

              *  * *  **          *       *            **                  * 
     319198 TTTCACCAAAGGCATCAAAGCGCTCCAAATGTCCACTTCCAGACACTACAAAAAGAGTGTTTCCA
          1 TTCCAACGAAATCATCAAAGCGATCCAAATATCCACTTCCAGATTCTACAAAAAGAGTGTTTCAA

             **       **   ***                 *    * *  * *         *       
     319263 ACGTGCTCTAAGAAAGCGAATGTTCAACTCTGTGACTTGAATGCAGATATCACAAAGTAGTTTCT
         66 AACTGCTCTATCAAAAGAAATGTTCAACTCTGTGAGTTGAGTACACACATCACAAAGAAGTTTCT

                **            *     *    *         * 
     319328 GAGAGGGCTTCTGTCTAGATTTTAGATGATGATATTCCCGT
        131 GAGAATGCTTCTGTCTAGTTTTTATATGAAGATATTCCCTT

                           * *   *                *   *                    * 
     319369 TTCCAACGAAATCATTAGAGCTATCCAAATATCCACTTACAGTTTCTACAAAAAGAGTGTTTCCA
          1 TTCCAACGAAATCATCAAAGCGATCCAAATATCCACTTCCAGATTCTACAAAAAGAGTGTTTCAA

                   **         * *    *       *  *                   ***      
     319434 AACTGCTGCATCAAAAGAGAGGTTCCACTCTGTTAGCTGAGTACACACATCACAAACTTGTTTCT
         66 AACTGCTCTATCAAAAGAAATGTTCAACTCTGTGAGTTGAGTACACACATCACAAAGAAGTTTCT

            *     *         *        **         **   
     319499 CAGAATCCTTCTGTCTCGTTTTTATGGGAAGATATTTACTT
        131 GAGAATGCTTCTGTCTAGTTTTTATATGAAGATATTCCCTT

              *  *  * **          *       *     *       *  *  *              
     319540 TTTCACCGTAGGCATCAAAGCGCTCCAAATGTCCACATCCAGATACTCCAGAAAGAGTGTTTCAA
          1 TTCCAACGAAATCATCAAAGCGATCCAAATATCCACTTCCAGATTCTACAAAAAGAGTGTTTCAA

             *         *   * *   *         *        * *  *      *      *     
     319605 ACCTGCTCTATGAAAGGGAATCTTCAACTCTATGAGTTGAATGCAGACATCAGAAAGAAATTTCT
         66 AACTGCTCTATCAAAAGAAATGTTCAACTCTGTGAGTTGAGTACACACATCACAAAGAAGTTTCT

                     *       **          *         **
     319670 GAGAATGCTGCTGTCTACCTTTTAT-T--TGA-ATTCCCGC
        131 GAGAATGCTTCTGTCTAGTTTTTATATGAAGATATTCCCTT

                         *  *    *                *       *                  
     319707 TTCCAACGAAATCCTCCAAGCTATCCAAATATCCACTTGCAGATTCCACAAAAAGAGTGTTTCAA
          1 TTCCAACGAAATCATCAAAGCGATCCAAATATCCACTTCCAGATTCTACAAAAAGAGTGTTTCAA

                            ** *  *            *       *     *     *  *      
     319772 AACTGCTCTCTATCAATGGCAAAGTTCAACTCTGTTAGTTGAGGACACATATCACCAACAAGTTT
         66 AACTG--CTCTATCAAAAGAAATGTTCAACTCTGTGAGTTGAGTACACACATCACAAAGAAGTTT

                               *       **         *    
     319837 CTGAGAATGCTTCTGTCTATTTTTTATGGGAAGATATTTCCTT
        129 CTGAGAATGCTTCTGTCTAGTTTTTATATGAAGATATTCCCTT

              *  *  * ** *    *      *    *          * **                    
     319880 TTTCACCGTAGGCGTCAAGGCGATCGAAATGTCCACTTCCACAAACTACAAAAAGAGTGTTTCAA
          1 TTCCAACGAAATCATCAAAGCGATCCAAATATCCACTTCCAGATTCTACAAAAAGAGTGTTTCAA

             *         *   * **       *    *        * ** * *   **      *     
     319945 ACCTGCTCTATGAAAGGCCATGTTCATCTCTATGAGTTGAATGGAAATATCCGAAAGAAATTTCT
         66 AACTGCTCTATCAAAAGAAATGTTCAACTCTGTGAGTTGAGTACACACATCACAAAGAAGTTTCT

             *       *         *      *            **
     320010 GGGAATGCTGCTGTCTAGTGTTTATACG-A-AT-TT-CCGC
        131 GAGAATGCTTCTGTCTAGTTTTTATATGAAGATATTCCCTT

                         *       *                *    *  *                  
     320047 TTCCAACGAAATCCTCAAAGCAATCCAAATATCCACTTGCAGAATCCACAAAAAGAGTGTTTCAA
          1 TTCCAACGAAATCATCAAAGCGATCCAAATATCCACTTCCAGATTCTACAAAAAGAGTGTTTCAA

                          *     *          * *                   *  *        
     320112 AACTGCTCTATCAATAGAAAGGTTCAACTCTTTTAGTTGAGTACACACATCACGAACAAGTTTCT
         66 AACTGCTCTATCAAAAGAAATGTTCAACTCTGTGAGTTGAGTACACACATCACAAAGAAGTTTCT

                            * *      **     **  *    
     320177 GAGAATGCTTCTGTCTGGCTTTTATTGGAAGACGTTTCCTT
        131 GAGAATGCTTCTGTCTAGTTTTTATATGAAGATATTCCCTT

                 * *  **          *       *                * *               
     320218 TT-CACCAAAGGCATCAAAGCGCTCCAAATGTCCACTTCCAGATTCTTCCAAAAGAGTGTTTCAA
          1 TTCCAACGAAATCATCAAAGCGATCCAAATATCCACTTCCAGATTCTACAAAAAGAGTGTTTCAA

             **     * ***  * *                 *    * *  * *     *   *       
     320282 ACGTGCTCAAAGTAAGGGAATGTTCAACTCTGTGACTTGAATGCAGATATCACCAAGTAGTTTCT
         66 AACTGCTCTATCAAAAGAAATGTTCAACTCTGTGAGTTGAGTACACACATCACAAAGAAGTTTCT

            * * *            **     *    *         * 
     320347 AATAGTGCTTCTGTCTACATTTTAGATGATGATATTCCCGT
        131 GAGAATGCTTCTGTCTAGTTTTTATATGAAGATATTCCCTT

               *         * * *   *             *  *   *      *     *       * 
     320388 TTCAAACGAAATCGTTAGAGCTATCCAAATATCCAGTTACAGTTTCTACCAAAAGGGTGTTTCCA
          1 TTCCAACGAAATCATCAAAGCGATCCAAATATCCACTTCCAGATTCTACAAAAAGAGTGTTTCAA

              *    **           *            *       *                     * 
     320453 AATTGCTGCATCAAAAGAAAGGTTCAACTCTGTTAGTTGAGGACACACATCACAAAGAAGTTTGT
         66 AACTGCTCTATCAAAAGAAATGTTCAACTCTGTGAGTTGAGTACACACATCACAAAGAAGTTTCT

                              *    *     *           
     320518 GAGAATGCTTCTGTCTAGATTTTGTATGACGATATTCCCTT
        131 GAGAATGCTTCTGTCTAGTTTTTATATGAAGATATTCCCTT

                     *   * *     *   *       * *  *    *  *       *  *       
     320559 TTCCAACGATATCGTTAAAGCAATCTAAATATCAATTTGCAGAATCCACAAAAATAGAGTTTCAA
          1 TTCCAACGAAATCATCAAAGCGATCCAAATATCCACTTCCAGATTCTACAAAAAGAGTGTTTCAA

             *       * *        *    *       *  *                   ***      
     320624 AGCTGCTCTGTAAAAAGAAAGGTTCCACTCTGTTAGCTGAGTACACACATCACAAACTTGTTTCT
         66 AACTGCTCTATCAAAAGAAATGTTCAACTCTGTGAGTTGAGTACACACATCACAAAGAAGTTTCT

            *     *         *        **         **   
     320689 CAGAATCCTTCTGTCTCGTTTTTATGGGAAGATATTTACTT
        131 GAGAATGCTTCTGTCTAGTTTTTATATGAAGATATTCCCTT

                 *  * **          *       *     *       *  *  *              
     320730 TTCCACCGTAGGCATCAAAGCGCTCCAAATGTCCACATCCAGATACTCCAGAAAGAGTGTTTCAA
          1 TTCCAACGAAATCATCAAAGCGATCCAAATATCCACTTCCAGATTCTACAAAAAGAGTGTTTCAA

             *         *   * *   *         *        * *  *      *      *     
     320795 ACCTGCTCTATGAAAGGGAATCTTCAACTCTATGAGTTGAATGCAGACATCAGAAAGAAATTTCT
         66 AACTGCTCTATCAAAAGAAATGTTCAACTCTGTGAGTTGAGTACACACATCACAAAGAAGTTTCT

                     *       **          *         **
     320860 GAGAATGCTGCTGTCTACCTTTTAT-T--TGA-ATTCCCGC
        131 GAGAATGCTTCTGTCTAGTTTTTATATGAAGATATTCCCTT

                         *  *    *                *  **   *                  
     320897 TTCCAACGAAATCCTCCAAGCTATCCAAATATCCACTTGCATTTTCCACAAAAAGAGTGTTTCAA
          1 TTCCAACGAAATCATCAAAGCGATCCAAATATCCACTTCCAGATTCTACAAAAAGAGTGTTTCAA

                          *              *  *    *  *                *       
     320962 AACTGCTCTATCAATAGAAATGTTCAACCCCTTTG-GCTGGGTACACACATCACAAACAAGTTTC
         66 AACTGCTCTATCAAAAGAAATGTTCAA-CTCTGTGAGTTGAGTACACACATCACAAAGAAGTTTC

                                      **     **       
     321026 TGAGAATGCTTCTGTCTAGTTTTTATGGGAAGACGTTCCCTT
        130 TGAGAATGCTTCTGTCTAGTTTTTATATGAAGATATTCCCTT

              *  * *  **          *       *        *   **                  * 
     321068 TTTCACCAAAGGCATCAAAGCGCTCCAAATGTCCACTTCAAGACACTACAAAAAGAGTGTTTCCA
          1 TTCCAACGAAATCATCAAAGCGATCCAAATATCCACTTCCAGATTCTACAAAAAGAGTGTTTCAA

             **       **   ***                 *    * *  * *         *       
     321133 ACGTGCTCTAAGAAAGCGAATGTTCAACTCTGTGACTTGAATGCAGATATCACAAAGTAGTTTCT
         66 AACTGCTCTATCAAAAGAAATGTTCAACTCTGTGAGTTGAGTACACACATCACAAAGAAGTTTCT

                **            *     *    *         * 
     321198 GAGAGGGCTTCTGTCTAGATTTTAGATGATGATATTCCCGT
        131 GAGAATGCTTCTGTCTAGTTTTTATATGAAGATATTCCCTT

                           * *   *                *   *                    * 
     321239 TTCCAACGAAATCATTAGAGCTATCCAAATATCCACTTACAGTTTCTACAAAAAGAGTGTTTCCA
          1 TTCCAACGAAATCATCAAAGCGATCCAAATATCCACTTCCAGATTCTACAAAAAGAGTGTTTCAA

                   **         * *    *       *  *                   ***      
     321304 AACTGCTGCATCAAAAGAGAGGTTCCACTCTGTTAGCTGAGTACACACATCACAAACTTGTTTCT
         66 AACTGCTCTATCAAAAGAAATGTTCAACTCTGTGAGTTGAGTACACACATCACAAAGAAGTTTCT

            *     *         *        **         **   
     321369 CAGAATCCTTCTGTCTCGTTTTTATGGGAAGATATTTACTT
        131 GAGAATGCTTCTGTCTAGTTTTTATATGAAGATATTCCCTT

              *  *  * **          *       *     *       *  *  *              
     321410 TTTCACCGTAGGCATCAAAGCGCTCCAAATGTCCACATCCAGATACTCCAGAAAGAGTGTTTCAA
          1 TTCCAACGAAATCATCAAAGCGATCCAAATATCCACTTCCAGATTCTACAAAAAGAGTGTTTCAA

             *         *   * *   *         *        * *  *      *      *     
     321475 ACCTGCTCTATGAAAGGGAATCTTCAACTCTATGAGTTGAATGCAGACATCAGAAAGAAATTTCT
         66 AACTGCTCTATCAAAAGAAATGTTCAACTCTGTGAGTTGAGTACACACATCACAAAGAAGTTTCT

                     *       **          *         **
     321540 GAGAATGCTGCTGTCTACCTTTTAT-T--TGA-ATTCCCGC
        131 GAGAATGCTTCTGTCTAGTTTTTATATGAAGATATTCCCTT

                         *  *    *                *       *                  
     321577 TTCCAACGAAATCCTCCAAGCTATCCAAATATCCACTTGCAGATTCCACAAAAAGAGTGTTTCAA
          1 TTCCAACGAAATCATCAAAGCGATCCAAATATCCACTTCCAGATTCTACAAAAAGAGTGTTTCAA

                            ** *  *            *       *     *     *  *      
     321642 AACTGCTCTCTATCAATGGCAAAGTTCAACTCTGTTAGTTGAGGACACATATCACCAACAAGTTT
         66 AACTG--CTCTATCAAAAGAAATGTTCAACTCTGTGAGTTGAGTACACACATCACAAAGAAGTTT

                               *       **         *    
     321707 CTGAGAATGCTTCTGTCTATTTTTTATGGGAAGATATTTCCTT
        129 CTGAGAATGCTTCTGTCTAGTTTTTATATGAAGATATTCCCTT

              *  *  * ** *    *      *    *          * **                    
     321750 TTTCACCGTAGGCGTCAAGGCGATCGAAATGTCCACTTCCACAAACTACAAAAAGAGTGTTTCAA
          1 TTCCAACGAAATCATCAAAGCGATCCAAATATCCACTTCCAGATTCTACAAAAAGAGTGTTTCAA

             *         *   * **       *    *        * ** * *   **      *     
     321815 ACCTGCTCTATGAAAGGCCATGTTCATCTCTATGAGTTGAATGGAAATATCCGAAAGAAATTTCT
         66 AACTGCTCTATCAAAAGAAATGTTCAACTCTGTGAGTTGAGTACACACATCACAAAGAAGTTTCT

             *       *          *        *         **
     321880 GGGAATGCTGCTGTCTAG-TGTT-TAT-ACGA-ATTCCCAC
        131 GAGAATGCTTCTGTCTAGTTTTTATATGAAGATATTCCCTT

                         *       *                *    *  *                  
     321917 TTCCAACGAAATCCTCAAAGCAATCCAAATATCCACTTGCAGAATCCACAAAAAGAGTGTTTCAA
          1 TTCCAACGAAATCATCAAAGCGATCCAAATATCCACTTCCAGATTCTACAAAAAGAGTGTTTCAA

                          *     *          * *                   *  *        
     321982 AACTGCTCTATCAATAGAAAGGTTCAACTCTTTTAGTTGAGTACACACATCACGAACAAGTTTCT
         66 AACTGCTCTATCAAAAGAAATGTTCAACTCTGTGAGTTGAGTACACACATCACAAAGAAGTTTCT

                            * *      **     **  *    
     322047 GAGAATGCTTCTGTCTGGCTTTTATTGGAAGACGTTTCCTT
        131 GAGAATGCTTCTGTCTAGTTTTTATATGAAGATATTCCCTT

                 * *  **          *       *                * *               
     322088 TT-CACCAAAGGCATCAAAGCGCTCCAAATGTCCACTTCCAGATTCTTCCAAAAGAGTGTTTCAA
          1 TTCCAACGAAATCATCAAAGCGATCCAAATATCCACTTCCAGATTCTACAAAAAGAGTGTTTCAA

             **     * ***  * *                 *    * *  * *     *   *       
     322152 ACGTGCTCAAAGTAAGGGAATGTTCAACTCTGTGACTTGAATGCAGATATCACCAAGTAGTTTCT
         66 AACTGCTCTATCAAAAGAAATGTTCAACTCTGTGAGTTGAGTACACACATCACAAAGAAGTTTCT

            * * *             *     *    *         * 
     322217 AATAGTGCTTCTGTCTAGATTTTAGATGATGATATTCCCGT
        131 GAGAATGCTTCTGTCTAGTTTTTATATGAAGATATTCCCTT

                         * * *   *                *   *                    * 
     322258 TTCCAACGAAATCGTTAGAGCTATCCAAATATCCACTTACAGTTTCTACAAAAAGAGTGTTTCCA
          1 TTCCAACGAAATCATCAAAGCGATCCAAATATCCACTTCCAGATTCTACAAAAAGAGTGTTTCAA

                   **           *            *       *                     * 
     322323 AACTGCTGCATCAAAAGAAAGGTTCAACTCTGTTAGTTGAGGACACACATCACAAAGAAGTTTGT
         66 AACTGCTCTATCAAAAGAAATGTTCAACTCTGTGAGTTGAGTACACACATCACAAAGAAGTTTCT

                              *    *     *           
     322388 GAGAATGCTTCTGTCTAGATTTTGTATGACGATATTCCCTT
        131 GAGAATGCTTCTGTCTAGTTTTTATATGAAGATATTCCCTT

                     *   * *     *   *       * *  *    *  *       *  *       
     322429 TTCCAACGATATCGTTAAAGCAATCTAAATATCAATTTGCAGAATCCACAAAAATAGAGTTTCAA
          1 TTCCAACGAAATCATCAAAGCGATCCAAATATCCACTTCCAGATTCTACAAAAAGAGTGTTTCAA

             *       * *        *    *       *  *                   ***      
     322494 AGCTGCTCTGTAAAAAGAAAGGTTCCACTCTGTTAGCTGAGTACACACATCACAAACTTGTTTCT
         66 AACTGCTCTATCAAAAGAAATGTTCAACTCTGTGAGTTGAGTACACACATCACAAAGAAGTTTCT

                  *         *        **         **   
     322559 GAGAATCCTTCTGTCTCGTTTTTATGGGAAGATATTTACTT
        131 GAGAATGCTTCTGTCTAGTTTTTATATGAAGATATTCCCTT

                 *  * **          *       *     *       *  *  *  *           
     322600 TTCCACCGTAGGCATCAAAGCGCTCCAAATGTCCACATCCAGATACTCCAGAACGAGTGTTTCAA
          1 TTCCAACGAAATCATCAAAGCGATCCAAATATCCACTTCCAGATTCTACAAAAAGAGTGTTTCAA

             *         *   * *   *         *        * *  *      *      *     
     322665 ACCTGCTCTATGAAAGGGAATCTTCAACTCTATGAGTTGAATGCAGACATCAGAAAGAAATTTCT
         66 AACTGCTCTATCAAAAGAAATGTTCAACTCTGTGAGTTGAGTACACACATCACAAAGAAGTTTCT

                     *       **          *         **
     322730 GAGAATGCTGCTGTCTACCTTTTAT-T--TGA-ATTCCCGC
        131 GAGAATGCTTCTGTCTAGTTTTTATATGAAGATATTCCCTT

                         *  *    *                *  **   *                  
     322767 TTCCAACGAAATCCTCCAAGCTATCCAAATATCCACTTGCATTTTCCACAAAAAGAGTGTTTCAA
          1 TTCCAACGAAATCATCAAAGCGATCCAAATATCCACTTCCAGATTCTACAAAAAGAGTGTTTCAA

                          *                 *    *  *                *       
     322832 AACTGCTCTATCAATAGAAATGTTCAACTCCTTTG-GCTGGGTACACACATCACAAACAAGTTTC
         66 AACTGCTCTATCAAAAGAAATGTTCAACT-CTGTGAGTTGAGTACACACATCACAAAGAAGTTTC

                                      **     **   *   
     322896 TGAGAATGCTTCTGTCTAGTTTTTATGGGAAGACGTTCGCTT
        130 TGAGAATGCTTCTGTCTAGTTTTTATATGAAGATATTCCCTT

              *  * *  **          *       *            **                  * 
     322938 TTTCACCAAAGGCATCAAAGCGCTCCAAATGTCCACTTCCAGACACTACAAAAAGAGTGTTTCCA
          1 TTCCAACGAAATCATCAAAGCGATCCAAATATCCACTTCCAGATTCTACAAAAAGAGTGTTTCAA

             **       **   ***                 *    * *  * *         *       
     323003 ACGTGCTCTAAGAAAGCGAATGTTCAACTCTGTGACTTGAATGCAGATATCACAAAGTAGTTTCT
         66 AACTGCTCTATCAAAAGAAATGTTCAACTCTGTGAGTTGAGTACACACATCACAAAGAAGTTTCT

                **            *     *    *         * 
     323068 GAGAGGGCTTCTGTCTAGATTTTAGATGATGATATTCCCGT
        131 GAGAATGCTTCTGTCTAGTTTTTATATGAAGATATTCCCTT

                           * *   *                *   *                    * 
     323109 TTCCAACGAAATCATTAGAGCTATCCAAATATCCACTTACAGTTTCTACAAAAAGAGTGTTTCCA
          1 TTCCAACGAAATCATCAAAGCGATCCAAATATCCACTTCCAGATTCTACAAAAAGAGTGTTTCAA

                   **         * *    *       *  *                   ***      
     323174 AACTGCTGCATCAAAAGAGAGGTTCCACTCTGTTAGCTGAGTACACACATCACAAACTTGTTTCT
         66 AACTGCTCTATCAAAAGAAATGTTCAACTCTGTGAGTTGAGTACACACATCACAAAGAAGTTTCT

            *     *         *        **         **   
     323239 CAGAATCCTTCTGTCTCGTTTTTATGGGAAGATATTTACTT
        131 GAGAATGCTTCTGTCTAGTTTTTATATGAAGATATTCCCTT

              *  *  * **          *       *     *       *  *  *              
     323280 TTTCACCGTAGGCATCAAAGCGCTCCAAATGTCCACATCCAGATACTCCAGAAAGAGTGTTTCAA
          1 TTCCAACGAAATCATCAAAGCGATCCAAATATCCACTTCCAGATTCTACAAAAAGAGTGTTTCAA

             *         *   * *   *         *        * *  *      *      *     
     323345 ACCTGCTCTATGAAAGGGAATCTTCAACTCTATGAGTTGAATGCAGACATCAGAAAGAAATTTCT
         66 AACTGCTCTATCAAAAGAAATGTTCAACTCTGTGAGTTGAGTACACACATCACAAAGAAGTTTCT

                     *       **          *         **
     323410 GAGAATGCTGCTGTCTACCTTTTAT-T--TGA-ATTCCCGC
        131 GAGAATGCTTCTGTCTAGTTTTTATATGAAGATATTCCCTT

                         *  *    *                *       *                  
     323447 TTCCAACGAAATCCTCCAAGCTATCCAAATATCCACTTGCAGATTCCACAAAAAGAGTGTTTCAA
          1 TTCCAACGAAATCATCAAAGCGATCCAAATATCCACTTCCAGATTCTACAAAAAGAGTGTTTCAA

                            ** *  *            * *     *     *     *  * *    
     323512 AACTGCTCTCTATCAATGGCAAAGTTCAACTCTGTTACTTGAGGACACATATCACCAACAGGTTT
         66 AACTG--CTCTATCAAAAGAAATGTTCAACTCTGTGAGTTGAGTACACACATCACAAAGAAGTTT

                               *       **         *    
     323577 CTGAGAATGCTTCTGTCTATTTTTTATGGGAAGATATTTCCTT
        129 CTGAGAATGCTTCTGTCTAGTTTTTATATGAAGATATTCCCTT

              *  *  * ** *    *      *    *          * **                    
     323620 TTTCACCGTAGGCGTCAAGGCGATCGAAATGTCCACTTCCACAAACTACAAAAAGAGTGTTTCAA
          1 TTCCAACGAAATCATCAAAGCGATCCAAATATCCACTTCCAGATTCTACAAAAAGAGTGTTTCAA

             *         *   * **       *    *        * ** * *   **      *     
     323685 ACCTGCTCTATGAAAGGCCATGTTCATCTCTATGAGTTGAATGGAAATATCCGAAAGAAATTTCT
         66 AACTGCTCTATCAAAAGAAATGTTCAACTCTGTGAGTTGAGTACACACATCACAAAGAAGTTTCT

             *       *                   *         **
     323750 GGGAATGCTGCTGTCTAG-TTTT-TAT-ACGA-ATTCCCGC
        131 GAGAATGCTTCTGTCTAGTTTTTATATGAAGATATTCCCTT

                         *       *                *    *  *                  
     323787 TTCCAACGAAATCCTCAAAGCAATCCAAATATCCACTTGCAGAATCCACAAAAAGAGTGTTTCAA
          1 TTCCAACGAAATCATCAAAGCGATCCAAATATCCACTTCCAGATTCTACAAAAAGAGTGTTTCAA

                          *     *          * *                   *  *        
     323852 AACTGCTCTATCAATAGAAAGGTTCAACTCTTTTAGTTGAGTACACACATCACGAACAAGTTTCT
         66 AACTGCTCTATCAAAAGAAATGTTCAACTCTGTGAGTTGAGTACACACATCACAAAGAAGTTTCT

            *          *    * *      **     **  *    
     323917 CAGAATGCTTCCGTCTGGCTTTTATTGGAAGACGTTTCCTT
        131 GAGAATGCTTCTGTCTAGTTTTTATATGAAGATATTCCCTT

                 * *  **          *       *                * *               
     323958 TT-CACCAAAGGCATCAAAGCGCTCCAAATGTCCACTTCCAGATTCTTCCAAAAGAGTGTTTCAA
          1 TTCCAACGAAATCATCAAAGCGATCCAAATATCCACTTCCAGATTCTACAAAAAGAGTGTTTCAA

             **     * ***  * *                 *    * *  * *     *   *       
     324022 ACGTGCTCAAAGTAAGGGAATGTTCAACTCTGTGACTTGAATGCAGATATCACCAAGTAGTTTCT
         66 AACTGCTCTATCAAAAGAAATGTTCAACTCTGTGAGTTGAGTACACACATCACAAAGAAGTTTCT

            * * *  *          *     *    *         * 
     324087 AATAGTGTTTCTGTCTAGATTTTAGATGATGATATTCCCGT
        131 GAGAATGCTTCTGTCTAGTTTTTATATGAAGATATTCCCTT

                         * * *   *                *   *            *       * 
     324128 TTCCAACGAAATCGTTAGAGCTATCCAAATATCCACTTACAGTTTCTACAAAAAGGGTGTTTCCA
          1 TTCCAACGAAATCATCAAAGCGATCCAAATATCCACTTCCAGATTCTACAAAAAGAGTGTTTCAA

                   **           *            *       *                     * 
     324193 AACTGCTGCATCAAAAGAAAGGTTCAACTCTGTTAGTTGAGGACACACATCACAAAGAAGTTTGT
         66 AACTGCTCTATCAAAAGAAATGTTCAACTCTGTGAGTTGAGTACACACATCACAAAGAAGTTTCT

                              *    *     *           
     324258 GAGAATGCTTCTGTCTAGATTTTGTATGACGATATTCCCTT
        131 GAGAATGCTTCTGTCTAGTTTTTATATGAAGATATTCCCTT

                     *   * *     *   *       * *  *    *  *       *  *       
     324299 TTCCAACGATATCGTTAAAGCAATCTAAATATCAATTTGCAGAATCCACAAAAATAGAGTTTCAA
          1 TTCCAACGAAATCATCAAAGCGATCCAAATATCCACTTCCAGATTCTACAAAAAGAGTGTTTCAA

             *       * *        *    *       *  *                   ***      
     324364 AGCTGCTCTGTAAAAAGAAAGGTTCCACTCTGTTAGCTGAGTACACACATCACAAACTTGTTTCT
         66 AACTGCTCTATCAAAAGAAATGTTCAACTCTGTGAGTTGAGTACACACATCACAAAGAAGTTTCT

                  *         *        **         **   
     324429 GAGAATCCTTCTGTCTTGTTTTTATGGGAAGATATTTACTT
        131 GAGAATGCTTCTGTCTAGTTTTTATATGAAGATATTCCCTT

              *  *  * **        * *       *     *       *  *  *              
     324470 TTTCACCGTAGGCATCAAAGGGCTCCAAATGTCCACATCCAGATACTCCAGAAAGAGTGTTTCAA
          1 TTCCAACGAAATCATCAAAGCGATCCAAATATCCACTTCCAGATTCTACAAAAAGAGTGTTTCAA

             *         *   * *   *         *        * *  *      *      *     
     324535 ACCTGCTCTATGAAAGGGAATCTTCAACTCTATGAGTTGAATGCAGACATCAGAAAGAAATTTCT
         66 AACTGCTCTATCAAAAGAAATGTTCAACTCTGTGAGTTGAGTACACACATCACAAAGAAGTTTCT

                     *        **         *         **
     324600 GAGAATGCTGCTGTCTA-CCTTT-TATG-TGA-ATTCCCGC
        131 GAGAATGCTTCTGTCTAGTTTTTATATGAAGATATTCCCTT

                  *      *  *    *              * *  **   *                  
     324637 TTCCAATGAAATCCTCCAAGCTATCCAAATATCCACCTGCATTTTCCACAAAAAGAGTGTTTCAA
          1 TTCCAACGAAATCATCAAAGCGATCCAAATATCCACTTCCAGATTCTACAAAAAGAGTGTTTCAA

                          *                 *    *  *                *       
     324702 AACTGCTCTATCAATAGAAATGTTCAACTCCTTTG-GCTGGGTACACACATCACAAACAAGTTTC
         66 AACTGCTCTATCAAAAGAAATGTTCAACT-CTGTGAGTTGAGTACACACATCACAAAGAAGTTTC

                                      **     *        
     324766 TGAGAATGCTTCTGTCTAGTTTTTATGGGAAGACATTCCCTT
        130 TGAGAATGCTTCTGTCTAGTTTTTATATGAAGATATTCCCTT

              *  * *  **          *       *            **                  * 
     324808 TTTCACCAAAGGCATCAAAGCGCTCCAAATGTCCACTTCCAGACACTACAAAAAGAGTGTTTCCA
          1 TTCCAACGAAATCATCAAAGCGATCCAAATATCCACTTCCAGATTCTACAAAAAGAGTGTTTCAA

             **       **   ***    *            *    * *  * *         *       
     324873 ACGTGCTCTAAGAAAGCGAATGATCAACTCTGTGACTTGAATGCAGATATCACAAAGTAGTTTCT
         66 AACTGCTCTATCAAAAGAAATGTTCAACTCTGTGAGTTGAGTACACACATCACAAAGAAGTTTCT

                **            *     * *  *         * 
     324938 GAGAGGGCTTCTGTCTAGATTTTAGACGATGATATTCCCGT
        131 GAGAATGCTTCTGTCTAGTTTTTATATGAAGATATTCCCTT

                           * *   *                *   *                    * 
     324979 TTCCAACGAAATCATTAGAGCTATCCAAATATCCACTTACAGTTTCTACAAAAAGAGTGTTTCCA
          1 TTCCAACGAAATCATCAAAGCGATCCAAATATCCACTTCCAGATTCTACAAAAAGAGTGTTTCAA

                   **    *    * *    *       *  *                   ***      
     325044 AACTGCTGCATCAGAAGAGAGGTTCCACTCTGTTAGCTGAGTACACACATCACAAACTTGTTTCT
         66 AACTGCTCTATCAAAAGAAATGTTCAACTCTGTGAGTTGAGTACACACATCACAAAGAAGTTTCT

            *     *         *        **         **   
     325109 CAGAATCCTTCTGTCTCGTTTTTATGGGAAGATATTTACTT
        131 GAGAATGCTTCTGTCTAGTTTTTATATGAAGATATTCCCTT

              *  *  * **          * *     *     *       *  *  *              
     325150 TTTCACCGTAGGCATCAAAGCGCTGCAAATGTCCACATCCAGATACTCCAGAAAGAGTGTTTCAA
          1 TTCCAACGAAATCATCAAAGCGATCCAAATATCCACTTCCAGATTCTACAAAAAGAGTGTTTCAA

             *         *   * *   *         *        * *  *      *      *     
     325215 ACCTGCTCTATGAAAGGGAATCTTCAACTCTATGAGTTGAATGCAGACATCAGAAAGAAATTTCT
         66 AACTGCTCTATCAAAAGAAATGTTCAACTCTGTGAGTTGAGTACACACATCACAAAGAAGTTTCT

                     *       **          *         **
     325280 GAGAATGCTGCTGTCTACCTTTTAT-T--TGA-ATTCCCGC
        131 GAGAATGCTTCTGTCTAGTTTTTATATGAAGATATTCCCTT

                         *  *    *                * *     *   *              
     325317 TTCCAACGAAATCCTCCAAGCTATCCAAATATCCACTTGCTGATTCCACAGAAAGAGTGTTTCAA
          1 TTCCAACGAAATCATCAAAGCGATCCAAATATCCACTTCCAGATTCTACAAAAAGAGTGTTTCAA

                            ** *  *            *       *     *     *  *      
     325382 AACTGCTCTCTATCAATGGCAAAGTTCAACTCTGTTAGTTGAGGACACATATCACCAACAAGTTT
         66 AACTG--CTCTATCAAAAGAAATGTTCAACTCTGTGAGTTGAGTACACACATCACAAAGAAGTTT

                               *       **         *    
     325447 CTGAGAATGCTTCTGTCTATTTTTTATGGGAAGATATTTCCTT
        129 CTGAGAATGCTTCTGTCTAGTTTTTATATGAAGATATTCCCTT

              *  *  * ** *    *      *    *          * **                    
     325490 TTTCACCGTAGGCGTCAAGGCGATCGAAATGTCCACTTCCACAAACTACAAAAAGAGTGTTTCAA
          1 TTCCAACGAAATCATCAAAGCGATCCAAATATCCACTTCCAGATTCTACAAAAAGAGTGTTTCAA

             *         *   * **       *    *        * ** * *   **      *     
     325555 ACCTGCTCTATGAAAGGCCATGTTCATCTCTATGAGTTGAATGGAAATATCCGAAAGAAATTTCT
         66 AACTGCTCTATCAAAAGAAATGTTCAACTCTGTGAGTTGAGTACACACATCACAAAGAAGTTTCT

             *       *                   *         **
     325620 GGGAATGCTGCTGTCTAG-TTTT-TAT-ACGA-ATTCCCGC
        131 GAGAATGCTTCTGTCTAGTTTTTATATGAAGATATTCCCTT

                         *     * *                *    *  *                  
     325657 TTCCAACGAAATCCTCAAAACAATCCAAATATCCACTTGCAGAATCCACAAAAAGAGTGTTTCAA
          1 TTCCAACGAAATCATCAAAGCGATCCAAATATCCACTTCCAGATTCTACAAAAAGAGTGTTTCAA

                          *     *       *  * *                   *  *        
     325722 AACTGCTCTATCAATAGAAAGGTTCAACACTTTTAGTTGAGTACACACATCACGAACAAGTTTCT
         66 AACTGCTCTATCAAAAGAAATGTTCAACTCTGTGAGTTGAGTACACACATCACAAAGAAGTTTCT

                            * *      **     **  *    
     325787 GAGAATGCTTCTGTCTGGCTTTTATTGGAAGACGTTTCCTT
        131 GAGAATGCTTCTGTCTAGTTTTTATATGAAGATATTCCCTT

                 * *  **          *       *                * *               
     325828 TT-CACCAAAGGCATCAAAGCGCTCCAAATGTCCACTTCCAGATTCTTCCAAAAGAGTGTTTCAA
          1 TTCCAACGAAATCATCAAAGCGATCCAAATATCCACTTCCAGATTCTACAAAAAGAGTGTTTCAA

             **     * ***  * *                 *    * *  * *     *   *       
     325892 ACGTGCTCAAAGTAAGGGAATGTTCAACTCTGTGACTTGAATGCAGATATCACCAAGTAGTTTCT
         66 AACTGCTCTATCAAAAGAAATGTTCAACTCTGTGAGTTGAGTACACACATCACAAAGAAGTTTCT

            * * *             *     *    *         * 
     325957 AATAGTGCTTCTGTCTAGATTTTAGATGATGATATTCCCGT
        131 GAGAATGCTTCTGTCTAGTTTTTATATGAAGATATTCCCTT

                         * * *   *                *   *      *     *       * 
     325998 TTCCAACGAAATCGTTAGAGCTATCCAAATATCCACTTACAGTTTCTACCAAAAGGGTGTTTCCA
          1 TTCCAACGAAATCATCAAAGCGATCCAAATATCCACTTCCAGATTCTACAAAAAGAGTGTTTCAA

                   **           *            *       *                     * 
     326063 AACTGCTGCATCAAAAGAAAGGTTCAACTCTGTTAGTTGAGGACACACATCACAAAGAAGTTTGT
         66 AACTGCTCTATCAAAAGAAATGTTCAACTCTGTGAGTTGAGTACACACATCACAAAGAAGTTTCT

                              *    *     *           
     326128 GAGAATGCTTCTGTCTAGATTTTGTATGACGATATTCCCTT
        131 GAGAATGCTTCTGTCTAGTTTTTATATGAAGATATTCCCTT

                     *   * *     *   *       * *  *    *  *       *  *       
     326169 TTCCAACGATATCGTTAAAGCAATCTAAATATCAATTTGCAGAATCCACAAAAATAGAGTTTCAA
          1 TTCCAACGAAATCATCAAAGCGATCCAAATATCCACTTCCAGATTCTACAAAAAGAGTGTTTCAA

             *       * *        *    *       *  *                   ***      
     326234 ACCTGCTCTGTAAAAAGAAAGGTTCCACTCTGTTAGCTGAGTACACACATCACAAACTTGTTTCT
         66 AACTGCTCTATCAAAAGAAATGTTCAACTCTGTGAGTTGAGTACACACATCACAAAGAAGTTTCT

                  *         *        **         **   
     326299 GAGAATCCTTCTGTCTCGTTTTTATGGGAAGATATTTACTT
        131 GAGAATGCTTCTGTCTAGTTTTTATATGAAGATATTCCCTT

              *  *  * **          *       *     *       * **  *              
     326340 TTTCACCGTAGGCATCAAAGCGCTCCAAATGTCCACATCCAGATACCCCAGAAAGAGTGTTTCAA
          1 TTCCAACGAAATCATCAAAGCGATCCAAATATCCACTTCCAGATTCTACAAAAAGAGTGTTTCAA

             *         *   * *   *        **        * *  *      *      *     
     326405 ACCTGCTCTATGAAAGGGAATCTTCAACTCAATGAGTTGAATGCAGACATCAGAAAGAACTTTCT
         66 AACTGCTCTATCAAAAGAAATGTTCAACTCTGTGAGTTGAGTACACACATCACAAAGAAGTTTCT

                     *       **          *         **
     326470 GAGAATGCTGCTGTCTACCTTTTAT-T--TGA-ATTCCCGC
        131 GAGAATGCTTCTGTCTAGTTTTTATATGAAGATATTCCCTT

                         *  *    *                *  **   *                  
     326507 TTCCAACGAAATCCTCCAAGCTATCCAAATATCCACTTGCATTTTCCACAAAAAGAGTGTTTCAA
          1 TTCCAACGAAATCATCAAAGCGATCCAAATATCCACTTCCAGATTCTACAAAAAGAGTGTTTCAA

                          *                 *    *  *                *       
     326572 AACTGCTCTATCAATAGAAATGTTCAACTCCTTTG-GCTGGGTACACACATCACAAACAAGTTTC
         66 AACTGCTCTATCAAAAGAAATGTTCAACT-CTGTGAGTTGAGTACACACATCACAAAGAAGTTTC

                                      **     *        
     326636 TGAGAATGCTTCTGTCTAGTTTTTATGGGAAGACATTCCCTT
        130 TGAGAATGCTTCTGTCTAGTTTTTATATGAAGATATTCCCTT

              *  * *  **          *       *            **            *     * 
     326678 TTTCACCAAAGGCATCAAAGCGCTCCAAATGTCCACTTCCAGACACTACAAAAAGAGGGTTTCCA
          1 TTCCAACGAAATCATCAAAGCGATCCAAATATCCACTTCCAGATTCTACAAAAAGAGTGTTTCAA

             **       **   ***                 *    * *  * *         *       
     326743 ACGTGCTCTAAGAAAGCGAATGTTCAACTCTGTGACTTGAATGCAGATATCACAAAGTAGTTTCT
         66 AACTGCTCTATCAAAAGAAATGTTCAACTCTGTGAGTTGAGTACACACATCACAAAGAAGTTTCT

                **          * *     *    *         * 
     326808 GAGAGGGCTTCTGTCTGGATTTTAGATGATGATATTCCCGT
        131 GAGAATGCTTCTGTCTAGTTTTTATATGAAGATATTCCCTT

                           * *   *                *   *                    * 
     326849 TTCCAACGAAATCATTAGAGCTATCCAAATATCCACTTACAGTTTCTACAAAAAGAGTGTTTCCA
          1 TTCCAACGAAATCATCAAAGCGATCCAAATATCCACTTCCAGATTCTACAAAAAGAGTGTTTCAA

                   **         * *    *       *  *                   ***      
     326914 AACTGCTGCATCAAAAGAGAGGTTCCACTCTGTTAGCTGAGTACACACATCACAAACTTGTTTCT
         66 AACTGCTCTATCAAAAGAAATGTTCAACTCTGTGAGTTGAGTACACACATCACAAAGAAGTTTCT

                  *         *        **         *    
     326979 GAGAATCCTTCTGTCTCGTTTTTATGGGAAGATATTTCCTT
        131 GAGAATGCTTCTGTCTAGTTTTTATATGAAGATATTCCCTT

              *  *  * **          *  *    *                * *               
     327020 TTTCACCGTAGGCATCAAAGCGCTCAAAATGTCCACTTCCAGATTCTTCCAAAAGAGTGTTTCAA
          1 TTCCAACGAAATCATCAAAGCGATCCAAATATCCACTTCCAGATTCTACAAAAAGAGTGTTTCAA

             **     * ***  * *                 *    * *  * *     *   *       
     327085 ACGTGCTCAAAGTAAGGGAATGTTCAACTCTGTGACTTGAATGCAGATATCACCAAGTAGTTTCT
         66 AACTGCTCTATCAAAAGAAATGTTCAACTCTGTGAGTTGAGTACACACATCACAAAGAAGTTTCT

            * * *            **     *    *         * 
     327150 AATAGTGCTTCTGTCTACATTTTAGATGATGATATTCCCGT
        131 GAGAATGCTTCTGTCTAGTTTTTATATGAAGATATTCCCTT

                  *      * *   * *                *   *                    * 
     327191 TTCCAATGAAATCGTTAAATCTATCCAAATATCCACTTACAGTTTCTACAAAAAGAGTGTTTCCA
          1 TTCCAACGAAATCATCAAAGCGATCCAAATATCCACTTCCAGATTCTACAAAAAGAGTGTTTCAA

                   **        *  *            *       *                *    * 
     327256 AACTGCTGCATCAAAAGGAAGGTTCAACTCTGTTAGTTGAGGACACACATCACAAAGATGTTTGT
         66 AACTGCTCTATCAAAAGAAATGTTCAACTCTGTGAGTTGAGTACACACATCACAAAGAAGTTTCT

                              *    *     **          
     327321 GAGAATGCTTCTGTCTAGATTTTGTATGACCATATTCCCTT
        131 GAGAATGCTTCTGTCTAGTTTTTATATGAAGATATTCCCTT

                     *   * *    **   *       * *  *    *  *       *  *       
     327362 TTCCAACGATATCGTTAAAGAAATCTAAATATCAATTTGCAGAATCCACAAAAATAGAGTTTCAA
          1 TTCCAACGAAATCATCAAAGCGATCCAAATATCCACTTCCAGATTCTACAAAAAGAGTGTTTCAA

             *       * *        *    *       *  *                   ***      
     327427 AGCTGCTCTGTAAAAAGAAAGGTTCCACTCTGTTAGCTGAGTACACACATCACAAACTTGTTTCT
         66 AACTGCTCTATCAAAAGAAATGTTCAACTCTGTGAGTTGAGTACACACATCACAAAGAAGTTTCT

                  *         *        **         **   
     327492 GAGAATCCTTCTGTCTCGTTTTTATGGGAAGATATTTACTT
        131 GAGAATGCTTCTGTCTAGTTTTTATATGAAGATATTCCCTT

              *  *  * **          *       *     *       *  *  *              
     327533 TTTCACCGTAGGCATCAAAGCGCTCCAAATGTCCACATCCAGATACTCCAGAAAGAGTGTTTCAA
          1 TTCCAACGAAATCATCAAAGCGATCCAAATATCCACTTCCAGATTCTACAAAAAGAGTGTTTCAA

             *         *   * *   *         *        * *  *      *      *     
     327598 ACCTGCTCTATGAAAGGGAATCTTCAACTCTATGAGTTGAATGCAGACATCAGAAAGAAATTTCT
         66 AACTGCTCTATCAAAAGAAATGTTCAACTCTGTGAGTTGAGTACACACATCACAAAGAAGTTTCT

                     *       **          *         **
     327663 GAGAATGCTGCTGTCTACCTTTTAT-T--TGA-ATTCCCGC
        131 GAGAATGCTTCTGTCTAGTTTTTATATGAAGATATTCCCTT

                         *  *    *              * *  **   *                  
     327700 TTCCAACGAAATCCTCCAAGCTATCCAAATATCCACCTGCATTTTCCACAAAAAGAGTGTTTCAA
          1 TTCCAACGAAATCATCAAAGCGATCCAAATATCCACTTCCAGATTCTACAAAAAGAGTGTTTCAA

                          *                 *    *  *                *       
     327765 AACTGCTCTATCAATAGAAATGTTCAACTCCTTTG-GCTGGGTACACACATCACAAACAAGTTTC
         66 AACTGCTCTATCAAAAGAAATGTTCAACT-CTGTGAGTTGAGTACACACATCACAAAGAAGTTTC

                                      **     *        
     327829 TGAGAATGCTTCTGTCTAGTTTTTATGGGAAGACATTCCCTT
        130 TGAGAATGCTTCTGTCTAGTTTTTATATGAAGATATTCCCTT

              *  * *  **          *       *            **                    
     327871 TTTCACCAAAGGCATCAAAGCGCTCCAAATGTCCACTTCCAGACACTACAAAAAGAGTGTTTCAA
          1 TTCCAACGAAATCATCAAAGCGATCCAAATATCCACTTCCAGATTCTACAAAAAGAGTGTTTCAA

             **       **   ***                 *    * *  * *         *       
     327936 ACGTGCTCTAAGAAAGCGAATGTTCAACTCTGTGACTTGAATGCAGATATCACAAAGTAGTTTCT
         66 AACTGCTCTATCAAAAGAAATGTTCAACTCTGTGAGTTGAGTACACACATCACAAAGAAGTTTCT

                **            *     *    *         **
     328001 GAGAGGGCTTCTGTCTAGATTTTAGATGATGATATTCCCGA
        131 GAGAATGCTTCTGTCTAGTTTTTATATGAAGATATTCCCTT

              *   *      * * *   *                *   *                    * 
     328042 TTACAAAGAAATCGTTAGAGCTATCCAAATATCCACTTACAGTTTCTACAAAAAGAGTGTTTCCA
          1 TTCCAACGAAATCATCAAAGCGATCCAAATATCCACTTCCAGATTCTACAAAAAGAGTGTTTCAA

                   **           **           *       *                     * 
     328107 AACTGCTGCATCAAAAGAAAGATTCAACTCTGTTAGTTGAGGACACACATCACAAAGAAGTTTGT
         66 AACTGCTCTATCAAAAGAAATGTTCAACTCTGTGAGTTGAGTACACACATCACAAAGAAGTTTCT

                              *    *     *           
     328172 GAGAATGCTTCTGTCTAGATTTTGTATGACGATATTCCCTT
        131 GAGAATGCTTCTGTCTAGTTTTTATATGAAGATATTCCCTT

                     *   * *     *   *       * *  *    *  *       *  *       
     328213 TTCCAACGATATCGTTAAAGCAATCTAAATATCAATTTGCAGAATCCACAAAAATAGAGTTTCAA
          1 TTCCAACGAAATCATCAAAGCGATCCAAATATCCACTTCCAGATTCTACAAAAAGAGTGTTTCAA

             *       * *        *    **      *  *                   ***      
     328278 AGCTGCTCTGTAAAAAGAAAGGTTCCGCTCTGTTAGCTGAGTACACACATCACAAACTTGTTTCT
         66 AACTGCTCTATCAAAAGAAATGTTCAACTCTGTGAGTTGAGTACACACATCACAAAGAAGTTTCT

                  *         *        **         **   
     328343 GAGAATCCTTCTGTCTCGTTTTTATGGGAAGATATTTACTT
        131 GAGAATGCTTCTGTCTAGTTTTTATATGAAGATATTCCCTT

              *  *  * **          *       *     *       *     *       *      
     328384 TTTCACCGTAGGCATCAAAGCGCTCCAAATGTCCACATCCAGATACTACAGAAAGAGTATTTCAA
          1 TTCCAACGAAATCATCAAAGCGATCCAAATATCCACTTCCAGATTCTACAAAAAGAGTGTTTCAA

             *         *   * *   *         *        * *  *      *      *     
     328449 ACCTGCTCTATGAAAGGGAATCTTCAACTCTATGAGTTGAATGCAGACATCAGAAAGAAATTTCT
         66 AACTGCTCTATCAAAAGAAATGTTCAACTCTGTGAGTTGAGTACACACATCACAAAGAAGTTTCT

                     *         *        **         * 
     328514 GAGAATGCTGCTGTCTA-TCTTT-TAT-TTGA-ATTCCCGT
        131 GAGAATGCTTCTGTCTAGTTTTTATATGAAGATATTCCCTT

                         *  *    *                *       *   *              
     328551 TTCCAACGAAATCCTCCAAGCTATCCAAATATCCACTTGCAGATTCCACAGAAAGAGTGTTTCAA
          1 TTCCAACGAAATCATCAAAGCGATCCAAATATCCACTTCCAGATTCTACAAAAAGAGTGTTTCAA

                            ** *  *            *       *     *   * *  *      
     328616 AACTGCTCTCTATCAATGGCAAAGTTCAACTCTGTCAGTTGAGGACACATATCTCCAACAAGTTT
         66 AACTG--CTCTATCAAAAGAAATGTTCAACTCTGTGAGTTGAGTACACACATCACAAAGAAGTTT

                               *       **         *    
     328681 CTGAGAATGCTTCTGTCTATTTTTTATGGGAAGATATTTCCTT
        129 CTGAGAATGCTTCTGTCTAGTTTTTATATGAAGATATTCCCTT

              *  *  * ** *    *      *    *          * **                    
     328724 TTTCACCGTAGGCGTCAAGGCGATCGAAATGTCCACTTCCACAAACTACAAAAAGAGTGTTTCAA
          1 TTCCAACGAAATCATCAAAGCGATCCAAATATCCACTTCCAGATTCTACAAAAAGAGTGTTTCAA

             *         *   * **       *    *        * ** * *   **      *     
     328789 ACCTGCTCTATGAAAGGCCATGTTCATCTCTATGAGTTGAATGGAAATATCCGAAAGAAATTTCT
         66 AACTGCTCTATCAAAAGAAATGTTCAACTCTGTGAGTTGAGTACACACATCACAAAGAAGTTTCT

             *       *                   *         **
     328854 GGGAATGCTGCTGTCTAG-TTTT-TAT-ACGA-ATTCCCGC
        131 GAGAATGCTTCTGTCTAGTTTTTATATGAAGATATTCCCTT

                         *       *                *    *  *                  
     328891 TTCCAACGAAATCCTCAAAGCAATCCAAATATCCACTTGCAGAATCCACAAAAAGAGTGTTTCAA
          1 TTCCAACGAAATCATCAAAGCGATCCAAATATCCACTTCCAGATTCTACAAAAAGAGTGTTTCAA

                          *     *          * *                   *  *        
     328956 AACTGCTCTATCAATAGAAAGGTTCAACTCTTTTAGTTGAGTACACACATCACGAACAAGTTTCT
         66 AACTGCTCTATCAAAAGAAATGTTCAACTCTGTGAGTTGAGTACACACATCACAAAGAAGTTTCT

            *               * *      **     **  *    
     329021 CAGAATGCTTCTGTCTGGCTTTTATTGGAAGACGTTTCCTT
        131 GAGAATGCTTCTGTCTAGTTTTTATATGAAGATATTCCCTT

                 * *  **          *       *                * *               
     329062 TT-CACCAAAGGCATCAAAGCGCTCCAAATGTCCACTTCCAGATTCTTCCAAAAGAGTGTTTCAA
          1 TTCCAACGAAATCATCAAAGCGATCCAAATATCCACTTCCAGATTCTACAAAAAGAGTGTTTCAA

             **     * ***  * *                 *    * *  * *     *   *       
     329126 ACGTGCTCAAAGTAAGGGAATGTTCAACTCTGTGACTTGAATGCAGATATCACCAAGTAGTTTCT
         66 AACTGCTCTATCAAAAGAAATGTTCAACTCTGTGAGTTGAGTACACACATCACAAAGAAGTTTCT

            * * *             *     *    *         * 
     329191 AATAGTGCTTCTGTCTAGATTTTAGATGATGATATTCCCGT
        131 GAGAATGCTTCTGTCTAGTTTTTATATGAAGATATTCCCTT

                         * * *   *                *   *                    * 
     329232 TTCCAACGAAATCGTTAGAGCTATCCAAATATCCACTTACAGTTTCTACAAAAAGAGTGTTTCCA
          1 TTCCAACGAAATCATCAAAGCGATCCAAATATCCACTTCCAGATTCTACAAAAAGAGTGTTTCAA

                   **           *            *       *                     * 
     329297 AACTGCTGCATCAAAAGAAAGGTTCAACTCTGTTAGTTGAGGACACACATCACAAAGAAGTTTGT
         66 AACTGCTCTATCAAAAGAAATGTTCAACTCTGTGAGTTGAGTACACACATCACAAAGAAGTTTCT

                      *       *    * *   **          
     329362 GAGAATGCTTTTGTCTAGATTTTGTGTGACCATATTCCCTT
        131 GAGAATGCTTCTGTCTAGTTTTTATATGAAGATATTCCCTT

                     *   * *     *   *       * *  *    *  *       *  *       
     329403 TTCCAACGATATCGTTAAAGCAATCTAAATATCAATTTGCAGAATCCACAAAAATAGAGTTTCAA
          1 TTCCAACGAAATCATCAAAGCGATCCAAATATCCACTTCCAGATTCTACAAAAAGAGTGTTTCAA

             *       * *       **    *       *  *                   ***      
     329468 AGCTGCTCTTTAAAAAGAAGGGTTCCACTCTGTTAGCTGAGTACACACATCACAAACTTGTTTCT
         66 AACTGCTCTATCAAAAGAAATGTTCAACTCTGTGAGTTGAGTACACACATCACAAAGAAGTTTCT

                 **         *        **         **   
     329533 GAGAAACCTTCTGTCTCGTTTTTATGGGAAGATATTTACTT
        131 GAGAATGCTTCTGTCTAGTTTTTATATGAAGATATTCCCTT

              *  *  * **          *       *     *       *  *  *              
     329574 TTTCACCGTAGGCATCAAAGCGCTCCAAATGTCCACATCCAGATACTCCAGAAAGAGTGTTTCAA
          1 TTCCAACGAAATCATCAAAGCGATCCAAATATCCACTTCCAGATTCTACAAAAAGAGTGTTTCAA

             *         *   * *   *         *        * *  *      *      *     
     329639 ACCTGCTCTATGAAAGGGAATCTTCAACTCTATGAGTTGAATGCAGACATCAGAAAGAAATTTCT
         66 AACTGCTCTATCAAAAGAAATGTTCAACTCTGTGAGTTGAGTACACACATCACAAAGAAGTTTCT

                     *       **          *         **
     329704 GAGAATGCTGCTGTCTACCTTTTAT-T--TGA-ATTCCCGC
        131 GAGAATGCTTCTGTCTAGTTTTTATATGAAGATATTCCCTT

                         *  *    *              * *  **   *       *          
     329741 TTCCAACGAAATCCTCCAAGCTATCCAAATATCCACCTGCATTTTCCACAAAAAAAGTGTTTCAA
          1 TTCCAACGAAATCATCAAAGCGATCCAAATATCCACTTCCAGATTCTACAAAAAGAGTGTTTCAA

                          *                 *   **  *                *       
     329806 AACTGCTCTATCAATAGAAATGTTCAACTCCTTTG-TCTGGGTACACACATCACAAACAAGTTTC
         66 AACTGCTCTATCAAAAGAAATGTTCAACT-CTGTGAGTTGAGTACACACATCACAAAGAAGTTTC

                                      **     *        
     329870 TGAGAATGCTTCTGTCTAGTTTTTATGGGAAGACATTCCCTT
        130 TGAGAATGCTTCTGTCTAGTTTTTATATGAAGATATTCCCTT

              *  * *  **          *       *            **                    
     329912 TTTCACCAAAGGCATCAAAGCGCTCCAAATGTCCACTTCCAGACACTACAAAAAGAGTGTTTCAA
          1 TTCCAACGAAATCATCAAAGCGATCCAAATATCCACTTCCAGATTCTACAAAAAGAGTGTTTCAA

             **       **   ***                 *    * *  * *     *   *       
     329977 ACGTGCTCTAAGAAAGCGAATGTTCAACTCTGTGACTTGAATGCAGATATCACCAAGTAGTTTCT
         66 AACTGCTCTATCAAAAGAAATGTTCAACTCTGTGAGTTGAGTACACACATCACAAAGAAGTTTCT

                **            *     *    *         **
     330042 GAGAGGGCTTCTGTCTAGATTTTAGATGATGATATTCCCGC
        131 GAGAATGCTTCTGTCTAGTTTTTATATGAAGATATTCCCTT

                           * *   *                *   *                    * 
     330083 TTCCAACGAAATCATTAGAGCTATCCAAATATCCACTTACAGTTTCTACAAAAAGAGTGTTTCCA
          1 TTCCAACGAAATCATCAAAGCGATCCAAATATCCACTTCCAGATTCTACAAAAAGAGTGTTTCAA

                   **          **            *       *                     * 
     330148 AACTGCTGCATCAAAAGAATGGTTCAACTCTGTTAGTTGAGGACACACATCACAAAGAAGTTTGT
         66 AACTGCTCTATCAAAAGAAATGTTCAACTCTGTGAGTTGAGTACACACATCACAAAGAAGTTTCT

                              *    *     *           
     330213 GAGAATGCTTCTGTCTAGATTTTGTATGACGATATTCCCTT
        131 GAGAATGCTTCTGTCTAGTTTTTATATGAAGATATTCCCTT

                     *   * *     *   *       * *  *    *  *       *  *       
     330254 TTCCAACGATATCGTTAAAGCAATCTAAATATCAATTTGCAGAATCCACAAAAATAGAGTTTCAA
          1 TTCCAACGAAATCATCAAAGCGATCCAAATATCCACTTCCAGATTCTACAAAAAGAGTGTTTCAA

             *      ** *        *    *       *  *                   ***      
     330319 AGCTGCTCGGTAAAAAGAAAGGTTCCACTCTGTTAGCTGAGTACACACATCACAAACTTGTTTCT
         66 AACTGCTCTATCAAAAGAAATGTTCAACTCTGTGAGTTGAGTACACACATCACAAAGAAGTTTCT

                  *         *        **         *    
     330384 GAGAATCCTTCTGTCTCGTTTTTATGGGAAGATATTTCCTT
        131 GAGAATGCTTCTGTCTAGTTTTTATATGAAGATATTCCCTT

              *  *  * **          *  *    *                * *               
     330425 TTTCACCGTAGGCATCAAAGCGCTCAAAATGTCCACTTCCAGATTCTTCCAAAAGAGTGTTTCAA
          1 TTCCAACGAAATCATCAAAGCGATCCAAATATCCACTTCCAGATTCTACAAAAAGAGTGTTTCAA

             **     * ***  * *                 *    * *  * *     *   *       
     330490 ACGTGCTCAAAGTAAGGGAATGTTCAACTCTGTGACTTGAATGCAGATATCACCAAGTAGTTTCT
         66 AACTGCTCTATCAAAAGAAATGTTCAACTCTGTGAGTTGAGTACACACATCACAAAGAAGTTTCT

            * * *             *     *    *         * 
     330555 AATAGTGCTTCTGTCTAGATTTTAGATGATGATATTCCCGT
        131 GAGAATGCTTCTGTCTAGTTTTTATATGAAGATATTCCCTT

                         * * *   *                *   *                   ** 
     330596 TTCCAACGAAATCGTTACAGCTATCCAAATATCCACTTACAGTTTCTACAAAAAGAGTGTTTGCA
          1 TTCCAACGAAATCATCAAAGCGATCCAAATATCCACTTCCAGATTCTACAAAAAGAGTGTTTCAA

                   **           *            *       *                     * 
     330661 AACTGCTGCATCAAAAGAAAGGTTCAACTCTGTTAGTTGAGGACACACATCACAAAGAAGTTTGT
         66 AACTGCTCTATCAAAAGAAATGTTCAACTCTGTGAGTTGAGTACACACATCACAAAGAAGTTTCT

                              *    *     **          
     330726 GAGAATGCTTCTGTCTAGATTTTGTATGACCATATTCCCTT
        131 GAGAATGCTTCTGTCTAGTTTTTATATGAAGATATTCCCTT

                     *     *     *   *         *  *    *  *       *  *       
     330767 TTCCAACGATATCATTAAAGCAATCTAAATATCCATTTGCAGAATCCACAAAAATAGAGTTTCAA
          1 TTCCAACGAAATCATCAAAGCGATCCAAATATCCACTTCCAGATTCTACAAAAAGAGTGTTTCAA

             *       * *        *    *       *  *                   ***      
     330832 AGCTGCTCTGTAAAAAGAAAAGTTCCACTCTGTTAGCTGAGTACACACATCACAAACTTGTTTCT
         66 AACTGCTCTATCAAAAGAAATGTTCAACTCTGTGAGTTGAGTACACACATCACAAAGAAGTTTCT

                  *         *        **         **   
     330897 GAGAATCCTTCTGTCTCGTTTTTATGGGAAGATATTTACTT
        131 GAGAATGCTTCTGTCTAGTTTTTATATGAAGATATTCCCTT

              *  *  * **          *       *     *       *  *  *              
     330938 TTTCACCGTAGGCATCAAAGCGCTCCAAATGTCCACATCCAGATACTCCAGAAAGAGTGTTTCAA
          1 TTCCAACGAAATCATCAAAGCGATCCAAATATCCACTTCCAGATTCTACAAAAAGAGTGTTTCAA

             *         *   * *   *         *        * *  *      *      *     
     331003 ACCTGCTCTATGAAAGGGAATCTTCAACTCTATGAGTTGAATGCAGACATCAGAAAGAAATTTCT
         66 AACTGCTCTATCAAAAGAAATGTTCAACTCTGTGAGTTGAGTACACACATCACAAAGAAGTTTCT

                     *       **   *      *         **
     331068 GAGAATGCTGCTGTCTACCTTTCAT-T--TGA-ATTCCCGC
        131 GAGAATGCTTCTGTCTAGTTTTTATATGAAGATATTCCCTT

                         *  * ** *                *       *                * 
     331105 TTCCAACGAAATCCTCCAGTCTATCCAAATATCCACTTGCAGATTCCACAAAAAGAGTGTTTCTA
          1 TTCCAACGAAATCATCAAAGCGATCCAAATATCCACTTCCAGATTCTACAAAAAGAGTGTTTCAA

                          ** *  *            *       * *            *        
     331170 AACTGCTCTATCAATGGCAAGGTTCAACTCTGTCAGTTGAGGATACACATCACAAACAAGTTTCT
         66 AACTGCTCTATCAAAAGAAATGTTCAACTCTGTGAGTTGAGTACACACATCACAAAGAAGTTTCT

                  *    **           ***     *   *    
     331235 GAGAATTCTTCAAT-T--TTTTTACGGGAAGACATTTCCTT
        131 GAGAATGCTTCTGTCTAGTTTTTATATGAAGATATTCCCTT

              *  *  * ** *           *    *  ** *    * ***         *         
     331273 TTTCACCGTAGGCGTCAAAGCGATCGAAATGTCTTCATCCATACAATACAAAAAGTGTGTTTCAA
          1 TTCCAACGAAATCATCAAAGCGATCCAAATATCCACTTCCAGATTCTACAAAAAGAGTGTTTCAA

             *         *   * *        *    *  *     * ** * *    *      *     
     331338 ACCTGCTCTATGAAAGGGAATGTTCATCTCTATGTGTTGAATGGAAATATCAGAAAGAAATTTCT
         66 AACTGCTCTATCAAAAGAAATGTTCAACTCTGTGAGTTGAGTACACACATCACAAAGAAGTTTCT

                             *                     **
     331403 GAGAATG---CTG-CTACTTTTT-TAT---GA-ATTCCCGC
        131 GAGAATGCTTCTGTCTAGTTTTTATATGAAGATATTCCCTT

                         *       *                *       *                  
     331435 TTCCAACGAAATCCTCAAAGCAATCCAAATATCCACTTGCAGATTCCACAAAAAGAGTGTTTCAA
          1 TTCCAACGAAATCATCAAAGCGATCCAAATATCCACTTCCAGATTCTACAAAAAGAGTGTTTCAA

                          *     *          * *                    * *        
     331500 AACTGCTCTATCAAGAGAAAGGTTCAACTCTTTTAGTTGAGTACACACATCACATACAAGTTTCT
         66 AACTGCTCTATCAAAAGAAATGTTCAACTCTGTGAGTTGAGTACACACATCACAAAGAAGTTTCT

                 *           *      ***     *   *    
     331565 GAGAAAGCTTCTGTCTACTTTTTAAGGGAAGACATTTCCTT
        131 GAGAATGCTTCTGTCTAGTTTTTATATGAAGATATTCCCTT

              *    *               *       *                                 
     331606 TTTC-ACCAAATGCATCAAAGCGCTCCAAATGTCCACTTCCAGATTCTACAAAAAGAGTGTTTCA
          1 TTCCAACGAAAT-CATCAAAGCGATCCAAATATCCACTTCCAGATTCTACAAAAAGAGTGTTTCA

              *        ***  * * * *             * *  * *  * *         *      
     331670 AACCTGCTCTAAGTAAGGGAGTTTTCAACTCTGTGACTGGAATGCAGATATCACAAAGTAGTTTC
         65 AAACTGCTCTATCAAAAGAAATGTTCAACTCTGTGAGTTGAGTACACACATCACAAAGAAGTTTC

                 *  *      *  ***    *      *       * 
     331735 TGAGACTGATTCTGTGTATACTTTAGATGAAGGTATTCCCGT
        130 TGAGAATGCTTCTGTCTAGTTTTTATATGAAGATATTCCCTT

                  *  *   * * * * * *              *   *                    * 
     331777 TTCCAATGATATCGTTAGACCTACCCAAATATCCACTTACAGTTTCTACAAAAAGAGTGTTTCCA
          1 TTCCAACGAAATCATCAAAGCGATCCAAATATCCACTTCCAGATTCTACAAAAAGAGTGTTTCAA

                   **   *       *                    *                       
     331842 AACTGCTGCATCTAAAGAAAGGTTCAACTCTGTGAGTTGAGGACACACATCACAAAGAAGTTTCT
         66 AACTGCTCTATCAAAAGAAATGTTCAACTCTGTGAGTTGAGTACACACATCACAAAGAAGTTTCT

                 *            *    *                 
     331907 GAGAAAGCTTCTGTCTAGATTTTGTATGAAGATATTCCCTT
        131 GAGAATGCTTCTGTCTAGTTTTTATATGAAGATATTCCCTT

                     *   * *   * * *         * *  *    *  *   *   *  *       
     331948 TTCCAACGATATCGTTAAATCAACCCAAATATCAATTTGCAGAATCCACAGAAATAGAGTTTCAA
          1 TTCCAACGAAATCATCAAAGCGATCCAAATATCCACTTCCAGATTCTACAAAAAGAGTGTTTCAA

             *       * *        * *  *       *  *                   ***      
     332013 AGCTGCTCTGTAAAAAGAAAGGATCCACTCTGTTAGCTGAGTACACACATCACAAACTTGTTTCT
         66 AACTGCTCTATCAAAAGAAATGTTCAACTCTGTGAGTTGAGTACACACATCACAAAGAAGTTTCT

                  *                  **         **   
     332078 GAGAATACTTCTGTCTAGTTTTTATGGGAAGATATTTACTT
        131 GAGAATGCTTCTGTCTAGTTTTTATATGAAGATATTCCCTT

              *  *  * ***         *       *     *       *     *              
     332119 TTTCACCGTAGGTATCAAAGCGCTCCAAATGTCCACATCCAGATACTACAGAAAGAGTGTTTCAA
          1 TTCCAACGAAATCATCAAAGCGATCCAAATATCCACTTCCAGATTCTACAAAAAGAGTGTTTCAA

             *         *   * *   *         *        * *  *      *    * *     
     332184 ACCTGCTCTATGAAAGGGAATCTTCAACTCTATGAGTTGAATGCAGACATCAGAAAGTAATTTCT
         66 AACTGCTCTATCAAAAGAAATGTTCAACTCTGTGAGTTGAGTACACACATCACAAAGAAGTTTCT

                     *       **   *      *         **
     332249 GAGAATGCTGCTGTCTACCTTTCAT-T--TGA-ATTCCCGC
        131 GAGAATGCTTCTGTCTAGTTTTTATATGAAGATATTCCCTT

                         *  *    *          *     *       *                  
     332286 TTCCAACGAAATCCTCCAAGCTATCCAAATATTCACTTGCAGATTCCACAAAAAGAGTGTTTCAA
          1 TTCCAACGAAATCATCAAAGCGATCCAAATATCCACTTCCAGATTCTACAAAAAGAGTGTTTCAA

                *         *     *  *     *   *       *              *        
     332351 AACTACTCTATCAATAGAAAGGTACAACTGTGTCAGTTGAGGACACACATCACAAACAAGTTTCT
         66 AACTGCTCTATCAAAAGAAATGTTCAACTCTGTGAGTTGAGTACACACATCACAAAGAAGTTTCT

                           * *       **     *   *    
     332416 GAGAA---TTCT-TCAATTTTTTATGGGAAGACATTTCCTT
        131 GAGAATGCTTCTGTCTAGTTTTTATATGAAGATATTCCCTT

              *  *  * ** *       **       *     *       **    *              
     332453 TTTCACCGTAGGCGTCAAAGCACTCCAAATGTCCACATCCAGATAGTACAGAAAGAGTGTTTCAA
          1 TTCCAACGAAATCATCAAAGCGATCCAAATATCCACTTCCAGATTCTACAAAAAGAGTGTTTCAA

             *         *   * *             *        * *  *      *      *     
     332518 ACCTGCTCTATTAAAGGGAATGTTCAACTCTATGAGTTGAATGCAAACATCAGAAAGAAATTTCT
         66 AACTGCTCTATCAAAAGAAATGTTCAACTCTGTGAGTTGAGTACACACATCACAAAGAAGTTTCT

                     *       **          *         **
     332583 GAGAATGCTGCTGTCTACCTTTTAT-T--TGA-ATTCCCGC
        131 GAGAATGCTTCTGTCTAGTTTTTATATGAAGATATTCCCTT

                       * *  *    *                *       *                  
     332620 TTCCAACGAAAACCTCCAAGCTATCCAAATATCCACTTGCAGATTCCACAAAAAGAGTGTTTCAA
          1 TTCCAACGAAATCATCAAAGCGATCCAAATATCCACTTCCAGATTCTACAAAAAGAGTGTTTCAA

                          *               ** ** *  *                *        
     332685 AACTGCTCTATCAATAGAAATGTTCAACTCCTTTCGCTGGGTACACACATCACAAACAAGTTTCT
         66 AACTGCTCTATCAAAAGAAATGTTCAACTCTGTGAGTTGAGTACACACATCACAAAGAAGTTTCT

                 *                   **     *   *    
     332750 GAGAAAGCTTCTGTCTAGTTTTTATGGGAAGACATTTCCTT
        131 GAGAATGCTTCTGTCTAGTTTTTATATGAAGATATTCCCTT

              *  * *  **        * *       *             *                    
     332791 TTTCACCAAAGGCATCAAAGAGCTCCAAATGTCCACTTCCAGATACTACAAAAAGAGTGTTTCAA
          1 TTCCAACGAAATCATCAAAGCGATCCAAATATCCACTTCCAGATTCTACAAAAAGAGTGTTTCAA

              *       **   ***                 *    * *  * *         *       
     332856 AAGTGCTCTAAGAAAGCGAATGTTCAACTCTGTGACTTGAATGCAGATATCACAAAGTAGTTTCT
         66 AACTGCTCTATCAAAAGAAATGTTCAACTCTGTGAGTTGAGTACACACATCACAAAGAAGTTTCT

                *                   *    *         * 
     332921 GAGAGTGCTTCTGTCTAGTTTTTACATGATGATATTCCCGT
        131 GAGAATGCTTCTGTCTAGTTTTTATATGAAGATATTCCCTT

                             *   *                *   *                    * 
     332962 TTCCAACGAAATCATCAGAGCTATCCAAATATCCACTTACAGTTTCTACAAAAAGAGTGTTTCCA
          1 TTCCAACGAAATCATCAAAGCGATCCAAATATCCACTTCCAGATTCTACAAAAAGAGTGTTTCAA

                   **         * *    *       *  *                   ***      
     333027 AACTGCTGCATCAAAAGAGAGGTTCCACTCTGTTAGCTGAGTACACACATCACAAACTTGTTTCT
         66 AACTGCTCTATCAAAAGAAATGTTCAACTCTGTGAGTTGAGTACACACATCACAAAGAAGTTTCT

                  *           *      **         **   
     333092 GAGAATCCTTCTGTCTAGCTTTTATGGGAAGATATTTTCTT
        131 GAGAATGCTTCTGTCTAGTTTTTATATGAAGATATTCCCTT

              *  *  * **          *       *     *       **    *      *       
     333133 TTTCACCGTAGGCATCAAAGCGTTCCAAATGTCCACATCCAGATAGTACAGAAAGAGCGTTTCAA
          1 TTCCAACGAAATCATCAAAGCGATCCAAATATCCACTTCCAGATTCTACAAAAAGAGTGTTTCAA

             *         *   * *             *        * *  *  *          *     
     333198 ACCTGCTCTATGAAAGGGAATGTTCAACTCTATGAGTTGAATGCAAACGTCACAAAGAAATTTCT
         66 AACTGCTCTATCAAAAGAAATGTTCAACTCTGTGAGTTGAGTACACACATCACAAAGAAGTTTCT

                     *       **   *      *         **
     333263 GAGAATGCTGCTGTCTACCTTTCAT-T--TGA-ATTCCCGC
        131 GAGAATGCTTCTGTCTAGTTTTTATATGAAGATATTCCCTT

                         *  * *  *                *       *                * 
     333300 TTCCAACGAAATCCTCCAGGCTATCCAAATATCCACTTGCAGATTCCACAAAAAGAGTGTTTCTA
          1 TTCCAACGAAATCATCAAAGCGATCCAAATATCCACTTCCAGATTCTACAAAAAGAGTGTTTCAA

                          ** *  *            *       * *            *        
     333365 AACTGCTCTATCAATGGCAAGGTTCAACTCTGTCAGTTGAGGATACACATCACAAACAAGTTTCT
         66 AACTGCTCTATCAAAAGAAATGTTCAACTCTGTGAGTTGAGTACACACATCACAAAGAAGTTTCT

                  *    **            **         *    
     333430 GAGAATTCTTCAAT-T--TTTTTATGGGAAGATATTTCCTT
        131 GAGAATGCTTCTGTCTAGTTTTTATATGAAGATATTCCCTT

              *  *  * ** *           *    *  ** *    * ***         *         
     333468 TTTCACCGTAGGCGTCAAAGCGATCGAAATGTCTTCATCCATACAATACAAAAAGTGTGTTTCAA
          1 TTCCAACGAAATCATCAAAGCGATCCAAATATCCACTTCCAGATTCTACAAAAAGAGTGTTTCAA

             *         *   * *        *    *  *     * ** * *    *      *     
     333533 ACCTGCTCTATGAAAGGGAATGTTCATCTCTATGTGTTGAATGGAAATATCAGAAAGAAATTTCT
         66 AACTGCTCTATCAAAAGAAATGTTCAACTCTGTGAGTTGAGTACACACATCACAAAGAAGTTTCT

                             *                     **
     333598 GAGAATG---CTG-CTACTTTTT-TAT---GA-ATTCCCGC
        131 GAGAATGCTTCTGTCTAGTTTTTATATGAAGATATTCCCTT

                         *       *                *       *                  
     333630 TTCCAACGAAATCCTCAAAGCAATCCAAATATCCACTTGCAGATTCCACAAAAAGAGTGTTTCAA
          1 TTCCAACGAAATCATCAAAGCGATCCAAATATCCACTTCCAGATTCTACAAAAAGAGTGTTTCAA

                          *     *          * *                    * *        
     333695 AACTGCTCTATCAAGAGAAAGGTTCAACTCTTTTAGTTGAGTACACACATCACATACAAGTTTCT
         66 AACTGCTCTATCAAAAGAAATGTTCAACTCTGTGAGTTGAGTACACACATCACAAAGAAGTTTCT

                 *           *      ***     *   *    
     333760 GAGAAAGCTTCTGTCTACTTTTTAAGGGAAGACATTTCCTT
        131 GAGAATGCTTCTGTCTAGTTTTTATATGAAGATATTCCCTT

              *    *               *       *                                 
     333801 TTTC-ACCAAATGCATCAAAGCGCTCCAAATGTCCACTTCCAGATTCTACAAAAAGAGTGTTTCA
          1 TTCCAACGAAAT-CATCAAAGCGATCCAAATATCCACTTCCAGATTCTACAAAAAGAGTGTTTCA

              *        ***  * * * *             * *  * *  * *         *      
     333865 AACCTGCTCTAAGTAAGGGAGTTTTCAACTCTGTGACTGGAATGCAGATATCACAAAGTAGTTTC
         65 AAACTGCTCTATCAAAAGAAATGTTCAACTCTGTGAGTTGAGTACACACATCACAAAGAAGTTTC

                 *  *      *  ***    *      *       * 
     333930 TGAGACTGATTCTGTGTATACTTTAGATGAAGGTATTCCCGT
        130 TGAGAATGCTTCTGTCTAGTTTTTATATGAAGATATTCCCTT

                  *  *   * * * * * *              *   *      *             * 
     333972 TTCCAATGATATCGTTAGATCTACCCAAATATCCACTTACAGTTTCTACTAAAAGAGTGTTTCCA
          1 TTCCAACGAAATCATCAAAGCGATCCAAATATCCACTTCCAGATTCTACAAAAAGAGTGTTTCAA

                   **   *       **                   *  *              *     
     334037 AACTGCTGCATCTAAAGAAAGTTTCAACTCTGTGAGTTGAGGACGCACATCACAAAGAAATTTCT
         66 AACTGCTCTATCAAAAGAAATGTTCAACTCTGTGAGTTGAGTACACACATCACAAAGAAGTTTCT

                 *            *    *                 
     334102 GAGAAAGCTTCTGTCTAGATTTTGTATGAAGATATTCCCTT
        131 GAGAATGCTTCTGTCTAGTTTTTATATGAAGATATTCCCTT

                     *   * *   * * *         * *  *    *  *   *   *  *       
     334143 TTCCAACGATATCGTTAAATCAACCCAAATATCAATTTGCAGAATCCACAGAAATAGAGTTTCAA
          1 TTCCAACGAAATCATCAAAGCGATCCAAATATCCACTTCCAGATTCTACAAAAAGAGTGTTTCAA

             *       * *        * *  *       *  *                   ***      
     334208 AGCTGCTCTGTAAAAAGAAAGGATCCACTCTGTTAGCTGAGTACACACATCACAAACTTGTTTCT
         66 AACTGCTCTATCAAAAGAAATGTTCAACTCTGTGAGTTGAGTACACACATCACAAAGAAGTTTCT

                  *                  **         **   
     334273 GAGAATCCTTCTGTCTAGTTTTTATGGGAAGATATTTACTT
        131 GAGAATGCTTCTGTCTAGTTTTTATATGAAGATATTCCCTT

              *  *  * ***         *       *     *       *     *              
     334314 TTTCACCGTAGGTATCAAAGCGCTCCAAATGTCCACATCCAGATACTACAGAAAGAGTGTTTCAA
          1 TTCCAACGAAATCATCAAAGCGATCCAAATATCCACTTCCAGATTCTACAAAAAGAGTGTTTCAA

             *         *   * *   *         *        * *  *      *    * *     
     334379 ACCTGCTCTATGAAAGGGAATCTTCAACTCTATGAGTTGAATGCAGACATCAGAAAGTAATTTCT
         66 AACTGCTCTATCAAAAGAAATGTTCAACTCTGTGAGTTGAGTACACACATCACAAAGAAGTTTCT

                     *       **   *      *         **
     334444 GAGAATGCTGCTGTCTACCTTTCAT-T--TGA-ATTCCCGC
        131 GAGAATGCTTCTGTCTAGTTTTTATATGAAGATATTCCCTT

                         *  *    *          *     *       *                  
     334481 TTCCAACGAAATCCTCCAAGCTATCCAAATATTCACTTGCAGATTCCACAAAAAGAGTGTTTCAA
          1 TTCCAACGAAATCATCAAAGCGATCCAAATATCCACTTCCAGATTCTACAAAAAGAGTGTTTCAA

                *         *     *  *     *   *       *              *        
     334546 AACTACTCTATCAATAGAAAGGTACAACTGTGTCAGTTGAGGACACACATCACAAACAAGTTTCT
         66 AACTGCTCTATCAAAAGAAATGTTCAACTCTGTGAGTTGAGTACACACATCACAAAGAAGTTTCT

            *              * *       **     *   *    
     334611 CAGAA---TTCT-TCAATTTTTTATGGGAAGACATTTCCTT
        131 GAGAATGCTTCTGTCTAGTTTTTATATGAAGATATTCCCTT

              *  *  * ** *       **       *     *       **    *              
     334648 TTTCACCGTAGGCGTCAAAGCACTCCAAATGTCCACATCCAGATAGTACAGAAAGAGTGTTTCAA
          1 TTCCAACGAAATCATCAAAGCGATCCAAATATCCACTTCCAGATTCTACAAAAAGAGTGTTTCAA

             *         *   * *             *        * *  *      *      *     
     334713 ACCTGCTCTATTAAAGGGAATGTTCAACTCTATGAGTTGAATGCAAACATCAGAAAGAAATTTCT
         66 AACTGCTCTATCAAAAGAAATGTTCAACTCTGTGAGTTGAGTACACACATCACAAAGAAGTTTCT

                 *   *       **          *         **
     334778 GAGAACGCTGCTGTCTACCTTTTAT-T--TGAT-TTCCCGC
        131 GAGAATGCTTCTGTCTAGTTTTTATATGAAGATATTCCCTT

                       * *  *    *                *       *                  
     334815 TTCCAACGAAAACCTCCAAGCTATCCAAATATCCACTTGCAGATTCCACAAAAAGAGTGTTTCAA
          1 TTCCAACGAAATCATCAAAGCGATCCAAATATCCACTTCCAGATTCTACAAAAAGAGTGTTTCAA

                          *               ** ** *  *                *        
     334880 AACTGCTCTATCAATAGAAATGTTCAACTCCTTTCGCTGGGTACACACATCACAAACAAGTTTCT
         66 AACTGCTCTATCAAAAGAAATGTTCAACTCTGTGAGTTGAGTACACACATCACAAAGAAGTTTCT

                 *                   **     *   *    
     334945 GAGAAAGCTTCTGTCTAGTTTTTATGGGAAGACATTTCCTT
        131 GAGAATGCTTCTGTCTAGTTTTTATATGAAGATATTCCCTT

              *  * *  **        * *       *             *                    
     334986 TTTCACCAAAGGCATCAAAGAGCTCCAAATGTCCACTTCCAGATACTACAAAAAGAGTGTTTCAA
          1 TTCCAACGAAATCATCAAAGCGATCCAAATATCCACTTCCAGATTCTACAAAAAGAGTGTTTCAA

              *       **   ***                 *    * *  * *         *       
     335051 AAGTGCTCTAAGAAAGCGAATGTTCAACTCTGTGACTTGAATGCAGATATCACAAAGTAGTTTCT
         66 AACTGCTCTATCAAAAGAAATGTTCAACTCTGTGAGTTGAGTACACACATCACAAAGAAGTTTCT

                *             *     *    *         * 
     335116 GAGAGTGCTTCTGTCTAGATTTTAGATGATGATATTCCCGT
        131 GAGAATGCTTCTGTCTAGTTTTTATATGAAGATATTCCCTT

                           * *   *   *            *   *                    * 
     335157 TTCCAACGAAATCATTAGAGCTATCGAAATATCCACTTACAGTTTCTACAAAAAGAGTGTTTCCA
          1 TTCCAACGAAATCATCAAAGCGATCCAAATATCCACTTCCAGATTCTACAAAAAGAGTGTTTCAA

                   **         * *    *       *  *                   ***      
     335222 AACTGCTGCATCAAAAGAGAGGTTCCACTCTGTTAGCTGAGTACACACATCACAAACTTGTTTCT
         66 AACTGCTCTATCAAAAGAAATGTTCAACTCTGTGAGTTGAGTACACACATCACAAAGAAGTTTCT

            *     *         *        **         **   
     335287 CAGAATCCTTCTGTCTCGTTTTTATGGGAAGATATTTACTT
        131 GAGAATGCTTCTGTCTAGTTTTTATATGAAGATATTCCCTT

              *  *  * **          *       *     *       *  *  *              
     335328 TTTCACCGTAGGCATCAAAGCGCTCCAAATGTCCACATCCAGATACTCCAGAAAGAGTGTTTCAA
          1 TTCCAACGAAATCATCAAAGCGATCCAAATATCCACTTCCAGATTCTACAAAAAGAGTGTTTCAA

             *         *   * *   *         *        * *  *      *      *     
     335393 ACCTGCTCTATGAAAGGGAATCTTCAACTCTATGAGTTGAATGCAGACATCAGAAAGAAATTTCT
         66 AACTGCTCTATCAAAAGAAATGTTCAACTCTGTGAGTTGAGTACACACATCACAAAGAAGTTTCT

                     *       **          *         **
     335458 GAGAATGCTGCTGTCTACCTTTTAT-T--TGA-ATTCCCGA
        131 GAGAATGCTTCTGTCTAGTTTTTATATGAAGATATTCCCTT

                         *  *    *                *       *                  
     335495 TTCCAACGAAATCCTCCAAGCTATCCAAATATCCACTTGCAGATTCCACAAAAAGAGTGTTTCAA
          1 TTCCAACGAAATCATCAAAGCGATCCAAATATCCACTTCCAGATTCTACAAAAAGAGTGTTTCAA

                            ** *  *            *       *     *     *  *      
     335560 AACTGCTCTCTATCAATGGCAAAGTTCAACTCTGTTAGTTGAGGACACATATCACCAACAAGTTT
         66 AACTG--CTCTATCAAAAGAAATGTTCAACTCTGTGAGTTGAGTACACACATCACAAAGAAGTTT

                               *       **         *    
     335625 CTGAGAATGCTTCTGTCTATTTTTTATGGGAAGATATTTCCTT
        129 CTGAGAATGCTTCTGTCTAGTTTTTATATGAAGATATTCCCTT

              *  *  * ** *    *      *    *          * **                    
     335668 TTTCACCGTAGGCGTCAAGGCGATCGAAATGTCCACTTCCACAAACTACAAAAAGAGTGTTTCAA
          1 TTCCAACGAAATCATCAAAGCGATCCAAATATCCACTTCCAGATTCTACAAAAAGAGTGTTTCAA

             *         *   * **       *    *        * ** * *   **      *     
     335733 ACCTGCTCTATGAAAGGCCATGTTCATCTCTATGAGTTGAATGGAAATATCCGAAAGAAATTTCT
         66 AACTGCTCTATCAAAAGAAATGTTCAACTCTGTGAGTTGAGTACACACATCACAAAGAAGTTTCT

             *       *                             **
     335798 GGGAATGCTGCTGTCTAGTTTTTATAT---GA-ATTCCCGC
        131 GAGAATGCTTCTGTCTAGTTTTTATATGAAGATATTCCCTT

                         *       *                *    *  *              *   
     335835 TTCCAACGAAATCCTCAAAGCAATCCAAATATCCACTTGCAGAATCCACAAAAAGAGTGTTGCAA
          1 TTCCAACGAAATCATCAAAGCGATCCAAATATCCACTTCCAGATTCTACAAAAAGAGTGTTTCAA

                          *     *          * *                      *        
     335900 AACTGCTCTATCAATAGAAAGGTTCAACTCTTTTAGTTGAGTACACACATCACAAACAAGTTTCT
         66 AACTGCTCTATCAAAAGAAATGTTCAACTCTGTGAGTTGAGTACACACATCACAAAGAAGTTTCT

                            * *      **     **  *    
     335965 GAGAATGCTTCTGTCTGGCTTTTATTGGAAGACGTTTCCTT
        131 GAGAATGCTTCTGTCTAGTTTTTATATGAAGATATTCCCTT

                 * *  **          * *     *                * *               
     336006 TT-CACCAAAGGCATCAAAGCGTTACAAATGTCCACTTCCAGATTCTTCCAAAAGAGTGTTTCAA
          1 TTCCAACGAAATCATCAAAGCGATCCAAATATCCACTTCCAGATTCTACAAAAAGAGTGTTTCAA

             **     * ***  * *                 *    * *  * *     *   *       
     336070 ACGTGCTCAAAGTAAGGGAATGTTCAACTCTGTGACTTGAATGCAGATATCACCAAGTAGTTTCT
         66 AACTGCTCTATCAAAAGAAATGTTCAACTCTGTGAGTTGAGTACACACATCACAAAGAAGTTTCT

            * ***             *     *  * *         * 
     336135 AATGGTGCTTCTGTCTAGATTTTAGATAATGATATTCCCGT
        131 GAGAATGCTTCTGTCTAGTTTTTATATGAAGATATTCCCTT

                   *     * * *   *                *   *              *     * 
     336176 TTCCAACAAAATCGTTAGAGCTATCCAAATATCCACTTACAGTTTCTACAAAAAGAGCGTTTCCA
          1 TTCCAACGAAATCATCAAAGCGATCCAAATATCCACTTCCAGATTCTACAAAAAGAGTGTTTCAA

                   **           *            *       *                     * 
     336241 AACTGCTGCATCAAAAGAAAGGTTCAACTCTGTTAGTTGAGGACACACATCACAAAGAAGTTTGT
         66 AACTGCTCTATCAAAAGAAATGTTCAACTCTGTGAGTTGAGTACACACATCACAAAGAAGTTTCT

                              *    *     **          
     336306 GAGAATGCTTCTGTCTAGATTTTGTATGACCATATTCCCTT
        131 GAGAATGCTTCTGTCTAGTTTTTATATGAAGATATTCCCTT

                     *     *     *   *       * *  *    *  *       *  *       
     336347 TTCCAACGATATCATTAAAGCAATCTAAATATCAATTTGCAGAATCCACAAAAATAGAGTTTCAA
          1 TTCCAACGAAATCATCAAAGCGATCCAAATATCCACTTCCAGATTCTACAAAAAGAGTGTTTCAA

             *       * *        *    *       *  *                   ***      
     336412 AGCTGCTCTGTAAAAAGAAAGGTTCCACTCTGTTAGCTGAGTACACACATCACAAACTTGTTTCT
         66 AACTGCTCTATCAAAAGAAATGTTCAACTCTGTGAGTTGAGTACACACATCACAAAGAAGTTTCT

                  *         *   *    **         **   
     336477 GAGAATCCTTCTGTCTCGTTATTATGGGAAGATATTTACTT
        131 GAGAATGCTTCTGTCTAGTTTTTATATGAAGATATTCCCTT

              *  *  * **          *       *     *       *  *  *              
     336518 TTTCACCGTAGGCATCAAAGCGCTCCAAATGTCCACATCCAGATACTCCAGAAAGAGTGTTTCAA
          1 TTCCAACGAAATCATCAAAGCGATCCAAATATCCACTTCCAGATTCTACAAAAAGAGTGTTTCAA

             *         *   * *   *         *        * *  *      *      *     
     336583 ACCTGCTCTATGAAAGGGAATCTTCAACTCTATGAGTTGAATGCAGACATCAGAAAGAAATTTCT
         66 AACTGCTCTATCAAAAGAAATGTTCAACTCTGTGAGTTGAGTACACACATCACAAAGAAGTTTCT

                     *       **          *         **
     336648 GAGAATGCTGCTGTCTACCTTTTAT-T--TGA-ATTCCCGC
        131 GAGAATGCTTCTGTCTAGTTTTTATATGAAGATATTCCCTT

                         *  *    *              * *  **   *          *       
     336685 TTCCAACGAAATCCTCCAAGCTATCCAAATATCCACCTGCATTTTCCACAAAAAGAGAGTTTCAA
          1 TTCCAACGAAATCATCAAAGCGATCCAAATATCCACTTCCAGATTCTACAAAAAGAGTGTTTCAA

                          *           *     *    *  *                *       
     336750 AACTGCTCTATCAATAGAAATGTTCATCTCCTTTG-GCTGGGTACACACATCACAAACAAGTTTC
         66 AACTGCTCTATCAAAAGAAATGTTCAACT-CTGTGAGTTGAGTACACACATCACAAAGAAGTTTC

                                      **     *        
     336814 TGAGAATGCTTCTGTCTAGTTTTTATGGGAAGACATTCCCTT
        130 TGAGAATGCTTCTGTCTAGTTTTTATATGAAGATATTCCCTT

              *  * *  **          *       *            **          *       * 
     336856 TTTCACCAAAGGCATCAAAGCGCTCCAAATGTCCACTTCCAGACACTACAAAAAGTGTGTTTCCA
          1 TTCCAACGAAATCATCAAAGCGATCCAAATATCCACTTCCAGATTCTACAAAAAGAGTGTTTCAA

             **       **   ***                 *    * *  * *         *       
     336921 ACGTGCTCTAAGAAAGCGAATGTTCAACTCTGTGACTTGAATGCAGATATCACAAAGTAGTTTCT
         66 AACTGCTCTATCAAAAGAAATGTTCAACTCTGTGAGTTGAGTACACACATCACAAAGAAGTTTCT

                **            * *   *    *         * 
     336986 GAGAGGGCTTCTGTCTAGATATTAGATGATGATATTCCCGT
        131 GAGAATGCTTCTGTCTAGTTTTTATATGAAGATATTCCCTT

                           * *   *                *   *                    * 
     337027 TTCCAACGAAATCATTAGAGCTATCCAAATATCCACTTACAGTTTCTACAAAAAGAGTGTTTCCA
          1 TTCCAACGAAATCATCAAAGCGATCCAAATATCCACTTCCAGATTCTACAAAAAGAGTGTTTCAA

                   **         * *    *       *  *                   ***      
     337092 AACTGCTGCATCAAAAGAGAGGTTCCACTCTGTTAGCTGAGTACACACATCACAAACTTGTTTCT
         66 AACTGCTCTATCAAAAGAAATGTTCAACTCTGTGAGTTGAGTACACACATCACAAAGAAGTTTCT

            *     *         *        **         **   
     337157 CAGAATCCTTCTGTCTCGTTTTTATGGGAAGATATTTACTT
        131 GAGAATGCTTCTGTCTAGTTTTTATATGAAGATATTCCCTT

             **  *  * **          *       *     *    *  *  *  *              
     337198 TCTCACCGCAGGCATCAAAGCGCTCCAAATGTCCACATCCATATACTCCAGAAAGAGTGTTTCAA
          1 TTCCAACGAAATCATCAAAGCGATCCAAATATCCACTTCCAGATTCTACAAAAAGAGTGTTTCAA

             *         *   * *   *         *        * *  *      *      *     
     337263 ACCTGCTCTATGAAAGGGAATCTTCAACTCTATGAGTTGAATGCAGACATCAGAAAGAAATTTCT
         66 AACTGCTCTATCAAAAGAAATGTTCAACTCTGTGAGTTGAGTACACACATCACAAAGAAGTTTCT

                     *       **          *         **
     337328 GAGAATGCTGCTGTCTACCTTTTAT-T--TGA-ATTCCCGC
        131 GAGAATGCTTCTGTCTAGTTTTTATATGAAGATATTCCCTT

                         *  *    *                *       *                  
     337365 TTCCAACGAAATCCTCCAAGCTATCCAAATATCCACTTGCAGATTCCACAAAAAGAGTGTTTCAA
          1 TTCCAACGAAATCATCAAAGCGATCCAAATATCCACTTCCAGATTCTACAAAAAGAGTGTTTCAA

                            ** *  *            *       *     *     *  *      
     337430 AACTGCTCTCTATCAATGGCAAAGTTCAACTCTGTTAGTTGAGGACACATATCACCAACAAGTTT
         66 AACTG--CTCTATCAAAAGAAATGTTCAACTCTGTGAGTTGAGTACACACATCACAAAGAAGTTT

                               *       **         *    
     337495 CTGAGAATGCTTCTGTCTATTTTTTATGGGAAGATATTTCCTT
        129 CTGAGAATGCTTCTGTCTAGTTTTTATATGAAGATATTCCCTT

              *  *  * ** *    *      *    *          * **                    
     337538 TTTCACCGTAGGCGTCAAGGCGATCGAAATGTCCACTTCCACAAACTACAAAAAGAGTGTTTCAA
          1 TTCCAACGAAATCATCAAAGCGATCCAAATATCCACTTCCAGATTCTACAAAAAGAGTGTTTCAA

             *         *   * **       *    *        * ** * *   **      *     
     337603 ACCTGCTCTATGAAAGGCCATGTTCATCTCTATGAGTTGAATGGAAATATCCGAAAGAAATTTCT
         66 AACTGCTCTATCAAAAGAAATGTTCAACTCTGTGAGTTGAGTACACACATCACAAAGAAGTTTCT

             *       *          *        *         **
     337668 GGGAATGCTGCTGTCTAG-TGTT-TAT-ACGA-ATTCCCGC
        131 GAGAATGCTTCTGTCTAGTTTTTATATGAAGATATTCCCTT

                         *       *                *    *  *                  
     337705 TTCCAACGAAATCCTCAAAGCAATCCAAATATCCACTTGCAGAATCCACAAAAAGAGTGTTTCAA
          1 TTCCAACGAAATCATCAAAGCGATCCAAATATCCACTTCCAGATTCTACAAAAAGAGTGTTTCAA

                          *     *          * *                      *        
     337770 AACTGCTCTATCAATAGAAAGGTTCAACTCTTTTAGTTGAGTACACACATCACAAACAAGTTTCT
         66 AACTGCTCTATCAAAAGAAATGTTCAACTCTGTGAGTTGAGTACACACATCACAAAGAAGTTTCT

                            * *      **     **  *    
     337835 GAGAATGCTTCTGTCTGGCTTTTATTGGAAGACGTTTCCTT
        131 GAGAATGCTTCTGTCTAGTTTTTATATGAAGATATTCCCTT

                   *  ***         *       *                * *               
     337876 TT-CAACAAAGGTATCAAAGCGCTCCAAATGTCCACTTCCAGATTCTTCCAAAAGAGTGTTTCAA
          1 TTCCAACGAAATCATCAAAGCGATCCAAATATCCACTTCCAGATTCTACAAAAAGAGTGTTTCAA

             **     * ***  * *                 *    * *  * *     *   *     * 
     337940 ACGTGCTCAAAGTAAGGGAATGTTCAACTCTGTGACTTGAATGCAGATATCACCAAGTAGTTTGT
         66 AACTGCTCTATCAAAAGAAATGTTCAACTCTGTGAGTTGAGTACACACATCACAAAGAAGTTTCT

            * * *             *     *    *         * 
     338005 AATAGTGCTTCTGTCTAGATTTTAGATGATGATATTCCCGT
        131 GAGAATGCTTCTGTCTAGTTTTTATATGAAGATATTCCCTT

                         * * *   *                *   *                    * 
     338046 TTCCAACGAAATCGTTAGAGCTATCCAAATATCCACTTACAGTTTCTACAAAAAGAGTGTTTCCA
          1 TTCCAACGAAATCATCAAAGCGATCCAAATATCCACTTCCAGATTCTACAAAAAGAGTGTTTCAA

                   **           *            *       *                     * 
     338111 AACTGCTGCATCAAAAGAAAGGTTCAACTCTGTTAGTTGAGGACACACATCACAAAGAAGTTTGT
         66 AACTGCTCTATCAAAAGAAATGTTCAACTCTGTGAGTTGAGTACACACATCACAAAGAAGTTTCT

                              *    *     *           
     338176 GAGAATGCTTCTGTCTAGATTTTGTATGACGATATTCCCTT
        131 GAGAATGCTTCTGTCTAGTTTTTATATGAAGATATTCCCTT

                     *   * *     *   *       * *  *    *  *       *  *       
     338217 TTCCAACGATATCGTTAAAGCAATCTAAATATCAATTTGCAGAATCCACAAAAATAGAGTTTCAA
          1 TTCCAACGAAATCATCAAAGCGATCCAAATATCCACTTCCAGATTCTACAAAAAGAGTGTTTCAA

             *       * *        *    *       *  *                   ***      
     338282 AGCTGCTCTGTAAAAAGAAAGGTTCCACTCTGTTAGCTGAGTACACACATCACAAACTTGTTTCT
         66 AACTGCTCTATCAAAAGAAATGTTCAACTCTGTGAGTTGAGTACACACATCACAAAGAAGTTTCT

            *     *         *        **         **   
     338347 CAGAATCCTTCTGTCTCGTTTTTATGGGAAGATATTTACTT
        131 GAGAATGCTTCTGTCTAGTTTTTATATGAAGATATTCCCTT

              *  *  ****         **       *     *       *  *  *              
     338388 TTTCACCGTGGGCATCAAAGCACTCCAAATGTCCACATCCAGATACTCCAGAAAGAGTGTTTCAA
          1 TTCCAACGAAATCATCAAAGCGATCCAAATATCCACTTCCAGATTCTACAAAAAGAGTGTTTCAA

             *         *   * *   *         *        * *  *      *      *     
     338453 ACCTGCTCTATGAAAGGGAATCTTCAACTCTATGAGTTGAATGCAGACATCAGAAAGAAATTTCT
         66 AACTGCTCTATCAAAAGAAATGTTCAACTCTGTGAGTTGAGTACACACATCACAAAGAAGTTTCT

                     *       **          *         **
     338518 GAGAATGCTGCTGTCTACCTTTTAT-T--TGA-ATTCCCGC
        131 GAGAATGCTTCTGTCTAGTTTTTATATGAAGATATTCCCTT

                         *  *    *                *  **   *                  
     338555 TTCCAACGAAATCCTCCAAGCTATCCAAATATCCACTTGCATTTTCCACAAAAAGAGTGTTTCAA
          1 TTCCAACGAAATCATCAAAGCGATCCAAATATCCACTTCCAGATTCTACAAAAAGAGTGTTTCAA

                          *                 *    *  *                *       
     338620 AACTGCTCTATCAATAGAAATGTTCAACTCCTTTG-GCTGGGTACACACATCACAAACAAGTTTC
         66 AACTGCTCTATCAAAAGAAATGTTCAACT-CTGTGAGTTGAGTACACACATCACAAAGAAGTTTC

                                      **     *        
     338684 TGAGAATGCTTCTGTCTAGTTTTTATGGGAAGACATTCCCTT
        130 TGAGAATGCTTCTGTCTAGTTTTTATATGAAGATATTCCCTT

              *  * *  **          *       *            **                    
     338726 TTTCACCAAAGGCATCAAAGCGCTCCAAATGTCCACTTCCAGACACTACAAAAAGAGTGTTTCAA
          1 TTCCAACGAAATCATCAAAGCGATCCAAATATCCACTTCCAGATTCTACAAAAAGAGTGTTTCAA

             **       **   ***                 *    * *  * *         *  *    
     338791 ACGTGCTCTAAGAAAGCGAATGTTCAACTCTGTGACTTGAATGCAGATATCACAAAGCAGATTCT
         66 AACTGCTCTATCAAAAGAAATGTTCAACTCTGTGAGTTGAGTACACACATCACAAAGAAGTTTCT

                **            *     *    *         * 
     338856 GAGAGGGCTTCTGTCTAGATTTTAGATGATGATATTCCCGT
        131 GAGAATGCTTCTGTCTAGTTTTTATATGAAGATATTCCCTT

                           * *   *   *            *   *                    * 
     338897 TTCCAACGAAATCATTAGAGCTATCGAAATATCCACTTACAGTTTCTACAAAAAGAGTGTTTCCA
          1 TTCCAACGAAATCATCAAAGCGATCCAAATATCCACTTCCAGATTCTACAAAAAGAGTGTTTCAA

                   **         * *    *       *  *                   ***      
     338962 AACTGCTGCATCAAAAGAGAGGTTCCACTCTGTTAGCTGAGTACACACATCACAAACTTGTTTCT
         66 AACTGCTCTATCAAAAGAAATGTTCAACTCTGTGAGTTGAGTACACACATCACAAAGAAGTTTCT

                  *         *        **         **   
     339027 GAGAATCCTTCTGTCTCGTTTTTATGGGAAGATATTTACTT
        131 GAGAATGCTTCTGTCTAGTTTTTATATGAAGATATTCCCTT

              *  *  * **          *       *     *       *  *  *              
     339068 TTTCACCGTAGGCATCAAAGCGCTCCAAATGTCCACATCCAGATACTCCAGAAAGAGTGTTTCAA
          1 TTCCAACGAAATCATCAAAGCGATCCAAATATCCACTTCCAGATTCTACAAAAAGAGTGTTTCAA

             *         *   * *   *         *        * *  *      *      *     
     339133 ACCTGCTCTATGAAAGGGAATCTTCAACTCTTTGAGTTGAATGCAGACATCAGAAAGAAATTTCT
         66 AACTGCTCTATCAAAAGAAATGTTCAACTCTGTGAGTTGAGTACACACATCACAAAGAAGTTTCT

                     *       **          *         **
     339198 GAGAATGCTGCTGTCTACCTTTTAT-T--TGA-ATTCCCGC
        131 GAGAATGCTTCTGTCTAGTTTTTATATGAAGATATTCCCTT

                         *  *    *                *       *                  
     339235 TTCCAACGAAATCCTCCAAGCTATCCAAATATCCACTTGCAGATTCCACAAAAAGAGTGTTTCAA
          1 TTCCAACGAAATCATCAAAGCGATCCAAATATCCACTTCCAGATTCTACAAAAAGAGTGTTTCAA

                       *    ** *  *            *       *     *     *  *  *   
     339300 AACTGCTCTCTGTCAATGGCAAAGTTCAACTCTGTTAGTTGAGGACACATATCACCAACAACTTT
         66 AACTG--CTCTATCAAAAGAAATGTTCAACTCTGTGAGTTGAGTACACACATCACAAAGAAGTTT

                               *       **         *    
     339365 CTGAGAATGCTTCTGTCTATTTTTTATGGGAAGATATTTCCTT
        129 CTGAGAATGCTTCTGTCTAGTTTTTATATGAAGATATTCCCTT

              *  * ** **      *      *    *          * **                    
     339408 TTTCACCTTAGGCATCAAGGCGATCGAAATGTCCACTTCCACAAACTACAAAAAGAGTGTTTCAA
          1 TTCCAACGAAATCATCAAAGCGATCCAAATATCCACTTCCAGATTCTACAAAAAGAGTGTTTCAA

             *         *   * **       *    *        * ** * *   **      *     
     339473 ACCTGCTCTATGAAAGGCCATGTTCATCTCTATGAGTTGAATGGAAATATCCGAAAGAAATTTCT
         66 AACTGCTCTATCAAAAGAAATGTTCAACTCTGTGAGTTGAGTACACACATCACAAAGAAGTTTCT

             *       *                   *         **
     339538 GGGAATGCTGCTGTCTAG-TTTT-TAT-ACGA-ATTCCCGC
        131 GAGAATGCTTCTGTCTAGTTTTTATATGAAGATATTCCCTT

                         *     * *                *    *  *                  
     339575 TTCCAACGAAATCCTCAAATCAATCCAAATATCCACTTGCAGAATCCACAAAAAGAGTGTTTCAA
          1 TTCCAACGAAATCATCAAAGCGATCCAAATATCCACTTCCAGATTCTACAAAAAGAGTGTTTCAA

                       *  *     *          * *                      *        
     339640 AACTGCTCTATGAATAGAAAGGTTCAACTCTTTTAGTTGAGTACACACATCACAAACAAGTTTCT
         66 AACTGCTCTATCAAAAGAAATGTTCAACTCTGTGAGTTGAGTACACACATCACAAAGAAGTTTCT

                     *    *** *      **     **  *    
     339705 GAGAATGCTGCTGTTGGGCTTTTATTGGAAGACGTTTCCTT
        131 GAGAATGCTTCTGTCTAGTTTTTATATGAAGATATTCCCTT

                 * *  **          *       *                * *               
     339746 TT-CACCAAAGGCATCAAAGCGCTCCAAATGTCCACTTCCAGATTCTTCCAAAAGAGTGTTTCAA
          1 TTCCAACGAAATCATCAAAGCGATCCAAATATCCACTTCCAGATTCTACAAAAAGAGTGTTTCAA

             **     * ***  * *                 *    * *  * *    *    *       
     339810 ACGTGCTCAAAGTAAGGGAATGTTCAACTCTGTGACTTGAATGCAGATATCAAAAAGTAGTTTCT
         66 AACTGCTCTATCAAAAGAAATGTTCAACTCTGTGAGTTGAGTACACACATCACAAAGAAGTTTCT

                *             *     *    *         * 
     339875 GAGAGTGCTTCTGTCTAGATTTTAGATGATGATATTCCCGT
        131 GAGAATGCTTCTGTCTAGTTTTTATATGAAGATATTCCCTT

                           * *   *                *   *                    * 
     339916 TTCCAACGAAATCATTAGAGCTATCCAAATATCCACTTACAGTTTCTACAAAAAGAGTGTTTCCA
          1 TTCCAACGAAATCATCAAAGCGATCCAAATATCCACTTCCAGATTCTACAAAAAGAGTGTTTCAA

                   **         * *    *       *  *                   ***      
     339981 AACTGCTGCATCAAAAGAGAGGTTCCACTCTGTTAGCTGAGTACACACATCACAAACTTGTTTCT
         66 AACTGCTCTATCAAAAGAAATGTTCAACTCTGTGAGTTGAGTACACACATCACAAAGAAGTTTCT

            *     *         *        **         **   
     340046 CAGAATCCTTCTGTCTCGTTTTTATGGGAAGATATTTACTT
        131 GAGAATGCTTCTGTCTAGTTTTTATATGAAGATATTCCCTT

              *  *  * **          *       *     *       *  *  *              
     340087 TTTCACCGTAGGCATCAAAGCGCTCCAAATGTCCACATCCAGATACTCCAGAAAGAGTGTTTCAA
          1 TTCCAACGAAATCATCAAAGCGATCCAAATATCCACTTCCAGATTCTACAAAAAGAGTGTTTCAA

             *         *   * *   *         *        * *  *      *      *     
     340152 ACCTGCTCTATGAAAGGGAATCTTCAACTCTATGAGTTGAATGCAGACATCAGAAAGAAATTTCT
         66 AACTGCTCTATCAAAAGAAATGTTCAACTCTGTGAGTTGAGTACACACATCACAAAGAAGTTTCT

                     *       **          *         **
     340217 GAGAATGCTGCTGTCTACCTTTTAT-T--TGA-ATTCCCGC
        131 GAGAATGCTTCTGTCTAGTTTTTATATGAAGATATTCCCTT

                         *  *    *              * *  **   *          *       
     340254 TTCCAACGAAATCCTCCAAGCTATCCAAATATCCACCTGCATTTTCCACAAAAAGAGAGTTTCAA
          1 TTCCAACGAAATCATCAAAGCGATCCAAATATCCACTTCCAGATTCTACAAAAAGAGTGTTTCAA

                          *                 *    *  *         *      *       
     340319 AACTGCTCTATCAATAGAAATGTTCAACTCCTTTG-GCTGGGTACACACAGCACAAACAAGTTTC
         66 AACTGCTCTATCAAAAGAAATGTTCAACT-CTGTGAGTTGAGTACACACATCACAAAGAAGTTTC

                                      **     *        
     340383 TGAGAATGCTTCTGTCTAGTTTTTATGGGAAGACATTCCCTT
        130 TGAGAATGCTTCTGTCTAGTTTTTATATGAAGATATTCCCTT

              *  * *  **     *    *      **       *    **                    
     340425 TTTCACCAAAGGCATCAGAGCGCTCCAAAGGTCCACTTTCAGACACTACAAAAAGAGTGTTTCAA
          1 TTCCAACGAAATCATCAAAGCGATCCAAATATCCACTTCCAGATTCTACAAAAAGAGTGTTTCAA

             **       **   ***                 *    * *  * *         *       
     340490 ACGTGCTCTAAGAAAGCGAATGTTCAACTCTGTGACTTGAATGCAGATATCACAAAGTAGTTTCT
         66 AACTGCTCTATCAAAAGAAATGTTCAACTCTGTGAGTTGAGTACACACATCACAAAGAAGTTTCT

                **            *     *    *         * 
     340555 GAGAGGGCTTCTGTCTAGATTTTAGATGATGATATTCCCGT
        131 GAGAATGCTTCTGTCTAGTTTTTATATGAAGATATTCCCTT

                           * *   *                *   *                    * 
     340596 TTCCAACGAAATCATTAGAGCTATCCAAATATCCACTTACAGTTTCTACAAAAAGAGTGTTTCCA
          1 TTCCAACGAAATCATCAAAGCGATCCAAATATCCACTTCCAGATTCTACAAAAAGAGTGTTTCAA

                   **         * *    *       *  *                   ***      
     340661 AACTGCTGCATCAAAAGAGAGGTTCCACTCTGTTAGCTGAGTACACACATCACAAACTTGTTTCT
         66 AACTGCTCTATCAAAAGAAATGTTCAACTCTGTGAGTTGAGTACACACATCACAAAGAAGTTTCT

            *     *         *        **         **   
     340726 CAGAATCCTTCTGTCTCGTTTTTATGGGAAGATATTTACTT
        131 GAGAATGCTTCTGTCTAGTTTTTATATGAAGATATTCCCTT

              *  *  * **          *       *     *       *  *  *              
     340767 TTTCACCGTAGGCATCAAAGCGCTCCAAATGTCCACATCCAGATACTCCAGAAAGAGTGTTTCAA
          1 TTCCAACGAAATCATCAAAGCGATCCAAATATCCACTTCCAGATTCTACAAAAAGAGTGTTTCAA

             *         *   * *   *         *        * *  *      *      *     
     340832 ACCTGCTCTATGAAAGGGAATCTTCAACTCTATGAGTTGAATGCAGACATCAGAAAGAAATTTCT
         66 AACTGCTCTATCAAAAGAAATGTTCAACTCTGTGAGTTGAGTACACACATCACAAAGAAGTTTCT

                     *       **          *         **
     340897 GAGAATGCTGCTGTCTACCTTTTAT-T--TGA-ATTCCCGC
        131 GAGAATGCTTCTGTCTAGTTTTTATATGAAGATATTCCCTT

                         *  *    *                *  **   *                  
     340934 TTCCAACGAAATCCTCCAAGCTATCCAAATATCCACTTGCATTTTCCACAAAAAGAGTGTTTCAA
          1 TTCCAACGAAATCATCAAAGCGATCCAAATATCCACTTCCAGATTCTACAAAAAGAGTGTTTCAA

               *            ****  *          * *                      *      
     340999 AACGGCTCTCTATCAATGTCAAAGTTCAACTCTTTTAGTTGAGTACACACATCACAAACAAGTTT
         66 AACTG--CTCTATCAAAAGAAATGTTCAACTCTGTGAGTTGAGTACACACATCACAAAGAAGTTT

                   *           *      ***     *   *    
     341064 CTGAGAAAGCTTCTGTCTACTTTTTAAGGGAAGACATTTCCTT
        129 CTGAGAATGCTTCTGTCTAGTTTTTATATGAAGATATTCCCTT

              *  * *  **          *       *                                  
     341107 TTTCACCAAAGGCATCAAAGCGCTCCAAATGTCCACTTCCAGATTCTACAAAAAGAGTGTTTCAA
          1 TTCCAACGAAATCATCAAAGCGATCCAAATATCCACTTCCAGATTCTACAAAAAGAGTGTTTCAA

             *         ***      * *             * *  * *  * *         *      
     341172 ACCTGCTCTA-AGTAAGAGAGTTTTCAACTCTGTGACTGGAATGCAGATATCACAAAGTAGTTTC
         66 AACTGCTCTATCAAAAGA-AATGTTCAACTCTGTGAGTTGAGTACACACATCACAAAGAAGTTTC

                 *         *  ***    *              * 
     341236 TGAGACTGCTTCTGTGTATACTTTAGATGAAGATATTCCCGT
        130 TGAGAATGCTTCTGTCTAGTTTTTATATGAAGATATTCCCTT

                     *   * * * * *                *   *                    * 
     341278 TTCCAACGATATCGTTAGACCTATCCAAATATCCACTTACAGTTTCTACAAAAAGAGTGTTTCCA
          1 TTCCAACGAAATCATCAAAGCGATCCAAATATCCACTTCCAGATTCTACAAAAAGAGTGTTTCAA

                   **           *      *     *       *                       
     341343 AACTGCTGCATCAAAAGAAAGGTTCAAGTCTGTTAGTTGAGGACACACATCACAAAGAAGTTTCT
         66 AACTGCTCTATCAAAAGAAATGTTCAACTCTGTGAGTTGAGTACACACATCACAAAGAAGTTTCT

                 *      *     *    *                 
     341408 GAGAAAGCTTCTCTCTAGATTTTGTATGAAGATATTCCCTT
        131 GAGAATGCTTCTGTCTAGTTTTTATATGAAGATATTCCCTT

                     *   * *   * * *         * *  *    *  *   *   *  *       
     341449 TTCCAACGATATCCTTAAATCAACCCAAATATCAATTTGCAGAATCCACAGAAATAGAGTTTCAA
          1 TTCCAACGAAATCATCAAAGCGATCCAAATATCCACTTCCAGATTCTACAAAAAGAGTGTTTCAA

             *       * *        * *  *       *  *                   ***      
     341514 AGCTGCTCTGTAAAAAGAAAGGATCCACTCTGTTAGCTGAGTACACACATCACAAACTTGTTTCT
         66 AACTGCTCTATCAAAAGAAATGTTCAACTCTGTGAGTTGAGTACACACATCACAAAGAAGTTTCT

                  *                  **         **   
     341579 GAGAATCCTTCTGTCTAGTTTTTATGGGAAGATATTTACTT
        131 GAGAATGCTTCTGTCTAGTTTTTATATGAAGATATTCCCTT

              *  ** * ***         *       *     *       *     *              
     341620 TTTCACAGTAGGTATCAAAGCGCTCCAAATGTCCACATCCAGATACTACAGAAAGAGTGTTTCAA
          1 TTCCAACGAAATCATCAAAGCGATCCAAATATCCACTTCCAGATTCTACAAAAAGAGTGTTTCAA

             *         *   * *   *         *        * *  * *    *    * *     
     341685 ACCTGCTCTATGAAAGGGAATCTTCAACTCTATGAGTTGAATGCAGATATCAGAAAGTAATTTCT
         66 AACTGCTCTATCAAAAGAAATGTTCAACTCTGTGAGTTGAGTACACACATCACAAAGAAGTTTCT

                     *       **   *      *         **
     341750 GAGAATGCTGCTGTCTACCTTTCAT-T--TGA-ATTCCCGC
        131 GAGAATGCTTCTGTCTAGTTTTTATATGAAGATATTCCCTT

                         *  *    *      *   *     *       *                  
     341787 TTCCAACGAAATCCTCCAAGCTATCCAAGTATTCACTTGCAGATTCCACAAAAAGAGTGTTTCAA
          1 TTCCAACGAAATCATCAAAGCGATCCAAATATCCACTTCCAGATTCTACAAAAAGAGTGTTTCAA

                *         *     *  *         *       *              *        
     341852 AACTACTCTATCAATAGAAAGGTACAACTCTGTCAGTTGAGGACACACATCACAAACAAGTTTCT
         66 AACTGCTCTATCAAAAGAAATGTTCAACTCTGTGAGTTGAGTACACACATCACAAAGAAGTTTCT

                           * *       **     *   *    
     341917 GAGAA---TTCT-TCAATTTTTTATGGGAAGACATTTCCTT
        131 GAGAATGCTTCTGTCTAGTTTTTATATGAAGATATTCCCTT

              *  *  * **          *       *     *       **    *              
     341954 TTTCACCGTAGGCATCAAAGCGCTCCAAATGTCCACATCCAGATAGTACAGAAAGAGTGTTTCAA
          1 TTCCAACGAAATCATCAAAGCGATCCAAATATCCACTTCCAGATTCTACAAAAAGAGTGTTTCAA

             *         *   * *             *        * *  *      *    * *     
     342019 ACCTGCTCTATTAAAGGGAATGTTCAACTCTATGAGTTGAATGCAAACATCAGAAAGGAATTTCT
         66 AACTGCTCTATCAAAAGAAATGTTCAACTCTGTGAGTTGAGTACACACATCACAAAGAAGTTTCT

                     *         *         *         **
     342084 GAGAATGCTGCTGTCTAG-CTTT-TATG-TGA-ATTCCCGC
        131 GAGAATGCTTCTGTCTAGTTTTTATATGAAGATATTCCCTT

                       ***  *    *                *       *                  
     342121 TTCCAACGAAAAACTCCAAGCTATCCAAATATCCACTTGCAGATTCCACAAAAAGAGTGTTTCAA
          1 TTCCAACGAAATCATCAAAGCGATCCAAATATCCACTTCCAGATTCTACAAAAAGAGTGTTTCAA

                          *               ** ** *  *       *        *        
     342186 AACTGCTCTATCAATAGAAATGTTCAACTCCTTTCGCTGGGTACACAGATCACAAACAAGTTTCT
         66 AACTGCTCTATCAAAAGAAATGTTCAACTCTGTGAGTTGAGTACACACATCACAAAGAAGTTTCT

                                     **     *   *    
     342251 GAGAATGCTTCTGTCTAGTTTTTATGGGAAGACATTTCCTT
        131 GAGAATGCTTCTGTCTAGTTTTTATATGAAGATATTCCCTT

              *  * *  **        * *       *              *                   
     342292 TTTCACCAAAGGCATCAAAGAGCTCCAAATGTCCACTTCCAGA-TATACAAAAAGAGTGTTTCAA
          1 TTCCAACGAAATCATCAAAGCGATCCAAATATCCACTTCCAGATTCTACAAAAAGAGTGTTTCAA

              *       **   ***                 *    * *  * *         *       
     342356 AAGTGCTCTAAGAAAGCGAATGTTCAACTCTGTGACTTGAATGCAGATATCACAAAGTAGTTTCT
         66 AACTGCTCTATCAAAAGAAATGTTCAACTCTGTGAGTTGAGTACACACATCACAAAGAAGTTTCT

                *       *     *  *  * *       * *  * 
     342421 GAGAGTGCTTCTCTCTAGATTCTAGACGAAGATACTGCCGT
        131 GAGAATGCTTCTGTCTAGTTTTTATATGAAGATATTCCCTT

                 *   *   * * *   *            *   * ***               *   ** 
     342462 TTCCATCGATATCGTTAGAGCTATCCAAATATCCGCTTGCTATTTCTACAAAAAGAGTATTTGCA
          1 TTCCAACGAAATCATCAAAGCGATCCAAATATCCACTTCCAGATTCTACAAAAAGAGTGTTTCAA

                   *          * *   **       *      **    * *        *       
     342527 AACTGCTGTATC-AAAGACAGGTTGTACTCTGTTAGTTGACGACACTCCTCACAAAGGAGTTTCT
         66 AACTGCTCTATCAAAAGAAATGTTCAACTCTGTGAGTTGAGTACACACATCACAAAGAAGTTTCT

                             *       **      * **    
     342591 GAGAATGCTTCTGTCTAATTTTTATTGGAAGATGTCTCCTT
        131 GAGAATGCTTCTGTCTAGTTTTTATATGAAGATATTCCCTT

              *  * ** ** * *      *  *    *   *         **    *              
     342632 TTTCACCATAGGCCTGAAAGCGCTCGAAATGTCCCCTTCCAGATAGTACAGAAAGAGTGTTTCAA
          1 TTCCAACGAAATCATCAAAGCGATCCAAATATCCACTTCCAGATTCTACAAAAAGAGTGTTTCAA

             *         *   *     *     *       *  * ***  *              *    
     342697 ACCTGCTCTATGAAAGGAAATTTTCAATTCTGTGACTTAAAAGCAAACATCACAAAGAAGCTTCT
         66 AACTGCTCTATCAAAAGAAATGTTCAACTCTGTGAGTTGAGTACACACATCACAAAGAAGTTTCT

                  *  *      **           **        * 
     342762 GAGAATTCTGCTGTCTTCTTTTT-TATG-TCATA---CCGT
        131 GAGAATGCTTCTGTCTAGTTTTTATATGAAGATATTCCCTT

               *         *       *      *         *       *               *  
     342798 TTCTAACGAAATCCTCAAAGCTATCCAATTATCCACTTGCAGATTCCACAAAAAGAGTGTTTGAA
          1 TTCCAACGAAATCATCAAAGCGATCCAAATATCCACTTCCAGATTCTACAAAAAGAGTGTTTCAA

                 *     *  *     *            **          *  *        *     * 
     342863 AACTGATCTATAAATAGAAAAGTTCAACTCTGTTGGTTGAGTACATACTTCACAAAGTAGTTTGT
         66 AACTGCTCTATCAAAAGAAATGTTCAACTCTGTGAGTTGAGTACACACATCACAAAGAAGTTTCT

            *      *                 **     *   *    
     342928 TAGAATGTTTCTGTCTAGTTTTTATCGGAAGACATTTCCTT
        131 GAGAATGCTTCTGTCTAGTTTTTATATGAAGATATTCCCTT

              *  * *  ** *        *       *                      *           
     342969 TTTCACCAAAGGCGTCAAAGCGCTCCAAATGTCCACTTCCAGATTCTACAAAATGAGT-TTTCAA
          1 TTCCAACGAAATCATCAAAGCGATCCAAATATCCACTTCCAGATTCTACAAAAAGAGTGTTTCAA

             *        **   * *                 *    * *  * *         *       
     343033 ACCTGCTCTAAGAAAGGGAATGTTCAACTCTGTGACTTGAATGCAGATATCACAAAGTAGTTTCT
         66 AACTGCTCTATCAAAAGAAATGTTCAACTCTGTGAGTTGAGTACACACATCACAAAGAAGTTTCT

                *         *  ***    *              * 
     343098 GAGAGTGCTTCTGTGTATACTTTAGATGAAGATATTCCCGT
        131 GAGAATGCTTCTGTCTAGTTTTTATATGAAGATATTCCCTT

                     *   * * * * *                *   *                    * 
     343139 TTCCAACGATATCGTTAGACCTATCCAAATATCCACTTACAGTTTCTACAAAAAGAGTGTTTCCA
          1 TTCCAACGAAATCATCAAAGCGATCCAAATATCCACTTCCAGATTCTACAAAAAGAGTGTTTCAA

                *  **           *            *       *   *      *            
     343204 AACTTCTGCATCAAAAGAAAAGTTCAACTCTGTTAGTTGAGGACAGACATCAGAAAGAAGTTTCT
         66 AACTGCTCTATCAAAAGAAATGTTCAACTCTGTGAGTTGAGTACACACATCACAAAGAAGTTTCT

                        *     *    *               * 
     343269 GAGAATGCTTCTCTCTAGATTTTGTATGAAGATATTCCCGT
        131 GAGAATGCTTCTGTCTAGTTTTTATATGAAGATATTCCCTT

                  *       * *     *           *    *       *  **   *  *     *
     343310 TTCC-AGGAATATCGTTAAAGCAATCCAAATATCAACTTGCAGATTCCACGGAAATAGAGTTTCT
          1 TTCCAACGAA-ATCATCAAAGCGATCCAAATATCCACTTCCAGATTCTACAAAAAGAGTGTTTCA

              *       * *        *    *       *  *       *           * **    
     343374 AAGCTGCTCTGTAAAAAGAAAGGTTCCACTCTGTTAGGTGAGTACGCACATCACAAACATATTTC
         65 AAACTGCTCTATCAAAAGAAATGTTCAACTCTGTGAGTTGAGTACACACATCACAAAGAAGTTTC

                   *                  **         *  * 
     343439 TGAGAATCCTTCTGTCTAGTTTTTATGGGAAGATATTTCCAT
        130 TGAGAATGCTTCTGTCTAGTTTTTATATGAAGATATTCCCTT

              *  * ** ****       **   *   *         *   *          *         
     343481 TTTCACCATAGGTGTCAAAGCACTCCTAATGTCCACTTCCCGATACTACAAAAAGGGTGTTTCAA
          1 TTCCAACGAAATCATCAAAGCGATCCAAATATCCACTTCCAGATTCTACAAAAAGAGTGTTTCAA

            ** *       *   ***             **       * ** * *    **           
     343546 CCCGGCTCTATGAAATTGAATGTTCAACTCTACGAGTTGAATGAAAAGATCATGAAGAAGTTTCT
         66 AACTGCTCTATCAAAAGAAATGTTCAACTCTGTGAGTTGAGTACACACATCACAAAGAAGTTTCT

                              *    *       *       * 
     343611 GAGAATGCTTCTGTCTAGATTTTGTATGAAGGTATTCCCGT
        131 GAGAATGCTTCTGTCTAGTTTTTATATGAAGATATTCCCTT

                       * *  *    *        *       *        *                 
     343652 TTCCAACGAAACCCTCTAAGCTATCCAAATTTCCACTTACAGATTCTTCAAAAAGAGTGTTTCAA
          1 TTCCAACGAAATCATCAAAGCGATCCAAATATCCACTTCCAGATTCTACAAAAAGAGTGTTTCAA

             *       *          *            *                       *       
     343717 ACCTGCTCTGTCAAAAGAAAGGTTCAACTCTGTTAGTTGAGGTA-ACACATCACAAACAAGTTTC
         66 AACTGCTCTATCAAAAGAAATGTTCAACTCTGTGAGTTGA-GTACACACATCACAAAGAAGTTTC

                             **       **         *    
     343781 TGAGAATGCTTCTGTCTTTTTTTTATTGGAAGATATTTCCTT
        130 TGAGAATGCTTCTGTCTAGTTTTTATATGAAGATATTCCCTT

              *    *   ** **     * *  *            *  *  *          *        
     343823 TTTC-ACAATAGGCGACAAAGTGCTCGAAATATCCACTTGCACATACTACAAAAAGTGTGTTTCA
          1 TTCCAACGA-AATCATCAAAGCGATCCAAATATCCACTTCCAGATTCTACAAAAAGAGTGTTTCA

              *         *   *               *        ***  *      *      *    
     343887 AACCTGCTCTATGAAAGGAAATGTTCAACTCTATGAGTTGAAAGCAAACATCAAAAAGAAATTTC
         65 AAACTGCTCTATCAAAAGAAATGTTCAACTCTGTGAGTTGAGTACACACATCACAAAGAAGTTTC

                      *       *                     * 
     343952 TGAGAATGCTGCTGTCTACTTTTTATAT---GA-ATTCCCGT
        130 TGAGAATGCTTCTGTCTAGTTTTTATATGAAGATATTCCCTT

                  *      *       *      *     *   **      *                  
     343990 TTCCAATGAAATCCTCAAAGCAATCCAAGTATCCCCTTGTAGATTCCACAAAAAGAGTGTTTCAA
          1 TTCCAACGAAATCATCAAAGCGATCCAAATATCCACTTCCAGATTCTACAAAAAGAGTGTTTCAA

                *      *  *  *  *            *       *                **     
     344055 AACTCCTCTATGAACAGCAAGGTTCAACTCTGTTAGTTG-GGACACACATCACAAAGAGTTTTCT
         66 AACTGCTCTATCAAAAGAAATGTTCAACTCTGTGAGTTGAGTACACACATCACAAAGAAGTTTCT

                        * *   *                    * 
     344119 GAGAATGCTTCTATGTAGATTTTATATGAAGATATTCCCGT
        131 GAGAATGCTTCTGTCTAGTTTTTATATGAAGATATTCCCTT

                         **      *       *    *   * *                        
     344160 TTCCAACGAAATCCACAAAGCCATCC-AACATCCCCTTGCCGATTCTACAAAAAGAGTGTTTCAA
          1 TTCCAACGAAATCATCAAAGCGATCCAAATATCCACTTCCAGATTCTACAAAAAGAGTGTTTCAA

                     * *        *            * *         *     *    * *      
     344224 AACTGCTCTGTAAAAAGAAAGGTTCAACTCTGTTAATTGAGTACAGACATCCCAAACATGTTTCT
         66 AACTGCTCTATCAAAAGAAATGTTCAACTCTGTGAGTTGAGTACACACATCACAAAGAAGTTTCT

                           *                     *    
     344289 GAGAATGCTTCTGTCCAGTTTTTA-ATGGAAGATATTTCCTT
        131 GAGAATGCTTCTGTCTAGTTTTTATAT-GAAGATATTCCCTT

              *  * ** ** *        *       *    *        *                    
     344330 TTTCACCATAGGCGTCAAAGCGCTCCAAATGTCCAATTCCAGATACTACAAAAAAGAGTGTTTCA
          1 TTCCAACGAAATCATCAAAGCGATCCAAATATCCACTTCCAGATTCTAC-AAAAAGAGTGTTTCA

              *         **  * *      * *    **  *    * ** *                  
     344395 AACCTGCTCTATGTAAGGGAATGTTTATCTCTACGAATTGAATGAAAACATCAC-AAGAAGTTTC
         65 AAACTGCTCTATCAAAAGAAATGTTCAACTCTGTGAGTTGAGTACACACATCACAAAGAAGTTTC

             **                *       *            * 
     344459 TAGGAATGCTTCTGTCTAGGTTTTATAGGAAGATATTCCCGT
        130 TGAGAATGCTTCTGTCTAGTTTTTATATGAAGATATTCCCTT

                         *    *  *                        *                  
     344501 TTCCAACGAAATCCTCAAGGCTATCCAAATATCCACTTCCAGATTCGACAAAAAGAGTGTTTCAA
          1 TTCCAACGAAATCATCAAAGCGATCCAAATATCCACTTCCAGATTCTACAAAAAGAGTGTTTCAA

                 *   *          **           *           * *        ***      
     344566 AACTGGTCTGTCAAAAGAAAATTTCAACTCTGTTAGTTGAGTACATATATCACAAACCGGTTTCT
         66 AACTGCTCTATCAAAAGAAATGTTCAACTCTGTGAGTTGAGTACACACATCACAAAGAAGTTTCT

                                     **         *    
     344631 GAGAATGCTTCTGTCTAGTTTTTATGGGAAGATATTTCCTT
        131 GAGAATGCTTCTGTCTAGTTTTTATATGAAGATATTCCCTT

              *  *    **       *  *                     *                    
     344672 TTTCACCGAAGGCATCAAAACGCTCCAAATATCCACTTCCAGATACTACAAAAAGAGTGTTTCAA
          1 TTCCAACGAAATCATCAAAGCGATCCAAATATCCACTTCCAGATTCTACAAAAAGAGTGTTTCAA

             *         *    *             *       *    * * ** *         **   
     344737 ACCTGCTCTATGAAAGGGAATGATGTTCAAATCTGTGACTTGAATGCGGATATCACAAAGCGGTT
         66 AACTGCTCTATCAAA-AGAA--ATGTTCAACTCTGTGAGTTGAGTACACACATCACAAAGAAGTT

                   *          *  *                    * 
     344802 TCTGAGAGTGCTTCTGTCGAGATTTTATATGAAGATATTCCCGT
        128 TCTGAGAATGCTTCTGTCTAGTTTTTATATGAAGATATTCCCTT

                  *     ** * **  *       *        *                        * 
     344846 TTCCAAAGAAATATTTAGCGCTATCCAAAGATCCACTTGCAGATTCTACAAAAAGAGTGTTTCCA
          1 TTCCAACGAAATCATCAAAGCGATCCAAATATCCACTTCCAGATTCTACAAAAAGAGTGTTTCAA

                   *            *        * *                          *      
     344911 AACTGCTGTATCAAAAGAAAGGTTCAAC-ATCT--G-T--G---A-ACAT---AAAGATGTTTCT
         66 AACTGCTCTATCAAAAGAAATGTTCAACTCTGTGAGTTGAGTACACACATCACAAAGAAGTTTCT

              *    *                 *  *          * 
     344963 GATAATGATTCTGTCTAGTTTTTATGTGGAGATATTCCCGT
        131 GAGAATGCTTCTGTCTAGTTTTTATATGAAGATATTCCCTT

               *      ** *      * * *     ** *    *        *                 
     345004 TTCGAACGAAGGCCTCAAAGTGCTACAAATGACAACTTGCAGATTCTGCAAAAAGAGTGTTTCAA
          1 TTCCAACGAAATCATCAAAGCGATCCAAATATCCACTTCCAGATTCTACAAAAAGAGTGTTTCAA

                 *     *                    *       * *  *  *                
     345069 AACTGTTCTATGAAAAGAAATGTTCAACTCTGAGAGTTGAATGCAAACGTCACAAAGAAGTTTCT
         66 AACTGCTCTATCAAAAGAAATGTTCAACTCTGTGAGTTGAGTACACACATCACAAAGAAGTTTCT

                            *        *          *  * 
     345134 GAGAATGCTTCTGTCTGGTTTTTATGTGAAGATATTACCGT
        131 GAGAATGCTTCTGTCTAGTTTTTATATGAAGATATTCCCTT

                       * *      ***   *           ****          *            
     345175 TTCCAACGAAAGCCTCAAAGATGTCCTAATATCCACTTATGAATTCTAC-AAGA-AGTGTTTCAA
          1 TTCCAACGAAATCATCAAAGCGATCCAAATATCCACTTCCAGATTCTACAAAAAGAGTGTTTCAA

              *      *     *    *   *   *                            *       
     345238 AAGTGCTCTTTCAAAGGAAAGGTTTAACACTGTGAGTTGAGTACACACATCACAAAGCAGTTTCT
         66 AACTGCTCTATCAAAAGAAATGTTCAACTCTGTGAGTTGAGTACACACATCACAAAGAAGTTTCT

            *  *                     *          *    
     345303 TAGCATGCTTCTGTCTAGTTTTTATGTGAAGATATTTCCTT
        131 GAGAATGCTTCTGTCTAGTTTTTATATGAAGATATTCCCTT

              *  * ** ** *        *       *          *  *  *             *   
     345344 TTTCACCATAGGCCTCAAAGCGCTCCAAATGTCCACTTCCAAATACTGCAAAAAGAGTGTTACAA
          1 TTCCAACGAAATCATCAAAGCGATCCAAATATCCACTTCCAGATTCTACAAAAAGAGTGTTTCAA

                *      *   * *                      * *  *                   
     345409 AACTACTCTATGAAAGGGAATGTTCAACTCTGTGAGTTGAATGCAAACATCACAAAGAAGTTTCT
         66 AACTGCTCTATCAAAAGAAATGTTCAACTCTGTGAGTTGAGTACACACATCACAAAGAAGTTTCT

                 *                   *             * 
     345474 GAGAACGCTTCTGTCTAGTTTTTATGTGAAGATATTCCCGT
        131 GAGAATGCTTCTGTCTAGTTTTTATATGAAGATATTCCCTT

               *      ** **      **       *     * *       * *    *           
     345515 TTCGAACGAAGGCCACAAAGCTCTCCAAATGTCCACCTGCAGATTCCAGAAAACGAGTGTTTCAA
          1 TTCCAACGAAATCATCAAAGCGATCCAAATATCCACTTCCAGATTCTACAAAAAGAGTGTTTCAA

             *  *           *  *    *                      *                 
     345580 ATCTCCTCTATCAAAAAAATTGTTAAACTCTGTGAGTTGAGTACACATATCACAAAGAAGTTTCT
         66 AACTGCTCTATCAAAAGAAATGTTCAACTCTGTGAGTTGAGTACACACATCACAAAGAAGTTTCT

             *    *   *   *  *       **         *    
     345645 GTGAATTCTTGTGTGTAATTTTTATGGGAAGATATTTCCTT
        131 GAGAATGCTTCTGTCTAGTTTTTATATGAAGATATTCCCTT

                     * *   *      * *      **       *                *       
     345686 TATCACAA-TACA-CCTCAAAGAGCTCCAAACGTCCACTTGCAGATTCTACAAAAAGTGTGTTTC
          1 T-TC-CAACGAAATCATCAAAGCGATCCAAATATCCACTTCCAGATTCTACAAAAAGAGTGTTTC

                    *    *     *            **        * *  *      *          
     345749 AAAACTGCCCTATGAAAAGGAATGTTCAACTCCATGAGTTGAATGCAAACATCAAAAAGAAGTTT
         64 AAAACTGCTCTATCAAAAGAAATGTTCAACTCTGTGAGTTGAGTACACACATCACAAAGAAGTTT

                          *            *   *         * 
     345814 CTGAGAATGCTTCTTTCTAGTTTTTATGTGAGGATATTCCCGT
        129 CTGAGAATGCTTCTGTCTAGTTTTTATATGAAGATATTCCCTT

                 **      *       *                *     *           *        
     345857 TTCCACGGAAATCCTCAAAGCTATCCAAATATCCACTTGCAGATACTACAAAAAGATTGTTTCAA
          1 TTCCAACGAAATCATCAAAGCGATCCAAATATCCACTTCCAGATTCTACAAAAAGAGTGTTTCAA

              *  *          *                  *                         *   
     345922 AATTGGTCTATCAAAATAAA-GTTCAACTCTGTGAATTGAGTACACACATCACAAAGAAGTCTCT
         66 AACTGCTCTATCAAAAGAAATGTTCAACTCTGTGAGTTGAGTACACACATCACAAAGAAGTTTCT

              *   *      *           *          *    
     345986 GATAATTCTTCTGACTAGTTTTTATGTGAAGATATTTCCTT
        131 GAGAATGCTTCTGTCTAGTTTTTATATGAAGATATTCCCTT

              *  * ** ** *     *  *       *             *          *         
     346027 TTTCACCATAGGCCTCAAATCGCTCCAAATGTCCACTTCCAGATACTACAAAAAGTGTGTTTCAA
          1 TTCCAACGAAATCATCAAAGCGATCCAAATATCCACTTCCAGATTCTACAAAAAGAGTGTTTCAA

                       **  * *   *     *            * *  *                   
     346092 AACTGCTCTATGTAAGGGAATTTTCAAATCTGTGAGTTGAATGCAAACATCACAAAGAAGTTTCT
         66 AACTGCTCTATCAAAAGAAATGTTCAACTCTGTGAGTTGAGTACACACATCACAAAGAAGTTTCT

                                     *     **      * 
     346157 GAGAATGCTTCTGTCTAGTTTTTATGTGAAGGCATTCCCGT
        131 GAGAATGCTTCTGTCTAGTTTTTATATGAAGATATTCCCTT

                *        *       *            *   *                *         
     346198 TTCCTACGAAATCCTCAAAGCTATCCAAATATCCTCTTGCAGATTCTACAAAAAGCGTGTTTCAA
          1 TTCCAACGAAATCATCAAAGCGATCCAAATATCCACTTCCAGATTCTACAAAAAGAGTGTTTCAA

                                *                           *                
     346263 AACTGCTCTATCAAAAGAAAAGTTCAACTCTGTGAGTTGAGTACACACCTCACAAAGAAGTTTCT
         66 AACTGCTCTATCAAAAGAAATGTTCAACTCTGTGAGTTGAGTACACACATCACAAAGAAGTTTCT

                  **  *      *       **         *    
     346328 GAGAATTTTTATGTCTATTTTTTATGGGAAGATATTTCCTT
        131 GAGAATGCTTCTGTCTAGTTTTTATATGAAGATATTCCCTT

              ** * ** ** *       **     * *       *  *                       
     346369 TTTTATCATAGACCTCAAAGCCCTCCAAGTTTCCACTTACACATTCTAC-AAAA-AGTGTTTCAA
          1 TTCCAACGAAATCATCAAAGCGATCCAAATATCCACTTCCAGATTCTACAAAAAGAGTGTTTCAA

                       *     *   *     *            * ** **                  
     346432 AACTGCTCTATGAAAAGGAATTTTCAATTCTGTGAGTTGAATGGAAGCATCACAAAGAAGTTTCT
         66 AACTGCTCTATCAAAAGAAATGTTCAACTCTGTGAGTTGAGTACACACATCACAAAGAAGTTTCT

            * *           * *        *         *   * 
     346497 TACAATGCTTCTGTGTGGTTTTTATGTGAAGATATACCCGT
        131 GAGAATGCTTCTGTCTAGTTTTTATATGAAGATATTCCCTT

                 **   ** *  *  * **                  *              *      * 
     346538 TTCCAGTGAAGGCCTCCAATCTCTCCAAATATCCACTTCCAAATTCTACAAAAAGATTGTTTCCA
          1 TTCCAACGAAATCATCAAAGCGATCCAAATATCCACTTCCAGATTCTACAAAAAGAGTGTTTCAA

             *  *               **                  * *             *        
     346603 ATCTCCTCTATCAAAAGAAAATTTCAACTCTGTGAGTTGAATGCACACATCACAAAAAAGTTTCT
         66 AACTGCTCTATCAAAAGAAATGTTCAACTCTGTGAGTTGAGTACACACATCACAAAGAAGTTTCT

                                     *        *    * 
     346668 GAGAATGCTTCTGTCTAGTTTTT-TGTGAAGATAATCCCGT
        131 GAGAATGCTTCTGTCTAGTTTTTATATGAAGATATTCCCTT

                  *      *    *  **  **           ** *         *             
     346708 TTCCAAAGAAATCCTCAATGCTGTCTTAATATCCACTTGTAAATTCTACAAGAAGAGTGTTTCAA
          1 TTCCAACGAAATCATCAAAGCGATCCAAATATCCACTTCCAGATTCTACAAAAAGAGTGTTTCAA

                   *            *   *                                *       
     346773 AACTGCTGTATCAAAAGAAAGGTTTAACTCTGTGAGTTGAGTACACACATCACAAAGTAGTTTCT
         66 AACTGCTCTATCAAAAGAAATGTTCAACTCTGTGAGTTGAGTACACACATCACAAAGAAGTTTCT

                             *       *          *    
     346838 GAGAATGCTTCTGTCTACTTTTTATGTGAAGATATTTCCTT
        131 GAGAATGCTTCTGTCTAGTTTTTATATGAAGATATTCCCTT

              *  *  * **        * **               *  *    **                
     346879 TTTCACCATAGGTC-TCAAATCTCTCCAAATATCCACTTGCATATTCCTCAAAAAGAGTGTTTCA
          1 TTCCAAC-GAAATCATCAAAGCGATCCAAATATCCACTTCCAGATTCTACAAAAAGAGTGTTTCA

                        *     *  *              **   * *  **         *    *  
     346943 AAACTGCTCTATGAAAAGGAACGTTCAACTCTGTGAAATGAATGCAAGCATCACAAATAAGTCTC
         65 AAACTGCTCTATCAAAAGAAATGTTCAACTCTGTGAGTTGAGTACACACATCACAAAGAAGTTTC

                         *                          * 
     347008 TGAGAATGCTTCTCTCTAGTTTTTATATGAAGATATTCCCGT
        130 TGAGAATGCTTCTGTCTAGTTTTTATATGAAGATATTCCCTT

                         *       *                *  * * **                  
     347050 TTCCAACGAAATCCTCAAAGCTATCCAAATATCCACTTGCACACTTGACAAAAAGAGTGTTTCAA
          1 TTCCAACGAAATCATCAAAGCGATCCAAATATCCACTTCCAGATTCTACAAAAAGAGTGTTTCAA

              *   **            *      *       *                * *      *   
     347115 AATTGCAGTATCAAAAGAAAAGTTCAAATCTGTGAATTGAGTACACACATCAGACAGAAGTATCT
         66 AACTGCTCTATCAAAAGAAATGTTCAACTCTGTGAGTTGAGTACACACATCACAAAGAAGTTTCT

                  *                  **         *    
     347180 GAGAATTCTTCTGTCTAGTTTTTATTGGAAGATATTTCCTT
        131 GAGAATGCTTCTGTCTAGTTTTTATATGAAGATATTCCCTT

              *  *   *           * *    ** *       *     *  *                
     347221 TTTCATC-ATATGCATCAAAGAGCTCCATGTTTCCACTTACAGATCCTTCAAAAAGAGTGTTTCA
          1 TTCCAACGAAAT-CATCAAAGCGATCCAAATATCCACTTCCAGATTCTACAAAAAGAGTGTTTCA

                        *     *                    * *  * **     *   *       
     347285 AAACTGCTCTATGAAAAGGAATGTTCAACTCTGTGAGTTAAATAAAAGCATCAAAAAAAAGTTTC
         65 AAACTGCTCTATCAAAAGAAATGTTCAACTCTGTGAGTTGAGTACACACATCACAAAGAAGTTTC

                                      *          *  * 
     347350 TGAGAATGCTTCTGTCTAGTTTTTATGTGAAGATATTTCCAT
        130 TGAGAATGCTTCTGTCTAGTTTTTATATGAAGATATTCCCTT

              *  * *    * *       **       *       *     *                   
     347392 TTTC-TCTATAAGCCTCAAAGCTGTCCAAATGTCCACTTGCAGATACTACAAAAAGAGTGTTTCA
          1 TTCCAACGA-AATCATCAAAGCGATCCAAATATCCACTTCCAGATTCTACAAAAAGAGTGTTTCA

               *     *  *     *        *           * * *  *          *       
     347456 AAAGTGCTCAATGAAAAGGAATGTTCAGCTCTGTGAGTTAAATGCAAACATCACAAATAAGTTTC
         65 AAACTGCTCTATCAAAAGAAATGTTCAACTCTGTGAGTTGAGTACACACATCACAAAGAAGTTTC

                                      **       * *  * 
     347521 TGAGAATGCTTCTGTCTAGTTTTTATGGGAAGATAATTCCGT
        130 TGAGAATGCTTCTGTCTAGTTTTTATATGAAGATATTCCCTT

            *    *    ** *       **  *            *  *                       
     347563 GTCCAGCGAAGGCTTCAAAGCTTTCAAAATATCCACTTGCAAATTCTACAAAAAGAGTGTTTCAA
          1 TTCCAACGAAATCATCAAAGCGATCCAAATATCCACTTCCAGATTCTACAAAAAGAGTGTTTCAA

             *     *            **                  * ***                    
     347628 AGCTGCTTTATCAAAAGAAAGTTTCAACTCTGTGAGTTGAATGTGCACATCACAAAGAAGTTTCT
         66 AACTGCTCTATCAAAAGAAATGTTCAACTCTGTGAGTTGAGTACACACATCACAAAGAAGTTTCT

                        *    *        *               
     347693 GAGAATGCCTTCAGTCTGGTTTTTATGTGAAGATATTCCCTT
        131 GAGAATG-CTTCTGTCTAGTTTTTATATGAAGATATTCCCTT

                       * *  *    **               ** ** *  *                 
     347735 TTCCAACGAAAGCCTCGAAGCTGTCCAAATATCCACTTGTAAGTGCTGCAAAAAGAGTGTTTCAA
          1 TTCCAACGAAATCATCAAAGCGATCCAAATATCCACTTCCAGATTCTACAAAAAGAGTGTTTCAA

                   ** *         *   * *                *        **     *     
     347800 AACTGCTACAGCAAAAGAAAGGTTTATCTCTGTGAGTTGAGTAGACACATCAAGAAGAAATTTCT
         66 AACTGCTCTATCAAAAGAAATGTTCAACTCTGTGAGTTGAGTACACACATCACAAAGAAGTTTCT

                                     *          *    
     347865 GAGAATGCTTCTGTCTAGTTTTTATGTGAAGATATTTCCTT
        131 GAGAATGCTTCTGTCTAGTTTTTATATGAAGATATTCCCTT

             **  * ** ** *  *    **       *       *     *                    
     347906 TGTCACCATAGGCCTCCAAGCCCTCCAAATGTCCACTTGCAGATGCTACAAAAAGAGTGTTTCAA
          1 TTCCAACGAAATCATCAAAGCGATCCAAATATCCACTTCCAGATTCTACAAAAAGAGTGTTTCAA

                   *   *          *    *        * * * *  *               * * 
     347971 AACTGCTGTATGAAAAGAAATGCTCAAATCTGTGAGATAAATGCATACATCACAAAGAAGTCTTT
         66 AACTGCTCTATCAAAAGAAATGTTCAACTCTGTGAGTTGAGTACACACATCACAAAGAAGTTTCT

                                     * *        *   *
     348036 GAGAATGCTTCTGTCTAGTTTTTATGTTAAGATATTTCCTA
        131 GAGAATGCTTCTGTCTAGTTTTTATATGAAGATATTCCCTT

              *  *  * **       *  *** *    * *     *     *       *           
     348077 TTTCACCATACGTC-TCAACGCACACAAAATGTACACTTGCAGATGCTACAAAGAGAGTGTTTCA
          1 TTCCAAC-GAAATCATCAAAGCGATCCAAATATCCACTTCCAGATTCTACAAAAAGAGTGTTTCA

                 ** **       *  *         *           *         **           
     348141 AAACTTGTAGATCAAAACAAGTGTTCAACTTTGTGAGTTGAGGACACACATCTGAAAGAAGTTTC
         65 AAACTGCTCTATCAAAAGAAATGTTCAACTCTGTGAGTTGAGTACACACATCACAAAGAAGTTTC

                                      *             * 
     348206 TGAGAATGCTTCTGTCTAGTTTTTATGTGAAGATATTCCCGT
        130 TGAGAATGCTTCTGTCTAGTTTTTATATGAAGATATTCCCTT

                 *     * **    * *                *  *                       
     348248 TTCCAGCGAAAGCCCCAAAACTATCCAAATATCCACTTGCACATTCTACAAAAAGAGTGTTTCAA
          1 TTCCAACGAAATCATCAAAGCGATCCAAATATCCACTTCCAGATTCTACAAAAAGAGTGTTTCAA

             *              *   *                   *                        
     348313 ATCTGCTCTATCAAAATAAAGGTTCAACTCTGTGAGTTGACTACACACATCACAAAGAAGTTTCT
         66 AACTGCTCTATCAAAAGAAATGTTCAACTCTGTGAGTTGAGTACACACATCACAAAGAAGTTTCT

            *               *        **         *    
     348378 AAGAATGCTTCTGTCTGGTTTTTATGGGAAGATATTTCCTT
        131 GAGAATGCTTCTGTCTAGTTTTTATATGAAGATATTCCCTT

              *    
     348419 TTTCAAC
          1 TTCCAAC

     348426 ATAGGCCTTG


Statistics
Matches: 252588,  Mismatches: 92378, Indels: 6542
        0.72            0.26        0.02

Matches are distributed among these distances:
 158  114  0.00
 159    1  0.00
 161    4  0.00
 162  207  0.00
 163   66  0.00
 164   20  0.00
 165   73  0.00
 166  1396  0.01
 167  52989  0.21
 168  2984  0.01
 169  13064  0.05
 170  27645  0.11
 171  142816  0.57
 172  612  0.00
 173  10467  0.04
 174  130  0.00

ACGTcount: A:0.31, C:0.22, G:0.17, T:0.30


Consensus pattern (171 bp):   
TTCCAACGAAATCATCAAAGCGATCCAAATATCCACTTCCAGATTCTACAAAAAGAGTGTTTCAA
AACTGCTCTATCAAAAGAAATGTTCAACTCTGTGAGTTGAGTACACACATCACAAAGAAGTTTCT
GAGAATGCTTCTGTCTAGTTTTTATATGAAGATATTCCCTT

Found at i:2887 original size:1190 final size:1193

Alignment explanation  
  

    Indices: 21--348425  Score: 307376
    Period size: 1190  Copynumber: 292.9  Consensus size: 1193

         11 TCCGTGCTTC

                *     *       *                *       *          *        * 
         21 CAACTAAATCCTCAAAGCAATCCAAATATCCACTTGCAGATTCCACAAAAAGAGGGTTTCAAATC
          1 CAACGAAATCATCAAAGCGATCCAAATATCCACTTCCAGATTCTACAAAAAGAGTGTTTCAAACC

                    *  ** *  *            *   **  *     *     *  *           
         86 TGCTCTATCAATAGCAAGGTTCAACTCTGTTAGTATAGGACACATATCACCAACAAGTTTCTGAG
         66 TGCTCTA-GAAAGGGAATGTTCAACTCTGTGAGTTGAGTACACACATCACAAAGAAGTTTCTGAG

                          *   *   **  *      *  *   *  ** * **  **         * 
        151 AATGCTTCTGTCTACTTTCTATGGGAGGATATTTCCTTTTTCACTGTAGGCGACAAAGCGATCAA
        130 AATGCTTCTGTCTAGTTTTTATATGAAGATATTCCCGTTTCCAACGAAATCGCTAAAGCGATCCA

             * *          *  *          *          *     *   *   * *   *    *
        216 ATTGTCCA-TTCCATATACTACAAAAAGTGTGTTTCAAACCTGCTTTATGAAAGGTAATATTCAT
        195 AATATCCACTTCCAGATTCTACAAAAAGAGTGTTTCAAAACTGCTCTATCAAAAGAAATGTTCAA

                * **     * ** * *    *      *  *        *  *       **        
        280 CTCTATGTGTTGAATGGAAATATCAGAAAGAAATTGCTGAGAATTCTGCTGTCTACATTTTATAT
        260 CTCTGTTAGTTGAGTACACACATCACAAAGAAGTTTCTGAGAATGCTTCTGTCTAGTTTTTATAT

                        **             *     * *                *       *    
        345 ---GA-ATTCCCGCTTCCAACGAAATCCTCAAATCAATCCAAATATCCACTTGCAGATTCCACAA
        325 GAAGATATTCCCTTTTCCAACGAAATCATCAAAGCGATCCAAATATCCACTTCCAGATTCTACAA

                                     *  *     *          *         *         
        406 AAAGAGTGTTTCAAAACTGCTCTATCAATAGAAAGGTTCAACTCTTTTAGTTGAGAACACACATC
        390 AAAGAGTGTTTCAAAACTGCTCTATGAAAAGAAATGTTCAACTCTGTTAGTTGAGTACACACATC

                 *                    *   * *             **  *         * *  
        471 ACAAACAAGTTTCTGAGAATGCTTCTCTCTCGCTTTTATGGGAAGACGTTTCC-TTTCCACCAAA
        455 ACAAAGAAGTTTCTGAGAATGCTTCTGTCTAGTTTTTATGGGAAGATATTCCCTTTTCCAACGAA

            *         * *       *                  *                **       
        535 GGCATCAAAGTGTTCCAAATGTCCACTTCCAGATTCTACCAAAAGAGTGTTTCAAACGTGCTC-A
        520 AGCATCAAAGCGATCCAAATATCCACTTCCAGATTCTACAAAAAGAGTGTTTCAAAACTGCTCTA

              **    *                 *    * *  * *     *   *       * * *    
        599 TAGTAAGGGAATGTTCAACTCTGTGACTTGAATGCAGATATCACCAAGTAGTTTCTAATAGTGCT
        585 T-CAAAGGAAATGTTCAACTCTGTGAGTTGAGTACACACATCACAAAGAAGTTTCTGAGAATGCT

                           *              *              * * *   *        *  
        664 TCTGTCTAGATTTTAGATGATGATATTCCCGTTTCCAACGAAATCGTTAGAGCTATCCAAATGTC
        649 TCTGTCTAG-TTTT-TATGATGATATTCCCTTTTCCAACGAAATCATCAAAGCGATCCAAATATC

              *  *   *      *     *       *   *     **      *    *           
        729 CAGTTACAGTTTCTACCAAAAGGGTGTTTCCAAATTG-CTGCATCAAAAGAAAGGTTCAACTCTG
        712 CACTTCCAGATTCTACAAAAAGAGTGTTTCAAAACTGCCTCTATCAAAGGAAATGTTCAACTCTG

                     *                     *                   *    * **  *  
        793 TTAGTTGAGGACACACATCACAAAGAAGTTTGTGAGAATGCTTCTGTCTAGATTTTGTATGACGA
        777 TTAGTTGAGTACACACATCACAAAGAAGTTTCTGAGAATGCTTCTGTCTAGTTTTTATGGGAAGA

                              *   * *     *   *       * *  *    *  *       * 
        858 TATTCCCTTTTCCAACGATATCGTTAAAGCAATCTAAATATCAATTTGCAGAATCCACAAAAATA
        842 TATTCCCTTTTCCAACGAAATCATCAAAGCGATCCAAATATCCACTTCCAGATTCTACAAAAAGA

             *        *       * *   *    *    *       *  *   * *  *          
        923 GAGTTTCAAAGCTGCTCTGTAAAAAGAAAGGTTCCACTCTGTTAGCTGAGTACACACATCACAAA
        907 GTGTTTCAAAACTGCTCTATCAAAGGAAATGTTCAACTCTGTGAGTTGAATGCAGACATCACAAA

            ***            *         *                   **        *  * **   
        988 CTTGTTTCTGAGAATCCTTCTGTCTCGTTTTTATGGGAAGATATTTACTTTTCCACCGTAGGCAT
        972 GAAGTTTCTGAGAATGCTTCTGTCTAGTTTTTAT--GAAGATATTCCCTTTTCCAACGAAATCAT

                   *       *     *       *  *  *               *         *   
       1053 CAAAGCGCTCCAAATGTCCACATCCAGATACTCCAGAAAGAGTGTTTCAAACCTGCTCTATGAAA
       1035 CAAAGCGATCCAAATATCCACTTCCAGATTCTACAAAAAGAGTGTTTCAAAACTGCTCTATCAAA

            * *   *         * *      * *  *      *      *              *     
       1118 GGGAATCTTCAACTCTATGAGTTGAATGCAGACATCAGAAAGAAATTTCTGAGAATGCTGCTGTC
       1100 AGAAATGTTCAACTCTGTTAGTTGAGTACACACATCACAAAGAAGTTTCTGAGAATGCTTCTGTC

              **         **          *  *
       1183 TACCTTTTAT---TTGA-ATTCCCGCTTC
       1165 TAGTTTTTATGGGAAGATATTCCCGTTTT

                      *  *    *              * *  **   *    *              * 
       1208 CAACGAAATCCTCCAAGCTATCCAAATATCCACCTGCATTTTCCACAACAAGAGTGTTTCAAAAC
          1 CAACGAAATCATCAAAGCGATCCAAATATCCACTTCCAGATTCTACAAAAAGAGTGTTTCAAACC

                    *  ** *              *    *  *                *          
       1273 TGCTCTATCAATAGAAATGTTCAACTCCTTTG-GCTGGGTACACACATCACAAACAAGTTTCTGA
         66 TGCTCTA-GAAAGGGAATGTTCAACT-CTGTGAGTTGAGTACACACATCACAAAGAAGTTTCTGA

                                   **     *      *   *    *    *           * 
       1337 GAATGCTTCTGTCTAGTTTTTATGGGAAGACATTCCCTTTTTC-ACCAAA-GGCATCAAAGCGCT
        129 GAATGCTTCTGTCTAGTTTTTATATGAAGATATTCCCGTTTCCAACGAAATCGC-T-AAAGCGAT

                  *            **                     **       **    **      
       1400 CCAAATGTCCACTTCCAGACACTACAAAAAGAGTGTTTCAAACGTGCTCTAAGAAAACGAATGTT
        192 CCAAATATCCACTTCCAGATTCTACAAAAAGAGTGTTTCAAAACTGCTCTATCAAAAGAAATGTT

                     * *    * *  * *      *  *           **            *     
       1465 CAACTCTGTGACTTGAATGCAGATATCACAGAGTAGTTTCTGAGAGGGCTTCTGTCTAGATTTTA
        257 CAACTCTGTTAGTTGAGTACACACATCACAAAGAAGTTTCTGAGAATGCTTCTGTCTAGTTTTTA

            *    *         *                * *   *                *   *     
       1530 GATGATGATATTCCCGTTTCCAACGAAATCATTAGAGCTATCCAAATATCCACTTACAGTTTCTA
        322 TATGAAGATATTCCCTTTTCCAACGAAATCATCAAAGCGATCCAAATATCCACTTCCAGATTCTA

                           *        **  *      * *    *          *           
       1595 CAAAAAGAGTGTTTCCAAACTGCTGCATCAAAAGAGAGGTTCCACTCTGTTAGCTGAGTACACAC
        387 CAAAAAGAGTGTTTCAAAACTGCTCTATGAAAAGAAATGTTCAACTCTGTTAGTTGAGTACACAC

                    ***      *     *         *                   **     *  * 
       1660 ATCACAAACTTGTTTCTCAGAATCCTTCTGTCTCGTTTTTATGGGAAGATATTTACTTTTTCACC
        452 ATCACAAAGAAGTTTCTGAGAATGCTTCTGTCTAGTTTTTATGGGAAGATATTCCCTTTTCCAAC

             * * *         *       *     *       **    *       *       *     
       1725 GTAGGTATCAAAGCGCTCCAAATGTCCACATCCAGATAGTACAGAAAGAGTTTTTCAAACCTGCT
        517 GAAAGCATCAAAGCGATCCAAATATCCACTTCCAGATTCTACAAAAAGAGTGTTTCAAAACTGCT

                *     *   *         *        * *  *      *      *            
       1790 CTATGAAAGGGAATCTTCAACTCTATGAGTTGAATGCAGACATCAGAAAGAAATTTCTGAGAATG
        582 CTATCAAAGGAAATGTTCAACTCTGTGAGTTGAGTACACACATCACAAAGAAGTTTCTGAGAATG

              *       **       *          **             *  *    *           
       1855 CTGCTGTCTACCTTTTAT-TTGA-ATTCCCGCTTCCAACGAAATCCTCCAAGCTATCCAAATATC
        647 CTTCTGTCTAGTTTTTATGATGATATTCCCTTTTCCAACGAAATCATCAAAGCGATCCAAATATC

                 *       *                                  *  *  *          
       1918 CACTTGCAGATTCCACAAAAAGAGTGTTTCAAAACTGCTCTCTATCAATGGCAAAGTTCAACTCT
        712 CACTTCCAGATTCTACAAAAAGAGTGTTTCAAAACTGC-CTCTATCAAAGGAAATGTTCAACTCT

                      *     *     *  *                         *             
       1983 GTTAGTTGAGGACACATATCACCAACAAGTTTCTGAGAATGCTTCTGTCTATTTTTTATGGGAAG
        776 GTTAGTTGAGTACACACATCACAAAGAAGTTTCTGAGAATGCTTCTGTCTAGTTTTTATGGGAAG

                 *      *  *  * ** *    *      *    *          * **          
       2048 ATATTTCCTTTTTCACCGTAGGCGTCAAGGCGATCGAAATGTCCACTTCCACAAACTACAAAAAG
        841 ATATTCCCTTTTCCAACGAAATCATCAAAGCGATCCAAATATCCACTTCCAGATTCTACAAAAAG

                       *         *     **       *    *           * * *   **  
       2113 AGTGTTTCAAACCTGCTCTATGAAAGGCCATGTTCATCTCTATGAGTTGAATGGAAATATCCGAA
        906 AGTGTTTCAAAACTGCTCTATCAAAGGAAATGTTCAACTCTGTGAGTTGAATGCAGACATCACAA

                *      *       *         *       *         **             *  
       2178 AGAAATTTCTGGGAATGCTGCTGTCTAGTGTTTAT-ACGA-ATTCCCGCTTCCAACGAAATCCTC
        971 AGAAGTTTCTGAGAATGCTTCTGTCTAGTTTTTATGAAGATATTCCCTTTTCCAACGAAATCATC

                 *                *    *  *                                * 
       2241 AAAGCAATCCAAATATCCACTTGCAGAATCCACAAAAAGAGTGTTTCAAAACTGCTCTATCAATA
       1036 AAAGCGATCCAAATATCCACTTCCAGATTCTACAAAAAGAGTGTTTCAAAACTGCTCTATCAAAA

                *          *                     *  *                        
       2306 GAAAGGTTCAACTCTTTTAGTTGAGTACACACATCACGAACAAGTTTCTGAGAATGCTTCTGTCT
       1101 GAAATGTTCAACTCTGTTAGTTGAGTACACACATCACAAAGAAGTTTCTGAGAATGCTTCTGTCT

            * *      *      **  *       
       2371 GGCTTTTATTGGAAGACGTTTCC-TTTT
       1166 AGTTTTTATGGGAAGATATTCCCGTTTT

              * *  **        * *       *                  *                 *
       2398 CACCAAAGGCATCAAAGTGCTCCAAATGTCCACTTCCAGATTCTACCAAAAGAGTGTTTCAAACG
          1 CAACGAAATCATCAAAGCGATCCAAATATCCACTTCCAGATTCTACAAAAAGAGTGTTTCAAACC

                     *                      *    * *  * *     *   *       * *
       2463 TGCTCATAGTAAGGGAATGTTCAACTCTGTGACTTGAATGCAGATATCACCAAGTAGTTTCTAAT
         66 TGCTC-TAGAAAGGGAATGTTCAACTCTGTGAGTTGAGTACACACATCACAAAGAAGTTTCTGAG

             *             *     *    *                            *   *     
       2528 AGTGCTTCTGTCTAGATTTTAGATGATGATATTCCCGTTTCCAACGAAATCGCTAGAGCTATCCA
        130 AATGCTTCTGTCTAGTTTTTATATGAAGATATTCCCGTTTCCAACGAAATCGCTAAAGCGATCCA

                    *  *   *      *     *       *   *    **           *      
       2593 AATATCCAGTTACAGTTTCTACCAAAAGGGTGTTTCCAAATTGCTGCATCAAAAGAAAGGTTCAA
        195 AATATCCACTTCCAGATTCTACAAAAAGAGTGTTTCAAAACTGCTCTATCAAAAGAAATGTTCAA

                          *      *              *                   *    *   
       2658 CTCTGTTAGTTGAGGACACACGTCACAAAGAAGTTTGTGAGAATGCTTCTGTCTAGATTTTGTAT
        260 CTCTGTTAGTTGAGTACACACATCACAAAGAAGTTTCTGAGAATGCTTCTGTCTAGTTTTTATAT

              *                    *   * *     *   *       * *  *    *  *    
       2723 GACGATATTCCCTTTTCCAACGATATCGTTAAAGCAATCTAAATATCAATTTGCAGAATCCACAA
        325 GAAGATATTCCCTTTTCCAACGAAATCATCAAAGCGATCCAAATATCCACTTCCAGATTCTACAA

               *  *        *       * *        *    *          *              
       2788 AAATAGAGTTTCAAAGCTGCTCTGTAAAAAGAAAGGTTCCACTCTGTTAGCTGAGTACACACATC
        390 AAAGAGTGTTTCAAAACTGCTCTATGAAAAGAAATGTTCAACTCTGTTAGTTGAGTACACACATC

                 ***            *         *                   **     *  *  * 
       2853 ACAAACTTGTTTCTGAGAATCCTTCTGTCTCGTTTTTATGGGAAGATATTTACTTTTTCACCGTA
        455 ACAAAGAAGTTTCTGAGAATGCTTCTGTCTAGTTTTTATGGGAAGATATTCCCTTTTCCAACGAA

            *           *       *     *       *  *  *               *        
       2918 GGCATCAAAGCGCTCCAAATGTCCACATCCAGATACTCCAGAAAGAGTGTTTCAAACCTGCTCTA
        520 AGCATCAAAGCGATCCAAATATCCACTTCCAGATTCTACAAAAAGAGTGTTTCAAAACTGCTCTA

             *     *   *         *        * *  *      *      *              *
       2983 TGAAAGGCAATCTTCAACTCTATGAGTTGAATGCAGACATCAGAAAGAAATTTCTGAGAATGCTG
        585 TCAAAGGAAATGTTCAACTCTGTGAGTTGAGTACACACATCACAAAGAAGTTTCTGAGAATGCTT

                   **       *          **             *  *    *              
       3048 CTGTCTACCTTTTAT-TTGA-ATTCCCGCTTCCAACGAAATCCTCCAAGCTATCCAAATATCCAC
        650 CTGTCTAGTTTTTATGATGATATTCCCTTTTCCAACGAAATCATCAAAGCGATCCAAATATCCAC

            * *  **   *    *                            **              **  *
       3111 CTGCATTTTCCACAACAAGAGTGTTTCAAAACTG-CTCTATCAATAGAAATGTTCAACTCCTTTG
        715 TTCCAGATTCTACAAAAAGAGTGTTTCAAAACTGCCTCTATCAAAGGAAATGTTCAACTCTGTTA

             *  *                *                                        ** 
       3175 GCTGGGTACACACATCACAAACAAGTTTCTGAGAATGCTTCTGTCTAGTTTTTATGGGAAGACGT
        780 GTTGAGTACACACATCACAAAGAAGTTTCTGAGAATGCTTCTGTCTAGTTTTTATGGGAAGATAT

                    *  * *  **          *       *            **              
       3240 TCCCTTTTTCACCAAAGGCATCAAAGCGCTCCAAATGTCCACTTCCAGACACTACAAAAAGAGTG
        845 TCCCTTTTCCAACGAAATCATCAAAGCGATCCAAATATCCACTTCCAGATTCTACAAAAAGAGTG

                *  **   *   **    **                 *           *         * 
       3305 TTTCCAACGTGCCCTAAGAAAGCGAATGTTCAACTCTGTGACTTGAATGCAGATATCACAAAGTA
        910 TTTCAAAACTGCTCTATCAAAGGAAATGTTCAACTCTGTGAGTTGAATGCAGACATCACAAAGAA

                      **                  *    *         *                * *
       3370 GTTTCTGAGAGGGCTTCTGTCTAGATTTTAGATGATGATATTCCCGTTTCCAACGAAATCATTAG
        975 GTTTCTGAGAATGCTTCTGTCTAG-TTTT-TATGAAGATATTCCCTTTTCCAACGAAATCATCAA

               *                *   *                    *        **         
       3435 AGCTATCCAAATATCCACTTACAGTTTCTACAAAAAGAGTGTTTCCAAACTGCTGCATCAAAAGA
       1038 AGCGATCCAAATATCCACTTCCAGATTCTACAAAAAGAGTGTTTCAAAACTGCTCTATCAAAAGA

            * *    *          *                   ***      *     *         * 
       3500 GAGGTTCCACTCTGTTAGCTGAGTACACACATCACAAACTTGTTTCTCAGAATCCTTCTGTCTCG
       1103 AATGTTCAACTCTGTTAGTTGAGTACACACATCACAAAGAAGTTTCTGAGAATGCTTCTGTCTAG

                              ** *    
       3565 TTTTTATGGGAAGATATTTACTTTTT
       1168 TTTTTATGGGAAGATATTCCCGTTTT

              *  * **          *       *     *       *  *  *                 
       3591 CACCGTAGGCATCAAAGCGCTCCAAATGTCCACATCCAGATACTCCAGAAAGAGTGTTTCAAACC
          1 CAACGAAATCATCAAAGCGATCCAAATATCCACTTCCAGATTCTACAAAAAGAGTGTTTCAAACC

                              *         *        * *  *      *      *        
       3656 TGCTCTATGAAAGGGAATCTTCAACTCTATGAGTTGAATGCAGACATCAGAAAGAAATTTCTGAG
         66 TGCTCTA-GAAAGGGAATGTTCAACTCTGTGAGTTGAGTACACACATCACAAAGAAGTTTCTGAG

                  *       **          *          *                 *    *    
       3721 AATGCTGCTGTCTACCTTTTAT-T--TGA-ATTCCCGCTTCCAACGAAATC-CTCCAAGCTATCC
        130 AATGCTTCTGTCTAGTTTTTATATGAAGATATTCCCGTTTCCAACGAAATCGCT-AAAGCGATCC

                        *       *                                  ** *  *   
       3781 AAATATCCACTTGCAGATTCCACAAAAAGAGTGTTTCAAAACTGCTCTCTATCAATGGCAAAGTT
        194 AAATATCCACTTCCAGATTCTACAAAAAGAGTGTTTCAAAACTG--CTCTATCAAAAGAAATGTT

                             *     *     *  *                         *      
       3846 CAACTCTGTTAGTTGAGGACACATATCACCAACAAGTTTCTGAGAATGCTTCTGTCTATTTTTTA
        257 CAACTCTGTTAGTTGAGTACACACATCACAAAGAAGTTTCTGAGAATGCTTCTGTCTAGTTTTTA

             **         *      *  *  * ** *    *      *    *          * **   
       3911 TGGGAAGATATTTCCTTTTTCACCGTAGGCGTCAAGGCGATCGAAATGTCCACTTCCACAAACTA
        322 TATGAAGATATTCCCTTTTCCAACGAAATCATCAAAGCGATCCAAATATCCACTTCCAGATTCTA

                              *             * **       *    * *      * ** * *
       3976 CAAAAAGAGTGTTTCAAACCTGCTCTATGAAAGGCCATGTTCATCTCTATGAGTTGAATGGAAAT
        387 CAAAAAGAGTGTTTCAAAACTGCTCTATGAAAAGAAATGTTCAACTCTGTTAGTTGAGTACACAC

               **      *      *       *         *         *         **       
       4041 ATCCGAAAGAAATTTCTGGGAATGCTGCTGTCTAGTGTTTAT---ACGA-ATTCCCGCTTCCAAC
        452 ATCACAAAGAAGTTTCTGAGAATGCTTCTGTCTAGTTTTTATGGGAAGATATTCCCTTTTCCAAC

                * *       *                *    *  *                         
       4102 GAAATCCTCAAAGCAATCCAAATATCCACTTGCAGAATCCACAAAAAGAGTGTTTCAAAACTGCT
        517 GAAAGCATCAAAGCGATCCAAATATCCACTTCCAGATTCTACAAAAAGAGTGTTTCAAAACTGCT

                   **    *          * *                   *  *               
       4167 CTATCAATAGAAAGGTTCAACTCTTTTAGTTGAGTACACACATCACGAACAAGTTTCTGAGAATG
        582 CTATCAAAGGAAATGTTCAACTCTGTGAGTTGAGTACACACATCACAAAGAAGTTTCTGAGAATG

                     * *          *  **  *         * *  **          *       *
       4232 CTTCTGTCTGGCTTTTATTGGAAGACGTTTCCTTTT-CACCAAAGGCATCAAAGCGCTCCAAATG
        647 CTTCTGTCTAGTTTTTA-T-GATGATATTCCCTTTTCCAACGAAATCATCAAAGCGATCCAAATA

                            * *                **      * ***    *            
       4296 TCCACTTCCAGATTCTTCCAAAAGAGTGTTTCAAACGTG-CTCAAAGTAAGGGAATGTTCAACTC
        710 TCCACTTCCAGATTCTACAAAAAGAGTGTTTCAAAACTGCCTCTATCAAAGGAAATGTTCAACTC

               * *    * *  * *     *   *       * * *             *   * ***  *
       4360 TGTGACTTGAATGCAGATATCACCAAGTAGTTTCTAATAGTGCTTCTGTCTAGATTTCAGATGAT
        775 TGTTAGTTGAGTACACACATCACAAAGAAGTTTCTGAGAATGCTTCTGTCTAGTTTTTATGGGAA

                     *              * * *   *                *   *           
       4425 GATATTCCCGTTTCCAACGAAATCGTTAGAGCTATCCAAATATCCACTTACAGTTTCTACAAAAA
        840 GATATTCCCTTTTCCAACGAAATCATCAAAGCGATCCAAATATCCACTTCCAGATTCTACAAAAA

                     *        **      *    *            *      ***  *        
       4490 GAGTGTTTCCAAACTGCTGCATCAAAAGAAAGGTTCAACTCTGTTAGTTGAGGACACACATCACA
        905 GAGTGTTTCAAAACTGCTCTATCAAAGGAAATGTTCAACTCTGTGAGTTGAATGCAGACATCACA

                     *                              *                    *   
       4555 AAGAAGTTTGTGAGAATGCTTCTGTCTAGATTTTGTATGACGATATTCCCTTTTCCAACGATATC
        970 AAGAAGTTTCTGAGAATGCTTCTGTCTAG-TTTT-TATGAAGATATTCCCTTTTCCAACGAAATC

            * *     *   *       * *  *    *  *       *  *        *       * * 
       4620 GTTAAAGCAATCTAAATATCAATTTGCAGAATCCACAAAAATAGAGTTTCAAAGCTGCTCTGTAA
       1033 ATCAAAGCGATCCAAATATCCACTTCCAGATTCTACAAAAAGAGTGTTTCAAAACTGCTCTATCA

               *   *    *          *                   ***            *      
       4685 AAATAAAGGTTCCACTCTGTTAGCTGAGTACACACATCACAAACTTGTTTCTGAGAATCCTTCTG
       1098 AAAGAAATGTTCAACTCTGTTAGTTGAGTACACACATCACAAAGAAGTTTCTGAGAATGCTTCTG

               *                   ** *    
       4750 TCTCGTTTTTATGGGAAGATATTTACTTTTT
       1163 TCTAGTTTTTATGGGAAGATATTCCCGTTTT

              *  * **          *       *     *       *  *  *     *           
       4781 CACCGTAGGCATCAAAGCGCTCCAAATGTCCACATCCAGATACTCCAGAAAGACTGTTTCAAACC
          1 CAACGAAATCATCAAAGCGATCCAAATATCCACTTCCAGATTCTACAAAAAGAGTGTTTCAAACC

                              *         *        * *  *      *      *        
       4846 TGCTCTATGAAAGGGAATCTTCAACTCTATGAGTTGAATGCAGACATCAGAAAGAAATTTCTGAG
         66 TGCTCTA-GAAAGGGAATGTTCAACTCTGTGAGTTGAGTACACACATCACAAAGAAGTTTCTGAG

                  *       **          *          *                 *    *    
       4911 AATGCTGCTGTCTACCTTTTAT-T--TGA-ATTCCCGCTTCCAACGAAATC-CTCCAAGCTATCC
        130 AATGCTTCTGTCTAGTTTTTATATGAAGATATTCCCGTTTCCAACGAAATCGCT-AAAGCGATCC

                        *  **   *                                *           
       4971 AAATATCCACTTGCATTTTCCACAAAAAGAGTGTTTCAAAACTGCTCTATCAATAGAAATGTTCA
        194 AAATATCCACTTCCAGATTCTACAAAAAGAGTGTTTCAAAACTGCTCTATCAAAAGAAATGTTCA

                **    *  *                *                                 *
       5036 ACTCCTTTAGCTGGGTACACACATCACAAACAAGTTTCTGAGAATGCTTCTGTCTAGTTTTTATG
        259 ACTCTGTTAGTTGAGTACACACATCACAAAGAAGTTTCTGAGAATGCTTCTGTCTAGTTTTTATA

            *     **         *  * *  **          *       *            **     
       5101 GGAAGACGTTCCCTTTTTCACCAAAGGCATCAAAGCGCTCCAAATGTCCACTTCCAGACACTACA
        324 TGAAGATATTCCCTTTTCCAACGAAATCATCAAAGCGATCCAAATATCCACTTCCAGATTCTACA

                            **       *    ***               * *    * *  * *  
       5166 AAAAGAGTGTTTCAAACGTGCTCTAAGAAAGCGAATGTTCAACTCTGTGACTTGAATGCAGATAT
        389 AAAAGAGTGTTTCAAAACTGCTCTATGAAAAGAAATGTTCAACTCTGTTAGTTGAGTACACACAT

                   *           **            *     ***  *         *          
       5231 CACAAAGTAGTTTCTGAGAGGGCTTCTGTCTAGATTTTAGATGATGATATTCCCGTTTCCAACGA
        454 CACAAAGAAGTTTCTGAGAATGCTTCTGTCTAGTTTTTATGGGAAGATATTCCCTTTTCCAACGA

              *   * *   *                *   *                    *        **
       5296 AATCATTAGAGCTATCCAAATATCCACTTACAGTTTCTACAAAAAGAGTGTTTCCAAACTGCTGC
        519 AAGCATCAAAGCGATCCAAATATCCACTTCCAGATTCTACAAAAAGAGTGTTTCAAAACTGCTCT

                  *  * *    *       *  *                   ***      *     *  
       5361 ATCAAAAGAGAGGTTCCACTCTGTTAGCTGAGTACACACATCACAAACTTGTTTCTCAGAATCCT
        584 ATCAAAGGAAATGTTCAACTCTGTGAGTTGAGTACACACATCACAAAGAAGTTTCTGAGAATGCT

                   *            *      **     *  *  * **          *       *  
       5426 TCTGTCTCGTTTTTATGGGAAGATATTTACTTTTTCACCGTAGGCATCAAAGCGCTCCAAATGTC
        649 TCTGTCTAGTTTTTAT--GATGATATTCCCTTTTCCAACGAAATCATCAAAGCGATCCAAATATC

               *       *  *  *               *          *     *   *         *
       5491 CACATCCAGATACTCCAGAAAGAGTGTTTCAAACCTG-CTCTATGAAAGGGAATCTTCAACTCTA
        712 CACTTCCAGATTCTACAAAAAGAGTGTTTCAAAACTGCCTCTATCAAAGGAAATGTTCAACTCTG

             *      * *  *      *      *              *       **         **  
       5555 TGAGTTGAATGCAGACATCAGAAAGAAATTTCTGAGAATGCTGCTGTCTACCTTTTAT---TTGA
        777 TTAGTTGAGTACACACATCACAAAGAAGTTTCTGAGAATGCTTCTGTCTAGTTTTTATGGGAAGA

                   **             *  *    *                *       *         
       5617 -ATTCCCGCTTCCAACGAAATCCTCCAAGCTATCCAAATATCCACTTGCAGATTCCACAAAAAGA
        842 TATTCCCTTTTCCAACGAAATCATCAAAGCGATCCAAATATCCACTTCCAGATTCTACAAAAAGA

                                     *  *  *            *      ***  * *     *
       5681 GTGTTTCAAAACTGCTCTCTATCAATGGCAAAGTTCAACTCTGTTAGTTGAGGACACATATCACC
        907 GTGTTTCAAAACTG--CTCTATCAAAGGAAATGTTCAACTCTGTGAGTTGAATGCAGACATCACA

              *                         *                  *      *  *  * ** 
       5746 AACAAGTTTCTGAGAATGCTTCTGTCTATTTTTTATGGGAAGATATTTCCTTTTTCACCGTAGGC
        970 AAGAAGTTTCTGAGAATGCTTCTGTCTAGTTTTTAT--GAAGATATTCCCTTTTCCAACGAAATC

            *    *      *    *          * **                     *         * 
       5811 GTCAAGGCGATCGAAATGTCCACTTCCACAAACTACAAAAAGAGTGTTTCAAACCTGCTCTATGA
       1033 ATCAAAGCGATCCAAATATCCACTTCCAGATTCTACAAAAAGAGTGTTTCAAAACTGCTCTATCA

              * **       *    * *      * ** * *   **      *      *       *   
       5876 AAGGCGATGTTCATCTCTATGAGTTGAATGGAAATATCCGAAAGAAATTTCTGGGAATGCTGCTG
       1098 AAAGAAATGTTCAACTCTGTTAGTTGAGTACACACATCACAAAGAAGTTTCTGAGAATGCTTCTG

                            *          *  *
       5941 TCTAGTTTTTAT---ATGA-ATTCCCGCTTC
       1163 TCTAGTTTTTATGGGAAGATATTCCCGTTTT

                      *       *                *    *  *         *         * 
       5968 CAACGAAATCCTCAAAGCAATCCAAATATCCACTTGCAGAATCCACAAAAAGATTGTTTCAAAAC
          1 CAACGAAATCATCAAAGCGATCCAAATATCCACTTCCAGATTCTACAAAAAGAGTGTTTCAAACC

                    *  ** *  *          * *                      *           
       6033 TGCTCTATCAATAGAAAGGTTCAACTCTTTTAGTTGAGTACACACATCACAAACAAGTTTCTGAG
         66 TGCTCTA-GAAAGGGAATGTTCAACTCTGTGAGTTGAGTACACACATCACAAAGAAGTTTCTGAG

                         * *      **     **  *      *    *    *         * *  
       6098 AATGCTTCTGTCTGGCTTTTATTGGAAGACGTTTCC-TTTTC-ACCAAA-GGCATCAAAGTGCTC
        130 AATGCTTCTGTCTAGTTTTTATATGAAGATATTCCCGTTTCCAACGAAATCGC-T-AAAGCGATC

                 *                * *                **  *  * ***  * *       
       6160 CAAATGTCCACTTCCAGATTCTTCCAAAAGAGTGTTTCAAACGTGGTCGAAGTAAGGGAATGTTC
        193 CAAATATCCACTTCCAGATTCTACAAAAAGAGTGTTTCAAAACTGCTCTATCAAAAGAAATGTTC

                    * *    * *  * *     *   *       * * *            **     *
       6225 AACTCTGTGACTTGAATGCAGATATCACCAAGTAGTTTCTAATAGTGCTTCTGTCTACATTTTAG
        258 AACTCTGTTAGTTGAGTACACACATCACAAAGAAGTTTCTGAGAATGCTTCTGTCTAGTTTTTAT

                *         *              * * *   *             *  *   *      
       6290 ATGATGATATTCCCGTTTCCAACGAAATCGTTAGAGCTATCCAAATATCCAGTTACAGTTTCTAC
        323 ATGAAGATATTCCCTTTTCCAACGAAATCATCAAAGCGATCCAAATATCCACTTCCAGATTCTAC

            *     *       *   *    **  *        *                    *       
       6355 CAAAAGGGTGTTTCCAAATTGCTGCATCAAAAGAAAGGTTCAACTCTGTTAGTTGAGGACACACA
        388 AAAAAGAGTGTTTCAAAACTGCTCTATGAAAAGAAATGTTCAACTCTGTTAGTTGAGTACACACA

                          *                   *    * **  *                   
       6420 TCACAAAGAAGTTTGTGAGAATGCTTCTGTCTAGATTTTGTATGACGATATTCCCTTTTCCAACG
        453 TCACAAAGAAGTTTCTGAGAATGCTTCTGTCTAGTTTTTATGGGAAGATATTCCCTTTTCCAACG

             * * * *     *   *       * *  *    *  *       *  *        *      
       6485 ATATCGTTAAAGCAATCTAAATATCAATTTGCAGAATCCACAAAAATAGAGTTTCAAAGCTGCTC
        518 AAAGCATCAAAGCGATCCAAATATCCACTTCCAGATTCTACAAAAAGAGTGTTTCAAAACTGCTC

             * *   *    *    *       *  *                   ***            * 
       6550 TGTAAAAAGAAAGGTTCCACTCTGTTAGCTGAGTACACACATCACAAACTTGTTTCTGAGAATCC
        583 TATCAAAGGAAATGTTCAACTCTGTGAGTTGAGTACACACATCACAAAGAAGTTTCTGAGAATGC

                    *            *      **        *  * **          *       * 
       6615 TTCTGTCTCGTTTTTATGGGAAGATATTTACTTTTCCACCGTAGGCATCAAAGCGCTCCAAATGT
        648 TTCTGTCTAGTTTTTAT--GATGATATTCCCTTTTCCAACGAAATCATCAAAGCGATCCAAATAT

                *       *  *  *  *            *          *     *   *         
       6680 CCACATCCAGATACTCCAGAACGAGTGTTTCAAACCTG-CTCTATGAAAGGGAATCTTCAACTCT
        711 CCACTTCCAGATTCTACAAAAAGAGTGTTTCAAAACTGCCTCTATCAAAGGAAATGTTCAACTCT

            * *      * *  *      *      *              *       **         ** 
       6744 ATGAGTTGAATGCAGACATCAGAAAGAAATTTCTGAGAATGCTGCTGTCTACCTTTTAT---TTG
        776 GTTAGTTGAGTACACACATCACAAAGAAGTTTCTGAGAATGCTTCTGTCTAGTTTTTATGGGAAG

                    **             *  *    *              * *  **   *    *   
       6806 A-ATTCCCGCTTCCAACGAAATCCTCCAAGCTATCCAAATATCCACCTGCATTTTCCACAACAAG
        841 ATATTCCCTTTTCCAACGAAATCATCAAAGCGATCCAAATATCCACTTCCAGATTCTACAAAAAG

                                    **                *    *  ** *  *        
       6870 AGTGTTTCAAAACTGCTCTATCAATAGAAATGTTCAACTCCTTTG-GCTGGGTACACACATCACA
        906 AGTGTTTCAAAACTGCTCTATCAAAGGAAATGTTCAACT-CTGTGAGTTGAATGCAGACATCACA

              *                                        **         *  * *  ** 
       6934 AACAAGTTTCTGAGAATGCTTCTGTCTAGTTTTTATGGGAAGACGTTCCCTTTTTCACCAAAGGC
        970 AAGAAGTTTCTGAGAATGCTTCTGTCTAGTTTTTAT--GAAGATATTCCCTTTTCCAACGAAATC

                     *       *            **                  *  **   *   ** 
       6999 ATCAAAGCGCTCCAAATGTCCACTTCCAGACACTACAAAAAGAGTGTTTCCAACGTGCCCTAAGA
       1033 ATCAAAGCGATCCAAATATCCACTTCCAGATTCTACAAAAAGAGTGTTTCAAAACTGCTCTATCA

              ***               * *    * *  * *         *           **       
       7064 AAGCGAATGTTCAACTCTGTGACTTGAATGCAGATATCACAAAGTAGTTTCTGAGAGGGCTTCTG
       1098 AAAGAAATGTTCAACTCTGTTAGTTGAGTACACACATCACAAAGAAGTTTCTGAGAATGCTTCTG

                 *     ***  *             *
       7129 TCTAGATTTTAGATGATGATATTCCCGTTTC
       1163 TCTAGTTTTTATGGGAAGATATTCCCGTTTT

                        * *   *                *   *                    *  * 
       7160 CAACGAAATCATTAGAGCTATCCAAATATCCACTTACAGTTTCTACAAAAAGAGTGTTTCCAAAC
          1 CAACGAAATCATCAAAGCGATCCAAATATCCACTTCCAGATTCTACAAAAAGAGTGTTTCAAACC

                    **    *   *    *       *  *                   ***      * 
       7225 TGCTGCATCAAAAGAG-AGGTTCCACTCTGTTAGCTGAGTACACACATCACAAACTTGTTTCTCA
         66 TGCT-C-TAGAAAGGGAATGTTCAACTCTGTGAGTTGAGTACACACATCACAAAGAAGTTTCTGA

                *         *        **         ** *   *  *  * ** ***      *   
       7289 GAATCCTTCTGTCTCGTTTTTATGGGAAGATATTTACTTTTTCACCGTAGGCATCAAAGCGCTCC
        129 GAATGCTTCTGTCTAGTTTTTATATGAAGATATTCCCGTTTCCAACGAAATCGCTAAAGCGATCC

                *     *       **            *       *         *   * *   *    
       7354 AAATGTCCACATCCAGATAGTAC----AGAGTTTTTCAAACCTGCTCTATGAAAGGGAATCTTCA
        194 AAATATCCACTTCCAGATTCTACAAAAAGAGTGTTTCAAAACTGCTCTATCAAAAGAAATGTTCA

                 * *      * *  *      *      *              *       **       
       7415 ACTCTATGAGTTGAATGCAGACATCAGAAAGAAATTTCTGAGAATGCTGCTGTCTACCTTTTAT-
        259 ACTCTGTTAGTTGAGTACACACATCACAAAGAAGTTTCTGAGAATGCTTCTGTCTAGTTTTTATA

               *         **             *  *    *                *       *   
       7479 T--TGA-ATTCCCGCTTCCAACGAAATCCTCCAAGCTATCCAAATATCCACTTGCAGATTCCACA
        324 TGAAGATATTCCCTTTTCCAACGAAATCATCAAAGCGATCCAAATATCCACTTCCAGATTCTACA

                                        *  ** *  *                    *     *
       7541 AAAAGAGTGTTTCAAAACTGCTCTCTATCAATGGCAAAGTTCAACTCTGTTAGTTGAGGACACAT
        389 AAAAGAGTGTTTCAAAACTG--CTCTATGAAAAGAAATGTTCAACTCTGTTAGTTGAGTACACAC

                 *  *                         *                  *      *  * 
       7606 ATCACCAACAAGTTTCTGAGAATGCTTCTGTCTATTTTTTATGGGAAGATATTTCCTTTTTCACC
        452 ATCACAAAGAAGTTTCTGAGAATGCTTCTGTCTAGTTTTTATGGGAAGATATTCCCTTTTCCAAC

             * *  *    *      *    *          * **                     *     
       7671 GTAGGCGTCAAGGCGATCGAAATGTCCACTTCCACAAACTACAAAAAGAGTGTTTCAAACCTGCT
        517 GAAAGCATCAAAGCGATCCAAATATCCACTTCCAGATTCTACAAAAAGAGTGTTTCAAAACTGCT

                *     **       *    *        * ** * *   **      *      *     
       7736 CTATGAAAGGCCATGTTCATCTCTATGAGTTGAATGGAAATATCCGAAAGAAATTTCTGGGAATG
        582 CTATCAAAGGAAATGTTCAACTCTGTGAGTTGAGTACACACATCACAAAGAAGTTTCTGAGAATG

              *         *       *         **             *       *           
       7801 CTGCTGTCTAGTGTTTAT-ACGA-ATTCCCGCTTCCAACGAAATCCTCAAAGCAATCCAAATATC
        647 CTTCTGTCTAGTTTTTATGATGATATTCCCTTTTCCAACGAAATCATCAAAGCGATCCAAATATC

                 *    *  *                                 **    *          *
       7864 CACTTGCAGAATCCACAAAAAGAGTGTTTCAAAACTG-CTCTATCAATAGAAAGGTTCAACTCTT
        712 CACTTCCAGATTCTACAAAAAGAGTGTTTCAAAACTGCCTCTATCAAAGGAAATGTTCAACTCTG

                                 *  *                        * *      *      
       7928 TTAGTTGAGTACACACATCACGAACAAGTTTCTGAGAATGCTTCTGTCTGGCTTTTATTGGAAGA
        777 TTAGTTGAGTACACACATCACAAAGAAGTTTCTGAGAATGCTTCTGTCTAGTTTTTATGGGAAGA

            **  *         * *  **        * *       *                  *      
       7993 CGTTTCCTTTT-CACCAAAGGCATCAAAGTGCTCCAAATGTCCACTTCCAGATTCTACCAAAAGA
        842 TATTCCCTTTTCCAACGAAATCATCAAAGCGATCCAAATATCCACTTCCAGATTCTACAAAAAGA

                      **         **    *                 *           *     * 
       8057 GTGTTTCAAACGTGCTC-ATAGTAAGGGAATGTTCAACTCTGTGACTTGAATGCAGATATCACCA
        907 GTGTTTCAAAACTGCTCTAT-CAAAGGAAATGTTCAACTCTGTGAGTTGAATGCAGACATCACAA

              *       * * *                   *    *         *              *
       8121 AGTAGTTTCTAATAGTGCTTCTGTCTAGATTTTAGATGATGATATTCCCGTTTCCAACGAAATCG
        971 AGAAGTTTCTGAGAATGCTTCTGTCTAG-TTTT-TATGAAGATATTCCCTTTTCCAACGAAATCA

            ** *   *             *  *   *      *     *       *   *    **     
       8186 CTAGAGCTATCCAAATATCCAGTTACAGTTTCTACCAAAAGGGTGTTTCCAAATTGCTGCATCAA
       1034 TCAAAGCGATCCAAATATCCACTTCCAGATTCTACAAAAAGAGTGTTTCAAAACTGCTCTATCAA

                  *                    *      *              *               
       8251 AAGAAAGGTTCAACTCTGTTAGTTGAGGACACACGTCACAAAGAAGTTTGTGAGAATGCTTCTGT
       1099 AAGAAATGTTCAACTCTGTTAGTTGAGTACACACATCACAAAGAAGTTTCTGAGAATGCTTCTGT

                *    * **  *         *   *
       8316 CTAGATTTTGTATGACGATATTCCCTTTTC
       1164 CTAGTTTTTATGGGAAGATATTCCCGTTTT

                  *   * *     *   *       * *  *    *  *       *  *        * 
       8346 CAACGATATCGTTAAAGCAATCTAAATATCAATTTGCAGAATCCACAAAAATAGAGTTTCAAAGC
          1 CAACGAAATCATCAAAGCGATCCAAATATCCACTTCCAGATTCTACAAAAAGAGTGTTTCAAACC

                   **   * *  *    *       *  *                   ***         
       8411 TGCTCTGTAAAAAGAAAGGTTCCACTCTGTTAGCTGAGTACACACATCACAAACTTGTTTCTGAG
         66 TGCTCT-AGAAAGGGAATGTTCAACTCTGTGAGTTGAGTACACACATCACAAAGAAGTTTCTGAG

               *         *        **         ** *      *  * ** ***      *    
       8476 AATCCTTCTGTCTCGTTTTTATGGGAAGATATTTACTTTTCCACCGTAGGCATCAAAGCGCTCCA
        130 AATGCTTCTGTCTAGTTTTTATATGAAGATATTCCCGTTTCCAACGAAATCGCTAAAGCGATCCA

               *     *       *  *  *  *            *         *   * *   *     
       8541 AATGTCCACATCCAGATACTCCAGAACGAGTGTTTCAAACCTGCTCTATGAAAGGGAATCTTCAA
        195 AATATCCACTTCCAGATTCTACAAAAAGAGTGTTTCAAAACTGCTCTATCAAAAGAAATGTTCAA

                * *      * *  *      *      *              *       **        
       8606 CTCTATGAGTTGAATGCAGACATCAGAAAGAAATTTCTGAGAATGCTGCTGTCTACCTTTTAT-T
        260 CTCTGTTAGTTGAGTACACACATCACAAAGAAGTTTCTGAGAATGCTTCTGTCTAGTTTTTATAT

              *         **             *  *    *              * *  **   *    
       8670 --TGA-ATTCCCGCTTCCAACGAAATCCTCCAAGCTATCCAAATATCCACCTGCATTTTCCACAA
        325 GAAGATATTCCCTTTTCCAACGAAATCATCAAAGCGATCCAAATATCCACTTCCAGATTCTACAA

            *                        *  *               **  * *  *           
       8732 CAAGAGTGTTTCAAAACTGCTCTATCAATAGAAATGTTCAACTCCTTTGGCTGGGTACACACATC
        390 AAAGAGTGTTTCAAAACTGCTCTATGAAAAGAAATGTTCAACTCTGTTAGTTGAGTACACACATC

                 *                                        **         *  * *  
       8797 ACAAACAAGTTTCTGAGAATGCTTCTGTCTAGTTTTTATGGGAAGACGTTCCCTTTTTCACCAAA
        455 ACAAAGAAGTTTCTGAGAATGCTTCTGTCTAGTTTTTATGGGAAGATATTCCCTTTTCCAACGAA

            *           *       *            **                  *  **   *   
       8862 GGCATCAAAGCGCTCCAAATGTCCACTTCCAGACACTACAAAAAGAGTGTTTCCAACGTGCCCTA
        520 AGCATCAAAGCGATCCAAATATCCACTTCCAGATTCTACAAAAAGAGTGTTTCAAAACTGCTCTA

            **    **                 *    * *  * *         *           **    
       8927 AGAAAGCGAATGTTCAACTCTGTGACTTGAATGCAGATATCACAAAGTAGTTTCTGAGAGGGCTT
        585 TCAAAGGAAATGTTCAACTCTGTGAGTTGAGTACACACATCACAAAGAAGTTTCTGAGAATGCTT

                          *              *                * *   *            
       8992 CTGTCTAGATTTTAGATGATGATATTCCCGTTTCCAACGAAATCATTAGAGCTATCCAAATATCC
        650 CTGTCTAG-TTTT-TATGATGATATTCCCTTTTCCAACGAAATCATCAAAGCGATCCAAATATCC

                *   *                    *         **      *  * *    *       
       9057 ACTTACAGTTTCTACAAAAAGAGTGTTTCCAAACTG-CTGCATCAAAAGAGAGGTTCCACTCTGT
        713 ACTTCCAGATTCTACAAAAAGAGTGTTTCAAAACTGCCTCTATCAAAGGAAATGTTCAACTCTGT

               *                   ***      *     *         *                
       9121 TAGCTGAGTACACACATCACAAACTTGTTTCTCAGAATCCTTCTGTCTCGTTTTTATGGGAAGAT
        778 TAGTTGAGTACACACATCACAAAGAAGTTTCTGAGAATGCTTCTGTCTAGTTTTTATGGGAAGAT

               **     *  *  * **          *       *     *       *  *  *      
       9186 ATTTACTTTTTCACCGTAGGCATCAAAGCGCTCCAAATGTCCACATCCAGATACTCCAGAAAGAG
        843 ATTCCCTTTTCCAACGAAATCATCAAAGCGATCCAAATATCCACTTCCAGATTCTACAAAAAGAG

                     *         *     *   *         *                    *    
       9251 TGTTTCAAACCTGCTCTATGAAAGGGAATCTTCAACTCTATGAGTTGAATGCAGACATCAGAAAG
        908 TGTTTCAAAACTGCTCTATCAAAGGAAATGTTCAACTCTGTGAGTTGAATGCAGACATCACAAAG

              *              *       **       **         **             *  * 
       9316 AAATTTCTGAGAATGCTGCTGTCTACCTTTTAT-TTGA-ATTCCCGCTTCCAACGAAATCCTCCA
        973 AAGTTTCTGAGAATGCTTCTGTCTAGTTTTTATGAAGATATTCCCTTTTCCAACGAAATCATCAA

               *                *       *                                  **
       9379 AGCTATCCAAATATCCACTTGCAGATTCCACAAAAAGAGTGTTTCAAAACTGCTCTCTATCAATG
       1038 AGCGATCCAAATATCCACTTCCAGATTCTACAAAAAGAGTGTTTCAAAACTG--CTCTATCAAAA

             *  *                    *     *     *  *                        
       9444 GCAAAGTTCAACTCTGTTAGTTGAGGACACATATCACCAACAAGTTTCTGAGAATGCTTCTGTCT
       1101 GAAATGTTCAACTCTGTTAGTTGAGTACACACATCACAAAGAAGTTTCTGAGAATGCTTCTGTCT

             *                  *  *    
       9509 ATTTTTTATGGGAAGATATTTCCTTTTT
       1166 AGTTTTTATGGGAAGATATTCCCGTTTT

              *  * ** *    *      *    *          * **          *            
       9537 CACCGTAGGCGTCAAGGCGATCGAAATGTCCACTTCCACAAACTACAAAAAGTGTGTTTCAAACC
          1 CAACGAAATCATCAAAGCGATCCAAATATCCACTTCCAGATTCTACAAAAAGAGTGTTTCAAACC

                          **       *    *        * ** * *   **      *      * 
       9602 TGCTCTATGAAAGGCCATGTTCATCTCTATGAGTTGAATGGAAATATCCGAAAGAAATTTCTGGG
         66 TGCTCTA-GAAAGGGAATGTTCAACTCTGTGAGTTGAGTACACACATCACAAAGAAGTTTCTGAG

                  *          *        *          *                      *    
       9667 AATGCTGCTGTCTAG-TGTT-TAT-ACGA-ATTCCCGCTTCCAACGAAATC-CTCAAAGCAATCC
        130 AATGCTTCTGTCTAGTTTTTATATGAAGATATTCCCGTTTCCAACGAAATCGCT-AAAGCGATCC

                        *    *  *                                *     *     
       9727 AAATATCCACTTGCAGAATCCACAAAAAGAGTGTTTCAAAACTGCTCTATCAATAGAAAGGTTCA
        194 AAATATCCACTTCCAGATTCTACAAAAAGAGTGTTTCAAAACTGCTCTATCAAAAGAAATGTTCA

                 *                     *  *                        * *      *
       9792 ACTCTTTTAGTTGAGTACACACATCACGAACAAGTTTCTGAGAATGCTTCTGTCTGGCTTTTATT
        259 ACTCTGTTAGTTGAGTACACACATCACAAAGAAGTTTCTGAGAATGCTTCTGTCTAGTTTTTATA

            *     **  *         * *  **          *       *                * *
       9857 GGAAGACGTTTCCTTTT-CACCAAAGGCATCAAAGCGCTCCAAATGTCCACTTCCAGATTCTTCC
        324 TGAAGATATTCCCTTTTCCAACGAAATCATCAAAGCGATCCAAATATCCACTTCCAGATTCTACA

                            **     * * *  * *               * *    * *  * *  
       9921 AAAAGAGTGTTTCAAACGTGCTCAAAGTAAGGGAATGTTCAACTCTGTGACTTGAATGCAGATAT
        389 AAAAGAGTGTTTCAAAACTGCTCTATGAAAAGAAATGTTCAACTCTGTTAGTTGAGTACACACAT

               *   *       * * *             *   * ***  *         *          
       9986 CACCAAGTAGTTTCTAATAGTGCTTCTGTCTAGATTTCAGATGATGATATTCCCGTTTCCAACGA
        454 CACAAAGAAGTTTCTGAGAATGCTTCTGTCTAGTTTTTATGGGAAGATATTCCCTTTTCCAACGA

              * * * *   * **          *  *   *      *     *       *   *    **
      10051 AATCGTTAGAGCTAAGCAAATATCCAGTTACAGTTTCTACCAAAAGGGTGTTTCCAAATTGCTGC
        519 AAGCATCAAAGCGATCCAAATATCCACTTCCAGATTCTACAAAAAGAGTGTTTCAAAACTGCTCT

                  *    *            *       *                     *          
      10116 ATCAAAAGAAAGGTTCAACTCTGTTAGTTGAGGACACACATCACAAAGAAGTTTGTGAGAATGCT
        584 ATCAAAGGAAATGTTCAACTCTGTGAGTTGAGTACACACATCACAAAGAAGTTTCTGAGAATGCT

                                *                    *   * *     *   *       
      10181 TCTGTCTAGATTTTGTATGACGATATTCCCTTTTCCAACGATATCGTTAAAGCAATCTAAATATC
        649 TCTGTCTAG-TTTT-TATGATGATATTCCCTTTTCCAACGAAATCATCAAAGCGATCCAAATATC

            * *  *    *  *       *  *        *        * *   *    *    *      
      10246 AATTTGCAGAATCCACAAAAATAGAGTTTCAAAGCTG-CTCTGTAAAAAGAAAGGTTCCACTCTG
        712 CACTTCCAGATTCTACAAAAAGAGTGTTTCAAAACTGCCTCTATCAAAGGAAATGTTCAACTCTG

                *                   ***            *         *               
      10310 TTAGCTGAGTACACACATCACAAACTTGTTTCTGAGAATCCTTCTGTCTCGTTTTTATGGGAAGA
        777 TTAGTTGAGTACACACATCACAAAGAAGTTTCTGAGAATGCTTCTGTCTAGTTTTTATGGGAAGA

                **        *  * **          *       *     *       *  *  *  *  
      10375 TATTTACTTTTCCACCGTAGGCATCAAAGCGCTCCAAATGTCCACATCCAGATACTCCAGAACGA
        842 TATTCCCTTTTCCAACGAAATCATCAAAGCGATCCAAATATCCACTTCCAGATTCTACAAAAAGA

                      *         *     *   *         *                    *   
      10440 GTGTTTCAAACCTGCTCTATGAAAGGGAATCTTCAACTCTATGAGTTGAATGCAGACATCAGAAA
        907 GTGTTTCAAAACTGCTCTATCAAAGGAAATGTTCAACTCTGTGAGTTGAATGCAGACATCACAAA

               *              *       **       **         **             *  *
      10505 GAAATTTCTGAGAATGCTGCTGTCTACCTTTTAT-TTGA-ATTCCCGCTTCCAACGAAATCCTCC
        972 GAAGTTTCTGAGAATGCTTCTGTCTAGTTTTTATGAAGATATTCCCTTTTCCAACGAAATCATCA

                *              * *  **   *    *                           *  
      10568 AAGCTATCCAAATATCCACCTGCATTTTCCACAACAAGAGTGTTTCAAAACTGCTCTATCAATAG
       1037 AAGCGATCCAAATATCCACTTCCAGATTCTACAAAAAGAGTGTTTCAAAACTGCTCTATCAAAAG

                         **  * *  *                *                         
      10633 AAATGTTCAACTCCTTTGGCTGGGTACACACATCACAAACAAGTTTCTGAGAATGCTTCTGTCTA
       1102 AAATGTTCAACTCTGTTAGTTGAGTACACACATCACAAAGAAGTTTCTGAGAATGCTTCTGTCTA

                           **     *    
      10698 GTTTTTATGGGAAGACGTTCCCTTTTT
       1167 GTTTTTATGGGAAGATATTCCCGTTTT

              * *  **          *       *            **                  *   *
      10725 CACCAAAGGCATCAAAGCGCTCCAAATGTCCACTTCCAGACACTACAAAAAGAGTGTTTCCAACG
          1 CAACGAAATCATCAAAGCGATCCAAATATCCACTTCCAGATTCTACAAAAAGAGTGTTTCAAACC

               *         *                  *    * *  * *         *          
      10790 TGCCCTAAGAAAGCGAATGTTCAACTCTGTGACTTGAATGCAGATATCACAAAGTAGTTTCTGAG
         66 TGCTCT-AGAAAGGGAATGTTCAACTCTGTGAGTTGAGTACACACATCACAAAGAAGTTTCTGAG

             **            *     *    *                        **  *   *     
      10855 AGGGCTTCTGTCTAGATTTTAGATGATGATATTCCCGTTTCCAACGAAATCATTAGAGCTATCCA
        130 AATGCTTCTGTCTAGTTTTTATATGAAGATATTCCCGTTTCCAACGAAATCGCTAAAGCGATCCA

                       *   *                    *        **         * *    * 
      10920 AATATCCACTTACAGTTTCTACAAAAAGAGTGTTTCCAAACTGCTGCATCAAAAGAGAGGTTCCA
        195 AATATCCACTTCCAGATTCTACAAAAAGAGTGTTTCAAAACTGCTCTATCAAAAGAAATGTTCAA

                     *                   ***      *     *         *        **
      10985 CTCTGTTAGCTGAGTACACACATCACAAACTTGTTTCTCAGAATCCTTCTGTCTCGTTTTTATGG
        260 CTCTGTTAGTTGAGTACACACATCACAAAGAAGTTTCTGAGAATGCTTCTGTCTAGTTTTTATAT

                     **     *  *  * **          *       *     *       *  *  *
      11050 GAAGATATTTACTTTTTCACCGTAGGCATCAAAGCGCTCCAAATGTCCACATCCAGATACTCCAG
        325 GAAGATATTCCCTTTTCCAACGAAATCATCAAAGCGATCCAAATATCCACTTCCAGATTCTACAA

                           *             * *   *         * *      * *  *     
      11115 AAAGAGTGTTTCAAACCTGCTCTATGAAAGGGAATCTTCAACTCTATGAGTTGAATGCAGACATC
        390 AAAGAGTGTTTCAAAACTGCTCTATGAAAAGAAATGTTCAACTCTGTTAGTTGAGTACACACATC

             *      *              *       **         **         **          
      11180 AGAAAGAAATTTCTGAGAATGCTGCTGTCTACCTTTTAT---TTGA-ATTCCCGCTTCCAACGAA
        455 ACAAAGAAGTTTCTGAGAATGCTTCTGTCTAGTTTTTATGGGAAGATATTCCCTTTTCCAACGAA

             * *  *    *                *       *                            
      11241 ATCCTCCAAGCTATCCAAATATCCACTTGCAGATTCCACAAAAAGAGTGTTTCAAAACTGCTCTC
        520 AGCATCAAAGCGATCCAAATATCCACTTCCAGATTCTACAAAAAGAGTGTTTCAAAACTG--CTC

                  *  *  *            *       *     *     *  *                
      11306 TATCAATGGCAAAGTTCAACTCTGTTAGTTGAGGACACATATCACCAACAAGTTTCTGAGAATGC
        583 TATCAAAGGAAATGTTCAACTCTGTGAGTTGAGTACACACATCACAAAGAAGTTTCTGAGAATGC

                     *           *      *      *  *  * ** *    *      *    * 
      11371 TTCTGTCTATTTTTTATGGGAAGATATTTCCTTTTTCACCGTAGGCGTCAAGGCGATCGAAATGT
        648 TTCTGTCTAGTTTTTAT--GATGATATTCCCTTTTCCAACGAAATCATCAAAGCGATCCAAATAT

                     * **                     *          *     **       *    
      11436 CCACTTCCACAAACTACAAAAAGAGTGTTTCAAACCTG-CTCTATGAAAGGCCATGTTCATCTCT
        711 CCACTTCCAGATTCTACAAAAAGAGTGTTTCAAAACTGCCTCTATCAAAGGAAATGTTCAACTCT

            * *      * ** * *   **      *      *       *                   * 
      11500 ATGAGTTGAATGGAAATATCCGAAAGAAATTTCTGGGAATGCTGCTGTCTAGTTTTTAT---ATG
        776 GTTAGTTGAGTACACACATCACAAAGAAGTTTCTGAGAATGCTTCTGTCTAGTTTTTATGGGAAG

                    **             *       *                *    *  *        
      11562 A-ATTCCCGCTTCCAACGAAATCCTCAAAGCAATCCAAATATCCACTTGCAGAATCCACAAAAAG
        841 ATATTCCCTTTTCCAACGAAATCATCAAAGCGATCCAAATATCCACTTCCAGATTCTACAAAAAG

                                    **    *          * *      * *  *         
      11626 AGTGTTTCAAAACTGCTCTATCAATAGAAAGGTTCAACTCTTTTAGTTGAGTACACACATCACAA
        906 AGTGTTTCAAAACTGCTCTATCAAAGGAAATGTTCAACTCTGTGAGTTGAATGCAGACATCACAA

             *                        * *             **  *         * *  **  
      11691 ACAAGTTTCTGAGAATGCTTCTGTCTGGCTTTTATTGGAAGACGTTTCCTTTT-CACCAAAGGCA
        971 AGAAGTTTCTGAGAATGCTTCTGTCTAGTTTTTA-T-GAAGATATTCCCTTTTCCAACGAAATCA

                    *       *                * *                **  *  * *** 
      11755 TCAAAGCGCTCCAAATGTCCACTTCCAGATTCTTCCAAAAGAGTGTTTCAAACGTGGTCGAAGTA
       1034 TCAAAGCGATCCAAATATCCACTTCCAGATTCTACAAAAAGAGTGTTTCAAAACTGCTCTATCAA

             * *               * *    * *  * *     *   *       * * *         
      11820 AGGGAATGTTCAACTCTGTGACTTGAATGCAGATATCACCAAGTAGTTTCTAATAGTGCTTCTGT
       1099 AAGAAATGTTCAACTCTGTTAGTTGAGTACACACATCACAAAGAAGTTTCTGAGAATGCTTCTGT

               **     ***  *             *
      11885 CTACATTTTAGATGATGATATTCCCGTTTC
       1164 CTAGTTTTTATGGGAAGATATTCCCGTTTT

                      * * *   * **          *  *   *      *     *       *  **
      11915 CAACGAAATCGTTAGAGCTAAGCAAATATCCAGTTACAGTTTCTACCAAAAGGGTGTTTCCAAAT
          1 CAACGAAATCATCAAAGCGATCCAAATATCCACTTCCAGATTCTACAAAAAGAGTGTTTCAAACC

                  ***   * *  *            *       *                     *    
      11980 TGCTGCATCAAAAGAAAGGTTCAACTCTGTTAGTTGAGGACACACATCACAAAGAAGTTTGTGAG
         66 TGCT-CTAGAAAGGGAATGTTCAACTCTGTGAGTTGAGTACACACATCACAAAGAAGTTTCTGAG

                           *    *     *         *          *    *      *   * 
      12045 AATGCTTCTGTCTAGATTTTGTATGACGATATTCCCTTTTCCAACGATATCGTTAAAGCAATCTA
        130 AATGCTTCTGTCTAGTTTTTATATGAAGATATTCCCGTTTCCAACGAAATCGCTAAAGCGATCCA

                  * *  *    *  *       *  *        *       * *        *    * 
      12110 AATATCAATTTGCAGAATCCACAAAAATAGAGTTTCAAAGCTGCTCTGTAAAAAGAAAGGTTCCA
        195 AATATCCACTTCCAGATTCTACAAAAAGAGTGTTTCAAAACTGCTCTATCAAAAGAAATGTTCAA

                     *                   ***            *         *        **
      12175 CTCTGTTAGCTGAGTACACACATCACAAACTTGTTTCTGAGAATCCTTCTGTCTCGTTTTTATGG
        260 CTCTGTTAGTTGAGTACACACATCACAAAGAAGTTTCTGAGAATGCTTCTGTCTAGTTTTTATAT

                     **        *  * **          *       *     *       *  *  *
      12240 GAAGATATTTACTTTTCCACCGTAGGCATCAAAGCGCTCCAAATGTCCACATCCAGATACTCCAG
        325 GAAGATATTCCCTTTTCCAACGAAATCATCAAAGCGATCCAAATATCCACTTCCAGATTCTACAA

                           *             * *   *         * *      * *  *     
      12305 AAAGAGTGTTTCAAACCTGCTCTATGAAAGGGAATCTTCAACTCTATGAGTTGAATGCAGACATC
        390 AAAGAGTGTTTCAAAACTGCTCTATGAAAAGAAATGTTCAACTCTGTTAGTTGAGTACACACATC

             *      *              *       **         **         **          
      12370 AGAAAGAAATTTCTGAGAATGCTGCTGTCTACCTTTTAT---TTGA-ATTCCCGCTTCCAACGAA
        455 ACAAAGAAGTTTCTGAGAATGCTTCTGTCTAGTTTTTATGGGAAGATATTCCCTTTTCCAACGAA

             * *  *    *              * *  **   *                            
      12431 ATCCTCCAAGCTATCCAAATATCCACCTGCATTTTCCACAAAAAGAGTGTTTCAAAACTGCTCTA
        520 AGCATCAAAGCGATCCAAATATCCACTTCCAGATTCTACAAAAAGAGTGTTTCAAAACTGCTCTA

                **                *    *  *                *                 
      12496 TCAATAGAAATGTTCAACTCCTTTG-GCTGGGTACACACATCACAAACAAGTTTCTGAGAATGCT
        585 TCAAAGGAAATGTTCAACT-CTGTGAGTTGAGTACACACATCACAAAGAAGTTTCTGAGAATGCT

                                *  **         *  * *  **          *       *  
      12560 TCTGTCTAGTTTTTATGGGAAGACGTTCCCTTTTTCACCAAAGGCATCAAAGCGCTCCAAATGTC
        649 TCTGTCTAGTTTTTAT--GATGATATTCCCTTTTCCAACGAAATCATCAAAGCGATCCAAATATC

                      **                     **        **    **              
      12625 CACTTCCAGACACTACAAAAAGAGTGTTTCAAACGTG-CTCTAAGAAAGCGAATGTTCAACTCTG
        712 CACTTCCAGATTCTACAAAAAGAGTGTTTCAAAACTGCCTCTATCAAAGGAAATGTTCAACTCTG

             * *    * *  * *         *           **            *     ***  *  
      12689 TGACTTGAATGCAGATATCACAAAGTAGTTTCTGAGAGGGCTTCTGTCTAGATTTTAGATGATGA
        777 TTAGTTGAGTACACACATCACAAAGAAGTTTCTGAGAATGCTTCTGTCTAGTTTTTATGGGAAGA

                   *                * *   *                *   *             
      12754 TATTCCCGTTTCCAACGAAATCATTAGAGCTATCCAAATATCCACTTACAGTTTCTACAAAAAGA
        842 TATTCCCTTTTCCAACGAAATCATCAAAGCGATCCAAATATCCACTTCCAGATTCTACAAAAAGA

                   *        **      *  * *    *       *  *   * *  *          
      12819 GTGTTTCCAAACTGCTGCATCAAAAGAGAGGTTCCACTCTGTTAGCTGAGTACACACATCACAAA
        907 GTGTTTCAAAACTGCTCTATCAAAGGAAATGTTCAACTCTGTGAGTTGAATGCAGACATCACAAA

            ***      *     *         *                   **     *  *  * **   
      12884 CTTGTTTCTCAGAATCCTTCTGTCTCGTTTTTATGGGAAGATATTTACTTTTTCACCGTAGGCAT
        972 GAAGTTTCTGAGAATGCTTCTGTCTAGTTTTTAT--GAAGATATTCCCTTTTCCAACGAAATCAT

                   *       *     *       **    *       *       *         *   
      12949 CAAAGCGCTCCAAATGTCCACATCCAGATAGTACAGAAAGAGTTTTTCAAACCTGCTCTATGAAA
       1035 CAAAGCGATCCAAATATCCACTTCCAGATTCTACAAAAAGAGTGTTTCAAAACTGCTCTATCAAA

            * *   *         * *      * *  *      *      *              *     
      13014 GGGAATCTTCAACTCTATGAGTTGAATGCAGACATCAGAAAGAAATTTCTGAGAATGCTGCTGTC
       1100 AGAAATGTTCAACTCTGTTAGTTGAGTACACACATCACAAAGAAGTTTCTGAGAATGCTTCTGTC

              **         **          *  *
      13079 TACCTTTTAT---TTGA-ATTCCCGCTTC
       1165 TAGTTTTTATGGGAAGATATTCCCGTTTT

                      *  *    *                *       *                   * 
      13104 CAACGAAATCCTCCAAGCTATCCAAATATCCACTTGCAGATTCCACAAAAAGAGTGTTTCAAAAC
          1 CAACGAAATCATCAAAGCGATCCAAATATCCACTTCCAGATTCTACAAAAAGAGTGTTTCAAACC

                      *  *  *  *            *       *     *     *  *         
      13169 TGCTCTCTATCAATGGCAAAGTTCAACTCTGTTAGTTGAGGACACATATCACCAACAAGTTTCTG
         66 TG--CTCTA-GAAAGGGAATGTTCAACTCTGTGAGTTGAGTACACACATCACAAAGAAGTTTCTG

                            *       **         *  *   *  *  * **  **  *      
      13234 AGAATGCTTCTGTCTATTTTTTATGGGAAGATATTTCCTTTTTCACCGTAGGCGTCAAGGCGATC
        128 AGAATGCTTCTGTCTAGTTTTTATATGAAGATATTCCCGTTTCCAACGAAATCGCTAAAGCGATC

            *    *          * **                     *         *   * **      
      13299 GAAATGTCCACTTCCACAAACTACAAAAAGAGTGTTTCAAACCTGCTCTATGAAAGGCCATGTTC
        193 CAAATATCCACTTCCAGATTCTACAAAAAGAGTGTTTCAAAACTGCTCTATCAAAAGAAATGTTC

             *    * *      * ** * *   **      *      *       *          *    
      13364 ATCTCTATGAGTTGAATGGAAATATCCGAAAGAAATTTCTGGGAATGCTGCTGTCTAG-TGTT-T
        258 AACTCTGTTAGTTGAGTACACACATCACAAAGAAGTTTCTGAGAATGCTTCTGTCTAGTTTTTAT

                *         **             *       *                *    *  *  
      13427 AT-ACGA-ATTCCCGCTTCCAACGAAATCCTCAAAGCAATCCAAATATCCACTTGCAGAATCCAC
        323 ATGAAGATATTCCCTTTTCCAACGAAATCATCAAAGCGATCCAAATATCCACTTCCAGATTCTAC

                                       *  *     *          *                 
      13490 AAAAAGAGTGTTTCAAAACTGCTCTATCAATAGAAAGGTTCAACTCTTTTAGTTGAGTACACACA
        388 AAAAAGAGTGTTTCAAAACTGCTCTATGAAAAGAAATGTTCAACTCTGTTAGTTGAGTACACACA

                *  *                        * *      *      **  *           *
      13555 TCACGAACAAGTTTCTGAGAATGCTTCTGTCTGGCTTTTATTGGAAGACGTTTCCTTTT-CAACA
        453 TCACAAAGAAGTTTCTGAGAATGCTTCTGTCTAGTTTTTATGGGAAGATATTCCCTTTTCCAACG

              *           *       *                * *                **     
      13619 AAGGCATCAAAGCGCTCCAAATGTCCACTTCCAGATTCTTCCAAAAGAGTGTTTCAAACGTGCTC
        518 AAAGCATCAAAGCGATCCAAATATCCACTTCCAGATTCTACAAAAAGAGTGTTTCAAAACTGCTC

            * ***    *                 *    * *  * *     *   *       * * *   
      13684 GAAGTAAGGGAATGTTCAACTCTGTGACTTGAATGCAGATATCACCAAGTAGTTTCTAATAGTGC
        583 TATCAAAGGAAATGTTCAACTCTGTGAGTTGAGTACACACATCACAAAGAAGTTTCTGAGAATGC

                      *     *              *              * * *   *          
      13749 TTCTGTCTACATTTTAGATGATGATATTCCCGTTTCCAACGAAATCGTTAGAGCTATCCAAATAT
        648 TTCTGTCTA-GTTTT-TATGATGATATTCCCTTTTCCAACGAAATCATCAAAGCGATCCAAATAT

               *  *   *      *     *       *   *     **      *    *          
      13814 CCAGTTACAGTTTCTACCAAAAGGGTGTTTCCAAATTG-CTGCATCAAAAGAAAGGTTCAACTCT
        711 CCACTTCCAGATTCTACAAAAAGAGTGTTTCAAAACTGCCTCTATCAAAGGAAATGTTCAACTCT

                      *                     *                   *    * **  * 
      13878 GTTAGTTGAGGACACACATCACAAAGAAGTTTGTGAGAATGCTTCTGTCTAGATTTTGTATGACG
        776 GTTAGTTGAGTACACACATCACAAAGAAGTTTCTGAGAATGCTTCTGTCTAGTTTTTATGGGAAG

                               *   * *     *   *       * *  *    *  *       *
      13943 ATATTCCCTTTTCCAACGATATCGTTAAAGCAATCTAAATATCAATTTGCAGAATCCACAAAAAT
        841 ATATTCCCTTTTCCAACGAAATCATCAAAGCGATCCAAATATCCACTTCCAGATTCTACAAAAAG

              *        *       * *   *    *    *       *  *   * *  *         
      14008 AGAGTTTCAAAGCTGCTCTGTAAAAAGAAAGGTTCCACTCTGTTAGCTGAGTACACACATCACAA
        906 AGTGTTTCAAAACTGCTCTATCAAAGGAAATGTTCAACTCTGTGAGTTGAATGCAGACATCACAA

             ***            *         *                   **     *  *  * **  
      14073 ACTTGTTTCTGAGAATCCTTCTGTCTCGTTTTTATGGGAAGATATTTACTTTTTCACCGTAGGCA
        971 AGAAGTTTCTGAGAATGCTTCTGTCTAGTTTTTAT--GAAGATATTCCCTTTTCCAACGAAATCA

                    *       *     *       *  *  *  *            *         *  
      14138 TCAAAGCGCTCCAAATGTCCACATCCAGATACTCCAGAACGAGTGTTTCAAACCTGCTCTATGAA
       1034 TCAAAGCGATCCAAATATCCACTTCCAGATTCTACAAAAAGAGTGTTTCAAAACTGCTCTATCAA

             * *   *         * *      * *  *      *      *              *    
      14203 AGGGAATCTTCAACTCTATGAGTTGAATGCAGACATCAGAAAGAAATTTCTGAGAATGCTGCTGT
       1099 AAGAAATGTTCAACTCTGTTAGTTGAGTACACACATCACAAAGAAGTTTCTGAGAATGCTTCTGT

               **         **          *  *
      14268 CTACCTTTTAT---TTGA-ATTCCCGCTTC
       1164 CTAGTTTTTATGGGAAGATATTCCCGTTTT

                      *  *    *              * *  **   *    *              * 
      14294 CAACGAAATCCTCCAAGCTATCCAAATATCCACCTGCATTTTCCACAACAAGAGTGTTTCAAAAC
          1 CAACGAAATCATCAAAGCGATCCAAATATCCACTTCCAGATTCTACAAAAAGAGTGTTTCAAACC

                    *  ** *              *    *  *                *          
      14359 TGCTCTATCAATAGAAATGTTCAACTCCTTTG-GCTGGGTACACACATCACAAACAAGTTTCTGA
         66 TGCTCTA-GAAAGGGAATGTTCAACT-CTGTGAGTTGAGTACACACATCACAAAGAAGTTTCTGA

                                   **     **     *   *    *    *           * 
      14423 GAATGCTTCTGTCTAGTTTTTATGGGAAGACGTTCCCTTTTTC-ACCAAA-GGCATCAAAGCGCT
        129 GAATGCTTCTGTCTAGTTTTTATATGAAGATATTCCCGTTTCCAACGAAATCGC-T-AAAGCGAT

                  *            **                  *  **   *   **   ***      
      14486 CCAAATGTCCACTTCCAGACACTACAAAAAGAGTGTTTCCAACGTGCCCTAAGAAAGCGAATGTT
        192 CCAAATATCCACTTCCAGATTCTACAAAAAGAGTGTTTCAAAACTGCTCTATCAAAAGAAATGTT

                     * *    * *  * *         *           **            *     
      14551 CAACTCTGTGACTTGAATGCAGATATCACAAAGTAGTTTCTGAGAGGGCTTCTGTCTAGATTTTA
        257 CAACTCTGTTAGTTGAGTACACACATCACAAAGAAGTTTCTGAGAATGCTTCTGTCTAGTTTTTA

            *    *         *                * *   *                *   *     
      14616 GATGATGATATTCCCGTTTCCAACGAAATCATTAGAGCTATCCAAATATCCACTTACAGTTTCTA
        322 TATGAAGATATTCCCTTTTCCAACGAAATCATCAAAGCGATCCAAATATCCACTTCCAGATTCTA

                           *        **  *      * *    *          *           
      14681 CAAAAAGAGTGTTTCCAAACTGCTGCATCAAAAGAGAGGTTCCACTCTGTTAGCTGAGTACACAC
        387 CAAAAAGAGTGTTTCAAAACTGCTCTATGAAAAGAAATGTTCAACTCTGTTAGTTGAGTACACAC

                    ***      *     *         *                   **     *  * 
      14746 ATCACAAACTTGTTTCTCAGAATCCTTCTGTCTCGTTTTTATGGGAAGATATTTACTTTTTCACC
        452 ATCACAAAGAAGTTTCTGAGAATGCTTCTGTCTAGTTTTTATGGGAAGATATTCCCTTTTCCAAC

             * *           *       *     *       *  *  *               *     
      14811 GTAGGCATCAAAGCGCTCCAAATGTCCACATCCAGATACTCCAGAAAGAGTGTTTCAAACCTGCT
        517 GAAAGCATCAAAGCGATCCAAATATCCACTTCCAGATTCTACAAAAAGAGTGTTTCAAAACTGCT

                *     *   *         *        * *  *      *      *            
      14876 CTATGAAAGGGAATCTTCAACTCTATGAGTTGAATGCAGACATCAGAAAGAAATTTCTGAGAATG
        582 CTATCAAAGGAAATGTTCAACTCTGTGAGTTGAGTACACACATCACAAAGAAGTTTCTGAGAATG

              *       **       *          **             *  *    *           
      14941 CTGCTGTCTACCTTTTAT-TTGA-ATTCCCGCTTCCAACGAAATCCTCCAAGCTATCCAAATATC
        647 CTTCTGTCTAGTTTTTATGATGATATTCCCTTTTCCAACGAAATCATCAAAGCGATCCAAATATC

                 *       *                                  *  *  *          
      15004 CACTTGCAGATTCCACAAAAAGAGTGTTTCAAAACTGCTCTCTATCAATGGCAAAGTTCAACTCT
        712 CACTTCCAGATTCTACAAAAAGAGTGTTTCAAAACTGC-CTCTATCAAAGGAAATGTTCAACTCT

                      *     *     *  *                         *             
      15069 GTTAGTTGAGGACACATATCACCAACAAGTTTCTGAGAATGCTTCTGTCTATTTTTTATGGGAAG
        776 GTTAGTTGAGTACACACATCACAAAGAAGTTTCTGAGAATGCTTCTGTCTAGTTTTTATGGGAAG

                 *      *  *  * ** *    *      *    *          * **          
      15134 ATATTTCCTTTTTCACCGTAGGCGTCAAGGCGATCGAAATGTCCACTTCCACAAACTACAAAAAG
        841 ATATTCCCTTTTCCAACGAAATCATCAAAGCGATCCAAATATCCACTTCCAGATTCTACAAAAAG

                       *         *     **       *    *           * * *   **  
      15199 AGTGTTTCAAACCTGCTCTATGAAAGGCCATGTTCATCTCTATGAGTTGAATGGAAATATCCGAA
        906 AGTGTTTCAAAACTGCTCTATCAAAGGAAATGTTCAACTCTGTGAGTTGAATGCAGACATCACAA

                *      *       *                 *         **             *  
      15264 AGAAATTTCTGGGAATGCTGCTGTCTAGTTTTTAT-ATGA-ATTCCCGCTTCCAACGAAATCCTC
        971 AGAAGTTTCTGAGAATGCTTCTGTCTAGTTTTTATGAAGATATTCCCTTTTCCAACGAAATCATC

                 *                *    *  *                                * 
      15327 AAAGCAATCCAAATATCCACTTGCAGAATCCACAAAAAGAGTGTTTCAAAACTGCTCTATCAATA
       1036 AAAGCGATCCAAATATCCACTTCCAGATTCTACAAAAAGAGTGTTTCAAAACTGCTCTATCAAAA

                *          *                        *                        
      15392 GAAAGGTTCAACTCTTTTAGTTGAGTACACACATCACAAACAAGTTTCTGAGAATGCTTCTGTCT
       1101 GAAATGTTCAACTCTGTTAGTTGAGTACACACATCACAAAGAAGTTTCTGAGAATGCTTCTGTCT

            * *      *      **  *       
      15457 GGCTTTTATTGGAAGACGTTTCC-TTTT
       1166 AGTTTTTATGGGAAGATATTCCCGTTTT

              * *  **          *       *                * *                 *
      15484 CACCAAAGGCATCAAAGCGCTCCAAATGTCCACTTCCAGATTCTTCCAAAAGAGTGTTTCAAACG
          1 CAACGAAATCATCAAAGCGATCCAAATATCCACTTCCAGATTCTACAAAAAGAGTGTTTCAAACC

              *   *  *                      *    * *  * *     *   *       * *
      15549 TGGTCGAAGTAAGGGAATGTTCAACTCTGTGACTTGAATGCAGATATCACCAAGTAGTTTCTAAT
         66 TGCTC-TAGAAAGGGAATGTTCAACTCTGTGAGTTGAGTACACACATCACAAAGAAGTTTCTGAG

             *             *     *    *                         *  *   *     
      15614 AGTGCTTCTGTCTAGATTTTAGATGATGATATTCCCGTTTCCAACGAAATCGTTAGAGCTATCCA
        130 AATGCTTCTGTCTAGTTTTTATATGAAGATATTCCCGTTTCCAACGAAATCGCTAAAGCGATCCA

                    *  *   *      *     *       *   *    **           *      
      15679 AATATCCAGTTACAGTTTCTACCAAAAGGGTGTTTCCAAATTGCTGCATCAAAAGAAAGGTTCAA
        195 AATATCCACTTCCAGATTCTACAAAAAGAGTGTTTCAAAACTGCTCTATCAAAAGAAATGTTCAA

                          *                     *                   *    *   
      15744 CTCTGTTAGTTGAGGACACACATCACAAAGAAGTTTGTGAGAATGCTTCTGTCTAGATTTTGTAT
        260 CTCTGTTAGTTGAGTACACACATCACAAAGAAGTTTCTGAGAATGCTTCTGTCTAGTTTTTATAT

              *                    *   * *     *   *       * *  *    *  *    
      15809 GACGATATTCCCTTTTCCAACGATATCGTTAAAGCAATCTAAATATCAATTTGCAGAATCCACAA
        325 GAAGATATTCCCTTTTCCAACGAAATCATCAAAGCGATCCAAATATCCACTTCCAGATTCTACAA

               *  *        *       * *        *    *          *              
      15874 AAATAGAGTTTCAAAGCTGCTCTGTAAAAAGAAAGGTTCCACTCTGTTAGCTGAGTACACACATC
        390 AAAGAGTGTTTCAAAACTGCTCTATGAAAAGAAATGTTCAACTCTGTTAGTTGAGTACACACATC

                 ***            *         *                   **        *  * 
      15939 ACAAACTTGTTTCTGAGAATCCTTCTGTCTCGTTTTTATGGGAAGATATTTACTTTTCCACCGTA
        455 ACAAAGAAGTTTCTGAGAATGCTTCTGTCTAGTTTTTATGGGAAGATATTCCCTTTTCCAACGAA

            *         * *       *     *       *  *  *               *        
      16004 GGCATCAAAGTGCTCCAAATGTCCACATCCAGATACTCCAGAAAGAGTGTTTCAAACCTGCTCTA
        520 AGCATCAAAGCGATCCAAATATCCACTTCCAGATTCTACAAAAAGAGTGTTTCAAAACTGCTCTA

             *     *   *         *        * *  *      *      *              *
      16069 TGAAAGGGAATCTTCAACTCTATGAGTTGAATGCAGACATCAGAAAGAAATTTCTGAGAATGCTG
        585 TCAAAGGAAATGTTCAACTCTGTGAGTTGAGTACACACATCACAAAGAAGTTTCTGAGAATGCTT

                   **       *          **             *  *    *              
      16134 CTGTCTACCTTTTAT-TTGA-ATTCCCGCTTCCAACGAAATCCTCCAAGCTATCCAAATATCCAC
        650 CTGTCTAGTTTTTATGATGATATTCCCTTTTCCAACGAAATCATCAAAGCGATCCAAATATCCAC

            * *  **   *    *                            **              **  *
      16197 CTGCATTTTCCACAACAAGAGTGTTTCAAAACTG-CTCTATCAATAGAAATGTTCAACTCCTTTG
        715 TTCCAGATTCTACAAAAAGAGTGTTTCAAAACTGCCTCTATCAAAGGAAATGTTCAACTCTGTTA

             *  *                *                                        ** 
      16261 GCTGGGTACACACATCACAAACAAGTTTCTGAGAATGCTTCTGTCTAGTTTTTATGGGAAGACGT
        780 GTTGAGTACACACATCACAAAGAAGTTTCTGAGAATGCTTCTGTCTAGTTTTTATGGGAAGATAT

                    *  * *  **          *       *            **              
      16326 TCCCTTTTTCACCAAAGGCATCAAAGCGCTCCAAATGTCCACTTCCAGACACTACAAAAAGAGTG
        845 TCCCTTTTCCAACGAAATCATCAAAGCGATCCAAATATCCACTTCCAGATTCTACAAAAAGAGTG

                   **       **    **                 *           *         * 
      16391 TTTCAAACGTGCTCTAAGAAAGCGAATGTTCAACTCTGTGACTTGAATGCAGATATCACAAAGTA
        910 TTTCAAAACTGCTCTATCAAAGGAAATGTTCAACTCTGTGAGTTGAATGCAGACATCACAAAGAA

                      *                   * *  *         *                * *
      16456 GTTTCTGAGAGTGCTTCTGTCTAGATTTTAGACGATGATATTCCCGTTTCCAACGAAATCATTAG
        975 GTTTCTGAGAATGCTTCTGTCTAG-TTTT-TATGAAGATATTCCCTTTTCCAACGAAATCATCAA

               *                *   *                    *        **         
      16521 AGCTATCCAAATATCCACTTACAGTTTCTACAAAAAGAGTGTTTCCAAACTGCTGCATCAAAAGA
       1038 AGCGATCCAAATATCCACTTCCAGATTCTACAAAAAGAGTGTTTCAAAACTGCTCTATCAAAAGA

            * *    *          *                   ***      *     *         * 
      16586 GAGGTTCCACTCTGTTAGCTGAGTACACACATCACAAACTTGTTTCTCAGAATCCTTCTGTCTCG
       1103 AATGTTCAACTCTGTTAGTTGAGTACACACATCACAAAGAAGTTTCTGAGAATGCTTCTGTCTAG

                              ** *    
      16651 TTTTTATGGGAAGATATTTACTTTTT
       1168 TTTTTATGGGAAGATATTCCCGTTTT

              *  * **          *       *     *       **    *       *         
      16677 CACCGTAGGCATCAAAGCGCTCCAAATGTCCACATCCAGATAGTACAGAAAGAGTTTTTCAAACC
          1 CAACGAAATCATCAAAGCGATCCAAATATCCACTTCCAGATTCTACAAAAAGAGTGTTTCAAACC

                              *         *        * *  *      *      *        
      16742 TGCTCTATGAAAGGGAATCTTCAACTCTATGAGTTGAATGCAGACATCAGAAAGAAATTTCTGAG
         66 TGCTCTA-GAAAGGGAATGTTCAACTCTGTGAGTTGAGTACACACATCACAAAGAAGTTTCTGAG

                  *       **          *          *                 *    *    
      16807 AATGCTGCTGTCTACCTTTTAT-T--TGA-ATTCCCGCTTCCAACGAAATC-CTCCAAGCTATCC
        130 AATGCTTCTGTCTAGTTTTTATATGAAGATATTCCCGTTTCCAACGAAATCGCT-AAAGCGATCC

                        *       *                                  ** *  *   
      16867 AAATATCCACTTGCAGATTCCACAAAAAGAGTGTTTCAAAACTGCTCTCTATCAATGGCAAAGTT
        194 AAATATCCACTTCCAGATTCTACAAAAAGAGTGTTTCAAAACTG--CTCTATCAAAAGAAATGTT

                             *     *     *  *                         *      
      16932 CAACTCTGTTAGTTGAGGACACATATCACCAACAAGTTTCTGAGAATGCTTCTGTCTATTTTTTA
        257 CAACTCTGTTAGTTGAGTACACACATCACAAAGAAGTTTCTGAGAATGCTTCTGTCTAGTTTTTA

             **         *      *  *  * ** *    *      *    *          * **   
      16997 TGGGAAGATATTTCCTTTTTCACCGTAGGCGTCAAGGCGATCGAAATGTCCACTTCCACAAACTA
        322 TATGAAGATATTCCCTTTTCCAACGAAATCATCAAAGCGATCCAAATATCCACTTCCAGATTCTA

                              *             * **       *    * *      * ** * *
      17062 CAAAAAGAGTGTTTCAAACCTGCTCTATGAAAGGCCATGTTCATCTCTATGAGTTGAATGGAAAT
        387 CAAAAAGAGTGTTTCAAAACTGCTCTATGAAAAGAAATGTTCAACTCTGTTAGTTGAGTACACAC

               **      *      *       *         *         *         **       
      17127 ATCCGAAAGAAATTTCTGGGAATGCTGCTGTCTAGTGTTTAT---ACGA-ATTCCCGCTTCCAAC
        452 ATCACAAAGAAGTTTCTGAGAATGCTTCTGTCTAGTTTTTATGGGAAGATATTCCCTTTTCCAAC

                * *       *                *    *  *                         
      17188 GAAATCCTCAAAGCAATCCAAATATCCACTTGCAGAATCCACAAAAAGAGTGTTTCAAAACTGCT
        517 GAAAGCATCAAAGCGATCCAAATATCCACTTCCAGATTCTACAAAAAGAGTGTTTCAAAACTGCT

                   **    *          * *                   *  *               
      17253 CTATCAATAGAAAGGTTCAACTCTTTTAGTTGAGTACACACATCACGAACAAGTTTCTGAGAATG
        582 CTATCAAAGGAAATGTTCAACTCTGTGAGTTGAGTACACACATCACAAAGAAGTTTCTGAGAATG

                     * *          *  **  *           *  **          *       *
      17318 CTTCTGTCTGGCTTTTATTGGAAGACGTTTCCTTTT-CAACAAAGGCATCAAAGCGCTCCAAATG
        647 CTTCTGTCTAGTTTTTA-T-GATGATATTCCCTTTTCCAACGAAATCATCAAAGCGATCCAAATA

                            * *                **      * ***    *            
      17382 TCCACTTCCAGATTCTTCCAAAAGAGTGTTTCAAACGTG-CTCGAAGTAAGGGAATGTTCAACTC
        710 TCCACTTCCAGATTCTACAAAAAGAGTGTTTCAAAACTGCCTCTATCAAAGGAAATGTTCAACTC

               * *    * *  * *     *   *       * * *            **     ***  *
      17446 TGTGACTTGAATGCAGATATCACCAAGTAGTTTCTAATAGTGCTTCTGTCTACATTTTAGATGAT
        775 TGTTAGTTGAGTACACACATCACAAAGAAGTTTCTGAGAATGCTTCTGTCTAGTTTTTATGGGAA

                     *              * * *   *             *  *   *      *    
      17511 GATATTCCCGTTTCCAACGAAATCGTTAGAGCTATCCAAATATCCAGTTACAGTTTCTACCAAAA
        840 GATATTCCCTTTTCCAACGAAATCATCAAAGCGATCCAAATATCCACTTCCAGATTCTACAAAAA

             *       *   *    **      *    *            *      ***  *        
      17576 GGGTGTTTCCAAATTGCTGCATCAAAAGAAAGGTTCAACTCTGTTAGTTGAGGACACACATCACA
        905 GAGTGTTTCAAAACTGCTCTATCAAAGGAAATGTTCAACTCTGTGAGTTGAATGCAGACATCACA

                     *                              *                    *   
      17641 AAGAAGTTTGTGAGAATGCTTCTGTCTAGATTTTGTATGACGATATTCCCTTTTCCAACGATATC
        970 AAGAAGTTTCTGAGAATGCTTCTGTCTAG-TTTT-TATGAAGATATTCCCTTTTCCAACGAAATC

            * *     *   *       * *  *    *  *       *  *        *       * * 
      17706 GTTAAAGCAATCTAAATATCAATTTGCAGAATCCACAAAAATAGAGTTTCAAAGCTGCTCTGTAA
       1033 ATCAAAGCGATCCAAATATCCACTTCCAGATTCTACAAAAAGAGTGTTTCAAAACTGCTCTATCA

                   *    *          *                   ***            *      
      17771 AAAGAAAGGTTCCACTCTGTTAGCTGAGTACACACATCACAAACTTGTTTCTGAGAATCCTTCTG
       1098 AAAGAAATGTTCAACTCTGTTAGTTGAGTACACACATCACAAAGAAGTTTCTGAGAATGCTTCTG

               *                   ** *   *
      17836 TCTCGTTTTTATGGGAAGATATTTACTTTTC
       1163 TCTAGTTTTTATGGGAAGATATTCCCGTTTT

              *  * **          *       *     *       *  *  *  *              
      17867 CACCGTAGGCATCAAAGCGCTCCAAATGTCCACATCCAGATACTCCAGAACGAGTGTTTCAAACC
          1 CAACGAAATCATCAAAGCGATCCAAATATCCACTTCCAGATTCTACAAAAAGAGTGTTTCAAACC

                              *         *        * *  *      *      *        
      17932 TGCTCTATGAAAGGGAATCTTCAACTCTATGAGTTGAATGCAGACATCAGAAAGAAATTTCTGAG
         66 TGCTCTA-GAAAGGGAATGTTCAACTCTGTGAGTTGAGTACACACATCACAAAGAAGTTTCTGAG

                  *       **          *          *                 *    *    
      17997 AATGCTGCTGTCTACCTTTTAT-T--TGA-ATTCCCGCTTCCAACGAAATC-CTCCAAGCTATCC
        130 AATGCTTCTGTCTAGTTTTTATATGAAGATATTCCCGTTTCCAACGAAATCGCT-AAAGCGATCC

                      * *  **   *    *                           *           
      18057 AAATATCCACCTGCATTTTCCACAACAAGAGTGTTTCAAAACTGCTCTATCAATAGAAATGTTCA
        194 AAATATCCACTTCCAGATTCTACAAAAAGAGTGTTTCAAAACTGCTCTATCAAAAGAAATGTTCA

                **  * *  *                *                                 *
      18122 ACTCCTTTGGCTGGGTACACACATCACAAACAAGTTTCTGAGAATGCTTCTGTCTAGTTTTTATG
        259 ACTCTGTTAGTTGAGTACACACATCACAAAGAAGTTTCTGAGAATGCTTCTGTCTAGTTTTTATA

            *     **         *  * *  **          *       *            **     
      18187 GGAAGACGTTCCCTTTTTCACCAAAGGCATCAAAGCGCTCCAAATGTCCACTTCCAGACACTACA
        324 TGAAGATATTCCCTTTTCCAACGAAATCATCAAAGCGATCCAAATATCCACTTCCAGATTCTACA

                         *  **       *     **               * *    * *  * *  
      18252 AAAAGAGTGTTTCCAACGTGCTCTAAGAAAACGAATGTTCAACTCTGTGACTTGAATGCAGATAT
        389 AAAAGAGTGTTTCAAAACTGCTCTATGAAAAGAAATGTTCAACTCTGTTAGTTGAGTACACACAT

                *  *           **            *     ***  *         *          
      18317 CACAGAGTAGTTTCTGAGAGGGCTTCTGTCTAGATTTTAGATGATGATATTCCCGTTTCCAACGA
        454 CACAAAGAAGTTTCTGAGAATGCTTCTGTCTAGTTTTTATGGGAAGATATTCCCTTTTCCAACGA

              *   * *   *                *   *                    *        **
      18382 AATCATTAGAGCTATCCAAATATCCACTTACAGTTTCTACAAAAAGAGTGTTTCCAAACTGCTGC
        519 AAGCATCAAAGCGATCCAAATATCCACTTCCAGATTCTACAAAAAGAGTGTTTCAAAACTGCTCT

                  *  * *    *       *  *                *  ***      *     *  
      18447 ATCAAAAGAGAGGTTCCACTCTGTTAGCTGAGTACACACATCACGAACTTGTTTCTCAGAATCCT
        584 ATCAAAGGAAATGTTCAACTCTGTGAGTTGAGTACACACATCACAAAGAAGTTTCTGAGAATGCT

                   *            *      **     *  *  * **          *       *  
      18512 TCTGTCTCGTTTTTATGGGAAGATATTTACTTTTTCACCGTAGGCATCAAAGCGCTCCAAATGTC
        649 TCTGTCTAGTTTTTAT--GATGATATTCCCTTTTCCAACGAAATCATCAAAGCGATCCAAATATC

               *       *  *  *               *          *     *   *         *
      18577 CACATCCAGATACTCCAGAAAGAGTGTTTCAAACCTG-CTCTATGAAAGGGAATCTTCAACTCTA
        712 CACTTCCAGATTCTACAAAAAGAGTGTTTCAAAACTGCCTCTATCAAAGGAAATGTTCAACTCTG

             *      * *  *      *      *              *       **         **  
      18641 TGAGTTGAATGCAGACATCAGAAAGAAATTTCTGAGAATGCTGCTGTCTACCTTTTAT---TTGA
        777 TTAGTTGAGTACACACATCACAAAGAAGTTTCTGAGAATGCTTCTGTCTAGTTTTTATGGGAAGA

                   **             *  *    *                *       *         
      18703 -ATTCCCGCTTCCAACGAAATCCTCCAAGCTATCCAAATATCCACTTGCAGATTCCACAAAAAGA
        842 TATTCCCTTTTCCAACGAAATCATCAAAGCGATCCAAATATCCACTTCCAGATTCTACAAAAAGA

                                     *  *  *            *      ***  * *     *
      18767 GTGTTTCAAAACTGCTCTCTATCAATGGCAAAGTTCAACTCTGTTAGTTGAGGACACATATCACC
        907 GTGTTTCAAAACTG--CTCTATCAAAGGAAATGTTCAACTCTGTGAGTTGAATGCAGACATCACA

              *                         *                  *      *  *  * ** 
      18832 AACAAGTTTCTGAGAATGCTTCTGTCTATTTTTTATGGGAAGATATTTCCTTTTTCACCGTAGGC
        970 AAGAAGTTTCTGAGAATGCTTCTGTCTAGTTTTTAT--GAAGATATTCCCTTTTCCAACGAAATC

            *    *      *    *          * **                     *         * 
      18897 GTCAAGGCGATCGAAATGTCCACTTCCACAAACTACAAAAAGAGTGTTTCAAACCTGCTCTATGA
       1033 ATCAAAGCGATCCAAATATCCACTTCCAGATTCTACAAAAAGAGTGTTTCAAAACTGCTCTATCA

              * **       *    * *      * ** * *   **      *      *       *   
      18962 AAGGCGATGTTCATCTCTATGAGTTGAATGGAAATATCCGAAAGAAATTTCTGGGAATGCTGCTG
       1098 AAAGAAATGTTCAACTCTGTTAGTTGAGTACACACATCACAAAGAAGTTTCTGAGAATGCTTCTG

                  *         *          *  *
      19027 TCTAGTGTTTAT---ACGA-ATTCCCGCTTC
       1163 TCTAGTTTTTATGGGAAGATATTCCCGTTTT

                      *       *                *    *  *                   * 
      19054 CAACGAAATCCTCAAAGCAATCCAAATATCCACTTGCAGAATCCACAAAAAGAGTGTTTCAAAAC
          1 CAACGAAATCATCAAAGCGATCCAAATATCCACTTCCAGATTCTACAAAAAGAGTGTTTCAAACC

                    *  ** *  *          * *                   *  *           
      19119 TGCTCTATCAATAGAAAGGTTCAACTCTTTTAGTTGAGTACACACATCACGAACAAGTTTCTGAG
         66 TGCTCTA-GAAAGGGAATGTTCAACTCTGTGAGTTGAGTACACACATCACAAAGAAGTTTCTGAG

                         * *      **     **  *      *    *    *           *  
      19184 AATGCTTCTGTCTGGCTTTTATTGGAAGACGTTTCC-TTTTC-ACCAAA-GGCATCAAAGCGCTC
        130 AATGCTTCTGTCTAGTTTTTATATGAAGATATTCCCGTTTCCAACGAAATCGC-T-AAAGCGATC

                 *                * *                **     * ***  * *       
      19246 CAAATGTCCACTTCCAGATTCTTCCAAAAGAGTGTTTCAAACGTGCTCAAAGTAAGGGAATGTTC
        193 CAAATATCCACTTCCAGATTCTACAAAAAGAGTGTTTCAAAACTGCTCTATCAAAAGAAATGTTC

                    * *    * *  * *     *   *       * * *             *     *
      19311 AACTCTGTGACTTGAATGCAGATATCACCAAGTAGTTTCTAATAGTGCTTCTGTCTAGATTTTAG
        258 AACTCTGTTAGTTGAGTACACACATCACAAAGAAGTTTCTGAGAATGCTTCTGTCTAGTTTTTAT

                *         *              * * *   *             *  *   *      
      19376 ATGATGATATTCCCGTTTCCAACGAAATCGTTAGAGCTATCCAAATATCCAGTTACAGTTTCTAC
        323 ATGAAGATATTCCCTTTTCCAACGAAATCATCAAAGCGATCCAAATATCCACTTCCAGATTCTAC

                          *        **  *        *                    *       
      19441 AAAAAGAGTGTTTCCAAACTGCTGCATCAAAAGAAAGGTTCAACTCTGTTAGTTGAGGACACACA
        388 AAAAAGAGTGTTTCAAAACTGCTCTATGAAAAGAAATGTTCAACTCTGTTAGTTGAGTACACACA

                          *                   *    * **  *                   
      19506 TCACAAAGAAGTTTGTGAGAATGCTTCTGTCTAGATTTTGTATGACGATATTCCCTTTTCCAACG
        453 TCACAAAGAAGTTTCTGAGAATGCTTCTGTCTAGTTTTTATGGGAAGATATTCCCTTTTCCAACG

             * * * *     *   *       * *  *    *  *       *  *        *      
      19571 ATATCGTTAAAGCAATCTAAATATCAATTTGCAGAATCCACAAAAATAGAGTTTCAAAGCTGCTC
        518 AAAGCATCAAAGCGATCCAAATATCCACTTCCAGATTCTACAAAAAGAGTGTTTCAAAACTGCTC

             * *   **   *    *       *  *                   ***            * 
      19636 TGTAAAAATAAAAGTTCCACTCTGTTAGCTGAGTACACACATCACAAACTTGTTTCTGAGAATCC
        583 TATCAAAGGAAATGTTCAACTCTGTGAGTTGAGTACACACATCACAAAGAAGTTTCTGAGAATGC

                    *            *      **     *  *  * **          *       * 
      19701 TTCTGTCTCGTTTTTATGGGAAGATATTTACTTTTTCACCGTAGGCATCAAAGCGCTCCAAATGT
        648 TTCTGTCTAGTTTTTAT--GATGATATTCCCTTTTCCAACGAAATCATCAAAGCGATCCAAATAT

                *       *  *  *     *         *          *     *   *         
      19766 CCACATCCAGATACTCCAGAAAGACTGTTTCAAACCTG-CTCTATGAAAGGGAATCTTCAACTCT
        711 CCACTTCCAGATTCTACAAAAAGAGTGTTTCAAAACTGCCTCTATCAAAGGAAATGTTCAACTCT

            * *      * *  *      *      *              *       **         ** 
      19830 ATGAGTTGAATGCAGACATCAGAAAGAAATTTCTGAGAATGCTGCTGTCTACCTTTTAT---TTG
        776 GTTAGTTGAGTACACACATCACAAAGAAGTTTCTGAGAATGCTTCTGTCTAGTTTTTATGGGAAG

                    **             *  *    *                *  **   *        
      19892 A-ATTCCCGCTTCCAACGAAATCCTCCAAGCTATCCAAATATCCACTTGCATTTTCCACAAAAAG
        841 ATATTCCCTTTTCCAACGAAATCATCAAAGCGATCCAAATATCCACTTCCAGATTCTACAAAAAG

                                    **              ** *  *  ** *  *         
      19956 AGTGTTTCAAAACTGCTCTATCAATAGAAATGTTCAACTCCTTTAGCTGGGTACACACATCACAA
        906 AGTGTTTCAAAACTGCTCTATCAAAGGAAATGTTCAACTCTGTGAGTTGAATGCAGACATCACAA

             *                                        **         *  * *  **  
      20021 ACAAGTTTCTGAGAATGCTTCTGTCTAGTTTTTATGGGAAGACGTTCCCTTTTTCACCAAAGGCA
        971 AGAAGTTTCTGAGAATGCTTCTGTCTAGTTTTTAT--GAAGATATTCCCTTTTCCAACGAAATCA

                    *       *            **                     **       **  
      20086 TCAAAGCGCTCCAAATGTCCACTTCCAGACACTACAAAAAGAGTGTTTCAAACGTGCTCTAAGAA
       1034 TCAAAGCGATCCAAATATCCACTTCCAGATTCTACAAAAAGAGTGTTTCAAAACTGCTCTATCAA

             ***               * *    * *  * *         *           **        
      20151 AGCGAATGTTCAACTCTGTGACTTGAATGCAGATATCACAAAGTAGTTTCTGAGAGGGCTTCTGT
       1099 AAGAAATGTTCAACTCTGTTAGTTGAGTACACACATCACAAAGAAGTTTCTGAGAATGCTTCTGT

                *     ***  *             *
      20216 CTAGATTTTAGATGATGATATTCCCGTTTC
       1164 CTAGTTTTTATGGGAAGATATTCCCGTTTT

                        * *   *                *   *                    *  * 
      20246 CAACGAAATCATTAGAGCTATCCAAATATCCACTTACAGTTTCTACAAAAAGAGTGTTTCCAAAC
          1 CAACGAAATCATCAAAGCGATCCAAATATCCACTTCCAGATTCTACAAAAAGAGTGTTTCAAACC

                    **    *   *    *       *  **                  ***      * 
      20311 TGCTGCATCAAAAGAG-AGGTTCCACTCTGTTAGCCGAGTACACACATCACAAACTTGTTTCTCA
         66 TGCT-C-TAGAAAGGGAATGTTCAACTCTGTGAGTTGAGTACACACATCACAAAGAAGTTTCTGA

                *         *        **         ** *   *  *  * ** ***      *   
      20375 GAATCCTTCTGTCTCGTTTTTATGGGAAGATATTTACTTTTTCACCGTAGGCATCAAAGCGCTCC
        129 GAATGCTTCTGTCTAGTTTTTATATGAAGATATTCCCGTTTCCAACGAAATCGCTAAAGCGATCC

                *     *       *  *  *               *         *   * *   *    
      20440 AAATGTCCACATCCAGATACTCCAGAAAGAGTGTTTCAAACCTGCTCTATGAAAGGGAATCTTCA
        194 AAATATCCACTTCCAGATTCTACAAAAAGAGTGTTTCAAAACTGCTCTATCAAAAGAAATGTTCA

                 * *      * *  *      *      *              *       **       
      20505 ACTCTATGAGTTGAATGCAGACATCAGAAAGAAATTTCTGAGAATGCTGCTGTCTACCTTTTAT-
        259 ACTCTGTTAGTTGAGTACACACATCACAAAGAAGTTTCTGAGAATGCTTCTGTCTAGTTTTTATA

               *         **             *  *    *                *       *   
      20569 T--TGA-ATTCCCGCTTCCAACGAAATCCTCCAAGCTATCCAAATATCCACTTGCAGATTCCACA
        324 TGAAGATATTCCCTTTTCCAACGAAATCATCAAAGCGATCCAAATATCCACTTCCAGATTCTACA

                                        *  ** *  *                    *     *
      20631 AAAAGAGTGTTTCAAAACTGCTCTCTATCAATGGCAAAGTTCAACTCTGTTAGTTGAGGACACAT
        389 AAAAGAGTGTTTCAAAACTG--CTCTATGAAAAGAAATGTTCAACTCTGTTAGTTGAGTACACAC

                 *  *                         *                  *      *  * 
      20696 ATCACCAACAAGTTTCTGAGAATGCTTCTGTCTATTTTTTATGGGAAGATATTTCCTTTTTCACC
        452 ATCACAAAGAAGTTTCTGAGAATGCTTCTGTCTAGTTTTTATGGGAAGATATTCCCTTTTCCAAC

             * *  *    *      *    *          * **                     *     
      20761 GTAGGCGTCAAGGCGATCGAAATGTCCACTTCCACAAACTACAAAAAGAGTGTTTCAAACCTGCT
        517 GAAAGCATCAAAGCGATCCAAATATCCACTTCCAGATTCTACAAAAAGAGTGTTTCAAAACTGCT

                *     **       *    *        * ** * *   **      *      *     
      20826 CTATGAAAGGCCATGTTCATCTCTATGAGTTGAATGGAAATATCCGAAAGAAATTTCTGGGAATG
        582 CTATCAAAGGAAATGTTCAACTCTGTGAGTTGAGTACACACATCACAAAGAAGTTTCTGAGAATG

              *         *       *         **             *       *           
      20891 CTGCTGTCTAGTGTTTAT-ACGA-ATTCCCGCTTCCAACGAAATCCTCAAAGCAATCCAAATATC
        647 CTTCTGTCTAGTTTTTATGATGATATTCCCTTTTCCAACGAAATCATCAAAGCGATCCAAATATC

                 *    *  *                                 **    *          *
      20954 CACTTGCAGAATCCACAAAAAGAGTGTTTCAAAACTG-CTCTATCAATAGAAAGGTTCAACTCTT
        712 CACTTCCAGATTCTACAAAAAGAGTGTTTCAAAACTGCCTCTATCAAAGGAAATGTTCAACTCTG

                                 *  *                        * *      *      
      21018 TTAGTTGAGTACACACATCACGAACAAGTTTCTGAGAATGCTTCTGTCTGGCTTTTATTGGAAGA
        777 TTAGTTGAGTACACACATCACAAAGAAGTTTCTGAGAATGCTTCTGTCTAGTTTTTATGGGAAGA

            **  *         * *  **          *       *                * *      
      21083 CGTTTCCTTTT-CACCAAAGGCATCAAAGCGCTCCAAATGTCCACTTCCAGATTCTTCCAAAAGA
        842 TATTCCCTTTTCCAACGAAATCATCAAAGCGATCCAAATATCCACTTCCAGATTCTACAAAAAGA

                      **     * ***    *                 *           *     *  
      21147 GTGTTTCAAACGTGCTCAAAGTAAGGGAATGTTCAACTCTGTGACTTGAATGCAGATATCACCAA
        907 GTGTTTCAAAACTGCTCTATCAAAGGAAATGTTCAACTCTGTGAGTTGAATGCAGACATCACAAA

             *       * * *                  **    *         *              * 
      21212 GTAGTTTCTAATAGTGCTTCTGTCTAGATTTCAGATGATGATATTCCCGTTTCCAACGAAATCGT
        972 GAAGTTTCTGAGAATGCTTCTGTCTAG-TTT-TTATGAAGATATTCCCTTTTCCAACGAAATCAT

            * *   * **          *  *   *      *     *       *   *    **      
      21277 TAGAGCTAAGCAAATATCCAGTTACAGTTTCTACCAAAAGGGTGTTTCCAAATTGCTGCATCAAA
       1035 CAAAGCGATCCAAATATCCACTTCCAGATTCTACAAAAAGAGTGTTTCAAAACTGCTCTATCAAA

                 *                    *                     *                
      21342 AGAAAGGTTCAACTCTGTTAGTTGAGGACACACATCACAAAGAAGTTTGTGAGAATGCTTCTGTC
       1100 AGAAATGTTCAACTCTGTTAGTTGAGTACACACATCACAAAGAAGTTTCTGAGAATGCTTCTGTC

               *    * **  *         *   *
      21407 TAGATTTTGTATGACGATATTCCCTTTTC
       1165 TAGTTTTTATGGGAAGATATTCCCGTTTT

                  *   * *     *   *       * *  *    *  *       *  *        * 
      21436 CAACGATATCGTTAAAGCAATCTAAATATCAATTTGCAGAATCCACAAAAATAGAGTTTCAAAGC
          1 CAACGAAATCATCAAAGCGATCCAAATATCCACTTCCAGATTCTACAAAAAGAGTGTTTCAAACC

                   **   * *  *    *       *  *                   ***         
      21501 TGCTCTGTAAAAAGAAAGGTTCCACTCTGTTAGCTGAGTACACACATCACAAACTTGTTTCTGAG
         66 TGCTCT-AGAAAGGGAATGTTCAACTCTGTGAGTTGAGTACACACATCACAAAGAAGTTTCTGAG

               *         *        **         ** *      *  * ** ***      *    
      21566 AATCCTTCTGTCTCGTTTTTATGGGAAGATATTTACTTTTCCACCGTAGGCATCAAAGCGCTCCA
        130 AATGCTTCTGTCTAGTTTTTATATGAAGATATTCCCGTTTCCAACGAAATCGCTAAAGCGATCCA

               *     *       *  *  *               *         *   * *   *     
      21631 AATGTCCACATCCAGATACTCCAGAAAGAGTGTTTCAAACCTGCTCTATGAAAGGCAATCTTCAA
        195 AATATCCACTTCCAGATTCTACAAAAAGAGTGTTTCAAAACTGCTCTATCAAAAGAAATGTTCAA

                * *      * *  *      *      *              *       **        
      21696 CTCTATGAGTTGAATGCAGACATCAGAAAGAAATTTCTGAGAATGCTGCTGTCTACCTTTTAT-T
        260 CTCTGTTAGTTGAGTACACACATCACAAAGAAGTTTCTGAGAATGCTTCTGTCTAGTTTTTATAT

              *         **             *  *    *              * *  **   *    
      21760 --TGA-ATTCCCGCTTCCAACGAAATCCTCCAAGCTATCCAAATATCCACCTGCATTTTCCACAA
        325 GAAGATATTCCCTTTTCCAACGAAATCATCAAAGCGATCCAAATATCCACTTCCAGATTCTACAA

                                     *  *               **  * *  *           
      21822 AAAGAGTGTTTCAAAACTGCTCTATCAATAGAAATGTTCAACTCCTTTGGCTGGGTACACACATC
        390 AAAGAGTGTTTCAAAACTGCTCTATGAAAAGAAATGTTCAACTCTGTTAGTTGAGTACACACATC

                 *           *                            *          *  * *  
      21887 ACAAACAAGTTTCTGAGCATGCTTCTGTCTAGTTTTTATGGGAAGACATTCCCTTTTTCACCAAA
        455 ACAAAGAAGTTTCTGAGAATGCTTCTGTCTAGTTTTTATGGGAAGATATTCCCTTTTCCAACGAA

            *           *       *            **                     **       
      21952 GGCATCAAAGCGCTCCAAATGTCCACTTCCAGACACTACAAAAAGAGTGTTTCAAACGTGCTCTA
        520 AGCATCAAAGCGATCCAAATATCCACTTCCAGATTCTACAAAAAGAGTGTTTCAAAACTGCTCTA

            **   ***                 *    * *  * *      *  *           **    
      22017 AGAAAACGAATGTTCAACTCTGTGACTTGAATGCAGATATCACAGAGTAGTTTCTGAGAGGGCTT
        585 TCAAAGGAAATGTTCAACTCTGTGAGTTGAGTACACACATCACAAAGAAGTTTCTGAGAATGCTT

                          *              *                * *   *            
      22082 CTGTCTAGATTTTAGATGATGATATTCCCGTTTCCAACGAAATCATTAGAGCTATCCAAATATCC
        650 CTGTCTAG-TTTT-TATGATGATATTCCCTTTTCCAACGAAATCATCAAAGCGATCCAAATATCC

                *   *                    *         **      *  * *    *       
      22147 ACTTACAGTTTCTACAAAAAGAGTGTTTCCAAACTG-CTGCATCAAAAGAGAGGTTCCACTCTGT
        713 ACTTCCAGATTCTACAAAAAGAGTGTTTCAAAACTGCCTCTATCAAAGGAAATGTTCAACTCTGT

               *                   ***      *     *         *                
      22211 TAGCTGAGTACACACATCACAAACTTGTTTCTCAGAATCCTTCTGTCTCGTTTTTATGGGAAGAT
        778 TAGTTGAGTACACACATCACAAAGAAGTTTCTGAGAATGCTTCTGTCTAGTTTTTATGGGAAGAT

               **     *  *  * **          *       *     *       *  *  *      
      22276 ATTTACTTTTTCACCGTAGGCATCAAAGCGCTCCAAATGTCCACATCCAGATACTCCAGAAAGAG
        843 ATTCCCTTTTCCAACGAAATCATCAAAGCGATCCAAATATCCACTTCCAGATTCTACAAAAAGAG

                     *         *     *   *         *                    *    
      22341 TGTTTCAAACCTGCTCTATGAAAGGGAATCTTCAACTCTATGAGTTGAATGCAGACATCAGAAAG
        908 TGTTTCAAAACTGCTCTATCAAAGGAAATGTTCAACTCTGTGAGTTGAATGCAGACATCACAAAG

              *              *       **       **         **             *  * 
      22406 AAATTTCTGAGAATGCTGCTGTCTACCTTTTAT-TTGA-ATTCCCGCTTCCAACGAAATCCTCCA
        973 AAGTTTCTGAGAATGCTTCTGTCTAGTTTTTATGAAGATATTCCCTTTTCCAACGAAATCATCAA

               *                *       *                                  **
      22469 AGCTATCCAAATATCCACTTGCAGATTCCACAAAAAGAGTGTTTCAAAACTGCTCTCTATCAATG
       1038 AGCGATCCAAATATCCACTTCCAGATTCTACAAAAAGAGTGTTTCAAAACTG--CTCTATCAAAA

             *  *                    *     *     *  *                        
      22534 GCAAAGTTCAACTCTGTTAGTTGAGGACACATATCACCAACAAGTTTCTGAGAATGCTTCTGTCT
       1101 GAAATGTTCAACTCTGTTAGTTGAGTACACACATCACAAAGAAGTTTCTGAGAATGCTTCTGTCT

             *                  *  *    
      22599 ATTTTTTATGGGAAGATATTTCCTTTTT
       1166 AGTTTTTATGGGAAGATATTCCCGTTTT

              *  * ** *    *      *    *          * **                       
      22627 CACCGTAGGCGTCAAGGCGATCGAAATGTCCACTTCCACAAACTACAAAAAGAGTGTTTCAAACC
          1 CAACGAAATCATCAAAGCGATCCAAATATCCACTTCCAGATTCTACAAAAAGAGTGTTTCAAACC

                          **       *    *        * ** * *   **      *      * 
      22692 TGCTCTATGAAAGGCCATGTTCATCTCTATGAGTTGAATGGAAATATCCGAAAGAAATTTCTGGG
         66 TGCTCTA-GAAAGGGAATGTTCAACTCTGTGAGTTGAGTACACACATCACAAAGAAGTTTCTGAG

                  *          *        *          *                      *    
      22757 AATGCTGCTGTCTAG-TGTT-TAT-ACGA-ATTCCCGCTTCCAACGAAATC-CTCAAAGCAATCC
        130 AATGCTTCTGTCTAGTTTTTATATGAAGATATTCCCGTTTCCAACGAAATCGCT-AAAGCGATCC

                        *    *  *                                *     *     
      22817 AAATATCCACTTGCAGAATCCACAAAAAGAGTGTTTCAAAACTGCTCTATCAATAGAAAGGTTCA
        194 AAATATCCACTTCCAGATTCTACAAAAAGAGTGTTTCAAAACTGCTCTATCAAAAGAAATGTTCA

                 *                     *  *                        * *      *
      22882 ACTCTTTTAGTTGAGTACACACATCACGAACAAGTTTCTGAGAATGCTTCTGTCTGGCTTTTATT
        259 ACTCTGTTAGTTGAGTACACACATCACAAAGAAGTTTCTGAGAATGCTTCTGTCTAGTTTTTATA

            *     **  *         * *  **          *       *                * *
      22947 GGAAGACGTTTCCTTTT-CACCAAAGGCATCAAAGCGCTCCAAATGTCCACTTCCAGATTCTTCC
        324 TGAAGATATTCCCTTTTCCAACGAAATCATCAAAGCGATCCAAATATCCACTTCCAGATTCTACA

                            **     * * *  * *               * *    * *  * *  
      23011 AAAAGAGTGTTTCAAACGTGCTCAAAGTAAGGGAATGTTCAACTCTGTGACTTGAATGCAGATAT
        389 AAAAGAGTGTTTCAAAACTGCTCTATGAAAAGAAATGTTCAACTCTGTTAGTTGAGTACACACAT

               *   *       * * *             *     ***  *         *          
      23076 CACCAAGTAGTTTCTAATAGTGCTTCTGTCTAGATTTTAGATGATGATATTCCCGTTTCCAACGA
        454 CACAAAGAAGTTTCTGAGAATGCTTCTGTCTAGTTTTTATGGGAAGATATTCCCTTTTCCAACGA

              * * * *   *             *  *   *                    *        **
      23141 AATCGTTAGAGCTATCCAAATATCCAGTTACAGTTTCTACAAAAAGAGTGTTTCCAAACTGCTGC
        519 AAGCATCAAAGCGATCCAAATATCCACTTCCAGATTCTACAAAAAGAGTGTTTCAAAACTGCTCT

                  *    *            *       *                     *          
      23206 ATCAAAAGAAAGGTTCAACTCTGTTAGTTGAGGACACACATCACAAAGAAGTTTGTGAGAATGCT
        584 ATCAAAGGAAATGTTCAACTCTGTGAGTTGAGTACACACATCACAAAGAAGTTTCTGAGAATGCT

                                *                    *   * *     *   *       
      23271 TCTGTCTAGATTTTGTATGACGATATTCCCTTTTCCAACGATATCGTTAAAGCAATCTAAATATC
        649 TCTGTCTAG-TTTT-TATGATGATATTCCCTTTTCCAACGAAATCATCAAAGCGATCCAAATATC

            * *  *    *  *       *  *        *        * *   **   *    *      
      23336 AATTTGCAGAATCCACAAAAATAGAGTTTCAAAGCTG-CTCTGTAAAAATAAAAGTTCCACTCTG
        712 CACTTCCAGATTCTACAAAAAGAGTGTTTCAAAACTGCCTCTATCAAAGGAAATGTTCAACTCTG

                *                   ***            *         *               
      23400 TTAGCTGAGTACACACATCACAAACTTGTTTCTGAGAATCCTTCTGTCTCGTTTTTATGGGAAGA
        777 TTAGTTGAGTACACACATCACAAAGAAGTTTCTGAGAATGCTTCTGTCTAGTTTTTATGGGAAGA

                **     *  *  * **          *       *     *       *  *  *     
      23465 TATTTACTTTTTCACCGTAGGCATCAAAGCGCTCCAAATGTCCACATCCAGATACTCCAGAAAGA
        842 TATTCCCTTTTCCAACGAAATCATCAAAGCGATCCAAATATCCACTTCCAGATTCTACAAAAAGA

            *         *         *     *   *         *                    *   
      23530 CTGTTTCAAACCTGCTCTATGAAAGGGAATCTTCAACTCTATGAGTTGAATGCAGACATCAGAAA
        907 GTGTTTCAAAACTGCTCTATCAAAGGAAATGTTCAACTCTGTGAGTTGAATGCAGACATCACAAA

               *              *       **       **         **             *  *
      23595 GAAATTTCTGAGAATGCTGCTGTCTACCTTTTAT-TTGA-ATTCCCGCTTCCAACGAAATCCTCC
        972 GAAGTTTCTGAGAATGCTTCTGTCTAGTTTTTATGAAGATATTCCCTTTTCCAACGAAATCATCA

                *                *  **   *                                *  
      23658 AAGCTATCCAAATATCCACTTGCATTTTCCACAAAAAGAGTGTTTCAAAACTGCTCTATCAATAG
       1037 AAGCGATCCAAATATCCACTTCCAGATTCTACAAAAAGAGTGTTTCAAAACTGCTCTATCAAAAG

                         **    *  *                *                         
      23723 AAATGTTCAACTCCTTTAGCTGGGTACACACATCACAAACAAGTTTCTGAGAATGCTTCTGTCTA
       1102 AAATGTTCAACTCTGTTAGTTGAGTACACACATCACAAAGAAGTTTCTGAGAATGCTTCTGTCTA

                           **     *    
      23788 GTTTTTATGGGAAGACGTTCCCTTTTT
       1167 GTTTTTATGGGAAGATATTCCCGTTTT

              * *  **          *       *            **                      *
      23815 CACCAAAGGCATCAAAGCGCTCCAAATGTCCACTTCCAGACACTACAAAAAGAGTGTTTCAAACG
          1 CAACGAAATCATCAAAGCGATCCAAATATCCACTTCCAGATTCTACAAAAAGAGTGTTTCAAACC

                         *                  *    * *  * *         *          
      23880 TGCTCTAAGAAAGCGAATGTTCAACTCTGTGACTTGAATGCAGATATCACAAAGTAGTTTCTGAG
         66 TGCTCT-AGAAAGGGAATGTTCAACTCTGTGAGTTGAGTACACACATCACAAAGAAGTTTCTGAG

             **            *     *    *                        **  *   *     
      23945 AGGGCTTCTGTCTAGATTTTAGATGATGATATTCCCGTTTCCAACGAAATCATTAGAGCTATCCA
        130 AATGCTTCTGTCTAGTTTTTATATGAAGATATTCCCGTTTCCAACGAAATCGCTAAAGCGATCCA

                       *   *                    *        **         * *    * 
      24010 AATATCCACTTACAGTTTCTACAAAAAGAGTGTTTCCAAACTGCTGCATCAAAAGAGAGGTTCCA
        195 AATATCCACTTCCAGATTCTACAAAAAGAGTGTTTCAAAACTGCTCTATCAAAAGAAATGTTCAA

                     **                  ***      *     *         *        **
      24075 CTCTGTTAGCCGAGTACACACATCACAAACTTGTTTCTCAGAATCCTTCTGTCTCGTTTTTATGG
        260 CTCTGTTAGTTGAGTACACACATCACAAAGAAGTTTCTGAGAATGCTTCTGTCTAGTTTTTATAT

                     **     *  *  * **          *       *     *       *  *  *
      24140 GAAGATATTTACTTTTTCACCGTAGGCATCAAAGCGCTCCAAATGTCCACATCCAGATACTCCAG
        325 GAAGATATTCCCTTTTCCAACGAAATCATCAAAGCGATCCAAATATCCACTTCCAGATTCTACAA

                           *             * *   *         * *      * *  *     
      24205 AAAGAGTGTTTCAAACCTGCTCTATGAAAGGGAATCTTCAACTCTATGAGTTGAATGCAGACATC
        390 AAAGAGTGTTTCAAAACTGCTCTATGAAAAGAAATGTTCAACTCTGTTAGTTGAGTACACACATC

             *      *              *       **         **         **          
      24270 AGAAAGAAATTTCTGAGAATGCTGCTGTCTACCTTTTAT---TTGA-ATTCCCGCTTCCAACGAA
        455 ACAAAGAAGTTTCTGAGAATGCTTCTGTCTAGTTTTTATGGGAAGATATTCCCTTTTCCAACGAA

             * *  *    *                *       *                            
      24331 ATCCTCCAAGCTATCCAAATATCCACTTGCAGATTCCACAAAAAGAGTGTTTCAAAACTGCTCTC
        520 AGCATCAAAGCGATCCAAATATCCACTTCCAGATTCTACAAAAAGAGTGTTTCAAAACTG--CTC

                  *  *  *            *       *     *     *  *                
      24396 TATCAATGGCAAAGTTCAACTCTGTTAGTTGAGGACACATATCACCAACAAGTTTCTGAGAATGC
        583 TATCAAAGGAAATGTTCAACTCTGTGAGTTGAGTACACACATCACAAAGAAGTTTCTGAGAATGC

                     *           *      *      *  *  * ** *    *   *  *    * 
      24461 TTCTGTCTATTTTTTATGGGAAGATATTTCCTTTTTCACCGTAGGCGTCAACGCGTTCGAAATGT
        648 TTCTGTCTAGTTTTTAT--GATGATATTCCCTTTTCCAACGAAATCATCAAAGCGATCCAAATAT

                     * **                     *          *     **       *    
      24526 CCACTTCCACAAACTACAAAAAGAGTGTTTCAAACCTG-CTCTATGAAAGGCCATGTTCATCTCT
        711 CCACTTCCAGATTCTACAAAAAGAGTGTTTCAAAACTGCCTCTATCAAAGGAAATGTTCAACTCT

            * *      * ** * *   **      *      *       *         *         * 
      24590 ATGAGTTGAATGGAAATATCCGAAAGAAATTTCTGGGAATGCTGCTGTCTAGTGTTTAT---ACG
        776 GTTAGTTGAGTACACACATCACAAAGAAGTTTCTGAGAATGCTTCTGTCTAGTTTTTATGGGAAG

                    **             *       *                *    *  *        
      24652 A-ATTCCCGCTTCCAACGAAATCCTCAAAGCAATCCAAATATCCACTTGCAGAATCCACAAAAAG
        841 ATATTCCCTTTTCCAACGAAATCATCAAAGCGATCCAAATATCCACTTCCAGATTCTACAAAAAG

                                    **    *          * *      * *  *       * 
      24716 AGTGTTTCAAAACTGCTCTATCAATAGAAAGGTTCAACTCTTTTAGTTGAGTACACACATCACGA
        906 AGTGTTTCAAAACTGCTCTATCAAAGGAAATGTTCAACTCTGTGAGTTGAATGCAGACATCACAA

             *                        * *             **  *         * *  **  
      24781 ACAAGTTTCTGAGAATGCTTCTGTCTGGCTTTTATTGGAAGACGTTTCCTTTT-CACCAAAGGCA
        971 AGAAGTTTCTGAGAATGCTTCTGTCTAGTTTTTA-T-GAAGATATTCCCTTTTCCAACGAAATCA

                    *       *                * *                **     * *** 
      24845 TCAAAGCGCTCCAAATGTCCACTTCCAGATTCTTCCAAAAGAGTGTTTCAAACGTGCTCAAAGTA
       1034 TCAAAGCGATCCAAATATCCACTTCCAGATTCTACAAAAAGAGTGTTTCAAAACTGCTCTATCAA

             * *               * *    * *  * *     *   *       * * *         
      24910 AGGGAATGTTCAACTCTGTGACTTGAATGCAGATATCACCAAGTAGTTTCTAATAGTGCTTCTGT
       1099 AAGAAATGTTCAACTCTGTTAGTTGAGTACACACATCACAAAGAAGTTTCTGAGAATGCTTCTGT

                *   * ***  *             *
      24975 CTAGATTTCAGATGATGATATTCCCGTTTC
       1164 CTAGTTTTTATGGGAAGATATTCCCGTTTT

                      * * *   * **          *  *   *      *     *       *  **
      25005 CAACGAAATCGTTAGAGCTAAGCAAATATCCAGTTACAGTTTCTACCAAAAGGGTGTTTCCAAAT
          1 CAACGAAATCATCAAAGCGATCCAAATATCCACTTCCAGATTCTACAAAAAGAGTGTTTCAAACC

                  ***   * *  *            *       *                     *    
      25070 TGCTGCATCAAAAGAAAGGTTCAACTCTGTTAGTTGAGGACACACATCACAAAGAAGTTTGTGAG
         66 TGCT-CTAGAAAGGGAATGTTCAACTCTGTGAGTTGAGTACACACATCACAAAGAAGTTTCTGAG

                           *    *     *         *          *    *      *   * 
      25135 AATGCTTCTGTCTAGATTTTGTATGACGATATTCCCTTTTCCAACGATATCGTTAAAGCAATCTA
        130 AATGCTTCTGTCTAGTTTTTATATGAAGATATTCCCGTTTCCAACGAAATCGCTAAAGCGATCCA

                  * *  *    *  *       *  *        *       * *        *    * 
      25200 AATATCAATTTGCAGAATCCACAAAAATAGAGTTTCAAAGCTGCTCTGTAAAAAGAAAGGTTCCA
        195 AATATCCACTTCCAGATTCTACAAAAAGAGTGTTTCAAAACTGCTCTATCAAAAGAAATGTTCAA

                     *                   ***            *         *        **
      25265 CTCTGTTAGCTGAGTACACACATCACAAACTTGTTTCTGAGAATCCTTCTGTCTCGTTTTTATGG
        260 CTCTGTTAGTTGAGTACACACATCACAAAGAAGTTTCTGAGAATGCTTCTGTCTAGTTTTTATAT

                     **        *  * **          *       *     *       *  *  *
      25330 GAAGATATTTACTTTTCCACCGTAGGCATCAAAGCGCTCCAAATGTCCACATCCAGATACTCCAG
        325 GAAGATATTCCCTTTTCCAACGAAATCATCAAAGCGATCCAAATATCCACTTCCAGATTCTACAA

                           *             * *   *         * *      * *  *     
      25395 AAAGAGTGTTTCAAACCTGCTCTATGAAAGGCAATCTTCAACTCTATGAGTTGAATGCAGACATC
        390 AAAGAGTGTTTCAAAACTGCTCTATGAAAAGAAATGTTCAACTCTGTTAGTTGAGTACACACATC

             *      *              *       **         **         **          
      25460 AGAAAGAAATTTCTGAGAATGCTGCTGTCTACCTTTTAT---TTGA-ATTCCCGCTTCCAACGAA
        455 ACAAAGAAGTTTCTGAGAATGCTTCTGTCTAGTTTTTATGGGAAGATATTCCCTTTTCCAACGAA

             * *  *    *              * *  **   *                            
      25521 ATCCTCCAAGCTATCCAAATATCCACCTGCATTTTCCACAAAAAGAGTGTTTCAAAACTGCTCTA
        520 AGCATCAAAGCGATCCAAATATCCACTTCCAGATTCTACAAAAAGAGTGTTTCAAAACTGCTCTA

                **                *    *  *                *           *     
      25586 TCAATAGAAATGTTCAACTCCTTTG-GCTGGGTACACACATCACAAACAAGTTTCTGAGCATGCT
        585 TCAAAGGAAATGTTCAACT-CTGTGAGTTGAGTACACACATCACAAAGAAGTTTCTGAGAATGCT

                                *  *          *  * *  **          *       *  
      25650 TCTGTCTAGTTTTTATGGGAAGACATTCCCTTTTTCACCAAAGGCATCAAAGCGCTCCAAATGTC
        649 TCTGTCTAGTTTTTAT--GATGATATTCCCTTTTCCAACGAAATCATCAAAGCGATCCAAATATC

                      **                     **        **   ***              
      25715 CACTTCCAGACACTACAAAAAGAGTGTTTCAAACGTG-CTCTAAGAAAACGAATGTTCAACTCTG
        712 CACTTCCAGATTCTACAAAAAGAGTGTTTCAAAACTGCCTCTATCAAAGGAAATGTTCAACTCTG

             * *    * *  * *      *  *           **            *     ***  *  
      25779 TGACTTGAATGCAGATATCACAGAGTAGTTTCTGAGAGGGCTTCTGTCTAGATTTTAGATGATGA
        777 TTAGTTGAGTACACACATCACAAAGAAGTTTCTGAGAATGCTTCTGTCTAGTTTTTATGGGAAGA

                   *                * *   *                *   *             
      25844 TATTCCCGTTTCCAACGAAATCATTAGAGCTATCCAAATATCCACTTACAGTTTCTACAAAAAGA
        842 TATTCCCTTTTCCAACGAAATCATCAAAGCGATCCAAATATCCACTTCCAGATTCTACAAAAAGA

                   *        **      *  * *    *       *  *   * *  *          
      25909 GTGTTTCCAAACTGCTGCATCAAAAGAGAGGTTCCACTCTGTTAGCTGAGTACACACATCACAAA
        907 GTGTTTCAAAACTGCTCTATCAAAGGAAATGTTCAACTCTGTGAGTTGAATGCAGACATCACAAA

            ***      *     *         *                   **     *  *  * **   
      25974 CTTGTTTCTCAGAATCCTTCTGTCTCGTTTTTATGGGAAGATATTTACTTTTTCACCGTAGGCAT
        972 GAAGTTTCTGAGAATGCTTCTGTCTAGTTTTTAT--GAAGATATTCCCTTTTCCAACGAAATCAT

                   *       *     *       *  *  *               *         *   
      26039 CAAAGCGCTCCAAATGTCCACATCCAGATACTCCAGAAAGAGTGTTTCAAACCTGCTCTATGAAA
       1035 CAAAGCGATCCAAATATCCACTTCCAGATTCTACAAAAAGAGTGTTTCAAAACTGCTCTATCAAA

            * *   *         * *      * *  *      *      *              *     
      26104 GGGAATCTTCAACTCTATGAGTTGAATGCAGACATCAGAAAGAAATTTCTGAGAATGCTGCTGTC
       1100 AGAAATGTTCAACTCTGTTAGTTGAGTACACACATCACAAAGAAGTTTCTGAGAATGCTTCTGTC

              **         **          *  *
      26169 TACCTTTTAT---TTGA-ATTCCCGCTTC
       1165 TAGTTTTTATGGGAAGATATTCCCGTTTT

                      *  *    *                *       *                   * 
      26194 CAACGAAATCCTCCAAGCTATCCAAATATCCACTTGCAGATTCCACAAAAAGAGTGTTTCAAAAC
          1 CAACGAAATCATCAAAGCGATCCAAATATCCACTTCCAGATTCTACAAAAAGAGTGTTTCAAACC

                      *  *  *  *            *       *     *     *  *         
      26259 TGCTCTCTATCAATGGCAAAGTTCAACTCTGTTAGTTGAGGACACATATCACCAACAAGTTTCTG
         66 TG--CTCTA-GAAAGGGAATGTTCAACTCTGTGAGTTGAGTACACACATCACAAAGAAGTTTCTG

                            *       **         *  *   *  *  * **  **  *      
      26324 AGAATGCTTCTGTCTATTTTTTATGGGAAGATATTTCCTTTTTCACCGTAGGCGTCAAGGCGATC
        128 AGAATGCTTCTGTCTAGTTTTTATATGAAGATATTCCCGTTTCCAACGAAATCGCTAAAGCGATC

            *    *          * **                     *         *   * **      
      26389 GAAATGTCCACTTCCACAAACTACAAAAAGAGTGTTTCAAACCTGCTCTATGAAAGGCCATGTTC
        193 CAAATATCCACTTCCAGATTCTACAAAAAGAGTGTTTCAAAACTGCTCTATCAAAAGAAATGTTC

             *    * *      * ** * *   **      *      *       *          *    
      26454 ATCTCTATGAGTTGAATGGAAATATCCGAAAGAAATTTCTGGGAATGCTGCTGTCTAG-TGTT-T
        258 AACTCTGTTAGTTGAGTACACACATCACAAAGAAGTTTCTGAGAATGCTTCTGTCTAGTTTTTAT

                *         **             *       *                *    *  *  
      26517 AT-ACGA-ATTCCCGCTTCCAACGAAATCCTCAAAGCAATCCAAATATCCACTTGCAGAATCCAC
        323 ATGAAGATATTCCCTTTTCCAACGAAATCATCAAAGCGATCCAAATATCCACTTCCAGATTCTAC

                                       *  *     *          *                 
      26580 AAAAAGAGTGTTTCAAAACTGCTCTATCAATAGAAAGGTTCAACTCTTTTAGTTGAGTACACACA
        388 AAAAAGAGTGTTTCAAAACTGCTCTATGAAAAGAAATGTTCAACTCTGTTAGTTGAGTACACACA

                *  *                        * *      *      **  *         * *
      26645 TCACGAACAAGTTTCTGAGAATGCTTCTGTCTGGCTTTTATTGGAAGACGTTTCCTTTT-CACCA
        453 TCACAAAGAAGTTTCTGAGAATGCTTCTGTCTAGTTTTTATGGGAAGATATTCCCTTTTCCAACG

              *           *       *                * *                **     
      26709 AAGGCATCAAAGCGCTCCAAATGTCCACTTCCAGATTCTTCCAAAAGAGTGTTTCAAACGTGCTC
        518 AAAGCATCAAAGCGATCCAAATATCCACTTCCAGATTCTACAAAAAGAGTGTTTCAAAACTGCTC

            * ***    *                 *    * *  * *     *   *       * * *   
      26774 AAAGTAAGGGAATGTTCAACTCTGTGACTTGAATGCAGATATCACCAAGTAGTTTCTAATAGTGC
        583 TATCAAAGGAAATGTTCAACTCTGTGAGTTGAGTACACACATCACAAAGAAGTTTCTGAGAATGC

                            *              *              * * *   *          
      26839 TTCTGTCTAGATTTTAGATGATGATATTCCCGTTTCCAACGAAATCGTTAGAGCTATCCAAATAT
        648 TTCTGTCTAG-TTTT-TATGATGATATTCCCTTTTCCAACGAAATCATCAAAGCGATCCAAATAT

               *  *   *                    *         **      *    *          
      26904 CCAGTTACAGTTTCTACAAAAAGAGTGTTTCCAAACTG-CTGCATCAAAAGAAAGGTTCAACTCT
        711 CCACTTCCAGATTCTACAAAAAGAGTGTTTCAAAACTGCCTCTATCAAAGGAAATGTTCAACTCT

                      *                     *                   *    * **  * 
      26968 GTTAGTTGAGGACACACATCACAAAGAAGTTTGTGAGAATGCTTCTGTCTAGATTTTGTATGACG
        776 GTTAGTTGAGTACACACATCACAAAGAAGTTTCTGAGAATGCTTCTGTCTAGTTTTTATGGGAAG

                               *   * *     *   *       * *  *    *  *       *
      27033 ATATTCCCTTTTCCAACGATATCGTTAAAGCAATCTAAATATCAATTTGCAGAATCCACAAAAAT
        841 ATATTCCCTTTTCCAACGAAATCATCAAAGCGATCCAAATATCCACTTCCAGATTCTACAAAAAG

              *        *       * *   **   *    *       *  *   * *  *         
      27098 AGAGTTTCAAAGCTGCTCTGTAAAAATAAAAGTTCCACTCTGTTAGCTGAGTACACACATCACAA
        906 AGTGTTTCAAAACTGCTCTATCAAAGGAAATGTTCAACTCTGTGAGTTGAATGCAGACATCACAA

             ***            *         *                   **     *  *  * **  
      27163 ACTTGTTTCTGAGAATCCTTCTGTCTCGTTTTTATGGGAAGATATTTACTTTTTCACCGTAGGCA
        971 AGAAGTTTCTGAGAATGCTTCTGTCTAGTTTTTAT--GAAGATATTCCCTTTTCCAACGAAATCA

                    *       *     *       *  *  *     *         *         *  
      27228 TCAAAGCGCTCCAAATGTCCACATCCAGATACTCCAGAAAGACTGTTTCAAACCTGCTCTATGAA
       1034 TCAAAGCGATCCAAATATCCACTTCCAGATTCTACAAAAAGAGTGTTTCAAAACTGCTCTATCAA

             * *   *         * *      * *  *      *      *              *    
      27293 AGGGAATCTTCAACTCTATGAGTTGAATGCAGACATCAGAAAGAAATTTCTGAGAATGCTGCTGT
       1099 AAGAAATGTTCAACTCTGTTAGTTGAGTACACACATCACAAAGAAGTTTCTGAGAATGCTTCTGT

               **         **          *  *
      27358 CTACCTTTTAT---TTGA-ATTCCCGCTTC
       1164 CTAGTTTTTATGGGAAGATATTCCCGTTTT

                      *  *    *                *  **   *                   * 
      27384 CAACGAAATCCTCCAAGCTATCCAAATATCCACTTGCATTTTCCACAAAAAGAGTGTTTCAAAAC
          1 CAACGAAATCATCAAAGCGATCCAAATATCCACTTCCAGATTCTACAAAAAGAGTGTTTCAAACC

                    *  ** *            ** *  *  *                *           
      27449 TGCTCTATCAATAGAAATGTTCAACTCCTTTAGCTGGGTACACACATCACAAACAAGTTTCTGAG
         66 TGCTCTA-GAAAGGGAATGTTCAACTCTGTGAGTTGAGTACACACATCACAAAGAAGTTTCTGAG

                                  **     **     *   *    *    *           *  
      27514 AATGCTTCTGTCTAGTTTTTATGGGAAGACGTTCCCTTTTTC-ACCAAA-GGCATCAAAGCGCTC
        130 AATGCTTCTGTCTAGTTTTTATATGAAGATATTCCCGTTTCCAACGAAATCGC-T-AAAGCGATC

                 *            **                  *  **       **   ***       
      27577 CAAATGTCCACTTCCAGACACTACAAAAAGAGTGTTTCCAACGTGCTCTAAGAAAGCGAATGTTC
        193 CAAATATCCACTTCCAGATTCTACAAAAAGAGTGTTTCAAAACTGCTCTATCAAAAGAAATGTTC

                    * *    * *  * *         *           **            *     *
      27642 AACTCTGTGACTTGAATGCAGATATCACAAAGTAGTTTCTGAGAGGGCTTCTGTCTAGATTTTAG
        258 AACTCTGTTAGTTGAGTACACACATCACAAAGAAGTTTCTGAGAATGCTTCTGTCTAGTTTTTAT

                *         *                * *   *                *   *      
      27707 ATGATGATATTCCCGTTTCCAACGAAATCATTAGAGCTATCCAAATATCCACTTACAGTTTCTAC
        323 ATGAAGATATTCCCTTTTCCAACGAAATCATCAAAGCGATCCAAATATCCACTTCCAGATTCTAC

                          *        **  *      * *    *          **           
      27772 AAAAAGAGTGTTTCCAAACTGCTGCATCAAAAGAGAGGTTCCACTCTGTTAGCCGAGTACACACA
        388 AAAAAGAGTGTTTCAAAACTGCTCTATGAAAAGAAATGTTCAACTCTGTTAGTTGAGTACACACA

                   ***      *     *         *                   **     *  *  
      27837 TCACAAACTTGTTTCTCAGAATCCTTCTGTCTCGTTTTTATGGGAAGATATTTACTTTTTCACCG
        453 TCACAAAGAAGTTTCTGAGAATGCTTCTGTCTAGTTTTTATGGGAAGATATTCCCTTTTCCAACG

            * *           *       *     *       **    *               *      
      27902 TAGGCATCAAAGCGCTCCAAATGTCCACATCCAGATAGTACAGAAAGAGTGTTTCAAACCTGCTC
        518 AAAGCATCAAAGCGATCCAAATATCCACTTCCAGATTCTACAAAAAGAGTGTTTCAAAACTGCTC

               *     *   *         *        * *  *      *      *             
      27967 TATGAAAGGGAATCTTCAACTCTATGAGTTGAATGCAGACATCAGAAAGAAATTTCTGAGAATGC
        583 TATCAAAGGAAATGTTCAACTCTGTGAGTTGAGTACACACATCACAAAGAAGTTTCTGAGAATGC

             *       **       *          **             *  *    *            
      28032 TGCTGTCTACCTTTTAT-TTGA-ATTCCCGCTTCCAACGAAATCCTCCAAGCTATCCAAATATCC
        648 TTCTGTCTAGTTTTTATGATGATATTCCCTTTTCCAACGAAATCATCAAAGCGATCCAAATATCC

                *       *                                  *  *  *           
      28095 ACTTGCAGATTCCACAAAAAGAGTGTTTCAAAACTGCTCTCTATCAATGGCAAAGTTCAACTCTG
        713 ACTTCCAGATTCTACAAAAAGAGTGTTTCAAAACTGC-CTCTATCAAAGGAAATGTTCAACTCTG

                     *     *     *  *                         *              
      28160 TTAGTTGAGGACACATATCACCAACAAGTTTCTGAGAATGCTTCTGTCTATTTTTTATGGGAAGA
        777 TTAGTTGAGTACACACATCACAAAGAAGTTTCTGAGAATGCTTCTGTCTAGTTTTTATGGGAAGA

                *      *  *  * ** *    *      *    *          * **           
      28225 TATTTCCTTTTTCACCGTAGGCGTCAAGGCGATCGAAATGTCCACTTCCACAAACTACAAAAAGA
        842 TATTCCCTTTTCCAACGAAATCATCAAAGCGATCCAAATATCCACTTCCAGATTCTACAAAAAGA

                      *         *     **       *    *           * * *   **   
      28290 GTGTTTCAAACCTGCTCTATGAAAGGCCATGTTCATCTCTATGAGTTGAATGGAAATATCCGAAA
        907 GTGTTTCAAAACTGCTCTATCAAAGGAAATGTTCAACTCTGTGAGTTGAATGCAGACATCACAAA

               *      *       *         *       *         **             *   
      28355 GAAATTTCTGGGAATGCTGCTGTCTAGTGTTTAT-ACGA-ATTCCCGCTTCCAACGAAATCCTCA
        972 GAAGTTTCTGAGAATGCTTCTGTCTAGTTTTTATGAAGATATTCCCTTTTCCAACGAAATCATCA

                *                *    *  *                                *  
      28418 AAGCAATCCAAATATCCACTTGCAGAATCCACAAAAAGAGTGTTTCAAAACTGCTCTATCAATAG
       1037 AAGCGATCCAAATATCCACTTCCAGATTCTACAAAAAGAGTGTTTCAAAACTGCTCTATCAAAAG

               *          *                     *  *                        *
      28483 AAAGGTTCAACTCTTTTAGTTGAGTACACACATCACGAACAAGTTTCTGAGAATGCTTCTGTCTG
       1102 AAATGTTCAACTCTGTTAGTTGAGTACACACATCACAAAGAAGTTTCTGAGAATGCTTCTGTCTA

             *      *      **  *       
      28548 GCTTTTATTGGAAGACGTTTCC-TTTT
       1167 GTTTTTATGGGAAGATATTCCCGTTTT

              * *  **          *       *                * *                 *
      28574 CACCAAAGGCATCAAAGCGCTCCAAATGTCCACTTCCAGATTCTTCCAAAAGAGTGTTTCAAACG
          1 CAACGAAATCATCAAAGCGATCCAAATATCCACTTCCAGATTCTACAAAAAGAGTGTTTCAAACC

                  *  *                      *    * *  * *     *   *       * *
      28639 TGCTCAAAGTAAGGGAATGTTCAACTCTGTGACTTGAATGCAGATATCACCAAGTAGTTTCTAAT
         66 TGCTC-TAGAAAGGGAATGTTCAACTCTGTGAGTTGAGTACACACATCACAAAGAAGTTTCTGAG

             *             *   * *    *                         *  *   * **  
      28704 AGTGCTTCTGTCTAGATTTCAGATGATGATATTCCCGTTTCCAACGAAATCGTTAGAGCTAAGCA
        130 AATGCTTCTGTCTAGTTTTTATATGAAGATATTCCCGTTTCCAACGAAATCGCTAAAGCGATCCA

                    *  *   *      *     *       *   *    **           *      
      28769 AATATCCAGTTACAGTTTCTACCAAAAGGGTGTTTCCAAATTGCTGCATCAAAAGAAAGGTTCAA
        195 AATATCCACTTCCAGATTCTACAAAAAGAGTGTTTCAAAACTGCTCTATCAAAAGAAATGTTCAA

                          *                     *                   *    *   
      28834 CTCTGTTAGTTGAGGACACACATCACAAAGAAGTTTGTGAGAATGCTTCTGTCTAGATTTTGTAT
        260 CTCTGTTAGTTGAGTACACACATCACAAAGAAGTTTCTGAGAATGCTTCTGTCTAGTTTTTATAT

              *                    *   * *     *   *       * *  *    *  *    
      28899 GACGATATTCCCTTTTCCAACGATATCGTTAAAGCAATCTAAATATCAATTTGCAGAATCCACAA
        325 GAAGATATTCCCTTTTCCAACGAAATCATCAAAGCGATCCAAATATCCACTTCCAGATTCTACAA

               *  *        *       * *        *    *          *              
      28964 AAATAGAGTTTCAAAGCTGCTCTGTAAAAAGAAAGGTTCCACTCTGTTAGCTGAGTACACACATC
        390 AAAGAGTGTTTCAAAACTGCTCTATGAAAAGAAATGTTCAACTCTGTTAGTTGAGTACACACATC

                 ***            *         *                   **        *  * 
      29029 ACAAACTTGTTTCTGAGAATCCTTCTGTCTCGTTTTTATGGGAAGATATTTACTTTTCCACCGTA
        455 ACAAAGAAGTTTCTGAGAATGCTTCTGTCTAGTTTTTATGGGAAGATATTCCCTTTTCCAACGAA

            *           *       *     *       *  *  *               *        
      29094 GGCATCAAAGCGCTCCAAATGTCCACATCCAGATACTCCAGAAAGAGTGTTTCAAACCTGCTCTA
        520 AGCATCAAAGCGATCCAAATATCCACTTCCAGATTCTACAAAAAGAGTGTTTCAAAACTGCTCTA

             *     *   *         *        * *  *      *      *              *
      29159 TGAAAGGCAATCTTCAACTCTATGAGTTGAATGCAGACATCAGAAAGAAATTTCTGAGAATGCTG
        585 TCAAAGGAAATGTTCAACTCTGTGAGTTGAGTACACACATCACAAAGAAGTTTCTGAGAATGCTT

                   **       *          **             *  *    *              
      29224 CTGTCTACCTTTTAT-TTGA-ATTCCCGCTTCCAACGAAATCCTCCAAGCTATCCAAATATCCAC
        650 CTGTCTAGTTTTTATGATGATATTCCCTTTTCCAACGAAATCATCAAAGCGATCCAAATATCCAC

            * *  **   *                                 **              **  *
      29287 CTGCATTTTCCACAAAAAGAGTGTTTCAAAACTG-CTCTATCAATAGAAATGTTCAACTCCTTTG
        715 TTCCAGATTCTACAAAAAGAGTGTTTCAAAACTGCCTCTATCAAAGGAAATGTTCAACTCTGTTA

             *  *                *           *                            *  
      29351 GCTGGGTACACACATCACAAACAAGTTTCTGAGCATGCTTCTGTCTAGTTTTTATGGGAAGACAT
        780 GTTGAGTACACACATCACAAAGAAGTTTCTGAGAATGCTTCTGTCTAGTTTTTATGGGAAGATAT

                    *  * *  **          *       *            **              
      29416 TCCCTTTTTCACCAAAGGCATCAAAGCGCTCCAAATGTCCACTTCCAGACACTACAAAAAGAGTG
        845 TCCCTTTTCCAACGAAATCATCAAAGCGATCCAAATATCCACTTCCAGATTCTACAAAAAGAGTG

                *  **       **   ***                 *           *      *  * 
      29481 TTTCCAACGTGCTCTAAGAAAACGAATGTTCAACTCTGTGACTTGAATGCAGATATCACAGAGTA
        910 TTTCAAAACTGCTCTATCAAAGGAAATGTTCAACTCTGTGAGTTGAATGCAGACATCACAAAGAA

                      **                  *    *         *                * *
      29546 GTTTCTGAGAGGGCTTCTGTCTAGATTTTAGATGATGATATTCCCGTTTCCAACGAAATCATTAG
        975 GTTTCTGAGAATGCTTCTGTCTAG-TTTT-TATGAAGATATTCCCTTTTCCAACGAAATCATCAA

               *                *   *                    *        **         
      29611 AGCTATCCAAATATCCACTTACAGTTTCTACAAAAAGAGTGTTTCCAAACTGCTGCATCAAAAGA
       1038 AGCGATCCAAATATCCACTTCCAGATTCTACAAAAAGAGTGTTTCAAAACTGCTCTATCAAAAGA

            * *    *          *                   ***      *     *         * 
      29676 GAGGTTCCACTCTGTTAGCTGAGTACACACATCACAAACTTGTTTCTCAGAATCCTTCTGTCTCG
       1103 AATGTTCAACTCTGTTAGTTGAGTACACACATCACAAAGAAGTTTCTGAGAATGCTTCTGTCTAG

                              ** *    
      29741 TTTTTATGGGAAGATATTTACTTTTT
       1168 TTTTTATGGGAAGATATTCCCGTTTT

              *  * **          *       *     *       *  *  *                 
      29767 CACCGTAGGCATCAAAGCGCTCCAAATGTCCACATCCAGATACTCCAGAAAGAGTGTTTCAAACC
          1 CAACGAAATCATCAAAGCGATCCAAATATCCACTTCCAGATTCTACAAAAAGAGTGTTTCAAACC

                              *         *        * *  *      *      *        
      29832 TGCTCTATGAAAGGGAATCTTCAACTCTATGAGTTGAATGCAGACATCAGAAAGAAATTTCTGAG
         66 TGCTCTA-GAAAGGGAATGTTCAACTCTGTGAGTTGAGTACACACATCACAAAGAAGTTTCTGAG

                  *       **          *          *                 *    *    
      29897 AATGCTGCTGTCTACCTTTTAT-T--TGA-ATTCCCGCTTCCAACGAAATC-CTCCAAGCTATCC
        130 AATGCTTCTGTCTAGTTTTTATATGAAGATATTCCCGTTTCCAACGAAATCGCT-AAAGCGATCC

                        *       *                                  ** *  *   
      29957 AAATATCCACTTGCAGATTCCACAAAAAGAGTGTTTCAAAACTGCTCTCTATCAATGGCAAAGTT
        194 AAATATCCACTTCCAGATTCTACAAAAAGAGTGTTTCAAAACTG--CTCTATCAAAAGAAATGTT

                             *     *     *  *                         *      
      30022 CAACTCTGTTAGTTGAGGACACATATCACCAACAAGTTTCTGAGAATGCTTCTGTCTATTTTTTA
        257 CAACTCTGTTAGTTGAGTACACACATCACAAAGAAGTTTCTGAGAATGCTTCTGTCTAGTTTTTA

             **         *      *  *  * ** *    *      *    *          * **   
      30087 TGGGAAGATATTTCCTTTTTCACCGTAGGCGTCAAGGCGATCGAAATGTCCACTTCCACAAACTA
        322 TATGAAGATATTCCCTTTTCCAACGAAATCATCAAAGCGATCCAAATATCCACTTCCAGATTCTA

                              *             * **       *    * *      * ** * *
      30152 CAAAAAGAGTGTTTCAAACCTGCTCTATGAAAGGCGATGTTCATCTCTATGAGTTGAATGGAAAT
        387 CAAAAAGAGTGTTTCAAAACTGCTCTATGAAAAGAAATGTTCAACTCTGTTAGTTGAGTACACAC

               **      *      *       *         *         *         **       
      30217 ATCCGAAAGAAATTTCTGGGAATGCTGCTGTCTAGTGTTTAT---ACGA-ATTCCCGCTTCCAAC
        452 ATCACAAAGAAGTTTCTGAGAATGCTTCTGTCTAGTTTTTATGGGAAGATATTCCCTTTTCCAAC

                * *       *                *    *  *                         
      30278 GAAATCCTCAAAGCAATCCAAATATCCACTTGCAGAATCCACAAAAAGAGTGTTTCAAAACTGCT
        517 GAAAGCATCAAAGCGATCCAAATATCCACTTCCAGATTCTACAAAAAGAGTGTTTCAAAACTGCT

                   **    *          * *                   *  *               
      30343 CTATCAATAGAAAGGTTCAACTCTTTTAGTTGAGTACACACATCACGAACAAGTTTCTGAGAATG
        582 CTATCAAAGGAAATGTTCAACTCTGTGAGTTGAGTACACACATCACAAAGAAGTTTCTGAGAATG

                     * *          *  **  *         * *  **          *       *
      30408 CTTCTGTCTGGCTTTTATTGGAAGACGTTTCCTTTT-CACCAAAGGCATCAAAGCGCTCCAAATG
        647 CTTCTGTCTAGTTTTTA-T-GATGATATTCCCTTTTCCAACGAAATCATCAAAGCGATCCAAATA

                            * *                **      * ***    *            
      30472 TCCACTTCCAGATTCTTCCAAAAGAGTGTTTCAAACGTG-CTCAAAGTAAGGGAATGTTCAACTC
        710 TCCACTTCCAGATTCTACAAAAAGAGTGTTTCAAAACTGCCTCTATCAAAGGAAATGTTCAACTC

               * *    * *  * *     *   *       * * *             *     ***  *
      30536 TGTGACTTGAATGCAGATATCACCAAGTAGTTTCTAATAGTGCTTCTGTCTAGATTTTAGATGAT
        775 TGTTAGTTGAGTACACACATCACAAAGAAGTTTCTGAGAATGCTTCTGTCTAGTTTTTATGGGAA

                     *              * * *   *             *  *   *           
      30601 GATATTCCCGTTTCCAACGAAATCGTTAGAGCTATCCAAATATCCAGTTACAGTTTCTACAAAAA
        840 GATATTCCCTTTTCCAACGAAATCATCAAAGCGATCCAAATATCCACTTCCAGATTCTACAAAAA

                     *        **      *    *            *      ***  *        
      30666 GAGTGTTTCCAAACTGCTGCATCAAAAGAAAGGTTCAACTCTGTTAGTTGAGGACACACATCACA
        905 GAGTGTTTCAAAACTGCTCTATCAAAGGAAATGTTCAACTCTGTGAGTTGAATGCAGACATCACA

                     *                              *                    *   
      30731 AAGAAGTTTGTGAGAATGCTTCTGTCTAGATTTTGTATGACGATATTCCCTTTTCCAACGATATC
        970 AAGAAGTTTCTGAGAATGCTTCTGTCTAG-TTTT-TATGAAGATATTCCCTTTTCCAACGAAATC

            * *     *   *       * *  *    *  *       *  *        *       * * 
      30796 GTTAAAGCAATCTAAATATCAATTTGCAGAATCCACAAAAATAGAGTTTCAAAGCTGCTCTGTAA
       1033 ATCAAAGCGATCCAAATATCCACTTCCAGATTCTACAAAAAGAGTGTTTCAAAACTGCTCTATCA

               *   *    *          *                   ***            *      
      30861 AAATAAAAGTTCCACTCTGTTAGCTGAGTACACACATCACAAACTTGTTTCTGAGAATCCTTCTG
       1098 AAAGAAATGTTCAACTCTGTTAGTTGAGTACACACATCACAAAGAAGTTTCTGAGAATGCTTCTG

               *                   ** *    
      30926 TCTCGTTTTTATGGGAAGATATTTACTTTTT
       1163 TCTAGTTTTTATGGGAAGATATTCCCGTTTT

              *  * **          *       *     *       *  *  *     *           
      30957 CACCGTAGGCATCAAAGCGCTCCAAATGTCCACATCCAGATACTCCAGAAAGACTGTTTCAAACC
          1 CAACGAAATCATCAAAGCGATCCAAATATCCACTTCCAGATTCTACAAAAAGAGTGTTTCAAACC

                              *         *        * *  *      *      *        
      31022 TGCTCTATGAAAGGGAATCTTCAACTCTATGAGTTGAATGCAGACATCAGAAAGAAATTTCTGAG
         66 TGCTCTA-GAAAGGGAATGTTCAACTCTGTGAGTTGAGTACACACATCACAAAGAAGTTTCTGAG

                  *       **          *          *                 *    *    
      31087 AATGCTGCTGTCTACCTTTTAT-T--TGA-ATTCCCGCTTCCAACGAAATC-CTCCAAGCTATCC
        130 AATGCTTCTGTCTAGTTTTTATATGAAGATATTCCCGTTTCCAACGAAATCGCT-AAAGCGATCC

                        *  **   *                                *           
      31147 AAATATCCACTTGCATTTTCCACAAAAAGAGTGTTTCAAAACTGCTCTATCAATAGAAATGTTCA
        194 AAATATCCACTTCCAGATTCTACAAAAAGAGTGTTTCAAAACTGCTCTATCAAAAGAAATGTTCA

                **    *  *                *                                 *
      31212 ACTCCTTTAGCTGGGTACACACATCACAAACAAGTTTCTGAGAATGCTTCTGTCTAGTTTTTATG
        259 ACTCTGTTAGTTGAGTACACACATCACAAAGAAGTTTCTGAGAATGCTTCTGTCTAGTTTTTATA

            *     **         *  * *  **          *       *            **     
      31277 GGAAGACGTTCCCTTTTTCACCAAAGGCATCAAAGCGCTCCAAATGTCCACTTCCAGACACTACA
        324 TGAAGATATTCCCTTTTCCAACGAAATCATCAAAGCGATCCAAATATCCACTTCCAGATTCTACA

                            **       *    ***               * *    * *  * *  
      31342 AAAAGAGTGTTTCAAACGTGCTCTAAGAAAGCGAATGTTCAACTCTGTGACTTGAATGCAGATAT
        389 AAAAGAGTGTTTCAAAACTGCTCTATGAAAAGAAATGTTCAACTCTGTTAGTTGAGTACACACAT

                   *           **            *     ***  *         *          
      31407 CACAAAGTAGTTTCTGAGAGGGCTTCTGTCTAGATTTTAGATGATGATATTCCCGTTTCCAACGA
        454 CACAAAGAAGTTTCTGAGAATGCTTCTGTCTAGTTTTTATGGGAAGATATTCCCTTTTCCAACGA

              *   * *   *                *   *                    *        **
      31472 AATCATTAGAGCTATCCAAATATCCACTTACAGTTTCTACAAAAAGAGTGTTTCCAAACTGCTGC
        519 AAGCATCAAAGCGATCCAAATATCCACTTCCAGATTCTACAAAAAGAGTGTTTCAAAACTGCTCT

                  *  * *    *       *  **                  ***      *     *  
      31537 ATCAAAAGAGAGGTTCCACTCTGTTAGCCGAGTACACACATCACAAACTTGTTTCTCAGAATCCT
        584 ATCAAAGGAAATGTTCAACTCTGTGAGTTGAGTACACACATCACAAAGAAGTTTCTGAGAATGCT

                   *            *      **     *  *  * **          *       *  
      31602 TCTGTCTCGTTTTTATGGGAAGATATTTACTTTTTCACCGTAGGCATCAAAGCGCTCCAAATGTC
        649 TCTGTCTAGTTTTTAT--GATGATATTCCCTTTTCCAACGAAATCATCAAAGCGATCCAAATATC

               *       *  *  *               *          *     *   *         *
      31667 CACATCCAGATACTCCAGAAAGAGTGTTTCAAACCTG-CTCTATGAAAGGGAATCTTCAACTCTA
        712 CACTTCCAGATTCTACAAAAAGAGTGTTTCAAAACTGCCTCTATCAAAGGAAATGTTCAACTCTG

             *      * *  *      *      *              *       **         **  
      31731 TGAGTTGAATGCAGACATCAGAAAGAAATTTCTGAGAATGCTGCTGTCTACCTTTTAT---TTGA
        777 TTAGTTGAGTACACACATCACAAAGAAGTTTCTGAGAATGCTTCTGTCTAGTTTTTATGGGAAGA

                   **             *  *    *                *       *         
      31793 -ATTCCCGCTTCCAACGAAATCCTCCAAGCTATCCAAATATCCACTTGCAGATTCCACAAAAAGA
        842 TATTCCCTTTTCCAACGAAATCATCAAAGCGATCCAAATATCCACTTCCAGATTCTACAAAAAGA

                                     *  *  *            *      ***  * *     *
      31857 GTGTTTCAAAACTGCTCTCTATCAATGGCAAAGTTCAACTCTGTTAGTTGAGGACACATATCACC
        907 GTGTTTCAAAACTG--CTCTATCAAAGGAAATGTTCAACTCTGTGAGTTGAATGCAGACATCACA

              *                         *                  *      *  *  * ** 
      31922 AACAAGTTTCTGAGAATGCTTCTGTCTATTTTTTATGGGAAGATATTTCCTTTTTCACCGTAGGC
        970 AAGAAGTTTCTGAGAATGCTTCTGTCTAGTTTTTAT--GAAGATATTCCCTTTTCCAACGAAATC

            *    *   *  *    *          * **                     *         * 
      31987 GTCAACGCGTTCGAAATGTCCACTTCCACAAACTACAAAAAGAGTGTTTCAAACCTGCTCTATGA
       1033 ATCAAAGCGATCCAAATATCCACTTCCAGATTCTACAAAAAGAGTGTTTCAAAACTGCTCTATCA

              * **       *    * *      * ** * *   **      *      *       *   
      32052 AAGGCCATGTTCATCTCTATGAGTTGAATGGAAATATCCGAAAGAAATTTCTGGGAATGCTGCTG
       1098 AAAGAAATGTTCAACTCTGTTAGTTGAGTACACACATCACAAAGAAGTTTCTGAGAATGCTTCTG

                  *         *          *  *
      32117 TCTAGTGTTTAT---ACGA-ATTCCCGCTTC
       1163 TCTAGTTTTTATGGGAAGATATTCCCGTTTT

                      *       *                *    *  *                   * 
      32144 CAACGAAATCCTCAAAGCAATCCAAATATCCACTTGCAGAATCCACAAAAAGAGTGTTTCAAAAC
          1 CAACGAAATCATCAAAGCGATCCAAATATCCACTTCCAGATTCTACAAAAAGAGTGTTTCAAACC

                    *  ** *  *          * *                   *  *           
      32209 TGCTCTATCAATAGAAAGGTTCAACTCTTTTAGTTGAGTACACACATCACGAACAAGTTTCTGAG
         66 TGCTCTA-GAAAGGGAATGTTCAACTCTGTGAGTTGAGTACACACATCACAAAGAAGTTTCTGAG

                         * *      **     **  *      *    *    *           *  
      32274 AATGCTTCTGTCTGGCTTTTATTGGAAGACGTTTCC-TTTTC-ACCAAA-GGCATCAAAGCGCTC
        130 AATGCTTCTGTCTAGTTTTTATATGAAGATATTCCCGTTTCCAACGAAATCGC-T-AAAGCGATC

                 *                * *                **     * ***  * *       
      32336 CAAATGTCCACTTCCAGATTCTTCCAAAAGAGTGTTTCAAACGTGCTCAAAGTAAGGGAATGTTC
        193 CAAATATCCACTTCCAGATTCTACAAAAAGAGTGTTTCAAAACTGCTCTATCAAAAGAAATGTTC

                    * *    * *  * *     *   *       * * *             *   * *
      32401 AACTCTGTGACTTGAATGCAGATATCACCAAGTAGTTTCTAATAGTGCTTCTGTCTAGATTTCAG
        258 AACTCTGTTAGTTGAGTACACACATCACAAAGAAGTTTCTGAGAATGCTTCTGTCTAGTTTTTAT

                *         *              * * *   * **          *  *   *      
      32466 ATGATGATATTCCCGTTTCCAACGAAATCGTTAGAGCTAAGCAAATATCCAGTTACAGTTTCTAC
        323 ATGAAGATATTCCCTTTTCCAACGAAATCATCAAAGCGATCCAAATATCCACTTCCAGATTCTAC

            *     *       *   *    **  *        *                    *       
      32531 CAAAAGGGTGTTTCCAAATTGCTGCATCAAAAGAAAGGTTCAACTCTGTTAGTTGAGGACACACA
        388 AAAAAGAGTGTTTCAAAACTGCTCTATGAAAAGAAATGTTCAACTCTGTTAGTTGAGTACACACA

                          *                   *    * **  *                   
      32596 TCACAAAGAAGTTTGTGAGAATGCTTCTGTCTAGATTTTGTATGACGATATTCCCTTTTCCAACG
        453 TCACAAAGAAGTTTCTGAGAATGCTTCTGTCTAGTTTTTATGGGAAGATATTCCCTTTTCCAACG

             * * * *     *   *       * *  *    *  *       *  *        *      
      32661 ATATCGTTAAAGCAATCTAAATATCAATTTGCAGAATCCACAAAAATAGAGTTTCAAAGCTGCTC
        518 AAAGCATCAAAGCGATCCAAATATCCACTTCCAGATTCTACAAAAAGAGTGTTTCAAAACTGCTC

             * *   *    *    *       *  *                   ***            * 
      32726 TGTAAAAAGAAAGGTTCCACTCTGTTAGCTGAGTACACACATCACAAACTTGTTTCTGAGAATCC
        583 TATCAAAGGAAATGTTCAACTCTGTGAGTTGAGTACACACATCACAAAGAAGTTTCTGAGAATGC

                    *            *      **        *  * **          *       * 
      32791 TTCTGTCTCGTTTTTATGGGAAGATATTTACTTTTCCACCGTAGGCATCAAAGCGCTCCAAATGT
        648 TTCTGTCTAGTTTTTAT--GATGATATTCCCTTTTCCAACGAAATCATCAAAGCGATCCAAATAT

                *       *  *  *               *          *     *   *         
      32856 CCACATCCAGATACTCCAGAAAGAGTGTTTCAAACCTG-CTCTATGAAAGGCAATCTTCAACTCT
        711 CCACTTCCAGATTCTACAAAAAGAGTGTTTCAAAACTGCCTCTATCAAAGGAAATGTTCAACTCT

            * *      * *  *      *      *              *       **         ** 
      32920 ATGAGTTGAATGCAGACATCAGAAAGAAATTTCTGAGAATGCTGCTGTCTACCTTTTAT---TTG
        776 GTTAGTTGAGTACACACATCACAAAGAAGTTTCTGAGAATGCTTCTGTCTAGTTTTTATGGGAAG

                    **             *  *    *              * *  **   *        
      32982 A-ATTCCCGCTTCCAACGAAATCCTCCAAGCTATCCAAATATCCACCTGCATTTTCCACAAAAAG
        841 ATATTCCCTTTTCCAACGAAATCATCAAAGCGATCCAAATATCCACTTCCAGATTCTACAAAAAG

                                    **                *    *  ** *  *        
      33046 AGTGTTTCAAAACTGCTCTATCAATAGAAATGTTCAACTCCTTTG-GCTGGGTACACACATCACA
        906 AGTGTTTCAAAACTGCTCTATCAAAGGAAATGTTCAACT-CTGTGAGTTGAATGCAGACATCACA

              *           *                            *          *  * *  ** 
      33110 AACAAGTTTCTGAGCATGCTTCTGTCTAGTTTTTATGGGAAGACATTCCCTTTTTCACCAAAGGC
        970 AAGAAGTTTCTGAGAATGCTTCTGTCTAGTTTTTAT--GAAGATATTCCCTTTTCCAACGAAATC

                     *       *            **                     **       ** 
      33175 ATCAAAGCGCTCCAAATGTCCACTTCCAGACACTACAAAAAGAGTGTTTCAAACGTGCTCTAAGA
       1033 ATCAAAGCGATCCAAATATCCACTTCCAGATTCTACAAAAAGAGTGTTTCAAAACTGCTCTATCA

               **               * *    * *  * *      *  *           **       
      33240 AAACGAATGTTCAACTCTGTGACTTGAATGCAGATATCACAGAGTAGTTTCTGAGAGGGCTTCTG
       1098 AAAGAAATGTTCAACTCTGTTAGTTGAGTACACACATCACAAAGAAGTTTCTGAGAATGCTTCTG

                 *     ***  *             *
      33305 TCTAGATTTTAGATGATGATATTCCCGTTTC
       1163 TCTAGTTTTTATGGGAAGATATTCCCGTTTT

                        * *   *                *   *                    *  * 
      33336 CAACGAAATCATTAGAGCTATCCAAATATCCACTTACAGTTTCTACAAAAAGAGTGTTTCCAAAC
          1 CAACGAAATCATCAAAGCGATCCAAATATCCACTTCCAGATTCTACAAAAAGAGTGTTTCAAACC

                    **    *   *    *       *  *                   ***      * 
      33401 TGCTGCATCAAAAGAG-AGGTTCCACTCTGTTAGCTGAGTACACACATCACAAACTTGTTTCTCA
         66 TGCT-C-TAGAAAGGGAATGTTCAACTCTGTGAGTTGAGTACACACATCACAAAGAAGTTTCTGA

                *         *        **         ** *   *  *  * ** ***      *   
      33465 GAATCCTTCTGTCTCGTTTTTATGGGAAGATATTTACTTTTTCACCGTAGGCATCAAAGCGCTCC
        129 GAATGCTTCTGTCTAGTTTTTATATGAAGATATTCCCGTTTCCAACGAAATCGCTAAAGCGATCC

                *     *       *  *  *               *         *   * *   *    
      33530 AAATGTCCACATCCAGATACTCCAGAAAGAGTGTTTCAAACCTGCTCTATGAAAGGGAATCTTCA
        194 AAATATCCACTTCCAGATTCTACAAAAAGAGTGTTTCAAAACTGCTCTATCAAAAGAAATGTTCA

                 * *      * *  *      *      *              *       **       
      33595 ACTCTATGAGTTGAATGCAGACATCAGAAAGAAATTTCTGAGAATGCTGCTGTCTACCTTTTAT-
        259 ACTCTGTTAGTTGAGTACACACATCACAAAGAAGTTTCTGAGAATGCTTCTGTCTAGTTTTTATA

               *         **             *  *    *                *       *   
      33659 T--TGA-ATTCCCGCTTCCAACGAAATCCTCCAAGCTATCCAAATATCCACTTGCAGATTCCACA
        324 TGAAGATATTCCCTTTTCCAACGAAATCATCAAAGCGATCCAAATATCCACTTCCAGATTCTACA

                                        *  ** *  *                    *     *
      33721 AAAAGAGTGTTTCAAAACTGCTCTCTATCAATGGCAAAGTTCAACTCTGTTAGTTGAGGACACAT
        389 AAAAGAGTGTTTCAAAACTG--CTCTATGAAAAGAAATGTTCAACTCTGTTAGTTGAGTACACAC

                 *  *                         *                  *      *  * 
      33786 ATCACCAACAAGTTTCTGAGAATGCTTCTGTCTATTTTTTATGGGAAGATATTTCCTTTTTCACC
        452 ATCACAAAGAAGTTTCTGAGAATGCTTCTGTCTAGTTTTTATGGGAAGATATTCCCTTTTCCAAC

             * *  *    *      *    *          * **                     *     
      33851 GTAGGCGTCAAGGCGATCGAAATGTCCACTTCCACAAACTACAAAAAGAGTGTTTCAAACCTGCT
        517 GAAAGCATCAAAGCGATCCAAATATCCACTTCCAGATTCTACAAAAAGAGTGTTTCAAAACTGCT

                *     **       *    *        * ** * *   **      *      *     
      33916 CTATGAAAGGCCATGTTCATCTCTATGAGTTGAATGGAAATATCCGAAAGAAATTTCTGGGAATG
        582 CTATCAAAGGAAATGTTCAACTCTGTGAGTTGAGTACACACATCACAAAGAAGTTTCTGAGAATG

              *         *       *         **             *       *           
      33981 CTGCTGTCTAGTGTTTAT-ACGA-ATTCCCGCTTCCAACGAAATCCTCAAAGCAATCCAAATATC
        647 CTTCTGTCTAGTTTTTATGATGATATTCCCTTTTCCAACGAAATCATCAAAGCGATCCAAATATC

                 *    *  *                                 **    *          *
      34044 CACTTGCAGAATCCACAAAAAGAGTGTTTCAAAACTG-CTCTATCAATAGAAAGGTTCAACTCTT
        712 CACTTCCAGATTCTACAAAAAGAGTGTTTCAAAACTGCCTCTATCAAAGGAAATGTTCAACTCTG

                                 *  *                        * *      *      
      34108 TTAGTTGAGTACACACATCACGAACAAGTTTCTGAGAATGCTTCTGTCTGGCTTTTATTGGAAGA
        777 TTAGTTGAGTACACACATCACAAAGAAGTTTCTGAGAATGCTTCTGTCTAGTTTTTATGGGAAGA

            **  *         * *  **          *       *                * *      
      34173 CGTTTCCTTTT-CACCAAAGGCATCAAAGCGCTCCAAATGTCCACTTCCAGATTCTTCCAAAAGA
        842 TATTCCCTTTTCCAACGAAATCATCAAAGCGATCCAAATATCCACTTCCAGATTCTACAAAAAGA

                      **     * ***    *                 *           *     *  
      34237 GTGTTTCAAACGTGCTCAAAGTAAGGGAATGTTCAACTCTGTGACTTGAATGCAGATATCACCAA
        907 GTGTTTCAAAACTGCTCTATCAAAGGAAATGTTCAACTCTGTGAGTTGAATGCAGACATCACAAA

             *       * * *                   *    *         *              * 
      34302 GTAGTTTCTAATAGTGCTTCTGTCTAGATTTTAGATGATGATATTCCCGTTTCCAACGAAATCGT
        972 GAAGTTTCTGAGAATGCTTCTGTCTAG-TTTT-TATGAAGATATTCCCTTTTCCAACGAAATCAT

            * *   *             *  *   *                    *        **      
      34367 TAGAGCTATCCAAATATCCAGTTACAGTTTCTACAAAAAGAGTGTTTCCAAACTGCTGCATCAAA
       1035 CAAAGCGATCCAAATATCCACTTCCAGATTCTACAAAAAGAGTGTTTCAAAACTGCTCTATCAAA

                 *                    *                     *                
      34432 AGAAAGGTTCAACTCTGTTAGTTGAGGACACACATCACAAAGAAGTTTGTGAGAATGCTTCTGTC
       1100 AGAAATGTTCAACTCTGTTAGTTGAGTACACACATCACAAAGAAGTTTCTGAGAATGCTTCTGTC

               *    * **  *         *   *
      34497 TAGATTTTGTATGACGATATTCCCTTTTC
       1165 TAGTTTTTATGGGAAGATATTCCCGTTTT

                  *   * *     *   *       * *  *    *  *       *  *        * 
      34526 CAACGATATCGTTAAAGCAATCTAAATATCAATTTGCAGAATCCACAAAAATAGAGTTTCAAAGC
          1 CAACGAAATCATCAAAGCGATCCAAATATCCACTTCCAGATTCTACAAAAAGAGTGTTTCAAACC

                   **   ***  *    *       *  *                   ***         
      34591 TGCTCTGTAAAAATAAAAGTTCCACTCTGTTAGCTGAGTACACACATCACAAACTTGTTTCTGAG
         66 TGCTCT-AGAAAGGGAATGTTCAACTCTGTGAGTTGAGTACACACATCACAAAGAAGTTTCTGAG

               *         *        **         ** *   *  *  * ** ***      *    
      34656 AATCCTTCTGTCTCGTTTTTATGGGAAGATATTTACTTTTTCACCGTAGGCATCAAAGCGCTCCA
        130 AATGCTTCTGTCTAGTTTTTATATGAAGATATTCCCGTTTCCAACGAAATCGCTAAAGCGATCCA

               *     *       *  *  *     *         *         *   * *   *     
      34721 AATGTCCACATCCAGATACTCCAGAAAGACTGTTTCAAACCTGCTCTATGAAAGGGAATCTTCAA
        195 AATATCCACTTCCAGATTCTACAAAAAGAGTGTTTCAAAACTGCTCTATCAAAAGAAATGTTCAA

                * *      * *  *      *      *              *       **        
      34786 CTCTATGAGTTGAATGCAGACATCAGAAAGAAATTTCTGAGAATGCTGCTGTCTACCTTTTAT-T
        260 CTCTGTTAGTTGAGTACACACATCACAAAGAAGTTTCTGAGAATGCTTCTGTCTAGTTTTTATAT

              *         **             *  *    *                *  **   *    
      34850 --TGA-ATTCCCGCTTCCAACGAAATCCTCCAAGCTATCCAAATATCCACTTGCATTTTCCACAA
        325 GAAGATATTCCCTTTTCCAACGAAATCATCAAAGCGATCCAAATATCCACTTCCAGATTCTACAA

                                     *  *               **    *  *           
      34912 AAAGAGTGTTTCAAAACTGCTCTATCAATAGAAATGTTCAACTCCTTTAGCTGGGTACACACATC
        390 AAAGAGTGTTTCAAAACTGCTCTATGAAAAGAAATGTTCAACTCTGTTAGTTGAGTACACACATC

                 *                                        **         *  * *  
      34977 ACAAACAAGTTTCTGAGAATGCTTCTGTCTAGTTTTTATGGGAAGACGTTCCCTTTTTCACCAAA
        455 ACAAAGAAGTTTCTGAGAATGCTTCTGTCTAGTTTTTATGGGAAGATATTCCCTTTTCCAACGAA

            *           *       *            **                     **       
      35042 GGCATCAAAGCGCTCCAAATGTCCACTTCCAGACACTACAAAAAGAGTGTTTCAAACGTGCTCTA
        520 AGCATCAAAGCGATCCAAATATCCACTTCCAGATTCTACAAAAAGAGTGTTTCAAAACTGCTCTA

            **    **                 *    * *  * *         *           **    
      35107 AGAAAGCGAATGTTCAACTCTGTGACTTGAATGCAGATATCACAAAGTAGTTTCTGAGAGGGCTT
        585 TCAAAGGAAATGTTCAACTCTGTGAGTTGAGTACACACATCACAAAGAAGTTTCTGAGAATGCTT

                          *              *                * *   *            
      35172 CTGTCTAGATTTTAGATGATGATATTCCCGTTTCCAACGAAATCATTAGAGCTATCCAAATATCC
        650 CTGTCTAG-TTTT-TATGATGATATTCCCTTTTCCAACGAAATCATCAAAGCGATCCAAATATCC

                *   *                    *         **      *  * *    *       
      35237 ACTTACAGTTTCTACAAAAAGAGTGTTTCCAAACTG-CTGCATCAAAAGAGAGGTTCCACTCTGT
        713 ACTTCCAGATTCTACAAAAAGAGTGTTTCAAAACTGCCTCTATCAAAGGAAATGTTCAACTCTGT

               **                  ***      *     *         *                
      35301 TAGCCGAGTACACACATCACAAACTTGTTTCTCAGAATCCTTCTGTCTCGTTTTTATGGGAAGAT
        778 TAGTTGAGTACACACATCACAAAGAAGTTTCTGAGAATGCTTCTGTCTAGTTTTTATGGGAAGAT

               **     *  *  * **          *       *     *       **    *      
      35366 ATTTACTTTTTCACCGTAGGCATCAAAGCGCTCCAAATGTCCACATCCAGATAGTACAGAAAGAG
        843 ATTCCCTTTTCCAACGAAATCATCAAAGCGATCCAAATATCCACTTCCAGATTCTACAAAAAGAG

                     *         *     *   *         *                    *    
      35431 TGTTTCAAACCTGCTCTATGAAAGGGAATCTTCAACTCTATGAGTTGAATGCAGACATCAGAAAG
        908 TGTTTCAAAACTGCTCTATCAAAGGAAATGTTCAACTCTGTGAGTTGAATGCAGACATCACAAAG

              *              *       **       **         **             *  * 
      35496 AAATTTCTGAGAATGCTGCTGTCTACCTTTTAT-TTGA-ATTCCCGCTTCCAACGAAATCCTCCA
        973 AAGTTTCTGAGAATGCTTCTGTCTAGTTTTTATGAAGATATTCCCTTTTCCAACGAAATCATCAA

               *                *       *                                  **
      35559 AGCTATCCAAATATCCACTTGCAGATTCCACAAAAAGAGTGTTTCAAAACTGCTCTCTATCAATG
       1038 AGCGATCCAAATATCCACTTCCAGATTCTACAAAAAGAGTGTTTCAAAACTG--CTCTATCAAAA

             *  *                    *     *     *  *                        
      35624 GCAAAGTTCAACTCTGTTAGTTGAGGACACATATCACCAACAAGTTTCTGAGAATGCTTCTGTCT
       1101 GAAATGTTCAACTCTGTTAGTTGAGTACACACATCACAAAGAAGTTTCTGAGAATGCTTCTGTCT

             *                  *  *    
      35689 ATTTTTTATGGGAAGATATTTCCTTTTT
       1166 AGTTTTTATGGGAAGATATTCCCGTTTT

              *  * ** *    *      *    *          * **                       
      35717 CACCGTAGGCGTCAAGGCGATCGAAATGTCCACTTCCACAAACTACAAAAAGAGTGTTTCAAACC
          1 CAACGAAATCATCAAAGCGATCCAAATATCCACTTCCAGATTCTACAAAAAGAGTGTTTCAAACC

                          **       *    *        * ** * *   **      *      * 
      35782 TGCTCTATGAAAGGCCATGTTCATCTCTATGAGTTGAATGGAAATATCCGAAAGAAATTTCTGGG
         66 TGCTCTA-GAAAGGGAATGTTCAACTCTGTGAGTTGAGTACACACATCACAAAGAAGTTTCTGAG

                  *          *        *          *                      *    
      35847 AATGCTGCTGTCTAG-TGTT-TAT-ACGA-ATTCCCGCTTCCAACGAAATC-CTCAAAGCAATCC
        130 AATGCTTCTGTCTAGTTTTTATATGAAGATATTCCCGTTTCCAACGAAATCGCT-AAAGCGATCC

                        *    *  *                                *     *     
      35907 AAATATCCACTTGCAGAATCCACAAAAAGAGTGTTTCAAAACTGCTCTATCAATAGAAAGGTTCA
        194 AAATATCCACTTCCAGATTCTACAAAAAGAGTGTTTCAAAACTGCTCTATCAAAAGAAATGTTCA

                 *                     *  *                        * *      *
      35972 ACTCTTTTAGTTGAGTACACACATCACGAACAAGTTTCTGAGAATGCTTCTGTCTGGCTTTTATT
        259 ACTCTGTTAGTTGAGTACACACATCACAAAGAAGTTTCTGAGAATGCTTCTGTCTAGTTTTTATA

            *     **  *         * *  **          *       *                * *
      36037 GGAAGACGTTTCCTTTT-CACCAAAGGCATCAAAGCGCTCCAAATGTCCACTTCCAGATTCTTCC
        324 TGAAGATATTCCCTTTTCCAACGAAATCATCAAAGCGATCCAAATATCCACTTCCAGATTCTACA

                            **     * * *  * *               * *    * *  * *  
      36101 AAAAGAGTGTTTCAAACGTGCTCAAAGTAAGGGAATGTTCAACTCTGTGACTTGAATGCAGATAT
        389 AAAAGAGTGTTTCAAAACTGCTCTATGAAAAGAAATGTTCAACTCTGTTAGTTGAGTACACACAT

               *   *       * * *             *   * ***  *         *          
      36166 CACCAAGTAGTTTCTAATAGTGCTTCTGTCTAGATTTCAGATGATGATATTCCCGTTTCCAACGA
        454 CACAAAGAAGTTTCTGAGAATGCTTCTGTCTAGTTTTTATGGGAAGATATTCCCTTTTCCAACGA

              * * * *   * **          *  *   *      *     *       *   *    **
      36231 AATCGTTAGAGCTAAGCAAATATCCAGTTACAGTTTCTACCAAAAGGGTGTTTCCAAATTGCTGC
        519 AAGCATCAAAGCGATCCAAATATCCACTTCCAGATTCTACAAAAAGAGTGTTTCAAAACTGCTCT

                  *    *            *       *                     *          
      36296 ATCAAAAGAAAGGTTCAACTCTGTTAGTTGAGGACACACATCACAAAGAAGTTTGTGAGAATGCT
        584 ATCAAAGGAAATGTTCAACTCTGTGAGTTGAGTACACACATCACAAAGAAGTTTCTGAGAATGCT

                                *                    *   * *     *   *       
      36361 TCTGTCTAGATTTTGTATGACGATATTCCCTTTTCCAACGATATCGTTAAAGCAATCTAAATATC
        649 TCTGTCTAG-TTTT-TATGATGATATTCCCTTTTCCAACGAAATCATCAAAGCGATCCAAATATC

            * *  *    *  *       *  *        *        * *   *    *    *      
      36426 AATTTGCAGAATCCACAAAAATAGAGTTTCAAAGCTG-CTCTGTAAAAAGAAAGGTTCCACTCTG
        712 CACTTCCAGATTCTACAAAAAGAGTGTTTCAAAACTGCCTCTATCAAAGGAAATGTTCAACTCTG

                *                   ***            *         *               
      36490 TTAGCTGAGTACACACATCACAAACTTGTTTCTGAGAATCCTTCTGTCTCGTTTTTATGGGAAGA
        777 TTAGTTGAGTACACACATCACAAAGAAGTTTCTGAGAATGCTTCTGTCTAGTTTTTATGGGAAGA

                **        *  * **          *       *     *       *  *  *     
      36555 TATTTACTTTTCCACCGTAGGCATCAAAGCGCTCCAAATGTCCACATCCAGATACTCCAGAAAGA
        842 TATTCCCTTTTCCAACGAAATCATCAAAGCGATCCAAATATCCACTTCCAGATTCTACAAAAAGA

                      *         *     *   *         *                    *   
      36620 GTGTTTCAAACCTGCTCTATGAAAGGCAATCTTCAACTCTATGAGTTGAATGCAGACATCAGAAA
        907 GTGTTTCAAAACTGCTCTATCAAAGGAAATGTTCAACTCTGTGAGTTGAATGCAGACATCACAAA

               *              *       **       **         **             *  *
      36685 GAAATTTCTGAGAATGCTGCTGTCTACCTTTTAT-TTGA-ATTCCCGCTTCCAACGAAATCCTCC
        972 GAAGTTTCTGAGAATGCTTCTGTCTAGTTTTTATGAAGATATTCCCTTTTCCAACGAAATCATCA

                *              * *  **   *                                *  
      36748 AAGCTATCCAAATATCCACCTGCATTTTCCACAAAAAGAGTGTTTCAAAACTGCTCTATCAATAG
       1037 AAGCGATCCAAATATCCACTTCCAGATTCTACAAAAAGAGTGTTTCAAAACTGCTCTATCAAAAG

                         **  * *  *                *           *             
      36813 AAATGTTCAACTCCTTTGGCTGGGTACACACATCACAAACAAGTTTCTGAGCATGCTTCTGTCTA
       1102 AAATGTTCAACTCTGTTAGTTGAGTACACACATCACAAAGAAGTTTCTGAGAATGCTTCTGTCTA

                           *      *    
      36878 GTTTTTATGGGAAGACATTCCCTTTTT
       1167 GTTTTTATGGGAAGATATTCCCGTTTT

              * *  **          *       *            **                  *   *
      36905 CACCAAAGGCATCAAAGCGCTCCAAATGTCCACTTCCAGACACTACAAAAAGAGTGTTTCCAACG
          1 CAACGAAATCATCAAAGCGATCCAAATATCCACTTCCAGATTCTACAAAAAGAGTGTTTCAAACC

                        **                  *    * *  * *      *  *          
      36970 TGCTCTAAGAAAACGAATGTTCAACTCTGTGACTTGAATGCAGATATCACAGAGTAGTTTCTGAG
         66 TGCTCT-AGAAAGGGAATGTTCAACTCTGTGAGTTGAGTACACACATCACAAAGAAGTTTCTGAG

             **            *     *    *                        **  *   *     
      37035 AGGGCTTCTGTCTAGATTTTAGATGATGATATTCCCGTTTCCAACGAAATCATTAGAGCTATCCA
        130 AATGCTTCTGTCTAGTTTTTATATGAAGATATTCCCGTTTCCAACGAAATCGCTAAAGCGATCCA

                       *   *                    *        **         * *    * 
      37100 AATATCCACTTACAGTTTCTACAAAAAGAGTGTTTCCAAACTGCTGCATCAAAAGAGAGGTTCCA
        195 AATATCCACTTCCAGATTCTACAAAAAGAGTGTTTCAAAACTGCTCTATCAAAAGAAATGTTCAA

                     *                   ***      *     *         *        **
      37165 CTCTGTTAGCTGAGTACACACATCACAAACTTGTTTCTCAGAATCCTTCTGTCTCGTTTTTATGG
        260 CTCTGTTAGTTGAGTACACACATCACAAAGAAGTTTCTGAGAATGCTTCTGTCTAGTTTTTATAT

                     **     *  *  * **          *       *     *       *  *  *
      37230 GAAGATATTTACTTTTTCACCGTAGGCATCAAAGCGCTCCAAATGTCCACATCCAGATACTCCAG
        325 GAAGATATTCCCTTTTCCAACGAAATCATCAAAGCGATCCAAATATCCACTTCCAGATTCTACAA

                           *             * *   *         * *      * *  *     
      37295 AAAGAGTGTTTCAAACCTGCTCTATGAAAGGGAATCTTCAACTCTATGAGTTGAATGCAGACATC
        390 AAAGAGTGTTTCAAAACTGCTCTATGAAAAGAAATGTTCAACTCTGTTAGTTGAGTACACACATC

             *      *              *       **         **         **          
      37360 AGAAAGAAATTTCTGAGAATGCTGCTGTCTACCTTTTAT---TTGA-ATTCCCGCTTCCAACGAA
        455 ACAAAGAAGTTTCTGAGAATGCTTCTGTCTAGTTTTTATGGGAAGATATTCCCTTTTCCAACGAA

             * *  *    *                *       *                            
      37421 ATCCTCCAAGCTATCCAAATATCCACTTGCAGATTCCACAAAAAGAGTGTTTCAAAACTGCTCTC
        520 AGCATCAAAGCGATCCAAATATCCACTTCCAGATTCTACAAAAAGAGTGTTTCAAAACTG--CTC

                  *  *  *            *       *     *     *  *                
      37486 TATCAATGGCAAAGTTCAACTCTGTTAGTTGAGGACACATATCACCAACAAGTTTCTGAGAATGC
        583 TATCAAAGGAAATGTTCAACTCTGTGAGTTGAGTACACACATCACAAAGAAGTTTCTGAGAATGC

                     *           *      *      *  *  * ** *    *      *    * 
      37551 TTCTGTCTATTTTTTATGGGAAGATATTTCCTTTTTCACCGTAGGCGTCAAGGCGATCGAAATGT
        648 TTCTGTCTAGTTTTTAT--GATGATATTCCCTTTTCCAACGAAATCATCAAAGCGATCCAAATAT

                     * **                     *          *     **       *    
      37616 CCACTTCCACAAACTACAAAAAGAGTGTTTCAAACCTG-CTCTATGAAAGGCGATGTTCATCTCT
        711 CCACTTCCAGATTCTACAAAAAGAGTGTTTCAAAACTGCCTCTATCAAAGGAAATGTTCAACTCT

            * *      * ** * *   **      *      *       *         *         * 
      37680 ATGAGTTGAATGGAAATATCCGAAAGAAATTTCTGGGAATGCTGCTGTCTAGTGTTTAT---ACG
        776 GTTAGTTGAGTACACACATCACAAAGAAGTTTCTGAGAATGCTTCTGTCTAGTTTTTATGGGAAG

                    **             *       *                *    *  *        
      37742 A-ATTCCCGCTTCCAACGAAATCCTCAAAGCAATCCAAATATCCACTTGCAGAATCCACAAAAAG
        841 ATATTCCCTTTTCCAACGAAATCATCAAAGCGATCCAAATATCCACTTCCAGATTCTACAAAAAG

                                    **    *          * *      * *  *       * 
      37806 AGTGTTTCAAAACTGCTCTATCAATAGAAAGGTTCAACTCTTTTAGTTGAGTACACACATCACGA
        906 AGTGTTTCAAAACTGCTCTATCAAAGGAAATGTTCAACTCTGTGAGTTGAATGCAGACATCACAA

             *                        * *             **  *         * *  **  
      37871 ACAAGTTTCTGAGAATGCTTCTGTCTGGCTTTTATTGGAAGACGTTTCCTTTT-CACCAAAGGCA
        971 AGAAGTTTCTGAGAATGCTTCTGTCTAGTTTTTA-T-GAAGATATTCCCTTTTCCAACGAAATCA

                    *       *                * *                **     * *** 
      37935 TCAAAGCGCTCCAAATGTCCACTTCCAGATTCTTCCAAAAGAGTGTTTCAAACGTGCTCAAAGTA
       1034 TCAAAGCGATCCAAATATCCACTTCCAGATTCTACAAAAAGAGTGTTTCAAAACTGCTCTATCAA

             * *               * *    * *  * *     *   *       * * *         
      38000 AGGGAATGTTCAACTCTGTGACTTGAATGCAGATATCACCAAGTAGTTTCTAATAGTGCTTCTGT
       1099 AAGAAATGTTCAACTCTGTTAGTTGAGTACACACATCACAAAGAAGTTTCTGAGAATGCTTCTGT

                *     ***  *             *
      38065 CTAGATTTTAGATGATGATATTCCCGTTTC
       1164 CTAGTTTTTATGGGAAGATATTCCCGTTTT

                      * * *   *             *  *   *                    *  * 
      38095 CAACGAAATCGTTAGAGCTATCCAAATATCCAGTTACAGTTTCTACAAAAAGAGTGTTTCCAAAC
          1 CAACGAAATCATCAAAGCGATCCAAATATCCACTTCCAGATTCTACAAAAAGAGTGTTTCAAACC

                  ***   * *  *            *       *                     *    
      38160 TGCTGCATCAAAAGAAAGGTTCAACTCTGTTAGTTGAGGACACACATCACAAAGAAGTTTGTGAG
         66 TGCT-CTAGAAAGGGAATGTTCAACTCTGTGAGTTGAGTACACACATCACAAAGAAGTTTCTGAG

                           *    *     *         *          *    *      *   * 
      38225 AATGCTTCTGTCTAGATTTTGTATGACGATATTCCCTTTTCCAACGATATCGTTAAAGCAATCTA
        130 AATGCTTCTGTCTAGTTTTTATATGAAGATATTCCCGTTTCCAACGAAATCGCTAAAGCGATCCA

                  * *  *    *  *       *  *        *       * *    *   *    * 
      38290 AATATCAATTTGCAGAATCCACAAAAATAGAGTTTCAAAGCTGCTCTGTAAAAATAAAAGTTCCA
        195 AATATCCACTTCCAGATTCTACAAAAAGAGTGTTTCAAAACTGCTCTATCAAAAGAAATGTTCAA

                     *                   ***            *         *        **
      38355 CTCTGTTAGCTGAGTACACACATCACAAACTTGTTTCTGAGAATCCTTCTGTCTCGTTTTTATGG
        260 CTCTGTTAGTTGAGTACACACATCACAAAGAAGTTTCTGAGAATGCTTCTGTCTAGTTTTTATAT

                     **     *  *  * **          *       *     *       *  *  *
      38420 GAAGATATTTACTTTTTCACCGTAGGCATCAAAGCGCTCCAAATGTCCACATCCAGATACTCCAG
        325 GAAGATATTCCCTTTTCCAACGAAATCATCAAAGCGATCCAAATATCCACTTCCAGATTCTACAA

                 *         *             * *   *         * *      * *  *     
      38485 AAAGACTGTTTCAAACCTGCTCTATGAAAGGGAATCTTCAACTCTATGAGTTGAATGCAGACATC
        390 AAAGAGTGTTTCAAAACTGCTCTATGAAAAGAAATGTTCAACTCTGTTAGTTGAGTACACACATC

             *      *              *       **         **         **          
      38550 AGAAAGAAATTTCTGAGAATGCTGCTGTCTACCTTTTAT---TTGA-ATTCCCGCTTCCAACGAA
        455 ACAAAGAAGTTTCTGAGAATGCTTCTGTCTAGTTTTTATGGGAAGATATTCCCTTTTCCAACGAA

             * *  *    *                *  **   *                            
      38611 ATCCTCCAAGCTATCCAAATATCCACTTGCATTTTCCACAAAAAGAGTGTTTCAAAACTGCTCTA
        520 AGCATCAAAGCGATCCAAATATCCACTTCCAGATTCTACAAAAAGAGTGTTTCAAAACTGCTCTA

                **              ** *  *  *                *                  
      38676 TCAATAGAAATGTTCAACTCCTTTAGCTGGGTACACACATCACAAACAAGTTTCTGAGAATGCTT
        585 TCAAAGGAAATGTTCAACTCTGTGAGTTGAGTACACACATCACAAAGAAGTTTCTGAGAATGCTT

                               *  **         *  * *  **          *       *   
      38741 CTGTCTAGTTTTTATGGGAAGACGTTCCCTTTTTCACCAAAGGCATCAAAGCGCTCCAAATGTCC
        650 CTGTCTAGTTTTTAT--GATGATATTCCCTTTTCCAACGAAATCATCAAAGCGATCCAAATATCC

                     **                     **        **    **               
      38806 ACTTCCAGACACTACAAAAAGAGTGTTTCAAACGTG-CTCTAAGAAAGCGAATGTTCAACTCTGT
        713 ACTTCCAGATTCTACAAAAAGAGTGTTTCAAAACTGCCTCTATCAAAGGAAATGTTCAACTCTGT

            * *    * *  * *         *           **            *     ***  *   
      38870 GACTTGAATGCAGATATCACAAAGTAGTTTCTGAGAGGGCTTCTGTCTAGATTTTAGATGATGAT
        778 TAGTTGAGTACACACATCACAAAGAAGTTTCTGAGAATGCTTCTGTCTAGTTTTTATGGGAAGAT

                  *                * *   *                *   *              
      38935 ATTCCCGTTTCCAACGAAATCATTAGAGCTATCCAAATATCCACTTACAGTTTCTACAAAAAGAG
        843 ATTCCCTTTTCCAACGAAATCATCAAAGCGATCCAAATATCCACTTCCAGATTCTACAAAAAGAG

                  *        **      *  * *    *       *  **  * *  *          *
      39000 TGTTTCCAAACTGCTGCATCAAAAGAGAGGTTCCACTCTGTTAGCCGAGTACACACATCACAAAC
        908 TGTTTCAAAACTGCTCTATCAAAGGAAATGTTCAACTCTGTGAGTTGAATGCAGACATCACAAAG

            **      *     *         *                   **     *  *  * **    
      39065 TTGTTTCTCAGAATCCTTCTGTCTCGTTTTTATGGGAAGATATTTACTTTTTCACCGTAGGCATC
        973 AAGTTTCTGAGAATGCTTCTGTCTAGTTTTTAT--GAAGATATTCCCTTTTCCAACGAAATCATC

                  *       *     *       *  *  *               *         *   *
      39130 AAAGCGCTCCAAATGTCCACATCCAGATACTCCAGAAAGAGTGTTTCAAACCTGCTCTATGAAAG
       1036 AAAGCGATCCAAATATCCACTTCCAGATTCTACAAAAAGAGTGTTTCAAAACTGCTCTATCAAAA

             *   *         * *      * *  *      *      *              *      
      39195 GGAATCTTCAACTCTATGAGTTGAATGCAGACATCAGAAAGAAATTTCTGAGAATGCTGCTGTCT
       1101 GAAATGTTCAACTCTGTTAGTTGAGTACACACATCACAAAGAAGTTTCTGAGAATGCTTCTGTCT

             **         **          *  *
      39260 ACCTTTTAT---TTGA-ATTCCCGCTTC
       1166 AGTTTTTATGGGAAGATATTCCCGTTTT

                      *  *    *                *       *                   * 
      39284 CAACGAAATCCTCCAAGCTATCCAAATATCCACTTGCAGATTCCACAAAAAGAGTGTTTCAAAAC
          1 CAACGAAATCATCAAAGCGATCCAAATATCCACTTCCAGATTCTACAAAAAGAGTGTTTCAAACC

                      *  *  *  *            *       *     *     *  *         
      39349 TGCTCTCTATCAATGGCAAAGTTCAACTCTGTTAGTTGAGGACACATATCACCAACAAGTTTCTG
         66 TG--CTCTA-GAAAGGGAATGTTCAACTCTGTGAGTTGAGTACACACATCACAAAGAAGTTTCTG

                            *       **         *  *   *  *  * **  **  *   *  
      39414 AGAATGCTTCTGTCTATTTTTTATGGGAAGATATTTCCTTTTTCACCGTAGGCGTCAACGCGTTC
        128 AGAATGCTTCTGTCTAGTTTTTATATGAAGATATTCCCGTTTCCAACGAAATCGCTAAAGCGATC

            *    *          * **                     *         *   * **      
      39479 GAAATGTCCACTTCCACAAACTACAAAAAGAGTGTTTCAAACCTGCTCTATGAAAGGCCATGTTC
        193 CAAATATCCACTTCCAGATTCTACAAAAAGAGTGTTTCAAAACTGCTCTATCAAAAGAAATGTTC

             *    * *      * ** * *   **      *      *       *          *    
      39544 ATCTCTATGAGTTGAATGGAAATATCCGAAAGAAATTTCTGGGAATGCTGCTGTCTAG-TGTT-T
        258 AACTCTGTTAGTTGAGTACACACATCACAAAGAAGTTTCTGAGAATGCTTCTGTCTAGTTTTTAT

                *         **             *       *                *    *  *  
      39607 AT-ACGA-ATTCCCGCTTCCAACGAAATCCTCAAAGCAATCCAAATATCCACTTGCAGAATCCAC
        323 ATGAAGATATTCCCTTTTCCAACGAAATCATCAAAGCGATCCAAATATCCACTTCCAGATTCTAC

                                       *  *     *          *                 
      39670 AAAAAGAGTGTTTCAAAACTGCTCTATCAATAGAAAGGTTCAACTCTTTTAGTTGAGTACACACA
        388 AAAAAGAGTGTTTCAAAACTGCTCTATGAAAAGAAATGTTCAACTCTGTTAGTTGAGTACACACA

                *  *                        * *      *      **  *         * *
      39735 TCACGAACAAGTTTCTGAGAATGCTTCTGTCTGGCTTTTATTGGAAGACGTTTCCTTTT-CACCA
        453 TCACAAAGAAGTTTCTGAGAATGCTTCTGTCTAGTTTTTATGGGAAGATATTCCCTTTTCCAACG

              *           *       *                * *                **     
      39799 AAGGCATCAAAGCGCTCCAAATGTCCACTTCCAGATTCTTCCAAAAGAGTGTTTCAAACGTGCTC
        518 AAAGCATCAAAGCGATCCAAATATCCACTTCCAGATTCTACAAAAAGAGTGTTTCAAAACTGCTC

            * ***    *                 *    * *  * *     *   *       * * *   
      39864 AAAGTAAGGGAATGTTCAACTCTGTGACTTGAATGCAGATATCACCAAGTAGTTTCTAATAGTGC
        583 TATCAAAGGAAATGTTCAACTCTGTGAGTTGAGTACACACATCACAAAGAAGTTTCTGAGAATGC

                           **              *              * * *   * **       
      39929 TTCTGTCTAGATTTCAGATGATGATATTCCCGTTTCCAACGAAATCGTTAGAGCTAAGCAAATAT
        648 TTCTGTCTAG-TTT-TTATGATGATATTCCCTTTTCCAACGAAATCATCAAAGCGATCCAAATAT

               *  *   *      *     *       *   *     **      *    *          
      39994 CCAGTTACAGTTTCTACCAAAAGGGTGTTTCCAAATTG-CTGCATCAAAAGAAAGGTTCAACTCT
        711 CCACTTCCAGATTCTACAAAAAGAGTGTTTCAAAACTGCCTCTATCAAAGGAAATGTTCAACTCT

                      *                     *                   *    * **  * 
      40058 GTTAGTTGAGGACACACATCACAAAGAAGTTTGTGAGAATGCTTCTGTCTAGATTTTGTATGACG
        776 GTTAGTTGAGTACACACATCACAAAGAAGTTTCTGAGAATGCTTCTGTCTAGTTTTTATGGGAAG

                               *   * *     *   *       * *  *    *  *       *
      40123 ATATTCCCTTTTCCAACGATATCGTTAAAGCAATCTAAATATCAATTTGCAGAATCCACAAAAAT
        841 ATATTCCCTTTTCCAACGAAATCATCAAAGCGATCCAAATATCCACTTCCAGATTCTACAAAAAG

              *        *       * *   *    *    *       *  *   * *  *         
      40188 AGAGTTTCAAAGCTGCTCTGTAAAAAGAAAGGTTCCACTCTGTTAGCTGAGTACACACATCACAA
        906 AGTGTTTCAAAACTGCTCTATCAAAGGAAATGTTCAACTCTGTGAGTTGAATGCAGACATCACAA

             ***            *         *                   **        *  * **  
      40253 ACTTGTTTCTGAGAATCCTTCTGTCTCGTTTTTATGGGAAGATATTTACTTTTCCACCGTAGGCA
        971 AGAAGTTTCTGAGAATGCTTCTGTCTAGTTTTTAT--GAAGATATTCCCTTTTCCAACGAAATCA

                    *       *     *       *  *  *               *         *  
      40318 TCAAAGCGCTCCAAATGTCCACATCCAGATACTCCAGAAAGAGTGTTTCAAACCTGCTCTATGAA
       1034 TCAAAGCGATCCAAATATCCACTTCCAGATTCTACAAAAAGAGTGTTTCAAAACTGCTCTATCAA

             * *   *         * *      * *  *      *      *              *    
      40383 AGGCAATCTTCAACTCTATGAGTTGAATGCAGACATCAGAAAGAAATTTCTGAGAATGCTGCTGT
       1099 AAGAAATGTTCAACTCTGTTAGTTGAGTACACACATCACAAAGAAGTTTCTGAGAATGCTTCTGT

               **         **          *  *
      40448 CTACCTTTTAT---TTGA-ATTCCCGCTTC
       1164 CTAGTTTTTATGGGAAGATATTCCCGTTTT

                      *  *    *              * *  **   *                   * 
      40474 CAACGAAATCCTCCAAGCTATCCAAATATCCACCTGCATTTTCCACAAAAAGAGTGTTTCAAAAC
          1 CAACGAAATCATCAAAGCGATCCAAATATCCACTTCCAGATTCTACAAAAAGAGTGTTTCAAACC

                    *  ** *              *    *  *                *          
      40539 TGCTCTATCAATAGAAATGTTCAACTCCTTTG-GCTGGGTACACACATCACAAACAAGTTTCTGA
         66 TGCTCTA-GAAAGGGAATGTTCAACT-CTGTGAGTTGAGTACACACATCACAAAGAAGTTTCTGA

             *                     **     *      *   *    *    *           * 
      40603 GCATGCTTCTGTCTAGTTTTTATGGGAAGACATTCCCTTTTTC-ACCAAA-GGCATCAAAGCGCT
        129 GAATGCTTCTGTCTAGTTTTTATATGAAGATATTCCCGTTTCCAACGAAATCGC-T-AAAGCGAT

                  *            **                     **       **    **      
      40666 CCAAATGTCCACTTCCAGACACTACAAAAAGAGTGTTTCAAACGTGCTCTAAGAAAACGAATGTT
        192 CCAAATATCCACTTCCAGATTCTACAAAAAGAGTGTTTCAAAACTGCTCTATCAAAAGAAATGTT

                     * *    * *  * *      *  *           **            *     
      40731 CAACTCTGTGACTTGAATGCAGATATCACAGAGTAGTTTCTGAGAGGGCTTCTGTCTAGATTTTA
        257 CAACTCTGTTAGTTGAGTACACACATCACAAAGAAGTTTCTGAGAATGCTTCTGTCTAGTTTTTA

            *    *         *                * *   *                *   *     
      40796 GATGATGATATTCCCGTTTCCAACGAAATCATTAGAGCTATCCAAATATCCACTTACAGTTTCTA
        322 TATGAAGATATTCCCTTTTCCAACGAAATCATCAAAGCGATCCAAATATCCACTTCCAGATTCTA

                           *        **  *      * *    *          *           
      40861 CAAAAAGAGTGTTTCCAAACTGCTGCATCAAAAGAGAGGTTCCACTCTGTTAGCTGAGTACACAC
        387 CAAAAAGAGTGTTTCAAAACTGCTCTATGAAAAGAAATGTTCAACTCTGTTAGTTGAGTACACAC

                    ***      *     *         *                   **     *  * 
      40926 ATCACAAACTTGTTTCTCAGAATCCTTCTGTCTCGTTTTTATGGGAAGATATTTACTTTTTCACC
        452 ATCACAAAGAAGTTTCTGAGAATGCTTCTGTCTAGTTTTTATGGGAAGATATTCCCTTTTCCAAC

             * *           *       *     *       *  *  *               *     
      40991 GTAGGCATCAAAGCGCTCCAAATGTCCACATCCAGATACTCCAGAAAGAGTGTTTCAAACCTGCT
        517 GAAAGCATCAAAGCGATCCAAATATCCACTTCCAGATTCTACAAAAAGAGTGTTTCAAAACTGCT

                *     *   *         *        * *  *      *      *            
      41056 CTATGAAAGGGAATCTTCAACTCTATGAGTTGAATGCAGACATCAGAAAGAAATTTCTGAGAATG
        582 CTATCAAAGGAAATGTTCAACTCTGTGAGTTGAGTACACACATCACAAAGAAGTTTCTGAGAATG

              *       **       *          **             *  *    *           
      41121 CTGCTGTCTACCTTTTAT-TTGA-ATTCCCGCTTCCAACGAAATCCTCCAAGCTATCCAAATATC
        647 CTTCTGTCTAGTTTTTATGATGATATTCCCTTTTCCAACGAAATCATCAAAGCGATCCAAATATC

                 *       *                                  *  *  *          
      41184 CACTTGCAGATTCCACAAAAAGAGTGTTTCAAAACTGCTCTCTATCAATGGCAAAGTTCAACTCT
        712 CACTTCCAGATTCTACAAAAAGAGTGTTTCAAAACTGC-CTCTATCAAAGGAAATGTTCAACTCT

                      *     *     *  *                         *             
      41249 GTTAGTTGAGGACACATATCACCAACAAGTTTCTGAGAATGCTTCTGTCTATTTTTTATGGGAAG
        776 GTTAGTTGAGTACACACATCACAAAGAAGTTTCTGAGAATGCTTCTGTCTAGTTTTTATGGGAAG

                 *      *  *  * ** *    *      *    *          * **          
      41314 ATATTTCCTTTTTCACCGTAGGCGTCAAGGCGATCGAAATGTCCACTTCCACAAACTACAAAAAG
        841 ATATTCCCTTTTCCAACGAAATCATCAAAGCGATCCAAATATCCACTTCCAGATTCTACAAAAAG

                       *         *     **       *    *           * * *   **  
      41379 AGTGTTTCAAACCTGCTCTATGAAAGGCCATGTTCATCTCTATGAGTTGAATGGAAATATCCGAA
        906 AGTGTTTCAAAACTGCTCTATCAAAGGAAATGTTCAACTCTGTGAGTTGAATGCAGACATCACAA

                *      *       *         *       *         **             *  
      41444 AGAAATTTCTGGGAATGCTGCTGTCTAGTGTTTAT-ACGA-ATTCCCGCTTCCAACGAAATCCTC
        971 AGAAGTTTCTGAGAATGCTTCTGTCTAGTTTTTATGAAGATATTCCCTTTTCCAACGAAATCATC

                 *                *    *  *                                * 
      41507 AAAGCAATCCAAATATCCACTTGCAGAATCCACAAAAAGAGTGTTTCAAAACTGCTCTATCAATA
       1036 AAAGCGATCCAAATATCCACTTCCAGATTCTACAAAAAGAGTGTTTCAAAACTGCTCTATCAAAA

                *          *                     *  *                        
      41572 GAAAGGTTCAACTCTTTTAGTTGAGTACACACATCACGAACAAGTTTCTGAGAATGCTTCTGTCT
       1101 GAAATGTTCAACTCTGTTAGTTGAGTACACACATCACAAAGAAGTTTCTGAGAATGCTTCTGTCT

            * *      *      **  *       
      41637 GGCTTTTATTGGAAGACGTTTCC-TTTT
       1166 AGTTTTTATGGGAAGATATTCCCGTTTT

                *  **          *       *                * *                 *
      41664 CAACAAAGGCATCAAAGCGCTCCAAATGTCCACTTCCAGATTCTTCCAAAAGAGTGTTTCAAACG
          1 CAACGAAATCATCAAAGCGATCCAAATATCCACTTCCAGATTCTACAAAAAGAGTGTTTCAAACC

                  *  *                      *    * *  * *    **   *       * *
      41729 TGCTCGAAGTAAGGGAATGTTCAACTCTGTGACTTGAATGCAGATATCATCAAGTAGTTTCTAAT
         66 TGCTC-TAGAAAGGGAATGTTCAACTCTGTGAGTTGAGTACACACATCACAAAGAAGTTTCTGAG

             *            **     *    *                         *  *   *     
      41794 AGTGCTTCTGTCTACATTTTAGATGATGATATTCCCGTTTCCAACGAAATCGTTAGAGCTATCCA
        130 AATGCTTCTGTCTAGTTTTTATATGAAGATATTCCCGTTTCCAACGAAATCGCTAAAGCGATCCA

                    *  *   *      *     *       *   *    **           *      
      41859 AATATCCAGTTACAGTTTCTACCAAAAGGGTGTTTCCAAATTGCTGCATCAAAAGAAAGGTTCAA
        195 AATATCCACTTCCAGATTCTACAAAAAGAGTGTTTCAAAACTGCTCTATCAAAAGAAATGTTCAA

                          *                     *                   *    *   
      41924 CTCTGTTAGTTGAGGACACACATCACAAAGAAGTTTGTGAGAATGCTTCTGTCTAGATTTTGTAT
        260 CTCTGTTAGTTGAGTACACACATCACAAAGAAGTTTCTGAGAATGCTTCTGTCTAGTTTTTATAT

              *                    *   * *     *   *       * *  *    *  *    
      41989 GACGATATTCCCTTTTCCAACGATATCGTTAAAGCAATCTAAATATCAATTTGCAGAATCCACAA
        325 GAAGATATTCCCTTTTCCAACGAAATCATCAAAGCGATCCAAATATCCACTTCCAGATTCTACAA

               *  *        *       * *        *    *          *              
      42054 AAATAGAGTTTCAAAGCTGCTCTGTAAAAAGAAAGGTTCCACTCTGTTAGCTGAGTACACACATC
        390 AAAGAGTGTTTCAAAACTGCTCTATGAAAAGAAATGTTCAACTCTGTTAGTTGAGTACACACATC

                 ***            *         *                   **        *  * 
      42119 ACAAACTTGTTTCTGAGAATCCTTCTGTCTCGTTTTTATGGGAAGATATTTACTTTTCCACCGTA
        455 ACAAAGAAGTTTCTGAGAATGCTTCTGTCTAGTTTTTATGGGAAGATATTCCCTTTTCCAACGAA

            *           *       *     *       *  *  *  *            *        
      42184 GGCATCAAAGCGCTCCAAATGTCCACATCCAGATACTCCAGAACGAGTGTTTCAAACCTGCTCTA
        520 AGCATCAAAGCGATCCAAATATCCACTTCCAGATTCTACAAAAAGAGTGTTTCAAAACTGCTCTA

             *     *   *         *        * *  *      *      *              *
      42249 TGAAAGGGAATCTTCAACTCTATGAGTTGAATGCAGACATCAGAAAGAAATTTCTGAGAATGCTG
        585 TCAAAGGAAATGTTCAACTCTGTGAGTTGAGTACACACATCACAAAGAAGTTTCTGAGAATGCTT

                   **       *          **             *  *    *              
      42314 CTGTCTACCTTTTAT-TTGA-ATTCCCGCTTCCAACGAAATCCTCCAAGCTATCCAAATATCCAC
        650 CTGTCTAGTTTTTATGATGATATTCCCTTTTCCAACGAAATCATCAAAGCGATCCAAATATCCAC

            * *  **   *    *                            **              **  *
      42377 CTGCATTTTCCACAACAAGAGTGTTTCAAAACTG-CTCTATCAATAGAAATGTTCAACTCCTTTG
        715 TTCCAGATTCTACAAAAAGAGTGTTTCAAAACTGCCTCTATCAAAGGAAATGTTCAACTCTGTTA

             *  *                *           *                            *  
      42441 GCTGGGTACACACATCACAAACAAGTTTCTGAGCATGCTTCTGTCTAGTTTTTATGGGAAGACAT
        780 GTTGAGTACACACATCACAAAGAAGTTTCTGAGAATGCTTCTGTCTAGTTTTTATGGGAAGATAT

                    *  * *  **          *       *            **              
      42506 TCCCTTTTTCACCAAAGGCATCAAAGCGCTCCAAATGTCCACTTCCAGACACTACAAAAAGAGTG
        845 TCCCTTTTCCAACGAAATCATCAAAGCGATCCAAATATCCACTTCCAGATTCTACAAAAAGAGTG

                   **       **   ***                 *           *      *  * 
      42571 TTTCAAACGTGCTCTAAGAAAACGAATGTTCAACTCTGTGACTTGAATGCAGATATCACAGAGTA
        910 TTTCAAAACTGCTCTATCAAAGGAAATGTTCAACTCTGTGAGTTGAATGCAGACATCACAAAGAA

                      **                  *    *         *                * *
      42636 GTTTCTGAGAGGGCTTCTGTCTAGATTTTAGATGATGATATTCCCGTTTCCAACGAAATCATTAG
        975 GTTTCTGAGAATGCTTCTGTCTAG-TTTT-TATGAAGATATTCCCTTTTCCAACGAAATCATCAA

               *                *   *                    *        **         
      42701 AGCTATCCAAATATCCACTTACAGTTTCTACAAAAAGAGTGTTTCCAAACTGCTGCATCAAAAGA
       1038 AGCGATCCAAATATCCACTTCCAGATTCTACAAAAAGAGTGTTTCAAAACTGCTCTATCAAAAGA

            * *    *          *                   ***      *     *         * 
      42766 GAGGTTCCACTCTGTTAGCTGAGTACACACATCACAAACTTGTTTCTCAGAATCCTTCTGTCTCG
       1103 AATGTTCAACTCTGTTAGTTGAGTACACACATCACAAAGAAGTTTCTGAGAATGCTTCTGTCTAG

                              ** *    
      42831 TTTTTATGGGAAGATATTTACTTTTT
       1168 TTTTTATGGGAAGATATTCCCGTTTT

              *  * **          *       *     *       *  *  *                 
      42857 CACCGTAGGCATCAAAGCGCTCCAAATGTCCACATCCAGATACTCCAGAAAGAGTGTTTCAAACC
          1 CAACGAAATCATCAAAGCGATCCAAATATCCACTTCCAGATTCTACAAAAAGAGTGTTTCAAACC

                              *         *        * *  *      *      *        
      42922 TGCTCTATGAAAGGGAATCTTCAACTCTATGAGTTGAATGCAGACATCAGAAAGAAATTTCTGAG
         66 TGCTCTA-GAAAGGGAATGTTCAACTCTGTGAGTTGAGTACACACATCACAAAGAAGTTTCTGAG

                  *       **          *          *                 *    *    
      42987 AATGCTGCTGTCTACCTTTTAT-T--TGA-ATTCCCGCTTCCAACGAAATC-CTCCAAGCTATCC
        130 AATGCTTCTGTCTAGTTTTTATATGAAGATATTCCCGTTTCCAACGAAATCGCT-AAAGCGATCC

                        *       *                                  ** *  *   
      43047 AAATATCCACTTGCAGATTCCACAAAAAGAGTGTTTCAAAACTGCTCTCTATCAATGGCAAAGTT
        194 AAATATCCACTTCCAGATTCTACAAAAAGAGTGTTTCAAAACTG--CTCTATCAAAAGAAATGTT

                             *     *     *  *                         *      
      43112 CAACTCTGTTAGTTGAGGACACATATCACCAACAAGTTTCTGAGAATGCTTCTGTCTATTTTTTA
        257 CAACTCTGTTAGTTGAGTACACACATCACAAAGAAGTTTCTGAGAATGCTTCTGTCTAGTTTTTA

             **         *      *  *  * ** *    *      *    *          * **   
      43177 TGGGAAGATATTTCCTTTTTCACCGTAGGCGTCAAGGCGATCGAAATGTCCACTTCCACAAACTA
        322 TATGAAGATATTCCCTTTTCCAACGAAATCATCAAAGCGATCCAAATATCCACTTCCAGATTCTA

                              *             * **       *    * *      * ** * *
      43242 CAAAAAGAGTGTTTCAAACCTGCTCTATGAAAGGCGATGTTCATCTCTATGAGTTGAATGGAAAT
        387 CAAAAAGAGTGTTTCAAAACTGCTCTATGAAAAGAAATGTTCAACTCTGTTAGTTGAGTACACAC

               **      *      *       *         *         *         **       
      43307 ATCCGAAAGAAATTTCTGGGAATGCTGCTGTCTAGTGTTTAT---ACGA-ATTCCCGCTTCCAAC
        452 ATCACAAAGAAGTTTCTGAGAATGCTTCTGTCTAGTTTTTATGGGAAGATATTCCCTTTTCCAAC

                * *       *                *    *  *                         
      43368 GAAATCCTCAAAGCAATCCAAATATCCACTTGCAGAATCCACAAAAAGAGTGTTTCAAAACTGCT
        517 GAAAGCATCAAAGCGATCCAAATATCCACTTCCAGATTCTACAAAAAGAGTGTTTCAAAACTGCT

                   **    *          * *                   *  *               
      43433 CTATCAATAGAAAGGTTCAACTCTTTTAGTTGAGTACACACATCACGAACAAGTTTCTGAGAATG
        582 CTATCAAAGGAAATGTTCAACTCTGTGAGTTGAGTACACACATCACAAAGAAGTTTCTGAGAATG

                     * *          *  **  *         * *  **          *       *
      43498 CTTCTGTCTGGCTTTTATTGGAAGACGTTTCCTTTT-CACCAAAGGCATCAAAGCGCTCCAAATG
        647 CTTCTGTCTAGTTTTTA-T-GATGATATTCCCTTTTCCAACGAAATCATCAAAGCGATCCAAATA

                            * *                **      * ***    *            
      43562 TCCACTTCCAGATTCTTCCAAAAGAGTGTTTCAAACGTG-CTCAAAGTAAGGGAATGTTCAACTC
        710 TCCACTTCCAGATTCTACAAAAAGAGTGTTTCAAAACTGCCTCTATCAAAGGAAATGTTCAACTC

               * *    * *  * *     *   *       * * *             *     ***  *
      43626 TGTGACTTGAATGCAGATATCACCAAGTAGTTTCTAATAGTGCTTCTGTCTAGATTTTAGATGAT
        775 TGTTAGTTGAGTACACACATCACAAAGAAGTTTCTGAGAATGCTTCTGTCTAGTTTTTATGGGAA

                     *              * * *   *                *   *           
      43691 GATATTCCCGTTTCCAACGAAATCGTTAGAGCTATCCAAATATCCACTTACAGTTTCTACAAAAA
        840 GATATTCCCTTTTCCAACGAAATCATCAAAGCGATCCAAATATCCACTTCCAGATTCTACAAAAA

                     *        **      *    *            *      ***  *        
      43756 GAGTGTTTCCAAACTGCTGCATCAAAAGAAAGGTTCAACTCTGTTAGTTGAGGACACACATCACA
        905 GAGTGTTTCAAAACTGCTCTATCAAAGGAAATGTTCAACTCTGTGAGTTGAATGCAGACATCACA

                     *                              *                    *   
      43821 AAGAAGTTTGTGAGAATGCTTCTGTCTAGATTTTGTATGACGATATTCCCTTTTCCAACGATATC
        970 AAGAAGTTTCTGAGAATGCTTCTGTCTAG-TTTT-TATGAAGATATTCCCTTTTCCAACGAAATC

            * *     *   *       * *  *    *  *       *  *        *       * * 
      43886 GTTAAAGCAATCTAAATATCAATTTGCAGAATCCACAAAAATAGAGTTTCAAAGCTGCTCTGTAA
       1033 ATCAAAGCGATCCAAATATCCACTTCCAGATTCTACAAAAAGAGTGTTTCAAAACTGCTCTATCA

               *   *    *          *                   ***            *      
      43951 AAATAAAAGTTCCACTCTGTTAGCTGAGTACACACATCACAAACTTGTTTCTGAGAATCCTTCTG
       1098 AAAGAAATGTTCAACTCTGTTAGTTGAGTACACACATCACAAAGAAGTTTCTGAGAATGCTTCTG

               *                   ** *    
      44016 TCTCGTTTTTATGGGAAGATATTTACTTTTT
       1163 TCTAGTTTTTATGGGAAGATATTCCCGTTTT

              *  * **          *       *     *       *  *  *     *           
      44047 CACCGTAGGCATCAAAGCGCTCCAAATGTCCACATCCAGATACTCCAGAAAGACTGTTTCAAACC
          1 CAACGAAATCATCAAAGCGATCCAAATATCCACTTCCAGATTCTACAAAAAGAGTGTTTCAAACC

                              *         *        * *  *      *      *        
      44112 TGCTCTATGAAAGGGAATCTTCAACTCTATGAGTTGAATGCAGACATCAGAAAGAAATTTCTGAG
         66 TGCTCTA-GAAAGGGAATGTTCAACTCTGTGAGTTGAGTACACACATCACAAAGAAGTTTCTGAG

                  *       **          *          *                 *    *    
      44177 AATGCTGCTGTCTACCTTTTAT-T--TGA-ATTCCCGCTTCCAACGAAATC-CTCCAAGCTATCC
        130 AATGCTTCTGTCTAGTTTTTATATGAAGATATTCCCGTTTCCAACGAAATCGCT-AAAGCGATCC

                      * *  **   *                                *           
      44237 AAATATCCACCTGCATTTTCCACAAAAAGAGTGTTTCAAAACTGCTCTATCAATAGAAATGTTCA
        194 AAATATCCACTTCCAGATTCTACAAAAAGAGTGTTTCAAAACTGCTCTATCAAAAGAAATGTTCA

                **  * *  *                *                                 *
      44302 ACTCCTTTGGCTGGGTACACACATCACAAACAAGTTTCTGAGAATGCTTCTGTCTAGTTTTTATG
        259 ACTCTGTTAGTTGAGTACACACATCACAAAGAAGTTTCTGAGAATGCTTCTGTCTAGTTTTTATA

            *     **         *  * *  **          *       *            **     
      44367 GGAAGACGTTCCCTTTTTCACCAAAGGCATCAAAGCGCTCCAAATGTCCACTTCCAGACACTACA
        324 TGAAGATATTCCCTTTTCCAACGAAATCATCAAAGCGATCCAAATATCCACTTCCAGATTCTACA

                            **       *    ***               * *    * *  * *  
      44432 AAAAGAGTGTTTCAAACGTGCTCTAAGAAAGCGAATGTTCAACTCTGTGACTTGAATGCAGATAT
        389 AAAAGAGTGTTTCAAAACTGCTCTATGAAAAGAAATGTTCAACTCTGTTAGTTGAGTACACACAT

                   *           *             *     ***  *         *          
      44497 CACAAAGTAGTTTCTGAGAGTGCTTCTGTCTAGATTTTAGACGATGATATTCCCGTTTCCAACGA
        454 CACAAAGAAGTTTCTGAGAATGCTTCTGTCTAGTTTTTATGGGAAGATATTCCCTTTTCCAACGA

              *   * *   *                *   *                    *        **
      44562 AATCATTAGAGCTATCCAAATATCCACTTACAGTTTCTACAAAAAGAGTGTTTCCAAACTGCTGC
        519 AAGCATCAAAGCGATCCAAATATCCACTTCCAGATTCTACAAAAAGAGTGTTTCAAAACTGCTCT

                  *  * *    *       *  *                   ***      *     *  
      44627 ATCAAAAGAGAGGTTCCACTCTGTTAGCTGAGTACACACATCACAAACTTGTTTCTCAGAATCCT
        584 ATCAAAGGAAATGTTCAACTCTGTGAGTTGAGTACACACATCACAAAGAAGTTTCTGAGAATGCT

                   *            *      **     *  *  * **          *       *  
      44692 TCTGTCTCGTTTTTATGGGAAGATATTTACTTTTTCACCGTAGGCATCAAAGCGCTCCAAATGTC
        649 TCTGTCTAGTTTTTAT--GATGATATTCCCTTTTCCAACGAAATCATCAAAGCGATCCAAATATC

               *       **    *       *       *          *     *   *         *
      44757 CACATCCAGATAGTACAGAAAGAGTTTTTCAAACCTG-CTCTATGAAAGGGAATCTTCAACTCTA
        712 CACTTCCAGATTCTACAAAAAGAGTGTTTCAAAACTGCCTCTATCAAAGGAAATGTTCAACTCTG

             *      * *  *      *      *              *       **         **  
      44821 TGAGTTGAATGCAGACATCAGAAAGAAATTTCTGAGAATGCTGCTGTCTACCTTTTAT---TTGA
        777 TTAGTTGAGTACACACATCACAAAGAAGTTTCTGAGAATGCTTCTGTCTAGTTTTTATGGGAAGA

                   **             *  *    *                *       *         
      44883 -ATTCCCGCTTCCAACGAAATCCTCCAAGCTATCCAAATATCCACTTGCAGATTCCACAAAAAGA
        842 TATTCCCTTTTCCAACGAAATCATCAAAGCGATCCAAATATCCACTTCCAGATTCTACAAAAAGA

                                     *  *  *            *      ***  * *     *
      44947 GTGTTTCAAAACTGCTCTCTATCAATGGCAAAGTTCAACTCTGTTAGTTGAGGACACATATCACC
        907 GTGTTTCAAAACTG--CTCTATCAAAGGAAATGTTCAACTCTGTGAGTTGAATGCAGACATCACA

              *                         *                  *      *  *  * ** 
      45012 AACAAGTTTCTGAGAATGCTTCTGTCTATTTTTTATGGGAAGATATTTCCTTTTTCACCGTAGGC
        970 AAGAAGTTTCTGAGAATGCTTCTGTCTAGTTTTTAT--GAAGATATTCCCTTTTCCAACGAAATC

            *    *      *    *          * **                     *         * 
      45077 GTCAAGGCGATCGAAATGTCCACTTCCACAAACTACAAAAAGAGTGTTTCAAACCTGCTCTATGA
       1033 ATCAAAGCGATCCAAATATCCACTTCCAGATTCTACAAAAAGAGTGTTTCAAAACTGCTCTATCA

              * **       *    * *      * ** * *   **      *      *       *   
      45142 AAGGCGATGTTCATCTCTATGAGTTGAATGGAAATATCCGAAAGAAATTTCTGGGAATGCTGCTG
       1098 AAAGAAATGTTCAACTCTGTTAGTTGAGTACACACATCACAAAGAAGTTTCTGAGAATGCTTCTG

                  *         *          *  *
      45207 TCTAGTGTTTAT---ACGA-ATTCCCGCTTC
       1163 TCTAGTTTTTATGGGAAGATATTCCCGTTTT

                      *       *                *    *  *                   * 
      45234 CAACGAAATCCTCAAAGCAATCCAAATATCCACTTGCAGAATCCACAAAAAGAGTGTTTCAAAAC
          1 CAACGAAATCATCAAAGCGATCCAAATATCCACTTCCAGATTCTACAAAAAGAGTGTTTCAAACC

                    *  ** *  *          * *                   *  *           
      45299 TGCTCTATCAATAGAAAGGTTCAACTCTTTTAGTTGAGTACACACATCACGAACAAGTTTCTGAG
         66 TGCTCTA-GAAAGGGAATGTTCAACTCTGTGAGTTGAGTACACACATCACAAAGAAGTTTCTGAG

                         * *      **     **  *      *    *    *           *  
      45364 AATGCTTCTGTCTGGCTTTTATTGGAAGACGTTTCC-TTTTC-ACCAAA-GGCATCAAAGCGCTC
        130 AATGCTTCTGTCTAGTTTTTATATGAAGATATTCCCGTTTCCAACGAAATCGC-T-AAAGCGATC

                 *                * *                **     * ***  * *       
      45426 CAAATGTCCACTTCCAGATTCTTCCAAAAGAGTGTTTCAAACGTGCTCAAAGTAAGGGAATGTTC
        193 CAAATATCCACTTCCAGATTCTACAAAAAGAGTGTTTCAAAACTGCTCTATCAAAAGAAATGTTC

                    * *    * *  * *     *   *       * * *             *     *
      45491 AACTCTGTGACTTGAATGCAGATATCACCAAGTAGTTTCTAATAGTGCTTCTGTCTAGATTTTAG
        258 AACTCTGTTAGTTGAGTACACACATCACAAAGAAGTTTCTGAGAATGCTTCTGTCTAGTTTTTAT

                *         *              * * *   *                *   *      
      45556 ATGATGATATTCCCGTTTCCAACGAAATCGTTAGAGCTATCCAAATATCCACTTACAGTTTCTAC
        323 ATGAAGATATTCCCTTTTCCAACGAAATCATCAAAGCGATCCAAATATCCACTTCCAGATTCTAC

                          *        **  *        *                    *       
      45621 AAAAAGAGTGTTTCCAAACTGCTGCATCAAAAGAAAGGTTCAACTCTGTTAGTTGAGGACACACA
        388 AAAAAGAGTGTTTCAAAACTGCTCTATGAAAAGAAATGTTCAACTCTGTTAGTTGAGTACACACA

                          *                   *    * **  *                   
      45686 TCACAAAGAAGTTTGTGAGAATGCTTCTGTCTAGATTTTGTATGACGATATTCCCTTTTCCAACG
        453 TCACAAAGAAGTTTCTGAGAATGCTTCTGTCTAGTTTTTATGGGAAGATATTCCCTTTTCCAACG

             * * * *     *   *       * *  *    *  *       *  *        *      
      45751 ATATCGTTAAAGCAATCTAAATATCAATTTGCAGAATCCACAAAAATAGAGTTTCAAAGCTGCTC
        518 AAAGCATCAAAGCGATCCAAATATCCACTTCCAGATTCTACAAAAAGAGTGTTTCAAAACTGCTC

             * *   **   *    *       *  *                   ***            * 
      45816 TGTAAAAATAAAAGTTCCACTCTGTTAGCTGAGTACACACATCACAAACTTGTTTCTGAGAATCC
        583 TATCAAAGGAAATGTTCAACTCTGTGAGTTGAGTACACACATCACAAAGAAGTTTCTGAGAATGC

                    *            *      **     *  *  * **          *       * 
      45881 TTCTGTCTCGTTTTTATGGGAAGATATTTACTTTTTCACCGTAGGCATCAAAGCGCTCCAAATGT
        648 TTCTGTCTAGTTTTTAT--GATGATATTCCCTTTTCCAACGAAATCATCAAAGCGATCCAAATAT

                *       *  *  *     *         *          *     *   *         
      45946 CCACATCCAGATACTCCAGAAAGACTGTTTCAAACCTG-CTCTATGAAAGGGAATCTTCAACTCT
        711 CCACTTCCAGATTCTACAAAAAGAGTGTTTCAAAACTGCCTCTATCAAAGGAAATGTTCAACTCT

            * *      * *  *      *      *              *       **         ** 
      46010 ATGAGTTGAATGCAGACATCAGAAAGAAATTTCTGAGAATGCTGCTGTCTACCTTTTAT---TTG
        776 GTTAGTTGAGTACACACATCACAAAGAAGTTTCTGAGAATGCTTCTGTCTAGTTTTTATGGGAAG

                    **             *  *    *              * *  **   *        
      46072 A-ATTCCCGCTTCCAACGAAATCCTCCAAGCTATCCAAATATCCACCTGCATTTTCCACAAAAAG
        841 ATATTCCCTTTTCCAACGAAATCATCAAAGCGATCCAAATATCCACTTCCAGATTCTACAAAAAG

                                    **                *    *  ** *  *        
      46136 AGTGTTTCAAAACTGCTCTATCAATAGAAATGTTCAACTCCTTTG-GCTGGGTACACACATCACA
        906 AGTGTTTCAAAACTGCTCTATCAAAGGAAATGTTCAACT-CTGTGAGTTGAATGCAGACATCACA

              *                                        **         *  * *  ** 
      46200 AACAAGTTTCTGAGAATGCTTCTGTCTAGTTTTTATGGGAAGACGTTCCCTTTTTCACCAAAGGC
        970 AAGAAGTTTCTGAGAATGCTTCTGTCTAGTTTTTAT--GAAGATATTCCCTTTTCCAACGAAATC

                     *       *            **                     **       ** 
      46265 ATCAAAGCGCTCCAAATGTCCACTTCCAGACACTACAAAAAGAGTGTTTCAAACGTGCTCTAAGA
       1033 ATCAAAGCGATCCAAATATCCACTTCCAGATTCTACAAAAAGAGTGTTTCAAAACTGCTCTATCA

              ***               * *    * *  * *         *           *        
      46330 AAGCGAATGTTCAACTCTGTGACTTGAATGCAGATATCACAAAGTAGTTTCTGAGAGTGCTTCTG
       1098 AAAGAAATGTTCAACTCTGTTAGTTGAGTACACACATCACAAAGAAGTTTCTGAGAATGCTTCTG

                 *     ***  *             *
      46395 TCTAGATTTTAGACGATGATATTCCCGTTTC
       1163 TCTAGTTTTTATGGGAAGATATTCCCGTTTT

                        * *   *                *   *                    *  * 
      46426 CAACGAAATCATTAGAGCTATCCAAATATCCACTTACAGTTTCTACAAAAAGAGTGTTTCCAAAC
          1 CAACGAAATCATCAAAGCGATCCAAATATCCACTTCCAGATTCTACAAAAAGAGTGTTTCAAACC

                    **    *   *    *       *  *                   ***      * 
      46491 TGCTGCATCAAAAGAG-AGGTTCCACTCTGTTAGCTGAGTACACACATCACAAACTTGTTTCTCA
         66 TGCT-C-TAGAAAGGGAATGTTCAACTCTGTGAGTTGAGTACACACATCACAAAGAAGTTTCTGA

                *         *        **         ** *   *  *  * ** ***      *   
      46555 GAATCCTTCTGTCTCGTTTTTATGGGAAGATATTTACTTTTTCACCGTAGGCATCAAAGCGCTCC
        129 GAATGCTTCTGTCTAGTTTTTATATGAAGATATTCCCGTTTCCAACGAAATCGCTAAAGCGATCC

                *     *       **    *       *       *         *   * *   *    
      46620 AAATGTCCACATCCAGATAGTACAGAAAGAGTTTTTCAAACCTGCTCTATGAAAGGGAATCTTCA
        194 AAATATCCACTTCCAGATTCTACAAAAAGAGTGTTTCAAAACTGCTCTATCAAAAGAAATGTTCA

                 * *      * *  *      *      *              *       **       
      46685 ACTCTATGAGTTGAATGCAGACATCAGAAAGAAATTTCTGAGAATGCTGCTGTCTACCTTTTAT-
        259 ACTCTGTTAGTTGAGTACACACATCACAAAGAAGTTTCTGAGAATGCTTCTGTCTAGTTTTTATA

               *         **             *  *    *                *       *   
      46749 T--TGA-ATTCCCGCTTCCAACGAAATCCTCCAAGCTATCCAAATATCCACTTGCAGATTCCACA
        324 TGAAGATATTCCCTTTTCCAACGAAATCATCAAAGCGATCCAAATATCCACTTCCAGATTCTACA

                                        *  ** *  *                    *     *
      46811 AAAAGAGTGTTTCAAAACTGCTCTCTATCAATGGCAAAGTTCAACTCTGTTAGTTGAGGACACAT
        389 AAAAGAGTGTTTCAAAACTG--CTCTATGAAAAGAAATGTTCAACTCTGTTAGTTGAGTACACAC

                 *  *                         *                  *      *  * 
      46876 ATCACCAACAAGTTTCTGAGAATGCTTCTGTCTATTTTTTATGGGAAGATATTTCCTTTTTCACC
        452 ATCACAAAGAAGTTTCTGAGAATGCTTCTGTCTAGTTTTTATGGGAAGATATTCCCTTTTCCAAC

             * *  *    *      *    *          * **                     *     
      46941 GTAGGCGTCAAGGCGATCGAAATGTCCACTTCCACAAACTACAAAAAGAGTGTTTCAAACCTGCT
        517 GAAAGCATCAAAGCGATCCAAATATCCACTTCCAGATTCTACAAAAAGAGTGTTTCAAAACTGCT

                *     **       *    *        * ** * *   **      *      *     
      47006 CTATGAAAGGCCATGTTCATCTCTATGAGTTGAATGGAAATATCCGAAAGAAATTTCTGGGAATG
        582 CTATCAAAGGAAATGTTCAACTCTGTGAGTTGAGTACACACATCACAAAGAAGTTTCTGAGAATG

              *         *       *         **             *       *           
      47071 CTGCTGTCTAGTGTTTAT-ACGA-ATTCCCGCTTCCAACGAAATCCTCAAAGCAATCCAAATATC
        647 CTTCTGTCTAGTTTTTATGATGATATTCCCTTTTCCAACGAAATCATCAAAGCGATCCAAATATC

                 *    *  *                                 **    *          *
      47134 CACTTGCAGAATCCACAAAAAGAGTGTTTCAAAACTG-CTCTATCAATAGAAAGGTTCAACTCTT
        712 CACTTCCAGATTCTACAAAAAGAGTGTTTCAAAACTGCCTCTATCAAAGGAAATGTTCAACTCTG

                                 *  *                        * *      *      
      47198 TTAGTTGAGTACACACATCACGAACAAGTTTCTGAGAATGCTTCTGTCTGGCTTTTATTGGAAGA
        777 TTAGTTGAGTACACACATCACAAAGAAGTTTCTGAGAATGCTTCTGTCTAGTTTTTATGGGAAGA

            **  *         * *  **          *       *                * *      
      47263 CGTTTCCTTTT-CACCAAAGGCATCAAAGCGCTCCAAATGTCCACTTCCAGATTCTTCCAAAAGA
        842 TATTCCCTTTTCCAACGAAATCATCAAAGCGATCCAAATATCCACTTCCAGATTCTACAAAAAGA

                      **     * ***    *                 *           *     *  
      47327 GTGTTTCAAACGTGCTCAAAGTAAGGGAATGTTCAACTCTGTGACTTGAATGCAGATATCACCAA
        907 GTGTTTCAAAACTGCTCTATCAAAGGAAATGTTCAACTCTGTGAGTTGAATGCAGACATCACAAA

             *       * * *                   *    *         *              * 
      47392 GTAGTTTCTAATAGTGCTTCTGTCTAGATTTTAGATGATGATATTCCCGTTTCCAACGAAATCGT
        972 GAAGTTTCTGAGAATGCTTCTGTCTAG-TTTT-TATGAAGATATTCCCTTTTCCAACGAAATCAT

            * *   *                *   *                    *        **      
      47457 TAGAGCTATCCAAATATCCACTTACAGTTTCTACAAAAAGAGTGTTTCCAAACTGCTGCATCAAA
       1035 CAAAGCGATCCAAATATCCACTTCCAGATTCTACAAAAAGAGTGTTTCAAAACTGCTCTATCAAA

                 *                    *                     *                
      47522 AGAAAGGTTCAACTCTGTTAGTTGAGGACACACATCACAAAGAAGTTTGTGAGAATGCTTCTGTC
       1100 AGAAATGTTCAACTCTGTTAGTTGAGTACACACATCACAAAGAAGTTTCTGAGAATGCTTCTGTC

               *    * **  *         *   *
      47587 TAGATTTTGTATGACGATATTCCCTTTTC
       1165 TAGTTTTTATGGGAAGATATTCCCGTTTT

                  *   * *     *   *       * *  *    *  *       *  *        * 
      47616 CAACGATATCGTTAAAGCAATCTAAATATCAATTTGCAGAATCCACAAAAATAGAGTTTCAAAGC
          1 CAACGAAATCATCAAAGCGATCCAAATATCCACTTCCAGATTCTACAAAAAGAGTGTTTCAAACC

                   **   ***  *    *       *  *                   ***         
      47681 TGCTCTGTAAAAATAAAAGTTCCACTCTGTTAGCTGAGTACACACATCACAAACTTGTTTCTGAG
         66 TGCTCT-AGAAAGGGAATGTTCAACTCTGTGAGTTGAGTACACACATCACAAAGAAGTTTCTGAG

               *         *        **         ** *   *  *  * ** ***      *    
      47746 AATCCTTCTGTCTCGTTTTTATGGGAAGATATTTACTTTTTCACCGTAGGCATCAAAGCGCTCCA
        130 AATGCTTCTGTCTAGTTTTTATATGAAGATATTCCCGTTTCCAACGAAATCGCTAAAGCGATCCA

               *     *       *  *  *     *         *         *   * *   *     
      47811 AATGTCCACATCCAGATACTCCAGAAAGACTGTTTCAAACCTGCTCTATGAAAGGGAATCTTCAA
        195 AATATCCACTTCCAGATTCTACAAAAAGAGTGTTTCAAAACTGCTCTATCAAAAGAAATGTTCAA

                * *      * *  *      *      *              *       **        
      47876 CTCTATGAGTTGAATGCAGACATCAGAAAGAAATTTCTGAGAATGCTGCTGTCTACCTTTTAT-T
        260 CTCTGTTAGTTGAGTACACACATCACAAAGAAGTTTCTGAGAATGCTTCTGTCTAGTTTTTATAT

              *         **             *  *    *              * *  **   *    
      47940 --TGA-ATTCCCGCTTCCAACGAAATCCTCCAAGCTATCCAAATATCCACCTGCATTTTCCACAA
        325 GAAGATATTCCCTTTTCCAACGAAATCATCAAAGCGATCCAAATATCCACTTCCAGATTCTACAA

                                     *  *               **  * *  *           
      48002 AAAGAGTGTTTCAAAACTGCTCTATCAATAGAAATGTTCAACTCCTTTGGCTGGGTACACACATC
        390 AAAGAGTGTTTCAAAACTGCTCTATGAAAAGAAATGTTCAACTCTGTTAGTTGAGTACACACATC

                 *                                        **         *  * *  
      48067 ACAAACAAGTTTCTGAGAATGCTTCTGTCTAGTTTTTATGGGAAGACGTTCCCTTTTTCACCAAA
        455 ACAAAGAAGTTTCTGAGAATGCTTCTGTCTAGTTTTTATGGGAAGATATTCCCTTTTCCAACGAA

            *           *       *            **                     **       
      48132 GGCATCAAAGCGCTCCAAATGTCCACTTCCAGACACTACAAAAAGAGTGTTTCAAACGTGCTCTA
        520 AGCATCAAAGCGATCCAAATATCCACTTCCAGATTCTACAAAAAGAGTGTTTCAAAACTGCTCTA

            **    **                 *    * *  * *         *           *     
      48197 AGAAAGCGAATGTTCAACTCTGTGACTTGAATGCAGATATCACAAAGTAGTTTCTGAGAGTGCTT
        585 TCAAAGGAAATGTTCAACTCTGTGAGTTGAGTACACACATCACAAAGAAGTTTCTGAGAATGCTT

                          * *            *                * *   *            
      48262 CTGTCTAGATTTTAGACGATGATATTCCCGTTTCCAACGAAATCATTAGAGCTATCCAAATATCC
        650 CTGTCTAG-TTTT-TATGATGATATTCCCTTTTCCAACGAAATCATCAAAGCGATCCAAATATCC

                *   *                    *         **      *  * *    *       
      48327 ACTTACAGTTTCTACAAAAAGAGTGTTTCCAAACTG-CTGCATCAAAAGAGAGGTTCCACTCTGT
        713 ACTTCCAGATTCTACAAAAAGAGTGTTTCAAAACTGCCTCTATCAAAGGAAATGTTCAACTCTGT

               *                   ***      *     *         *                
      48391 TAGCTGAGTACACACATCACAAACTTGTTTCTCAGAATCCTTCTGTCTCGTTTTTATGGGAAGAT
        778 TAGTTGAGTACACACATCACAAAGAAGTTTCTGAGAATGCTTCTGTCTAGTTTTTATGGGAAGAT

               **     *  *  * **          *       *     *       **    *      
      48456 ATTTACTTTTTCACCGTAGGCATCAAAGCGCTCCAAATGTCCACATCCAGATAGTACAGAAAGAG
        843 ATTCCCTTTTCCAACGAAATCATCAAAGCGATCCAAATATCCACTTCCAGATTCTACAAAAAGAG

             *       *         *     *   *         *                    *    
      48521 TTTTTCAAACCTGCTCTATGAAAGGGAATCTTCAACTCTATGAGTTGAATGCAGACATCAGAAAG
        908 TGTTTCAAAACTGCTCTATCAAAGGAAATGTTCAACTCTGTGAGTTGAATGCAGACATCACAAAG

              *              *       **       **         **             *  * 
      48586 AAATTTCTGAGAATGCTGCTGTCTACCTTTTAT-TTGA-ATTCCCGCTTCCAACGAAATCCTCCA
        973 AAGTTTCTGAGAATGCTTCTGTCTAGTTTTTATGAAGATATTCCCTTTTCCAACGAAATCATCAA

               *                *       *                                  **
      48649 AGCTATCCAAATATCCACTTGCAGATTCCACAAAAAGAGTGTTTCAAAACTGCTCTCTATCAATG
       1038 AGCGATCCAAATATCCACTTCCAGATTCTACAAAAAGAGTGTTTCAAAACTG--CTCTATCAAAA

             *  *                    *     *     *  *                        
      48714 GCAAAGTTCAACTCTGTTAGTTGAGGACACATATCACCAACAAGTTTCTGAGAATGCTTCTGTCT
       1101 GAAATGTTCAACTCTGTTAGTTGAGTACACACATCACAAAGAAGTTTCTGAGAATGCTTCTGTCT

             *                  *  *    
      48779 ATTTTTTATGGGAAGATATTTCCTTTTT
       1166 AGTTTTTATGGGAAGATATTCCCGTTTT

              *  * ** *    *      *    *          * **                       
      48807 CACCGTAGGCGTCAAGGCGATCGAAATGTCCACTTCCACAAACTACAAAAAGAGTGTTTCAAACC
          1 CAACGAAATCATCAAAGCGATCCAAATATCCACTTCCAGATTCTACAAAAAGAGTGTTTCAAACC

                          **       *    *        * ** * *   **      *      * 
      48872 TGCTCTATGAAAGGCGATGTTCATCTCTATGAGTTGAATGGAAATATCCGAAAGAAATTTCTGGG
         66 TGCTCTA-GAAAGGGAATGTTCAACTCTGTGAGTTGAGTACACACATCACAAAGAAGTTTCTGAG

                  *          *        *          *                      *    
      48937 AATGCTGCTGTCTAG-TGTT-TAT-ACGA-ATTCCCGCTTCCAACGAAATC-CTCAAAGCAATCC
        130 AATGCTTCTGTCTAGTTTTTATATGAAGATATTCCCGTTTCCAACGAAATCGCT-AAAGCGATCC

                        *    *  *                                *     *     
      48997 AAATATCCACTTGCAGAATCCACAAAAAGAGTGTTTCAAAACTGCTCTATCAATAGAAAGGTTCA
        194 AAATATCCACTTCCAGATTCTACAAAAAGAGTGTTTCAAAACTGCTCTATCAAAAGAAATGTTCA

                 *                     *  *                        * *      *
      49062 ACTCTTTTAGTTGAGTACACACATCACGAACAAGTTTCTGAGAATGCTTCTGTCTGGCTTTTATT
        259 ACTCTGTTAGTTGAGTACACACATCACAAAGAAGTTTCTGAGAATGCTTCTGTCTAGTTTTTATA

            *     **  *         * *  **          *       *                * *
      49127 GGAAGACGTTTCCTTTT-CACCAAAGGCATCAAAGCGCTCCAAATGTCCACTTCCAGATTCTTCC
        324 TGAAGATATTCCCTTTTCCAACGAAATCATCAAAGCGATCCAAATATCCACTTCCAGATTCTACA

                            **     * * *  * *               * *    * *  * *  
      49191 AAAAGAGTGTTTCAAACGTGCTCAAAGTAAGGGAATGTTCAACTCTGTGACTTGAATGCAGATAT
        389 AAAAGAGTGTTTCAAAACTGCTCTATGAAAAGAAATGTTCAACTCTGTTAGTTGAGTACACACAT

               *   *       * * *             *     ***  *         *          
      49256 CACCAAGTAGTTTCTAATAGTGCTTCTGTCTAGATTTTAGATGATGATATTCCCGTTTCCAACGA
        454 CACAAAGAAGTTTCTGAGAATGCTTCTGTCTAGTTTTTATGGGAAGATATTCCCTTTTCCAACGA

              * * * *   *                *   *                    *        **
      49321 AATCGTTAGAGCTATCCAAATATCCACTTACAGTTTCTACAAAAAGAGTGTTTCCAAACTGCTGC
        519 AAGCATCAAAGCGATCCAAATATCCACTTCCAGATTCTACAAAAAGAGTGTTTCAAAACTGCTCT

                  *    *            *       *                     *          
      49386 ATCAAAAGAAAGGTTCAACTCTGTTAGTTGAGGACACACATCACAAAGAAGTTTGTGAGAATGCT
        584 ATCAAAGGAAATGTTCAACTCTGTGAGTTGAGTACACACATCACAAAGAAGTTTCTGAGAATGCT

                                *                    *   * *     *   *       
      49451 TCTGTCTAGATTTTGTATGACGATATTCCCTTTTCCAACGATATCGTTAAAGCAATCTAAATATC
        649 TCTGTCTAG-TTTT-TATGATGATATTCCCTTTTCCAACGAAATCATCAAAGCGATCCAAATATC

            * *  *    *  *       *  *        *        * *   **   *    *      
      49516 AATTTGCAGAATCCACAAAAATAGAGTTTCAAAGCTG-CTCTGTAAAAATAAAAGTTCCACTCTG
        712 CACTTCCAGATTCTACAAAAAGAGTGTTTCAAAACTGCCTCTATCAAAGGAAATGTTCAACTCTG

                *                   ***            *         *               
      49580 TTAGCTGAGTACACACATCACAAACTTGTTTCTGAGAATCCTTCTGTCTCGTTTTTATGGGAAGA
        777 TTAGTTGAGTACACACATCACAAAGAAGTTTCTGAGAATGCTTCTGTCTAGTTTTTATGGGAAGA

                **     *  *  * **          *       *     *       *  *  *     
      49645 TATTTACTTTTTCACCGTAGGCATCAAAGCGCTCCAAATGTCCACATCCAGATACTCCAGAAAGA
        842 TATTCCCTTTTCCAACGAAATCATCAAAGCGATCCAAATATCCACTTCCAGATTCTACAAAAAGA

            *         *         *     *   *         *                    *   
      49710 CTGTTTCAAACCTGCTCTATGAAAGGGAATCTTCAACTCTATGAGTTGAATGCAGACATCAGAAA
        907 GTGTTTCAAAACTGCTCTATCAAAGGAAATGTTCAACTCTGTGAGTTGAATGCAGACATCACAAA

               *              *       **       **         **             *  *
      49775 GAAATTTCTGAGAATGCTGCTGTCTACCTTTTAT-TTGA-ATTCCCGCTTCCAACGAAATCCTCC
        972 GAAGTTTCTGAGAATGCTTCTGTCTAGTTTTTATGAAGATATTCCCTTTTCCAACGAAATCATCA

                *              * *  **   *                                *  
      49838 AAGCTATCCAAATATCCACCTGCATTTTCCACAAAAAGAGTGTTTCAAAACTGCTCTATCAATAG
       1037 AAGCGATCCAAATATCCACTTCCAGATTCTACAAAAAGAGTGTTTCAAAACTGCTCTATCAAAAG

                         **  * *  *                *                         
      49903 AAATGTTCAACTCCTTTGGCTGGGTACACACATCACAAACAAGTTTCTGAGAATGCTTCTGTCTA
       1102 AAATGTTCAACTCTGTTAGTTGAGTACACACATCACAAAGAAGTTTCTGAGAATGCTTCTGTCTA

                           **     *    
      49968 GTTTTTATGGGAAGACGTTCCCTTTTT
       1167 GTTTTTATGGGAAGATATTCCCGTTTT

              * *  **          *       *            **                      *
      49995 CACCAAAGGCATCAAAGCGCTCCAAATGTCCACTTCCAGACACTACAAAAAGAGTGTTTCAAACG
          1 CAACGAAATCATCAAAGCGATCCAAATATCCACTTCCAGATTCTACAAAAAGAGTGTTTCAAACC

                         *                  *    * *  * *         *          
      50060 TGCTCTAAGAAAGCGAATGTTCAACTCTGTGACTTGAATGCAGATATCACAAAGTAGTTTCTGAG
         66 TGCTCT-AGAAAGGGAATGTTCAACTCTGTGAGTTGAGTACACACATCACAAAGAAGTTTCTGAG

             *             *     * *  *                        **  *   *     
      50125 AGTGCTTCTGTCTAGATTTTAGACGATGATATTCCCGTTTCCAACGAAATCATTAGAGCTATCCA
        130 AATGCTTCTGTCTAGTTTTTATATGAAGATATTCCCGTTTCCAACGAAATCGCTAAAGCGATCCA

                       *   *                    *        **         * *    * 
      50190 AATATCCACTTACAGTTTCTACAAAAAGAGTGTTTCCAAACTGCTGCATCAAAAGAGAGGTTCCA
        195 AATATCCACTTCCAGATTCTACAAAAAGAGTGTTTCAAAACTGCTCTATCAAAAGAAATGTTCAA

                     *                   ***      *     *         *        **
      50255 CTCTGTTAGCTGAGTACACACATCACAAACTTGTTTCTCAGAATCCTTCTGTCTCGTTTTTATGG
        260 CTCTGTTAGTTGAGTACACACATCACAAAGAAGTTTCTGAGAATGCTTCTGTCTAGTTTTTATAT

                     **     *  *  * **          *       *     *       **    *
      50320 GAAGATATTTACTTTTTCACCGTAGGCATCAAAGCGCTCCAAATGTCCACATCCAGATAGTACAG
        325 GAAGATATTCCCTTTTCCAACGAAATCATCAAAGCGATCCAAATATCCACTTCCAGATTCTACAA

                   *       *             * *   *         * *      * *  *     
      50385 AAAGAGTTTTTCAAACCTGCTCTATGAAAGGGAATCTTCAACTCTATGAGTTGAATGCAGACATC
        390 AAAGAGTGTTTCAAAACTGCTCTATGAAAAGAAATGTTCAACTCTGTTAGTTGAGTACACACATC

             *      *              *       **         **         **          
      50450 AGAAAGAAATTTCTGAGAATGCTGCTGTCTACCTTTTAT---TTGA-ATTCCCGCTTCCAACGAA
        455 ACAAAGAAGTTTCTGAGAATGCTTCTGTCTAGTTTTTATGGGAAGATATTCCCTTTTCCAACGAA

             * *  *    *                *       *                            
      50511 ATCCTCCAAGCTATCCAAATATCCACTTGCAGATTCCACAAAAAGAGTGTTTCAAAACTGCTCTC
        520 AGCATCAAAGCGATCCAAATATCCACTTCCAGATTCTACAAAAAGAGTGTTTCAAAACTG--CTC

                  *  *  *            *       *     *     *  *                
      50576 TATCAATGGCAAAGTTCAACTCTGTTAGTTGAGGACACATATCACCAACAAGTTTCTGAGAATGC
        583 TATCAAAGGAAATGTTCAACTCTGTGAGTTGAGTACACACATCACAAAGAAGTTTCTGAGAATGC

                     *           *      *      *  *  * ** *    *      *    * 
      50641 TTCTGTCTATTTTTTATGGGAAGATATTTCCTTTTTCACCGTAGGCGTCAAGGCGATCGAAATGT
        648 TTCTGTCTAGTTTTTAT--GATGATATTCCCTTTTCCAACGAAATCATCAAAGCGATCCAAATAT

                     * **                     *          *     **       *    
      50706 CCACTTCCACAAACTACAAAAAGAGTGTTTCAAACCTG-CTCTATGAAAGGCGATGTTCATCTCT
        711 CCACTTCCAGATTCTACAAAAAGAGTGTTTCAAAACTGCCTCTATCAAAGGAAATGTTCAACTCT

            * *      * ** * *   **      *      *       *         *         * 
      50770 ATGAGTTGAATGGAAATATCCGAAAGAAATTTCTGGGAATGCTGCTGTCTAGTGTTTAT---ACG
        776 GTTAGTTGAGTACACACATCACAAAGAAGTTTCTGAGAATGCTTCTGTCTAGTTTTTATGGGAAG

                    **             *       *                *    *  *        
      50832 A-ATTCCCGCTTCCAACGAAATCCTCAAAGCAATCCAAATATCCACTTGCAGAATCCACAAAAAG
        841 ATATTCCCTTTTCCAACGAAATCATCAAAGCGATCCAAATATCCACTTCCAGATTCTACAAAAAG

                                    **    *          * *      * *  *       * 
      50896 AGTGTTTCAAAACTGCTCTATCAATAGAAAGGTTCAACTCTTTTAGTTGAGTACACACATCACGA
        906 AGTGTTTCAAAACTGCTCTATCAAAGGAAATGTTCAACTCTGTGAGTTGAATGCAGACATCACAA

             *                        * *             **  *         * *  **  
      50961 ACAAGTTTCTGAGAATGCTTCTGTCTGGCTTTTATTGGAAGACGTTTCCTTTT-CACCAAAGGCA
        971 AGAAGTTTCTGAGAATGCTTCTGTCTAGTTTTTA-T-GAAGATATTCCCTTTTCCAACGAAATCA

                    *       *                * *                **     * *** 
      51025 TCAAAGCGCTCCAAATGTCCACTTCCAGATTCTTCCAAAAGAGTGTTTCAAACGTGCTCAAAGTA
       1034 TCAAAGCGATCCAAATATCCACTTCCAGATTCTACAAAAAGAGTGTTTCAAAACTGCTCTATCAA

             * *               * *    * *  * *     *   *       * * *         
      51090 AGGGAATGTTCAACTCTGTGACTTGAATGCAGATATCACCAAGTAGTTTCTAATAGTGCTTCTGT
       1099 AAGAAATGTTCAACTCTGTTAGTTGAGTACACACATCACAAAGAAGTTTCTGAGAATGCTTCTGT

                *     ***  *             *
      51155 CTAGATTTTAGATGATGATATTCCCGTTTC
       1164 CTAGTTTTTATGGGAAGATATTCCCGTTTT

                      * * *   *                *   *                    *  * 
      51185 CAACGAAATCGTTAGAGCTATCCAAATATCCACTTACAGTTTCTACAAAAAGAGTGTTTCCAAAC
          1 CAACGAAATCATCAAAGCGATCCAAATATCCACTTCCAGATTCTACAAAAAGAGTGTTTCAAACC

                  ***   * *  *            *       *                     *    
      51250 TGCTGCATCAAAAGAAAGGTTCAACTCTGTTAGTTGAGGACACACATCACAAAGAAGTTTGTGAG
         66 TGCT-CTAGAAAGGGAATGTTCAACTCTGTGAGTTGAGTACACACATCACAAAGAAGTTTCTGAG

                           *    *     *         *          *    *      *   * 
      51315 AATGCTTCTGTCTAGATTTTGTATGACGATATTCCCTTTTCCAACGATATCGTTAAAGCAATCTA
        130 AATGCTTCTGTCTAGTTTTTATATGAAGATATTCCCGTTTCCAACGAAATCGCTAAAGCGATCCA

                  * *  *    *  *       *  *        *       * *    *   *    * 
      51380 AATATCAATTTGCAGAATCCACAAAAATAGAGTTTCAAAGCTGCTCTGTAAAAATAAAAGTTCCA
        195 AATATCCACTTCCAGATTCTACAAAAAGAGTGTTTCAAAACTGCTCTATCAAAAGAAATGTTCAA

                     *                   ***            *         *        **
      51445 CTCTGTTAGCTGAGTACACACATCACAAACTTGTTTCTGAGAATCCTTCTGTCTCGTTTTTATGG
        260 CTCTGTTAGTTGAGTACACACATCACAAAGAAGTTTCTGAGAATGCTTCTGTCTAGTTTTTATAT

                     **     *  *  * **          *       *     *       *  *  *
      51510 GAAGATATTTACTTTTTCACCGTAGGCATCAAAGCGCTCCAAATGTCCACATCCAGATACTCCAG
        325 GAAGATATTCCCTTTTCCAACGAAATCATCAAAGCGATCCAAATATCCACTTCCAGATTCTACAA

                 *         *             * *   *         * *      * *  *     
      51575 AAAGACTGTTTCAAACCTGCTCTATGAAAGGGAATCTTCAACTCTATGAGTTGAATGCAGACATC
        390 AAAGAGTGTTTCAAAACTGCTCTATGAAAAGAAATGTTCAACTCTGTTAGTTGAGTACACACATC

             *      *              *       **         **         **          
      51640 AGAAAGAAATTTCTGAGAATGCTGCTGTCTACCTTTTAT---TTGA-ATTCCCGCTTCCAACGAA
        455 ACAAAGAAGTTTCTGAGAATGCTTCTGTCTAGTTTTTATGGGAAGATATTCCCTTTTCCAACGAA

             * *  *    *              * *  **   *                            
      51701 ATCCTCCAAGCTATCCAAATATCCACCTGCATTTTCCACAAAAAGAGTGTTTCAAAACTGCTCTA
        520 AGCATCAAAGCGATCCAAATATCCACTTCCAGATTCTACAAAAAGAGTGTTTCAAAACTGCTCTA

                **                *    *  *                *                 
      51766 TCAATAGAAATGTTCAACTCCTTTG-GCTGGGTACACACATCACAAACAAGTTTCTGAGAATGCT
        585 TCAAAGGAAATGTTCAACT-CTGTGAGTTGAGTACACACATCACAAAGAAGTTTCTGAGAATGCT

                                *  **         *  * *  **          *       *  
      51830 TCTGTCTAGTTTTTATGGGAAGACGTTCCCTTTTTCACCAAAGGCATCAAAGCGCTCCAAATGTC
        649 TCTGTCTAGTTTTTAT--GATGATATTCCCTTTTCCAACGAAATCATCAAAGCGATCCAAATATC

                      **                     **        **    **              
      51895 CACTTCCAGACACTACAAAAAGAGTGTTTCAAACGTG-CTCTAAGAAAGCGAATGTTCAACTCTG
        712 CACTTCCAGATTCTACAAAAAGAGTGTTTCAAAACTGCCTCTATCAAAGGAAATGTTCAACTCTG

             * *    * *  * *         *           *             *     ***  *  
      51959 TGACTTGAATGCAGATATCACAAAGTAGTTTCTGAGAGTGCTTCTGTCTAGATTTTAGACGATGA
        777 TTAGTTGAGTACACACATCACAAAGAAGTTTCTGAGAATGCTTCTGTCTAGTTTTTATGGGAAGA

                   *                * *   *                *   *             
      52024 TATTCCCGTTTCCAACGAAATCATTAGAGCTATCCAAATATCCACTTACAGTTTCTACAAAAAGA
        842 TATTCCCTTTTCCAACGAAATCATCAAAGCGATCCAAATATCCACTTCCAGATTCTACAAAAAGA

                   *        **      *  * *    *       *  *   * *  *          
      52089 GTGTTTCCAAACTGCTGCATCAAAAGAGAGGTTCCACTCTGTTAGCTGAGTACACACATCACAAA
        907 GTGTTTCAAAACTGCTCTATCAAAGGAAATGTTCAACTCTGTGAGTTGAATGCAGACATCACAAA

            ***      *     *         *                   **     *  *  * **   
      52154 CTTGTTTCTCAGAATCCTTCTGTCTCGTTTTTATGGGAAGATATTTACTTTTTCACCGTAGGCAT
        972 GAAGTTTCTGAGAATGCTTCTGTCTAGTTTTTAT--GAAGATATTCCCTTTTCCAACGAAATCAT

                   *       *     *       **    *       *       *         *   
      52219 CAAAGCGCTCCAAATGTCCACATCCAGATAGTACAGAAAGAGTTTTTCAAACCTGCTCTATGAAA
       1035 CAAAGCGATCCAAATATCCACTTCCAGATTCTACAAAAAGAGTGTTTCAAAACTGCTCTATCAAA

            * *   *         * *      * *  *      *      *              *     
      52284 GGGAATCTTCAACTCTATGAGTTGAATGCAGACATCAGAAAGAAATTTCTGAGAATGCTGCTGTC
       1100 AGAAATGTTCAACTCTGTTAGTTGAGTACACACATCACAAAGAAGTTTCTGAGAATGCTTCTGTC

              **         **          *  *
      52349 TACCTTTTAT---TTGA-ATTCCCGCTTC
       1165 TAGTTTTTATGGGAAGATATTCCCGTTTT

                      *  *    *                *       *                   * 
      52374 CAACGAAATCCTCCAAGCTATCCAAATATCCACTTGCAGATTCCACAAAAAGAGTGTTTCAAAAC
          1 CAACGAAATCATCAAAGCGATCCAAATATCCACTTCCAGATTCTACAAAAAGAGTGTTTCAAACC

                      *  *  *  *            *       *     *     *  *         
      52439 TGCTCTCTATCAATGGCAAAGTTCAACTCTGTTAGTTGAGGACACATATCACCAACAAGTTTCTG
         66 TG--CTCTA-GAAAGGGAATGTTCAACTCTGTGAGTTGAGTACACACATCACAAAGAAGTTTCTG

                            *       **         *  *   *  *  * **  **  *      
      52504 AGAATGCTTCTGTCTATTTTTTATGGGAAGATATTTCCTTTTTCACCGTAGGCGTCAAGGCGATC
        128 AGAATGCTTCTGTCTAGTTTTTATATGAAGATATTCCCGTTTCCAACGAAATCGCTAAAGCGATC

            *    *          * **                     *         *   * **      
      52569 GAAATGTCCACTTCCACAAACTACAAAAAGAGTGTTTCAAACCTGCTCTATGAAAGGCCATGTTC
        193 CAAATATCCACTTCCAGATTCTACAAAAAGAGTGTTTCAAAACTGCTCTATCAAAAGAAATGTTC

             *    * *      * ** * *   **      *      *       *          *    
      52634 ATCTCTATGAGTTGAATGGAAATATCCGAAAGAAATTTCTGGGAATGCTGCTGTCTAG-TGTT-T
        258 AACTCTGTTAGTTGAGTACACACATCACAAAGAAGTTTCTGAGAATGCTTCTGTCTAGTTTTTAT

                *         **             *       *                *    *  *  
      52697 AT-ACGA-ATTCCCGCTTCCAACGAAATCCTCAAAGCAATCCAAATATCCACTTGCAGAATCCAC
        323 ATGAAGATATTCCCTTTTCCAACGAAATCATCAAAGCGATCCAAATATCCACTTCCAGATTCTAC

                                       *  *     *          *                 
      52760 AAAAAGAGTGTTTCAAAACTGCTCTATCAATAGAAAGGTTCAACTCTTTTAGTTGAGTACACACA
        388 AAAAAGAGTGTTTCAAAACTGCTCTATGAAAAGAAATGTTCAACTCTGTTAGTTGAGTACACACA

                *  *                        * *      *      **  *           *
      52825 TCACGAACAAGTTTCTGAGAATGCTTCTGTCTGGCTTTTATTGGAAGACGTTTCCTTTT-CAACA
        453 TCACAAAGAAGTTTCTGAGAATGCTTCTGTCTAGTTTTTATGGGAAGATATTCCCTTTTCCAACG

              *           *       *                * *                **     
      52889 AAGGCATCAAAGCGCTCCAAATGTCCACTTCCAGATTCTTCCAAAAGAGTGTTTCAAACGTGCTC
        518 AAAGCATCAAAGCGATCCAAATATCCACTTCCAGATTCTACAAAAAGAGTGTTTCAAAACTGCTC

            * ***    *                 *    * *  * *     *   *       * * *   
      52954 GAAGTAAGGGAATGTTCAACTCTGTGACTTGAATGCAGATATCACCAAGTAGTTTCTAATAGTGC
        583 TATCAAAGGAAATGTTCAACTCTGTGAGTTGAGTACACACATCACAAAGAAGTTTCTGAGAATGC

                      *     *              *              * * *   *          
      53019 TTCTGTCTACATTTTAGATGATGATATTCCCGTTTCCAACGAAATCGTTAGAGCTATCCAAATAT
        648 TTCTGTCTA-GTTTT-TATGATGATATTCCCTTTTCCAACGAAATCATCAAAGCGATCCAAATAT

               *  *   *      *     *       *   *     **      *    *          
      53084 CCAGTTACAGTTTCTACCAAAAGGGTGTTTCCAAATTG-CTGCATCAAAAGAAAGGTTCAACTCT
        711 CCACTTCCAGATTCTACAAAAAGAGTGTTTCAAAACTGCCTCTATCAAAGGAAATGTTCAACTCT

                      *                     *                   *    * **  * 
      53148 GTTAGTTGAGGACACACATCACAAAGAAGTTTGTGAGAATGCTTCTGTCTAGATTTTGTATGACG
        776 GTTAGTTGAGTACACACATCACAAAGAAGTTTCTGAGAATGCTTCTGTCTAGTTTTTATGGGAAG

                               *   * *     *   *       * *  *    *  *       *
      53213 ATATTCCCTTTTCCAACGATATCGTTAAAGCAATCTAAATATCAATTTGCAGAATCCACAAAAAT
        841 ATATTCCCTTTTCCAACGAAATCATCAAAGCGATCCAAATATCCACTTCCAGATTCTACAAAAAG

              *        *       * *   *    *    *       *  *   * *  *         
      53278 AGAGTTTCAAAGCTGCTCTGTAAAAAGAAAGGTTCCACTCTGTTAGCTGAGTACACACATCACAA
        906 AGTGTTTCAAAACTGCTCTATCAAAGGAAATGTTCAACTCTGTGAGTTGAATGCAGACATCACAA

             ***            *         *                   **        *  * **  
      53343 ACTTGTTTCTGAGAATCCTTCTGTCTCGTTTTTATGGGAAGATATTTACTTTTCCACCGTAGGCA
        971 AGAAGTTTCTGAGAATGCTTCTGTCTAGTTTTTAT--GAAGATATTCCCTTTTCCAACGAAATCA

                    *       *     *       *  *  *  *            *         *  
      53408 TCAAAGCGCTCCAAATGTCCACATCCAGATACTCCAGAACGAGTGTTTCAAACCTGCTCTATGAA
       1034 TCAAAGCGATCCAAATATCCACTTCCAGATTCTACAAAAAGAGTGTTTCAAAACTGCTCTATCAA

             * *   *         * *      * *  *      *      *              *    
      53473 AGGGAATCTTCAACTCTATGAGTTGAATGCAGACATCAGAAAGAAATTTCTGAGAATGCTGCTGT
       1099 AAGAAATGTTCAACTCTGTTAGTTGAGTACACACATCACAAAGAAGTTTCTGAGAATGCTTCTGT

               **         **          *  *
      53538 CTACCTTTTAT---TTGA-ATTCCCGCTTC
       1164 CTAGTTTTTATGGGAAGATATTCCCGTTTT

                      *  *    *              * *  **   *    *              * 
      53564 CAACGAAATCCTCCAAGCTATCCAAATATCCACCTGCATTTTCCACAACAAGAGTGTTTCAAAAC
          1 CAACGAAATCATCAAAGCGATCCAAATATCCACTTCCAGATTCTACAAAAAGAGTGTTTCAAACC

                    *  ** *              *    *  *                *          
      53629 TGCTCTATCAATAGAAATGTTCAACTCCTTTG-GCTGGGTACACACATCACAAACAAGTTTCTGA
         66 TGCTCTA-GAAAGGGAATGTTCAACT-CTGTGAGTTGAGTACACACATCACAAAGAAGTTTCTGA

             *                     **     *      *   *    *    *           * 
      53693 GCATGCTTCTGTCTAGTTTTTATGGGAAGACATTCCCTTTTTC-ACCAAA-GGCATCAAAGCGCT
        129 GAATGCTTCTGTCTAGTTTTTATATGAAGATATTCCCGTTTCCAACGAAATCGC-T-AAAGCGAT

                  *            **                     **       **    **      
      53756 CCAAATGTCCACTTCCAGACACTACAAAAAGAGTGTTTCAAACGTGCTCTAAGAAAACGAATGTT
        192 CCAAATATCCACTTCCAGATTCTACAAAAAGAGTGTTTCAAAACTGCTCTATCAAAAGAAATGTT

                     * *    * *  * *      *  *           **            *     
      53821 CAACTCTGTGACTTGAATGCAGATATCACAGAGTAGTTTCTGAGAGGGCTTCTGTCTAGATTTTA
        257 CAACTCTGTTAGTTGAGTACACACATCACAAAGAAGTTTCTGAGAATGCTTCTGTCTAGTTTTTA

            *    *         *                * *   *                *   *     
      53886 GATGATGATATTCCCGTTTCCAACGAAATCATTAGAGCTATCCAAATATCCACTTACAGTTTCTA
        322 TATGAAGATATTCCCTTTTCCAACGAAATCATCAAAGCGATCCAAATATCCACTTCCAGATTCTA

                           *        **  *      * *    *          *           
      53951 CAAAAAGAGTGTTTCCAAACTGCTGCATCAAAAGAGAGGTTCCACTCTGTTAGCTGAGTACACAC
        387 CAAAAAGAGTGTTTCAAAACTGCTCTATGAAAAGAAATGTTCAACTCTGTTAGTTGAGTACACAC

                    ***      *     *         *                   **     *  * 
      54016 ATCACAAACTTGTTTCTCAGAATCCTTCTGTCTCGTTTTTATGGGAAGATATTTACTTTTTCACC
        452 ATCACAAAGAAGTTTCTGAGAATGCTTCTGTCTAGTTTTTATGGGAAGATATTCCCTTTTCCAAC

             * *           *       *     *       *  *  *               *     
      54081 GTAGGCATCAAAGCGCTCCAAATGTCCACATCCAGATACTCCAGAAAGAGTGTTTCAAACCTGCT
        517 GAAAGCATCAAAGCGATCCAAATATCCACTTCCAGATTCTACAAAAAGAGTGTTTCAAAACTGCT

                *     *   *         *        * *  *      *      *            
      54146 CTATGAAAGGGAATCTTCAACTCTATGAGTTGAATGCAGACATCAGAAAGAAATTTCTGAGAATG
        582 CTATCAAAGGAAATGTTCAACTCTGTGAGTTGAGTACACACATCACAAAGAAGTTTCTGAGAATG

              *       **       *          **             *  *    *           
      54211 CTGCTGTCTACCTTTTAT-TTGA-ATTCCCGCTTCCAACGAAATCCTCCAAGCTATCCAAATATC
        647 CTTCTGTCTAGTTTTTATGATGATATTCCCTTTTCCAACGAAATCATCAAAGCGATCCAAATATC

                 *       *                                  *  *  *          
      54274 CACTTGCAGATTCCACAAAAAGAGTGTTTCAAAACTGCTCTCTATCAATGGCAAAGTTCAACTCT
        712 CACTTCCAGATTCTACAAAAAGAGTGTTTCAAAACTGC-CTCTATCAAAGGAAATGTTCAACTCT

                      *     *     *  *                         *             
      54339 GTTAGTTGAGGACACATATCACCAACAAGTTTCTGAGAATGCTTCTGTCTATTTTTTATGGGAAG
        776 GTTAGTTGAGTACACACATCACAAAGAAGTTTCTGAGAATGCTTCTGTCTAGTTTTTATGGGAAG

                 *      *  *  * ** *    *      *    *          * **          
      54404 ATATTTCCTTTTTCACCGTAGGCGTCAAGGCGATCGAAATGTCCACTTCCACAAACTACAAAAAG
        841 ATATTCCCTTTTCCAACGAAATCATCAAAGCGATCCAAATATCCACTTCCAGATTCTACAAAAAG

                       *         *     **       *    *           * * *   **  
      54469 AGTGTTTCAAACCTGCTCTATGAAAGGCGATGTTCATCTCTATGAGTTGAATGGAAATATCCGAA
        906 AGTGTTTCAAAACTGCTCTATCAAAGGAAATGTTCAACTCTGTGAGTTGAATGCAGACATCACAA

                *      *       *         *       *         **             *  
      54534 AGAAATTTCTGGGAATGCTGCTGTCTAGTGTTTAT-ACGA-ATTCCCGCTTCCAACGAAATCCTC
        971 AGAAGTTTCTGAGAATGCTTCTGTCTAGTTTTTATGAAGATATTCCCTTTTCCAACGAAATCATC

                 *                *    *  *                                * 
      54597 AAAGCAATCCAAATATCCACTTGCAGAATCCACAAAAAGAGTGTTTCAAAACTGCTCTATCAATA
       1036 AAAGCGATCCAAATATCCACTTCCAGATTCTACAAAAAGAGTGTTTCAAAACTGCTCTATCAAAA

                *          *                     *  *                        
      54662 GAAAGGTTCAACTCTTTTAGTTGAGTACACACATCACGAACAAGTTTCTGAGAATGCTTCTGTCT
       1101 GAAATGTTCAACTCTGTTAGTTGAGTACACACATCACAAAGAAGTTTCTGAGAATGCTTCTGTCT

            * *      *      **  *       
      54727 GGCTTTTATTGGAAGACGTTTCC-TTTT
       1166 AGTTTTTATGGGAAGATATTCCCGTTTT

              * *  **          *       *                * *                 *
      54754 CACCAAAGGCATCAAAGCGCTCCAAATGTCCACTTCCAGATTCTTCCAAAAGAGTGTTTCAAACG
          1 CAACGAAATCATCAAAGCGATCCAAATATCCACTTCCAGATTCTACAAAAAGAGTGTTTCAAACC

                  *  *                      *    * *  * *     *   *       * *
      54819 TGCTCGAAGTAAGGGAATGTTCAACTCTGTGACTTGAATGCAGATATCACCAAGTAGTTTCTAAT
         66 TGCTC-TAGAAAGGGAATGTTCAACTCTGTGAGTTGAGTACACACATCACAAAGAAGTTTCTGAG

             *             *     *    *                         *  *   *     
      54884 AGTGCTTCTGTCTAGATTTTAGATGATGATATTCCCGTTTCCAACGAAATCGTTAGAGCTATCCA
        130 AATGCTTCTGTCTAGTTTTTATATGAAGATATTCCCGTTTCCAACGAAATCGCTAAAGCGATCCA

                       *   *                    *        **           *      
      54949 AATATCCACTTACAGTTTCTACAAAAAGAGTGTTTCCAAACTGCTGCATCAAAAGAAAGGTTCAA
        195 AATATCCACTTCCAGATTCTACAAAAAGAGTGTTTCAAAACTGCTCTATCAAAAGAAATGTTCAA

                          *                     *                   *    *   
      55014 CTCTGTTAGTTGAGGACACACATCACAAAGAAGTTTGTGAGAATGCTTCTGTCTAGATTTTGTAT
        260 CTCTGTTAGTTGAGTACACACATCACAAAGAAGTTTCTGAGAATGCTTCTGTCTAGTTTTTATAT

              *                    *   * *     *   *       * *  *    *  *    
      55079 GACGATATTCCCTTTTCCAACGATATCGTTAAAGCAATCTAAATATCAATTTGCAGAATCCACAA
        325 GAAGATATTCCCTTTTCCAACGAAATCATCAAAGCGATCCAAATATCCACTTCCAGATTCTACAA

               *  *        *       * *    *   *    *          *              
      55144 AAATAGAGTTTCAAAGCTGCTCTGTAAAAATAAAAGTTCCACTCTGTTAGCTGAGTACACACATC
        390 AAAGAGTGTTTCAAAACTGCTCTATGAAAAGAAATGTTCAACTCTGTTAGTTGAGTACACACATC

                 ***            *         *                   **     *  *  * 
      55209 ACAAACTTGTTTCTGAGAATCCTTCTGTCTCGTTTTTATGGGAAGATATTTACTTTTTCACCGTA
        455 ACAAAGAAGTTTCTGAGAATGCTTCTGTCTAGTTTTTATGGGAAGATATTCCCTTTTCCAACGAA

            *           *       *     *       *  *  *     *         *        
      55274 GGCATCAAAGCGCTCCAAATGTCCACATCCAGATACTCCAGAAAGACTGTTTCAAACCTGCTCTA
        520 AGCATCAAAGCGATCCAAATATCCACTTCCAGATTCTACAAAAAGAGTGTTTCAAAACTGCTCTA

             *     *   *         *        * *  *      *      *              *
      55339 TGAAAGGGAATCTTCAACTCTATGAGTTGAATGCAGACATCAGAAAGAAATTTCTGAGAATGCTG
        585 TCAAAGGAAATGTTCAACTCTGTGAGTTGAGTACACACATCACAAAGAAGTTTCTGAGAATGCTT

                   **       *          **             *  *    *              
      55404 CTGTCTACCTTTTAT-TTGA-ATTCCCGCTTCCAACGAAATCCTCCAAGCTATCCAAATATCCAC
        650 CTGTCTAGTTTTTATGATGATATTCCCTTTTCCAACGAAATCATCAAAGCGATCCAAATATCCAC

            * *  **   *                                 **              **  *
      55467 CTGCATTTTCCACAAAAAGAGTGTTTCAAAACTG-CTCTATCAATAGAAATGTTCAACTCCTTTG
        715 TTCCAGATTCTACAAAAAGAGTGTTTCAAAACTGCCTCTATCAAAGGAAATGTTCAACTCTGTTA

             *  *                *                                        ** 
      55531 GCTGGGTACACACATCACAAACAAGTTTCTGAGAATGCTTCTGTCTAGTTTTTATGGGAAGACGT
        780 GTTGAGTACACACATCACAAAGAAGTTTCTGAGAATGCTTCTGTCTAGTTTTTATGGGAAGATAT

                    *  * *  **          *       *            **              
      55596 TCCCTTTTTCACCAAAGGCATCAAAGCGCTCCAAATGTCCACTTCCAGACACTACAAAAAGAGTG
        845 TCCCTTTTCCAACGAAATCATCAAAGCGATCCAAATATCCACTTCCAGATTCTACAAAAAGAGTG

                   **       **    **                 *           *         * 
      55661 TTTCAAACGTGCTCTAAGAAAGCGAATGTTCAACTCTGTGACTTGAATGCAGATATCACAAAGTA
        910 TTTCAAAACTGCTCTATCAAAGGAAATGTTCAACTCTGTGAGTTGAATGCAGACATCACAAAGAA

                      *                   * *  *         *                * *
      55726 GTTTCTGAGAGTGCTTCTGTCTAGATTTTAGACGATGATATTCCCGTTTCCAACGAAATCATTAG
        975 GTTTCTGAGAATGCTTCTGTCTAG-TTTT-TATGAAGATATTCCCTTTTCCAACGAAATCATCAA

               *                *   *                    *        **         
      55791 AGCTATCCAAATATCCACTTACAGTTTCTACAAAAAGAGTGTTTCCAAACTGCTGCATCAAAAGA
       1038 AGCGATCCAAATATCCACTTCCAGATTCTACAAAAAGAGTGTTTCAAAACTGCTCTATCAAAAGA

            * *    *          *                   ***      *     *         * 
      55856 GAGGTTCCACTCTGTTAGCTGAGTACACACATCACAAACTTGTTTCTCAGAATCCTTCTGTCTCG
       1103 AATGTTCAACTCTGTTAGTTGAGTACACACATCACAAAGAAGTTTCTGAGAATGCTTCTGTCTAG

                              ** *    
      55921 TTTTTATGGGAAGATATTTACTTTTT
       1168 TTTTTATGGGAAGATATTCCCGTTTT

              *  * **          *       *     *       *  *  *                 
      55947 CACCGTAGGCATCAAAGCGCTCCAAATGTCCACATCCAGATACTCCAGAAAGAGTGTTTCAAACC
          1 CAACGAAATCATCAAAGCGATCCAAATATCCACTTCCAGATTCTACAAAAAGAGTGTTTCAAACC

                              *         *        * *  *      *      *        
      56012 TGCTCTATGAAAGGGAATCTTCAACTCTATGAGTTGAATGCAGACATCAGAAAGAAATTTCTGAG
         66 TGCTCTA-GAAAGGGAATGTTCAACTCTGTGAGTTGAGTACACACATCACAAAGAAGTTTCTGAG

                  *       **          *          *                 *    *    
      56077 AATGCTGCTGTCTACCTTTTAT-T--TGA-ATTCCCGCTTCCAACGAAATC-CTCCAAGCTATCC
        130 AATGCTTCTGTCTAGTTTTTATATGAAGATATTCCCGTTTCCAACGAAATCGCT-AAAGCGATCC

                        *       *                                  ** *  *   
      56137 AAATATCCACTTGCAGATTCCACAAAAAGAGTGTTTCAAAACTGCTCTCTATCAATGGCAAAGTT
        194 AAATATCCACTTCCAGATTCTACAAAAAGAGTGTTTCAAAACTG--CTCTATCAAAAGAAATGTT

                             *     *     *  *                         *      
      56202 CAACTCTGTTAGTTGAGGACACATATCACCAACAAGTTTCTGAGAATGCTTCTGTCTATTTTTTA
        257 CAACTCTGTTAGTTGAGTACACACATCACAAAGAAGTTTCTGAGAATGCTTCTGTCTAGTTTTTA

             **         *      *  *  * ** *    *      *    *          * **   
      56267 TGGGAAGATATTTCCTTTTTCACCGTAGGCGTCAAGGCGATCGAAATGTCCACTTCCACAAACTA
        322 TATGAAGATATTCCCTTTTCCAACGAAATCATCAAAGCGATCCAAATATCCACTTCCAGATTCTA

                              *             * **       *    * *      * ** * *
      56332 CAAAAAGAGTGTTTCAAACCTGCTCTATGAAAGGCCATGTTCATCTCTATGAGTTGAATGGAAAT
        387 CAAAAAGAGTGTTTCAAAACTGCTCTATGAAAAGAAATGTTCAACTCTGTTAGTTGAGTACACAC

               **      *      *       *         *         *         **       
      56397 ATCCGAAAGAAATTTCTGGGAATGCTGCTGTCTAGTGTTTAT---ACGA-ATTCCCGCTTCCAAC
        452 ATCACAAAGAAGTTTCTGAGAATGCTTCTGTCTAGTTTTTATGGGAAGATATTCCCTTTTCCAAC

                * *       *                *    *  *                         
      56458 GAAATCCTCAAAGCAATCCAAATATCCACTTGCAGAATCCACAAAAAGAGTGTTTCAAAACTGCT
        517 GAAAGCATCAAAGCGATCCAAATATCCACTTCCAGATTCTACAAAAAGAGTGTTTCAAAACTGCT

                   **    *          * *                   *  *               
      56523 CTATCAATAGAAAGGTTCAACTCTTTTAGTTGAGTACACACATCACGAACAAGTTTCTGAGAATG
        582 CTATCAAAGGAAATGTTCAACTCTGTGAGTTGAGTACACACATCACAAAGAAGTTTCTGAGAATG

                     * *          *  **  *           *  **          *       *
      56588 CTTCTGTCTGGCTTTTATTGGAAGACGTTTCCTTTT-CAACAAAGGCATCAAAGCGCTCCAAATG
        647 CTTCTGTCTAGTTTTTA-T-GATGATATTCCCTTTTCCAACGAAATCATCAAAGCGATCCAAATA

                            * *                **      * ***    *            
      56652 TCCACTTCCAGATTCTTCCAAAAGAGTGTTTCAAACGTG-CTCGAAGTAAGGGAATGTTCAACTC
        710 TCCACTTCCAGATTCTACAAAAAGAGTGTTTCAAAACTGCCTCTATCAAAGGAAATGTTCAACTC

               * *    * *  * *     *   *       * * *            **     ***  *
      56716 TGTGACTTGAATGCAGATATCACCAAGTAGTTTCTAATAGTGCTTCTGTCTACATTTTAGATGAT
        775 TGTTAGTTGAGTACACACATCACAAAGAAGTTTCTGAGAATGCTTCTGTCTAGTTTTTATGGGAA

                     *              * * *   *             *  *   *      *    
      56781 GATATTCCCGTTTCCAACGAAATCGTTAGAGCTATCCAAATATCCAGTTACAGTTTCTACCAAAA
        840 GATATTCCCTTTTCCAACGAAATCATCAAAGCGATCCAAATATCCACTTCCAGATTCTACAAAAA

             *       *   *    **      *    *            *      ***  *        
      56846 GGGTGTTTCCAAATTGCTGCATCAAAAGAAAGGTTCAACTCTGTTAGTTGAGGACACACATCACA
        905 GAGTGTTTCAAAACTGCTCTATCAAAGGAAATGTTCAACTCTGTGAGTTGAATGCAGACATCACA

                     *                              *                    *   
      56911 AAGAAGTTTGTGAGAATGCTTCTGTCTAGATTTTGTATGACGATATTCCCTTTTCCAACGATATC
        970 AAGAAGTTTCTGAGAATGCTTCTGTCTAG-TTTT-TATGAAGATATTCCCTTTTCCAACGAAATC

            * *     *   *       * *  *    *  *       *  *        *       * * 
      56976 GTTAAAGCAATCTAAATATCAATTTGCAGAATCCACAAAAATAGAGTTTCAAAGCTGCTCTGTAA
       1033 ATCAAAGCGATCCAAATATCCACTTCCAGATTCTACAAAAAGAGTGTTTCAAAACTGCTCTATCA

                   *    *          *                   ***            *      
      57041 AAAGAAAGGTTCCACTCTGTTAGCTGAGTACACACATCACAAACTTGTTTCTGAGAATCCTTCTG
       1098 AAAGAAATGTTCAACTCTGTTAGTTGAGTACACACATCACAAAGAAGTTTCTGAGAATGCTTCTG

               *                   ** *   *
      57106 TCTCGTTTTTATGGGAAGATATTTACTTTTC
       1163 TCTAGTTTTTATGGGAAGATATTCCCGTTTT

              *  * **          *       *     *       *  *  *  *              
      57137 CACCGTAGGCATCAAAGCGCTCCAAATGTCCACATCCAGATACTCCAGAACGAGTGTTTCAAACC
          1 CAACGAAATCATCAAAGCGATCCAAATATCCACTTCCAGATTCTACAAAAAGAGTGTTTCAAACC

                              *         *        * *  *      *      *        
      57202 TGCTCTATGAAAGGGAATCTTCAACTCTATGAGTTGAATGCAGACATCAGAAAGAAATTTCTGAG
         66 TGCTCTA-GAAAGGGAATGTTCAACTCTGTGAGTTGAGTACACACATCACAAAGAAGTTTCTGAG

                  *       **          *          *                 *    *    
      57267 AATGCTGCTGTCTACCTTTTAT-T--TGA-ATTCCCGCTTCCAACGAAATC-CTCCAAGCTATCC
        130 AATGCTTCTGTCTAGTTTTTATATGAAGATATTCCCGTTTCCAACGAAATCGCT-AAAGCGATCC

                      * *  **   *    *                           *           
      57327 AAATATCCACCTGCATTTTCCACAACAAGAGTGTTTCAAAACTGCTCTATCAATAGAAATGTTCA
        194 AAATATCCACTTCCAGATTCTACAAAAAGAGTGTTTCAAAACTGCTCTATCAAAAGAAATGTTCA

                **  * *  *                *           *                     *
      57392 ACTCCTTTGGCTGGGTACACACATCACAAACAAGTTTCTGAGCATGCTTCTGTCTAGTTTTTATG
        259 ACTCTGTTAGTTGAGTACACACATCACAAAGAAGTTTCTGAGAATGCTTCTGTCTAGTTTTTATA

            *     *          *  * *  **          *       *            **     
      57457 GGAAGACATTCCCTTTTTCACCAAAGGCATCAAAGCGCTCCAAATGTCCACTTCCAGACACTACA
        324 TGAAGATATTCCCTTTTCCAACGAAATCATCAAAGCGATCCAAATATCCACTTCCAGATTCTACA

                            **       *     **               * *    * *  * *  
      57522 AAAAGAGTGTTTCAAACGTGCTCTAAGAAAACGAATGTTCAACTCTGTGACTTGAATGCAGATAT
        389 AAAAGAGTGTTTCAAAACTGCTCTATGAAAAGAAATGTTCAACTCTGTTAGTTGAGTACACACAT

                *  *           **            *     ***  *         *          
      57587 CACAGAGTAGTTTCTGAGAGGGCTTCTGTCTAGATTTTAGATGATGATATTCCCGTTTCCAACGA
        454 CACAAAGAAGTTTCTGAGAATGCTTCTGTCTAGTTTTTATGGGAAGATATTCCCTTTTCCAACGA

              *   * *   *                *   *                    *        **
      57652 AATCATTAGAGCTATCCAAATATCCACTTACAGTTTCTACAAAAAGAGTGTTTCCAAACTGCTGC
        519 AAGCATCAAAGCGATCCAAATATCCACTTCCAGATTCTACAAAAAGAGTGTTTCAAAACTGCTCT

                  *  * *    *       *  *                   ***      *     *  
      57717 ATCAAAAGAGAGGTTCCACTCTGTTAGCTGAGTACACACATCACAAACTTGTTTCTCAGAATCCT
        584 ATCAAAGGAAATGTTCAACTCTGTGAGTTGAGTACACACATCACAAAGAAGTTTCTGAGAATGCT

                   *            *      **     *  *  * **          *       *  
      57782 TCTGTCTCGTTTTTATGGGAAGATATTTACTTTTTCACCGTAGGCATCAAAGCGCTCCAAATGTC
        649 TCTGTCTAGTTTTTAT--GATGATATTCCCTTTTCCAACGAAATCATCAAAGCGATCCAAATATC

               *       *  *  *               *          *     *   *         *
      57847 CACATCCAGATACTCCAGAAAGAGTGTTTCAAACCTG-CTCTATGAAAGGGAATCTTCAACTCTA
        712 CACTTCCAGATTCTACAAAAAGAGTGTTTCAAAACTGCCTCTATCAAAGGAAATGTTCAACTCTG

             *      * *  *      *      *              *       **         **  
      57911 TGAGTTGAATGCAGACATCAGAAAGAAATTTCTGAGAATGCTGCTGTCTACCTTTTAT---TTGA
        777 TTAGTTGAGTACACACATCACAAAGAAGTTTCTGAGAATGCTTCTGTCTAGTTTTTATGGGAAGA

                   **             *  *    *                *       *         
      57973 -ATTCCCGCTTCCAACGAAATCCTCCAAGCTATCCAAATATCCACTTGCAGATTCCACAAAAAGA
        842 TATTCCCTTTTCCAACGAAATCATCAAAGCGATCCAAATATCCACTTCCAGATTCTACAAAAAGA

                                     *  *  *            *      ***  * *     *
      58037 GTGTTTCAAAACTGCTCTCTATCAATGGCAAAGTTCAACTCTGTTAGTTGAGGACACATATCACC
        907 GTGTTTCAAAACTG--CTCTATCAAAGGAAATGTTCAACTCTGTGAGTTGAATGCAGACATCACA

              *                         *                  *      *  *  * ** 
      58102 AACAAGTTTCTGAGAATGCTTCTGTCTATTTTTTATGGGAAGATATTTCCTTTTTCACCGTAGGC
        970 AAGAAGTTTCTGAGAATGCTTCTGTCTAGTTTTTAT--GAAGATATTCCCTTTTCCAACGAAATC

            *    *      *    *          * **                     *         * 
      58167 GTCAAGGCGATCGAAATGTCCACTTCCACAAACTACAAAAAGAGTGTTTCAAACCTGCTCTATGA
       1033 ATCAAAGCGATCCAAATATCCACTTCCAGATTCTACAAAAAGAGTGTTTCAAAACTGCTCTATCA

              * **       *    * *      * ** * *   **      *      *       *   
      58232 AAGGCCATGTTCATCTCTATGAGTTGAATGGAAATATCCGAAAGAAATTTCTGGGAATGCTGCTG
       1098 AAAGAAATGTTCAACTCTGTTAGTTGAGTACACACATCACAAAGAAGTTTCTGAGAATGCTTCTG

                  *         *          *  *
      58297 TCTAGTGTTTAT---ACGA-ATTCCCGCTTC
       1163 TCTAGTTTTTATGGGAAGATATTCCCGTTTT

                      *       *                *    *  *                   * 
      58324 CAACGAAATCCTCAAAGCAATCCAAATATCCACTTGCAGAATCCACAAAAAGAGTGTTTCAAAAC
          1 CAACGAAATCATCAAAGCGATCCAAATATCCACTTCCAGATTCTACAAAAAGAGTGTTTCAAACC

                    *  ** *  *          * *                   *  *           
      58389 TGCTCTATCAATAGAAAGGTTCAACTCTTTTAGTTGAGTACACACATCACGAACAAGTTTCTGAG
         66 TGCTCTA-GAAAGGGAATGTTCAACTCTGTGAGTTGAGTACACACATCACAAAGAAGTTTCTGAG

                         * *      **     **  *      *    *    *           *  
      58454 AATGCTTCTGTCTGGCTTTTATTGGAAGACGTTTCC-TTTTC-ACCAAA-GGCATCAAAGCGCTC
        130 AATGCTTCTGTCTAGTTTTTATATGAAGATATTCCCGTTTCCAACGAAATCGC-T-AAAGCGATC

                 *                * *                **     * ***  * *       
      58516 CAAATGTCCACTTCCAGATTCTTCCAAAAGAGTGTTTCAAACGTGCTCAAAGTAAGGGAATGTTC
        193 CAAATATCCACTTCCAGATTCTACAAAAAGAGTGTTTCAAAACTGCTCTATCAAAAGAAATGTTC

                    * *    * *  * *     *   *       * * *             *   * *
      58581 AACTCTGTGACTTGAATGCAGATATCACCAAGTAGTTTCTAATAGTGCTTCTGTCTAGATTTCAG
        258 AACTCTGTTAGTTGAGTACACACATCACAAAGAAGTTTCTGAGAATGCTTCTGTCTAGTTTTTAT

                *         *              * * *   *             *  *   *      
      58646 ATGATGATATTCCCGTTTCCAACGAAATCGTTAGAGCTATCCAAATATCCAGTTACAGTTTCTAC
        323 ATGAAGATATTCCCTTTTCCAACGAAATCATCAAAGCGATCCAAATATCCACTTCCAGATTCTAC

                          *        **  *        *                    *       
      58711 AAAAAGAGTGTTTCCAAACTGCTGCATCAAAAGAAAGGTTCAACTCTGTTAGTTGAGGACACACA
        388 AAAAAGAGTGTTTCAAAACTGCTCTATGAAAAGAAATGTTCAACTCTGTTAGTTGAGTACACACA

                          *                   *    * **  *                   
      58776 TCACAAAGAAGTTTGTGAGAATGCTTCTGTCTAGATTTTGTATGACGATATTCCCTTTTCCAACG
        453 TCACAAAGAAGTTTCTGAGAATGCTTCTGTCTAGTTTTTATGGGAAGATATTCCCTTTTCCAACG

             * * * *     *   *       * *  *    *  *       *  *        *      
      58841 ATATCGTTAAAGCAATCTAAATATCAATTTGCAGAATCCACAAAAATAGAGTTTCAAAGCTGCTC
        518 AAAGCATCAAAGCGATCCAAATATCCACTTCCAGATTCTACAAAAAGAGTGTTTCAAAACTGCTC

             * *   *    *    *       *  *                   ***            * 
      58906 TGTAAAAAGAAAGGTTCCACTCTGTTAGCTGAGTACACACATCACAAACTTGTTTCTGAGAATCC
        583 TATCAAAGGAAATGTTCAACTCTGTGAGTTGAGTACACACATCACAAAGAAGTTTCTGAGAATGC

                    *            *      **     *  *  * **          *       * 
      58971 TTCTGTCTCGTTTTTATGGGAAGATATTTACTTTTTCACCGTAGGCATCAAAGCGCTCCAAATGT
        648 TTCTGTCTAGTTTTTAT--GATGATATTCCCTTTTCCAACGAAATCATCAAAGCGATCCAAATAT

                *       *  *  *     *         *          *     *   *         
      59036 CCACATCCAGATACTCCAGAAAGACTGTTTCAAACCTG-CTCTATGAAAGGGAATCTTCAACTCT
        711 CCACTTCCAGATTCTACAAAAAGAGTGTTTCAAAACTGCCTCTATCAAAGGAAATGTTCAACTCT

            * *      * *  *      *      *              *       **         ** 
      59100 ATGAGTTGAATGCAGACATCAGAAAGAAATTTCTGAGAATGCTGCTGTCTACCTTTTAT---TTG
        776 GTTAGTTGAGTACACACATCACAAAGAAGTTTCTGAGAATGCTTCTGTCTAGTTTTTATGGGAAG

                    **             *  *    *                *  **   *        
      59162 A-ATTCCCGCTTCCAACGAAATCCTCCAAGCTATCCAAATATCCACTTGCATTTTCCACAAAAAG
        841 ATATTCCCTTTTCCAACGAAATCATCAAAGCGATCCAAATATCCACTTCCAGATTCTACAAAAAG

                                    **              ** *  *  ** *  *         
      59226 AGTGTTTCAAAACTGCTCTATCAATAGAAATGTTCAACTCCTTTAGCTGGGTACACACATCACAA
        906 AGTGTTTCAAAACTGCTCTATCAAAGGAAATGTTCAACTCTGTGAGTTGAATGCAGACATCACAA

             *                                        **         *  * *  **  
      59291 ACAAGTTTCTGAGAATGCTTCTGTCTAGTTTTTATGGGAAGACGTTCCCTTTTTCACCAAAGGCA
        971 AGAAGTTTCTGAGAATGCTTCTGTCTAGTTTTTAT--GAAGATATTCCCTTTTCCAACGAAATCA

                    *       *            **                     **       **  
      59356 TCAAAGCGCTCCAAATGTCCACTTCCAGACACTACAAAAAGAGTGTTTCAAACGTGCTCTAAGAA
       1034 TCAAAGCGATCCAAATATCCACTTCCAGATTCTACAAAAAGAGTGTTTCAAAACTGCTCTATCAA

             ***               * *    * *  * *         *           **        
      59421 AGCGAATGTTCAACTCTGTGACTTGAATGCAGATATCACAAAGTAGTTTCTGAGAGGGCTTCTGT
       1099 AAGAAATGTTCAACTCTGTTAGTTGAGTACACACATCACAAAGAAGTTTCTGAGAATGCTTCTGT

                *     ***  *             *
      59486 CTAGATTTTAGATGATGATATTCCCGTTTC
       1164 CTAGTTTTTATGGGAAGATATTCCCGTTTT

                        * *   *                *   *                    *  * 
      59516 CAACGAAATCATTAGAGCTATCCAAATATCCACTTACAGTTTCTACAAAAAGAGTGTTTCCAAAC
          1 CAACGAAATCATCAAAGCGATCCAAATATCCACTTCCAGATTCTACAAAAAGAGTGTTTCAAACC

                    **    *   *    *       *  **                  ***      * 
      59581 TGCTGCATCAAAAGAG-AGGTTCCACTCTGTTAGCCGAGTACACACATCACAAACTTGTTTCTCA
         66 TGCT-C-TAGAAAGGGAATGTTCAACTCTGTGAGTTGAGTACACACATCACAAAGAAGTTTCTGA

                *         *        **         ** *   *  *  * ** ***      *   
      59645 GAATCCTTCTGTCTCGTTTTTATGGGAAGATATTTACTTTTTCACCGTAGGCATCAAAGCGCTCC
        129 GAATGCTTCTGTCTAGTTTTTATATGAAGATATTCCCGTTTCCAACGAAATCGCTAAAGCGATCC

                *     *       *  *  *               *         *   * *   *    
      59710 AAATGTCCACATCCAGATACTCCAGAAAGAGTGTTTCAAACCTGCTCTATGAAAGGGAATCTTCA
        194 AAATATCCACTTCCAGATTCTACAAAAAGAGTGTTTCAAAACTGCTCTATCAAAAGAAATGTTCA

                 * *      * *  *      *      *              *       **       
      59775 ACTCTATGAGTTGAATGCAGACATCAGAAAGAAATTTCTGAGAATGCTGCTGTCTACCTTTTAT-
        259 ACTCTGTTAGTTGAGTACACACATCACAAAGAAGTTTCTGAGAATGCTTCTGTCTAGTTTTTATA

               *         **             *  *    *                *       *   
      59839 T--TGA-ATTCCCGCTTCCAACGAAATCCTCCAAGCTATCCAAATATCCACTTGCAGATTCCACA
        324 TGAAGATATTCCCTTTTCCAACGAAATCATCAAAGCGATCCAAATATCCACTTCCAGATTCTACA

                                        *  ** *  *                    *     *
      59901 AAAAGAGTGTTTCAAAACTGCTCTCTATCAATGGCAAAGTTCAACTCTGTTAGTTGAGGACACAT
        389 AAAAGAGTGTTTCAAAACTG--CTCTATGAAAAGAAATGTTCAACTCTGTTAGTTGAGTACACAC

                 *  *                         *                  *      *  * 
      59966 ATCACCAACAAGTTTCTGAGAATGCTTCTGTCTATTTTTTATGGGAAGATATTTCCTTTTTCACC
        452 ATCACAAAGAAGTTTCTGAGAATGCTTCTGTCTAGTTTTTATGGGAAGATATTCCCTTTTCCAAC

             * *  *    *      *    *          * **                     *     
      60031 GTAGGCGTCAAGGCGATCGAAATGTCCACTTCCACAAACTACAAAAAGAGTGTTTCAAACCTGCT
        517 GAAAGCATCAAAGCGATCCAAATATCCACTTCCAGATTCTACAAAAAGAGTGTTTCAAAACTGCT

                *     **       *    *        * ** * *   **      *      *     
      60096 CTATGAAAGGCCATGTTCATCTCTATGAGTTGAATGGAAATATCCGAAAGAAATTTCTGGGAATG
        582 CTATCAAAGGAAATGTTCAACTCTGTGAGTTGAGTACACACATCACAAAGAAGTTTCTGAGAATG

              *         *       *         **             *       *           
      60161 CTGCTGTCTAGTGTTTAT-ACGA-ATTCCCGCTTCCAACGAAATCCTCAAAGCAATCCAAATATC
        647 CTTCTGTCTAGTTTTTATGATGATATTCCCTTTTCCAACGAAATCATCAAAGCGATCCAAATATC

                 *    *  *                                 **    *          *
      60224 CACTTGCAGAATCCACAAAAAGAGTGTTTCAAAACTG-CTCTATCAATAGAAAGGTTCAACTCTT
        712 CACTTCCAGATTCTACAAAAAGAGTGTTTCAAAACTGCCTCTATCAAAGGAAATGTTCAACTCTG

                                 *  *                        * *      *      
      60288 TTAGTTGAGTACACACATCACGAACAAGTTTCTGAGAATGCTTCTGTCTGGCTTTTATTGGAAGA
        777 TTAGTTGAGTACACACATCACAAAGAAGTTTCTGAGAATGCTTCTGTCTAGTTTTTATGGGAAGA

            **  *           *  **          *       *                * *      
      60353 CGTTTCCTTTT-CAACAAAGGCATCAAAGCGCTCCAAATGTCCACTTCCAGATTCTTCCAAAAGA
        842 TATTCCCTTTTCCAACGAAATCATCAAAGCGATCCAAATATCCACTTCCAGATTCTACAAAAAGA

                      **     * ***    *                 *           *     *  
      60417 GTGTTTCAAACGTGCTCGAAGTAAGGGAATGTTCAACTCTGTGACTTGAATGCAGATATCACCAA
        907 GTGTTTCAAAACTGCTCTATCAAAGGAAATGTTCAACTCTGTGAGTTGAATGCAGACATCACAAA

             *       * * *             *     *    *         *              * 
      60482 GTAGTTTCTAATAGTGCTTCTGTCTACATTTTAGATGATGATATTCCCGTTTCCAACGAAATCGT
        972 GAAGTTTCTGAGAATGCTTCTGTCTA-GTTTT-TATGAAGATATTCCCTTTTCCAACGAAATCAT

            * *   * **          *  *   *      *     *       *   *    **      
      60547 TAGAGCTAAGCAAATATCCAGTTACAGTTTCTACCAAAAGGGTGTTTCCAAATTGCTGCATCAAA
       1035 CAAAGCGATCCAAATATCCACTTCCAGATTCTACAAAAAGAGTGTTTCAAAACTGCTCTATCAAA

                 *                    *                     *                
      60612 AGAAAGGTTCAACTCTGTTAGTTGAGGACACACATCACAAAGAAGTTTGTGAGAATGCTTCTGTC
       1100 AGAAATGTTCAACTCTGTTAGTTGAGTACACACATCACAAAGAAGTTTCTGAGAATGCTTCTGTC

               *    * **  *         *   *
      60677 TAGATTTTGTATGACGATATTCCCTTTTC
       1165 TAGTTTTTATGGGAAGATATTCCCGTTTT

                  *   * *     *   *       * *  *    *  *       *  *        * 
      60706 CAACGATATCGTTAAAGCAATCTAAATATCAATTTGCAGAATCCACAAAAATAGAGTTTCAAAGC
          1 CAACGAAATCATCAAAGCGATCCAAATATCCACTTCCAGATTCTACAAAAAGAGTGTTTCAAACC

                   **   * *  *    *       *  *                   ***         
      60771 TGCTCTGTAAAAAGAAAGGTTCCACTCTGTTAGCTGAGTACACACATCACAAACTTGTTTCTGAG
         66 TGCTCT-AGAAAGGGAATGTTCAACTCTGTGAGTTGAGTACACACATCACAAAGAAGTTTCTGAG

               *         *        **         ** *      *  * ** ***      *    
      60836 AATCCTTCTGTCTCGTTTTTATGGGAAGATATTTACTTTTCCACCGTAGGCATCAAAGCGCTCCA
        130 AATGCTTCTGTCTAGTTTTTATATGAAGATATTCCCGTTTCCAACGAAATCGCTAAAGCGATCCA

               *     *       *  *  *  *            *         *   * *   *     
      60901 AATGTCCACATCCAGATACTCCAGAACGAGTGTTTCAAACCTGCTCTATGAAAGGGAATCTTCAA
        195 AATATCCACTTCCAGATTCTACAAAAAGAGTGTTTCAAAACTGCTCTATCAAAAGAAATGTTCAA

                * *      * *  *      *      *              *       **        
      60966 CTCTATGAGTTGAATGCAGACATCAGAAAGAAATTTCTGAGAATGCTGCTGTCTACCTTTTAT-T
        260 CTCTGTTAGTTGAGTACACACATCACAAAGAAGTTTCTGAGAATGCTTCTGTCTAGTTTTTATAT

              *         **             *  *    *              * *  **   *    
      61030 --TGA-ATTCCCGCTTCCAACGAAATCCTCCAAGCTATCCAAATATCCACCTGCATTTTCCACAA
        325 GAAGATATTCCCTTTTCCAACGAAATCATCAAAGCGATCCAAATATCCACTTCCAGATTCTACAA

            *                        *  *               **  * *  *           
      61092 CAAGAGTGTTTCAAAACTGCTCTATCAATAGAAATGTTCAACTCCTTTGGCTGGGTACACACATC
        390 AAAGAGTGTTTCAAAACTGCTCTATGAAAAGAAATGTTCAACTCTGTTAGTTGAGTACACACATC

                 *           *                            *          *  * *  
      61157 ACAAACAAGTTTCTGAGCATGCTTCTGTCTAGTTTTTATGGGAAGACATTCCCTTTTTCACCAAA
        455 ACAAAGAAGTTTCTGAGAATGCTTCTGTCTAGTTTTTATGGGAAGATATTCCCTTTTCCAACGAA

            *           *       *            **                     **       
      61222 GGCATCAAAGCGCTCCAAATGTCCACTTCCAGACACTACAAAAAGAGTGTTTCAAACGTGCTCTA
        520 AGCATCAAAGCGATCCAAATATCCACTTCCAGATTCTACAAAAAGAGTGTTTCAAAACTGCTCTA

            **   ***                 *    * *  * *      *  *           **    
      61287 AGAAAACGAATGTTCAACTCTGTGACTTGAATGCAGATATCACAGAGTAGTTTCTGAGAGGGCTT
        585 TCAAAGGAAATGTTCAACTCTGTGAGTTGAGTACACACATCACAAAGAAGTTTCTGAGAATGCTT

                          *              *                * *   *            
      61352 CTGTCTAGATTTTAGATGATGATATTCCCGTTTCCAACGAAATCATTAGAGCTATCCAAATATCC
        650 CTGTCTAG-TTTT-TATGATGATATTCCCTTTTCCAACGAAATCATCAAAGCGATCCAAATATCC

                *   *                    *         **      *  * *    *       
      61417 ACTTACAGTTTCTACAAAAAGAGTGTTTCCAAACTG-CTGCATCAAAAGAGAGGTTCCACTCTGT
        713 ACTTCCAGATTCTACAAAAAGAGTGTTTCAAAACTGCCTCTATCAAAGGAAATGTTCAACTCTGT

               *                   ***      *     *         *                
      61481 TAGCTGAGTACACACATCACAAACTTGTTTCTCAGAATCCTTCTGTCTCGTTTTTATGGGAAGAT
        778 TAGTTGAGTACACACATCACAAAGAAGTTTCTGAGAATGCTTCTGTCTAGTTTTTATGGGAAGAT

               **     *  *  * **          *       *     *       *  *  *      
      61546 ATTTACTTTTTCACCGTAGGCATCAAAGCGCTCCAAATGTCCACATCCAGATACTCCAGAAAGAG
        843 ATTCCCTTTTCCAACGAAATCATCAAAGCGATCCAAATATCCACTTCCAGATTCTACAAAAAGAG

                     *         *     *   *         *                    *    
      61611 TGTTTCAAACCTGCTCTATGAAAGGGAATCTTCAACTCTATGAGTTGAATGCAGACATCAGAAAG
        908 TGTTTCAAAACTGCTCTATCAAAGGAAATGTTCAACTCTGTGAGTTGAATGCAGACATCACAAAG

              *              *       **       **         **             *  * 
      61676 AAATTTCTGAGAATGCTGCTGTCTACCTTTTAT-TTGA-ATTCCCGCTTCCAACGAAATCCTCCA
        973 AAGTTTCTGAGAATGCTTCTGTCTAGTTTTTATGAAGATATTCCCTTTTCCAACGAAATCATCAA

               *                *       *                                  **
      61739 AGCTATCCAAATATCCACTTGCAGATTCCACAAAAAGAGTGTTTCAAAACTGCTCTCTATCAATG
       1038 AGCGATCCAAATATCCACTTCCAGATTCTACAAAAAGAGTGTTTCAAAACTG--CTCTATCAAAA

             *  *                    *     *     *  *                        
      61804 GCAAAGTTCAACTCTGTTAGTTGAGGACACATATCACCAACAAGTTTCTGAGAATGCTTCTGTCT
       1101 GAAATGTTCAACTCTGTTAGTTGAGTACACACATCACAAAGAAGTTTCTGAGAATGCTTCTGTCT

             *                  *  *    
      61869 ATTTTTTATGGGAAGATATTTCCTTTTT
       1166 AGTTTTTATGGGAAGATATTCCCGTTTT

              *  * ** *    *      *    *          * **                       
      61897 CACCGTAGGCGTCAAGGCGATCGAAATGTCCACTTCCACAAACTACAAAAAGAGTGTTTCAAACC
          1 CAACGAAATCATCAAAGCGATCCAAATATCCACTTCCAGATTCTACAAAAAGAGTGTTTCAAACC

                          **       *    *        * ** * *   **      *      * 
      61962 TGCTCTATGAAAGGCCATGTTCATCTCTATGAGTTGAATGGAAATATCCGAAAGAAATTTCTGGG
         66 TGCTCTA-GAAAGGGAATGTTCAACTCTGTGAGTTGAGTACACACATCACAAAGAAGTTTCTGAG

                  *          *        *          *                      *    
      62027 AATGCTGCTGTCTAG-TGTT-TAT-ACGA-ATTCCCGCTTCCAACGAAATC-CTCAAAGCAATCC
        130 AATGCTTCTGTCTAGTTTTTATATGAAGATATTCCCGTTTCCAACGAAATCGCT-AAAGCGATCC

                        *    *  *                                *     *     
      62087 AAATATCCACTTGCAGAATCCACAAAAAGAGTGTTTCAAAACTGCTCTATCAATAGAAAGGTTCA
        194 AAATATCCACTTCCAGATTCTACAAAAAGAGTGTTTCAAAACTGCTCTATCAAAAGAAATGTTCA

                 *                     *  *                        * *      *
      62152 ACTCTTTTAGTTGAGTACACACATCACGAACAAGTTTCTGAGAATGCTTCTGTCTGGCTTTTATT
        259 ACTCTGTTAGTTGAGTACACACATCACAAAGAAGTTTCTGAGAATGCTTCTGTCTAGTTTTTATA

            *     **  *         * *  **          *       *                * *
      62217 GGAAGACGTTTCCTTTT-CACCAAAGGCATCAAAGCGCTCCAAATGTCCACTTCCAGATTCTTCC
        324 TGAAGATATTCCCTTTTCCAACGAAATCATCAAAGCGATCCAAATATCCACTTCCAGATTCTACA

                            **     * * *  * *               * *    * *  * *  
      62281 AAAAGAGTGTTTCAAACGTGCTCAAAGTAAGGGAATGTTCAACTCTGTGACTTGAATGCAGATAT
        389 AAAAGAGTGTTTCAAAACTGCTCTATGAAAAGAAATGTTCAACTCTGTTAGTTGAGTACACACAT

               *   *       * * *             *     ***  *         *          
      62346 CACCAAGTAGTTTCTAATAGTGCTTCTGTCTAGATTTTAGATGATGATATTCCCGTTTCCAACGA
        454 CACAAAGAAGTTTCTGAGAATGCTTCTGTCTAGTTTTTATGGGAAGATATTCCCTTTTCCAACGA

              * * * *   *                *   *                    *        **
      62411 AATCGTTAGAGCTATCCAAATATCCACTTACAGTTTCTACAAAAAGAGTGTTTCCAAACTGCTGC
        519 AAGCATCAAAGCGATCCAAATATCCACTTCCAGATTCTACAAAAAGAGTGTTTCAAAACTGCTCT

                  *    *            *       *                     *          
      62476 ATCAAAAGAAAGGTTCAACTCTGTTAGTTGAGGACACACATCACAAAGAAGTTTGTGAGAATGCT
        584 ATCAAAGGAAATGTTCAACTCTGTGAGTTGAGTACACACATCACAAAGAAGTTTCTGAGAATGCT

                                *                    *   * *     *   *       
      62541 TCTGTCTAGATTTTGTATGACGATATTCCCTTTTCCAACGATATCGTTAAAGCAATCTAAATATC
        649 TCTGTCTAG-TTTT-TATGATGATATTCCCTTTTCCAACGAAATCATCAAAGCGATCCAAATATC

            * *  *    *  *       *  *        *        * *   **   *    *      
      62606 AATTTGCAGAATCCACAAAAATAGAGTTTCAAAGCTG-CTCTGTAAAAATAAAAGTTCCACTCTG
        712 CACTTCCAGATTCTACAAAAAGAGTGTTTCAAAACTGCCTCTATCAAAGGAAATGTTCAACTCTG

                *                   ***            *         *               
      62670 TTAGCTGAGTACACACATCACAAACTTGTTTCTGAGAATCCTTCTGTCTCGTTTTTATGGGAAGA
        777 TTAGTTGAGTACACACATCACAAAGAAGTTTCTGAGAATGCTTCTGTCTAGTTTTTATGGGAAGA

                **     *  *  * **          *       *     *       *  *  *     
      62735 TATTTACTTTTTCACCGTAGGCATCAAAGCGCTCCAAATGTCCACATCCAGATACTCCAGAAAGA
        842 TATTCCCTTTTCCAACGAAATCATCAAAGCGATCCAAATATCCACTTCCAGATTCTACAAAAAGA

            *         *         *     *   *         *                    *   
      62800 CTGTTTCAAACCTGCTCTATGAAAGGGAATCTTCAACTCTATGAGTTGAATGCAGACATCAGAAA
        907 GTGTTTCAAAACTGCTCTATCAAAGGAAATGTTCAACTCTGTGAGTTGAATGCAGACATCACAAA

               *              *       **       **         **             *  *
      62865 GAAATTTCTGAGAATGCTGCTGTCTACCTTTTAT-TTGA-ATTCCCGCTTCCAACGAAATCCTCC
        972 GAAGTTTCTGAGAATGCTTCTGTCTAGTTTTTATGAAGATATTCCCTTTTCCAACGAAATCATCA

                *              * *  **   *                                *  
      62928 AAGCTATCCAAATATCCACCTGCATTTTCCACAAAAAGAGTGTTTCAAAACTGCTCTATCAATAG
       1037 AAGCGATCCAAATATCCACTTCCAGATTCTACAAAAAGAGTGTTTCAAAACTGCTCTATCAAAAG

                         **  * *  *                *                         
      62993 AAATGTTCAACTCCTTTGGCTGGGTACACACATCACAAACAAGTTTCTGAGAATGCTTCTGTCTA
       1102 AAATGTTCAACTCTGTTAGTTGAGTACACACATCACAAAGAAGTTTCTGAGAATGCTTCTGTCTA

                           **     *    
      63058 GTTTTTATGGGAAGACGTTCCCTTTTT
       1167 GTTTTTATGGGAAGATATTCCCGTTTT

              * *  **          *       *            **                      *
      63085 CACCAAAGGCATCAAAGCGCTCCAAATGTCCACTTCCAGACACTACAAAAAGAGTGTTTCAAACG
          1 CAACGAAATCATCAAAGCGATCCAAATATCCACTTCCAGATTCTACAAAAAGAGTGTTTCAAACC

                         *                  *    * *  * *         *          
      63150 TGCTCTAAGAAAGCGAATGTTCAACTCTGTGACTTGAATGCAGATATCACAAAGTAGTTTCTGAG
         66 TGCTCT-AGAAAGGGAATGTTCAACTCTGTGAGTTGAGTACACACATCACAAAGAAGTTTCTGAG

             *             *     * *  *                        **  *   *     
      63215 AGTGCTTCTGTCTAGATTTTAGACGATGATATTCCCGTTTCCAACGAAATCATTAGAGCTATCCA
        130 AATGCTTCTGTCTAGTTTTTATATGAAGATATTCCCGTTTCCAACGAAATCGCTAAAGCGATCCA

                       *   *                    *        **         * *    * 
      63280 AATATCCACTTACAGTTTCTACAAAAAGAGTGTTTCCAAACTGCTGCATCAAAAGAGAGGTTCCA
        195 AATATCCACTTCCAGATTCTACAAAAAGAGTGTTTCAAAACTGCTCTATCAAAAGAAATGTTCAA

                     *                   ***      *     *         *        **
      63345 CTCTGTTAGCTGAGTACACACATCACAAACTTGTTTCTCAGAATCCTTCTGTCTCGTTTTTATGG
        260 CTCTGTTAGTTGAGTACACACATCACAAAGAAGTTTCTGAGAATGCTTCTGTCTAGTTTTTATAT

                     **     *  *  * **          *       *     *       **    *
      63410 GAAGATATTTACTTTTTCACCGTAGGCATCAAAGCGCTCCAAATGTCCACATCCAGATAGTACAG
        325 GAAGATATTCCCTTTTCCAACGAAATCATCAAAGCGATCCAAATATCCACTTCCAGATTCTACAA

                   *       *             * *   *         * *      * *  *     
      63475 AAAGAGTTTTTCAAACCTGCTCTATGAAAGGGAATCTTCAACTCTATGAGTTGAATGCAGACATC
        390 AAAGAGTGTTTCAAAACTGCTCTATGAAAAGAAATGTTCAACTCTGTTAGTTGAGTACACACATC

             *      *              *       **         **         **          
      63540 AGAAAGAAATTTCTGAGAATGCTGCTGTCTACCTTTTAT---TTGA-ATTCCCGCTTCCAACGAA
        455 ACAAAGAAGTTTCTGAGAATGCTTCTGTCTAGTTTTTATGGGAAGATATTCCCTTTTCCAACGAA

             * *  *    *                *       *                            
      63601 ATCCTCCAAGCTATCCAAATATCCACTTGCAGATTCCACAAAAAGAGTGTTTCAAAACTGCTCTC
        520 AGCATCAAAGCGATCCAAATATCCACTTCCAGATTCTACAAAAAGAGTGTTTCAAAACTG--CTC

                  *  *  *            *       *     *     *  *                
      63666 TATCAATGGCAAAGTTCAACTCTGTTAGTTGAGGACACATATCACCAACAAGTTTCTGAGAATGC
        583 TATCAAAGGAAATGTTCAACTCTGTGAGTTGAGTACACACATCACAAAGAAGTTTCTGAGAATGC

                     *           *      *      *  *  * ** *    *      *    * 
      63731 TTCTGTCTATTTTTTATGGGAAGATATTTCCTTTTTCACCGTAGGCGTCAAGGCGATCGAAATGT
        648 TTCTGTCTAGTTTTTAT--GATGATATTCCCTTTTCCAACGAAATCATCAAAGCGATCCAAATAT

                     * **                     *          *     **       *    
      63796 CCACTTCCACAAACTACAAAAAGAGTGTTTCAAACCTG-CTCTATGAAAGGCCATGTTCATCTCT
        711 CCACTTCCAGATTCTACAAAAAGAGTGTTTCAAAACTGCCTCTATCAAAGGAAATGTTCAACTCT

            * *      * ** * *   **      *      *       *         *         * 
      63860 ATGAGTTGAATGGAAATATCCGAAAGAAATTTCTGGGAATGCTGCTGTCTAGTGTTTAT---ACG
        776 GTTAGTTGAGTACACACATCACAAAGAAGTTTCTGAGAATGCTTCTGTCTAGTTTTTATGGGAAG

                    **             *       *                *    *  *        
      63922 A-ATTCCCGCTTCCAACGAAATCCTCAAAGCAATCCAAATATCCACTTGCAGAATCCACAAAAAG
        841 ATATTCCCTTTTCCAACGAAATCATCAAAGCGATCCAAATATCCACTTCCAGATTCTACAAAAAG

                                    **    *          * *      * *  *       * 
      63986 AGTGTTTCAAAACTGCTCTATCAATAGAAAGGTTCAACTCTTTTAGTTGAGTACACACATCACGA
        906 AGTGTTTCAAAACTGCTCTATCAAAGGAAATGTTCAACTCTGTGAGTTGAATGCAGACATCACAA

             *                        * *             **  *           *  **  
      64051 ACAAGTTTCTGAGAATGCTTCTGTCTGGCTTTTATTGGAAGACGTTTCCTTTT-CAACAAAGGCA
        971 AGAAGTTTCTGAGAATGCTTCTGTCTAGTTTTTA-T-GAAGATATTCCCTTTTCCAACGAAATCA

                    *       *                * *                **     * *** 
      64115 TCAAAGCGCTCCAAATGTCCACTTCCAGATTCTTCCAAAAGAGTGTTTCAAACGTGCTCGAAGTA
       1034 TCAAAGCGATCCAAATATCCACTTCCAGATTCTACAAAAAGAGTGTTTCAAAACTGCTCTATCAA

             * *               * *    * *  * *     *   *       * * *         
      64180 AGGGAATGTTCAACTCTGTGACTTGAATGCAGATATCACCAAGTAGTTTCTAATAGTGCTTCTGT
       1099 AAGAAATGTTCAACTCTGTTAGTTGAGTACACACATCACAAAGAAGTTTCTGAGAATGCTTCTGT

               **     ***  *             *
      64245 CTACATTTTAGATGATGATATTCCCGTTTC
       1164 CTAGTTTTTATGGGAAGATATTCCCGTTTT

                      * * *   *             *  *   *      *     *       *  **
      64275 CAACGAAATCGTTAGAGCTATCCAAATATCCAGTTACAGTTTCTACCAAAAGGGTGTTTCCAAAT
          1 CAACGAAATCATCAAAGCGATCCAAATATCCACTTCCAGATTCTACAAAAAGAGTGTTTCAAACC

                  ***   * *  *            *       *                     *    
      64340 TGCTGCATCAAAAGAAAGGTTCAACTCTGTTAGTTGAGGACACACATCACAAAGAAGTTTGTGAG
         66 TGCT-CTAGAAAGGGAATGTTCAACTCTGTGAGTTGAGTACACACATCACAAAGAAGTTTCTGAG

                           *    *     *         *          *    *      *   * 
      64405 AATGCTTCTGTCTAGATTTTGTATGACGATATTCCCTTTTCCAACGATATCGTTAAAGCAATCTA
        130 AATGCTTCTGTCTAGTTTTTATATGAAGATATTCCCGTTTCCAACGAAATCGCTAAAGCGATCCA

                  * *  *    *  *       *  *        *       * *        *    * 
      64470 AATATCAATTTGCAGAATCCACAAAAATAGAGTTTCAAAGCTGCTCTGTAAAAAGAAAGGTTCCA
        195 AATATCCACTTCCAGATTCTACAAAAAGAGTGTTTCAAAACTGCTCTATCAAAAGAAATGTTCAA

                     *                   ***            *         *        **
      64535 CTCTGTTAGCTGAGTACACACATCACAAACTTGTTTCTGAGAATCCTTCTGTCTCGTTTTTATGG
        260 CTCTGTTAGTTGAGTACACACATCACAAAGAAGTTTCTGAGAATGCTTCTGTCTAGTTTTTATAT

                     **        *  * **          *       *     *       *  *  *
      64600 GAAGATATTTACTTTTCCACCGTAGGCATCAAAGCGCTCCAAATGTCCACATCCAGATACTCCAG
        325 GAAGATATTCCCTTTTCCAACGAAATCATCAAAGCGATCCAAATATCCACTTCCAGATTCTACAA

              *            *             * *   *         * *      * *  *     
      64665 AACGAGTGTTTCAAACCTGCTCTATGAAAGGGAATCTTCAACTCTATGAGTTGAATGCAGACATC
        390 AAAGAGTGTTTCAAAACTGCTCTATGAAAAGAAATGTTCAACTCTGTTAGTTGAGTACACACATC

             *      *              *       **         **         **          
      64730 AGAAAGAAATTTCTGAGAATGCTGCTGTCTACCTTTTAT---TTGA-ATTCCCGCTTCCAACGAA
        455 ACAAAGAAGTTTCTGAGAATGCTTCTGTCTAGTTTTTATGGGAAGATATTCCCTTTTCCAACGAA

             * *  *    *              * *  **   *    *                       
      64791 ATCCTCCAAGCTATCCAAATATCCACCTGCATTTTCCACAACAAGAGTGTTTCAAAACTGCTCTA
        520 AGCATCAAAGCGATCCAAATATCCACTTCCAGATTCTACAAAAAGAGTGTTTCAAAACTGCTCTA

                **                *    *  *                *           *     
      64856 TCAATAGAAATGTTCAACTCCTTTG-GCTGGGTACACACATCACAAACAAGTTTCTGAGCATGCT
        585 TCAAAGGAAATGTTCAACT-CTGTGAGTTGAGTACACACATCACAAAGAAGTTTCTGAGAATGCT

                                *  *          *  * *  **          *       *  
      64920 TCTGTCTAGTTTTTATGGGAAGACATTCCCTTTTTCACCAAAGGCATCAAAGCGCTCCAAATGTC
        649 TCTGTCTAGTTTTTAT--GATGATATTCCCTTTTCCAACGAAATCATCAAAGCGATCCAAATATC

                      **                     **        **   ***              
      64985 CACTTCCAGACACTACAAAAAGAGTGTTTCAAACGTG-CTCTAAGAAAACGAATGTTCAACTCTG
        712 CACTTCCAGATTCTACAAAAAGAGTGTTTCAAAACTGCCTCTATCAAAGGAAATGTTCAACTCTG

             * *    * *  * *      *  *           **            *     ***  *  
      65049 TGACTTGAATGCAGATATCACAGAGTAGTTTCTGAGAGGGCTTCTGTCTAGATTTTAGATGATGA
        777 TTAGTTGAGTACACACATCACAAAGAAGTTTCTGAGAATGCTTCTGTCTAGTTTTTATGGGAAGA

                   *                * *   *                *   *             
      65114 TATTCCCGTTTCCAACGAAATCATTAGAGCTATCCAAATATCCACTTACAGTTTCTACAAAAAGA
        842 TATTCCCTTTTCCAACGAAATCATCAAAGCGATCCAAATATCCACTTCCAGATTCTACAAAAAGA

                   *        **      *  * *    *       *  *   * *  *          
      65179 GTGTTTCCAAACTGCTGCATCAAAAGAGAGGTTCCACTCTGTTAGCTGAGTACACACATCACAAA
        907 GTGTTTCAAAACTGCTCTATCAAAGGAAATGTTCAACTCTGTGAGTTGAATGCAGACATCACAAA

            ***      *     *         *                   **     *  *  * **   
      65244 CTTGTTTCTCAGAATCCTTCTGTCTCGTTTTTATGGGAAGATATTTACTTTTTCACCGTAGGCAT
        972 GAAGTTTCTGAGAATGCTTCTGTCTAGTTTTTAT--GAAGATATTCCCTTTTCCAACGAAATCAT

                   *       *     *       *  *  *               *         *   
      65309 CAAAGCGCTCCAAATGTCCACATCCAGATACTCCAGAAAGAGTGTTTCAAACCTGCTCTATGAAA
       1035 CAAAGCGATCCAAATATCCACTTCCAGATTCTACAAAAAGAGTGTTTCAAAACTGCTCTATCAAA

            * *   *         * *      * *  *      *      *              *     
      65374 GGGAATCTTCAACTCTATGAGTTGAATGCAGACATCAGAAAGAAATTTCTGAGAATGCTGCTGTC
       1100 AGAAATGTTCAACTCTGTTAGTTGAGTACACACATCACAAAGAAGTTTCTGAGAATGCTTCTGTC

              **         **          *  *
      65439 TACCTTTTAT---TTGA-ATTCCCGCTTC
       1165 TAGTTTTTATGGGAAGATATTCCCGTTTT

                      *  *    *                *       *                   * 
      65464 CAACGAAATCCTCCAAGCTATCCAAATATCCACTTGCAGATTCCACAAAAAGAGTGTTTCAAAAC
          1 CAACGAAATCATCAAAGCGATCCAAATATCCACTTCCAGATTCTACAAAAAGAGTGTTTCAAACC

                      *  *  *  *            *       *     *     *  *         
      65529 TGCTCTCTATCAATGGCAAAGTTCAACTCTGTTAGTTGAGGACACATATCACCAACAAGTTTCTG
         66 TG--CTCTA-GAAAGGGAATGTTCAACTCTGTGAGTTGAGTACACACATCACAAAGAAGTTTCTG

                            *       **         *  *   *  *  * **  **  *      
      65594 AGAATGCTTCTGTCTATTTTTTATGGGAAGATATTTCCTTTTTCACCGTAGGCGTCAAGGCGATC
        128 AGAATGCTTCTGTCTAGTTTTTATATGAAGATATTCCCGTTTCCAACGAAATCGCTAAAGCGATC

            *    *          * **                     *         *   * **      
      65659 GAAATGTCCACTTCCACAAACTACAAAAAGAGTGTTTCAAACCTGCTCTATGAAAGGCCATGTTC
        193 CAAATATCCACTTCCAGATTCTACAAAAAGAGTGTTTCAAAACTGCTCTATCAAAAGAAATGTTC

             *    * *      * ** * *   **      *      *       *          *    
      65724 ATCTCTATGAGTTGAATGGAAATATCCGAAAGAAATTTCTGGGAATGCTGCTGTCTAG-TGTT-T
        258 AACTCTGTTAGTTGAGTACACACATCACAAAGAAGTTTCTGAGAATGCTTCTGTCTAGTTTTTAT

                *         **             *       *                *    *  *  
      65787 AT-ACGA-ATTCCCGCTTCCAACGAAATCCTCAAAGCAATCCAAATATCCACTTGCAGAATCCAC
        323 ATGAAGATATTCCCTTTTCCAACGAAATCATCAAAGCGATCCAAATATCCACTTCCAGATTCTAC

                                       *  *     *          *                 
      65850 AAAAAGAGTGTTTCAAAACTGCTCTATCAATAGAAAGGTTCAACTCTTTTAGTTGAGTACACACA
        388 AAAAAGAGTGTTTCAAAACTGCTCTATGAAAAGAAATGTTCAACTCTGTTAGTTGAGTACACACA

                *  *                        * *      *      **  *         * *
      65915 TCACGAACAAGTTTCTGAGAATGCTTCTGTCTGGCTTTTATTGGAAGACGTTTCCTTTT-CACCA
        453 TCACAAAGAAGTTTCTGAGAATGCTTCTGTCTAGTTTTTATGGGAAGATATTCCCTTTTCCAACG

              *           *       *                * *                **     
      65979 AAGGCATCAAAGCGCTCCAAATGTCCACTTCCAGATTCTTCCAAAAGAGTGTTTCAAACGTGCTC
        518 AAAGCATCAAAGCGATCCAAATATCCACTTCCAGATTCTACAAAAAGAGTGTTTCAAAACTGCTC

            * ***    *                 *    * *  * *     *   *       * * *   
      66044 AAAGTAAGGGAATGTTCAACTCTGTGACTTGAATGCAGATATCACCAAGTAGTTTCTAATAGTGC
        583 TATCAAAGGAAATGTTCAACTCTGTGAGTTGAGTACACACATCACAAAGAAGTTTCTGAGAATGC

                           **              *              * * *   *          
      66109 TTCTGTCTAGATTTCAGATGATGATATTCCCGTTTCCAACGAAATCGTTAGAGCTATCCAAATAT
        648 TTCTGTCTAG-TTT-TTATGATGATATTCCCTTTTCCAACGAAATCATCAAAGCGATCCAAATAT

               *  *   *                    *         **      *    *          
      66174 CCAGTTACAGTTTCTACAAAAAGAGTGTTTCCAAACTG-CTGCATCAAAAGAAAGGTTCAACTCT
        711 CCACTTCCAGATTCTACAAAAAGAGTGTTTCAAAACTGCCTCTATCAAAGGAAATGTTCAACTCT

                      *                     *                   *    * **  * 
      66238 GTTAGTTGAGGACACACATCACAAAGAAGTTTGTGAGAATGCTTCTGTCTAGATTTTGTATGACG
        776 GTTAGTTGAGTACACACATCACAAAGAAGTTTCTGAGAATGCTTCTGTCTAGTTTTTATGGGAAG

                               *   * *     *   *       * *  *    *  *       *
      66303 ATATTCCCTTTTCCAACGATATCGTTAAAGCAATCTAAATATCAATTTGCAGAATCCACAAAAAT
        841 ATATTCCCTTTTCCAACGAAATCATCAAAGCGATCCAAATATCCACTTCCAGATTCTACAAAAAG

              *        *       * *   *    *    *       *  *   * *  *         
      66368 AGAGTTTCAAAGCTGCTCTGTAAAAAGAAAGGTTCCACTCTGTTAGCTGAGTACACACATCACAA
        906 AGTGTTTCAAAACTGCTCTATCAAAGGAAATGTTCAACTCTGTGAGTTGAATGCAGACATCACAA

             ***            *         *                   **     *  *  * **  
      66433 ACTTGTTTCTGAGAATCCTTCTGTCTCGTTTTTATGGGAAGATATTTACTTTTTCACCGTAGGCA
        971 AGAAGTTTCTGAGAATGCTTCTGTCTAGTTTTTAT--GAAGATATTCCCTTTTCCAACGAAATCA

                    *       *     *       *  *  *     *         *         *  
      66498 TCAAAGCGCTCCAAATGTCCACATCCAGATACTCCAGAAAGACTGTTTCAAACCTGCTCTATGAA
       1034 TCAAAGCGATCCAAATATCCACTTCCAGATTCTACAAAAAGAGTGTTTCAAAACTGCTCTATCAA

             * *   *         * *      * *  *      *      *              *    
      66563 AGGGAATCTTCAACTCTATGAGTTGAATGCAGACATCAGAAAGAAATTTCTGAGAATGCTGCTGT
       1099 AAGAAATGTTCAACTCTGTTAGTTGAGTACACACATCACAAAGAAGTTTCTGAGAATGCTTCTGT

               **         **          *  *
      66628 CTACCTTTTAT---TTGA-ATTCCCGCTTC
       1164 CTAGTTTTTATGGGAAGATATTCCCGTTTT

                      *  *    *                *  **   *                   * 
      66654 CAACGAAATCCTCCAAGCTATCCAAATATCCACTTGCATTTTCCACAAAAAGAGTGTTTCAAAAC
          1 CAACGAAATCATCAAAGCGATCCAAATATCCACTTCCAGATTCTACAAAAAGAGTGTTTCAAACC

                    *  ** *            ** *  *  *                *           
      66719 TGCTCTATCAATAGAAATGTTCAACTCCTTTAGCTGGGTACACACATCACAAACAAGTTTCTGAG
         66 TGCTCTA-GAAAGGGAATGTTCAACTCTGTGAGTTGAGTACACACATCACAAAGAAGTTTCTGAG

                                  **     **     *   *    *    *           *  
      66784 AATGCTTCTGTCTAGTTTTTATGGGAAGACGTTCCCTTTTTC-ACCAAA-GGCATCAAAGCGCTC
        130 AATGCTTCTGTCTAGTTTTTATATGAAGATATTCCCGTTTCCAACGAAATCGC-T-AAAGCGATC

                 *            **                     **       **   ***       
      66847 CAAATGTCCACTTCCAGACACTACAAAAAGAGTGTTTCAAACGTGCTCTAAGAAAGCGAATGTTC
        193 CAAATATCCACTTCCAGATTCTACAAAAAGAGTGTTTCAAAACTGCTCTATCAAAAGAAATGTTC

                    * *    * *  * *         *           **            *     *
      66912 AACTCTGTGACTTGAATGCAGATATCACAAAGTAGTTTCTGAGAGGGCTTCTGTCTAGATTTTAG
        258 AACTCTGTTAGTTGAGTACACACATCACAAAGAAGTTTCTGAGAATGCTTCTGTCTAGTTTTTAT

                *         *                * *   *                *   *      
      66977 ATGATGATATTCCCGTTTCCAACGAAATCATTAGAGCTATCCAAATATCCACTTACAGTTTCTAC
        323 ATGAAGATATTCCCTTTTCCAACGAAATCATCAAAGCGATCCAAATATCCACTTCCAGATTCTAC

                          *        **  *      * *    *          **           
      67042 AAAAAGAGTGTTTCCAAACTGCTGCATCAAAAGAGAGGTTCCACTCTGTTAGCCGAGTACACACA
        388 AAAAAGAGTGTTTCAAAACTGCTCTATGAAAAGAAATGTTCAACTCTGTTAGTTGAGTACACACA

                   ***      *     *         *                   **     *  *  
      67107 TCACAAACTTGTTTCTCAGAATCCTTCTGTCTCGTTTTTATGGGAAGATATTTACTTTTTCACCG
        453 TCACAAAGAAGTTTCTGAGAATGCTTCTGTCTAGTTTTTATGGGAAGATATTCCCTTTTCCAACG

            * *           *       * *   *       *  *  *               *      
      67172 TAGGCATCAAAGCGCTCCAAATGTTCACATCCAGATACTCCAGAAAGAGTGTTTCAAACCTGCTC
        518 AAAGCATCAAAGCGATCCAAATATCCACTTCCAGATTCTACAAAAAGAGTGTTTCAAAACTGCTC

               *     *   *         *        * *  *      *      *             
      67237 TATGAAAGGGAATCTTCAACTCTATGAGTTGAATGCAGACATCAGAAAGAAATTTCTGAGAATGC
        583 TATCAAAGGAAATGTTCAACTCTGTGAGTTGAGTACACACATCACAAAGAAGTTTCTGAGAATGC

             *       **       *          **             *  *    *            
      67302 TGCTGTCTACCTTTTAT-TTGA-ATTCCCGCTTCCAACGAAATCCTCCAAGCTATCCAAATATCC
        648 TTCTGTCTAGTTTTTATGATGATATTCCCTTTTCCAACGAAATCATCAAAGCGATCCAAATATCC

                *       *                                  *  *  *           
      67365 ACTTGCAGATTCCACAAAAAGAGTGTTTCAAAACTGCTCTCTATCAATGGCAAAGTTCAACTCTG
        713 ACTTCCAGATTCTACAAAAAGAGTGTTTCAAAACTGC-CTCTATCAAAGGAAATGTTCAACTCTG

                     *     *     *  *                         *              
      67430 TTAGTTGAGGACACATATCACCAACAAGTTTCTGAGAATGCTTCTGTCTATTTTTTATGGGAAGA
        777 TTAGTTGAGTACACACATCACAAAGAAGTTTCTGAGAATGCTTCTGTCTAGTTTTTATGGGAAGA

                *      *  *  * ** *    *   *  *    *          * **           
      67495 TATTTCCTTTTTCACCGTAGGCGTCAAGGCGTTCGAAATGTCCACTTCCACAAACTACAAAAAGA
        842 TATTCCCTTTTCCAACGAAATCATCAAAGCGATCCAAATATCCACTTCCAGATTCTACAAAAAGA

                      *         *     **       *    *           * * *   **   
      67560 GTGTTTCAAACCTGCTCTATGAAAGGCCATGTTCATCTCTATGAGTTGAATGGAAATATCCGAAA
        907 GTGTTTCAAAACTGCTCTATCAAAGGAAATGTTCAACTCTGTGAGTTGAATGCAGACATCACAAA

               *      *       *         *       *         **             *   
      67625 GAAATTTCTGGGAATGCTGCTGTCTAGTGTTTAT-ACGA-ATTCCCGCTTCCAACGAAATCCTCA
        972 GAAGTTTCTGAGAATGCTTCTGTCTAGTTTTTATGAAGATATTCCCTTTTCCAACGAAATCATCA

                *                *    *  *                                *  
      67688 AAGCAATCCAAATATCCACTTGCAGAATCCACAAAAAGAGTGTTTCAAAACTGCTCTATCAATAG
       1037 AAGCGATCCAAATATCCACTTCCAGATTCTACAAAAAGAGTGTTTCAAAACTGCTCTATCAAAAG

               *          *                     *  *                        *
      67753 AAAGGTTCAACTCTTTTAGTTGAGTACACACATCACGAACAAGTTTCTGAGAATGCTTCTGTCTG
       1102 AAATGTTCAACTCTGTTAGTTGAGTACACACATCACAAAGAAGTTTCTGAGAATGCTTCTGTCTA

             *      *      **  *       
      67818 GCTTTTATTGGAAGACGTTTCC-TTTT
       1167 GTTTTTATGGGAAGATATTCCCGTTTT

              * *  **          *       *                * *                 *
      67844 CACCAAAGGCATCAAAGCGCTCCAAATGTCCACTTCCAGATTCTTCCAAAAGAGTGTTTCAAACG
          1 CAACGAAATCATCAAAGCGATCCAAATATCCACTTCCAGATTCTACAAAAAGAGTGTTTCAAACC

                  *  *                      *    * *  * *     *   *       * *
      67909 TGCTCAAAGTAAGGGAATGTTCAACTCTGTGACTTGAATGCAGATATCACCAAGTAGTTTCTAAT
         66 TGCTC-TAGAAAGGGAATGTTCAACTCTGTGAGTTGAGTACACACATCACAAAGAAGTTTCTGAG

             *             *   * *    *                         *  *   * **  
      67974 AGTGCTTCTGTCTAGATTTCAGATGATGATATTCCCGTTTCCAACGAAATCGTTAGAGCTAAGCA
        130 AATGCTTCTGTCTAGTTTTTATATGAAGATATTCCCGTTTCCAACGAAATCGCTAAAGCGATCCA

                    *  *   *      *     *       *   *    **           *      
      68039 AATATCCAGTTACAGTTTCTACCAAAAGGGTGTTTCCAAATTGCTGCATCAAAAGAAAGGTTCAA
        195 AATATCCACTTCCAGATTCTACAAAAAGAGTGTTTCAAAACTGCTCTATCAAAAGAAATGTTCAA

                          *                     *                   *    *   
      68104 CTCTGTTAGTTGAGGACACACATCACAAAGAAGTTTGTGAGAATGCTTCTGTCTAGATTTTGTAT
        260 CTCTGTTAGTTGAGTACACACATCACAAAGAAGTTTCTGAGAATGCTTCTGTCTAGTTTTTATAT

              *                    *   * *     *   *       * *  *    *  *    
      68169 GACGATATTCCCTTTTCCAACGATATCGTTAAAGCAATCTAAATATCAATTTGCAGAATCCACAA
        325 GAAGATATTCCCTTTTCCAACGAAATCATCAAAGCGATCCAAATATCCACTTCCAGATTCTACAA

               *  *        *       * *        *    *          *              
      68234 AAATAGAGTTTCAAAGCTGCTCTGTAAAAAGAAAGGTTCCACTCTGTTAGCTGAGTACACACATC
        390 AAAGAGTGTTTCAAAACTGCTCTATGAAAAGAAATGTTCAACTCTGTTAGTTGAGTACACACATC

                 ***            *         *                   **        *  * 
      68299 ACAAACTTGTTTCTGAGAATCCTTCTGTCTCGTTTTTATGGGAAGATATTTACTTTTCCACCGTA
        455 ACAAAGAAGTTTCTGAGAATGCTTCTGTCTAGTTTTTATGGGAAGATATTCCCTTTTCCAACGAA

            *           *       *     *       *  *  *  *            *        
      68364 GGCATCAAAGCGCTCCAAATGTCCACATCCAGATACTCCAGAACGAGTGTTTCAAACCTGCTCTA
        520 AGCATCAAAGCGATCCAAATATCCACTTCCAGATTCTACAAAAAGAGTGTTTCAAAACTGCTCTA

             *     *   *         *        * *  *      *      *              *
      68429 TGAAAGGGAATCTTCAACTCTATGAGTTGAATGCAGACATCAGAAAGAAATTTCTGAGAATGCTG
        585 TCAAAGGAAATGTTCAACTCTGTGAGTTGAGTACACACATCACAAAGAAGTTTCTGAGAATGCTT

                   **       *          **             *  *    *              
      68494 CTGTCTACCTTTTAT-TTGA-ATTCCCGCTTCCAACGAAATCCTCCAAGCTATCCAAATATCCAC
        650 CTGTCTAGTTTTTATGATGATATTCCCTTTTCCAACGAAATCATCAAAGCGATCCAAATATCCAC

            * *  **   *    *                            **              **  *
      68557 CTGCATTTTCCACAACAAGAGTGTTTCAAAACTG-CTCTATCAATAGAAATGTTCAACTCCTTTG
        715 TTCCAGATTCTACAAAAAGAGTGTTTCAAAACTGCCTCTATCAAAGGAAATGTTCAACTCTGTTA

             *  *                *           *                            *  
      68621 GCTGGGTACACACATCACAAACAAGTTTCTGAGCATGCTTCTGTCTAGTTTTTATGGGAAGACAT
        780 GTTGAGTACACACATCACAAAGAAGTTTCTGAGAATGCTTCTGTCTAGTTTTTATGGGAAGATAT

                    *  * *  **          *       *            **              
      68686 TCCCTTTTTCACCAAAGGCATCAAAGCGCTCCAAATGTCCACTTCCAGACACTACAAAAAGAGTG
        845 TCCCTTTTCCAACGAAATCATCAAAGCGATCCAAATATCCACTTCCAGATTCTACAAAAAGAGTG

                   **       **   ***                 *           *      *  * 
      68751 TTTCAAACGTGCTCTAAGAAAACGAATGTTCAACTCTGTGACTTGAATGCAGATATCACAGAGTA
        910 TTTCAAAACTGCTCTATCAAAGGAAATGTTCAACTCTGTGAGTTGAATGCAGACATCACAAAGAA

                      **                  *    *         *                * *
      68816 GTTTCTGAGAGGGCTTCTGTCTAGATTTTAGATGATGATATTCCCGTTTCCAACGAAATCATTAG
        975 GTTTCTGAGAATGCTTCTGTCTAG-TTTT-TATGAAGATATTCCCTTTTCCAACGAAATCATCAA

               *                *   *                    *        **         
      68881 AGCTATCCAAATATCCACTTACAGTTTCTACAAAAAGAGTGTTTCCAAACTGCTGCATCAAAAGA
       1038 AGCGATCCAAATATCCACTTCCAGATTCTACAAAAAGAGTGTTTCAAAACTGCTCTATCAAAAGA

            * *    *          *                   ***      *     *         * 
      68946 GAGGTTCCACTCTGTTAGCTGAGTACACACATCACAAACTTGTTTCTCAGAATCCTTCTGTCTCG
       1103 AATGTTCAACTCTGTTAGTTGAGTACACACATCACAAAGAAGTTTCTGAGAATGCTTCTGTCTAG

                              ** *    
      69011 TTTTTATGGGAAGATATTTACTTTTT
       1168 TTTTTATGGGAAGATATTCCCGTTTT

              *  * **          *       *     *       *  *  *                 
      69037 CACCGTAGGCATCAAAGCGCTCCAAATGTCCACATCCAGATACTCCAGAAAGAGTGTTTCAAACC
          1 CAACGAAATCATCAAAGCGATCCAAATATCCACTTCCAGATTCTACAAAAAGAGTGTTTCAAACC

                              *         *        * *  *      *      *        
      69102 TGCTCTATGAAAGGGAATCTTCAACTCTATGAGTTGAATGCAGACATCAGAAAGAAATTTCTGAG
         66 TGCTCTA-GAAAGGGAATGTTCAACTCTGTGAGTTGAGTACACACATCACAAAGAAGTTTCTGAG

                  *       **          *          *                 *    *    
      69167 AATGCTGCTGTCTACCTTTTAT-T--TGA-ATTCCCGCTTCCAACGAAATC-CTCCAAGCTATCC
        130 AATGCTTCTGTCTAGTTTTTATATGAAGATATTCCCGTTTCCAACGAAATCGCT-AAAGCGATCC

                        *       *                                  ** *  *   
      69227 AAATATCCACTTGCAGATTCCACAAAAAGAGTGTTTCAAAACTGCTCTCTATCAATGGCAAAGTT
        194 AAATATCCACTTCCAGATTCTACAAAAAGAGTGTTTCAAAACTG--CTCTATCAAAAGAAATGTT

                             *     *     *  *                         *      
      69292 CAACTCTGTTAGTTGAGGACACATATCACCAACAAGTTTCTGAGAATGCTTCTGTCTATTTTTTA
        257 CAACTCTGTTAGTTGAGTACACACATCACAAAGAAGTTTCTGAGAATGCTTCTGTCTAGTTTTTA

             **         *      *  *  * ** *    *      *    *          * **   
      69357 TGGGAAGATATTTCCTTTTTCACCGTAGGCGTCAAGGCGATCGAAATGTCCACTTCCACAAACTA
        322 TATGAAGATATTCCCTTTTCCAACGAAATCATCAAAGCGATCCAAATATCCACTTCCAGATTCTA

                              *             * **       *    * *      * ** * *
      69422 CAAAAAGAGTGTTTCAAACCTGCTCTATGAAAGGCCATGTTCATCTCTATGAGTTGAATGGAAAT
        387 CAAAAAGAGTGTTTCAAAACTGCTCTATGAAAAGAAATGTTCAACTCTGTTAGTTGAGTACACAC

               **      *      *       *         *         *         **       
      69487 ATCCGAAAGAAATTTCTGGGAATGCTGCTGTCTAGTGTTTAT---ACGA-ATTCCCGCTTCCAAC
        452 ATCACAAAGAAGTTTCTGAGAATGCTTCTGTCTAGTTTTTATGGGAAGATATTCCCTTTTCCAAC

                * *       *                *    *  *                         
      69548 GAAATCCTCAAAGCAATCCAAATATCCACTTGCAGAATCCACAAAAAGAGTGTTTCAAAACTGCT
        517 GAAAGCATCAAAGCGATCCAAATATCCACTTCCAGATTCTACAAAAAGAGTGTTTCAAAACTGCT

                   **    *          * *                   *  *               
      69613 CTATCAATAGAAAGGTTCAACTCTTTTAGTTGAGTACACACATCACGAACAAGTTTCTGAGAATG
        582 CTATCAAAGGAAATGTTCAACTCTGTGAGTTGAGTACACACATCACAAAGAAGTTTCTGAGAATG

                     * *          *  **  *         * *  **          *       *
      69678 CTTCTGTCTGGCTTTTATTGGAAGACGTTTCCTTTT-CACCAAAGGCATCAAAGCGCTCCAAATG
        647 CTTCTGTCTAGTTTTTA-T-GATGATATTCCCTTTTCCAACGAAATCATCAAAGCGATCCAAATA

                            * *                **      * ***    *            
      69742 TCCACTTCCAGATTCTTCCAAAAGAGTGTTTCAAACGTG-CTCAAAGTAAGGGAATGTTCAACTC
        710 TCCACTTCCAGATTCTACAAAAAGAGTGTTTCAAAACTGCCTCTATCAAAGGAAATGTTCAACTC

               * *    * *  * *     *   *       * * *             *   * ***  *
      69806 TGTGACTTGAATGCAGATATCACCAAGTAGTTTCTAATAGTGCTTCTGTCTAGATTTCAGATGAT
        775 TGTTAGTTGAGTACACACATCACAAAGAAGTTTCTGAGAATGCTTCTGTCTAGTTTTTATGGGAA

                     *              * * *   *             *  *   *      *    
      69871 GATATTCCCGTTTCCAACGAAATCGTTAGAGCTATCCAAATATCCAGTTACAGTTTCTACCAAAA
        840 GATATTCCCTTTTCCAACGAAATCATCAAAGCGATCCAAATATCCACTTCCAGATTCTACAAAAA

             *       *   *    **      *    *            *      ***  *        
      69936 GGGTGTTTCCAAATTGCTGCATCAAAAGAAAGGTTCAACTCTGTTAGTTGAGGACACACATCACA
        905 GAGTGTTTCAAAACTGCTCTATCAAAGGAAATGTTCAACTCTGTGAGTTGAATGCAGACATCACA

                     *                              *                    *   
      70001 AAGAAGTTTGTGAGAATGCTTCTGTCTAGATTTTGTATGACGATATTCCCTTTTCCAACGATATC
        970 AAGAAGTTTCTGAGAATGCTTCTGTCTAG-TTTT-TATGAAGATATTCCCTTTTCCAACGAAATC

            * *     *   *       * *  *    *  *       *  *        *       * * 
      70066 GTTAAAGCAATCTAAATATCAATTTGCAGAATCCACAAAAATAGAGTTTCAAAGCTGCTCTGTAA
       1033 ATCAAAGCGATCCAAATATCCACTTCCAGATTCTACAAAAAGAGTGTTTCAAAACTGCTCTATCA

                   *    *          *                   ***            *      
      70131 AAAGAAAGGTTCCACTCTGTTAGCTGAGTACACACATCACAAACTTGTTTCTGAGAATCCTTCTG
       1098 AAAGAAATGTTCAACTCTGTTAGTTGAGTACACACATCACAAAGAAGTTTCTGAGAATGCTTCTG

               *                   ** *   *
      70196 TCTCGTTTTTATGGGAAGATATTTACTTTTC
       1163 TCTAGTTTTTATGGGAAGATATTCCCGTTTT

              *  * **          *       *     *       *  *  *     *           
      70227 CACCGTAGGCATCAAAGCGCTCCAAATGTCCACATCCAGATACTCCAGAAAGACTGTTTCAAACC
          1 CAACGAAATCATCAAAGCGATCCAAATATCCACTTCCAGATTCTACAAAAAGAGTGTTTCAAACC

                              *         *        * *  *      *      *        
      70292 TGCTCTATGAAAGGGAATCTTCAACTCTATGAGTTGAATGCAGACATCAGAAAGAAATTTCTGAG
         66 TGCTCTA-GAAAGGGAATGTTCAACTCTGTGAGTTGAGTACACACATCACAAAGAAGTTTCTGAG

                  *       **          *          *                 *    *    
      70357 AATGCTGCTGTCTACCTTTTAT-T--TGA-ATTCCCGCTTCCAACGAAATC-CTCCAAGCTATCC
        130 AATGCTTCTGTCTAGTTTTTATATGAAGATATTCCCGTTTCCAACGAAATCGCT-AAAGCGATCC

                      * *  **   *                                *           
      70417 AAATATCCACCTGCATTTTCCACAAAAAGAGTGTTTCAAAACTGCTCTATCAATAGAAATGTTCA
        194 AAATATCCACTTCCAGATTCTACAAAAAGAGTGTTTCAAAACTGCTCTATCAAAAGAAATGTTCA

                **  * *  *                *           *                     *
      70482 ACTCCTTTGGCTGGGTACACACATCACAAACAAGTTTCTGAGCATGCTTCTGTCTAGTTTTTATG
        259 ACTCTGTTAGTTGAGTACACACATCACAAAGAAGTTTCTGAGAATGCTTCTGTCTAGTTTTTATA

            *     *          *  * *  **          *       *            **     
      70547 GGAAGACATTCCCTTTTTCACCAAAGGCATCAAAGCGCTCCAAATGTCCACTTCCAGACACTACA
        324 TGAAGATATTCCCTTTTCCAACGAAATCATCAAAGCGATCCAAATATCCACTTCCAGATTCTACA

                            **       *     **               * *    * *  * *  
      70612 AAAAGAGTGTTTCAAACGTGCTCTAAGAAAACGAATGTTCAACTCTGTGACTTGAATGCAGATAT
        389 AAAAGAGTGTTTCAAAACTGCTCTATGAAAAGAAATGTTCAACTCTGTTAGTTGAGTACACACAT

                *  *           **            *     ***  *         *          
      70677 CACAGAGTAGTTTCTGAGAGGGCTTCTGTCTAGATTTTAGATGATGATATTCCCGTTTCCAACGA
        454 CACAAAGAAGTTTCTGAGAATGCTTCTGTCTAGTTTTTATGGGAAGATATTCCCTTTTCCAACGA

              *   * *   *                *   *                    *        **
      70742 AATCATTAGAGCTATCCAAATATCCACTTACAGTTTCTACAAAAAGAGTGTTTCCAAACTGCTGC
        519 AAGCATCAAAGCGATCCAAATATCCACTTCCAGATTCTACAAAAAGAGTGTTTCAAAACTGCTCT

                  *  * *    *       *  *                   ***      *     *  
      70807 ATCAAAAGAGAGGTTCCACTCTGTTAGCTGAGTACACACATCACAAACTTGTTTCTCAGAATCCT
        584 ATCAAAGGAAATGTTCAACTCTGTGAGTTGAGTACACACATCACAAAGAAGTTTCTGAGAATGCT

                   *            *      **     *  *  * **          *       *  
      70872 TCTGTCTCGTTTTTATGGGAAGATATTTACTTTTTCACCGTAGGCATCAAAGCGCTCCAAATGTC
        649 TCTGTCTAGTTTTTAT--GATGATATTCCCTTTTCCAACGAAATCATCAAAGCGATCCAAATATC

               *       *  *  *               *          *     *   *         *
      70937 CACATCCAGATACTCCAGAAAGAGTGTTTCAAACCTG-CTCTATGAAAGGGAATCTTCAACTCTA
        712 CACTTCCAGATTCTACAAAAAGAGTGTTTCAAAACTGCCTCTATCAAAGGAAATGTTCAACTCTG

             *      * *  *      *      *              *       **         **  
      71001 TGAGTTGAATGCAGACATCAGAAAGAAATTTCTGAGAATGCTGCTGTCTACCTTTTAT---TTGA
        777 TTAGTTGAGTACACACATCACAAAGAAGTTTCTGAGAATGCTTCTGTCTAGTTTTTATGGGAAGA

                   **             *  *    *                *       *         
      71063 -ATTCCCGCTTCCAACGAAATCCTCCAAGCTATCCAAATATCCACTTGCAGATTCCACAAAAAGA
        842 TATTCCCTTTTCCAACGAAATCATCAAAGCGATCCAAATATCCACTTCCAGATTCTACAAAAAGA

                                     *  *  *            *      ***  * *     *
      71127 GTGTTTCAAAACTGCTCTCTATCAATGGCAAAGTTCAACTCTGTTAGTTGAGGACACATATCACC
        907 GTGTTTCAAAACTG--CTCTATCAAAGGAAATGTTCAACTCTGTGAGTTGAATGCAGACATCACA

              *                         *                  *      *  *  * ** 
      71192 AACAAGTTTCTGAGAATGCTTCTGTCTATTTTTTATGGGAAGATATTTCCTTTTTCACCGTAGGC
        970 AAGAAGTTTCTGAGAATGCTTCTGTCTAGTTTTTAT--GAAGATATTCCCTTTTCCAACGAAATC

            *    *      *    *          * **                     *         * 
      71257 GTCAAGGCGATCGAAATGTCCACTTCCACAAACTACAAAAAGAGTGTTTCAAACCTGCTCTATGA
       1033 ATCAAAGCGATCCAAATATCCACTTCCAGATTCTACAAAAAGAGTGTTTCAAAACTGCTCTATCA

              * **       *    * *      * ** * *   **      *      *       *   
      71322 AAGGCCATGTTCATCTCTATGAGTTGAATGGAAATATCCGAAAGAAATTTCTGGGAATGCTGCTG
       1098 AAAGAAATGTTCAACTCTGTTAGTTGAGTACACACATCACAAAGAAGTTTCTGAGAATGCTTCTG

                  *         *          *  *
      71387 TCTAGTGTTTAT---ACGA-ATTCCCGCTTC
       1163 TCTAGTTTTTATGGGAAGATATTCCCGTTTT

                      *       *                *    *  *                   * 
      71414 CAACGAAATCCTCAAAGCAATCCAAATATCCACTTGCAGAATCCACAAAAAGAGTGTTTCAAAAC
          1 CAACGAAATCATCAAAGCGATCCAAATATCCACTTCCAGATTCTACAAAAAGAGTGTTTCAAACC

                    *  ** *  *          * *                   *  *           
      71479 TGCTCTATCAATAGAAAGGTTCAACTCTTTTAGTTGAGTACACACATCACGAACAAGTTTCTGAG
         66 TGCTCTA-GAAAGGGAATGTTCAACTCTGTGAGTTGAGTACACACATCACAAAGAAGTTTCTGAG

                         * *      **     **  *      *    *    *           *  
      71544 AATGCTTCTGTCTGGCTTTTATTGGAAGACGTTTCC-TTTTC-ACCAAA-GGCATCAAAGCGCTC
        130 AATGCTTCTGTCTAGTTTTTATATGAAGATATTCCCGTTTCCAACGAAATCGC-T-AAAGCGATC

                 *                * *                **  *  * ***  * *       
      71606 CAAATGTCCACTTCCAGATTCTTCCAAAAGAGTGTTTCAAACGTGGTCGAAGTAAGGGAATGTTC
        193 CAAATATCCACTTCCAGATTCTACAAAAAGAGTGTTTCAAAACTGCTCTATCAAAAGAAATGTTC

                    * *    * *  * *     *   *       * * *             *     *
      71671 AACTCTGTGACTTGAATGCAGATATCACCAAGTAGTTTCTAATAGTGCTTCTGTCTAGATTTTAG
        258 AACTCTGTTAGTTGAGTACACACATCACAAAGAAGTTTCTGAGAATGCTTCTGTCTAGTTTTTAT

                *         *              * * *   * **          *  *   *      
      71736 ATGATGATATTCCCGTTTCCAACGAAATCGTTAGAGCTAAGCAAATATCCAGTTACAGTTTCTAC
        323 ATGAAGATATTCCCTTTTCCAACGAAATCATCAAAGCGATCCAAATATCCACTTCCAGATTCTAC

            *     *       *   *    **  *        *                    *       
      71801 CAAAAGGGTGTTTCCAAATTGCTGCATCAAAAGAAAGGTTCAACTCTGTTAGTTGAGGACACACA
        388 AAAAAGAGTGTTTCAAAACTGCTCTATGAAAAGAAATGTTCAACTCTGTTAGTTGAGTACACACA

                          *                   *    * **  *                   
      71866 TCACAAAGAAGTTTGTGAGAATGCTTCTGTCTAGATTTTGTATGACGATATTCCCTTTTCCAACG
        453 TCACAAAGAAGTTTCTGAGAATGCTTCTGTCTAGTTTTTATGGGAAGATATTCCCTTTTCCAACG

             * * * *     *   *       * *  *    *  *       *  *        *      
      71931 ATATCGTTAAAGCAATCTAAATATCAATTTGCAGAATCCACAAAAATAGCGTTTCAAAGCTGCTC
        518 AAAGCATCAAAGCGATCCAAATATCCACTTCCAGATTCTACAAAAAGAGTGTTTCAAAACTGCTC

             * *   *    *    *       *  *                   ***            * 
      71996 TGTAAAAAGAAAGGTTCCACTCTGTTAGCTGAGTACACACATCACAAACTTGTTTCTGAGAATCC
        583 TATCAAAGGAAATGTTCAACTCTGTGAGTTGAGTACACACATCACAAAGAAGTTTCTGAGAATGC

                    *            *      **        *  * **          *       * 
      72061 TTCTGTCTCGTTTTTATGGGAAGATATTTACTTTTCCACCGTAGGCATCAAAGCGCTCCAAATGT
        648 TTCTGTCTAGTTTTTAT--GATGATATTCCCTTTTCCAACGAAATCATCAAAGCGATCCAAATAT

                *       *  *  *  *            *          *     *   *         
      72126 CCACATCCAGATACTCCAGAACGAGTGTTTCAAACCTG-CTCTATGAAAGGGAATCTTCAACTCT
        711 CCACTTCCAGATTCTACAAAAAGAGTGTTTCAAAACTGCCTCTATCAAAGGAAATGTTCAACTCT

            * *      * *  *      *      *              *       **         ** 
      72190 ATGAGTTGAATGCAGACATCAGAAAGAAATTTCTGAGAATGCTGCTGTCTACCTTTTAT---TTG
        776 GTTAGTTGAGTACACACATCACAAAGAAGTTTCTGAGAATGCTTCTGTCTAGTTTTTATGGGAAG

                    **             *  *    *              * *  **   *    *   
      72252 A-ATTCCCGCTTCCAACGAAATCCTCCAAGCTATCCAAATATCCACCTGCATTTTCCACAACAAG
        841 ATATTCCCTTTTCCAACGAAATCATCAAAGCGATCCAAATATCCACTTCCAGATTCTACAAAAAG

                                    **                *    *  ** *  *        
      72316 AGTGTTTCAAAACTGCTCTATCAATAGAAATGTTCAACTCCTTTG-GCTGGGTACACACATCACA
        906 AGTGTTTCAAAACTGCTCTATCAAAGGAAATGTTCAACT-CTGTGAGTTGAATGCAGACATCACA

              *                                        **         *  * *  ** 
      72380 AACAAGTTTCTGAGAATGCTTCTGTCTAGTTTTTATGGGAAGACGTTCCCTTTTTCACCAAAGGC
        970 AAGAAGTTTCTGAGAATGCTTCTGTCTAGTTTTTAT--GAAGATATTCCCTTTTCCAACGAAATC

                     *       *            **                  *  **   *   ** 
      72445 ATCAAAGCGCTCCAAATGTCCACTTCCAGACACTACAAAAAGAGTGTTTCCAACGTGCCCTAAGA
       1033 ATCAAAGCGATCCAAATATCCACTTCCAGATTCTACAAAAAGAGTGTTTCAAAACTGCTCTATCA

              ***               * *    * *  * *         *           **       
      72510 AAGCGAATGTTCAACTCTGTGACTTGAATGCAGATATCACAAAGTAGTTTCTGAGAGGGCTTCTG
       1098 AAAGAAATGTTCAACTCTGTTAGTTGAGTACACACATCACAAAGAAGTTTCTGAGAATGCTTCTG

                 *     ***  *             *
      72575 TCTAGATTTTAGATGATGATATTCCCGTTTC
       1163 TCTAGTTTTTATGGGAAGATATTCCCGTTTT

                        * *   *                *   *                    *  * 
      72606 CAACGAAATCATTAGAGCTATCCAAATATCCACTTACAGTTTCTACAAAAAGAGTGTTTCCAAAC
          1 CAACGAAATCATCAAAGCGATCCAAATATCCACTTCCAGATTCTACAAAAAGAGTGTTTCAAACC

                    **    *   *    *       *  *                   ***      * 
      72671 TGCTGCATCAAAAGAG-AGGTTCCACTCTGTTAGCTGAGTACACACATCACAAACTTGTTTCTCA
         66 TGCT-C-TAGAAAGGGAATGTTCAACTCTGTGAGTTGAGTACACACATCACAAAGAAGTTTCTGA

                *         *        **         ** *   *  *  * ** ***      *   
      72735 GAATCCTTCTGTCTCGTTTTTATGGGAAGATATTTACTTTTTCACCGTAGGCATCAAAGCGCTCC
        129 GAATGCTTCTGTCTAGTTTTTATATGAAGATATTCCCGTTTCCAACGAAATCGCTAAAGCGATCC

                *     *       *  *  *               *         *   * *   *    
      72800 AAATGTCCACATCCAGATACTCCAGAAAGAGTGTTTCAAACCTGCTCTATGAAAGGGAATCTTCA
        194 AAATATCCACTTCCAGATTCTACAAAAAGAGTGTTTCAAAACTGCTCTATCAAAAGAAATGTTCA

                 * *      * *  *      *      *              *       **       
      72865 ACTCTATGAGTTGAATGCAGACATCAGAAAGAAATTTCTGAGAATGCTGCTGTCTACCTTTTAT-
        259 ACTCTGTTAGTTGAGTACACACATCACAAAGAAGTTTCTGAGAATGCTTCTGTCTAGTTTTTATA

               *         **             *  *    *                *       *   
      72929 T--TGA-ATTCCCGCTTCCAACGAAATCCTCCAAGCTATCCAAATATCCACTTGCAGATTCCACA
        324 TGAAGATATTCCCTTTTCCAACGAAATCATCAAAGCGATCCAAATATCCACTTCCAGATTCTACA

                                        *  ** *  *                    *     *
      72991 AAAAGAGTGTTTCAAAACTGCTCTCTATCAATGGCAAAGTTCAACTCTGTTAGTTGAGGACACAT
        389 AAAAGAGTGTTTCAAAACTG--CTCTATGAAAAGAAATGTTCAACTCTGTTAGTTGAGTACACAC

                 *  *                         *                  *      *  * 
      73056 ATCACCAACAAGTTTCTGAGAATGCTTCTGTCTATTTTTTATGGGAAGATATTTCCTTTTTCACC
        452 ATCACAAAGAAGTTTCTGAGAATGCTTCTGTCTAGTTTTTATGGGAAGATATTCCCTTTTCCAAC

             * *  *    *      *    *          * **                     *     
      73121 GTAGGCGTCAAGGCGATCGAAATGTCCACTTCCACAAACTACAAAAAGAGTGTTTCAAACCTGCT
        517 GAAAGCATCAAAGCGATCCAAATATCCACTTCCAGATTCTACAAAAAGAGTGTTTCAAAACTGCT

                *     **       *    *        * ** * *   **      *      *     
      73186 CTATGAAAGGCGATGTTCATCTCTATGAGTTGAATGGAAATATCCGAAAGAAATTTCTGGGAATG
        582 CTATCAAAGGAAATGTTCAACTCTGTGAGTTGAGTACACACATCACAAAGAAGTTTCTGAGAATG

              *         *       *         **             *       *           
      73251 CTGCTGTCTAGTGTTTAT-ACGA-ATTCCCGCTTCCAACGAAATCCTCAAAGCAATCCAAATATC
        647 CTTCTGTCTAGTTTTTATGATGATATTCCCTTTTCCAACGAAATCATCAAAGCGATCCAAATATC

                 *    *  *                                 **    *          *
      73314 CACTTGCAGAATCCACAAAAAGAGTGTTTCAAAACTG-CTCTATCAATAGAAAGGTTCAACTCTT
        712 CACTTCCAGATTCTACAAAAAGAGTGTTTCAAAACTGCCTCTATCAAAGGAAATGTTCAACTCTG

                                 *  *                        * *      *      
      73378 TTAGTTGAGTACACACATCACGAACAAGTTTCTGAGAATGCTTCTGTCTGGCTTTTATTGGAAGA
        777 TTAGTTGAGTACACACATCACAAAGAAGTTTCTGAGAATGCTTCTGTCTAGTTTTTATGGGAAGA

            **  *    *    * *  **          *       *                * *      
      73443 CGTTTCCTTAT-CACCAAAGGCATCAAAGCGCTCCAAATGTCCACTTCCAGATTCTTCCAAAAGA
        842 TATTCCCTTTTCCAACGAAATCATCAAAGCGATCCAAATATCCACTTCCAGATTCTACAAAAAGA

                      **     * ***    *                 *           *     *  
      73507 GTGTTTCAAACGTGCTCAAAGTAAGGGAATGTTCAACTCTGTGACTTGAATGCAGATATCACCAA
        907 GTGTTTCAAAACTGCTCTATCAAAGGAAATGTTCAACTCTGTGAGTTGAATGCAGACATCACAAA

             *       * * *                   *    *         *              * 
      73572 GTAGTTTCTAATAGTGCTTCTGTCTAGATTTTAGATGATGATATTCCCGTTTCCAACGAAATCGT
        972 GAAGTTTCTGAGAATGCTTCTGTCTAG-TTTT-TATGAAGATATTCCCTTTTCCAACGAAATCAT

            * *   *             *  *   *                    *        **      
      73637 TAGAGCTATCCAAATATCCAGTTACAGTTTCTACAAAAAGAGTGTTTCCAAACTGCTGCATCAAA
       1035 CAAAGCGATCCAAATATCCACTTCCAGATTCTACAAAAAGAGTGTTTCAAAACTGCTCTATCAAA

                 *                    *                     *                
      73702 AGAAAGGTTCAACTCTGTTAGTTGAGGACACACATCACAAAGAAGTTTGTGAGAATGCTTCTGTC
       1100 AGAAATGTTCAACTCTGTTAGTTGAGTACACACATCACAAAGAAGTTTCTGAGAATGCTTCTGTC

               *    * **  *         *   *
      73767 TAGATTTTGTATGACGATATTCCCTTTTC
       1165 TAGTTTTTATGGGAAGATATTCCCGTTTT

                  *   * *     *   *       * *  *    *  *       *  *        * 
      73796 CAACGATATCGTTAAAGCAATCTAAATATCAATTTGCAGAATCCACAAAAATAGAGTTTCAAAGC
          1 CAACGAAATCATCAAAGCGATCCAAATATCCACTTCCAGATTCTACAAAAAGAGTGTTTCAAACC

                   **   ***  *    *       *  *                   ***         
      73861 TGCTCTGTAAAAATAAAAGTTCCACTCTGTTAGCTGAGTACACACATCACAAACTTGTTTCTGAG
         66 TGCTCT-AGAAAGGGAATGTTCAACTCTGTGAGTTGAGTACACACATCACAAAGAAGTTTCTGAG

               *         *        **         ** *   *  *  * ** ***      *    
      73926 AATCCTTCTGTCTCGTTTTTATGGGAAGATATTTACTTTTTCACCGTAGGCATCAAAGCGCTCCA
        130 AATGCTTCTGTCTAGTTTTTATATGAAGATATTCCCGTTTCCAACGAAATCGCTAAAGCGATCCA

               *     *       *  *  *     *         *         *   * *   *     
      73991 AATGTCCACATCCAGATACTCCAGAAAGACTGTTTCAAACCTGCTCTATGAAAGGGAATCTTCAA
        195 AATATCCACTTCCAGATTCTACAAAAAGAGTGTTTCAAAACTGCTCTATCAAAAGAAATGTTCAA

                * *      * *  *      *      *              *       **        
      74056 CTCTATGAGTTGAATGCAGACATCAGAAAGAAATTTCTGAGAATGCTGCTGTCTACCTTTTAT-T
        260 CTCTGTTAGTTGAGTACACACATCACAAAGAAGTTTCTGAGAATGCTTCTGTCTAGTTTTTATAT

              *         **             *  *    *                *  **   *    
      74120 --TGA-ATTCCCGCTTCCAACGAAATCCTCCAAGCTATCCAAATATCCACTTGCATTTTCCACAA
        325 GAAGATATTCCCTTTTCCAACGAAATCATCAAAGCGATCCAAATATCCACTTCCAGATTCTACAA

                                     *  *               **    *  *           
      74182 AAAGAGTGTTTCAAAACTGCTCTATCAATAGAAATGTTCAACTCCTTTAGCTGGGTACACACATC
        390 AAAGAGTGTTTCAAAACTGCTCTATGAAAAGAAATGTTCAACTCTGTTAGTTGAGTACACACATC

                 *                                        **         *  * *  
      74247 ACAAACAAGTTTCTGAGAATGCTTCTGTCTAGTTTTTATGGGAAGACGTTCCCTTTTTCACCAAA
        455 ACAAAGAAGTTTCTGAGAATGCTTCTGTCTAGTTTTTATGGGAAGATATTCCCTTTTCCAACGAA

            *           *       *            **                     **       
      74312 GGCATCAAAGCGCTCCAAATGTCCACTTCCAGACACTACAAAAAGAGTGTTTCAAACGTGCTCTA
        520 AGCATCAAAGCGATCCAAATATCCACTTCCAGATTCTACAAAAAGAGTGTTTCAAAACTGCTCTA

            **    **                 *    * *  * *         *           **    
      74377 AGAAAGCGAATGTTCAACTCTGTGACTTGAATGCAGATATCACAAAGTAGTTTCTGAGAGGGCTT
        585 TCAAAGGAAATGTTCAACTCTGTGAGTTGAGTACACACATCACAAAGAAGTTTCTGAGAATGCTT

                          *              *                * *   *            
      74442 CTGTCTAGATTTTAGATGATGATATTCCCGTTTCCAACGAAATCATTAGAGCTATCCAAATATCC
        650 CTGTCTAG-TTTT-TATGATGATATTCCCTTTTCCAACGAAATCATCAAAGCGATCCAAATATCC

                *   *     *              *         **      *  * *    *       
      74507 ACTTACAGTTTCTATAAAAAGAGTGTTTCCAAACTG-CTGCATCAAAAGAGAGGTTCCACTCTGT
        713 ACTTCCAGATTCTACAAAAAGAGTGTTTCAAAACTGCCTCTATCAAAGGAAATGTTCAACTCTGT

               **                  ***      *     *         *                
      74571 TAGCCGAGTACACACATCACAAACTTGTTTCTCAGAATCCTTCTGTCTCGTTTTTATGGGAAGAT
        778 TAGTTGAGTACACACATCACAAAGAAGTTTCTGAGAATGCTTCTGTCTAGTTTTTATGGGAAGAT

               **        *  * **          *       *     *       *  *  *      
      74636 ATTTACTTTTCCACCGTAGGCATCAAAGCGCTCCAAATGTCCACATCCAGATACTCCAGAAAGAG
        843 ATTCCCTTTTCCAACGAAATCATCAAAGCGATCCAAATATCCACTTCCAGATTCTACAAAAAGAG

                     *         *     *   *         *                    *    
      74701 TGTTTCAAACCTGCTCTATGAAAGGGAATCTTCAACTCTATGAGTTGAATGCAGACATCAGAAAG
        908 TGTTTCAAAACTGCTCTATCAAAGGAAATGTTCAACTCTGTGAGTTGAATGCAGACATCACAAAG

              *              *       **       **         **             *  * 
      74766 AAATTTCTGAGAATGCTGCTGTCTACCTTTTAT-TTGA-ATTCCCGCTTCCAACGAAATCCTCCA
        973 AAGTTTCTGAGAATGCTTCTGTCTAGTTTTTATGAAGATATTCCCTTTTCCAACGAAATCATCAA

               *              * *  **   *    *                           *   
      74829 AGCTATCCAAATATCCACCTGCATTTTCCACAACAAGAGTGTTTCAAAACTGCTCTATCAATAGA
       1038 AGCGATCCAAATATCCACTTCCAGATTCTACAAAAAGAGTGTTTCAAAACTGCTCTATCAAAAGA

                        **  * *  *                *           *              
      74894 AATGTTCAACTCCTTTGGCTGGGTACACACATCACAAACAAGTTTCTGAGCATGCTTCTGTCTAG
       1103 AATGTTCAACTCTGTTAGTTGAGTACACACATCACAAAGAAGTTTCTGAGAATGCTTCTGTCTAG

                          *      *    
      74959 TTTTTATGGGAAGACATTCCCTTTTT
       1168 TTTTTATGGGAAGATATTCCCGTTTT

              * *  **          *       *            **                      *
      74985 CACCAAAGGCATCAAAGCGCTCCAAATGTCCACTTCCAGACACTACAAAAAGAGTGTTTCAAACG
          1 CAACGAAATCATCAAAGCGATCCAAATATCCACTTCCAGATTCTACAAAAAGAGTGTTTCAAACC

                        **                  *    * *  * *      *  *          
      75050 TGCTCTAAGAAAACGAATGTTCAACTCTGTGACTTGAATGCAGATATCACAGAGTAGTTTCTGAG
         66 TGCTCT-AGAAAGGGAATGTTCAACTCTGTGAGTTGAGTACACACATCACAAAGAAGTTTCTGAG

             **            *     *    *                        **  *   *     
      75115 AGGGCTTCTGTCTAGATTTTAGATGATGATATTCCCGTTTCCAACGAAATCATTAGAGCTATCCA
        130 AATGCTTCTGTCTAGTTTTTATATGAAGATATTCCCGTTTCCAACGAAATCGCTAAAGCGATCCA

                       *   *                    *        **         * *    * 
      75180 AATATCCACTTACAGTTTCTACAAAAAGAGTGTTTCCAAACTGCTGCATCAAAAGAGAGGTTCCA
        195 AATATCCACTTCCAGATTCTACAAAAAGAGTGTTTCAAAACTGCTCTATCAAAAGAAATGTTCAA

                     *                   ***      *     *         *        **
      75245 CTCTGTTAGCTGAGTACACACATCACAAACTTGTTTCTCAGAATCCTTCTGTCTCGTTTTTATGG
        260 CTCTGTTAGTTGAGTACACACATCACAAAGAAGTTTCTGAGAATGCTTCTGTCTAGTTTTTATAT

                     **     *  *  * **          *       *     *       *  *  *
      75310 GAAGATATTTACTTTTTCACCGTAGGCATCAAAGCGCTCCAAATGTCCACATCCAGATACTCCAG
        325 GAAGATATTCCCTTTTCCAACGAAATCATCAAAGCGATCCAAATATCCACTTCCAGATTCTACAA

                           *             * *   *         * *      * *  *     
      75375 AAAGAGTGTTTCAAACCTGCTCTATGAAAGGGAATCTTCAACTCTATGAGTTGAATGCAGACATC
        390 AAAGAGTGTTTCAAAACTGCTCTATGAAAAGAAATGTTCAACTCTGTTAGTTGAGTACACACATC

             *      *              *       **         **         **          
      75440 AGAAAGAAATTTCTGAGAATGCTGCTGTCTACCTTTTAT---TTGA-ATTCCCGCTTCCAACGAA
        455 ACAAAGAAGTTTCTGAGAATGCTTCTGTCTAGTTTTTATGGGAAGATATTCCCTTTTCCAACGAA

             * *  *    *                *       *                            
      75501 ATCCTCCAAGCTATCCAAATATCCACTTGCAGATTCCACAAAAAGAGTGTTTCAAAACTGCTCTC
        520 AGCATCAAAGCGATCCAAATATCCACTTCCAGATTCTACAAAAAGAGTGTTTCAAAACTG--CTC

                  *  *  *            *       *     *     *  *                
      75566 TATCAATGGCAAAGTTCAACTCTGTTAGTTGAGGACACATATCACCAACAAGTTTCTGAGAATGC
        583 TATCAAAGGAAATGTTCAACTCTGTGAGTTGAGTACACACATCACAAAGAAGTTTCTGAGAATGC

                     *           *      *      *  *  * ** *    *      *    * 
      75631 TTCTGTCTATTTTTTATGGGAAGATATTTCCTTTTTCACCGTAGGCGTCAAGGCGATCGAAATGT
        648 TTCTGTCTAGTTTTTAT--GATGATATTCCCTTTTCCAACGAAATCATCAAAGCGATCCAAATAT

                     * **                     *          *     **       *    
      75696 CCACTTCCACAAACTACAAAAAGAGTGTTTCAAACCTG-CTCTATGAAAGGCCATGTTCATCTCT
        711 CCACTTCCAGATTCTACAAAAAGAGTGTTTCAAAACTGCCTCTATCAAAGGAAATGTTCAACTCT

            * *      * ** * *   **      *      *       *         *         * 
      75760 ATGAGTTGAATGGAAATATCCGAAAGAAATTTCTGGGAATGCTGCTGTCTAGTGTTTAT---ACG
        776 GTTAGTTGAGTACACACATCACAAAGAAGTTTCTGAGAATGCTTCTGTCTAGTTTTTATGGGAAG

                    **             *       *                *    *  *        
      75822 A-ATTCCCGCTTCCAACGAAATCCTCAAAGCAATCCAAATATCCACTTGCAGAATCCACAAAAAG
        841 ATATTCCCTTTTCCAACGAAATCATCAAAGCGATCCAAATATCCACTTCCAGATTCTACAAAAAG

                                    **    *          * *      * *  *       * 
      75886 AGTGTTTCAAAACTGCTCTATCAATAGAAAGGTTCAACTCTTTTAGTTGAGTACACACATCACGA
        906 AGTGTTTCAAAACTGCTCTATCAAAGGAAATGTTCAACTCTGTGAGTTGAATGCAGACATCACAA

             *                        * *             **  *         * *  **  
      75951 ACAAGTTTCTGAGAATGCTTCTGTCTGGCTTTTATTGGAAGACGTTTCCTTTT-CACCAAAGGCA
        971 AGAAGTTTCTGAGAATGCTTCTGTCTAGTTTTTA-T-GAAGATATTCCCTTTTCCAACGAAATCA

                    *       *                * *                **  *  * *** 
      76015 TCAAAGCGCTCCAAATGTCCACTTCCAGATTCTTCCAAAAGAGTGTTTCAAACGTGGTCGAAGTA
       1034 TCAAAGCGATCCAAATATCCACTTCCAGATTCTACAAAAAGAGTGTTTCAAAACTGCTCTATCAA

             * *               * *    * *  * *     *   *       * * *         
      76080 AGGGAATGTTCAACTCTGTGACTTGAATGCAGATATCACCAAGTAGTTTCTAATAGTGCTTCTGT
       1099 AAGAAATGTTCAACTCTGTTAGTTGAGTACACACATCACAAAGAAGTTTCTGAGAATGCTTCTGT

                *     ***  *             *
      76145 CTAGATTTTAGATGATGATATTCCCGTTTC
       1164 CTAGTTTTTATGGGAAGATATTCCCGTTTT

                      * * *   * **          *  *   *      *     *       *  **
      76175 CAACGAAATCGTTAGAGCTAAGCAAATATCCAGTTACAGTTTCTACCAAAAGGGTGTTTCCAAAT
          1 CAACGAAATCATCAAAGCGATCCAAATATCCACTTCCAGATTCTACAAAAAGAGTGTTTCAAACC

                  ***   * *  *            *       *                     *    
      76240 TGCTGCATCAAAAGAAAGGTTCAACTCTGTTAGTTGAGGACACACATCACAAAGAAGTTTGTGAG
         66 TGCT-CTAGAAAGGGAATGTTCAACTCTGTGAGTTGAGTACACACATCACAAAGAAGTTTCTGAG

                           *    *     *         *          *    *      *   * 
      76305 AATGCTTCTGTCTAGATTTTGTATGACGATATTCCCTTTTCCAACGATATCGTTAAAGCAATCTA
        130 AATGCTTCTGTCTAGTTTTTATATGAAGATATTCCCGTTTCCAACGAAATCGCTAAAGCGATCCA

                  * *  *    *  *       *  *        *       * *        *    * 
      76370 AATATCAATTTGCAGAATCCACAAAAATAGCGTTTCAAAGCTGCTCTGTAAAAAGAAAGGTTCCA
        195 AATATCCACTTCCAGATTCTACAAAAAGAGTGTTTCAAAACTGCTCTATCAAAAGAAATGTTCAA

                     *                   ***            *         *        **
      76435 CTCTGTTAGCTGAGTACACACATCACAAACTTGTTTCTGAGAATCCTTCTGTCTCGTTTTTATGG
        260 CTCTGTTAGTTGAGTACACACATCACAAAGAAGTTTCTGAGAATGCTTCTGTCTAGTTTTTATAT

                     **        *  * **          *       *     *       *  *  *
      76500 GAAGATATTTACTTTTCCACCGTAGGCATCAAAGCGCTCCAAATGTCCACATCCAGATACTCCAG
        325 GAAGATATTCCCTTTTCCAACGAAATCATCAAAGCGATCCAAATATCCACTTCCAGATTCTACAA

              *            *             * *   *         * *      * *  *     
      76565 AACGAGTGTTTCAAACCTGCTCTATGAAAGGGAATCTTCAACTCTATGAGTTGAATGCAGACATC
        390 AAAGAGTGTTTCAAAACTGCTCTATGAAAAGAAATGTTCAACTCTGTTAGTTGAGTACACACATC

             *      *              *       **         **         **          
      76630 AGAAAGAAATTTCTGAGAATGCTGCTGTCTACCTTTTAT---TTGA-ATTCCCGCTTCCAACGAA
        455 ACAAAGAAGTTTCTGAGAATGCTTCTGTCTAGTTTTTATGGGAAGATATTCCCTTTTCCAACGAA

             * *  *    *              * *  **   *    *                       
      76691 ATCCTCCAAGCTATCCAAATATCCACCTGCATTTTCCACAACAAGAGTGTTTCAAAACTGCTCTA
        520 AGCATCAAAGCGATCCAAATATCCACTTCCAGATTCTACAAAAAGAGTGTTTCAAAACTGCTCTA

                **                *    *  *                *                 
      76756 TCAATAGAAATGTTCAACTCCTTTG-GCTGGGTACACACATCACAAACAAGTTTCTGAGAATGCT
        585 TCAAAGGAAATGTTCAACT-CTGTGAGTTGAGTACACACATCACAAAGAAGTTTCTGAGAATGCT

                                *  **         *  * *  **          *       *  
      76820 TCTGTCTAGTTTTTATGGGAAGACGTTCCCTTTTTCACCAAAGGCATCAAAGCGCTCCAAATGTC
        649 TCTGTCTAGTTTTTAT--GATGATATTCCCTTTTCCAACGAAATCATCAAAGCGATCCAAATATC

                      **                  *  **        **    **              
      76885 CACTTCCAGACACTACAAAAAGAGTGTTTCCAACGTGCC-CTAAGAAAGCGAATGTTCAACTCTG
        712 CACTTCCAGATTCTACAAAAAGAGTGTTTCAAAACTGCCTCTATCAAAGGAAATGTTCAACTCTG

             * *    * *  * *         *           **            *     ***  *  
      76949 TGACTTGAATGCAGATATCACAAAGTAGTTTCTGAGAGGGCTTCTGTCTAGATTTTAGATGATGA
        777 TTAGTTGAGTACACACATCACAAAGAAGTTTCTGAGAATGCTTCTGTCTAGTTTTTATGGGAAGA

                   *                * *   *                *   *             
      77014 TATTCCCGTTTCCAACGAAATCATTAGAGCTATCCAAATATCCACTTACAGTTTCTACAAAAAGA
        842 TATTCCCTTTTCCAACGAAATCATCAAAGCGATCCAAATATCCACTTCCAGATTCTACAAAAAGA

                   *        **      *  * *    *       *  *   * *  *          
      77079 GTGTTTCCAAACTGCTGCATCAAAAGAGAGGTTCCACTCTGTTAGCTGAGTACACACATCACAAA
        907 GTGTTTCAAAACTGCTCTATCAAAGGAAATGTTCAACTCTGTGAGTTGAATGCAGACATCACAAA

            ***      *     *         *                   **     *  *  * **   
      77144 CTTGTTTCTCAGAATCCTTCTGTCTCGTTTTTATGGGAAGATATTTACTTTTTCACCGTAGGCAT
        972 GAAGTTTCTGAGAATGCTTCTGTCTAGTTTTTAT--GAAGATATTCCCTTTTCCAACGAAATCAT

                   *       *     *       *  *  *               *         *   
      77209 CAAAGCGCTCCAAATGTCCACATCCAGATACTCCAGAAAGAGTGTTTCAAACCTGCTCTATGAAA
       1035 CAAAGCGATCCAAATATCCACTTCCAGATTCTACAAAAAGAGTGTTTCAAAACTGCTCTATCAAA

            * *   *         * *      * *  *      *      *              *     
      77274 GGGAATCTTCAACTCTATGAGTTGAATGCAGACATCAGAAAGAAATTTCTGAGAATGCTGCTGTC
       1100 AGAAATGTTCAACTCTGTTAGTTGAGTACACACATCACAAAGAAGTTTCTGAGAATGCTTCTGTC

              **         **          *  *
      77339 TACCTTTTAT---TTGA-ATTCCCGCTTC
       1165 TAGTTTTTATGGGAAGATATTCCCGTTTT

                      *  *    *                *       *                   * 
      77364 CAACGAAATCCTCCAAGCTATCCAAATATCCACTTGCAGATTCCACAAAAAGAGTGTTTCAAAAC
          1 CAACGAAATCATCAAAGCGATCCAAATATCCACTTCCAGATTCTACAAAAAGAGTGTTTCAAACC

                      *  *  *  *            *       *     *     *  *         
      77429 TGCTCTCTATCAATGGCAAAGTTCAACTCTGTTAGTTGAGGACACATATCACCAACAAGTTTCTG
         66 TG--CTCTA-GAAAGGGAATGTTCAACTCTGTGAGTTGAGTACACACATCACAAAGAAGTTTCTG

                            *       **         *  *   *  *  * **  **  *      
      77494 AGAATGCTTCTGTCTATTTTTTATGGGAAGATATTTCCTTTTTCACCGTAGGCGTCAAGGCGATC
        128 AGAATGCTTCTGTCTAGTTTTTATATGAAGATATTCCCGTTTCCAACGAAATCGCTAAAGCGATC

            *    *          * **                     *         *   * **      
      77559 GAAATGTCCACTTCCACAAACTACAAAAAGAGTGTTTCAAACCTGCTCTATGAAAGGCGATGTTC
        193 CAAATATCCACTTCCAGATTCTACAAAAAGAGTGTTTCAAAACTGCTCTATCAAAAGAAATGTTC

             *    * *      * ** * *   **      *      *       *          *    
      77624 ATCTCTATGAGTTGAATGGAAATATCCGAAAGAAATTTCTGGGAATGCTGCTGTCTAG-TGTT-T
        258 AACTCTGTTAGTTGAGTACACACATCACAAAGAAGTTTCTGAGAATGCTTCTGTCTAGTTTTTAT

                *         **             *       *                *    *  *  
      77687 AT-ACGA-ATTCCCGCTTCCAACGAAATCCTCAAAGCAATCCAAATATCCACTTGCAGAATCCAC
        323 ATGAAGATATTCCCTTTTCCAACGAAATCATCAAAGCGATCCAAATATCCACTTCCAGATTCTAC

                                       *  *     *          *                 
      77750 AAAAAGAGTGTTTCAAAACTGCTCTATCAATAGAAAGGTTCAACTCTTTTAGTTGAGTACACACA
        388 AAAAAGAGTGTTTCAAAACTGCTCTATGAAAAGAAATGTTCAACTCTGTTAGTTGAGTACACACA

                *  *                        * *      *      **  *    *    * *
      77815 TCACGAACAAGTTTCTGAGAATGCTTCTGTCTGGCTTTTATTGGAAGACGTTTCCTTAT-CACCA
        453 TCACAAAGAAGTTTCTGAGAATGCTTCTGTCTAGTTTTTATGGGAAGATATTCCCTTTTCCAACG

              *           *       *                * *                **     
      77879 AAGGCATCAAAGCGCTCCAAATGTCCACTTCCAGATTCTTCCAAAAGAGTGTTTCAAACGTGCTC
        518 AAAGCATCAAAGCGATCCAAATATCCACTTCCAGATTCTACAAAAAGAGTGTTTCAAAACTGCTC

            * ***    *                 *    * *  * *     *   *       * * *   
      77944 AAAGTAAGGGAATGTTCAACTCTGTGACTTGAATGCAGATATCACCAAGTAGTTTCTAATAGTGC
        583 TATCAAAGGAAATGTTCAACTCTGTGAGTTGAGTACACACATCACAAAGAAGTTTCTGAGAATGC

                            *              *              * * *   *          
      78009 TTCTGTCTAGATTTTAGATGATGATATTCCCGTTTCCAACGAAATCGTTAGAGCTATCCAAATAT
        648 TTCTGTCTAG-TTTT-TATGATGATATTCCCTTTTCCAACGAAATCATCAAAGCGATCCAAATAT

               *  *   *                    *         **      *    *          
      78074 CCAGTTACAGTTTCTACAAAAAGAGTGTTTCCAAACTG-CTGCATCAAAAGAAAGGTTCAACTCT
        711 CCACTTCCAGATTCTACAAAAAGAGTGTTTCAAAACTGCCTCTATCAAAGGAAATGTTCAACTCT

                      *                     *                   *    * **  * 
      78138 GTTAGTTGAGGACACACATCACAAAGAAGTTTGTGAGAATGCTTCTGTCTAGATTTTGTATGACG
        776 GTTAGTTGAGTACACACATCACAAAGAAGTTTCTGAGAATGCTTCTGTCTAGTTTTTATGGGAAG

                               *   * *     *   *       * *  *    *  *       *
      78203 ATATTCCCTTTTCCAACGATATCGTTAAAGCAATCTAAATATCAATTTGCAGAATCCACAAAAAT
        841 ATATTCCCTTTTCCAACGAAATCATCAAAGCGATCCAAATATCCACTTCCAGATTCTACAAAAAG

              *        *       * *   **   *    *       *  *   * *  *         
      78268 AGAGTTTCAAAGCTGCTCTGTAAAAATAAAAGTTCCACTCTGTTAGCTGAGTACACACATCACAA
        906 AGTGTTTCAAAACTGCTCTATCAAAGGAAATGTTCAACTCTGTGAGTTGAATGCAGACATCACAA

             ***            *         *                   **     *  *  * **  
      78333 ACTTGTTTCTGAGAATCCTTCTGTCTCGTTTTTATGGGAAGATATTTACTTTTTCACCGTAGGCA
        971 AGAAGTTTCTGAGAATGCTTCTGTCTAGTTTTTAT--GAAGATATTCCCTTTTCCAACGAAATCA

                    *       *     *       *  *  *     *         *         *  
      78398 TCAAAGCGCTCCAAATGTCCACATCCAGATACTCCAGAAAGACTGTTTCAAACCTGCTCTATGAA
       1034 TCAAAGCGATCCAAATATCCACTTCCAGATTCTACAAAAAGAGTGTTTCAAAACTGCTCTATCAA

             * *   *         * *      * *  *      *      *              *    
      78463 AGGGAATCTTCAACTCTATGAGTTGAATGCAGACATCAGAAAGAAATTTCTGAGAATGCTGCTGT
       1099 AAGAAATGTTCAACTCTGTTAGTTGAGTACACACATCACAAAGAAGTTTCTGAGAATGCTTCTGT

               **         **          *  *
      78528 CTACCTTTTAT---TTGA-ATTCCCGCTTC
       1164 CTAGTTTTTATGGGAAGATATTCCCGTTTT

                      *  *    *                *  **   *                   * 
      78554 CAACGAAATCCTCCAAGCTATCCAAATATCCACTTGCATTTTCCACAAAAAGAGTGTTTCAAAAC
          1 CAACGAAATCATCAAAGCGATCCAAATATCCACTTCCAGATTCTACAAAAAGAGTGTTTCAAACC

                    *  ** *            ** *  *  *                *           
      78619 TGCTCTATCAATAGAAATGTTCAACTCCTTTAGCTGGGTACACACATCACAAACAAGTTTCTGAG
         66 TGCTCTA-GAAAGGGAATGTTCAACTCTGTGAGTTGAGTACACACATCACAAAGAAGTTTCTGAG

                                  **     **     *   *    *    *           *  
      78684 AATGCTTCTGTCTAGTTTTTATGGGAAGACGTTCCCTTTTTC-ACCAAA-GGCATCAAAGCGCTC
        130 AATGCTTCTGTCTAGTTTTTATATGAAGATATTCCCGTTTCCAACGAAATCGC-T-AAAGCGATC

                 *            **                     **       **   ***       
      78747 CAAATGTCCACTTCCAGACACTACAAAAAGAGTGTTTCAAACGTGCTCTAAGAAAGCGAATGTTC
        193 CAAATATCCACTTCCAGATTCTACAAAAAGAGTGTTTCAAAACTGCTCTATCAAAAGAAATGTTC

                    * *    * *  * *         *           **            *     *
      78812 AACTCTGTGACTTGAATGCAGATATCACAAAGTAGTTTCTGAGAGGGCTTCTGTCTAGATTTTAG
        258 AACTCTGTTAGTTGAGTACACACATCACAAAGAAGTTTCTGAGAATGCTTCTGTCTAGTTTTTAT

                *         *                * *   *                *   *     *
      78877 ATGATGATATTCCCGTTTCCAACGAAATCATTAGAGCTATCCAAATATCCACTTACAGTTTCTAT
        323 ATGAAGATATTCCCTTTTCCAACGAAATCATCAAAGCGATCCAAATATCCACTTCCAGATTCTAC

                          *        **  *      * *    *          **           
      78942 AAAAAGAGTGTTTCCAAACTGCTGCATCAAAAGAGAGGTTCCACTCTGTTAGCCGAGTACACACA
        388 AAAAAGAGTGTTTCAAAACTGCTCTATGAAAAGAAATGTTCAACTCTGTTAGTTGAGTACACACA

                   ***      *     *         *                   **        *  
      79007 TCACAAACTTGTTTCTCAGAATCCTTCTGTCTCGTTTTTATGGGAAGATATTTACTTTTCCACCG
        453 TCACAAAGAAGTTTCTGAGAATGCTTCTGTCTAGTTTTTATGGGAAGATATTCCCTTTTCCAACG

            * *           *       *     *       *  *  *               *      
      79072 TAGGCATCAAAGCGCTCCAAATGTCCACATCCAGATACTCCAGAAAGAGTGTTTCAAACCTGCTC
        518 AAAGCATCAAAGCGATCCAAATATCCACTTCCAGATTCTACAAAAAGAGTGTTTCAAAACTGCTC

               *     *   *         *        * *  *      *      *             
      79137 TATGAAAGGGAATCTTCAACTCTATGAGTTGAATGCAGACATCAGAAAGAAATTTCTGAGAATGC
        583 TATCAAAGGAAATGTTCAACTCTGTGAGTTGAGTACACACATCACAAAGAAGTTTCTGAGAATGC

             *       **       *          **             *  *    *            
      79202 TGCTGTCTACCTTTTAT-TTGA-ATTCCCGCTTCCAACGAAATCCTCCAAGCTATCCAAATATCC
        648 TTCTGTCTAGTTTTTATGATGATATTCCCTTTTCCAACGAAATCATCAAAGCGATCCAAATATCC

              * *  **   *    *                            **              ** 
      79265 ACCTGCATTTTCCACAACAAGAGTGTTTCAAAACTG-CTCTATCAATAGAAATGTTCAACTCCTT
        713 ACTTCCAGATTCTACAAAAAGAGTGTTTCAAAACTGCCTCTATCAAAGGAAATGTTCAACTCTGT

             * *  *                *           *                            *
      79329 TGGCTGGGTACACACATCACAAACAAGTTTCTGAGCATGCTTCTGTCTAGTTTTTATGGGAAGAC
        778 TAGTTGAGTACACACATCACAAAGAAGTTTCTGAGAATGCTTCTGTCTAGTTTTTATGGGAAGAT

                      *  * *  **          *       *            **            
      79394 ATTCCCTTTTTCACCAAAGGCATCAAAGCGCTCCAAATGTCCACTTCCAGACACTACAAAAAGAG
        843 ATTCCCTTTTCCAACGAAATCATCAAAGCGATCCAAATATCCACTTCCAGATTCTACAAAAAGAG

                     **       **   ***                 *           *      *  
      79459 TGTTTCAAACGTGCTCTAAGAAAACGAATGTTCAACTCTGTGACTTGAATGCAGATATCACAGAG
        908 TGTTTCAAAACTGCTCTATCAAAGGAAATGTTCAACTCTGTGAGTTGAATGCAGACATCACAAAG

            *           **                  *    *         *                *
      79524 TAGTTTCTGAGAGGGCTTCTGTCTAGATTTTAGATGATGATATTCCCGTTTCCAACGAAATCATT
        973 AAGTTTCTGAGAATGCTTCTGTCTAG-TTTT-TATGAAGATATTCCCTTTTCCAACGAAATCATC

             *   *                *   *                    *        **       
      79589 AGAGCTATCCAAATATCCACTTACAGTTTCTACAAAAAGAGTGTTTCCAAACTGCTGCATCAAAA
       1036 AAAGCGATCCAAATATCCACTTCCAGATTCTACAAAAAGAGTGTTTCAAAACTGCTCTATCAAAA

              * *    *          *                   ***      *     *         
      79654 GAGAGGTTCCACTCTGTTAGCTGAGTACACACATCACAAACTTGTTTCTCAGAATCCTTCTGTCT
       1101 GAAATGTTCAACTCTGTTAGTTGAGTACACACATCACAAAGAAGTTTCTGAGAATGCTTCTGTCT

            *                   ** *    
      79719 CGTTTTTATGGGAAGATATTTACTTTTT
       1166 AGTTTTTATGGGAAGATATTCCCGTTTT

              *  * **          *       *     *       *  *  *                 
      79747 CACCGTAGGCATCAAAGCGCTCCAAATGTCCACATCCAGATACTCCAGAAAGAGTGTTTCAAACC
          1 CAACGAAATCATCAAAGCGATCCAAATATCCACTTCCAGATTCTACAAAAAGAGTGTTTCAAACC

                              *         *        * *  *      *      *        
      79812 TGCTCTATGAAAGGGAATCTTCAACTCTATGAGTTGAATGCAGACATCAGAAAGAAATTTCTGAG
         66 TGCTCTA-GAAAGGGAATGTTCAACTCTGTGAGTTGAGTACACACATCACAAAGAAGTTTCTGAG

                  *       **          *          *                 *    *    
      79877 AATGCTGCTGTCTACCTTTTAT-T--TGA-ATTCCCGCTTCCAACGAAATC-CTCCAAGCTATCC
        130 AATGCTTCTGTCTAGTTTTTATATGAAGATATTCCCGTTTCCAACGAAATCGCT-AAAGCGATCC

                        *       *                                  ** *  *   
      79937 AAATATCCACTTGCAGATTCCACAAAAAGAGTGTTTCAAAACTGCTCTCTATCAATGGCAAAGTT
        194 AAATATCCACTTCCAGATTCTACAAAAAGAGTGTTTCAAAACTG--CTCTATCAAAAGAAATGTT

                             *     *     *  *                         *      
      80002 CAACTCTGTTAGTTGAGGACACATATCACCAACAAGTTTCTGAGAATGCTTCTGTCTATTTTTTA
        257 CAACTCTGTTAGTTGAGTACACACATCACAAAGAAGTTTCTGAGAATGCTTCTGTCTAGTTTTTA

             **         *      *  *  * ** *    *      *    *          * **   
      80067 TGGGAAGATATTTCCTTTTTCACCGTAGGCGTCAAGGCGATCGAAATGTCCACTTCCACAAACTA
        322 TATGAAGATATTCCCTTTTCCAACGAAATCATCAAAGCGATCCAAATATCCACTTCCAGATTCTA

                              *             * **       *    * *      * ** * *
      80132 CAAAAAGAGTGTTTCAAACCTGCTCTATGAAAGGCCATGTTCATCTCTATGAGTTGAATGGAAAT
        387 CAAAAAGAGTGTTTCAAAACTGCTCTATGAAAAGAAATGTTCAACTCTGTTAGTTGAGTACACAC

               **      *      *       *         *         *         **       
      80197 ATCCGAAAGAAATTTCTGGGAATGCTGCTGTCTAGTGTTTAT---ACGA-ATTCCCGCTTCCAAC
        452 ATCACAAAGAAGTTTCTGAGAATGCTTCTGTCTAGTTTTTATGGGAAGATATTCCCTTTTCCAAC

                * *       *                *    *  *                         
      80258 GAAATCCTCAAAGCAATCCAAATATCCACTTGCAGAATCCACAAAAAGAGTGTTTCAAAACTGCT
        517 GAAAGCATCAAAGCGATCCAAATATCCACTTCCAGATTCTACAAAAAGAGTGTTTCAAAACTGCT

                   **    *          * *                   *  *               
      80323 CTATCAATAGAAAGGTTCAACTCTTTTAGTTGAGTACACACATCACGAACAAGTTTCTGAGAATG
        582 CTATCAAAGGAAATGTTCAACTCTGTGAGTTGAGTACACACATCACAAAGAAGTTTCTGAGAATG

                     * *          *  **  *         * *  **          *       *
      80388 CTTCTGTCTGGCTTTTATTGGAAGACGTTTCCTTTT-CACCAAAGGCATCAAAGCGCTCCAAATG
        647 CTTCTGTCTAGTTTTTA-T-GATGATATTCCCTTTTCCAACGAAATCATCAAAGCGATCCAAATA

                            * *                **      * ***    *            
      80452 TCCACTTCCAGATTCTTCCAAAAGAGTGTTTCAAACGTG-CTCAAAGTAAGGGAATGTTCAACTC
        710 TCCACTTCCAGATTCTACAAAAAGAGTGTTTCAAAACTGCCTCTATCAAAGGAAATGTTCAACTC

               * *    * *  * *     *   *       * * *             *   * ***  *
      80516 TGTGACTTGAATGCAGATATCACCAAGTAGTTTCTAATAGTGCTTCTGTCTAGATTTCAGATGAT
        775 TGTTAGTTGAGTACACACATCACAAAGAAGTTTCTGAGAATGCTTCTGTCTAGTTTTTATGGGAA

                     *              * * *   * **          *  *   *      *    
      80581 GATATTCCCGTTTCCAACGAAATCGTTAGAGCTAAGCAAATATCCAGTTACAGTTTCTACCAAAA
        840 GATATTCCCTTTTCCAACGAAATCATCAAAGCGATCCAAATATCCACTTCCAGATTCTACAAAAA

             *       *   *    **      *    *            *      ***  *        
      80646 GGGTGTTTCCAAATTGCTGCATCAAAAGAAAGGTTCAACTCTGTTAGTTGAGGACACACATCACA
        905 GAGTGTTTCAAAACTGCTCTATCAAAGGAAATGTTCAACTCTGTGAGTTGAATGCAGACATCACA

                     *                              *                    *   
      80711 AAGAAGTTTGTGAGAATGCTTCTGTCTAGATTTTGTATGACGATATTCCCTTTTCCAACGATATC
        970 AAGAAGTTTCTGAGAATGCTTCTGTCTAG-TTTT-TATGAAGATATTCCCTTTTCCAACGAAATC

            * *     *   *       * *  *    *  *       *  *        *       * * 
      80776 GTTAAAGCAATCTAAATATCAATTTGCAGAATCCACAAAAATAGAGTTTCAAAGCTGCTCTGTAA
       1033 ATCAAAGCGATCCAAATATCCACTTCCAGATTCTACAAAAAGAGTGTTTCAAAACTGCTCTATCA

                   *    *          *                   ***            *      
      80841 AAAGAAAGGTTCCACTCTGTTAGCTGAGTACACACATCACAAACTTGTTTCTGAGAATCCTTCTG
       1098 AAAGAAATGTTCAACTCTGTTAGTTGAGTACACACATCACAAAGAAGTTTCTGAGAATGCTTCTG

               *                   ** *   *
      80906 TCTCGTTTTTATGGGAAGATATTTACTTTTC
       1163 TCTAGTTTTTATGGGAAGATATTCCCGTTTT

              *  * **          *       *     *       *  *  *                 
      80937 CACCGTAGGCATCAAAGCGCTCCAAATGTCCACATCCAGATACTCCAGAAAGAGTGTTTCAAACC
          1 CAACGAAATCATCAAAGCGATCCAAATATCCACTTCCAGATTCTACAAAAAGAGTGTTTCAAACC

                              *         *        * *  *      *      *        
      81002 TGCTCTATGAAAGGGAATCTTCAACTCTATGAGTTGAATGCAGACATCAGAAAGAAATTTCTGAG
         66 TGCTCTA-GAAAGGGAATGTTCAACTCTGTGAGTTGAGTACACACATCACAAAGAAGTTTCTGAG

                  *       **          *          *                 *    *    
      81067 AATGCTGCTGTCTACCTTTTAT-T--TGA-ATTCCCGCTTCCAACGAAATC-CTCCAAGCTATCC
        130 AATGCTTCTGTCTAGTTTTTATATGAAGATATTCCCGTTTCCAACGAAATCGCT-AAAGCGATCC

                      * *  **   *                                *           
      81127 AAATATCCACCTGCATTTTCCACAAAAAGAGTGTTTCAAAACTGCTCTATCAATAGAAATGTTCA
        194 AAATATCCACTTCCAGATTCTACAAAAAGAGTGTTTCAAAACTGCTCTATCAAAAGAAATGTTCA

                **  * *  *                *           *                     *
      81192 ACTCCTTTGGCTGGGTACACACATCACAAACAAGTTTCTGAGCATGCTTCTGTCTAGTTTTTATG
        259 ACTCTGTTAGTTGAGTACACACATCACAAAGAAGTTTCTGAGAATGCTTCTGTCTAGTTTTTATA

            *     *          *  * *  **          *       *            **     
      81257 GGAAGACATTCCCTTTTTCACCAAAGGCATCAAAGCGCTCCAAATGTCCACTTCCAGACACTACA
        324 TGAAGATATTCCCTTTTCCAACGAAATCATCAAAGCGATCCAAATATCCACTTCCAGATTCTACA

                            **       *     **               * *    * *  * *  
      81322 AAAAGAGTGTTTCAAACGTGCTCTAAGAAAACGAATGTTCAACTCTGTGACTTGAATGCAGATAT
        389 AAAAGAGTGTTTCAAAACTGCTCTATGAAAAGAAATGTTCAACTCTGTTAGTTGAGTACACACAT

                *  *           **            *     ***  *         *          
      81387 CACAGAGTAGTTTCTGAGAGGGCTTCTGTCTAGATTTTAGATGATGATATTCCCGTTTCCAACGA
        454 CACAAAGAAGTTTCTGAGAATGCTTCTGTCTAGTTTTTATGGGAAGATATTCCCTTTTCCAACGA

              *   * *   *                *   *                    *        **
      81452 AATCATTAGAGCTATCCAAATATCCACTTACAGTTTCTACAAAAAGAGTGTTTCCAAACTGCTGC
        519 AAGCATCAAAGCGATCCAAATATCCACTTCCAGATTCTACAAAAAGAGTGTTTCAAAACTGCTCT

                  *  * *    *       *  *                   ***      *     *  
      81517 ATCAAAAGAGAGGTTCCACTCTGTTAGCTGAGTACACACATCACAAACTTGTTTCTCAGAATCCT
        584 ATCAAAGGAAATGTTCAACTCTGTGAGTTGAGTACACACATCACAAAGAAGTTTCTGAGAATGCT

                   *            *      **     *  *  * **          *       *  
      81582 TCTGTCTCGTTTTTATGGGAAGATATTTACTTTTTCACCGTAGGCATCAAAGCGCTCCAAATGTC
        649 TCTGTCTAGTTTTTAT--GATGATATTCCCTTTTCCAACGAAATCATCAAAGCGATCCAAATATC

               *       **    *       *       *          *     *   *         *
      81647 CACATCCAGATAGTACAGAAAGAGTTTTTCAAACCTG-CTCTATGAAAGGGAATCTTCAACTCTA
        712 CACTTCCAGATTCTACAAAAAGAGTGTTTCAAAACTGCCTCTATCAAAGGAAATGTTCAACTCTG

             *      * *  *      *      *              *       **         **  
      81711 TGAGTTGAATGCAGACATCAGAAAGAAATTTCTGAGAATGCTGCTGTCTACCTTTTAT---TTGA
        777 TTAGTTGAGTACACACATCACAAAGAAGTTTCTGAGAATGCTTCTGTCTAGTTTTTATGGGAAGA

                   **             *  *    *                *       *         
      81773 -ATTCCCGCTTCCAACGAAATCCTCCAAGCTATCCAAATATCCACTTGCAGATTCCACAAAAAGA
        842 TATTCCCTTTTCCAACGAAATCATCAAAGCGATCCAAATATCCACTTCCAGATTCTACAAAAAGA

                                     *  *  *            *      ***  * *     *
      81837 GTGTTTCAAAACTGCTCTCTATCAATGGCAAAGTTCAACTCTGTTAGTTGAGGACACATATCACC
        907 GTGTTTCAAAACTG--CTCTATCAAAGGAAATGTTCAACTCTGTGAGTTGAATGCAGACATCACA

              *                         *                  *      *  *  * ** 
      81902 AACAAGTTTCTGAGAATGCTTCTGTCTATTTTTTATGGGAAGATATTTCCTTTTTCACCGTAGGC
        970 AAGAAGTTTCTGAGAATGCTTCTGTCTAGTTTTTAT--GAAGATATTCCCTTTTCCAACGAAATC

            *    *      *    *          * **                     *         * 
      81967 GTCAAGGCGATCGAAATGTCCACTTCCACAAACTACAAAAAGAGTGTTTCAAACCTGCTCTATGA
       1033 ATCAAAGCGATCCAAATATCCACTTCCAGATTCTACAAAAAGAGTGTTTCAAAACTGCTCTATCA

              * **       *    * *      * ** * *   **      *      *       *   
      82032 AAGGCCATGTTCATCTCTATGAGTTGAATGGAAATATCCGAAAGAAATTTCTGGGAATGCTGCTG
       1098 AAAGAAATGTTCAACTCTGTTAGTTGAGTACACACATCACAAAGAAGTTTCTGAGAATGCTTCTG

                  *         *          *  *
      82097 TCTAGTGTTTAT---ACGA-ATTCCCGCTTC
       1163 TCTAGTTTTTATGGGAAGATATTCCCGTTTT

                      *       *                *    *  *                   * 
      82124 CAACGAAATCCTCAAAGCAATCCAAATATCCACTTGCAGAATCCACAAAAAGAGTGTTTCAAAAC
          1 CAACGAAATCATCAAAGCGATCCAAATATCCACTTCCAGATTCTACAAAAAGAGTGTTTCAAACC

                    *  ** *  *          * *                   *  *           
      82189 TGCTCTATCAATAGAAAGGTTCAACTCTTTTAGTTGAGTACACACATCACGAACAAGTTTCTGAG
         66 TGCTCTA-GAAAGGGAATGTTCAACTCTGTGAGTTGAGTACACACATCACAAAGAAGTTTCTGAG

                         * *      **     **  *      *    *    *           *  
      82254 AATGCTTCTGTCTGGCTTTTATTGGAAGACGTTTCC-TTTTC-ACCAAA-GGCATCAAAGCGCTC
        130 AATGCTTCTGTCTAGTTTTTATATGAAGATATTCCCGTTTCCAACGAAATCGC-T-AAAGCGATC

                 *                * *                **     * ***  * *       
      82316 CAAATGTCCACTTCCAGATTCTTCCAAAAGAGTGTTTCAAACGTGCTCAAAGTAAGGGAATGTTC
        193 CAAATATCCACTTCCAGATTCTACAAAAAGAGTGTTTCAAAACTGCTCTATCAAAAGAAATGTTC

                    * *    * *  * *     *   *       * * *             *   * *
      82381 AACTCTGTGACTTGAATGCAGATATCACCAAGTAGTTTCTAATAGTGCTTCTGTCTAGATTTCAG
        258 AACTCTGTTAGTTGAGTACACACATCACAAAGAAGTTTCTGAGAATGCTTCTGTCTAGTTTTTAT

                *         *              * * *   * **          *  *   *      
      82446 ATGATGATATTCCCGTTTCCAACGAAATCGTTAGAGCTAAGCAAATATCCAGTTACAGTTTCTAC
        323 ATGAAGATATTCCCTTTTCCAACGAAATCATCAAAGCGATCCAAATATCCACTTCCAGATTCTAC

            *     *       *   *    **  *        *                    *       
      82511 CAAAAGGGTGTTTCCAAATTGCTGCATCAAAAGAAAGGTTCAACTCTGTTAGTTGAGGACACACA
        388 AAAAAGAGTGTTTCAAAACTGCTCTATGAAAAGAAATGTTCAACTCTGTTAGTTGAGTACACACA

                          *                   *    * **  *                   
      82576 TCACAAAGAAGTTTGTGAGAATGCTTCTGTCTAGATTTTGTATGACGATATTCCCTTTTCCAACG
        453 TCACAAAGAAGTTTCTGAGAATGCTTCTGTCTAGTTTTTATGGGAAGATATTCCCTTTTCCAACG

             * * * *     *   *       * *  *    *  *       *  *        *      
      82641 ATATCGTTAAAGCAATCTAAATATCAATTTGCAGAATCCACAAAAATAGAGTTTCAAAGCTGCTC
        518 AAAGCATCAAAGCGATCCAAATATCCACTTCCAGATTCTACAAAAAGAGTGTTTCAAAACTGCTC

             * *   *    *    *       *  *                   ***            * 
      82706 TGTAAAAAGAAAGGTTCCACTCTGTTAGCTGAGTACACACATCACAAACTTGTTTCTGAGAATCC
        583 TATCAAAGGAAATGTTCAACTCTGTGAGTTGAGTACACACATCACAAAGAAGTTTCTGAGAATGC

                    *            *      **        *  * **          *       * 
      82771 TTCTGTCTCGTTTTTATGGGAAGATATTTACTTTTCCACCGTAGGCATCAAAGCGCTCCAAATGT
        648 TTCTGTCTAGTTTTTAT--GATGATATTCCCTTTTCCAACGAAATCATCAAAGCGATCCAAATAT

                *       *  *  *  *            *          *     *   *         
      82836 CCACATCCAGATACTCCAGAACGAGTGTTTCAAACCTG-CTCTATGAAAGGGAATCTTCAACTCT
        711 CCACTTCCAGATTCTACAAAAAGAGTGTTTCAAAACTGCCTCTATCAAAGGAAATGTTCAACTCT

            * *      * *  *      *      *              *       **         ** 
      82900 ATGAGTTGAATGCAGACATCAGAAAGAAATTTCTGAGAATGCTGCTGTCTACCTTTTAT---TTG
        776 GTTAGTTGAGTACACACATCACAAAGAAGTTTCTGAGAATGCTTCTGTCTAGTTTTTATGGGAAG

                    **             *  *    *              * *  **   *    *   
      82962 A-ATTCCCGCTTCCAACGAAATCCTCCAAGCTATCCAAATATCCACCTGCATTTTCCACAACAAG
        841 ATATTCCCTTTTCCAACGAAATCATCAAAGCGATCCAAATATCCACTTCCAGATTCTACAAAAAG

                                    **                *    *  ** *  *        
      83026 AGTGTTTCAAAACTGCTCTATCAATAGAAATGTTCAACTCCTTTG-GCTGGGTACACACATCACA
        906 AGTGTTTCAAAACTGCTCTATCAAAGGAAATGTTCAACT-CTGTGAGTTGAATGCAGACATCACA

              *                                        **         *  * *  ** 
      83090 AACAAGTTTCTGAGAATGCTTCTGTCTAGTTTTTATGGGAAGACGTTCCCTTTTTCACCAAAGGC
        970 AAGAAGTTTCTGAGAATGCTTCTGTCTAGTTTTTAT--GAAGATATTCCCTTTTCCAACGAAATC

                     *       *            **                     **       ** 
      83155 ATCAAAGCGCTCCAAATGTCCACTTCCAGACACTACAAAAAGAGTGTTTCAAACGTGCTCTAAGA
       1033 ATCAAAGCGATCCAAATATCCACTTCCAGATTCTACAAAAAGAGTGTTTCAAAACTGCTCTATCA

              ***               * *    * *  * *         *           **       
      83220 AAGCGAATGTTCAACTCTGTGACTTGAATGCAGATATCACAAAGTAGTTTCTGAGAGGGCTTCTG
       1098 AAAGAAATGTTCAACTCTGTTAGTTGAGTACACACATCACAAAGAAGTTTCTGAGAATGCTTCTG

                 *     ***  *             *
      83285 TCTAGATTTTAGATGATGATATTCCCGTTTC
       1163 TCTAGTTTTTATGGGAAGATATTCCCGTTTT

                        * *   *                *   *                    *  * 
      83316 CAACGAAATCATTAGAGCTATCCAAATATCCACTTACAGTTTCTACAAAAAGAGTGTTTCCAAAC
          1 CAACGAAATCATCAAAGCGATCCAAATATCCACTTCCAGATTCTACAAAAAGAGTGTTTCAAACC

                    **    *   *    *       *  *                   ***      * 
      83381 TGCTGCATCAAAAGAG-AGGTTCCACTCTGTTAGCTGAGTACACACATCACAAACTTGTTTCTCA
         66 TGCT-C-TAGAAAGGGAATGTTCAACTCTGTGAGTTGAGTACACACATCACAAAGAAGTTTCTGA

                *         *        **         ** *   *  *  * ** ***      *   
      83445 GAATCCTTCTGTCTCGTTTTTATGGGAAGATATTTACTTTTTCACCGTAGGCATCAAAGCGCTCC
        129 GAATGCTTCTGTCTAGTTTTTATATGAAGATATTCCCGTTTCCAACGAAATCGCTAAAGCGATCC

                *     *       *  *  *               *         *   * *   *    
      83510 AAATGTCCACATCCAGATACTCCAGAAAGAGTGTTTCAAACCTGCTCTATGAAAGGGAATCTTCA
        194 AAATATCCACTTCCAGATTCTACAAAAAGAGTGTTTCAAAACTGCTCTATCAAAAGAAATGTTCA

                 * *      * *  *      *      *              *       **       
      83575 ACTCTATGAGTTGAATGCAGACATCAGAAAGAAATTTCTGAGAATGCTGCTGTCTACCTTTTAT-
        259 ACTCTGTTAGTTGAGTACACACATCACAAAGAAGTTTCTGAGAATGCTTCTGTCTAGTTTTTATA

               *         **             *  *    *                *       *   
      83639 T--TGA-ATTCCCGCTTCCAACGAAATCCTCCAAGCTATCCAAATATCCACTTGCAGATTCCACA
        324 TGAAGATATTCCCTTTTCCAACGAAATCATCAAAGCGATCCAAATATCCACTTCCAGATTCTACA

                                        *  ** *  *                    *     *
      83701 AAAAGAGTGTTTCAAAACTGCTCTCTATCAATGGCAAAGTTCAACTCTGTTAGTTGAGGACACAT
        389 AAAAGAGTGTTTCAAAACTG--CTCTATGAAAAGAAATGTTCAACTCTGTTAGTTGAGTACACAC

                 *  *                         *                  *      *  * 
      83766 ATCACCAACAAGTTTCTGAGAATGCTTCTGTCTATTTTTTATGGGAAGATATTTCCTTTTTCACC
        452 ATCACAAAGAAGTTTCTGAGAATGCTTCTGTCTAGTTTTTATGGGAAGATATTCCCTTTTCCAAC

             * *  *    *      *    *          * **                     *     
      83831 GTAGGCGTCAAGGCGATCGAAATGTCCACTTCCACAAACTACAAAAAGAGTGTTTCAAACCTGCT
        517 GAAAGCATCAAAGCGATCCAAATATCCACTTCCAGATTCTACAAAAAGAGTGTTTCAAAACTGCT

                *     **       *    *        * ** * *   **      *      *     
      83896 CTATGAAAGGCCATGTTCATCTCTATGAGTTGAATGGAAATATCCGAAAGAAATTTCTGGGAATG
        582 CTATCAAAGGAAATGTTCAACTCTGTGAGTTGAGTACACACATCACAAAGAAGTTTCTGAGAATG

              *         *       *         **             *       *           
      83961 CTGCTGTCTAGTGTTTAT-ACGA-ATTCCCGCTTCCAACGAAATCCTCAAAGCAATCCAAATATC
        647 CTTCTGTCTAGTTTTTATGATGATATTCCCTTTTCCAACGAAATCATCAAAGCGATCCAAATATC

                 *    *  *                                 **    *          *
      84024 CACTTGCAGAATCCACAAAAAGAGTGTTTCAAAACTG-CTCTATCAATAGAAAGGTTCAACTCTT
        712 CACTTCCAGATTCTACAAAAAGAGTGTTTCAAAACTGCCTCTATCAAAGGAAATGTTCAACTCTG

                                 *  *                        * *      *      
      84088 TTAGTTGAGTACACACATCACGAACAAGTTTCTGAGAATGCTTCTGTCTGGCTTTTATTGGAAGA
        777 TTAGTTGAGTACACACATCACAAAGAAGTTTCTGAGAATGCTTCTGTCTAGTTTTTATGGGAAGA

            **  *         * *  **          *       *                * *      
      84153 CGTTTCCTTTT-CACCAAAGGCATCAAAGCGCTCCAAATGTCCACTTCCAGATTCTTCCAAAAGA
        842 TATTCCCTTTTCCAACGAAATCATCAAAGCGATCCAAATATCCACTTCCAGATTCTACAAAAAGA

                      **     * ***    *                 *           *     *  
      84217 GTGTTTCAAACGTGCTCAAAGTAAGGGAATGTTCAACTCTGTGACTTGAATGCAGATATCACCAA
        907 GTGTTTCAAAACTGCTCTATCAAAGGAAATGTTCAACTCTGTGAGTTGAATGCAGACATCACAAA

             *       * * *                  **    *         *              * 
      84282 GTAGTTTCTAATAGTGCTTCTGTCTAGATTTCAGATGATGATATTCCCGTTTCCAACGAAATCGT
        972 GAAGTTTCTGAGAATGCTTCTGTCTAG-TTT-TTATGAAGATATTCCCTTTTCCAACGAAATCAT

            * *   * **          *  *   *      *     *       *   *    **      
      84347 TAGAGCTAAGCAAATATCCAGTTACAGTTTCTACCAAAAGGGTGTTTCCAAATTGCTGCATCAAA
       1035 CAAAGCGATCCAAATATCCACTTCCAGATTCTACAAAAAGAGTGTTTCAAAACTGCTCTATCAAA

                 *                    *                     *                
      84412 AGAAAGGTTCAACTCTGTTAGTTGAGGACACACATCACAAAGAAGTTTGTGAGAATGCTTCTGTC
       1100 AGAAATGTTCAACTCTGTTAGTTGAGTACACACATCACAAAGAAGTTTCTGAGAATGCTTCTGTC

               *    * **  *         *   *
      84477 TAGATTTTGTATGACGATATTCCCTTTTC
       1165 TAGTTTTTATGGGAAGATATTCCCGTTTT

                  *   * *     *   *       * *  *    *  *       *  *        * 
      84506 CAACGATATCGTTAAAGCAATCTAAATATCAATTTGCAGAATCCACAAAAATAGCGTTTCAAAGC
          1 CAACGAAATCATCAAAGCGATCCAAATATCCACTTCCAGATTCTACAAAAAGAGTGTTTCAAACC

                   **   * *  *    *       *  *                   ***         
      84571 TGCTCTGTAAAAAGAAAGGTTCCACTCTGTTAGCTGAGTACACACATCACAAACTTGTTTCTGAG
         66 TGCTCT-AGAAAGGGAATGTTCAACTCTGTGAGTTGAGTACACACATCACAAAGAAGTTTCTGAG

               *         *        **         ** *      *  * ** ***      *    
      84636 AATCCTTCTGTCTCGTTTTTATGGGAAGATATTTACTTTTCCACCGTAGGCATCAAAGCGCTCCA
        130 AATGCTTCTGTCTAGTTTTTATATGAAGATATTCCCGTTTCCAACGAAATCGCTAAAGCGATCCA

               *     *       *  *  *  *            *         *   * *   *     
      84701 AATGTCCACATCCAGATACTCCAGAACGAGTGTTTCAAACCTGCTCTATGAAAGGGAATCTTCAA
        195 AATATCCACTTCCAGATTCTACAAAAAGAGTGTTTCAAAACTGCTCTATCAAAAGAAATGTTCAA

                * *      * *  *      *      *              *       **        
      84766 CTCTATGAGTTGAATGCAGACATCAGAAAGAAATTTCTGAGAATGCTGCTGTCTACCTTTTAT-T
        260 CTCTGTTAGTTGAGTACACACATCACAAAGAAGTTTCTGAGAATGCTTCTGTCTAGTTTTTATAT

              *         **             *  *    *              * *  **   *    
      84830 --TGA-ATTCCCGCTTCCAACGAAATCCTCCAAGCTATCCAAATATCCACCTGCATTTTCCACAA
        325 GAAGATATTCCCTTTTCCAACGAAATCATCAAAGCGATCCAAATATCCACTTCCAGATTCTACAA

            *                        *  *               **  * *  *           
      84892 CAAGAGTGTTTCAAAACTGCTCTATCAATAGAAATGTTCAACTCCTTTGGCTGGGTACACACATC
        390 AAAGAGTGTTTCAAAACTGCTCTATGAAAAGAAATGTTCAACTCTGTTAGTTGAGTACACACATC

                 *                                        **         *  * *  
      84957 ACAAACAAGTTTCTGAGAATGCTTCTGTCTAGTTTTTATGGGAAGACGTTCCCTTTTTCACCAAA
        455 ACAAAGAAGTTTCTGAGAATGCTTCTGTCTAGTTTTTATGGGAAGATATTCCCTTTTCCAACGAA

            *           *       *            **                     **       
      85022 GGCATCAAAGCGCTCCAAATGTCCACTTCCAGACACTACAAAAAGAGTGTTTCAAACGTGCTCTA
        520 AGCATCAAAGCGATCCAAATATCCACTTCCAGATTCTACAAAAAGAGTGTTTCAAAACTGCTCTA

            **    **                 *    * *  * *         *           **    
      85087 AGAAAGCGAATGTTCAACTCTGTGACTTGAATGCAGATATCACAAAGTAGTTTCTGAGAGGGCTT
        585 TCAAAGGAAATGTTCAACTCTGTGAGTTGAGTACACACATCACAAAGAAGTTTCTGAGAATGCTT

                          *              *                * *   *            
      85152 CTGTCTAGATTTTAGATGATGATATTCCCGTTTCCAACGAAATCATTAGAGCTATCCAAATATCC
        650 CTGTCTAG-TTTT-TATGATGATATTCCCTTTTCCAACGAAATCATCAAAGCGATCCAAATATCC

                *   *                    *         **      *  * *    *       
      85217 ACTTACAGTTTCTACAAAAAGAGTGTTTCCAAACTG-CTGCATCAAAAGAGAGGTTCCACTCTGT
        713 ACTTCCAGATTCTACAAAAAGAGTGTTTCAAAACTGCCTCTATCAAAGGAAATGTTCAACTCTGT

               **                  ***      *     *         *                
      85281 TAGCCGAGTACACACATCACAAACTTGTTTCTCAGAATCCTTCTGTCTCGTTTTTATGGGAAGAT
        778 TAGTTGAGTACACACATCACAAAGAAGTTTCTGAGAATGCTTCTGTCTAGTTTTTATGGGAAGAT

               **     *  *  * **          *       *     *       *  *  *      
      85346 ATTTACTTTTTCACCGTAGGCATCAAAGCGCTCCAAATGTCCACATCCAGATACTCCAGAAAGAG
        843 ATTCCCTTTTCCAACGAAATCATCAAAGCGATCCAAATATCCACTTCCAGATTCTACAAAAAGAG

                     *         *     *   *         *                    *    
      85411 TGTTTCAAACCTGCTCTATGAAAGGGAATCTTCAACTCTATGAGTTGAATGCAGACATCAGAAAG
        908 TGTTTCAAAACTGCTCTATCAAAGGAAATGTTCAACTCTGTGAGTTGAATGCAGACATCACAAAG

              *              *       **       **         **             *  * 
      85476 AAATTTCTGAGAATGCTGCTGTCTACCTTTTAT-TTGA-ATTCCCGCTTCCAACGAAATCCTCCA
        973 AAGTTTCTGAGAATGCTTCTGTCTAGTTTTTATGAAGATATTCCCTTTTCCAACGAAATCATCAA

               *                *       *                                ** *
      85539 AGCTATCCAAATATCCACTTGCAGATTCCACAAAAAGAGTGTTTCAAAACTG--CTATCAATGGC
       1038 AGCGATCCAAATATCCACTTCCAGATTCTACAAAAAGAGTGTTTCAAAACTGCTCTATCAAAAGA

              *                    *     *     *  *                         *
      85602 AAAGTTCAACTCTGTTAGTTGAGGACACATATCACCAACAAGTTTCTGAGAATGCTTCTGTCTAT
       1103 AATGTTCAACTCTGTTAGTTGAGTACACACATCACAAAGAAGTTTCTGAGAATGCTTCTGTCTAG

              *               *  *    
      85667 TTCTTATGGGAAGATATTTCCTTTTT
       1168 TTTTTATGGGAAGATATTCCCGTTTT

              *  * ** *    *      *    *          * **                       
      85693 CAGCGTAGGCGTCAAGGCGATCGAAATGTCCACTTCCACAAACTACAAAAAGAGTGTTTCAAACC
          1 CAACGAAATCATCAAAGCGATCCAAATATCCACTTCCAGATTCTACAAAAAGAGTGTTTCAAACC

                          **       *    *        * ** * *   **      *      * 
      85758 TGCTCTATGAAAGGCCATGTTCATCTCTATGAGTTGAATGGAAATATCCGAAAGAAATTTCTGGG
         66 TGCTCTA-GAAAGGGAATGTTCAACTCTGTGAGTTGAGTACACACATCACAAAGAAGTTTCTGAG

                  *          *        *          *                      *    
      85823 AATGCTGCTGTCTAG-TGTT-TAT-ACGA-ATTCCCGCTTCCAACGAAATC-CTCAAAGCAATCC
        130 AATGCTTCTGTCTAGTTTTTATATGAAGATATTCCCGTTTCCAACGAAATCGCT-AAAGCGATCC

                        *    *                                   *     *     
      85883 AAATATCCACTTGCAGAATCTACAAAAAGAGTGTTTCAAAACTGCTCTATCAATAGAAAGGTTCA
        194 AAATATCCACTTCCAGATTCTACAAAAAGAGTGTTTCAAAACTGCTCTATCAAAAGAAATGTTCA

             *   *                     *  *                        * *      *
      85948 AATCTTTTAGTTGAGTACACACATCACGAACAAGTTTCTGAGAATGCTTCTGTCTGGCTTTTATT
        259 ACTCTGTTAGTTGAGTACACACATCACAAAGAAGTTTCTGAGAATGCTTCTGTCTAGTTTTTATA

            *     **  *         * *  **        * *       *                  *
      86013 GGAAGACGTTTCCTTTT-CACCAAAGGCATCAAAGTGCTCCAAATGTCCACTTCCAGATTCTACC
        324 TGAAGATATTCCCTTTTCCAACGAAATCATCAAAGCGATCCAAATATCCACTTCCAGATTCTACA

                            **          *  * *               * *    * *  * * 
      86077 AAAAGAGTGTTTCAAACGTGCTC-ATAGTAAGGGAATGTTCAACTCTGTGACTTGAATGCAGATA
        389 AAAAGAGTGTTTCAAAACTGCTCTAT-GAAAAGAAATGTTCAACTCTGTTAGTTGAGTACACACA

                *   *       * * *             *     ***  *         *         
      86141 TCACCAAGTAGTTTCTAATAGTGCTTCTGTCTAGATTTTAGATGATGATATTCCCGTTTCCAACG
        453 TCACAAAGAAGTTTCTGAGAATGCTTCTGTCTAGTTTTTATGGGAAGATATTCCCTTTTCCAACG

               * *** *   *             *  *   *      *     *       *   *    *
      86206 AAATCGCTAGAGCTATCCAAATATCCAGTTACAGTTTCTACCAAAAGGGTGTTTCCAAATTGCTG
        518 AAAGCATCAAAGCGATCCAAATATCCACTTCCAGATTCTACAAAAAGAGTGTTTCAAAACTGCTC

            *      *    *            *       *                     *         
      86271 CATCAAAAGAAAGGTTCAACTCTGTTAGTTGAGGACACACATCACAAAGAAGTTTGTGAGAATGC
        583 TATCAAAGGAAATGTTCAACTCTGTGAGTTGAGTACACACATCACAAAGAAGTTTCTGAGAATGC

                                 *                    *   * *     *   *      
      86336 TTCTGTCTAGATTTTGTATGACGATATTCCCTTTTCCAACGATATCGTTAAAGCAATCTAAATAT
        648 TTCTGTCTAG-TTTT-TATGATGATATTCCCTTTTCCAACGAAATCATCAAAGCGATCCAAATAT

             * *  *    *  *       *  *        *        * *   *    *    *     
      86401 CAATTTGCAGAATCCACAAAAATAGAGTTTCAAAGCTG-CTCTGTAAAAAGAAAGGTTCCACTCT
        711 CCACTTCCAGATTCTACAAAAAGAGTGTTTCAAAACTGCCTCTATCAAAGGAAATGTTCAACTCT

                 *                   ***            *         *              
      86465 GTTAGCTGAGTACACACATCACAAACTTGTTTCTGAGAATCCTTCTGTCTCGTTTTTATGGGAAG
        776 GTTAGTTGAGTACACACATCACAAAGAAGTTTCTGAGAATGCTTCTGTCTAGTTTTTATGGGAAG

                 **        *  * **          *       *     *       *  *  *  * 
      86530 ATATTTACTTTTCCACCGTAGGCATCAAAGCGCTCCAAATGTCCACATCCAGATACTCCAGAACG
        841 ATATTCCCTTTTCCAACGAAATCATCAAAGCGATCCAAATATCCACTTCCAGATTCTACAAAAAG

                       *         *     *   *         *                    *  
      86595 AGTGTTTCAAACCTGCTCTATGAAAGGGAATCTTCAACTCTATGAGTTGAATGCAGACATCAGAA
        906 AGTGTTTCAAAACTGCTCTATCAAAGGAAATGTTCAACTCTGTGAGTTGAATGCAGACATCACAA

                *              *       **       **         **             *  
      86660 AGAAATTTCTGAGAATGCTGCTGTCTACCTTTTAT-TTGA-ATTCCCGCTTCCAACGAAATCCTC
        971 AGAAGTTTCTGAGAATGCTTCTGTCTAGTTTTTATGAAGATATTCCCTTTTCCAACGAAATCATC

            *    *              * *  **   *    *                           * 
      86723 CAAGCTATCCAAATATCCACCTGCATTTTCCACAACAAGAGTGTTTCAAAACTGCTCTATCAATA
       1036 AAAGCGATCCAAATATCCACTTCCAGATTCTACAAAAAGAGTGTTTCAAAACTGCTCTATCAAAA

                          **  * *  *                *                        
      86788 GAAATGTTCAACTCCTTTGGCTGGGTACACACATCACAAACAAGTTTCTGAGAATGCTTCTGTCT
       1101 GAAATGTTCAACTCTGTTAGTTGAGTACACACATCACAAAGAAGTTTCTGAGAATGCTTCTGTCT

                            **     *    
      86853 AGTTTTTATGGGAAGACGTTCCCTTTTT
       1166 AGTTTTTATGGGAAGATATTCCCGTTTT

              * *  **          *       *            **                  *   *
      86881 CACCAAAGGCATCAAAGCGCTCCAAATGTCCACTTCCAGACACTACAAAAAGAGTGTTTCCAACG
          1 CAACGAAATCATCAAAGCGATCCAAATATCCACTTCCAGATTCTACAAAAAGAGTGTTTCAAACC

               *         *                  *    * *  * *         *          
      86946 TGCCCTAAGAAAGCGAATGTTCAACTCTGTGACTTGAATGCAGATATCACAAAGTAGTTTCTGAG
         66 TGCTCT-AGAAAGGGAATGTTCAACTCTGTGAGTTGAGTACACACATCACAAAGAAGTTTCTGAG

             **            *     *    *                        **  *   *     
      87011 AGGGCTTCTGTCTAGATTTTAGATGATGATATTCCCGTTTCCAACGAAATCATTAGAGCTATCCA
        130 AATGCTTCTGTCTAGTTTTTATATGAAGATATTCCCGTTTCCAACGAAATCGCTAAAGCGATCCA

                       *   *                    *        **         * *    * 
      87076 AATATCCACTTACAGTTTCTACAAAAAGAGTGTTTCCAAACTGCTGCATCAAAAGAGAGGTTCCA
        195 AATATCCACTTCCAGATTCTACAAAAAGAGTGTTTCAAAACTGCTCTATCAAAAGAAATGTTCAA

                     *                   ***      *     *         *        **
      87141 CTCTGTTAGCTGAGTACACACATCACAAACTTGTTTCTCAGAATCCTTCTGTCTCGTTTTTATGG
        260 CTCTGTTAGTTGAGTACACACATCACAAAGAAGTTTCTGAGAATGCTTCTGTCTAGTTTTTATAT

                     **     *  *  * **          *       *     *       *  *  *
      87206 GAAGATATTTACTTTTTCACCGTAGGCATCAAAGCGCTCCAAATGTCCACATCCAGATACTCCAG
        325 GAAGATATTCCCTTTTCCAACGAAATCATCAAAGCGATCCAAATATCCACTTCCAGATTCTACAA

                           *             * *   *         * *      * *  *     
      87271 AAAGAGTGTTTCAAACCTGCTCTATGAAAGGGAATCTTCAACTCTATGAGTTGAATGCAGACATC
        390 AAAGAGTGTTTCAAAACTGCTCTATGAAAAGAAATGTTCAACTCTGTTAGTTGAGTACACACATC

             *      *              *       **         **         **          
      87336 AGAAAGAAATTTCTGAGAATGCTGCTGTCTACCTTTTAT---TTGA-ATTCCCGCTTCCAACGAA
        455 ACAAAGAAGTTTCTGAGAATGCTTCTGTCTAGTTTTTATGGGAAGATATTCCCTTTTCCAACGAA

             * *  *    *                *       *                            
      87397 ATCCTCCAAGCTATCCAAATATCCACTTGCAGATTCCACAAAAAGAGTGTTTCAAAACTGCTCTC
        520 AGCATCAAAGCGATCCAAATATCCACTTCCAGATTCTACAAAAAGAGTGTTTCAAAACTG--CTC

                  *  *  *            *       *     *     *  *                
      87462 TATCAATGGCAAAGTTCAACTCTGTTAGTTGAGGACACATATCACCAACAAGTTTCTGAGAATGC
        583 TATCAAAGGAAATGTTCAACTCTGTGAGTTGAGTACACACATCACAAAGAAGTTTCTGAGAATGC

                     *           *      *      *  *  * ** *    *      *    * 
      87527 TTCTGTCTATTTTTTATGGGAAGATATTTCCTTTTTCACCGTAGGCGTCAAGGCGATCGAAATGT
        648 TTCTGTCTAGTTTTTAT--GATGATATTCCCTTTTCCAACGAAATCATCAAAGCGATCCAAATAT

                     * **          *          *          *     **       *    
      87592 CCACTTCCACAAACTACAAAAAGTGTGTTTCAAACCTG-CTCTATGAAAGGCCATGTTCATCTCT
        711 CCACTTCCAGATTCTACAAAAAGAGTGTTTCAAAACTGCCTCTATCAAAGGAAATGTTCAACTCT

            * *      * ** * *   **      *      *       *         *         * 
      87656 ATGAGTTGAATGGAAATATCCGAAAGAAATTTCTGGGAATGCTGCTGTCTAGTGTTTAT---ACG
        776 GTTAGTTGAGTACACACATCACAAAGAAGTTTCTGAGAATGCTTCTGTCTAGTTTTTATGGGAAG

                    **             *       *                *    *  *        
      87718 A-ATTCCCGCTTCCAACGAAATCCTCAAAGCAATCCAAATATCCACTTGCAGAATCCACAAAAAG
        841 ATATTCCCTTTTCCAACGAAATCATCAAAGCGATCCAAATATCCACTTCCAGATTCTACAAAAAG

                                    **    *          * *      * *  *       * 
      87782 AGTGTTTCAAAACTGCTCTATCAATAGAAAGGTTCAACTCTTTTAGTTGAGTACACACATCACGA
        906 AGTGTTTCAAAACTGCTCTATCAAAGGAAATGTTCAACTCTGTGAGTTGAATGCAGACATCACAA

             *                        * *             **  *         * *  **  
      87847 ACAAGTTTCTGAGAATGCTTCTGTCTGGCTTTTATTGGAAGACGTTTCCTTTT-CACCAAAGGCA
        971 AGAAGTTTCTGAGAATGCTTCTGTCTAGTTTTTA-T-GAAGATATTCCCTTTTCCAACGAAATCA

                    *       *                * *                **     * *** 
      87911 TCAAAGCGCTCCAAATGTCCACTTCCAGATTCTTCCAAAAGAGTGTTTCAAACGTGCTCAAAGTA
       1034 TCAAAGCGATCCAAATATCCACTTCCAGATTCTACAAAAAGAGTGTTTCAAAACTGCTCTATCAA

             * *               * *    * *  * *     *   *       * * *         
      87976 AGGGAATGTTCAACTCTGTGACTTGAATGCAGATATCACCAAGTAGTTTCTAATAGTGCTTCTGT
       1099 AAGAAATGTTCAACTCTGTTAGTTGAGTACACACATCACAAAGAAGTTTCTGAGAATGCTTCTGT

                *     ***  *             *
      88041 CTAGATTTTAGATGATGATATTCCCGTTTC
       1164 CTAGTTTTTATGGGAAGATATTCCCGTTTT

                 *    * * *   *          *  *  *   *      *     *       *  **
      88071 CAACGGAATCGTTAGAGCTATCCAAATATACAGTTACAGTTTCTACCAAAAGGGTGTTTCCAAAT
          1 CAACGAAATCATCAAAGCGATCCAAATATCCACTTCCAGATTCTACAAAAAGAGTGTTTCAAACC

                  ***   * *  *            *       *                     *    
      88136 TGCTGCATCAAAAGAAAGGTTCAACTCTGTTAGTTGAGGACACACATCACAAAGAAGTTTGTGAG
         66 TGCT-CTAGAAAGGGAATGTTCAACTCTGTGAGTTGAGTACACACATCACAAAGAAGTTTCTGAG

                           *    *     *         *          *    *      *   * 
      88201 AATGCTTCTGTCTAGATTTTGTATGACGATATTCCCTTTTCCAACGATATCGTTAAAGCAATCTA
        130 AATGCTTCTGTCTAGTTTTTATATGAAGATATTCCCGTTTCCAACGAAATCGCTAAAGCGATCCA

                  * *  *    *  *       *  *        *       * *        *    * 
      88266 AATATCAATTTGCAGAATCCACAAAAATAGAGTTTCAAAGCTGCTCTGTAAAAAGAAAGGTTCCA
        195 AATATCCACTTCCAGATTCTACAAAAAGAGTGTTTCAAAACTGCTCTATCAAAAGAAATGTTCAA

                     *                   *** *          *         *        **
      88331 CTCTGTTAGCTGAGTACACACATCACAAACTTGATTCTGAGAATCCTTCTGTCTCGTTTTTATGG
        260 CTCTGTTAGTTGAGTACACACATCACAAAGAAGTTTCTGAGAATGCTTCTGTCTAGTTTTTATAT

                     **        *  * **          *       *     *       *  *  *
      88396 GAAGATATTTACTTTTCCACCGTAGGCATCAAAGCGCTCCAAATGTCCACATCCAGATACTCCAG
        325 GAAGATATTCCCTTTTCCAACGAAATCATCAAAGCGATCCAAATATCCACTTCCAGATTCTACAA

              *            *             * *   *         * *      * *  *     
      88461 AACGAGTGTTTCAAACCTGCTCTATGAAAGGGAATCTTCAACTCTATGAGTTGAATGCAGACATC
        390 AAAGAGTGTTTCAAAACTGCTCTATGAAAAGAAATGTTCAACTCTGTTAGTTGAGTACACACATC

             *      *              *       **         **         **          
      88526 AGAAAGAAATTTCTGAGAATGCTGCTGTCTACCTTTTAT---TTGA-ATTCCCGCTTCCAACGAA
        455 ACAAAGAAGTTTCTGAGAATGCTTCTGTCTAGTTTTTATGGGAAGATATTCCCTTTTCCAACGAA

             * *  *    *              * *  **   *    *                       
      88587 ATCCTCCAAGCTATCCAAATATCCACCTGCATTTTCCACAACAAGAGTGTTTCAAAACTGCTCTA
        520 AGCATCAAAGCGATCCAAATATCCACTTCCAGATTCTACAAAAAGAGTGTTTCAAAACTGCTCTA

                **                *    *  *                *                 
      88652 TCAATAGAAATGTTCAACTCCTTTG-GCTGGGTACACACATCACAAACAAGTTTCTGAGAATGCT
        585 TCAAAGGAAATGTTCAACT-CTGTGAGTTGAGTACACACATCACAAAGAAGTTTCTGAGAATGCT

                                *  **         *  * *  **          *       *  
      88716 TCTGTCTAGTTTTTATGGGAAGACGTTCCCTTTTTCACCAAAGGCATCAAAGCGCTCCAAATGTC
        649 TCTGTCTAGTTTTTAT--GATGATATTCCCTTTTCCAACGAAATCATCAAAGCGATCCAAATATC

                      **                  *  **        **    **              
      88781 CACTTCCAGACACTACAAAAAGAGTGTTTCCAACGTGCC-CTAAGAAAGCGAATGTTCAACTCTG
        712 CACTTCCAGATTCTACAAAAAGAGTGTTTCAAAACTGCCTCTATCAAAGGAAATGTTCAACTCTG

             * *    * *  * *         *           **            *     ***  *  
      88845 TGACTTGAATGCAGATATCACAAAGTAGTTTCTGAGAGGGCTTCTGTCTAGATTTTAGATGATGA
        777 TTAGTTGAGTACACACATCACAAAGAAGTTTCTGAGAATGCTTCTGTCTAGTTTTTATGGGAAGA

                   *                * *   *                *   *             
      88910 TATTCCCGTTTCCAACGAAATCATTAGAGCTATCCAAATATCCACTTACAGTTTCTACAAAAAGA
        842 TATTCCCTTTTCCAACGAAATCATCAAAGCGATCCAAATATCCACTTCCAGATTCTACAAAAAGA

                   *        **      *  * *    *       *  *   * *  *          
      88975 GTGTTTCCAAACTGCTGCATCAAAAGAGAGGTTCCACTCTGTTAGCTGAGTACACACATCACAAA
        907 GTGTTTCAAAACTGCTCTATCAAAGGAAATGTTCAACTCTGTGAGTTGAATGCAGACATCACAAA

            ***      *     *         *                   **     *  *  * **   
      89040 CTTGTTTCTCAGAATCCTTCTGTCTCGTTTTTATGGGAAGATATTTACTTTTTCACCGTAGGCAT
        972 GAAGTTTCTGAGAATGCTTCTGTCTAGTTTTTAT--GAAGATATTCCCTTTTCCAACGAAATCAT

                   *       *     *       *  *  *               *         *   
      89105 CAAAGCGCTCCAAATGTCCACATCCAGATACTCCAGAAAGAGTGTTTCAAACCTGCTCTATGAAA
       1035 CAAAGCGATCCAAATATCCACTTCCAGATTCTACAAAAAGAGTGTTTCAAAACTGCTCTATCAAA

            * *   *         * *      * *  *      *      *              *     
      89170 GGGAATCTTCAACTCTATGAGTTGAATGCAGACATCAGAAAGAAATTTCTGAGAATGCTGCTGTC
       1100 AGAAATGTTCAACTCTGTTAGTTGAGTACACACATCACAAAGAAGTTTCTGAGAATGCTTCTGTC

              **         **          *  *
      89235 TACCTTTTAT---TTGA-ATTCCCGCTTC
       1165 TAGTTTTTATGGGAAGATATTCCCGTTTT

                      *  *    *                *       *                   * 
      89260 CAACGAAATCCTCCAAGCTATCCAAATATCCACTTGCAGATTCCACAAAAAGAGTGTTTCAAAAC
          1 CAACGAAATCATCAAAGCGATCCAAATATCCACTTCCAGATTCTACAAAAAGAGTGTTTCAAACC

                      *  *  *  *            *       *     *     *  *         
      89325 TGCTCTCTATCAATGGCAAAGTTCAACTCTGTTAGTTGAGGACACATATCACCAACAAGTTTCTG
         66 TG--CTCTA-GAAAGGGAATGTTCAACTCTGTGAGTTGAGTACACACATCACAAAGAAGTTTCTG

                            *       **         *  *   *  *  * **  **  *      
      89390 AGAATGCTTCTGTCTATTTTTTATGGGAAGATATTTCCTTTTTCACCGTAGGCGTCAAGGCGATC
        128 AGAATGCTTCTGTCTAGTTTTTATATGAAGATATTCCCGTTTCCAACGAAATCGCTAAAGCGATC

            *    *          * **          *          *         *   * **      
      89455 GAAATGTCCACTTCCACAAACTACAAAAAGTGTGTTTCAAACCTGCTCTATGAAAGGCCATGTTC
        193 CAAATATCCACTTCCAGATTCTACAAAAAGAGTGTTTCAAAACTGCTCTATCAAAAGAAATGTTC

             *    * *      * ** * *   **      *      *       *          *    
      89520 ATCTCTATGAGTTGAATGGAAATATCCGAAAGAAATTTCTGGGAATGCTGCTGTCTAG-TGTT-T
        258 AACTCTGTTAGTTGAGTACACACATCACAAAGAAGTTTCTGAGAATGCTTCTGTCTAGTTTTTAT

                *         **             *       *                *    *  *  
      89583 AT-ACGA-ATTCCCGCTTCCAACGAAATCCTCAAAGCAATCCAAATATCCACTTGCAGAATCCAC
        323 ATGAAGATATTCCCTTTTCCAACGAAATCATCAAAGCGATCCAAATATCCACTTCCAGATTCTAC

                                       *  *     *          *                 
      89646 AAAAAGAGTGTTTCAAAACTGCTCTATCAATAGAAAGGTTCAACTCTTTTAGTTGAGTACACACA
        388 AAAAAGAGTGTTTCAAAACTGCTCTATGAAAAGAAATGTTCAACTCTGTTAGTTGAGTACACACA

                *  *                        * *      *      **  *         * *
      89711 TCACGAACAAGTTTCTGAGAATGCTTCTGTCTGGCTTTTATTGGAAGACGTTTCCTTTT-CACCA
        453 TCACAAAGAAGTTTCTGAGAATGCTTCTGTCTAGTTTTTATGGGAAGATATTCCCTTTTCCAACG

              *           *       *                * *                **     
      89775 AAGGCATCAAAGCGCTCCAAATGTCCACTTCCAGATTCTTCCAAAAGAGTGTTTCAAACGTGCTC
        518 AAAGCATCAAAGCGATCCAAATATCCACTTCCAGATTCTACAAAAAGAGTGTTTCAAAACTGCTC

            * ***    *                 *    * *  * *     *   *       * * *   
      89840 AAAGTAAGGGAATGTTCAACTCTGTGACTTGAATGCAGATATCACCAAGTAGTTTCTAATAGTGC
        583 TATCAAAGGAAATGTTCAACTCTGTGAGTTGAGTACACACATCACAAAGAAGTTTCTGAGAATGC

                            *              *         *    * * *   *          
      89905 TTCTGTCTAGATTTTAGATGATGATATTCCCGTTTCCAACGGAATCGTTAGAGCTATCCAAATAT
        648 TTCTGTCTAG-TTTT-TATGATGATATTCCCTTTTCCAACGAAATCATCAAAGCGATCCAAATAT

            *  *  *   *      *     *       *   *     **      *    *          
      89970 ACAGTTACAGTTTCTACCAAAAGGGTGTTTCCAAATTG-CTGCATCAAAAGAAAGGTTCAACTCT
        711 CCACTTCCAGATTCTACAAAAAGAGTGTTTCAAAACTGCCTCTATCAAAGGAAATGTTCAACTCT

                      *                     *                  **    * **  * 
      90034 GTTAGTTGAGGACACACATCACAAAGAAGTTTGTGAGAATGCTTCTGTCTACATTTTGTATGACG
        776 GTTAGTTGAGTACACACATCACAAAGAAGTTTCTGAGAATGCTTCTGTCTAGTTTTTATGGGAAG

                               *   * *     *   *       * *  *    *  *       *
      90099 ATATTCCCTTTTCCAACGATATCGTTAAAGCAATCTAAATATCAATTTGCAGAATCCACAAAAAT
        841 ATATTCCCTTTTCCAACGAAATCATCAAAGCGATCCAAATATCCACTTCCAGATTCTACAAAAAG

              *        *       * *   *    *    *       *  *   * *  *         
      90164 AGAGTTTCAAAGCTGCTCTGTAAAAAGAAAGGTTCCACTCTGTTAGCTGAGTACACACATCACAA
        906 AGTGTTTCAAAACTGCTCTATCAAAGGAAATGTTCAACTCTGTGAGTTGAATGCAGACATCACAA

             *** *          *         *                   **        *  * **  
      90229 ACTTGATTCTGAGAATCCTTCTGTCTCGTTTTTATGGGAAGATATTTACTTTTCCACCGTAGGCA
        971 AGAAGTTTCTGAGAATGCTTCTGTCTAGTTTTTAT--GAAGATATTCCCTTTTCCAACGAAATCA

                    *       *     *       *  *  *  *            *         *  
      90294 TCAAAGCGCTCCAAATGTCCACATCCAGATACTCCAGAACGAGTGTTTCAAACCTGCTCTATGAA
       1034 TCAAAGCGATCCAAATATCCACTTCCAGATTCTACAAAAAGAGTGTTTCAAAACTGCTCTATCAA

             * *   *         * *      * *  *      *      *              *    
      90359 AGGGAATCTTCAACTCTATGAGTTGAATGCAGACATCAGAAAGAAATTTCTGAGAATGCTGCTGT
       1099 AAGAAATGTTCAACTCTGTTAGTTGAGTACACACATCACAAAGAAGTTTCTGAGAATGCTTCTGT

               **         **          *  *
      90424 CTACCTTTTAT---TTGA-ATTCCCGCTTC
       1164 CTAGTTTTTATGGGAAGATATTCCCGTTTT

                      *  *    *              * *  **   *    *              * 
      90450 CAACGAAATCCTCCAAGCTATCCAAATATCCACCTGCATTTTCCACAACAAGAGTGTTTCAAAAC
          1 CAACGAAATCATCAAAGCGATCCAAATATCCACTTCCAGATTCTACAAAAAGAGTGTTTCAAACC

                    *  ** *              *    *  *                *          
      90515 TGCTCTATCAATAGAAATGTTCAACTCCTTTG-GCTGGGTACACACATCACAAACAAGTTTCTGA
         66 TGCTCTA-GAAAGGGAATGTTCAACT-CTGTGAGTTGAGTACACACATCACAAAGAAGTTTCTGA

                                   **     **     *   *    *    *           * 
      90579 GAATGCTTCTGTCTAGTTTTTATGGGAAGACGTTCCCTTTTTC-ACCAAA-GGCATCAAAGCGCT
        129 GAATGCTTCTGTCTAGTTTTTATATGAAGATATTCCCGTTTCCAACGAAATCGC-T-AAAGCGAT

                  *            **                  *  **   *   **   ***      
      90642 CCAAATGTCCACTTCCAGACACTACAAAAAGAGTGTTTCCAACGTGCCCTAAGAAAGCGAATGTT
        192 CCAAATATCCACTTCCAGATTCTACAAAAAGAGTGTTTCAAAACTGCTCTATCAAAAGAAATGTT

                     * *    * *  * *         *           **            *     
      90707 CAACTCTGTGACTTGAATGCAGATATCACAAAGTAGTTTCTGAGAGGGCTTCTGTCTAGATTTTA
        257 CAACTCTGTTAGTTGAGTACACACATCACAAAGAAGTTTCTGAGAATGCTTCTGTCTAGTTTTTA

            *    *         *                * *   *                *   *     
      90772 GATGATGATATTCCCGTTTCCAACGAAATCATTAGAGCTATCCAAATATCCACTTACAGTTTCTA
        322 TATGAAGATATTCCCTTTTCCAACGAAATCATCAAAGCGATCCAAATATCCACTTCCAGATTCTA

                           *        **  *      * *    *          *           
      90837 CAAAAAGAGTGTTTCCAAACTGCTGCATCAAAAGAGAGGTTCCACTCTGTTAGCTGAGTACACAC
        387 CAAAAAGAGTGTTTCAAAACTGCTCTATGAAAAGAAATGTTCAACTCTGTTAGTTGAGTACACAC

                    ***      *     *         *                   **     *  * 
      90902 ATCACAAACTTGTTTCTCAGAATCCTTCTGTCTCGTTTTTATGGGAAGATATTTACTTTTTCACC
        452 ATCACAAAGAAGTTTCTGAGAATGCTTCTGTCTAGTTTTTATGGGAAGATATTCCCTTTTCCAAC

             * *           *       *     *       *  *  *               *     
      90967 GTAGGCATCAAAGCGCTCCAAATGTCCACATCCAGATACTCCAGAAAGAGTGTTTCAAACCTGCT
        517 GAAAGCATCAAAGCGATCCAAATATCCACTTCCAGATTCTACAAAAAGAGTGTTTCAAAACTGCT

                *     *   *         *        * *  *      *      *            
      91032 CTATGAAAGGGAATCTTCAACTCTATGAGTTGAATGCAGACATCAGAAAGAAATTTCTGAGAATG
        582 CTATCAAAGGAAATGTTCAACTCTGTGAGTTGAGTACACACATCACAAAGAAGTTTCTGAGAATG

              *       **       *          **             *  *    *           
      91097 CTGCTGTCTACCTTTTAT-TTGA-ATTCCCGCTTCCAACGAAATCCTCCAAGCTATCCAAATATC
        647 CTTCTGTCTAGTTTTTATGATGATATTCCCTTTTCCAACGAAATCATCAAAGCGATCCAAATATC

                 *       *                                  *  *  *          
      91160 CACTTGCAGATTCCACAAAAAGAGTGTTTCAAAACTGCTCTCTATCAATGGCAAAGTTCAACTCT
        712 CACTTCCAGATTCTACAAAAAGAGTGTTTCAAAACTGC-CTCTATCAAAGGAAATGTTCAACTCT

                      *     *     *  *              *         *              
      91225 GTTAGTTGAGGACACATATCACCAACAAGTTTCTGAGAATCCTTCTGTCTCGTTTTTATGGGAAG
        776 GTTAGTTGAGTACACACATCACAAAGAAGTTTCTGAGAATGCTTCTGTCTAGTTTTTATGGGAAG

                 **        *  * **          *       *     *       *  *  *  * 
      91290 ATATTTACTTTTCCACCGTAGGCATCAAAGCGCTCCAAATGTCCACATCCAGATACTCCAGAACG
        841 ATATTCCCTTTTCCAACGAAATCATCAAAGCGATCCAAATATCCACTTCCAGATTCTACAAAAAG

                       *         *     **       *    *           * * *   **  
      91355 AGTGTTTCAAACCTGCTCTATGAAAGGCCATGTTCATCTCTATGAGTTGAATGGAAATATCCGAA
        906 AGTGTTTCAAAACTGCTCTATCAAAGGAAATGTTCAACTCTGTGAGTTGAATGCAGACATCACAA

                *      *       *         *       *         **             *  
      91420 AGAAATTTCTGGGAATGCTGCTGTCTAGTGTTTAT-ACGA-ATTCCCGCTTCCAACGAAATCCTC
        971 AGAAGTTTCTGAGAATGCTTCTGTCTAGTTTTTATGAAGATATTCCCTTTTCCAACGAAATCATC

                 *                *    *  *                     *          * 
      91483 AAAGCAATCCAAATATCCACTTGCAGAATCCACAAAAAGAGTGTTTCAAAACCGCTCTATCAATA
       1036 AAAGCGATCCAAATATCCACTTCCAGATTCTACAAAAAGAGTGTTTCAAAACTGCTCTATCAAAA

                *          *                        *                        
      91548 GAAAGGTTCAACTCTTTTAGTTGAGTACACACATCACAAACAAGTTTCTGAGAATGCTTCTGTCT
       1101 GAAATGTTCAACTCTGTTAGTTGAGTACACACATCACAAAGAAGTTTCTGAGAATGCTTCTGTCT

            * *      *      **  *       
      91613 GGCTTTTATTGGAAGACGTTTCC-TTTT
       1166 AGTTTTTATGGGAAGATATTCCCGTTTT

              * *  **          *       *                * *                 *
      91640 CACCAAAGGCATCAAAGCGCTCCAAATGTCCACTTCCAGATTCTTCCAAAAGAGTGTTTCAAACG
          1 CAACGAAATCATCAAAGCGATCCAAATATCCACTTCCAGATTCTACAAAAAGAGTGTTTCAAACC

              *   *  *                      *    * *  * *     *   *       * *
      91705 TGGTCGAAGTAAGGGAATGTTCAACTCTGTGACTTGAATGCAGATATCACCAAGTAGTTTCTAAT
         66 TGCTC-TAGAAAGGGAATGTTCAACTCTGTGAGTTGAGTACACACATCACAAAGAAGTTTCTGAG

             *             *     *    *                         *  *   *     
      91770 AGTGCTTCTGTCTAGATTTTAGATGATGATATTCCCGTTTCCAACGAAATCGTTAGAGCTATCCA
        130 AATGCTTCTGTCTAGTTTTTATATGAAGATATTCCCGTTTCCAACGAAATCGCTAAAGCGATCCA

                    *  *   *      *     *       *   *    **           *      
      91835 AATATCCAGTTACAGTTTCTACCAAAAGGGTGTTTCCAAATTGCTGCATCAAAAGAAAGGTTCAA
        195 AATATCCACTTCCAGATTCTACAAAAAGAGTGTTTCAAAACTGCTCTATCAAAAGAAATGTTCAA

                          *                     *                   *    *   
      91900 CTCTGTTAGTTGAGGACACACATCACAAAGAAGTTTGTGAGAATGCTTCTGTCTAGATTTTGTAT
        260 CTCTGTTAGTTGAGTACACACATCACAAAGAAGTTTCTGAGAATGCTTCTGTCTAGTTTTTATAT

              *                    *   * *     *   *       * *  *    *  *    
      91965 GACGATATTCCCTTTTCCAACGATATCGTTAAAGCAATCTAAATATCAATTTGCAGAATCCACAA
        325 GAAGATATTCCCTTTTCCAACGAAATCATCAAAGCGATCCAAATATCCACTTCCAGATTCTACAA

               *  *        *       * *        *    *          *              
      92030 AAATAGAGTTTCAAAGCTGCTCTGTAAAAAGAAAGGTTCCACTCTGTTAGCTGAGTACACACATC
        390 AAAGAGTGTTTCAAAACTGCTCTATGAAAAGAAATGTTCAACTCTGTTAGTTGAGTACACACATC

                 ***           **         *                   **        ** * 
      92095 ACAAACTTGTTTCTGAGAAACCTTCTGTCTCGTTTTTATGGGAAGATATTTACTTTTCCACTGTA
        455 ACAAAGAAGTTTCTGAGAATGCTTCTGTCTAGTTTTTATGGGAAGATATTCCCTTTTCCAACGAA

            *           *       *     *       *  *  *  *            *        
      92160 GGCATCAAAGCGCTCCAAATGTCCACATCCAGATACTCCAGAACGAGTGTTTCAAACCTGCTCTA
        520 AGCATCAAAGCGATCCAAATATCCACTTCCAGATTCTACAAAAAGAGTGTTTCAAAACTGCTCTA

             *     *   *         *        * *  *      *      *              *
      92225 TGAAAGGGAATCTTCAACTCTATGAGTTGAATGCAGA-ATCAGAAAGAAATTTCTGAGAATGCTG
        585 TCAAAGGAAATGTTCAACTCTGTGAGTTGAGTACACACATCACAAAGAAGTTTCTGAGAATGCTT

                   **       *          **             *  *    *              
      92289 CTGTCTACCTTTTAT-TTGA-ATTCCCGCTTCCAACGAAATCCTCCAAGCTATCCAAATATCCAC
        650 CTGTCTAGTTTTTATGATGATATTCCCTTTTCCAACGAAATCATCAAAGCGATCCAAATATCCAC

            * *  **   *    *                            **              **  *
      92352 CTGCATTTTCCACAACAAGAGTGTTTCAAAACTG-CTCTATCAATAGAAATGTTCAACTCCTTTG
        715 TTCCAGATTCTACAAAAAGAGTGTTTCAAAACTGCCTCTATCAAAGGAAATGTTCAACTCTGTTA

             *  *                *                                        ** 
      92416 GCTGGGTACACACATCACAAACAAGTTTCTGAGAATGCTTCTGTCTAGTTTTTATGGGAAGACGT
        780 GTTGAGTACACACATCACAAAGAAGTTTCTGAGAATGCTTCTGTCTAGTTTTTATGGGAAGATAT

                    *  * *  **          *       *            **              
      92481 TCCCTTTTTCACCAAAGGCATCAAAGCGCTCCAAATGTCCACTTCCAGACACTACAAAAAGAGTG
        845 TCCCTTTTCCAACGAAATCATCAAAGCGATCCAAATATCCACTTCCAGATTCTACAAAAAGAGTG

                   **       **   ***                 *           *         * 
      92546 TTTCAAACGTGCTCTAAGAAAACGAATGTTCAACTCTGTGACTTGAATGCAGATATCACAAAGTA
        910 TTTCAAAACTGCTCTATCAAAGGAAATGTTCAACTCTGTGAGTTGAATGCAGACATCACAAAGAA

                      **                  *    *         *                * *
      92611 GTTTCTGAGAGGGCTTCTGTCTAGATTTTAGATGATGATATTCCCGTTTCCAACGAAATCATTAG
        975 GTTTCTGAGAATGCTTCTGTCTAG-TTTT-TATGAAGATATTCCCTTTTCCAACGAAATCATCAA

               *                *   *                    *        **         
      92676 AGCTATCCAAATATCCACTTACAGTTTCTACAAAAAGAGTGTTTCCAAACTGCTGCATCAAAAGA
       1038 AGCGATCCAAATATCCACTTCCAGATTCTACAAAAAGAGTGTTTCAAAACTGCTCTATCAAAAGA

            * *    *          **                  ***      *     *         * 
      92741 GAGGTTCCACTCTGTTAGCCGAGTACACACATCACAAACTTGTTTCTCAGAATCCTTCTGTCTCG
       1103 AATGTTCAACTCTGTTAGTTGAGTACACACATCACAAAGAAGTTTCTGAGAATGCTTCTGTCTAG

                              ** *    
      92806 TTTTTATGGGAAGATATTTACTTTTT
       1168 TTTTTATGGGAAGATATTCCCGTTTT

              *  * **          *       *     *       *  *  *                 
      92832 CACCGTAGGCATCAAAGCGCTCCAAATGTCCACATCCAGATACTCCAGAAAGAGTGTTTCAAACC
          1 CAACGAAATCATCAAAGCGATCCAAATATCCACTTCCAGATTCTACAAAAAGAGTGTTTCAAACC

                              *         *        * *  *      *      *        
      92897 TGCTCTATGAAAGGGAATCTTCAACTCTATGAGTTGAATGCAGACATCAGAAAGAAATTTCTGAG
         66 TGCTCTA-GAAAGGGAATGTTCAACTCTGTGAGTTGAGTACACACATCACAAAGAAGTTTCTGAG

                  *       **          *          *                 *    *    
      92962 AATGCTGCTGTCTACCTTTTAT-T--TGA-ATTCCCGCTTCCAACGAAATC-CTCCAAGCTATCC
        130 AATGCTTCTGTCTAGTTTTTATATGAAGATATTCCCGTTTCCAACGAAATCGCT-AAAGCGATCC

                        *       *                                  ** *  *   
      93022 AAATATCCACTTGCAGATTCCACAAAAAGAGTGTTTCAAAACTGCTCTCTATCAATGGCAAAGTT
        194 AAATATCCACTTCCAGATTCTACAAAAAGAGTGTTTCAAAACTG--CTCTATCAAAAGAAATGTT

                             *     *     *  *                         *      
      93087 CAACTCTGTTAGTTGAGGACACATATCACCAACAAGTTTCTGAGAATGCTTCTGTCTATTTTTTA
        257 CAACTCTGTTAGTTGAGTACACACATCACAAAGAAGTTTCTGAGAATGCTTCTGTCTAGTTTTTA

             **         *      *  *  * ** *    *      *    *          * **   
      93152 TGGGAAGATATTTCCTTTTTCACCGTAGGCGTCAAGGCGATCGAAATGTCCACTTCCACAAACTA
        322 TATGAAGATATTCCCTTTTCCAACGAAATCATCAAAGCGATCCAAATATCCACTTCCAGATTCTA

                              *             * **       *    * *      * ** * *
      93217 CAAAAAGAGTGTTTCAAACCTGCTCTATGAAAGGCCATGTTCATCTCTATGAGTTGAATGGAAAT
        387 CAAAAAGAGTGTTTCAAAACTGCTCTATGAAAAGAAATGTTCAACTCTGTTAGTTGAGTACACAC

               **      *      *       *                   *         **       
      93282 ATCCGAAAGAAATTTCTGGGAATGCTGCTGTCTAGTTTTTAT---ATGA-ATTCCCGCTTCCAAC
        452 ATCACAAAGAAGTTTCTGAGAATGCTTCTGTCTAGTTTTTATGGGAAGATATTCCCTTTTCCAAC

                * *       *                *    *  *                         
      93343 GAAATCCTCAAAGCAATCCAAATATCCACTTGCAGAATCCACAAAAAGAGTGTTTCAAAACTGCT
        517 GAAAGCATCAAAGCGATCCAAATATCCACTTCCAGATTCTACAAAAAGAGTGTTTCAAAACTGCT

                   **    *          * *                      *               
      93408 CTATCAATAGAAAGGTTCAACTCTTTTAGTTGAGTACACACATCACAAACAAGTTTCTGAGAATG
        582 CTATCAAAGGAAATGTTCAACTCTGTGAGTTGAGTACACACATCACAAAGAAGTTTCTGAGAATG

                     * *          *  **  *         * *  **          *      **
      93473 CTTCTGTCTGGCTTTTATTGGAAGACGTTTCCTTTT-CACCAAAGGCATCAAAGCGCTCCAAAGG
        647 CTTCTGTCTAGTTTTTA-T-GATGATATTCCCTTTTCCAACGAAATCATCAAAGCGATCCAAATA

                            * *                **           *    *           
      93537 TCCACTTCCAGATTCTTCCAAAAGAGTGTTTCAAACGTG-CTC-ATACTAAGGGAATGTTCAACT
        710 TCCACTTCCAGATTCTACAAAAAGAGTGTTTCAAAACTGCCTCTAT-CAAAGGAAATGTTCAACT

                * *    * *  * *     *   *       * * *             *   * ***  
      93600 CTGTGACTTGAATGCAGATATCACCAAGTAGTTTCTAATAGTGCTTCTGTCTAGATTTCAGATGA
        774 CTGTTAGTTGAGTACACACATCACAAAGAAGTTTCTGAGAATGCTTCTGTCTAGTTTTTATGGGA

            *         *              *** *   *             *  *   *      *   
      93665 TGATATTCCCGTTTCCAACGAAATCGCTAGAGCTATCCAAATATCCAGTTACAGTTTCTACCAAA
        839 AGATATTCCCTTTTCCAACGAAATCATCAAAGCGATCCAAATATCCACTTCCAGATTCTACAAAA

              *       *   *    **      *    *            *      ***  *       
      93730 AGGGTGTTTCCAAATTGCTGCATCAAAAGAAAGGTTCAACTCTGTTAGTTGAGGACACACATCAC
        904 AGAGTGTTTCAAAACTGCTCTATCAAAGGAAATGTTCAACTCTGTGAGTTGAATGCAGACATCAC

                      *                   *          *                    *  
      93795 AAAGAAGTTTGTGAGAATGCTTCTGTCTACATTTTGTATGACGATATTCCCTTTTCCAACGATAT
        969 AAAGAAGTTTCTGAGAATGCTTCTGTCTA-GTTTT-TATGAAGATATTCCCTTTTCCAACGAAAT

             * *     *   *       * *  *    *  *       *  *        *       * *
      93860 CGTTAAAGCAATCTAAATATCAATTTGCAGAATCCACAAAAATAGAGTTTCAAAGCTGCTCTGTA
       1032 CATCAAAGCGATCCAAATATCCACTTCCAGATTCTACAAAAAGAGTGTTTCAAAACTGCTCTATC

                    *    *          *                   ***            *     
      93925 AAAAGAAAGGTTCCACTCTGTTAGCTGAGTACACACATCACAAACTTGTTTCTGAGAATCCTTCT
       1097 AAAAGAAATGTTCAACTCTGTTAGTTGAGTACACACATCACAAAGAAGTTTCTGAGAATGCTTCT

                *                   ** *   *
      93990 GTCTCGTTTTTATGGGAAGATATTTACTTTTC
       1162 GTCTAGTTTTTATGGGAAGATATTCCCGTTTT

              *  * **          *       *                * *                 *
      94022 CACCGTAGGCATCAAAGCGCTCCAAATGTCCACTTCCAGATTCTTCCAAAAGAGTGTTTCAAACG
          1 CAACGAAATCATCAAAGCGATCCAAATATCCACTTCCAGATTCTACAAAAAGAGTGTTTCAAACC

              *   *  *                      *    * *  * *     *   *       * *
      94087 TGGTCGAAGTAAGGGAATGTTCAACTCTGTGACTTGAATGCAGATATCACCAAGTAGTTTCTAAT
         66 TGCTC-TAGAAAGGGAATGTTCAACTCTGTGAGTTGAGTACACACATCACAAAGAAGTTTCTGAG

             *             *     *    *                         *  *   *     
      94152 AGTGCTTCTGTCTAGATTTTAGATGATGATATTCCCGTTTCCAACGAAATCGTTAGAGCTATCCA
        130 AATGCTTCTGTCTAGTTTTTATATGAAGATATTCCCGTTTCCAACGAAATCGCTAAAGCGATCCA

                    *  *   *      *     *       *   *    **           *      
      94217 AATATCCAGTTACAGTTTCTACCAAAAGGGTGTTTCCAAATTGCTGCATCAAAAGAAAGGTTCAA
        195 AATATCCACTTCCAGATTCTACAAAAAGAGTGTTTCAAAACTGCTCTATCAAAAGAAATGTTCAA

                          *                     *                   *    *   
      94282 CTCTGTTAGTTGAGGACACACATCACAAAGAAGTTTGTGAGAATGCTTCTGTCTAGATTTTGTAT
        260 CTCTGTTAGTTGAGTACACACATCACAAAGAAGTTTCTGAGAATGCTTCTGTCTAGTTTTTATAT

              *                    *   * *     *   *       * *  *    *  *    
      94347 GACGATATTCCCTTTTCCAACGATATCGTTAAAGCAATCTAAATATCAATTTGCAGAATCCACAA
        325 GAAGATATTCCCTTTTCCAACGAAATCATCAAAGCGATCCAAATATCCACTTCCAGATTCTACAA

               *  *        *       * *        *    *          *              
      94412 AAATAGAGTTTCAAAGCTGCTCTGTAAAAAGAAAGGTTCCACTCTGTTAGCTGAGTACACACATC
        390 AAAGAGTGTTTCAAAACTGCTCTATGAAAAGAAATGTTCAACTCTGTTAGTTGAGTACACACATC

                 ***           **         *                   **        ** * 
      94477 ACAAACTTGTTTCTGAGAAACCTTCTGTCTCGTTTTTATGGGAAGATATTTACTTTTCCACTGTA
        455 ACAAAGAAGTTTCTGAGAATGCTTCTGTCTAGTTTTTATGGGAAGATATTCCCTTTTCCAACGAA

            *           *       *     *       *  *  *  *            *        
      94542 GGCATCAAAGCGCTCCAAATGTCCACATCCAGATACTCCAGAACGAGTGTTTCAAACCTGCTCTA
        520 AGCATCAAAGCGATCCAAATATCCACTTCCAGATTCTACAAAAAGAGTGTTTCAAAACTGCTCTA

             *     *   *         *        * *  *      *      *              *
      94607 TGAAAGGGAATCTTCAACTCTATGAGTTGAATGCAGA-ATCAGAAAGAAATTTCTGAGAATGCTG
        585 TCAAAGGAAATGTTCAACTCTGTGAGTTGAGTACACACATCACAAAGAAGTTTCTGAGAATGCTT

                   **       *          **             *  *    *              
      94671 CTGTCTACCTTTTAT-TTGA-ATTCCCGCTTCCAACGAAATCCTCCAAGCTATCCAAATATCCAC
        650 CTGTCTAGTTTTTATGATGATATTCCCTTTTCCAACGAAATCATCAAAGCGATCCAAATATCCAC

            * *  **   *    *                            **              **  *
      94734 CTGCATTTTCCACAACAAGAGTGTTTCAAAACTG-CTCTATCAATAGAAATGTTCAACTCCTTTG
        715 TTCCAGATTCTACAAAAAGAGTGTTTCAAAACTGCCTCTATCAAAGGAAATGTTCAACTCTGTTA

             *  *                *                                        ** 
      94798 GCTGGGTACACACATCACAAACAAGTTTCTGAGAATGCTTCTGTCTAGTTTTTATGGGAAGACGT
        780 GTTGAGTACACACATCACAAAGAAGTTTCTGAGAATGCTTCTGTCTAGTTTTTATGGGAAGATAT

                    *  * *  **          *       *            **              
      94863 TCCCTTTTTCACCAAAGGCATCAAAGCGCTCCAAATGTCCACTTCCAGACACTACAAAAAGAGTG
        845 TCCCTTTTCCAACGAAATCATCAAAGCGATCCAAATATCCACTTCCAGATTCTACAAAAAGAGTG

                   **       **   ***                 *           *         * 
      94928 TTTCAAACGTGCTCTAAGAAAACGAATGTTCAACTCTGTGACTTGAATGCAGATATCACAAAGTA
        910 TTTCAAAACTGCTCTATCAAAGGAAATGTTCAACTCTGTGAGTTGAATGCAGACATCACAAAGAA

                      **                  *    *         *                * *
      94993 GTTTCTGAGAGGGCTTCTGTCTAGATTTTAGATGATGATATTCCCGTTTCCAACGAAATCATTAG
        975 GTTTCTGAGAATGCTTCTGTCTAG-TTTT-TATGAAGATATTCCCTTTTCCAACGAAATCATCAA

               *                *   *                    *        **         
      95058 AGCTATCCAAATATCCACTTACAGTTTCTACAAAAAGAGTGTTTCCAAACTGCTGCATCAAAAGA
       1038 AGCGATCCAAATATCCACTTCCAGATTCTACAAAAAGAGTGTTTCAAAACTGCTCTATCAAAAGA

            * *    *          **                  ***      *     *         * 
      95123 GAGGTTCCACTCTGTTAGCCGAGTACACACATCACAAACTTGTTTCTCAGAATCCTTCTGTCTCG
       1103 AATGTTCAACTCTGTTAGTTGAGTACACACATCACAAAGAAGTTTCTGAGAATGCTTCTGTCTAG

                              ** *    
      95188 TTTTTATGGGAAGATATTTACTTTTT
       1168 TTTTTATGGGAAGATATTCCCGTTTT

              *  * **          *       *     *       *  *  *                 
      95214 CACCGTAGGCATCAAAGCGCTCCAAATGTCCACATCCAGATACTCCAGAAAGAGTGTTTCAAACC
          1 CAACGAAATCATCAAAGCGATCCAAATATCCACTTCCAGATTCTACAAAAAGAGTGTTTCAAACC

                              *         *        * *  *      *      *        
      95279 TGCTCTATGAAAGGGAATCTTCAACTCTATGAGTTGAATGCAGACATCAGAAAGAAATTTCTGAG
         66 TGCTCTA-GAAAGGGAATGTTCAACTCTGTGAGTTGAGTACACACATCACAAAGAAGTTTCTGAG

                  *       **          *          *                 *    *    
      95344 AATGCTGCTGTCTACCTTTTAT-T--TGA-ATTCCCGCTTCCAACGAAATC-CTCCAAGCTATCC
        130 AATGCTTCTGTCTAGTTTTTATATGAAGATATTCCCGTTTCCAACGAAATCGCT-AAAGCGATCC

                        *       *                                  ** *  *   
      95404 AAATATCCACTTGCAGATTCCACAAAAAGAGTGTTTCAAAACTGCTCTCTATCAATGGCAAAGTT
        194 AAATATCCACTTCCAGATTCTACAAAAAGAGTGTTTCAAAACTG--CTCTATCAAAAGAAATGTT

                             *     *     *  *                         *      
      95469 CAACTCTGTTAGTTGAGGACACATATCACCAACAAGTTTCTGAGAATGCTTCTGTCTATTTTTTA
        257 CAACTCTGTTAGTTGAGTACACACATCACAAAGAAGTTTCTGAGAATGCTTCTGTCTAGTTTTTA

             **         *      *  *  * ** *    * *    *    *          * **   
      95534 TGGGAAGATATTTCCTTTTTCACCGTAGGCGTCAAGGTGATCGAAATGTCCACTTCCACAAACTA
        322 TATGAAGATATTCCCTTTTCCAACGAAATCATCAAAGCGATCCAAATATCCACTTCCAGATTCTA

                              *             * **       *    * *      * ** * *
      95599 CAAAAAGAGTGTTTCAAACCTGCTCTATGAAAGGCCATGTTCATCTCTATGAGTTGAATGGAAAT
        387 CAAAAAGAGTGTTTCAAAACTGCTCTATGAAAAGAAATGTTCAACTCTGTTAGTTGAGTACACAC

               **      *      *       *                   *         **       
      95664 ATCCGAAAGAAATTTCTGGGAATGCTGCTGTCTAGTTTTTAT---ATGA-ATTCCCGCTTCCAAC
        452 ATCACAAAGAAGTTTCTGAGAATGCTTCTGTCTAGTTTTTATGGGAAGATATTCCCTTTTCCAAC

                * *       *                *    *  *                         
      95725 GAAATCCTCAAAGCAATCCAAATATCCACTTGCAGAATCCACAAAAAGAGTGTTTCAAAACTGCT
        517 GAAAGCATCAAAGCGATCCAAATATCCACTTCCAGATTCTACAAAAAGAGTGTTTCAAAACTGCT

                   **    *          * *                      *               
      95790 CTATCAATAGAAAGGTTCAACTCTTTTAGTTGAGTACACACATCACAAACAAGTTTCTGAGAATG
        582 CTATCAAAGGAAATGTTCAACTCTGTGAGTTGAGTACACACATCACAAAGAAGTTTCTGAGAATG

                     * *          *  **  *         * *  **          *      **
      95855 CTTCTGTCTGGCTTTTATTGGAAGACGTTTCCTTTT-CACCAAAGGCATCAAAGCGCTCCAAAGG
        647 CTTCTGTCTAGTTTTTA-T-GATGATATTCCCTTTTCCAACGAAATCATCAAAGCGATCCAAATA

                            * *                **          **    *           
      95919 TCCACTTCCAGATTCTTCCAAAAGAGTGTTTCAAACGTG-CTC-ATAGTAAGGGAATGTTCAACT
        710 TCCACTTCCAGATTCTACAAAAAGAGTGTTTCAAAACTGCCTCTAT-CAAAGGAAATGTTCAACT

                * *    * *  * *     *   *       * * *             *     ***  
      95982 CTGTGACTTGAATGCAGATATCACCAAGTAGTTTCTAATAGTGCTTCTGTCTAGATTTTAGATGA
        774 CTGTTAGTTGAGTACACACATCACAAAGAAGTTTCTGAGAATGCTTCTGTCTAGTTTTTATGGGA

            *         *              *** *   *             *  *   *      *   
      96047 TGATATTCCCGTTTCCAACGAAATCGCTAGAGCTATCCAAATATCCAGTTACAGTTTCTACCAAA
        839 AGATATTCCCTTTTCCAACGAAATCATCAAAGCGATCCAAATATCCACTTCCAGATTCTACAAAA

              *       *   *    **      *    *            *      ***  *       
      96112 AGGGTGTTTCCAAATTGCTGCATCAAAAGAAAGGTTCAACTCTGTTAGTTGAGGACACACATCAC
        904 AGAGTGTTTCAAAACTGCTCTATCAAAGGAAATGTTCAACTCTGTGAGTTGAATGCAGACATCAC

                      *                   *          *                    *  
      96177 AAAGAAGTTTGTGAGAATGCTTCTGTCTACATTTTGTATGACGATATTCCCTTTTCCAACGATAT
        969 AAAGAAGTTTCTGAGAATGCTTCTGTCTA-GTTTT-TATGAAGATATTCCCTTTTCCAACGAAAT

             * *     *   *       * *  *    *  *       *  *        *       * *
      96242 CGTTAAAGCAATCTAAATATCAATTTGCAGAATCCACAAAAATAGAGTTTCAAAGCTGCTCTGTA
       1032 CATCAAAGCGATCCAAATATCCACTTCCAGATTCTACAAAAAGAGTGTTTCAAAACTGCTCTATC

                    *    *          *                   ***            *     
      96307 AAAAGAAAGGTTCCACTCTGTTAGCTGAGTACACACATCACAAACTTGTTTCTGAGAATCCTTCT
       1097 AAAAGAAATGTTCAACTCTGTTAGTTGAGTACACACATCACAAAGAAGTTTCTGAGAATGCTTCT

                *                   ** *   *
      96372 GTCTCGTTTTTATGGGAAGATATTTACTTTTC
       1162 GTCTAGTTTTTATGGGAAGATATTCCCGTTTT

              *  * **          *       *     *       *  *  *  *              
      96404 CACCGTAGGCATCAAAGCGCTCCAAATGTCCACATCCAGATACTCCAGAACGAGTGTTTCAAACC
          1 CAACGAAATCATCAAAGCGATCCAAATATCCACTTCCAGATTCTACAAAAAGAGTGTTTCAAACC

                              *         *        * *  *      *      *        
      96469 TGCTCTATGAAAGGGAATCTTCAACTCTATGAGTTGAATGCAGACATCAGAAAGAAATTTCTGAG
         66 TGCTCTA-GAAAGGGAATGTTCAACTCTGTGAGTTGAGTACACACATCACAAAGAAGTTTCTGAG

                  *       **          *          *                 *    *    
      96534 AATGCTGCTGTCTACCTTTTAT-T--TGA-ATTCCCGCTTCCAACGAAATC-CTCCAAGCTATCC
        130 AATGCTTCTGTCTAGTTTTTATATGAAGATATTCCCGTTTCCAACGAAATCGCT-AAAGCGATCC

                      * *  **   *    *                           *           
      96594 AAATATCCACCTGCATTTTCCACAACAAGAGTGTTTCAAAACTGCTCTATCAATAGAAATGTTCA
        194 AAATATCCACTTCCAGATTCTACAAAAAGAGTGTTTCAAAACTGCTCTATCAAAAGAAATGTTCA

                **  * *  *                *                                 *
      96659 ACTCCTTTGGCTGGGTACACACATCACAAACAAGTTTCTGAGAATGCTTCTGTCTAGTTTTTATG
        259 ACTCTGTTAGTTGAGTACACACATCACAAAGAAGTTTCTGAGAATGCTTCTGTCTAGTTTTTATA

            *     **         *  * *  **          *       *            **     
      96724 GGAAGACGTTCCCTTTTTCACCAAAGGCATCAAAGCGCTCCAAATGTCCACTTCCAGACACTACA
        324 TGAAGATATTCCCTTTTCCAACGAAATCATCAAAGCGATCCAAATATCCACTTCCAGATTCTACA

                            **       *    ***               * *    * *  * *  
      96789 AAAAGAGTGTTTCAAACGTGCTCTAAGAAAGCGAATGTTCAACTCTGTGACTTGAATGCAGATAT
        389 AAAAGAGTGTTTCAAAACTGCTCTATGAAAAGAAATGTTCAACTCTGTTAGTTGAGTACACACAT

                   *           **            *     ***  *         *          
      96854 CACAAAGTAGTTTCTGAGAGGGCTTCTGTCTAGATTTTAGATGATGATATTCCCGTTTCCAACGA
        454 CACAAAGAAGTTTCTGAGAATGCTTCTGTCTAGTTTTTATGGGAAGATATTCCCTTTTCCAACGA

              *   * *   *                *   *                    *        **
      96919 AATCATTAGAGCTATCCAAATATCCACTTACAGTTTCTACAAAAAGAGTGTTTCCAAACTGCTGC
        519 AAGCATCAAAGCGATCCAAATATCCACTTCCAGATTCTACAAAAAGAGTGTTTCAAAACTGCTCT

                  *  * *    *       *  *                   ***      *     *  
      96984 ATCAAAAGAGAGGTTCCACTCTGTTAGCTGAGTACACACATCACAAACTTGTTTCTCAGAATCCT
        584 ATCAAAGGAAATGTTCAACTCTGTGAGTTGAGTACACACATCACAAAGAAGTTTCTGAGAATGCT

                   *            *      **     *  *  * **          *       *  
      97049 TCTGTCTCGTTTTTATGGGAAGATATTTACTTTTTCACCGTAGGCATCAAAGCGCTCCAAATGTC
        649 TCTGTCTAGTTTTTAT--GATGATATTCCCTTTTCCAACGAAATCATCAAAGCGATCCAAATATC

               *       *  *  *               *          *     *   *         *
      97114 CACATCCAGATACTCCAGAAAGAGTGTTTCAAACCTG-CTCTATGAAAGGGAATCTTCAACTCTA
        712 CACTTCCAGATTCTACAAAAAGAGTGTTTCAAAACTGCCTCTATCAAAGGAAATGTTCAACTCTG

             *      * *  *      *      *              *       **         **  
      97178 TGAGTTGAATGCAGACATCAGAAAGAAATTTCTGAGAATGCTGCTGTCTACCTTTTAT---TTGA
        777 TTAGTTGAGTACACACATCACAAAGAAGTTTCTGAGAATGCTTCTGTCTAGTTTTTATGGGAAGA

                   **             *  *    *                *       *         
      97240 -ATTCCCGCTTCCAACGAAATCCTCCAAGCTATCCAAATATCCACTTGCAGATTCCACAAAAAGA
        842 TATTCCCTTTTCCAACGAAATCATCAAAGCGATCCAAATATCCACTTCCAGATTCTACAAAAAGA

                                     *  *  *            *      ***  * *     *
      97304 GTGTTTCAAAACTGCTCTCTATCAATGGCAAAGTTCAACTCTGTTAGTTGAGGACACATATCACC
        907 GTGTTTCAAAACTG--CTCTATCAAAGGAAATGTTCAACTCTGTGAGTTGAATGCAGACATCACA

              *                         *                  *      *  *  * ** 
      97369 AACAAGTTTCTGAGAATGCTTCTGTCTATTTTTTATGGGAAGATATTTCCTTTTTCACCGTAGGC
        970 AAGAAGTTTCTGAGAATGCTTCTGTCTAGTTTTTAT--GAAGATATTCCCTTTTCCAACGAAATC

            *    *      *    *          * **                     *         * 
      97434 GTCAAGGCGATCGAAATGTCCACTTCCACAAACTACAAAAAGAGTGTTTCAAACCTGCTCTATGA
       1033 ATCAAAGCGATCCAAATATCCACTTCCAGATTCTACAAAAAGAGTGTTTCAAAACTGCTCTATCA

              * **       *    * *      * ** * *   **      *      *       *   
      97499 AAGGCCATGTTCATCTCTATGAGTTGAATGGAAATATCCGAAAGAAATTTCTGGGAATGCTGCTG
       1098 AAAGAAATGTTCAACTCTGTTAGTTGAGTACACACATCACAAAGAAGTTTCTGAGAATGCTTCTG

                  *         *          *  *
      97564 TCTAGTGTTTAT---ACGA-ATTCCCGCTTC
       1163 TCTAGTTTTTATGGGAAGATATTCCCGTTTT

                      *       *                *    *  *                   * 
      97591 CAACGAAATCCTCAAAGCAATCCAAATATCCACTTGCAGAATCCACAAAAAGAGTGTTTCAAAAC
          1 CAACGAAATCATCAAAGCGATCCAAATATCCACTTCCAGATTCTACAAAAAGAGTGTTTCAAACC

            *       *  ** *  *          * *                      *           
      97656 CGCTCTATCAATAGAAAGGTTCAACTCTTTTAGTTGAGTACACACATCACAAACAAGTTTCTGAG
         66 TGCTCTA-GAAAGGGAATGTTCAACTCTGTGAGTTGAGTACACACATCACAAAGAAGTTTCTGAG

                         * *      **     **  *      *    *    *           *  
      97721 AATGCTTCTGTCTGGCTTTTATTGGAAGACGTTTCC-TTTTC-ACCAAA-GGCATCAAAGCGCTC
        130 AATGCTTCTGTCTAGTTTTTATATGAAGATATTCCCGTTTCCAACGAAATCGC-T-AAAGCGATC

                 *                * *                **  *  * ***  * *       
      97783 CAAATGTCCACTTCCAGATTCTTCCAAAAGAGTGTTTCAAACGTGGTCGAAGTAAGGGAATGTTC
        193 CAAATATCCACTTCCAGATTCTACAAAAAGAGTGTTTCAAAACTGCTCTATCAAAAGAAATGTTC

                    * *    * *  * *     *   *       * * *             *     *
      97848 AACTCTGTGACTTGAATGCAGATATCACCAAGTAGTTTCTAATAGTGCTTCTGTCTAGATTTTAG
        258 AACTCTGTTAGTTGAGTACACACATCACAAAGAAGTTTCTGAGAATGCTTCTGTCTAGTTTTTAT

                *         *              * * *   *             *  *   *      
      97913 ATGATGATATTCCCGTTTCCAACGAAATCGTTAGAGCTATCCAAATATCCAGTTACAGTTTCTAC
        323 ATGAAGATATTCCCTTTTCCAACGAAATCATCAAAGCGATCCAAATATCCACTTCCAGATTCTAC

            *     *       *   *    **  *        *                    *       
      97978 CAAAAGGGTGTTTCCAAATTGCTGCATCAAAAGAAAGGTTCAACTCTGTTAGTTGAGGACACACA
        388 AAAAAGAGTGTTTCAAAACTGCTCTATGAAAAGAAATGTTCAACTCTGTTAGTTGAGTACACACA

                          *                   *    * **  *                   
      98043 TCACAAAGAAGTTTGTGAGAATGCTTCTGTCTAGATTTTGTATGACGATATTCCCTTTTCCAACG
        453 TCACAAAGAAGTTTCTGAGAATGCTTCTGTCTAGTTTTTATGGGAAGATATTCCCTTTTCCAACG

             * * * *     *   *       * *  *    *  *       *  *        *      
      98108 ATATCGTTAAAGCAATCTAAATATCAATTTGCAGAATCCACAAAAATAGAGTTTCAAAGCTGCTC
        518 AAAGCATCAAAGCGATCCAAATATCCACTTCCAGATTCTACAAAAAGAGTGTTTCAAAACTGCTC

             * *   *    *    *       *  *                   ***           ** 
      98173 TGTAAAAAGAAAGGTTCCACTCTGTTAGCTGAGTACACACATCACAAACTTGTTTCTGAGAAACC
        583 TATCAAAGGAAATGTTCAACTCTGTGAGTTGAGTACACACATCACAAAGAAGTTTCTGAGAATGC

                    *            *      **        ** * **          *       * 
      98238 TTCTGTCTCGTTTTTATGGGAAGATATTTACTTTTCCACTGTAGGCATCAAAGCGCTCCAAATGT
        648 TTCTGTCTAGTTTTTAT--GATGATATTCCCTTTTCCAACGAAATCATCAAAGCGATCCAAATAT

                *       *  *  *  *            *          *     *   *         
      98303 CCACATCCAGATACTCCAGAACGAGTGTTTCAAACCTG-CTCTATGAAAGGGAATCTTCAACTCT
        711 CCACTTCCAGATTCTACAAAAAGAGTGTTTCAAAACTGCCTCTATCAAAGGAAATGTTCAACTCT

            * *      * *  *      *      *              *       **         ** 
      98367 ATGAGTTGAATGCAGA-ATCAGAAAGAAATTTCTGAGAATGCTGCTGTCTACCTTTTAT---TTG
        776 GTTAGTTGAGTACACACATCACAAAGAAGTTTCTGAGAATGCTTCTGTCTAGTTTTTATGGGAAG

                    **             *  *    *              * *  **   *    *   
      98428 A-ATTCCCGCTTCCAACGAAATCCTCCAAGCTATCCAAATATCCACCTGCATTTTCCACAACAAG
        841 ATATTCCCTTTTCCAACGAAATCATCAAAGCGATCCAAATATCCACTTCCAGATTCTACAAAAAG

                                    **                *    *  ** *  *        
      98492 AGTGTTTCAAAACTGCTCTATCAATAGAAATGTTCAACTCCTTTG-GCTGGGTACACACATCACA
        906 AGTGTTTCAAAACTGCTCTATCAAAGGAAATGTTCAACT-CTGTGAGTTGAATGCAGACATCACA

              *                                        **         *  * *  ** 
      98556 AACAAGTTTCTGAGAATGCTTCTGTCTAGTTTTTATGGGAAGACGTTCCCTTTTTCACCAAAGGC
        970 AAGAAGTTTCTGAGAATGCTTCTGTCTAGTTTTTAT--GAAGATATTCCCTTTTCCAACGAAATC

                     *       *            **                     **       ** 
      98621 ATCAAAGCGCTCCAAATGTCCACTTCCAGACACTACAAAAAGAGTGTTTCAAACGTGCTCTAAGA
       1033 ATCAAAGCGATCCAAATATCCACTTCCAGATTCTACAAAAAGAGTGTTTCAAAACTGCTCTATCA

               **               * *    * *  * *         *           **       
      98686 AAACGAATGTTCAACTCTGTGACTTGAATGCAGATATCACAAAGTAGTTTCTGAGAGGGCTTCTG
       1098 AAAGAAATGTTCAACTCTGTTAGTTGAGTACACACATCACAAAGAAGTTTCTGAGAATGCTTCTG

                 *     ***  *             *
      98751 TCTAGATTTTAGATGATGATATTCCCGTTTC
       1163 TCTAGTTTTTATGGGAAGATATTCCCGTTTT

                        * *   *                *   *     *              *  * 
      98782 CAACGAAATCATTAGAGCTATCCAAATATCCACTTACAGTTTCTATAAAAAGAGTGTTTCCAAAC
          1 CAACGAAATCATCAAAGCGATCCAAATATCCACTTCCAGATTCTACAAAAAGAGTGTTTCAAACC

                    **    *   *    *       *  **                  ***      * 
      98847 TGCTGCATCAAAAGAG-AGGTTCCACTCTGTTAGCCGAGTACACACATCACAAACTTGTTTCTCA
         66 TGCT-C-TAGAAAGGGAATGTTCAACTCTGTGAGTTGAGTACACACATCACAAAGAAGTTTCTGA

                *         *        **         ** *   *  *  * ** ***      *   
      98911 GAATCCTTCTGTCTCGTTTTTATGGGAAGATATTTACTTTTTCACCGTAGGCATCAAAGCGCTCC
        129 GAATGCTTCTGTCTAGTTTTTATATGAAGATATTCCCGTTTCCAACGAAATCGCTAAAGCGATCC

                *     *       *  *  *               *         *   * *   *    
      98976 AAATGTCCACATCCAGATACTCCAGAAAGAGTGTTTCAAACCTGCTCTATGAAAGGGAATCTTCA
        194 AAATATCCACTTCCAGATTCTACAAAAAGAGTGTTTCAAAACTGCTCTATCAAAAGAAATGTTCA

                 * *      * *  *      *      *              *       **       
      99041 ACTCTATGAGTTGAATGCAGACATCAGAAAGAAATTTCTGAGAATGCTGCTGTCTACCTTTTAT-
        259 ACTCTGTTAGTTGAGTACACACATCACAAAGAAGTTTCTGAGAATGCTTCTGTCTAGTTTTTATA

               *         **             *  *    *                *       *   
      99105 T--TGA-ATTCCCGCTTCCAACGAAATCCTCCAAGCTATCCAAATATCCACTTGCAGATTCCACA
        324 TGAAGATATTCCCTTTTCCAACGAAATCATCAAAGCGATCCAAATATCCACTTCCAGATTCTACA

                                        *  ** *  *                    *     *
      99167 AAAAGAGTGTTTCAAAACTGCTCTCTATCAATGGCAAAGTTCAACTCTGTTAGTTGAGGACACAT
        389 AAAAGAGTGTTTCAAAACTG--CTCTATGAAAAGAAATGTTCAACTCTGTTAGTTGAGTACACAC

                 *  *                         *                  *      *  * 
      99232 ATCACCAACAAGTTTCTGAGAATGCTTCTGTCTATTTTTTATGGGAAGATATTTCCTTTTTCACC
        452 ATCACAAAGAAGTTTCTGAGAATGCTTCTGTCTAGTTTTTATGGGAAGATATTCCCTTTTCCAAC

             * *  *    *      *    *          * **                     *     
      99297 GTAGGCGTCAAGGCGATCGAAATGTCCACTTCCACAAACTACAAAAAGAGTGTTTCAAACCTGCT
        517 GAAAGCATCAAAGCGATCCAAATATCCACTTCCAGATTCTACAAAAAGAGTGTTTCAAAACTGCT

                *     **       *    *        * ** * *   **      *      *     
      99362 CTATGAAAGGCCATGTTCATCTCTATGAGTTGAATGGAAATATCCGAAAGAAATTTCTGGGAATG
        582 CTATCAAAGGAAATGTTCAACTCTGTGAGTTGAGTACACACATCACAAAGAAGTTTCTGAGAATG

              *                           **             *       *           
      99427 CTGCTGTCTAGTTTTTAT-ATGA-ATTCCCGCTTCCAACGAAATCCTCAAAGCAATCCAAATATC
        647 CTTCTGTCTAGTTTTTATGATGATATTCCCTTTTCCAACGAAATCATCAAAGCGATCCAAATATC

                 *    *  *                                 **    *          *
      99490 CACTTGCAGAATCCACAAAAAGAGTGTTTCAAAACTG-CTCTATCAATAGAAAGGTTCAACTCTT
        712 CACTTCCAGATTCTACAAAAAGAGTGTTTCAAAACTGCCTCTATCAAAGGAAATGTTCAACTCTG

                                    *                        * *      *      
      99554 TTAGTTGAGTACACACATCACAAACAAGTTTCTGAGAATGCTTCTGTCTGGCTTTTATTGGAAGA
        777 TTAGTTGAGTACACACATCACAAAGAAGTTTCTGAGAATGCTTCTGTCTAGTTTTTATGGGAAGA

            **  *         * *  **          *      **                * *      
      99619 CGTTTCCTTTT-CACCAAAGGCATCAAAGCGCTCCAAAGGTCCACTTCCAGATTCTTCCAAAAGA
        842 TATTCCCTTTTCCAACGAAATCATCAAAGCGATCCAAATATCCACTTCCAGATTCTACAAAAAGA

                      **         **    *                 *           *     * 
      99683 GTGTTTCAAACGTGCTC-ATAGTAAGGGAATGTTCAACTCTGTGACTTGAATGCAGATATCACCA
        907 GTGTTTCAAAACTGCTCTAT-CAAAGGAAATGTTCAACTCTGTGAGTTGAATGCAGACATCACAA

              *       * * *                   *    *         *              *
      99747 AGTAGTTTCTAATAGTGCTTCTGTCTAGATTTTAGATGATGATATTCCCGTTTCCAACGAAATCG
        971 AGAAGTTTCTGAGAATGCTTCTGTCTAG-TTTT-TATGAAGATATTCCCTTTTCCAACGAAATCA

            ** *   *             *  *   *      *     *       *   *    **     
      99812 CTAGAGCTATCCAAATATCCAGTTACAGTTTCTACCAAAAGGGTGTTTCCAAATTGCTGCATCAA
       1034 TCAAAGCGATCCAAATATCCACTTCCAGATTCTACAAAAAGAGTGTTTCAAAACTGCTCTATCAA

                  *                    *                     *               
      99877 AAGAAAGGTTCAACTCTGTTAGTTGAGGACACACATCACAAAGAAGTTTGTGAGAATGCTTCTGT
       1099 AAGAAATGTTCAACTCTGTTAGTTGAGTACACACATCACAAAGAAGTTTCTGAGAATGCTTCTGT

               **    * **  *         *   *
      99942 CTACATTTTGTATGACGATATTCCCTTTTC
       1164 CTAGTTTTTATGGGAAGATATTCCCGTTTT

                  *   * *     *   *       * *  *    *  *       *  *        * 
      99972 CAACGATATCGTTAAAGCAATCTAAATATCAATTTGCAGAATCCACAAAAATAGAGTTTCAAAGC
          1 CAACGAAATCATCAAAGCGATCCAAATATCCACTTCCAGATTCTACAAAAAGAGTGTTTCAAACC

                   **   * *  *    *       *  *                   ***         
     100037 TGCTCTGTAAAAAGAAAGGTTCCACTCTGTTAGCTGAGTACACACATCACAAACTTGTTTCTGAG
         66 TGCTCT-AGAAAGGGAATGTTCAACTCTGTGAGTTGAGTACACACATCACAAAGAAGTTTCTGAG

               *         *        **         ** *      *  * ** ***      *    
     100102 AATCCTTCTGTCTCGTTTTTATGGGAAGATATTTACTTTTCCACCGTAGGCATCAAAGCGCTCCA
        130 AATGCTTCTGTCTAGTTTTTATATGAAGATATTCCCGTTTCCAACGAAATCGCTAAAGCGATCCA

               *     *       *  *  *  *            *         *   * *   *     
     100167 AATGTCCACATCCAGATACTCCAGAACGAGTGTTTCAAACCTGCTCTATGAAAGGGAATCTTCAA
        195 AATATCCACTTCCAGATTCTACAAAAAGAGTGTTTCAAAACTGCTCTATCAAAAGAAATGTTCAA

              * * *      * *  *      *      *              *       **        
     100232 CTGTATGAGTTGAATGCAGACATCAGAAAGAAATTTCTGAGAATGCTGCTGTCTACCTTTTAT-T
        260 CTCTGTTAGTTGAGTACACACATCACAAAGAAGTTTCTGAGAATGCTTCTGTCTAGTTTTTATAT

              *         **             *  *    *              * *  **   *    
     100296 --TGA-ATTCCCGCTTCCAACGAAATCCTCCAAGCTATCCAAATATCCACCTGCATTTTCCACAA
        325 GAAGATATTCCCTTTTCCAACGAAATCATCAAAGCGATCCAAATATCCACTTCCAGATTCTACAA

            *                        *  *               **  * *  *           
     100358 CAAGAGTGTTTCAAAACTGCTCTATCAATAGAAATGTTCAACTCCTTTGGCTGGGTACACACATC
        390 AAAGAGTGTTTCAAAACTGCTCTATGAAAAGAAATGTTCAACTCTGTTAGTTGAGTACACACATC

                 *                                        **         *  * *  
     100423 ACAAACAAGTTTCTGAGAATGCTTCTGTCTAGTTTTTATGGGAAGACGTTCCCTTTTTCACCAAA
        455 ACAAAGAAGTTTCTGAGAATGCTTCTGTCTAGTTTTTATGGGAAGATATTCCCTTTTCCAACGAA

            *           *       *            **                     **       
     100488 GGCATCAAAGCGCTCCAAATGTCCACTTCCAGACACTACAAAAAGAGTGTTTCAAACGTGCTCTA
        520 AGCATCAAAGCGATCCAAATATCCACTTCCAGATTCTACAAAAAGAGTGTTTCAAAACTGCTCTA

            **    **                 *    * *  * *         *           **    
     100553 AGAAAGCGAATGTTCAACTCTGTGACTTGAATGCAGATATCACAAAGTAGTTTCTGAGAGGGCTT
        585 TCAAAGGAAATGTTCAACTCTGTGAGTTGAGTACACACATCACAAAGAAGTTTCTGAGAATGCTT

                          *              *                * *   *            
     100618 CTGTCTAGATTTTAGATGATGATATTCCCGTTTCCAACGAAATCATTAGAGCTATCCAAATATCC
        650 CTGTCTAG-TTTT-TATGATGATATTCCCTTTTCCAACGAAATCATCAAAGCGATCCAAATATCC

                *   *                    *         **      *  * *    *       
     100683 ACTTACAGTTTCTACAAAAAGAGTGTTTCCAAACTG-CTGCATCAAAAGAGAGGTTCCACTCTGT
        713 ACTTCCAGATTCTACAAAAAGAGTGTTTCAAAACTGCCTCTATCAAAGGAAATGTTCAACTCTGT

               *                   ***      *     *         *                
     100747 TAGCTGAGTACACACATCACAAACTTGTTTCTCAGAATCCTTCTGTCTCGTTTTTATGGGAAGAT
        778 TAGTTGAGTACACACATCACAAAGAAGTTTCTGAGAATGCTTCTGTCTAGTTTTTATGGGAAGAT

               **     *  *  * **          *       *     *       *  *  *      
     100812 ATTTACTTTTTCACCGTAGGCATCAAAGCGCTCCAAATGTCCACATCCAGATACTCCAGAAAGAG
        843 ATTCCCTTTTCCAACGAAATCATCAAAGCGATCCAAATATCCACTTCCAGATTCTACAAAAAGAG

                     *         *     *   *         *                    *    
     100877 TGTTTCAAACCTGCTCTATGAAAGGGAATCTTCAACTCTATGAGTTGAATGCAGACATCAGAAAG
        908 TGTTTCAAAACTGCTCTATCAAAGGAAATGTTCAACTCTGTGAGTTGAATGCAGACATCACAAAG

              *              *       **       **         **             *  * 
     100942 AAATTTCTGAGAATGCTGCTGTCTACCTTTTAT-TTGA-ATTCCCGCTTCCAACGAAATCCTCCA
        973 AAGTTTCTGAGAATGCTTCTGTCTAGTTTTTATGAAGATATTCCCTTTTCCAACGAAATCATCAA

               *                *       *                                  **
     101005 AGCTATCCAAATATCCACTTGCAGATTCCACAAAAAGAGTGTTTCAAAACTGCTCTCTATCAATG
       1038 AGCGATCCAAATATCCACTTCCAGATTCTACAAAAAGAGTGTTTCAAAACTG--CTCTATCAAAA

             *  *                    *     *     *  *                        
     101070 GCAAAGTTCAACTCTGTTAGTTGAGGACACATATCACCAACAAGTTTCTGAGAATGCTTCTGTCT
       1101 GAAATGTTCAACTCTGTTAGTTGAGTACACACATCACAAAGAAGTTTCTGAGAATGCTTCTGTCT

             *                  *  *    
     101135 ATTTTTTATGGGAAGATATTTCCTTTTT
       1166 AGTTTTTATGGGAAGATATTCCCGTTTT

              *  * ** *    *      *    *          * **                       
     101163 CACCGTAGGCGTCAAGGCGATCGAAATGTCCACTTCCACAAACTACAAAAAGAGTGTTTCAAACC
          1 CAACGAAATCATCAAAGCGATCCAAATATCCACTTCCAGATTCTACAAAAAGAGTGTTTCAAACC

                          **       *    *        * ** * *   **      *      * 
     101228 TGCTCTATGAAAGGCCATGTTCATCTCTATGAGTTGAATGGAAATATCCGAAAGAAATTTCTGGG
         66 TGCTCTA-GAAAGGGAATGTTCAACTCTGTGAGTTGAGTACACACATCACAAAGAAGTTTCTGAG

                  *          *        *          *                      *    
     101293 AATGCTGCTGTCTAG-TGTT-TAT-ACGA-ATTCCCGCTTCCAACGAAATC-CTCAAAGCAATCC
        130 AATGCTTCTGTCTAGTTTTTATATGAAGATATTCCCGTTTCCAACGAAATCGCT-AAAGCGATCC

                        *    *  *                                *     *     
     101353 AAATATCCACTTGCAGAATCCACAAAAAGAGTGTTTCAAAACTGCTCTATCAATAGAAAGGTTCA
        194 AAATATCCACTTCCAGATTCTACAAAAAGAGTGTTTCAAAACTGCTCTATCAAAAGAAATGTTCA

                 *                        *                        * *      *
     101418 ACTCTTTTAGTTGAGTACACACATCACAAACAAGTTTCTGAGAATGCTTCTGTCTGGCTTTTATT
        259 ACTCTGTTAGTTGAGTACACACATCACAAAGAAGTTTCTGAGAATGCTTCTGTCTAGTTTTTATA

            *     **  *         * *  **          *       *                * *
     101483 GGAAGACGTTTCCTTTT-CACCAAAGGCATCAAAGCGCTCCAAATGTCCACTTCCAGATTCTTCC
        324 TGAAGATATTCCCTTTTCCAACGAAATCATCAAAGCGATCCAAATATCCACTTCCAGATTCTACA

                            **  *  * * *  * *               * *    * *  * *  
     101547 AAAAGAGTGTTTCAAACGTGGTCGAAGTAAGGGAATGTTCAACTCTGTGACTTGAATGCAGATAT
        389 AAAAGAGTGTTTCAAAACTGCTCTATGAAAAGAAATGTTCAACTCTGTTAGTTGAGTACACACAT

               *   *       * * *             *     ***  *         *          
     101612 CACCAAGTAGTTTCTAATAGTGCTTCTGTCTAGATTTTAGATGATGATATTCCCGTTTCCAACGA
        454 CACAAAGAAGTTTCTGAGAATGCTTCTGTCTAGTTTTTATGGGAAGATATTCCCTTTTCCAACGA

              * * * *   *             *  *   *      *     *       *   *    **
     101677 AATCGTTAGAGCTATCCAAATATCCAGTTACAGTTTCTACCAAAAGGGTGTTTCCAAATTGCTGC
        519 AAGCATCAAAGCGATCCAAATATCCACTTCCAGATTCTACAAAAAGAGTGTTTCAAAACTGCTCT

                  *    *            *       *                     *          
     101742 ATCAAAAGAAAGGTTCAACTCTGTTAGTTGAGGACACACATCACAAAGAAGTTTGTGAGAATGCT
        584 ATCAAAGGAAATGTTCAACTCTGTGAGTTGAGTACACACATCACAAAGAAGTTTCTGAGAATGCT

                                *                    *   * *     *   *       
     101807 TCTGTCTAGATTTTGTATGACGATATTCCCTTTTCCAACGATATCGTTAAAGCAATCTAAATATC
        649 TCTGTCTAG-TTTT-TATGATGATATTCCCTTTTCCAACGAAATCATCAAAGCGATCCAAATATC

            * *  *    *  *       *  *        *        * *   *    *    *      
     101872 AATTTGCAGAATCCACAAAAATAGAGTTTCAAAGCTG-CTCTGTAAAAAGAAAGGTTCCACTCTG
        712 CACTTCCAGATTCTACAAAAAGAGTGTTTCAAAACTGCCTCTATCAAAGGAAATGTTCAACTCTG

                *                   ***           **         *               
     101936 TTAGCTGAGTACACACATCACAAACTTGTTTCTGAGAAACCTTCTGTCTCGTTTTTATGGGAAGA
        777 TTAGTTGAGTACACACATCACAAAGAAGTTTCTGAGAATGCTTCTGTCTAGTTTTTATGGGAAGA

                **        *  * **          *       *     *       *  *  *  *  
     102001 TATTTACTTTTCCACCGTAGGCATCAAAGCGCTCCAAATGTCCACATCCAGATACTCCAGAACGA
        842 TATTCCCTTTTCCAACGAAATCATCAAAGCGATCCAAATATCCACTTCCAGATTCTACAAAAAGA

                      *         *     *   *         *                    *   
     102066 GTGTTTCAAACCTGCTCTATGAAAGGGAATCTTCAACTCTATGAGTTGAATGCAGA-ATCAGAAA
        907 GTGTTTCAAAACTGCTCTATCAAAGGAAATGTTCAACTCTGTGAGTTGAATGCAGACATCACAAA

               *              *       **       **         **             *  *
     102130 GAAATTTCTGAGAATGCTGCTGTCTACCTTTTAT-TTGA-ATTCCCGCTTCCAACGAAATCCTCC
        972 GAAGTTTCTGAGAATGCTTCTGTCTAGTTTTTATGAAGATATTCCCTTTTCCAACGAAATCATCA

                *              * *  **   *    *                           *  
     102193 AAGCTATCCAAATATCCACCTGCATTTTCCACAACAAGAGTGTTTCAAAACTGCTCTATCAATAG
       1037 AAGCGATCCAAATATCCACTTCCAGATTCTACAAAAAGAGTGTTTCAAAACTGCTCTATCAAAAG

                         **  * *  *                *                         
     102258 AAATGTTCAACTCCTTTGGCTGGGTACACACATCACAAACAAGTTTCTGAGAATGCTTCTGTCTA
       1102 AAATGTTCAACTCTGTTAGTTGAGTACACACATCACAAAGAAGTTTCTGAGAATGCTTCTGTCTA

                           **     *    
     102323 GTTTTTATGGGAAGACGTTCCCTTTTT
       1167 GTTTTTATGGGAAGATATTCCCGTTTT

              * *  **          *       *            **                      *
     102350 CACCAAAGGCATCAAAGCGCTCCAAATGTCCACTTCCAGACACTACAAAAAGAGTGTTTCAAACG
          1 CAACGAAATCATCAAAGCGATCCAAATATCCACTTCCAGATTCTACAAAAAGAGTGTTTCAAACC

                        **                  *    * *  * *         *          
     102415 TGCTCTAAGAAAACGAATGTTCAACTCTGTGACTTGAATGCAGATATCACAAAGTAGTTTCTGAG
         66 TGCTCT-AGAAAGGGAATGTTCAACTCTGTGAGTTGAGTACACACATCACAAAGAAGTTTCTGAG

             **            *     *    *                        **  *   *     
     102480 AGGGCTTCTGTCTAGATTTTAGATGATGATATTCCCGTTTCCAACGAAATCATTAGAGCTATCCA
        130 AATGCTTCTGTCTAGTTTTTATATGAAGATATTCCCGTTTCCAACGAAATCGCTAAAGCGATCCA

                       *   *     *              *        **         * *    * 
     102545 AATATCCACTTACAGTTTCTATAAAAAGAGTGTTTCCAAACTGCTGCATCAAAAGAGAGGTTCCA
        195 AATATCCACTTCCAGATTCTACAAAAAGAGTGTTTCAAAACTGCTCTATCAAAAGAAATGTTCAA

                     **                  ***      *     *         *        **
     102610 CTCTGTTAGCCGAGTACACACATCACAAACTTGTTTCTCAGAATCCTTCTGTCTCGTTTTTATGG
        260 CTCTGTTAGTTGAGTACACACATCACAAAGAAGTTTCTGAGAATGCTTCTGTCTAGTTTTTATAT

                     **     *  *  * **          *       *     *       *  *  *
     102675 GAAGATATTTACTTTTTCACCGTAGGCATCAAAGCGCTCCAAATGTCCACATCCAGATACTCCAG
        325 GAAGATATTCCCTTTTCCAACGAAATCATCAAAGCGATCCAAATATCCACTTCCAGATTCTACAA

                           *             * *   *         * *      * *  *     
     102740 AAAGAGTGTTTCAAACCTGCTCTATGAAAGGGAATCTTCAACTCTATGAGTTGAATGCAGACATC
        390 AAAGAGTGTTTCAAAACTGCTCTATGAAAAGAAATGTTCAACTCTGTTAGTTGAGTACACACATC

             *      *              *  *    **         **         **          
     102805 AGAAAGAAATTTCTGAGAATGCTGCTCTCTACCTTTTAT---TTGA-ATTCCCGCTTCCAACGAA
        455 ACAAAGAAGTTTCTGAGAATGCTTCTGTCTAGTTTTTATGGGAAGATATTCCCTTTTCCAACGAA

             * *  *    *                *       *                            
     102866 ATCCTCCAAGCTATCCAAATATCCACTTGCAGATTCCACAAAAAGAGTGTTTCAAAACTGCTCTC
        520 AGCATCAAAGCGATCCAAATATCCACTTCCAGATTCTACAAAAAGAGTGTTTCAAAACTG--CTC

                  *  *  *            *       *     *     *  *                
     102931 TATCAATGGCAAAGTTCAACTCTGTTAGTTGAGGACACATATCACCAACAAGTTTCTGAGAATGC
        583 TATCAAAGGAAATGTTCAACTCTGTGAGTTGAGTACACACATCACAAAGAAGTTTCTGAGAATGC

                     *           *      *      *  *  * ** *    *      *    * 
     102996 TTCTGTCTATTTTTTATGGGAAGATATTTCCTTTTTCACCGTAGGCGTCAAGGCGATCGAAATGT
        648 TTCTGTCTAGTTTTTAT--GATGATATTCCCTTTTCCAACGAAATCATCAAAGCGATCCAAATAT

                     * **                     *          *     **       *    
     103061 CCACTTCCACAAACTACAAAAAGAGTGTTTCAAACCTG-CTCTATGAAAGGCCATGTTCATCTCT
        711 CCACTTCCAGATTCTACAAAAAGAGTGTTTCAAAACTGCCTCTATCAAAGGAAATGTTCAACTCT

            * *      * ** * *   **      *      *       *                   * 
     103125 ATGAGTTGAATGGAAATATCCGAAAGAAATTTCTGGGAATGCTGCTGTCTAGTTTTTAT---ATG
        776 GTTAGTTGAGTACACACATCACAAAGAAGTTTCTGAGAATGCTTCTGTCTAGTTTTTATGGGAAG

                    **             *       *                *    *  *        
     103187 A-ATTCCCGCTTCCAACGAAATCCTCAAAGCAATCCAAATATCCACTTGCAGAATCCACAAAAAG
        841 ATATTCCCTTTTCCAACGAAATCATCAAAGCGATCCAAATATCCACTTCCAGATTCTACAAAAAG

                                    **    *          * *      * *  *         
     103251 AGTGTTTCAAAACTGCTCTATCAATAGAAAGGTTCAACTCTTTTAGTTGAGTACACACATCACAA
        906 AGTGTTTCAAAACTGCTCTATCAAAGGAAATGTTCAACTCTGTGAGTTGAATGCAGACATCACAA

             *                        * *             **  *         * *  **  
     103316 ACAAGTTTCTGAGAATGCTTCTGTCTGGCTTTTATTGGAAGACGTTTCCTTTT-CACCAAAGGCA
        971 AGAAGTTTCTGAGAATGCTTCTGTCTAGTTTTTA-T-GAAGATATTCCCTTTTCCAACGAAATCA

                    *      **                * *                **         **
     103380 TCAAAGCGCTCCAAAGGTCCACTTCCAGATTCTTCCAAAAGAGTGTTTCAAACGTGCTC-ATAGT
       1034 TCAAAGCGATCCAAATATCCACTTCCAGATTCTACAAAAAGAGTGTTTCAAAACTGCTCTAT-CA

              * *               * *    * *  * *     *   *       * * *        
     103444 AAGGGAATGTTCAACTCTGTGACTTGAATGCAGATATCACCAAGTAGTTTCTAATAGTGCTTCTG
       1098 AAAGAAATGTTCAACTCTGTTAGTTGAGTACACACATCACAAAGAAGTTTCTGAGAATGCTTCTG

                 *     ***  *             *
     103509 TCTAGATTTTAGATGATGATATTCCCGTTTC
       1163 TCTAGTTTTTATGGGAAGATATTCCCGTTTT

                      *** *   *             *  *   *      *     *       *  **
     103540 CAACGAAATCGCTAGAGCTATCCAAATATCCAGTTACAGTTTCTACCAAAAGGGTGTTTCCAAAT
          1 CAACGAAATCATCAAAGCGATCCAAATATCCACTTCCAGATTCTACAAAAAGAGTGTTTCAAACC

                  ***   * *  *            *       *                     *    
     103605 TGCTGCATCAAAAGAAAGGTTCAACTCTGTTAGTTGAGGACACACATCACAAAGAAGTTTGTGAG
         66 TGCT-CTAGAAAGGGAATGTTCAACTCTGTGAGTTGAGTACACACATCACAAAGAAGTTTCTGAG

                          **    *     *         *          *    *      *   * 
     103670 AATGCTTCTGTCTACATTTTGTATGACGATATTCCCTTTTCCAACGATATCGTTAAAGCAATCTA
        130 AATGCTTCTGTCTAGTTTTTATATGAAGATATTCCCGTTTCCAACGAAATCGCTAAAGCGATCCA

                  * *  *    *  *       *  *        *       * *        *    * 
     103735 AATATCAATTTGCAGAATCCACAAAAATAGAGTTTCAAAGCTGCTCTGTAAAAAGAAAGGTTCCA
        195 AATATCCACTTCCAGATTCTACAAAAAGAGTGTTTCAAAACTGCTCTATCAAAAGAAATGTTCAA

                     *                   ***            *         *        **
     103800 CTCTGTTAGCTGAGTACACACATCACAAACTTGTTTCTGAGAATCCTTCTGTCTCGTTTTTATGG
        260 CTCTGTTAGTTGAGTACACACATCACAAAGAAGTTTCTGAGAATGCTTCTGTCTAGTTTTTATAT

                     **        *  * **          *       *     *       *  *  *
     103865 GAAGATATTTACTTTTCCACCGTAGGCATCAAAGCGCTCCAAATGTCCACATCCAGATACTCCAG
        325 GAAGATATTCCCTTTTCCAACGAAATCATCAAAGCGATCCAAATATCCACTTCCAGATTCTACAA

              *            *             * *   *         * *      * *  *     
     103930 AACGAGTGTTTCAAACCTGCTCTATGAAAGGGAATCTTCAACTCTATGAGTTGAATGCAGACATC
        390 AAAGAGTGTTTCAAAACTGCTCTATGAAAAGAAATGTTCAACTCTGTTAGTTGAGTACACACATC

             *      *              *       **         **         **          
     103995 AGAAAGAAATTTCTGAGAATGCTGCTGTCTACCTTTTAT---TTGA-ATTCCCGCTTCCAACGAA
        455 ACAAAGAAGTTTCTGAGAATGCTTCTGTCTAGTTTTTATGGGAAGATATTCCCTTTTCCAACGAA

             * *  *    *              * *  **   *    *                       
     104056 ATCCTCCAAGCTATCCAAATATCCACCTGCATTTTCCACAACAAGAGTGTTTCAAAACTGCTCTA
        520 AGCATCAAAGCGATCCAAATATCCACTTCCAGATTCTACAAAAAGAGTGTTTCAAAACTGCTCTA

                **                *    *  *                *                 
     104121 TCAATAGAAATGTTCAACTCCTTTG-GCTGGGTACACACATCACAAACAAGTTTCTGAGAATGCT
        585 TCAAAGGAAATGTTCAACT-CTGTGAGTTGAGTACACACATCACAAAGAAGTTTCTGAGAATGCT

                                *  **         *  * *  **          *       *  
     104185 TCTGTCTAGTTTTTATGGGAAGACGTTCCCTTTTTCACCAAAGGCATCAAAGCGCTCCAAATGTC
        649 TCTGTCTAGTTTTTAT--GATGATATTCCCTTTTCCAACGAAATCATCAAAGCGATCCAAATATC

                      **                     **        **    **              
     104250 CACTTCCAGACACTACAAAAAGAGTGTTTCAAACGTG-CTCTAAGAAAGCGAATGTTCAACTCTG
        712 CACTTCCAGATTCTACAAAAAGAGTGTTTCAAAACTGCCTCTATCAAAGGAAATGTTCAACTCTG

             * *    * *  * *         *           **            *     ***  *  
     104314 TGACTTGAATGCAGATATCACAAAGTAGTTTCTGAGAGGGCTTCTGTCTAGATTTTAGATGATGA
        777 TTAGTTGAGTACACACATCACAAAGAAGTTTCTGAGAATGCTTCTGTCTAGTTTTTATGGGAAGA

                   *                * *   *                *   *             
     104379 TATTCCCGTTTCCAACGAAATCATTAGAGCTATCCAAATATCCACTTACAGTTTCTACAAAAAGA
        842 TATTCCCTTTTCCAACGAAATCATCAAAGCGATCCAAATATCCACTTCCAGATTCTACAAAAAGA

                   *        **      *  * *    *       *  *   * *  *          
     104444 GTGTTTCCAAACTGCTGCATCAAAAGAGAGGTTCCACTCTGTTAGCTGAGTACACACATCACAAA
        907 GTGTTTCAAAACTGCTCTATCAAAGGAAATGTTCAACTCTGTGAGTTGAATGCAGACATCACAAA

            ***      *     *         *                   **     *  *  * **   
     104509 CTTGTTTCTCAGAATCCTTCTGTCTCGTTTTTATGGGAAGATATTTACTTTTTCACCGTAGGCAT
        972 GAAGTTTCTGAGAATGCTTCTGTCTAGTTTTTAT--GAAGATATTCCCTTTTCCAACGAAATCAT

                   *       *     *       *  *  *               *         *   
     104574 CAAAGCGCTCCAAATGTCCACATCCAGATACTCCAGAAAGAGTGTTTCAAACCTGCTCTATGAAA
       1035 CAAAGCGATCCAAATATCCACTTCCAGATTCTACAAAAAGAGTGTTTCAAAACTGCTCTATCAAA

            * *   *         * *      * *  *      *      *              *     
     104639 GGGAATCTTCAACTCTATGAGTTGAATGCAGACATCAGAAAGAAATTTCTGAGAATGCTGCTGTC
       1100 AGAAATGTTCAACTCTGTTAGTTGAGTACACACATCACAAAGAAGTTTCTGAGAATGCTTCTGTC

              **         **          *  *
     104704 TACCTTTTAT---TTGA-ATTCCCGCTTC
       1165 TAGTTTTTATGGGAAGATATTCCCGTTTT

                      *  *    *                *       *                   * 
     104729 CAACGAAATCCTCCAAGCTATCCAAATATCCACTTGCAGATTCCACAAAAAGAGTGTTTCAAAAC
          1 CAACGAAATCATCAAAGCGATCCAAATATCCACTTCCAGATTCTACAAAAAGAGTGTTTCAAACC

                      *  *  *  *            *       *     *     *  *         
     104794 TGCTCTCTATCAATGGCAAAGTTCAACTCTGTTAGTTGAGGACACATATCACCAACAAGTTTCTG
         66 TG--CTCTA-GAAAGGGAATGTTCAACTCTGTGAGTTGAGTACACACATCACAAAGAAGTTTCTG

                            *       **         *  *   *  *  * **  **  *      
     104859 AGAATGCTTCTGTCTATTTTTTATGGGAAGATATTTCCTTTTTCACCGTAGGCGTCAAGGCGATC
        128 AGAATGCTTCTGTCTAGTTTTTATATGAAGATATTCCCGTTTCCAACGAAATCGCTAAAGCGATC

            *    *          * **                     *         *   * **      
     104924 GAAATGTCCACTTCCACAAACTACAAAAAGAGTGTTTCAAACCTGCTCTATGAAAGGCCATGTTC
        193 CAAATATCCACTTCCAGATTCTACAAAAAGAGTGTTTCAAAACTGCTCTATCAAAAGAAATGTTC

             *    * *      * ** * *   **      *      *       *          *    
     104989 ATCTCTATGAGTTGAATGGAAATATCCGAAAGAAATTTCTGGGAATGCTGCTGTCTAG-TGTT-T
        258 AACTCTGTTAGTTGAGTACACACATCACAAAGAAGTTTCTGAGAATGCTTCTGTCTAGTTTTTAT

                *         **             *       *                *    *  *  
     105052 AT-ACGA-ATTCCCGCTTCCAACGAAATCCTCAAAGCAATCCAAATATCCACTTGCAGAATCCAC
        323 ATGAAGATATTCCCTTTTCCAACGAAATCATCAAAGCGATCCAAATATCCACTTCCAGATTCTAC

                               *       *  *     *          *                 
     105115 AAAAAGAGTGTTTCAAAACCGCTCTATCAATAGAAAGGTTCAACTCTTTTAGTTGAGTACACACA
        388 AAAAAGAGTGTTTCAAAACTGCTCTATGAAAAGAAATGTTCAACTCTGTTAGTTGAGTACACACA

                   *                        * *      *      **  *         * *
     105180 TCACAAACAAGTTTCTGAGAATGCTTCTGTCTGGCTTTTATTGGAAGACGTTTCCTTTT-CACCA
        453 TCACAAAGAAGTTTCTGAGAATGCTTCTGTCTAGTTTTTATGGGAAGATATTCCCTTTTCCAACG

              *           *       *                * *                **  *  
     105244 AAGGCATCAAAGCGCTCCAAATGTCCACTTCCAGATTCTTCCAAAAGAGTGTTTCAAACGTGGTC
        518 AAAGCATCAAAGCGATCCAAATATCCACTTCCAGATTCTACAAAAAGAGTGTTTCAAAACTGCTC

            * ***    *                 *    * *  * *     *   *       * * *   
     105309 GAAGTAAGGGAATGTTCAACTCTGTGACTTGAATGCAGATATCACCAAGTAGTTTCTAATAGTGC
        583 TATCAAAGGAAATGTTCAACTCTGTGAGTTGAGTACACACATCACAAAGAAGTTTCTGAGAATGC

                            *              *              * * *   *          
     105374 TTCTGTCTAGATTTTAGATGATGATATTCCCGTTTCCAACGAAATCGTTAGAGCTATCCAAATAT
        648 TTCTGTCTAG-TTTT-TATGATGATATTCCCTTTTCCAACGAAATCATCAAAGCGATCCAAATAT

               *  *   *      *     *       *   *     **      *    *          
     105439 CCAGTTACAGTTTCTACCAAAAGGGTGTTTCCAAATTG-CTGCATCAAAAGAAAGGTTCAACTCT
        711 CCACTTCCAGATTCTACAAAAAGAGTGTTTCAAAACTGCCTCTATCAAAGGAAATGTTCAACTCT

                      *                     *                   *    * **  * 
     105503 GTTAGTTGAGGACACACATCACAAAGAAGTTTGTGAGAATGCTTCTGTCTAGATTTTGTATGACG
        776 GTTAGTTGAGTACACACATCACAAAGAAGTTTCTGAGAATGCTTCTGTCTAGTTTTTATGGGAAG

                               *   * *     *   *       * *  *    *  *       *
     105568 ATATTCCCTTTTCCAACGATATCGTTAAAGCAATCTAAATATCAATTTGCAGAATCCACAAAAAT
        841 ATATTCCCTTTTCCAACGAAATCATCAAAGCGATCCAAATATCCACTTCCAGATTCTACAAAAAG

              *        *       * *   *    *    *       *  *   * *  *         
     105633 AGAGTTTCAAAGCTGCTCTGTAAAAAGAAAGGTTCCACTCTGTTAGCTGAGTACACACATCACAA
        906 AGTGTTTCAAAACTGCTCTATCAAAGGAAATGTTCAACTCTGTGAGTTGAATGCAGACATCACAA

             ***           **         *                   **        ** * **  
     105698 ACTTGTTTCTGAGAAACCTTCTGTCTCGTTTTTATGGGAAGATATTTACTTTTCCACTGTAGGCA
        971 AGAAGTTTCTGAGAATGCTTCTGTCTAGTTTTTAT--GAAGATATTCCCTTTTCCAACGAAATCA

                    *       *     *       *  *  *  *            *         *  
     105763 TCAAAGCGCTCCAAATGTCCACATCCAGATACTCCAGAACGAGTGTTTCAAACCTGCTCTATGAA
       1034 TCAAAGCGATCCAAATATCCACTTCCAGATTCTACAAAAAGAGTGTTTCAAAACTGCTCTATCAA

             * *   *         * *      * *  *      *      *              *    
     105828 AGGGAATCTTCAACTCTATGAGTTGAATGCAGA-ATCAGAAAGAAATTTCTGAGAATGCTGCTGT
       1099 AAGAAATGTTCAACTCTGTTAGTTGAGTACACACATCACAAAGAAGTTTCTGAGAATGCTTCTGT

               **         **          *  *
     105892 CTACCTTTTAT---TTGA-ATTCCCGCTTC
       1164 CTAGTTTTTATGGGAAGATATTCCCGTTTT

                      *  *    *              * *  **   *    *              * 
     105918 CAACGAAATCCTCCAAGCTATCCAAATATCCACCTGCATTTTCCACAACAAGAGTGTTTCAAAAC
          1 CAACGAAATCATCAAAGCGATCCAAATATCCACTTCCAGATTCTACAAAAAGAGTGTTTCAAACC

                    *  ** *              *    *  *                *          
     105983 TGCTCTATCAATAGAAATGTTCAACTCCTTTG-GCTGGGTACACACATCACAAACAAGTTTCTGA
         66 TGCTCTA-GAAAGGGAATGTTCAACT-CTGTGAGTTGAGTACACACATCACAAAGAAGTTTCTGA

                                   **     **     *   *    *    *           * 
     106047 GAATGCTTCTGTCTAGTTTTTATGGGAAGACGTTCCCTTTTTC-ACCAAA-GGCATCAAAGCGCT
        129 GAATGCTTCTGTCTAGTTTTTATATGAAGATATTCCCGTTTCCAACGAAATCGC-T-AAAGCGAT

                  *            **                     **       **    **      
     106110 CCAAATGTCCACTTCCAGACACTACAAAAAGAGTGTTTCAAACGTGCTCTAAGAAAACGAATGTT
        192 CCAAATATCCACTTCCAGATTCTACAAAAAGAGTGTTTCAAAACTGCTCTATCAAAAGAAATGTT

                     * *    * *  * *         *           **            *     
     106175 CAACTCTGTGACTTGAATGCAGATATCACAAAGTAGTTTCTGAGAGGGCTTCTGTCTAGATTTTA
        257 CAACTCTGTTAGTTGAGTACACACATCACAAAGAAGTTTCTGAGAATGCTTCTGTCTAGTTTTTA

            *    *         *                * *   *                *   *     
     106240 GATGATGATATTCCCGTTTCCAACGAAATCATTAGAGCTATCCAAATATCCACTTACAGTTTCTA
        322 TATGAAGATATTCCCTTTTCCAACGAAATCATCAAAGCGATCCAAATATCCACTTCCAGATTCTA

                           *        **  *      * *    *          **          
     106305 CAAAAAGAGTGTTTCCAAACTGCTGCATCAAAAGAGAGGTTCCACTCTGTTAGCCGAGTACACAC
        387 CAAAAAGAGTGTTTCAAAACTGCTCTATGAAAAGAAATGTTCAACTCTGTTAGTTGAGTACACAC

                    ***      *     *         *                   **     *  * 
     106370 ATCACAAACTTGTTTCTCAGAATCCTTCTGTCTCGTTTTTATGGGAAGATATTTACTTTTTCACC
        452 ATCACAAAGAAGTTTCTGAGAATGCTTCTGTCTAGTTTTTATGGGAAGATATTCCCTTTTCCAAC

             * *           *       *     *       *  *  *               *     
     106435 GTAGGCATCAAAGCGCTCCAAATGTCCACATCCAGATACTCCAGAAAGAGTGTTTCAAACCTGCT
        517 GAAAGCATCAAAGCGATCCAAATATCCACTTCCAGATTCTACAAAAAGAGTGTTTCAAAACTGCT

                *     *   *         *        * *  *      *      *            
     106500 CTATGAAAGGGAATCTTCAACTCTATGAGTTGAATGCAGACATCAGAAAGAAATTTCTGAGAATG
        582 CTATCAAAGGAAATGTTCAACTCTGTGAGTTGAGTACACACATCACAAAGAAGTTTCTGAGAATG

              *       **       *          **             *  *    *           
     106565 CTGCTGTCTACCTTTTAT-TTGA-ATTCCCGCTTCCAACGAAATCCTCCAAGCTATCCAAATATC
        647 CTTCTGTCTAGTTTTTATGATGATATTCCCTTTTCCAACGAAATCATCAAAGCGATCCAAATATC

                 *       *                                  *  *  *          
     106628 CACTTGCAGATTCCACAAAAAGAGTGTTTCAAAACTGCTCTCTATCAATGGCAAAGTTCAACTCT
        712 CACTTCCAGATTCTACAAAAAGAGTGTTTCAAAACTGC-CTCTATCAAAGGAAATGTTCAACTCT

                      *     *     *  *                         *             
     106693 GTTAGTTGAGGACACATATCACCAACAAGTTTCTGAGAATGCTTCTGTCTATTTTTTATGGGAAG
        776 GTTAGTTGAGTACACACATCACAAAGAAGTTTCTGAGAATGCTTCTGTCTAGTTTTTATGGGAAG

                 *      *  *  * ** *    *      *    *          * **          
     106758 ATATTTCCTTTTTCACCGTAGGCGTCAAGGCGATCGAAATGTCCACTTCCACAAACTACAAAAAG
        841 ATATTCCCTTTTCCAACGAAATCATCAAAGCGATCCAAATATCCACTTCCAGATTCTACAAAAAG

                       *         *     **       *    *           * * *   **  
     106823 AGTGTTTCAAACCTGCTCTATGAAAGGCCATGTTCATCTCTATGAGTTGAATGGAAATATCCGAA
        906 AGTGTTTCAAAACTGCTCTATCAAAGGAAATGTTCAACTCTGTGAGTTGAATGCAGACATCACAA

                *      *       *                 *         **             *  
     106888 AGAAATTTCTGGGAATGCTGCTGTCTAGTTTTTAT-ATGA-ATTCCCGCTTCCAACGAAATCCTC
        971 AGAAGTTTCTGAGAATGCTTCTGTCTAGTTTTTATGAAGATATTCCCTTTTCCAACGAAATCATC

                 *                *    *  *                                * 
     106951 AAAGCAATCCAAATATCCACTTGCAGAATCCACAAAAAGAGTGTTTCAAAACTGCTCTATCAATA
       1036 AAAGCGATCCAAATATCCACTTCCAGATTCTACAAAAAGAGTGTTTCAAAACTGCTCTATCAAAA

                *          *                        *                        
     107016 GAAAGGTTCAACTCTTTTAGTTGAGTACACACATCACAAACAAGTTTCTGAGAATGCTTCTGTCT
       1101 GAAATGTTCAACTCTGTTAGTTGAGTACACACATCACAAAGAAGTTTCTGAGAATGCTTCTGTCT

            * *      *      **  *       
     107081 GGCTTTTATTGGAAGACGTTTCC-TTTT
       1166 AGTTTTTATGGGAAGATATTCCCGTTTT

              * *  **          *      **                * *                 *
     107108 CACCAAAGGCATCAAAGCGCTCCAAAGGTCCACTTCCAGATTCTTCCAAAAGAGTGTTTCAAACG
          1 CAACGAAATCATCAAAGCGATCCAAATATCCACTTCCAGATTCTACAAAAAGAGTGTTTCAAACC

                    **                      *    * *  * *     *   *       * *
     107173 TGCTCATACTAAGGGAATGTTCAACTCTGTGACTTGAATGCAGATATCACCAAGTAGTTTCTAAT
         66 TGCTC-TAGAAAGGGAATGTTCAACTCTGTGAGTTGAGTACACACATCACAAAGAAGTTTCTGAG

             *            **     *    *                            *   *     
     107238 AGTGCTTCTGTCTATATTTTAGATGATGATATTCCCGTTTCCAACGAAATCGCTAGAGCTATCCA
        130 AATGCTTCTGTCTAGTTTTTATATGAAGATATTCCCGTTTCCAACGAAATCGCTAAAGCGATCCA

                    *  *   *      *     *       *   *    **           *      
     107303 AATATCCAGTTACAGTTTCTACCAAAAGGGTGTTTCCAAATTGCTGCATCAAAAGAAAGGTTCAA
        195 AATATCCACTTCCAGATTCTACAAAAAGAGTGTTTCAAAACTGCTCTATCAAAAGAAATGTTCAA

                          *                     *                   *    *   
     107368 CTCTGTTAGTTGAGGACACACATCACAAAGAAGTTTGTGAGAATGCTTCTGTCTAGATTTTGTAT
        260 CTCTGTTAGTTGAGTACACACATCACAAAGAAGTTTCTGAGAATGCTTCTGTCTAGTTTTTATAT

              *                    *   * *     *   *       * *  *    *  *    
     107433 GACGATATTCCCTTTTCCAACGATATCGTTAAAGCAATCTAAATATCAATTTGCAGAATCCACAA
        325 GAAGATATTCCCTTTTCCAACGAAATCATCAAAGCGATCCAAATATCCACTTCCAGATTCTACAA

               *  *        *       * *        *    *          *              
     107498 AAATAGAGTTTCAAAGCTGCTCTGTAAAAAGAAAGGTTCCACTCTGTTAGCTGAGTACACACATC
        390 AAAGAGTGTTTCAAAACTGCTCTATGAAAAGAAATGTTCAACTCTGTTAGTTGAGTACACACATC

                 ***            *         *                   **        *  * 
     107563 ACAAACTTGTTTCTGAGAATCCTTCTGTCTCGTTTTTATGGGAAGATATTTACTTTTCCACCGTA
        455 ACAAAGAAGTTTCTGAGAATGCTTCTGTCTAGTTTTTATGGGAAGATATTCCCTTTTCCAACGAA

            *           *       *     *       *  *  *  *            *        
     107628 GGCATCAAAGCGCTCCAAATGTCCACATCCAGATACTCCAGAACGAGTGTTTCAAACCTGCTCTA
        520 AGCATCAAAGCGATCCAAATATCCACTTCCAGATTCTACAAAAAGAGTGTTTCAAAACTGCTCTA

             *     *   *         *        * *  *      *      *              *
     107693 TGAAAGGGAATCTTCAACTCTATGAGTTGAATGCAGACATCAGAAAGAAATTTCTGAGAATGCTG
        585 TCAAAGGAAATGTTCAACTCTGTGAGTTGAGTACACACATCACAAAGAAGTTTCTGAGAATGCTT

                   **       *          **             *  *    *              
     107758 CTGTCTACCTTTTAT-TTGA-ATTCCCGCTTCCAACGAAATCCTCCAAGCTATCCAAATATCCAC
        650 CTGTCTAGTTTTTATGATGATATTCCCTTTTCCAACGAAATCATCAAAGCGATCCAAATATCCAC

            * *  **   *    *                            **              **  *
     107821 CTGCATTTTCCACAACAAGAGTGTTTCAAAACTG-CTCTATCAATAGAAATGTTCAACTCCTTTG
        715 TTCCAGATTCTACAAAAAGAGTGTTTCAAAACTGCCTCTATCAAAGGAAATGTTCAACTCTGTTA

             *  *                *                                        ** 
     107885 GCTGGGTACACACATCACAAACAAGTTTCTGAGAATGCTTCTGTCTAGTTTTTATGGGAAGACGT
        780 GTTGAGTACACACATCACAAAGAAGTTTCTGAGAATGCTTCTGTCTAGTTTTTATGGGAAGATAT

                    *  * *  **          *       *            **              
     107950 TCCCTTTTTCACCAAAGGCATCAAAGCGCTCCAAATGTCCACTTCCAGACACTACAAAAAGAGTG
        845 TCCCTTTTCCAACGAAATCATCAAAGCGATCCAAATATCCACTTCCAGATTCTACAAAAAGAGTG

                   **       **    **                 *           *         * 
     108015 TTTCAAACGTGCTCTAAGAAAGCGAATGTTCAACTCTGTGACTTGAATGCAGATATCACAAAGTA
        910 TTTCAAAACTGCTCTATCAAAGGAAATGTTCAACTCTGTGAGTTGAATGCAGACATCACAAAGAA

                      **                  *    *         *                * *
     108080 GTTTCTGAGAGGGCTTCTGTCTAGATTTTAGATGATGATATTCCCGTTTCCAACGAAATCATTAG
        975 GTTTCTGAGAATGCTTCTGTCTAG-TTTT-TATGAAGATATTCCCTTTTCCAACGAAATCATCAA

               *                *   *                    *        **         
     108145 AGCTATCCAAATATCCACTTACAGTTTCTACAAAAAGAGTGTTTCCAAACTGCTGCATCAAAAGA
       1038 AGCGATCCAAATATCCACTTCCAGATTCTACAAAAAGAGTGTTTCAAAACTGCTCTATCAAAAGA

            * *    *          *                   ***      *     *         * 
     108210 GAGGTTCCACTCTGTTAGCTGAGTACACACATCACAAACTTGTTTCTCAGAATCCTTCTGTCTCG
       1103 AATGTTCAACTCTGTTAGTTGAGTACACACATCACAAAGAAGTTTCTGAGAATGCTTCTGTCTAG

                              ** *    
     108275 TTTTTATGGGAAGATATTTACTTTTT
       1168 TTTTTATGGGAAGATATTCCCGTTTT

              *  * **          *       *     *       *  *  *                 
     108301 CACCGTAGGCATCAAAGCGCTCCAAATGTCCACATCCAGATACTCCAGAAAGAGTGTTTCAAACC
          1 CAACGAAATCATCAAAGCGATCCAAATATCCACTTCCAGATTCTACAAAAAGAGTGTTTCAAACC

                              *         *        * *  *      *      *        
     108366 TGCTCTATGAAAGGGAATCTTCAACTCTATGAGTTGAATGCAGACATCAGAAAGAAATTTCTGAG
         66 TGCTCTA-GAAAGGGAATGTTCAACTCTGTGAGTTGAGTACACACATCACAAAGAAGTTTCTGAG

                  *       **          *          *                 *    *    
     108431 AATGCTGCTGTCTACCTTTTAT-T--TGA-ATTCCCGCTTCCAACGAAATC-CTCCAAGCTATCC
        130 AATGCTTCTGTCTAGTTTTTATATGAAGATATTCCCGTTTCCAACGAAATCGCT-AAAGCGATCC

                        *       *                                  ** *  *   
     108491 AAATATCCACTTGCAGATTCCACAAAAAGAGTGTTTCAAAACTGCTCTCTATCAATGGCAAAGTT
        194 AAATATCCACTTCCAGATTCTACAAAAAGAGTGTTTCAAAACTG--CTCTATCAAAAGAAATGTT

                             *     *     *  *                         *      
     108556 CAACTCTGTTAGTTGAGGACACATATCACCAACAAGTTTCTGAGAATGCTTCTGTCTATTTTTTA
        257 CAACTCTGTTAGTTGAGTACACACATCACAAAGAAGTTTCTGAGAATGCTTCTGTCTAGTTTTTA

             **         *      *  *  * ** *    *      *    *          * **   
     108621 TGGGAAGATATTTCCTTTTTCACCGTAGGCGTCAAGGCGATCGAAATGTCCACTTCCACAAACTA
        322 TATGAAGATATTCCCTTTTCCAACGAAATCATCAAAGCGATCCAAATATCCACTTCCAGATTCTA

                              *             * **       *    * *      * ** * *
     108686 CAAAAAGAGTGTTTCAAACCTGCTCTATGAAAGGCCATGTTCATCTCTATGAGTTGAATGGAAAT
        387 CAAAAAGAGTGTTTCAAAACTGCTCTATGAAAAGAAATGTTCAACTCTGTTAGTTGAGTACACAC

               **      *      *       *         *         *         **       
     108751 ATCCGAAAGAAATTTCTGGGAATGCTGCTGTCTAGTGTTTAT---ACGA-ATTCCCGCTTCCAAC
        452 ATCACAAAGAAGTTTCTGAGAATGCTTCTGTCTAGTTTTTATGGGAAGATATTCCCTTTTCCAAC

                * *       *                *    *  *                     *   
     108812 GAAATCCTCAAAGCAATCCAAATATCCACTTGCAGAATCCACAAAAAGAGTGTTTCAAAACCGCT
        517 GAAAGCATCAAAGCGATCCAAATATCCACTTCCAGATTCTACAAAAAGAGTGTTTCAAAACTGCT

                   **    *          * *                      *               
     108877 CTATCAATAGAAAGGTTCAACTCTTTTAGTTGAGTACACACATCACAAACAAGTTTCTGAGAATG
        582 CTATCAAAGGAAATGTTCAACTCTGTGAGTTGAGTACACACATCACAAAGAAGTTTCTGAGAATG

                     * *          *  **  *         * *  **          *       *
     108942 CTTCTGTCTGGCTTTTATTGGAAGACGTTTCCTTTT-CACCAAAGGCATCAAAGCGCTCCAAATG
        647 CTTCTGTCTAGTTTTTA-T-GATGATATTCCCTTTTCCAACGAAATCATCAAAGCGATCCAAATA

                            * *                **   *  * ***    *            
     109006 TCCACTTCCAGATTCTTCCAAAAGAGTGTTTCAAACGTG-GTCGAAGTAAGGGAATGTTCAACTC
        710 TCCACTTCCAGATTCTACAAAAAGAGTGTTTCAAAACTGCCTCTATCAAAGGAAATGTTCAACTC

               * *    * *  * *     *   *       * * *             *     ***  *
     109070 TGTGACTTGAATGCAGATATCACCAAGTAGTTTCTAATAGTGCTTCTGTCTAGATTTTAGATGAT
        775 TGTTAGTTGAGTACACACATCACAAAGAAGTTTCTGAGAATGCTTCTGTCTAGTTTTTATGGGAA

                     *              * * *   *             *  *   *      *    
     109135 GATATTCCCGTTTCCAACGAAATCGTTAGAGCTATCCAAATATCCAGTTACAGTTTCTACCAAAA
        840 GATATTCCCTTTTCCAACGAAATCATCAAAGCGATCCAAATATCCACTTCCAGATTCTACAAAAA

             *       *   *    **      *    *            *      ***  *        
     109200 GGGTGTTTCCAAATTGCTGCATCAAAAGAAAGGTTCAACTCTGTTAGTTGAGGACACACATCACA
        905 GAGTGTTTCAAAACTGCTCTATCAAAGGAAATGTTCAACTCTGTGAGTTGAATGCAGACATCACA

                     *                              *                    *   
     109265 AAGAAGTTTGTGAGAATGCTTCTGTCTAGATTTTGTATGACGATATTCCCTTTTCCAACGATATC
        970 AAGAAGTTTCTGAGAATGCTTCTGTCTAG-TTTT-TATGAAGATATTCCCTTTTCCAACGAAATC

            * *     *   *       * *  *    *  *       *  *        *       * * 
     109330 GTTAAAGCAATCTAAATATCAATTTGCAGAATCCACAAAAATAGAGTTTCAAAGCTGCTCTGTAA
       1033 ATCAAAGCGATCCAAATATCCACTTCCAGATTCTACAAAAAGAGTGTTTCAAAACTGCTCTATCA

                   *    *          *                   ***           **      
     109395 AAAGAAAGGTTCCACTCTGTTAGCTGAGTACACACATCACAAACTTGTTTCTGAGAAACCTTCTG
       1098 AAAGAAATGTTCAACTCTGTTAGTTGAGTACACACATCACAAAGAAGTTTCTGAGAATGCTTCTG

               *                   **      
     109460 TCTCGTTTTTATGGGAAGATATTTAC-TTTT
       1163 TCTAGTTTTTATGGGAAGATATTCCCGTTTT

             *    * **          *       *     *       *  *  *  *             
     109490 CCACTGTAGGCATCAAAGCGCTCCAAATGTCCACATCCAGATACTCCAGAACGAGTGTTTCAAAC
          1 CAAC-GAAATCATCAAAGCGATCCAAATATCCACTTCCAGATTCTACAAAAAGAGTGTTTCAAAC

                               *         *        * *  *      *      *       
     109555 CTGCTCTATGAAAGGGAATCTTCAACTCTATGAGTTGAATGCAGA-ATCAGAAAGAAATTTCTGA
         65 CTGCTCTA-GAAAGGGAATGTTCAACTCTGTGAGTTGAGTACACACATCACAAAGAAGTTTCTGA

                   *       **          *          *                 *    *   
     109619 GAATGCTGCTGTCTACCTTTTAT-T--TGA-ATTCCCGCTTCCAACGAAATC-CTCCAAGCTATC
        129 GAATGCTTCTGTCTAGTTTTTATATGAAGATATTCCCGTTTCCAACGAAATCGCT-AAAGCGATC

                       * *  **   *    *                           *          
     109679 CAAATATCCACCTGCATTTTCCACAACAAGAGTGTTTCAAAACTGCTCTATCAATAGAAATGTTC
        193 CAAATATCCACTTCCAGATTCTACAAAAAGAGTGTTTCAAAACTGCTCTATCAAAAGAAATGTTC

                 **  * *  *                *                                 
     109744 AACTCCTTTGGCTGGGTACACACATCACAAACAAGTTTCTGAGAATGCTTCTGTCTAGTTTTTAT
        258 AACTCTGTTAGTTGAGTACACACATCACAAAGAAGTTTCTGAGAATGCTTCTGTCTAGTTTTTAT

            **     **         *  * *  **          *       *            **    
     109809 GGGAAGACGTTCCCTTTTTCACCAAAGGCATCAAAGCGCTCCAAATGTCCACTTCCAGACACTAC
        323 ATGAAGATATTCCCTTTTCCAACGAAATCATCAAAGCGATCCAAATATCCACTTCCAGATTCTAC

                             **       *     **               * *    * *  * * 
     109874 AAAAAGAGTGTTTCAAACGTGCTCTAAGAAAACGAATGTTCAACTCTGTGACTTGAATGCAGATA
        388 AAAAAGAGTGTTTCAAAACTGCTCTATGAAAAGAAATGTTCAACTCTGTTAGTTGAGTACACACA

                    *           **            *     ***  *         *         
     109939 TCACAAAGTAGTTTCTGAGAGGGCTTCTGTCTAGATTTTAGATGATGATATTCCCGTTTCCAACG
        453 TCACAAAGAAGTTTCTGAGAATGCTTCTGTCTAGTTTTTATGGGAAGATATTCCCTTTTCCAACG

               *   * *   *                *   *                    *        *
     110004 AAATCATTAGAGCTATCCAAATATCCACTTACAGTTTCTACAAAAAGAGTGTTTCCAAACTGCTG
        518 AAAGCATCAAAGCGATCCAAATATCCACTTCCAGATTCTACAAAAAGAGTGTTTCAAAACTGCTC

            *      *  * *    *       *  **                  ***      *     * 
     110069 CATCAAAAGAGAGGTTCCACTCTGTTAGCCGAGTACACACATCACAAACTTGTTTCTCAGAATCC
        583 TATCAAAGGAAATGTTCAACTCTGTGAGTTGAGTACACACATCACAAAGAAGTTTCTGAGAATGC

                    *            *      **     *  *  * **          *       * 
     110134 TTCTGTCTCGTTTTTATGGGAAGATATTTACTTTTTCACCGTAGGCATCAAAGCGCTCCAAATGT
        648 TTCTGTCTAGTTTTTAT--GATGATATTCCCTTTTCCAACGAAATCATCAAAGCGATCCAAATAT

                *       *  *  *               *          *     *   *         
     110199 CCACATCCAGATACTCCAGAAAGAGTGTTTCAAACCTG-CTCTATGAAAGGGAATCTTCAACTCT
        711 CCACTTCCAGATTCTACAAAAAGAGTGTTTCAAAACTGCCTCTATCAAAGGAAATGTTCAACTCT

            * *      * *  *      *      *              *       **         ** 
     110263 ATGAGTTGAATGCAGACATCAGAAAGAAATTTCTGAGAATGCTGCTGTCTACCTTTTAT---TTG
        776 GTTAGTTGAGTACACACATCACAAAGAAGTTTCTGAGAATGCTTCTGTCTAGTTTTTATGGGAAG

                    **             *  *    *                *       *        
     110325 A-ATTCCCGCTTCCAACGAAATCCTCCAAGCTATCCAAATATCCACTTGCAGATTCCACAAAAAG
        841 ATATTCCCTTTTCCAACGAAATCATCAAAGCGATCCAAATATCCACTTCCAGATTCTACAAAAAG

                                      *  *  *            *      ***  * *     
     110389 AGTGTTTCAAAACTGCTCTCTATCAATGGCAAAGTTCAACTCTGTTAGTTGAGGACACATATCAC
        906 AGTGTTTCAAAACTG--CTCTATCAAAGGAAATGTTCAACTCTGTGAGTTGAATGCAGACATCAC

            *  *                         *                  *      *  *  * **
     110454 CAACAAGTTTCTGAGAATGCTTCTGTCTATTTTTTATGGGAAGATATTTCCTTTTTCACCGTAGG
        969 AAAGAAGTTTCTGAGAATGCTTCTGTCTAGTTTTTAT--GAAGATATTCCCTTTTCCAACGAAAT

             *    *      *    *          * **                     *         *
     110519 CGTCAAGGCGATCGAAATGTCCACTTCCACAAACTACAAAAAGAGTGTTTCAAACCTGCTCTATG
       1032 CATCAAAGCGATCCAAATATCCACTTCCAGATTCTACAAAAAGAGTGTTTCAAAACTGCTCTATC

               * **       *    * *      * ** * *   **      *      *       *  
     110584 AAAGGCCATGTTCATCTCTATGAGTTGAATGGAAATATCCGAAAGAAATTTCTGGGAATGCTGCT
       1097 AAAAGAAATGTTCAACTCTGTTAGTTGAGTACACACATCACAAAGAAGTTTCTGAGAATGCTTCT

                             *          *  *
     110649 GTCTAGTTTTTAT---ATGA-ATTCCCGCTTC
       1162 GTCTAGTTTTTATGGGAAGATATTCCCGTTTT

                      *       *                *    *  *                   * 
     110677 CAACGAAATCCTCAAAGCAATCCAAATATCCACTTGCAGAATCCACAAAAAGAGTGTTTCAAAAC
          1 CAACGAAATCATCAAAGCGATCCAAATATCCACTTCCAGATTCTACAAAAAGAGTGTTTCAAACC

                    *  ** *  *          * *                      *           
     110742 TGCTCTATCAATAGAAAGGTTCAACTCTTTTAGTTGAGTACACACATCACAAACAAGTTTCTGAG
         66 TGCTCTA-GAAAGGGAATGTTCAACTCTGTGAGTTGAGTACACACATCACAAAGAAGTTTCTGAG

                         * *      **     **  *      *    *    *           *  
     110807 AATGCTTCTGTCTGGCTTTTATTGGAAGACGTTTCC-TTTTC-ACCAAA-GGCATCAAAGCGCTC
        130 AATGCTTCTGTCTAGTTTTTATATGAAGATATTCCCGTTTCCAACGAAATCGC-T-AAAGCGATC

                **                * *                **         **  * *      
     110869 CAAAGGTCCACTTCCAGATTCTTCCAAAAGAGTGTTTCAAACGTGCTC-ATAGTAAGGGAATGTT
        193 CAAATATCCACTTCCAGATTCTACAAAAAGAGTGTTTCAAAACTGCTCTAT-CAAAAGAAATGTT

                     * *    * *  * *     *   *       * * *             *     
     110933 CAACTCTGTGACTTGAATGCAGATATCACCAAGTAGTTTCTAATAGTGCTTCTGTCTAGATTTTA
        257 CAACTCTGTTAGTTGAGTACACACATCACAAAGAAGTTTCTGAGAATGCTTCTGTCTAGTTTTTA

            *    *         *              *** *   *             *  *   *     
     110998 GATGATGATATTCCCGTTTCCAACGAAATCGCTAGAGCTATCCAAATATCCAGTTACAGTTTCTA
        322 TATGAAGATATTCCCTTTTCCAACGAAATCATCAAAGCGATCCAAATATCCACTTCCAGATTCTA

             *     *       *   *    **  *        *                    *      
     111063 CCAAAAGGGTGTTTCCAAATTGCTGCATCAAAAGAAAGGTTCAACTCTGTTAGTTGAGGACACAC
        387 CAAAAAGAGTGTTTCAAAACTGCTCTATGAAAAGAAATGTTCAACTCTGTTAGTTGAGTACACAC

                           *                  **    * **  *                  
     111128 ATCACAAAGAAGTTTGTGAGAATGCTTCTGTCTACATTTTGTATGACGATATTCCCTTTTCCAAC
        452 ATCACAAAGAAGTTTCTGAGAATGCTTCTGTCTAGTTTTTATGGGAAGATATTCCCTTTTCCAAC

              * * * *     *   *       * *  *    *  *       *  *        *     
     111193 GATATCGTTAAAGCAATCTAAATATCAATTTGCAGAATCCACAAAAATAGAGTTTCAAAGCTGCT
        517 GAAAGCATCAAAGCGATCCAAATATCCACTTCCAGATTCTACAAAAAGAGTGTTTCAAAACTGCT

              * *   *    *    *       *  *                   ***            *
     111258 CTGTAAAAAGAAAGGTTCCACTCTGTTAGCTGAGTACACACATCACAAACTTGTTTCTGAGAATC
        582 CTATCAAAGGAAATGTTCAACTCTGTGAGTTGAGTACACACATCACAAAGAAGTTTCTGAGAATG

                     *            *      **        *  * **          *       *
     111323 CTTCTGTCTCGTTTTTATGGGAAGATATTTACTTTTCCACCGTAGGCATCAAAGCGCTCCAAATG
        647 CTTCTGTCTAGTTTTTAT--GATGATATTCCCTTTTCCAACGAAATCATCAAAGCGATCCAAATA

                 *       *  *  *  *            *          *     *   *        
     111388 TCCACATCCAGATACTCCAGAACGAGTGTTTCAAACCTG-CTCTATGAAAGGGAATCTTCAACTC
        710 TCCACTTCCAGATTCTACAAAAAGAGTGTTTCAAAACTGCCTCTATCAAAGGAAATGTTCAACTC

             * *      * *  *      *      *              *       **         **
     111452 TATGAGTTGAATGCAGACATCAGAAAGAAATTTCTGAGAATGCTGCTGTCTACCTTTTAT---TT
        775 TGTTAGTTGAGTACACACATCACAAAGAAGTTTCTGAGAATGCTTCTGTCTAGTTTTTATGGGAA

                     **             *  *    *              * *  **   *    *  
     111514 GA-ATTCCCGCTTCCAACGAAATCCTCCAAGCTATCCAAATATCCACCTGCATTTTCCACAACAA
        840 GATATTCCCTTTTCCAACGAAATCATCAAAGCGATCCAAATATCCACTTCCAGATTCTACAAAAA

                                     **                *    *  ** *  *       
     111578 GAGTGTTTCAAAACTGCTCTATCAATAGAAATGTTCAACTCCTTTG-GCTGGGTACACACATCAC
        905 GAGTGTTTCAAAACTGCTCTATCAAAGGAAATGTTCAACT-CTGTGAGTTGAATGCAGACATCAC

               *                                        **         *  * *  **
     111642 AAACAAGTTTCTGAGAATGCTTCTGTCTAGTTTTTATGGGAAGACGTTCCCTTTTTCACCAAAGG
        969 AAAGAAGTTTCTGAGAATGCTTCTGTCTAGTTTTTAT--GAAGATATTCCCTTTTCCAACGAAAT

                      *       *            **                  *  **   *   **
     111707 CATCAAAGCGCTCCAAATGTCCACTTCCAGACACTACAAAAAGAGTGTTTCCAACGTGCCCTAAG
       1032 CATCAAAGCGATCCAAATATCCACTTCCAGATTCTACAAAAAGAGTGTTTCAAAACTGCTCTATC

               ***               * *    * *  * *         *           **      
     111772 AAAGCGAATGTTCAACTCTGTGACTTGAATGCAGATATCACAAAGTAGTTTCTGAGAGGGCTTCT
       1097 AAAAGAAATGTTCAACTCTGTTAGTTGAGTACACACATCACAAAGAAGTTTCTGAGAATGCTTCT

                  *     ***  *             *
     111837 GTCTAGATTTTAGATGATGATATTCCCGTTTC
       1162 GTCTAGTTTTTATGGGAAGATATTCCCGTTTT

                        * *   *                *   *                    *  * 
     111869 CAACGAAATCATTAGAGCTATCCAAATATCCACTTACAGTTTCTACAAAAAGAGTGTTTCCAAAC
          1 CAACGAAATCATCAAAGCGATCCAAATATCCACTTCCAGATTCTACAAAAAGAGTGTTTCAAACC

                    **    *   *    *       *  *                   ***      * 
     111934 TGCTGCATCAAAAGAG-AGGTTCCACTCTGTTAGCTGAGTACACACATCACAAACTTGTTTCTCA
         66 TGCT-C-TAGAAAGGGAATGTTCAACTCTGTGAGTTGAGTACACACATCACAAAGAAGTTTCTGA

                *         *        **         ** *   *  *  * ** ***      *   
     111998 GAATCCTTCTGTCTCGTTTTTATGGGAAGATATTTACTTTTTCACCGTAGGCATCAAAGCGCTCC
        129 GAATGCTTCTGTCTAGTTTTTATATGAAGATATTCCCGTTTCCAACGAAATCGCTAAAGCGATCC

                *     *       *  *  *               *         *   * *   *    
     112063 AAATGTCCACATCCAGATACTCCAGAAAGAGTGTTTCAAACCTGCTCTATGAAAGGGAATCTTCA
        194 AAATATCCACTTCCAGATTCTACAAAAAGAGTGTTTCAAAACTGCTCTATCAAAAGAAATGTTCA

                 * *      * *  *      *      *              *       **       
     112128 ACTCTATGAGTTGAATGCAGACATCAGAAAGAAATTTCTGAGAATGCTGCTGTCTACCTTTTAT-
        259 ACTCTGTTAGTTGAGTACACACATCACAAAGAAGTTTCTGAGAATGCTTCTGTCTAGTTTTTATA

               *         **             *  *    *                *       *   
     112192 T--TGA-ATTCCCGCTTCCAACGAAATCCTCCAAGCTATCCAAATATCCACTTGCAGATTCCACA
        324 TGAAGATATTCCCTTTTCCAACGAAATCATCAAAGCGATCCAAATATCCACTTCCAGATTCTACA

                                        *  ** *  *                    *     *
     112254 AAAAGAGTGTTTCAAAACTGCTCTCTATCAATGGCAAAGTTCAACTCTGTTAGTTGAGGACACAT
        389 AAAAGAGTGTTTCAAAACTG--CTCTATGAAAAGAAATGTTCAACTCTGTTAGTTGAGTACACAC

                 *  *                         *                  *      *  * 
     112319 ATCACCAACAAGTTTCTGAGAATGCTTCTGTCTATTTTTTATGGGAAGATATTTCCTTTTTCACC
        452 ATCACAAAGAAGTTTCTGAGAATGCTTCTGTCTAGTTTTTATGGGAAGATATTCCCTTTTCCAAC

             * *  *    *      *    *          * **          *          *     
     112384 GTAGGCGTCAAGGCGATCGAAATGTCCACTTCCACAAACTACAAAAAGTGTGTTTCAAACCTGCT
        517 GAAAGCATCAAAGCGATCCAAATATCCACTTCCAGATTCTACAAAAAGAGTGTTTCAAAACTGCT

                *     **       *    *        * ** * *   **      *      *     
     112449 CTATGAAAGGCCATGTTCATCTCTATGAGTTGAATGGAAATATCCGAAAGAAATTTCTGGGAATG
        582 CTATCAAAGGAAATGTTCAACTCTGTGAGTTGAGTACACACATCACAAAGAAGTTTCTGAGAATG

              *         *       *         **             *       *           
     112514 CTGCTGTCTAGTGTTTAT-ACGA-ATTCCCGCTTCCAACGAAATCCTCAAAGCAATCCAAATATC
        647 CTTCTGTCTAGTTTTTATGATGATATTCCCTTTTCCAACGAAATCATCAAAGCGATCCAAATATC

                 *    *  *                                 **    *          *
     112577 CACTTGCAGAATCCACAAAAAGAGTGTTTCAAAACTG-CTCTATCAATAGAAAGGTTCAACTCTT
        712 CACTTCCAGATTCTACAAAAAGAGTGTTTCAAAACTGCCTCTATCAAAGGAAATGTTCAACTCTG

                                 *  *                        * *      *      
     112641 TTAGTTGAGTACACACATCACGAACAAGTTTCTGAGAATGCTTCTGTCTGGCTTTTATTGGAAGA
        777 TTAGTTGAGTACACACATCACAAAGAAGTTTCTGAGAATGCTTCTGTCTAGTTTTTATGGGAAGA

            **  *         * *  **          *       *                * *      
     112706 CGTTTCCTTTT-CACCAAAGGCATCAAAGCGCTCCAAATGTCCACTTCCAGATTCTTCCAAAAGA
        842 TATTCCCTTTTCCAACGAAATCATCAAAGCGATCCAAATATCCACTTCCAGATTCTACAAAAAGA

                      **     * ***    *                 *           *     *  
     112770 GTGTTTCAAACGTGCTCAAAGTAAGGGAATGTTCAACTCTGTGACTTGAATGCAGATATCACCAA
        907 GTGTTTCAAAACTGCTCTATCAAAGGAAATGTTCAACTCTGTGAGTTGAATGCAGACATCACAAA

             *       * * *                   *    *         *              * 
     112835 GTAGTTTCTAATAGTGCTTCTGTCTAGATTTTAGATGATGATATTCCCGTTTCCAACGAAATCGT
        972 GAAGTTTCTGAGAATGCTTCTGTCTAG-TTTT-TATGAAGATATTCCCTTTTCCAACGAAATCAT

            * *   * **          *  *   *      *     *       *   *    **      
     112900 TAGAGCTAAGCAAATATCCAGTTACAGTTTCTACCAAAAGGGTGTTTCCAAATTGCTGCATCAAA
       1035 CAAAGCGATCCAAATATCCACTTCCAGATTCTACAAAAAGAGTGTTTCAAAACTGCTCTATCAAA

                 *                    *                     *                
     112965 AGAAAGGTTCAACTCTGTTAGTTGAGGACACACATCACAAAGAAGTTTGTGAGAATGCTTCTGTC
       1100 AGAAATGTTCAACTCTGTTAGTTGAGTACACACATCACAAAGAAGTTTCTGAGAATGCTTCTGTC

               *    * **  *         *   *
     113030 TAGATTTTGTATGACGATATTCCCTTTTC
       1165 TAGTTTTTATGGGAAGATATTCCCGTTTT

                  *   * *     *   *       * *  *    *  *       *  *        * 
     113059 CAACGATATCGTTAAAGCAATCTAAATATCAATTTGCAGAATCCACAAAAATAGAGTTTCAAAGC
          1 CAACGAAATCATCAAAGCGATCCAAATATCCACTTCCAGATTCTACAAAAAGAGTGTTTCAAACC

                   **   * *  *    *       *  *                   *** *       
     113124 TGCTCTGTAAAAAGAAAGGTTCCACTCTGTTAGCTGAGTACACACATCACAAACTTGATTCTGAG
         66 TGCTCT-AGAAAGGGAATGTTCAACTCTGTGAGTTGAGTACACACATCACAAAGAAGTTTCTGAG

               *         *        **         ** *      *  * ** ***      *    
     113189 AATCCTTCTGTCTCGTTTTTATGGGAAGATATTTACTTTTCCACCGTAGGCATCAAAGCGCTCCA
        130 AATGCTTCTGTCTAGTTTTTATATGAAGATATTCCCGTTTCCAACGAAATCGCTAAAGCGATCCA

               *     *       *  *  *  *            *         *   * *   *     
     113254 AATGTCCACATCCAGATACTCCAGAACGAGTGTTTCAAACCTGCTCTATGAAAGGGAATCTTCAA
        195 AATATCCACTTCCAGATTCTACAAAAAGAGTGTTTCAAAACTGCTCTATCAAAAGAAATGTTCAA

                * *      * *  *      *      *              *       **        
     113319 CTCTATGAGTTGAATGCAGACATCAGAAAGAAATTTCTGAGAATGCTGCTGTCTACCTTTTAT-T
        260 CTCTGTTAGTTGAGTACACACATCACAAAGAAGTTTCTGAGAATGCTTCTGTCTAGTTTTTATAT

              *         **             *  *    *              * *  **   *    
     113383 --TGA-ATTCCCGCTTCCAACGAAATCCTCCAAGCTATCCAAATATCCACCTGCATTTTCCACAA
        325 GAAGATATTCCCTTTTCCAACGAAATCATCAAAGCGATCCAAATATCCACTTCCAGATTCTACAA

            *                        *  *               **  * *  *           
     113445 CAAGAGTGTTTCAAAACTGCTCTATCAATAGAAATGTTCAACTCCTTTGGCTGGGTACACACATC
        390 AAAGAGTGTTTCAAAACTGCTCTATGAAAAGAAATGTTCAACTCTGTTAGTTGAGTACACACATC

                 *                                        **         *  * *  
     113510 ACAAACAAGTTTCTGAGAATGCTTCTGTCTAGTTTTTATGGGAAGACGTTCCCTTTTTCACCAAA
        455 ACAAAGAAGTTTCTGAGAATGCTTCTGTCTAGTTTTTATGGGAAGATATTCCCTTTTCCAACGAA

            *           *       *            **                     **       
     113575 GGCATCAAAGCGCTCCAAATGTCCACTTCCAGACACTACAAAAAGAGTGTTTCAAACGTGCTCTA
        520 AGCATCAAAGCGATCCAAATATCCACTTCCAGATTCTACAAAAAGAGTGTTTCAAAACTGCTCTA

            **    **                 *    * *  * *         *           **    
     113640 AGAAAGCGAATGTTCAACTCTGTGACTTGAATGCAGATATCACAAAGTAGTTTCTGAGAGGGCTT
        585 TCAAAGGAAATGTTCAACTCTGTGAGTTGAGTACACACATCACAAAGAAGTTTCTGAGAATGCTT

                          *              *                * *   *            
     113705 CTGTCTAGATTTTAGATGATGATATTCCCGTTTCCAACGAAATCATTAGAGCTATCCAAATATCC
        650 CTGTCTAG-TTTT-TATGATGATATTCCCTTTTCCAACGAAATCATCAAAGCGATCCAAATATCC

                *   *                    *         **      *  * *    *       
     113770 ACTTACAGTTTCTACAAAAAGAGTGTTTCCAAACTG-CTGCATCAAAAGAGAGGTTCCACTCTGT
        713 ACTTCCAGATTCTACAAAAAGAGTGTTTCAAAACTGCCTCTATCAAAGGAAATGTTCAACTCTGT

               **                  ***      *     *      *  *                
     113834 TAGCCGAGTACACACATCACAAACTTGTTTCTCAGAATCCTTCTGACTCGTTTTTATGGGAAGAT
        778 TAGTTGAGTACACACATCACAAAGAAGTTTCTGAGAATGCTTCTGTCTAGTTTTTATGGGAAGAT

               **     *  *  * **          *       *     *       *  *  *      
     113899 ATTTACTTTTTCACCGTAGGCATCAAAGCGCTCCAAATGTCCACATCCAGATACTCCAGAAAGAG
        843 ATTCCCTTTTCCAACGAAATCATCAAAGCGATCCAAATATCCACTTCCAGATTCTACAAAAAGAG

                     *         *     *   *         *                    *    
     113964 TGTTTCAAACCTGCTCTATGAAAGGGAATCTTCAACTCTATGAGTTGAATGCAGACATCAGAAAG
        908 TGTTTCAAAACTGCTCTATCAAAGGAAATGTTCAACTCTGTGAGTTGAATGCAGACATCACAAAG

              *              *       **       **         **             *  * 
     114029 AAATTTCTGAGAATGCTGCTGTCTACCTTTTAT-TTGA-ATTCCCGCTTCCAACGAAATCCTCCA
        973 AAGTTTCTGAGAATGCTTCTGTCTAGTTTTTATGAAGATATTCCCTTTTCCAACGAAATCATCAA

               *                *       *                                  **
     114092 AGCTATCCAAATATCCACTTGCAGATTCCACAAAAAGAGTGTTTCAAAACTGCTCTCTATCAATG
       1038 AGCGATCCAAATATCCACTTCCAGATTCTACAAAAAGAGTGTTTCAAAACTG--CTCTATCAAAA

             *  *                    *     *     *  *               *        
     114157 GCAAAGTTCAACTCTGTTAGTTGAGGACACATATCACCAACAAGTTTCTGAGAATGTTTCTGTCT
       1101 GAAATGTTCAACTCTGTTAGTTGAGTACACACATCACAAAGAAGTTTCTGAGAATGCTTCTGTCT

             *                  *  *    
     114222 ATTTTTTATGGGAAGATATTTCCTTTTT
       1166 AGTTTTTATGGGAAGATATTCCCGTTTT

              *  * ** *    *      *    *          * **                       
     114250 CACCGTAGGCGTCAAGGCGATCGAAATGTCCACTTCCACAAACTACAAAAAGAGTGTTTCAAACC
          1 CAACGAAATCATCAAAGCGATCCAAATATCCACTTCCAGATTCTACAAAAAGAGTGTTTCAAACC

                          **       *    *        * ** * *   **      *      * 
     114315 TGCTCTATGAAAGGCCATGTTCATCTCTATGAGTTGAATGGAAATATCCGAAAGAAATTTCTGGG
         66 TGCTCTA-GAAAGGGAATGTTCAACTCTGTGAGTTGAGTACACACATCACAAAGAAGTTTCTGAG

                  *          *        *          *                      *    
     114380 AATGCTGCTGTCTAG-TGTT-TAT-ACGA-ATTCCCGCTTCCAACGAAATC-CTCAAAGCAATCC
        130 AATGCTTCTGTCTAGTTTTTATATGAAGATATTCCCGTTTCCAACGAAATCGCT-AAAGCGATCC

                        *    *  *                                *     *     
     114440 AAATATCCACTTGCAGAATCCACAAAAAGAGTGTTTCAAAACTGCTCTATCAATAGAAAGGTTCA
        194 AAATATCCACTTCCAGATTCTACAAAAAGAGTGTTTCAAAACTGCTCTATCAAAAGAAATGTTCA

                 *                        *                        * *      *
     114505 ACTCTTTTAGTTGAGTACACACATCACAAACAAGTTTCTGAGAATGCTTCTGTCTGGCTTTTATT
        259 ACTCTGTTAGTTGAGTACACACATCACAAAGAAGTTTCTGAGAATGCTTCTGTCTAGTTTTTATA

            *     **  *           *  **          *       *                * *
     114570 GGAAGACGTTTCCTTTT-CAACAAAGGCATCAAAGCGCTCCAAATGTCCACTTCCAGATTCTTCC
        324 TGAAGATATTCCCTTTTCCAACGAAATCATCAAAGCGATCCAAATATCCACTTCCAGATTCTACA

                            **  *  * * *  * *               * *    * *  * *  
     114634 AAAAGAGTGTTTCAAACGTGGTCGAAGTAAGGGAATGTTCAACTCTGTGACTTGAATGCAGATAT
        389 AAAAGAGTGTTTCAAAACTGCTCTATGAAAAGAAATGTTCAACTCTGTTAGTTGAGTACACACAT

               *   *       * * *            **     ***  *         *          
     114699 CACCAAGTAGTTTCTAATAGTGCTTCTGTCTACATTTTAGATGATGATATTCCCGTTTCCAACGA
        454 CACAAAGAAGTTTCTGAGAATGCTTCTGTCTAGTTTTTATGGGAAGATATTCCCTTTTCCAACGA

              * * * *   *             *  *   *      *     *       *   *    **
     114764 AATCGTTAGAGCTATCCAAATATCCAGTTACAGTTTCTACCAAAAGGGTGTTTCCAAATTGCTGC
        519 AAGCATCAAAGCGATCCAAATATCCACTTCCAGATTCTACAAAAAGAGTGTTTCAAAACTGCTCT

                  *    *            *       *                     *          
     114829 ATCAAAAGAAAGGTTCAACTCTGTTAGTTGAGGACACACATCACAAAGAAGTTTGTGAGAATGCT
        584 ATCAAAGGAAATGTTCAACTCTGTGAGTTGAGTACACACATCACAAAGAAGTTTCTGAGAATGCT

                                *                    *   * *     *   *       
     114894 TCTGTCTAGATTTTGTATGACGATATTCCCTTTTCCAACGATATCGTTAAAGCAATCTAAATATC
        649 TCTGTCTAG-TTTT-TATGATGATATTCCCTTTTCCAACGAAATCATCAAAGCGATCCAAATATC

            * *  *    *  *       *  *        *        * *   *    *    *      
     114959 AATTTGCAGAATCCACAAAAATAGAGTTTCAAAGCTG-CTCTGTAAAAAGAAAGGTTCCACTCTG
        712 CACTTCCAGATTCTACAAAAAGAGTGTTTCAAAACTGCCTCTATCAAAGGAAATGTTCAACTCTG

                *                   ***           **         *               
     115023 TTAGCTGAGTACACACATCACAAACTTGTTTCTGAGAAACCTTCTGTCTCGTTTTTATGGGAAGA
        777 TTAGTTGAGTACACACATCACAAAGAAGTTTCTGAGAATGCTTCTGTCTAGTTTTTATGGGAAGA

                **        ** * **          *       *     *       *  *  *  *  
     115088 TATTTACTTTTCCACTGTAGGCATCAAAGCGCTCCAAATGTCCACATCCAGATACTCCAGAACGA
        842 TATTCCCTTTTCCAACGAAATCATCAAAGCGATCCAAATATCCACTTCCAGATTCTACAAAAAGA

                      *         *     *   *         *                    *   
     115153 GTGTTTCAAACCTGCTCTATGAAAGGGAATCTTCAACTCTATGAGTTGAATGCAGA-ATCAGAAA
        907 GTGTTTCAAAACTGCTCTATCAAAGGAAATGTTCAACTCTGTGAGTTGAATGCAGACATCACAAA

               *              *       **       **         **             *  *
     115217 GAAATTTCTGAGAATGCTGCTGTCTACCTTTTAT-TTGA-ATTCCCGCTTCCAACGAAATCCTCC
        972 GAAGTTTCTGAGAATGCTTCTGTCTAGTTTTTATGAAGATATTCCCTTTTCCAACGAAATCATCA

                *              * *  **   *                                *  
     115280 AAGCTATCCAAATATCCACCTGCATTTTCCACAAAAAGAGTGTTTCAAAACTGCTCTATCAATAG
       1037 AAGCGATCCAAATATCCACTTCCAGATTCTACAAAAAGAGTGTTTCAAAACTGCTCTATCAAAAG

                         **  * *  *                *                         
     115345 AAATGTTCAACTCCTTTGGCTGGGTACACACATCACAAACAAGTTTCTGAGAATGCTTCTGTCTA
       1102 AAATGTTCAACTCTGTTAGTTGAGTACACACATCACAAAGAAGTTTCTGAGAATGCTTCTGTCTA

                           **     *    
     115410 GTTTTTATGGGAAGACGTTCCCTTTTT
       1167 GTTTTTATGGGAAGATATTCCCGTTTT

              * *  **          *       *            **                      *
     115437 CACCAAAGGCATCAAAGCGCTCCAAATGTCCACTTCCAGACACTACAAAAAGAGTGTTTCAAACG
          1 CAACGAAATCATCAAAGCGATCCAAATATCCACTTCCAGATTCTACAAAAAGAGTGTTTCAAACC

                         *                  *    * *  * *         *          
     115502 TGCTCTAAGAAAGCGAATGTTCAACTCTGTGACTTGAATGCAGATATCACAAAGTAGTTTCTGAG
         66 TGCTCT-AGAAAGGGAATGTTCAACTCTGTGAGTTGAGTACACACATCACAAAGAAGTTTCTGAG

             **            *     *    *                        **  *   *     
     115567 AGGGCTTCTGTCTAGATTTTAGATGATGATATTCCCGTTTCCAACGAAATCATTAGAGCTATCCA
        130 AATGCTTCTGTCTAGTTTTTATATGAAGATATTCCCGTTTCCAACGAAATCGCTAAAGCGATCCA

                       *   *                    *        **         * *    * 
     115632 AATATCCACTTACAGTTTCTACAAAAAGAGTGTTTCCAAACTGCTGCATCAAAAGAGAGGTTCCA
        195 AATATCCACTTCCAGATTCTACAAAAAGAGTGTTTCAAAACTGCTCTATCAAAAGAAATGTTCAA

                     **                  ***      *     *         *        **
     115697 CTCTGTTAGCCGAGTACACACATCACAAACTTGTTTCTCAGAATCCTTCTGTCTCGTTTTTATGG
        260 CTCTGTTAGTTGAGTACACACATCACAAAGAAGTTTCTGAGAATGCTTCTGTCTAGTTTTTATAT

                     **     *  *  * **          *       *     *       *  *  *
     115762 GAAGATATTTACTTTTTCACCGTAGGCATCAAAGCGCTCCAAATGTCCACATCCAGATACTCCAG
        325 GAAGATATTCCCTTTTCCAACGAAATCATCAAAGCGATCCAAATATCCACTTCCAGATTCTACAA

                           *             * *   *         * *      * *  *     
     115827 AAAGAGTGTTTCAAACCTGCTCTATGAAAGGGAATCTTCAACTCTATGAGTTGAATGCAGACATC
        390 AAAGAGTGTTTCAAAACTGCTCTATGAAAAGAAATGTTCAACTCTGTTAGTTGAGTACACACATC

             *      *              *       **         **         **          
     115892 AGAAAGAAATTTCTGAGAATGCTGCTGTCTACCTTTTAT---TTGA-ATTCCCGCTTCCAACGAA
        455 ACAAAGAAGTTTCTGAGAATGCTTCTGTCTAGTTTTTATGGGAAGATATTCCCTTTTCCAACGAA

             * *  *    *                *       *                            
     115953 ATCCTCCAAGCTATCCAAATATCCACTTGCAGATTCCACAAAAAGAGTGTTTCAAAACTGCTCTC
        520 AGCATCAAAGCGATCCAAATATCCACTTCCAGATTCTACAAAAAGAGTGTTTCAAAACTG--CTC

                  *  *  *            *       *     *     *  *                
     116018 TATCAATGGCAAAGTTCAACTCTGTTAGTTGAGGACACATATCACCAACAAGTTTCTGAGAATGC
        583 TATCAAAGGAAATGTTCAACTCTGTGAGTTGAGTACACACATCACAAAGAAGTTTCTGAGAATGC

                     *           *      *      *  *  * ** *    *      *    * 
     116083 TTCTGTCTATTTTTTATGGGAAGATATTTCCTTTTTCACCGTAGGCGTCAAGGCGATCGAAATGT
        648 TTCTGTCTAGTTTTTAT--GATGATATTCCCTTTTCCAACGAAATCATCAAAGCGATCCAAATAT

                     * **                     *          *     **       *    
     116148 CCACTTCCACAAACTACAAAAAGAGTGTTTCAAACCTG-CTCTATGAAAGGCCATGTTCATCTCT
        711 CCACTTCCAGATTCTACAAAAAGAGTGTTTCAAAACTGCCTCTATCAAAGGAAATGTTCAACTCT

            * *      * ** * *   **      *      *       *         *         * 
     116212 ATGAGTTGAATGGAAATATCCGAAAGAAATTTCTGGGAATGCTGCTGTCTAGTGTTTAT---ACG
        776 GTTAGTTGAGTACACACATCACAAAGAAGTTTCTGAGAATGCTTCTGTCTAGTTTTTATGGGAAG

                    **             *       *                *    *  *        
     116274 A-ATTCCCGCTTCCAACGAAATCCTCAAAGCAATCCAAATATCCACTTGCAGAATCCACAAAAAG
        841 ATATTCCCTTTTCCAACGAAATCATCAAAGCGATCCAAATATCCACTTCCAGATTCTACAAAAAG

                         *          **    *          * *      * *  *         
     116338 AGTGTTTCAAAACCGCTCTATCAATAGAAAGGTTCAACTCTTTTAGTTGAGTACACACATCACAA
        906 AGTGTTTCAAAACTGCTCTATCAAAGGAAATGTTCAACTCTGTGAGTTGAATGCAGACATCACAA

             *                        * *             **  *         * *  **  
     116403 ACAAGTTTCTGAGAATGCTTCTGTCTGGCTTTTATTGGAAGACGTTTCCTTTT-CACCAAAGGCA
        971 AGAAGTTTCTGAGAATGCTTCTGTCTAGTTTTTA-T-GAAGATATTCCCTTTTCCAACGAAATCA

                    *       *                * *                **  *  * *** 
     116467 TCAAAGCGCTCCAAATGTCCACTTCCAGATTCTTCCAAAAGAGTGTTTCAAACGTGGTCGAAGTA
       1034 TCAAAGCGATCCAAATATCCACTTCCAGATTCTACAAAAAGAGTGTTTCAAAACTGCTCTATCAA

             * *               * *    * *  * *     *   *       * * *         
     116532 AGGGAATGTTCAACTCTGTGACTTGAATGCAGATATCACCAAGTAGTTTCTAATAGTGCTTCTGT
       1099 AAGAAATGTTCAACTCTGTTAGTTGAGTACACACATCACAAAGAAGTTTCTGAGAATGCTTCTGT

                *     ***  *             *
     116597 CTAGATTTTAGATGATGATATTCCCGTTTC
       1164 CTAGTTTTTATGGGAAGATATTCCCGTTTT

                      * * *   *             *  *   *      *     *       *  **
     116627 CAACGAAATCGTTAGAGCTATCCAAATATCCAGTTACAGTTTCTACCAAAAGGGTGTTTCCAAAT
          1 CAACGAAATCATCAAAGCGATCCAAATATCCACTTCCAGATTCTACAAAAAGAGTGTTTCAAACC

                  ***   * *  *            *       *                     *    
     116692 TGCTGCATCAAAAGAAAGGTTCAACTCTGTTAGTTGAGGACACACATCACAAAGAAGTTTGTGAG
         66 TGCT-CTAGAAAGGGAATGTTCAACTCTGTGAGTTGAGTACACACATCACAAAGAAGTTTCTGAG

                           *    *     *         *          *    *      *   * 
     116757 AATGCTTCTGTCTAGATTTTGTATGACGATATTCCCTTTTCCAACGATATCGTTAAAGCAATCTA
        130 AATGCTTCTGTCTAGTTTTTATATGAAGATATTCCCGTTTCCAACGAAATCGCTAAAGCGATCCA

                  * *  *    *  *       *  *        *       * *        *    * 
     116822 AATATCAATTTGCAGAATCCACAAAAATAGAGTTTCAAAGCTGCTCTGTAAAAAGAAAGGTTCCA
        195 AATATCCACTTCCAGATTCTACAAAAAGAGTGTTTCAAAACTGCTCTATCAAAAGAAATGTTCAA

                     *                   ***           **         *        **
     116887 CTCTGTTAGCTGAGTACACACATCACAAACTTGTTTCTGAGAAACCTTCTGTCTCGTTTTTATGG
        260 CTCTGTTAGTTGAGTACACACATCACAAAGAAGTTTCTGAGAATGCTTCTGTCTAGTTTTTATAT

                     **        ** * **          *       *     *       *  *  *
     116952 GAAGATATTTACTTTTCCACTGTAGGCATCAAAGCGCTCCAAATGTCCACATCCAGATACTCCAG
        325 GAAGATATTCCCTTTTCCAACGAAATCATCAAAGCGATCCAAATATCCACTTCCAGATTCTACAA

              *            *             * *   *         * *      * *  *     
     117017 AACGAGTGTTTCAAACCTGCTCTATGAAAGGGAATCTTCAACTCTATGAGTTGAATGCAGA-ATC
        390 AAAGAGTGTTTCAAAACTGCTCTATGAAAAGAAATGTTCAACTCTGTTAGTTGAGTACACACATC

             *      *              *       **         **         **          
     117081 AGAAAGAAATTTCTGAGAATGCTGCTGTCTACCTTTTAT---TTGA-ATTCCCGCTTCCAACGAA
        455 ACAAAGAAGTTTCTGAGAATGCTTCTGTCTAGTTTTTATGGGAAGATATTCCCTTTTCCAACGAA

             * *  *    *              * *  **   *    *                       
     117142 ATCCTCCAAGCTATCCAAATATCCACCTGCATTTTCCACAACAAGAGTGTTTCAAAACTGCTCTA
        520 AGCATCAAAGCGATCCAAATATCCACTTCCAGATTCTACAAAAAGAGTGTTTCAAAACTGCTCTA

                **                *    *  *                *                 
     117207 TCAATAGAAATGTTCAACTCCTTTG-GCTGGGTACACACATCACAAACAAGTTTCTGAGAATGCT
        585 TCAAAGGAAATGTTCAACT-CTGTGAGTTGAGTACACACATCACAAAGAAGTTTCTGAGAATGCT

                                *  **         *  * *  **          *       *  
     117271 TCTGTCTAGTTTTTATGGGAAGACGTTCCCTTTTTCACCAAAGGCATCAAAGCGCTCCAAATGTC
        649 TCTGTCTAGTTTTTAT--GATGATATTCCCTTTTCCAACGAAATCATCAAAGCGATCCAAATATC

                      **                     **        **   ***              
     117336 CACTTCCAGACACTACAAAAAGAGTGTTTCAAACGTG-CTCTAAGAAAACGAATGTTCAACTCTG
        712 CACTTCCAGATTCTACAAAAAGAGTGTTTCAAAACTGCCTCTATCAAAGGAAATGTTCAACTCTG

             * *    * *  * *         *           **            *     ***  *  
     117400 TGACTTGAATGCAGATATCACAAAGTAGTTTCTGAGAGGGCTTCTGTCTAGATTTTAGATGATGA
        777 TTAGTTGAGTACACACATCACAAAGAAGTTTCTGAGAATGCTTCTGTCTAGTTTTTATGGGAAGA

                   *                * *   *                *   *             
     117465 TATTCCCGTTTCCAACGAAATCATTAGAGCTATCCAAATATCCACTTACAGTTTCTACAAAAAGA
        842 TATTCCCTTTTCCAACGAAATCATCAAAGCGATCCAAATATCCACTTCCAGATTCTACAAAAAGA

                   *        **      *  * *    *       *  **  * *  *          
     117530 GTGTTTCCAAACTGCTGCATCAAAAGAGAGGTTCCACTCTGTTAGCCGAGTACACACATCACAAA
        907 GTGTTTCAAAACTGCTCTATCAAAGGAAATGTTCAACTCTGTGAGTTGAATGCAGACATCACAAA

            ***      *     *         *                   **     *  *  * **   
     117595 CTTGTTTCTCAGAATCCTTCTGTCTCGTTTTTATGGGAAGATATTTACTTTTTCACCGTAGGCAT
        972 GAAGTTTCTGAGAATGCTTCTGTCTAGTTTTTAT--GAAGATATTCCCTTTTCCAACGAAATCAT

                   *       *     *       *  *  *               *         *   
     117660 CAAAGCGCTCCAAATGTCCACATCCAGATACTCCAGAAAGAGTGTTTCAAACCTGCTCTATGAAA
       1035 CAAAGCGATCCAAATATCCACTTCCAGATTCTACAAAAAGAGTGTTTCAAAACTGCTCTATCAAA

            * *   *         * *      * *  *      *      *              *     
     117725 GGGAATCTTCAACTCTATGAGTTGAATGCAGACATCAGAAAGAAATTTCTGAGAATGCTGCTGTC
       1100 AGAAATGTTCAACTCTGTTAGTTGAGTACACACATCACAAAGAAGTTTCTGAGAATGCTTCTGTC

              **         **          *  *
     117790 TACCTTTTAT---TTGA-ATTCCCGCTTC
       1165 TAGTTTTTATGGGAAGATATTCCCGTTTT

                      *  *    *                *       *                   * 
     117815 CAACGAAATCCTCCAAGCTATCCAAATATCCACTTGCAGATTCCACAAAAAGAGTGTTTCAAAAC
          1 CAACGAAATCATCAAAGCGATCCAAATATCCACTTCCAGATTCTACAAAAAGAGTGTTTCAAACC

                      *  *  *  *            *       *     *     *  *         
     117880 TGCTCTCTATCAATGGCAAAGTTCAACTCTGTTAGTTGAGGACACATATCACCAACAAGTTTCTG
         66 TG--CTCTA-GAAAGGGAATGTTCAACTCTGTGAGTTGAGTACACACATCACAAAGAAGTTTCTG

                            *       **         *  *   *  *  * **  **  *      
     117945 AGAATGCTTCTGTCTATTTTTTATGGGAAGATATTTCCTTTTTCACCGTAGGCGTCAAGGCGATC
        128 AGAATGCTTCTGTCTAGTTTTTATATGAAGATATTCCCGTTTCCAACGAAATCGCTAAAGCGATC

            *    *          * **                     *         *   * **      
     118010 GAAATGTCCACTTCCACAAACTACAAAAAGAGTGTTTCAAACCTGCTCTATGAAAGGCCATGTTC
        193 CAAATATCCACTTCCAGATTCTACAAAAAGAGTGTTTCAAAACTGCTCTATCAAAAGAAATGTTC

             *    * *      * ** * *   **      *      *       *               
     118075 ATCTCTATGAGTTGAATGGAAATATCCGAAAGAAATTTCTGGGAATGCTGCTGTCTAGTTTTTAT
        258 AACTCTGTTAGTTGAGTACACACATCACAAAGAAGTTTCTGAGAATGCTTCTGTCTAGTTTTTAT

                          **             *       *                *    *  *  
     118140 AT---GA-ATTCCCGCTTCCAACGAAATCCTCAAAGCAATCCAAATATCCACTTGCAGAATCCAC
        323 ATGAAGATATTCCCTTTTCCAACGAAATCATCAAAGCGATCCAAATATCCACTTCCAGATTCTAC

                                       *  *     *          *                 
     118201 AAAAAGAGTGTTTCAAAACTGCTCTATCAATAGAAAGGTTCAACTCTTTTAGTTGAGTACACACA
        388 AAAAAGAGTGTTTCAAAACTGCTCTATGAAAAGAAATGTTCAACTCTGTTAGTTGAGTACACACA

                   *                        * *      *      **  *         * *
     118266 TCACAAACAAGTTTCTGAGAATGCTTCTGTCTGGCTTTTATTGGAAGACGTTTCCTTTT-CACCA
        453 TCACAAAGAAGTTTCTGAGAATGCTTCTGTCTAGTTTTTATGGGAAGATATTCCCTTTTCCAACG

              *           *       *                * *                **     
     118330 AAGGCATCAAAGCGCTCCAAATGTCCACTTCCAGATTCTTCCAAAAGAGTGTTTCAAACGTGCTC
        518 AAAGCATCAAAGCGATCCAAATATCCACTTCCAGATTCTACAAAAAGAGTGTTTCAAAACTGCTC

                **    *                 *    * *  * *     *   *       * * *  
     118395 -ATAGTAAGGGAATGTTCAACTCTGTGACTTGAATGCAGATATCACCAAGTAGTTTCTAATAGTG
        583 TAT-CAAAGGAAATGTTCAACTCTGTGAGTTGAGTACACACATCACAAAGAAGTTTCTGAGAATG

                             *              *              *** *   *         
     118459 CTTCTGTCTAGATTTTAGATGATGATATTCCCGTTTCCAACGAAATCGCTAGAGCTATCCAAATA
        647 CTTCTGTCTAG-TTTT-TATGATGATATTCCCTTTTCCAACGAAATCATCAAAGCGATCCAAATA

                *  *   *      *     *       *   *     **      *    *         
     118524 TCCAGTTACAGTTTCTACCAAAAGGGTGTTTCCAAATTG-CTGCATCAAAAGAAAGGTTCAACTC
        710 TCCACTTCCAGATTCTACAAAAAGAGTGTTTCAAAACTGCCTCTATCAAAGGAAATGTTCAACTC

                       *                     *                   *    * **  *
     118588 TGTTAGTTGAGGACACACATCACAAAGAAGTTTGTGAGAATGCTTCTGTCTAGATTTTGTATGAC
        775 TGTTAGTTGAGTACACACATCACAAAGAAGTTTCTGAGAATGCTTCTGTCTAGTTTTTATGGGAA

                                *   * *     *   *       * *  *    *  *       
     118653 GATATTCCCTTTTCCAACGATATCGTTAAAGCAATCTAAATATCAATTTGCAGAATCCACAAAAA
        840 GATATTCCCTTTTCCAACGAAATCATCAAAGCGATCCAAATATCCACTTCCAGATTCTACAAAAA

            *  *        *       * *   *    *    *       *  *   * *  *        
     118718 TAGAGTTTCAAAGCTGCTCTGTAAAAAGAAAGGTTCCACTCTGTTAGCTGAGTACACACATCACA
        905 GAGTGTTTCAAAACTGCTCTATCAAAGGAAATGTTCAACTCTGTGAGTTGAATGCAGACATCACA

              ***            *         *                   **        *  * ** 
     118783 AACTTGTTTCTGAGAATCCTTCTGTCTCGTTTTTATGGGAAGATATTTACTTTTCCACCGTAGGC
        970 AAGAAGTTTCTGAGAATGCTTCTGTCTAGTTTTTAT--GAAGATATTCCCTTTTCCAACGAAATC

                     *       *     *       *  *  *  *            *         * 
     118848 ATCAAAGCGCTCCAAATGTCCACATCCAGATACTCCAGAACGAGTGTTTCAAACCTGCTCTATGA
       1033 ATCAAAGCGATCCAAATATCCACTTCCAGATTCTACAAAAAGAGTGTTTCAAAACTGCTCTATCA

              * *   *         * *      * *  *      *      *              *   
     118913 AAGGGAATCTTCAACTCTATGAGTTGAATGCAGACATCAGAAAGAAATTTCTGAGAATGCTGCTG
       1098 AAAGAAATGTTCAACTCTGTTAGTTGAGTACACACATCACAAAGAAGTTTCTGAGAATGCTTCTG

                **         **          *  *
     118978 TCTACCTTTTAT---TTGA-ATTCCCGCTTC
       1163 TCTAGTTTTTATGGGAAGATATTCCCGTTTT

                      *  *    *              * *  **   *    *              * 
     119005 CAACGAAATCCTCCAAGCTATCCAAATATCCACCTGCATTTTCCACAACAAGAGTGTTTCAAAAC
          1 CAACGAAATCATCAAAGCGATCCAAATATCCACTTCCAGATTCTACAAAAAGAGTGTTTCAAACC

                    *  ** *              *    *  *                *          
     119070 TGCTCTATCAATAGAAATGTTCAACTCCTTTG-GCTGGGTACACACATCACAAACAAGTTTCTGA
         66 TGCTCTA-GAAAGGGAATGTTCAACT-CTGTGAGTTGAGTACACACATCACAAAGAAGTTTCTGA

                                   **     **     *   *    *    *           * 
     119134 GAATGCTTCTGTCTAGTTTTTATGGGAAGACGTTCCCTTTTTC-ACCAAA-GGCATCAAAGCGCT
        129 GAATGCTTCTGTCTAGTTTTTATATGAAGATATTCCCGTTTCCAACGAAATCGC-T-AAAGCGAT

                  *            **                  *  **   *   **   ***      
     119197 CCAAATGTCCACTTCCAGACACTACAAAAAGAGTGTTTCCAACGTGCCCTAAGAAAGCGAATGTT
        192 CCAAATATCCACTTCCAGATTCTACAAAAAGAGTGTTTCAAAACTGCTCTATCAAAAGAAATGTT

                     * *    * *  * *         *           **            *     
     119262 CAACTCTGTGACTTGAATGCAGATATCACAAAGTAGTTTCTGAGAGGGCTTCTGTCTAGATTTTA
        257 CAACTCTGTTAGTTGAGTACACACATCACAAAGAAGTTTCTGAGAATGCTTCTGTCTAGTTTTTA

            *    *         *                * *   *             *  *   *     
     119327 GATGATGATATTCCCGTTTCCAACGAAATCATTAGAGCTATCCAAATATCCAATTACAGTTTCTA
        322 TATGAAGATATTCCCTTTTCCAACGAAATCATCAAAGCGATCCAAATATCCACTTCCAGATTCTA

                           *        **  *      * *    *          *           
     119392 CAAAAAGAGTGTTTCCAAACTGCTGCATCAAAAGAGAGGTTCCACTCTGTTAGCTGAGTACACAC
        387 CAAAAAGAGTGTTTCAAAACTGCTCTATGAAAAGAAATGTTCAACTCTGTTAGTTGAGTACACAC

                    ***      *     *         *                   **     *  * 
     119457 ATCACAAACTTGTTTCTCAGAATCCTTCTGTCTCGTTTTTATGGGAAGATATTTACTTTTTCACC
        452 ATCACAAAGAAGTTTCTGAGAATGCTTCTGTCTAGTTTTTATGGGAAGATATTCCCTTTTCCAAC

             * *           *       *     *       *  *  *               *     
     119522 GTAGGCATCAAAGCGCTCCAAATGTCCACATCCAGATACTCCAGAAAGAGTGTTTCAAACCTGCT
        517 GAAAGCATCAAAGCGATCCAAATATCCACTTCCAGATTCTACAAAAAGAGTGTTTCAAAACTGCT

                *     *   *         *        * *  *      *      *            
     119587 CTATGAAAGGGAATCTTCAACTCTATGAGTTGAATGCAGACATCAGAAAGAAATTTCTGAGAATG
        582 CTATCAAAGGAAATGTTCAACTCTGTGAGTTGAGTACACACATCACAAAGAAGTTTCTGAGAATG

              *       **       *          **             *  *    *           
     119652 CTGCTGTCTACCTTTTAT-TTGA-ATTCCCGCTTCCAACGAAATCCTCCAAGCTATCCAAATATC
        647 CTTCTGTCTAGTTTTTATGATGATATTCCCTTTTCCAACGAAATCATCAAAGCGATCCAAATATC

                 *       *                                  *  *  *          
     119715 CACTTGCAGATTCCACAAAAAGAGTGTTTCAAAACTGCTCTCTATCAATGGCAAAGTTCAACTCT
        712 CACTTCCAGATTCTACAAAAAGAGTGTTTCAAAACTGC-CTCTATCAAAGGAAATGTTCAACTCT

                      *     *     *  *                         *             
     119780 GTTAGTTGAGGACACATATCACCAACAAGTTTCTGAGAATGCTTCTGTCTATTTTTTATGGGAAG
        776 GTTAGTTGAGTACACACATCACAAAGAAGTTTCTGAGAATGCTTCTGTCTAGTTTTTATGGGAAG

                 *      *  *  * ** *    *      *    *          * **          
     119845 ATATTTCCTTTTTCACCGTAGGCGTCAAGGCGATCGAAATGTCCACTTCCACAAACTACAAAAAG
        841 ATATTCCCTTTTCCAACGAAATCATCAAAGCGATCCAAATATCCACTTCCAGATTCTACAAAAAG

                       *         *     **       *    *           * * *   **  
     119910 AGTGTTTCAAACCTGCTCTATGAAAGGCCATGTTCATCTCTATGAGTTGAATGGAAATATCCGAA
        906 AGTGTTTCAAAACTGCTCTATCAAAGGAAATGTTCAACTCTGTGAGTTGAATGCAGACATCACAA

                *      *       *                 *         **             *  
     119975 AGAAATTTCTGGGAATGCTGCTGTCTAGTTTTTAT-ATGA-ATTCCCGCTTCCAACGAAATCCTC
        971 AGAAGTTTCTGAGAATGCTTCTGTCTAGTTTTTATGAAGATATTCCCTTTTCCAACGAAATCATC

                 *                *    *  *                                * 
     120038 AAAGCAATCCAAATATCCACTTGCAGAATCCACAAAAAGAGTGTTTCAAAACTGCTCTATCAATA
       1036 AAAGCGATCCAAATATCCACTTCCAGATTCTACAAAAAGAGTGTTTCAAAACTGCTCTATCAAAA

                *          *                        *                        
     120103 GAAAGGTTCAACTCTTTTAGTTGAGTACACACATCACAAACAAGTTTCTGAGAATGCTTCTGTCT
       1101 GAAATGTTCAACTCTGTTAGTTGAGTACACACATCACAAAGAAGTTTCTGAGAATGCTTCTGTCT

            * *      *      **  *       
     120168 GGCTTTTATTGGAAGACGTTTCC-TTTT
       1166 AGTTTTTATGGGAAGATATTCCCGTTTT

              * *  **          *      **                * *                 *
     120195 CACCAAAGGCATCAAAGCGCTCCAAAGGTCCACTTCCAGATTCTTCCAAAAGAGTGTTTCAAACG
          1 CAACGAAATCATCAAAGCGATCCAAATATCCACTTCCAGATTCTACAAAAAGAGTGTTTCAAACC

                     *                      *    * *  * *     *   *       * *
     120260 TGCTCATAGTAAGGGAATGTTCAACTCTGTGACTTGAATGCAGATATCACCAAGTAGTTTCTAAT
         66 TGCTC-TAGAAAGGGAATGTTCAACTCTGTGAGTTGAGTACACACATCACAAAGAAGTTTCTGAG

             *             *     *    *                            *   *     
     120325 AGTGCTTCTGTCTAGATTTTAGATGATGATATTCCCGTTTCCAACGAAATCGCTAGAGCTATCCA
        130 AATGCTTCTGTCTAGTTTTTATATGAAGATATTCCCGTTTCCAACGAAATCGCTAAAGCGATCCA

                    *  *   *      *     *       *   *    **           *      
     120390 AATATCCAGTTACAGTTTCTACCAAAAGGGTGTTTCCAAATTGCTGCATCAAAAGAAAGGTTCAA
        195 AATATCCACTTCCAGATTCTACAAAAAGAGTGTTTCAAAACTGCTCTATCAAAAGAAATGTTCAA

                          *                     *                  **    *   
     120455 CTCTGTTAGTTGAGGACACACATCACAAAGAAGTTTGTGAGAATGCTTCTGTCTACATTTTGTAT
        260 CTCTGTTAGTTGAGTACACACATCACAAAGAAGTTTCTGAGAATGCTTCTGTCTAGTTTTTATAT

              *                    *   * *     *   *       * *  *    *  *    
     120520 GACGATATTCCCTTTTCCAACGATATCGTTAAAGCAATCTAAATATCAATTTGCAGAATCCACAA
        325 GAAGATATTCCCTTTTCCAACGAAATCATCAAAGCGATCCAAATATCCACTTCCAGATTCTACAA

               *  *        *       * *        *    *          *              
     120585 AAATAGAGTTTCAAAGCTGCTCTGTAAAAAGAAAGGTTCCACTCTGTTAGCTGAGTACACACATC
        390 AAAGAGTGTTTCAAAACTGCTCTATGAAAAGAAATGTTCAACTCTGTTAGTTGAGTACACACATC

                 ***            *         *                   **        *  * 
     120650 ACAAACTTGTTTCTGAGAATCCTTCTGTCTCGTTTTTATGGGAAGATATTTACTTTTCCACCGTA
        455 ACAAAGAAGTTTCTGAGAATGCTTCTGTCTAGTTTTTATGGGAAGATATTCCCTTTTCCAACGAA

            *           *       *     *       *  *  *  *            *        
     120715 GGCATCAAAGCGCTCCAAATGTCCACATCCAGATACTCCAGAACGAGTGTTTCAAACCTGCTCTA
        520 AGCATCAAAGCGATCCAAATATCCACTTCCAGATTCTACAAAAAGAGTGTTTCAAAACTGCTCTA

             *     *   *         *        * *  *      *      *              *
     120780 TGAAAGGGAATCTTCAACTCTATGAGTTGAATGCAGACATCAGAAAGAAATTTCTGAGAATGCTG
        585 TCAAAGGAAATGTTCAACTCTGTGAGTTGAGTACACACATCACAAAGAAGTTTCTGAGAATGCTT

                   **       *          **             *  *    *              
     120845 CTGTCTACCTTTTAT-TTGA-ATTCCCGCTTCCAACGAAATCCTCCAAGCTATCCAAATATCCAC
        650 CTGTCTAGTTTTTATGATGATATTCCCTTTTCCAACGAAATCATCAAAGCGATCCAAATATCCAC

            * *  **   *    *                            **              **  *
     120908 CTGCATTTTCCACAACAAGAGTGTTTCAAAACTG-CTCTATCAATAGAAATGTTCAACTCCTTTG
        715 TTCCAGATTCTACAAAAAGAGTGTTTCAAAACTGCCTCTATCAAAGGAAATGTTCAACTCTGTTA

             *  *                *                                        ** 
     120972 GCTGGGTACACACATCACAAACAAGTTTCTGAGAATGCTTCTGTCTAGTTTTTATGGGAAGACGT
        780 GTTGAGTACACACATCACAAAGAAGTTTCTGAGAATGCTTCTGTCTAGTTTTTATGGGAAGATAT

                    *  * *  **          *       *            **              
     121037 TCCCTTTTTCACCAAAGGCATCAAAGCGCTCCAAATGTCCACTTCCAGACACTACAAAAAGAGTG
        845 TCCCTTTTCCAACGAAATCATCAAAGCGATCCAAATATCCACTTCCAGATTCTACAAAAAGAGTG

                *  **   *   **    **                 *           *         * 
     121102 TTTCCAACGTGCCCTAAGAAAGCGAATGTTCAACTCTGTGACTTGAATGCAGATATCACAAAGTA
        910 TTTCAAAACTGCTCTATCAAAGGAAATGTTCAACTCTGTGAGTTGAATGCAGACATCACAAAGAA

                      **                  *    *         *                * *
     121167 GTTTCTGAGAGGGCTTCTGTCTAGATTTTAGATGATGATATTCCCGTTTCCAACGAAATCATTAG
        975 GTTTCTGAGAATGCTTCTGTCTAG-TTTT-TATGAAGATATTCCCTTTTCCAACGAAATCATCAA

               *                *   *                    *        **         
     121232 AGCTATCCAAATATCCACTTACAGTTTCTACAAAAAGAGTGTTTCCAAACTGCTGCATCAAAAGA
       1038 AGCGATCCAAATATCCACTTCCAGATTCTACAAAAAGAGTGTTTCAAAACTGCTCTATCAAAAGA

            * *    *          *                   ***      *     *         * 
     121297 GAGGTTCCACTCTGTTAGCTGAGTACACACATCACAAACTTGTTTCTCAGAATCCTTCTGTCTCG
       1103 AATGTTCAACTCTGTTAGTTGAGTACACACATCACAAAGAAGTTTCTGAGAATGCTTCTGTCTAG

                              ** *    
     121362 TTTTTATGGGAAGATATTTACTTTTT
       1168 TTTTTATGGGAAGATATTCCCGTTTT

              *  * **          *       *     *       *  *  *                 
     121388 CACCGTAGGCATCAAAGCGCTCCAAATGTCCACATCCAGATACTCCAGAAAGAGTGTTTCAAACC
          1 CAACGAAATCATCAAAGCGATCCAAATATCCACTTCCAGATTCTACAAAAAGAGTGTTTCAAACC

                              *         *        * *  *      *      *        
     121453 TGCTCTATGAAAGGGAATCTTCAACTCTATGAGTTGAATGCAGACATCAGAAAGAAATTTCTGAG
         66 TGCTCTA-GAAAGGGAATGTTCAACTCTGTGAGTTGAGTACACACATCACAAAGAAGTTTCTGAG

                  *       **          *          *                 *    *    
     121518 AATGCTGCTGTCTACCTTTTAT-T--TGA-ATTCCCGCTTCCAACGAAATC-CTCCAAGCTATCC
        130 AATGCTTCTGTCTAGTTTTTATATGAAGATATTCCCGTTTCCAACGAAATCGCT-AAAGCGATCC

                        *       *                                  ** *  *   
     121578 AAATATCCACTTGCAGATTCCACAAAAAGAGTGTTTCAAAACTGCTCTCTATCAATGGCAAAGTT
        194 AAATATCCACTTCCAGATTCTACAAAAAGAGTGTTTCAAAACTG--CTCTATCAAAAGAAATGTT

                             *     *     *  *                         *      
     121643 CAACTCTGTTAGTTGAGGACACATATCACCAACAAGTTTCTGAGAATGCTTCTGTCTATTTTTTA
        257 CAACTCTGTTAGTTGAGTACACACATCACAAAGAAGTTTCTGAGAATGCTTCTGTCTAGTTTTTA

             **         *      *  *  * ** *    *      *    *          * **   
     121708 TGGGAAGATATTTCCTTTTTCACCGTAGGCGTCAAGGCGATCGAAATGTCCACTTCCACAAACTA
        322 TATGAAGATATTCCCTTTTCCAACGAAATCATCAAAGCGATCCAAATATCCACTTCCAGATTCTA

                   *          *             * **       *    * *      * ** * *
     121773 CAAAAAGTGTGTTTCAAACCTGCTCTATGAAAGGCCATGTTCATCTCTATGAGTTGAATGGAAAT
        387 CAAAAAGAGTGTTTCAAAACTGCTCTATGAAAAGAAATGTTCAACTCTGTTAGTTGAGTACACAC

               **      *      *       *         *         *         **       
     121838 ATCCGAAAGAAATTTCTGGGAATGCTGCTGTCTAGTGTTTAT---ACGA-ATTCCCGCTTCCAAC
        452 ATCACAAAGAAGTTTCTGAGAATGCTTCTGTCTAGTTTTTATGGGAAGATATTCCCTTTTCCAAC

                * *       *                *    *  *                         
     121899 GAAATCCTCAAAGCAATCCAAATATCCACTTGCAGAATCCACAAAAAGAGTGTTTCAAAACTGCT
        517 GAAAGCATCAAAGCGATCCAAATATCCACTTCCAGATTCTACAAAAAGAGTGTTTCAAAACTGCT

                   **    *          * *                   *  *               
     121964 CTATCAATAGAAAGGTTCAACTCTTTTAGTTGAGTACACACATCACGAACAAGTTTCTGAGAATG
        582 CTATCAAAGGAAATGTTCAACTCTGTGAGTTGAGTACACACATCACAAAGAAGTTTCTGAGAATG

                     * *          *  **  *         * *  **          *       *
     122029 CTTCTGTCTGGCTTTTATTGGAAGACGTTTCCTTTT-CACCAAAGGCATCAAAGCGCTCCAAATG
        647 CTTCTGTCTAGTTTTTA-T-GATGATATTCCCTTTTCCAACGAAATCATCAAAGCGATCCAAATA

                            * *                **      * ***    *            
     122093 TCCACTTCCAGATTCTTCCAAAAGAGTGTTTCAAACGTG-CTCAAAGTAAGGGAATGTTCAACTC
        710 TCCACTTCCAGATTCTACAAAAAGAGTGTTTCAAAACTGCCTCTATCAAAGGAAATGTTCAACTC

               * *    * *  * *     *   *       * * *             *     ***  *
     122157 TGTGACTTGAATGCAGATATCACCAAGTAGTTTCTAATAGTGCTTCTGTCTAGATTTTAGATGAT
        775 TGTTAGTTGAGTACACACATCACAAAGAAGTTTCTGAGAATGCTTCTGTCTAGTTTTTATGGGAA

                     *              * * *   * **          *  *   *      *    
     122222 GATATTCCCGTTTCCAACGAAATCGTTAGAGCTAAGCAAATATCCAGTTACAGTTTCTACCAAAA
        840 GATATTCCCTTTTCCAACGAAATCATCAAAGCGATCCAAATATCCACTTCCAGATTCTACAAAAA

             *       *   *    **      *    *            *      ***  *        
     122287 GGGTGTTTCCAAATTGCTGCATCAAAAGAAAGGTTCAACTCTGTTAGTTGAGGACACACATCACA
        905 GAGTGTTTCAAAACTGCTCTATCAAAGGAAATGTTCAACTCTGTGAGTTGAATGCAGACATCACA

                     *                              *                    *   
     122352 AAGAAGTTTGTGAGAATGCTTCTGTCTAGATTTTGTATGACGATATTCCCTTTTCCAACGATATC
        970 AAGAAGTTTCTGAGAATGCTTCTGTCTAG-TTTT-TATGAAGATATTCCCTTTTCCAACGAAATC

            * *     *   *       * *  *    *  *       *  *        *       * * 
     122417 GTTAAAGCAATCTAAATATCAATTTGCAGAATCCACAAAAATAGAGTTTCAAAGCTGCTCTGTAA
       1033 ATCAAAGCGATCCAAATATCCACTTCCAGATTCTACAAAAAGAGTGTTTCAAAACTGCTCTATCA

                   *    *          *                   *** *          *      
     122482 AAAGAAAGGTTCCACTCTGTTAGCTGAGTACACACATCACAAACTTGATTCTGAGAATCCTTCTG
       1098 AAAGAAATGTTCAACTCTGTTAGTTGAGTACACACATCACAAAGAAGTTTCTGAGAATGCTTCTG

               *                   ** *   *
     122547 TCTCGTTTTTATGGGAAGATATTTACTTTTC
       1163 TCTAGTTTTTATGGGAAGATATTCCCGTTTT

              *  * **          *       *     *       *  *  *  *              
     122578 CACCGTAGGCATCAAAGCGCTCCAAATGTCCACATCCAGATACTCCAGAACGAGTGTTTCAAACC
          1 CAACGAAATCATCAAAGCGATCCAAATATCCACTTCCAGATTCTACAAAAAGAGTGTTTCAAACC

                              *         *        * *  *      *      *        
     122643 TGCTCTATGAAAGGGAATCTTCAACTCTATGAGTTGAATGCAGACATCAGAAAGAAATTTCTGAG
         66 TGCTCTA-GAAAGGGAATGTTCAACTCTGTGAGTTGAGTACACACATCACAAAGAAGTTTCTGAG

                  *       **          *          *                 *    *    
     122708 AATGCTGCTGTCTACCTTTTAT-T--TGA-ATTCCCGCTTCCAACGAAATC-CTCCAAGCTATCC
        130 AATGCTTCTGTCTAGTTTTTATATGAAGATATTCCCGTTTCCAACGAAATCGCT-AAAGCGATCC

                      * *  **   *    *                           *           
     122768 AAATATCCACCTGCATTTTCCACAACAAGAGTGTTTCAAAACTGCTCTATCAATAGAAATGTTCA
        194 AAATATCCACTTCCAGATTCTACAAAAAGAGTGTTTCAAAACTGCTCTATCAAAAGAAATGTTCA

                **  * *  *                *                                 *
     122833 ACTCCTTTGGCTGGGTACACACATCACAAACAAGTTTCTGAGAATGCTTCTGTCTAGTTTTTATG
        259 ACTCTGTTAGTTGAGTACACACATCACAAAGAAGTTTCTGAGAATGCTTCTGTCTAGTTTTTATA

            *     **         *  * *  **          *       *            **     
     122898 GGAAGACGTTCCCTTTTTCACCAAAGGCATCAAAGCGCTCCAAATGTCCACTTCCAGACACTACA
        324 TGAAGATATTCCCTTTTCCAACGAAATCATCAAAGCGATCCAAATATCCACTTCCAGATTCTACA

                            **       *    ***               * *    * *  * *  
     122963 AAAAGAGTGTTTCAAACGTGCTCTAAGAAAGCGAATGTTCAACTCTGTGACTTGAATGCAGATAT
        389 AAAAGAGTGTTTCAAAACTGCTCTATGAAAAGAAATGTTCAACTCTGTTAGTTGAGTACACACAT

                   *           **            *     ***  *         *          
     123028 CACAAAGTAGTTTCTGAGAGGGCTTCTGTCTAGATTTTAGATGATGATATTCCCGTTTCCAACGA
        454 CACAAAGAAGTTTCTGAGAATGCTTCTGTCTAGTTTTTATGGGAAGATATTCCCTTTTCCAACGA

              *   * *   *                *   *                    *        **
     123093 AATCATTAGAGCTATCCAAATATCCACTTACAGTTTCTACAAAAAGAGTGTTTCCAAACTGCTGC
        519 AAGCATCAAAGCGATCCAAATATCCACTTCCAGATTCTACAAAAAGAGTGTTTCAAAACTGCTCT

                  *  * *    *       *  **                  ***      *     *  
     123158 ATCAAAAGAGAGGTTCCACTCTGTTAGCCGAGTACACACATCACAAACTTGTTTCTCAGAATCCT
        584 ATCAAAGGAAATGTTCAACTCTGTGAGTTGAGTACACACATCACAAAGAAGTTTCTGAGAATGCT

                *  *            *      **     *  *  * **          *       *  
     123223 TCTGACTCGTTTTTATGGGAAGATATTTACTTTTTCACCGTAGGCATCAAAGCGCTCCAAATGTC
        649 TCTGTCTAGTTTTTAT--GATGATATTCCCTTTTCCAACGAAATCATCAAAGCGATCCAAATATC

               *       *  *  *               *          *     *   *         *
     123288 CACATCCAGATACTCCAGAAAGAGTGTTTCAAACCTG-CTCTATGAAAGGGAATCTTCAACTCTA
        712 CACTTCCAGATTCTACAAAAAGAGTGTTTCAAAACTGCCTCTATCAAAGGAAATGTTCAACTCTG

             *      * *  *      *      *              *       **         **  
     123352 TGAGTTGAATGCAGACATCAGAAAGAAATTTCTGAGAATGCTGCTGTCTACCTTTTAT---TTGA
        777 TTAGTTGAGTACACACATCACAAAGAAGTTTCTGAGAATGCTTCTGTCTAGTTTTTATGGGAAGA

                   **             *  *    *                *       *         
     123414 -ATTCCCGCTTCCAACGAAATCCTCCAAGCTATCCAAATATCCACTTGCAGATTCCACAAAAAGA
        842 TATTCCCTTTTCCAACGAAATCATCAAAGCGATCCAAATATCCACTTCCAGATTCTACAAAAAGA

                                     *  *  *            *      ***  * *     *
     123478 GTGTTTCAAAACTGCTCTCTATCAATGGCAAAGTTCAACTCTGTTAGTTGAGGACACATATCACC
        907 GTGTTTCAAAACTG--CTCTATCAAAGGAAATGTTCAACTCTGTGAGTTGAATGCAGACATCACA

              *                         *                  *      *  *  * ** 
     123543 AACAAGTTTCTGAGAATGCTTCTGTCTATTTTTTATGGGAAGATATTTCCTTTTTCACCGTAGGC
        970 AAGAAGTTTCTGAGAATGCTTCTGTCTAGTTTTTAT--GAAGATATTCCCTTTTCCAACGAAATC

            *    *      *    *          * **                     *         * 
     123608 GTCAAGGCGATCGAAATGTCCACTTCCACAAACTACAAAAAGAGTGTTTCAAACCTGCTCTATGA
       1033 ATCAAAGCGATCCAAATATCCACTTCCAGATTCTACAAAAAGAGTGTTTCAAAACTGCTCTATCA

              * **       *    * *      * ** * *   **      *      *       *   
     123673 AAGGCCATGTTCATCTCTATGAGTTGAATGGAAATATCCGAAAGAAATTTCTGGGAATGCTGCTG
       1098 AAAGAAATGTTCAACTCTGTTAGTTGAGTACACACATCACAAAGAAGTTTCTGAGAATGCTTCTG

                  *         *          *  *
     123738 TCTAGTGTTTAT---ACGA-ATTCCCGCTTC
       1163 TCTAGTTTTTATGGGAAGATATTCCCGTTTT

                      *       *                *    *  *                   * 
     123765 CAACGAAATCCTCAAAGCAATCCAAATATCCACTTGCAGAATCCACAAAAAGAGTGTTTCAAAAC
          1 CAACGAAATCATCAAAGCGATCCAAATATCCACTTCCAGATTCTACAAAAAGAGTGTTTCAAACC

                    *  ** *  *          * *                      *           
     123830 TGCTCTATCAATAGAAAGGTTCAACTCTTTTAGTTGAGTACACACATCACAAACAAGTTTCTGAG
         66 TGCTCTA-GAAAGGGAATGTTCAACTCTGTGAGTTGAGTACACACATCACAAAGAAGTTTCTGAG

                         * *      **     **  *      *         *           *  
     123895 AATGCTTCTGTCTGGCTTTTATTGGAAGACGTTTCC-TTTTCAAC-AAA-GGCATCAAAGCGCTC
        130 AATGCTTCTGTCTAGTTTTTATATGAAGATATTCCCGTTTCCAACGAAATCGC-T-AAAGCGATC

                 *                * *                **  *  * ***  * *       
     123957 CAAATGTCCACTTCCAGATTCTTCCAAAAGAGTGTTTCAAACGTGGTCGAAGTAAGGGAATGTTC
        193 CAAATATCCACTTCCAGATTCTACAAAAAGAGTGTTTCAAAACTGCTCTATCAAAAGAAATGTTC

                    * *    * *  * *     *   *       * * *            **     *
     124022 AACTCTGTGACTTGAATGCAGATATCACCAAGTAGTTTCTAATAGTGCTTCTGTCTACATTTTAG
        258 AACTCTGTTAGTTGAGTACACACATCACAAAGAAGTTTCTGAGAATGCTTCTGTCTAGTTTTTAT

                *         *              * * *   *             *  *   *      
     124087 ATGATGATATTCCCGTTTCCAACGAAATCGTTAGAGCTATCCAAATATCCAGTTACAGTTTCTAC
        323 ATGAAGATATTCCCTTTTCCAACGAAATCATCAAAGCGATCCAAATATCCACTTCCAGATTCTAC

            *     *       *   *    **  *        *                    *       
     124152 CAAAAGGGTGTTTCCAAATTGCTGCATCAAAAGAAAGGTTCAACTCTGTTAGTTGAGGACACACA
        388 AAAAAGAGTGTTTCAAAACTGCTCTATGAAAAGAAATGTTCAACTCTGTTAGTTGAGTACACACA

                          *                   *    * **  *                   
     124217 TCACAAAGAAGTTTGTGAGAATGCTTCTGTCTAGATTTTGTATGACGATATTCCCTTTTCCAACG
        453 TCACAAAGAAGTTTCTGAGAATGCTTCTGTCTAGTTTTTATGGGAAGATATTCCCTTTTCCAACG

             * * * *     *   *       * *  *    *  *       *  *        *      
     124282 ATATCGTTAAAGCAATCTAAATATCAATTTGCAGAATCCACAAAAATAGAGTTTCAAAGCTGCTC
        518 AAAGCATCAAAGCGATCCAAATATCCACTTCCAGATTCTACAAAAAGAGTGTTTCAAAACTGCTC

             * *   *    *    *       *  *                   ***           ** 
     124347 TGTAAAAAGAAAGGTTCCACTCTGTTAGCTGAGTACACACATCACAAACTTGTTTCTGAGAAACC
        583 TATCAAAGGAAATGTTCAACTCTGTGAGTTGAGTACACACATCACAAAGAAGTTTCTGAGAATGC

                    *            *      **        ** * **          *       * 
     124412 TTCTGTCTCGTTTTTATGGGAAGATATTTACTTTTCCACTGTAGGCATCAAAGCGCTCCAAATGT
        648 TTCTGTCTAGTTTTTAT--GATGATATTCCCTTTTCCAACGAAATCATCAAAGCGATCCAAATAT

                *       *  *  *  *            *          *     *   *         
     124477 CCACATCCAGATACTCCAGAACGAGTGTTTCAAACCTG-CTCTATGAAAGGGAATCTTCAACTCT
        711 CCACTTCCAGATTCTACAAAAAGAGTGTTTCAAAACTGCCTCTATCAAAGGAAATGTTCAACTCT

            * *      * *  *      *      *              *       **         ** 
     124541 ATGAGTTGAATGCAGA-ATCAGAAAGAAATTTCTGAGAATGCTGCTGTCTACCTTTTAT---TTG
        776 GTTAGTTGAGTACACACATCACAAAGAAGTTTCTGAGAATGCTTCTGTCTAGTTTTTATGGGAAG

                    **             *  *    *              * *  **   *        
     124602 A-ATTCCCGCTTCCAACGAAATCCTCCAAGCTATCCAAATATCCACCTGCATTTTCCACAAAAAG
        841 ATATTCCCTTTTCCAACGAAATCATCAAAGCGATCCAAATATCCACTTCCAGATTCTACAAAAAG

                                    **                *    *  ** *  *        
     124666 AGTGTTTCAAAACTGCTCTATCAATAGAAATGTTCAACTCCTTTG-GCTGGGTACACACATCACA
        906 AGTGTTTCAAAACTGCTCTATCAAAGGAAATGTTCAACT-CTGTGAGTTGAATGCAGACATCACA

              *                                        **         *  * *  ** 
     124730 AACAAGTTTCTGAGAATGCTTCTGTCTAGTTTTTATGGGAAGACGTTCCCTTTTTCACCAAAGGC
        970 AAGAAGTTTCTGAGAATGCTTCTGTCTAGTTTTTAT--GAAGATATTCCCTTTTCCAACGAAATC

                     *       *            **                  *  **       ** 
     124795 ATCAAAGCGCTCCAAATGTCCACTTCCAGACACTACAAAAAGAGTGTTTCCAACGTGCTCTAAGA
       1033 ATCAAAGCGATCCAAATATCCACTTCCAGATTCTACAAAAAGAGTGTTTCAAAACTGCTCTATCA

              ***               * *    * *  * *         *           **       
     124860 AAGCGAATGTTCAACTCTGTGACTTGAATGCAGATATCACAAAGTAGTTTCTGAGAGGGCTTCTG
       1098 AAAGAAATGTTCAACTCTGTTAGTTGAGTACACACATCACAAAGAAGTTTCTGAGAATGCTTCTG

                 *     ***  *             *
     124925 TCTAGATTTTAGATGATGATATTCCCGTTTC
       1163 TCTAGTTTTTATGGGAAGATATTCCCGTTTT

                        * *   *                *   *                    *  * 
     124956 CAACGAAATCATTAGAGCTATCCAAATATCCACTTACAGTTTCTACAAAAAGAGTGTTTCCAAAC
          1 CAACGAAATCATCAAAGCGATCCAAATATCCACTTCCAGATTCTACAAAAAGAGTGTTTCAAACC

                    **    *   *    *       *  **                  ***      * 
     125021 TGCTGCATCAAAAGAG-AGGTTCCACTCTGTTAGCCGAGTACACACATCACAAACTTGTTTCTCA
         66 TGCT-C-TAGAAAGGGAATGTTCAACTCTGTGAGTTGAGTACACACATCACAAAGAAGTTTCTGA

                *         *        **         ** *   *  *  * ** ***      *   
     125085 GAATCCTTCTGTCTCGTTTTTATGGGAAGATATTTACTTTTTCACCGTAGGCATCAAAGCGCTCC
        129 GAATGCTTCTGTCTAGTTTTTATATGAAGATATTCCCGTTTCCAACGAAATCGCTAAAGCGATCC

                *     *       *  *  *               *         *   * *   *    
     125150 AAATGTCCACATCCAGATACTCCAGAAAGAGTGTTTCAAACCTGCTCTATGAAAGGGAATCTTCA
        194 AAATATCCACTTCCAGATTCTACAAAAAGAGTGTTTCAAAACTGCTCTATCAAAAGAAATGTTCA

                 * *      * *  *      *      *              *       **       
     125215 ACTCTATGAGTTGAATGCAGACATCAGAAAGAAATTTCTGAGAATGCTGCTGTCTACCTTTTAT-
        259 ACTCTGTTAGTTGAGTACACACATCACAAAGAAGTTTCTGAGAATGCTTCTGTCTAGTTTTTATA

               *         **             *  *    *                *       *   
     125279 T--TGA-ATTCCCGCTTCCAACGAAATCCTCCAAGCTATCCAAATATCCACTTGCAGATTCCACA
        324 TGAAGATATTCCCTTTTCCAACGAAATCATCAAAGCGATCCAAATATCCACTTCCAGATTCTACA

                                        *  ** *  *                    *     *
     125341 AAAAGAGTGTTTCAAAACTGCTCTCTATCAATGGCAAAGTTCAACTCTGTTAGTTGAGGACACAT
        389 AAAAGAGTGTTTCAAAACTG--CTCTATGAAAAGAAATGTTCAACTCTGTTAGTTGAGTACACAC

                 *  *                         *                  *      *  * 
     125406 ATCACCAACAAGTTTCTGAGAATGCTTCTGTCTATTTTTTATGGGAAGATATTTCCTTTTTCACC
        452 ATCACAAAGAAGTTTCTGAGAATGCTTCTGTCTAGTTTTTATGGGAAGATATTCCCTTTTCCAAC

             * *  *    *      *    *          * **                     *     
     125471 GTAGGCGTCAAGGCGATCGAAATGTCCACTTCCACAAACTACAAAAAGAGTGTTTCAAACCTGCT
        517 GAAAGCATCAAAGCGATCCAAATATCCACTTCCAGATTCTACAAAAAGAGTGTTTCAAAACTGCT

                *     **       *    *        * ** * *   **      *      *     
     125536 CTATGAAAGGCCATGTTCATCTCTATGAGTTGAATGGAAATATCCGAAAGAAATTTCTGGGAATG
        582 CTATCAAAGGAAATGTTCAACTCTGTGAGTTGAGTACACACATCACAAAGAAGTTTCTGAGAATG

              *         *       *         **             *       *           
     125601 CTGCTGTCTAGTGTTTAT-ACGA-ATTCCCGCTTCCAACGAAATCCTCAAAGCAATCCAAATATC
        647 CTTCTGTCTAGTTTTTATGATGATATTCCCTTTTCCAACGAAATCATCAAAGCGATCCAAATATC

                 *    *  *                     *           **    *          *
     125664 CACTTGCAGAATCCACAAAAAGAGTGTTTCAAAACCG-CTCTATCAATAGAAAGGTTCAACTCTT
        712 CACTTCCAGATTCTACAAAAAGAGTGTTTCAAAACTGCCTCTATCAAAGGAAATGTTCAACTCTG

                                    *                        * *      *      
     125728 TTAGTTGAGTACACACATCACAAACAAGTTTCTGAGAATGCTTCTGTCTGGCTTTTATTGGAAGA
        777 TTAGTTGAGTACACACATCACAAAGAAGTTTCTGAGAATGCTTCTGTCTAGTTTTTATGGGAAGA

            **  *         * *  **          *       *                * *      
     125793 CGTTTCCTTTT-CACCAAAGGCATCAAAGCGCTCCAAATGTCCACTTCCAGATTCTTCCAAAAGA
        842 TATTCCCTTTTCCAACGAAATCATCAAAGCGATCCAAATATCCACTTCCAGATTCTACAAAAAGA

                      **  *  * ***    *                 *           *     *  
     125857 GTGTTTCAAACGTGGTCGAAGTAAGGGAATGTTCAACTCTGTGACTTGAATGCAGATATCACCAA
        907 GTGTTTCAAAACTGCTCTATCAAAGGAAATGTTCAACTCTGTGAGTTGAATGCAGACATCACAAA

             *       * * *                   *    *         *              * 
     125922 GTAGTTTCTAATAGTGCTTCTGTCTAGATTTTAGATGATGATATTCCCGTTTCCAACGAAATCGT
        972 GAAGTTTCTGAGAATGCTTCTGTCTAG-TTTT-TATGAAGATATTCCCTTTTCCAACGAAATCAT

            * *   *             *  *   *      *     *       *   *    **      
     125987 TAGAGCTATCCAAATATCCAGTTACAGTTTCTACCAAAAGGGTGTTTCCAAATTGCTGCATCAAA
       1035 CAAAGCGATCCAAATATCCACTTCCAGATTCTACAAAAAGAGTGTTTCAAAACTGCTCTATCAAA

                 *                    *                     *                
     126052 AGAAAGGTTCAACTCTGTTAGTTGAGGACACACATCACAAAGAAGTTTGTGAGAATGCTTCTGTC
       1100 AGAAATGTTCAACTCTGTTAGTTGAGTACACACATCACAAAGAAGTTTCTGAGAATGCTTCTGTC

               *    * **  *         *   *
     126117 TAGATTTTGTATGACGATATTCCCTTTTC
       1165 TAGTTTTTATGGGAAGATATTCCCGTTTT

                  *   * *     *   *       * *  *    *  *       *  *        * 
     126146 CAACGATATCGTTAAAGCAATCTAAATATCAATTTGCAGAATCCACAAAAATAGAGTTTCAAAGC
          1 CAACGAAATCATCAAAGCGATCCAAATATCCACTTCCAGATTCTACAAAAAGAGTGTTTCAAACC

                   **   * *  *    *       *  *                   ***         
     126211 TGCTCTGTAAAAAGAAAGGTTCCACTCTGTTAGCTGAGTACACACATCACAAACTTGTTTCTGAG
         66 TGCTCT-AGAAAGGGAATGTTCAACTCTGTGAGTTGAGTACACACATCACAAAGAAGTTTCTGAG

              **         *        **         ** *      ** * ** ***      *    
     126276 AAACCTTCTGTCTCGTTTTTATGGGAAGATATTTACTTTTCCACTGTAGGCATCAAAGCGCTCCA
        130 AATGCTTCTGTCTAGTTTTTATATGAAGATATTCCCGTTTCCAACGAAATCGCTAAAGCGATCCA

               *     *       *  *  *  *            *         *   * *   *     
     126341 AATGTCCACATCCAGATACTCCAGAACGAGTGTTTCAAACCTGCTCTATGAAAGGGAATCTTCAA
        195 AATATCCACTTCCAGATTCTACAAAAAGAGTGTTTCAAAACTGCTCTATCAAAAGAAATGTTCAA

                * *      * *  *      *      *              *       **        
     126406 CTCTATGAGTTGAATGCAGA-ATCAGAAAGAAATTTCTGAGAATGCTGCTGTCTACCTTTTAT-T
        260 CTCTGTTAGTTGAGTACACACATCACAAAGAAGTTTCTGAGAATGCTTCTGTCTAGTTTTTATAT

              *         **             *  *    *              * *  **   *    
     126469 --TGA-ATTCCCGCTTCCAACGAAATCCTCCAAGCTATCCAAATATCCACCTGCATTTTCCACAA
        325 GAAGATATTCCCTTTTCCAACGAAATCATCAAAGCGATCCAAATATCCACTTCCAGATTCTACAA

            *                        *  *               **  * *  *           
     126531 CAAGAGTGTTTCAAAACTGCTCTATCAATAGAAATGTTCAACTCCTTTGGCTGGGTACACACATC
        390 AAAGAGTGTTTCAAAACTGCTCTATGAAAAGAAATGTTCAACTCTGTTAGTTGAGTACACACATC

                 *                                        **         *  * *  
     126596 ACAAACAAGTTTCTGAGAATGCTTCTGTCTAGTTTTTATGGGAAGACGTTCCCTTTTTCACCAAA
        455 ACAAAGAAGTTTCTGAGAATGCTTCTGTCTAGTTTTTATGGGAAGATATTCCCTTTTCCAACGAA

            *           *       *            **                     **       
     126661 GGCATCAAAGCGCTCCAAATGTCCACTTCCAGACACTACAAAAAGAGTGTTTCAAACGTGCTCTA
        520 AGCATCAAAGCGATCCAAATATCCACTTCCAGATTCTACAAAAAGAGTGTTTCAAAACTGCTCTA

            **   ***                 *    * *  * *         *           **    
     126726 AGAAAACGAATGTTCAACTCTGTGACTTGAATGCAGATATCACAAAGTAGTTTCTGAGAGGGCTT
        585 TCAAAGGAAATGTTCAACTCTGTGAGTTGAGTACACACATCACAAAGAAGTTTCTGAGAATGCTT

                          *              *                * *   *            
     126791 CTGTCTAGATTTTAGATGATGATATTCCCGTTTCCAACGAAATCATTAGAGCTATCCAAATATCC
        650 CTGTCTAG-TTTT-TATGATGATATTCCCTTTTCCAACGAAATCATCAAAGCGATCCAAATATCC

                *   *                    *         **      *  * *    *       
     126856 ACTTACAGTTTCTACAAAAAGAGTGTTTCCAAACTG-CTGCATCAAAAGAGAGGTTCCACTCTGT
        713 ACTTCCAGATTCTACAAAAAGAGTGTTTCAAAACTGCCTCTATCAAAGGAAATGTTCAACTCTGT

               **                  ***      *     *         *                
     126920 TAGCCGAGTACACACATCACAAACTTGTTTCTCAGAATCCTTCTGTCTCGTTTTTATGGGAAGAT
        778 TAGTTGAGTACACACATCACAAAGAAGTTTCTGAGAATGCTTCTGTCTAGTTTTTATGGGAAGAT

               **     *  *  * **          *       *     *       *  *  *      
     126985 ATTTACTTTTTCACCGTAGGCATCAAAGCGCTCCAAATGTCCACATCCAGATACTCCAGAAAGAG
        843 ATTCCCTTTTCCAACGAAATCATCAAAGCGATCCAAATATCCACTTCCAGATTCTACAAAAAGAG

                     *         *     *   *         *                    *    
     127050 TGTTTCAAACCTGCTCTATGAAAGGGAATCTTCAACTCTATGAGTTGAATGCAGACATCAGAAAG
        908 TGTTTCAAAACTGCTCTATCAAAGGAAATGTTCAACTCTGTGAGTTGAATGCAGACATCACAAAG

              *              *       **       **         **             *  * 
     127115 AAATTTCTGAGAATGCTGCTGTCTACCTTTTAT-TTGA-ATTCCCGCTTCCAACGAAATCCTCCA
        973 AAGTTTCTGAGAATGCTTCTGTCTAGTTTTTATGAAGATATTCCCTTTTCCAACGAAATCATCAA

               *                *       *                                  **
     127178 AGCTATCCAAATATCCACTTGCAGATTCCACAAAAAGAGTGTTTCAAAACTGCTCTCTATCAATG
       1038 AGCGATCCAAATATCCACTTCCAGATTCTACAAAAAGAGTGTTTCAAAACTG--CTCTATCAAAA

             *  *                    *     *     *  *                        
     127243 GCAAAGTTCAACTCTGTTAGTTGAGGACACATATCACCAACAAGTTTCTGAGAATGCTTCTGTCT
       1101 GAAATGTTCAACTCTGTTAGTTGAGTACACACATCACAAAGAAGTTTCTGAGAATGCTTCTGTCT

             *                  *  *    
     127308 ATTTTTTATGGGAAGATATTTCCTTTTT
       1166 AGTTTTTATGGGAAGATATTCCCGTTTT

              *  * ** *    *      *    *          * **                       
     127336 CACCGTAGGCGTCAAGGCGATCGAAATGTCCACTTCCACAAACTACAAAAAGAGTGTTTCAAACC
          1 CAACGAAATCATCAAAGCGATCCAAATATCCACTTCCAGATTCTACAAAAAGAGTGTTTCAAACC

                          **       *    *        * ** * *   **      *      * 
     127401 TGCTCTATGAAAGGCCATGTTCATCTCTATGAGTTGAATGGAAATATCCGAAAGAAATTTCTGGG
         66 TGCTCTA-GAAAGGGAATGTTCAACTCTGTGAGTTGAGTACACACATCACAAAGAAGTTTCTGAG

                  *                              *                      *    
     127466 AATGCTGCTGTCTAGTTTTTATAT---GA-ATTCCCGCTTCCAACGAAATC-CTCAAAGCAATCC
        130 AATGCTTCTGTCTAGTTTTTATATGAAGATATTCCCGTTTCCAACGAAATCGCT-AAAGCGATCC

                        *    *  *                                *     *     
     127526 AAATATCCACTTGCAGAATCCACAAAAAGAGTGTTTCAAAACTGCTCTATCAATAGAAAGGTTCA
        194 AAATATCCACTTCCAGATTCTACAAAAAGAGTGTTTCAAAACTGCTCTATCAAAAGAAATGTTCA

                 *                        *                        * *      *
     127591 ACTCTTTTAGTTGAGTACACACATCACAAACAAGTTTCTGAGAATGCTTCTGTCTGGCTTTTATT
        259 ACTCTGTTAGTTGAGTACACACATCACAAAGAAGTTTCTGAGAATGCTTCTGTCTAGTTTTTATA

            *     **  *         * *  **          *      **                * *
     127656 GGAAGACGTTTCCTTTT-CACCAAAGGCATCAAAGCGCTCCAAAGGTCCACTTCCAGATTCTTCC
        324 TGAAGATATTCCCTTTTCCAACGAAATCATCAAAGCGATCCAAATATCCACTTCCAGATTCTACA

                            **          *  * *               * *    * *  * * 
     127720 AAAAGAGTGTTTCAAACGTGCTC-ATAGTAAGGGAATGTTCAACTCTGTGACTTGAATGCAGATA
        389 AAAAGAGTGTTTCAAAACTGCTCTAT-GAAAAGAAATGTTCAACTCTGTTAGTTGAGTACACACA

                *   *       * * *             *     ***  *         *         
     127784 TCACCAAGTAGTTTCTAATAGTGCTTCTGTCTAGATTTTAGATGATGATATTCCCGTTTCCAACG
        453 TCACAAAGAAGTTTCTGAGAATGCTTCTGTCTAGTTTTTATGGGAAGATATTCCCTTTTCCAACG

               * *** *   *             *  *   *      *     *       *   *    *
     127849 AAATCGCTAGAGCTATCCAAATATCCAGTTACAGTTTCTACCAAAAGGGTGTTTCCAAATTGCTG
        518 AAAGCATCAAAGCGATCCAAATATCCACTTCCAGATTCTACAAAAAGAGTGTTTCAAAACTGCTC

            *      *    *            *       *                     *         
     127914 CATCAAAAGAAAGGTTCAACTCTGTTAGTTGAGGACACACATCACAAAGAAGTTTGTGAGAATGC
        583 TATCAAAGGAAATGTTCAACTCTGTGAGTTGAGTACACACATCACAAAGAAGTTTCTGAGAATGC

                                 *                    *   * *     *   *      
     127979 TTCTGTCTAGATTTTGTATGACGATATTCCCTTTTCCAACGATATCGTTAAAGCAATCTAAATAT
        648 TTCTGTCTAG-TTTT-TATGATGATATTCCCTTTTCCAACGAAATCATCAAAGCGATCCAAATAT

             * *  *    *  *       *  *        *        * *   *    *    *     
     128044 CAATTTGCAGAATCCACAAAAATAGAGTTTCAAAGCTG-CTCTGTAAAAAGAAAGGTTCCACTCT
        711 CCACTTCCAGATTCTACAAAAAGAGTGTTTCAAAACTGCCTCTATCAAAGGAAATGTTCAACTCT

                 *                   ***            *         *              
     128108 GTTAGCTGAGTACACACATCACAAACTTGTTTCTGAGAATCCTTCTGTCTCGTTTTTATGGGAAG
        776 GTTAGTTGAGTACACACATCACAAAGAAGTTTCTGAGAATGCTTCTGTCTAGTTTTTATGGGAAG

                 **        *  * **          *       *     *       *  *  *  * 
     128173 ATATTTACTTTTCCACCGTAGGCATCAAAGCGCTCCAAATGTCCACATCCAGATACTCCAGAACG
        841 ATATTCCCTTTTCCAACGAAATCATCAAAGCGATCCAAATATCCACTTCCAGATTCTACAAAAAG

                       *         *     *   *         *                    *  
     128238 AGTGTTTCAAACCTGCTCTATGAAAGGGAATCTTCAACTCTATGAGTTGAATGCAGACATCAGAA
        906 AGTGTTTCAAAACTGCTCTATCAAAGGAAATGTTCAACTCTGTGAGTTGAATGCAGACATCACAA

                *              *       **       **         **             *  
     128303 AGAAATTTCTGAGAATGCTGCTGTCTACCTTTTAT-TTGA-ATTCCCGCTTCCAACGAAATCCTC
        971 AGAAGTTTCTGAGAATGCTTCTGTCTAGTTTTTATGAAGATATTCCCTTTTCCAACGAAATCATC

            *    *              * *  **   *    *                           * 
     128366 CAAGCTATCCAAATATCCACCTGCATTTTCCACAACAAGAGTGTTTCAAAACTGCTCTATCAATA
       1036 AAAGCGATCCAAATATCCACTTCCAGATTCTACAAAAAGAGTGTTTCAAAACTGCTCTATCAAAA

                          **  * *  *                *                        
     128431 GAAATGTTCAACTCCTTTGGCTGGGTACACACATCACAAACAAGTTTCTGAGAATGCTTCTGTCT
       1101 GAAATGTTCAACTCTGTTAGTTGAGTACACACATCACAAAGAAGTTTCTGAGAATGCTTCTGTCT

                            **     *    
     128496 AGTTTTTATGGGAAGACGTTCCCTTTTT
       1166 AGTTTTTATGGGAAGATATTCCCGTTTT

              * *  **          *       *            **                  *   *
     128524 CACCAAAGGCATCAAAGCGCTCCAAATGTCCACTTCCAGACACTACAAAAAGAGTGTTTCCAACG
          1 CAACGAAATCATCAAAGCGATCCAAATATCCACTTCCAGATTCTACAAAAAGAGTGTTTCAAACC

               *         *                  *    * *  * *         *          
     128589 TGCCCTAAGAAAGCGAATGTTCAACTCTGTGACTTGAATGCAGATATCACAAAGTAGTTTCTGAG
         66 TGCTCT-AGAAAGGGAATGTTCAACTCTGTGAGTTGAGTACACACATCACAAAGAAGTTTCTGAG

             **            *     *    *                        **  *   *     
     128654 AGGGCTTCTGTCTAGATTTTAGATGATGATATTCCCGTTTCCAACGAAATCATTAGAGCTATCCA
        130 AATGCTTCTGTCTAGTTTTTATATGAAGATATTCCCGTTTCCAACGAAATCGCTAAAGCGATCCA

                    *  *   *                    *        **         * *    * 
     128719 AATATCCAATTACAGTTTCTACAAAAAGAGTGTTTCCAAACTGCTGCATCAAAAGAGAGGTTCCA
        195 AATATCCACTTCCAGATTCTACAAAAAGAGTGTTTCAAAACTGCTCTATCAAAAGAAATGTTCAA

                     *                   ***      *     *         *        **
     128784 CTCTGTTAGCTGAGTACACACATCACAAACTTGTTTCTCAGAATCCTTCTGTCTCGTTTTTATGG
        260 CTCTGTTAGTTGAGTACACACATCACAAAGAAGTTTCTGAGAATGCTTCTGTCTAGTTTTTATAT

                     **     *  *  * **          *       *     *       *  *  *
     128849 GAAGATATTTACTTTTTCACCGTAGGCATCAAAGCGCTCCAAATGTCCACATCCAGATACTCCAG
        325 GAAGATATTCCCTTTTCCAACGAAATCATCAAAGCGATCCAAATATCCACTTCCAGATTCTACAA

                           *             * *   *         * *      * *  *     
     128914 AAAGAGTGTTTCAAACCTGCTCTATGAAAGGGAATCTTCAACTCTATGAGTTGAATGCAGACATC
        390 AAAGAGTGTTTCAAAACTGCTCTATGAAAAGAAATGTTCAACTCTGTTAGTTGAGTACACACATC

             *      *              *       **         **         **          
     128979 AGAAAGAAATTTCTGAGAATGCTGCTGTCTACCTTTTAT---TTGA-ATTCCCGCTTCCAACGAA
        455 ACAAAGAAGTTTCTGAGAATGCTTCTGTCTAGTTTTTATGGGAAGATATTCCCTTTTCCAACGAA

             * *  *    *                *       *                            
     129040 ATCCTCCAAGCTATCCAAATATCCACTTGCAGATTCCACAAAAAGAGTGTTTCAAAACTGCTCTC
        520 AGCATCAAAGCGATCCAAATATCCACTTCCAGATTCTACAAAAAGAGTGTTTCAAAACTG--CTC

                  *  *  *            *       *     *     *  *                
     129105 TATCAATGGCAAAGTTCAACTCTGTTAGTTGAGGACACATATCACCAACAAGTTTCTGAGAATGC
        583 TATCAAAGGAAATGTTCAACTCTGTGAGTTGAGTACACACATCACAAAGAAGTTTCTGAGAATGC

                     *           *      *      *  *  * ** *    *      *    * 
     129170 TTCTGTCTATTTTTTATGGGAAGATATTTCCTTTTTCACCGTAGGCGTCAAGGCGATCGAAATGT
        648 TTCTGTCTAGTTTTTAT--GATGATATTCCCTTTTCCAACGAAATCATCAAAGCGATCCAAATAT

                     * **          *          *          *     **       *    
     129235 CCACTTCCACAAACTACAAAAAGTGTGTTTCAAACCTG-CTCTATGAAAGGCCATGTTCATCTCT
        711 CCACTTCCAGATTCTACAAAAAGAGTGTTTCAAAACTGCCTCTATCAAAGGAAATGTTCAACTCT

            * *      * ** * *   **      *      *       *         *         * 
     129299 ATGAGTTGAATGGAAATATCCGAAAGAAATTTCTGGGAATGCTGCTGTCTAGTGTTTAT---ACG
        776 GTTAGTTGAGTACACACATCACAAAGAAGTTTCTGAGAATGCTTCTGTCTAGTTTTTATGGGAAG

                    **             *       *                *    *  *        
     129361 A-ATTCCCGCTTCCAACGAAATCCTCAAAGCAATCCAAATATCCACTTGCAGAATCCACAAAAAG
        841 ATATTCCCTTTTCCAACGAAATCATCAAAGCGATCCAAATATCCACTTCCAGATTCTACAAAAAG

                                    **    *          * *      * *  *       * 
     129425 AGTGTTTCAAAACTGCTCTATCAATAGAAAGGTTCAACTCTTTTAGTTGAGTACACACATCACGA
        906 AGTGTTTCAAAACTGCTCTATCAAAGGAAATGTTCAACTCTGTGAGTTGAATGCAGACATCACAA

             *                        * *             **  *         * *  **  
     129490 ACAAGTTTCTGAGAATGCTTCTGTCTGGCTTTTATTGGAAGACGTTTCCTTTT-CACCAAAGGCA
        971 AGAAGTTTCTGAGAATGCTTCTGTCTAGTTTTTA-T-GAAGATATTCCCTTTTCCAACGAAATCA

                    *       *                * *                **     * *** 
     129554 TCAAAGCGCTCCAAATGTCCACTTCCAGATTCTTCCAAAAGAGTGTTTCAAACGTGCTCAAAGTA
       1034 TCAAAGCGATCCAAATATCCACTTCCAGATTCTACAAAAAGAGTGTTTCAAAACTGCTCTATCAA

             * *               * *    * *  * *     *   *       * * *         
     129619 AGGGAATGTTCAACTCTGTGACTTGAATGCAGATATCACCAAGTAGTTTCTAATAGTGCTTCTGT
       1099 AAGAAATGTTCAACTCTGTTAGTTGAGTACACACATCACAAAGAAGTTTCTGAGAATGCTTCTGT

                *     ***  *             *
     129684 CTAGATTTTAGATGATGATATTCCCGTTTC
       1164 CTAGTTTTTATGGGAAGATATTCCCGTTTT

                      * * *   * **          *  *   *      *     *       *  **
     129714 CAACGAAATCGTTAGAGCTAAGCAAATATCCAGTTACAGTTTCTACCAAAAGGGTGTTTCCAAAT
          1 CAACGAAATCATCAAAGCGATCCAAATATCCACTTCCAGATTCTACAAAAAGAGTGTTTCAAACC

                  ***   * *  *            *       *                     *    
     129779 TGCTGCATCAAAAGAAAGGTTCAACTCTGTTAGTTGAGGACACACATCACAAAGAAGTTTGTGAG
         66 TGCT-CTAGAAAGGGAATGTTCAACTCTGTGAGTTGAGTACACACATCACAAAGAAGTTTCTGAG

                           *    *     *         *          *    *      *   * 
     129844 AATGCTTCTGTCTAGATTTTGTATGACGATATTCCCTTTTCCAACGATATCGTTAAAGCAATCTA
        130 AATGCTTCTGTCTAGTTTTTATATGAAGATATTCCCGTTTCCAACGAAATCGCTAAAGCGATCCA

                  * *  *    *  *       *  *        *       * *        *    * 
     129909 AATATCAATTTGCAGAATCCACAAAAATAGAGTTTCAAAGCTGCTCTGTAAAAAGAAAGGTTCCA
        195 AATATCCACTTCCAGATTCTACAAAAAGAGTGTTTCAAAACTGCTCTATCAAAAGAAATGTTCAA

                     *                   *** *          *         *        **
     129974 CTCTGTTAGCTGAGTACACACATCACAAACTTGATTCTGAGAATCCTTCTGTCTCGTTTTTATGG
        260 CTCTGTTAGTTGAGTACACACATCACAAAGAAGTTTCTGAGAATGCTTCTGTCTAGTTTTTATAT

                     **        *  * **          *       *     *       *  *  *
     130039 GAAGATATTTACTTTTCCACCGTAGGCATCAAAGCGCTCCAAATGTCCACATCCAGATACTCCAG
        325 GAAGATATTCCCTTTTCCAACGAAATCATCAAAGCGATCCAAATATCCACTTCCAGATTCTACAA

              *            *             * *   *         * *      * *  *     
     130104 AACGAGTGTTTCAAACCTGCTCTATGAAAGGGAATCTTCAACTCTATGAGTTGAATGCAGACATC
        390 AAAGAGTGTTTCAAAACTGCTCTATGAAAAGAAATGTTCAACTCTGTTAGTTGAGTACACACATC

             *      *              *       **         **         **          
     130169 AGAAAGAAATTTCTGAGAATGCTGCTGTCTACCTTTTAT---TTGA-ATTCCCGCTTCCAACGAA
        455 ACAAAGAAGTTTCTGAGAATGCTTCTGTCTAGTTTTTATGGGAAGATATTCCCTTTTCCAACGAA

             * *  *    *              * *  **   *    *                       
     130230 ATCCTCCAAGCTATCCAAATATCCACCTGCATTTTCCACAACAAGAGTGTTTCAAAACTGCTCTA
        520 AGCATCAAAGCGATCCAAATATCCACTTCCAGATTCTACAAAAAGAGTGTTTCAAAACTGCTCTA

                **                *    *  *                *                 
     130295 TCAATAGAAATGTTCAACTCCTTTG-GCTGGGTACACACATCACAAACAAGTTTCTGAGAATGCT
        585 TCAAAGGAAATGTTCAACT-CTGTGAGTTGAGTACACACATCACAAAGAAGTTTCTGAGAATGCT

                                *  **         *  * *  **          *       *  
     130359 TCTGTCTAGTTTTTATGGGAAGACGTTCCCTTTTTCACCAAAGGCATCAAAGCGCTCCAAATGTC
        649 TCTGTCTAGTTTTTAT--GATGATATTCCCTTTTCCAACGAAATCATCAAAGCGATCCAAATATC

                      **                     **        **    **              
     130424 CACTTCCAGACACTACAAAAAGAGTGTTTCAAACGTG-CTCTAAGAAAGCGAATGTTCAACTCTG
        712 CACTTCCAGATTCTACAAAAAGAGTGTTTCAAAACTGCCTCTATCAAAGGAAATGTTCAACTCTG

             * *    * *  * *         *           **            *     ***  *  
     130488 TGACTTGAATGCAGATATCACAAAGTAGTTTCTGAGAGGGCTTCTGTCTAGATTTTAGATGATGA
        777 TTAGTTGAGTACACACATCACAAAGAAGTTTCTGAGAATGCTTCTGTCTAGTTTTTATGGGAAGA

                   *                * *   *                *   *             
     130553 TATTCCCGTTTCCAACGAAATCATTAGAGCTATCCAAATATCCACTTACAGTTTCTACAAAAAGA
        842 TATTCCCTTTTCCAACGAAATCATCAAAGCGATCCAAATATCCACTTCCAGATTCTACAAAAAGA

                   *        **      *  * *    *       *  **  * *  *          
     130618 GTGTTTCCAAACTGCTGCATCAAAAGAGAGGTTCCACTCTGTTAGCCGAGTACACACATCACAAA
        907 GTGTTTCAAAACTGCTCTATCAAAGGAAATGTTCAACTCTGTGAGTTGAATGCAGACATCACAAA

            ***      *     *      *  *                   **     *  *  * **   
     130683 CTTGTTTCTCAGAATCCTTCTGACTCGTTTTTATGGGAAGATATTTACTTTTTCACCGTAGGCAT
        972 GAAGTTTCTGAGAATGCTTCTGTCTAGTTTTTAT--GAAGATATTCCCTTTTCCAACGAAATCAT

                   *       *     *       *  *  *               *         *   
     130748 CAAAGCGCTCCAAATGTCCACATCCAGATACTCCAGAAAGAGTGTTTCAAACCTGCTCTATGAAA
       1035 CAAAGCGATCCAAATATCCACTTCCAGATTCTACAAAAAGAGTGTTTCAAAACTGCTCTATCAAA

            * *   *         * *      * *  *      *      *              *     
     130813 GGGAATCTTCAACTCTATGAGTTGAATGCAGACATCAGAAAGAAATTTCTGAGAATGCTGCTGTC
       1100 AGAAATGTTCAACTCTGTTAGTTGAGTACACACATCACAAAGAAGTTTCTGAGAATGCTTCTGTC

              **         **          *  *
     130878 TACCTTTTAT---TTGA-ATTCCCGCTTC
       1165 TAGTTTTTATGGGAAGATATTCCCGTTTT

                      *  *    *                *       *             *     * 
     130903 CAACGAAATCCTCCAAGCTATCCAAATATCCACTTGCAGATTCCACAAAAAGAGTGTATCAAAAC
          1 CAACGAAATCATCAAAGCGATCCAAATATCCACTTCCAGATTCTACAAAAAGAGTGTTTCAAACC

                      *  *  *  *            *       *     *     *  *         
     130968 TGCTCTCTATCAATGGCAAAGTTCAACTCTGTTAGTTGAGGACACATATCACCAACAAGTTTCTG
         66 TG--CTCTA-GAAAGGGAATGTTCAACTCTGTGAGTTGAGTACACACATCACAAAGAAGTTTCTG

                            *       **         *  *   *  *  * **  **  *      
     131033 AGAATGCTTCTGTCTATTTTTTATGGGAAGATATTTCCTTTTTCACCGTAGGCGTCAAGGCGATC
        128 AGAATGCTTCTGTCTAGTTTTTATATGAAGATATTCCCGTTTCCAACGAAATCGCTAAAGCGATC

            *    *          * **                     *         *   * **      
     131098 GAAATGTCCACTTCCACAAACTACAAAAAGAGTGTTTCAAACCTGCTCTATGAAAGGCCATGTTC
        193 CAAATATCCACTTCCAGATTCTACAAAAAGAGTGTTTCAAAACTGCTCTATCAAAAGAAATGTTC

             *    * *      * ** * *   **      *      *       *          *    
     131163 ATCTCTATGAGTTGAATGGAAATATCCGAAAGAAATTTCTGGGAATGCTGCTGTCTAG-TGTT-T
        258 AACTCTGTTAGTTGAGTACACACATCACAAAGAAGTTTCTGAGAATGCTTCTGTCTAGTTTTTAT

                *         **             *       *                *    *  *  
     131226 AT-ACGA-ATTCCCGCTTCCAACGAAATCCTCAAAGCAATCCAAATATCCACTTGCAGAATCCAC
        323 ATGAAGATATTCCCTTTTCCAACGAAATCATCAAAGCGATCCAAATATCCACTTCCAGATTCTAC

                                       *  *     *          *                 
     131289 AAAAAGAGTGTTTCAAAACTGCTCTATCAATAGAAAGGTTCAACTCTTTTAGTTGAGTACACACA
        388 AAAAAGAGTGTTTCAAAACTGCTCTATGAAAAGAAATGTTCAACTCTGTTAGTTGAGTACACACA

                *  *                        * *      *      **  *         * *
     131354 TCACGAACAAGTTTCTGAGAATGCTTCTGTCTGGCTTTTATTGGAAGACGTTTCCTTTT-CACCA
        453 TCACAAAGAAGTTTCTGAGAATGCTTCTGTCTAGTTTTTATGGGAAGATATTCCCTTTTCCAACG

              *           *       *                * *                **     
     131418 AAGGCATCAAAGCGCTCCAAATGTCCACTTCCAGATTCTTCCAAAAGAGTGTTTCAAACGTGCTC
        518 AAAGCATCAAAGCGATCCAAATATCCACTTCCAGATTCTACAAAAAGAGTGTTTCAAAACTGCTC

            * ***    *                 *    * *  * *     *   *       * * *   
     131483 AAAGTAAGGGAATGTTCAACTCTGTGACTTGAATGCAGATATCACCAAGTAGTTTCTAATAGTGC
        583 TATCAAAGGAAATGTTCAACTCTGTGAGTTGAGTACACACATCACAAAGAAGTTTCTGAGAATGC

                            *              *              *** *   *          
     131548 TTCTGTCTAGATTTTAGATGATGATATTCCCGTTTCCAACGAAATCGCTAGAGCTATCCAAATAT
        648 TTCTGTCTAG-TTTT-TATGATGATATTCCCTTTTCCAACGAAATCATCAAAGCGATCCAAATAT

               *  *   *      *     *       *   *     **      *    *          
     131613 CCAGTTACAGTTTCTACCAAAAGGGTGTTTCCAAATTG-CTGCATCAAAAGAAAGGTTCAACTCT
        711 CCACTTCCAGATTCTACAAAAAGAGTGTTTCAAAACTGCCTCTATCAAAGGAAATGTTCAACTCT

                      *                     *                   *    * **  * 
     131677 GTTAGTTGAGGACACACATCACAAAGAAGTTTGTGAGAATGCTTCTGTCTAGATTTTGTATGACG
        776 GTTAGTTGAGTACACACATCACAAAGAAGTTTCTGAGAATGCTTCTGTCTAGTTTTTATGGGAAG

                               *   * *     *   *       * *  *    *  *       *
     131742 ATATTCCCTTTTCCAACGATATCGTTAAAGCAATCTAAATATCAATTTGCAGAATCCACAAAAAT
        841 ATATTCCCTTTTCCAACGAAATCATCAAAGCGATCCAAATATCCACTTCCAGATTCTACAAAAAG

              *        *       * *   *    *    *       *  *   * *  *         
     131807 AGAGTTTCAAAGCTGCTCTGTAAAAAGAAAGGTTCCACTCTGTTAGCTGAGTACACACATCACAA
        906 AGTGTTTCAAAACTGCTCTATCAAAGGAAATGTTCAACTCTGTGAGTTGAATGCAGACATCACAA

             ***           **         *                   **        ** * **  
     131872 ACTTGTTTCTGAGAAACCTTCTGTCTCGTTTTTATGGGAAGATATTTACTTTTCCACTGTAGGCA
        971 AGAAGTTTCTGAGAATGCTTCTGTCTAGTTTTTAT--GAAGATATTCCCTTTTCCAACGAAATCA

                    *       *     *       *  *  *  *            *         *  
     131937 TCAAAGCGCTCCAAATGTCCACATCCAGATACTCCAGAACGAGTGTTTCAAACCTGCTCTATGAA
       1034 TCAAAGCGATCCAAATATCCACTTCCAGATTCTACAAAAAGAGTGTTTCAAAACTGCTCTATCAA

             * *   *         * *      * *  *      *      *              *    
     132002 AGGGAATCTTCAACTCTATGAGTTGAATGCAGA-ATCAGAAAGAAATTTCTGAGAATGCTGCTGT
       1099 AAGAAATGTTCAACTCTGTTAGTTGAGTACACACATCACAAAGAAGTTTCTGAGAATGCTTCTGT

               **         **          *  *
     132066 CTACCTTTTAT---TTGA-ATTCCCGCTTC
       1164 CTAGTTTTTATGGGAAGATATTCCCGTTTT

                      *  *    *              * *  **   *                   * 
     132092 CAACGAAATCCTCCAAGCTATCCAAATATCCACCTGCATTTTCCACAAAAAGAGTGTTTCAAAAC
          1 CAACGAAATCATCAAAGCGATCCAAATATCCACTTCCAGATTCTACAAAAAGAGTGTTTCAAACC

                    *  ** *              *    *  *                *          
     132157 TGCTCTATCAATAGAAATGTTCAACTCCTTTG-GCTGGGTACACACATCACAAACAAGTTTCTGA
         66 TGCTCTA-GAAAGGGAATGTTCAACT-CTGTGAGTTGAGTACACACATCACAAAGAAGTTTCTGA

                                   **     **     *   *    *    *           * 
     132221 GAATGCTTCTGTCTAGTTTTTATGGGAAGACGTTCCCTTTTTC-ACCAAA-GGCATCAAAGCGCT
        129 GAATGCTTCTGTCTAGTTTTTATATGAAGATATTCCCGTTTCCAACGAAATCGC-T-AAAGCGAT

                  *            **                     **       **   ***      
     132284 CCAAATGTCCACTTCCAGACACTACAAAAAGAGTGTTTCAAACGTGCTCTAAGAAAGCGAATGTT
        192 CCAAATATCCACTTCCAGATTCTACAAAAAGAGTGTTTCAAAACTGCTCTATCAAAAGAAATGTT

                     * *    * *  * *         *           **            *     
     132349 CAACTCTGTGACTTGAATGCAGATATCACAAAGTAGTTTCTGAGAGGGCTTCTGTCTAGATTTTA
        257 CAACTCTGTTAGTTGAGTACACACATCACAAAGAAGTTTCTGAGAATGCTTCTGTCTAGTTTTTA

            *    *         *                * *   *                *   *     
     132414 GATGATGATATTCCCGTTTCCAACGAAATCATTAGAGCTATCCAAATATCCACTTACAGTTTCTA
        322 TATGAAGATATTCCCTTTTCCAACGAAATCATCAAAGCGATCCAAATATCCACTTCCAGATTCTA

                           *        **  *      * *    *          **          
     132479 CAAAAAGAGTGTTTCCAAACTGCTGCATCAAAAGAGAGGTTCCACTCTGTTAGCCGAGTACACAC
        387 CAAAAAGAGTGTTTCAAAACTGCTCTATGAAAAGAAATGTTCAACTCTGTTAGTTGAGTACACAC

                    ***      *     *         *                   **     *  * 
     132544 ATCACAAACTTGTTTCTCAGAATCCTTCTGTCTCGTTTTTATGGGAAGATATTTACTTTTTCACC
        452 ATCACAAAGAAGTTTCTGAGAATGCTTCTGTCTAGTTTTTATGGGAAGATATTCCCTTTTCCAAC

             * *           *       *     *       *  *  *               *     
     132609 GTAGGCATCAAAGCGCTCCAAATGTCCACATCCAGATACTCCAGAAAGAGTGTTTCAAACCTGCT
        517 GAAAGCATCAAAGCGATCCAAATATCCACTTCCAGATTCTACAAAAAGAGTGTTTCAAAACTGCT

                *     *   *         *        * *  *      *      *            
     132674 CTATGAAAGGGAATCTTCAACTCTATGAGTTGAATGCAGACATCAGAAAGAAATTTCTGAGAATG
        582 CTATCAAAGGAAATGTTCAACTCTGTGAGTTGAGTACACACATCACAAAGAAGTTTCTGAGAATG

              *       **       *          **             *  *    *           
     132739 CTGCTGTCTACCTTTTAT-TTGA-ATTCCCGCTTCCAACGAAATCCTCCAAGCTATCCAAATATC
        647 CTTCTGTCTAGTTTTTATGATGATATTCCCTTTTCCAACGAAATCATCAAAGCGATCCAAATATC

                 *       *                                  *  *  *          
     132802 CACTTGCAGATTCCACAAAAAGAGTGTTTCAAAACTGCTCTCTATCAATGGCAAAGTTCAACTCT
        712 CACTTCCAGATTCTACAAAAAGAGTGTTTCAAAACTGC-CTCTATCAAAGGAAATGTTCAACTCT

                      *     *     *  *                         *             
     132867 GTTAGTTGAGGACACATATCACCAACAAGTTTCTGAGAATGCTTCTGTCTATTTTTTATGGGAAG
        776 GTTAGTTGAGTACACACATCACAAAGAAGTTTCTGAGAATGCTTCTGTCTAGTTTTTATGGGAAG

                 *      *  *  * ** *    *      *    *          * **          
     132932 ATATTTCCTTTTTCACCGTAGGCGTCAAGGCGATCGAAATGTCCACTTCCACAAACTACAAAAAG
        841 ATATTCCCTTTTCCAACGAAATCATCAAAGCGATCCAAATATCCACTTCCAGATTCTACAAAAAG

                       *         *     **       *    *           * * *   **  
     132997 AGTGTTTCAAACCTGCTCTATGAAAGGCCATGTTCATCTCTATGAGTTGAATGGAAATATCCGAA
        906 AGTGTTTCAAAACTGCTCTATCAAAGGAAATGTTCAACTCTGTGAGTTGAATGCAGACATCACAA

                *      *       *         *       *         **             *  
     133062 AGAAATTTCTGGGAATGCTGCTGTCTAGTGTTTAT-ACGA-ATTCCCGCTTCCAACGAAATCCTC
        971 AGAAGTTTCTGAGAATGCTTCTGTCTAGTTTTTATGAAGATATTCCCTTTTCCAACGAAATCATC

                 *                *    *  *                                * 
     133125 AAAGCAATCCAAATATCCACTTGCAGAATCCACAAAAAGAGTGTTTCAAAACTGCTCTATCAATA
       1036 AAAGCGATCCAAATATCCACTTCCAGATTCTACAAAAAGAGTGTTTCAAAACTGCTCTATCAAAA

                *          *                        *                        
     133190 GAAAGGTTCAACTCTTTTAGTTGAGTACACACATCACAAACAAGTTTCTGAGAATGCTTCTGTCT
       1101 GAAATGTTCAACTCTGTTAGTTGAGTACACACATCACAAAGAAGTTTCTGAGAATGCTTCTGTCT

            * *      *      **  *       
     133255 GGCTTTTATTGGAAGACGTTTCC-TTTT
       1166 AGTTTTTATGGGAAGATATTCCCGTTTT

              * *  **          *       *                * *                 *
     133282 CACCAAAGGCATCAAAGCGCTCCAAATGTCCACTTCCAGATTCTTCCAAAAGAGTGTTTCAAACG
          1 CAACGAAATCATCAAAGCGATCCAAATATCCACTTCCAGATTCTACAAAAAGAGTGTTTCAAACC

              *   *  *                      *    * *  * *     *   *       * *
     133347 TGGTCGAAGTAAGGGAATGTTCAACTCTGTGACTTGAATGCAGATATCACCAAGTAGTTTCTAAT
         66 TGCTC-TAGAAAGGGAATGTTCAACTCTGTGAGTTGAGTACACACATCACAAAGAAGTTTCTGAG

             *            **     *    *                         *  *   *     
     133412 AGTGCTTCTGTCTACATTTTAGATGATGATATTCCCGTTTCCAACGAAATCGTTAGAGCTATCCA
        130 AATGCTTCTGTCTAGTTTTTATATGAAGATATTCCCGTTTCCAACGAAATCGCTAAAGCGATCCA

                    *  *   *      *     *       *   *    **           *      
     133477 AATATCCAGTTACAGTTTCTACCAAAAGGGTGTTTCCAAATTGCTGCATCAAAAGAAAGGTTCAA
        195 AATATCCACTTCCAGATTCTACAAAAAGAGTGTTTCAAAACTGCTCTATCAAAAGAAATGTTCAA

                          *                     *                   *    *   
     133542 CTCTGTTAGTTGAGGACACACATCACAAAGAAGTTTGTGAGAATGCTTCTGTCTAGATTTTGTAT
        260 CTCTGTTAGTTGAGTACACACATCACAAAGAAGTTTCTGAGAATGCTTCTGTCTAGTTTTTATAT

              *                    *   * *     *   *       * *  *    *  *    
     133607 GACGATATTCCCTTTTCCAACGATATCGTTAAAGCAATCTAAATATCAATTTGCAGAATCCACAA
        325 GAAGATATTCCCTTTTCCAACGAAATCATCAAAGCGATCCAAATATCCACTTCCAGATTCTACAA

               *  *        *       * *        *    *          *              
     133672 AAATAGAGTTTCAAAGCTGCTCTGTAAAAAGAAAGGTTCCACTCTGTTAGCTGAGTACACACATC
        390 AAAGAGTGTTTCAAAACTGCTCTATGAAAAGAAATGTTCAACTCTGTTAGTTGAGTACACACATC

                 ***           **         *                   **        ** * 
     133737 ACAAACTTGTTTCTGAGAAACCTTCTGTCTCGTTTTTATGGGAAGATATTTACTTTTCCACTGTA
        455 ACAAAGAAGTTTCTGAGAATGCTTCTGTCTAGTTTTTATGGGAAGATATTCCCTTTTCCAACGAA

            *           *       *     *       *  *  *  *            *        
     133802 GGCATCAAAGCGCTCCAAATGTCCACATCCAGATACTCCAGAACGAGTGTTTCAAACCTGCTCTA
        520 AGCATCAAAGCGATCCAAATATCCACTTCCAGATTCTACAAAAAGAGTGTTTCAAAACTGCTCTA

             *     *   *         *        * *  *      *      *              *
     133867 TGAAAGGGAATCTTCAACTCTATGAGTTGAATGCAGA-ATCAGAAAGAAATTTCTGAGAATGCTG
        585 TCAAAGGAAATGTTCAACTCTGTGAGTTGAGTACACACATCACAAAGAAGTTTCTGAGAATGCTT

                   **       *          **             *  *    *              
     133931 CTGTCTACCTTTTAT-TTGA-ATTCCCGCTTCCAACGAAATCCTCCAAGCTATCCAAATATCCAC
        650 CTGTCTAGTTTTTATGATGATATTCCCTTTTCCAACGAAATCATCAAAGCGATCCAAATATCCAC

            * *  **   *                                 **              **  *
     133994 CTGCATTTTCCACAAAAAGAGTGTTTCAAAACTG-CTCTATCAATAGAAATGTTCAACTCCTTTG
        715 TTCCAGATTCTACAAAAAGAGTGTTTCAAAACTGCCTCTATCAAAGGAAATGTTCAACTCTGTTA

             *  *                *                                        ** 
     134058 GCTGGGTACACACATCACAAACAAGTTTCTGAGAATGCTTCTGTCTAGTTTTTATGGGAAGACGT
        780 GTTGAGTACACACATCACAAAGAAGTTTCTGAGAATGCTTCTGTCTAGTTTTTATGGGAAGATAT

                    *  * *  **          *       *            **              
     134123 TCCCTTTTTCACCAAAGGCATCAAAGCGCTCCAAATGTCCACTTCCAGACACTACAAAAAGAGTG
        845 TCCCTTTTCCAACGAAATCATCAAAGCGATCCAAATATCCACTTCCAGATTCTACAAAAAGAGTG

                   **       **    **                 *           *         * 
     134188 TTTCAAACGTGCTCTAAGAAAGCGAATGTTCAACTCTGTGACTTGAATGCAGATATCACAAAGTA
        910 TTTCAAAACTGCTCTATCAAAGGAAATGTTCAACTCTGTGAGTTGAATGCAGACATCACAAAGAA

                      **                  *    *         *                * *
     134253 GTTTCTGAGAGGGCTTCTGTCTAGATTTTAGATGATGATATTCCCGTTTCCAACGAAATCATTAG
        975 GTTTCTGAGAATGCTTCTGTCTAG-TTTT-TATGAAGATATTCCCTTTTCCAACGAAATCATCAA

               *                *   *                    *        **         
     134318 AGCTATCCAAATATCCACTTACAGTTTCTACAAAAAGAGTGTTTCCAAACTGCTGCATCAAAAGA
       1038 AGCGATCCAAATATCCACTTCCAGATTCTACAAAAAGAGTGTTTCAAAACTGCTCTATCAAAAGA

            * *    *          **                  ***      *     *         * 
     134383 GAGGTTCCACTCTGTTAGCCGAGTACACACATCACAAACTTGTTTCTCAGAATCCTTCTGTCTCG
       1103 AATGTTCAACTCTGTTAGTTGAGTACACACATCACAAAGAAGTTTCTGAGAATGCTTCTGTCTAG

                              ** *    
     134448 TTTTTATGGGAAGATATTTACTTTTT
       1168 TTTTTATGGGAAGATATTCCCGTTTT

              *  * **          *       *     *       *  *  *                 
     134474 CACCGTAGGCATCAAAGCGCTCCAAATGTCCACATCCAGATACTCCAGAAAGAGTGTTTCAAACC
          1 CAACGAAATCATCAAAGCGATCCAAATATCCACTTCCAGATTCTACAAAAAGAGTGTTTCAAACC

                              *         *        * *  *      *      *        
     134539 TGCTCTATGAAAGGGAATCTTCAACTCTATGAGTTGAATGCAGACATCAGAAAGAAATTTCTGAG
         66 TGCTCTA-GAAAGGGAATGTTCAACTCTGTGAGTTGAGTACACACATCACAAAGAAGTTTCTGAG

                  *       **          *          *                 *    *    
     134604 AATGCTGCTGTCTACCTTTTAT-T--TGA-ATTCCCGCTTCCAACGAAATC-CTCCAAGCTATCC
        130 AATGCTTCTGTCTAGTTTTTATATGAAGATATTCCCGTTTCCAACGAAATCGCT-AAAGCGATCC

                        *       *                                  ** *  *   
     134664 AAATATCCACTTGCAGATTCCACAAAAAGAGTGTTTCAAAACTGCTCTCTATCAATGGCAAAGTT
        194 AAATATCCACTTCCAGATTCTACAAAAAGAGTGTTTCAAAACTG--CTCTATCAAAAGAAATGTT

                             *     *     *  *                         *      
     134729 CAACTCTGTTAGTTGAGGACACATATCACCAACAAGTTTCTGAGAATGCTTCTGTCTATTTTTTA
        257 CAACTCTGTTAGTTGAGTACACACATCACAAAGAAGTTTCTGAGAATGCTTCTGTCTAGTTTTTA

             **         *      *  *  * ** *    *      *    *          * **   
     134794 TGGGAAGATATTTCCTTTTTCACCGTAGGCGTCAAGGCGATCGAAATGTCCACTTCCACAAACTA
        322 TATGAAGATATTCCCTTTTCCAACGAAATCATCAAAGCGATCCAAATATCCACTTCCAGATTCTA

                              *             * **       *    * *      * ** * *
     134859 CAAAAAGAGTGTTTCAAACCTGCTCTATGAAAGGCCATGTTCATCTCTATGAGTTGAATGGAAAT
        387 CAAAAAGAGTGTTTCAAAACTGCTCTATGAAAAGAAATGTTCAACTCTGTTAGTTGAGTACACAC

               **      *      *       *                   *        ***       
     134924 ATCCGAAAGAAATTTCTGGGAATGCTGCTGTCTAGTTTTTAT---ATGA-ATTCCGGCTTCCAAC
        452 ATCACAAAGAAGTTTCTGAGAATGCTTCTGTCTAGTTTTTATGGGAAGATATTCCCTTTTCCAAC

                * *       *                *    *  *                         
     134985 GAAATCCTCAAAGCAATCCAAATATCCACTTGCAGAATCCACAAAAAGAGTGTTTCAAAACTGCT
        517 GAAAGCATCAAAGCGATCCAAATATCCACTTCCAGATTCTACAAAAAGAGTGTTTCAAAACTGCT

                   **    *          * *                      *               
     135050 CTATCAATAGAAAGGTTCAACTCTTTTAGTTGAGTACACACATCACAAACAAGTTTCTGAGAATG
        582 CTATCAAAGGAAATGTTCAACTCTGTGAGTTGAGTACACACATCACAAAGAAGTTTCTGAGAATG

                     * *          *  **  *         * *  **          *       *
     135115 CTTCTGTCTGGCTTTTATTGGAAGACGTTTCCTTTT-CACCAAAGGCATCAAAGCGCTCCAAATG
        647 CTTCTGTCTAGTTTTTA-T-GATGATATTCCCTTTTCCAACGAAATCATCAAAGCGATCCAAATA

                            * *                **      * ***    *       *    
     135179 TCCACTTCCAGATTCTTCCAAAAGAGTGTTTCAAACGTG-CTCGAAGTAAGGGAATGTTCTACTC
        710 TCCACTTCCAGATTCTACAAAAAGAGTGTTTCAAAACTGCCTCTATCAAAGGAAATGTTCAACTC

               * *    * *  * *     *   *       * * *             *     ***  *
     135243 TGTGACTTGAATGCAGATATCACCAAGTAGTTTCTAATAGTGCTTCTGTCTAGATTTTAGATGAT
        775 TGTTAGTTGAGTACACACATCACAAAGAAGTTTCTGAGAATGCTTCTGTCTAGTTTTTATGGGAA

                     *              *** *   *             *  *   *      *    
     135308 GATATTCCCGTTTCCAACGAAATCGCTAGAGCTATCCAAATATCCAGTTACAGTTTCTACCAAAA
        840 GATATTCCCTTTTCCAACGAAATCATCAAAGCGATCCAAATATCCACTTCCAGATTCTACAAAAA

             *       *   *    **      *    *            *      ***  *        
     135373 GGGTGTTTCCAAATTGCTGCATCAAAAGAAAGGTTCAACTCTGTTAGTTGAGGACACACATCACA
        905 GAGTGTTTCAAAACTGCTCTATCAAAGGAAATGTTCAACTCTGTGAGTTGAATGCAGACATCACA

                     *                              *                    *   
     135438 AAGAAGTTTGTGAGAATGCTTCTGTCTAGATTTTGTATGACGATATTCCCTTTTCCAACGATATC
        970 AAGAAGTTTCTGAGAATGCTTCTGTCTAG-TTTT-TATGAAGATATTCCCTTTTCCAACGAAATC

            * *     *   *       * *  *    *  *       *  *        *       * * 
     135503 GTTAAAGCAATCTAAATATCAATTTGCAGAATCCACAAAAATAGAGTTTCAAAGCTGCTCTGTAA
       1033 ATCAAAGCGATCCAAATATCCACTTCCAGATTCTACAAAAAGAGTGTTTCAAAACTGCTCTATCA

                   *    *          *                   ***      *     *      
     135568 AAAGAAAGGTTCCACTCTGTTAGCTGAGTACACACATCACAAACTTGTTTCTCAGAATCCTTCTG
       1098 AAAGAAATGTTCAACTCTGTTAGTTGAGTACACACATCACAAAGAAGTTTCTGAGAATGCTTCTG

               *                 * ** *   *
     135633 TCTCGTTTTTATGGGAAGATACTTACTTTTC
       1163 TCTAGTTTTTATGGGAAGATATTCCCGTTTT

              *  * **          *       *     *       *  *  *  *              
     135664 CACCGTAGGCATCAAAGCGCTCCAAATGTCCACATCCAGATACTCCAGAACGAGTGTTTCAAACC
          1 CAACGAAATCATCAAAGCGATCCAAATATCCACTTCCAGATTCTACAAAAAGAGTGTTTCAAACC

                              *         *        * *  *      *      *        
     135729 TGCTCTATGAAAGGGAATCTTCAACTCTATGAGTTGAATGCAGACATCAGAAAGAAATTTCTGAG
         66 TGCTCTA-GAAAGGGAATGTTCAACTCTGTGAGTTGAGTACACACATCACAAAGAAGTTTCTGAG

                  *       **          *          *                 *    *    
     135794 AATGCTGCTGTCTACCTTTTAT-T--TGA-ATTCCCGCTTCCAACGAAATC-CTCCAAGCTATCC
        130 AATGCTTCTGTCTAGTTTTTATATGAAGATATTCCCGTTTCCAACGAAATCGCT-AAAGCGATCC

                      * *  **   *    *                           *           
     135854 AAATATCCACCTGCATTTTCCACAACAAGAGTGTTTCAAAACTGCTCTATCAATAGAAATGTTCA
        194 AAATATCCACTTCCAGATTCTACAAAAAGAGTGTTTCAAAACTGCTCTATCAAAAGAAATGTTCA

                **  * *  *                *                                 *
     135919 ACTCCTTTGGCTGGGTACACACATCACAAACAAGTTTCTGAGAATGCTTCTGTCTAGTTTTTATG
        259 ACTCTGTTAGTTGAGTACACACATCACAAAGAAGTTTCTGAGAATGCTTCTGTCTAGTTTTTATA

            *     **         *  * *  **          *       *            **     
     135984 GGAAGACGTTCCCTTTTTCACCAAAGGCATCAAAGCGCTCCAAATGTCCACTTCCAGACACTACA
        324 TGAAGATATTCCCTTTTCCAACGAAATCATCAAAGCGATCCAAATATCCACTTCCAGATTCTACA

                         *  **       *    ***               * *    * *  * *  
     136049 AAAAGAGTGTTTCCAACGTGCTCTAAGAAAGCGAATGTTCAACTCTGTGACTTGAATGCAGATAT
        389 AAAAGAGTGTTTCAAAACTGCTCTATGAAAAGAAATGTTCAACTCTGTTAGTTGAGTACACACAT

                   *           **            *     ***  *         *        * 
     136114 CACAAAGTAGTTTCTGAGAGGGCTTCTGTCTAGATTTTAGATGATGATATTCCCGTTTCCAACAA
        454 CACAAAGAAGTTTCTGAGAATGCTTCTGTCTAGTTTTTATGGGAAGATATTCCCTTTTCCAACGA

              *   * *    *               *   *                    *        **
     136179 AATCATTAGAGC-TTCCAAATATCCACTTACAGTTTCTACAAAAAGAGTGTTTCCAAACTGCTGC
        519 AAGCATCAAAGCGATCCAAATATCCACTTCCAGATTCTACAAAAAGAGTGTTTCAAAACTGCTCT

                  *  * *    *       *  **                  ***      *     *  
     136243 ATCAAAAGAGAGGTTCCACTCTGTTAGCCGAGTACACACATCACAAACTTGTTTCTCAGAATCCT
        584 ATCAAAGGAAATGTTCAACTCTGTGAGTTGAGTACACACATCACAAAGAAGTTTCTGAGAATGCT

                   *            *      **     *  *  * **          *       *  
     136308 TCTGTCTCGTTTTTATGGGAAGATATTTACTTTTTCACCGTAGGCATCAAAGCGCTCCAAATGTC
        649 TCTGTCTAGTTTTTAT--GATGATATTCCCTTTTCCAACGAAATCATCAAAGCGATCCAAATATC

               *       *  *  *               *          *     *   *         *
     136373 CACATCCAGATACTCCAGAAAGAGTGTTTCAAACCTG-CTCTATGAAAGGGAATCTTCAACTCTA
        712 CACTTCCAGATTCTACAAAAAGAGTGTTTCAAAACTGCCTCTATCAAAGGAAATGTTCAACTCTG

             *      * *  *      *      *              *       **         **  
     136437 TGAGTTGAATGCAGACATCAGAAAGAAATTTCTGAGAATGCTGCTGTCTACCTTTTAT---TTGA
        777 TTAGTTGAGTACACACATCACAAAGAAGTTTCTGAGAATGCTTCTGTCTAGTTTTTATGGGAAGA

                   **             *  *    *                *       *         
     136499 -ATTCCCGCTTCCAACGAAATCCTCCAAGCTATCCAAATATCCACTTGCAGATTCCACAAAAAGA
        842 TATTCCCTTTTCCAACGAAATCATCAAAGCGATCCAAATATCCACTTCCAGATTCTACAAAAAGA

                                     *  *  *            *      ***  * *     *
     136563 GTGTTTCAAAACTGCTCTCTATCAATGGCAAAGTTCAACTCTGTTAGTTGAGGACACATATCACC
        907 GTGTTTCAAAACTG--CTCTATCAAAGGAAATGTTCAACTCTGTGAGTTGAATGCAGACATCACA

              *                         *                  *      *  *  * ** 
     136628 AACAAGTTTCTGAGAATGCTTCTGTCTATTTTTTATGGGAAGATATTTCCTTTTTCACCGTAGGC
        970 AAGAAGTTTCTGAGAATGCTTCTGTCTAGTTTTTAT--GAAGATATTCCCTTTTCCAACGAAATC

            *    *      *    *          * **                     *         * 
     136693 GTCAAGGCGATCGAAATGTCCACTTCCACAAACTACAAAAAGAGTGTTTCAAACCTGCTCTATGA
       1033 ATCAAAGCGATCCAAATATCCACTTCCAGATTCTACAAAAAGAGTGTTTCAAAACTGCTCTATCA

              * **       *    * *      * ** * *   **      *      *       *   
     136758 AAGGCCATGTTCATCTCTATGAGTTGAATGGAAATATCCGAAAGAAATTTCTGGGAATGCTGCTG
       1098 AAAGAAATGTTCAACTCTGTTAGTTGAGTACACACATCACAAAGAAGTTTCTGAGAATGCTTCTG

                            *          *  *
     136823 TCTAGTTTTTAT---ATGA-ATTCCCGCTTC
       1163 TCTAGTTTTTATGGGAAGATATTCCCGTTTT

                      *       *                *    *  *                   * 
     136850 CAACGAAATCCTCAAAGCAATCCAAATATCCACTTGCAGAATCCACAAAAAGAGTGTTTCAAAAC
          1 CAACGAAATCATCAAAGCGATCCAAATATCCACTTCCAGATTCTACAAAAAGAGTGTTTCAAACC

                    *  ** *  *          * *                      *           
     136915 TGCTCTATCAATAGAAAGGTTCAACTCTTTTAGTTGAGTACACACATCACAAACAAGTTTCTGAG
         66 TGCTCTA-GAAAGGGAATGTTCAACTCTGTGAGTTGAGTACACACATCACAAAGAAGTTTCTGAG

                         * *      **     **  *      *    *    *           *  
     136980 AATGCTTCTGTCTGGCTTTTATTGGAAGACGTTTCC-TTTTC-ACCAAA-GGCATCAAAGCGCTC
        130 AATGCTTCTGTCTAGTTTTTATATGAAGATATTCCCGTTTCCAACGAAATCGC-T-AAAGCGATC

                **                * *                **         **  * *      
     137042 CAAAGGTCCACTTCCAGATTCTTCCAAAAGAGTGTTTCAAACGTGCTC-ATAGTAAGGGAATGTT
        193 CAAATATCCACTTCCAGATTCTACAAAAAGAGTGTTTCAAAACTGCTCTAT-CAAAAGAAATGTT

                     * *    * *  * *     *   *       * * *             *     
     137106 CAACTCTGTGACTTGAATGCAGATATCACCAAGTAGTTTCTAATAGTGCTTCTGTCTAGATTTTA
        257 CAACTCTGTTAGTTGAGTACACACATCACAAAGAAGTTTCTGAGAATGCTTCTGTCTAGTTTTTA

            *    *         *              *** *   *             *  *   *     
     137171 GATGATGATATTCCCGTTTCCAACGAAATCGCTAGAGCTATCCAAATATCCAGTTACAGTTTCTA
        322 TATGAAGATATTCCCTTTTCCAACGAAATCATCAAAGCGATCCAAATATCCACTTCCAGATTCTA

             *     *       *   *    **  *        *                    *      
     137236 CCAAAAGGGTGTTTCCAAATTGCTGCATCAAAAGAAAGGTTCAACTCTGTTAGTTGAGGACACAC
        387 CAAAAAGAGTGTTTCAAAACTGCTCTATGAAAAGAAATGTTCAACTCTGTTAGTTGAGTACACAC

                           *                   *    * **  *                  
     137301 ATCACAAAGAAGTTTGTGAGAATGCTTCTGTCTAGATTTTGTATGACGATATTCCCTTTTCCAAC
        452 ATCACAAAGAAGTTTCTGAGAATGCTTCTGTCTAGTTTTTATGGGAAGATATTCCCTTTTCCAAC

              * * * *     *   *       * *  *    *  *       *  *        *     
     137366 GATATCGTTAAAGCAATCTAAATATCAATTTGCAGAATCCACAAAAATAGAGTTTCAAAGCTGCT
        517 GAAAGCATCAAAGCGATCCAAATATCCACTTCCAGATTCTACAAAAAGAGTGTTTCAAAACTGCT

              * *   *    *    *       *  *                   ***            *
     137431 CTGTAAAAAGAAAGGTTCCACTCTGTTAGCTGAGTACACACATCACAAACTTGTTTCTGAGAATC
        582 CTATCAAAGGAAATGTTCAACTCTGTGAGTTGAGTACACACATCACAAAGAAGTTTCTGAGAATG

                     *            *      **        *  * **          *       *
     137496 CTTCTGTCTCGTTTTTATGGGAAGATATTTACTTTTCCACCGTAGGCATCAAAGCGCTCCAAATG
        647 CTTCTGTCTAGTTTTTAT--GATGATATTCCCTTTTCCAACGAAATCATCAAAGCGATCCAAATA

                 *       *  *  *  *            *          *     *   *        
     137561 TCCACATCCAGATACTCCAGAACGAGTGTTTCAAACCTG-CTCTATGAAAGGGAATCTTCAACTC
        710 TCCACTTCCAGATTCTACAAAAAGAGTGTTTCAAAACTGCCTCTATCAAAGGAAATGTTCAACTC

             * *      * *  *      *      *              *       **         **
     137625 TATGAGTTGAATGCAGACATCAGAAAGAAATTTCTGAGAATGCTGCTGTCTACCTTTTAT---TT
        775 TGTTAGTTGAGTACACACATCACAAAGAAGTTTCTGAGAATGCTTCTGTCTAGTTTTTATGGGAA

                     **             *  *    *              * *  **   *    *  
     137687 GA-ATTCCCGCTTCCAACGAAATCCTCCAAGCTATCCAAATATCCACCTGCATTTTCCACAACAA
        840 GATATTCCCTTTTCCAACGAAATCATCAAAGCGATCCAAATATCCACTTCCAGATTCTACAAAAA

                                     **                *    *  ** *  *       
     137751 GAGTGTTTCAAAACTGCTCTATCAATAGAAATGTTCAACTCCTTTG-GCTGGGTACACACATCAC
        905 GAGTGTTTCAAAACTGCTCTATCAAAGGAAATGTTCAACT-CTGTGAGTTGAATGCAGACATCAC

               *                                        **         *  * *  **
     137815 AAACAAGTTTCTGAGAATGCTTCTGTCTAGTTTTTATGGGAAGACGTTCCCTTTTTCACCAAAGG
        969 AAAGAAGTTTCTGAGAATGCTTCTGTCTAGTTTTTAT--GAAGATATTCCCTTTTCCAACGAAAT

                      *       *            **                  *  **   *   **
     137880 CATCAAAGCGCTCCAAATGTCCACTTCCAGACACTACAAAAAGAGTGTTTCCAACGTGCCCTAAG
       1032 CATCAAAGCGATCCAAATATCCACTTCCAGATTCTACAAAAAGAGTGTTTCAAAACTGCTCTATC

               ***               * *    * *  * *         *           **      
     137945 AAAGCGAATGTTCAACTCTGTGACTTGAATGCAGATATCACAAAGTAGTTTCTGAGAGGGCTTCT
       1097 AAAAGAAATGTTCAACTCTGTTAGTTGAGTACACACATCACAAAGAAGTTTCTGAGAATGCTTCT

                  *     ***  *             *
     138010 GTCTAGATTTTAGATGATGATATTCCCGTTTC
       1162 GTCTAGTTTTTATGGGAAGATATTCCCGTTTT

                        * *   *             *  *   *                    *  * 
     138042 CAACGAAATCATTAGAGCTATCCAAATATCCAATTACAGTTTCTACAAAAAGAGTGTTTCCAAAC
          1 CAACGAAATCATCAAAGCGATCCAAATATCCACTTCCAGATTCTACAAAAAGAGTGTTTCAAACC

                    **    *   *    *       *  *                   ***      * 
     138107 TGCTGCATCAAAAGAG-AGGTTCCACTCTGTTAGCTGAGTACACACATCACAAACTTGTTTCTCA
         66 TGCT-C-TAGAAAGGGAATGTTCAACTCTGTGAGTTGAGTACACACATCACAAAGAAGTTTCTGA

                *         *        **         ** *   *  *  * ** ***      *   
     138171 GAATCCTTCTGTCTCGTTTTTATGGGAAGATATTTACTTTTTCACCGTAGGCATCAAAGCGCTCC
        129 GAATGCTTCTGTCTAGTTTTTATATGAAGATATTCCCGTTTCCAACGAAATCGCTAAAGCGATCC

                *     *       *  *  *               *         *   * *   *    
     138236 AAATGTCCACATCCAGATACTCCAGAAAGAGTGTTTCAAACCTGCTCTATGAAAGGGAATCTTCA
        194 AAATATCCACTTCCAGATTCTACAAAAAGAGTGTTTCAAAACTGCTCTATCAAAAGAAATGTTCA

                 * *      * *  *      *      *              *       **       
     138301 ACTCTATGAGTTGAATGCAGACATCAGAAAGAAATTTCTGAGAATGCTGCTGTCTACCTTTTAT-
        259 ACTCTGTTAGTTGAGTACACACATCACAAAGAAGTTTCTGAGAATGCTTCTGTCTAGTTTTTATA

               *         **             *  *    *                *       *   
     138365 T--TGA-ATTCCCGCTTCCAACGAAATCCTCCAAGCTATCCAAATATCCACTTGCAGATTCCACA
        324 TGAAGATATTCCCTTTTCCAACGAAATCATCAAAGCGATCCAAATATCCACTTCCAGATTCTACA

                                        *  ** *  *                    *     *
     138427 AAAAGAGTGTTTCAAAACTGCTCTCTATCAATGGCAAAGTTCAACTCTGTTAGTTGAGGACACAT
        389 AAAAGAGTGTTTCAAAACTG--CTCTATGAAAAGAAATGTTCAACTCTGTTAGTTGAGTACACAC

                 *  *                         *                  *      *  * 
     138492 ATCACCAACAAGTTTCTGAGAATGCTTCTGTCTATTTTTTATGGGAAGATATTTCCTTTTTCACC
        452 ATCACAAAGAAGTTTCTGAGAATGCTTCTGTCTAGTTTTTATGGGAAGATATTCCCTTTTCCAAC

             * *  *    *      *    *          * **                     *     
     138557 GTAGGCGTCAAGGCGATCGAAATGTCCACTTCCACAAACTACAAAAAGAGTGTTTCAAACCTGCT
        517 GAAAGCATCAAAGCGATCCAAATATCCACTTCCAGATTCTACAAAAAGAGTGTTTCAAAACTGCT

                *     **       *    *        * ** * *   **      *      *     
     138622 CTATGAAAGGCCATGTTCATCTCTATGAGTTGAATGGAAATATCCGAAAGAAATTTCTGGGAATG
        582 CTATCAAAGGAAATGTTCAACTCTGTGAGTTGAGTACACACATCACAAAGAAGTTTCTGAGAATG

              *         *       *         **             *       *           
     138687 CTGCTGTCTAGTGTTTAT-ACGA-ATTCCCGCTTCCAACGAAATCCTCAAAGCAATCCAAATATC
        647 CTTCTGTCTAGTTTTTATGATGATATTCCCTTTTCCAACGAAATCATCAAAGCGATCCAAATATC

                 *    *  *                                 **    *          *
     138750 CACTTGCAGAATCCACAAAAAGAGTGTTTCAAAACTG-CTCTATCAATAGAAAGGTTCAACTCTT
        712 CACTTCCAGATTCTACAAAAAGAGTGTTTCAAAACTGCCTCTATCAAAGGAAATGTTCAACTCTG

                                 *  *                        * *      *      
     138814 TTAGTTGAGTACACACATCACGAACAAGTTTCTGAGAATGCTTCTGTCTGGCTTTTATTGGAAGA
        777 TTAGTTGAGTACACACATCACAAAGAAGTTTCTGAGAATGCTTCTGTCTAGTTTTTATGGGAAGA

            **  *         * *  **          *       *                * *      
     138879 CGTTTCCTTTT-CACCAAAGGCATCAAAGCGCTCCAAATGTCCACTTCCAGATTCTTCCAAAAGA
        842 TATTCCCTTTTCCAACGAAATCATCAAAGCGATCCAAATATCCACTTCCAGATTCTACAAAAAGA

                      **     * ***    *                 *           *     *  
     138943 GTGTTTCAAACGTGCTCAAAGTAAGGGAATGTTCAACTCTGTGACTTGAATGCAGATATCACCAA
        907 GTGTTTCAAAACTGCTCTATCAAAGGAAATGTTCAACTCTGTGAGTTGAATGCAGACATCACAAA

             *       * * *                   *    *         *              * 
     139008 GTAGTTTCTAATAGTGCTTCTGTCTAGATTTTAGATGATGATATTCCCGTTTCCAACGAAATCGT
        972 GAAGTTTCTGAGAATGCTTCTGTCTAG-TTTT-TATGAAGATATTCCCTTTTCCAACGAAATCAT

            * *   * **          *  *   *      *     *       *   *    **      
     139073 TAGAGCTAAGCAAATATCCAGTTACAGTTTCTACCAAAAGGGTGTTTCCAAATTGCTGCATCAAA
       1035 CAAAGCGATCCAAATATCCACTTCCAGATTCTACAAAAAGAGTGTTTCAAAACTGCTCTATCAAA

                 *                    *                     *                
     139138 AGAAAGGTTCAACTCTGTTAGTTGAGGACACACATCACAAAGAAGTTTGTGAGAATGCTTCTGTC
       1100 AGAAATGTTCAACTCTGTTAGTTGAGTACACACATCACAAAGAAGTTTCTGAGAATGCTTCTGTC

               *    * **  *         *   *
     139203 TAGATTTTGTATGACGATATTCCCTTTTC
       1165 TAGTTTTTATGGGAAGATATTCCCGTTTT

                  *   * *     *   *       * *  *    *  *       *  *        * 
     139232 CAACGATATCGTTAAAGCAATCTAAATATCAATTTGCAGAATCCACAAAAATAGAGTTTCAAAGC
          1 CAACGAAATCATCAAAGCGATCCAAATATCCACTTCCAGATTCTACAAAAAGAGTGTTTCAAACC

                   **   * *  *    *       *  *                   *** *       
     139297 TGCTCTGTAAAAAGAAAGGTTCCACTCTGTTAGCTGAGTACACACATCACAAACTTGATTCTGAG
         66 TGCTCT-AGAAAGGGAATGTTCAACTCTGTGAGTTGAGTACACACATCACAAAGAAGTTTCTGAG

               *         *        **         ** *      *  * ** ***      *    
     139362 AATCCTTCTGTCTCGTTTTTATGGGAAGATATTTACTTTTCCACCGTAGGCATCAAAGCGCTCCA
        130 AATGCTTCTGTCTAGTTTTTATATGAAGATATTCCCGTTTCCAACGAAATCGCTAAAGCGATCCA

               *     *       *  *  *  *            *         *   * *   *     
     139427 AATGTCCACATCCAGATACTCCAGAACGAGTGTTTCAAACCTGCTCTATGAAAGGGAATCTTCAA
        195 AATATCCACTTCCAGATTCTACAAAAAGAGTGTTTCAAAACTGCTCTATCAAAAGAAATGTTCAA

                * *      * *  *      *      *              *       **        
     139492 CTCTATGAGTTGAATGCAGACATCAGAAAGAAATTTCTGAGAATGCTGCTGTCTACCTTTTAT-T
        260 CTCTGTTAGTTGAGTACACACATCACAAAGAAGTTTCTGAGAATGCTTCTGTCTAGTTTTTATAT

              *         **             *  *    *              * *  **   *    
     139556 --TGA-ATTCCCGCTTCCAACGAAATCCTCCAAGCTATCCAAATATCCACCTGCATTTTCCACAA
        325 GAAGATATTCCCTTTTCCAACGAAATCATCAAAGCGATCCAAATATCCACTTCCAGATTCTACAA

            *                        *  *               **  * *  *           
     139618 CAAGAGTGTTTCAAAACTGCTCTATCAATAGAAATGTTCAACTCCTTTGGCTGGGTACACACATC
        390 AAAGAGTGTTTCAAAACTGCTCTATGAAAAGAAATGTTCAACTCTGTTAGTTGAGTACACACATC

                 *                                        **         *  * *  
     139683 ACAAACAAGTTTCTGAGAATGCTTCTGTCTAGTTTTTATGGGAAGACGTTCCCTTTTTCACCAAA
        455 ACAAAGAAGTTTCTGAGAATGCTTCTGTCTAGTTTTTATGGGAAGATATTCCCTTTTCCAACGAA

            *           *       *            **                     **       
     139748 GGCATCAAAGCGCTCCAAATGTCCACTTCCAGACACTACAAAAAGAGTGTTTCAAACGTGCTCTA
        520 AGCATCAAAGCGATCCAAATATCCACTTCCAGATTCTACAAAAAGAGTGTTTCAAAACTGCTCTA

            **    **                 *    * *  * *         *           **    
     139813 AGAAAGCGAATGTTCAACTCTGTGACTTGAATGCAGATATCACAAAGTAGTTTCTGAGAGGGCTT
        585 TCAAAGGAAATGTTCAACTCTGTGAGTTGAGTACACACATCACAAAGAAGTTTCTGAGAATGCTT

                          *              *                * *   *            
     139878 CTGTCTAGATTTTAGATGATGATATTCCCGTTTCCAACGAAATCATTAGAGCTATCCAAATATCC
        650 CTGTCTAG-TTTT-TATGATGATATTCCCTTTTCCAACGAAATCATCAAAGCGATCCAAATATCC

                *   *                    *         **      *  * *    *       
     139943 ACTTACAGTTTCTACAAAAAGAGTGTTTCCAAACTG-CTGCATCAAAAGAGAGGTTCCACTCTGT
        713 ACTTCCAGATTCTACAAAAAGAGTGTTTCAAAACTGCCTCTATCAAAGGAAATGTTCAACTCTGT

               **                  ***      *     *      *  *                
     140007 TAGCCGAGTACACACATCACAAACTTGTTTCTCAGAATCCTTCTGACTCGTTTTTATGGGAAGAT
        778 TAGTTGAGTACACACATCACAAAGAAGTTTCTGAGAATGCTTCTGTCTAGTTTTTATGGGAAGAT

               **     *  *  * **          *       *     *       *  *  *      
     140072 ATTTACTTTTTCACCGTAGGCATCAAAGCGCTCCAAATGTCCACATCCAGATACTCCAGAAAGAG
        843 ATTCCCTTTTCCAACGAAATCATCAAAGCGATCCAAATATCCACTTCCAGATTCTACAAAAAGAG

                     *         *     *   *         *                    *    
     140137 TGTTTCAAACCTGCTCTATGAAAGGGAATCTTCAACTCTATGAGTTGAATGCAGACATCAGAAAG
        908 TGTTTCAAAACTGCTCTATCAAAGGAAATGTTCAACTCTGTGAGTTGAATGCAGACATCACAAAG

              *              *       **       **         **             *  * 
     140202 AAATTTCTGAGAATGCTGCTGTCTACCTTTTAT-TTGA-ATTCCCGCTTCCAACGAAATCCTCCA
        973 AAGTTTCTGAGAATGCTTCTGTCTAGTTTTTATGAAGATATTCCCTTTTCCAACGAAATCATCAA

               *                *       *             *                    **
     140265 AGCTATCCAAATATCCACTTGCAGATTCCACAAAAAGAGTGTATCAAAACTGCTCTCTATCAATG
       1038 AGCGATCCAAATATCCACTTCCAGATTCTACAAAAAGAGTGTTTCAAAACTG--CTCTATCAAAA

             *  *                    *     *     *  *                        
     140330 GCAAAGTTCAACTCTGTTAGTTGAGGACACATATCACCAACAAGTTTCTGAGAATGCTTCTGTCT
       1101 GAAATGTTCAACTCTGTTAGTTGAGTACACACATCACAAAGAAGTTTCTGAGAATGCTTCTGTCT

             *                  *  *    
     140395 ATTTTTTATGGGAAGATATTTCCTTTTT
       1166 AGTTTTTATGGGAAGATATTCCCGTTTT

              *  * ** *    *      *    *          * **                       
     140423 CACCGTAGGCGTCAAGGCGATCGAAATGTCCACTTCCACAAACTACAAAAAGAGTGTTTCAAACC
          1 CAACGAAATCATCAAAGCGATCCAAATATCCACTTCCAGATTCTACAAAAAGAGTGTTTCAAACC

                          **       *    *        * ** * *   **      *      * 
     140488 TGCTCTATGAAAGGCCATGTTCATCTCTATGAGTTGAATGGAAATATCCGAAAGAAATTTCTGGG
         66 TGCTCTA-GAAAGGGAATGTTCAACTCTGTGAGTTGAGTACACACATCACAAAGAAGTTTCTGAG

                  *          *        *          *                      *    
     140553 AATGCTGCTGTCTAG-TGTT-TAT-ACGA-ATTCCCGCTTCCAACGAAATC-CTCAAAGCAATCC
        130 AATGCTTCTGTCTAGTTTTTATATGAAGATATTCCCGTTTCCAACGAAATCGCT-AAAGCGATCC

                        *    *  *                                *     *     
     140613 AAATATCCACTTGCAGAATCCACAAAAAGAGTGTTTCAAAACTGCTCTATCAATAGAAAGGTTCA
        194 AAATATCCACTTCCAGATTCTACAAAAAGAGTGTTTCAAAACTGCTCTATCAAAAGAAATGTTCA

                 *                     *  *                        * *      *
     140678 ACTCTTTTAGTTGAGTACACACATCACGAACAAGTTTCTGAGAATGCTTCTGTCTGGCTTTTATT
        259 ACTCTGTTAGTTGAGTACACACATCACAAAGAAGTTTCTGAGAATGCTTCTGTCTAGTTTTTATA

            *     **  *         * *  **          *       *                * *
     140743 GGAAGACGTTTCCTTTT-CACCAAAGGCATCAAAGCGCTCCAAATGTCCACTTCCAGATTCTTCC
        324 TGAAGATATTCCCTTTTCCAACGAAATCATCAAAGCGATCCAAATATCCACTTCCAGATTCTACA

                            **     * * *  * *               * *    * *  * *  
     140807 AAAAGAGTGTTTCAAACGTGCTCAAAGTAAGGGAATGTTCAACTCTGTGACTTGAATGCAGATAT
        389 AAAAGAGTGTTTCAAAACTGCTCTATGAAAAGAAATGTTCAACTCTGTTAGTTGAGTACACACAT

               *   *       * * *             *     ***  *         *          
     140872 CACCAAGTAGTTTCTAATAGTGCTTCTGTCTAGATTTTAGATGATGATATTCCCGTTTCCAACGA
        454 CACAAAGAAGTTTCTGAGAATGCTTCTGTCTAGTTTTTATGGGAAGATATTCCCTTTTCCAACGA

              * *** *   *             *  *   *      *     *       *   *    **
     140937 AATCGCTAGAGCTATCCAAATATCCAGTTACAGTTTCTACCAAAAGGGTGTTTCCAAATTGCTGC
        519 AAGCATCAAAGCGATCCAAATATCCACTTCCAGATTCTACAAAAAGAGTGTTTCAAAACTGCTCT

                  *    *            *       *                     *          
     141002 ATCAAAAGAAAGGTTCAACTCTGTTAGTTGAGGACACACATCACAAAGAAGTTTGTGAGAATGCT
        584 ATCAAAGGAAATGTTCAACTCTGTGAGTTGAGTACACACATCACAAAGAAGTTTCTGAGAATGCT

                                *                    *   * *     *   *       
     141067 TCTGTCTAGATTTTGTATGACGATATTCCCTTTTCCAACGATATCGTTAAAGCAATCTAAATATC
        649 TCTGTCTAG-TTTT-TATGATGATATTCCCTTTTCCAACGAAATCATCAAAGCGATCCAAATATC

            * *  *    *  *       *  *        *        * *   *    *    *      
     141132 AATTTGCAGAATCCACAAAAATAGAGTTTCAAAGCTG-CTCTGTAAAAAGAAAGGTTCCACTCTG
        712 CACTTCCAGATTCTACAAAAAGAGTGTTTCAAAACTGCCTCTATCAAAGGAAATGTTCAACTCTG

                *                   ***           **         *               
     141196 TTAGCTGAGTACACACATCACAAACTTGTTTCTGAGAAACCTTCTGTCTCGTTTTTATGGGAAGA
        777 TTAGTTGAGTACACACATCACAAAGAAGTTTCTGAGAATGCTTCTGTCTAGTTTTTATGGGAAGA

                **        ** * **          *       *     *       *  *  *  *  
     141261 TATTTACTTTTCCACTGTAGGCATCAAAGCGCTCCAAATGTCCACATCCAGATACTCCAGAACGA
        842 TATTCCCTTTTCCAACGAAATCATCAAAGCGATCCAAATATCCACTTCCAGATTCTACAAAAAGA

                      *         *     *   *         *                    *   
     141326 GTGTTTCAAACCTGCTCTATGAAAGGGAATCTTCAACTCTATGAGTTGAATGCAGA-ATCAGAAA
        907 GTGTTTCAAAACTGCTCTATCAAAGGAAATGTTCAACTCTGTGAGTTGAATGCAGACATCACAAA

               *              *       **       **         **             *  *
     141390 GAAATTTCTGAGAATGCTGCTGTCTACCTTTTAT-TTGA-ATTCCCGCTTCCAACGAAATCCTCC
        972 GAAGTTTCTGAGAATGCTTCTGTCTAGTTTTTATGAAGATATTCCCTTTTCCAACGAAATCATCA

                *              * *  **   *                                *  
     141453 AAGCTATCCAAATATCCACCTGCATTTTCCACAAAAAGAGTGTTTCAAAACTGCTCTATCAATAG
       1037 AAGCGATCCAAATATCCACTTCCAGATTCTACAAAAAGAGTGTTTCAAAACTGCTCTATCAAAAG

                         **  * *  *                *                         
     141518 AAATGTTCAACTCCTTTGGCTGGGTACACACATCACAAACAAGTTTCTGAGAATGCTTCTGTCTA
       1102 AAATGTTCAACTCTGTTAGTTGAGTACACACATCACAAAGAAGTTTCTGAGAATGCTTCTGTCTA

                           **     *    
     141583 GTTTTTATGGGAAGACGTTCCCTTTTT
       1167 GTTTTTATGGGAAGATATTCCCGTTTT

              * *  **          *       *            **                      *
     141610 CACCAAAGGCATCAAAGCGCTCCAAATGTCCACTTCCAGACACTACAAAAAGAGTGTTTCAAACG
          1 CAACGAAATCATCAAAGCGATCCAAATATCCACTTCCAGATTCTACAAAAAGAGTGTTTCAAACC

                         *                  *    * *  * *         *          
     141675 TGCTCTAAGAAAGCGAATGTTCAACTCTGTGACTTGAATGCAGATATCACAAAGTAGTTTCTGAG
         66 TGCTCT-AGAAAGGGAATGTTCAACTCTGTGAGTTGAGTACACACATCACAAAGAAGTTTCTGAG

             **            *     *    *                        **  *   *     
     141740 AGGGCTTCTGTCTAGATTTTAGATGATGATATTCCCGTTTCCAACGAAATCATTAGAGCTATCCA
        130 AATGCTTCTGTCTAGTTTTTATATGAAGATATTCCCGTTTCCAACGAAATCGCTAAAGCGATCCA

                       *   *                    *        **         * *    * 
     141805 AATATCCACTTACAGTTTCTACAAAAAGAGTGTTTCCAAACTGCTGCATCAAAAGAGAGGTTCCA
        195 AATATCCACTTCCAGATTCTACAAAAAGAGTGTTTCAAAACTGCTCTATCAAAAGAAATGTTCAA

                     **                  ***      *     *         *        **
     141870 CTCTGTTAGCCGAGTACACACATCACAAACTTGTTTCTCAGAATCCTTCTGTCTCGTTTTTATGG
        260 CTCTGTTAGTTGAGTACACACATCACAAAGAAGTTTCTGAGAATGCTTCTGTCTAGTTTTTATAT

                     **     *  *  * **          *       *     *       *  *  *
     141935 GAAGATATTTACTTTTTCACCGTAGGCATCAAAGCGCTCCAAATGTCCACATCCAGATACTCCAG
        325 GAAGATATTCCCTTTTCCAACGAAATCATCAAAGCGATCCAAATATCCACTTCCAGATTCTACAA

                           *             * *   *         * *      * *  *     
     142000 AAAGAGTGTTTCAAACCTGCTCTATGAAAGGGAATCTTCAACTCTATGAGTTGAATGCAGACATC
        390 AAAGAGTGTTTCAAAACTGCTCTATGAAAAGAAATGTTCAACTCTGTTAGTTGAGTACACACATC

             *      *              *       **         **         **          
     142065 AGAAAGAAATTTCTGAGAATGCTGCTGTCTACCTTTTAT---TTGA-ATTCCCGCTTCCAACGAA
        455 ACAAAGAAGTTTCTGAGAATGCTTCTGTCTAGTTTTTATGGGAAGATATTCCCTTTTCCAACGAA

             * *  *    *                *       *                            
     142126 ATCCTCCAAGCTATCCAAATATCCACTTGCAGATTCCACAAAAAGAGTGTTTCAAAACTGCTCTC
        520 AGCATCAAAGCGATCCAAATATCCACTTCCAGATTCTACAAAAAGAGTGTTTCAAAACTG--CTC

                  *  *  *            *       *     *     *  *                
     142191 TATCAATGGCAAAGTTCAACTCTGTTAGTTGAGGACACATATCACCAACAAGTTTCTGAGAATGC
        583 TATCAAAGGAAATGTTCAACTCTGTGAGTTGAGTACACACATCACAAAGAAGTTTCTGAGAATGC

                     *           *      *      *  *  * ** *    *      *    * 
     142256 TTCTGTCTATTTTTTATGGGAAGATATTTCCTTTTTCACCGTAGGCGTCAAGGCGATCGAAATGT
        648 TTCTGTCTAGTTTTTAT--GATGATATTCCCTTTTCCAACGAAATCATCAAAGCGATCCAAATAT

                     * **                     *          *     **       *    
     142321 CCACTTCCACAAACTACAAAAAGAGTGTTTCAAACCTG-CTCTATGAAAGGCCATGTTCATCTCT
        711 CCACTTCCAGATTCTACAAAAAGAGTGTTTCAAAACTGCCTCTATCAAAGGAAATGTTCAACTCT

            * *      * ** * *   **      *      *       *         *         * 
     142385 ATGAGTTGAATGGAAATATCCGAAAGAAATTTCTGGGAATGCTGCTGTCTAGTGTTTAT---ACG
        776 GTTAGTTGAGTACACACATCACAAAGAAGTTTCTGAGAATGCTTCTGTCTAGTTTTTATGGGAAG

                    **             *       *                *    *  *        
     142447 A-ATTCCCGCTTCCAACGAAATCCTCAAAGCAATCCAAATATCCACTTGCAGAATCCACAAAAAG
        841 ATATTCCCTTTTCCAACGAAATCATCAAAGCGATCCAAATATCCACTTCCAGATTCTACAAAAAG

                                    **    *          * *      * *  *         
     142511 AGTGTTTCAAAACTGCTCTATCAATAGAAAGGTTCAACTCTTTTAGTTGAGTACACACATCACAA
        906 AGTGTTTCAAAACTGCTCTATCAAAGGAAATGTTCAACTCTGTGAGTTGAATGCAGACATCACAA

             *                        * *             **  *         * *  **  
     142576 ACAAGTTTCTGAGAATGCTTCTGTCTGGCTTTTATTGGAAGACGTTTCCTTTT-CACCAAAGGCA
        971 AGAAGTTTCTGAGAATGCTTCTGTCTAGTTTTTA-T-GAAGATATTCCCTTTTCCAACGAAATCA

                    *       *                * *                **  *  * *** 
     142640 TCAAAGCGCTCCAAATGTCCACTTCCAGATTCTTCCAAAAGAGTGTTTCAAACGTGGTCGAAGTA
       1034 TCAAAGCGATCCAAATATCCACTTCCAGATTCTACAAAAAGAGTGTTTCAAAACTGCTCTATCAA

             * *               * *    * *  * *     *   *       * * *         
     142705 AGGGAATGTTCAACTCTGTGACTTGAATGCAGATATCACCAAGTAGTTTCTAATAGTGCTTCTGT
       1099 AAGAAATGTTCAACTCTGTTAGTTGAGTACACACATCACAAAGAAGTTTCTGAGAATGCTTCTGT

               **     ***  *             *
     142770 CTACATTTTAGATGATGATATTCCCGTTTC
       1164 CTAGTTTTTATGGGAAGATATTCCCGTTTT

                      * * *   *             *  *   *      *     *       *  **
     142800 CAACGAAATCGTTAGAGCTATCCAAATATCCAGTTACAGTTTCTACCAAAAGGGTGTTTCCAAAT
          1 CAACGAAATCATCAAAGCGATCCAAATATCCACTTCCAGATTCTACAAAAAGAGTGTTTCAAACC

                  ***   * *  *            *       *                     *    
     142865 TGCTGCATCAAAAGAAAGGTTCAACTCTGTTAGTTGAGGACACACATCACAAAGAAGTTTGTGAG
         66 TGCT-CTAGAAAGGGAATGTTCAACTCTGTGAGTTGAGTACACACATCACAAAGAAGTTTCTGAG

                           *    *     *         *          *    *      *   * 
     142930 AATGCTTCTGTCTAGATTTTGTATGACGATATTCCCTTTTCCAACGATATCGTTAAAGCAATCTA
        130 AATGCTTCTGTCTAGTTTTTATATGAAGATATTCCCGTTTCCAACGAAATCGCTAAAGCGATCCA

                  * *  *    *  *       *  *        *       * *        *    * 
     142995 AATATCAATTTGCAGAATCCACAAAAATAGAGTTTCAAAGCTGCTCTGTAAAAAGAAAGGTTCCA
        195 AATATCCACTTCCAGATTCTACAAAAAGAGTGTTTCAAAACTGCTCTATCAAAAGAAATGTTCAA

                     *                   ***           **         *        **
     143060 CTCTGTTAGCTGAGTACACACATCACAAACTTGTTTCTGAGAAACCTTCTGTCTCGTTTTTATGG
        260 CTCTGTTAGTTGAGTACACACATCACAAAGAAGTTTCTGAGAATGCTTCTGTCTAGTTTTTATAT

                     **        ** * **          *       *     *       *  *  *
     143125 GAAGATATTTACTTTTCCACTGTAGGCATCAAAGCGCTCCAAATGTCCACATCCAGATACTCCAG
        325 GAAGATATTCCCTTTTCCAACGAAATCATCAAAGCGATCCAAATATCCACTTCCAGATTCTACAA

              *            *             * *   *         * *      * *  *     
     143190 AACGAGTGTTTCAAACCTGCTCTATGAAAGGGAATCTTCAACTCTATGAGTTGAATGCAGA-ATC
        390 AAAGAGTGTTTCAAAACTGCTCTATGAAAAGAAATGTTCAACTCTGTTAGTTGAGTACACACATC

             *      *              *       **         **         **          
     143254 AGAAAGAAATTTCTGAGAATGCTGCTGTCTACCTTTTAT---TTGA-ATTCCCGCTTCCAACGAA
        455 ACAAAGAAGTTTCTGAGAATGCTTCTGTCTAGTTTTTATGGGAAGATATTCCCTTTTCCAACGAA

             * *  *    *              * *  **   *                            
     143315 ATCCTCCAAGCTATCCAAATATCCACCTGCATTTTCCACAAAAAGAGTGTTTCAAAACTGCTCTA
        520 AGCATCAAAGCGATCCAAATATCCACTTCCAGATTCTACAAAAAGAGTGTTTCAAAACTGCTCTA

                **                *    *  *                *                 
     143380 TCAATAGAAATGTTCAACTCCTTTG-GCTGGGTACACACATCACAAACAAGTTTCTGAGAATGCT
        585 TCAAAGGAAATGTTCAACT-CTGTGAGTTGAGTACACACATCACAAAGAAGTTTCTGAGAATGCT

                                *  **         *  * *  **          *       *  
     143444 TCTGTCTAGTTTTTATGGGAAGACGTTCCCTTTTTCACCAAAGGCATCAAAGCGCTCCAAATGTC
        649 TCTGTCTAGTTTTTAT--GATGATATTCCCTTTTCCAACGAAATCATCAAAGCGATCCAAATATC

                      **                     **        **    **              
     143509 CACTTCCAGACACTACAAAAAGAGTGTTTCAAACGTG-CTCTAAGAAAGCGAATGTTCAACTCTG
        712 CACTTCCAGATTCTACAAAAAGAGTGTTTCAAAACTGCCTCTATCAAAGGAAATGTTCAACTCTG

             * *    * *  * *         *           **            *     ***  *  
     143573 TGACTTGAATGCAGATATCACAAAGTAGTTTCTGAGAGGGCTTCTGTCTAGATTTTAGATGATGA
        777 TTAGTTGAGTACACACATCACAAAGAAGTTTCTGAGAATGCTTCTGTCTAGTTTTTATGGGAAGA

                   *                * *   *                *   *             
     143638 TATTCCCGTTTCCAACGAAATCATTAGAGCTATCCAAATATCCACTTACAGTTTCTACAAAAAGA
        842 TATTCCCTTTTCCAACGAAATCATCAAAGCGATCCAAATATCCACTTCCAGATTCTACAAAAAGA

                   *        **      *  * *    *       *  **  * *  *          
     143703 GTGTTTCCAAACTGCTGCATCAAAAGAGAGGTTCCACTCTGTTAGCCGAGTACACACATCACAAA
        907 GTGTTTCAAAACTGCTCTATCAAAGGAAATGTTCAACTCTGTGAGTTGAATGCAGACATCACAAA

            ***      *     *         *                   **     *  *  * **   
     143768 CTTGTTTCTCAGAATCCTTCTGTCTCGTTTTTATGGGAAGATATTTACTTTTTCACCGTAGGCAT
        972 GAAGTTTCTGAGAATGCTTCTGTCTAGTTTTTAT--GAAGATATTCCCTTTTCCAACGAAATCAT

                   *       *     *       *  *  *               *         *   
     143833 CAAAGCGCTCCAAATGTCCACATCCAGATACTCCAGAAAGAGTGTTTCAAACCTGCTCTATGAAA
       1035 CAAAGCGATCCAAATATCCACTTCCAGATTCTACAAAAAGAGTGTTTCAAAACTGCTCTATCAAA

            * *   *         * *      * *  *      *      *              *     
     143898 GGGAATCTTCAACTCTATGAGTTGAATGCAGACATCAGAAAGAAATTTCTGAGAATGCTGCTGTC
       1100 AGAAATGTTCAACTCTGTTAGTTGAGTACACACATCACAAAGAAGTTTCTGAGAATGCTTCTGTC

              **         **          *  *
     143963 TACCTTTTAT---TTGA-ATTCCCGCTTC
       1165 TAGTTTTTATGGGAAGATATTCCCGTTTT

                      *  *    *                *       *                   * 
     143988 CAACGAAATCCTCCAAGCTATCCAAATATCCACTTGCAGATTCCACAAAAAGAGTGTTTCAAAAC
          1 CAACGAAATCATCAAAGCGATCCAAATATCCACTTCCAGATTCTACAAAAAGAGTGTTTCAAACC

                      *  *  *  *            *       *     *     *  *         
     144053 TGCTCTCTATCAATGGCAAAGTTCAACTCTGTTAGTTGAGGACACATATCACCAACAAGTTTCTG
         66 TG--CTCTA-GAAAGGGAATGTTCAACTCTGTGAGTTGAGTACACACATCACAAAGAAGTTTCTG

                            *       **         *  *   *  *  * **  **  *      
     144118 AGAATGCTTCTGTCTATTTTTTATGGGAAGATATTTCCTTTTTCACCGTAGGCGTCAAGGCGATC
        128 AGAATGCTTCTGTCTAGTTTTTATATGAAGATATTCCCGTTTCCAACGAAATCGCTAAAGCGATC

            *    *          * **                     *         *   * **      
     144183 GAAATGTCCACTTCCACAAACTACAAAAAGAGTGTTTCAAACCTGCTCTATGAAAGGCCATGTTC
        193 CAAATATCCACTTCCAGATTCTACAAAAAGAGTGTTTCAAAACTGCTCTATCAAAAGAAATGTTC

             *    * *      * ** * *   **      *      *       *               
     144248 ATCTCTATGAGTTGAATGGAAATATCCGAAAGAAATTTCTGGGAATGCTGCTGTCTAGTTTTTAT
        258 AACTCTGTTAGTTGAGTACACACATCACAAAGAAGTTTCTGAGAATGCTTCTGTCTAGTTTTTAT

                         ***             *       *                *    *  *  
     144313 AT---GA-ATTCCGGCTTCCAACGAAATCCTCAAAGCAATCCAAATATCCACTTGCAGAATCCAC
        323 ATGAAGATATTCCCTTTTCCAACGAAATCATCAAAGCGATCCAAATATCCACTTCCAGATTCTAC

                                       *  *     *          *                 
     144374 AAAAAGAGTGTTTCAAAACTGCTCTATCAATAGAAAGGTTCAACTCTTTTAGTTGAGTACACACA
        388 AAAAAGAGTGTTTCAAAACTGCTCTATGAAAAGAAATGTTCAACTCTGTTAGTTGAGTACACACA

                   *                        * *      *      **  *         * *
     144439 TCACAAACAAGTTTCTGAGAATGCTTCTGTCTGGCTTTTATTGGAAGACGTTTCCTTTT-CACCA
        453 TCACAAAGAAGTTTCTGAGAATGCTTCTGTCTAGTTTTTATGGGAAGATATTCCCTTTTCCAACG

              *           *       *                * *                **     
     144503 AAGGCATCAAAGCGCTCCAAATGTCCACTTCCAGATTCTTCCAAAAGAGTGTTTCAAACGTGCTC
        518 AAAGCATCAAAGCGATCCAAATATCCACTTCCAGATTCTACAAAAAGAGTGTTTCAAAACTGCTC

            * ***    *       *         *    * *  * *     *   *       * * *   
     144568 GAAGTAAGGGAATGTTCTACTCTGTGACTTGAATGCAGATATCACCAAGTAGTTTCTAATAGTGC
        583 TATCAAAGGAAATGTTCAACTCTGTGAGTTGAGTACACACATCACAAAGAAGTTTCTGAGAATGC

                            *              *              *** *   *          
     144633 TTCTGTCTAGATTTTAGATGATGATATTCCCGTTTCCAACGAAATCGCTAGAGCTATCCAAATAT
        648 TTCTGTCTAG-TTTT-TATGATGATATTCCCTTTTCCAACGAAATCATCAAAGCGATCCAAATAT

               *  *   *      *     *       *   *     **      *    *          
     144698 CCAGTTACAGTTTCTACCAAAAGGGTGTTTCCAAATTG-CTGCATCAAAAGAAAGGTTCAACTCT
        711 CCACTTCCAGATTCTACAAAAAGAGTGTTTCAAAACTGCCTCTATCAAAGGAAATGTTCAACTCT

                      *                     *                   *    * **  * 
     144762 GTTAGTTGAGGACACACATCACAAAGAAGTTTGTGAGAATGCTTCTGTCTAGATTTTGTATGACG
        776 GTTAGTTGAGTACACACATCACAAAGAAGTTTCTGAGAATGCTTCTGTCTAGTTTTTATGGGAAG

                               *   * *     *   *       * *  *    *  *       *
     144827 ATATTCCCTTTTCCAACGATATCGTTAAAGCAATCTAAATATCAATTTGCAGAATCCACAAAAAT
        841 ATATTCCCTTTTCCAACGAAATCATCAAAGCGATCCAAATATCCACTTCCAGATTCTACAAAAAG

              *        *       * *   *    *    *       *  *   * *  *         
     144892 AGAGTTTCAAAGCTGCTCTGTAAAAAGAAAGGTTCCACTCTGTTAGCTGAGTACACACATCACAA
        906 AGTGTTTCAAAACTGCTCTATCAAAGGAAATGTTCAACTCTGTGAGTTGAATGCAGACATCACAA

             ***      *     *         *                 * **        *  * **  
     144957 ACTTGTTTCTCAGAATCCTTCTGTCTCGTTTTTATGGGAAGATACTTACTTTTCCACCGTAGGCA
        971 AGAAGTTTCTGAGAATGCTTCTGTCTAGTTTTTAT--GAAGATATTCCCTTTTCCAACGAAATCA

                    *       *     *       *  *  *  *            *         *  
     145022 TCAAAGCGCTCCAAATGTCCACATCCAGATACTCCAGAACGAGTGTTTCAAACCTGCTCTATGAA
       1034 TCAAAGCGATCCAAATATCCACTTCCAGATTCTACAAAAAGAGTGTTTCAAAACTGCTCTATCAA

             * *   *         * *      * *  *      *      *              *    
     145087 AGGGAATCTTCAACTCTATGAGTTGAATGCAGACATCAGAAAGAAATTTCTGAGAATGCTGCTGT
       1099 AAGAAATGTTCAACTCTGTTAGTTGAGTACACACATCACAAAGAAGTTTCTGAGAATGCTTCTGT

               **         **          *  *
     145152 CTACCTTTTAT---TTGA-ATTCCCGCTTC
       1164 CTAGTTTTTATGGGAAGATATTCCCGTTTT

                      *  *    *              * *  **   *    *              * 
     145178 CAACGAAATCCTCCAAGCTATCCAAATATCCACCTGCATTTTCCACAACAAGAGTGTTTCAAAAC
          1 CAACGAAATCATCAAAGCGATCCAAATATCCACTTCCAGATTCTACAAAAAGAGTGTTTCAAACC

                    *  ** *              *    *  *                *          
     145243 TGCTCTATCAATAGAAATGTTCAACTCCTTTG-GCTGGGTACACACATCACAAACAAGTTTCTGA
         66 TGCTCTA-GAAAGGGAATGTTCAACT-CTGTGAGTTGAGTACACACATCACAAAGAAGTTTCTGA

                                   **     **     *   *    *    *           * 
     145307 GAATGCTTCTGTCTAGTTTTTATGGGAAGACGTTCCCTTTTTC-ACCAAA-GGCATCAAAGCGCT
        129 GAATGCTTCTGTCTAGTTTTTATATGAAGATATTCCCGTTTCCAACGAAATCGC-T-AAAGCGAT

                  *            **                  *  **       **   ***      
     145370 CCAAATGTCCACTTCCAGACACTACAAAAAGAGTGTTTCCAACGTGCTCTAAGAAAGCGAATGTT
        192 CCAAATATCCACTTCCAGATTCTACAAAAAGAGTGTTTCAAAACTGCTCTATCAAAAGAAATGTT

                     * *    * *  * *         *           **            *     
     145435 CAACTCTGTGACTTGAATGCAGATATCACAAAGTAGTTTCTGAGAGGGCTTCTGTCTAGATTTTA
        257 CAACTCTGTTAGTTGAGTACACACATCACAAAGAAGTTTCTGAGAATGCTTCTGTCTAGTTTTTA

            *    *         *        *       * *    *               *   *     
     145500 GATGATGATATTCCCGTTTCCAACAAAATCATTAGAGC-TTCCAAATATCCACTTACAGTTTCTA
        322 TATGAAGATATTCCCTTTTCCAACGAAATCATCAAAGCGATCCAAATATCCACTTCCAGATTCTA

                           *        **  *      * *    *          *           
     145564 CAAAAAGAGTGTTTCCAAACTGCTGCATCAAAAGAGAGGTTCCACTCTGTTAGCTGAGTACACAC
        387 CAAAAAGAGTGTTTCAAAACTGCTCTATGAAAAGAAATGTTCAACTCTGTTAGTTGAGTACACAC

                    ***      *     *         *                   **     *  * 
     145629 ATCACAAACTTGTTTCTCAGAATCCTTCTGTCTCGTTTTTATGGGAAGATATTTACTTTTTCACC
        452 ATCACAAAGAAGTTTCTGAGAATGCTTCTGTCTAGTTTTTATGGGAAGATATTCCCTTTTCCAAC

             * *           *       *     *       *  *  *               *     
     145694 GTAGGCATCAAAGCGCTCCAAATGTCCACATCCAGATACTCCAGAAAGAGTGTTTCAAACCTGCT
        517 GAAAGCATCAAAGCGATCCAAATATCCACTTCCAGATTCTACAAAAAGAGTGTTTCAAAACTGCT

                *     *   *         *        * *  *      *      *            
     145759 CTATGAAAGGGAATCTTCAACTCTATGAGTTGAATGCAGACATCAGAAAGAAATTTCTGAGAATG
        582 CTATCAAAGGAAATGTTCAACTCTGTGAGTTGAGTACACACATCACAAAGAAGTTTCTGAGAATG

              *       **       *          **             *  *    *           
     145824 CTGCTGTCTACCTTTTAT-TTGA-ATTCCCGCTTCCAACGAAATCCTCCAAGCTATCCAAATATC
        647 CTTCTGTCTAGTTTTTATGATGATATTCCCTTTTCCAACGAAATCATCAAAGCGATCCAAATATC

                 *       *                                  *  *  *          
     145887 CACTTGCAGATTCCACAAAAAGAGTGTTTCAAAACTGCTCTCTATCAATGGCAAAGTTCAACTCT
        712 CACTTCCAGATTCTACAAAAAGAGTGTTTCAAAACTGC-CTCTATCAAAGGAAATGTTCAACTCT

                      *     *     *  *                         *             
     145952 GTTAGTTGAGGACACATATCACCAACAAGTTTCTGAGAATGCTTCTGTCTATTTTTTATGGGAAG
        776 GTTAGTTGAGTACACACATCACAAAGAAGTTTCTGAGAATGCTTCTGTCTAGTTTTTATGGGAAG

                 *      *  *  * ** *    *      *    *          * **          
     146017 ATATTTCCTTTTTCACCGTAGGCGTCAAGGCGATCGAAATGTCCACTTCCACAAACTACAAAAAG
        841 ATATTCCCTTTTCCAACGAAATCATCAAAGCGATCCAAATATCCACTTCCAGATTCTACAAAAAG

                       *         *     **       *    *           * * *   **  
     146082 AGTGTTTCAAACCTGCTCTATGAAAGGCCATGTTCATCTCTATGAGTTGAATGGAAATATCCGAA
        906 AGTGTTTCAAAACTGCTCTATCAAAGGAAATGTTCAACTCTGTGAGTTGAATGCAGACATCACAA

                *      *       *         *       *         **             *  
     146147 AGAAATTTCTGGGAATGCTGCTGTCTAGTGTTTAT-ACGA-ATTCCCGCTTCCAACGAAATCCTC
        971 AGAAGTTTCTGAGAATGCTTCTGTCTAGTTTTTATGAAGATATTCCCTTTTCCAACGAAATCATC

                 *                *    *  *                                * 
     146210 AAAGCAATCCAAATATCCACTTGCAGAATCCACAAAAAGAGTGTTTCAAAACTGCTCTATCAATA
       1036 AAAGCGATCCAAATATCCACTTCCAGATTCTACAAAAAGAGTGTTTCAAAACTGCTCTATCAAAA

                *          *                     *  *                        
     146275 GAAAGGTTCAACTCTTTTAGTTGAGTACACACATCACGAACAAGTTTCTGAGAATGCTTCTGTCT
       1101 GAAATGTTCAACTCTGTTAGTTGAGTACACACATCACAAAGAAGTTTCTGAGAATGCTTCTGTCT

            * *      *      **  *       
     146340 GGCTTTTATTGGAAGACGTTTCC-TTTT
       1166 AGTTTTTATGGGAAGATATTCCCGTTTT

              * *  **          *       *                * *                 *
     146367 CACCAAAGGCATCAAAGCGCTCCAAATGTCCACTTCCAGATTCTTCCAAAAGAGTGTTTCAAACG
          1 CAACGAAATCATCAAAGCGATCCAAATATCCACTTCCAGATTCTACAAAAAGAGTGTTTCAAACC

                  *  *                      *    * *  * *     *   *       * *
     146432 TGCTCAAAGTAAGGGAATGTTCAACTCTGTGACTTGAATGCAGATATCACCAAGTAGTTTCTAAT
         66 TGCTC-TAGAAAGGGAATGTTCAACTCTGTGAGTTGAGTACACACATCACAAAGAAGTTTCTGAG

             *             *     *    *                         *  *   *     
     146497 AGTGCTTCTGTCTAGATTTTAGATGATGATATTCCCGTTTCCAACGAAATCGTTAGAGCTATCCA
        130 AATGCTTCTGTCTAGTTTTTATATGAAGATATTCCCGTTTCCAACGAAATCGCTAAAGCGATCCA

                    *  *   *      *     *       *   *    **           *      
     146562 AATATCCAGTTACAGTTTCTACCAAAAGGGTGTTTCCAAATTGCTGCATCAAAAGAAAGGTTCAA
        195 AATATCCACTTCCAGATTCTACAAAAAGAGTGTTTCAAAACTGCTCTATCAAAAGAAATGTTCAA

                          *                     *                   *    *   
     146627 CTCTGTTAGTTGAGGACACACATCACAAAGAAGTTTGTGAGAATGCTTCTGTCTAGATTTTGTAT
        260 CTCTGTTAGTTGAGTACACACATCACAAAGAAGTTTCTGAGAATGCTTCTGTCTAGTTTTTATAT

              *                    *   * *     *   *       * *  *    *  *    
     146692 GACGATATTCCCTTTTCCAACGATATCGTTAAAGCAATCTAAATATCAATTTGCAGAATCCACAA
        325 GAAGATATTCCCTTTTCCAACGAAATCATCAAAGCGATCCAAATATCCACTTCCAGATTCTACAA

               *  *        *       * *        *    *          *              
     146757 AAATAGAGTTTCAAAGCTGCTCTGTAAAAAGAAAGGTTCCACTCTGTTAGCTGAGTACACACATC
        390 AAAGAGTGTTTCAAAACTGCTCTATGAAAAGAAATGTTCAACTCTGTTAGTTGAGTACACACATC

                 ***            *         *                   **        *  * 
     146822 ACAAACTTGTTTCTGAGAATCCTTCTGTCTCGTTTTTATGGGAAGATATTTACTTTTCCACCGTA
        455 ACAAAGAAGTTTCTGAGAATGCTTCTGTCTAGTTTTTATGGGAAGATATTCCCTTTTCCAACGAA

            *           *       *     *       *  *  *  *            *        
     146887 GGCATCAAAGCGCTCCAAATGTCCACATCCAGATACTCCAGAACGAGTGTTTCAAACCTGCTCTA
        520 AGCATCAAAGCGATCCAAATATCCACTTCCAGATTCTACAAAAAGAGTGTTTCAAAACTGCTCTA

             *     *   *         *        * *  *      *      *              *
     146952 TGAAAGGGAATCTTCAACTCTATGAGTTGAATGCAGACATCAGAAAGAAATTTCTGAGAATGCTG
        585 TCAAAGGAAATGTTCAACTCTGTGAGTTGAGTACACACATCACAAAGAAGTTTCTGAGAATGCTT

                   **       *          **             *  *    *              
     147017 CTGTCTACCTTTTAT-TTGA-ATTCCCGCTTCCAACGAAATCCTCCAAGCTATCCAAATATCCAC
        650 CTGTCTAGTTTTTATGATGATATTCCCTTTTCCAACGAAATCATCAAAGCGATCCAAATATCCAC

              *  **   *    *                            **              **  *
     147080 TTGCATTTTCCACAACAAGAGTGTTTCAAAACTG-CTCTATCAATAGAAATGTTCAACTCCTTTG
        715 TTCCAGATTCTACAAAAAGAGTGTTTCAAAACTGCCTCTATCAAAGGAAATGTTCAACTCTGTTA

             *  *                *                                        *  
     147144 GCTGGGTACACACATCACAAACAAGTTTCTGAGAATGCTTCTGTCTAGTTTTTATGGGAAGACAT
        780 GTTGAGTACACACATCACAAAGAAGTTTCTGAGAATGCTTCTGTCTAGTTTTTATGGGAAGATAT

                    *  * *  **          *       *            **              
     147209 TCCCTTTTTCACCAAAGGCATCAAAGCGCTCCAAATGTCCACTTCCAGACACTACAAAAAGAGTG
        845 TCCCTTTTCCAACGAAATCATCAAAGCGATCCAAATATCCACTTCCAGATTCTACAAAAAGAGTG

                   **       **    **                 *           *         * 
     147274 TTTCAAACGTGCTCTAAGAAAGCGAATGTTCAACTCTGTGACTTGAATGCAGATATCACAAAGTA
        910 TTTCAAAACTGCTCTATCAAAGGAAATGTTCAACTCTGTGAGTTGAATGCAGACATCACAAAGAA

                      *                   *    *         *       *        * *
     147339 GTTTCTGAGAGTGCTTCTGTCTAGATTTTAGATGATGATATTCCCGTTTCCAAAGAAATCATTAG
        975 GTTTCTGAGAATGCTTCTGTCTAG-TTTT-TATGAAGATATTCCCTTTTCCAACGAAATCATCAA

               *                *   *                    *        **         
     147404 AGCTATCCAAATATCCACTTACAGTTTCTACAAAAAGAGTGTTTCCAAACTGCTGCATCAAAAGA
       1038 AGCGATCCAAATATCCACTTCCAGATTCTACAAAAAGAGTGTTTCAAAACTGCTCTATCAAAAGA

            * *    *          *                   ***      *     *         * 
     147469 GAGGTTCCACTCTGTTAGCTGAGTACACACATCACAAACTTGTTTCTCAGAATCCTTCTGTCTCG
       1103 AATGTTCAACTCTGTTAGTTGAGTACACACATCACAAAGAAGTTTCTGAGAATGCTTCTGTCTAG

                              ** *    
     147534 TTTTTATGGGAAGATATTTACTTTTT
       1168 TTTTTATGGGAAGATATTCCCGTTTT

              *  * **          *       *     *       *  *  *                 
     147560 CACCGTAGGCATCAAAGCGCTCCAAATGTCCACATCCAGATACTCCAGAAAGAGTGTTTCAAACC
          1 CAACGAAATCATCAAAGCGATCCAAATATCCACTTCCAGATTCTACAAAAAGAGTGTTTCAAACC

                              *         *        * *  *      *      *        
     147625 TGCTCTATGAAAGGGAATCTTCAACTCTATGAGTTGAATGCAGACATCAGAAAGAAATTTCTGAG
         66 TGCTCTA-GAAAGGGAATGTTCAACTCTGTGAGTTGAGTACACACATCACAAAGAAGTTTCTGAG

                  *       **          *          *                 *    *    
     147690 AATGCTGCTGTCTACCTTTTAT-T--TGA-ATTCCCGCTTCCAACGAAATC-CTCCAAGCTATCC
        130 AATGCTTCTGTCTAGTTTTTATATGAAGATATTCCCGTTTCCAACGAAATCGCT-AAAGCGATCC

                        *       *                                  ** *  *   
     147750 AAATATCCACTTGCAGATTCCACAAAAAGAGTGTTTCAAAACTGCTCTCTATCAATGGCAAAGTT
        194 AAATATCCACTTCCAGATTCTACAAAAAGAGTGTTTCAAAACTG--CTCTATCAAAAGAAATGTT

                             *     *     *  *                         *      
     147815 CAACTCTGTTAGTTGAGGACACATATCACCAACAAGTTTCTGAGAATGCTTCTGTCTATTTTTTA
        257 CAACTCTGTTAGTTGAGTACACACATCACAAAGAAGTTTCTGAGAATGCTTCTGTCTAGTTTTTA

             **         *      *  *  * ** *    *  *   *    *         ** **   
     147880 TGGGAAGATATTTCCTTTTTCACCGTAGGCGTCAAGGCAATCGAAATGTCCACTTCCGCAAACTA
        322 TATGAAGATATTCCCTTTTCCAACGAAATCATCAAAGCGATCCAAATATCCACTTCCAGATTCTA

                              *             * **       *    * *      * ** * *
     147945 CAAAAAGAGTGTTTCAAACCTGCTCTATGAAAGGCCATGTTCATCTCTATGAGTTGAATGGAAAT
        387 CAAAAAGAGTGTTTCAAAACTGCTCTATGAAAAGAAATGTTCAACTCTGTTAGTTGAGTACACAC

               **      *      *       *                   *         **       
     148010 ATCCGAAAGAAATTTCTGGGAATGCTGCTGTCTAGTTTTTAT---ATGA-ATTCCCGCTTCCAAC
        452 ATCACAAAGAAGTTTCTGAGAATGCTTCTGTCTAGTTTTTATGGGAAGATATTCCCTTTTCCAAC

                * *       *                *    *  *                         
     148071 GAAATCCTCAAAGCAATCCAAATATCCACTTGCAGAATCCACAAAAAGAGTGTTTCAAAACTGCT
        517 GAAAGCATCAAAGCGATCCAAATATCCACTTCCAGATTCTACAAAAAGAGTGTTTCAAAACTGCT

                   **    *          * *                      *               
     148136 CTATCAATAGAAAGGTTCAACTCTTTTAGTTGAGTACACACATCACAAACAAGTTTCTGAGAATG
        582 CTATCAAAGGAAATGTTCAACTCTGTGAGTTGAGTACACACATCACAAAGAAGTTTCTGAGAATG

                     * *          *  **  *         * *  **          *       *
     148201 CTTCTGTCTGGCTTTTATTGGAAGACGTTTCCTTTT-CACCAAAGGCATCAAAGCGCTCCAAATG
        647 CTTCTGTCTAGTTTTTA-T-GATGATATTCCCTTTTCCAACGAAATCATCAAAGCGATCCAAATA

                            * *                **   *  * ***    *            
     148265 TCCACTTCCAGATTCTTCCAAAAGAGTGTTTCAAACGTG-GTCGAAGTAAGGGAATGTTCAACTC
        710 TCCACTTCCAGATTCTACAAAAAGAGTGTTTCAAAACTGCCTCTATCAAAGGAAATGTTCAACTC

               * *    * *  * *     *   *       * * *             *     ***  *
     148329 TGTGACTTGAATGCAGATATCACCAAGTAGTTTCTAATAGTGCTTCTGTCTAGATTTTAGATGAT
        775 TGTTAGTTGAGTACACACATCACAAAGAAGTTTCTGAGAATGCTTCTGTCTAGTTTTTATGGGAA

                     *              * * *   *             *  *   *      *    
     148394 GATATTCCCGTTTCCAACGAAATCGTTAGAGCTATCCAAATATCCAGTTACAGTTTCTACCAAAA
        840 GATATTCCCTTTTCCAACGAAATCATCAAAGCGATCCAAATATCCACTTCCAGATTCTACAAAAA

             *       *   *    **      *    *            *      ***  *        
     148459 GGGTGTTTCCAAATTGCTGCATCAAAAGAAAGGTTCAACTCTGTTAGTTGAGGACACACATCACA
        905 GAGTGTTTCAAAACTGCTCTATCAAAGGAAATGTTCAACTCTGTGAGTTGAATGCAGACATCACA

                     *                              *                    *   
     148524 AAGAAGTTTGTGAGAATGCTTCTGTCTAGATTTTGTATGACGATATTCCCTTTTCCAACGATATC
        970 AAGAAGTTTCTGAGAATGCTTCTGTCTAG-TTTT-TATGAAGATATTCCCTTTTCCAACGAAATC

            * *     *   *       * *  *    *  *       *  *        *       * * 
     148589 GTTAAAGCAATCTAAATATCAATTTGCAGAATCCACAAAAATAGAGTTTCAAAGCTGCTCTGTAA
       1033 ATCAAAGCGATCCAAATATCCACTTCCAGATTCTACAAAAAGAGTGTTTCAAAACTGCTCTATCA

                   *    *          *                   ***           **      
     148654 AAAGAAAGGTTCCACTCTGTTAGCTGAGTACACACATCACAAACTTGTTTCTGAGAAACCTTCTG
       1098 AAAGAAATGTTCAACTCTGTTAGTTGAGTACACACATCACAAAGAAGTTTCTGAGAATGCTTCTG

               *                   **      
     148719 TCTCGTTTTTATGGGAAGATATTTAC-TTTT
       1163 TCTAGTTTTTATGGGAAGATATTCCCGTTTT

             *    * **          *       *     *       *  *  *  *             
     148749 CCACTGTAGGCATCAAAGCGCTCCAAATGTCCACATCCAGATACTCCAGAACGAGTGTTTCAAAC
          1 CAAC-GAAATCATCAAAGCGATCCAAATATCCACTTCCAGATTCTACAAAAAGAGTGTTTCAAAC

                               *         *        * *  *      *      *       
     148814 CTGCTCTATGAAAGGGAATCTTCAACTCTATGAGTTGAATGCAGA-ATCAGAAAGAAATTTCTGA
         65 CTGCTCTA-GAAAGGGAATGTTCAACTCTGTGAGTTGAGTACACACATCACAAAGAAGTTTCTGA

                   *       **          *          *                 *    *   
     148878 GAATGCTGCTGTCTACCTTTTAT-T--TGA-ATTCCCGCTTCCAACGAAATC-CTCCAAGCTATC
        129 GAATGCTTCTGTCTAGTTTTTATATGAAGATATTCCCGTTTCCAACGAAATCGCT-AAAGCGATC

                       * *  **   *    *                           *          
     148938 CAAATATCCACCTGCATTTTCCACAACAAGAGTGTTTCAAAACTGCTCTATCAATAGAAATGTTC
        193 CAAATATCCACTTCCAGATTCTACAAAAAGAGTGTTTCAAAACTGCTCTATCAAAAGAAATGTTC

                 **  * *  *                *                                 
     149003 AACTCCTTTGGCTGGGTACACACATCACAAACAAGTTTCTGAGAATGCTTCTGTCTAGTTTTTAT
        258 AACTCTGTTAGTTGAGTACACACATCACAAAGAAGTTTCTGAGAATGCTTCTGTCTAGTTTTTAT

            **     **         *  * *  **          *       *            **    
     149068 GGGAAGACGTTCCCTTTTTCACCAAAGGCATCAAAGCGCTCCAAATGTCCACTTCCAGACACTAC
        323 ATGAAGATATTCCCTTTTCCAACGAAATCATCAAAGCGATCCAAATATCCACTTCCAGATTCTAC

                             **       *    ***               * *    * *    * 
     149133 AAAAAGAGTGTTTCAAACGTGCTCTAAGAAAGCGAATGTTCAACTCTGTGACTTGAATGCACATA
        388 AAAAAGAGTGTTTCAAAACTGCTCTATGAAAAGAAATGTTCAACTCTGTTAGTTGAGTACACACA

                    *           **            *     ***  *         *        *
     149198 TCACAAAGTAGTTTCTGAGAGGGCTTCTGTCTAGATTTTAGATGATGATATTCCCGTTTCCAACA
        453 TCACAAAGAAGTTTCTGAGAATGCTTCTGTCTAGTTTTTATGGGAAGATATTCCCTTTTCCAACG

               *   * *    *               *   *                    *        *
     149263 AAATCATTAGAGC-TTCCAAATATCCACTTACAGTTTCTACAAAAAGAGTGTTTCCAAACTGCTG
        518 AAAGCATCAAAGCGATCCAAATATCCACTTCCAGATTCTACAAAAAGAGTGTTTCAAAACTGCTC

            *      *  * *    *       *  *                   ***      *     * 
     149327 CATCAAAAGAGAGGTTCCACTCTGTTAGCTGAGTACACACATCACAAACTTGTTTCTCAGAATCC
        583 TATCAAAGGAAATGTTCAACTCTGTGAGTTGAGTACACACATCACAAAGAAGTTTCTGAGAATGC

                    *         *** *    **     *  *  * **          *       *  
     149392 TTCTGTCTCGTTTTTATGGGAAGATTATACTTTTTCACCGTAGGCATCAAAGCGCTCCAAATGTC
        648 TTCTGTCTAGTTTTTATGATGATATT-CCCTTTTCCAACGAAATCATCAAAGCGATCCAAATATC

               *       *  *  *               *          *     *   *         *
     149457 CACATCCAGATACTCCAGAAAGAGTGTTTCAAACCTG-CTCTATGAAAGGGAATCTTCAACTCTA
        712 CACTTCCAGATTCTACAAAAAGAGTGTTTCAAAACTGCCTCTATCAAAGGAAATGTTCAACTCTG

             *      * *  *      *      *              *       **         **  
     149521 TGAGTTGAATGCAGACATCAGAAAGAAATTTCTGAGAATGCTGCTGTCTACCTTTTAT---TTGA
        777 TTAGTTGAGTACACACATCACAAAGAAGTTTCTGAGAATGCTTCTGTCTAGTTTTTATGGGAAGA

                   **             *  *    *                *       *         
     149583 -ATTCCCGCTTCCAACGAAATCCTCCAAGCTATCCAAATATCCACTTGCAGATTCCACAAAAAGA
        842 TATTCCCTTTTCCAACGAAATCATCAAAGCGATCCAAATATCCACTTCCAGATTCTACAAAAAGA

                                     *  *  *            *      ***  * *     *
     149647 GTGTTTCAAAACTGCTCTCTATCAATGGCAAAGTTCAACTCTGTTAGTTGAGGACACATATCACC
        907 GTGTTTCAAAACTG--CTCTATCAAAGGAAATGTTCAACTCTGTGAGTTGAATGCAGACATCACA

              *                         *                  *      *  *  * ** 
     149712 AACAAGTTTCTGAGAATGCTTCTGTCTATTTTTTATGGGAAGATATTTCCTTTTTCACCGTAGGC
        970 AAGAAGTTTCTGAGAATGCTTCTGTCTAGTTTTTAT--GAAGATATTCCCTTTTCCAACGAAATC

            *    *      *    *          * **                     *         * 
     149777 GTCAAGGCGATCGAAATGTCCACTTCCACAAACTACAAAAAGAGTGTTTCAAACCTGCTCTATGA
       1033 ATCAAAGCGATCCAAATATCCACTTCCAGATTCTACAAAAAGAGTGTTTCAAAACTGCTCTATCA

              * **       *    * *      * ** * *   **      *      *       *   
     149842 AAGGCCATGTTCATCTCTATGAGTTGAATGGAAATATCCGAAAGAAATTTCTGGGAATGCTGCTG
       1098 AAAGAAATGTTCAACTCTGTTAGTTGAGTACACACATCACAAAGAAGTTTCTGAGAATGCTTCTG

                  *         *          *  *
     149907 TCTAGTGTTTAT---ACGA-ATTCCCGCTTC
       1163 TCTAGTTTTTATGGGAAGATATTCCCGTTTT

                      *       *                *    *  *                   * 
     149934 CAACGAAATCCTCAAAGCAATCCAAATATCCACTTGCAGAATCCACAAAAAGAGTGTTTCAAAAC
          1 CAACGAAATCATCAAAGCGATCCAAATATCCACTTCCAGATTCTACAAAAAGAGTGTTTCAAACC

                    *  ** *  *          * *                   *  *           
     149999 TGCTCTATCAATAGAAAGGTTCAACTCTTTTAGTTGAGTACACACATCACGAACAAGTTTCTGAG
         66 TGCTCTA-GAAAGGGAATGTTCAACTCTGTGAGTTGAGTACACACATCACAAAGAAGTTTCTGAG

                         * *      **     **  *      *    *    *           *  
     150064 AATGCTTCTGTCTGGCTTTTATTGGAAGACGTTTCC-TTTTC-ACCAAA-GGCATCAAAGCGCTC
        130 AATGCTTCTGTCTAGTTTTTATATGAAGATATTCCCGTTTCCAACGAAATCGC-T-AAAGCGATC

                 *                * *                **     * ***  * *       
     150126 CAAATGTCCACTTCCAGATTCTTCCAAAAGAGTGTTTCAAACGTGCTCAAAGTAAGGGAATGTTC
        193 CAAATATCCACTTCCAGATTCTACAAAAAGAGTGTTTCAAAACTGCTCTATCAAAAGAAATGTTC

                    * *    * *  * *     *   *       * * *            **     *
     150191 AACTCTGTGACTTGAATGCAGATATCACCAAGTAGTTTCTAATAGTGCTTCTGTCTACATTTTAG
        258 AACTCTGTTAGTTGAGTACACACATCACAAAGAAGTTTCTGAGAATGCTTCTGTCTAGTTTTTAT

                *         *              * * *   *             *  *   *      
     150256 ATGATGATATTCCCGTTTCCAACGAAATCGTTAGAGCTATCCAAATATCCAGTTACAGTTTCTAC
        323 ATGAAGATATTCCCTTTTCCAACGAAATCATCAAAGCGATCCAAATATCCACTTCCAGATTCTAC

            *     *       *   *    **  *        *                    *       
     150321 CAAAAGGGTGTTTCCAAATTGCTGCATCAAAAGAAAGGTTCAACTCTGTTAGTTGAGGACACACA
        388 AAAAAGAGTGTTTCAAAACTGCTCTATGAAAAGAAATGTTCAACTCTGTTAGTTGAGTACACACA

                          *                   *    * **  *                   
     150386 TCACAAAGAAGTTTGTGAGAATGCTTCTGTCTAGATTTTGTATGACGATATTCCCTTTTCCAACG
        453 TCACAAAGAAGTTTCTGAGAATGCTTCTGTCTAGTTTTTATGGGAAGATATTCCCTTTTCCAACG

             * * * *     *   *       * *  *    *  *       *  *        *      
     150451 ATATCGTTAAAGCAATCTAAATATCAATTTGCAGAATCCACAAAAATAGAGTTTCAAAGCTGCTC
        518 AAAGCATCAAAGCGATCCAAATATCCACTTCCAGATTCTACAAAAAGAGTGTTTCAAAACTGCTC

             * *   *    *    *       *  *                   ***            * 
     150516 TGTAAAAAGAAAGGTTCCACTCTGTTAGCTGAGTACACACATCACAAACTTGTTTCTGAGAATCC
        583 TATCAAAGGAAATGTTCAACTCTGTGAGTTGAGTACACACATCACAAAGAAGTTTCTGAGAATGC

                    *            *      **        *  * **          *       * 
     150581 TTCTGTCTCGTTTTTATGGGAAGATATTTACTTTTCCACCGTAGGCATCAAAGCGCTCCAAATGT
        648 TTCTGTCTAGTTTTTAT--GATGATATTCCCTTTTCCAACGAAATCATCAAAGCGATCCAAATAT

                *       *  *  *  *            *          *     *   *         
     150646 CCACATCCAGATACTCCAGAACGAGTGTTTCAAACCTG-CTCTATGAAAGGGAATCTTCAACTCT
        711 CCACTTCCAGATTCTACAAAAAGAGTGTTTCAAAACTGCCTCTATCAAAGGAAATGTTCAACTCT

            * *      * *  *      *      *              *       **         ** 
     150710 ATGAGTTGAATGCAGACATCAGAAAGAAATTTCTGAGAATGCTGCTGTCTACCTTTTAT---TTG
        776 GTTAGTTGAGTACACACATCACAAAGAAGTTTCTGAGAATGCTTCTGTCTAGTTTTTATGGGAAG

                    **             *  *    *              * *  **   *    *   
     150772 A-ATTCCCGCTTCCAACGAAATCCTCCAAGCTATCCAAATATCCACCTGCATTTTCCACAACAAG
        841 ATATTCCCTTTTCCAACGAAATCATCAAAGCGATCCAAATATCCACTTCCAGATTCTACAAAAAG

                       *            **                *    *  ** *  *        
     150836 AGTGTTTCAAACCTGCTCTATCAATAGAAATGTTCAACTCCTTTG-GCTGGGTACACACATCACA
        906 AGTGTTTCAAAACTGCTCTATCAAAGGAAATGTTCAACT-CTGTGAGTTGAATGCAGACATCACA

              *                                        **         *  * *  ** 
     150900 AACAAGTTTCTGAGAATGCTTCTGTCTAGTTTTTATGGGAAGACGTTCCCTTTTTCACCAAAGGC
        970 AAGAAGTTTCTGAGAATGCTTCTGTCTAGTTTTTAT--GAAGATATTCCCTTTTCCAACGAAATC

                     *       *            **                     **       ** 
     150965 ATCAAAGCGCTCCAAATGTCCACTTCCAGACACTACAAAAAGAGTGTTTCAAACGTGCTCTAAGA
       1033 ATCAAAGCGATCCAAATATCCACTTCCAGATTCTACAAAAAGAGTGTTTCAAAACTGCTCTATCA

              ***               * *    * *  * *         *           **       
     151030 AAGCGAATGTTCAACTCTGTGACTTGAATGCAGATATCACAAAGTAGTTTCTGAGAGGGCTTCTG
       1098 AAAGAAATGTTCAACTCTGTTAGTTGAGTACACACATCACAAAGAAGTTTCTGAGAATGCTTCTG

                 *     ***  *             *
     151095 TCTAGATTTTAGATGATGATATTCCCGTTTC
       1163 TCTAGTTTTTATGGGAAGATATTCCCGTTTT

                        * *   *                *   *                    *  * 
     151126 CAACGAAATCATTAGAGCTATCCAAATATCCACTTACAGTTTCTACAAAAAGAGTGTTTCCAAAC
          1 CAACGAAATCATCAAAGCGATCCAAATATCCACTTCCAGATTCTACAAAAAGAGTGTTTCAAACC

                    **    *   *    *       *  *                   ***      * 
     151191 TGCTGCATCAAAAGAG-AGGTTCCACTCTGTTAGCTGAGTACACACATCACAAACTTGTTTCTCA
         66 TGCT-C-TAGAAAGGGAATGTTCAACTCTGTGAGTTGAGTACACACATCACAAAGAAGTTTCTGA

                *         *        **         ** *   *  *  * ** ***      *   
     151255 GAATCCTTCTGTCTCGTTTTTATGGGAAGATATTTACTTTTTCACCGTAGGCATCAAAGCGCTCC
        129 GAATGCTTCTGTCTAGTTTTTATATGAAGATATTCCCGTTTCCAACGAAATCGCTAAAGCGATCC

                *     *       *  *  *               *         *   * *   *    
     151320 AAATGTCCACATCCAGATACTCCAGAAAGAGTGTTTCAAACCTGCTCTATGAAAGGGAATCTTCA
        194 AAATATCCACTTCCAGATTCTACAAAAAGAGTGTTTCAAAACTGCTCTATCAAAAGAAATGTTCA

                 * *      * *  *      *      *              *       **       
     151385 ACTCTATGAGTTGAATGCAGACATCAGAAAGAAATTTCTGAGAATGCTGCTGTCTACCTTTTAT-
        259 ACTCTGTTAGTTGAGTACACACATCACAAAGAAGTTTCTGAGAATGCTTCTGTCTAGTTTTTATA

               *         **             *  *    *                *       *   
     151449 T--TGA-ATTCCCGCTTCCAACGAAATCCTCCAAGCTATCCAAATATCCACTTGCAGATTCCACA
        324 TGAAGATATTCCCTTTTCCAACGAAATCATCAAAGCGATCCAAATATCCACTTCCAGATTCTACA

                                        *  ** *  *                    *     *
     151511 AAAAGAGTGTTTCAAAACTGCTCTCTATCAATGGCAAAGTTCAACTCTGTTAGTTGAGGACACAT
        389 AAAAGAGTGTTTCAAAACTG--CTCTATGAAAAGAAATGTTCAACTCTGTTAGTTGAGTACACAC

                 *  *                         *                  *      *  * 
     151576 ATCACCAACAAGTTTCTGAGAATGCTTCTGTCTATTTTTTATGGGAAGATATTTCCTTTTTCACC
        452 ATCACAAAGAAGTTTCTGAGAATGCTTCTGTCTAGTTTTTATGGGAAGATATTCCCTTTTCCAAC

             * *  *    *      *    *          * **                     *     
     151641 GTAGGCGTCAAGGCGATCGAAATGTCCACTTCCACAAACTACAAAAAGAGTGTTTCAAACCTGCT
        517 GAAAGCATCAAAGCGATCCAAATATCCACTTCCAGATTCTACAAAAAGAGTGTTTCAAAACTGCT

                *     **       *    *        * ** * *   **      *      *     
     151706 CTATGAAAGGCCATGTTCATCTCTATGAGTTGAATGGAAATATCCGAAAGAAATTTCTGGGAATG
        582 CTATCAAAGGAAATGTTCAACTCTGTGAGTTGAGTACACACATCACAAAGAAGTTTCTGAGAATG

              *         *       *         **             *       *           
     151771 CTGCTGTCTAGTGTTTAT-ACGA-ATTCCCGCTTCCAACGAAATCCTCAAAGCAATCCAAATATC
        647 CTTCTGTCTAGTTTTTATGATGATATTCCCTTTTCCAACGAAATCATCAAAGCGATCCAAATATC

                 *    *  *                                 **    *          *
     151834 CACTTGCAGAATCCACAAAAAGAGTGTTTCAAAACTG-CTCTATCAATAGAAAGGTTCAACTCTT
        712 CACTTCCAGATTCTACAAAAAGAGTGTTTCAAAACTGCCTCTATCAAAGGAAATGTTCAACTCTG

                                 *  *                        * *      *      
     151898 TTAGTTGAGTACACACATCACGAACAAGTTTCTGAGAATGCTTCTGTCTGGCTTTTATTGGAAGA
        777 TTAGTTGAGTACACACATCACAAAGAAGTTTCTGAGAATGCTTCTGTCTAGTTTTTATGGGAAGA

            **  *         * *  **          *       *                * *      
     151963 CGTTTCCTTTT-CACCAAAGGCATCAAAGCGCTCCAAATGTCCACTTCCAGATTCTTCCAAAAGA
        842 TATTCCCTTTTCCAACGAAATCATCAAAGCGATCCAAATATCCACTTCCAGATTCTACAAAAAGA

                      **     * ***    *                 *           *     *  
     152027 GTGTTTCAAACGTGCTCAAAGTAAGGGAATGTTCAACTCTGTGACTTGAATGCAGATATCACCAA
        907 GTGTTTCAAAACTGCTCTATCAAAGGAAATGTTCAACTCTGTGAGTTGAATGCAGACATCACAAA

             *       * * *             *     *    *         *              * 
     152092 GTAGTTTCTAATAGTGCTTCTGTCTACATTTTAGATGATGATATTCCCGTTTCCAACGAAATCGT
        972 GAAGTTTCTGAGAATGCTTCTGTCTA-GTTTT-TATGAAGATATTCCCTTTTCCAACGAAATCAT

            * *   *             *  *   *      *     *       *   *    **      
     152157 TAGAGCTATCCAAATATCCAGTTACAGTTTCTACCAAAAGGGTGTTTCCAAATTGCTGCATCAAA
       1035 CAAAGCGATCCAAATATCCACTTCCAGATTCTACAAAAAGAGTGTTTCAAAACTGCTCTATCAAA

                 *                    *                     *                
     152222 AGAAAGGTTCAACTCTGTTAGTTGAGGACACACATCACAAAGAAGTTTGTGAGAATGCTTCTGTC
       1100 AGAAATGTTCAACTCTGTTAGTTGAGTACACACATCACAAAGAAGTTTCTGAGAATGCTTCTGTC

               *    * **  *         *   *
     152287 TAGATTTTGTATGACGATATTCCCTTTTC
       1165 TAGTTTTTATGGGAAGATATTCCCGTTTT

                  *   * *     *   *       * *  *    *  *       *  *        * 
     152316 CAACGATATCGTTAAAGCAATCTAAATATCAATTTGCAGAATCCACAAAAATAGAGTTTCAAAGC
          1 CAACGAAATCATCAAAGCGATCCAAATATCCACTTCCAGATTCTACAAAAAGAGTGTTTCAAACC

                   **   * *  *    *       *  *                   ***         
     152381 TGCTCTGTAAAAAGAAAGGTTCCACTCTGTTAGCTGAGTACACACATCACAAACTTGTTTCTGAG
         66 TGCTCT-AGAAAGGGAATGTTCAACTCTGTGAGTTGAGTACACACATCACAAAGAAGTTTCTGAG

               *         *        **         ** *      *  * ** ***      *    
     152446 AATCCTTCTGTCTCGTTTTTATGGGAAGATATTTACTTTTCCACCGTAGGCATCAAAGCGCTCCA
        130 AATGCTTCTGTCTAGTTTTTATATGAAGATATTCCCGTTTCCAACGAAATCGCTAAAGCGATCCA

               *     *       *  *  *  *            *         *   * *   *     
     152511 AATGTCCACATCCAGATACTCCAGAACGAGTGTTTCAAACCTGCTCTATGAAAGGGAATCTTCAA
        195 AATATCCACTTCCAGATTCTACAAAAAGAGTGTTTCAAAACTGCTCTATCAAAAGAAATGTTCAA

                * *      * *  *      *      *              *       **        
     152576 CTCTATGAGTTGAATGCAGACATCAGAAAGAAATTTCTGAGAATGCTGCTGTCTACCTTTTAT-T
        260 CTCTGTTAGTTGAGTACACACATCACAAAGAAGTTTCTGAGAATGCTTCTGTCTAGTTTTTATAT

              *         **             *  *    *              * *  **   *    
     152640 --TGA-ATTCCCGCTTCCAACGAAATCCTCCAAGCTATCCAAATATCCACCTGCATTTTCCACAA
        325 GAAGATATTCCCTTTTCCAACGAAATCATCAAAGCGATCCAAATATCCACTTCCAGATTCTACAA

            *                        *  *               **  * *  *           
     152702 CAAGAGTGTTTCAAAACTGCTCTATCAATAGAAATGTTCAACTCCTTTGGCTGGGTACACACATC
        390 AAAGAGTGTTTCAAAACTGCTCTATGAAAAGAAATGTTCAACTCTGTTAGTTGAGTACACACATC

                 *                                        **         *  * *  
     152767 ACAAACAAGTTTCTGAGAATGCTTCTGTCTAGTTTTTATGGGAAGACGTTCCCTTTTTCACCAAA
        455 ACAAAGAAGTTTCTGAGAATGCTTCTGTCTAGTTTTTATGGGAAGATATTCCCTTTTCCAACGAA

            *           *       *            **                     **       
     152832 GGCATCAAAGCGCTCCAAATGTCCACTTCCAGACACTACAAAAAGAGTGTTTCAAACGTGCTCTA
        520 AGCATCAAAGCGATCCAAATATCCACTTCCAGATTCTACAAAAAGAGTGTTTCAAAACTGCTCTA

            **    **                 *    * *  * *         *           **    
     152897 AGAAAGCGAATGTTCAACTCTGTGACTTGAATGCAGATATCACAAAGTAGTTTCTGAGAGGGCTT
        585 TCAAAGGAAATGTTCAACTCTGTGAGTTGAGTACACACATCACAAAGAAGTTTCTGAGAATGCTT

                          *              *                * *   *            
     152962 CTGTCTAGATTTTAGATGATGATATTCCCGTTTCCAACGAAATCATTAGAGCTATCCAAATATCC
        650 CTGTCTAG-TTTT-TATGATGATATTCCCTTTTCCAACGAAATCATCAAAGCGATCCAAATATCC

                *   *                    *         **      *  * *    *       
     153027 ACTTACAGTTTCTACAAAAAGAGTGTTTCCAAACTG-CTGCATCAAAAGAGAGGTTCCACTCTGT
        713 ACTTCCAGATTCTACAAAAAGAGTGTTTCAAAACTGCCTCTATCAAAGGAAATGTTCAACTCTGT

               *                   ***      *     *         *             *  
     153091 TAGCTGAGTACACACATCACAAACTTGTTTCTCAGAATCCTTCTGTCTCGTTTTTATGGGAAAAT
        778 TAGTTGAGTACACACATCACAAAGAAGTTTCTGAGAATGCTTCTGTCTAGTTTTTATGGGAAGAT

               **     *  *  * **          *       *     *       *  *  *      
     153156 ATTTACTTTTTCACCGTAGGCATCAAAGCGCTCCAAATGTCCACATCCAGATACTCCAGAAAGAG
        843 ATTCCCTTTTCCAACGAAATCATCAAAGCGATCCAAATATCCACTTCCAGATTCTACAAAAAGAG

                     *         *     *   *         *                    *    
     153221 TGTTTCAAACCTGCTCTATGAAAGGGAATCTTCAACTCTATGAGTTGAATGCAGACATCAGAAAG
        908 TGTTTCAAAACTGCTCTATCAAAGGAAATGTTCAACTCTGTGAGTTGAATGCAGACATCACAAAG

              *              *       **       **         **             *  * 
     153286 AAATTTCTGAGAATGCTGCTGTCTACCTTTTAT-TTGA-ATTCCCGCTTCCAACGAAATCCTCCA
        973 AAGTTTCTGAGAATGCTTCTGTCTAGTTTTTATGAAGATATTCCCTTTTCCAACGAAATCATCAA

               *                *       *                                  **
     153349 AGCTATCCAAATATCCACTTGCAGATTCCACAAAAAGAGTGTTTCAAAACTGCTCTCTATCAATG
       1038 AGCGATCCAAATATCCACTTCCAGATTCTACAAAAAGAGTGTTTCAAAACTG--CTCTATCAAAA

             *  *                    *     *     *  *                        
     153414 GCAAAGTTCAACTCTGTTAGTTGAGGACACATATCACCAACAAGTTTCTGAGAATGCTTCTGTCT
       1101 GAAATGTTCAACTCTGTTAGTTGAGTACACACATCACAAAGAAGTTTCTGAGAATGCTTCTGTCT

             *                  *  *    
     153479 ATTTTTTATGGGAAGATATTTCCTTTTT
       1166 AGTTTTTATGGGAAGATATTCCCGTTTT

              *  * ** *    *      *    *          * **                       
     153507 CACCGTAGGCGTCAAGGCGATCGAAATGTCCACTTCCACAAACTACAAAAAGAGTGTTTCAAACC
          1 CAACGAAATCATCAAAGCGATCCAAATATCCACTTCCAGATTCTACAAAAAGAGTGTTTCAAACC

                          **       *    *        * ** * *   **      *      * 
     153572 TGCTCTATGAAAGGCCATGTTCATCTCTATGAGTTGAATGGAAATATCCGAAAGAAATTTCTGGG
         66 TGCTCTA-GAAAGGGAATGTTCAACTCTGTGAGTTGAGTACACACATCACAAAGAAGTTTCTGAG

                  *          *        *          *                      *    
     153637 AATGCTGCTGTCTAG-TGTT-TAT-ACGA-ATTCCCGCTTCCAACGAAATC-CTCAAAGCAATCC
        130 AATGCTTCTGTCTAGTTTTTATATGAAGATATTCCCGTTTCCAACGAAATCGCT-AAAGCGATCC

                        *    *  *                                *     *     
     153697 AAATATCCACTTGCAGAATCCACAAAAAGAGTGTTTCAAAACTGCTCTATCAATAGAAAGGTTCA
        194 AAATATCCACTTCCAGATTCTACAAAAAGAGTGTTTCAAAACTGCTCTATCAAAAGAAATGTTCA

                 *                        *                        * *      *
     153762 ACTCTTTTAGTTGAGTACACACATCACAAACAAGTTTCTGAGAATGCTTCTGTCTGGCTTTTATT
        259 ACTCTGTTAGTTGAGTACACACATCACAAAGAAGTTTCTGAGAATGCTTCTGTCTAGTTTTTATA

            *     **  *         * *  **          *       *                * *
     153827 GGAAGACGTTTCCTTTT-CACCAAAGGCATCAAAGCGCTCCAAATGTCCACTTCCAGATTCTTCC
        324 TGAAGATATTCCCTTTTCCAACGAAATCATCAAAGCGATCCAAATATCCACTTCCAGATTCTACA

                            **     * * *  * *               * *    * *  * *  
     153891 AAAAGAGTGTTTCAAACGTGCTCAAAGTAAGGGAATGTTCAACTCTGTGACTTGAATGCAGATAT
        389 AAAAGAGTGTTTCAAAACTGCTCTATGAAAAGAAATGTTCAACTCTGTTAGTTGAGTACACACAT

               *   *       * * *             *     ***  *         *          
     153956 CACCAAGTAGTTTCTAATAGTGCTTCTGTCTAGATTTTAGATGATGATATTCCCGTTTCCAACGA
        454 CACAAAGAAGTTTCTGAGAATGCTTCTGTCTAGTTTTTATGGGAAGATATTCCCTTTTCCAACGA

              * * * *   *             *  *   *                    *        **
     154021 AATCGTTAGAGCTATCCAAATATCCAGTTACAGTTTCTACAAAAAGAGTGTTTCCAAACTGCTGC
        519 AAGCATCAAAGCGATCCAAATATCCACTTCCAGATTCTACAAAAAGAGTGTTTCAAAACTGCTCT

                  *    *            *       *                     *          
     154086 ATCAAAAGAAAGGTTCAACTCTGTTAGTTGAGGACACACATCACAAAGAAGTTTGTGAGAATGCT
        584 ATCAAAGGAAATGTTCAACTCTGTGAGTTGAGTACACACATCACAAAGAAGTTTCTGAGAATGCT

                                *                    *   * *     *   *       
     154151 TCTGTCTAGATTTTGTATGACGATATTCCCTTTTCCAACGATATCGTTAAAGCAATCTAAATATC
        649 TCTGTCTAG-TTTT-TATGATGATATTCCCTTTTCCAACGAAATCATCAAAGCGATCCAAATATC

            * *  *    *  *       *  *        *        * *   **   *    *      
     154216 AATTTGCAGAATCCACAAAAATAGAGTTTCAAAGCTG-CTCTGTAAAAATAAAAGTTCCACTCTG
        712 CACTTCCAGATTCTACAAAAAGAGTGTTTCAAAACTGCCTCTATCAAAGGAAATGTTCAACTCTG

                *                   ***            *         *               
     154280 TTAGCTGAGTACACACATCACAAACTTGTTTCTGAGAATCCTTCTGTCTCGTTTTTATGGGAAGA
        777 TTAGTTGAGTACACACATCACAAAGAAGTTTCTGAGAATGCTTCTGTCTAGTTTTTATGGGAAGA

                **     *  *  * **          *       *     *       *  *  *     
     154345 TATTTACTTTTTCACCGTAGGCATCAAAGCGCTCCAAATGTCCACATCCAGATACTCCAGAAAGA
        842 TATTCCCTTTTCCAACGAAATCATCAAAGCGATCCAAATATCCACTTCCAGATTCTACAAAAAGA

            *         *         *     *   *         *                    *   
     154410 CTGTTTCAAACCTGCTCTATGAAAGGGAATCTTCAACTCTATGAGTTGAATGCAGACATCAGAAA
        907 GTGTTTCAAAACTGCTCTATCAAAGGAAATGTTCAACTCTGTGAGTTGAATGCAGACATCACAAA

               *              *       **       **         **             *  *
     154475 GAAATTTCTGAGAATGCTGCTGTCTACCTTTTAT-TTGA-ATTCCCGCTTCCAACGAAATCCTCC
        972 GAAGTTTCTGAGAATGCTTCTGTCTAGTTTTTATGAAGATATTCCCTTTTCCAACGAAATCATCA

                *                *  **   *    *                           *  
     154538 AAGCTATCCAAATATCCACTTGCATTTTCCACAACAAGAGTGTTTCAAAACTGCTCTATCAATAG
       1037 AAGCGATCCAAATATCCACTTCCAGATTCTACAAAAAGAGTGTTTCAAAACTGCTCTATCAAAAG

                         **    *  *                *                         
     154603 AAATGTTCAACTCCTTTAGCTGGGTACACACATCACAAACAAGTTTCTGAGAATGCTTCTGTCTA
       1102 AAATGTTCAACTCTGTTAGTTGAGTACACACATCACAAAGAAGTTTCTGAGAATGCTTCTGTCTA

                           **     *    
     154668 GTTTTTATGGGAAGACGTTCCCTTTTT
       1167 GTTTTTATGGGAAGATATTCCCGTTTT

              * *  **          *       *            **                      *
     154695 CACCAAAGGCATCAAAGCGCTCCAAATGTCCACTTCCAGACACTACAAAAAGAGTGTTTCAAACG
          1 CAACGAAATCATCAAAGCGATCCAAATATCCACTTCCAGATTCTACAAAAAGAGTGTTTCAAACC

                         *                  *    * *  * *         *    *     
     154760 TGCTCTAAGAAAGCGAATGTTCAACTCTGTGACTTGAATGCAGATATCACAAAGTAGTTACTGAG
         66 TGCTCT-AGAAAGGGAATGTTCAACTCTGTGAGTTGAGTACACACATCACAAAGAAGTTTCTGAG

             **            *     *    *                        **  *   *     
     154825 AGGGCTTCTGTCTAGATTTTAGATGATGATATTCCCGTTTCCAACGAAATCATTAGAGCTATCCA
        130 AATGCTTCTGTCTAGTTTTTATATGAAGATATTCCCGTTTCCAACGAAATCGCTAAAGCGATCCA

                       *   *                    *        **         * *    * 
     154890 AATATCCACTTACAGTTTCTACAAAAAGAGTGTTTCCAAACTGCTGCATCAAAAGAGAGGTTCCA
        195 AATATCCACTTCCAGATTCTACAAAAAGAGTGTTTCAAAACTGCTCTATCAAAAGAAATGTTCAA

                     **                  ***      *     *         *        **
     154955 CTCTGTTAGCCGAGTACACACATCACAAACTTGTTTCTCAGAATCCTTCTGTCTCGTTTTTATGG
        260 CTCTGTTAGTTGAGTACACACATCACAAAGAAGTTTCTGAGAATGCTTCTGTCTAGTTTTTATAT

                     **     *  *  * **          *       *     *       *  *  *
     155020 GAAGATATTTACTTTTTCACCGTAGGCATCAAAGCGCTCCAAATGTCCACATCCAGATACTCCAG
        325 GAAGATATTCCCTTTTCCAACGAAATCATCAAAGCGATCCAAATATCCACTTCCAGATTCTACAA

                           *             * *   *         * *      * *  *     
     155085 AAAGAGTGTTTCAAACCTGCTCTATGAAAGGGAATCTTCAACTCTATGAGTTGAATGCAGACATC
        390 AAAGAGTGTTTCAAAACTGCTCTATGAAAAGAAATGTTCAACTCTGTTAGTTGAGTACACACATC

             *      *              *       **         **         **          
     155150 AGAAAGAAATTTCTGAGAATGCTGCTGTCTACCTTTTAT---TTGA-ATTCCCGCTTCCAACGAA
        455 ACAAAGAAGTTTCTGAGAATGCTTCTGTCTAGTTTTTATGGGAAGATATTCCCTTTTCCAACGAA

             * *  *    *                *       *                            
     155211 ATCCTCCAAGCTATCCAAATATCCACTTGCAGATTCCACAAAAAGAGTGTTTCAAAACTGCTCTC
        520 AGCATCAAAGCGATCCAAATATCCACTTCCAGATTCTACAAAAAGAGTGTTTCAAAACTG--CTC

                  *  *  *            *       *     *     *  *                
     155276 TATCAATGGCAAAGTTCAACTCTGTTAGTTGAGGACACATATCACCAACAAGTTTCTGAGAATGC
        583 TATCAAAGGAAATGTTCAACTCTGTGAGTTGAGTACACACATCACAAAGAAGTTTCTGAGAATGC

                     *           *      *      *  *  * ** *    *      *    * 
     155341 TTCTGTCTATTTTTTATGGGAAGATATTTCCTTTTTCACCGTAGGCGTCAAGGCGATCGAAATGT
        648 TTCTGTCTAGTTTTTAT--GATGATATTCCCTTTTCCAACGAAATCATCAAAGCGATCCAAATAT

                     * **                     *          *     **       *    
     155406 CCACTTCCACAAACTACAAAAAGAGTGTTTCAAACCTG-CTCTATGAAAGGCCATGTTCATCTCT
        711 CCACTTCCAGATTCTACAAAAAGAGTGTTTCAAAACTGCCTCTATCAAAGGAAATGTTCAACTCT

            * *      * ** * *   **      *      *       *         *         * 
     155470 ATGAGTTGAATGGAAATATCCGAAAGAAATTTCTGGGAATGCTGCTGTCTAGTGTTTAT---ACG
        776 GTTAGTTGAGTACACACATCACAAAGAAGTTTCTGAGAATGCTTCTGTCTAGTTTTTATGGGAAG

                    **             *       *                *    *  *        
     155532 A-ATTCCCGCTTCCAACGAAATCCTCAAAGCAATCCAAATATCCACTTGCAGAATCCACAAAAAG
        841 ATATTCCCTTTTCCAACGAAATCATCAAAGCGATCCAAATATCCACTTCCAGATTCTACAAAAAG

                                    **    *          * *      * *  *       * 
     155596 AGTGTTTCAAAACTGCTCTATCAATAGAAAGGTTCAACTCTTTTAGTTGAGTACACACATCACGA
        906 AGTGTTTCAAAACTGCTCTATCAAAGGAAATGTTCAACTCTGTGAGTTGAATGCAGACATCACAA

             *                        * *             **  *         * *  **  
     155661 ACAAGTTTCTGAGAATGCTTCTGTCTGGCTTTTATTGGAAGACGTTTCCTTTT-CACCAAAGGCA
        971 AGAAGTTTCTGAGAATGCTTCTGTCTAGTTTTTA-T-GAAGATATTCCCTTTTCCAACGAAATCA

                    *       *                * *                **     * *** 
     155725 TCAAAGCGCTCCAAATGTCCACTTCCAGATTCTTCCAAAAGAGTGTTTCAAACGTGCTCAAAGTA
       1034 TCAAAGCGATCCAAATATCCACTTCCAGATTCTACAAAAAGAGTGTTTCAAAACTGCTCTATCAA

             * *               * *    * *  * *     *   *       * * *         
     155790 AGGGAATGTTCAACTCTGTGACTTGAATGCAGATATCACCAAGTAGTTTCTAATAGTGCTTCTGT
       1099 AAGAAATGTTCAACTCTGTTAGTTGAGTACACACATCACAAAGAAGTTTCTGAGAATGCTTCTGT

               **     ***  *         *   *
     155855 CTACATTTTAGATGATGATATTCCCTTTTC
       1164 CTAGTTTTTATGGGAAGATATTCCCGTTTT

                      * * *   *             *  *   *      *     *       *  **
     155885 CAACGAAATCGTTAGAGCTATCCAAATATCCAGTTACAGTTTCTACCAAAAGGGTGTTTCCAAAT
          1 CAACGAAATCATCAAAGCGATCCAAATATCCACTTCCAGATTCTACAAAAAGAGTGTTTCAAACC

                  ***   * *  *            *       *                     *    
     155950 TGCTGCATCAAAAGAAAGGTTCAACTCTGTTAGTTGAGGACACACATCACAAAGAAGTTTGTGAG
         66 TGCT-CTAGAAAGGGAATGTTCAACTCTGTGAGTTGAGTACACACATCACAAAGAAGTTTCTGAG

                           *    *     *         *          *    *      *   * 
     156015 AATGCTTCTGTCTAGATTTTGTATGACGATATTCCCTTTTCCAACGATATCGTTAAAGCAATCTA
        130 AATGCTTCTGTCTAGTTTTTATATGAAGATATTCCCGTTTCCAACGAAATCGCTAAAGCGATCCA

                  * *  *    *  *       *  *        *       * *        *    * 
     156080 AATATCAATTTGCAGAATCCACAAAAATAGAGTTTCAAAGCTGCTCTGTAAAAAGAAAGGTTCCA
        195 AATATCCACTTCCAGATTCTACAAAAAGAGTGTTTCAAAACTGCTCTATCAAAAGAAATGTTCAA

                     *                   ***            *         *        **
     156145 CTCTGTTAGCTGAGTACACACATCACAAACTTGTTTCTGAGAATCCTTCTGTCTCGTTTTTATGG
        260 CTCTGTTAGTTGAGTACACACATCACAAAGAAGTTTCTGAGAATGCTTCTGTCTAGTTTTTATAT

                     **     *  *  * **          *       *     *       *  *  *
     156210 GAAGATATTTACTTTTTCACCGTAGGCATCAAAGCGCTCCAAATGTCCACATCCAGATACTCCAG
        325 GAAGATATTCCCTTTTCCAACGAAATCATCAAAGCGATCCAAATATCCACTTCCAGATTCTACAA

              *            *             * *   *         * *      * *  *     
     156275 AACGAGTGTTTCAAACCTGCTCTATGAAAGGGAATCTTCAACTCTATGAGTTGAATGCAGACATC
        390 AAAGAGTGTTTCAAAACTGCTCTATGAAAAGAAATGTTCAACTCTGTTAGTTGAGTACACACATC

             *      *              *       **         **         **          
     156340 AGAAAGAAATTTCTGAGAATGCTGCTGTCTACCTTTTAT---TTGA-ATTCCCGCTTCCAACGAA
        455 ACAAAGAAGTTTCTGAGAATGCTTCTGTCTAGTTTTTATGGGAAGATATTCCCTTTTCCAACGAA

             * *  *    *                *       *                            
     156401 ATCCTCCAAGCTATCCAAATATCCACTTGCAGATTCCACAAAAAGAGTGTTTCAAAACTGCTCTC
        520 AGCATCAAAGCGATCCAAATATCCACTTCCAGATTCTACAAAAAGAGTGTTTCAAAACTG--CTC

                  *  *  *            *       *     *     *  *                
     156466 TATCAATGGCAAAGTTCAACTCTGTTAGTTGAGGACACATATCACCAACAAGTTTCTGAGAATGC
        583 TATCAAAGGAAATGTTCAACTCTGTGAGTTGAGTACACACATCACAAAGAAGTTTCTGAGAATGC

                     *           *      *      *  *  * ** *    *      *    * 
     156531 TTCTGTCTATTTTTTATGGGAAGATATTTCCTTTTTCAGCGTAGGCGTCAAGGCGATCGAAATGT
        648 TTCTGTCTAGTTTTTAT--GATGATATTCCCTTTTCCAACGAAATCATCAAAGCGATCCAAATAT

                     * **                     *          *     **       *    
     156596 CCACTTCCACAAACTACAAAAAGAGTGTTTCAAACCTG-CTCTATGAAAGGCCATGTTCATCTCT
        711 CCACTTCCAGATTCTACAAAAAGAGTGTTTCAAAACTGCCTCTATCAAAGGAAATGTTCAACTCT

            * *      * ** * *   **      *      *       *         *         * 
     156660 ATGAGTTGAATGGAAATATCCGAAAGAAATTTCTGGGAATGCTGCTGTCTAGTGTTTAT---ACG
        776 GTTAGTTGAGTACACACATCACAAAGAAGTTTCTGAGAATGCTTCTGTCTAGTTTTTATGGGAAG

                    **             *       *                *    *  *        
     156722 A-ATTCCCGCTTCCAACGAAATCCTCAAAGCAATCCAAATATCCACTTGCAGAATCCACAAAAAG
        841 ATATTCCCTTTTCCAACGAAATCATCAAAGCGATCCAAATATCCACTTCCAGATTCTACAAAAAG

                                    **    *          * *      * *  *       * 
     156786 AGTGTTTCAAAACTGCTCTATCAATAGAAAGGTTCAACTCTTTTAGTTGAGTACACACATCACGA
        906 AGTGTTTCAAAACTGCTCTATCAAAGGAAATGTTCAACTCTGTGAGTTGAATGCAGACATCACAA

             *                        * *             **  *         * *  **  
     156851 ACAAGTTTCTGAGAATGCTTCTGTCTGGCTTTTATTGGAAGACGTTTCCTTTT-CACCAAAGGCA
        971 AGAAGTTTCTGAGAATGCTTCTGTCTAGTTTTTA-T-GAAGATATTCCCTTTTCCAACGAAATCA

                    *       *                * *                **     * *** 
     156915 TCAAAGCGCTCCAAATGTCCACTTCCAGATTCTTCCAAAAGAGTGTTTCAAACGTGCTCAAAGTA
       1034 TCAAAGCGATCCAAATATCCACTTCCAGATTCTACAAAAAGAGTGTTTCAAAACTGCTCTATCAA

             * *               * *    * *  * *     *   *       * * *         
     156980 AGGGAATGTTCAACTCTGTGACTTGAATGCAGATATCACCAAGTAGTTTCTAATAGTGCTTCTGT
       1099 AAGAAATGTTCAACTCTGTTAGTTGAGTACACACATCACAAAGAAGTTTCTGAGAATGCTTCTGT

               **     ***  *             *
     157045 CTACATTTTAGATGATGATATTCCCGTTTC
       1164 CTAGTTTTTATGGGAAGATATTCCCGTTTT

                      * * *   *             *  *   *      *     *       *  **
     157075 CAACGAAATCGTTAGAGCTATCCAAATATCCAGTTACAGTTTCTACCAAAAGGGTGTTTCCAAAT
          1 CAACGAAATCATCAAAGCGATCCAAATATCCACTTCCAGATTCTACAAAAAGAGTGTTTCAAACC

                  ***   * *  *            *       *                     *    
     157140 TGCTGCATCAAAAGAAAGGTTCAACTCTGTTAGTTGAGGACACACATCACAAAGAAGTTTGTGAG
         66 TGCT-CTAGAAAGGGAATGTTCAACTCTGTGAGTTGAGTACACACATCACAAAGAAGTTTCTGAG

                           *    *     *         *          *    *      *   * 
     157205 AATGCTTCTGTCTAGATTTTGTATGACGATATTCCCTTTTCCAACGATATCGTTAAAGCAATCTA
        130 AATGCTTCTGTCTAGTTTTTATATGAAGATATTCCCGTTTCCAACGAAATCGCTAAAGCGATCCA

                  * *  *    *  *       *  *        *       * *        *    * 
     157270 AATATCAATTTGCAGAATCCACAAAAATAGAGTTTCAAAGCTGCTCTGTAAAAAGAAAGGTTCCA
        195 AATATCCACTTCCAGATTCTACAAAAAGAGTGTTTCAAAACTGCTCTATCAAAAGAAATGTTCAA

                     *                   ***            *         *        **
     157335 CTCTGTTAGCTGAGTACACACATCACAAACTTGTTTCTGAGAATCCTTCTGTCTCGTTTTTATGG
        260 CTCTGTTAGTTGAGTACACACATCACAAAGAAGTTTCTGAGAATGCTTCTGTCTAGTTTTTATAT

                     **        *  * **          *       *     *       *  *  *
     157400 GAAGATATTTACTTTTCCACCGTAGGCATCAAAGCGCTCCAAATGTCCACATCCAGATACTCCAG
        325 GAAGATATTCCCTTTTCCAACGAAATCATCAAAGCGATCCAAATATCCACTTCCAGATTCTACAA

              *            *             * *   *         * *      * *  *     
     157465 AACGAGTGTTTCAAACCTGCTCTATGAAAGGGAATCTTCAACTCTATGAGTTGAATGCAGACATC
        390 AAAGAGTGTTTCAAAACTGCTCTATGAAAAGAAATGTTCAACTCTGTTAGTTGAGTACACACATC

             *      *              *       **         **         **          
     157530 AGAAAGAAATTTCTGAGAATGCTGCTGTCTACCTTTTAT---TTGA-ATTCCCGCTTCCAACGAA
        455 ACAAAGAAGTTTCTGAGAATGCTTCTGTCTAGTTTTTATGGGAAGATATTCCCTTTTCCAACGAA

             * *  *    *              * *  **   *    *                       
     157591 ATCCTCCAAGCTATCCAAATATCCACCTGCATTTTCCACAACAAGAGTGTTTCAAAACTGCTCTA
        520 AGCATCAAAGCGATCCAAATATCCACTTCCAGATTCTACAAAAAGAGTGTTTCAAAACTGCTCTA

                **                *    *  *                *                 
     157656 TCAATAGAAATGTTCAACTCCTTTG-GCTGGGTACACACATCACAAACAAGTTTCTGAGAATGCT
        585 TCAAAGGAAATGTTCAACT-CTGTGAGTTGAGTACACACATCACAAAGAAGTTTCTGAGAATGCT

                                *  **         *  * *  **          *       *  
     157720 TCTGTCTAGTTTTTATGGGAAGACGTTCCCTTTTTCACCAAAGGCATCAAAGCGCTCCAAATGTC
        649 TCTGTCTAGTTTTTAT--GATGATATTCCCTTTTCCAACGAAATCATCAAAGCGATCCAAATATC

                      **                     **        **    **              
     157785 CACTTCCAGACACTACAAAAAGAGTGTTTCAAACGTG-CTCTAAGAAAGCGAATGTTCAACTCTG
        712 CACTTCCAGATTCTACAAAAAGAGTGTTTCAAAACTGCCTCTATCAAAGGAAATGTTCAACTCTG

             * *    * *  * *         *           **            *     ***  *  
     157849 TGACTTGAATGCAGATATCACAAAGTAGTTTCTGAGAGGGCTTCTGTCTAGATTTTAGATGATGA
        777 TTAGTTGAGTACACACATCACAAAGAAGTTTCTGAGAATGCTTCTGTCTAGTTTTTATGGGAAGA

                   *                * *   *                *   *             
     157914 TATTCCCGTTTCCAACGAAATCATTAGAGCTATCCAAATATCCACTTACAGTTTCTACAAAAAGA
        842 TATTCCCTTTTCCAACGAAATCATCAAAGCGATCCAAATATCCACTTCCAGATTCTACAAAAAGA

                   *        **      *  * *    *       *  *   * *  *          
     157979 GTGTTTCCAAACTGCTGCATCAAAAGAGAGGTTCCACTCTGTTAGCTGAGTACACACATCACAAA
        907 GTGTTTCAAAACTGCTCTATCAAAGGAAATGTTCAACTCTGTGAGTTGAATGCAGACATCACAAA

            ***      *     *         *                   **     *  *  * **   
     158044 CTTGTTTCTCAGAATCCTTCTGTCTCGTTTTTATGGGAAGATATTTACTTTTTCACCGTAGGCAT
        972 GAAGTTTCTGAGAATGCTTCTGTCTAGTTTTTAT--GAAGATATTCCCTTTTCCAACGAAATCAT

                   *       *     *       *  *  *               *         *   
     158109 CAAAGCGCTCCAAATGTCCACATCCAGATACTCCAGAAAGAGTGTTTCAAACCTGCTCTATGAAA
       1035 CAAAGCGATCCAAATATCCACTTCCAGATTCTACAAAAAGAGTGTTTCAAAACTGCTCTATCAAA

            * *   *         * *      * *  *      *      *              *     
     158174 GGGAATCTTCAACTCTATGAGTTGAATGCAGACATCAGAAAGAAATTTCTGAGAATGCTGCTGTC
       1100 AGAAATGTTCAACTCTGTTAGTTGAGTACACACATCACAAAGAAGTTTCTGAGAATGCTTCTGTC

              **         **          *  *
     158239 TACCTTTTAT---TTGA-ATTCCCGCTTC
       1165 TAGTTTTTATGGGAAGATATTCCCGTTTT

                      *  *    *                *       *                   * 
     158264 CAACGAAATCCTCCAAGCTATCCAAATATCCACTTGCAGATTCCACAAAAAGAGTGTTTCAAAAC
          1 CAACGAAATCATCAAAGCGATCCAAATATCCACTTCCAGATTCTACAAAAAGAGTGTTTCAAACC

                      *  *  *  *            *       *     *     *  *         
     158329 TGCTCTCTATCAATGGCAAAGTTCAACTCTGTTAGTTGAGGACACATATCACCAACAAGTTTCTG
         66 TG--CTCTA-GAAAGGGAATGTTCAACTCTGTGAGTTGAGTACACACATCACAAAGAAGTTTCTG

                            *       **         *  *   *  *  * **  **  *      
     158394 AGAATGCTTCTGTCTATTTTTTATGGGAAGATATTTCCTTTTTCACCGTAGGCGTCAAGGCGATC
        128 AGAATGCTTCTGTCTAGTTTTTATATGAAGATATTCCCGTTTCCAACGAAATCGCTAAAGCGATC

            *    *          * **                     *         *   * **      
     158459 GAAATGTCCACTTCCACAAACTACAAAAAGAGTGTTTCAAACCTGCTCTATGAAAGGCCATGTTC
        193 CAAATATCCACTTCCAGATTCTACAAAAAGAGTGTTTCAAAACTGCTCTATCAAAAGAAATGTTC

             *    * *      * ** * *   **      *      *       *          *    
     158524 ATCTCTATGAGTTGAATGGAAATATCCGAAAGAAATTTCTGGGAATGCTGCTGTCTAG-TGTT-T
        258 AACTCTGTTAGTTGAGTACACACATCACAAAGAAGTTTCTGAGAATGCTTCTGTCTAGTTTTTAT

                *         **        *    *       *                *    *  *  
     158587 AT-ACGA-ATTCCCGCTTCCAACGTAATCCTCAAAGCAATCCAAATATCCACTTGCAGAATCCAC
        323 ATGAAGATATTCCCTTTTCCAACGAAATCATCAAAGCGATCCAAATATCCACTTCCAGATTCTAC

                                       *  *     *          *                 
     158650 AAAAAGAGTGTTTCAAAACTGCTCTATCAATAGAAAGGTTCAACTCTTTTAGTTGAGTACACACA
        388 AAAAAGAGTGTTTCAAAACTGCTCTATGAAAAGAAATGTTCAACTCTGTTAGTTGAGTACACACA

                *  *                        * *      *      **  *         * *
     158715 TCACGAACAAGTTTCTGAGAATGCTTCTGTCTGGCTTTTATTGGAAGACGTTTCCTTTT-CACCA
        453 TCACAAAGAAGTTTCTGAGAATGCTTCTGTCTAGTTTTTATGGGAAGATATTCCCTTTTCCAACG

              *           *       *                * *                **     
     158779 AAGGCATCAAAGCGCTCCAAATGTCCACTTCCAGATTCTTCCAAAAGAGTGTTTCAAACGTGCTC
        518 AAAGCATCAAAGCGATCCAAATATCCACTTCCAGATTCTACAAAAAGAGTGTTTCAAAACTGCTC

            * ***    *                 *    * *  * *     *   *       * * *   
     158844 AAAGTAAGGGAATGTTCAACTCTGTGACTTGAATGCAGATATCACCAAGTAGTTTCTAATAGTGC
        583 TATCAAAGGAAATGTTCAACTCTGTGAGTTGAGTACACACATCACAAAGAAGTTTCTGAGAATGC

                      *     *              *              *** *   *          
     158909 TTCTGTCTACATTTTAGATGATGATATTCCCGTTTCCAACGAAATCGCTAGAGCTATCCAAATAT
        648 TTCTGTCTA-GTTTT-TATGATGATATTCCCTTTTCCAACGAAATCATCAAAGCGATCCAAATAT

               *  *   *      *     *       *   *     **      *    *          
     158974 CCAGTTACAGTTTCTACCAAAAGGGTGTTTCCAAATTG-CTGCATCAAAAGAAAGGTTCAACTCT
        711 CCACTTCCAGATTCTACAAAAAGAGTGTTTCAAAACTGCCTCTATCAAAGGAAATGTTCAACTCT

                      *                     *                   *    * **  * 
     159038 GTTAGTTGAGGACACACATCACAAAGAAGTTTGTGAGAATGCTTCTGTCTAGGTTTTGTATGACG
        776 GTTAGTTGAGTACACACATCACAAAGAAGTTTCTGAGAATGCTTCTGTCTAGTTTTTATGGGAAG

                               *   * *     *   *       * *  *    *  *       *
     159103 ATATTCCCTTTTCCAACGATATCGTTAAAGCAATCTAAATATCAATTTGCAGAATCCACAAAAAT
        841 ATATTCCCTTTTCCAACGAAATCATCAAAGCGATCCAAATATCCACTTCCAGATTCTACAAAAAG

              *        *       * *   *    *    *       *  *   * *  *         
     159168 AGAGTTTCAAAGCTGCTCTGTAAAAAGAAAGGTTCCACTCTGTTAGCTGAGTACACACATCACAA
        906 AGTGTTTCAAAACTGCTCTATCAAAGGAAATGTTCAACTCTGTGAGTTGAATGCAGACATCACAA

             ***      *     *         *                 * **        *  * **  
     159233 ACTTGTTTCTCAGAATCCTTCTGTCTCGTTTTTATGGGAAGATACTTACTTTTCCACCGTAGGCA
        971 AGAAGTTTCTGAGAATGCTTCTGTCTAGTTTTTAT--GAAGATATTCCCTTTTCCAACGAAATCA

                    *       *     *       *  *  *  *            *         *  
     159298 TCAAAGCGCTCCAAATGTCCACATCCAGATACTCCAGAACGAGTGTTTCAAACCTGCTCTATGAA
       1034 TCAAAGCGATCCAAATATCCACTTCCAGATTCTACAAAAAGAGTGTTTCAAAACTGCTCTATCAA

             * *   *         * *      * *  *      *      *              *    
     159363 AGGGAATCTTCAACTCTATGAGTTGAATGCAGACATCAGAAAGAAATTTCTGAGAATGCTGCTGT
       1099 AAGAAATGTTCAACTCTGTTAGTTGAGTACACACATCACAAAGAAGTTTCTGAGAATGCTTCTGT

               **         **          *  *
     159428 CTACCTTTTAT---TTGA-ATTCCCGCTTC
       1164 CTAGTTTTTATGGGAAGATATTCCCGTTTT

                      *  *    *              * *  **   *    *              * 
     159454 CAACGAAATCCTCCAAGCTATCCAAATATCCACCTGCATTTTCCACAACAAGAGTGTTTCAAAAC
          1 CAACGAAATCATCAAAGCGATCCAAATATCCACTTCCAGATTCTACAAAAAGAGTGTTTCAAACC

                    *  ** *              *    *  *                *          
     159519 TGCTCTATCAATAGAAATGTTCAACTCCTTTG-GCTGGGTACACACATCACAAACAAGTTTCTGA
         66 TGCTCTA-GAAAGGGAATGTTCAACT-CTGTGAGTTGAGTACACACATCACAAAGAAGTTTCTGA

                                   **     **     *   *    *    *           * 
     159583 GAATGCTTCTGTCTAGTTTTTATGGGAAGACGTTCCCTTTTTC-ACCAAA-GGCATCAAAGCGCT
        129 GAATGCTTCTGTCTAGTTTTTATATGAAGATATTCCCGTTTCCAACGAAATCGC-T-AAAGCGAT

                  *            **                     **       **   ***      
     159646 CCAAATGTCCACTTCCAGACACTACAAAAAGAGTGTTTCAAACGTGCTCTAAGAAAGCGAATGTT
        192 CCAAATATCCACTTCCAGATTCTACAAAAAGAGTGTTTCAAAACTGCTCTATCAAAAGAAATGTT

                     * *    * *    *         *           **            *     
     159711 CAACTCTGTGACTTGAATGCACATATCACAAAGTAGTTTCTGAGAGGGCTTCTGTCTAGATTTTA
        257 CAACTCTGTTAGTTGAGTACACACATCACAAAGAAGTTTCTGAGAATGCTTCTGTCTAGTTTTTA

            *    *         *        *       * *    *               *   *     
     159776 GATGATGATATTCCCGTTTCCAACAAAATCATTAGAGC-TTCCAAATATCCACTTACAGTTTCTA
        322 TATGAAGATATTCCCTTTTCCAACGAAATCATCAAAGCGATCCAAATATCCACTTCCAGATTCTA

                           *        **  *      * *    *          *           
     159840 CAAAAAGAGTGTTTCCAAACTGCTGCATCAAAAGAGAGGTTCCACTCTGTTAGCTGAGTACACAC
        387 CAAAAAGAGTGTTTCAAAACTGCTCTATGAAAAGAAATGTTCAACTCTGTTAGTTGAGTACACAC

                    ***      *     *         *                   **     *  * 
     159905 ATCACAAACTTGTTTCTCAGAATCCTTCTGTCTCGTTTTTATGGGAAGATATTTACTTTTTCACC
        452 ATCACAAAGAAGTTTCTGAGAATGCTTCTGTCTAGTTTTTATGGGAAGATATTCCCTTTTCCAAC

             * *           *       *     *       *  *  *               *    *
     159970 GTAGGCATCAAAGCGCTCCAAATGTCCACATCCAGATACTCCAGAAAGAGTGTTTCAAACCTGCC
        517 GAAAGCATCAAAGCGATCCAAATATCCACTTCCAGATTCTACAAAAAGAGTGTTTCAAAACTGCT

                *     *   *         *        * *  *      *      *            
     160035 CTATGAAAGGGAATCTTCAACTCTATGAGTTGAATGCAGACATCAGAAAGAAATTTCTGAGAATG
        582 CTATCAAAGGAAATGTTCAACTCTGTGAGTTGAGTACACACATCACAAAGAAGTTTCTGAGAATG

              *       **       *          **             *  *    *           
     160100 CTGCTGTCTACCTTTTAT-TTGA-ATTCCCGCTTCCAACGAAATCCTCCAAGCTATCCAAATATC
        647 CTTCTGTCTAGTTTTTATGATGATATTCCCTTTTCCAACGAAATCATCAAAGCGATCCAAATATC

                 *       *                                  *  *  *          
     160163 CACTTGCAGATTCCACAAAAAGAGTGTTTCAAAACTGCTCTCTATCAATGGCAAAGTTCAACTCT
        712 CACTTCCAGATTCTACAAAAAGAGTGTTTCAAAACTGC-CTCTATCAAAGGAAATGTTCAACTCT

                      *     *     *  *                         *             
     160228 GTTAGTTGAGGACACATATCACCAACAAGTTTCTGAGAATGCTTCTGTCTATTTTTTATGGGAAG
        776 GTTAGTTGAGTACACACATCACAAAGAAGTTTCTGAGAATGCTTCTGTCTAGTTTTTATGGGAAG

                 *      *  *  * ** *    *      *    *          * **          
     160293 ATATTTCCTTTTTCACCGTAGGCGTCAAGGCGATCGAAATGTCCACTTCCACAAACTACAAAAAG
        841 ATATTCCCTTTTCCAACGAAATCATCAAAGCGATCCAAATATCCACTTCCAGATTCTACAAAAAG

                       *         *     **       *    *           * * *   **  
     160358 AGTGTTTCAAACCTGCTCTATGAAAGGCCATGTTCATCTCTATGAGTTGAATGGAAATATCCGAA
        906 AGTGTTTCAAAACTGCTCTATCAAAGGAAATGTTCAACTCTGTGAGTTGAATGCAGACATCACAA

                *      *       *         *       *         **             *  
     160423 AGAAATTTCTGGGAATGCTGCTGTCTAGTGTTTAT-ACGA-ATTCCCGCTTCCAACGAAATCCTC
        971 AGAAGTTTCTGAGAATGCTTCTGTCTAGTTTTTATGAAGATATTCCCTTTTCCAACGAAATCATC

                 *                *    *  *                                * 
     160486 AAAGCAATCCAAATATCCACTTGCAGAATCCACAAAAAGAGTGTTTCAAAACTGCTCTATCAATA
       1036 AAAGCGATCCAAATATCCACTTCCAGATTCTACAAAAAGAGTGTTTCAAAACTGCTCTATCAAAA

                *          *                     *  *                        
     160551 GAAAGGTTCAACTCTTTTAGTTGAGTACACACATCACGAACAAGTTTCTGAGAATGCTTCTGTCT
       1101 GAAATGTTCAACTCTGTTAGTTGAGTACACACATCACAAAGAAGTTTCTGAGAATGCTTCTGTCT

            * *      *      **  *       
     160616 GGCTTTTATTGGAAGACGTTTCC-TTTT
       1166 AGTTTTTATGGGAAGATATTCCCGTTTT

                *  **          *       *                * *                 *
     160643 CAACAAAGGCATCAAAGCGCTCCAAATGTCCACTTCCAGATTCTTCCAAAAGAGTGTTTCAAACG
          1 CAACGAAATCATCAAAGCGATCCAAATATCCACTTCCAGATTCTACAAAAAGAGTGTTTCAAACC

                  *  *  *                   *    * *  * *     *   *       * *
     160708 TGCTCAAAGTAAAGGAATGTTCAACTCTGTGACTTGAATGCAGATATCACCAAGTAGTTTCTAAT
         66 TGCTC-TAGAAAGGGAATGTTCAACTCTGTGAGTTGAGTACACACATCACAAAGAAGTTTCTGAG

             *        *   **     *    *         *               *  *   *     
     160773 AGTGCTTCTGCCTACATTTTAGATGATGATATTCCCTTTTCCAACGAAATCGTTAGAGCTATCCA
        130 AATGCTTCTGTCTAGTTTTTATATGAAGATATTCCCGTTTCCAACGAAATCGCTAAAGCGATCCA

                    *  *   *      *     *       *   *    **           *      
     160838 AATATCCAGTTACAGTTTCTACCAAAAGGGTGTTTCCAAATTGCTGCATCAAAAGAAAGGTTCAA
        195 AATATCCACTTCCAGATTCTACAAAAAGAGTGTTTCAAAACTGCTCTATCAAAAGAAATGTTCAA

                          *                     *                   *    *   
     160903 CTCTGTTAGTTGAGGACACACATCACAAAGAAGTTTGTGAGAATGCTTCTGTCTAGATTTTGTAT
        260 CTCTGTTAGTTGAGTACACACATCACAAAGAAGTTTCTGAGAATGCTTCTGTCTAGTTTTTATAT

              *                    *   * *     *   *       * *  *    *  *    
     160968 GACGATATTCCCTTTTCCAACGATATCGTTAAAGCAATCTAAATATCAATTTGCAGAATCCACAA
        325 GAAGATATTCCCTTTTCCAACGAAATCATCAAAGCGATCCAAATATCCACTTCCAGATTCTACAA

               *  *        *       * *        *    *          *              
     161033 AAATAGAGTTTCAAAGCTGCTCTGTAAAAAGAAAGGTTCCACTCTGTTAGCTGAGTACACACATC
        390 AAAGAGTGTTTCAAAACTGCTCTATGAAAAGAAATGTTCAACTCTGTTAGTTGAGTACACACATC

                 ***            *         *                   **        *  * 
     161098 ACAAACTTGTTTCTGAGAATCCTTCTGTCTCGTTTTTATGGGAAGATATTTACTTTTCCACCGTA
        455 ACAAAGAAGTTTCTGAGAATGCTTCTGTCTAGTTTTTATGGGAAGATATTCCCTTTTCCAACGAA

            *           *       *     *       *  *  *  *            *        
     161163 GGCATCAAAGCGCTCCAAATGTCCACATCCAGATACTCCAGAACGAGTGTTTCAAACCTGCTCTA
        520 AGCATCAAAGCGATCCAAATATCCACTTCCAGATTCTACAAAAAGAGTGTTTCAAAACTGCTCTA

             *     *   *         *        * *  *      *      *              *
     161228 TGAAAGGGAATCTTCAACTCTATGAGTTGAATGCAGACATCAGAAAGAAATTTCTGAGAATGCTG
        585 TCAAAGGAAATGTTCAACTCTGTGAGTTGAGTACACACATCACAAAGAAGTTTCTGAGAATGCTT

                   **       *          **             *  *    *              
     161293 CTGTCTACCTTTTAT-TTGA-ATTCCCGCTTCCAACGAAATCCTCCAAGCTATCCAAATATCCAC
        650 CTGTCTAGTTTTTATGATGATATTCCCTTTTCCAACGAAATCATCAAAGCGATCCAAATATCCAC

            * *  **   *                                 **              **  *
     161356 CTGCATTTTCCACAAAAAGAGTGTTTCAAAACTG-CTCTATCAATAGAAATGTTCAACTCCTTTG
        715 TTCCAGATTCTACAAAAAGAGTGTTTCAAAACTGCCTCTATCAAAGGAAATGTTCAACTCTGTTA

             *  *                *                                        *  
     161420 GCTGGGTACACACATCACAAACAAGTTTCTGAGAATGCTTCTGTCTAGTTTTTATGGGAAGACAT
        780 GTTGAGTACACACATCACAAAGAAGTTTCTGAGAATGCTTCTGTCTAGTTTTTATGGGAAGATAT

                    *  * *  **          *       *            **              
     161485 TCCCTTTTTCACCAAAGACATCAAAGCGCTCCAAATGTCCACTTCCAGACACTACAAAAAGAGTG
        845 TCCCTTTTCCAACGAAATCATCAAAGCGATCCAAATATCCACTTCCAGATTCTACAAAAAGAGTG

                   **       **    **           *     *           *         * 
     161550 TTTCAAACGTGCTCTAAGAAAGCGAATGTTCAACTTTGTGACTTGAATGCAGATATCACAAAGTA
        910 TTTCAAAACTGCTCTATCAAAGGAAATGTTCAACTCTGTGAGTTGAATGCAGACATCACAAAGAA

                      **                  *    *         *                * *
     161615 GTTTCTGAGAGGGCTTCTGTCTAGATTTTAGATGATGATATTCCCGTTTCCAACGAAATCATTAG
        975 GTTTCTGAGAATGCTTCTGTCTAG-TTTT-TATGAAGATATTCCCTTTTCCAACGAAATCATCAA

               *                *   *                    *        **         
     161680 AGCTATCCAAATATCCACTTACAGTTTCTACAAAAAGAGTGTTTCCAAACTGCTGCATCAAAAGA
       1038 AGCGATCCAAATATCCACTTCCAGATTCTACAAAAAGAGTGTTTCAAAACTGCTCTATCAAAAGA

            * *    *          *                   ***      *     *         * 
     161745 GAGGTTCCACTCTGTTAGCTGAGTACACACATCACAAACTTGTTTCTCAGAATCCTTCTGTCTCG
       1103 AATGTTCAACTCTGTTAGTTGAGTACACACATCACAAAGAAGTTTCTGAGAATGCTTCTGTCTAG

                              ** *    
     161810 TTTTTATGGGAAGATATTTACTTTTT
       1168 TTTTTATGGGAAGATATTCCCGTTTT

              *  * **          *       *     *       *  *  *                 
     161836 CACCGTAGGCATCAAAGCGCTCCAAATGTCCACATCCAGATACTCCAGAAAGAGTGTTTCAAACC
          1 CAACGAAATCATCAAAGCGATCCAAATATCCACTTCCAGATTCTACAAAAAGAGTGTTTCAAACC

                              *         *        * *  *      *      *        
     161901 TGCTCTATGAAAGGGAATCTTCAACTCTATGAGTTGAATGCAGACATCAGAAAGAAATTTCTGAG
         66 TGCTCTA-GAAAGGGAATGTTCAACTCTGTGAGTTGAGTACACACATCACAAAGAAGTTTCTGAG

                  *       **          *          *                 *    *    
     161966 AATGCTGCTGTCTACCTTTTAT-T--TGA-ATTCCCGCTTCCAACGAAATC-CTCCAAGCTATCC
        130 AATGCTTCTGTCTAGTTTTTATATGAAGATATTCCCGTTTCCAACGAAATCGCT-AAAGCGATCC

                        *       *                                  ** *  *   
     162026 AAATATCCACTTGCAGATTCCACAAAAAGAGTGTTTCAAAACTGCTCTCTATCAATGGCAAAGTT
        194 AAATATCCACTTCCAGATTCTACAAAAAGAGTGTTTCAAAACTG--CTCTATCAAAAGAAATGTT

                             *     *     *  *                         *      
     162091 CAACTCTGTTAGTTGAGGACACATATCACCAACAAGTTTCTGAGAATGCTTCTGTCTATTTTTTA
        257 CAACTCTGTTAGTTGAGTACACACATCACAAAGAAGTTTCTGAGAATGCTTCTGTCTAGTTTTTA

             **         *      *  *  * ** *    *      *    *          * **   
     162156 TGGGAAGATATTTCCTTTTTCACCGTAGGCGTCAAGGCGATCGAAATGTCCACTTCCACAAACTA
        322 TATGAAGATATTCCCTTTTCCAACGAAATCATCAAAGCGATCCAAATATCCACTTCCAGATTCTA

                              *             * **       *    * *      * ** * *
     162221 CAAAAAGAGTGTTTCAAACCTGCTCTATGAAAGGCCATGTTCATCTCTATGAGTTGAATGGAAAT
        387 CAAAAAGAGTGTTTCAAAACTGCTCTATGAAAAGAAATGTTCAACTCTGTTAGTTGAGTACACAC

               **      *      *       *         *         *         **       
     162286 ATCCGAAAGAAATTTCTGGGAATGCTGCTGTCTAGTGTTTAT---ACGA-ATTCCCGCTTCCAAC
        452 ATCACAAAGAAGTTTCTGAGAATGCTTCTGTCTAGTTTTTATGGGAAGATATTCCCTTTTCCAAC

             *  * *       *                *    *  *                         
     162347 GTAATCCTCAAAGCAATCCAAATATCCACTTGCAGAATCCACAAAAAGAGTGTTTCAAAACTGCT
        517 GAAAGCATCAAAGCGATCCAAATATCCACTTCCAGATTCTACAAAAAGAGTGTTTCAAAACTGCT

                   **    *          * *                   *  *               
     162412 CTATCAATAGAAAGGTTCAACTCTTTTAGTTGAGTACACACATCACGAACAAGTTTCTGAGAATG
        582 CTATCAAAGGAAATGTTCAACTCTGTGAGTTGAGTACACACATCACAAAGAAGTTTCTGAGAATG

                     * *          *  **  *         * *  **          *       *
     162477 CTTCTGTCTGGCTTTTATTGGAAGACGTTTCCTTTT-CACCAAAGGCATCAAAGCGCTCCAAATG
        647 CTTCTGTCTAGTTTTTA-T-GATGATATTCCCTTTTCCAACGAAATCATCAAAGCGATCCAAATA

                            * *                **      * ***    *            
     162541 TCCACTTCCAGATTCTTCCAAAAGAGTGTTTCAAACGTG-CTCAAAGTAAGGGAATGTTCAACTC
        710 TCCACTTCCAGATTCTACAAAAAGAGTGTTTCAAAACTGCCTCTATCAAAGGAAATGTTCAACTC

               * *    * *  * *     *   *       * * *            **     ***  *
     162605 TGTGACTTGAATGCAGATATCACCAAGTAGTTTCTAATAGTGCTTCTGTCTACATTTTAGATGAT
        775 TGTTAGTTGAGTACACACATCACAAAGAAGTTTCTGAGAATGCTTCTGTCTAGTTTTTATGGGAA

                     *              *** *   *             *  *   *      *    
     162670 GATATTCCCGTTTCCAACGAAATCGCTAGAGCTATCCAAATATCCAGTTACAGTTTCTACCAAAA
        840 GATATTCCCTTTTCCAACGAAATCATCAAAGCGATCCAAATATCCACTTCCAGATTCTACAAAAA

             *       *   *    **      *    *            *      ***  *        
     162735 GGGTGTTTCCAAATTGCTGCATCAAAAGAAAGGTTCAACTCTGTTAGTTGAGGACACACATCACA
        905 GAGTGTTTCAAAACTGCTCTATCAAAGGAAATGTTCAACTCTGTGAGTTGAATGCAGACATCACA

                     *                              *                    *   
     162800 AAGAAGTTTGTGAGAATGCTTCTGTCTAGGTTTTGTATGACGATATTCCCTTTTCCAACGATATC
        970 AAGAAGTTTCTGAGAATGCTTCTGTCTA-GTTTT-TATGAAGATATTCCCTTTTCCAACGAAATC

            * *     *   *       * *  *    *  *       *  *        *       * * 
     162865 GTTAAAGCAATCTAAATATCAATTTGCAGAATCCACAAAAATAGAGTTTCAAAGCTGCTCTGTAA
       1033 ATCAAAGCGATCCAAATATCCACTTCCAGATTCTACAAAAAGAGTGTTTCAAAACTGCTCTATCA

                   *    *          *                   ***      *     *      
     162930 AAAGAAAGGTTCCACTCTGTTAGCTGAGTACACACATCACAAACTTGTTTCTCAGAATCCTTCTG
       1098 AAAGAAATGTTCAACTCTGTTAGTTGAGTACACACATCACAAAGAAGTTTCTGAGAATGCTTCTG

               *                 * ** *   *
     162995 TCTCGTTTTTATGGGAAGATACTTACTTTTC
       1163 TCTAGTTTTTATGGGAAGATATTCCCGTTTT

              *  * **          *       *     *       *  *  *  *              
     163026 CACCGTAGGCATCAAAGCGCTCCAAATGTCCACATCCAGATACTCCAGAACGAGTGTTTCAAACC
          1 CAACGAAATCATCAAAGCGATCCAAATATCCACTTCCAGATTCTACAAAAAGAGTGTTTCAAACC

                              *         *        * *  *      *      *        
     163091 TGCTCTATGAAAGGGAATCTTCAACTCTATGAGTTGAATGCAGACATCAGAAAGAAATTTCTGAG
         66 TGCTCTA-GAAAGGGAATGTTCAACTCTGTGAGTTGAGTACACACATCACAAAGAAGTTTCTGAG

                  *       **          *          *                 *    *    
     163156 AATGCTGCTGTCTACCTTTTAT-T--TGA-ATTCCCGCTTCCAACGAAATC-CTCCAAGCTATCC
        130 AATGCTTCTGTCTAGTTTTTATATGAAGATATTCCCGTTTCCAACGAAATCGCT-AAAGCGATCC

                      * *  **   *    *                           *           
     163216 AAATATCCACCTGCATTTTCCACAACAAGAGTGTTTCAAAACTGCTCTATCAATAGAAATGTTCA
        194 AAATATCCACTTCCAGATTCTACAAAAAGAGTGTTTCAAAACTGCTCTATCAAAAGAAATGTTCA

                **  * *  *                *                                 *
     163281 ACTCCTTTGGCTGGGTACACACATCACAAACAAGTTTCTGAGAATGCTTCTGTCTAGTTTTTATG
        259 ACTCTGTTAGTTGAGTACACACATCACAAAGAAGTTTCTGAGAATGCTTCTGTCTAGTTTTTATA

            *     **         *  * *  **          *       *            **     
     163346 GGAAGACGTTCCCTTTTTCACCAAAGGCATCAAAGCGCTCCAAATGTCCACTTCCAGACACTACA
        324 TGAAGATATTCCCTTTTCCAACGAAATCATCAAAGCGATCCAAATATCCACTTCCAGATTCTACA

                            **       *    ***               * *    * *    *  
     163411 AAAAGAGTGTTTCAAACGTGCTCTAAGAAAGCGAATGTTCAACTCTGTGACTTGAATGCACATAT
        389 AAAAGAGTGTTTCAAAACTGCTCTATGAAAAGAAATGTTCAACTCTGTTAGTTGAGTACACACAT

                   *           **            *     ***  *         *        * 
     163476 CACAAAGTAGTTTCTGAGAGGGCTTCTGTCTAGATTTTAGATGATGATATTCCCGTTTCCAACAA
        454 CACAAAGAAGTTTCTGAGAATGCTTCTGTCTAGTTTTTATGGGAAGATATTCCCTTTTCCAACGA

              *   * *    *               *   *                    *        **
     163541 AATCATTAGAGC-TTCCAAATATCCACTTACAGTTTCTACAAAAAGAGTGTTTCCAAACTGCTGC
        519 AAGCATCAAAGCGATCCAAATATCCACTTCCAGATTCTACAAAAAGAGTGTTTCAAAACTGCTCT

                  *  * *    *       *  *                   ***      *     *  
     163605 ATCAAAAGAGAGGTTCCACTCTGTTAGCTGAGTACACACATCACAAACTTGTTTCTCAGAATCCT
        584 ATCAAAGGAAATGTTCAACTCTGTGAGTTGAGTACACACATCACAAAGAAGTTTCTGAGAATGCT

                   *            *      **     *  *  * **          *       *  
     163670 TCTGTCTCGTTTTTATGGGAAGATATTTACTTTTTCACCGTAGGCATCAAAGCGCTCCAAATGTC
        649 TCTGTCTAGTTTTTAT--GATGATATTCCCTTTTCCAACGAAATCATCAAAGCGATCCAAATATC

               *       *  *  *               *          *     *   *         *
     163735 CACATCCAGATACTCCAGAAAGAGTGTTTCAAACCTGCC-CTATGAAAGGGAATCTTCAACTCTA
        712 CACTTCCAGATTCTACAAAAAGAGTGTTTCAAAACTGCCTCTATCAAAGGAAATGTTCAACTCTG

             *      * *  *      *      *              *       **         **  
     163799 TGAGTTGAATGCAGACATCAGAAAGAAATTTCTGAGAATGCTGCTGTCTACCTTTTAT---TTGA
        777 TTAGTTGAGTACACACATCACAAAGAAGTTTCTGAGAATGCTTCTGTCTAGTTTTTATGGGAAGA

                   **             *  *    *                *       *         
     163861 -ATTCCCGCTTCCAACGAAATCCTCCAAGCTATCCAAATATCCACTTGCAGATTCCACAAAAAGA
        842 TATTCCCTTTTCCAACGAAATCATCAAAGCGATCCAAATATCCACTTCCAGATTCTACAAAAAGA

                                     *  *  *            *      ***  * *     *
     163925 GTGTTTCAAAACTGCTCTCTATCAATGGCAAAGTTCAACTCTGTTAGTTGAGGACACATATCACC
        907 GTGTTTCAAAACTG--CTCTATCAAAGGAAATGTTCAACTCTGTGAGTTGAATGCAGACATCACA

              *                         *                  *      *  *  * ** 
     163990 AACAAGTTTCTGAGAATGCTTCTGTCTATTTTTTATGGGAAGATATTTCCTTTTTCACCGTAGGC
        970 AAGAAGTTTCTGAGAATGCTTCTGTCTAGTTTTTAT--GAAGATATTCCCTTTTCCAACGAAATC

            *    *      *    *          * **                     *         * 
     164055 GTCAAGGCGATCGAAATGTCCACTTCCACAAACTACAAAAAGAGTGTTTCAAACCTGCTCTATGA
       1033 ATCAAAGCGATCCAAATATCCACTTCCAGATTCTACAAAAAGAGTGTTTCAAAACTGCTCTATCA

              * **       *    * *      * ** * *   **      *      *       *   
     164120 AAGGCCATGTTCATCTCTATGAGTTGAATGGAAATATCCGAAAGAAATTTCTGGGAATGCTGCTG
       1098 AAAGAAATGTTCAACTCTGTTAGTTGAGTACACACATCACAAAGAAGTTTCTGAGAATGCTTCTG

                  *         *          *  *
     164185 TCTAGTGTTTAT---ACGA-ATTCCCGCTTC
       1163 TCTAGTTTTTATGGGAAGATATTCCCGTTTT

                      *       *                *    *  *                   * 
     164212 CAACGAAATCCTCAAAGCAATCCAAATATCCACTTGCAGAATCCACAAAAAGAGTGTTTCAAAAC
          1 CAACGAAATCATCAAAGCGATCCAAATATCCACTTCCAGATTCTACAAAAAGAGTGTTTCAAACC

                    *  ** *  *          * *                   *  *           
     164277 TGCTCTATCAATAGAAAGGTTCAACTCTTTTAGTTGAGTACACACATCACGAACAAGTTTCTGAG
         66 TGCTCTA-GAAAGGGAATGTTCAACTCTGTGAGTTGAGTACACACATCACAAAGAAGTTTCTGAG

                         * *      **     **  *      *         *           *  
     164342 AATGCTTCTGTCTGGCTTTTATTGGAAGACGTTTCC-TTTTCAAC-AAA-GGCATCAAAGCGCTC
        130 AATGCTTCTGTCTAGTTTTTATATGAAGATATTCCCGTTTCCAACGAAATCGC-T-AAAGCGATC

                 *                * *                **     * ***    *       
     164404 CAAATGTCCACTTCCAGATTCTTCCAAAAGAGTGTTTCAAACGTGCTCAAAGTAAAGGAATGTTC
        193 CAAATATCCACTTCCAGATTCTACAAAAAGAGTGTTTCAAAACTGCTCTATCAAAAGAAATGTTC

                    * *    * *  * *     *   *       * * *        *   **     *
     164469 AACTCTGTGACTTGAATGCAGATATCACCAAGTAGTTTCTAATAGTGCTTCTGCCTACATTTTAG
        258 AACTCTGTTAGTTGAGTACACACATCACAAAGAAGTTTCTGAGAATGCTTCTGTCTAGTTTTTAT

                *                        * * *   *             *  *   *      
     164534 ATGATGATATTCCCTTTTCCAACGAAATCGTTAGAGCTATCCAAATATCCAGTTACAGTTTCTAC
        323 ATGAAGATATTCCCTTTTCCAACGAAATCATCAAAGCGATCCAAATATCCACTTCCAGATTCTAC

            *     *       *   *    **  *        *                    *       
     164599 CAAAAGGGTGTTTCCAAATTGCTGCATCAAAAGAAAGGTTCAACTCTGTTAGTTGAGGACACACA
        388 AAAAAGAGTGTTTCAAAACTGCTCTATGAAAAGAAATGTTCAACTCTGTTAGTTGAGTACACACA

                          *                   *    * **  *                   
     164664 TCACAAAGAAGTTTGTGAGAATGCTTCTGTCTAGATTTTGTATGACGATATTCCCTTTTCCAACG
        453 TCACAAAGAAGTTTCTGAGAATGCTTCTGTCTAGTTTTTATGGGAAGATATTCCCTTTTCCAACG

             * * * *     *   *       * *  *    *  *       *  *        *      
     164729 ATATCGTTAAAGCAATCTAAATATCAATTTGCAGAATCCACAAAAATAGAGTTTCAAAGCTGCTC
        518 AAAGCATCAAAGCGATCCAAATATCCACTTCCAGATTCTACAAAAAGAGTGTTTCAAAACTGCTC

             * *   *    *    *       *  *                   ***            * 
     164794 TGTAAAAAGAAAGGTTCCACTCTGTTAGCTGAGTACACACATCACAAACTTGTTTCTGAGAATCC
        583 TATCAAAGGAAATGTTCAACTCTGTGAGTTGAGTACACACATCACAAAGAAGTTTCTGAGAATGC

                    *            *      **        *  * **          *       * 
     164859 TTCTGTCTCGTTTTTATGGGAAGATATTTACTTTTCCACCGTAGGCATCAAAGCGCTCCAAATGT
        648 TTCTGTCTAGTTTTTAT--GATGATATTCCCTTTTCCAACGAAATCATCAAAGCGATCCAAATAT

                *       *  *  *  *            *          *     *   *         
     164924 CCACATCCAGATACTCCAGAACGAGTGTTTCAAACCTG-CTCTATGAAAGGGAATCTTCAACTCT
        711 CCACTTCCAGATTCTACAAAAAGAGTGTTTCAAAACTGCCTCTATCAAAGGAAATGTTCAACTCT

            * *      * *  *      *      *              *       **         ** 
     164988 ATGAGTTGAATGCAGACATCAGAAAGAAATTTCTGAGAATGCTGCTGTCTACCTTTTAT---TTG
        776 GTTAGTTGAGTACACACATCACAAAGAAGTTTCTGAGAATGCTTCTGTCTAGTTTTTATGGGAAG

                    **             *  *    *              * *  **   *        
     165050 A-ATTCCCGCTTCCAACGAAATCCTCCAAGCTATCCAAATATCCACCTGCATTTTCCACAAAAAG
        841 ATATTCCCTTTTCCAACGAAATCATCAAAGCGATCCAAATATCCACTTCCAGATTCTACAAAAAG

                                    **                *    *  ** *  *        
     165114 AGTGTTTCAAAACTGCTCTATCAATAGAAATGTTCAACTCCTTTG-GCTGGGTACACACATCACA
        906 AGTGTTTCAAAACTGCTCTATCAAAGGAAATGTTCAACT-CTGTGAGTTGAATGCAGACATCACA

              *                                        *          *  * *  ** 
     165178 AACAAGTTTCTGAGAATGCTTCTGTCTAGTTTTTATGGGAAGACATTCCCTTTTTCACCAAAGAC
        970 AAGAAGTTTCTGAGAATGCTTCTGTCTAGTTTTTAT--GAAGATATTCCCTTTTCCAACGAAATC

                     *       *            **                     **       ** 
     165243 ATCAAAGCGCTCCAAATGTCCACTTCCAGACACTACAAAAAGAGTGTTTCAAACGTGCTCTAAGA
       1033 ATCAAAGCGATCCAAATATCCACTTCCAGATTCTACAAAAAGAGTGTTTCAAAACTGCTCTATCA

              ***           *   * *    * *  * *         *           **       
     165308 AAGCGAATGTTCAACTTTGTGACTTGAATGCAGATATCACAAAGTAGTTTCTGAGAGGGCTTCTG
       1098 AAAGAAATGTTCAACTCTGTTAGTTGAGTACACACATCACAAAGAAGTTTCTGAGAATGCTTCTG

                 *     ***  *             *
     165373 TCTAGATTTTAGATGATGATATTCCCGTTTC
       1163 TCTAGTTTTTATGGGAAGATATTCCCGTTTT

                        * *   *                *   *                    *  * 
     165404 CAACGAAATCATTAGAGCTATCCAAATATCCACTTACAGTTTCTACAAAAAGAGTGTTTCCAAAC
          1 CAACGAAATCATCAAAGCGATCCAAATATCCACTTCCAGATTCTACAAAAAGAGTGTTTCAAACC

                    **    *   *    *       *  *                   ***      * 
     165469 TGCTGCATCAAAAGAG-AGGTTCCACTCTGTTAGCTGAGTACACACATCACAAACTTGTTTCTCA
         66 TGCT-C-TAGAAAGGGAATGTTCAACTCTGTGAGTTGAGTACACACATCACAAAGAAGTTTCTGA

                *         *        **         ** *   *  *  * ** ***      *   
     165533 GAATCCTTCTGTCTCGTTTTTATGGGAAGATATTTACTTTTTCACCGTAGGCATCAAAGCGCTCC
        129 GAATGCTTCTGTCTAGTTTTTATATGAAGATATTCCCGTTTCCAACGAAATCGCTAAAGCGATCC

                *     *       *  *  *               *         *   * *   *    
     165598 AAATGTCCACATCCAGATACTCCAGAAAGAGTGTTTCAAACCTGCTCTATGAAAGGGAATCTTCA
        194 AAATATCCACTTCCAGATTCTACAAAAAGAGTGTTTCAAAACTGCTCTATCAAAAGAAATGTTCA

                 * *      * *  *      *      *              *       **       
     165663 ACTCTATGAGTTGAATGCAGACATCAGAAAGAAATTTCTGAGAATGCTGCTGTCTACCTTTTAT-
        259 ACTCTGTTAGTTGAGTACACACATCACAAAGAAGTTTCTGAGAATGCTTCTGTCTAGTTTTTATA

               *         **             *  *    *                *       *   
     165727 T--TGA-ATTCCCGCTTCCAACGAAATCCTCCAAGCTATCCAAATATCCACTTGCAGATTCCACA
        324 TGAAGATATTCCCTTTTCCAACGAAATCATCAAAGCGATCCAAATATCCACTTCCAGATTCTACA

                                        *  ** *  *                    *     *
     165789 AAAAGAGTGTTTCAAAACTGCTCTCTATCAATGGCAAAGTTCAACTCTGTTAGTTGAGGACACAT
        389 AAAAGAGTGTTTCAAAACTG--CTCTATGAAAAGAAATGTTCAACTCTGTTAGTTGAGTACACAC

                 *  *                         *                  *      *  * 
     165854 ATCACCAACAAGTTTCTGAGAATGCTTCTGTCTATTTTTTATGGGAAGATATTTCCTTTTTCACC
        452 ATCACAAAGAAGTTTCTGAGAATGCTTCTGTCTAGTTTTTATGGGAAGATATTCCCTTTTCCAAC

             * *  *    *      *    *          * **                     *     
     165919 GTAGGCGTCAAGGCGATCGAAATGTCCACTTCCACAAACTACAAAAAGAGTGTTTCAAACCTGCT
        517 GAAAGCATCAAAGCGATCCAAATATCCACTTCCAGATTCTACAAAAAGAGTGTTTCAAAACTGCT

                *     **       *    *        * ** * *   **      *      *     
     165984 CTATGAAAGGCCATGTTCATCTCTATGAGTTGAATGGAAATATCCGAAAGAAATTTCTGGGAATG
        582 CTATCAAAGGAAATGTTCAACTCTGTGAGTTGAGTACACACATCACAAAGAAGTTTCTGAGAATG

              *         *       *         **        *    *       *           
     166049 CTGCTGTCTAGTGTTTAT-ACGA-ATTCCCGCTTCCAACGTAATCCTCAAAGCAATCCAAATATC
        647 CTTCTGTCTAGTTTTTATGATGATATTCCCTTTTCCAACGAAATCATCAAAGCGATCCAAATATC

                 *    *  *                                 **    *          *
     166112 CACTTGCAGAATCCACAAAAAGAGTGTTTCAAAACTG-CTCTATCAATAGAAAGGTTCAACTCTT
        712 CACTTCCAGATTCTACAAAAAGAGTGTTTCAAAACTGCCTCTATCAAAGGAAATGTTCAACTCTG

                                 *  *                        * *      *      
     166176 TTAGTTGAGTACACACATCACGAACAAGTTTCTGAGAATGCTTCTGTCTGGCTTTTATTGGAAGA
        777 TTAGTTGAGTACACACATCACAAAGAAGTTTCTGAGAATGCTTCTGTCTAGTTTTTATGGGAAGA

            **  *         * *  **          *       *                * *      
     166241 CGTTTCCTTTT-CACCAAAGGCATCAAAGCGCTCCAAATGTCCACTTCCAGATTCTTCCAAAAGA
        842 TATTCCCTTTTCCAACGAAATCATCAAAGCGATCCAAATATCCACTTCCAGATTCTACAAAAAGA

                      **     * ***    *                 *           *     *  
     166305 GTGTTTCAAACGTGCTCAAAGTAAGGGAATGTTCAACTCTGTGACTTGAATGCAGATATCACCAA
        907 GTGTTTCAAAACTGCTCTATCAAAGGAAATGTTCAACTCTGTGAGTTGAATGCAGACATCACAAA

             *       * * *             *     *    *         *              **
     166370 GTAGTTTCTAATAGTGCTTCTGTCTACATTTTAGATGATGATATTCCCGTTTCCAACGAAATCGC
        972 GAAGTTTCTGAGAATGCTTCTGTCTA-GTTTT-TATGAAGATATTCCCTTTTCCAACGAAATCAT

            * *   *             *  *   *      *     *       *   *    **      
     166435 TAGAGCTATCCAAATATCCAGTTACAGTTTCTACCAAAAGGGTGTTTCCAAATTGCTGCATCAAA
       1035 CAAAGCGATCCAAATATCCACTTCCAGATTCTACAAAAAGAGTGTTTCAAAACTGCTCTATCAAA

                 *                    *                     *                
     166500 AGAAAGGTTCAACTCTGTTAGTTGAGGACACACATCACAAAGAAGTTTGTGAGAATGCTTCTGTC
       1100 AGAAATGTTCAACTCTGTTAGTTGAGTACACACATCACAAAGAAGTTTCTGAGAATGCTTCTGTC

               *    * **  *         *   *
     166565 TAGGTTTTGTATGACGATATTCCCTTTTC
       1165 TAGTTTTTATGGGAAGATATTCCCGTTTT

                  *   * *     *   *       * *  *    *  *       *  *        * 
     166594 CAACGATATCGTTAAAGCAATCTAAATATCAATTTGCAGAATCCACAAAAATAGAGTTTCAAAGC
          1 CAACGAAATCATCAAAGCGATCCAAATATCCACTTCCAGATTCTACAAAAAGAGTGTTTCAAACC

                   **   * *  *    *       *  *                   ***      *  
     166659 TGCTCTGTAAAAAGAAAGGTTCCACTCTGTTAGCTGAGTACACACATCACAAACTTGTTTCTCAG
         66 TGCTCT-AGAAAGGGAATGTTCAACTCTGTGAGTTGAGTACACACATCACAAAGAAGTTTCTGAG

               *         *        **       * ** *      *  * ** ***      *    
     166724 AATCCTTCTGTCTCGTTTTTATGGGAAGATACTTACTTTTCCACCGTAGGCATCAAAGCGCTCCA
        130 AATGCTTCTGTCTAGTTTTTATATGAAGATATTCCCGTTTCCAACGAAATCGCTAAAGCGATCCA

               *     *       *  *  *  *            *         *   * *   *     
     166789 AATGTCCACATCCAGATACTCCAGAACGAGTGTTTCAAACCTGCTCTATGAAAGGGAATCTTCAA
        195 AATATCCACTTCCAGATTCTACAAAAAGAGTGTTTCAAAACTGCTCTATCAAAAGAAATGTTCAA

                * *      * *  *      *      *              *       **        
     166854 CTCTATGAGTTGAATGCAGACATCAGAAAGAAATTTCTGAGAATGCTGCTGTCTACCTTTTAT-T
        260 CTCTGTTAGTTGAGTACACACATCACAAAGAAGTTTCTGAGAATGCTTCTGTCTAGTTTTTATAT

              *         **             *  *    *              * *  **   *    
     166918 --TGA-ATTCCCGCTTCCAACGAAATCCTCCAAGCTATCCAAATATCCACCTGCATTTTCCACAA
        325 GAAGATATTCCCTTTTCCAACGAAATCATCAAAGCGATCCAAATATCCACTTCCAGATTCTACAA

            *                        *  *               **  * *  *           
     166980 CAAGAGTGTTTCAAAACTGCTCTATCAATAGAAATGTTCAACTCCTTTGGCTGGGTACACACATC
        390 AAAGAGTGTTTCAAAACTGCTCTATGAAAAGAAATGTTCAACTCTGTTAGTTGAGTACACACATC

                 *                                        **         *  * *  
     167045 ACAAACAAGTTTCTGAGAATGCTTCTGTCTAGTTTTTATGGGAAGACGTTCCCTTTTTCACCAAA
        455 ACAAAGAAGTTTCTGAGAATGCTTCTGTCTAGTTTTTATGGGAAGATATTCCCTTTTCCAACGAA

            *           *       *            **                     **       
     167110 GGCATCAAAGCGCTCCAAATGTCCACTTCCAGACACTACAAAAAGAGTGTTTCAAACGTGCTCTA
        520 AGCATCAAAGCGATCCAAATATCCACTTCCAGATTCTACAAAAAGAGTGTTTCAAAACTGCTCTA

            **    **                 *    * *    *         *           **    
     167175 AGAAAGCGAATGTTCAACTCTGTGACTTGAATGCACATATCACAAAGTAGTTTCTGAGAGGGCTT
        585 TCAAAGGAAATGTTCAACTCTGTGAGTTGAGTACACACATCACAAAGAAGTTTCTGAGAATGCTT

                          *              *        *       * *    *           
     167240 CTGTCTAGATTTTAGATGATGATATTCCCGTTTCCAACAAAATCATTAGAGC-TTCCAAATATCC
        650 CTGTCTAG-TTTT-TATGATGATATTCCCTTTTCCAACGAAATCATCAAAGCGATCCAAATATCC

                *   *                    *         **      *  * *    *       
     167304 ACTTACAGTTTCTACAAAAAGAGTGTTTCCAAACTG-CTGCATCAAAAGAGAGGTTCCACTCTGT
        713 ACTTCCAGATTCTACAAAAAGAGTGTTTCAAAACTGCCTCTATCAAAGGAAATGTTCAACTCTGT

               *                   ***      *     *         *                
     167368 TAGCTGAGTACACACATCACAAACTTGTTTCTCAGAATCCTTCTGTCTCGTTTTTATGGGAAGAT
        778 TAGTTGAGTACACACATCACAAAGAAGTTTCTGAGAATGCTTCTGTCTAGTTTTTATGGGAAGAT

               **     *  *  * **          *       *     *       *  *  *      
     167433 ATTTACTTTTTCACCGTAGGCATCAAAGCGCTCCAAATGTCCACATCCAGATACTCCAGAAAGAG
        843 ATTCCCTTTTCCAACGAAATCATCAAAGCGATCCAAATATCCACTTCCAGATTCTACAAAAAGAG

                     *    *    *     *   *         *                    *    
     167498 TGTTTCAAACCTGCCCTATGAAAGGGAATCTTCAACTCTATGAGTTGAATGCAGACATCAGAAAG
        908 TGTTTCAAAACTGCTCTATCAAAGGAAATGTTCAACTCTGTGAGTTGAATGCAGACATCACAAAG

              *              *       **       **         **             *  * 
     167563 AAATTTCTGAGAATGCTGCTGTCTACCTTTTAT-TTGA-ATTCCCGCTTCCAACGAAATCCTCCA
        973 AAGTTTCTGAGAATGCTTCTGTCTAGTTTTTATGAAGATATTCCCTTTTCCAACGAAATCATCAA

               *                *       *                                  **
     167626 AGCTATCCAAATATCCACTTGCAGATTCCACAAAAAGAGTGTTTCAAAACTGCTCTCTATCAATG
       1038 AGCGATCCAAATATCCACTTCCAGATTCTACAAAAAGAGTGTTTCAAAACTG--CTCTATCAAAA

             *  *                    *     *     *  *                        
     167691 GCAAAGTTCAACTCTGTTAGTTGAGGACACATATCACCAACAAGTTTCTGAGAATGCTTCTGTCT
       1101 GAAATGTTCAACTCTGTTAGTTGAGTACACACATCACAAAGAAGTTTCTGAGAATGCTTCTGTCT

             *                  *  *    
     167756 ATTTTTTATGGGAAGATATTTCCTTTTT
       1166 AGTTTTTATGGGAAGATATTCCCGTTTT

              *  * ** *    *      *    *          * **                       
     167784 CACCGTAGGCGTCAAGGCGATCGAAATGTCCACTTCCACAAACTACAAAAAGAGTGTTTCAAACC
          1 CAACGAAATCATCAAAGCGATCCAAATATCCACTTCCAGATTCTACAAAAAGAGTGTTTCAAACC

                          **       *    *        * ** * *   **      *      * 
     167849 TGCTCTATGAAAGGCCATGTTCATCTCTATGAGTTGAATGGAAATATCCGAAAGAAATTTCTGGG
         66 TGCTCTA-GAAAGGGAATGTTCAACTCTGTGAGTTGAGTACACACATCACAAAGAAGTTTCTGAG

                  *          *        *          *                      *    
     167914 AATGCTGCTGTCTAG-TGTT-TAT-ACGA-ATTCCCGCTTCCAACGAAATC-CTCAAAGCAATCC
        130 AATGCTTCTGTCTAGTTTTTATATGAAGATATTCCCGTTTCCAACGAAATCGCT-AAAGCGATCC

                        *    *  *                                *     *     
     167974 AAATATCCACTTGCAGAATCCACAAAAAGAGTGTTTCAAAACTGCTCTATCAATAGAAAGGTTCA
        194 AAATATCCACTTCCAGATTCTACAAAAAGAGTGTTTCAAAACTGCTCTATCAAAAGAAATGTTCA

                 *                     *  *                        * *      *
     168039 ACTCTTTTAGTTGAGTACACACATCACGAACAAGTTTCTGAGAATGCTTCTGTCTGGCTTTTATT
        259 ACTCTGTTAGTTGAGTACACACATCACAAAGAAGTTTCTGAGAATGCTTCTGTCTAGTTTTTATA

            *     **  *           *  **          *       *                * *
     168104 GGAAGACGTTTCCTTTT-CAACAAAGGCATCAAAGCGCTCCAAATGTCCACTTCCAGATTCTTCC
        324 TGAAGATATTCCCTTTTCCAACGAAATCATCAAAGCGATCCAAATATCCACTTCCAGATTCTACA

                            **     * * *  * *               * *    * *  * *  
     168168 AAAAGAGTGTTTCAAACGTGCTCAAAGTAAGGGAATGTTCAACTCTGTGACTTGAATGCAGATAT
        389 AAAAGAGTGTTTCAAAACTGCTCTATGAAAAGAAATGTTCAACTCTGTTAGTTGAGTACACACAT

               *   *       * * *        *   **     ***  *                    
     168233 CACCAAGTAGTTTCTAATAGTGCTTCTGCCTACATTTTAGATGATGATATTCCCTTTTCCAACGA
        454 CACAAAGAAGTTTCTGAGAATGCTTCTGTCTAGTTTTTATGGGAAGATATTCCCTTTTCCAACGA

              * * * *   *             *  *   *      *     *       *   *    **
     168298 AATCGTTAGAGCTATCCAAATATCCAGTTACAGTTTCTACCAAAAGGGTGTTTCCAAATTGCTGC
        519 AAGCATCAAAGCGATCCAAATATCCACTTCCAGATTCTACAAAAAGAGTGTTTCAAAACTGCTCT

                  *    *            *       *                     *          
     168363 ATCAAAAGAAAGGTTCAACTCTGTTAGTTGAGGACACACATCACAAAGAAGTTTGTGAGAATGCT
        584 ATCAAAGGAAATGTTCAACTCTGTGAGTTGAGTACACACATCACAAAGAAGTTTCTGAGAATGCT

                                *                    *   * *     *   *       
     168428 TCTGTCTAGATTTTGTATGACGATATTCCCTTTTCCAACGATATCGTTAAAGCAATCTAAATATC
        649 TCTGTCTAG-TTTT-TATGATGATATTCCCTTTTCCAACGAAATCATCAAAGCGATCCAAATATC

            * *  *    *  *       *  *        *        * *   *    *    *      
     168493 AATTTGCAGAATCCACAAAAATAGAGTTTCAAAGCTG-CTCTGTAAAAAGAAAGGTTCCACTCTG
        712 CACTTCCAGATTCTACAAAAAGAGTGTTTCAAAACTGCCTCTATCAAAGGAAATGTTCAACTCTG

                *                   ***            *         *               
     168557 TTAGCTGAGTACACACATCACAAACTTGTTTCTGAGAATCCTTCTGTCTCGTTTTTATGGGAAGA
        777 TTAGTTGAGTACACACATCACAAAGAAGTTTCTGAGAATGCTTCTGTCTAGTTTTTATGGGAAGA

                **        *  * **          *       *     *       *  *  *  *  
     168622 TATTTACTTTTCCACCGTAGGCATCAAAGCGCTCCAAATGTCCACATCCAGATACTCCAGAACGA
        842 TATTCCCTTTTCCAACGAAATCATCAAAGCGATCCAAATATCCACTTCCAGATTCTACAAAAAGA

                      *         *     *   *         *                    *   
     168687 GTGTTTCAAACCTGCTCTATGAAAGGGAATCTTCAACTCTATGAGTTGAATGCAGACATCAGAAA
        907 GTGTTTCAAAACTGCTCTATCAAAGGAAATGTTCAACTCTGTGAGTTGAATGCAGACATCACAAA

               *              *       **       **         **             *  *
     168752 GAAATTTCTGAGAATGCTGCTGTCTACCTTTTAT-TTGA-ATTCCCGCTTCCAACGAAATCCTCC
        972 GAAGTTTCTGAGAATGCTTCTGTCTAGTTTTTATGAAGATATTCCCTTTTCCAACGAAATCATCA

                *              * *  **   *                                *  
     168815 AAGCTATCCAAATATCCACCTGCATTTTCCACAAAAAGAGTGTTTCAAAACTGCTCTATCAATAG
       1037 AAGCGATCCAAATATCCACTTCCAGATTCTACAAAAAGAGTGTTTCAAAACTGCTCTATCAAAAG

                         **  * *  *                *                         
     168880 AAATGTTCAACTCCTTTGGCTGGGTACACACATCACAAACAAGTTTCTGAGAATGCTTCTGTCTA
       1102 AAATGTTCAACTCTGTTAGTTGAGTACACACATCACAAAGAAGTTTCTGAGAATGCTTCTGTCTA

                           *      *    
     168945 GTTTTTATGGGAAGACATTCCCTTTTT
       1167 GTTTTTATGGGAAGATATTCCCGTTTT

              * *  **          *       *            **                      *
     168972 CACCAAAGACATCAAAGCGCTCCAAATGTCCACTTCCAGACACTACAAAAAGAGTGTTTCAAACG
          1 CAACGAAATCATCAAAGCGATCCAAATATCCACTTCCAGATTCTACAAAAAGAGTGTTTCAAACC

                         *                  *    * *  * *         *          
     169037 TGCTCTAAGAAAGCGAATGTTCAACTCTGTGACTTGAATGCAGATATCACAAAGTAGTTTCTGAG
         66 TGCTCT-AGAAAGGGAATGTTCAACTCTGTGAGTTGAGTACACACATCACAAAGAAGTTTCTGAG

             **            *     *    *                        **  *   *     
     169102 AGGGCTTCTGTCTAGATTTTAGATGATGATATTCCCGTTTCCAACGAAATCATTAGAGCTATCCA
        130 AATGCTTCTGTCTAGTTTTTATATGAAGATATTCCCGTTTCCAACGAAATCGCTAAAGCGATCCA

                       *   *                    *        **         * *    * 
     169167 AATATCCACTTACAGTTTCTACAAAAAGAGTGTTTCCAAACTGCTGCATCAAAAGAGAGGTTCCA
        195 AATATCCACTTCCAGATTCTACAAAAAGAGTGTTTCAAAACTGCTCTATCAAAAGAAATGTTCAA

                     *                   ***      *     *         *        **
     169232 CTCTGTTAGCTGAGTACACACATCACAAACTTGTTTCTCAGAATCCTTCTGTCTCGTTTTTATGG
        260 CTCTGTTAGTTGAGTACACACATCACAAAGAAGTTTCTGAGAATGCTTCTGTCTAGTTTTTATAT

                     **     *  *  * **          *       *     *       *  *  *
     169297 GAAGATATTTACTTTTTCACCGTAGGCATCAAAGCGCTCCAAATGTCCACATCCAGATACTCCAG
        325 GAAGATATTCCCTTTTCCAACGAAATCATCAAAGCGATCCAAATATCCACTTCCAGATTCTACAA

                           *             * *   *         * *      * *  *     
     169362 AAAGAGTGTTTCAAACCTGCTCTATGAAAGGGAATCTTCAACTCTATGAGTTGAATGCAGACATC
        390 AAAGAGTGTTTCAAAACTGCTCTATGAAAAGAAATGTTCAACTCTGTTAGTTGAGTACACACATC

             *      *              *       **         **         **          
     169427 AGAAAGAAATTTCTGAGAATGCTGCTGTCTACCTTTTAT---TTGA-ATTCCCGCTTCCAACGAA
        455 ACAAAGAAGTTTCTGAGAATGCTTCTGTCTAGTTTTTATGGGAAGATATTCCCTTTTCCAACGAA

             * *  *    *                *       *                            
     169488 ATCCTCCAAGCTATCCAAATATCCACTTGCAGATTCCACAAAAAGAGTGTTTCAAAACTGCTCTC
        520 AGCATCAAAGCGATCCAAATATCCACTTCCAGATTCTACAAAAAGAGTGTTTCAAAACTG--CTC

                  *  *  *            *       *     *     *  *                
     169553 TATCAATGGCAAAGTTCAACTCTGTTAGTTGAGGACACATATCACCAACAAGTTTCTGAGAATGC
        583 TATCAAAGGAAATGTTCAACTCTGTGAGTTGAGTACACACATCACAAAGAAGTTTCTGAGAATGC

                     *           *      *      *  *  * ** *    *      *    * 
     169618 TTCTGTCTATTTTTTATGGGAAGATATTTCCTTTTTCACCGTAGGCGTCAAGGCGATCGAAATGT
        648 TTCTGTCTAGTTTTTAT--GATGATATTCCCTTTTCCAACGAAATCATCAAAGCGATCCAAATAT

                     * **                     *          *     **       *    
     169683 CCACTTCCACAAACTACAAAAAGAGTGTTTCAAACCTG-CTCTATGAAAGGCCATGTTCATCTCT
        711 CCACTTCCAGATTCTACAAAAAGAGTGTTTCAAAACTGCCTCTATCAAAGGAAATGTTCAACTCT

            * *      * ** * *   **      *      *       *         *         * 
     169747 ATGAGTTGAATGGAAATATCCGAAAGAAATTTCTGGGAATGCTGCTGTCTAGTGTTTAT---ACG
        776 GTTAGTTGAGTACACACATCACAAAGAAGTTTCTGAGAATGCTTCTGTCTAGTTTTTATGGGAAG

                    **        *    *       *                *    *  *        
     169809 A-ATTCCCGCTTCCAACGTAATCCTCAAAGCAATCCAAATATCCACTTGCAGAATCCACAAAAAG
        841 ATATTCCCTTTTCCAACGAAATCATCAAAGCGATCCAAATATCCACTTCCAGATTCTACAAAAAG

                                    **    *          * *      * *  *       * 
     169873 AGTGTTTCAAAACTGCTCTATCAATAGAAAGGTTCAACTCTTTTAGTTGAGTACACACATCACGA
        906 AGTGTTTCAAAACTGCTCTATCAAAGGAAATGTTCAACTCTGTGAGTTGAATGCAGACATCACAA

             *                        * *             **  *         * *  **  
     169938 ACAAGTTTCTGAGAATGCTTCTGTCTGGCTTTTATTGGAAGACGTTTCCTTTT-CACCAAAGGCA
        971 AGAAGTTTCTGAGAATGCTTCTGTCTAGTTTTTA-T-GAAGATATTCCCTTTTCCAACGAAATCA

                    *       *                * *                **     * *** 
     170002 TCAAAGCGCTCCAAATGTCCACTTCCAGATTCTTCCAAAAGAGTGTTTCAAACGTGCTCAAAGTA
       1034 TCAAAGCGATCCAAATATCCACTTCCAGATTCTACAAAAAGAGTGTTTCAAAACTGCTCTATCAA

             * *               * *    * *  * *     *   *       * * *         
     170067 AGGGAATGTTCAACTCTGTGACTTGAATGCAGATATCACCAAGTAGTTTCTAATAGTGCTTCTGT
       1099 AAGAAATGTTCAACTCTGTTAGTTGAGTACACACATCACAAAGAAGTTTCTGAGAATGCTTCTGT

               **     ***  *             *
     170132 CTACATTTTAGATGATGATATTCCCGTTTC
       1164 CTAGTTTTTATGGGAAGATATTCCCGTTTT

                      *** *   *             *  *   *      *     *       *  **
     170162 CAACGAAATCGCTAGAGCTATCCAAATATCCAGTTACAGTTTCTACCAAAAGGGTGTTTCCAAAT
          1 CAACGAAATCATCAAAGCGATCCAAATATCCACTTCCAGATTCTACAAAAAGAGTGTTTCAAACC

                  ***   * *  *            *       *                     *    
     170227 TGCTGCATCAAAAGAAAGGTTCAACTCTGTTAGTTGAGGACACACATCACAAAGAAGTTTGTGAG
         66 TGCT-CTAGAAAGGGAATGTTCAACTCTGTGAGTTGAGTACACACATCACAAAGAAGTTTCTGAG

                           *    *     *         *          *    *      *   * 
     170292 AATGCTTCTGTCTAGATTTTGTATGACGATATTCCCTTTTCCAACGATATCGTTAAAGCAATCTA
        130 AATGCTTCTGTCTAGTTTTTATATGAAGATATTCCCGTTTCCAACGAAATCGCTAAAGCGATCCA

                  * *  *    *  *       *  *        *       * *        *    * 
     170357 AATATCAATTTGCAGAATCCACAAAAATAGAGTTTCAAAGCTGCTCTGTAAAAAGAAAGGTTCCA
        195 AATATCCACTTCCAGATTCTACAAAAAGAGTGTTTCAAAACTGCTCTATCAAAAGAAATGTTCAA

                     *                   ***      *     *         *        **
     170422 CTCTGTTAGCTGAGTACACACATCACAAACTTGTTTCTCAGAATCCTTCTGTCTCGTTTTTATGG
        260 CTCTGTTAGTTGAGTACACACATCACAAAGAAGTTTCTGAGAATGCTTCTGTCTAGTTTTTATAT

                   * **        *  * **          *       *     *       *  *  *
     170487 GAAGATACTTACTTTTCCACCGTAGGCATCAAAGCGCTCCAAATGTCCACATCCAGATACTCCAG
        325 GAAGATATTCCCTTTTCCAACGAAATCATCAAAGCGATCCAAATATCCACTTCCAGATTCTACAA

              *            *             * *   *         * *      * *  *     
     170552 AACGAGTGTTTCAAACCTGCTCTATGAAAGGGAATCTTCAACTCTATGAGTTGAATGCAGACATC
        390 AAAGAGTGTTTCAAAACTGCTCTATGAAAAGAAATGTTCAACTCTGTTAGTTGAGTACACACATC

             *      *              *       **         **         **          
     170617 AGAAAGAAATTTCTGAGAATGCTGCTGTCTACCTTTTAT---TTGA-ATTCCCGCTTCCAACGAA
        455 ACAAAGAAGTTTCTGAGAATGCTTCTGTCTAGTTTTTATGGGAAGATATTCCCTTTTCCAACGAA

             * *  *    *              * *  **   *    *                       
     170678 ATCCTCCAAGCTATCCAAATATCCACCTGCATTTTCCACAACAAGAGTGTTTCAAAACTGCTCTA
        520 AGCATCAAAGCGATCCAAATATCCACTTCCAGATTCTACAAAAAGAGTGTTTCAAAACTGCTCTA

                **                *    *  *                *                 
     170743 TCAATAGAAATGTTCAACTCCTTTG-GCTGGGTACACACATCACAAACAAGTTTCTGAGAATGCT
        585 TCAAAGGAAATGTTCAACT-CTGTGAGTTGAGTACACACATCACAAAGAAGTTTCTGAGAATGCT

                                *  **         *  * *  **    *     *       *  
     170807 TCTGTCTAGTTTTTATGGGAAGACGTTCCCTTTTTCACCAAAGGCATCCAAGCGCTCCAAATGTC
        649 TCTGTCTAGTTTTTAT--GATGATATTCCCTTTTCCAACGAAATCATCAAAGCGATCCAAATATC

               *       *  *  *               *          *     *   *         *
     170872 CACATCCAGATACTCCAGAAAGAGTGTTTCAAACCTG-CTCTATGAAAGGGAATCTTCAACTCTA
        712 CACTTCCAGATTCTACAAAAAGAGTGTTTCAAAACTGCCTCTATCAAAGGAAATGTTCAACTCTG

             *      * *  *      *      *              *       **         **  
     170936 TGAGTTGAATGCAGACATCAGAAAGAAATTTCTGAGAATGCTGCTGTCTACCTTTTAT---TTGA
        777 TTAGTTGAGTACACACATCACAAAGAAGTTTCTGAGAATGCTTCTGTCTAGTTTTTATGGGAAGA

                   **             *  *    *                *       *         
     170998 -ATTCCCGCTTCCAACGAAATCCTCCAAGCTATCCAAATATCCACTTGCAGATTCCACAAAAAGA
        842 TATTCCCTTTTCCAACGAAATCATCAAAGCGATCCAAATATCCACTTCCAGATTCTACAAAAAGA

                                     *  *  *            *      ***  * *     *
     171062 GTGTTTCAAAACTGCTCTCTATCAATGGCAAAGTTCAACTCTGTTAGTTGAGGACACATATCACC
        907 GTGTTTCAAAACTG--CTCTATCAAAGGAAATGTTCAACTCTGTGAGTTGAATGCAGACATCACA

              *                         *                  *      *  * ** ** 
     171127 AACAAGTTTCTGAGAATGCTTCTGTCTATTTTTTATGGGAAGATATTTCCTTTTTCACCATAGGC
        970 AAGAAGTTTCTGAGAATGCTTCTGTCTAGTTTTTAT--GAAGATATTCCCTTTTCCAACGAAATC

            *    *      *    *          * **                     *         * 
     171192 GTCAAGGCGATCGAAATGTCCACTTCCACAAACTACAAAAAGAGTGTTTCAAACCTGCTCTATGA
       1033 ATCAAAGCGATCCAAATATCCACTTCCAGATTCTACAAAAAGAGTGTTTCAAAACTGCTCTATCA

              * **       *    * *      * ** * *   **      *      *       *   
     171257 AAGGCCATGTTCATCTCTATGAGTTGAATGGAAATATCCGAAAGAAATTTCTGGGAATGCTGCTG
       1098 AAAGAAATGTTCAACTCTGTTAGTTGAGTACACACATCACAAAGAAGTTTCTGAGAATGCTTCTG

                  *         *          *  *
     171322 TCTAGTGTTTAT---ACGA-ATTCCCGCTTC
       1163 TCTAGTTTTTATGGGAAGATATTCCCGTTTT

                      *       *                *    *  *                   * 
     171349 CAACGAAATCCTCAAAGCAATCCAAATATCCACTTGCAGAATCCACAAAAAGAGTGTTTCAAAAC
          1 CAACGAAATCATCAAAGCGATCCAAATATCCACTTCCAGATTCTACAAAAAGAGTGTTTCAAACC

                    *  ** *  *          * *                   *  *           
     171414 TGCTCTATCAATAGAAAGGTTCAACTCTTTTAGTTGAGTACACACATCACGAACAAGTTTCTGAG
         66 TGCTCTA-GAAAGGGAATGTTCAACTCTGTGAGTTGAGTACACACATCACAAAGAAGTTTCTGAG

                         * *      **     **  *      *         *           *  
     171479 AATGCTTCTGTCTGGCTTTTATTGGAAGACGTTTCC-TTTTCAAC-AAA-GGCATCAAAGCGCTC
        130 AATGCTTCTGTCTAGTTTTTATATGAAGATATTCCCGTTTCCAACGAAATCGC-T-AAAGCGATC

                 *                * *                **     * ***  * *       
     171541 CAAATGTCCACTTCCAGATTCTTCCAAAAGAGTGTTTCAAACGTGCTCAAAGTAAGGGAATGTTC
        193 CAAATATCCACTTCCAGATTCTACAAAAAGAGTGTTTCAAAACTGCTCTATCAAAAGAAATGTTC

                    * *    * *  * *     *   *       * * *        *   **     *
     171606 AACTCTGTGACTTGAATGCAGATATCACCAAGTAGTTTCTAATAGTGCTTCTGCCTACATTTTAG
        258 AACTCTGTTAGTTGAGTACACACATCACAAAGAAGTTTCTGAGAATGCTTCTGTCTAGTTTTTAT

                *                        * * *   *             *  *   *      
     171671 ATGATGATATTCCCTTTTCCAACGAAATCGTTAGAGCTATCCAAATATCCAGTTACAGTTTCTAC
        323 ATGAAGATATTCCCTTTTCCAACGAAATCATCAAAGCGATCCAAATATCCACTTCCAGATTCTAC

            *     *       *   *    **  *        *                    *       
     171736 CAAAAGGGTGTTTCCAAATTGCTGCATCAAAAGAAAGGTTCAACTCTGTTAGTTGAGGACACACA
        388 AAAAAGAGTGTTTCAAAACTGCTCTATGAAAAGAAATGTTCAACTCTGTTAGTTGAGTACACACA

                          *                   *    * **  *                   
     171801 TCACAAAGAAGTTTGTGAGAATGCTTCTGTCTAGATTTTGTATGACGATATTCCCTTTTCCAACG
        453 TCACAAAGAAGTTTCTGAGAATGCTTCTGTCTAGTTTTTATGGGAAGATATTCCCTTTTCCAACG

             * * * *     *   *       * *  *    *  *       *  *        *      
     171866 ATATCGTTAAAGCAATCTAAATATCAATTTGCAGAATCCACAAAAATAGAGTTTCAAAGCTGCTC
        518 AAAGCATCAAAGCGATCCAAATATCCACTTCCAGATTCTACAAAAAGAGTGTTTCAAAACTGCTC

             * *   *    *    *       *  *                   ***            * 
     171931 TGTAAAAAGAAAGGTTCCACTCTGTTAGCTGAGTACACACATCACAAACTTGTTTCTGAGAATCC
        583 TATCAAAGGAAATGTTCAACTCTGTGAGTTGAGTACACACATCACAAAGAAGTTTCTGAGAATGC

                    *            *      **        *  * **          *       * 
     171996 TTCTGTCTCGTTTTTATGGGAAGATATTTACTTTTCCACCGTAGGCATCAAAGCGCTCCAAATGT
        648 TTCTGTCTAGTTTTTAT--GATGATATTCCCTTTTCCAACGAAATCATCAAAGCGATCCAAATAT

                *       *  *  *  *            *          *     *   *         
     172061 CCACATCCAGATACTCCAGAACGAGTGTTTCAAACCTG-CTCTATGAAAGGGAATCTTCAACTCT
        711 CCACTTCCAGATTCTACAAAAAGAGTGTTTCAAAACTGCCTCTATCAAAGGAAATGTTCAACTCT

            * *      * *  *      *      *              *       **         ** 
     172125 ATGAGTTGAATGCAGACATCAGAAAGAAATTTCTGAGAATGCTGCTGTCTACCTTTTAT---TTG
        776 GTTAGTTGAGTACACACATCACAAAGAAGTTTCTGAGAATGCTTCTGTCTAGTTTTTATGGGAAG

                    **             *  *    *              * *  **   *        
     172187 A-ATTCCCGCTTCCAACGAAATCCTCCAAGCTATCCAAATATCCACCTGCATTTTCCACAAAAAG
        841 ATATTCCCTTTTCCAACGAAATCATCAAAGCGATCCAAATATCCACTTCCAGATTCTACAAAAAG

                                    **                *    *  ** *  *        
     172251 AGTGTTTCAAAACTGCTCTATCAATAGAAATGTTCAACTCCTTTG-GCTGGGTACACACATCACA
        906 AGTGTTTCAAAACTGCTCTATCAAAGGAAATGTTCAACT-CTGTGAGTTGAATGCAGACATCACA

              *                                        *          *  * *  ** 
     172315 AACAAGTTTCTGAGAATGCTTCTGTCTAGTTTTTATGGGAAGACATTCCCTTTTTCACCAAAGAC
        970 AAGAAGTTTCTGAGAATGCTTCTGTCTAGTTTTTAT--GAAGATATTCCCTTTTCCAACGAAATC

                     *       *            **                     **       ** 
     172380 ATCAAAGCGCTCCAAATGTCCACTTCCAGACACTACAAAAAGAGTGTTTCAAACGTGCTCTAAGA
       1033 ATCAAAGCGATCCAAATATCCACTTCCAGATTCTACAAAAAGAGTGTTTCAAAACTGCTCTATCA

              ***               * *    * *  * *         *           **       
     172445 AAGCGAATGTTCAACTCTGTGACTTGAATGCAGATATCACAAAGTAGTTTCTGAGAGGGCTTCTG
       1098 AAAGAAATGTTCAACTCTGTTAGTTGAGTACACACATCACAAAGAAGTTTCTGAGAATGCTTCTG

                 *     ***  *             *
     172510 TCTAGATTTTAGATGATGATATTCCCGTTTC
       1163 TCTAGTTTTTATGGGAAGATATTCCCGTTTT

                        * *   *                *   *                    *  * 
     172541 CAACGAAATCATTAGAGCTATCCAAATATCCACTTACAGTTTCTACAAAAAGAGTGTTTCCAAAC
          1 CAACGAAATCATCAAAGCGATCCAAATATCCACTTCCAGATTCTACAAAAAGAGTGTTTCAAACC

                    **    *   *    *       *  *                   ***      * 
     172606 TGCTGCATCAAAAGAG-AGGTTCCACTCTGTTAGCTGAGTACACACATCACAAACTTGTTTCTCA
         66 TGCT-C-TAGAAAGGGAATGTTCAACTCTGTGAGTTGAGTACACACATCACAAAGAAGTTTCTGA

                *         *        **         ** *   *  *  * ** ***      *   
     172670 GAATCCTTCTGTCTCGTTTTTATGGGAAGATATTTACTTTTTCACCGTAGGCATCAAAGCGCTCC
        129 GAATGCTTCTGTCTAGTTTTTATATGAAGATATTCCCGTTTCCAACGAAATCGCTAAAGCGATCC

                *     *       *  *  *               *         *   * *   *    
     172735 AAATGTCCACATCCAGATACTCCAGAAAGAGTGTTTCAAACCTGCTCTATGAAAGGGAATCTTCA
        194 AAATATCCACTTCCAGATTCTACAAAAAGAGTGTTTCAAAACTGCTCTATCAAAAGAAATGTTCA

                 * *      * *  *      *      *              *       **       
     172800 ACTCTATGAGTTGAATGCAGACATCAGAAAGAAATTTCTGAGAATGCTGCTGTCTACCTTTTAT-
        259 ACTCTGTTAGTTGAGTACACACATCACAAAGAAGTTTCTGAGAATGCTTCTGTCTAGTTTTTATA

               *         **             *  *    *                *       *   
     172864 T--TGA-ATTCCCGCTTCCAACGAAATCCTCCAAGCTATCCAAATATCCACTTGCAGATTCCACA
        324 TGAAGATATTCCCTTTTCCAACGAAATCATCAAAGCGATCCAAATATCCACTTCCAGATTCTACA

                                        *  ** *  *                    *     *
     172926 AAAAGAGTGTTTCAAAACTGCTCTCTATCAATGGCAAAGTTCAACTCTGTTAGTTGAGGACACAT
        389 AAAAGAGTGTTTCAAAACTG--CTCTATGAAAAGAAATGTTCAACTCTGTTAGTTGAGTACACAC

                 *  *                         *                  *      *  * 
     172991 ATCACCAACAAGTTTCTGAGAATGCTTCTGTCTATTTTTTATGGGAAGATATTTCCTTTTTCACC
        452 ATCACAAAGAAGTTTCTGAGAATGCTTCTGTCTAGTTTTTATGGGAAGATATTCCCTTTTCCAAC

             * *  *    *      *    *          * **                     *     
     173056 GTAGGCGTCAAGGCGATCGAAATGTCCACTTCCACAAACTACAAAAAGAGTGTTTCAAACCTGCT
        517 GAAAGCATCAAAGCGATCCAAATATCCACTTCCAGATTCTACAAAAAGAGTGTTTCAAAACTGCT

                *     **       *    *        * ** * *   **      *      *     
     173121 CTATGAAAGGCCATGTTCATCTCTATGAGTTGAATGGAAATATCCGAAAGAAATTTCTGGGAATG
        582 CTATCAAAGGAAATGTTCAACTCTGTGAGTTGAGTACACACATCACAAAGAAGTTTCTGAGAATG

              *         *       *         **        *    *       *           
     173186 CTGCTGTCTAGTGTTTAT-ACGA-ATTCCCGCTTCCAACGTAATCCTCAAAGCAATCCAAATATC
        647 CTTCTGTCTAGTTTTTATGATGATATTCCCTTTTCCAACGAAATCATCAAAGCGATCCAAATATC

                 *    *  *                                 **    *          *
     173249 CACTTGCAGAATCCACAAAAAGAGTGTTTCAAAACTG-CTCTATCAATAGAAAGGTTCAACTCTT
        712 CACTTCCAGATTCTACAAAAAGAGTGTTTCAAAACTGCCTCTATCAAAGGAAATGTTCAACTCTG

                                 *  *                        * *      *      
     173313 TTAGTTGAGTACACACATCACGAACAAGTTTCTGAGAATGCTTCTGTCTGGCTTTTATTGGAAGA
        777 TTAGTTGAGTACACACATCACAAAGAAGTTTCTGAGAATGCTTCTGTCTAGTTTTTATGGGAAGA

            **  *         * *  **          *       *                * *      
     173378 CGTTTCCTTTT-CACCAAAGGCATCAAAGCGCTCCAAATGTCCACTTCCAGATTCTTCCAAAAGA
        842 TATTCCCTTTTCCAACGAAATCATCAAAGCGATCCAAATATCCACTTCCAGATTCTACAAAAAGA

                      **     * ***    *                 *           *     *  
     173442 GTGTTTCAAACGTGCTCAAAGTAAGGGAATGTTCAACTCTGTGACTTGAATGCAGATATCACCAA
        907 GTGTTTCAAAACTGCTCTATCAAAGGAAATGTTCAACTCTGTGAGTTGAATGCAGACATCACAAA

             *       * * *             *     *    *         *              **
     173507 GTAGTTTCTAATAGTGCTTCTGTCTACATTTTAGATGATGATATTCCCGTTTCCAACGAAATCGC
        972 GAAGTTTCTGAGAATGCTTCTGTCTA-GTTTT-TATGAAGATATTCCCTTTTCCAACGAAATCAT

            * *   *             *  *   *      *     *       *   *    **      
     173572 TAGAGCTATCCAAATATCCAGTTACAGTTTCTACCAAAAGGGTGTTTCCAAATTGCTGCATCAAA
       1035 CAAAGCGATCCAAATATCCACTTCCAGATTCTACAAAAAGAGTGTTTCAAAACTGCTCTATCAAA

                 *                    *                     *                
     173637 AGAAAGGTTCAACTCTGTTAGTTGAGGACACACATCACAAAGAAGTTTGTGAGAATGCTTCTGTC
       1100 AGAAATGTTCAACTCTGTTAGTTGAGTACACACATCACAAAGAAGTTTCTGAGAATGCTTCTGTC

               *    * **  *         *   *
     173702 TAGGTTTTGTATGACGATATTCCCTTTTC
       1165 TAGTTTTTATGGGAAGATATTCCCGTTTT

                  *   * *     *   *       * *  *    *  *       *  *        * 
     173731 CAACGATATCGTTAAAGCAATCTAAATATCAATTTGCAGAATCCACAAAAATAGAGTTTCAAAGC
          1 CAACGAAATCATCAAAGCGATCCAAATATCCACTTCCAGATTCTACAAAAAGAGTGTTTCAAACC

                   **   * *  *    *       *  *                   ***      *  
     173796 TGCTCTGTAAAAAGAAAGGTTCCACTCTGTTAGCTGAGTACACACATCACAAACTTGTTTCTCAG
         66 TGCTCT-AGAAAGGGAATGTTCAACTCTGTGAGTTGAGTACACACATCACAAAGAAGTTTCTGAG

               *         *        **       * ** *      *  * ** ***      *    
     173861 AATCCTTCTGTCTCGTTTTTATGGGAAGATACTTACTTTTCCACCGTAGGCATCAAAGCGCTCCA
        130 AATGCTTCTGTCTAGTTTTTATATGAAGATATTCCCGTTTCCAACGAAATCGCTAAAGCGATCCA

               *     *       *  *  *  *            *         *   * *   *     
     173926 AATGTCCACATCCAGATACTCCAGAACGAGTGTTTCAAACCTGCTCTATGAAAGGGAATCTTCAA
        195 AATATCCACTTCCAGATTCTACAAAAAGAGTGTTTCAAAACTGCTCTATCAAAAGAAATGTTCAA

                * *      * *  *      *      *              *       **        
     173991 CTCTATGAGTTGAATGCAGACATCAGAAAGAAATTTCTGAGAATGCTGCTGTCTACCTTTTAT-T
        260 CTCTGTTAGTTGAGTACACACATCACAAAGAAGTTTCTGAGAATGCTTCTGTCTAGTTTTTATAT

              *         **             *  *    *              * *  **   *    
     174055 --TGA-ATTCCCGCTTCCAACGAAATCCTCCAAGCTATCCAAATATCCACCTGCATTTTCCACAA
        325 GAAGATATTCCCTTTTCCAACGAAATCATCAAAGCGATCCAAATATCCACTTCCAGATTCTACAA

            *                        *  *               **  * *  *           
     174117 CAAGAGTGTTTCAAAACTGCTCTATCAATAGAAATGTTCAACTCCTTTGGCTGGGTACACACATC
        390 AAAGAGTGTTTCAAAACTGCTCTATGAAAAGAAATGTTCAACTCTGTTAGTTGAGTACACACATC

                 *                                        **         *  * *  
     174182 ACAAACAAGTTTCTGAGAATGCTTCTGTCTAGTTTTTATGGGAAGACGTTCCCTTTTTCACCAAA
        455 ACAAAGAAGTTTCTGAGAATGCTTCTGTCTAGTTTTTATGGGAAGATATTCCCTTTTCCAACGAA

            *           *       *            **                     **       
     174247 GGCATCAAAGCGCTCCAAATGTCCACTTCCAGACACTACAAAAAGAGTGTTTCAAACGTGCTCTA
        520 AGCATCAAAGCGATCCAAATATCCACTTCCAGATTCTACAAAAAGAGTGTTTCAAAACTGCTCTA

            **    **                 *    * *    *         *           **    
     174312 AGAAAGCGAATGTTCAACTCTGTGACTTGAATGCACATATCACAAAGTAGTTTCTGAGAGGGCTT
        585 TCAAAGGAAATGTTCAACTCTGTGAGTTGAGTACACACATCACAAAGAAGTTTCTGAGAATGCTT

                          *              *        *       * *    *           
     174377 CTGTCTAGATTTTAGATGATGATATTCCCGTTTCCAACAAAATCATTAGAGC-TTCCAAATATCC
        650 CTGTCTAG-TTTT-TATGATGATATTCCCTTTTCCAACGAAATCATCAAAGCGATCCAAATATCC

                *   *                    *         **      *  * *    *       
     174441 ACTTACAGTTTCTACAAAAAGAGTGTTTCCAAACTG-CTGCATCAAAAGAGAGGTTCCACTCTGT
        713 ACTTCCAGATTCTACAAAAAGAGTGTTTCAAAACTGCCTCTATCAAAGGAAATGTTCAACTCTGT

               *                   ***      *     *         *                
     174505 TAGCTGAGTACACACATCACAAACTTGTTTCTCAGAATCCTTCTGTCTCGTTTTTATGGGAAGAT
        778 TAGTTGAGTACACACATCACAAAGAAGTTTCTGAGAATGCTTCTGTCTAGTTTTTATGGGAAGAT

              ***     *  *  * **          *       *     *       *  *  *      
     174570 -TATACTTTTTCACCGTAGGCATCAAAGCGCTCCAAATGTCCACATCCAGATACTCCAGAAAGAG
        843 ATTCCCTTTTCCAACGAAATCATCAAAGCGATCCAAATATCCACTTCCAGATTCTACAAAAAGAG

                     *    *    *     *   *         *                    *    
     174634 TGTTTCAAACCTGCCCTATGAAAGGGAATCTTCAACTCTATGAGTTGAATGCAGACATCAGAAAG
        908 TGTTTCAAAACTGCTCTATCAAAGGAAATGTTCAACTCTGTGAGTTGAATGCAGACATCACAAAG

              *              *       **       **         **             *  * 
     174699 AAATTTCTGAGAATGCTGCTGTCTACCTTTTAT-TTGA-ATTCCCGCTTCCAACGAAATCCTCCA
        973 AAGTTTCTGAGAATGCTTCTGTCTAGTTTTTATGAAGATATTCCCTTTTCCAACGAAATCATCAA

               *                *       *                                  **
     174762 AGCTATCCAAATATCCACTTGCAGATTCCACAAAAAGAGTGTTTCAAAACTGCTCTCTATCAATG
       1038 AGCGATCCAAATATCCACTTCCAGATTCTACAAAAAGAGTGTTTCAAAACTG--CTCTATCAAAA

             *  *                    *     *     *  *                        
     174827 GCAAAGTTCAACTCTGTTAGTTGAGGACACATATCACCAACAAGTTTCTGAGAATGCTTCTGTCT
       1101 GAAATGTTCAACTCTGTTAGTTGAGTACACACATCACAAAGAAGTTTCTGAGAATGCTTCTGTCT

             *                  *  *    
     174892 ATTTTTTATGGGAAGATATTTCCTTTTT
       1166 AGTTTTTATGGGAAGATATTCCCGTTTT

              *  * ** *    *      *    *          * **                       
     174920 CACCGTAGGCGTCAAGGCGATCGAAATGTCCACTTCCACAAACTACAAAAAGAGTGTTTCAAACC
          1 CAACGAAATCATCAAAGCGATCCAAATATCCACTTCCAGATTCTACAAAAAGAGTGTTTCAAACC

                          **       *    *        * ** * *   **      *      * 
     174985 TGCTCTATGAAAGGCCATGTTCATCTCTATGAGTTGAATGGAAATATCCGAAAGAAATTTCTGGG
         66 TGCTCTA-GAAAGGGAATGTTCAACTCTGTGAGTTGAGTACACACATCACAAAGAAGTTTCTGAG

                  *          *        *          *                      *    
     175050 AATGCTGCTGTCTAG-TGTT-TAT-ACGA-ATTCCCGCTTCCAACGAAATC-CTCAAAGCAATCC
        130 AATGCTTCTGTCTAGTTTTTATATGAAGATATTCCCGTTTCCAACGAAATCGCT-AAAGCGATCC

                        *    *  *                                *     *     
     175110 AAATATCCACTTGCAGAATCCACAAAAAGAGTGTTTCAAAACTGCTCTATCAATAGAAAGGTTCA
        194 AAATATCCACTTCCAGATTCTACAAAAAGAGTGTTTCAAAACTGCTCTATCAAAAGAAATGTTCA

                 *                     *  *                        * *      *
     175175 ACTCTTTTAGTTGAGTACACACATCACGAACAAGTTTCTGAGAATGCTTCTGTCTGGCTTTTATT
        259 ACTCTGTTAGTTGAGTACACACATCACAAAGAAGTTTCTGAGAATGCTTCTGTCTAGTTTTTATA

            *     **  *           *  **          *       *                * *
     175240 GGAAGACGTTTCCTTTT-CAACAAAGGCATCAAAGCGCTCCAAATGTCCACTTCCAGATTCTTCC
        324 TGAAGATATTCCCTTTTCCAACGAAATCATCAAAGCGATCCAAATATCCACTTCCAGATTCTACA

                            **     * * *  * *               * *    * *  * *  
     175304 AAAAGAGTGTTTCAAACGTGCTCAAAGTAAGGGAATGTTCAACTCTGTGACTTGAATGCAGATAT
        389 AAAAGAGTGTTTCAAAACTGCTCTATGAAAAGAAATGTTCAACTCTGTTAGTTGAGTACACACAT

               *   *       * * *        *   **     ***  *                    
     175369 CACCAAGTAGTTTCTAATAGTGCTTCTGCCTACATTTTAGATGATGATATTCCCTTTTCCAACGA
        454 CACAAAGAAGTTTCTGAGAATGCTTCTGTCTAGTTTTTATGGGAAGATATTCCCTTTTCCAACGA

              * * * *   *             *  *   *      *     *       *   *    **
     175434 AATCGTTAGAGCTATCCAAATATCCAGTTACAGTTTCTACCAAAAGGGTGTTTCCAAATTGCTGC
        519 AAGCATCAAAGCGATCCAAATATCCACTTCCAGATTCTACAAAAAGAGTGTTTCAAAACTGCTCT

                  *    *            *       *                     *          
     175499 ATCAAAAGAAAGGTTCAACTCTGTTAGTTGAGGACACACATCACAAAGAAGTTTGTGAGAATGCT
        584 ATCAAAGGAAATGTTCAACTCTGTGAGTTGAGTACACACATCACAAAGAAGTTTCTGAGAATGCT

                                *                    *   * *     *   *       
     175564 TCTGTCTAGATTTTGTATGACGATATTCCCTTTTCCAACGATATCGTTAAAGCAATCTAAATATC
        649 TCTGTCTAG-TTTT-TATGATGATATTCCCTTTTCCAACGAAATCATCAAAGCGATCCAAATATC

            * *  *    *  *       *  *        *        * *   *    *    *      
     175629 AATTTGCAGAATCCACAAAAATAGAGTTTCAAAGCTG-CTCTGTAAAAAGAAAGGTTCCACTCTG
        712 CACTTCCAGATTCTACAAAAAGAGTGTTTCAAAACTGCCTCTATCAAAGGAAATGTTCAACTCTG

                *                   ***            *         *               
     175693 TTAGCTGAGTACACACATCACAAACTTGTTTCTGAGAATCCTTCTGTCTCGTTTTTATGGGAAGA
        777 TTAGTTGAGTACACACATCACAAAGAAGTTTCTGAGAATGCTTCTGTCTAGTTTTTATGGGAAGA

                **        *  * **          *       *     *       *  *  *  *  
     175758 TATTTACTTTTCCACCGTAGGCATCAAAGCGCTCCAAATGTCCACATCCAGATACTCCAGAACGA
        842 TATTCCCTTTTCCAACGAAATCATCAAAGCGATCCAAATATCCACTTCCAGATTCTACAAAAAGA

                      *         *     *   *         *                    *   
     175823 GTGTTTCAAACCTGCTCTATGAAAGGGAATCTTCAACTCTATGAGTTGAATGCAGACATCAGAAA
        907 GTGTTTCAAAACTGCTCTATCAAAGGAAATGTTCAACTCTGTGAGTTGAATGCAGACATCACAAA

               *              *       **       **         **             *  *
     175888 GAAATTTCTGAGAATGCTGCTGTCTACCTTTTAT-TTGA-ATTCCCGCTTCCAACGAAATCCTCC
        972 GAAGTTTCTGAGAATGCTTCTGTCTAGTTTTTATGAAGATATTCCCTTTTCCAACGAAATCATCA

                *              * *  **   *                                *  
     175951 AAGCTATCCAAATATCCACCTGCATTTTCCACAAAAAGAGTGTTTCAAAACTGCTCTATCAATAG
       1037 AAGCGATCCAAATATCCACTTCCAGATTCTACAAAAAGAGTGTTTCAAAACTGCTCTATCAAAAG

                         **  * *  *                *                         
     176016 AAATGTTCAACTCCTTTGGCTGGGTACACACATCACAAACAAGTTTCTGAGAATGCTTCTGTCTA
       1102 AAATGTTCAACTCTGTTAGTTGAGTACACACATCACAAAGAAGTTTCTGAGAATGCTTCTGTCTA

                           *      *    
     176081 GTTTTTATGGGAAGACATTCCCTTTTT
       1167 GTTTTTATGGGAAGATATTCCCGTTTT

              * *  **          *       *            **                      *
     176108 CACCAAAGACATCAAAGCGCTCCAAATGTCCACTTCCAGACACTACAAAAAGAGTGTTTCAAACG
          1 CAACGAAATCATCAAAGCGATCCAAATATCCACTTCCAGATTCTACAAAAAGAGTGTTTCAAACC

                         *                  *    * *  * *         *          
     176173 TGCTCTAAGAAAGCGAATGTTCAACTCTGTGACTTGAATGCAGATATCACAAAGTAGTTTCTGAG
         66 TGCTCT-AGAAAGGGAATGTTCAACTCTGTGAGTTGAGTACACACATCACAAAGAAGTTTCTGAG

             **            *     *    *                        **  *   *     
     176238 AGGGCTTCTGTCTAGATTTTAGATGATGATATTCCCGTTTCCAACGAAATCATTAGAGCTATCCA
        130 AATGCTTCTGTCTAGTTTTTATATGAAGATATTCCCGTTTCCAACGAAATCGCTAAAGCGATCCA

                       *   *                    *        **         * *    * 
     176303 AATATCCACTTACAGTTTCTACAAAAAGAGTGTTTCCAAACTGCTGCATCAAAAGAGAGGTTCCA
        195 AATATCCACTTCCAGATTCTACAAAAAGAGTGTTTCAAAACTGCTCTATCAAAAGAAATGTTCAA

                     **                  ***      *     *         *        **
     176368 CTCTGTTAGCCGAGTACACACATCACAAACTTGTTTCTCAGAATCCTTCTGTCTCGTTTTTATGG
        260 CTCTGTTAGTTGAGTACACACATCACAAAGAAGTTTCTGAGAATGCTTCTGTCTAGTTTTTATAT

                     **     *  *  * **          *       *     *       *  *  *
     176433 GAAGATATTTACTTTTTCACCGTAGGCATCAAAGCGCTCCAAATGTCCACATCCAGATACTCCAG
        325 GAAGATATTCCCTTTTCCAACGAAATCATCAAAGCGATCCAAATATCCACTTCCAGATTCTACAA

                           *             * *   *         * *      * *  *     
     176498 AAAGAGTGTTTCAAACCTGCTCTATGAAAGGGAATCTTCAACTCTATGAGTTGAATGCAGACATC
        390 AAAGAGTGTTTCAAAACTGCTCTATGAAAAGAAATGTTCAACTCTGTTAGTTGAGTACACACATC

             *      *              *       **         **         **          
     176563 AGAAAGAAATTTCTGAGAATGCTGCTGTCTACCTTTTAT---TTGA-ATTCCCGCTTCCAACGAA
        455 ACAAAGAAGTTTCTGAGAATGCTTCTGTCTAGTTTTTATGGGAAGATATTCCCTTTTCCAACGAA

             * *  *    *                *       *                            
     176624 ATCCTCCAAGCTATCCAAATATCCACTTGCAGATTCCACAAAAAGAGTGTTTCAAAACTGCTCTC
        520 AGCATCAAAGCGATCCAAATATCCACTTCCAGATTCTACAAAAAGAGTGTTTCAAAACTG--CTC

                  *  *  *            *       *     *     *  *                
     176689 TATCAATGGCAAAGTTCAACTCTGTTAGTTGAGGACACATATCACCAACAAGTTTCTGAGAATGC
        583 TATCAAAGGAAATGTTCAACTCTGTGAGTTGAGTACACACATCACAAAGAAGTTTCTGAGAATGC

                     *           *      *      *  *  * ** *    *      *    * 
     176754 TTCTGTCTATTTTTTATGGGAAGATATTTCCTTTTTCACCGTAGGCGTCAAGGCGATCGAAATGT
        648 TTCTGTCTAGTTTTTAT--GATGATATTCCCTTTTCCAACGAAATCATCAAAGCGATCCAAATAT

                     * **                     *          *     **       *    
     176819 CCACTTCCACAAACTACAAAAAGAGTGTTTCAAACCTG-CTCTATGAAAGGCCATGTTCATCTCT
        711 CCACTTCCAGATTCTACAAAAAGAGTGTTTCAAAACTGCCTCTATCAAAGGAAATGTTCAACTCT

            * *      * ** * *   **      *      *       *         *         * 
     176883 ATGAGTTGAATGGAAATATCCGAAAGAAATTTCTGGGAATGCTGCTGTCTAGTGTTTAT---ACG
        776 GTTAGTTGAGTACACACATCACAAAGAAGTTTCTGAGAATGCTTCTGTCTAGTTTTTATGGGAAG

                    **             *       *                *    *  *        
     176945 A-ATTCCCGCTTCCAACGAAATCCTCAAAGCAATCCAAATATCCACTTGCAGAATCCACAAAAAG
        841 ATATTCCCTTTTCCAACGAAATCATCAAAGCGATCCAAATATCCACTTCCAGATTCTACAAAAAG

                                    **    *          * *      * *  *       * 
     177009 AGTGTTTCAAAACTGCTCTATCAATAGAAAGGTTCAACTCTTTTAGTTGAGTACACACATCACGA
        906 AGTGTTTCAAAACTGCTCTATCAAAGGAAATGTTCAACTCTGTGAGTTGAATGCAGACATCACAA

             *                        * *             **  *         * *  **  
     177074 ACAAGTTTCTGAGAATGCTTCTGTCTGGCTTTTATTGGAAGACGTTTCCTTTT-CACCAAAGGCA
        971 AGAAGTTTCTGAGAATGCTTCTGTCTAGTTTTTA-T-GAAGATATTCCCTTTTCCAACGAAATCA

                    *       *                * *                **     * *** 
     177138 TCAAAGCGCTCCAAATGTCCACTTCCAGATTCTTCCAAAAGAGTGTTTCAAACGTGCTCAAAGTA
       1034 TCAAAGCGATCCAAATATCCACTTCCAGATTCTACAAAAAGAGTGTTTCAAAACTGCTCTATCAA

             * *               * *    * *  * *     *   *       * * *         
     177203 AGGGAATGTTCAACTCTGTGACTTGAATGCAGATATCACCAAGTAGTTTCTAATAGTGCTTCTGT
       1099 AAGAAATGTTCAACTCTGTTAGTTGAGTACACACATCACAAAGAAGTTTCTGAGAATGCTTCTGT

               **     ***  *             *
     177268 CTACATTTTAGATGATGATATTCCCGTTTC
       1164 CTAGTTTTTATGGGAAGATATTCCCGTTTT

                      *** *   *             *  *   *      *     *       *  **
     177298 CAACGAAATCGCTAGAGCTATCCAAATATCCAGTTACAGTTTCTACCAAAAGGGTGTTTCCAAAT
          1 CAACGAAATCATCAAAGCGATCCAAATATCCACTTCCAGATTCTACAAAAAGAGTGTTTCAAACC

                  ***   * *  *            *       *                     *    
     177363 TGCTGCATCAAAAGAAAGGTTCAACTCTGTTAGTTGAGGACACACATCACAAAGAAGTTTGTGAG
         66 TGCT-CTAGAAAGGGAATGTTCAACTCTGTGAGTTGAGTACACACATCACAAAGAAGTTTCTGAG

                           *    *     *         *          *    *      *   * 
     177428 AATGCTTCTGTCTAGATTTTGTATGACGATATTCCCTTTTCCAACGATATCGTTAAAGCAATCTA
        130 AATGCTTCTGTCTAGTTTTTATATGAAGATATTCCCGTTTCCAACGAAATCGCTAAAGCGATCCA

                  * *  *    *  *       *  *        *       * *        *    * 
     177493 AATATCAATTTGCAGAATCCACAAAAATAGAGTTTCAAAGCTGCTCTGTAAAAAGAAAGGTTCCA
        195 AATATCCACTTCCAGATTCTACAAAAAGAGTGTTTCAAAACTGCTCTATCAAAAGAAATGTTCAA

                     *                   ***      *     *         *        **
     177558 CTCTGTTAGCTGAGTACACACATCACAAACTTGTTTCTCAGAATCCTTCTGTCTCGTTTTTATGG
        260 CTCTGTTAGTTGAGTACACACATCACAAAGAAGTTTCTGAGAATGCTTCTGTCTAGTTTTTATAT

                   * **        *  * **          *       *     *       *  *  *
     177623 GAAGATACTTACTTTTCCACCGTAGGCATCAAAGCGCTCCAAATGTCCACATCCAGATACTCCAG
        325 GAAGATATTCCCTTTTCCAACGAAATCATCAAAGCGATCCAAATATCCACTTCCAGATTCTACAA

              *            *             * *   *         * *      * *  *     
     177688 AACGAGTGTTTCAAACCTGCTCTATGAAAGGGAATCTTCAACTCTATGAGTTGAATGCAGACATC
        390 AAAGAGTGTTTCAAAACTGCTCTATGAAAAGAAATGTTCAACTCTGTTAGTTGAGTACACACATC

             *      *              *       **         **         **          
     177753 AGAAAGAAATTTCTGAGAATGCTGCTGTCTACCTTTTAT---TTGA-ATTCCCGCTTCCAACGAA
        455 ACAAAGAAGTTTCTGAGAATGCTTCTGTCTAGTTTTTATGGGAAGATATTCCCTTTTCCAACGAA

             * *  *    *              * *  **   *    *                       
     177814 ATCCTCCAAGCTATCCAAATATCCACCTGCATTTTCCACAACAAGAGTGTTTCAAAACTGCTCTA
        520 AGCATCAAAGCGATCCAAATATCCACTTCCAGATTCTACAAAAAGAGTGTTTCAAAACTGCTCTA

                **                *    *  *                *                 
     177879 TCAATAGAAATGTTCAACTCCTTTG-GCTGGGTACACACATCACAAACAAGTTTCTGAGAATGCT
        585 TCAAAGGAAATGTTCAACT-CTGTGAGTTGAGTACACACATCACAAAGAAGTTTCTGAGAATGCT

                                *  **         *  * *  **    *     *       *  
     177943 TCTGTCTAGTTTTTATGGGAAGACGTTCCCTTTTTCACCAAAGGCATCCAAGCGCTCCAAATGTC
        649 TCTGTCTAGTTTTTAT--GATGATATTCCCTTTTCCAACGAAATCATCAAAGCGATCCAAATATC

               *       *  *  *               *          *     *   *         *
     178008 CACATCCAGATACTCCAGAAAGAGTGTTTCAAACCTG-CTCTATGAAAGGGAATCTTCAACTCTA
        712 CACTTCCAGATTCTACAAAAAGAGTGTTTCAAAACTGCCTCTATCAAAGGAAATGTTCAACTCTG

             *      * *  *      *      *              *       **         **  
     178072 TGAGTTGAATGCAGACATCAGAAAGAAATTTCTGAGAATGCTGCTGTCTACCTTTTAT---TTGA
        777 TTAGTTGAGTACACACATCACAAAGAAGTTTCTGAGAATGCTTCTGTCTAGTTTTTATGGGAAGA

                   **             *  *    *                *       *         
     178134 -ATTCCCGCTTCCAACGAAATCCTCCAAGCTATCCAAATATCCACTTGCAGATTCCACAAAAAGA
        842 TATTCCCTTTTCCAACGAAATCATCAAAGCGATCCAAATATCCACTTCCAGATTCTACAAAAAGA

                                     *  *  *            *      ***  * *     *
     178198 GTGTTTCAAAACTGCTCTCTATCAATGGCAAAGTTCAACTCTGTTAGTTGAGGACACATATCACC
        907 GTGTTTCAAAACTG--CTCTATCAAAGGAAATGTTCAACTCTGTGAGTTGAATGCAGACATCACA

              *                         *                  *      *  * ** ** 
     178263 AACAAGTTTCTGAGAATGCTTCTGTCTATTTTTTATGGGAAGATATTTCCTTTTTCACCATAGGC
        970 AAGAAGTTTCTGAGAATGCTTCTGTCTAGTTTTTAT--GAAGATATTCCCTTTTCCAACGAAATC

            *    *      *    *          * **                     *         * 
     178328 GTCAAGGCGATCGAAATGTCCACTTCCACAAACTACAAAAAGAGTGTTTCAAACCTGCTCTATGA
       1033 ATCAAAGCGATCCAAATATCCACTTCCAGATTCTACAAAAAGAGTGTTTCAAAACTGCTCTATCA

              * **       *    * *      * ** * *   **      *      *       *   
     178393 AAGGCCATGTTCATCTCTATGAGTTGAATGGAAATATCCGAAAGAAATTTCTGGGAATGCTGCTG
       1098 AAAGAAATGTTCAACTCTGTTAGTTGAGTACACACATCACAAAGAAGTTTCTGAGAATGCTTCTG

                  *         *          *  *
     178458 TCTAGTGTTTAT---ACGA-ATTCCCGCTTC
       1163 TCTAGTTTTTATGGGAAGATATTCCCGTTTT

                      *       *                *    *  *                   * 
     178485 CAACGAAATCCTCAAAGCAATCCAAATATCCACTTGCAGAATCCACAAAAAGAGTGTTTCAAAAC
          1 CAACGAAATCATCAAAGCGATCCAAATATCCACTTCCAGATTCTACAAAAAGAGTGTTTCAAACC

                    *  ** *  *          * *                   *  *           
     178550 TGCTCTATCAATAGAAAGGTTCAACTCTTTTAGTTGAGTACACACATCACGAACAAGTTTCTGAG
         66 TGCTCTA-GAAAGGGAATGTTCAACTCTGTGAGTTGAGTACACACATCACAAAGAAGTTTCTGAG

                         * *      **     **  *      *         *           *  
     178615 AATGCTTCTGTCTGGCTTTTATTGGAAGACGTTTCC-TTTTCAAC-AAA-GGCATCAAAGCGCTC
        130 AATGCTTCTGTCTAGTTTTTATATGAAGATATTCCCGTTTCCAACGAAATCGC-T-AAAGCGATC

                 *                * *                **     * ***  * *       
     178677 CAAATGTCCACTTCCAGATTCTTCCAAAAGAGTGTTTCAAACGTGCTCAAAGTAAGGGAATGTTC
        193 CAAATATCCACTTCCAGATTCTACAAAAAGAGTGTTTCAAAACTGCTCTATCAAAAGAAATGTTC

                    * *    * *  * *     *   *       * * *        *   **     *
     178742 AACTCTGTGACTTGAATGCAGATATCACCAAGTAGTTTCTAATAGTGCTTCTGCCTACATTTTAG
        258 AACTCTGTTAGTTGAGTACACACATCACAAAGAAGTTTCTGAGAATGCTTCTGTCTAGTTTTTAT

                *                        * * *   *             *  *   *      
     178807 ATGATGATATTCCCTTTTCCAACGAAATCGTTAGAGCTATCCAAATATCCAGTTACAGTTTCTAC
        323 ATGAAGATATTCCCTTTTCCAACGAAATCATCAAAGCGATCCAAATATCCACTTCCAGATTCTAC

            *     *       *   *    **  *        *                    *       
     178872 CAAAAGGGTGTTTCCAAATTGCTGCATCAAAAGAAAGGTTCAACTCTGTTAGTTGAGGACACACA
        388 AAAAAGAGTGTTTCAAAACTGCTCTATGAAAAGAAATGTTCAACTCTGTTAGTTGAGTACACACA

                          *                   *    * **  *                   
     178937 TCACAAAGAAGTTTGTGAGAATGCTTCTGTCTAGATTTTGTATGACGATATTCCCTTTTCCAACG
        453 TCACAAAGAAGTTTCTGAGAATGCTTCTGTCTAGTTTTTATGGGAAGATATTCCCTTTTCCAACG

             * * * *     *   *       * *  *    *  *       *  *        *      
     179002 ATATCGTTAAAGCAATCTAAATATCAATTTGCAGAATCCACAAAAATAGAGTTTCAAAGCTGCTC
        518 AAAGCATCAAAGCGATCCAAATATCCACTTCCAGATTCTACAAAAAGAGTGTTTCAAAACTGCTC

             * *   *    *    *       *  *                   ***            * 
     179067 TGTAAAAAGAAAGGTTCCACTCTGTTAGCTGAGTACACACATCACAAACTTGTTTCTGAGAATCC
        583 TATCAAAGGAAATGTTCAACTCTGTGAGTTGAGTACACACATCACAAAGAAGTTTCTGAGAATGC

                    *            *      **        *  * **          *       * 
     179132 TTCTGTCTCGTTTTTATGGGAAGATATTTACTTTTCCACCGTAGGCATCAAAGCGCTCCAAATGT
        648 TTCTGTCTAGTTTTTAT--GATGATATTCCCTTTTCCAACGAAATCATCAAAGCGATCCAAATAT

                *       *  *  *  *            *          *     *   *         
     179197 CCACATCCAGATACTCCAGAACGAGTGTTTCAAACCTG-CTCTATGAAAGGGAATCTTCAACTCT
        711 CCACTTCCAGATTCTACAAAAAGAGTGTTTCAAAACTGCCTCTATCAAAGGAAATGTTCAACTCT

            * *      * *  *      *      *              *       **         ** 
     179261 ATGAGTTGAATGCAGACATCAGAAAGAAATTTCTGAGAATGCTGCTGTCTACCTTTTAT---TTG
        776 GTTAGTTGAGTACACACATCACAAAGAAGTTTCTGAGAATGCTTCTGTCTAGTTTTTATGGGAAG

                    **             *  *    *              * *  **   *        
     179323 A-ATTCCCGCTTCCAACGAAATCCTCCAAGCTATCCAAATATCCACCTGCATTTTCCACAAAAAG
        841 ATATTCCCTTTTCCAACGAAATCATCAAAGCGATCCAAATATCCACTTCCAGATTCTACAAAAAG

                                    **                *    *  ** *  *        
     179387 AGTGTTTCAAAACTGCTCTATCAATAGAAATGTTCAACTCCTTTG-GCTGGGTACACACATCACA
        906 AGTGTTTCAAAACTGCTCTATCAAAGGAAATGTTCAACT-CTGTGAGTTGAATGCAGACATCACA

              *                                        *          *  * *  ** 
     179451 AACAAGTTTCTGAGAATGCTTCTGTCTAGTTTTTATGGGAAGACATTCCCTTTTTCACCAAAGAC
        970 AAGAAGTTTCTGAGAATGCTTCTGTCTAGTTTTTAT--GAAGATATTCCCTTTTCCAACGAAATC

                     *       *            **                     **       ** 
     179516 ATCAAAGCGCTCCAAATGTCCACTTCCAGACACTACAAAAAGAGTGTTTCAAACGTGCTCTAAGA
       1033 ATCAAAGCGATCCAAATATCCACTTCCAGATTCTACAAAAAGAGTGTTTCAAAACTGCTCTATCA

              ***               * *    * *  * *         *           **       
     179581 AAGCGAATGTTCAACTCTGTGACTTGAATGCAGATATCACAAAGTAGTTTCTGAGAGGGCTTCTG
       1098 AAAGAAATGTTCAACTCTGTTAGTTGAGTACACACATCACAAAGAAGTTTCTGAGAATGCTTCTG

                 *     ***  *             *
     179646 TCTAGATTTTAGATGATGATATTCCCGTTTC
       1163 TCTAGTTTTTATGGGAAGATATTCCCGTTTT

                        * *   *                *   *                    *  * 
     179677 CAACGAAATCATTAGAGCTATCCAAATATCCACTTACAGTTTCTACAAAAAGAGTGTTTCCAAAC
          1 CAACGAAATCATCAAAGCGATCCAAATATCCACTTCCAGATTCTACAAAAAGAGTGTTTCAAACC

                    **    *   *    *       *  *                   ***      * 
     179742 TGCTGCATCAAAAGAG-AGGTTCCACTCTGTTAGCTGAGTACACACATCACAAACTTGTTTCTCA
         66 TGCT-C-TAGAAAGGGAATGTTCAACTCTGTGAGTTGAGTACACACATCACAAAGAAGTTTCTGA

                *         *        **         ** *   *  *  * ** ***      *   
     179806 GAATCCTTCTGTCTCGTTTTTATGGGAAGATATTTACTTTTTCACCGTAGGCATCAAAGCGCTCC
        129 GAATGCTTCTGTCTAGTTTTTATATGAAGATATTCCCGTTTCCAACGAAATCGCTAAAGCGATCC

                *     *       *  *  *               *         *   * *   *    
     179871 AAATGTCCACATCCAGATACTCCAGAAAGAGTGTTTCAAACCTGCTCTATGAAAGGGAATCTTCA
        194 AAATATCCACTTCCAGATTCTACAAAAAGAGTGTTTCAAAACTGCTCTATCAAAAGAAATGTTCA

                 * *      * *  *      *      *              *       **       
     179936 ACTCTATGAGTTGAATGCAGACATCAGAAAGAAATTTCTGAGAATGCTGCTGTCTACCTTTTAT-
        259 ACTCTGTTAGTTGAGTACACACATCACAAAGAAGTTTCTGAGAATGCTTCTGTCTAGTTTTTATA

               *         **             *  *    *                *       *   
     180000 T--TGA-ATTCCCGCTTCCAACGAAATCCTCCAAGCTATCCAAATATCCACTTGCAGATTCCACA
        324 TGAAGATATTCCCTTTTCCAACGAAATCATCAAAGCGATCCAAATATCCACTTCCAGATTCTACA

                                        *  ** *  *                    *     *
     180062 AAAAGAGTGTTTCAAAACTGCTCTCTATCAATGGCAAAGTTCAACTCTGTTAGTTGAGGACACAT
        389 AAAAGAGTGTTTCAAAACTG--CTCTATGAAAAGAAATGTTCAACTCTGTTAGTTGAGTACACAC

                 *  *                         *                  *      *  * 
     180127 ATCACCAACAAGTTTCTGAGAATGCTTCTGTCTATTTTTTATGGGAAGATATTTCCTTTTTCACC
        452 ATCACAAAGAAGTTTCTGAGAATGCTTCTGTCTAGTTTTTATGGGAAGATATTCCCTTTTCCAAC

             * *  *    *      *    *          * **                     *     
     180192 GTAGGCGTCAAGGCGATCGAAATGTCCACTTCCACAAACTACAAAAAGAGTGTTTCAAACCTGCT
        517 GAAAGCATCAAAGCGATCCAAATATCCACTTCCAGATTCTACAAAAAGAGTGTTTCAAAACTGCT

                *     **       *    *        * ** * *   **      *      *     
     180257 CTATGAAAGGCCATGTTCATCTCTATGAGTTGAATGGAAATATCCGAAAGAAATTTCTGGGAATG
        582 CTATCAAAGGAAATGTTCAACTCTGTGAGTTGAGTACACACATCACAAAGAAGTTTCTGAGAATG

              *         *       *         **        *    *       *           
     180322 CTGCTGTCTAGTGTTTAT-ACGA-ATTCCCGCTTCCAACGTAATCCTCAAAGCAATCCAAATATC
        647 CTTCTGTCTAGTTTTTATGATGATATTCCCTTTTCCAACGAAATCATCAAAGCGATCCAAATATC

                 *    *  *                                 **    *          *
     180385 CACTTGCAGAATCCACAAAAAGAGTGTTTCAAAACTG-CTCTATCAATAGAAAGGTTCAACTCTT
        712 CACTTCCAGATTCTACAAAAAGAGTGTTTCAAAACTGCCTCTATCAAAGGAAATGTTCAACTCTG

                                 *  *                        * *      *      
     180449 TTAGTTGAGTACACACATCACGAACAAGTTTCTGAGAATGCTTCTGTCTGGCTTTTATTGGAAGA
        777 TTAGTTGAGTACACACATCACAAAGAAGTTTCTGAGAATGCTTCTGTCTAGTTTTTATGGGAAGA

            **  *         * *  **          *       *                * *      
     180514 CGTTTCCTTTT-CACCAAAGGCATCAAAGCGCTCCAAATGTCCACTTCCAGATTCTTCCAAAAGA
        842 TATTCCCTTTTCCAACGAAATCATCAAAGCGATCCAAATATCCACTTCCAGATTCTACAAAAAGA

                      **     * ***    *                 *           *     *  
     180578 GTGTTTCAAACGTGCTCAAAGTAAGGGAATGTTCAACTCTGTGACTTGAATGCAGATATCACCAA
        907 GTGTTTCAAAACTGCTCTATCAAAGGAAATGTTCAACTCTGTGAGTTGAATGCAGACATCACAAA

             *       * * *             *     *    *         *              **
     180643 GTAGTTTCTAATAGTGCTTCTGTCTACATTTTAGATGATGATATTCCCGTTTCCAACGAAATCGC
        972 GAAGTTTCTGAGAATGCTTCTGTCTA-GTTTT-TATGAAGATATTCCCTTTTCCAACGAAATCAT

            * *   *             *  *   *      *     *       *   *    **      
     180708 TAGAGCTATCCAAATATCCAGTTACAGTTTCTACCAAAAGGGTGTTTCCAAATTGCTGCATCAAA
       1035 CAAAGCGATCCAAATATCCACTTCCAGATTCTACAAAAAGAGTGTTTCAAAACTGCTCTATCAAA

                 *                    *                     *                
     180773 AGAAAGGTTCAACTCTGTTAGTTGAGGACACACATCACAAAGAAGTTTGTGAGAATGCTTCTGTC
       1100 AGAAATGTTCAACTCTGTTAGTTGAGTACACACATCACAAAGAAGTTTCTGAGAATGCTTCTGTC

               *    * **  *         *   *
     180838 TAGGTTTTGTATGACGATATTCCCTTTTC
       1165 TAGTTTTTATGGGAAGATATTCCCGTTTT

                  *   * *     *   *       * *  *    *  *       *  *        * 
     180867 CAACGATATCGTTAAAGCAATCTAAATATCAATTTGCAGAATCCACAAAAATAGAGTTTCAAAGC
          1 CAACGAAATCATCAAAGCGATCCAAATATCCACTTCCAGATTCTACAAAAAGAGTGTTTCAAACC

                   **   * *  *    *       *  *                   ***         
     180932 TGCTCTGTAAAAAGAAAGGTTCCACTCTGTTAGCTGAGTACACACATCACAAACTTGTTTCTGAG
         66 TGCTCT-AGAAAGGGAATGTTCAACTCTGTGAGTTGAGTACACACATCACAAAGAAGTTTCTGAG

               *         *        **       * ** *      *  * ** ***      *    
     180997 AATCCTTCTGTCTCGTTTTTATGGGAAGATACTTACTTTTCCACCGTAGGCATCAAAGCGCTCCA
        130 AATGCTTCTGTCTAGTTTTTATATGAAGATATTCCCGTTTCCAACGAAATCGCTAAAGCGATCCA

               *     *       *  *  *  *            *         *   * *   *     
     181062 AATGTCCACATCCAGATACTCCAGAACGAGTGTTTCAAACCTGCTCTATGAAAGGGAATCTTCAA
        195 AATATCCACTTCCAGATTCTACAAAAAGAGTGTTTCAAAACTGCTCTATCAAAAGAAATGTTCAA

                * *      * *  *      *      *              *       **        
     181127 CTCTATGAGTTGAATGCAGACATCAGAAAGAAATTTCTGAGAATGCTGCTGTCTACCTTTTAT-T
        260 CTCTGTTAGTTGAGTACACACATCACAAAGAAGTTTCTGAGAATGCTTCTGTCTAGTTTTTATAT

              *         **             *  *    *              * *  **   *    
     181191 --TGA-ATTCCCGCTTCCAACGAAATCCTCCAAGCTATCCAAATATCCACCTGCATTTTCCACAA
        325 GAAGATATTCCCTTTTCCAACGAAATCATCAAAGCGATCCAAATATCCACTTCCAGATTCTACAA

            *                        *  *               **  * *  *           
     181253 CAAGAGTGTTTCAAAACTGCTCTATCAATAGAAATGTTCAACTCCTTTGGCTGGGTACACACATC
        390 AAAGAGTGTTTCAAAACTGCTCTATGAAAAGAAATGTTCAACTCTGTTAGTTGAGTACACACATC

                 *                                        **         *  * *  
     181318 ACAAACAAGTTTCTGAGAATGCTTCTGTCTAGTTTTTATGGGAAGACGTTCCCTTTTTCACCAAA
        455 ACAAAGAAGTTTCTGAGAATGCTTCTGTCTAGTTTTTATGGGAAGATATTCCCTTTTCCAACGAA

            *           *       *            **                     **       
     181383 GGCATCAAAGCGCTCCAAATGTCCACTTCCAGACACTACAAAAAGAGTGTTTCAAACGTGCTCTA
        520 AGCATCAAAGCGATCCAAATATCCACTTCCAGATTCTACAAAAAGAGTGTTTCAAAACTGCTCTA

            **    **                 *    * *    *         *    *      **    
     181448 AGAAAGCGAATGTTCAACTCTGTGACTTGAATGCACATATCACAAAGTAGTTCCTGAGAGGGCTT
        585 TCAAAGGAAATGTTCAACTCTGTGAGTTGAGTACACACATCACAAAGAAGTTTCTGAGAATGCTT

                          *              *        *       * *    *           
     181513 CTGTCTAGATTTTAGATGATGATATTCCCGTTTCCAACAAAATCATTAGAGC-TTCCAAATATCC
        650 CTGTCTAG-TTTT-TATGATGATATTCCCTTTTCCAACGAAATCATCAAAGCGATCCAAATATCC

                *   *                    *         **      *  * *    *       
     181577 ACTTACAGTTTCTACAAAAAGAGTGTTTCCAAACTG-CTGCATCAAAAGAGAGGTTCCACTCTGT
        713 ACTTCCAGATTCTACAAAAAGAGTGTTTCAAAACTGCCTCTATCAAAGGAAATGTTCAACTCTGT

               *                   ***      *     *         *                
     181641 TAGCTGAGTACACACATCACAAACTTGTTTCTCAGAATCCTTCTGTCTCGTTTTTATGGGAAGAT
        778 TAGTTGAGTACACACATCACAAAGAAGTTTCTGAGAATGCTTCTGTCTAGTTTTTATGGGAAGAT

              ***     *  *  * **          *       *     *       *  *  *      
     181706 -TATACTTTTTCACCGTAGGCATCAAAGCGCTCCAAATGTCCACATCCAGATACTCCAGAAAGAG
        843 ATTCCCTTTTCCAACGAAATCATCAAAGCGATCCAAATATCCACTTCCAGATTCTACAAAAAGAG

                     *    *    *     *   *         *                    *    
     181770 TGTTTCAAACCTGCCCTATGAAAGGGAATCTTCAACTCTATGAGTTGAATGCAGACATCAGAAAG
        908 TGTTTCAAAACTGCTCTATCAAAGGAAATGTTCAACTCTGTGAGTTGAATGCAGACATCACAAAG

              *              *       **       **         **             *  * 
     181835 AAATTTCTGAGAATGCTGCTGTCTACCTTTTAT-TTGA-ATTCCCGCTTCCAACGAAATCCTCCA
        973 AAGTTTCTGAGAATGCTTCTGTCTAGTTTTTATGAAGATATTCCCTTTTCCAACGAAATCATCAA

               *                *       *                                  **
     181898 AGCTATCCAAATATCCACTTGCAGATTCCACAAAAAGAGTGTTTCAAAACTGCTCTCTATCAATG
       1038 AGCGATCCAAATATCCACTTCCAGATTCTACAAAAAGAGTGTTTCAAAACTG--CTCTATCAAAA

             *  *                    *     *     *  *                        
     181963 GCAAAGTTCAACTCTGTTAGTTGAGGACACATATCACCAACAAGTTTCTGAGAATGCTTCTGTCT
       1101 GAAATGTTCAACTCTGTTAGTTGAGTACACACATCACAAAGAAGTTTCTGAGAATGCTTCTGTCT

             *                  *  *    
     182028 ATTTTTTATGGGAAGATATTTCCTTTTT
       1166 AGTTTTTATGGGAAGATATTCCCGTTTT

              *  * ** *    *      *    *          * **                       
     182056 CACCGTAGGCGTCAAGGCGATCGAAATGTCCACTTCCACAAACTACAAAAAGAGTGTTTCAAACC
          1 CAACGAAATCATCAAAGCGATCCAAATATCCACTTCCAGATTCTACAAAAAGAGTGTTTCAAACC

                          **       *    *        * ** * *   **      *      * 
     182121 TGCTCTATGAAAGGCCATGTTCATCTCTATGAGTTGAATGGAAATATCCGAAAGAAATTTCTGGG
         66 TGCTCTA-GAAAGGGAATGTTCAACTCTGTGAGTTGAGTACACACATCACAAAGAAGTTTCTGAG

                  *          *        *          *                      *    
     182186 AATGCTGCTGTCTAG-TGTT-TAT-ACGA-ATTCCCGCTTCCAACGAAATC-CTCAAAGCAATCC
        130 AATGCTTCTGTCTAGTTTTTATATGAAGATATTCCCGTTTCCAACGAAATCGCT-AAAGCGATCC

                        *    *  *                                *     *     
     182246 AAATATCCACTTGCAGAATCCACAAAAAGAGTGTTTCAAAACTGCTCTATCAATAGAAAGGTTCA
        194 AAATATCCACTTCCAGATTCTACAAAAAGAGTGTTTCAAAACTGCTCTATCAAAAGAAATGTTCA

                 *                     *  *                        * *      *
     182311 ACTCTTTTAGTTGAGTACACACATCACGAACAAGTTTCTGAGAATGCTTCTGTCTGGCTTTTATT
        259 ACTCTGTTAGTTGAGTACACACATCACAAAGAAGTTTCTGAGAATGCTTCTGTCTAGTTTTTATA

            *     **  *         * *  **          *       *                * *
     182376 GGAAGACGTTTCCTTTT-CACCAAAGGCATCAAAGCGCTCCAAATGTCCACTTCCAGATTCTTCC
        324 TGAAGATATTCCCTTTTCCAACGAAATCATCAAAGCGATCCAAATATCCACTTCCAGATTCTACA

                            **     * * *  * *               * *    * *  * *  
     182440 AAAAGAGTGTTTCAAACGTGCTCAAAGTAAGGGAATGTTCAACTCTGTGACTTGAATGCAGATAT
        389 AAAAGAGTGTTTCAAAACTGCTCTATGAAAAGAAATGTTCAACTCTGTTAGTTGAGTACACACAT

               *   *       * * *        *   **     ***  *                    
     182505 CACCAAGTAGTTTCTAATAGTGCTTCTGCCTACATTTTAGATGATGATATTCCCTTTTCCAACGA
        454 CACAAAGAAGTTTCTGAGAATGCTTCTGTCTAGTTTTTATGGGAAGATATTCCCTTTTCCAACGA

              * * * *   *             *  *   *      *     *       *   *    **
     182570 AATCGTTAGAGCTATCCAAATATCCAGTTACAGTTTCTACCAAAAGGGTGTTTCCAAATTGCTGC
        519 AAGCATCAAAGCGATCCAAATATCCACTTCCAGATTCTACAAAAAGAGTGTTTCAAAACTGCTCT

                  *    *            *       *                     *          
     182635 ATCAAAAGAAAGGTTCAACTCTGTTAGTTGAGGACACACATCACAAAGAAGTTTGTGAGAATGCT
        584 ATCAAAGGAAATGTTCAACTCTGTGAGTTGAGTACACACATCACAAAGAAGTTTCTGAGAATGCT

                                *                    *   * *     *   *       
     182700 TCTGTCTAGATTTTGTATGACGATATTCCCTTTTCCAACGATATCGTTAAAGCAATCTAAATATC
        649 TCTGTCTAG-TTTT-TATGATGATATTCCCTTTTCCAACGAAATCATCAAAGCGATCCAAATATC

            * *  *    *  *       *  *        *        * *   *    *    *      
     182765 AATTTGCAGAATCCACAAAAATAGAGTTTCAAAGCTG-CTCTGTAAAAAGAAAGGTTCCACTCTG
        712 CACTTCCAGATTCTACAAAAAGAGTGTTTCAAAACTGCCTCTATCAAAGGAAATGTTCAACTCTG

                *                   ***            *         *               
     182829 TTAGCTGAGTACACACATCACAAACTTGTTTCTGAGAATCCTTCTGTCTCGTTTTTATGGGAAGA
        777 TTAGTTGAGTACACACATCACAAAGAAGTTTCTGAGAATGCTTCTGTCTAGTTTTTATGGGAAGA

                **        *  * **          *       *     *       *  *  *  *  
     182894 TATTTACTTTTCCACCGTAGGCATCAAAGCGCTCCAAATGTCCACATCCAGATACTCCAGAACGA
        842 TATTCCCTTTTCCAACGAAATCATCAAAGCGATCCAAATATCCACTTCCAGATTCTACAAAAAGA

                      *         *     *   *         *                    *   
     182959 GTGTTTCAAACCTGCTCTATGAAAGGGAATCTTCAACTCTATGAGTTGAATGCAGACATCAGAAA
        907 GTGTTTCAAAACTGCTCTATCAAAGGAAATGTTCAACTCTGTGAGTTGAATGCAGACATCACAAA

               *              *       **       **         **             *  *
     183024 GAAATTTCTGAGAATGCTGCTGTCTACCTTTTAT-TTGA-ATTCCCGCTTCCAACGAAATCCTCC
        972 GAAGTTTCTGAGAATGCTTCTGTCTAGTTTTTATGAAGATATTCCCTTTTCCAACGAAATCATCA

                *              * *  **   *                                *  
     183087 AAGCTATCCAAATATCCACCTGCATTTTCCACAAAAAGAGTGTTTCAAAACTGCTCTATCAATAG
       1037 AAGCGATCCAAATATCCACTTCCAGATTCTACAAAAAGAGTGTTTCAAAACTGCTCTATCAAAAG

                         **  * *  *                *                         
     183152 AAATGTTCAACTCCTTTGGCTGGGTACACACATCACAAACAAGTTTCTGAGAATGCTTCTGTCTA
       1102 AAATGTTCAACTCTGTTAGTTGAGTACACACATCACAAAGAAGTTTCTGAGAATGCTTCTGTCTA

                           *      *    
     183217 GTTTTTATGGGAAGACATTCCCTTTTT
       1167 GTTTTTATGGGAAGATATTCCCGTTTT

              * *  **          *       *            **                      *
     183244 CACCAAAGACATCAAAGCGCTCCAAATGTCCACTTCCAGACACTACAAAAAGAGTGTTTCAAACG
          1 CAACGAAATCATCAAAGCGATCCAAATATCCACTTCCAGATTCTACAAAAAGAGTGTTTCAAACC

                         *                  *    * *  * *         *          
     183309 TGCTCTAAGAAAGCGAATGTTCAACTCTGTGACTTGAATGCAGATATCACAAAGTAGTTTCTGAG
         66 TGCTCT-AGAAAGGGAATGTTCAACTCTGTGAGTTGAGTACACACATCACAAAGAAGTTTCTGAG

             **            *     *    *                        **  *   *     
     183374 AGGGCTTCTGTCTAGATTTTAGATGATGATATTCCCGTTTCCAACGAAATCATTAGAGCTATCCA
        130 AATGCTTCTGTCTAGTTTTTATATGAAGATATTCCCGTTTCCAACGAAATCGCTAAAGCGATCCA

                       *   *                    *        **         * *    * 
     183439 AATATCCACTTACAGTTTCTACAAAAAGAGTGTTTCCAAACTGCTGCATCAAAAGAGAGGTTCCA
        195 AATATCCACTTCCAGATTCTACAAAAAGAGTGTTTCAAAACTGCTCTATCAAAAGAAATGTTCAA

                     **                  ***      *     *         *        **
     183504 CTCTGTTAGCCGAGTACACACATCACAAACTTGTTTCTCAGAATCCTTCTGTCTCGTTTTTATGG
        260 CTCTGTTAGTTGAGTACACACATCACAAAGAAGTTTCTGAGAATGCTTCTGTCTAGTTTTTATAT

                     **     *  *  * **          *       *     *       *  *  *
     183569 GAAGATATTTACTTTTTCACCGTAGGCATCAAAGCGCTCCAAATGTCCACATCCAGATACTCCAG
        325 GAAGATATTCCCTTTTCCAACGAAATCATCAAAGCGATCCAAATATCCACTTCCAGATTCTACAA

                           *             * *   *         * *      * *  *     
     183634 AAAGAGTGTTTCAAACCTGCTCTATGAAAGGGAATCTTCAACTCTATGAGTTGAATGCAGACATC
        390 AAAGAGTGTTTCAAAACTGCTCTATGAAAAGAAATGTTCAACTCTGTTAGTTGAGTACACACATC

             *      *              *       **         **         **          
     183699 AGAAAGAAATTTCTGAGAATGCTGCTGTCTACCTTTTAT---TTGA-ATTCCCGCTTCCAACGAA
        455 ACAAAGAAGTTTCTGAGAATGCTTCTGTCTAGTTTTTATGGGAAGATATTCCCTTTTCCAACGAA

             * *  *    *                *       *                            
     183760 ATCCTCCAAGCTATCCAAATATCCACTTGCAGATTCCACAAAAAGAGTGTTTCAAAACTGCTCTC
        520 AGCATCAAAGCGATCCAAATATCCACTTCCAGATTCTACAAAAAGAGTGTTTCAAAACTG--CTC

                  *  *  *            *       *     *     *  *                
     183825 TATCAATGGCAAAGTTCAACTCTGTTAGTTGAGGACACATATCACCAACAAGTTTCTGAGAATGC
        583 TATCAAAGGAAATGTTCAACTCTGTGAGTTGAGTACACACATCACAAAGAAGTTTCTGAGAATGC

                     *           *      *      *  *  * ** *    *      *    * 
     183890 TTCTGTCTATTTTTTATGGGAAGATATTTCCTTTTTCACCGTAGGCGTCAAGGCGATCGAAATGT
        648 TTCTGTCTAGTTTTTAT--GATGATATTCCCTTTTCCAACGAAATCATCAAAGCGATCCAAATAT

                     * **                     *          *     **       *    
     183955 CCACTTCCACAAACTACAAAAAGAGTGTTTCAAACCTG-CTCTATGAAAGGCCATGTTCATCTCT
        711 CCACTTCCAGATTCTACAAAAAGAGTGTTTCAAAACTGCCTCTATCAAAGGAAATGTTCAACTCT

            * *      * ** * *   **      *      *       *         *         * 
     184019 ATGAGTTGAATGGAAATATCCGAAAGAAATTTCTGGGAATGCTGCTGTCTAGTGTTTAT---ACG
        776 GTTAGTTGAGTACACACATCACAAAGAAGTTTCTGAGAATGCTTCTGTCTAGTTTTTATGGGAAG

                    **        *    *       *                *    *  *        
     184081 A-ATTCCCGCTTCCAACGTAATCCTCAAAGCAATCCAAATATCCACTTGCAGAATCCACAAAAAG
        841 ATATTCCCTTTTCCAACGAAATCATCAAAGCGATCCAAATATCCACTTCCAGATTCTACAAAAAG

                                    **    *          * *      * *  *       * 
     184145 AGTGTTTCAAAACTGCTCTATCAATAGAAAGGTTCAACTCTTTTAGTTGAGTACACACATCACGA
        906 AGTGTTTCAAAACTGCTCTATCAAAGGAAATGTTCAACTCTGTGAGTTGAATGCAGACATCACAA

             *                        * *             **  *         * *  **  
     184210 ACAAGTTTCTGAGAATGCTTCTGTCTGGCTTTTATTGGAAGACGTTTCCTTTT-CACCAAAGGCA
        971 AGAAGTTTCTGAGAATGCTTCTGTCTAGTTTTTA-T-GAAGATATTCCCTTTTCCAACGAAATCA

                    *       *                * *                **     * *** 
     184274 TCAAAGCGCTCCAAATGTCCACTTCCAGATTCTTCCAAAAGAGTGTTTCAAACGTGCTCAAAGTA
       1034 TCAAAGCGATCCAAATATCCACTTCCAGATTCTACAAAAAGAGTGTTTCAAAACTGCTCTATCAA

             * *               * *    * *  * *     *   *       * * *         
     184339 AGGGAATGTTCAACTCTGTGACTTGAATGCAGATATCACCAAGTAGTTTCTAATAGTGCTTCTGT
       1099 AAGAAATGTTCAACTCTGTTAGTTGAGTACACACATCACAAAGAAGTTTCTGAGAATGCTTCTGT

               **     ***  *             *
     184404 CTACATTTTAGATGATGATATTCCCGTTTC
       1164 CTAGTTTTTATGGGAAGATATTCCCGTTTT

                      *** *   *             *  *   *      *     *       *  **
     184434 CAACGAAATCGCTAGAGCTATCCAAATATCCAGTTACAGTTTCTACCAAAAGGGTGTTTCCAAAT
          1 CAACGAAATCATCAAAGCGATCCAAATATCCACTTCCAGATTCTACAAAAAGAGTGTTTCAAACC

                  ***   * *  *            *       *                     *    
     184499 TGCTGCATCAAAAGAAAGGTTCAACTCTGTTAGTTGAGGACACACATCACAAAGAAGTTTGTGAG
         66 TGCT-CTAGAAAGGGAATGTTCAACTCTGTGAGTTGAGTACACACATCACAAAGAAGTTTCTGAG

                           *    *     *         *          *    *      *   * 
     184564 AATGCTTCTGTCTAGATTTTGTATGACGATATTCCCTTTTCCAACGATATCGTTAAAGCAATCTA
        130 AATGCTTCTGTCTAGTTTTTATATGAAGATATTCCCGTTTCCAACGAAATCGCTAAAGCGATCCA

                  * *  *    *  *       *  *        *       * *        *    * 
     184629 AATATCAATTTGCAGAATCCACAAAAATAGAGTTTCAAAGCTGCTCTGTAAAAAGAAAGGTTCCA
        195 AATATCCACTTCCAGATTCTACAAAAAGAGTGTTTCAAAACTGCTCTATCAAAAGAAATGTTCAA

                     *                   ***      *     *         *        **
     184694 CTCTGTTAGCTGAGTACACACATCACAAACTTGTTTCTCAGAATCCTTCTGTCTCGTTTTTATGG
        260 CTCTGTTAGTTGAGTACACACATCACAAAGAAGTTTCTGAGAATGCTTCTGTCTAGTTTTTATAT

                   * **        *  * **          *       *     *       *  *  *
     184759 GAAGATACTTACTTTTCCACCGTAGGCATCAAAGCGCTCCAAATGTCCACATCCAGATACTCCAG
        325 GAAGATATTCCCTTTTCCAACGAAATCATCAAAGCGATCCAAATATCCACTTCCAGATTCTACAA

              *            *             * *   *         * *      * *  *     
     184824 AACGAGTGTTTCAAACCTGCTCTATGAAAGGGAATCTTCAACTCTATGAGTTGAATGCAGACATC
        390 AAAGAGTGTTTCAAAACTGCTCTATGAAAAGAAATGTTCAACTCTGTTAGTTGAGTACACACATC

             *      *              *       **         **         **          
     184889 AGAAAGAAATTTCTGAGAATGCTGCTGTCTACCTTTTAT---TTGA-ATTCCCGCTTCCAACGAA
        455 ACAAAGAAGTTTCTGAGAATGCTTCTGTCTAGTTTTTATGGGAAGATATTCCCTTTTCCAACGAA

             * *  *    *              * *  **   *    *                       
     184950 ATCCTCCAAGCTATCCAAATATCCACCTGCATTTTCCACAACAAGAGTGTTTCAAAACTGCTCTA
        520 AGCATCAAAGCGATCCAAATATCCACTTCCAGATTCTACAAAAAGAGTGTTTCAAAACTGCTCTA

                **                *    *  *                *                 
     185015 TCAATAGAAATGTTCAACTCCTTTG-GCTGGGTACACACATCACAAACAAGTTTCTGAGAATGCT
        585 TCAAAGGAAATGTTCAACT-CTGTGAGTTGAGTACACACATCACAAAGAAGTTTCTGAGAATGCT

                                *  **         *  * *  **    *     *       *  
     185079 TCTGTCTAGTTTTTATGGGAAGACGTTCCCTTTTTCACCAAAGGCATCCAAGCGCTCCAAATGTC
        649 TCTGTCTAGTTTTTAT--GATGATATTCCCTTTTCCAACGAAATCATCAAAGCGATCCAAATATC

               *       *  *  *               *          *     *   *         *
     185144 CACATCCAGATACTCCAGAAAGAGTGTTTCAAACCTG-CTCTATGAAAGGGAATCTTCAACTCTA
        712 CACTTCCAGATTCTACAAAAAGAGTGTTTCAAAACTGCCTCTATCAAAGGAAATGTTCAACTCTG

             *      * *  *      *      *              *       **         **  
     185208 TGAGTTGAATGCAGACATCAGAAAGAAATTTCTGAGAATGCTGCTGTCTACCTTTTAT---TTGA
        777 TTAGTTGAGTACACACATCACAAAGAAGTTTCTGAGAATGCTTCTGTCTAGTTTTTATGGGAAGA

                   **             *  *    *                *       *         
     185270 -ATTCCCGCTTCCAACGAAATCCTCCAAGCTATCCAAATATCCACTTGCAGATTCCACAAAAAGA
        842 TATTCCCTTTTCCAACGAAATCATCAAAGCGATCCAAATATCCACTTCCAGATTCTACAAAAAGA

                                     *  *  *            *      ***  * *     *
     185334 GTGTTTCAAAACTGCTCTCTATCAATGGCAAAGTTCAACTCTGTTAGTTGAGGACACATATCACC
        907 GTGTTTCAAAACTG--CTCTATCAAAGGAAATGTTCAACTCTGTGAGTTGAATGCAGACATCACA

              *                         *                  *      *  * ** ** 
     185399 AACAAGTTTCTGAGAATGCTTCTGTCTATTTTTTATGGGAAGATATTTCCTTTTTCACCATAGGC
        970 AAGAAGTTTCTGAGAATGCTTCTGTCTAGTTTTTAT--GAAGATATTCCCTTTTCCAACGAAATC

            *    *      *    *          * **                     *         * 
     185464 GTCAAGGCGATCGAAATGTCCACTTCCACAAACTACAAAAAGAGTGTTTCAAACCTGCTCTATGA
       1033 ATCAAAGCGATCCAAATATCCACTTCCAGATTCTACAAAAAGAGTGTTTCAAAACTGCTCTATCA

              * **       *    * *      * ** * *   **      *      *       *   
     185529 AAGGCCATGTTCATCTCTATGAGTTGAATGGAAATATCCGAAAGAAATTTCTGGGAATGCTGCTG
       1098 AAAGAAATGTTCAACTCTGTTAGTTGAGTACACACATCACAAAGAAGTTTCTGAGAATGCTTCTG

                  *         *          *  *
     185594 TCTAGTGTTTAT---ACGA-ATTCCCGCTTC
       1163 TCTAGTTTTTATGGGAAGATATTCCCGTTTT

                      *       *                *    *  *                   * 
     185621 CAACGAAATCCTCAAAGCAATCCAAATATCCACTTGCAGAATCCACAAAAAGAGTGTTTCAAAAC
          1 CAACGAAATCATCAAAGCGATCCAAATATCCACTTCCAGATTCTACAAAAAGAGTGTTTCAAACC

                    *  ** *  *          * *                   *  *           
     185686 TGCTCTATCAATAGAAAGGTTCAACTCTTTTAGTTGAGTACACACATCACGAACAAGTTTCTGAG
         66 TGCTCTA-GAAAGGGAATGTTCAACTCTGTGAGTTGAGTACACACATCACAAAGAAGTTTCTGAG

                         * *      **     **  *      *         *           *  
     185751 AATGCTTCTGTCTGGCTTTTATTGGAAGACGTTTCC-TTTTCAAC-AAA-GGCATCAAAGCGCTC
        130 AATGCTTCTGTCTAGTTTTTATATGAAGATATTCCCGTTTCCAACGAAATCGC-T-AAAGCGATC

                 *                * *                **     * ***  * *       
     185813 CAAATGTCCACTTCCAGATTCTTCCAAAAGAGTGTTTCAAACGTGCTCAAAGTAAGGGAATGTTC
        193 CAAATATCCACTTCCAGATTCTACAAAAAGAGTGTTTCAAAACTGCTCTATCAAAAGAAATGTTC

                    * *    * *  * *     *   *       * * *        *   **     *
     185878 AACTCTGTGACTTGAATGCAGATATCACCAAGTAGTTTCTAATAGTGCTTCTGCCTACATTTTAG
        258 AACTCTGTTAGTTGAGTACACACATCACAAAGAAGTTTCTGAGAATGCTTCTGTCTAGTTTTTAT

                *                        * * *   *             *  *   *      
     185943 ATGATGATATTCCCTTTTCCAACGAAATCGTTAGAGCTATCCAAATATCCAGTTACAGTTTCTAC
        323 ATGAAGATATTCCCTTTTCCAACGAAATCATCAAAGCGATCCAAATATCCACTTCCAGATTCTAC

            *     *       *   *    **  *        *                    *       
     186008 CAAAAGGGTGTTTCCAAATTGCTGCATCAAAAGAAAGGTTCAACTCTGTTAGTTGAGGACACACA
        388 AAAAAGAGTGTTTCAAAACTGCTCTATGAAAAGAAATGTTCAACTCTGTTAGTTGAGTACACACA

                          *                   *    * **  *                   
     186073 TCACAAAGAAGTTTGTGAGAATGCTTCTGTCTAGATTTTGTATGACGATATTCCCTTTTCCAACG
        453 TCACAAAGAAGTTTCTGAGAATGCTTCTGTCTAGTTTTTATGGGAAGATATTCCCTTTTCCAACG

             * * * *     *   *       * *  *    *  *       *  *        *      
     186138 ATATCGTTAAAGCAATCTAAATATCAATTTGCAGAATCCACAAAAATAGAGTTTCAAAGCTGCTC
        518 AAAGCATCAAAGCGATCCAAATATCCACTTCCAGATTCTACAAAAAGAGTGTTTCAAAACTGCTC

             * *   *    *    *       *  *                   ***            * 
     186203 TGTAAAAAGAAAGGTTCCACTCTGTTAGCTGAGTACACACATCACAAACTTGTTTCTGAGAATCC
        583 TATCAAAGGAAATGTTCAACTCTGTGAGTTGAGTACACACATCACAAAGAAGTTTCTGAGAATGC

                    *            *      **        *  * **          *       * 
     186268 TTCTGTCTCGTTTTTATGGGAAGATATTTACTTTTCCACCGTAGGCATCAAAGCGCTCCAAATGT
        648 TTCTGTCTAGTTTTTAT--GATGATATTCCCTTTTCCAACGAAATCATCAAAGCGATCCAAATAT

                *       *  *  *  *            *          *     *   *         
     186333 CCACATCCAGATACTCCAGAACGAGTGTTTCAAACCTG-CTCTATGAAAGGGAATCTTCAACTCT
        711 CCACTTCCAGATTCTACAAAAAGAGTGTTTCAAAACTGCCTCTATCAAAGGAAATGTTCAACTCT

            * *      * *  *      *      *              *       **         ** 
     186397 ATGAGTTGAATGCAGACATCAGAAAGAAATTTCTGAGAATGCTGCTGTCTACCTTTTAT---TTG
        776 GTTAGTTGAGTACACACATCACAAAGAAGTTTCTGAGAATGCTTCTGTCTAGTTTTTATGGGAAG

                    **             *  *    *              * *  **   *        
     186459 A-ATTCCCGCTTCCAACGAAATCCTCCAAGCTATCCAAATATCCACCTGCATTTTCCACAAAAAG
        841 ATATTCCCTTTTCCAACGAAATCATCAAAGCGATCCAAATATCCACTTCCAGATTCTACAAAAAG

                                    **                *    *  ** *  *        
     186523 AGTGTTTCAAAACTGCTCTATCAATAGAAATGTTCAACTCCTTTG-GCTGGGTACACACATCACA
        906 AGTGTTTCAAAACTGCTCTATCAAAGGAAATGTTCAACT-CTGTGAGTTGAATGCAGACATCACA

              *                                        *          *  * *  ** 
     186587 AACAAGTTTCTGAGAATGCTTCTGTCTAGTTTTTATGGGAAGACATTCCCTTTTTCACCAAAGAC
        970 AAGAAGTTTCTGAGAATGCTTCTGTCTAGTTTTTAT--GAAGATATTCCCTTTTCCAACGAAATC

                     *       *            **                     **       ** 
     186652 ATCAAAGCGCTCCAAATGTCCACTTCCAGACACTACAAAAAGAGTGTTTCAAACGTGCTCTAAGA
       1033 ATCAAAGCGATCCAAATATCCACTTCCAGATTCTACAAAAAGAGTGTTTCAAAACTGCTCTATCA

              ***               * *    * *    *         *           **       
     186717 AAGCGAATGTTCAACTCTGTGACTTGAATGCACATATCACAAAGTAGTTTCTGAGAGGGCTTCTG
       1098 AAAGAAATGTTCAACTCTGTTAGTTGAGTACACACATCACAAAGAAGTTTCTGAGAATGCTTCTG

                 *     ***  *             *
     186782 TCTAGATTTTAGATGATGATATTCCCGTTTC
       1163 TCTAGTTTTTATGGGAAGATATTCCCGTTTT

                *       * *    *               *   *                    *  * 
     186813 CAACAAAATCATTAGAGC-TTCCAAATATCCACTTACAGTTTCTACAAAAAGAGTGTTTCCAAAC
          1 CAACGAAATCATCAAAGCGATCCAAATATCCACTTCCAGATTCTACAAAAAGAGTGTTTCAAACC

                    **    *   *    *       *  *                   ***      * 
     186877 TGCTGCATCAAAAGAG-AGGTTCCACTCTGTTAGCTGAGTACACACATCACAAACTTGTTTCTCA
         66 TGCT-C-TAGAAAGGGAATGTTCAACTCTGTGAGTTGAGTACACACATCACAAAGAAGTTTCTGA

                *         *        **        *** *   *  *  * ** ***      *   
     186941 GAATCCTTCTGTCTCGTTTTTATGGGAAGAT-TATACTTTTTCACCGTAGGCATCAAAGCGCTCC
        129 GAATGCTTCTGTCTAGTTTTTATATGAAGATATTCCCGTTTCCAACGAAATCGCTAAAGCGATCC

                *     *       *  *  *               *    *    *   * *   *    
     187005 AAATGTCCACATCCAGATACTCCAGAAAGAGTGTTTCAAACCTGCCCTATGAAAGGGAATCTTCA
        194 AAATATCCACTTCCAGATTCTACAAAAAGAGTGTTTCAAAACTGCTCTATCAAAAGAAATGTTCA

                 * *      * *  *      *      *              *       **       
     187070 ACTCTATGAGTTGAATGCAGACATCAGAAAGAAATTTCTGAGAATGCTGCTGTCTACCTTTTAT-
        259 ACTCTGTTAGTTGAGTACACACATCACAAAGAAGTTTCTGAGAATGCTTCTGTCTAGTTTTTATA

               *         **             *  *    *                *       *   
     187134 T--TGA-ATTCCCGCTTCCAACGAAATCCTCCAAGCTATCCAAATATCCACTTGCAGATTCCACA
        324 TGAAGATATTCCCTTTTCCAACGAAATCATCAAAGCGATCCAAATATCCACTTCCAGATTCTACA

                                        *  ** *  *                    *     *
     187196 AAAAGAGTGTTTCAAAACTGCTCTCTATCAATGGCAAAGTTCAACTCTGTTAGTTGAGGACACAT
        389 AAAAGAGTGTTTCAAAACTG--CTCTATGAAAAGAAATGTTCAACTCTGTTAGTTGAGTACACAC

                 *  *                         *                  *      *  * 
     187261 ATCACCAACAAGTTTCTGAGAATGCTTCTGTCTATTTTTTATGGGAAGATATTTCCTTTTTCACC
        452 ATCACAAAGAAGTTTCTGAGAATGCTTCTGTCTAGTTTTTATGGGAAGATATTCCCTTTTCCAAC

             * *  *    *      *    *          * **                     *     
     187326 GTAGGCGTCAAGGCGATCGAAATGTCCACTTCCACAAACTACAAAAAGAGTGTTTCAAACCTGCT
        517 GAAAGCATCAAAGCGATCCAAATATCCACTTCCAGATTCTACAAAAAGAGTGTTTCAAAACTGCT

                *     **       *    *        * ** * *   **      *      *     
     187391 CTATGAAAGGCCATGTTCATCTCTATGAGTTGAATGGAAATATCCGAAAGAAATTTCTGGGAATG
        582 CTATCAAAGGAAATGTTCAACTCTGTGAGTTGAGTACACACATCACAAAGAAGTTTCTGAGAATG

              *         *       *         **             *       *           
     187456 CTGCTGTCTAGTGTTTAT-ACGA-ATTCCCGCTTCCAACGAAATCCTCAAAGCAATCCAAATATC
        647 CTTCTGTCTAGTTTTTATGATGATATTCCCTTTTCCAACGAAATCATCAAAGCGATCCAAATATC

                 *    *  *                                 **    *          *
     187519 CACTTGCAGAATCCACAAAAAGAGTGTTTCAAAACTG-CTCTATCAATAGAAAGGTTCAACTCTT
        712 CACTTCCAGATTCTACAAAAAGAGTGTTTCAAAACTGCCTCTATCAAAGGAAATGTTCAACTCTG

                                 *  *                        * *      *      
     187583 TTAGTTGAGTACACACATCACGAACAAGTTTCTGAGAATGCTTCTGTCTGGCTTTTATTGGAAGA
        777 TTAGTTGAGTACACACATCACAAAGAAGTTTCTGAGAATGCTTCTGTCTAGTTTTTATGGGAAGA

            **  *           *  **          *       *                * *      
     187648 CGTTTCCTTTT-CAACAAAGGCATCAAAGCGCTCCAAATGTCCACTTCCAGATTCTTCCAAAAGA
        842 TATTCCCTTTTCCAACGAAATCATCAAAGCGATCCAAATATCCACTTCCAGATTCTACAAAAAGA

                      **     * ***    *                 *           *     *  
     187712 GTGTTTCAAACGTGCTCAAAGTAAGGGAATGTTCAACTCTGTGACTTGAATGCAGATATCACCAA
        907 GTGTTTCAAAACTGCTCTATCAAAGGAAATGTTCAACTCTGTGAGTTGAATGCAGACATCACAAA

             *       * * *        *    *     *    *                        * 
     187777 GTAGTTTCTAATAGTGCTTCTGCCTACATTTTAGATGATGATATTCCCTTTTCCAACGAAATCGT
        972 GAAGTTTCTGAGAATGCTTCTGTCTA-GTTTT-TATGAAGATATTCCCTTTTCCAACGAAATCAT

            * *   *             *  *   *      *     *       *   *    **      
     187842 TAGAGCTATCCAAATATCCAGTTACAGTTTCTACCAAAAGGGTGTTTCCAAATTGCTGCATCAAA
       1035 CAAAGCGATCCAAATATCCACTTCCAGATTCTACAAAAAGAGTGTTTCAAAACTGCTCTATCAAA

                 *                    *                     *                
     187907 AGAAAGGTTCAACTCTGTTAGTTGAGGACACACATCACAAAGAAGTTTGTGAGAATGCTTCTGTC
       1100 AGAAATGTTCAACTCTGTTAGTTGAGTACACACATCACAAAGAAGTTTCTGAGAATGCTTCTGTC

               *    * **  *         *   *
     187972 TAGATTTTGTATGACGATATTCCCTTTTC
       1165 TAGTTTTTATGGGAAGATATTCCCGTTTT

                  *   * *     *   *       * *  *    *  *       *  *        * 
     188001 CAACGATATCGTTAAAGCAATCTAAATATCAATTTGCAGAATCCACAAAAATAGAGTTTCAAAGC
          1 CAACGAAATCATCAAAGCGATCCAAATATCCACTTCCAGATTCTACAAAAAGAGTGTTTCAAACC

                   **   * *  *    *       *  *                   ***         
     188066 TGCTCTGTAAAAAGAAAGGTTCCACTCTGTTAGCTGAGTACACACATCACAAACTTGTTTCTGAG
         66 TGCTCT-AGAAAGGGAATGTTCAACTCTGTGAGTTGAGTACACACATCACAAAGAAGTTTCTGAG

               *         *        **         ** *      *  * ** ***      *    
     188131 AATCCTTCTGTCTCGTTTTTATGGGAAGATATTTACTTTTCCACCGTAGGCATCAAAGCGCTCCA
        130 AATGCTTCTGTCTAGTTTTTATATGAAGATATTCCCGTTTCCAACGAAATCGCTAAAGCGATCCA

               *     *       *  *  *  *            *         *   * *   *     
     188196 AATGTCCACATCCAGATACTCCAGAACGAGTGTTTCAAACCTGCTCTATGAAAGGGAATCTTCAA
        195 AATATCCACTTCCAGATTCTACAAAAAGAGTGTTTCAAAACTGCTCTATCAAAAGAAATGTTCAA

                * *      * *  *      *      *              *       **        
     188261 CTCTATGAGTTGAATGCAGACATCAGAAAGAAATTTCTGAGAATGCTGCTGTCTACCTTTTAT-T
        260 CTCTGTTAGTTGAGTACACACATCACAAAGAAGTTTCTGAGAATGCTTCTGTCTAGTTTTTATAT

              *         **             *  *    *              * *  **   *    
     188325 --TGA-ATTCCCGCTTCCAACGAAATCCTCCAAGCTATCCAAATATCCACCTGCATTTTCCACAA
        325 GAAGATATTCCCTTTTCCAACGAAATCATCAAAGCGATCCAAATATCCACTTCCAGATTCTACAA

                                     *  *               **  * *  *           
     188387 AAAGAGTGTTTCAAAACTGCTCTATCAATAGAAATGTTCAACTCCTTTGGCTGGGTACACACATC
        390 AAAGAGTGTTTCAAAACTGCTCTATGAAAAGAAATGTTCAACTCTGTTAGTTGAGTACACACATC

                 *                                        *          *    *  
     188452 ACAAACAAGTTTCTGAGAATGCTTCTGTCTAGTTTTTATGGGAAGACATTCCCTTTTTC-ACCAA
        455 ACAAAGAAGTTTCTGAGAATGCTTCTGTCTAGTTTTTATGGGAAGATATTCCCTTTTCCAACGAA

                         *       *            **                     **      
     188516 AGACATCAAAGCGCTCCAAATGTCCACTTCCAGACACTACAAAAAGAGTGTTTCAAACGTGCTCT
        520 AG-CATCAAAGCGATCCAAATATCCACTTCCAGATTCTACAAAAAGAGTGTTTCAAAACTGCTCT

             **    **                 *    * *  * *         *           **   
     188581 AAGAAAGCGAATGTTCAACTCTGTGACTTGAATGCAGATATCACAAAGTAGTTTCTGAGAGGGCT
        584 ATCAAAGGAAATGTTCAACTCTGTGAGTTGAGTACACACATCACAAAGAAGTTTCTGAGAATGCT

                           *              *                * *   *           
     188646 TCTGTCTAGATTTTAGATGATGATATTCCCGTTTCCAACGAAATCATTAGAGCTATCCAAATATC
        649 TCTGTCTAG-TTTT-TATGATGATATTCCCTTTTCCAACGAAATCATCAAAGCGATCCAAATATC

                 *   *                    *         **      *  * *    *      
     188711 CACTTACAGTTTCTACAAAAAGAGTGTTTCCAAACTG-CTGCATCAAAAGAGAGGTTCCACTCTG
        712 CACTTCCAGATTCTACAAAAAGAGTGTTTCAAAACTGCCTCTATCAAAGGAAATGTTCAACTCTG

                **                  ***      *     *         *               
     188775 TTAGCCGAGTACACACATCACAAACTTGTTTCTCAGAATCCTTCTGTCTCGTTTTTATGGGAAGA
        777 TTAGTTGAGTACACACATCACAAAGAAGTTTCTGAGAATGCTTCTGTCTAGTTTTTATGGGAAGA

                **     *  *  * **          *       *     *       *  *  *     
     188840 TATTTACTTTTTCACCGTAGGCATCAAAGCGCTCCAAATGTCCACATCCAGATACTCCAGAAAGA
        842 TATTCCCTTTTCCAACGAAATCATCAAAGCGATCCAAATATCCACTTCCAGATTCTACAAAAAGA

                      *         *     *   *         *                    *   
     188905 GTGTTTCAAACCTGCTCTATGAAAGGGAATCTTCAACTCTATGAGTTGAATGCAGACATCAGAAA
        907 GTGTTTCAAAACTGCTCTATCAAAGGAAATGTTCAACTCTGTGAGTTGAATGCAGACATCACAAA

               *              *       **       **         **             *  *
     188970 GAAATTTCTGAGAATGCTGCTGTCTACCTTTTAT-TTGA-ATTCCCGCTTCCAACGAAATCCTCC
        972 GAAGTTTCTGAGAATGCTTCTGTCTAGTTTTTATGAAGATATTCCCTTTTCCAACGAAATCATCA

                *                *       *                                  *
     189033 AAGCTATCCAAATATCCACTTGCAGATTCCACAAAAAGAGTGTTTCAAAACTGCTCTCTATCAAT
       1037 AAGCGATCCAAATATCCACTTCCAGATTCTACAAAAAGAGTGTTTCAAAACTG--CTCTATCAAA

            * *  *                    *     *     *  *                       
     189098 GGCAAAGTTCAACTCTGTTAGTTGAGGACACATATCACCAACAAGTTTCTGAGAATGCTTCTGTC
       1100 AGAAATGTTCAACTCTGTTAGTTGAGTACACACATCACAAAGAAGTTTCTGAGAATGCTTCTGTC

              *                  *  *    
     189163 TATTTTTTATGGGAAGATATTTCCTTTTT
       1165 TAGTTTTTATGGGAAGATATTCCCGTTTT

              *  * ** *    *      *    *          * **                       
     189192 CACCGTAGGCGTCAAGGCGATCGAAATGTCCACTTCCACAAACTACAAAAAGAGTGTTTCAAACC
          1 CAACGAAATCATCAAAGCGATCCAAATATCCACTTCCAGATTCTACAAAAAGAGTGTTTCAAACC

                          **       *    *        * ** * *   **      *      * 
     189257 TGCTCTATGAAAGGCCATGTTCATCTCTATGAGTTGAATGGAAATATCCGAAAGAAATTTCTGGG
         66 TGCTCTA-GAAAGGGAATGTTCAACTCTGTGAGTTGAGTACACACATCACAAAGAAGTTTCTGAG

                  *          *        *          *                      *    
     189322 AATGCTGCTGTCTAG-TGTT-TAT-ACGA-ATTCCCGCTTCCAACGAAATC-CTCAAAGCAATCC
        130 AATGCTTCTGTCTAGTTTTTATATGAAGATATTCCCGTTTCCAACGAAATCGCT-AAAGCGATCC

                        *    *  *                                *     *     
     189382 AAATATCCACTTGCAGAATCCACAAAAAGAGTGTTTCAAAACTGCTCTATCAATAGAAAGGTTCA
        194 AAATATCCACTTCCAGATTCTACAAAAAGAGTGTTTCAAAACTGCTCTATCAAAAGAAATGTTCA

                 *                     *  *                        * *      *
     189447 ACTCTTTTAGTTGAGTACACACATCACGAACAAGTTTCTGAGAATGCTTCTGTCTGGCTTTTATT
        259 ACTCTGTTAGTTGAGTACACACATCACAAAGAAGTTTCTGAGAATGCTTCTGTCTAGTTTTTATA

            *     **  *         * *  **          *       *                * *
     189512 GGAAGACGTTTCCTTTT-CACCAAAGGCATCAAAGCGCTCCAAATGTCCACTTCCAGATTCTTCC
        324 TGAAGATATTCCCTTTTCCAACGAAATCATCAAAGCGATCCAAATATCCACTTCCAGATTCTACA

                            **     * * *  * *               * *    * *  * *  
     189576 AAAAGAGTGTTTCAAACGTGCTCAAAGTAAGGGAATGTTCAACTCTGTGACTTGAATGCAGATAT
        389 AAAAGAGTGTTTCAAAACTGCTCTATGAAAAGAAATGTTCAACTCTGTTAGTTGAGTACACACAT

               *   *       * * *            **     ***  *         *          
     189641 CACCAAGTAGTTTCTAATAGTGCTTCTGTCTACATTTTAGATGATGATATTCCCGTTTCCAACGA
        454 CACAAAGAAGTTTCTGAGAATGCTTCTGTCTAGTTTTTATGGGAAGATATTCCCTTTTCCAACGA

              * *** *   *             *  *   *      *     *       *   *    **
     189706 AATCGCTAGAGCTATCCAAATATCCAGTTACAGTTTCTACCAAAAGGGTGTTTCCAAATTGCTGC
        519 AAGCATCAAAGCGATCCAAATATCCACTTCCAGATTCTACAAAAAGAGTGTTTCAAAACTGCTCT

                  *    *            *       *                     *          
     189771 ATCAAAAGAAAGGTTCAACTCTGTTAGTTGAGGACACACATCACAAAGAAGTTTGTGAGAATGCT
        584 ATCAAAGGAAATGTTCAACTCTGTGAGTTGAGTACACACATCACAAAGAAGTTTCTGAGAATGCT

                                *                    *   * *     *   *       
     189836 TCTGTCTAGATTTTGTATGACGATATTCCCTTTTCCAACGATATCGTTAAAGCAATCTAAATATC
        649 TCTGTCTAG-TTTT-TATGATGATATTCCCTTTTCCAACGAAATCATCAAAGCGATCCAAATATC

            * *  *    *  *       *  *        *        * *   *    *    *      
     189901 AATTTGCAGAATCCACAAAAATAGAGTTTCAAAGCTG-CTCTGTAAAAAGAAAGGTTCCACTCTG
        712 CACTTCCAGATTCTACAAAAAGAGTGTTTCAAAACTGCCTCTATCAAAGGAAATGTTCAACTCTG

                *                   ***      *     *         *               
     189965 TTAGCTGAGTACACACATCACAAACTTGTTTCTCAGAATCCTTCTGTCTCGTTTTTATGGGAAGA
        777 TTAGTTGAGTACACACATCACAAAGAAGTTTCTGAGAATGCTTCTGTCTAGTTTTTATGGGAAGA

              * **        *  * **          *       *     *       *  *  *  *  
     190030 TACTTACTTTTCCACCGTAGGCATCAAAGCGCTCCAAATGTCCACATCCAGATACTCCAGAACGA
        842 TATTCCCTTTTCCAACGAAATCATCAAAGCGATCCAAATATCCACTTCCAGATTCTACAAAAAGA

                      *         *     *   *         *                    *   
     190095 GTGTTTCAAACCTGCTCTATGAAAGGGAATCTTCAACTCTATGAGTTGAATGCAGACATCAGAAA
        907 GTGTTTCAAAACTGCTCTATCAAAGGAAATGTTCAACTCTGTGAGTTGAATGCAGACATCACAAA

               *              *       **       **         **             *  *
     190160 GAAATTTCTGAGAATGCTGCTGTCTACCTTTTAT-TTGA-ATTCCCGCTTCCAACGAAATCCTCC
        972 GAAGTTTCTGAGAATGCTTCTGTCTAGTTTTTATGAAGATATTCCCTTTTCCAACGAAATCATCA

                *              * *  **   *    *                           *  
     190223 AAGCTATCCAAATATCCACCTGCATTTTCCACAACAAGAGTGTTTCAAAACTGCTCTATCAATAG
       1037 AAGCGATCCAAATATCCACTTCCAGATTCTACAAAAAGAGTGTTTCAAAACTGCTCTATCAAAAG

                         **  * *  *                *                         
     190288 AAATGTTCAACTCCTTTGGCTGGGTACACACATCACAAACAAGTTTCTGAGAATGCTTCTGTCTA
       1102 AAATGTTCAACTCTGTTAGTTGAGTACACACATCACAAAGAAGTTTCTGAGAATGCTTCTGTCTA

                           **     *    
     190353 GTTTTTATGGGAAGACGTTCCCTTTTT
       1167 GTTTTTATGGGAAGATATTCCCGTTTT

              * *  **    *     *       *     *       *  *  *                 
     190380 CACCAAAGGCATCCAAGCGCTCCAAATGTCCACATCCAGATACTCCAGAAAGAGTGTTTCAAACC
          1 CAACGAAATCATCAAAGCGATCCAAATATCCACTTCCAGATTCTACAAAAAGAGTGTTTCAAACC

                              *         *        * *  *      *      *        
     190445 TGCTCTATGAAAGGGAATCTTCAACTCTATGAGTTGAATGCAGACATCAGAAAGAAATTTCTGAG
         66 TGCTCTA-GAAAGGGAATGTTCAACTCTGTGAGTTGAGTACACACATCACAAAGAAGTTTCTGAG

                  *       **          *          *                 *    *    
     190510 AATGCTGCTGTCTACCTTTTAT-T--TGA-ATTCCCGCTTCCAACGAAATC-CTCCAAGCTATCC
        130 AATGCTTCTGTCTAGTTTTTATATGAAGATATTCCCGTTTCCAACGAAATCGCT-AAAGCGATCC

                        *       *                                  ** *  *   
     190570 AAATATCCACTTGCAGATTCCACAAAAAGAGTGTTTCAAAACTGCTCTCTATCAATGGCAAAGTT
        194 AAATATCCACTTCCAGATTCTACAAAAAGAGTGTTTCAAAACTG--CTCTATCAAAAGAAATGTT

                             *     *     *  *                         *      
     190635 CAACTCTGTTAGTTGAGGACACATATCACCAACAAGTTTCTGAGAATGCTTCTGTCTATTTTTTA
        257 CAACTCTGTTAGTTGAGTACACACATCACAAAGAAGTTTCTGAGAATGCTTCTGTCTAGTTTTTA

             **         *      *  * ** ** *    *      *    *          * **   
     190700 TGGGAAGATATTTCCTTTTTCACCATAGGCGTCAAGGCGATCGAAATGTCCACTTCCACAAACTA
        322 TATGAAGATATTCCCTTTTCCAACGAAATCATCAAAGCGATCCAAATATCCACTTCCAGATTCTA

                              *             * **       *    * *      * ** * *
     190765 CAAAAAGAGTGTTTCAAACCTGCTCTATGAAAGGCCATGTTCATCTCTATGAGTTGAATGGAAAT
        387 CAAAAAGAGTGTTTCAAAACTGCTCTATGAAAAGAAATGTTCAACTCTGTTAGTTGAGTACACAC

               **      *      *       *         *         *         **       
     190830 ATCCGAAAGAAATTTCTGGGAATGCTGCTGTCTAGTGTTTAT---ACGA-ATTCCCGCTTCCAAC
        452 ATCACAAAGAAGTTTCTGAGAATGCTTCTGTCTAGTTTTTATGGGAAGATATTCCCTTTTCCAAC
[truncated: 1,851,510 more chars]
