# Supplementary material for: Tigerfish designs oligonucleotide-based in situ hybridization probes targeting intervals of highly repetitive DNA at the scale of genomes
Source: Nat Commun. 2024 Feb 3;15:1027. doi: 10.1038/s41467-024-45385-x (PMC10838309; doi:10.1038/s41467-024-45385-x)
Supplement: Supplementary file 10 — Source Data [file 41467_2024_45385_MOESM10_ESM.gz › Tigerfish_full_data/Figure_S1_HOR_TRF_Analysis/TRF/trf_output/chr2_8_21_Y_hor.fa.s1.2.5.7.80.10.50.2000.1.html]

chr2\_8\_21\_Y\_hor.fa.s1.2.5.7.80.10.50.2000.1.html  

```
Tandem Repeats Finder Program written by:
```

```
Gary Benson  
Program in Bioinformatics  
Boston University  
Version 4.09  

Please cite:
G. Benson,
"Tandem repeats finder: a program to analyze DNA sequences"
Nucleic Acid Research(1999)
Vol. 27, No. 2, pp. 573-580.

Sequence: chr2:92297386-94695067
Parameters: 2 5 7 80 10 50 2000
Length:  2397681
```

```
Tables:   1   

This is table  1  of  1  ( 9 repeats found )
```

```
Click on indices to view alignment
```

Table Explanation  
  

|  |  |  |  |  |  |  |  |  |  |  |  |
| --- | --- | --- | --- | --- | --- | --- | --- | --- | --- | --- | --- |
| Indices | Period Size | Copy Number | Consensus Size | Percent Matches | Percent Indels | Score | A | C | G | T | Entropy (0-2) |
| 41--1342 | 171 | 7.6 | 171 | 71 | 3 | 1191 | 32 | 16 | 20 | 31 | 1.94 |
| 1851--2397681 | 171 | 14098.2 | 171 | 71 | 2 | 2246883 | 29 | 16 | 21 | 32 | 1.96 |
| 2347--2397681 | 680 | 3523.8 | 683 | 92 | 0 | 2681449 | 29 | 16 | 21 | 32 | 1.96 |
| 27235--27288 | 27 | 2.0 | 27 | 77 | 0 | 66 | 24 | 14 | 20 | 40 | 1.90 |
| 32004--32057 | 27 | 2.0 | 27 | 77 | 0 | 66 | 24 | 14 | 20 | 40 | 1.90 |
| 1711509--1711545 | 1 | 37.0 | 1 | 100 | 0 | 74 | 0 | 0 | 0 | 100 | 0.00 |
| 1723851--1723887 | 1 | 37.0 | 1 | 100 | 0 | 74 | 0 | 0 | 0 | 100 | 0.00 |
| 2000291--2000326 | 1 | 36.0 | 1 | 100 | 0 | 72 | 0 | 0 | 0 | 100 | 0.00 |
| 2005834--2005870 | 1 | 37.0 | 1 | 100 | 0 | 74 | 0 | 0 | 0 | 100 | 0.00 |

```
Tables:   1
```

The End!
