# Supplementary material for: Tigerfish designs oligonucleotide-based in situ hybridization probes targeting intervals of highly repetitive DNA at the scale of genomes
Source: Nat Commun. 2024 Feb 3;15:1027. doi: 10.1038/s41467-024-45385-x (PMC10838309; doi:10.1038/s41467-024-45385-x)
Supplement: Supplementary file 10 — Source Data [file 41467_2024_45385_MOESM10_ESM.gz › Tigerfish_full_data/Figure_S1_HOR_TRF_Analysis/TRF/trf_output/chr2_8_21_Y_hor.fa.s4.2.5.7.80.10.50.2000.1.txt.html]

chr2\_8\_21\_Y\_hor.fa.s4.2.5.7.80.10.50.2000.txt.html

```
Tandem Repeats Finder Program written by:

                 Gary Benson
      Program in Bioinformatics
          Boston University

Version 4.09

Sequence: chrY:10565750-10883085

Parameters: 2 5 7 80 10 50 2000

Pmatch=0.80,Pindel=0.10
tuple sizes 0,4,5,7
tuple distances 0, 29, 159, 2000

Length: 317335
ACGTcount: A:0.33, C:0.20, G:0.17, T:0.30


Found at i:286 original size:171 final size:171

Alignment explanation  
  

    Indices: 1--316722  Score: 378844
    Period size: 171  Copynumber: 1861.4  Consensus size: 171

                          *  * * * * ***             *            *     * *  
          1 CTTTTCCACCATAGACCGCCAGGGGACACAAATATCCACTTTCAGATTCTACAACAAGAGAGGTT
          1 CTTTTCCACCATAGGCCTCAAAGCGCTCCAAATATCCACTTGCAGATTCTACAAAAAGAGTGTTT

                   *   *     *   ** *       *      *                      * *
         66 CAAAACTACTCGATCAAGAGATGGTTTCAACTATGTGAGTTGAATGCACACATCACAAAGAACTA
         66 CAAAACTGCTCAATCAAAAGAAAGGTTCAACTCTGTGAGATGAATGCACACATCACAAAGAAGTT

             *  *    *                               
        131 TGTCGGAATTCTTCTGTGTAGTTTTTATGTGAAGATATTTC
        131 TCTCAGAATGCTTCTGTGTAGTTTTTATGTGAAGATATTTC

                     *    * *      * *    *                  *               
        172 CTTTTCCACAATAGACGTCAAAGTGATCCAGATATCCACTTGCAGATTCCACAAAAAGAGTGTTT
          1 CTTTTCCACCATAGGCCTCAAAGCGCTCCAAATATCCACTTGCAGATTCTACAAAAAGAGTGTTT

                 *   *   *                  **                     *  *      
        237 CAAAAGTGCACAACCAAAAGAAAGGTTCAACTAGGTGAGATGAATGCACACATCAGAAGGAAGTT
         66 CAAAACTGCTCAATCAAAAGAAAGGTTCAACTCTGTGAGATGAATGCACACATCACAAAGAAGTT

                            **   *     * *       *   
        302 TCTCAGAATGCTTCTGCATAGCTTTTAAGGGAAGATACTTC
        131 TCTCAGAATGCTTCTGTGTAGTTTTTATGTGAAGATATTTC

                    *               *            * *  *    * *               
        343 CTTTTCCAACATAGGCCTCAAAGCACTCCAAATATCCTCCTGGAGATACCACAAAAAGAGTGTTT
          1 CTTTTCCACCATAGGCCTCAAAGCGCTCCAAATATCCACTTGCAGATTCTACAAAAAGAGTGTTT

            **                      *  *                 *       *           
        408 GCAAACTGCTCAATCAAAAGAAAGATTTAACTCTGTGAGATGAATCCACACATGACAAAGAAGTT
         66 CAAAACTGCTCAATCAAAAGAAAGGTTCAACTCTGTGAGATGAATGCACACATCACAAAGAAGTT

                                                     
        473 TCTCAGAATGCTTCTGTGTAGTTTTTATGTGAAGATATTTC
        131 TCTCAGAATGCTTCTGTGTAGTTTTTATGTGAAGATATTTC

                     *   * * *    **           *                *     *      
        514 CTTTTCCACAATAAGACCCAAAAGGCTCCAAATATTCACTTGCAGATTCTAAAAAAAACAGTGTT
          1 CTTTTCCACCATAGGCCTCAAAGCGCTCCAAATATCCACTTGCAGATTCT-ACAAAAAGAGTGTT

                                  *                  *      *        **      
        579 TCAAAACTGCTCAATCAAAAGATA-GTTCAACTCTGTGAGAAGAATGCTCACATCACTGAGAAGT
         65 TCAAAACTGCTCAATCAAAAGAAAGGTTCAACTCTGTGAGATGAATGCACACATCACAAAGAAGT

                                         *            
        643 TTCTCAGAATGCTTCTGTGTAGTTTTTATATGAAGATATTTC
        130 TTCTCAGAATGCTTCTGTGTAGTTTTTATGTGAAGATATTTC

                *     *      *    **                       *   **       *    
        685 CTTTCCCACCGTAGGCCACAAAAGGCTCCAAATATCCACTTGCAGATACTATGAAAAGAGAGTTT
          1 CTTTTCCACCATAGGCCTCAAAGCGCTCCAAATATCCACTTGCAGATTCTACAAAAAGAGTGTTT

                        *        *               ***            *            
        750 CAAAACTGCTCATTCAAAAGATAGGTTCAACTCTGTGGTTTGAATGCACACAGCACAAAGAAGTT
         66 CAAAACTGCTCAATCAAAAGAAAGGTTCAACTCTGTGAGATGAATGCACACATCACAAAGAAGTT

              *       **                   **   *    
        815 TCACAGAATGTGTCTGTGTAGTTTTTATGTGCGGATGTTTC
        131 TCTCAGAATGCTTCTGTGTAGTTTTTATGTGAAGATATTTC

                         *        ****   *   *                    *          
        856 CTTTTCCACCATATGCCT-AAATATTTCCCAATTTCCACTTGCAGATTCTACAAGAAGAGTGTTT
          1 CTTTTCCACCATAGGCCTCAAAGCGCTCCAAATATCCACTTGCAGATTCTACAAAAAGAGTGTTT

                      **       *       *           *            *      ***   
        920 CAAAACTGCTGTATC-AAATAAA-GTTGAACTCTGTGAGGTGAATGCACACAGCACAAAATGGTT
         66 CAAAACTGCTCAATCAAAAGAAAGGTTCAACTCTGTGAGATGAATGCACACATCACAAAGAAGTT

                          **   *        *       *    
        983 TCTCAGAATGCTTCCTTGTTGTTTTTATATGAAGATGTTTC
        131 TCTCAGAATGCTTCTGTGTAGTTTTTATGTGAAGATATTTC

                  *  *             *   *     *                               
       1024 CTTTTCAACAATAGGCCTCAAAGTGCTTCAAATGTCCACTTGCAGATTCTACAAAAAGAGTGTTT
          1 CTTTTCCACCATAGGCCTCAAAGCGCTCCAAATATCCACTTGCAGATTCTACAAAAAGAGTGTTT

                                        *      *  *  *                       
       1089 CAAAACTGCTCAATCAAAAGAAAGGTTCGACTCTGGGAAATTAATGCACACATCACAAAGAAGTT
         66 CAAAACTGCTCAATCAAAAGAAAGGTTCAACTCTGTGAGATGAATGCACACATCACAAAGAAGTT

                                     *        *      
       1154 TCTC---A-GCTTCTGTGTAGTTTTCATGTGAAGTTATTTC
        131 TCTCAGAATGCTTCTGTGTAGTTTTTATGTGAAGATATTTC

                     *       *     *            *    *         **       *    
       1191 CTTTTCCACAATAGGCCGCAAAGGGCTCCAAATATCAACTTACAGATTCTAGGAAAAGAGAGTTT
          1 CTTTTCCACCATAGGCCTCAAAGCGCTCCAAATATCCACTTGCAGATTCTACAAAAAGAGTGTTT

                       * **      *     *                                     
       1256 CAAAACTGCTCTACGAAAAGATAGGTTGAACTCTGTGAGATGAATGCACACATCACAAAGAAGTT
         66 CAAAACTGCTCAATCAAAAGAAAGGTTCAACTCTGTGAGATGAATGCACACATCACAAAGAAGTT

                       *               * *           
       1321 TCTCAGAATGCATCTGTGTAGTTTTTACGGGAAGATATTTC
        131 TCTCAGAATGCTTCTGTGTAGTTTTTATGTGAAGATATTTC

                        ***  *     **      *  *                  *     ***   
       1362 CTTTTCCACCATCTTCCACAAAGGTCTCCAAGTAACCACTTGCAGATTCTACAGAAAGACACTTT
          1 CTTTTCCACCATAGGCCTCAAAGCGCTCCAAATATCCACTTGCAGATTCTACAAAAAGAGTGTTT

            *          *         ***      *      ***                      *  
       1427 AAAAACTGCTCTATCAAAAGATCAGTTCAAGTCTGTGGTTTGAATGCACACATCACAAAGAATTT
         66 CAAAACTGCTCAATCAAAAGAAAGGTTCAACTCTGTGAGATGAATGCACACATCACAAAGAAGTT

                                     *  *           *
       1492 TCTCAGAATGCTTCTGTGTAGTTTTCATATGAAGATATTTT
        131 TCTCAGAATGCTTCTGTGTAGTTTTTATGTGAAGATATTTC

                                   *                                         
       1533 CTTTTCCACCATAGGCCTCAAAGTGCTCCAAATATCCACTTGCAGATTCTACAAAAAGAGTGTTT
          1 CTTTTCCACCATAGGCCTCAAAGCGCTCCAAATATCCACTTGCAGATTCTACAAAAAGAGTGTTT

                                      *       *  *       *   **  *       *   
       1598 CAAAACTGCTCAATCAAAAGAAAGGTACAACTCTTTGTGATGAATTCACTAATAACAAAGACGTT
         66 CAAAACTGCTCAATCAAAAGAAAGGTTCAACTCTGTGAGATGAATGCACACATCACAAAGAAGTT

                 *         *                    *    
       1663 TCTCACAATGCTTCTCTGTAGTTTTTATGTGAAGATCTTTC
        131 TCTCAGAATGCTTCTGTGTAGTTTTTATGTGAAGATATTTC

                  *         *       *  *          * *                        
       1704 CTTTTCTACCATAGGCATCAAAGCACTACAAATATCCAATAGCAGATTCTACAAAAAGAGTGTTT
          1 CTTTTCCACCATAGGCCTCAAAGCGCTCCAAATATCCACTTGCAGATTCTACAAAAAGAGTGTTT

                              *    *       *                *    *           
       1769 CAAAACTGCTCAATCAAATGAAACGTTCAACCCTGTGAGATGAATGCAGACATTA-AAAGATAGT
         66 CAAAACTGCTCAATCAAAAGAAAGGTTCAACTCTGTGAGATGAATGCACACATCACAAAGA-AGT

                  **   *     *                **      
       1833 TTCTCATTATGGTTCTGGGTAGTTTTTATGTGAACTTATTTC
        130 TTCTCAGAATGCTTCTGTGTAGTTTTTATGTGAAGATATTTC

                    **       **    *       *                              *  
       1875 CTTTTCCAAAATAGGCCGGAAAGGGCTCCAAGTATCCACTTGCAGATTCTACAAAAAGA--GATT
          1 CTTTTCCACCATAGGCCTCAAAGCGCTCCAAATATCCACTTGCAGATTCTACAAAAAGAGTGTTT

                   *             *     *     *       *      *         *      
       1938 CAAAACTCCTCAATCAAAAGATAGGTTGAACTCCGTGAGATTAATGCATACATCACAACGAAGTT
         66 CAAAACTGCTCAATCAAAAGAAAGGTTCAACTCTGTGAGATGAATGCACACATCACAAAGAAGTT

                   **     **             *           
       2003 TCTCAGAGGGCTTCATTGTAG-TTTTATGGGAAGATATTTC
        131 TCTCAGAATGCTTCTGTGTAGTTTTTATGTGAAGATATTTC

                     *   * *       ***         *                    *   *    
       2043 CTTTTCCACTATATGTCTCAAAGATATCCAAATATACACTTGCAGATTCTACAAAATGAGAGTTT
          1 CTTTTCCACCATAGGCCTCAAAGCGCTCCAAATATCCACTTGCAGATTCTACAAAAAGAGTGTTT

                       **      *          *  ***    *                        
       2108 CAAAACTGCTCTGTCAAAAAAAGAGGTTCAGCTGCATGAGTTGAATGCACACATCACAAAGAAGT
         66 CAAAACTGCTCAATCAAAAGAA-AGGTTCAACTCTGTGAGATGAATGCACACATCACAAAGAAGT

                                 *         *         *
       2173 TTCTCAGAATGCTTCTGTGTATTTTTTATGTTAAGATATTTT
        130 TTCTCAGAATGCTTCTGTGTAGTTTTTATGTGAAGATATTTC

                            **     *                                    **   
       2215 CTTTTCCACCATAGGCTGCAAAGGGCTCCAAATATCCACTTGCAGATTCTACAAAAAGAGATTTT
          1 CTTTTCCACCATAGGCCTCAAAGCGCTCCAAATATCCACTTGCAGATTCTACAAAAAGAGTGTTT

                                     *                          **      *    
       2280 CAAAACTGCTCAATCAAAAGAAAGGCTCAACTCTGTGAGATGAATGCACACAAAACAAAGGAGTT
         66 CAAAACTGCTCAATCAAAAGAAAGGTTCAACTCTGTGAGATGAATGCACACATCACAAAGAAGTT

                                        *         *   
       2345 TCTGCA-AATGCTTCTGTGTAGTTTTTAAGTGAAGATACTTC
        131 TCT-CAGAATGCTTCTGTGTAGTTTTTATGTGAAGATATTTC

                     *     *   *    *             *   **                     
       2386 CTTTTCCACAATAGGTCTCGAAGCCCTCCAAATATCCAATTGTGGATTCTACAAAAAGAGTGTTT
          1 CTTTTCCACCATAGGCCTCAAAGCGCTCCAAATATCCACTTGCAGATTCTACAAAAAGAGTGTTT

                              * *   *         **  *      *     * *           
       2451 CAAAACTGCTCAATCAAATGGAAGCTTCAACTCTAAGAAATGAATACACACCTGACAAAGAAGTT
         66 CAAAACTGCTCAATCAAAAGAAAGGTTCAACTCTGTGAGATGAATGCACACATCACAAAGAAGTT

                         *            ** *    ***   *
       2516 TCTCAGAATGCTTTTGTGTAGTTTTTTGGGGAAGGCGTTTT
        131 TCTCAGAATGCTTCTGTGTAGTTTTTATGTGAAGATATTTC

             *          *  * *     **         *    *             *  *   *    
       2557 CCTTTCCACCATCGGTCACAAAGGCCTCCAAATAACCACATGCAGATTCTACACAAGGAGAGTTT
          1 CTTTTCCACCATAGGCCTCAAAGCGCTCCAAATATCCACTTGCAGATTCTACAAAAAGAGTGTTT

                       **        *       *     *   *                 * *     
       2622 CAAAACTGCTCTGTCAAAAGATAGGTTCAGCTCTGCGAGTTGAATGCACACATCACAGACAAGTT
         66 CAAAACTGCTCAATCAAAAGAAAGGTTCAACTCTGTGAGATGAATGCACACATCACAAAGAAGTT

                                     *               
       2687 TCTCAGAATGCTTCTGTGTAGTTTTCATGTGAAGATATTTC
        131 TCTCAGAATGCTTCTGTGTAGTTTTTATGTGAAGATATTTC

                     **      *      *                     **             *   
       2728 CTTTTCCACTTTAGGCCACAAAGCACTCCAAATATCCACTTGCAGAAACTACAAAAAGAGTCTTT
          1 CTTTTCCACCATAGGCCTCAAAGCGCTCCAAATATCCACTTGCAGATTCTACAAAAAGAGTGTTT

                         *         *                                    *    
       2793 CAAAACTGCTCAAGCAAAAGAAAAGTTCAACTCTGTGAGATGAATGCACACATCACAAAGGAGTT
         66 CAAAACTGCTCAATCAAAAGAAAGGTTCAACTCTGTGAGATGAATGCACACATCACAAAGAAGTT

               *                                     
       2858 TCTGAGAATGCTTCTGTGTAGTTTTTATGTGAA-ATATTTC
        131 TCTCAGAATGCTTCTGTGTAGTTTTTATGTGAAGATATTTC

               *            *      *           *        *   *  *   *   * *   
       2898 CTTATCCACCATAGGCTTCAAAGTGCTCCAAATATTCACTTGCAAATTATAAAAACAGAATTTTT
          1 CTTTTCCACCATAGGCCTCAAAGCGCTCCAAATATCCACTTGCAGATTCTACAAAAAGAGTGTTT

                 *      * *       * *      *                                 
       2963 CAAAAATGCTCAGTTAAAAGAATGTTTCAACACTGTGAGATGAATGCACACATCACAAA-AAGTT
         66 CAAAACTGCTCAATCAAAAGAAAGGTTCAACTCTGTGAGATGAATGCACACATCACAAAGAAGTT

               **              *            *        
       3027 TCTGGGAATGCTTCTGTGTGGTTTTTATGTGAGGATATTTC
        131 TCTCAGAATGCTTCTGTGTAGTTTTTATGTGAAGATATTTC

                          * **     *                                    *    
       3068 CTTTTCCACCATAGACTACAAAGGGCTCCAAATATCCACTTGCAGATTCTACAAAAAGAGAGTTT
          1 CTTTTCCACCATAGGCCTCAAAGCGCTCCAAATATCCACTTGCAGATTCTACAAAAAGAGTGTTT

             *         *         *        * *     *                       *  
       3133 CGAAA-TGCTCTATCAAAAGATAGGTTCAAATATGTGATATGAATGCACACATCACAAAGAATTT
         66 CAAAACTGCTCAATCAAAAGAAAGGTTCAACTCTGTGAGATGAATGCACACATCACAAAGAAGTT

                                     *  *            
       3197 TCTCAGAATGCTTCTGTGTAGTTTTCATATGAAGATATTTC
        131 TCTCAGAATGCTTCTGTGTAGTTTTTATGTGAAGATATTTC

                      *             *                                   **   
       3238 CTTTTCCACCGTAGGCCTCAAAGCACTCCAAATATCCACTTGCAGATTCTACAAAAAGAGATTTT
          1 CTTTTCCACCATAGGCCTCAAAGCGCTCCAAATATCCACTTGCAGATTCTACAAAAAGAGTGTTT

                   **                           *  *          *        *     
       3303 CAAAACTAGTCAATCAAAAGAAAGGTTCAACTCTGTCAGTTGAATGCACATATCACAAACAAGTT
         66 CAAAACTGCTCAATCAAAAGAAAGGTTCAACTCTGTGAGATGAATGCACACATCACAAAGAAGTT

                *      *                             
       3368 TCTCGGAATGCGTCTGTGTAGTTTTTATGTGAAGATATTTC
        131 TCTCAGAATGCTTCTGTGTAGTTTTTATGTGAAGATATTTC

               *     * *           *     *                  *    *           
       3409 CTTCTCCACAACAGGCCTCAAAGTGCTCCGAATATCCACTTGCAGATTTTAC-TAAAGAGTGTTT
          1 CTTTTCCACCATAGGCCTCAAAGCGCTCCAAATATCCACTTGCAGATTCTACAAAAAGAGTGTTT

             *               *  *   *     *        *    *               *    
       3473 CCAAACTGCTCAATCAAGAGGAAGTTTCAAGTCTGTGAGCTGAACGCACACATCACAAAGTAGTT
         66 CAAAACTGCTCAATCAAAAGAAAGGTTCAACTCTGTGAGATGAATGCACACATCACAAAGAAGTT

               *                                *    
       3538 TCTGAGAATGCTTCTGTGTAGTTTTTATGTGAAGATGTTTC
        131 TCTCAGAATGCTTCTGTGTAGTTTTTATGTGAAGATATTTC

                            **     *                                    *    
       3579 CTTTTCCACCATAGGCTGCAAAGGGCTCCAAATATCCACTTGCAGATTCTACAAAAAGAGAGTTT
          1 CTTTTCCACCATAGGCCTCAAAGCGCTCCAAATATCCACTTGCAGATTCTACAAAAAGAGTGTTT

                 *     *         *          *     *                     *    
       3644 CAAAAGTGCTCTATCAAAAGATAGGTTCAACTATGTGATATGAATGCACACATCACAAAGTAGTT
         66 CAAAACTGCTCAATCAAAAGAAAGGTTCAACTCTGTGAGATGAATGCACACATCACAAAGAAGTT

                                          *          
       3709 TCTCAGAATGCTTCTGTGTAGTTTTTATGTAAAGATATTTC
        131 TCTCAGAATGCTTCTGTGTAGTTTTTATGTGAAGATATTTC

                                    *                                   **   
       3750 CTTTTCCACCATAGGCCTCAAAGCACTCCAAATATCCACTTGCAGATTCTACAAAAAGAGATTTT
          1 CTTTTCCACCATAGGCCTCAAAGCGCTCCAAATATCCACTTGCAGATTCTACAAAAAGAGTGTTT

                   ** *                   *     *  *    * *   *        *     
       3815 CAAAACTATTTAATCAAAAGAAAGGTTCAAATCTGTCAGTTGAAGGTACATATCACAAACAAGTT
         66 CAAAACTGCTCAATCAAAAGAAAGGTTCAACTCTGTGAGATGAATGCACACATCACAAAGAAGTT

             * **                                    
       3880 TATTGGAATGCTTCTGTGTAGTTTTTATGTGAAGATATTTC
        131 TCTCAGAATGCTTCTGTGTAGTTTTTATGTGAAGATATTTC

                     * *         * *                        **  *     *      
       3921 CTTTTCCACAACAGGCCTCAAGGTGCTCCAAATATCCACTTGCAGATTTCACTAAAAGTGTGTTT
          1 CTTTTCCACCATAGGCCTCAAAGCGCTCCAAATATCCACTTGCAGATTCTACAAAAAGAGTGTTT

             *  *            *  *   *     *        *             *           
       3986 CCAAGCTGCTCAATCAAGAGGAAGTTTCAAGTCTGTGAGGTGAATGCACACATTACAAAGAAGTT
         66 CAAAACTGCTCAATCAAAAGAAAGGTTCAACTCTGTGAGATGAATGCACACATCACAAAGAAGTT

            *  *                                     
       4051 ACTGAGAATGCTTCTGTGTAGTTTTTATGTGAAGATATTTC
        131 TCTCAGAATGCTTCTGTGTAGTTTTTATGTGAAGATATTTC

                      **               *                                *    
       4092 CTTTTCCACCGCAGGCCTCAAAGCGCTGCAAATATCCACTTGCAGATTCTACAAAAAGAGAGTTT
          1 CTTTTCCACCATAGGCCTCAAAGCGCTCCAAATATCCACTTGCAGATTCTACAAAAAGAGTGTTT

                      **         *   *         *   *     **            *     
       4157 CAAAACTGCTGTATCAAAAGATAGGGTCAACTCTGCGAGTTGAATAAACACATCACAAATAAGTT
         66 CAAAACTGCTCAATCAAAAGAAAGGTTCAACTCTGTGAGATGAATGCACACATCACAAAGAAGTT

               **   *        *               *       
       4222 TCTGGGAACGCTTCTGTATAG-TTTTATGTGAATATATTTC
        131 TCTCAGAATGCTTCTGTGTAGTTTTTATGTGAAGATATTTC

                         *          *                   *   *  *   * *   *   
       4262 CTTTTCCACCATATGCCTCAAAGCACTCCAAATATCCACTTGCACATTATAGAAACATAGTCTTT
          1 CTTTTCCACCATAGGCCTCAAAGCGCTCCAAATATCCACTTGCAGATTCTACAAAAAGAGTGTTT

                   **                        *         *             *       
       4327 CAAAACTTGTCAATC-AAAGAAAGGTTCAACTCCGTGAGATGAGTGCACACATCACAGAGAAGTT
         66 CAAAACTGCTCAATCAAAAGAAAGGTTCAACTCTGTGAGATGAATGCACACATCACAAAGAAGTT

                *     *                            * 
       4391 TCTCGGAATGTTTCTGTGTAGTTTTTATGTGAAGATATTGC
        131 TCTCAGAATGCTTCTGTGTAGTTTTTATGTGAAGATATTTC

                     *               *            *          *       *   *   
       4432 CTTTTCCACAATAGGCCTCAAAGCGTTCCAAATATCCAATTGCAGATTCCACAAAAAAAGTTTTT
          1 CTTTTCCACCATAGGCCTCAAAGCGCTCCAAATATCCACTTGCAGATTCTACAAAAAGAGTGTTT

            *                 *  *  *  *             * *         **    *     
       4497 TAAAACTGCTCAATCAAATGATAGATTAAACTCTGTGAGATTAGTGCACACATGTCAAAAAAGTT
         66 CAAAACTGCTCAATCAAAAGAAAGGTTCAACTCTGTGAGATGAATGCACACATCACAAAGAAGTT

                                *      * *           
       4562 TCTCAGAATGCTTCTGTGTACTTTTTAGGGGAAGATATTTC
        131 TCTCAGAATGCTTCTGTGTAGTTTTTATGTGAAGATATTTC

                        *    *     **         *    *           **  * *  *    
       4603 CTTTTCCACCATCGGCCACAAAGGACTCCAAATAACCACATGCAGATTCTAGTAACACAGAGTTT
          1 CTTTTCCACCATAGGCCTCAAAGCGCTCCAAATATCCACTTGCAGATTCTACAAAAAGAGTGTTT

                       *         * *           *   * * *    **    ***        
       4668 CAAAACTGCTCTATCAAAAGATAAGTTCAACTCTGAGAGTTTAGTGCAACCATCGTGAAGAAGTT
         66 CAAAACTGCTCAATCAAAAGAAAGGTTCAACTCTGTGAGATGAATGCACACATCACAAAGAAGTT

                            *     *                  
       4733 TCTCAGAATGCTTCTGAGTAGTGTTTATGTGAAGATATTTC
        131 TCTCAGAATGCTTCTGTGTAGTTTTTATGTGAAGATATTTC

                              *     *                      *           **    
       4774 CTTTTCCACCATAGGCCTGAAAGCCCTCCAAATATCCACTTGCAGATCCTACAAAAAGAAAGTTT
          1 CTTTTCCACCATAGGCCTCAAAGCGCTCCAAATATCCACTTGCAGATTCTACAAAAAGAGTGTTT

             *         **     *  *  *   *        **      *                   
       4839 CGAAA-TGCTCTCTCAAACGATAGTTTCGACTCTGTGGTATGAATACACACATCACAAAGAAGTT
         66 CAAAACTGCTCAATCAAAAGAAAGGTTCAACTCTGTGAGATGAATGCACACATCACAAAGAAGTT

                                       **            
       4903 TCTCAGAATGCTTCTGTGTAGTTTTTAAATGAAGATATTTC
        131 TCTCAGAATGCTTCTGTGTAGTTTTTATGTGAAGATATTTC

            *                       *          *     *                       
       4944 TTTTTCCACCATAGGCCTCAAAGCACTCCAAATATGCACTTCCAGATTCTACAAAAAGAGTGTTT
          1 CTTTTCCACCATAGGCCTCAAAGCGCTCCAAATATCCACTTGCAGATTCTACAAAAAGAGTGTTT

              *                 *       * *         **   *         *  * *    
       5009 CAGAACTGCTCAATCAAAAGGAAGGTTCCAGTC--TGAGACAAATACACACATCAAAAGGTAGTT
         66 CAAAACTGCTCAATCAAAAGAAAGGTTCAACTCTGTGAGATGAATGCACACATCACAAAGAAGTT

                                                    *
       5072 TCTCAGAATGCTTCTGTGTAGTTTTTATGTGAAGATATTTT
        131 TCTCAGAATGCTTCTGTGTAGTTTTTATGTGAAGATATTTC

             *               *    **    *     *      *  **   *          *    
       5113 CCTTTCCACCATAGGCCACAAATGGCTCTAAATACCCACTTACATTTTCCACAAAAAGAGAGTTT
          1 CTTTTCCACCATAGGCCTCAAAGCGCTCCAAATATCCACTTGCAGATTCTACAAAAAGAGTGTTT

                       * *    * *      *   *         *                       
       5178 CAAAACTGCTCTACCAAAGGTAA-GTTTAACGCTGTGAG-TTAA-G-A-ACATCACAAAGAAGTT
         66 CAAAACTGCTCAATCAAAAGAAAGGTTCAACTCTGTGAGATGAATGCACACATCACAAAGAAGTT

                                   *   *  *          
       5238 TCTCAGAATGCTTCTGTGTAGTTCTTACGTAAAGATATTTC
        131 TCTCAGAATGCTTCTGTGTAGTTTTTATGTGAAGATATTTC

                 *   *      ***    *                  *              **      
       5279 CTTTTACACAATAGGCAGAAAAGTGCTCCAAATATCCACTTGAAGATTCTACAAAAACCGTGTTT
          1 CTTTTCCACCATAGGCCTCAAAGCGCTCCAAATATCCACTTGCAGATTCTACAAAAAGAGTGTTT

                     **                                          *      *    
       5344 CAAAACTGCCGAATCAAAAGAAAGGTTCAACTCTGTGAGATGAATGCACACATAACAAAGGAGTT
         66 CAAAACTGCTCAATCAAAAGAAAGGTTCAACTCTGTGAGATGAATGCACACATCACAAAGAAGTT

                                 *      *      *    *
       5409 TCTCAGAATGCTTCTGTGTAGCTTTTATATGAAGACATTTA
        131 TCTCAGAATGCTTCTGTGTAGTTTTTATGTGAAGATATTTC

            *        * *            *   ***                     **        *  
       5450 GTTTTCCACAACAGGCCTCAAAGCTCTCTCCATATCCACTTGCAGATTCTACCGAAAGAGTGCTT
          1 CTTTTCCACCATAGGCCTCAAAGCGCTCCAAATATCCACTTGCAGATTCTACAAAAAGAGTGTTT

             *                   * **     *        *        *     **         
       5515 CCAAACTGCTCAATCAAAAGAGACATTCAAATCTGTGAGGTGAATGCAGACATCGTAAAGAAGTT
         66 CAAAACTGCTCAATCAAAAGAAAGGTTCAACTCTGTGAGATGAATGCACACATCACAAAGAAGTT

                                *     *       *    **
       5580 TCTCAGAATGCTTCTGTGTATTTTTTGTGTGAAGTTATTCG
        131 TCTCAGAATGCTTCTGTGTAGTTTTTATGTGAAGATATTTC

            *    *             *     *  *            **                 *    
       5621 TTTTTGCACCATAGGCCTCCAAGCGTTCTAAATATCCACTTCTAGATTCTACAAAAAGAGAGTTT
          1 CTTTTCCACCATAGGCCTCAAAGCGCTCCAAATATCCACTTGCAGATTCTACAAAAAGAGTGTTT

                   *         *            *        *    *   *                
       5686 CAAAACTACTC-A--AACA-AAAGGTTCAATTCTGTGAGTTGAAAGCAAACATCACAAAGAAGTT
         66 CAAAACTGCTCAATCAAAAGAAAGGTTCAACTCTGTGAGATGAATGCACACATCACAAAGAAGTT

                       *             *               
       5747 TCTCAGAATGCGTCTGTGTAGTTTTGATGTGAAGATATTTC
        131 TCTCAGAATGCTTCTGTGTAGTTTTTATGTGAAGATATTTC

                     **   ****     *                  *                      
       5788 CTTTT-CACAGTAGAATGCAAAGGGCTCCAAATATCCACTTGGAGATTCTACAAAAAGA--GTTT
          1 CTTTTCCACCATAGGCCTCAAAGCGCTCCAAATATCCACTTGCAGATTCTACAAAAAGAGTGTTT

                  *    **     *  *     *     ***   *     *               *   
       5850 CAAAACCGCTCTGTCAAATGATAGGTTGAACTCCCGGAGGTGAATACACACATCACAAAGAGGTT
         66 CAAAACTGCTCAATCAAAAGAAAGGTTCAACTCTGTGAGATGAATGCACACATCACAAAGAAGTT

                  *                       *  *       
       5915 TCTCAGCATGCTTCTGTGTAGTTTTTATGTAAACATATTTC
        131 TCTCAGAATGCTTCTGTGTAGTTTTTATGTGAAGATATTTC

             *    * *              *           *      * *       *   *    *   
       5956 CGTTTCTATCATAGGCCTCAAAGTGCTCCAAATATTCACTTGTACATTCTACCAAACGAGTATTT
          1 CTTTTCCACCATAGGCCTCAAAGCGCTCCAAATATCCACTTGCAGATTCTACAAAAAGAGTGTTT

                              * *         ** *    *        *            *    
       6021 CAAAACTGCTCAATCAAATGGAAGGTTCAAAACCGTGACATGAATGCCCACATCACAAAGTAGTT
         66 CAAAACTGCTCAATCAAAAGAAAGGTTCAACTCTGTGAGATGAATGCACACATCACAAAGAAGTT

                                                     
       6086 TCTCAGAATGCTTCTGTGTAGTTTTTATGTGAAGATATTTC
        131 TCTCAGAATGCTTCTGTGTAGTTTTTATGTGAAGATATTTC

                     * *    *      *    *       * *   **      *              
       6127 CTTTTCCACAACA-GCGTGCAAAACGCTTCAAATATGCCCTTAGAGATTCCACAAAAAGAGTGTT
          1 CTTTTCCACCATAGGCCT-CAAAGCGCTCCAAATATCCACTTGCAGATTCTACAAAAAGAGTGTT

              *     *               *  *                                  ** 
       6191 TCCAAACTACTCAAATCAAAA-AATGATTTCAACTCTGTGAGATGAATGCACACATCACAAACTA
         65 TCAAAACTGCTC-AATCAAAAGAAAG-GTTCAACTCTGTGAGATGAATGCACACATCACAAAGAA

                         *     ** *   * *   *       *   
       6255 GTTTCTCAGAATGTTTCTGCCTGGTTCTCATGCGAAGATAGTTC
        128 GTTTCTCAGAATGCTTCTGTGTAGTTTTTATGTGAAGATATTTC

                 *           *   * **              *                  * *    
       6299 CTTTTTCACCATAGGCCGCAATGTACTCCAAATATCCACCTGCAGATTCTAC-AAAAGTGAGTTT
          1 CTTTTCCACCATAGGCCTCAAAGCGCTCCAAATATCCACTTGCAGATTCTACAAAAAGAGTGTTT

                       *         ***    **         *        *      *       * 
       6363 CAAAACTGCTCTATCAAAAGATCAGTTCGTCTCTGTGAGTTGAATGCATACATCAAAAAGAAGCT
         66 CAAAACTGCTCAATCAAAAGAAAGGTTCAACTCTGTGAGATGAATGCACACATCACAAAGAAGTT

                 *             *      **         *   
       6428 TCTCAAAATGCTTCTGTGTGGTTTTTCGGTGAAGATAGTTC
        131 TCTCAGAATGCTTCTGTGTAGTTTTTATGTGAAGATATTTC

            *     *        *      * *                 *        *        *    
       6469 TTTTTCTACCATAGGTCTCAAACCACTCCAAATATCCACTTGTAGATTCTATAAAAAGGAATG-T
          1 CTTTTCCACCATAGGCCTCAAAGCGCTCCAAATATCCACTTGCAGATTCTACAAAAA-GAGTGTT

                  *        *    *    *      * *         *      *         *   
       6533 TCAAAATTGCTCAATAAAAATAAAGTTTCAACACCGTGAGATGAGTGCACAAATCACAAAGGAGT
         65 TCAAAACTGCTCAATCAAAAGAAAGGTTCAACTCTGTGAGATGAATGCACACATCACAAAGAAGT

                  *          *         *          *   
       6598 TTCTCAAAATGCTTCTGGGTAGTTTTTCTGTGAAGATAGTTC
        130 TTCTCAGAATGCTTCTGTGTAGTTTTTATGTGAAGATATTTC

                  *     *    *     *          *                         *    
       6640 CTTTTCTACCATGGGCCACAAAGGGCTCCAAATACCCACTTGCAGATTCTACAAAAAGAGAGTTT
          1 CTTTTCCACCATAGGCCTCAAAGCGCTCCAAATATCCACTTGCAGATTCTACAAAAAGAGTGTTT

              *        *      ** * *        *    * *    **  * *           *  
       6705 CACAACTGCTCTATCAAACAATATGTTCAACTTTGTGGGTTGAACACAAATATCAC-AAGAATTT
         66 CAAAACTGCTCAATCAAAAGAAAGGTTCAACTCTGTGAGATGAATGCACACATCACAAAGAAGTT

                **                             *     
       6769 TCTCCCAATGCTTCTGTGTAGTTTTTATGTGAAGACATTTC
        131 TCTCAGAATGCTTCTGTGTAGTTTTTATGTGAAGATATTTC

            *   *  *      *  *     *                 *  *      *       *  *  
       6810 TTTTCCCTCCATAGTCCACAAAGTGCTCCAAATATCCACTTACATATTCTAGAAAAAGATTGCTT
          1 CTTTTCCACCATAGGCCTCAAAGCGCTCCAAATATCCACTTGCAGATTCTACAAAAAGAGTGTTT

            **       *    *               * * *                              
       6875 GGAAACTGCACAATGAAAAGAAAGGTTCAAATATATGAGATGAATGCACACATCACAAAGAAGTT
         66 CAAAACTGCTCAATCAAAAGAAAGGTTCAACTCTGTGAGATGAATGCACACATCACAAAGAAGTT

                                 *                    
       6940 TCTCAGAAT-CTCTCTGTGTAATTTTTATGTGAAGATATTTC
        131 TCTCAGAATGCT-TCTGTGTAGTTTTTATGTGAAGATATTTC

                *     *    *  *   *                        *      *    *     
       6981 CTTTCCCACCTTAGGTCTTAAAACGCTCCAAATATCCACTTGCAGATACTACAAGAAGATTGTTT
          1 CTTTTCCACCATAGGCCTCAAAGCGCTCCAAATATCCACTTGCAGATTCTACAAAAAGAGTGTTT

                     *    *        *      *     **                           
       7046 CAAAACTGCACAA-AAAAAGAAATGTTCAATTCTGTTTGATGAATGCACACATCACAAAGAAGTT
         66 CAAAACTGCTCAATCAAAAGAAAGGTTCAACTCTGTGAGATGAATGCACACATCACAAAGAAGTT

                           *                         
       7110 TCTCAGAATGCTTCTCTGTAGTTTTTATGTGAAGATATTTC
        131 TCTCAGAATGCTTCTGTGTAGTTTTTATGTGAAGATATTTC

                     *             *                 *         **      * *   
       7151 CTTTTCCACAATAGGCCTCAAAGGGCTCCAAATATCCACTTCCAGATTCTATGAAAAGAATATTT
          1 CTTTTCCACCATAGGCCTCAAAGCGCTCCAAATATCCACTTGCAGATTCTACAAAAAGAGTGTTT

             *              * *    *                                      *  
       7216 CCAAACTGCTCAATCATAGGAAATGTTCAACTCTGTGAGATGAATGCACACATCAC-AAGAAATT
         66 CAAAACTGCTCAATCAAAAGAAAGGTTCAACTCTGTGAGATGAATGCACACATCACAAAGAAGTT

                     *    *      *       *       *   
       7280 TCTCAGAATCCTTCAGTGTAGGTTTTATGAGAAGATAATTC
        131 TCTCAGAATGCTTCTGTGTAGTTTTTATGTGAAGATATTTC

                     *    **        *   *                           *    *   
       7321 CTTTTCCACAATAGTTCTCAAAGCACTCAAAATATCCACTTGCAGATTCTACAAAAGGAGTATTT
          1 CTTTTCCACCATAGGCCTCAAAGCGCTCCAAATATCCACTTGCAGATTCTACAAAAAGAGTGTTT

                                                          *                  
       7386 CAAAACTGCTCAATCAAAAGAAAGGTTCAACTCTGTGAGATGAATGGACACATCACAAAGAAGTT
         66 CAAAACTGCTCAATCAAAAGAAAGGTTCAACTCTGTGAGATGAATGCACACATCACAAAGAAGTT

                                  *   *              
       7451 TCTCAGAATGCTTCTGTGTAGTATTTTTGTGAAGATATTT-
        131 TCTCAGAATGCTTCTGTGTAGTTTTTATGTGAAGATATTTC

                          *  * * * * ***             *            *     * *  
       7491 CTTTTCCACCATAGACCGCCAGGGGACACAAATATCCACTTTCAGATTCTACAACAAGAGAGGTT
          1 CTTTTCCACCATAGGCCTCAAAGCGCTCCAAATATCCACTTGCAGATTCTACAAAAAGAGTGTTT

                   *   *     *   ** *       *      *                      * *
       7556 CAAAACTACTCGATCAAGAGATGGTTTCAACTATGTGAGTTGAATGCACACATCACAAAGAACTA
         66 CAAAACTGCTCAATCAAAAGAAAGGTTCAACTCTGTGAGATGAATGCACACATCACAAAGAAGTT

             *  *    *                               
       7621 TGTCGGAATTCTTCTGTGTAGTTTTTATGTGAAGATATTTC
        131 TCTCAGAATGCTTCTGTGTAGTTTTTATGTGAAGATATTTC

                     *    * *      * *    *                  *               
       7662 CTTTTCCACAATAGACGTCAAAGTGATCCAGATATCCACTTGCAGATTCCACAAAAAGAGTGTTT
          1 CTTTTCCACCATAGGCCTCAAAGCGCTCCAAATATCCACTTGCAGATTCTACAAAAAGAGTGTTT

                 *   *   *                  **                     *  *      
       7727 CAAAAGTGCACAACCAAAAGAAAGGTTCAACTAGGTGAGATGAATGCACACATCAGAAGGAAGTT
         66 CAAAACTGCTCAATCAAAAGAAAGGTTCAACTCTGTGAGATGAATGCACACATCACAAAGAAGTT

                            **   *     * *       *   
       7792 TCTCAGAATGCTTCTGCATAGCTTTTAAGGGAAGATACTTC
        131 TCTCAGAATGCTTCTGTGTAGTTTTTATGTGAAGATATTTC

                    *               *            * *  *    * *               
       7833 CTTTTCCAACATAGGCCTCAAAGCACTCCAAATATCCTCCTGGAGATACCACAAAAAGAGTGTTT
          1 CTTTTCCACCATAGGCCTCAAAGCGCTCCAAATATCCACTTGCAGATTCTACAAAAAGAGTGTTT

            **                      *  *                 *       *           
       7898 GCAAACTGCTCAATCAAAAGAAAGATTTAACTCTGTGAGATGAATCCACACATGACAAAGAAGTT
         66 CAAAACTGCTCAATCAAAAGAAAGGTTCAACTCTGTGAGATGAATGCACACATCACAAAGAAGTT

                                                     
       7963 TCTCAGAATGCTTCTGTGTAGTTTTTATGTGAAGATATTTC
        131 TCTCAGAATGCTTCTGTGTAGTTTTTATGTGAAGATATTTC

                     *   * * *    **           *                *     *      
       8004 CTTTTCCACAATAAGACCCAAAAGGCTCCAAATATTCACTTGCAGATTCTAAAAAAAACAGTGTT
          1 CTTTTCCACCATAGGCCTCAAAGCGCTCCAAATATCCACTTGCAGATTCT-ACAAAAAGAGTGTT

                                  *                  *      *        **      
       8069 TCAAAACTGCTCAATCAAAAGATA-GTTCAACTCTGTGAGAAGAATGCTCACATCACTGAGAAGT
         65 TCAAAACTGCTCAATCAAAAGAAAGGTTCAACTCTGTGAGATGAATGCACACATCACAAAGAAGT

                                         *            
       8133 TTCTCAGAATGCTTCTGTGTAGTTTTTATATGAAGATATTTC
        130 TTCTCAGAATGCTTCTGTGTAGTTTTTATGTGAAGATATTTC

                *     *      *    **                       *   **       *    
       8175 CTTTCCCACCGTAGGCCACAAAAGGCTCCAAATATCCACTTGCAGATACTATGAAAAGAGAGTTT
          1 CTTTTCCACCATAGGCCTCAAAGCGCTCCAAATATCCACTTGCAGATTCTACAAAAAGAGTGTTT

                        *        *               ***            *            
       8240 CAAAACTGCTCATTCAAAAGATAGGTTCAACTCTGTGGTTTGAATGCACACAGCACAAAGAAGTT
         66 CAAAACTGCTCAATCAAAAGAAAGGTTCAACTCTGTGAGATGAATGCACACATCACAAAGAAGTT

              *       **                   **   *    
       8305 TCACAGAATGTGTCTGTGTAGTTTTTATGTGCGGATGTTTC
        131 TCTCAGAATGCTTCTGTGTAGTTTTTATGTGAAGATATTTC

                         *        ****   *   *                    *          
       8346 CTTTTCCACCATATGCCT-AAATATTTCCCAATTTCCACTTGCAGATTCTACAAGAAGAGTGTTT
          1 CTTTTCCACCATAGGCCTCAAAGCGCTCCAAATATCCACTTGCAGATTCTACAAAAAGAGTGTTT

                      **       *       *           *            *      ***   
       8410 CAAAACTGCTGTATC-AAATAAA-GTTGAACTCTGTGAGGTGAATGCACACAGCACAAAATGGTT
         66 CAAAACTGCTCAATCAAAAGAAAGGTTCAACTCTGTGAGATGAATGCACACATCACAAAGAAGTT

                          **   *        *       *    
       8473 TCTCAGAATGCTTCCTTGTTGTTTTTATATGAAGATGTTTC
        131 TCTCAGAATGCTTCTGTGTAGTTTTTATGTGAAGATATTTC

                  *  *             *   *     *                               
       8514 CTTTTCAACAATAGGCCTCAAAGTGCTTCAAATGTCCACTTGCAGATTCTACAAAAAGAGTGTTT
          1 CTTTTCCACCATAGGCCTCAAAGCGCTCCAAATATCCACTTGCAGATTCTACAAAAAGAGTGTTT

                                        *      *  *  *                       
       8579 CAAAACTGCTCAATCAAAAGAAAGGTTCGACTCTGGGAAATTAATGCACACATCACAAAGAAGTT
         66 CAAAACTGCTCAATCAAAAGAAAGGTTCAACTCTGTGAGATGAATGCACACATCACAAAGAAGTT

                                     *        *      
       8644 TCTC---A-GCTTCTGTGTAGTTTTCATGTGAAGTTATTTC
        131 TCTCAGAATGCTTCTGTGTAGTTTTTATGTGAAGATATTTC

                     *       *     *            *    *         **       *    
       8681 CTTTTCCACAATAGGCCGCAAAGGGCTCCAAATATCAACTTACAGATTCTAGGAAAAGAGAGTTT
          1 CTTTTCCACCATAGGCCTCAAAGCGCTCCAAATATCCACTTGCAGATTCTACAAAAAGAGTGTTT

                       * **      *     *                                     
       8746 CAAAACTGCTCTACGAAAAGATAGGTTGAACTCTGTGAGATGAATGCACACATCACAAAGAAGTT
         66 CAAAACTGCTCAATCAAAAGAAAGGTTCAACTCTGTGAGATGAATGCACACATCACAAAGAAGTT

                       *               * *           
       8811 TCTCAGAATGCATCTGTGTAGTTTTTACGGGAAGATATTTC
        131 TCTCAGAATGCTTCTGTGTAGTTTTTATGTGAAGATATTTC

                        ***  *     **      *  *                  *     ***   
       8852 CTTTTCCACCATCTTCCACAAAGGTCTCCAAGTAACCACTTGCAGATTCTACAGAAAGACACTTT
          1 CTTTTCCACCATAGGCCTCAAAGCGCTCCAAATATCCACTTGCAGATTCTACAAAAAGAGTGTTT

            *          *         ***      *      ***                      *  
       8917 AAAAACTGCTCTATCAAAAGATCAGTTCAAGTCTGTGGTTTGAATGCACACATCACAAAGAATTT
         66 CAAAACTGCTCAATCAAAAGAAAGGTTCAACTCTGTGAGATGAATGCACACATCACAAAGAAGTT

                                     *  *            
       8982 TCTCAGAATGCTTCTGTGTAGTTTTCATATGAAGATATTTC
        131 TCTCAGAATGCTTCTGTGTAGTTTTTATGTGAAGATATTTC

                                    *                                   **   
       9023 CTTTTCCACCATAGGCCTCAAAGCACTCCAAATATCCACTTGCAGATTCTACAAAAAGAGATTTT
          1 CTTTTCCACCATAGGCCTCAAAGCGCTCCAAATATCCACTTGCAGATTCTACAAAAAGAGTGTTT

                   **                           *  *          *        *     
       9088 CAAAACTAGTCAATCAAAAGAAAGGTTCAACTCTGTCAGTTGAATGCACATATCACAAACAAGTT
         66 CAAAACTGCTCAATCAAAAGAAAGGTTCAACTCTGTGAGATGAATGCACACATCACAAAGAAGTT

                *      *                             
       9153 TCTCGGAATGCGTCTGTGTAGTTTTTATGTGAAGATATTTC
        131 TCTCAGAATGCTTCTGTGTAGTTTTTATGTGAAGATATTTC

               *     * *           *     *                  *    *           
       9194 CTTCTCCACAACAGGCCTCAAAGTGCTCCGAATATCCACTTGCAGATTTTAC-TAAAGAGTGTTT
          1 CTTTTCCACCATAGGCCTCAAAGCGCTCCAAATATCCACTTGCAGATTCTACAAAAAGAGTGTTT

             *               *  *   *     *        *    *               *    
       9258 CCAAACTGCTCAATCAAGAGGAAGTTTCAAGTCTGTGAGCTGAACGCACACATCACAAAGTAGTT
         66 CAAAACTGCTCAATCAAAAGAAAGGTTCAACTCTGTGAGATGAATGCACACATCACAAAGAAGTT

               *                                *    
       9323 TCTGAGAATGCTTCTGTGTAGTTTTTATGTGAAGATGTTTC
        131 TCTCAGAATGCTTCTGTGTAGTTTTTATGTGAAGATATTTC

                            **     *                                    *    
       9364 CTTTTCCACCATAGGCTGCAAAGGGCTCCAAATATCCACTTGCAGATTCTACAAAAAGAGAGTTT
          1 CTTTTCCACCATAGGCCTCAAAGCGCTCCAAATATCCACTTGCAGATTCTACAAAAAGAGTGTTT

                 *     *         *          *     *                     *    
       9429 CAAAAGTGCTCTATCAAAAGATAGGTTCAACTATGTGATATGAATGCACACATCACAAAGTAGTT
         66 CAAAACTGCTCAATCAAAAGAAAGGTTCAACTCTGTGAGATGAATGCACACATCACAAAGAAGTT

                                          *          
       9494 TCTCAGAATGCTTCTGTGTAGTTTTTATGTAAAGATATTTC
        131 TCTCAGAATGCTTCTGTGTAGTTTTTATGTGAAGATATTTC

                                    *                                   **   
       9535 CTTTTCCACCATAGGCCTCAAAGCACTCCAAATATCCACTTGCAGATTCTACAAAAAGAGATTTT
          1 CTTTTCCACCATAGGCCTCAAAGCGCTCCAAATATCCACTTGCAGATTCTACAAAAAGAGTGTTT

                   ** *                   *     *  *    * *   *        *     
       9600 CAAAACTATTTAATCAAAAGAAAGGTTCAAATCTGTCAGTTGAAGGTACATATCACAAACAAGTT
         66 CAAAACTGCTCAATCAAAAGAAAGGTTCAACTCTGTGAGATGAATGCACACATCACAAAGAAGTT

             * **                                    
       9665 TATTGGAATGCTTCTGTGTAGTTTTTATGTGAAGATATTTC
        131 TCTCAGAATGCTTCTGTGTAGTTTTTATGTGAAGATATTTC

                     * *         * *                        **  *     *      
       9706 CTTTTCCACAACAGGCCTCAAGGTGCTCCAAATATCCACTTGCAGATTTCACTAAAAGTGTGTTT
          1 CTTTTCCACCATAGGCCTCAAAGCGCTCCAAATATCCACTTGCAGATTCTACAAAAAGAGTGTTT

             *  *            *  *   *     *        *             *           
       9771 CCAAGCTGCTCAATCAAGAGGAAGTTTCAAGTCTGTGAGGTGAATGCACACATTACAAAGAAGTT
         66 CAAAACTGCTCAATCAAAAGAAAGGTTCAACTCTGTGAGATGAATGCACACATCACAAAGAAGTT

            *  *                                     
       9836 ACTGAGAATGCTTCTGTGTAGTTTTTATGTGAAGATATTTC
        131 TCTCAGAATGCTTCTGTGTAGTTTTTATGTGAAGATATTTC

                      **               *                                *    
       9877 CTTTTCCACCGCAGGCCTCAAAGCGCTGCAAATATCCACTTGCAGATTCTACAAAAAGAGAGTTT
          1 CTTTTCCACCATAGGCCTCAAAGCGCTCCAAATATCCACTTGCAGATTCTACAAAAAGAGTGTTT

                      **         *   *         *   *     **            *     
       9942 CAAAACTGCTGTATCAAAAGATAGGGTCAACTCTGCGAGTTGAATAAACACATCACAAATAAGTT
         66 CAAAACTGCTCAATCAAAAGAAAGGTTCAACTCTGTGAGATGAATGCACACATCACAAAGAAGTT

               **   *        *               *       
      10007 TCTGGGAACGCTTCTGTATAG-TTTTATGTGAATATATTTC
        131 TCTCAGAATGCTTCTGTGTAGTTTTTATGTGAAGATATTTC

                         *          *                   *   *  *   * *   *   
      10047 CTTTTCCACCATATGCCTCAAAGCACTCCAAATATCCACTTGCACATTATAGAAACATAGTCTTT
          1 CTTTTCCACCATAGGCCTCAAAGCGCTCCAAATATCCACTTGCAGATTCTACAAAAAGAGTGTTT

                   **                        *         *             *       
      10112 CAAAACTTGTCAATC-AAAGAAAGGTTCAACTCCGTGAGATGAGTGCACACATCACAGAGAAGTT
         66 CAAAACTGCTCAATCAAAAGAAAGGTTCAACTCTGTGAGATGAATGCACACATCACAAAGAAGTT

                *     *                            * 
      10176 TCTCGGAATGTTTCTGTGTAGTTTTTATGTGAAGATATTGC
        131 TCTCAGAATGCTTCTGTGTAGTTTTTATGTGAAGATATTTC

                     *               *            *          *       *   *   
      10217 CTTTTCCACAATAGGCCTCAAAGCGTTCCAAATATCCAATTGCAGATTCCACAAAAAAAGTTTTT
          1 CTTTTCCACCATAGGCCTCAAAGCGCTCCAAATATCCACTTGCAGATTCTACAAAAAGAGTGTTT

            *                 *  *  *  *             * *         **    *     
      10282 TAAAACTGCTCAATCAAATGATAGATTAAACTCTGTGAGATTAGTGCACACATGTCAAAAAAGTT
         66 CAAAACTGCTCAATCAAAAGAAAGGTTCAACTCTGTGAGATGAATGCACACATCACAAAGAAGTT

                                *      * *           
      10347 TCTCAGAATGCTTCTGTGTACTTTTTAGGGGAAGATATTTC
        131 TCTCAGAATGCTTCTGTGTAGTTTTTATGTGAAGATATTTC

                        *    *     **         *    *           **  * *  *    
      10388 CTTTTCCACCATCGGCCACAAAGGACTCCAAATAACCACATGCAGATTCTAGTAACACAGAGTTT
          1 CTTTTCCACCATAGGCCTCAAAGCGCTCCAAATATCCACTTGCAGATTCTACAAAAAGAGTGTTT

                       *         * *           *   * * *    **    ***        
      10453 CAAAACTGCTCTATCAAAAGATAAGTTCAACTCTGAGAGTTTAGTGCAACCATCGTGAAGAAGTT
         66 CAAAACTGCTCAATCAAAAGAAAGGTTCAACTCTGTGAGATGAATGCACACATCACAAAGAAGTT

                            *     *                  
      10518 TCTCAGAATGCTTCTGAGTAGTGTTTATGTGAAGATATTTC
        131 TCTCAGAATGCTTCTGTGTAGTTTTTATGTGAAGATATTTC

                              *     *                      *           **    
      10559 CTTTTCCACCATAGGCCTGAAAGCCCTCCAAATATCCACTTGCAGATCCTACAAAAAGAAAGTTT
          1 CTTTTCCACCATAGGCCTCAAAGCGCTCCAAATATCCACTTGCAGATTCTACAAAAAGAGTGTTT

             *         **     *  *  *   *        **      *                   
      10624 CGAAA-TGCTCTCTCAAACGATAGTTTCGACTCTGTGGTATGAATACACACATCACAAAGAAGTT
         66 CAAAACTGCTCAATCAAAAGAAAGGTTCAACTCTGTGAGATGAATGCACACATCACAAAGAAGTT

                                       **            
      10688 TCTCAGAATGCTTCTGTGTAGTTTTTAAATGAAGATATTTC
        131 TCTCAGAATGCTTCTGTGTAGTTTTTATGTGAAGATATTTC

            *                       *          *     *                       
      10729 TTTTTCCACCATAGGCCTCAAAGCACTCCAAATATGCACTTCCAGATTCTACAAAAAGAGTGTTT
          1 CTTTTCCACCATAGGCCTCAAAGCGCTCCAAATATCCACTTGCAGATTCTACAAAAAGAGTGTTT

              *                 *       * *         **   *         *  * *    
      10794 CAGAACTGCTCAATCAAAAGGAAGGTTCCAGTC--TGAGACAAATACACACATCAAAAGGTAGTT
         66 CAAAACTGCTCAATCAAAAGAAAGGTTCAACTCTGTGAGATGAATGCACACATCACAAAGAAGTT

                                                    *
      10857 TCTCAGAATGCTTCTGTGTAGTTTTTATGTGAAGATATTTT
        131 TCTCAGAATGCTTCTGTGTAGTTTTTATGTGAAGATATTTC

             *               *    **    *     *      *  **   *          *    
      10898 CCTTTCCACCATAGGCCACAAATGGCTCTAAATACCCACTTACATTTTCCACAAAAAGAGAGTTT
          1 CTTTTCCACCATAGGCCTCAAAGCGCTCCAAATATCCACTTGCAGATTCTACAAAAAGAGTGTTT

                       * *    * *      *   *         *                       
      10963 CAAAACTGCTCTACCAAAGGTAA-GTTTAACGCTGTGAG-TTAA-G-A-ACATCACAAAGAAGTT
         66 CAAAACTGCTCAATCAAAAGAAAGGTTCAACTCTGTGAGATGAATGCACACATCACAAAGAAGTT

                                   *   *  *          
      11023 TCTCAGAATGCTTCTGTGTAGTTCTTACGTAAAGATATTTC
        131 TCTCAGAATGCTTCTGTGTAGTTTTTATGTGAAGATATTTC

                 *   *      ***    *                  *              **      
      11064 CTTTTACACAATAGGCAGAAAAGTGCTCCAAATATCCACTTGAAGATTCTACAAAAACCGTGTTT
          1 CTTTTCCACCATAGGCCTCAAAGCGCTCCAAATATCCACTTGCAGATTCTACAAAAAGAGTGTTT

                     **                                          *      *    
      11129 CAAAACTGCCGAATCAAAAGAAAGGTTCAACTCTGTGAGATGAATGCACACATAACAAAGGAGTT
         66 CAAAACTGCTCAATCAAAAGAAAGGTTCAACTCTGTGAGATGAATGCACACATCACAAAGAAGTT

                                 *      *      *    *
      11194 TCTCAGAATGCTTCTGTGTAGCTTTTATATGAAGACATTTA
        131 TCTCAGAATGCTTCTGTGTAGTTTTTATGTGAAGATATTTC

            *        * *            *   ***                     **        *  
      11235 GTTTTCCACAACAGGCCTCAAAGCTCTCTCCATATCCACTTGCAGATTCTACCGAAAGAGTGCTT
          1 CTTTTCCACCATAGGCCTCAAAGCGCTCCAAATATCCACTTGCAGATTCTACAAAAAGAGTGTTT

             *                   * **     *        *        *     **         
      11300 CCAAACTGCTCAATCAAAAGAGACATTCAAATCTGTGAGGTGAATGCAGACATCGTAAAGAAGTT
         66 CAAAACTGCTCAATCAAAAGAAAGGTTCAACTCTGTGAGATGAATGCACACATCACAAAGAAGTT

                                *     *       *    **
      11365 TCTCAGAATGCTTCTGTGTATTTTTTGTGTGAAGTTATTCG
        131 TCTCAGAATGCTTCTGTGTAGTTTTTATGTGAAGATATTTC

            *    *             *     *  *            **                 *    
      11406 TTTTTGCACCATAGGCCTCCAAGCGTTCTAAATATCCACTTCTAGATTCTACAAAAAGAGAGTTT
          1 CTTTTCCACCATAGGCCTCAAAGCGCTCCAAATATCCACTTGCAGATTCTACAAAAAGAGTGTTT

                   *         *            *        *    *   *                
      11471 CAAAACTACTC-A--AACA-AAAGGTTCAATTCTGTGAGTTGAAAGCAAACATCACAAAGAAGTT
         66 CAAAACTGCTCAATCAAAAGAAAGGTTCAACTCTGTGAGATGAATGCACACATCACAAAGAAGTT

                       *             *               
      11532 TCTCAGAATGCGTCTGTGTAGTTTTGATGTGAAGATATTTC
        131 TCTCAGAATGCTTCTGTGTAGTTTTTATGTGAAGATATTTC

                     **   ****     *                  *                      
      11573 CTTTT-CACAGTAGAATGCAAAGGGCTCCAAATATCCACTTGGAGATTCTACAAAAAGA--GTTT
          1 CTTTTCCACCATAGGCCTCAAAGCGCTCCAAATATCCACTTGCAGATTCTACAAAAAGAGTGTTT

                  *    **     *  *     *     ***   *     *               *   
      11635 CAAAACCGCTCTGTCAAATGATAGGTTGAACTCCCGGAGGTGAATACACACATCACAAAGAGGTT
         66 CAAAACTGCTCAATCAAAAGAAAGGTTCAACTCTGTGAGATGAATGCACACATCACAAAGAAGTT

                  *                       *  *       
      11700 TCTCAGCATGCTTCTGTGTAGTTTTTATGTAAACATATTTC
        131 TCTCAGAATGCTTCTGTGTAGTTTTTATGTGAAGATATTTC

             *    * *              *           *      * *       *   *    *   
      11741 CGTTTCTATCATAGGCCTCAAAGTGCTCCAAATATTCACTTGTACATTCTACCAAACGAGTATTT
          1 CTTTTCCACCATAGGCCTCAAAGCGCTCCAAATATCCACTTGCAGATTCTACAAAAAGAGTGTTT

                              * *         ** *    *        *            *    
      11806 CAAAACTGCTCAATCAAATGGAAGGTTCAAAACCGTGACATGAATGCCCACATCACAAAGTAGTT
         66 CAAAACTGCTCAATCAAAAGAAAGGTTCAACTCTGTGAGATGAATGCACACATCACAAAGAAGTT

                                                     
      11871 TCTCAGAATGCTTCTGTGTAGTTTTTATGTGAAGATATTTC
        131 TCTCAGAATGCTTCTGTGTAGTTTTTATGTGAAGATATTTC

                     * *    *      *    *       * *   **      *              
      11912 CTTTTCCACAACA-GCGTGCAAAACGCTTCAAATATGCCCTTAGAGATTCCACAAAAAGAGTGTT
          1 CTTTTCCACCATAGGCCT-CAAAGCGCTCCAAATATCCACTTGCAGATTCTACAAAAAGAGTGTT

              *     *               *  *                                  ** 
      11976 TCCAAACTACTCAAATCAAAA-AATGATTTCAACTCTGTGAGATGAATGCACACATCACAAACTA
         65 TCAAAACTGCTC-AATCAAAAGAAAG-GTTCAACTCTGTGAGATGAATGCACACATCACAAAGAA

                         *     ** *   * *   *       *   
      12040 GTTTCTCAGAATGTTTCTGCCTGGTTCTCATGCGAAGATAGTTC
        128 GTTTCTCAGAATGCTTCTGTGTAGTTTTTATGTGAAGATATTTC

                 *           *   * **              *                  * *    
      12084 CTTTTTCACCATAGGCCGCAATGTACTCCAAATATCCACCTGCAGATTCTAC-AAAAGTGAGTTT
          1 CTTTTCCACCATAGGCCTCAAAGCGCTCCAAATATCCACTTGCAGATTCTACAAAAAGAGTGTTT

                       *         ***    **         *        *      *       * 
      12148 CAAAACTGCTCTATCAAAAGATCAGTTCGTCTCTGTGAGTTGAATGCATACATCAAAAAGAAGCT
         66 CAAAACTGCTCAATCAAAAGAAAGGTTCAACTCTGTGAGATGAATGCACACATCACAAAGAAGTT

                 *             *      **         *   
      12213 TCTCAAAATGCTTCTGTGTGGTTTTTCGGTGAAGATAGTTC
        131 TCTCAGAATGCTTCTGTGTAGTTTTTATGTGAAGATATTTC

            *     *        *      * *                 *        *        *    
      12254 TTTTTCTACCATAGGTCTCAAACCACTCCAAATATCCACTTGTAGATTCTATAAAAAGGAATG-T
          1 CTTTTCCACCATAGGCCTCAAAGCGCTCCAAATATCCACTTGCAGATTCTACAAAAA-GAGTGTT

                  *        *    *    *      * *         *      *         *   
      12318 TCAAAATTGCTCAATAAAAATAAAGTTTCAACACCGTGAGATGAGTGCACAAATCACAAAGGAGT
         65 TCAAAACTGCTCAATCAAAAGAAAGGTTCAACTCTGTGAGATGAATGCACACATCACAAAGAAGT

                  *          *         *          *   
      12383 TTCTCAAAATGCTTCTGGGTAGTTTTTCTGTGAAGATAGTTC
        130 TTCTCAGAATGCTTCTGTGTAGTTTTTATGTGAAGATATTTC

                  *     *    *     *          *                         *    
      12425 CTTTTCTACCATGGGCCACAAAGGGCTCCAAATACCCACTTGCAGATTCTACAAAAAGAGAGTTT
          1 CTTTTCCACCATAGGCCTCAAAGCGCTCCAAATATCCACTTGCAGATTCTACAAAAAGAGTGTTT

              *        *      ** * *        *    * *    **  * *           *  
      12490 CACAACTGCTCTATCAAACAATATGTTCAACTTTGTGGGTTGAACACAAATATCAC-AAGAATTT
         66 CAAAACTGCTCAATCAAAAGAAAGGTTCAACTCTGTGAGATGAATGCACACATCACAAAGAAGTT

                **                             *     
      12554 TCTCCCAATGCTTCTGTGTAGTTTTTATGTGAAGACATTTC
        131 TCTCAGAATGCTTCTGTGTAGTTTTTATGTGAAGATATTTC

            *   *  *      *  *     *                 *  *      *       *  *  
      12595 TTTTCCCTCCATAGTCCACAAAGTGCTCCAAATATCCACTTACATATTCTAGAAAAAGATTGCTT
          1 CTTTTCCACCATAGGCCTCAAAGCGCTCCAAATATCCACTTGCAGATTCTACAAAAAGAGTGTTT

            **       *    *               * * *                              
      12660 GGAAACTGCACAATGAAAAGAAAGGTTCAAATATATGAGATGAATGCACACATCACAAAGAAGTT
         66 CAAAACTGCTCAATCAAAAGAAAGGTTCAACTCTGTGAGATGAATGCACACATCACAAAGAAGTT

                                 *                    
      12725 TCTCAGAAT-CTCTCTGTGTAATTTTTATGTGAAGATATTTC
        131 TCTCAGAATGCT-TCTGTGTAGTTTTTATGTGAAGATATTTC

                *     *    *  *   *                        *      *    *     
      12766 CTTTCCCACCTTAGGTCTTAAAACGCTCCAAATATCCACTTGCAGATACTACAAGAAGATTGTTT
          1 CTTTTCCACCATAGGCCTCAAAGCGCTCCAAATATCCACTTGCAGATTCTACAAAAAGAGTGTTT

                     *    *        *      *     **                           
      12831 CAAAACTGCACAA-AAAAAGAAATGTTCAATTCTGTTTGATGAATGCACACATCACAAAGAAGTT
         66 CAAAACTGCTCAATCAAAAGAAAGGTTCAACTCTGTGAGATGAATGCACACATCACAAAGAAGTT

                           *                         
      12895 TCTCAGAATGCTTCTCTGTAGTTTTTATGTGAAGATATTTC
        131 TCTCAGAATGCTTCTGTGTAGTTTTTATGTGAAGATATTTC

                     *             *                 *         **      * *   
      12936 CTTTTCCACAATAGGCCTCAAAGGGCTCCAAATATCCACTTCCAGATTCTATGAAAAGAATATTT
          1 CTTTTCCACCATAGGCCTCAAAGCGCTCCAAATATCCACTTGCAGATTCTACAAAAAGAGTGTTT

             *              * *    *                                      *  
      13001 CCAAACTGCTCAATCATAGGAAATGTTCAACTCTGTGAGATGAATGCACACATCAC-AAGAAATT
         66 CAAAACTGCTCAATCAAAAGAAAGGTTCAACTCTGTGAGATGAATGCACACATCACAAAGAAGTT

                     *    *      *       *       *   
      13065 TCTCAGAATCCTTCAGTGTAGGTTTTATGAGAAGATAATTC
        131 TCTCAGAATGCTTCTGTGTAGTTTTTATGTGAAGATATTTC

                     *    **        *   *                           *    *   
      13106 CTTTTCCACAATAGTTCTCAAAGCACTCAAAATATCCACTTGCAGATTCTACAAAAGGAGTATTT
          1 CTTTTCCACCATAGGCCTCAAAGCGCTCCAAATATCCACTTGCAGATTCTACAAAAAGAGTGTTT

                                                          *                  
      13171 CAAAACTGCTCAATCAAAAGAAAGGTTCAACTCTGTGAGATGAATGGACACATCACAAAGAAGTT
         66 CAAAACTGCTCAATCAAAAGAAAGGTTCAACTCTGTGAGATGAATGCACACATCACAAAGAAGTT

                                  *   *              
      13236 TCTCAGAATGCTTCTGTGTAGTATTTTTGTGAAGATATTT-
        131 TCTCAGAATGCTTCTGTGTAGTTTTTATGTGAAGATATTTC

                          *  * * * * ***             *            *     * *  
      13276 CTTTTCCACCATAGACCGCCAGGGGACACAAATATCCACTTTCAGATTCTACAACAAGAGAGGTT
          1 CTTTTCCACCATAGGCCTCAAAGCGCTCCAAATATCCACTTGCAGATTCTACAAAAAGAGTGTTT

                   *   *     *   ** *       *      *                      * *
      13341 CAAAACTACTCGATCAAGAGATGGTTTCAACTATGTGAGTTGAATGCACACATCACAAAGAACTA
         66 CAAAACTGCTCAATCAAAAGAAAGGTTCAACTCTGTGAGATGAATGCACACATCACAAAGAAGTT

             *  *    *                               
      13406 TGTCGGAATTCTTCTGTGTAGTTTTTATGTGAAGATATTTC
        131 TCTCAGAATGCTTCTGTGTAGTTTTTATGTGAAGATATTTC

                     *    * *      * *    *                  *               
      13447 CTTTTCCACAATAGACGTCAAAGTGATCCAGATATCCACTTGCAGATTCCACAAAAAGAGTGTTT
          1 CTTTTCCACCATAGGCCTCAAAGCGCTCCAAATATCCACTTGCAGATTCTACAAAAAGAGTGTTT

                 *   *   *                  **                     *  *      
      13512 CAAAAGTGCACAACCAAAAGAAAGGTTCAACTAGGTGAGATGAATGCACACATCAGAAGGAAGTT
         66 CAAAACTGCTCAATCAAAAGAAAGGTTCAACTCTGTGAGATGAATGCACACATCACAAAGAAGTT

                            **   *     * *       *   
      13577 TCTCAGAATGCTTCTGCATAGCTTTTAAGGGAAGATACTTC
        131 TCTCAGAATGCTTCTGTGTAGTTTTTATGTGAAGATATTTC

                    *               *            * *  *    * *               
      13618 CTTTTCCAACATAGGCCTCAAAGCACTCCAAATATCCTCCTGGAGATACCACAAAAAGAGTGTTT
          1 CTTTTCCACCATAGGCCTCAAAGCGCTCCAAATATCCACTTGCAGATTCTACAAAAAGAGTGTTT

            **                      *  *                 *       *           
      13683 GCAAACTGCTCAATCAAAAGAAAGATTTAACTCTGTGAGATGAATCCACACATGACAAAGAAGTT
         66 CAAAACTGCTCAATCAAAAGAAAGGTTCAACTCTGTGAGATGAATGCACACATCACAAAGAAGTT

                                                     
      13748 TCTCAGAATGCTTCTGTGTAGTTTTTATGTGAAGATATTTC
        131 TCTCAGAATGCTTCTGTGTAGTTTTTATGTGAAGATATTTC

                     *   * * *    **           *                *     *      
      13789 CTTTTCCACAATAAGACCCAAAAGGCTCCAAATATTCACTTGCAGATTCTAAAAAAAACAGTGTT
          1 CTTTTCCACCATAGGCCTCAAAGCGCTCCAAATATCCACTTGCAGATTCT-ACAAAAAGAGTGTT

                                  *                  *      *        **      
      13854 TCAAAACTGCTCAATCAAAAGATA-GTTCAACTCTGTGAGAAGAATGCTCACATCACTGAGAAGT
         65 TCAAAACTGCTCAATCAAAAGAAAGGTTCAACTCTGTGAGATGAATGCACACATCACAAAGAAGT

                                         *            
      13918 TTCTCAGAATGCTTCTGTGTAGTTTTTATATGAAGATATTTC
        130 TTCTCAGAATGCTTCTGTGTAGTTTTTATGTGAAGATATTTC

                *     *      *    **                       *   **       *    
      13960 CTTTCCCACCGTAGGCCACAAAAGGCTCCAAATATCCACTTGCAGATACTATGAAAAGAGAGTTT
          1 CTTTTCCACCATAGGCCTCAAAGCGCTCCAAATATCCACTTGCAGATTCTACAAAAAGAGTGTTT

                        *        *               ***            *            
      14025 CAAAACTGCTCATTCAAAAGATAGGTTCAACTCTGTGGTTTGAATGCACACAGCACAAAGAAGTT
         66 CAAAACTGCTCAATCAAAAGAAAGGTTCAACTCTGTGAGATGAATGCACACATCACAAAGAAGTT

              *       **                   **   *    
      14090 TCACAGAATGTGTCTGTGTAGTTTTTATGTGCGGATGTTTC
        131 TCTCAGAATGCTTCTGTGTAGTTTTTATGTGAAGATATTTC

                         *        ****   *   *               *    *          
      14131 CTTTTCCACCATATGCCT-AAATATTTCCCAATTTCCACTTGCAGATTCCACAAGAAGAGTGTTT
          1 CTTTTCCACCATAGGCCTCAAAGCGCTCCAAATATCCACTTGCAGATTCTACAAAAAGAGTGTTT

                      **       *       *           *            *      ***   
      14195 CAAAACTGCTGTATC-AAATAAA-GTTGAACTCTGTGAGGTGAATGCACACAGCACAAAATGGTT
         66 CAAAACTGCTCAATCAAAAGAAAGGTTCAACTCTGTGAGATGAATGCACACATCACAAAGAAGTT

                          **   *        *       *    
      14258 TCTCAGAATGCTTCCTTGTTGTTTTTATATGAAGATGTTTC
        131 TCTCAGAATGCTTCTGTGTAGTTTTTATGTGAAGATATTTC

                  *  *             *   *     *                               
      14299 CTTTTCAACAATAGGCCTCAAAGTGCTTCAAATGTCCACTTGCAGATTCTACAAAAAGAGTGTTT
          1 CTTTTCCACCATAGGCCTCAAAGCGCTCCAAATATCCACTTGCAGATTCTACAAAAAGAGTGTTT

                                        *      *  *  *                       
      14364 CAAAACTGCTCAATCAAAAGAAAGGTTCGACTCTGGGAAATTAATGCACACATCACAAAGAAGTT
         66 CAAAACTGCTCAATCAAAAGAAAGGTTCAACTCTGTGAGATGAATGCACACATCACAAAGAAGTT

                                     *        *      
      14429 TCTC---A-GCTTCTGTGTAGTTTTCATGTGAAGTTATTTC
        131 TCTCAGAATGCTTCTGTGTAGTTTTTATGTGAAGATATTTC

                     *       *     *            *    *         **       *    
      14466 CTTTTCCACAATAGGCCGCAAAGGGCTCCAAATATCAACTTACAGATTCTAGGAAAAGAGAGTTT
          1 CTTTTCCACCATAGGCCTCAAAGCGCTCCAAATATCCACTTGCAGATTCTACAAAAAGAGTGTTT

                       * **      *     *                                     
      14531 CAAAACTGCTCTACGAAAAGATAGGTTGAACTCTGTGAGATGAATGCACACATCACAAAGAAGTT
         66 CAAAACTGCTCAATCAAAAGAAAGGTTCAACTCTGTGAGATGAATGCACACATCACAAAGAAGTT

                       *               * *     *     
      14596 TCTCAGAATGCATCTGTGTAGTTTTTACGGGAAGACATTTC
        131 TCTCAGAATGCTTCTGTGTAGTTTTTATGTGAAGATATTTC

                        ***  *     **      *  *                  *     ***   
      14637 CTTTTCCACCATCTTCCACAAAGGTCTCCAAGTAACCACTTGCAGATTCTACAGAAAGACACTTT
          1 CTTTTCCACCATAGGCCTCAAAGCGCTCCAAATATCCACTTGCAGATTCTACAAAAAGAGTGTTT

            *          *         ***      *      ***                      *  
      14702 AAAAACTGCTCTATCAAAAGATCAGTTCAAGTCTGTGGTTTGAATGCACACATCACAAAGAATTT
         66 CAAAACTGCTCAATCAAAAGAAAGGTTCAACTCTGTGAGATGAATGCACACATCACAAAGAAGTT

                                     *  *            
      14767 TCTCAGAATGCTTCTGTGTAGTTTTCATATGAAGATATTTC
        131 TCTCAGAATGCTTCTGTGTAGTTTTTATGTGAAGATATTTC

                                    *                                   **   
      14808 CTTTTCCACCATAGGCCTCAAAGCACTCCAAATATCCACTTGCAGATTCTACAAAAAGAGATTTT
          1 CTTTTCCACCATAGGCCTCAAAGCGCTCCAAATATCCACTTGCAGATTCTACAAAAAGAGTGTTT

                   **                           *  *          *        *     
      14873 CAAAACTAGTCAATCAAAAGAAAGGTTCAACTCTGTCAGTTGAATGCACATATCACAAACAAGTT
         66 CAAAACTGCTCAATCAAAAGAAAGGTTCAACTCTGTGAGATGAATGCACACATCACAAAGAAGTT

                *      *                             
      14938 TCTCGGAATGCGTCTGTGTAGTTTTTATGTGAAGATATTTC
        131 TCTCAGAATGCTTCTGTGTAGTTTTTATGTGAAGATATTTC

               *     * *           *     *                  *    *           
      14979 CTTCTCCACAACAGGCCTCAAAGTGCTCCGAATATCCACTTGCAGATTTTAC-TAAAGAGTGTTT
          1 CTTTTCCACCATAGGCCTCAAAGCGCTCCAAATATCCACTTGCAGATTCTACAAAAAGAGTGTTT

             *               *  *   *     *        *    *               *    
      15043 CCAAACTGCTCAATCAAGAGGAAGTTTCAAGTCTGTGAGCTGAACGCACACATCACAAAGTAGTT
         66 CAAAACTGCTCAATCAAAAGAAAGGTTCAACTCTGTGAGATGAATGCACACATCACAAAGAAGTT

               *                                *    
      15108 TCTGAGAATGCTTCTGTGTAGTTTTTATGTGAAGATGTTTC
        131 TCTCAGAATGCTTCTGTGTAGTTTTTATGTGAAGATATTTC

                            **     *                                    *    
      15149 CTTTTCCACCATAGGCTGCAAAGGGCTCCAAATATCCACTTGCAGATTCTACAAAAAGAGAGTTT
          1 CTTTTCCACCATAGGCCTCAAAGCGCTCCAAATATCCACTTGCAGATTCTACAAAAAGAGTGTTT

                 *     *         *          *     *                     *    
      15214 CAAAAGTGCTCTATCAAAAGATAGGTTCAACTATGTGATATGAATGCACACATCACAAAGTAGTT
         66 CAAAACTGCTCAATCAAAAGAAAGGTTCAACTCTGTGAGATGAATGCACACATCACAAAGAAGTT

                                          *          
      15279 TCTCAGAATGCTTCTGTGTAGTTTTTATGTAAAGATATTTC
        131 TCTCAGAATGCTTCTGTGTAGTTTTTATGTGAAGATATTTC

                                    *                                   **   
      15320 CTTTTCCACCATAGGCCTCAAAGCACTCCAAATATCCACTTGCAGATTCTACAAAAAGAGATTTT
          1 CTTTTCCACCATAGGCCTCAAAGCGCTCCAAATATCCACTTGCAGATTCTACAAAAAGAGTGTTT

                   ** *                   *     *  *    * *   *        *     
      15385 CAAAACTATTTAATCAAAAGAAAGGTTCAAATCTGTCAGTTGAAGGTACATATCACAAACAAGTT
         66 CAAAACTGCTCAATCAAAAGAAAGGTTCAACTCTGTGAGATGAATGCACACATCACAAAGAAGTT

             * **                                    
      15450 TATTGGAATGCTTCTGTGTAGTTTTTATGTGAAGATATTTC
        131 TCTCAGAATGCTTCTGTGTAGTTTTTATGTGAAGATATTTC

                     * *       * * *                        **  *     *      
      15491 CTTTTCCACAACAGGCCTCCAGGTGCTCCAAATATCCACTTGCAGATTTCACTAAAAGTGTGTTT
          1 CTTTTCCACCATAGGCCTCAAAGCGCTCCAAATATCCACTTGCAGATTCTACAAAAAGAGTGTTT

             *  *            *  *   *     *        *             *           
      15556 CCAAGCTGCTCAATCAAGAGGAAGTTTCAAGTCTGTGAGGTGAATGCACACATTACAAAGAAGTT
         66 CAAAACTGCTCAATCAAAAGAAAGGTTCAACTCTGTGAGATGAATGCACACATCACAAAGAAGTT

            *  *                                     
      15621 ACTGAGAATGCTTCTGTGTAGTTTTTATGTGAAGATATTTC
        131 TCTCAGAATGCTTCTGTGTAGTTTTTATGTGAAGATATTTC

                      **               *                                *    
      15662 CTTTTCCACCGCAGGCCTCAAAGCGCTGCAAATATCCACTTGCAGATTCTACAAAAAGAGAGTTT
          1 CTTTTCCACCATAGGCCTCAAAGCGCTCCAAATATCCACTTGCAGATTCTACAAAAAGAGTGTTT

                      **         *   *         *   *     **            *     
      15727 CAAAACTGCTGTATCAAAAGATAGGGTCAACTCTGCGAGTTGAATAAACACATCACAAATAAGTT
         66 CAAAACTGCTCAATCAAAAGAAAGGTTCAACTCTGTGAGATGAATGCACACATCACAAAGAAGTT

               **   *        *               *       
      15792 TCTGGGAACGCTTCTGTATAG-TTTTATGTGAATATATTTC
        131 TCTCAGAATGCTTCTGTGTAGTTTTTATGTGAAGATATTTC

                         *          *                   *   *  *   * *   *   
      15832 CTTTTCCACCATATGCCTCAAAGCACTCCAAATATCCACTTGCACATTATAGAAACATAGTCTTT
          1 CTTTTCCACCATAGGCCTCAAAGCGCTCCAAATATCCACTTGCAGATTCTACAAAAAGAGTGTTT

                   **                        *         *             *       
      15897 CAAAACTTGTCAATC-AAAGAAAGGTTCAACTCCGTGAGATGAGTGCACACATCACAGAGAAGTT
         66 CAAAACTGCTCAATCAAAAGAAAGGTTCAACTCTGTGAGATGAATGCACACATCACAAAGAAGTT

                *     *                            * 
      15961 TCTCGGAATGTTTCTGTGTAGTTTTTATGTGAAGATATTGC
        131 TCTCAGAATGCTTCTGTGTAGTTTTTATGTGAAGATATTTC

                     *               *            *          *       *   *   
      16002 CTTTTCCACAATAGGCCTCAAAGCGTTCCAAATATCCAATTGCAGATTCCACAAAAAAAGTTTTT
          1 CTTTTCCACCATAGGCCTCAAAGCGCTCCAAATATCCACTTGCAGATTCTACAAAAAGAGTGTTT

            *                 *  *  *  *             * *         **    *     
      16067 TAAAACTGCTCAATCAAATGATAGATTAAACTCTGTGAGATTAGTGCACACATGTCAAAAAAGTT
         66 CAAAACTGCTCAATCAAAAGAAAGGTTCAACTCTGTGAGATGAATGCACACATCACAAAGAAGTT

                                *      * *           
      16132 TCTCAGAATGCTTCTGTGTACTTTTTAGGGGAAGATATTTC
        131 TCTCAGAATGCTTCTGTGTAGTTTTTATGTGAAGATATTTC

                        *    *     **         *    *           **  * *  *    
      16173 CTTTTCCACCATCGGCCACAAAGGACTCCAAATAACCACATGCAGATTCTAGTAACACAGAGTTT
          1 CTTTTCCACCATAGGCCTCAAAGCGCTCCAAATATCCACTTGCAGATTCTACAAAAAGAGTGTTT

                       *         * *           *   * * *    **    ***        
      16238 CAAAACTGCTCTATCAAAAGATAAGTTCAACTCTGAGAGTTTAGTGCAACCATCGTGAAGAAGTT
         66 CAAAACTGCTCAATCAAAAGAAAGGTTCAACTCTGTGAGATGAATGCACACATCACAAAGAAGTT

                            *     *                  
      16303 TCTCAGAATGCTTCTGAGTAGTGTTTATGTGAAGATATTTC
        131 TCTCAGAATGCTTCTGTGTAGTTTTTATGTGAAGATATTTC

                              *     *                      *           **    
      16344 CTTTTCCACCATAGGCCTGAAAGCCCTCCAAATATCCACTTGCAGATCCTACAAAAAGAAAGTTT
          1 CTTTTCCACCATAGGCCTCAAAGCGCTCCAAATATCCACTTGCAGATTCTACAAAAAGAGTGTTT

             *         **     *  *  *   *        **      *                   
      16409 CGAAA-TGCTCTCTCAAACGATAGTTTCGACTCTGTGGTATGAATACACACATCACAAAGAAGTT
         66 CAAAACTGCTCAATCAAAAGAAAGGTTCAACTCTGTGAGATGAATGCACACATCACAAAGAAGTT

                                       **            
      16473 TCTCAGAATGCTTCTGTGTAGTTTTTAAATGAAGATATTTC
        131 TCTCAGAATGCTTCTGTGTAGTTTTTATGTGAAGATATTTC

            *                       *          *     *                       
      16514 TTTTTCCACCATAGGCCTCAAAGCACTCCAAATATGCACTTCCAGATTCTACAAAAAGAGTGTTT
          1 CTTTTCCACCATAGGCCTCAAAGCGCTCCAAATATCCACTTGCAGATTCTACAAAAAGAGTGTTT

              *                 *       * *         **   *         *  * *    
      16579 CAGAACTGCTCAATCAAAAGGAAGGTTCCAGTC--TGAGACAAATACACACATCAAAAGGTAGTT
         66 CAAAACTGCTCAATCAAAAGAAAGGTTCAACTCTGTGAGATGAATGCACACATCACAAAGAAGTT

                                                    *
      16642 TCTCAGAATGCTTCTGTGTAGTTTTTATGTGAAGATATTTT
        131 TCTCAGAATGCTTCTGTGTAGTTTTTATGTGAAGATATTTC

             *               *    **    *     *      *  **   *          *    
      16683 CCTTTCCACCATAGGCCACAAATGGCTCTAAATACCCACTTACATTTTCCACAAAAAGAGAGTTT
          1 CTTTTCCACCATAGGCCTCAAAGCGCTCCAAATATCCACTTGCAGATTCTACAAAAAGAGTGTTT

                       * *    * *      *   *         *                       
      16748 CAAAACTGCTCTACCAAAGGTAA-GTTTAACGCTGTGAG-TTAA-G-A-ACATCACAAAGAAGTT
         66 CAAAACTGCTCAATCAAAAGAAAGGTTCAACTCTGTGAGATGAATGCACACATCACAAAGAAGTT

                                   *   *  *          
      16808 TCTCAGAATGCTTCTGTGTAGTTCTTACGTAAAGATATTTC
        131 TCTCAGAATGCTTCTGTGTAGTTTTTATGTGAAGATATTTC

                 *   *      ***    *                  *              **      
      16849 CTTTTACACAATAGGCAGAAAAGTGCTCCAAATATCCACTTGAAGATTCTACAAAAACCGTGTTT
          1 CTTTTCCACCATAGGCCTCAAAGCGCTCCAAATATCCACTTGCAGATTCTACAAAAAGAGTGTTT

                     **                                          *      *    
      16914 CAAAACTGCCGAATCAAAAGAAAGGTTCAACTCTGTGAGATGAATGCACACATAACAAAGGAGTT
         66 CAAAACTGCTCAATCAAAAGAAAGGTTCAACTCTGTGAGATGAATGCACACATCACAAAGAAGTT

                                 *      *      *    *
      16979 TCTCAGAATGCTTCTGTGTAGCTTTTATATGAAGACATTTA
        131 TCTCAGAATGCTTCTGTGTAGTTTTTATGTGAAGATATTTC

            *        * *            *   ***                     **        *  
      17020 GTTTTCCACAACAGGCCTCAAAGCTCTCTCCATATCCACTTGCAGATTCTACCGAAAGAGTGCTT
          1 CTTTTCCACCATAGGCCTCAAAGCGCTCCAAATATCCACTTGCAGATTCTACAAAAAGAGTGTTT

             *                   * **     *        *        *     **         
      17085 CCAAACTGCTCAATCAAAAGAGACATTCAAATCTGTGAGGTGAATGCAGACATCGTAAAGAAGTT
         66 CAAAACTGCTCAATCAAAAGAAAGGTTCAACTCTGTGAGATGAATGCACACATCACAAAGAAGTT

                                *     *       *    **
      17150 TCTCAGAATGCTTCTGTGTATTTTTTGTGTGAAGTTATTCG
        131 TCTCAGAATGCTTCTGTGTAGTTTTTATGTGAAGATATTTC

            *    *             *     *  *            **                 *    
      17191 TTTTTGCACCATAGGCCTCCAAGCGTTCTAAATATCCACTTCTAGATTCTACAAAAAGAGAGTTT
          1 CTTTTCCACCATAGGCCTCAAAGCGCTCCAAATATCCACTTGCAGATTCTACAAAAAGAGTGTTT

                   *         *            *        *    *   *                
      17256 CAAAACTACTC-A--AACA-AAAGGTTCAATTCTGTGAGTTGAAAGCAAACATCACAAAGAAGTT
         66 CAAAACTGCTCAATCAAAAGAAAGGTTCAACTCTGTGAGATGAATGCACACATCACAAAGAAGTT

                       *             *               
      17317 TCTCAGAATGCGTCTGTGTAGTTTTGATGTGAAGATATTTC
        131 TCTCAGAATGCTTCTGTGTAGTTTTTATGTGAAGATATTTC

                     **   ****     *                  *                      
      17358 CTTTT-CACAGTAGAATGCAAAGGGCTCCAAATATCCACTTGGAGATTCTACAAAAAGA--GTTT
          1 CTTTTCCACCATAGGCCTCAAAGCGCTCCAAATATCCACTTGCAGATTCTACAAAAAGAGTGTTT

                  *    **     *  *     *     ***   *     *               *   
      17420 CAAAACCGCTCTGTCAAATGATAGGTTGAACTCCCGGAGGTGAATACACACATCACAAAGAGGTT
         66 CAAAACTGCTCAATCAAAAGAAAGGTTCAACTCTGTGAGATGAATGCACACATCACAAAGAAGTT

                  *                       *  *       
      17485 TCTCAGCATGCTTCTGTGTAGTTTTTATGTAAACATATTTC
        131 TCTCAGAATGCTTCTGTGTAGTTTTTATGTGAAGATATTTC

             *    * *              *           *      * *       *   *    *   
      17526 CGTTTCTATCATAGGCCTCAAAGTGCTCCAAATATTCACTTGTACATTCTACCAAACGAGTATTT
          1 CTTTTCCACCATAGGCCTCAAAGCGCTCCAAATATCCACTTGCAGATTCTACAAAAAGAGTGTTT

                              * *         ** *    *        *            *    
      17591 CAAAACTGCTCAATCAAATGGAAGGTTCAAAACCGTGACATGAATGCCCACATCACAAAGTAGTT
         66 CAAAACTGCTCAATCAAAAGAAAGGTTCAACTCTGTGAGATGAATGCACACATCACAAAGAAGTT

                                                     
      17656 TCTCAGAATGCTTCTGTGTAGTTTTTATGTGAAGATATTTC
        131 TCTCAGAATGCTTCTGTGTAGTTTTTATGTGAAGATATTTC

                     * *    *      *    *       * *   **      *              
      17697 CTTTTCCACAACA-GCGTGCAAAACGCTTCAAATATGCCCTTAGAGATTCCACAAAAAGAGTGTT
          1 CTTTTCCACCATAGGCCT-CAAAGCGCTCCAAATATCCACTTGCAGATTCTACAAAAAGAGTGTT

              *     *               *  *                                  ** 
      17761 TCCAAACTACTCAAATCAAAA-AATGATTTCAACTCTGTGAGATGAATGCACACATCACAAACTA
         65 TCAAAACTGCTC-AATCAAAAGAAAG-GTTCAACTCTGTGAGATGAATGCACACATCACAAAGAA

                         *     ** *   * *   *       *   
      17825 GTTTCTCAGAATGTTTCTGCCTGGTTCTCATGCGAAGATAGTTC
        128 GTTTCTCAGAATGCTTCTGTGTAGTTTTTATGTGAAGATATTTC

                 *           *   * **              *                  * *    
      17869 CTTTTTCACCATAGGCCGCAATGTACTCCAAATATCCACCTGCAGATTCTAC-AAAAGTGAGTTT
          1 CTTTTCCACCATAGGCCTCAAAGCGCTCCAAATATCCACTTGCAGATTCTACAAAAAGAGTGTTT

                       *         ***    **         *        *      *       * 
      17933 CAAAACTGCTCTATCAAAAGATCAGTTCGTCTCTGTGAGTTGAATGCATACATCAAAAAGAAGCT
         66 CAAAACTGCTCAATCAAAAGAAAGGTTCAACTCTGTGAGATGAATGCACACATCACAAAGAAGTT

                 *             *      **         *   
      17998 TCTCAAAATGCTTCTGTGTGGTTTTTCGGTGAAGATAGTTC
        131 TCTCAGAATGCTTCTGTGTAGTTTTTATGTGAAGATATTTC

            *     *        *      * *                 *        *        *    
      18039 TTTTTCTACCATAGGTCTCAAACCACTCCAAATATCCACTTGTAGATTCTATAAAAAGGAATG-T
          1 CTTTTCCACCATAGGCCTCAAAGCGCTCCAAATATCCACTTGCAGATTCTACAAAAA-GAGTGTT

                  *        *    *    *      * *         *      *         *   
      18103 TCAAAATTGCTCAATAAAAATAAAGTTTCAACACCGTGAGATGAGTGCACAAATCACAAAGGAGT
         65 TCAAAACTGCTCAATCAAAAGAAAGGTTCAACTCTGTGAGATGAATGCACACATCACAAAGAAGT

                  *          *         *          *   
      18168 TTCTCAAAATGCTTCTGGGTAGTTTTTCTGTGAAGATAGTTC
        130 TTCTCAGAATGCTTCTGTGTAGTTTTTATGTGAAGATATTTC

                  *     *    *     *          *                         *    
      18210 CTTTTCTACCATGGGCCACAAAGGGCTCCAAATACCCACTTGCAGATTCTACAAAAAGAGAGTTT
          1 CTTTTCCACCATAGGCCTCAAAGCGCTCCAAATATCCACTTGCAGATTCTACAAAAAGAGTGTTT

              *        *      ** * *        *    * *    **  * *           *  
      18275 CACAACTGCTCTATCAAACAATATGTTCAACTTTGTGGGTTGAACACAAATATCAC-AAGAATTT
         66 CAAAACTGCTCAATCAAAAGAAAGGTTCAACTCTGTGAGATGAATGCACACATCACAAAGAAGTT

                **                             *     
      18339 TCTCCCAATGCTTCTGTGTAGTTTTTATGTGAAGACATTTC
        131 TCTCAGAATGCTTCTGTGTAGTTTTTATGTGAAGATATTTC

            *   *  *      *  *     *                 *  *      *       *  *  
      18380 TTTTCCCTCCATAGTCCACAAAGTGCTCCAAATATCCACTTACATATTCTAGAAAAAGATTGCTT
          1 CTTTTCCACCATAGGCCTCAAAGCGCTCCAAATATCCACTTGCAGATTCTACAAAAAGAGTGTTT

            **       *    *               * * *                              
      18445 GGAAACTGCACAATGAAAAGAAAGGTTCAAATATATGAGATGAATGCACACATCACAAAGAAGTT
         66 CAAAACTGCTCAATCAAAAGAAAGGTTCAACTCTGTGAGATGAATGCACACATCACAAAGAAGTT

                                 *                    
      18510 TCTCAGAAT-CTCTCTGTGTAATTTTTATGTGAAGATATTTC
        131 TCTCAGAATGCT-TCTGTGTAGTTTTTATGTGAAGATATTTC

                *     *    *  *   *                        *      *    *     
      18551 CTTTCCCACCTTAGGTCTTAAAACGCTCCAAATATCCACTTGCAGATACTACAAGAAGATTGTTT
          1 CTTTTCCACCATAGGCCTCAAAGCGCTCCAAATATCCACTTGCAGATTCTACAAAAAGAGTGTTT

                     *    *        *      *     **                           
      18616 CAAAACTGCACAA-AAAAAGAAATGTTCAATTCTGTTTGATGAATGCACACATCACAAAGAAGTT
         66 CAAAACTGCTCAATCAAAAGAAAGGTTCAACTCTGTGAGATGAATGCACACATCACAAAGAAGTT

                           *                         
      18680 TCTCAGAATGCTTCTCTGTAGTTTTTATGTGAAGATATTTC
        131 TCTCAGAATGCTTCTGTGTAGTTTTTATGTGAAGATATTTC

                     *             *                 *         **      * *   
      18721 CTTTTCCACAATAGGCCTCAAAGGGCTCCAAATATCCACTTCCAGATTCTATGAAAAGAATATTT
          1 CTTTTCCACCATAGGCCTCAAAGCGCTCCAAATATCCACTTGCAGATTCTACAAAAAGAGTGTTT

             *              * *    *                                      *  
      18786 CCAAACTGCTCAATCATAGGAAATGTTCAACTCTGTGAGATGAATGCACACATCAC-AAGAAATT
         66 CAAAACTGCTCAATCAAAAGAAAGGTTCAACTCTGTGAGATGAATGCACACATCACAAAGAAGTT

                     *    *      *       *       *   
      18850 TCTCAGAATCCTTCAGTGTAGGTTTTATGAGAAGATAATTC
        131 TCTCAGAATGCTTCTGTGTAGTTTTTATGTGAAGATATTTC

                     *    **        *   *                           *    *   
      18891 CTTTTCCACAATAGTTCTCAAAGCACTCAAAATATCCACTTGCAGATTCTACAAAAGGAGTATTT
          1 CTTTTCCACCATAGGCCTCAAAGCGCTCCAAATATCCACTTGCAGATTCTACAAAAAGAGTGTTT

                                                          *                  
      18956 CAAAACTGCTCAATCAAAAGAAAGGTTCAACTCTGTGAGATGAATGGACACATCACAAAGAAGTT
         66 CAAAACTGCTCAATCAAAAGAAAGGTTCAACTCTGTGAGATGAATGCACACATCACAAAGAAGTT

                                  *   *              
      19021 TCTCAGAATGCTTCTGTGTAGTATTTTTGTGAAGATATTT-
        131 TCTCAGAATGCTTCTGTGTAGTTTTTATGTGAAGATATTTC

                          *  * * * * ***             *            *     * *  
      19061 CTTTTCCACCATAGACCGCCAGGGGACACAAATATCCACTTTCAGATTCTACAACAAGAGAGGTT
          1 CTTTTCCACCATAGGCCTCAAAGCGCTCCAAATATCCACTTGCAGATTCTACAAAAAGAGTGTTT

                   *   *     *   ** *       *      *                      * *
      19126 CAAAACTACTCGATCAAGAGATGGTTTCAACTATGTGAGTTGAATGCACACATCACAAAGAACTA
         66 CAAAACTGCTCAATCAAAAGAAAGGTTCAACTCTGTGAGATGAATGCACACATCACAAAGAAGTT

             *  *    *                               
      19191 TGTCGGAATTCTTCTGTGTAGTTTTTATGTGAAGATATTTC
        131 TCTCAGAATGCTTCTGTGTAGTTTTTATGTGAAGATATTTC

                     *    * *      * *    *                  *               
      19232 CTTTTCCACAATAGACGTCAAAGTGATCCAGATATCCACTTGCAGATTCCACAAAAAGAGTGTTT
          1 CTTTTCCACCATAGGCCTCAAAGCGCTCCAAATATCCACTTGCAGATTCTACAAAAAGAGTGTTT

                 *   *   *                  **                     *  *      
      19297 CAAAAGTGCACAACCAAAAGAAAGGTTCAACTAGGTGAGATGAATGCACACATCAGAAGGAAGTT
         66 CAAAACTGCTCAATCAAAAGAAAGGTTCAACTCTGTGAGATGAATGCACACATCACAAAGAAGTT

                            **   *     * *       *   
      19362 TCTCAGAATGCTTCTGCATAGCTTTTAAGGGAAGATACTTC
        131 TCTCAGAATGCTTCTGTGTAGTTTTTATGTGAAGATATTTC

                    *               *            * *  *    * *               
      19403 CTTTTCCAACATAGGCCTCAAAGCACTCCAAATATCCTCCTGGAGATACCACAAAAAGAGTGTTT
          1 CTTTTCCACCATAGGCCTCAAAGCGCTCCAAATATCCACTTGCAGATTCTACAAAAAGAGTGTTT

            **                      *  *                 *       *           
      19468 GCAAACTGCTCAATCAAAAGAAAGATTTAACTCTGTGAGATGAATCCACACATGACAAAGAAGTT
         66 CAAAACTGCTCAATCAAAAGAAAGGTTCAACTCTGTGAGATGAATGCACACATCACAAAGAAGTT

                                                     
      19533 TCTCAGAATGCTTCTGTGTAGTTTTTATGTGAAGATATTTC
        131 TCTCAGAATGCTTCTGTGTAGTTTTTATGTGAAGATATTTC

                     *   * * *    **           *                *     *      
      19574 CTTTTCCACAATAAGACCCAAAAGGCTCCAAATATTCACTTGCAGATTCTAAAAAAAACAGTGTT
          1 CTTTTCCACCATAGGCCTCAAAGCGCTCCAAATATCCACTTGCAGATTCT-ACAAAAAGAGTGTT

                                  *                  *      *        **      
      19639 TCAAAACTGCTCAATCAAAAGATA-GTTCAACTCTGTGAGAAGAATGCTCACATCACTGAGAAGT
         65 TCAAAACTGCTCAATCAAAAGAAAGGTTCAACTCTGTGAGATGAATGCACACATCACAAAGAAGT

                                         *            
      19703 TTCTCAGAATGCTTCTGTGTAGTTTTTATATGAAGATATTTC
        130 TTCTCAGAATGCTTCTGTGTAGTTTTTATGTGAAGATATTTC

                *     *      *    **   *                   *   **       *    
      19745 CTTTCCCACCGTAGGCCACAAAAGGCTTCAAATATCCACTTGCAGATACTATGAAAAGAGAGTTT
          1 CTTTTCCACCATAGGCCTCAAAGCGCTCCAAATATCCACTTGCAGATTCTACAAAAAGAGTGTTT

                        *        *               ***            *            
      19810 CAAAACTGCTCATTCAAAAGATAGGTTCAACTCTGTGGTTTGAATGCACACAGCACAAAGAAGTT
         66 CAAAACTGCTCAATCAAAAGAAAGGTTCAACTCTGTGAGATGAATGCACACATCACAAAGAAGTT

              *       **                   **   *    
      19875 TCACAGAATGTGTCTGTGTAGTTTTTATGTGCGGATGTTTC
        131 TCTCAGAATGCTTCTGTGTAGTTTTTATGTGAAGATATTTC

                         *        ****   *   *               *    *          
      19916 CTTTTCCACCATATGCCT-AAATATTTCCCAATTTCCACTTGCAGATTCCACAAGAAGAGTGTTT
          1 CTTTTCCACCATAGGCCTCAAAGCGCTCCAAATATCCACTTGCAGATTCTACAAAAAGAGTGTTT

                      **       *       *           *            *      ***   
      19980 CAAAACTGCTGTATC-AAATAAA-GTTGAACTCTGTGAGGTGAATGCACACAGCACAAAATGGTT
         66 CAAAACTGCTCAATCAAAAGAAAGGTTCAACTCTGTGAGATGAATGCACACATCACAAAGAAGTT

                          **   *        *       *    
      20043 TCTCAGAATGCTTCCTTGTTGTTTTTATATGAAGATGTTTC
        131 TCTCAGAATGCTTCTGTGTAGTTTTTATGTGAAGATATTTC

                  *  *             *   *     *                               
      20084 CTTTTCAACAATAGGCCTCAAAGTGCTTCAAATGTCCACTTGCAGATTCTACAAAAAGAGTGTTT
          1 CTTTTCCACCATAGGCCTCAAAGCGCTCCAAATATCCACTTGCAGATTCTACAAAAAGAGTGTTT

                                        *      *  *  *                       
      20149 CAAAACTGCTCAATCAAAAGAAAGGTTCGACTCTGGGAAATTAATGCACACATCACAAAGAAGTT
         66 CAAAACTGCTCAATCAAAAGAAAGGTTCAACTCTGTGAGATGAATGCACACATCACAAAGAAGTT

                                     *        *      
      20214 TCTC---A-GCTTCTGTGTAGTTTTCATGTGAAGTTATTTC
        131 TCTCAGAATGCTTCTGTGTAGTTTTTATGTGAAGATATTTC

                     *       *     *            *    *         **       *    
      20251 CTTTTCCACAATAGGCCGCAAAGGGCTCCAAATATCAACTTACAGATTCTAGGAAAAGAGAGTTT
          1 CTTTTCCACCATAGGCCTCAAAGCGCTCCAAATATCCACTTGCAGATTCTACAAAAAGAGTGTTT

                       * **      *     *                                     
      20316 CAAAACTGCTCTACGAAAAGATAGGTTGAACTCTGTGAGATGAATGCACACATCACAAAGAAGTT
         66 CAAAACTGCTCAATCAAAAGAAAGGTTCAACTCTGTGAGATGAATGCACACATCACAAAGAAGTT

                       *               * *           
      20381 TCTCAGAATGCATCTGTGTAGTTTTTACGGGAAGATATTTC
        131 TCTCAGAATGCTTCTGTGTAGTTTTTATGTGAAGATATTTC

                        ***  *  *  **      *  *                  *     ***   
      20422 CTTTTCCACCATCTTCCACAGAGGTCTCCAAGTAACCACTTGCAGATTCTACAGAAAGACACTTT
          1 CTTTTCCACCATAGGCCTCAAAGCGCTCCAAATATCCACTTGCAGATTCTACAAAAAGAGTGTTT

            *          *         ***      *      ***                      *  
      20487 AAAAACTGCTCTATCAAAAGATCAGTTCAAGTCTGTGGTTTGAATGCACACATCACAAAGAATTT
         66 CAAAACTGCTCAATCAAAAGAAAGGTTCAACTCTGTGAGATGAATGCACACATCACAAAGAAGTT

                                     *  *            
      20552 TCTCAGAATGCTTCTGTGTAGTTTTCATATGAAGATATTTC
        131 TCTCAGAATGCTTCTGTGTAGTTTTTATGTGAAGATATTTC

                                    *                                   **   
      20593 CTTTTCCACCATAGGCCTCAAAGCACTCCAAATATCCACTTGCAGATTCTACAAAAAGAGATTTT
          1 CTTTTCCACCATAGGCCTCAAAGCGCTCCAAATATCCACTTGCAGATTCTACAAAAAGAGTGTTT

                   **                           *  *          *        *     
      20658 CAAAACTAGTCAATCAAAAGAAAGGTTCAACTCTGTCAGTTGAATGCACATATCACAAACAAGTT
         66 CAAAACTGCTCAATCAAAAGAAAGGTTCAACTCTGTGAGATGAATGCACACATCACAAAGAAGTT

                *      *                             
      20723 TCTCGGAATGCGTCTGTGTAGTTTTTATGTGAAGATATTTC
        131 TCTCAGAATGCTTCTGTGTAGTTTTTATGTGAAGATATTTC

               *     * *           *     *                  *    *           
      20764 CTTCTCCACAACAGGCCTCAAAGTGCTCCGAATATCCACTTGCAGATTTTAC-TAAAGAGTGTTT
          1 CTTTTCCACCATAGGCCTCAAAGCGCTCCAAATATCCACTTGCAGATTCTACAAAAAGAGTGTTT

             *               *  *   *     *        *    *               *    
      20828 CCAAACTGCTCAATCAAGAGGAAGTTTCAAGTCTGTGAGCTGAACGCACACATCACAAAGTAGTT
         66 CAAAACTGCTCAATCAAAAGAAAGGTTCAACTCTGTGAGATGAATGCACACATCACAAAGAAGTT

               *                                *    
      20893 TCTGAGAATGCTTCTGTGTAGTTTTTATGTGAAGATGTTTC
        131 TCTCAGAATGCTTCTGTGTAGTTTTTATGTGAAGATATTTC

                            **     *                                    *    
      20934 CTTTTCCACCATAGGCTGCAAAGGGCTCCAAATATCCACTTGCAGATTCTACAAAAAGAGAGTTT
          1 CTTTTCCACCATAGGCCTCAAAGCGCTCCAAATATCCACTTGCAGATTCTACAAAAAGAGTGTTT

                 *     *         *          *     *                     *    
      20999 CAAAAGTGCTCTATCAAAAGATAGGTTCAACTATGTGATATGAATGCACACATCACAAAGTAGTT
         66 CAAAACTGCTCAATCAAAAGAAAGGTTCAACTCTGTGAGATGAATGCACACATCACAAAGAAGTT

                                          *          
      21064 TCTCAGAATGCTTCTGTGTAGTTTTTATGTAAAGATATTTC
        131 TCTCAGAATGCTTCTGTGTAGTTTTTATGTGAAGATATTTC

                                    *                                   **   
      21105 CTTTTCCACCATAGGCCTCAAAGCACTCCAAATATCCACTTGCAGATTCTACAAAAAGAGATTTT
          1 CTTTTCCACCATAGGCCTCAAAGCGCTCCAAATATCCACTTGCAGATTCTACAAAAAGAGTGTTT

                   ** *                   *     *  *    * *   *        *     
      21170 CAAAACTATTTAATCAAAAGAAAGGTTCAAATCTGTCAGTTGAAGGTACATATCACAAACAAGTT
         66 CAAAACTGCTCAATCAAAAGAAAGGTTCAACTCTGTGAGATGAATGCACACATCACAAAGAAGTT

             * **                                    
      21235 TATTGGAATGCTTCTGTGTAGTTTTTATGTGAAGATATTTC
        131 TCTCAGAATGCTTCTGTGTAGTTTTTATGTGAAGATATTTC

                     * *         * *                        **  *     *      
      21276 CTTTTCCACAACAGGCCTCAAGGTGCTCCAAATATCCACTTGCAGATTTCACTAAAAGTGTGTTT
          1 CTTTTCCACCATAGGCCTCAAAGCGCTCCAAATATCCACTTGCAGATTCTACAAAAAGAGTGTTT

             *  *            *  *   *     *        *             *           
      21341 CCAAGCTGCTCAATCAAGAGGAAGTTTCAAGTCTGTGAGGTGAATGCACACATTACAAAGAAGTT
         66 CAAAACTGCTCAATCAAAAGAAAGGTTCAACTCTGTGAGATGAATGCACACATCACAAAGAAGTT

            *  *                                     
      21406 ACTGAGAATGCTTCTGTGTAGTTTTTATGTGAAGATATTTC
        131 TCTCAGAATGCTTCTGTGTAGTTTTTATGTGAAGATATTTC

                      **               *                                *    
      21447 CTTTTCCACCGCAGGCCTCAAAGCGCTGCAAATATCCACTTGCAGATTCTACAAAAAGAGAGTTT
          1 CTTTTCCACCATAGGCCTCAAAGCGCTCCAAATATCCACTTGCAGATTCTACAAAAAGAGTGTTT

                      **         *   *         *   *     ***           *     
      21512 CAAAACTGCTGTATCAAAAGATAGGGTCAACTCTGCGAGTTGAATAAGCACATCACAAATAAGTT
         66 CAAAACTGCTCAATCAAAAGAAAGGTTCAACTCTGTGAGATGAATGCACACATCACAAAGAAGTT

               **   *        *               *       
      21577 TCTGGGAACGCTTCTGTATAG-TTTTATGTGAATATATTTC
        131 TCTCAGAATGCTTCTGTGTAGTTTTTATGTGAAGATATTTC

                         *          *                   *   *  *   * *   *   
      21617 CTTTTCCACCATATGCCTCAAAGCACTCCAAATATCCACTTGCACATTATAGAAACATAGTCTTT
          1 CTTTTCCACCATAGGCCTCAAAGCGCTCCAAATATCCACTTGCAGATTCTACAAAAAGAGTGTTT

                   **                        *         *             *       
      21682 CAAAACTTGTCAATC-AAAGAAAGGTTCAACTCCGTGAGATGAGTGCACACATCACAGAGAAGTT
         66 CAAAACTGCTCAATCAAAAGAAAGGTTCAACTCTGTGAGATGAATGCACACATCACAAAGAAGTT

                *     *                            * 
      21746 TCTCGGAATGTTTCTGTGTAGTTTTTATGTGAAGATATTGC
        131 TCTCAGAATGCTTCTGTGTAGTTTTTATGTGAAGATATTTC

                     *               *            *          *       *   *   
      21787 CTTTTCCACAATAGGCCTCAAAGCGTTCCAAATATCCAATTGCAGATTCCACAAAAAAAGTTTTT
          1 CTTTTCCACCATAGGCCTCAAAGCGCTCCAAATATCCACTTGCAGATTCTACAAAAAGAGTGTTT

            *                 *  *  *  *             * *         **    *     
      21852 TAAAACTGCTCAATCAAATGATAGATTAAACTCTGTGAGATTAGTGCACACATGTCAAAAAAGTT
         66 CAAAACTGCTCAATCAAAAGAAAGGTTCAACTCTGTGAGATGAATGCACACATCACAAAGAAGTT

                                *      * *           
      21917 TCTCAGAATGCTTCTGTGTACTTTTTAGGGGAAGATATTTC
        131 TCTCAGAATGCTTCTGTGTAGTTTTTATGTGAAGATATTTC

                        *    *     **         *    *           **  * *  *    
      21958 CTTTTCCACCATCGGCCACAAAGGACTCCAAATAACCACATGCAGATTCTAGTAACACAGAGTTT
          1 CTTTTCCACCATAGGCCTCAAAGCGCTCCAAATATCCACTTGCAGATTCTACAAAAAGAGTGTTT

                       *         * *           *   * * *    **    ***        
      22023 CAAAACTGCTCTATCAAAAGATAAGTTCAACTCTGAGAGTTTAGTGCAACCATCGTGAAGAAGTT
         66 CAAAACTGCTCAATCAAAAGAAAGGTTCAACTCTGTGAGATGAATGCACACATCACAAAGAAGTT

                            *     *                  
      22088 TCTCAGAATGCTTCTGAGTAGTGTTTATGTGAAGATATTTC
        131 TCTCAGAATGCTTCTGTGTAGTTTTTATGTGAAGATATTTC

                              *     *                      *           **    
      22129 CTTTTCCACCATAGGCCTGAAAGCCCTCCAAATATCCACTTGCAGATCCTACAAAAAGAAAGTTT
          1 CTTTTCCACCATAGGCCTCAAAGCGCTCCAAATATCCACTTGCAGATTCTACAAAAAGAGTGTTT

             *         **     *  *  *   *        **      *                   
      22194 CGAAA-TGCTCTCTCAAACGATAGTTTCGACTCTGTGGTATGAATACACACATCACAAAGAAGTT
         66 CAAAACTGCTCAATCAAAAGAAAGGTTCAACTCTGTGAGATGAATGCACACATCACAAAGAAGTT

                                       **            
      22258 TCTCAGAATGCTTCTGTGTAGTTTTTAAATGAAGATATTTC
        131 TCTCAGAATGCTTCTGTGTAGTTTTTATGTGAAGATATTTC

            *                       *          *     *                       
      22299 TTTTTCCACCATAGGCCTCAAAGCACTCCAAATATGCACTTCCAGATTCTACAAAAAGAGTGTTT
          1 CTTTTCCACCATAGGCCTCAAAGCGCTCCAAATATCCACTTGCAGATTCTACAAAAAGAGTGTTT

              *                 *       * *         **   *         *  * *    
      22364 CAGAACTGCTCAATCAAAAGGAAGGTTCCAGTC--TGAGACAAATACACACATCAAAAGGTAGTT
         66 CAAAACTGCTCAATCAAAAGAAAGGTTCAACTCTGTGAGATGAATGCACACATCACAAAGAAGTT

                                                    *
      22427 TCTCAGAATGCTTCTGTGTAGTTTTTATGTGAAGATATTTT
        131 TCTCAGAATGCTTCTGTGTAGTTTTTATGTGAAGATATTTC

             *               *    **    *     *      *  **   *          *    
      22468 CCTTTCCACCATAGGCCACAAATGGCTCTAAATACCCACTTACATTTTCCACAAAAAGAGAGTTT
          1 CTTTTCCACCATAGGCCTCAAAGCGCTCCAAATATCCACTTGCAGATTCTACAAAAAGAGTGTTT

                       * *    * *      *   *         *                       
      22533 CAAAACTGCTCTACCAAAGGTAA-GTTTAACGCTGTGAG-TTAA-G-A-ACATCACAAAGAAGTT
         66 CAAAACTGCTCAATCAAAAGAAAGGTTCAACTCTGTGAGATGAATGCACACATCACAAAGAAGTT

                                   *   *  *          
      22593 TCTCAGAATGCTTCTGTGTAGTTCTTACGTAAAGATATTTC
        131 TCTCAGAATGCTTCTGTGTAGTTTTTATGTGAAGATATTTC

                 *   *      ***    *                  *          *   **      
      22634 CTTTTACACAATAGGCAGAAAAGTGCTCCAAATATCCACTTGAAGATTCTACAGAAACCGTGTTT
          1 CTTTTCCACCATAGGCCTCAAAGCGCTCCAAATATCCACTTGCAGATTCTACAAAAAGAGTGTTT

                     **                                          *      *    
      22699 CAAAACTGCCGAATCAAAAGAAAGGTTCAACTCTGTGAGATGAATGCACACATAACAAAGGAGTT
         66 CAAAACTGCTCAATCAAAAGAAAGGTTCAACTCTGTGAGATGAATGCACACATCACAAAGAAGTT

                                 *      *      *    *
      22764 TCTCAGAATGCTTCTGTGTAGCTTTTATATGAAGACATTTA
        131 TCTCAGAATGCTTCTGTGTAGTTTTTATGTGAAGATATTTC

            *        * *            *   ***                     **        *  
      22805 GTTTTCCACAACAGGCCTCAAAGCTCTCTCCATATCCACTTGCAGATTCTACCGAAAGAGTGCTT
          1 CTTTTCCACCATAGGCCTCAAAGCGCTCCAAATATCCACTTGCAGATTCTACAAAAAGAGTGTTT

             *                   * **     *        *        *     **         
      22870 CCAAACTGCTCAATCAAAAGAGACATTCAAATCTGTGAGGTGAATGCAGACATCGTAAAGAAGTT
         66 CAAAACTGCTCAATCAAAAGAAAGGTTCAACTCTGTGAGATGAATGCACACATCACAAAGAAGTT

                                *     *       *    **
      22935 TCTCAGAATGCTTCTGTGTATTTTTTGTGTGAAGTTATTCG
        131 TCTCAGAATGCTTCTGTGTAGTTTTTATGTGAAGATATTTC

            *    *             *     *  *            **                 *    
      22976 TTTTTGCACCATAGGCCTCCAAGCGTTCTAAATATCCACTTCTAGATTCTACAAAAAGAGAGTTT
          1 CTTTTCCACCATAGGCCTCAAAGCGCTCCAAATATCCACTTGCAGATTCTACAAAAAGAGTGTTT

                   *         *            *        *    *   *                
      23041 CAAAACTACTC-A--AACA-AAAGGTTCAATTCTGTGAGTTGAAAGCAAACATCACAAAGAAGTT
         66 CAAAACTGCTCAATCAAAAGAAAGGTTCAACTCTGTGAGATGAATGCACACATCACAAAGAAGTT

                       *             *               
      23102 TCTCAGAATGCGTCTGTGTAGTTTTGATGTGAAGATATTTC
        131 TCTCAGAATGCTTCTGTGTAGTTTTTATGTGAAGATATTTC

                     **   ****     *                  *                      
      23143 CTTTT-CACAGTAGAATGCAAAGGGCTCCAAATATCCACTTGGAGATTCTACAAAAAGA--GTTT
          1 CTTTTCCACCATAGGCCTCAAAGCGCTCCAAATATCCACTTGCAGATTCTACAAAAAGAGTGTTT

                  *    **     *  *     *     ***   *     *               *   
      23205 CAAAACCGCTCTGTCAAATGATAGGTTGAACTCCCGGAGGTGAATACACACATCACAAAGAGGTT
         66 CAAAACTGCTCAATCAAAAGAAAGGTTCAACTCTGTGAGATGAATGCACACATCACAAAGAAGTT

                  *                       *  *       
      23270 TCTCAGCATGCTTCTGTGTAGTTTTTATGTAAACATATTTC
        131 TCTCAGAATGCTTCTGTGTAGTTTTTATGTGAAGATATTTC

             *    * *              *           *      * *       *   *    *   
      23311 CGTTTCTATCATAGGCCTCAAAGTGCTCCAAATATTCACTTGTACATTCTACCAAACGAGTATTT
          1 CTTTTCCACCATAGGCCTCAAAGCGCTCCAAATATCCACTTGCAGATTCTACAAAAAGAGTGTTT

                              * *         ** *    *        *            *    
      23376 CAAAACTGCTCAATCAAATGGAAGGTTCAAAACCGTGACATGAATGCCCACATCACAAAGTAGTT
         66 CAAAACTGCTCAATCAAAAGAAAGGTTCAACTCTGTGAGATGAATGCACACATCACAAAGAAGTT

                                                     
      23441 TCTCAGAATGCTTCTGTGTAGTTTTTATGTGAAGATATTTC
        131 TCTCAGAATGCTTCTGTGTAGTTTTTATGTGAAGATATTTC

                     * *    *      *    *       * *   **      *              
      23482 CTTTTCCACAACA-GCGTGCAAAACGCTTCAAATATGCCCTTAGAGATTCCACAAAAAGAGTGTT
          1 CTTTTCCACCATAGGCCT-CAAAGCGCTCCAAATATCCACTTGCAGATTCTACAAAAAGAGTGTT

              *     *               *  *                                  ** 
      23546 TCCAAACTACTCAAATCAAAA-AATGATTTCAACTCTGTGAGATGAATGCACACATCACAAACTA
         65 TCAAAACTGCTC-AATCAAAAGAAAG-GTTCAACTCTGTGAGATGAATGCACACATCACAAAGAA

                         *     ** *   * *   *       *   
      23610 GTTTCTCAGAATGTTTCTGCCTGGTTCTCATGCGAAGATAGTTC
        128 GTTTCTCAGAATGCTTCTGTGTAGTTTTTATGTGAAGATATTTC

                 *           *   * **              *                  * *    
      23654 CTTTTTCACCATAGGCCGCAATGTACTCCAAATATCCACCTGCAGATTCTAC-AAAAGTGAGTTT
          1 CTTTTCCACCATAGGCCTCAAAGCGCTCCAAATATCCACTTGCAGATTCTACAAAAAGAGTGTTT

                       *         ***    **         *        *      *       * 
      23718 CAAAACTGCTCTATCAAAAGATCAGTTCGTCTCTGTGAGTTGAATGCATACATCAAAAAGAAGCT
         66 CAAAACTGCTCAATCAAAAGAAAGGTTCAACTCTGTGAGATGAATGCACACATCACAAAGAAGTT

                 *             *      **         *   
      23783 TCTCAAAATGCTTCTGTGTGGTTTTTCGGTGAAGATAGTTC
        131 TCTCAGAATGCTTCTGTGTAGTTTTTATGTGAAGATATTTC

            *     *        *      * *                 *        *        *    
      23824 TTTTTCTACCATAGGTCTCAAACCACTCCAAATATCCACTTGTAGATTCTATAAAAAGGAATG-T
          1 CTTTTCCACCATAGGCCTCAAAGCGCTCCAAATATCCACTTGCAGATTCTACAAAAA-GAGTGTT

                  *        *    *    *      * *         *      *         *   
      23888 TCAAAATTGCTCAATAAAAATAAAGTTTCAACACCGTGAGATGAGTGCACAAATCACAAAGGAGT
         65 TCAAAACTGCTCAATCAAAAGAAAGGTTCAACTCTGTGAGATGAATGCACACATCACAAAGAAGT

                  *          *         *          *   
      23953 TTCTCAAAATGCTTCTGGGTAGTTTTTCTGTGAAGATAGTTC
        130 TTCTCAGAATGCTTCTGTGTAGTTTTTATGTGAAGATATTTC

                  *     *    *     *          *                         *    
      23995 CTTTTCTACCATGGGCCACAAAGGGCTCCAAATACCCACTTGCAGATTCTACAAAAAGAGAGTTT
          1 CTTTTCCACCATAGGCCTCAAAGCGCTCCAAATATCCACTTGCAGATTCTACAAAAAGAGTGTTT

              *        *      ** * *        *    * *    **  * *           *  
      24060 CACAACTGCTCTATCAAACAATATGTTCAACTTTGTGGGTTGAACACAAATATCAC-AAGAATTT
         66 CAAAACTGCTCAATCAAAAGAAAGGTTCAACTCTGTGAGATGAATGCACACATCACAAAGAAGTT

                **                             *     
      24124 TCTCCCAATGCTTCTGTGTAGTTTTTATGTGAAGACATTTC
        131 TCTCAGAATGCTTCTGTGTAGTTTTTATGTGAAGATATTTC

            *   *  *      *  *     *                 *  *      *       *  *  
      24165 TTTTCCCTCCATAGTCCACAAAGTGCTCCAAATATCCACTTACATATTCTAGAAAAAGATTGCTT
          1 CTTTTCCACCATAGGCCTCAAAGCGCTCCAAATATCCACTTGCAGATTCTACAAAAAGAGTGTTT

            **       *    *               * * *                              
      24230 GGAAACTGCACAATGAAAAGAAAGGTTCAAATATATGAGATGAATGCACACATCACAAAGAAGTT
         66 CAAAACTGCTCAATCAAAAGAAAGGTTCAACTCTGTGAGATGAATGCACACATCACAAAGAAGTT

                                 *                    
      24295 TCTCAGAAT-CTCTCTGTGTAATTTTTATGTGAAGATATTTC
        131 TCTCAGAATGCT-TCTGTGTAGTTTTTATGTGAAGATATTTC

                *     *    *  *   *                        *      *    *     
      24336 CTTTCCCACCTTAGGTCTTAAAACGCTCCAAATATCCACTTGCAGATACTACAAGAAGATTGTTT
          1 CTTTTCCACCATAGGCCTCAAAGCGCTCCAAATATCCACTTGCAGATTCTACAAAAAGAGTGTTT

                     *    *        *      *     **                           
      24401 CAAAACTGCACAA-AAAAAGAAATGTTCAATTCTGTTTGATGAATGCACACATCACAAAGAAGTT
         66 CAAAACTGCTCAATCAAAAGAAAGGTTCAACTCTGTGAGATGAATGCACACATCACAAAGAAGTT

                           *                         
      24465 TCTCAGAATGCTTCTCTGTAGTTTTTATGTGAAGATATTTC
        131 TCTCAGAATGCTTCTGTGTAGTTTTTATGTGAAGATATTTC

                     *             *                 *         **      * *   
      24506 CTTTTCCACAATAGGCCTCAAAGGGCTCCAAATATCCACTTCCAGATTCTATGAAAAGAATATTT
          1 CTTTTCCACCATAGGCCTCAAAGCGCTCCAAATATCCACTTGCAGATTCTACAAAAAGAGTGTTT

             *              * *    *                                      *  
      24571 CCAAACTGCTCAATCATAGGAAATGTTCAACTCTGTGAGATGAATGCACACATCAC-AAGAAATT
         66 CAAAACTGCTCAATCAAAAGAAAGGTTCAACTCTGTGAGATGAATGCACACATCACAAAGAAGTT

                     *    *      *       *       *   
      24635 TCTCAGAATCCTTCAGTGTAGGTTTTATGAGAAGATAATTC
        131 TCTCAGAATGCTTCTGTGTAGTTTTTATGTGAAGATATTTC

                     *    **        *   *                           *    *   
      24676 CTTTTCCACAATAGTTCTCAAAGCACTCAAAATATCCACTTGCAGATTCTACAAAAGGAGTATTT
          1 CTTTTCCACCATAGGCCTCAAAGCGCTCCAAATATCCACTTGCAGATTCTACAAAAAGAGTGTTT

                                                          *                  
      24741 CAAAACTGCTCAATCAAAAGAAAGGTTCAACTCTGTGAGATGAATGGACACATCACAAAGAAGTT
         66 CAAAACTGCTCAATCAAAAGAAAGGTTCAACTCTGTGAGATGAATGCACACATCACAAAGAAGTT

                                  *   *              
      24806 TCTCAGAATGCTTCTGTGTAGTATTTTTGTGAAGATATTT-
        131 TCTCAGAATGCTTCTGTGTAGTTTTTATGTGAAGATATTTC

                          *  * * * * ***             *            *     * *  
      24846 CTTTTCCACCATAGACCGCCAGGGGACACAAATATCCACTTTCAGATTCTACAACAAGAGAGGTT
          1 CTTTTCCACCATAGGCCTCAAAGCGCTCCAAATATCCACTTGCAGATTCTACAAAAAGAGTGTTT

                   *   *     *   ** *       *      *                      * *
      24911 CAAAACTACTCGATCAAGAGATGGTTTCAACTATGTGAGTTGAATGCACACATCACAAAGAACTA
         66 CAAAACTGCTCAATCAAAAGAAAGGTTCAACTCTGTGAGATGAATGCACACATCACAAAGAAGTT

             *  *    *                               
      24976 TGTCGGAATTCTTCTGTGTAGTTTTTATGTGAAGATATTTC
        131 TCTCAGAATGCTTCTGTGTAGTTTTTATGTGAAGATATTTC

                     *    * *      * *    *                  *               
      25017 CTTTTCCACAATAGACGTCAAAGTGATCCAGATATCCACTTGCAGATTCCACAAAAAGAGTGTTT
          1 CTTTTCCACCATAGGCCTCAAAGCGCTCCAAATATCCACTTGCAGATTCTACAAAAAGAGTGTTT

                 *   *   *                  **                     *  *      
      25082 CAAAAGTGCACAACCAAAAGAAAGGTTCAACTAGGTGAGATGAATGCACACATCAGAAGGAAGTT
         66 CAAAACTGCTCAATCAAAAGAAAGGTTCAACTCTGTGAGATGAATGCACACATCACAAAGAAGTT

                            **   *     * *       *   
      25147 TCTCAGAATGCTTCTGCATAGCTTTTAAGGGAAGATACTTC
        131 TCTCAGAATGCTTCTGTGTAGTTTTTATGTGAAGATATTTC

                    *               *            * *  *    * *               
      25188 CTTTTCCAACATAGGCCTCAAAGCACTCCAAATATCCTCCTGGAGATACCACAAAAAGAGTGTTT
          1 CTTTTCCACCATAGGCCTCAAAGCGCTCCAAATATCCACTTGCAGATTCTACAAAAAGAGTGTTT

            **                      *  *                 *       *           
      25253 GCAAACTGCTCAATCAAAAGAAAGATTTAACTCTGTGAGATGAATCCACACATGACAAAGAAGTT
         66 CAAAACTGCTCAATCAAAAGAAAGGTTCAACTCTGTGAGATGAATGCACACATCACAAAGAAGTT

                                                     
      25318 TCTCAGAATGCTTCTGTGTAGTTTTTATGTGAAGATATTTC
        131 TCTCAGAATGCTTCTGTGTAGTTTTTATGTGAAGATATTTC

                     *   * * *    **           *                *     *      
      25359 CTTTTCCACAATAAGACCCAAAAGGCTCCAAATATTCACTTGCAGATTCTAAAAAAAACAGTGTT
          1 CTTTTCCACCATAGGCCTCAAAGCGCTCCAAATATCCACTTGCAGATTCT-ACAAAAAGAGTGTT

                                  *                  *      *        **      
      25424 TCAAAACTGCTCAATCAAAAGATA-GTTCAACTCTGTGAGAAGAATGCTCACATCACTGAGAAGT
         65 TCAAAACTGCTCAATCAAAAGAAAGGTTCAACTCTGTGAGATGAATGCACACATCACAAAGAAGT

                                         *            
      25488 TTCTCAGAATGCTTCTGTGTAGTTTTTATATGAAGATATTTC
        130 TTCTCAGAATGCTTCTGTGTAGTTTTTATGTGAAGATATTTC

                *     *      *    **                       *   **       *    
      25530 CTTTCCCACCGTAGGCCACAAAAGGCTCCAAATATCCACTTGCAGATACTATGAAAAGAGAGTTT
          1 CTTTTCCACCATAGGCCTCAAAGCGCTCCAAATATCCACTTGCAGATTCTACAAAAAGAGTGTTT

                        *        *               ***            *            
      25595 CAAAACTGCTCATTCAAAAGATAGGTTCAACTCTGTGGTTTGAATGCACACAGCACAAAGAAGTT
         66 CAAAACTGCTCAATCAAAAGAAAGGTTCAACTCTGTGAGATGAATGCACACATCACAAAGAAGTT

              *       **                   **   *    
      25660 TCACAGAATGTGTCTGTGTAGTTTTTATGTGCGGATGTTTC
        131 TCTCAGAATGCTTCTGTGTAGTTTTTATGTGAAGATATTTC

                         *        ****   *   *               *    *          
      25701 CTTTTCCACCATATGCCT-AAATATTTCCCAATTTCCACTTGCAGATTCCACAAGAAGAGTGTTT
          1 CTTTTCCACCATAGGCCTCAAAGCGCTCCAAATATCCACTTGCAGATTCTACAAAAAGAGTGTTT

                      **       *       *           *            *      ***   
      25765 CAAAACTGCTGTATC-AAATAAA-GTTGAACTCTGTGAGGTGAATGCACACAGCACAAAATGGTT
         66 CAAAACTGCTCAATCAAAAGAAAGGTTCAACTCTGTGAGATGAATGCACACATCACAAAGAAGTT

                          **   *        *       *    
      25828 TCTCAGAATGCTTCCTTGTTGTTTTTATATGAAGATGTTTC
        131 TCTCAGAATGCTTCTGTGTAGTTTTTATGTGAAGATATTTC

                  *  *             *   *     *                               
      25869 CTTTTCAACAATAGGCCTCAAAGTGCTTCAAATGTCCACTTGCAGATTCTACAAAAAGAGTGTTT
          1 CTTTTCCACCATAGGCCTCAAAGCGCTCCAAATATCCACTTGCAGATTCTACAAAAAGAGTGTTT

                                        *      *  *  *                       
      25934 CAAAACTGCTCAATCAAAAGAAAGGTTCGACTCTGGGAAATTAATGCACACATCACAAAGAAGTT
         66 CAAAACTGCTCAATCAAAAGAAAGGTTCAACTCTGTGAGATGAATGCACACATCACAAAGAAGTT

                                     *        *      
      25999 TCTC---A-GCTTCTGTGTAGTTTTCATGTGAAGTTATTTC
        131 TCTCAGAATGCTTCTGTGTAGTTTTTATGTGAAGATATTTC

                     *       *     *            *    *         **       *    
      26036 CTTTTCCACAATAGGCCGCAAAGGGCTCCAAATATCAACTTACAGATTCTAGGAAAAGAGAGTTT
          1 CTTTTCCACCATAGGCCTCAAAGCGCTCCAAATATCCACTTGCAGATTCTACAAAAAGAGTGTTT

                       * **      *     *                                     
      26101 CAAAACTGCTCTACGAAAAGATAGGTTGAACTCTGTGAGATGAATGCACACATCACAAAGAAGTT
         66 CAAAACTGCTCAATCAAAAGAAAGGTTCAACTCTGTGAGATGAATGCACACATCACAAAGAAGTT

                       *               * *     *     
      26166 TCTCAGAATGCATCTGTGTAGTTTTTACGGGAAGACATTTC
        131 TCTCAGAATGCTTCTGTGTAGTTTTTATGTGAAGATATTTC

                        ***  *     **      *  *                  *     ***   
      26207 CTTTTCCACCATCTTCCACAAAGGTCTCCAAGTAACCACTTGCAGATTCTACAGAAAGACACTTT
          1 CTTTTCCACCATAGGCCTCAAAGCGCTCCAAATATCCACTTGCAGATTCTACAAAAAGAGTGTTT

            *          *         ***      *      ***                      *  
      26272 AAAAACTGCTCTATCAAAAGATCAGTTCAAGTCTGTGGTTTGAATGCACACATCACAAAGAATTT
         66 CAAAACTGCTCAATCAAAAGAAAGGTTCAACTCTGTGAGATGAATGCACACATCACAAAGAAGTT

                                     *  *            
      26337 TCTCAGAATGCTTCTGTGTAGTTTTCATATGAAGATATTTC
        131 TCTCAGAATGCTTCTGTGTAGTTTTTATGTGAAGATATTTC

                                    *                                   **   
      26378 CTTTTCCACCATAGGCCTCAAAGCACTCCAAATATCCACTTGCAGATTCTACAAAAAGAGATTTT
          1 CTTTTCCACCATAGGCCTCAAAGCGCTCCAAATATCCACTTGCAGATTCTACAAAAAGAGTGTTT

                   **                           *  *          *        *     
      26443 CAAAACTAGTCAATCAAAAGAAAGGTTCAACTCTGTCAGTTGAATGCACATATCACAAACAAGTT
         66 CAAAACTGCTCAATCAAAAGAAAGGTTCAACTCTGTGAGATGAATGCACACATCACAAAGAAGTT

                *      *                             
      26508 TCTCGGAATGCGTCTGTGTAGTTTTTATGTGAAGATATTTC
        131 TCTCAGAATGCTTCTGTGTAGTTTTTATGTGAAGATATTTC

               *     * *           *     *                  *    *           
      26549 CTTCTCCACAACAGGCCTCAAAGTGCTCCGAATATCCACTTGCAGATTTTAC-TAAAGAGTGTTT
          1 CTTTTCCACCATAGGCCTCAAAGCGCTCCAAATATCCACTTGCAGATTCTACAAAAAGAGTGTTT

             *               *  *   *     *        *    *               *    
      26613 CCAAACTGCTCAATCAAGAGGAAGTTTCAAGTCTGTGAGCTGAACGCACACATCACAAAGTAGTT
         66 CAAAACTGCTCAATCAAAAGAAAGGTTCAACTCTGTGAGATGAATGCACACATCACAAAGAAGTT

               *                                *    
      26678 TCTGAGAATGCTTCTGTGTAGTTTTTATGTGAAGATGTTTC
        131 TCTCAGAATGCTTCTGTGTAGTTTTTATGTGAAGATATTTC

                            **     *                                    *    
      26719 CTTTTCCACCATAGGCTGCAAAGGGCTCCAAATATCCACTTGCAGATTCTACAAAAAGAGAGTTT
          1 CTTTTCCACCATAGGCCTCAAAGCGCTCCAAATATCCACTTGCAGATTCTACAAAAAGAGTGTTT

                 *     *         *          *     *                     *    
      26784 CAAAAGTGCTCTATCAAAAGATAGGTTCAACTATGTGATATGAATGCACACATCACAAAGTAGTT
         66 CAAAACTGCTCAATCAAAAGAAAGGTTCAACTCTGTGAGATGAATGCACACATCACAAAGAAGTT

                                          *          
      26849 TCTCAGAATGCTTCTGTGTAGTTTTTATGTAAAGATATTTC
        131 TCTCAGAATGCTTCTGTGTAGTTTTTATGTGAAGATATTTC

                                    *                                   **   
      26890 CTTTTCCACCATAGGCCTCAAAGCACTCCAAATATCCACTTGCAGATTCTACAAAAAGAGATTTT
          1 CTTTTCCACCATAGGCCTCAAAGCGCTCCAAATATCCACTTGCAGATTCTACAAAAAGAGTGTTT

                   ** *                   *     *  *    * *   *        *     
      26955 CAAAACTATTTAATCAAAAGAAAGGTTCAAATCTGTCAGTTGAAGGTACATATCACAAACAAGTT
         66 CAAAACTGCTCAATCAAAAGAAAGGTTCAACTCTGTGAGATGAATGCACACATCACAAAGAAGTT

             * **                                    
      27020 TATTGGAATGCTTCTGTGTAGTTTTTATGTGAAGATATTTC
        131 TCTCAGAATGCTTCTGTGTAGTTTTTATGTGAAGATATTTC

                     * *         * *                        **  *     *      
      27061 CTTTTCCACAACAGGCCTCAAGGTGCTCCAAATATCCACTTGCAGATTTCACTAAAAGTGTGTTT
          1 CTTTTCCACCATAGGCCTCAAAGCGCTCCAAATATCCACTTGCAGATTCTACAAAAAGAGTGTTT

             *  *            *  *   *     *        *             *           
      27126 CCAAGCTGCTCAATCAAGAGGAAGTTTCAAGTCTGTGAGGTGAATGCACACATTACAAAGAAGTT
         66 CAAAACTGCTCAATCAAAAGAAAGGTTCAACTCTGTGAGATGAATGCACACATCACAAAGAAGTT

            *  *                                     
      27191 ACTGAGAATGCTTCTGTGTAGTTTTTATGTGAAGATATTTC
        131 TCTCAGAATGCTTCTGTGTAGTTTTTATGTGAAGATATTTC

                      **               *                                *    
      27232 CTTTTCCACCGCAGGCCTCAAAGCGCTGCAAATATCCACTTGCAGATTCTACAAAAAGAGAGTTT
          1 CTTTTCCACCATAGGCCTCAAAGCGCTCCAAATATCCACTTGCAGATTCTACAAAAAGAGTGTTT

                      **         *   *         *   *     **            *     
      27297 CAAAACTGCTGTATCAAAAGATAGGGTCAACTCTGCGAGTTGAATAAACACATCACAAATAAGTT
         66 CAAAACTGCTCAATCAAAAGAAAGGTTCAACTCTGTGAGATGAATGCACACATCACAAAGAAGTT

            *  **   *        *               *       
      27362 CCTGGGAACGCTTCTGTATAG-TTTTATGTGAATATATTTC
        131 TCTCAGAATGCTTCTGTGTAGTTTTTATGTGAAGATATTTC

                         *          *                   *   *  *   * *   *   
      27402 CTTTTCCACCATATGCCTCAAAGCACTCCAAATATCCACTTGCACATTATAGAAACATAGTCTTT
          1 CTTTTCCACCATAGGCCTCAAAGCGCTCCAAATATCCACTTGCAGATTCTACAAAAAGAGTGTTT

                   **                        *         *             *       
      27467 CAAAACTTGTCAATC-AAAGAAAGGTTCAACTCCGTGAGATGAGTGCACACATCACAGAGAAGTT
         66 CAAAACTGCTCAATCAAAAGAAAGGTTCAACTCTGTGAGATGAATGCACACATCACAAAGAAGTT

                *     *                            * 
      27531 TCTCGGAATGTTTCTGTGTAGTTTTTATGTGAAGATATTGC
        131 TCTCAGAATGCTTCTGTGTAGTTTTTATGTGAAGATATTTC

                     *               *            *          *       *   *   
      27572 CTTTTCCACAATAGGCCTCAAAGCGTTCCAAATATCCAATTGCAGATTCCACAAAAAAAGTTTTT
          1 CTTTTCCACCATAGGCCTCAAAGCGCTCCAAATATCCACTTGCAGATTCTACAAAAAGAGTGTTT

            *                 *  *  *  *             * *         **    *     
      27637 TAAAACTGCTCAATCAAATGATAGATTAAACTCTGTGAGATTAGTGCACACATGTCAAAAAAGTT
         66 CAAAACTGCTCAATCAAAAGAAAGGTTCAACTCTGTGAGATGAATGCACACATCACAAAGAAGTT

                                *      * *           
      27702 TCTCAGAATGCTTCTGTGTACTTTTTAGGGGAAGATATTTC
        131 TCTCAGAATGCTTCTGTGTAGTTTTTATGTGAAGATATTTC

                        *    *     **         *    *           **  * *  *    
      27743 CTTTTCCACCATCGGCCACAAAGGACTCCAAATAACCACATGCAGATTCTAGTAACACAGAGTTT
          1 CTTTTCCACCATAGGCCTCAAAGCGCTCCAAATATCCACTTGCAGATTCTACAAAAAGAGTGTTT

                       *         * *           *   * * *    **    ***        
      27808 CAAAACTGCTCTATCAAAAGATAAGTTCAACTCTGAGAGTTTAGTGCAACCATCGTGAAGAAGTT
         66 CAAAACTGCTCAATCAAAAGAAAGGTTCAACTCTGTGAGATGAATGCACACATCACAAAGAAGTT

                            *     *                  
      27873 TCTCAGAATGCTTCTGAGTAGTGTTTATGTGAAGATATTTC
        131 TCTCAGAATGCTTCTGTGTAGTTTTTATGTGAAGATATTTC

                              *     *                      *           **    
      27914 CTTTTCCACCATAGGCCTGAAAGCCCTCCAAATATCCACTTGCAGATCCTACAAAAAGAAAGTTT
          1 CTTTTCCACCATAGGCCTCAAAGCGCTCCAAATATCCACTTGCAGATTCTACAAAAAGAGTGTTT

             *         **     *  *  *   *        **      *                   
      27979 CGAAA-TGCTCTCTCAAACGATAGTTTCGACTCTGTGGTATGAATACACACATCACAAAGAAGTT
         66 CAAAACTGCTCAATCAAAAGAAAGGTTCAACTCTGTGAGATGAATGCACACATCACAAAGAAGTT

                                       **            
      28043 TCTCAGAATGCTTCTGTGTAGTTTTTAAATGAAGATATTTC
        131 TCTCAGAATGCTTCTGTGTAGTTTTTATGTGAAGATATTTC

            *                       *          *     *                       
      28084 TTTTTCCACCATAGGCCTCAAAGCACTCCAAATATGCACTTCCAGATTCTACAAAAAGAGTGTTT
          1 CTTTTCCACCATAGGCCTCAAAGCGCTCCAAATATCCACTTGCAGATTCTACAAAAAGAGTGTTT

              *                 *       * *         **   *         *  * *    
      28149 CAGAACTGCTCAATCAAAAGGAAGGTTCCAGTC--TGAGACAAATACACACATCAAAAGGTAGTT
         66 CAAAACTGCTCAATCAAAAGAAAGGTTCAACTCTGTGAGATGAATGCACACATCACAAAGAAGTT

                                                    *
      28212 TCTCAGAATGCTTCTGTGTAGTTTTTATGTGAAGATATTTT
        131 TCTCAGAATGCTTCTGTGTAGTTTTTATGTGAAGATATTTC

             *               *    **    *     *      *  **   *          *    
      28253 CCTTTCCACCATAGGCCACAAATGGCTCTAAATACCCACTTACATTTTCCACAAAAAGAGAGTTT
          1 CTTTTCCACCATAGGCCTCAAAGCGCTCCAAATATCCACTTGCAGATTCTACAAAAAGAGTGTTT

                       * *    * *      *   *         *                       
      28318 CAAAACTGCTCTACCAAAGGTAA-GTTTAACGCTGTGAG-TTAA-G-A-ACATCACAAAGAAGTT
         66 CAAAACTGCTCAATCAAAAGAAAGGTTCAACTCTGTGAGATGAATGCACACATCACAAAGAAGTT

                                   *   *  *          
      28378 TCTCAGAATGCTTCTGTGTAGTTCTTACGTAAAGATATTTC
        131 TCTCAGAATGCTTCTGTGTAGTTTTTATGTGAAGATATTTC

                 *   *      ***    *                  *              **      
      28419 CTTTTACACAATAGGCAGAAAAGTGCTCCAAATATCCACTTGAAGATTCTACAAAAACCGTGTTT
          1 CTTTTCCACCATAGGCCTCAAAGCGCTCCAAATATCCACTTGCAGATTCTACAAAAAGAGTGTTT

                     **                                          *      *    
      28484 CAAAACTGCCGAATCAAAAGAAAGGTTCAACTCTGTGAGATGAATGCACACATAACAAAGGAGTT
         66 CAAAACTGCTCAATCAAAAGAAAGGTTCAACTCTGTGAGATGAATGCACACATCACAAAGAAGTT

                                 *      *      *    *
      28549 TCTCAGAATGCTTCTGTGTAGCTTTTATATGAAGACATTTA
        131 TCTCAGAATGCTTCTGTGTAGTTTTTATGTGAAGATATTTC

            *        * *            *   ***                     **        *  
      28590 GTTTTCCACAACAGGCCTCAAAGCTCTCTCCATATCCACTTGCAGATTCTACCGAAAGAGTGCTT
          1 CTTTTCCACCATAGGCCTCAAAGCGCTCCAAATATCCACTTGCAGATTCTACAAAAAGAGTGTTT

             *                   * **     *        *        *     **         
      28655 CCAAACTGCTCAATCAAAAGAGACATTCAAATCTGTGAGGTGAATGCAGACATCGTAAAGAAGTT
         66 CAAAACTGCTCAATCAAAAGAAAGGTTCAACTCTGTGAGATGAATGCACACATCACAAAGAAGTT

                                *     *       *    **
      28720 TCTCAGAATGCTTCTGTGTATTTTTTGTGTGAAGTTATTCG
        131 TCTCAGAATGCTTCTGTGTAGTTTTTATGTGAAGATATTTC

            *    *             *     *  *            **                 *    
      28761 TTTTTGCACCATAGGCCTCCAAGCGTTCTAAATATCCACTTCTAGATTCTACAAAAAGAGAGTTT
          1 CTTTTCCACCATAGGCCTCAAAGCGCTCCAAATATCCACTTGCAGATTCTACAAAAAGAGTGTTT

                   *         *            *        *    *   *                
      28826 CAAAACTACTC-A--AACA-AAAGGTTCAATTCTGTGAGTTGAAAGCAAACATCACAAAGAAGTT
         66 CAAAACTGCTCAATCAAAAGAAAGGTTCAACTCTGTGAGATGAATGCACACATCACAAAGAAGTT

                       *             *               
      28887 TCTCAGAATGCGTCTGTGTAGTTTTGATGTGAAGATATTTC
        131 TCTCAGAATGCTTCTGTGTAGTTTTTATGTGAAGATATTTC

                     **   ****     *                  *                      
      28928 CTTTT-CACAGTAGAATGCAAAGGGCTCCAAATATCCACTTGGAGATTCTACAAAAAGA--GTTT
          1 CTTTTCCACCATAGGCCTCAAAGCGCTCCAAATATCCACTTGCAGATTCTACAAAAAGAGTGTTT

                  *    **     *  *     *     ***   *     *               *   
      28990 CAAAACCGCTCTGTCAAATGATAGGTTGAACTCCCGGAGGTGAATACACACATCACAAAGAGGTT
         66 CAAAACTGCTCAATCAAAAGAAAGGTTCAACTCTGTGAGATGAATGCACACATCACAAAGAAGTT

                  *                       *  *       
      29055 TCTCAGCATGCTTCTGTGTAGTTTTTATGTAAACATATTTC
        131 TCTCAGAATGCTTCTGTGTAGTTTTTATGTGAAGATATTTC

             *    * *              *           *      * *       *   *    *   
      29096 CGTTTCTATCATAGGCCTCAAAGTGCTCCAAATATTCACTTGTACATTCTACCAAACGAGTATTT
          1 CTTTTCCACCATAGGCCTCAAAGCGCTCCAAATATCCACTTGCAGATTCTACAAAAAGAGTGTTT

                              * *         ** *    *        *            *    
      29161 CAAAACTGCTCAATCAAATGGAAGGTTCAAAACCGTGACATGAATGCCCACATCACAAAGTAGTT
         66 CAAAACTGCTCAATCAAAAGAAAGGTTCAACTCTGTGAGATGAATGCACACATCACAAAGAAGTT

                                                     
      29226 TCTCAGAATGCTTCTGTGTAGTTTTTATGTGAAGATATTTC
        131 TCTCAGAATGCTTCTGTGTAGTTTTTATGTGAAGATATTTC

                     * *    *      *    *       * *   **      *              
      29267 CTTTTCCACAACA-GCGTGCAAAACGCTTCAAATATGCCCTTAGAGATTCCACAAAAAGAGTGTT
          1 CTTTTCCACCATAGGCCT-CAAAGCGCTCCAAATATCCACTTGCAGATTCTACAAAAAGAGTGTT

              *     *               *  *                                  ** 
      29331 TCCAAACTACTCAAATCAAAA-AATGATTTCAACTCTGTGAGATGAATGCACACATCACAAACTA
         65 TCAAAACTGCTC-AATCAAAAGAAAG-GTTCAACTCTGTGAGATGAATGCACACATCACAAAGAA

                         *     ** *   * *   *       *   
      29395 GTTTCTCAGAATGTTTCTGCCTGGTTCTCATGCGAAGATAGTTC
        128 GTTTCTCAGAATGCTTCTGTGTAGTTTTTATGTGAAGATATTTC

                 *           *   * **              *                  * *    
      29439 CTTTTTCACCATAGGCCGCAATGTACTCCAAATATCCACCTGCAGATTCTAC-AAAAGTGAGTTT
          1 CTTTTCCACCATAGGCCTCAAAGCGCTCCAAATATCCACTTGCAGATTCTACAAAAAGAGTGTTT

                       *         ***    **         *        *      *       * 
      29503 CAAAACTGCTCTATCAAAAGATCAGTTCGTCTCTGTGAGTTGAATGCATACATCAAAAAGAAGCT
         66 CAAAACTGCTCAATCAAAAGAAAGGTTCAACTCTGTGAGATGAATGCACACATCACAAAGAAGTT

                 *             *      **         *   
      29568 TCTCAAAATGCTTCTGTGTGGTTTTTCGGTGAAGATAGTTC
        131 TCTCAGAATGCTTCTGTGTAGTTTTTATGTGAAGATATTTC

            *     *        *      * *                 *        *        *    
      29609 TTTTTCTACCATAGGTCTCAAACCACTCCAAATATCCACTTGTAGATTCTATAAAAAGGAATG-T
          1 CTTTTCCACCATAGGCCTCAAAGCGCTCCAAATATCCACTTGCAGATTCTACAAAAA-GAGTGTT

                  *        *    *    *      * *         *      *         *   
      29673 TCAAAATTGCTCAATAAAAATAAAGTTTCAACACCGTGAGATGAGTGCACAAATCACAAAGGAGT
         65 TCAAAACTGCTCAATCAAAAGAAAGGTTCAACTCTGTGAGATGAATGCACACATCACAAAGAAGT

                  *          *         *          *   
      29738 TTCTCAAAATGCTTCTGGGTAGTTTTTCTGTGAAGATAGTTC
        130 TTCTCAGAATGCTTCTGTGTAGTTTTTATGTGAAGATATTTC

                  *     *    *     *          *                         *    
      29780 CTTTTCTACCATGGGCCACAAAGGGCTCCAAATACCCACTTGCAGATTCTACAAAAAGAGAGTTT
          1 CTTTTCCACCATAGGCCTCAAAGCGCTCCAAATATCCACTTGCAGATTCTACAAAAAGAGTGTTT

              *        *      ** * *        *    * *    **  * *           *  
      29845 CACAACTGCTCTATCAAACAATATGTTCAACTTTGTGGGTTGAACACAAATATCAC-AAGAATTT
         66 CAAAACTGCTCAATCAAAAGAAAGGTTCAACTCTGTGAGATGAATGCACACATCACAAAGAAGTT

                **                             *     
      29909 TCTCCCAATGCTTCTGTGTAGTTTTTATGTGAAGACATTTC
        131 TCTCAGAATGCTTCTGTGTAGTTTTTATGTGAAGATATTTC

            *   *  *      *  *     *                 *  *      *       *  *  
      29950 TTTTCCCTCCATAGTCCACAAAGTGCTCCAAATATCCACTTACATATTCTAGAAAAAGATTGCTT
          1 CTTTTCCACCATAGGCCTCAAAGCGCTCCAAATATCCACTTGCAGATTCTACAAAAAGAGTGTTT

            **       *    *               * * *                              
      30015 GGAAACTGCACAATGAAAAGAAAGGTTCAAATATATGAGATGAATGCACACATCACAAAGAAGTT
         66 CAAAACTGCTCAATCAAAAGAAAGGTTCAACTCTGTGAGATGAATGCACACATCACAAAGAAGTT

                                 *                    
      30080 TCTCAGAAT-CTCTCTGTGTAATTTTTATGTGAAGATATTTC
        131 TCTCAGAATGCT-TCTGTGTAGTTTTTATGTGAAGATATTTC

                *     *    *  *   *                        *      *    *     
      30121 CTTTCCCACCTTAGGTCTTAAAACGCTCCAAATATCCACTTGCAGATACTACAAGAAGATTGTTT
          1 CTTTTCCACCATAGGCCTCAAAGCGCTCCAAATATCCACTTGCAGATTCTACAAAAAGAGTGTTT

                     *    *        *      *     **                           
      30186 CAAAACTGCACAA-AAAAAGAAATGTTCAATTCTGTTTGATGAATGCACACATCACAAAGAAGTT
         66 CAAAACTGCTCAATCAAAAGAAAGGTTCAACTCTGTGAGATGAATGCACACATCACAAAGAAGTT

                           *                         
      30250 TCTCAGAATGCTTCTCTGTAGTTTTTATGTGAAGATATTTC
        131 TCTCAGAATGCTTCTGTGTAGTTTTTATGTGAAGATATTTC

                     *             *                 *         **      * *   
      30291 CTTTTCCACAATAGGCCTCAAAGGGCTCCAAATATCCACTTCCAGATTCTATGAAAAGAATATTT
          1 CTTTTCCACCATAGGCCTCAAAGCGCTCCAAATATCCACTTGCAGATTCTACAAAAAGAGTGTTT

             *              * *    *                                      *  
      30356 CCAAACTGCTCAATCATAGGAAATGTTCAACTCTGTGAGATGAATGCACACATCAC-AAGAAATT
         66 CAAAACTGCTCAATCAAAAGAAAGGTTCAACTCTGTGAGATGAATGCACACATCACAAAGAAGTT

                     *    *      *       *       *   
      30420 TCTCAGAATCCTTCAGTGTAGGTTTTATGAGAAGATAATTC
        131 TCTCAGAATGCTTCTGTGTAGTTTTTATGTGAAGATATTTC

                     *    **        *   *                           *    *   
      30461 CTTTTCCACAATAGTTCTCAAAGCACTCAAAATATCCACTTGCAGATTCTACAAAAGGAGTATTT
          1 CTTTTCCACCATAGGCCTCAAAGCGCTCCAAATATCCACTTGCAGATTCTACAAAAAGAGTGTTT

                                                          *                  
      30526 CAAAACTGCTCAATCAAAAGAAAGGTTCAACTCTGTGAGATGAATGGACACATCACAAAGAAGTT
         66 CAAAACTGCTCAATCAAAAGAAAGGTTCAACTCTGTGAGATGAATGCACACATCACAAAGAAGTT

                                  *   *              
      30591 TCTCAGAATGCTTCTGTGTAGTATTTTTGTGAAGATATTT-
        131 TCTCAGAATGCTTCTGTGTAGTTTTTATGTGAAGATATTTC

                          *  * * * * ***             *            *     * *  
      30631 CTTTTCCACCATAGACCGCCAGGGGACACAAATATCCACTTTCAGATTCTACAACAAGAGAGGTT
          1 CTTTTCCACCATAGGCCTCAAAGCGCTCCAAATATCCACTTGCAGATTCTACAAAAAGAGTGTTT

                   *   *     *   ** *       *      *                      * *
      30696 CAAAACTACTCGATCAAGAGATGGTTTCAACTATGTGAGTTGAATGCACACATCACAAAGAACTA
         66 CAAAACTGCTCAATCAAAAGAAAGGTTCAACTCTGTGAGATGAATGCACACATCACAAAGAAGTT

             *  *    *                               
      30761 TGTCGGAATTCTTCTGTGTAGTTTTTATGTGAAGATATTTC
        131 TCTCAGAATGCTTCTGTGTAGTTTTTATGTGAAGATATTTC

                     *    * *      * *    *                  *               
      30802 CTTTTCCACAATAGACGTCAAAGTGATCCAGATATCCACTTGCAGATTCCACAAAAAGAGTGTTT
          1 CTTTTCCACCATAGGCCTCAAAGCGCTCCAAATATCCACTTGCAGATTCTACAAAAAGAGTGTTT

                 *   *   *                  **                     *  *      
      30867 CAAAAGTGCACAACCAAAAGAAAGGTTCAACTAGGTGAGATGAATGCACACATCAGAAGGAAGTT
         66 CAAAACTGCTCAATCAAAAGAAAGGTTCAACTCTGTGAGATGAATGCACACATCACAAAGAAGTT

                            **   *     * *       *   
      30932 TCTCAGAATGCTTCTGCATAGCTTTTAAGGGAAGATACTTC
        131 TCTCAGAATGCTTCTGTGTAGTTTTTATGTGAAGATATTTC

                    *               *            * *  *    * *               
      30973 CTTTTCCAACATAGGCCTCAAAGCACTCCAAATATCCTCCTGGAGATACCACAAAAAGAGTGTTT
          1 CTTTTCCACCATAGGCCTCAAAGCGCTCCAAATATCCACTTGCAGATTCTACAAAAAGAGTGTTT

            **                      *  *                 *       *           
      31038 GCAAACTGCTCAATCAAAAGAAAGATTTAACTCTGTGAGATGAATCCACACATGACAAAGAAGTT
         66 CAAAACTGCTCAATCAAAAGAAAGGTTCAACTCTGTGAGATGAATGCACACATCACAAAGAAGTT

                                                     
      31103 TCTCAGAATGCTTCTGTGTAGTTTTTATGTGAAGATATTTC
        131 TCTCAGAATGCTTCTGTGTAGTTTTTATGTGAAGATATTTC

                     *   * * *    **           *                *     *      
      31144 CTTTTCCACAATAAGACCCAAAAGGCTCCAAATATTCACTTGCAGATTCTAAAAAAAACAGTGTT
          1 CTTTTCCACCATAGGCCTCAAAGCGCTCCAAATATCCACTTGCAGATTCT-ACAAAAAGAGTGTT

                                  *                  *      *        **      
      31209 TCAAAACTGCTCAATCAAAAGATA-GTTCAACTCTGTGAGAAGAATGCTCACATCACTGAGAAGT
         65 TCAAAACTGCTCAATCAAAAGAAAGGTTCAACTCTGTGAGATGAATGCACACATCACAAAGAAGT

                                         *            
      31273 TTCTCAGAATGCTTCTGTGTAGTTTTTATATGAAGATATTTC
        130 TTCTCAGAATGCTTCTGTGTAGTTTTTATGTGAAGATATTTC

                *     *      *    **                       *   **       *    
      31315 CTTTCCCACCGTAGGCCACAAAAGGCTCCAAATATCCACTTGCAGATACTATGAAAAGAGAGTTT
          1 CTTTTCCACCATAGGCCTCAAAGCGCTCCAAATATCCACTTGCAGATTCTACAAAAAGAGTGTTT

                        *        *               ***            *            
      31380 CAAAACTGCTCATTCAAAAGATAGGTTCAACTCTGTGGTTTGAATGCACACAGCACAAAGAAGTT
         66 CAAAACTGCTCAATCAAAAGAAAGGTTCAACTCTGTGAGATGAATGCACACATCACAAAGAAGTT

              *       **                   **   *    
      31445 TCACAGAATGTGTCTGTGTAGTTTTTATGTGCGGATGTTTC
        131 TCTCAGAATGCTTCTGTGTAGTTTTTATGTGAAGATATTTC

                         *        ****   *   *               *    *          
      31486 CTTTTCCACCATATGCCT-AAATATTTCCCAATTTCCACTTGCAGATTCCACAAGAAGAGTGTTT
          1 CTTTTCCACCATAGGCCTCAAAGCGCTCCAAATATCCACTTGCAGATTCTACAAAAAGAGTGTTT

                      **       *       *           *            *      ***   
      31550 CAAAACTGCTGTATC-AAATAAA-GTTGAACTCTGTGAGGTGAATGCACACAGCACAAAATGGTT
         66 CAAAACTGCTCAATCAAAAGAAAGGTTCAACTCTGTGAGATGAATGCACACATCACAAAGAAGTT

                          **   *        *       *    
      31613 TCTCAGAATGCTTCCTTGTTGTTTTTATATGAAGATGTTTC
        131 TCTCAGAATGCTTCTGTGTAGTTTTTATGTGAAGATATTTC

                  *  *             *   *     *                               
      31654 CTTTTCAACAATAGGCCTCAAAGTGCTTCAAATGTCCACTTGCAGATTCTACAAAAAGAGTGTTT
          1 CTTTTCCACCATAGGCCTCAAAGCGCTCCAAATATCCACTTGCAGATTCTACAAAAAGAGTGTTT

                                        *      *  *  *                       
      31719 CAAAACTGCTCAATCAAAAGAAAGGTTCGACTCTGGGAAATTAATGCACACATCACAAAGAAGTT
         66 CAAAACTGCTCAATCAAAAGAAAGGTTCAACTCTGTGAGATGAATGCACACATCACAAAGAAGTT

                                     *        *      
      31784 TCTC---A-GCTTCTGTGTAGTTTTCATGTGAAGTTATTTC
        131 TCTCAGAATGCTTCTGTGTAGTTTTTATGTGAAGATATTTC

                     *       *     *            *    *         **       *    
      31821 CTTTTCCACAATAGGCCGCAAAGGGCTCCAAATATCAACTTACAGATTCTAGGAAAAGAGAGTTT
          1 CTTTTCCACCATAGGCCTCAAAGCGCTCCAAATATCCACTTGCAGATTCTACAAAAAGAGTGTTT

                       * **      *     *                                     
      31886 CAAAACTGCTCTACGAAAAGATAGGTTGAACTCTGTGAGATGAATGCACACATCACAAAGAAGTT
         66 CAAAACTGCTCAATCAAAAGAAAGGTTCAACTCTGTGAGATGAATGCACACATCACAAAGAAGTT

                       *               * *     *     
      31951 TCTCAGAATGCATCTGTGTAGTTTTTACGGGAAGACATTTC
        131 TCTCAGAATGCTTCTGTGTAGTTTTTATGTGAAGATATTTC

                        ***  *     **      *  *                  *     ***   
      31992 CTTTTCCACCATCTTCCACAAAGGTCTCCAAGTAACCACTTGCAGATTCTACAGAAAGACACTTT
          1 CTTTTCCACCATAGGCCTCAAAGCGCTCCAAATATCCACTTGCAGATTCTACAAAAAGAGTGTTT

            *          *         ***      *      ***                      *  
      32057 AAAAACTGCTCTATCAAAAGATCAGTTCAAGTCTGTGGTTTGAATGCACACATCACAAAGAATTT
         66 CAAAACTGCTCAATCAAAAGAAAGGTTCAACTCTGTGAGATGAATGCACACATCACAAAGAAGTT

                                     *  *            
      32122 TCTCAGAATGCTTCTGTGTAGTTTTCATATGAAGATATTTC
        131 TCTCAGAATGCTTCTGTGTAGTTTTTATGTGAAGATATTTC

                                    *                                   **   
      32163 CTTTTCCACCATAGGCCTCAAAGCACTCCAAATATCCACTTGCAGATTCTACAAAAAGAGATTTT
          1 CTTTTCCACCATAGGCCTCAAAGCGCTCCAAATATCCACTTGCAGATTCTACAAAAAGAGTGTTT

                   **                           *  *          *        *     
      32228 CAAAACTAGTCAATCAAAAGAAAGGTTCAACTCTGTCAGTTGAATGCACATATCACAAACAAGTT
         66 CAAAACTGCTCAATCAAAAGAAAGGTTCAACTCTGTGAGATGAATGCACACATCACAAAGAAGTT

                *      *                             
      32293 TCTCGGAATGCGTCTGTGTAGTTTTTATGTGAAGATATTTC
        131 TCTCAGAATGCTTCTGTGTAGTTTTTATGTGAAGATATTTC

               *     * *           *     *                  *    *           
      32334 CTTCTCCACAACAGGCCTCAAAGTGCTCCGAATATCCACTTGCAGATTTTAC-TAAAGAGTGTTT
          1 CTTTTCCACCATAGGCCTCAAAGCGCTCCAAATATCCACTTGCAGATTCTACAAAAAGAGTGTTT

             *               *  *   *     *        *    *               *    
      32398 CCAAACTGCTCAATCAAGAGGAAGTTTCAAGTCTGTGAGCTGAACGCACACATCACAAAGTAGTT
         66 CAAAACTGCTCAATCAAAAGAAAGGTTCAACTCTGTGAGATGAATGCACACATCACAAAGAAGTT

               *                                *    
      32463 TCTGAGAATGCTTCTGTGTAGTTTTTATGTGAAGATGTTTC
        131 TCTCAGAATGCTTCTGTGTAGTTTTTATGTGAAGATATTTC

                            **     *                                    *    
      32504 CTTTTCCACCATAGGCTGCAAAGGGCTCCAAATATCCACTTGCAGATTCTACAAAAAGAGAGTTT
          1 CTTTTCCACCATAGGCCTCAAAGCGCTCCAAATATCCACTTGCAGATTCTACAAAAAGAGTGTTT

                 *     *         *          *     *                     *    
      32569 CAAAAGTGCTCTATCAAAAGATAGGTTCAACTATGTGATATGAATGCACACATCACAAAGTAGTT
         66 CAAAACTGCTCAATCAAAAGAAAGGTTCAACTCTGTGAGATGAATGCACACATCACAAAGAAGTT

                                          *          
      32634 TCTCAGAATGCTTCTGTGTAGTTTTTATGTAAAGATATTTC
        131 TCTCAGAATGCTTCTGTGTAGTTTTTATGTGAAGATATTTC

                                    *                                   **   
      32675 CTTTTCCACCATAGGCCTCAAAGCACTCCAAATATCCACTTGCAGATTCTACAAAAAGAGATTTT
          1 CTTTTCCACCATAGGCCTCAAAGCGCTCCAAATATCCACTTGCAGATTCTACAAAAAGAGTGTTT

                   ** *                   *     *  *    * *   *        *     
      32740 CAAAACTATTTAATCAAAAGAAAGGTTCAAATCTGTCAGTTGAAGGTACATATCACAAACAAGTT
         66 CAAAACTGCTCAATCAAAAGAAAGGTTCAACTCTGTGAGATGAATGCACACATCACAAAGAAGTT

             * **                                    
      32805 TATTGGAATGCTTCTGTGTAGTTTTTATGTGAAGATATTTC
        131 TCTCAGAATGCTTCTGTGTAGTTTTTATGTGAAGATATTTC

                     * *       * * *                        **  *     *      
      32846 CTTTTCCACAACAGGCCTCCAGGTGCTCCAAATATCCACTTGCAGATTTCACTAAAAGTGTGTTT
          1 CTTTTCCACCATAGGCCTCAAAGCGCTCCAAATATCCACTTGCAGATTCTACAAAAAGAGTGTTT

             *  *            *  *   *     *     *  *             *           
      32911 CCAAGCTGCTCAATCAAGAGGAAGTTTCAAGTCTGTCAGGTGAATGCACACATTACAAAGAAGTT
         66 CAAAACTGCTCAATCAAAAGAAAGGTTCAACTCTGTGAGATGAATGCACACATCACAAAGAAGTT

            *  *                                     
      32976 ACTGAGAATGCTTCTGTGTAGTTTTTATGTGAAGATATTTC
        131 TCTCAGAATGCTTCTGTGTAGTTTTTATGTGAAGATATTTC

                      **               *                                *    
      33017 CTTTTCCACCGCAGGCCTCAAAGCGCTGCAAATATCCACTTGCAGATTCTACAAAAAGAGAGTTT
          1 CTTTTCCACCATAGGCCTCAAAGCGCTCCAAATATCCACTTGCAGATTCTACAAAAAGAGTGTTT

                      **         *   *         *   *     **            *     
      33082 CAAAACTGCTGTATCAAAAGATAGGGTCAACTCTGCGAGTTGAATAAACACATCACAAATAAGTT
         66 CAAAACTGCTCAATCAAAAGAAAGGTTCAACTCTGTGAGATGAATGCACACATCACAAAGAAGTT

               **   *        *               *       
      33147 TCTGGGAACGCTTCTGTATAG-TTTTATGTGAATATATTTC
        131 TCTCAGAATGCTTCTGTGTAGTTTTTATGTGAAGATATTTC

                         *          *                   *   *  *   * *   *   
      33187 CTTTTCCACCATATGCCTCAAAGCACTCCAAATATCCACTTGCACATTATAGAAACATAGTCTTT
          1 CTTTTCCACCATAGGCCTCAAAGCGCTCCAAATATCCACTTGCAGATTCTACAAAAAGAGTGTTT

                   **                        *         *             *       
      33252 CAAAACTTGTCAATC-AAAGAAAGGTTCAACTCCGTGAGATGAGTGCACACATCACAGAGAAGTT
         66 CAAAACTGCTCAATCAAAAGAAAGGTTCAACTCTGTGAGATGAATGCACACATCACAAAGAAGTT

                *     *                            * 
      33316 TCTCGGAATGTTTCTGTGTAGTTTTTATGTGAAGATATTGC
        131 TCTCAGAATGCTTCTGTGTAGTTTTTATGTGAAGATATTTC

                     *               *            *          *       *   *   
      33357 CTTTTCCACAATAGGCCTCAAAGCGTTCCAAATATCCAATTGCAGATTCCACAAAAAAAGTTTTT
          1 CTTTTCCACCATAGGCCTCAAAGCGCTCCAAATATCCACTTGCAGATTCTACAAAAAGAGTGTTT

            *                 *  *  *  *             * *         **    *     
      33422 TAAAACTGCTCAATCAAATGATAGATTAAACTCTGTGAGATTAGTGCACACATGTCAAAAAAGTT
         66 CAAAACTGCTCAATCAAAAGAAAGGTTCAACTCTGTGAGATGAATGCACACATCACAAAGAAGTT

                                *      * *           
      33487 TCTCAGAATGCTTCTGTGTACTTTTTAGGGGAAGATATTTC
        131 TCTCAGAATGCTTCTGTGTAGTTTTTATGTGAAGATATTTC

                        *    *     **         *    *           **  * *  *    
      33528 CTTTTCCACCATCGGCCACAAAGGACTCCAAATAACCACATGCAGATTCTAGTAACACAGAGTTT
          1 CTTTTCCACCATAGGCCTCAAAGCGCTCCAAATATCCACTTGCAGATTCTACAAAAAGAGTGTTT

                       *         * *           *   * * *    **    ***        
      33593 CAAAACTGCTCTATCAAAAGATAAGTTCAACTCTGAGAGTTTAGTGCAACCATCGTGAAGAAGTT
         66 CAAAACTGCTCAATCAAAAGAAAGGTTCAACTCTGTGAGATGAATGCACACATCACAAAGAAGTT

                            *     *                  
      33658 TCTCAGAATGCTTCTGAGTAGTGTTTATGTGAAGATATTTC
        131 TCTCAGAATGCTTCTGTGTAGTTTTTATGTGAAGATATTTC

                              *     *                      *           **    
      33699 CTTTTCCACCATAGGCCTGAAAGCCCTCCAAATATCCACTTGCAGATCCTACAAAAAGAAAGTTT
          1 CTTTTCCACCATAGGCCTCAAAGCGCTCCAAATATCCACTTGCAGATTCTACAAAAAGAGTGTTT

             *         **     *  *  *   *        **      *                   
      33764 CGAAA-TGCTCTCTCAAACGATAGTTTCGACTCTGTGGTATGAATACACACATCACAAAGAAGTT
         66 CAAAACTGCTCAATCAAAAGAAAGGTTCAACTCTGTGAGATGAATGCACACATCACAAAGAAGTT

                                       **            
      33828 TCTCAGAATGCTTCTGTGTAGTTTTTAAATGAAGATATTTC
        131 TCTCAGAATGCTTCTGTGTAGTTTTTATGTGAAGATATTTC

            *                       *          *     *                       
      33869 TTTTTCCACCATAGGCCTCAAAGCACTCCAAATATGCACTTCCAGATTCTACAAAAAGAGTGTTT
          1 CTTTTCCACCATAGGCCTCAAAGCGCTCCAAATATCCACTTGCAGATTCTACAAAAAGAGTGTTT

              *                 *       * *         **   *         *  * *    
      33934 CAGAACTGCTCAATCAAAAGGAAGGTTCCAGTC--TGAGACAAATACACACATCAAAAGGTAGTT
         66 CAAAACTGCTCAATCAAAAGAAAGGTTCAACTCTGTGAGATGAATGCACACATCACAAAGAAGTT

                                                    *
      33997 TCTCAGAATGCTTCTGTGTAGTTTTTATGTGAAGATATTTT
        131 TCTCAGAATGCTTCTGTGTAGTTTTTATGTGAAGATATTTC

             *               *    **    *     *      *  **   *          *    
      34038 CCTTTCCACCATAGGCCACAAATGGCTCTAAATACCCACTTACATTTTCCACAAAAAGAGAGTTT
          1 CTTTTCCACCATAGGCCTCAAAGCGCTCCAAATATCCACTTGCAGATTCTACAAAAAGAGTGTTT

                       * *    * *      *   *         *                       
      34103 CAAAACTGCTCTACCAAAGGTAA-GTTTAACGCTGTGAG-TTAA-G-A-ACATCACAAAGAAGTT
         66 CAAAACTGCTCAATCAAAAGAAAGGTTCAACTCTGTGAGATGAATGCACACATCACAAAGAAGTT

                                   *   *  *          
      34163 TCTCAGAATGCTTCTGTGTAGTTCTTACGTAAAGATATTTC
        131 TCTCAGAATGCTTCTGTGTAGTTTTTATGTGAAGATATTTC

                 *   *      ***    *                  *              **      
      34204 CTTTTACACAATAGGCAGAAAAGTGCTCCAAATATCCACTTGAAGATTCTACAAAAACCGTGTTT
          1 CTTTTCCACCATAGGCCTCAAAGCGCTCCAAATATCCACTTGCAGATTCTACAAAAAGAGTGTTT

                     **                                          *      *    
      34269 CAAAACTGCCGAATCAAAAGAAAGGTTCAACTCTGTGAGATGAATGCACACATAACAAAGGAGTT
         66 CAAAACTGCTCAATCAAAAGAAAGGTTCAACTCTGTGAGATGAATGCACACATCACAAAGAAGTT

                                 *      *      *    *
      34334 TCTCAGAATGCTTCTGTGTAGCTTTTATATGAAGACATTTA
        131 TCTCAGAATGCTTCTGTGTAGTTTTTATGTGAAGATATTTC

            *        * *            *   ***                     **        *  
      34375 GTTTTCCACAACAGGCCTCAAAGCTCTCTCCATATCCACTTGCAGATTCTACCGAAAGAGTGCTT
          1 CTTTTCCACCATAGGCCTCAAAGCGCTCCAAATATCCACTTGCAGATTCTACAAAAAGAGTGTTT

             *                   * **     *        *        *     **         
      34440 CCAAACTGCTCAATCAAAAGAGACATTCAAATCTGTGAGGTGAATGCAGACATCGTAAAGAAGTT
         66 CAAAACTGCTCAATCAAAAGAAAGGTTCAACTCTGTGAGATGAATGCACACATCACAAAGAAGTT

                                *     *       *    **
      34505 TCTCAGAATGCTTCTGTGTATTTTTTGTGTGAAGTTATTCG
        131 TCTCAGAATGCTTCTGTGTAGTTTTTATGTGAAGATATTTC

            *    *             *     *  *            **                 *    
      34546 TTTTTGCACCATAGGCCTCCAAGCGTTCTAAATATCCACTTCTAGATTCTACAAAAAGAGAGTTT
          1 CTTTTCCACCATAGGCCTCAAAGCGCTCCAAATATCCACTTGCAGATTCTACAAAAAGAGTGTTT

                   *         *            *        *    *   *                
      34611 CAAAACTACTC-A--AACA-AAAGGTTCAATTCTGTGAGTTGAAAGCAAACATCACAAAGAAGTT
         66 CAAAACTGCTCAATCAAAAGAAAGGTTCAACTCTGTGAGATGAATGCACACATCACAAAGAAGTT

                       *             *               
      34672 TCTCAGAATGCGTCTGTGTAGTTTTGATGTGAAGATATTTC
        131 TCTCAGAATGCTTCTGTGTAGTTTTTATGTGAAGATATTTC

                     *    ****     *                  *                      
      34713 CTTTT-CACAATAGAATGCAAAGGGCTCCAAATATCCACTTGGAGATTCTACAAAAAGA--GTTT
          1 CTTTTCCACCATAGGCCTCAAAGCGCTCCAAATATCCACTTGCAGATTCTACAAAAAGAGTGTTT

                  *    **     *  *     *     ***   *     *               *   
      34775 CAAAACCGCTCTGTCAAATGATAGGTTGAACTCCCGGAGGTGAATACACACATCACAAAGAGGTT
         66 CAAAACTGCTCAATCAAAAGAAAGGTTCAACTCTGTGAGATGAATGCACACATCACAAAGAAGTT

                  *                       *  *       
      34840 TCTCAGCATGCTTCTGTGTAGTTTTTATGTAAACATATTTC
        131 TCTCAGAATGCTTCTGTGTAGTTTTTATGTGAAGATATTTC

             *    * *              *           *      * *       *   *    *   
      34881 CGTTTCTATCATAGGCCTCAAAGTGCTCCAAATATTCACTTGTACATTCTACCAAACGAGTATTT
          1 CTTTTCCACCATAGGCCTCAAAGCGCTCCAAATATCCACTTGCAGATTCTACAAAAAGAGTGTTT

                              * *         ** *    *        *            *    
      34946 CAAAACTGCTCAATCAAATGGAAGGTTCAAAACCGTGACATGAATGCCCACATCACAAAGTAGTT
         66 CAAAACTGCTCAATCAAAAGAAAGGTTCAACTCTGTGAGATGAATGCACACATCACAAAGAAGTT

                                                     
      35011 TCTCAGAATGCTTCTGTGTAGTTTTTATGTGAAGATATTTC
        131 TCTCAGAATGCTTCTGTGTAGTTTTTATGTGAAGATATTTC

                     * *    *      *    *       * *   **      *              
      35052 CTTTTCCACAACA-GCGTGCAAAACGCTTCAAATATGCCCTTAGAGATTCCACAAAAAGAGTGTT
          1 CTTTTCCACCATAGGCCT-CAAAGCGCTCCAAATATCCACTTGCAGATTCTACAAAAAGAGTGTT

              *     *               *  *                                  ** 
      35116 TCCAAACTACTCAAATCAAAA-AATGATTTCAACTCTGTGAGATGAATGCACACATCACAAACTA
         65 TCAAAACTGCTC-AATCAAAAGAAAG-GTTCAACTCTGTGAGATGAATGCACACATCACAAAGAA

                         *     ** *   * *   *       *   
      35180 GTTTCTCAGAATGTTTCTGCCTGGTTCTCATGCGAAGATAGTTC
        128 GTTTCTCAGAATGCTTCTGTGTAGTTTTTATGTGAAGATATTTC

                 *           *   * **              *                  * *    
      35224 CTTTTTCACCATAGGCCGCAATGTACTCCAAATATCCACCTGCAGATTCTAC-AAAAGTGAGTTT
          1 CTTTTCCACCATAGGCCTCAAAGCGCTCCAAATATCCACTTGCAGATTCTACAAAAAGAGTGTTT

                       *         ***    **         *        *      *       * 
      35288 CAAAACTGCTCTATCAAAAGATCAGTTCGTCTCTGTGAGTTGAATGCATACATCAAAAAGAAGCT
         66 CAAAACTGCTCAATCAAAAGAAAGGTTCAACTCTGTGAGATGAATGCACACATCACAAAGAAGTT

                 *             *      **         *   
      35353 TCTCAAAATGCTTCTGTGTGGTTTTTCGGTGAAGATAGTTC
        131 TCTCAGAATGCTTCTGTGTAGTTTTTATGTGAAGATATTTC

            *     *        *      * *                 *        *        *    
      35394 TTTTTCTACCATAGGTCTCAAACCACTCCAAATATCCACTTGTAGATTCTATAAAAAGGAATG-T
          1 CTTTTCCACCATAGGCCTCAAAGCGCTCCAAATATCCACTTGCAGATTCTACAAAAA-GAGTGTT

                  *        *    *    *      * *         *      *         *   
      35458 TCAAAATTGCTCAATAAAAATAAAGTTTCAACACCGTGAGATGAGTGCACAAATCACAAAGGAGT
         65 TCAAAACTGCTCAATCAAAAGAAAGGTTCAACTCTGTGAGATGAATGCACACATCACAAAGAAGT

                  *          *         *          *   
      35523 TTCTCAAAATGCTTCTGGGTAGTTTTTCTGTGAAGATAGTTC
        130 TTCTCAGAATGCTTCTGTGTAGTTTTTATGTGAAGATATTTC

                  *     *    *     *          *                         *    
      35565 CTTTTCTACCATGGGCCACAAAGGGCTCCAAATACCCACTTGCAGATTCTACAAAAAGAGAGTTT
          1 CTTTTCCACCATAGGCCTCAAAGCGCTCCAAATATCCACTTGCAGATTCTACAAAAAGAGTGTTT

              *        *      ** * *        *    * *    **  * *           *  
      35630 CACAACTGCTCTATCAAACAATATGTTCAACTTTGTGGGTTGAACACAAATATCAC-AAGAATTT
         66 CAAAACTGCTCAATCAAAAGAAAGGTTCAACTCTGTGAGATGAATGCACACATCACAAAGAAGTT

                **                             *     
      35694 TCTCCCAATGCTTCTGTGTAGTTTTTATGTGAAGACATTTC
        131 TCTCAGAATGCTTCTGTGTAGTTTTTATGTGAAGATATTTC

            *   *  *      *  *     *                 *  *      *       *  *  
      35735 TTTTCCCTCCATAGTCCACAAAGTGCTCCAAATATCCACTTACATATTCTAGAAAAAGATTGCTT
          1 CTTTTCCACCATAGGCCTCAAAGCGCTCCAAATATCCACTTGCAGATTCTACAAAAAGAGTGTTT

            **       *    *               * * *                              
      35800 GGAAACTGCACAATGAAAAGAAAGGTTCAAATATATGAGATGAATGCACACATCACAAAGAAGTT
         66 CAAAACTGCTCAATCAAAAGAAAGGTTCAACTCTGTGAGATGAATGCACACATCACAAAGAAGTT

                                 *                    
      35865 TCTCAGAAT-CTCTCTGTGTAATTTTTATGTGAAGATATTTC
        131 TCTCAGAATGCT-TCTGTGTAGTTTTTATGTGAAGATATTTC

                *     *    *  *   *                        *      *    *     
      35906 CTTTCCCACCTTAGGTCTTAAAACGCTCCAAATATCCACTTGCAGATACTACAAGAAGATTGTTT
          1 CTTTTCCACCATAGGCCTCAAAGCGCTCCAAATATCCACTTGCAGATTCTACAAAAAGAGTGTTT

                     *    *        *      *     **                           
      35971 CAAAACTGCACAA-AAAAAGAAATGTTCAATTCTGTTTGATGAATGCACACATCACAAAGAAGTT
         66 CAAAACTGCTCAATCAAAAGAAAGGTTCAACTCTGTGAGATGAATGCACACATCACAAAGAAGTT

                           *                         
      36035 TCTCAGAATGCTTCTCTGTAGTTTTTATGTGAAGATATTTC
        131 TCTCAGAATGCTTCTGTGTAGTTTTTATGTGAAGATATTTC

                     *             *                 *         **      * *   
      36076 CTTTTCCACAATAGGCCTCAAAGGGCTCCAAATATCCACTTCCAGATTCTATGAAAAGAATATTT
          1 CTTTTCCACCATAGGCCTCAAAGCGCTCCAAATATCCACTTGCAGATTCTACAAAAAGAGTGTTT

             *              * *    *                                      *  
      36141 CCAAACTGCTCAATCATAGGAAATGTTCAACTCTGTGAGATGAATGCACACATCAC-AAGAAATT
         66 CAAAACTGCTCAATCAAAAGAAAGGTTCAACTCTGTGAGATGAATGCACACATCACAAAGAAGTT

                     *    *      *       *       *   
      36205 TCTCAGAATCCTTCAGTGTAGGTTTTATGAGAAGATAATTC
        131 TCTCAGAATGCTTCTGTGTAGTTTTTATGTGAAGATATTTC

                     *    **        *   *                           *    *   
      36246 CTTTTCCACAATAGTTCTCAAAGCACTCAAAATATCCACTTGCAGATTCTACAAAAGGAGTATTT
          1 CTTTTCCACCATAGGCCTCAAAGCGCTCCAAATATCCACTTGCAGATTCTACAAAAAGAGTGTTT

                                                          *                  
      36311 CAAAACTGCTCAATCAAAAGAAAGGTTCAACTCTGTGAGATGAATGGACACATCACAAAGAAGTT
         66 CAAAACTGCTCAATCAAAAGAAAGGTTCAACTCTGTGAGATGAATGCACACATCACAAAGAAGTT

                                  *   *              
      36376 TCTCAGAATGCTTCTGTGTAGTATTTTTGTGAAGATATTT-
        131 TCTCAGAATGCTTCTGTGTAGTTTTTATGTGAAGATATTTC

                          *  * * * * ***             *            *     * *  
      36416 CTTTTCCACCATAGACCGCCAGGGGACACAAATATCCACTTTCAGATTCTACAACAAGAGAGGTT
          1 CTTTTCCACCATAGGCCTCAAAGCGCTCCAAATATCCACTTGCAGATTCTACAAAAAGAGTGTTT

                   *   *     *   ** *       *      *                      * *
      36481 CAAAACTACTCGATCAAGAGATGGTTTCAACTATGTGAGTTGAATGCACACATCACAAAGAACTA
         66 CAAAACTGCTCAATCAAAAGAAAGGTTCAACTCTGTGAGATGAATGCACACATCACAAAGAAGTT

             *  *    *                               
      36546 TGTCGGAATTCTTCTGTGTAGTTTTTATGTGAAGATATTTC
        131 TCTCAGAATGCTTCTGTGTAGTTTTTATGTGAAGATATTTC

                     *    * *      * *    *                  *               
      36587 CTTTTCCACAATAGACGTCAAAGTGATCCAGATATCCACTTGCAGATTCCACAAAAAGAGTGTTT
          1 CTTTTCCACCATAGGCCTCAAAGCGCTCCAAATATCCACTTGCAGATTCTACAAAAAGAGTGTTT

                 *   *   *                  **                     *  *      
      36652 CAAAAGTGCACAACCAAAAGAAAGGTTCAACTAGGTGAGATGAATGCACACATCAGAAGGAAGTT
         66 CAAAACTGCTCAATCAAAAGAAAGGTTCAACTCTGTGAGATGAATGCACACATCACAAAGAAGTT

                            **   *     * *       *   
      36717 TCTCAGAATGCTTCTGCATAGCTTTTAAGGGAAGATACTTC
        131 TCTCAGAATGCTTCTGTGTAGTTTTTATGTGAAGATATTTC

                    *               *            * *  *    * *               
      36758 CTTTTCCAACATAGGCCTCAAAGCACTCCAAATATCCTCCTGGAGATACCACAAAAAGAGTGTTT
          1 CTTTTCCACCATAGGCCTCAAAGCGCTCCAAATATCCACTTGCAGATTCTACAAAAAGAGTGTTT

            **                      *  *                 *       *           
      36823 GCAAACTGCTCAATCAAAAGAAAGATTTAACTCTGTGAGATGAATCCACACATGACAAAGAAGTT
         66 CAAAACTGCTCAATCAAAAGAAAGGTTCAACTCTGTGAGATGAATGCACACATCACAAAGAAGTT

                                                     
      36888 TCTCAGAATGCTTCTGTGTAGTTTTTATGTGAAGATATTTC
        131 TCTCAGAATGCTTCTGTGTAGTTTTTATGTGAAGATATTTC

                     *   * * *    **           *                *     *      
      36929 CTTTTCCACAATAAGACCCAAAAGGCTCCAAATATTCACTTGCAGATTCTAAAAAAAACAGTGTT
          1 CTTTTCCACCATAGGCCTCAAAGCGCTCCAAATATCCACTTGCAGATTCT-ACAAAAAGAGTGTT

                                  *                  *      *        **      
      36994 TCAAAACTGCTCAATCAAAAGATA-GTTCAACTCTGTGAGAAGAATGCTCACATCACTGAGAAGT
         65 TCAAAACTGCTCAATCAAAAGAAAGGTTCAACTCTGTGAGATGAATGCACACATCACAAAGAAGT

                                         *            
      37058 TTCTCAGAATGCTTCTGTGTAGTTTTTATATGAAGATATTTC
        130 TTCTCAGAATGCTTCTGTGTAGTTTTTATGTGAAGATATTTC

                *     *      *    **                       *   **       *    
      37100 CTTTCCCACCGTAGGCCACAAAAGGCTCCAAATATCCACTTGCAGATACTATGAAAAGAGAGTTT
          1 CTTTTCCACCATAGGCCTCAAAGCGCTCCAAATATCCACTTGCAGATTCTACAAAAAGAGTGTTT

                        *        *               ***            *            
      37165 CAAAACTGCTCATTCAAAAGATAGGTTCAACTCTGTGGTTTGAATGCACACAGCACAAAGAAGTT
         66 CAAAACTGCTCAATCAAAAGAAAGGTTCAACTCTGTGAGATGAATGCACACATCACAAAGAAGTT

              *       **                   **   *    
      37230 TCACAGAATGTGTCTGTGTAGTTTTTATGTGCGGATGTTTC
        131 TCTCAGAATGCTTCTGTGTAGTTTTTATGTGAAGATATTTC

                         *        ****   *   *               *    *          
      37271 CTTTTCCACCATATGCCT-AAATATTTCCCAATTTCCACTTGCAGATTCCACAAGAAGAGTGTTT
          1 CTTTTCCACCATAGGCCTCAAAGCGCTCCAAATATCCACTTGCAGATTCTACAAAAAGAGTGTTT

                      **       *       *           *            *      ***   
      37335 CAAAACTGCTGTATC-AAATAAA-GTTGAACTCTGTGAGGTGAATGCACACAGCACAAAATGGTT
         66 CAAAACTGCTCAATCAAAAGAAAGGTTCAACTCTGTGAGATGAATGCACACATCACAAAGAAGTT

                          **   *        *       *    
      37398 TCTCAGAATGCTTCCTTGTTGTTTTTATATGAAGATGTTTC
        131 TCTCAGAATGCTTCTGTGTAGTTTTTATGTGAAGATATTTC

                  *  *             *   *     *                               
      37439 CTTTTCAACAATAGGCCTCAAAGTGCTTCAAATGTCCACTTGCAGATTCTACAAAAAGAGTGTTT
          1 CTTTTCCACCATAGGCCTCAAAGCGCTCCAAATATCCACTTGCAGATTCTACAAAAAGAGTGTTT

                                        *      *  *  *                       
      37504 CAAAACTGCTCAATCAAAAGAAAGGTTCGACTCTGGGAAATTAATGCACACATCACAAAGAAGTT
         66 CAAAACTGCTCAATCAAAAGAAAGGTTCAACTCTGTGAGATGAATGCACACATCACAAAGAAGTT

                                     *        *      
      37569 TCTC---A-GCTTCTGTGTAGTTTTCATGTGAAGTTATTTC
        131 TCTCAGAATGCTTCTGTGTAGTTTTTATGTGAAGATATTTC

                     *       *     *            *    *         **       *    
      37606 CTTTTCCACAATAGGCCGCAAAGGGCTCCAAATATCAACTTACAGATTCTAGGAAAAGAGAGTTT
          1 CTTTTCCACCATAGGCCTCAAAGCGCTCCAAATATCCACTTGCAGATTCTACAAAAAGAGTGTTT

                       * **      *     *                                     
      37671 CAAAACTGCTCTACGAAAAGATAGGTTGAACTCTGTGAGATGAATGCACACATCACAAAGAAGTT
         66 CAAAACTGCTCAATCAAAAGAAAGGTTCAACTCTGTGAGATGAATGCACACATCACAAAGAAGTT

                       *               * *     *     
      37736 TCTCAGAATGCATCTGTGTAGTTTTTACGGGAAGACATTTC
        131 TCTCAGAATGCTTCTGTGTAGTTTTTATGTGAAGATATTTC

                        ***  *     **      *  *                  *     ***   
      37777 CTTTTCCACCATCTTCCACAAAGGTCTCCAAGTAACCACTTGCAGATTCTACAGAAAGACACTTT
          1 CTTTTCCACCATAGGCCTCAAAGCGCTCCAAATATCCACTTGCAGATTCTACAAAAAGAGTGTTT

            *          *         ***      *      ***                      *  
      37842 AAAAACTGCTCTATCAAAAGATCAGTTCAAGTCTGTGGTTTGAATGCACACATCACAAAGAATTT
         66 CAAAACTGCTCAATCAAAAGAAAGGTTCAACTCTGTGAGATGAATGCACACATCACAAAGAAGTT

                                     *  *            
      37907 TCTCAGAATGCTTCTGTGTAGTTTTCATATGAAGATATTTC
        131 TCTCAGAATGCTTCTGTGTAGTTTTTATGTGAAGATATTTC

                                    *                                   **   
      37948 CTTTTCCACCATAGGCCTCAAAGCACTCCAAATATCCACTTGCAGATTCTACAAAAAGAGATTTT
          1 CTTTTCCACCATAGGCCTCAAAGCGCTCCAAATATCCACTTGCAGATTCTACAAAAAGAGTGTTT

                   **                           *  *          *        *     
      38013 CAAAACTAGTCAATCAAAAGAAAGGTTCAACTCTGTCAGTTGAATGCACATATCACAAACAAGTT
         66 CAAAACTGCTCAATCAAAAGAAAGGTTCAACTCTGTGAGATGAATGCACACATCACAAAGAAGTT

                *      *                             
      38078 TCTCGGAATGCGTCTGTGTAGTTTTTATGTGAAGATATTTC
        131 TCTCAGAATGCTTCTGTGTAGTTTTTATGTGAAGATATTTC

               *     * *           *     *                  *    *           
      38119 CTTCTCCACAACAGGCCTCAAAGTGCTCCGAATATCCACTTGCAGATTTTAC-TAAAGAGTGTTT
          1 CTTTTCCACCATAGGCCTCAAAGCGCTCCAAATATCCACTTGCAGATTCTACAAAAAGAGTGTTT

             *               *  *   *     *        *    *               *    
      38183 CCAAACTGCTCAATCAAGAGGAAGTTTCAAGTCTGTGAGCTGAACGCACACATCACAAAGTAGTT
         66 CAAAACTGCTCAATCAAAAGAAAGGTTCAACTCTGTGAGATGAATGCACACATCACAAAGAAGTT

               *                                *    
      38248 TCTGAGAATGCTTCTGTGTAGTTTTTATGTGAAGATGTTTC
        131 TCTCAGAATGCTTCTGTGTAGTTTTTATGTGAAGATATTTC

                            **     *                                    *    
      38289 CTTTTCCACCATAGGCTGCAAAGGGCTCCAAATATCCACTTGCAGATTCTACAAAAAGAGAGTTT
          1 CTTTTCCACCATAGGCCTCAAAGCGCTCCAAATATCCACTTGCAGATTCTACAAAAAGAGTGTTT

                 *     *         *          *     *                     *    
      38354 CAAAAGTGCTCTATCAAAAGATAGGTTCAACTATGTGATATGAATGCACACATCACAAAGTAGTT
         66 CAAAACTGCTCAATCAAAAGAAAGGTTCAACTCTGTGAGATGAATGCACACATCACAAAGAAGTT

                                          *          
      38419 TCTCAGAATGCTTCTGTGTAGTTTTTATGTAAAGATATTTC
        131 TCTCAGAATGCTTCTGTGTAGTTTTTATGTGAAGATATTTC

                                    *                                   **   
      38460 CTTTTCCACCATAGGCCTCAAAGCACTCCAAATATCCACTTGCAGATTCTACAAAAAGAGATTTT
          1 CTTTTCCACCATAGGCCTCAAAGCGCTCCAAATATCCACTTGCAGATTCTACAAAAAGAGTGTTT

                   ** *                   *     *  *    * *   *        *     
      38525 CAAAACTATTTAATCAAAAGAAAGGTTCAAATCTGTCAGTTGAAGGTACATATCACAAACAAGTT
         66 CAAAACTGCTCAATCAAAAGAAAGGTTCAACTCTGTGAGATGAATGCACACATCACAAAGAAGTT

             * **                                    
      38590 TATTGGAATGCTTCTGTGTAGTTTTTATGTGAAGATATTTC
        131 TCTCAGAATGCTTCTGTGTAGTTTTTATGTGAAGATATTTC

                     * *       * * *                        **  *     *      
      38631 CTTTTCCACAACAGGCCTCCAGGTGCTCCAAATATCCACTTGCAGATTTCACTAAAAGTGTGTTT
          1 CTTTTCCACCATAGGCCTCAAAGCGCTCCAAATATCCACTTGCAGATTCTACAAAAAGAGTGTTT

             *  *            *  *   *     *        *             *           
      38696 CCAAGCTGCTCAATCAAGAGGAAGTTTCAAGTCTGTGAGGTGAATGCACACATTACAAAGAAGTT
         66 CAAAACTGCTCAATCAAAAGAAAGGTTCAACTCTGTGAGATGAATGCACACATCACAAAGAAGTT

            *  *                                     
      38761 ACTGAGAATGCTTCTGTGTAGTTTTTATGTGAAGATATTTC
        131 TCTCAGAATGCTTCTGTGTAGTTTTTATGTGAAGATATTTC

                      **               *                                *    
      38802 CTTTTCCACCGCAGGCCTCAAAGCGCTGCAAATATCCACTTGCAGATTCTACAAAAAGAGAGTTT
          1 CTTTTCCACCATAGGCCTCAAAGCGCTCCAAATATCCACTTGCAGATTCTACAAAAAGAGTGTTT

                      **         *   *         *   *     **            *     
      38867 CAAAACTGCTGTATCAAAAGATAGGGTCAACTCTGCGAGTTGAATAAACACATCACAAATAAGTT
         66 CAAAACTGCTCAATCAAAAGAAAGGTTCAACTCTGTGAGATGAATGCACACATCACAAAGAAGTT

               **   *        *               *       
      38932 TCTGGGAACGCTTCTGTATAG-TTTTATGTGAATATATTTC
        131 TCTCAGAATGCTTCTGTGTAGTTTTTATGTGAAGATATTTC

                         *          *                   *   *  *   * *   *   
      38972 CTTTTCCACCATATGCCTCAAAGCACTCCAAATATCCACTTGCACATTATAGAAACATAGTCTTT
          1 CTTTTCCACCATAGGCCTCAAAGCGCTCCAAATATCCACTTGCAGATTCTACAAAAAGAGTGTTT

                   **                        *         *             *       
      39037 CAAAACTTGTCAATC-AAAGAAAGGTTCAACTCCGTGAGATGAGTGCACACATCACAGAGAAGTT
         66 CAAAACTGCTCAATCAAAAGAAAGGTTCAACTCTGTGAGATGAATGCACACATCACAAAGAAGTT

                *     *                            * 
      39101 TCTCGGAATGTTTCTGTGTAGTTTTTATGTGAAGATATTGC
        131 TCTCAGAATGCTTCTGTGTAGTTTTTATGTGAAGATATTTC

                     *               *            *          *       *   *   
      39142 CTTTTCCACAATAGGCCTCAAAGCGTTCCAAATATCCAATTGCAGATTCCACAAAAAAAGTTTTT
          1 CTTTTCCACCATAGGCCTCAAAGCGCTCCAAATATCCACTTGCAGATTCTACAAAAAGAGTGTTT

            *                 *  *  *  *             * *         **    *     
      39207 TAAAACTGCTCAATCAAATGATAGATTAAACTCTGTGAGATTAGTGCACACATGTCAAAAAAGTT
         66 CAAAACTGCTCAATCAAAAGAAAGGTTCAACTCTGTGAGATGAATGCACACATCACAAAGAAGTT

                                *      * *           
      39272 TCTCAGAATGCTTCTGTGTACTTTTTAGGGGAAGATATTTC
        131 TCTCAGAATGCTTCTGTGTAGTTTTTATGTGAAGATATTTC

                        *    *     **         *    *           **  * *  *    
      39313 CTTTTCCACCATCGGCCACAAAGGACTCCAAATAACCACATGCAGATTCTAGTAACACAGAGTTT
          1 CTTTTCCACCATAGGCCTCAAAGCGCTCCAAATATCCACTTGCAGATTCTACAAAAAGAGTGTTT

                       *         * *           *   * * *    **    ***        
      39378 CAAAACTGCTCTATCAAAAGATAAGTTCAACTCTGAGAGTTTAGTGCAACCATCGTGAAGAAGTT
         66 CAAAACTGCTCAATCAAAAGAAAGGTTCAACTCTGTGAGATGAATGCACACATCACAAAGAAGTT

                            *     *                  
      39443 TCTCAGAATGCTTCTGAGTAGTGTTTATGTGAAGATATTTC
        131 TCTCAGAATGCTTCTGTGTAGTTTTTATGTGAAGATATTTC

                              *     *                      *           **    
      39484 CTTTTCCACCATAGGCCTGAAAGCCCTCCAAATATCCACTTGCAGATCCTACAAAAAGAAAGTTT
          1 CTTTTCCACCATAGGCCTCAAAGCGCTCCAAATATCCACTTGCAGATTCTACAAAAAGAGTGTTT

             *         **     *  *  *   *        **      *                   
      39549 CGAAA-TGCTCTCTCAAACGATAGTTTCGACTCTGTGGTATGAATACACACATCACAAAGAAGTT
         66 CAAAACTGCTCAATCAAAAGAAAGGTTCAACTCTGTGAGATGAATGCACACATCACAAAGAAGTT

                                       **            
      39613 TCTCAGAATGCTTCTGTGTAGTTTTTAAATGAAGATATTTC
        131 TCTCAGAATGCTTCTGTGTAGTTTTTATGTGAAGATATTTC

            *                       *          *     *                       
      39654 TTTTTCCACCATAGGCCTCAAAGCACTCCAAATATGCACTTCCAGATTCTACAAAAAGAGTGTTT
          1 CTTTTCCACCATAGGCCTCAAAGCGCTCCAAATATCCACTTGCAGATTCTACAAAAAGAGTGTTT

              *                 *       * *         **   *         *  * *    
      39719 CAGAACTGCTCAATCAAAAGGAAGGTTCCAGTC--TGAGACAAATACACACATCAAAAGGTAGTT
         66 CAAAACTGCTCAATCAAAAGAAAGGTTCAACTCTGTGAGATGAATGCACACATCACAAAGAAGTT

                                                    *
      39782 TCTCAGAATGCTTCTGTGTAGTTTTTATGTGAAGATATTTT
        131 TCTCAGAATGCTTCTGTGTAGTTTTTATGTGAAGATATTTC

             *               *    **    *     *      *  **   *          *    
      39823 CCTTTCCACCATAGGCCACAAATGGCTCTAAATACCCACTTACATTTTCCACAAAAAGAGAGTTT
          1 CTTTTCCACCATAGGCCTCAAAGCGCTCCAAATATCCACTTGCAGATTCTACAAAAAGAGTGTTT

                       * *    * *      *   *         *                       
      39888 CAAAACTGCTCTACCAAAGGTAA-GTTTAACGCTGTGAG-TTAA-G-A-ACATCACAAAGAAGTT
         66 CAAAACTGCTCAATCAAAAGAAAGGTTCAACTCTGTGAGATGAATGCACACATCACAAAGAAGTT

                                   *   *  *          
      39948 TCTCAGAATGCTTCTGTGTAGTTCTTACGTAAAGATATTTC
        131 TCTCAGAATGCTTCTGTGTAGTTTTTATGTGAAGATATTTC

                 *   *      ***    *                  *              **      
      39989 CTTTTACACAATAGGCAGAAAAGTGCTCCAAATATCCACTTGAAGATTCTACAAAAACCGTGTTT
          1 CTTTTCCACCATAGGCCTCAAAGCGCTCCAAATATCCACTTGCAGATTCTACAAAAAGAGTGTTT

                     **                                          *      *    
      40054 CAAAACTGCCGAATCAAAAGAAAGGTTCAACTCTGTGAGATGAATGCACACATAACAAAGGAGTT
         66 CAAAACTGCTCAATCAAAAGAAAGGTTCAACTCTGTGAGATGAATGCACACATCACAAAGAAGTT

                                 *      *      *    *
      40119 TCTCAGAATGCTTCTGTGTAGCTTTTATATGAAGACATTTA
        131 TCTCAGAATGCTTCTGTGTAGTTTTTATGTGAAGATATTTC

            *        * *            *   ***                     **        *  
      40160 GTTTTCCACAACAGGCCTCAAAGCTCTCTCCATATCCACTTGCAGATTCTACCGAAAGAGTGCTT
          1 CTTTTCCACCATAGGCCTCAAAGCGCTCCAAATATCCACTTGCAGATTCTACAAAAAGAGTGTTT

             *                   * **     *        *        *     **         
      40225 CCAAACTGCTCAATCAAAAGAGACATTCAAATCTGTGAGGTGAATGCAGACATCGTAAAGAAGTT
         66 CAAAACTGCTCAATCAAAAGAAAGGTTCAACTCTGTGAGATGAATGCACACATCACAAAGAAGTT

                                *     *       *    **
      40290 TCTCAGAATGCTTCTGTGTATTTTTTGTGTGAAGTTATTCG
        131 TCTCAGAATGCTTCTGTGTAGTTTTTATGTGAAGATATTTC

            *    *             *     *  *            **                 *    
      40331 TTTTTGCACCATAGGCCTCCAAGCGTTCTAAATATCCACTTCTAGATTCTACAAAAAGAGAGTTT
          1 CTTTTCCACCATAGGCCTCAAAGCGCTCCAAATATCCACTTGCAGATTCTACAAAAAGAGTGTTT

                   *         *            *        *    *   *                
      40396 CAAAACTACTC-A--AACA-AAAGGTTCAATTCTGTGAGTTGAAAGCAAACATCACAAAGAAGTT
         66 CAAAACTGCTCAATCAAAAGAAAGGTTCAACTCTGTGAGATGAATGCACACATCACAAAGAAGTT

                       *             *               
      40457 TCTCAGAATGCGTCTGTGTAGTTTTGATGTGAAGATATTTC
        131 TCTCAGAATGCTTCTGTGTAGTTTTTATGTGAAGATATTTC

                     **   ****     *                  *                      
      40498 CTTTT-CACAGTAGAATGCAAAGGGCTCCAAATATCCACTTGGAGATTCTACAAAAAGA--GTTT
          1 CTTTTCCACCATAGGCCTCAAAGCGCTCCAAATATCCACTTGCAGATTCTACAAAAAGAGTGTTT

                  *    **     *  *     *     ***   *     *               *   
      40560 CAAAACCGCTCTGTCAAATGATAGGTTGAACTCCCGGAGGTGAATACACACATCACAAAGAGGTT
         66 CAAAACTGCTCAATCAAAAGAAAGGTTCAACTCTGTGAGATGAATGCACACATCACAAAGAAGTT

                  *                       *  *       
      40625 TCTCAGCATGCTTCTGTGTAGTTTTTATGTAAACATATTTC
        131 TCTCAGAATGCTTCTGTGTAGTTTTTATGTGAAGATATTTC

             *    * *              *           *      * *       *   *    *   
      40666 CGTTTCTATCATAGGCCTCAAAGTGCTCCAAATATTCACTTGTACATTCTACCAAACGAGTATTT
          1 CTTTTCCACCATAGGCCTCAAAGCGCTCCAAATATCCACTTGCAGATTCTACAAAAAGAGTGTTT

                              * *         ** *    *        *            *    
      40731 CAAAACTGCTCAATCAAATGGAAGGTTCAAAACCGTGACATGAATGCCCACATCACAAAGTAGTT
         66 CAAAACTGCTCAATCAAAAGAAAGGTTCAACTCTGTGAGATGAATGCACACATCACAAAGAAGTT

                                                     
      40796 TCTCAGAATGCTTCTGTGTAGTTTTTATGTGAAGATATTTC
        131 TCTCAGAATGCTTCTGTGTAGTTTTTATGTGAAGATATTTC

                     * *    *      *    *       * *   **      *              
      40837 CTTTTCCACAACA-GCGTGCAAAACGCTTCAAATATGCCCTTAGAGATTCCACAAAAAGAGTGTT
          1 CTTTTCCACCATAGGCCT-CAAAGCGCTCCAAATATCCACTTGCAGATTCTACAAAAAGAGTGTT

              *     *               *  *                                  ** 
      40901 TCCAAACTACTCAAATCAAAA-AATGATTTCAACTCTGTGAGATGAATGCACACATCACAAACTA
         65 TCAAAACTGCTC-AATCAAAAGAAAG-GTTCAACTCTGTGAGATGAATGCACACATCACAAAGAA

                         *     ** *   * *   *       *   
      40965 GTTTCTCAGAATGTTTCTGCCTGGTTCTCATGCGAAGATAGTTC
        128 GTTTCTCAGAATGCTTCTGTGTAGTTTTTATGTGAAGATATTTC

                 *           *   * **              *                  * *    
      41009 CTTTTTCACCATAGGCCGCAATGTACTCCAAATATCCACCTGCAGATTCTAC-AAAAGTGAGTTT
          1 CTTTTCCACCATAGGCCTCAAAGCGCTCCAAATATCCACTTGCAGATTCTACAAAAAGAGTGTTT

                       *         ***    **         *        *      *       * 
      41073 CAAAACTGCTCTATCAAAAGATCAGTTCGTCTCTGTGAGTTGAATGCATACATCAAAAAGAAGCT
         66 CAAAACTGCTCAATCAAAAGAAAGGTTCAACTCTGTGAGATGAATGCACACATCACAAAGAAGTT

                 *             *      **         *   
      41138 TCTCAAAATGCTTCTGTGTGGTTTTTCGGTGAAGATAGTTC
        131 TCTCAGAATGCTTCTGTGTAGTTTTTATGTGAAGATATTTC

            *     *        *      * *                 *        *        *    
      41179 TTTTTCTACCATAGGTCTCAAACCACTCCAAATATCCACTTGTAGATTCTATAAAAAGGAATG-T
          1 CTTTTCCACCATAGGCCTCAAAGCGCTCCAAATATCCACTTGCAGATTCTACAAAAA-GAGTGTT

                  *        *    *    *      * *         *      *         *   
      41243 TCAAAATTGCTCAATAAAAATAAAGTTTCAACACCGTGAGATGAGTGCACAAATCACAAAGGAGT
         65 TCAAAACTGCTCAATCAAAAGAAAGGTTCAACTCTGTGAGATGAATGCACACATCACAAAGAAGT

                  *          *         *          *   
      41308 TTCTCAAAATGCTTCTGGGTAGTTTTTCTGTGAAGATAGTTC
        130 TTCTCAGAATGCTTCTGTGTAGTTTTTATGTGAAGATATTTC

                  *     *    *     *          *                         *    
      41350 CTTTTCTACCATGGGCCACAAAGGGCTCCAAATACCCACTTGCAGATTCTACAAAAAGAGAGTTT
          1 CTTTTCCACCATAGGCCTCAAAGCGCTCCAAATATCCACTTGCAGATTCTACAAAAAGAGTGTTT

              *        *      ** * *        *    * *    **  * *           *  
      41415 CACAACTGCTCTATCAAACAATATGTTCAACTTTGTGGGTTGAACACAAATATCAC-AAGAATTT
         66 CAAAACTGCTCAATCAAAAGAAAGGTTCAACTCTGTGAGATGAATGCACACATCACAAAGAAGTT

                **                             *     
      41479 TCTCCCAATGCTTCTGTGTAGTTTTTATGTGAAGACATTTC
        131 TCTCAGAATGCTTCTGTGTAGTTTTTATGTGAAGATATTTC

            *   *  *      *  *     *                 *  *      *       *  *  
      41520 TTTTCCCTCCATAGTCCACAAAGTGCTCCAAATATCCACTTACATATTCTAGAAAAAGATTGCTT
          1 CTTTTCCACCATAGGCCTCAAAGCGCTCCAAATATCCACTTGCAGATTCTACAAAAAGAGTGTTT

            **       *    *               * * *                              
      41585 GGAAACTGCACAATGAAAAGAAAGGTTCAAATATATGAGATGAATGCACACATCACAAAGAAGTT
         66 CAAAACTGCTCAATCAAAAGAAAGGTTCAACTCTGTGAGATGAATGCACACATCACAAAGAAGTT

                                 *                    
      41650 TCTCAGAAT-CTCTCTGTGTAATTTTTATGTGAAGATATTTC
        131 TCTCAGAATGCT-TCTGTGTAGTTTTTATGTGAAGATATTTC

                *     *    *  *   *                        *      *    *     
      41691 CTTTCCCACCTTAGGTCTTAAAACGCTCCAAATATCCACTTGCAGATACTACAAGAAGATTGTTT
          1 CTTTTCCACCATAGGCCTCAAAGCGCTCCAAATATCCACTTGCAGATTCTACAAAAAGAGTGTTT

                     *    *        *      *     **                           
      41756 CAAAACTGCACAA-AAAAAGAAATGTTCAATTCTGTTTGATGAATGCACACATCACAAAGAAGTT
         66 CAAAACTGCTCAATCAAAAGAAAGGTTCAACTCTGTGAGATGAATGCACACATCACAAAGAAGTT

                           *                         
      41820 TCTCAGAATGCTTCTCTGTAGTTTTTATGTGAAGATATTTC
        131 TCTCAGAATGCTTCTGTGTAGTTTTTATGTGAAGATATTTC

                     *             *                 *         **      * *   
      41861 CTTTTCCACAATAGGCCTCAAAGGGCTCCAAATATCCACTTCCAGATTCTATGAAAAGAATATTT
          1 CTTTTCCACCATAGGCCTCAAAGCGCTCCAAATATCCACTTGCAGATTCTACAAAAAGAGTGTTT

             *              * *    *                                      *  
      41926 CCAAACTGCTCAATCATAGGAAATGTTCAACTCTGTGAGATGAATGCACACATCAC-AAGAAATT
         66 CAAAACTGCTCAATCAAAAGAAAGGTTCAACTCTGTGAGATGAATGCACACATCACAAAGAAGTT

                     *    *      *       *       *   
      41990 TCTCAGAATCCTTCAGTGTAGGTTTTATGAGAAGATAATTC
        131 TCTCAGAATGCTTCTGTGTAGTTTTTATGTGAAGATATTTC

                     *    **        *   *                           *    *   
      42031 CTTTTCCACAATAGTTCTCAAAGCACTCAAAATATCCACTTGCAGATTCTACAAAAGGAGTATTT
          1 CTTTTCCACCATAGGCCTCAAAGCGCTCCAAATATCCACTTGCAGATTCTACAAAAAGAGTGTTT

                                                          *                  
      42096 CAAAACTGCTCAATCAAAAGAAAGGTTCAACTCTGTGAGATGAATGGACACATCACAAAGAAGTT
         66 CAAAACTGCTCAATCAAAAGAAAGGTTCAACTCTGTGAGATGAATGCACACATCACAAAGAAGTT

                                  *   *              
      42161 TCTCAGAATGCTTCTGTGTAGTATTTTTGTGAAGATATTT-
        131 TCTCAGAATGCTTCTGTGTAGTTTTTATGTGAAGATATTTC

                          *  * * * * ***             *            *     * *  
      42201 CTTTTCCACCATAGACCGCCAGGGGACACAAATATCCACTTTCAGATTCTACAACAAGAGAGGTT
          1 CTTTTCCACCATAGGCCTCAAAGCGCTCCAAATATCCACTTGCAGATTCTACAAAAAGAGTGTTT

                   *   *     *   ** *       *      *                      * *
      42266 CAAAACTACTCGATCAAGAGATGGTTTCAACTATGTGAGTTGAATGCACACATCACAAAGAACTA
         66 CAAAACTGCTCAATCAAAAGAAAGGTTCAACTCTGTGAGATGAATGCACACATCACAAAGAAGTT

             *  *    *                               
      42331 TGTCGGAATTCTTCTGTGTAGTTTTTATGTGAAGATATTTC
        131 TCTCAGAATGCTTCTGTGTAGTTTTTATGTGAAGATATTTC

                     *    * *      * *    *                  *               
      42372 CTTTTCCACAATAGACGTCAAAGTGATCCAGATATCCACTTGCAGATTCCACAAAAAGAGTGTTT
          1 CTTTTCCACCATAGGCCTCAAAGCGCTCCAAATATCCACTTGCAGATTCTACAAAAAGAGTGTTT

                 *   *   *                  **                     *  *      
      42437 CAAAAGTGCACAACCAAAAGAAAGGTTCAACTAGGTGAGATGAATGCACACATCAGAAGGAAGTT
         66 CAAAACTGCTCAATCAAAAGAAAGGTTCAACTCTGTGAGATGAATGCACACATCACAAAGAAGTT

                            **   *     * *       *   
      42502 TCTCAGAATGCTTCTGCATAGCTTTTAAGGGAAGATACTTC
        131 TCTCAGAATGCTTCTGTGTAGTTTTTATGTGAAGATATTTC

                    *               *            * *  *    * *               
      42543 CTTTTCCAACATAGGCCTCAAAGCACTCCAAATATCCTCCTGGAGATACCACAAAAAGAGTGTTT
          1 CTTTTCCACCATAGGCCTCAAAGCGCTCCAAATATCCACTTGCAGATTCTACAAAAAGAGTGTTT

            **                      *  *                 *       *           
      42608 GCAAACTGCTCAATCAAAAGAAAGATTTAACTCTGTGAGATGAATCCACACATGACAAAGAAGTT
         66 CAAAACTGCTCAATCAAAAGAAAGGTTCAACTCTGTGAGATGAATGCACACATCACAAAGAAGTT

                                                     
      42673 TCTCAGAATGCTTCTGTGTAGTTTTTATGTGAAGATATTTC
        131 TCTCAGAATGCTTCTGTGTAGTTTTTATGTGAAGATATTTC

                     *   * * *    **           *                *     *      
      42714 CTTTTCCACAATAAGACCCAAAAGGCTCCAAATATTCACTTGCAGATTCTAAAAAAAACAGTGTT
          1 CTTTTCCACCATAGGCCTCAAAGCGCTCCAAATATCCACTTGCAGATTCT-ACAAAAAGAGTGTT

                                  *                  *      *        **      
      42779 TCAAAACTGCTCAATCAAAAGATA-GTTCAACTCTGTGAGAAGAATGCTCACATCACTGAGAAGT
         65 TCAAAACTGCTCAATCAAAAGAAAGGTTCAACTCTGTGAGATGAATGCACACATCACAAAGAAGT

                                         *            
      42843 TTCTCAGAATGCTTCTGTGTAGTTTTTATATGAAGATATTTC
        130 TTCTCAGAATGCTTCTGTGTAGTTTTTATGTGAAGATATTTC

                *     *      *    **                       *   **       *    
      42885 CTTTCCCACCGTAGGCCACAAAAGGCTCCAAATATCCACTTGCAGATACTATGAAAAGAGAGTTT
          1 CTTTTCCACCATAGGCCTCAAAGCGCTCCAAATATCCACTTGCAGATTCTACAAAAAGAGTGTTT

                        *        *               ***            *            
      42950 CAAAACTGCTCATTCAAAAGATAGGTTCAACTCTGTGGTTTGAATGCACACAGCACAAAGAAGTT
         66 CAAAACTGCTCAATCAAAAGAAAGGTTCAACTCTGTGAGATGAATGCACACATCACAAAGAAGTT

              *       **                   **   *    
      43015 TCACAGAATGTGTCTGTGTAGTTTTTATGTGCGGATGTTTC
        131 TCTCAGAATGCTTCTGTGTAGTTTTTATGTGAAGATATTTC

                         *        ****   *   *               *    *          
      43056 CTTTTCCACCATATGCCT-AAATATTTCCCAATTTCCACTTGCAGATTCCACAAGAAGAGTGTTT
          1 CTTTTCCACCATAGGCCTCAAAGCGCTCCAAATATCCACTTGCAGATTCTACAAAAAGAGTGTTT

                      **       *       *           *            *      ***   
      43120 CAAAACTGCTGTATC-AAATAAA-GTTGAACTCTGTGAGGTGAATGCACACAGCACAAAATGGTT
         66 CAAAACTGCTCAATCAAAAGAAAGGTTCAACTCTGTGAGATGAATGCACACATCACAAAGAAGTT

                          **   *        *       *    
      43183 TCTCAGAATGCTTCCTTGTTGTTTTTATATGAAGATGTTTC
        131 TCTCAGAATGCTTCTGTGTAGTTTTTATGTGAAGATATTTC

                  *  *             *   *     *                               
      43224 CTTTTCAACAATAGGCCTCAAAGTGCTTCAAATGTCCACTTGCAGATTCTACAAAAAGAGTGTTT
          1 CTTTTCCACCATAGGCCTCAAAGCGCTCCAAATATCCACTTGCAGATTCTACAAAAAGAGTGTTT

                                        *      *  *  *                       
      43289 CAAAACTGCTCAATCAAAAGAAAGGTTCGACTCTGGGAAATTAATGCACACATCACAAAGAAGTT
         66 CAAAACTGCTCAATCAAAAGAAAGGTTCAACTCTGTGAGATGAATGCACACATCACAAAGAAGTT

                                     *        *      
      43354 TCTC---A-GCTTCTGTGTAGTTTTCATGTGAAGTTATTTC
        131 TCTCAGAATGCTTCTGTGTAGTTTTTATGTGAAGATATTTC

                     *       *     *            *    *         **       *    
      43391 CTTTTCCACAATAGGCCGCAAAGGGCTCCAAATATCAACTTACAGATTCTAGGAAAAGAGAGTTT
          1 CTTTTCCACCATAGGCCTCAAAGCGCTCCAAATATCCACTTGCAGATTCTACAAAAAGAGTGTTT

                       * **      *     *                                     
      43456 CAAAACTGCTCTACGAAAAGATAGGTTGAACTCTGTGAGATGAATGCACACATCACAAAGAAGTT
         66 CAAAACTGCTCAATCAAAAGAAAGGTTCAACTCTGTGAGATGAATGCACACATCACAAAGAAGTT

                       *               * *           
      43521 TCTCAGAATGCATCTGTGTAGTTTTTACGGGAAGATATTTC
        131 TCTCAGAATGCTTCTGTGTAGTTTTTATGTGAAGATATTTC

                        ***  *  *  **      *  *                  *     ***   
      43562 CTTTTCCACCATCTTCCACAGAGGTCTCCAAGTAACCACTTGCAGATTCTACAGAAAGACACTTT
          1 CTTTTCCACCATAGGCCTCAAAGCGCTCCAAATATCCACTTGCAGATTCTACAAAAAGAGTGTTT

            *          *         ***      *      ***                      *  
      43627 AAAAACTGCTCTATCAAAAGATCAGTTCAAGTCTGTGGTTTGAATGCACACATCACAAAGAATTT
         66 CAAAACTGCTCAATCAAAAGAAAGGTTCAACTCTGTGAGATGAATGCACACATCACAAAGAAGTT

                                     *  *            
      43692 TCTCAGAATGCTTCTGTGTAGTTTTCATATGAAGATATTTC
        131 TCTCAGAATGCTTCTGTGTAGTTTTTATGTGAAGATATTTC

                                    *                                   **   
      43733 CTTTTCCACCATAGGCCTCAAAGCACTCCAAATATCCACTTGCAGATTCTACAAAAAGAGATTTT
          1 CTTTTCCACCATAGGCCTCAAAGCGCTCCAAATATCCACTTGCAGATTCTACAAAAAGAGTGTTT

                   **                           *  *          *        *     
      43798 CAAAACTAGTCAATCAAAAGAAAGGTTCAACTCTGTCAGTTGAATGCACATATCACAAACAAGTT
         66 CAAAACTGCTCAATCAAAAGAAAGGTTCAACTCTGTGAGATGAATGCACACATCACAAAGAAGTT

                *      *                             
      43863 TCTCGGAATGCGTCTGTGTAGTTTTTATGTGAAGATATTTC
        131 TCTCAGAATGCTTCTGTGTAGTTTTTATGTGAAGATATTTC

               *     * *           *     *                  *    *           
      43904 CTTCTCCACAACAGGCCTCAAAGTGCTCCGAATATCCACTTGCAGATTTTAC-TAAAGAGTGTTT
          1 CTTTTCCACCATAGGCCTCAAAGCGCTCCAAATATCCACTTGCAGATTCTACAAAAAGAGTGTTT

             *               *  *   *     *        *    *               *    
      43968 CCAAACTGCTCAATCAAGAGGAAGTTTCAAGTCTGTGAGCTGAACGCACACATCACAAAGTAGTT
         66 CAAAACTGCTCAATCAAAAGAAAGGTTCAACTCTGTGAGATGAATGCACACATCACAAAGAAGTT

               *                                *    
      44033 TCTGAGAATGCTTCTGTGTAGTTTTTATGTGAAGATGTTTC
        131 TCTCAGAATGCTTCTGTGTAGTTTTTATGTGAAGATATTTC

                            **     *                                    *    
      44074 CTTTTCCACCATAGGCTGCAAAGGGCTCCAAATATCCACTTGCAGATTCTACAAAAAGAGAGTTT
          1 CTTTTCCACCATAGGCCTCAAAGCGCTCCAAATATCCACTTGCAGATTCTACAAAAAGAGTGTTT

                 *     *         *          *     *                     *    
      44139 CAAAAGTGCTCTATCAAAAGATAGGTTCAACTATGTGATATGAATGCACACATCACAAAGTAGTT
         66 CAAAACTGCTCAATCAAAAGAAAGGTTCAACTCTGTGAGATGAATGCACACATCACAAAGAAGTT

                                          *          
      44204 TCTCAGAATGCTTCTGTGTAGTTTTTATGTAAAGATATTTC
        131 TCTCAGAATGCTTCTGTGTAGTTTTTATGTGAAGATATTTC

                                    *                                   **   
      44245 CTTTTCCACCATAGGCCTCAAAGCACTCCAAATATCCACTTGCAGATTCTACAAAAAGAGATTTT
          1 CTTTTCCACCATAGGCCTCAAAGCGCTCCAAATATCCACTTGCAGATTCTACAAAAAGAGTGTTT

                   ** *                   *     *  *    * *   *        *     
      44310 CAAAACTATTTAATCAAAAGAAAGGTTCAAATCTGTCAGTTGAAGGTACATATCACAAACAAGTT
         66 CAAAACTGCTCAATCAAAAGAAAGGTTCAACTCTGTGAGATGAATGCACACATCACAAAGAAGTT

             * **                                    
      44375 TATTGGAATGCTTCTGTGTAGTTTTTATGTGAAGATATTTC
        131 TCTCAGAATGCTTCTGTGTAGTTTTTATGTGAAGATATTTC

                     * *         * *                        **  *     *      
      44416 CTTTTCCACAACAGGCCTCAAGGTGCTCCAAATATCCACTTGCAGATTTCACTAAAAGTGTGTTT
          1 CTTTTCCACCATAGGCCTCAAAGCGCTCCAAATATCCACTTGCAGATTCTACAAAAAGAGTGTTT

             *  *            *  *   *     *        *             *           
      44481 CCAAGCTGCTCAATCAAGAGGAAGTTTCAAGTCTGTGAGGTGAATGCACACATTACAAAGAAGTT
         66 CAAAACTGCTCAATCAAAAGAAAGGTTCAACTCTGTGAGATGAATGCACACATCACAAAGAAGTT

            *  *                                     
      44546 ACTGAGAATGCTTCTGTGTAGTTTTTATGTGAAGATATTTC
        131 TCTCAGAATGCTTCTGTGTAGTTTTTATGTGAAGATATTTC

                      **               *                                *    
      44587 CTTTTCCACCGCAGGCCTCAAAGCGCTGCAAATATCCACTTGCAGATTCTACAAAAAGAGAGTTT
          1 CTTTTCCACCATAGGCCTCAAAGCGCTCCAAATATCCACTTGCAGATTCTACAAAAAGAGTGTTT

                      **         *   *         *   *     ***           *     
      44652 CAAAACTGCTGTATCAAAAGATAGGGTCAACTCTGCGAGTTGAATAAGCACATCACAAATAAGTT
         66 CAAAACTGCTCAATCAAAAGAAAGGTTCAACTCTGTGAGATGAATGCACACATCACAAAGAAGTT

               **   *        *               *       
      44717 TCTGGGAACGCTTCTGTATAG-TTTTATGTGAATATATTTC
        131 TCTCAGAATGCTTCTGTGTAGTTTTTATGTGAAGATATTTC

                         *          *                   *   *  *   * *   *   
      44757 CTTTTCCACCATATGCCTCAAAGCACTCCAAATATCCACTTGCACATTATAGAAACATAGTCTTT
          1 CTTTTCCACCATAGGCCTCAAAGCGCTCCAAATATCCACTTGCAGATTCTACAAAAAGAGTGTTT

                   **                        *         *             *       
      44822 CAAAACTTGTCAATC-AAAGAAAGGTTCAACTCCGTGAGATGAGTGCACACATCACAGAGAAGTT
         66 CAAAACTGCTCAATCAAAAGAAAGGTTCAACTCTGTGAGATGAATGCACACATCACAAAGAAGTT

                *     *                            * 
      44886 TCTCGGAATGTTTCTGTGTAGTTTTTATGTGAAGATATTGC
        131 TCTCAGAATGCTTCTGTGTAGTTTTTATGTGAAGATATTTC

                     *               *            *          *       *   *   
      44927 CTTTTCCACAATAGGCCTCAAAGCGTTCCAAATATCCAATTGCAGATTCCACAAAAAAAGTTTTT
          1 CTTTTCCACCATAGGCCTCAAAGCGCTCCAAATATCCACTTGCAGATTCTACAAAAAGAGTGTTT

            *                 *  *  *  *             * *         **    *     
      44992 TAAAACTGCTCAATCAAATGATAGATTAAACTCTGTGAGATTAGTGCACACATGTCAAAAAAGTT
         66 CAAAACTGCTCAATCAAAAGAAAGGTTCAACTCTGTGAGATGAATGCACACATCACAAAGAAGTT

                                *      * *           
      45057 TCTCAGAATGCTTCTGTGTACTTTTTAGGGGAAGATATTTC
        131 TCTCAGAATGCTTCTGTGTAGTTTTTATGTGAAGATATTTC

                        *    *     **         *    *           **  * *  *    
      45098 CTTTTCCACCATCGGCCACAAAGGACTCCAAATAACCACATGCAGATTCTAGTAACACAGAGTTT
          1 CTTTTCCACCATAGGCCTCAAAGCGCTCCAAATATCCACTTGCAGATTCTACAAAAAGAGTGTTT

                       *         * *           *   * * *    **    ***        
      45163 CAAAACTGCTCTATCAAAAGATAAGTTCAACTCTGAGAGTTTAGTGCAACCATCGTGAAGAAGTT
         66 CAAAACTGCTCAATCAAAAGAAAGGTTCAACTCTGTGAGATGAATGCACACATCACAAAGAAGTT

                            *     *                  
      45228 TCTCAGAATGCTTCTGAGTAGTGTTTATGTGAAGATATTTC
        131 TCTCAGAATGCTTCTGTGTAGTTTTTATGTGAAGATATTTC

                              *     *                      *           **    
      45269 CTTTTCCACCATAGGCCTGAAAGCCCTCCAAATATCCACTTGCAGATCCTACAAAAAGAAAGTTT
          1 CTTTTCCACCATAGGCCTCAAAGCGCTCCAAATATCCACTTGCAGATTCTACAAAAAGAGTGTTT

             *         **     *  *  *   *        **      *                   
      45334 CGAAA-TGCTCTCTCAAACGATAGTTTCGACTCTGTGGTATGAATACACACATCACAAAGAAGTT
         66 CAAAACTGCTCAATCAAAAGAAAGGTTCAACTCTGTGAGATGAATGCACACATCACAAAGAAGTT

                                       **            
      45398 TCTCAGAATGCTTCTGTGTAGTTTTTAAATGAAGATATTTC
        131 TCTCAGAATGCTTCTGTGTAGTTTTTATGTGAAGATATTTC

            *                       *          *     *                       
      45439 TTTTTCCACCATAGGCCTCAAAGCACTCCAAATATGCACTTCCAGATTCTACAAAAAGAGTGTTT
          1 CTTTTCCACCATAGGCCTCAAAGCGCTCCAAATATCCACTTGCAGATTCTACAAAAAGAGTGTTT

              *                 *       * *         **   *         *  * *    
      45504 CAGAACTGCTCAATCAAAAGGAAGGTTCCAGTC--TGAGACAAATACACACATCAAAAGGTAGTT
         66 CAAAACTGCTCAATCAAAAGAAAGGTTCAACTCTGTGAGATGAATGCACACATCACAAAGAAGTT

                                                    *
      45567 TCTCAGAATGCTTCTGTGTAGTTTTTATGTGAAGATATTTT
        131 TCTCAGAATGCTTCTGTGTAGTTTTTATGTGAAGATATTTC

             *               *    **    *     *      *  **   *          *    
      45608 CCTTTCCACCATAGGCCACAAATGGCTCTAAATACCCACTTACATTTTCCACAAAAAGAGAGTTT
          1 CTTTTCCACCATAGGCCTCAAAGCGCTCCAAATATCCACTTGCAGATTCTACAAAAAGAGTGTTT

                       * *    * *      *   *         *                       
      45673 CAAAACTGCTCTACCAAAGGTAA-GTTTAACGCTGTGAG-TTAA-G-A-ACATCACAAAGAAGTT
         66 CAAAACTGCTCAATCAAAAGAAAGGTTCAACTCTGTGAGATGAATGCACACATCACAAAGAAGTT

                                   *   *  *          
      45733 TCTCAGAATGCTTCTGTGTAGTTCTTACGTAAAGATATTTC
        131 TCTCAGAATGCTTCTGTGTAGTTTTTATGTGAAGATATTTC

                 *   *      ***    *                  *              **      
      45774 CTTTTACACAATAGGCAGAAAAGTGCTCCAAATATCCACTTGAAGATTCTACAAAAACCGTGTTT
          1 CTTTTCCACCATAGGCCTCAAAGCGCTCCAAATATCCACTTGCAGATTCTACAAAAAGAGTGTTT

                     **                                          *      *    
      45839 CAAAACTGCCGAATCAAAAGAAAGGTTCAACTCTGTGAGATGAATGCACACATAACAAAGGAGTT
         66 CAAAACTGCTCAATCAAAAGAAAGGTTCAACTCTGTGAGATGAATGCACACATCACAAAGAAGTT

                                 *      *      *    *
      45904 TCTCAGAATGCTTCTGTGTAGCTTTTATATGAAGACATTTA
        131 TCTCAGAATGCTTCTGTGTAGTTTTTATGTGAAGATATTTC

            *        * *            *   ***                     **        *  
      45945 GTTTTCCACAACAGGCCTCAAAGCTCTCTCCATATCCACTTGCAGATTCTACCGAAAGAGTGCTT
          1 CTTTTCCACCATAGGCCTCAAAGCGCTCCAAATATCCACTTGCAGATTCTACAAAAAGAGTGTTT

             *                   * **     *        *        *     **         
      46010 CCAAACTGCTCAATCAAAAGAGACATTCAAATCTGTGAGGTGAATGCAGACATCGTAAAGAAGTT
         66 CAAAACTGCTCAATCAAAAGAAAGGTTCAACTCTGTGAGATGAATGCACACATCACAAAGAAGTT

                                *     *       *    **
      46075 TCTCAGAATGCTTCTGTGTATTTTTTGTGTGAAGTTATTCG
        131 TCTCAGAATGCTTCTGTGTAGTTTTTATGTGAAGATATTTC

            *    *             *     *  *            **                 *    
      46116 TTTTTGCACCATAGGCCTCCAAGCGTTCTAAATATCCACTTCTAGATTCTACAAAAAGAGAGTTT
          1 CTTTTCCACCATAGGCCTCAAAGCGCTCCAAATATCCACTTGCAGATTCTACAAAAAGAGTGTTT

                   *         *            *        *    *   *                
      46181 CAAAACTACTC-A--AACA-AAAGGTTCAATTCTGTGAGTTGAAAGCAAACATCACAAAGAAGTT
         66 CAAAACTGCTCAATCAAAAGAAAGGTTCAACTCTGTGAGATGAATGCACACATCACAAAGAAGTT

                       *             *               
      46242 TCTCAGAATGCGTCTGTGTAGTTTTGATGTGAAGATATTTC
        131 TCTCAGAATGCTTCTGTGTAGTTTTTATGTGAAGATATTTC

                     **   ****     *                  *                      
      46283 CTTTT-CACAGTAGAATGCAAAGGGCTCCAAATATCCACTTGGAGATTCTACAAAAAGA--GTTT
          1 CTTTTCCACCATAGGCCTCAAAGCGCTCCAAATATCCACTTGCAGATTCTACAAAAAGAGTGTTT

                  *    **     *  *     *     ***   *     *               *   
      46345 CAAAACCGCTCTGTCAAATGATAGGTTGAACTCCCGGAGGTGAATACACACATCACAAAGAGGTT
         66 CAAAACTGCTCAATCAAAAGAAAGGTTCAACTCTGTGAGATGAATGCACACATCACAAAGAAGTT

                  *                       *  *       
      46410 TCTCAGCATGCTTCTGTGTAGTTTTTATGTAAACATATTTC
        131 TCTCAGAATGCTTCTGTGTAGTTTTTATGTGAAGATATTTC

             *    * *              *           *      * *       *   *    *   
      46451 CGTTTCTATCATAGGCCTCAAAGTGCTCCAAATATTCACTTGTACATTCTACCAAACGAGTATTT
          1 CTTTTCCACCATAGGCCTCAAAGCGCTCCAAATATCCACTTGCAGATTCTACAAAAAGAGTGTTT

                              * *         ** *    *        *            *    
      46516 CAAAACTGCTCAATCAAATGGAAGGTTCAAAACCGTGACATGAATGCCCACATCACAAAGTAGTT
         66 CAAAACTGCTCAATCAAAAGAAAGGTTCAACTCTGTGAGATGAATGCACACATCACAAAGAAGTT

                                                     
      46581 TCTCAGAATGCTTCTGTGTAGTTTTTATGTGAAGATATTTC
        131 TCTCAGAATGCTTCTGTGTAGTTTTTATGTGAAGATATTTC

                     * *    *      *    *       * *   **      *              
      46622 CTTTTCCACAACA-GCGTGCAAAACGCTTCAAATATGCCCTTAGAGATTCCACAAAAAGAGTGTT
          1 CTTTTCCACCATAGGCCT-CAAAGCGCTCCAAATATCCACTTGCAGATTCTACAAAAAGAGTGTT

              *     *               *  *                                  ** 
      46686 TCCAAACTACTCAAATCAAAA-AATGATTTCAACTCTGTGAGATGAATGCACACATCACAAACTA
         65 TCAAAACTGCTC-AATCAAAAGAAAG-GTTCAACTCTGTGAGATGAATGCACACATCACAAAGAA

                         *     ** *   * *   *       *   
      46750 GTTTCTCAGAATGTTTCTGCCTGGTTCTCATGCGAAGATAGTTC
        128 GTTTCTCAGAATGCTTCTGTGTAGTTTTTATGTGAAGATATTTC

                 *           *   * **              *                  * *    
      46794 CTTTTTCACCATAGGCCGCAATGTACTCCAAATATCCACCTGCAGATTCTAC-AAAAGTGAGTTT
          1 CTTTTCCACCATAGGCCTCAAAGCGCTCCAAATATCCACTTGCAGATTCTACAAAAAGAGTGTTT

                       *         ***    **         *        *      *       * 
      46858 CAAAACTGCTCTATCAAAAGATCAGTTCGTCTCTGTGAGTTGAATGCATACATCAAAAAGAAGCT
         66 CAAAACTGCTCAATCAAAAGAAAGGTTCAACTCTGTGAGATGAATGCACACATCACAAAGAAGTT

                 *             *      **         *   
      46923 TCTCAAAATGCTTCTGTGTGGTTTTTCGGTGAAGATAGTTC
        131 TCTCAGAATGCTTCTGTGTAGTTTTTATGTGAAGATATTTC

            *     *        *      * *                 *        *        *    
      46964 TTTTTCTACCATAGGTCTCAAACCACTCCAAATATCCACTTGTAGATTCTATAAAAAGGAATG-T
          1 CTTTTCCACCATAGGCCTCAAAGCGCTCCAAATATCCACTTGCAGATTCTACAAAAA-GAGTGTT

                  *        *    *    *      * *         *      *         *   
      47028 TCAAAATTGCTCAATAAAAATAAAGTTTCAACACCGTGAGATGAGTGCACAAATCACAAAGGAGT
         65 TCAAAACTGCTCAATCAAAAGAAAGGTTCAACTCTGTGAGATGAATGCACACATCACAAAGAAGT

                  *          *         *          *   
      47093 TTCTCAAAATGCTTCTGGGTAGTTTTTCTGTGAAGATAGTTC
        130 TTCTCAGAATGCTTCTGTGTAGTTTTTATGTGAAGATATTTC

                  *     *    *     *          *                         *    
      47135 CTTTTCTACCATGGGCCACAAAGGGCTCCAAATACCCACTTGCAGATTCTACAAAAAGAGAGTTT
          1 CTTTTCCACCATAGGCCTCAAAGCGCTCCAAATATCCACTTGCAGATTCTACAAAAAGAGTGTTT

              *        *      ** * *        *    * *    **  * *           *  
      47200 CACAACTGCTCTATCAAACAATATGTTCAACTTTGTGGGTTGAACACAAATATCAC-AAGAATTT
         66 CAAAACTGCTCAATCAAAAGAAAGGTTCAACTCTGTGAGATGAATGCACACATCACAAAGAAGTT

                **                             *     
      47264 TCTCCCAATGCTTCTGTGTAGTTTTTATGTGAAGACATTTC
        131 TCTCAGAATGCTTCTGTGTAGTTTTTATGTGAAGATATTTC

            *   *  *      *  *     *                 *  *      *       *  *  
      47305 TTTTCCCTCCATAGTCCACAAAGTGCTCCAAATATCCACTTACATATTCTAGAAAAAGATTGCTT
          1 CTTTTCCACCATAGGCCTCAAAGCGCTCCAAATATCCACTTGCAGATTCTACAAAAAGAGTGTTT

            **       *    *               * * *                              
      47370 GGAAACTGCACAATGAAAAGAAAGGTTCAAATATATGAGATGAATGCACACATCACAAAGAAGTT
         66 CAAAACTGCTCAATCAAAAGAAAGGTTCAACTCTGTGAGATGAATGCACACATCACAAAGAAGTT

                                 *                    
      47435 TCTCAGAAT-CTCTCTGTGTAATTTTTATGTGAAGATATTTC
        131 TCTCAGAATGCT-TCTGTGTAGTTTTTATGTGAAGATATTTC

                *     *    *  *   *                        *      *    *     
      47476 CTTTCCCACCTTAGGTCTTAAAACGCTCCAAATATCCACTTGCAGATACTACAAGAAGATTGTTT
          1 CTTTTCCACCATAGGCCTCAAAGCGCTCCAAATATCCACTTGCAGATTCTACAAAAAGAGTGTTT

                     *    *        *      *     **                           
      47541 CAAAACTGCACAA-AAAAAGAAATGTTCAATTCTGTTTGATGAATGCACACATCACAAAGAAGTT
         66 CAAAACTGCTCAATCAAAAGAAAGGTTCAACTCTGTGAGATGAATGCACACATCACAAAGAAGTT

                           *                         
      47605 TCTCAGAATGCTTCTCTGTAGTTTTTATGTGAAGATATTTC
        131 TCTCAGAATGCTTCTGTGTAGTTTTTATGTGAAGATATTTC

                     *             *                 *         **      * *   
      47646 CTTTTCCACAATAGGCCTCAAAGGGCTCCAAATATCCACTTCCAGATTCTATGAAAAGAATATTT
          1 CTTTTCCACCATAGGCCTCAAAGCGCTCCAAATATCCACTTGCAGATTCTACAAAAAGAGTGTTT

             *              * *    *                                      *  
      47711 CCAAACTGCTCAATCATAGGAAATGTTCAACTCTGTGAGATGAATGCACACATCAC-AAGAAATT
         66 CAAAACTGCTCAATCAAAAGAAAGGTTCAACTCTGTGAGATGAATGCACACATCACAAAGAAGTT

                     *    *      *       *       *   
      47775 TCTCAGAATCCTTCAGTGTAGGTTTTATGAGAAGATAATTC
        131 TCTCAGAATGCTTCTGTGTAGTTTTTATGTGAAGATATTTC

                     *    **        *   *                           *    *   
      47816 CTTTTCCACAATAGTTCTCAAAGCACTCAAAATATCCACTTGCAGATTCTACAAAAGGAGTATTT
          1 CTTTTCCACCATAGGCCTCAAAGCGCTCCAAATATCCACTTGCAGATTCTACAAAAAGAGTGTTT

                                                          *                  
      47881 CAAAACTGCTCAATCAAAAGAAAGGTTCAACTCTGTGAGATGAATGGACACATCACAAAGAAGTT
         66 CAAAACTGCTCAATCAAAAGAAAGGTTCAACTCTGTGAGATGAATGCACACATCACAAAGAAGTT

                                  *   *              
      47946 TCTCAGAATGCTTCTGTGTAGTATTTTTGTGAAGATATTT-
        131 TCTCAGAATGCTTCTGTGTAGTTTTTATGTGAAGATATTTC

                          *  * * * * ***             *            *     * *  
      47986 CTTTTCCACCATAGACCGCCAGGGGACACAAATATCCACTTTCAGATTCTACAACAAGAGAGGTT
          1 CTTTTCCACCATAGGCCTCAAAGCGCTCCAAATATCCACTTGCAGATTCTACAAAAAGAGTGTTT

                   *   *     *   ** *       *      *                      * *
      48051 CAAAACTACTCGATCAAGAGATGGTTTCAACTATGTGAGTTGAATGCACACATCACAAAGAACTA
         66 CAAAACTGCTCAATCAAAAGAAAGGTTCAACTCTGTGAGATGAATGCACACATCACAAAGAAGTT

             *  *    *                               
      48116 TGTCGGAATTCTTCTGTGTAGTTTTTATGTGAAGATATTTC
        131 TCTCAGAATGCTTCTGTGTAGTTTTTATGTGAAGATATTTC

                     *    * *      * *    *                  *               
      48157 CTTTTCCACAATAGACGTCAAAGTGATCCAGATATCCACTTGCAGATTCCACAAAAAGAGTGTTT
          1 CTTTTCCACCATAGGCCTCAAAGCGCTCCAAATATCCACTTGCAGATTCTACAAAAAGAGTGTTT

                 *   *   *                  **                     *  *      
      48222 CAAAAGTGCACAACCAAAAGAAAGGTTCAACTAGGTGAGATGAATGCACACATCAGAAGGAAGTT
         66 CAAAACTGCTCAATCAAAAGAAAGGTTCAACTCTGTGAGATGAATGCACACATCACAAAGAAGTT

                            **   *     * *       *   
      48287 TCTCAGAATGCTTCTGCATAGCTTTTAAGGGAAGATACTTC
        131 TCTCAGAATGCTTCTGTGTAGTTTTTATGTGAAGATATTTC

                    *               *            * *  *    * *               
      48328 CTTTTCCAACATAGGCCTCAAAGCACTCCAAATATCCTCCTGGAGATACCACAAAAAGAGTGTTT
          1 CTTTTCCACCATAGGCCTCAAAGCGCTCCAAATATCCACTTGCAGATTCTACAAAAAGAGTGTTT

            **                      *  *                 *       *           
      48393 GCAAACTGCTCAATCAAAAGAAAGATTTAACTCTGTGAGATGAATCCACACATGACAAAGAAGTT
         66 CAAAACTGCTCAATCAAAAGAAAGGTTCAACTCTGTGAGATGAATGCACACATCACAAAGAAGTT

                                                     
      48458 TCTCAGAATGCTTCTGTGTAGTTTTTATGTGAAGATATTTC
        131 TCTCAGAATGCTTCTGTGTAGTTTTTATGTGAAGATATTTC

                     *   * * *    **           *                *     *      
      48499 CTTTTCCACAATAAGACCCAAAAGGCTCCAAATATTCACTTGCAGATTCTAAAAAAAACAGTGTT
          1 CTTTTCCACCATAGGCCTCAAAGCGCTCCAAATATCCACTTGCAGATTCT-ACAAAAAGAGTGTT

                                  *                  *      *        **      
      48564 TCAAAACTGCTCAATCAAAAGATA-GTTCAACTCTGTGAGAAGAATGCTCACATCACTGAGAAGT
         65 TCAAAACTGCTCAATCAAAAGAAAGGTTCAACTCTGTGAGATGAATGCACACATCACAAAGAAGT

                                         *            
      48628 TTCTCAGAATGCTTCTGTGTAGTTTTTATATGAAGATATTTC
        130 TTCTCAGAATGCTTCTGTGTAGTTTTTATGTGAAGATATTTC

                *     *      *    **                       *   **       *    
      48670 CTTTCCCACCGTAGGCCACAAAAGGCTCCAAATATCCACTTGCAGATACTATGAAAAGAGAGTTT
          1 CTTTTCCACCATAGGCCTCAAAGCGCTCCAAATATCCACTTGCAGATTCTACAAAAAGAGTGTTT

                        *        *               ***            *            
      48735 CAAAACTGCTCATTCAAAAGATAGGTTCAACTCTGTGGTTTGAATGCACACAGCACAAAGAAGTT
         66 CAAAACTGCTCAATCAAAAGAAAGGTTCAACTCTGTGAGATGAATGCACACATCACAAAGAAGTT

              *       **                   **   *    
      48800 TCACAGAATGTGTCTGTGTAGTTTTTATGTGCGGATGTTTC
        131 TCTCAGAATGCTTCTGTGTAGTTTTTATGTGAAGATATTTC

                         *        ****   *   *               *    *          
      48841 CTTTTCCACCATATGCCT-AAATATTTCCCAATTTCCACTTGCAGATTCCACAAGAAGAGTGTTT
          1 CTTTTCCACCATAGGCCTCAAAGCGCTCCAAATATCCACTTGCAGATTCTACAAAAAGAGTGTTT

                      **       *       *           *            *      ***   
      48905 CAAAACTGCTGTATC-AAATAAA-GTTGAACTCTGTGAGGTGAATGCACACAGCACAAAATGGTT
         66 CAAAACTGCTCAATCAAAAGAAAGGTTCAACTCTGTGAGATGAATGCACACATCACAAAGAAGTT

                          **   *        *       *    
      48968 TCTCAGAATGCTTCCTTGTTGTTTTTATATGAAGATGTTTC
        131 TCTCAGAATGCTTCTGTGTAGTTTTTATGTGAAGATATTTC

                  *  *             *   *     *                               
      49009 CTTTTCAACAATAGGCCTCAAAGTGCTTCAAATGTCCACTTGCAGATTCTACAAAAAGAGTGTTT
          1 CTTTTCCACCATAGGCCTCAAAGCGCTCCAAATATCCACTTGCAGATTCTACAAAAAGAGTGTTT

                                        *      *  *  *                       
      49074 CAAAACTGCTCAATCAAAAGAAAGGTTCGACTCTGGGAAATTAATGCACACATCACAAAGAAGTT
         66 CAAAACTGCTCAATCAAAAGAAAGGTTCAACTCTGTGAGATGAATGCACACATCACAAAGAAGTT

                                     *        *      
      49139 TCTC---A-GCTTCTGTGTAGTTTTCATGTGAAGTTATTTC
        131 TCTCAGAATGCTTCTGTGTAGTTTTTATGTGAAGATATTTC

                     *       *     *            *    *         **       *    
      49176 CTTTTCCACAATAGGCCGCAAAGGGCTCCAAATATCAACTTACAGATTCTAGGAAAAGAGAGTTT
          1 CTTTTCCACCATAGGCCTCAAAGCGCTCCAAATATCCACTTGCAGATTCTACAAAAAGAGTGTTT

                       * **      *     *                                     
      49241 CAAAACTGCTCTACGAAAAGATAGGTTGAACTCTGTGAGATGAATGCACACATCACAAAGAAGTT
         66 CAAAACTGCTCAATCAAAAGAAAGGTTCAACTCTGTGAGATGAATGCACACATCACAAAGAAGTT

                       *               * *     *     
      49306 TCTCAGAATGCATCTGTGTAGTTTTTACGGGAAGACATTTC
        131 TCTCAGAATGCTTCTGTGTAGTTTTTATGTGAAGATATTTC

                        ***  *     **      *  *                  *     ***   
      49347 CTTTTCCACCATCTTCCACAAAGGTCTCCAAGTAACCACTTGCAGATTCTACAGAAAGACACTTT
          1 CTTTTCCACCATAGGCCTCAAAGCGCTCCAAATATCCACTTGCAGATTCTACAAAAAGAGTGTTT

            *          *         ***      *      ***                      *  
      49412 AAAAACTGCTCTATCAAAAGATCAGTTCAAGTCTGTGGTTTGAATGCACACATCACAAAGAATTT
         66 CAAAACTGCTCAATCAAAAGAAAGGTTCAACTCTGTGAGATGAATGCACACATCACAAAGAAGTT

                                     *  *            
      49477 TCTCAGAATGCTTCTGTGTAGTTTTCATATGAAGATATTTC
        131 TCTCAGAATGCTTCTGTGTAGTTTTTATGTGAAGATATTTC

                                    *                                   **   
      49518 CTTTTCCACCATAGGCCTCAAAGCACTCCAAATATCCACTTGCAGATTCTACAAAAAGAGATTTT
          1 CTTTTCCACCATAGGCCTCAAAGCGCTCCAAATATCCACTTGCAGATTCTACAAAAAGAGTGTTT

                   **                           *  *          *        *     
      49583 CAAAACTAGTCAATCAAAAGAAAGGTTCAACTCTGTCAGTTGAATGCACATATCACAAACAAGTT
         66 CAAAACTGCTCAATCAAAAGAAAGGTTCAACTCTGTGAGATGAATGCACACATCACAAAGAAGTT

                *      *                             
      49648 TCTCGGAATGCGTCTGTGTAGTTTTTATGTGAAGATATTTC
        131 TCTCAGAATGCTTCTGTGTAGTTTTTATGTGAAGATATTTC

               *     * *           *     *                  *    *           
      49689 CTTCTCCACAACAGGCCTCAAAGTGCTCCGAATATCCACTTGCAGATTTTAC-TAAAGAGTGTTT
          1 CTTTTCCACCATAGGCCTCAAAGCGCTCCAAATATCCACTTGCAGATTCTACAAAAAGAGTGTTT

             *               *  *   *     *        *    *               *    
      49753 CCAAACTGCTCAATCAAGAGGAAGTTTCAAGTCTGTGAGCTGAACGCACACATCACAAAGTAGTT
         66 CAAAACTGCTCAATCAAAAGAAAGGTTCAACTCTGTGAGATGAATGCACACATCACAAAGAAGTT

               *                                *    
      49818 TCTGAGAATGCTTCTGTGTAGTTTTTATGTGAAGATGTTTC
        131 TCTCAGAATGCTTCTGTGTAGTTTTTATGTGAAGATATTTC

                            **     *                                    *    
      49859 CTTTTCCACCATAGGCTGCAAAGGGCTCCAAATATCCACTTGCAGATTCTACAAAAAGAGAGTTT
          1 CTTTTCCACCATAGGCCTCAAAGCGCTCCAAATATCCACTTGCAGATTCTACAAAAAGAGTGTTT

                 *     *         *          *     *                     *    
      49924 CAAAAGTGCTCTATCAAAAGATAGGTTCAACTATGTGATATGAATGCACACATCACAAAGTAGTT
         66 CAAAACTGCTCAATCAAAAGAAAGGTTCAACTCTGTGAGATGAATGCACACATCACAAAGAAGTT

                                          *          
      49989 TCTCAGAATGCTTCTGTGTAGTTTTTATGTAAAGATATTTC
        131 TCTCAGAATGCTTCTGTGTAGTTTTTATGTGAAGATATTTC

                                    *                                   **   
      50030 CTTTTCCACCATAGGCCTCAAAGCACTCCAAATATCCACTTGCAGATTCTACAAAAAGAGATTTT
          1 CTTTTCCACCATAGGCCTCAAAGCGCTCCAAATATCCACTTGCAGATTCTACAAAAAGAGTGTTT

                   ** *                   *     *  *    * *   *        *     
      50095 CAAAACTATTTAATCAAAAGAAAGGTTCAAATCTGTCAGTTGAAGGTACATATCACAAACAAGTT
         66 CAAAACTGCTCAATCAAAAGAAAGGTTCAACTCTGTGAGATGAATGCACACATCACAAAGAAGTT

             * **                                    
      50160 TATTGGAATGCTTCTGTGTAGTTTTTATGTGAAGATATTTC
        131 TCTCAGAATGCTTCTGTGTAGTTTTTATGTGAAGATATTTC

                     * *         * *                        **  *     *      
      50201 CTTTTCCACAACAGGCCTCAAGGTGCTCCAAATATCCACTTGCAGATTTCACTAAAAGTGTGTTT
          1 CTTTTCCACCATAGGCCTCAAAGCGCTCCAAATATCCACTTGCAGATTCTACAAAAAGAGTGTTT

             *  *            *  *   *     *        *             *           
      50266 CCAAGCTGCTCAATCAAGAGGAAGTTTCAAGTCTGTGAGGTGAATGCACACATTACAAAGAAGTT
         66 CAAAACTGCTCAATCAAAAGAAAGGTTCAACTCTGTGAGATGAATGCACACATCACAAAGAAGTT

            *  *                                     
      50331 ACTGAGAATGCTTCTGTGTAGTTTTTATGTGAAGATATTTC
        131 TCTCAGAATGCTTCTGTGTAGTTTTTATGTGAAGATATTTC

                      **               *                                *    
      50372 CTTTTCCACCGCAGGCCTCAAAGCGCTGCAAATATCCACTTGCAGATTCTACAAAAAGAGAGTTT
          1 CTTTTCCACCATAGGCCTCAAAGCGCTCCAAATATCCACTTGCAGATTCTACAAAAAGAGTGTTT

                      **         *   *         *   *     **            *     
      50437 CAAAACTGCTGTATCAAAAGATAGGGTCAACTCTGCGAGTTGAATAAACACATCACAAATAAGTT
         66 CAAAACTGCTCAATCAAAAGAAAGGTTCAACTCTGTGAGATGAATGCACACATCACAAAGAAGTT

               **   *        *               *       
      50502 TCTGGGAACGCTTCTGTATAG-TTTTATGTGAATATATTTC
        131 TCTCAGAATGCTTCTGTGTAGTTTTTATGTGAAGATATTTC

                         *          *                   *   *  *   * *   *   
      50542 CTTTTCCACCATATGCCTCAAAGCACTCCAAATATCCACTTGCACATTATAGAAACATAGTCTTT
          1 CTTTTCCACCATAGGCCTCAAAGCGCTCCAAATATCCACTTGCAGATTCTACAAAAAGAGTGTTT

                   **                        *         *             *       
      50607 CAAAACTTGTCAATC-AAAGAAAGGTTCAACTCCGTGAGATGAGTGCACACATCACAGAGAAGTT
         66 CAAAACTGCTCAATCAAAAGAAAGGTTCAACTCTGTGAGATGAATGCACACATCACAAAGAAGTT

                *     *                            * 
      50671 TCTCGGAATGTTTCTGTGTAGTTTTTATGTGAAGATATTGC
        131 TCTCAGAATGCTTCTGTGTAGTTTTTATGTGAAGATATTTC

                     *               *            *          *       *   *   
      50712 CTTTTCCACAATAGGCCTCAAAGCGTTCCAAATATCCAATTGCAGATTCCACAAAAAAAGTTTTT
          1 CTTTTCCACCATAGGCCTCAAAGCGCTCCAAATATCCACTTGCAGATTCTACAAAAAGAGTGTTT

            *                 *  *  *  *             * *         **    *     
      50777 TAAAACTGCTCAATCAAATGATAGATTAAACTCTGTGAGATTAGTGCACACATGTCAAAAAAGTT
         66 CAAAACTGCTCAATCAAAAGAAAGGTTCAACTCTGTGAGATGAATGCACACATCACAAAGAAGTT

                                *      * *           
      50842 TCTCAGAATGCTTCTGTGTACTTTTTAGGGGAAGATATTTC
        131 TCTCAGAATGCTTCTGTGTAGTTTTTATGTGAAGATATTTC

                        *    *     **         *    *           **  * *  *    
      50883 CTTTTCCACCATCGGCCACAAAGGACTCCAAATAACCACATGCAGATTCTAGTAACACAGAGTTT
          1 CTTTTCCACCATAGGCCTCAAAGCGCTCCAAATATCCACTTGCAGATTCTACAAAAAGAGTGTTT

                       *         * *           *   * * *    **    ***        
      50948 CAAAACTGCTCTATCAAAAGATAAGTTCAACTCTGAGAGTTTAGTGCAACCATCGTGAAGAAGTT
         66 CAAAACTGCTCAATCAAAAGAAAGGTTCAACTCTGTGAGATGAATGCACACATCACAAAGAAGTT

                            *     *                  
      51013 TCTCAGAATGCTTCTGAGTAGTGTTTATGTGAAGATATTTC
        131 TCTCAGAATGCTTCTGTGTAGTTTTTATGTGAAGATATTTC

                              *     *                      *           **    
      51054 CTTTTCCACCATAGGCCTGAAAGCCCTCCAAATATCCACTTGCAGATCCTACAAAAAGAAAGTTT
          1 CTTTTCCACCATAGGCCTCAAAGCGCTCCAAATATCCACTTGCAGATTCTACAAAAAGAGTGTTT

             *         **     *  *  *   *        **      *                   
      51119 CGAAA-TGCTCTCTCAAACGATAGTTTCGACTCTGTGGTATGAATACACACATCACAAAGAAGTT
         66 CAAAACTGCTCAATCAAAAGAAAGGTTCAACTCTGTGAGATGAATGCACACATCACAAAGAAGTT

                                       **            
      51183 TCTCAGAATGCTTCTGTGTAGTTTTTAAATGAAGATATTTC
        131 TCTCAGAATGCTTCTGTGTAGTTTTTATGTGAAGATATTTC

            *                       *          *     *                       
      51224 TTTTTCCACCATAGGCCTCAAAGCACTCCAAATATGCACTTCCAGATTCTACAAAAAGAGTGTTT
          1 CTTTTCCACCATAGGCCTCAAAGCGCTCCAAATATCCACTTGCAGATTCTACAAAAAGAGTGTTT

              *                 *       * *         **   *         *  * *    
      51289 CAGAACTGCTCAATCAAAAGGAAGGTTCCAGTC--TGAGACAAATACACACATCAAAAGGTAGTT
         66 CAAAACTGCTCAATCAAAAGAAAGGTTCAACTCTGTGAGATGAATGCACACATCACAAAGAAGTT

                                                    *
      51352 TCTCAGAATGCTTCTGTGTAGTTTTTATGTGAAGATATTTT
        131 TCTCAGAATGCTTCTGTGTAGTTTTTATGTGAAGATATTTC

             *               *    **    *     *      *  **   *          *    
      51393 CCTTTCCACCATAGGCCACAAATGGCTCTAAATACCCACTTACATTTTCCACAAAAAGAGAGTTT
          1 CTTTTCCACCATAGGCCTCAAAGCGCTCCAAATATCCACTTGCAGATTCTACAAAAAGAGTGTTT

                       * *    * *      *   *         *                       
      51458 CAAAACTGCTCTACCAAAGGTAA-GTTTAACGCTGTGAG-TTAA-G-A-ACATCACAAAGAAGTT
         66 CAAAACTGCTCAATCAAAAGAAAGGTTCAACTCTGTGAGATGAATGCACACATCACAAAGAAGTT

                                   *   *  *          
      51518 TCTCAGAATGCTTCTGTGTAGTTCTTACGTAAAGATATTTC
        131 TCTCAGAATGCTTCTGTGTAGTTTTTATGTGAAGATATTTC

                 *   *      ***    *                  *              **      
      51559 CTTTTACACAATAGGCAGAAAAGTGCTCCAAATATCCACTTGAAGATTCTACAAAAACCGTGTTT
          1 CTTTTCCACCATAGGCCTCAAAGCGCTCCAAATATCCACTTGCAGATTCTACAAAAAGAGTGTTT

                     **                                          *      *    
      51624 CAAAACTGCCGAATCAAAAGAAAGGTTCAACTCTGTGAGATGAATGCACACATAACAAAGGAGTT
         66 CAAAACTGCTCAATCAAAAGAAAGGTTCAACTCTGTGAGATGAATGCACACATCACAAAGAAGTT

                                 *      *      *    *
      51689 TCTCAGAATGCTTCTGTGTAGCTTTTATATGAAGACATTTA
        131 TCTCAGAATGCTTCTGTGTAGTTTTTATGTGAAGATATTTC

            *        * *            *   ***                     **        *  
      51730 GTTTTCCACAACAGGCCTCAAAGCTCTCTCCATATCCACTTGCAGATTCTACCGAAAGAGTGCTT
          1 CTTTTCCACCATAGGCCTCAAAGCGCTCCAAATATCCACTTGCAGATTCTACAAAAAGAGTGTTT

             *                   * **     *        *        *     **         
      51795 CCAAACTGCTCAATCAAAAGAGACATTCAAATCTGTGAGGTGAATGCAGACATCGTAAAGAAGTT
         66 CAAAACTGCTCAATCAAAAGAAAGGTTCAACTCTGTGAGATGAATGCACACATCACAAAGAAGTT

                                *     *       *    **
      51860 TCTCAGAATGCTTCTGTGTATTTTTTGTGTGAAGTTATTCG
        131 TCTCAGAATGCTTCTGTGTAGTTTTTATGTGAAGATATTTC

            *    *             *     *  *            **                 *    
      51901 TTTTTGCACCATAGGCCTCCAAGCGTTCTAAATATCCACTTCTAGATTCTACAAAAAGAGAGTTT
          1 CTTTTCCACCATAGGCCTCAAAGCGCTCCAAATATCCACTTGCAGATTCTACAAAAAGAGTGTTT

                   *         *            *        *    *   *                
      51966 CAAAACTACTC-A--AACA-AAAGGTTCAATTCTGTGAGTTGAAAGCAAACATCACAAAGAAGTT
         66 CAAAACTGCTCAATCAAAAGAAAGGTTCAACTCTGTGAGATGAATGCACACATCACAAAGAAGTT

                       *             *               
      52027 TCTCAGAATGCGTCTGTGTAGTTTTGATGTGAAGATATTTC
        131 TCTCAGAATGCTTCTGTGTAGTTTTTATGTGAAGATATTTC

                     **   ****     *                  *                      
      52068 CTTTT-CACAGTAGAATGCAAAGGGCTCCAAATATCCACTTGGAGATTCTACAAAAAGA--GTTT
          1 CTTTTCCACCATAGGCCTCAAAGCGCTCCAAATATCCACTTGCAGATTCTACAAAAAGAGTGTTT

                  *    **     *  *     *     ***   *     *               *   
      52130 CAAAACCGCTCTGTCAAATGATAGGTTGAACTCCCGGAGGTGAATACACACATCACAAAGAGGTT
         66 CAAAACTGCTCAATCAAAAGAAAGGTTCAACTCTGTGAGATGAATGCACACATCACAAAGAAGTT

                  *                       *  *       
      52195 TCTCAGCATGCTTCTGTGTAGTTTTTATGTAAACATATTTC
        131 TCTCAGAATGCTTCTGTGTAGTTTTTATGTGAAGATATTTC

             *    * *              *           *      * *       *   *    *   
      52236 CGTTTCTATCATAGGCCTCAAAGTGCTCCAAATATTCACTTGTACATTCTACCAAACGAGTATTT
          1 CTTTTCCACCATAGGCCTCAAAGCGCTCCAAATATCCACTTGCAGATTCTACAAAAAGAGTGTTT

                              * *         ** *    *        *            *    
      52301 CAAAACTGCTCAATCAAACGGAAGGTTCAAAACCGTGACATGAATGCCCACATCACAAAGTAGTT
         66 CAAAACTGCTCAATCAAAAGAAAGGTTCAACTCTGTGAGATGAATGCACACATCACAAAGAAGTT

                                                     
      52366 TCTCAGAATGCTTCTGTGTAGTTTTTATGTGAAGATATTTC
        131 TCTCAGAATGCTTCTGTGTAGTTTTTATGTGAAGATATTTC

                     * *    *      *    *       * *   **      *              
      52407 CTTTTCCACAACA-GCGTGCAAAACGCTTCAAATATGCCCTTAGAGATTCCACAAAAAGAGTGTT
          1 CTTTTCCACCATAGGCCT-CAAAGCGCTCCAAATATCCACTTGCAGATTCTACAAAAAGAGTGTT

              *     *               *  *                                  ** 
      52471 TCCAAACTACTCAAATCAAAA-AATGATTTCAACTCTGTGAGATGAATGCACACATCACAAACTA
         65 TCAAAACTGCTC-AATCAAAAGAAAG-GTTCAACTCTGTGAGATGAATGCACACATCACAAAGAA

                         *     ** *   * *   *       *   
      52535 GTTTCTCAGAATGTTTCTGCCTGGTTCTCATGCGAAGATAGTTC
        128 GTTTCTCAGAATGCTTCTGTGTAGTTTTTATGTGAAGATATTTC

                 *           *   * **              *                  * *    
      52579 CTTTTTCACCATAGGCCGCAATGTACTCCAAATATCCACCTGCAGATTCTAC-AAAAGTGAGTTT
          1 CTTTTCCACCATAGGCCTCAAAGCGCTCCAAATATCCACTTGCAGATTCTACAAAAAGAGTGTTT

                       *         ***    **         *        *      *       * 
      52643 CAAAACTGCTCTATCAAAAGATCAGTTCGTCTCTGTGAGTTGAATGCATACATCAAAAAGAAGCT
         66 CAAAACTGCTCAATCAAAAGAAAGGTTCAACTCTGTGAGATGAATGCACACATCACAAAGAAGTT

                 *             *      **         *   
      52708 TCTCAAAATGCTTCTGTGTGGTTTTTCGGTGAAGATAGTTC
        131 TCTCAGAATGCTTCTGTGTAGTTTTTATGTGAAGATATTTC

            *     *        *      * *                 *        *        *    
      52749 TTTTTCTACCATAGGTCTCAAACCACTCCAAATATCCACTTGTAGATTCTATAAAAAGGAATG-T
          1 CTTTTCCACCATAGGCCTCAAAGCGCTCCAAATATCCACTTGCAGATTCTACAAAAA-GAGTGTT

                  *        *    *    *      * *         *      *         *   
      52813 TCAAAATTGCTCAATAAAAATAAAGTTTCAACACCGTGAGATGAGTGCACAAATCACAAAGGAGT
         65 TCAAAACTGCTCAATCAAAAGAAAGGTTCAACTCTGTGAGATGAATGCACACATCACAAAGAAGT

                  *          *         *          *   
      52878 TTCTCAAAATGCTTCTGGGTAGTTTTTCTGTGAAGATAGTTC
        130 TTCTCAGAATGCTTCTGTGTAGTTTTTATGTGAAGATATTTC

                  *     *    *     *          *                         *    
      52920 CTTTTCTACCATGGGCCACAAAGGGCTCCAAATACCCACTTGCAGATTCTACAAAAAGAGAGTTT
          1 CTTTTCCACCATAGGCCTCAAAGCGCTCCAAATATCCACTTGCAGATTCTACAAAAAGAGTGTTT

              *        *      ** * *        *    * *    **  * *           *  
      52985 CACAACTGCTCTATCAAACAATATGTTCAACTTTGTGGGTTGAACACAAATATCAC-AAGAATTT
         66 CAAAACTGCTCAATCAAAAGAAAGGTTCAACTCTGTGAGATGAATGCACACATCACAAAGAAGTT

                **                             *     
      53049 TCTCCCAATGCTTCTGTGTAGTTTTTATGTGAAGACATTTC
        131 TCTCAGAATGCTTCTGTGTAGTTTTTATGTGAAGATATTTC

            *   *  *      *  *     *                 *  *      *       *  *  
      53090 TTTTCCCTCCATAGTCCACAAAGTGCTCCAAATATCCACTTACATATTCTAGAAAAAGATTGCTT
          1 CTTTTCCACCATAGGCCTCAAAGCGCTCCAAATATCCACTTGCAGATTCTACAAAAAGAGTGTTT

            **       *    *               * * *                              
      53155 GGAAACTGCACAATGAAAAGAAAGGTTCAAATATATGAGATGAATGCACACATCACAAAGAAGTT
         66 CAAAACTGCTCAATCAAAAGAAAGGTTCAACTCTGTGAGATGAATGCACACATCACAAAGAAGTT

                                 *                    
      53220 TCTCAGAAT-CTCTCTGTGTAATTTTTATGTGAAGATATTTC
        131 TCTCAGAATGCT-TCTGTGTAGTTTTTATGTGAAGATATTTC

                *     *    *  *   *                        *      *    *     
      53261 CTTTCCCACCTTAGGTCTTAAAACGCTCCAAATATCCACTTGCAGATACTACAAGAAGATTGTTT
          1 CTTTTCCACCATAGGCCTCAAAGCGCTCCAAATATCCACTTGCAGATTCTACAAAAAGAGTGTTT

                     *    *        *      *     **                           
      53326 CAAAACTGCACAA-AAAAAGAAATGTTCAATTCTGTTTGATGAATGCACACATCACAAAGAAGTT
         66 CAAAACTGCTCAATCAAAAGAAAGGTTCAACTCTGTGAGATGAATGCACACATCACAAAGAAGTT

                           *                         
      53390 TCTCAGAATGCTTCTCTGTAGTTTTTATGTGAAGATATTTC
        131 TCTCAGAATGCTTCTGTGTAGTTTTTATGTGAAGATATTTC

                     *             *                 *         **      * *   
      53431 CTTTTCCACAATAGGCCTCAAAGGGCTCCAAATATCCACTTCCAGATTCTATGAAAAGAATATTT
          1 CTTTTCCACCATAGGCCTCAAAGCGCTCCAAATATCCACTTGCAGATTCTACAAAAAGAGTGTTT

             *              * *    *                                      *  
      53496 CCAAACTGCTCAATCATAGGAAATGTTCAACTCTGTGAGATGAATGCACACATCAC-AAGAAATT
         66 CAAAACTGCTCAATCAAAAGAAAGGTTCAACTCTGTGAGATGAATGCACACATCACAAAGAAGTT

                     *    *      *       *       *   
      53560 TCTCAGAATCCTTCAGTGTAGGTTTTATGAGAAGATAATTC
        131 TCTCAGAATGCTTCTGTGTAGTTTTTATGTGAAGATATTTC

                     *    **        *   *                           *    *   
      53601 CTTTTCCACAATAGTTCTCAAAGCACTCAAAATATCCACTTGCAGATTCTACAAAAGGAGTATTT
          1 CTTTTCCACCATAGGCCTCAAAGCGCTCCAAATATCCACTTGCAGATTCTACAAAAAGAGTGTTT

                                                          *                  
      53666 CAAAACTGCTCAATCAAAAGAAAGGTTCAACTCTGTGAGATGAATGGACACATCACAAAGAAGTT
         66 CAAAACTGCTCAATCAAAAGAAAGGTTCAACTCTGTGAGATGAATGCACACATCACAAAGAAGTT

                                  *   *              
      53731 TCTCAGAATGCTTCTGTGTAGTATTTTTGTGAAGATATTT-
        131 TCTCAGAATGCTTCTGTGTAGTTTTTATGTGAAGATATTTC

                          *  * * * * ***             *            *     * *  
      53771 CTTTTCCACCATAGACCGCCAGGGGACACAAATATCCACTTTCAGATTCTACAACAAGAGAGGTT
          1 CTTTTCCACCATAGGCCTCAAAGCGCTCCAAATATCCACTTGCAGATTCTACAAAAAGAGTGTTT

                   *   *     *   ** *       *      *                      * *
      53836 CAAAACTACTCGATCAAGAGATGGTTTCAACTATGTGAGTTGAATGCACACATCACAAAGAACTA
         66 CAAAACTGCTCAATCAAAAGAAAGGTTCAACTCTGTGAGATGAATGCACACATCACAAAGAAGTT

             *  *    *                               
      53901 TGTCGGAATTCTTCTGTGTAGTTTTTATGTGAAGATATTTC
        131 TCTCAGAATGCTTCTGTGTAGTTTTTATGTGAAGATATTTC

                     *    * *      * *    *                  *               
      53942 CTTTTCCACAATAGACGTCAAAGTGATCCAGATATCCACTTGCAGATTCCACAAAAAGAGTGTTT
          1 CTTTTCCACCATAGGCCTCAAAGCGCTCCAAATATCCACTTGCAGATTCTACAAAAAGAGTGTTT

                 *   *   *                  **                     *  *      
      54007 CAAAAGTGCACAACCAAAAGAAAGGTTCAACTAGGTGAGATGAATGCACACATCAGAAGGAAGTT
         66 CAAAACTGCTCAATCAAAAGAAAGGTTCAACTCTGTGAGATGAATGCACACATCACAAAGAAGTT

                            **   *     * *       *   
      54072 TCTCAGAATGCTTCTGCATAGCTTTTAAGGGAAGATACTTC
        131 TCTCAGAATGCTTCTGTGTAGTTTTTATGTGAAGATATTTC

                    *               *            * *  *    * *               
      54113 CTTTTCCAACATAGGCCTCAAAGCACTCCAAATATCCTCCTGGAGATACCACAAAAAGAGTGTTT
          1 CTTTTCCACCATAGGCCTCAAAGCGCTCCAAATATCCACTTGCAGATTCTACAAAAAGAGTGTTT

            **                      *  *                 *       *           
      54178 GCAAACTGCTCAATCAAAAGAAAGATTTAACTCTGTGAGATGAATCCACACATGACAAAGAAGTT
         66 CAAAACTGCTCAATCAAAAGAAAGGTTCAACTCTGTGAGATGAATGCACACATCACAAAGAAGTT

                                                     
      54243 TCTCAGAATGCTTCTGTGTAGTTTTTATGTGAAGATATTTC
        131 TCTCAGAATGCTTCTGTGTAGTTTTTATGTGAAGATATTTC

                     *   * * *    **           *                *     *      
      54284 CTTTTCCACAATAAGACCCAAAAGGCTCCAAATATTCACTTGCAGATTCTAAAAAAAACAGTGTT
          1 CTTTTCCACCATAGGCCTCAAAGCGCTCCAAATATCCACTTGCAGATTCT-ACAAAAAGAGTGTT

                                  *                  *      *        **      
      54349 TCAAAACTGCTCAATCAAAAGATA-GTTCAACTCTGTGAGAAGAATGCTCACATCACTGAGAAGT
         65 TCAAAACTGCTCAATCAAAAGAAAGGTTCAACTCTGTGAGATGAATGCACACATCACAAAGAAGT

                                         *            
      54413 TTCTCAGAATGCTTCTGTGTAGTTTTTATATGAAGATATTTC
        130 TTCTCAGAATGCTTCTGTGTAGTTTTTATGTGAAGATATTTC

                *     *      *    **                       *   **       *    
      54455 CTTTCCCACCGTAGGCCACAAAAGGCTCCAAATATCCACTTGCAGATACTATGAAAAGAGAGTTT
          1 CTTTTCCACCATAGGCCTCAAAGCGCTCCAAATATCCACTTGCAGATTCTACAAAAAGAGTGTTT

                        *        *               ***            *            
      54520 CAAAACTGCTCATTCAAAAGATAGGTTCAACTCTGTGGTTTGAATGCACACAGCACAAAGAAGTT
         66 CAAAACTGCTCAATCAAAAGAAAGGTTCAACTCTGTGAGATGAATGCACACATCACAAAGAAGTT

              *       **                   **   *    
      54585 TCACAGAATGTGTCTGTGTAGTTTTTATGTGCGGATGTTTC
        131 TCTCAGAATGCTTCTGTGTAGTTTTTATGTGAAGATATTTC

                         *        ****   *   *               *    *          
      54626 CTTTTCCACCATATGCCT-AAATATTTCCCAATTTCCACTTGCAGATTCCACAAGAAGAGTGTTT
          1 CTTTTCCACCATAGGCCTCAAAGCGCTCCAAATATCCACTTGCAGATTCTACAAAAAGAGTGTTT

                      **       *       *           *            *      ***   
      54690 CAAAACTGCTGTATC-AAATAAA-GTTGAACTCTGTGAGGTGAATGCACACAGCACAAAATGGTT
         66 CAAAACTGCTCAATCAAAAGAAAGGTTCAACTCTGTGAGATGAATGCACACATCACAAAGAAGTT

                          **   *        *       *    
      54753 TCTCAGAATGCTTCCTTGTTGTTTTTATATGAAGATGTTTC
        131 TCTCAGAATGCTTCTGTGTAGTTTTTATGTGAAGATATTTC

                  *  *             *   *     *                               
      54794 CTTTTCAACAATAGGCCTCAAAGTGCTTCAAATGTCCACTTGCAGATTCTACAAAAAGAGTGTTT
          1 CTTTTCCACCATAGGCCTCAAAGCGCTCCAAATATCCACTTGCAGATTCTACAAAAAGAGTGTTT

                                        *      *  *  *                       
      54859 CAAAACTGCTCAATCAAAAGAAAGGTTCGACTCTGGGAAATTAATGCACACATCACAAAGAAGTT
         66 CAAAACTGCTCAATCAAAAGAAAGGTTCAACTCTGTGAGATGAATGCACACATCACAAAGAAGTT

                                     *        *      
      54924 TCTC---A-GCTTCTGTGTAGTTTTCATGTGAAGTTATTTC
        131 TCTCAGAATGCTTCTGTGTAGTTTTTATGTGAAGATATTTC

                     *       *     *            *    *         **       *    
      54961 CTTTTCCACAATAGGCCGCAAAGGGCTCCAAATATCAACTTACAGATTCTAGGAAAAGAGAGTTT
          1 CTTTTCCACCATAGGCCTCAAAGCGCTCCAAATATCCACTTGCAGATTCTACAAAAAGAGTGTTT

                       * **      *     *                                     
      55026 CAAAACTGCTCTACGAAAAGATAGGTTGAACTCTGTGAGATGAATGCACACATCACAAAGAAGTT
         66 CAAAACTGCTCAATCAAAAGAAAGGTTCAACTCTGTGAGATGAATGCACACATCACAAAGAAGTT

                       *               * *     *     
      55091 TCTCAGAATGCATCTGTGTAGTTTTTACGGGAAGACATTTC
        131 TCTCAGAATGCTTCTGTGTAGTTTTTATGTGAAGATATTTC

                        ***  *     **      *  *                  *     ***   
      55132 CTTTTCCACCATCTTCCACAAAGGTCTCCAAGTAACCACTTGCAGATTCTACAGAAAGACACTTT
          1 CTTTTCCACCATAGGCCTCAAAGCGCTCCAAATATCCACTTGCAGATTCTACAAAAAGAGTGTTT

            *          *         ***      *      ***                      *  
      55197 AAAAACTGCTCTATCAAAAGATCAGTTCAAGTCTGTGGTTTGAATGCACACATCACAAAGAATTT
         66 CAAAACTGCTCAATCAAAAGAAAGGTTCAACTCTGTGAGATGAATGCACACATCACAAAGAAGTT

                                     *  *            
      55262 TCTCAGAATGCTTCTGTGTAGTTTTCATATGAAGATATTTC
        131 TCTCAGAATGCTTCTGTGTAGTTTTTATGTGAAGATATTTC

                                    *                                   **   
      55303 CTTTTCCACCATAGGCCTCAAAGCACTCCAAATATCCACTTGCAGATTCTACAAAAAGAGATTTT
          1 CTTTTCCACCATAGGCCTCAAAGCGCTCCAAATATCCACTTGCAGATTCTACAAAAAGAGTGTTT

                   **                           *  *          *        *     
      55368 CAAAACTAGTCAATCAAAAGAAAGGTTCAACTCTGTCAGTTGAATGCACATATCACAAACAAGTT
         66 CAAAACTGCTCAATCAAAAGAAAGGTTCAACTCTGTGAGATGAATGCACACATCACAAAGAAGTT

                *      *                             
      55433 TCTCGGAATGCGTCTGTGTAGTTTTTATGTGAAGATATTTC
        131 TCTCAGAATGCTTCTGTGTAGTTTTTATGTGAAGATATTTC

               *     * *           *     *                  *    *           
      55474 CTTCTCCACAACAGGCCTCAAAGTGCTCCGAATATCCACTTGCAGATTTTAC-TAAAGAGTGTTT
          1 CTTTTCCACCATAGGCCTCAAAGCGCTCCAAATATCCACTTGCAGATTCTACAAAAAGAGTGTTT

             *               *  *   *     *        *    *               *    
      55538 CCAAACTGCTCAATCAAGAGGAAGTTTCAAGTCTGTGAGCTGAACGCACACATCACAAAGTAGTT
         66 CAAAACTGCTCAATCAAAAGAAAGGTTCAACTCTGTGAGATGAATGCACACATCACAAAGAAGTT

               *                                *    
      55603 TCTGAGAATGCTTCTGTGTAGTTTTTATGTGAAGATGTTTC
        131 TCTCAGAATGCTTCTGTGTAGTTTTTATGTGAAGATATTTC

                            **     *                                    *    
      55644 CTTTTCCACCATAGGCTGCAAAGGGCTCCAAATATCCACTTGCAGATTCTACAAAAAGAGAGTTT
          1 CTTTTCCACCATAGGCCTCAAAGCGCTCCAAATATCCACTTGCAGATTCTACAAAAAGAGTGTTT

                 *     *         *          *     *                     *    
      55709 CAAAAGTGCTCTATCAAAAGATAGGTTCAACTATGTGATATGAATGCACACATCACAAAGTAGTT
         66 CAAAACTGCTCAATCAAAAGAAAGGTTCAACTCTGTGAGATGAATGCACACATCACAAAGAAGTT

                                          *          
      55774 TCTCAGAATGCTTCTGTGTAGTTTTTATGTAAAGATATTTC
        131 TCTCAGAATGCTTCTGTGTAGTTTTTATGTGAAGATATTTC

                                    *                                   **   
      55815 CTTTTCCACCATAGGCCTCAAAGCACTCCAAATATCCACTTGCAGATTCTACAAAAAGAGATTTT
          1 CTTTTCCACCATAGGCCTCAAAGCGCTCCAAATATCCACTTGCAGATTCTACAAAAAGAGTGTTT

                   ** *                   *     *  *    * *   *        *     
      55880 CAAAACTATTTAATCAAAAGAAAGGTTCAAATCTGTCAGTTGAAGGTACATATCACAAACAAGTT
         66 CAAAACTGCTCAATCAAAAGAAAGGTTCAACTCTGTGAGATGAATGCACACATCACAAAGAAGTT

             * **                                    
      55945 TATTGGAATGCTTCTGTGTAGTTTTTATGTGAAGATATTTC
        131 TCTCAGAATGCTTCTGTGTAGTTTTTATGTGAAGATATTTC

                     * *       * * *                        **  *     *      
      55986 CTTTTCCACAACAGGCCTCCAGGTGCTCCAAATATCCACTTGCAGATTTCACTAAAAGTGTGTTT
          1 CTTTTCCACCATAGGCCTCAAAGCGCTCCAAATATCCACTTGCAGATTCTACAAAAAGAGTGTTT

             *  *            *  *   *     *     *  *             *           
      56051 CCAAGCTGCTCAATCAAGAGGAAGTTTCAAGTCTGTCAGGTGAATGCACACATTACAAAGAAGTT
         66 CAAAACTGCTCAATCAAAAGAAAGGTTCAACTCTGTGAGATGAATGCACACATCACAAAGAAGTT

            *  *                                     
      56116 ACTGAGAATGCTTCTGTGTAGTTTTTATGTGAAGATATTTC
        131 TCTCAGAATGCTTCTGTGTAGTTTTTATGTGAAGATATTTC

                      **               *                                *    
      56157 CTTTTCCACCGCAGGCCTCAAAGCGCTGCAAATATCCACTTGCAGATTCTACAAAAAGAGAGTTT
          1 CTTTTCCACCATAGGCCTCAAAGCGCTCCAAATATCCACTTGCAGATTCTACAAAAAGAGTGTTT

                      **         *   *         *   *     **            *     
      56222 CAAAACTGCTGTATCAAAAGATAGGGTCAACTCTGCGAGTTGAATAAACACATCACAAATAAGTT
         66 CAAAACTGCTCAATCAAAAGAAAGGTTCAACTCTGTGAGATGAATGCACACATCACAAAGAAGTT

               **   *        *               *       
      56287 TCTGGGAACGCTTCTGTATAG-TTTTATGTGAATATATTTC
        131 TCTCAGAATGCTTCTGTGTAGTTTTTATGTGAAGATATTTC

                         *          *                   *   *  *   * *   *   
      56327 CTTTTCCACCATATGCCTCAAAGCACTCCAAATATCCACTTGCACATTATAGAAACATAGTCTTT
          1 CTTTTCCACCATAGGCCTCAAAGCGCTCCAAATATCCACTTGCAGATTCTACAAAAAGAGTGTTT

                   **                        *         *             *       
      56392 CAAAACTTGTCAATC-AAAGAAAGGTTCAACTCCGTGAGATGAGTGCACACATCACAGAGAAGTT
         66 CAAAACTGCTCAATCAAAAGAAAGGTTCAACTCTGTGAGATGAATGCACACATCACAAAGAAGTT

                *     *                            * 
      56456 TCTCGGAATGTTTCTGTGTAGTTTTTATGTGAAGATATTGC
        131 TCTCAGAATGCTTCTGTGTAGTTTTTATGTGAAGATATTTC

                     *               *            *          *       *   *   
      56497 CTTTTCCACAATAGGCCTCAAAGCGTTCCAAATATCCAATTGCAGATTCCACAAAAAAAGTTTTT
          1 CTTTTCCACCATAGGCCTCAAAGCGCTCCAAATATCCACTTGCAGATTCTACAAAAAGAGTGTTT

            *                 *  *  *  *             * *         **    *     
      56562 TAAAACTGCTCAATCAAATGATAGATTAAACTCTGTGAGATTAGTGCACACATGTCAAAAAAGTT
         66 CAAAACTGCTCAATCAAAAGAAAGGTTCAACTCTGTGAGATGAATGCACACATCACAAAGAAGTT

                                *      * *           
      56627 TCTCAGAATGCTTCTGTGTACTTTTTAGGGGAAGATATTTC
        131 TCTCAGAATGCTTCTGTGTAGTTTTTATGTGAAGATATTTC

                        *    *     **         *    *           **  * *  *    
      56668 CTTTTCCACCATCGGCCACAAAGGACTCCAAATAACCACATGCAGATTCTAGTAACACAGAGTTT
          1 CTTTTCCACCATAGGCCTCAAAGCGCTCCAAATATCCACTTGCAGATTCTACAAAAAGAGTGTTT

                       *         * *           *   * * *    **    ***        
      56733 CAAAACTGCTCTATCAAAAGATAAGTTCAACTCTGAGAGTTTAGTGCAACCATCGTGAAGAAGTT
         66 CAAAACTGCTCAATCAAAAGAAAGGTTCAACTCTGTGAGATGAATGCACACATCACAAAGAAGTT

                            *     *                  
      56798 TCTCAGAATGCTTCTGAGTAGTGTTTATGTGAAGATATTTC
        131 TCTCAGAATGCTTCTGTGTAGTTTTTATGTGAAGATATTTC

                              *     *                      *           **    
      56839 CTTTTCCACCATAGGCCTGAAAGCCCTCCAAATATCCACTTGCAGATCCTACAAAAAGAAAGTTT
          1 CTTTTCCACCATAGGCCTCAAAGCGCTCCAAATATCCACTTGCAGATTCTACAAAAAGAGTGTTT

             *         **     *  *  *   *        **      *                   
      56904 CGAAA-TGCTCTCTCAAACGATAGTTTCGACTCTGTGGTATGAATACACACATCACAAAGAAGTT
         66 CAAAACTGCTCAATCAAAAGAAAGGTTCAACTCTGTGAGATGAATGCACACATCACAAAGAAGTT

                                       **            
      56968 TCTCAGAATGCTTCTGTGTAGTTTTTAAATGAAGATATTTC
        131 TCTCAGAATGCTTCTGTGTAGTTTTTATGTGAAGATATTTC

            *                       *          *     *                       
      57009 TTTTTCCACCATAGGCCTCAAAGCACTCCAAATATGCACTTCCAGATTCTACAAAAAGAGTGTTT
          1 CTTTTCCACCATAGGCCTCAAAGCGCTCCAAATATCCACTTGCAGATTCTACAAAAAGAGTGTTT

              *                 *       * *         **   *         *  * *    
      57074 CAGAACTGCTCAATCAAAAGGAAGGTTCCAGTC--TGAGACAAATACACACATCAAAAGGTAGTT
         66 CAAAACTGCTCAATCAAAAGAAAGGTTCAACTCTGTGAGATGAATGCACACATCACAAAGAAGTT

                                                    *
      57137 TCTCAGAATGCTTCTGTGTAGTTTTTATGTGAAGATATTTT
        131 TCTCAGAATGCTTCTGTGTAGTTTTTATGTGAAGATATTTC

             *               *    **    *     *      *  **   *          *    
      57178 CCTTTCCACCATAGGCCACAAATGGCTCTAAATACCCACTTACATTTTCCACAAAAAGAGAGTTT
          1 CTTTTCCACCATAGGCCTCAAAGCGCTCCAAATATCCACTTGCAGATTCTACAAAAAGAGTGTTT

                       * *    * *      *   *         *                       
      57243 CAAAACTGCTCTACCAAAGGTAA-GTTTAACGCTGTGAG-TTAA-G-A-ACATCACAAAGAAGTT
         66 CAAAACTGCTCAATCAAAAGAAAGGTTCAACTCTGTGAGATGAATGCACACATCACAAAGAAGTT

                                   *   *  *          
      57303 TCTCAGAATGCTTCTGTGTAGTTCTTACGTAAAGATATTTC
        131 TCTCAGAATGCTTCTGTGTAGTTTTTATGTGAAGATATTTC

                 *   *      ***    *                  *              **      
      57344 CTTTTACACAATAGGCAGAAAAGTGCTCCAAATATCCACTTGAAGATTCTACAAAAACCGTGTTT
          1 CTTTTCCACCATAGGCCTCAAAGCGCTCCAAATATCCACTTGCAGATTCTACAAAAAGAGTGTTT

                     **                                          *      *    
      57409 CAAAACTGCCGAATCAAAAGAAAGGTTCAACTCTGTGAGATGAATGCACACATAACAAAGGAGTT
         66 CAAAACTGCTCAATCAAAAGAAAGGTTCAACTCTGTGAGATGAATGCACACATCACAAAGAAGTT

                                 *      *      *    *
      57474 TCTCAGAATGCTTCTGTGTAGCTTTTATATGAAGACATTTA
        131 TCTCAGAATGCTTCTGTGTAGTTTTTATGTGAAGATATTTC

            *        * *            *   ***                     **        *  
      57515 GTTTTCCACAACAGGCCTCAAAGCTCTCTCCATATCCACTTGCAGATTCTACCGAAAGAGTGCTT
          1 CTTTTCCACCATAGGCCTCAAAGCGCTCCAAATATCCACTTGCAGATTCTACAAAAAGAGTGTTT

             *                   * **     *        *        *     **         
      57580 CCAAACTGCTCAATCAAAAGAGACATTCAAATCTGTGAGGTGAATGCAGACATCGTAAAGAAGTT
         66 CAAAACTGCTCAATCAAAAGAAAGGTTCAACTCTGTGAGATGAATGCACACATCACAAAGAAGTT

                                *     *       *    **
      57645 TCTCAGAATGCTTCTGTGTATTTTTTGTGTGAAGTTATTCG
        131 TCTCAGAATGCTTCTGTGTAGTTTTTATGTGAAGATATTTC

            *    *             *     *  *            **                 *    
      57686 TTTTTGCACCATAGGCCTCCAAGCGTTCTAAATATCCACTTCTAGATTCTACAAAAAGAGAGTTT
          1 CTTTTCCACCATAGGCCTCAAAGCGCTCCAAATATCCACTTGCAGATTCTACAAAAAGAGTGTTT

                   *         *            *        *    *   *                
      57751 CAAAACTACTC-A--AACA-AAAGGTTCAATTCTGTGAGTTGAAAGCAAACATCACAAAGAAGTT
         66 CAAAACTGCTCAATCAAAAGAAAGGTTCAACTCTGTGAGATGAATGCACACATCACAAAGAAGTT

                       *             *               
      57812 TCTCAGAATGCGTCTGTGTAGTTTTGATGTGAAGATATTTC
        131 TCTCAGAATGCTTCTGTGTAGTTTTTATGTGAAGATATTTC

                     **   ****     *                  *                      
      57853 CTTTT-CACAGTAGAATGCAAAGGGCTCCAAATATCCACTTGGAGATTCTACAAAAAGA--GTTT
          1 CTTTTCCACCATAGGCCTCAAAGCGCTCCAAATATCCACTTGCAGATTCTACAAAAAGAGTGTTT

                  *    **     *  *     *     ***   *     *               *   
      57915 CAAAACCGCTCTGTCAAATGATAGGTTGAACTCCCGGAGGTGAATACACACATCACAAAGAGGTT
         66 CAAAACTGCTCAATCAAAAGAAAGGTTCAACTCTGTGAGATGAATGCACACATCACAAAGAAGTT

                  *                       *  *       
      57980 TCTCAGCATGCTTCTGTGTAGTTTTTATGTAAACATATTTC
        131 TCTCAGAATGCTTCTGTGTAGTTTTTATGTGAAGATATTTC

             *    * *              *           *      * *       *   *    *   
      58021 CGTTTCTATCATAGGCCTCAAAGTGCTCCAAATATTCACTTGTACATTCTACCAAACGAGTATTT
          1 CTTTTCCACCATAGGCCTCAAAGCGCTCCAAATATCCACTTGCAGATTCTACAAAAAGAGTGTTT

                              * *         ** *    *        *            *    
      58086 CAAAACTGCTCAATCAAATGGAAGGTTCAAAACCGTGACATGAATGCCCACATCACAAAGTAGTT
         66 CAAAACTGCTCAATCAAAAGAAAGGTTCAACTCTGTGAGATGAATGCACACATCACAAAGAAGTT

                                                     
      58151 TCTCAGAATGCTTCTGTGTAGTTTTTATGTGAAGATATTTC
        131 TCTCAGAATGCTTCTGTGTAGTTTTTATGTGAAGATATTTC

                     * *    *      *    *       * *   **      *              
      58192 CTTTTCCACAACA-GCGTGCAAAACGCTTCAAATATGCCCTTAGAGATTCCACAAAAAGAGTGTT
          1 CTTTTCCACCATAGGCCT-CAAAGCGCTCCAAATATCCACTTGCAGATTCTACAAAAAGAGTGTT

              *     *               *  *                                  ** 
      58256 TCCAAACTACTCAAATCAAAA-AATGATTTCAACTCTGTGAGATGAATGCACACATCACAAACTA
         65 TCAAAACTGCTC-AATCAAAAGAAAG-GTTCAACTCTGTGAGATGAATGCACACATCACAAAGAA

                         *     ** *   * *   *       *   
      58320 GTTTCTCAGAATGTTTCTGCCTGGTTCTCATGCGAAGATAGTTC
        128 GTTTCTCAGAATGCTTCTGTGTAGTTTTTATGTGAAGATATTTC

                 *           *   * **              *                  * *    
      58364 CTTTTTCACCATAGGCCGCAATGTACTCCAAATATCCACCTGCAGATTCTAC-AAAAGTGAGTTT
          1 CTTTTCCACCATAGGCCTCAAAGCGCTCCAAATATCCACTTGCAGATTCTACAAAAAGAGTGTTT

                       *         ***    **         *        *      *       * 
      58428 CAAAACTGCTCTATCAAAAGATCAGTTCGTCTCTGTGAGTTGAATGCATACATCAAAAAGAAGCT
         66 CAAAACTGCTCAATCAAAAGAAAGGTTCAACTCTGTGAGATGAATGCACACATCACAAAGAAGTT

                 *             *      **         *   
      58493 TCTCAAAATGCTTCTGTGTGGTTTTTCGGTGAAGATAGTTC
        131 TCTCAGAATGCTTCTGTGTAGTTTTTATGTGAAGATATTTC

            *     *        *      * *                 *        *        *    
      58534 TTTTTCTACCATAGGTCTCAAACCACTCCAAATATCCACTTGTAGATTCTATAAAAAGGAATG-T
          1 CTTTTCCACCATAGGCCTCAAAGCGCTCCAAATATCCACTTGCAGATTCTACAAAAA-GAGTGTT

                  *        *    *    *      * *         *      *         *   
      58598 TCAAAATTGCTCAATAAAAATAAAGTTTCAACACCGTGAGATGAGTGCACAAATCACAAAGGAGT
         65 TCAAAACTGCTCAATCAAAAGAAAGGTTCAACTCTGTGAGATGAATGCACACATCACAAAGAAGT

                  *          *         *          *   
      58663 TTCTCAAAATGCTTCTGGGTAGTTTTTCTGTGAAGATAGTTC
        130 TTCTCAGAATGCTTCTGTGTAGTTTTTATGTGAAGATATTTC

                  *     *    *     *          *                         *    
      58705 CTTTTCTACCATGGGCCACAAAGGGCTCCAAATACCCACTTGCAGATTCTACAAAAAGAGAGTTT
          1 CTTTTCCACCATAGGCCTCAAAGCGCTCCAAATATCCACTTGCAGATTCTACAAAAAGAGTGTTT

              *        *      ** * *        *    * *    **  * *           *  
      58770 CACAACTGCTCTATCAAACAATATGTTCAACTTTGTGGGTTGAACACAAATATCAC-AAGAATTT
         66 CAAAACTGCTCAATCAAAAGAAAGGTTCAACTCTGTGAGATGAATGCACACATCACAAAGAAGTT

                **                             *     
      58834 TCTCCCAATGCTTCTGTGTAGTTTTTATGTGAAGACATTTC
        131 TCTCAGAATGCTTCTGTGTAGTTTTTATGTGAAGATATTTC

            *   *  *      *  *     *                 *  *      *       *  *  
      58875 TTTTCCCTCCATAGTCCACAAAGTGCTCCAAATATCCACTTACATATTCTAGAAAAAGATTGCTT
          1 CTTTTCCACCATAGGCCTCAAAGCGCTCCAAATATCCACTTGCAGATTCTACAAAAAGAGTGTTT

            **       *    *               * * *                              
      58940 GGAAACTGCACAATGAAAAGAAAGGTTCAAATATATGAGATGAATGCACACATCACAAAGAAGTT
         66 CAAAACTGCTCAATCAAAAGAAAGGTTCAACTCTGTGAGATGAATGCACACATCACAAAGAAGTT

                                 *                    
      59005 TCTCAGAAT-CTCTCTGTGTAATTTTTATGTGAAGATATTTC
        131 TCTCAGAATGCT-TCTGTGTAGTTTTTATGTGAAGATATTTC

                *     *    *  *   *                        *      *    *     
      59046 CTTTCCCACCTTAGGTCTTAAAACGCTCCAAATATCCACTTGCAGATACTACAAGAAGATTGTTT
          1 CTTTTCCACCATAGGCCTCAAAGCGCTCCAAATATCCACTTGCAGATTCTACAAAAAGAGTGTTT

                     *    *        *      *     **                           
      59111 CAAAACTGCACAA-AAAAAGAAATGTTCAATTCTGTTTGATGAATGCACACATCACAAAGAAGTT
         66 CAAAACTGCTCAATCAAAAGAAAGGTTCAACTCTGTGAGATGAATGCACACATCACAAAGAAGTT

                           *                         
      59175 TCTCAGAATGCTTCTCTGTAGTTTTTATGTGAAGATATTTC
        131 TCTCAGAATGCTTCTGTGTAGTTTTTATGTGAAGATATTTC

                     *             *                 *         **      * *   
      59216 CTTTTCCACAATAGGCCTCAAAGGGCTCCAAATATCCACTTCCAGATTCTATGAAAAGAATATTT
          1 CTTTTCCACCATAGGCCTCAAAGCGCTCCAAATATCCACTTGCAGATTCTACAAAAAGAGTGTTT

             *              * *    *                                      *  
      59281 CCAAACTGCTCAATCATAGGAAATGTTCAACTCTGTGAGATGAATGCACACATCAC-AAGAAATT
         66 CAAAACTGCTCAATCAAAAGAAAGGTTCAACTCTGTGAGATGAATGCACACATCACAAAGAAGTT

                     *    *      *       *     * *   
      59345 TCTCAGAATCCTTCAGTGTAGGTTTTATGAGAAGACAATTC
        131 TCTCAGAATGCTTCTGTGTAGTTTTTATGTGAAGATATTTC

                     *    **        *   *                           *    *   
      59386 CTTTTCCACAATAGTTCTCAAAGCACTCAAAATATCCACTTGCAGATTCTACAAAAGGAGTATTT
          1 CTTTTCCACCATAGGCCTCAAAGCGCTCCAAATATCCACTTGCAGATTCTACAAAAAGAGTGTTT

                                                          *                  
      59451 CAAAACTGCTCAATCAAAAGAAAGGTTCAACTCTGTGAGATGAATGGACACATCACAAAGAAGTT
         66 CAAAACTGCTCAATCAAAAGAAAGGTTCAACTCTGTGAGATGAATGCACACATCACAAAGAAGTT

                                  *   *              
      59516 TCTCAGAATGCTTCTGTGTAGTATTTTTGTGAAGATATTT-
        131 TCTCAGAATGCTTCTGTGTAGTTTTTATGTGAAGATATTTC

                          *  * * * * ***             *            *     * *  
      59556 CTTTTCCACCATAGACCGCCAGGGGACACAAATATCCACTTTCAGATTCTACAACAAGAGAGGTT
          1 CTTTTCCACCATAGGCCTCAAAGCGCTCCAAATATCCACTTGCAGATTCTACAAAAAGAGTGTTT

                   *   *     *   ** *       *      *                      * *
      59621 CAAAACTACTCGATCAAGAGATGGTTTCAACTATGTGAGTTGAATGCACACATCACAAAGAACTA
         66 CAAAACTGCTCAATCAAAAGAAAGGTTCAACTCTGTGAGATGAATGCACACATCACAAAGAAGTT

             *  *    *                               
      59686 TGTCGGAATTCTTCTGTGTAGTTTTTATGTGAAGATATTTC
        131 TCTCAGAATGCTTCTGTGTAGTTTTTATGTGAAGATATTTC

                     *    * *      * *    *                  *               
      59727 CTTTTCCACAATAGACGTCAAAGTGATCCAGATATCCACTTGCAGATTCCACAAAAAGAGTGTTT
          1 CTTTTCCACCATAGGCCTCAAAGCGCTCCAAATATCCACTTGCAGATTCTACAAAAAGAGTGTTT

                 *   *   *                  **                     *  *      
      59792 CAAAAGTGCACAACCAAAAGAAAGGTTCAACTAGGTGAGATGAATGCACACATCAGAAGGAAGTT
         66 CAAAACTGCTCAATCAAAAGAAAGGTTCAACTCTGTGAGATGAATGCACACATCACAAAGAAGTT

                            **   *     * *       *   
      59857 TCTCAGAATGCTTCTGCATAGCTTTTAAGGGAAGATACTTC
        131 TCTCAGAATGCTTCTGTGTAGTTTTTATGTGAAGATATTTC

                    *               *            * *  *    * *               
      59898 CTTTTCCAACATAGGCCTCAAAGCACTCCAAATATCCTCCTGGAGATACCACAAAAAGAGTGTTT
          1 CTTTTCCACCATAGGCCTCAAAGCGCTCCAAATATCCACTTGCAGATTCTACAAAAAGAGTGTTT

            **                      *  *                 *       *           
      59963 GCAAACTGCTCAATCAAAAGAAAGATTTAACTCTGTGAGATGAATCCACACATGACAAAGAAGTT
         66 CAAAACTGCTCAATCAAAAGAAAGGTTCAACTCTGTGAGATGAATGCACACATCACAAAGAAGTT

                                                     
      60028 TCTCAGAATGCTTCTGTGTAGTTTTTATGTGAAGATATTTC
        131 TCTCAGAATGCTTCTGTGTAGTTTTTATGTGAAGATATTTC

                     *   * * *    **           *                *     *      
      60069 CTTTTCCACAATAAGACCCAAAAGGCTCCAAATATTCACTTGCAGATTCTAAAAAAAACAGTGTT
          1 CTTTTCCACCATAGGCCTCAAAGCGCTCCAAATATCCACTTGCAGATTCT-ACAAAAAGAGTGTT

                                  *                  *      *        **      
      60134 TCAAAACTGCTCAATCAAAAGATA-GTTCAACTCTGTGAGAAGAATGCTCACATCACTGAGAAGT
         65 TCAAAACTGCTCAATCAAAAGAAAGGTTCAACTCTGTGAGATGAATGCACACATCACAAAGAAGT

                                         *            
      60198 TTCTCAGAATGCTTCTGTGTAGTTTTTATATGAAGATATTTC
        130 TTCTCAGAATGCTTCTGTGTAGTTTTTATGTGAAGATATTTC

                *     *      *    **                       *   **       *    
      60240 CTTTCCCACCGTAGGCCACAAAAGGCTCCAAATATCCACTTGCAGATACTATGAAAAGAGAGTTT
          1 CTTTTCCACCATAGGCCTCAAAGCGCTCCAAATATCCACTTGCAGATTCTACAAAAAGAGTGTTT

                        *        *               ***            *            
      60305 CAAAACTGCTCATTCAAAAGATAGGTTCAACTCTGTGGTTTGAATGCACACAGCACAAAGAAGTT
         66 CAAAACTGCTCAATCAAAAGAAAGGTTCAACTCTGTGAGATGAATGCACACATCACAAAGAAGTT

              *       **                   **   *    
      60370 TCACAGAATGTGTCTGTGTAGTTTTTATGTGCGGATGTTTC
        131 TCTCAGAATGCTTCTGTGTAGTTTTTATGTGAAGATATTTC

                         *        ****   *   *               *    *          
      60411 CTTTTCCACCATATGCCT-AAATATTTCCCAATTTCCACTTGCAGATTCCACAAGAAGAGTGTTT
          1 CTTTTCCACCATAGGCCTCAAAGCGCTCCAAATATCCACTTGCAGATTCTACAAAAAGAGTGTTT

                      **       *       *           *            *      ***   
      60475 CAAAACTGCTGTATC-AAATAAA-GTTGAACTCTGTGAGGTGAATGCACACAGCACAAAATGGTT
         66 CAAAACTGCTCAATCAAAAGAAAGGTTCAACTCTGTGAGATGAATGCACACATCACAAAGAAGTT

                          **   *        *       *    
      60538 TCTCAGAATGCTTCCTTGTTGTTTTTATATGAAGATGTTTC
        131 TCTCAGAATGCTTCTGTGTAGTTTTTATGTGAAGATATTTC

                  *  *             *   *     *                               
      60579 CTTTTCAACAATAGGCCTCAAAGTGCTTCAAATGTCCACTTGCAGATTCTACAAAAAGAGTGTTT
          1 CTTTTCCACCATAGGCCTCAAAGCGCTCCAAATATCCACTTGCAGATTCTACAAAAAGAGTGTTT

                                   *    *      *  *  *                       
      60644 CAAAACTGCTCAATCAAAAGAAAAGTTCGACTCTGGGAAATTAATGCACACATCACAAAGAAGTT
         66 CAAAACTGCTCAATCAAAAGAAAGGTTCAACTCTGTGAGATGAATGCACACATCACAAAGAAGTT

                                     *        *      
      60709 TCTC---A-GCTTCTGTGTAGTTTTCATGTGAAGTTATTTC
        131 TCTCAGAATGCTTCTGTGTAGTTTTTATGTGAAGATATTTC

                     *       *     *            *    *         **       *    
      60746 CTTTTCCACAATAGGCCGCAAAGGGCTCCAAATATCAACTTACAGATTCTAGGAAAAGAGAGTTT
          1 CTTTTCCACCATAGGCCTCAAAGCGCTCCAAATATCCACTTGCAGATTCTACAAAAAGAGTGTTT

                       * **      *     *                                     
      60811 CAAAACTGCTCTACGAAAAGATAGGTTGAACTCTGTGAGATGAATGCACACATCACAAAGAAGTT
         66 CAAAACTGCTCAATCAAAAGAAAGGTTCAACTCTGTGAGATGAATGCACACATCACAAAGAAGTT

                       *               * *     *     
      60876 TCTCAGAATGCATCTGTGTAGTTTTTACGGGAAGACATTTC
        131 TCTCAGAATGCTTCTGTGTAGTTTTTATGTGAAGATATTTC

                        ***  *     **      *  *                  *     ***   
      60917 CTTTTCCACCATCTTCCACAAAGGTCTCCAAGTAACCACTTGCAGATTCTACAGAAAGACACTTT
          1 CTTTTCCACCATAGGCCTCAAAGCGCTCCAAATATCCACTTGCAGATTCTACAAAAAGAGTGTTT

            *          *         ***      *      ***                      *  
      60982 AAAAACTGCTCTATCAAAAGATCAGTTCAAGTCTGTGGTTTGAATGCACACATCACAAAGAATTT
         66 CAAAACTGCTCAATCAAAAGAAAGGTTCAACTCTGTGAGATGAATGCACACATCACAAAGAAGTT

                                     *  *            
      61047 TCTCAGAATGCTTCTGTGTAGTTTTCATATGAAGATATTTC
        131 TCTCAGAATGCTTCTGTGTAGTTTTTATGTGAAGATATTTC

                                    *                                   **   
      61088 CTTTTCCACCATAGGCCTCAAAGCACTCCAAATATCCACTTGCAGATTCTACAAAAAGAGATTTT
          1 CTTTTCCACCATAGGCCTCAAAGCGCTCCAAATATCCACTTGCAGATTCTACAAAAAGAGTGTTT

                   **                           *  *          *        *     
      61153 CAAAACTAGTCAATCAAAAGAAAGGTTCAACTCTGTCAGTTGAATGCACATATCACAAACAAGTT
         66 CAAAACTGCTCAATCAAAAGAAAGGTTCAACTCTGTGAGATGAATGCACACATCACAAAGAAGTT

                *      *                             
      61218 TCTCGGAATGCGTCTGTGTAGTTTTTATGTGAAGATATTTC
        131 TCTCAGAATGCTTCTGTGTAGTTTTTATGTGAAGATATTTC

               *     * *           *     *                  *    *           
      61259 CTTCTCCACAACAGGCCTCAAAGTGCTCCGAATATCCACTTGCAGATTTTAC-TAAAGAGTGTTT
          1 CTTTTCCACCATAGGCCTCAAAGCGCTCCAAATATCCACTTGCAGATTCTACAAAAAGAGTGTTT

             *               *  *   *     *        *    *               *    
      61323 CCAAACTGCTCAATCAAGAGGAAGTTTCAAGTCTGTGAGCTGAACGCACACATCACAAAGTAGTT
         66 CAAAACTGCTCAATCAAAAGAAAGGTTCAACTCTGTGAGATGAATGCACACATCACAAAGAAGTT

               *                                *    
      61388 TCTGAGAATGCTTCTGTGTAGTTTTTATGTGAAGATGTTTC
        131 TCTCAGAATGCTTCTGTGTAGTTTTTATGTGAAGATATTTC

                            **     *                                    *    
      61429 CTTTTCCACCATAGGCTGCAAAGGGCTCCAAATATCCACTTGCAGATTCTACAAAAAGAGAGTTT
          1 CTTTTCCACCATAGGCCTCAAAGCGCTCCAAATATCCACTTGCAGATTCTACAAAAAGAGTGTTT

                 *     *         *          *     *                     *    
      61494 CAAAAGTGCTCTATCAAAAGATAGGTTCAACTATGTGATATGAATGCACACATCACAAAGTAGTT
         66 CAAAACTGCTCAATCAAAAGAAAGGTTCAACTCTGTGAGATGAATGCACACATCACAAAGAAGTT

                                          *          
      61559 TCTCAGAATGCTTCTGTGTAGTTTTTATGTAAAGATATTTC
        131 TCTCAGAATGCTTCTGTGTAGTTTTTATGTGAAGATATTTC

                                    *                                   **   
      61600 CTTTTCCACCATAGGCCTCAAAGCACTCCAAATATCCACTTGCAGATTCTACAAAAAGAGATTTT
          1 CTTTTCCACCATAGGCCTCAAAGCGCTCCAAATATCCACTTGCAGATTCTACAAAAAGAGTGTTT

                   ** *                   *     *  *    * *   *        *     
      61665 CAAAACTATTTAATCAAAAGAAAGGTTCAAATCTGTCAGTTGAAGGTACATATCACAAACAAGTT
         66 CAAAACTGCTCAATCAAAAGAAAGGTTCAACTCTGTGAGATGAATGCACACATCACAAAGAAGTT

             * **                                    
      61730 TATTGGAATGCTTCTGTGTAGTTTTTATGTGAAGATATTTC
        131 TCTCAGAATGCTTCTGTGTAGTTTTTATGTGAAGATATTTC

                     * *         * *                        **  *     *      
      61771 CTTTTCCACAACAGGCCTCAAGGTGCTCCAAATATCCACTTGCAGATTTCACTAAAAGTGTGTTT
          1 CTTTTCCACCATAGGCCTCAAAGCGCTCCAAATATCCACTTGCAGATTCTACAAAAAGAGTGTTT

             *  *            *  *   *     *        *             *           
      61836 CCAAGCTGCTCAATCAAGAGGAAGTTTCAAGTCTGTGAGGTGAATGCACACATTACAAAGAAGTT
         66 CAAAACTGCTCAATCAAAAGAAAGGTTCAACTCTGTGAGATGAATGCACACATCACAAAGAAGTT

            *  *                                     
      61901 ACTGAGAATGCTTCTGTGTAGTTTTTATGTGAAGATATTTC
        131 TCTCAGAATGCTTCTGTGTAGTTTTTATGTGAAGATATTTC

                      **               *                                *    
      61942 CTTTTCCACCGCAGGCCTCAAAGCGCTGCAAATATCCACTTGCAGATTCTACAAAAAGAGAGTTT
          1 CTTTTCCACCATAGGCCTCAAAGCGCTCCAAATATCCACTTGCAGATTCTACAAAAAGAGTGTTT

                      **         *   *         *   *     **            *     
      62007 CAAAACTGCTGTATCAAAAGATAGGGTCAACTCTGCGAGTTGAATAAACACATCACAAATAAGTT
         66 CAAAACTGCTCAATCAAAAGAAAGGTTCAACTCTGTGAGATGAATGCACACATCACAAAGAAGTT

               **   *        *               *       
      62072 TCTGGGAACGCTTCTGTATAG-TTTTATGTGAATATATTTC
        131 TCTCAGAATGCTTCTGTGTAGTTTTTATGTGAAGATATTTC

                         *          *                   *   *  *   * *   *   
      62112 CTTTTCCACCATATGCCTCAAAGCACTCCAAATATCCACTTGCACATTATAGAAACATAGTCTTT
          1 CTTTTCCACCATAGGCCTCAAAGCGCTCCAAATATCCACTTGCAGATTCTACAAAAAGAGTGTTT

                   **                        *         *             *       
      62177 CAAAACTTGTCAATC-AAAGAAAGGTTCAACTCCGTGAGATGAGTGCACACATCACAGAGAAGTT
         66 CAAAACTGCTCAATCAAAAGAAAGGTTCAACTCTGTGAGATGAATGCACACATCACAAAGAAGTT

                *     *                            * 
      62241 TCTCGGAATGTTTCTGTGTAGTTTTTATGTGAAGATATTGC
        131 TCTCAGAATGCTTCTGTGTAGTTTTTATGTGAAGATATTTC

                     *               *            *          *       *   *   
      62282 CTTTTCCACAATAGGCCTCAAAGCGTTCCAAATATCCAATTGCAGATTCCACAAAAAAAGTTTTT
          1 CTTTTCCACCATAGGCCTCAAAGCGCTCCAAATATCCACTTGCAGATTCTACAAAAAGAGTGTTT

            *                 *  *  *  *             * *         **    *     
      62347 TAAAACTGCTCAATCAAATGATAGATTAAACTCTGTGAGATTAGTGCACACATGTCAAAAAAGTT
         66 CAAAACTGCTCAATCAAAAGAAAGGTTCAACTCTGTGAGATGAATGCACACATCACAAAGAAGTT

                                *      * *           
      62412 TCTCAGAATGCTTCTGTGTACTTTTTAGGGGAAGATATTTC
        131 TCTCAGAATGCTTCTGTGTAGTTTTTATGTGAAGATATTTC

                        *    *     **         *    *           **  * *  *    
      62453 CTTTTCCACCATCGGCCACAAAGGACTCCAAATAACCACATGCAGATTCTAGTAACACAGAGTTT
          1 CTTTTCCACCATAGGCCTCAAAGCGCTCCAAATATCCACTTGCAGATTCTACAAAAAGAGTGTTT

                       *         * *           *   * * *    **    ***        
      62518 CAAAACTGCTCTATCAAAAGATAAGTTCAACTCTGAGAGTTTAGTGCAACCATCGTGAAGAAGTT
         66 CAAAACTGCTCAATCAAAAGAAAGGTTCAACTCTGTGAGATGAATGCACACATCACAAAGAAGTT

                            *     *                  
      62583 TCTCAGAATGCTTCTGAGTAGTGTTTATGTGAAGATATTTC
        131 TCTCAGAATGCTTCTGTGTAGTTTTTATGTGAAGATATTTC

                              *     *                      *           **    
      62624 CTTTTCCACCATAGGCCTGAAAGCCCTCCAAATATCCACTTGCAGATCCTACAAAAAGAAAGTTT
          1 CTTTTCCACCATAGGCCTCAAAGCGCTCCAAATATCCACTTGCAGATTCTACAAAAAGAGTGTTT

             *         **     *  *  *   *        **      *                   
      62689 CGAAA-TGCTCTCTCAAACGATAGTTTCGACTCTGTGGTATGAATACACACATCACAAAGAAGTT
         66 CAAAACTGCTCAATCAAAAGAAAGGTTCAACTCTGTGAGATGAATGCACACATCACAAAGAAGTT

                                       **            
      62753 TCTCAGAATGCTTCTGTGTAGTTTTTAAATGAAGATATTTC
        131 TCTCAGAATGCTTCTGTGTAGTTTTTATGTGAAGATATTTC

            *                       *          *     *                       
      62794 TTTTTCCACCATAGGCCTCAAAGCACTCCAAATATGCACTTCCAGATTCTACAAAAAGAGTGTTT
          1 CTTTTCCACCATAGGCCTCAAAGCGCTCCAAATATCCACTTGCAGATTCTACAAAAAGAGTGTTT

              *                 *       * *         **   *         *  * *    
      62859 CAGAACTGCTCAATCAAAAGGAAGGTTCCAGTC--TGAGACAAATACACACATCAAAAGGTAGTT
         66 CAAAACTGCTCAATCAAAAGAAAGGTTCAACTCTGTGAGATGAATGCACACATCACAAAGAAGTT

                                                    *
      62922 TCTCAGAATGCTTCTGTGTAGTTTTTATGTGAAGATATTTT
        131 TCTCAGAATGCTTCTGTGTAGTTTTTATGTGAAGATATTTC

             *               *    **    *     *      *  **   *          *    
      62963 CCTTTCCACCATAGGCCACAAATGGCTCTAAATACCCACTTACATTTTCCACAAAAAGAGAGTTT
          1 CTTTTCCACCATAGGCCTCAAAGCGCTCCAAATATCCACTTGCAGATTCTACAAAAAGAGTGTTT

                       * *    * *      *   *         *                       
      63028 CAAAACTGCTCTACCAAAGGTAA-GTTTAACGCTGTGAG-TTAA-G-A-ACATCACAAAGAAGTT
         66 CAAAACTGCTCAATCAAAAGAAAGGTTCAACTCTGTGAGATGAATGCACACATCACAAAGAAGTT

                                   *   *  *          
      63088 TCTCAGAATGCTTCTGTGTAGTTCTTACGTAAAGATATTTC
        131 TCTCAGAATGCTTCTGTGTAGTTTTTATGTGAAGATATTTC

                 *   *      ***    *                  *              **      
      63129 CTTTTACACAATAGGCAGAAAAGTGCTCCAAATATCCACTTGAAGATTCTACAAAAACCGTGTTT
          1 CTTTTCCACCATAGGCCTCAAAGCGCTCCAAATATCCACTTGCAGATTCTACAAAAAGAGTGTTT

                     **                                          *      *    
      63194 CAAAACTGCCGAATCAAAAGAAAGGTTCAACTCTGTGAGATGAATGCACACATAACAAAGGAGTT
         66 CAAAACTGCTCAATCAAAAGAAAGGTTCAACTCTGTGAGATGAATGCACACATCACAAAGAAGTT

                                 *      *      *    *
      63259 TCTCAGAATGCTTCTGTGTAGCTTTTATATGAAGACATTTA
        131 TCTCAGAATGCTTCTGTGTAGTTTTTATGTGAAGATATTTC

            *        * *            *   ***                     **        *  
      63300 GTTTTCCACAACAGGCCTCAAAGCTCTCTCCATATCCACTTGCAGATTCTACCGAAAGAGTGCTT
          1 CTTTTCCACCATAGGCCTCAAAGCGCTCCAAATATCCACTTGCAGATTCTACAAAAAGAGTGTTT

             *                   * **     *        *        *     **         
      63365 CCAAACTGCTCAATCAAAAGAGACATTCAAATCTGTGAGGTGAATGCAGACATCGTAAAGAAGTT
         66 CAAAACTGCTCAATCAAAAGAAAGGTTCAACTCTGTGAGATGAATGCACACATCACAAAGAAGTT

                                *     *       *    **
      63430 TCTCAGAATGCTTCTGTGTATTTTTTGTGTGAAGTTATTCG
        131 TCTCAGAATGCTTCTGTGTAGTTTTTATGTGAAGATATTTC

            *    *             *     *  *            **                 *    
      63471 TTTTTGCACCATAGGCCTCCAAGCGTTCTAAATATCCACTTCTAGATTCTACAAAAAGAGAGTTT
          1 CTTTTCCACCATAGGCCTCAAAGCGCTCCAAATATCCACTTGCAGATTCTACAAAAAGAGTGTTT

                   *         *            *        *    *   *                
      63536 CAAAACTACTC-A--AACA-AAAGGTTCAATTCTGTGAGTTGAAAGCAAACATCACAAAGAAGTT
         66 CAAAACTGCTCAATCAAAAGAAAGGTTCAACTCTGTGAGATGAATGCACACATCACAAAGAAGTT

                       *                             
      63597 TCTCAGAATGCGTCTGTGTAGTTTTTATGTGAAGATATTTC
        131 TCTCAGAATGCTTCTGTGTAGTTTTTATGTGAAGATATTTC

                     **   ****     *                  *                      
      63638 CTTTT-CACAGTAGAATGCAAAGGGCTCCAAATATCCACTTGGAGATTCTACAAAAAGA--GTTT
          1 CTTTTCCACCATAGGCCTCAAAGCGCTCCAAATATCCACTTGCAGATTCTACAAAAAGAGTGTTT

                  *    **     *  *     *     ***   *     *               *   
      63700 CAAAACCGCTCTGTCAAATGATAGGTTGAACTCCCGGAGGTGAATACACACATCACAAAGAGGTT
         66 CAAAACTGCTCAATCAAAAGAAAGGTTCAACTCTGTGAGATGAATGCACACATCACAAAGAAGTT

                  *                       *  *       
      63765 TCTCAGCATGCTTCTGTGTAGTTTTTATGTAAACATATTTC
        131 TCTCAGAATGCTTCTGTGTAGTTTTTATGTGAAGATATTTC

             *    * *              *           *      * *       *   *    *   
      63806 CGTTTCTATCATAGGCCTCAAAGTGCTCCAAATATTCACTTGTACATTCTACCAAACGAGTATTT
          1 CTTTTCCACCATAGGCCTCAAAGCGCTCCAAATATCCACTTGCAGATTCTACAAAAAGAGTGTTT

                              * *         ** *    *        *            *    
      63871 CAAAACTGCTCAATCAAACGGAAGGTTCAAAACCGTGACATGAATGCCCACATCACAAAGTAGTT
         66 CAAAACTGCTCAATCAAAAGAAAGGTTCAACTCTGTGAGATGAATGCACACATCACAAAGAAGTT

                                                     
      63936 TCTCAGAATGCTTCTGTGTAGTTTTTATGTGAAGATATTTC
        131 TCTCAGAATGCTTCTGTGTAGTTTTTATGTGAAGATATTTC

                     * *    *      *    *       * *   **      *              
      63977 CTTTTCCACAACA-GCGTGCAAAACGCTTCAAATATGCCCTTAGAGATTCCACAAAAAGAGTGTT
          1 CTTTTCCACCATAGGCCT-CAAAGCGCTCCAAATATCCACTTGCAGATTCTACAAAAAGAGTGTT

              *     *               *  *                                  ** 
      64041 TCCAAACTACTCAAATCAAAA-AATGATTTCAACTCTGTGAGATGAATGCACACATCACAAACTA
         65 TCAAAACTGCTC-AATCAAAAGAAAG-GTTCAACTCTGTGAGATGAATGCACACATCACAAAGAA

                         *     ** *   * *   *       *   
      64105 GTTTCTCAGAATGTTTCTGCCTGGTTCTCATGCGAAGATAGTTC
        128 GTTTCTCAGAATGCTTCTGTGTAGTTTTTATGTGAAGATATTTC

                 *           *   * **              *                  * *    
      64149 CTTTTTCACCATAGGCCGCAATGTACTCCAAATATCCACCTGCAGATTCTAC-AAAAGTGAGTTT
          1 CTTTTCCACCATAGGCCTCAAAGCGCTCCAAATATCCACTTGCAGATTCTACAAAAAGAGTGTTT

                       *         ***    **         *        *      *       * 
      64213 CAAAACTGCTCTATCAAAAGATCAGTTCGTCTCTGTGAGTTGAATGCATACATCAAAAAGAAGCT
         66 CAAAACTGCTCAATCAAAAGAAAGGTTCAACTCTGTGAGATGAATGCACACATCACAAAGAAGTT

                 *             *      **         *   
      64278 TCTCAAAATGCTTCTGTGTGGTTTTTCGGTGAAGATAGTTC
        131 TCTCAGAATGCTTCTGTGTAGTTTTTATGTGAAGATATTTC

            *     *        *      * *                 *        *        *    
      64319 TTTTTCTACCATAGGTCTCAAACCACTCCAAATATCCACTTGTAGATTCTATAAAAAGGAATG-T
          1 CTTTTCCACCATAGGCCTCAAAGCGCTCCAAATATCCACTTGCAGATTCTACAAAAA-GAGTGTT

                  *        *    *    *      * *         *      *         *   
      64383 TCAAAATTGCTCAATAAAAATAAAGTTTCAACACCGTGAGATGAGTGCACAAATCACAAAGGAGT
         65 TCAAAACTGCTCAATCAAAAGAAAGGTTCAACTCTGTGAGATGAATGCACACATCACAAAGAAGT

                  *          *         *          *   
      64448 TTCTCAAAATGCTTCTGGGTAGTTTTTCTGTGAAGATAGTTC
        130 TTCTCAGAATGCTTCTGTGTAGTTTTTATGTGAAGATATTTC

                  *     *    *     *          *                         *    
      64490 CTTTTCTACCATGGGCCACAAAGGGCTCCAAATACCCACTTGCAGATTCTACAAAAAGAGAGTTT
          1 CTTTTCCACCATAGGCCTCAAAGCGCTCCAAATATCCACTTGCAGATTCTACAAAAAGAGTGTTT

              *        *      ** * *        *    * *    **  * *           *  
      64555 CACAACTGCTCTATCAAACAATATGTTCAACTTTGTGGGTTGAACACAAATATCAC-AAGAATTT
         66 CAAAACTGCTCAATCAAAAGAAAGGTTCAACTCTGTGAGATGAATGCACACATCACAAAGAAGTT

                **                             *     
      64619 TCTCCCAATGCTTCTGTGTAGTTTTTATGTGAAGACATTTC
        131 TCTCAGAATGCTTCTGTGTAGTTTTTATGTGAAGATATTTC

            *   *  *      *  *     *                 *  *      *       *  *  
      64660 TTTTCCCTCCATAGTCCACAAAGTGCTCCAAATATCCACTTACATATTCTAGAAAAAGATTGCTT
          1 CTTTTCCACCATAGGCCTCAAAGCGCTCCAAATATCCACTTGCAGATTCTACAAAAAGAGTGTTT

            **       *    *               * * *                              
      64725 GGAAACTGCACAATGAAAAGAAAGGTTCAAATATATGAGATGAATGCACACATCACAAAGAAGTT
         66 CAAAACTGCTCAATCAAAAGAAAGGTTCAACTCTGTGAGATGAATGCACACATCACAAAGAAGTT

                                 *                    
      64790 TCTCAGAAT-CTCTCTGTGTAATTTTTATGTGAAGATATTTC
        131 TCTCAGAATGCT-TCTGTGTAGTTTTTATGTGAAGATATTTC

                *     *    *  *   *                        *      *    *     
      64831 CTTTCCCACCTTAGGTCTTAAAACGCTCCAAATATCCACTTGCAGATACTACAAGAAGATTGTTT
          1 CTTTTCCACCATAGGCCTCAAAGCGCTCCAAATATCCACTTGCAGATTCTACAAAAAGAGTGTTT

                     *    *        *      *     **                           
      64896 CAAAACTGCACAA-AAAAAGAAATGTTCAATTCTGTTTGATGAATGCACACATCACAAAGAAGTT
         66 CAAAACTGCTCAATCAAAAGAAAGGTTCAACTCTGTGAGATGAATGCACACATCACAAAGAAGTT

                           *                         
      64960 TCTCAGAATGCTTCTCTGTAGTTTTTATGTGAAGATATTTC
        131 TCTCAGAATGCTTCTGTGTAGTTTTTATGTGAAGATATTTC

                     *             *                 *         **      * *   
      65001 CTTTTCCACAATAGGCCTCAAAGGGCTCCAAATATCCACTTCCAGATTCTATGAAAAGAATATTT
          1 CTTTTCCACCATAGGCCTCAAAGCGCTCCAAATATCCACTTGCAGATTCTACAAAAAGAGTGTTT

             *              * *    *                                      *  
      65066 CCAAACTGCTCAATCATAGGAAATGTTCAACTCTGTGAGATGAATGCACACATCAC-AAGAAATT
         66 CAAAACTGCTCAATCAAAAGAAAGGTTCAACTCTGTGAGATGAATGCACACATCACAAAGAAGTT

                     *    *      *       *       *   
      65130 TCTCAGAATCCTTCAGTGTAGGTTTTATGAGAAGATAATTC
        131 TCTCAGAATGCTTCTGTGTAGTTTTTATGTGAAGATATTTC

                     *    **        *   *                           *    *   
      65171 CTTTTCCACAATAGTTCTCAAAGCACTCAAAATATCCACTTGCAGATTCTACAAAAGGAGTATTT
          1 CTTTTCCACCATAGGCCTCAAAGCGCTCCAAATATCCACTTGCAGATTCTACAAAAAGAGTGTTT

                                                          *                  
      65236 CAAAACTGCTCAATCAAAAGAAAGGTTCAACTCTGTGAGATGAATGGACACATCACAAAGAAGTT
         66 CAAAACTGCTCAATCAAAAGAAAGGTTCAACTCTGTGAGATGAATGCACACATCACAAAGAAGTT

                                  *   *              
      65301 TCTCAGAATGCTTCTGTGTAGTATTTTTGTGAAGATATTT-
        131 TCTCAGAATGCTTCTGTGTAGTTTTTATGTGAAGATATTTC

                          *  * * * * ***             *            *     * *  
      65341 CTTTTCCACCATAGACCGCCAGGGGACACAAATATCCACTTTCAGATTCTACAACAAGAGAGGTT
          1 CTTTTCCACCATAGGCCTCAAAGCGCTCCAAATATCCACTTGCAGATTCTACAAAAAGAGTGTTT

                   *   *     *   ** *       *      *                      * *
      65406 CAAAACTACTCGATCAAGAGATGGTTTCAACTATGTGAGTTGAATGCACACATCACAAAGAACTA
         66 CAAAACTGCTCAATCAAAAGAAAGGTTCAACTCTGTGAGATGAATGCACACATCACAAAGAAGTT

             *  *    *                               
      65471 TGTCGGAATTCTTCTGTGTAGTTTTTATGTGAAGATATTTC
        131 TCTCAGAATGCTTCTGTGTAGTTTTTATGTGAAGATATTTC

                     *    * *      * *    *                  *               
      65512 CTTTTCCACAATAGACGTCAAAGTGATCCAGATATCCACTTGCAGATTCCACAAAAAGAGTGTTT
          1 CTTTTCCACCATAGGCCTCAAAGCGCTCCAAATATCCACTTGCAGATTCTACAAAAAGAGTGTTT

                 *   *   *                  **                     *  *      
      65577 CAAAAGTGCACAACCAAAAGAAAGGTTCAACTAGGTGAGATGAATGCACACATCAGAAGGAAGTT
         66 CAAAACTGCTCAATCAAAAGAAAGGTTCAACTCTGTGAGATGAATGCACACATCACAAAGAAGTT

                            **   *     * *       *   
      65642 TCTCAGAATGCTTCTGCATAGCTTTTAAGGGAAGATACTTC
        131 TCTCAGAATGCTTCTGTGTAGTTTTTATGTGAAGATATTTC

                    *               *            * *  *    * *               
      65683 CTTTTCCAACATAGGCCTCAAAGCACTCCAAATATCCTCCTGGAGATACCACAAAAAGAGTGTTT
          1 CTTTTCCACCATAGGCCTCAAAGCGCTCCAAATATCCACTTGCAGATTCTACAAAAAGAGTGTTT

            **                      *  *                 *       *           
      65748 GCAAACTGCTCAATCAAAAGAAAGATTTAACTCTGTGAGATGAATCCACACATGACAAAGAAGTT
         66 CAAAACTGCTCAATCAAAAGAAAGGTTCAACTCTGTGAGATGAATGCACACATCACAAAGAAGTT

                                                     
      65813 TCTCAGAATGCTTCTGTGTAGTTTTTATGTGAAGATATTTC
        131 TCTCAGAATGCTTCTGTGTAGTTTTTATGTGAAGATATTTC

                     *   * * *    **           *                *     *      
      65854 CTTTTCCACAATAAGACCCAAAAGGCTCCAAATATTCACTTGCAGATTCTAAAAAAAACAGTGTT
          1 CTTTTCCACCATAGGCCTCAAAGCGCTCCAAATATCCACTTGCAGATTCT-ACAAAAAGAGTGTT

                                  *                  *      *        **      
      65919 TCAAAACTGCTCAATCAAAAGATA-GTTCAACTCTGTGAGAAGAATGCTCACATCACTGAGAAGT
         65 TCAAAACTGCTCAATCAAAAGAAAGGTTCAACTCTGTGAGATGAATGCACACATCACAAAGAAGT

                                         *            
      65983 TTCTCAGAATGCTTCTGTGTAGTTTTTATATGAAGATATTTC
        130 TTCTCAGAATGCTTCTGTGTAGTTTTTATGTGAAGATATTTC

                *     *      *    **                       *   **       *    
      66025 CTTTCCCACCGTAGGCCACAAAAGGCTCCAAATATCCACTTGCAGATACTATGAAAAGAGAGTTT
          1 CTTTTCCACCATAGGCCTCAAAGCGCTCCAAATATCCACTTGCAGATTCTACAAAAAGAGTGTTT

                        *        *               ***            *            
      66090 CAAAACTGCTCATTCAAAAGATAGGTTCAACTCTGTGGTTTGAATGCACACAGCACAAAGAAGTT
         66 CAAAACTGCTCAATCAAAAGAAAGGTTCAACTCTGTGAGATGAATGCACACATCACAAAGAAGTT

              *       **                   **   *    
      66155 TCACAGAATGTGTCTGTGTAGTTTTTATGTGCGGATGTTTC
        131 TCTCAGAATGCTTCTGTGTAGTTTTTATGTGAAGATATTTC

                         *        ****   *   *                    *          
      66196 CTTTTCCACCATATGCCT-AAATATTTCCCAATTTCCACTTGCAGATTCTACAAGAAGAGTGTTT
          1 CTTTTCCACCATAGGCCTCAAAGCGCTCCAAATATCCACTTGCAGATTCTACAAAAAGAGTGTTT

                      **       *       *           *            *      ***   
      66260 CAAAACTGCTGTATC-AAATAAA-GTTGAACTCTGTGAGGTGAATGCACACAGCACAAAATGGTT
         66 CAAAACTGCTCAATCAAAAGAAAGGTTCAACTCTGTGAGATGAATGCACACATCACAAAGAAGTT

                          **   *        *       *    
      66323 TCTCAGAATGCTTCCTTGTTGTTTTTATATGAAGATGTTTC
        131 TCTCAGAATGCTTCTGTGTAGTTTTTATGTGAAGATATTTC

                  *  *             *   *     *                               
      66364 CTTTTCAACAATAGGCCTCAAAGTGCTTCAAATGTCCACTTGCAGATTCTACAAAAAGAGTGTTT
          1 CTTTTCCACCATAGGCCTCAAAGCGCTCCAAATATCCACTTGCAGATTCTACAAAAAGAGTGTTT

                                        *      *  *  *                       
      66429 CAAAACTGCTCAATCAAAAGAAAGGTTCGACTCTGGGAAATTAATGCACACATCACAAAGAAGTT
         66 CAAAACTGCTCAATCAAAAGAAAGGTTCAACTCTGTGAGATGAATGCACACATCACAAAGAAGTT

                                     *        *      
      66494 TCTC---A-GCTTCTGTGTAGTTTTCATGTGAAGTTATTTC
        131 TCTCAGAATGCTTCTGTGTAGTTTTTATGTGAAGATATTTC

                     *       *     *            *    *         **       *    
      66531 CTTTTCCACAATAGGCCGCAAAGGGCTCCAAATATCAACTTACAGATTCTAGGAAAAGAGAGTTT
          1 CTTTTCCACCATAGGCCTCAAAGCGCTCCAAATATCCACTTGCAGATTCTACAAAAAGAGTGTTT

                       * **      *     *                                     
      66596 CAAAACTGCTCTACGAAAAGATAGGTTGAACTCTGTGAGATGAATGCACACATCACAAAGAAGTT
         66 CAAAACTGCTCAATCAAAAGAAAGGTTCAACTCTGTGAGATGAATGCACACATCACAAAGAAGTT

                       *               * *     *     
      66661 TCTCAGAATGCATCTGTGTAGTTTTTACGGGAAGACATTTC
        131 TCTCAGAATGCTTCTGTGTAGTTTTTATGTGAAGATATTTC

                        ***  *     **      *  *                  *     ***   
      66702 CTTTTCCACCATCTTCCACAAAGGTCTCCAAGTAACCACTTGCAGATTCTACAGAAAGACACTTT
          1 CTTTTCCACCATAGGCCTCAAAGCGCTCCAAATATCCACTTGCAGATTCTACAAAAAGAGTGTTT

            *          *         ***      *      ***                      *  
      66767 AAAAACTGCTCTATCAAAAGATCAGTTCAAGTCTGTGGTTTGAATGCACACATCACAAAGAATTT
         66 CAAAACTGCTCAATCAAAAGAAAGGTTCAACTCTGTGAGATGAATGCACACATCACAAAGAAGTT

                                     *  *            
      66832 TCTCAGAATGCTTCTGTGTAGTTTTCATATGAAGATATTTC
        131 TCTCAGAATGCTTCTGTGTAGTTTTTATGTGAAGATATTTC

                                    *                                   **   
      66873 CTTTTCCACCATAGGCCTCAAAGCACTCCAAATATCCACTTGCAGATTCTACAAAAAGAGATTTT
          1 CTTTTCCACCATAGGCCTCAAAGCGCTCCAAATATCCACTTGCAGATTCTACAAAAAGAGTGTTT

                   **                           *  *          *        *     
      66938 CAAAACTAGTCAATCAAAAGAAAGGTTCAACTCTGTCAGTTGAATGCACATATCACAAACAAGTT
         66 CAAAACTGCTCAATCAAAAGAAAGGTTCAACTCTGTGAGATGAATGCACACATCACAAAGAAGTT

                *      *                             
      67003 TCTCGGAATGCGTCTGTGTAGTTTTTATGTGAAGATATTTC
        131 TCTCAGAATGCTTCTGTGTAGTTTTTATGTGAAGATATTTC

               *     * *           *     *                  *    *           
      67044 CTTCTCCACAACAGGCCTCAAAGTGCTCCGAATATCCACTTGCAGATTTTAC-TAAAGAGTGTTT
          1 CTTTTCCACCATAGGCCTCAAAGCGCTCCAAATATCCACTTGCAGATTCTACAAAAAGAGTGTTT

             *               *  *   *     *        *    *               *    
      67108 CCAAACTGCTCAATCAAGAGGAAGTTTCAAGTCTGTGAGCTGAACGCACACATCACAAAGTAGTT
         66 CAAAACTGCTCAATCAAAAGAAAGGTTCAACTCTGTGAGATGAATGCACACATCACAAAGAAGTT

               *                                *    
      67173 TCTGAGAATGCTTCTGTGTAGTTTTTATGTGAAGATGTTTC
        131 TCTCAGAATGCTTCTGTGTAGTTTTTATGTGAAGATATTTC

                            **     *                                    *    
      67214 CTTTTCCACCATAGGCTGCAAAGGGCTCCAAATATCCACTTGCAGATTCTACAAAAAGAGAGTTT
          1 CTTTTCCACCATAGGCCTCAAAGCGCTCCAAATATCCACTTGCAGATTCTACAAAAAGAGTGTTT

                 *     *         *          *     *                     *    
      67279 CAAAAGTGCTCTATCAAAAGATAGGTTCAACTATGTGATATGAATGCACACATCACAAAGTAGTT
         66 CAAAACTGCTCAATCAAAAGAAAGGTTCAACTCTGTGAGATGAATGCACACATCACAAAGAAGTT

                                          *          
      67344 TCTCAGAATGCTTCTGTGTAGTTTTTATGTAAAGATATTTC
        131 TCTCAGAATGCTTCTGTGTAGTTTTTATGTGAAGATATTTC

                                    *                                   **   
      67385 CTTTTCCACCATAGGCCTCAAAGCACTCCAAATATCCACTTGCAGATTCTACAAAAAGAGATTTT
          1 CTTTTCCACCATAGGCCTCAAAGCGCTCCAAATATCCACTTGCAGATTCTACAAAAAGAGTGTTT

                   ** *                   *     *  *    * *   *        *     
      67450 CAAAACTATTTAATCAAAAGAAAGGTTCAAATCTGTCAGTTGAAGGTACATATCACAAACAAGTT
         66 CAAAACTGCTCAATCAAAAGAAAGGTTCAACTCTGTGAGATGAATGCACACATCACAAAGAAGTT

             * **                                    
      67515 TATTGGAATGCTTCTGTGTAGTTTTTATGTGAAGATATTTC
        131 TCTCAGAATGCTTCTGTGTAGTTTTTATGTGAAGATATTTC

                     * *         * *                        **  *     *      
      67556 CTTTTCCACAACAGGCCTCAAGGTGCTCCAAATATCCACTTGCAGATTTCACTAAAAGTGTGTTT
          1 CTTTTCCACCATAGGCCTCAAAGCGCTCCAAATATCCACTTGCAGATTCTACAAAAAGAGTGTTT

             *  *            *  *   *     *        *             *           
      67621 CCAAGCTGCTCAATCAAGAGGAAGTTTCAAGTCTGTGAGGTGAATGCACACATTACAAAGAAGTT
         66 CAAAACTGCTCAATCAAAAGAAAGGTTCAACTCTGTGAGATGAATGCACACATCACAAAGAAGTT

            *  *                                     
      67686 ACTGAGAATGCTTCTGTGTAGTTTTTATGTGAAGATATTTC
        131 TCTCAGAATGCTTCTGTGTAGTTTTTATGTGAAGATATTTC

                      **               *                                *    
      67727 CTTTTCCACCGCAGGCCTCAAAGCGCTGCAAATATCCACTTGCAGATTCTACAAAAAGAGAGTTT
          1 CTTTTCCACCATAGGCCTCAAAGCGCTCCAAATATCCACTTGCAGATTCTACAAAAAGAGTGTTT

                      **         *   *         *   *     **            *     
      67792 CAAAACTGCTGTATCAAAAGATAGGGTCAACTCTGCGAGTTGAATAAACACATCACAAATAAGTT
         66 CAAAACTGCTCAATCAAAAGAAAGGTTCAACTCTGTGAGATGAATGCACACATCACAAAGAAGTT

            *  **   *        *               *       
      67857 CCTGGGAACGCTTCTGTATAG-TTTTATGTGAATATATTTC
        131 TCTCAGAATGCTTCTGTGTAGTTTTTATGTGAAGATATTTC

                         *          *                   *   *  *   * *   *   
      67897 CTTTTCCACCATATGCCTCAAAGCACTCCAAATATCCACTTGCACATTATAGAAACATAGTCTTT
          1 CTTTTCCACCATAGGCCTCAAAGCGCTCCAAATATCCACTTGCAGATTCTACAAAAAGAGTGTTT

                   **                        *         *             *       
      67962 CAAAACTTGTCAATC-AAAGAAAGGTTCAACTCCGTGAGATGAGTGCACACATCACAGAGAAGTT
         66 CAAAACTGCTCAATCAAAAGAAAGGTTCAACTCTGTGAGATGAATGCACACATCACAAAGAAGTT

                *     *                            * 
      68026 TCTCGGAATGTTTCTGTGTAGTTTTTATGTGAAGATATTGC
        131 TCTCAGAATGCTTCTGTGTAGTTTTTATGTGAAGATATTTC

                     *               *            *          *       *   *   
      68067 CTTTTCCACAATAGGCCTCAAAGCGTTCCAAATATCCAATTGCAGATTCCACAAAAAAAGTTTTT
          1 CTTTTCCACCATAGGCCTCAAAGCGCTCCAAATATCCACTTGCAGATTCTACAAAAAGAGTGTTT

            *                 *  *  *  *             * *         **    *     
      68132 TAAAACTGCTCAATCAAATGATAGATTAAACTCTGTGAGATTAGTGCACACATGTCAAAAAAGTT
         66 CAAAACTGCTCAATCAAAAGAAAGGTTCAACTCTGTGAGATGAATGCACACATCACAAAGAAGTT

                                *      * *           
      68197 TCTCAGAATGCTTCTGTGTACTTTTTAGGGGAAGATATTTC
        131 TCTCAGAATGCTTCTGTGTAGTTTTTATGTGAAGATATTTC

                        *    *     **         *    *           **  * *  *    
      68238 CTTTTCCACCATCGGCCACAAAGGACTCCAAATAACCACATGCAGATTCTAGTAACACAGAGTTT
          1 CTTTTCCACCATAGGCCTCAAAGCGCTCCAAATATCCACTTGCAGATTCTACAAAAAGAGTGTTT

                       *         * *           *   * * *    **    ***        
      68303 CAAAACTGCTCTATCAAAAGATAAGTTCAACTCTGAGAGTTTAGTGCAACCATCGTGAAGAAGTT
         66 CAAAACTGCTCAATCAAAAGAAAGGTTCAACTCTGTGAGATGAATGCACACATCACAAAGAAGTT

                            *     *                  
      68368 TCTCAGAATGCTTCTGAGTAGTGTTTATGTGAAGATATTTC
        131 TCTCAGAATGCTTCTGTGTAGTTTTTATGTGAAGATATTTC

                              *     *                      *           **    
      68409 CTTTTCCACCATAGGCCTGAAAGCCCTCCAAATATCCACTTGCAGATCCTACAAAAAGAAAGTTT
          1 CTTTTCCACCATAGGCCTCAAAGCGCTCCAAATATCCACTTGCAGATTCTACAAAAAGAGTGTTT

             *         **     *  *  *   *        **      *                   
      68474 CGAAA-TGCTCTCTCAAACGATAGTTTCGACTCTGTGGTATGAATACACACATCACAAAGAAGTT
         66 CAAAACTGCTCAATCAAAAGAAAGGTTCAACTCTGTGAGATGAATGCACACATCACAAAGAAGTT

                                       **            
      68538 TCTCAGAATGCTTCTGTGTAGTTTTTAAATGAAGATATTTC
        131 TCTCAGAATGCTTCTGTGTAGTTTTTATGTGAAGATATTTC

            *                       *          *     *                       
      68579 TTTTTCCACCATAGGCCTCAAAGCACTCCAAATATGCACTTCCAGATTCTACAAAAAGAGTGTTT
          1 CTTTTCCACCATAGGCCTCAAAGCGCTCCAAATATCCACTTGCAGATTCTACAAAAAGAGTGTTT

              *                 *       * *         **   *         *  * *    
      68644 CAGAACTGCTCAATCAAAAGGAAGGTTCCAGTC--TGAGACAAATACACACATCAAAAGGTAGTT
         66 CAAAACTGCTCAATCAAAAGAAAGGTTCAACTCTGTGAGATGAATGCACACATCACAAAGAAGTT

                                                    *
      68707 TCTCAGAATGCTTCTGTGTAGTTTTTATGTGAAGATATTTT
        131 TCTCAGAATGCTTCTGTGTAGTTTTTATGTGAAGATATTTC

             *               *    **    *     *      *  **   *          *    
      68748 CCTTTCCACCATAGGCCACAAATGGCTCTAAATACCCACTTACATTTTCCACAAAAAGAGAGTTT
          1 CTTTTCCACCATAGGCCTCAAAGCGCTCCAAATATCCACTTGCAGATTCTACAAAAAGAGTGTTT

                       * *    * *      *   *         *                       
      68813 CAAAACTGCTCTACCAAAGGTAA-GTTTAACGCTGTGAG-TTAA-G-A-ACATCACAAAGAAGTT
         66 CAAAACTGCTCAATCAAAAGAAAGGTTCAACTCTGTGAGATGAATGCACACATCACAAAGAAGTT

                                   *   *  *          
      68873 TCTCAGAATGCTTCTGTGTAGTTCTTACGTAAAGATATTTC
        131 TCTCAGAATGCTTCTGTGTAGTTTTTATGTGAAGATATTTC

                 *   *      ***    *                  *              **      
      68914 CTTTTACACAATAGGCAGAAAAGTGCTCCAAATATCCACTTGAAGATTCTACAAAAACCGTGTTT
          1 CTTTTCCACCATAGGCCTCAAAGCGCTCCAAATATCCACTTGCAGATTCTACAAAAAGAGTGTTT

                     **                                          *      *    
      68979 CAAAACTGCCGAATCAAAAGAAAGGTTCAACTCTGTGAGATGAATGCACACATAACAAAGGAGTT
         66 CAAAACTGCTCAATCAAAAGAAAGGTTCAACTCTGTGAGATGAATGCACACATCACAAAGAAGTT

                                 *      *      *    *
      69044 TCTCAGAATGCTTCTGTGTAGCTTTTATATGAAGACATTTA
        131 TCTCAGAATGCTTCTGTGTAGTTTTTATGTGAAGATATTTC

            *        * *            *   ***                     **        *  
      69085 GTTTTCCACAACAGGCCTCAAAGCTCTCTCCATATCCACTTGCAGATTCTACCGAAAGAGTGCTT
          1 CTTTTCCACCATAGGCCTCAAAGCGCTCCAAATATCCACTTGCAGATTCTACAAAAAGAGTGTTT

             *                   * **     *        *        *     **         
      69150 CCAAACTGCTCAATCAAAAGAGACATTCAAATCTGTGAGGTGAATGCAGACATCGTAAAGAAGTT
         66 CAAAACTGCTCAATCAAAAGAAAGGTTCAACTCTGTGAGATGAATGCACACATCACAAAGAAGTT

                                *     *       *    **
      69215 TCTCAGAATGCTTCTGTGTATTTTTTGTGTGAAGTTATTCG
        131 TCTCAGAATGCTTCTGTGTAGTTTTTATGTGAAGATATTTC

            *    *             *     *  *            **                 *    
      69256 TTTTTGCACCATAGGCCTCCAAGCGTTCTAAATATCCACTTCTAGATTCTACAAAAAGAGAGTTT
          1 CTTTTCCACCATAGGCCTCAAAGCGCTCCAAATATCCACTTGCAGATTCTACAAAAAGAGTGTTT

                   *         *            *        *    *   *                
      69321 CAAAACTACTC-A--AACA-AAAGGTTCAATTCTGTGAGTTGAAAGCAAACATCACAAAGAAGTT
         66 CAAAACTGCTCAATCAAAAGAAAGGTTCAACTCTGTGAGATGAATGCACACATCACAAAGAAGTT

                       *             *               
      69382 TCTCAGAATGCGTCTGTGTAGTTTTGATGTGAAGATATTTC
        131 TCTCAGAATGCTTCTGTGTAGTTTTTATGTGAAGATATTTC

                     **   ****     *                  *                      
      69423 CTTTT-CACAGTAGAATGCAAAGGGCTCCAAATATCCACTTGGAGATTCTACAAAAAGA--GTTT
          1 CTTTTCCACCATAGGCCTCAAAGCGCTCCAAATATCCACTTGCAGATTCTACAAAAAGAGTGTTT

                  *    **     *  *     *     ***   *     *               *   
      69485 CAAAACCGCTCTGTCAAATGATAGGTTGAACTCCCAGAGGTGAATACACACATCACAAAGAGGTT
         66 CAAAACTGCTCAATCAAAAGAAAGGTTCAACTCTGTGAGATGAATGCACACATCACAAAGAAGTT

                  *                       *  *       
      69550 TCTCAGCATGCTTCTGTGTAGTTTTTATGTAAACATATTTC
        131 TCTCAGAATGCTTCTGTGTAGTTTTTATGTGAAGATATTTC

             *    * *              *           *      * *       *   *    *   
      69591 CGTTTCTATCATAGGCCTCAAAGTGCTCCAAATATTCACTTGTACATTCTACCAAACGAGTATTT
          1 CTTTTCCACCATAGGCCTCAAAGCGCTCCAAATATCCACTTGCAGATTCTACAAAAAGAGTGTTT

                              * *         ** *    *        *            *    
      69656 CAAAACTGCTCAATCAAATGGAAGGTTCAAAACCGTGACATGAATGCCCACATCACAAAGTAGTT
         66 CAAAACTGCTCAATCAAAAGAAAGGTTCAACTCTGTGAGATGAATGCACACATCACAAAGAAGTT

                                                     
      69721 TCTCAGAATGCTTCTGTGTAGTTTTTATGTGAAGATATTTC
        131 TCTCAGAATGCTTCTGTGTAGTTTTTATGTGAAGATATTTC

                     * *    *      *    *       * *   **      *              
      69762 CTTTTCCACAACA-GCGTGCAAAACGCTTCAAATATGCCCTTAGAGATTCCACAAAAAGAGTGTT
          1 CTTTTCCACCATAGGCCT-CAAAGCGCTCCAAATATCCACTTGCAGATTCTACAAAAAGAGTGTT

              *     *               *  *                                  ** 
      69826 TCCAAACTACTCAAATCAAAA-AATGATTTCAACTCTGTGAGATGAATGCACACATCACAAACTA
         65 TCAAAACTGCTC-AATCAAAAGAAAG-GTTCAACTCTGTGAGATGAATGCACACATCACAAAGAA

                         *     ** *   * *   *       *   
      69890 GTTTCTCAGAATGTTTCTGCCTGGTTCTCATGCGAAGATAGTTC
        128 GTTTCTCAGAATGCTTCTGTGTAGTTTTTATGTGAAGATATTTC

              *  *           *   * **              *                  * *    
      69934 CTCTTTCACCATAGGCCGCAATGTACTCCAAATATCCACCTGCAGATTCTAC-AAAAGTGAGTTT
          1 CTTTTCCACCATAGGCCTCAAAGCGCTCCAAATATCCACTTGCAGATTCTACAAAAAGAGTGTTT

                       *         ***    **         *        *      *       * 
      69998 CAAAACTGCTCTATCAAAAGATCAGTTCGTCTCTGTGAGTTGAATGCATACATCAAAAAGAAGCT
         66 CAAAACTGCTCAATCAAAAGAAAGGTTCAACTCTGTGAGATGAATGCACACATCACAAAGAAGTT

                 *             *      **         *   
      70063 TCTCAAAATGCTTCTGTGTGGTTTTTCGGTGAAGATAGTTC
        131 TCTCAGAATGCTTCTGTGTAGTTTTTATGTGAAGATATTTC

            *     *        *      * *                 *        *        *    
      70104 TTTTTCTACCATAGGTCTCAAACCACTCCAAATATCCACTTGTAGATTCTATAAAAAGGAATG-T
          1 CTTTTCCACCATAGGCCTCAAAGCGCTCCAAATATCCACTTGCAGATTCTACAAAAA-GAGTGTT

                  *        *    *    *      * *         *      *         *   
      70168 TCAAAATTGCTCAATAAAAATAAAGTTTCAACACCGTGAGATGAGTGCACAAATCACAAAGGAGT
         65 TCAAAACTGCTCAATCAAAAGAAAGGTTCAACTCTGTGAGATGAATGCACACATCACAAAGAAGT

                  *          *         *          *   
      70233 TTCTCAAAATGCTTCTGGGTAGTTTTTCTGTGAAGATAGTTC
        130 TTCTCAGAATGCTTCTGTGTAGTTTTTATGTGAAGATATTTC

                  *     *    *     *          *                         *    
      70275 CTTTTCTACCATGGGCCACAAAGGGCTCCAAATACCCACTTGCAGATTCTACAAAAAGAGAGTTT
          1 CTTTTCCACCATAGGCCTCAAAGCGCTCCAAATATCCACTTGCAGATTCTACAAAAAGAGTGTTT

              *        *      ** * *        *    * *    **  * *           *  
      70340 CACAACTGCTCTATCAAACAATATGTTCAACTTTGTGGGTTGAACACAAATATCAC-AAGAATTT
         66 CAAAACTGCTCAATCAAAAGAAAGGTTCAACTCTGTGAGATGAATGCACACATCACAAAGAAGTT

                **                             *     
      70404 TCTCCCAATGCTTCTGTGTAGTTTTTATGTGAAGACATTTC
        131 TCTCAGAATGCTTCTGTGTAGTTTTTATGTGAAGATATTTC

            *   *  *      *  *     *                 *  *      *       *  *  
      70445 TTTTCCCTCCATAGTCCACAAAGTGCTCCAAATATCCACTTACATATTCTAGAAAAAGATTGCTT
          1 CTTTTCCACCATAGGCCTCAAAGCGCTCCAAATATCCACTTGCAGATTCTACAAAAAGAGTGTTT

            **       *    *               * * *                              
      70510 GGAAACTGCACAATGAAAAGAAAGGTTCAAATATATGAGATGAATGCACACATCACAAAGAAGTT
         66 CAAAACTGCTCAATCAAAAGAAAGGTTCAACTCTGTGAGATGAATGCACACATCACAAAGAAGTT

                                 *                    
      70575 TCTCAGAAT-CTCTCTGTGTAATTTTTATGTGAAGATATTTC
        131 TCTCAGAATGCT-TCTGTGTAGTTTTTATGTGAAGATATTTC

                *     *    *  *   *                        *      *    *     
      70616 CTTTCCCACCTTAGGTCTTAAAACGCTCCAAATATCCACTTGCAGATACTACAAGAAGATTGTTT
          1 CTTTTCCACCATAGGCCTCAAAGCGCTCCAAATATCCACTTGCAGATTCTACAAAAAGAGTGTTT

                     *    *        *      *     **                           
      70681 CAAAACTGCACAA-AAAAAGAAATGTTCAATTCTGTTTGATGAATGCACACATCACAAAGAAGTT
         66 CAAAACTGCTCAATCAAAAGAAAGGTTCAACTCTGTGAGATGAATGCACACATCACAAAGAAGTT

                           *                         
      70745 TCTCAGAATGCTTCTCTGTAGTTTTTATGTGAAGATATTTC
        131 TCTCAGAATGCTTCTGTGTAGTTTTTATGTGAAGATATTTC

                     *             *                 *         **      * *   
      70786 CTTTTCCACAATAGGCCTCAAAGGGCTCCAAATATCCACTTCCAGATTCTATGAAAAGAATATTT
          1 CTTTTCCACCATAGGCCTCAAAGCGCTCCAAATATCCACTTGCAGATTCTACAAAAAGAGTGTTT

             *              * *    *                                      *  
      70851 CCAAACTGCTCAATCATAGGAAATGTTCAACTCTGTGAGATGAATGCACACATCAC-AAGAAATT
         66 CAAAACTGCTCAATCAAAAGAAAGGTTCAACTCTGTGAGATGAATGCACACATCACAAAGAAGTT

                     *    *      *       *       *   
      70915 TCTCAGAATCCTTCAGTGTAGGTTTTATGAGAAGATAATTC
        131 TCTCAGAATGCTTCTGTGTAGTTTTTATGTGAAGATATTTC

                     *    **        *   *                           *    *   
      70956 CTTTTCCACAATAGTTCTCAAAGCACTCAAAATATCCACTTGCAGATTCTACAAAAGGAGTATTT
          1 CTTTTCCACCATAGGCCTCAAAGCGCTCCAAATATCCACTTGCAGATTCTACAAAAAGAGTGTTT

                                                          *                  
      71021 CAAAACTGCTCAATCAAAAGAAAGGTTCAACTCTGTGAGATGAATGGACACATCACAAAGAAGTT
         66 CAAAACTGCTCAATCAAAAGAAAGGTTCAACTCTGTGAGATGAATGCACACATCACAAAGAAGTT

                                  *   *              
      71086 TCTCAGAATGCTTCTGTGTAGTATTTTTGTGAAGATATTT-
        131 TCTCAGAATGCTTCTGTGTAGTTTTTATGTGAAGATATTTC

                          *  * * * * ***             *            *     * *  
      71126 CTTTTCCACCATAGACCGCCAGGGGACACAAATATCCACTTTCAGATTCTACAACAAGAGAGGTT
          1 CTTTTCCACCATAGGCCTCAAAGCGCTCCAAATATCCACTTGCAGATTCTACAAAAAGAGTGTTT

                   *   *     *   ** *       *      *                      * *
      71191 CAAAACTACTCGATCAAGAGATGGTTTCAACTATGTGAGTTGAATGCACACATCACAAAGAACTA
         66 CAAAACTGCTCAATCAAAAGAAAGGTTCAACTCTGTGAGATGAATGCACACATCACAAAGAAGTT

             *  *    *                               
      71256 TGTCGGAATTCTTCTGTGTAGTTTTTATGTGAAGATATTTC
        131 TCTCAGAATGCTTCTGTGTAGTTTTTATGTGAAGATATTTC

                     *    * *      * *    *                  *               
      71297 CTTTTCCACAATAGACGTCAAAGTGATCCAGATATCCACTTGCAGATTCCACAAAAAGAGTGTTT
          1 CTTTTCCACCATAGGCCTCAAAGCGCTCCAAATATCCACTTGCAGATTCTACAAAAAGAGTGTTT

                 *   *   *                  **                     *  *      
      71362 CAAAAGTGCACAACCAAAAGAAAGGTTCAACTAGGTGAGATGAATGCACACATCAGAAGGAAGTT
         66 CAAAACTGCTCAATCAAAAGAAAGGTTCAACTCTGTGAGATGAATGCACACATCACAAAGAAGTT

                            **   *     * *       *   
      71427 TCTCAGAATGCTTCTGCATAGCTTTTAAGGGAAGATACTTC
        131 TCTCAGAATGCTTCTGTGTAGTTTTTATGTGAAGATATTTC

                    *               *            * *  *    * *               
      71468 CTTTTCCAACATAGGCCTCAAAGCACTCCAAATATCCTCCTGGAGATACCACAAAAAGAGTGTTT
          1 CTTTTCCACCATAGGCCTCAAAGCGCTCCAAATATCCACTTGCAGATTCTACAAAAAGAGTGTTT

            **                      *  *                 *       *           
      71533 GCAAACTGCTCAATCAAAAGAAAGATTTAACTCTGTGAGATGAATCCACACATGACAAAGAAGTT
         66 CAAAACTGCTCAATCAAAAGAAAGGTTCAACTCTGTGAGATGAATGCACACATCACAAAGAAGTT

                                                     
      71598 TCTCAGAATGCTTCTGTGTAGTTTTTATGTGAAGATATTTC
        131 TCTCAGAATGCTTCTGTGTAGTTTTTATGTGAAGATATTTC

                     *   * * *    **           *                *     *      
      71639 CTTTTCCACAATAAGACCCAAAAGGCTCCAAATATTCACTTGCAGATTCTAAAAAAAACAGTGTT
          1 CTTTTCCACCATAGGCCTCAAAGCGCTCCAAATATCCACTTGCAGATTCT-ACAAAAAGAGTGTT

                                  *                  *      *        **      
      71704 TCAAAACTGCTCAATCAAAAGATA-GTTCAACTCTGTGAGAAGAATGCTCACATCACTGAGAAGT
         65 TCAAAACTGCTCAATCAAAAGAAAGGTTCAACTCTGTGAGATGAATGCACACATCACAAAGAAGT

                                         *            
      71768 TTCTCAGAATGCTTCTGTGTAGTTTTTATATGAAGATATTTC
        130 TTCTCAGAATGCTTCTGTGTAGTTTTTATGTGAAGATATTTC

                *     *      *    **                       *   **       *    
      71810 CTTTCCCACCGTAGGCCACAAAAGGCTCCAAATATCCACTTGCAGATACTATGAAAAGAGAGTTT
          1 CTTTTCCACCATAGGCCTCAAAGCGCTCCAAATATCCACTTGCAGATTCTACAAAAAGAGTGTTT

                        *        *               ***            *            
      71875 CAAAACTGCTCATTCAAAAGATAGGTTCAACTCTGTGGTTTGAATGCACACAGCACAAAGAAGTT
         66 CAAAACTGCTCAATCAAAAGAAAGGTTCAACTCTGTGAGATGAATGCACACATCACAAAGAAGTT

              *       **                   **   *    
      71940 TCACAGAATGTGTCTGTGTAGTTTTTATGTGCGGATGTTTC
        131 TCTCAGAATGCTTCTGTGTAGTTTTTATGTGAAGATATTTC

                         *        ****   *   *                    *          
      71981 CTTTTCCACCATATGCCT-AAATATTTCCCAATTTCCACTTGCAGATTCTACAAGAAGAGTGTTT
          1 CTTTTCCACCATAGGCCTCAAAGCGCTCCAAATATCCACTTGCAGATTCTACAAAAAGAGTGTTT

                      **       *       *           *            *      ***   
      72045 CAAAACTGCTGTATC-AAATAAA-GTTGAACTCTGTGAGGTGAATGCACACAGCACAAAATGGTT
         66 CAAAACTGCTCAATCAAAAGAAAGGTTCAACTCTGTGAGATGAATGCACACATCACAAAGAAGTT

                          **   *        *       *    
      72108 TCTCAGAATGCTTCCTTGTTGTTTTTATATGAAGATGTTTC
        131 TCTCAGAATGCTTCTGTGTAGTTTTTATGTGAAGATATTTC

                  *  *             *   *     *                               
      72149 CTTTTCAACAATAGGCCTCAAAGTGCTTCAAATGTCCACTTGCAGATTCTACAAAAAGAGTGTTT
          1 CTTTTCCACCATAGGCCTCAAAGCGCTCCAAATATCCACTTGCAGATTCTACAAAAAGAGTGTTT

                                        *      *  *  *                       
      72214 CAAAACTGCTCAATCAAAAGAAAGGTTCGACTCTGGGAAATTAATGCACACATCACAAAGAAGTT
         66 CAAAACTGCTCAATCAAAAGAAAGGTTCAACTCTGTGAGATGAATGCACACATCACAAAGAAGTT

                                     *        *      
      72279 TCTC---A-GCTTCTGTGTAGTTTTCATGTGAAGTTATTTC
        131 TCTCAGAATGCTTCTGTGTAGTTTTTATGTGAAGATATTTC

                     *       *     *            *    *         **       *    
      72316 CTTTTCCACAATAGGCCGCAAAGGGCTCCAAATATCAACTTACAGATTCTAGGAAAAGAGAGTTT
          1 CTTTTCCACCATAGGCCTCAAAGCGCTCCAAATATCCACTTGCAGATTCTACAAAAAGAGTGTTT

                       * **      *     *                                     
      72381 CAAAACTGCTCTACGAAAAGATAGGTTGAACTCTGTGAGATGAATGCACACATCACAAAGAAGTT
         66 CAAAACTGCTCAATCAAAAGAAAGGTTCAACTCTGTGAGATGAATGCACACATCACAAAGAAGTT

                       *               * *     *     
      72446 TCTCAGAATGCATCTGTGTAGTTTTTACGGGAAGACATTTC
        131 TCTCAGAATGCTTCTGTGTAGTTTTTATGTGAAGATATTTC

                        ***  *     **      *  *                  *     ***   
      72487 CTTTTCCACCATCTTCCACAAAGGTCTCCAAGTAACCACTTGCAGATTCTACAGAAAGACACTTT
          1 CTTTTCCACCATAGGCCTCAAAGCGCTCCAAATATCCACTTGCAGATTCTACAAAAAGAGTGTTT

            *          *         ***      *      ***                      *  
      72552 AAAAACTGCTCTATCAAAAGATCAGTTCAAGTCTGTGGTTTGAATGCACACATCACAAAGAATTT
         66 CAAAACTGCTCAATCAAAAGAAAGGTTCAACTCTGTGAGATGAATGCACACATCACAAAGAAGTT

                                     *  *            
      72617 TCTCAGAATGCTTCTGTGTAGTTTTCATATGAAGATATTTC
        131 TCTCAGAATGCTTCTGTGTAGTTTTTATGTGAAGATATTTC

                                    *                                   **   
      72658 CTTTTCCACCATAGGCCTCAAAGCACTCCAAATATCCACTTGCAGATTCTACAAAAAGAGATTTT
          1 CTTTTCCACCATAGGCCTCAAAGCGCTCCAAATATCCACTTGCAGATTCTACAAAAAGAGTGTTT

                   **                           *  *          *        *     
      72723 CAAAACTAGTCAATCAAAAGAAAGGTTCAACTCTGTCAGTTGAATGCACATATCACAAACAAGTT
         66 CAAAACTGCTCAATCAAAAGAAAGGTTCAACTCTGTGAGATGAATGCACACATCACAAAGAAGTT

                *      *                             
      72788 TCTCGGAATGCGTCTGTGTAGTTTTTATGTGAAGATATTTC
        131 TCTCAGAATGCTTCTGTGTAGTTTTTATGTGAAGATATTTC

               *     * *           *     *                  *    *           
      72829 CTTCTCCACAACAGGCCTCAAAGTGCTCCGAATATCCACTTGCAGATTTTAC-TAAAGAGTGTTT
          1 CTTTTCCACCATAGGCCTCAAAGCGCTCCAAATATCCACTTGCAGATTCTACAAAAAGAGTGTTT

             *               *  *   *     *        *    *               *    
      72893 CCAAACTGCTCAATCAAGAGGAAGTTTCAAGTCTGTGAGCTGAACGCACACATCACAAAGTAGTT
         66 CAAAACTGCTCAATCAAAAGAAAGGTTCAACTCTGTGAGATGAATGCACACATCACAAAGAAGTT

               *                                *    
      72958 TCTGAGAATGCTTCTGTGTAGTTTTTATGTGAAGATGTTTC
        131 TCTCAGAATGCTTCTGTGTAGTTTTTATGTGAAGATATTTC

                            **     *                                    *    
      72999 CTTTTCCACCATAGGCTGCAAAGGGCTCCAAATATCCACTTGCAGATTCTACAAAAAGAGAGTTT
          1 CTTTTCCACCATAGGCCTCAAAGCGCTCCAAATATCCACTTGCAGATTCTACAAAAAGAGTGTTT

                 *     *         *          *     *                     *    
      73064 CAAAAGTGCTCTATCAAAAGATAGGTTCAACTATGTGATATGAATGCACACATCACAAAGTAGTT
         66 CAAAACTGCTCAATCAAAAGAAAGGTTCAACTCTGTGAGATGAATGCACACATCACAAAGAAGTT

                                          *          
      73129 TCTCAGAATGCTTCTGTGTAGTTTTTATGTAAAGATATTTC
        131 TCTCAGAATGCTTCTGTGTAGTTTTTATGTGAAGATATTTC

                                    *                                   **   
      73170 CTTTTCCACCATAGGCCTCAAAGCACTCCAAATATCCACTTGCAGATTCTACAAAAAGAGATTTT
          1 CTTTTCCACCATAGGCCTCAAAGCGCTCCAAATATCCACTTGCAGATTCTACAAAAAGAGTGTTT

                   ** *                   *     *  *    * *   *        *     
      73235 CAAAACTATTTAATCAAAAGAAAGGTTCAAATCTGTCAGTTGAAGGTACATATCACAAACAAGTT
         66 CAAAACTGCTCAATCAAAAGAAAGGTTCAACTCTGTGAGATGAATGCACACATCACAAAGAAGTT

             * **                                    
      73300 TATTGGAATGCTTCTGTGTAGTTTTTATGTGAAGATATTTC
        131 TCTCAGAATGCTTCTGTGTAGTTTTTATGTGAAGATATTTC

                     * *         * *                        **  *     *      
      73341 CTTTTCCACAACAGGCCTCAAGGTGCTCCAAATATCCACTTGCAGATTTCACTAAAAGTGTGTTT
          1 CTTTTCCACCATAGGCCTCAAAGCGCTCCAAATATCCACTTGCAGATTCTACAAAAAGAGTGTTT

             *  *            *  *   *     *        *             *           
      73406 CCAAGCTGCTCAATCAAGAGGAAGTTTCAAGTCTGTGAGGTGAATGCACACATTACAAAGAAGTT
         66 CAAAACTGCTCAATCAAAAGAAAGGTTCAACTCTGTGAGATGAATGCACACATCACAAAGAAGTT

            *  *                                     
      73471 ACTGAGAATGCTTCTGTGTAGTTTTTATGTGAAGATATTTC
        131 TCTCAGAATGCTTCTGTGTAGTTTTTATGTGAAGATATTTC

                      **               *                                *    
      73512 CTTTTCCACCGCAGGCCTCAAAGCGCTGCAAATATCCACTTGCAGATTCTACAAAAAGAGAGTTT
          1 CTTTTCCACCATAGGCCTCAAAGCGCTCCAAATATCCACTTGCAGATTCTACAAAAAGAGTGTTT

                      **         *   *         *   *     **            *     
      73577 CAAAACTGCTGTATCAAAAGATAGGGTCAACTCTGCGAGTTGAATAAACACATCACAAATAAGTT
         66 CAAAACTGCTCAATCAAAAGAAAGGTTCAACTCTGTGAGATGAATGCACACATCACAAAGAAGTT

            *  **   *        *               *       
      73642 CCTGGGAACGCTTCTGTATAG-TTTTATGTGAATATATTTC
        131 TCTCAGAATGCTTCTGTGTAGTTTTTATGTGAAGATATTTC

                         *          *                   *   *  *   * *   *   
      73682 CTTTTCCACCATATGCCTCAAAGCACTCCAAATATCCACTTGCACATTATAGAAACATAGTCTTT
          1 CTTTTCCACCATAGGCCTCAAAGCGCTCCAAATATCCACTTGCAGATTCTACAAAAAGAGTGTTT

                   **                        *         *             *       
      73747 CAAAACTTGTCAATC-AAAGAAAGGTTCAACTCCGTGAGATGAGTGCACACATCACAGAGAAGTT
         66 CAAAACTGCTCAATCAAAAGAAAGGTTCAACTCTGTGAGATGAATGCACACATCACAAAGAAGTT

                *     *                            * 
      73811 TCTCGGAATGTTTCTGTGTAGTTTTTATGTGAAGATATTGC
        131 TCTCAGAATGCTTCTGTGTAGTTTTTATGTGAAGATATTTC

                     *               *            *          *       *   *   
      73852 CTTTTCCACAATAGGCCTCAAAGCGTTCCAAATATCCAATTGCAGATTCCACAAAAAAAGTTTTT
          1 CTTTTCCACCATAGGCCTCAAAGCGCTCCAAATATCCACTTGCAGATTCTACAAAAAGAGTGTTT

            *                 *  *  *  *             * *         **    *     
      73917 TAAAACTGCTCAATCAAATGATAGATTAAACTCTGTGAGATTAGTGCACACATGTCAAAAAAGTT
         66 CAAAACTGCTCAATCAAAAGAAAGGTTCAACTCTGTGAGATGAATGCACACATCACAAAGAAGTT

                                *      * *           
      73982 TCTCAGAATGCTTCTGTGTACTTTTTAGGGGAAGATATTTC
        131 TCTCAGAATGCTTCTGTGTAGTTTTTATGTGAAGATATTTC

                        *    *     **         *    *           **  * *  *    
      74023 CTTTTCCACCATCGGCCACAAAGGACTCCAAATAACCACATGCAGATTCTAGTAACACAGAGTTT
          1 CTTTTCCACCATAGGCCTCAAAGCGCTCCAAATATCCACTTGCAGATTCTACAAAAAGAGTGTTT

                       *         * *           *   * * *    **    ***        
      74088 CAAAACTGCTCTATCAAAAGATAAGTTCAACTCTGAGAGTTTAGTGCAACCATCGTGAAGAAGTT
         66 CAAAACTGCTCAATCAAAAGAAAGGTTCAACTCTGTGAGATGAATGCACACATCACAAAGAAGTT

                            *     *                  
      74153 TCTCAGAATGCTTCTGAGTAGTGTTTATGTGAAGATATTTC
        131 TCTCAGAATGCTTCTGTGTAGTTTTTATGTGAAGATATTTC

                              *     *                      *           **    
      74194 CTTTTCCACCATAGGCCTGAAAGCCCTCCAAATATCCACTTGCAGATCCTACAAAAAGAAAGTTT
          1 CTTTTCCACCATAGGCCTCAAAGCGCTCCAAATATCCACTTGCAGATTCTACAAAAAGAGTGTTT

             *         **     *  *  *   *        **      *                   
      74259 CGAAA-TGCTCTCTCAAACGATAGTTTCGACTCTGTGGTATGAATACACACATCACAAAGAAGTT
         66 CAAAACTGCTCAATCAAAAGAAAGGTTCAACTCTGTGAGATGAATGCACACATCACAAAGAAGTT

                                       **            
      74323 TCTCAGAATGCTTCTGTGTAGTTTTTAAATGAAGATATTTC
        131 TCTCAGAATGCTTCTGTGTAGTTTTTATGTGAAGATATTTC

            *                       *          *     *                       
      74364 TTTTTCCACCATAGGCCTCAAAGCACTCCAAATATGCACTTCCAGATTCTACAAAAAGAGTGTTT
          1 CTTTTCCACCATAGGCCTCAAAGCGCTCCAAATATCCACTTGCAGATTCTACAAAAAGAGTGTTT

              *                 *       * *         **   *         *  * *    
      74429 CAGAACTGCTCAATCAAAAGGAAGGTTCCAGTC--TGAGACAAATACACACATCAAAAGGTAGTT
         66 CAAAACTGCTCAATCAAAAGAAAGGTTCAACTCTGTGAGATGAATGCACACATCACAAAGAAGTT

                                                    *
      74492 TCTCAGAATGCTTCTGTGTAGTTTTTATGTGAAGATATTTT
        131 TCTCAGAATGCTTCTGTGTAGTTTTTATGTGAAGATATTTC

             *               *    **    *     *      *  **   *          *    
      74533 CCTTTCCACCATAGGCCACAAATGGCTCTAAATACCCACTTACATTTTCCACAAAAAGAGAGTTT
          1 CTTTTCCACCATAGGCCTCAAAGCGCTCCAAATATCCACTTGCAGATTCTACAAAAAGAGTGTTT

                       * *    * *      *   *         *                       
      74598 CAAAACTGCTCTACCAAAGGTAA-GTTTAACGCTGTGAG-TTAA-G-A-ACATCACAAAGAAGTT
         66 CAAAACTGCTCAATCAAAAGAAAGGTTCAACTCTGTGAGATGAATGCACACATCACAAAGAAGTT

                                   *   *  *          
      74658 TCTCAGAATGCTTCTGTGTAGTTCTTACGTAAAGATATTTC
        131 TCTCAGAATGCTTCTGTGTAGTTTTTATGTGAAGATATTTC

                 *   *      ***    *                  *              **      
      74699 CTTTTACACAATAGGCAGAAAAGTGCTCCAAATATCCACTTGAAGATTCTACAAAAACCGTGTTT
          1 CTTTTCCACCATAGGCCTCAAAGCGCTCCAAATATCCACTTGCAGATTCTACAAAAAGAGTGTTT

                     **                                          *      *    
      74764 CAAAACTGCCGAATCAAAAGAAAGGTTCAACTCTGTGAGATGAATGCACACATAACAAAGGAGTT
         66 CAAAACTGCTCAATCAAAAGAAAGGTTCAACTCTGTGAGATGAATGCACACATCACAAAGAAGTT

                                 *      *      *    *
      74829 TCTCAGAATGCTTCTGTGTAGCTTTTATATGAAGACATTTA
        131 TCTCAGAATGCTTCTGTGTAGTTTTTATGTGAAGATATTTC

            *        * *            *   ***                     **        *  
      74870 GTTTTCCACAACAGGCCTCAAAGCTCTCTCCATATCCACTTGCAGATTCTACCGAAAGAGTGCTT
          1 CTTTTCCACCATAGGCCTCAAAGCGCTCCAAATATCCACTTGCAGATTCTACAAAAAGAGTGTTT

             *                   * **     *        *        *     **         
      74935 CCAAACTGCTCAATCAAAAGAGACATTCAAATCTGTGAGGTGAATGCAGACATCGTAAAGAAGTT
         66 CAAAACTGCTCAATCAAAAGAAAGGTTCAACTCTGTGAGATGAATGCACACATCACAAAGAAGTT

                                *     *       *    **
      75000 TCTCAGAATGCTTCTGTGTATTTTTTGTGTGAAGTTATTCG
        131 TCTCAGAATGCTTCTGTGTAGTTTTTATGTGAAGATATTTC

            *    *             *     *  *            **                 *    
      75041 TTTTTGCACCATAGGCCTCCAAGCGTTCTAAATATCCACTTCTAGATTCTACAAAAAGAGAGTTT
          1 CTTTTCCACCATAGGCCTCAAAGCGCTCCAAATATCCACTTGCAGATTCTACAAAAAGAGTGTTT

                   *         *            *        *    *   *                
      75106 CAAAACTACTC-A--AACA-AAAGGTTCAATTCTGTGAGTTGAAAGCAAACATCACAAAGAAGTT
         66 CAAAACTGCTCAATCAAAAGAAAGGTTCAACTCTGTGAGATGAATGCACACATCACAAAGAAGTT

                       *             *               
      75167 TCTCAGAATGCGTCTGTGTAGTTTTGATGTGAAGATATTTC
        131 TCTCAGAATGCTTCTGTGTAGTTTTTATGTGAAGATATTTC

                     **   ****     *                  *                      
      75208 CTTTT-CACAGTAGAATGCAAAGGGCTCCAAATATCCACTTGGAGATTCTACAAAAAGA--GTTT
          1 CTTTTCCACCATAGGCCTCAAAGCGCTCCAAATATCCACTTGCAGATTCTACAAAAAGAGTGTTT

                  *    **     *  *     *     ***   *     *               *   
      75270 CAAAACCGCTCTGTCAAATGATAGGTTGAACTCCCGGAGGTGAATACACACATCACAAAGAGGTT
         66 CAAAACTGCTCAATCAAAAGAAAGGTTCAACTCTGTGAGATGAATGCACACATCACAAAGAAGTT

                  *                       *  *       
      75335 TCTCAGCATGCTTCTGTGTAGTTTTTATGTAAACATATTTC
        131 TCTCAGAATGCTTCTGTGTAGTTTTTATGTGAAGATATTTC

             *    * *              *           *      * *       *   *    *   
      75376 CGTTTCTATCATAGGCCTCAAAGTGCTCCAAATATTCACTTGTACATTCTACCAAACGAGTATTT
          1 CTTTTCCACCATAGGCCTCAAAGCGCTCCAAATATCCACTTGCAGATTCTACAAAAAGAGTGTTT

                              * *         ** *    *        *            *    
      75441 CAAAACTGCTCAATCAAACGGAAGGTTCAAAACCGTGACATGAATGCCCACATCACAAAGTAGTT
         66 CAAAACTGCTCAATCAAAAGAAAGGTTCAACTCTGTGAGATGAATGCACACATCACAAAGAAGTT

                                                     
      75506 TCTCAGAATGCTTCTGTGTAGTTTTTATGTGAAGATATTTC
        131 TCTCAGAATGCTTCTGTGTAGTTTTTATGTGAAGATATTTC

                     * *    *      *    *       * *   **      *              
      75547 CTTTTCCACAACA-GCGTGCAAAACGCTTCAAATATGCCCTTAGAGATTCCACAAAAAGAGTGTT
          1 CTTTTCCACCATAGGCCT-CAAAGCGCTCCAAATATCCACTTGCAGATTCTACAAAAAGAGTGTT

              *     *               *  *                                  ** 
      75611 TCCAAACTACTCAAATCAAAA-AATGATTTCAACTCTGTGAGATGAATGCACACATCACAAACTA
         65 TCAAAACTGCTC-AATCAAAAGAAAG-GTTCAACTCTGTGAGATGAATGCACACATCACAAAGAA

                         *     ** *   * *   *       *   
      75675 GTTTCTCAGAATGTTTCTGCCTGGTTCTCATGCGAAGATAGTTC
        128 GTTTCTCAGAATGCTTCTGTGTAGTTTTTATGTGAAGATATTTC

                 *           *   * **              *                  * *    
      75719 CTTTTTCACCATAGGCCGCAATGTACTCCAAATATCCACCTGCAGATTCTAC-AAAAGTGAGTTT
          1 CTTTTCCACCATAGGCCTCAAAGCGCTCCAAATATCCACTTGCAGATTCTACAAAAAGAGTGTTT

                       *         ***    **         *        *      *       * 
      75783 CAAAACTGCTCTATCAAAAGATCAGTTCGTCTCTGTGAGTTGAATGCATACATCAAAAAGAAGCT
         66 CAAAACTGCTCAATCAAAAGAAAGGTTCAACTCTGTGAGATGAATGCACACATCACAAAGAAGTT

                 *             *      **         *   
      75848 TCTCAAAATGCTTCTGTGTGGTTTTTCGGTGAAGATAGTTC
        131 TCTCAGAATGCTTCTGTGTAGTTTTTATGTGAAGATATTTC

            *     *        *      * *                 *        *        *    
      75889 TTTTTCTACCATAGGTCTCAAACCACTCCAAATATCCACTTGTAGATTCTATAAAAAGGAATG-T
          1 CTTTTCCACCATAGGCCTCAAAGCGCTCCAAATATCCACTTGCAGATTCTACAAAAA-GAGTGTT

                  *        *    *    *      * *         *      *         *   
      75953 TCAAAATTGCTCAATAAAAATAAAGTTTCAACACCGTGAGATGAGTGCACAAATCACAAAGGAGT
         65 TCAAAACTGCTCAATCAAAAGAAAGGTTCAACTCTGTGAGATGAATGCACACATCACAAAGAAGT

                  *          *         *          *   
      76018 TTCTCAAAATGCTTCTGGGTAGTTTTTCTGTGAAGATAGTTC
        130 TTCTCAGAATGCTTCTGTGTAGTTTTTATGTGAAGATATTTC

                  *     *    *     *          *                         *    
      76060 CTTTTCTACCATGGGCCACAAAGGGCTCCAAATACCCACTTGCAGATTCTACAAAAAGAGAGTTT
          1 CTTTTCCACCATAGGCCTCAAAGCGCTCCAAATATCCACTTGCAGATTCTACAAAAAGAGTGTTT

              *        *      ** * *        *    * *    **  * *           *  
      76125 CACAACTGCTCTATCAAACAATATGTTCAACTTTGTGGGTTGAACACAAATATCAC-AAGAATTT
         66 CAAAACTGCTCAATCAAAAGAAAGGTTCAACTCTGTGAGATGAATGCACACATCACAAAGAAGTT

                **                             *     
      76189 TCTCCCAATGCTTCTGTGTAGTTTTTATGTGAAGACATTTC
        131 TCTCAGAATGCTTCTGTGTAGTTTTTATGTGAAGATATTTC

            *   *  *      *  *     *                 *  *      *       *  *  
      76230 TTTTCCCTCCATAGTCCACAAAGTGCTCCAAATATCCACTTACATATTCTAGAAAAAGATTGCTT
          1 CTTTTCCACCATAGGCCTCAAAGCGCTCCAAATATCCACTTGCAGATTCTACAAAAAGAGTGTTT

            **       *    *               * * *                              
      76295 GGAAACTGCACAATGAAAAGAAAGGTTCAAATATATGAGATGAATGCACACATCACAAAGAAGTT
         66 CAAAACTGCTCAATCAAAAGAAAGGTTCAACTCTGTGAGATGAATGCACACATCACAAAGAAGTT

                                 *                    
      76360 TCTCAGAAT-CTCTCTGTGTAATTTTTATGTGAAGATATTTC
        131 TCTCAGAATGCT-TCTGTGTAGTTTTTATGTGAAGATATTTC

                *     *    *  *   *                        *      *    *     
      76401 CTTTCCCACCTTAGGTCTTAAAACGCTCCAAATATCCACTTGCAGATACTACAAGAAGATTGTTT
          1 CTTTTCCACCATAGGCCTCAAAGCGCTCCAAATATCCACTTGCAGATTCTACAAAAAGAGTGTTT

                     *    *        *      *     **                           
      76466 CAAAACTGCACAA-AAAAAGAAATGTTCAATTCTGTTTGATGAATGCACACATCACAAAGAAGTT
         66 CAAAACTGCTCAATCAAAAGAAAGGTTCAACTCTGTGAGATGAATGCACACATCACAAAGAAGTT

                           *                         
      76530 TCTCAGAATGCTTCTCTGTAGTTTTTATGTGAAGATATTTC
        131 TCTCAGAATGCTTCTGTGTAGTTTTTATGTGAAGATATTTC

                     *             *                 *         **      * *   
      76571 CTTTTCCACAATAGGCCTCAAAGGGCTCCAAATATCCACTTCCAGATTCTATGAAAAGAATATTT
          1 CTTTTCCACCATAGGCCTCAAAGCGCTCCAAATATCCACTTGCAGATTCTACAAAAAGAGTGTTT

             *              * *    *                                      *  
      76636 CCAAACTGCTCAATCATAGGAAATGTTCAACTCTGTGAGATGAATGCACACATCAC-AAGAAATT
         66 CAAAACTGCTCAATCAAAAGAAAGGTTCAACTCTGTGAGATGAATGCACACATCACAAAGAAGTT

                     *    *      *       *       *   
      76700 TCTCAGAATCCTTCAGTGTAGGTTTTATGAGAAGATAATTC
        131 TCTCAGAATGCTTCTGTGTAGTTTTTATGTGAAGATATTTC

                     *    **        *   *                           *    *   
      76741 CTTTTCCACAATAGTTCTCAAAGCACTCAAAATATCCACTTGCAGATTCTACAAAAGGAGTATTT
          1 CTTTTCCACCATAGGCCTCAAAGCGCTCCAAATATCCACTTGCAGATTCTACAAAAAGAGTGTTT

                                                          *                  
      76806 CAAAACTGCTCAATCAAAAGAAAGGTTCAACTCTGTGAGATGAATGGACACATCACAAAGAAGTT
         66 CAAAACTGCTCAATCAAAAGAAAGGTTCAACTCTGTGAGATGAATGCACACATCACAAAGAAGTT

                                  *   *              
      76871 TCTCAGAATGCTTCTGTGTAGTATTTTTGTGAAGATATTT-
        131 TCTCAGAATGCTTCTGTGTAGTTTTTATGTGAAGATATTTC

                          *  * * * * ***             *            *     * *  
      76911 CTTTTCCACCATAGACCGCCAGGGGACACAAATATCCACTTTCAGATTCTACAACAAGAGAGGTT
          1 CTTTTCCACCATAGGCCTCAAAGCGCTCCAAATATCCACTTGCAGATTCTACAAAAAGAGTGTTT

                   *   *     *   ** *       *      *                      * *
      76976 CAAAACTACTCGATCAAGAGATGGTTTCAACTATGTGAGTTGAATGCACACATCACAAAGAACTA
         66 CAAAACTGCTCAATCAAAAGAAAGGTTCAACTCTGTGAGATGAATGCACACATCACAAAGAAGTT

             *  *    *                               
      77041 TGTCGGAATTCTTCTGTGTAGTTTTTATGTGAAGATATTTC
        131 TCTCAGAATGCTTCTGTGTAGTTTTTATGTGAAGATATTTC

                     *    * *      * *    *                  *               
      77082 CTTTTCCACAATAGACGTCAAAGTGATCCAGATATCCACTTGCAGATTCCACAAAAAGAGTGTTT
          1 CTTTTCCACCATAGGCCTCAAAGCGCTCCAAATATCCACTTGCAGATTCTACAAAAAGAGTGTTT

                 *   *   *                  **                     *  *      
      77147 CAAAAGTGCACAACCAAAAGAAAGGTTCAACTAGGTGAGATGAATGCACACATCAGAAGGAAGTT
         66 CAAAACTGCTCAATCAAAAGAAAGGTTCAACTCTGTGAGATGAATGCACACATCACAAAGAAGTT

                            **   *     * *       *   
      77212 TCTCAGAATGCTTCTGCATAGCTTTTAAGGGAAGATACTTC
        131 TCTCAGAATGCTTCTGTGTAGTTTTTATGTGAAGATATTTC

                    *               *            * *  *    * *               
      77253 CTTTTCCAACATAGGCCTCAAAGCACTCCAAATATCCTCCTGGAGATACCACAAAAAGAGTGTTT
          1 CTTTTCCACCATAGGCCTCAAAGCGCTCCAAATATCCACTTGCAGATTCTACAAAAAGAGTGTTT

            **                      *  *                 *       *           
      77318 GCAAACTGCTCAATCAAAAGAAAGATTTAACTCTGTGAGATGAATCCACACATGACAAAGAAGTT
         66 CAAAACTGCTCAATCAAAAGAAAGGTTCAACTCTGTGAGATGAATGCACACATCACAAAGAAGTT

                                                     
      77383 TCTCAGAATGCTTCTGTGTAGTTTTTATGTGAAGATATTTC
        131 TCTCAGAATGCTTCTGTGTAGTTTTTATGTGAAGATATTTC

                     *   * * *    **           *                *     *      
      77424 CTTTTCCACAATAAGACCCAAAAGGCTCCAAATATTCACTTGCAGATTCTAAAAAAAACAGTGTT
          1 CTTTTCCACCATAGGCCTCAAAGCGCTCCAAATATCCACTTGCAGATTCT-ACAAAAAGAGTGTT

                                  *                  *      *        **      
      77489 TCAAAACTGCTCAATCAAAAGATA-GTTCAACTCTGTGAGAAGAATGCTCACATCACTGAGAAGT
         65 TCAAAACTGCTCAATCAAAAGAAAGGTTCAACTCTGTGAGATGAATGCACACATCACAAAGAAGT

                                         *            
      77553 TTCTCAGAATGCTTCTGTGTAGTTTTTATATGAAGATATTTC
        130 TTCTCAGAATGCTTCTGTGTAGTTTTTATGTGAAGATATTTC

                *     *      *    **                       *   **       *    
      77595 CTTTCCCACCGTAGGCCACAAAAGGCTCCAAATATCCACTTGCAGATACTATGAAAAGAGAGTTT
          1 CTTTTCCACCATAGGCCTCAAAGCGCTCCAAATATCCACTTGCAGATTCTACAAAAAGAGTGTTT

                        *        *               ***            *            
      77660 CAAAACTGCTCATTCAAAAGATAGGTTCAACTCTGTGGTTTGAATGCACACAGCACAAAGAAGTT
         66 CAAAACTGCTCAATCAAAAGAAAGGTTCAACTCTGTGAGATGAATGCACACATCACAAAGAAGTT

              *       **                   **   *    
      77725 TCACAGAATGTGTCTGTGTAGTTTTTATGTGCGGATGTTTC
        131 TCTCAGAATGCTTCTGTGTAGTTTTTATGTGAAGATATTTC

                         *        ****   *   *                    *          
      77766 CTTTTCCACCATATGCCT-AAATATTTCCCAATTTCCACTTGCAGATTCTACAAGAAGAGTGTTT
          1 CTTTTCCACCATAGGCCTCAAAGCGCTCCAAATATCCACTTGCAGATTCTACAAAAAGAGTGTTT

                      **       *       *           *            *      ***   
      77830 CAAAACTGCTGTATC-AAATAAA-GTTGAACTCTGTGAGGTGAATGCACACAGCACAAAATGGTT
         66 CAAAACTGCTCAATCAAAAGAAAGGTTCAACTCTGTGAGATGAATGCACACATCACAAAGAAGTT

                          **   *        *       *    
      77893 TCTCAGAATGCTTCCTTGTTGTTTTTATATGAAGATGTTTC
        131 TCTCAGAATGCTTCTGTGTAGTTTTTATGTGAAGATATTTC

                  *  *             *   *     *                               
      77934 CTTTTCAACAATAGGCCTCAAAGTGCTTCAAATGTCCACTTGCAGATTCTACAAAAAGAGTGTTT
          1 CTTTTCCACCATAGGCCTCAAAGCGCTCCAAATATCCACTTGCAGATTCTACAAAAAGAGTGTTT

                                        *      *  *  *                       
      77999 CAAAACTGCTCAATCAAAAGAAAGGTTCGACTCTGGGAAATTAATGCACACATCACAAAGAAGTT
         66 CAAAACTGCTCAATCAAAAGAAAGGTTCAACTCTGTGAGATGAATGCACACATCACAAAGAAGTT

                                     *        *      
      78064 TCTC---A-GCTTCTGTGTAGTTTTCATGTGAAGTTATTTC
        131 TCTCAGAATGCTTCTGTGTAGTTTTTATGTGAAGATATTTC

                     *       *     *            *    *         **       *    
      78101 CTTTTCCACAATAGGCCGCAAAGGGCTCCAAATATCAACTTACAGATTCTAGGAAAAGAGAGTTT
          1 CTTTTCCACCATAGGCCTCAAAGCGCTCCAAATATCCACTTGCAGATTCTACAAAAAGAGTGTTT

                       * **      *     *                                     
      78166 CAAAACTGCTCTACGAAAAGATAGGTTGAACTCTGTGAGATGAATGCACACATCACAAAGAAGTT
         66 CAAAACTGCTCAATCAAAAGAAAGGTTCAACTCTGTGAGATGAATGCACACATCACAAAGAAGTT

                       *               * *     *     
      78231 TCTCAGAATGCATCTGTGTAGTTTTTACGGGAAGACATTTC
        131 TCTCAGAATGCTTCTGTGTAGTTTTTATGTGAAGATATTTC

                        ***  *     **      *  *                  *     ***   
      78272 CTTTTCCACCATCTTCCACAAAGGTCTCCAAGTAACCACTTGCAGATTCTACAGAAAGACACTTT
          1 CTTTTCCACCATAGGCCTCAAAGCGCTCCAAATATCCACTTGCAGATTCTACAAAAAGAGTGTTT

            *          *         ***      *      ***                      *  
      78337 AAAAACTGCTCTATCAAAAGATCAGTTCAAGTCTGTGGTTTGAATGCACACATCACAAAGAATTT
         66 CAAAACTGCTCAATCAAAAGAAAGGTTCAACTCTGTGAGATGAATGCACACATCACAAAGAAGTT

                                     *  *            
      78402 TCTCAGAATGCTTCTGTGTAGTTTTCATATGAAGATATTTC
        131 TCTCAGAATGCTTCTGTGTAGTTTTTATGTGAAGATATTTC

                                    *                                   **   
      78443 CTTTTCCACCATAGGCCTCAAAGCACTCCAAATATCCACTTGCAGATTCTACAAAAAGAGATTTT
          1 CTTTTCCACCATAGGCCTCAAAGCGCTCCAAATATCCACTTGCAGATTCTACAAAAAGAGTGTTT

                   **                           *  *          *        *     
      78508 CAAAACTAGTCAATCAAAAGAAAGGTTCAACTCTGTCAGTTGAATGCACATATCACAAACAAGTT
         66 CAAAACTGCTCAATCAAAAGAAAGGTTCAACTCTGTGAGATGAATGCACACATCACAAAGAAGTT

                *      *                             
      78573 TCTCGGAATGCGTCTGTGTAGTTTTTATGTGAAGATATTTC
        131 TCTCAGAATGCTTCTGTGTAGTTTTTATGTGAAGATATTTC

               *     * *           *     *                  *    *           
      78614 CTTCTCCACAACAGGCCTCAAAGTGCTCCGAATATCCACTTGCAGATTTTAC-TAAAGAGTGTTT
          1 CTTTTCCACCATAGGCCTCAAAGCGCTCCAAATATCCACTTGCAGATTCTACAAAAAGAGTGTTT

             *               *  *   *     *        *    *               *    
      78678 CCAAACTGCTCAATCAAGAGGAAGTTTCAAGTCTGTGAGCTGAACGCACACATCACAAAGTAGTT
         66 CAAAACTGCTCAATCAAAAGAAAGGTTCAACTCTGTGAGATGAATGCACACATCACAAAGAAGTT

               *                                *    
      78743 TCTGAGAATGCTTCTGTGTAGTTTTTATGTGAAGATGTTTC
        131 TCTCAGAATGCTTCTGTGTAGTTTTTATGTGAAGATATTTC

                            **     *                                    *    
      78784 CTTTTCCACCATAGGCTGCAAAGGGCTCCAAATATCCACTTGCAGATTCTACAAAAAGAGAGTTT
          1 CTTTTCCACCATAGGCCTCAAAGCGCTCCAAATATCCACTTGCAGATTCTACAAAAAGAGTGTTT

                 *     *         *          *     *                     *    
      78849 CAAAAGTGCTCTATCAAAAGATAGGTTCAACTATGTGATATGAATGCACACATCACAAAGTAGTT
         66 CAAAACTGCTCAATCAAAAGAAAGGTTCAACTCTGTGAGATGAATGCACACATCACAAAGAAGTT

                                          *          
      78914 TCTCAGAATGCTTCTGTGTAGTTTTTATGTAAAGATATTTC
        131 TCTCAGAATGCTTCTGTGTAGTTTTTATGTGAAGATATTTC

                                    *                                   **   
      78955 CTTTTCCACCATAGGCCTCAAAGCACTCCAAATATCCACTTGCAGATTCTACAAAAAGAGATTTT
          1 CTTTTCCACCATAGGCCTCAAAGCGCTCCAAATATCCACTTGCAGATTCTACAAAAAGAGTGTTT

                   ** *                   *     *  *    * *   *        *     
      79020 CAAAACTATTTAATCAAAAGAAAGGTTCAAATCTGTCAGTTGAAGGTACATATCACAAACAAGTT
         66 CAAAACTGCTCAATCAAAAGAAAGGTTCAACTCTGTGAGATGAATGCACACATCACAAAGAAGTT

             * **                                    
      79085 TATTGGAATGCTTCTGTGTAGTTTTTATGTGAAGATATTTC
        131 TCTCAGAATGCTTCTGTGTAGTTTTTATGTGAAGATATTTC

                     * *         * *                        **  *     *      
      79126 CTTTTCCACAACAGGCCTCAAGGTGCTCCAAATATCCACTTGCAGATTTCACTAAAAGTGTGTTT
          1 CTTTTCCACCATAGGCCTCAAAGCGCTCCAAATATCCACTTGCAGATTCTACAAAAAGAGTGTTT

             *  *            *  *   *     *        *             *           
      79191 CCAAGCTGCTCAATCAAGAGGAAGTTTCAAGTCTGTGAGGTGAATGCACACATTACAAAGAAGTT
         66 CAAAACTGCTCAATCAAAAGAAAGGTTCAACTCTGTGAGATGAATGCACACATCACAAAGAAGTT

            *  *                                     
      79256 ACTGAGAATGCTTCTGTGTAGTTTTTATGTGAAGATATTTC
        131 TCTCAGAATGCTTCTGTGTAGTTTTTATGTGAAGATATTTC

                      **               *                                *    
      79297 CTTTTCCACCGCAGGCCTCAAAGCGCTGCAAATATCCACTTGCAGATTCTACAAAAAGAGAGTTT
          1 CTTTTCCACCATAGGCCTCAAAGCGCTCCAAATATCCACTTGCAGATTCTACAAAAAGAGTGTTT

                      **         *   *         *   *     **            *     
      79362 CAAAACTGCTGTATCAAAAGATAGGGTCAACTCTGCGAGTTGAATAAACACATCACAAATAAGTT
         66 CAAAACTGCTCAATCAAAAGAAAGGTTCAACTCTGTGAGATGAATGCACACATCACAAAGAAGTT

            *  **   *        *               *       
      79427 CCTGGGAACGCTTCTGTATAG-TTTTATGTGAATATATTTC
        131 TCTCAGAATGCTTCTGTGTAGTTTTTATGTGAAGATATTTC

                         *          *                   *   *  *   * *   *   
      79467 CTTTTCCACCATATGCCTCAAAGCACTCCAAATATCCACTTGCACATTATAGAAACATAGTCTTT
          1 CTTTTCCACCATAGGCCTCAAAGCGCTCCAAATATCCACTTGCAGATTCTACAAAAAGAGTGTTT

                   **                        *         *             *       
      79532 CAAAACTTGTCAATC-AAAGAAAGGTTCAACTCCGTGAGATGAGTGCACACATCACAGAGAAGTT
         66 CAAAACTGCTCAATCAAAAGAAAGGTTCAACTCTGTGAGATGAATGCACACATCACAAAGAAGTT

                *     *                            * 
      79596 TCTCGGAATGTTTCTGTGTAGTTTTTATGTGAAGATATTGC
        131 TCTCAGAATGCTTCTGTGTAGTTTTTATGTGAAGATATTTC

                     *               *            *          *       *   *   
      79637 CTTTTCCACAATAGGCCTCAAAGCGTTCCAAATATCCAATTGCAGATTCCACAAAAAAAGTTTTT
          1 CTTTTCCACCATAGGCCTCAAAGCGCTCCAAATATCCACTTGCAGATTCTACAAAAAGAGTGTTT

            *                 *  *  *  *             * *         **    *     
      79702 TAAAACTGCTCAATCAAATGATAGATTAAACTCTGTGAGATTAGTGCACACATGTCAAAAAAGTT
         66 CAAAACTGCTCAATCAAAAGAAAGGTTCAACTCTGTGAGATGAATGCACACATCACAAAGAAGTT

                                *      * *           
      79767 TCTCAGAATGCTTCTGTGTACTTTTTACGGGAAGATATTTC
        131 TCTCAGAATGCTTCTGTGTAGTTTTTATGTGAAGATATTTC

                        *    *     **         *    *           **  * *  *    
      79808 CTTTTCCACCATCGGCCACAAAGGACTCCAAATAACCACATGCAGATTCTAGTAACACAGAGTTT
          1 CTTTTCCACCATAGGCCTCAAAGCGCTCCAAATATCCACTTGCAGATTCTACAAAAAGAGTGTTT

                       *         * *           *   * * *    **    ***        
      79873 CAAAACTGCTCTATCAAAAGATAAGTTCAACTCTGAGAGTTTAGTGCAACCATCGTGAAGAAGTT
         66 CAAAACTGCTCAATCAAAAGAAAGGTTCAACTCTGTGAGATGAATGCACACATCACAAAGAAGTT

                            *     *                  
      79938 TCTCAGAATGCTTCTGAGTAGTGTTTATGTGAAGATATTTC
        131 TCTCAGAATGCTTCTGTGTAGTTTTTATGTGAAGATATTTC

                              *     *                      *           **    
      79979 CTTTTCCACCATAGGCCTGAAAGCCCTCCAAATATCCACTTGCAGATCCTACAAAAAGAAAGTTT
          1 CTTTTCCACCATAGGCCTCAAAGCGCTCCAAATATCCACTTGCAGATTCTACAAAAAGAGTGTTT

             *         **     *  *  *   *        **      *                   
      80044 CGAAA-TGCTCTCTCAAACGATAGTTTCGACTCTGTGGTATGAATACACACATCACAAAGAAGTT
         66 CAAAACTGCTCAATCAAAAGAAAGGTTCAACTCTGTGAGATGAATGCACACATCACAAAGAAGTT

                                       **            
      80108 TCTCAGAATGCTTCTGTGTAGTTTTTAAATGAAGATATTTC
        131 TCTCAGAATGCTTCTGTGTAGTTTTTATGTGAAGATATTTC

            *                       *          *     *                       
      80149 TTTTTCCACCATAGGCCTCAAAGCACTCCAAATATGCACTTCCAGATTCTACAAAAAGAGTGTTT
          1 CTTTTCCACCATAGGCCTCAAAGCGCTCCAAATATCCACTTGCAGATTCTACAAAAAGAGTGTTT

              *                 *       * *         **   *         *  * *    
      80214 CAGAACTGCTCAATCAAAAGGAAGGTTCCAGTC--TGAGACAAATACACACATCAAAAGGTAGTT
         66 CAAAACTGCTCAATCAAAAGAAAGGTTCAACTCTGTGAGATGAATGCACACATCACAAAGAAGTT

                                                    *
      80277 TCTCAGAATGCTTCTGTGTAGTTTTTATGTGAAGATATTTT
        131 TCTCAGAATGCTTCTGTGTAGTTTTTATGTGAAGATATTTC

             *               *    **    *     *      *  **   *          *    
      80318 CCTTTCCACCATAGGCCACAAATGGCTCTAAATACCCACTTACATTTTCCACAAAAAGAGAGTTT
          1 CTTTTCCACCATAGGCCTCAAAGCGCTCCAAATATCCACTTGCAGATTCTACAAAAAGAGTGTTT

                       * *    * *      *   *         *                       
      80383 CAAAACTGCTCTACCAAAGGTAA-GTTTAACGCTGTGAG-TTAA-G-A-ACATCACAAAGAAGTT
         66 CAAAACTGCTCAATCAAAAGAAAGGTTCAACTCTGTGAGATGAATGCACACATCACAAAGAAGTT

                                   *   *  *          
      80443 TCTCAGAATGCTTCTGTGTAGTTCTTACGTAAAGATATTTC
        131 TCTCAGAATGCTTCTGTGTAGTTTTTATGTGAAGATATTTC

                 *   *      ***    *                  *              **      
      80484 CTTTTACACAATAGGCAGAAAAGTGCTCCAAATATCCACTTGAAGATTCTACAAAAACCGTGTTT
          1 CTTTTCCACCATAGGCCTCAAAGCGCTCCAAATATCCACTTGCAGATTCTACAAAAAGAGTGTTT

                     **                                          *      *    
      80549 CAAAACTGCCGAATCAAAAGAAAGGTTCAACTCTGTGAGATGAATGCACACATAACAAAGGAGTT
         66 CAAAACTGCTCAATCAAAAGAAAGGTTCAACTCTGTGAGATGAATGCACACATCACAAAGAAGTT

                                 *      *      *    *
      80614 TCTCAGAATGCTTCTGTGTAGCTTTTATATGAAGACATTTA
        131 TCTCAGAATGCTTCTGTGTAGTTTTTATGTGAAGATATTTC

            *        * *            *   ***                     **        *  
      80655 GTTTTCCACAACAGGCCTCAAAGCTCTCTCCATATCCACTTGCAGATTCTACCGAAAGAGTGCTT
          1 CTTTTCCACCATAGGCCTCAAAGCGCTCCAAATATCCACTTGCAGATTCTACAAAAAGAGTGTTT

             *                   * **     *        *        *     **         
      80720 CCAAACTGCTCAATCAAAAGAGACATTCAAATCTGTGAGGTGAATGCAGACATCGTAAAGAAGTT
         66 CAAAACTGCTCAATCAAAAGAAAGGTTCAACTCTGTGAGATGAATGCACACATCACAAAGAAGTT

                                *     *       *    **
      80785 TCTCAGAATGCTTCTGTGTATTTTTTGTGTGAAGTTATTCG
        131 TCTCAGAATGCTTCTGTGTAGTTTTTATGTGAAGATATTTC

            *    *             *     *  *            **                 *    
      80826 TTTTTGCACCATAGGCCTCCAAGCGTTCTAAATATCCACTTCTAGATTCTACAAAAAGAGAGTTT
          1 CTTTTCCACCATAGGCCTCAAAGCGCTCCAAATATCCACTTGCAGATTCTACAAAAAGAGTGTTT

                   *         *            *        *    *   *                
      80891 CAAAACTACTC-A--AACA-AAAGGTTCAATTCTGTGAGTTGAAAGCAAACATCACAAAGAAGTT
         66 CAAAACTGCTCAATCAAAAGAAAGGTTCAACTCTGTGAGATGAATGCACACATCACAAAGAAGTT

                       *             *               
      80952 TCTCAGAATGCGTCTGTGTAGTTTTGATGTGAAGATATTTC
        131 TCTCAGAATGCTTCTGTGTAGTTTTTATGTGAAGATATTTC

                     **   ****     *                  *                      
      80993 CTTTT-CACAGTAGAATGCAAAGGGCTCCAAATATCCACTTGGAGATTCTACAAAAAGA--GTTT
          1 CTTTTCCACCATAGGCCTCAAAGCGCTCCAAATATCCACTTGCAGATTCTACAAAAAGAGTGTTT

                  *    **     *  *     *     ***   *     *               *   
      81055 CAAAACCGCTCTGTCAAATGATAGGTTGAACTCCCGGAGGTGAATACACACATCACAAAGAGGTT
         66 CAAAACTGCTCAATCAAAAGAAAGGTTCAACTCTGTGAGATGAATGCACACATCACAAAGAAGTT

                  *                       *  *       
      81120 TCTCAGCATGCTTCTGTGTAGTTTTTATGTAAACATATTTC
        131 TCTCAGAATGCTTCTGTGTAGTTTTTATGTGAAGATATTTC

             *    * *              *           *      * *       *   *    *   
      81161 CGTTTCTATCATAGGCCTCAAAGTGCTCCAAATATTCACTTGTACATTCTACCAAACGAGTATTT
          1 CTTTTCCACCATAGGCCTCAAAGCGCTCCAAATATCCACTTGCAGATTCTACAAAAAGAGTGTTT

                              * *         ** *    *        *            *    
      81226 CAAAACTGCTCAATCAAATGGAAGGTTCAAAACCGTGACATGAATGCCCACATCACAAAGTAGTT
         66 CAAAACTGCTCAATCAAAAGAAAGGTTCAACTCTGTGAGATGAATGCACACATCACAAAGAAGTT

                                                     
      81291 TCTCAGAATGCTTCTGTGTAGTTTTTATGTGAAGATATTTC
        131 TCTCAGAATGCTTCTGTGTAGTTTTTATGTGAAGATATTTC

                     * *    *      *    *       * *   **      *              
      81332 CTTTTCCACAACA-GCGTGCAAAACGCTTCAAATATGCCCTTAGAGATTCCACAAAAAGAGTGTT
          1 CTTTTCCACCATAGGCCT-CAAAGCGCTCCAAATATCCACTTGCAGATTCTACAAAAAGAGTGTT

              *     *               *  *                                  ** 
      81396 TCCAAACTACTCAAATCAAAA-AATGATTTCAACTCTGTGAGATGAATGCACACATCACAAACTA
         65 TCAAAACTGCTC-AATCAAAAGAAAG-GTTCAACTCTGTGAGATGAATGCACACATCACAAAGAA

                         *     ** *   * *   *       *   
      81460 GTTTCTCAGAATGTTTCTGCCTGGTTCTCATGCGAAGATAGTTC
        128 GTTTCTCAGAATGCTTCTGTGTAGTTTTTATGTGAAGATATTTC

                 *           *   * **              *                  * *    
      81504 CTTTTTCACCATAGGCCGCAATGTACTCCAAATATCCACCTGCAGATTCTAC-AAAAGTGAGTTT
          1 CTTTTCCACCATAGGCCTCAAAGCGCTCCAAATATCCACTTGCAGATTCTACAAAAAGAGTGTTT

                       *         ***    **         *        *      *       * 
      81568 CAAAACTGCTCTATCAAAAGATCAGTTCGTCTCTGTGAGTTGAATGCATACATCAAAAAGAAGCT
         66 CAAAACTGCTCAATCAAAAGAAAGGTTCAACTCTGTGAGATGAATGCACACATCACAAAGAAGTT

                 *             *      **         *   
      81633 TCTCAAAATGCTTCTGTGTGGTTTTTCGGTGAAGATAGTTC
        131 TCTCAGAATGCTTCTGTGTAGTTTTTATGTGAAGATATTTC

            *     *        *      * *                 *        *        *    
      81674 TTTTTCTACCATAGGTCTCAAACCACTCCAAATATCCACTTGTAGATTCTATAAAAAGGAATG-T
          1 CTTTTCCACCATAGGCCTCAAAGCGCTCCAAATATCCACTTGCAGATTCTACAAAAA-GAGTGTT

                  *        *    *    *      * *         *      *         *   
      81738 TCAAAATTGCTCAATAAAAATAAAGTTTCAACACCGTGAGATGAGTGCACAAATCACAAAGGAGT
         65 TCAAAACTGCTCAATCAAAAGAAAGGTTCAACTCTGTGAGATGAATGCACACATCACAAAGAAGT

                  *          *         *          *   
      81803 TTCTCAAAATGCTTCTGGGTAGTTTTTCTGTGAAGATAGTTC
        130 TTCTCAGAATGCTTCTGTGTAGTTTTTATGTGAAGATATTTC

                  *     *    *     *          *                         *    
      81845 CTTTTCTACCATGGGCCACAAAGGGCTCCAAATACCCACTTGCAGATTCTACAAAAAGAGAGTTT
          1 CTTTTCCACCATAGGCCTCAAAGCGCTCCAAATATCCACTTGCAGATTCTACAAAAAGAGTGTTT

              *        *      ** * *        *    * *    **  * *           *  
      81910 CACAACTGCTCTATCAAACAATATGTTCAACTTTGTGGGTTGAACACAAATATCAC-AAGAATTT
         66 CAAAACTGCTCAATCAAAAGAAAGGTTCAACTCTGTGAGATGAATGCACACATCACAAAGAAGTT

                **                             *     
      81974 TCTCCCAATGCTTCTGTGTAGTTTTTATGTGAAGACATTTC
        131 TCTCAGAATGCTTCTGTGTAGTTTTTATGTGAAGATATTTC

            *   *  *      *  *     *                 *  *      *       *  *  
      82015 TTTTCCCTCCATAGTCCACAAAGTGCTCCAAATATCCACTTACATATTCTAGAAAAAGATTGCTT
          1 CTTTTCCACCATAGGCCTCAAAGCGCTCCAAATATCCACTTGCAGATTCTACAAAAAGAGTGTTT

            **       *    *               * * *                              
      82080 GGAAACTGCACAATGAAAAGAAAGGTTCAAATATATGAGATGAATGCACACATCACAAAGAAGTT
         66 CAAAACTGCTCAATCAAAAGAAAGGTTCAACTCTGTGAGATGAATGCACACATCACAAAGAAGTT

                                 *                    
      82145 TCTCAGAAT-CTCTCTGTGTAATTTTTATGTGAAGATATTTC
        131 TCTCAGAATGCT-TCTGTGTAGTTTTTATGTGAAGATATTTC

                *     *    *  *   *                        *      *    *     
      82186 CTTTCCCACCTTAGGTCTTAAAACGCTCCAAATATCCACTTGCAGATACTACAAGAAGATTGTTT
          1 CTTTTCCACCATAGGCCTCAAAGCGCTCCAAATATCCACTTGCAGATTCTACAAAAAGAGTGTTT

                     *    *        *      *     **                           
      82251 CAAAACTGCACAA-AAAAAGAAATGTTCAATTCTGTTTGATGAATGCACACATCACAAAGAAGTT
         66 CAAAACTGCTCAATCAAAAGAAAGGTTCAACTCTGTGAGATGAATGCACACATCACAAAGAAGTT

                           *                         
      82315 TCTCAGAATGCTTCTCTGTAGTTTTTATGTGAAGATATTTC
        131 TCTCAGAATGCTTCTGTGTAGTTTTTATGTGAAGATATTTC

                     *             *                 *         **      * *   
      82356 CTTTTCCACAATAGGCCTCAAAGGGCTCCAAATATCCACTTCCAGATTCTATGAAAAGAATATTT
          1 CTTTTCCACCATAGGCCTCAAAGCGCTCCAAATATCCACTTGCAGATTCTACAAAAAGAGTGTTT

             *              * *    *                                      *  
      82421 CCAAACTGCTCAATCATAGGAAATGTTCAACTCTGTGAGATGAATGCACACATCAC-AAGAAATT
         66 CAAAACTGCTCAATCAAAAGAAAGGTTCAACTCTGTGAGATGAATGCACACATCACAAAGAAGTT

                     *    *      *       *       *   
      82485 TCTCAGAATCCTTCAGTGTAGGTTTTATGAGAAGATAATTC
        131 TCTCAGAATGCTTCTGTGTAGTTTTTATGTGAAGATATTTC

                     *    **        *   *                           *    *   
      82526 CTTTTCCACAATAGTTCTCAAAGCACTCAAAATATCCACTTGCAGATTCTACAAAAGGAGTATTT
          1 CTTTTCCACCATAGGCCTCAAAGCGCTCCAAATATCCACTTGCAGATTCTACAAAAAGAGTGTTT

                                                          *                  
      82591 CAAAACTGCTCAATCAAAAGAAAGGTTCAACTCTGTGAGATGAATGGACACATCACAAAGAAGTT
         66 CAAAACTGCTCAATCAAAAGAAAGGTTCAACTCTGTGAGATGAATGCACACATCACAAAGAAGTT

                                  *   *              
      82656 TCTCAGAATGCTTCTGTGTAGTATTTTTGTGAAGATATTT-
        131 TCTCAGAATGCTTCTGTGTAGTTTTTATGTGAAGATATTTC

                          *  * * * * ***             *            *     * *  
      82696 CTTTTCCACCATAGACCGCCAGGGGACACAAATATCCACTTTCAGATTCTACAACAAGAGAGGTT
          1 CTTTTCCACCATAGGCCTCAAAGCGCTCCAAATATCCACTTGCAGATTCTACAAAAAGAGTGTTT

                   *   *     *   ** *       *      *                      * *
      82761 CAAAACTACTCGATCAAGAGATGGTTTCAACTATGTGAGTTGAATGCACACATCACAAAGAACTA
         66 CAAAACTGCTCAATCAAAAGAAAGGTTCAACTCTGTGAGATGAATGCACACATCACAAAGAAGTT

             *  *    *                               
      82826 TGTCGGAATTCTTCTGTGTAGTTTTTATGTGAAGATATTTC
        131 TCTCAGAATGCTTCTGTGTAGTTTTTATGTGAAGATATTTC

                     *    * *      * *    *                  *               
      82867 CTTTTCCACAATAGACGTCAAAGTGATCCAGATATCCACTTGCAGATTCCACAAAAAGAGTGTTT
          1 CTTTTCCACCATAGGCCTCAAAGCGCTCCAAATATCCACTTGCAGATTCTACAAAAAGAGTGTTT

                 *   *   *                  **                     *  *      
      82932 CAAAAGTGCACAACCAAAAGAAAGGTTCAACTAGGTGAGATGAATGCACACATCAGAAGGAAGTT
         66 CAAAACTGCTCAATCAAAAGAAAGGTTCAACTCTGTGAGATGAATGCACACATCACAAAGAAGTT

                            **   *     * *       *   
      82997 TCTCAGAATGCTTCTGCATAGCTTTTAAGGGAAGATACTTC
        131 TCTCAGAATGCTTCTGTGTAGTTTTTATGTGAAGATATTTC

                    *               *            * *  *    * *               
      83038 CTTTTCCAACATAGGCCTCAAAGCACTCCAAATATCCTCCTGGAGATACCACAAAAAGAGTGTTT
          1 CTTTTCCACCATAGGCCTCAAAGCGCTCCAAATATCCACTTGCAGATTCTACAAAAAGAGTGTTT

            **                      *  *                 *       *           
      83103 GCAAACTGCTCAATCAAAAGAAAGATTTAACTCTGTGAGATGAATCCACACATGACAAAGAAGTT
         66 CAAAACTGCTCAATCAAAAGAAAGGTTCAACTCTGTGAGATGAATGCACACATCACAAAGAAGTT

                                                     
      83168 TCTCAGAATGCTTCTGTGTAGTTTTTATGTGAAGATATTTC
        131 TCTCAGAATGCTTCTGTGTAGTTTTTATGTGAAGATATTTC

                     *   * * *    **           *                *     *      
      83209 CTTTTCCACAATAAGACCCAAAAGGCTCCAAATATTCACTTGCAGATTCTAAAAAAAACAGTGTT
          1 CTTTTCCACCATAGGCCTCAAAGCGCTCCAAATATCCACTTGCAGATTCT-ACAAAAAGAGTGTT

                                  *                  *      *        **      
      83274 TCAAAACTGCTCAATCAAAAGATA-GTTCAACTCTGTGAGAAGAATGCTCACATCACTGAGAAGT
         65 TCAAAACTGCTCAATCAAAAGAAAGGTTCAACTCTGTGAGATGAATGCACACATCACAAAGAAGT

                                         *            
      83338 TTCTCAGAATGCTTCTGTGTAGTTTTTATATGAAGATATTTC
        130 TTCTCAGAATGCTTCTGTGTAGTTTTTATGTGAAGATATTTC

                *     *      *    **                       *   **       *    
      83380 CTTTCCCACCGTAGGCCACAAAAGGCTCCAAATATCCACTTGCAGATACTATGAAAAGAGAGTTT
          1 CTTTTCCACCATAGGCCTCAAAGCGCTCCAAATATCCACTTGCAGATTCTACAAAAAGAGTGTTT

                        *        *               ***            *            
      83445 CAAAACTGCTCATTCAAAAGATAGGTTCAACTCTGTGGTTTGAATGCACACAGCACAAAGAAGTT
         66 CAAAACTGCTCAATCAAAAGAAAGGTTCAACTCTGTGAGATGAATGCACACATCACAAAGAAGTT

              *       **                   **   *    
      83510 TCACAGAATGTGTCTGTGTAGTTTTTATGTGCGGATGTTTC
        131 TCTCAGAATGCTTCTGTGTAGTTTTTATGTGAAGATATTTC

                         *        ****   *   *               *    *          
      83551 CTTTTCCACCATATGCCT-AAATATTTCCCAATTTCCACTTGCAGATTCCACAAGAAGAGTGTTT
          1 CTTTTCCACCATAGGCCTCAAAGCGCTCCAAATATCCACTTGCAGATTCTACAAAAAGAGTGTTT

                      **       *       *           *            *      ***   
      83615 CAAAACTGCTGTATC-AAATAAA-GTTGAACTCTGTGAGGTGAATGCACACAGCACAAAATGGTT
         66 CAAAACTGCTCAATCAAAAGAAAGGTTCAACTCTGTGAGATGAATGCACACATCACAAAGAAGTT

                          **   *        *       *    
      83678 TCTCAGAATGCTTCCTTGTTGTTTTTATATGAAGATGTTTC
        131 TCTCAGAATGCTTCTGTGTAGTTTTTATGTGAAGATATTTC

                  *  *             *   *     *                               
      83719 CTTTTCAACAATAGGCCTCAAAGTGCTTCAAATGTCCACTTGCAGATTCTACAAAAAGAGTGTTT
          1 CTTTTCCACCATAGGCCTCAAAGCGCTCCAAATATCCACTTGCAGATTCTACAAAAAGAGTGTTT

                                        *      *  *  *                       
      83784 CAAAACTGCTCAATCAAAAGAAAGGTTCGACTCTGGGAAATTAATGCACACATCACAAAGAAGTT
         66 CAAAACTGCTCAATCAAAAGAAAGGTTCAACTCTGTGAGATGAATGCACACATCACAAAGAAGTT

                                     *        *      
      83849 TCTC---A-GCTTCTGTGTAGTTTTCATGTGAAGTTATTTC
        131 TCTCAGAATGCTTCTGTGTAGTTTTTATGTGAAGATATTTC

                     *       *     *            *    *         **       *    
      83886 CTTTTCCACAATAGGCCGCAAAGGGCTCCAAATATCAACTTACAGATTCTAGGAAAAGAGAGTTT
          1 CTTTTCCACCATAGGCCTCAAAGCGCTCCAAATATCCACTTGCAGATTCTACAAAAAGAGTGTTT

                       * **      *     *                                     
      83951 CAAAACTGCTCTACGAAAAGATAGGTTGAACTCTGTGAGATGAATGCACACATCACAAAGAAGTT
         66 CAAAACTGCTCAATCAAAAGAAAGGTTCAACTCTGTGAGATGAATGCACACATCACAAAGAAGTT

                       *               * *     *     
      84016 TCTCAGAATGCATCTGTGTAGTTTTTACGGGAAGACATTTC
        131 TCTCAGAATGCTTCTGTGTAGTTTTTATGTGAAGATATTTC

                        ***  *     **      *  *                  *     ***   
      84057 CTTTTCCACCATCTTCCACAAAGGTCTCCAAGTAACCACTTGCAGATTCTACAGAAAGACACTTT
          1 CTTTTCCACCATAGGCCTCAAAGCGCTCCAAATATCCACTTGCAGATTCTACAAAAAGAGTGTTT

            *          *         ***      *      ***                      *  
      84122 AAAAACTGCTCTATCAAAAGATCAGTTCAAGTCTGTGGTTTGAATGCACACATCACAAAGAATTT
         66 CAAAACTGCTCAATCAAAAGAAAGGTTCAACTCTGTGAGATGAATGCACACATCACAAAGAAGTT

                                     *  *            
      84187 TCTCAGAATGCTTCTGTGTAGTTTTCATATGAAGATATTTC
        131 TCTCAGAATGCTTCTGTGTAGTTTTTATGTGAAGATATTTC

                                    *                                   **   
      84228 CTTTTCCACCATAGGCCTCAAAGCACTCCAAATATCCACTTGCAGATTCTACAAAAAGAGATTTT
          1 CTTTTCCACCATAGGCCTCAAAGCGCTCCAAATATCCACTTGCAGATTCTACAAAAAGAGTGTTT

                   **                           *  *          *        *     
      84293 CAAAACTAGTCAATCAAAAGAAAGGTTCAACTCTGTCAGTTGAATGCACATATCACAAACAAGTT
         66 CAAAACTGCTCAATCAAAAGAAAGGTTCAACTCTGTGAGATGAATGCACACATCACAAAGAAGTT

                *      *                             
      84358 TCTCGGAATGCGTCTGTGTAGTTTTTATGTGAAGATATTTC
        131 TCTCAGAATGCTTCTGTGTAGTTTTTATGTGAAGATATTTC

               *     * *           *     *                  *    *           
      84399 CTTCTCCACAACAGGCCTCAAAGTGCTCCGAATATCCACTTGCAGATTTTAC-TAAAGAGTGTTT
          1 CTTTTCCACCATAGGCCTCAAAGCGCTCCAAATATCCACTTGCAGATTCTACAAAAAGAGTGTTT

             *               *  *   *     *        *    *               *    
      84463 CCAAACTGCTCAATCAAGAGGAAGTTTCAAGTCTGTGAGCTGAACGCACACATCACAAAGTAGTT
         66 CAAAACTGCTCAATCAAAAGAAAGGTTCAACTCTGTGAGATGAATGCACACATCACAAAGAAGTT

               *                                *    
      84528 TCTGAGAATGCTTCTGTGTAGTTTTTATGTGAAGATGTTTC
        131 TCTCAGAATGCTTCTGTGTAGTTTTTATGTGAAGATATTTC

                            **     *                                    *    
      84569 CTTTTCCACCATAGGCTGCAAAGGGCTCCAAATATCCACTTGCAGATTCTACAAAAAGAGAGTTT
          1 CTTTTCCACCATAGGCCTCAAAGCGCTCCAAATATCCACTTGCAGATTCTACAAAAAGAGTGTTT

                 *     *         *          *     *                     *    
      84634 CAAAAGTGCTCTATCAAAAGATAGGTTCAACTATGTGATATGAATGCACACATCACAAAGTAGTT
         66 CAAAACTGCTCAATCAAAAGAAAGGTTCAACTCTGTGAGATGAATGCACACATCACAAAGAAGTT

                                          *          
      84699 TCTCAGAATGCTTCTGTGTAGTTTTTATGTAAAGATATTTC
        131 TCTCAGAATGCTTCTGTGTAGTTTTTATGTGAAGATATTTC

                                    *                                   **   
      84740 CTTTTCCACCATAGGCCTCAAAGCACTCCAAATATCCACTTGCAGATTCTACAAAAAGAGATTTT
          1 CTTTTCCACCATAGGCCTCAAAGCGCTCCAAATATCCACTTGCAGATTCTACAAAAAGAGTGTTT

                   ** *                   *     *  *    * *   *        *     
      84805 CAAAACTATTTAATCAAAAGAAAGGTTCAAATCTGTCAGTTGAAGGTACATATCACAAACAAGTT
         66 CAAAACTGCTCAATCAAAAGAAAGGTTCAACTCTGTGAGATGAATGCACACATCACAAAGAAGTT

             * **                                    
      84870 TATTGGAATGCTTCTGTGTAGTTTTTATGTGAAGATATTTC
        131 TCTCAGAATGCTTCTGTGTAGTTTTTATGTGAAGATATTTC

                     * *         * *                        **  *     *      
      84911 CTTTTCCACAACAGGCCTCAAGGTGCTCCAAATATCCACTTGCAGATTTCACTAAAAGTGTGTTT
          1 CTTTTCCACCATAGGCCTCAAAGCGCTCCAAATATCCACTTGCAGATTCTACAAAAAGAGTGTTT

             *  *            *  *   *     *        *             *           
      84976 CCAAGCTGCTCAATCAAGAGGAAGTTTCAAGTCTGTGAGGTGAATGCACACATTACAAAGAAGTT
         66 CAAAACTGCTCAATCAAAAGAAAGGTTCAACTCTGTGAGATGAATGCACACATCACAAAGAAGTT

            *  *                                     
      85041 ACTGAGAATGCTTCTGTGTAGTTTTTATGTGAAGATATTTC
        131 TCTCAGAATGCTTCTGTGTAGTTTTTATGTGAAGATATTTC

                      **               *                                *    
      85082 CTTTTCCACCGCAGGCCTCAAAGCGCTGCAAATATCCACTTGCAGATTCTACAAAAAGAGAGTTT
          1 CTTTTCCACCATAGGCCTCAAAGCGCTCCAAATATCCACTTGCAGATTCTACAAAAAGAGTGTTT

                      **         *   *         *   *     **            *     
      85147 CAAAACTGCTGTATCAAAAGATAGGGTCAACTCTGCGAGTTGAATAAACACATCACAAATAAGTT
         66 CAAAACTGCTCAATCAAAAGAAAGGTTCAACTCTGTGAGATGAATGCACACATCACAAAGAAGTT

               **   *        *               *       
      85212 TCTGGGAACGCTTCTGTATAG-TTTTATGTGAATATATTTC
        131 TCTCAGAATGCTTCTGTGTAGTTTTTATGTGAAGATATTTC

                         *          *                   *   *  *   * *   *   
      85252 CTTTTCCACCATATGCCTCAAAGCACTCCAAATATCCACTTGCACATTATAGAAACATAGTCTTT
          1 CTTTTCCACCATAGGCCTCAAAGCGCTCCAAATATCCACTTGCAGATTCTACAAAAAGAGTGTTT

                   **                        *         *             *       
      85317 CAAAACTTGTCAATC-AAAGAAAGGTTCAACTCCGTGAGATGAGTGCACACATCACAGAGAAGTT
         66 CAAAACTGCTCAATCAAAAGAAAGGTTCAACTCTGTGAGATGAATGCACACATCACAAAGAAGTT

                *     *                            * 
      85381 TCTCGGAATGTTTCTGTGTAGTTTTTATGTGAAGATATTGC
        131 TCTCAGAATGCTTCTGTGTAGTTTTTATGTGAAGATATTTC

                     *               *            *          *       *   *   
      85422 CTTTTCCACAATAGGCCTCAAAGCGTTCCAAATATCCAATTGCAGATTCCACAAAAAAAGTTTTT
          1 CTTTTCCACCATAGGCCTCAAAGCGCTCCAAATATCCACTTGCAGATTCTACAAAAAGAGTGTTT

            *                 *  *  *  *             * *         **    *     
      85487 TAAAACTGCTCAATCAAATGATAGATTAAACTCTGTGAGATTAGTGCACACATGTCAAAAAAGTT
         66 CAAAACTGCTCAATCAAAAGAAAGGTTCAACTCTGTGAGATGAATGCACACATCACAAAGAAGTT

                                *      * *           
      85552 TCTCAGAATGCTTCTGTGTACTTTTTAGGGGAAGATATTTC
        131 TCTCAGAATGCTTCTGTGTAGTTTTTATGTGAAGATATTTC

                        *    *     **         *    *           **  * *  *    
      85593 CTTTTCCACCATCGGCCACAAAGGACTCCAAATAACCACATGCAGATTCTAGTAACACAGAGTTT
          1 CTTTTCCACCATAGGCCTCAAAGCGCTCCAAATATCCACTTGCAGATTCTACAAAAAGAGTGTTT

                       *         * *           *   * * *    **    ***        
      85658 CAAAACTGCTCTATCAAAAGATAAGTTCAACTCTGAGAGTTTAGTGCAACCATCGTGAAGAAGTT
         66 CAAAACTGCTCAATCAAAAGAAAGGTTCAACTCTGTGAGATGAATGCACACATCACAAAGAAGTT

                            *     *                  
      85723 TCTCAGAATGCTTCTGAGTAGTGTTTATGTGAAGATATTTC
        131 TCTCAGAATGCTTCTGTGTAGTTTTTATGTGAAGATATTTC

                              *     *                      *           **    
      85764 CTTTTCCACCATAGGCCTGAAAGCCCTCCAAATATCCACTTGCAGATCCTACAAAAAGAAAGTTT
          1 CTTTTCCACCATAGGCCTCAAAGCGCTCCAAATATCCACTTGCAGATTCTACAAAAAGAGTGTTT

             *         **     *  *  *   *        **      *                   
      85829 CGAAA-TGCTCTCTCAAACGATAGTTTCGACTCTGTGGTATGAATACACACATCACAAAGAAGTT
         66 CAAAACTGCTCAATCAAAAGAAAGGTTCAACTCTGTGAGATGAATGCACACATCACAAAGAAGTT

                                       **            
      85893 TCTCAGAATGCTTCTGTGTAGTTTTTAAATGAAGATATTTC
        131 TCTCAGAATGCTTCTGTGTAGTTTTTATGTGAAGATATTTC

            *                       *          *     *                       
      85934 TTTTTCCACCATAGGCCTCAAAGCACTCCAAATATGCACTTCCAGATTCTACAAAAAGAGTGTTT
          1 CTTTTCCACCATAGGCCTCAAAGCGCTCCAAATATCCACTTGCAGATTCTACAAAAAGAGTGTTT

              *                 *       * *         **   *         *  * *    
      85999 CAGAACTGCTCAATCAAAAGGAAGGTTCCAGTC--TGAGACAAATACACACATCAAAAGGTAGTT
         66 CAAAACTGCTCAATCAAAAGAAAGGTTCAACTCTGTGAGATGAATGCACACATCACAAAGAAGTT

                                                    *
      86062 TCTCAGAATGCTTCTGTGTAGTTTTTATGTGAAGATATTTT
        131 TCTCAGAATGCTTCTGTGTAGTTTTTATGTGAAGATATTTC

             *               *    **    *     *      *  **   *          *    
      86103 CCTTTCCACCATAGGCCACAAATGGCTCTAAATACCCACTTACATTTTCCACAAAAAGAGAGTTT
          1 CTTTTCCACCATAGGCCTCAAAGCGCTCCAAATATCCACTTGCAGATTCTACAAAAAGAGTGTTT

                       * *    * *      *   *         *                       
      86168 CAAAACTGCTCTACCAAAGGTAA-GTTTAACGCTGTGAG-TTAA-G-A-ACATCACAAAGAAGTT
         66 CAAAACTGCTCAATCAAAAGAAAGGTTCAACTCTGTGAGATGAATGCACACATCACAAAGAAGTT

                                   *   *  *          
      86228 TCTCAGAATGCTTCTGTGTAGTTCTTACGTAAAGATATTTC
        131 TCTCAGAATGCTTCTGTGTAGTTTTTATGTGAAGATATTTC

                 *   *      ***    *                  *              **      
      86269 CTTTTACACAATAGGCAGAAAAGTGCTCCAAATATCCACTTGAAGATTCTACAAAAACCGTGTTT
          1 CTTTTCCACCATAGGCCTCAAAGCGCTCCAAATATCCACTTGCAGATTCTACAAAAAGAGTGTTT

                     **                                          *      *    
      86334 CAAAACTGCCGAATCAAAAGAAAGGTTCAACTCTGTGAGATGAATGCACACATAACAAAGGAGTT
         66 CAAAACTGCTCAATCAAAAGAAAGGTTCAACTCTGTGAGATGAATGCACACATCACAAAGAAGTT

                                 *      *      *    *
      86399 TCTCAGAATGCTTCTGTGTAGCTTTTATATGAAGACATTTA
        131 TCTCAGAATGCTTCTGTGTAGTTTTTATGTGAAGATATTTC

            *        * *            *   ***                     **        *  
      86440 GTTTTCCACAACAGGCCTCAAAGCTCTCTCCATATCCACTTGCAGATTCTACCGAAAGAGTGCTT
          1 CTTTTCCACCATAGGCCTCAAAGCGCTCCAAATATCCACTTGCAGATTCTACAAAAAGAGTGTTT

             *                   * **     *        *        *     **         
      86505 CCAAACTGCTCAATCAAAAGAGACATTCAAATCTGTGAGGTGAATGCAGACATCGTAAAGAAGTT
         66 CAAAACTGCTCAATCAAAAGAAAGGTTCAACTCTGTGAGATGAATGCACACATCACAAAGAAGTT

                                *     *       *    **
      86570 TCTCAGAATGCTTCTGTGTATTTTTTGTGTGAAGTTATTCG
        131 TCTCAGAATGCTTCTGTGTAGTTTTTATGTGAAGATATTTC

            *    *             *     *  *            **                 *    
      86611 TTTTTGCACCATAGGCCTCCAAGCGTTCTAAATATCCACTTCTAGATTCTACAAAAAGAGAGTTT
          1 CTTTTCCACCATAGGCCTCAAAGCGCTCCAAATATCCACTTGCAGATTCTACAAAAAGAGTGTTT

                   *         *            *        *    *   *                
      86676 CAAAACTACTC-A--AACA-AAAGGTTCAATTCTGTGAGTTGAAAGCAAACATCACAAAGAAGTT
         66 CAAAACTGCTCAATCAAAAGAAAGGTTCAACTCTGTGAGATGAATGCACACATCACAAAGAAGTT

                       *             *               
      86737 TCTCAGAATGCGTCTGTGTAGTTTTGATGTGAAGATATTTC
        131 TCTCAGAATGCTTCTGTGTAGTTTTTATGTGAAGATATTTC

                     **   ****     *                  *                      
      86778 CTTTT-CACAGTAGAATGCAAAGGGCTCCAAATATCCACTTGGAGATTCTACAAAAAGA--GTTT
          1 CTTTTCCACCATAGGCCTCAAAGCGCTCCAAATATCCACTTGCAGATTCTACAAAAAGAGTGTTT

                  *    **     *  *     *     ***   *     *               *   
      86840 CAAAACCGCTCTGTCAAATGATAGGTTGAACTCCCGGAGGTGAATACACACATCACAAAGAGGTT
         66 CAAAACTGCTCAATCAAAAGAAAGGTTCAACTCTGTGAGATGAATGCACACATCACAAAGAAGTT

                  *                       *  *       
      86905 TCTCAGCATGCTTCTGTGTAGTTTTTATGTAAACATATTTC
        131 TCTCAGAATGCTTCTGTGTAGTTTTTATGTGAAGATATTTC

             *    * *              *           *      * *       *   *    *   
      86946 CGTTTCTATCATAGGCCTCAAAGTGCTCCAAATATTCACTTGTACATTCTACCAAACGAGTATTT
          1 CTTTTCCACCATAGGCCTCAAAGCGCTCCAAATATCCACTTGCAGATTCTACAAAAAGAGTGTTT

                              * *         ** *    *        *            *    
      87011 CAAAACTGCTCAATCAAATGGAAGGTTCAAAACCGTGACATGAATGCCCACATCACAAAGTAGTT
         66 CAAAACTGCTCAATCAAAAGAAAGGTTCAACTCTGTGAGATGAATGCACACATCACAAAGAAGTT

                                                     
      87076 TCTCAGAATGCTTCTGTGTAGTTTTTATGTGAAGATATTTC
        131 TCTCAGAATGCTTCTGTGTAGTTTTTATGTGAAGATATTTC

                     * *    *      *    *       * *   **      *              
      87117 CTTTTCCACAACA-GCGTGCAAAACGCTTCAAATATGCCCTTAGAGATTCCACAAAAAGAGTGTT
          1 CTTTTCCACCATAGGCCT-CAAAGCGCTCCAAATATCCACTTGCAGATTCTACAAAAAGAGTGTT

              *     *               *  *                                  ** 
      87181 TCCAAACTACTCAAATCAAAA-AATGATTTCAACTCTGTGAGATGAATGCACACATCACAAACTA
         65 TCAAAACTGCTC-AATCAAAAGAAAG-GTTCAACTCTGTGAGATGAATGCACACATCACAAAGAA

                         *     ** *   * *   *       *   
      87245 GTTTCTCAGAATGTTTCTGCCTGGTTCTCATGCGAAGATAGTTC
        128 GTTTCTCAGAATGCTTCTGTGTAGTTTTTATGTGAAGATATTTC

                 *           *   * **              *                  * *    
      87289 CTTTTTCACCATAGGCCGCAATGTACTCCAAATATCCACCTGCAGATTCTAC-AAAAGTGAGTTT
          1 CTTTTCCACCATAGGCCTCAAAGCGCTCCAAATATCCACTTGCAGATTCTACAAAAAGAGTGTTT

                       *         ***    **         *        *      *       * 
      87353 CAAAACTGCTCTATCAAAAGATCAGTTCGTCTCTGTGAGTTGAATGCATACATCAAAAAGAAGCT
         66 CAAAACTGCTCAATCAAAAGAAAGGTTCAACTCTGTGAGATGAATGCACACATCACAAAGAAGTT

                 *             *      **         *   
      87418 TCTCAAAATGCTTCTGTGTGGTTTTTCGGTGAAGATAGTTC
        131 TCTCAGAATGCTTCTGTGTAGTTTTTATGTGAAGATATTTC

            *     *        *      * *                 *        *        *    
      87459 TTTTTCTACCATAGGTCTCAAACCACTCCAAATATCCACTTGTAGATTCTATAAAAAGGAATG-T
          1 CTTTTCCACCATAGGCCTCAAAGCGCTCCAAATATCCACTTGCAGATTCTACAAAAA-GAGTGTT

                  *        *    *    *      * *         *      *         *   
      87523 TCAAAATTGCTCAATAAAAATAAAGTTTCAACACCGTGAGATGAGTGCACAAATCACAAAGGAGT
         65 TCAAAACTGCTCAATCAAAAGAAAGGTTCAACTCTGTGAGATGAATGCACACATCACAAAGAAGT

                  *          *         *          *   
      87588 TTCTCAAAATGCTTCTGGGTAGTTTTTCTGTGAAGATAGTTC
        130 TTCTCAGAATGCTTCTGTGTAGTTTTTATGTGAAGATATTTC

                  *     *    *     *          *                         *    
      87630 CTTTTCTACCATGGGCCACAAAGGGCTCCAAATACCCACTTGCAGATTCTACAAAAAGAGAGTTT
          1 CTTTTCCACCATAGGCCTCAAAGCGCTCCAAATATCCACTTGCAGATTCTACAAAAAGAGTGTTT

              *        *      ** * *        *    * *    **  * *           *  
      87695 CACAACTGCTCTATCAAACAATATGTTCAACTTTGTGGGTTGAACACAAATATCAC-AAGAATTT
         66 CAAAACTGCTCAATCAAAAGAAAGGTTCAACTCTGTGAGATGAATGCACACATCACAAAGAAGTT

                **                             *     
      87759 TCTCCCAATGCTTCTGTGTAGTTTTTATGTGAAGACATTTC
        131 TCTCAGAATGCTTCTGTGTAGTTTTTATGTGAAGATATTTC

            *   *  *      *  *     *                 *  *      *       *  *  
      87800 TTTTCCCTCCATAGTCCACAAAGTGCTCCAAATATCCACTTACATATTCTAGAAAAAGATTGCTT
          1 CTTTTCCACCATAGGCCTCAAAGCGCTCCAAATATCCACTTGCAGATTCTACAAAAAGAGTGTTT

            **       *    *               * * *                              
      87865 GGAAACTGCACAATGAAAAGAAAGGTTCAAATATATGAGATGAATGCACACATCACAAAGAAGTT
         66 CAAAACTGCTCAATCAAAAGAAAGGTTCAACTCTGTGAGATGAATGCACACATCACAAAGAAGTT

                                 *                    
      87930 TCTCAGAAT-CTCTCTGTGTAATTTTTATGTGAAGATATTTC
        131 TCTCAGAATGCT-TCTGTGTAGTTTTTATGTGAAGATATTTC

                *     *    *  *   *                        *      *    *     
      87971 CTTTCCCACCTTAGGTCTTAAAACGCTCCAAATATCCACTTGCAGATACTACAAGAAGATTGTTT
          1 CTTTTCCACCATAGGCCTCAAAGCGCTCCAAATATCCACTTGCAGATTCTACAAAAAGAGTGTTT

                     *    *        *      *     **                           
      88036 CAAAACTGCACAA-AAAAAGAAATGTTCAATTCTGTTTGATGAATGCACACATCACAAAGAAGTT
         66 CAAAACTGCTCAATCAAAAGAAAGGTTCAACTCTGTGAGATGAATGCACACATCACAAAGAAGTT

                           *                         
      88100 TCTCAGAATGCTTCTCTGTAGTTTTTATGTGAAGATATTTC
        131 TCTCAGAATGCTTCTGTGTAGTTTTTATGTGAAGATATTTC

                     *             *                 *         **      * *   
      88141 CTTTTCCACAATAGGCCTCAAAGGGCTCCAAATATCCACTTCCAGATTCTATGAAAAGAATATTT
          1 CTTTTCCACCATAGGCCTCAAAGCGCTCCAAATATCCACTTGCAGATTCTACAAAAAGAGTGTTT

             *              * *    *                                      *  
      88206 CCAAACTGCTCAATCATAGGAAATGTTCAACTCTGTGAGATGAATGCACACATCAC-AAGAAATT
         66 CAAAACTGCTCAATCAAAAGAAAGGTTCAACTCTGTGAGATGAATGCACACATCACAAAGAAGTT

                     *    *      *       *       *   
      88270 TCTCAGAATCCTTCAGTGTAGGTTTTATGAGAAGATAATTC
        131 TCTCAGAATGCTTCTGTGTAGTTTTTATGTGAAGATATTTC

                     *    **        *   *                           *    *   
      88311 CTTTTCCACAATAGTTCTCAAAGCACTCAAAATATCCACTTGCAGATTCTACAAAAGGAGTATTT
          1 CTTTTCCACCATAGGCCTCAAAGCGCTCCAAATATCCACTTGCAGATTCTACAAAAAGAGTGTTT

                                                          *                  
      88376 CAAAACTGCTCAATCAAAAGAAAGGTTCAACTCTGTGAGATGAATGGACACATCACAAAGAAGTT
         66 CAAAACTGCTCAATCAAAAGAAAGGTTCAACTCTGTGAGATGAATGCACACATCACAAAGAAGTT

                                  *   *              
      88441 TCTCAGAATGCTTCTGTGTAGTATTTTTGTGAAGATATTT-
        131 TCTCAGAATGCTTCTGTGTAGTTTTTATGTGAAGATATTTC

                          *  * * * * ***             *            *     * *  
      88481 CTTTTCCACCATAGACCGCCAGGGGACACAAATATCCACTTTCAGATTCTACAACAAGAGAGGTT
          1 CTTTTCCACCATAGGCCTCAAAGCGCTCCAAATATCCACTTGCAGATTCTACAAAAAGAGTGTTT

                   *   *     *   ** *       *      *                      * *
      88546 CAAAACTACTCGATCAAGAGATGGTTTCAACTATGTGAGTTGAATGCACACATCACAAAGAACTA
         66 CAAAACTGCTCAATCAAAAGAAAGGTTCAACTCTGTGAGATGAATGCACACATCACAAAGAAGTT

             *  *    *                               
      88611 TGTCGGAATTCTTCTGTGTAGTTTTTATGTGAAGATATTTC
        131 TCTCAGAATGCTTCTGTGTAGTTTTTATGTGAAGATATTTC

                     *    * *      * *    *                  *               
      88652 CTTTTCCACAATAGACGTCAAAGTGATCCAGATATCCACTTGCAGATTCCACAAAAAGAGTGTTT
          1 CTTTTCCACCATAGGCCTCAAAGCGCTCCAAATATCCACTTGCAGATTCTACAAAAAGAGTGTTT

                 *   *   *                  **                     *  *      
      88717 CAAAAGTGCACAACCAAAAGAAAGGTTCAACTAGGTGAGATGAATGCACACATCAGAAGGAAGTT
         66 CAAAACTGCTCAATCAAAAGAAAGGTTCAACTCTGTGAGATGAATGCACACATCACAAAGAAGTT

                            **   *     * *       *   
      88782 TCTCAGAATGCTTCTGCATAGCTTTTAAGGGAAGATACTTC
        131 TCTCAGAATGCTTCTGTGTAGTTTTTATGTGAAGATATTTC

                    *               *            * *  *    * *               
      88823 CTTTTCCAACATAGGCCTCAAAGCACTCCAAATATCCTCCTGGAGATACCACAAAAAGAGTGTTT
          1 CTTTTCCACCATAGGCCTCAAAGCGCTCCAAATATCCACTTGCAGATTCTACAAAAAGAGTGTTT

            **                      *  *                 *       *           
      88888 GCAAACTGCTCAATCAAAAGAAAGATTTAACTCTGTGAGATGAATCCACACATGACAAAGAAGTT
         66 CAAAACTGCTCAATCAAAAGAAAGGTTCAACTCTGTGAGATGAATGCACACATCACAAAGAAGTT

                                                     
      88953 TCTCAGAATGCTTCTGTGTAGTTTTTATGTGAAGATATTTC
        131 TCTCAGAATGCTTCTGTGTAGTTTTTATGTGAAGATATTTC

                     *   * * *    **           *                *     *      
      88994 CTTTTCCACAATAAGACCCAAAAGGCTCCAAATATTCACTTGCAGATTCTAAAAAAAACAGTGTT
          1 CTTTTCCACCATAGGCCTCAAAGCGCTCCAAATATCCACTTGCAGATTCT-ACAAAAAGAGTGTT

                                  *                  *      *        **      
      89059 TCAAAACTGCTCAATCAAAAGATA-GTTCAACTCTGTGAGAAGAATGCTCACATCACTGAGAAGT
         65 TCAAAACTGCTCAATCAAAAGAAAGGTTCAACTCTGTGAGATGAATGCACACATCACAAAGAAGT

                                         *            
      89123 TTCTCAGAATGCTTCTGTGTAGTTTTTATATGAAGATATTTC
        130 TTCTCAGAATGCTTCTGTGTAGTTTTTATGTGAAGATATTTC

                *     *      *    **                       *   **       *    
      89165 CTTTCCCACCGTAGGCCACAAAAGGCTCCAAATATCCACTTGCAGATACTATGAAAAGAGAGTTT
          1 CTTTTCCACCATAGGCCTCAAAGCGCTCCAAATATCCACTTGCAGATTCTACAAAAAGAGTGTTT

                        *        *               ***            *            
      89230 CAAAACTGCTCATTCAAAAGATAGGTTCAACTCTGTGGTTTGAATGCACACAGCACAAAGAAGTT
         66 CAAAACTGCTCAATCAAAAGAAAGGTTCAACTCTGTGAGATGAATGCACACATCACAAAGAAGTT

              *       **                   **   *    
      89295 TCACAGAATGTGTCTGTGTAGTTTTTATGTGCGGATGTTTC
        131 TCTCAGAATGCTTCTGTGTAGTTTTTATGTGAAGATATTTC

                         *        ****   *   *                    *          
      89336 CTTTTCCACCATATGCCT-AAATATTTCCCAATTTCCACTTGCAGATTCTACAAGAAGAGTGTTT
          1 CTTTTCCACCATAGGCCTCAAAGCGCTCCAAATATCCACTTGCAGATTCTACAAAAAGAGTGTTT

                      **       *       *           *            *      ***   
      89400 CAAAACTGCTGTATC-AAATAAA-GTTGAACTCTGTGAGGTGAATGCACACAGCACAAAATGGTT
         66 CAAAACTGCTCAATCAAAAGAAAGGTTCAACTCTGTGAGATGAATGCACACATCACAAAGAAGTT

                          **   *        *       *    
      89463 TCTCAGAATGCTTCCTTGTTGTTTTTATATGAAGATGTTTC
        131 TCTCAGAATGCTTCTGTGTAGTTTTTATGTGAAGATATTTC

                  *  *             *   *     *                               
      89504 CTTTTCAACAATAGGCCTCAAAGTGCTTCAAATGTCCACTTGCAGATTCTACAAAAAGAGTGTTT
          1 CTTTTCCACCATAGGCCTCAAAGCGCTCCAAATATCCACTTGCAGATTCTACAAAAAGAGTGTTT

                                        *      *  *  *                       
      89569 CAAAACTGCTCAATCAAAAGAAAGGTTCGACTCTGGGAAATTAATGCACACATCACAAAGAAGTT
         66 CAAAACTGCTCAATCAAAAGAAAGGTTCAACTCTGTGAGATGAATGCACACATCACAAAGAAGTT

                                     *        *      
      89634 TCTC---A-GCTTCTGTGTAGTTTTCATGTGAAGTTATTTC
        131 TCTCAGAATGCTTCTGTGTAGTTTTTATGTGAAGATATTTC

                     *       *     *            *    *         **       *    
      89671 CTTTTCCACAATAGGCCGCAAAGGGCTCCAAATATCAACTTACAGATTCTAGGAAAAGAGAGTTT
          1 CTTTTCCACCATAGGCCTCAAAGCGCTCCAAATATCCACTTGCAGATTCTACAAAAAGAGTGTTT

                       * **      *     *                                     
      89736 CAAAACTGCTCTACGAAAAGATAGGTTGAACTCTGTGAGATGAATGCACACATCACAAAGAAGTT
         66 CAAAACTGCTCAATCAAAAGAAAGGTTCAACTCTGTGAGATGAATGCACACATCACAAAGAAGTT

                       *               * *     *     
      89801 TCTCAGAATGCATCTGTGTAGTTTTTACGGGAAGACATTTC
        131 TCTCAGAATGCTTCTGTGTAGTTTTTATGTGAAGATATTTC

                        ***  *     **      *  *                  *     ***   
      89842 CTTTTCCACCATCTTCCACAAAGGTCTCCAAGTAACCACTTGCAGATTCTACAGAAAGACACTTT
          1 CTTTTCCACCATAGGCCTCAAAGCGCTCCAAATATCCACTTGCAGATTCTACAAAAAGAGTGTTT

            *          *         ***      *      ***                      *  
      89907 AAAAACTGCTCTATCAAAAGATCAGTTCAAGTCTGTGGTTTGAATGCACACATCACAAAGAATTT
         66 CAAAACTGCTCAATCAAAAGAAAGGTTCAACTCTGTGAGATGAATGCACACATCACAAAGAAGTT

                                     *  *            
      89972 TCTCAGAATGCTTCTGTGTAGTTTTCATATGAAGATATTTC
        131 TCTCAGAATGCTTCTGTGTAGTTTTTATGTGAAGATATTTC

                                    *                                   **   
      90013 CTTTTCCACCATAGGCCTCAAAGCACTCCAAATATCCACTTGCAGATTCTACAAAAAGAGATTTT
          1 CTTTTCCACCATAGGCCTCAAAGCGCTCCAAATATCCACTTGCAGATTCTACAAAAAGAGTGTTT

                   **                           *  *          *        *     
      90078 CAAAACTAGTCAATCAAAAGAAAGGTTCAACTCTGTCAGTTGAATGCACATATCACAAACAAGTT
         66 CAAAACTGCTCAATCAAAAGAAAGGTTCAACTCTGTGAGATGAATGCACACATCACAAAGAAGTT

                *      *                             
      90143 TCTCGGAATGCGTCTGTGTAGTTTTTATGTGAAGATATTTC
        131 TCTCAGAATGCTTCTGTGTAGTTTTTATGTGAAGATATTTC

               *     * *           *     *                  *    *           
      90184 CTTCTCCACAACAGGCCTCAAAGTGCTCCGAATATCCACTTGCAGATTTTAC-TAAAGAGTGTTT
          1 CTTTTCCACCATAGGCCTCAAAGCGCTCCAAATATCCACTTGCAGATTCTACAAAAAGAGTGTTT

             *               *  *   *     *        *    *               *    
      90248 CCAAACTGCTCAATCAAGAGGAAGTTTCAAGTCTGTGAGCTGAACGCACACATCACAAAGTAGTT
         66 CAAAACTGCTCAATCAAAAGAAAGGTTCAACTCTGTGAGATGAATGCACACATCACAAAGAAGTT

               *                                *    
      90313 TCTGAGAATGCTTCTGTGTAGTTTTTATGTGAAGATGTTTC
        131 TCTCAGAATGCTTCTGTGTAGTTTTTATGTGAAGATATTTC

                            **     *                                    *    
      90354 CTTTTCCACCATAGGCTGCAAAGGGCTCCAAATATCCACTTGCAGATTCTACAAAAAGAGAGTTT
          1 CTTTTCCACCATAGGCCTCAAAGCGCTCCAAATATCCACTTGCAGATTCTACAAAAAGAGTGTTT

                 *     *         *          *     *                     *    
      90419 CAAAAGTGCTCTATCAAAAGATAGGTTCAACTATGTGATATGAATGCACACATCACAAAGTAGTT
         66 CAAAACTGCTCAATCAAAAGAAAGGTTCAACTCTGTGAGATGAATGCACACATCACAAAGAAGTT

                                          *          
      90484 TCTCAGAATGCTTCTGTGTAGTTTTTATGTAAAGATATTTC
        131 TCTCAGAATGCTTCTGTGTAGTTTTTATGTGAAGATATTTC

                                    *                                   **   
      90525 CTTTTCCACCATAGGCCTCAAAGCACTCCAAATATCCACTTGCAGATTCTACAAAAAGAGATTTT
          1 CTTTTCCACCATAGGCCTCAAAGCGCTCCAAATATCCACTTGCAGATTCTACAAAAAGAGTGTTT

                   ** *                   *     *  *    * *   *        *     
      90590 CAAAACTATTTAATCAAAAGAAAGGTTCAAATCTGTCAGTTGAAGGTACATATCACAAACAAGTT
         66 CAAAACTGCTCAATCAAAAGAAAGGTTCAACTCTGTGAGATGAATGCACACATCACAAAGAAGTT

             * **                                    
      90655 TATTGGAATGCTTCTGTGTAGTTTTTATGTGAAGATATTTC
        131 TCTCAGAATGCTTCTGTGTAGTTTTTATGTGAAGATATTTC

                     * *         * *                        **  *     *      
      90696 CTTTTCCACAACAGGCCTCAAGGTGCTCCAAATATCCACTTGCAGATTTCACTAAAAGTGTGTTT
          1 CTTTTCCACCATAGGCCTCAAAGCGCTCCAAATATCCACTTGCAGATTCTACAAAAAGAGTGTTT

             *  *            *  *   *     *        *             *           
      90761 CCAAGCTGCTCAATCAAGAGGAAGTTTCAAGTCTGTGAGGTGAATGCACACATTACAAAGAAGTT
         66 CAAAACTGCTCAATCAAAAGAAAGGTTCAACTCTGTGAGATGAATGCACACATCACAAAGAAGTT

            *  *                                     
      90826 ACTGAGAATGCTTCTGTGTAGTTTTTATGTGAAGATATTTC
        131 TCTCAGAATGCTTCTGTGTAGTTTTTATGTGAAGATATTTC

                      **               *                                *    
      90867 CTTTTCCACCGCAGGCCTCAAAGCGCTGCAAATATCCACTTGCAGATTCTACAAAAAGAGAGTTT
          1 CTTTTCCACCATAGGCCTCAAAGCGCTCCAAATATCCACTTGCAGATTCTACAAAAAGAGTGTTT

                      **         *   *         *   *     **            *     
      90932 CAAAACTGCTGTATCAAAAGATAGGGTCAACTCTGCGAGTTGAATAAACACATCACAAATAAGTT
         66 CAAAACTGCTCAATCAAAAGAAAGGTTCAACTCTGTGAGATGAATGCACACATCACAAAGAAGTT

            *  **   *        *               *       
      90997 CCTGGGAACGCTTCTGTATAG-TTTTATGTGAATATATTTC
        131 TCTCAGAATGCTTCTGTGTAGTTTTTATGTGAAGATATTTC

                         *          *                   *   *  *   * *   *   
      91037 CTTTTCCACCATATGCCTCAAAGCACTCCAAATATCCACTTGCACATTATAGAAACATAGTCTTT
          1 CTTTTCCACCATAGGCCTCAAAGCGCTCCAAATATCCACTTGCAGATTCTACAAAAAGAGTGTTT

                   **                        *         *             *       
      91102 CAAAACTTGTCAATC-AAAGAAAGGTTCAACTCCGTGAGATGAGTGCACACATCACAGAGAAGTT
         66 CAAAACTGCTCAATCAAAAGAAAGGTTCAACTCTGTGAGATGAATGCACACATCACAAAGAAGTT

                *     *                            * 
      91166 TCTCGGAATGTTTCTGTGTAGTTTTTATGTGAAGATATTGC
        131 TCTCAGAATGCTTCTGTGTAGTTTTTATGTGAAGATATTTC

                     *               *            *          *       *   *   
      91207 CTTTTCCACAATAGGCCTCAAAGCGTTCCAAATATCCAATTGCAGATTCCACAAAAAAAGTTTTT
          1 CTTTTCCACCATAGGCCTCAAAGCGCTCCAAATATCCACTTGCAGATTCTACAAAAAGAGTGTTT

            *                 *  *  *  *             * *         **    *     
      91272 TAAAACTGCTCAATCAAATGATAGATTAAACTCTGTGAGATTAGTGCACACATGTCAAAAAAGTT
         66 CAAAACTGCTCAATCAAAAGAAAGGTTCAACTCTGTGAGATGAATGCACACATCACAAAGAAGTT

                                *      * *           
      91337 TCTCAGAATGCTTCTGTGTACTTTTTACGGGAAGATATTTC
        131 TCTCAGAATGCTTCTGTGTAGTTTTTATGTGAAGATATTTC

                        *    *     **         *    *           **  * *  *    
      91378 CTTTTCCACCATCGGCCACAAAGGACTCCAAATAACCACATGCAGATTCTAGTAACACAGAGTTT
          1 CTTTTCCACCATAGGCCTCAAAGCGCTCCAAATATCCACTTGCAGATTCTACAAAAAGAGTGTTT

                       *         * *           *   * * *    **    ***        
      91443 CAAAACTGCTCTATCAAAAGATAAGTTCAACTCTGAGAGTTTAGTGCAACCATCGTGAAGAAGTT
         66 CAAAACTGCTCAATCAAAAGAAAGGTTCAACTCTGTGAGATGAATGCACACATCACAAAGAAGTT

                            *     *                  
      91508 TCTCAGAATGCTTCTGAGTAGTGTTTATGTGAAGATATTTC
        131 TCTCAGAATGCTTCTGTGTAGTTTTTATGTGAAGATATTTC

                              *     *                      *           **    
      91549 CTTTTCCACCATAGGCCTGAAAGCCCTCCAAATATCCACTTGCAGATCCTACAAAAAGAAAGTTT
          1 CTTTTCCACCATAGGCCTCAAAGCGCTCCAAATATCCACTTGCAGATTCTACAAAAAGAGTGTTT

             *         **     *  *  *   *        **      *                   
      91614 CGAAA-TGCTCTCTCAAACGATAGTTTCGACTCTGTGGTATGAATACACACATCACAAAGAAGTT
         66 CAAAACTGCTCAATCAAAAGAAAGGTTCAACTCTGTGAGATGAATGCACACATCACAAAGAAGTT

                                       **            
      91678 TCTCAGAATGCTTCTGTGTAGTTTTTAAATGAAGATATTTC
        131 TCTCAGAATGCTTCTGTGTAGTTTTTATGTGAAGATATTTC

            *                       *          *     *                       
      91719 TTTTTCCACCATAGGCCTCAAAGCACTCCAAATATGCACTTCCAGATTCTACAAAAAGAGTGTTT
          1 CTTTTCCACCATAGGCCTCAAAGCGCTCCAAATATCCACTTGCAGATTCTACAAAAAGAGTGTTT

              *                 *       * *         **   *         *  * *    
      91784 CAGAACTGCTCAATCAAAAGGAAGGTTCCAGTC--TGAGACAAATACACACATCAAAAGGTAGTT
         66 CAAAACTGCTCAATCAAAAGAAAGGTTCAACTCTGTGAGATGAATGCACACATCACAAAGAAGTT

                                                    *
      91847 TCTCAGAATGCTTCTGTGTAGTTTTTATGTGAAGATATTTT
        131 TCTCAGAATGCTTCTGTGTAGTTTTTATGTGAAGATATTTC

             *               *    **    *     *      *  **   *          *    
      91888 CCTTTCCACCATAGGCCACAAATGGCTCTAAATACCCACTTACATTTTCCACAAAAAGAGAGTTT
          1 CTTTTCCACCATAGGCCTCAAAGCGCTCCAAATATCCACTTGCAGATTCTACAAAAAGAGTGTTT

                       * *    * *      *   *         *                       
      91953 CAAAACTGCTCTACCAAAGGTAA-GTTTAACGCTGTGAG-TTAA-G-A-ACATCACAAAGAAGTT
         66 CAAAACTGCTCAATCAAAAGAAAGGTTCAACTCTGTGAGATGAATGCACACATCACAAAGAAGTT

                                   *   *  *          
      92013 TCTCAGAATGCTTCTGTGTAGTTCTTACGTAAAGATATTTC
        131 TCTCAGAATGCTTCTGTGTAGTTTTTATGTGAAGATATTTC

                 *   *      ***    *                  *              **      
      92054 CTTTTACACAATAGGCAGAAAAGTGCTCCAAATATCCACTTGAAGATTCTACAAAAACCGTGTTT
          1 CTTTTCCACCATAGGCCTCAAAGCGCTCCAAATATCCACTTGCAGATTCTACAAAAAGAGTGTTT

                     **                                          *      *    
      92119 CAAAACTGCCGAATCAAAAGAAAGGTTCAACTCTGTGAGATGAATGCACACATAACAAAGGAGTT
         66 CAAAACTGCTCAATCAAAAGAAAGGTTCAACTCTGTGAGATGAATGCACACATCACAAAGAAGTT

                                 *      *      *    *
      92184 TCTCAGAATGCTTCTGTGTAGCTTTTATATGAAGACATTTA
        131 TCTCAGAATGCTTCTGTGTAGTTTTTATGTGAAGATATTTC

            *        * *            *   ***                     **        *  
      92225 GTTTTCCACAACAGGCCTCAAAGCTCTCTCCATATCCACTTGCAGATTCTACCGAAAGAGTGCTT
          1 CTTTTCCACCATAGGCCTCAAAGCGCTCCAAATATCCACTTGCAGATTCTACAAAAAGAGTGTTT

             *                   * **     *        *        *     **         
      92290 CCAAACTGCTCAATCAAAAGAGACATTCAAATCTGTGAGGTGAATGCAGACATCGTAAAGAAGTT
         66 CAAAACTGCTCAATCAAAAGAAAGGTTCAACTCTGTGAGATGAATGCACACATCACAAAGAAGTT

                                *     *       *    **
      92355 TCTCAGAATGCTTCTGTGTATTTTTTGTGTGAAGTTATTCG
        131 TCTCAGAATGCTTCTGTGTAGTTTTTATGTGAAGATATTTC

            *    *             *     *  *            **                 *    
      92396 TTTTTGCACCATAGGCCTCCAAGCGTTCTAAATATCCACTTCTAGATTCTACAAAAAGAGAGTTT
          1 CTTTTCCACCATAGGCCTCAAAGCGCTCCAAATATCCACTTGCAGATTCTACAAAAAGAGTGTTT

                   *         *            *        *    *   *                
      92461 CAAAACTACTC-A--AACA-AAAGGTTCAATTCTGTGAGTTGAAAGCAAACATCACAAAGAAGTT
         66 CAAAACTGCTCAATCAAAAGAAAGGTTCAACTCTGTGAGATGAATGCACACATCACAAAGAAGTT

                       *             *               
      92522 TCTCAGAATGCGTCTGTGTAGTTTTGATGTGAAGATATTTC
        131 TCTCAGAATGCTTCTGTGTAGTTTTTATGTGAAGATATTTC

                     **   ****     *                  *                      
      92563 CTTTT-CACAGTAGAATGCAAAGGGCTCCAAATATCCACTTGGAGATTCTACAAAAAGA--GTTT
          1 CTTTTCCACCATAGGCCTCAAAGCGCTCCAAATATCCACTTGCAGATTCTACAAAAAGAGTGTTT

                  *    **     *  *     *     ***   *     *               *   
      92625 CAAAACCGCTCTGTCAAATGATAGGTTGAACTCCCGGAGGTGAATACACACATCACAAAGAGGTT
         66 CAAAACTGCTCAATCAAAAGAAAGGTTCAACTCTGTGAGATGAATGCACACATCACAAAGAAGTT

                  *                       *  *       
      92690 TCTCAGCATGCTTCTGTGTAGTTTTTATGTAAACATATTTC
        131 TCTCAGAATGCTTCTGTGTAGTTTTTATGTGAAGATATTTC

             *    * *              *           *      * *       *   *    *   
      92731 CGTTTCTATCATAGGCCTCAAAGTGCTCCAAATATTCACTTGTACATTCTACCAAACGAGTATTT
          1 CTTTTCCACCATAGGCCTCAAAGCGCTCCAAATATCCACTTGCAGATTCTACAAAAAGAGTGTTT

                              * *         ** *    *        *            *    
      92796 CAAAACTGCTCAATCAAATGGAAGGTTCAAAACCGTGACATGAATGCCCACATCACAAAGTAGTT
         66 CAAAACTGCTCAATCAAAAGAAAGGTTCAACTCTGTGAGATGAATGCACACATCACAAAGAAGTT

                                                     
      92861 TCTCAGAATGCTTCTGTGTAGTTTTTATGTGAAGATATTTC
        131 TCTCAGAATGCTTCTGTGTAGTTTTTATGTGAAGATATTTC

                     * *    *      *    *       * *   **      *              
      92902 CTTTTCCACAACA-GCGTGCAAAACGCTTCAAATATGCCCTTAGAGATTCCACAAAAAGAGTGTT
          1 CTTTTCCACCATAGGCCT-CAAAGCGCTCCAAATATCCACTTGCAGATTCTACAAAAAGAGTGTT

              *     *               *  *                                  ** 
      92966 TCCAAACTACTCAAATCAAAA-AATGATTTCAACTCTGTGAGATGAATGCACACATCACAAACTA
         65 TCAAAACTGCTC-AATCAAAAGAAAG-GTTCAACTCTGTGAGATGAATGCACACATCACAAAGAA

                         *     ** *   * *   *       *   
      93030 GTTTCTCAGAATGTTTCTGCCTGGTTCTCATGCGAAGATAGTTC
        128 GTTTCTCAGAATGCTTCTGTGTAGTTTTTATGTGAAGATATTTC

                 *           *   * **              *                  * *    
      93074 CTTTTTCACCATAGGCCGCAATGTACTCCAAATATCCACCTGCAGATTCTAC-AAAAGTGAGTTT
          1 CTTTTCCACCATAGGCCTCAAAGCGCTCCAAATATCCACTTGCAGATTCTACAAAAAGAGTGTTT

                       *         ***    **         *        *      *       * 
      93138 CAAAACTGCTCTATCAAAAGATCAGTTCGTCTCTGTGAGTTGAATGCATACATCAAAAAGAAGCT
         66 CAAAACTGCTCAATCAAAAGAAAGGTTCAACTCTGTGAGATGAATGCACACATCACAAAGAAGTT

                 *             *      **         *   
      93203 TCTCAAAATGCTTCTGTGTGGTTTTTCGGTGAAGATAGTTC
        131 TCTCAGAATGCTTCTGTGTAGTTTTTATGTGAAGATATTTC

            *     *        *      * *                 *        *        *    
      93244 TTTTTCTACCATAGGTCTCAAACCACTCCAAATATCCACTTGTAGATTCTATAAAAAGGAATG-T
          1 CTTTTCCACCATAGGCCTCAAAGCGCTCCAAATATCCACTTGCAGATTCTACAAAAA-GAGTGTT

                  *        *    *    *      * *         *      *         *   
      93308 TCAAAATTGCTCAATAAAAATAAAGTTTCAACACCGTGAGATGAGTGCACAAATCACAAAGGAGT
         65 TCAAAACTGCTCAATCAAAAGAAAGGTTCAACTCTGTGAGATGAATGCACACATCACAAAGAAGT

                  *          *         *          *   
      93373 TTCTCAAAATGCTTCTGGGTAGTTTTTCTGTGAAGATAGTTC
        130 TTCTCAGAATGCTTCTGTGTAGTTTTTATGTGAAGATATTTC

                  *     *    *     *          *                         *    
      93415 CTTTTCTACCATGGGCCACAAAGGGCTCCAAATACCCACTTGCAGATTCTACAAAAAGAGAGTTT
          1 CTTTTCCACCATAGGCCTCAAAGCGCTCCAAATATCCACTTGCAGATTCTACAAAAAGAGTGTTT

              *        *      ** * *        *    * *    **  * *           *  
      93480 CACAACTGCTCTATCAAACAATATGTTCAACTTTGTGGGTTGAACACAAATATCAC-AAGAATTT
         66 CAAAACTGCTCAATCAAAAGAAAGGTTCAACTCTGTGAGATGAATGCACACATCACAAAGAAGTT

                **                             *     
      93544 TCTCCCAATGCTTCTGTGTAGTTTTTATGTGAAGACATTTC
        131 TCTCAGAATGCTTCTGTGTAGTTTTTATGTGAAGATATTTC

            *   *  *      *  *     *                 *  *      *       *  *  
      93585 TTTTCCCTCCATAGTCCACAAAGTGCTCCAAATATCCACTTACATATTCTAGAAAAAGATTGCTT
          1 CTTTTCCACCATAGGCCTCAAAGCGCTCCAAATATCCACTTGCAGATTCTACAAAAAGAGTGTTT

            **       *    *               * * *                              
      93650 GGAAACTGCACAATGAAAAGAAAGGTTCAAATATATGAGATGAATGCACACATCACAAAGAAGTT
         66 CAAAACTGCTCAATCAAAAGAAAGGTTCAACTCTGTGAGATGAATGCACACATCACAAAGAAGTT

                                 *                    
      93715 TCTCAGAAT-CTCTCTGTGTAATTTTTATGTGAAGATATTTC
        131 TCTCAGAATGCT-TCTGTGTAGTTTTTATGTGAAGATATTTC

                *     *    *  *   *                        *      *    *     
      93756 CTTTCCCACCTTAGGTCTTAAAACGCTCCAAATATCCACTTGCAGATACTACAAGAAGATTGTTT
          1 CTTTTCCACCATAGGCCTCAAAGCGCTCCAAATATCCACTTGCAGATTCTACAAAAAGAGTGTTT

                     *    *        *      *     **                           
      93821 CAAAACTGCACAA-AAAAAGAAATGTTCAATTCTGTTTGATGAATGCACACATCACAAAGAAGTT
         66 CAAAACTGCTCAATCAAAAGAAAGGTTCAACTCTGTGAGATGAATGCACACATCACAAAGAAGTT

                           *                         
      93885 TCTCAGAATGCTTCTCTGTAGTTTTTATGTGAAGATATTTC
        131 TCTCAGAATGCTTCTGTGTAGTTTTTATGTGAAGATATTTC

                     *             *                 *         **      * *   
      93926 CTTTTCCACAATAGGCCTCAAAGGGCTCCAAATATCCACTTCCAGATTCTATGAAAAGAATATTT
          1 CTTTTCCACCATAGGCCTCAAAGCGCTCCAAATATCCACTTGCAGATTCTACAAAAAGAGTGTTT

             *              * *    *                                      *  
      93991 CCAAACTGCTCAATCATAGGAAATGTTCAACTCTGTGAGATGAATGCACACATCAC-AAGAAATT
         66 CAAAACTGCTCAATCAAAAGAAAGGTTCAACTCTGTGAGATGAATGCACACATCACAAAGAAGTT

                     *    *      *       *       *   
      94055 TCTCAGAATCCTTCAGTGTAGGTTTTATGAGAAGATAATTC
        131 TCTCAGAATGCTTCTGTGTAGTTTTTATGTGAAGATATTTC

                     *    **        *   *                           *    *   
      94096 CTTTTCCACAATAGTTCTCAAAGCACTCAAAATATCCACTTGCAGATTCTACAAAAGGAGTATTT
          1 CTTTTCCACCATAGGCCTCAAAGCGCTCCAAATATCCACTTGCAGATTCTACAAAAAGAGTGTTT

                                                          *                  
      94161 CAAAACTGCTCAATCAAAAGAAAGGTTCAACTCTGTGAGATGAATGGACACATCACAAAGAAGTT
         66 CAAAACTGCTCAATCAAAAGAAAGGTTCAACTCTGTGAGATGAATGCACACATCACAAAGAAGTT

                                  *   *              
      94226 TCTCAGAATGCTTCTGTGTAGTATTTTTGTGAAGATATTT-
        131 TCTCAGAATGCTTCTGTGTAGTTTTTATGTGAAGATATTTC

                          *  * * * * ***             *            *     * *  
      94266 CTTTTCCACCATAGACCGCCAGGGGACACAAATATCCACTTTCAGATTCTACAACAAGAGAGGTT
          1 CTTTTCCACCATAGGCCTCAAAGCGCTCCAAATATCCACTTGCAGATTCTACAAAAAGAGTGTTT

                   *   *     *   ** *       *      *                      * *
      94331 CAAAACTACTCGATCAAGAGATGGTTTCAACTATGTGAGTTGAATGCACACATCACAAAGAACTA
         66 CAAAACTGCTCAATCAAAAGAAAGGTTCAACTCTGTGAGATGAATGCACACATCACAAAGAAGTT

             *  *    *                               
      94396 TGTCGGAATTCTTCTGTGTAGTTTTTATGTGAAGATATTTC
        131 TCTCAGAATGCTTCTGTGTAGTTTTTATGTGAAGATATTTC

                     *    * *      * *    *                  *               
      94437 CTTTTCCACAATAGACGTCAAAGTGATCCAGATATCCACTTGCAGATTCCACAAAAAGAGTGTTT
          1 CTTTTCCACCATAGGCCTCAAAGCGCTCCAAATATCCACTTGCAGATTCTACAAAAAGAGTGTTT

                 *   *   *                  **                     *  *      
      94502 CAAAAGTGCACAACCAAAAGAAAGGTTCAACTAGGTGAGATGAATGCACACATCAGAAGGAAGTT
         66 CAAAACTGCTCAATCAAAAGAAAGGTTCAACTCTGTGAGATGAATGCACACATCACAAAGAAGTT

                            **   *     * *       *   
      94567 TCTCAGAATGCTTCTGCATAGCTTTTAAGGGAAGATACTTC
        131 TCTCAGAATGCTTCTGTGTAGTTTTTATGTGAAGATATTTC

                    *               *            * *  *    * *               
      94608 CTTTTCCAACATAGGCCTCAAAGCACTCCAAATATCCTCCTGGAGATACCACAAAAAGAGTGTTT
          1 CTTTTCCACCATAGGCCTCAAAGCGCTCCAAATATCCACTTGCAGATTCTACAAAAAGAGTGTTT

            **                      *  *                 *       *           
      94673 GCAAACTGCTCAATCAAAAGAAAGATTTAACTCTGTGAGATGAATCCACACATGACAAAGAAGTT
         66 CAAAACTGCTCAATCAAAAGAAAGGTTCAACTCTGTGAGATGAATGCACACATCACAAAGAAGTT

                                                     
      94738 TCTCAGAATGCTTCTGTGTAGTTTTTATGTGAAGATATTTC
        131 TCTCAGAATGCTTCTGTGTAGTTTTTATGTGAAGATATTTC

                     *   * * *    **           *                *     *      
      94779 CTTTTCCACAATAAGACCCAAAAGGCTCCAAATATTCACTTGCAGATTCTAAAAAAAACAGTGTT
          1 CTTTTCCACCATAGGCCTCAAAGCGCTCCAAATATCCACTTGCAGATTCT-ACAAAAAGAGTGTT

                                  *                  *      *        **      
      94844 TCAAAACTGCTCAATCAAAAGATA-GTTCAACTCTGTGAGAAGAATGCTCACATCACTGAGAAGT
         65 TCAAAACTGCTCAATCAAAAGAAAGGTTCAACTCTGTGAGATGAATGCACACATCACAAAGAAGT

                                         *            
      94908 TTCTCAGAATGCTTCTGTGTAGTTTTTATATGAAGATATTTC
        130 TTCTCAGAATGCTTCTGTGTAGTTTTTATGTGAAGATATTTC

                *     *      *    **                       *   **       *    
      94950 CTTTCCCACCGTAGGCCACAAAAGGCTCCAAATATCCACTTGCAGATACTATGAAAAGAGAGTTT
          1 CTTTTCCACCATAGGCCTCAAAGCGCTCCAAATATCCACTTGCAGATTCTACAAAAAGAGTGTTT

                        *        *               ***            *            
      95015 CAAAACTGCTCATTCAAAAGATAGGTTCAACTCTGTGGTTTGAATGCACACAGCACAAAGAAGTT
         66 CAAAACTGCTCAATCAAAAGAAAGGTTCAACTCTGTGAGATGAATGCACACATCACAAAGAAGTT

              *       **                   **   *    
      95080 TCACAGAATGTGTCTGTGTAGTTTTTATGTGCGGATGTTTC
        131 TCTCAGAATGCTTCTGTGTAGTTTTTATGTGAAGATATTTC

                         *        ****   *   *               *    *          
      95121 CTTTTCCACCATATGCCT-AAATATTTCCCAATTTCCACTTGCAGATTCCACAAGAAGAGTGTTT
          1 CTTTTCCACCATAGGCCTCAAAGCGCTCCAAATATCCACTTGCAGATTCTACAAAAAGAGTGTTT

                      **       *       *           *            *      ***   
      95185 CAAAACTGCTGTATC-AAATAAA-GTTGAACTCTGTGAGGTGAATGCACACAGCACAAAATGGTT
         66 CAAAACTGCTCAATCAAAAGAAAGGTTCAACTCTGTGAGATGAATGCACACATCACAAAGAAGTT

                          **   *        *       *    
      95248 TCTCAGAATGCTTCCTTGTTGTTTTTATATGAAGATGTTTC
        131 TCTCAGAATGCTTCTGTGTAGTTTTTATGTGAAGATATTTC

                  *  *             *   *     *                               
      95289 CTTTTCAACAATAGGCCTCAAAGTGCTTCAAATGTCCACTTGCAGATTCTACAAAAAGAGTGTTT
          1 CTTTTCCACCATAGGCCTCAAAGCGCTCCAAATATCCACTTGCAGATTCTACAAAAAGAGTGTTT

                                        *      *  *  *                       
      95354 CAAAACTGCTCAATCAAAAGAAAGGTTCGACTCTGGGAAATTAATGCACACATCACAAAGAAGTT
         66 CAAAACTGCTCAATCAAAAGAAAGGTTCAACTCTGTGAGATGAATGCACACATCACAAAGAAGTT

                                     *        *      
      95419 TCTC---A-GCTTCTGTGTAGTTTTCATGTGAAGTTATTTC
        131 TCTCAGAATGCTTCTGTGTAGTTTTTATGTGAAGATATTTC

                     *       *     *            *    *         **       *    
      95456 CTTTTCCACAATAGGCCGCAAAGGGCTCCAAATATCAACTTACAGATTCTAGGAAAAGAGAGTTT
          1 CTTTTCCACCATAGGCCTCAAAGCGCTCCAAATATCCACTTGCAGATTCTACAAAAAGAGTGTTT

                       * **      *     *                                     
      95521 CAAAACTGCTCTACGAAAAGATAGGTTGAACTCTGTGAGATGAATGCACACATCACAAAGAAGTT
         66 CAAAACTGCTCAATCAAAAGAAAGGTTCAACTCTGTGAGATGAATGCACACATCACAAAGAAGTT

                       *               * *     *     
      95586 TCTCAGAATGCATCTGTGTAGTTTTTACGGGAAGACATTTC
        131 TCTCAGAATGCTTCTGTGTAGTTTTTATGTGAAGATATTTC

                        ***  *     **      *  *                  *     ***   
      95627 CTTTTCCACCATCTTCCACAAAGGTCTCCAAGTAACCACTTGCAGATTCTACAGAAAGACACTTT
          1 CTTTTCCACCATAGGCCTCAAAGCGCTCCAAATATCCACTTGCAGATTCTACAAAAAGAGTGTTT

            *          *         ***      *      ***                      *  
      95692 AAAAACTGCTCTATCAAAAGATCAGTTCAAGTCTGTGGTTTGAATGCACACATCACAAAGAATTT
         66 CAAAACTGCTCAATCAAAAGAAAGGTTCAACTCTGTGAGATGAATGCACACATCACAAAGAAGTT

                                     *  *            
      95757 TCTCAGAATGCTTCTGTGTAGTTTTCATATGAAGATATTTC
        131 TCTCAGAATGCTTCTGTGTAGTTTTTATGTGAAGATATTTC

                                    *                                   **   
      95798 CTTTTCCACCATAGGCCTCAAAGCACTCCAAATATCCACTTGCAGATTCTACAAAAAGAGATTTT
          1 CTTTTCCACCATAGGCCTCAAAGCGCTCCAAATATCCACTTGCAGATTCTACAAAAAGAGTGTTT

                   **                           *  *          *        *     
      95863 CAAAACTAGTCAATCAAAAGAAAGGTTCAACTCTGTCAGTTGAATGCACATATCACAAACAAGTT
         66 CAAAACTGCTCAATCAAAAGAAAGGTTCAACTCTGTGAGATGAATGCACACATCACAAAGAAGTT

                *      *                             
      95928 TCTCGGAATGCGTCTGTGTAGTTTTTATGTGAAGATATTTC
        131 TCTCAGAATGCTTCTGTGTAGTTTTTATGTGAAGATATTTC

               *     * *           *     *                  *    *           
      95969 CTTCTCCACAACAGGCCTCAAAGTGCTCCGAATATCCACTTGCAGATTTTAC-TAAAGAGTGTTT
          1 CTTTTCCACCATAGGCCTCAAAGCGCTCCAAATATCCACTTGCAGATTCTACAAAAAGAGTGTTT

             *               *  *   *     *        *    *               *    
      96033 CCAAACTGCTCAATCAAGAGGAAGTTTCAAGTCTGTGAGCTGAACGCACACATCACAAAGTAGTT
         66 CAAAACTGCTCAATCAAAAGAAAGGTTCAACTCTGTGAGATGAATGCACACATCACAAAGAAGTT

               *                                *    
      96098 TCTGAGAATGCTTCTGTGTAGTTTTTATGTGAAGATGTTTC
        131 TCTCAGAATGCTTCTGTGTAGTTTTTATGTGAAGATATTTC

                            **     *                                    *    
      96139 CTTTTCCACCATAGGCTGCAAAGGGCTCCAAATATCCACTTGCAGATTCTACAAAAAGAGAGTTT
          1 CTTTTCCACCATAGGCCTCAAAGCGCTCCAAATATCCACTTGCAGATTCTACAAAAAGAGTGTTT

                 *     *         *          *     *                     *    
      96204 CAAAAGTGCTCTATCAAAAGATAGGTTCAACTATGTGATATGAATGCACACATCACAAAGTAGTT
         66 CAAAACTGCTCAATCAAAAGAAAGGTTCAACTCTGTGAGATGAATGCACACATCACAAAGAAGTT

                                          *          
      96269 TCTCAGAATGCTTCTGTGTAGTTTTTATGTAAAGATATTTC
        131 TCTCAGAATGCTTCTGTGTAGTTTTTATGTGAAGATATTTC

                                    *                                   **   
      96310 CTTTTCCACCATAGGCCTCAAAGCACTCCAAATATCCACTTGCAGATTCTACAAAAAGAGATTTT
          1 CTTTTCCACCATAGGCCTCAAAGCGCTCCAAATATCCACTTGCAGATTCTACAAAAAGAGTGTTT

                   ** *                   *     *  *    * *   *        *     
      96375 CAAAACTATTTAATCAAAAGAAAGGTTCAAATCTGTCAGTTGAAGGTACATATCACAAACAAGTT
         66 CAAAACTGCTCAATCAAAAGAAAGGTTCAACTCTGTGAGATGAATGCACACATCACAAAGAAGTT

             * **                                    
      96440 TATTGGAATGCTTCTGTGTAGTTTTTATGTGAAGATATTTC
        131 TCTCAGAATGCTTCTGTGTAGTTTTTATGTGAAGATATTTC

                     * *       * * *                        **  *     *      
      96481 CTTTTCCACAACAGGCCTCCAGGTGCTCCAAATATCCACTTGCAGATTTCACTAAAAGTGTGTTT
          1 CTTTTCCACCATAGGCCTCAAAGCGCTCCAAATATCCACTTGCAGATTCTACAAAAAGAGTGTTT

             *  *            *  *   *     *        *             *           
      96546 CCAAGCTGCTCAATCAAGAGGAAGTTTCAAGTCTGTGAGGTGAATGCACACATTACAAAGAAGTT
         66 CAAAACTGCTCAATCAAAAGAAAGGTTCAACTCTGTGAGATGAATGCACACATCACAAAGAAGTT

            *  *                                     
      96611 ACTGAGAATGCTTCTGTGTAGTTTTTATGTGAAGATATTTC
        131 TCTCAGAATGCTTCTGTGTAGTTTTTATGTGAAGATATTTC

                      **               *                                *    
      96652 CTTTTCCACCGCAGGCCTCAAAGCGCTGCAAATATCCACTTGCAGATTCTACAAAAAGAGAGTTT
          1 CTTTTCCACCATAGGCCTCAAAGCGCTCCAAATATCCACTTGCAGATTCTACAAAAAGAGTGTTT

                      **         *   *         *   *     **            *     
      96717 CAAAACTGCTGTATCAAAAGATAGGGTCAACTCTGCGAGTTGAATAAACACATCACAAATAAGTT
         66 CAAAACTGCTCAATCAAAAGAAAGGTTCAACTCTGTGAGATGAATGCACACATCACAAAGAAGTT

               **   *        *               *       
      96782 TCTGGGAACGCTTCTGTATAG-TTTTATGTGAATATATTTC
        131 TCTCAGAATGCTTCTGTGTAGTTTTTATGTGAAGATATTTC

                         *          *                   *   *  *   * *   *   
      96822 CTTTTCCACCATATGCCTCAAAGCACTCCAAATATCCACTTGCACATTATAGAAACATAGTCTTT
          1 CTTTTCCACCATAGGCCTCAAAGCGCTCCAAATATCCACTTGCAGATTCTACAAAAAGAGTGTTT

                   **                        *         *             *       
      96887 CAAAACTTGTCAATC-AAAGAAAGGTTCAACTCCGTGAGATGAGTGCACACATCACAGAGAAGTT
         66 CAAAACTGCTCAATCAAAAGAAAGGTTCAACTCTGTGAGATGAATGCACACATCACAAAGAAGTT

                *     *                            * 
      96951 TCTCGGAATGTTTCTGTGTAGTTTTTATGTGAAGATATTGC
        131 TCTCAGAATGCTTCTGTGTAGTTTTTATGTGAAGATATTTC

                     *               *            *          *       *   *   
      96992 CTTTTCCACAATAGGCCTCAAAGCGTTCCAAATATCCAATTGCAGATTCCACAAAAAAAGTTTTT
          1 CTTTTCCACCATAGGCCTCAAAGCGCTCCAAATATCCACTTGCAGATTCTACAAAAAGAGTGTTT

            *                 *  *  *  *             * *         **    *     
      97057 TAAAACTGCTCAATCAAATGATAGATTAAACTCTGTGAGATTAGTGCACACATGTCAAAAAAGTT
         66 CAAAACTGCTCAATCAAAAGAAAGGTTCAACTCTGTGAGATGAATGCACACATCACAAAGAAGTT

                                *      * *           
      97122 TCTCAGAATGCTTCTGTGTACTTTTTAGGGGAAGATATTTC
        131 TCTCAGAATGCTTCTGTGTAGTTTTTATGTGAAGATATTTC

                        *    *     **         *    *           **  * *  *    
      97163 CTTTTCCACCATCGGCCACAAAGGACTCCAAATAACCACATGCAGATTCTAGTAACACAGAGTTT
          1 CTTTTCCACCATAGGCCTCAAAGCGCTCCAAATATCCACTTGCAGATTCTACAAAAAGAGTGTTT

                       *         * *           *   * * *    **    ***        
      97228 CAAAACTGCTCTATCAAAAGATAAGTTCAACTCTGAGAGTTTAGTGCAACCATCGTGAAGAAGTT
         66 CAAAACTGCTCAATCAAAAGAAAGGTTCAACTCTGTGAGATGAATGCACACATCACAAAGAAGTT

                            *     *                  
      97293 TCTCAGAATGCTTCTGAGTAGTGTTTATGTGAAGATATTTC
        131 TCTCAGAATGCTTCTGTGTAGTTTTTATGTGAAGATATTTC

                              *     *                      *           **    
      97334 CTTTTCCACCATAGGCCTGAAAGCCCTCCAAATATCCACTTGCAGATCCTACAAAAAGAAAGTTT
          1 CTTTTCCACCATAGGCCTCAAAGCGCTCCAAATATCCACTTGCAGATTCTACAAAAAGAGTGTTT

             *         **     *  *  *   *        ** *    *                   
      97399 CGAAA-TGCTCTCTCAAACGATAGTTTCGACTCTGTGGTACGAATACACACATCACAAAGAAGTT
         66 CAAAACTGCTCAATCAAAAGAAAGGTTCAACTCTGTGAGATGAATGCACACATCACAAAGAAGTT

                                       **            
      97463 TCTCAGAATGCTTCTGTGTAGTTTTTAAATGAAGATATTTC
        131 TCTCAGAATGCTTCTGTGTAGTTTTTATGTGAAGATATTTC

            *                       *          *     *                       
      97504 TTTTTCCACCATAGGCCTCAAAGCACTCCAAATATGCACTTCCAGATTCTACAAAAAGAGTGTTT
          1 CTTTTCCACCATAGGCCTCAAAGCGCTCCAAATATCCACTTGCAGATTCTACAAAAAGAGTGTTT

              *                 *       * *         **   *         *  * *    
      97569 CAGAACTGCTCAATCAAAAGGAAGGTTCCAGTC--TGAGACAAATACACACATCAAAAGGTAGTT
         66 CAAAACTGCTCAATCAAAAGAAAGGTTCAACTCTGTGAGATGAATGCACACATCACAAAGAAGTT

                                                    *
      97632 TCTCAGAATGCTTCTGTGTAGTTTTTATGTGAAGATATTTT
        131 TCTCAGAATGCTTCTGTGTAGTTTTTATGTGAAGATATTTC

             *               *    **    *     *      *  **   *          *    
      97673 CCTTTCCACCATAGGCCACAAATGGCTCTAAATACCCACTTACATTTTCCACAAAAAGAGAGTTT
          1 CTTTTCCACCATAGGCCTCAAAGCGCTCCAAATATCCACTTGCAGATTCTACAAAAAGAGTGTTT

                       * *    * *      *   *         *                       
      97738 CAAAACTGCTCTACCAAAGGTAA-GTTTAACGCTGTGAG-TTAA-G-A-ACATCACAAAGAAGTT
         66 CAAAACTGCTCAATCAAAAGAAAGGTTCAACTCTGTGAGATGAATGCACACATCACAAAGAAGTT

                                   *   *  *          
      97798 TCTCAGAATGCTTCTGTGTAGTTCTTACGTAAAGATATTTC
        131 TCTCAGAATGCTTCTGTGTAGTTTTTATGTGAAGATATTTC

                 *   *      ***    *                  *              **      
      97839 CTTTTACACAATAGGCAGAAAAGTGCTCCAAATATCCACTTGAAGATTCTACAAAAACCGTGTTT
          1 CTTTTCCACCATAGGCCTCAAAGCGCTCCAAATATCCACTTGCAGATTCTACAAAAAGAGTGTTT

                     **                                          *      *    
      97904 CAAAACTGCCGAATCAAAAGAAAGGTTCAACTCTGTGAGATGAATGCACACATAACAAAGGAGTT
         66 CAAAACTGCTCAATCAAAAGAAAGGTTCAACTCTGTGAGATGAATGCACACATCACAAAGAAGTT

                                 *      *      *    *
      97969 TCTCAGAATGCTTCTGTGTAGCTTTTATATGAAGACATTTA
        131 TCTCAGAATGCTTCTGTGTAGTTTTTATGTGAAGATATTTC

            *        * *            *   ***                     **        *  
      98010 GTTTTCCACAACAGGCCTCAAAGCTCTCTCCATATCCACTTGCAGATTCTACCGAAAGAGTGCTT
          1 CTTTTCCACCATAGGCCTCAAAGCGCTCCAAATATCCACTTGCAGATTCTACAAAAAGAGTGTTT

             *                   * **     *        *        *     **         
      98075 CGAAACTGCTCAATCAAAAGAGACATTCAAATCTGTGAGGTGAATGCAGACATCGTAAAGAAGTT
         66 CAAAACTGCTCAATCAAAAGAAAGGTTCAACTCTGTGAGATGAATGCACACATCACAAAGAAGTT

                                *     *       *    **
      98140 TCTCAGAATGCTTCTGTGTATTTTTTGTGTGAAGTTATTCG
        131 TCTCAGAATGCTTCTGTGTAGTTTTTATGTGAAGATATTTC

            *    *             *     *  *            **                 *    
      98181 TTTTTGCACCATAGGCCTCCAAGCGTTCTAAATATCCACTTCTAGATTCTACAAAAAGAGAGTTT
          1 CTTTTCCACCATAGGCCTCAAAGCGCTCCAAATATCCACTTGCAGATTCTACAAAAAGAGTGTTT

                   *         *            *        *    *   *                
      98246 CAAAACTACTC-A--AACA-AAAGGTTCAATTCTGTGAGTTGAAAGCAAACATCACAAAGAAGTT
         66 CAAAACTGCTCAATCAAAAGAAAGGTTCAACTCTGTGAGATGAATGCACACATCACAAAGAAGTT

                       *                             
      98307 TCTCAGAATGCGTCTGTGTAGTTTTTATGTGAAGATATTTC
        131 TCTCAGAATGCTTCTGTGTAGTTTTTATGTGAAGATATTTC

                     **   ****     *                  *                      
      98348 CTTTT-CACAGTAGAATGCAAAGGGCTCCAAATATCCACTTGGAGATTCTACAAAAAGA--GTTT
          1 CTTTTCCACCATAGGCCTCAAAGCGCTCCAAATATCCACTTGCAGATTCTACAAAAAGAGTGTTT

                  *    **     *  *     *     ***   *     *               *   
      98410 CAAAACCGCTCTGTCAAATGATAGGTTGAACTCCCGGAGGTGAATACACACATCACAAAGAGGTT
         66 CAAAACTGCTCAATCAAAAGAAAGGTTCAACTCTGTGAGATGAATGCACACATCACAAAGAAGTT

                  *                       *  *       
      98475 TCTCAGCATGCTTCTGTGTAGTTTTTATGTAAACATATTTC
        131 TCTCAGAATGCTTCTGTGTAGTTTTTATGTGAAGATATTTC

             *    * *              *           *      * *       *   *    *   
      98516 CGTTTCTATCATAGGCCTCAAAGTGCTCCAAATATTCACTTGTACATTCTACCAAACGAGTATTT
          1 CTTTTCCACCATAGGCCTCAAAGCGCTCCAAATATCCACTTGCAGATTCTACAAAAAGAGTGTTT

                              * *         ** *    *        *            *    
      98581 CAAAACTGCTCAATCAAACGGAAGGTTCAAAACCGTGACATGAATGCCCACATCACAAAGTAGTT
         66 CAAAACTGCTCAATCAAAAGAAAGGTTCAACTCTGTGAGATGAATGCACACATCACAAAGAAGTT

                                                     
      98646 TCTCAGAATGCTTCTGTGTAGTTTTTATGTGAAGATATTTC
        131 TCTCAGAATGCTTCTGTGTAGTTTTTATGTGAAGATATTTC

                     * *    *      *    *       * *   **      *              
      98687 CTTTTCCACAACA-GCGTGCAAAACGCTTCAAATATGCCCTTAGAGATTCCACAAAAAGAGTGTT
          1 CTTTTCCACCATAGGCCT-CAAAGCGCTCCAAATATCCACTTGCAGATTCTACAAAAAGAGTGTT

              *     *               *  *                                  ** 
      98751 TCCAAACTACTCAAATCAAAA-AATGATTTCAACTCTGTGAGATGAATGCACACATCACAAACTA
         65 TCAAAACTGCTC-AATCAAAAGAAAG-GTTCAACTCTGTGAGATGAATGCACACATCACAAAGAA

                         *     ** *   * *   *       *   
      98815 GTTTCTCAGAATGTTTCTGCCTGGTTCTCATGCGAAGATAGTTC
        128 GTTTCTCAGAATGCTTCTGTGTAGTTTTTATGTGAAGATATTTC

                 *           *   * **              *                  * *    
      98859 CTTTTTCACCATAGGCCGCAATGTACTCCAAATATCCACCTGCAGATTCTAC-AAAAGTGAGTTT
          1 CTTTTCCACCATAGGCCTCAAAGCGCTCCAAATATCCACTTGCAGATTCTACAAAAAGAGTGTTT

                       *         ***    **         *        *      *       * 
      98923 CAAAACTGCTCTATCAAAAGATCAGTTCGTCTCTGTGAGTTGAATGCATACATCAAAAAGAAGCT
         66 CAAAACTGCTCAATCAAAAGAAAGGTTCAACTCTGTGAGATGAATGCACACATCACAAAGAAGTT

                 *             *      **         *   
      98988 TCTCAAAATGCTTCTGTGTGGTTTTTCGGTGAAGATAGTTC
        131 TCTCAGAATGCTTCTGTGTAGTTTTTATGTGAAGATATTTC

            *     *        *      * *                 *        *        *    
      99029 TTTTTCTACCATAGGTCTCAAACCACTCCAAATATCCACTTGTAGATTCTATAAAAAGGAATG-T
          1 CTTTTCCACCATAGGCCTCAAAGCGCTCCAAATATCCACTTGCAGATTCTACAAAAA-GAGTGTT

                  *        *    *    *      * *         *      *         *   
      99093 TCAAAATTGCTCAATAAAAATAAAGTTTCAACACCGTGAGATGAGTGCACAAATCACAAAGGAGT
         65 TCAAAACTGCTCAATCAAAAGAAAGGTTCAACTCTGTGAGATGAATGCACACATCACAAAGAAGT

                  *          *         *          *   
      99158 TTCTCAAAATGCTTCTGGGTAGTTTTTCTGTGAAGATAGTTC
        130 TTCTCAGAATGCTTCTGTGTAGTTTTTATGTGAAGATATTTC

                  *     *    *     *          *                         *    
      99200 CTTTTCTACCATGGGCCACAAAGGGCTCCAAATACCCACTTGCAGATTCTACAAAAAGAGAGTTT
          1 CTTTTCCACCATAGGCCTCAAAGCGCTCCAAATATCCACTTGCAGATTCTACAAAAAGAGTGTTT

              *        *      ** * *        *    * *    **  * *           *  
      99265 CACAACTGCTCTATCAAACAATATGTTCAACTTTGTGGGTTGAACACAAATATCAC-AAGAATTT
         66 CAAAACTGCTCAATCAAAAGAAAGGTTCAACTCTGTGAGATGAATGCACACATCACAAAGAAGTT

                **                             *     
      99329 TCTCCCAATGCTTCTGTGTAGTTTTTATGTGAAGACATTTC
        131 TCTCAGAATGCTTCTGTGTAGTTTTTATGTGAAGATATTTC

            *   *  *      *  *     *                 *  *      *       *  *  
      99370 TTTTCCCTCCATAGTCCACAAAGTGCTCCAAATATCCACTTACATATTCTAGAAAAAGATTGCTT
          1 CTTTTCCACCATAGGCCTCAAAGCGCTCCAAATATCCACTTGCAGATTCTACAAAAAGAGTGTTT

            **       *    *               * * *                              
      99435 GGAAACTGCACAATGAAAAGAAAGGTTCAAATATATGAGATGAATGCACACATCACAAAGAAGTT
         66 CAAAACTGCTCAATCAAAAGAAAGGTTCAACTCTGTGAGATGAATGCACACATCACAAAGAAGTT

                                 *                    
      99500 TCTCAGAAT-CTCTCTGTGTAATTTTTATGTGAAGATATTTC
        131 TCTCAGAATGCT-TCTGTGTAGTTTTTATGTGAAGATATTTC

                *     *    *  *   *                        *      *    *     
      99541 CTTTCCCACCTTAGGTCTTAAAACGCTCCAAATATCCACTTGCAGATACTACAAGAAGATTGTTT
          1 CTTTTCCACCATAGGCCTCAAAGCGCTCCAAATATCCACTTGCAGATTCTACAAAAAGAGTGTTT

                     *    *        *      *     **                           
      99606 CAAAACTGCACAA-AAAAAGAAATGTTCAATTCTGTTTGATGAATGCACACATCACAAAGAAGTT
         66 CAAAACTGCTCAATCAAAAGAAAGGTTCAACTCTGTGAGATGAATGCACACATCACAAAGAAGTT

                           *                         
      99670 TCTCAGAATGCTTCTCTGTAGTTTTTATGTGAAGATATTTC
        131 TCTCAGAATGCTTCTGTGTAGTTTTTATGTGAAGATATTTC

                     *             *                 *         **      * *   
      99711 CTTTTCCACAATAGGCCTCAAAGGGCTCCAAATATCCACTTCCAGATTCTATGAAAAGAATATTT
          1 CTTTTCCACCATAGGCCTCAAAGCGCTCCAAATATCCACTTGCAGATTCTACAAAAAGAGTGTTT

             *              * *    *                                      *  
      99776 CCAAACTGCTCAATCATAGGAAATGTTCAACTCTGTGAGATGAATGCACACATCAC-AAGAAATT
         66 CAAAACTGCTCAATCAAAAGAAAGGTTCAACTCTGTGAGATGAATGCACACATCACAAAGAAGTT

                     *    *      *       *       *   
      99840 TCTCAGAATCCTTCAGTGTAGGTTTTATGAGAAGATAATTC
        131 TCTCAGAATGCTTCTGTGTAGTTTTTATGTGAAGATATTTC

                     *    **        *   *                           *    *   
      99881 CTTTTCCACAATAGTTCTCAAAGCACTCAAAATATCCACTTGCAGATTCTACAAAAGGAGTATTT
          1 CTTTTCCACCATAGGCCTCAAAGCGCTCCAAATATCCACTTGCAGATTCTACAAAAAGAGTGTTT

                                                          *                  
      99946 CAAAACTGCTCAATCAAAAGAAAGGTTCAACTCTGTGAGATGAATGGACACATCACAAAGAAGTT
         66 CAAAACTGCTCAATCAAAAGAAAGGTTCAACTCTGTGAGATGAATGCACACATCACAAAGAAGTT

                                  *   *              
     100011 TCTCAGAATGCTTCTGTGTAGTATTTTTGTGAAGATATTT-
        131 TCTCAGAATGCTTCTGTGTAGTTTTTATGTGAAGATATTTC

                          *  * * * * ***             *            *     * *  
     100051 CTTTTCCACCATAGACCGCCAGGGGACACAAATATCCACTTTCAGATTCTACAACAAGAGAGGTT
          1 CTTTTCCACCATAGGCCTCAAAGCGCTCCAAATATCCACTTGCAGATTCTACAAAAAGAGTGTTT

                   *   *     *   ** *       *      *                      * *
     100116 CAAAACTACTCGATCAAGAGATGGTTTCAACTATGTGAGTTGAATGCACACATCACAAAGAACTA
         66 CAAAACTGCTCAATCAAAAGAAAGGTTCAACTCTGTGAGATGAATGCACACATCACAAAGAAGTT

             *  *    *                               
     100181 TGTCGGAATTCTTCTGTGTAGTTTTTATGTGAAGATATTTC
        131 TCTCAGAATGCTTCTGTGTAGTTTTTATGTGAAGATATTTC

                     *    * *      * *    *                  *               
     100222 CTTTTCCACAATAGACGTCAAAGTGATCCAGATATCCACTTGCAGATTCCACAAAAAGAGTGTTT
          1 CTTTTCCACCATAGGCCTCAAAGCGCTCCAAATATCCACTTGCAGATTCTACAAAAAGAGTGTTT

                 *   *   *                  **                     *  *      
     100287 CAAAAGTGCACAACCAAAAGAAAGGTTCAACTAGGTGAGATGAATGCACACATCAGAAGGAAGTT
         66 CAAAACTGCTCAATCAAAAGAAAGGTTCAACTCTGTGAGATGAATGCACACATCACAAAGAAGTT

                            **   *     * *       *   
     100352 TCTCAGAATGCTTCTGCATAGCTTTTAAGGGAAGATACTTC
        131 TCTCAGAATGCTTCTGTGTAGTTTTTATGTGAAGATATTTC

                    *               *            * *  *    * *               
     100393 CTTTTCCAACATAGGCCTCAAAGCACTCCAAATATCCTCCTGGAGATACCACAAAAAGAGTGTTT
          1 CTTTTCCACCATAGGCCTCAAAGCGCTCCAAATATCCACTTGCAGATTCTACAAAAAGAGTGTTT

            **                      *  *                 *       *           
     100458 GCAAACTGCTCAATCAAAAGAAAGATTTAACTCTGTGAGATGAATCCACACATGACAAAGAAGTT
         66 CAAAACTGCTCAATCAAAAGAAAGGTTCAACTCTGTGAGATGAATGCACACATCACAAAGAAGTT

                                                     
     100523 TCTCAGAATGCTTCTGTGTAGTTTTTATGTGAAGATATTTC
        131 TCTCAGAATGCTTCTGTGTAGTTTTTATGTGAAGATATTTC

                     *   * * *    **           *                *     *      
     100564 CTTTTCCACAATAAGACCCAAAAGGCTCCAAATATTCACTTGCAGATTCTAAAAAAAACAGTGTT
          1 CTTTTCCACCATAGGCCTCAAAGCGCTCCAAATATCCACTTGCAGATTCT-ACAAAAAGAGTGTT

                                  *  *               *      *        **      
     100629 TCAAAACTGCTCAATCAAAAGATA-TTTCAACTCTGTGAGAAGAATGCTCACATCACTGAGAAGT
         65 TCAAAACTGCTCAATCAAAAGAAAGGTTCAACTCTGTGAGATGAATGCACACATCACAAAGAAGT

                                         *            
     100693 TTCTCAGAATGCTTCTGTGTAGTTTTTATATGAAGATATTTC
        130 TTCTCAGAATGCTTCTGTGTAGTTTTTATGTGAAGATATTTC

                *     *      *    **                       *   **       *    
     100735 CTTTCCCACCGTAGGCCACAAAAGGCTCCAAATATCCACTTGCAGATACTATGAAAAGAGAGTTT
          1 CTTTTCCACCATAGGCCTCAAAGCGCTCCAAATATCCACTTGCAGATTCTACAAAAAGAGTGTTT

                        *        *               ***            *            
     100800 CAAAACTGCTCATTCAAAAGATAGGTTCAACTCTGTGGTTTGAATGCACACAGCACAAAGAAGTT
         66 CAAAACTGCTCAATCAAAAGAAAGGTTCAACTCTGTGAGATGAATGCACACATCACAAAGAAGTT

              *       **                   **   *    
     100865 TCACAGAATGTGTCTGTGTAGTTTTTATGTGCGGATGTTTC
        131 TCTCAGAATGCTTCTGTGTAGTTTTTATGTGAAGATATTTC

                         *        ****   *   *                    *          
     100906 CTTTTCCACCATATGCCT-AAATATTTCCCAATTTCCACTTGCAGATTCTACAAGAAGAGTGTTT
          1 CTTTTCCACCATAGGCCTCAAAGCGCTCCAAATATCCACTTGCAGATTCTACAAAAAGAGTGTTT

                      **       *       *           *            *      ***   
     100970 CAAAACTGCTGTATC-AAATAAA-GTTGAACTCTGTGAGGTGAATGCACACAGCACAAAATGGTT
         66 CAAAACTGCTCAATCAAAAGAAAGGTTCAACTCTGTGAGATGAATGCACACATCACAAAGAAGTT

                          **   *        *       *    
     101033 TCTCAGAATGCTTCCTTGTTGTTTTTATATGAAGATGTTTC
        131 TCTCAGAATGCTTCTGTGTAGTTTTTATGTGAAGATATTTC

                  *  *             *   *     *                               
     101074 CTTTTCAACAATAGGCCTCAAAGTGCTTCAAATGTCCACTTGCAGATTCTACAAAAAGAGTGTTT
          1 CTTTTCCACCATAGGCCTCAAAGCGCTCCAAATATCCACTTGCAGATTCTACAAAAAGAGTGTTT

                                        *      *  *  *                       
     101139 CAAAACTGCTCAATCAAAAGAAAGGTTCGACTCTGGGAAATTAATGCACACATCACAAAGAAGTT
         66 CAAAACTGCTCAATCAAAAGAAAGGTTCAACTCTGTGAGATGAATGCACACATCACAAAGAAGTT

                                     *        *      
     101204 TCTC---A-GCTTCTGTGTAGTTTTCATGTGAAGTTATTTC
        131 TCTCAGAATGCTTCTGTGTAGTTTTTATGTGAAGATATTTC

                     *       *     *            *    *         **       *    
     101241 CTTTTCCACAATAGGCCGCAAAGGGCTCCAAATATCAACTTACAGATTCTAGGAAAAGAGAGTTT
          1 CTTTTCCACCATAGGCCTCAAAGCGCTCCAAATATCCACTTGCAGATTCTACAAAAAGAGTGTTT

                       * **      *     *                                     
     101306 CAAAACTGCTCTACGAAAAGATAGGTTGAACTCTGTGAGATGAATGCACACATCACAAAGAAGTT
         66 CAAAACTGCTCAATCAAAAGAAAGGTTCAACTCTGTGAGATGAATGCACACATCACAAAGAAGTT

                       *               * *     *     
     101371 TCTCAGAATGCATCTGTGTAGTTTTTACGGGAAGACATTTC
        131 TCTCAGAATGCTTCTGTGTAGTTTTTATGTGAAGATATTTC

                        ***  *     **      *  *                  *     ***   
     101412 CTTTTCCACCATCTTCCACAAAGGTCTCCAAGTAACCACTTGCAGATTCTACAGAAAGACACTTT
          1 CTTTTCCACCATAGGCCTCAAAGCGCTCCAAATATCCACTTGCAGATTCTACAAAAAGAGTGTTT

            *          *         ***      *      ***                      *  
     101477 AAAAACTGCTCTATCAAAAGATCAGTTCAAGTCTGTGGTTTGAATGCACACATCACAAAGAATTT
         66 CAAAACTGCTCAATCAAAAGAAAGGTTCAACTCTGTGAGATGAATGCACACATCACAAAGAAGTT

                                     *  *            
     101542 TCTCAGAATGCTTCTGTGTAGTTTTCATATGAAGATATTTC
        131 TCTCAGAATGCTTCTGTGTAGTTTTTATGTGAAGATATTTC

                                    *                                   **   
     101583 CTTTTCCACCATAGGCCTCAAAGCACTCCAAATATCCACTTGCAGATTCTACAAAAAGAGATTTT
          1 CTTTTCCACCATAGGCCTCAAAGCGCTCCAAATATCCACTTGCAGATTCTACAAAAAGAGTGTTT

                   **                           *  *          *        *     
     101648 CAAAACTAGTCAATCAAAAGAAAGGTTCAACTCTGTCAGTTGAATGCACATATCACAAACAAGTT
         66 CAAAACTGCTCAATCAAAAGAAAGGTTCAACTCTGTGAGATGAATGCACACATCACAAAGAAGTT

                *      *                             
     101713 TCTCGGAATGCGTCTGTGTAGTTTTTATGTGAAGATATTTC
        131 TCTCAGAATGCTTCTGTGTAGTTTTTATGTGAAGATATTTC

               *     * *           *     *                  *    *           
     101754 CTTCTCCACAACAGGCCTCAAAGTGCTCCGAATATCCACTTGCAGATTTTAC-TAAAGAGTGTTT
          1 CTTTTCCACCATAGGCCTCAAAGCGCTCCAAATATCCACTTGCAGATTCTACAAAAAGAGTGTTT

             *               *  *   *     *        *    *               *    
     101818 CCAAACTGCTCAATCAAGAGGAAGTTTCAAGTCTGTGAGCTGAACGCACACATCACAAAGTAGTT
         66 CAAAACTGCTCAATCAAAAGAAAGGTTCAACTCTGTGAGATGAATGCACACATCACAAAGAAGTT

               *                                *    
     101883 TCTGAGAATGCTTCTGTGTAGTTTTTATGTGAAGATGTTTC
        131 TCTCAGAATGCTTCTGTGTAGTTTTTATGTGAAGATATTTC

                            **     *                                    *    
     101924 CTTTTCCACCATAGGCTGCAAAGGGCTCCAAATATCCACTTGCAGATTCTACAAAAAGAGAGTTT
          1 CTTTTCCACCATAGGCCTCAAAGCGCTCCAAATATCCACTTGCAGATTCTACAAAAAGAGTGTTT

                 *     *         *          *     *                     *    
     101989 CAAAAGTGCTCTATCAAAAGATAGGTTCAACTATGTGATATGAATGCACACATCACAAAGTAGTT
         66 CAAAACTGCTCAATCAAAAGAAAGGTTCAACTCTGTGAGATGAATGCACACATCACAAAGAAGTT

                                          *          
     102054 TCTCAGAATGCTTCTGTGTAGTTTTTATGTAAAGATATTTC
        131 TCTCAGAATGCTTCTGTGTAGTTTTTATGTGAAGATATTTC

                                    *                                   **   
     102095 CTTTTCCACCATAGGCCTCAAAGCACTCCAAATATCCACTTGCAGATTCTACAAAAAGAGATTTT
          1 CTTTTCCACCATAGGCCTCAAAGCGCTCCAAATATCCACTTGCAGATTCTACAAAAAGAGTGTTT

                   ** *                   *     *  *    * *   *        *     
     102160 CAAAACTATTTAATCAAAAGAAAGGTTCAAATCTGTCAGTTGAAGGTACATATCACAAACAAGTT
         66 CAAAACTGCTCAATCAAAAGAAAGGTTCAACTCTGTGAGATGAATGCACACATCACAAAGAAGTT

             * **                                    
     102225 TATTGGAATGCTTCTGTGTAGTTTTTATGTGAAGATATTTC
        131 TCTCAGAATGCTTCTGTGTAGTTTTTATGTGAAGATATTTC

                     * *         * *                        **  *     *      
     102266 CTTTTCCACAACAGGCCTCAAGGTGCTCCAAATATCCACTTGCAGATTTCACTAAAAGTGTGTTT
          1 CTTTTCCACCATAGGCCTCAAAGCGCTCCAAATATCCACTTGCAGATTCTACAAAAAGAGTGTTT

             *  *            *  *   *     *        *             *           
     102331 CCAAGCTGCTCAATCAAGAGGAAGTTTCAAGTCTGTGAGGTGAATGCACACATTACAAAGAAGTT
         66 CAAAACTGCTCAATCAAAAGAAAGGTTCAACTCTGTGAGATGAATGCACACATCACAAAGAAGTT

            *  *                                     
     102396 ACTGAGAATGCTTCTGTGTAGTTTTTATGTGAAGATATTTC
        131 TCTCAGAATGCTTCTGTGTAGTTTTTATGTGAAGATATTTC

                      **               *                                *    
     102437 CTTTTCCACCGCAGGCCTCAAAGCGCTGCAAATATCCACTTGCAGATTCTACAAAAAGAGAGTTT
          1 CTTTTCCACCATAGGCCTCAAAGCGCTCCAAATATCCACTTGCAGATTCTACAAAAAGAGTGTTT

                      **         *   *         *   *     **            *     
     102502 CAAAACTGCTGTATCAAAAGATAGGGTCAACTCTGCGAGTTGAATAAACACATCACAAATAAGTT
         66 CAAAACTGCTCAATCAAAAGAAAGGTTCAACTCTGTGAGATGAATGCACACATCACAAAGAAGTT

            *  **   *        *               *       
     102567 CCTGGGAACGCTTCTGTATAG-TTTTATGTGAATATATTTC
        131 TCTCAGAATGCTTCTGTGTAGTTTTTATGTGAAGATATTTC

                         *          *                   *   *  *   * *   *   
     102607 CTTTTCCACCATATGCCTCAAAGCACTCCAAATATCCACTTGCACATTATAGAAACATAGTCTTT
          1 CTTTTCCACCATAGGCCTCAAAGCGCTCCAAATATCCACTTGCAGATTCTACAAAAAGAGTGTTT

                   **                        *         *             *       
     102672 CAAAACTTGTCAATC-AAAGAAAGGTTCAACTCCGTGAGATGAGTGCACACATCACAGAGAAGTT
         66 CAAAACTGCTCAATCAAAAGAAAGGTTCAACTCTGTGAGATGAATGCACACATCACAAAGAAGTT

                *     *                            * 
     102736 TCTCGGAATGTTTCTGTGTAGTTTTTATGTGAAGATATTGC
        131 TCTCAGAATGCTTCTGTGTAGTTTTTATGTGAAGATATTTC

                     *               *            *          *       *   *   
     102777 CTTTTCCACAATAGGCCTCAAAGCGTTCCAAATATCCAATTGCAGATTCCACAAAAAAAGTTTTT
          1 CTTTTCCACCATAGGCCTCAAAGCGCTCCAAATATCCACTTGCAGATTCTACAAAAAGAGTGTTT

            *                 *  *  *  *             * *         **    *     
     102842 TAAAACTGCTCAATCAAATGATAGATTAAACTCTGTGAGATTAGTGCACACATGTCAAAAAAGTT
         66 CAAAACTGCTCAATCAAAAGAAAGGTTCAACTCTGTGAGATGAATGCACACATCACAAAGAAGTT

                                *      * *           
     102907 TCTCAGAATGCTTCTGTGTACTTTTTACGGGAAGATATTTC
        131 TCTCAGAATGCTTCTGTGTAGTTTTTATGTGAAGATATTTC

                        *    *     **         *    *           **  * *  *    
     102948 CTTTTCCACCATCGGCCACAAAGGACTCCAAATAACCACATGCAGATTCTAGTAACACAGAGTTT
          1 CTTTTCCACCATAGGCCTCAAAGCGCTCCAAATATCCACTTGCAGATTCTACAAAAAGAGTGTTT

                       *         * *           *   * * *    **    ***        
     103013 CAAAACTGCTCTATCAAAAGATAAGTTCAACTCTGAGAGTTTAGTGCAACCATCGTGAAGAAGTT
         66 CAAAACTGCTCAATCAAAAGAAAGGTTCAACTCTGTGAGATGAATGCACACATCACAAAGAAGTT

                            *     *                  
     103078 TCTCAGAATGCTTCTGAGTAGTGTTTATGTGAAGATATTTC
        131 TCTCAGAATGCTTCTGTGTAGTTTTTATGTGAAGATATTTC

                              *     *                      *           **    
     103119 CTTTTCCACCATAGGCCTGAAAGCCCTCCAAATATCCACTTGCAGATCCTACAAAAAGAAAGTTT
          1 CTTTTCCACCATAGGCCTCAAAGCGCTCCAAATATCCACTTGCAGATTCTACAAAAAGAGTGTTT

             *         **     *  *  *   *        **      *                   
     103184 CGAAA-TGCTCTCTCAAACGATAGTTTCGACTCTGTGGTATGAATACACACATCACAAAGAAGTT
         66 CAAAACTGCTCAATCAAAAGAAAGGTTCAACTCTGTGAGATGAATGCACACATCACAAAGAAGTT

                                       **            
     103248 TCTCAGAATGCTTCTGTGTAGTTTTTAAATGAAGATATTTC
        131 TCTCAGAATGCTTCTGTGTAGTTTTTATGTGAAGATATTTC

            *                       *          *     *                       
     103289 TTTTTCCACCATAGGCCTCAAAGCACTCCAAATATGCACTTCCAGATTCTACAAAAAGAGTGTTT
          1 CTTTTCCACCATAGGCCTCAAAGCGCTCCAAATATCCACTTGCAGATTCTACAAAAAGAGTGTTT

              *                 *       * *         **   *         *  * *    
     103354 CAGAACTGCTCAATCAAAAGGAAGGTTCCAGTC--TGAGACAAATACACACATCAAAAGGTAGTT
         66 CAAAACTGCTCAATCAAAAGAAAGGTTCAACTCTGTGAGATGAATGCACACATCACAAAGAAGTT

                                                    *
     103417 TCTCAGAATGCTTCTGTGTAGTTTTTATGTGAAGATATTTT
        131 TCTCAGAATGCTTCTGTGTAGTTTTTATGTGAAGATATTTC

             *               *    **    *     *      *  **   *          *    
     103458 CCTTTCCACCATAGGCCACAAATGGCTCTAAATACCCACTTACATTTTCCACAAAAAGAGAGTTT
          1 CTTTTCCACCATAGGCCTCAAAGCGCTCCAAATATCCACTTGCAGATTCTACAAAAAGAGTGTTT

                       * *    * *      *   *         *                       
     103523 CAAAACTGCTCTACCAAAGGTAA-GTTTAACGCTGTGAG-TTAA-G-A-ACATCACAAAGAAGTT
         66 CAAAACTGCTCAATCAAAAGAAAGGTTCAACTCTGTGAGATGAATGCACACATCACAAAGAAGTT

                                   *   *  *          
     103583 TCTCAGAATGCTTCTGTGTAGTTCTTACGTAAAGATATTTC
        131 TCTCAGAATGCTTCTGTGTAGTTTTTATGTGAAGATATTTC

                 *   *      ***    *                  *              **      
     103624 CTTTTACACAATAGGCAGAAAAGTGCTCCAAATATCCACTTGAAGATTCTACAAAAACCGTGTTT
          1 CTTTTCCACCATAGGCCTCAAAGCGCTCCAAATATCCACTTGCAGATTCTACAAAAAGAGTGTTT

                     **                                          *      *    
     103689 CAAAACTGCCGAATCAAAAGAAAGGTTCAACTCTGTGAGATGAATGCACACATAACAAAGGAGTT
         66 CAAAACTGCTCAATCAAAAGAAAGGTTCAACTCTGTGAGATGAATGCACACATCACAAAGAAGTT

                                 *      *      *    *
     103754 TCTCAGAATGCTTCTGTGTAGCTTTTATATGAAGACATTTA
        131 TCTCAGAATGCTTCTGTGTAGTTTTTATGTGAAGATATTTC

            *        * *            *   ***                     **        *  
     103795 GTTTTCCACAACAGGCCTCAAAGCTCTCTCCATATCCACTTGCAGATTCTACCGAAAGAGTGCTT
          1 CTTTTCCACCATAGGCCTCAAAGCGCTCCAAATATCCACTTGCAGATTCTACAAAAAGAGTGTTT

             *                   * **     *        *        *    ***         
     103860 CCAAACTGCTCAATCAAAAGAGACATTCAAATCTGTGAGGTGAATGCAGACATGGTAAAGAAGTT
         66 CAAAACTGCTCAATCAAAAGAAAGGTTCAACTCTGTGAGATGAATGCACACATCACAAAGAAGTT

                                *     *       *    **
     103925 TCTCAGAATGCTTCTGTGTATTTTTTGTGTGAAGTTATTCG
        131 TCTCAGAATGCTTCTGTGTAGTTTTTATGTGAAGATATTTC

            *    *             *     *  *            **                 *    
     103966 TTTTTGCACCATAGGCCTCCAAGCGTTCTAAATATCCACTTCTAGATTCTACAAAAAGAGAGTTT
          1 CTTTTCCACCATAGGCCTCAAAGCGCTCCAAATATCCACTTGCAGATTCTACAAAAAGAGTGTTT

                   *         *            *        *    *   *                
     104031 CAAAACTACTC-A--AACA-AAAGGTTCAATTCTGTGAGTTGAAAGCAAACATCACAAAGAAGTT
         66 CAAAACTGCTCAATCAAAAGAAAGGTTCAACTCTGTGAGATGAATGCACACATCACAAAGAAGTT

                       *             *               
     104092 TCTCAGAATGCGTCTGTGTAGTTTTGATGTGAAGATATTTC
        131 TCTCAGAATGCTTCTGTGTAGTTTTTATGTGAAGATATTTC

                     **   ****     *                  *                      
     104133 CTTTT-CACAGTAGAATGCAAAGGGCTCCAAATATCCACTTGGAGATTCTACAAAAAGA--GTTT
          1 CTTTTCCACCATAGGCCTCAAAGCGCTCCAAATATCCACTTGCAGATTCTACAAAAAGAGTGTTT

                  *    **     *  *     *     ***   *     *               *   
     104195 CAAAACCGCTCTGTCAAATGATAGGTTGAACTCCCGGAGGTGAATACACACATCACAAAGAGGTT
         66 CAAAACTGCTCAATCAAAAGAAAGGTTCAACTCTGTGAGATGAATGCACACATCACAAAGAAGTT

                  *                       *  *       
     104260 TCTCAGCATGCTTCTGTGTAGTTTTTATGTAAACATATTTC
        131 TCTCAGAATGCTTCTGTGTAGTTTTTATGTGAAGATATTTC

             *    * *              *           *      * *       *   *    *   
     104301 CGTTTCTATCATAGGCCTCAAAGTGCTCCAAATATTCACTTGTACATTCTACCAAACGAGTATTT
          1 CTTTTCCACCATAGGCCTCAAAGCGCTCCAAATATCCACTTGCAGATTCTACAAAAAGAGTGTTT

                              * *         ** *    *        *            *    
     104366 CAAAACTGCTCAATCAAATGGAAGGTTCAAAACCGTGACATGAATGCCCACATCACAAAGTAGTT
         66 CAAAACTGCTCAATCAAAAGAAAGGTTCAACTCTGTGAGATGAATGCACACATCACAAAGAAGTT

                                                     
     104431 TCTCAGAATGCTTCTGTGTAGTTTTTATGTGAAGATATTTC
        131 TCTCAGAATGCTTCTGTGTAGTTTTTATGTGAAGATATTTC

                     * *    *      *    *       * *   **      *              
     104472 CTTTTCCACAACA-GCGTGCAAAACGCTTCAAATATGCCCTTAGAGATTCCACAAAAAGAGTGTT
          1 CTTTTCCACCATAGGCCT-CAAAGCGCTCCAAATATCCACTTGCAGATTCTACAAAAAGAGTGTT

              *     *               *  *                                  ** 
     104536 TCCAAACTACTCAAATCAAAA-AATGATTTCAACTCTGTGAGATGAATGCACACATCACAAACTA
         65 TCAAAACTGCTC-AATCAAAAGAAAG-GTTCAACTCTGTGAGATGAATGCACACATCACAAAGAA

                         *     ** *   * *   *       *   
     104600 GTTTCTCAGAATGTTTCTGCCTGGTTCTCATGCGAAGATAGTTC
        128 GTTTCTCAGAATGCTTCTGTGTAGTTTTTATGTGAAGATATTTC

                 *           *   * **              *                  * *    
     104644 CTTTTTCACCATAGGCCGCAATGTACTCCAAATATCCACCTGCAGATTCTAC-AAAAGTGAGTTT
          1 CTTTTCCACCATAGGCCTCAAAGCGCTCCAAATATCCACTTGCAGATTCTACAAAAAGAGTGTTT

                       *         ***    **         *        *      *       * 
     104708 CAAAACTGCTCTATCAAAAGATCAGTTCGTCTCTGTGAGTTGAATGCATACATCAAAAAGAAGCT
         66 CAAAACTGCTCAATCAAAAGAAAGGTTCAACTCTGTGAGATGAATGCACACATCACAAAGAAGTT

                 *             *      **         *   
     104773 TCTCAAAATGCTTCTGTGTGGTTTTTCGGTGAAGATAGTTC
        131 TCTCAGAATGCTTCTGTGTAGTTTTTATGTGAAGATATTTC

            *     *        *      * *                 *        *        *    
     104814 TTTTTCTACCATAGGTCTCAAACCACTCCAAATATCCACTTGTAGATTCTATAAAAAGGAATG-T
          1 CTTTTCCACCATAGGCCTCAAAGCGCTCCAAATATCCACTTGCAGATTCTACAAAAA-GAGTGTT

                  *        *    *    *      * *         *      *         *   
     104878 TCAAAATTGCTCAATAAAAATAAAGTTTCAACACCGTGAGATGAGTGCACAAATCACAAAGGAGT
         65 TCAAAACTGCTCAATCAAAAGAAAGGTTCAACTCTGTGAGATGAATGCACACATCACAAAGAAGT

                  *          *         *          *   
     104943 TTCTCAAAATGCTTCTGGGTAGTTTTTCTGTGAAGATAGTTC
        130 TTCTCAGAATGCTTCTGTGTAGTTTTTATGTGAAGATATTTC

                  *     *    *     *          *                         *    
     104985 CTTTTCTACCATGGGCCACAAAGGGCTCCAAATACCCACTTGCAGATTCTACAAAAAGAGAGTTT
          1 CTTTTCCACCATAGGCCTCAAAGCGCTCCAAATATCCACTTGCAGATTCTACAAAAAGAGTGTTT

              *        *      ** * *        *    * *    **  * *           *  
     105050 CACAACTGCTCTATCAAACAATATGTTCAACTTTGTGGGTTGAACACAAATATCAC-AAGAATTT
         66 CAAAACTGCTCAATCAAAAGAAAGGTTCAACTCTGTGAGATGAATGCACACATCACAAAGAAGTT

                **                             *     
     105114 TCTCCCAATGCTTCTGTGTAGTTTTTATGTGAAGACATTTC
        131 TCTCAGAATGCTTCTGTGTAGTTTTTATGTGAAGATATTTC

            *   *  *      *  *     *                 *  *      *       *  *  
     105155 TTTTCCCTCCATAGTCCACAAAGTGCTCCAAATATCCACTTACATATTCTAGAAAAAGATTGCTT
          1 CTTTTCCACCATAGGCCTCAAAGCGCTCCAAATATCCACTTGCAGATTCTACAAAAAGAGTGTTT

            **       *    *               * * *                              
     105220 GGAAACTGCACAATGAAAAGAAAGGTTCAAATATATGAGATGAATGCACACATCACAAAGAAGTT
         66 CAAAACTGCTCAATCAAAAGAAAGGTTCAACTCTGTGAGATGAATGCACACATCACAAAGAAGTT

                                 *                    
     105285 TCTCAGAAT-CTCTCTGTGTAATTTTTATGTGAAGATATTTC
        131 TCTCAGAATGCT-TCTGTGTAGTTTTTATGTGAAGATATTTC

                *     *    *  *   *                        *      *    *     
     105326 CTTTCCCACCTTAGGTCTTAAAACGCTCCAAATATCCACTTGCAGATACTACAAGAAGATTGTTT
          1 CTTTTCCACCATAGGCCTCAAAGCGCTCCAAATATCCACTTGCAGATTCTACAAAAAGAGTGTTT

                     *    *        *      *     **                           
     105391 CAAAACTGCACAA-AAAAAGAAATGTTCAATTCTGTTTGATGAATGCACACATCACAAAGAAGTT
         66 CAAAACTGCTCAATCAAAAGAAAGGTTCAACTCTGTGAGATGAATGCACACATCACAAAGAAGTT

                           *                         
     105455 TCTCAGAATGCTTCTCTGTAGTTTTTATGTGAAGATATTTC
        131 TCTCAGAATGCTTCTGTGTAGTTTTTATGTGAAGATATTTC

                     *             *                 *         **      * *   
     105496 CTTTTCCACAATAGGCCTCAAAGGGCTCCAAATATCCACTTCCAGATTCTATGAAAAGAATATTT
          1 CTTTTCCACCATAGGCCTCAAAGCGCTCCAAATATCCACTTGCAGATTCTACAAAAAGAGTGTTT

             *              * *    *                                      *  
     105561 CCAAACTGCTCAATCATAGGAAATGTTCAACTCTGTGAGATGAATGCACACATCAC-AAGAAATT
         66 CAAAACTGCTCAATCAAAAGAAAGGTTCAACTCTGTGAGATGAATGCACACATCACAAAGAAGTT

                     *    *      *       *       *   
     105625 TCTCAGAATCCTTCAGTGTAGGTTTTATGAGAAGATAATTC
        131 TCTCAGAATGCTTCTGTGTAGTTTTTATGTGAAGATATTTC

                     *    **        *   *                           *    *   
     105666 CTTTTCCACAATAGTTCTCAAAGCACTCAAAATATCCACTTGCAGATTCTACAAAAGGAGTATTT
          1 CTTTTCCACCATAGGCCTCAAAGCGCTCCAAATATCCACTTGCAGATTCTACAAAAAGAGTGTTT

                                                          *                  
     105731 CAAAACTGCTCAATCAAAAGAAAGGTTCAACTCTGTGAGATGAATGGACACATCACAAAGAAGTT
         66 CAAAACTGCTCAATCAAAAGAAAGGTTCAACTCTGTGAGATGAATGCACACATCACAAAGAAGTT

                                  *   *              
     105796 TCTCAGAATGCTTCTGTGTAGTATTTTTGTGAAGATATTT-
        131 TCTCAGAATGCTTCTGTGTAGTTTTTATGTGAAGATATTTC

                          *  * * * * ***             *            *     * *  
     105836 CTTTTCCACCATAGACCGCCAGGGGACACAAATATCCACTTTCAGATTCTACAACAAGAGAGGTT
          1 CTTTTCCACCATAGGCCTCAAAGCGCTCCAAATATCCACTTGCAGATTCTACAAAAAGAGTGTTT

                   *   *     *   ** *       *      *                      * *
     105901 CAAAACTACTCGATCAAGAGATGGTTTCAACTATGTGAGTTGAATGCACACATCACAAAGAACTA
         66 CAAAACTGCTCAATCAAAAGAAAGGTTCAACTCTGTGAGATGAATGCACACATCACAAAGAAGTT

             *  *    *                               
     105966 TGTCGGAATTCTTCTGTGTAGTTTTTATGTGAAGATATTTC
        131 TCTCAGAATGCTTCTGTGTAGTTTTTATGTGAAGATATTTC

                     *    * *      * *    *                  *               
     106007 CTTTTCCACAATAGACGTCAAAGTGATCCAGATATCCACTTGCAGATTCCACAAAAAGAGTGTTT
          1 CTTTTCCACCATAGGCCTCAAAGCGCTCCAAATATCCACTTGCAGATTCTACAAAAAGAGTGTTT

                 *   *   *                  **                     *  *      
     106072 CAAAAGTGCACAACCAAAAGAAAGGTTCAACTAGGTGAGATGAATGCACACATCAGAAGGAAGTT
         66 CAAAACTGCTCAATCAAAAGAAAGGTTCAACTCTGTGAGATGAATGCACACATCACAAAGAAGTT

                            **   *     * *       *   
     106137 TCTCAGAATGCTTCTGCATAGCTTTTAAGGGAAGATACTTC
        131 TCTCAGAATGCTTCTGTGTAGTTTTTATGTGAAGATATTTC

                    *               *            * *  *    * *               
     106178 CTTTTCCAACATAGGCCTCAAAGCACTCCAAATATCCTCCTGGAGATACCACAAAAAGAGTGTTT
          1 CTTTTCCACCATAGGCCTCAAAGCGCTCCAAATATCCACTTGCAGATTCTACAAAAAGAGTGTTT

            **                      *  *                 *       *           
     106243 GCAAACTGCTCAATCAAAAGAAAGATTTAACTCTGTGAGATGAATCCACACATGACAAAGAAGTT
         66 CAAAACTGCTCAATCAAAAGAAAGGTTCAACTCTGTGAGATGAATGCACACATCACAAAGAAGTT

                                                     
     106308 TCTCAGAATGCTTCTGTGTAGTTTTTATGTGAAGATATTTC
        131 TCTCAGAATGCTTCTGTGTAGTTTTTATGTGAAGATATTTC

                     *   * * *    **           *                *     *      
     106349 CTTTTCCACAATAAGACCCAAAAGGCTCCAAATATTCACTTGCAGATTCTAAAAAAAACAGTGTT
          1 CTTTTCCACCATAGGCCTCAAAGCGCTCCAAATATCCACTTGCAGATTCT-ACAAAAAGAGTGTT

                                  *                  *      *        **      
     106414 TCAAAACTGCTCAATCAAAAGATA-GTTCAACTCTGTGAGAAGAATGCTCACATCACTGAGAAGT
         65 TCAAAACTGCTCAATCAAAAGAAAGGTTCAACTCTGTGAGATGAATGCACACATCACAAAGAAGT

                                         *            
     106478 TTCTCAGAATGCTTCTGTGTAGTTTTTATATGAAGATATTTC
        130 TTCTCAGAATGCTTCTGTGTAGTTTTTATGTGAAGATATTTC

                *     *      *    **                       *   **       *    
     106520 CTTTCCCACCGTAGGCCACAAAAGGCTCCAAATATCCACTTGCAGATACTATGAAAAGAGAGTTT
          1 CTTTTCCACCATAGGCCTCAAAGCGCTCCAAATATCCACTTGCAGATTCTACAAAAAGAGTGTTT

                        *        *               ***            *            
     106585 CAAAACTGCTCATTCAAAAGATAGGTTCAACTCTGTGGTTTGAATGCACACAGCACAAAGAAGTT
         66 CAAAACTGCTCAATCAAAAGAAAGGTTCAACTCTGTGAGATGAATGCACACATCACAAAGAAGTT

              *       **                   **   *    
     106650 TCACAGAATGTGTCTGTGTAGTTTTTATGTGCGGATGTTTC
        131 TCTCAGAATGCTTCTGTGTAGTTTTTATGTGAAGATATTTC

                         *        ****   *   *               *    *          
     106691 CTTTTCCACCATATGCCT-AAATATTTCCCAATTTCCACTTGCAGATTCCACAAGAAGAGTGTTT
          1 CTTTTCCACCATAGGCCTCAAAGCGCTCCAAATATCCACTTGCAGATTCTACAAAAAGAGTGTTT

                      **       *       *           *            *      ***   
     106755 CAAAACTGCTGTATC-AAATAAA-GTTGAACTCTGTGAGGTGAATGCACACAGCACAAAATGGTT
         66 CAAAACTGCTCAATCAAAAGAAAGGTTCAACTCTGTGAGATGAATGCACACATCACAAAGAAGTT

                          **   *        *       *    
     106818 TCTCAGAATGCTTCCTTGTTGTTTTTATATGAAGATGTTTC
        131 TCTCAGAATGCTTCTGTGTAGTTTTTATGTGAAGATATTTC

                  *  *             *   *     *                               
     106859 CTTTTCAACAATAGGCCTCAAAGTGCTTCAAATGTCCACTTGCAGATTCTACAAAAAGAGTGTTT
          1 CTTTTCCACCATAGGCCTCAAAGCGCTCCAAATATCCACTTGCAGATTCTACAAAAAGAGTGTTT

                                        *      *  *  *                       
     106924 CAAAACTGCTCAATCAAAAGAAAGGTTCGACTCTGGGAAATTAATGCACACATCACAAAGAAGTT
         66 CAAAACTGCTCAATCAAAAGAAAGGTTCAACTCTGTGAGATGAATGCACACATCACAAAGAAGTT

                                     *        *      
     106989 TCTC---A-GCTTCTGTGTAGTTTTCATGTGAAGTTATTTC
        131 TCTCAGAATGCTTCTGTGTAGTTTTTATGTGAAGATATTTC

                     *       *     *            *    *         **       *    
     107026 CTTTTCCACAATAGGCCGCAAAGGGCTCCAAATATCAACTTACAGATTCTAGGAAAAGAGAGTTT
          1 CTTTTCCACCATAGGCCTCAAAGCGCTCCAAATATCCACTTGCAGATTCTACAAAAAGAGTGTTT

                       * **      *     *                                     
     107091 CAAAACTGCTCTACGAAAAGATAGGTTGAACTCTGTGAGATGAATGCACACATCACAAAGAAGTT
         66 CAAAACTGCTCAATCAAAAGAAAGGTTCAACTCTGTGAGATGAATGCACACATCACAAAGAAGTT

                       *               * *     *     
     107156 TCTCAGAATGCATCTGTGTAGTTTTTACGGGAAGACATTTC
        131 TCTCAGAATGCTTCTGTGTAGTTTTTATGTGAAGATATTTC

                        ***  *     **      *  *                  *     ***   
     107197 CTTTTCCACCATCTTCCACAAAGGTCTCCAAGTAACCACTTGCAGATTCTACAGAAAGACACTTT
          1 CTTTTCCACCATAGGCCTCAAAGCGCTCCAAATATCCACTTGCAGATTCTACAAAAAGAGTGTTT

            *          *         ***      *      ***                      *  
     107262 AAAAACTGCTCTATCAAAAGATCAGTTCAAGTCTGTGGTTTGAATGCACACATCACAAAGAATTT
         66 CAAAACTGCTCAATCAAAAGAAAGGTTCAACTCTGTGAGATGAATGCACACATCACAAAGAAGTT

                                     *  *            
     107327 TCTCAGAATGCTTCTGTGTAGTTTTCATATGAAGATATTTC
        131 TCTCAGAATGCTTCTGTGTAGTTTTTATGTGAAGATATTTC

                                    *                                   **   
     107368 CTTTTCCACCATAGGCCTCAAAGCACTCCAAATATCCACTTGCAGATTCTACAAAAAGAGATTTT
          1 CTTTTCCACCATAGGCCTCAAAGCGCTCCAAATATCCACTTGCAGATTCTACAAAAAGAGTGTTT

                   **                           *  *          *        *     
     107433 CAAAACTAGTCAATCAAAAGAAAGGTTCAACTCTGTCAGTTGAATGCACATATCACAAACAAGTT
         66 CAAAACTGCTCAATCAAAAGAAAGGTTCAACTCTGTGAGATGAATGCACACATCACAAAGAAGTT

                *      *                             
     107498 TCTCGGAATGCGTCTGTGTAGTTTTTATGTGAAGATATTTC
        131 TCTCAGAATGCTTCTGTGTAGTTTTTATGTGAAGATATTTC

               *     * *           *     *                  *    *           
     107539 CTTCTCCACAACAGGCCTCAAAGTGCTCCGAATATCCACTTGCAGATTTTAC-TAAAGAGTGTTT
          1 CTTTTCCACCATAGGCCTCAAAGCGCTCCAAATATCCACTTGCAGATTCTACAAAAAGAGTGTTT

             *               *  *   *     *        *    *               *    
     107603 CCAAACTGCTCAATCAAGAGGAAGTTTCAAGTCTGTGAGCTGAACGCACACATCACAAAGTAGTT
         66 CAAAACTGCTCAATCAAAAGAAAGGTTCAACTCTGTGAGATGAATGCACACATCACAAAGAAGTT

               *                                *    
     107668 TCTGAGAATGCTTCTGTGTAGTTTTTATGTGAAGATGTTTC
        131 TCTCAGAATGCTTCTGTGTAGTTTTTATGTGAAGATATTTC

                            **     *                                    *    
     107709 CTTTTCCACCATAGGCTGCAAAGGGCTCCAAATATCCACTTGCAGATTCTACAAAAAGAGAGTTT
          1 CTTTTCCACCATAGGCCTCAAAGCGCTCCAAATATCCACTTGCAGATTCTACAAAAAGAGTGTTT

                 *     *         *          *     *                     *    
     107774 CAAAAGTGCTCTATCAAAAGATAGGTTCAACTATGTGATATGAATGCACACATCACAAAGTAGTT
         66 CAAAACTGCTCAATCAAAAGAAAGGTTCAACTCTGTGAGATGAATGCACACATCACAAAGAAGTT

                                          *          
     107839 TCTCAGAATGCTTCTGTGTAGTTTTTATGTAAAGATATTTC
        131 TCTCAGAATGCTTCTGTGTAGTTTTTATGTGAAGATATTTC

                                    *                                   **   
     107880 CTTTTCCACCATAGGCCTCAAAGCACTCCAAATATCCACTTGCAGATTCTACAAAAAGAGATTTT
          1 CTTTTCCACCATAGGCCTCAAAGCGCTCCAAATATCCACTTGCAGATTCTACAAAAAGAGTGTTT

                   ** *                   *     *  *    * *   *        *     
     107945 CAAAACTATTTAATCAAAAGAAAGGTTCAAATCTGTCAGTTGAAGGTACATATCACAAACAAGTT
         66 CAAAACTGCTCAATCAAAAGAAAGGTTCAACTCTGTGAGATGAATGCACACATCACAAAGAAGTT

             * **                                    
     108010 TATTGGAATGCTTCTGTGTAGTTTTTATGTGAAGATATTTC
        131 TCTCAGAATGCTTCTGTGTAGTTTTTATGTGAAGATATTTC

                     * *       * * *                        **  *     *      
     108051 CTTTTCCACAACAGGCCTCCAGGTGCTCCAAATATCCACTTGCAGATTTCACTAAAAGTGTGTTT
          1 CTTTTCCACCATAGGCCTCAAAGCGCTCCAAATATCCACTTGCAGATTCTACAAAAAGAGTGTTT

             *  *            *  *   *     *        *             *           
     108116 CCAAGCTGCTCAATCAAGAGGAAGTTTCAAGTCTGTGAGGTGAATGCACACATTACAAAGAAGTT
         66 CAAAACTGCTCAATCAAAAGAAAGGTTCAACTCTGTGAGATGAATGCACACATCACAAAGAAGTT

            *  *                                     
     108181 ACTGAGAATGCTTCTGTGTAGTTTTTATGTGAAGATATTTC
        131 TCTCAGAATGCTTCTGTGTAGTTTTTATGTGAAGATATTTC

                      **               *                                * *  
     108222 CTTTTCCACCGCAGGCCTCAAAGCGCTGCAAATATCCACTTGCAGATTCTACAAAAAGAGAGCTT
          1 CTTTTCCACCATAGGCCTCAAAGCGCTCCAAATATCCACTTGCAGATTCTACAAAAAGAGTGTTT

                      **         *   *         *   *     **            *     
     108287 CAAAACTGCTGTATCAAAAGATAGGGTCAACTCTGCGAGTTGAATAAACACATCACAAATAAGTT
         66 CAAAACTGCTCAATCAAAAGAAAGGTTCAACTCTGTGAGATGAATGCACACATCACAAAGAAGTT

               **   *        *               *       
     108352 TCTGGGAACGCTTCTGTATAG-TTTTATGTGAATATATTTC
        131 TCTCAGAATGCTTCTGTGTAGTTTTTATGTGAAGATATTTC

                         *          *                   *   *  *   * *   *   
     108392 CTTTTCCACCATATGCCTCAAAGCACTCCAAATATCCACTTGCACATTATAGAAACATAGTCTTT
          1 CTTTTCCACCATAGGCCTCAAAGCGCTCCAAATATCCACTTGCAGATTCTACAAAAAGAGTGTTT

                   **                        *         *             *       
     108457 CAAAACTTGTCAATC-AAAGAAAGGTTCAACTCCGTGAGATGAGTGCACACATCACAGAGAAGTT
         66 CAAAACTGCTCAATCAAAAGAAAGGTTCAACTCTGTGAGATGAATGCACACATCACAAAGAAGTT

                *     *                            * 
     108521 TCTCGGAATGTTTCTGTGTAGTTTTTATGTGAAGATATTGC
        131 TCTCAGAATGCTTCTGTGTAGTTTTTATGTGAAGATATTTC

                     *               *            *          *       *   *   
     108562 CTTTTCCACAATAGGCCTCAAAGCGTTCCAAATATCCAATTGCAGATTCCACAAAAAAAGTTTTT
          1 CTTTTCCACCATAGGCCTCAAAGCGCTCCAAATATCCACTTGCAGATTCTACAAAAAGAGTGTTT

            *                 *  *  *  *             * *         **    *     
     108627 TAAAACTGCTCAATCAAATGATAGATTAAACTCTGTGAGATTAGTGCACACATGTCAAAAAAGTT
         66 CAAAACTGCTCAATCAAAAGAAAGGTTCAACTCTGTGAGATGAATGCACACATCACAAAGAAGTT

                                *      * *           
     108692 TCTCAGAATGCTTCTGTGTACTTTTTAGGGGAAGATATTTC
        131 TCTCAGAATGCTTCTGTGTAGTTTTTATGTGAAGATATTTC

                        *    *     **         *    *           **  * *  *    
     108733 CTTTTCCACCATCGGCCACAAAGGACTCCAAATAACCACATGCAGATTCTAGTAACACAGAGTTT
          1 CTTTTCCACCATAGGCCTCAAAGCGCTCCAAATATCCACTTGCAGATTCTACAAAAAGAGTGTTT

                       *         * *           *   * * *    **    ***        
     108798 CAAAACTGCTCTATCAAAAGATAAGTTCAACTCTGAGAGTTTAGTGCAACCATCGTGAAGAAGTT
         66 CAAAACTGCTCAATCAAAAGAAAGGTTCAACTCTGTGAGATGAATGCACACATCACAAAGAAGTT

                            *     *                  
     108863 TCTCAGAATGCTTCTGAGTAGTGTTTATGTGAAGATATTTC
        131 TCTCAGAATGCTTCTGTGTAGTTTTTATGTGAAGATATTTC

                              *     *                      *           **    
     108904 CTTTTCCACCATAGGCCTGAAAGCCCTCCAAATATCCACTTGCAGATCCTACAAAAAGAAAGTTT
          1 CTTTTCCACCATAGGCCTCAAAGCGCTCCAAATATCCACTTGCAGATTCTACAAAAAGAGTGTTT

             *         **     *  *  *   *        ** *    *                   
     108969 CGAAA-TGCTCTCTCAAACGATAGTTTCGACTCTGTGGTACGAATACACACATCACAAAGAAGTT
         66 CAAAACTGCTCAATCAAAAGAAAGGTTCAACTCTGTGAGATGAATGCACACATCACAAAGAAGTT

                                       **            
     109033 TCTCAGAATGCTTCTGTGTAGTTTTTAAATGAAGATATTTC
        131 TCTCAGAATGCTTCTGTGTAGTTTTTATGTGAAGATATTTC

            *                       *          *     *                       
     109074 TTTTTCCACCATAGGCCTCAAAGCACTCCAAATATGCACTTCCAGATTCTACAAAAAGAGTGTTT
          1 CTTTTCCACCATAGGCCTCAAAGCGCTCCAAATATCCACTTGCAGATTCTACAAAAAGAGTGTTT

              *                 *       * *         **   *         *  * *    
     109139 CAGAACTGCTCAATCAAAAGGAAGGTTCCAGTC--TGAGACAAATACACACATCAAAAGGTAGTT
         66 CAAAACTGCTCAATCAAAAGAAAGGTTCAACTCTGTGAGATGAATGCACACATCACAAAGAAGTT

                                                    *
     109202 TCTCAGAATGCTTCTGTGTAGTTTTTATGTGAAGATATTTT
        131 TCTCAGAATGCTTCTGTGTAGTTTTTATGTGAAGATATTTC

             *               *    **    *     *      *  **   *          *    
     109243 CCTTTCCACCATAGGCCACAAATGGCTCTAAATACCCACTTACATTTTCCACAAAAAGAGAGTTT
          1 CTTTTCCACCATAGGCCTCAAAGCGCTCCAAATATCCACTTGCAGATTCTACAAAAAGAGTGTTT

                       * *    * *      *   *         *                       
     109308 CAAAACTGCTCTACCAAAGGTAA-GTTTAACGCTGTGAG-TTAA-G-A-ACATCACAAAGAAGTT
         66 CAAAACTGCTCAATCAAAAGAAAGGTTCAACTCTGTGAGATGAATGCACACATCACAAAGAAGTT

                                   *   *  *          
     109368 TCTCAGAATGCTTCTGTGTAGTTCTTACGTAAAGATATTTC
        131 TCTCAGAATGCTTCTGTGTAGTTTTTATGTGAAGATATTTC

                 *   *      ***    *                  *              **      
     109409 CTTTTACACAATAGGCAGAAAAGTGCTCCAAATATCCACTTGAAGATTCTACAAAAACCGTGTTT
          1 CTTTTCCACCATAGGCCTCAAAGCGCTCCAAATATCCACTTGCAGATTCTACAAAAAGAGTGTTT

                     **                                          *      *    
     109474 CAAAACTGCCGAATCAAAAGAAAGGTTCAACTCTGTGAGATGAATGCACACATAACAAAGGAGTT
         66 CAAAACTGCTCAATCAAAAGAAAGGTTCAACTCTGTGAGATGAATGCACACATCACAAAGAAGTT

                                 *      *      *    *
     109539 TCTCAGAATGCTTCTGTGTAGCTTTTATATGAAGACATTTA
        131 TCTCAGAATGCTTCTGTGTAGTTTTTATGTGAAGATATTTC

            *        * *            *   ***                     **        *  
     109580 GTTTTCCACAACAGGCCTCAAAGCTCTCTCCATATCCACTTGCAGATTCTACCGAAAGAGTGCTT
          1 CTTTTCCACCATAGGCCTCAAAGCGCTCCAAATATCCACTTGCAGATTCTACAAAAAGAGTGTTT

             *                   * **     *        *        *     **         
     109645 CCAAACTGCTCAATCAAAAGAGACATTCAAATCTGTGAGGTGAATGCAGACATCGTAAAGAAGTT
         66 CAAAACTGCTCAATCAAAAGAAAGGTTCAACTCTGTGAGATGAATGCACACATCACAAAGAAGTT

                                *     *       *    **
     109710 TCTCAGAATGCTTCTGTGTATTTTTTGTGTGAAGTTATTCG
        131 TCTCAGAATGCTTCTGTGTAGTTTTTATGTGAAGATATTTC

            *    *             *     *  *            **                 *    
     109751 TTTTTGCACCATAGGCCTCCAAGCGTTCTAAATATCCACTTCTAGATTCTACAAAAAGAGAGTTT
          1 CTTTTCCACCATAGGCCTCAAAGCGCTCCAAATATCCACTTGCAGATTCTACAAAAAGAGTGTTT

                   *         *            *        *    *   *                
     109816 CAAAACTACTC-A--AACA-AAAGGTTCAATTCTGTGAGTTGAAAGCAAACATCACAAAGAAGTT
         66 CAAAACTGCTCAATCAAAAGAAAGGTTCAACTCTGTGAGATGAATGCACACATCACAAAGAAGTT

                       *             *               
     109877 TCTCAGAATGCGTCTGTGTAGTTTTGATGTGAAGATATTTC
        131 TCTCAGAATGCTTCTGTGTAGTTTTTATGTGAAGATATTTC

                     **   ****     *                  *                      
     109918 CTTTT-CACAGTAGAATGCAAAGGGCTCCAAATATCCACTTGGAGATTCTACAAAAAGA--GTTT
          1 CTTTTCCACCATAGGCCTCAAAGCGCTCCAAATATCCACTTGCAGATTCTACAAAAAGAGTGTTT

                  *    **     *  *     *     ***   *     *               *   
     109980 CAAAACCGCTCTGTCAAATGATAGGTTGAACTCCCGGAGGTGAATACACACATCACAAAGAGGTT
         66 CAAAACTGCTCAATCAAAAGAAAGGTTCAACTCTGTGAGATGAATGCACACATCACAAAGAAGTT

                  *                       *  *       
     110045 TCTCAGCATGCTTCTGTGTAGTTTTTATGTAAACATATTTC
        131 TCTCAGAATGCTTCTGTGTAGTTTTTATGTGAAGATATTTC

             *    * *              *           *      * *       *   *    *   
     110086 CGTTTCTATCATAGGCCTCAAAGTGCTCCAAATATTCACTTGTACATTCTACCAAACGAGTATTT
          1 CTTTTCCACCATAGGCCTCAAAGCGCTCCAAATATCCACTTGCAGATTCTACAAAAAGAGTGTTT

                              * *         ** *    *        *            *    
     110151 CAAAACTGCTCAATCAAACGGAAGGTTCAAAACCGTGACATGAATGCCCACATCACAAAGTAGTT
         66 CAAAACTGCTCAATCAAAAGAAAGGTTCAACTCTGTGAGATGAATGCACACATCACAAAGAAGTT

                                                     
     110216 TCTCAGAATGCTTCTGTGTAGTTTTTATGTGAAGATATTTC
        131 TCTCAGAATGCTTCTGTGTAGTTTTTATGTGAAGATATTTC

                     * *    *      *    *       * *   **      *              
     110257 CTTTTCCACAACA-GCGTGCAAAACGCTTCAAATATGCCCTTAGAGATTCCACAAAAAGAGTGTT
          1 CTTTTCCACCATAGGCCT-CAAAGCGCTCCAAATATCCACTTGCAGATTCTACAAAAAGAGTGTT

              *     *               *  *                                  ** 
     110321 TCCAAACTACTCAAATCAAAA-AATGATTTCAACTCTGTGAGATGAATGCACACATCACAAACTA
         65 TCAAAACTGCTC-AATCAAAAGAAAG-GTTCAACTCTGTGAGATGAATGCACACATCACAAAGAA

                         *     ** *   * *   *       *   
     110385 GTTTCTCAGAATGTTTCTGCCTGGTTCTCATGCGAAGATAGTTC
        128 GTTTCTCAGAATGCTTCTGTGTAGTTTTTATGTGAAGATATTTC

                 *           *   * **              *                  * *    
     110429 CTTTTTCACCATAGGCCGCAATGTACTCCAAATATCCACCTGCAGATTCTAC-AAAAGTGAGTTT
          1 CTTTTCCACCATAGGCCTCAAAGCGCTCCAAATATCCACTTGCAGATTCTACAAAAAGAGTGTTT

                       *         ***    **         *        *      *       * 
     110493 CAAAACTGCTCTATCAAAAGATCAGTTCGTCTCTGTGAGTTGAATGCATACATCAAAAAGAAGCT
         66 CAAAACTGCTCAATCAAAAGAAAGGTTCAACTCTGTGAGATGAATGCACACATCACAAAGAAGTT

                 *             *      **         *   
     110558 TCTCAAAATGCTTCTGTGTGGTTTTTCGGTGAAGATAGTTC
        131 TCTCAGAATGCTTCTGTGTAGTTTTTATGTGAAGATATTTC

            *     *        *      * *                 *        *        *    
     110599 TTTTTCTACCATAGGTCTCAAACCACTCCAAATATCCACTTGTAGATTCTATAAAAAGGAATG-T
          1 CTTTTCCACCATAGGCCTCAAAGCGCTCCAAATATCCACTTGCAGATTCTACAAAAA-GAGTGTT

                  *        *    *    *      * *         *      *         *   
     110663 TCAAAATTGCTCAATAAAAATAAAGTTTCAACACCGTGAGATGAGTGCACAAATCACAAAGGAGT
         65 TCAAAACTGCTCAATCAAAAGAAAGGTTCAACTCTGTGAGATGAATGCACACATCACAAAGAAGT

                  *          *         *          *   
     110728 TTCTCAAAATGCTTCTGGGTAGTTTTTCTGTGAAGATAGTTC
        130 TTCTCAGAATGCTTCTGTGTAGTTTTTATGTGAAGATATTTC

                  *     *    *     *          *                         *    
     110770 CTTTTCTACCATGGGCCACAAAGGGCTCCAAATACCCACTTGCAGATTCTACAAAAAGAGAGTTT
          1 CTTTTCCACCATAGGCCTCAAAGCGCTCCAAATATCCACTTGCAGATTCTACAAAAAGAGTGTTT

              *        *      ** * *        *    * *    **  * *           *  
     110835 CACAACTGCTCTATCAAACAATATGTTCAACTTTGTGGGTTGAACACAAATATCAC-AAGAATTT
         66 CAAAACTGCTCAATCAAAAGAAAGGTTCAACTCTGTGAGATGAATGCACACATCACAAAGAAGTT

                **                             *     
     110899 TCTCCCAATGCTTCTGTGTAGTTTTTATGTGAAGACATTTC
        131 TCTCAGAATGCTTCTGTGTAGTTTTTATGTGAAGATATTTC

            *   *  *      *  *     *                 *  *      *       *  *  
     110940 TTTTCCCTCCATAGTCCACAAAGTGCTCCAAATATCCACTTACATATTCTAGAAAAAGATTGCTT
          1 CTTTTCCACCATAGGCCTCAAAGCGCTCCAAATATCCACTTGCAGATTCTACAAAAAGAGTGTTT

            **       *    *               * * *                              
     111005 GGAAACTGCACAATGAAAAGAAAGGTTCAAATATATGAGATGAATGCACACATCACAAAGAAGTT
         66 CAAAACTGCTCAATCAAAAGAAAGGTTCAACTCTGTGAGATGAATGCACACATCACAAAGAAGTT

                                 *                    
     111070 TCTCAGAAT-CTCTCTGTGTAATTTTTATGTGAAGATATTTC
        131 TCTCAGAATGCT-TCTGTGTAGTTTTTATGTGAAGATATTTC

                *     *    *  *   *                        *      *    *     
     111111 CTTTCCCACCTTAGGTCTTAAAACGCTCCAAATATCCACTTGCAGATACTACAAGAAGATTGTTT
          1 CTTTTCCACCATAGGCCTCAAAGCGCTCCAAATATCCACTTGCAGATTCTACAAAAAGAGTGTTT

                     *    *        *      *     **                           
     111176 CAAAACTGCACAA-AAAAAGAAATGTTCAATTCTGTTTGATGAATGCACACATCACAAAGAAGTT
         66 CAAAACTGCTCAATCAAAAGAAAGGTTCAACTCTGTGAGATGAATGCACACATCACAAAGAAGTT

                           *                         
     111240 TCTCAGAATGCTTCTCTGTAGTTTTTATGTGAAGATATTTC
        131 TCTCAGAATGCTTCTGTGTAGTTTTTATGTGAAGATATTTC

                     *             *                 *         **      * *   
     111281 CTTTTCCACAATAGGCCTCAAAGGGCTCCAAATATCCACTTCCAGATTCTATGAAAAGAATATTT
          1 CTTTTCCACCATAGGCCTCAAAGCGCTCCAAATATCCACTTGCAGATTCTACAAAAAGAGTGTTT

             *              * *    *                                      *  
     111346 CCAAACTGCTCAATCATAGGAAATGTTCAACTCTGTGAGATGAATGCACACATCAC-AAGAAATT
         66 CAAAACTGCTCAATCAAAAGAAAGGTTCAACTCTGTGAGATGAATGCACACATCACAAAGAAGTT

                     *    *      *       *       *   
     111410 TCTCAGAATCCTTCAGTGTAGGTTTTATGAGAAGATAATTC
        131 TCTCAGAATGCTTCTGTGTAGTTTTTATGTGAAGATATTTC

                     *    **        *   *                           *    *   
     111451 CTTTTCCACAATAGTTCTCAAAGCACTCAAAATATCCACTTGCAGATTCTACAAAAGGAGTATTT
          1 CTTTTCCACCATAGGCCTCAAAGCGCTCCAAATATCCACTTGCAGATTCTACAAAAAGAGTGTTT

                                                          *                  
     111516 CAAAACTGCTCAATCAAAAGAAAGGTTCAACTCTGTGAGATGAATGGACACATCACAAAGAAGTT
         66 CAAAACTGCTCAATCAAAAGAAAGGTTCAACTCTGTGAGATGAATGCACACATCACAAAGAAGTT

                                  *   *              
     111581 TCTCAGAATGCTTCTGTGTAGTATTTTTGTGAAGATATTT-
        131 TCTCAGAATGCTTCTGTGTAGTTTTTATGTGAAGATATTTC

                          *  * * * * ***             *            *     * *  
     111621 CTTTTCCACCATAGACCGCCAGGGGACACAAATATCCACTTTCAGATTCTACAACAAGAGAGGTT
          1 CTTTTCCACCATAGGCCTCAAAGCGCTCCAAATATCCACTTGCAGATTCTACAAAAAGAGTGTTT

                   *   *     *   ** *       *      *                      * *
     111686 CAAAACTACTCGATCAAGAGATGGTTTCAACTATGTGAGTTGAATGCACACATCACAAAGAACTA
         66 CAAAACTGCTCAATCAAAAGAAAGGTTCAACTCTGTGAGATGAATGCACACATCACAAAGAAGTT

             *  *    *                               
     111751 TGTCGGAATTCTTCTGTGTAGTTTTTATGTGAAGATATTTC
        131 TCTCAGAATGCTTCTGTGTAGTTTTTATGTGAAGATATTTC

                     *    * *      * *    *                  *               
     111792 CTTTTCCACAATAGACGTCAAAGTGATCCAGATATCCACTTGCAGATTCCACAAAAAGAGTGTTT
          1 CTTTTCCACCATAGGCCTCAAAGCGCTCCAAATATCCACTTGCAGATTCTACAAAAAGAGTGTTT

                 *   *   *                  **                     *  *      
     111857 CAAAAGTGCACAACCAAAAGAAAGGTTCAACTAGGTGAGATGAATGCACACATCAGAAGGAAGTT
         66 CAAAACTGCTCAATCAAAAGAAAGGTTCAACTCTGTGAGATGAATGCACACATCACAAAGAAGTT

                            **   *     * *       *   
     111922 TCTCAGAATGCTTCTGCATAGCTTTTAAGGGAAGATACTTC
        131 TCTCAGAATGCTTCTGTGTAGTTTTTATGTGAAGATATTTC

                    *               *            * *  *    * *               
     111963 CTTTTCCAACATAGGCCTCAAAGCACTCCAAATATCCTCCTGGAGATACCACAAAAAGAGTGTTT
          1 CTTTTCCACCATAGGCCTCAAAGCGCTCCAAATATCCACTTGCAGATTCTACAAAAAGAGTGTTT

            **                      *  *                 *       *           
     112028 GCAAACTGCTCAATCAAAAGAAAGATTTAACTCTGTGAGATGAATCCACACATGACAAAGAAGTT
         66 CAAAACTGCTCAATCAAAAGAAAGGTTCAACTCTGTGAGATGAATGCACACATCACAAAGAAGTT

                                                     
     112093 TCTCAGAATGCTTCTGTGTAGTTTTTATGTGAAGATATTTC
        131 TCTCAGAATGCTTCTGTGTAGTTTTTATGTGAAGATATTTC

                     *   * * *    **           *                *     *      
     112134 CTTTTCCACAATAAGACCCAAAAGGCTCCAAATATTCACTTGCAGATTCTAAAAAAAACAGTGTT
          1 CTTTTCCACCATAGGCCTCAAAGCGCTCCAAATATCCACTTGCAGATTCT-ACAAAAAGAGTGTT

                                  *                  *      *        **      
     112199 TCAAAACTGCTCAATCAAAAGATA-GTTCAACTCTGTGAGAAGAATGCTCACATCACTGAGAAGT
         65 TCAAAACTGCTCAATCAAAAGAAAGGTTCAACTCTGTGAGATGAATGCACACATCACAAAGAAGT

                                         *            
     112263 TTCTCAGAATGCTTCTGTGTAGTTTTTATATGAAGATATTTC
        130 TTCTCAGAATGCTTCTGTGTAGTTTTTATGTGAAGATATTTC

                *     *      *    **                       *   **       *    
     112305 CTTTCCCACCGTAGGCCACAAAAGGCTCCAAATATCCACTTGCAGATACTATGAAAAGAGAGTTT
          1 CTTTTCCACCATAGGCCTCAAAGCGCTCCAAATATCCACTTGCAGATTCTACAAAAAGAGTGTTT

                        *        *               ***            *            
     112370 CAAAACTGCTCATTCAAAAGATAGGTTCAACTCTGTGGTTTGAATGCACACAGCACAAAGAAGTT
         66 CAAAACTGCTCAATCAAAAGAAAGGTTCAACTCTGTGAGATGAATGCACACATCACAAAGAAGTT

              *       **                   **   *    
     112435 TCACAGAATGTGTCTGTGTAGTTTTTATGTGCGGATGTTTC
        131 TCTCAGAATGCTTCTGTGTAGTTTTTATGTGAAGATATTTC

                         *        ****   *   *               *    *          
     112476 CTTTTCCACCATATGCCT-AAATATTTCCCAATTTCCACTTGCAGATTCCACAAGAAGAGTGTTT
          1 CTTTTCCACCATAGGCCTCAAAGCGCTCCAAATATCCACTTGCAGATTCTACAAAAAGAGTGTTT

                      **       *       *           *            *      ***   
     112540 CAAAACTGCTGTATC-AAATAAA-GTTGAACTCTGTGAGGTGAATGCACACAGCACAAAATGGTT
         66 CAAAACTGCTCAATCAAAAGAAAGGTTCAACTCTGTGAGATGAATGCACACATCACAAAGAAGTT

                          **   *        *       *    
     112603 TCTCAGAATGCTTCCTTGTTGTTTTTATATGAAGATGTTTC
        131 TCTCAGAATGCTTCTGTGTAGTTTTTATGTGAAGATATTTC

                  *  *             *   *     *                               
     112644 CTTTTCAACAATAGGCCTCAAAGTGCTTCAAATGTCCACTTGCAGATTCTACAAAAAGAGTGTTT
          1 CTTTTCCACCATAGGCCTCAAAGCGCTCCAAATATCCACTTGCAGATTCTACAAAAAGAGTGTTT

                                        *      *  *  *                       
     112709 CAAAACTGCTCAATCAAAAGAAAGGTTCGACTCTGGGAAATTAATGCACACATCACAAAGAAGTT
         66 CAAAACTGCTCAATCAAAAGAAAGGTTCAACTCTGTGAGATGAATGCACACATCACAAAGAAGTT

                                     *        *      
     112774 TCTC---A-GCTTCTGTGTAGTTTTCATGTGAAGTTATTTC
        131 TCTCAGAATGCTTCTGTGTAGTTTTTATGTGAAGATATTTC

                     *       *     *            *    *         **       *    
     112811 CTTTTCCACAATAGGCCGCAAAGGGCTCCAAATATCAACTTACAGATTCTAGGAAAAGAGAGTTT
          1 CTTTTCCACCATAGGCCTCAAAGCGCTCCAAATATCCACTTGCAGATTCTACAAAAAGAGTGTTT

                       * **      *     *                                     
     112876 CAAAACTGCTCTACGAAAAGATAGGTTGAACTCTGTGAGATGAATGCACACATCACAAAGAAGTT
         66 CAAAACTGCTCAATCAAAAGAAAGGTTCAACTCTGTGAGATGAATGCACACATCACAAAGAAGTT

                       *               * *     *     
     112941 TCTCAGAATGCATCTGTGTAGTTTTTACGGGAAGACATTTC
        131 TCTCAGAATGCTTCTGTGTAGTTTTTATGTGAAGATATTTC

                        ***  *     **      *  *                  *     ***   
     112982 CTTTTCCACCATCTTCCACAAAGGTCTCCAAGTAACCACTTGCAGATTCTACAGAAAGACACTTT
          1 CTTTTCCACCATAGGCCTCAAAGCGCTCCAAATATCCACTTGCAGATTCTACAAAAAGAGTGTTT

            *          *         ***      *      ***                      *  
     113047 AAAAACTGCTCTATCAAAAGATCAGTTCAAGTCTGTGGTTTGAATGCACACATCACAAAGAATTT
         66 CAAAACTGCTCAATCAAAAGAAAGGTTCAACTCTGTGAGATGAATGCACACATCACAAAGAAGTT

                                     *  *            
     113112 TCTCAGAATGCTTCTGTGTAGTTTTCATATGAAGATATTTC
        131 TCTCAGAATGCTTCTGTGTAGTTTTTATGTGAAGATATTTC

                                    *                                   **   
     113153 CTTTTCCACCATAGGCCTCAAAGCACTCCAAATATCCACTTGCAGATTCTACAAAAAGAGATTTT
          1 CTTTTCCACCATAGGCCTCAAAGCGCTCCAAATATCCACTTGCAGATTCTACAAAAAGAGTGTTT

                   **                           *  *          *        *     
     113218 CAAAACTAGTCAATCAAAAGAAAGGTTCAACTCTGTCAGTTGAATGCACATATCACAAACAAGTT
         66 CAAAACTGCTCAATCAAAAGAAAGGTTCAACTCTGTGAGATGAATGCACACATCACAAAGAAGTT

                *      *                             
     113283 TCTCGGAATGCGTCTGTGTAGTTTTTATGTGAAGATATTTC
        131 TCTCAGAATGCTTCTGTGTAGTTTTTATGTGAAGATATTTC

               *     * *           *     *                  *    *           
     113324 CTTCTCCACAACAGGCCTCAAAGTGCTCCGAATATCCACTTGCAGATTTTAC-TAAAGAGTGTTT
          1 CTTTTCCACCATAGGCCTCAAAGCGCTCCAAATATCCACTTGCAGATTCTACAAAAAGAGTGTTT

             *               *  *   *     *        *    *               *    
     113388 CCAAACTGCTCAATCAAGAGGAAGTTTCAAGTCTGTGAGCTGAACGCACACATCACAAAGTAGTT
         66 CAAAACTGCTCAATCAAAAGAAAGGTTCAACTCTGTGAGATGAATGCACACATCACAAAGAAGTT

               *                                *    
     113453 TCTGAGAATGCTTCTGTGTAGTTTTTATGTGAAGATGTTTC
        131 TCTCAGAATGCTTCTGTGTAGTTTTTATGTGAAGATATTTC

                            **     *                                    *    
     113494 CTTTTCCACCATAGGCTGCAAAGGGCTCCAAATATCCACTTGCAGATTCTACAAAAAGAGAGTTT
          1 CTTTTCCACCATAGGCCTCAAAGCGCTCCAAATATCCACTTGCAGATTCTACAAAAAGAGTGTTT

                 *     *         *          *     *                     *    
     113559 CAAAAGTGCTCTATCAAAAGATAGGTTCAACTATGTGATATGAATGCACACATCACAAAGTAGTT
         66 CAAAACTGCTCAATCAAAAGAAAGGTTCAACTCTGTGAGATGAATGCACACATCACAAAGAAGTT

                                          *          
     113624 TCTCAGAATGCTTCTGTGTAGTTTTTATGTAAAGATATTTC
        131 TCTCAGAATGCTTCTGTGTAGTTTTTATGTGAAGATATTTC

                                    *                                   **   
     113665 CTTTTCCACCATAGGCCTCAAAGCACTCCAAATATCCACTTGCAGATTCTACAAAAAGAGATTTT
          1 CTTTTCCACCATAGGCCTCAAAGCGCTCCAAATATCCACTTGCAGATTCTACAAAAAGAGTGTTT

                   ** *                   *     *  *    * *   *        *     
     113730 CAAAACTATTTAATCAAAAGAAAGGTTCAAATCTGTCAGTTGAAGGTACATATCACAAACAAGTT
         66 CAAAACTGCTCAATCAAAAGAAAGGTTCAACTCTGTGAGATGAATGCACACATCACAAAGAAGTT

             * **                                    
     113795 TATTGGAATGCTTCTGTGTAGTTTTTATGTGAAGATATTTC
        131 TCTCAGAATGCTTCTGTGTAGTTTTTATGTGAAGATATTTC

                     * *         * *                        **  *     *      
     113836 CTTTTCCACAACAGGCCTCAAGGTGCTCCAAATATCCACTTGCAGATTTCACTAAAAGTGTGTTT
          1 CTTTTCCACCATAGGCCTCAAAGCGCTCCAAATATCCACTTGCAGATTCTACAAAAAGAGTGTTT

             *  *            *  *   *     *        *             *           
     113901 CCAAGCTGCTCAATCAAGAGGAAGTTTCAAGTCTGTGAGGTGAATGCACACATTACAAAGAAGTT
         66 CAAAACTGCTCAATCAAAAGAAAGGTTCAACTCTGTGAGATGAATGCACACATCACAAAGAAGTT

            *  *                                     
     113966 ACTGAGAATGCTTCTGTGTAGTTTTTATGTGAAGATATTTC
        131 TCTCAGAATGCTTCTGTGTAGTTTTTATGTGAAGATATTTC

                      **               *                                *    
     114007 CTTTTCCACCGCAGGCCTCAAAGCGCTGCAAATATCCACTTGCAGATTCTACAAAAAGAGAGTTT
          1 CTTTTCCACCATAGGCCTCAAAGCGCTCCAAATATCCACTTGCAGATTCTACAAAAAGAGTGTTT

                      **         *   *         *   *     **            *     
     114072 CAAAACTGCTGTATCAAAAGATAGGGTCAACTCTGCGAGTTGAATAAACACATCACAAATAAGTT
         66 CAAAACTGCTCAATCAAAAGAAAGGTTCAACTCTGTGAGATGAATGCACACATCACAAAGAAGTT

            *  **   *        *               *       
     114137 CCTGGGAACGCTTCTGTATAG-TTTTATGTGAATATATTTC
        131 TCTCAGAATGCTTCTGTGTAGTTTTTATGTGAAGATATTTC

                         *          *                   *   *  *   * *   *   
     114177 CTTTTCCACCATATGCCTCAAAGCACTCCAAATATCCACTTGCACATTATAGAAACATAGTCTTT
          1 CTTTTCCACCATAGGCCTCAAAGCGCTCCAAATATCCACTTGCAGATTCTACAAAAAGAGTGTTT

                   **                        *         *             *       
     114242 CAAAACTTGTCAATC-AAAGAAAGGTTCAACTCCGTGAGATGAGTGCACACATCACAGAGAAGTT
         66 CAAAACTGCTCAATCAAAAGAAAGGTTCAACTCTGTGAGATGAATGCACACATCACAAAGAAGTT

                *     *                            * 
     114306 TCTCGGAATGTTTCTGTGTAGTTTTTATGTGAAGATATTGC
        131 TCTCAGAATGCTTCTGTGTAGTTTTTATGTGAAGATATTTC

                     *               *            *          *       *   *   
     114347 CTTTTCCACAATAGGCCTCAAAGCGTTCCAAATATCCAATTGCAGATTCCACAAAAAAAGTTTTT
          1 CTTTTCCACCATAGGCCTCAAAGCGCTCCAAATATCCACTTGCAGATTCTACAAAAAGAGTGTTT

            *                 *  *  *  *             * *         **    *     
     114412 TAAAACTGCTCAATCAAATGATAGATTAAACTCTGTGAGATTAGTGCACACATGTCAAAAAAGTT
         66 CAAAACTGCTCAATCAAAAGAAAGGTTCAACTCTGTGAGATGAATGCACACATCACAAAGAAGTT

                                *      * *           
     114477 TCTCAGAATGCTTCTGTGTACTTTTTAGGGGAAGATATTTC
        131 TCTCAGAATGCTTCTGTGTAGTTTTTATGTGAAGATATTTC

                        *    *     **         *    *           **  * *  *    
     114518 CTTTTCCACCATCGGCCACAAAGGACTCCAAATAACCACATGCAGATTCTAGTAACACAGAGTTT
          1 CTTTTCCACCATAGGCCTCAAAGCGCTCCAAATATCCACTTGCAGATTCTACAAAAAGAGTGTTT

                       *         * *           *   * * *    **    ***        
     114583 CAAAACTGCTCTATCAAAAGATAAGTTCAACTCTGAGAGTTTAGTGCAACCATCGTGAAGAAGTT
         66 CAAAACTGCTCAATCAAAAGAAAGGTTCAACTCTGTGAGATGAATGCACACATCACAAAGAAGTT

                            *     *                  
     114648 TCTCAGAATGCTTCTGAGTAGTGTTTATGTGAAGATATTTC
        131 TCTCAGAATGCTTCTGTGTAGTTTTTATGTGAAGATATTTC

                              *     *                      *           **    
     114689 CTTTTCCACCATAGGCCTGAAAGCCCTCCAAATATCCACTTGCAGATCCTACAAAAAGAAAGTTT
          1 CTTTTCCACCATAGGCCTCAAAGCGCTCCAAATATCCACTTGCAGATTCTACAAAAAGAGTGTTT

             *         **     *  *  *   *        **      *                   
     114754 CGAAA-TGCTCTCTCAAACGATAGTTTCGACTCTGTGGTATGAATACACACATCACAAAGAAGTT
         66 CAAAACTGCTCAATCAAAAGAAAGGTTCAACTCTGTGAGATGAATGCACACATCACAAAGAAGTT

                                       **            
     114818 TCTCAGAATGCTTCTGTGTAGTTTTTAAATGAAGATATTTC
        131 TCTCAGAATGCTTCTGTGTAGTTTTTATGTGAAGATATTTC

            *                       *          *     *                       
     114859 TTTTTCCACCATAGGCCTCAAAGCACTCCAAATATGCACTTCCAGATTCTACAAAAAGAGTGTTT
          1 CTTTTCCACCATAGGCCTCAAAGCGCTCCAAATATCCACTTGCAGATTCTACAAAAAGAGTGTTT

              *                 *       * *         **   *         *  * *    
     114924 CAGAACTGCTCAATCAAAAGGAAGGTTCCAGTC--TGAGACAAATACACACATCAAAAGGTAGTT
         66 CAAAACTGCTCAATCAAAAGAAAGGTTCAACTCTGTGAGATGAATGCACACATCACAAAGAAGTT

                                                    *
     114987 TCTCAGAATGCTTCTGTGTAGTTTTTATGTGAAGATATTTT
        131 TCTCAGAATGCTTCTGTGTAGTTTTTATGTGAAGATATTTC

             *               *    **    *     *      *  **   *          *    
     115028 CCTTTCCACCATAGGCCACAAATGGCTCTAAATACCCACTTACATTTTCCACAAAAAGAGAGTTT
          1 CTTTTCCACCATAGGCCTCAAAGCGCTCCAAATATCCACTTGCAGATTCTACAAAAAGAGTGTTT

                       * *    * *      *   *         *                       
     115093 CAAAACTGCTCTACCAAAGGTAA-GTTTAACGCTGTGAG-TTAA-G-A-ACATCACAAAGAAGTT
         66 CAAAACTGCTCAATCAAAAGAAAGGTTCAACTCTGTGAGATGAATGCACACATCACAAAGAAGTT

                                   *   *  *          
     115153 TCTCAGAATGCTTCTGTGTAGTTCTTACGTAAAGATATTTC
        131 TCTCAGAATGCTTCTGTGTAGTTTTTATGTGAAGATATTTC

                 *   *      ***    *                  *              **      
     115194 CTTTTACACAATAGGCAGAAAAGTGCTCCAAATATCCACTTGAAGATTCTACAAAAACCGTGTTT
          1 CTTTTCCACCATAGGCCTCAAAGCGCTCCAAATATCCACTTGCAGATTCTACAAAAAGAGTGTTT

                     **                                          *      *    
     115259 CAAAACTGCCGAATCAAAAGAAAGGTTCAACTCTGTGAGATGAATGCACACATAACAAAGGAGTT
         66 CAAAACTGCTCAATCAAAAGAAAGGTTCAACTCTGTGAGATGAATGCACACATCACAAAGAAGTT

                                 *      *      *    *
     115324 TCTCAGAATGCTTCTGTGTAGCTTTTATATGAAGACATTTA
        131 TCTCAGAATGCTTCTGTGTAGTTTTTATGTGAAGATATTTC

            *        * *            *   ***                     **        *  
     115365 GTTTTCCACAACAGGCCTCAAAGCTCTCTCCATATCCACTTGCAGATTCTACCGAAAGAGTGCTT
          1 CTTTTCCACCATAGGCCTCAAAGCGCTCCAAATATCCACTTGCAGATTCTACAAAAAGAGTGTTT

             *                   * **     *        *        *     **         
     115430 CCAAACTGCTCAATCAAAAGAGACATTCAAATCTGTGAGGTGAATGCAGACATCGTAAAGAAGTT
         66 CAAAACTGCTCAATCAAAAGAAAGGTTCAACTCTGTGAGATGAATGCACACATCACAAAGAAGTT

                                *     *       *    **
     115495 TCTCAGAATGCTTCTGTGTATTTTTTGTGTGAAGTTATTCG
        131 TCTCAGAATGCTTCTGTGTAGTTTTTATGTGAAGATATTTC

            *    *             *     *  *            **                 *    
     115536 TTTTTGCACCATAGGCCTCCAAGCGTTCTAAATATCCACTTCTAGATTCTACAAAAAGAGAGTTT
          1 CTTTTCCACCATAGGCCTCAAAGCGCTCCAAATATCCACTTGCAGATTCTACAAAAAGAGTGTTT

                   *         *            *        *    *   *                
     115601 CAAAACTACTC-A--AACA-AAAGGTTCAATTCTGTGAGTTGAAAGCAAACATCACAAAGAAGTT
         66 CAAAACTGCTCAATCAAAAGAAAGGTTCAACTCTGTGAGATGAATGCACACATCACAAAGAAGTT

                       *             *               
     115662 TCTCAGAATGCGTCTGTGTAGTTTTGATGTGAAGATATTTC
        131 TCTCAGAATGCTTCTGTGTAGTTTTTATGTGAAGATATTTC

                     **   ****     *                  *                      
     115703 CTTTT-CACAGTAGAATGCAAAGGGCTCCAAATATCCACTTGGAGATTCTACAAAAAGA--GTTT
          1 CTTTTCCACCATAGGCCTCAAAGCGCTCCAAATATCCACTTGCAGATTCTACAAAAAGAGTGTTT

                  *    **     *  *     *     ***   *     *               *   
     115765 CAAAACCGCTCTGTCAAATGATAGGTTGAACTCCCGGAGGTGAATACACACATCACAAAGAGGTT
         66 CAAAACTGCTCAATCAAAAGAAAGGTTCAACTCTGTGAGATGAATGCACACATCACAAAGAAGTT

                  *                       *  *       
     115830 TCTCAGCATGCTTCTGTGTAGTTTTTATGTAAACATATTTC
        131 TCTCAGAATGCTTCTGTGTAGTTTTTATGTGAAGATATTTC

             *    * *              *           *      * *       *   *    *   
     115871 CGTTTCTATCATAGGCCTCAAAGTGCTCCAAATATTCACTTGTACATTCTACCAAACGAGTATTT
          1 CTTTTCCACCATAGGCCTCAAAGCGCTCCAAATATCCACTTGCAGATTCTACAAAAAGAGTGTTT

                              * *         ** *    *        *            *    
     115936 CAAAACTGCTCAATCAAATGGAAGGTTCAAAACCGTGACATGAATGCCCACATCACAAAGTAGTT
         66 CAAAACTGCTCAATCAAAAGAAAGGTTCAACTCTGTGAGATGAATGCACACATCACAAAGAAGTT

                                                     
     116001 TCTCAGAATGCTTCTGTGTAGTTTTTATGTGAAGATATTTC
        131 TCTCAGAATGCTTCTGTGTAGTTTTTATGTGAAGATATTTC

                     * *    *      *    *       * *   **      *              
     116042 CTTTTCCACAACA-GCGTGCAAAACGCTTCAAATATGCCCTTAGAGATTCCACAAAAAGAGTGTT
          1 CTTTTCCACCATAGGCCT-CAAAGCGCTCCAAATATCCACTTGCAGATTCTACAAAAAGAGTGTT

              *     *               *  *                                  ** 
     116106 TCCAAACTACTCAAATCAAAA-AATGATTTCAACTCTGTGAGATGAATGCACACATCACAAACTA
         65 TCAAAACTGCTC-AATCAAAAGAAAG-GTTCAACTCTGTGAGATGAATGCACACATCACAAAGAA

                         *     ** *   * *   *       *   
     116170 GTTTCTCAGAATGTTTCTGCCTGGTTCTCATGCGAAGATAGTTC
        128 GTTTCTCAGAATGCTTCTGTGTAGTTTTTATGTGAAGATATTTC

                 *           *   * **              *                  * *    
     116214 CTTTTTCACCATAGGCCGCAATGTACTCCAAATATCCACCTGCAGATTCTAC-AAAAGTGAGTTT
          1 CTTTTCCACCATAGGCCTCAAAGCGCTCCAAATATCCACTTGCAGATTCTACAAAAAGAGTGTTT

                       *         ***    **         *        *      *       * 
     116278 CAAAACTGCTCTATCAAAAGATCAGTTCGTCTCTGTGAGTTGAATGCATACATCAAAAAGAAGCT
         66 CAAAACTGCTCAATCAAAAGAAAGGTTCAACTCTGTGAGATGAATGCACACATCACAAAGAAGTT

                 *             *      **         *   
     116343 TCTCAAAATGCTTCTGTGTGGTTTTTCGGTGAAGATAGTTC
        131 TCTCAGAATGCTTCTGTGTAGTTTTTATGTGAAGATATTTC

            *     *        *      * *                 *        *        *    
     116384 TTTTTCTACCATAGGTCTCAAACCACTCCAAATATCCACTTGTAGATTCTATAAAAAGGAATG-T
          1 CTTTTCCACCATAGGCCTCAAAGCGCTCCAAATATCCACTTGCAGATTCTACAAAAA-GAGTGTT

                  *        *    *    *      * *         *      *         *   
     116448 TCAAAATTGCTCAATAAAAATAAAGTTTCAACACCGTGAGATGAGTGCACAAATCACAAAGGAGT
         65 TCAAAACTGCTCAATCAAAAGAAAGGTTCAACTCTGTGAGATGAATGCACACATCACAAAGAAGT

                  *          *         *          *   
     116513 TTCTCAAAATGCTTCTGGGTAGTTTTTCTGTGAAGATAGTTC
        130 TTCTCAGAATGCTTCTGTGTAGTTTTTATGTGAAGATATTTC

                  *     *    *     *          *                         *    
     116555 CTTTTCTACCATGGGCCACAAAGGGCTCCAAATACCCACTTGCAGATTCTACAAAAAGAGAGTTT
          1 CTTTTCCACCATAGGCCTCAAAGCGCTCCAAATATCCACTTGCAGATTCTACAAAAAGAGTGTTT

              *        *      ** * *        *    * *    **  * *           *  
     116620 CACAACTGCTCTATCAAACAATATGTTCAACTTTGTGGGTTGAACACAAATATCAC-AAGAATTT
         66 CAAAACTGCTCAATCAAAAGAAAGGTTCAACTCTGTGAGATGAATGCACACATCACAAAGAAGTT

                **                             *     
     116684 TCTCCCAATGCTTCTGTGTAGTTTTTATGTGAAGACATTTC
        131 TCTCAGAATGCTTCTGTGTAGTTTTTATGTGAAGATATTTC

            *   *  *      *  *     *                 *  *      *       *  *  
     116725 TTTTCCCTCCATAGTCCACAAAGTGCTCCAAATATCCACTTACATATTCTAGAAAAAGATTGCTT
          1 CTTTTCCACCATAGGCCTCAAAGCGCTCCAAATATCCACTTGCAGATTCTACAAAAAGAGTGTTT

            **       *    *               * * *                              
     116790 GGAAACTGCACAATGAAAAGAAAGGTTCAAATATATGAGATGAATGCACACATCACAAAGAAGTT
         66 CAAAACTGCTCAATCAAAAGAAAGGTTCAACTCTGTGAGATGAATGCACACATCACAAAGAAGTT

                                 *                    
     116855 TCTCAGAAT-CTCTCTGTGTAATTTTTATGTGAAGATATTTC
        131 TCTCAGAATGCT-TCTGTGTAGTTTTTATGTGAAGATATTTC

                *     *    *  *   *                        *      *    *     
     116896 CTTTCCCACCTTAGGTCTTAAAACGCTCCAAATATCCACTTGCAGATACTACAAGAAGATTGTTT
          1 CTTTTCCACCATAGGCCTCAAAGCGCTCCAAATATCCACTTGCAGATTCTACAAAAAGAGTGTTT

                     *    *        *      *     **                           
     116961 CAAAACTGCACAA-AAAAAGAAATGTTCAATTCTGTTTGATGAATGCACACATCACAAAGAAGTT
         66 CAAAACTGCTCAATCAAAAGAAAGGTTCAACTCTGTGAGATGAATGCACACATCACAAAGAAGTT

                           *                         
     117025 TCTCAGAATGCTTCTCTGTAGTTTTTATGTGAAGATATTTC
        131 TCTCAGAATGCTTCTGTGTAGTTTTTATGTGAAGATATTTC

                     *             *                 *         **      * *   
     117066 CTTTTCCACAATAGGCCTCAAAGGGCTCCAAATATCCACTTCCAGATTCTATGAAAAGAATATTT
          1 CTTTTCCACCATAGGCCTCAAAGCGCTCCAAATATCCACTTGCAGATTCTACAAAAAGAGTGTTT

             *              * *    *                                      *  
     117131 CCAAACTGCTCAATCATAGGAAATGTTCAACTCTGTGAGATGAATGCACACATCAC-AAGAAATT
         66 CAAAACTGCTCAATCAAAAGAAAGGTTCAACTCTGTGAGATGAATGCACACATCACAAAGAAGTT

                     *    *      *       *     * *   
     117195 TCTCAGAATCCTTCAGTGTAGGTTTTATGAGAAGACAATTC
        131 TCTCAGAATGCTTCTGTGTAGTTTTTATGTGAAGATATTTC

                     *    **        *   *                           *    *   
     117236 CTTTTCCACAATAGTTCTCAAAGCACTCAAAATATCCACTTGCAGATTCTACAAAAGGAGTATTT
          1 CTTTTCCACCATAGGCCTCAAAGCGCTCCAAATATCCACTTGCAGATTCTACAAAAAGAGTGTTT

                                                          *                  
     117301 CAAAACTGCTCAATCAAAAGAAAGGTTCAACTCTGTGAGATGAATGGACACATCACAAAGAAGTT
         66 CAAAACTGCTCAATCAAAAGAAAGGTTCAACTCTGTGAGATGAATGCACACATCACAAAGAAGTT

                                  *   *              
     117366 TCTCAGAATGCTTCTGTGTAGTATTTTTGTGAAGATATTT-
        131 TCTCAGAATGCTTCTGTGTAGTTTTTATGTGAAGATATTTC

                          *  * * * * ***             *            *     * *  
     117406 CTTTTCCACCATAGACCGCCAGGGGACACAAATATCCACTTTCAGATTCTACAACAAGAGAGGTT
          1 CTTTTCCACCATAGGCCTCAAAGCGCTCCAAATATCCACTTGCAGATTCTACAAAAAGAGTGTTT

                   *   *     *   ** *       *      *                      * *
     117471 CAAAACTACTCGATCAAGAGATGGTTTCAACTATGTGAGTTGAATGCACACATCACAAAGAACTA
         66 CAAAACTGCTCAATCAAAAGAAAGGTTCAACTCTGTGAGATGAATGCACACATCACAAAGAAGTT

             *  *    *                               
     117536 TGTCGGAATTCTTCTGTGTAGTTTTTATGTGAAGATATTTC
        131 TCTCAGAATGCTTCTGTGTAGTTTTTATGTGAAGATATTTC

                     *    * *      * *    *                  *               
     117577 CTTTTCCACAATAGACGTCAAAGTGATCCAGATATCCACTTGCAGATTCCACAAAAAGAGTGTTT
          1 CTTTTCCACCATAGGCCTCAAAGCGCTCCAAATATCCACTTGCAGATTCTACAAAAAGAGTGTTT

                 *   *   *                  **                     *  *      
     117642 CAAAAGTGCACAACCAAAAGAAAGGTTCAACTAGGTGAGATGAATGCACACATCAGAAGGAAGTT
         66 CAAAACTGCTCAATCAAAAGAAAGGTTCAACTCTGTGAGATGAATGCACACATCACAAAGAAGTT

                            **   *     * *       *   
     117707 TCTCAGAATGCTTCTGCATAGCTTTTAAGGGAAGATACTTC
        131 TCTCAGAATGCTTCTGTGTAGTTTTTATGTGAAGATATTTC

                    *               *            * *  *    * *               
     117748 CTTTTCCAACATAGGCCTCAAAGCACTCCAAATATCCTCCTGGAGATACCACAAAAAGAGTGTTT
          1 CTTTTCCACCATAGGCCTCAAAGCGCTCCAAATATCCACTTGCAGATTCTACAAAAAGAGTGTTT

            **                      *  *                 *       *           
     117813 GCAAACTGCTCAATCAAAAGAAAGATTTAACTCTGTGAGATGAATCCACACATGACAAAGAAGTT
         66 CAAAACTGCTCAATCAAAAGAAAGGTTCAACTCTGTGAGATGAATGCACACATCACAAAGAAGTT

                                                     
     117878 TCTCAGAATGCTTCTGTGTAGTTTTTATGTGAAGATATTTC
        131 TCTCAGAATGCTTCTGTGTAGTTTTTATGTGAAGATATTTC

                     *   * * *    **           *                *     *      
     117919 CTTTTCCACAATAAGACCCAAAAGGCTCCAAATATTCACTTGCAGATTCTAAAAAAAACAGTGTT
          1 CTTTTCCACCATAGGCCTCAAAGCGCTCCAAATATCCACTTGCAGATTCT-ACAAAAAGAGTGTT

                                  *                  *      *        **      
     117984 TCAAAACTGCTCAATCAAAAGATA-GTTCAACTCTGTGAGAAGAATGCTCACATCACTGAGAAGT
         65 TCAAAACTGCTCAATCAAAAGAAAGGTTCAACTCTGTGAGATGAATGCACACATCACAAAGAAGT

                                         *            
     118048 TTCTCAGAATGCTTCTGTGTAGTTTTTATATGAAGATATTTC
        130 TTCTCAGAATGCTTCTGTGTAGTTTTTATGTGAAGATATTTC

                *     *      *    **                       *   **       *    
     118090 CTTTCCCACCGTAGGCCACAAAAGGCTCCAAATATCCACTTGCAGATACTATGAAAAGAGAGTTT
          1 CTTTTCCACCATAGGCCTCAAAGCGCTCCAAATATCCACTTGCAGATTCTACAAAAAGAGTGTTT

                        *        *               ***            *            
     118155 CAAAACTGCTCATTCAAAAGATAGGTTCAACTCTGTGGTTTGAATGCACACAGCACAAAGAAGTT
         66 CAAAACTGCTCAATCAAAAGAAAGGTTCAACTCTGTGAGATGAATGCACACATCACAAAGAAGTT

              *       **                   **   *    
     118220 TCACAGAATGTGTCTGTGTAGTTTTTATGTGCGGATGTTTC
        131 TCTCAGAATGCTTCTGTGTAGTTTTTATGTGAAGATATTTC

                         *        ****   *   *               *    *          
     118261 CTTTTCCACCATATGCCT-AAATATTTCCCAATTTCCACTTGCAGATTCCACAAGAAGAGTGTTT
          1 CTTTTCCACCATAGGCCTCAAAGCGCTCCAAATATCCACTTGCAGATTCTACAAAAAGAGTGTTT

                      **       *       *           *            *      ***   
     118325 CAAAACTGCTGTATC-AAATAAA-GTTGAACTCTGTGAGGTGAATGCACACAGCACAAAATGGTT
         66 CAAAACTGCTCAATCAAAAGAAAGGTTCAACTCTGTGAGATGAATGCACACATCACAAAGAAGTT

                          **   *        *       *    
     118388 TCTCAGAATGCTTCCTTGTTGTTTTTATATGAAGATGTTTC
        131 TCTCAGAATGCTTCTGTGTAGTTTTTATGTGAAGATATTTC

                  *  *             *   *     *                  *            
     118429 CTTTTCAACAATAGGCCTCAAAGTGCTTCAAATGTCCACTTGCAGATTCTACCAAAAGAGTGTTT
          1 CTTTTCCACCATAGGCCTCAAAGCGCTCCAAATATCCACTTGCAGATTCTACAAAAAGAGTGTTT

                                        *      *  *  *                       
     118494 CAAAACTGCTCAATCAAAAGAAAGGTTCGACTCTGGGAAATTAATGCACACATCACAAAGAAGTT
         66 CAAAACTGCTCAATCAAAAGAAAGGTTCAACTCTGTGAGATGAATGCACACATCACAAAGAAGTT

                                     *        *      
     118559 TCTC---A-GCTTCTGTGTAGTTTTCATGTGAAGTTATTTC
        131 TCTCAGAATGCTTCTGTGTAGTTTTTATGTGAAGATATTTC

                     *       *     *            *    *         **       *    
     118596 CTTTTCCACAATAGGCCGCAAAGGGCTCCAAATATCAACTTACAGATTCTAGGAAAAGAGAGTTT
          1 CTTTTCCACCATAGGCCTCAAAGCGCTCCAAATATCCACTTGCAGATTCTACAAAAAGAGTGTTT

                       * **      *     *                                     
     118661 CAAAACTGCTCTACGAAAAGATAGGTTGAACTCTGTGAGATGAATGCACACATCACAAAGAAGTT
         66 CAAAACTGCTCAATCAAAAGAAAGGTTCAACTCTGTGAGATGAATGCACACATCACAAAGAAGTT

                       *               * *     *     
     118726 TCTCAGAATGCATCTGTGTAGTTTTTACGGGAAGACATTTC
        131 TCTCAGAATGCTTCTGTGTAGTTTTTATGTGAAGATATTTC

                        ***  *     **      *  *                  *     ***   
     118767 CTTTTCCACCATCTTCCACAAAGGTCTCCAAGTAACCACTTGCAGATTCTACAGAAAGACACTTT
          1 CTTTTCCACCATAGGCCTCAAAGCGCTCCAAATATCCACTTGCAGATTCTACAAAAAGAGTGTTT

            *          *         ***      *      ***                      *  
     118832 AAAAACTGCTCTATCAAAAGATCAGTTCAAGTCTGTGGTTTGAATGCACACATCACAAAGAATTT
         66 CAAAACTGCTCAATCAAAAGAAAGGTTCAACTCTGTGAGATGAATGCACACATCACAAAGAAGTT

                                     *  *            
     118897 TCTCAGAATGCTTCTGTGTAGTTTTCATATGAAGATATTTC
        131 TCTCAGAATGCTTCTGTGTAGTTTTTATGTGAAGATATTTC

                                    *                                   **   
     118938 CTTTTCCACCATAGGCCTCAAAGCACTCCAAATATCCACTTGCAGATTCTACAAAAAGAGATTTT
          1 CTTTTCCACCATAGGCCTCAAAGCGCTCCAAATATCCACTTGCAGATTCTACAAAAAGAGTGTTT

                   **                           *  *          *        *     
     119003 CAAAACTAGTCAATCAAAAGAAAGGTTCAACTCTGTCAGTTGAATGCACATATCACAAACAAGTT
         66 CAAAACTGCTCAATCAAAAGAAAGGTTCAACTCTGTGAGATGAATGCACACATCACAAAGAAGTT

                *      *                             
     119068 TCTCGGAATGCGTCTGTGTAGTTTTTATGTGAAGATATTTC
        131 TCTCAGAATGCTTCTGTGTAGTTTTTATGTGAAGATATTTC

               *     * *           *     *                  *    *           
     119109 CTTCTCCACAACAGGCCTCAAAGTGCTCCGAATATCCACTTGCAGATTTTAC-TAAAGAGTGTTT
          1 CTTTTCCACCATAGGCCTCAAAGCGCTCCAAATATCCACTTGCAGATTCTACAAAAAGAGTGTTT

             *               *  *   *     *        *    *               *    
     119173 CCAAACTGCTCAATCAAGAGGAAGTTTCAAGTCTGTGAGCTGAACGCACACATCACAAAGTAGTT
         66 CAAAACTGCTCAATCAAAAGAAAGGTTCAACTCTGTGAGATGAATGCACACATCACAAAGAAGTT

               *                                *    
     119238 TCTGAGAATGCTTCTGTGTAGTTTTTATGTGAAGATGTTTC
        131 TCTCAGAATGCTTCTGTGTAGTTTTTATGTGAAGATATTTC

                            **     *                                    *    
     119279 CTTTTCCACCATAGGCTGCAAAGGGCTCCAAATATCCACTTGCAGATTCTACAAAAAGAGAGTTT
          1 CTTTTCCACCATAGGCCTCAAAGCGCTCCAAATATCCACTTGCAGATTCTACAAAAAGAGTGTTT

                 *     *         *          *     *                     *    
     119344 CAAAAGTGCTCTATCAAAAGATAGGTTCAACTATGTGATATGAATGCACACATCACAAAGTAGTT
         66 CAAAACTGCTCAATCAAAAGAAAGGTTCAACTCTGTGAGATGAATGCACACATCACAAAGAAGTT

                                          *          
     119409 TCTCAGAATGCTTCTGTGTAGTTTTTATGTAAAGATATTTC
        131 TCTCAGAATGCTTCTGTGTAGTTTTTATGTGAAGATATTTC

                                    *                                   **   
     119450 CTTTTCCACCATAGGCCTCAAAGCACTCCAAATATCCACTTGCAGATTCTACAAAAAGAGATTTT
          1 CTTTTCCACCATAGGCCTCAAAGCGCTCCAAATATCCACTTGCAGATTCTACAAAAAGAGTGTTT

                   ** *                   *     *  *    * *   *        *     
     119515 CAAAACTATTTAATCAAAAGAAAGGTTCAAATCTGTCAGTTGAAGGTACATATCACAAACAAGTT
         66 CAAAACTGCTCAATCAAAAGAAAGGTTCAACTCTGTGAGATGAATGCACACATCACAAAGAAGTT

             * **                                    
     119580 TATTGGAATGCTTCTGTGTAGTTTTTATGTGAAGATATTTC
        131 TCTCAGAATGCTTCTGTGTAGTTTTTATGTGAAGATATTTC

                     * *         * *                        **  *     *      
     119621 CTTTTCCACAACAGGCCTCAAGGTGCTCCAAATATCCACTTGCAGATTTCACTAAAAGTGTGTTT
          1 CTTTTCCACCATAGGCCTCAAAGCGCTCCAAATATCCACTTGCAGATTCTACAAAAAGAGTGTTT

             *  *            *  *   *     *        *             *           
     119686 CCAAGCTGCTCAATCAAGAGGAAGTTTCAAGTCTGTGAGGTGAATGCACACATTACAAAGAAGTT
         66 CAAAACTGCTCAATCAAAAGAAAGGTTCAACTCTGTGAGATGAATGCACACATCACAAAGAAGTT

            *  *                                     
     119751 ACTGAGAATGCTTCTGTGTAGTTTTTATGTGAAGATATTTC
        131 TCTCAGAATGCTTCTGTGTAGTTTTTATGTGAAGATATTTC

                      **               *                                *    
     119792 CTTTTCCACCGCAGGCCTCAAAGCGCTGCAAATATCCACTTGCAGATTCTACAAAAAGAGAGTTT
          1 CTTTTCCACCATAGGCCTCAAAGCGCTCCAAATATCCACTTGCAGATTCTACAAAAAGAGTGTTT

                      **         *   *         *   *     **            *     
     119857 CAAAACTGCTGTATCAAAAGATAGGGTCAACTCTGCGAGTTGAATAAACACATCACAAATAAGTT
         66 CAAAACTGCTCAATCAAAAGAAAGGTTCAACTCTGTGAGATGAATGCACACATCACAAAGAAGTT

               **   *        *               *       
     119922 TCTGGGAACGCTTCTGTATAG-TTTTATGTGAATATATTTC
        131 TCTCAGAATGCTTCTGTGTAGTTTTTATGTGAAGATATTTC

                         *          *                   *   *  *   * *   *   
     119962 CTTTTCCACCATATGCCTCAAAGCACTCCAAATATCCACTTGCACATTATAGAAACATAGTCTTT
          1 CTTTTCCACCATAGGCCTCAAAGCGCTCCAAATATCCACTTGCAGATTCTACAAAAAGAGTGTTT

                   **                        *         *             *       
     120027 CAAAACTTGTCAATC-AAAGAAAGGTTCAACTCCGTGAGATGAGTGCACACATCACAGAGAAGTT
         66 CAAAACTGCTCAATCAAAAGAAAGGTTCAACTCTGTGAGATGAATGCACACATCACAAAGAAGTT

                *     *                            * 
     120091 TCTCGGAATGTTTCTGTGTAGTTTTTATGTGAAGATATTGC
        131 TCTCAGAATGCTTCTGTGTAGTTTTTATGTGAAGATATTTC

                     *               *            *          *       *   *   
     120132 CTTTTCCACAATAGGCCTCAAAGCGTTCCAAATATCCAATTGCAGATTCCACAAAAAAAGTTTTT
          1 CTTTTCCACCATAGGCCTCAAAGCGCTCCAAATATCCACTTGCAGATTCTACAAAAAGAGTGTTT

            *                 *  *  *  *             * *         **    *     
     120197 TAAAACTGCTCAATCAAATGATAGATTAAACTCTGTGAGATTAGTGCACACATGTCAAAAAAGTT
         66 CAAAACTGCTCAATCAAAAGAAAGGTTCAACTCTGTGAGATGAATGCACACATCACAAAGAAGTT

                                *      * *           
     120262 TCTCAGAATGCTTCTGTGTACTTTTTAGGGGAAGATATTTC
        131 TCTCAGAATGCTTCTGTGTAGTTTTTATGTGAAGATATTTC

                        *    *     **         *    *           **  * *  *    
     120303 CTTTTCCACCATCGGCCACAAAGGACTCCAAATAACCACATGCAGATTCTAGTAACACAGAGTTT
          1 CTTTTCCACCATAGGCCTCAAAGCGCTCCAAATATCCACTTGCAGATTCTACAAAAAGAGTGTTT

                       *         * *           *   * * *    **    ***        
     120368 CAAAACTGCTCTATCAAAAGATAAGTTCAACTCTGAGAGTTTAGTGCAACCATCGTGAAGAAGTT
         66 CAAAACTGCTCAATCAAAAGAAAGGTTCAACTCTGTGAGATGAATGCACACATCACAAAGAAGTT

                            *     *                  
     120433 TCTCAGAATGCTTCTGAGTAGTGTTTATGTGAAGATATTTC
        131 TCTCAGAATGCTTCTGTGTAGTTTTTATGTGAAGATATTTC

                              *     *                      *           **    
     120474 CTTTTCCACCATAGGCCTGAAAGCCCTCCAAATATCCACTTGCAGATCCTACAAAAAGAAAGTTT
          1 CTTTTCCACCATAGGCCTCAAAGCGCTCCAAATATCCACTTGCAGATTCTACAAAAAGAGTGTTT

             *         **     *  *  *   *        **      *                   
     120539 CGAAA-TGCTCTCTCAAACGATAGTTTCGACTCTGTGGTATGAATACACACATCACAAAGAAGTT
         66 CAAAACTGCTCAATCAAAAGAAAGGTTCAACTCTGTGAGATGAATGCACACATCACAAAGAAGTT

                                       **            
     120603 TCTCAGAATGCTTCTGTGTAGTTTTTAAATGAAGATATTTC
        131 TCTCAGAATGCTTCTGTGTAGTTTTTATGTGAAGATATTTC

            *                       *          *     *                       
     120644 TTTTTCCACCATAGGCCTCAAAGCACTCCAAATATGCACTTCCAGATTCTACAAAAAGAGTGTTT
          1 CTTTTCCACCATAGGCCTCAAAGCGCTCCAAATATCCACTTGCAGATTCTACAAAAAGAGTGTTT

              *                 *       * *         **   *         *  * *    
     120709 CAGAACTGCTCAATCAAAAGGAAGGTTCCAGTC--TGAGACAAATACACACATCAAAAGGTAGTT
         66 CAAAACTGCTCAATCAAAAGAAAGGTTCAACTCTGTGAGATGAATGCACACATCACAAAGAAGTT

                                                    *
     120772 TCTCAGAATGCTTCTGTGTAGTTTTTATGTGAAGATATTTT
        131 TCTCAGAATGCTTCTGTGTAGTTTTTATGTGAAGATATTTC

             *               *    **    *     *      *  **   *          *    
     120813 CCTTTCCACCATAGGCCACAAATGGCTCTAAATACCCACTTACATTTTCCACAAAAAGAGAGTTT
          1 CTTTTCCACCATAGGCCTCAAAGCGCTCCAAATATCCACTTGCAGATTCTACAAAAAGAGTGTTT

                       * *    * *      *   *         *                       
     120878 CAAAACTGCTCTACCAAAGGTAA-GTTTAACGCTGTGAG-TTAA-G-A-ACATCACAAAGAAGTT
         66 CAAAACTGCTCAATCAAAAGAAAGGTTCAACTCTGTGAGATGAATGCACACATCACAAAGAAGTT

                                   *   *  *          
     120938 TCTCAGAATGCTTCTGTGTAGTTCTTACGTAAAGATATTTC
        131 TCTCAGAATGCTTCTGTGTAGTTTTTATGTGAAGATATTTC

                 *   *      ***    *                  *              **      
     120979 CTTTTACACAATAGGCAGAAAAGTGCTCCAAATATCCACTTGAAGATTCTACAAAAACCGTGTTT
          1 CTTTTCCACCATAGGCCTCAAAGCGCTCCAAATATCCACTTGCAGATTCTACAAAAAGAGTGTTT

                     **                                          *      *    
     121044 CAAAACTGCCGAATCAAAAGAAAGGTTCAACTCTGTGAGATGAATGCACACATAACAAAGGAGTT
         66 CAAAACTGCTCAATCAAAAGAAAGGTTCAACTCTGTGAGATGAATGCACACATCACAAAGAAGTT

                                 *      *      *    *
     121109 TCTCAGAATGCTTCTGTGTAGCTTTTATATGAAGACATTTA
        131 TCTCAGAATGCTTCTGTGTAGTTTTTATGTGAAGATATTTC

            *        * *            *   ***                     **        *  
     121150 GTTTTCCACAACAGGCCTCAAAGCTCTCTCCATATCCACTTGCAGATTCTACCGAAAGAGTGCTT
          1 CTTTTCCACCATAGGCCTCAAAGCGCTCCAAATATCCACTTGCAGATTCTACAAAAAGAGTGTTT

             *                   * **     *        *        *     **         
     121215 CCAAACTGCTCAATCAAAAGAGACATTCAAATCTGTGAGGTGAATGCAGACATCGTAAAGAAGTT
         66 CAAAACTGCTCAATCAAAAGAAAGGTTCAACTCTGTGAGATGAATGCACACATCACAAAGAAGTT

                                *     *       *    **
     121280 TCTCAGAATGCTTCTGTGTATTTTTTGTGTGAAGTTATTCG
        131 TCTCAGAATGCTTCTGTGTAGTTTTTATGTGAAGATATTTC

            *    *             *     *  *            **                 *    
     121321 TTTTTGCACCATAGGCCTCCAAGCGTTCTAAATATCCACTTCTAGATTCTACAAAAAGAGAGTTT
          1 CTTTTCCACCATAGGCCTCAAAGCGCTCCAAATATCCACTTGCAGATTCTACAAAAAGAGTGTTT

                   *         *            *        *    *   *                
     121386 CAAAACTACTC-A--AACA-AAAGGTTCAATTCTGTGAGTTGAAAGCAAACATCACAAAGAAGTT
         66 CAAAACTGCTCAATCAAAAGAAAGGTTCAACTCTGTGAGATGAATGCACACATCACAAAGAAGTT

                       *             *               
     121447 TCTCAGAATGCGTCTGTGTAGTTTTGATGTGAAGATATTTC
        131 TCTCAGAATGCTTCTGTGTAGTTTTTATGTGAAGATATTTC

                     **   ****     *                  *                      
     121488 CTTTT-CACAGTAGAATGCAAAGGGCTCCAAATATCCACTTGGAGATTCTACAAAAAGA--GTTT
          1 CTTTTCCACCATAGGCCTCAAAGCGCTCCAAATATCCACTTGCAGATTCTACAAAAAGAGTGTTT

                  *    **     *  *     *     ***   *     *               *   
     121550 CAAAACCGCTCTGTCAAATGATAGGTTGAACTCCCGGAGGTGAATACACACATCACAAAGAGGTT
         66 CAAAACTGCTCAATCAAAAGAAAGGTTCAACTCTGTGAGATGAATGCACACATCACAAAGAAGTT

                  *                       *  *       
     121615 TCTCAGCATGCTTCTGTGTAGTTTTTATGTAAACATATTTC
        131 TCTCAGAATGCTTCTGTGTAGTTTTTATGTGAAGATATTTC

             *    * *              *           *      * *       *   *    *   
     121656 CGTTTCTATCATAGGCCTCAAAGTGCTCCAAATATTCACTTGTACATTCTACCAAACGAGTATTT
          1 CTTTTCCACCATAGGCCTCAAAGCGCTCCAAATATCCACTTGCAGATTCTACAAAAAGAGTGTTT

                              * *         ** *    *        *            *    
     121721 CAAAACTGCTCAATCAAACGGAAGGTTCAAAACCGTGACATGAATGCCCACATCACAAAGTAGTT
         66 CAAAACTGCTCAATCAAAAGAAAGGTTCAACTCTGTGAGATGAATGCACACATCACAAAGAAGTT

                                                     
     121786 TCTCAGAATGCTTCTGTGTAGTTTTTATGTGAAGATATTTC
        131 TCTCAGAATGCTTCTGTGTAGTTTTTATGTGAAGATATTTC

                     * *    *      *    *       * *   **      *              
     121827 CTTTTCCACAACA-GCGTGCAAAACGCTTCAAATATGCCCTTAGAGATTCCACAAAAAGAGTGTT
          1 CTTTTCCACCATAGGCCT-CAAAGCGCTCCAAATATCCACTTGCAGATTCTACAAAAAGAGTGTT

              *     *               *  *                                  ** 
     121891 TCCAAACTACTCAAATCAAAA-AATGATTTCAACTCTGTGAGATGAATGCACACATCACAAACTA
         65 TCAAAACTGCTC-AATCAAAAGAAAG-GTTCAACTCTGTGAGATGAATGCACACATCACAAAGAA

                         *     ** *   * *   *       *   
     121955 GTTTCTCAGAATGTTTCTGCCTGGTTCTCATGCGAAGATAGTTC
        128 GTTTCTCAGAATGCTTCTGTGTAGTTTTTATGTGAAGATATTTC

                 *           *   * **              *                  * *    
     121999 CTTTTTCACCATAGGCCGCAATGTACTCCAAATATCCACCTGCAGATTCTAC-AAAAGTGAGTTT
          1 CTTTTCCACCATAGGCCTCAAAGCGCTCCAAATATCCACTTGCAGATTCTACAAAAAGAGTGTTT

                       *         ***    **         *        *      *       * 
     122063 CAAAACTGCTCTATCAAAAGATCAGTTCGTCTCTGTGAGTTGAATGCATACATCAAAAAGAAGCT
         66 CAAAACTGCTCAATCAAAAGAAAGGTTCAACTCTGTGAGATGAATGCACACATCACAAAGAAGTT

                 *             *      **         *   
     122128 TCTCAAAATGCTTCTGTGTGGTTTTTCGGTGAAGATAGTTC
        131 TCTCAGAATGCTTCTGTGTAGTTTTTATGTGAAGATATTTC

            *     *        *      * *                 *        *        *    
     122169 TTTTTCTACCATAGGTCTCAAACCACTCCAAATATCCACTTGTAGATTCTATAAAAAGGAATG-T
          1 CTTTTCCACCATAGGCCTCAAAGCGCTCCAAATATCCACTTGCAGATTCTACAAAAA-GAGTGTT

                  *        *    *    *      * *         *      *         *   
     122233 TCAAAATTGCTCAATAAAAATAAAGTTTCAACACCGTGAGATGAGTGCACAAATCACAAAGGAGT
         65 TCAAAACTGCTCAATCAAAAGAAAGGTTCAACTCTGTGAGATGAATGCACACATCACAAAGAAGT

                  *          *         *          *   
     122298 TTCTCAAAATGCTTCTGGGTAGTTTTTCTGTGAAGATAGTTC
        130 TTCTCAGAATGCTTCTGTGTAGTTTTTATGTGAAGATATTTC

                  *     *    *     *          *                         *    
     122340 CTTTTCTACCATGGGCCACAAAGGGCTCCAAATACCCACTTGCAGATTCTACAAAAAGAGAGTTT
          1 CTTTTCCACCATAGGCCTCAAAGCGCTCCAAATATCCACTTGCAGATTCTACAAAAAGAGTGTTT

              *        *      ** * *        *    * *    **  * *           *  
     122405 CACAACTGCTCTATCAAACAATATGTTCAACTTTGTGGGTTGAACACAAATATCAC-AAGAATTT
         66 CAAAACTGCTCAATCAAAAGAAAGGTTCAACTCTGTGAGATGAATGCACACATCACAAAGAAGTT

                **                             *     
     122469 TCTCCCAATGCTTCTGTGTAGTTTTTATGTGAAGACATTTC
        131 TCTCAGAATGCTTCTGTGTAGTTTTTATGTGAAGATATTTC

            *   *  *      *  *     *                 *  *      *       *  *  
     122510 TTTTCCCTCCATAGTCCACAAAGTGCTCCAAATATCCACTTACATATTCTAGAAAAAGATTGCTT
          1 CTTTTCCACCATAGGCCTCAAAGCGCTCCAAATATCCACTTGCAGATTCTACAAAAAGAGTGTTT

            **       *    *               * * *                              
     122575 GGAAACTGCACAATGAAAAGAAAGGTTCAAATATATGAGATGAATGCACACATCACAAAGAAGTT
         66 CAAAACTGCTCAATCAAAAGAAAGGTTCAACTCTGTGAGATGAATGCACACATCACAAAGAAGTT

                                 *                    
     122640 TCTCAGAAT-CTCTCTGTGTAATTTTTATGTGAAGATATTTC
        131 TCTCAGAATGCT-TCTGTGTAGTTTTTATGTGAAGATATTTC

                *     *    *  *   *                        *      *    *     
     122681 CTTTCCCACCTTAGGTCTTAAAACGCTCCAAATATCCACTTGCAGATACTACAAGAAGATTGTTT
          1 CTTTTCCACCATAGGCCTCAAAGCGCTCCAAATATCCACTTGCAGATTCTACAAAAAGAGTGTTT

                     *    *        *      *     **                           
     122746 CAAAACTGCACAA-AAAAAGAAATGTTCAATTCTGTTTGATGAATGCACACATCACAAAGAAGTT
         66 CAAAACTGCTCAATCAAAAGAAAGGTTCAACTCTGTGAGATGAATGCACACATCACAAAGAAGTT

                           *                         
     122810 TCTCAGAATGCTTCTCTGTAGTTTTTATGTGAAGATATTTC
        131 TCTCAGAATGCTTCTGTGTAGTTTTTATGTGAAGATATTTC

                     *             *                 *         **      * *   
     122851 CTTTTCCACAATAGGCCTCAAAGGGCTCCAAATATCCACTTCCAGATTCTATGAAAAGAATATTT
          1 CTTTTCCACCATAGGCCTCAAAGCGCTCCAAATATCCACTTGCAGATTCTACAAAAAGAGTGTTT

             *              * *    *                                      *  
     122916 CCAAACTGCTCAATCATAGGAAATGTTCAACTCTGTGAGATGAATGCACACATCAC-AAGAAATT
         66 CAAAACTGCTCAATCAAAAGAAAGGTTCAACTCTGTGAGATGAATGCACACATCACAAAGAAGTT

                     *    *      *       *       *   
     122980 TCTCAGAATCCTTCAGTGTAGGTTTTATGAGAAGATAATTC
        131 TCTCAGAATGCTTCTGTGTAGTTTTTATGTGAAGATATTTC

                     *    **        *   *                           *    *   
     123021 CTTTTCCACAATAGTTCTCAAAGCACTCAAAATATCCACTTGCAGATTCTACAAAAGGAGTATTT
          1 CTTTTCCACCATAGGCCTCAAAGCGCTCCAAATATCCACTTGCAGATTCTACAAAAAGAGTGTTT

                                                          *                  
     123086 CAAAACTGCTCAATCAAAAGAAAGGTTCAACTCTGTGAGATGAATGGACACATCACAAAGAAGTT
         66 CAAAACTGCTCAATCAAAAGAAAGGTTCAACTCTGTGAGATGAATGCACACATCACAAAGAAGTT

                                  *   *              
     123151 TCTCAGAATGCTTCTGTGTAGTATTTTTGTGAAGATATTT-
        131 TCTCAGAATGCTTCTGTGTAGTTTTTATGTGAAGATATTTC

                          *  * * * * ***             *            *     * *  
     123191 CTTTTCCACCATAGACCGCCAGGGGACACAAATATCCACTTTCAGATTCTACAACAAGAGAGGTT
          1 CTTTTCCACCATAGGCCTCAAAGCGCTCCAAATATCCACTTGCAGATTCTACAAAAAGAGTGTTT

                   *   *     *   ** *       *      *                      * *
     123256 CAAAACTACTCGATCAAGAGATGGTTTCAACTATGTGAGTTGAATGCACACATCACAAAGAACTA
         66 CAAAACTGCTCAATCAAAAGAAAGGTTCAACTCTGTGAGATGAATGCACACATCACAAAGAAGTT

             *  *    *                               
     123321 TGTCGGAATTCTTCTGTGTAGTTTTTATGTGAAGATATTTC
        131 TCTCAGAATGCTTCTGTGTAGTTTTTATGTGAAGATATTTC

                     *    * *      * *    *                  *               
     123362 CTTTTCCACAATAGACGTCAAAGTGATCCAGATATCCACTTGCAGATTCCACAAAAAGAGTGTTT
          1 CTTTTCCACCATAGGCCTCAAAGCGCTCCAAATATCCACTTGCAGATTCTACAAAAAGAGTGTTT

                 *   *   *                  **                     *  *      
     123427 CAAAAGTGCACAACCAAAAGAAAGGTTCAACTAGGTGAGATGAATGCACACATCAGAAGGAAGTT
         66 CAAAACTGCTCAATCAAAAGAAAGGTTCAACTCTGTGAGATGAATGCACACATCACAAAGAAGTT

                            **   *     * *       *   
     123492 TCTCAGAATGCTTCTGCATAGCTTTTAAGGGAAGATACTTC
        131 TCTCAGAATGCTTCTGTGTAGTTTTTATGTGAAGATATTTC

                    *               *            * *  *    * *               
     123533 CTTTTCCAACATAGGCCTCAAAGCACTCCAAATATCCTCCTGGAGATACCACAAAAAGAGTGTTT
          1 CTTTTCCACCATAGGCCTCAAAGCGCTCCAAATATCCACTTGCAGATTCTACAAAAAGAGTGTTT

            **                      *  *                 *       *           
     123598 GCAAACTGCTCAATCAAAAGAAAGATTTAACTCTGTGAGATGAATCCACACATGACAAAGAAGTT
         66 CAAAACTGCTCAATCAAAAGAAAGGTTCAACTCTGTGAGATGAATGCACACATCACAAAGAAGTT

                                                     
     123663 TCTCAGAATGCTTCTGTGTAGTTTTTATGTGAAGATATTTC
        131 TCTCAGAATGCTTCTGTGTAGTTTTTATGTGAAGATATTTC

                     *   * * *    **           *                *     *      
     123704 CTTTTCCACAATAAGACCCAAAAGGCTCCAAATATTCACTTGCAGATTCTAAAAAAAACAGTGTT
          1 CTTTTCCACCATAGGCCTCAAAGCGCTCCAAATATCCACTTGCAGATTCT-ACAAAAAGAGTGTT

                                  *                  *      *        **      
     123769 TCAAAACTGCTCAATCAAAAGATA-GTTCAACTCTGTGAGAAGAATGCTCACATCACTGAGAAGT
         65 TCAAAACTGCTCAATCAAAAGAAAGGTTCAACTCTGTGAGATGAATGCACACATCACAAAGAAGT

                                         *            
     123833 TTCTCAGAATGCTTCTGTGTAGTTTTTATATGAAGATATTTC
        130 TTCTCAGAATGCTTCTGTGTAGTTTTTATGTGAAGATATTTC

                *     *      *    **                       *   **       *    
     123875 CTTTCCCACCGTAGGCCACAAAAGGCTCCAAATATCCACTTGCAGATACTATGAAAAGAGAGTTT
          1 CTTTTCCACCATAGGCCTCAAAGCGCTCCAAATATCCACTTGCAGATTCTACAAAAAGAGTGTTT

                        *        *               ***            *            
     123940 CAAAACTGCTCATTCAAAAGATAGGTTCAACTCTGTGGTTTGAATGCACACAGCACAAAGAAGTT
         66 CAAAACTGCTCAATCAAAAGAAAGGTTCAACTCTGTGAGATGAATGCACACATCACAAAGAAGTT

              *       **                   **   *    
     124005 TCACAGAATGTGTCTGTGTAGTTTTTATGTGCGGATGTTTC
        131 TCTCAGAATGCTTCTGTGTAGTTTTTATGTGAAGATATTTC

                         *        ****   *   *               *    *          
     124046 CTTTTCCACCATATGCCT-AAATATTTCCCAATTTCCACTTGCAGATTCCACAAGAAGAGTGTTT
          1 CTTTTCCACCATAGGCCTCAAAGCGCTCCAAATATCCACTTGCAGATTCTACAAAAAGAGTGTTT

                      **       *       *           *            *      ***   
     124110 CAAAACTGCTGTATC-AAATAAA-GTTGAACTCTGTGAGGTGAATGCACACAGCACAAAATGGTT
         66 CAAAACTGCTCAATCAAAAGAAAGGTTCAACTCTGTGAGATGAATGCACACATCACAAAGAAGTT

                          **   *        *       *    
     124173 TCTCAGAATGCTTCCTTGTTGTTTTTATATGAAGATGTTTC
        131 TCTCAGAATGCTTCTGTGTAGTTTTTATGTGAAGATATTTC

                  *  *             *   *     *                               
     124214 CTTTTCAACAATAGGCCTCAAAGTGCTTCAAATGTCCACTTGCAGATTCTACAAAAAGAGTGTTT
          1 CTTTTCCACCATAGGCCTCAAAGCGCTCCAAATATCCACTTGCAGATTCTACAAAAAGAGTGTTT

                                        *      *  *  *                       
     124279 CAAAACTGCTCAATCAAAAGAAAGGTTCGACTCTGGGAAATTAATGCACACATCACAAAGAAGTT
         66 CAAAACTGCTCAATCAAAAGAAAGGTTCAACTCTGTGAGATGAATGCACACATCACAAAGAAGTT

                                     *        *      
     124344 TCTC---A-GCTTCTGTGTAGTTTTCATGTGAAGTTATTTC
        131 TCTCAGAATGCTTCTGTGTAGTTTTTATGTGAAGATATTTC

                     *       *     *            *    *         **       *    
     124381 CTTTTCCACAATAGGCCGCAAAGGGCTCCAAATATCAACTTACAGATTCTAGGAAAAGAGAGTTT
          1 CTTTTCCACCATAGGCCTCAAAGCGCTCCAAATATCCACTTGCAGATTCTACAAAAAGAGTGTTT

                       * **      *     *                                     
     124446 CAAAACTGCTCTACGAAAAGATAGGTTGAACTCTGTGAGATGAATGCACACATCACAAAGAAGTT
         66 CAAAACTGCTCAATCAAAAGAAAGGTTCAACTCTGTGAGATGAATGCACACATCACAAAGAAGTT

                       *               * *           
     124511 TCTCAGAATGCATCTGTGTAGTTTTTACGGGAAGATATTTC
        131 TCTCAGAATGCTTCTGTGTAGTTTTTATGTGAAGATATTTC

                        ***  *     **      *  *           *      *     ***   
     124552 CTTTTCCACCATCTTCCACAAAGGTCTCCAAGTAACCACTTGCAGACTCTACAGAAAGACACTTT
          1 CTTTTCCACCATAGGCCTCAAAGCGCTCCAAATATCCACTTGCAGATTCTACAAAAAGAGTGTTT

            *          *         ***      *      ***                      *  
     124617 AAAAACTGCTCTATCAAAAGATCAGTTCAAGTCTGTGGTTTGAATGCACACATCACAAAGAATTT
         66 CAAAACTGCTCAATCAAAAGAAAGGTTCAACTCTGTGAGATGAATGCACACATCACAAAGAAGTT

                                     *  *            
     124682 TCTCAGAATGCTTCTGTGTAGTTTTCATATGAAGATATTTC
        131 TCTCAGAATGCTTCTGTGTAGTTTTTATGTGAAGATATTTC

                                    *                                   **   
     124723 CTTTTCCACCATAGGCCTCAAAGCACTCCAAATATCCACTTGCAGATTCTACAAAAAGAGATTTT
          1 CTTTTCCACCATAGGCCTCAAAGCGCTCCAAATATCCACTTGCAGATTCTACAAAAAGAGTGTTT

                   **                           *  *          *        *     
     124788 CAAAACTAGTCAATCAAAAGAAAGGTTCAACTCTGTCAGTTGAATGCACATATCACAAACAAGTT
         66 CAAAACTGCTCAATCAAAAGAAAGGTTCAACTCTGTGAGATGAATGCACACATCACAAAGAAGTT

                *      *                             
     124853 TCTCGGAATGCGTCTGTGTAGTTTTTATGTGAAGATATTTC
        131 TCTCAGAATGCTTCTGTGTAGTTTTTATGTGAAGATATTTC

               *     * *           *     *                  *    *           
     124894 CTTCTCCACAACAGGCCTCAAAGTGCTCCGAATATCCACTTGCAGATTTTAC-TAAAGAGTGTTT
          1 CTTTTCCACCATAGGCCTCAAAGCGCTCCAAATATCCACTTGCAGATTCTACAAAAAGAGTGTTT

             *               *  *   *     *        *    *               *    
     124958 CCAAACTGCTCAATCAAGAGGAAGTTTCAAGTCTGTGAGCTGAACGCACACATCACAAAGTAGTT
         66 CAAAACTGCTCAATCAAAAGAAAGGTTCAACTCTGTGAGATGAATGCACACATCACAAAGAAGTT

               *                                *    
     125023 TCTGAGAATGCTTCTGTGTAGTTTTTATGTGAAGATGTTTC
        131 TCTCAGAATGCTTCTGTGTAGTTTTTATGTGAAGATATTTC

                            **     *                                    *    
     125064 CTTTTCCACCATAGGCTGCAAAGGGCTCCAAATATCCACTTGCAGATTCTACAAAAAGAGAGTTT
          1 CTTTTCCACCATAGGCCTCAAAGCGCTCCAAATATCCACTTGCAGATTCTACAAAAAGAGTGTTT

                 *     *         *          *     *                     *    
     125129 CAAAAGTGCTCTATCAAAAGATAGGTTCAACTATGTGATATGAATGCACACATCACAAAGTAGTT
         66 CAAAACTGCTCAATCAAAAGAAAGGTTCAACTCTGTGAGATGAATGCACACATCACAAAGAAGTT

                                          *          
     125194 TCTCAGAATGCTTCTGTGTAGTTTTTATGTAAAGATATTTC
        131 TCTCAGAATGCTTCTGTGTAGTTTTTATGTGAAGATATTTC

                                    *                                   **   
     125235 CTTTTCCACCATAGGCCTCAAAGCACTCCAAATATCCACTTGCAGATTCTACAAAAAGAGATTTT
          1 CTTTTCCACCATAGGCCTCAAAGCGCTCCAAATATCCACTTGCAGATTCTACAAAAAGAGTGTTT

                   ** *                   *     *  *    * *   *        *     
     125300 CAAAACTATTTAATCAAAAGAAAGGTTCAAATCTGTCAGTTGAAGGTACATATCACAAACAAGTT
         66 CAAAACTGCTCAATCAAAAGAAAGGTTCAACTCTGTGAGATGAATGCACACATCACAAAGAAGTT

             * **                                    
     125365 TATTGGAATGCTTCTGTGTAGTTTTTATGTGAAGATATTTC
        131 TCTCAGAATGCTTCTGTGTAGTTTTTATGTGAAGATATTTC

                     * *         * *                        **  *     *      
     125406 CTTTTCCACAACAGGCCTCAAGGTGCTCCAAATATCCACTTGCAGATTTCACTAAAAGTGTGTTT
          1 CTTTTCCACCATAGGCCTCAAAGCGCTCCAAATATCCACTTGCAGATTCTACAAAAAGAGTGTTT

             *  *            *  *   *     *        *             *           
     125471 CCAAGCTGCTCAATCAAGAGGAAGTTTCAAGTCTGTGAGGTGAATGCACACATTACAAAGAAGTT
         66 CAAAACTGCTCAATCAAAAGAAAGGTTCAACTCTGTGAGATGAATGCACACATCACAAAGAAGTT

            *  *                                     
     125536 ACTGAGAATGCTTCTGTGTAGTTTTTATGTGAAGATATTTC
        131 TCTCAGAATGCTTCTGTGTAGTTTTTATGTGAAGATATTTC

                      **               *                                *    
     125577 CTTTTCCACCGCAGGCCTCAAAGCGCTGCAAATATCCACTTGCAGATTCTACAAAAAGAGAGTTT
          1 CTTTTCCACCATAGGCCTCAAAGCGCTCCAAATATCCACTTGCAGATTCTACAAAAAGAGTGTTT

                      **         *   *         *   *     **            *     
     125642 CAAAACTGCTGTATCAAAAGATAGGGTCAACTCTGCGAGTTGAATAAACACATCACAAATAAGTT
         66 CAAAACTGCTCAATCAAAAGAAAGGTTCAACTCTGTGAGATGAATGCACACATCACAAAGAAGTT

               **   *        *               *       
     125707 TCTGGGAACGCTTCTGTATAG-TTTTATGTGAATATATTTC
        131 TCTCAGAATGCTTCTGTGTAGTTTTTATGTGAAGATATTTC

                         *          *                   *   *  *   * *   *   
     125747 CTTTTCCACCATATGCCTCAAAGCACTCCAAATATCCACTTGCACATTATAGAAACATAGTCTTT
          1 CTTTTCCACCATAGGCCTCAAAGCGCTCCAAATATCCACTTGCAGATTCTACAAAAAGAGTGTTT

                   **                        *         *             *       
     125812 CAAAACTTGTCAATC-AAAGAAAGGTTCAACTCCGTGAGATGAGTGCACACATCACAGAGAAGTT
         66 CAAAACTGCTCAATCAAAAGAAAGGTTCAACTCTGTGAGATGAATGCACACATCACAAAGAAGTT

                *     *                            * 
     125876 TCTCGGAATGTTTCTGTGTAGTTTTTATGTGAAGATATTGC
        131 TCTCAGAATGCTTCTGTGTAGTTTTTATGTGAAGATATTTC

                     *               *            *          *       *   *   
     125917 CTTTTCCACAATAGGCCTCAAAGCGTTCCAAATATCCAATTGCAGATTCCACAAAAAAAGTTTTT
          1 CTTTTCCACCATAGGCCTCAAAGCGCTCCAAATATCCACTTGCAGATTCTACAAAAAGAGTGTTT

            *                 *  *  *  *             * *         **    *     
     125982 TAAAACTGCTCAATCAAATGATAGATTAAACTCTGTGAGATTAGTGCACACATGTCAAAAAAGTT
         66 CAAAACTGCTCAATCAAAAGAAAGGTTCAACTCTGTGAGATGAATGCACACATCACAAAGAAGTT

                                *      * *           
     126047 TCTCAGAATGCTTCTGTGTACTTTTTAGGGGAAGATATTTC
        131 TCTCAGAATGCTTCTGTGTAGTTTTTATGTGAAGATATTTC

                        *    *     **         *    *           **  * *  *    
     126088 CTTTTCCACCATCGGCCACAAAGGACTCCAAATAACCACATGCAGATTCTAGTAACACAGAGTTT
          1 CTTTTCCACCATAGGCCTCAAAGCGCTCCAAATATCCACTTGCAGATTCTACAAAAAGAGTGTTT

                       *         * *           *   * * *    **    ***        
     126153 CAAAACTGCTCTATCAAAAGATAAGTTCAACTCTGAGAGTTTAGTGCAACCATCGTGAAGAAGTT
         66 CAAAACTGCTCAATCAAAAGAAAGGTTCAACTCTGTGAGATGAATGCACACATCACAAAGAAGTT

                            *     *                  
     126218 TCTCAGAATGCTTCTGAGTAGTGTTTATGTGAAGATATTTC
        131 TCTCAGAATGCTTCTGTGTAGTTTTTATGTGAAGATATTTC

                              *     *                      *           **    
     126259 CTTTTCCACCATAGGCCTGAAAGCCCTCCAAATATCCACTTGCAGATCCTACAAAAAGAAAGTTT
          1 CTTTTCCACCATAGGCCTCAAAGCGCTCCAAATATCCACTTGCAGATTCTACAAAAAGAGTGTTT

             *         **     *  *  *   *        **      *                   
     126324 CGAAA-TGCTCTCTCAAACGATAGTTTCGACTCTGTGGTATGAATACACACATCACAAAGAAGTT
         66 CAAAACTGCTCAATCAAAAGAAAGGTTCAACTCTGTGAGATGAATGCACACATCACAAAGAAGTT

                                       **            
     126388 TCTCAGAATGCTTCTGTGTAGTTTTTAAATGAAGATATTTC
        131 TCTCAGAATGCTTCTGTGTAGTTTTTATGTGAAGATATTTC

            *                       *          *     *                       
     126429 TTTTTCCACCATAGGCCTCAAAGCACTCCAAATATGCACTTCCAGATTCTACAAAAAGAGTGTTT
          1 CTTTTCCACCATAGGCCTCAAAGCGCTCCAAATATCCACTTGCAGATTCTACAAAAAGAGTGTTT

              *                 *       * *         **   *         *  * *    
     126494 CAGAACTGCTCAATCAAAAGGAAGGTTCCAGTC--TGAGACAAATACACACATCAAAAGGTAGTT
         66 CAAAACTGCTCAATCAAAAGAAAGGTTCAACTCTGTGAGATGAATGCACACATCACAAAGAAGTT

                                                    *
     126557 TCTCAGAATGCTTCTGTGTAGTTTTTATGTGAAGATATTTT
        131 TCTCAGAATGCTTCTGTGTAGTTTTTATGTGAAGATATTTC

             *               *    **    *     *      *  **   *          *    
     126598 CCTTTCCACCATAGGCCACAAATGGCTCTAAATACCCACTTACATTTTCCACAAAAAGAGAGTTT
          1 CTTTTCCACCATAGGCCTCAAAGCGCTCCAAATATCCACTTGCAGATTCTACAAAAAGAGTGTTT

                       * *    * *      *   *         *                       
     126663 CAAAACTGCTCTACCAAAGGTAA-GTTTAACGCTGTGAG-TTAA-G-A-ACATCACAAAGAAGTT
         66 CAAAACTGCTCAATCAAAAGAAAGGTTCAACTCTGTGAGATGAATGCACACATCACAAAGAAGTT

                                   *   *  *          
     126723 TCTCAGAATGCTTCTGTGTAGTTCTTACGTAAAGATATTTC
        131 TCTCAGAATGCTTCTGTGTAGTTTTTATGTGAAGATATTTC

                 *   *      ***    *                  *              **      
     126764 CTTTTACACAATAGGCAGAAAAGTGCTCCAAATATCCACTTGAAGATTCTACAAAAACCGTGTTT
          1 CTTTTCCACCATAGGCCTCAAAGCGCTCCAAATATCCACTTGCAGATTCTACAAAAAGAGTGTTT

                     **                                          *      *    
     126829 CAAAACTGCCGAATCAAAAGAAAGGTTCAACTCTGTGAGATGAATGCACACATAACAAAGGAGTT
         66 CAAAACTGCTCAATCAAAAGAAAGGTTCAACTCTGTGAGATGAATGCACACATCACAAAGAAGTT

                                 *      *      *    *
     126894 TCTCAGAATGCTTCTGTGTAGCTTTTATATGAAGACATTTA
        131 TCTCAGAATGCTTCTGTGTAGTTTTTATGTGAAGATATTTC

            *        * *            *   ***                     **        *  
     126935 GTTTTCCACAACAGGCCTCAAAGCTCTCTCCATATCCACTTGCAGATTCTACCGAAAGAGTGCTT
          1 CTTTTCCACCATAGGCCTCAAAGCGCTCCAAATATCCACTTGCAGATTCTACAAAAAGAGTGTTT

             *                   * **     *        *        *     **         
     127000 CCAAACTGCTCAATCAAAAGAGACATTCAAATCTGTGAGGTGAATGCAGACATCGTAAAGAAGTT
         66 CAAAACTGCTCAATCAAAAGAAAGGTTCAACTCTGTGAGATGAATGCACACATCACAAAGAAGTT

                                *     *       *    **
     127065 TCTCAGAATGCTTCTGTGTATTTTTTGTGTGAAGTTATTCG
        131 TCTCAGAATGCTTCTGTGTAGTTTTTATGTGAAGATATTTC

            *    *             *     *  *            **                 *    
     127106 TTTTTGCACCATAGGCCTCCAAGCGTTCTAAATATCCACTTCTAGATTCTACAAAAAGAGAGTTT
          1 CTTTTCCACCATAGGCCTCAAAGCGCTCCAAATATCCACTTGCAGATTCTACAAAAAGAGTGTTT

                   *         *            *        *    *   *                
     127171 CAAAACTACTC-A--AACA-AAAGGTTCAATTCTGTGAGTTGAAAGCAAACATCACAAAGAAGTT
         66 CAAAACTGCTCAATCAAAAGAAAGGTTCAACTCTGTGAGATGAATGCACACATCACAAAGAAGTT

                       *                             
     127232 TCTCAGAATGCGTCTGTGTAGTTTTTATGTGAAGATATTTC
        131 TCTCAGAATGCTTCTGTGTAGTTTTTATGTGAAGATATTTC

                     *    ****     *                  *                      
     127273 CTTTT-CACAATAGAATGCAAAGGGCTCCAAATATCCACTTGGAGATTCTACAAAAAGA--GTTT
          1 CTTTTCCACCATAGGCCTCAAAGCGCTCCAAATATCCACTTGCAGATTCTACAAAAAGAGTGTTT

                  *    **     *  *     *     ***   *     *               *   
     127335 CAAAACCGCTCTGTCAAATGATAGGTTGAACTCCCGGAGGTGAATACACACATCACAAAGAGGTT
         66 CAAAACTGCTCAATCAAAAGAAAGGTTCAACTCTGTGAGATGAATGCACACATCACAAAGAAGTT

                  *                       *  *       
     127400 TCTCAGCATGCTTCTGTGTAGTTTTTATGTAAACATATTTC
        131 TCTCAGAATGCTTCTGTGTAGTTTTTATGTGAAGATATTTC

             *    * *              *           *      * *       *   *    *   
     127441 CGTTTCTATCATAGGCCTCAAAGTGCTCCAAATATTCACTTGTACATTCTACCAAACGAGTATTT
          1 CTTTTCCACCATAGGCCTCAAAGCGCTCCAAATATCCACTTGCAGATTCTACAAAAAGAGTGTTT

                              * *         ** *    *        *            *    
     127506 CAAAACTGCTCAATCAAATGGAAGGTTCAAAACCGTGACATGAATGCCCACATCACAAAGTAGTT
         66 CAAAACTGCTCAATCAAAAGAAAGGTTCAACTCTGTGAGATGAATGCACACATCACAAAGAAGTT

                                                     
     127571 TCTCAGAATGCTTCTGTGTAGTTTTTATGTGAAGATATTTC
        131 TCTCAGAATGCTTCTGTGTAGTTTTTATGTGAAGATATTTC

                     * *    *      *    *       * *   **      *              
     127612 CTTTTCCACAACA-GCGTGCAAAACGCTTCAAATATGCCCTTAGAGATTCCACAAAAAGAGTGTT
          1 CTTTTCCACCATAGGCCT-CAAAGCGCTCCAAATATCCACTTGCAGATTCTACAAAAAGAGTGTT

              *     *               *  *                                  ** 
     127676 TCCAAACTACTCAAATCAAAA-AATGATTTCAACTCTGTGAGATGAATGCACACATCACAAACTA
         65 TCAAAACTGCTC-AATCAAAAGAAAG-GTTCAACTCTGTGAGATGAATGCACACATCACAAAGAA

                         *     ** *   * *   *       *   
     127740 GTTTCTCAGAATGTTTCTGCCTGGTTCTCATGCGAAGATAGTTC
        128 GTTTCTCAGAATGCTTCTGTGTAGTTTTTATGTGAAGATATTTC

                 *           *   * **              *                  * *    
     127784 CTTTTTCACCATAGGCCGCAATGTACTCCAAATATCCACCTGCAGATTCTAC-AAAAGTGAGTTT
          1 CTTTTCCACCATAGGCCTCAAAGCGCTCCAAATATCCACTTGCAGATTCTACAAAAAGAGTGTTT

                       *         ***    **         *        *      *       * 
     127848 CAAAACTGCTCTATCAAAAGATCAGTTCGTCTCTGTGAGTTGAATGCATACATCAAAAAGAAGCT
         66 CAAAACTGCTCAATCAAAAGAAAGGTTCAACTCTGTGAGATGAATGCACACATCACAAAGAAGTT

                 *             *      **         *   
     127913 TCTCAAAATGCTTCTGTGTGGTTTTTCGGTGAAGATAGTTC
        131 TCTCAGAATGCTTCTGTGTAGTTTTTATGTGAAGATATTTC

            *     *        *      * *                 *        *        *    
     127954 TTTTTCTACCATAGGTCTCAAACCACTCCAAATATCCACTTGTAGATTCTATAAAAAGGAATG-T
          1 CTTTTCCACCATAGGCCTCAAAGCGCTCCAAATATCCACTTGCAGATTCTACAAAAA-GAGTGTT

                  *        *    *    *      * *         *      *             
     128018 TCAAAATTGCTCAATAAAAATAAAGTTTCAACACCGTGAGATGAGTGCACAAATCACAAAGAAGT
         65 TCAAAACTGCTCAATCAAAAGAAAGGTTCAACTCTGTGAGATGAATGCACACATCACAAAGAAGT

                  *          *         *          *   
     128083 TTCTCAAAATGCTTCTGGGTAGTTTTTCTGTGAAGATAGTTC
        130 TTCTCAGAATGCTTCTGTGTAGTTTTTATGTGAAGATATTTC

                  *     *    *     *          *                         *    
     128125 CTTTTCTACCATGGGCCACAAAGGGCTCCAAATACCCACTTGCAGATTCTACAAAAAGAGAGTTT
          1 CTTTTCCACCATAGGCCTCAAAGCGCTCCAAATATCCACTTGCAGATTCTACAAAAAGAGTGTTT

              *        *      ** * *        *    * *    **  * *           *  
     128190 CACAACTGCTCTATCAAACAATATGTTCAACTTTGTGGGTTGAACACAAATATCAC-AAGAATTT
         66 CAAAACTGCTCAATCAAAAGAAAGGTTCAACTCTGTGAGATGAATGCACACATCACAAAGAAGTT

                **                             *     
     128254 TCTCCCAATGCTTCTGTGTAGTTTTTATGTGAAGACATTTC
        131 TCTCAGAATGCTTCTGTGTAGTTTTTATGTGAAGATATTTC

            *   *  *      *  *     *                 *  *      *       *  *  
     128295 TTTTCCCTCCATAGTCCACAAAGTGCTCCAAATATCCACTTACATATTCTAGAAAAAGATTGCTT
          1 CTTTTCCACCATAGGCCTCAAAGCGCTCCAAATATCCACTTGCAGATTCTACAAAAAGAGTGTTT

            **       *    *               * * *                              
     128360 GGAAACTGCACAATGAAAAGAAAGGTTCAAATATATGAGATGAATGCACACATCACAAAGAAGTT
         66 CAAAACTGCTCAATCAAAAGAAAGGTTCAACTCTGTGAGATGAATGCACACATCACAAAGAAGTT

                                 *                    
     128425 TCTCAGAAT-CTCTCTGTGTAATTTTTATGTGAAGATATTTC
        131 TCTCAGAATGCT-TCTGTGTAGTTTTTATGTGAAGATATTTC

                *     *    *  *   *                        *      *    *     
     128466 CTTTCCCACCTTAGGTCTTAAAACGCTCCAAATATCCACTTGCAGATACTACAAGAAGATTGTTT
          1 CTTTTCCACCATAGGCCTCAAAGCGCTCCAAATATCCACTTGCAGATTCTACAAAAAGAGTGTTT

                     *    *        *      *     **                           
     128531 CAAAACTGCACAA-AAAAAGAAATGTTCAATTCTGTTTGATGAATGCACACATCACAAAGAAGTT
         66 CAAAACTGCTCAATCAAAAGAAAGGTTCAACTCTGTGAGATGAATGCACACATCACAAAGAAGTT

                           *                         
     128595 TCTCAGAATGCTTCTCTGTAGTTTTTATGTGAAGATATTTC
        131 TCTCAGAATGCTTCTGTGTAGTTTTTATGTGAAGATATTTC

                     *             *                 *         **      * *   
     128636 CTTTTCCACAATAGGCCTCAAAGGGCTCCAAATATCCACTTCCAGATTCTATGAAAAGAATATTT
          1 CTTTTCCACCATAGGCCTCAAAGCGCTCCAAATATCCACTTGCAGATTCTACAAAAAGAGTGTTT

             *              * *    *                                      *  
     128701 CCAAACTGCTCAATCATAGGAAATGTTCAACTCTGTGAGATGAATGCACACATCAC-AAGAAATT
         66 CAAAACTGCTCAATCAAAAGAAAGGTTCAACTCTGTGAGATGAATGCACACATCACAAAGAAGTT

                     *    *      *       *       *   
     128765 TCTCAGAATCCTTCAGTGTAGGTTTTATGAGAAGATAATTC
        131 TCTCAGAATGCTTCTGTGTAGTTTTTATGTGAAGATATTTC

                     *    **        *   *                           *    *   
     128806 CTTTTCCACAATAGTTCTCAAAGCACTCAAAATATCCACTTGCAGATTCTACAAAAGGAGTATTT
          1 CTTTTCCACCATAGGCCTCAAAGCGCTCCAAATATCCACTTGCAGATTCTACAAAAAGAGTGTTT

                                                 *        *                  
     128871 CAAAACTGCTCAATCAAAAGAAAGGTTCAACTCTGTGGGATGAATGGACACATCACAAAGAAGTT
         66 CAAAACTGCTCAATCAAAAGAAAGGTTCAACTCTGTGAGATGAATGCACACATCACAAAGAAGTT

                                  *   *              
     128936 TCTCAGAATGCTTCTGTGTAGTATTTTTGTGAAGATATTT-
        131 TCTCAGAATGCTTCTGTGTAGTTTTTATGTGAAGATATTTC

                          *  * * * * ***             *            *     * *  
     128976 CTTTTCCACCATAGACCGCCAGGGGACACAAATATCCACTTTCAGATTCTACAACAAGAGAGGTT
          1 CTTTTCCACCATAGGCCTCAAAGCGCTCCAAATATCCACTTGCAGATTCTACAAAAAGAGTGTTT

                   *   *     *   ** *       *      *                      * *
     129041 CAAAACTACTCGATCAAGAGATGGTTTCAACTATGTGAGTTGAATGCACACATCACAAAGAACTA
         66 CAAAACTGCTCAATCAAAAGAAAGGTTCAACTCTGTGAGATGAATGCACACATCACAAAGAAGTT

             *  *    *                               
     129106 TGTCGGAATTCTTCTGTGTAGTTTTTATGTGAAGATATTTC
        131 TCTCAGAATGCTTCTGTGTAGTTTTTATGTGAAGATATTTC

                     *    * *      * *    *                  *               
     129147 CTTTTCCACAATAGACGTCAAAGTGATCCAGATATCCACTTGCAGATTCCACAAAAAGAGTGTTT
          1 CTTTTCCACCATAGGCCTCAAAGCGCTCCAAATATCCACTTGCAGATTCTACAAAAAGAGTGTTT

                 *   *   *                  **                     *  *      
     129212 CAAAAGTGCACAACCAAAAGAAAGGTTCAACTAGGTGAGATGAATGCACACATCAGAAGGAAGTT
         66 CAAAACTGCTCAATCAAAAGAAAGGTTCAACTCTGTGAGATGAATGCACACATCACAAAGAAGTT

                            **   *     * *       *   
     129277 TCTCAGAATGCTTCTGCATAGCTTTTAAGGGAAGATACTTC
        131 TCTCAGAATGCTTCTGTGTAGTTTTTATGTGAAGATATTTC

                    *               *            * *  *    * *               
     129318 CTTTTCCAACATAGGCCTCAAAGCACTCCAAATATCCTCCTGGAGATACCACAAAAAGAGTGTTT
          1 CTTTTCCACCATAGGCCTCAAAGCGCTCCAAATATCCACTTGCAGATTCTACAAAAAGAGTGTTT

            **                      *  *                 *       *           
     129383 GCAAACTGCTCAATCAAAAGAAAGATTTAACTCTGTGAGATGAATCCACACATGACAAAGAAGTT
         66 CAAAACTGCTCAATCAAAAGAAAGGTTCAACTCTGTGAGATGAATGCACACATCACAAAGAAGTT

                                                     
     129448 TCTCAGAATGCTTCTGTGTAGTTTTTATGTGAAGATATTTC
        131 TCTCAGAATGCTTCTGTGTAGTTTTTATGTGAAGATATTTC

                     *   * * *    **           *                *     *      
     129489 CTTTTCCACAATAAGACCCAAAAGGCTCCAAATATTCACTTGCAGATTCTAAAAAAAACAGTGTT
          1 CTTTTCCACCATAGGCCTCAAAGCGCTCCAAATATCCACTTGCAGATTCT-ACAAAAAGAGTGTT

                                  *                  *      *        **      
     129554 TCAAAACTGCTCAATCAAAAGATA-GTTCAACTCTGTGAGAAGAATGCTCACATCACTGAGAAGT
         65 TCAAAACTGCTCAATCAAAAGAAAGGTTCAACTCTGTGAGATGAATGCACACATCACAAAGAAGT

                                         *            
     129618 TTCTCAGAATGCTTCTGTGTAGTTTTTATATGAAGATATTTC
        130 TTCTCAGAATGCTTCTGTGTAGTTTTTATGTGAAGATATTTC

                *     *      *    **                       *   **       *    
     129660 CTTTCCCACCGTAGGCCACAAAAGGCTCCAAATATCCACTTGCAGATACTATGAAAAGAGAGTTT
          1 CTTTTCCACCATAGGCCTCAAAGCGCTCCAAATATCCACTTGCAGATTCTACAAAAAGAGTGTTT

                        *        *               ***            *            
     129725 CAAAACTGCTCATTCAAAAGATAGGTTCAACTCTGTGGTTTGAATGCACACAGCACAAAGAAGTT
         66 CAAAACTGCTCAATCAAAAGAAAGGTTCAACTCTGTGAGATGAATGCACACATCACAAAGAAGTT

              *       **                   **   *    
     129790 TCACAGAATGTGTCTGTGTAGTTTTTATGTGCGGATGTTTC
        131 TCTCAGAATGCTTCTGTGTAGTTTTTATGTGAAGATATTTC

                         *        ****   *   *                    *          
     129831 CTTTTCCACCATATGCCT-AAATATTTCCCAATTTCCACTTGCAGATTCTACAAGAAGAGTGTTT
          1 CTTTTCCACCATAGGCCTCAAAGCGCTCCAAATATCCACTTGCAGATTCTACAAAAAGAGTGTTT

                      **       *       *           *            *      ***   
     129895 CAAAACTGCTGTATC-AAATAAA-GTTGAACTCTGTGAGGTGAATGCACACAGCACAAAATGGTT
         66 CAAAACTGCTCAATCAAAAGAAAGGTTCAACTCTGTGAGATGAATGCACACATCACAAAGAAGTT

                          **   *        *       *    
     129958 TCTCAGAATGCTTCCTTGTTGTTTTTATATGAAGATGTTTC
        131 TCTCAGAATGCTTCTGTGTAGTTTTTATGTGAAGATATTTC

                  *  *             *   *     *                  *            
     129999 CTTTTCAACAATAGGCCTCAAAGTGCTTCAAATGTCCACTTGCAGATTCTACCAAAAGAGTGTTT
          1 CTTTTCCACCATAGGCCTCAAAGCGCTCCAAATATCCACTTGCAGATTCTACAAAAAGAGTGTTT

                                        *      *  *  *                       
     130064 CAAAACTGCTCAATCAAAAGAAAGGTTCGACTCTGGGAAATTAATGCACACATCACAAAGAAGTT
         66 CAAAACTGCTCAATCAAAAGAAAGGTTCAACTCTGTGAGATGAATGCACACATCACAAAGAAGTT

                                     *        *      
     130129 TCTC---A-GCTTCTGTGTAGTTTTCATGTGAAGTTATTTC
        131 TCTCAGAATGCTTCTGTGTAGTTTTTATGTGAAGATATTTC

                     *       *     *            *    *         **       *    
     130166 CTTTTCCACAATAGGCCGCAAAGGGCTCCAAATATCAACTTACAGATTCTAGGAAAAGAGAGTTT
          1 CTTTTCCACCATAGGCCTCAAAGCGCTCCAAATATCCACTTGCAGATTCTACAAAAAGAGTGTTT

                       * **      *     *                                     
     130231 CAAAACTGCTCTACGAAAAGATAGGTTGAACTCTGTGAGATGAATGCACACATCACAAAGAAGTT
         66 CAAAACTGCTCAATCAAAAGAAAGGTTCAACTCTGTGAGATGAATGCACACATCACAAAGAAGTT

                       *               * *     *     
     130296 TCTCAGAATGCATCTGTGTAGTTTTTACGGGAAGACATTTC
        131 TCTCAGAATGCTTCTGTGTAGTTTTTATGTGAAGATATTTC

                        ***  *     **      *  *                  *     ***   
     130337 CTTTTCCACCATCTTCCACAAAGGTCTCCAAGTAACCACTTGCAGATTCTACAGAAAGACACTTT
          1 CTTTTCCACCATAGGCCTCAAAGCGCTCCAAATATCCACTTGCAGATTCTACAAAAAGAGTGTTT

            *          *         ***      *      ***                      *  
     130402 AAAAACTGCTCTATCAAAAGATCAGTTCAAGTCTGTGGTTTGAATGCACACATCACAAAGAATTT
         66 CAAAACTGCTCAATCAAAAGAAAGGTTCAACTCTGTGAGATGAATGCACACATCACAAAGAAGTT

                                     *  *            
     130467 TCTCAGAATGCTTCTGTGTAGTTTTCATATGAAGATATTTC
        131 TCTCAGAATGCTTCTGTGTAGTTTTTATGTGAAGATATTTC

                                    *                                   **   
     130508 CTTTTCCACCATAGGCCTCAAAGCACTCCAAATATCCACTTGCAGATTCTACAAAAAGAGATTTT
          1 CTTTTCCACCATAGGCCTCAAAGCGCTCCAAATATCCACTTGCAGATTCTACAAAAAGAGTGTTT

                   **                           *  *          *        *     
     130573 CAAAACTAGTCAATCAAAAGAAAGGTTCAACTCTGTCAGTTGAATGCACATATCACAAACAAGTT
         66 CAAAACTGCTCAATCAAAAGAAAGGTTCAACTCTGTGAGATGAATGCACACATCACAAAGAAGTT

                *      *                             
     130638 TCTCGGAATGCGTCTGTGTAGTTTTTATGTGAAGATATTTC
        131 TCTCAGAATGCTTCTGTGTAGTTTTTATGTGAAGATATTTC

               *     * *           *     *                  *    *           
     130679 CTTCTCCACAACAGGCCTCAAAGTGCTCCGAATATCCACTTGCAGATTTTAC-TAAAGAGTGTTT
          1 CTTTTCCACCATAGGCCTCAAAGCGCTCCAAATATCCACTTGCAGATTCTACAAAAAGAGTGTTT

             *               *  *   *     *        *    *               *    
     130743 CCAAACTGCTCAATCAAGAGGAAGTTTCAAGTCTGTGAGCTGAACGCACACATCACAAAGTAGTT
         66 CAAAACTGCTCAATCAAAAGAAAGGTTCAACTCTGTGAGATGAATGCACACATCACAAAGAAGTT

               *                                *    
     130808 TCTGAGAATGCTTCTGTGTAGTTTTTATGTGAAGATGTTTC
        131 TCTCAGAATGCTTCTGTGTAGTTTTTATGTGAAGATATTTC

                            **     *                                    *    
     130849 CTTTTCCACCATAGGCTGCAAAGGGCTCCAAATATCCACTTGCAGATTCTACAAAAAGAGAGTTT
          1 CTTTTCCACCATAGGCCTCAAAGCGCTCCAAATATCCACTTGCAGATTCTACAAAAAGAGTGTTT

                 *     *         *          *     *                     *    
     130914 CAAAAGTGCTCTATCAAAAGATAGGTTCAACTATGTGATATGAATGCACACATCACAAAGTAGTT
         66 CAAAACTGCTCAATCAAAAGAAAGGTTCAACTCTGTGAGATGAATGCACACATCACAAAGAAGTT

                                          *          
     130979 TCTCAGAATGCTTCTGTGTAGTTTTTATGTAAAGATATTTC
        131 TCTCAGAATGCTTCTGTGTAGTTTTTATGTGAAGATATTTC

                                    *                                   **   
     131020 CTTTTCCACCATAGGCCTCAAAGCACTCCAAATATCCACTTGCAGATTCTACAAAAAGAGATTTT
          1 CTTTTCCACCATAGGCCTCAAAGCGCTCCAAATATCCACTTGCAGATTCTACAAAAAGAGTGTTT

                   ** *                   *     *  *    * *   *        *     
     131085 CAAAACTATTTAATCAAAAGAAAGGTTCAAATCTGTCAGTTGAAGGTACATATCACAAACAAGTT
         66 CAAAACTGCTCAATCAAAAGAAAGGTTCAACTCTGTGAGATGAATGCACACATCACAAAGAAGTT

             * **                                    
     131150 TATTGGAATGCTTCTGTGTAGTTTTTATGTGAAGATATTTC
        131 TCTCAGAATGCTTCTGTGTAGTTTTTATGTGAAGATATTTC

                     * *         * *                        **  *     *      
     131191 CTTTTCCACAACAGGCCTCAAGGTGCTCCAAATATCCACTTGCAGATTTCACTAAAAGTGTGTTT
          1 CTTTTCCACCATAGGCCTCAAAGCGCTCCAAATATCCACTTGCAGATTCTACAAAAAGAGTGTTT

             *  *            *  *   *     *        *             *           
     131256 CCAAGCTGCTCAATCAAGAGGAAGTTTCAAGTCTGTGAGGTGAATGCACACATTACAAAGAAGTT
         66 CAAAACTGCTCAATCAAAAGAAAGGTTCAACTCTGTGAGATGAATGCACACATCACAAAGAAGTT

            *  *                                     
     131321 ACTGAGAATGCTTCTGTGTAGTTTTTATGTGAAGATATTTC
        131 TCTCAGAATGCTTCTGTGTAGTTTTTATGTGAAGATATTTC

                      **               *                                *    
     131362 CTTTTCCACCGCAGGCCTCAAAGCGCTGCAAATATCCACTTGCAGATTCTACAAAAAGAGAGTTT
          1 CTTTTCCACCATAGGCCTCAAAGCGCTCCAAATATCCACTTGCAGATTCTACAAAAAGAGTGTTT

                      **         *   *         *   *     **            *     
     131427 CAAAACTGCTGTATCAAAAGATAGGGTCAACTCTGCGAGTTGAATAAACACATCACAAATAAGTT
         66 CAAAACTGCTCAATCAAAAGAAAGGTTCAACTCTGTGAGATGAATGCACACATCACAAAGAAGTT

               **   *     *  *               *       
     131492 TCTGGGAACGCTTCCGTATAG-TTTTATGTGAATATATTTC
        131 TCTCAGAATGCTTCTGTGTAGTTTTTATGTGAAGATATTTC

                         *          *                   *   *  *   * *   *   
     131532 CTTTTCCACCATATGCCTCAAAGCACTCCAAATATCCACTTGCACATTATAGAAACATAGTCTTT
          1 CTTTTCCACCATAGGCCTCAAAGCGCTCCAAATATCCACTTGCAGATTCTACAAAAAGAGTGTTT

                   **                        *         *             *       
     131597 CAAAACTTGTCAATC-AAAGAAAGGTTCAACTCCGTGAGATGAGTGCACACATCACAGAGAAGTT
         66 CAAAACTGCTCAATCAAAAGAAAGGTTCAACTCTGTGAGATGAATGCACACATCACAAAGAAGTT

                *     *                            * 
     131661 TCTCGGAATGTTTCTGTGTAGTTTTTATGTGAAGATATTGC
        131 TCTCAGAATGCTTCTGTGTAGTTTTTATGTGAAGATATTTC

                     *               *            *          *       *   *   
     131702 CTTTTCCACAATAGGCCTCAAAGCGTTCCAAATATCCAATTGCAGATTCCACAAAAAAAGTTTTT
          1 CTTTTCCACCATAGGCCTCAAAGCGCTCCAAATATCCACTTGCAGATTCTACAAAAAGAGTGTTT

            *                 *  *  *  *             * *         **    *     
     131767 TAAAACTGCTCAATCAAATGATAGATTAAACTCTGTGAGATTAGTGCACACATGTCAAAAAAGTT
         66 CAAAACTGCTCAATCAAAAGAAAGGTTCAACTCTGTGAGATGAATGCACACATCACAAAGAAGTT

                                *      * *           
     131832 TCTCAGAATGCTTCTGTGTACTTTTTAGGGGAAGATATTTC
        131 TCTCAGAATGCTTCTGTGTAGTTTTTATGTGAAGATATTTC

                        *    *     **         *    *           **  * *  *    
     131873 CTTTTCCACCATCGGCCACAAAGGACTCCAAATAACCACATGCAGATTCTAGTAACACAGAGTTT
          1 CTTTTCCACCATAGGCCTCAAAGCGCTCCAAATATCCACTTGCAGATTCTACAAAAAGAGTGTTT

                       *         * *           *   * * *    **    ***        
     131938 CAAAACTGCTCTATCAAAAGATAAGTTCAACTCTGAGAGTTTAGTGCAACCATCGTGAAGAAGTT
         66 CAAAACTGCTCAATCAAAAGAAAGGTTCAACTCTGTGAGATGAATGCACACATCACAAAGAAGTT

                            *     *                  
     132003 TCTCAGAATGCTTCTGAGTAGTGTTTATGTGAAGATATTTC
        131 TCTCAGAATGCTTCTGTGTAGTTTTTATGTGAAGATATTTC

                              *     *                      *           **    
     132044 CTTTTCCACCATAGGCCTGAAAGCCCTCCAAATATCCACTTGCAGATCCTACAAAAAGAAAGTTT
          1 CTTTTCCACCATAGGCCTCAAAGCGCTCCAAATATCCACTTGCAGATTCTACAAAAAGAGTGTTT

             *         **     *  *  *   *        **      *                   
     132109 CGAAA-TGCTCTCTCAAACGATAGTTTCGACTCTGTGGTATGAATACACACATCACAAAGAAGTT
         66 CAAAACTGCTCAATCAAAAGAAAGGTTCAACTCTGTGAGATGAATGCACACATCACAAAGAAGTT

                                       **            
     132173 TCTCAGAATGCTTCTGTGTAGTTTTTAAATGAAGATATTTC
        131 TCTCAGAATGCTTCTGTGTAGTTTTTATGTGAAGATATTTC

            *                       *          *     *                       
     132214 TTTTTCCACCATAGGCCTCAAAGCACTCCAAATATGCACTTCCAGATTCTACAAAAAGAGTGTTT
          1 CTTTTCCACCATAGGCCTCAAAGCGCTCCAAATATCCACTTGCAGATTCTACAAAAAGAGTGTTT

              *                 *       * *         **   *         *  * *    
     132279 CAGAACTGCTCAATCAAAAGGAAGGTTCCAGTC--TGAGACAAATACACACATCAAAAGGTAGTT
         66 CAAAACTGCTCAATCAAAAGAAAGGTTCAACTCTGTGAGATGAATGCACACATCACAAAGAAGTT

                                                    *
     132342 TCTCAGAATGCTTCTGTGTAGTTTTTATGTGAAGATATTTT
        131 TCTCAGAATGCTTCTGTGTAGTTTTTATGTGAAGATATTTC

             *               *    **    *     *      *  **   *          *    
     132383 CCTTTCCACCATAGGCCACAAATGGCTCTAAATACCCACTTACATTTTCCACAAAAAGAGAGTTT
          1 CTTTTCCACCATAGGCCTCAAAGCGCTCCAAATATCCACTTGCAGATTCTACAAAAAGAGTGTTT

                       * *    * *      *   *         *                       
     132448 CAAAACTGCTCTACCAAAGGTAA-GTTTAACGCTGTGAG-TTAA-G-A-ACATCACAAAGAAGTT
         66 CAAAACTGCTCAATCAAAAGAAAGGTTCAACTCTGTGAGATGAATGCACACATCACAAAGAAGTT

                                   *   *  *          
     132508 TCTCAGAATGCTTCTGTGTAGTTCTTACGTAAAGATATTTC
        131 TCTCAGAATGCTTCTGTGTAGTTTTTATGTGAAGATATTTC

                 *   *      ***    *                  *          *   **      
     132549 CTTTTACACAATAGGCAGAAAAGTGCTCCAAATATCCACTTGAAGATTCTACAGAAACCGTGTTT
          1 CTTTTCCACCATAGGCCTCAAAGCGCTCCAAATATCCACTTGCAGATTCTACAAAAAGAGTGTTT

                     **                                          *      *    
     132614 CAAAACTGCCGAATCAAAAGAAAGGTTCAACTCTGTGAGATGAATGCACACATAACAAAGGAGTT
         66 CAAAACTGCTCAATCAAAAGAAAGGTTCAACTCTGTGAGATGAATGCACACATCACAAAGAAGTT

                                 *      *      *    *
     132679 TCTCAGAATGCTTCTGTGTAGCTTTTATATGAAGACATTTA
        131 TCTCAGAATGCTTCTGTGTAGTTTTTATGTGAAGATATTTC

            *        * *            *   ***                     **        *  
     132720 GTTTTCCACAACAGGCCTCAAAGCTCTCTCCATATCCACTTGCAGATTCTACCGAAAGAGTGCTT
          1 CTTTTCCACCATAGGCCTCAAAGCGCTCCAAATATCCACTTGCAGATTCTACAAAAAGAGTGTTT

             *                   * **     *        *        *     **         
     132785 CCAAACTGCTCAATCAAAAGAGACATTCAAATCTGTGAGGTGAATGCAGACATCGTAAAGAAGTT
         66 CAAAACTGCTCAATCAAAAGAAAGGTTCAACTCTGTGAGATGAATGCACACATCACAAAGAAGTT

                                *     *       *    **
     132850 TCTCAGAATGCTTCTGTGTATTTTTTGTGTGAAGTTATTCG
        131 TCTCAGAATGCTTCTGTGTAGTTTTTATGTGAAGATATTTC

            *    *             *     *  *            **                 *    
     132891 TTTTTGCACCATAGGCCTCCAAGCGTTCTAAATATCCACTTCTAGATTCTACAAAAAGAGAGTTT
          1 CTTTTCCACCATAGGCCTCAAAGCGCTCCAAATATCCACTTGCAGATTCTACAAAAAGAGTGTTT

                   *         *            *        *    *   *                
     132956 CAAAACTACTC-A--AACA-AAAGGTTCAATTCTGTGAGTTGAAAGCAAACATCACAAAGAAGTT
         66 CAAAACTGCTCAATCAAAAGAAAGGTTCAACTCTGTGAGATGAATGCACACATCACAAAGAAGTT

                       *             *               
     133017 TCTCAGAATGCGTCTGTGTAGTTTTGATGTGAAGATATTTC
        131 TCTCAGAATGCTTCTGTGTAGTTTTTATGTGAAGATATTTC

                     **   ****     *                  *                      
     133058 CTTTT-CACAGTAGAATGCAAAGGGCTCCAAATATCCACTTGGAGATTCTACAAAAAGA--GTTT
          1 CTTTTCCACCATAGGCCTCAAAGCGCTCCAAATATCCACTTGCAGATTCTACAAAAAGAGTGTTT

                  *    **     *  *     *     ***   *     *               *   
     133120 CAAAACCGCTCTGTCAAATGATAGGTTGAACTCCCGGAGGTGAATACACACATCACAAAGAGGTT
         66 CAAAACTGCTCAATCAAAAGAAAGGTTCAACTCTGTGAGATGAATGCACACATCACAAAGAAGTT

                  *                       *  *       
     133185 TCTCAGCATGCTTCTGTGTAGTTTTTATGTAAACATATTTC
        131 TCTCAGAATGCTTCTGTGTAGTTTTTATGTGAAGATATTTC

             *    * *              *           *      * *       *   *    *   
     133226 CGTTTCTATCATAGGCCTCAAAGTGCTCCAAATATTCACTTGTACATTCTACCAAACGAGTATTT
          1 CTTTTCCACCATAGGCCTCAAAGCGCTCCAAATATCCACTTGCAGATTCTACAAAAAGAGTGTTT

                              * *         ** *    *        *            *    
     133291 CAAAACTGCTCAATCAAATGGAAGGTTCAAAACCGTGACATGAATGCCCACATCACAAAGTAGTT
         66 CAAAACTGCTCAATCAAAAGAAAGGTTCAACTCTGTGAGATGAATGCACACATCACAAAGAAGTT

                                                     
     133356 TCTCAGAATGCTTCTGTGTAGTTTTTATGTGAAGATATTTC
        131 TCTCAGAATGCTTCTGTGTAGTTTTTATGTGAAGATATTTC

                     * *    *      *    *       * *   **      *              
     133397 CTTTTCCACAACA-GCGTGCAAAACGCTTCAAATATGCCCTTAGAGATTCCACAAAAAGAGTGTT
          1 CTTTTCCACCATAGGCCT-CAAAGCGCTCCAAATATCCACTTGCAGATTCTACAAAAAGAGTGTT

              *     *               *  *                                  ** 
     133461 TCCAAACTACTCAAATCAAAA-AATGATTTCAACTCTGTGAGATGAATGCACACATCACAAACTA
         65 TCAAAACTGCTC-AATCAAAAGAAAG-GTTCAACTCTGTGAGATGAATGCACACATCACAAAGAA

                         *     ** *   * *   *       *   
     133525 GTTTCTCAGAATGTTTCTGCCTGGTTCTCATGCGAAGATAGTTC
        128 GTTTCTCAGAATGCTTCTGTGTAGTTTTTATGTGAAGATATTTC

                 *           *   * **              *                  * *    
     133569 CTTTTTCACCATAGGCCGCAATGTACTCCAAATATCCACCTGCAGATTCTAC-AAAAGTGAGTTT
          1 CTTTTCCACCATAGGCCTCAAAGCGCTCCAAATATCCACTTGCAGATTCTACAAAAAGAGTGTTT

                       *         ***    **         *        *      *       * 
     133633 CAAAACTGCTCTATCAAAAGATCAGTTCGTCTCTGTGAGTTGAATGCATACATCAAAAAGAAGCT
         66 CAAAACTGCTCAATCAAAAGAAAGGTTCAACTCTGTGAGATGAATGCACACATCACAAAGAAGTT

                 *             *      **         *   
     133698 TCTCAAAATGCTTCTGTGTGGTTTTTCGGTGAAGATAGTTC
        131 TCTCAGAATGCTTCTGTGTAGTTTTTATGTGAAGATATTTC

            *     *        *      * *                 *        *        *    
     133739 TTTTTCTACCATAGGTCTCAAACCACTCCAAATATCCACTTGTAGATTCTATAAAAAGGAATG-T
          1 CTTTTCCACCATAGGCCTCAAAGCGCTCCAAATATCCACTTGCAGATTCTACAAAAA-GAGTGTT

                  *        *    *    *      * *         *      *             
     133803 TCAAAATTGCTCAATAAAAATAAAGTTTCAACACCGTGAGATGAGTGCACAAATCACAAAGAAGT
         65 TCAAAACTGCTCAATCAAAAGAAAGGTTCAACTCTGTGAGATGAATGCACACATCACAAAGAAGT

                  *          *         *          *   
     133868 TTCTCAAAATGCTTCTGGGTAGTTTTTCTGTGAAGATAGTTC
        130 TTCTCAGAATGCTTCTGTGTAGTTTTTATGTGAAGATATTTC

                  *     *    *     *          *                         *    
     133910 CTTTTCTACCATGGGCCACAAAGGGCTCCAAATACCCACTTGCAGATTCTACAAAAAGAGAGTTT
          1 CTTTTCCACCATAGGCCTCAAAGCGCTCCAAATATCCACTTGCAGATTCTACAAAAAGAGTGTTT

              *        *      ** * *        *    * *    **  * *           *  
     133975 CACAACTGCTCTATCAAACAATATGTTCAACTTTGTGGGTTGAACACAAATATCAC-AAGAATTT
         66 CAAAACTGCTCAATCAAAAGAAAGGTTCAACTCTGTGAGATGAATGCACACATCACAAAGAAGTT

                **                             *     
     134039 TCTCCCAATGCTTCTGTGTAGTTTTTATGTGAAGACATTTC
        131 TCTCAGAATGCTTCTGTGTAGTTTTTATGTGAAGATATTTC

            *   *  *      *  *     *                 *  *      *       *  *  
     134080 TTTTCCCTCCATAGTCCACAAAGTGCTCCAAATATCCACTTACATATTCTAGAAAAAGATTGCTT
          1 CTTTTCCACCATAGGCCTCAAAGCGCTCCAAATATCCACTTGCAGATTCTACAAAAAGAGTGTTT

            **       *    *               * * *                              
     134145 GGAAACTGCACAATGAAAAGAAAGGTTCAAATATATGAGATGAATGCACACATCACAAAGAAGTT
         66 CAAAACTGCTCAATCAAAAGAAAGGTTCAACTCTGTGAGATGAATGCACACATCACAAAGAAGTT

                                 *                    
     134210 TCTCAGAAT-CTCTCTGTGTAATTTTTATGTGAAGATATTTC
        131 TCTCAGAATGCT-TCTGTGTAGTTTTTATGTGAAGATATTTC

                *     *    *  *   *                        *      *    *     
     134251 CTTTCCCACCTTAGGTCTTAAAACGCTCCAAATATCCACTTGCAGATACTACAAGAAGATTGTTT
          1 CTTTTCCACCATAGGCCTCAAAGCGCTCCAAATATCCACTTGCAGATTCTACAAAAAGAGTGTTT

                     *    *        *      *     **                           
     134316 CAAAACTGCACAA-AAAAAGAAATGTTCAATTCTGTTTGATGAATGCACACATCACAAAGAAGTT
         66 CAAAACTGCTCAATCAAAAGAAAGGTTCAACTCTGTGAGATGAATGCACACATCACAAAGAAGTT

                           *                         
     134380 TCTCAGAATGCTTCTCTGTAGTTTTTATGTGAAGATATTTC
        131 TCTCAGAATGCTTCTGTGTAGTTTTTATGTGAAGATATTTC

                     *             *                 *         **      * *   
     134421 CTTTTCCACAATAGGCCTCAAAGGGCTCCAAATATCCACTTCCAGATTCTATGAAAAGAATATTT
          1 CTTTTCCACCATAGGCCTCAAAGCGCTCCAAATATCCACTTGCAGATTCTACAAAAAGAGTGTTT

             *              * *    *                                      *  
     134486 CCAAACTGCTCAATCATAGGAAATGTTCAACTCTGTGAGATGAATGCACACATCAC-AAGAAATT
         66 CAAAACTGCTCAATCAAAAGAAAGGTTCAACTCTGTGAGATGAATGCACACATCACAAAGAAGTT

                     *    *      *       *       *   
     134550 TCTCAGAATCCTTCAGTGTAGGTTTTATGAGAAGATAATTC
        131 TCTCAGAATGCTTCTGTGTAGTTTTTATGTGAAGATATTTC

                     *    **        *   *                           *    *   
     134591 CTTTTCCACAATAGTTCTCAAAGCACTCAAAATATCCACTTGCAGATTCTACAAAAGGAGTATTT
          1 CTTTTCCACCATAGGCCTCAAAGCGCTCCAAATATCCACTTGCAGATTCTACAAAAAGAGTGTTT

                                                          *                  
     134656 CAAAACTGCTCAATCAAAAGAAAGGTTCAACTCTGTGAGATGAATGGACACATCACAAAGAAGTT
         66 CAAAACTGCTCAATCAAAAGAAAGGTTCAACTCTGTGAGATGAATGCACACATCACAAAGAAGTT

                                  *   *              
     134721 TCTCAGAATGCTTCTGTGTAGTATTTTTGTGAAGATATTT-
        131 TCTCAGAATGCTTCTGTGTAGTTTTTATGTGAAGATATTTC

                          *  * * * * ***             *            *     * *  
     134761 CTTTTCCACCATAGACCGCCAGGGGACACAAATATCCACTTTCAGATTCTACAACAAGAGAGGTT
          1 CTTTTCCACCATAGGCCTCAAAGCGCTCCAAATATCCACTTGCAGATTCTACAAAAAGAGTGTTT

                   *   *     *   ** *       *      *                      * *
     134826 CAAAACTACTCGATCAAGAGATGGTTTCAACTATGTGAGTTGAATGCACACATCACAAAGAACTA
         66 CAAAACTGCTCAATCAAAAGAAAGGTTCAACTCTGTGAGATGAATGCACACATCACAAAGAAGTT

             *  *    *                               
     134891 TGTCGGAATTCTTCTGTGTAGTTTTTATGTGAAGATATTTC
        131 TCTCAGAATGCTTCTGTGTAGTTTTTATGTGAAGATATTTC

                     *    * *      * *    *                  *               
     134932 CTTTTCCACAATAGACGTCAAAGTGATCCAGATATCCACTTGCAGATTCCACAAAAAGAGTGTTT
          1 CTTTTCCACCATAGGCCTCAAAGCGCTCCAAATATCCACTTGCAGATTCTACAAAAAGAGTGTTT

                 *   *   *                  **                     *  *      
     134997 CAAAAGTGCACAACCAAAAGAAAGGTTCAACTAGGTGAGATGAATGCACACATCAGAAGGAAGTT
         66 CAAAACTGCTCAATCAAAAGAAAGGTTCAACTCTGTGAGATGAATGCACACATCACAAAGAAGTT

                            **   *     * *       *   
     135062 TCTCAGAATGCTTCTGCATAGCTTTTAAGGGAAGATACTTC
        131 TCTCAGAATGCTTCTGTGTAGTTTTTATGTGAAGATATTTC

                    *               *            * *  *    * *               
     135103 CTTTTCCAACATAGGCCTCAAAGCACTCCAAATATCCTCCTGGAGATACCACAAAAAGAGTGTTT
          1 CTTTTCCACCATAGGCCTCAAAGCGCTCCAAATATCCACTTGCAGATTCTACAAAAAGAGTGTTT

            **                      *  *                 *       *           
     135168 GCAAACTGCTCAATCAAAAGAAAGATTTAACTCTGTGAGATGAATCCACACATGACAAAGAAGTT
         66 CAAAACTGCTCAATCAAAAGAAAGGTTCAACTCTGTGAGATGAATGCACACATCACAAAGAAGTT

                                                     
     135233 TCTCAGAATGCTTCTGTGTAGTTTTTATGTGAAGATATTTC
        131 TCTCAGAATGCTTCTGTGTAGTTTTTATGTGAAGATATTTC

                     *   * * *    **           *                *     *      
     135274 CTTTTCCACAATAAGACCCAAAAGGCTCCAAATATTCACTTGCAGATTCTAAAAAAAACAGTGTT
          1 CTTTTCCACCATAGGCCTCAAAGCGCTCCAAATATCCACTTGCAGATTCT-ACAAAAAGAGTGTT

                                  *                  *      *        **      
     135339 TCAAAACTGCTCAATCAAAAGATA-GTTCAACTCTGTGAGAAGAATGCTCACATCACTGAGAAGT
         65 TCAAAACTGCTCAATCAAAAGAAAGGTTCAACTCTGTGAGATGAATGCACACATCACAAAGAAGT

                                         *            
     135403 TTCTCAGAATGCTTCTGTGTAGTTTTTATATGAAGATATTTC
        130 TTCTCAGAATGCTTCTGTGTAGTTTTTATGTGAAGATATTTC

                *     *      *    **   *                   *   **       *    
     135445 CTTTCCCACCGTAGGCCACAAAAGGCTTCAAATATCCACTTGCAGATACTATGAAAAGAGAGTTT
          1 CTTTTCCACCATAGGCCTCAAAGCGCTCCAAATATCCACTTGCAGATTCTACAAAAAGAGTGTTT

                        *        *               ***            *            
     135510 CAAAACTGCTCATTCAAAAGATAGGTTCAACTCTGTGGTTTGAATGCACACAGCACAAAGAAGTT
         66 CAAAACTGCTCAATCAAAAGAAAGGTTCAACTCTGTGAGATGAATGCACACATCACAAAGAAGTT

              *       **                   **   *    
     135575 TCACAGAATGTGTCTGTGTAGTTTTTATGTGCGGATGTTTC
        131 TCTCAGAATGCTTCTGTGTAGTTTTTATGTGAAGATATTTC

                         *        ****   *   *               *    *          
     135616 CTTTTCCACCATATGCCT-AAATATTTCCCAATTTCCACTTGCAGATTCCACAAGAAGAGTGTTT
          1 CTTTTCCACCATAGGCCTCAAAGCGCTCCAAATATCCACTTGCAGATTCTACAAAAAGAGTGTTT

                      **       *       *           *            *      ***   
     135680 CAAAACTGCTGTATC-AAATAAA-GTTGAACTCTGTGAGGTGAATGCACACAGCACAAAATGGTT
         66 CAAAACTGCTCAATCAAAAGAAAGGTTCAACTCTGTGAGATGAATGCACACATCACAAAGAAGTT

                          **   *        *       *    
     135743 TCTCAGAATGCTTCCTTGTTGTTTTTATATGAAGATGTTTC
        131 TCTCAGAATGCTTCTGTGTAGTTTTTATGTGAAGATATTTC

                  *  *             *   *     *                               
     135784 CTTTTCAACAATAGGCCTCAAAGTGCTTCAAATGTCCACTTGCAGATTCTACAAAAAGAGTGTTT
          1 CTTTTCCACCATAGGCCTCAAAGCGCTCCAAATATCCACTTGCAGATTCTACAAAAAGAGTGTTT

                                        *      *  *  *                       
     135849 CAAAACTGCTCAATCAAAAGAAAGGTTCGACTCTGGGAAATTAATGCACACATCACAAAGAAGTT
         66 CAAAACTGCTCAATCAAAAGAAAGGTTCAACTCTGTGAGATGAATGCACACATCACAAAGAAGTT

                                     *        *      
     135914 TCTC---A-GCTTCTGTGTAGTTTTCATGTGAAGTTATTTC
        131 TCTCAGAATGCTTCTGTGTAGTTTTTATGTGAAGATATTTC

                     *       *     **           *    *         **       *    
     135951 CTTTTCCACAATAGGCCGCAAAGGCCTCCAAATATCAACTTACAGATTCTAGGAAAAGAGAGTTT
          1 CTTTTCCACCATAGGCCTCAAAGCGCTCCAAATATCCACTTGCAGATTCTACAAAAAGAGTGTTT

                       * **      *     *                                     
     136016 CAAAACTGCTCTACGAAAAGATAGGTTGAACTCTGTGAGATGAATGCACACATCACAAAGAAGTT
         66 CAAAACTGCTCAATCAAAAGAAAGGTTCAACTCTGTGAGATGAATGCACACATCACAAAGAAGTT

                       *               * *           
     136081 TCTCAGAATGCATCTGTGTAGTTTTTACGGGAAGATATTTC
        131 TCTCAGAATGCTTCTGTGTAGTTTTTATGTGAAGATATTTC

                        ***  *     **      *  *                  *     ***   
     136122 CTTTTCCACCATCTTCCACAAAGGTCTCCAAGTAACCACTTGCAGATTCTACAGAAAGACACTTT
          1 CTTTTCCACCATAGGCCTCAAAGCGCTCCAAATATCCACTTGCAGATTCTACAAAAAGAGTGTTT

            *          *         ***      *      ***                      *  
     136187 AAAAACTGCTCTATCAAAAGATCAGTTCAAGTCTGTGGTTTGAATGCACACATCACAAAGAATTT
         66 CAAAACTGCTCAATCAAAAGAAAGGTTCAACTCTGTGAGATGAATGCACACATCACAAAGAAGTT

                                     *  *            
     136252 TCTCAGAATGCTTCTGTGTAGTTTTCATATGAAGATATTTC
        131 TCTCAGAATGCTTCTGTGTAGTTTTTATGTGAAGATATTTC

                                    *                                   **   
     136293 CTTTTCCACCATAGGCCTCAAAGCACTCCAAATATCCACTTGCAGATTCTACAAAAAGAGATTTT
          1 CTTTTCCACCATAGGCCTCAAAGCGCTCCAAATATCCACTTGCAGATTCTACAAAAAGAGTGTTT

                   **                           *  *          *        *     
     136358 CAAAACTAGTCAATCAAAAGAAAGGTTCAACTCTGTCAGTTGAATGCACATATCACAAACAAGTT
         66 CAAAACTGCTCAATCAAAAGAAAGGTTCAACTCTGTGAGATGAATGCACACATCACAAAGAAGTT

                *      *                             
     136423 TCTCGGAATGCGTCTGTGTAGTTTTTATGTGAAGATATTTC
        131 TCTCAGAATGCTTCTGTGTAGTTTTTATGTGAAGATATTTC

               *     * *           *     *                  *    *           
     136464 CTTCTCCACAACAGGCCTCAAAGTGCTCCGAATATCCACTTGCAGATTTTAC-TAAAGAGTGTTT
          1 CTTTTCCACCATAGGCCTCAAAGCGCTCCAAATATCCACTTGCAGATTCTACAAAAAGAGTGTTT

             *               *  *   *     *        *    *               *    
     136528 CCAAACTGCTCAATCAAGAGGAAGTTTCAAGTCTGTGAGCTGAACGCACACATCACAAAGTAGTT
         66 CAAAACTGCTCAATCAAAAGAAAGGTTCAACTCTGTGAGATGAATGCACACATCACAAAGAAGTT

               *                                *    
     136593 TCTGAGAATGCTTCTGTGTAGTTTTTATGTGAAGATGTTTC
        131 TCTCAGAATGCTTCTGTGTAGTTTTTATGTGAAGATATTTC

                            **     *                                    *    
     136634 CTTTTCCACCATAGGCTGCAAAGGGCTCCAAATATCCACTTGCAGATTCTACAAAAAGAGAGTTT
          1 CTTTTCCACCATAGGCCTCAAAGCGCTCCAAATATCCACTTGCAGATTCTACAAAAAGAGTGTTT

                 *     *         *          *     *                     *    
     136699 CAAAAGTGCTCTATCAAAAGATAGGTTCAACTATGTGATATGAATGCACACATCACAAAGTAGTT
         66 CAAAACTGCTCAATCAAAAGAAAGGTTCAACTCTGTGAGATGAATGCACACATCACAAAGAAGTT

                                          *          
     136764 TCTCAGAATGCTTCTGTGTAGTTTTTATGTAAAGATATTTC
        131 TCTCAGAATGCTTCTGTGTAGTTTTTATGTGAAGATATTTC

                                    *                                   **   
     136805 CTTTTCCACCATAGGCCTCAAAGCACTCCAAATATCCACTTGCAGATTCTACAAAAAGAGATTTT
          1 CTTTTCCACCATAGGCCTCAAAGCGCTCCAAATATCCACTTGCAGATTCTACAAAAAGAGTGTTT

                   ** *                   *     *  *    * *   *        *     
     136870 CAAAACTATTTAATCAAAAGAAAGGTTCAAATCTGTCAGTTGAAGGTACATATCACAAACAAGTT
         66 CAAAACTGCTCAATCAAAAGAAAGGTTCAACTCTGTGAGATGAATGCACACATCACAAAGAAGTT

             * **                                    
     136935 TATTGGAATGCTTCTGTGTAGTTTTTATGTGAAGATATTTC
        131 TCTCAGAATGCTTCTGTGTAGTTTTTATGTGAAGATATTTC

                     * *       * * *                        **  *     *      
     136976 CTTTTCCACAACAGGCCTCCAGGTGCTCCAAATATCCACTTGCAGATTTCACTAAAAGTGTGTTT
          1 CTTTTCCACCATAGGCCTCAAAGCGCTCCAAATATCCACTTGCAGATTCTACAAAAAGAGTGTTT

             *  *            *  *   *     *        *             *           
     137041 CCAAGCTGCTCAATCAAGAGGAAGTTTCAAGTCTGTGAGGTGAATGCACACATTACAAAGAAGTT
         66 CAAAACTGCTCAATCAAAAGAAAGGTTCAACTCTGTGAGATGAATGCACACATCACAAAGAAGTT

            *  *                                     
     137106 ACTGAGAATGCTTCTGTGTAGTTTTTATGTGAAGATATTTC
        131 TCTCAGAATGCTTCTGTGTAGTTTTTATGTGAAGATATTTC

                      **               *                                *    
     137147 CTTTTCCACCGCAGGCCTCAAAGCGCTGCAAATATCCACTTGCAGATTCTACAAAAAGAGAGTTT
          1 CTTTTCCACCATAGGCCTCAAAGCGCTCCAAATATCCACTTGCAGATTCTACAAAAAGAGTGTTT

                      **         *   *         *   *     **            *     
     137212 CAAAACTGCTGTATCAAAAGATAGGGTCAACTCTGCGAGTTGAATAAACACATCACAAATAAGTT
         66 CAAAACTGCTCAATCAAAAGAAAGGTTCAACTCTGTGAGATGAATGCACACATCACAAAGAAGTT

               **   *        *               *       
     137277 TCTGGGAACGCTTCTGTATAG-TTTTATGTGAATATATTTC
        131 TCTCAGAATGCTTCTGTGTAGTTTTTATGTGAAGATATTTC

                         *          *                   *   *  *   * *   *   
     137317 CTTTTCCACCATATGCCTCAAAGCACTCCAAATATCCACTTGCACATTATAGAAACATAGTCTTT
          1 CTTTTCCACCATAGGCCTCAAAGCGCTCCAAATATCCACTTGCAGATTCTACAAAAAGAGTGTTT

                   **                        *         *             *       
     137382 CAAAACTTGTCAATC-AAAGAAAGGTTCAACTCCGTGAGATGAGTGCACACATCACAGAGAAGTT
         66 CAAAACTGCTCAATCAAAAGAAAGGTTCAACTCTGTGAGATGAATGCACACATCACAAAGAAGTT

                *     *                            * 
     137446 TCTCGGAATGTTTCTGTGTAGTTTTTATGTGAAGATATTGC
        131 TCTCAGAATGCTTCTGTGTAGTTTTTATGTGAAGATATTTC

                     *               *            *          *       *   *   
     137487 CTTTTCCACAATAGGCCTCAAAGCGTTCCAAATATCCAATTGCAGATTCCACAAAAAAAGTTTTT
          1 CTTTTCCACCATAGGCCTCAAAGCGCTCCAAATATCCACTTGCAGATTCTACAAAAAGAGTGTTT

            *                 *  *  *  *             * *         **    *     
     137552 TAAAACTGCTCAATCAAATGATAGATTAAACTCTGTGAGATTAGTGCACACATGTCAAAAAAGTT
         66 CAAAACTGCTCAATCAAAAGAAAGGTTCAACTCTGTGAGATGAATGCACACATCACAAAGAAGTT

                                *      * *           
     137617 TCTCAGAATGCTTCTGTGTACTTTTTAGGGGAAGATATTTC
        131 TCTCAGAATGCTTCTGTGTAGTTTTTATGTGAAGATATTTC

                        *    *     **         *    *           **  * *  *    
     137658 CTTTTCCACCATCGGCCACAAAGGACTCCAAATAACCACATGCAGATTCTAGTAACACAGAGTTT
          1 CTTTTCCACCATAGGCCTCAAAGCGCTCCAAATATCCACTTGCAGATTCTACAAAAAGAGTGTTT

                       *         * *           *   * * *    **    ***        
     137723 CAAAACTGCTCTATCAAAAGATAAGTTCAACTCTGAGAGTTTAGTGCAACCATCGTGAAGAAGTT
         66 CAAAACTGCTCAATCAAAAGAAAGGTTCAACTCTGTGAGATGAATGCACACATCACAAAGAAGTT

                            *     *                  
     137788 TCTCAGAATGCTTCTGAGTAGTGTTTATGTGAAGATATTTC
        131 TCTCAGAATGCTTCTGTGTAGTTTTTATGTGAAGATATTTC

                              *     *                      *           **    
     137829 CTTTTCCACCATAGGCCTGAAAGCCCTCCAAATATCCACTTGCAGATCCTACAAAAAGAAAGTTT
          1 CTTTTCCACCATAGGCCTCAAAGCGCTCCAAATATCCACTTGCAGATTCTACAAAAAGAGTGTTT

             *         **     *  *  *   *        **      *                   
     137894 CGAAA-TGCTCTCTCAAACGATAGTTTCGACTCTGTGGTATGAATACACACATCACAAAGAAGTT
         66 CAAAACTGCTCAATCAAAAGAAAGGTTCAACTCTGTGAGATGAATGCACACATCACAAAGAAGTT

                                       **            
     137958 TCTCAGAATGCTTCTGTGTAGTTTTTAAATGAAGATATTTC
        131 TCTCAGAATGCTTCTGTGTAGTTTTTATGTGAAGATATTTC

            *                       *          *     *                       
     137999 TTTTTCCACCATAGGCCTCAAAGCACTCCAAATATGCACTTCCAGATTCTACAAAAAGAGTGTTT
          1 CTTTTCCACCATAGGCCTCAAAGCGCTCCAAATATCCACTTGCAGATTCTACAAAAAGAGTGTTT

              *                 *       * *         **   *         *  * *    
     138064 CAGAACTGCTCAATCAAAAGGAAGGTTCCAGTC--TGAGACAAATACACACATCAAAAGGTAGTT
         66 CAAAACTGCTCAATCAAAAGAAAGGTTCAACTCTGTGAGATGAATGCACACATCACAAAGAAGTT

                                                    *
     138127 TCTCAGAATGCTTCTGTGTAGTTTTTATGTGAAGATATTTT
        131 TCTCAGAATGCTTCTGTGTAGTTTTTATGTGAAGATATTTC

             *               *    **    *     *      *  **   *          *    
     138168 CCTTTCCACCATAGGCCACAAATGGCTCTAAATACCCACTTACATTTTCCACAAAAAGAGAGTTT
          1 CTTTTCCACCATAGGCCTCAAAGCGCTCCAAATATCCACTTGCAGATTCTACAAAAAGAGTGTTT

                       * *    * *      *   *         *                       
     138233 CAAAACTGCTCTACCAAAGGTAA-GTTTAACGCTGTGAG-TTAA-G-A-ACATCACAAAGAAGTT
         66 CAAAACTGCTCAATCAAAAGAAAGGTTCAACTCTGTGAGATGAATGCACACATCACAAAGAAGTT

                                   *   *  *          
     138293 TCTCAGAATGCTTCTGTGTAGTTCTTACGTAAAGATATTTC
        131 TCTCAGAATGCTTCTGTGTAGTTTTTATGTGAAGATATTTC

                 *   *      ***    *                  *              **      
     138334 CTTTTACACAATAGGCAGAAAAGTGCTCCAAATATCCACTTGAAGATTCTACAAAAACCGTGTTT
          1 CTTTTCCACCATAGGCCTCAAAGCGCTCCAAATATCCACTTGCAGATTCTACAAAAAGAGTGTTT

                     **                                          *      *    
     138399 CAAAACTGCCGAATCAAAAGAAAGGTTCAACTCTGTGAGATGAATGCACACATAACAAAGGAGTT
         66 CAAAACTGCTCAATCAAAAGAAAGGTTCAACTCTGTGAGATGAATGCACACATCACAAAGAAGTT

                                 *      *      *    *
     138464 TCTCAGAATGCTTCTGTGTAGCTTTTATATGAAGACATTTA
        131 TCTCAGAATGCTTCTGTGTAGTTTTTATGTGAAGATATTTC

            *        * *            *   ***                     **        *  
     138505 GTTTTCCACAACAGGCCTCAAAGCTCTCTCCATATCCACTTGCAGATTCTACCGAAAGAGTGCTT
          1 CTTTTCCACCATAGGCCTCAAAGCGCTCCAAATATCCACTTGCAGATTCTACAAAAAGAGTGTTT

             *                   * **     *        *        *     **         
     138570 CCAAACTGCTCAATCAAAAGAGACATTCAAATCTGTGAGGTGAATGCAGACATCGTAAAGAAGTT
         66 CAAAACTGCTCAATCAAAAGAAAGGTTCAACTCTGTGAGATGAATGCACACATCACAAAGAAGTT

                                *     *       *    **
     138635 TCTCAGAATGCTTCTGTGTATTTTTTGTGTGAAGTTATTCG
        131 TCTCAGAATGCTTCTGTGTAGTTTTTATGTGAAGATATTTC

            *    *             *     *  *            **                 *    
     138676 TTTTTGCACCATAGGCCTCCAAGCGTTCTAAATATCCACTTCTAGATTCTACAAAAAGAGAGTTT
          1 CTTTTCCACCATAGGCCTCAAAGCGCTCCAAATATCCACTTGCAGATTCTACAAAAAGAGTGTTT

                   *         *            *        *    *   *                
     138741 CAAAACTACTC-A--AACA-AAAGGTTCAATTCTGTGAGTTGAAAGCAAACATCACAAAGAAGTT
         66 CAAAACTGCTCAATCAAAAGAAAGGTTCAACTCTGTGAGATGAATGCACACATCACAAAGAAGTT

                       *             *               
     138802 TCTCAGAATGCGTCTGTGTAGTTTTGATGTGAAGATATTTC
        131 TCTCAGAATGCTTCTGTGTAGTTTTTATGTGAAGATATTTC

                     *    ****     *                  *                      
     138843 CTTTT-CACAATAGAATGCAAAGGGCTCCAAATATCCACTTGGAGATTCTACAAAAAGA--GTTT
          1 CTTTTCCACCATAGGCCTCAAAGCGCTCCAAATATCCACTTGCAGATTCTACAAAAAGAGTGTTT

                  *    **     *  *     *     ***   *     *               *   
     138905 CAAAACCGCTCTGTCAAATGATAGGTTGAACTCCCGGAGGTGAATACACACATCACAAAGAGGTT
         66 CAAAACTGCTCAATCAAAAGAAAGGTTCAACTCTGTGAGATGAATGCACACATCACAAAGAAGTT

                  *                       *  *       
     138970 TCTCAGCATGCTTCTGTGTAGTTTTTATGTAAACATATTTC
        131 TCTCAGAATGCTTCTGTGTAGTTTTTATGTGAAGATATTTC

             *    * *              *           *      * *       *   *    *   
     139011 CGTTTCTATCATAGGCCTCAAAGTGCTCCAAATATTCACTTGTACATTCTACCAAACGAGTATTT
          1 CTTTTCCACCATAGGCCTCAAAGCGCTCCAAATATCCACTTGCAGATTCTACAAAAAGAGTGTTT

                              * *         **      *        *            *    
     139076 CAAAACTGCTCAATCAAATGGAAGGTTCAAAACTGTGACATGAATGCCCACATCACAAAGTAGTT
         66 CAAAACTGCTCAATCAAAAGAAAGGTTCAACTCTGTGAGATGAATGCACACATCACAAAGAAGTT

                                                     
     139141 TCTCAGAATGCTTCTGTGTAGTTTTTATGTGAAGATATTTC
        131 TCTCAGAATGCTTCTGTGTAGTTTTTATGTGAAGATATTTC

                     * *    *      *    *       * *   **      *              
     139182 CTTTTCCACAACA-GCGTGCAAAACGCTTCAAATATGCCCTTAGAGATTCCACAAAAAGAGTGTT
          1 CTTTTCCACCATAGGCCT-CAAAGCGCTCCAAATATCCACTTGCAGATTCTACAAAAAGAGTGTT

              *     *               *  *                                  ** 
     139246 TCCAAACTACTCAAATCAAAA-AATGATTTCAACTCTGTGAGATGAATGCACACATCACAAACTA
         65 TCAAAACTGCTC-AATCAAAAGAAAG-GTTCAACTCTGTGAGATGAATGCACACATCACAAAGAA

                         *     ** *   * *   *       *   
     139310 GTTTCTCAGAATGTTTCTGCCTGGTTCTCATGCGAAGATAGTTC
        128 GTTTCTCAGAATGCTTCTGTGTAGTTTTTATGTGAAGATATTTC

                 *           *   * **              *                  * *    
     139354 CTTTTTCACCATAGGCCGCAATGTACTCCAAATATCCACCTGCAGATTCTAC-AAAAGTGAGTTT
          1 CTTTTCCACCATAGGCCTCAAAGCGCTCCAAATATCCACTTGCAGATTCTACAAAAAGAGTGTTT

                       *         ***    **         *        *      *       * 
     139418 CAAAACTGCTCTATCAAAAGATCAGTTCGTCTCTGTGAGTTGAATGCATACATCAAAAAGAAGCT
         66 CAAAACTGCTCAATCAAAAGAAAGGTTCAACTCTGTGAGATGAATGCACACATCACAAAGAAGTT

                 *             *      **         *   
     139483 TCTCAAAATGCTTCTGTGTGGTTTTTCGGTGAAGATAGTTC
        131 TCTCAGAATGCTTCTGTGTAGTTTTTATGTGAAGATATTTC

            *     *        *      * *                 *        *        *    
     139524 TTTTTCTACCATAGGTCTCAAACCACTCCAAATATCCACTTGTAGATTCTATAAAAAGGAATG-T
          1 CTTTTCCACCATAGGCCTCAAAGCGCTCCAAATATCCACTTGCAGATTCTACAAAAA-GAGTGTT

                  *        *    *    *      * *         *      *         *   
     139588 TCAAAATTGCTCAATAAAAATAAAGTTTCAACACCGTGAGATGAGTGCACAAATCACAAAGGAGT
         65 TCAAAACTGCTCAATCAAAAGAAAGGTTCAACTCTGTGAGATGAATGCACACATCACAAAGAAGT

                  *          *         *          *   
     139653 TTCTCAAAATGCTTCTGGGTAGTTTTTCTGTGAAGATAGTTC
        130 TTCTCAGAATGCTTCTGTGTAGTTTTTATGTGAAGATATTTC

                  *     *    *     *          *                         *    
     139695 CTTTTCTACCATGGGCCACAAAGGGCTCCAAATACCCACTTGCAGATTCTACAAAAAGAGAGTTT
          1 CTTTTCCACCATAGGCCTCAAAGCGCTCCAAATATCCACTTGCAGATTCTACAAAAAGAGTGTTT

              *        *      ** * *        *    * *    **  * *           *  
     139760 CACAACTGCTCTATCAAACAATATGTTCAACTTTGTGGGTTGAACACAAATATCAC-AAGAATTT
         66 CAAAACTGCTCAATCAAAAGAAAGGTTCAACTCTGTGAGATGAATGCACACATCACAAAGAAGTT

                **                             *     
     139824 TCTCCCAATGCTTCTGTGTAGTTTTTATGTGAAGACATTTC
        131 TCTCAGAATGCTTCTGTGTAGTTTTTATGTGAAGATATTTC

            *   *  *      *  *     *                 *  *      *       *  *  
     139865 TTTTCCCTCCATAGTCCACAAAGTGCTCCAAATATCCACTTACATATTCTAGAAAAAGATTGCTT
          1 CTTTTCCACCATAGGCCTCAAAGCGCTCCAAATATCCACTTGCAGATTCTACAAAAAGAGTGTTT

            **       *    *               * * *                              
     139930 GGAAACTGCACAATGAAAAGAAAGGTTCAAATATATGAGATGAATGCACACATCACAAAGAAGTT
         66 CAAAACTGCTCAATCAAAAGAAAGGTTCAACTCTGTGAGATGAATGCACACATCACAAAGAAGTT

                                 *                    
     139995 TCTCAGAAT-CTCTCTGTGTAATTTTTATGTGAAGATATTTC
        131 TCTCAGAATGCT-TCTGTGTAGTTTTTATGTGAAGATATTTC

                *     *    *  *   *                        *      *    *     
     140036 CTTTCCCACCTTAGGTCTTAAAACGCTCCAAATATCCACTTGCAGATACTACAAGAAGATTGTTT
          1 CTTTTCCACCATAGGCCTCAAAGCGCTCCAAATATCCACTTGCAGATTCTACAAAAAGAGTGTTT

                     *    *        *      *     **                           
     140101 CAAAACTGCACAA-AAAAAGAAATGTTCAATTCTGTTTGATGAATGCACACATCACAAAGAAGTT
         66 CAAAACTGCTCAATCAAAAGAAAGGTTCAACTCTGTGAGATGAATGCACACATCACAAAGAAGTT

                           *                         
     140165 TCTCAGAATGCTTCTCTGTAGTTTTTATGTGAAGATATTTC
        131 TCTCAGAATGCTTCTGTGTAGTTTTTATGTGAAGATATTTC

                     *             *                 *         **      * *   
     140206 CTTTTCCACAATAGGCCTCAAAGGGCTCCAAATATCCACTTCCAGATTCTATGAAAAGAATATTT
          1 CTTTTCCACCATAGGCCTCAAAGCGCTCCAAATATCCACTTGCAGATTCTACAAAAAGAGTGTTT

             *              * *    *                                      *  
     140271 CCAAACTGCTCAATCATAGGAAATGTTCAACTCTGTGAGATGAATGCACACATCAC-AAGAAATT
         66 CAAAACTGCTCAATCAAAAGAAAGGTTCAACTCTGTGAGATGAATGCACACATCACAAAGAAGTT

                     *    *      *       *       *   
     140335 TCTCAGAATCCTTCAGTGTAGGTTTTATGAGAAGATAATTC
        131 TCTCAGAATGCTTCTGTGTAGTTTTTATGTGAAGATATTTC

                     *    **        *   *                           *    *   
     140376 CTTTTCCACAATAGTTCTCAAAGCACTCAAAATATCCACTTGCAGATTCTACAAAAGGAGTATTT
          1 CTTTTCCACCATAGGCCTCAAAGCGCTCCAAATATCCACTTGCAGATTCTACAAAAAGAGTGTTT

                                                          *                  
     140441 CAAAACTGCTCAATCAAAAGAAAGGTTCAACTCTGTGAGATGAATGGACACATCACAAAGAAGTT
         66 CAAAACTGCTCAATCAAAAGAAAGGTTCAACTCTGTGAGATGAATGCACACATCACAAAGAAGTT

                                  *   *              
     140506 TCTCAGAATGCTTCTGTGTAGTATTTTTGTGAAGATATTT-
        131 TCTCAGAATGCTTCTGTGTAGTTTTTATGTGAAGATATTTC

                          *  * * * * ***             *            *     * *  
     140546 CTTTTCCACCATAGACCGCCAGGGGACACAAATATCCACTTTCAGATTCTACAACAAGAGAGGTT
          1 CTTTTCCACCATAGGCCTCAAAGCGCTCCAAATATCCACTTGCAGATTCTACAAAAAGAGTGTTT

                   *   *     *   ** *       *      *                      * *
     140611 CAAAACTACTCGATCAAGAGATGGTTTCAACTATGTGAGTTGAATGCACACATCACAAAGAACTA
         66 CAAAACTGCTCAATCAAAAGAAAGGTTCAACTCTGTGAGATGAATGCACACATCACAAAGAAGTT

             *  *    *                               
     140676 TGTCGGAATTCTTCTGTGTAGTTTTTATGTGAAGATATTTC
        131 TCTCAGAATGCTTCTGTGTAGTTTTTATGTGAAGATATTTC

                     *    * *      * *    *                  *               
     140717 CTTTTCCACAATAGACGTCAAAGTGATCCAGATATCCACTTGCAGATTCCACAAAAAGAGTGTTT
          1 CTTTTCCACCATAGGCCTCAAAGCGCTCCAAATATCCACTTGCAGATTCTACAAAAAGAGTGTTT

                 *   *   *                  **                     *  *      
     140782 CAAAAGTGCACAACCAAAAGAAAGGTTCAACTAGGTGAGATGAATGCACACATCAGAAGGAAGTT
         66 CAAAACTGCTCAATCAAAAGAAAGGTTCAACTCTGTGAGATGAATGCACACATCACAAAGAAGTT

                            **   *     * *       *   
     140847 TCTCAGAATGCTTCTGCATAGCTTTTAAGGGAAGATACTTC
        131 TCTCAGAATGCTTCTGTGTAGTTTTTATGTGAAGATATTTC

                    *               *            * *  *    * *               
     140888 CTTTTCCAACATAGGCCTCAAAGCACTCCAAATATCCTCCTGGAGATACCACAAAAAGAGTGTTT
          1 CTTTTCCACCATAGGCCTCAAAGCGCTCCAAATATCCACTTGCAGATTCTACAAAAAGAGTGTTT

            **                      *  *                 *       *           
     140953 GCAAACTGCTCAATCAAAAGAAAGATTTAACTCTGTGAGATGAATCCACACATGACAAAGAAGTT
         66 CAAAACTGCTCAATCAAAAGAAAGGTTCAACTCTGTGAGATGAATGCACACATCACAAAGAAGTT

                                                     
     141018 TCTCAGAATGCTTCTGTGTAGTTTTTATGTGAAGATATTTC
        131 TCTCAGAATGCTTCTGTGTAGTTTTTATGTGAAGATATTTC

                     *   * * *    **           *                *     *      
     141059 CTTTTCCACAATAAGACCCAAAAGGCTCCAAATATTCACTTGCAGATTCTAAAAAAAACAGTGTT
          1 CTTTTCCACCATAGGCCTCAAAGCGCTCCAAATATCCACTTGCAGATTCT-ACAAAAAGAGTGTT

                                  *                  *      *        **      
     141124 TCAAAACTGCTCAATCAAAAGATA-GTTCAACTCTGTGAGAAGAATGCTCACATCACTGAGAAGT
         65 TCAAAACTGCTCAATCAAAAGAAAGGTTCAACTCTGTGAGATGAATGCACACATCACAAAGAAGT

                                         *            
     141188 TTCTCAGAATGCTTCTGTGTAGTTTTTATATGAAGATATTTC
        130 TTCTCAGAATGCTTCTGTGTAGTTTTTATGTGAAGATATTTC

                *     *      *    **   *                   *   **       *    
     141230 CTTTCCCACCGTAGGCCACAAAAGGCTTCAAATATCCACTTGCAGATACTATGAAAAGAGAGTTT
          1 CTTTTCCACCATAGGCCTCAAAGCGCTCCAAATATCCACTTGCAGATTCTACAAAAAGAGTGTTT

                        *        *               ***            *            
     141295 CAAAACTGCTCATTCAAAAGATAGGTTCAACTCTGTGGTTTGAATGCACACAGCACAAAGAAGTT
         66 CAAAACTGCTCAATCAAAAGAAAGGTTCAACTCTGTGAGATGAATGCACACATCACAAAGAAGTT

              *       **                   **   *    
     141360 TCACAGAATGTGTCTGTGTAGTTTTTATGTGCGGATGTTTC
        131 TCTCAGAATGCTTCTGTGTAGTTTTTATGTGAAGATATTTC

                         *        ****   *   *               *    *          
     141401 CTTTTCCACCATATGCCT-AAATATTTCCCAATTTCCACTTGCAGATTCCACAAGAAGAGTGTTT
          1 CTTTTCCACCATAGGCCTCAAAGCGCTCCAAATATCCACTTGCAGATTCTACAAAAAGAGTGTTT

                      **       *       *           *            *      ***   
     141465 CAAAACTGCTGTATC-AAATAAA-GTTGAACTCTGTGAGGTGAATGCACACAGCACAAAATGGTT
         66 CAAAACTGCTCAATCAAAAGAAAGGTTCAACTCTGTGAGATGAATGCACACATCACAAAGAAGTT

                          **   *        *       *    
     141528 TCTCAGAATGCTTCCTTGTTGTTTTTATATGAAGATGTTTC
        131 TCTCAGAATGCTTCTGTGTAGTTTTTATGTGAAGATATTTC

                  *  *             *   *     *                               
     141569 CTTTTCAACAATAGGCCTCAAAGTGCTTCAAATGTCCACTTGCAGATTCTACAAAAAGAGTGTTT
          1 CTTTTCCACCATAGGCCTCAAAGCGCTCCAAATATCCACTTGCAGATTCTACAAAAAGAGTGTTT

                                        *      *  *  *                       
     141634 CAAAACTGCTCAATCAAAAGAAAGGTTCGACTCTGGGAAATTAATGCACACATCACAAAGAAGTT
         66 CAAAACTGCTCAATCAAAAGAAAGGTTCAACTCTGTGAGATGAATGCACACATCACAAAGAAGTT

                                     *        *      
     141699 TCTC---A-GCTTCTGTGTAGTTTTCATGTGAAGTTATTTC
        131 TCTCAGAATGCTTCTGTGTAGTTTTTATGTGAAGATATTTC

                     *       *     *            *    *         **       *    
     141736 CTTTTCCACAATAGGCCGCAAAGGGCTCCAAATATCAACTTACAGATTCTAGGAAAAGAGAGTTT
          1 CTTTTCCACCATAGGCCTCAAAGCGCTCCAAATATCCACTTGCAGATTCTACAAAAAGAGTGTTT

                       * **      *     *                                     
     141801 CAAAACTGCTCTACGAAAAGATAGGTTGAACTCTGTGAGATGAATGCACACATCACAAAGAAGTT
         66 CAAAACTGCTCAATCAAAAGAAAGGTTCAACTCTGTGAGATGAATGCACACATCACAAAGAAGTT

                       *               * *           
     141866 TCTCAGAATGCATCTGTGTAGTTTTTACGGGAAGATATTTC
        131 TCTCAGAATGCTTCTGTGTAGTTTTTATGTGAAGATATTTC

                        ***  *     **      *  *                  *     ***   
     141907 CTTTTCCACCATCTTCCACAAAGGTCTCCAAGTAACCACTTGCAGATTCTACAGAAAGACACTTT
          1 CTTTTCCACCATAGGCCTCAAAGCGCTCCAAATATCCACTTGCAGATTCTACAAAAAGAGTGTTT

            *          *         ***      *      ***                      *  
     141972 AAAAACTGCTCTATCAAAAGATCAGTTCAAGTCTGTGGTTTGAATGCACACATCACAAAGAATTT
         66 CAAAACTGCTCAATCAAAAGAAAGGTTCAACTCTGTGAGATGAATGCACACATCACAAAGAAGTT

                                     *  *            
     142037 TCTCAGAATGCTTCTGTGTAGTTTTCATATGAAGATATTTC
        131 TCTCAGAATGCTTCTGTGTAGTTTTTATGTGAAGATATTTC

                                    *                                   **   
     142078 CTTTTCCACCATAGGCCTCAAAGCACTCCAAATATCCACTTGCAGATTCTACAAAAAGAGATTTT
          1 CTTTTCCACCATAGGCCTCAAAGCGCTCCAAATATCCACTTGCAGATTCTACAAAAAGAGTGTTT

                   **                           *  *          *        *     
     142143 CAAAACTAGTCAATCAAAAGAAAGGTTCAACTCTGTCAGTTGAATGCACATATCACAAACAAGTT
         66 CAAAACTGCTCAATCAAAAGAAAGGTTCAACTCTGTGAGATGAATGCACACATCACAAAGAAGTT

                *      *                             
     142208 TCTCGGAATGCGTCTGTGTAGTTTTTATGTGAAGATATTTC
        131 TCTCAGAATGCTTCTGTGTAGTTTTTATGTGAAGATATTTC

               *     * *           *     *                  *    *           
     142249 CTTCTCCACAACAGGCCTCAAAGTGCTCCGAATATCCACTTGCAGATTTTAC-TAAAGAGTGTTT
          1 CTTTTCCACCATAGGCCTCAAAGCGCTCCAAATATCCACTTGCAGATTCTACAAAAAGAGTGTTT

             *               *  *   *     *        *    *               *    
     142313 CCAAACTGCTCAATCAAGAGGAAGTTTCAAGTCTGTGAGCTGAACGCACACATCACAAAGTAGTT
         66 CAAAACTGCTCAATCAAAAGAAAGGTTCAACTCTGTGAGATGAATGCACACATCACAAAGAAGTT

               *                                *    
     142378 TCTGAGAATGCTTCTGTGTAGTTTTTATGTGAAGATGTTTC
        131 TCTCAGAATGCTTCTGTGTAGTTTTTATGTGAAGATATTTC

                            **     *                                    *    
     142419 CTTTTCCACCATAGGCTGCAAAGGGCTCCAAATATCCACTTGCAGATTCTACAAAAAGAGAGTTT
          1 CTTTTCCACCATAGGCCTCAAAGCGCTCCAAATATCCACTTGCAGATTCTACAAAAAGAGTGTTT

                 *     *         *          *     *                     *    
     142484 CAAAAGTGCTCTATCAAAAGATAGGTTCAACTATGTGATATGAATGCACACATCACAAAGTAGTT
         66 CAAAACTGCTCAATCAAAAGAAAGGTTCAACTCTGTGAGATGAATGCACACATCACAAAGAAGTT

                                          *          
     142549 TCTCAGAATGCTTCTGTGTAGTTTTTATGTAAAGATATTTC
        131 TCTCAGAATGCTTCTGTGTAGTTTTTATGTGAAGATATTTC

                                    *                                   **   
     142590 CTTTTCCACCATAGGCCTCAAAGCACTCCAAATATCCACTTGCAGATTCTACAAAAAGAGATTTT
          1 CTTTTCCACCATAGGCCTCAAAGCGCTCCAAATATCCACTTGCAGATTCTACAAAAAGAGTGTTT

                   ** *                   *     *  *    * *   *        *     
     142655 CAAAACTATTTAATCAAAAGAAAGGTTCAAATCTGTCAGTTGAAGGTACATATCACAAACAAGTT
         66 CAAAACTGCTCAATCAAAAGAAAGGTTCAACTCTGTGAGATGAATGCACACATCACAAAGAAGTT

             * **                                    
     142720 TATTGGAATGCTTCTGTGTAGTTTTTATGTGAAGATATTTC
        131 TCTCAGAATGCTTCTGTGTAGTTTTTATGTGAAGATATTTC

                     * *       * * *                        **  *     *      
     142761 CTTTTCCACAACAGGCCTCCAGGTGCTCCAAATATCCACTTGCAGATTTCACTAAAAGTGTGTTT
          1 CTTTTCCACCATAGGCCTCAAAGCGCTCCAAATATCCACTTGCAGATTCTACAAAAAGAGTGTTT

             *  *            *  *   *     *        *             *           
     142826 CCAAGCTGCTCAATCAAGAGGAAGTTTCAAGTCTGTGAGGTGAATGCACACATTACAAAGAAGTT
         66 CAAAACTGCTCAATCAAAAGAAAGGTTCAACTCTGTGAGATGAATGCACACATCACAAAGAAGTT

            *  *                                     
     142891 ACTGAGAATGCTTCTGTGTAGTTTTTATGTGAAGATATTTC
        131 TCTCAGAATGCTTCTGTGTAGTTTTTATGTGAAGATATTTC

                      **               *                                *    
     142932 CTTTTCCACCGCAGGCCTCAAAGCGCTGCAAATATCCACTTGCAGATTCTACAAAAAGAGAGTTT
          1 CTTTTCCACCATAGGCCTCAAAGCGCTCCAAATATCCACTTGCAGATTCTACAAAAAGAGTGTTT

                      **         *   *         *   *     **            *     
     142997 CAAAACTGCTGTATCAAAAGATAGGGTCAACTCTGCGAGTTGAATAAACACATCACAAATAAGTT
         66 CAAAACTGCTCAATCAAAAGAAAGGTTCAACTCTGTGAGATGAATGCACACATCACAAAGAAGTT

               **   *        *               *       
     143062 TCTGGGAACGCTTCTGTATAG-TTTTATGTGAATATATTTC
        131 TCTCAGAATGCTTCTGTGTAGTTTTTATGTGAAGATATTTC

                         *          *                   *   *  *   * *   *   
     143102 CTTTTCCACCATATGCCTCAAAGCACTCCAAATATCCACTTGCACATTATAGAAACATAGTCTTT
          1 CTTTTCCACCATAGGCCTCAAAGCGCTCCAAATATCCACTTGCAGATTCTACAAAAAGAGTGTTT

                   **                        *         *             *       
     143167 CAAAACTTGTCAATC-AAAGAAAGGTTCAACTCCGTGAGATGAGTGCACACATCACAGAGAAGTT
         66 CAAAACTGCTCAATCAAAAGAAAGGTTCAACTCTGTGAGATGAATGCACACATCACAAAGAAGTT

                *     *                            * 
     143231 TCTCGGAATGTTTCTGTGTAGTTTTTATGTGAAGATATTGC
        131 TCTCAGAATGCTTCTGTGTAGTTTTTATGTGAAGATATTTC

                     *               *            *          *       *   *   
     143272 CTTTTCCACAATAGGCCTCAAAGCGTTCCAAATATCCAATTGCAGATTCCACAAAAAAAGTTTTT
          1 CTTTTCCACCATAGGCCTCAAAGCGCTCCAAATATCCACTTGCAGATTCTACAAAAAGAGTGTTT

            *                 *  *  *  *             * *         **    *     
     143337 TAAAACTGCTCAATCAAATGATAGATTAAACTCTGTGAGATTAGTGCACACATGTCAAAAAAGTT
         66 CAAAACTGCTCAATCAAAAGAAAGGTTCAACTCTGTGAGATGAATGCACACATCACAAAGAAGTT

                                *      * *           
     143402 TCTCAGAATGCTTCTGTGTACTTTTTAGGGGAAGATATTTC
        131 TCTCAGAATGCTTCTGTGTAGTTTTTATGTGAAGATATTTC

                        *    *     **         *    *           **  * *  *    
     143443 CTTTTCCACCATCGGCCACAAAGGACTCCAAATAACCACATGCAGATTCTAGTAACACAGAGTTT
          1 CTTTTCCACCATAGGCCTCAAAGCGCTCCAAATATCCACTTGCAGATTCTACAAAAAGAGTGTTT

                       *         * *           *   * * *    **    ***        
     143508 CAAAACTGCTCTATCAAAAGATAAGTTCAACTCTGAGAGTTTAGTGCAACCATCGTGAAGAAGTT
         66 CAAAACTGCTCAATCAAAAGAAAGGTTCAACTCTGTGAGATGAATGCACACATCACAAAGAAGTT

                            *     *                  
     143573 TCTCAGAATGCTTCTGAGTAGTGTTTATGTGAAGATATTTC
        131 TCTCAGAATGCTTCTGTGTAGTTTTTATGTGAAGATATTTC

                              *     *                      *           **    
     143614 CTTTTCCACCATAGGCCTGAAAGCCCTCCAAATATCCACTTGCAGATCCTACAAAAAGAAAGTTT
          1 CTTTTCCACCATAGGCCTCAAAGCGCTCCAAATATCCACTTGCAGATTCTACAAAAAGAGTGTTT

             *         **     *  *  *   *        **      *                   
     143679 CGAAA-TGCTCTCTCAAACGATAGTTTCGACTCTGTGGTATGAATACACACATCACAAAGAAGTT
         66 CAAAACTGCTCAATCAAAAGAAAGGTTCAACTCTGTGAGATGAATGCACACATCACAAAGAAGTT

                                       **            
     143743 TCTCAGAATGCTTCTGTGTAGTTTTTAAATGAAGATATTTC
        131 TCTCAGAATGCTTCTGTGTAGTTTTTATGTGAAGATATTTC

            *                       *          *     *                       
     143784 TTTTTCCACCATAGGCCTCAAAGCACTCCAAATATGCACTTCCAGATTCTACAAAAAGAGTGTTT
          1 CTTTTCCACCATAGGCCTCAAAGCGCTCCAAATATCCACTTGCAGATTCTACAAAAAGAGTGTTT

              *                 *       * *         **   *         *  * *    
     143849 CAGAACTGCTCAATCAAAAGGAAGGTTCCAGTC--TGAGACAAATACACACATCAAAAGGTAGTT
         66 CAAAACTGCTCAATCAAAAGAAAGGTTCAACTCTGTGAGATGAATGCACACATCACAAAGAAGTT

                                                    *
     143912 TCTCAGAATGCTTCTGTGTAGTTTTTATGTGAAGATATTTT
        131 TCTCAGAATGCTTCTGTGTAGTTTTTATGTGAAGATATTTC

             *               *    **    *     *      *  **   *          *    
     143953 CCTTTCCACCATAGGCCACAAATGGCTCTAAATACCCACTTACATTTTCCACAAAAAGAGAGTTT
          1 CTTTTCCACCATAGGCCTCAAAGCGCTCCAAATATCCACTTGCAGATTCTACAAAAAGAGTGTTT

                       * *    * *      *   *         *                       
     144018 CAAAACTGCTCTACCAAAGGTAA-GTTTAACGCTGTGAG-TTAA-G-A-ACATCACAAAGAAGTT
         66 CAAAACTGCTCAATCAAAAGAAAGGTTCAACTCTGTGAGATGAATGCACACATCACAAAGAAGTT

                                   *   *  *          
     144078 TCTCAGAATGCTTCTGTGTAGTTCTTACGTAAAGATATTTC
        131 TCTCAGAATGCTTCTGTGTAGTTTTTATGTGAAGATATTTC

                 *   *      ***    *                  *              **      
     144119 CTTTTACACAATAGGCAGAAAAGTGCTCCAAATATCCACTTGAAGATTCTACAAAAACCGTGTTT
          1 CTTTTCCACCATAGGCCTCAAAGCGCTCCAAATATCCACTTGCAGATTCTACAAAAAGAGTGTTT

                     **                                          *      *    
     144184 CAAAACTGCCGAATCAAAAGAAAGGTTCAACTCTGTGAGATGAATGCACACATAACAAAGGAGTT
         66 CAAAACTGCTCAATCAAAAGAAAGGTTCAACTCTGTGAGATGAATGCACACATCACAAAGAAGTT

                                 *      *      *    *
     144249 TCTCAGAATGCTTCTGTGTAGCTTTTATATGAAGACATTTA
        131 TCTCAGAATGCTTCTGTGTAGTTTTTATGTGAAGATATTTC

            *        * *            *   ***                     **        *  
     144290 GTTTTCCACAACAGGCCTCAAAGCTCTCTCCATATCCACTTGCAGATTCTACCGAAAGAGTGCTT
          1 CTTTTCCACCATAGGCCTCAAAGCGCTCCAAATATCCACTTGCAGATTCTACAAAAAGAGTGTTT

             *                   * **     *        *        *     **         
     144355 CCAAACTGCTCAATCAAAAGAGACATTCAAATCTGTGAGGTGAATGCAGACATCGTAAAGAAGTT
         66 CAAAACTGCTCAATCAAAAGAAAGGTTCAACTCTGTGAGATGAATGCACACATCACAAAGAAGTT

                                *     *       *    **
     144420 TCTCAGAATGCTTCTGTGTATTTTTTGTGTGAAGTTATTCG
        131 TCTCAGAATGCTTCTGTGTAGTTTTTATGTGAAGATATTTC

            *    *             *     *  *            **                 *    
     144461 TTTTTGCACCATAGGCCTCCAAGCGTTCTAAATATCCACTTCTAGATTCTACAAAAAGAGAGTTT
          1 CTTTTCCACCATAGGCCTCAAAGCGCTCCAAATATCCACTTGCAGATTCTACAAAAAGAGTGTTT

                   *         *            *        *    *   *                
     144526 CAAAACTACTC-A--AACA-AAAGGTTCAATTCTGTGAGTTGAAAGCAAACATCACAAAGAAGTT
         66 CAAAACTGCTCAATCAAAAGAAAGGTTCAACTCTGTGAGATGAATGCACACATCACAAAGAAGTT

                       *             *               
     144587 TCTCAGAATGCGTCTGTGTAGTTTTGATGTGAAGATATTTC
        131 TCTCAGAATGCTTCTGTGTAGTTTTTATGTGAAGATATTTC

                     **   ****     *                  *                      
     144628 CTTTT-CACAGTAGAATGCAAAGGGCTCCAAATATCCACTTGGAGATTCTACAAAAAGA--GTTT
          1 CTTTTCCACCATAGGCCTCAAAGCGCTCCAAATATCCACTTGCAGATTCTACAAAAAGAGTGTTT

                  *    **     *  *     *     ***   *     *               *   
     144690 CAAAACCGCTCTGTCAAATGATAGGTTGAACTCCCGGAGGTGAATACACACATCACAAAGAGGTT
         66 CAAAACTGCTCAATCAAAAGAAAGGTTCAACTCTGTGAGATGAATGCACACATCACAAAGAAGTT

                  *                       *  *       
     144755 TCTCAGCATGCTTCTGTGTAGTTTTTATGTAAACATATTTC
        131 TCTCAGAATGCTTCTGTGTAGTTTTTATGTGAAGATATTTC

             *    * *              *           *      * *       *   *    *   
     144796 CGTTTCTATCATAGGCCTCAAAGTGCTCCAAATATTCACTTGTACATTCTACCAAACGAGTATTT
          1 CTTTTCCACCATAGGCCTCAAAGCGCTCCAAATATCCACTTGCAGATTCTACAAAAAGAGTGTTT

                              * *         **      *        *            *    
     144861 CAAAACTGCTCAATCAAATGGAAGGTTCAAAACTGTGACATGAATGCCCACATCACAAAGTAGTT
         66 CAAAACTGCTCAATCAAAAGAAAGGTTCAACTCTGTGAGATGAATGCACACATCACAAAGAAGTT

                                                     
     144926 TCTCAGAATGCTTCTGTGTAGTTTTTATGTGAAGATATTTC
        131 TCTCAGAATGCTTCTGTGTAGTTTTTATGTGAAGATATTTC

                     * *    *      *    *       * *   **      *              
     144967 CTTTTCCACAACA-GCGTGCAAAACGCTTCAAATATGCCCTTAGAGATTCCACAAAAAGAGTGTT
          1 CTTTTCCACCATAGGCCT-CAAAGCGCTCCAAATATCCACTTGCAGATTCTACAAAAAGAGTGTT

              *     *               *  *                                  ** 
     145031 TCCAAACTACTCAAATCAAAA-AATGATTTCAACTCTGTGAGATGAATGCACACATCACAAACTA
         65 TCAAAACTGCTC-AATCAAAAGAAAG-GTTCAACTCTGTGAGATGAATGCACACATCACAAAGAA

                         *     ** *   * *   *       *   
     145095 GTTTCTCAGAATGTTTCTGCCTGGTTCTCATGCGAAGATAGTTC
        128 GTTTCTCAGAATGCTTCTGTGTAGTTTTTATGTGAAGATATTTC

                 *           *   * **              *                  * *    
     145139 CTTTTTCACCATAGGCCGCAATGTACTCCAAATATCCACCTGCAGATTCTAC-AAAAGTGAGTTT
          1 CTTTTCCACCATAGGCCTCAAAGCGCTCCAAATATCCACTTGCAGATTCTACAAAAAGAGTGTTT

                       *         ***    **         *        *      *       * 
     145203 CAAAACTGCTCTATCAAAAGATCAGTTCGTCTCTGTGAGTTGAATGCATACATCAAAAAGAAGCT
         66 CAAAACTGCTCAATCAAAAGAAAGGTTCAACTCTGTGAGATGAATGCACACATCACAAAGAAGTT

                 *             *      **         *   
     145268 TCTCAAAATGCTTCTGTGTGGTTTTTCGGTGAAGATAGTTC
        131 TCTCAGAATGCTTCTGTGTAGTTTTTATGTGAAGATATTTC

            *     *        *      * *                 *        *        *    
     145309 TTTTTCTACCATAGGTCTCAAACCACTCCAAATATCCACTTGTAGATTCTATAAAAAGGAATG-T
          1 CTTTTCCACCATAGGCCTCAAAGCGCTCCAAATATCCACTTGCAGATTCTACAAAAA-GAGTGTT

                  *        *    *    *      * *         *      *             
     145373 TCAAAATTGCTCAATAAAAATAAAGTTTCAACACCGTGAGATGAGTGCACAAATCACAAAGAAGT
         65 TCAAAACTGCTCAATCAAAAGAAAGGTTCAACTCTGTGAGATGAATGCACACATCACAAAGAAGT

                  *          *         *          *   
     145438 TTCTCAAAATGCTTCTGGGTAGTTTTTCTGTGAAGATAGTTC
        130 TTCTCAGAATGCTTCTGTGTAGTTTTTATGTGAAGATATTTC

                  *     *    *     *          *                         *    
     145480 CTTTTCTACCATGGGCCACAAAGGGCTCCAAATACCCACTTGCAGATTCTACAAAAAGAGAGTTT
          1 CTTTTCCACCATAGGCCTCAAAGCGCTCCAAATATCCACTTGCAGATTCTACAAAAAGAGTGTTT

              *        *      ** * *        *    * *    **  * *           *  
     145545 CACAACTGCTCTATCAAACAATATGTTCAACTTTGTGGGTTGAACACAAATATCAC-AAGAATTT
         66 CAAAACTGCTCAATCAAAAGAAAGGTTCAACTCTGTGAGATGAATGCACACATCACAAAGAAGTT

                **                             *     
     145609 TCTCCCAATGCTTCTGTGTAGTTTTTATGTGAAGACATTTC
        131 TCTCAGAATGCTTCTGTGTAGTTTTTATGTGAAGATATTTC

            *   *  *      *  *     *                 *  *      *       *  *  
     145650 TTTTCCCTCCATAGTCCACAAAGTGCTCCAAATATCCACTTACATATTCTAGAAAAAGATTGCTT
          1 CTTTTCCACCATAGGCCTCAAAGCGCTCCAAATATCCACTTGCAGATTCTACAAAAAGAGTGTTT

            **       *    *               * * *                              
     145715 GGAAACTGCACAATGAAAAGAAAGGTTCAAATATATGAGATGAATGCACACATCACAAAGAAGTT
         66 CAAAACTGCTCAATCAAAAGAAAGGTTCAACTCTGTGAGATGAATGCACACATCACAAAGAAGTT

                                 *                    
     145780 TCTCAGAAT-CTCTCTGTGTAATTTTTATGTGAAGATATTTC
        131 TCTCAGAATGCT-TCTGTGTAGTTTTTATGTGAAGATATTTC

                *     *    *  *   *                        *      *    *     
     145821 CTTTCCCACCTTAGGTCTTAAAACGCTCCAAATATCCACTTGCAGATACTACAAGAAGATTGTTT
          1 CTTTTCCACCATAGGCCTCAAAGCGCTCCAAATATCCACTTGCAGATTCTACAAAAAGAGTGTTT

                     *    *        *      *     **                           
     145886 CAAAACTGCACAA-AAAAAGAAATGTTCAATTCTGTTTGATGAATGCACACATCACAAAGAAGTT
         66 CAAAACTGCTCAATCAAAAGAAAGGTTCAACTCTGTGAGATGAATGCACACATCACAAAGAAGTT

                           *                         
     145950 TCTCAGAATGCTTCTCTGTAGTTTTTATGTGAAGATATTTC
        131 TCTCAGAATGCTTCTGTGTAGTTTTTATGTGAAGATATTTC

                     *             *                 *         **      * *   
     145991 CTTTTCCACAATAGGCCTCAAAGGGCTCCAAATATCCACTTCCAGATTCTATGAAAAGAATATTT
          1 CTTTTCCACCATAGGCCTCAAAGCGCTCCAAATATCCACTTGCAGATTCTACAAAAAGAGTGTTT

             *              * *    *                                      *  
     146056 CCAAACTGCTCAATCATAGGAAATGTTCAACTCTGTGAGATGAATGCACACATCAC-AAGAAATT
         66 CAAAACTGCTCAATCAAAAGAAAGGTTCAACTCTGTGAGATGAATGCACACATCACAAAGAAGTT

                     *    *      *       *       *   
     146120 TCTCAGAATCCTTCAGTGTAGGTTTTATGAGAAGATAATTC
        131 TCTCAGAATGCTTCTGTGTAGTTTTTATGTGAAGATATTTC

                     *    **        *   *                           *    *   
     146161 CTTTTCCACAATAGTTCTCAAAGCACTCAAAATATCCACTTGCAGATTCTACAAAAGGAGTATTT
          1 CTTTTCCACCATAGGCCTCAAAGCGCTCCAAATATCCACTTGCAGATTCTACAAAAAGAGTGTTT

                                                          *                  
     146226 CAAAACTGCTCAATCAAAAGAAAGGTTCAACTCTGTGAGATGAATGGACACATCACAAAGAAGTT
         66 CAAAACTGCTCAATCAAAAGAAAGGTTCAACTCTGTGAGATGAATGCACACATCACAAAGAAGTT

                                  *   *              
     146291 TCTCAGAATGCTTCTGTGTAGTATTTTTGTGAAGATATTT-
        131 TCTCAGAATGCTTCTGTGTAGTTTTTATGTGAAGATATTTC

                          *  * * * * ***             *            *     * *  
     146331 CTTTTCCACCATAGACCGCCAGGGGACACAAATATCCACTTTCAGATTCTACAACAAGAGAGGTT
          1 CTTTTCCACCATAGGCCTCAAAGCGCTCCAAATATCCACTTGCAGATTCTACAAAAAGAGTGTTT

                   *   *     *   ** *       *      *                      * *
     146396 CAAAACTACTCGATCAAGAGATGGTTTCAACTATGTGAGTTGAATGCACACATCACAAAGAACTA
         66 CAAAACTGCTCAATCAAAAGAAAGGTTCAACTCTGTGAGATGAATGCACACATCACAAAGAAGTT

             *  *    *                               
     146461 TGTCGGAATTCTTCTGTGTAGTTTTTATGTGAAGATATTTC
        131 TCTCAGAATGCTTCTGTGTAGTTTTTATGTGAAGATATTTC

                     *    * *      * *    *                  *               
     146502 CTTTTCCACAATAGACGTCAAAGTGATCCAGATATCCACTTGCAGATTCCACAAAAAGAGTGTTT
          1 CTTTTCCACCATAGGCCTCAAAGCGCTCCAAATATCCACTTGCAGATTCTACAAAAAGAGTGTTT

                 *   *   *                  **                     *  *      
     146567 CAAAAGTGCACAACCAAAAGAAAGGTTCAACTAGGTGAGATGAATGCACACATCAGAAGGAAGTT
         66 CAAAACTGCTCAATCAAAAGAAAGGTTCAACTCTGTGAGATGAATGCACACATCACAAAGAAGTT

                            **   *     * *       *   
     146632 TCTCAGAATGCTTCTGCATAGCTTTTAAGGGAAGATACTTC
        131 TCTCAGAATGCTTCTGTGTAGTTTTTATGTGAAGATATTTC

                    *               *            * *  *    * *               
     146673 CTTTTCCAACATAGGCCTCAAAGCACTCCAAATATCCTCCTGGAGATACCACAAAAAGAGTGTTT
          1 CTTTTCCACCATAGGCCTCAAAGCGCTCCAAATATCCACTTGCAGATTCTACAAAAAGAGTGTTT

            **                      *  *                 *       *           
     146738 GCAAACTGCTCAATCAAAAGAAAGATTTAACTCTGTGAGATGAATCCACACATGACAAAGAAGTT
         66 CAAAACTGCTCAATCAAAAGAAAGGTTCAACTCTGTGAGATGAATGCACACATCACAAAGAAGTT

                                                     
     146803 TCTCAGAATGCTTCTGTGTAGTTTTTATGTGAAGATATTTC
        131 TCTCAGAATGCTTCTGTGTAGTTTTTATGTGAAGATATTTC

                     *   * * *    **           *                *     *      
     146844 CTTTTCCACAATAAGACCCAAAAGGCTCCAAATATTCACTTGCAGATTCTAAAAAAAACAGTGTT
          1 CTTTTCCACCATAGGCCTCAAAGCGCTCCAAATATCCACTTGCAGATTCT-ACAAAAAGAGTGTT

                                  *                  *      *        **      
     146909 TCAAAACTGCTCAATCAAAAGATA-GTTCAACTCTGTGAGAAGAATGCTCACATCACTGAGAAGT
         65 TCAAAACTGCTCAATCAAAAGAAAGGTTCAACTCTGTGAGATGAATGCACACATCACAAAGAAGT

                                         *            
     146973 TTCTCAGAATGCTTCTGTGTAGTTTTTATATGAAGATATTTC
        130 TTCTCAGAATGCTTCTGTGTAGTTTTTATGTGAAGATATTTC

                *     *      *    **   *                   *   **       *    
     147015 CTTTCCCACCGTAGGCCACAAAAGGCTTCAAATATCCACTTGCAGATACTATGAAAAGAGAGTTT
          1 CTTTTCCACCATAGGCCTCAAAGCGCTCCAAATATCCACTTGCAGATTCTACAAAAAGAGTGTTT

                        *        *               ***            *            
     147080 CAAAACTGCTCATTCAAAAGATAGGTTCAACTCTGTGGTTTGAATGCACACAGCACAAAGAAGTT
         66 CAAAACTGCTCAATCAAAAGAAAGGTTCAACTCTGTGAGATGAATGCACACATCACAAAGAAGTT

              *       **                   **   *    
     147145 TCACAGAATGTGTCTGTGTAGTTTTTATGTGCGGATGTTTC
        131 TCTCAGAATGCTTCTGTGTAGTTTTTATGTGAAGATATTTC

                         *        ****   *   *               *    *          
     147186 CTTTTCCACCATATGCCT-AAATATTTCCCAATTTCCACTTGCAGATTCCACAAGAAGAGTGTTT
          1 CTTTTCCACCATAGGCCTCAAAGCGCTCCAAATATCCACTTGCAGATTCTACAAAAAGAGTGTTT

                      **       *       *           *            *      ***   
     147250 CAAAACTGCTGTATC-AAATAAA-GTTGAACTCTGTGAGGTGAATGCACACAGCACAAAATGGTT
         66 CAAAACTGCTCAATCAAAAGAAAGGTTCAACTCTGTGAGATGAATGCACACATCACAAAGAAGTT

                          **   *        *       *    
     147313 TCTCAGAATGCTTCCTTGTTGTTTTTATATGAAGATGTTTC
        131 TCTCAGAATGCTTCTGTGTAGTTTTTATGTGAAGATATTTC

                  *  *             *   *     *                               
     147354 CTTTTCAACAATAGGCCTCAAAGTGCTTCAAATGTCCACTTGCAGATTCTACAAAAAGAGTGTTT
          1 CTTTTCCACCATAGGCCTCAAAGCGCTCCAAATATCCACTTGCAGATTCTACAAAAAGAGTGTTT

                                        *      *  *  *                       
     147419 CAAAACTGCTCAATCAAAAGAAAGGTTCGACTCTGGGAAATTAATGCACACATCACAAAGAAGTT
         66 CAAAACTGCTCAATCAAAAGAAAGGTTCAACTCTGTGAGATGAATGCACACATCACAAAGAAGTT

                                     *        *      
     147484 TCTC---A-GCTTCTGTGTAGTTTTCATGTGAAGTTATTTC
        131 TCTCAGAATGCTTCTGTGTAGTTTTTATGTGAAGATATTTC

                     *       *     *            *    *         **       *    
     147521 CTTTTCCACAATAGGCCGCAAAGGGCTCCAAATATCAACTTACAGATTCTAGGAAAAGAGAGTTT
          1 CTTTTCCACCATAGGCCTCAAAGCGCTCCAAATATCCACTTGCAGATTCTACAAAAAGAGTGTTT

                       * **      *     *                                     
     147586 CAAAACTGCTCTACGAAAAGATAGGTTGAACTCTGTGAGATGAATGCACACATCACAAAGAAGTT
         66 CAAAACTGCTCAATCAAAAGAAAGGTTCAACTCTGTGAGATGAATGCACACATCACAAAGAAGTT

                       *               * *           
     147651 TCTCAGAATGCATCTGTGTAGTTTTTACGGGAAGATATTTC
        131 TCTCAGAATGCTTCTGTGTAGTTTTTATGTGAAGATATTTC

                        ***  *     **      *  *                  *     ***   
     147692 CTTTTCCACCATCTTCCACAAAGGTCTCCAAGTAACCACTTGCAGATTCTACAGAAAGACACTTT
          1 CTTTTCCACCATAGGCCTCAAAGCGCTCCAAATATCCACTTGCAGATTCTACAAAAAGAGTGTTT

            *          *         ***      *      ***                      *  
     147757 AAAAACTGCTCTATCAAAAGATCAGTTCAAGTCTGTGGTTTGAATGCACACATCACAAAGAATTT
         66 CAAAACTGCTCAATCAAAAGAAAGGTTCAACTCTGTGAGATGAATGCACACATCACAAAGAAGTT

                                     *  *            
     147822 TCTCAGAATGCTTCTGTGTAGTTTTCATATGAAGATATTTC
        131 TCTCAGAATGCTTCTGTGTAGTTTTTATGTGAAGATATTTC

                                    *                                   **   
     147863 CTTTTCCACCATAGGCCTCAAAGCACTCCAAATATCCACTTGCAGATTCTACAAAAAGAGATTTT
          1 CTTTTCCACCATAGGCCTCAAAGCGCTCCAAATATCCACTTGCAGATTCTACAAAAAGAGTGTTT

                   **                           *  *          *        *     
     147928 CAAAACTAGTCAATCAAAAGAAAGGTTCAACTCTGTCAGTTGAATGCACATATCACAAACAAGTT
         66 CAAAACTGCTCAATCAAAAGAAAGGTTCAACTCTGTGAGATGAATGCACACATCACAAAGAAGTT

                *      *                             
     147993 TCTCGGAATGCGTCTGTGTAGTTTTTATGTGAAGATATTTC
        131 TCTCAGAATGCTTCTGTGTAGTTTTTATGTGAAGATATTTC

               *     * *           *     *                  *    *           
     148034 CTTCTCCACAACAGGCCTCAAAGTGCTCCGAATATCCACTTGCAGATTTTAC-TAAAGAGTGTTT
          1 CTTTTCCACCATAGGCCTCAAAGCGCTCCAAATATCCACTTGCAGATTCTACAAAAAGAGTGTTT

             *               *  *   *     *        *    *               *    
     148098 CCAAACTGCTCAATCAAGAGGAAGTTTCAAGTCTGTGAGCTGAACGCACACATCACAAAGTAGTT
         66 CAAAACTGCTCAATCAAAAGAAAGGTTCAACTCTGTGAGATGAATGCACACATCACAAAGAAGTT

               *                                *    
     148163 TCTGAGAATGCTTCTGTGTAGTTTTTATGTGAAGATGTTTC
        131 TCTCAGAATGCTTCTGTGTAGTTTTTATGTGAAGATATTTC

                            **     *                                    *    
     148204 CTTTTCCACCATAGGCTGCAAAGGGCTCCAAATATCCACTTGCAGATTCTACAAAAAGAGAGTTT
          1 CTTTTCCACCATAGGCCTCAAAGCGCTCCAAATATCCACTTGCAGATTCTACAAAAAGAGTGTTT

                 *     *         *          *     *                     *    
     148269 CAAAAGTGCTCTATCAAAAGATAGGTTCAACTATGTGATATGAATGCACACATCACAAAGTAGTT
         66 CAAAACTGCTCAATCAAAAGAAAGGTTCAACTCTGTGAGATGAATGCACACATCACAAAGAAGTT

                                          *          
     148334 TCTCAGAATGCTTCTGTGTAGTTTTTATGTAAAGATATTTC
        131 TCTCAGAATGCTTCTGTGTAGTTTTTATGTGAAGATATTTC

                                    *                                   **   
     148375 CTTTTCCACCATAGGCCTCAAAGCACTCCAAATATCCACTTGCAGATTCTACAAAAAGAGATTTT
          1 CTTTTCCACCATAGGCCTCAAAGCGCTCCAAATATCCACTTGCAGATTCTACAAAAAGAGTGTTT

                   ** *                   *     *  *    * *   *        *     
     148440 CAAAACTATTTAATCAAAAGAAAGGTTCAAATCTGTCAGTTGAAGGTACATATCACAAACAAGTT
         66 CAAAACTGCTCAATCAAAAGAAAGGTTCAACTCTGTGAGATGAATGCACACATCACAAAGAAGTT

             * **                                    
     148505 TATTGGAATGCTTCTGTGTAGTTTTTATGTGAAGATATTTC
        131 TCTCAGAATGCTTCTGTGTAGTTTTTATGTGAAGATATTTC

                     * *       * * *                        **  *     *      
     148546 CTTTTCCACAACAGGCCTCCAGGTGCTCCAAATATCCACTTGCAGATTTCACTAAAAGTGTGTTT
          1 CTTTTCCACCATAGGCCTCAAAGCGCTCCAAATATCCACTTGCAGATTCTACAAAAAGAGTGTTT

             *  *            *  *   *     *        *             *           
     148611 CCAAGCTGCTCAATCAAGAGGAAGTTTCAAGTCTGTGAGGTGAATGCACACATTACAAAGAAGTT
         66 CAAAACTGCTCAATCAAAAGAAAGGTTCAACTCTGTGAGATGAATGCACACATCACAAAGAAGTT

            *  *                                     
     148676 ACTGAGAATGCTTCTGTGTAGTTTTTATGTGAAGATATTTC
        131 TCTCAGAATGCTTCTGTGTAGTTTTTATGTGAAGATATTTC

                      **               *                                *    
     148717 CTTTTCCACCGCAGGCCTCAAAGCGCTGCAAATATCCACTTGCAGATTCTACAAAAAGAGAGTTT
          1 CTTTTCCACCATAGGCCTCAAAGCGCTCCAAATATCCACTTGCAGATTCTACAAAAAGAGTGTTT

                      **         *   *         *   *     **            *     
     148782 CAAAACTGCTGTATCAAAAGATAGGGTCAACTCTGCGAGTTGAATAAACACATCACAAATAAGTT
         66 CAAAACTGCTCAATCAAAAGAAAGGTTCAACTCTGTGAGATGAATGCACACATCACAAAGAAGTT

               **   *        *               *       
     148847 TCTGGGAACGCTTCTGTATAG-TTTTATGTGAATATATTTC
        131 TCTCAGAATGCTTCTGTGTAGTTTTTATGTGAAGATATTTC

                         *          *                   *   *  *   * *   *   
     148887 CTTTTCCACCATATGCCTCAAAGCACTCCAAATATCCACTTGCACATTATAGAAACATAGTCTTT
          1 CTTTTCCACCATAGGCCTCAAAGCGCTCCAAATATCCACTTGCAGATTCTACAAAAAGAGTGTTT

                   **                        *         *             *       
     148952 CAAAACTTGTCAATC-AAAGAAAGGTTCAACTCCGTGAGATGAGTGCACACATCACAGAGAAGTT
         66 CAAAACTGCTCAATCAAAAGAAAGGTTCAACTCTGTGAGATGAATGCACACATCACAAAGAAGTT

                *     *                            * 
     149016 TCTCGGAATGTTTCTGTGTAGTTTTTATGTGAAGATATTGC
        131 TCTCAGAATGCTTCTGTGTAGTTTTTATGTGAAGATATTTC

                     *               *            *          *       *   *   
     149057 CTTTTCCACAATAGGCCTCAAAGCGTTCCAAATATCCAATTGCAGATTCCACAAAAAAAGTTTTT
          1 CTTTTCCACCATAGGCCTCAAAGCGCTCCAAATATCCACTTGCAGATTCTACAAAAAGAGTGTTT

            *                 *  *  *  *             * *         **    *     
     149122 TAAAACTGCTCAATCAAATGATAGATTAAACTCTGTGAGATTAGTGCACACATGTCAAAAAAGTT
         66 CAAAACTGCTCAATCAAAAGAAAGGTTCAACTCTGTGAGATGAATGCACACATCACAAAGAAGTT

                                *      * *           
     149187 TCTCAGAATGCTTCTGTGTACTTTTTAGGGGAAGATATTTC
        131 TCTCAGAATGCTTCTGTGTAGTTTTTATGTGAAGATATTTC

                        *    *     **         *    *           **  * *  *    
     149228 CTTTTCCACCATCGGCCACAAAGGACTCCAAATAACCACATGCAGATTCTAGTAACACAGAGTTT
          1 CTTTTCCACCATAGGCCTCAAAGCGCTCCAAATATCCACTTGCAGATTCTACAAAAAGAGTGTTT

                       *         * *           *   * * *    **    ***        
     149293 CAAAACTGCTCTATCAAAAGATAAGTTCAACTCTGAGAGTTTAGTGCAACCATCGTGAAGAAGTT
         66 CAAAACTGCTCAATCAAAAGAAAGGTTCAACTCTGTGAGATGAATGCACACATCACAAAGAAGTT

                            *     *                  
     149358 TCTCAGAATGCTTCTGAGTAGTGTTTATGTGAAGATATTTC
        131 TCTCAGAATGCTTCTGTGTAGTTTTTATGTGAAGATATTTC

                              *     *                      *           **    
     149399 CTTTTCCACCATAGGCCTGAAAGCCCTCCAAATATCCACTTGCAGATCCTACAAAAAGAAAGTTT
          1 CTTTTCCACCATAGGCCTCAAAGCGCTCCAAATATCCACTTGCAGATTCTACAAAAAGAGTGTTT

             *         **     *  *  *   *        **      *                   
     149464 CGAAA-TGCTCTCTCAAACGATAGTTTCGACTCTGTGGTATGAATACACACATCACAAAGAAGTT
         66 CAAAACTGCTCAATCAAAAGAAAGGTTCAACTCTGTGAGATGAATGCACACATCACAAAGAAGTT

                                       **            
     149528 TCTCAGAATGCTTCTGTGTAGTTTTTAAATGAAGATATTTC
        131 TCTCAGAATGCTTCTGTGTAGTTTTTATGTGAAGATATTTC

            *                       *          *     *                       
     149569 TTTTTCCACCATAGGCCTCAAAGCACTCCAAATATGCACTTCCAGATTCTACAAAAAGAGTGTTT
          1 CTTTTCCACCATAGGCCTCAAAGCGCTCCAAATATCCACTTGCAGATTCTACAAAAAGAGTGTTT

              *                 *       * *         **   *         *  * *    
     149634 CAGAACTGCTCAATCAAAAGGAAGGTTCCAGTC--TGAGACAAATACACACATCAAAAGGTAGTT
         66 CAAAACTGCTCAATCAAAAGAAAGGTTCAACTCTGTGAGATGAATGCACACATCACAAAGAAGTT

                                                    *
     149697 TCTCAGAATGCTTCTGTGTAGTTTTTATGTGAAGATATTTT
        131 TCTCAGAATGCTTCTGTGTAGTTTTTATGTGAAGATATTTC

             *               *    **    *     *      *  **   *          *    
     149738 CCTTTCCACCATAGGCCACAAATGGCTCTAAATACCCACTTACATTTTCCACAAAAAGAGAGTTT
          1 CTTTTCCACCATAGGCCTCAAAGCGCTCCAAATATCCACTTGCAGATTCTACAAAAAGAGTGTTT

                       * *    * *      *   *         *                       
     149803 CAAAACTGCTCTACCAAAGGTAA-GTTTAACGCTGTGAG-TTAA-G-A-ACATCACAAAGAAGTT
         66 CAAAACTGCTCAATCAAAAGAAAGGTTCAACTCTGTGAGATGAATGCACACATCACAAAGAAGTT

                                   *   *  *          
     149863 TCTCAGAATGCTTCTGTGTAGTTCTTACGTAAAGATATTTC
        131 TCTCAGAATGCTTCTGTGTAGTTTTTATGTGAAGATATTTC

                 *   *      ***    *                  *              **      
     149904 CTTTTACACAATAGGCAGAAAAGTGCTCCAAATATCCACTTGAAGATTCTACAAAAACCGTGTTT
          1 CTTTTCCACCATAGGCCTCAAAGCGCTCCAAATATCCACTTGCAGATTCTACAAAAAGAGTGTTT

                     **                                          *      *    
     149969 CAAAACTGCCGAATCAAAAGAAAGGTTCAACTCTGTGAGATGAATGCACACATAACAAAGGAGTT
         66 CAAAACTGCTCAATCAAAAGAAAGGTTCAACTCTGTGAGATGAATGCACACATCACAAAGAAGTT

                                 *      *      *    *
     150034 TCTCAGAATGCTTCTGTGTAGCTTTTATATGAAGACATTTA
        131 TCTCAGAATGCTTCTGTGTAGTTTTTATGTGAAGATATTTC

            *        * *            *   ***                     **        *  
     150075 GTTTTCCACAACAGGCCTCAAAGCTCTCTCCATATCCACTTGCAGATTCTACCGAAAGAGTGCTT
          1 CTTTTCCACCATAGGCCTCAAAGCGCTCCAAATATCCACTTGCAGATTCTACAAAAAGAGTGTTT

             *                   * **     *        *        *     **         
     150140 CCAAACTGCTCAATCAAAAGAGACATTCAAATCTGTGAGGTGAATGCAGACATCGTAAAGAAGTT
         66 CAAAACTGCTCAATCAAAAGAAAGGTTCAACTCTGTGAGATGAATGCACACATCACAAAGAAGTT

                                *     *       *    **
     150205 TCTCAGAATGCTTCTGTGTATTTTTTGTGTGAAGTTATTCG
        131 TCTCAGAATGCTTCTGTGTAGTTTTTATGTGAAGATATTTC

            *    *             *     *  *            **                 *    
     150246 TTTTTGCACCATAGGCCTCCAAGCGTTCTAAATATCCACTTCTAGATTCTACAAAAAGAGAGTTT
          1 CTTTTCCACCATAGGCCTCAAAGCGCTCCAAATATCCACTTGCAGATTCTACAAAAAGAGTGTTT

                   *         *            *        *    *   *                
     150311 CAAAACTACTC-A--AACA-AAAGGTTCAATTCTGTGAGTTGAAAGCAAACATCACAAAGAAGTT
         66 CAAAACTGCTCAATCAAAAGAAAGGTTCAACTCTGTGAGATGAATGCACACATCACAAAGAAGTT

                       *             *               
     150372 TCTCAGAATGCGTCTGTGTAGTTTTGATGTGAAGATATTTC
        131 TCTCAGAATGCTTCTGTGTAGTTTTTATGTGAAGATATTTC

                     **   ****     *                  *                      
     150413 CTTTT-CACAGTAGAATGCAAAGGGCTCCAAATATCCACTTGGAGATTCTACAAAAAGA--GTTT
          1 CTTTTCCACCATAGGCCTCAAAGCGCTCCAAATATCCACTTGCAGATTCTACAAAAAGAGTGTTT

                  *    **     *  *     *     ***   *     *               *   
     150475 CAAAACCGCTCTGTCAAATGATAGGTTGAACTCCCGGAGGTGAATACACACATCACAAAGAGGTT
         66 CAAAACTGCTCAATCAAAAGAAAGGTTCAACTCTGTGAGATGAATGCACACATCACAAAGAAGTT

                  *                       *  *       
     150540 TCTCAGCATGCTTCTGTGTAGTTTTTATGTAAACATATTTC
        131 TCTCAGAATGCTTCTGTGTAGTTTTTATGTGAAGATATTTC

             *    * *              *           *      * *       *   *    *   
     150581 CGTTTCTATCATAGGCCTCAAAGTGCTCCAAATATTCACTTGTACATTCTACCAAACGAGTATTT
          1 CTTTTCCACCATAGGCCTCAAAGCGCTCCAAATATCCACTTGCAGATTCTACAAAAAGAGTGTTT

                              * *         **      *        *            *    
     150646 CAAAACTGCTCAATCAAATGGAAGGTTCAAAACTGTGACATGAATGCCCACATCACAAAGTAGTT
         66 CAAAACTGCTCAATCAAAAGAAAGGTTCAACTCTGTGAGATGAATGCACACATCACAAAGAAGTT

                                                     
     150711 TCTCAGAATGCTTCTGTGTAGTTTTTATGTGAAGATATTTC
        131 TCTCAGAATGCTTCTGTGTAGTTTTTATGTGAAGATATTTC

                     * *    *      *    *       * *   **      *              
     150752 CTTTTCCACAACA-GCGTGCAAAACGCTTCAAATATGCCCTTAGAGATTCCACAAAAAGAGTGTT
          1 CTTTTCCACCATAGGCCT-CAAAGCGCTCCAAATATCCACTTGCAGATTCTACAAAAAGAGTGTT

              *     *               *  *                                  ** 
     150816 TCCAAACTACTCAAATCAAAA-AATGATTTCAACTCTGTGAGATGAATGCACACATCACAAACTA
         65 TCAAAACTGCTC-AATCAAAAGAAAG-GTTCAACTCTGTGAGATGAATGCACACATCACAAAGAA

                         *     ** *   * *   *       *   
     150880 GTTTCTCAGAATGTTTCTGCCTGGTTCTCATGCGAAGATAGTTC
        128 GTTTCTCAGAATGCTTCTGTGTAGTTTTTATGTGAAGATATTTC

                 *           *   * **              *                  * *    
     150924 CTTTTTCACCATAGGCCGCAATGTACTCCAAATATCCACCTGCAGATTCTAC-AAAAGTGAGTTT
          1 CTTTTCCACCATAGGCCTCAAAGCGCTCCAAATATCCACTTGCAGATTCTACAAAAAGAGTGTTT

                       *         ***    **         *        *      *       * 
     150988 CAAAACTGCTCTATCAAAAGATCAGTTCGTCTCTGTGAGTTGAATGCATACATCAAAAAGAAGCT
         66 CAAAACTGCTCAATCAAAAGAAAGGTTCAACTCTGTGAGATGAATGCACACATCACAAAGAAGTT

                 *             *      **         *   
     151053 TCTCAAAATGCTTCTGTGTGGTTTTTCGGTGAAGATAGTTC
        131 TCTCAGAATGCTTCTGTGTAGTTTTTATGTGAAGATATTTC

            *     *        *      * *                 *        *        *    
     151094 TTTTTCTACCATAGGTCTCAAACCACTCCAAATATCCACTTGTAGATTCTATAAAAAGGAATG-T
          1 CTTTTCCACCATAGGCCTCAAAGCGCTCCAAATATCCACTTGCAGATTCTACAAAAA-GAGTGTT

                  *        *    *    *      * *         *      *             
     151158 TCAAAATTGCTCAATAAAAATAAAGTTTCAACACCGTGAGATGAGTGCACAAATCACAAAGAAGT
         65 TCAAAACTGCTCAATCAAAAGAAAGGTTCAACTCTGTGAGATGAATGCACACATCACAAAGAAGT

                  *          *         *          *   
     151223 TTCTCAAAATGCTTCTGGGTAGTTTTTCTGTGAAGATAGTTC
        130 TTCTCAGAATGCTTCTGTGTAGTTTTTATGTGAAGATATTTC

                  *     *    *     *          *                         *    
     151265 CTTTTCTACCATGGGCCACAAAGGGCTCCAAATACCCACTTGCAGATTCTACAAAAAGAGAGTTT
          1 CTTTTCCACCATAGGCCTCAAAGCGCTCCAAATATCCACTTGCAGATTCTACAAAAAGAGTGTTT

              *        *      ** * *        *    * *    **  * *           *  
     151330 CACAACTGCTCTATCAAACAATATGTTCAACTTTGTGGGTTGAACACAAATATCAC-AAGAATTT
         66 CAAAACTGCTCAATCAAAAGAAAGGTTCAACTCTGTGAGATGAATGCACACATCACAAAGAAGTT

                **                             *     
     151394 TCTCCCAATGCTTCTGTGTAGTTTTTATGTGAAGACATTTC
        131 TCTCAGAATGCTTCTGTGTAGTTTTTATGTGAAGATATTTC

            *   *  *      *  *     *                 *  *      *       *  *  
     151435 TTTTCCCTCCATAGTCCACAAAGTGCTCCAAATATCCACTTACATATTCTAGAAAAAGATTGCTT
          1 CTTTTCCACCATAGGCCTCAAAGCGCTCCAAATATCCACTTGCAGATTCTACAAAAAGAGTGTTT

            **       *    *               * * *                              
     151500 GGAAACTGCACAATGAAAAGAAAGGTTCAAATATATGAGATGAATGCACACATCACAAAGAAGTT
         66 CAAAACTGCTCAATCAAAAGAAAGGTTCAACTCTGTGAGATGAATGCACACATCACAAAGAAGTT

                                 *                    
     151565 TCTCAGAAT-CTCTCTGTGTAATTTTTATGTGAAGATATTTC
        131 TCTCAGAATGCT-TCTGTGTAGTTTTTATGTGAAGATATTTC

                *     *    *  *   *                        *      *    *     
     151606 CTTTCCCACCTTAGGTCTTAAAACGCTCCAAATATCCACTTGCAGATACTACAAGAAGATTGTTT
          1 CTTTTCCACCATAGGCCTCAAAGCGCTCCAAATATCCACTTGCAGATTCTACAAAAAGAGTGTTT

                     *    *        *      *     **                           
     151671 CAAAACTGCACAA-AAAAAGAAATGTTCAATTCTGTTTGATGAATGCACACATCACAAAGAAGTT
         66 CAAAACTGCTCAATCAAAAGAAAGGTTCAACTCTGTGAGATGAATGCACACATCACAAAGAAGTT

                           *                         
     151735 TCTCAGAATGCTTCTCTGTAGTTTTTATGTGAAGATATTTC
        131 TCTCAGAATGCTTCTGTGTAGTTTTTATGTGAAGATATTTC

                     *             *                 *         **      * *   
     151776 CTTTTCCACAATAGGCCTCAAAGGGCTCCAAATATCCACTTCCAGATTCTATGAAAAGAATATTT
          1 CTTTTCCACCATAGGCCTCAAAGCGCTCCAAATATCCACTTGCAGATTCTACAAAAAGAGTGTTT

             *              * *    *                                      *  
     151841 CCAAACTGCTCAATCATAGGAAATGTTCAACTCTGTGAGATGAATGCACACATCAC-AAGAAATT
         66 CAAAACTGCTCAATCAAAAGAAAGGTTCAACTCTGTGAGATGAATGCACACATCACAAAGAAGTT

                     *    *      *       *       *   
     151905 TCTCAGAATCCTTCAGTGTAGGTTTTATGAGAAGATAATTC
        131 TCTCAGAATGCTTCTGTGTAGTTTTTATGTGAAGATATTTC

                     *    **        *   *                           *    *   
     151946 CTTTTCCACAATAGTTCTCAAAGCACTCAAAATATCCACTTGCAGATTCTACAAAAGGAGTATTT
          1 CTTTTCCACCATAGGCCTCAAAGCGCTCCAAATATCCACTTGCAGATTCTACAAAAAGAGTGTTT

                                                          *                  
     152011 CAAAACTGCTCAATCAAAAGAAAGGTTCAACTCTGTGAGATGAATGGACACATCACAAAGAAGTT
         66 CAAAACTGCTCAATCAAAAGAAAGGTTCAACTCTGTGAGATGAATGCACACATCACAAAGAAGTT

                                  *   *              
     152076 TCTCAGAATGCTTCTGTGTAGTATTTTTGTGAAGATATTT-
        131 TCTCAGAATGCTTCTGTGTAGTTTTTATGTGAAGATATTTC

                          *  * * * * ***             *            *     * *  
     152116 CTTTTCCACCATAGACCGCCAGGGGACACAAATATCCACTTTCAGATTCTACAACAAGAGAGGTT
          1 CTTTTCCACCATAGGCCTCAAAGCGCTCCAAATATCCACTTGCAGATTCTACAAAAAGAGTGTTT

                   *   *     *   ** *       *      *                      * *
     152181 CAAAACTACTCGATCAAGAGATGGTTTCAACTATGTGAGTTGAATGCACACATCACAAAGAACTA
         66 CAAAACTGCTCAATCAAAAGAAAGGTTCAACTCTGTGAGATGAATGCACACATCACAAAGAAGTT

             *  *    *                               
     152246 TGTCGGAATTCTTCTGTGTAGTTTTTATGTGAAGATATTTC
        131 TCTCAGAATGCTTCTGTGTAGTTTTTATGTGAAGATATTTC

                     *    * *      * *    *                  *               
     152287 CTTTTCCACAATAGACGTCAAAGTGATCCAGATATCCACTTGCAGATTCCACAAAAAGAGTGTTT
          1 CTTTTCCACCATAGGCCTCAAAGCGCTCCAAATATCCACTTGCAGATTCTACAAAAAGAGTGTTT

                 *   *   *                  **                     *  *      
     152352 CAAAAGTGCACAACCAAAAGAAAGGTTCAACTAGGTGAGATGAATGCACACATCAGAAGGAAGTT
         66 CAAAACTGCTCAATCAAAAGAAAGGTTCAACTCTGTGAGATGAATGCACACATCACAAAGAAGTT

                            **   *     * *       *   
     152417 TCTCAGAATGCTTCTGCATAGCTTTTAAGGGAAGATACTTC
        131 TCTCAGAATGCTTCTGTGTAGTTTTTATGTGAAGATATTTC

                    *               *            * *  *    * *               
     152458 CTTTTCCAACATAGGCCTCAAAGCACTCCAAATATCCTCCTGGAGATACCACAAAAAGAGTGTTT
          1 CTTTTCCACCATAGGCCTCAAAGCGCTCCAAATATCCACTTGCAGATTCTACAAAAAGAGTGTTT

            **                      *  *                 *       *           
     152523 GCAAACTGCTCAATCAAAAGAAAGATTTAACTCTGTGAGATGAATCCACACATGACAAAGAAGTT
         66 CAAAACTGCTCAATCAAAAGAAAGGTTCAACTCTGTGAGATGAATGCACACATCACAAAGAAGTT

                                                     
     152588 TCTCAGAATGCTTCTGTGTAGTTTTTATGTGAAGATATTTC
        131 TCTCAGAATGCTTCTGTGTAGTTTTTATGTGAAGATATTTC

                     *   * * *    **           *                *     *      
     152629 CTTTTCCACAATAAGACCCAAAAGGCTCCAAATATTCACTTGCAGATTCTAAAAAAAACAGTGTT
          1 CTTTTCCACCATAGGCCTCAAAGCGCTCCAAATATCCACTTGCAGATTCT-ACAAAAAGAGTGTT

                                  *                  *      *        **      
     152694 TCAAAACTGCTCAATCAAAAGATA-GTTCAACTCTGTGAGAAGAATGCTCACATCACTGAGAAGT
         65 TCAAAACTGCTCAATCAAAAGAAAGGTTCAACTCTGTGAGATGAATGCACACATCACAAAGAAGT

                                         *            
     152758 TTCTCAGAATGCTTCTGTGTAGTTTTTATATGAAGATATTTC
        130 TTCTCAGAATGCTTCTGTGTAGTTTTTATGTGAAGATATTTC

                *     *      *    **   *                   *   **       *    
     152800 CTTTCCCACCGTAGGCCACAAAAGGCTTCAAATATCCACTTGCAGATACTATGAAAAGAGAGTTT
          1 CTTTTCCACCATAGGCCTCAAAGCGCTCCAAATATCCACTTGCAGATTCTACAAAAAGAGTGTTT

                        *        *               ***            *            
     152865 CAAAACTGCTCATTCAAAAGATAGGTTCAACTCTGTGGTTTGAATGCACACAGCACAAAGAAGTT
         66 CAAAACTGCTCAATCAAAAGAAAGGTTCAACTCTGTGAGATGAATGCACACATCACAAAGAAGTT

              *       **                   **   *    
     152930 TCACAGAATGTGTCTGTGTAGTTTTTATGTGCGGATGTTTC
        131 TCTCAGAATGCTTCTGTGTAGTTTTTATGTGAAGATATTTC

                         *        ****   *   *               *    *          
     152971 CTTTTCCACCATATGCCT-AAATATTTCCCAATTTCCACTTGCAGATTCCACAAGAAGAGTGTTT
          1 CTTTTCCACCATAGGCCTCAAAGCGCTCCAAATATCCACTTGCAGATTCTACAAAAAGAGTGTTT

                      **       *       *           *            *      ***   
     153035 CAAAACTGCTGTATC-AAATAAA-GTTGAACTCTGTGAGGTGAATGCACACAGCACAAAATGGTT
         66 CAAAACTGCTCAATCAAAAGAAAGGTTCAACTCTGTGAGATGAATGCACACATCACAAAGAAGTT

                          **   *        *       *    
     153098 TCTCAGAATGCTTCCTTGTTGTTTTTATATGAAGATGTTTC
        131 TCTCAGAATGCTTCTGTGTAGTTTTTATGTGAAGATATTTC

                  *  *             *   *     *                               
     153139 CTTTTCAACAATAGGCCTCAAAGTGCTTCAAATGTCCACTTGCAGATTCTACAAAAAGAGTGTTT
          1 CTTTTCCACCATAGGCCTCAAAGCGCTCCAAATATCCACTTGCAGATTCTACAAAAAGAGTGTTT

                                        *      *  *  *                       
     153204 CAAAACTGCTCAATCAAAAGAAAGGTTCGACTCTGGGAAATTAATGCACACATCACAAAGAAGTT
         66 CAAAACTGCTCAATCAAAAGAAAGGTTCAACTCTGTGAGATGAATGCACACATCACAAAGAAGTT

                                     *        *      
     153269 TCTC---A-GCTTCTGTGTAGTTTTCATGTGAAGTTATTTC
        131 TCTCAGAATGCTTCTGTGTAGTTTTTATGTGAAGATATTTC

                     *       *     *            *    *         **       *    
     153306 CTTTTCCACAATAGGCCGCAAAGGGCTCCAAATATCAACTTACAGATTCTAGGAAAAGAGAGTTT
          1 CTTTTCCACCATAGGCCTCAAAGCGCTCCAAATATCCACTTGCAGATTCTACAAAAAGAGTGTTT

                       * **      *     *                                     
     153371 CAAAACTGCTCTACGAAAAGATAGGTTGAACTCTGTGAGATGAATGCACACATCACAAAGAAGTT
         66 CAAAACTGCTCAATCAAAAGAAAGGTTCAACTCTGTGAGATGAATGCACACATCACAAAGAAGTT

                       *               * *           
     153436 TCTCAGAATGCATCTGTGTAGTTTTTACGGGAAGATATTTC
        131 TCTCAGAATGCTTCTGTGTAGTTTTTATGTGAAGATATTTC

                        ***  *     **      *  *                  *     ***   
     153477 CTTTTCCACCATCTTCCACAAAGGTCTCCAAGTAACCACTTGCAGATTCTACAGAAAGACACTTT
          1 CTTTTCCACCATAGGCCTCAAAGCGCTCCAAATATCCACTTGCAGATTCTACAAAAAGAGTGTTT

            *          *         ***      *      ***                      *  
     153542 AAAAACTGCTCTATCAAAAGATCAGTTCAAGTCTGTGGTTTGAATGCACACATCACAAAGAATTT
         66 CAAAACTGCTCAATCAAAAGAAAGGTTCAACTCTGTGAGATGAATGCACACATCACAAAGAAGTT

                                     *  *            
     153607 TCTCAGAATGCTTCTGTGTAGTTTTCATATGAAGATATTTC
        131 TCTCAGAATGCTTCTGTGTAGTTTTTATGTGAAGATATTTC

                                    *                                   **   
     153648 CTTTTCCACCATAGGCCTCAAAGCACTCCAAATATCCACTTGCAGATTCTACAAAAAGAGATTTT
          1 CTTTTCCACCATAGGCCTCAAAGCGCTCCAAATATCCACTTGCAGATTCTACAAAAAGAGTGTTT

                   **                           *  *          *        *     
     153713 CAAAACTAGTCAATCAAAAGAAAGGTTCAACTCTGTCAGTTGAATGCACATATCACAAACAAGTT
         66 CAAAACTGCTCAATCAAAAGAAAGGTTCAACTCTGTGAGATGAATGCACACATCACAAAGAAGTT

                *      *                             
     153778 TCTCGGAATGCGTCTGTGTAGTTTTTATGTGAAGATATTTC
        131 TCTCAGAATGCTTCTGTGTAGTTTTTATGTGAAGATATTTC

               *     * *           *     *                  *    *           
     153819 CTTCTCCACAACAGGCCTCAAAGTGCTCCGAATATCCACTTGCAGATTTTAC-TAAAGAGTGTTT
          1 CTTTTCCACCATAGGCCTCAAAGCGCTCCAAATATCCACTTGCAGATTCTACAAAAAGAGTGTTT

             *               *  *   *     *        *    *               *    
     153883 CCAAACTGCTCAATCAAGAGGAAGTTTCAAGTCTGTGAGCTGAACGCACACATCACAAAGTAGTT
         66 CAAAACTGCTCAATCAAAAGAAAGGTTCAACTCTGTGAGATGAATGCACACATCACAAAGAAGTT

               *                                *    
     153948 TCTGAGAATGCTTCTGTGTAGTTTTTATGTGAAGATGTTTC
        131 TCTCAGAATGCTTCTGTGTAGTTTTTATGTGAAGATATTTC

                            **     *                                    *    
     153989 CTTTTCCACCATAGGCTGCAAAGGGCTCCAAATATCCACTTGCAGATTCTACAAAAAGAGAGTTT
          1 CTTTTCCACCATAGGCCTCAAAGCGCTCCAAATATCCACTTGCAGATTCTACAAAAAGAGTGTTT

                 *     *         *          *     *                     *    
     154054 CAAAAGTGCTCTATCAAAAGATAGGTTCAACTATGTGATATGAATGCACACATCACAAAGTAGTT
         66 CAAAACTGCTCAATCAAAAGAAAGGTTCAACTCTGTGAGATGAATGCACACATCACAAAGAAGTT

                                          *          
     154119 TCTCAGAATGCTTCTGTGTAGTTTTTATGTAAAGATATTTC
        131 TCTCAGAATGCTTCTGTGTAGTTTTTATGTGAAGATATTTC

                                    *                                   **   
     154160 CTTTTCCACCATAGGCCTCAAAGCACTCCAAATATCCACTTGCAGATTCTACAAAAAGAGATTTT
          1 CTTTTCCACCATAGGCCTCAAAGCGCTCCAAATATCCACTTGCAGATTCTACAAAAAGAGTGTTT

                   ** *                   *     *  *    * *   *        *     
     154225 CAAAACTATTTAATCAAAAGAAAGGTTCAAATCTGTCAGTTGAAGGTACATATCACAAACAAGTT
         66 CAAAACTGCTCAATCAAAAGAAAGGTTCAACTCTGTGAGATGAATGCACACATCACAAAGAAGTT

             * **                                    
     154290 TATTGGAATGCTTCTGTGTAGTTTTTATGTGAAGATATTTC
        131 TCTCAGAATGCTTCTGTGTAGTTTTTATGTGAAGATATTTC

                     * *       * * *                        **  *     *      
     154331 CTTTTCCACAACAGGCCTCCAGGTGCTCCAAATATCCACTTGCAGATTTCACTAAAAGTGTGTTT
          1 CTTTTCCACCATAGGCCTCAAAGCGCTCCAAATATCCACTTGCAGATTCTACAAAAAGAGTGTTT

             *  *            *  *   *     *        *             *           
     154396 CCAAGCTGCTCAATCAAGAGGAAGTTTCAAGTCTGTGAGGTGAATGCACACATTACAAAGAAGTT
         66 CAAAACTGCTCAATCAAAAGAAAGGTTCAACTCTGTGAGATGAATGCACACATCACAAAGAAGTT

            *  *                                     
     154461 ACTGAGAATGCTTCTGTGTAGTTTTTATGTGAAGATATTTC
        131 TCTCAGAATGCTTCTGTGTAGTTTTTATGTGAAGATATTTC

                      **               *                                *    
     154502 CTTTTCCACCGCAGGCCTCAAAGCGCTGCAAATATCCACTTGCAGATTCTACAAAAAGAGAGTTT
          1 CTTTTCCACCATAGGCCTCAAAGCGCTCCAAATATCCACTTGCAGATTCTACAAAAAGAGTGTTT

                      **         *   *         *   *     **            *     
     154567 CAAAACTGCTGTATCAAAAGATAGGGTCAACTCTGCGAGTTGAATAAACACATCACAAATAAGTT
         66 CAAAACTGCTCAATCAAAAGAAAGGTTCAACTCTGTGAGATGAATGCACACATCACAAAGAAGTT

               **   *        *               *       
     154632 TCTGGGAACGCTTCTGTATAG-TTTTATGTGAATATATTTC
        131 TCTCAGAATGCTTCTGTGTAGTTTTTATGTGAAGATATTTC

                         *          *                   *   *  *   * *   *   
     154672 CTTTTCCACCATATGCCTCAAAGCACTCCAAATATCCACTTGCACATTATAGAAACATAGTCTTT
          1 CTTTTCCACCATAGGCCTCAAAGCGCTCCAAATATCCACTTGCAGATTCTACAAAAAGAGTGTTT

                   **                        *         *             *       
     154737 CAAAACTTGTCAATC-AAAGAAAGGTTCAACTCCGTGAGATGAGTGCACACATCACAGAGAAGTT
         66 CAAAACTGCTCAATCAAAAGAAAGGTTCAACTCTGTGAGATGAATGCACACATCACAAAGAAGTT

                *     *                            * 
     154801 TCTCGGAATGTTTCTGTGTAGTTTTTATGTGAAGATATTGC
        131 TCTCAGAATGCTTCTGTGTAGTTTTTATGTGAAGATATTTC

                     *               *            *          *       *   *   
     154842 CTTTTCCACAATAGGCCTCAAAGCGTTCCAAATATCCAATTGCAGATTCCACAAAAAAAGTTTTT
          1 CTTTTCCACCATAGGCCTCAAAGCGCTCCAAATATCCACTTGCAGATTCTACAAAAAGAGTGTTT

            *                 *  *  *  *             * *         **    *     
     154907 TAAAACTGCTCAATCAAATGATAGATTAAACTCTGTGAGATTAGTGCACACATGTCAAAAAAGTT
         66 CAAAACTGCTCAATCAAAAGAAAGGTTCAACTCTGTGAGATGAATGCACACATCACAAAGAAGTT

                                *      * *           
     154972 TCTCAGAATGCTTCTGTGTACTTTTTAGGGGAAGATATTTC
        131 TCTCAGAATGCTTCTGTGTAGTTTTTATGTGAAGATATTTC

                        *    *     **         *    *           **  * *  *    
     155013 CTTTTCCACCATCGGCCACAAAGGACTCCAAATAACCACATGCAGATTCTAGTAACACAGAGTTT
          1 CTTTTCCACCATAGGCCTCAAAGCGCTCCAAATATCCACTTGCAGATTCTACAAAAAGAGTGTTT

                       *         * *           *   * * *    **    ***        
     155078 CAAAACTGCTCTATCAAAAGATAAGTTCAACTCTGAGAGTTTAGTGCAACCATCGTGAAGAAGTT
         66 CAAAACTGCTCAATCAAAAGAAAGGTTCAACTCTGTGAGATGAATGCACACATCACAAAGAAGTT

                            *     *                  
     155143 TCTCAGAATGCTTCTGAGTAGTGTTTATGTGAAGATATTTC
        131 TCTCAGAATGCTTCTGTGTAGTTTTTATGTGAAGATATTTC

                              *     *                      *           **    
     155184 CTTTTCCACCATAGGCCTGAAAGCCCTCCAAATATCCACTTGCAGATCCTACAAAAAGAAAGTTT
          1 CTTTTCCACCATAGGCCTCAAAGCGCTCCAAATATCCACTTGCAGATTCTACAAAAAGAGTGTTT

             *         **     *  *  *   *        **      *                   
     155249 CGAAA-TGCTCTCTCAAACGATAGTTTCGACTCTGTGGTATGAATACACACATCACAAAGAAGTT
         66 CAAAACTGCTCAATCAAAAGAAAGGTTCAACTCTGTGAGATGAATGCACACATCACAAAGAAGTT

                                       **            
     155313 TCTCAGAATGCTTCTGTGTAGTTTTTAAATGAAGATATTTC
        131 TCTCAGAATGCTTCTGTGTAGTTTTTATGTGAAGATATTTC

            *                       *          *     *                       
     155354 TTTTTCCACCATAGGCCTCAAAGCACTCCAAATATGCACTTCCAGATTCTACAAAAAGAGTGTTT
          1 CTTTTCCACCATAGGCCTCAAAGCGCTCCAAATATCCACTTGCAGATTCTACAAAAAGAGTGTTT

              *                 *       * *         **   *         *  * *    
     155419 CAGAACTGCTCAATCAAAAGGAAGGTTCCAGTC--TGAGACAAATACACACATCAAAAGGTAGTT
         66 CAAAACTGCTCAATCAAAAGAAAGGTTCAACTCTGTGAGATGAATGCACACATCACAAAGAAGTT

                                                    *
     155482 TCTCAGAATGCTTCTGTGTAGTTTTTATGTGAAGATATTTT
        131 TCTCAGAATGCTTCTGTGTAGTTTTTATGTGAAGATATTTC

             *               *    **    *     *      *  **   *          *    
     155523 CCTTTCCACCATAGGCCACAAATGGCTCTAAATACCCACTTACATTTTCCACAAAAAGAGAGTTT
          1 CTTTTCCACCATAGGCCTCAAAGCGCTCCAAATATCCACTTGCAGATTCTACAAAAAGAGTGTTT

                       * *    * *      *   *         *                       
     155588 CAAAACTGCTCTACCAAAGGTAA-GTTTAACGCTGTGAG-TTAA-G-A-ACATCACAAAGAAGTT
         66 CAAAACTGCTCAATCAAAAGAAAGGTTCAACTCTGTGAGATGAATGCACACATCACAAAGAAGTT

                                   *   *  *          
     155648 TCTCAGAATGCTTCTGTGTAGTTCTTACGTAAAGATATTTC
        131 TCTCAGAATGCTTCTGTGTAGTTTTTATGTGAAGATATTTC

                 *   *      ***    *                  *              **      
     155689 CTTTTACACAATAGGCAGAAAAGTGCTCCAAATATCCACTTGAAGATTCTACAAAAACCGTGTTT
          1 CTTTTCCACCATAGGCCTCAAAGCGCTCCAAATATCCACTTGCAGATTCTACAAAAAGAGTGTTT

                     **                                          *      *    
     155754 CAAAACTGCCGAATCAAAAGAAAGGTTCAACTCTGTGAGATGAATGCACACATAACAAAGGAGTT
         66 CAAAACTGCTCAATCAAAAGAAAGGTTCAACTCTGTGAGATGAATGCACACATCACAAAGAAGTT

                                 *      *      *    *
     155819 TCTCAGAATGCTTCTGTGTAGCTTTTATATGAAGACATTTA
        131 TCTCAGAATGCTTCTGTGTAGTTTTTATGTGAAGATATTTC

            *        * *            *   ***                     **        *  
     155860 GTTTTCCACAACAGGCCTCAAAGCTCTCTCCATATCCACTTGCAGATTCTACCGAAAGAGTGCTT
          1 CTTTTCCACCATAGGCCTCAAAGCGCTCCAAATATCCACTTGCAGATTCTACAAAAAGAGTGTTT

             *                   * **     *        *        *     **         
     155925 CCAAACTGCTCAATCAAAAGAGACATTCAAATCTGTGAGGTGAATGCAGACATCGTAAAGAAGTT
         66 CAAAACTGCTCAATCAAAAGAAAGGTTCAACTCTGTGAGATGAATGCACACATCACAAAGAAGTT

                                *     *       *    **
     155990 TCTCAGAATGCTTCTGTGTATTTTTTGTGTGAAGTTATTCG
        131 TCTCAGAATGCTTCTGTGTAGTTTTTATGTGAAGATATTTC

            *    *             *     *  *            **                 *    
     156031 TTTTTGCACCATAGGCCTCCAAGCGTTCTAAATATCCACTTCTAGATTCTACAAAAAGAGAGTTT
          1 CTTTTCCACCATAGGCCTCAAAGCGCTCCAAATATCCACTTGCAGATTCTACAAAAAGAGTGTTT

                   *         *            *        *    *   *                
     156096 CAAAACTACTC-A--AACA-AAAGGTTCAATTCTGTGAGTTGAAAGCAAACATCACAAAGAAGTT
         66 CAAAACTGCTCAATCAAAAGAAAGGTTCAACTCTGTGAGATGAATGCACACATCACAAAGAAGTT

                       *                             
     156157 TCTCAGAATGCGTCTGTGTAGTTTTTATGTGAAGATATTTC
        131 TCTCAGAATGCTTCTGTGTAGTTTTTATGTGAAGATATTTC

                     **   ****     *                  *                      
     156198 CTTTT-CACAGTAGAATGCAAAGGGCTCCAAATATCCACTTGGAGATTCTACAAAAAGA--GTTT
          1 CTTTTCCACCATAGGCCTCAAAGCGCTCCAAATATCCACTTGCAGATTCTACAAAAAGAGTGTTT

                  *    **     *  *     *     ***   *     *               *   
     156260 CAAAACCGCTCTGTCAAATGATAGGTTGAACTCCCGGAGGTGAATACACACATCACAAAGAGGTT
         66 CAAAACTGCTCAATCAAAAGAAAGGTTCAACTCTGTGAGATGAATGCACACATCACAAAGAAGTT

                  *                       *  * *     
     156325 TCTCAGCATGCTTCTGTGTAGTTTTTATGTAAACACATTTC
        131 TCTCAGAATGCTTCTGTGTAGTTTTTATGTGAAGATATTTC

             *    * *              *           *      * *       *   *    *   
     156366 CGTTTCTATCATAGGCCTCAAAGTGCTCCAAATATTCACTTGTACATTCTACCAAACGAGTATTT
          1 CTTTTCCACCATAGGCCTCAAAGCGCTCCAAATATCCACTTGCAGATTCTACAAAAAGAGTGTTT

                              * *         **      *        *            *    
     156431 CAAAACTGCTCAATCAAATGGAAGGTTCAAAACTGTGACATGAATGCCCACATCACAAAGTAGTT
         66 CAAAACTGCTCAATCAAAAGAAAGGTTCAACTCTGTGAGATGAATGCACACATCACAAAGAAGTT

                                                     
     156496 TCTCAGAATGCTTCTGTGTAGTTTTTATGTGAAGATATTTC
        131 TCTCAGAATGCTTCTGTGTAGTTTTTATGTGAAGATATTTC

                     * *    *      *    *       * *   **      *              
     156537 CTTTTCCACAACA-GCGTGCAAAACGCTTCAAATATGCCCTTAGAGATTCCACAAAAAGAGTGTT
          1 CTTTTCCACCATAGGCCT-CAAAGCGCTCCAAATATCCACTTGCAGATTCTACAAAAAGAGTGTT

              *     *               *  *                                  ** 
     156601 TCCAAACTACTCAAATCAAAA-AATGATTTCAACTCTGTGAGATGAATGCACACATCACAAACTA
         65 TCAAAACTGCTC-AATCAAAAGAAAG-GTTCAACTCTGTGAGATGAATGCACACATCACAAAGAA

                         *     ** *   * *   *       *   
     156665 GTTTCTCAGAATGTTTCTGCCTGGTTCTCATGCGAAGATAGTTC
        128 GTTTCTCAGAATGCTTCTGTGTAGTTTTTATGTGAAGATATTTC

                 *           *   * **              *                  * *    
     156709 CTTTTTCACCATAGGCCGCAATGTACTCCAAATATCCACCTGCAGATTCTAC-AAAAGTGAGTTT
          1 CTTTTCCACCATAGGCCTCAAAGCGCTCCAAATATCCACTTGCAGATTCTACAAAAAGAGTGTTT

                       *         ***    **         *        *      *       * 
     156773 CAAAACTGCTCTATCAAAAGATCAGTTCGTCTCTGTGAGTTGAATGCATACATCAAAAAGAAGCT
         66 CAAAACTGCTCAATCAAAAGAAAGGTTCAACTCTGTGAGATGAATGCACACATCACAAAGAAGTT

                 *             *      **         *   
     156838 TCTCAAAATGCTTCTGTGTGGTTTTTCGGTGAAGATAGTTC
        131 TCTCAGAATGCTTCTGTGTAGTTTTTATGTGAAGATATTTC

            *     *        *      * *                 *        *        *    
     156879 TTTTTCTACCATAGGTCTCAAACCACTCCAAATATCCACTTGTAGATTCTATAAAAAGGAATG-T
          1 CTTTTCCACCATAGGCCTCAAAGCGCTCCAAATATCCACTTGCAGATTCTACAAAAA-GAGTGTT

                  *        *    *    *      * *         *      *             
     156943 TCAAAATTGCTCAATAAAAATAAAGTTTCAACACCGTGAGATGAGTGCACAAATCACAAAGAAGT
         65 TCAAAACTGCTCAATCAAAAGAAAGGTTCAACTCTGTGAGATGAATGCACACATCACAAAGAAGT

                  *          *         *          *   
     157008 TTCTCAAAATGCTTCTGGGTAGTTTTTCTGTGAAGATAGTTC
        130 TTCTCAGAATGCTTCTGTGTAGTTTTTATGTGAAGATATTTC

                  *     *    *     *          *                         *    
     157050 CTTTTCTACCATGGGCCACAAAGGGCTCCAAATACCCACTTGCAGATTCTACAAAAAGAGAGTTT
          1 CTTTTCCACCATAGGCCTCAAAGCGCTCCAAATATCCACTTGCAGATTCTACAAAAAGAGTGTTT

              *        *      ** * *        *    * *    **  * *           *  
     157115 CACAACTGCTCTATCAAACAATATGTTCAACTTTGTGGGTTGAACACAAATATCAC-AAGAATTT
         66 CAAAACTGCTCAATCAAAAGAAAGGTTCAACTCTGTGAGATGAATGCACACATCACAAAGAAGTT

                **                             *     
     157179 TCTCCCAATGCTTCTGTGTAGTTTTTATGTGAAGACATTTC
        131 TCTCAGAATGCTTCTGTGTAGTTTTTATGTGAAGATATTTC

            *   *  *      *  *     *                 *  *      *       *  *  
     157220 TTTTCCCTCCATAGTCCACAAAGTGCTCCAAATATCCACTTACATATTCTAGAAAAAGATTGCTT
          1 CTTTTCCACCATAGGCCTCAAAGCGCTCCAAATATCCACTTGCAGATTCTACAAAAAGAGTGTTT

            **       *    *               * * *                              
     157285 GGAAACTGCACAATGAAAAGAAAGGTTCAAATATATGAGATGAATGCACACATCACAAAGAAGTT
         66 CAAAACTGCTCAATCAAAAGAAAGGTTCAACTCTGTGAGATGAATGCACACATCACAAAGAAGTT

                                 *                    
     157350 TCTCAGAAT-CTCTCTGTGTAATTTTTATGTGAAGATATTTC
        131 TCTCAGAATGCT-TCTGTGTAGTTTTTATGTGAAGATATTTC

                *     *    *  *   *                        *      *    *     
     157391 CTTTCCCACCTTAGGTCTTAAAACGCTCCAAATATCCACTTGCAGATACTACAAGAAGATTGTTT
          1 CTTTTCCACCATAGGCCTCAAAGCGCTCCAAATATCCACTTGCAGATTCTACAAAAAGAGTGTTT

                     *    *        *      *     **                           
     157456 CAAAACTGCACAA-AAAAAGAAATGTTCAATTCTGTTTGATGAATGCACACATCACAAAGAAGTT
         66 CAAAACTGCTCAATCAAAAGAAAGGTTCAACTCTGTGAGATGAATGCACACATCACAAAGAAGTT

                           *                         
     157520 TCTCAGAATGCTTCTCTGTAGTTTTTATGTGAAGATATTTC
        131 TCTCAGAATGCTTCTGTGTAGTTTTTATGTGAAGATATTTC

                     *             *                 *         **      * *   
     157561 CTTTTCCACAATAGGCCTCAAAGGGCTCCAAATATCCACTTCCAGATTCTATGAAAAGAATATTT
          1 CTTTTCCACCATAGGCCTCAAAGCGCTCCAAATATCCACTTGCAGATTCTACAAAAAGAGTGTTT

             *              * *    *                                      *  
     157626 CCAAACTGCTCAATCATAGGAAATGTTCAACTCTGTGAGATGAATGCACACATCAC-AAGAAATT
         66 CAAAACTGCTCAATCAAAAGAAAGGTTCAACTCTGTGAGATGAATGCACACATCACAAAGAAGTT

                     *    *      *       *       *   
     157690 TCTCAGAATCCTTCAGTGTAGGTTTTATGAGAAGATAATTC
        131 TCTCAGAATGCTTCTGTGTAGTTTTTATGTGAAGATATTTC

                     *    **        *   *                           *    *   
     157731 CTTTTCCACAATAGTTCTCAAAGCACTCAAAATATCCACTTGCAGATTCTACAAAAGGAGTATTT
          1 CTTTTCCACCATAGGCCTCAAAGCGCTCCAAATATCCACTTGCAGATTCTACAAAAAGAGTGTTT

                                                          *                  
     157796 CAAAACTGCTCAATCAAAAGAAAGGTTCAACTCTGTGAGATGAATGGACACATCACAAAGAAGTT
         66 CAAAACTGCTCAATCAAAAGAAAGGTTCAACTCTGTGAGATGAATGCACACATCACAAAGAAGTT

                                  *   *              
     157861 TCTCAGAATGCTTCTGTGTAGTATTTTTGTGAAGATATTT-
        131 TCTCAGAATGCTTCTGTGTAGTTTTTATGTGAAGATATTTC

                          *  * * * * ***             *            *     * *  
     157901 CTTTTCCACCATAGACCGCCAGGGGACACAAATATCCACTTTCAGATTCTACAACAAGAGAGGTT
          1 CTTTTCCACCATAGGCCTCAAAGCGCTCCAAATATCCACTTGCAGATTCTACAAAAAGAGTGTTT

                   *   *     *   ** *       *      *                      * *
     157966 CAAAACTACTCGATCAAGAGATGGTTTCAACTATGTGAGTTGAATGCACACATCACAAAGAACTA
         66 CAAAACTGCTCAATCAAAAGAAAGGTTCAACTCTGTGAGATGAATGCACACATCACAAAGAAGTT

             *  *    *                               
     158031 TGTCGGAATTCTTCTGTGTAGTTTTTATGTGAAGATATTTC
        131 TCTCAGAATGCTTCTGTGTAGTTTTTATGTGAAGATATTTC

                     *    * *      * *    *                  *               
     158072 CTTTTCCACAATAGACGTCAAAGTGATCCAGATATCCACTTGCAGATTCCACAAAAAGAGTGTTT
          1 CTTTTCCACCATAGGCCTCAAAGCGCTCCAAATATCCACTTGCAGATTCTACAAAAAGAGTGTTT

                 *   *   *                  **                     *  *      
     158137 CAAAAGTGCACAACCAAAAGAAAGGTTCAACTAGGTGAGATGAATGCACACATCAGAAGGAAGTT
         66 CAAAACTGCTCAATCAAAAGAAAGGTTCAACTCTGTGAGATGAATGCACACATCACAAAGAAGTT

                            **   *     * *       *   
     158202 TCTCAGAATGCTTCTGCATAGCTTTTAAGGGAAGATACTTC
        131 TCTCAGAATGCTTCTGTGTAGTTTTTATGTGAAGATATTTC

                    *               *            * *  *    * *               
     158243 CTTTTCCAACATAGGCCTCAAAGCACTCCAAATATCCTCCTGGAGATACCACAAAAAGAGTGTTT
          1 CTTTTCCACCATAGGCCTCAAAGCGCTCCAAATATCCACTTGCAGATTCTACAAAAAGAGTGTTT

            **                      *  *                 *       *           
     158308 GCAAACTGCTCAATCAAAAGAAAGATTTAACTCTGTGAGATGAATCCACACATGACAAAGAAGTT
         66 CAAAACTGCTCAATCAAAAGAAAGGTTCAACTCTGTGAGATGAATGCACACATCACAAAGAAGTT

                                                     
     158373 TCTCAGAATGCTTCTGTGTAGTTTTTATGTGAAGATATTTC
        131 TCTCAGAATGCTTCTGTGTAGTTTTTATGTGAAGATATTTC

                     *   * * *    **           *                *     *      
     158414 CTTTTCCACAATAAGACCCAAAAGGCTCCAAATATTCACTTGCAGATTCTAAAAAAAACAGTGTT
          1 CTTTTCCACCATAGGCCTCAAAGCGCTCCAAATATCCACTTGCAGATTCT-ACAAAAAGAGTGTT

                                  *                  *      *        **      
     158479 TCAAAACTGCTCAATCAAAAGATA-GTTCAACTCTGTGAGAAGAATGCTCACATCACTGAGAAGT
         65 TCAAAACTGCTCAATCAAAAGAAAGGTTCAACTCTGTGAGATGAATGCACACATCACAAAGAAGT

                                         *            
     158543 TTCTCAGAATGCTTCTGTGTAGTTTTTATATGAAGATATTTC
        130 TTCTCAGAATGCTTCTGTGTAGTTTTTATGTGAAGATATTTC

                *     *      *    **   *                   *   **       *    
     158585 CTTTCCCACCGTAGGCCACAAAAGGCTTCAAATATCCACTTGCAGATACTATGAAAAGAGAGTTT
          1 CTTTTCCACCATAGGCCTCAAAGCGCTCCAAATATCCACTTGCAGATTCTACAAAAAGAGTGTTT

                        *        *               ***            *            
     158650 CAAAACTGCTCATTCAAAAGATAGGTTCAACTCTGTGGTTTGAATGCACACAGCACAAAGAAGTT
         66 CAAAACTGCTCAATCAAAAGAAAGGTTCAACTCTGTGAGATGAATGCACACATCACAAAGAAGTT

              *       **                   **   *    
     158715 TCACAGAATGTGTCTGTGTAGTTTTTATGTGCGGATGTTTC
        131 TCTCAGAATGCTTCTGTGTAGTTTTTATGTGAAGATATTTC

                         *        ****   *   *               *    *          
     158756 CTTTTCCACCATATGCCT-AAATATTTCCCAATTTCCACTTGCAGATTCCACAAGAAGAGTGTTT
          1 CTTTTCCACCATAGGCCTCAAAGCGCTCCAAATATCCACTTGCAGATTCTACAAAAAGAGTGTTT

                      **       *       *           *            *      ***   
     158820 CAAAACTGCTGTATC-AAATAAA-GTTGAACTCTGTGAGGTGAATGCACACAGCACAAAATGGTT
         66 CAAAACTGCTCAATCAAAAGAAAGGTTCAACTCTGTGAGATGAATGCACACATCACAAAGAAGTT

                          **   *        *       *    
     158883 TCTCAGAATGCTTCCTTGTTGTTTTTATATGAAGATGTTTC
        131 TCTCAGAATGCTTCTGTGTAGTTTTTATGTGAAGATATTTC

                  *  *             *   *     *                               
     158924 CTTTTCAACAATAGGCCTCAAAGTGCTTCAAATGTCCACTTGCAGATTCTACAAAAAGAGTGTTT
          1 CTTTTCCACCATAGGCCTCAAAGCGCTCCAAATATCCACTTGCAGATTCTACAAAAAGAGTGTTT

                                        *      *  *  *                       
     158989 CAAAACTGCTCAATCAAAAGAAAGGTTCGACTCTGGGAAATTAATGCACACATCACAAAGAAGTT
         66 CAAAACTGCTCAATCAAAAGAAAGGTTCAACTCTGTGAGATGAATGCACACATCACAAAGAAGTT

                                     *        *      
     159054 TCTC---A-GCTTCTGTGTAGTTTTCATGTGAAGTTATTTC
        131 TCTCAGAATGCTTCTGTGTAGTTTTTATGTGAAGATATTTC

                     *       *     *            *    *         **       *    
     159091 CTTTTCCACAATAGGCCGCAAAGGGCTCCAAATATCAACTTACAGATTCTAGGAAAAGAGAGTTT
          1 CTTTTCCACCATAGGCCTCAAAGCGCTCCAAATATCCACTTGCAGATTCTACAAAAAGAGTGTTT

                       * **      *     *                                     
     159156 CAAAACTGCTCTACGAAAAGATAGGTTGAACTCTGTGAGATGAATGCACACATCACAAAGAAGTT
         66 CAAAACTGCTCAATCAAAAGAAAGGTTCAACTCTGTGAGATGAATGCACACATCACAAAGAAGTT

                       *               * *  *        
     159221 TCTCAGAATGCATCTGTGTAGTTTTTACGGGATGATATTTC
        131 TCTCAGAATGCTTCTGTGTAGTTTTTATGTGAAGATATTTC

                        ***  *     **      *  *                  *     ***   
     159262 CTTTTCCACCATCTTCCACAAAGGTCTCCAAGTAACCACTTGCAGATTCTACAGAAAGACACTTT
          1 CTTTTCCACCATAGGCCTCAAAGCGCTCCAAATATCCACTTGCAGATTCTACAAAAAGAGTGTTT

            *          *         ***      *      ***                      *  
     159327 AAAAACTGCTCTATCAAAAGATCAGTTCAAGTCTGTGGTTTGAATGCACACATCACAAAGAATTT
         66 CAAAACTGCTCAATCAAAAGAAAGGTTCAACTCTGTGAGATGAATGCACACATCACAAAGAAGTT

                                     *  *            
     159392 TCTCAGAATGCTTCTGTGTAGTTTTCATATGAAGATATTTC
        131 TCTCAGAATGCTTCTGTGTAGTTTTTATGTGAAGATATTTC

                                    *                                   **   
     159433 CTTTTCCACCATAGGCCTCAAAGCACTCCAAATATCCACTTGCAGATTCTACAAAAAGAGATTTT
          1 CTTTTCCACCATAGGCCTCAAAGCGCTCCAAATATCCACTTGCAGATTCTACAAAAAGAGTGTTT

                   **                           *  *          *        *     
     159498 CAAAACTAGTCAATCAAAAGAAAGGTTCAACTCTGTCAGTTGAATGCACATATCACAAACAAGTT
         66 CAAAACTGCTCAATCAAAAGAAAGGTTCAACTCTGTGAGATGAATGCACACATCACAAAGAAGTT

                *      *                             
     159563 TCTCGGAATGCGTCTGTGTAGTTTTTATGTGAAGATATTTC
        131 TCTCAGAATGCTTCTGTGTAGTTTTTATGTGAAGATATTTC

               *     * *           *     *                  *    *           
     159604 CTTCTCCACAACAGGCCTCAAAGTGCTCCGAATATCCACTTGCAGATTTTAC-TAAAGAGTGTTT
          1 CTTTTCCACCATAGGCCTCAAAGCGCTCCAAATATCCACTTGCAGATTCTACAAAAAGAGTGTTT

             *               *  *   *     *        *    *               *    
     159668 CCAAACTGCTCAATCAAGAGGAAGTTTCAAGTCTGTGAGCTGAACGCACACATCACAAAGTAGTT
         66 CAAAACTGCTCAATCAAAAGAAAGGTTCAACTCTGTGAGATGAATGCACACATCACAAAGAAGTT

               *                                *    
     159733 TCTGAGAATGCTTCTGTGTAGTTTTTATGTGAAGATGTTTC
        131 TCTCAGAATGCTTCTGTGTAGTTTTTATGTGAAGATATTTC

                            **     *                                    *    
     159774 CTTTTCCACCATAGGCTGCAAAGGGCTCCAAATATCCACTTGCAGATTCTACAAAAAGAGAGTTT
          1 CTTTTCCACCATAGGCCTCAAAGCGCTCCAAATATCCACTTGCAGATTCTACAAAAAGAGTGTTT

                 *     *         *          *     *                     *    
     159839 CAAAAGTGCTCTATCAAAAGATAGGTTCAACTATGTGATATGAATGCACACATCACAAAGTAGTT
         66 CAAAACTGCTCAATCAAAAGAAAGGTTCAACTCTGTGAGATGAATGCACACATCACAAAGAAGTT

                                          *          
     159904 TCTCAGAATGCTTCTGTGTAGTTTTTATGTAAAGATATTTC
        131 TCTCAGAATGCTTCTGTGTAGTTTTTATGTGAAGATATTTC

                                    *                                   **   
     159945 CTTTTCCACCATAGGCCTCAAAGCACTCCAAATATCCACTTGCAGATTCTACAAAAAGAGATTTT
          1 CTTTTCCACCATAGGCCTCAAAGCGCTCCAAATATCCACTTGCAGATTCTACAAAAAGAGTGTTT

                   ** *                   *     *  *    * *   *        *     
     160010 CAAAACTATTTAATCAAAAGAAAGGTTCAAATCTGTCAGTTGAAGGTACATATCACAAACAAGTT
         66 CAAAACTGCTCAATCAAAAGAAAGGTTCAACTCTGTGAGATGAATGCACACATCACAAAGAAGTT

             * **                                    
     160075 TATTGGAATGCTTCTGTGTAGTTTTTATGTGAAGATATTTC
        131 TCTCAGAATGCTTCTGTGTAGTTTTTATGTGAAGATATTTC

                     * *       * * *                        **  *     *      
     160116 CTTTTCCACAACAGGCCTCCAGGTGCTCCAAATATCCACTTGCAGATTTCACTAAAAGTGTGTTT
          1 CTTTTCCACCATAGGCCTCAAAGCGCTCCAAATATCCACTTGCAGATTCTACAAAAAGAGTGTTT

             *  *            *  *   *     *        *             *           
     160181 CCAAGCTGCTCAATCAAGAGGAAGTTTCAAGTCTGTGAGGTGAATGCACACATTACAAAGAAGTT
         66 CAAAACTGCTCAATCAAAAGAAAGGTTCAACTCTGTGAGATGAATGCACACATCACAAAGAAGTT

            *  *                                     
     160246 ACTGAGAATGCTTCTGTGTAGTTTTTATGTGAAGATATTTC
        131 TCTCAGAATGCTTCTGTGTAGTTTTTATGTGAAGATATTTC

                      **               *                                *    
     160287 CTTTTCCACCGCAGGCCTCAAAGCGCTGCAAATATCCACTTGCAGATTCTACAAAAAGAGAGTTT
          1 CTTTTCCACCATAGGCCTCAAAGCGCTCCAAATATCCACTTGCAGATTCTACAAAAAGAGTGTTT

                      **         *   *         *   *     **            *     
     160352 CAAAACTGCTGTATCAAAAGATAGGGTCAACTCTGCGAGTTGAATAAACACATCACAAATAAGTT
         66 CAAAACTGCTCAATCAAAAGAAAGGTTCAACTCTGTGAGATGAATGCACACATCACAAAGAAGTT

               **   *        *               *       
     160417 TCTGGGAACGCTTCTGTATAG-TTTTATGTGAATATATTTC
        131 TCTCAGAATGCTTCTGTGTAGTTTTTATGTGAAGATATTTC

                         *          *                   *   *  *   * *   *   
     160457 CTTTTCCACCATATGCCTCAAAGCACTCCAAATATCCACTTGCACATTATAGAAACATAGTCTTT
          1 CTTTTCCACCATAGGCCTCAAAGCGCTCCAAATATCCACTTGCAGATTCTACAAAAAGAGTGTTT

                   **                        *         *             *       
     160522 CAAAACTTGTCAATC-AAAGAAAGGTTCAACTCCGTGAGATGAGTGCACACATCACAGAGAAGTT
         66 CAAAACTGCTCAATCAAAAGAAAGGTTCAACTCTGTGAGATGAATGCACACATCACAAAGAAGTT

                *     *                            * 
     160586 TCTCGGAATGTTTCTGTGTAGTTTTTATGTGAAGATATTGC
        131 TCTCAGAATGCTTCTGTGTAGTTTTTATGTGAAGATATTTC

                     *               *            *          *       *   *   
     160627 CTTTTCCACAATAGGCCTCAAAGCGTTCCAAATATCCAATTGCAGATTCCACAAAAAAAGTTTTT
          1 CTTTTCCACCATAGGCCTCAAAGCGCTCCAAATATCCACTTGCAGATTCTACAAAAAGAGTGTTT

            *                 *  *  *  *             * *         **    *     
     160692 TAAAACTGCTCAATCAAATGATAGATTAAACTCTGTGAGATTAGTGCACACATGTCAAAAAAGTT
         66 CAAAACTGCTCAATCAAAAGAAAGGTTCAACTCTGTGAGATGAATGCACACATCACAAAGAAGTT

                                *      * *           
     160757 TCTCAGAATGCTTCTGTGTACTTTTTAGGGGAAGATATTTC
        131 TCTCAGAATGCTTCTGTGTAGTTTTTATGTGAAGATATTTC

                        *    *     **         *    *           **  * *  *    
     160798 CTTTTCCACCATCGGCCACAAAGGACTCCAAATAACCACATGCAGATTCTAGTAACACAGAGTTT
          1 CTTTTCCACCATAGGCCTCAAAGCGCTCCAAATATCCACTTGCAGATTCTACAAAAAGAGTGTTT

                       *         * *           *   * * *    **    ***        
     160863 CAAAACTGCTCTATCAAAAGATAAGTTCAACTCTGAGAGTTTAGTGCAACCATCGTGAAGAAGTT
         66 CAAAACTGCTCAATCAAAAGAAAGGTTCAACTCTGTGAGATGAATGCACACATCACAAAGAAGTT

                            *     *                  
     160928 TCTCAGAATGCTTCTGAGTAGTGTTTATGTGAAGATATTTC
        131 TCTCAGAATGCTTCTGTGTAGTTTTTATGTGAAGATATTTC

                              *     *                      *           **    
     160969 CTTTTCCACCATAGGCCTGAAAGCCCTCCAAATATCCACTTGCAGATCCTACAAAAAGAAAGTTT
          1 CTTTTCCACCATAGGCCTCAAAGCGCTCCAAATATCCACTTGCAGATTCTACAAAAAGAGTGTTT

             *         **     *  *  *   *        **      *                   
     161034 CGAAA-TGCTCTCTCAAACGATAGTTTCGACTCTGTGGTATGAATACACACATCACAAAGAAGTT
         66 CAAAACTGCTCAATCAAAAGAAAGGTTCAACTCTGTGAGATGAATGCACACATCACAAAGAAGTT

                                       **            
     161098 TCTCAGAATGCTTCTGTGTAGTTTTTAAATGAAGATATTTC
        131 TCTCAGAATGCTTCTGTGTAGTTTTTATGTGAAGATATTTC

            *                       *          *     *                       
     161139 TTTTTCCACCATAGGCCTCAAAGCACTCCAAATATGCACTTCCAGATTCTACAAAAAGAGTGTTT
          1 CTTTTCCACCATAGGCCTCAAAGCGCTCCAAATATCCACTTGCAGATTCTACAAAAAGAGTGTTT

              *                 *       * *         **   *         *  * *    
     161204 CAGAACTGCTCAATCAAAAGGAAGGTTCCAGTC--TGAGACAAATACACACATCAAAAGGTAGTT
         66 CAAAACTGCTCAATCAAAAGAAAGGTTCAACTCTGTGAGATGAATGCACACATCACAAAGAAGTT

                                                    *
     161267 TCTCAGAATGCTTCTGTGTAGTTTTTATGTGAAGATATTTT
        131 TCTCAGAATGCTTCTGTGTAGTTTTTATGTGAAGATATTTC

             *               *    **    *     *      *  **   *          *    
     161308 CCTTTCCACCATAGGCCACAAATGGCTCTAAATACCCACTTACATTTTCCACAAAAAGAGAGTTT
          1 CTTTTCCACCATAGGCCTCAAAGCGCTCCAAATATCCACTTGCAGATTCTACAAAAAGAGTGTTT

                       * *    * *      *   *         *                       
     161373 CAAAACTGCTCTACCAAAGGTAA-GTTTAACGCTGTGAG-TTAA-G-A-ACATCACAAAGAAGTT
         66 CAAAACTGCTCAATCAAAAGAAAGGTTCAACTCTGTGAGATGAATGCACACATCACAAAGAAGTT

                                   *   *  *          
     161433 TCTCAGAATGCTTCTGTGTAGTTCTTACGTAAAGATATTTC
        131 TCTCAGAATGCTTCTGTGTAGTTTTTATGTGAAGATATTTC

                 *   *      ***    *                  *              **      
     161474 CTTTTACACAATAGGCAGAAAAGTGCTCCAAATATCCACTTGAAGATTCTACAAAAACCGTGTTT
          1 CTTTTCCACCATAGGCCTCAAAGCGCTCCAAATATCCACTTGCAGATTCTACAAAAAGAGTGTTT

                     **                                          *      *    
     161539 CAAAACTGCCGAATCAAAAGAAAGGTTCAACTCTGTGAGATGAATGCACACATAACAAAGGAGTT
         66 CAAAACTGCTCAATCAAAAGAAAGGTTCAACTCTGTGAGATGAATGCACACATCACAAAGAAGTT

                                 *      *      *    *
     161604 TCTCAGAATGCTTCTGTGTAGCTTTTATATGAAGACATTTA
        131 TCTCAGAATGCTTCTGTGTAGTTTTTATGTGAAGATATTTC

            *        * *            *   ***                     **        *  
     161645 GTTTTCCACAACAGGCCTCAAAGCTCTCTCCATATCCACTTGCAGATTCTACCGAAAGAGTGCTT
          1 CTTTTCCACCATAGGCCTCAAAGCGCTCCAAATATCCACTTGCAGATTCTACAAAAAGAGTGTTT

             *                   * **     *        *        *     **         
     161710 CCAAACTGCTCAATCAAAAGAGACATTCAAATCTGTGAGGTGAATGCAGACATCGTAAAGAAGTT
         66 CAAAACTGCTCAATCAAAAGAAAGGTTCAACTCTGTGAGATGAATGCACACATCACAAAGAAGTT

                                *     *       *    **
     161775 TCTCAGAATGCTTCTGTGTATTTTTTGTGTGAAGTTATTCG
        131 TCTCAGAATGCTTCTGTGTAGTTTTTATGTGAAGATATTTC

            *    *             *     *  *            **                 *    
     161816 TTTTTGCACCATAGGCCTCCAAGCGTTCTAAATATCCACTTCTAGATTCTACAAAAAGAGAGTTT
          1 CTTTTCCACCATAGGCCTCAAAGCGCTCCAAATATCCACTTGCAGATTCTACAAAAAGAGTGTTT

                   *         *            *        *    *   *                
     161881 CAAAACTACTC-A--AACA-AAAGGTTCAATTCTGTGAGTTGAAAGCAAACATCACAAAGAAGTT
         66 CAAAACTGCTCAATCAAAAGAAAGGTTCAACTCTGTGAGATGAATGCACACATCACAAAGAAGTT

                       *                             
     161942 TCTCAGAATGCGTCTGTGTAGTTTTTATGTGAAGATATTTC
        131 TCTCAGAATGCTTCTGTGTAGTTTTTATGTGAAGATATTTC

                     *    ****     *                  *                      
     161983 CTTTT-CACAATAGAATGCAAAGGGCTCCAAATATCCACTTGGAGATTCTACAAAAAGA--GTTT
          1 CTTTTCCACCATAGGCCTCAAAGCGCTCCAAATATCCACTTGCAGATTCTACAAAAAGAGTGTTT

                  *    **     *  *     *     ***   *     *               *   
     162045 CAAAACCGCTCTGTCAAATGATAGGTTGAACTCCCGGAGGTGAATACACACATCACAAAGAGGTT
         66 CAAAACTGCTCAATCAAAAGAAAGGTTCAACTCTGTGAGATGAATGCACACATCACAAAGAAGTT

                  *                       *  *       
     162110 TCTCAGCATGCTTCTGTGTAGTTTTTATGTAAACATATTTC
        131 TCTCAGAATGCTTCTGTGTAGTTTTTATGTGAAGATATTTC

             *    * *              *           *      * *       *   *    *   
     162151 CGTTTCTATCATAGGCCTCAAAGTGCTCCAAATATTCACTTGTACATTCTACCAAACGAGTATTT
          1 CTTTTCCACCATAGGCCTCAAAGCGCTCCAAATATCCACTTGCAGATTCTACAAAAAGAGTGTTT

                              * *         **      *        *            *    
     162216 CAAAACTGCTCAATCAAATGGAAGGTTCAAAACTGTGACATGAATGCCCACATCACAAAGTAGTT
         66 CAAAACTGCTCAATCAAAAGAAAGGTTCAACTCTGTGAGATGAATGCACACATCACAAAGAAGTT

                                                     
     162281 TCTCAGAATGCTTCTGTGTAGTTTTTATGTGAAGATATTTC
        131 TCTCAGAATGCTTCTGTGTAGTTTTTATGTGAAGATATTTC

                     * *    *      *    *       * *   **      *              
     162322 CTTTTCCACAACA-GCGTGCAAAACGCTTCAAATATGCCCTTAGAGATTCCACAAAAAGAGTGTT
          1 CTTTTCCACCATAGGCCT-CAAAGCGCTCCAAATATCCACTTGCAGATTCTACAAAAAGAGTGTT

              *     *               *  *                                  ** 
     162386 TCCAAACTACTCAAATCAAAA-AATGATTTCAACTCTGTGAGATGAATGCACACATCACAAACTA
         65 TCAAAACTGCTC-AATCAAAAGAAAG-GTTCAACTCTGTGAGATGAATGCACACATCACAAAGAA

                         *     ** *   * *   *       *   
     162450 GTTTCTCAGAATGTTTCTGCCTGGTTCTCATGCGAAGATAGTTC
        128 GTTTCTCAGAATGCTTCTGTGTAGTTTTTATGTGAAGATATTTC

                 *           *   * **              *                  * *    
     162494 CTTTTTCACCATAGGCCGCAATGTACTCCAAATATCCACCTGCAGATTCTAC-AAAAGTGAGTTT
          1 CTTTTCCACCATAGGCCTCAAAGCGCTCCAAATATCCACTTGCAGATTCTACAAAAAGAGTGTTT

                       *         ***    **         *        *      *       * 
     162558 CAAAACTGCTCTATCAAAAGATCAGTTCGTCTCTGTGAGTTGAATGCATACATCAAAAAGAAGCT
         66 CAAAACTGCTCAATCAAAAGAAAGGTTCAACTCTGTGAGATGAATGCACACATCACAAAGAAGTT

                 *             *      **         *   
     162623 TCTCAAAATGCTTCTGTGTGGTTTTTCGGTGAAGATAGTTC
        131 TCTCAGAATGCTTCTGTGTAGTTTTTATGTGAAGATATTTC

            *     *        *      * *                 *        *        *    
     162664 TTTTTCTACCATAGGTCTCAAACCACTCCAAATATCCACTTGTAGATTCTATAAAAAGGAATG-T
          1 CTTTTCCACCATAGGCCTCAAAGCGCTCCAAATATCCACTTGCAGATTCTACAAAAA-GAGTGTT

                  *        *    *    *      * *         *      *             
     162728 TCAAAATTGCTCAATAAAAATAAAGTTTCAACACCGTGAGATGAGTGCACAAATCACAAAGAAGT
         65 TCAAAACTGCTCAATCAAAAGAAAGGTTCAACTCTGTGAGATGAATGCACACATCACAAAGAAGT

                  *          *         *          *   
     162793 TTCTCAAAATGCTTCTGGGTAGTTTTTCTGTGAAGATAGTTC
        130 TTCTCAGAATGCTTCTGTGTAGTTTTTATGTGAAGATATTTC

                  *     *    *     *          *                         *    
     162835 CTTTTCTACCATGGGCCACAAAGGGCTCCAAATACCCACTTGCAGATTCTACAAAAAGAGAGTTT
          1 CTTTTCCACCATAGGCCTCAAAGCGCTCCAAATATCCACTTGCAGATTCTACAAAAAGAGTGTTT

              *        *      ** * *        *    * *    **  * *           *  
     162900 CACAACTGCTCTATCAAACAATATGTTCAACTTTGTGGGTTGAACACAAATATCAC-AAGAATTT
         66 CAAAACTGCTCAATCAAAAGAAAGGTTCAACTCTGTGAGATGAATGCACACATCACAAAGAAGTT

                **                             *     
     162964 TCTCCCAATGCTTCTGTGTAGTTTTTATGTGAAGACATTTC
        131 TCTCAGAATGCTTCTGTGTAGTTTTTATGTGAAGATATTTC

            *   *  *      *  *     *                 *  *      *       *  *  
     163005 TTTTCCCTCCATAGTCCACAAAGTGCTCCAAATATCCACTTACATATTCTAGAAAAAGATTGCTT
          1 CTTTTCCACCATAGGCCTCAAAGCGCTCCAAATATCCACTTGCAGATTCTACAAAAAGAGTGTTT

            **       *    *               * * *                              
     163070 GGAAACTGCACAATGAAAAGAAAGGTTCAAATATATGAGATGAATGCACACATCACAAAGAAGTT
         66 CAAAACTGCTCAATCAAAAGAAAGGTTCAACTCTGTGAGATGAATGCACACATCACAAAGAAGTT

                                 *                    
     163135 TCTCAGAAT-CTCTCTGTGTAATTTTTATGTGAAGATATTTC
        131 TCTCAGAATGCT-TCTGTGTAGTTTTTATGTGAAGATATTTC

                *     *    *  *   *                        *      *    *     
     163176 CTTTCCCACCTTAGGTCTTAAAACGCTCCAAATATCCACTTGCAGATACTACAAGAAGATTGTTT
          1 CTTTTCCACCATAGGCCTCAAAGCGCTCCAAATATCCACTTGCAGATTCTACAAAAAGAGTGTTT

                     *    *        *      *     **                           
     163241 CAAAACTGCACAA-AAAAAGAAATGTTCAATTCTGTTTGATGAATGCACACATCACAAAGAAGTT
         66 CAAAACTGCTCAATCAAAAGAAAGGTTCAACTCTGTGAGATGAATGCACACATCACAAAGAAGTT

                           *                         
     163305 TCTCAGAATGCTTCTCTGTAGTTTTTATGTGAAGATATTTC
        131 TCTCAGAATGCTTCTGTGTAGTTTTTATGTGAAGATATTTC

                     *             *                 *         **      * *   
     163346 CTTTTCCACAATAGGCCTCAAAGGGCTCCAAATATCCACTTCCAGATTCTATGAAAAGAATATTT
          1 CTTTTCCACCATAGGCCTCAAAGCGCTCCAAATATCCACTTGCAGATTCTACAAAAAGAGTGTTT

             *              * *    *                                      *  
     163411 CCAAACTGCTCAATCATAGGAAATGTTCAACTCTGTGAGATGAATGCACACATCAC-AAGAAATT
         66 CAAAACTGCTCAATCAAAAGAAAGGTTCAACTCTGTGAGATGAATGCACACATCACAAAGAAGTT

                     *    *      *       *       *   
     163475 TCTCAGAATCCTTCAGTGTAGGTTTTATGAGAAGATAATTC
        131 TCTCAGAATGCTTCTGTGTAGTTTTTATGTGAAGATATTTC

                     *    **        *   *                           *    *   
     163516 CTTTTCCACAATAGTTCTCAAAGCACTCAAAATATCCACTTGCAGATTCTACAAAAGGAGTATTT
          1 CTTTTCCACCATAGGCCTCAAAGCGCTCCAAATATCCACTTGCAGATTCTACAAAAAGAGTGTTT

                                                          *                  
     163581 CAAAACTGCTCAATCAAAAGAAAGGTTCAACTCTGTGAGATGAATGGACACATCACAAAGAAGTT
         66 CAAAACTGCTCAATCAAAAGAAAGGTTCAACTCTGTGAGATGAATGCACACATCACAAAGAAGTT

                                  *   *              
     163646 TCTCAGAATGCTTCTGTGTAGTATTTTTGTGAAGATATTT-
        131 TCTCAGAATGCTTCTGTGTAGTTTTTATGTGAAGATATTTC

                          *  * * * * ***             *            *     * *  
     163686 CTTTTCCACCATAGACCGCCAGGGGACACAAATATCCACTTTCAGATTCTACAACAAGAGAGGTT
          1 CTTTTCCACCATAGGCCTCAAAGCGCTCCAAATATCCACTTGCAGATTCTACAAAAAGAGTGTTT

                   *   *     *   ** *       *      *                      * *
     163751 CAAAACTACTCGATCAAGAGATGGTTTCAACTATGTGAGTTGAATGCACACATCACAAAGAACTA
         66 CAAAACTGCTCAATCAAAAGAAAGGTTCAACTCTGTGAGATGAATGCACACATCACAAAGAAGTT

             *  *    *                               
     163816 TGTCGGAATTCTTCTGTGTAGTTTTTATGTGAAGATATTTC
        131 TCTCAGAATGCTTCTGTGTAGTTTTTATGTGAAGATATTTC

                     *    * *      * *    *                  *               
     163857 CTTTTCCACAATAGACGTCAAAGTGATCCAGATATCCACTTGCAGATTCCACAAAAAGAGTGTTT
          1 CTTTTCCACCATAGGCCTCAAAGCGCTCCAAATATCCACTTGCAGATTCTACAAAAAGAGTGTTT

                 *   *   *                  **                     *  *      
     163922 CAAAAGTGCACAACCAAAAGAAAGGTTCAACTAGGTGAGATGAATGCACACATCAGAAGGAAGTT
         66 CAAAACTGCTCAATCAAAAGAAAGGTTCAACTCTGTGAGATGAATGCACACATCACAAAGAAGTT

                            **   *     * *       *   
     163987 TCTCAGAATGCTTCTGCATAGCTTTTAAGGGAAGATACTTC
        131 TCTCAGAATGCTTCTGTGTAGTTTTTATGTGAAGATATTTC

                    *               *            * *  *    * *               
     164028 CTTTTCCAACATAGGCCTCAAAGCACTCCAAATATCCTCCTGGAGATACCACAAAAAGAGTGTTT
          1 CTTTTCCACCATAGGCCTCAAAGCGCTCCAAATATCCACTTGCAGATTCTACAAAAAGAGTGTTT

            **                      *  *                 *       *           
     164093 GCAAACTGCTCAATCAAAAGAAAGATTTAACTCTGTGAGATGAATCCACACATGACAAAGAAGTT
         66 CAAAACTGCTCAATCAAAAGAAAGGTTCAACTCTGTGAGATGAATGCACACATCACAAAGAAGTT

                                                     
     164158 TCTCAGAATGCTTCTGTGTAGTTTTTATGTGAAGATATTTC
        131 TCTCAGAATGCTTCTGTGTAGTTTTTATGTGAAGATATTTC

                     *   * * *    **           *                *     *      
     164199 CTTTTCCACAATAAGACCCAAAAGGCTCCAAATATTCACTTGCAGATTCTAAAAAAAACAGTGTT
          1 CTTTTCCACCATAGGCCTCAAAGCGCTCCAAATATCCACTTGCAGATTCT-ACAAAAAGAGTGTT

                                  *                  *      *        **      
     164264 TCAAAACTGCTCAATCAAAAGATA-GTTCAACTCTGTGAGAAGAATGCTCACATCACTGAGAAGT
         65 TCAAAACTGCTCAATCAAAAGAAAGGTTCAACTCTGTGAGATGAATGCACACATCACAAAGAAGT

                                         *            
     164328 TTCTCAGAATGCTTCTGTGTAGTTTTTATATGAAGATATTTC
        130 TTCTCAGAATGCTTCTGTGTAGTTTTTATGTGAAGATATTTC

                *     *      *    **   *                   *   **       *    
     164370 CTTTCCCACCGTAGGCCACAAAAGGCTTCAAATATCCACTTGCAGATACTATGAAAAGAGAGTTT
          1 CTTTTCCACCATAGGCCTCAAAGCGCTCCAAATATCCACTTGCAGATTCTACAAAAAGAGTGTTT

                        *        *               ***            *            
     164435 CAAAACTGCTCATTCAAAAGATAGGTTCAACTCTGTGGTTTGAATGCACACAGCACAAAGAAGTT
         66 CAAAACTGCTCAATCAAAAGAAAGGTTCAACTCTGTGAGATGAATGCACACATCACAAAGAAGTT

              *       **                   **   *    
     164500 TCACAGAATGTGTCTGTGTAGTTTTTATGTGCGGATGTTTC
        131 TCTCAGAATGCTTCTGTGTAGTTTTTATGTGAAGATATTTC

                         *        ****   *   *               *    *          
     164541 CTTTTCCACCATATGCCT-AAATATTTCCCAATTTCCACTTGCAGATTCCACAAGAAGAGTGTTT
          1 CTTTTCCACCATAGGCCTCAAAGCGCTCCAAATATCCACTTGCAGATTCTACAAAAAGAGTGTTT

                      **       *       *           *            *      ***   
     164605 CAAAACTGCTGTATC-AAATAAA-GTTGAACTCTGTGAGGTGAATGCACACAGCACAAAATGGTT
         66 CAAAACTGCTCAATCAAAAGAAAGGTTCAACTCTGTGAGATGAATGCACACATCACAAAGAAGTT

                          **   *        *       *    
     164668 TCTCAGAATGCTTCCTTGTTGTTTTTATATGAAGATGTTTC
        131 TCTCAGAATGCTTCTGTGTAGTTTTTATGTGAAGATATTTC

                  *  *             *   *     *                               
     164709 CTTTTCAACAATAGGCCTCAAAGTGCTTCAAATGTCCACTTGCAGATTCTACAAAAAGAGTGTTT
          1 CTTTTCCACCATAGGCCTCAAAGCGCTCCAAATATCCACTTGCAGATTCTACAAAAAGAGTGTTT

                                        *      *  *  *                       
     164774 CAAAACTGCTCAATCAAAAGAAAGGTTCGACTCTGGGAAATTAATGCACACATCACAAAGAAGTT
         66 CAAAACTGCTCAATCAAAAGAAAGGTTCAACTCTGTGAGATGAATGCACACATCACAAAGAAGTT

                                     *        *      
     164839 TCTC---A-GCTTCTGTGTAGTTTTCATGTGAAGTTATTTC
        131 TCTCAGAATGCTTCTGTGTAGTTTTTATGTGAAGATATTTC

                     *       *     *            *    *         **       *    
     164876 CTTTTCCACAATAGGCCGCAAAGGGCTCCAAATATCAACTTACAGATTCTAGGAAAAGAGAGTTT
          1 CTTTTCCACCATAGGCCTCAAAGCGCTCCAAATATCCACTTGCAGATTCTACAAAAAGAGTGTTT

                       * **      *     *                                     
     164941 CAAAACTGCTCTACGAAAAGATAGGTTGAACTCTGTGAGATGAATGCACACATCACAAAGAAGTT
         66 CAAAACTGCTCAATCAAAAGAAAGGTTCAACTCTGTGAGATGAATGCACACATCACAAAGAAGTT

                       *               * *           
     165006 TCTCAGAATGCATCTGTGTAGTTTTTACGGGAAGATATTTC
        131 TCTCAGAATGCTTCTGTGTAGTTTTTATGTGAAGATATTTC

                        ***  *     **      *  *                  *     ***   
     165047 CTTTTCCACCATCTTCCACAAAGGTCTCCAAGTAACCACTTGCAGATTCTACAGAAAGACACTTT
          1 CTTTTCCACCATAGGCCTCAAAGCGCTCCAAATATCCACTTGCAGATTCTACAAAAAGAGTGTTT

            *          *         ***      *      ***                      *  
     165112 AAAAACTGCTCTATCAAAAGATCAGTTCAAGTCTGTGGTTTGAATGCACACATCACAAAGAATTT
         66 CAAAACTGCTCAATCAAAAGAAAGGTTCAACTCTGTGAGATGAATGCACACATCACAAAGAAGTT

                                     *  *            
     165177 TCTCAGAATGCTTCTGTGTAGTTTTCATATGAAGATATTTC
        131 TCTCAGAATGCTTCTGTGTAGTTTTTATGTGAAGATATTTC

                                    *                                   **   
     165218 CTTTTCCACCATAGGCCTCAAAGCACTCCAAATATCCACTTGCAGATTCTACAAAAAGAGATTTT
          1 CTTTTCCACCATAGGCCTCAAAGCGCTCCAAATATCCACTTGCAGATTCTACAAAAAGAGTGTTT

                   **                           *  *          *        *     
     165283 CAAAACTAGTCAATCAAAAGAAAGGTTCAACTCTGTCAGTTGAATGCACATATCACAAACAAGTT
         66 CAAAACTGCTCAATCAAAAGAAAGGTTCAACTCTGTGAGATGAATGCACACATCACAAAGAAGTT

                *      *                             
     165348 TCTCGGAATGCGTCTGTGTAGTTTTTATGTGAAGATATTTC
        131 TCTCAGAATGCTTCTGTGTAGTTTTTATGTGAAGATATTTC

               *     * *           *     *                  *    *           
     165389 CTTCTCCACAACAGGCCTCAAAGTGCTCCGAATATCCACTTGCAGATTTTAC-TAAAGAGTGTTT
          1 CTTTTCCACCATAGGCCTCAAAGCGCTCCAAATATCCACTTGCAGATTCTACAAAAAGAGTGTTT

             *               *  *   *     *        *    *               *    
     165453 CCAAACTGCTCAATCAAGAGGAAGTTTCAAGTCTGTGAGCTGAACGCACACATCACAAAGTAGTT
         66 CAAAACTGCTCAATCAAAAGAAAGGTTCAACTCTGTGAGATGAATGCACACATCACAAAGAAGTT

               *                                *    
     165518 TCTGAGAATGCTTCTGTGTAGTTTTTATGTGAAGATGTTTC
        131 TCTCAGAATGCTTCTGTGTAGTTTTTATGTGAAGATATTTC

                            **     *                                    *    
     165559 CTTTTCCACCATAGGCTGCAAAGGGCTCCAAATATCCACTTGCAGATTCTACAAAAAGAGAGTTT
          1 CTTTTCCACCATAGGCCTCAAAGCGCTCCAAATATCCACTTGCAGATTCTACAAAAAGAGTGTTT

                 *     *         *          *     *                     *    
     165624 CAAAAGTGCTCTATCAAAAGATAGGTTCAACTATGTGATATGAATGCACACATCACAAAGTAGTT
         66 CAAAACTGCTCAATCAAAAGAAAGGTTCAACTCTGTGAGATGAATGCACACATCACAAAGAAGTT

                                          *          
     165689 TCTCAGAATGCTTCTGTGTAGTTTTTATGTAAAGATATTTC
        131 TCTCAGAATGCTTCTGTGTAGTTTTTATGTGAAGATATTTC

                                    *                                   **   
     165730 CTTTTCCACCATAGGCCTCAAAGCACTCCAAATATCCACTTGCAGATTCTACAAAAAGAGATTTT
          1 CTTTTCCACCATAGGCCTCAAAGCGCTCCAAATATCCACTTGCAGATTCTACAAAAAGAGTGTTT

                   ** *                   *     *  *    * *   *        *     
     165795 CAAAACTATTTAATCAAAAGAAAGGTTCAAATCTGTCAGTTGAAGGTACATATCACAAACAAGTT
         66 CAAAACTGCTCAATCAAAAGAAAGGTTCAACTCTGTGAGATGAATGCACACATCACAAAGAAGTT

             * **                                    
     165860 TATTGGAATGCTTCTGTGTAGTTTTTATGTGAAGATATTTC
        131 TCTCAGAATGCTTCTGTGTAGTTTTTATGTGAAGATATTTC

                     * *       * * *                        **  *     *      
     165901 CTTTTCCACAACAGGCCTCCAGGTGCTCCAAATATCCACTTGCAGATTTCACTAAAAGTGTGTTT
          1 CTTTTCCACCATAGGCCTCAAAGCGCTCCAAATATCCACTTGCAGATTCTACAAAAAGAGTGTTT

             *  *            *  *   *     *        *             *           
     165966 CCAAGCTGCTCAATCAAGAGGAAGTTTCAAGTCTGTGAGGTGAATGCACACATTACAAAGAAGTT
         66 CAAAACTGCTCAATCAAAAGAAAGGTTCAACTCTGTGAGATGAATGCACACATCACAAAGAAGTT

            *  *                                     
     166031 ACTGAGAATGCTTCTGTGTAGTTTTTATGTGAAGATATTTC
        131 TCTCAGAATGCTTCTGTGTAGTTTTTATGTGAAGATATTTC

                      **               *                                *    
     166072 CTTTTCCACCGCAGGCCTCAAAGCGCTGCAAATATCCACTTGCAGATTCTACAAAAAGAGAGTTT
          1 CTTTTCCACCATAGGCCTCAAAGCGCTCCAAATATCCACTTGCAGATTCTACAAAAAGAGTGTTT

                      **         *   *         *   *     **            *     
     166137 CAAAACTGCTGTATCAAAAGATAGGGTCAACTCTGCGAGTTGAATAAACACATCACAAATAAGTT
         66 CAAAACTGCTCAATCAAAAGAAAGGTTCAACTCTGTGAGATGAATGCACACATCACAAAGAAGTT

               **   *        *               *       
     166202 TCTGGGAACGCTTCTGTATAG-TTTTATGTGAATATATTTC
        131 TCTCAGAATGCTTCTGTGTAGTTTTTATGTGAAGATATTTC

                         *          *                   *   *  *   * *   *   
     166242 CTTTTCCACCATATGCCTCAAAGCACTCCAAATATCCACTTGCACATTATAGAAACATAGTCTTT
          1 CTTTTCCACCATAGGCCTCAAAGCGCTCCAAATATCCACTTGCAGATTCTACAAAAAGAGTGTTT

                   **                        *         *             *       
     166307 CAAAACTTGTCAATC-AAAGAAAGGTTCAACTCCGTGAGATGAGTGCACACATCACAGAGAAGTT
         66 CAAAACTGCTCAATCAAAAGAAAGGTTCAACTCTGTGAGATGAATGCACACATCACAAAGAAGTT

                *     *                            * 
     166371 TCTCGGAATGTTTCTGTGTAGTTTTTATGTGAAGATATTGC
        131 TCTCAGAATGCTTCTGTGTAGTTTTTATGTGAAGATATTTC

                     *               *            *          *       *   *   
     166412 CTTTTCCACAATAGGCCTCAAAGCGTTCCAAATATCCAATTGCAGATTCCACAAAAAAAGTTTTT
          1 CTTTTCCACCATAGGCCTCAAAGCGCTCCAAATATCCACTTGCAGATTCTACAAAAAGAGTGTTT

            *                 *  *  *  *             * *         **    *     
     166477 TAAAACTGCTCAATCAAATGATAGATTAAACTCTGTGAGATTAGTGCACACATGTCAAAAAAGTT
         66 CAAAACTGCTCAATCAAAAGAAAGGTTCAACTCTGTGAGATGAATGCACACATCACAAAGAAGTT

                                *      * *           
     166542 TCTCAGAATGCTTCTGTGTACTTTTTAGGGGAAGATATTTC
        131 TCTCAGAATGCTTCTGTGTAGTTTTTATGTGAAGATATTTC

                        *    *     **         *    *           **  * *  *    
     166583 CTTTTCCACCATCGGCCACAAAGGACTCCAAATAACCACATGCAGATTCTAGTAACACAGAGTTT
          1 CTTTTCCACCATAGGCCTCAAAGCGCTCCAAATATCCACTTGCAGATTCTACAAAAAGAGTGTTT

                       *         * *           *   * * *    **    ***        
     166648 CAAAACTGCTCTATCAAAAGATAAGTTCAACTCTGAGAGTTTAGTGCAACCATCGTGAAGAAGTT
         66 CAAAACTGCTCAATCAAAAGAAAGGTTCAACTCTGTGAGATGAATGCACACATCACAAAGAAGTT

                            *     *                  
     166713 TCTCAGAATGCTTCTGAGTAGTGTTTATGTGAAGATATTTC
        131 TCTCAGAATGCTTCTGTGTAGTTTTTATGTGAAGATATTTC

                              *     *                      *           **    
     166754 CTTTTCCACCATAGGCCTGAAAGCCCTCCAAATATCCACTTGCAGATCCTACAAAAAGAAAGTTT
          1 CTTTTCCACCATAGGCCTCAAAGCGCTCCAAATATCCACTTGCAGATTCTACAAAAAGAGTGTTT

             *         **     *  *  *   *        **      *                   
     166819 CGAAA-TGCTCTCTCAAACGATAGTTTCGACTCTGTGGTATGAATACACACATCACAAAGAAGTT
         66 CAAAACTGCTCAATCAAAAGAAAGGTTCAACTCTGTGAGATGAATGCACACATCACAAAGAAGTT

                                       **            
     166883 TCTCAGAATGCTTCTGTGTAGTTTTTAAATGAAGATATTTC
        131 TCTCAGAATGCTTCTGTGTAGTTTTTATGTGAAGATATTTC

            *                       *          *     *                       
     166924 TTTTTCCACCATAGGCCTCAAAGCACTCCAAATATGCACTTCCAGATTCTACAAAAAGAGTGTTT
          1 CTTTTCCACCATAGGCCTCAAAGCGCTCCAAATATCCACTTGCAGATTCTACAAAAAGAGTGTTT

              *                 *       * *         **   *         *  * *    
     166989 CAGAACTGCTCAATCAAAAGGAAGGTTCCAGTC--TGAGACAAATACACACATCAAAAGGTAGTT
         66 CAAAACTGCTCAATCAAAAGAAAGGTTCAACTCTGTGAGATGAATGCACACATCACAAAGAAGTT

                                                    *
     167052 TCTCAGAATGCTTCTGTGTAGTTTTTATGTGAAGATATTTT
        131 TCTCAGAATGCTTCTGTGTAGTTTTTATGTGAAGATATTTC

             *               *    **    *     *      *  **   *          *    
     167093 CCTTTCCACCATAGGCCACAAATGGCTCTAAATACCCACTTACATTTTCCACAAAAAGAGAGTTT
          1 CTTTTCCACCATAGGCCTCAAAGCGCTCCAAATATCCACTTGCAGATTCTACAAAAAGAGTGTTT

                       * *    * *      *   *         *                       
     167158 CAAAACTGCTCTACCAAAGGTAA-GTTTAACGCTGTGAG-TTAA-G-A-ACATCACAAAGAAGTT
         66 CAAAACTGCTCAATCAAAAGAAAGGTTCAACTCTGTGAGATGAATGCACACATCACAAAGAAGTT

                                   *   *  *          
     167218 TCTCAGAATGCTTCTGTGTAGTTCTTACGTAAAGATATTTC
        131 TCTCAGAATGCTTCTGTGTAGTTTTTATGTGAAGATATTTC

                 *   *      ***    *                  *              **      
     167259 CTTTTACACAATAGGCAGAAAAGTGCTCCAAATATCCACTTGAAGATTCTACAAAAACCGTGTTT
          1 CTTTTCCACCATAGGCCTCAAAGCGCTCCAAATATCCACTTGCAGATTCTACAAAAAGAGTGTTT

                     **                                          *      *    
     167324 CAAAACTGCCGAATCAAAAGAAAGGTTCAACTCTGTGAGATGAATGCACACATAACAAAGGAGTT
         66 CAAAACTGCTCAATCAAAAGAAAGGTTCAACTCTGTGAGATGAATGCACACATCACAAAGAAGTT

                                 *      *      *    *
     167389 TCTCAGAATGCTTCTGTGTAGCTTTTATATGAAGACATTTA
        131 TCTCAGAATGCTTCTGTGTAGTTTTTATGTGAAGATATTTC

            *        * *            *   ***                     **        *  
     167430 GTTTTCCACAACAGGCCTCAAAGCTCTCTCCATATCCACTTGCAGATTCTACCGAAAGAGTGCTT
          1 CTTTTCCACCATAGGCCTCAAAGCGCTCCAAATATCCACTTGCAGATTCTACAAAAAGAGTGTTT

             *                   * **     *        *        *     **         
     167495 CCAAACTGCTCAATCAAAAGAGACATTCAAATCTGTGAGGTGAATGCAGACATCGTAAAGAAGTT
         66 CAAAACTGCTCAATCAAAAGAAAGGTTCAACTCTGTGAGATGAATGCACACATCACAAAGAAGTT

                                *     *       *    **
     167560 TCTCAGAATGCTTCTGTGTATTTTTTGTGTGAAGTTATTCG
        131 TCTCAGAATGCTTCTGTGTAGTTTTTATGTGAAGATATTTC

            *    *             *     *  *            **                 *    
     167601 TTTTTGCACCATAGGCCTCCAAGCGTTCTAAATATCCACTTCTAGATTCTACAAAAAGAGAGTTT
          1 CTTTTCCACCATAGGCCTCAAAGCGCTCCAAATATCCACTTGCAGATTCTACAAAAAGAGTGTTT

                   *         *            *        *    *   *                
     167666 CAAAACTACTC-A--AACA-AAAGGTTCAATTCTGTGAGTTGAAAGCAAACATCACAAAGAAGTT
         66 CAAAACTGCTCAATCAAAAGAAAGGTTCAACTCTGTGAGATGAATGCACACATCACAAAGAAGTT

                       *             *               
     167727 TCTCAGAATGCGTCTGTGTAGTTTTGATGTGAAGATATTTC
        131 TCTCAGAATGCTTCTGTGTAGTTTTTATGTGAAGATATTTC

                     **   ****     *                  *                      
     167768 CTTTT-CACAGTAGAATGCAAAGGGCTCCAAATATCCACTTGGAGATTCTACAAAAAGA--GTTT
          1 CTTTTCCACCATAGGCCTCAAAGCGCTCCAAATATCCACTTGCAGATTCTACAAAAAGAGTGTTT

                  *    **     *  *     *     ***   *     *               *   
     167830 CAAAACCGCTCTGTCAAATGATAGGTTGAACTCCCGGAGGTGAATACACACATCACAAAGAGGTT
         66 CAAAACTGCTCAATCAAAAGAAAGGTTCAACTCTGTGAGATGAATGCACACATCACAAAGAAGTT

                  *                       *  *       
     167895 TCTCAGCATGCTTCTGTGTAGTTTTTATGTAAACATATTTC
        131 TCTCAGAATGCTTCTGTGTAGTTTTTATGTGAAGATATTTC

             *    * *              *           *      * *       *   *    *   
     167936 CGTTTCTATCATAGGCCTCAAAGTGCTCCAAATATTCACTTGTACATTCTACCAAACGAGTATTT
          1 CTTTTCCACCATAGGCCTCAAAGCGCTCCAAATATCCACTTGCAGATTCTACAAAAAGAGTGTTT

                              * *         **      *        *            *    
     168001 CAAAACTGCTCAATCAAATGGAAGGTTCAAAACTGTGACATGAATGCCCACATCACAAAGTAGTT
         66 CAAAACTGCTCAATCAAAAGAAAGGTTCAACTCTGTGAGATGAATGCACACATCACAAAGAAGTT

                                                     
     168066 TCTCAGAATGCTTCTGTGTAGTTTTTATGTGAAGATATTTC
        131 TCTCAGAATGCTTCTGTGTAGTTTTTATGTGAAGATATTTC

                     * *    *      *    *       * *   **      *              
     168107 CTTTTCCACAACA-GCGTGCAAAACGCTTCAAATATGCCCTTAGAGATTCCACAAAAAGAGTGTT
          1 CTTTTCCACCATAGGCCT-CAAAGCGCTCCAAATATCCACTTGCAGATTCTACAAAAAGAGTGTT

              *     *               *  *                                  ** 
     168171 TCCAAACTACTCAAATCAAAA-AATGATTTCAACTCTGTGAGATGAATGCACACATCACAAACTA
         65 TCAAAACTGCTC-AATCAAAAGAAAG-GTTCAACTCTGTGAGATGAATGCACACATCACAAAGAA

                         *     ** *   * *   *       *   
     168235 GTTTCTCAGAATGTTTCTGCCTGGTTCTCATGCGAAGATAGTTC
        128 GTTTCTCAGAATGCTTCTGTGTAGTTTTTATGTGAAGATATTTC

                 *           *   * **              *                  * *    
     168279 CTTTTTCACCATAGGCCGCAATGTACTCCAAATATCCACCTGCAGATTCTAC-AAAAGTGAGTTT
          1 CTTTTCCACCATAGGCCTCAAAGCGCTCCAAATATCCACTTGCAGATTCTACAAAAAGAGTGTTT

                       *         ***    **         *        *      *       * 
     168343 CAAAACTGCTCTATCAAAAGATCAGTTCGTCTCTGTGAGTTGAATGCATACATCAAAAAGAAGCT
         66 CAAAACTGCTCAATCAAAAGAAAGGTTCAACTCTGTGAGATGAATGCACACATCACAAAGAAGTT

                 *             *      **         *   
     168408 TCTCAAAATGCTTCTGTGTGGTTTTTCGGTGAAGATAGTTC
        131 TCTCAGAATGCTTCTGTGTAGTTTTTATGTGAAGATATTTC

            *     *        *      * *                 *        *        *    
     168449 TTTTTCTACCATAGGTCTCAAACCACTCCAAATATCCACTTGTAGATTCTATAAAAAGGAATG-T
          1 CTTTTCCACCATAGGCCTCAAAGCGCTCCAAATATCCACTTGCAGATTCTACAAAAA-GAGTGTT

                  *        *    *    *      * *         *      *             
     168513 TCAAAATTGCTCAATAAAAATAAAGTTTCAACACCGTGAGATGAGTGCACAAATCACAAAGAAGT
         65 TCAAAACTGCTCAATCAAAAGAAAGGTTCAACTCTGTGAGATGAATGCACACATCACAAAGAAGT

                  *          *         *          *   
     168578 TTCTCAAAATGCTTCTGGGTAGTTTTTCTGTGAAGATAGTTC
        130 TTCTCAGAATGCTTCTGTGTAGTTTTTATGTGAAGATATTTC

                  *     *    *     *          *                         *    
     168620 CTTTTCTACCATGGGCCACAAAGGGCTCCAAATACCCACTTGCAGATTCTACAAAAAGAGAGTTT
          1 CTTTTCCACCATAGGCCTCAAAGCGCTCCAAATATCCACTTGCAGATTCTACAAAAAGAGTGTTT

              *        *      ** * *        *    * *    **  * *           *  
     168685 CACAACTGCTCTATCAAACAATATGTTCAACTTTGTGGGTTGAACACAAATATCAC-AAGAATTT
         66 CAAAACTGCTCAATCAAAAGAAAGGTTCAACTCTGTGAGATGAATGCACACATCACAAAGAAGTT

                **                             *     
     168749 TCTCCCAATGCTTCTGTGTAGTTTTTATGTGAAGACATTTC
        131 TCTCAGAATGCTTCTGTGTAGTTTTTATGTGAAGATATTTC

            *   *  *      *  *     *                 *  *      *       *  *  
     168790 TTTTCCCTCCATAGTCCACAAAGTGCTCCAAATATCCACTTACATATTCTAGAAAAAGATTGCTT
          1 CTTTTCCACCATAGGCCTCAAAGCGCTCCAAATATCCACTTGCAGATTCTACAAAAAGAGTGTTT

            **       *    *               * * *                              
     168855 GGAAACTGCACAATGAAAAGAAAGGTTCAAATATATGAGATGAATGCACACATCACAAAGAAGTT
         66 CAAAACTGCTCAATCAAAAGAAAGGTTCAACTCTGTGAGATGAATGCACACATCACAAAGAAGTT

                                 *                    
     168920 TCTCAGAAT-CTCTCTGTGTAATTTTTATGTGAAGATATTTC
        131 TCTCAGAATGCT-TCTGTGTAGTTTTTATGTGAAGATATTTC

                *     *    *  *   *                        *      *    *     
     168961 CTTTCCCACCTTAGGTCTTAAAACGCTCCAAATATCCACTTGCAGATACTACAAGAAGATTGTTT
          1 CTTTTCCACCATAGGCCTCAAAGCGCTCCAAATATCCACTTGCAGATTCTACAAAAAGAGTGTTT

                     *    *        *      *     **                           
     169026 CAAAACTGCACAA-AAAAAGAAATGTTCAATTCTGTTTGATGAATGCACACATCACAAAGAAGTT
         66 CAAAACTGCTCAATCAAAAGAAAGGTTCAACTCTGTGAGATGAATGCACACATCACAAAGAAGTT

                           *                         
     169090 TCTCAGAATGCTTCTCTGTAGTTTTTATGTGAAGATATTTC
        131 TCTCAGAATGCTTCTGTGTAGTTTTTATGTGAAGATATTTC

                     *             *                 *         **      * *   
     169131 CTTTTCCACAATAGGCCTCAAAGGGCTCCAAATATCCACTTCCAGATTCTATGAAAAGAATATTT
          1 CTTTTCCACCATAGGCCTCAAAGCGCTCCAAATATCCACTTGCAGATTCTACAAAAAGAGTGTTT

             *              * *    *                                      *  
     169196 CCAAACTGCTCAATCATAGGAAATGTTCAACTCTGTGAGATGAATGCACACATCAC-AAGAAATT
         66 CAAAACTGCTCAATCAAAAGAAAGGTTCAACTCTGTGAGATGAATGCACACATCACAAAGAAGTT

                     *    *      *       *       *   
     169260 TCTCAGAATCCTTCAGTGTAGGTTTTATGAGAAGATAATTC
        131 TCTCAGAATGCTTCTGTGTAGTTTTTATGTGAAGATATTTC

                     *    **        *   *                           *    *   
     169301 CTTTTCCACAATAGTTCTCAAAGCACTCAAAATATCCACTTGCAGATTCTACAAAAGGAGTATTT
          1 CTTTTCCACCATAGGCCTCAAAGCGCTCCAAATATCCACTTGCAGATTCTACAAAAAGAGTGTTT

                                                          *                  
     169366 CAAAACTGCTCAATCAAAAGAAAGGTTCAACTCTGTGAGATGAATGGACACATCACAAAGAAGTT
         66 CAAAACTGCTCAATCAAAAGAAAGGTTCAACTCTGTGAGATGAATGCACACATCACAAAGAAGTT

                                  *   *              
     169431 TCTCAGAATGCTTCTGTGTAGTATTTTTGTGAAGATATTT-
        131 TCTCAGAATGCTTCTGTGTAGTTTTTATGTGAAGATATTTC

                          *  * * * * ***             *            *     * *  
     169471 CTTTTCCACCATAGACCGCCAGGGGACACAAATATCCACTTTCAGATTCTACAACAAGAGAGGTT
          1 CTTTTCCACCATAGGCCTCAAAGCGCTCCAAATATCCACTTGCAGATTCTACAAAAAGAGTGTTT

                   *   *     *   ** *       *      *                      * *
     169536 CAAAACTACTCGATCAAGAGATGGTTTCAACTATGTGAGTTGAATGCACACATCACAAAGAACTA
         66 CAAAACTGCTCAATCAAAAGAAAGGTTCAACTCTGTGAGATGAATGCACACATCACAAAGAAGTT

             *  *    *                               
     169601 TGTCGGAATTCTTCTGTGTAGTTTTTATGTGAAGATATTTC
        131 TCTCAGAATGCTTCTGTGTAGTTTTTATGTGAAGATATTTC

                     *    * *      * *    *                  *               
     169642 CTTTTCCACAATAGACGTCAAAGTGATCCAGATATCCACTTGCAGATTCCACAAAAAGAGTGTTT
          1 CTTTTCCACCATAGGCCTCAAAGCGCTCCAAATATCCACTTGCAGATTCTACAAAAAGAGTGTTT

                 *   *   *                  **                     *  *      
     169707 CAAAAGTGCACAACCAAAAGAAAGGTTCAACTAGGTGAGATGAATGCACACATCAGAAGGAAGTT
         66 CAAAACTGCTCAATCAAAAGAAAGGTTCAACTCTGTGAGATGAATGCACACATCACAAAGAAGTT

                            **   *     * *       *   
     169772 TCTCAGAATGCTTCTGCATAGCTTTTAAGGGAAGATACTTC
        131 TCTCAGAATGCTTCTGTGTAGTTTTTATGTGAAGATATTTC

                    *               *            * *  *    * *               
     169813 CTTTTCCAACATAGGCCTCAAAGCACTCCAAATATCCTCCTGGAGATACCACAAAAAGAGTGTTT
          1 CTTTTCCACCATAGGCCTCAAAGCGCTCCAAATATCCACTTGCAGATTCTACAAAAAGAGTGTTT

            **                      *  *                 *       *           
     169878 GCAAACTGCTCAATCAAAAGAAAGATTTAACTCTGTGAGATGAATCCACACATGACAAAGAAGTT
         66 CAAAACTGCTCAATCAAAAGAAAGGTTCAACTCTGTGAGATGAATGCACACATCACAAAGAAGTT

                                                     
     169943 TCTCAGAATGCTTCTGTGTAGTTTTTATGTGAAGATATTTC
        131 TCTCAGAATGCTTCTGTGTAGTTTTTATGTGAAGATATTTC

                     *   * * *    **           *                *     *      
     169984 CTTTTCCACAATAAGACCCAAAAGGCTCCAAATATTCACTTGCAGATTCTAAAAAAAACAGTGTT
          1 CTTTTCCACCATAGGCCTCAAAGCGCTCCAAATATCCACTTGCAGATTCT-ACAAAAAGAGTGTT

                                  *                  *      *        **      
     170049 TCAAAACTGCTCAATCAAAAGATA-GTTCAACTCTGTGAGAAGAATGCTCACATCACTGAGAAGT
         65 TCAAAACTGCTCAATCAAAAGAAAGGTTCAACTCTGTGAGATGAATGCACACATCACAAAGAAGT

                                         *            
     170113 TTCTCAGAATGCTTCTGTGTAGTTTTTATATGAAGATATTTC
        130 TTCTCAGAATGCTTCTGTGTAGTTTTTATGTGAAGATATTTC

                *     *      *    **   *                   *   **       *    
     170155 CTTTCCCACCGTAGGCCACAAAAGGCTTCAAATATCCACTTGCAGATACTATGAAAAGAGAGTTT
          1 CTTTTCCACCATAGGCCTCAAAGCGCTCCAAATATCCACTTGCAGATTCTACAAAAAGAGTGTTT

                        *        *               ***            *            
     170220 CAAAACTGCTCATTCAAAAGATAGGTTCAACTCTGTGGTTTGAATGCACACAGCACAAAGAAGTT
         66 CAAAACTGCTCAATCAAAAGAAAGGTTCAACTCTGTGAGATGAATGCACACATCACAAAGAAGTT

              *       **                   **   *    
     170285 TCACAGAATGTGTCTGTGTAGTTTTTATGTGCGGATGTTTC
        131 TCTCAGAATGCTTCTGTGTAGTTTTTATGTGAAGATATTTC

                         *        ****   *   *               *    *          
     170326 CTTTTCCACCATATGCCT-AAATATTTCCCAATTTCCACTTGCAGATTCCACAAGAAGAGTGTTT
          1 CTTTTCCACCATAGGCCTCAAAGCGCTCCAAATATCCACTTGCAGATTCTACAAAAAGAGTGTTT

                      **       *       *           *            *      ***   
     170390 CAAAACTGCTGTATC-AAATAAA-GTTGAACTCTGTGAGGTGAATGCACACAGCACAAAATGGTT
         66 CAAAACTGCTCAATCAAAAGAAAGGTTCAACTCTGTGAGATGAATGCACACATCACAAAGAAGTT

                          **   *        *       *    
     170453 TCTCAGAATGCTTCCTTGTTGTTTTTATATGAAGATGTTTC
        131 TCTCAGAATGCTTCTGTGTAGTTTTTATGTGAAGATATTTC

                  *  *             *   *     *                               
     170494 CTTTTCAACAATAGGCCTCAAAGTGCTTCAAATGTCCACTTGCAGATTCTACAAAAAGAGTGTTT
          1 CTTTTCCACCATAGGCCTCAAAGCGCTCCAAATATCCACTTGCAGATTCTACAAAAAGAGTGTTT

                                        *      *  *  *                       
     170559 CAAAACTGCTCAATCAAAAGAAAGGTTCGACTCTGGGAAATTAATGCACACATCACAAAGAAGTT
         66 CAAAACTGCTCAATCAAAAGAAAGGTTCAACTCTGTGAGATGAATGCACACATCACAAAGAAGTT

                                     *        *      
     170624 TCTC---A-GCTTCTGTGTAGTTTTCATGTGAAGTTATTTC
        131 TCTCAGAATGCTTCTGTGTAGTTTTTATGTGAAGATATTTC

                     *       *     *            *    *         **       *    
     170661 CTTTTCCACAATAGGCCGCAAAGGGCTCCAAATATCAACTTACAGATTCTAGGAAAAGAGAGTTT
          1 CTTTTCCACCATAGGCCTCAAAGCGCTCCAAATATCCACTTGCAGATTCTACAAAAAGAGTGTTT

                       * **      *     *                                     
     170726 CAAAACTGCTCTACGAAAAGATAGGTTGAACTCTGTGAGATGAATGCACACATCACAAAGAAGTT
         66 CAAAACTGCTCAATCAAAAGAAAGGTTCAACTCTGTGAGATGAATGCACACATCACAAAGAAGTT

                       *               * *           
     170791 TCTCAGAATGCATCTGTGTAGTTTTTACGGGAAGATATTTC
        131 TCTCAGAATGCTTCTGTGTAGTTTTTATGTGAAGATATTTC

                        ***  *     **      *  *                  *     ***   
     170832 CTTTTCCACCATCTTCCACAAAGGTCTCCAAGTAACCACTTGCAGATTCTACAGAAAGACACTTT
          1 CTTTTCCACCATAGGCCTCAAAGCGCTCCAAATATCCACTTGCAGATTCTACAAAAAGAGTGTTT

            *          *         ***      *      ***                      *  
     170897 AAAAACTGCTCTATCAAAAGATCAGTTCAAGTCTGTGGTTTGAATGCACACATCACAAAGAATTT
         66 CAAAACTGCTCAATCAAAAGAAAGGTTCAACTCTGTGAGATGAATGCACACATCACAAAGAAGTT

                                     *  *            
     170962 TCTCAGAATGCTTCTGTGTAGTTTTCATATGAAGATATTTC
        131 TCTCAGAATGCTTCTGTGTAGTTTTTATGTGAAGATATTTC

                                    *                                   **   
     171003 CTTTTCCACCATAGGCCTCAAAGCACTCCAAATATCCACTTGCAGATTCTACAAAAAGAGATTTT
          1 CTTTTCCACCATAGGCCTCAAAGCGCTCCAAATATCCACTTGCAGATTCTACAAAAAGAGTGTTT

                   **                           *  *          *        *     
     171068 CAAAACTAGTCAATCAAAAGAAAGGTTCAACTCTGTCAGTTGAATGCACATATCACAAACAAGTT
         66 CAAAACTGCTCAATCAAAAGAAAGGTTCAACTCTGTGAGATGAATGCACACATCACAAAGAAGTT

                *      *                             
     171133 TCTCGGAATGCGTCTGTGTAGTTTTTATGTGAAGATATTTC
        131 TCTCAGAATGCTTCTGTGTAGTTTTTATGTGAAGATATTTC

               *     * *           *     *                  *    *           
     171174 CTTCTCCACAACAGGCCTCAAAGTGCTCCGAATATCCACTTGCAGATTTTAC-TAAAGAGTGTTT
          1 CTTTTCCACCATAGGCCTCAAAGCGCTCCAAATATCCACTTGCAGATTCTACAAAAAGAGTGTTT

             *               *  *   *     *        *    *               *    
     171238 CCAAACTGCTCAATCAAGAGGAAGTTTCAAGTCTGTGAGCTGAACGCACACATCACAAAGTAGTT
         66 CAAAACTGCTCAATCAAAAGAAAGGTTCAACTCTGTGAGATGAATGCACACATCACAAAGAAGTT

               *                                *    
     171303 TCTGAGAATGCTTCTGTGTAGTTTTTATGTGAAGATGTTTC
        131 TCTCAGAATGCTTCTGTGTAGTTTTTATGTGAAGATATTTC

                            **     *                                    *    
     171344 CTTTTCCACCATAGGCTGCAAAGGGCTCCAAATATCCACTTGCAGATTCTACAAAAAGAGAGTTT
          1 CTTTTCCACCATAGGCCTCAAAGCGCTCCAAATATCCACTTGCAGATTCTACAAAAAGAGTGTTT

                 *     *         *          *     *                     *    
     171409 CAAAAGTGCTCTATCAAAAGATAGGTTCAACTATGTGATATGAATGCACACATCACAAAGTAGTT
         66 CAAAACTGCTCAATCAAAAGAAAGGTTCAACTCTGTGAGATGAATGCACACATCACAAAGAAGTT

                                          *          
     171474 TCTCAGAATGCTTCTGTGTAGTTTTTATGTAAAGATATTTC
        131 TCTCAGAATGCTTCTGTGTAGTTTTTATGTGAAGATATTTC

                                    *                                   **   
     171515 CTTTTCCACCATAGGCCTCAAAGCACTCCAAATATCCACTTGCAGATTCTACAAAAAGAGATTTT
          1 CTTTTCCACCATAGGCCTCAAAGCGCTCCAAATATCCACTTGCAGATTCTACAAAAAGAGTGTTT

                   ** *                   *     *  *    * *   *        *     
     171580 CAAAACTATTTAATCAAAAGAAAGGTTCAAATCTGTCAGTTGAAGGTACATATCACAAACAAGTT
         66 CAAAACTGCTCAATCAAAAGAAAGGTTCAACTCTGTGAGATGAATGCACACATCACAAAGAAGTT

             * **                                    
     171645 TATTGGAATGCTTCTGTGTAGTTTTTATGTGAAGATATTTC
        131 TCTCAGAATGCTTCTGTGTAGTTTTTATGTGAAGATATTTC

                     * *         * *                        **  *     *      
     171686 CTTTTCCACAACAGGCCTCAAGGTGCTCCAAATATCCACTTGCAGATTTCACTAAAAGTGTGTTT
          1 CTTTTCCACCATAGGCCTCAAAGCGCTCCAAATATCCACTTGCAGATTCTACAAAAAGAGTGTTT

             *  *            *  *   *     *        *             *           
     171751 CCAAGCTGCTCAATCAAGAGGAAGTTTCAAGTCTGTGAGGTGAATGCACACATTACAAAGAAGTT
         66 CAAAACTGCTCAATCAAAAGAAAGGTTCAACTCTGTGAGATGAATGCACACATCACAAAGAAGTT

            *  *                                     
     171816 ACTGAGAATGCTTCTGTGTAGTTTTTATGTGAAGATATTTC
        131 TCTCAGAATGCTTCTGTGTAGTTTTTATGTGAAGATATTTC

                      **               *                                *    
     171857 CTTTTCCACCGCAGGCCTCAAAGCGCTGCAAATATCCACTTGCAGATTCTACAAAAAGAGAGTTT
          1 CTTTTCCACCATAGGCCTCAAAGCGCTCCAAATATCCACTTGCAGATTCTACAAAAAGAGTGTTT

                      **         *   *         *   *     **            *     
     171922 CAAAACTGCTGTATCAAAAGATAGGGTCAACTCTGCGAGTTGAATAAACACATCACAAATAAGTT
         66 CAAAACTGCTCAATCAAAAGAAAGGTTCAACTCTGTGAGATGAATGCACACATCACAAAGAAGTT

               **   *        *               *       
     171987 TCTGGGAACGCTTCTGTATAG-TTTTATGTGAATATATTTC
        131 TCTCAGAATGCTTCTGTGTAGTTTTTATGTGAAGATATTTC

                         *          *                   *   *  *   * *   *   
     172027 CTTTTCCACCATATGCCTCAAAGCACTCCAAATATCCACTTGCACATTATAGAAACATAGTCTTT
          1 CTTTTCCACCATAGGCCTCAAAGCGCTCCAAATATCCACTTGCAGATTCTACAAAAAGAGTGTTT

                   **                        *         *             *       
     172092 CAAAACTTGTCAATC-AAAGAAAGGTTCAACTCCGTGAGATGAGTGCACACATCACAGAGAAGTT
         66 CAAAACTGCTCAATCAAAAGAAAGGTTCAACTCTGTGAGATGAATGCACACATCACAAAGAAGTT

                *     *                            * 
     172156 TCTCGGAATGTTTCTGTGTAGTTTTTATGTGAAGATATTGC
        131 TCTCAGAATGCTTCTGTGTAGTTTTTATGTGAAGATATTTC

                     *               *            *          *       *   *   
     172197 CTTTTCCACAATAGGCCTCAAAGCGTTCCAAATATCCAATTGCAGATTCCACAAAAAAAGTTTTT
          1 CTTTTCCACCATAGGCCTCAAAGCGCTCCAAATATCCACTTGCAGATTCTACAAAAAGAGTGTTT

            *                 *  *  *  *             * *         **    *     
     172262 TAAAACTGCTCAATCAAATGATAGATTAAACTCTGTGAGATTAGTGCACACATGTCAAAAAAGTT
         66 CAAAACTGCTCAATCAAAAGAAAGGTTCAACTCTGTGAGATGAATGCACACATCACAAAGAAGTT

                                *      * *           
     172327 TCTCAGAATGCTTCTGTGTACTTTTTAGGGGAAGATATTTC
        131 TCTCAGAATGCTTCTGTGTAGTTTTTATGTGAAGATATTTC

                        *    *     **         *    *           **  * *  *    
     172368 CTTTTCCACCATCGGCCACAAAGGACTCCAAATAACCACATGCAGATTCTAGTAACACAGAGTTT
          1 CTTTTCCACCATAGGCCTCAAAGCGCTCCAAATATCCACTTGCAGATTCTACAAAAAGAGTGTTT

                       *         * *           *   * * *    **    ***        
     172433 CAAAACTGCTCTATCAAAAGATAAGTTCAACTCTGAGAGTTTAGTGCAACCATCGTGAAGAAGTT
         66 CAAAACTGCTCAATCAAAAGAAAGGTTCAACTCTGTGAGATGAATGCACACATCACAAAGAAGTT

                            *     *                  
     172498 TCTCAGAATGCTTCTGAGTAGTGTTTATGTGAAGATATTTC
        131 TCTCAGAATGCTTCTGTGTAGTTTTTATGTGAAGATATTTC

                              *     *                      *           **    
     172539 CTTTTCCACCATAGGCCTGAAAGCCCTCCAAATATCCACTTGCAGATCCTACAAAAAGAAAGTTT
          1 CTTTTCCACCATAGGCCTCAAAGCGCTCCAAATATCCACTTGCAGATTCTACAAAAAGAGTGTTT

             *         **     *  *  *   *        **      *                   
     172604 CGAAA-TGCTCTCTCAAACGATAGTTTCGACTCTGTGGTATGAATACACACATCACAAAGAAGTT
         66 CAAAACTGCTCAATCAAAAGAAAGGTTCAACTCTGTGAGATGAATGCACACATCACAAAGAAGTT

                                       **            
     172668 TCTCAGAATGCTTCTGTGTAGTTTTTAAATGAAGATATTTC
        131 TCTCAGAATGCTTCTGTGTAGTTTTTATGTGAAGATATTTC

            *                       *          *     *                       
     172709 TTTTTCCACCATAGGCCTCAAAGCACTCCAAATATGCACTTCCAGATTCTACAAAAAGAGTGTTT
          1 CTTTTCCACCATAGGCCTCAAAGCGCTCCAAATATCCACTTGCAGATTCTACAAAAAGAGTGTTT

              *                 *       * *         **   *         *  * *    
     172774 CAGAACTGCTCAATCAAAAGGAAGGTTCCAGTC--TGAGACAAATACACACATCAAAAGGTAGTT
         66 CAAAACTGCTCAATCAAAAGAAAGGTTCAACTCTGTGAGATGAATGCACACATCACAAAGAAGTT

                                                    *
     172837 TCTCAGAATGCTTCTGTGTAGTTTTTATGTGAAGATATTTT
        131 TCTCAGAATGCTTCTGTGTAGTTTTTATGTGAAGATATTTC

             *               *    **    *     *      *  **   *          *    
     172878 CCTTTCCACCATAGGCCACAAATGGCTCTAAATACCCACTTACATTTTCCACAAAAAGAGAGTTT
          1 CTTTTCCACCATAGGCCTCAAAGCGCTCCAAATATCCACTTGCAGATTCTACAAAAAGAGTGTTT

                       * *    * *      *   *         *                       
     172943 CAAAACTGCTCTACCAAAGGTAA-GTTTAACGCTGTGAG-TTAA-G-A-ACATCACAAAGAAGTT
         66 CAAAACTGCTCAATCAAAAGAAAGGTTCAACTCTGTGAGATGAATGCACACATCACAAAGAAGTT

                                   *   *  *          
     173003 TCTCAGAATGCTTCTGTGTAGTTCTTACGTAAAGATATTTC
        131 TCTCAGAATGCTTCTGTGTAGTTTTTATGTGAAGATATTTC

                 *   *      ***    *                  *              **      
     173044 CTTTTACACAATAGGCAGAAAAGTGCTCCAAATATCCACTTGAAGATTCTACAAAAACCGTGTTT
          1 CTTTTCCACCATAGGCCTCAAAGCGCTCCAAATATCCACTTGCAGATTCTACAAAAAGAGTGTTT

                     **                                          *      *    
     173109 CAAAACTGCCGAATCAAAAGAAAGGTTCAACTCTGTGAGATGAATGCACACATAACAAAGGAGTT
         66 CAAAACTGCTCAATCAAAAGAAAGGTTCAACTCTGTGAGATGAATGCACACATCACAAAGAAGTT

                                 *      *      *    *
     173174 TCTCAGAATGCTTCTGTGTAGCTTTTATATGAAGACATTTA
        131 TCTCAGAATGCTTCTGTGTAGTTTTTATGTGAAGATATTTC

            *        * *            *   ***                     **        *  
     173215 GTTTTCCACAACAGGCCTCAAAGCTCTCTCCATATCCACTTGCAGATTCTACCGAAAGAGTGCTT
          1 CTTTTCCACCATAGGCCTCAAAGCGCTCCAAATATCCACTTGCAGATTCTACAAAAAGAGTGTTT

             *                   * **     *        *        *     **         
     173280 CCAAACTGCTCAATCAAAAGAGACATTCAAATCTGTGAGGTGAATGCAGACATCGTAAAGAAGTT
         66 CAAAACTGCTCAATCAAAAGAAAGGTTCAACTCTGTGAGATGAATGCACACATCACAAAGAAGTT

                                *     *       *    **
     173345 TCTCAGAATGCTTCTGTGTATTTTTTGTGTGAAGTTATTCG
        131 TCTCAGAATGCTTCTGTGTAGTTTTTATGTGAAGATATTTC

            *    *             *     *  *            **                 *    
     173386 TTTTTGCACCATAGGCCTCCAAGCGTTCTAAATATCCACTTCTAGATTCTACAAAAAGAGAGTTT
          1 CTTTTCCACCATAGGCCTCAAAGCGCTCCAAATATCCACTTGCAGATTCTACAAAAAGAGTGTTT

                   *         *            *        *    *   *                
     173451 CAAAACTACTC-A--AACA-AAAGGTTCAATTCTGTGAGTTGAAAGCAAACATCACAAAGAAGTT
         66 CAAAACTGCTCAATCAAAAGAAAGGTTCAACTCTGTGAGATGAATGCACACATCACAAAGAAGTT

                       *                             
     173512 TCTCAGAATGCGTCTGTGTAGTTTTTATGTGAAGATATTTC
        131 TCTCAGAATGCTTCTGTGTAGTTTTTATGTGAAGATATTTC

                     **   ****     *                  *                      
     173553 CTTTT-CACAGTAGAATGCAAAGGGCTCCAAATATCCACTTGGAGATTCTACAAAAAGA--GTTT
          1 CTTTTCCACCATAGGCCTCAAAGCGCTCCAAATATCCACTTGCAGATTCTACAAAAAGAGTGTTT

                  *    **     *  *     *     ***   *     *               *   
     173615 CAAAACCGCTCTGTCAAATGATAGGTTGAACTCCCGGAGGTGAATACACACATCACAAAGAGGTT
         66 CAAAACTGCTCAATCAAAAGAAAGGTTCAACTCTGTGAGATGAATGCACACATCACAAAGAAGTT

                  *                       *  * *     
     173680 TCTCAGCATGCTTCTGTGTAGTTTTTATGTAAACACATTTC
        131 TCTCAGAATGCTTCTGTGTAGTTTTTATGTGAAGATATTTC

             *    * *              *           *      * *       *   *    *   
     173721 CGTTTCTATCATAGGCCTCAAAGTGCTCCAAATATTCACTTGTACATTCTACCAAACGAGTATTT
          1 CTTTTCCACCATAGGCCTCAAAGCGCTCCAAATATCCACTTGCAGATTCTACAAAAAGAGTGTTT

                              * *         **      *        *            *    
     173786 CAAAACTGCTCAATCAAATGGAAGGTTCAAAACTGTGACATGAATGCCCACATCACAAAGTAGTT
         66 CAAAACTGCTCAATCAAAAGAAAGGTTCAACTCTGTGAGATGAATGCACACATCACAAAGAAGTT

                                                     
     173851 TCTCAGAATGCTTCTGTGTAGTTTTTATGTGAAGATATTTC
        131 TCTCAGAATGCTTCTGTGTAGTTTTTATGTGAAGATATTTC

                     * *    *      *    *       * *   **      *              
     173892 CTTTTCCACAACA-GCGTGCAAAACGCTTCAAATATGCCCTTAGAGATTCCACAAAAAGAGTGTT
          1 CTTTTCCACCATAGGCCT-CAAAGCGCTCCAAATATCCACTTGCAGATTCTACAAAAAGAGTGTT

              *     *               *  *                                  ** 
     173956 TCCAAACTACTCAAATCAAAA-AATGATTTCAACTCTGTGAGATGAATGCACACATCACAAACTA
         65 TCAAAACTGCTC-AATCAAAAGAAAG-GTTCAACTCTGTGAGATGAATGCACACATCACAAAGAA

                         *     ** *   * *   *       *   
     174020 GTTTCTCAGAATGTTTCTGCCTGGTTCTCATGCGAAGATAGTTC
        128 GTTTCTCAGAATGCTTCTGTGTAGTTTTTATGTGAAGATATTTC

                 *           *   * **              *                  * *    
     174064 CTTTTTCACCATAGGCCGCAATGTACTCCAAATATCCACCTGCAGATTCTAC-AAAAGTGAGTTT
          1 CTTTTCCACCATAGGCCTCAAAGCGCTCCAAATATCCACTTGCAGATTCTACAAAAAGAGTGTTT

                       *         ***    **         *        *      *       * 
     174128 CAAAACTGCTCTATCAAAAGATCAGTTCGTCTCTGTGAGTTGAATGCATACATCAAAAAGAAGCT
         66 CAAAACTGCTCAATCAAAAGAAAGGTTCAACTCTGTGAGATGAATGCACACATCACAAAGAAGTT

                 *             *      **         *   
     174193 TCTCAAAATGCTTCTGTGTGGTTTTTCGGTGAAGATAGTTC
        131 TCTCAGAATGCTTCTGTGTAGTTTTTATGTGAAGATATTTC

            *     *        *      * *                 *        *        *    
     174234 TTTTTCTACCATAGGTCTCAAACCACTCCAAATATCCACTTGTAGATTCTATAAAAAGGAATG-T
          1 CTTTTCCACCATAGGCCTCAAAGCGCTCCAAATATCCACTTGCAGATTCTACAAAAA-GAGTGTT

                  *        *    *    *      * *         *      *             
     174298 TCAAAATTGCTCAATAAAAATAAAGTTTCAACACCGTGAGATGAGTGCACAAATCACAAAGAAGT
         65 TCAAAACTGCTCAATCAAAAGAAAGGTTCAACTCTGTGAGATGAATGCACACATCACAAAGAAGT

                  *          *         *          *   
     174363 TTCTCAAAATGCTTCTGGGTAGTTTTTCTGTGAAGATAGTTC
        130 TTCTCAGAATGCTTCTGTGTAGTTTTTATGTGAAGATATTTC

                  *     *    *     *          *                         *    
     174405 CTTTTCTACCATGGGCCACAAAGGGCTCCAAATACCCACTTGCAGATTCTACAAAAAGAGAGTTT
          1 CTTTTCCACCATAGGCCTCAAAGCGCTCCAAATATCCACTTGCAGATTCTACAAAAAGAGTGTTT

              *        *      ** * *        *    * *    **  * *           *  
     174470 CACAACTGCTCTATCAAACAATATGTTCAACTTTGTGGGTTGAACACAAATATCAC-AAGAATTT
         66 CAAAACTGCTCAATCAAAAGAAAGGTTCAACTCTGTGAGATGAATGCACACATCACAAAGAAGTT

                **                             *     
     174534 TCTCCCAATGCTTCTGTGTAGTTTTTATGTGAAGACATTTC
        131 TCTCAGAATGCTTCTGTGTAGTTTTTATGTGAAGATATTTC

            *   *  *      *  *     *                 *  *      *       *  *  
     174575 TTTTCCCTCCATAGTCCACAAAGTGCTCCAAATATCCACTTACATATTCTAGAAAAAGATTGCTT
          1 CTTTTCCACCATAGGCCTCAAAGCGCTCCAAATATCCACTTGCAGATTCTACAAAAAGAGTGTTT

            **       *    *               * * *                              
     174640 GGAAACTGCACAATGAAAAGAAAGGTTCAAATATATGAGATGAATGCACACATCACAAAGAAGTT
         66 CAAAACTGCTCAATCAAAAGAAAGGTTCAACTCTGTGAGATGAATGCACACATCACAAAGAAGTT

                                 *                    
     174705 TCTCAGAAT-CTCTCTGTGTAATTTTTATGTGAAGATATTTC
        131 TCTCAGAATGCT-TCTGTGTAGTTTTTATGTGAAGATATTTC

                *     *    *  *   *                        *      *    *     
     174746 CTTTCCCACCTTAGGTCTTAAAACGCTCCAAATATCCACTTGCAGATACTACAAGAAGATTGTTT
          1 CTTTTCCACCATAGGCCTCAAAGCGCTCCAAATATCCACTTGCAGATTCTACAAAAAGAGTGTTT

                     *    *        *      *     **                           
     174811 CAAAACTGCACAA-AAAAAGAAATGTTCAATTCTGTTTGATGAATGCACACATCACAAAGAAGTT
         66 CAAAACTGCTCAATCAAAAGAAAGGTTCAACTCTGTGAGATGAATGCACACATCACAAAGAAGTT

                           *                         
     174875 TCTCAGAATGCTTCTCTGTAGTTTTTATGTGAAGATATTTC
        131 TCTCAGAATGCTTCTGTGTAGTTTTTATGTGAAGATATTTC

                     *             *                 *         **      * *   
     174916 CTTTTCCACAATAGGCCTCAAAGGGCTCCAAATATCCACTTCCAGATTCTATGAAAAGAATATTT
          1 CTTTTCCACCATAGGCCTCAAAGCGCTCCAAATATCCACTTGCAGATTCTACAAAAAGAGTGTTT

             *              * *    *                                      *  
     174981 CCAAACTGCTCAATCATAGGAAATGTTCAACTCTGTGAGATGAATGCACACATCAC-AAGAAATT
         66 CAAAACTGCTCAATCAAAAGAAAGGTTCAACTCTGTGAGATGAATGCACACATCACAAAGAAGTT

                     *    *      *       *       *   
     175045 TCTCAGAATCCTTCAGTGTAGGTTTTATGAGAAGATAATTC
        131 TCTCAGAATGCTTCTGTGTAGTTTTTATGTGAAGATATTTC

                     *    **        *   *                           *    *   
     175086 CTTTTCCACAATAGTTCTCAAAGCACTCAAAATATCCACTTGCAGATTCTACAAAAGGAGTATTT
          1 CTTTTCCACCATAGGCCTCAAAGCGCTCCAAATATCCACTTGCAGATTCTACAAAAAGAGTGTTT

                                                          *                  
     175151 CAAAACTGCTCAATCAAAAGAAAGGTTCAACTCTGTGAGATGAATGGACACATCACAAAGAAGTT
         66 CAAAACTGCTCAATCAAAAGAAAGGTTCAACTCTGTGAGATGAATGCACACATCACAAAGAAGTT

                                  *   *              
     175216 TCTCAGAATGCTTCTGTGTAGTATTTTTGTGAAGATATTT-
        131 TCTCAGAATGCTTCTGTGTAGTTTTTATGTGAAGATATTTC

                          *  * * * * ***             *            *     * *  
     175256 CTTTTCCACCATAGACCGCCAGGGGACACAAATATCCACTTTCAGATTCTACAACAAGAGAGGTT
          1 CTTTTCCACCATAGGCCTCAAAGCGCTCCAAATATCCACTTGCAGATTCTACAAAAAGAGTGTTT

                   *   *     *   ** *       *      *                      * *
     175321 CAAAACTACTCGATCAAGAGATGGTTTCAACTATGTGAGTTGAATGCACACATCACAAAGAACTA
         66 CAAAACTGCTCAATCAAAAGAAAGGTTCAACTCTGTGAGATGAATGCACACATCACAAAGAAGTT

             *  *    *                               
     175386 TGTCGGAATTCTTCTGTGTAGTTTTTATGTGAAGATATTTC
        131 TCTCAGAATGCTTCTGTGTAGTTTTTATGTGAAGATATTTC

                     *    * *      * *    *                  *               
     175427 CTTTTCCACAATAGACGTCAAAGTGATCCAGATATCCACTTGCAGATTCCACAAAAAGAGTGTTT
          1 CTTTTCCACCATAGGCCTCAAAGCGCTCCAAATATCCACTTGCAGATTCTACAAAAAGAGTGTTT

                 *   *   *                  **                     *  *      
     175492 CAAAAGTGCACAACCAAAAGAAAGGTTCAACTAGGTGAGATGAATGCACACATCAGAAGGAAGTT
         66 CAAAACTGCTCAATCAAAAGAAAGGTTCAACTCTGTGAGATGAATGCACACATCACAAAGAAGTT

                            **   *     * *       *   
     175557 TCTCAGAATGCTTCTGCATAGCTTTTAAGGGAAGATACTTC
        131 TCTCAGAATGCTTCTGTGTAGTTTTTATGTGAAGATATTTC

                    *               *            * *  *    * *               
     175598 CTTTTCCAACATAGGCCTCAAAGCACTCCAAATATCCTCCTGGAGATACCACAAAAAGAGTGTTT
          1 CTTTTCCACCATAGGCCTCAAAGCGCTCCAAATATCCACTTGCAGATTCTACAAAAAGAGTGTTT

            **                      *  *                 *       *           
     175663 GCAAACTGCTCAATCAAAAGAAAGATTTAACTCTGTGAGATGAATCCACACATGACAAAGAAGTT
         66 CAAAACTGCTCAATCAAAAGAAAGGTTCAACTCTGTGAGATGAATGCACACATCACAAAGAAGTT

                                                     
     175728 TCTCAGAATGCTTCTGTGTAGTTTTTATGTGAAGATATTTC
        131 TCTCAGAATGCTTCTGTGTAGTTTTTATGTGAAGATATTTC

                     *   * * *    **           *                *     *      
     175769 CTTTTCCACAATAAGACCCAAAAGGCTCCAAATATTCACTTGCAGATTCTAAAAAAAACAGTGTT
          1 CTTTTCCACCATAGGCCTCAAAGCGCTCCAAATATCCACTTGCAGATTCT-ACAAAAAGAGTGTT

                                  *                  *      *        **      
     175834 TCAAAACTGCTCAATCAAAAGATA-GTTCAACTCTGTGAGAAGAATGCTCACATCACTGAGAAGT
         65 TCAAAACTGCTCAATCAAAAGAAAGGTTCAACTCTGTGAGATGAATGCACACATCACAAAGAAGT

                                         *            
     175898 TTCTCAGAATGCTTCTGTGTAGTTTTTATATGAAGATATTTC
        130 TTCTCAGAATGCTTCTGTGTAGTTTTTATGTGAAGATATTTC

                *     *      *    **   *                   *   **       *    
     175940 CTTTCCCACCGTAGGCCACAAAAGGCTTCAAATATCCACTTGCAGATACTATGAAAAGAGAGTTT
          1 CTTTTCCACCATAGGCCTCAAAGCGCTCCAAATATCCACTTGCAGATTCTACAAAAAGAGTGTTT

                        *        *               ***            *            
     176005 CAAAACTGCTCATTCAAAAGATAGGTTCAACTCTGTGGTTTGAATGCACACAGCACAAAGAAGTT
         66 CAAAACTGCTCAATCAAAAGAAAGGTTCAACTCTGTGAGATGAATGCACACATCACAAAGAAGTT

              *       **                   **   *    
     176070 TCACAGAATGTGTCTGTGTAGTTTTTATGTGCGGATGTTTC
        131 TCTCAGAATGCTTCTGTGTAGTTTTTATGTGAAGATATTTC

                         *        ****   *   *               *    *          
     176111 CTTTTCCACCATATGCCT-AAATATTTCCCAATTTCCACTTGCAGATTCCACAAGAAGAGTGTTT
          1 CTTTTCCACCATAGGCCTCAAAGCGCTCCAAATATCCACTTGCAGATTCTACAAAAAGAGTGTTT

                      **       *       *           *            *      ***   
     176175 CAAAACTGCTGTATC-AAATAAA-GTTGAACTCTGTGAGGTGAATGCACACAGCACAAAATGGTT
         66 CAAAACTGCTCAATCAAAAGAAAGGTTCAACTCTGTGAGATGAATGCACACATCACAAAGAAGTT

                          **   *        *       *    
     176238 TCTCAGAATGCTTCCTTGTTGTTTTTATATGAAGATGTTTC
        131 TCTCAGAATGCTTCTGTGTAGTTTTTATGTGAAGATATTTC

                  *  *             *   *     *                               
     176279 CTTTTCAACAATAGGCCTCAAAGTGCTTCAAATGTCCACTTGCAGATTCTACAAAAAGAGTGTTT
          1 CTTTTCCACCATAGGCCTCAAAGCGCTCCAAATATCCACTTGCAGATTCTACAAAAAGAGTGTTT

                                        *      *  *  *                       
     176344 CAAAACTGCTCAATCAAAAGAAAGGTTCGACTCTGGGAAATTAATGCACACATCACAAAGAAGTT
         66 CAAAACTGCTCAATCAAAAGAAAGGTTCAACTCTGTGAGATGAATGCACACATCACAAAGAAGTT

                                     *        *      
     176409 TCTC---A-GCTTCTGTGTAGTTTTCATGTGAAGTTATTTC
        131 TCTCAGAATGCTTCTGTGTAGTTTTTATGTGAAGATATTTC

                     *       *     *            *    *         **       *    
     176446 CTTTTCCACAATAGGCCGCAAAGGGCTCCAAATATCAACTTACAGATTCTAGGAAAAGAGAGTTT
          1 CTTTTCCACCATAGGCCTCAAAGCGCTCCAAATATCCACTTGCAGATTCTACAAAAAGAGTGTTT

                       * **      *     *                                     
     176511 CAAAACTGCTCTACGAAAAGATAGGTTGAACTCTGTGAGATGAATGCACACATCACAAAGAAGTT
         66 CAAAACTGCTCAATCAAAAGAAAGGTTCAACTCTGTGAGATGAATGCACACATCACAAAGAAGTT

                       *               * *  *        
     176576 TCTCAGAATGCATCTGTGTAGTTTTTACGGGATGATATTTC
        131 TCTCAGAATGCTTCTGTGTAGTTTTTATGTGAAGATATTTC

                        ***  *     **      *  *                  *     ***   
     176617 CTTTTCCACCATCTTCCACAAAGGTCTCCAAGTAACCACTTGCAGATTCTACAGAAAGACACTTT
          1 CTTTTCCACCATAGGCCTCAAAGCGCTCCAAATATCCACTTGCAGATTCTACAAAAAGAGTGTTT

            *          *         ***      *      ***                      *  
     176682 AAAAACTGCTCTATCAAAAGATCAGTTCAAGTCTGTGGTTTGAATGCACACATCACAAAGAATTT
         66 CAAAACTGCTCAATCAAAAGAAAGGTTCAACTCTGTGAGATGAATGCACACATCACAAAGAAGTT

                                     *  *            
     176747 TCTCAGAATGCTTCTGTGTAGTTTTCATATGAAGATATTTC
        131 TCTCAGAATGCTTCTGTGTAGTTTTTATGTGAAGATATTTC

                                    *                                   **   
     176788 CTTTTCCACCATAGGCCTCAAAGCACTCCAAATATCCACTTGCAGATTCTACAAAAAGAGATTTT
          1 CTTTTCCACCATAGGCCTCAAAGCGCTCCAAATATCCACTTGCAGATTCTACAAAAAGAGTGTTT

                   **                           *  *          *        *     
     176853 CAAAACTAGTCAATCAAAAGAAAGGTTCAACTCTGTCAGTTGAATGCACATATCACAAACAAGTT
         66 CAAAACTGCTCAATCAAAAGAAAGGTTCAACTCTGTGAGATGAATGCACACATCACAAAGAAGTT

                *      *                             
     176918 TCTCGGAATGCGTCTGTGTAGTTTTTATGTGAAGATATTTC
        131 TCTCAGAATGCTTCTGTGTAGTTTTTATGTGAAGATATTTC

               *     * *           *     *                  *    *           
     176959 CTTCTCCACAACAGGCCTCAAAGTGCTCCGAATATCCACTTGCAGATTTTAC-TAAAGAGTGTTT
          1 CTTTTCCACCATAGGCCTCAAAGCGCTCCAAATATCCACTTGCAGATTCTACAAAAAGAGTGTTT

             *               *  *   *     *        *    *               *    
     177023 CCAAACTGCTCAATCAAGAGGAAGTTTCAAGTCTGTGAGCTGAACGCACACATCACAAAGTAGTT
         66 CAAAACTGCTCAATCAAAAGAAAGGTTCAACTCTGTGAGATGAATGCACACATCACAAAGAAGTT

               *                                *    
     177088 TCTGAGAATGCTTCTGTGTAGTTTTTATGTGAAGATGTTTC
        131 TCTCAGAATGCTTCTGTGTAGTTTTTATGTGAAGATATTTC

                            **     *                                    *    
     177129 CTTTTCCACCATAGGCTGCAAAGGGCTCCAAATATCCACTTGCAGATTCTACAAAAAGAGAGTTT
          1 CTTTTCCACCATAGGCCTCAAAGCGCTCCAAATATCCACTTGCAGATTCTACAAAAAGAGTGTTT

                 *     *         *          *     *                     *    
     177194 CAAAAGTGCTCTATCAAAAGATAGGTTCAACTATGTGATATGAATGCACACATCACAAAGTAGTT
         66 CAAAACTGCTCAATCAAAAGAAAGGTTCAACTCTGTGAGATGAATGCACACATCACAAAGAAGTT

                                          *          
     177259 TCTCAGAATGCTTCTGTGTAGTTTTTATGTAAAGATATTTC
        131 TCTCAGAATGCTTCTGTGTAGTTTTTATGTGAAGATATTTC

                                    *                                   **   
     177300 CTTTTCCACCATAGGCCTCAAAGCACTCCAAATATCCACTTGCAGATTCTACAAAAAGAGATTTT
          1 CTTTTCCACCATAGGCCTCAAAGCGCTCCAAATATCCACTTGCAGATTCTACAAAAAGAGTGTTT

                   ** *                   *     *  *    * *   *        *     
     177365 CAAAACTATTTAATCAAAAGAAAGGTTCAAATCTGTCAGTTGAAGGTACATATCACAAACAAGTT
         66 CAAAACTGCTCAATCAAAAGAAAGGTTCAACTCTGTGAGATGAATGCACACATCACAAAGAAGTT

             * **                                    
     177430 TATTGGAATGCTTCTGTGTAGTTTTTATGTGAAGATATTTC
        131 TCTCAGAATGCTTCTGTGTAGTTTTTATGTGAAGATATTTC

                     * *       * * *                        **  *     *      
     177471 CTTTTCCACAACAGGCCTCCAGGTGCTCCAAATATCCACTTGCAGATTTCACTAAAAGTGTGTTT
          1 CTTTTCCACCATAGGCCTCAAAGCGCTCCAAATATCCACTTGCAGATTCTACAAAAAGAGTGTTT

             *  *            *  *   *     *        *             *           
     177536 CCAAGCTGCTCAATCAAGAGGAAGTTTCAAGTCTGTGAGGTGAATGCACACATTACAAAGAAGTT
         66 CAAAACTGCTCAATCAAAAGAAAGGTTCAACTCTGTGAGATGAATGCACACATCACAAAGAAGTT

            *  *                                     
     177601 ACTGAGAATGCTTCTGTGTAGTTTTTATGTGAAGATATTTC
        131 TCTCAGAATGCTTCTGTGTAGTTTTTATGTGAAGATATTTC

                      **               *                                *    
     177642 CTTTTCCACCGCAGGCCTCAAAGCGCTGCAAATATCCACTTGCAGATTCTACAAAAAGAGAGTTT
          1 CTTTTCCACCATAGGCCTCAAAGCGCTCCAAATATCCACTTGCAGATTCTACAAAAAGAGTGTTT

                      **         *   *         *   *     **            *     
     177707 CAAAACTGCTGTATCAAAAGATAGGGTCAACTCTGCGAGTTGAATAAACACATCACAAATAAGTT
         66 CAAAACTGCTCAATCAAAAGAAAGGTTCAACTCTGTGAGATGAATGCACACATCACAAAGAAGTT

               **   *        *               *       
     177772 TCTGGGAACGCTTCTGTATAG-TTTTATGTGAATATATTTC
        131 TCTCAGAATGCTTCTGTGTAGTTTTTATGTGAAGATATTTC

                         *          *                   *   *  *   * *   *   
     177812 CTTTTCCACCATATGCCTCAAAGCACTCCAAATATCCACTTGCACATTATAGAAACATAGTCTTT
          1 CTTTTCCACCATAGGCCTCAAAGCGCTCCAAATATCCACTTGCAGATTCTACAAAAAGAGTGTTT

                   **                        *         *             *       
     177877 CAAAACTTGTCAATC-AAAGAAAGGTTCAACTCCGTGAGATGAGTGCACACATCACAGAGAAGTT
         66 CAAAACTGCTCAATCAAAAGAAAGGTTCAACTCTGTGAGATGAATGCACACATCACAAAGAAGTT

                *     *                            * 
     177941 TCTCGGAATGTTTCTGTGTAGTTTTTATGTGAAGATATTGC
        131 TCTCAGAATGCTTCTGTGTAGTTTTTATGTGAAGATATTTC

                     *               *            *          *       *   *   
     177982 CTTTTCCACAATAGGCCTCAAAGCGTTCCAAATATCCAATTGCAGATTCCACAAAAAAAGTTTTT
          1 CTTTTCCACCATAGGCCTCAAAGCGCTCCAAATATCCACTTGCAGATTCTACAAAAAGAGTGTTT

            *                 *  *  *  *             * *         **    *     
     178047 TAAAACTGCTCAATCAAATGATAGATTAAACTCTGTGAGATTAGTGCACACATGTCAAAAAAGTT
         66 CAAAACTGCTCAATCAAAAGAAAGGTTCAACTCTGTGAGATGAATGCACACATCACAAAGAAGTT

                                *      * *           
     178112 TCTCAGAATGCTTCTGTGTACTTTTTAGGGGAAGATATTTC
        131 TCTCAGAATGCTTCTGTGTAGTTTTTATGTGAAGATATTTC

                        *    *     **         *    *           **  * *  *    
     178153 CTTTTCCACCATCGGCCACAAAGGACTCCAAATAACCACATGCAGATTCTAGTAACACAGAGTTT
          1 CTTTTCCACCATAGGCCTCAAAGCGCTCCAAATATCCACTTGCAGATTCTACAAAAAGAGTGTTT

                       *         * *           *   * * *    **    ***        
     178218 CAAAACTGCTCTATCAAAAGATAAGTTCAACTCTGAGAGTTTAGTGCAACCATCGTGAAGAAGTT
         66 CAAAACTGCTCAATCAAAAGAAAGGTTCAACTCTGTGAGATGAATGCACACATCACAAAGAAGTT

                            *     *                  
     178283 TCTCAGAATGCTTCTGAGTAGTGTTTATGTGAAGATATTTC
        131 TCTCAGAATGCTTCTGTGTAGTTTTTATGTGAAGATATTTC

                              *     *                      *           **    
     178324 CTTTTCCACCATAGGCCTGAAAGCCCTCCAAATATCCACTTGCAGATCCTACAAAAAGAAAGTTT
          1 CTTTTCCACCATAGGCCTCAAAGCGCTCCAAATATCCACTTGCAGATTCTACAAAAAGAGTGTTT

             *         **     *  *  *   *        **      *                   
     178389 CGAAA-TGCTCTCTCAAACGATAGTTTCGACTCTGTGGTATGAATACACACATCACAAAGAAGTT
         66 CAAAACTGCTCAATCAAAAGAAAGGTTCAACTCTGTGAGATGAATGCACACATCACAAAGAAGTT

                                       **            
     178453 TCTCAGAATGCTTCTGTGTAGTTTTTAAATGAAGATATTTC
        131 TCTCAGAATGCTTCTGTGTAGTTTTTATGTGAAGATATTTC

            *                       *          *     *                       
     178494 TTTTTCCACCATAGGCCTCAAAGCACTCCAAATATGCACTTCCAGATTCTACAAAAAGAGTGTTT
          1 CTTTTCCACCATAGGCCTCAAAGCGCTCCAAATATCCACTTGCAGATTCTACAAAAAGAGTGTTT

              *                 *       * *         **   *         *  * *    
     178559 CAGAACTGCTCAATCAAAAGGAAGGTTCCAGTC--TGAGACAAATACACACATCAAAAGGTAGTT
         66 CAAAACTGCTCAATCAAAAGAAAGGTTCAACTCTGTGAGATGAATGCACACATCACAAAGAAGTT

                                                    *
     178622 TCTCAGAATGCTTCTGTGTAGTTTTTATGTGAAGATATTTT
        131 TCTCAGAATGCTTCTGTGTAGTTTTTATGTGAAGATATTTC

             *               *    **    *     *      *  **   *          *    
     178663 CCTTTCCACCATAGGCCACAAATGGCTCTAAATACCCACTTACATTTTCCACAAAAAGAGAGTTT
          1 CTTTTCCACCATAGGCCTCAAAGCGCTCCAAATATCCACTTGCAGATTCTACAAAAAGAGTGTTT

                       * *    * *      *   *         *                       
     178728 CAAAACTGCTCTACCAAAGGTAA-GTTTAACGCTGTGAG-TTAA-G-A-ACATCACAAAGAAGTT
         66 CAAAACTGCTCAATCAAAAGAAAGGTTCAACTCTGTGAGATGAATGCACACATCACAAAGAAGTT

                                   *   *  *          
     178788 TCTCAGAATGCTTCTGTGTAGTTCTTACGTAAAGATATTTC
        131 TCTCAGAATGCTTCTGTGTAGTTTTTATGTGAAGATATTTC

                 *   *      ***    *                  *              **      
     178829 CTTTTACACAATAGGCAGAAAAGTGCTCCAAATATCCACTTGAAGATTCTACAAAAACCGTGTTT
          1 CTTTTCCACCATAGGCCTCAAAGCGCTCCAAATATCCACTTGCAGATTCTACAAAAAGAGTGTTT

                     **                                          *      *    
     178894 CAAAACTGCCGAATCAAAAGAAAGGTTCAACTCTGTGAGATGAATGCACACATAACAAAGGAGTT
         66 CAAAACTGCTCAATCAAAAGAAAGGTTCAACTCTGTGAGATGAATGCACACATCACAAAGAAGTT

                                 *      *      *    *
     178959 TCTCAGAATGCTTCTGTGTAGCTTTTATATGAAGACATTTA
        131 TCTCAGAATGCTTCTGTGTAGTTTTTATGTGAAGATATTTC

            *        * *            *   ***                     **        *  
     179000 GTTTTCCACAACAGGCCTCAAAGCTCTCTCCATATCCACTTGCAGATTCTACCGAAAGAGTGCTT
          1 CTTTTCCACCATAGGCCTCAAAGCGCTCCAAATATCCACTTGCAGATTCTACAAAAAGAGTGTTT

             *                   * **     *        *        *     **         
     179065 CCAAACTGCTCAATCAAAAGAGACATTCAAATCTGTGAGGTGAATGCAGACATCGTAAAGAAGTT
         66 CAAAACTGCTCAATCAAAAGAAAGGTTCAACTCTGTGAGATGAATGCACACATCACAAAGAAGTT

                                *     *       *    **
     179130 TCTCAGAATGCTTCTGTGTATTTTTTGTGTGAAGTTATTCG
        131 TCTCAGAATGCTTCTGTGTAGTTTTTATGTGAAGATATTTC

            *    *             *     *  *            **                 *    
     179171 TTTTTGCACCATAGGCCTCCAAGCGTTCTAAATATCCACTTCTAGATTCTACAAAAAGAGAGTTT
          1 CTTTTCCACCATAGGCCTCAAAGCGCTCCAAATATCCACTTGCAGATTCTACAAAAAGAGTGTTT

                   *         *            *        *    *   *                
     179236 CAAAACTACTC-A--AACA-AAAGGTTCAATTCTGTGAGTTGAAAGCAAACATCACAAAGAAGTT
         66 CAAAACTGCTCAATCAAAAGAAAGGTTCAACTCTGTGAGATGAATGCACACATCACAAAGAAGTT

                       *                             
     179297 TCTCAGAATGCGTCTGTGTAGTTTTTATGTGAAGATATTTC
        131 TCTCAGAATGCTTCTGTGTAGTTTTTATGTGAAGATATTTC

                     *    ****     *                  *                      
     179338 CTTTT-CACAATAGAATGCAAAGGGCTCCAAATATCCACTTGGAGATTCTACAAAAAGA--GTTT
          1 CTTTTCCACCATAGGCCTCAAAGCGCTCCAAATATCCACTTGCAGATTCTACAAAAAGAGTGTTT

                  *    **     *  *     *     ***   *     *               *   
     179400 CAAAACCGCTCTGTCAAATGATAGGTTGAACTCCCGGAGGTGAATACACACATCACAAAGAGGTT
         66 CAAAACTGCTCAATCAAAAGAAAGGTTCAACTCTGTGAGATGAATGCACACATCACAAAGAAGTT

                  *                       *  *       
     179465 TCTCAGCATGCTTCTGTGTAGTTTTTATGTAAACATATTTC
        131 TCTCAGAATGCTTCTGTGTAGTTTTTATGTGAAGATATTTC

             *    * *              *           *      * *       *   *    *   
     179506 CGTTTCTATCATAGGCCTCAAAGTGCTCCAAATATTCACTTGTACATTCTACCAAACGAGTATTT
          1 CTTTTCCACCATAGGCCTCAAAGCGCTCCAAATATCCACTTGCAGATTCTACAAAAAGAGTGTTT

                              * *         **      *        *            *    
     179571 CAAAACTGCTCAATCAAATGGAAGGTTCAAAACTGTGACATGAATGCCCACATCACAAAGTAGTT
         66 CAAAACTGCTCAATCAAAAGAAAGGTTCAACTCTGTGAGATGAATGCACACATCACAAAGAAGTT

                                                     
     179636 TCTCAGAATGCTTCTGTGTAGTTTTTATGTGAAGATATTTC
        131 TCTCAGAATGCTTCTGTGTAGTTTTTATGTGAAGATATTTC

                     * *    *      *    *       * *   **      *              
     179677 CTTTTCCACAACA-GCGTGCAAAACGCTTCAAATATGCCCTTAGAGATTCCACAAAAAGAGTGTT
          1 CTTTTCCACCATAGGCCT-CAAAGCGCTCCAAATATCCACTTGCAGATTCTACAAAAAGAGTGTT

              *     *               *  *                                  ** 
     179741 TCCAAACTACTCAAATCAAAA-AATGATTTCAACTCTGTGAGATGAATGCACACATCACAAACTA
         65 TCAAAACTGCTC-AATCAAAAGAAAG-GTTCAACTCTGTGAGATGAATGCACACATCACAAAGAA

                         *     ** *   * *   *       *   
     179805 GTTTCTCAGAATGTTTCTGCCTGGTTCTCATGCGAAGATAGTTC
        128 GTTTCTCAGAATGCTTCTGTGTAGTTTTTATGTGAAGATATTTC

                 *           *   * **              *                  * *    
     179849 CTTTTTCACCATAGGCCGCAATGTACTCCAAATATCCACCTGCAGATTCTAC-AAAAGTGAGTTT
          1 CTTTTCCACCATAGGCCTCAAAGCGCTCCAAATATCCACTTGCAGATTCTACAAAAAGAGTGTTT

                       *         ***    **         *        *      *       * 
     179913 CAAAACTGCTCTATCAAAAGATCAGTTCGTCTCTGTGAGTTGAATGCATACATCAAAAAGAAGCT
         66 CAAAACTGCTCAATCAAAAGAAAGGTTCAACTCTGTGAGATGAATGCACACATCACAAAGAAGTT

                 *             *      **         *   
     179978 TCTCAAAATGCTTCTGTGTGGTTTTTCGGTGAAGATAGTTC
        131 TCTCAGAATGCTTCTGTGTAGTTTTTATGTGAAGATATTTC

            *     *        *      * *                 *        *        *    
     180019 TTTTTCTACCATAGGTCTCAAACCACTCCAAATATCCACTTGTAGATTCTATAAAAAGGAATG-T
          1 CTTTTCCACCATAGGCCTCAAAGCGCTCCAAATATCCACTTGCAGATTCTACAAAAA-GAGTGTT

                  *        *    *    *      * *         *      *             
     180083 TCAAAATTGCTCAATAAAAATAAAGTTTCAACACCGTGAGATGAGTGCACAAATCACAAAGAAGT
         65 TCAAAACTGCTCAATCAAAAGAAAGGTTCAACTCTGTGAGATGAATGCACACATCACAAAGAAGT

                  *          *         *          *   
     180148 TTCTCAAAATGCTTCTGGGTAGTTTTTCTGTGAAGATAGTTC
        130 TTCTCAGAATGCTTCTGTGTAGTTTTTATGTGAAGATATTTC

                  *     *    *     *          *                         *    
     180190 CTTTTCTACCATGGGCCACAAAGGGCTCCAAATACCCACTTGCAGATTCTACAAAAAGAGAGTTT
          1 CTTTTCCACCATAGGCCTCAAAGCGCTCCAAATATCCACTTGCAGATTCTACAAAAAGAGTGTTT

              *        *      ** * *        *    * *    **  * *           *  
     180255 CACAACTGCTCTATCAAACAATATGTTCAACTTTGTGGGTTGAACACAAATATCAC-AAGAATTT
         66 CAAAACTGCTCAATCAAAAGAAAGGTTCAACTCTGTGAGATGAATGCACACATCACAAAGAAGTT

                **                             *     
     180319 TCTCCCAATGCTTCTGTGTAGTTTTTATGTGAAGACATTTC
        131 TCTCAGAATGCTTCTGTGTAGTTTTTATGTGAAGATATTTC

            *   *  *      *  *     *                 *  *      *       *  *  
     180360 TTTTCCCTCCATAGTCCACAAAGTGCTCCAAATATCCACTTACATATTCTAGAAAAAGATTGCTT
          1 CTTTTCCACCATAGGCCTCAAAGCGCTCCAAATATCCACTTGCAGATTCTACAAAAAGAGTGTTT

            **       *    *               * * *                              
     180425 GGAAACTGCACAATGAAAAGAAAGGTTCAAATATATGAGATGAATGCACACATCACAAAGAAGTT
         66 CAAAACTGCTCAATCAAAAGAAAGGTTCAACTCTGTGAGATGAATGCACACATCACAAAGAAGTT

                                 *                    
     180490 TCTCAGAAT-CTCTCTGTGTAATTTTTATGTGAAGATATTTC
        131 TCTCAGAATGCT-TCTGTGTAGTTTTTATGTGAAGATATTTC

                *     *    *  *   *                        *      *    *     
     180531 CTTTCCCACCTTAGGTCTTAAAACGCTCCAAATATCCACTTGCAGATACTACAAGAAGATTGTTT
          1 CTTTTCCACCATAGGCCTCAAAGCGCTCCAAATATCCACTTGCAGATTCTACAAAAAGAGTGTTT

                     *    *        *      *     **                           
     180596 CAAAACTGCACAA-AAAAAGAAATGTTCAATTCTGTTTGATGAATGCACACATCACAAAGAAGTT
         66 CAAAACTGCTCAATCAAAAGAAAGGTTCAACTCTGTGAGATGAATGCACACATCACAAAGAAGTT

                           *                         
     180660 TCTCAGAATGCTTCTCTGTAGTTTTTATGTGAAGATATTTC
        131 TCTCAGAATGCTTCTGTGTAGTTTTTATGTGAAGATATTTC

                     *             *                 *         **      * *   
     180701 CTTTTCCACAATAGGCCTCAAAGGGCTCCAAATATCCACTTCCAGATTCTATGAAAAGAATATTT
          1 CTTTTCCACCATAGGCCTCAAAGCGCTCCAAATATCCACTTGCAGATTCTACAAAAAGAGTGTTT

             *              * *    *                                      *  
     180766 CCAAACTGCTCAATCATAGGAAATGTTCAACTCTGTGAGATGAATGCACACATCAC-AAGAAATT
         66 CAAAACTGCTCAATCAAAAGAAAGGTTCAACTCTGTGAGATGAATGCACACATCACAAAGAAGTT

                     *    *      *       *       *   
     180830 TCTCAGAATCCTTCAGTGTAGGTTTTATGAGAAGATAATTC
        131 TCTCAGAATGCTTCTGTGTAGTTTTTATGTGAAGATATTTC

                     *    **        *   *                           *    *   
     180871 CTTTTCCACAATAGTTCTCAAAGCACTCAAAATATCCACTTGCAGATTCTACAAAAGGAGTATTT
          1 CTTTTCCACCATAGGCCTCAAAGCGCTCCAAATATCCACTTGCAGATTCTACAAAAAGAGTGTTT

                                                          *                  
     180936 CAAAACTGCTCAATCAAAAGAAAGGTTCAACTCTGTGAGATGAATGGACACATCACAAAGAAGTT
         66 CAAAACTGCTCAATCAAAAGAAAGGTTCAACTCTGTGAGATGAATGCACACATCACAAAGAAGTT

                                  *   *              
     181001 TCTCAGAATGCTTCTGTGTAGTATTTTTGTGAAGATATTT-
        131 TCTCAGAATGCTTCTGTGTAGTTTTTATGTGAAGATATTTC

                          *  * * * * ***             *            *     * *  
     181041 CTTTTCCACCATAGACCGCCAGGGGACACAAATATCCACTTTCAGATTCTACAACAAGAGAGGTT
          1 CTTTTCCACCATAGGCCTCAAAGCGCTCCAAATATCCACTTGCAGATTCTACAAAAAGAGTGTTT

                   *   *     *   ** *       *      *                      * *
     181106 CAAAACTACTCGATCAAGAGATGGTTTCAACTATGTGAGTTGAATGCACACATCACAAAGAACTA
         66 CAAAACTGCTCAATCAAAAGAAAGGTTCAACTCTGTGAGATGAATGCACACATCACAAAGAAGTT

             *  *    *                               
     181171 TGTCGGAATTCTTCTGTGTAGTTTTTATGTGAAGATATTTC
        131 TCTCAGAATGCTTCTGTGTAGTTTTTATGTGAAGATATTTC

                     *    * *      * *    *                  *               
     181212 CTTTTCCACAATAGACGTCAAAGTGATCCAGATATCCACTTGCAGATTCCACAAAAAGAGTGTTT
          1 CTTTTCCACCATAGGCCTCAAAGCGCTCCAAATATCCACTTGCAGATTCTACAAAAAGAGTGTTT

                 *   *   *                  **                     *  *      
     181277 CAAAAGTGCACAACCAAAAGAAAGGTTCAACTAGGTGAGATGAATGCACACATCAGAAGGAAGTT
         66 CAAAACTGCTCAATCAAAAGAAAGGTTCAACTCTGTGAGATGAATGCACACATCACAAAGAAGTT

                            **   *     * *       *   
     181342 TCTCAGAATGCTTCTGCATAGCTTTTAAGGGAAGATACTTC
        131 TCTCAGAATGCTTCTGTGTAGTTTTTATGTGAAGATATTTC

                    *               *            * *  *    * *               
     181383 CTTTTCCAACATAGGCCTCAAAGCACTCCAAATATCCTCCTGGAGATACCACAAAAAGAGTGTTT
          1 CTTTTCCACCATAGGCCTCAAAGCGCTCCAAATATCCACTTGCAGATTCTACAAAAAGAGTGTTT

            **                      *  *                 *       *           
     181448 GCAAACTGCTCAATCAAAAGAAAGATTTAACTCTGTGAGATGAATCCACACATGACAAAGAAGTT
         66 CAAAACTGCTCAATCAAAAGAAAGGTTCAACTCTGTGAGATGAATGCACACATCACAAAGAAGTT

                                                     
     181513 TCTCAGAATGCTTCTGTGTAGTTTTTATGTGAAGATATTTC
        131 TCTCAGAATGCTTCTGTGTAGTTTTTATGTGAAGATATTTC

                     *   * * *    **           *                *     *      
     181554 CTTTTCCACAATAAGACCCAAAAGGCTCCAAATATTCACTTGCAGATTCTAAAAAAAACAGTGTT
          1 CTTTTCCACCATAGGCCTCAAAGCGCTCCAAATATCCACTTGCAGATTCT-ACAAAAAGAGTGTT

                                  *                  *      *        **      
     181619 TCAAAACTGCTCAATCAAAAGATA-GTTCAACTCTGTGAGAAGAATGCTCACATCACTGAGAAGT
         65 TCAAAACTGCTCAATCAAAAGAAAGGTTCAACTCTGTGAGATGAATGCACACATCACAAAGAAGT

                                         *            
     181683 TTCTCAGAATGCTTCTGTGTAGTTTTTATATGAAGATATTTC
        130 TTCTCAGAATGCTTCTGTGTAGTTTTTATGTGAAGATATTTC

                *     *      *    **                       *   **       *    
     181725 CTTTCCCACCGTAGGCCACAAAAGGCTCCAAATATCCACTTGCAGATACTATGAAAAGAGAGTTT
          1 CTTTTCCACCATAGGCCTCAAAGCGCTCCAAATATCCACTTGCAGATTCTACAAAAAGAGTGTTT

                        *        *               ***            *            
     181790 CAAAACTGCTCATTCAAAAGATAGGTTCAACTCTGTGGTTTGAATGCACACAGCACAAAGAAGTT
         66 CAAAACTGCTCAATCAAAAGAAAGGTTCAACTCTGTGAGATGAATGCACACATCACAAAGAAGTT

              *       **                   **   *    
     181855 TCACAGAATGTGTCTGTGTAGTTTTTATGTGCGGATGTTTC
        131 TCTCAGAATGCTTCTGTGTAGTTTTTATGTGAAGATATTTC

                         *        ****   *   *               *    *          
     181896 CTTTTCCACCATATGCCT-AAATATTTCCCAATTTCCACTTGCAGATTCCACAAGAAGAGTGTTT
          1 CTTTTCCACCATAGGCCTCAAAGCGCTCCAAATATCCACTTGCAGATTCTACAAAAAGAGTGTTT

                      **       *       *           *            *      ***   
     181960 CAAAACTGCTGTATC-AAATAAA-GTTGAACTCTGTGAGGTGAATGCACACAGCACAAAATGGTT
         66 CAAAACTGCTCAATCAAAAGAAAGGTTCAACTCTGTGAGATGAATGCACACATCACAAAGAAGTT

                          **   *        *       *    
     182023 TCTCAGAATGCTTCCTTGTTGTTTTTATATGAAGATGTTTC
        131 TCTCAGAATGCTTCTGTGTAGTTTTTATGTGAAGATATTTC

                  *  *             *   *     *                               
     182064 CTTTTCAACAATAGGCCTCAAAGTGCTTCAAATGTCCACTTGCAGATTCTACAAAAAGAGTGTTT
          1 CTTTTCCACCATAGGCCTCAAAGCGCTCCAAATATCCACTTGCAGATTCTACAAAAAGAGTGTTT

                                        *      *  *  *                       
     182129 CAAAACTGCTCAATCAAAAGAAAGGTTCGACTCTGGGAAATTAATGCACACATCACAAAGAAGTT
         66 CAAAACTGCTCAATCAAAAGAAAGGTTCAACTCTGTGAGATGAATGCACACATCACAAAGAAGTT

                                     *        *      
     182194 TCTC---A-GCTTCTGTGTAGTTTTCATGTGAAGTTATTTC
        131 TCTCAGAATGCTTCTGTGTAGTTTTTATGTGAAGATATTTC

                     *       *     *            *    *         **       *    
     182231 CTTTTCCACAATAGGCCGCAAAGGGCTCCAAATATCAACTTACAGATTCTAGGAAAAGAGAGTTT
          1 CTTTTCCACCATAGGCCTCAAAGCGCTCCAAATATCCACTTGCAGATTCTACAAAAAGAGTGTTT

                       * **      *     *                                     
     182296 CAAAACTGCTCTACGAAAAGATAGGTTGAACTCTGTGAGATGAATGCACACATCACAAAGAAGTT
         66 CAAAACTGCTCAATCAAAAGAAAGGTTCAACTCTGTGAGATGAATGCACACATCACAAAGAAGTT

                       *               * *           
     182361 TCTCAGAATGCATCTGTGTAGTTTTTACGGGAAGATATTTC
        131 TCTCAGAATGCTTCTGTGTAGTTTTTATGTGAAGATATTTC

                        ***  *     **      *  *                  *     ***   
     182402 CTTTTCCACCATCTTCCACAAAGGTCTCCAAGTAACCACTTGCAGATTCTACAGAAAGACACTTT
          1 CTTTTCCACCATAGGCCTCAAAGCGCTCCAAATATCCACTTGCAGATTCTACAAAAAGAGTGTTT

            *          *         ***      *      ***                      *  
     182467 AAAAACTGCTCTATCAAAAGATCAGTTCAAGTCTGTGGTTTGAATGCACACATCACAAAGAATTT
         66 CAAAACTGCTCAATCAAAAGAAAGGTTCAACTCTGTGAGATGAATGCACACATCACAAAGAAGTT

                                     *  *            
     182532 TCTCAGAATGCTTCTGTGTAGTTTTCATATGAAGATATTTC
        131 TCTCAGAATGCTTCTGTGTAGTTTTTATGTGAAGATATTTC

                                    *                                   **   
     182573 CTTTTCCACCATAGGCCTCAAAGCACTCCAAATATCCACTTGCAGATTCTACAAAAAGAGATTTT
          1 CTTTTCCACCATAGGCCTCAAAGCGCTCCAAATATCCACTTGCAGATTCTACAAAAAGAGTGTTT

                   **                           *  *          *        *     
     182638 CAAAACTAGTCAATCAAAAGAAAGGTTCAACTCTGTCAGTTGAATGCACATATCACAAACAAGTT
         66 CAAAACTGCTCAATCAAAAGAAAGGTTCAACTCTGTGAGATGAATGCACACATCACAAAGAAGTT

                *      *                             
     182703 TCTCGGAATGCGTCTGTGTAGTTTTTATGTGAAGATATTTC
        131 TCTCAGAATGCTTCTGTGTAGTTTTTATGTGAAGATATTTC

               *     * *           *     *                  *    *           
     182744 CTTCTCCACAACAGGCCTCAAAGTGCTCCGAATATCCACTTGCAGATTTTAC-TAAAGAGTGTTT
          1 CTTTTCCACCATAGGCCTCAAAGCGCTCCAAATATCCACTTGCAGATTCTACAAAAAGAGTGTTT

             *               *  *   *     *        *    *               *    
     182808 CCAAACTGCTCAATCAAGAGGAAGTTTCAAGTCTGTGAGCTGAACGCACACATCACAAAGTAGTT
         66 CAAAACTGCTCAATCAAAAGAAAGGTTCAACTCTGTGAGATGAATGCACACATCACAAAGAAGTT

               *                                *    
     182873 TCTGAGAATGCTTCTGTGTAGTTTTTATGTGAAGATGTTTC
        131 TCTCAGAATGCTTCTGTGTAGTTTTTATGTGAAGATATTTC

                            **     *                                    *    
     182914 CTTTTCCACCATAGGCTGCAAAGGGCTCCAAATATCCACTTGCAGATTCTACAAAAAGAGAGTTT
          1 CTTTTCCACCATAGGCCTCAAAGCGCTCCAAATATCCACTTGCAGATTCTACAAAAAGAGTGTTT

                 *     *         *          *     *                     *    
     182979 CAAAAGTGCTCTATCAAAAGATAGGTTCAACTATGTGATATGAATGCACACATCACAAAGTAGTT
         66 CAAAACTGCTCAATCAAAAGAAAGGTTCAACTCTGTGAGATGAATGCACACATCACAAAGAAGTT

                                          *          
     183044 TCTCAGAATGCTTCTGTGTAGTTTTTATGTAAAGATATTTC
        131 TCTCAGAATGCTTCTGTGTAGTTTTTATGTGAAGATATTTC

                                    *                                   **   
     183085 CTTTTCCACCATAGGCCTCAAAGCACTCCAAATATCCACTTGCAGATTCTACAAAAAGAGATTTT
          1 CTTTTCCACCATAGGCCTCAAAGCGCTCCAAATATCCACTTGCAGATTCTACAAAAAGAGTGTTT

                   ** *                   *     *  *    * *   *        *     
     183150 CAAAACTATTTAATCAAAAGAAAGGTTCAAATCTGTCAGTTGAAGGTACATATCACAAACAAGTT
         66 CAAAACTGCTCAATCAAAAGAAAGGTTCAACTCTGTGAGATGAATGCACACATCACAAAGAAGTT

             * **                                    
     183215 TATTGGAATGCTTCTGTGTAGTTTTTATGTGAAGATATTTC
        131 TCTCAGAATGCTTCTGTGTAGTTTTTATGTGAAGATATTTC

                     * *       * * *                        **  *     *      
     183256 CTTTTCCACAACAGGCCTCCAGGTGCTCCAAATATCCACTTGCAGATTTCACTAAAAGTGTGTTT
          1 CTTTTCCACCATAGGCCTCAAAGCGCTCCAAATATCCACTTGCAGATTCTACAAAAAGAGTGTTT

             *  *            *  *   *     *        *             *           
     183321 CCAAGCTGCTCAATCAAGAGGAAGTTTCAAGTCTGTGAGGTGAATGCACACATTACAAAGAAGTT
         66 CAAAACTGCTCAATCAAAAGAAAGGTTCAACTCTGTGAGATGAATGCACACATCACAAAGAAGTT

            *  *                                     
     183386 ACTGAGAATGCTTCTGTGTAGTTTTTATGTGAAGATATTTC
        131 TCTCAGAATGCTTCTGTGTAGTTTTTATGTGAAGATATTTC

                      **               *                                *    
     183427 CTTTTCCACCGCAGGCCTCAAAGCGCTGCAAATATCCACTTGCAGATTCTACAAAAAGAGAGTTT
          1 CTTTTCCACCATAGGCCTCAAAGCGCTCCAAATATCCACTTGCAGATTCTACAAAAAGAGTGTTT

                      **         *   *         *   *     **        *   *     
     183492 CAAAACTGCTGTATCAAAAGATAGGGTCAACTCTGCGAGTTGAATAAACACATCAGAAATAAGTT
         66 CAAAACTGCTCAATCAAAAGAAAGGTTCAACTCTGTGAGATGAATGCACACATCACAAAGAAGTT

               **   *        *               *       
     183557 TCTGGGAACGCTTCTGTATAG-TTTTATGTGAATATATTTC
        131 TCTCAGAATGCTTCTGTGTAGTTTTTATGTGAAGATATTTC

                         *          *                   *   *  *   * *   *   
     183597 CTTTTCCACCATATGCCTCAAAGCACTCCAAATATCCACTTGCACATTATAGAAACATAGTCTTT
          1 CTTTTCCACCATAGGCCTCAAAGCGCTCCAAATATCCACTTGCAGATTCTACAAAAAGAGTGTTT

                   **                        *         *             *       
     183662 CAAAACTTGTCAATC-AAAGAAAGGTTCAACTCCGTGAGATGAGTGCACACATCACAGAGAAGTT
         66 CAAAACTGCTCAATCAAAAGAAAGGTTCAACTCTGTGAGATGAATGCACACATCACAAAGAAGTT

                *     *                            * 
     183726 TCTCGGAATGTTTCTGTGTAGTTTTTATGTGAAGATATTGC
        131 TCTCAGAATGCTTCTGTGTAGTTTTTATGTGAAGATATTTC

                     *               *            *          *       *   *   
     183767 CTTTTCCACAATAGGCCTCAAAGCGTTCCAAATATCCAATTGCAGATTCCACAAAAAAAGTTTTT
          1 CTTTTCCACCATAGGCCTCAAAGCGCTCCAAATATCCACTTGCAGATTCTACAAAAAGAGTGTTT

            *                 *  *  *  *             * *         **    *     
     183832 TAAAACTGCTCAATCAAATGATAGATTAAACTCTGTGAGATTAGTGCACACATGTCAAAAAAGTT
         66 CAAAACTGCTCAATCAAAAGAAAGGTTCAACTCTGTGAGATGAATGCACACATCACAAAGAAGTT

                                *      * *           
     183897 TCTCAGAATGCTTCTGTGTACTTTTTAGGGGAAGATATTTC
        131 TCTCAGAATGCTTCTGTGTAGTTTTTATGTGAAGATATTTC

                        *    *     **         *    *           **  * *  *    
     183938 CTTTTCCACCATCGGCCACAAAGGACTCCAAATAACCACATGCAGATTCTAGTAACACAGAGTTT
          1 CTTTTCCACCATAGGCCTCAAAGCGCTCCAAATATCCACTTGCAGATTCTACAAAAAGAGTGTTT

                       *         * *           *   * * *    **    ***        
     184003 CAAAACTGCTCTATCAAAAGATAAGTTCAACTCTGAGAGTTTAGTGCAACCATCGTGAAGAAGTT
         66 CAAAACTGCTCAATCAAAAGAAAGGTTCAACTCTGTGAGATGAATGCACACATCACAAAGAAGTT

                            *     *                  
     184068 TCTCAGAATGCTTCTGAGTAGTGTTTATGTGAAGATATTTC
        131 TCTCAGAATGCTTCTGTGTAGTTTTTATGTGAAGATATTTC

                              *     *                      *           **    
     184109 CTTTTCCACCATAGGCCTGAAAGCCCTCCAAATATCCACTTGCAGATCCTACAAAAAGAAAGTTT
          1 CTTTTCCACCATAGGCCTCAAAGCGCTCCAAATATCCACTTGCAGATTCTACAAAAAGAGTGTTT

             *         **     *  *  *   *        **      *                   
     184174 CGAAA-TGCTCTCTCAAACGATAGTTTCGACTCTGTGGTATGAATACACACATCACAAAGAAGTT
         66 CAAAACTGCTCAATCAAAAGAAAGGTTCAACTCTGTGAGATGAATGCACACATCACAAAGAAGTT

                                       **            
     184238 TCTCAGAATGCTTCTGTGTAGTTTTTAAATGAAGATATTTC
        131 TCTCAGAATGCTTCTGTGTAGTTTTTATGTGAAGATATTTC

            *                       *          *     *                       
     184279 TTTTTCCACCATAGGCCTCAAAGCACTCCAAATATGCACTTCCAGATTCTACAAAAAGAGTGTTT
          1 CTTTTCCACCATAGGCCTCAAAGCGCTCCAAATATCCACTTGCAGATTCTACAAAAAGAGTGTTT

              *                 *       * *         **   *         *  * *    
     184344 CAGAACTGCTCAATCAAAAGGAAGGTTCCAGTC--TGAGACAAATACACACATCAAAAGGTAGTT
         66 CAAAACTGCTCAATCAAAAGAAAGGTTCAACTCTGTGAGATGAATGCACACATCACAAAGAAGTT

                                                    *
     184407 TCTCAGAATGCTTCTGTGTAGTTTTTATGTGAAGATATTTT
        131 TCTCAGAATGCTTCTGTGTAGTTTTTATGTGAAGATATTTC

             *               *    **    *     *      *  **   *          *    
     184448 CCTTTCCACCATAGGCCACAAATGGCTCTAAATACCCACTTACATTTTCCACAAAAAGAGAGTTT
          1 CTTTTCCACCATAGGCCTCAAAGCGCTCCAAATATCCACTTGCAGATTCTACAAAAAGAGTGTTT

                       * *    * *      *   *         *                       
     184513 CAAAACTGCTCTACCAAAGGTAA-GTTTAACGCTGTGAG-TTAA-G-A-ACATCACAAAGAAGTT
         66 CAAAACTGCTCAATCAAAAGAAAGGTTCAACTCTGTGAGATGAATGCACACATCACAAAGAAGTT

                                   *   *  *          
     184573 TCTCAGAATGCTTCTGTGTAGTTCTTACGTAAAGATATTTC
        131 TCTCAGAATGCTTCTGTGTAGTTTTTATGTGAAGATATTTC

                 *   *      ***    *                  *              **      
     184614 CTTTTACACAATAGGCAGAAAAGTGCTCCAAATATCCACTTGAAGATTCTACAAAAACCGTGTTT
          1 CTTTTCCACCATAGGCCTCAAAGCGCTCCAAATATCCACTTGCAGATTCTACAAAAAGAGTGTTT

                     **                                          *      *    
     184679 CAAAACTGCCGAATCAAAAGAAAGGTTCAACTCTGTGAGATGAATGCACACATAACAAAGGAGTT
         66 CAAAACTGCTCAATCAAAAGAAAGGTTCAACTCTGTGAGATGAATGCACACATCACAAAGAAGTT

                                 *      *      *    *
     184744 TCTCAGAATGCTTCTGTGTAGCTTTTATATGAAGACATTTA
        131 TCTCAGAATGCTTCTGTGTAGTTTTTATGTGAAGATATTTC

            *        * *            *   ***                     **        *  
     184785 GTTTTCCACAACAGGCCTCAAAGCTCTCTCCATATCCACTTGCAGATTCTACCGAAAGAGTGCTT
          1 CTTTTCCACCATAGGCCTCAAAGCGCTCCAAATATCCACTTGCAGATTCTACAAAAAGAGTGTTT

             *                   * **     *        *        *     **         
     184850 CCAAACTGCTCAATCAAAAGAGACATTCAAATCTGTGAGGTGAATGCAGACATCGTAAAGAAGTT
         66 CAAAACTGCTCAATCAAAAGAAAGGTTCAACTCTGTGAGATGAATGCACACATCACAAAGAAGTT

                                *     *       *    **
     184915 TCTCAGAATGCTTCTGTGTATTTTTTGTGTGAAGTTATTCG
        131 TCTCAGAATGCTTCTGTGTAGTTTTTATGTGAAGATATTTC

            *    *             *     *  *            **                 *    
     184956 TTTTTGCACCATAGGCCTCCAAGCGTTCTAAATATCCACTTCTAGATTCTACAAAAAGAGAGTTT
          1 CTTTTCCACCATAGGCCTCAAAGCGCTCCAAATATCCACTTGCAGATTCTACAAAAAGAGTGTTT

                   *         *            *        *    *   *                
     185021 CAAAACTACTC-A--AACA-AAAGGTTCAATTCTGTGAGTTGAAAGCAAACATCACAAAGAAGTT
         66 CAAAACTGCTCAATCAAAAGAAAGGTTCAACTCTGTGAGATGAATGCACACATCACAAAGAAGTT

                       *             *               
     185082 TCTCAGAATGCGTCTGTGTAGTTTTGATGTGAAGATATTTC
        131 TCTCAGAATGCTTCTGTGTAGTTTTTATGTGAAGATATTTC

                     *    ****     *                  *                      
     185123 CTTTT-CACAATAGAATGCAAAGGGCTCCAAATATCCACTTGGAGATTCTACAAAAAGA--GTTT
          1 CTTTTCCACCATAGGCCTCAAAGCGCTCCAAATATCCACTTGCAGATTCTACAAAAAGAGTGTTT

                  *    **     *  *     *     ***   *     *               *   
     185185 CAAAACCGCTCTGTCAAATGATAGGTTGAACTCCCGGAGGTGAATACACACATCACAAAGAGGTT
         66 CAAAACTGCTCAATCAAAAGAAAGGTTCAACTCTGTGAGATGAATGCACACATCACAAAGAAGTT

                  *                       *  *       
     185250 TCTCAGCATGCTTCTGTGTAGTTTTTATGTAAACATATTTC
        131 TCTCAGAATGCTTCTGTGTAGTTTTTATGTGAAGATATTTC

             *    * *              *           *      * *       *   *    *   
     185291 CGTTTCTATCATAGGCCTCAAAGTGCTCCAAATATTCACTTGTACATTCTACCAAACGAGTATTT
          1 CTTTTCCACCATAGGCCTCAAAGCGCTCCAAATATCCACTTGCAGATTCTACAAAAAGAGTGTTT

                              * *         **      *        *            *    
     185356 CAAAACTGCTCAATCAAATGGAAGGTTCAAAACTGTGACATGAATGCCCACATCACAAAGTAGTT
         66 CAAAACTGCTCAATCAAAAGAAAGGTTCAACTCTGTGAGATGAATGCACACATCACAAAGAAGTT

                                                     
     185421 TCTCAGAATGCTTCTGTGTAGTTTTTATGTGAAGATATTTC
        131 TCTCAGAATGCTTCTGTGTAGTTTTTATGTGAAGATATTTC

                     * *    *      *    *       * *   **      *              
     185462 CTTTTCCACAACA-GCGTGCAAAACGCTTCAAATATGCCCTTAGAGATTCCACAAAAAGAGTGTT
          1 CTTTTCCACCATAGGCCT-CAAAGCGCTCCAAATATCCACTTGCAGATTCTACAAAAAGAGTGTT

              *     *               *  *                                  ** 
     185526 TCCAAACTACTCAAATCAAAA-AATGATTTCAACTCTGTGAGATGAATGCACACATCACAAACTA
         65 TCAAAACTGCTC-AATCAAAAGAAAG-GTTCAACTCTGTGAGATGAATGCACACATCACAAAGAA

                         *     ** *   * *   *       *   
     185590 GTTTCTCAGAATGTTTCTGCCTGGTTCTCATGCGAAGATAGTTC
        128 GTTTCTCAGAATGCTTCTGTGTAGTTTTTATGTGAAGATATTTC

                 *           *   * **              *                  * *    
     185634 CTTTTTCACCATAGGCCGCAATGTACTCCAAATATCCACCTGCAGATTCTAC-AAAAGTGAGTTT
          1 CTTTTCCACCATAGGCCTCAAAGCGCTCCAAATATCCACTTGCAGATTCTACAAAAAGAGTGTTT

                       *         ***    **         *        *      *       * 
     185698 CAAAACTGCTCTATCAAAAGATCAGTTCGTCTCTGTGAGTTGAATGCATACATCAAAAAGAAGCT
         66 CAAAACTGCTCAATCAAAAGAAAGGTTCAACTCTGTGAGATGAATGCACACATCACAAAGAAGTT

                 *             *      **         *   
     185763 TCTCAAAATGCTTCTGTGTGGTTTTTCGGTGAAGATAGTTC
        131 TCTCAGAATGCTTCTGTGTAGTTTTTATGTGAAGATATTTC

            *     *        *      * *                 *        *        *    
     185804 TTTTTCTACCATAGGTCTCAAACCACTCCAAATATCCACTTGTAGATTCTATAAAAAGGAATG-T
          1 CTTTTCCACCATAGGCCTCAAAGCGCTCCAAATATCCACTTGCAGATTCTACAAAAA-GAGTGTT

                  *        *    *    *      * *         *      *             
     185868 TCAAAATTGCTCAATAAAAATAAAGTTTCAACACCGTGAGATGAGTGCACAAATCACAAAGAAGT
         65 TCAAAACTGCTCAATCAAAAGAAAGGTTCAACTCTGTGAGATGAATGCACACATCACAAAGAAGT

                  *          *         *          *   
     185933 TTCTCAAAATGCTTCTGGGTAGTTTTTCTGTGAAGATAGTTC
        130 TTCTCAGAATGCTTCTGTGTAGTTTTTATGTGAAGATATTTC

                  *     *    *     *          *                         *    
     185975 CTTTTCTACCATGGGCCACAAAGGGCTCCAAATACCCACTTGCAGATTCTACAAAAAGAGAGTTT
          1 CTTTTCCACCATAGGCCTCAAAGCGCTCCAAATATCCACTTGCAGATTCTACAAAAAGAGTGTTT

              *        *      ** * *        *    * *    **  * *           *  
     186040 CACAACTGCTCTATCAAACAATATGTTCAACTTTGTGGGTTGAACACAAATATCAC-AAGAATTT
         66 CAAAACTGCTCAATCAAAAGAAAGGTTCAACTCTGTGAGATGAATGCACACATCACAAAGAAGTT

                **                             *     
     186104 TCTCCCAATGCTTCTGTGTAGTTTTTATGTGAAGACATTTC
        131 TCTCAGAATGCTTCTGTGTAGTTTTTATGTGAAGATATTTC

            *   *  *      *  *     *                 *  *      *       *  *  
     186145 TTTTCCCTCCATAGTCCACAAAGTGCTCCAAATATCCACTTACATATTCTAGAAAAAGATTGCTT
          1 CTTTTCCACCATAGGCCTCAAAGCGCTCCAAATATCCACTTGCAGATTCTACAAAAAGAGTGTTT

            **       *    *               * * *                              
     186210 GGAAACTGCACAATGAAAAGAAAGGTTCAAATATATGAGATGAATGCACACATCACAAAGAAGTT
         66 CAAAACTGCTCAATCAAAAGAAAGGTTCAACTCTGTGAGATGAATGCACACATCACAAAGAAGTT

                                 *                    
     186275 TCTCAGAAT-CTCTCTGTGTAATTTTTATGTGAAGATATTTC
        131 TCTCAGAATGCT-TCTGTGTAGTTTTTATGTGAAGATATTTC

                *     *    *  *   *                        *      *    *     
     186316 CTTTCCCACCTTAGGTCTTAAAACGCTCCAAATATCCACTTGCAGATACTACAAGAAGATTGTTT
          1 CTTTTCCACCATAGGCCTCAAAGCGCTCCAAATATCCACTTGCAGATTCTACAAAAAGAGTGTTT

                     *    *        *      *     **                           
     186381 CAAAACTGCACAA-AAAAAGAAATGTTCAATTCTGTTTGATGAATGCACACATCACAAAGAAGTT
         66 CAAAACTGCTCAATCAAAAGAAAGGTTCAACTCTGTGAGATGAATGCACACATCACAAAGAAGTT

                           *                         
     186445 TCTCAGAATGCTTCTCTGTAGTTTTTATGTGAAGATATTTC
        131 TCTCAGAATGCTTCTGTGTAGTTTTTATGTGAAGATATTTC

                     *             *                 *         **      * *   
     186486 CTTTTCCACAATAGGCCTCAAAGGGCTCCAAATATCCACTTCCAGATTCTATGAAAAGAATATTT
          1 CTTTTCCACCATAGGCCTCAAAGCGCTCCAAATATCCACTTGCAGATTCTACAAAAAGAGTGTTT

             *              * *    *                                      *  
     186551 CCAAACTGCTCAATCATAGGAAATGTTCAACTCTGTGAGATGAATGCACACATCAC-AAGAAATT
         66 CAAAACTGCTCAATCAAAAGAAAGGTTCAACTCTGTGAGATGAATGCACACATCACAAAGAAGTT

                     *    *      *       *       *   
     186615 TCTCAGAATCCTTCAGTGTAGGTTTTATGAGAAGATAATTC
        131 TCTCAGAATGCTTCTGTGTAGTTTTTATGTGAAGATATTTC

                     *    **        *   *                           *    *   
     186656 CTTTTCCACAATAGTTCTCAAAGCACTCAAAATATCCACTTGCAGATTCTACAAAAGGAGTATTT
          1 CTTTTCCACCATAGGCCTCAAAGCGCTCCAAATATCCACTTGCAGATTCTACAAAAAGAGTGTTT

                                                          *                  
     186721 CAAAACTGCTCAATCAAAAGAAAGGTTCAACTCTGTGAGATGAATGGACACATCACAAAGAAGTT
         66 CAAAACTGCTCAATCAAAAGAAAGGTTCAACTCTGTGAGATGAATGCACACATCACAAAGAAGTT

                                  *   *              
     186786 TCTCAGAATGCTTCTGTGTAGTATTTTTGTGAAGATATTT-
        131 TCTCAGAATGCTTCTGTGTAGTTTTTATGTGAAGATATTTC

                          *  * * * * ***             *            *     * *  
     186826 CTTTTCCACCATAGACCGCCAGGGGACACAAATATCCACTTTCAGATTCTACAACAAGAGAGGTT
          1 CTTTTCCACCATAGGCCTCAAAGCGCTCCAAATATCCACTTGCAGATTCTACAAAAAGAGTGTTT

                   *   *     *   ** *       *      *                      * *
     186891 CAAAACTACTCGATCAAGAGATGGTTTCAACTATGTGAGTTGAATGCACACATCACAAAGAACTA
         66 CAAAACTGCTCAATCAAAAGAAAGGTTCAACTCTGTGAGATGAATGCACACATCACAAAGAAGTT

             *  *    *                               
     186956 TGTCGGAATTCTTCTGTGTAGTTTTTATGTGAAGATATTTC
        131 TCTCAGAATGCTTCTGTGTAGTTTTTATGTGAAGATATTTC

                     *    * *      * *    *                  *               
     186997 CTTTTCCACAATAGACGTCAAAGTGATCCAGATATCCACTTGCAGATTCCACAAAAAGAGTGTTT
          1 CTTTTCCACCATAGGCCTCAAAGCGCTCCAAATATCCACTTGCAGATTCTACAAAAAGAGTGTTT

                 *   *   *                  **                     *  *      
     187062 CAAAAGTGCACAACCAAAAGAAAGGTTCAACTAGGTGAGATGAATGCACACATCAGAAGGAAGTT
         66 CAAAACTGCTCAATCAAAAGAAAGGTTCAACTCTGTGAGATGAATGCACACATCACAAAGAAGTT

                            **   *     * *       *   
     187127 TCTCAGAATGCTTCTGCATAGCTTTTAAGGGAAGATACTTC
        131 TCTCAGAATGCTTCTGTGTAGTTTTTATGTGAAGATATTTC

                    *               *            * *  *    * *               
     187168 CTTTTCCAACATAGGCCTCAAAGCACTCCAAATATCCTCCTGGAGATACCACAAAAAGAGTGTTT
          1 CTTTTCCACCATAGGCCTCAAAGCGCTCCAAATATCCACTTGCAGATTCTACAAAAAGAGTGTTT

            **                      *  *                 *       *           
     187233 GCAAACTGCTCAATCAAAAGAAAGATTTAACTCTGTGAGATGAATCCACACATGACAAAGAAGTT
         66 CAAAACTGCTCAATCAAAAGAAAGGTTCAACTCTGTGAGATGAATGCACACATCACAAAGAAGTT

                                                     
     187298 TCTCAGAATGCTTCTGTGTAGTTTTTATGTGAAGATATTTC
        131 TCTCAGAATGCTTCTGTGTAGTTTTTATGTGAAGATATTTC

                     *   * * *    **           *                *     *      
     187339 CTTTTCCACAATAAGACCCAAAAGGCTCCAAATATTCACTTGCAGATTCTAAAAAAAACAGTGTT
          1 CTTTTCCACCATAGGCCTCAAAGCGCTCCAAATATCCACTTGCAGATTCT-ACAAAAAGAGTGTT

                                  *                  *      *        **      
     187404 TCAAAACTGCTCAATCAAAAGATA-GTTCAACTCTGTGAGAAGAATGCTCACATCACTGAGAAGT
         65 TCAAAACTGCTCAATCAAAAGAAAGGTTCAACTCTGTGAGATGAATGCACACATCACAAAGAAGT

                                         *            
     187468 TTCTCAGAATGCTTCTGTGTAGTTTTTATATGAAGATATTTC
        130 TTCTCAGAATGCTTCTGTGTAGTTTTTATGTGAAGATATTTC

                *     *      *    **                       *   **       *    
     187510 CTTTCCCACCGTAGGCCACAAAAGGCTCCAAATATCCACTTGCAGATACTATGAAAAGAGAGTTT
          1 CTTTTCCACCATAGGCCTCAAAGCGCTCCAAATATCCACTTGCAGATTCTACAAAAAGAGTGTTT

                        *        *               ***            *            
     187575 CAAAACTGCTCATTCAAAAGATAGGTTCAACTCTGTGGTTTGAATGCACACAGCACAAAGAAGTT
         66 CAAAACTGCTCAATCAAAAGAAAGGTTCAACTCTGTGAGATGAATGCACACATCACAAAGAAGTT

              *       **                   **   *    
     187640 TCACAGAATGTGTCTGTGTAGTTTTTATGTGCGGATGTTTC
        131 TCTCAGAATGCTTCTGTGTAGTTTTTATGTGAAGATATTTC

                         *        ****   *   *               *    *          
     187681 CTTTTCCACCATATGCCT-AAATATTTCCCAATTTCCACTTGCAGATTCCACAAGAAGAGTGTTT
          1 CTTTTCCACCATAGGCCTCAAAGCGCTCCAAATATCCACTTGCAGATTCTACAAAAAGAGTGTTT

                      **       *       *           *            *      ***   
     187745 CAAAACTGCTGTATC-AAATAAA-GTTGAACTCTGTGAGGTGAATGCACACAGCACAAAATGGTT
         66 CAAAACTGCTCAATCAAAAGAAAGGTTCAACTCTGTGAGATGAATGCACACATCACAAAGAAGTT

                          **   *        *       *    
     187808 TCTCAGAATGCTTCCTTGTTGTTTTTATATGAAGATGTTTC
        131 TCTCAGAATGCTTCTGTGTAGTTTTTATGTGAAGATATTTC

                  *  *             *   *     *                               
     187849 CTTTTCAACAATAGGCCTCAAAGTGCTTCAAATGTCCACTTGCAGATTCTACAAAAAGAGTGTTT
          1 CTTTTCCACCATAGGCCTCAAAGCGCTCCAAATATCCACTTGCAGATTCTACAAAAAGAGTGTTT

                                        *      *  *  *                       
     187914 CAAAACTGCTCAATCAAAAGAAAGGTTCGACTCTGGGAAATTAATGCACACATCACAAAGAAGTT
         66 CAAAACTGCTCAATCAAAAGAAAGGTTCAACTCTGTGAGATGAATGCACACATCACAAAGAAGTT

                                     *        *      
     187979 TCTC---A-GCTTCTGTGTAGTTTTCATGTGAAGTTATTTC
        131 TCTCAGAATGCTTCTGTGTAGTTTTTATGTGAAGATATTTC

                     *       *     *            *    *         **       *    
     188016 CTTTTCCACAATAGGCCGCAAAGGGCTCCAAATATCAACTTACAGATTCTAGGAAAAGAGAGTTT
          1 CTTTTCCACCATAGGCCTCAAAGCGCTCCAAATATCCACTTGCAGATTCTACAAAAAGAGTGTTT

                       * **      *     *                                     
     188081 CAAAACTGCTCTACGAAAAGATAGGTTGAACTCTGTGAGATGAATGCACACATCACAAAGAAGTT
         66 CAAAACTGCTCAATCAAAAGAAAGGTTCAACTCTGTGAGATGAATGCACACATCACAAAGAAGTT

                       *               * *           
     188146 TCTCAGAATGCATCTGTGTAGTTTTTACGGGAAGATATTTC
        131 TCTCAGAATGCTTCTGTGTAGTTTTTATGTGAAGATATTTC

                        ***  *     **      *  *                  *     ***   
     188187 CTTTTCCACCATCTTCCACAAAGGTCTCCAAGTAACCACTTGCAGATTCTACAGAAAGACACTTT
          1 CTTTTCCACCATAGGCCTCAAAGCGCTCCAAATATCCACTTGCAGATTCTACAAAAAGAGTGTTT

            *          *         ***      *      ***                      *  
     188252 AAAAACTGCTCTATCAAAAGATCAGTTCAAGTCTGTGGTTTGAATGCACACATCACAAAGAATTT
         66 CAAAACTGCTCAATCAAAAGAAAGGTTCAACTCTGTGAGATGAATGCACACATCACAAAGAAGTT

                                     *  *            
     188317 TCTCAGAATGCTTCTGTGTAGTTTTCATATGAAGATATTTC
        131 TCTCAGAATGCTTCTGTGTAGTTTTTATGTGAAGATATTTC

                                    *                                   **   
     188358 CTTTTCCACCATAGGCCTCAAAGCACTCCAAATATCCACTTGCAGATTCTACAAAAAGAGATTTT
          1 CTTTTCCACCATAGGCCTCAAAGCGCTCCAAATATCCACTTGCAGATTCTACAAAAAGAGTGTTT

                   **                           *  *          *        *     
     188423 CAAAACTAGTCAATCAAAAGAAAGGTTCAACTCTGTCAGTTGAATGCACATATCACAAACAAGTT
         66 CAAAACTGCTCAATCAAAAGAAAGGTTCAACTCTGTGAGATGAATGCACACATCACAAAGAAGTT

                *      *                             
     188488 TCTCGGAATGCGTCTGTGTAGTTTTTATGTGAAGATATTTC
        131 TCTCAGAATGCTTCTGTGTAGTTTTTATGTGAAGATATTTC

               *     * *           *     *                  *    *           
     188529 CTTCTCCACAACAGGCCTCAAAGTGCTCCGAATATCCACTTGCAGATTTTAC-TAAAGAGTGTTT
          1 CTTTTCCACCATAGGCCTCAAAGCGCTCCAAATATCCACTTGCAGATTCTACAAAAAGAGTGTTT

             *               *  *   *     *        *    *               *    
     188593 CCAAACTGCTCAATCAAGAGGAAGTTTCAAGTCTGTGAGCTGAACGCACACATCACAAAGTAGTT
         66 CAAAACTGCTCAATCAAAAGAAAGGTTCAACTCTGTGAGATGAATGCACACATCACAAAGAAGTT

               *                                *    
     188658 TCTGAGAATGCTTCTGTGTAGTTTTTATGTGAAGATGTTTC
        131 TCTCAGAATGCTTCTGTGTAGTTTTTATGTGAAGATATTTC

                            **     *                                    *    
     188699 CTTTTCCACCATAGGCTGCAAAGGGCTCCAAATATCCACTTGCAGATTCTACAAAAAGAGAGTTT
          1 CTTTTCCACCATAGGCCTCAAAGCGCTCCAAATATCCACTTGCAGATTCTACAAAAAGAGTGTTT

                 *     *         *          *     *                     *    
     188764 CAAAAGTGCTCTATCAAAAGATAGGTTCAACTATGTGATATGAATGCACACATCACAAAGTAGTT
         66 CAAAACTGCTCAATCAAAAGAAAGGTTCAACTCTGTGAGATGAATGCACACATCACAAAGAAGTT

                                          *          
     188829 TCTCAGAATGCTTCTGTGTAGTTTTTATGTAAAGATATTTC
        131 TCTCAGAATGCTTCTGTGTAGTTTTTATGTGAAGATATTTC

                                    *                                   **   
     188870 CTTTTCCACCATAGGCCTCAAAGCACTCCAAATATCCACTTGCAGATTCTACAAAAAGAGATTTT
          1 CTTTTCCACCATAGGCCTCAAAGCGCTCCAAATATCCACTTGCAGATTCTACAAAAAGAGTGTTT

                   ** *                   *     *  *    * *   *        *     
     188935 CAAAACTATTTAATCAAAAGAAAGGTTCAAATCTGTCAGTTGAAGGTACATATCACAAACAAGTT
         66 CAAAACTGCTCAATCAAAAGAAAGGTTCAACTCTGTGAGATGAATGCACACATCACAAAGAAGTT

             * **                                    
     189000 TATTGGAATGCTTCTGTGTAGTTTTTATGTGAAGATATTTC
        131 TCTCAGAATGCTTCTGTGTAGTTTTTATGTGAAGATATTTC

                     * *       * * *                        **  *     *      
     189041 CTTTTCCACAACAGGCCTCCAGGTGCTCCAAATATCCACTTGCAGATTTCACTAAAAGTGTGTTT
          1 CTTTTCCACCATAGGCCTCAAAGCGCTCCAAATATCCACTTGCAGATTCTACAAAAAGAGTGTTT

             *  *            *  *   *     *        *             *           
     189106 CCAAGCTGCTCAATCAAGAGGAAGTTTCAAGTCTGTGAGGTGAATGCACACATTACAAAGAAGTT
         66 CAAAACTGCTCAATCAAAAGAAAGGTTCAACTCTGTGAGATGAATGCACACATCACAAAGAAGTT

            *  *                                     
     189171 ACTGAGAATGCTTCTGTGTAGTTTTTATGTGAAGATATTTC
        131 TCTCAGAATGCTTCTGTGTAGTTTTTATGTGAAGATATTTC

                      **               *                                *    
     189212 CTTTTCCACCGCAGGCCTCAAAGCGCTGCAAATATCCACTTGCAGATTCTACAAAAAGAGAGTTT
          1 CTTTTCCACCATAGGCCTCAAAGCGCTCCAAATATCCACTTGCAGATTCTACAAAAAGAGTGTTT

                      **         *   *         *   *     **            *     
     189277 CAAAACTGCTGTATCAAAAGATAGGGTCAACTCTGCGAGTTGAATAAACACATCACAAATAAGTT
         66 CAAAACTGCTCAATCAAAAGAAAGGTTCAACTCTGTGAGATGAATGCACACATCACAAAGAAGTT

               **   *        *               *       
     189342 TCTGGGAACGCTTCTGTATAG-TTTTATGTGAATATATTTC
        131 TCTCAGAATGCTTCTGTGTAGTTTTTATGTGAAGATATTTC

                         *          *                   *   *  *   * *   *   
     189382 CTTTTCCACCATATGCCTCAAAGCACTCCAAATATCCACTTGCACATTATAGAAACATAGTCTTT
          1 CTTTTCCACCATAGGCCTCAAAGCGCTCCAAATATCCACTTGCAGATTCTACAAAAAGAGTGTTT

                   **                        *         *             *       
     189447 CAAAACTTGTCAATC-AAAGAAAGGTTCAACTCCGTGAGATGAGTGCACACATCACAGAGAAGTT
         66 CAAAACTGCTCAATCAAAAGAAAGGTTCAACTCTGTGAGATGAATGCACACATCACAAAGAAGTT

                *     *                            * 
     189511 TCTCGGAATGTTTCTGTGTAGTTTTTATGTGAAGATATTGC
        131 TCTCAGAATGCTTCTGTGTAGTTTTTATGTGAAGATATTTC

                     *               *            *          *       *   *   
     189552 CTTTTCCACAATAGGCCTCAAAGCGTTCCAAATATCCAATTGCAGATTCCACAAAAAAAGTTTTT
          1 CTTTTCCACCATAGGCCTCAAAGCGCTCCAAATATCCACTTGCAGATTCTACAAAAAGAGTGTTT

            *                 *  *  *  *             * *         **    *     
     189617 TAAAACTGCTCAATCAAATGATAGATTAAACTCTGTGAGATTAGTGCACACATGTCAAAAAAGTT
         66 CAAAACTGCTCAATCAAAAGAAAGGTTCAACTCTGTGAGATGAATGCACACATCACAAAGAAGTT

                                **     * *           
     189682 TCTCAGAATGCTTCTGTGTACATTTTAGGGGAAGATATTTC
        131 TCTCAGAATGCTTCTGTGTAGTTTTTATGTGAAGATATTTC

                        *    *     **         *    *           **  * *  *    
     189723 CTTTTCCACCATCGGCCACAAAGGACTCCAAATAACCACATGCAGATTCTAGTAACACAGAGTTT
          1 CTTTTCCACCATAGGCCTCAAAGCGCTCCAAATATCCACTTGCAGATTCTACAAAAAGAGTGTTT

                       *         * *           *   * * *    **    ***        
     189788 CAAAACTGCTCTATCAAAAGATAAGTTCAACTCTGAGAGTTTAGTGCAACCATCGTGAAGAAGTT
         66 CAAAACTGCTCAATCAAAAGAAAGGTTCAACTCTGTGAGATGAATGCACACATCACAAAGAAGTT

                            *     *                  
     189853 TCTCAGAATGCTTCTGAGTAGTGTTTATGTGAAGATATTTC
        131 TCTCAGAATGCTTCTGTGTAGTTTTTATGTGAAGATATTTC

                              *     *                      *           **    
     189894 CTTTTCCACCATAGGCCTGAAAGCCCTCCAAATATCCACTTGCAGATCCTACAAAAAGAAAGTTT
          1 CTTTTCCACCATAGGCCTCAAAGCGCTCCAAATATCCACTTGCAGATTCTACAAAAAGAGTGTTT

             *         **     *  *  *   *        **      *                   
     189959 CGAAA-TGCTCTCTCAAACGATAGTTTCGACTCTGTGGTATGAATACACACATCACAAAGAAGTT
         66 CAAAACTGCTCAATCAAAAGAAAGGTTCAACTCTGTGAGATGAATGCACACATCACAAAGAAGTT

                                       **            
     190023 TCTCAGAATGCTTCTGTGTAGTTTTTAAATGAAGATATTTC
        131 TCTCAGAATGCTTCTGTGTAGTTTTTATGTGAAGATATTTC

            *                       *          *     *                       
     190064 TTTTTCCACCATAGGCCTCAAAGCACTCCAAATATGCACTTCCAGATTCTACAAAAAGAGTGTTT
          1 CTTTTCCACCATAGGCCTCAAAGCGCTCCAAATATCCACTTGCAGATTCTACAAAAAGAGTGTTT

              *                 *       * *         **   *               *   
     190129 CAGAACTGCTCAATCAAAAGGAAGGTTCCAGTC--TGAGACAAATACACACATCA-AAAGTTAGT
         66 CAAAACTGCTCAATCAAAAGAAAGGTTCAACTCTGTGAGATGAATGCACACATCACAAAG-AAGT

                                                     *
     190191 TTCTCAGAATGCTTCTGTGTAGTTTTTATGTGAAGATATTTT
        130 TTCTCAGAATGCTTCTGTGTAGTTTTTATGTGAAGATATTTC

             *               *    **    *     *      *  **   *          *    
     190233 CCTTTCCACCATAGGCCACAAATGGCTCTAAATACCCACTTACATTTTCCACAAAAAGAGAGTTT
          1 CTTTTCCACCATAGGCCTCAAAGCGCTCCAAATATCCACTTGCAGATTCTACAAAAAGAGTGTTT

                       * *    * *      *   *         *                       
     190298 CAAAACTGCTCTACCAAAGGTAA-GTTTAACGCTGTGAG-TTAA-G-A-ACATCACAAAGAAGTT
         66 CAAAACTGCTCAATCAAAAGAAAGGTTCAACTCTGTGAGATGAATGCACACATCACAAAGAAGTT

                                   *   *  *          
     190358 TCTCAGAATGCTTCTGTGTAGTTCTTACGTAAAGATATTTC
        131 TCTCAGAATGCTTCTGTGTAGTTTTTATGTGAAGATATTTC

                 *   *      ***    *                  *              **      
     190399 CTTTTACACAATAGGCAGAAAAGTGCTCCAAATATCCACTTGAAGATTCTACAAAAACCGTGTTT
          1 CTTTTCCACCATAGGCCTCAAAGCGCTCCAAATATCCACTTGCAGATTCTACAAAAAGAGTGTTT

                     **                                          *      *    
     190464 CAAAACTGCCGAATCAAAAGAAAGGTTCAACTCTGTGAGATGAATGCACACATAACAAAGGAGTT
         66 CAAAACTGCTCAATCAAAAGAAAGGTTCAACTCTGTGAGATGAATGCACACATCACAAAGAAGTT

                                 *      *      *    *
     190529 TCTCAGAATGCTTCTGTGTAGCTTTTATATGAAGACATTTA
        131 TCTCAGAATGCTTCTGTGTAGTTTTTATGTGAAGATATTTC

            *        * *            *   ***                     **        *  
     190570 GTTTTCCACAACAGGCCTCAAAGCTCTCTCCATATCCACTTGCAGATTCTACCGAAAGAGTGCTT
          1 CTTTTCCACCATAGGCCTCAAAGCGCTCCAAATATCCACTTGCAGATTCTACAAAAAGAGTGTTT

             *                   * **     *        *        *     **         
     190635 CCAAACTGCTCAATCAAAAGAGACATTCAAATCTGTGAGGTGAATGCAGACATCGTAAAGAAGTT
         66 CAAAACTGCTCAATCAAAAGAAAGGTTCAACTCTGTGAGATGAATGCACACATCACAAAGAAGTT

                                *     *       *    **
     190700 TCTCAGAATGCTTCTGTGTATTTTTTGTGTGAAGTTATTCG
        131 TCTCAGAATGCTTCTGTGTAGTTTTTATGTGAAGATATTTC

            *    *             *     *  *            **                 *    
     190741 TTTTTGCACCATAGGCCTCCAAGCGTTCTAAATATCCACTTCTAGATTCTACAAAAAGAGAGTTT
          1 CTTTTCCACCATAGGCCTCAAAGCGCTCCAAATATCCACTTGCAGATTCTACAAAAAGAGTGTTT

                   *         *            *        *    *   *                
     190806 CAAAACTACTC-A--AACA-AAAGGTTCAATTCTGTGAGTTGAAAGCAAACATCACAAAGAAGTT
         66 CAAAACTGCTCAATCAAAAGAAAGGTTCAACTCTGTGAGATGAATGCACACATCACAAAGAAGTT

                       *                             
     190867 TCTCAGAATGCGTCTGTGTAGTTTTTATGTGAAGATATTTC
        131 TCTCAGAATGCTTCTGTGTAGTTTTTATGTGAAGATATTTC

                     *    ****     *                  *                      
     190908 CTTTT-CACAATAGAATGCAAAGGGCTCCAAATATCCACTTGGAGATTCTACAAAAAGA--GTTT
          1 CTTTTCCACCATAGGCCTCAAAGCGCTCCAAATATCCACTTGCAGATTCTACAAAAAGAGTGTTT

                  *    **     *  *     *     ***   *     *               *   
     190970 CAAAACCGCTCTGTCAAATGATAGGTTGAACTCCCGGAGGTGAATACACACATCACAAAGAGGTT
         66 CAAAACTGCTCAATCAAAAGAAAGGTTCAACTCTGTGAGATGAATGCACACATCACAAAGAAGTT

                  *                       *  *       
     191035 TCTCAGCATGCTTCTGTGTAGTTTTTATGTAAACATATTTC
        131 TCTCAGAATGCTTCTGTGTAGTTTTTATGTGAAGATATTTC

             *    * *              *           *      * *       *   *    *   
     191076 CGTTTCTATCATAGGCCTCAAAGTGCTCCAAATATTCACTTGTACATTCTACCAAACGAGTATTT
          1 CTTTTCCACCATAGGCCTCAAAGCGCTCCAAATATCCACTTGCAGATTCTACAAAAAGAGTGTTT

                              * *         **      *        *            *    
     191141 CAAAACTGCTCAATCAAATGGAAGGTTCAAAACTGTGACATGAATGCCCACATCACAAAGTAGTT
         66 CAAAACTGCTCAATCAAAAGAAAGGTTCAACTCTGTGAGATGAATGCACACATCACAAAGAAGTT

                                                     
     191206 TCTCAGAATGCTTCTGTGTAGTTTTTATGTGAAGATATTTC
        131 TCTCAGAATGCTTCTGTGTAGTTTTTATGTGAAGATATTTC

                     * *    *      *    *       * *   **      *              
     191247 CTTTTCCACAACA-GCGTGCAAAACGCTTCAAATATGCCCTTAGAGATTCCACAAAAAGAGTGTT
          1 CTTTTCCACCATAGGCCT-CAAAGCGCTCCAAATATCCACTTGCAGATTCTACAAAAAGAGTGTT

              *     *               *  *                                  ** 
     191311 TCCAAACTACTCAAATCAAAA-AATGATTTCAACTCTGTGAGATGAATGCACACATCACAAACTA
         65 TCAAAACTGCTC-AATCAAAAGAAAG-GTTCAACTCTGTGAGATGAATGCACACATCACAAAGAA

                         *     ** *   * *   *       *   
     191375 GTTTCTCAGAATGTTTCTGCCTGGTTCTCATGCGAAGATAGTTC
        128 GTTTCTCAGAATGCTTCTGTGTAGTTTTTATGTGAAGATATTTC

                 *           *   * **              *                  * *    
     191419 CTTTTTCACCATAGGCCGCAATGTACTCCAAATATCCACCTGCAGATTCTAC-AAAAGTGAGTTT
          1 CTTTTCCACCATAGGCCTCAAAGCGCTCCAAATATCCACTTGCAGATTCTACAAAAAGAGTGTTT

                       *         ***    **         *        *      *       * 
     191483 CAAAACTGCTCTATCAAAAGATCAGTTCGTCTCTGTGAGTTGAATGCATACATCAAAAAGAAGCT
         66 CAAAACTGCTCAATCAAAAGAAAGGTTCAACTCTGTGAGATGAATGCACACATCACAAAGAAGTT

                 *             *      **         *   
     191548 TCTCAAAATGCTTCTGTGTGGTTTTTCGGTGAAGATAGTTC
        131 TCTCAGAATGCTTCTGTGTAGTTTTTATGTGAAGATATTTC

            *     *        *      * *                 *        *        *    
     191589 TTTTTCTACCATAGGTCTCAAACCACTCCAAATATCCACTTGTAGATTCTATAAAAAGGAATG-T
          1 CTTTTCCACCATAGGCCTCAAAGCGCTCCAAATATCCACTTGCAGATTCTACAAAAA-GAGTGTT

                  *        *    *    *      * *         *      *             
     191653 TCAAAATTGCTCAATAAAAATAAAGTTTCAACACCGTGAGATGAGTGCACAAATCACAAAGAAGT
         65 TCAAAACTGCTCAATCAAAAGAAAGGTTCAACTCTGTGAGATGAATGCACACATCACAAAGAAGT

                  *          *         *          *   
     191718 TTCTCAAAATGCTTCTGGGTAGTTTTTCTGTGAAGATAGTTC
        130 TTCTCAGAATGCTTCTGTGTAGTTTTTATGTGAAGATATTTC

                  *     *    *     *          *                         *    
     191760 CTTTTCTACCATGGGCCACAAAGGGCTCCAAATACCCACTTGCAGATTCTACAAAAAGAGAGTTT
          1 CTTTTCCACCATAGGCCTCAAAGCGCTCCAAATATCCACTTGCAGATTCTACAAAAAGAGTGTTT

              *        *      ** * *        *    * *    **  * *           *  
     191825 CACAACTGCTCTATCAAACAATATGTTCAACTTTGTGGGTTGAACACAAATATCAC-AAGAATTT
         66 CAAAACTGCTCAATCAAAAGAAAGGTTCAACTCTGTGAGATGAATGCACACATCACAAAGAAGTT

                **                             *     
     191889 TCTCCCAATGCTTCTGTGTAGTTTTTATGTGAAGACATTTC
        131 TCTCAGAATGCTTCTGTGTAGTTTTTATGTGAAGATATTTC

            *   *  *      *  *     *                 *  *      *       *  *  
     191930 TTTTCCCTCCATAGTCCACAAAGTGCTCCAAATATCCACTTACATATTCTAGAAAAAGATTGCTT
          1 CTTTTCCACCATAGGCCTCAAAGCGCTCCAAATATCCACTTGCAGATTCTACAAAAAGAGTGTTT

            **       *    *               * * *                              
     191995 GGAAACTGCACAATGAAAAGAAAGGTTCAAATATATGAGATGAATGCACACATCACAAAGAAGTT
         66 CAAAACTGCTCAATCAAAAGAAAGGTTCAACTCTGTGAGATGAATGCACACATCACAAAGAAGTT

                                 *                    
     192060 TCTCAGAAT-CTCTCTGTGTAATTTTTATGTGAAGATATTTC
        131 TCTCAGAATGCT-TCTGTGTAGTTTTTATGTGAAGATATTTC

                *     *    *  *   *                        *      *    *     
     192101 CTTTCCCACCTTAGGTCTTAAAACGCTCCAAATATCCACTTGCAGATACTACAAGAAGATTGTTT
          1 CTTTTCCACCATAGGCCTCAAAGCGCTCCAAATATCCACTTGCAGATTCTACAAAAAGAGTGTTT

                     *    *        *      *     **                           
     192166 CAAAACTGCACAA-AAAAAGAAATGTTCAATTCTGTTTGATGAATGCACACATCACAAAGAAGTT
         66 CAAAACTGCTCAATCAAAAGAAAGGTTCAACTCTGTGAGATGAATGCACACATCACAAAGAAGTT

                           *                         
     192230 TCTCAGAATGCTTCTCTGTAGTTTTTATGTGAAGATATTTC
        131 TCTCAGAATGCTTCTGTGTAGTTTTTATGTGAAGATATTTC

                     *             *                 *         **      * *   
     192271 CTTTTCCACAATAGGCCTCAAAGGGCTCCAAATATCCACTTCCAGATTCTATGAAAAGAATATTT
          1 CTTTTCCACCATAGGCCTCAAAGCGCTCCAAATATCCACTTGCAGATTCTACAAAAAGAGTGTTT

             *              * *    *                                      *  
     192336 CCAAACTGCTCAATCATAGGAAATGTTCAACTCTGTGAGATGAATGCACACATCAC-AAGAAATT
         66 CAAAACTGCTCAATCAAAAGAAAGGTTCAACTCTGTGAGATGAATGCACACATCACAAAGAAGTT

                     *    *      *       *       *   
     192400 TCTCAGAATCCTTCAGTGTAGGTTTTATGAGAAGATAATTC
        131 TCTCAGAATGCTTCTGTGTAGTTTTTATGTGAAGATATTTC

                     *    **        *   *                           *    *   
     192441 CTTTTCCACAATAGTTCTCAAAGCACTCAAAATATCCACTTGCAGATTCTACAAAAGGAGTATTT
          1 CTTTTCCACCATAGGCCTCAAAGCGCTCCAAATATCCACTTGCAGATTCTACAAAAAGAGTGTTT

                                                 *        *                  
     192506 CAAAACTGCTCAATCAAAAGAAAGGTTCAACTCTGTGGGATGAATGGACACATCACAAAGAAGTT
         66 CAAAACTGCTCAATCAAAAGAAAGGTTCAACTCTGTGAGATGAATGCACACATCACAAAGAAGTT

                                  *   *              
     192571 TCTCAGAATGCTTCTGTGTAGTATTTTTGTGAAGATATTT-
        131 TCTCAGAATGCTTCTGTGTAGTTTTTATGTGAAGATATTTC

                          *  * * * * ***             *            *     * *  
     192611 CTTTTCCACCATAGACCGCCAGGGGACACAAATATCCACTTTCAGATTCTACAACAAGAGAGGTT
          1 CTTTTCCACCATAGGCCTCAAAGCGCTCCAAATATCCACTTGCAGATTCTACAAAAAGAGTGTTT

                   *   *     *   ** *       *      *                      * *
     192676 CAAAACTACTCGATCAAGAGATGGTTTCAACTATGTGAGTTGAATGCACACATCACAAAGAACTA
         66 CAAAACTGCTCAATCAAAAGAAAGGTTCAACTCTGTGAGATGAATGCACACATCACAAAGAAGTT

             *  *    *                               
     192741 TGTCGGAATTCTTCTGTGTAGTTTTTATGTGAAGATATTTC
        131 TCTCAGAATGCTTCTGTGTAGTTTTTATGTGAAGATATTTC

                     *    * *      * *    *                  *               
     192782 CTTTTCCACAATAGACGTCAAAGTGATCCAGATATCCACTTGCAGATTCCACAAAAAGAGTGTTT
          1 CTTTTCCACCATAGGCCTCAAAGCGCTCCAAATATCCACTTGCAGATTCTACAAAAAGAGTGTTT

                 *   *   *                  **                     *  *      
     192847 CAAAAGTGCACAACCAAAAGAAAGGTTCAACTAGGTGAGATGAATGCACACATCAGAAGGAAGTT
         66 CAAAACTGCTCAATCAAAAGAAAGGTTCAACTCTGTGAGATGAATGCACACATCACAAAGAAGTT

                            **   *     * *       *   
     192912 TCTCAGAATGCTTCTGCATAGCTTTTAAGGGAAGATACTTC
        131 TCTCAGAATGCTTCTGTGTAGTTTTTATGTGAAGATATTTC

                    *               *            * *  *    * *               
     192953 CTTTTCCAACATAGGCCTCAAAGCACTCCAAATATCCTCCTGGAGATACCACAAAAAGAGTGTTT
          1 CTTTTCCACCATAGGCCTCAAAGCGCTCCAAATATCCACTTGCAGATTCTACAAAAAGAGTGTTT

            **                      *  *                 *       *           
     193018 GCAAACTGCTCAATCAAAAGAAAGATTTAACTCTGTGAGATGAATCCACACATGACAAAGAAGTT
         66 CAAAACTGCTCAATCAAAAGAAAGGTTCAACTCTGTGAGATGAATGCACACATCACAAAGAAGTT

                                                     
     193083 TCTCAGAATGCTTCTGTGTAGTTTTTATGTGAAGATATTTC
        131 TCTCAGAATGCTTCTGTGTAGTTTTTATGTGAAGATATTTC

                     *   * * *    **           *               *      *      
     193124 CTTTTCCACAATAAGACCCAAAAGGCTCCAAATATTCACTTGCAGATTCTAAAAAAGACAGTGTT
          1 CTTTTCCACCATAGGCCTCAAAGCGCTCCAAATATCCACTTGCAGATTCTACAAAA-AGAGTGTT

                      *           *                  *      *        **      
     193189 TCAAAACTGCACAATCAAAAGATA-GTTCAACTCTGTGAGAAGAATGCTCACATCACTGAGAAGT
         65 TCAAAACTGCTCAATCAAAAGAAAGGTTCAACTCTGTGAGATGAATGCACACATCACAAAGAAGT

                                         *            
     193253 TTCTCAGAATGCTTCTGTGTAGTTTTTATATGAAGATATTTC
        130 TTCTCAGAATGCTTCTGTGTAGTTTTTATGTGAAGATATTTC

                *     *      *    **                       *   **       *    
     193295 CTTTCCCACCGTAGGCCACAAAAGGCTCCAAATATCCACTTGCAGATACTATGAAAAGAGAGTTT
          1 CTTTTCCACCATAGGCCTCAAAGCGCTCCAAATATCCACTTGCAGATTCTACAAAAAGAGTGTTT

                        *        *               ***            *            
     193360 CAAAACTGCTCATTCAAAAGATAGGTTCAACTCTGTGGTTTGAATGCACACAGCACAAAGAAGTT
         66 CAAAACTGCTCAATCAAAAGAAAGGTTCAACTCTGTGAGATGAATGCACACATCACAAAGAAGTT

              *       **                   **   *    
     193425 TCACAGAATGTGTCTGTGTAGTTTTTATGTGCGGATGTTTC
        131 TCTCAGAATGCTTCTGTGTAGTTTTTATGTGAAGATATTTC

                         *        ****   *   *               *    *          
     193466 CTTTTCCACCATATGCCT-AAATATTTCCCAATTTCCACTTGCAGATTCCACAAGAAGAGTGTTT
          1 CTTTTCCACCATAGGCCTCAAAGCGCTCCAAATATCCACTTGCAGATTCTACAAAAAGAGTGTTT

                      **       *       *           *            *      ***   
     193530 CAAAACTGCTGTATC-AAATAAA-GTTGAACTCTGTGAGGTGAATGCACACAGCACAAAATGGTT
         66 CAAAACTGCTCAATCAAAAGAAAGGTTCAACTCTGTGAGATGAATGCACACATCACAAAGAAGTT

                          **   *        *       *    
     193593 TCTCAGAATGCTTCCTTGTTGTTTTTATATGAAGATGTTTC
        131 TCTCAGAATGCTTCTGTGTAGTTTTTATGTGAAGATATTTC

                  *  *             *   *     *                               
     193634 CTTTTCAACAATAGGCCTCAAAGTGCTTCAAATGTCCACTTGCAGATTCTACAAAAAGAGTGTTT
          1 CTTTTCCACCATAGGCCTCAAAGCGCTCCAAATATCCACTTGCAGATTCTACAAAAAGAGTGTTT

                                        *      *  *  *                       
     193699 CAAAACTGCTCAATCAAAAGAAAGGTTCGACTCTGGGAAATTAATGCACACATCACAAAGAAGTT
         66 CAAAACTGCTCAATCAAAAGAAAGGTTCAACTCTGTGAGATGAATGCACACATCACAAAGAAGTT

                                     *        *      
     193764 TCTC---A-GCTTCTGTGTAGTTTTCATGTGAAGTTATTTC
        131 TCTCAGAATGCTTCTGTGTAGTTTTTATGTGAAGATATTTC

                     *       *     *            *    *         **       *    
     193801 CTTTTCCACAATAGGCCGCAAAGGGCTCCAAATATCAACTTACAGATTCTAGGAAAAGAGAGTTT
          1 CTTTTCCACCATAGGCCTCAAAGCGCTCCAAATATCCACTTGCAGATTCTACAAAAAGAGTGTTT

                       *  *      *     *                                     
     193866 CAAAACTGCTCTATGAAAAGATAGGTTGAACTCTGTGAGATGAATGCACACATCACAAAGAAGTT
         66 CAAAACTGCTCAATCAAAAGAAAGGTTCAACTCTGTGAGATGAATGCACACATCACAAAGAAGTT

                       *               * *           
     193931 TCTCAGAATGCATCTGTGTAGTTTTTACGGGAAGATATTTC
        131 TCTCAGAATGCTTCTGTGTAGTTTTTATGTGAAGATATTTC

                        ***  *     **      *  *                  *     ***   
     193972 CTTTTCCACCATCTTCCACAAAGGTCTCCAAGTAACCACTTGCAGATTCTACAGAAAGACACTTT
          1 CTTTTCCACCATAGGCCTCAAAGCGCTCCAAATATCCACTTGCAGATTCTACAAAAAGAGTGTTT

            *          *         ***      *      ***                      *  
     194037 AAAAACTGCTCTATCAAAAGATCAGTTCAAGTCTGTGGTTTGAATGCACACATCACAAAGAATTT
         66 CAAAACTGCTCAATCAAAAGAAAGGTTCAACTCTGTGAGATGAATGCACACATCACAAAGAAGTT

                                     *  *            
     194102 TCTCAGAATGCTTCTGTGTAGTTTTCATATGAAGATATTTC
        131 TCTCAGAATGCTTCTGTGTAGTTTTTATGTGAAGATATTTC

                                    *                                   **   
     194143 CTTTTCCACCATAGGCCTCAAAGCACTCCAAATATCCACTTGCAGATTCTACAAAAAGAGATTTT
          1 CTTTTCCACCATAGGCCTCAAAGCGCTCCAAATATCCACTTGCAGATTCTACAAAAAGAGTGTTT

                   **                           *  *          *        *     
     194208 CAAAACTAGTCAATCAAAAGAAAGGTTCAACTCTGTCAGTTGAATGCACATATCACAAACAAGTT
         66 CAAAACTGCTCAATCAAAAGAAAGGTTCAACTCTGTGAGATGAATGCACACATCACAAAGAAGTT

                *      *                             
     194273 TCTCGGAATGCGTCTGTGTAGTTTTTATGTGAAGATATTTC
        131 TCTCAGAATGCTTCTGTGTAGTTTTTATGTGAAGATATTTC

               *     * *           *     *                  *    *           
     194314 CTTCTCCACAACAGGCCTCAAAGTGCTCCGAATATCCACTTGCAGATTTTAC-TAAAGAGTGTTT
          1 CTTTTCCACCATAGGCCTCAAAGCGCTCCAAATATCCACTTGCAGATTCTACAAAAAGAGTGTTT

             *               *  *   *     *        *    *               *    
     194378 CCAAACTGCTCAATCAAGAGGAAGTTTCAAGTCTGTGAGCTGAACGCACACATCACAAAGTAGTT
         66 CAAAACTGCTCAATCAAAAGAAAGGTTCAACTCTGTGAGATGAATGCACACATCACAAAGAAGTT

               *                                *    
     194443 TCTGAGAATGCTTCTGTGTAGTTTTTATGTGAAGATGTTTC
        131 TCTCAGAATGCTTCTGTGTAGTTTTTATGTGAAGATATTTC

                            **     *                                    *    
     194484 CTTTTCCACCATAGGCTGCAAAGGGCTCCAAATATCCACTTGCAGATTCTACAAAAAGAGAGTTT
          1 CTTTTCCACCATAGGCCTCAAAGCGCTCCAAATATCCACTTGCAGATTCTACAAAAAGAGTGTTT

                 *     *         *          *     *                     *    
     194549 CAAAAGTGCTCTATCAAAAGATAGGTTCAACTATGTGATATGAATGCACACATCACAAAGTAGTT
         66 CAAAACTGCTCAATCAAAAGAAAGGTTCAACTCTGTGAGATGAATGCACACATCACAAAGAAGTT

                                          *          
     194614 TCTCAGAATGCTTCTGTGTAGTTTTTATGTAAAGATATTTC
        131 TCTCAGAATGCTTCTGTGTAGTTTTTATGTGAAGATATTTC

                                    *           *                       **   
     194655 CTTTTCCACCATAGGCCTCAAAGCACTCCAAATATCTACTTGCAGATTCTACAAAAAGAGATTTT
          1 CTTTTCCACCATAGGCCTCAAAGCGCTCCAAATATCCACTTGCAGATTCTACAAAAAGAGTGTTT

                   ** *                   *     *  *    * *   *        *     
     194720 CAAAACTATTTAATCAAAAGAAAGGTTCAAATCTGTCAGTTGAAGGTACATATCACAAACAAGTT
         66 CAAAACTGCTCAATCAAAAGAAAGGTTCAACTCTGTGAGATGAATGCACACATCACAAAGAAGTT

             * **                                    
     194785 TATTGGAATGCTTCTGTGTAGTTTTTATGTGAAGATATTTC
        131 TCTCAGAATGCTTCTGTGTAGTTTTTATGTGAAGATATTTC

                     * *         * *                        **  *     *      
     194826 CTTTTCCACAACAGGCCTCAAGGTGCTCCAAATATCCACTTGCAGATTTCACTAAAAGTGTGTTT
          1 CTTTTCCACCATAGGCCTCAAAGCGCTCCAAATATCCACTTGCAGATTCTACAAAAAGAGTGTTT

             *  *            *  *   *     *        *             *           
     194891 CCAAGCTGCTCAATCAAGAGGAAGTTTCAAGTCTGTGAGGTGAATGCACACATTACAAAGAAGTT
         66 CAAAACTGCTCAATCAAAAGAAAGGTTCAACTCTGTGAGATGAATGCACACATCACAAAGAAGTT

            *  *                                     
     194956 ACTGAGAATGCTTCTGTGTAGTTTTTATGTGAAGATATTTC
        131 TCTCAGAATGCTTCTGTGTAGTTTTTATGTGAAGATATTTC

                      **               *                                *    
     194997 CTTTTCCACCGCAGGCCTCAAAGCGCTGCAAATATCCACTTGCAGATTCTACAAAAAGAGAGTTT
          1 CTTTTCCACCATAGGCCTCAAAGCGCTCCAAATATCCACTTGCAGATTCTACAAAAAGAGTGTTT

                      **         *   *         *   *     **            *     
     195062 CAAAACTGCTGTATCAAAAGATAGGGTCAACTCTGCGAGTTGAATAAACACATCACAAATAAGTT
         66 CAAAACTGCTCAATCAAAAGAAAGGTTCAACTCTGTGAGATGAATGCACACATCACAAAGAAGTT

               **   *        *               *       
     195127 TCTGGGAACGCTTCTGTATAG-TTTTATGTGAATATATTTC
        131 TCTCAGAATGCTTCTGTGTAGTTTTTATGTGAAGATATTTC

                         *          *                   *   *  *   * *   *   
     195167 CTTTTCCACCATATGCCTCAAAGCACTCCAAATATCCACTTGCACATTATAGAAACATAGTCTTT
          1 CTTTTCCACCATAGGCCTCAAAGCGCTCCAAATATCCACTTGCAGATTCTACAAAAAGAGTGTTT

                   **                        *         *             *       
     195232 CAAAACTTGTCAATC-AAAGAAAGGTTCAACTCCGTGAGATGAGTGCACACATCACAGAGAAGTT
         66 CAAAACTGCTCAATCAAAAGAAAGGTTCAACTCTGTGAGATGAATGCACACATCACAAAGAAGTT

                *     *                            * 
     195296 TCTCGGAATGTTTCTGTGTAGTTTTTATGTGAAGATATTGC
        131 TCTCAGAATGCTTCTGTGTAGTTTTTATGTGAAGATATTTC

                     *               *            *          *       *   *   
     195337 CTTTTCCACAATAGGCCTCAAAGCGTTCCAAATATCCAATTGCAGATTCCACAAAAAAAGTTTTT
          1 CTTTTCCACCATAGGCCTCAAAGCGCTCCAAATATCCACTTGCAGATTCTACAAAAAGAGTGTTT

            *                 *  *  *  *             * *         **    *     
     195402 TAAAACTGCTCAATCAAATGATAGATTAAACTCTGTGAGATTAGTGCACACATGTCAAAAAAGTT
         66 CAAAACTGCTCAATCAAAAGAAAGGTTCAACTCTGTGAGATGAATGCACACATCACAAAGAAGTT

                                *      * *           
     195467 TCTCAGAATGCTTCTGTGTACTTTTTAGGGGAAGATATTTC
        131 TCTCAGAATGCTTCTGTGTAGTTTTTATGTGAAGATATTTC

                        *    *     **         *    *           **  * *  *    
     195508 CTTTTCCACCATCGGCCACAAAGGACTCCAAATAACCACATGCAGATTCTAGTAACACAGAGTTT
          1 CTTTTCCACCATAGGCCTCAAAGCGCTCCAAATATCCACTTGCAGATTCTACAAAAAGAGTGTTT

                       *         * *           *   * * *    **    ***        
     195573 CAAAACTGCTCTATCAAAAGATAAGTTCAACTCTGAGAGTTTAGTGCAACCATCGTGAAGAAGTT
         66 CAAAACTGCTCAATCAAAAGAAAGGTTCAACTCTGTGAGATGAATGCACACATCACAAAGAAGTT

                            *     *                  
     195638 TCTCAGAATGCTTCTGAGTAGTGTTTATGTGAAGATATTTC
        131 TCTCAGAATGCTTCTGTGTAGTTTTTATGTGAAGATATTTC

                              *     *                      *           **    
     195679 CTTTTCCACCATAGGCCTGAAAGCCCTCCAAATATCCACTTGCAGATCCTACAAAAAGAAAGTTT
          1 CTTTTCCACCATAGGCCTCAAAGCGCTCCAAATATCCACTTGCAGATTCTACAAAAAGAGTGTTT

             *         **     *  *  *   *        **      *                   
     195744 CGAAA-TGCTCTCTCAAACGATAGTTTCGACTCTGTGGTATGAATACACACATCACAAAGAAGTT
         66 CAAAACTGCTCAATCAAAAGAAAGGTTCAACTCTGTGAGATGAATGCACACATCACAAAGAAGTT

                                       **            
     195808 TCTCAGAATGCTTCTGTGTAGTTTTTAAATGAAGATATTTC
        131 TCTCAGAATGCTTCTGTGTAGTTTTTATGTGAAGATATTTC

            *                       *          *     *                       
     195849 TTTTTCCACCATAGGCCTCAAAGCACTCCAAATATGCACTTCCAGATTCTACAAAAAGAGTGTTT
          1 CTTTTCCACCATAGGCCTCAAAGCGCTCCAAATATCCACTTGCAGATTCTACAAAAAGAGTGTTT

              *                 *       * *         **   *         *  * *    
     195914 CAGAACTGCTCAATCAAAAGGAAGGTTCCAGTC--TGAGACAAATACACACATCAAAAGGTAGTT
         66 CAAAACTGCTCAATCAAAAGAAAGGTTCAACTCTGTGAGATGAATGCACACATCACAAAGAAGTT

                                                    *
     195977 TCTCAGAATGCTTCTGTGTAGTTTTTATGTGAAGATATTTT
        131 TCTCAGAATGCTTCTGTGTAGTTTTTATGTGAAGATATTTC

             *               *    **    *     *      *  **   *          *    
     196018 CCTTTCCACCATAGGCCACAAATGGCTCTAAATACCCACTTACATTTTCCACAAAAAGAGAGTTT
          1 CTTTTCCACCATAGGCCTCAAAGCGCTCCAAATATCCACTTGCAGATTCTACAAAAAGAGTGTTT

                       * *    * *      *   *         *                       
     196083 CAAAACTGCTCTACCAAAGGTAA-GTTTAACGCTGTGAG-TTAA-G-A-ACATCACAAAGAAGTT
         66 CAAAACTGCTCAATCAAAAGAAAGGTTCAACTCTGTGAGATGAATGCACACATCACAAAGAAGTT

                                   *   *  *          
     196143 TCTCAGAATGCTTCTGTGTAGTTCTTACGTAAAGATATTTC
        131 TCTCAGAATGCTTCTGTGTAGTTTTTATGTGAAGATATTTC

                 *   *      ***    *                  *              **      
     196184 CTTTTACACAATAGGCAGAAAAGTGCTCCAAATATCCACTTGAAGATTCTACAAAAACCGTGTTT
          1 CTTTTCCACCATAGGCCTCAAAGCGCTCCAAATATCCACTTGCAGATTCTACAAAAAGAGTGTTT

                     **                                          *      *    
     196249 CAAAACTGCCGAATCAAAAGAAAGGTTCAACTCTGTGAGATGAATGCACACATAACAAAGGAGTT
         66 CAAAACTGCTCAATCAAAAGAAAGGTTCAACTCTGTGAGATGAATGCACACATCACAAAGAAGTT

                                 *      *      *    *
     196314 TCTCAGAATGCTTCTGTGTAGCTTTTATATGAAGACATTTA
        131 TCTCAGAATGCTTCTGTGTAGTTTTTATGTGAAGATATTTC

            *        * *            *   ***                     **        *  
     196355 GTTTTCCACAACAGGCCTCAAAGCTCTCTCCATATCCACTTGCAGATTCTACCGAAAGAGTGCTT
          1 CTTTTCCACCATAGGCCTCAAAGCGCTCCAAATATCCACTTGCAGATTCTACAAAAAGAGTGTTT

             *                   * **     *        *        *     **         
     196420 CCAAACTGCTCAATCAAAAGAGACATTCAAATCTGTGAGGTGAATGCAGACATCGTAAAGAAGTT
         66 CAAAACTGCTCAATCAAAAGAAAGGTTCAACTCTGTGAGATGAATGCACACATCACAAAGAAGTT

                                *     *       *    **
     196485 TCTCAGAATGCTTCTGTGTATTTTTTGTGTGAAGTTATTCG
        131 TCTCAGAATGCTTCTGTGTAGTTTTTATGTGAAGATATTTC

            *    *             *     *  *            **                 *    
     196526 TTTTTGCACCATAGGCCTCCAAGCGTTCTAAATATCCACTTCTAGATTCTACAAAAAGAGAGTTT
          1 CTTTTCCACCATAGGCCTCAAAGCGCTCCAAATATCCACTTGCAGATTCTACAAAAAGAGTGTTT

                   *         *            *        *    *   *                
     196591 CAAAACTACTC-A--AACA-AAAGGTTCAATTCTGTGAGTTGAAAGCAAACATCACAAAGAAGTT
         66 CAAAACTGCTCAATCAAAAGAAAGGTTCAACTCTGTGAGATGAATGCACACATCACAAAGAAGTT

                       *                             
     196652 TCTCAGAATGCGTCTGTGTAGTTTTTATGTGAAGATATTTC
        131 TCTCAGAATGCTTCTGTGTAGTTTTTATGTGAAGATATTTC

                     *    ****     *                  *                      
     196693 CTTTT-CACAATAGAATGCAAAGGGCTCCAAATATCCACTTGGAGATTCTACAAAAAGA--GTTT
          1 CTTTTCCACCATAGGCCTCAAAGCGCTCCAAATATCCACTTGCAGATTCTACAAAAAGAGTGTTT

                  *    **     *  *     *     ***   *     *               *   
     196755 CAAAACCGCTCTGTCAAATGATAGGTTGAACTCCCGGAGGTGAATACACACATCACAAAGAGGTT
         66 CAAAACTGCTCAATCAAAAGAAAGGTTCAACTCTGTGAGATGAATGCACACATCACAAAGAAGTT

                  *                       *  *       
     196820 TCTCAGCATGCTTCTGTGTAGTTTTTATGTAAACATATTTC
        131 TCTCAGAATGCTTCTGTGTAGTTTTTATGTGAAGATATTTC

             *    * *              *           *      * *       *   *    *   
     196861 CGTTTCTATCATAGGCCTCAAAGTGCTCCAAATATTCACTTGTACATTCTACCAAACGAGTATTT
          1 CTTTTCCACCATAGGCCTCAAAGCGCTCCAAATATCCACTTGCAGATTCTACAAAAAGAGTGTTT

                              * *         **      *        *            *    
     196926 CAAAACTGCTCAATCAAATGGAAGGTTCAAAACTGTGACATGAATGCCCACATCACAAAGTAGTT
         66 CAAAACTGCTCAATCAAAAGAAAGGTTCAACTCTGTGAGATGAATGCACACATCACAAAGAAGTT

                                                     
     196991 TCTCAGAATGCTTCTGTGTAGTTTTTATGTGAAGATATTTC
        131 TCTCAGAATGCTTCTGTGTAGTTTTTATGTGAAGATATTTC

                     * *    *      *    *       * *   **      *              
     197032 CTTTTCCACAACA-GCGTGCAAAACGCTTCAAATATGCCCTTAGAGATTCCACAAAAAGAGTGTT
          1 CTTTTCCACCATAGGCCT-CAAAGCGCTCCAAATATCCACTTGCAGATTCTACAAAAAGAGTGTT

              *     *               *  *                                  ** 
     197096 TCCAAACTACTCAAATCAAAA-AATGATTTCAACTCTGTGAGATGAATGCACACATCACAAACTA
         65 TCAAAACTGCTC-AATCAAAAGAAAG-GTTCAACTCTGTGAGATGAATGCACACATCACAAAGAA

                         *     ** *   * *   *       *   
     197160 GTTTCTCAGAATGTTTCTGCCTGGTTCTCATGCGAAGATAGTTC
        128 GTTTCTCAGAATGCTTCTGTGTAGTTTTTATGTGAAGATATTTC

                 *           *   * **              *                  * *    
     197204 CTTTTTCACCATAGGCCGCAATGTACTCCAAATATCCACCTGCAGATTCTAC-AAAAGTGAGTTT
          1 CTTTTCCACCATAGGCCTCAAAGCGCTCCAAATATCCACTTGCAGATTCTACAAAAAGAGTGTTT

                       *         ***    **         *        *      *       * 
     197268 CAAAACTGCTCTATCAAAAGATCAGTTCGTCTCTGTGAGTTGAATGCATACATCAAAAAGAAGCT
         66 CAAAACTGCTCAATCAAAAGAAAGGTTCAACTCTGTGAGATGAATGCACACATCACAAAGAAGTT

                 *             *      **         *   
     197333 TCTCAAAATGCTTCTGTGTGGTTTTTCGGTGAAGATAGTTC
        131 TCTCAGAATGCTTCTGTGTAGTTTTTATGTGAAGATATTTC

            *     *        *      * *                 *        *        *    
     197374 TTTTTCTACCATAGGTCTCAAACCACTCCAAATATCCACTTGTAGATTCTATAAAAAGGAATG-T
          1 CTTTTCCACCATAGGCCTCAAAGCGCTCCAAATATCCACTTGCAGATTCTACAAAAA-GAGTGTT

                  *        *    *    *      * *         *      *             
     197438 TCAAAATTGCTCAATAAAAATAAAGTTTCAACACCGTGAGATGAGTGCACAAATCACAAAGAAGT
         65 TCAAAACTGCTCAATCAAAAGAAAGGTTCAACTCTGTGAGATGAATGCACACATCACAAAGAAGT

                  *          *         *          *   
     197503 TTCTCAAAATGCTTCTGGGTAGTTTTTCTGTGAAGATAGTTC
        130 TTCTCAGAATGCTTCTGTGTAGTTTTTATGTGAAGATATTTC

                  *     *    *     *          *                         *    
     197545 CTTTTCTACCATGGGCCACAAAGGGCTCCAAATACCCACTTGCAGATTCTACAAAAAGAGAGTTT
          1 CTTTTCCACCATAGGCCTCAAAGCGCTCCAAATATCCACTTGCAGATTCTACAAAAAGAGTGTTT

              *        *      ** * *        *    * *    **  * *           *  
     197610 CACAACTGCTCTATCAAACAATATGTTCAACTTTGTGGGTTGAACACAAATATCAC-AAGAATTT
         66 CAAAACTGCTCAATCAAAAGAAAGGTTCAACTCTGTGAGATGAATGCACACATCACAAAGAAGTT

                **                             *     
     197674 TCTCCCAATGCTTCTGTGTAGTTTTTATGTGAAGACATTTC
        131 TCTCAGAATGCTTCTGTGTAGTTTTTATGTGAAGATATTTC

            *   *  *      *  *     *                 *  *      *       *  *  
     197715 TTTTCCCTCCATAGTCCACAAAGTGCTCCAAATATCCACTTACATATTCTAGAAAAAGATTGCTT
          1 CTTTTCCACCATAGGCCTCAAAGCGCTCCAAATATCCACTTGCAGATTCTACAAAAAGAGTGTTT

            **       *    *               * * *                              
     197780 GGAAACTGCACAATGAAAAGAAAGGTTCAAATATATGAGATGAATGCACACATCACAAAGAAGTT
         66 CAAAACTGCTCAATCAAAAGAAAGGTTCAACTCTGTGAGATGAATGCACACATCACAAAGAAGTT

                                 *                    
     197845 TCTCAGAAT-CTCTCTGTGTAATTTTTATGTGAAGATATTTC
        131 TCTCAGAATGCT-TCTGTGTAGTTTTTATGTGAAGATATTTC

                *     *    *  *   *                        *      *    *     
     197886 CTTTCCCACCTTAGGTCTTAAAACGCTCCAAATATCCACTTGCAGATACTACAAGAAGATTGTTT
          1 CTTTTCCACCATAGGCCTCAAAGCGCTCCAAATATCCACTTGCAGATTCTACAAAAAGAGTGTTT

                     *    *        *      *     **                           
     197951 CAAAACTGCACAA-AAAAAGAAATGTTCAATTCTGTTTGATGAATGCACACATCACAAAGAAGTT
         66 CAAAACTGCTCAATCAAAAGAAAGGTTCAACTCTGTGAGATGAATGCACACATCACAAAGAAGTT

                           *                         
     198015 TCTCAGAATGCTTCTCTGTAGTTTTTATGTGAAGATATTTC
        131 TCTCAGAATGCTTCTGTGTAGTTTTTATGTGAAGATATTTC

                     *             *                 *         **      * *   
     198056 CTTTTCCACAATAGGCCTCAAAGGGCTCCAAATATCCACTTCCAGATTCTATGAAAAGAATATTT
          1 CTTTTCCACCATAGGCCTCAAAGCGCTCCAAATATCCACTTGCAGATTCTACAAAAAGAGTGTTT

             *              * *    *                                      *  
     198121 CCAAACTGCTCAATCATAGGAAATGTTCAACTCTGTGAGATGAATGCACACATCAC-AAGAAATT
         66 CAAAACTGCTCAATCAAAAGAAAGGTTCAACTCTGTGAGATGAATGCACACATCACAAAGAAGTT

                     *    *      *       *       *   
     198185 TCTCAGAATCCTTCAGTGTAGGTTTTATGAGAAGATAATTC
        131 TCTCAGAATGCTTCTGTGTAGTTTTTATGTGAAGATATTTC

                     *    **        *   *                           *    *   
     198226 CTTTTCCACAATAGTTCTCAAAGCACTCAAAATATCCACTTGCAGATTCTACAAAAGGAGTATTT
          1 CTTTTCCACCATAGGCCTCAAAGCGCTCCAAATATCCACTTGCAGATTCTACAAAAAGAGTGTTT

                                                 *        *                  
     198291 CAAAACTGCTCAATCAAAAGAAAGGTTCAACTCTGTGGGATGAATGGACACATCACAAAGAAGTT
         66 CAAAACTGCTCAATCAAAAGAAAGGTTCAACTCTGTGAGATGAATGCACACATCACAAAGAAGTT

                                  *   *              
     198356 TCTCAGAATGCTTCTGTGTAGTATTTTTGTGAAGATATTT-
        131 TCTCAGAATGCTTCTGTGTAGTTTTTATGTGAAGATATTTC

                          *  * * * * ***             *            *     * *  
     198396 CTTTTCCACCATAGACCGCCAGGGGACACAAATATCCACTTTCAGATTCTACAACAAGAGAGGTT
          1 CTTTTCCACCATAGGCCTCAAAGCGCTCCAAATATCCACTTGCAGATTCTACAAAAAGAGTGTTT

                   *   *     *   ** *       *      *                      * *
     198461 CAAAACTACTCGATCAAGAGATGGTTTCAACTATGTGAGTTGAATGCACACATCACAAAGAACTA
         66 CAAAACTGCTCAATCAAAAGAAAGGTTCAACTCTGTGAGATGAATGCACACATCACAAAGAAGTT

             *  *    *                               
     198526 TGTCGGAATTCTTCTGTGTAGTTTTTATGTGAAGATATTTC
        131 TCTCAGAATGCTTCTGTGTAGTTTTTATGTGAAGATATTTC

                     *    * *      * *    *                  *               
     198567 CTTTTCCACAATAGACGTCAAAGTGATCCAGATATCCACTTGCAGATTCCACAAAAAGAGTGTTT
          1 CTTTTCCACCATAGGCCTCAAAGCGCTCCAAATATCCACTTGCAGATTCTACAAAAAGAGTGTTT

                 *   *   *                  **                     *  *      
     198632 CAAAAGTGCACAACCAAAAGAAAGGTTCAACTAGGTGAGATGAATGCACACATCAGAAGGAAGTT
         66 CAAAACTGCTCAATCAAAAGAAAGGTTCAACTCTGTGAGATGAATGCACACATCACAAAGAAGTT

                            **   *     * *       *   
     198697 TCTCAGAATGCTTCTGCATAGCTTTTAAGGGAAGATACTTC
        131 TCTCAGAATGCTTCTGTGTAGTTTTTATGTGAAGATATTTC

                    *               *            * *  *    * *               
     198738 CTTTTCCAACATAGGCCTCAAAGCACTCCAAATATCCTCCTGGAGATACCACAAAAAGAGTGTTT
          1 CTTTTCCACCATAGGCCTCAAAGCGCTCCAAATATCCACTTGCAGATTCTACAAAAAGAGTGTTT

            **                      *  *                 *       *           
     198803 GCAAACTGCTCAATCAAAAGAAAGATTTAACTCTGTGAGATGAATCCACACATGACAAAGAAGTT
         66 CAAAACTGCTCAATCAAAAGAAAGGTTCAACTCTGTGAGATGAATGCACACATCACAAAGAAGTT

                                                     
     198868 TCTCAGAATGCTTCTGTGTAGTTTTTATGTGAAGATATTTC
        131 TCTCAGAATGCTTCTGTGTAGTTTTTATGTGAAGATATTTC

                     *   * * *    **           *                *     *      
     198909 CTTTTCCACAATAAGACCCAAAAGGCTCCAAATATTCACTTGCAGATTCTAAAAAAAACAGTGTT
          1 CTTTTCCACCATAGGCCTCAAAGCGCTCCAAATATCCACTTGCAGATTCT-ACAAAAAGAGTGTT

                                  *                  *      *        **      
     198974 TCAAAACTGCTCAATCAAAAGATA-GTTCAACTCTGTGAGAAGAATGCTCACATCACTGAGAAGT
         65 TCAAAACTGCTCAATCAAAAGAAAGGTTCAACTCTGTGAGATGAATGCACACATCACAAAGAAGT

                                         *            
     199038 TTCTCAGAATGCTTCTGTGTAGTTTTTATATGAAGATATTTC
        130 TTCTCAGAATGCTTCTGTGTAGTTTTTATGTGAAGATATTTC

                *     *      *    **                       *   **       *    
     199080 CTTTCCCACCGTAGGCCACAAAAGGCTCCAAATATCCACTTGCAGATACTATGAAAAGAGAGTTT
          1 CTTTTCCACCATAGGCCTCAAAGCGCTCCAAATATCCACTTGCAGATTCTACAAAAAGAGTGTTT

                        *        *               ***            *            
     199145 CAAAACTGCTCATTCAAAAGATAGGTTCAACTCTGTGGTTTGAATGCACACAGCACAAAGAAGTT
         66 CAAAACTGCTCAATCAAAAGAAAGGTTCAACTCTGTGAGATGAATGCACACATCACAAAGAAGTT

              *       **                   **   *    
     199210 TCACAGAATGTGTCTGTGTAGTTTTTATGTGCGGATGTTTC
        131 TCTCAGAATGCTTCTGTGTAGTTTTTATGTGAAGATATTTC

                         *        ****   *   *               *    *          
     199251 CTTTTCCACCATATGCCT-AAATATTTCCCAATTTCCACTTGCAGATTCCACAAGAAGAGTGTTT
          1 CTTTTCCACCATAGGCCTCAAAGCGCTCCAAATATCCACTTGCAGATTCTACAAAAAGAGTGTTT

                      **       *       *           *            *      ***   
     199315 CAAAACTGCTGTATC-AAATAAA-GTTGAACTCTGTGAGGTGAATGCACACAGCACAAAATGGTT
         66 CAAAACTGCTCAATCAAAAGAAAGGTTCAACTCTGTGAGATGAATGCACACATCACAAAGAAGTT

                          **   *        *       *    
     199378 TCTCAGAATGCTTCCTTGTTGTTTTTATATGAAGATGTTTC
        131 TCTCAGAATGCTTCTGTGTAGTTTTTATGTGAAGATATTTC

                  *  *             *   *     *                               
     199419 CTTTTCAACAATAGGCCTCAAAGTGCTTCAAATGTCCACTTGCAGATTCTACAAAAAGAGTGTTT
          1 CTTTTCCACCATAGGCCTCAAAGCGCTCCAAATATCCACTTGCAGATTCTACAAAAAGAGTGTTT

                                        *      *  *  *                       
     199484 CAAAACTGCTCAATCAAAAGAAAGGTTCGACTCTGGGAAATTAATGCACACATCACAAAGAAGTT
         66 CAAAACTGCTCAATCAAAAGAAAGGTTCAACTCTGTGAGATGAATGCACACATCACAAAGAAGTT

                                     *        *      
     199549 TCTC---A-GCTTCTGTGTAGTTTTCATGTGAAGTTATTTC
        131 TCTCAGAATGCTTCTGTGTAGTTTTTATGTGAAGATATTTC

                     *       *     *            *    *         **       *    
     199586 CTTTTCCACAATAGGCCGCAAAGGGCTCCAAATATCAACTTACAGATTCTAGGAAAAGAGAGTTT
          1 CTTTTCCACCATAGGCCTCAAAGCGCTCCAAATATCCACTTGCAGATTCTACAAAAAGAGTGTTT

                       *  *      *     *                                     
     199651 CAAAACTGCTCTATGAAAAGATAGGTTGAACTCTGTGAGATGAATGCACACATCACAAAGAAGTT
         66 CAAAACTGCTCAATCAAAAGAAAGGTTCAACTCTGTGAGATGAATGCACACATCACAAAGAAGTT

                       *               * *           
     199716 TCTCAGAATGCATCTGTGTAGTTTTTACGGGAAGATATTTC
        131 TCTCAGAATGCTTCTGTGTAGTTTTTATGTGAAGATATTTC

                        ***  *     **      *  *                  *     ***   
     199757 CTTTTCCACCATCTTCCACAAAGGTCTCCAAGTAACCACTTGCAGATTCTACAGAAAGACACTTT
          1 CTTTTCCACCATAGGCCTCAAAGCGCTCCAAATATCCACTTGCAGATTCTACAAAAAGAGTGTTT

            *          *         ***      *      ***                      *  
     199822 AAAAACTGCTCTATCAAAAGATCAGTTCAAGTCTGTGGTTTGAATGCACACATCACAAAGAATTT
         66 CAAAACTGCTCAATCAAAAGAAAGGTTCAACTCTGTGAGATGAATGCACACATCACAAAGAAGTT

                                     *  *            
     199887 TCTCAGAATGCTTCTGTGTAGTTTTCATATGAAGATATTTC
        131 TCTCAGAATGCTTCTGTGTAGTTTTTATGTGAAGATATTTC

                                    *                                   **   
     199928 CTTTTCCACCATAGGCCTCAAAGCACTCCAAATATCCACTTGCAGATTCTACAAAAAGAGATTTT
          1 CTTTTCCACCATAGGCCTCAAAGCGCTCCAAATATCCACTTGCAGATTCTACAAAAAGAGTGTTT

                   **                           *  *          *        *     
     199993 CAAAACTAGTCAATCAAAAGAAAGGTTCAACTCTGTCAGTTGAATGCACATATCACAAACAAGTT
         66 CAAAACTGCTCAATCAAAAGAAAGGTTCAACTCTGTGAGATGAATGCACACATCACAAAGAAGTT

                *      *                             
     200058 TCTCGGAATGCGTCTGTGTAGTTTTTATGTGAAGATATTTC
        131 TCTCAGAATGCTTCTGTGTAGTTTTTATGTGAAGATATTTC

               *     * *           *     *                  *    *           
     200099 CTTCTCCACAACAGGCCTCAAAGTGCTCCGAATATCCACTTGCAGATTTTAC-TAAAGAGTGTTT
          1 CTTTTCCACCATAGGCCTCAAAGCGCTCCAAATATCCACTTGCAGATTCTACAAAAAGAGTGTTT

             *               *  *   *     *        *    *               *    
     200163 CCAAACTGCTCAATCAAGAGGAAGTTTCAAGTCTGTGAGCTGAACGCACACATCACAAAGTAGTT
         66 CAAAACTGCTCAATCAAAAGAAAGGTTCAACTCTGTGAGATGAATGCACACATCACAAAGAAGTT

               *                                *    
     200228 TCTGAGAATGCTTCTGTGTAGTTTTTATGTGAAGATGTTTC
        131 TCTCAGAATGCTTCTGTGTAGTTTTTATGTGAAGATATTTC

                            **     *                                    *    
     200269 CTTTTCCACCATAGGCTGCAAAGGGCTCCAAATATCCACTTGCAGATTCTACAAAAAGAGAGTTT
          1 CTTTTCCACCATAGGCCTCAAAGCGCTCCAAATATCCACTTGCAGATTCTACAAAAAGAGTGTTT

                 *     *         *          *     *                     *    
     200334 CAAAAGTGCTCTATCAAAAGATAGGTTCAACTATGTGATATGAATGCACACATCACAAAGTAGTT
         66 CAAAACTGCTCAATCAAAAGAAAGGTTCAACTCTGTGAGATGAATGCACACATCACAAAGAAGTT

                                          *          
     200399 TCTCAGAATGCTTCTGTGTAGTTTTTATGTAAAGATATTTC
        131 TCTCAGAATGCTTCTGTGTAGTTTTTATGTGAAGATATTTC

                                    *                                   **   
     200440 CTTTTCCACCATAGGCCTCAAAGCACTCCAAATATCCACTTGCAGATTCTACAAAAAGAGATTTT
          1 CTTTTCCACCATAGGCCTCAAAGCGCTCCAAATATCCACTTGCAGATTCTACAAAAAGAGTGTTT

                   ** *                   *     *  *    * *   *        *     
     200505 CAAAACTATTTAATCAAAAGAAAGGTTCAAATCTGTCAGTTGAAGGTACATATCACAAACAAGTT
         66 CAAAACTGCTCAATCAAAAGAAAGGTTCAACTCTGTGAGATGAATGCACACATCACAAAGAAGTT

             * **                                    
     200570 TATTGGAATGCTTCTGTGTAGTTTTTATGTGAAGATATTTC
        131 TCTCAGAATGCTTCTGTGTAGTTTTTATGTGAAGATATTTC

                     * *       * * *                        **  *     *      
     200611 CTTTTCCACAACAGGCCTCCAGGTGCTCCAAATATCCACTTGCAGATTTCACTAAAAGTGTGTTT
          1 CTTTTCCACCATAGGCCTCAAAGCGCTCCAAATATCCACTTGCAGATTCTACAAAAAGAGTGTTT

             *  *            *  *   *     *        *             *           
     200676 CCAAGCTGCTCAATCAAGAGGAAGTTTCAAGTCTGTGAGGTGAATGCACACATTACAAAGAAGTT
         66 CAAAACTGCTCAATCAAAAGAAAGGTTCAACTCTGTGAGATGAATGCACACATCACAAAGAAGTT

            *  *                                     
     200741 ACTGAGAATGCTTCTGTGTAGTTTTTATGTGAAGATATTTC
        131 TCTCAGAATGCTTCTGTGTAGTTTTTATGTGAAGATATTTC

                      **               *                                *    
     200782 CTTTTCCACCGCAGGCCTCAAAGCGCTGCAAATATCCACTTGCAGATTCTACAAAAAGAGAGTTT
          1 CTTTTCCACCATAGGCCTCAAAGCGCTCCAAATATCCACTTGCAGATTCTACAAAAAGAGTGTTT

                      **         *   *         *   *     **            *     
     200847 CAAAACTGCTGTATCAAAAGATAGGGTCAACTCTGCGAGTTGAATAAACACATCACAAATAAGTT
         66 CAAAACTGCTCAATCAAAAGAAAGGTTCAACTCTGTGAGATGAATGCACACATCACAAAGAAGTT

               **   *        *               *       
     200912 TCTGGGAACGCTTCTGTATAG-TTTTATGTGAATATATTTC
        131 TCTCAGAATGCTTCTGTGTAGTTTTTATGTGAAGATATTTC

                         *          *                   *   *  *   * *   *   
     200952 CTTTTCCACCATATGCCTCAAAGCACTCCAAATATCCACTTGCACATTATAGAAACATAGTCTTT
          1 CTTTTCCACCATAGGCCTCAAAGCGCTCCAAATATCCACTTGCAGATTCTACAAAAAGAGTGTTT

                   **                        *         *             *       
     201017 CAAAACTTGTCAATC-AAAGAAAGGTTCAACTCCGTGAGATGAGTGCACACATCACAGAGAAGTT
         66 CAAAACTGCTCAATCAAAAGAAAGGTTCAACTCTGTGAGATGAATGCACACATCACAAAGAAGTT

                *     *                            * 
     201081 TCTCGGAATGTTTCTGTGTAGTTTTTATGTGAAGATATTGC
        131 TCTCAGAATGCTTCTGTGTAGTTTTTATGTGAAGATATTTC

                     *               *            *          *       *   *   
     201122 CTTTTCCACAATAGGCCTCAAAGCGTTCCAAATATCCAATTGCAGATTCCACAAAAAAAGTTTTT
          1 CTTTTCCACCATAGGCCTCAAAGCGCTCCAAATATCCACTTGCAGATTCTACAAAAAGAGTGTTT

            *                 *  *  *  *             * *         **    *     
     201187 TAAAACTGCTCAATCAAATGATAGATTAAACTCTGTGAGATTAGTGCACACATGTCAAAAAAGTT
         66 CAAAACTGCTCAATCAAAAGAAAGGTTCAACTCTGTGAGATGAATGCACACATCACAAAGAAGTT

                                *      * *           
     201252 TCTCAGAATGCTTCTGTGTACTTTTTAGGGGAAGATATTTC
        131 TCTCAGAATGCTTCTGTGTAGTTTTTATGTGAAGATATTTC

                        *    *     **         *    *           **  * *  *    
     201293 CTTTTCCACCATCGGCCACAAAGGACTCCAAATAACCACATGCAGATTCTAGTAACACAGAGTTT
          1 CTTTTCCACCATAGGCCTCAAAGCGCTCCAAATATCCACTTGCAGATTCTACAAAAAGAGTGTTT

                       *         * *           *   * * *    **    ***        
     201358 CAAAACTGCTCTATCAAAAGATAAGTTCAACTCTGAGAGTTTAGTGCAACCATCGTGAAGAAGTT
         66 CAAAACTGCTCAATCAAAAGAAAGGTTCAACTCTGTGAGATGAATGCACACATCACAAAGAAGTT

                            *     *                  
     201423 TCTCAGAATGCTTCTGAGTAGTGTTTATGTGAAGATATTTC
        131 TCTCAGAATGCTTCTGTGTAGTTTTTATGTGAAGATATTTC

                              *     *                      *           **    
     201464 CTTTTCCACCATAGGCCTGAAAGCCCTCCAAATATCCACTTGCAGATCCTACAAAAAGAAAGTTT
          1 CTTTTCCACCATAGGCCTCAAAGCGCTCCAAATATCCACTTGCAGATTCTACAAAAAGAGTGTTT

             *         **     *  *  *   *        **      *                   
     201529 CGAAA-TGCTCTCTCAAACGATAGTTTCGACTCTGTGGTATGAATACACACATCACAAAGAAGTT
         66 CAAAACTGCTCAATCAAAAGAAAGGTTCAACTCTGTGAGATGAATGCACACATCACAAAGAAGTT

                                       **            
     201593 TCTCAGAATGCTTCTGTGTAGTTTTTAAATGAAGATATTTC
        131 TCTCAGAATGCTTCTGTGTAGTTTTTATGTGAAGATATTTC

            *                       *          *     *                       
     201634 TTTTTCCACCATAGGCCTCAAAGCACTCCAAATATGCACTTCCAGATTCTACAAAAAGAGTGTTT
          1 CTTTTCCACCATAGGCCTCAAAGCGCTCCAAATATCCACTTGCAGATTCTACAAAAAGAGTGTTT

              *                 *       * *         **   *         *  * *    
     201699 CAGAACTGCTCAATCAAAAGGAAGGTTCCAGTC--TGAGACAAATACACACATCAAAAGGTAGTT
         66 CAAAACTGCTCAATCAAAAGAAAGGTTCAACTCTGTGAGATGAATGCACACATCACAAAGAAGTT

                                                    *
     201762 TCTCAGAATGCTTCTGTGTAGTTTTTATGTGAAGATATTTT
        131 TCTCAGAATGCTTCTGTGTAGTTTTTATGTGAAGATATTTC

             *               *    **    *     *      *  **   *          *    
     201803 CCTTTCCACCATAGGCCACAAATGGCTCTAAATACCCACTTACATTTTCCACAAAAAGAGAGTTT
          1 CTTTTCCACCATAGGCCTCAAAGCGCTCCAAATATCCACTTGCAGATTCTACAAAAAGAGTGTTT

                       * *    * *      *   *         *                       
     201868 CAAAACTGCTCTACCAAAGGTAA-GTTTAACGCTGTGAG-TTAA-G-A-ACATCACAAAGAAGTT
         66 CAAAACTGCTCAATCAAAAGAAAGGTTCAACTCTGTGAGATGAATGCACACATCACAAAGAAGTT

                                   *   *  *          
     201928 TCTCAGAATGCTTCTGTGTAGTTCTTACGTAAAGATATTTC
        131 TCTCAGAATGCTTCTGTGTAGTTTTTATGTGAAGATATTTC

                 *   *      ***    *                  *              **      
     201969 CTTTTACACAATAGGCAGAAAAGTGCTCCAAATATCCACTTGAAGATTCTACAAAAACCGTGTTT
          1 CTTTTCCACCATAGGCCTCAAAGCGCTCCAAATATCCACTTGCAGATTCTACAAAAAGAGTGTTT

                     **                                          *      *    
     202034 CAAAACTGCCGAATCAAAAGAAAGGTTCAACTCTGTGAGATGAATGCACACATAACAAAGGAGTT
         66 CAAAACTGCTCAATCAAAAGAAAGGTTCAACTCTGTGAGATGAATGCACACATCACAAAGAAGTT

                                 *      *      *    *
     202099 TCTCAGAATGCTTCTGTGTAGCTTTTATATGAAGACATTTA
        131 TCTCAGAATGCTTCTGTGTAGTTTTTATGTGAAGATATTTC

            *        * *            *   ***                     **        *  
     202140 GTTTTCCACAACAGGCCTCAAAGCTCTCTCCATATCCACTTGCAGATTCTACCGAAAGAGTGCTT
          1 CTTTTCCACCATAGGCCTCAAAGCGCTCCAAATATCCACTTGCAGATTCTACAAAAAGAGTGTTT

             *                   * **     *        *        *     **         
     202205 CCAAACTGCTCAATCAAAAGAGACATTCAAATCTGTGAGGTGAATGCAGACATCGTAAAGAAGTT
         66 CAAAACTGCTCAATCAAAAGAAAGGTTCAACTCTGTGAGATGAATGCACACATCACAAAGAAGTT

                                *     *       *    **
     202270 TCTCAGAATGCTTCTGTGTATTTTTTGTGTGAAGTTATTCG
        131 TCTCAGAATGCTTCTGTGTAGTTTTTATGTGAAGATATTTC

            *    *             *     *  *            **                 *    
     202311 TTTTTGCACCATAGGCCTCCAAGCGTTCTAAATATCCACTTCTAGATTCTACAAAAAGAGAGTTT
          1 CTTTTCCACCATAGGCCTCAAAGCGCTCCAAATATCCACTTGCAGATTCTACAAAAAGAGTGTTT

                   *         *            *        *    *   *                
     202376 CAAAACTACTC-A--AACA-AAAGGTTCAATTCTGTGAGTTGAAAGCAAACATCACAAAGAAGTT
         66 CAAAACTGCTCAATCAAAAGAAAGGTTCAACTCTGTGAGATGAATGCACACATCACAAAGAAGTT

                       *                             
     202437 TCTCAGAATGCGTCTGTGTAGTTTTTATGTGAAGATATTTC
        131 TCTCAGAATGCTTCTGTGTAGTTTTTATGTGAAGATATTTC

                     *    ****     *                  *                      
     202478 CTTTT-CACAATAGAATGCAAAGGGCTCCAAATATCCACTTGGAGATTCTACAAAAAGA--GTTT
          1 CTTTTCCACCATAGGCCTCAAAGCGCTCCAAATATCCACTTGCAGATTCTACAAAAAGAGTGTTT

                  *    **     *  *     *     ***   *     *               *   
     202540 CAAAACCGCTCTGTCAAATGATAGGTTGAACTCCCGGAGGTGAATACACACATCACAAAGAGGTT
         66 CAAAACTGCTCAATCAAAAGAAAGGTTCAACTCTGTGAGATGAATGCACACATCACAAAGAAGTT

                  *                       *  *       
     202605 TCTCAGCATGCTTCTGTGTAGTTTTTATGTAAACATATTTC
        131 TCTCAGAATGCTTCTGTGTAGTTTTTATGTGAAGATATTTC

             *    * *              *           *      * *       *   *    *   
     202646 CGTTTCTATCATAGGCCTCAAAGTGCTCCAAATATTCACTTGTACATTCTACCAAACGAGTATTT
          1 CTTTTCCACCATAGGCCTCAAAGCGCTCCAAATATCCACTTGCAGATTCTACAAAAAGAGTGTTT

                              * *         **      *        *            *    
     202711 CAAAACTGCTCAATCAAATGGAAGGTTCAAAACTGTGACATGAATGCCCACATCACAAAGTAGTT
         66 CAAAACTGCTCAATCAAAAGAAAGGTTCAACTCTGTGAGATGAATGCACACATCACAAAGAAGTT

                                                     
     202776 TCTCAGAATGCTTCTGTGTAGTTTTTATGTGAAGATATTTC
        131 TCTCAGAATGCTTCTGTGTAGTTTTTATGTGAAGATATTTC

                     * *    *      *    *       * *   **      *              
     202817 CTTTTCCACAACA-GCGTGCAAAACGCTTCAAATATGCCCTTAGAGATTCCACAAAAAGAGTGTT
          1 CTTTTCCACCATAGGCCT-CAAAGCGCTCCAAATATCCACTTGCAGATTCTACAAAAAGAGTGTT

              *     *               *  *                                  ** 
     202881 TCCAAACTACTCAAATCAAAA-AATGATTTCAACTCTGTGAGATGAATGCACACATCACAAACTA
         65 TCAAAACTGCTC-AATCAAAAGAAAG-GTTCAACTCTGTGAGATGAATGCACACATCACAAAGAA

                         *     ** *   * *   *       *   
     202945 GTTTCTCAGAATGTTTCTGCCTGGTTCTCATGCGAAGATAGTTC
        128 GTTTCTCAGAATGCTTCTGTGTAGTTTTTATGTGAAGATATTTC

                 *           *   * **              *                  * *    
     202989 CTTTTTCACCATAGGCCGCAATGTACTCCAAATATCCACCTGCAGATTCTAC-AAAAGTGAGTTT
          1 CTTTTCCACCATAGGCCTCAAAGCGCTCCAAATATCCACTTGCAGATTCTACAAAAAGAGTGTTT

                       *         ***    **         *        *      *       * 
     203053 CAAAACTGCTCTATCAAAAGATCAGTTCGTCTCTGTGAGTTGAATGCATACATCAAAAAGAAGCT
         66 CAAAACTGCTCAATCAAAAGAAAGGTTCAACTCTGTGAGATGAATGCACACATCACAAAGAAGTT

                 *             *      **         *   
     203118 TCTCAAAATGCTTCTGTGTGGTTTTTCGGTGAAGATAGTTC
        131 TCTCAGAATGCTTCTGTGTAGTTTTTATGTGAAGATATTTC

            *     *        *      * *                 *        *        *    
     203159 TTTTTCTACCATAGGTCTCAAACCACTCCAAATATCCACTTGTAGATTCTATAAAAAGGAATG-T
          1 CTTTTCCACCATAGGCCTCAAAGCGCTCCAAATATCCACTTGCAGATTCTACAAAAA-GAGTGTT

                  *        *    *    *      * *         *      *             
     203223 TCAAAATTGCTCAATAAAAATAAAGTTTCAACACCGTGAGATGAGTGCACAAATCACAAAGAAGT
         65 TCAAAACTGCTCAATCAAAAGAAAGGTTCAACTCTGTGAGATGAATGCACACATCACAAAGAAGT

                  *          *         *          *   
     203288 TTCTCAAAATGCTTCTGGGTAGTTTTTCTGTGAAGATAGTTC
        130 TTCTCAGAATGCTTCTGTGTAGTTTTTATGTGAAGATATTTC

                  *     *    *     *          *                         *    
     203330 CTTTTCTACCATGGGCCACAAAGGGCTCCAAATACCCACTTGCAGATTCTACAAAAAGAGAGTTT
          1 CTTTTCCACCATAGGCCTCAAAGCGCTCCAAATATCCACTTGCAGATTCTACAAAAAGAGTGTTT

              *        *      ** * *        *    * *    **  * *           *  
     203395 CACAACTGCTCTATCAAACAATATGTTCAACTTTGTGGGTTGAACACAAATATCAC-AAGAATTT
         66 CAAAACTGCTCAATCAAAAGAAAGGTTCAACTCTGTGAGATGAATGCACACATCACAAAGAAGTT

                **                             *     
     203459 TCTCCCAATGCTTCTGTGTAGTTTTTATGTGAAGACATTTC
        131 TCTCAGAATGCTTCTGTGTAGTTTTTATGTGAAGATATTTC

            *   *  *      *  *     *                 *  *      *       *  *  
     203500 TTTTCCCTCCATAGTCCACAAAGTGCTCCAAATATCCACTTACATATTCTAGAAAAAGATTGCTT
          1 CTTTTCCACCATAGGCCTCAAAGCGCTCCAAATATCCACTTGCAGATTCTACAAAAAGAGTGTTT

            **       *    *               * * *                              
     203565 GGAAACTGCACAATGAAAAGAAAGGTTCAAATATATGAGATGAATGCACACATCACAAAGAAGTT
         66 CAAAACTGCTCAATCAAAAGAAAGGTTCAACTCTGTGAGATGAATGCACACATCACAAAGAAGTT

                                 *                    
     203630 TCTCAGAAT-CTCTCTGTGTAATTTTTATGTGAAGATATTTC
        131 TCTCAGAATGCT-TCTGTGTAGTTTTTATGTGAAGATATTTC

                *     *    *  *   *                        *      *    *     
     203671 CTTTCCCACCTTAGGTCTTAAAACGCTCCAAATATCCACTTGCAGATACTACAAGAAGATTGTTT
          1 CTTTTCCACCATAGGCCTCAAAGCGCTCCAAATATCCACTTGCAGATTCTACAAAAAGAGTGTTT

                     *    *        *      *     **                           
     203736 CAAAACTGCACAA-AAAAAGAAATGTTCAATTCTGTTTGATGAATGCACACATCACAAAGAAGTT
         66 CAAAACTGCTCAATCAAAAGAAAGGTTCAACTCTGTGAGATGAATGCACACATCACAAAGAAGTT

                           *                         
     203800 TCTCAGAATGCTTCTCTGTAGTTTTTATGTGAAGATATTTC
        131 TCTCAGAATGCTTCTGTGTAGTTTTTATGTGAAGATATTTC

                     *             *                 *         **      * *   
     203841 CTTTTCCACAATAGGCCTCAAAGGGCTCCAAATATCCACTTCCAGATTCTATGAAAAGAATATTT
          1 CTTTTCCACCATAGGCCTCAAAGCGCTCCAAATATCCACTTGCAGATTCTACAAAAAGAGTGTTT

             *              * *    *                                      *  
     203906 CCAAACTGCTCAATCATAGGAAATGTTCAACTCTGTGAGATGAATGCACACATCAC-AAGAAATT
         66 CAAAACTGCTCAATCAAAAGAAAGGTTCAACTCTGTGAGATGAATGCACACATCACAAAGAAGTT

                     *    *      *       *       *   
     203970 TCTCAGAATCCTTCAGTGTAGGTTTTATGAGAAGATAATTC
        131 TCTCAGAATGCTTCTGTGTAGTTTTTATGTGAAGATATTTC

                     *    **        *   *                           *    *   
     204011 CTTTTCCACAATAGTTCTCAAAGCACTCAAAATATCCACTTGCAGATTCTACAAAAGGAGTATTT
          1 CTTTTCCACCATAGGCCTCAAAGCGCTCCAAATATCCACTTGCAGATTCTACAAAAAGAGTGTTT

                                                          *                  
     204076 CAAAACTGCTCAATCAAAAGAAAGGTTCAACTCTGTGAGATGAATGGACACATCACAAAGAAGTT
         66 CAAAACTGCTCAATCAAAAGAAAGGTTCAACTCTGTGAGATGAATGCACACATCACAAAGAAGTT

                                  *   *              
     204141 TCTCAGAATGCTTCTGTGTAGTATTTTTGTGAAGATATTT-
        131 TCTCAGAATGCTTCTGTGTAGTTTTTATGTGAAGATATTTC

                          *  * * * * ***             *            *     * *  
     204181 CTTTTCCACCATAGACCGCCAGGGGACACAAATATCCACTTTCAGATTCTACAACAAGAGAGGTT
          1 CTTTTCCACCATAGGCCTCAAAGCGCTCCAAATATCCACTTGCAGATTCTACAAAAAGAGTGTTT

                   *   *     *   ** *       *      *                      * *
     204246 CAAAACTACTCGATCAAGAGATGGTTTCAACTATGTGAGTTGAATGCACACATCACAAAGAACTA
         66 CAAAACTGCTCAATCAAAAGAAAGGTTCAACTCTGTGAGATGAATGCACACATCACAAAGAAGTT

             *  *    *                               
     204311 TGTCGGAATTCTTCTGTGTAGTTTTTATGTGAAGATATTTC
        131 TCTCAGAATGCTTCTGTGTAGTTTTTATGTGAAGATATTTC

                     *    * *      * *    *                  *               
     204352 CTTTTCCACAATAGACGTCAAAGTGATCCAGATATCCACTTGCAGATTCCACAAAAAGAGTGTTT
          1 CTTTTCCACCATAGGCCTCAAAGCGCTCCAAATATCCACTTGCAGATTCTACAAAAAGAGTGTTT

                 *   *   *                  **                     *  *      
     204417 CAAAAGTGCACAACCAAAAGAAAGGTTCAACTAGGTGAGATGAATGCACACATCAGAAGGAAGTT
         66 CAAAACTGCTCAATCAAAAGAAAGGTTCAACTCTGTGAGATGAATGCACACATCACAAAGAAGTT

                            **   *     * *       *   
     204482 TCTCAGAATGCTTCTGCATAGCTTTTAAGGGAAGATACTTC
        131 TCTCAGAATGCTTCTGTGTAGTTTTTATGTGAAGATATTTC

                    *               *            * *  *    * *               
     204523 CTTTTCCAACATAGGCCTCAAAGCACTCCAAATATCCTCCTGGAGATACCACAAAAAGAGTGTTT
          1 CTTTTCCACCATAGGCCTCAAAGCGCTCCAAATATCCACTTGCAGATTCTACAAAAAGAGTGTTT

            **                      *  *                 *       *           
     204588 GCAAACTGCTCAATCAAAAGAAAGATTTAACTCTGTGAGATGAATCCACACATGACAAAGAAGTT
         66 CAAAACTGCTCAATCAAAAGAAAGGTTCAACTCTGTGAGATGAATGCACACATCACAAAGAAGTT

                                                     
     204653 TCTCAGAATGCTTCTGTGTAGTTTTTATGTGAAGATATTTC
        131 TCTCAGAATGCTTCTGTGTAGTTTTTATGTGAAGATATTTC

                     *   * * *    **           *                *     *      
     204694 CTTTTCCACAATAAGACCCAAAAGGCTCCAAATATTCACTTGCAGATTCTAAAAAAAACAGTGTT
          1 CTTTTCCACCATAGGCCTCAAAGCGCTCCAAATATCCACTTGCAGATTCT-ACAAAAAGAGTGTT

                                  *                  *      *        **      
     204759 TCAAAACTGCTCAATCAAAAGATA-GTTCAACTCTGTGAGAAGAATGCTCACATCACTGAGAAGT
         65 TCAAAACTGCTCAATCAAAAGAAAGGTTCAACTCTGTGAGATGAATGCACACATCACAAAGAAGT

                                         *            
     204823 TTCTCAGAATGCTTCTGTGTAGTTTTTATATGAAGATATTTC
        130 TTCTCAGAATGCTTCTGTGTAGTTTTTATGTGAAGATATTTC

                *     *      *    **                       *   **       *    
     204865 CTTTCCCACCGTAGGCCACAAAAGGCTCCAAATATCCACTTGCAGATACTATGAAAAGAGAGTTT
          1 CTTTTCCACCATAGGCCTCAAAGCGCTCCAAATATCCACTTGCAGATTCTACAAAAAGAGTGTTT

                        *        *               ***            *            
     204930 CAAAACTGCTCATTCAAAAGATAGGTTCAACTCTGTGGTTTGAATGCACACAGCACAAAGAAGTT
         66 CAAAACTGCTCAATCAAAAGAAAGGTTCAACTCTGTGAGATGAATGCACACATCACAAAGAAGTT

              *       **                   **   *    
     204995 TCACAGAATGTGTCTGTGTAGTTTTTATGTGCGGATGTTTC
        131 TCTCAGAATGCTTCTGTGTAGTTTTTATGTGAAGATATTTC

                         *        ****   *   *               *    *          
     205036 CTTTTCCACCATATGCCT-AAATATTTCCCAATTTCCACTTGCAGATTCCACAAGAAGAGTGTTT
          1 CTTTTCCACCATAGGCCTCAAAGCGCTCCAAATATCCACTTGCAGATTCTACAAAAAGAGTGTTT

                      **       *       *           *            *      ***   
     205100 CAAAACTGCTGTATC-AAATAAA-GTTGAACTCTGTGAGGTGAATGCACACAGCACAAAATGGTT
         66 CAAAACTGCTCAATCAAAAGAAAGGTTCAACTCTGTGAGATGAATGCACACATCACAAAGAAGTT

                          **   *        *       *    
     205163 TCTCAGAATGCTTCCTTGTTGTTTTTATATGAAGATGTTTC
        131 TCTCAGAATGCTTCTGTGTAGTTTTTATGTGAAGATATTTC

                  *  *             *   *     *                               
     205204 CTTTTCAACAATAGGCCTCAAAGTGCTTCAAATGTCCACTTGCAGATTCTACAAAAAGAGTGTTT
          1 CTTTTCCACCATAGGCCTCAAAGCGCTCCAAATATCCACTTGCAGATTCTACAAAAAGAGTGTTT

                                        *      *  *  *                       
     205269 CAAAACTGCTCAATCAAAAGAAAGGTTCGACTCTGGGAAATTAATGCACACATCACAAAGAAGTT
         66 CAAAACTGCTCAATCAAAAGAAAGGTTCAACTCTGTGAGATGAATGCACACATCACAAAGAAGTT

                                     *        *      
     205334 TCTC---A-GCTTCTGTGTAGTTTTCATGTGAAGTTATTTC
        131 TCTCAGAATGCTTCTGTGTAGTTTTTATGTGAAGATATTTC

                     *       *     *            *    *         **       *    
     205371 CTTTTCCACAATAGGCCGCAAAGGGCTCCAAATATCAACTTACAGATTCTAGGAAAAGAGAGTTT
          1 CTTTTCCACCATAGGCCTCAAAGCGCTCCAAATATCCACTTGCAGATTCTACAAAAAGAGTGTTT

                       * **      *     *                                     
     205436 CAAAACTGCTCTACGAAAAGATAGGTTGAACTCTGTGAGATGAATGCACACATCACAAAGAAGTT
         66 CAAAACTGCTCAATCAAAAGAAAGGTTCAACTCTGTGAGATGAATGCACACATCACAAAGAAGTT

                       *         *     * *           
     205501 TCTCAGAATGCATCTGTGTAGATTTTACGGGAAGATATTTC
        131 TCTCAGAATGCTTCTGTGTAGTTTTTATGTGAAGATATTTC

                        ***  *     **      *  *                  *     ***   
     205542 CTTTTCCACCATCTTCCACAAAGGTCTCCAAGTAACCACTTGCAGATTCTACAGAAAGACACTTT
          1 CTTTTCCACCATAGGCCTCAAAGCGCTCCAAATATCCACTTGCAGATTCTACAAAAAGAGTGTTT

            *          *         ***      *      ***                      *  
     205607 AAAAACTGCTCTATCAAAAGATCAGTTCAAGTCTGTGGTTTGAATGCACACATCACAAAGAATTT
         66 CAAAACTGCTCAATCAAAAGAAAGGTTCAACTCTGTGAGATGAATGCACACATCACAAAGAAGTT

                                     *  *            
     205672 TCTCAGAATGCTTCTGTGTAGTTTTCATATGAAGATATTTC
        131 TCTCAGAATGCTTCTGTGTAGTTTTTATGTGAAGATATTTC

                                    *                                   **   
     205713 CTTTTCCACCATAGGCCTCAAAGCACTCCAAATATCCACTTGCAGATTCTACAAAAAGAGATTTT
          1 CTTTTCCACCATAGGCCTCAAAGCGCTCCAAATATCCACTTGCAGATTCTACAAAAAGAGTGTTT

                   **                           *  *          *        *     
     205778 CAAAACTAGTCAATCAAAAGAAAGGTTCAACTCTGTCAGTTGAATGCACATATCACAAACAAGTT
         66 CAAAACTGCTCAATCAAAAGAAAGGTTCAACTCTGTGAGATGAATGCACACATCACAAAGAAGTT

                *      *                             
     205843 TCTCGGAATGCGTCTGTGTAGTTTTTATGTGAAGATATTTC
        131 TCTCAGAATGCTTCTGTGTAGTTTTTATGTGAAGATATTTC

              **     * *           *     *                  *    *           
     205884 CTCCTCCACAACAGGCCTCAAAGTGCTCCGAATATCCACTTGCAGATTTTAC-TAAAGAGTGTTT
          1 CTTTTCCACCATAGGCCTCAAAGCGCTCCAAATATCCACTTGCAGATTCTACAAAAAGAGTGTTT

             *               *  *   *     *        *    *               *    
     205948 CCAAACTGCTCAATCAAGAGGAAGTTTCAAGTCTGTGAGCTGAACGCACACATCACAAAGTAGTT
         66 CAAAACTGCTCAATCAAAAGAAAGGTTCAACTCTGTGAGATGAATGCACACATCACAAAGAAGTT

               *                                *    
     206013 TCTGAGAATGCTTCTGTGTAGTTTTTATGTGAAGATGTTTC
        131 TCTCAGAATGCTTCTGTGTAGTTTTTATGTGAAGATATTTC

                            **     *                                    *    
     206054 CTTTTCCACCATAGGCTGCAAAGGGCTCCAAATATCCACTTGCAGATTCTACAAAAAGAGAGTTT
          1 CTTTTCCACCATAGGCCTCAAAGCGCTCCAAATATCCACTTGCAGATTCTACAAAAAGAGTGTTT

                 *     *         *          *     *                     *    
     206119 CAAAAGTGCTCTATCAAAAGATAGGTTCAACTATGTGATATGAATGCACACATCACAAAGTAGTT
         66 CAAAACTGCTCAATCAAAAGAAAGGTTCAACTCTGTGAGATGAATGCACACATCACAAAGAAGTT

                                          *          
     206184 TCTCAGAATGCTTCTGTGTAGTTTTTATGTAAAGATATTTC
        131 TCTCAGAATGCTTCTGTGTAGTTTTTATGTGAAGATATTTC

                                    *                                   **   
     206225 CTTTTCCACCATAGGCCTCAAAGCACTCCAAATATCCACTTGCAGATTCTACAAAAAGAGATTTT
          1 CTTTTCCACCATAGGCCTCAAAGCGCTCCAAATATCCACTTGCAGATTCTACAAAAAGAGTGTTT

                   ** *                   *     *  *    * *   *        *     
     206290 CAAAACTATTTAATCAAAAGAAAGGTTCAAATCTGTCAGTTGAAGGTACATATCACAAACAAGTT
         66 CAAAACTGCTCAATCAAAAGAAAGGTTCAACTCTGTGAGATGAATGCACACATCACAAAGAAGTT

             * **                                    
     206355 TATTGGAATGCTTCTGTGTAGTTTTTATGTGAAGATATTTC
        131 TCTCAGAATGCTTCTGTGTAGTTTTTATGTGAAGATATTTC

                     * *         * *                        **  *     *      
     206396 CTTTTCCACAACAGGCCTCAAGGTGCTCCAAATATCCACTTGCAGATTTCACTAAAAGTGTGTTT
          1 CTTTTCCACCATAGGCCTCAAAGCGCTCCAAATATCCACTTGCAGATTCTACAAAAAGAGTGTTT

             *  *            *  *   *     *        *             *           
     206461 CCAAGCTGCTCAATCAAGAGGAAGTTTCAAGTCTGTGAGGTGAATGCACACATTACAAAGAAGTT
         66 CAAAACTGCTCAATCAAAAGAAAGGTTCAACTCTGTGAGATGAATGCACACATCACAAAGAAGTT

            *  *                                     
     206526 ACTGAGAATGCTTCTGTGTAGTTTTTATGTGAAGATATTTC
        131 TCTCAGAATGCTTCTGTGTAGTTTTTATGTGAAGATATTTC

                      **               *                                *    
     206567 CTTTTCCACCGCAGGCCTCAAAGCGCTGCAAATATCCACTTGCAGATTCTACAAAAAGAGAGTTT
          1 CTTTTCCACCATAGGCCTCAAAGCGCTCCAAATATCCACTTGCAGATTCTACAAAAAGAGTGTTT

                      **         *   *         *   *     ***           *     
     206632 CAAAACTGCTGTATCAAAAGATAGGGTCAACTCTGCGAGTTGAATAAGCACATCACAAATAAGTT
         66 CAAAACTGCTCAATCAAAAGAAAGGTTCAACTCTGTGAGATGAATGCACACATCACAAAGAAGTT

               **   *        *               *       
     206697 TCTGGGAACGCTTCTGTATAG-TTTTATGTGAATATATTTC
        131 TCTCAGAATGCTTCTGTGTAGTTTTTATGTGAAGATATTTC

                         *          *                   *   *  *   * *   *   
     206737 CTTTTCCACCATATGCCTCAAAGCACTCCAAATATCCACTTGCACATTATAGAAACATAGTCTTT
          1 CTTTTCCACCATAGGCCTCAAAGCGCTCCAAATATCCACTTGCAGATTCTACAAAAAGAGTGTTT

                   **                        *         *             *       
     206802 CAAAACTTGTCAATC-AAAGAAAGGTTCAACTCCGTGAGATGAGTGCACACATCACAGAGAAGTT
         66 CAAAACTGCTCAATCAAAAGAAAGGTTCAACTCTGTGAGATGAATGCACACATCACAAAGAAGTT

                *     *                            * 
     206866 TCTCGGAATGTTTCTGTGTAGTTTTTATGTGAAGATATTGC
        131 TCTCAGAATGCTTCTGTGTAGTTTTTATGTGAAGATATTTC

                     *               *            *          *       *   *   
     206907 CTTTTCCACAATAGGCCTCAAAGCGTTCCAAATATCCAATTGCAGATTCCACAAAAAAAGTTTTT
          1 CTTTTCCACCATAGGCCTCAAAGCGCTCCAAATATCCACTTGCAGATTCTACAAAAAGAGTGTTT

            *                 *  *  *  *             * *         **    *     
     206972 TAAAACTGCTCAATCAAATGATAGATTAAACTCTGTGAGATTAGTGCACACATGTCAAAAAAGTT
         66 CAAAACTGCTCAATCAAAAGAAAGGTTCAACTCTGTGAGATGAATGCACACATCACAAAGAAGTT

                                *      * *           
     207037 TCTCAGAATGCTTCTGTGTACTTTTTAGGGGAAGATATTTC
        131 TCTCAGAATGCTTCTGTGTAGTTTTTATGTGAAGATATTTC

                        *    *     **         *    **          **  * *  *    
     207078 CTTTTCCACCATCGGCCACAAAGGACTCCAAATAACCACACGCAGATTCTAGTAACACAGAGTTT
          1 CTTTTCCACCATAGGCCTCAAAGCGCTCCAAATATCCACTTGCAGATTCTACAAAAAGAGTGTTT

                       *         * *           *   * * *    **    ***        
     207143 CAAAACTGCTCTATCAAAAGATAAGTTCAACTCTGAGAGTTTAGTGCAACCATCGTGAAGAAGTT
         66 CAAAACTGCTCAATCAAAAGAAAGGTTCAACTCTGTGAGATGAATGCACACATCACAAAGAAGTT

                            *     *                  
     207208 TCTCAGAATGCTTCTGAGTAGTGTTTATGTGAAGATATTTC
        131 TCTCAGAATGCTTCTGTGTAGTTTTTATGTGAAGATATTTC

                              *     *                      *           **    
     207249 CTTTTCCACCATAGGCCTGAAAGCCCTCCAAATATCCACTTGCAGATCCTACAAAAAGAAAGTTT
          1 CTTTTCCACCATAGGCCTCAAAGCGCTCCAAATATCCACTTGCAGATTCTACAAAAAGAGTGTTT

             *         **     *  *  *   *        **      *                   
     207314 CGAAA-TGCTCTCTCAAACGATAGTTTCGACTCTGTGGTATGAATACACACATCACAAAGAAGTT
         66 CAAAACTGCTCAATCAAAAGAAAGGTTCAACTCTGTGAGATGAATGCACACATCACAAAGAAGTT

                                       **            
     207378 TCTCAGAATGCTTCTGTGTAGTTTTTAAATGAAGATATTTC
        131 TCTCAGAATGCTTCTGTGTAGTTTTTATGTGAAGATATTTC

            *                       *          *     *                       
     207419 TTTTTCCACCATAGGCCTCAAAGCACTCCAAATATGCACTTCCAGATTCTACAAAAAGAGTGTTT
          1 CTTTTCCACCATAGGCCTCAAAGCGCTCCAAATATCCACTTGCAGATTCTACAAAAAGAGTGTTT

              *                 *       * *         **   *         *  * *    
     207484 CAGAACTGCTCAATCAAAAGGAAGGTTCCAGTC--TGAGACAAATACACACATCAAAAGGTAGTT
         66 CAAAACTGCTCAATCAAAAGAAAGGTTCAACTCTGTGAGATGAATGCACACATCACAAAGAAGTT

                                                    *
     207547 TCTCAGAATGCTTCTGTGTAGTTTTTATGTGAAGATATTTT
        131 TCTCAGAATGCTTCTGTGTAGTTTTTATGTGAAGATATTTC

             *               *    **    *     *      *  **   *          *    
     207588 CCTTTCCACCATAGGCCACAAATGGCTCTAAATACCCACTTACATTTTCCACAAAAAGAGAGTTT
          1 CTTTTCCACCATAGGCCTCAAAGCGCTCCAAATATCCACTTGCAGATTCTACAAAAAGAGTGTTT

                       * *    * *      *   *         *                       
     207653 CAAAACTGCTCTACCAAAGGTAA-GTTTAACGCTGTGAG-TTAA-G-A-ACATCACAAAGAAGTT
         66 CAAAACTGCTCAATCAAAAGAAAGGTTCAACTCTGTGAGATGAATGCACACATCACAAAGAAGTT

                                   *   *  *          
     207713 TCTCAGAATGCTTCTGTGTAGTTCTTACGTAAAGATATTTC
        131 TCTCAGAATGCTTCTGTGTAGTTTTTATGTGAAGATATTTC

                 *   *      ***    *                  *              **      
     207754 CTTTTACACAATAGGCAGAAAAGTGCTCCAAATATCCACTTGAAGATTCTACAAAAACCGTGTTT
          1 CTTTTCCACCATAGGCCTCAAAGCGCTCCAAATATCCACTTGCAGATTCTACAAAAAGAGTGTTT

                     **                                          *      *    
     207819 CAAAACTGCCGAATCAAAAGAAAGGTTCAACTCTGTGAGATGAATGCACACATAACAAAGGAGTT
         66 CAAAACTGCTCAATCAAAAGAAAGGTTCAACTCTGTGAGATGAATGCACACATCACAAAGAAGTT

                                 *      *      *    *
     207884 TCTCAGAATGCTTCTGTGTAGCTTTTATATGAAGACATTTA
        131 TCTCAGAATGCTTCTGTGTAGTTTTTATGTGAAGATATTTC

            *        * *            *   ***                     **        *  
     207925 GTTTTCCACAACAGGCCTCAAAGCTCTCTCCATATCCACTTGCAGATTCTACCGAAAGAGTGCTT
          1 CTTTTCCACCATAGGCCTCAAAGCGCTCCAAATATCCACTTGCAGATTCTACAAAAAGAGTGTTT

             *                   * **     *        *        *     **         
     207990 CCAAACTGCTCAATCAAAAGAGACATTCAAATCTGTGAGGTGAATGCAGACATCGTAAAGAAGTT
         66 CAAAACTGCTCAATCAAAAGAAAGGTTCAACTCTGTGAGATGAATGCACACATCACAAAGAAGTT

                                *     *       *    **
     208055 TCTCAGAATGCTTCTGTGTATTTTTTGTGTGAAGTTATTCG
        131 TCTCAGAATGCTTCTGTGTAGTTTTTATGTGAAGATATTTC

            *    *             *     *  *            **                 *    
     208096 TTTTTGCACCATAGGCCTCCAAGCGTTCTAAATATCCACTTCTAGATTCTACAAAAAGAGAGTTT
          1 CTTTTCCACCATAGGCCTCAAAGCGCTCCAAATATCCACTTGCAGATTCTACAAAAAGAGTGTTT

                   *         *            *        *    *   *                
     208161 CAAAACTACTC-A--AACA-AAAGGTTCAATTCTGTGAGTTGAAAGCAAACATCACAAAGAAGTT
         66 CAAAACTGCTCAATCAAAAGAAAGGTTCAACTCTGTGAGATGAATGCACACATCACAAAGAAGTT

                       *             *               
     208222 TCTCAGAATGCGTCTGTGTAGTTTTGATGTGAAGATATTTC
        131 TCTCAGAATGCTTCTGTGTAGTTTTTATGTGAAGATATTTC

                     **   ****     *                  *                      
     208263 CTTTT-CACAGTAGAATGCAAAGGGCTCCAAATATCCACTTGGAGATTCTACAAAAAGA--GTTT
          1 CTTTTCCACCATAGGCCTCAAAGCGCTCCAAATATCCACTTGCAGATTCTACAAAAAGAGTGTTT

                  *    **     *  *     *     ***   *     *               *   
     208325 CAAAACCGCTCTGTCAAATGATAGGTTGAACTCCCGGAGGTGAATACACACATCACAAAGAGGTT
         66 CAAAACTGCTCAATCAAAAGAAAGGTTCAACTCTGTGAGATGAATGCACACATCACAAAGAAGTT

                  *                       *  *       
     208390 TCTCAGCATGCTTCTGTGTAGTTTTTATGTAAACATATTTC
        131 TCTCAGAATGCTTCTGTGTAGTTTTTATGTGAAGATATTTC

             *    * *              *           *      * *       *   *    *   
     208431 CGTTTCTATCATAGGCCTCAAAGTGCTCCAAATATTCACTTGTACATTCTACCAAACGAGTATTT
          1 CTTTTCCACCATAGGCCTCAAAGCGCTCCAAATATCCACTTGCAGATTCTACAAAAAGAGTGTTT

                              * *         ** *    *        *            *    
     208496 CAAAACTGCTCAATCAAATGGAAGGTTCAAAACCGTGACATGAATGCCCACATCACAAAGTAGTT
         66 CAAAACTGCTCAATCAAAAGAAAGGTTCAACTCTGTGAGATGAATGCACACATCACAAAGAAGTT

                                                     
     208561 TCTCAGAATGCTTCTGTGTAGTTTTTATGTGAAGATATTTC
        131 TCTCAGAATGCTTCTGTGTAGTTTTTATGTGAAGATATTTC

                     * *    *      *    *       * *   **      *              
     208602 CTTTTCCACAACA-GCGTGCAAAACGCTTCAAATATGCCCTTAGAGATTCCACAAAAAGAGTGTT
          1 CTTTTCCACCATAGGCCT-CAAAGCGCTCCAAATATCCACTTGCAGATTCTACAAAAAGAGTGTT

              *     *               *  *                                  ** 
     208666 TCCAAACTACTCAAATCAAAA-AATGATTTCAACTCTGTGAGATGAATGCACACATCACAAACTA
         65 TCAAAACTGCTC-AATCAAAAGAAAG-GTTCAACTCTGTGAGATGAATGCACACATCACAAAGAA

                         *     ** *   * *   *       *   
     208730 GTTTCTCAGAATGTTTCTGCCTGGTTCTCATGCGAAGATAGTTC
        128 GTTTCTCAGAATGCTTCTGTGTAGTTTTTATGTGAAGATATTTC

                 *           *   * **              *                  * *    
     208774 CTTTTTCACCATAGGCCGCAATGTACTCCAAATATCCACCTGCAGATTCTAC-AAAAGTGAGTTT
          1 CTTTTCCACCATAGGCCTCAAAGCGCTCCAAATATCCACTTGCAGATTCTACAAAAAGAGTGTTT

                       *         ***    **         *        *      *       * 
     208838 CAAAACTGCTCTATCAAAAGATCAGTTCGTCTCTGTGAGTTGAATGCATACATCAAAAAGAAGCT
         66 CAAAACTGCTCAATCAAAAGAAAGGTTCAACTCTGTGAGATGAATGCACACATCACAAAGAAGTT

                 *             *      **         *   
     208903 TCTCAAAATGCTTCTGTGTGGTTTTTCGGTGAAGATAGTTC
        131 TCTCAGAATGCTTCTGTGTAGTTTTTATGTGAAGATATTTC

            *     *        *      * *                 *        *        *    
     208944 TTTTTCTACCATAGGTCTCAAACCACTCCAAATATCCACTTGTAGATTCTATAAAAAGGAATG-T
          1 CTTTTCCACCATAGGCCTCAAAGCGCTCCAAATATCCACTTGCAGATTCTACAAAAA-GAGTGTT

                  *        *    *    *      * *         *      *         *   
     209008 TCAAAATTGCTCAATAAAAATAAAGTTTCAACACCGTGAGATGAGTGCACAAATCACAAAGGAGT
         65 TCAAAACTGCTCAATCAAAAGAAAGGTTCAACTCTGTGAGATGAATGCACACATCACAAAGAAGT

                  *          *         *          *   
     209073 TTCTCAAAATGCTTCTGGGTAGTTTTTCTGTGAAGATAGTTC
        130 TTCTCAGAATGCTTCTGTGTAGTTTTTATGTGAAGATATTTC

                  *     *    *     *          *                         *    
     209115 CTTTTCTACCATGGGCCACAAAGGGCTCCAAATACCCACTTGCAGATTCTACAAAAAGAGAGTTT
          1 CTTTTCCACCATAGGCCTCAAAGCGCTCCAAATATCCACTTGCAGATTCTACAAAAAGAGTGTTT

              *        *      ** * *        *    * *    **  * *           *  
     209180 CACAACTGCTCTATCAAACAATATGTTCAACTTTGTGGGTTGAACACAAATATCAC-AAGAATTT
         66 CAAAACTGCTCAATCAAAAGAAAGGTTCAACTCTGTGAGATGAATGCACACATCACAAAGAAGTT

                **                             *     
     209244 TCTCCCAATGCTTCTGTGTAGTTTTTATGTGAAGACATTTC
        131 TCTCAGAATGCTTCTGTGTAGTTTTTATGTGAAGATATTTC

            *   *  *      *  *     *                 *  *      *       *  *  
     209285 TTTTCCCTCCATAGTCCACAAAGTGCTCCAAATATCCACTTACATATTCTAGAAAAAGATTGCTT
          1 CTTTTCCACCATAGGCCTCAAAGCGCTCCAAATATCCACTTGCAGATTCTACAAAAAGAGTGTTT

            **       *    *               * * *                              
     209350 GGAAACTGCACAATGAAAAGAAAGGTTCAAATATATGAGATGAATGCACACATCACAAAGAAGTT
         66 CAAAACTGCTCAATCAAAAGAAAGGTTCAACTCTGTGAGATGAATGCACACATCACAAAGAAGTT

                                 *                    
     209415 TCTCAGAAT-CTCTCTGTGTAATTTTTATGTGAAGATATTTC
        131 TCTCAGAATGCT-TCTGTGTAGTTTTTATGTGAAGATATTTC

                *     *    *  *   *                        *      *    *     
     209456 CTTTCCCACCTTAGGTCTTAAAACGCTCCAAATATCCACTTGCAGATACTACAAGAAGATTGTTT
          1 CTTTTCCACCATAGGCCTCAAAGCGCTCCAAATATCCACTTGCAGATTCTACAAAAAGAGTGTTT

                     *    *        *      *     **                           
     209521 CAAAACTGCACAA-AAAAAGAAATGTTCAATTCTGTTTGATGAATGCACACATCACAAAGAAGTT
         66 CAAAACTGCTCAATCAAAAGAAAGGTTCAACTCTGTGAGATGAATGCACACATCACAAAGAAGTT

                           *                         
     209585 TCTCAGAATGCTTCTCTGTAGTTTTTATGTGAAGATATTTC
        131 TCTCAGAATGCTTCTGTGTAGTTTTTATGTGAAGATATTTC

                     *             *                 *         **      * *   
     209626 CTTTTCCACAATAGGCCTCAAAGGGCTCCAAATATCCACTTCCAGATTCTATGAAAAGAATATTT
          1 CTTTTCCACCATAGGCCTCAAAGCGCTCCAAATATCCACTTGCAGATTCTACAAAAAGAGTGTTT

             *              * *    *                                      *  
     209691 CCAAACTGCTCAATCATAGGAAATGTTCAACTCTGTGAGATGAATGCACACATCAC-AAGAAATT
         66 CAAAACTGCTCAATCAAAAGAAAGGTTCAACTCTGTGAGATGAATGCACACATCACAAAGAAGTT

                     *    *      *       *       *   
     209755 TCTCAGAATCCTTCAGTGTAGGTTTTATGAGAAGATAATTC
        131 TCTCAGAATGCTTCTGTGTAGTTTTTATGTGAAGATATTTC

                     *    **        *   *                           *    *   
     209796 CTTTTCCACAATAGTTCTCAAAGCACTCAAAATATCCACTTGCAGATTCTACAAAAGGAGTATTT
          1 CTTTTCCACCATAGGCCTCAAAGCGCTCCAAATATCCACTTGCAGATTCTACAAAAAGAGTGTTT

                                                 *        *                  
     209861 CAAAACTGCTCAATCAAAAGAAAGGTTCAACTCTGTGGGATGAATGGACACATCACAAAGAAGTT
         66 CAAAACTGCTCAATCAAAAGAAAGGTTCAACTCTGTGAGATGAATGCACACATCACAAAGAAGTT

                                  *   *              
     209926 TCTCAGAATGCTTCTGTGTAGTATTTTTGTGAAGATATTT-
        131 TCTCAGAATGCTTCTGTGTAGTTTTTATGTGAAGATATTTC

                          *  * * * * ***             *            *     * *  
     209966 CTTTTCCACCATAGACCGCCAGGGGACACAAATATCCACTTTCAGATTCTACAACAAGAGAGGTT
          1 CTTTTCCACCATAGGCCTCAAAGCGCTCCAAATATCCACTTGCAGATTCTACAAAAAGAGTGTTT

                   *   *     *   ** *       *      *                      * *
     210031 CAAAACTACTCGATCAAGAGATGGTTTCAACTATGTGAGTTGAATGCACACATCACAAAGAACTA
         66 CAAAACTGCTCAATCAAAAGAAAGGTTCAACTCTGTGAGATGAATGCACACATCACAAAGAAGTT

             *  *    *                               
     210096 TGTCGGAATTCTTCTGTGTAGTTTTTATGTGAAGATATTTC
        131 TCTCAGAATGCTTCTGTGTAGTTTTTATGTGAAGATATTTC

                     *    * *      * *    *                  *               
     210137 CTTTTCCACAATAGACGTCAAAGTGATCCAGATATCCACTTGCAGATTCCACAAAAAGAGTGTTT
          1 CTTTTCCACCATAGGCCTCAAAGCGCTCCAAATATCCACTTGCAGATTCTACAAAAAGAGTGTTT

                 *   *   *                  **                     *  *      
     210202 CAAAAGTGCACAACCAAAAGAAAGGTTCAACTAGGTGAGATGAATGCACACATCAGAAGGAAGTT
         66 CAAAACTGCTCAATCAAAAGAAAGGTTCAACTCTGTGAGATGAATGCACACATCACAAAGAAGTT

                            **   *     * *       *   
     210267 TCTCAGAATGCTTCTGCATAGCTTTTAAGGGAAGATACTTC
        131 TCTCAGAATGCTTCTGTGTAGTTTTTATGTGAAGATATTTC

                    *               *            * *  *    * *               
     210308 CTTTTCCAACATAGGCCTCAAAGCACTCCAAATATCCTCCTGGAGATACCACAAAAAGAGTGTTT
          1 CTTTTCCACCATAGGCCTCAAAGCGCTCCAAATATCCACTTGCAGATTCTACAAAAAGAGTGTTT

            **                      *  *                 *       *           
     210373 GCAAACTGCTCAATCAAAAGAAAGATTTAACTCTGTGAGATGAATCCACACATGACAAAGAAGTT
         66 CAAAACTGCTCAATCAAAAGAAAGGTTCAACTCTGTGAGATGAATGCACACATCACAAAGAAGTT

                                                     
     210438 TCTCAGAATGCTTCTGTGTAGTTTTTATGTGAAGATATTTC
        131 TCTCAGAATGCTTCTGTGTAGTTTTTATGTGAAGATATTTC

                     *   * * *    **           *                *     *      
     210479 CTTTTCCACAATAAGACCCAAAAGGCTCCAAATATTCACTTGCAGATTCTAAAAAAAACAGTGTT
          1 CTTTTCCACCATAGGCCTCAAAGCGCTCCAAATATCCACTTGCAGATTCT-ACAAAAAGAGTGTT

                                  *                  *      *        **      
     210544 TCAAAACTGCTCAATCAAAAGATA-GTTCAACTCTGTGAGAAGAATGCTCACATCACTGAGAAGT
         65 TCAAAACTGCTCAATCAAAAGAAAGGTTCAACTCTGTGAGATGAATGCACACATCACAAAGAAGT

                                         *            
     210608 TTCTCAGAATGCTTCTGTGTAGTTTTTATATGAAGATATTTC
        130 TTCTCAGAATGCTTCTGTGTAGTTTTTATGTGAAGATATTTC

                *     *      *    **                       *   **       *    
     210650 CTTTCCCACCGTAGGCCACAAAAGGCTCCAAATATCCACTTGCAGATACTATGAAAAGAGAGTTT
          1 CTTTTCCACCATAGGCCTCAAAGCGCTCCAAATATCCACTTGCAGATTCTACAAAAAGAGTGTTT

                        *        *               ***            *            
     210715 CAAAACTGCTCATTCAAAAGATAGGTTCAACTCTGTGGTTTGAATGCACACAGCACAAAGAAGTT
         66 CAAAACTGCTCAATCAAAAGAAAGGTTCAACTCTGTGAGATGAATGCACACATCACAAAGAAGTT

              *       **                   **   *    
     210780 TCACAGAATGTGTCTGTGTAGTTTTTATGTGCGGATGTTTC
        131 TCTCAGAATGCTTCTGTGTAGTTTTTATGTGAAGATATTTC

                         *        ****   *   *               *    *          
     210821 CTTTTCCACCATATGCCT-AAATATTTCCCAATTTCCACTTGCAGATTCCACAAGAAGAGTGTTT
          1 CTTTTCCACCATAGGCCTCAAAGCGCTCCAAATATCCACTTGCAGATTCTACAAAAAGAGTGTTT

                      **       *       *           *            *      ***   
     210885 CAAAACTGCTGTATC-AAATAAA-GTTGAACTCTGTGAGGTGAATGCACACAGCACAAAATGGTT
         66 CAAAACTGCTCAATCAAAAGAAAGGTTCAACTCTGTGAGATGAATGCACACATCACAAAGAAGTT

                          **   *        *       *    
     210948 TCTCAGAATGCTTCCTTGTTGTTTTTATATGAAGATGTTTC
        131 TCTCAGAATGCTTCTGTGTAGTTTTTATGTGAAGATATTTC

                  *  *             *   *     *                               
     210989 CTTTTCAACAATAGGCCTCAAAGTGCTTCAAATGTCCACTTGCAGATTCTACAAAAAGAGTGTTT
          1 CTTTTCCACCATAGGCCTCAAAGCGCTCCAAATATCCACTTGCAGATTCTACAAAAAGAGTGTTT

                                        *      *  *  *                       
     211054 CAAAACTGCTCAATCAAAAGAAAGGTTCGACTCTGGGAAATTAATGCACACATCACAAAGAAGTT
         66 CAAAACTGCTCAATCAAAAGAAAGGTTCAACTCTGTGAGATGAATGCACACATCACAAAGAAGTT

                                     *        *      
     211119 TCTC---A-GCTTCTGTGTAGTTTTCATGTGAAGTTATTTC
        131 TCTCAGAATGCTTCTGTGTAGTTTTTATGTGAAGATATTTC

                     *       *     *            *    *         **       *    
     211156 CTTTTCCACAATAGGCCGCAAAGGGCTCCAAATATCAACTTACAGATTCTAGGAAAAGAGAGTTT
          1 CTTTTCCACCATAGGCCTCAAAGCGCTCCAAATATCCACTTGCAGATTCTACAAAAAGAGTGTTT

                       * **      *     *                                     
     211221 CAAAACTGCTCTACGAAAAGATAGGTTGAACTCTGTGAGATGAATGCACACATCACAAAGAAGTT
         66 CAAAACTGCTCAATCAAAAGAAAGGTTCAACTCTGTGAGATGAATGCACACATCACAAAGAAGTT

                       *               * *           
     211286 TCTCAGAATGCATCTGTGTAGTTTTTACGGGAAGATATTTC
        131 TCTCAGAATGCTTCTGTGTAGTTTTTATGTGAAGATATTTC

                        ***  *     **      *  *           *      *     ***   
     211327 CTTTTCCACCATCTTCCACAAAGGTCTCCAAGTAACCACTTGCAGACTCTACAGAAAGACACTTT
          1 CTTTTCCACCATAGGCCTCAAAGCGCTCCAAATATCCACTTGCAGATTCTACAAAAAGAGTGTTT

            *          *         ***      *      ***                      *  
     211392 AAAAACTGCTCTATCAAAAGATCAGTTCAAGTCTGTGGTTTGAATGCACACATCACAAAGAATTT
         66 CAAAACTGCTCAATCAAAAGAAAGGTTCAACTCTGTGAGATGAATGCACACATCACAAAGAAGTT

                                     *  *            
     211457 TCTCAGAATGCTTCTGTGTAGTTTTCATATGAAGATATTTC
        131 TCTCAGAATGCTTCTGTGTAGTTTTTATGTGAAGATATTTC

                                    *                                   **   
     211498 CTTTTCCACCATAGGCCTCAAAGCACTCCAAATATCCACTTGCAGATTCTACAAAAAGAGATTTT
          1 CTTTTCCACCATAGGCCTCAAAGCGCTCCAAATATCCACTTGCAGATTCTACAAAAAGAGTGTTT

                   **                           *  *          *        *     
     211563 CAAAACTAGTCAATCAAAAGAAAGGTTCAACTCTGTCAGTTGAATGCACATATCACAAACAAGTT
         66 CAAAACTGCTCAATCAAAAGAAAGGTTCAACTCTGTGAGATGAATGCACACATCACAAAGAAGTT

                *      *                             
     211628 TCTCGGAATGCGTCTGTGTAGTTTTTATGTGAAGATATTTC
        131 TCTCAGAATGCTTCTGTGTAGTTTTTATGTGAAGATATTTC

               *     * *           *     *                  *    *           
     211669 CTTCTCCACAACAGGCCTCAAAGTGCTCCGAATATCCACTTGCAGATTTTAC-TAAAGAGTGTTT
          1 CTTTTCCACCATAGGCCTCAAAGCGCTCCAAATATCCACTTGCAGATTCTACAAAAAGAGTGTTT

             *               *  *   *     *        *    *               *    
     211733 CCAAACTGCTCAATCAAGAGGAAGTTTCAAGTCTGTGAGCTGAACGCACACATCACAAAGTAGTT
         66 CAAAACTGCTCAATCAAAAGAAAGGTTCAACTCTGTGAGATGAATGCACACATCACAAAGAAGTT

               *                                *    
     211798 TCTGAGAATGCTTCTGTGTAGTTTTTATGTGAAGATGTTTC
        131 TCTCAGAATGCTTCTGTGTAGTTTTTATGTGAAGATATTTC

                            **     *                                    *    
     211839 CTTTTCCACCATAGGCTGCAAAGGGCTCCAAATATCCACTTGCAGATTCTACAAAAAGAGAGTTT
          1 CTTTTCCACCATAGGCCTCAAAGCGCTCCAAATATCCACTTGCAGATTCTACAAAAAGAGTGTTT

                 *     *         *          *     *                     *    
     211904 CAAAAGTGCTCTATCAAAAGATAGGTTCAACTATGTGATATGAATGCACACATCACAAAGTAGTT
         66 CAAAACTGCTCAATCAAAAGAAAGGTTCAACTCTGTGAGATGAATGCACACATCACAAAGAAGTT

                                          *          
     211969 TCTCAGAATGCTTCTGTGTAGTTTTTATGTAAAGATATTTC
        131 TCTCAGAATGCTTCTGTGTAGTTTTTATGTGAAGATATTTC

                                    *                                   **   
     212010 CTTTTCCACCATAGGCCTCAAAGCACTCCAAATATCCACTTGCAGATTCTACAAAAAGAGATTTT
          1 CTTTTCCACCATAGGCCTCAAAGCGCTCCAAATATCCACTTGCAGATTCTACAAAAAGAGTGTTT

                   ** *                   *     *  *    * *   *        *     
     212075 CAAAACTATTTAATCAAAAGAAAGGTTCAAATCTGTCAGTTGAAGGTACATATCACAAATAAGTT
         66 CAAAACTGCTCAATCAAAAGAAAGGTTCAACTCTGTGAGATGAATGCACACATCACAAAGAAGTT

             * **                                    
     212140 TATTGGAATGCTTCTGTGTAGTTTTTATGTGAAGATATTTC
        131 TCTCAGAATGCTTCTGTGTAGTTTTTATGTGAAGATATTTC

                     * *         * *                        **  *     *      
     212181 CTTTTCCACAACAGGCCTCAAGGTGCTCCAAATATCCACTTGCAGATTTCACTAAAAGTGTGTTT
          1 CTTTTCCACCATAGGCCTCAAAGCGCTCCAAATATCCACTTGCAGATTCTACAAAAAGAGTGTTT

             *  *            *  *   *     *        *             *           
     212246 CCAAGCTGCTCAATCAAGAGGAAGTTTCAAGTCTGTGAGGTGAATGCACACATTACAAAGAAGTT
         66 CAAAACTGCTCAATCAAAAGAAAGGTTCAACTCTGTGAGATGAATGCACACATCACAAAGAAGTT

            *  *                                     
     212311 ACTGAGAATGCTTCTGTGTAGTTTTTATGTGAAGATATTTC
        131 TCTCAGAATGCTTCTGTGTAGTTTTTATGTGAAGATATTTC

                       *               *                                *    
     212352 CTTTTCCACCACAGGCCTCAAAGCGCTGCAAATATCCACTTGCAGATTCTACAAAAAGAGAGTTT
          1 CTTTTCCACCATAGGCCTCAAAGCGCTCCAAATATCCACTTGCAGATTCTACAAAAAGAGTGTTT

                      **         *   *         *   *     **            *     
     212417 CAAAACTGCTGTATCAAAAGATAGGGTCAACTCTGCGAGTTGAATAAACACATCACAAATAAGTT
         66 CAAAACTGCTCAATCAAAAGAAAGGTTCAACTCTGTGAGATGAATGCACACATCACAAAGAAGTT

               **   *        *               *       
     212482 TCTGGGAACGCTTCTGTATAG-TTTTATGTGAATATATTTC
        131 TCTCAGAATGCTTCTGTGTAGTTTTTATGTGAAGATATTTC

                         *          *                   *   *  *   * *   *   
     212522 CTTTTCCACCATATGCCTCAAAGCACTCCAAATATCCACTTGCACATTATAGAAACATAGTCTTT
          1 CTTTTCCACCATAGGCCTCAAAGCGCTCCAAATATCCACTTGCAGATTCTACAAAAAGAGTGTTT

                   **                        *         *             *       
     212587 CAAAACTTGTCAATC-AAAGAAAGGTTCAACTCCGTGAGATGAGTGCACACATCACAGAGAAGTT
         66 CAAAACTGCTCAATCAAAAGAAAGGTTCAACTCTGTGAGATGAATGCACACATCACAAAGAAGTT

                *     *                            * 
     212651 TCTCGGAATGTTTCTGTGTAGTTTTTATGTGAAGATATTGC
        131 TCTCAGAATGCTTCTGTGTAGTTTTTATGTGAAGATATTTC

                     *               *            *          *       *   *   
     212692 CTTTTCCACAATAGGCCTCAAAGCGTTCCAAATATCCAATTGCAGATTCCACAAAAAAAGTTTTT
          1 CTTTTCCACCATAGGCCTCAAAGCGCTCCAAATATCCACTTGCAGATTCTACAAAAAGAGTGTTT

            *                 *  *  *  *             * *         **    *     
     212757 TAAAACTGCTCAATCAAATGATAGATTAAACTCTGTGAGATTAGTGCACACATGTCAAAAAAGTT
         66 CAAAACTGCTCAATCAAAAGAAAGGTTCAACTCTGTGAGATGAATGCACACATCACAAAGAAGTT

                                *      * *           
     212822 TCTCAGAATGCTTCTGTGTACTTTTTAGGGGAAGATATTTC
        131 TCTCAGAATGCTTCTGTGTAGTTTTTATGTGAAGATATTTC

                        *    *     **         *    *           **  * *  *    
     212863 CTTTTCCACCATCGGCCACAAAGGACTCCAAATAACCACATGCAGATTCTAGTAACACAGAGTTT
          1 CTTTTCCACCATAGGCCTCAAAGCGCTCCAAATATCCACTTGCAGATTCTACAAAAAGAGTGTTT

                       *         * *           *   * * *    **    ***        
     212928 CAAAACTGCTCTATCAAAAGATAAGTTCAACTCTGAGAGTTTAGTGCAACCATCGTGAAGAAGTT
         66 CAAAACTGCTCAATCAAAAGAAAGGTTCAACTCTGTGAGATGAATGCACACATCACAAAGAAGTT

                            *     *                  
     212993 TCTCAGAATGCTTCTGAGTAGTGTTTATGTGAAGATATTTC
        131 TCTCAGAATGCTTCTGTGTAGTTTTTATGTGAAGATATTTC

                              *     *                      *           **    
     213034 CTTTTCCACCATAGGCCTGAAAGCCCTCCAAATATCCACTTGCAGATCCTACAAAAAGAAAGTTT
          1 CTTTTCCACCATAGGCCTCAAAGCGCTCCAAATATCCACTTGCAGATTCTACAAAAAGAGTGTTT

             *         **     *  *  *   *        **      *                   
     213099 CGAAA-TGCTCTCTCAAACGATAGTTTCGACTCTGTGGTATGAATACACACATCACAAAGAAGTT
         66 CAAAACTGCTCAATCAAAAGAAAGGTTCAACTCTGTGAGATGAATGCACACATCACAAAGAAGTT

                                       **            
     213163 TCTCAGAATGCTTCTGTGTAGTTTTTAAATGAAGATATTTC
        131 TCTCAGAATGCTTCTGTGTAGTTTTTATGTGAAGATATTTC

            *                       *          *     *                       
     213204 TTTTTCCACCATAGGCCTCAAAGCACTCCAAATATGCACTTCCAGATTCTACAAAAAGAGTGTTT
          1 CTTTTCCACCATAGGCCTCAAAGCGCTCCAAATATCCACTTGCAGATTCTACAAAAAGAGTGTTT

              *                 *       * *         **   *         *  * *    
     213269 CAGAACTGCTCAATCAAAAGGAAGGTTCCAGTC--TGAGACAAATACACACATCAAAAGGTAGTT
         66 CAAAACTGCTCAATCAAAAGAAAGGTTCAACTCTGTGAGATGAATGCACACATCACAAAGAAGTT

                                                    *
     213332 TCTCAGAATGCTTCTGTGTAGTTTTTATGTGAAGATATTTT
        131 TCTCAGAATGCTTCTGTGTAGTTTTTATGTGAAGATATTTC

             *               *    **    *     *      *  **   *          *    
     213373 CCTTTCCACCATAGGCCACAAATGGCTCTAAATACCCACTTACATTTTCCACAAAAAGAGAGTTT
          1 CTTTTCCACCATAGGCCTCAAAGCGCTCCAAATATCCACTTGCAGATTCTACAAAAAGAGTGTTT

                       * *    * *      *   *         *                       
     213438 CAAAACTGCTCTACCAAAGGTAA-GTTTAACGCTGTGAG-TTAA-G-A-ACATCACAAAGAAGTT
         66 CAAAACTGCTCAATCAAAAGAAAGGTTCAACTCTGTGAGATGAATGCACACATCACAAAGAAGTT

                                   *   *  *          
     213498 TCTCAGAATGCTTCTGTGTAGTTCTTACGTAAAGATATTTC
        131 TCTCAGAATGCTTCTGTGTAGTTTTTATGTGAAGATATTTC

                 *   *      ***    *                  *              **      
     213539 CTTTTACACAATAGGCAGAAAAGTGCTCCAAATATCCACTTGAAGATTCTACAAAAACCGTGTTT
          1 CTTTTCCACCATAGGCCTCAAAGCGCTCCAAATATCCACTTGCAGATTCTACAAAAAGAGTGTTT

                     **                                          *      *    
     213604 CAAAACTGCCGAATCAAAAGAAAGGTTCAACTCTGTGAGATGAATGCACACATAACAAAGGAGTT
         66 CAAAACTGCTCAATCAAAAGAAAGGTTCAACTCTGTGAGATGAATGCACACATCACAAAGAAGTT

                                 *      *      *    *
     213669 TCTCAGAATGCTTCTGTGTAGCTTTTATATGAAGACATTTA
        131 TCTCAGAATGCTTCTGTGTAGTTTTTATGTGAAGATATTTC

            *        * *            *   ***                     **        *  
     213710 GTTTTCCACAACAGGCCTCAAAGCTCTCTCCATATCCACTTGCAGATTCTACCGAAAGAGTGCTT
          1 CTTTTCCACCATAGGCCTCAAAGCGCTCCAAATATCCACTTGCAGATTCTACAAAAAGAGTGTTT

             *                   * **     *        *        *     **         
     213775 CCAAACTGCTCAATCAAAAGAGACATTCAAATCTGTGAGGTGAATGCAGACATCGTAAAGAAGTT
         66 CAAAACTGCTCAATCAAAAGAAAGGTTCAACTCTGTGAGATGAATGCACACATCACAAAGAAGTT

                                *     *       *    **
     213840 TCTCAGAATGCTTCTGTGTATTTTTTGTGTGAAGTTATTCG
        131 TCTCAGAATGCTTCTGTGTAGTTTTTATGTGAAGATATTTC

            *    *             *     *  *            **                 *    
     213881 TTTTTGCACCATAGGCCTCCAAGCGTTCTAAATATCCACTTCTAGATTCTACAAAAAGAGAGTTT
          1 CTTTTCCACCATAGGCCTCAAAGCGCTCCAAATATCCACTTGCAGATTCTACAAAAAGAGTGTTT

                   *         *            *        *    *   *                
     213946 CAAAACTACTC-A--AACA-AAAGGTTCAATTCTGTGAGTTGAAAGCAAACATCACAAAGAAGTT
         66 CAAAACTGCTCAATCAAAAGAAAGGTTCAACTCTGTGAGATGAATGCACACATCACAAAGAAGTT

                       *                             
     214007 TCTCAGAATGCGTCTGTGTAGTTTTTATGTGAAGATATTTC
        131 TCTCAGAATGCTTCTGTGTAGTTTTTATGTGAAGATATTTC

                     **   ****     *                  *                      
     214048 CTTTT-CACAGTAGAATGCAAAGGGCTCCAAATATCCACTTGGAGATTCTACAAAAAGA--GTTT
          1 CTTTTCCACCATAGGCCTCAAAGCGCTCCAAATATCCACTTGCAGATTCTACAAAAAGAGTGTTT

                  *    **     *  *     *     ***   *     *               *   
     214110 CAAAACCGCTCTGTCAAATGATAGGTTGAACTCCCGGAGGTGAATACACACATCACAAAGAGGTT
         66 CAAAACTGCTCAATCAAAAGAAAGGTTCAACTCTGTGAGATGAATGCACACATCACAAAGAAGTT

                  *                       *  *       
     214175 TCTCAGCATGCTTCTGTGTAGTTTTTATGTAAACATATTTC
        131 TCTCAGAATGCTTCTGTGTAGTTTTTATGTGAAGATATTTC

             *    * *              *           *      * *       *   *    *   
     214216 CGTTTCTATCATAGGCCTCAAAGTGCTCCAAATATTCACTTGTACATTCTACCAAACGAGTATTT
          1 CTTTTCCACCATAGGCCTCAAAGCGCTCCAAATATCCACTTGCAGATTCTACAAAAAGAGTGTTT

                              * *         **      *        *            *    
     214281 CAAAACTGCTCAATCAAATGGAAGGTTCAAAACTGTGACATGAATGCCCACATCACAAAGTAGTT
         66 CAAAACTGCTCAATCAAAAGAAAGGTTCAACTCTGTGAGATGAATGCACACATCACAAAGAAGTT

                                                     
     214346 TCTCAGAATGCTTCTGTGTAGTTTTTATGTGAAGATATTTC
        131 TCTCAGAATGCTTCTGTGTAGTTTTTATGTGAAGATATTTC

                     * *    *      *    *       * *   **      *              
     214387 CTTTTCCACAACA-GCGTGCAAAACGCTTCAAATATGCCCTTAGAGATTCCACAAAAAGAGTGTT
          1 CTTTTCCACCATAGGCCT-CAAAGCGCTCCAAATATCCACTTGCAGATTCTACAAAAAGAGTGTT

              *     *               *  *                                  ** 
     214451 TCCAAACTACTCAAATCAAAA-AATGATTTCAACTCTGTGAGATGAATGCACACATCACAAACTA
         65 TCAAAACTGCTC-AATCAAAAGAAAG-GTTCAACTCTGTGAGATGAATGCACACATCACAAAGAA

                         *     ** *   * *   *       *   
     214515 GTTTCTCAGAATGTTTCTGCCTGGTTCTCATGCGAAGATAGTTC
        128 GTTTCTCAGAATGCTTCTGTGTAGTTTTTATGTGAAGATATTTC

                 *           *   * **              *                  * *    
     214559 CTTTTTCACCATAGGCCGCAATGTACTCCAAATATCCACCTGCAGATTCTAC-AAAAGTGAGTTT
          1 CTTTTCCACCATAGGCCTCAAAGCGCTCCAAATATCCACTTGCAGATTCTACAAAAAGAGTGTTT

                       *         ***    **         *        *      *       * 
     214623 CAAAACTGCTCTATCAAAAGATCAGTTCGTCTCTGTGAGTTGAATGCATACATCAAAAAGAAGCT
         66 CAAAACTGCTCAATCAAAAGAAAGGTTCAACTCTGTGAGATGAATGCACACATCACAAAGAAGTT

                 *             *      **         *   
     214688 TCTCAAAATGCTTCTGTGTGGTTTTTCGGTGAAGATAGTTC
        131 TCTCAGAATGCTTCTGTGTAGTTTTTATGTGAAGATATTTC

            *     *        *      * *                 *        *        *    
     214729 TTTTTCTACCATAGGTCTCAAACCACTCCAAATATCCACTTGTAGATTCTATAAAAAGGAATG-T
          1 CTTTTCCACCATAGGCCTCAAAGCGCTCCAAATATCCACTTGCAGATTCTACAAAAA-GAGTGTT

                  *        *    *    *      * *         *      *             
     214793 TCAAAATTGCTCAATAAAAATAAAGTTTCAACACCGTGAGATGAGTGCACAAATCACAAAGAAGT
         65 TCAAAACTGCTCAATCAAAAGAAAGGTTCAACTCTGTGAGATGAATGCACACATCACAAAGAAGT

                  *          *         *          *   
     214858 TTCTCAAAATGCTTCTGGGTAGTTTTTCTGTGAAGATAGTTC
        130 TTCTCAGAATGCTTCTGTGTAGTTTTTATGTGAAGATATTTC

                  *     *    *     *          *                         *    
     214900 CTTTTCTACCATGGGCCACAAAGGGCTCCAAATACCCACTTGCAGATTCTACAAAAAGAGAGTTT
          1 CTTTTCCACCATAGGCCTCAAAGCGCTCCAAATATCCACTTGCAGATTCTACAAAAAGAGTGTTT

              *        *      ** * *        *    * *    **  * *           *  
     214965 CACAACTGCTCTATCAAACAATATGTTCAACTTTGTGGGTTGAACACAAATATCAC-AAGAATTT
         66 CAAAACTGCTCAATCAAAAGAAAGGTTCAACTCTGTGAGATGAATGCACACATCACAAAGAAGTT

                **                             *     
     215029 TCTCCCAATGCTTCTGTGTAGTTTTTATGTGAAGACATTTC
        131 TCTCAGAATGCTTCTGTGTAGTTTTTATGTGAAGATATTTC

            *   *  *      *  *     *                 *  *      *       *  *  
     215070 TTTTCCCTCCATAGTCCACAAAGTGCTCCAAATATCCACTTACATATTCTAGAAAAAGATTGCTT
          1 CTTTTCCACCATAGGCCTCAAAGCGCTCCAAATATCCACTTGCAGATTCTACAAAAAGAGTGTTT

            **       *    *               * * *                              
     215135 GGAAACTGCACAATGAAAAGAAAGGTTCAAATATATGAGATGAATGCACACATCACAAAGAAGTT
         66 CAAAACTGCTCAATCAAAAGAAAGGTTCAACTCTGTGAGATGAATGCACACATCACAAAGAAGTT

                                 *                    
     215200 TCTCAGAAT-CTCTCTGTGTAATTTTTATGTGAAGATATTTC
        131 TCTCAGAATGCT-TCTGTGTAGTTTTTATGTGAAGATATTTC

                *    **    *  *   *                        *      *    *     
     215241 CTTTCCCACTTTAGGTCTTAAAACGCTCCAAATATCCACTTGCAGATACTACAAGAAGATTGTTT
          1 CTTTTCCACCATAGGCCTCAAAGCGCTCCAAATATCCACTTGCAGATTCTACAAAAAGAGTGTTT

                     *    *        *      *     **                           
     215306 CAAAACTGCACAA-AAAAAGAAATGTTCAATTCTGTTTGATGAATGCACACATCACAAAGAAGTT
         66 CAAAACTGCTCAATCAAAAGAAAGGTTCAACTCTGTGAGATGAATGCACACATCACAAAGAAGTT

                           *                         
     215370 TCTCAGAATGCTTCTCTGTAGTTTTTATGTGAAGATATTTC
        131 TCTCAGAATGCTTCTGTGTAGTTTTTATGTGAAGATATTTC

                     *             *                 *         **      * *   
     215411 CTTTTCCACAATAGGCCTCAAAGGGCTCCAAATATCCACTTCCAGATTCTATGAAAAGAATATTT
          1 CTTTTCCACCATAGGCCTCAAAGCGCTCCAAATATCCACTTGCAGATTCTACAAAAAGAGTGTTT

             *              * *    *                                      *  
     215476 CCAAACTGCTCAATCATAGGAAATGTTCAACTCTGTGAGATGAATGCACACATCAC-AAGAAATT
         66 CAAAACTGCTCAATCAAAAGAAAGGTTCAACTCTGTGAGATGAATGCACACATCACAAAGAAGTT

                     *    *      *       *       *   
     215540 TCTCAGAATCCTTCAGTGTAGGTTTTATGAGAAGATAATTC
        131 TCTCAGAATGCTTCTGTGTAGTTTTTATGTGAAGATATTTC

                     *    **        *   *                           *    *   
     215581 CTTTTCCACAATAGTTCTCAAAGCACTCAAAATATCCACTTGCAGATTCTACAAAAGGAGTATTT
          1 CTTTTCCACCATAGGCCTCAAAGCGCTCCAAATATCCACTTGCAGATTCTACAAAAAGAGTGTTT

                                                          *                  
     215646 CAAAACTGCTCAATCAAAAGAAAGGTTCAACTCTGTGAGATGAATGGACACATCACAAAGAAGTT
         66 CAAAACTGCTCAATCAAAAGAAAGGTTCAACTCTGTGAGATGAATGCACACATCACAAAGAAGTT

                                  *   *              
     215711 TCTCAGAATGCTTCTGTGTAGTATTTTTGTGAAGATATTT-
        131 TCTCAGAATGCTTCTGTGTAGTTTTTATGTGAAGATATTTC

                          *  * * * * ***             *            *     * *  
     215751 CTTTTCCACCATAGACCGCCAGGGGACACAAATATCCACTTTCAGATTCTACAACAAGAGAGGTT
          1 CTTTTCCACCATAGGCCTCAAAGCGCTCCAAATATCCACTTGCAGATTCTACAAAAAGAGTGTTT

                   *   *     *   ** *       *      *                      * *
     215816 CAAAACTACTCGATCAAGAGATGGTTTCAACTATGTGAGTTGAATGCACACATCACAAAGAACTA
         66 CAAAACTGCTCAATCAAAAGAAAGGTTCAACTCTGTGAGATGAATGCACACATCACAAAGAAGTT

             *  *    *                               
     215881 TGTCGGAATTCTTCTGTGTAGTTTTTATGTGAAGATATTTC
        131 TCTCAGAATGCTTCTGTGTAGTTTTTATGTGAAGATATTTC

                     *    * *      * *    *                  *               
     215922 CTTTTCCACAATAGACGTCAAAGTGATCCAGATATCCACTTGCAGATTCCACAAAAAGAGTGTTT
          1 CTTTTCCACCATAGGCCTCAAAGCGCTCCAAATATCCACTTGCAGATTCTACAAAAAGAGTGTTT

                 *   *   *                  **                     *  *      
     215987 CAAAAGTGCACAACCAAAAGAAAGGTTCAACTAGGTGAGATGAATGCACACATCAGAAGGAAGTT
         66 CAAAACTGCTCAATCAAAAGAAAGGTTCAACTCTGTGAGATGAATGCACACATCACAAAGAAGTT

                            **   *     * *       *   
     216052 TCTCAGAATGCTTCTGCATAGCTTTTAAGGGAAGATACTTC
        131 TCTCAGAATGCTTCTGTGTAGTTTTTATGTGAAGATATTTC

                    *               *            * *  *    * *               
     216093 CTTTTCCAACATAGGCCTCAAAGCACTCCAAATATCCTCCTGGAGATACCACAAAAAGAGTGTTT
          1 CTTTTCCACCATAGGCCTCAAAGCGCTCCAAATATCCACTTGCAGATTCTACAAAAAGAGTGTTT

            **                      *  *                 *       *           
     216158 GCAAACTGCTCAATCAAAAGAAAGATTTAACTCTGTGAGATGAATCCACACATGACAAAGAAGTT
         66 CAAAACTGCTCAATCAAAAGAAAGGTTCAACTCTGTGAGATGAATGCACACATCACAAAGAAGTT

                                                     
     216223 TCTCAGAATGCTTCTGTGTAGTTTTTATGTGAAGATATTTC
        131 TCTCAGAATGCTTCTGTGTAGTTTTTATGTGAAGATATTTC

                     *   * * *    **           *                *     *      
     216264 CTTTTCCACAATAAGACCCAAAAGGCTCCAAATATTCACTTGCAGATTCTAAAAAAAACAGTGTT
          1 CTTTTCCACCATAGGCCTCAAAGCGCTCCAAATATCCACTTGCAGATTCT-ACAAAAAGAGTGTT

                                  *                  *      *        **      
     216329 TCAAAACTGCTCAATCAAAAGATA-GTTCAACTCTGTGAGAAGAATGCTCACATCACTGAGAAGT
         65 TCAAAACTGCTCAATCAAAAGAAAGGTTCAACTCTGTGAGATGAATGCACACATCACAAAGAAGT

                                         *            
     216393 TTCTCAGAATGCTTCTGTGTAGTTTTTATATGAAGATATTTC
        130 TTCTCAGAATGCTTCTGTGTAGTTTTTATGTGAAGATATTTC

                *     *      *    **                       *   **       *    
     216435 CTTTCCCACCGTAGGCCACAAAAGGCTCCAAATATCCACTTGCAGATACTATGAAAAGAGAGTTT
          1 CTTTTCCACCATAGGCCTCAAAGCGCTCCAAATATCCACTTGCAGATTCTACAAAAAGAGTGTTT

                        *        *               ***            *            
     216500 CAAAACTGCTCATTCAAAAGATAGGTTCAACTCTGTGGTTTGAATGCACACAGCACAAAGAAGTT
         66 CAAAACTGCTCAATCAAAAGAAAGGTTCAACTCTGTGAGATGAATGCACACATCACAAAGAAGTT

              *       **                   **   *    
     216565 TCACAGAATGTGTCTGTGTAGTTTTTATGTGCGGATGTTTC
        131 TCTCAGAATGCTTCTGTGTAGTTTTTATGTGAAGATATTTC

                         *        ****   *   *               *    *      *   
     216606 CTTTTCCACCATATGCCT-AAATATTTCCCAATTTCCACTTGCAGATTCCACAAGAAGAGTTTTT
          1 CTTTTCCACCATAGGCCTCAAAGCGCTCCAAATATCCACTTGCAGATTCTACAAAAAGAGTGTTT

                      **       *       *           *            *      ***   
     216670 CAAAACTGCTGTATC-AAATAAA-GTTGAACTCTGTGAGGTGAATGCACACAGCACAAAATGGTT
         66 CAAAACTGCTCAATCAAAAGAAAGGTTCAACTCTGTGAGATGAATGCACACATCACAAAGAAGTT

                          **   *        *       *    
     216733 TCTCAGAATGCTTCCTTGTTGTTTTTATATGAAGATGTTTC
        131 TCTCAGAATGCTTCTGTGTAGTTTTTATGTGAAGATATTTC

                  *  *             *   *     *                               
     216774 CTTTTCAACAATAGGCCTCAAAGTGCTTCAAATGTCCACTTGCAGATTCTACAAAAAGAGTGTTT
          1 CTTTTCCACCATAGGCCTCAAAGCGCTCCAAATATCCACTTGCAGATTCTACAAAAAGAGTGTTT

                                        *      *  *  *                       
     216839 CAAAACTGCTCAATCAAAAGAAAGGTTCGACTCTGGGAAATTAATGCACACATCACAAAGAAGTT
         66 CAAAACTGCTCAATCAAAAGAAAGGTTCAACTCTGTGAGATGAATGCACACATCACAAAGAAGTT

                                     *        *      
     216904 TCTC---A-GCTTCTGTGTAGTTTTCATGTGAAGTTATTTC
        131 TCTCAGAATGCTTCTGTGTAGTTTTTATGTGAAGATATTTC

                     *       *     *            *    *         **       *    
     216941 CTTTTCCACAATAGGCCGCAAAGGGCTCCAAATATCAACTTACAGATTCTAGGAAAAGAGAGTTT
          1 CTTTTCCACCATAGGCCTCAAAGCGCTCCAAATATCCACTTGCAGATTCTACAAAAAGAGTGTTT

                       * **      *     *                                     
     217006 CAAAACTGCTCTACGAAAAGATAGGTTGAACTCTGTGAGATGAATGCACACATCACAAAGAAGTT
         66 CAAAACTGCTCAATCAAAAGAAAGGTTCAACTCTGTGAGATGAATGCACACATCACAAAGAAGTT

                       *               * *           
     217071 TCTCAGAATGCATCTGTGTAGTTTTTACGGGAAGATATTTC
        131 TCTCAGAATGCTTCTGTGTAGTTTTTATGTGAAGATATTTC

                        ***  *     **      *  *           *      *     ***   
     217112 CTTTTCCACCATCTTCCACAAAGGTCTCCAAGTAACCACTTGCAGACTCTACAGAAAGACACTTT
          1 CTTTTCCACCATAGGCCTCAAAGCGCTCCAAATATCCACTTGCAGATTCTACAAAAAGAGTGTTT

            *          *         ***      *      ***                      *  
     217177 AAAAACTGCTCTATCAAAAGATCAGTTCAAGTCTGTGGTTTGAATGCACACATCACAAAGAATTT
         66 CAAAACTGCTCAATCAAAAGAAAGGTTCAACTCTGTGAGATGAATGCACACATCACAAAGAAGTT

                                     *  *            
     217242 TCTCAGAATGCTTCTGTGTAGTTTTCATATGAAGATATTTC
        131 TCTCAGAATGCTTCTGTGTAGTTTTTATGTGAAGATATTTC

                                    *                                   **   
     217283 CTTTTCCACCATAGGCCTCAAAGCACTCCAAATATCCACTTGCAGATTCTACAAAAAGAGATTTT
          1 CTTTTCCACCATAGGCCTCAAAGCGCTCCAAATATCCACTTGCAGATTCTACAAAAAGAGTGTTT

                   **                           *  *          *        *     
     217348 CAAAACTAGTCAATCAAAAGAAAGGTTCAACTCTGTCAGTTGAATGCACATATCACAAACAAGTT
         66 CAAAACTGCTCAATCAAAAGAAAGGTTCAACTCTGTGAGATGAATGCACACATCACAAAGAAGTT

                *      *                             
     217413 TCTCGGAATGCGTCTGTGTAGTTTTTATGTGAAGATATTTC
        131 TCTCAGAATGCTTCTGTGTAGTTTTTATGTGAAGATATTTC

               *     * *           *     *                  *    *           
     217454 CTTCTCCACAACAGGCCTCAAAGTGCTCCGAATATCCACTTGCAGATTTTAC-TAAAGAGTGTTT
          1 CTTTTCCACCATAGGCCTCAAAGCGCTCCAAATATCCACTTGCAGATTCTACAAAAAGAGTGTTT

             *               *  *   *     *        *    *               *    
     217518 CCAAACTGCTCAATCAAGAGGAAGTTTCAAGTCTGTGAGCTGAACGCACACATCACAAAGTAGTT
         66 CAAAACTGCTCAATCAAAAGAAAGGTTCAACTCTGTGAGATGAATGCACACATCACAAAGAAGTT

               *                                *    
     217583 TCTGAGAATGCTTCTGTGTAGTTTTTATGTGAAGATGTTTC
        131 TCTCAGAATGCTTCTGTGTAGTTTTTATGTGAAGATATTTC

                            **     *                                    *  * 
     217624 CTTTTCCACCATAGGCTGCAAAGGGCTCCAAATATCCACTTGCAGATTCTACAAAAAGAGAGTCT
          1 CTTTTCCACCATAGGCCTCAAAGCGCTCCAAATATCCACTTGCAGATTCTACAAAAAGAGTGTTT

                 *     *         *          *     *                     *    
     217689 CAAAAGTGCTCTATCAAAAGATAGGTTCAACTATGTGATATGAATGCACACATCACAAAGTAGTT
         66 CAAAACTGCTCAATCAAAAGAAAGGTTCAACTCTGTGAGATGAATGCACACATCACAAAGAAGTT

                                          *          
     217754 TCTCAGAATGCTTCTGTGTAGTTTTTATGTAAAGATATTTC
        131 TCTCAGAATGCTTCTGTGTAGTTTTTATGTGAAGATATTTC

                                    *                                        
     217795 CTTTTCCACCATAGGCCTCAAAGCACTCCAAATATCCACTTGCAGATTCTACAAAAAGAGATG-T
          1 CTTTTCCACCATAGGCCTCAAAGCGCTCCAAATATCCACTTGCAGATTCTACAAAAAGAG-TGTT

                    ** *                   *     *  *    * *   *        *    
     217859 TCAAAACTATTTAATCAAAAGAAAGGTTCAAATCTGTCAGTTGAAGGTACATATCACAAACAAGT
         65 TCAAAACTGCTCAATCAAAAGAAAGGTTCAACTCTGTGAGATGAATGCACACATCACAAAGAAGT

              * **                                    
     217924 TTATTGGAATGCTTCTGTGTAGTTTTTATGTGAAGATATTTC
        130 TTCTCAGAATGCTTCTGTGTAGTTTTTATGTGAAGATATTTC

                     * *         * *                        **  *     *      
     217966 CTTTTCCACAACAGGCCTCAAGGTGCTCCAAATATCCACTTGCAGATTTCACTAAAAGTGTGTTT
          1 CTTTTCCACCATAGGCCTCAAAGCGCTCCAAATATCCACTTGCAGATTCTACAAAAAGAGTGTTT

             *  *            *  *   *     *        *             *           
     218031 CCAAGCTGCTCAATCAAGAGGAAGTTTCAAGTCTGTGAGGTGAATGCACACATTACAAAGAAGTT
         66 CAAAACTGCTCAATCAAAAGAAAGGTTCAACTCTGTGAGATGAATGCACACATCACAAAGAAGTT

            *  *                                     
     218096 ACTGAGAATGCTTCTGTGTAGTTTTTATGTGAAGATATTTC
        131 TCTCAGAATGCTTCTGTGTAGTTTTTATGTGAAGATATTTC

                      **               *                                *    
     218137 CTTTTCCACCGCAGGCCTCAAAGCGCTGCAAATATCCACTTGCAGATTCTACAAAAAGAGAGTTT
          1 CTTTTCCACCATAGGCCTCAAAGCGCTCCAAATATCCACTTGCAGATTCTACAAAAAGAGTGTTT

                      **         *   *         *   *     **            *     
     218202 CAAAACTGCTGTATCAAAAGATAGGGTCAACTCTGCGAGTTGAATAAACACATCACAAATAAGTT
         66 CAAAACTGCTCAATCAAAAGAAAGGTTCAACTCTGTGAGATGAATGCACACATCACAAAGAAGTT

               **   *        *               *       
     218267 TCTGGGAACGCTTCTGTATAG-TTTTATGTGAATATATTTC
        131 TCTCAGAATGCTTCTGTGTAGTTTTTATGTGAAGATATTTC

                         *          *                   *   *  *   * *   *   
     218307 CTTTTCCACCATATGCCTCAAAGCACTCCAAATATCCACTTGCACATTATAGAAACATAGTCTTT
          1 CTTTTCCACCATAGGCCTCAAAGCGCTCCAAATATCCACTTGCAGATTCTACAAAAAGAGTGTTT

                   **                        *         *             *       
     218372 CAAAACTTGTCAATC-AAAGAAAGGTTCAACTCCGTGAGATGAGTGCACACATCACAGAGAAGTT
         66 CAAAACTGCTCAATCAAAAGAAAGGTTCAACTCTGTGAGATGAATGCACACATCACAAAGAAGTT

                *     *                            * 
     218436 TCTCGGAATGTTTCTGTGTAGTTTTTATGTGAAGATATTGC
        131 TCTCAGAATGCTTCTGTGTAGTTTTTATGTGAAGATATTTC

                     *               *            *          *       *   *   
     218477 CTTTTCCACAATAGGCCTCAAAGCGTTCCAAATATCCAATTGCAGATTCCACAAAAAAAGTTTTT
          1 CTTTTCCACCATAGGCCTCAAAGCGCTCCAAATATCCACTTGCAGATTCTACAAAAAGAGTGTTT

            *                 *  *  *  *             * *         **    *     
     218542 TAAAACTGCTCAATCAAATGATAGATTAAACTCTGTGAGATTAGTGCACACATGTCAAAAAAGTT
         66 CAAAACTGCTCAATCAAAAGAAAGGTTCAACTCTGTGAGATGAATGCACACATCACAAAGAAGTT

                                *      * *           
     218607 TCTCAGAATGCTTCTGTGTACTTTTTAGGGGAAGATATTTC
        131 TCTCAGAATGCTTCTGTGTAGTTTTTATGTGAAGATATTTC

                        *    *     **         *    *           **  * *  *    
     218648 CTTTTCCACCATCGGCCACAAAGGACTCCAAATAACCACATGCAGATTCTAGTAACACAGAGTTT
          1 CTTTTCCACCATAGGCCTCAAAGCGCTCCAAATATCCACTTGCAGATTCTACAAAAAGAGTGTTT

                       *         * *           *   * * *    **    ***        
     218713 CAAAACTGCTCTATCAAAAGATAAGTTCAACTCTGAGAGTTTAGTGCAACCATCGTGAAGAAGTT
         66 CAAAACTGCTCAATCAAAAGAAAGGTTCAACTCTGTGAGATGAATGCACACATCACAAAGAAGTT

                            *     *                  
     218778 TCTCAGAATGCTTCTGAGTAGTGTTTATGTGAAGATATTTC
        131 TCTCAGAATGCTTCTGTGTAGTTTTTATGTGAAGATATTTC

                              *     *                      *           **    
     218819 CTTTTCCACCATAGGCCTGAAAGCCCTCCAAATATCCACTTGCAGATCCTACAAAAAGAAAGTTT
          1 CTTTTCCACCATAGGCCTCAAAGCGCTCCAAATATCCACTTGCAGATTCTACAAAAAGAGTGTTT

             *         **     *  *  *   *        **      *                   
     218884 CGAAA-TGCTCTCTCAAACGATAGTTTCGACTCTGTGGTATGAATACACACATCACAAAGAAGTT
         66 CAAAACTGCTCAATCAAAAGAAAGGTTCAACTCTGTGAGATGAATGCACACATCACAAAGAAGTT

                                       **            
     218948 TCTCAGAATGCTTCTGTGTAGTTTTTAAATGAAGATATTTC
        131 TCTCAGAATGCTTCTGTGTAGTTTTTATGTGAAGATATTTC

            *                       *          *     *                       
     218989 TTTTTCCACCATAGGCCTCAAAGCACTCCAAATATGCACTTCCAGATTCTACAAAAAGAGTGTTT
          1 CTTTTCCACCATAGGCCTCAAAGCGCTCCAAATATCCACTTGCAGATTCTACAAAAAGAGTGTTT

              *                 *       * *         **   *         *  * *    
     219054 CAGAACTGCTCAATCAAAAGGAAGGTTCCAGTC--TGAGACAAATACACACATCAAAAGGTAGTT
         66 CAAAACTGCTCAATCAAAAGAAAGGTTCAACTCTGTGAGATGAATGCACACATCACAAAGAAGTT

                                                    *
     219117 TCTCAGAATGCTTCTGTGTAGTTTTTATGTGAAGATATTTT
        131 TCTCAGAATGCTTCTGTGTAGTTTTTATGTGAAGATATTTC

             *               *    **    *     *      *  **   *          *    
     219158 CCTTTCCACCATAGGCCACAAATGGCTCTAAATACCCACTTACATTTTCCACAAAAAGAGAGTTT
          1 CTTTTCCACCATAGGCCTCAAAGCGCTCCAAATATCCACTTGCAGATTCTACAAAAAGAGTGTTT

                       * *    * *      *   *         *                       
     219223 CAAAACTGCTCTACCAAAGGTAA-GTTTAACGCTGTGAG-TTAA-G-A-ACATCACAAAGAAGTT
         66 CAAAACTGCTCAATCAAAAGAAAGGTTCAACTCTGTGAGATGAATGCACACATCACAAAGAAGTT

                                   *   *  *          
     219283 TCTCAGAATGCTTCTGTGTAGTTCTTACGTAAAGATATTTC
        131 TCTCAGAATGCTTCTGTGTAGTTTTTATGTGAAGATATTTC

                 *   *      ***    **                 *              **      
     219324 CTTTTACACAATAGGCAGAAAAGTACTCCAAATATCCACTTGAAGATTCTACAAAAACCGTGTTT
          1 CTTTTCCACCATAGGCCTCAAAGCGCTCCAAATATCCACTTGCAGATTCTACAAAAAGAGTGTTT

                     **                                          *      *    
     219389 CAAAACTGCCGAATCAAAAGAAAGGTTCAACTCTGTGAGATGAATGCACACATAACAAAGGAGTT
         66 CAAAACTGCTCAATCAAAAGAAAGGTTCAACTCTGTGAGATGAATGCACACATCACAAAGAAGTT

                                 *      *      *    *
     219454 TCTCAGAATGCTTCTGTGTAGCTTTTATATGAAGACATTTA
        131 TCTCAGAATGCTTCTGTGTAGTTTTTATGTGAAGATATTTC

            *        * *            *   ***                     **        *  
     219495 GTTTTCCACAACAGGCCTCAAAGCTCTCTCCATATCCACTTGCAGATTCTACCGAAAGAGTGCTT
          1 CTTTTCCACCATAGGCCTCAAAGCGCTCCAAATATCCACTTGCAGATTCTACAAAAAGAGTGTTT

             *                   * **     *        *        *     **         
     219560 CCAAACTGCTCAATCAAAAGAGACATTCAAATCTGTGAGGTGAATGCAGACATCGTAAAGAAGTT
         66 CAAAACTGCTCAATCAAAAGAAAGGTTCAACTCTGTGAGATGAATGCACACATCACAAAGAAGTT

                                *     *       *    **
     219625 TCTCAGAATGCTTCTGTGTATTTTTTGTGTGAAGTTATTCG
        131 TCTCAGAATGCTTCTGTGTAGTTTTTATGTGAAGATATTTC

            *    *             *     *  *            **                 *    
     219666 TTTTTGCACCATAGGCCTCCAAGCGTTCTAAATATCCACTTCTAGATTCTACAAAAAGAGAGTTT
          1 CTTTTCCACCATAGGCCTCAAAGCGCTCCAAATATCCACTTGCAGATTCTACAAAAAGAGTGTTT

                   *         *            *        *    *   *                
     219731 CAAAACTACTC-A--AACA-AAAGGTTCAATTCTGTGAGTTGAAAGCAAACATCACAAAGAAGTT
         66 CAAAACTGCTCAATCAAAAGAAAGGTTCAACTCTGTGAGATGAATGCACACATCACAAAGAAGTT

                       *                             
     219792 TCTCAGAATGCGTCTGTGTAGTTTTTATGTGAAGATATTTC
        131 TCTCAGAATGCTTCTGTGTAGTTTTTATGTGAAGATATTTC

                     *    ****     *                  *                      
     219833 CTTTT-CACAATAGAATGCAAAGGGCTCCAAATATCCACTTGGAGATTCTACAAAAAGA--GTTT
          1 CTTTTCCACCATAGGCCTCAAAGCGCTCCAAATATCCACTTGCAGATTCTACAAAAAGAGTGTTT

                  *    **     *  *     *     ***   *     *               *   
     219895 CAAAACCGCTCTGTCAAATGATAGGTTGAACTCCCGGAGGTGAATACACACATCACAAAGAGGTT
         66 CAAAACTGCTCAATCAAAAGAAAGGTTCAACTCTGTGAGATGAATGCACACATCACAAAGAAGTT

                  *                       *  *       
     219960 TCTCAGCATGCTTCTGTGTAGTTTTTATGTAAACATATTTC
        131 TCTCAGAATGCTTCTGTGTAGTTTTTATGTGAAGATATTTC

             *    * *              *           *      * *       *   *    *   
     220001 CGTTTCTATCATAGGCCTCAAAGTGCTCCAAATATTCACTTGTACATTCTACCAAACGAGTATTT
          1 CTTTTCCACCATAGGCCTCAAAGCGCTCCAAATATCCACTTGCAGATTCTACAAAAAGAGTGTTT

                              * *         **      *        *            *    
     220066 CAAAACTGCTCAATCAAATGGAAGGTTCAAAACTGTGACATGAATGCCCACATCACAAAGTAGTT
         66 CAAAACTGCTCAATCAAAAGAAAGGTTCAACTCTGTGAGATGAATGCACACATCACAAAGAAGTT

                                                     
     220131 TCTCAGAATGCTTCTGTGTAGTTTTTATGTGAAGATATTTC
        131 TCTCAGAATGCTTCTGTGTAGTTTTTATGTGAAGATATTTC

                     * *    *      *    *       * *   **      *              
     220172 CTTTTCCACAACA-GCGTGCAAAACGCTTCAAATATGCCCTTAGAGATTCCACAAAAAGAGTGTT
          1 CTTTTCCACCATAGGCCT-CAAAGCGCTCCAAATATCCACTTGCAGATTCTACAAAAAGAGTGTT

              *     *               *  *                                  ** 
     220236 TCCAAACTACTCAAATCAAAA-AATGATTTCAACTCTGTGAGATGAATGCACACATCACAAACTA
         65 TCAAAACTGCTC-AATCAAAAGAAAG-GTTCAACTCTGTGAGATGAATGCACACATCACAAAGAA

                         *     ** *   * *   *       *   
     220300 GTTTCTCAGAATGTTTCTGCCTGGTTCTCATGCGAAGATAGTTC
        128 GTTTCTCAGAATGCTTCTGTGTAGTTTTTATGTGAAGATATTTC

                 *           *   * **              *                  * *    
     220344 CTTTTTCACCATAGGCCGCAATGTACTCCAAATATCCACCTGCAGATTCTAC-AAAAGTGAGTTT
          1 CTTTTCCACCATAGGCCTCAAAGCGCTCCAAATATCCACTTGCAGATTCTACAAAAAGAGTGTTT

                       *         ***    **         *        *      *       * 
     220408 CAAAACTGCTCTATCAAAAGATCAGTTCGTCTCTGTGAGTTGAATGCATACATCAAAAAGAAGCT
         66 CAAAACTGCTCAATCAAAAGAAAGGTTCAACTCTGTGAGATGAATGCACACATCACAAAGAAGTT

                 *             *      **         *   
     220473 TCTCAAAATGCTTCTGTGTGGTTTTTCGGTGAAGATAGTTC
        131 TCTCAGAATGCTTCTGTGTAGTTTTTATGTGAAGATATTTC

            *     *        *      * *                 *        *        *    
     220514 TTTTTCTACCATAGGTCTCAAACCACTCCAAATATCCACTTGTAGATTCTATAAAAAGGAATG-T
          1 CTTTTCCACCATAGGCCTCAAAGCGCTCCAAATATCCACTTGCAGATTCTACAAAAA-GAGTGTT

                  *        *    *    *      * *         *      *         *   
     220578 TCAAAATTGCTCAATAAAAATAAAGTTTCAACACCGTGAGATGAGTGCACAAATCACAAAGGAGT
         65 TCAAAACTGCTCAATCAAAAGAAAGGTTCAACTCTGTGAGATGAATGCACACATCACAAAGAAGT

                  *          *         *          *   
     220643 TTCTCAAAATGCTTCTGGGTAGTTTTTCTGTGAAGATAGTTC
        130 TTCTCAGAATGCTTCTGTGTAGTTTTTATGTGAAGATATTTC

                  *     *    *     *          *                         *    
     220685 CTTTTCTACCATGGGCCACAAAGGGCTCCAAATACCCACTTGCAGATTCTACAAAAAGAGAGTTT
          1 CTTTTCCACCATAGGCCTCAAAGCGCTCCAAATATCCACTTGCAGATTCTACAAAAAGAGTGTTT

              *        *      ** * *        *    * *    **  * *           *  
     220750 CACAACTGCTCTATCAAACAATATGTTCAACTTTGTGGGTTGAACACAAATATCAC-AAGAATTT
         66 CAAAACTGCTCAATCAAAAGAAAGGTTCAACTCTGTGAGATGAATGCACACATCACAAAGAAGTT

                **                             *     
     220814 TCTCCCAATGCTTCTGTGTAGTTTTTATGTGAAGACATTTC
        131 TCTCAGAATGCTTCTGTGTAGTTTTTATGTGAAGATATTTC

            *   *  *      *  *     *                 *  *      *       *  *  
     220855 TTTTCCCTCCATAGTCCACAAAGTGCTCCAAATATCCACTTACATATTCTAGAAAAAGATTGCTT
          1 CTTTTCCACCATAGGCCTCAAAGCGCTCCAAATATCCACTTGCAGATTCTACAAAAAGAGTGTTT

            **       *    *               * * *                              
     220920 GGAAACTGCACAATGAAAAGAAAGGTTCAAATATATGAGATGAATGCACACATCACAAAGAAGTT
         66 CAAAACTGCTCAATCAAAAGAAAGGTTCAACTCTGTGAGATGAATGCACACATCACAAAGAAGTT

                                 *                    
     220985 TCTCAGAAT-CTCTCTGTGTAATTTTTATGTGAAGATATTTC
        131 TCTCAGAATGCT-TCTGTGTAGTTTTTATGTGAAGATATTTC

                *     *    *  *   *                        *      *    *     
     221026 CTTTCCCACCTTAGGTCTTAAAACGCTCCAAATATCCACTTGCAGATACTACAAGAAGATTGTTT
          1 CTTTTCCACCATAGGCCTCAAAGCGCTCCAAATATCCACTTGCAGATTCTACAAAAAGAGTGTTT

                     *    *        *      *     **                           
     221091 CAAAACTGCACAA-AAAAAGAAATGTTCAATTCTGTTTGATGAATGCACACATCACAAAGAAGTT
         66 CAAAACTGCTCAATCAAAAGAAAGGTTCAACTCTGTGAGATGAATGCACACATCACAAAGAAGTT

                           *                         
     221155 TCTCAGAATGCTTCTCTGTAGTTTTTATGTGAAGATATTTC
        131 TCTCAGAATGCTTCTGTGTAGTTTTTATGTGAAGATATTTC

                     *             *                 *         **      * *   
     221196 CTTTTCCACAATAGGCCTCAAAGGGCTCCAAATATCCACTTCCAGATTCTATGAAAAGAATATTT
          1 CTTTTCCACCATAGGCCTCAAAGCGCTCCAAATATCCACTTGCAGATTCTACAAAAAGAGTGTTT

             *              * *    *                                      *  
     221261 CCAAACTGCTCAATCATAGGAAATGTTCAACTCTGTGAGATGAATGCACACATCAC-AAGAAATT
         66 CAAAACTGCTCAATCAAAAGAAAGGTTCAACTCTGTGAGATGAATGCACACATCACAAAGAAGTT

                     *    *      *       *       *   
     221325 TCTCAGAATCCTTCAGTGTAGGTTTTATGAGAAGATAATTC
        131 TCTCAGAATGCTTCTGTGTAGTTTTTATGTGAAGATATTTC

                     *    **        *   *                           *    *   
     221366 CTTTTCCACAATAGTTCTCAAAGCACTCAAAATATCCACTTGCAGATTCTACAAAAGGAGTATTT
          1 CTTTTCCACCATAGGCCTCAAAGCGCTCCAAATATCCACTTGCAGATTCTACAAAAAGAGTGTTT

                                                 *        *                  
     221431 CAAAACTGCTCAATCAAAAGAAAGGTTCAACTCTGTGGGATGAATGGACACATCACAAAGAAGTT
         66 CAAAACTGCTCAATCAAAAGAAAGGTTCAACTCTGTGAGATGAATGCACACATCACAAAGAAGTT

                                  *   *              
     221496 TCTCAGAATGCTTCTGTGTAGTATTTTTGTGAAGATATTT-
        131 TCTCAGAATGCTTCTGTGTAGTTTTTATGTGAAGATATTTC

                          *  * * * * ***             *            *     * *  
     221536 CTTTTCCACCATAGACCGCCAGGGGACACAAATATCCACTTTCAGATTCTACAACAAGAGAGGTT
          1 CTTTTCCACCATAGGCCTCAAAGCGCTCCAAATATCCACTTGCAGATTCTACAAAAAGAGTGTTT

                   *   *     *   ** *       *      *                      * *
     221601 CAAAACTACTCGATCAAGAGATGGTTTCAACTATGTGAGTTGAATGCACACATCACAAAGAACTA
         66 CAAAACTGCTCAATCAAAAGAAAGGTTCAACTCTGTGAGATGAATGCACACATCACAAAGAAGTT

             *  *    *                               
     221666 TGTCGGAATTCTTCTGTGTAGTTTTTATGTGAAGATATTTC
        131 TCTCAGAATGCTTCTGTGTAGTTTTTATGTGAAGATATTTC

                     *    * *      * *    *                  *               
     221707 CTTTTCCACAATAGACGTCAAAGTGATCCAGATATCCACTTGCAGATTCCACAAAAAGAGTGTTT
          1 CTTTTCCACCATAGGCCTCAAAGCGCTCCAAATATCCACTTGCAGATTCTACAAAAAGAGTGTTT

                 *   *   *                  **                     *  *      
     221772 CAAAAGTGCACAACCAAAAGAAAGGTTCAACTAGGTGAGATGAATGCACACATCAGAAGGAAGTT
         66 CAAAACTGCTCAATCAAAAGAAAGGTTCAACTCTGTGAGATGAATGCACACATCACAAAGAAGTT

                            **   *     * *       *   
     221837 TCTCAGAATGCTTCTGCATAGCTTTTAAGGGAAGATACTTC
        131 TCTCAGAATGCTTCTGTGTAGTTTTTATGTGAAGATATTTC

                    *               *            * *  *    * *               
     221878 CTTTTCCAACATAGGCCTCAAAGCACTCCAAATATCCTCCTGGAGATACCACAAAAAGAGTGTTT
          1 CTTTTCCACCATAGGCCTCAAAGCGCTCCAAATATCCACTTGCAGATTCTACAAAAAGAGTGTTT

            **                      *  *                 * *     *           
     221943 GCAAACTGCTCAATCAAAAGAAAGATTTAACTCTGTGAGATGAATCCGCACATGACAAAGAAGTT
         66 CAAAACTGCTCAATCAAAAGAAAGGTTCAACTCTGTGAGATGAATGCACACATCACAAAGAAGTT

                                                     
     222008 TCTCAGAATGCTTCTGTGTAGTTTTTATGTGAAGATATTTC
        131 TCTCAGAATGCTTCTGTGTAGTTTTTATGTGAAGATATTTC

                     *   * * *    **           *                *     *      
     222049 CTTTTCCACAATAAGACCCAAAAGGCTCCAAATATTCACTTGCAGATTCTAAAAAAAACAGTGTT
          1 CTTTTCCACCATAGGCCTCAAAGCGCTCCAAATATCCACTTGCAGATTCT-ACAAAAAGAGTGTT

                                  *                  *      *        **      
     222114 TCAAAACTGCTCAATCAAAAGATA-GTTCAACTCTGTGAGAAGAATGCTCACATCACTGAGAAGT
         65 TCAAAACTGCTCAATCAAAAGAAAGGTTCAACTCTGTGAGATGAATGCACACATCACAAAGAAGT

                                         *            
     222178 TTCTCAGAATGCTTCTGTGTAGTTTTTATATGAAGATATTTC
        130 TTCTCAGAATGCTTCTGTGTAGTTTTTATGTGAAGATATTTC

                *     *      *    **                       *   **       *    
     222220 CTTTCCCACCGTAGGCCACAAAAGGCTCCAAATATCCACTTGCAGATACTATGAAAAGAGAGTTT
          1 CTTTTCCACCATAGGCCTCAAAGCGCTCCAAATATCCACTTGCAGATTCTACAAAAAGAGTGTTT

                        *        *               ***            *            
     222285 CAAAACTGCTCATTCAAAAGATAGGTTCAACTCTGTGGTTTGAATGCACACAGCACAAAGAAGTT
         66 CAAAACTGCTCAATCAAAAGAAAGGTTCAACTCTGTGAGATGAATGCACACATCACAAAGAAGTT

              *       **                   **   *    
     222350 TCACAGAATGTGTCTGTGTAGTTTTTATGTGCGGATGTTTC
        131 TCTCAGAATGCTTCTGTGTAGTTTTTATGTGAAGATATTTC

                         *        ****   *   *               *    *          
     222391 CTTTTCCACCATATGCCT-AAATATTTCCCAATTTCCACTTGCAGATTCCACAAGAAGAGTGTTT
          1 CTTTTCCACCATAGGCCTCAAAGCGCTCCAAATATCCACTTGCAGATTCTACAAAAAGAGTGTTT

                      **       *       *           *            *      ***   
     222455 CAAAACTGCTGTATC-AAATAAA-GTTGAACTCTGTGAGGTGAATGCACACAGCACAAAATGGTT
         66 CAAAACTGCTCAATCAAAAGAAAGGTTCAACTCTGTGAGATGAATGCACACATCACAAAGAAGTT

                          **   *        *       *    
     222518 TCTCAGAATGCTTCCTTGTTGTTTTTATATGAAGATGTTTC
        131 TCTCAGAATGCTTCTGTGTAGTTTTTATGTGAAGATATTTC

                  *  *             *   *     *                               
     222559 CTTTTCAACAATAGGCCTCAAAGTGCTTCAAATGTCCACTTGCAGATTCTACAAAAAGAGTGTTT
          1 CTTTTCCACCATAGGCCTCAAAGCGCTCCAAATATCCACTTGCAGATTCTACAAAAAGAGTGTTT

                                        *      *  *  *                       
     222624 CAAAACTGCTCAATCAAAAGAAAGGTTCGACTCTGGGAAATTAATGCACACATCACAAAGAAGTT
         66 CAAAACTGCTCAATCAAAAGAAAGGTTCAACTCTGTGAGATGAATGCACACATCACAAAGAAGTT

                                     *        *      
     222689 TCTC---A-GCTTCTGTGTAGTTTTCATGTGAAGTTATTTC
        131 TCTCAGAATGCTTCTGTGTAGTTTTTATGTGAAGATATTTC

                     *       *     *            *    *         **       *    
     222726 CTTTTCCACAATAGGCCGCAAAGGGCTCCAAATATCAACTTACAGATTCTAGGAAAAGAGAGTTT
          1 CTTTTCCACCATAGGCCTCAAAGCGCTCCAAATATCCACTTGCAGATTCTACAAAAAGAGTGTTT

                       * **      *     *                                     
     222791 CAAAACTGCTCTACGAAAAGATAGGTTGAACTCTGTGAGATGAATGCACACATCACAAAGAAGTT
         66 CAAAACTGCTCAATCAAAAGAAAGGTTCAACTCTGTGAGATGAATGCACACATCACAAAGAAGTT

                       *               * *           
     222856 TCTCAGAATGCATCTGTGTAGTTTTTACGGGAAGATATTTC
        131 TCTCAGAATGCTTCTGTGTAGTTTTTATGTGAAGATATTTC

                        ***  *     **      *  *           *      *     ***   
     222897 CTTTTCCACCATCTTCCACAAAGGTCTCCAAGTAACCACTTGCAGACTCTACAGAAAGACACTTT
          1 CTTTTCCACCATAGGCCTCAAAGCGCTCCAAATATCCACTTGCAGATTCTACAAAAAGAGTGTTT

            *          *         ***      *      ***                      *  
     222962 AAAAACTGCTCTATCAAAAGATCAGTTCAAGTCTGTGGTTTGAATGCACACATCACAAAGAATTT
         66 CAAAACTGCTCAATCAAAAGAAAGGTTCAACTCTGTGAGATGAATGCACACATCACAAAGAAGTT

                                     *  *            
     223027 TCTCAGAATGCTTCTGTGTAGTTTTCATATGAAGATATTTC
        131 TCTCAGAATGCTTCTGTGTAGTTTTTATGTGAAGATATTTC

                                    *                                   **   
     223068 CTTTTCCACCATAGGCCTCAAAGCACTCCAAATATCCACTTGCAGATTCTACAAAAAGAGATTTT
          1 CTTTTCCACCATAGGCCTCAAAGCGCTCCAAATATCCACTTGCAGATTCTACAAAAAGAGTGTTT

                   **                           *  *          *        *     
     223133 CAAAACTAGTCAATCAAAAGAAAGGTTCAACTCTGTCAGTTGAATGCACATATCACAAACAAGTT
         66 CAAAACTGCTCAATCAAAAGAAAGGTTCAACTCTGTGAGATGAATGCACACATCACAAAGAAGTT

                *      *                             
     223198 TCTCGGAATGCGTCTGTGTAGTTTTTATGTGAAGATATTTC
        131 TCTCAGAATGCTTCTGTGTAGTTTTTATGTGAAGATATTTC

               *     * *           *     *                  *    *           
     223239 CTTCTCCACAACAGGCCTCAAAGTGCTCCGAATATCCACTTGCAGATTTTAC-TAAAGAGTGTTT
          1 CTTTTCCACCATAGGCCTCAAAGCGCTCCAAATATCCACTTGCAGATTCTACAAAAAGAGTGTTT

             *               *  *   *     *        *    *               *    
     223303 CCAAACTGCTCAATCAAGAGGAAGTTTCAAGTCTGTGAGCTGAACGCACACATCACAAAGTAGTT
         66 CAAAACTGCTCAATCAAAAGAAAGGTTCAACTCTGTGAGATGAATGCACACATCACAAAGAAGTT

               *                                *    
     223368 TCTGAGAATGCTTCTGTGTAGTTTTTATGTGAAGATGTTTC
        131 TCTCAGAATGCTTCTGTGTAGTTTTTATGTGAAGATATTTC

                            **   * *                                    *  * 
     223409 CTTTTCCACCATAGGCTGCAAGGGGCTCCAAATATCCACTTGCAGATTCTACAAAAAGAGAGTCT
          1 CTTTTCCACCATAGGCCTCAAAGCGCTCCAAATATCCACTTGCAGATTCTACAAAAAGAGTGTTT

                 *     *         *          *     *                     *    
     223474 CAAAAGTGCTCTATCAAAAGATAGGTTCAACTATGTGATATGAATGCACACATCACAAAGTAGTT
         66 CAAAACTGCTCAATCAAAAGAAAGGTTCAACTCTGTGAGATGAATGCACACATCACAAAGAAGTT

                                          *          
     223539 TCTCAGAATGCTTCTGTGTAGTTTTTATGTAAAGATATTTC
        131 TCTCAGAATGCTTCTGTGTAGTTTTTATGTGAAGATATTTC

                                    *                                        
     223580 CTTTTCCACCATAGGCCTCAAAGCACTCCAAATATCCACTTGCAGATTCTACAAAAAGAGATG-T
          1 CTTTTCCACCATAGGCCTCAAAGCGCTCCAAATATCCACTTGCAGATTCTACAAAAAGAG-TGTT

                    ** *                   *     *  *    * *   *        *    
     223644 TCAAAACTATTTAATCAAAAGAAAGGTTCAAATCTGTCAGTTGAAGGTACATATCACAAACAAGT
         65 TCAAAACTGCTCAATCAAAAGAAAGGTTCAACTCTGTGAGATGAATGCACACATCACAAAGAAGT

              * **                                    
     223709 TTATTGGAATGCTTCTGTGTAGTTTTTATGTGAAGATATTTC
        130 TTCTCAGAATGCTTCTGTGTAGTTTTTATGTGAAGATATTTC

                     * *         * *                        **  *     *      
     223751 CTTTTCCACAACAGGCCTCAAGGTGCTCCAAATATCCACTTGCAGATTTCACTAAAAGTGTGTTT
          1 CTTTTCCACCATAGGCCTCAAAGCGCTCCAAATATCCACTTGCAGATTCTACAAAAAGAGTGTTT

             *  *            *  *   *     *        *             *           
     223816 CCAAGCTGCTCAATCAAGAGGAAGTTTCAAGTCTGTGAGGTGAATGCACACATTACAAAGAAGTT
         66 CAAAACTGCTCAATCAAAAGAAAGGTTCAACTCTGTGAGATGAATGCACACATCACAAAGAAGTT

            *  *                                     
     223881 GCTGAGAATGCTTCTGTGTAGTTTTTATGTGAAGATATTTC
        131 TCTCAGAATGCTTCTGTGTAGTTTTTATGTGAAGATATTTC

                       *            *  *                                *    
     223922 CTTTTCCACCACAGGCCTCAAAGCACTGCAAATATCCACTTGCAGATTCTACAAAAAGAGAGTTT
          1 CTTTTCCACCATAGGCCTCAAAGCGCTCCAAATATCCACTTGCAGATTCTACAAAAAGAGTGTTT

                      **         *   *         *   *     **            *     
     223987 CAAAACTGCTGTATCAAAAGATAGGGTCAACTCTGCGAGTTGAATAAACACATCACAAATAAGTT
         66 CAAAACTGCTCAATCAAAAGAAAGGTTCAACTCTGTGAGATGAATGCACACATCACAAAGAAGTT

               **   *        *               *       
     224052 TCTGGGAACGCTTCTGTATAG-TTTTATGTGAATATATTTC
        131 TCTCAGAATGCTTCTGTGTAGTTTTTATGTGAAGATATTTC

                         *          *                   *   *  *   * *   *   
     224092 CTTTTCCACCATATGCCTCAAAGCACTCCAAATATCCACTTGCACATTATAGAAACATAGTCTTT
          1 CTTTTCCACCATAGGCCTCAAAGCGCTCCAAATATCCACTTGCAGATTCTACAAAAAGAGTGTTT

                   **                        *         *             *       
     224157 CAAAACTTGTCAATC-AAAGAAAGGTTCAACTCCGTGAGATGAGTGCACACATCACAGAGAAGTT
         66 CAAAACTGCTCAATCAAAAGAAAGGTTCAACTCTGTGAGATGAATGCACACATCACAAAGAAGTT

                *     *                            * 
     224221 TCTCGGAATGTTTCTGTGTAGTTTTTATGTGAAGATATTGC
        131 TCTCAGAATGCTTCTGTGTAGTTTTTATGTGAAGATATTTC

                     *               *            *          *       *   *   
     224262 CTTTTCCACAATAGGCCTCAAAGCGTTCCAAATATCCAATTGCAGATTCCACAAAAAAAGTTTTT
          1 CTTTTCCACCATAGGCCTCAAAGCGCTCCAAATATCCACTTGCAGATTCTACAAAAAGAGTGTTT

            *                 *  *  *  *             * *         **    *     
     224327 TAAAACTGCTCAATCAAATGATAGATTAAACTCTGTGAGATTAGTGCACACATGTCAAAAAAGTT
         66 CAAAACTGCTCAATCAAAAGAAAGGTTCAACTCTGTGAGATGAATGCACACATCACAAAGAAGTT

                                *      * *           
     224392 TCTCAGAATGCTTCTGTGTACTTTTTAGGGGAAGATATTTC
        131 TCTCAGAATGCTTCTGTGTAGTTTTTATGTGAAGATATTTC

                        *    *     **         *    *           **  * *  *    
     224433 CTTTTCCACCATCGGCCACAAAGGACTCCAAATAACCACATGCAGATTCTAGTAACACAGAGTTT
          1 CTTTTCCACCATAGGCCTCAAAGCGCTCCAAATATCCACTTGCAGATTCTACAAAAAGAGTGTTT

                       *         * *           *   * * *    **    ***        
     224498 CAAAACTGCTCTATCAAAAGATAAGTTCAACTCTGAGAGTTTAGTGCAACCATCGTGAAGAAGTT
         66 CAAAACTGCTCAATCAAAAGAAAGGTTCAACTCTGTGAGATGAATGCACACATCACAAAGAAGTT

                            *     *                  
     224563 TCTCAGAATGCTTCTGAGTAGTGTTTATGTGAAGATATTTC
        131 TCTCAGAATGCTTCTGTGTAGTTTTTATGTGAAGATATTTC

                              *     *                      *           **    
     224604 CTTTTCCACCATAGGCCTGAAAGCCCTCCAAATATCCACTTGCAGATCCTACAAAAAGAAAGTTT
          1 CTTTTCCACCATAGGCCTCAAAGCGCTCCAAATATCCACTTGCAGATTCTACAAAAAGAGTGTTT

             *         **     *  *  *   *        **      *                   
     224669 CGAAA-TGCTCTCTCAAACGATAGTTTCGACTCTGTGGTATGAATACACACATCACAAAGAAGTT
         66 CAAAACTGCTCAATCAAAAGAAAGGTTCAACTCTGTGAGATGAATGCACACATCACAAAGAAGTT

                                       **            
     224733 TCTCAGAATGCTTCTGTGTAGTTTTTAAATGAAGATATTTC
        131 TCTCAGAATGCTTCTGTGTAGTTTTTATGTGAAGATATTTC

            *                       *          *     *                       
     224774 TTTTTCCACCATAGGCCTCAAAGCACTCCAAATATGCACTTCCAGATTCTACAAAAAGAGTGTTT
          1 CTTTTCCACCATAGGCCTCAAAGCGCTCCAAATATCCACTTGCAGATTCTACAAAAAGAGTGTTT

              *                 *       * *         **   *         *  * *    
     224839 CAGAACTGCTCAATCAAAAGGAAGGTTCCAGTC--TGAGACAAATACACACATCAAAAGGTAGTT
         66 CAAAACTGCTCAATCAAAAGAAAGGTTCAACTCTGTGAGATGAATGCACACATCACAAAGAAGTT

                                                    *
     224902 TCTCAGAATGCTTCTGTGTAGTTTTTATGTGAAGATATTTT
        131 TCTCAGAATGCTTCTGTGTAGTTTTTATGTGAAGATATTTC

             *               *    **    *     *      *  **   *          *    
     224943 CCTTTCCACCATAGGCCACAAATGGCTCTAAATACCCACTTACATTTTCCACAAAAAGAGAGTTT
          1 CTTTTCCACCATAGGCCTCAAAGCGCTCCAAATATCCACTTGCAGATTCTACAAAAAGAGTGTTT

                       * *    * *      *   *         *                       
     225008 CAAAACTGCTCTACCAAAGGTAA-GTTTAACGCTGTGAG-TTAA-G-A-ACATCACAAAGAAGTT
         66 CAAAACTGCTCAATCAAAAGAAAGGTTCAACTCTGTGAGATGAATGCACACATCACAAAGAAGTT

                                   *   *  *          
     225068 TCTCAGAATGCTTCTGTGTAGTTCTTACGTAAAGATATTTC
        131 TCTCAGAATGCTTCTGTGTAGTTTTTATGTGAAGATATTTC

                 *   *      ***    *                  *              **      
     225109 CTTTTACACAATAGGCAGAAAAGTGCTCCAAATATCCACTTGAAGATTCTACAAAAACCGTGTTT
          1 CTTTTCCACCATAGGCCTCAAAGCGCTCCAAATATCCACTTGCAGATTCTACAAAAAGAGTGTTT

                     **                                          *      *    
     225174 CAAAACTGCCGAATCAAAAGAAAGGTTCAACTCTGTGAGATGAATGCACACATAACAAAGGAGTT
         66 CAAAACTGCTCAATCAAAAGAAAGGTTCAACTCTGTGAGATGAATGCACACATCACAAAGAAGTT

                                 *   *  *      *    *
     225239 TCTCAGAATGCTTCTGTGTAGCTTTAATATGAAGACATTTA
        131 TCTCAGAATGCTTCTGTGTAGTTTTTATGTGAAGATATTTC

            *        * *            *   ***                     **        *  
     225280 GTTTTCCACAACAGGCCTCAAAGCTCTCTCCATATCCACTTGCAGATTCTACCGAAAGAGTGCTT
          1 CTTTTCCACCATAGGCCTCAAAGCGCTCCAAATATCCACTTGCAGATTCTACAAAAAGAGTGTTT

             *                   * **     *        *        *     **         
     225345 CCAAACTGCTCAATCAAAAGAGACATTCAAATCTGTGAGGTGAATGCAGACATCGTAAAGAAGTT
         66 CAAAACTGCTCAATCAAAAGAAAGGTTCAACTCTGTGAGATGAATGCACACATCACAAAGAAGTT

                                *     *       *    **
     225410 TCTCAGAATGCTTCTGTGTATTTTTTGTGTGAAGTTATTCG
        131 TCTCAGAATGCTTCTGTGTAGTTTTTATGTGAAGATATTTC

            *    *             *     *  *            **                 *    
     225451 TTTTTGCACCATAGGCCTCCAAGCGTTCTAAATATCCACTTCTAGATTCTACAAAAAGAGAGTTT
          1 CTTTTCCACCATAGGCCTCAAAGCGCTCCAAATATCCACTTGCAGATTCTACAAAAAGAGTGTTT

                   *         *            *        *    *   *                
     225516 CAAAACTACTC-A--AACA-AAAGGTTCAATTCTGTGAGTTGAAAGCAAACATCACAAAGAAGTT
         66 CAAAACTGCTCAATCAAAAGAAAGGTTCAACTCTGTGAGATGAATGCACACATCACAAAGAAGTT

                       *                             
     225577 TCTCAGAATGCGTCTGTGTAGTTTTTATGTGAAGATATTTC
        131 TCTCAGAATGCTTCTGTGTAGTTTTTATGTGAAGATATTTC

                     **   ****     *                  *                      
     225618 CTTTT-CACAGTAGAATGCAAAGGGCTCCAAATATCCACTTGGAGATTCTACAAAAAGA--GTTT
          1 CTTTTCCACCATAGGCCTCAAAGCGCTCCAAATATCCACTTGCAGATTCTACAAAAAGAGTGTTT

                  *    **     *  *     *     ***   *     *               *   
     225680 CAAAACCGCTCTGTCAAATGATAGGTTGAACTCCCGGAGGTGAATACACACATCACAAAGAGGTT
         66 CAAAACTGCTCAATCAAAAGAAAGGTTCAACTCTGTGAGATGAATGCACACATCACAAAGAAGTT

                  *                       *  *       
     225745 TCTCAGCATGCTTCTGTGTAGTTTTTATGTAAACATATTTC
        131 TCTCAGAATGCTTCTGTGTAGTTTTTATGTGAAGATATTTC

             *    * *              *           *      * *       *   *    *   
     225786 CGTTTCTATCATAGGCCTCAAAGTGCTCCAAATATTCACTTGTACATTCTACCAAACGAGTATTT
          1 CTTTTCCACCATAGGCCTCAAAGCGCTCCAAATATCCACTTGCAGATTCTACAAAAAGAGTGTTT

                              * *         **      *        *            *    
     225851 CAAAACTGCTCAATCAAATGGAAGGTTCAAAACTGTGACATGAATGCCCACATCACAAAGTAGTT
         66 CAAAACTGCTCAATCAAAAGAAAGGTTCAACTCTGTGAGATGAATGCACACATCACAAAGAAGTT

                                                     
     225916 TCTCAGAATGCTTCTGTGTAGTTTTTATGTGAAGATATTTC
        131 TCTCAGAATGCTTCTGTGTAGTTTTTATGTGAAGATATTTC

                     * *    *      *    *       * *   **      *              
     225957 CTTTTCCACAACA-GCGTGCAAAACGCTTCAAATATGCCCTTAGAGATTCCACAAAAAGAGTGTT
          1 CTTTTCCACCATAGGCCT-CAAAGCGCTCCAAATATCCACTTGCAGATTCTACAAAAAGAGTGTT

              *     *               *  *                                  ** 
     226021 TCCAAACTACTCAAATCAAAA-AATGATTTCAACTCTGTGAGATGAATGCACACATCACAAACTA
         65 TCAAAACTGCTC-AATCAAAAGAAAG-GTTCAACTCTGTGAGATGAATGCACACATCACAAAGAA

                         *     ** *   * *   *       *   
     226085 GTTTCTCAGAATGTTTCTGCCTGGTTCTCATGCGAAGATAGTTC
        128 GTTTCTCAGAATGCTTCTGTGTAGTTTTTATGTGAAGATATTTC

                 *           *   * **              *                  * *    
     226129 CTTTTTCACCATAGGCCGCAATGTACTCCAAATATCCACCTGCAGATTCTAC-AAAAGTGAGTTT
          1 CTTTTCCACCATAGGCCTCAAAGCGCTCCAAATATCCACTTGCAGATTCTACAAAAAGAGTGTTT

                       *         ***    **         *        *      *       * 
     226193 CAAAACTGCTCTATCAAAAGATCAGTTCGTCTCTGTGAGTTGAATGCATACATCAAAAAGAAGCT
         66 CAAAACTGCTCAATCAAAAGAAAGGTTCAACTCTGTGAGATGAATGCACACATCACAAAGAAGTT

                 *             *      **         *   
     226258 TCTCAAAATGCTTCTGTGTGGTTTTTCGGTGAAGATAGTTC
        131 TCTCAGAATGCTTCTGTGTAGTTTTTATGTGAAGATATTTC

            *     *        *      * *                 *        *        *    
     226299 TTTTTCTACCATAGGTCTCAAACCACTCCAAATATCCACTTGTAGATTCTATAAAAAGGAATG-T
          1 CTTTTCCACCATAGGCCTCAAAGCGCTCCAAATATCCACTTGCAGATTCTACAAAAA-GAGTGTT

                  *        *    *    *      * *         *      *             
     226363 TCAAAATTGCTCAATAAAAATAAAGTTTCAACACCGTGAGATGAGTGCACAAATCACAAAGAAGT
         65 TCAAAACTGCTCAATCAAAAGAAAGGTTCAACTCTGTGAGATGAATGCACACATCACAAAGAAGT

                  *          *         *          *   
     226428 TTCTCAAAATGCTTCTGGGTAGTTTTTCTGTGAAGATAGTTC
        130 TTCTCAGAATGCTTCTGTGTAGTTTTTATGTGAAGATATTTC

                  *     *    *     *          *                         *    
     226470 CTTTTCTACCATGGGCCACAAAGGGCTCCAAATACCCACTTGCAGATTCTACAAAAAGAGAGTTT
          1 CTTTTCCACCATAGGCCTCAAAGCGCTCCAAATATCCACTTGCAGATTCTACAAAAAGAGTGTTT

              *        *      ** * *        *    * *    **  * *           *  
     226535 CACAACTGCTCTATCAAACAATATGTTCAACTTTGTGGGTTGAACACAAATATCAC-AAGAATTT
         66 CAAAACTGCTCAATCAAAAGAAAGGTTCAACTCTGTGAGATGAATGCACACATCACAAAGAAGTT

                **                             *     
     226599 TCTCCCAATGCTTCTGTGTAGTTTTTATGTGAAGACATTTC
        131 TCTCAGAATGCTTCTGTGTAGTTTTTATGTGAAGATATTTC

            *   *  *      *  *     *                 *  *      *       *  *  
     226640 TTTTCCCTCCATAGTCCACAAAGTGCTCCAAATATCCACTTACATATTCTAGAAAAAGATTGCTT
          1 CTTTTCCACCATAGGCCTCAAAGCGCTCCAAATATCCACTTGCAGATTCTACAAAAAGAGTGTTT

            **       *    *           *   * * *                              
     226705 GGAAACTGCACAATGAAAAGAAAGGTCCAAATATATGAGATGAATGCACACATCACAAAGAAGTT
         66 CAAAACTGCTCAATCAAAAGAAAGGTTCAACTCTGTGAGATGAATGCACACATCACAAAGAAGTT

                                 *                    
     226770 TCTCAGAAT-CTCTCTGTGTAATTTTTATGTGAAGATATTTC
        131 TCTCAGAATGCT-TCTGTGTAGTTTTTATGTGAAGATATTTC

                *     *    *  *   *                        *      *    *     
     226811 CTTTCCCACCTTAGGTCTTAAAACGCTCCAAATATCCACTTGCAGATACTACAAGAAGATTGTTT
          1 CTTTTCCACCATAGGCCTCAAAGCGCTCCAAATATCCACTTGCAGATTCTACAAAAAGAGTGTTT

                     *    *        *      *     **                           
     226876 CAAAACTGCACAA-AAAAAGAAATGTTCAATTCTGTTTGATGAATGCACACATCACAAAGAAGTT
         66 CAAAACTGCTCAATCAAAAGAAAGGTTCAACTCTGTGAGATGAATGCACACATCACAAAGAAGTT

                           *                         
     226940 TCTCAGAATGCTTCTCTGTAGTTTTTATGTGAAGATATTTC
        131 TCTCAGAATGCTTCTGTGTAGTTTTTATGTGAAGATATTTC

                     *             *                 *         **      * *   
     226981 CTTTTCCACAATAGGCCTCAAAGGGCTCCAAATATCCACTTCCAGATTCTATGAAAAGAATATTT
          1 CTTTTCCACCATAGGCCTCAAAGCGCTCCAAATATCCACTTGCAGATTCTACAAAAAGAGTGTTT

             *              * *    *                                      *  
     227046 CCAAACTGCTCAATCATAGGAAATGTTCAACTCTGTGAGATGAATGCACACATCAC-AAGAAATT
         66 CAAAACTGCTCAATCAAAAGAAAGGTTCAACTCTGTGAGATGAATGCACACATCACAAAGAAGTT

                     *    *      *       *       *   
     227110 TCTCAGAATCCTTCAGTGTAGGTTTTATGAGAAGATAATTC
        131 TCTCAGAATGCTTCTGTGTAGTTTTTATGTGAAGATATTTC

                     *    **        *   *                           *    *   
     227151 CTTTTCCACAATAGTTCTCAAAGCACTCAAAATATCCACTTGCAGATTCTACAAAAGGAGTATTT
          1 CTTTTCCACCATAGGCCTCAAAGCGCTCCAAATATCCACTTGCAGATTCTACAAAAAGAGTGTTT

                                                 *        *                  
     227216 CAAAACTGCTCAATCAAAAGAAAGGTTCAACTCTGTGGGATGAATGGACACATCACAAAGAAGTT
         66 CAAAACTGCTCAATCAAAAGAAAGGTTCAACTCTGTGAGATGAATGCACACATCACAAAGAAGTT

                                  *   *              
     227281 TCTCAGAATGCTTCTGTGTAGTATTTTTGTGAAGATATTT-
        131 TCTCAGAATGCTTCTGTGTAGTTTTTATGTGAAGATATTTC

                          *  * * * * ***             *            *     * *  
     227321 CTTTTCCACCATAGACCGCCAGGGGACACAAATATCCACTTTCAGATTCTACAACAAGAGAGGTT
          1 CTTTTCCACCATAGGCCTCAAAGCGCTCCAAATATCCACTTGCAGATTCTACAAAAAGAGTGTTT

                   *   *     *   ** *       *      *                      * *
     227386 CAAAACTACTCGATCAAGAGATGGTTTCAACTATGTGAGTTGAATGCACACATCACAAAGAACTA
         66 CAAAACTGCTCAATCAAAAGAAAGGTTCAACTCTGTGAGATGAATGCACACATCACAAAGAAGTT

             *  *    *                               
     227451 TGTCGGAATTCTTCTGTGTAGTTTTTATGTGAAGATATTTC
        131 TCTCAGAATGCTTCTGTGTAGTTTTTATGTGAAGATATTTC

                     *    * *      * *    *                  *               
     227492 CTTTTCCACAATAGACGTCAAAGTGATCCAGATATCCACTTGCAGATTCCACAAAAAGAGTGTTT
          1 CTTTTCCACCATAGGCCTCAAAGCGCTCCAAATATCCACTTGCAGATTCTACAAAAAGAGTGTTT

                 *   *   *                  **                     *  *      
     227557 CAAAAGTGCACAACCAAAAGAAAGGTTCAACTAGGTGAGATGAATGCACACATCAGAAGGAAGTT
         66 CAAAACTGCTCAATCAAAAGAAAGGTTCAACTCTGTGAGATGAATGCACACATCACAAAGAAGTT

                            **   *     * *       *   
     227622 TCTCAGAATGCTTCTGCATAGCTTTTAAGGGAAGATACTTC
        131 TCTCAGAATGCTTCTGTGTAGTTTTTATGTGAAGATATTTC

                    *               *            * *  *    * *               
     227663 CTTTTCCAACATAGGCCTCAAAGCACTCCAAATATCCTCCTGGAGATACCACAAAAAGAGTGTTT
          1 CTTTTCCACCATAGGCCTCAAAGCGCTCCAAATATCCACTTGCAGATTCTACAAAAAGAGTGTTT

            **                      *  *                 *       *           
     227728 GCAAACTGCTCAATCAAAAGAAAGATTTAACTCTGTGAGATGAATCCACACATGACAAAGAAGTT
         66 CAAAACTGCTCAATCAAAAGAAAGGTTCAACTCTGTGAGATGAATGCACACATCACAAAGAAGTT

                                                     
     227793 TCTCAGAATGCTTCTGTGTAGTTTTTATGTGAAGATATTTC
        131 TCTCAGAATGCTTCTGTGTAGTTTTTATGTGAAGATATTTC

                     *   * * *    **           *                *     *      
     227834 CTTTTCCACAATAAGACCCAAAAGGCTCCAAATATTCACTTGCAGATTCTAAAAAAAACAGTGTT
          1 CTTTTCCACCATAGGCCTCAAAGCGCTCCAAATATCCACTTGCAGATTCT-ACAAAAAGAGTGTT

                                  *                  *      *        **      
     227899 TCAAAACTGCTCAATCAAAAGATA-GTTCAACTCTGTGAGAAGAATGCTCACATCACTGAGAAGT
         65 TCAAAACTGCTCAATCAAAAGAAAGGTTCAACTCTGTGAGATGAATGCACACATCACAAAGAAGT

                                         *            
     227963 TTCTCAGAATGCTTCTGTGTAGTTTTTATATGAAGATATTTC
        130 TTCTCAGAATGCTTCTGTGTAGTTTTTATGTGAAGATATTTC

                *     *      *    **                       *   **       *    
     228005 CTTTCCCACCGTAGGCCACAAAAGGCTCCAAATATCCACTTGCAGATACTATGAAAAGAGAGTTT
          1 CTTTTCCACCATAGGCCTCAAAGCGCTCCAAATATCCACTTGCAGATTCTACAAAAAGAGTGTTT

                        *        *               ***            *            
     228070 CAAAACTGCTCATTCAAAAGATAGGTTCAACTCTGTGGTTTGAATGCACACAGCACAAAGAAGTT
         66 CAAAACTGCTCAATCAAAAGAAAGGTTCAACTCTGTGAGATGAATGCACACATCACAAAGAAGTT

              *       **                   **   *    
     228135 TCACAGAATGTGTCTGTGTAGTTTTTATGTGCGGATGTTTC
        131 TCTCAGAATGCTTCTGTGTAGTTTTTATGTGAAGATATTTC

                         *        ****   *   *               *    *          
     228176 CTTTTCCACCATATGCCT-AAATATTTCCCAATTTCCACTTGCAGATTCCACAAGAAGAGTGTTT
          1 CTTTTCCACCATAGGCCTCAAAGCGCTCCAAATATCCACTTGCAGATTCTACAAAAAGAGTGTTT

                      **       *       *           *            *      ***   
     228240 CAAAACTGCTGTATC-AAATAAA-GTTGAACTCTGTGAGGTGAATGCACACAGCACAAAATGGTT
         66 CAAAACTGCTCAATCAAAAGAAAGGTTCAACTCTGTGAGATGAATGCACACATCACAAAGAAGTT

                          **   *        *       *    
     228303 TCTCAGAATGCTTCCTTGTTGTTTTTATATGAAGATGTTTC
        131 TCTCAGAATGCTTCTGTGTAGTTTTTATGTGAAGATATTTC

                  *  *             *   *     *                               
     228344 CTTTTCAACAATAGGCCTCAAAGTGCTTCAAATGTCCACTTGCAGATTCTACAAAAAGAGTGTTT
          1 CTTTTCCACCATAGGCCTCAAAGCGCTCCAAATATCCACTTGCAGATTCTACAAAAAGAGTGTTT

                                        *      *  *  *                       
     228409 CAAAACTGCTCAATCAAAAGAAAGGTTCGACTCTGGGAAATTAATGCACACATCACAAAGAAGTT
         66 CAAAACTGCTCAATCAAAAGAAAGGTTCAACTCTGTGAGATGAATGCACACATCACAAAGAAGTT

                                     *        *      
     228474 TCTC---A-GCTTCTGTGTAGTTTTCATGTGAAGTTATTTC
        131 TCTCAGAATGCTTCTGTGTAGTTTTTATGTGAAGATATTTC

                     *       *     *            *    *         **       *    
     228511 CTTTTCCACAATAGGCCGCAAAGGGCTCCAAATATCAACTTACAGATTCTAGGAAAAGAGAGTTT
          1 CTTTTCCACCATAGGCCTCAAAGCGCTCCAAATATCCACTTGCAGATTCTACAAAAAGAGTGTTT

                       * **      *     *                                     
     228576 CAAAACTGCTCTACGAAAAGATAGGTTGAACTCTGTGAGATGAATGCACACATCACAAAGAAGTT
         66 CAAAACTGCTCAATCAAAAGAAAGGTTCAACTCTGTGAGATGAATGCACACATCACAAAGAAGTT

                       *               * *           
     228641 TCTCAGAATGCATCTGTGTAGTTTTTACGGGAAGATATTTC
        131 TCTCAGAATGCTTCTGTGTAGTTTTTATGTGAAGATATTTC

                        ***  *     **      *  *           *      *     ***   
     228682 CTTTTCCACCATCTTCCACAAAGGTCTCCAAGTAACCACTTGCAGACTCTACAGAAAGACACTTT
          1 CTTTTCCACCATAGGCCTCAAAGCGCTCCAAATATCCACTTGCAGATTCTACAAAAAGAGTGTTT

            *          *         ***      *      ***                      *  
     228747 AAAAACTGCTCTATCAAAAGATCAGTTCAAGTCTGTGGTTTGAATGCACACATCACAAAGAATTT
         66 CAAAACTGCTCAATCAAAAGAAAGGTTCAACTCTGTGAGATGAATGCACACATCACAAAGAAGTT

                                     *  *            
     228812 TCTCAGAATGCTTCTGTGTAGTTTTCATATGAAGATATTTC
        131 TCTCAGAATGCTTCTGTGTAGTTTTTATGTGAAGATATTTC

                                    *                                   **   
     228853 CTTTTCCACCATAGGCCTCAAAGCACTCCAAATATCCACTTGCAGATTCTACAAAAAGAGATTTT
          1 CTTTTCCACCATAGGCCTCAAAGCGCTCCAAATATCCACTTGCAGATTCTACAAAAAGAGTGTTT

                   **                           *  *          *        *     
     228918 CAAAACTAGTCAATCAAAAGAAAGGTTCAACTCTGTCAGTTGAATGCACATATCACAAACAAGTT
         66 CAAAACTGCTCAATCAAAAGAAAGGTTCAACTCTGTGAGATGAATGCACACATCACAAAGAAGTT

                *      *                             
     228983 TCTCGGAATGCGTCTGTGTAGTTTTTATGTGAAGATATTTC
        131 TCTCAGAATGCTTCTGTGTAGTTTTTATGTGAAGATATTTC

               *     * *           *     *                  *    *           
     229024 CTTCTCCACAACAGGCCTCAAAGTGCTCCGAATATCCACTTGCAGATTTTAC-TAAAGAGTGTTT
          1 CTTTTCCACCATAGGCCTCAAAGCGCTCCAAATATCCACTTGCAGATTCTACAAAAAGAGTGTTT

             *               *  *   *     *        *    *               *    
     229088 CCAAACTGCTCAATCAAGAGGAAGTTTCAAGTCTGTGAGCTGAACGCACACATCACAAAGTAGTT
         66 CAAAACTGCTCAATCAAAAGAAAGGTTCAACTCTGTGAGATGAATGCACACATCACAAAGAAGTT

               *                                *    
     229153 TCTGAGAATGCTTCTGTGTAGTTTTTATGTGAAGATGTTTC
        131 TCTCAGAATGCTTCTGTGTAGTTTTTATGTGAAGATATTTC

                            **     *                                    *    
     229194 CTTTTCCACCATAGGCTGCAAAGGGCTCCAAATATCCACTTGCAGATTCTACAAAAAGAGAGTTT
          1 CTTTTCCACCATAGGCCTCAAAGCGCTCCAAATATCCACTTGCAGATTCTACAAAAAGAGTGTTT

                 *     *         *          *     *                     *    
     229259 CAAAAGTGCTCTATCAAAAGATAGGTTCAACTATGTGATATGAATGCACACATCACAAAGTAGTT
         66 CAAAACTGCTCAATCAAAAGAAAGGTTCAACTCTGTGAGATGAATGCACACATCACAAAGAAGTT

                                          *          
     229324 TCTCAGAATGCTTCTGTGTAGTTTTTATGTAAAGATATTTC
        131 TCTCAGAATGCTTCTGTGTAGTTTTTATGTGAAGATATTTC

                                    *                                   **   
     229365 CTTTTCCACCATAGGCCTCAAAGCACTCCAAATATCCACTTGCAGATTCTACAAAAAGAGATTTT
          1 CTTTTCCACCATAGGCCTCAAAGCGCTCCAAATATCCACTTGCAGATTCTACAAAAAGAGTGTTT

                   ** *                   *     *  *    * *   *        *     
     229430 CAAAACTATTTAATCAAAAGAAAGGTTCAAATCTGTCAGTTGAAGGTACATATCACAAACAAGTT
         66 CAAAACTGCTCAATCAAAAGAAAGGTTCAACTCTGTGAGATGAATGCACACATCACAAAGAAGTT

             * **                                    
     229495 TATTGGAATGCTTCTGTGTAGTTTTTATGTGAAGATATTTC
        131 TCTCAGAATGCTTCTGTGTAGTTTTTATGTGAAGATATTTC

                     * *         * *                        **  *     *      
     229536 CTTTTCCACAACAGGCCTCAAGGTGCTCCAAATATCCACTTGCAGATTTCACTAAAAGTGTGTTT
          1 CTTTTCCACCATAGGCCTCAAAGCGCTCCAAATATCCACTTGCAGATTCTACAAAAAGAGTGTTT

             *  *            *  *   *     *        *             *           
     229601 CCAAGCTGCTCAATCAAGAGGAAGTTTCAAGTCTGTGAGGTGAATGCACACATTACAAAGAAGTT
         66 CAAAACTGCTCAATCAAAAGAAAGGTTCAACTCTGTGAGATGAATGCACACATCACAAAGAAGTT

            *  *                                     
     229666 ACTGAGAATGCTTCTGTGTAGTTTTTATGTGAAGATATTTC
        131 TCTCAGAATGCTTCTGTGTAGTTTTTATGTGAAGATATTTC

                      **               *                                *    
     229707 CTTTTCCACCGCAGGCCTCAAAGCGCTGCAAATATCCACTTGCAGATTCTACAAAAAGAGAGTTT
          1 CTTTTCCACCATAGGCCTCAAAGCGCTCCAAATATCCACTTGCAGATTCTACAAAAAGAGTGTTT

                      **         *   *         *   *     **            *     
     229772 CAAAACTGCTGTATCAAAAGATAGGGTCAACTCTGCGAGTTGAATAAACACATCACAAATAAGTT
         66 CAAAACTGCTCAATCAAAAGAAAGGTTCAACTCTGTGAGATGAATGCACACATCACAAAGAAGTT

               **   *        *               *       
     229837 TCTGGGAACGCTTCTGTATAG-TTTTATGTGAATATATTTC
        131 TCTCAGAATGCTTCTGTGTAGTTTTTATGTGAAGATATTTC

                         *          *                   *   *  *   * *   *   
     229877 CTTTTCCACCATATGCCTCAAAGCACTCCAAATATCCACTTGCACATTATAGAAACATAGTCTTT
          1 CTTTTCCACCATAGGCCTCAAAGCGCTCCAAATATCCACTTGCAGATTCTACAAAAAGAGTGTTT

                   **                        *         *             *       
     229942 CAAAACTTGTCAATC-AAAGAAAGGTTCAACTCCGTGAGATGAGTGCACACATCACAGAGAAGTT
         66 CAAAACTGCTCAATCAAAAGAAAGGTTCAACTCTGTGAGATGAATGCACACATCACAAAGAAGTT

                *     *                            * 
     230006 TCTCGGAATGTTTCTGTGTAGTTTTTATGTGAAGATATTGC
        131 TCTCAGAATGCTTCTGTGTAGTTTTTATGTGAAGATATTTC

                     *               *            *          *       *   *   
     230047 CTTTTCCACAATAGGCCTCAAAGCGTTCCAAATATCCAATTGCAGATTCCACAAAAAAAGTTTTT
          1 CTTTTCCACCATAGGCCTCAAAGCGCTCCAAATATCCACTTGCAGATTCTACAAAAAGAGTGTTT

            *                 *  *  *  *             * *         **    *     
     230112 TAAAACTGCTCAATCAAATGATAGATTAAACTCTGTGAGATTAGTGCACACATGTCAAAAAAGTT
         66 CAAAACTGCTCAATCAAAAGAAAGGTTCAACTCTGTGAGATGAATGCACACATCACAAAGAAGTT

                                *      * *           
     230177 TCTCAGAATGCTTCTGTGTACTTTTTAGGGGAAGATATTTC
        131 TCTCAGAATGCTTCTGTGTAGTTTTTATGTGAAGATATTTC

                        *    *     **         *    *           **  * *  *    
     230218 CTTTTCCACCATCGGCCACAAAGGACTCCAAATAACCACATGCAGATTCTAGTAACACAGAGTTT
          1 CTTTTCCACCATAGGCCTCAAAGCGCTCCAAATATCCACTTGCAGATTCTACAAAAAGAGTGTTT

                       *         * *           *   * * *    **    ***        
     230283 CAAAACTGCTCTATCAAAAGATAAGTTCAACTCTGAGAGTTTAGTGCAACCATCGTGAAGAAGTT
         66 CAAAACTGCTCAATCAAAAGAAAGGTTCAACTCTGTGAGATGAATGCACACATCACAAAGAAGTT

                            *     *                  
     230348 TCTCAGAATGCTTCTGAGTAGTGTTTATGTGAAGATATTTC
        131 TCTCAGAATGCTTCTGTGTAGTTTTTATGTGAAGATATTTC

                              *     *                      *           **    
     230389 CTTTTCCACCATAGGCCTGAAAGCCCTCCAAATATCCACTTGCAGATCCTACAAAAAGAAAGTTT
          1 CTTTTCCACCATAGGCCTCAAAGCGCTCCAAATATCCACTTGCAGATTCTACAAAAAGAGTGTTT

             *         **     *  *  *   *        **      *                   
     230454 CGAAA-TGCTCTCTCAAACGATAGTTTCGACTCTGTGGTATGAATACACACATCACAAAGAAGTT
         66 CAAAACTGCTCAATCAAAAGAAAGGTTCAACTCTGTGAGATGAATGCACACATCACAAAGAAGTT

                                       **            
     230518 TCTCAGAATGCTTCTGTGTAGTTTTTAAATGAAGATATTTC
        131 TCTCAGAATGCTTCTGTGTAGTTTTTATGTGAAGATATTTC

            *                       *          *     *                       
     230559 TTTTTCCACCATAGGCCTCAAAGCACTCCAAATATGCACTTCCAGATTCTACAAAAAGAGTGTTT
          1 CTTTTCCACCATAGGCCTCAAAGCGCTCCAAATATCCACTTGCAGATTCTACAAAAAGAGTGTTT

              *                 *       * *         **   *         *  * *    
     230624 CAGAACTGCTCAATCAAAAGGAAGGTTCCAGTC--TGAGACAAATACACACATCAAAAGGTAGTT
         66 CAAAACTGCTCAATCAAAAGAAAGGTTCAACTCTGTGAGATGAATGCACACATCACAAAGAAGTT

                                                    *
     230687 TCTCAGAATGCTTCTGTGTAGTTTTTATGTGAAGATATTTT
        131 TCTCAGAATGCTTCTGTGTAGTTTTTATGTGAAGATATTTC

             *               *    **    *     *      *  **   *          *    
     230728 CCTTTCCACCATAGGCCACAAATGGCTCTAAATACCCACTTACATTTTCCACAAAAAGAGAGTTT
          1 CTTTTCCACCATAGGCCTCAAAGCGCTCCAAATATCCACTTGCAGATTCTACAAAAAGAGTGTTT

                       * *    * *      *   *         *                       
     230793 CAAAACTGCTCTACCAAAGGTAA-GTTTAACGCTGTGAG-TTAA-G-A-ACATCACAAAGAAGTT
         66 CAAAACTGCTCAATCAAAAGAAAGGTTCAACTCTGTGAGATGAATGCACACATCACAAAGAAGTT

                                   *   *  *          
     230853 TCTCAGAATGCTTCTGTGTAGTTCTTACGTAAAGATATTTC
        131 TCTCAGAATGCTTCTGTGTAGTTTTTATGTGAAGATATTTC

                 *   *      ***    *                  *              **      
     230894 CTTTTACACAATAGGCAGAAAAGTGCTCCAAATATCCACTTGAAGATTCTACAAAAACCGTGTTT
          1 CTTTTCCACCATAGGCCTCAAAGCGCTCCAAATATCCACTTGCAGATTCTACAAAAAGAGTGTTT

                     **                                          *      *    
     230959 CAAAACTGCCGAATCAAAAGAAAGGTTCAACTCTGTGAGATGAATGCACACATAACAAAGGAGTT
         66 CAAAACTGCTCAATCAAAAGAAAGGTTCAACTCTGTGAGATGAATGCACACATCACAAAGAAGTT

                                 *   *  *      *    *
     231024 TCTCAGAATGCTTCTGTGTAGCTTTAATATGAAGACATTTA
        131 TCTCAGAATGCTTCTGTGTAGTTTTTATGTGAAGATATTTC

            *        * *            *   ***                     **        *  
     231065 GTTTTCCACAACAGGCCTCAAAGCTCTCTCCATATCCACTTGCAGATTCTACCGAAAGAGTGCTT
          1 CTTTTCCACCATAGGCCTCAAAGCGCTCCAAATATCCACTTGCAGATTCTACAAAAAGAGTGTTT

             *                   * **     *        *        *     **         
     231130 CCAAACTGCTCAATCAAAAGAGACATTCAAATCTGTGAGGTGAATGCAGACATCGTAAAGAAGTT
         66 CAAAACTGCTCAATCAAAAGAAAGGTTCAACTCTGTGAGATGAATGCACACATCACAAAGAAGTT

                                *     *       *    **
     231195 TCTCAGAATGCTTCTGTGTATTTTTTGTGTGAAGTTATTCG
        131 TCTCAGAATGCTTCTGTGTAGTTTTTATGTGAAGATATTTC

            *    *             *     *  *            **                 *    
     231236 TTTTTGCACCATAGGCCTCCAAGCGTTCTAAATATCCACTTCTAGATTCTACAAAAAGAGAGTTT
          1 CTTTTCCACCATAGGCCTCAAAGCGCTCCAAATATCCACTTGCAGATTCTACAAAAAGAGTGTTT

                   *         *            *        *    *   *                
     231301 CAAAACTACTC-A--AACA-AAAGGTTCAATTCTGTGAGTTGAAAGCAAACATCACAAAGAAGTT
         66 CAAAACTGCTCAATCAAAAGAAAGGTTCAACTCTGTGAGATGAATGCACACATCACAAAGAAGTT

                       *             *               
     231362 TCTCAGAATGCGTCTGTGTAGTTTTGATGTGAAGATATTTC
        131 TCTCAGAATGCTTCTGTGTAGTTTTTATGTGAAGATATTTC

                     **   ****     *                  *                      
     231403 CTTTT-CACAGTAGAATGCAAAGGGCTCCAAATATCCACTTGGAGATTCTACAAAAAGA--GTTT
          1 CTTTTCCACCATAGGCCTCAAAGCGCTCCAAATATCCACTTGCAGATTCTACAAAAAGAGTGTTT

                  *    **     *  *     *     ***   *     *               *   
     231465 CAAAACCGCTCTGTCAAATGATAGGTTGAACTCCCGGAGGTGAATACACACATCACAAAGAGGTT
         66 CAAAACTGCTCAATCAAAAGAAAGGTTCAACTCTGTGAGATGAATGCACACATCACAAAGAAGTT

                  *                       *  *       
     231530 TCTCAGCATGCTTCTGTGTAGTTTTTATGTAAACATATTTC
        131 TCTCAGAATGCTTCTGTGTAGTTTTTATGTGAAGATATTTC

             *    * *              *           *      * *       *   *    *   
     231571 CGTTTCTATCATAGGCCTCAAAGTGCTCCAAATATTCACTTGTACATTCTACCAAACGAGTATTT
          1 CTTTTCCACCATAGGCCTCAAAGCGCTCCAAATATCCACTTGCAGATTCTACAAAAAGAGTGTTT

                              * *         **      *        *            *    
     231636 CAAAACTGCTCAATCAAATGGAAGGTTCAAAACTGTGACATGAATGCCCACATCACAAAGTAGTT
         66 CAAAACTGCTCAATCAAAAGAAAGGTTCAACTCTGTGAGATGAATGCACACATCACAAAGAAGTT

                                                     
     231701 TCTCAGAATGCTTCTGTGTAGTTTTTATGTGAAGATATTTC
        131 TCTCAGAATGCTTCTGTGTAGTTTTTATGTGAAGATATTTC

                     * *    *      *    *       * *   **      *              
     231742 CTTTTCCACAACA-GCGTGCAAAACGCTTCAAATATGCCCTTAGAGATTCCACAAAAAGAGTGTT
          1 CTTTTCCACCATAGGCCT-CAAAGCGCTCCAAATATCCACTTGCAGATTCTACAAAAAGAGTGTT

              *     *               *  *                                  ** 
     231806 TCCAAACTACTCAAATCAAAA-AATGATTTCAACTCTGTGAGATGAATGCACACATCACAAACTA
         65 TCAAAACTGCTC-AATCAAAAGAAAG-GTTCAACTCTGTGAGATGAATGCACACATCACAAAGAA

                         *     ** *   * *   *       *   
     231870 GTTTCTCAGAATGTTTCTGCCTGGTTCTCATGCGAAGATAGTTC
        128 GTTTCTCAGAATGCTTCTGTGTAGTTTTTATGTGAAGATATTTC

                 *           *   * **              *                  * *    
     231914 CTTTTTCACCATAGGCCGCAATGTACTCCAAATATCCACCTGCAGATTCTAC-AAAAGTGAGTTT
          1 CTTTTCCACCATAGGCCTCAAAGCGCTCCAAATATCCACTTGCAGATTCTACAAAAAGAGTGTTT

                       *         ***    **         *        *      *       * 
     231978 CAAAACTGCTCTATCAAAAGATCAGTTCGTCTCTGTGAGTTGAATGCATACATCAAAAAGAAGCT
         66 CAAAACTGCTCAATCAAAAGAAAGGTTCAACTCTGTGAGATGAATGCACACATCACAAAGAAGTT

                 *             *      **         *   
     232043 TCTCAAAATGCTTCTGTGTGGTTTTTCGGTGAAGATAGTTC
        131 TCTCAGAATGCTTCTGTGTAGTTTTTATGTGAAGATATTTC

            *     *        *      * *                 *        *        *    
     232084 TTTTTCTACCATAGGTCTCAAACCACTCCAAATATCCACTTGTAGATTCTATAAAAAGGAATG-T
          1 CTTTTCCACCATAGGCCTCAAAGCGCTCCAAATATCCACTTGCAGATTCTACAAAAA-GAGTGTT

                  *        *    *    *      * *         *      *             
     232148 TCAAAATTGCTCAATAAAAATAAAGTTTCAACACCGTGAGATGAGTGCACAAATCACAAAGAAGT
         65 TCAAAACTGCTCAATCAAAAGAAAGGTTCAACTCTGTGAGATGAATGCACACATCACAAAGAAGT

                  *          *         *          *   
     232213 TTCTCAAAATGCTTCTGGGTAGTTTTTCTGTGAAGATAGTTC
        130 TTCTCAGAATGCTTCTGTGTAGTTTTTATGTGAAGATATTTC

                  *     *    *     *          *                         *    
     232255 CTTTTCTACCATGGGCCACAAAGGGCTCCAAATACCCACTTGCAGATTCTACAAAAAGAGAGTTT
          1 CTTTTCCACCATAGGCCTCAAAGCGCTCCAAATATCCACTTGCAGATTCTACAAAAAGAGTGTTT

              *        *      ** * *        *    * *    **  * *           *  
     232320 CACAACTGCTCTATCAAACAATATGTTCAACTTTGTGGGTTGAACACAAATATCAC-AAGAATTT
         66 CAAAACTGCTCAATCAAAAGAAAGGTTCAACTCTGTGAGATGAATGCACACATCACAAAGAAGTT

                **                             *     
     232384 TCTCCCAATGCTTCTGTGTAGTTTTTATGTGAAGACATTTC
        131 TCTCAGAATGCTTCTGTGTAGTTTTTATGTGAAGATATTTC

            *   *  *      *  *     *                 *  *      *       *  *  
     232425 TTTTCCCTCCATAGTCCACAAAGTGCTCCAAATATCCACTTACATATTCTAGAAAAAGATTGCTT
          1 CTTTTCCACCATAGGCCTCAAAGCGCTCCAAATATCCACTTGCAGATTCTACAAAAAGAGTGTTT

            **       *    *               * * *                              
     232490 GGAAACTGCACAATGAAAAGAAAGGTTCAAATATATGAGATGAATGCACACATCACAAAGAAGTT
         66 CAAAACTGCTCAATCAAAAGAAAGGTTCAACTCTGTGAGATGAATGCACACATCACAAAGAAGTT

                                 *                    
     232555 TCTCAGAAT-CTCTCTGTGTAATTTTTATGTGAAGATATTTC
        131 TCTCAGAATGCT-TCTGTGTAGTTTTTATGTGAAGATATTTC

                *     *    *  *   *                        *      *    *     
     232596 CTTTCCCACCTTAGGTCTTAAAACGCTCCAAATATCCACTTGCAGATACTACAAGAAGATTGTTT
          1 CTTTTCCACCATAGGCCTCAAAGCGCTCCAAATATCCACTTGCAGATTCTACAAAAAGAGTGTTT

                     *    *        *      *     **                           
     232661 CAAAACTGCACAA-AAAAAGAAATGTTCAATTCTGTTTGATGAATGCACACATCACAAAGAAGTT
         66 CAAAACTGCTCAATCAAAAGAAAGGTTCAACTCTGTGAGATGAATGCACACATCACAAAGAAGTT

                           *                         
     232725 TCTCAGAATGCTTCTCTGTAGTTTTTATGTGAAGATATTTC
        131 TCTCAGAATGCTTCTGTGTAGTTTTTATGTGAAGATATTTC

                     *             *                 *         **      * *   
     232766 CTTTTCCACAATAGGCCTCAAAGGGCTCCAAATATCCACTTCCAGATTCTATGAAAAGAATATTT
          1 CTTTTCCACCATAGGCCTCAAAGCGCTCCAAATATCCACTTGCAGATTCTACAAAAAGAGTGTTT

             *              * *    *                                      *  
     232831 CCAAACTGCTCAATCATAGGAAATGTTCAACTCTGTGAGATGAATGCACACATCAC-AAGAAATT
         66 CAAAACTGCTCAATCAAAAGAAAGGTTCAACTCTGTGAGATGAATGCACACATCACAAAGAAGTT

                     *    *      *       *       *   
     232895 TCTCAGAATCCTTCAGTGTAGGTTTTATGAGAAGATAATTC
        131 TCTCAGAATGCTTCTGTGTAGTTTTTATGTGAAGATATTTC

                     *    **        *   *                           *    *   
     232936 CTTTTCCACAATAGTTCTCAAAGCACTCAAAATATCCACTTGCAGATTCTACAAAAGGAGTATTT
          1 CTTTTCCACCATAGGCCTCAAAGCGCTCCAAATATCCACTTGCAGATTCTACAAAAAGAGTGTTT

                                                 *        *                  
     233001 CAAAACTGCTCAATCAAAAGAAAGGTTCAACTCTGTGGGATGAATGGACACATCACAAAGAAGTT
         66 CAAAACTGCTCAATCAAAAGAAAGGTTCAACTCTGTGAGATGAATGCACACATCACAAAGAAGTT

                                  *   *              
     233066 TCTCAGAATGCTTCTGTGTAGTATTTTTGTGAAGATATTT-
        131 TCTCAGAATGCTTCTGTGTAGTTTTTATGTGAAGATATTTC

                          *  * * * * ***             *            *     * *  
     233106 CTTTTCCACCATAGACCGCCAGGGGACACAAATATCCACTTTCAGATTCTACAACAAGAGAGGTT
          1 CTTTTCCACCATAGGCCTCAAAGCGCTCCAAATATCCACTTGCAGATTCTACAAAAAGAGTGTTT

                   *   *     *   ** *       *      *                      * *
     233171 CAAAACTACTCGATCAAGAGATGGTTTCAACTATGTGAGTTGAATGCACACATCACAAAGAACTA
         66 CAAAACTGCTCAATCAAAAGAAAGGTTCAACTCTGTGAGATGAATGCACACATCACAAAGAAGTT

             *  *    *                               
     233236 TGTCGGAATTCTTCTGTGTAGTTTTTATGTGAAGATATTTC
        131 TCTCAGAATGCTTCTGTGTAGTTTTTATGTGAAGATATTTC

                     *    * *      * *    *                  *               
     233277 CTTTTCCACAATAGACGTCAAAGTGATCCAGATATCCACTTGCAGATTCCACAAAAAGAGTGTTT
          1 CTTTTCCACCATAGGCCTCAAAGCGCTCCAAATATCCACTTGCAGATTCTACAAAAAGAGTGTTT

                 *   *   *                  **                     *  *      
     233342 CAAAAGTGCACAACCAAAAGAAAGGTTCAACTAGGTGAGATGAATGCACACATCAGAAGGAAGTT
         66 CAAAACTGCTCAATCAAAAGAAAGGTTCAACTCTGTGAGATGAATGCACACATCACAAAGAAGTT

                            **   *     * *       *   
     233407 TCTCAGAATGCTTCTGCATAGCTTTTAAGGGAAGATACTTC
        131 TCTCAGAATGCTTCTGTGTAGTTTTTATGTGAAGATATTTC

                    *               *            * *  *    * *               
     233448 CTTTTCCAACATAGGCCTCAAAGCACTCCAAATATCCTCCTGGAGATACCACAAAAAGAGTGTTT
          1 CTTTTCCACCATAGGCCTCAAAGCGCTCCAAATATCCACTTGCAGATTCTACAAAAAGAGTGTTT

            **                      *  *                 *       *           
     233513 GCAAACTGCTCAATCAAAAGAAAGATTTAACTCTGTGAGATGAATCCACACATGACAAAGAAGTT
         66 CAAAACTGCTCAATCAAAAGAAAGGTTCAACTCTGTGAGATGAATGCACACATCACAAAGAAGTT

                                                     
     233578 TCTCAGAATGCTTCTGTGTAGTTTTTATGTGAAGATATTTC
        131 TCTCAGAATGCTTCTGTGTAGTTTTTATGTGAAGATATTTC

                     *   * * *    **           *                *     *      
     233619 CTTTTCCACAATAAGACCCAAAAGGCTCCAAATATTCACTTGCAGATTCTAAAAAAAACAGTGTT
          1 CTTTTCCACCATAGGCCTCAAAGCGCTCCAAATATCCACTTGCAGATTCT-ACAAAAAGAGTGTT

                                  *                  *      *        **      
     233684 TCAAAACTGCTCAATCAAAAGATA-GTTCAACTCTGTGAGAAGAATGCTCACATCACTGAGAAGT
         65 TCAAAACTGCTCAATCAAAAGAAAGGTTCAACTCTGTGAGATGAATGCACACATCACAAAGAAGT

                                         *            
     233748 TTCTCAGAATGCTTCTGTGTAGTTTTTATATGAAGATATTTC
        130 TTCTCAGAATGCTTCTGTGTAGTTTTTATGTGAAGATATTTC

                *     *      *    **                       *   **       *    
     233790 CTTTCCCACCGTAGGCCACAAAAGGCTCCAAATATCCACTTGCAGATACTATGAAAAGAGAGTTT
          1 CTTTTCCACCATAGGCCTCAAAGCGCTCCAAATATCCACTTGCAGATTCTACAAAAAGAGTGTTT

                        *        *               ***            *            
     233855 CAAAACTGCTCATTCAAAAGATAGGTTCAACTCTGTGGTTTGAATGCACACAGCACAAAGAAGTT
         66 CAAAACTGCTCAATCAAAAGAAAGGTTCAACTCTGTGAGATGAATGCACACATCACAAAGAAGTT

              *       **                   **   *    
     233920 TCACAGAATGTGTCTGTGTAGTTTTTATGTGCGGATGTTTC
        131 TCTCAGAATGCTTCTGTGTAGTTTTTATGTGAAGATATTTC

                         *        ****   *   *               *    *          
     233961 CTTTTCCACCATATGCCT-AAATATTTCCCAATTTCCACTTGCAGATTCCACAAGAAGAGTGTTT
          1 CTTTTCCACCATAGGCCTCAAAGCGCTCCAAATATCCACTTGCAGATTCTACAAAAAGAGTGTTT

                      **       *       *           *            *      ***   
     234025 CAAAACTGCTGTATC-AAATAAA-GTTGAACTCTGTGAGGTGAATGCACACAGCACAAAATGGTT
         66 CAAAACTGCTCAATCAAAAGAAAGGTTCAACTCTGTGAGATGAATGCACACATCACAAAGAAGTT

                          **   *        *       *    
     234088 TCTCAGAATGCTTCCTTGTTGTTTTTATATGAAGATGTTTC
        131 TCTCAGAATGCTTCTGTGTAGTTTTTATGTGAAGATATTTC

                  *  *             *   *     *                               
     234129 CTTTTCAACAATAGGCCTCAAAGTGCTTCAAATGTCCACTTGCAGATTCTACAAAAAGAGTGTTT
          1 CTTTTCCACCATAGGCCTCAAAGCGCTCCAAATATCCACTTGCAGATTCTACAAAAAGAGTGTTT

                                        *      *  *  *                       
     234194 CAAAACTGCTCAATCAAAAGAAAGGTTCGACTCTGGGAAATTAATGCACACATCACAAAGAAGTT
         66 CAAAACTGCTCAATCAAAAGAAAGGTTCAACTCTGTGAGATGAATGCACACATCACAAAGAAGTT

                                     *        *      
     234259 TCTC---A-GCTTCTGTGTAGTTTTCATGTGAAGTTATTTC
        131 TCTCAGAATGCTTCTGTGTAGTTTTTATGTGAAGATATTTC

                     *       *     *            *    *         **       *    
     234296 CTTTTCCACAATAGGCCGCAAAGGGCTCCAAATATCAACTTACAGATTCTAGGAAAAGAGAGTTT
          1 CTTTTCCACCATAGGCCTCAAAGCGCTCCAAATATCCACTTGCAGATTCTACAAAAAGAGTGTTT

                       * **      *     *                                     
     234361 CAAAACTGCTCTACGAAAAGATAGGTTGAACTCTGTGAGATGAATGCACACATCACAAAGAAGTT
         66 CAAAACTGCTCAATCAAAAGAAAGGTTCAACTCTGTGAGATGAATGCACACATCACAAAGAAGTT

                       *               * *           
     234426 TCTCAGAATGCATCTGTGTAGTTTTTACGGGAAGATATTTC
        131 TCTCAGAATGCTTCTGTGTAGTTTTTATGTGAAGATATTTC

                        ***  *     **      *  *           *      *     ***   
     234467 CTTTTCCACCATCTTCCACAAAGGTCTCCAAGTAACCACTTGCAGACTCTACAGAAAGACACTTT
          1 CTTTTCCACCATAGGCCTCAAAGCGCTCCAAATATCCACTTGCAGATTCTACAAAAAGAGTGTTT

            *          *         ***      *      ***                      *  
     234532 AAAAACTGCTCTATCAAAAGATCAGTTCAAGTCTGTGGTTTGAATGCACACATCACAAAGAATTT
         66 CAAAACTGCTCAATCAAAAGAAAGGTTCAACTCTGTGAGATGAATGCACACATCACAAAGAAGTT

                                     *  *            
     234597 TCTCAGAATGCTTCTGTGTAGTTTTCATATGAAGATATTTC
        131 TCTCAGAATGCTTCTGTGTAGTTTTTATGTGAAGATATTTC

                                    *                                   **   
     234638 CTTTTCCACCATAGGCCTCAAAGCACTCCAAATATCCACTTGCAGATTCTACAAAAAGAGATTTT
          1 CTTTTCCACCATAGGCCTCAAAGCGCTCCAAATATCCACTTGCAGATTCTACAAAAAGAGTGTTT

                   **                           *  *          *        *     
     234703 CAAAACTAGTCAATCAAAAGAAAGGTTCAACTCTGTCAGTTGAATGCACATATCACAAACAAGTT
         66 CAAAACTGCTCAATCAAAAGAAAGGTTCAACTCTGTGAGATGAATGCACACATCACAAAGAAGTT

                *      *                             
     234768 TCTCGGAATGCGTCTGTGTAGTTTTTATGTGAAGATATTTC
        131 TCTCAGAATGCTTCTGTGTAGTTTTTATGTGAAGATATTTC

               *     * *           *     *                  *    *           
     234809 CTTCTCCACAACAGGCCTCAAAGTGCTCCGAATATCCACTTGCAGATTTTAC-TAAAGAGTGTTT
          1 CTTTTCCACCATAGGCCTCAAAGCGCTCCAAATATCCACTTGCAGATTCTACAAAAAGAGTGTTT

             *               *  *   *     *        *    *               *    
     234873 CCAAACTGCTCAATCAAGAGGAAGTTTCAAGTCTGTGAGCTGAACGCACACATCACAAAGTAGTT
         66 CAAAACTGCTCAATCAAAAGAAAGGTTCAACTCTGTGAGATGAATGCACACATCACAAAGAAGTT

               *                                *    
     234938 TCTGAGAATGCTTCTGTGTAGTTTTTATGTGAAGATGTTTC
        131 TCTCAGAATGCTTCTGTGTAGTTTTTATGTGAAGATATTTC

                            **     *                                    *    
     234979 CTTTTCCACCATAGGCTGCAAAGGGCTCCAAATATCCACTTGCAGATTCTACAAAAAGAGAGTTT
          1 CTTTTCCACCATAGGCCTCAAAGCGCTCCAAATATCCACTTGCAGATTCTACAAAAAGAGTGTTT

                 *     *         *          *     *                     *    
     235044 CAAAAGTGCTCTATCAAAAGATAGGTTCAACTATGTGATATGAATGCACACATCACAAAGTAGTT
         66 CAAAACTGCTCAATCAAAAGAAAGGTTCAACTCTGTGAGATGAATGCACACATCACAAAGAAGTT

                                          *          
     235109 TCTCAGAATGCTTCTGTGTAGTTTTTATGTAAAGATATTTC
        131 TCTCAGAATGCTTCTGTGTAGTTTTTATGTGAAGATATTTC

                                    *                                   **   
     235150 CTTTTCCACCATAGGCCTCAAAGCACTCCAAATATCCACTTGCAGATTCTACAAAAAGAGATTTT
          1 CTTTTCCACCATAGGCCTCAAAGCGCTCCAAATATCCACTTGCAGATTCTACAAAAAGAGTGTTT

                   ** *                   *     *  *    * *   *        *     
     235215 CAAAACTATTTAATCAAAAGAAAGGTTCAAATCTGTCAGTTGAAGGTACATATCACAAACAAGTT
         66 CAAAACTGCTCAATCAAAAGAAAGGTTCAACTCTGTGAGATGAATGCACACATCACAAAGAAGTT

             * **                                    
     235280 TATTGGAATGCTTCTGTGTAGTTTTTATGTGAAGATATTTC
        131 TCTCAGAATGCTTCTGTGTAGTTTTTATGTGAAGATATTTC

                     * *         * *                        **  *     *      
     235321 CTTTTCCACAACAGGCCTCAAGGTGCTCCAAATATCCACTTGCAGATTTCACTAAAAGTGTGTTT
          1 CTTTTCCACCATAGGCCTCAAAGCGCTCCAAATATCCACTTGCAGATTCTACAAAAAGAGTGTTT

             *  *            *  *   *     *        *             *           
     235386 CCAAGCTGCTCAATCAAGAGGAAGTTTCAAGTCTGTGAGGTGAATGCACACATTACAAAGAAGTT
         66 CAAAACTGCTCAATCAAAAGAAAGGTTCAACTCTGTGAGATGAATGCACACATCACAAAGAAGTT

            *  *                                     
     235451 ACTGAGAATGCTTCTGTGTAGTTTTTATGTGAAGATATTTC
        131 TCTCAGAATGCTTCTGTGTAGTTTTTATGTGAAGATATTTC

                      **               *                                *    
     235492 CTTTTCCACCGCAGGCCTCAAAGCGCTGCAAATATCCACTTGCAGATTCTACAAAAAGAGAGTTT
          1 CTTTTCCACCATAGGCCTCAAAGCGCTCCAAATATCCACTTGCAGATTCTACAAAAAGAGTGTTT

                      **         *   *         *   *     **            *     
     235557 CAAAACTGCTGTATCAAAAGATAGGGTCAACTCTGCGAGTTGAATAAACACATCACAAATAAGTT
         66 CAAAACTGCTCAATCAAAAGAAAGGTTCAACTCTGTGAGATGAATGCACACATCACAAAGAAGTT

               **   *        *               *       
     235622 TCTGGGAACGCTTCTGTATAG-TTTTATGTGAATATATTTC
        131 TCTCAGAATGCTTCTGTGTAGTTTTTATGTGAAGATATTTC

                         *          *                   *   *  *   * *   *   
     235662 CTTTTCCACCATATGCCTCAAAGCACTCCAAATATCCACTTGCACATTATAGAAACATAGTCTTT
          1 CTTTTCCACCATAGGCCTCAAAGCGCTCCAAATATCCACTTGCAGATTCTACAAAAAGAGTGTTT

                   **                        *         *             *       
     235727 CAAAACTTGTCAATC-AAAGAAAGGTTCAACTCCGTGAGATGAGTGCACACATCACAGAGAAGTT
         66 CAAAACTGCTCAATCAAAAGAAAGGTTCAACTCTGTGAGATGAATGCACACATCACAAAGAAGTT

                *     *                            * 
     235791 TCTCGGAATGTTTCTGTGTAGTTTTTATGTGAAGATATTGC
        131 TCTCAGAATGCTTCTGTGTAGTTTTTATGTGAAGATATTTC

                     *               *            *          *       *   *   
     235832 CTTTTCCACAATAGGCCTCAAAGCGTTCCAAATATCCAATTGCAGATTCCACAAAAAAAGTTTTT
          1 CTTTTCCACCATAGGCCTCAAAGCGCTCCAAATATCCACTTGCAGATTCTACAAAAAGAGTGTTT

            *                 *  *  *  *             * *         **    *     
     235897 TAAAACTGCTCAATCAAATGATAGATTAAACTCTGTGAGATTAGTGCACACATGTCAAAAAAGTT
         66 CAAAACTGCTCAATCAAAAGAAAGGTTCAACTCTGTGAGATGAATGCACACATCACAAAGAAGTT

                                *      * *           
     235962 TCTCAGAATGCTTCTGTGTACTTTTTAGGGGAAGATATTTC
        131 TCTCAGAATGCTTCTGTGTAGTTTTTATGTGAAGATATTTC

                        *    *     **         *    *           **  * *  *    
     236003 CTTTTCCACCATCGGCCACAAAGGACTCCAAATAACCACATGCAGATTCTAGTAACACAGAGTTT
          1 CTTTTCCACCATAGGCCTCAAAGCGCTCCAAATATCCACTTGCAGATTCTACAAAAAGAGTGTTT

                       *         * *           *   * * *    **    ***        
     236068 CAAAACTGCTCTATCAAAAGATAAGTTCAACTCTGAGAGTTTAGTGCAACCATCGTGAAGAAGTT
         66 CAAAACTGCTCAATCAAAAGAAAGGTTCAACTCTGTGAGATGAATGCACACATCACAAAGAAGTT

                            *     *                  
     236133 TCTCAGAATGCTTCTGAGTAGTGTTTATGTGAAGATATTTC
        131 TCTCAGAATGCTTCTGTGTAGTTTTTATGTGAAGATATTTC

                              *     *                      *           **    
     236174 CTTTTCCACCATAGGCCTGAAAGCCCTCCAAATATCCACTTGCAGATCCTACAAAAAGAAAGTTT
          1 CTTTTCCACCATAGGCCTCAAAGCGCTCCAAATATCCACTTGCAGATTCTACAAAAAGAGTGTTT

             *         **     *  *  *   *        **      *                   
     236239 CGAAA-TGCTCTCTCAAACGATAGTTTCGACTCTGTGGTATGAATACACACATCACAAAGAAGTT
         66 CAAAACTGCTCAATCAAAAGAAAGGTTCAACTCTGTGAGATGAATGCACACATCACAAAGAAGTT

                                       **            
     236303 TCTCAGAATGCTTCTGTGTAGTTTTTAAATGAAGATATTTC
        131 TCTCAGAATGCTTCTGTGTAGTTTTTATGTGAAGATATTTC

            *                       *          *     *                       
     236344 TTTTTCCACCATAGGCCTCAAAGCACTCCAAATATGCACTTCCAGATTCTACAAAAAGAGTGTTT
          1 CTTTTCCACCATAGGCCTCAAAGCGCTCCAAATATCCACTTGCAGATTCTACAAAAAGAGTGTTT

              *                 *       * *         **   *         *  * *    
     236409 CAGAACTGCTCAATCAAAAGGAAGGTTCCAGTC--TGAGACAAATACACACATCAAAAGGTAGTT
         66 CAAAACTGCTCAATCAAAAGAAAGGTTCAACTCTGTGAGATGAATGCACACATCACAAAGAAGTT

                                                    *
     236472 TCTCAGAATGCTTCTGTGTAGTTTTTATGTGAAGATATTTT
        131 TCTCAGAATGCTTCTGTGTAGTTTTTATGTGAAGATATTTC

             *               *    **    *     *      *  **   *          *    
     236513 CCTTTCCACCATAGGCCACAAATGGCTCTAAATACCCACTTACATTTTCCACAAAAAGAGAGTTT
          1 CTTTTCCACCATAGGCCTCAAAGCGCTCCAAATATCCACTTGCAGATTCTACAAAAAGAGTGTTT

                       * *    * *      *   *         *                       
     236578 CAAAACTGCTCTACCAAAGGTAA-GTTTAACGCTGTGAG-TTAA-G-A-ACATCACAAAGAAGTT
         66 CAAAACTGCTCAATCAAAAGAAAGGTTCAACTCTGTGAGATGAATGCACACATCACAAAGAAGTT

                                   *   *  *          
     236638 TCTCAGAATGCTTCTGTGTAGTTCTTACGTAAAGATATTTC
        131 TCTCAGAATGCTTCTGTGTAGTTTTTATGTGAAGATATTTC

                 *   *      ***    *                  *              **      
     236679 CTTTTACACAATAGGCAGAAAAGTGCTCCAAATATCCACTTGAAGATTCTACAAAAACCGTGTTT
          1 CTTTTCCACCATAGGCCTCAAAGCGCTCCAAATATCCACTTGCAGATTCTACAAAAAGAGTGTTT

                     **                                          *      *    
     236744 CAAAACTGCCGAATCAAAAGAAAGGTTCAACTCTGTGAGATGAATGCACACATAACAAAGGAGTT
         66 CAAAACTGCTCAATCAAAAGAAAGGTTCAACTCTGTGAGATGAATGCACACATCACAAAGAAGTT

                                 *      *      *    *
     236809 TCTCAGAATGCTTCTGTGTAGCTTTTATATGAAGACATTTA
        131 TCTCAGAATGCTTCTGTGTAGTTTTTATGTGAAGATATTTC

            *        * *            *   ***                     **        *  
     236850 GTTTTCCACAACAGGCCTCAAAGCTCTCTCCATATCCACTTGCAGATTCTACCGAAAGAGTGCTT
          1 CTTTTCCACCATAGGCCTCAAAGCGCTCCAAATATCCACTTGCAGATTCTACAAAAAGAGTGTTT

             *                   * **     *        *        *     **         
     236915 CCAAACTGCTCAATCAAAAGAGACATTCAAATCTGTGAGGTGAATGCAGACATCGTAAAGAAGTT
         66 CAAAACTGCTCAATCAAAAGAAAGGTTCAACTCTGTGAGATGAATGCACACATCACAAAGAAGTT

                                *     *       *    **
     236980 TCTCAGAATGCTTCTGTGTATTTTTTGTGTGAAGTTATTCG
        131 TCTCAGAATGCTTCTGTGTAGTTTTTATGTGAAGATATTTC

            *    *             *     *  *            **                 *    
     237021 TTTTTGCACCATAGGCCTCCAAGCGTTCTAAATATCCACTTCTAGATTCTACAAAAAGAGAGTTT
          1 CTTTTCCACCATAGGCCTCAAAGCGCTCCAAATATCCACTTGCAGATTCTACAAAAAGAGTGTTT

                   *         *            *        *    *   *                
     237086 CAAAACTACTC-A--AACA-AAAGGTTCAATTCTGTGAGTTGAAAGCAAACATCACAAAGAAGTT
         66 CAAAACTGCTCAATCAAAAGAAAGGTTCAACTCTGTGAGATGAATGCACACATCACAAAGAAGTT

                       *                             
     237147 TCTCAGAATGCGTCTGTGTAGTTTTTATGTGAAGATATTTC
        131 TCTCAGAATGCTTCTGTGTAGTTTTTATGTGAAGATATTTC

                     **   ****     *                  *                      
     237188 CTTTT-CACAGTAGAATGCAAAGGGCTCCAAATATCCACTTGGAGATTCTACAAAAAGA--GTTT
          1 CTTTTCCACCATAGGCCTCAAAGCGCTCCAAATATCCACTTGCAGATTCTACAAAAAGAGTGTTT

                  *    **     *  *     *     ***   *     *               *   
     237250 CAAAACCGCTCTGTCAAATGATAGGTTGAACTCCCGGAGGTGAATACACACATCACAAAGAGGTT
         66 CAAAACTGCTCAATCAAAAGAAAGGTTCAACTCTGTGAGATGAATGCACACATCACAAAGAAGTT

                  *                       *  *       
     237315 TCTCAGCATGCTTCTGTGTAGTTTTTATGTAAACATATTTC
        131 TCTCAGAATGCTTCTGTGTAGTTTTTATGTGAAGATATTTC

             *    * *              *           *      * *       *   *    *   
     237356 CGTTTCTATCATAGGCCTCAAAGTGCTCCAAATATTCACTTGTACATTCTACCAAACGAGTATTT
          1 CTTTTCCACCATAGGCCTCAAAGCGCTCCAAATATCCACTTGCAGATTCTACAAAAAGAGTGTTT

                              * *         **      *        *            *    
     237421 CAAAACTGCTCAATCAAATGGAAGGTTCAAAACTGTGACATGAATGCCCACATCACAAAGTAGTT
         66 CAAAACTGCTCAATCAAAAGAAAGGTTCAACTCTGTGAGATGAATGCACACATCACAAAGAAGTT

                                                     
     237486 TCTCAGAATGCTTCTGTGTAGTTTTTATGTGAAGATATTTC
        131 TCTCAGAATGCTTCTGTGTAGTTTTTATGTGAAGATATTTC

                     * *    *      *    *       * *   **      *              
     237527 CTTTTCCACAACA-GCGTGCAAAACGCTTCAAATATGCCCTTAGAGATTCCACAAAAAGAGTGTT
          1 CTTTTCCACCATAGGCCT-CAAAGCGCTCCAAATATCCACTTGCAGATTCTACAAAAAGAGTGTT

              *     *               *  *                                  ** 
     237591 TCCAAACTACTCAAATCAAAA-AATGATTTCAACTCTGTGAGATGAATGCACACATCACAAACTA
         65 TCAAAACTGCTC-AATCAAAAGAAAG-GTTCAACTCTGTGAGATGAATGCACACATCACAAAGAA

                         *     ** *   * *   *       *   
     237655 GTTTCTCAGAATGTTTCTGCCTGGTTCTCATGCGAAGATAGTTC
        128 GTTTCTCAGAATGCTTCTGTGTAGTTTTTATGTGAAGATATTTC

                 *           *   * **              *                  * *    
     237699 CTTTTTCACCATAGGCCGCAATGTACTCCAAATATCCACCTGCAGATTCTAC-AAAAGTGAGTTT
          1 CTTTTCCACCATAGGCCTCAAAGCGCTCCAAATATCCACTTGCAGATTCTACAAAAAGAGTGTTT

                       *         ***    **         *        *      *       * 
     237763 CAAAACTGCTCTATCAAAAGATCAGTTCGTCTCTGTGAGTTGAATGCATACATCAAAAAGAAGCT
         66 CAAAACTGCTCAATCAAAAGAAAGGTTCAACTCTGTGAGATGAATGCACACATCACAAAGAAGTT

                 *             *      **         *   
     237828 TCTCAAAATGCTTCTGTGTGGTTTTTCGGTGAAGATAGTTC
        131 TCTCAGAATGCTTCTGTGTAGTTTTTATGTGAAGATATTTC

            *     *        *      * *                 *        *        *    
     237869 TTTTTCTACCATAGGTCTCAAACCACTCCAAATATCCACTTGTAGATTCTATAAAAAGGAATG-T
          1 CTTTTCCACCATAGGCCTCAAAGCGCTCCAAATATCCACTTGCAGATTCTACAAAAA-GAGTGTT

                  *        *    *    *      * *         *      *             
     237933 TCAAAATTGCTCAATAAAAATAAAGTTTCAACACCGTGAGATGAGTGCACAAATCACAAAGAAGT
         65 TCAAAACTGCTCAATCAAAAGAAAGGTTCAACTCTGTGAGATGAATGCACACATCACAAAGAAGT

                  *          *         *          *   
     237998 TTCTCAAAATGCTTCTGGGTAGTTTTTCTGTGAAGATAGTTC
        130 TTCTCAGAATGCTTCTGTGTAGTTTTTATGTGAAGATATTTC

                  *     *    *     *          *                         *    
     238040 CTTTTCTACCATGGGCCACAAAGGGCTCCAAATACCCACTTGCAGATTCTACAAAAAGAGAGTTT
          1 CTTTTCCACCATAGGCCTCAAAGCGCTCCAAATATCCACTTGCAGATTCTACAAAAAGAGTGTTT

              *        *      ** * *        *    * *    **  * *           *  
     238105 CACAACTGCTCTATCAAACAATATGTTCAACTTTGTGGGTTGAACACAAATATCAC-AAGAATTT
         66 CAAAACTGCTCAATCAAAAGAAAGGTTCAACTCTGTGAGATGAATGCACACATCACAAAGAAGTT

                **                             *     
     238169 TCTCCCAATGCTTCTGTGTAGTTTTTATGTGAAGACATTTC
        131 TCTCAGAATGCTTCTGTGTAGTTTTTATGTGAAGATATTTC

            *   *  *      *  *     *                 *  *      *       *  *  
     238210 TTTTCCCTCCATAGTCCACAAAGTGCTCCAAATATCCACTTACATATTCTAGAAAAAGATTGCTT
          1 CTTTTCCACCATAGGCCTCAAAGCGCTCCAAATATCCACTTGCAGATTCTACAAAAAGAGTGTTT

            **       *    **          *   * * *                              
     238275 GGAAACTGCACAATTGAAAGAAAGGTCCAAATATATGAGATGAATGCACACATCACAAAGAAGTT
         66 CAAAACTGCTCAATCAAAAGAAAGGTTCAACTCTGTGAGATGAATGCACACATCACAAAGAAGTT

                                 *                    
     238340 TCTCAGAAT-CTCTCTGTGTAATTTTTATGTGAAGATATTTC
        131 TCTCAGAATGCT-TCTGTGTAGTTTTTATGTGAAGATATTTC

                *     *    *  *   *                        *      *    *     
     238381 CTTTCCCACCTTAGGTCTTAAAACGCTCCAAATATCCACTTGCAGATACTACAAGAAGATTGTTT
          1 CTTTTCCACCATAGGCCTCAAAGCGCTCCAAATATCCACTTGCAGATTCTACAAAAAGAGTGTTT

                     *    *        *      *     **                           
     238446 CAAAACTGCACAA-AAAAAGAAATGTTCAATTCTGTTTGATGAATGCACACATCACAAAGAAGTT
         66 CAAAACTGCTCAATCAAAAGAAAGGTTCAACTCTGTGAGATGAATGCACACATCACAAAGAAGTT

                           *                         
     238510 TCTCAGAATGCTTCTCTGTAGTTTTTATGTGAAGATATTTC
        131 TCTCAGAATGCTTCTGTGTAGTTTTTATGTGAAGATATTTC

                     *             *                 *         **      * *   
     238551 CTTTTCCACAATAGGCCTCAAAGGGCTCCAAATATCCACTTCCAGATTCTATGAAAAGAATATTT
          1 CTTTTCCACCATAGGCCTCAAAGCGCTCCAAATATCCACTTGCAGATTCTACAAAAAGAGTGTTT

             *              * *    *                                      *  
     238616 CCAAACTGCTCAATCATAGGAAATGTTCAACTCTGTGAGATGAATGCACACATCAC-AAGAAATT
         66 CAAAACTGCTCAATCAAAAGAAAGGTTCAACTCTGTGAGATGAATGCACACATCACAAAGAAGTT

                     *    *      *       *       *   
     238680 TCTCAGAATCCTTCAGTGTAGGTTTTATGAGAAGATAATTC
        131 TCTCAGAATGCTTCTGTGTAGTTTTTATGTGAAGATATTTC

                     *    **        *   *                           *    *   
     238721 CTTTTCCACAATAGTTCTCAAAGCACTCAAAATATCCACTTGCAGATTCTACAAAAGGAGTATTT
          1 CTTTTCCACCATAGGCCTCAAAGCGCTCCAAATATCCACTTGCAGATTCTACAAAAAGAGTGTTT

                                                 *        *                  
     238786 CAAAACTGCTCAATCAAAAGAAAGGTTCAACTCTGTGGGATGAATGGACACATCACAAAGAAGTT
         66 CAAAACTGCTCAATCAAAAGAAAGGTTCAACTCTGTGAGATGAATGCACACATCACAAAGAAGTT

                                  *   *              
     238851 TCTCAGAATGCTTCTGTGTAGTATTTTTGTGAAGATATTT-
        131 TCTCAGAATGCTTCTGTGTAGTTTTTATGTGAAGATATTTC

                          *  * * * * ***             *            *     * *  
     238891 CTTTTCCACCATAGACCGCCAGGGGACACAAATATCCACTTTCAGATTCTACAACAAGAGAGGTT
          1 CTTTTCCACCATAGGCCTCAAAGCGCTCCAAATATCCACTTGCAGATTCTACAAAAAGAGTGTTT

                   *   *     *   ** *       *      *                      * *
     238956 CAAAACTACTCGATCAAGAGATGGTTTCAACTATGTGAGTTGAATGCACACATCACAAAGAACTA
         66 CAAAACTGCTCAATCAAAAGAAAGGTTCAACTCTGTGAGATGAATGCACACATCACAAAGAAGTT

             *  *    *                               
     239021 TGTCGGAATTCTTCTGTGTAGTTTTTATGTGAAGATATTTC
        131 TCTCAGAATGCTTCTGTGTAGTTTTTATGTGAAGATATTTC

                     *    * *      * *    *                  *               
     239062 CTTTTCCACAATAGACGTCAAAGTGATCCAGATATCCACTTGCAGATTCCACAAAAAGAGTGTTT
          1 CTTTTCCACCATAGGCCTCAAAGCGCTCCAAATATCCACTTGCAGATTCTACAAAAAGAGTGTTT

                 *   *   *                  **                     *  *      
     239127 CAAAAGTGCACAACCAAAAGAAAGGTTCAACTAGGTGAGATGAATGCACACATCAGAAGGAAGTT
         66 CAAAACTGCTCAATCAAAAGAAAGGTTCAACTCTGTGAGATGAATGCACACATCACAAAGAAGTT

                            **   *     * *       *   
     239192 TCTCAGAATGCTTCTGCATAGCTTTTAAGGGAAGATACTTC
        131 TCTCAGAATGCTTCTGTGTAGTTTTTATGTGAAGATATTTC

                    *               *            * *  *    * *               
     239233 CTTTTCCAACATAGGCCTCAAAGCACTCCAAATATCCTCCTGGAGATACCACAAAAAGAGTGTTT
          1 CTTTTCCACCATAGGCCTCAAAGCGCTCCAAATATCCACTTGCAGATTCTACAAAAAGAGTGTTT

            **                      *  *                 *       *           
     239298 GCAAACTGCTCAATCAAAAGAAAGATTTAACTCTGTGAGATGAATCCACACATGACAAAGAAGTT
         66 CAAAACTGCTCAATCAAAAGAAAGGTTCAACTCTGTGAGATGAATGCACACATCACAAAGAAGTT

                                                     
     239363 TCTCAGAATGCTTCTGTGTAGTTTTTATGTGAAGATATTTC
        131 TCTCAGAATGCTTCTGTGTAGTTTTTATGTGAAGATATTTC

            *        *                  *            *  *                    
     239404 TTTTTCCACAATAGGCCTCAAAGCGCTCAAAATATCCACTTTCACATTCTACAAAAAGAGTGTTT
          1 CTTTTCCACCATAGGCCTCAAAGCGCTCCAAATATCCACTTGCAGATTCTACAAAAAGAGTGTTT

                     *           *                 *       *    *  *     *   
     239469 CAAAACTGCACAATCAAAAGATA-GTTCAACTCTGTGAGTTGAATGCGCACAACAAAAAGATGTT
         66 CAAAACTGCTCAATCAAAAGAAAGGTTCAACTCTGTGAGATGAATGCACACATCACAAAGAAGTT

                      **                              
     239533 TCTCAGAATTATTTCTGTGTAGTTTTTATGTGAAGATATTTC
        131 TCTCAGAA-TGCTTCTGTGTAGTTTTTATGTGAAGATATTTC

                     *  *          *                                     *  *
     239575 CTTTTCCACAATGGGCCTCAAAGTGCTCCAAATATCCACTTGCAGATTCTACAAAAAGAGTTTTC
          1 CTTTTCCACCATAGGCCTCAAAGCGCTCCAAATATCCACTTGCAGATTCTACAAAAAGAGTGTTT

                       *            *    *                    * *           *
     239640 CAAAACTGCTCCATCAAAAGAAAGCTTCACCTCTGTGAGATGAATGCACATACCACAAAGAAGTG
         66 CAAAACTGCTCAATCAAAAGAAAGGTTCAACTCTGTGAGATGAATGCACACATCACAAAGAAGTT

                                        *            
     239705 TCTCAGAATGCTTCTGTGTAGTTTTTATATGAAGATATTTC
        131 TCTCAGAATGCTTCTGTGTAGTTTTTATGTGAAGATATTTC

                     *   * * *    **           *                *     *      
     239746 CTTTTCCACAATAAGACCCAAAAGGCTCCAAATATTCACTTGCAGATTCTAAAAAAAACAGTGTT
          1 CTTTTCCACCATAGGCCTCAAAGCGCTCCAAATATCCACTTGCAGATTCT-ACAAAAAGAGTGTT

                                  *                  *      *        **      
     239811 TCAAAACTGCTCAATCAAAAGATA-GTTCAACTCTGTGAGAAGAATGCTCACATCACTGAGAAGT
         65 TCAAAACTGCTCAATCAAAAGAAAGGTTCAACTCTGTGAGATGAATGCACACATCACAAAGAAGT

                                         *            
     239875 TTCTCAGAATGCTTCTGTGTAGTTTTTATATGAAGATATTTC
        130 TTCTCAGAATGCTTCTGTGTAGTTTTTATGTGAAGATATTTC

                *     *      *    **                       *   **       *    
     239917 CTTTCCCACCGTAGGCCACAAAAGGCTCCAAATATCCACTTGCAGATACTATGAAAAGAGAGTTT
          1 CTTTTCCACCATAGGCCTCAAAGCGCTCCAAATATCCACTTGCAGATTCTACAAAAAGAGTGTTT

                        *        *               ***            *            
     239982 CAAAACTGCTCATTCAAAAGATAGGTTCAACTCTGTGGTTTGAATGCACACAGCACAAAGAAGTT
         66 CAAAACTGCTCAATCAAAAGAAAGGTTCAACTCTGTGAGATGAATGCACACATCACAAAGAAGTT

              *       **                   **   *    
     240047 TCACAGAATGTGTCTGTGTAGTTTTTATGTGCGGATGTTTC
        131 TCTCAGAATGCTTCTGTGTAGTTTTTATGTGAAGATATTTC

                         *        ****   *   *               *    *          
     240088 CTTTTCCACCATATGCCT-AAATATTTCCCAATTTCCACTTGCAGATTCCACAAGAAGAGTGTTT
          1 CTTTTCCACCATAGGCCTCAAAGCGCTCCAAATATCCACTTGCAGATTCTACAAAAAGAGTGTTT

                      **       *       *           *            *      ***   
     240152 CAAAACTGCTGTATC-AAATAAA-GTTGAACTCTGTGAGGTGAATGCACACAGCACAAAATGGTT
         66 CAAAACTGCTCAATCAAAAGAAAGGTTCAACTCTGTGAGATGAATGCACACATCACAAAGAAGTT

                          **   *        *       *    
     240215 TCTCAGAATGCTTCCTTGTTGTTTTTATATGAAGATGTTTC
        131 TCTCAGAATGCTTCTGTGTAGTTTTTATGTGAAGATATTTC

                  *  *             *   *     *                               
     240256 CTTTTCAACAATAGGCCTCAAAGTGCTTCAAATGTCCACTTGCAGATTCTACAAAAAGAGTGTTT
          1 CTTTTCCACCATAGGCCTCAAAGCGCTCCAAATATCCACTTGCAGATTCTACAAAAAGAGTGTTT

                                        *      *  *  *                       
     240321 CAAAACTGCTCAATCAAAAGAAAGGTTCGACTCTGGGAAATTAATGCACACATCACAAAGAAGTT
         66 CAAAACTGCTCAATCAAAAGAAAGGTTCAACTCTGTGAGATGAATGCACACATCACAAAGAAGTT

                                     *        *      
     240386 TCTC---A-GCTTCTGTGTAGTTTTCATGTGAAGTTATTTC
        131 TCTCAGAATGCTTCTGTGTAGTTTTTATGTGAAGATATTTC

                     *       *     *            *    *         **       *    
     240423 CTTTTCCACAATAGGCCGCAAAGGGCTCCAAATATCAACTTACAGATTCTAGGAAAAGAGAGTTT
          1 CTTTTCCACCATAGGCCTCAAAGCGCTCCAAATATCCACTTGCAGATTCTACAAAAAGAGTGTTT

                       * **      *     *                                     
     240488 CAAAACTGCTCTACGAAAAGATAGGTTGAACTCTGTGAGATGAATGCACACATCACAAAGAAGTT
         66 CAAAACTGCTCAATCAAAAGAAAGGTTCAACTCTGTGAGATGAATGCACACATCACAAAGAAGTT

                       *               * *           
     240553 TCTCAGAATGCATCTGTGTAGTTTTTACGGGAAGATATTTC
        131 TCTCAGAATGCTTCTGTGTAGTTTTTATGTGAAGATATTTC

                        ***  *     **      *  *           *      *     ***   
     240594 CTTTTCCACCATCTTCCACAAAGGTCTCCAAGTAACCACTTGCAGACTCTACAGAAAGACACTTT
          1 CTTTTCCACCATAGGCCTCAAAGCGCTCCAAATATCCACTTGCAGATTCTACAAAAAGAGTGTTT

            *          *         ***      *      ***                      *  
     240659 AAAAACTGCTCTATCAAAAGATCAGTTCAAGTCTGTGGTTTGAATGCACACATCACAAAGAATTT
         66 CAAAACTGCTCAATCAAAAGAAAGGTTCAACTCTGTGAGATGAATGCACACATCACAAAGAAGTT

                                     *  *            
     240724 TCTCAGAATGCTTCTGTGTAGTTTTCATATGAAGATATTTC
        131 TCTCAGAATGCTTCTGTGTAGTTTTTATGTGAAGATATTTC

                                    *                                   **   
     240765 CTTTTCCACCATAGGCCTCAAAGCACTCCAAATATCCACTTGCAGATTCTACAAAAAGAGATTTT
          1 CTTTTCCACCATAGGCCTCAAAGCGCTCCAAATATCCACTTGCAGATTCTACAAAAAGAGTGTTT

                   **                           *  *          *        *     
     240830 CAAAACTAGTCAATCAAAAGAAAGGTTCAACTCTGTCAGTTGAATGCACATATCACAAACAAGTT
         66 CAAAACTGCTCAATCAAAAGAAAGGTTCAACTCTGTGAGATGAATGCACACATCACAAAGAAGTT

                *      *                             
     240895 TCTCGGAATGCGTCTGTGTAGTTTTTATGTGAAGATATTTC
        131 TCTCAGAATGCTTCTGTGTAGTTTTTATGTGAAGATATTTC

               *     * *           *     *                  *    *           
     240936 CTTCTCCACAACAGGCCTCAAAGTGCTCCGAATATCCACTTGCAGATTTTAC-TAAAGAGTGTTT
          1 CTTTTCCACCATAGGCCTCAAAGCGCTCCAAATATCCACTTGCAGATTCTACAAAAAGAGTGTTT

             *               *  *   *     *        *    *               *    
     241000 CCAAACTGCTCAATCAAGAGGAAGTTTCAAGTCTGTGAGCTGAACGCACACATCACAAAGTAGTT
         66 CAAAACTGCTCAATCAAAAGAAAGGTTCAACTCTGTGAGATGAATGCACACATCACAAAGAAGTT

               *                                *    
     241065 TCTGAGAATGCTTCTGTGTAGTTTTTATGTGAAGATGTTTC
        131 TCTCAGAATGCTTCTGTGTAGTTTTTATGTGAAGATATTTC

                            **     *                                    *  * 
     241106 CTTTTCCACCATAGGCTGCAAAGGGCTCCAAATATCCACTTGCAGATTCTACAAAAAGAGAGTCT
          1 CTTTTCCACCATAGGCCTCAAAGCGCTCCAAATATCCACTTGCAGATTCTACAAAAAGAGTGTTT

                 *     *         *          *     *                     *    
     241171 CAAAAGTGCTCTATCAAAAGATAGGTTCAACTATGTGATATGAATGCACACATCACAAAGTAGTT
         66 CAAAACTGCTCAATCAAAAGAAAGGTTCAACTCTGTGAGATGAATGCACACATCACAAAGAAGTT

                                          *          
     241236 TCTCAGAATGCTTCTGTGTAGTTTTTATGTAAAGATATTTC
        131 TCTCAGAATGCTTCTGTGTAGTTTTTATGTGAAGATATTTC

                                    *                                        
     241277 CTTTTCCACCATAGGCCTCAAAGCACTCCAAATATCCACTTGCAGATTCTACAAAAAGAGATG-T
          1 CTTTTCCACCATAGGCCTCAAAGCGCTCCAAATATCCACTTGCAGATTCTACAAAAAGAG-TGTT

                    ** *                   *     *  *    * *   *        *    
     241341 TCAAAACTATTTAATCAAAAGAAAGGTTCAAATCTGTCAGTTGAAGGTACATATCACAAACAAGT
         65 TCAAAACTGCTCAATCAAAAGAAAGGTTCAACTCTGTGAGATGAATGCACACATCACAAAGAAGT

              * **                                    
     241406 TTATTGGAATGCTTCTGTGTAGTTTTTATGTGAAGATATTTC
        130 TTCTCAGAATGCTTCTGTGTAGTTTTTATGTGAAGATATTTC

                     * *       * * *                        **  *     *      
     241448 CTTTTCCACAACAGGCCTCCAGGTGCTCCAAATATCCACTTGCAGATTTCACTAAAAGTGTGTTT
          1 CTTTTCCACCATAGGCCTCAAAGCGCTCCAAATATCCACTTGCAGATTCTACAAAAAGAGTGTTT

             *  *            *  *   *     *        *             *           
     241513 CCAAGCTGCTCAATCAAGAGGAAGTTTCAAGTCTGTGAGGTGAATGCACACATTACAAAGAAGTT
         66 CAAAACTGCTCAATCAAAAGAAAGGTTCAACTCTGTGAGATGAATGCACACATCACAAAGAAGTT

            *  *                                     
     241578 ACTGAGAATGCTTCTGTGTAGTTTTTATGTGAAGATATTTC
        131 TCTCAGAATGCTTCTGTGTAGTTTTTATGTGAAGATATTTC

                      **               *                                *    
     241619 CTTTTCCACCGCAGGCCTCAAAGCGCTGCAAATATCCACTTGCAGATTCTACAAAAAGAGAGTTT
          1 CTTTTCCACCATAGGCCTCAAAGCGCTCCAAATATCCACTTGCAGATTCTACAAAAAGAGTGTTT

                      **         *   *         *   *     **            *     
     241684 CAAAACTGCTGTATCAAAAGATAGGGTCAACTCTGCGAGTTGAATAAACACATCACAAATAAGTT
         66 CAAAACTGCTCAATCAAAAGAAAGGTTCAACTCTGTGAGATGAATGCACACATCACAAAGAAGTT

               **   *        *               *       
     241749 TCTGGGAACGCTTCTGTATAG-TTTTATGTGAATATATTTC
        131 TCTCAGAATGCTTCTGTGTAGTTTTTATGTGAAGATATTTC

                         *          *                   *   *  *   * *   *   
     241789 CTTTTCCACCATATGCCTCAAAGCACTCCAAATATCCACTTGCACATTATAGAAACATAGTCTTT
          1 CTTTTCCACCATAGGCCTCAAAGCGCTCCAAATATCCACTTGCAGATTCTACAAAAAGAGTGTTT

                   **                        *         *             *       
     241854 CAAAACTTGTCAATC-AAAGAAAGGTTCAACTCCGTGAGATGAGTGCACACATCACAGAGAAGTT
         66 CAAAACTGCTCAATCAAAAGAAAGGTTCAACTCTGTGAGATGAATGCACACATCACAAAGAAGTT

                *     *                            * 
     241918 TCTCGGAATGTTTCTGTGTAGTTTTTATGTGAAGATATTGC
        131 TCTCAGAATGCTTCTGTGTAGTTTTTATGTGAAGATATTTC

                     *               *            *          *       *   *   
     241959 CTTTTCCACAATAGGCCTCAAAGCGTTCCAAATATCCAATTGCAGATTCCACAAAAAAAGTTTTT
          1 CTTTTCCACCATAGGCCTCAAAGCGCTCCAAATATCCACTTGCAGATTCTACAAAAAGAGTGTTT

            *                 *  *  *  *             * *         **    *     
     242024 TAAAACTGCTCAATCAAATGATAGATTAAACTCTGTGAGATTAGTGCACACATGTCAAAAAAGTT
         66 CAAAACTGCTCAATCAAAAGAAAGGTTCAACTCTGTGAGATGAATGCACACATCACAAAGAAGTT

                                *      * *           
     242089 TCTCAGAATGCTTCTGTGTACTTTTTAGGGGAAGATATTTC
        131 TCTCAGAATGCTTCTGTGTAGTTTTTATGTGAAGATATTTC

                        *    *     **         *    *           **  * *  *    
     242130 CTTTTCCACCATCGGCCACAAAGGACTCCAAATAACCACATGCAGATTCTAGTAACACAGAGTTT
          1 CTTTTCCACCATAGGCCTCAAAGCGCTCCAAATATCCACTTGCAGATTCTACAAAAAGAGTGTTT

                       *         * *           *   * * *    **    ***        
     242195 CAAAACTGCTCTATCAAAAGATAAGTTCAACTCTGAGAGTTTAGTGCAACCATCGTGAAGAAGTT
         66 CAAAACTGCTCAATCAAAAGAAAGGTTCAACTCTGTGAGATGAATGCACACATCACAAAGAAGTT

                            *     *                  
     242260 TCTCAGAATGCTTCTGAGTAGTGTTTATGTGAAGATATTTC
        131 TCTCAGAATGCTTCTGTGTAGTTTTTATGTGAAGATATTTC

                              *     *                      *           **    
     242301 CTTTTCCACCATAGGCCTGAAAGCCCTCCAAATATCCACTTGCAGATCCTACAAAAAGAAAGTTT
          1 CTTTTCCACCATAGGCCTCAAAGCGCTCCAAATATCCACTTGCAGATTCTACAAAAAGAGTGTTT

             *         **     *  *  *   *        **      *                   
     242366 CGAAA-TGCTCTCTCAAACGATAGTTTCGACTCTGTGGTATGAATACACACATCACAAAGAAGTT
         66 CAAAACTGCTCAATCAAAAGAAAGGTTCAACTCTGTGAGATGAATGCACACATCACAAAGAAGTT

                                       **            
     242430 TCTCAGAATGCTTCTGTGTAGTTTTTAAATGAAGATATTTC
        131 TCTCAGAATGCTTCTGTGTAGTTTTTATGTGAAGATATTTC

            *                       *          *     *                       
     242471 TTTTTCCACCATAGGCCTCAAAGCACTCCAAATATGCACTTCCAGATTCTACAAAAAGAGTGTTT
          1 CTTTTCCACCATAGGCCTCAAAGCGCTCCAAATATCCACTTGCAGATTCTACAAAAAGAGTGTTT

              *                 *       * *         **   *         *  * *    
     242536 CAGAACTGCTCAATCAAAAGGAAGGTTCCAGTC--TGAGACAAATACACACATCAAAAGGTAGTT
         66 CAAAACTGCTCAATCAAAAGAAAGGTTCAACTCTGTGAGATGAATGCACACATCACAAAGAAGTT

                                                    *
     242599 TCTCAGAATGCTTCTGTGTAGTTTTTATGTGAAGATATTTT
        131 TCTCAGAATGCTTCTGTGTAGTTTTTATGTGAAGATATTTC

             *               *    **    *     *      *  **   *          *    
     242640 CCTTTCCACCATAGGCCACAAATGGCTCTAAATACCCACTTACATTTTCCACAAAAAGAGAGTTT
          1 CTTTTCCACCATAGGCCTCAAAGCGCTCCAAATATCCACTTGCAGATTCTACAAAAAGAGTGTTT

                       * *    * *      *   *         *                       
     242705 CAAAACTGCTCTACCAAAGGTAA-GTTTAACGCTGTGAG-TTAA-G-A-ACATCACAAAGAAGTT
         66 CAAAACTGCTCAATCAAAAGAAAGGTTCAACTCTGTGAGATGAATGCACACATCACAAAGAAGTT

                                   *   *  *          
     242765 TCTCAGAATGCTTCTGTGTAGTTCTTACGTAAAGATATTTC
        131 TCTCAGAATGCTTCTGTGTAGTTTTTATGTGAAGATATTTC

                 *   *      ***    *                  *              **      
     242806 CTTTTACACAATAGGCAGAAAAGTGCTCCAAATATCCACTTGAAGATTCTACAAAAACCGTGTTT
          1 CTTTTCCACCATAGGCCTCAAAGCGCTCCAAATATCCACTTGCAGATTCTACAAAAAGAGTGTTT

                     **                                          *      *    
     242871 CAAAACTGCCGAATCAAAAGAAAGGTTCAACTCTGTGAGATGAATGCACACATAACAAAGGAGTT
         66 CAAAACTGCTCAATCAAAAGAAAGGTTCAACTCTGTGAGATGAATGCACACATCACAAAGAAGTT

                                 *      *      *    *
     242936 TCTCAGAATGCTTCTGTGTAGCTTTTATATGAAGACATTTA
        131 TCTCAGAATGCTTCTGTGTAGTTTTTATGTGAAGATATTTC

            *        * *            *   ***                     **        *  
     242977 GTTTTCCACAACAGGCCTCAAAGCTCTCTCCATATCCACTTGCAGATTCTACCGAAAGAGTGCTT
          1 CTTTTCCACCATAGGCCTCAAAGCGCTCCAAATATCCACTTGCAGATTCTACAAAAAGAGTGTTT

             *                   * **     *        *        *     **         
     243042 CCAAACTGCTCAATCAAAAGAGACATTCAAATCTGTGAGGTGAATGCAGACATCGTAAAGAAGTT
         66 CAAAACTGCTCAATCAAAAGAAAGGTTCAACTCTGTGAGATGAATGCACACATCACAAAGAAGTT

                                *     *       *    **
     243107 TCTCAGAATGCTTCTGTGTATTTTTTGTGTGAAGTTATTCG
        131 TCTCAGAATGCTTCTGTGTAGTTTTTATGTGAAGATATTTC

            *    *             *     *  *            **                 *    
     243148 TTTTTGCACCATAGGCCTCCAAGCGTTCTAAATATCCACTTCTAGATTCTACAAAAAGAGAGTTT
          1 CTTTTCCACCATAGGCCTCAAAGCGCTCCAAATATCCACTTGCAGATTCTACAAAAAGAGTGTTT

                   *         *            *        *    *   *                
     243213 CAAAACTACTC-A--AACA-AAAGGTTCAATTCTGTGAGTTGAAAGCAAACATCACAAAGAAGTT
         66 CAAAACTGCTCAATCAAAAGAAAGGTTCAACTCTGTGAGATGAATGCACACATCACAAAGAAGTT

                       *                             
     243274 TCTCAGAATGCGTCTGTGTAGTTTTTATGTGAAGATATTTC
        131 TCTCAGAATGCTTCTGTGTAGTTTTTATGTGAAGATATTTC

                     *    ****     *                  *                      
     243315 CTTTT-CACAATAGAATGCAAAGGGCTCCAAATATCCACTTGGAGATTCTACAAAAAGA--GTTT
          1 CTTTTCCACCATAGGCCTCAAAGCGCTCCAAATATCCACTTGCAGATTCTACAAAAAGAGTGTTT

                  *    **     *  *     *     ***   *     *               *   
     243377 CAAAACCGCTCTGTCAAATGATAGGTTGAACTCCCGGAGGTGAATACACACATCACAAAGAGGTT
         66 CAAAACTGCTCAATCAAAAGAAAGGTTCAACTCTGTGAGATGAATGCACACATCACAAAGAAGTT

                  *                       *  *       
     243442 TCTCAGCATGCTTCTGTGTAGTTTTTATGTAAACATATTTC
        131 TCTCAGAATGCTTCTGTGTAGTTTTTATGTGAAGATATTTC

             *    * *              *           *      * *       *   *    *   
     243483 CGTTTCTATCATAGGCCTCAAAGTGCTCCAAATATTCACTTGTACATTCTACCAAACGAGTATTT
          1 CTTTTCCACCATAGGCCTCAAAGCGCTCCAAATATCCACTTGCAGATTCTACAAAAAGAGTGTTT

                              * *         **      *        *            *    
     243548 CAAAACTGCTCAATCAAATGGAAGGTTCAAAACTGTGACATGAATGCCCACATCACAAAGTAGTT
         66 CAAAACTGCTCAATCAAAAGAAAGGTTCAACTCTGTGAGATGAATGCACACATCACAAAGAAGTT

                                                     
     243613 TCTCAGAATGCTTCTGTGTAGTTTTTATGTGAAGATATTTC
        131 TCTCAGAATGCTTCTGTGTAGTTTTTATGTGAAGATATTTC

                     * *    *      *    *       * *   **      *              
     243654 CTTTTCCACAACA-GCGTGCAAAACGCTTCAAATATGCCCTTAGAGATTCCACAAAAAGAGTGTT
          1 CTTTTCCACCATAGGCCT-CAAAGCGCTCCAAATATCCACTTGCAGATTCTACAAAAAGAGTGTT

              *     *               *  *                                  ** 
     243718 TCCAAACTACTCAAATCAAAA-AATGATTTCAACTCTGTGAGATGAATGCACACATCACAAACTA
         65 TCAAAACTGCTC-AATCAAAAGAAAG-GTTCAACTCTGTGAGATGAATGCACACATCACAAAGAA

                         *     ** *   * *   *       *   
     243782 GTTTCTCAGAATGTTTCTGCCTGGTTCTCATGCGAAGATAGTTC
        128 GTTTCTCAGAATGCTTCTGTGTAGTTTTTATGTGAAGATATTTC

                 *           *   * **              *                  * *    
     243826 CTTTTTCACCATAGGCCGCAATGTACTCCAAATATCCACCTGCAGATTCTAC-AAAAGTGAGTTT
          1 CTTTTCCACCATAGGCCTCAAAGCGCTCCAAATATCCACTTGCAGATTCTACAAAAAGAGTGTTT

                       *         ***    **         *        *      *       * 
     243890 CAAAACTGCTCTATCAAAAGATCAGTTCGTCTCTGTGAGTTGAATGCATACATCAAAAAGAAGCT
         66 CAAAACTGCTCAATCAAAAGAAAGGTTCAACTCTGTGAGATGAATGCACACATCACAAAGAAGTT

                 *             *      **         *   
     243955 TCTCAAAATGCTTCTGTGTGGTTTTTCGGTGAAGATAGTTC
        131 TCTCAGAATGCTTCTGTGTAGTTTTTATGTGAAGATATTTC

            *     *        *      * *                 *        *        *    
     243996 TTTTTCTACCATAGGTCTCAAACCACTCCAAATATCCACTTGTAGATTCTATAAAAAGGAATG-T
          1 CTTTTCCACCATAGGCCTCAAAGCGCTCCAAATATCCACTTGCAGATTCTACAAAAA-GAGTGTT

                  *        *    *    *      * *         *      *             
     244060 TCAAAATTGCTCAATAAAAATAAAGTTTCAACACCGTGAGATGAGTGCACAAATCACAAAGAAGT
         65 TCAAAACTGCTCAATCAAAAGAAAGGTTCAACTCTGTGAGATGAATGCACACATCACAAAGAAGT

                  *          *         *          *   
     244125 TTCTCAAAATGCTTCTGGGTAGTTTTTCTGTGAAGATAGTTC
        130 TTCTCAGAATGCTTCTGTGTAGTTTTTATGTGAAGATATTTC

                  *     *    *     *          *                         *    
     244167 CTTTTCTACCATGGGCCACAAAGGGCTCCAAATACCCACTTGCAGATTCTACAAAAAGAGAGTTT
          1 CTTTTCCACCATAGGCCTCAAAGCGCTCCAAATATCCACTTGCAGATTCTACAAAAAGAGTGTTT

              *        *      ** * *        *    * *    **  * *           *  
     244232 CACAACTGCTCTATCAAACAATATGTTCAACTTTGTGGGTTGAACACAAATATCAC-AAGAATTT
         66 CAAAACTGCTCAATCAAAAGAAAGGTTCAACTCTGTGAGATGAATGCACACATCACAAAGAAGTT

                **                             *     
     244296 TCTCCCAATGCTTCTGTGTAGTTTTTATGTGAAGACATTTC
        131 TCTCAGAATGCTTCTGTGTAGTTTTTATGTGAAGATATTTC

            *   *  *      *  *     *                 *  *      *       *  *  
     244337 TTTTCCCTCCATAGTCCACAAAGTGCTCCAAATATCCACTTACATATTCTAGAAAAAGATTGCTT
          1 CTTTTCCACCATAGGCCTCAAAGCGCTCCAAATATCCACTTGCAGATTCTACAAAAAGAGTGTTT

            **       *    *           *   * * *                              
     244402 GGAAACTGCACAATGAAAAGAAAGGTCCAAATATATGAGATGAATGCACACATCACAAAGAAGTT
         66 CAAAACTGCTCAATCAAAAGAAAGGTTCAACTCTGTGAGATGAATGCACACATCACAAAGAAGTT

                                 *                    
     244467 TCTCAGAAT-CTCTCTGTGTAATTTTTATGTGAAGATATTTC
        131 TCTCAGAATGCT-TCTGTGTAGTTTTTATGTGAAGATATTTC

                *     *    *  *   *                        *      *    *     
     244508 CTTTCCCACCTTAGGTCTTAAAACGCTCCAAATATCCACTTGCAGATACTACAAGAAGATTGTTT
          1 CTTTTCCACCATAGGCCTCAAAGCGCTCCAAATATCCACTTGCAGATTCTACAAAAAGAGTGTTT

                     *    *        *      *     **                           
     244573 CAAAACTGCACAA-AAAAAGAAATGTTCAATTCTGTTTGATGAATGCACACATCACAAAGAAGTT
         66 CAAAACTGCTCAATCAAAAGAAAGGTTCAACTCTGTGAGATGAATGCACACATCACAAAGAAGTT

                           *                         
     244637 TCTCAGAATGCTTCTCTGTAGTTTTTATGTGAAGATATTTC
        131 TCTCAGAATGCTTCTGTGTAGTTTTTATGTGAAGATATTTC

                     *             *                 *         **      * *   
     244678 CTTTTCCACAATAGGCCTCAAAGGGCTCCAAATATCCACTTCCAGATTCTATGAAAAGAATATTT
          1 CTTTTCCACCATAGGCCTCAAAGCGCTCCAAATATCCACTTGCAGATTCTACAAAAAGAGTGTTT

             *              * *    *                                      *  
     244743 CCAAACTGCTCAATCATAGGAAATGTTCAACTCTGTGAGATGAATGCACACATCAC-AAGAAATT
         66 CAAAACTGCTCAATCAAAAGAAAGGTTCAACTCTGTGAGATGAATGCACACATCACAAAGAAGTT

                     *    *      *       *       *   
     244807 TCTCAGAATCCTTCAGTGTAGGTTTTATGAGAAGATAATTC
        131 TCTCAGAATGCTTCTGTGTAGTTTTTATGTGAAGATATTTC

                     *    **        *   *                           *    *   
     244848 CTTTTCCACAATAGTTCTCAAAGCACTCAAAATATCCACTTGCAGATTCTACAAAAGGAGTATTT
          1 CTTTTCCACCATAGGCCTCAAAGCGCTCCAAATATCCACTTGCAGATTCTACAAAAAGAGTGTTT

                                                 *        *                  
     244913 CAAAACTGCTCAATCAAAAGAAAGGTTCAACTCTGTGGGATGAATGGACACATCACAAAGAAGTT
         66 CAAAACTGCTCAATCAAAAGAAAGGTTCAACTCTGTGAGATGAATGCACACATCACAAAGAAGTT

                                  *   *              
     244978 TCTCAGAATGCTTCTGTGTAGTATTTTTGTGAAGATATTT-
        131 TCTCAGAATGCTTCTGTGTAGTTTTTATGTGAAGATATTTC

                          *  * * * * ***             *            *     * *  
     245018 CTTTTCCACCATAGACCGCCAGGGGACACAAATATCCACTTTCAGATTCTACAACAAGAGAGGTT
          1 CTTTTCCACCATAGGCCTCAAAGCGCTCCAAATATCCACTTGCAGATTCTACAAAAAGAGTGTTT

                   *   *     *   ** *       *      *                      * *
     245083 CAAAACTACTCGATCAAGAGATGGTTTCAACTATGTGAGTTGAATGCACACATCACAAAGAACTA
         66 CAAAACTGCTCAATCAAAAGAAAGGTTCAACTCTGTGAGATGAATGCACACATCACAAAGAAGTT

             *  *    *                               
     245148 TGTCGGAATTCTTCTGTGTAGTTTTTATGTGAAGATATTTC
        131 TCTCAGAATGCTTCTGTGTAGTTTTTATGTGAAGATATTTC

                     *    * *      * *    *                  *               
     245189 CTTTTCCACAATAGACGTCAAAGTGATCCAGATATCCACTTGCAGATTCCACAAAAAGAGTGTTT
          1 CTTTTCCACCATAGGCCTCAAAGCGCTCCAAATATCCACTTGCAGATTCTACAAAAAGAGTGTTT

                 *   *   *                  **                     *  *      
     245254 CAAAAGTGCACAACCAAAAGAAAGGTTCAACTAGGTGAGATGAATGCACACATCAGAAGGAAGTT
         66 CAAAACTGCTCAATCAAAAGAAAGGTTCAACTCTGTGAGATGAATGCACACATCACAAAGAAGTT

                            **   *     * *       *   
     245319 TCTCAGAATGCTTCTGCATAGCTTTTAAGGGAAGATACTTC
        131 TCTCAGAATGCTTCTGTGTAGTTTTTATGTGAAGATATTTC

                    *               *            * *  *    * *               
     245360 CTTTTCCAACATAGGCCTCAAAGCACTCCAAATATCCTCCTGGAGATACCACAAAAAGAGTGTTT
          1 CTTTTCCACCATAGGCCTCAAAGCGCTCCAAATATCCACTTGCAGATTCTACAAAAAGAGTGTTT

            **                      *  *                 *       *           
     245425 GCAAACTGCTCAATCAAAAGAAAGATTTAACTCTGTGAGATGAATCCACACATGACAAAGAAGTT
         66 CAAAACTGCTCAATCAAAAGAAAGGTTCAACTCTGTGAGATGAATGCACACATCACAAAGAAGTT

                                       **            
     245490 TCTCAGAATGCTTCTGTGTAGTTTTTAATTGAAGATATTTC
        131 TCTCAGAATGCTTCTGTGTAGTTTTTATGTGAAGATATTTC

            *        *                  *            *  *                    
     245531 TTTTTCCACAATAGGCCTCAAAGCGCTCAAAATATCCACTTTCACATTCTACAAAAAGAGTGTTT
          1 CTTTTCCACCATAGGCCTCAAAGCGCTCCAAATATCCACTTGCAGATTCTACAAAAAGAGTGTTT

                     *           *                 *       *    *  *     *   
     245596 CAAAACTGCACAATCAAAAGATA-GTTCAACTCTGTGAGTTGAATGCGCACAACAAAAAGATGTT
         66 CAAAACTGCTCAATCAAAAGAAAGGTTCAACTCTGTGAGATGAATGCACACATCACAAAGAAGTT

                      **                              
     245660 TCTCAGAATTATTTCTGTGTAGTTTTTATGTGAAGATATTTC
        131 TCTCAGAA-TGCTTCTGTGTAGTTTTTATGTGAAGATATTTC

                     *  *          *                                     *  *
     245702 CTTTTCCACAATGGGCCTCAAAGTGCTCCAAATATCCACTTGCAGATTCTACAAAAAGAGTTTTC
          1 CTTTTCCACCATAGGCCTCAAAGCGCTCCAAATATCCACTTGCAGATTCTACAAAAAGAGTGTTT

                       *            *    *                    * *           *
     245767 CAAAACTGCTCCATCAAAAGAAAGCTTCACCTCTGTGAGATGAATGCACATACCACAAAGAAGTG
         66 CAAAACTGCTCAATCAAAAGAAAGGTTCAACTCTGTGAGATGAATGCACACATCACAAAGAAGTT

                                        *            
     245832 TCTCAGAATGCTTCTGTGTAGTTTTTATATGAAGATATTTC
        131 TCTCAGAATGCTTCTGTGTAGTTTTTATGTGAAGATATTTC

                     *   * * *    **           *                *     *      
     245873 CTTTTCCACAATAAGACCCAAAAGGCTCCAAATATTCACTTGCAGATTCTAAAAAAAACAGTGTT
          1 CTTTTCCACCATAGGCCTCAAAGCGCTCCAAATATCCACTTGCAGATTCT-ACAAAAAGAGTGTT

                                  *                  *      *        **      
     245938 TCAAAACTGCTCAATCAAAAGATA-GTTCAACTCTGTGAGAAGAATGCTCACATCACTGAGAAGT
         65 TCAAAACTGCTCAATCAAAAGAAAGGTTCAACTCTGTGAGATGAATGCACACATCACAAAGAAGT

                                         *            
     246002 TTCTCAGAATGCTTCTGTGTAGTTTTTATATGAAGATATTTC
        130 TTCTCAGAATGCTTCTGTGTAGTTTTTATGTGAAGATATTTC

                *     *      *    **                       *   **       *    
     246044 CTTTCCCACCGTAGGCCACAAAAGGCTCCAAATATCCACTTGCAGATACTATGAAAAGAGAGTTT
          1 CTTTTCCACCATAGGCCTCAAAGCGCTCCAAATATCCACTTGCAGATTCTACAAAAAGAGTGTTT

                        *        *               ***            *            
     246109 CAAAACTGCTCATTCAAAAGATAGGTTCAACTCTGTGGTTTGAATGCACACAGCACAAAGAAGTT
         66 CAAAACTGCTCAATCAAAAGAAAGGTTCAACTCTGTGAGATGAATGCACACATCACAAAGAAGTT

              *       **                   **   *    
     246174 TCACAGAATGTGTCTGTGTAGTTTTTATGTGCGGATGTTTC
        131 TCTCAGAATGCTTCTGTGTAGTTTTTATGTGAAGATATTTC

                         *        ****   *   *               *    *          
     246215 CTTTTCCACCATATGCCT-AAATATTTCCCAATTTCCACTTGCAGATTCCACAAGAAGAGTGTTT
          1 CTTTTCCACCATAGGCCTCAAAGCGCTCCAAATATCCACTTGCAGATTCTACAAAAAGAGTGTTT

                      **       *       *           *            *      ***   
     246279 CAAAACTGCTGTATC-AAATAAA-GTTGAACTCTGTGAGGTGAATGCACACAGCACAAAATGGTT
         66 CAAAACTGCTCAATCAAAAGAAAGGTTCAACTCTGTGAGATGAATGCACACATCACAAAGAAGTT

                          **   *        *       *    
     246342 TCTCAGAATGCTTCCTTGTTGTTTTTATATGAAGATGTTTC
        131 TCTCAGAATGCTTCTGTGTAGTTTTTATGTGAAGATATTTC

                  *  *             *   *     *                               
     246383 CTTTTCAACAATAGGCCTCAAAGTGCTTCAAATGTCCACTTGCAGATTCTACAAAAAGAGTGTTT
          1 CTTTTCCACCATAGGCCTCAAAGCGCTCCAAATATCCACTTGCAGATTCTACAAAAAGAGTGTTT

                                        *      *  *  *                       
     246448 CAAAACTGCTCAATCAAAAGAAAGGTTCGACTCTGGGAAATTAATGCACACATCACAAAGAAGTT
         66 CAAAACTGCTCAATCAAAAGAAAGGTTCAACTCTGTGAGATGAATGCACACATCACAAAGAAGTT

                                     *        *      
     246513 TCTC---A-GCTTCTGTGTAGTTTTCATGTGAAGTTATTTC
        131 TCTCAGAATGCTTCTGTGTAGTTTTTATGTGAAGATATTTC

                     *       *     *            *    *         **       *    
     246550 CTTTTCCACAATAGGCCGCAAAGGGCTCCAAATATCAACTTACAGATTCTAGGAAAAGAGAGTTT
          1 CTTTTCCACCATAGGCCTCAAAGCGCTCCAAATATCCACTTGCAGATTCTACAAAAAGAGTGTTT

                       * **      *     *                                     
     246615 CAAAACTGCTCTACGAAAAGATAGGTTGAACTCTGTGAGATGAATGCACACATCACAAAGAAGTT
         66 CAAAACTGCTCAATCAAAAGAAAGGTTCAACTCTGTGAGATGAATGCACACATCACAAAGAAGTT

                       *               * *           
     246680 TCTCAGAATGCATCTGTGTAGTTTTTACGGGAAGATATTTC
        131 TCTCAGAATGCTTCTGTGTAGTTTTTATGTGAAGATATTTC

                        ***  *     **      *  *                  *     ***   
     246721 CTTTTCCACCATCTTCCACAAAGGTCTCCAAGTAACCACTTGCAGATTCTACAGAAAGACACTTT
          1 CTTTTCCACCATAGGCCTCAAAGCGCTCCAAATATCCACTTGCAGATTCTACAAAAAGAGTGTTT

            *          *         ***      *      ***                      *  
     246786 AAAAACTGCTCTATCAAAAGATCAGTTCAAGTCTGTGGTTTGAATGCACACATCACAAAGAATTT
         66 CAAAACTGCTCAATCAAAAGAAAGGTTCAACTCTGTGAGATGAATGCACACATCACAAAGAAGTT

                                     *  *            
     246851 TCTCAGAATGCTTCTGTGTAGTTTTCATATGAAGATATTTC
        131 TCTCAGAATGCTTCTGTGTAGTTTTTATGTGAAGATATTTC

                                    *                                   **   
     246892 CTTTTCCACCATAGGCCTCAAAGCACTCCAAATATCCACTTGCAGATTCTACAAAAAGAGATTTT
          1 CTTTTCCACCATAGGCCTCAAAGCGCTCCAAATATCCACTTGCAGATTCTACAAAAAGAGTGTTT

                   **                           *  *          *        *     
     246957 CAAAACTAGTCAATCAAAAGAAAGGTTCAACTCTGTCAGTTGAATGCACATATCACAAACAAGTT
         66 CAAAACTGCTCAATCAAAAGAAAGGTTCAACTCTGTGAGATGAATGCACACATCACAAAGAAGTT

                *      *                             
     247022 TCTCGGAATGCGTCTGTGTAGTTTTTATGTGAAGATATTTC
        131 TCTCAGAATGCTTCTGTGTAGTTTTTATGTGAAGATATTTC

               *     * *           *     *                  *    *           
     247063 CTTCTCCACAACAGGCCTCAAAGTGCTCCGAATATCCACTTGCAGATTTTAC-TAAAGAGTGTTT
          1 CTTTTCCACCATAGGCCTCAAAGCGCTCCAAATATCCACTTGCAGATTCTACAAAAAGAGTGTTT

             *               *  *   *     *        *    *               *    
     247127 CCAAACTGCTCAATCAAGAGGAAGTTTCAAGTCTGTGAGCTGAACGCACACATCACAAAGTAGTT
         66 CAAAACTGCTCAATCAAAAGAAAGGTTCAACTCTGTGAGATGAATGCACACATCACAAAGAAGTT

               *                                *    
     247192 TCTGAGAATGCTTCTGTGTAGTTTTTATGTGAAGATGTTTC
        131 TCTCAGAATGCTTCTGTGTAGTTTTTATGTGAAGATATTTC

                            **     *                                    *    
     247233 CTTTTCCACCATAGGCTGCAAAGGGCTCCAAATATCCACTTGCAGATTCTACAAAAAGAGAGTTT
          1 CTTTTCCACCATAGGCCTCAAAGCGCTCCAAATATCCACTTGCAGATTCTACAAAAAGAGTGTTT

                 *     *         *          *     *                     *    
     247298 CAAAAGTGCTCTATCAAAAGATAGGTTCAACTATGTGATATGAATGCACACATCACAAAGTAGTT
         66 CAAAACTGCTCAATCAAAAGAAAGGTTCAACTCTGTGAGATGAATGCACACATCACAAAGAAGTT

                                          *          
     247363 TCTCAGAATGCTTCTGTGTAGTTTTTATGTAAAGATATTTC
        131 TCTCAGAATGCTTCTGTGTAGTTTTTATGTGAAGATATTTC

                                    *                                        
     247404 CTTTTCCACCATAGGCCTCAAAGCACTCCAAATATCCACTTGCAGATTCTACAAAAAGAGATG-T
          1 CTTTTCCACCATAGGCCTCAAAGCGCTCCAAATATCCACTTGCAGATTCTACAAAAAGAG-TGTT

                    ** *                   *     *  *    * *   *        *    
     247468 TCAAAACTATTTAATCAAAAGAAAGGTTCAAATCTGTCAGTTGAAGGTACATATCACAAACAAGT
         65 TCAAAACTGCTCAATCAAAAGAAAGGTTCAACTCTGTGAGATGAATGCACACATCACAAAGAAGT

              * **                                    
     247533 TTATTGGAATGCTTCTGTGTAGTTTTTATGTGAAGATATTTC
        130 TTCTCAGAATGCTTCTGTGTAGTTTTTATGTGAAGATATTTC

                     * *         * *                        **  *     *      
     247575 CTTTTCCACAACAGGCCTCAAGGTGCTCCAAATATCCACTTGCAGATTTCACTAAAAGTGTGTTT
          1 CTTTTCCACCATAGGCCTCAAAGCGCTCCAAATATCCACTTGCAGATTCTACAAAAAGAGTGTTT

             *  *            *  *   *     *        *             *           
     247640 CCAAGCTGCTCAATCAAGAGGAAGTTTCAAGTCTGTGAGGTGAATGCACACATTACAAAGAAGTT
         66 CAAAACTGCTCAATCAAAAGAAAGGTTCAACTCTGTGAGATGAATGCACACATCACAAAGAAGTT

            *  *                                     
     247705 ACTGAGAATGCTTCTGTGTAGTTTTTATGTGAAGATATTTC
        131 TCTCAGAATGCTTCTGTGTAGTTTTTATGTGAAGATATTTC

                      **               *                                *    
     247746 CTTTTCCACCGCAGGCCTCAAAGCGCTGCAAATATCCACTTGCAGATTCTACAAAAAGAGAGTTT
          1 CTTTTCCACCATAGGCCTCAAAGCGCTCCAAATATCCACTTGCAGATTCTACAAAAAGAGTGTTT

                      **         *   *         *   *     **            *     
     247811 CAAAACTGCTGTATCAAAAGATAGGGTCAACTCTGCGAGTTGAATAAACACATCACAAATAAGTT
         66 CAAAACTGCTCAATCAAAAGAAAGGTTCAACTCTGTGAGATGAATGCACACATCACAAAGAAGTT

               **   *        *               *       
     247876 TCTGGGAACGCTTCTGTATAG-TTTTATGTGAATATATTTC
        131 TCTCAGAATGCTTCTGTGTAGTTTTTATGTGAAGATATTTC

                         *          *                   *   *  *   * *   *   
     247916 CTTTTCCACCATATGCCTCAAAGCACTCCAAATATCCACTTGCACATTATAGAAACATAGTCTTT
          1 CTTTTCCACCATAGGCCTCAAAGCGCTCCAAATATCCACTTGCAGATTCTACAAAAAGAGTGTTT

                   **                        *         *             *       
     247981 CAAAACTTGTCAATC-AAAGAAAGGTTCAACTCCGTGAGATGAGTGCACACATCACAGAGAAGTT
         66 CAAAACTGCTCAATCAAAAGAAAGGTTCAACTCTGTGAGATGAATGCACACATCACAAAGAAGTT

                *     *                            * 
     248045 TCTCGGAATGTTTCTGTGTAGTTTTTATGTGAAGATATTGC
        131 TCTCAGAATGCTTCTGTGTAGTTTTTATGTGAAGATATTTC

                     *               *            *          *       *   *   
     248086 CTTTTCCACAATAGGCCTCAAAGCGTTCCAAATATCCAATTGCAGATTCCACAAAAAAAGTTTTT
          1 CTTTTCCACCATAGGCCTCAAAGCGCTCCAAATATCCACTTGCAGATTCTACAAAAAGAGTGTTT

            *                 *  *  *  *             * *         **    *     
     248151 TAAAACTGCTCAATCAAATGATAGATTAAACTCTGTGAGATTAGTGCACACATGTCAAAAAAGTT
         66 CAAAACTGCTCAATCAAAAGAAAGGTTCAACTCTGTGAGATGAATGCACACATCACAAAGAAGTT

                                *      * *           
     248216 TCTCAGAATGCTTCTGTGTACTTTTTAGGGGAAGATATTTC
        131 TCTCAGAATGCTTCTGTGTAGTTTTTATGTGAAGATATTTC

                        *    *     **         *    *           **  * *  *    
     248257 CTTTTCCACCATCGGCCACAAAGGACTCCAAATAACCACATGCAGATTCTAGTAACACAGAGTTT
          1 CTTTTCCACCATAGGCCTCAAAGCGCTCCAAATATCCACTTGCAGATTCTACAAAAAGAGTGTTT

                       *         * *           *   * * *    **    ***        
     248322 CAAAACTGCTCTATCAAAAGATAAGTTCAACTCTGAGAGTTTAGTGCAACCATCGTGAAGAAGTT
         66 CAAAACTGCTCAATCAAAAGAAAGGTTCAACTCTGTGAGATGAATGCACACATCACAAAGAAGTT

                            *     *                  
     248387 TCTCAGAATGCTTCTGAGTAGTGTTTATGTGAAGATATTTC
        131 TCTCAGAATGCTTCTGTGTAGTTTTTATGTGAAGATATTTC

                              *     *                      *           **    
     248428 CTTTTCCACCATAGGCCTGAAAGCCCTCCAAATATCCACTTGCAGATCCTACAAAAAGAAAGTTT
          1 CTTTTCCACCATAGGCCTCAAAGCGCTCCAAATATCCACTTGCAGATTCTACAAAAAGAGTGTTT

             *         **     *  *  *   *        **      *                   
     248493 CGAAA-TGCTCTCTCAAACGATAGTTTCGACTCTGTGGTATGAATACACACATCACAAAGAAGTT
         66 CAAAACTGCTCAATCAAAAGAAAGGTTCAACTCTGTGAGATGAATGCACACATCACAAAGAAGTT

                                       **            
     248557 TCTCAGAATGCTTCTGTGTAGTTTTTAAATGAAGATATTTC
        131 TCTCAGAATGCTTCTGTGTAGTTTTTATGTGAAGATATTTC

            *                       *          *     *                       
     248598 TTTTTCCACCATAGGCCTCAAAGCACTCCAAATATGCACTTCCAGATTCTACAAAAAGAGTGTTT
          1 CTTTTCCACCATAGGCCTCAAAGCGCTCCAAATATCCACTTGCAGATTCTACAAAAAGAGTGTTT

              *                 *       * *         **   *         *  * *    
     248663 CAGAACTGCTCAATCAAAAGGAAGGTTCCAGTC--TGAGACAAATACACACATCAAAAGGTAGTT
         66 CAAAACTGCTCAATCAAAAGAAAGGTTCAACTCTGTGAGATGAATGCACACATCACAAAGAAGTT

                                                    *
     248726 TCTCAGAATGCTTCTGTGTAGTTTTTATGTGAAGATATTTT
        131 TCTCAGAATGCTTCTGTGTAGTTTTTATGTGAAGATATTTC

             *               *    **    *     *      *  **   *          *    
     248767 CCTTTCCACCATAGGCCACAAATGGCTCTAAATACCCACTTACATTTTCCACAAAAAGAGAGTTT
          1 CTTTTCCACCATAGGCCTCAAAGCGCTCCAAATATCCACTTGCAGATTCTACAAAAAGAGTGTTT

                       * *    * *      *   *         *                       
     248832 CAAAACTGCTCTACCAAAGGTAA-GTTTAACGCTGTGAG-TTAA-G-A-ACATCACAAAGAAGTT
         66 CAAAACTGCTCAATCAAAAGAAAGGTTCAACTCTGTGAGATGAATGCACACATCACAAAGAAGTT

                                   *   *  *          
     248892 TCTCAGAATGCTTCTGTGTAGTTCTTACGTAAAGATATTTC
        131 TCTCAGAATGCTTCTGTGTAGTTTTTATGTGAAGATATTTC

                 *   *      ***    *                  *              **      
     248933 CTTTTACACAATAGGCAGAAAAGTGCTCCAAATATCCACTTGAAGATTCTACAAAAACCGTGTTT
          1 CTTTTCCACCATAGGCCTCAAAGCGCTCCAAATATCCACTTGCAGATTCTACAAAAAGAGTGTTT

                     **                                          *      *    
     248998 CAAAACTGCCGAATCAAAAGAAAGGTTCAACTCTGTGAGATGAATGCACACATAACAAAGGAGTT
         66 CAAAACTGCTCAATCAAAAGAAAGGTTCAACTCTGTGAGATGAATGCACACATCACAAAGAAGTT

                                 *      *      *    *
     249063 TCTCAGAATGCTTCTGTGTAGCTTTTATATGAAGACATTTA
        131 TCTCAGAATGCTTCTGTGTAGTTTTTATGTGAAGATATTTC

            *        * *            *   ***                     **        *  
     249104 GTTTTCCACAACAGGCCTCAAAGCTCTCTCCATATCCACTTGCAGATTCTACCGAAAGAGTGCTT
          1 CTTTTCCACCATAGGCCTCAAAGCGCTCCAAATATCCACTTGCAGATTCTACAAAAAGAGTGTTT

             *                   * **     *        *        *     **         
     249169 CCAAACTGCTCAATCAAAAGAGACATTCAAATCTGTGAGGTGAATGCAGACATCGTAAAGAAGTT
         66 CAAAACTGCTCAATCAAAAGAAAGGTTCAACTCTGTGAGATGAATGCACACATCACAAAGAAGTT

                                *     *       *    **
     249234 TCTCAGAATGCTTCTGTGTATTTTTTGTGTGAAGTTATTCG
        131 TCTCAGAATGCTTCTGTGTAGTTTTTATGTGAAGATATTTC

            *    *             *     *  *            **                 *    
     249275 TTTTTGCACCATAGGCCTCCAAGCGTTCTAAATATCCACTTCTAGATTCTACAAAAAGAGAGTTT
          1 CTTTTCCACCATAGGCCTCAAAGCGCTCCAAATATCCACTTGCAGATTCTACAAAAAGAGTGTTT

                   *         *            *        *    *   *                
     249340 CAAAACTACTC-A--AACA-AAAGGTTCAATTCTGTGAGTTGAAAGCAAACATCACAAAGAAGTT
         66 CAAAACTGCTCAATCAAAAGAAAGGTTCAACTCTGTGAGATGAATGCACACATCACAAAGAAGTT

                       *                             
     249401 TCTCAGAATGCGTCTGTGTAGTTTTTATGTGAAGATATTTC
        131 TCTCAGAATGCTTCTGTGTAGTTTTTATGTGAAGATATTTC

                     **   ****     *                  *                      
     249442 CTTTT-CACAGTAGAATGCAAAGGGCTCCAAATATCCACTTGGAGATTCTACAAAAAGA--GTTT
          1 CTTTTCCACCATAGGCCTCAAAGCGCTCCAAATATCCACTTGCAGATTCTACAAAAAGAGTGTTT

                  *    **     *  *     *     ***   *     *               *   
     249504 CAAAACCGCTCTGTCAAATGATAGGTTGAACTCCCGGAGGTGAATACACACATCACAAAGAGGTT
         66 CAAAACTGCTCAATCAAAAGAAAGGTTCAACTCTGTGAGATGAATGCACACATCACAAAGAAGTT

                  *                       *  *       
     249569 TCTCAGCATGCTTCTGTGTAGTTTTTATGTAAACATATTTC
        131 TCTCAGAATGCTTCTGTGTAGTTTTTATGTGAAGATATTTC

             *    * *              *           *      * *       *   *    *   
     249610 CGTTTCTATCATAGGCCTCAAAGTGCTCCAAATATTCACTTGTACATTCTACCAAACGAGTATTT
          1 CTTTTCCACCATAGGCCTCAAAGCGCTCCAAATATCCACTTGCAGATTCTACAAAAAGAGTGTTT

                              * *         **      *        *            *    
     249675 CAAAACTGCTCAATCAAATGGAAGGTTCAAAACTGTGACATGAATGCCCACATCACAAAGTAGTT
         66 CAAAACTGCTCAATCAAAAGAAAGGTTCAACTCTGTGAGATGAATGCACACATCACAAAGAAGTT

                                                     
     249740 TCTCAGAATGCTTCTGTGTAGTTTTTATGTGAAGATATTTC
        131 TCTCAGAATGCTTCTGTGTAGTTTTTATGTGAAGATATTTC

                     * *    *      *    *       * *   **      *              
     249781 CTTTTCCACAACA-GCGTGCAAAACGCTTCAAATATGCCCTTAGAGATTCCACAAAAAGAGTGTT
          1 CTTTTCCACCATAGGCCT-CAAAGCGCTCCAAATATCCACTTGCAGATTCTACAAAAAGAGTGTT

              *     *               *  *                                  ** 
     249845 TCCAAACTACTCAAATCAAAA-AATGATTTCAACTCTGTGAGATGAATGCACACATCACAAACTA
         65 TCAAAACTGCTC-AATCAAAAGAAAG-GTTCAACTCTGTGAGATGAATGCACACATCACAAAGAA

                         *     ** *   * *   *       *   
     249909 GTTTCTCAGAATGTTTCTGCCTGGTTCTCATGCGAAGATAGTTC
        128 GTTTCTCAGAATGCTTCTGTGTAGTTTTTATGTGAAGATATTTC

                 *           *   * **              *                  * *    
     249953 CTTTTTCACCATAGGCCGCAATGTACTCCAAATATCCACCTGCAGATTCTAC-AAAAGTGAGTTT
          1 CTTTTCCACCATAGGCCTCAAAGCGCTCCAAATATCCACTTGCAGATTCTACAAAAAGAGTGTTT

                       *         ***    **         *        *      *       * 
     250017 CAAAACTGCTCTATCAAAAGATCAGTTCGTCTCTGTGAGTTGAATGCATACATCAAAAAGAAGCT
         66 CAAAACTGCTCAATCAAAAGAAAGGTTCAACTCTGTGAGATGAATGCACACATCACAAAGAAGTT

                 *             *      **         *   
     250082 TCTCAAAATGCTTCTGTGTGGTTTTTCGGTGAAGATAGTTC
        131 TCTCAGAATGCTTCTGTGTAGTTTTTATGTGAAGATATTTC

            *     *        *      * *                 *        *        *    
     250123 TTTTTCTACCATAGGTCTCAAACCACTCCAAATATCCACTTGTAGATTCTATAAAAAGGAATG-T
          1 CTTTTCCACCATAGGCCTCAAAGCGCTCCAAATATCCACTTGCAGATTCTACAAAAA-GAGTGTT

                  *        *    *    *      * *         *      *             
     250187 TCAAAATTGCTCAATAAAAATAAAGTTTCAACACCGTGAGATGAGTGCACAAATCACAAAGAAGT
         65 TCAAAACTGCTCAATCAAAAGAAAGGTTCAACTCTGTGAGATGAATGCACACATCACAAAGAAGT

                  *          *         *          *   
     250252 TTCTCAAAATGCTTCTGGGTAGTTTTTCTGTGAAGATAGTTC
        130 TTCTCAGAATGCTTCTGTGTAGTTTTTATGTGAAGATATTTC

                  *     *    *     *          *                         *    
     250294 CTTTTCTACCATGGGCCACAAAGGGCTCCAAATACCCACTTGCAGATTCTACAAAAAGAGAGTTT
          1 CTTTTCCACCATAGGCCTCAAAGCGCTCCAAATATCCACTTGCAGATTCTACAAAAAGAGTGTTT

              *        *      ** * *        *    * *    **  * *           *  
     250359 CACAACTGCTCTATCAAACAATATGTTCAACTTTGTGGGTTGAACACAAATATCAC-AAGAATTT
         66 CAAAACTGCTCAATCAAAAGAAAGGTTCAACTCTGTGAGATGAATGCACACATCACAAAGAAGTT

                **                             *     
     250423 TCTCCCAATGCTTCTGTGTAGTTTTTATGTGAAGACATTTC
        131 TCTCAGAATGCTTCTGTGTAGTTTTTATGTGAAGATATTTC

            *   *  *      *  *     *                 *  *      *       *  *  
     250464 TTTTCCCTCCATAGTCCACAAAGTGCTCCAAATATCCACTTACATATTCTAGAAAAAGATTGCTT
          1 CTTTTCCACCATAGGCCTCAAAGCGCTCCAAATATCCACTTGCAGATTCTACAAAAAGAGTGTTT

            **       *    *           *   * * *                              
     250529 GGAAACTGCACAATGAAAAGAAAGGTCCAAATATATGAGATGAATGCACACATCACAAAGAAGTT
         66 CAAAACTGCTCAATCAAAAGAAAGGTTCAACTCTGTGAGATGAATGCACACATCACAAAGAAGTT

                                 *                    
     250594 TCTCAGAAT-CTCTCTGTGTAATTTTTATGTGAAGATATTTC
        131 TCTCAGAATGCT-TCTGTGTAGTTTTTATGTGAAGATATTTC

                *     *    *  *   *                        *      *    *     
     250635 CTTTCCCACCTTAGGTCTTAAAACGCTCCAAATATCCACTTGCAGATACTACAAGAAGATTGTTT
          1 CTTTTCCACCATAGGCCTCAAAGCGCTCCAAATATCCACTTGCAGATTCTACAAAAAGAGTGTTT

                     *    *        *      *     **                           
     250700 CAAAACTGCACAA-AAAAAGAAATGTTCAATTCTGTTTGATGAATGCACACATCACAAAGAAGTT
         66 CAAAACTGCTCAATCAAAAGAAAGGTTCAACTCTGTGAGATGAATGCACACATCACAAAGAAGTT

                           *                         
     250764 TCTCAGAATGCTTCTCTGTAGTTTTTATGTGAAGATATTTC
        131 TCTCAGAATGCTTCTGTGTAGTTTTTATGTGAAGATATTTC

                     *             *                 *         **      * *   
     250805 CTTTTCCACAATAGGCCTCAAAGGGCTCCAAATATCCACTTCCAGATTCTATGAAAAGAATATTT
          1 CTTTTCCACCATAGGCCTCAAAGCGCTCCAAATATCCACTTGCAGATTCTACAAAAAGAGTGTTT

             *              * *    *                                      *  
     250870 CCAAACTGCTCAATCATAGGAAATGTTCAACTCTGTGAGATGAATGCACACATCAC-AAGAAATT
         66 CAAAACTGCTCAATCAAAAGAAAGGTTCAACTCTGTGAGATGAATGCACACATCACAAAGAAGTT

                     *    *      *       *       *   
     250934 TCTCAGAATCCTTCAGTGTAGGTTTTATGAGAAGATAATTC
        131 TCTCAGAATGCTTCTGTGTAGTTTTTATGTGAAGATATTTC

                     *    **        *   *                           *    *   
     250975 CTTTTCCACAATAGTTCTCAAAGCACTCAAAATATCCACTTGCAGATTCTACAAAAGGAGTATTT
          1 CTTTTCCACCATAGGCCTCAAAGCGCTCCAAATATCCACTTGCAGATTCTACAAAAAGAGTGTTT

                                                 *        *                  
     251040 CAAAACTGCTCAATCAAAAGAAAGGTTCAACTCTGTGGGATGAATGGACACATCACAAAGAAGTT
         66 CAAAACTGCTCAATCAAAAGAAAGGTTCAACTCTGTGAGATGAATGCACACATCACAAAGAAGTT

                                  *   *              
     251105 TCTCAGAATGCTTCTGTGTAGTATTTTTGTGAAGATATTT-
        131 TCTCAGAATGCTTCTGTGTAGTTTTTATGTGAAGATATTTC

                          *  * * * * ***             *            *     * *  
     251145 CTTTTCCACCATAGACCGCCAGGGGACACAAATATCCACTTTCAGATTCTACAACAAGAGAGGTT
          1 CTTTTCCACCATAGGCCTCAAAGCGCTCCAAATATCCACTTGCAGATTCTACAAAAAGAGTGTTT

                   *   *     *   ** *       *      *                      * *
     251210 CAAAACTACTCGATCAAGAGATGGTTTCAACTATGTGAGTTGAATGCACACATCACAAAGAACTA
         66 CAAAACTGCTCAATCAAAAGAAAGGTTCAACTCTGTGAGATGAATGCACACATCACAAAGAAGTT

             *  *    *                               
     251275 TGTCGGAATTCTTCTGTGTAGTTTTTATGTGAAGATATTTC
        131 TCTCAGAATGCTTCTGTGTAGTTTTTATGTGAAGATATTTC

                     *    * *      * *    *                  *               
     251316 CTTTTCCACAATAGACGTCAAAGTGATCCAGATATCCACTTGCAGATTCCACAAAAAGAGTGTTT
          1 CTTTTCCACCATAGGCCTCAAAGCGCTCCAAATATCCACTTGCAGATTCTACAAAAAGAGTGTTT

                 *   *   *                  **                     *  *      
     251381 CAAAAGTGCACAACCAAAAGAAAGGTTCAACTAGGTGAGATGAATGCACACATCAGAAGGAAGTT
         66 CAAAACTGCTCAATCAAAAGAAAGGTTCAACTCTGTGAGATGAATGCACACATCACAAAGAAGTT

                            **   *     * *       *   
     251446 TCTCAGAATGCTTCTGCATAGCTTTTAAGGGAAGATACTTC
        131 TCTCAGAATGCTTCTGTGTAGTTTTTATGTGAAGATATTTC

                    *               *            * *  *    * *               
     251487 CTTTTCCAACATAGGCCTCAAAGCACTCCAAATATCCTCCTGGAGATACCACAAAAAGAGTGTTT
          1 CTTTTCCACCATAGGCCTCAAAGCGCTCCAAATATCCACTTGCAGATTCTACAAAAAGAGTGTTT

            **                      *  *                 *       *           
     251552 GCAAACTGCTCAATCAAAAGAAAGATTTAACTCTGTGAGATGAATCCACACATGACAAAGAAGTT
         66 CAAAACTGCTCAATCAAAAGAAAGGTTCAACTCTGTGAGATGAATGCACACATCACAAAGAAGTT

                                       **            
     251617 TCTCAGAATGCTTCTGTGTAGTTTTTAATTGAAGATATTTC
        131 TCTCAGAATGCTTCTGTGTAGTTTTTATGTGAAGATATTTC

            *        *                  *            *  *                    
     251658 TTTTTCCACAATAGGCCTCAAAGCGCTCAAAATATCCACTTTCACATTCTACAAAAAGAGTGTTT
          1 CTTTTCCACCATAGGCCTCAAAGCGCTCCAAATATCCACTTGCAGATTCTACAAAAAGAGTGTTT

                     *           *                 *       *    *  *     *   
     251723 CAAAACTGCACAATCAAAAGATA-GTTCAACTCTGTGAGTTGAATGCGCACAACAAAAAGATGTT
         66 CAAAACTGCTCAATCAAAAGAAAGGTTCAACTCTGTGAGATGAATGCACACATCACAAAGAAGTT

                      **                              
     251787 TCTCAGAATTATTTCTGTGTAGTTTTTATGTGAAGATATTTC
        131 TCTCAGAA-TGCTTCTGTGTAGTTTTTATGTGAAGATATTTC

                     *  *          *                                     *  *
     251829 CTTTTCCACAATGGGCCTCAAAGTGCTCCAAATATCCACTTGCAGATTCTACAAAAAGAGTTTTC
          1 CTTTTCCACCATAGGCCTCAAAGCGCTCCAAATATCCACTTGCAGATTCTACAAAAAGAGTGTTT

                       *            *    *                    * *           *
     251894 CAAAACTGCTCCATCAAAAGAAAGCTTCACCTCTGTGAGATGAATGCACATACCACAAAGAAGTG
         66 CAAAACTGCTCAATCAAAAGAAAGGTTCAACTCTGTGAGATGAATGCACACATCACAAAGAAGTT

                                        *            
     251959 TCTCAGAATGCTTCTGTGTAGTTTTTATATGAAGATATTTC
        131 TCTCAGAATGCTTCTGTGTAGTTTTTATGTGAAGATATTTC

                     *   * * *    **           *                *     *      
     252000 CTTTTCCACAATAAGACCCAAAAGGCTCCAAATATTCACTTGCAGATTCTAAAAAAAACAGTGTT
          1 CTTTTCCACCATAGGCCTCAAAGCGCTCCAAATATCCACTTGCAGATTCT-ACAAAAAGAGTGTT

                                  *                  *      *        **      
     252065 TCAAAACTGCTCAATCAAAAGATA-GTTCAACTCTGTGAGAAGAATGCTCACATCACTGAGAAGT
         65 TCAAAACTGCTCAATCAAAAGAAAGGTTCAACTCTGTGAGATGAATGCACACATCACAAAGAAGT

                                         *            
     252129 TTCTCAGAATGCTTCTGTGTAGTTTTTATATGAAGATATTTC
        130 TTCTCAGAATGCTTCTGTGTAGTTTTTATGTGAAGATATTTC

                *     *      *    **                       *   **       *    
     252171 CTTTCCCACCGTAGGCCACAAAAGGCTCCAAATATCCACTTGCAGATACTATGAAAAGAGAGTTT
          1 CTTTTCCACCATAGGCCTCAAAGCGCTCCAAATATCCACTTGCAGATTCTACAAAAAGAGTGTTT

                        *        *               ***            *            
     252236 CAAAACTGCTCATTCAAAAGATAGGTTCAACTCTGTGGTTTGAATGCACACAGCACAAAGAAGTT
         66 CAAAACTGCTCAATCAAAAGAAAGGTTCAACTCTGTGAGATGAATGCACACATCACAAAGAAGTT

              *       **                   **   *    
     252301 TCACAGAATGTGTCTGTGTAGTTTTTATGTGCGGATGTTTC
        131 TCTCAGAATGCTTCTGTGTAGTTTTTATGTGAAGATATTTC

                         *        ****   *   *               *    *          
     252342 CTTTTCCACCATATGCCT-AAATATTTCCCAATTTCCACTTGCAGATTCCACAAGAAGAGTGTTT
          1 CTTTTCCACCATAGGCCTCAAAGCGCTCCAAATATCCACTTGCAGATTCTACAAAAAGAGTGTTT

                      **       *       *           *            *      ***   
     252406 CAAAACTGCTGTATC-AAATAAA-GTTGAACTCTGTGAGGTGAATGCACACAGCACAAAATGGTT
         66 CAAAACTGCTCAATCAAAAGAAAGGTTCAACTCTGTGAGATGAATGCACACATCACAAAGAAGTT

                          **   *        *       *    
     252469 TCTCAGAATGCTTCCTTGTTGTTTTTATATGAAGATGTTTC
        131 TCTCAGAATGCTTCTGTGTAGTTTTTATGTGAAGATATTTC

                  *  *             *   *     *                               
     252510 CTTTTCAACAATAGGCCTCAAAGTGCTTCAAATGTCCACTTGCAGATTCTACAAAAAGAGTGTTT
          1 CTTTTCCACCATAGGCCTCAAAGCGCTCCAAATATCCACTTGCAGATTCTACAAAAAGAGTGTTT

                                        *      *  *  *                       
     252575 CAAAACTGCTCAATCAAAAGAAAGGTTCGACTCTGGGAAATTAATGCACACATCACAAAGAAGTT
         66 CAAAACTGCTCAATCAAAAGAAAGGTTCAACTCTGTGAGATGAATGCACACATCACAAAGAAGTT

                                     *        *      
     252640 TCTC---A-GCTTCTGTGTAGTTTTCATGTGAAGTTATTTC
        131 TCTCAGAATGCTTCTGTGTAGTTTTTATGTGAAGATATTTC

                     *       *     *            *    *         **       *    
     252677 CTTTTCCACAATAGGCCGCAAAGGGCTCCAAATATCAACTTACAGATTCTAGGAAAAGAGAGTTT
          1 CTTTTCCACCATAGGCCTCAAAGCGCTCCAAATATCCACTTGCAGATTCTACAAAAAGAGTGTTT

                       * **      *     *                                     
     252742 CAAAACTGCTCTACGAAAAGATAGGTTGAACTCTGTGAGATGAATGCACACATCACAAAGAAGTT
         66 CAAAACTGCTCAATCAAAAGAAAGGTTCAACTCTGTGAGATGAATGCACACATCACAAAGAAGTT

                       *               * *          *
     252807 TCTCAGAATGCATCTGTGTAGTTTTTACGGGAAGATATTTT
        131 TCTCAGAATGCTTCTGTGTAGTTTTTATGTGAAGATATTTC

                        ***  *     **      *  *           *      *     ***   
     252848 CTTTTCCACCATCTTCCACAAAGGTCTCCAAGTAACCACTTGCAGACTCTACAGAAAGACACTTT
          1 CTTTTCCACCATAGGCCTCAAAGCGCTCCAAATATCCACTTGCAGATTCTACAAAAAGAGTGTTT

            *          *         ***      *      ***                      *  
     252913 AAAAACTGCTCTATCAAAAGATCAGTTCAAGTCTGTGGTTTGAATGCACACATCACAAAGAATTT
         66 CAAAACTGCTCAATCAAAAGAAAGGTTCAACTCTGTGAGATGAATGCACACATCACAAAGAAGTT

                                     *  *            
     252978 TCTCAGAATGCTTCTGTGTAGTTTTCATATGAAGATATTTC
        131 TCTCAGAATGCTTCTGTGTAGTTTTTATGTGAAGATATTTC

                                    *                                   **   
     253019 CTTTTCCACCATAGGCCTCAAAGCACTCCAAATATCCACTTGCAGATTCTACAAAAAGAGATTTT
          1 CTTTTCCACCATAGGCCTCAAAGCGCTCCAAATATCCACTTGCAGATTCTACAAAAAGAGTGTTT

                   **                           *  *          *        *     
     253084 CAAAACTAGTCAATCAAAAGAAAGGTTCAACTCTGTCAGTTGAATGCACATATCACAAACAAGTT
         66 CAAAACTGCTCAATCAAAAGAAAGGTTCAACTCTGTGAGATGAATGCACACATCACAAAGAAGTT

                *      *                             
     253149 TCTCGGAATGCGTCTGTGTAGTTTTTATGTGAAGATATTTC
        131 TCTCAGAATGCTTCTGTGTAGTTTTTATGTGAAGATATTTC

               *     * *           *     *                  *    *           
     253190 CTTCTCCACAACAGGCCTCAAAGTGCTCCGAATATCCACTTGCAGATTTTAC-TAAAGAGTGTTT
          1 CTTTTCCACCATAGGCCTCAAAGCGCTCCAAATATCCACTTGCAGATTCTACAAAAAGAGTGTTT

             *               *  *   *     *        *    *               *    
     253254 CCAAACTGCTCAATCAAGAGGAAGTTTCAAGTCTGTGAGCTGAACGCACACATCACAAAGTAGTT
         66 CAAAACTGCTCAATCAAAAGAAAGGTTCAACTCTGTGAGATGAATGCACACATCACAAAGAAGTT

               *                                *    
     253319 TCTGAGAATGCTTCTGTGTAGTTTTTATGTGAAGATGTTTC
        131 TCTCAGAATGCTTCTGTGTAGTTTTTATGTGAAGATATTTC

                            **     *                                    *  * 
     253360 CTTTTCCACCATAGGCTGCAAAGGGCTCCAAATATCCACTTGCAGATTCTACAAAAAGAGAGTCT
          1 CTTTTCCACCATAGGCCTCAAAGCGCTCCAAATATCCACTTGCAGATTCTACAAAAAGAGTGTTT

                 *     *         *          *     *                     *    
     253425 CAAAAGTGCTCTATCAAAAGATAGGTTCAACTATGTGATATGAATGCACACATCACAAAGTAGTT
         66 CAAAACTGCTCAATCAAAAGAAAGGTTCAACTCTGTGAGATGAATGCACACATCACAAAGAAGTT

                                          *          
     253490 TCTCAGAATGCTTCTGTGTAGTTTTTATGTAAAGATATTTC
        131 TCTCAGAATGCTTCTGTGTAGTTTTTATGTGAAGATATTTC

                                    *                                        
     253531 CTTTTCCACCATAGGCCTCAAAGCACTCCAAATATCCACTTGCAGATTCTACAAAAAGAGATG-T
          1 CTTTTCCACCATAGGCCTCAAAGCGCTCCAAATATCCACTTGCAGATTCTACAAAAAGAG-TGTT

                    ** *                   *     *  *    * *   *        *    
     253595 TCAAAACTATTTAATCAAAAGAAAGGTTCAAATCTGTCAGTTGAAGGTACATATCACAAACAAGT
         65 TCAAAACTGCTCAATCAAAAGAAAGGTTCAACTCTGTGAGATGAATGCACACATCACAAAGAAGT

              * **                                    
     253660 TTATTGGAATGCTTCTGTGTAGTTTTTATGTGAAGATATTTC
        130 TTCTCAGAATGCTTCTGTGTAGTTTTTATGTGAAGATATTTC

                     * *         * *                        **  *     *      
     253702 CTTTTCCACAACAGGCCTCAAGGTGCTCCAAATATCCACTTGCAGATTTCACTAAAAGTGTGTTT
          1 CTTTTCCACCATAGGCCTCAAAGCGCTCCAAATATCCACTTGCAGATTCTACAAAAAGAGTGTTT

             *  *            *  *   *     *        *             *      *    
     253767 CCAAGCTGCTCAATCAAGAGGAAGTTTCAAGTCTGTGAGGTGAATGCACACATTACAAAGTAGTT
         66 CAAAACTGCTCAATCAAAAGAAAGGTTCAACTCTGTGAGATGAATGCACACATCACAAAGAAGTT

            *  *                                     
     253832 ACTGAGAATGCTTCTGTGTAGTTTTTATGTGAAGATATTTC
        131 TCTCAGAATGCTTCTGTGTAGTTTTTATGTGAAGATATTTC

                      **               *                       *        *    
     253873 CTTTTCCACCGCAGGCCTCAAAGCGCTGCAAATATCCACTTGCAGATTCTATAAAAAGAGAGTTT
          1 CTTTTCCACCATAGGCCTCAAAGCGCTCCAAATATCCACTTGCAGATTCTACAAAAAGAGTGTTT

                      **         *   *         *   *     **            *     
     253938 CAAAACTGCTGTATCAAAAGATAGGGTCAACTCTGCGAGTTGAATAAACACATCACAAATAAGTT
         66 CAAAACTGCTCAATCAAAAGAAAGGTTCAACTCTGTGAGATGAATGCACACATCACAAAGAAGTT

               **   *        *               *       
     254003 TCTGGGAACGCTTCTGTATAG-TTTTATGTGAATATATTTC
        131 TCTCAGAATGCTTCTGTGTAGTTTTTATGTGAAGATATTTC

                         *          *                   *   *  *   * *   *   
     254043 CTTTTCCACCATATGCCTCAAAGCACTCCAAATATCCACTTGCACATTATAGAAACATAGTCTTT
          1 CTTTTCCACCATAGGCCTCAAAGCGCTCCAAATATCCACTTGCAGATTCTACAAAAAGAGTGTTT

                   **                        *         *             *       
     254108 CAAAACTTGTCAATC-AAAGAAAGGTTCAACTCCGTGAGATGAGTGCACACATCACAGAGAAGTT
         66 CAAAACTGCTCAATCAAAAGAAAGGTTCAACTCTGTGAGATGAATGCACACATCACAAAGAAGTT

                *     *                            * 
     254172 TCTCGGAATGTTTCTGTGTAGTTTTTATGTGAAGATATTGC
        131 TCTCAGAATGCTTCTGTGTAGTTTTTATGTGAAGATATTTC

                     *               *            *          *       *   *   
     254213 CTTTTCCACAATAGGCCTCAAAGCGTTCCAAATATCCAATTGCAGATTCCACAAAAAAAGTTTTT
          1 CTTTTCCACCATAGGCCTCAAAGCGCTCCAAATATCCACTTGCAGATTCTACAAAAAGAGTGTTT

            *                 *  *  *  *             * *         **    *     
     254278 TAAAACTGCTCAATCAAATGATAGATTAAACTCTGTGAGATTAGTGCACACATGTCAAAAAAGTT
         66 CAAAACTGCTCAATCAAAAGAAAGGTTCAACTCTGTGAGATGAATGCACACATCACAAAGAAGTT

                                *      * *           
     254343 TCTCAGAATGCTTCTGTGTACTTTTTAGGGGAAGATATTTC
        131 TCTCAGAATGCTTCTGTGTAGTTTTTATGTGAAGATATTTC

                        *    *     **         *    *           **  * *  *    
     254384 CTTTTCCACCATCGGCCACAAAGGACTCCAAATAACCACATGCAGATTCTAGTAACACAGAGTTT
          1 CTTTTCCACCATAGGCCTCAAAGCGCTCCAAATATCCACTTGCAGATTCTACAAAAAGAGTGTTT

                       *         * *           *   * * *    **    ***        
     254449 CAAAACTGCTCTATCAAAAGATAAGTTCAACTCTGAGAGTTTAGTGCAACCATCGTGAAGAAGTT
         66 CAAAACTGCTCAATCAAAAGAAAGGTTCAACTCTGTGAGATGAATGCACACATCACAAAGAAGTT

                            *     *                  
     254514 TCTCAGAATGCTTCTGAGTAGTGTTTATGTGAAGATATTTC
        131 TCTCAGAATGCTTCTGTGTAGTTTTTATGTGAAGATATTTC

                              *     *                      *           **    
     254555 CTTTTCCACCATAGGCCTGAAAGCCCTCCAAATATCCACTTGCAGATCCTACAAAAAGAAAGTTT
          1 CTTTTCCACCATAGGCCTCAAAGCGCTCCAAATATCCACTTGCAGATTCTACAAAAAGAGTGTTT

             *         **     *  *  *   *        **      *                   
     254620 CGAAA-TGCTCTCTCAAACGATAGTTTCGACTCTGTGGTATGAATACACACATCACAAAGAAGTT
         66 CAAAACTGCTCAATCAAAAGAAAGGTTCAACTCTGTGAGATGAATGCACACATCACAAAGAAGTT

                                       **            
     254684 TCTCAGAATGCTTCTGTGTAGTTTTTAAATGAAGATATTTC
        131 TCTCAGAATGCTTCTGTGTAGTTTTTATGTGAAGATATTTC

            *                       *          *     *                       
     254725 TTTTTCCACCATAGGCCTCAAAGCACTCCAAATATGCACTTCCAGATTCTACAAAAAGAGTGTTT
          1 CTTTTCCACCATAGGCCTCAAAGCGCTCCAAATATCCACTTGCAGATTCTACAAAAAGAGTGTTT

              *                 *       * *         **   *         *  * *    
     254790 CAGAACTGCTCAATCAAAAGGAAGGTTCCAGTC--TGAGACAAATACACACATCAAAAGGTAGTT
         66 CAAAACTGCTCAATCAAAAGAAAGGTTCAACTCTGTGAGATGAATGCACACATCACAAAGAAGTT

                                                    *
     254853 TCTCAGAATGCTTCTGTGTAGTTTTTATGTGAAGATATTTT
        131 TCTCAGAATGCTTCTGTGTAGTTTTTATGTGAAGATATTTC

             *               *    **    *     *      *  **   *          *    
     254894 CCTTTCCACCATAGGCCACAAATGGCTCTAAATACCCACTTACATTTTCCACAAAAAGAGAGTTT
          1 CTTTTCCACCATAGGCCTCAAAGCGCTCCAAATATCCACTTGCAGATTCTACAAAAAGAGTGTTT

                       * *    * *      *   *         *                       
     254959 CAAAACTGCTCTACCAAAGGTAA-GTTTAACGCTGTGAG-TTAA-G-A-ACATCACAAAGAAGTT
         66 CAAAACTGCTCAATCAAAAGAAAGGTTCAACTCTGTGAGATGAATGCACACATCACAAAGAAGTT

                                   *   *  *          
     255019 TCTCAGAATGCTTCTGTGTAGTTCTTACGTAAAGATATTTC
        131 TCTCAGAATGCTTCTGTGTAGTTTTTATGTGAAGATATTTC

                 *   *      ***    *                  *              **      
     255060 CTTTTACACAATAGGCAGAAAAGTGCTCCAAATATCCACTTGAAGATTCTACAAAAACCGTGTTT
          1 CTTTTCCACCATAGGCCTCAAAGCGCTCCAAATATCCACTTGCAGATTCTACAAAAAGAGTGTTT

                     **                                          *      *    
     255125 CAAAACTGCCGAATCAAAAGAAAGGTTCAACTCTGTGAGATGAATGCACACATAACAAAGGAGTT
         66 CAAAACTGCTCAATCAAAAGAAAGGTTCAACTCTGTGAGATGAATGCACACATCACAAAGAAGTT

                                 *      *      *    *
     255190 TCTCAGAATGCTTCTGTGTAGCTTTTATATGAAGACATTTA
        131 TCTCAGAATGCTTCTGTGTAGTTTTTATGTGAAGATATTTC

            *        * *            *   ***                     **        *  
     255231 GTTTTCCACAACAGGCCTCAAAGCTCTCTCCATATCCACTTGCAGATTCTACCGAAAGAGTGCTT
          1 CTTTTCCACCATAGGCCTCAAAGCGCTCCAAATATCCACTTGCAGATTCTACAAAAAGAGTGTTT

             *                   * **     *        *        *     **         
     255296 CCAAACTGCTCAATCAAAAGAGACATTCAAATCTGTGAGGTGAATGCAGACATCGTAAAGAAGTT
         66 CAAAACTGCTCAATCAAAAGAAAGGTTCAACTCTGTGAGATGAATGCACACATCACAAAGAAGTT

                                *     *       *    **
     255361 TCTCAGAATGCTTCTGTGTATTTTTTGTGTGAAGTTATTCG
        131 TCTCAGAATGCTTCTGTGTAGTTTTTATGTGAAGATATTTC

            *    *             *     *  *            **                 *    
     255402 TTTTTGCACCATAGGCCTCCAAGCGTTCTAAATATCCACTTCTAGATTCTACAAAAAGAGAGTTT
          1 CTTTTCCACCATAGGCCTCAAAGCGCTCCAAATATCCACTTGCAGATTCTACAAAAAGAGTGTTT

                   *         *            *        *    *   *                
     255467 CAAAACTACTC-A--AACA-AAAGGTTCAATTCTGTGAGTTGAAAGCAAACATCACAAAGAAGTT
         66 CAAAACTGCTCAATCAAAAGAAAGGTTCAACTCTGTGAGATGAATGCACACATCACAAAGAAGTT

                       *                             
     255528 TCTCAGAATGCGTCTGTGTAGTTTTTATGTGAAGATATTTC
        131 TCTCAGAATGCTTCTGTGTAGTTTTTATGTGAAGATATTTC

                     **   ****     *                  *                      
     255569 CTTTT-CACAGTAGAATGCAAAGGGCTCCAAATATCCACTTGGAGATTCTACAAAAAGA--GTTT
          1 CTTTTCCACCATAGGCCTCAAAGCGCTCCAAATATCCACTTGCAGATTCTACAAAAAGAGTGTTT

                  *    **     *  *     *     ***   *     *               *   
     255631 CAAAACCGCTCTGTCAAATGATAGGTTGAACTCCCGGAGGTGAATACACACATCACAAAGAGGTT
         66 CAAAACTGCTCAATCAAAAGAAAGGTTCAACTCTGTGAGATGAATGCACACATCACAAAGAAGTT

                  *                       *  *       
     255696 TCTCAGCATGCTTCTGTGTAGTTTTTATGTAAACATATTTC
        131 TCTCAGAATGCTTCTGTGTAGTTTTTATGTGAAGATATTTC

             *    * *              *           *      * *       *   *    *   
     255737 CGTTTCTATCATAGGCCTCAAAGTGCTCCAAATATTCACTTGTACATTCTACCAAACGAGTATTT
          1 CTTTTCCACCATAGGCCTCAAAGCGCTCCAAATATCCACTTGCAGATTCTACAAAAAGAGTGTTT

                              * *         **      *        *            *    
     255802 CAAAACTGCTCAATCAAATGGAAGGTTCAAAACTGTGACATGAATGCCCACATCACAAAGTAGTT
         66 CAAAACTGCTCAATCAAAAGAAAGGTTCAACTCTGTGAGATGAATGCACACATCACAAAGAAGTT

                                                     
     255867 TCTCAGAATGCTTCTGTGTAGTTTTTATGTGAAGATATTTC
        131 TCTCAGAATGCTTCTGTGTAGTTTTTATGTGAAGATATTTC

                     * *    *      *    *       * *   **      *              
     255908 CTTTTCCACAACA-GCGTGCAAAACGCTTCAAATATGCCCTTAGAGATTCCACAAAAAGAGTGTT
          1 CTTTTCCACCATAGGCCT-CAAAGCGCTCCAAATATCCACTTGCAGATTCTACAAAAAGAGTGTT

              *     *               *  *                                  ** 
     255972 TCCAAACTACTCAAATCAAAA-AATGATTTCAACTCTGTGAGATGAATGCACACATCACAAACTA
         65 TCAAAACTGCTC-AATCAAAAGAAAG-GTTCAACTCTGTGAGATGAATGCACACATCACAAAGAA

                         *     ** *   * *   *       *   
     256036 GTTTCTCAGAATGTTTCTGCCTGGTTCTCATGCGAAGATAGTTC
        128 GTTTCTCAGAATGCTTCTGTGTAGTTTTTATGTGAAGATATTTC

                 *           *   * **              *                  * *    
     256080 CTTTTTCACCATAGGCCGCAATGTACTCCAAATATCCACCTGCAGATTCTAC-AAAAGTGAGTTT
          1 CTTTTCCACCATAGGCCTCAAAGCGCTCCAAATATCCACTTGCAGATTCTACAAAAAGAGTGTTT

                       *         ***    **         *        *      *       * 
     256144 CAAAACTGCTCTATCAAAAGATCAGTTCGTCTCTGTGAGTTGAATGCATACATCAAAAAGAAGCT
         66 CAAAACTGCTCAATCAAAAGAAAGGTTCAACTCTGTGAGATGAATGCACACATCACAAAGAAGTT

                 *             *      **         *   
     256209 TCTCAAAATGCTTCTGTGTGGTTTTTCGGTGAAGATAGTTC
        131 TCTCAGAATGCTTCTGTGTAGTTTTTATGTGAAGATATTTC

            *     *        *      * *                 *        *        *    
     256250 TTTTTCTACCATAGGTCTCAAACCACTCCAAATATCCACTTGTAGATTCTATAAAAAGGAATG-T
          1 CTTTTCCACCATAGGCCTCAAAGCGCTCCAAATATCCACTTGCAGATTCTACAAAAA-GAGTGTT

                  *        *    *    *      * *         *      *             
     256314 TCAAAATTGCTCAATAAAAATAAAGTTTCAACACCGTGAGATGAGTGCACAAATCACAAAGAAGT
         65 TCAAAACTGCTCAATCAAAAGAAAGGTTCAACTCTGTGAGATGAATGCACACATCACAAAGAAGT

                  *          *         *          *   
     256379 TTCTCAAAATGCTTCTGGGTAGTTTTTCTGTGAAGATAGTTC
        130 TTCTCAGAATGCTTCTGTGTAGTTTTTATGTGAAGATATTTC

                  *     *    *     *          *                         *    
     256421 CTTTTCTACCATGGGCCACAAAGGGCTCCAAATACCCACTTGCAGATTCTACAAAAAGAGAGTTT
          1 CTTTTCCACCATAGGCCTCAAAGCGCTCCAAATATCCACTTGCAGATTCTACAAAAAGAGTGTTT

              *        *      ** * *        *    * *    **  * *           *  
     256486 CACAACTGCTCTATCAAACAATATGTTCAACTTTGTGGGTTGAACACAAATATCAC-AAGAATTT
         66 CAAAACTGCTCAATCAAAAGAAAGGTTCAACTCTGTGAGATGAATGCACACATCACAAAGAAGTT

                **                             *     
     256550 TCTCCCAATGCTTCTGTGTAGTTTTTATGTGAAGACATTTC
        131 TCTCAGAATGCTTCTGTGTAGTTTTTATGTGAAGATATTTC

            *   *  *      *  *     *                 *  *      *       *  *  
     256591 TTTTCCCTCCATAGTCCACAAAGTGCTCCAAATATCCACTTACATATTCTAGAAAAAGATTGCTT
          1 CTTTTCCACCATAGGCCTCAAAGCGCTCCAAATATCCACTTGCAGATTCTACAAAAAGAGTGTTT

            **       *    *           *   * * *                              
     256656 GGAAACTGCACAATGAAAAGAAAGGTCCAAATATATGAGATGAATGCACACATCACAAAGAAGTT
         66 CAAAACTGCTCAATCAAAAGAAAGGTTCAACTCTGTGAGATGAATGCACACATCACAAAGAAGTT

                                 *                    
     256721 TCTCAGAAT-CTCTCTGTGTAATTTTTATGTGAAGATATTTC
        131 TCTCAGAATGCT-TCTGTGTAGTTTTTATGTGAAGATATTTC

                *     *    *  *   *                        *      *    *     
     256762 CTTTCCCACCTTAGGTCTTAAAACGCTCCAAATATCCACTTGCAGATACTACAAGAAGATTGTTT
          1 CTTTTCCACCATAGGCCTCAAAGCGCTCCAAATATCCACTTGCAGATTCTACAAAAAGAGTGTTT

                     *    *        *      *     **                           
     256827 CAAAACTGCACAA-AAAAAGAAATGTTCAATTCTGTTTGATGAATGCACACATCACAAAGAAGTT
         66 CAAAACTGCTCAATCAAAAGAAAGGTTCAACTCTGTGAGATGAATGCACACATCACAAAGAAGTT

                           *                         
     256891 TCTCAGAATGCTTCTCTGTAGTTTTTATGTGAAGATATTTC
        131 TCTCAGAATGCTTCTGTGTAGTTTTTATGTGAAGATATTTC

                     *             *                 *         **      * *   
     256932 CTTTTCCACAATAGGCCTCAAAGGGCTCCAAATATCCACTTCCAGATTCTATGAAAAGAATATTT
          1 CTTTTCCACCATAGGCCTCAAAGCGCTCCAAATATCCACTTGCAGATTCTACAAAAAGAGTGTTT

             *              * *    *                                      *  
     256997 CCAAACTGCTCAATCATAGGAAATGTTCAACTCTGTGAGATGAATGCACACATCAC-AAGAAATT
         66 CAAAACTGCTCAATCAAAAGAAAGGTTCAACTCTGTGAGATGAATGCACACATCACAAAGAAGTT

                     *    *      *       *       *   
     257061 TCTCAGAATCCTTCAGTGTAGGTTTTATGAGAAGATAATTC
        131 TCTCAGAATGCTTCTGTGTAGTTTTTATGTGAAGATATTTC

                     *    **        *   *                           *    *   
     257102 CTTTTCCACAATAGTTCTCAAAGCACTCAAAATATCCACTTGCAGATTCTACAAAAGGAGTATTT
          1 CTTTTCCACCATAGGCCTCAAAGCGCTCCAAATATCCACTTGCAGATTCTACAAAAAGAGTGTTT

                                                 *        *                  
     257167 CAAAACTGCTCAATCAAAAGAAAGGTTCAACTCTGTGGGATGAATGGACACATCACAAAGAAGTT
         66 CAAAACTGCTCAATCAAAAGAAAGGTTCAACTCTGTGAGATGAATGCACACATCACAAAGAAGTT

                                  *   *              
     257232 TCTCAGAATGCTTCTGTGTAGTATTTTTGTGAAGATATTT-
        131 TCTCAGAATGCTTCTGTGTAGTTTTTATGTGAAGATATTTC

                          *  * * * * ***             *            *     * *  
     257272 CTTTTCCACCATAGACCGCCAGGGGACACAAATATCCACTTTCAGATTCTACAACAAGAGAGGTT
          1 CTTTTCCACCATAGGCCTCAAAGCGCTCCAAATATCCACTTGCAGATTCTACAAAAAGAGTGTTT

                   *   *     *   ** *       *      *                      * *
     257337 CAAAACTACTCGATCAAGAGATGGTTTCAACTATGTGAGTTGAATGCACACATCACAAAGAACTA
         66 CAAAACTGCTCAATCAAAAGAAAGGTTCAACTCTGTGAGATGAATGCACACATCACAAAGAAGTT

             *  *    *                               
     257402 TGTCGGAATTCTTCTGTGTAGTTTTTATGTGAAGATATTTC
        131 TCTCAGAATGCTTCTGTGTAGTTTTTATGTGAAGATATTTC

                     *    * *      * *    *                  *               
     257443 CTTTTCCACAATAGACGTCAAAGTGATCCAGATATCCACTTGCAGATTCCACAAAAAGAGTGTTT
          1 CTTTTCCACCATAGGCCTCAAAGCGCTCCAAATATCCACTTGCAGATTCTACAAAAAGAGTGTTT

                 *   *   *                  **                     *  *      
     257508 CAAAAGTGCACAACCAAAAGAAAGGTTCAACTAGGTGAGATGAATGCACACATCAGAAGGAAGTT
         66 CAAAACTGCTCAATCAAAAGAAAGGTTCAACTCTGTGAGATGAATGCACACATCACAAAGAAGTT

                            **   *     * *       *   
     257573 TCTCAGAATGCTTCTGCATAGCTTTTAAGGGAAGATACTTC
        131 TCTCAGAATGCTTCTGTGTAGTTTTTATGTGAAGATATTTC

                    *               *            * *  *    * *               
     257614 CTTTTCCAACATAGGCCTCAAAGCACTCCAAATATCCTCCTGGAGATACCACAAAAAGAGTGTTT
          1 CTTTTCCACCATAGGCCTCAAAGCGCTCCAAATATCCACTTGCAGATTCTACAAAAAGAGTGTTT

            **                      *  *                 *       *           
     257679 GCAAACTGCTCAATCAAAAGAAAGATTTAACTCTGTGAGATGAATCCACACATGACAAAGAAGTT
         66 CAAAACTGCTCAATCAAAAGAAAGGTTCAACTCTGTGAGATGAATGCACACATCACAAAGAAGTT

                                       **            
     257744 TCTCAGAATGCTTCTGTGTAGTTTTTAATTGAAGATATTTC
        131 TCTCAGAATGCTTCTGTGTAGTTTTTATGTGAAGATATTTC

            *        *                  *            *  *                    
     257785 TTTTTCCACAATAGGCCTCAAAGCGCTCAAAATATCCACTTTCACATTCTACAAAAAGAGTGTTT
          1 CTTTTCCACCATAGGCCTCAAAGCGCTCCAAATATCCACTTGCAGATTCTACAAAAAGAGTGTTT

                     *           *                 *       *    *  *     *   
     257850 CAAAACTGCACAATCAAAAGATA-GTTCAACTCTGTGAGTTGAATGCGCACAACAAAAAGATGTT
         66 CAAAACTGCTCAATCAAAAGAAAGGTTCAACTCTGTGAGATGAATGCACACATCACAAAGAAGTT

                      **                              
     257914 TCTCAGAATTATTTCTGTGTAGTTTTTATGTGAAGATATTTC
        131 TCTCAGAA-TGCTTCTGTGTAGTTTTTATGTGAAGATATTTC

                     *  *          *                                     *  *
     257956 CTTTTCCACAATGGGCCTCAAAGTGCTCCAAATATCCACTTGCAGATTCTACAAAAAGAGTTTTC
          1 CTTTTCCACCATAGGCCTCAAAGCGCTCCAAATATCCACTTGCAGATTCTACAAAAAGAGTGTTT

                       *            *    *                    * *           *
     258021 CAAAACTGCTCCATCAAAAGAAAGCTTCACCTCTGTGAGATGAATGCACATACCACAAAGAAGTG
         66 CAAAACTGCTCAATCAAAAGAAAGGTTCAACTCTGTGAGATGAATGCACACATCACAAAGAAGTT

                                        *            
     258086 TCTCAGAATGCTTCTGTGTAGTTTTTATATGAAGATATTTC
        131 TCTCAGAATGCTTCTGTGTAGTTTTTATGTGAAGATATTTC

                     *   * * *    **           *                *     *      
     258127 CTTTTCCACAATAAGACCCAAAAGGCTCCAAATATTCACTTGCAGATTCTAAAAAAAACAGTGTT
          1 CTTTTCCACCATAGGCCTCAAAGCGCTCCAAATATCCACTTGCAGATTCT-ACAAAAAGAGTGTT

                                  *                  *      *        **      
     258192 TCAAAACTGCTCAATCAAAAGATA-GTTCAACTCTGTGAGAAGAATGCTCACATCACTGAGAAGT
         65 TCAAAACTGCTCAATCAAAAGAAAGGTTCAACTCTGTGAGATGAATGCACACATCACAAAGAAGT

                                         *            
     258256 TTCTCAGAATGCTTCTGTGTAGTTTTTATATGAAGATATTTC
        130 TTCTCAGAATGCTTCTGTGTAGTTTTTATGTGAAGATATTTC

                *     *      *    **                       *   **       *    
     258298 CTTTCCCACCGTAGGCCACAAAAGGCTCCAAATATCCACTTGCAGATACTATGAAAAGAGAGTTT
          1 CTTTTCCACCATAGGCCTCAAAGCGCTCCAAATATCCACTTGCAGATTCTACAAAAAGAGTGTTT

                        *        *               ***            *            
     258363 CAAAACTGCTCATTCAAAAGATAGGTTCAACTCTGTGGTTTGAATGCACACAGCACAAAGAAGTT
         66 CAAAACTGCTCAATCAAAAGAAAGGTTCAACTCTGTGAGATGAATGCACACATCACAAAGAAGTT

              *       **                   **   *    
     258428 TCACAGAATGTGTCTGTGTAGTTTTTATGTGCGGATGTTTC
        131 TCTCAGAATGCTTCTGTGTAGTTTTTATGTGAAGATATTTC

                         *        ****   *   *               *    *          
     258469 CTTTTCCACCATATGCCT-AAATATTTCCCAATTTCCACTTGCAGATTCCACAAGAAGAGTGTTT
          1 CTTTTCCACCATAGGCCTCAAAGCGCTCCAAATATCCACTTGCAGATTCTACAAAAAGAGTGTTT

                      **       *       *           *            *      ***   
     258533 CAAAACTGCTGTATC-AAATAAA-GTTGAACTCTGTGAGGTGAATGCACACAGCACAAAATGGTT
         66 CAAAACTGCTCAATCAAAAGAAAGGTTCAACTCTGTGAGATGAATGCACACATCACAAAGAAGTT

                          **   *        *       *    
     258596 TCTCAGAATGCTTCCTTGTTGTTTTTATATGAAGATGTTTC
        131 TCTCAGAATGCTTCTGTGTAGTTTTTATGTGAAGATATTTC

                  *  *             *   *     *                               
     258637 CTTTTCAACAATAGGCCTCAAAGTGCTTCAAATGTCCACTTGCAGATTCTACAAAAAGAGTGTTT
          1 CTTTTCCACCATAGGCCTCAAAGCGCTCCAAATATCCACTTGCAGATTCTACAAAAAGAGTGTTT

                                        *      *  *  *                       
     258702 CAAAACTGCTCAATCAAAAGAAAGGTTCGACTCTGGGAAATTAATGCACACATCACAAAGAAGTT
         66 CAAAACTGCTCAATCAAAAGAAAGGTTCAACTCTGTGAGATGAATGCACACATCACAAAGAAGTT

                                     *        *      
     258767 TCTC---A-GCTTCTGTGTAGTTTTCATGTGAAGTTATTTC
        131 TCTCAGAATGCTTCTGTGTAGTTTTTATGTGAAGATATTTC

                     *       *     *            *    *         **       *    
     258804 CTTTTCCACAATAGGCCGCAAAGGGCTCCAAATATCAACTTACAGATTCTAGGAAAAGAGAGTTT
          1 CTTTTCCACCATAGGCCTCAAAGCGCTCCAAATATCCACTTGCAGATTCTACAAAAAGAGTGTTT

                       * **      *     *                                     
     258869 CAAAACTGCTCTACGAAAAGATAGGTTGAACTCTGTGAGATGAATGCACACATCACAAAGAAGTT
         66 CAAAACTGCTCAATCAAAAGAAAGGTTCAACTCTGTGAGATGAATGCACACATCACAAAGAAGTT

                       *               * *           
     258934 TCTCAGAATGCATCTGTGTAGTTTTTACGGGAAGATATTTC
        131 TCTCAGAATGCTTCTGTGTAGTTTTTATGTGAAGATATTTC

                        ***  *     **      *  *           *      *     ***   
     258975 CTTTTCCACCATCTTCCACAAAGGTCTCCAAGTAACCACTTGCAGACTCTACAGAAAGACACTTT
          1 CTTTTCCACCATAGGCCTCAAAGCGCTCCAAATATCCACTTGCAGATTCTACAAAAAGAGTGTTT

            *          *         ***      *      ***                      *  
     259040 AAAAACTGCTCTATCAAAAGATCAGTTCAAGTCTGTGGTTTGAATGCACACATCACAAAGAATTT
         66 CAAAACTGCTCAATCAAAAGAAAGGTTCAACTCTGTGAGATGAATGCACACATCACAAAGAAGTT

                                     *  *            
     259105 TCTCAGAATGCTTCTGTGTAGTTTTCATATGAAGATATTTC
        131 TCTCAGAATGCTTCTGTGTAGTTTTTATGTGAAGATATTTC

                                    *                                   **   
     259146 CTTTTCCACCATAGGCCTCAAAGCACTCCAAATATCCACTTGCAGATTCTACAAAAAGAGATTTT
          1 CTTTTCCACCATAGGCCTCAAAGCGCTCCAAATATCCACTTGCAGATTCTACAAAAAGAGTGTTT

                   **                           *  *          *        *     
     259211 CAAAACTAGTCAATCAAAAGAAAGGTTCAACTCTGTCAGTTGAATGCACATATCACAAACAAGTT
         66 CAAAACTGCTCAATCAAAAGAAAGGTTCAACTCTGTGAGATGAATGCACACATCACAAAGAAGTT

                *      *                             
     259276 TCTCGGAATGCGTCTGTGTAGTTTTTATGTGAAGATATTTC
        131 TCTCAGAATGCTTCTGTGTAGTTTTTATGTGAAGATATTTC

               *     * *           *     *                  *    *           
     259317 CTTCTCCACAACAGGCCTCAAAGTGCTCCGAATATCCACTTGCAGATTTTAC-TAAAGAGTGTTT
          1 CTTTTCCACCATAGGCCTCAAAGCGCTCCAAATATCCACTTGCAGATTCTACAAAAAGAGTGTTT

             *               *  *   *     *        *    *               *    
     259381 CCAAACTGCTCAATCAAGAGGAAGTTTCAAGTCTGTGAGCTGAACGCACACATCACAAAGTAGTT
         66 CAAAACTGCTCAATCAAAAGAAAGGTTCAACTCTGTGAGATGAATGCACACATCACAAAGAAGTT

               *                                *    
     259446 TCTGAGAATGCTTCTGTGTAGTTTTTATGTGAAGATGTTTC
        131 TCTCAGAATGCTTCTGTGTAGTTTTTATGTGAAGATATTTC

                            **     *                                    *  * 
     259487 CTTTTCCACCATAGGCTGCAAAGGGCTCCAAATATCCACTTGCAGATTCTACAAAAAGAGAGTCT
          1 CTTTTCCACCATAGGCCTCAAAGCGCTCCAAATATCCACTTGCAGATTCTACAAAAAGAGTGTTT

                 *     *         *          *     *                     *    
     259552 CAAAAGTGCTCTATCAAAAGATAGGTTCAACTATGTGATATGAATGCACACATCACAAAGTAGTT
         66 CAAAACTGCTCAATCAAAAGAAAGGTTCAACTCTGTGAGATGAATGCACACATCACAAAGAAGTT

                                          *          
     259617 TCTCAGAATGCTTCTGTGTAGTTTTTATGTAAAGATATTTC
        131 TCTCAGAATGCTTCTGTGTAGTTTTTATGTGAAGATATTTC

                                    *                                        
     259658 CTTTTCCACCATAGGCCTCAAAGCACTCCAAATATCCACTTGCAGATTCTACAAAAAGAGATG-T
          1 CTTTTCCACCATAGGCCTCAAAGCGCTCCAAATATCCACTTGCAGATTCTACAAAAAGAG-TGTT

                    ** *                   *     *  *    * *   *        *    
     259722 TCAAAACTATTTAATCAAAAGAAAGGTTCAAATCTGTCAGTTGAAGGTACATATCACAAACAAGT
         65 TCAAAACTGCTCAATCAAAAGAAAGGTTCAACTCTGTGAGATGAATGCACACATCACAAAGAAGT

              * **                                    
     259787 TTATTGGAATGCTTCTGTGTAGTTTTTATGTGAAGATATTTC
        130 TTCTCAGAATGCTTCTGTGTAGTTTTTATGTGAAGATATTTC

                     * *         * *                        **  *     *      
     259829 CTTTTCCACAACAGGCCTCAAGGTGCTCCAAATATCCACTTGCAGATTTCACTAAAAGTGTGTTT
          1 CTTTTCCACCATAGGCCTCAAAGCGCTCCAAATATCCACTTGCAGATTCTACAAAAAGAGTGTTT

             *  *            *  *   *     *        *             *           
     259894 CCAAGCTGCTCAATCAAGAGGAAGTTTCAAGTCTGTGAGGTGAATGCACACATTACAAAGAAGTT
         66 CAAAACTGCTCAATCAAAAGAAAGGTTCAACTCTGTGAGATGAATGCACACATCACAAAGAAGTT

            *  *                                     
     259959 ACTGAGAATGCTTCTGTGTAGTTTTTATGTGAAGATATTTC
        131 TCTCAGAATGCTTCTGTGTAGTTTTTATGTGAAGATATTTC

                      **               *                                *    
     260000 CTTTTCCACCGCAGGCCTCAAAGCGCTGCAAATATCCACTTGCAGATTCTACAAAAAGAGAGTTT
          1 CTTTTCCACCATAGGCCTCAAAGCGCTCCAAATATCCACTTGCAGATTCTACAAAAAGAGTGTTT

                      **         *   *         *   *     **            *     
     260065 CAAAACTGCTGTATCAAAAGATAGGGTCAACTCTGCGAGTTGAATAAACACATCACAAATAAGTT
         66 CAAAACTGCTCAATCAAAAGAAAGGTTCAACTCTGTGAGATGAATGCACACATCACAAAGAAGTT

               **   *        *               *       
     260130 TCTGGGAACGCTTCTGTATAG-TTTTATGTGAATATATTTC
        131 TCTCAGAATGCTTCTGTGTAGTTTTTATGTGAAGATATTTC

                         *          *                   *   *  *   * *   *   
     260170 CTTTTCCACCATATGCCTCAAAGCACTCCAAATATCCACTTGCACATTATAGAAACATAGTCTTT
          1 CTTTTCCACCATAGGCCTCAAAGCGCTCCAAATATCCACTTGCAGATTCTACAAAAAGAGTGTTT

                   **                        *         *             *       
     260235 CAAAACTTGTCAATC-AAAGAAAGGTTCAACTCCGTGAGATGAGTGCACACATCACAGAGAAGTT
         66 CAAAACTGCTCAATCAAAAGAAAGGTTCAACTCTGTGAGATGAATGCACACATCACAAAGAAGTT

                *     *                            * 
     260299 TCTCGGAATGTTTCTGTGTAGTTTTTATGTGAAGATATTGC
        131 TCTCAGAATGCTTCTGTGTAGTTTTTATGTGAAGATATTTC

                     *               *            *          *       *   *   
     260340 CTTTTCCACAATAGGCCTCAAAGCGTTCCAAATATCCAATTGCAGATTCCACAAAAAAAGTTTTT
          1 CTTTTCCACCATAGGCCTCAAAGCGCTCCAAATATCCACTTGCAGATTCTACAAAAAGAGTGTTT

            *                 *  *  *  *             * *         **    *     
     260405 TAAAACTGCTCAATCAAATGATAGATTAAACTCTGTGAGATTAGTGCACACATGTCAAAAAAGTT
         66 CAAAACTGCTCAATCAAAAGAAAGGTTCAACTCTGTGAGATGAATGCACACATCACAAAGAAGTT

                                *      * *           
     260470 TCTCAGAATGCTTCTGTGTACTTTTTAGGGGAAGATATTTC
        131 TCTCAGAATGCTTCTGTGTAGTTTTTATGTGAAGATATTTC

                        *    *     **         *    *           **  * *  *    
     260511 CTTTTCCACCATCGGCCACAAAGGACTCCAAATAACCACATGCAGATTCTAGTAACACAGAGTTT
          1 CTTTTCCACCATAGGCCTCAAAGCGCTCCAAATATCCACTTGCAGATTCTACAAAAAGAGTGTTT

                       *         * *           *   * * *    **    ***        
     260576 CAAAACTGCTCTATCAAAAGATAAGTTCAACTCTGAGAGTTTAGTGCAACCATCGTGAAGAAGTT
         66 CAAAACTGCTCAATCAAAAGAAAGGTTCAACTCTGTGAGATGAATGCACACATCACAAAGAAGTT

                            *     *                  
     260641 TCTCAGAATGCTTCTGAGTAGTGTTTATGTGAAGATATTTC
        131 TCTCAGAATGCTTCTGTGTAGTTTTTATGTGAAGATATTTC

                              *     *                      *            *    
     260682 CTTTTCCACCATAGGCCTGAAAGCCCTCCAAATATCCACTTGCAGATCCTACAAAAAGAGAGTTT
          1 CTTTTCCACCATAGGCCTCAAAGCGCTCCAAATATCCACTTGCAGATTCTACAAAAAGAGTGTTT

             *         **     *  *  *   *        **      *                   
     260747 CGAAA-TGCTCTCTCAAACGATAGTTTCGACTCTGTGGTATGAATACACACATCACAAAGAAGTT
         66 CAAAACTGCTCAATCAAAAGAAAGGTTCAACTCTGTGAGATGAATGCACACATCACAAAGAAGTT

                                       **            
     260811 TCTCAGAATGCTTCTGTGTAGTTTTTAAATGAAGATATTTC
        131 TCTCAGAATGCTTCTGTGTAGTTTTTATGTGAAGATATTTC

            *                       *          *     *                       
     260852 TTTTTCCACCATAGGCCTCAAAGCACTCCAAATATGCACTTCCAGATTCTACAAAAAGAGTGTTT
          1 CTTTTCCACCATAGGCCTCAAAGCGCTCCAAATATCCACTTGCAGATTCTACAAAAAGAGTGTTT

              *                 *       * *         **   *         *  * *    
     260917 CAGAACTGCTCAATCAAAAGGAAGGTTCCAGTC--TGAGACAAATACACACATCAAAAGGTAGTT
         66 CAAAACTGCTCAATCAAAAGAAAGGTTCAACTCTGTGAGATGAATGCACACATCACAAAGAAGTT

                                                    *
     260980 TCTCAGAATGCTTCTGTGTAGTTTTTATGTGAAGATATTTT
        131 TCTCAGAATGCTTCTGTGTAGTTTTTATGTGAAGATATTTC

             *               *    **    *     *      *  **   *          *    
     261021 CCTTTCCACCATAGGCCACAAATGGCTCTAAATACCCACTTACATTTTCCACAAAAAGAGAGTTT
          1 CTTTTCCACCATAGGCCTCAAAGCGCTCCAAATATCCACTTGCAGATTCTACAAAAAGAGTGTTT

                       * *    * *      *   *         *                       
     261086 CAAAACTGCTCTACCAAAGGTAA-GTTTAACGCTGTGAG-TTAA-G-A-ACATCACAAAGAAGTT
         66 CAAAACTGCTCAATCAAAAGAAAGGTTCAACTCTGTGAGATGAATGCACACATCACAAAGAAGTT

                                   *   *  *          
     261146 TCTCAGAATGCTTCTGTGTAGTTCTTACGTAAAGATATTTC
        131 TCTCAGAATGCTTCTGTGTAGTTTTTATGTGAAGATATTTC

                 *   *      ***    *                  *              **      
     261187 CTTTTACACAATAGGCAGAAAAGTGCTCCAAATATCCACTTGAAGATTCTACAAAAACCGTGTTT
          1 CTTTTCCACCATAGGCCTCAAAGCGCTCCAAATATCCACTTGCAGATTCTACAAAAAGAGTGTTT

                     **                                          *      *    
     261252 CAAAACTGCCGAATCAAAAGAAAGGTTCAACTCTGTGAGATGAATGCACACATAACAAAGGAGTT
         66 CAAAACTGCTCAATCAAAAGAAAGGTTCAACTCTGTGAGATGAATGCACACATCACAAAGAAGTT

                                 *      *      *    *
     261317 TCTCAGAATGCTTCTGTGTAGCTTTTATATGAAGACATTTA
        131 TCTCAGAATGCTTCTGTGTAGTTTTTATGTGAAGATATTTC

            *        * *            *   ***                     **        *  
     261358 GTTTTCCACAACAGGCCTCAAAGCTCTCTCCATATCCACTTGCAGATTCTACCGAAAGAGTGCTT
          1 CTTTTCCACCATAGGCCTCAAAGCGCTCCAAATATCCACTTGCAGATTCTACAAAAAGAGTGTTT

             *                   * **     *        *        *     **         
     261423 CCAAACTGCTCAATCAAAAGAGACATTCAAATCTGTGAGGTGAATGCAGACATCGTAAAGAAGTT
         66 CAAAACTGCTCAATCAAAAGAAAGGTTCAACTCTGTGAGATGAATGCACACATCACAAAGAAGTT

                                *     *       *    **
     261488 TCTCAGAATGCTTCTGTGTATTTTTTGTGTGAAGTTATTCG
        131 TCTCAGAATGCTTCTGTGTAGTTTTTATGTGAAGATATTTC

            *    *             *     *  *            **                 *    
     261529 TTTTTGCACCATAGGCCTCCAAGCGTTCTAAATATCCACTTCTAGATTCTACAAAAAGAGAGTTT
          1 CTTTTCCACCATAGGCCTCAAAGCGCTCCAAATATCCACTTGCAGATTCTACAAAAAGAGTGTTT

                   *         *            *        *    *   *                
     261594 CAAAACTACTC-A--AACA-AAAGGTTCAATTCTGTGAGTTGAAAGCAAACATCACAAAGAAGTT
         66 CAAAACTGCTCAATCAAAAGAAAGGTTCAACTCTGTGAGATGAATGCACACATCACAAAGAAGTT

                       *             *               
     261655 TCTCAGAATGCGTCTGTGTAGTTTTGATGTGAAGATATTTC
        131 TCTCAGAATGCTTCTGTGTAGTTTTTATGTGAAGATATTTC

                     **   ****     *                  *                      
     261696 CTTTT-CACAGTAGAATGCAAAGGGCTCCAAATATCCACTTGGAGATTCTACAAAAAGA--GTTT
          1 CTTTTCCACCATAGGCCTCAAAGCGCTCCAAATATCCACTTGCAGATTCTACAAAAAGAGTGTTT

                  *    **     *  *     *     ***   *     *               *   
     261758 CAAAACCGCTCTGTCAAATGATAGGTTGAACTCCCGGAGGTGAATACACACATCACAAAGAGGTT
         66 CAAAACTGCTCAATCAAAAGAAAGGTTCAACTCTGTGAGATGAATGCACACATCACAAAGAAGTT

                  *                       *  *       
     261823 TCTCAGCATGCTTCTGTGTAGTTTTTATGTAAACATATTTC
        131 TCTCAGAATGCTTCTGTGTAGTTTTTATGTGAAGATATTTC

             *    * *              *           *      * *       *   *    *   
     261864 CGTTTCTATCATAGGCCTCAAAGTGCTCCAAATATTCACTTGTACATTCTACCAAACGAGTATTT
          1 CTTTTCCACCATAGGCCTCAAAGCGCTCCAAATATCCACTTGCAGATTCTACAAAAAGAGTGTTT

                              * *         **      *        *            *    
     261929 CAAAACTGCTCAATCAAATGGAAGGTTCAAAACTGTGACATGAATGCCCACATCACAAAGTAGTT
         66 CAAAACTGCTCAATCAAAAGAAAGGTTCAACTCTGTGAGATGAATGCACACATCACAAAGAAGTT

                                                     
     261994 TCTCAGAATGCTTCTGTGTAGTTTTTATGTGAAGATATTTC
        131 TCTCAGAATGCTTCTGTGTAGTTTTTATGTGAAGATATTTC

                     * *    *      *    *       * *   **      *              
     262035 CTTTTCCACAACA-GCGTGCAAAACGCTTCAAATATGCCCTTAGAGATTCCACAAAAAGAGTGTT
          1 CTTTTCCACCATAGGCCT-CAAAGCGCTCCAAATATCCACTTGCAGATTCTACAAAAAGAGTGTT

              *     *               *  *                                  ** 
     262099 TCCAAACTACTCAAATCAAAA-AATGATTTCAACTCTGTGAGATGAATGCACACATCACAAACTA
         65 TCAAAACTGCTC-AATCAAAAGAAAG-GTTCAACTCTGTGAGATGAATGCACACATCACAAAGAA

                         *     ** *   * *   *       *   
     262163 GTTTCTCAGAATGTTTCTGCCTGGTTCTCATGCGAAGATAGTTC
        128 GTTTCTCAGAATGCTTCTGTGTAGTTTTTATGTGAAGATATTTC

                 *           *   * **              *                  * *    
     262207 CTTTTTCACCATAGGCCGCAATGTACTCCAAATATCCACCTGCAGATTCTAC-AAAAGTGAGTTT
          1 CTTTTCCACCATAGGCCTCAAAGCGCTCCAAATATCCACTTGCAGATTCTACAAAAAGAGTGTTT

                       *         ***    **         *        *      *       * 
     262271 CAAAACTGCTCTATCAAAAGATCAGTTCGTCTCTGTGAGTTGAATGCATACATCAAAAAGAAGCT
         66 CAAAACTGCTCAATCAAAAGAAAGGTTCAACTCTGTGAGATGAATGCACACATCACAAAGAAGTT

                 *             *      **         *   
     262336 TCTCAAAATGCTTCTGTGTGGTTTTTCGGTGAAGATAGTTC
        131 TCTCAGAATGCTTCTGTGTAGTTTTTATGTGAAGATATTTC

            *     *        *      * *                 *        *        *    
     262377 TTTTTCTACCATAGGTCTCAAACCACTCCAAATATCCACTTGTAGATTCTATAAAAAGGAATG-T
          1 CTTTTCCACCATAGGCCTCAAAGCGCTCCAAATATCCACTTGCAGATTCTACAAAAA-GAGTGTT

                  *        *    *    *      * *         *      *         *   
     262441 TCAAAATTGCTCAATAAAAATAAAGTTTCAACACCGTGAGATGAGTGCACAAATCACAAAGGAGT
         65 TCAAAACTGCTCAATCAAAAGAAAGGTTCAACTCTGTGAGATGAATGCACACATCACAAAGAAGT

                  *          *         *          *   
     262506 TTCTCAAAATGCTTCTGGGTAGTTTTTCTGTGAAGATAGTTC
        130 TTCTCAGAATGCTTCTGTGTAGTTTTTATGTGAAGATATTTC

                  *     *    *     *          *                         *    
     262548 CTTTTCTACCATGGGCCACAAAGGGCTCCAAATACCCACTTGCAGATTCTACAAAAAGAGAGTTT
          1 CTTTTCCACCATAGGCCTCAAAGCGCTCCAAATATCCACTTGCAGATTCTACAAAAAGAGTGTTT

              *        *      ** * *        *    * *    **  * *           *  
     262613 CACAACTGCTCTATCAAACAATATGTTCAACTTTGTGGGTTGAACACAAATATCAC-AAGAATTT
         66 CAAAACTGCTCAATCAAAAGAAAGGTTCAACTCTGTGAGATGAATGCACACATCACAAAGAAGTT

                **                             *     
     262677 TCTCCCAATGCTTCTGTGTAGTTTTTATGTGAAGACATTTC
        131 TCTCAGAATGCTTCTGTGTAGTTTTTATGTGAAGATATTTC

            *   *  *      *  *     *                 *  *      *       *  *  
     262718 TTTTCCCTCCATAGTCCACAAAGTGCTCCAAATATCCACTTACATATTCTAGAAAAAGATTGCTT
          1 CTTTTCCACCATAGGCCTCAAAGCGCTCCAAATATCCACTTGCAGATTCTACAAAAAGAGTGTTT

            **       *    *               * * *                              
     262783 GGAAACTGCACAATGAAAAGAAAGGTTCAAATATATGAGATGAATGCACACATCACAAAGAAGTT
         66 CAAAACTGCTCAATCAAAAGAAAGGTTCAACTCTGTGAGATGAATGCACACATCACAAAGAAGTT

                                 *                    
     262848 TCTCAGAAT-CTCTCTGTGTAATTTTTATGTGAAGATATTTC
        131 TCTCAGAATGCT-TCTGTGTAGTTTTTATGTGAAGATATTTC

                *     *    *  *   *                        *      *    *     
     262889 CTTTCCCACCTTAGGTCTTAAAACGCTCCAAATATCCACTTGCAGATACTACAAGAAGATTGTTT
          1 CTTTTCCACCATAGGCCTCAAAGCGCTCCAAATATCCACTTGCAGATTCTACAAAAAGAGTGTTT

                     *    *        *      *     **                           
     262954 CAAAACTGCACAA-AAAAAGAAATGTTCAATTCTGTTTGATGAATGCACACATCACAAAGAAGTT
         66 CAAAACTGCTCAATCAAAAGAAAGGTTCAACTCTGTGAGATGAATGCACACATCACAAAGAAGTT

                           *                         
     263018 TCTCAGAATGCTTCTCTGTAGTTTTTATGTGAAGATATTTC
        131 TCTCAGAATGCTTCTGTGTAGTTTTTATGTGAAGATATTTC

                     *             *                 *         **      * *   
     263059 CTTTTCCACAATAGGCCTCAAAGGGCTCCAAATATCCACTTCCAGATTCTATGAAAAGAATATTT
          1 CTTTTCCACCATAGGCCTCAAAGCGCTCCAAATATCCACTTGCAGATTCTACAAAAAGAGTGTTT

             *              * *    *                                      *  
     263124 CCAAACTGCTCAATCATAGGAAATGTTCAACTCTGTGAGATGAATGCACACATCAC-AAGAAATT
         66 CAAAACTGCTCAATCAAAAGAAAGGTTCAACTCTGTGAGATGAATGCACACATCACAAAGAAGTT

                     *    *      *       *       *   
     263188 TCTCAGAATCCTTCAGTGTAGGTTTTATGAG-AGATAATTC
        131 TCTCAGAATGCTTCTGTGTAGTTTTTATGTGAAGATATTTC

                     *    **        *   *                           *    *   
     263228 CTTTTCCACAATAGTTCTCAAAGCACTCAAAATATCCACTTGCAGATTCTACAAAAGGAGTATTT
          1 CTTTTCCACCATAGGCCTCAAAGCGCTCCAAATATCCACTTGCAGATTCTACAAAAAGAGTGTTT

                                                 *        *                  
     263293 CAAAACTGCTCAATCAAAAGAAAGGTTCAACTCTGTGGGATGAATGGACACATCACAAAGAAGTT
         66 CAAAACTGCTCAATCAAAAGAAAGGTTCAACTCTGTGAGATGAATGCACACATCACAAAGAAGTT

                                  *   *              
     263358 TCTCAGAATGCTTCTGTGTAGTATTTTTGTGAAGATATTT-
        131 TCTCAGAATGCTTCTGTGTAGTTTTTATGTGAAGATATTTC

                          *  * * * * ***             *            *     * *  
     263398 CTTTTCCACCATAGACCGCCAGGGGACACAAATATCCACTTTCAGATTCTACAACAAGAGAGGTT
          1 CTTTTCCACCATAGGCCTCAAAGCGCTCCAAATATCCACTTGCAGATTCTACAAAAAGAGTGTTT

                   *   *     *   ** *       *      *                      * *
     263463 CAAAACTACTCGATCAAGAGATGGTTTCAACTATGTGAGTTGAATGCACACATCACAAAGAACTA
         66 CAAAACTGCTCAATCAAAAGAAAGGTTCAACTCTGTGAGATGAATGCACACATCACAAAGAAGTT

             *  *    *                               
     263528 TGTCGGAATTCTTCTGTGTAGTTTTTATGTGAAGATATTTC
        131 TCTCAGAATGCTTCTGTGTAGTTTTTATGTGAAGATATTTC

                     *    * *      * *    *                  *               
     263569 CTTTTCCACAATAGACGTCAAAGTGATCCAGATATCCACTTGCAGATTCCACAAAAAGAGTGTTT
          1 CTTTTCCACCATAGGCCTCAAAGCGCTCCAAATATCCACTTGCAGATTCTACAAAAAGAGTGTTT

                 *   *   *                  **                     *  *      
     263634 CAAAAGTGCACAACCAAAAGAAAGGTTCAACTAGGTGAGATGAATGCACACATCAGAAGGAAGTT
         66 CAAAACTGCTCAATCAAAAGAAAGGTTCAACTCTGTGAGATGAATGCACACATCACAAAGAAGTT

                            **   *     * *       *   
     263699 TCTCAGAATGCTTCTGCATAGCTTTTAAGGGAAGATACTTC
        131 TCTCAGAATGCTTCTGTGTAGTTTTTATGTGAAGATATTTC

                    *               *            * *  *    * *               
     263740 CTTTTCCAACATAGGCCTCAAAGCACTCCAAATATCCTCCTGGAGATACCACAAAAAGAGTGTTT
          1 CTTTTCCACCATAGGCCTCAAAGCGCTCCAAATATCCACTTGCAGATTCTACAAAAAGAGTGTTT

            **                      *  *                 * *     *           
     263805 GCAAACTGCTCAATCAAAAGAAAGATTTAACTCTGTGAGATGAATCCGCACATGACAAAGAAGTT
         66 CAAAACTGCTCAATCAAAAGAAAGGTTCAACTCTGTGAGATGAATGCACACATCACAAAGAAGTT

                                                     
     263870 TCTCAGAATGCTTCTGTGTAGTTTTTATGTGAAGATATTTC
        131 TCTCAGAATGCTTCTGTGTAGTTTTTATGTGAAGATATTTC

                     *   * * *    **           *                *     *      
     263911 CTTTTCCACAATAAGACCCAAAAGGCTCCAAATATTCACTTGCAGATTCTAAAAAAAACAGTGTT
          1 CTTTTCCACCATAGGCCTCAAAGCGCTCCAAATATCCACTTGCAGATTCT-ACAAAAAGAGTGTT

                                  *                  *      *        **      
     263976 TCAAAACTGCTCAATCAAAAGATA-GTTCAACTCTGTGAGAAGAATGCTCACATCACTGAGAAGT
         65 TCAAAACTGCTCAATCAAAAGAAAGGTTCAACTCTGTGAGATGAATGCACACATCACAAAGAAGT

                                         *            
     264040 TTCTCAGAATGCTTCTGTGTAGTTTTTATATGAAGATATTTC
        130 TTCTCAGAATGCTTCTGTGTAGTTTTTATGTGAAGATATTTC

                *     *      *    **                       *   **       *    
     264082 CTTTCCCACCGTAGGCCACAAAAGGCTCCAAATATCCACTTGCAGATACTATGAAAAGAGAGTTT
          1 CTTTTCCACCATAGGCCTCAAAGCGCTCCAAATATCCACTTGCAGATTCTACAAAAAGAGTGTTT

                        *        *               ***            *            
     264147 CAAAACTGCTCATTCAAAAGATAGGTTCAACTCTGTGGTTTGAATGCACACAGCACAAAGAAGTT
         66 CAAAACTGCTCAATCAAAAGAAAGGTTCAACTCTGTGAGATGAATGCACACATCACAAAGAAGTT

              *       **                   **   *    
     264212 TCACAGAATGTGTCTGTGTAGTTTTTATGTGCGGATGTTTC
        131 TCTCAGAATGCTTCTGTGTAGTTTTTATGTGAAGATATTTC

                         *        ****   *   *               *    *          
     264253 CTTTTCCACCATATGCCT-AAATATTTCCCAATTTCCACTTGCAGATTCCACAAGAAGAGTGTTT
          1 CTTTTCCACCATAGGCCTCAAAGCGCTCCAAATATCCACTTGCAGATTCTACAAAAAGAGTGTTT

                      **       *       *           *            *      ***   
     264317 CAAAACTGCTGTATC-AAATAAA-GTTGAACTCTGTGAGGTGAATGCACACAGCACAAAATGGTT
         66 CAAAACTGCTCAATCAAAAGAAAGGTTCAACTCTGTGAGATGAATGCACACATCACAAAGAAGTT

                          **   *        *       *    
     264380 TCTCAGAATGCTTCCTTGTTGTTTTTATATGAAGATGTTTC
        131 TCTCAGAATGCTTCTGTGTAGTTTTTATGTGAAGATATTTC

                  *  *             *   *     *                               
     264421 CTTTTCAACAATAGGCCTCAAAGTGCTTCAAATGTCCACTTGCAGATTCTACAAAAAGAGTGTTT
          1 CTTTTCCACCATAGGCCTCAAAGCGCTCCAAATATCCACTTGCAGATTCTACAAAAAGAGTGTTT

                                        *      *  *  *                       
     264486 CAAAACTGCTCAATCAAAAGAAAGGTTCGACTCTGGGAAATTAATGCACACATCACAAAGAAGTT
         66 CAAAACTGCTCAATCAAAAGAAAGGTTCAACTCTGTGAGATGAATGCACACATCACAAAGAAGTT

                                     *        *      
     264551 TCTC---A-GCTTCTGTGTAGTTTTCATGTGAAGTTATTTC
        131 TCTCAGAATGCTTCTGTGTAGTTTTTATGTGAAGATATTTC

                     *       *     *            *    *         **       *    
     264588 CTTTTCCACAATAGGCCGCAAAGGGCTCCAAATATCAACTTACAGATTCTAGGAAAAGAGAGTTT
          1 CTTTTCCACCATAGGCCTCAAAGCGCTCCAAATATCCACTTGCAGATTCTACAAAAAGAGTGTTT

                       * **      *     *                                     
     264653 CAAAACTGCTCTACGAAAAGATAGGTTGAACTCTGTGAGATGAATGCACACATCACAAAGAAGTT
         66 CAAAACTGCTCAATCAAAAGAAAGGTTCAACTCTGTGAGATGAATGCACACATCACAAAGAAGTT

                       *               * *           
     264718 TCTCAGAATGCATCTGTGTAGTTTTTACGGGAAGATATTTC
        131 TCTCAGAATGCTTCTGTGTAGTTTTTATGTGAAGATATTTC

                        ***  *     **      *  *           *      *     ***   
     264759 CTTTTCCACCATCTTCCACAAAGGTCTCCAAGTAACCACTTGCAGACTCTACAGAAAGACACTTT
          1 CTTTTCCACCATAGGCCTCAAAGCGCTCCAAATATCCACTTGCAGATTCTACAAAAAGAGTGTTT

            *          *         ***      *      ***                      *  
     264824 AAAAACTGCTCTATCAAAAGATCAGTTCAAGTCTGTGGTTTGAATGCACACATCACAAAGAATTT
         66 CAAAACTGCTCAATCAAAAGAAAGGTTCAACTCTGTGAGATGAATGCACACATCACAAAGAAGTT

                                     *  *            
     264889 TCTCAGAATGCTTCTGTGTAGTTTTCATATGAAGATATTTC
        131 TCTCAGAATGCTTCTGTGTAGTTTTTATGTGAAGATATTTC

                                    *                                   **   
     264930 CTTTTCCACCATAGGCCTCAAAGCACTCCAAATATCCACTTGCAGATTCTACAAAAAGAGATTTT
          1 CTTTTCCACCATAGGCCTCAAAGCGCTCCAAATATCCACTTGCAGATTCTACAAAAAGAGTGTTT

                   **                           *  *          *        *     
     264995 CAAAACTAGTCAATCAAAAGAAAGGTTCAACTCTGTCAGTTGAATGCACATATCACAAACAAGTT
         66 CAAAACTGCTCAATCAAAAGAAAGGTTCAACTCTGTGAGATGAATGCACACATCACAAAGAAGTT

                *      *                             
     265060 TCTCGGAATGCGTCTGTGTAGTTTTTATGTGAAGATATTTC
        131 TCTCAGAATGCTTCTGTGTAGTTTTTATGTGAAGATATTTC

               *     * *           *     *                  *    *           
     265101 CTTCTCCACAACAGGCCTCAAAGTGCTCCGAATATCCACTTGCAGATTTTAC-TAAAGAGTGTTT
          1 CTTTTCCACCATAGGCCTCAAAGCGCTCCAAATATCCACTTGCAGATTCTACAAAAAGAGTGTTT

             *               *  *   *     *        *    *               *    
     265165 CCAAACTGCTCAATCAAGAGGAAGTTTCAAGTCTGTGAGCTGAACGCACACATCACAAAGTAGTT
         66 CAAAACTGCTCAATCAAAAGAAAGGTTCAACTCTGTGAGATGAATGCACACATCACAAAGAAGTT

               *                                *    
     265230 TCTGAGAATGCTTCTGTGTAGTTTTTATGTGAAGATGTTTC
        131 TCTCAGAATGCTTCTGTGTAGTTTTTATGTGAAGATATTTC

                            **     *                                    *    
     265271 CTTTTCCACCATAGGCTGCAAAGGGCTCCAAATATCCACTTGCAGATTCTACAAAAAGAGAGTTT
          1 CTTTTCCACCATAGGCCTCAAAGCGCTCCAAATATCCACTTGCAGATTCTACAAAAAGAGTGTTT

                 *     *         *          *     *                     *    
     265336 CAAAAGTGCTCTATCAAAAGATAGGTTCAACTATGTGATATGAATGCACACATCACAAAGTAGTT
         66 CAAAACTGCTCAATCAAAAGAAAGGTTCAACTCTGTGAGATGAATGCACACATCACAAAGAAGTT

                                          *          
     265401 TCTCAGAATGCTTCTGTGTAGTTTTTATGTAAAGATATTTC
        131 TCTCAGAATGCTTCTGTGTAGTTTTTATGTGAAGATATTTC

                                    *                                   **   
     265442 CTTTTCCACCATAGGCCTCAAAGCACTCCAAATATCCACTTGCAGATTCTACAAAAAGAGATTTT
          1 CTTTTCCACCATAGGCCTCAAAGCGCTCCAAATATCCACTTGCAGATTCTACAAAAAGAGTGTTT

                   ** *                   *     *  *    * *   *        *     
     265507 CAAAACTATTTAATCAAAAGAAAGGTTCAAATCTGTCAGTTGAAGGTACATATCACAAACAAGTT
         66 CAAAACTGCTCAATCAAAAGAAAGGTTCAACTCTGTGAGATGAATGCACACATCACAAAGAAGTT

             * **                                    
     265572 TATTGGAATGCTTCTGTGTAGTTTTTATGTGAAGATATTTC
        131 TCTCAGAATGCTTCTGTGTAGTTTTTATGTGAAGATATTTC

                     * *         * *                        **  *     *      
     265613 CTTTTCCACAACAGGCCTCAAGGTGCTCCAAATATCCACTTGCAGATTTCACTAAAAGTGTGTTT
          1 CTTTTCCACCATAGGCCTCAAAGCGCTCCAAATATCCACTTGCAGATTCTACAAAAAGAGTGTTT

             *  *            *  *   *     *        *             *           
     265678 CCAAGCTGCTCAATCAAGAGGAAGTTTCAAGTCTGTGAGGTGAATGCACACATTACAAAGAAGTT
         66 CAAAACTGCTCAATCAAAAGAAAGGTTCAACTCTGTGAGATGAATGCACACATCACAAAGAAGTT

            *  *                                     
     265743 ACTGAGAATGCTTCTGTGTAGTTTTTATGTGAAGATATTTC
        131 TCTCAGAATGCTTCTGTGTAGTTTTTATGTGAAGATATTTC

                      **               *                                *    
     265784 CTTTTCCACCGCAGGCCTCAAAGCGCTGCAAATATCCACTTGCAGATTCTACAAAAAGAGAGTTT
          1 CTTTTCCACCATAGGCCTCAAAGCGCTCCAAATATCCACTTGCAGATTCTACAAAAAGAGTGTTT

                      **         *   *         *   *     **            *     
     265849 CAAAACTGCTGTATCAAAAGATAGGGTCAACTCTGCGAGTTGAATAAACACATCACAAATAAGTT
         66 CAAAACTGCTCAATCAAAAGAAAGGTTCAACTCTGTGAGATGAATGCACACATCACAAAGAAGTT

               **   *        *               *       
     265914 TCTGGGAACGCTTCTGTATAG-TTTTATGTGAATATATTTC
        131 TCTCAGAATGCTTCTGTGTAGTTTTTATGTGAAGATATTTC

                         *          *                   *   *  *   * *   *   
     265954 CTTTTCCACCATATGCCTCAAAGCACTCCAAATATCCACTTGCACATTATAGAAACATAGTCTTT
          1 CTTTTCCACCATAGGCCTCAAAGCGCTCCAAATATCCACTTGCAGATTCTACAAAAAGAGTGTTT

                   **                        *         *             *       
     266019 CAAAACTTGTCAATC-AAAGAAAGGTTCAACTCCGTGAGATGAGTGCACACATCACAGAGAAGTT
         66 CAAAACTGCTCAATCAAAAGAAAGGTTCAACTCTGTGAGATGAATGCACACATCACAAAGAAGTT

                *     *                            * 
     266083 TCTCGGAATGTTTCTGTGTAGTTTTTATGTGAAGATATTGC
        131 TCTCAGAATGCTTCTGTGTAGTTTTTATGTGAAGATATTTC

                     *               *            *          *       *   *   
     266124 CTTTTCCACAATAGGCCTCAAAGCGTTCCAAATATCCAATTGCAGATTCCACAAAAAAAGTTTTT
          1 CTTTTCCACCATAGGCCTCAAAGCGCTCCAAATATCCACTTGCAGATTCTACAAAAAGAGTGTTT

            *                 *  *  *  *             * *         **    *     
     266189 TAAAACTGCTCAATCAAATGATAGATTAAACTCTGTGAGATTAGTGCACACATGTCAAAAAAGTT
         66 CAAAACTGCTCAATCAAAAGAAAGGTTCAACTCTGTGAGATGAATGCACACATCACAAAGAAGTT

                                *      * *           
     266254 TCTCAGAATGCTTCTGTGTACTTTTTAGGGGAAGATATTTC
        131 TCTCAGAATGCTTCTGTGTAGTTTTTATGTGAAGATATTTC

                        *    *     **         *    *           **  * *  *    
     266295 CTTTTCCACCATCGGCCACAAAGGACTCCAAATAACCACATGCAGATTCTAGTAACACAGAGTTT
          1 CTTTTCCACCATAGGCCTCAAAGCGCTCCAAATATCCACTTGCAGATTCTACAAAAAGAGTGTTT

                       *         * *           *   * * *    **    ***        
     266360 CAAAACTGCTCTATCAAAAGATAAGTTCAACTCTGAGAGTTTAGTGCAACCATCGTGAAGAAGTT
         66 CAAAACTGCTCAATCAAAAGAAAGGTTCAACTCTGTGAGATGAATGCACACATCACAAAGAAGTT

                            *     *                  
     266425 TCTCAGAATGCTTCTGAGTAGTGTTTATGTGAAGATATTTC
        131 TCTCAGAATGCTTCTGTGTAGTTTTTATGTGAAGATATTTC

                              *     *                      *           **    
     266466 CTTTTCCACCATAGGCCTGAAAGCCCTCCAAATATCCACTTGCAGATCCTACAAAAAGAAAGTTT
          1 CTTTTCCACCATAGGCCTCAAAGCGCTCCAAATATCCACTTGCAGATTCTACAAAAAGAGTGTTT

             *         **     *  *  *   *        **      *                   
     266531 CGAAA-TGCTCTCTCAAACGATAGTTTCGACTCTGTGGTATGAATACACACATCACAAAGAAGTT
         66 CAAAACTGCTCAATCAAAAGAAAGGTTCAACTCTGTGAGATGAATGCACACATCACAAAGAAGTT

                                       **            
     266595 TCTCAGAATGCTTCTGTGTAGTTTTTAAATGAAGATATTTC
        131 TCTCAGAATGCTTCTGTGTAGTTTTTATGTGAAGATATTTC

            *                       *          *     *                       
     266636 TTTTTCCACCATAGGCCTCAAAGCACTCCAAATATGCACTTCCAGATTCTACAAAAAGAGTGTTT
          1 CTTTTCCACCATAGGCCTCAAAGCGCTCCAAATATCCACTTGCAGATTCTACAAAAAGAGTGTTT

              *                 *       * *         **   *         *  * *    
     266701 CAGAACTGCTCAATCAAAAGGAAGGTTCCAGTC--TGAGACAAATACACACATCAAAAGGTAGTT
         66 CAAAACTGCTCAATCAAAAGAAAGGTTCAACTCTGTGAGATGAATGCACACATCACAAAGAAGTT

                                                    *
     266764 TCTCAGAATGCTTCTGTGTAGTTTTTATGTGAAGATATTTT
        131 TCTCAGAATGCTTCTGTGTAGTTTTTATGTGAAGATATTTC

             *               *    **    *     *      *  **   *          *    
     266805 CCTTTCCACCATAGGCCACAAATGGCTCTAAATACCCACTTACATTTTCCACAAAAAGAGAGTTT
          1 CTTTTCCACCATAGGCCTCAAAGCGCTCCAAATATCCACTTGCAGATTCTACAAAAAGAGTGTTT

                       * *    * *      *   *         *                       
     266870 CAAAACTGCTCTACCAAAGGTAA-GTTTAACGCTGTGAG-TTAA-G-A-ACATCACAAAGAAGTT
         66 CAAAACTGCTCAATCAAAAGAAAGGTTCAACTCTGTGAGATGAATGCACACATCACAAAGAAGTT

                                   *   *  *          
     266930 TCTCAGAATGCTTCTGTGTAGTTCTTACGTAAAGATATTTC
        131 TCTCAGAATGCTTCTGTGTAGTTTTTATGTGAAGATATTTC

                 *   *      ***    *                  *              **      
     266971 CTTTTACACAATAGGCAGAAAAGTGCTCCAAATATCCACTTGAAGATTCTACAAAAACCGTGTTT
          1 CTTTTCCACCATAGGCCTCAAAGCGCTCCAAATATCCACTTGCAGATTCTACAAAAAGAGTGTTT

                     **                                          *      *    
     267036 CAAAACTGCCGAATCAAAAGAAAGGTTCAACTCTGTGAGATGAATGCACACATAACAAAGGAGTT
         66 CAAAACTGCTCAATCAAAAGAAAGGTTCAACTCTGTGAGATGAATGCACACATCACAAAGAAGTT

                                 *      *      *    *
     267101 TCTCAGAATGCTTCTGTGTAGCTTTTATATGAAGACATTTA
        131 TCTCAGAATGCTTCTGTGTAGTTTTTATGTGAAGATATTTC

            *        * *            *   ***                     **        *  
     267142 GTTTTCCACAACAGGCCTCAAAGCTCTCTCCATATCCACTTGCAGATTCTACCGAAAGAGTGCTT
          1 CTTTTCCACCATAGGCCTCAAAGCGCTCCAAATATCCACTTGCAGATTCTACAAAAAGAGTGTTT

             *                   * **     *        *        *     **         
     267207 CCAAACTGCTCAATCAAAAGAGACATTCAAATCTGTGAGGTGAATGCAGACATCGTAAAGAAGTT
         66 CAAAACTGCTCAATCAAAAGAAAGGTTCAACTCTGTGAGATGAATGCACACATCACAAAGAAGTT

                                *     *       *    **
     267272 TCTCAGAATGCTTCTGTGTATTTTTTGTGTGAAGTTATTCG
        131 TCTCAGAATGCTTCTGTGTAGTTTTTATGTGAAGATATTTC

            *    *             *     *  *            **                 *    
     267313 TTTTTGCACCATAGGCCTCCAAGCGTTCTAAATATCCACTTCTAGATTCTACAAAAAGAGAGTTT
          1 CTTTTCCACCATAGGCCTCAAAGCGCTCCAAATATCCACTTGCAGATTCTACAAAAAGAGTGTTT

                   *         *            *        *    *   *                
     267378 CAAAACTACTC-A--AACA-AAAGGTTCAATTCTGTGAGTTGAAAGCAAACATCACAAAGAAGTT
         66 CAAAACTGCTCAATCAAAAGAAAGGTTCAACTCTGTGAGATGAATGCACACATCACAAAGAAGTT

                       *             *               
     267439 TCTCAGAATGCGTCTGTGTAGTTTTGATGTGAAGATATTTC
        131 TCTCAGAATGCTTCTGTGTAGTTTTTATGTGAAGATATTTC

                     **   ****     *                  *                      
     267480 CTTTT-CACAGTAGAATGCAAAGGGCTCCAAATATCCACTTGGAGATTCTACAAAAAGA--GTTT
          1 CTTTTCCACCATAGGCCTCAAAGCGCTCCAAATATCCACTTGCAGATTCTACAAAAAGAGTGTTT

                  *    **     *  *     *     ***   *     *               *   
     267542 CAAAACCGCTCTGTCAAATGATAGGTTGAACTCCCGGAGGTGAATACACACATCACAAAGAGGTT
         66 CAAAACTGCTCAATCAAAAGAAAGGTTCAACTCTGTGAGATGAATGCACACATCACAAAGAAGTT

                  *                       *  *       
     267607 TCTCAGCATGCTTCTGTGTAGTTTTTATGTAAACATATTTC
        131 TCTCAGAATGCTTCTGTGTAGTTTTTATGTGAAGATATTTC

             *    * *              *           *      * *       *   *    *   
     267648 CGTTTCTATCATAGGCCTCAAAGTGCTCCAAATATTCACTTGTACATTCTACCAAACGAGTATTT
          1 CTTTTCCACCATAGGCCTCAAAGCGCTCCAAATATCCACTTGCAGATTCTACAAAAAGAGTGTTT

                              * *         **      *        *            *    
     267713 CAAAACTGCTCAATCAAATGGAAGGTTCAAAACTGTGACATGAATGCCCACATCACAAAGTAGTT
         66 CAAAACTGCTCAATCAAAAGAAAGGTTCAACTCTGTGAGATGAATGCACACATCACAAAGAAGTT

                                                     
     267778 TCTCAGAATGCTTCTGTGTAGTTTTTATGTGAAGATATTTC
        131 TCTCAGAATGCTTCTGTGTAGTTTTTATGTGAAGATATTTC

                     * *    *      *    *       * *   **      *              
     267819 CTTTTCCACAACA-GCGTGCAAAACGCTTCAAATATGCCCTTAGAGATTCCACAAAAAGAGTGTT
          1 CTTTTCCACCATAGGCCT-CAAAGCGCTCCAAATATCCACTTGCAGATTCTACAAAAAGAGTGTT

              *     *               *  *                                  ** 
     267883 TCCAAACTACTCAAATCAAAA-AATGATTTCAACTCTGTGAGATGAATGCACACATCACAAACTA
         65 TCAAAACTGCTC-AATCAAAAGAAAG-GTTCAACTCTGTGAGATGAATGCACACATCACAAAGAA

                         *     ** *   * *   *       *   
     267947 GTTTCTCAGAATGTTTCTGCCTGGTTCTCATGCGAAGATAGTTC
        128 GTTTCTCAGAATGCTTCTGTGTAGTTTTTATGTGAAGATATTTC

                 *           *   * **              *                  * *    
     267991 CTTTTTCACCATAGGCCGCAATGTACTCCAAATATCCACCTGCAGATTCTAC-AAAAGTGAGTTT
          1 CTTTTCCACCATAGGCCTCAAAGCGCTCCAAATATCCACTTGCAGATTCTACAAAAAGAGTGTTT

                       *         ***    **         *        *      *       * 
     268055 CAAAACTGCTCTATCAAAAGATCAGTTCGTCTCTGTGAGTTGAATGCATACATCAAAAAGAAGCT
         66 CAAAACTGCTCAATCAAAAGAAAGGTTCAACTCTGTGAGATGAATGCACACATCACAAAGAAGTT

                 *             *      **         *   
     268120 TCTCAAAATGCTTCTGTGTGGTTTTTCGGTGAAGATAGTTC
        131 TCTCAGAATGCTTCTGTGTAGTTTTTATGTGAAGATATTTC

            *     *        *      * *                 *        *        *    
     268161 TTTTTCTACCATAGGTCTCAAACCACTCCAAATATCCACTTGTAGATTCTATAAAAAGGAATG-T
          1 CTTTTCCACCATAGGCCTCAAAGCGCTCCAAATATCCACTTGCAGATTCTACAAAAA-GAGTGTT

                  *        *    *    *      * *         *      *             
     268225 TCAAAATTGCTCAATAAAAATAAAGTTTCAACACCGTGAGATGAGTGCACAAATCACAAAGAAGT
         65 TCAAAACTGCTCAATCAAAAGAAAGGTTCAACTCTGTGAGATGAATGCACACATCACAAAGAAGT

                  *          *         *          *   
     268290 TTCTCAAAATGCTTCTGGGTAGTTTTTCTGTGAAGATAGTTC
        130 TTCTCAGAATGCTTCTGTGTAGTTTTTATGTGAAGATATTTC

                  *     *    *     *          *                         *    
     268332 CTTTTCTACCATGGGCCACAAAGGGCTCCAAATACCCACTTGCAGATTCTACAAAAAGAGAGTTT
          1 CTTTTCCACCATAGGCCTCAAAGCGCTCCAAATATCCACTTGCAGATTCTACAAAAAGAGTGTTT

              *        *      ** * *        *    * *    **  * *           *  
     268397 CACAACTGCTCTATCAAACAATATGTTCAACTTTGTGGGTTGAACACAAATATCAC-AAGAATTT
         66 CAAAACTGCTCAATCAAAAGAAAGGTTCAACTCTGTGAGATGAATGCACACATCACAAAGAAGTT

                **                             *     
     268461 TCTCCCAATGCTTCTGTGTAGTTTTTATGTGAAGACATTTC
        131 TCTCAGAATGCTTCTGTGTAGTTTTTATGTGAAGATATTTC

            *   *  *      *  *     *                 *  *      *       *  *  
     268502 TTTTCCCTCCATAGTCCACAAAGTGCTCCAAATATCCACTTACATATTCTAGAAAAAGATTGCTT
          1 CTTTTCCACCATAGGCCTCAAAGCGCTCCAAATATCCACTTGCAGATTCTACAAAAAGAGTGTTT

            **       *    *               * * *                              
     268567 GGAAACTGCACAATGAAAAGAAAGGTTCAAATATATGAGATGAATGCACACATCACAAAGAAGTT
         66 CAAAACTGCTCAATCAAAAGAAAGGTTCAACTCTGTGAGATGAATGCACACATCACAAAGAAGTT

                                 *                    
     268632 TCTCAGAAT-CTCTCTGTGTAATTTTTATGTGAAGATATTTC
        131 TCTCAGAATGCT-TCTGTGTAGTTTTTATGTGAAGATATTTC

                *     *    *  *   *                        *      *    *     
     268673 CTTTCCCACCTTAGGTCTTAAAACGCTCCAAATATCCACTTGCAGATACTACAAGAAGATTGTTT
          1 CTTTTCCACCATAGGCCTCAAAGCGCTCCAAATATCCACTTGCAGATTCTACAAAAAGAGTGTTT

                     *    *        *      *     **                           
     268738 CAAAACTGCACAA-AAAAAGAAATGTTCAATTCTGTTTGATGAATGCACACATCACAAAGAAGTT
         66 CAAAACTGCTCAATCAAAAGAAAGGTTCAACTCTGTGAGATGAATGCACACATCACAAAGAAGTT

                           *                         
     268802 TCTCAGAATGCTTCTCTGTAGTTTTTATGTGAAGATATTTC
        131 TCTCAGAATGCTTCTGTGTAGTTTTTATGTGAAGATATTTC

                     *             *                 *         **      * *   
     268843 CTTTTCCACAATAGGCCTCAAAGGGCTCCAAATATCCACTTCCAGATTCTATGAAAAGAATATTT
          1 CTTTTCCACCATAGGCCTCAAAGCGCTCCAAATATCCACTTGCAGATTCTACAAAAAGAGTGTTT

             *              * *    *                                      *  
     268908 CCAAACTGCTCAATCATAGGAAATGTTCAACTCTGTGAGATGAATGCACACATCAC-AAGAAATT
         66 CAAAACTGCTCAATCAAAAGAAAGGTTCAACTCTGTGAGATGAATGCACACATCACAAAGAAGTT

              *      *    *      *       *       *   
     268972 TCCCAGAATCCTTCAGTGTAGGTTTTATGAGAAGATAATTC
        131 TCTCAGAATGCTTCTGTGTAGTTTTTATGTGAAGATATTTC

                     *    **        *   *                           *    *   
     269013 CTTTTCCACAATAGTTCTCAAAGCACTCAAAATATCCACTTGCAGATTCTACAAAAGGAGTATTT
          1 CTTTTCCACCATAGGCCTCAAAGCGCTCCAAATATCCACTTGCAGATTCTACAAAAAGAGTGTTT

                                                          *                  
     269078 CAAAACTGCTCAATCAAAAGAAAGGTTCAACTCTGTGAGATGAATGGACACATCACAAAGAAGTT
         66 CAAAACTGCTCAATCAAAAGAAAGGTTCAACTCTGTGAGATGAATGCACACATCACAAAGAAGTT

                                  *   *              
     269143 TCTCAGAATGCTTCTGTGTAGTATTTTTGTGAAGATATTT-
        131 TCTCAGAATGCTTCTGTGTAGTTTTTATGTGAAGATATTTC

                          *  * * * * ***             *            *     * *  
     269183 CTTTTCCACCATAGACCGCCAGGGGACACAAATATCCACTTTCAGATTCTACAACAAGAGAGGTT
          1 CTTTTCCACCATAGGCCTCAAAGCGCTCCAAATATCCACTTGCAGATTCTACAAAAAGAGTGTTT

                   *   *     *   ** *       *      *                      * *
     269248 CAAAACTACTCGATCAAGAGATGGTTTCAACTATGTGAGTTGAATGCACACATCACAAAGAACTA
         66 CAAAACTGCTCAATCAAAAGAAAGGTTCAACTCTGTGAGATGAATGCACACATCACAAAGAAGTT

             *  *    *                               
     269313 TGTCGGAATTCTTCTGTGTAGTTTTTATGTGAAGATATTTC
        131 TCTCAGAATGCTTCTGTGTAGTTTTTATGTGAAGATATTTC

                     *    * *      * *    *                  *               
     269354 CTTTTCCACAATAGACGTCAAAGTGATCCAGATATCCACTTGCAGATTCCACAAAAAGAGTGTTT
          1 CTTTTCCACCATAGGCCTCAAAGCGCTCCAAATATCCACTTGCAGATTCTACAAAAAGAGTGTTT

                 *   *   *                  **                     *  *      
     269419 CAAAAGTGCACAACCAAAAGAAAGGTTCAACTAGGTGAGATGAATGCACACATCAGAAGGAAGTT
         66 CAAAACTGCTCAATCAAAAGAAAGGTTCAACTCTGTGAGATGAATGCACACATCACAAAGAAGTT

                            **   *     * *       *   
     269484 TCTCAGAATGCTTCTGCATAGCTTTTAAGGGAAGATACTTC
        131 TCTCAGAATGCTTCTGTGTAGTTTTTATGTGAAGATATTTC

                    *               *            * *  *    * *               
     269525 CTTTTCCAACATAGGCCTCAAAGCACTCCAAATATCCTCCTGGAGATACCACAAAAAGAGTGTTT
          1 CTTTTCCACCATAGGCCTCAAAGCGCTCCAAATATCCACTTGCAGATTCTACAAAAAGAGTGTTT

            **                      *  *                 *       *           
     269590 GCAAACTGCTCAATCAAAAGAAAGATTTAACTCTGTGAGATGAATCCACACATGACAAAGAAGTT
         66 CAAAACTGCTCAATCAAAAGAAAGGTTCAACTCTGTGAGATGAATGCACACATCACAAAGAAGTT

                                                     
     269655 TCTCAGAATGCTTCTGTGTAGTTTTTATGTGAAGATATTTC
        131 TCTCAGAATGCTTCTGTGTAGTTTTTATGTGAAGATATTTC

                     *   * * *    **           *                *     *      
     269696 CTTTTCCACAATAAGACCCAAAAGGCTCCAAATATTCACTTGCAGATTCTAAAAAAAACAGTGTT
          1 CTTTTCCACCATAGGCCTCAAAGCGCTCCAAATATCCACTTGCAGATTCT-ACAAAAAGAGTGTT

                                  *                  *      *        **      
     269761 TCAAAACTGCTCAATCAAAAGATA-GTTCAACTCTGTGAGAAGAATGCTCACATCACTGAGAAGT
         65 TCAAAACTGCTCAATCAAAAGAAAGGTTCAACTCTGTGAGATGAATGCACACATCACAAAGAAGT

                                         *            
     269825 TTCTCAGAATGCTTCTGTGTAGTTTTTATATGAAGATATTTC
        130 TTCTCAGAATGCTTCTGTGTAGTTTTTATGTGAAGATATTTC

                *     *      *    **                       *   **       *    
     269867 CTTTCCCACCGTAGGCCACAAAAGGCTCCAAATATCCACTTGCAGATACTATGAAAAGAGAGTTT
          1 CTTTTCCACCATAGGCCTCAAAGCGCTCCAAATATCCACTTGCAGATTCTACAAAAAGAGTGTTT

                        *        *               ***            *            
     269932 CAAAACTGCTCATTCAAAAGATAGGTTCAACTCTGTGGTTTGAATGCACACAGCACAAAGAAGTT
         66 CAAAACTGCTCAATCAAAAGAAAGGTTCAACTCTGTGAGATGAATGCACACATCACAAAGAAGTT

              *       **                   **   *    
     269997 TCACAGAATGTGTCTGTGTAGTTTTTATGTGCGGATGTTTC
        131 TCTCAGAATGCTTCTGTGTAGTTTTTATGTGAAGATATTTC

                         *        ****   *   *               *    *          
     270038 CTTTTCCACCATATGCCT-AAATATTTCCCAATTTCCACTTGCAGATTCCACAAGAAGAGTGTTT
          1 CTTTTCCACCATAGGCCTCAAAGCGCTCCAAATATCCACTTGCAGATTCTACAAAAAGAGTGTTT

                      **       *       *           *            *      ***   
     270102 CAAAACTGCTGTATC-AAATAAA-GTTGAACTCTGTGAGGTGAATGCACACAGCACAAAATGGTT
         66 CAAAACTGCTCAATCAAAAGAAAGGTTCAACTCTGTGAGATGAATGCACACATCACAAAGAAGTT

                          **   *        *       *    
     270165 TCTCAGAATGCTTCCTTGTTGTTTTTATATGAAGATGTTTC
        131 TCTCAGAATGCTTCTGTGTAGTTTTTATGTGAAGATATTTC

                  *  *             *   *     *                               
     270206 CTTTTCAACAATAGGCCTCAAAGTGCTTCAAATGTCCACTTGCAGATTCTACAAAAAGAGTGTTT
          1 CTTTTCCACCATAGGCCTCAAAGCGCTCCAAATATCCACTTGCAGATTCTACAAAAAGAGTGTTT

                                        *      *  *  *                       
     270271 CAAAACTGCTCAATCAAAAGAAAGGTTCGACTCTGGGAAATTAATGCACACATCACAAAGAAGTT
         66 CAAAACTGCTCAATCAAAAGAAAGGTTCAACTCTGTGAGATGAATGCACACATCACAAAGAAGTT

                                     *        *      
     270336 TCTC---A-GCTTCTGTGTAGTTTTCATGTGAAGTTATTTC
        131 TCTCAGAATGCTTCTGTGTAGTTTTTATGTGAAGATATTTC

                     *       *     *            *    *         **       *    
     270373 CTTTTCCACAATAGGCCGCAAAGGGCTCCAAATATCAACTTACAGATTCTAGGAAAAGAGAGTTT
          1 CTTTTCCACCATAGGCCTCAAAGCGCTCCAAATATCCACTTGCAGATTCTACAAAAAGAGTGTTT

                       * **      *     *                                     
     270438 CAAAACTGCTCTACGAAAAGATAGGTTGAACTCTGTGAGATGAATGCACACATCACAAAGAAGTT
         66 CAAAACTGCTCAATCAAAAGAAAGGTTCAACTCTGTGAGATGAATGCACACATCACAAAGAAGTT

                       *               * *           
     270503 TCTCAGAATGCATCTGTGTAGTTTTTACGGGAAGATATTTC
        131 TCTCAGAATGCTTCTGTGTAGTTTTTATGTGAAGATATTTC

                        ***  *     **      *  *           *      *     ***   
     270544 CTTTTCCACCATCTTCCACAAAGGTCTCCAAGTAACCACTTGCAGACTCTACAGAAAGACACTTT
          1 CTTTTCCACCATAGGCCTCAAAGCGCTCCAAATATCCACTTGCAGATTCTACAAAAAGAGTGTTT

            *          *         ***      *      ***                      *  
     270609 AAAAACTGCTCTATCAAAAGATCAGTTCAAGTCTGTGGTTTGAATGCACACATCACAAAGAATTT
         66 CAAAACTGCTCAATCAAAAGAAAGGTTCAACTCTGTGAGATGAATGCACACATCACAAAGAAGTT

                                     *  *            
     270674 TCTCAGAATGCTTCTGTGTAGTTTTCATATGAAGATATTTC
        131 TCTCAGAATGCTTCTGTGTAGTTTTTATGTGAAGATATTTC

                                    *                                   **   
     270715 CTTTTCCACCATAGGCCTCAAAGCACTCCAAATATCCACTTGCAGATTCTACAAAAAGAGATTTT
          1 CTTTTCCACCATAGGCCTCAAAGCGCTCCAAATATCCACTTGCAGATTCTACAAAAAGAGTGTTT

                   **                           *  *          *        *     
     270780 CAAAACTAGTCAATCAAAAGAAAGGTTCAACTCTGTCAGTTGAATGCACATATCACAAACAAGTT
         66 CAAAACTGCTCAATCAAAAGAAAGGTTCAACTCTGTGAGATGAATGCACACATCACAAAGAAGTT

                *      *                             
     270845 TCTCGGAATGCGTCTGTGTAGTTTTTATGTGAAGATATTTC
        131 TCTCAGAATGCTTCTGTGTAGTTTTTATGTGAAGATATTTC

               *     * *           *     *                  *    *           
     270886 CTTCTCCACAACAGGCCTCAAAGTGCTCCGAATATCCACTTGCAGATTTTAC-TAAAGAGTGTTT
          1 CTTTTCCACCATAGGCCTCAAAGCGCTCCAAATATCCACTTGCAGATTCTACAAAAAGAGTGTTT

             *               *  *   *     *        *    *               *    
     270950 CCAAACTGCTCAATCAAGAGGAAGTTTCAAGTCTGTGAGCTGAACGCACACATCACAAAGTAGTT
         66 CAAAACTGCTCAATCAAAAGAAAGGTTCAACTCTGTGAGATGAATGCACACATCACAAAGAAGTT

               *                                *    
     271015 TCTGAGAATGCTTCTGTGTAGTTTTTATGTGAAGATGTTTC
        131 TCTCAGAATGCTTCTGTGTAGTTTTTATGTGAAGATATTTC

                            **     *                                    *    
     271056 CTTTTCCACCATAGGCTGCAAAGGGCTCCAAATATCCACTTGCAGATTCTACAAAAAGAGAGTTT
          1 CTTTTCCACCATAGGCCTCAAAGCGCTCCAAATATCCACTTGCAGATTCTACAAAAAGAGTGTTT

                 *     *         *          *     *                     *    
     271121 CAAAAGTGCTCTATCAAAAGATAGGTTCAACTATGTGATATGAATGCACACATCACAAAGTAGTT
         66 CAAAACTGCTCAATCAAAAGAAAGGTTCAACTCTGTGAGATGAATGCACACATCACAAAGAAGTT

                                          *          
     271186 TCTCAGAATGCTTCTGTGTAGTTTTTATGTAAAGATATTTC
        131 TCTCAGAATGCTTCTGTGTAGTTTTTATGTGAAGATATTTC

                                    *                                   **   
     271227 CTTTTCCACCATAGGCCTCAAAGCACTCCAAATATCCACTTGCAGATTCTACAAAAAGAGATTTT
          1 CTTTTCCACCATAGGCCTCAAAGCGCTCCAAATATCCACTTGCAGATTCTACAAAAAGAGTGTTT

                   ** *                   *     *  *    * *   *        *     
     271292 CAAAACTATTTAATCAAAAGAAAGGTTCAAATCTGTCAGTTGAAGGTACATATCACAAACAAGTT
         66 CAAAACTGCTCAATCAAAAGAAAGGTTCAACTCTGTGAGATGAATGCACACATCACAAAGAAGTT

             * **                                    
     271357 TATTGGAATGCTTCTGTGTAGTTTTTATGTGAAGATATTTC
        131 TCTCAGAATGCTTCTGTGTAGTTTTTATGTGAAGATATTTC

                     * *         * *                        **  *     *      
     271398 CTTTTCCACAACAGGCCTCAAGGTGCTCCAAATATCCACTTGCAGATTTCACTAAAAGTGTGTTT
          1 CTTTTCCACCATAGGCCTCAAAGCGCTCCAAATATCCACTTGCAGATTCTACAAAAAGAGTGTTT

             *  *            *  *   *     *        *             *           
     271463 CCAAGCTGCTCAATCAAGAGGAAGTTTCAAGTCTGTGAGGTGAATGCACACATTACAAAGAAGTT
         66 CAAAACTGCTCAATCAAAAGAAAGGTTCAACTCTGTGAGATGAATGCACACATCACAAAGAAGTT

            *  *                                     
     271528 ACTGAGAATGCTTCTGTGTAGTTTTTATGTGAAGATATTTC
        131 TCTCAGAATGCTTCTGTGTAGTTTTTATGTGAAGATATTTC

                      **               *                                *    
     271569 CTTTTCCACCGCAGGCCTCAAAGCGCTGCAAATATCCACTTGCAGATTCTACAAAAAGAGAGTTT
          1 CTTTTCCACCATAGGCCTCAAAGCGCTCCAAATATCCACTTGCAGATTCTACAAAAAGAGTGTTT

                      **         *   *         *   *     **            *     
     271634 CAAAACTGCTGTATCAAAAGATAGGGTCAACTCTGCGAGTTGAATAAACACATCACAAATAAGTT
         66 CAAAACTGCTCAATCAAAAGAAAGGTTCAACTCTGTGAGATGAATGCACACATCACAAAGAAGTT

               **   *        *               *       
     271699 TCTGGGAACGCTTCTGTATAG-TTTTATGTGAATATATTTC
        131 TCTCAGAATGCTTCTGTGTAGTTTTTATGTGAAGATATTTC

                         *          *                   *   *  *   * *   *   
     271739 CTTTTCCACCATATGCCTCAAAGCACTCCAAATATCCACTTGCACATTATAGAAACATAGTCTTT
          1 CTTTTCCACCATAGGCCTCAAAGCGCTCCAAATATCCACTTGCAGATTCTACAAAAAGAGTGTTT

                   **                        *         *             *       
     271804 CAAAACTTGTCAATC-AAAGAAAGGTTCAACTCCGTGAGATGAGTGCACACATCACAGAGAAGTT
         66 CAAAACTGCTCAATCAAAAGAAAGGTTCAACTCTGTGAGATGAATGCACACATCACAAAGAAGTT

                *     *                            * 
     271868 TCTCGGAATGTTTCTGTGTAGTTTTTATGTGAAGATATTGC
        131 TCTCAGAATGCTTCTGTGTAGTTTTTATGTGAAGATATTTC

                     *               *            *          *       *   *   
     271909 CTTTTCCACAATAGGCCTCAAAGCGTTCCAAATATCCAATTGCAGATTCCACAAAAAAAGTTTTT
          1 CTTTTCCACCATAGGCCTCAAAGCGCTCCAAATATCCACTTGCAGATTCTACAAAAAGAGTGTTT

            *                 *  *  *  *             * *         **    *     
     271974 TAAAACTGCTCAATCAAATGATAGATTAAACTCTGTGAGATTAGTGCACACATGTCAAAAAAGTT
         66 CAAAACTGCTCAATCAAAAGAAAGGTTCAACTCTGTGAGATGAATGCACACATCACAAAGAAGTT

                                *      * *           
     272039 TCTCAGAATGCTTCTGTGTACTTTTTAGGGGAAGATATTTC
        131 TCTCAGAATGCTTCTGTGTAGTTTTTATGTGAAGATATTTC

                        *    *     **         *    *           **  * *  *    
     272080 CTTTTCCACCATCGGCCACAAAGGACTCCAAATAACCACATGCAGATTCTAGTAACACAGAGTTT
          1 CTTTTCCACCATAGGCCTCAAAGCGCTCCAAATATCCACTTGCAGATTCTACAAAAAGAGTGTTT

                       *         * *           *   * * *    **    ***        
     272145 CAAAACTGCTCTATCAAAAGATAAGTTCAACTCTGAGAGTTTAGTGCAACCATCGTGAAGAAGTT
         66 CAAAACTGCTCAATCAAAAGAAAGGTTCAACTCTGTGAGATGAATGCACACATCACAAAGAAGTT

                            *     *                  
     272210 TCTCAGAATGCTTCTGAGTAGTGTTTATGTGAAGATATTTC
        131 TCTCAGAATGCTTCTGTGTAGTTTTTATGTGAAGATATTTC

                              *     *                      *            *    
     272251 CTTTTCCACCATAGGCCTGAAAGCCCTCCAAATATCCACTTGCAGATCCTACAAAAAGAGAGTTT
          1 CTTTTCCACCATAGGCCTCAAAGCGCTCCAAATATCCACTTGCAGATTCTACAAAAAGAGTGTTT

             *         **     *  *  *   *        **      *                   
     272316 CGAAA-TGCTCTCTCAAACGATAGTTTCGACTCTGTGGTATGAATACACACATCACAAAGAAGTT
         66 CAAAACTGCTCAATCAAAAGAAAGGTTCAACTCTGTGAGATGAATGCACACATCACAAAGAAGTT

                                       **            
     272380 TCTCAGAATGCTTCTGTGTAGTTTTTAAATGAAGATATTTC
        131 TCTCAGAATGCTTCTGTGTAGTTTTTATGTGAAGATATTTC

            *                       *          *     *                       
     272421 TTTTTCCACCATAGGCCTCAAAGCACTCCAAATATGCACTTCCAGATTCTACAAAAAGAGTGTTT
          1 CTTTTCCACCATAGGCCTCAAAGCGCTCCAAATATCCACTTGCAGATTCTACAAAAAGAGTGTTT

              *                 *       * *         **   *         *  * *    
     272486 CAGAACTGCTCAATCAAAAGGAAGGTTCCAGTC--TGAGACAAATACACACATCAAAAGGTAGTT
         66 CAAAACTGCTCAATCAAAAGAAAGGTTCAACTCTGTGAGATGAATGCACACATCACAAAGAAGTT

                                                    *
     272549 TCTCAGAATGCTTCTGTGTAGTTTTTATGTGAAGATATTTT
        131 TCTCAGAATGCTTCTGTGTAGTTTTTATGTGAAGATATTTC

             *               *    **    *     *      *  **   *          *    
     272590 CCTTTCCACCATAGGCCACAAATGGCTCTAAATACCCACTTACATTTTCCACAAAAAGAGAGTTT
          1 CTTTTCCACCATAGGCCTCAAAGCGCTCCAAATATCCACTTGCAGATTCTACAAAAAGAGTGTTT

                       * *    * *      *   *         *                       
     272655 CAAAACTGCTCTACCAAAGGTAA-GTTTAACGCTGTGAG-TTAA-G-A-ACATCACAAAGAAGTT
         66 CAAAACTGCTCAATCAAAAGAAAGGTTCAACTCTGTGAGATGAATGCACACATCACAAAGAAGTT

                                   *   *  *          
     272715 TCTCAGAATGCTTCTGTGTAGTTCTTACGTAAAGATATTTC
        131 TCTCAGAATGCTTCTGTGTAGTTTTTATGTGAAGATATTTC

                 *   *      ***    *                  *              **      
     272756 CTTTTACACAATAGGCAGAAAAGTGCTCCAAATATCCACTTGAAGATTCTACAAAAACCGTGTTT
          1 CTTTTCCACCATAGGCCTCAAAGCGCTCCAAATATCCACTTGCAGATTCTACAAAAAGAGTGTTT

                     **                                          *      *    
     272821 CAAAACTGCCGAATCAAAAGAAAGGTTCAACTCTGTGAGATGAATGCACACATAACAAAGGAGTT
         66 CAAAACTGCTCAATCAAAAGAAAGGTTCAACTCTGTGAGATGAATGCACACATCACAAAGAAGTT

                                 *      *      *    *
     272886 TCTCAGAATGCTTCTGTGTAGCTTTTATATGAAGACATTTA
        131 TCTCAGAATGCTTCTGTGTAGTTTTTATGTGAAGATATTTC

            *        * *            *   ***                     **        *  
     272927 GTTTTCCACAACAGGCCTCAAAGCTCTCTCCATATCCACTTGCAGATTCTACCGAAAGAGTGCTT
          1 CTTTTCCACCATAGGCCTCAAAGCGCTCCAAATATCCACTTGCAGATTCTACAAAAAGAGTGTTT

             *                   * **     *        *        *     **         
     272992 CCAAACTGCTCAATCAAAAGAGACATTCAAATCTGTGAGGTGAATGCAGACATCGTAAAGAAGTT
         66 CAAAACTGCTCAATCAAAAGAAAGGTTCAACTCTGTGAGATGAATGCACACATCACAAAGAAGTT

                                *     *       *    **
     273057 TCTCAGAATGCTTCTGTGTATTTTTTGTGTGAAGTTATTCG
        131 TCTCAGAATGCTTCTGTGTAGTTTTTATGTGAAGATATTTC

            *    *             *     *  *            **                 *    
     273098 TTTTTGCACCATAGGCCTCCAAGCGTTCTAAATATCCACTTCTAGATTCTACAAAAAGAGAGTTT
          1 CTTTTCCACCATAGGCCTCAAAGCGCTCCAAATATCCACTTGCAGATTCTACAAAAAGAGTGTTT

                   *         *            *        *    *   *                
     273163 CAAAACTACTC-A--AACA-AAAGGTTCAATTCTGTGAGTTGAAAGCAAACATCACAAAGAAGTT
         66 CAAAACTGCTCAATCAAAAGAAAGGTTCAACTCTGTGAGATGAATGCACACATCACAAAGAAGTT

                       *             *               
     273224 TCTCAGAATGCGTCTGTGTAGTTTTGATGTGAAGATATTTC
        131 TCTCAGAATGCTTCTGTGTAGTTTTTATGTGAAGATATTTC

                     **   ****     *                  *                      
     273265 CTTTT-CACAGTAGAATGCAAAGGGCTCCAAATATCCACTTGGAGATTCTACAAAAAGA--GTTT
          1 CTTTTCCACCATAGGCCTCAAAGCGCTCCAAATATCCACTTGCAGATTCTACAAAAAGAGTGTTT

                  *    **     *  *     *     ***   *     *               *   
     273327 CAAAACCGCTCTGTCAAATGATAGGTTGAACTCCCGGAGGTGAATACACACATCACAAAGAGGTT
         66 CAAAACTGCTCAATCAAAAGAAAGGTTCAACTCTGTGAGATGAATGCACACATCACAAAGAAGTT

                  *                       *  *       
     273392 TCTCAGCATGCTTCTGTGTAGTTTTTATGTAAACATATTTC
        131 TCTCAGAATGCTTCTGTGTAGTTTTTATGTGAAGATATTTC

             *    * *              *           *      * *       *   *    *   
     273433 CGTTTCTATCATAGGCCTCAAAGTGCTCCAAATATTCACTTGTACATTCTACCAAACGAGTATTT
          1 CTTTTCCACCATAGGCCTCAAAGCGCTCCAAATATCCACTTGCAGATTCTACAAAAAGAGTGTTT

                              * *         **      *        *            *    
     273498 CAAAACTGCTCAATCAAATGGAAGGTTCAAAACTGTGACATGAATGCCCACATCACAAAGTAGTT
         66 CAAAACTGCTCAATCAAAAGAAAGGTTCAACTCTGTGAGATGAATGCACACATCACAAAGAAGTT

                                                     
     273563 TCTCAGAATGCTTCTGTGTAGTTTTTATGTGAAGATATTTC
        131 TCTCAGAATGCTTCTGTGTAGTTTTTATGTGAAGATATTTC

                     * *    *      *    *       * *   **      *              
     273604 CTTTTCCACAACA-GCGTGCAAAACGCTTCAAATATGCCCTTAGAGATTCCACAAAAAGAGTGTT
          1 CTTTTCCACCATAGGCCT-CAAAGCGCTCCAAATATCCACTTGCAGATTCTACAAAAAGAGTGTT

              *     *               *  *                                  ** 
     273668 TCCAAACTACTCAAATCAAAA-AATGATTTCAACTCTGTGAGATGAATGCACACATCACAAACTA
         65 TCAAAACTGCTC-AATCAAAAGAAAG-GTTCAACTCTGTGAGATGAATGCACACATCACAAAGAA

                         *     ** *   * *   *       *   
     273732 GTTTCTCAGAATGTTTCTGCCTGGTTCTCATGCGAAGATAGTTC
        128 GTTTCTCAGAATGCTTCTGTGTAGTTTTTATGTGAAGATATTTC

                 *           *   * **              *                  * *    
     273776 CTTTTTCACCATAGGCCGCAATGTACTCCAAATATCCACCTGCAGATTCTAC-AAAAGTGAGTTT
          1 CTTTTCCACCATAGGCCTCAAAGCGCTCCAAATATCCACTTGCAGATTCTACAAAAAGAGTGTTT

                       *         ***    **         *        *      *       * 
     273840 CAAAACTGCTCTATCAAAAGATCAGTTCGTCTCTGTGAGTTGAATGCATACATCAAAAAGAAGCT
         66 CAAAACTGCTCAATCAAAAGAAAGGTTCAACTCTGTGAGATGAATGCACACATCACAAAGAAGTT

                 *             *      **         *   
     273905 TCTCAAAATGCTTCTGTGTGGTTTTTCGGTGAAGATAGTTC
        131 TCTCAGAATGCTTCTGTGTAGTTTTTATGTGAAGATATTTC

            *     *        *      * *                 *        *        *    
     273946 TTTTTCTACCATAGGTCTCAAACCACTCCAAATATCCACTTGTAGATTCTATAAAAAGGAATG-T
          1 CTTTTCCACCATAGGCCTCAAAGCGCTCCAAATATCCACTTGCAGATTCTACAAAAA-GAGTGTT

                  *        *    *    *      * *         *      *             
     274010 TCAAAATTGCTCAATAAAAATAAAGTTTCAACACCGTGAGATGAGTGCACAAATCACAAAGAAGT
         65 TCAAAACTGCTCAATCAAAAGAAAGGTTCAACTCTGTGAGATGAATGCACACATCACAAAGAAGT

                  *          *         *          *   
     274075 TTCTCAAAATGCTTCTGGGTAGTTTTTCTGTGAAGATAGTTC
        130 TTCTCAGAATGCTTCTGTGTAGTTTTTATGTGAAGATATTTC

                  *     *    *     *          *                         *    
     274117 CTTTTCTACCATGGGCCACAAAGGGCTCCAAATACCCACTTGCAGATTCTACAAAAAGAGAGTTT
          1 CTTTTCCACCATAGGCCTCAAAGCGCTCCAAATATCCACTTGCAGATTCTACAAAAAGAGTGTTT

              *        *      ** * *        *    * *    **  * *           *  
     274182 CACAACTGCTCTATCAAACAATATGTTCAACTTTGTGGGTTGAACACAAATATCAC-AAGAATTT
         66 CAAAACTGCTCAATCAAAAGAAAGGTTCAACTCTGTGAGATGAATGCACACATCACAAAGAAGTT

                **                             *     
     274246 TCTCCCAATGCTTCTGTGTAGTTTTTATGTGAAGACATTTC
        131 TCTCAGAATGCTTCTGTGTAGTTTTTATGTGAAGATATTTC

            *   *  *      *  *     *                 *  *      *       *  *  
     274287 TTTTCCCTCCATAGTCCACAAAGTGCTCCAAATATCCACTTACATATTCTAGAAAAAGATTGCTT
          1 CTTTTCCACCATAGGCCTCAAAGCGCTCCAAATATCCACTTGCAGATTCTACAAAAAGAGTGTTT

            **       *    *               * * *                              
     274352 GGAAACTGCACAATGAAAAGAAAGGTTCAAATATATGAGATGAATGCACACATCACAAAGAAGTT
         66 CAAAACTGCTCAATCAAAAGAAAGGTTCAACTCTGTGAGATGAATGCACACATCACAAAGAAGTT

                                 *                    
     274417 TCTCAGAAT-CTCTCTGTGTAATTTTTATGTGAAGATATTTC
        131 TCTCAGAATGCT-TCTGTGTAGTTTTTATGTGAAGATATTTC

                *     *    *  *   *                        *      *    *     
     274458 CTTTCCCACCTTAGGTCTTAAAACGCTCCAAATATCCACTTGCAGATACTACAAGAAGATTGTTT
          1 CTTTTCCACCATAGGCCTCAAAGCGCTCCAAATATCCACTTGCAGATTCTACAAAAAGAGTGTTT

                     *    *        *      *     **                           
     274523 CAAAACTGCACAA-AAAAAGAAATGTTCAATTCTGTTTGATGAATGCACACATCACAAAGAAGTT
         66 CAAAACTGCTCAATCAAAAGAAAGGTTCAACTCTGTGAGATGAATGCACACATCACAAAGAAGTT

                           *                         
     274587 TCTCAGAATGCTTCTCTGTAGTTTTTATGTGAAGATATTTC
        131 TCTCAGAATGCTTCTGTGTAGTTTTTATGTGAAGATATTTC

                     *             *                 *         **      * *   
     274628 CTTTTCCACAATAGGCCTCAAAGGGCTCCAAATATCCACTTCCAGATTCTATGAAAAGAATATTT
          1 CTTTTCCACCATAGGCCTCAAAGCGCTCCAAATATCCACTTGCAGATTCTACAAAAAGAGTGTTT

             *              * *    *                                      *  
     274693 CCAAACTGCTCAATCATAGGAAATGTTCAACTCTGTGAGATGAATGCACACATCAC-AAGAAATT
         66 CAAAACTGCTCAATCAAAAGAAAGGTTCAACTCTGTGAGATGAATGCACACATCACAAAGAAGTT

                     *    *      *       *       *   
     274757 TCTCAGAATCCTTCAGTGTAGGTTTTATGAGAAGATAATTC
        131 TCTCAGAATGCTTCTGTGTAGTTTTTATGTGAAGATATTTC

                     *    **        *   *                           *    *   
     274798 CTTTTCCACAATAGTTCTCAAAGCACTCAAAATATCCACTTGCAGATTCTACAAAAGGAGTATTT
          1 CTTTTCCACCATAGGCCTCAAAGCGCTCCAAATATCCACTTGCAGATTCTACAAAAAGAGTGTTT

                                                          *                  
     274863 CAAAACTGCTCAATCAAAAGAAAGGTTCAACTCTGTGAGATGAATGGACACATCACAAAGAAGTT
         66 CAAAACTGCTCAATCAAAAGAAAGGTTCAACTCTGTGAGATGAATGCACACATCACAAAGAAGTT

                                  *   *              
     274928 TCTCAGAATGCTTCTGTGTAGTATTTTTGTGAAGATATTT-
        131 TCTCAGAATGCTTCTGTGTAGTTTTTATGTGAAGATATTTC

                          *  * * * * ***             *            *     * *  
     274968 CTTTTCCACCATAGACCGCCAGGGGACACAAATATCCACTTTCAGATTCTACAACAAGAGAGGTT
          1 CTTTTCCACCATAGGCCTCAAAGCGCTCCAAATATCCACTTGCAGATTCTACAAAAAGAGTGTTT

                   *   *     *   ** *       *      *                      * *
     275033 CAAAACTACTCGATCAAGAGATGGTTTCAACTATGTGAGTTGAATGCACACATCACAAAGAACTA
         66 CAAAACTGCTCAATCAAAAGAAAGGTTCAACTCTGTGAGATGAATGCACACATCACAAAGAAGTT

             *  *    *                               
     275098 TGTCGGAATTCTTCTGTGTAGTTTTTATGTGAAGATATTTC
        131 TCTCAGAATGCTTCTGTGTAGTTTTTATGTGAAGATATTTC

                     *    * *      * *    *                  *               
     275139 CTTTTCCACAATAGACGTCAAAGTGATCCAGATATCCACTTGCAGATTCCACAAAAAGAGTGTTT
          1 CTTTTCCACCATAGGCCTCAAAGCGCTCCAAATATCCACTTGCAGATTCTACAAAAAGAGTGTTT

                 *   *   *                  **                     *  *      
     275204 CAAAAGTGCACAACCAAAAGAAAGGTTCAACTAGGTGAGATGAATGCACACATCAGAAGGAAGTT
         66 CAAAACTGCTCAATCAAAAGAAAGGTTCAACTCTGTGAGATGAATGCACACATCACAAAGAAGTT

                            **   *     * *       *   
     275269 TCTCAGAATGCTTCTGCATAGCTTTTAAGGGAAGATACTTC
        131 TCTCAGAATGCTTCTGTGTAGTTTTTATGTGAAGATATTTC

                    *               *            * *  *    * *               
     275310 CTTTTCCAACATAGGCCTCAAAGCACTCCAAATATCCTCCTGGAGATACCACAAAAAGAGTGTTT
          1 CTTTTCCACCATAGGCCTCAAAGCGCTCCAAATATCCACTTGCAGATTCTACAAAAAGAGTGTTT

            **                      *  *                 *       *           
     275375 GCAAACTGCTCAATCAAAAGAAAGATTTAACTCTGTGAGATGAATCCACACATGACAAAGAAGTT
         66 CAAAACTGCTCAATCAAAAGAAAGGTTCAACTCTGTGAGATGAATGCACACATCACAAAGAAGTT

                                                     
     275440 TCTCAGAATGCTTCTGTGTAGTTTTTATGTGAAGATATTTC
        131 TCTCAGAATGCTTCTGTGTAGTTTTTATGTGAAGATATTTC

                     *   * * *    **           *                *     *      
     275481 CTTTTCCACAATAAGACCCAAAAGGCTCCAAATATTCACTTGCAGATTCTAAAAAAAACAGTGTT
          1 CTTTTCCACCATAGGCCTCAAAGCGCTCCAAATATCCACTTGCAGATTCT-ACAAAAAGAGTGTT

                                  *                  *      *        **      
     275546 TCAAAACTGCTCAATCAAAAGATA-GTTCAACTCTGTGAGAAGAATGCTCACATCACTGAGAAGT
         65 TCAAAACTGCTCAATCAAAAGAAAGGTTCAACTCTGTGAGATGAATGCACACATCACAAAGAAGT

                                         *            
     275610 TTCTCAGAATGCTTCTGTGTAGTTTTTATATGAAGATATTTC
        130 TTCTCAGAATGCTTCTGTGTAGTTTTTATGTGAAGATATTTC

                *     *      *    **                       *   **       *    
     275652 CTTTCCCACCGTAGGCCACAAAAGGCTCCAAATATCCACTTGCAGATACTATGAAAAGAGAGTTT
          1 CTTTTCCACCATAGGCCTCAAAGCGCTCCAAATATCCACTTGCAGATTCTACAAAAAGAGTGTTT

                        *        *               ***            *            
     275717 CAAAACTGCTCATTCAAAAGATAGGTTCAACTCTGTGGTTTGAATGCACACAGCACAAAGAAGTT
         66 CAAAACTGCTCAATCAAAAGAAAGGTTCAACTCTGTGAGATGAATGCACACATCACAAAGAAGTT

              *       **                   **   *    
     275782 TCACAGAATGTGTCTGTGTAGTTTTTATGTGCGGATGTTTC
        131 TCTCAGAATGCTTCTGTGTAGTTTTTATGTGAAGATATTTC

                         *        ****   *   *               *    *          
     275823 CTTTTCCACCATATGCCT-AAATATTTCCCAATTTCCACTTGCAGATTCCACAAGAAGAGTGTTT
          1 CTTTTCCACCATAGGCCTCAAAGCGCTCCAAATATCCACTTGCAGATTCTACAAAAAGAGTGTTT

                      **       *       *           *            *      ***   
     275887 CAAAACTGCTGTATC-AAATAAA-GTTGAACTCTGTGAGGTGAATGCACACAGCACAAAATGGTT
         66 CAAAACTGCTCAATCAAAAGAAAGGTTCAACTCTGTGAGATGAATGCACACATCACAAAGAAGTT

                          **   *        *       *    
     275950 TCTCAGAATGCTTCCTTGTTGTTTTTATATGAAGATGTTTC
        131 TCTCAGAATGCTTCTGTGTAGTTTTTATGTGAAGATATTTC

                  *  *             *   *     *                               
     275991 CTTTTCAACAATAGGCCTCAAAGTGCTTCAAATGTCCACTTGCAGATTCTACAAAAAGAGTGTTT
          1 CTTTTCCACCATAGGCCTCAAAGCGCTCCAAATATCCACTTGCAGATTCTACAAAAAGAGTGTTT

                                        *      *  *  *                       
     276056 CAAAACTGCTCAATCAAAAGAAAGGTTCGACTCTGGGAAATTAATGCACACATCACAAAGAAGTT
         66 CAAAACTGCTCAATCAAAAGAAAGGTTCAACTCTGTGAGATGAATGCACACATCACAAAGAAGTT

                                     *        *      
     276121 TCTC---A-GCTTCTGTGTAGTTTTCATGTGAAGTTATTTC
        131 TCTCAGAATGCTTCTGTGTAGTTTTTATGTGAAGATATTTC

                     *       *     *            *    *         **       *    
     276158 CTTTTCCACAATAGGCCGCAAAGGGCTCCAAATATCAACTTACAGATTCTAGGAAAAGAGAGTTT
          1 CTTTTCCACCATAGGCCTCAAAGCGCTCCAAATATCCACTTGCAGATTCTACAAAAAGAGTGTTT

                       * **      *     *                                     
     276223 CAAAACTGCTCTACGAAAAGATAGGTTGAACTCTGTGAGATGAATGCACACATCACAAAGAAGTT
         66 CAAAACTGCTCAATCAAAAGAAAGGTTCAACTCTGTGAGATGAATGCACACATCACAAAGAAGTT

                       *               * *           
     276288 TCTCAGAATGCATCTGTGTAGTTTTTACGGGAAGATATTTC
        131 TCTCAGAATGCTTCTGTGTAGTTTTTATGTGAAGATATTTC

                        ***  *     **      *  *           *      *     ***   
     276329 CTTTTCCACCATCTTCCACAAAGGTCTCCAAGTAACCACTTGCAGACTCTACAGAAAGACACTTT
          1 CTTTTCCACCATAGGCCTCAAAGCGCTCCAAATATCCACTTGCAGATTCTACAAAAAGAGTGTTT

            *          *         ***      *      ***                      *  
     276394 AAAAACTGCTCTATCAAAAGATCAGTTCAAGTCTGTGGTTTGAATGCACACATCACAAAGAATTT
         66 CAAAACTGCTCAATCAAAAGAAAGGTTCAACTCTGTGAGATGAATGCACACATCACAAAGAAGTT

                                     *  *            
     276459 TCTCAGAATGCTTCTGTGTAGTTTTCATATGAAGATATTTC
        131 TCTCAGAATGCTTCTGTGTAGTTTTTATGTGAAGATATTTC

                                    *                                   **   
     276500 CTTTTCCACCATAGGCCTCAAAGCACTCCAAATATCCACTTGCAGATTCTACAAAAAGAGATTTT
          1 CTTTTCCACCATAGGCCTCAAAGCGCTCCAAATATCCACTTGCAGATTCTACAAAAAGAGTGTTT

                   **                           *  *          *        *     
     276565 CAAAACTAGTCAATCAAAAGAAAGGTTCAACTCTGTCAGTTGAATGCACATATCACAAACAAGTT
         66 CAAAACTGCTCAATCAAAAGAAAGGTTCAACTCTGTGAGATGAATGCACACATCACAAAGAAGTT

                *      *                             
     276630 TCTCGGAATGCGTCTGTGTAGTTTTTATGTGAAGATATTTC
        131 TCTCAGAATGCTTCTGTGTAGTTTTTATGTGAAGATATTTC

               *     * *           *     *                  *    *           
     276671 CTTCTCCACAACAGGCCTCAAAGTGCTCCGAATATCCACTTGCAGATTTTAC-TAAAGAGTGTTT
          1 CTTTTCCACCATAGGCCTCAAAGCGCTCCAAATATCCACTTGCAGATTCTACAAAAAGAGTGTTT

             *               *  *   *     *        *    *               *    
     276735 CCAAACTGCTCAATCAAGAGGAAGTTTCAAGTCTGTGAGCTGAACGCACACATCACAAAGTAGTT
         66 CAAAACTGCTCAATCAAAAGAAAGGTTCAACTCTGTGAGATGAATGCACACATCACAAAGAAGTT

               *                                *    
     276800 TCTGAGAATGCTTCTGTGTAGTTTTTATGTGAAGATGTTTC
        131 TCTCAGAATGCTTCTGTGTAGTTTTTATGTGAAGATATTTC

                            **     *                                    *  * 
     276841 CTTTTCCACCATAGGCTGCAAAGGGCTCCAAATATCCACTTGCAGATTCTACAAAAAGAGAGTCT
          1 CTTTTCCACCATAGGCCTCAAAGCGCTCCAAATATCCACTTGCAGATTCTACAAAAAGAGTGTTT

                 *     *         *          *     *                     *    
     276906 CAAAAGTGCTCTATCAAAAGATAGGTTCAACTATGTGATATGAATGCACACATCACAAAGTAGTT
         66 CAAAACTGCTCAATCAAAAGAAAGGTTCAACTCTGTGAGATGAATGCACACATCACAAAGAAGTT

                                          *          
     276971 TCTCAGAATGCTTCTGTGTAGTTTTTATGTAAAGATATTTC
        131 TCTCAGAATGCTTCTGTGTAGTTTTTATGTGAAGATATTTC

                                    *                                        
     277012 CTTTTCCACCATAGGCCTCAAAGCACTCCAAATATCCACTTGCAGATTCTACAAAAAGAGATG-T
          1 CTTTTCCACCATAGGCCTCAAAGCGCTCCAAATATCCACTTGCAGATTCTACAAAAAGAG-TGTT

                    ** *                   *     *  *    * *   *        *    
     277076 TCAAAACTATTTAATCAAAAGAAAGGTTCAAATCTGTCAGTTGAAGGTACATATCACAAACAAGT
         65 TCAAAACTGCTCAATCAAAAGAAAGGTTCAACTCTGTGAGATGAATGCACACATCACAAAGAAGT

              * **                                    
     277141 TTATTGGAATGCTTCTGTGTAGTTTTTATGTGAAGATATTTC
        130 TTCTCAGAATGCTTCTGTGTAGTTTTTATGTGAAGATATTTC

                     * *         * *                        **  *     *      
     277183 CTTTTCCACAACAGGCCTCAAGGTGCTCCAAATATCCACTTGCAGATTTCACTAAAAGTGTGTTT
          1 CTTTTCCACCATAGGCCTCAAAGCGCTCCAAATATCCACTTGCAGATTCTACAAAAAGAGTGTTT

             *  *            *  *   *     *        *             *           
     277248 CCAAGCTGCTCAATCAAGAGGAAGTTTCAAGTCTGTGAGGTGAATGCACACATTACAAAGAAGTT
         66 CAAAACTGCTCAATCAAAAGAAAGGTTCAACTCTGTGAGATGAATGCACACATCACAAAGAAGTT

            *  *                                     
     277313 ACTGAGAATGCTTCTGTGTAGTTTTTATGTGAAGATATTTC
        131 TCTCAGAATGCTTCTGTGTAGTTTTTATGTGAAGATATTTC

                      **               *                                *    
     277354 CTTTTCCACCGCAGGCCTCAAAGCGCTGCAAATATCCACTTGCAGATTCTACAAAAAGAGAGTTT
          1 CTTTTCCACCATAGGCCTCAAAGCGCTCCAAATATCCACTTGCAGATTCTACAAAAAGAGTGTTT

                      **         *   *         *   *     **            *     
     277419 CAAAACTGCTGTATCAAAAGATAGGGTCAACTCTGCGAGTTGAATAAACACATCACAAATAAGTT
         66 CAAAACTGCTCAATCAAAAGAAAGGTTCAACTCTGTGAGATGAATGCACACATCACAAAGAAGTT

               **   *        *               *       
     277484 TCTGGGAACGCTTCTGTATAG-TTTTATGTGAATATATTTC
        131 TCTCAGAATGCTTCTGTGTAGTTTTTATGTGAAGATATTTC

                         *          *                   *   *  *   * *   *   
     277524 CTTTTCCACCATATGCCTCAAAGCACTCCAAATATCCACTTGCACATTATAGAAACATAGTCTTT
          1 CTTTTCCACCATAGGCCTCAAAGCGCTCCAAATATCCACTTGCAGATTCTACAAAAAGAGTGTTT

                   **                        *         *             *       
     277589 CAAAACTTGTCAATC-AAAGAAAGGTTCAACTCCGTGAGATGAGTGCACACATCACAGAGAAGTT
         66 CAAAACTGCTCAATCAAAAGAAAGGTTCAACTCTGTGAGATGAATGCACACATCACAAAGAAGTT

                *     *                            * 
     277653 TCTCGGAATGTTTCTGTGTAGTTTTTATGTGAAGATATTGC
        131 TCTCAGAATGCTTCTGTGTAGTTTTTATGTGAAGATATTTC

                     *               *            *          *       *   *   
     277694 CTTTTCCACAATAGGCCTCAAAGCGTTCCAAATATCCAATTGCAGATTCCACAAAAAAAGTTTTT
          1 CTTTTCCACCATAGGCCTCAAAGCGCTCCAAATATCCACTTGCAGATTCTACAAAAAGAGTGTTT

            *                 *  *  *  *             * *         **    *     
     277759 TAAAACTGCTCAATCAAATGATAGATTAAACTCTGTGAGATTAGTGCACACATGTCAAAAAAGTT
         66 CAAAACTGCTCAATCAAAAGAAAGGTTCAACTCTGTGAGATGAATGCACACATCACAAAGAAGTT

                                *      * *           
     277824 TCTCAGAATGCTTCTGTGTACTTTTTAGGGGAAGATATTTC
        131 TCTCAGAATGCTTCTGTGTAGTTTTTATGTGAAGATATTTC

                        *    *     **         *    *           **  * *  *    
     277865 CTTTTCCACCATCGGCCACAAAGGACTCCAAATAACCACATGCAGATTCTAGTAACACAGAGTTT
          1 CTTTTCCACCATAGGCCTCAAAGCGCTCCAAATATCCACTTGCAGATTCTACAAAAAGAGTGTTT

                       *         * *           *   * * *    **    ***        
     277930 CAAAACTGCTCTATCAAAAGATAAGTTCAACTCTGAGAGTTTAGTGCAACCATCGTGAAGAAGTT
         66 CAAAACTGCTCAATCAAAAGAAAGGTTCAACTCTGTGAGATGAATGCACACATCACAAAGAAGTT

                            *     *                  
     277995 TCTCAGAATGCTTCTGAGTAGTGTTTATGTGAAGATATTTC
        131 TCTCAGAATGCTTCTGTGTAGTTTTTATGTGAAGATATTTC

                              *     *                      *            *    
     278036 CTTTTCCACCATAGGCCTGAAAGCCCTCCAAATATCCACTTGCAGATCCTACAAAAAGAGAGTTT
          1 CTTTTCCACCATAGGCCTCAAAGCGCTCCAAATATCCACTTGCAGATTCTACAAAAAGAGTGTTT

             *         **     *  *  *   *        **      *                   
     278101 CGAAA-TGCTCTCTCAAACGATAGTTTCGACTCTGTGGTATGAATACACACATCACAAAGAAGTT
         66 CAAAACTGCTCAATCAAAAGAAAGGTTCAACTCTGTGAGATGAATGCACACATCACAAAGAAGTT

                                       **            
     278165 TCTCAGAATGCTTCTGTGTAGTTTTTAAATGAAGATATTTC
        131 TCTCAGAATGCTTCTGTGTAGTTTTTATGTGAAGATATTTC

            *                       *          *     *                       
     278206 TTTTTCCACCATAGGCCTCAAAGCACTCCAAATATGCACTTCCAGATTCTACAAAAAGAGTGTTT
          1 CTTTTCCACCATAGGCCTCAAAGCGCTCCAAATATCCACTTGCAGATTCTACAAAAAGAGTGTTT

              *                 *       * *         **   *         *  * *    
     278271 CAGAACTGCTCAATCAAAAGGAAGGTTCCAGTC--TGAGACAAATACACACATCAAAAGGTAGTT
         66 CAAAACTGCTCAATCAAAAGAAAGGTTCAACTCTGTGAGATGAATGCACACATCACAAAGAAGTT

                                                    *
     278334 TCTCAGAATGCTTCTGTGTAGTTTTTATGTGAAGATATTTT
        131 TCTCAGAATGCTTCTGTGTAGTTTTTATGTGAAGATATTTC

             *               *    **    *     *      *  **   *          *    
     278375 CCTTTCCACCATAGGCCACAAATGGCTCTAAATACCCACTTACATTTTCCACAAAAAGAGAGTTT
          1 CTTTTCCACCATAGGCCTCAAAGCGCTCCAAATATCCACTTGCAGATTCTACAAAAAGAGTGTTT

                       * *    * *      *   *         *                       
     278440 CAAAACTGCTCTACCAAAGGTAA-GTTTAACGCTGTGAG-TTAA-G-A-ACATCACAAAGAAGTT
         66 CAAAACTGCTCAATCAAAAGAAAGGTTCAACTCTGTGAGATGAATGCACACATCACAAAGAAGTT

                                   *   *  *          
     278500 TCTCAGAATGCTTCTGTGTAGTTCTTACGTAAAGATATTTC
        131 TCTCAGAATGCTTCTGTGTAGTTTTTATGTGAAGATATTTC

                 *   *      ***    *                  *              **      
     278541 CTTTTACACAATAGGCAGAAAAGTGCTCCAAATATCCACTTGAAGATTCTACAAAAACCGTGTTT
          1 CTTTTCCACCATAGGCCTCAAAGCGCTCCAAATATCCACTTGCAGATTCTACAAAAAGAGTGTTT

                     **                                          *      *    
     278606 CAAAACTGCCGAATCAAAAGAAAGGTTCAACTCTGTGAGATGAATGCACACATAACAAAGGAGTT
         66 CAAAACTGCTCAATCAAAAGAAAGGTTCAACTCTGTGAGATGAATGCACACATCACAAAGAAGTT

                                 *      *      *    *
     278671 TCTCAGAATGCTTCTGTGTAGCTTTTATATGAAGACATTTA
        131 TCTCAGAATGCTTCTGTGTAGTTTTTATGTGAAGATATTTC

            *        * *            *   ***                     **        *  
     278712 GTTTTCCACAACAGGCCTCAAAGCTCTCTCCATATCCACTTGCAGATTCTACCGAAAGAGTGCTT
          1 CTTTTCCACCATAGGCCTCAAAGCGCTCCAAATATCCACTTGCAGATTCTACAAAAAGAGTGTTT

             *                   * **     *        *        *     **         
     278777 CCAAACTGCTCAATCAAAAGAGACATTCAAATCTGTGAGGTGAATGCAGACATCGTAAAGAAGTT
         66 CAAAACTGCTCAATCAAAAGAAAGGTTCAACTCTGTGAGATGAATGCACACATCACAAAGAAGTT

                                *     *       *    **
     278842 TCTCAGAATGCTTCTGTGTATTTTTTGTGTGAAGTTATTCG
        131 TCTCAGAATGCTTCTGTGTAGTTTTTATGTGAAGATATTTC

            *    *             *     *  *            **                 *    
     278883 TTTTTGCACCATAGGCCTCCAAGCGTTCTAAATATCCACTTCTAGATTCTACAAAAAGAGAGTTT
          1 CTTTTCCACCATAGGCCTCAAAGCGCTCCAAATATCCACTTGCAGATTCTACAAAAAGAGTGTTT

                   *         *            *        *    *   *                
     278948 CAAAACTACTC-A--AACA-AAAGGTTCAATTCTGTGAGTTGAAAGCAAACATCACAAAGAAGTT
         66 CAAAACTGCTCAATCAAAAGAAAGGTTCAACTCTGTGAGATGAATGCACACATCACAAAGAAGTT

                       *                             
     279009 TCTCAGAATGCGTCTGTGTAGTTTTTATGTGAAGATATTTC
        131 TCTCAGAATGCTTCTGTGTAGTTTTTATGTGAAGATATTTC

                     **   ****     *                  *                      
     279050 CTTTT-CACAGTAGAATGCAAAGGGCTCCAAATATCCACTTGGAGATTCTACAAAAAGA--GTTT
          1 CTTTTCCACCATAGGCCTCAAAGCGCTCCAAATATCCACTTGCAGATTCTACAAAAAGAGTGTTT

                  *    **     *  *     *     ***   *     *               *   
     279112 CAAAACCGCTCTGTCAAATGATAGGTTGAACTCCCGGAGGTGAATACACACATCACAAAGAGGTT
         66 CAAAACTGCTCAATCAAAAGAAAGGTTCAACTCTGTGAGATGAATGCACACATCACAAAGAAGTT

                  *                       *  *   *   
     279177 TCTCAGCATGCTTCTGTGTAGTTTTTATGTAAACATACTTC
        131 TCTCAGAATGCTTCTGTGTAGTTTTTATGTGAAGATATTTC

             *    * *              *           *      * *       *   *    *   
     279218 CGTTTCTATCATAGGCCTCAAAGTGCTCCAAATATTCACTTGTACATTCTACCAAACGAGTATTT
          1 CTTTTCCACCATAGGCCTCAAAGCGCTCCAAATATCCACTTGCAGATTCTACAAAAAGAGTGTTT

                              * *         **      *        *            *    
     279283 C-AAACTGCTCAATCAAATGGAAGGTTCAAAACTGTGACATGAATGCCCACATCACAAAGTAGTT
         66 CAAAACTGCTCAATCAAAAGAAAGGTTCAACTCTGTGAGATGAATGCACACATCACAAAGAAGTT

                                                     
     279347 TCTCAGAATGCTTCTGTGTAGTTTTTATGTGAAGATATTTC
        131 TCTCAGAATGCTTCTGTGTAGTTTTTATGTGAAGATATTTC

                     * *    *      *    *       * *   **      *              
     279388 CTTTTCCACAACA-GCGTGCAAAACGCTTCAAATATGCCCTTAGAGATTCCACAAAAAGAGTGTT
          1 CTTTTCCACCATAGGCCT-CAAAGCGCTCCAAATATCCACTTGCAGATTCTACAAAAAGAGTGTT

              *     *               *  *                                  ** 
     279452 TCCAAACTACTCAAATCAAAA-AATGATTTCAACTCTGTGAGATGAATGCACACATCACAAACTA
         65 TCAAAACTGCTC-AATCAAAAGAAAG-GTTCAACTCTGTGAGATGAATGCACACATCACAAAGAA

                         *     ** *   * *   *       *   
     279516 GTTTCTCAGAATGTTTCTGCCTGGTTCTCATGCGAAGATAGTTC
        128 GTTTCTCAGAATGCTTCTGTGTAGTTTTTATGTGAAGATATTTC

                 *           *   * **              *                  * *    
     279560 CTTTTTCACCATAGGCCGCAATGTACTCCAAATATCCACCTGCAGATTCTAC-AAAAGTGAGTTT
          1 CTTTTCCACCATAGGCCTCAAAGCGCTCCAAATATCCACTTGCAGATTCTACAAAAAGAGTGTTT

                       *         ***    **         *        *      *       * 
     279624 CAAAACTGCTCTATCAAAAGATCAGTTCGTCTCTGTGAGTTGAATGCATACATCAAAAAGAAGCT
         66 CAAAACTGCTCAATCAAAAGAAAGGTTCAACTCTGTGAGATGAATGCACACATCACAAAGAAGTT

                 *             *      **         *   
     279689 TCTCAAAATGCTTCTGTGTGGTTTTTCGGTGAAGATAGTTC
        131 TCTCAGAATGCTTCTGTGTAGTTTTTATGTGAAGATATTTC

            *     *        *      * *                 *        *        *    
     279730 TTTTTCTACCATAGGTCTCAAACCACTCCAAATATCCACTTGTAGATTCTATAAAAAGGAATG-T
          1 CTTTTCCACCATAGGCCTCAAAGCGCTCCAAATATCCACTTGCAGATTCTACAAAAA-GAGTGTT

                  *        *    *    *      * *         *      *             
     279794 TCAAAATTGCTCAATAAAAATAAAGTTTCAACACCGTGAGATGAGTGCACAAATCACAAAGAAGT
         65 TCAAAACTGCTCAATCAAAAGAAAGGTTCAACTCTGTGAGATGAATGCACACATCACAAAGAAGT

                  *          *         *          *   
     279859 TTCTCAAAATGCTTCTGGGTAGTTTTTCTGTGAAGATAGTTC
        130 TTCTCAGAATGCTTCTGTGTAGTTTTTATGTGAAGATATTTC

                  *     *    *     *          *                         *    
     279901 CTTTTCTACCATGGGCCACAAAGGGCTCCAAATACCCACTTGCAGATTCTACAAAAAGAGAGTTT
          1 CTTTTCCACCATAGGCCTCAAAGCGCTCCAAATATCCACTTGCAGATTCTACAAAAAGAGTGTTT

              *        *      ** * *        *    * *    **  * *           *  
     279966 CACAACTGCTCTATCAAACAATATGTTCAACTTTGTGGGTTGAACACAAATATCAC-AAGAATTT
         66 CAAAACTGCTCAATCAAAAGAAAGGTTCAACTCTGTGAGATGAATGCACACATCACAAAGAAGTT

                **                             *     
     280030 TCTCCCAATGCTTCTGTGTAGTTTTTATGTGAAGACATTTC
        131 TCTCAGAATGCTTCTGTGTAGTTTTTATGTGAAGATATTTC

            *   *  *      *  *     *                 *  *      *       *  *  
     280071 TTTTCCCTCCATAGTCCACAAAGTGCTCCAAATATCCACTTACATATTCTAGAAAAAGATTGCTT
          1 CTTTTCCACCATAGGCCTCAAAGCGCTCCAAATATCCACTTGCAGATTCTACAAAAAGAGTGTTT

            **       *    *               * * *                              
     280136 GGAAACTGCACAATGAAAAGAAAGGTTCAAATATATGAGATGAATGCACACATCACAAAGAAGTT
         66 CAAAACTGCTCAATCAAAAGAAAGGTTCAACTCTGTGAGATGAATGCACACATCACAAAGAAGTT

                                 *                    
     280201 TCTCAGAAT-CTCTCTGTGTAATTTTTATGTGAAGATATTTC
        131 TCTCAGAATGCT-TCTGTGTAGTTTTTATGTGAAGATATTTC

                *     *    *  *   *                        *      *    *     
     280242 CTTTCCCACCTTAGGTCTTAAAACGCTCCAAATATCCACTTGCAGATACTACAAGAAGATTGTTT
          1 CTTTTCCACCATAGGCCTCAAAGCGCTCCAAATATCCACTTGCAGATTCTACAAAAAGAGTGTTT

                     *    *        *      *     **                           
     280307 CAAAACTGCACAA-AAAAAGAAATGTTCAATTCTGTTTGATGAATGCACACATCACAAAGAAGTT
         66 CAAAACTGCTCAATCAAAAGAAAGGTTCAACTCTGTGAGATGAATGCACACATCACAAAGAAGTT

                           *                         
     280371 TCTCAGAATGCTTCTCTGTAGTTTTTATGTGAAGATATTTC
        131 TCTCAGAATGCTTCTGTGTAGTTTTTATGTGAAGATATTTC

                     *             *                 *         **      * *   
     280412 CTTTTCCACAATAGGCCTCAAAGGGCTCCAAATATCCACTTCCAGATTCTATGAAAAGAATATTT
          1 CTTTTCCACCATAGGCCTCAAAGCGCTCCAAATATCCACTTGCAGATTCTACAAAAAGAGTGTTT

             *              * *    *                                      *  
     280477 CCAAACTGCTCAATCATAGGAAATGTTCAACTCTGTGAGATGAATGCACACATCAC-AAGAAATT
         66 CAAAACTGCTCAATCAAAAGAAAGGTTCAACTCTGTGAGATGAATGCACACATCACAAAGAAGTT

                     *    *      *       *       *   
     280541 TCTCAGAATCCTTCAGTGTAGGTTTTATGAGAAGATAATTC
        131 TCTCAGAATGCTTCTGTGTAGTTTTTATGTGAAGATATTTC

                     *    **        *   *                           *    *   
     280582 CTTTTCCACAATAGTTCTCAAAGCACTCAAAATATCCACTTGCAGATTCTACAAAAGGAGTATTT
          1 CTTTTCCACCATAGGCCTCAAAGCGCTCCAAATATCCACTTGCAGATTCTACAAAAAGAGTGTTT

                                                          *                  
     280647 CAAAACTGCTCAATCAAAAGAAAGGTTCAACTCTGTGAGATGAATGGACACATCACAAAGAAGTT
         66 CAAAACTGCTCAATCAAAAGAAAGGTTCAACTCTGTGAGATGAATGCACACATCACAAAGAAGTT

                                  *   *              
     280712 TCTCAGAATGCTTCTGTGTAGTATTTTTGTGAAGATATTT-
        131 TCTCAGAATGCTTCTGTGTAGTTTTTATGTGAAGATATTTC

                          *  * * * * ***             *            *     * *  
     280752 CTTTTCCACCATAGACCGCCAGGGGACACAAATATCCACTTTCAGATTCTACAACAAGAGAGGTT
          1 CTTTTCCACCATAGGCCTCAAAGCGCTCCAAATATCCACTTGCAGATTCTACAAAAAGAGTGTTT

                   *   *     *   ** *       *      *                      * *
     280817 CAAAACTACTCGATCAAGAGATGGTTTCAACTATGTGAGTTGAATGCACACATCACAAAGAACTA
         66 CAAAACTGCTCAATCAAAAGAAAGGTTCAACTCTGTGAGATGAATGCACACATCACAAAGAAGTT

             *  *    *                               
     280882 TGTCGGAATTCTTCTGTGTAGTTTTTATGTGAAGATATTTC
        131 TCTCAGAATGCTTCTGTGTAGTTTTTATGTGAAGATATTTC

                     *    * *      * *    *                  *               
     280923 CTTTTCCACAATAGACGTCAAAGTGATCCAGATATCCACTTGCAGATTCCACAAAAAGAGTGTTT
          1 CTTTTCCACCATAGGCCTCAAAGCGCTCCAAATATCCACTTGCAGATTCTACAAAAAGAGTGTTT

                 *   *   *                  **                     *  *      
     280988 CAAAAGTGCACAACCAAAAGAAAGGTTCAACTAGGTGAGATGAATGCACACATCAGAAGGAAGTT
         66 CAAAACTGCTCAATCAAAAGAAAGGTTCAACTCTGTGAGATGAATGCACACATCACAAAGAAGTT

                            **   *     * *       *   
     281053 TCTCAGAATGCTTCTGCATAGCTTTTAAGGGAAGATACTTC
        131 TCTCAGAATGCTTCTGTGTAGTTTTTATGTGAAGATATTTC

                    *               *            * *  *    * *               
     281094 CTTTTCCAACATAGGCCTCAAAGCACTCCAAATATCCTCCTGGAGATACCACAAAAAGAGTGTTT
          1 CTTTTCCACCATAGGCCTCAAAGCGCTCCAAATATCCACTTGCAGATTCTACAAAAAGAGTGTTT

            **                      *  *                 *       *           
     281159 GCAAACTGCTCAATCAAAAGAAAGATTTAACTCTGTGAGATGAATCCACACATGACAAAGAAGTT
         66 CAAAACTGCTCAATCAAAAGAAAGGTTCAACTCTGTGAGATGAATGCACACATCACAAAGAAGTT

                                                     
     281224 TCTCAGAATGCTTCTGTGTAGTTTTTATGTGAAGATATTTC
        131 TCTCAGAATGCTTCTGTGTAGTTTTTATGTGAAGATATTTC

                     *   * * *    **           *                *     *      
     281265 CTTTTCCACAATAAGACCCAAAAGGCTCCAAATATTCACTTGCAGATTCTAAAAAAAACAGTGTT
          1 CTTTTCCACCATAGGCCTCAAAGCGCTCCAAATATCCACTTGCAGATTCT-ACAAAAAGAGTGTT

                                  *                  *      *        **      
     281330 TCAAAACTGCTCAATCAAAAGATA-GTTCAACTCTGTGAGAAGAATGCTCACATCACTGAGAAGT
         65 TCAAAACTGCTCAATCAAAAGAAAGGTTCAACTCTGTGAGATGAATGCACACATCACAAAGAAGT

                                         *            
     281394 TTCTCAGAATGCTTCTGTGTAGTTTTTATATGAAGATATTTC
        130 TTCTCAGAATGCTTCTGTGTAGTTTTTATGTGAAGATATTTC

                *     *      *    **                       *   **       *    
     281436 CTTTCCCACCGTAGGCCACAAAAGGCTCCAAATATCCACTTGCAGATACTATGAAAAGAGAGTTT
          1 CTTTTCCACCATAGGCCTCAAAGCGCTCCAAATATCCACTTGCAGATTCTACAAAAAGAGTGTTT

                        *        *               ***            *            
     281501 CAAAACTGCTCATTCAAAAGATAGGTTCAACTCTGTGGTTTGAATGCACACAGCACAAAGAAGTT
         66 CAAAACTGCTCAATCAAAAGAAAGGTTCAACTCTGTGAGATGAATGCACACATCACAAAGAAGTT

              *       **                   **   *    
     281566 TCACAGAATGTGTCTGTGTAGTTTTTATGTGCGGATGTTTC
        131 TCTCAGAATGCTTCTGTGTAGTTTTTATGTGAAGATATTTC

                         *        ****   *   *               *    *          
     281607 CTTTTCCACCATATGCCT-AAATATTTCCCAATTTCCACTTGCAGATTCCACAAGAAGAGTGTTT
          1 CTTTTCCACCATAGGCCTCAAAGCGCTCCAAATATCCACTTGCAGATTCTACAAAAAGAGTGTTT

                      **       *       *           *            *      ***   
     281671 CAAAACTGCTGTATC-AAATAAA-GTTGAACTCTGTGAGGTGAATGCACACAGCACAAAATGGTT
         66 CAAAACTGCTCAATCAAAAGAAAGGTTCAACTCTGTGAGATGAATGCACACATCACAAAGAAGTT

                          **   *        *       *    
     281734 TCTCAGAATGCTTCCTTGTTGTTTTTATATGAAGATGTTTC
        131 TCTCAGAATGCTTCTGTGTAGTTTTTATGTGAAGATATTTC

                  *  *             *   *     *                               
     281775 CTTTTCAACAATAGGCCTCAAAGTGCTTCAAATGTCCACTTGCAGATTCTACAAAAAGAGTGTTT
          1 CTTTTCCACCATAGGCCTCAAAGCGCTCCAAATATCCACTTGCAGATTCTACAAAAAGAGTGTTT

                                        *      *  *  *                       
     281840 CAAAACTGCTCAATCAAAAGAAAGGTTCGACTCTGGGAAATTAATGCACACATCACAAAGAAGTT
         66 CAAAACTGCTCAATCAAAAGAAAGGTTCAACTCTGTGAGATGAATGCACACATCACAAAGAAGTT

                                     *        *      
     281905 TCTC---A-GCTTCTGTGTAGTTTTCATGTGAAGTTATTTC
        131 TCTCAGAATGCTTCTGTGTAGTTTTTATGTGAAGATATTTC

                     *       *     *            *    *         **       *    
     281942 CTTTTCCACAATAGGCCGCAAAGGGCTCCAAATATCAACTTACAGATTCTAGGAAAAGAGAGTTT
          1 CTTTTCCACCATAGGCCTCAAAGCGCTCCAAATATCCACTTGCAGATTCTACAAAAAGAGTGTTT

                       * **      *     *                                     
     282007 CAAAACTGCTCTACGAAAAGATAGGTTGAACTCTGTGAGATGAATGCACACATCACAAAGAAGTT
         66 CAAAACTGCTCAATCAAAAGAAAGGTTCAACTCTGTGAGATGAATGCACACATCACAAAGAAGTT

                       *               * *           
     282072 TCTCAGAATGCATCTGTGTAGTTTTTACGGGAAGATATTTC
        131 TCTCAGAATGCTTCTGTGTAGTTTTTATGTGAAGATATTTC

                        ***  *     **      *  *           *      *     ***   
     282113 CTTTTCCACCATCTTCCACAAAGGTCTCCAAGTAACCACTTGCAGACTCTACAGAAAGACACTTT
          1 CTTTTCCACCATAGGCCTCAAAGCGCTCCAAATATCCACTTGCAGATTCTACAAAAAGAGTGTTT

            *          *         ***      *      ***                      *  
     282178 AAAAACTGCTCTATCAAAAGATCAGTTCAAGTCTGTGGTTTGAATGCACACATCACAAAGAATTT
         66 CAAAACTGCTCAATCAAAAGAAAGGTTCAACTCTGTGAGATGAATGCACACATCACAAAGAAGTT

                                     *  *            
     282243 TCTCAGAATGCTTCTGTGTAGTTTTCATATGAAGATATTTC
        131 TCTCAGAATGCTTCTGTGTAGTTTTTATGTGAAGATATTTC

                                    *                                   **   
     282284 CTTTTCCACCATAGGCCTCAAAGCACTCCAAATATCCACTTGCAGATTCTACAAAAAGAGATTTT
          1 CTTTTCCACCATAGGCCTCAAAGCGCTCCAAATATCCACTTGCAGATTCTACAAAAAGAGTGTTT

                   **                           *  *          *        *     
     282349 CAAAACTAGTCAATCAAAAGAAAGGTTCAACTCTGTCAGTTGAATGCACATATCACAAACAAGTT
         66 CAAAACTGCTCAATCAAAAGAAAGGTTCAACTCTGTGAGATGAATGCACACATCACAAAGAAGTT

                *      *                             
     282414 TCTCGGAATGCGTCTGTGTAGTTTTTATGTGAAGATATTTC
        131 TCTCAGAATGCTTCTGTGTAGTTTTTATGTGAAGATATTTC

               *     * *           *     *                  *    *           
     282455 CTTCTCCACAACAGGCCTCAAAGTGCTCCGAATATCCACTTGCAGATTTTAC-TAAAGAGTGTTT
          1 CTTTTCCACCATAGGCCTCAAAGCGCTCCAAATATCCACTTGCAGATTCTACAAAAAGAGTGTTT

             *               *  *   *     *        *    *               *    
     282519 CCAAACTGCTCAATCAAGAGGAAGTTTCAAGTCTGTGAGCTGAACGCACACATCACAAAGTAGTT
         66 CAAAACTGCTCAATCAAAAGAAAGGTTCAACTCTGTGAGATGAATGCACACATCACAAAGAAGTT

               *                                *    
     282584 TCTGAGAATGCTTCTGTGTAGTTTTTATGTGAAGATGTTTC
        131 TCTCAGAATGCTTCTGTGTAGTTTTTATGTGAAGATATTTC

                            **     *                                    *  * 
     282625 CTTTTCCACCATAGGCTGCAAAGGGCTCCAAATATCCACTTGCAGATTCTACAAAAAGAGAGTCT
          1 CTTTTCCACCATAGGCCTCAAAGCGCTCCAAATATCCACTTGCAGATTCTACAAAAAGAGTGTTT

                 *     *         *          *     *                     *    
     282690 CAAAAGTGCTCTATCAAAAGATAGGTTCAACTATGTGATATGAATGCACACATCACAAAGTAGTT
         66 CAAAACTGCTCAATCAAAAGAAAGGTTCAACTCTGTGAGATGAATGCACACATCACAAAGAAGTT

                                          *          
     282755 TCTCAGAATGCTTCTGTGTAGTTTTTATGTAAAGATATTTC
        131 TCTCAGAATGCTTCTGTGTAGTTTTTATGTGAAGATATTTC

                                    *                                        
     282796 CTTTTCCACCATAGGCCTCAAAGCACTCCAAATATCCACTTGCAGATTCTACAAAAAGAGATG-T
          1 CTTTTCCACCATAGGCCTCAAAGCGCTCCAAATATCCACTTGCAGATTCTACAAAAAGAG-TGTT

                    ** *                   *     *  *    * *   *        *    
     282860 TCAAAACTATTTAATCAAAAGAAAGGTTCAAATCTGTCAGTTGAAGGTACATATCACAAACAAGT
         65 TCAAAACTGCTCAATCAAAAGAAAGGTTCAACTCTGTGAGATGAATGCACACATCACAAAGAAGT

              * **                                    
     282925 TTATTGGAATGCTTCTGTGTAGTTTTTATGTGAAGATATTTC
        130 TTCTCAGAATGCTTCTGTGTAGTTTTTATGTGAAGATATTTC

                     * *         * *                        **  *     *      
     282967 CTTTTCCACAACAGGCCTCAAGGTGCTCCAAATATCCACTTGCAGATTTCACTAAAAGTGTGTTT
          1 CTTTTCCACCATAGGCCTCAAAGCGCTCCAAATATCCACTTGCAGATTCTACAAAAAGAGTGTTT

             *  *            *  *   *     *        *             *           
     283032 CCAAGCTGCTCAATCAAGAGGAAGTTTCAAGTCTGTGAGGTGAATGCACACATTACAAAGAAGTT
         66 CAAAACTGCTCAATCAAAAGAAAGGTTCAACTCTGTGAGATGAATGCACACATCACAAAGAAGTT

            *  *                                     
     283097 ACTGAGAATGCTTCTGTGTAGTTTTTATGTGAAGATATTTC
        131 TCTCAGAATGCTTCTGTGTAGTTTTTATGTGAAGATATTTC

                       *               *                                *    
     283138 CTTTTCCACCACAGGCCTCAAAGCGCTGCAAATATCCACTTGCAGATTCTACAAAAAGAGAGTTT
          1 CTTTTCCACCATAGGCCTCAAAGCGCTCCAAATATCCACTTGCAGATTCTACAAAAAGAGTGTTT

                      **         *   *         *   *     **            *     
     283203 CAAAACTGCTGTATCAAAAGATAGGGTCAACTCTGCGAGTTGAATAAACACATCACAAATAAGTT
         66 CAAAACTGCTCAATCAAAAGAAAGGTTCAACTCTGTGAGATGAATGCACACATCACAAAGAAGTT

               **   *        *               *       
     283268 TCTGGGAACGCTTCTGTATAG-TTTTATGTGAATATATTTC
        131 TCTCAGAATGCTTCTGTGTAGTTTTTATGTGAAGATATTTC

                         *          *                   *   *  *   * *   *   
     283308 CTTTTCCACCATATGCCTCAAAGCACTCCAAATATCCACTTGCACATTATAGAAACATAGTCTTT
          1 CTTTTCCACCATAGGCCTCAAAGCGCTCCAAATATCCACTTGCAGATTCTACAAAAAGAGTGTTT

                   **                        *         *             *       
     283373 CAAAACTTGTCAATC-AAAGAAAGGTTCAACTCCGTGAGATGAGTGCACACATCACAGAGAAGTT
         66 CAAAACTGCTCAATCAAAAGAAAGGTTCAACTCTGTGAGATGAATGCACACATCACAAAGAAGTT

                *     *                            * 
     283437 TCTCGGAATGTTTCTGTGTAGTTTTTATGTGAAGATATTGC
        131 TCTCAGAATGCTTCTGTGTAGTTTTTATGTGAAGATATTTC

                     *               *            *          *       *   *   
     283478 CTTTTCCACAATAGGCCTCAAAGCGTTCCAAATATCCAATTGCAGATTCCACAAAAAAAGTTTTT
          1 CTTTTCCACCATAGGCCTCAAAGCGCTCCAAATATCCACTTGCAGATTCTACAAAAAGAGTGTTT

            *                 *  *  *  *             * *         **    *     
     283543 TAAAACTGCTCAATCAAATGATAGATTAAACTCTGTGAGATTAGTGCACACATGTCAAAAAAGTT
         66 CAAAACTGCTCAATCAAAAGAAAGGTTCAACTCTGTGAGATGAATGCACACATCACAAAGAAGTT

                                *      * *           
     283608 TCTCAGAATGCTTCTGTGTACTTTTTAGGGGAAGATATTTC
        131 TCTCAGAATGCTTCTGTGTAGTTTTTATGTGAAGATATTTC

                        *    *     **         *    *           **  * *  *    
     283649 CTTTTCCACCATCGGCCACAAAGGACTCCAAATAACCACATGCAGATTCTAGTAACACAGAGTTT
          1 CTTTTCCACCATAGGCCTCAAAGCGCTCCAAATATCCACTTGCAGATTCTACAAAAAGAGTGTTT

                       *         * *           *   * * *    **    ***        
     283714 CAAAACTGCTCTATCAAAAGATAAGTTCAACTCTGAGAGTTTAGTGCAACCATCGTGAAGAAGTT
         66 CAAAACTGCTCAATCAAAAGAAAGGTTCAACTCTGTGAGATGAATGCACACATCACAAAGAAGTT

                            *     *                  
     283779 TCTCAGAATGCTTCTGAGTAGTGTTTATGTGAAGATATTTC
        131 TCTCAGAATGCTTCTGTGTAGTTTTTATGTGAAGATATTTC

                              *     *                      *           **    
     283820 CTTTTCCACCATAGGCCTGAAAGCCCTCCAAATATCCACTTGCAGATCCTACAAAAAGAAAGTTT
          1 CTTTTCCACCATAGGCCTCAAAGCGCTCCAAATATCCACTTGCAGATTCTACAAAAAGAGTGTTT

             *         **     *  *  *   *        **      *                   
     283885 CGAAA-TGCTCTCTCAAACGATAGTTTCGACTCTGTGGTATGAATACACACATCACAAAGAAGTT
         66 CAAAACTGCTCAATCAAAAGAAAGGTTCAACTCTGTGAGATGAATGCACACATCACAAAGAAGTT

                                       **            
     283949 TCTCAGAATGCTTCTGTGTAGTTTTTAAATGAAGATATTTC
        131 TCTCAGAATGCTTCTGTGTAGTTTTTATGTGAAGATATTTC

            *                       *          *     *                       
     283990 TTTTTCCACCATAGGCCTCAAAGCACTCCAAATATGCACTTCCAGATTCTACAAAAAGAGTGTTT
          1 CTTTTCCACCATAGGCCTCAAAGCGCTCCAAATATCCACTTGCAGATTCTACAAAAAGAGTGTTT

              *                 *       * *         **   *         *  * *    
     284055 CAGAACTGCTCAATCAAAAGGAAGGTTCCAGTC--TGAGACAAATACACACATCAAAAGGTAGTT
         66 CAAAACTGCTCAATCAAAAGAAAGGTTCAACTCTGTGAGATGAATGCACACATCACAAAGAAGTT

                                                    *
     284118 TCTCAGAATGCTTCTGTGTAGTTTTTATGTGAAGATATTTT
        131 TCTCAGAATGCTTCTGTGTAGTTTTTATGTGAAGATATTTC

             *               *    **    *     *      *  **   *          *    
     284159 CCTTTCCACCATAGGCCACAAATGGCTCTAAATACCCACTTACATTTTCCACAAAAAGAGAGTTT
          1 CTTTTCCACCATAGGCCTCAAAGCGCTCCAAATATCCACTTGCAGATTCTACAAAAAGAGTGTTT

                       * *    * *      *   *         *                       
     284224 CAAAACTGCTCTACCAAAGGTAA-GTTTAACGCTGTGAG-TTAA-G-A-ACATCACAAAGAAGTT
         66 CAAAACTGCTCAATCAAAAGAAAGGTTCAACTCTGTGAGATGAATGCACACATCACAAAGAAGTT

                                   *   *  *          
     284284 TCTCAGAATGCTTCTGTGTAGTTCTTACGTAAAGATATTTC
        131 TCTCAGAATGCTTCTGTGTAGTTTTTATGTGAAGATATTTC

                 *   *      ***    *                  *              **      
     284325 CTTTTACACAATAGGCAGAAAAGTGCTCCAAATATCCACTTGAAGATTCTACAAAAACCGTGTTT
          1 CTTTTCCACCATAGGCCTCAAAGCGCTCCAAATATCCACTTGCAGATTCTACAAAAAGAGTGTTT

                     **                                          *      *    
     284390 CAAAACTGCCGAATCAAAAGAAAGGTTCAACTCTGTGAGATGAATGCACACATAACAAAGGAGTT
         66 CAAAACTGCTCAATCAAAAGAAAGGTTCAACTCTGTGAGATGAATGCACACATCACAAAGAAGTT

                                 *      *      *    *
     284455 TCTCAGAATGCTTCTGTGTAGCTTTTATATGAAGACATTTA
        131 TCTCAGAATGCTTCTGTGTAGTTTTTATGTGAAGATATTTC

            *        * *            *   ***                     **        *  
     284496 GTTTTCCACAACAGGCCTCAAAGCTCTCTCCATATCCACTTGCAGATTCTACCGAAAGAGTGCTT
          1 CTTTTCCACCATAGGCCTCAAAGCGCTCCAAATATCCACTTGCAGATTCTACAAAAAGAGTGTTT

             *                   * **     *        *        *     **         
     284561 CCAAACTGCTCAATCAAAAGAGACATTCAAATCTGTGAGGTGAATGCAGACATCGTAAAGAAGTT
         66 CAAAACTGCTCAATCAAAAGAAAGGTTCAACTCTGTGAGATGAATGCACACATCACAAAGAAGTT

                                *     *       *    **
     284626 TCTCAGAATGCTTCTGTGTATTTTTTGTGTGAAGTTATTCG
        131 TCTCAGAATGCTTCTGTGTAGTTTTTATGTGAAGATATTTC

            *    *             *     *  *            **                 *    
     284667 TTTTTGCACCATAGGCCTCCAAGCGTTCTAAATATCCACTTCTAGATTCTACAAAAAGAGAGTTT
          1 CTTTTCCACCATAGGCCTCAAAGCGCTCCAAATATCCACTTGCAGATTCTACAAAAAGAGTGTTT

                   *         *            *        *    *   *                
     284732 CAAAACTACTC-A--AACA-AAAGGTTCAATTCTGTGAGTTGAAAGCAAACATCACAAAGAAGTT
         66 CAAAACTGCTCAATCAAAAGAAAGGTTCAACTCTGTGAGATGAATGCACACATCACAAAGAAGTT

                       *             *               
     284793 TCTCAGAATGCGTCTGTGTAGTTTTGATGTGAAGATATTTC
        131 TCTCAGAATGCTTCTGTGTAGTTTTTATGTGAAGATATTTC

                     **   ****     *                  *                      
     284834 CTTTT-CACAGTAGAATGCAAAGGGCTCCAAATATCCACTTGGAGATTCTACAAAAAGA--GTTT
          1 CTTTTCCACCATAGGCCTCAAAGCGCTCCAAATATCCACTTGCAGATTCTACAAAAAGAGTGTTT

                  *    **     *  *     *     ***   *     *               *   
     284896 CAAAACCGCTCTGTCAAATGATAGGTTGAACTCCCGGAGGTGAATACACACATCACAAAGAGGTT
         66 CAAAACTGCTCAATCAAAAGAAAGGTTCAACTCTGTGAGATGAATGCACACATCACAAAGAAGTT

                  *                       *  *       
     284961 TCTCAGCATGCTTCTGTGTAGTTTTTATGTAAACATATTTC
        131 TCTCAGAATGCTTCTGTGTAGTTTTTATGTGAAGATATTTC

             *    * *              *           *      * *       *   *    *   
     285002 CGTTTCTATCATAGGCCTCAAAGTGCTCCAAATATTCACTTGTACATTCTACCAAACGAGTATTT
          1 CTTTTCCACCATAGGCCTCAAAGCGCTCCAAATATCCACTTGCAGATTCTACAAAAAGAGTGTTT

                              * *         ** *    *        *            *    
     285067 CAAAACTGCTCAATCAAATGGAAGGTTCAAAACCGTGACATGAATGCCCACATCACAAAGTAGTT
         66 CAAAACTGCTCAATCAAAAGAAAGGTTCAACTCTGTGAGATGAATGCACACATCACAAAGAAGTT

                                                     
     285132 TCTCAGAATGCTTCTGTGTAGTTTTTATGTGAAGATATTTC
        131 TCTCAGAATGCTTCTGTGTAGTTTTTATGTGAAGATATTTC

                     * *    *      *    *       * *   **      *              
     285173 CTTTTCCACAACA-GCGTGCAAAACGCTTCAAATATGCCCTTAGAGATTCCACAAAAAGAGTGTT
          1 CTTTTCCACCATAGGCCT-CAAAGCGCTCCAAATATCCACTTGCAGATTCTACAAAAAGAGTGTT

              *     *               *  *                                  ** 
     285237 TCCAAACTACTCAAATCAAAA-AATGATTTCAACTCTGTGAGATGAATGCACACATCACAAACTA
         65 TCAAAACTGCTC-AATCAAAAGAAAG-GTTCAACTCTGTGAGATGAATGCACACATCACAAAGAA

                         *     ** *   * *   *       *   
     285301 GTTTCTCAGAATGTTTCTGCCTGGTTCTCATGCGAAGATAGTTC
        128 GTTTCTCAGAATGCTTCTGTGTAGTTTTTATGTGAAGATATTTC

                 *           *   * **              *                  * *    
     285345 CTTTTTCACCATAGGCCGCAATGTACTCCAAATATCCACCTGCAGATTCTAC-AAAAGTGAGTTT
          1 CTTTTCCACCATAGGCCTCAAAGCGCTCCAAATATCCACTTGCAGATTCTACAAAAAGAGTGTTT

                       *         ***    **         *        *      *       * 
     285409 CAAAACTGCTCTATCAAAAGATCAGTTCGTCTCTGTGAGTTGAATGCATACATCAAAAAGAAGCT
         66 CAAAACTGCTCAATCAAAAGAAAGGTTCAACTCTGTGAGATGAATGCACACATCACAAAGAAGTT

                 *             *      **         *   
     285474 TCTCAAAATGCTTCTGTGTGGTTTTTCGGTGAAGATAGTTC
        131 TCTCAGAATGCTTCTGTGTAGTTTTTATGTGAAGATATTTC

            *     *        *      * *                 *        *        *    
     285515 TTTTTCTACCATAGGTCTCAAACCACTCCAAATATCCACTTGTAGATTCTATAAAAAGGAATG-T
          1 CTTTTCCACCATAGGCCTCAAAGCGCTCCAAATATCCACTTGCAGATTCTACAAAAA-GAGTGTT

                  *        *    *    *      * *         *      *             
     285579 TCAAAATTGCTCAATAAAAATAAAGTTTCAACACCGTGAGATGAGTGCACAAATCACAAAGAAGT
         65 TCAAAACTGCTCAATCAAAAGAAAGGTTCAACTCTGTGAGATGAATGCACACATCACAAAGAAGT

                  *          *         *          *   
     285644 TTCTCAAAATGCTTCTGGGTAGTTTTTCTGTGAAGATAGTTC
        130 TTCTCAGAATGCTTCTGTGTAGTTTTTATGTGAAGATATTTC

                  *     *    *     *          *                         *    
     285686 CTTTTCTACCATGGGCCACAAAGGGCTCCAAATACCCACTTGCAGATTCTACAAAAAGAGAGTTT
          1 CTTTTCCACCATAGGCCTCAAAGCGCTCCAAATATCCACTTGCAGATTCTACAAAAAGAGTGTTT

              *        *      ** * *        *    * *    **  * *           *  
     285751 CACAACTGCTCTATCAAACAATATGTTCAACTTTGTGGGTTGAACACAAATATCAC-AAGAATTT
         66 CAAAACTGCTCAATCAAAAGAAAGGTTCAACTCTGTGAGATGAATGCACACATCACAAAGAAGTT

                **                             *     
     285815 TCTCCCAATGCTTCTGTGTAGTTTTTATGTGAAGACATTTC
        131 TCTCAGAATGCTTCTGTGTAGTTTTTATGTGAAGATATTTC

            *   *  *      *  *     *                 *  *      *       *  *  
     285856 TTTTCCCTCCATAGTCCACAAAGTGCTCCAAATATCCACTTACATATTCTAGAAAAAGATTGCTT
          1 CTTTTCCACCATAGGCCTCAAAGCGCTCCAAATATCCACTTGCAGATTCTACAAAAAGAGTGTTT

            **       *    *               * * *                              
     285921 GGAAACTGCACAATGAAAAGAAAGGTTCAAATATATGAGATGAATGCACACATCACAAAGAAGTT
         66 CAAAACTGCTCAATCAAAAGAAAGGTTCAACTCTGTGAGATGAATGCACACATCACAAAGAAGTT

                                 *                    
     285986 TCTCAGAAT-CTCTCTGTGTAATTTTTATGTGAAGATATTTC
        131 TCTCAGAATGCT-TCTGTGTAGTTTTTATGTGAAGATATTTC

                *     *    *  *   *                        *      *    *     
     286027 CTTTCCCACCTTAGGTCTTAAAACGCTCCAAATATCCACTTGCAGATACTACAAGAAGATTGTTT
          1 CTTTTCCACCATAGGCCTCAAAGCGCTCCAAATATCCACTTGCAGATTCTACAAAAAGAGTGTTT

                     *    *        *      *     **                           
     286092 CAAAACTGCACAA-AAAAAGAAATGTTCAATTCTGTTTGATGAATGCACACATCACAAAGAAGTT
         66 CAAAACTGCTCAATCAAAAGAAAGGTTCAACTCTGTGAGATGAATGCACACATCACAAAGAAGTT

                           *                         
     286156 TCTCAGAATGCTTCTCTGTAGTTTTTATGTGAAGATATTTC
        131 TCTCAGAATGCTTCTGTGTAGTTTTTATGTGAAGATATTTC

                     *             *                 *         **      * *   
     286197 CTTTTCCACAATAGGCCTCAAAGGGCTCCAAATATCCACTTCCAGATTCTATGAAAAGAATATTT
          1 CTTTTCCACCATAGGCCTCAAAGCGCTCCAAATATCCACTTGCAGATTCTACAAAAAGAGTGTTT

             *              * *    *                                      *  
     286262 CCAAACTGCTCAATCATAGGAAATGTTCAACTCTGTGAGATGAATGCACACATCAC-AAGAAATT
         66 CAAAACTGCTCAATCAAAAGAAAGGTTCAACTCTGTGAGATGAATGCACACATCACAAAGAAGTT

                     *    *      *       *       *   
     286326 TCTCAGAATCCTTCAGTGTAGGTTTTATGAGAAGATAATTC
        131 TCTCAGAATGCTTCTGTGTAGTTTTTATGTGAAGATATTTC

                     *    **        *   *                           *    *   
     286367 CTTTTCCACAATAGTTCTCAAAGCACTCAAAATATCCACTTGCAGATTCTACAAAAGGAGTATTT
          1 CTTTTCCACCATAGGCCTCAAAGCGCTCCAAATATCCACTTGCAGATTCTACAAAAAGAGTGTTT

                                    *                     *                  
     286432 CAAAACTGCTCAATCAAAAGAAAGTTTCAACTCTGTGAGATGAATGGACACATCACAAAGAAGTT
         66 CAAAACTGCTCAATCAAAAGAAAGGTTCAACTCTGTGAGATGAATGCACACATCACAAAGAAGTT

                                  *   *              
     286497 TCTCAGAATGCTTCTGTGTAGTATTTTTGTGAAGATATTT-
        131 TCTCAGAATGCTTCTGTGTAGTTTTTATGTGAAGATATTTC

                          *  * * * * ***             *            *     * *  
     286537 CTTTTCCACCATAGACCGCCAGGGGACACAAATATCCACTTTCAGATTCTACAACAAGAGAGGTT
          1 CTTTTCCACCATAGGCCTCAAAGCGCTCCAAATATCCACTTGCAGATTCTACAAAAAGAGTGTTT

                   *   *     *   ** *       *      *                      * *
     286602 CAAAACTACTCGATCAAGAGATGGTTTCAACTATGTGAGTTGAATGCACACATCACAAAGAACTA
         66 CAAAACTGCTCAATCAAAAGAAAGGTTCAACTCTGTGAGATGAATGCACACATCACAAAGAAGTT

             *  *    *                               
     286667 TGTCGGAATTCTTCTGTGTAGTTTTTATGTGAAGATATTTC
        131 TCTCAGAATGCTTCTGTGTAGTTTTTATGTGAAGATATTTC

                     *    * *      * *    *                  *               
     286708 CTTTTCCACAATAGACGTCAAAGTGATCCAGATATCCACTTGCAGATTCCACAAAAAGAGTGTTT
          1 CTTTTCCACCATAGGCCTCAAAGCGCTCCAAATATCCACTTGCAGATTCTACAAAAAGAGTGTTT

                 *   *   *                  **                     *  *      
     286773 CAAAAGTGCACAACCAAAAGAAAGGTTCAACTAGGTGAGATGAATGCACACATCAGAAGGAAGTT
         66 CAAAACTGCTCAATCAAAAGAAAGGTTCAACTCTGTGAGATGAATGCACACATCACAAAGAAGTT

                            **   *     * *       *   
     286838 TCTCAGAATGCTTCTGCATAGCTTTTAAGGGAAGATACTTC
        131 TCTCAGAATGCTTCTGTGTAGTTTTTATGTGAAGATATTTC

                    *               *            * *  *    * *               
     286879 CTTTTCCAACATAGGCCTCAAAGCACTCCAAATATCCTCCTGGAGATACCACAAAAAGAGTGTTT
          1 CTTTTCCACCATAGGCCTCAAAGCGCTCCAAATATCCACTTGCAGATTCTACAAAAAGAGTGTTT

            **                      *  *                 *       *           
     286944 GCAAACTGCTCAATCAAAAGAAAGATTTAACTCTGTGAGATGAATCCACACATGACAAAGAAGTT
         66 CAAAACTGCTCAATCAAAAGAAAGGTTCAACTCTGTGAGATGAATGCACACATCACAAAGAAGTT

                                                     
     287009 TCTCAGAATGCTTCTGTGTAGTTTTTATGTGAAGATATTTC
        131 TCTCAGAATGCTTCTGTGTAGTTTTTATGTGAAGATATTTC

                     *   * * *    **           *                *     *      
     287050 CTTTTCCACAATAAGACCCAAAAGGCTCCAAATATTCACTTGCAGATTCTAAAAAAAACAGTGTT
          1 CTTTTCCACCATAGGCCTCAAAGCGCTCCAAATATCCACTTGCAGATTCT-ACAAAAAGAGTGTT

                                  *                  *      *        **      
     287115 TCAAAACTGCTCAATCAAAAGATA-GTTCAACTCTGTGAGAAGAATGCTCACATCACTGAGAAGT
         65 TCAAAACTGCTCAATCAAAAGAAAGGTTCAACTCTGTGAGATGAATGCACACATCACAAAGAAGT

                                         *            
     287179 TTCTCAGAATGCTTCTGTGTAGTTTTTATATGAAGATATTTC
        130 TTCTCAGAATGCTTCTGTGTAGTTTTTATGTGAAGATATTTC

                *     *      *    **                       *   **       *    
     287221 CTTTCCCACCGTAGGCCACAAAAGGCTCCAAATATCCACTTGCAGATACTATGAAAAGAGAGTTT
          1 CTTTTCCACCATAGGCCTCAAAGCGCTCCAAATATCCACTTGCAGATTCTACAAAAAGAGTGTTT

                        *        *               ***            *            
     287286 CAAAACTGCTCATTCAAAAGATAGGTTCAACTCTGTGGTTTGAATGCACACAGCACAAAGAAGTT
         66 CAAAACTGCTCAATCAAAAGAAAGGTTCAACTCTGTGAGATGAATGCACACATCACAAAGAAGTT

              *       **                   **   *    
     287351 TCACAGAATGTGTCTGTGTAGTTTTTATGTGCGGATGTTTC
        131 TCTCAGAATGCTTCTGTGTAGTTTTTATGTGAAGATATTTC

                         *        ****   *   *               *    *          
     287392 CTTTTCCACCATATGCCT-AAATATTTCCCAATTTCCACTTGCAGATTCCACAAGAAGAGTGTTT
          1 CTTTTCCACCATAGGCCTCAAAGCGCTCCAAATATCCACTTGCAGATTCTACAAAAAGAGTGTTT

                      **       *       *           *            *      ***   
     287456 CAAAACTGCTGTATC-AAATAAA-GTTGAACTCTGTGAGGTGAATGCACACAGCACAAAATGGTT
         66 CAAAACTGCTCAATCAAAAGAAAGGTTCAACTCTGTGAGATGAATGCACACATCACAAAGAAGTT

                          **   *        *       *    
     287519 TCTCAGAATGCTTCCTTGTTGTTTTTATATGAAGATGTTTC
        131 TCTCAGAATGCTTCTGTGTAGTTTTTATGTGAAGATATTTC

                  *  *             *   *     *                               
     287560 CTTTTCAACAATAGGCCTCAAAGTGCTTCAAATGTCCACTTGCAGATTCTACAAAAAGAGTGTTT
          1 CTTTTCCACCATAGGCCTCAAAGCGCTCCAAATATCCACTTGCAGATTCTACAAAAAGAGTGTTT

                                        *      *  *  *                       
     287625 CAAAACTGCTCAATCAAAAGAAAGGTTCGACTCTGGGAAATTAATGCACACATCACAAAGAAGTT
         66 CAAAACTGCTCAATCAAAAGAAAGGTTCAACTCTGTGAGATGAATGCACACATCACAAAGAAGTT

                                     *        *      
     287690 TCTC---A-GCTTCTGTGTAGTTTTCATGTGAAGTTATTTC
        131 TCTCAGAATGCTTCTGTGTAGTTTTTATGTGAAGATATTTC

                     *       *     *            *    *         **       *    
     287727 CTTTTCCACAATAGGCCGCAAAGGGCTCCAAATATCAACTTACAGATTCTAGGAAAAGAGAGTTT
          1 CTTTTCCACCATAGGCCTCAAAGCGCTCCAAATATCCACTTGCAGATTCTACAAAAAGAGTGTTT

                       * **      *     *                                     
     287792 CAAAACTGCTCTACGAAAAGATAGGTTGAACTCTGTGAGATGAATGCACACATCACAAAGAAGTT
         66 CAAAACTGCTCAATCAAAAGAAAGGTTCAACTCTGTGAGATGAATGCACACATCACAAAGAAGTT

                       *               * *     *     
     287857 TCTCAGAATGCATCTGTGTAGTTTTTACGGGAAGACATTTC
        131 TCTCAGAATGCTTCTGTGTAGTTTTTATGTGAAGATATTTC

                        ***  *     **      *  *                  *     ***   
     287898 CTTTTCCACCATCTTCCACAAAGGTCTCCAAGTAACCACTTGCAGATTCTACAGAAAGACACTTT
          1 CTTTTCCACCATAGGCCTCAAAGCGCTCCAAATATCCACTTGCAGATTCTACAAAAAGAGTGTTT

            *          *         ***      *      ***                      *  
     287963 AAAAACTGCTCTATCAAAAGATCAGTTCAAGTCTGTGGTTTGAATGCACACATCACAAAGAATTT
         66 CAAAACTGCTCAATCAAAAGAAAGGTTCAACTCTGTGAGATGAATGCACACATCACAAAGAAGTT

                                     *  *            
     288028 TCTCAGAATGCTTCTGTGTAGTTTTCATATGAAGATATTTC
        131 TCTCAGAATGCTTCTGTGTAGTTTTTATGTGAAGATATTTC

                                    *                                   **   
     288069 CTTTTCCACCATAGGCCTCAAAGCACTCCAAATATCCACTTGCAGATTCTACAAAAAGAGATTTT
          1 CTTTTCCACCATAGGCCTCAAAGCGCTCCAAATATCCACTTGCAGATTCTACAAAAAGAGTGTTT

                   **                           *  *          *        *     
     288134 CAAAACTAGTCAATCAAAAGAAAGGTTCAACTCTGTCAGTTGAATGCACATATCACAAACAAGTT
         66 CAAAACTGCTCAATCAAAAGAAAGGTTCAACTCTGTGAGATGAATGCACACATCACAAAGAAGTT

                *      *                             
     288199 TCTCGGAATGCGTCTGTGTAGTTTTTATGTGAAGATATTTC
        131 TCTCAGAATGCTTCTGTGTAGTTTTTATGTGAAGATATTTC

               *     * *           *     *                  *    *           
     288240 CTTCTCCACAACAGGCCTCAAAGTGCTCCGAATATCCACTTGCAGATTTTAC-TAAAGAGTGTTT
          1 CTTTTCCACCATAGGCCTCAAAGCGCTCCAAATATCCACTTGCAGATTCTACAAAAAGAGTGTTT

             *               *  *   *     *        *    *               *    
     288304 CCAAACTGCTCAATCAAGAGGAAGTTTCAAGTCTGTGAGCTGAACGCACACATCACAAAGTAGTT
         66 CAAAACTGCTCAATCAAAAGAAAGGTTCAACTCTGTGAGATGAATGCACACATCACAAAGAAGTT

               *                                *    
     288369 TCTGAGAATGCTTCTGTGTAGTTTTTATGTGAAGATGTTTC
        131 TCTCAGAATGCTTCTGTGTAGTTTTTATGTGAAGATATTTC

                            **     *                                    *    
     288410 CTTTTCCACCATAGGCTGCAAAGGGCTCCAAATATCCACTTGCAGATTCTACAAAAAGAGAGTTT
          1 CTTTTCCACCATAGGCCTCAAAGCGCTCCAAATATCCACTTGCAGATTCTACAAAAAGAGTGTTT

                 *     *         *          *     *                     *    
     288475 CAAAAGTGCTCTATCAAAAGATAGGTTCAACTATGTGATATGAATGCACACATCACAAAGTAGTT
         66 CAAAACTGCTCAATCAAAAGAAAGGTTCAACTCTGTGAGATGAATGCACACATCACAAAGAAGTT

                                          *          
     288540 TCTCAGAATGCTTCTGTGTAGTTTTTATGTAAAGATATTTC
        131 TCTCAGAATGCTTCTGTGTAGTTTTTATGTGAAGATATTTC

                                    *                                   **   
     288581 CTTTTCCACCATAGGCCTCAAAGCACTCCAAATATCCACTTGCAGATTCTACAAAAAGAGATTTT
          1 CTTTTCCACCATAGGCCTCAAAGCGCTCCAAATATCCACTTGCAGATTCTACAAAAAGAGTGTTT

                   ** *                   *     *  *    * *   *        *     
     288646 CAAAACTATTTAATCAAAAGAAAGGTTCAAATCTGTCAGTTGAAGGTACATATCACAAACAAGTT
         66 CAAAACTGCTCAATCAAAAGAAAGGTTCAACTCTGTGAGATGAATGCACACATCACAAAGAAGTT

             * **                                    
     288711 TATTGGAATGCTTCTGTGTAGTTTTTATGTGAAGATATTTC
        131 TCTCAGAATGCTTCTGTGTAGTTTTTATGTGAAGATATTTC

                     * *       * * *                        **  *     *      
     288752 CTTTTCCACAACAGGCCTCCAGGTGCTCCAAATATCCACTTGCAGATTTCACTAAAAGTGTGTTT
          1 CTTTTCCACCATAGGCCTCAAAGCGCTCCAAATATCCACTTGCAGATTCTACAAAAAGAGTGTTT

             *  *            *  *   *     *        *             *           
     288817 CCAAGCTGCTCAATCAAGAGGAAGTTTCAAGTCTGTGAGGTGAATGCACACATTACAAAGAAGTT
         66 CAAAACTGCTCAATCAAAAGAAAGGTTCAACTCTGTGAGATGAATGCACACATCACAAAGAAGTT

            *  *                                     
     288882 ACTGAGAATGCTTCTGTGTAGTTTTTATGTGAAGATATTTC
        131 TCTCAGAATGCTTCTGTGTAGTTTTTATGTGAAGATATTTC

                      **               *                                *    
     288923 CTTTTCCACCGCAGGCCTCAAAGCGCTGCAAATATCCACTTGCAGATTCTACAAAAAGAGAGTTT
          1 CTTTTCCACCATAGGCCTCAAAGCGCTCCAAATATCCACTTGCAGATTCTACAAAAAGAGTGTTT

                      **         *   *         *   *     **            *     
     288988 CAAAACTGCTGTATCAAAAGATAGGGTCAACTCTGCGAGTTGAATAAACACATCACAAATAAGTT
         66 CAAAACTGCTCAATCAAAAGAAAGGTTCAACTCTGTGAGATGAATGCACACATCACAAAGAAGTT

               **   *        *               *       
     289053 TCTGGGAACGCTTCTGTATAG-TTTTATGTGAATATATTTC
        131 TCTCAGAATGCTTCTGTGTAGTTTTTATGTGAAGATATTTC

                         *          *                   *   *  *   * *   *   
     289093 CTTTTCCACCATATGCCTCAAAGCACTCCAAATATCCACTTGCACATTATAGAAACATAGTCTTT
          1 CTTTTCCACCATAGGCCTCAAAGCGCTCCAAATATCCACTTGCAGATTCTACAAAAAGAGTGTTT

                   **                        *         *             *       
     289158 CAAAACTTGTCAATC-AAAGAAAGGTTCAACTCCGTGAGATGAGTGCACACATCACAGAGAAGTT
         66 CAAAACTGCTCAATCAAAAGAAAGGTTCAACTCTGTGAGATGAATGCACACATCACAAAGAAGTT

                *     *                            * 
     289222 TCTCGGAATGTTTCTGTGTAGTTTTTATGTGAAGATATTGC
        131 TCTCAGAATGCTTCTGTGTAGTTTTTATGTGAAGATATTTC

                     *               *            *          *       *   *   
     289263 CTTTTCCACAATAGGCCTCAAAGCGTTCCAAATATCCAATTGCAGATTCCACAAAAAAAGTTTTT
          1 CTTTTCCACCATAGGCCTCAAAGCGCTCCAAATATCCACTTGCAGATTCTACAAAAAGAGTGTTT

            *                 *  *  *  *             * *         **    *     
     289328 TAAAACTGCTCAATCAAATGATAGATTAAACTCTGTGAGATTAGTGCACACATGTCAAAAAAGTT
         66 CAAAACTGCTCAATCAAAAGAAAGGTTCAACTCTGTGAGATGAATGCACACATCACAAAGAAGTT

                                *      * *           
     289393 TCTCAGAATGCTTCTGTGTACTTTTTAGGGGAAGATATTTC
        131 TCTCAGAATGCTTCTGTGTAGTTTTTATGTGAAGATATTTC

                        *    *     **         *    *           **  * *  *    
     289434 CTTTTCCACCATCGGCCACAAAGGACTCCAAATAACCACATGCAGATTCTAGTAACACAGAGTTT
          1 CTTTTCCACCATAGGCCTCAAAGCGCTCCAAATATCCACTTGCAGATTCTACAAAAAGAGTGTTT

                       *         * *           *   * * *    **    ***        
     289499 CAAAACTGCTCTATCAAAAGATAAGTTCAACTCTGAGAGTTTAGTGCAACCATCGTGAAGAAGTT
         66 CAAAACTGCTCAATCAAAAGAAAGGTTCAACTCTGTGAGATGAATGCACACATCACAAAGAAGTT

                            *     *                  
     289564 TCTCAGAATGCTTCTGAGTAGTGTTTATGTGAAGATATTTC
        131 TCTCAGAATGCTTCTGTGTAGTTTTTATGTGAAGATATTTC

                              *     *                      *           **    
     289605 CTTTTCCACCATAGGCCTGAAAGCCCTCCAAATATCCACTTGCAGATCCTACAAAAAGAAAGTTT
          1 CTTTTCCACCATAGGCCTCAAAGCGCTCCAAATATCCACTTGCAGATTCTACAAAAAGAGTGTTT

             *         **     *  *  *   *        **      *                   
     289670 CGAAA-TGCTCTCTCAAACGATAGTTTCGACTCTGTGGTATGAATACACACATCACAAAGAAGTT
         66 CAAAACTGCTCAATCAAAAGAAAGGTTCAACTCTGTGAGATGAATGCACACATCACAAAGAAGTT

                                       **            
     289734 TCTCAGAATGCTTCTGTGTAGTTTTTAAATGAAGATATTTC
        131 TCTCAGAATGCTTCTGTGTAGTTTTTATGTGAAGATATTTC

            *                       *          *     *                       
     289775 TTTTTCCACCATAGGCCTCAAAGCACTCCAAATATGCACTTCCAGATTCTACAAAAAGAGTGTTT
          1 CTTTTCCACCATAGGCCTCAAAGCGCTCCAAATATCCACTTGCAGATTCTACAAAAAGAGTGTTT

              *                 *       * *         **   *         *  * *    
     289840 CAGAACTGCTCAATCAAAAGGAAGGTTCCAGTC--TGAGACAAATACACACATCAAAAGGTAGTT
         66 CAAAACTGCTCAATCAAAAGAAAGGTTCAACTCTGTGAGATGAATGCACACATCACAAAGAAGTT

                                                    *
     289903 TCTCAGAATGCTTCTGTGTAGTTTTTATGTGAAGATATTTT
        131 TCTCAGAATGCTTCTGTGTAGTTTTTATGTGAAGATATTTC

             *               *    **    *     *      *  **   *          *    
     289944 CCTTTCCACCATAGGCCACAAATGGCTCTAAATACCCACTTACATTTTCCACAAAAAGAGAGTTT
          1 CTTTTCCACCATAGGCCTCAAAGCGCTCCAAATATCCACTTGCAGATTCTACAAAAAGAGTGTTT

                       * *    * *      *   *         *                       
     290009 CAAAACTGCTCTACCAAAGGTAA-GTTTAACGCTGTGAG-TTAA-G-A-ACATCACAAAGAAGTT
         66 CAAAACTGCTCAATCAAAAGAAAGGTTCAACTCTGTGAGATGAATGCACACATCACAAAGAAGTT

                                   *   *  *          
     290069 TCTCAGAATGCTTCTGTGTAGTTCTTACGTAAAGATATTTC
        131 TCTCAGAATGCTTCTGTGTAGTTTTTATGTGAAGATATTTC

                 *   *      ***    *                  *              **      
     290110 CTTTTACACAATAGGCAGAAAAGTGCTCCAAATATCCACTTGAAGATTCTACAAAAACCGTGTTT
          1 CTTTTCCACCATAGGCCTCAAAGCGCTCCAAATATCCACTTGCAGATTCTACAAAAAGAGTGTTT

                     **                                          *      *    
     290175 CAAAACTGCCGAATCAAAAGAAAGGTTCAACTCTGTGAGATGAATGCACACATAACAAAGGAGTT
         66 CAAAACTGCTCAATCAAAAGAAAGGTTCAACTCTGTGAGATGAATGCACACATCACAAAGAAGTT

                                 *      *      *    *
     290240 TCTCAGAATGCTTCTGTGTAGCTTTTATATGAAGACATTTA
        131 TCTCAGAATGCTTCTGTGTAGTTTTTATGTGAAGATATTTC

            *        * *            *   ***                     **        *  
     290281 GTTTTCCACAACAGGCCTCAAAGCTCTCTCCATATCCACTTGCAGATTCTACCGAAAGAGTGCTT
          1 CTTTTCCACCATAGGCCTCAAAGCGCTCCAAATATCCACTTGCAGATTCTACAAAAAGAGTGTTT

             *                   * **     *        *        *     **         
     290346 CCAAACTGCTCAATCAAAAGAGACATTCAAATCTGTGAGGTGAATGCAGACATCGTAAAGAAGTT
         66 CAAAACTGCTCAATCAAAAGAAAGGTTCAACTCTGTGAGATGAATGCACACATCACAAAGAAGTT

                                *     *       *    **
     290411 TCTCAGAATGCTTCTGTGTATTTTTTGTGTGAAGTTATTCG
        131 TCTCAGAATGCTTCTGTGTAGTTTTTATGTGAAGATATTTC

            *    *             *     *  *            **                 *    
     290452 TTTTTGCACCATAGGCCTCCAAGCGTTCTAAATATCCACTTCTAGATTCTACAAAAAGAGAGTTT
          1 CTTTTCCACCATAGGCCTCAAAGCGCTCCAAATATCCACTTGCAGATTCTACAAAAAGAGTGTTT

                   *         *            *        *    *   *                
     290517 CAAAACTACTC-A--AACA-AAAGGTTCAATTCTGTGAGTTGAAAGCAAACATCACAAAGAAGTT
         66 CAAAACTGCTCAATCAAAAGAAAGGTTCAACTCTGTGAGATGAATGCACACATCACAAAGAAGTT

                       *                             
     290578 TCTCAGAATGCGTCTGTGTAGTTTTTATGTGAAGATATTTC
        131 TCTCAGAATGCTTCTGTGTAGTTTTTATGTGAAGATATTTC

                     **   ****     *                  *                      
     290619 CTTTT-CACAGTAGAATGCAAAGGGCTCCAAATATCCACTTGGAGATTCTACAAAAAGA--GTTT
          1 CTTTTCCACCATAGGCCTCAAAGCGCTCCAAATATCCACTTGCAGATTCTACAAAAAGAGTGTTT

                  *    **     *  *     *     ***   *     *               *   
     290681 CAAAACCGCTCTGTCAAATGATAGGTTGAACTCCCGGAGGTGAATACACACATCACAAAGAGGTT
         66 CAAAACTGCTCAATCAAAAGAAAGGTTCAACTCTGTGAGATGAATGCACACATCACAAAGAAGTT

                  *                       *  *       
     290746 TCTCAGCATGCTTCTGTGTAGTTTTTATGTAAACATATTTC
        131 TCTCAGAATGCTTCTGTGTAGTTTTTATGTGAAGATATTTC

             *    * *              *           *      * *       *   *    *   
     290787 CGTTTCTATCATAGGCCTCAAAGTGCTCCAAATATTCACTTGTACATTCTACCAAACGAGTATTT
          1 CTTTTCCACCATAGGCCTCAAAGCGCTCCAAATATCCACTTGCAGATTCTACAAAAAGAGTGTTT

                              * *         ** *    *        *            *    
     290852 CAAAACTGCTCAATCAAATGGAAGGTTCAAAACCGTGACATGAATGCCCACATCACAAAGTAGTT
         66 CAAAACTGCTCAATCAAAAGAAAGGTTCAACTCTGTGAGATGAATGCACACATCACAAAGAAGTT

                                                     
     290917 TCTCAGAATGCTTCTGTGTAGTTTTTATGTGAAGATATTTC
        131 TCTCAGAATGCTTCTGTGTAGTTTTTATGTGAAGATATTTC

                     * *    *      *    *       * *   **      *              
     290958 CTTTTCCACAACA-GCGTGCAAAACGCTTCAAATATGCCCTTAGAGATTCCACAAAAAGAGTGTT
          1 CTTTTCCACCATAGGCCT-CAAAGCGCTCCAAATATCCACTTGCAGATTCTACAAAAAGAGTGTT

              *     *               *  *                                  ** 
     291022 TCCAAACTACTCAAATCAAAA-AATGATTTCAACTCTGTGAGATGAATGCACACATCACAAACTA
         65 TCAAAACTGCTC-AATCAAAAGAAAG-GTTCAACTCTGTGAGATGAATGCACACATCACAAAGAA

                         *     ** *   * *   *       *   
     291086 GTTTCTCAGAATGTTTCTGCCTGGTTCTCATGCGAAGATAGTTC
        128 GTTTCTCAGAATGCTTCTGTGTAGTTTTTATGTGAAGATATTTC

                 *           *   * **              *                  * *    
     291130 CTTTTTCACCATAGGCCGCAATGTACTCCAAATATCCACCTGCAGATTCTAC-AAAAGTGAGTTT
          1 CTTTTCCACCATAGGCCTCAAAGCGCTCCAAATATCCACTTGCAGATTCTACAAAAAGAGTGTTT

                       *         ***    **         *        *      *       * 
     291194 CAAAACTGCTCTATCAAAAGATCAGTTCGTCTCTGTGAGTTGAATGCATACATCAAAAAGAAGCT
         66 CAAAACTGCTCAATCAAAAGAAAGGTTCAACTCTGTGAGATGAATGCACACATCACAAAGAAGTT

                 *             *      **         *   
     291259 TCTCAAAATGCTTCTGTGTGGTTTTTCGGTGAAGATAGTTC
        131 TCTCAGAATGCTTCTGTGTAGTTTTTATGTGAAGATATTTC

            *     *        *      * *                 *        *        *    
     291300 TTTTTCTACCATAGGTCTCAAACCACTCCAAATATCCACTTGTAGATTCTATAAAAAGGAATG-T
          1 CTTTTCCACCATAGGCCTCAAAGCGCTCCAAATATCCACTTGCAGATTCTACAAAAA-GAGTGTT

                  *        *    *    *      * *         *      *             
     291364 TCAAAATTGCTCAATAAAAATAAAGTTTCAACACCGTGAGATGAGTGCACAAATCACAAAGAAGT
         65 TCAAAACTGCTCAATCAAAAGAAAGGTTCAACTCTGTGAGATGAATGCACACATCACAAAGAAGT

                  *          *         *          *   
     291429 TTCTCAAAATGCTTCTGGGTAGTTTTTCTGTGAAGATAGTTC
        130 TTCTCAGAATGCTTCTGTGTAGTTTTTATGTGAAGATATTTC

                  *     *    *     *          *                         *    
     291471 CTTTTCTACCATGGGCCACAAAGGGCTCCAAATACCCACTTGCAGATTCTACAAAAAGAGAGTTT
          1 CTTTTCCACCATAGGCCTCAAAGCGCTCCAAATATCCACTTGCAGATTCTACAAAAAGAGTGTTT

              *        *      ** * *        *    * *    **  * *           *  
     291536 CACAACTGCTCTATCAAACAATATGTTCAACTTTGTGGGTTGAACACAAATATCAC-AAGAATTT
         66 CAAAACTGCTCAATCAAAAGAAAGGTTCAACTCTGTGAGATGAATGCACACATCACAAAGAAGTT

                **                             *     
     291600 TCTCCCAATGCTTCTGTGTAGTTTTTATGTGAAGACATTTC
        131 TCTCAGAATGCTTCTGTGTAGTTTTTATGTGAAGATATTTC

            *   *  *      *  *     *                 *  *      *       *  *  
     291641 TTTTCCCTCCATAGTCCACAAAGTGCTCCAAATATCCACTTACATATTCTAGAAAAAGATTGCTT
          1 CTTTTCCACCATAGGCCTCAAAGCGCTCCAAATATCCACTTGCAGATTCTACAAAAAGAGTGTTT

            **       *    *               * * *                              
     291706 GGAAACTGCACAATGAAAAGAAAGGTTCAAATATATGAGATGAATGCACACATCACAAAGAAGTT
         66 CAAAACTGCTCAATCAAAAGAAAGGTTCAACTCTGTGAGATGAATGCACACATCACAAAGAAGTT

                                 *                    
     291771 TCTCAGAAT-CTCTCTGTGTAATTTTTATGTGAAGATATTTC
        131 TCTCAGAATGCT-TCTGTGTAGTTTTTATGTGAAGATATTTC

                *     *    *  *   *                        *      *    *     
     291812 CTTTCCCACCTTAGGTCTTAAAACGCTCCAAATATCCACTTGCAGATACTACAAGAAGATTGTTT
          1 CTTTTCCACCATAGGCCTCAAAGCGCTCCAAATATCCACTTGCAGATTCTACAAAAAGAGTGTTT

                     *    *        *      *     **                           
     291877 CAAAACTGCACAA-AAAAAGAAATGTTCAATTCTGTTTGATGAATGCACACATCACAAAGAAGTT
         66 CAAAACTGCTCAATCAAAAGAAAGGTTCAACTCTGTGAGATGAATGCACACATCACAAAGAAGTT

                           *                         
     291941 TCTCAGAATGCTTCTCTGTAGTTTTTATGTGAAGATATTTC
        131 TCTCAGAATGCTTCTGTGTAGTTTTTATGTGAAGATATTTC

                     *             *                 *         **      * *   
     291982 CTTTTCCACAATAGGCCTCAAAGGGCTCCAAATATCCACTTCCAGATTCTATGAAAAGAATATTT
          1 CTTTTCCACCATAGGCCTCAAAGCGCTCCAAATATCCACTTGCAGATTCTACAAAAAGAGTGTTT

             *              * *    *                                      *  
     292047 CCAAACTGCTCAATCATAGGAAATGTTCAACTCTGTGAGATGAATGCACACATCAC-AAGAAATT
         66 CAAAACTGCTCAATCAAAAGAAAGGTTCAACTCTGTGAGATGAATGCACACATCACAAAGAAGTT

                     *    *      *       *       *   
     292111 TCTCAGAATCCTTCAGTGTAGGTTTTATGAGAAGATAATTC
        131 TCTCAGAATGCTTCTGTGTAGTTTTTATGTGAAGATATTTC

                     *    **        *   *                           *    *   
     292152 CTTTTCCACAATAGTTCTCAAAGCACTCAAAATATCCACTTGCAGATTCTACAAAAGGAGTATTT
          1 CTTTTCCACCATAGGCCTCAAAGCGCTCCAAATATCCACTTGCAGATTCTACAAAAAGAGTGTTT

                                    *                     *                  
     292217 CAAAACTGCTCAATCAAAAGAAAGTTTCAACTCTGTGAGATGAATGGACACATCACAAAGAAGTT
         66 CAAAACTGCTCAATCAAAAGAAAGGTTCAACTCTGTGAGATGAATGCACACATCACAAAGAAGTT

                                  *   *              
     292282 TCTCAGAATGCTTCTGTGTAGTATTTTTGTGAAGATATTT-
        131 TCTCAGAATGCTTCTGTGTAGTTTTTATGTGAAGATATTTC

                          *  * * * * ***             *            *     * *  
     292322 CTTTTCCACCATAGACCGCCAGGGGACACAAATATCCACTTTCAGATTCTACAACAAGAGAGGTT
          1 CTTTTCCACCATAGGCCTCAAAGCGCTCCAAATATCCACTTGCAGATTCTACAAAAAGAGTGTTT

                   *   *     *   ** *       *      *                      * *
     292387 CAAAACTACTCGATCAAGAGATGGTTTCAACTATGTGAGTTGAATGCACACATCACAAAGAACTA
         66 CAAAACTGCTCAATCAAAAGAAAGGTTCAACTCTGTGAGATGAATGCACACATCACAAAGAAGTT

             *  *    *                               
     292452 TGTCGGAATTCTTCTGTGTAGTTTTTATGTGAAGATATTTC
        131 TCTCAGAATGCTTCTGTGTAGTTTTTATGTGAAGATATTTC

                     *    * *      * *    *                  *               
     292493 CTTTTCCACAATAGACGTCAAAGTGATCCAGATATCCACTTGCAGATTCCACAAAAAGAGTGTTT
          1 CTTTTCCACCATAGGCCTCAAAGCGCTCCAAATATCCACTTGCAGATTCTACAAAAAGAGTGTTT

                 *   *   *                  **                     *  *      
     292558 CAAAAGTGCACAACCAAAAGAAAGGTTCAACTAGGTGAGATGAATGCACACATCAGAAGGAAGTT
         66 CAAAACTGCTCAATCAAAAGAAAGGTTCAACTCTGTGAGATGAATGCACACATCACAAAGAAGTT

                            **   *     * *       *   
     292623 TCTCAGAATGCTTCTGCATAGCTTTTAAGGGAAGATACTTC
        131 TCTCAGAATGCTTCTGTGTAGTTTTTATGTGAAGATATTTC

                    *               *            * *  *    * *               
     292664 CTTTTCCAACATAGGCCTCAAAGCACTCCAAATATCCTCCTGGAGATACCACAAAAAGAGTGTTT
          1 CTTTTCCACCATAGGCCTCAAAGCGCTCCAAATATCCACTTGCAGATTCTACAAAAAGAGTGTTT

            **                      *  *                 *       *           
     292729 GCAAACTGCTCAATCAAAAGAAAGATTTAACTCTGTGAGATGAATCCACACATGACAAAGAAGTT
         66 CAAAACTGCTCAATCAAAAGAAAGGTTCAACTCTGTGAGATGAATGCACACATCACAAAGAAGTT

                                                     
     292794 TCTCAGAATGCTTCTGTGTAGTTTTTATGTGAAGATATTTC
        131 TCTCAGAATGCTTCTGTGTAGTTTTTATGTGAAGATATTTC

                     *   * * *    **           *                *     *      
     292835 CTTTTCCACAATAAGACCCAAAAGGCTCCAAATATTCACTTGCAGATTCTAAAAAAAACAGTGTT
          1 CTTTTCCACCATAGGCCTCAAAGCGCTCCAAATATCCACTTGCAGATTCT-ACAAAAAGAGTGTT

                                  *                  *      *        **      
     292900 TCAAAACTGCTCAATCAAAAGATA-GTTCAACTCTGTGAGAAGAATGCTCACATCACTGAGAAGT
         65 TCAAAACTGCTCAATCAAAAGAAAGGTTCAACTCTGTGAGATGAATGCACACATCACAAAGAAGT

                                         *            
     292964 TTCTCAGAATGCTTCTGTGTAGTTTTTATATGAAGATATTTC
        130 TTCTCAGAATGCTTCTGTGTAGTTTTTATGTGAAGATATTTC

                *     *      *    **                       *   **       *    
     293006 CTTTCCCACCGTAGGCCACAAAAGGCTCCAAATATCCACTTGCAGATACTATGAAAAGAGAGTTT
          1 CTTTTCCACCATAGGCCTCAAAGCGCTCCAAATATCCACTTGCAGATTCTACAAAAAGAGTGTTT

                        *        *               ***            *            
     293071 CAAAACTGCTCATTCAAAAGATAGGTTCAACTCTGTGGTTTGAATGCACACAGCACAAAGAAGTT
         66 CAAAACTGCTCAATCAAAAGAAAGGTTCAACTCTGTGAGATGAATGCACACATCACAAAGAAGTT

              *       **                   **   *    
     293136 TCACAGAATGTGTCTGTGTAGTTTTTATGTGCGGATGTTTC
        131 TCTCAGAATGCTTCTGTGTAGTTTTTATGTGAAGATATTTC

                         *        ****   *   *               *    *          
     293177 CTTTTCCACCATATGCCT-AAATATTTCCCAATTTCCACTTGCAGATTCCACAAGAAGAGTGTTT
          1 CTTTTCCACCATAGGCCTCAAAGCGCTCCAAATATCCACTTGCAGATTCTACAAAAAGAGTGTTT

                      **       *       *           *            *      ***   
     293241 CAAAACTGCTGTATC-AAATAAA-GTTGAACTCTGTGAGGTGAATGCACACAGCACAAAATGGTT
         66 CAAAACTGCTCAATCAAAAGAAAGGTTCAACTCTGTGAGATGAATGCACACATCACAAAGAAGTT

                          **   *        *       *    
     293304 TCTCAGAATGCTTCCTTGTTGTTTTTATATGAAGATGTTTC
        131 TCTCAGAATGCTTCTGTGTAGTTTTTATGTGAAGATATTTC

                  *  *             *   *     *                               
     293345 CTTTTCAACAATAGGCCTCAAAGTGCTTCAAATGTCCACTTGCAGATTCTACAAAAAGAGTGTTT
          1 CTTTTCCACCATAGGCCTCAAAGCGCTCCAAATATCCACTTGCAGATTCTACAAAAAGAGTGTTT

                                        *      *  *  *                       
     293410 CAAAACTGCTCAATCAAAAGAAAGGTTCGACTCTGGGAAATTAATGCACACATCACAAAGAAGTT
         66 CAAAACTGCTCAATCAAAAGAAAGGTTCAACTCTGTGAGATGAATGCACACATCACAAAGAAGTT

                                     *        *      
     293475 TCTC---A-GCTTCTGTGTAGTTTTCATGTGAAGTTATTTC
        131 TCTCAGAATGCTTCTGTGTAGTTTTTATGTGAAGATATTTC

                     *       *     *            *    *         **       *    
     293512 CTTTTCCACAATAGGCCGCAAAGGGCTCCAAATATCAACTTACAGATTCTAGGAAAAGAGAGTTT
          1 CTTTTCCACCATAGGCCTCAAAGCGCTCCAAATATCCACTTGCAGATTCTACAAAAAGAGTGTTT

                       * **      *     *                                     
     293577 CAAAACTGCTCTACGAAAAGATAGGTTGAACTCTGTGAGATGAATGCACACATCACAAAGAAGTT
         66 CAAAACTGCTCAATCAAAAGAAAGGTTCAACTCTGTGAGATGAATGCACACATCACAAAGAAGTT

                       *               * *     *     
     293642 TCTCAGAATGCATCTGTGTAGTTTTTACGGGAAGACATTTC
        131 TCTCAGAATGCTTCTGTGTAGTTTTTATGTGAAGATATTTC

                        ***  *     **      *  *                  *     ***   
     293683 CTTTTCCACCATCTTCCACAAAGGTCTCCAAGTAACCACTTGCAGATTCTACAGAAAGACACTTT
          1 CTTTTCCACCATAGGCCTCAAAGCGCTCCAAATATCCACTTGCAGATTCTACAAAAAGAGTGTTT

            *          *         ***      *      ***                      *  
     293748 AAAAACTGCTCTATCAAAAGATCAGTTCAAGTCTGTGGTTTGAATGCACACATCACAAAGAATTT
         66 CAAAACTGCTCAATCAAAAGAAAGGTTCAACTCTGTGAGATGAATGCACACATCACAAAGAAGTT

                                     *  *            
     293813 TCTCAGAATGCTTCTGTGTAGTTTTCATATGAAGATATTTC
        131 TCTCAGAATGCTTCTGTGTAGTTTTTATGTGAAGATATTTC

                                    *                                   **   
     293854 CTTTTCCACCATAGGCCTCAAAGCACTCCAAATATCCACTTGCAGATTCTACAAAAAGAGATTTT
          1 CTTTTCCACCATAGGCCTCAAAGCGCTCCAAATATCCACTTGCAGATTCTACAAAAAGAGTGTTT

                   **                           *  *          *        *     
     293919 CAAAACTAGTCAATCAAAAGAAAGGTTCAACTCTGTCAGTTGAATGCACATATCACAAACAAGTT
         66 CAAAACTGCTCAATCAAAAGAAAGGTTCAACTCTGTGAGATGAATGCACACATCACAAAGAAGTT

                *      *                             
     293984 TCTCGGAATGCGTCTGTGTAGTTTTTATGTGAAGATATTTC
        131 TCTCAGAATGCTTCTGTGTAGTTTTTATGTGAAGATATTTC

               *     * *           *     *                  *    *           
     294025 CTTCTCCACAACAGGCCTCAAAGTGCTCCGAATATCCACTTGCAGATTTTAC-TAAAGAGTGTTT
          1 CTTTTCCACCATAGGCCTCAAAGCGCTCCAAATATCCACTTGCAGATTCTACAAAAAGAGTGTTT

             *               *  *   *     *        *    *               *    
     294089 CCAAACTGCTCAATCAAGAGGAAGTTTCAAGTCTGTGAGCTGAACGCACACATCACAAAGTAGTT
         66 CAAAACTGCTCAATCAAAAGAAAGGTTCAACTCTGTGAGATGAATGCACACATCACAAAGAAGTT

               *                                *    
     294154 TCTGAGAATGCTTCTGTGTAGTTTTTATGTGAAGATGTTTC
        131 TCTCAGAATGCTTCTGTGTAGTTTTTATGTGAAGATATTTC

                            **     *                                    *    
     294195 CTTTTCCACCATAGGCTGCAAAGGGCTCCAAATATCCACTTGCAGATTCTACAAAAAGAGAGTTT
          1 CTTTTCCACCATAGGCCTCAAAGCGCTCCAAATATCCACTTGCAGATTCTACAAAAAGAGTGTTT

                 *     *         *          *     *                     *    
     294260 CAAAAGTGCTCTATCAAAAGATAGGTTCAACTATGTGATATGAATGCACACATCACAAAGTAGTT
         66 CAAAACTGCTCAATCAAAAGAAAGGTTCAACTCTGTGAGATGAATGCACACATCACAAAGAAGTT

                                          *          
     294325 TCTCAGAATGCTTCTGTGTAGTTTTTATGTAAAGATATTTC
        131 TCTCAGAATGCTTCTGTGTAGTTTTTATGTGAAGATATTTC

                                    *                                   **   
     294366 CTTTTCCACCATAGGCCTCAAAGCACTCCAAATATCCACTTGCAGATTCTACAAAAAGAGATTTT
          1 CTTTTCCACCATAGGCCTCAAAGCGCTCCAAATATCCACTTGCAGATTCTACAAAAAGAGTGTTT

                   ** *                   *     *  *    * *   *        *     
     294431 CAAAACTATTTAATCAAAAGAAAGGTTCAAATCTGTCAGTTGAAGGTACATATCACAAACAAGTT
         66 CAAAACTGCTCAATCAAAAGAAAGGTTCAACTCTGTGAGATGAATGCACACATCACAAAGAAGTT

             * **                                    
     294496 TATTGGAATGCTTCTGTGTAGTTTTTATGTGAAGATATTTC
        131 TCTCAGAATGCTTCTGTGTAGTTTTTATGTGAAGATATTTC

                     * *         * *                        **  *     *      
     294537 CTTTTCCACAACAGGCCTCAAGGTGCTCCAAATATCCACTTGCAGATTTCACTAAAAGTGTGTTT
          1 CTTTTCCACCATAGGCCTCAAAGCGCTCCAAATATCCACTTGCAGATTCTACAAAAAGAGTGTTT

             *  *            *  *   *     *        *             *           
     294602 CCAAGCTGCTCAATCAAGAGGAAGTTTCAAGTCTGTGAGGTGAATGCACACATTACAAAGAAGTT
         66 CAAAACTGCTCAATCAAAAGAAAGGTTCAACTCTGTGAGATGAATGCACACATCACAAAGAAGTT

            *  *                                     
     294667 ACTGAGAATGCTTCTGTGTAGTTTTTATGTGAAGATATTTC
        131 TCTCAGAATGCTTCTGTGTAGTTTTTATGTGAAGATATTTC

                      **               *                                *    
     294708 CTTTTCCACCGCAGGCCTCAAAGCGCTGCAAATATCCACTTGCAGATTCTACAAAAAGAGAGTTT
          1 CTTTTCCACCATAGGCCTCAAAGCGCTCCAAATATCCACTTGCAGATTCTACAAAAAGAGTGTTT

                      **         *   *         *   *     **            *     
     294773 CAAAACTGCTGTATCAAAAGATAGGGTCAACTCTGCGAGTTGAATAAACACATCACAAATAAGTT
         66 CAAAACTGCTCAATCAAAAGAAAGGTTCAACTCTGTGAGATGAATGCACACATCACAAAGAAGTT

               **   *        *               *       
     294838 TCTGGGAACGCTTCTGTATAG-TTTTATGTGAATATATTTC
        131 TCTCAGAATGCTTCTGTGTAGTTTTTATGTGAAGATATTTC

                         *          *                   *   *  *   * *   *   
     294878 CTTTTCCACCATATGCCTCAAAGCACTCCAAATATCCACTTGCACATTATAGAAACATAGTCTTT
          1 CTTTTCCACCATAGGCCTCAAAGCGCTCCAAATATCCACTTGCAGATTCTACAAAAAGAGTGTTT

                   **                        *         *             *       
     294943 CAAAACTTGTCAATC-AAAGAAAGGTTCAACTCCGTGAGATGAGTGCACACATCACAGAGAAGTT
         66 CAAAACTGCTCAATCAAAAGAAAGGTTCAACTCTGTGAGATGAATGCACACATCACAAAGAAGTT

                *     *                            * 
     295007 TCTCGGAATGTTTCTGTGTAGTTTTTATGTGAAGATATTGC
        131 TCTCAGAATGCTTCTGTGTAGTTTTTATGTGAAGATATTTC

                     *       *       *       *    *          *       *   *   
     295048 CTTTTCCACAATAGGCCCCAAAGCGTTCCAAATGTCCAATTGCAGATTCCACAAAAAAAGTTTTT
          1 CTTTTCCACCATAGGCCTCAAAGCGCTCCAAATATCCACTTGCAGATTCTACAAAAAGAGTGTTT

            *                 *  *  *  *             * *         **    *     
     295113 TAAAACTGCTCAATCAAATGATAGATTAAACTCTGTGAGATTAGTGCACACATGTCAAAAAAGTT
         66 CAAAACTGCTCAATCAAAAGAAAGGTTCAACTCTGTGAGATGAATGCACACATCACAAAGAAGTT

                                *      * *           
     295178 TCTCAGAATGCTTCTGTGTACTTTTTAGGGGAAGATATTTC
        131 TCTCAGAATGCTTCTGTGTAGTTTTTATGTGAAGATATTTC

                        *    *     **         *    *           **  * *  *    
     295219 CTTTTCCACCATCGGCCACAAAGGACTCCAAATAACCACATGCAGATTCTAGTAACACAGAGTTT
          1 CTTTTCCACCATAGGCCTCAAAGCGCTCCAAATATCCACTTGCAGATTCTACAAAAAGAGTGTTT

                       *         * *           *   * * *    **    ***        
     295284 CAAAACTGCTCTATCAAAAGATAAGTTCAACTCTGAGAGTTTAGTGCAACCATCGTGAAGAAGTT
         66 CAAAACTGCTCAATCAAAAGAAAGGTTCAACTCTGTGAGATGAATGCACACATCACAAAGAAGTT

                            *     *                  
     295349 TCTCAGAATGCTTCTGAGTAGTGTTTATGTGAAGATATTTC
        131 TCTCAGAATGCTTCTGTGTAGTTTTTATGTGAAGATATTTC

                              *     *                      *           **    
     295390 CTTTTCCACCATAGGCCTGAAAGCCCTCCAAATATCCACTTGCAGATCCTACAAAAAGAAAGTTT
          1 CTTTTCCACCATAGGCCTCAAAGCGCTCCAAATATCCACTTGCAGATTCTACAAAAAGAGTGTTT

             *         **     *  *  *   *        **      *                   
     295455 CGAAA-TGCTCTCTCAAACGATAGTTTCGACTCTGTGGTATGAATACACACATCACAAAGAAGTT
         66 CAAAACTGCTCAATCAAAAGAAAGGTTCAACTCTGTGAGATGAATGCACACATCACAAAGAAGTT

                                       **            
     295519 TCTCAGAATGCTTCTGTGTAGTTTTTAAATGAAGATATTTC
        131 TCTCAGAATGCTTCTGTGTAGTTTTTATGTGAAGATATTTC

            *                       *          *     *                       
     295560 TTTTTCCACCATAGGCCTCAAAGCACTCCAAATATGCACTTCCAGATTCTACAAAAAGAGTGTTT
          1 CTTTTCCACCATAGGCCTCAAAGCGCTCCAAATATCCACTTGCAGATTCTACAAAAAGAGTGTTT

              *                 *       * *         **   *         *  * *    
     295625 CAGAACTGCTCAATCAAAAGGAAGGTTCCAGTC--TGAGACAAATACACACATCAAAAGGTAGTT
         66 CAAAACTGCTCAATCAAAAGAAAGGTTCAACTCTGTGAGATGAATGCACACATCACAAAGAAGTT

                                                    *
     295688 TCTCAGAATGCTTCTGTGTAGTTTTTATGTGAAGATATTTT
        131 TCTCAGAATGCTTCTGTGTAGTTTTTATGTGAAGATATTTC

             *               *    **    *     *      *  **   *          *    
     295729 CCTTTCCACCATAGGCCACAAATGGCTCTAAATACCCACTTACATTTTCCACAAAAAGAGAGTTT
          1 CTTTTCCACCATAGGCCTCAAAGCGCTCCAAATATCCACTTGCAGATTCTACAAAAAGAGTGTTT

                       * *    * *      *   *         *                       
     295794 CAAAACTGCTCTACCAAAGGTAA-GTTTAACGCTGTGAG-TTAA-G-A-ACATCACAAAGAAGTT
         66 CAAAACTGCTCAATCAAAAGAAAGGTTCAACTCTGTGAGATGAATGCACACATCACAAAGAAGTT

                                   *   *  *          
     295854 TCTCAGAATGCTTCTGTGTAGTTCTTACGTAAAGATATTTC
        131 TCTCAGAATGCTTCTGTGTAGTTTTTATGTGAAGATATTTC

                 *   *      ***    *                  *              **      
     295895 CTTTTACACAATAGGCAGAAAAGTGCTCCAAATATCCACTTGAAGATTCTACAAAAACCGTGTTT
          1 CTTTTCCACCATAGGCCTCAAAGCGCTCCAAATATCCACTTGCAGATTCTACAAAAAGAGTGTTT

                     **                                          *      *    
     295960 CAAAACTGCCGAATCAAAAGAAAGGTTCAACTCTGTGAGATGAATGCACACATAACAAAGGAGTT
         66 CAAAACTGCTCAATCAAAAGAAAGGTTCAACTCTGTGAGATGAATGCACACATCACAAAGAAGTT

                                 *      *      *    *
     296025 TCTCAGAATGCTTCTGTGTAGCTTTTATATGAAGACATTTA
        131 TCTCAGAATGCTTCTGTGTAGTTTTTATGTGAAGATATTTC

            *        * *            *   ***                     **        *  
     296066 GTTTTCCACAACAGGCCTCAAAGCTCTCTCCATATCCACTTGCAGATTCTACCGAAAGAGTGCTT
          1 CTTTTCCACCATAGGCCTCAAAGCGCTCCAAATATCCACTTGCAGATTCTACAAAAAGAGTGTTT

             *                   * **     *        *        *     **         
     296131 CCAAACTGCTCAATCAAAAGAGACATTCAAATCTGTGAGGTGAATGCAGACATCGTAAAGAAGTT
         66 CAAAACTGCTCAATCAAAAGAAAGGTTCAACTCTGTGAGATGAATGCACACATCACAAAGAAGTT

                                *     *       *    **
     296196 TCTCAGAATGCTTCTGTGTATTTTTTGTGTGAAGTTATTCG
        131 TCTCAGAATGCTTCTGTGTAGTTTTTATGTGAAGATATTTC

            *    *             *     *  *            **                 *    
     296237 TTTTTGCACCATAGGCCTCCAAGCGTTCTAAATATCCACTTCTAGATTCTACAAAAAGAGAGTTT
          1 CTTTTCCACCATAGGCCTCAAAGCGCTCCAAATATCCACTTGCAGATTCTACAAAAAGAGTGTTT

                   *         *            *        *    *   *                
     296302 CAAAACTACTC-A--AACA-AAAGGTTCAATTCTGTGAGTTGAAAGCAAACATCACAAAGAAGTT
         66 CAAAACTGCTCAATCAAAAGAAAGGTTCAACTCTGTGAGATGAATGCACACATCACAAAGAAGTT

                       *                             
     296363 TCTCAGAATGCGTCTGTGTAGTTTTTATGTGAAGATATTTC
        131 TCTCAGAATGCTTCTGTGTAGTTTTTATGTGAAGATATTTC

                     *    ****     *                  *                      
     296404 CTTTT-CACAATAGAATGCAAAGGGCTCCAAATATCCACTTGGAGATTCTACAAAAAGA--GTTT
          1 CTTTTCCACCATAGGCCTCAAAGCGCTCCAAATATCCACTTGCAGATTCTACAAAAAGAGTGTTT

                  *    **     *  *     *     ***   *     *               *   
     296466 CAAAACCGCTCTGTCAAATGATAGGTTGAACTCCCGGAGGTGAATACACACATCACAAAGAGGTT
         66 CAAAACTGCTCAATCAAAAGAAAGGTTCAACTCTGTGAGATGAATGCACACATCACAAAGAAGTT

                  *                       *  *       
     296531 TCTCAGCATGCTTCTGTGTAGTTTTTATGTAAACATATTTC
        131 TCTCAGAATGCTTCTGTGTAGTTTTTATGTGAAGATATTTC

             *    * *              *           *      * *       *   *    *   
     296572 CGTTTCTATCATAGGCCTCAAAGTGCTCCAAATATTCACTTGTACATTCTACCAAACGAGTATTT
          1 CTTTTCCACCATAGGCCTCAAAGCGCTCCAAATATCCACTTGCAGATTCTACAAAAAGAGTGTTT

                              * *         **      *        *            *    
     296637 CAAAACTGCTCAATCAAATGGAAGGTTCAAAACTGTGACATGAATGCCCACATCACAAAGTAGTT
         66 CAAAACTGCTCAATCAAAAGAAAGGTTCAACTCTGTGAGATGAATGCACACATCACAAAGAAGTT

                                                     
     296702 TCTCAGAATGCTTCTGTGTAGTTTTTATGTGAAGATATTTC
        131 TCTCAGAATGCTTCTGTGTAGTTTTTATGTGAAGATATTTC

                     * *    *      *    *       * *   **      *              
     296743 CTTTTCCACAACA-GCGTGCAAAACGCTTCAAATATGCCCTTAGAGATTCCACAAAAAGAGTGTT
          1 CTTTTCCACCATAGGCCT-CAAAGCGCTCCAAATATCCACTTGCAGATTCTACAAAAAGAGTGTT

              *     *               *  *                                  ** 
     296807 TCCAAACTACTCAAATCAAAA-AATGATTTCAACTCTGTGAGATGAATGCACACATCACAAACTA
         65 TCAAAACTGCTC-AATCAAAAGAAAG-GTTCAACTCTGTGAGATGAATGCACACATCACAAAGAA

                         *     ** *   * *   *       *   
     296871 GTTTCTCAGAATGTTTCTGCCTGGTTCTCATGCGAAGATAGTTC
        128 GTTTCTCAGAATGCTTCTGTGTAGTTTTTATGTGAAGATATTTC

                 *           *   * **              *                  * *    
     296915 CTTTTTCACCATAGGCCGCAATGTACTCCAAATATCCACCTGCAGATTCTAC-AAAAGTGAGTTT
          1 CTTTTCCACCATAGGCCTCAAAGCGCTCCAAATATCCACTTGCAGATTCTACAAAAAGAGTGTTT

                       *         ***    **         *        *      *       * 
     296979 CAAAACTGCTCTATCAAAAGATCAGTTCGTCTCTGTGAGTTGAATGCATACATCAAAAAGAAGCT
         66 CAAAACTGCTCAATCAAAAGAAAGGTTCAACTCTGTGAGATGAATGCACACATCACAAAGAAGTT

                 *             *      **         *   
     297044 TCTCAAAATGCTTCTGTGTGGTTTTTCGGTGAAGATAGTTC
        131 TCTCAGAATGCTTCTGTGTAGTTTTTATGTGAAGATATTTC

            *     *        *      * *                 *        *        *    
     297085 TTTTTCTACCATAGGTCTCAAACCACTCCAAATATCCACTTGTAGATTCTATAAAAAGGAATG-T
          1 CTTTTCCACCATAGGCCTCAAAGCGCTCCAAATATCCACTTGCAGATTCTACAAAAA-GAGTGTT

                  *        *    *    *      * *         *      *             
     297149 TCAAAATTGCTCAATAAAAATAAAGTTTCAACACCGTGAGATGAGTGCACAAATCACAAAGAAGT
         65 TCAAAACTGCTCAATCAAAAGAAAGGTTCAACTCTGTGAGATGAATGCACACATCACAAAGAAGT

                  *          *         *          *   
     297214 TTCTCAAAATGCTTCTGGGTAGTTTTTCTGTGAAGATAGTTC
        130 TTCTCAGAATGCTTCTGTGTAGTTTTTATGTGAAGATATTTC

                  *     *    *     *          *                         *    
     297256 CTTTTCTACCATGGGCCACAAAGGGCTCCAAATACCCACTTGCAGATTCTACAAAAAGAGAGTTT
          1 CTTTTCCACCATAGGCCTCAAAGCGCTCCAAATATCCACTTGCAGATTCTACAAAAAGAGTGTTT

              *        *      ** * *        *    * *    **  * *           *  
     297321 CACAACTGCTCTATCAAACAATATGTTCAACTTTGTGGGTTGAACACAAATATCAC-AAGAATTT
         66 CAAAACTGCTCAATCAAAAGAAAGGTTCAACTCTGTGAGATGAATGCACACATCACAAAGAAGTT

                **                             *     
     297385 TCTCCCAATGCTTCTGTGTAGTTTTTATGTGAAGACATTTC
        131 TCTCAGAATGCTTCTGTGTAGTTTTTATGTGAAGATATTTC

            *   *  *      *  *     *                 *  *      *       *  *  
     297426 TTTTCCCTCCATAGTCCACAAAGTGCTCCAAATATCCACTTACATATTCTAGAAAAAGATTGCTT
          1 CTTTTCCACCATAGGCCTCAAAGCGCTCCAAATATCCACTTGCAGATTCTACAAAAAGAGTGTTT

            **       *    *               * * *                              
     297491 GGAAACTGCACAATGAAAAGAAAGGTTCAAATATATGAGATGAATGCACACATCACAAAGAAGTT
         66 CAAAACTGCTCAATCAAAAGAAAGGTTCAACTCTGTGAGATGAATGCACACATCACAAAGAAGTT

                                 *                    
     297556 TCTCAGAAT-CTCTCTGTGTAATTTTTATGTGAAGATATTTC
        131 TCTCAGAATGCT-TCTGTGTAGTTTTTATGTGAAGATATTTC

                *     *    *  *   *                        *      *    *     
     297597 CTTTCCCACCTTAGGTCTTAAAACGCTCCAAATATCCACTTGCAGATACTACAAGAAGATTGTTT
          1 CTTTTCCACCATAGGCCTCAAAGCGCTCCAAATATCCACTTGCAGATTCTACAAAAAGAGTGTTT

                     *    *        *      *     **                           
     297662 CAAAACTGCACAA-AAAAAGAAATGTTCAATTCTGTTTGATGAATGCACACATCACAAAGAAGTT
         66 CAAAACTGCTCAATCAAAAGAAAGGTTCAACTCTGTGAGATGAATGCACACATCACAAAGAAGTT

                           *                         
     297726 TCTCAGAATGCTTCTCTGTAGTTTTTATGTGAAGATATTTC
        131 TCTCAGAATGCTTCTGTGTAGTTTTTATGTGAAGATATTTC

                     *             *                 *         **      * *   
     297767 CTTTTCCACAATAGGCCTCAAAGGGCTCCAAATATCCACTTCCAGATTCTATGAAAAGAATATTT
          1 CTTTTCCACCATAGGCCTCAAAGCGCTCCAAATATCCACTTGCAGATTCTACAAAAAGAGTGTTT

             *              * *    *                                      *  
     297832 CCAAACTGCTCAATCATAGGAAATGTTCAACTCTGTGAGATGAATGCACACATCAC-AAGAAATT
         66 CAAAACTGCTCAATCAAAAGAAAGGTTCAACTCTGTGAGATGAATGCACACATCACAAAGAAGTT

                     *    *      *       *       *   
     297896 TCTCAGAATCCTTCAGTGTAGGTTTTATGAGAAGATAATTC
        131 TCTCAGAATGCTTCTGTGTAGTTTTTATGTGAAGATATTTC

                     *    **        *   *                           *    *   
     297937 CTTTTCCACAATAGTTCTCAAAGCACTCAAAATATCCACTTGCAGATTCTACAAAAGGAGTATTT
          1 CTTTTCCACCATAGGCCTCAAAGCGCTCCAAATATCCACTTGCAGATTCTACAAAAAGAGTGTTT

                                                          *                  
     298002 CAAAACTGCTCAATCAAAAGAAAGGTTCAACTCTGTGAGATGAATGGACACATCACAAAGAAGTT
         66 CAAAACTGCTCAATCAAAAGAAAGGTTCAACTCTGTGAGATGAATGCACACATCACAAAGAAGTT

                                  *   *              
     298067 TCTCAGAATGCTTCTGTGTAGTATTTTTGTGAAGATATTT-
        131 TCTCAGAATGCTTCTGTGTAGTTTTTATGTGAAGATATTTC

                          *  * * * * ***             *            *     * *  
     298107 CTTTTCCACCATAGACCGCCAGGGGACACAAATATCCACTTTCAGATTCTACAACAAGAGAGGTT
          1 CTTTTCCACCATAGGCCTCAAAGCGCTCCAAATATCCACTTGCAGATTCTACAAAAAGAGTGTTT

                   *   *     *   ** *       *      *                      * *
     298172 CAAAACTACTCGATCAAGAGATGGTTTCAACTATGTGAGTTGAATGCACACATCACAAAGAACTA
         66 CAAAACTGCTCAATCAAAAGAAAGGTTCAACTCTGTGAGATGAATGCACACATCACAAAGAAGTT

             *  *    *                               
     298237 TGTCGGAATTCTTCTGTGTAGTTTTTATGTGAAGATATTTC
        131 TCTCAGAATGCTTCTGTGTAGTTTTTATGTGAAGATATTTC

                     *    * *      * *    *                  *               
     298278 CTTTTCCACAATAGACGTCAAAGTGATCCAGATATCCACTTGCAGATTCCACAAAAAGAGTGTTT
          1 CTTTTCCACCATAGGCCTCAAAGCGCTCCAAATATCCACTTGCAGATTCTACAAAAAGAGTGTTT

                 *   *   *                  **                     *  *      
     298343 CAAAAGTGCACAACCAAAAGAAAGGTTCAACTAGGTGAGATGAATGCACACATCAGAAGGAAGTT
         66 CAAAACTGCTCAATCAAAAGAAAGGTTCAACTCTGTGAGATGAATGCACACATCACAAAGAAGTT

                            **   *     * *       *   
     298408 TCTCAGAATGCTTCTGCATAGCTTTTAAGGGAAGATACTTC
        131 TCTCAGAATGCTTCTGTGTAGTTTTTATGTGAAGATATTTC

                    *               *            * *  *    * *               
     298449 CTTTTCCAACATAGGCCTCAAAGCACTCCAAATATCCTCCTGGAGATACCACAAAAAGAGTGTTT
          1 CTTTTCCACCATAGGCCTCAAAGCGCTCCAAATATCCACTTGCAGATTCTACAAAAAGAGTGTTT

            **                      *  *                 *       *           
     298514 GCAAACTGCTCAATCAAAAGAAAGATTTAACTCTGTGAGATGAATCCACACATGACAAAGAAGTT
         66 CAAAACTGCTCAATCAAAAGAAAGGTTCAACTCTGTGAGATGAATGCACACATCACAAAGAAGTT

                                                     
     298579 TCTCAGAATGCTTCTGTGTAGTTTTTATGTGAAGATATTTC
        131 TCTCAGAATGCTTCTGTGTAGTTTTTATGTGAAGATATTTC

                     *   * * *    **           *                *     *      
     298620 CTTTTCCACAATAAGACCCAAAAGGCTCCAAATATTCACTTGCAGATTCTAAAAAAAACAGTGTT
          1 CTTTTCCACCATAGGCCTCAAAGCGCTCCAAATATCCACTTGCAGATTCT-ACAAAAAGAGTGTT

                                  *                  *      *        **      
     298685 TCAAAACTGCTCAATCAAAAGATA-GTTCAACTCTGTGAGAAGAATGCTCACATCACTGAGAAGT
         65 TCAAAACTGCTCAATCAAAAGAAAGGTTCAACTCTGTGAGATGAATGCACACATCACAAAGAAGT

                                         *            
     298749 TTCTCAGAATGCTTCTGTGTAGTTTTTATATGAAGATATTTC
        130 TTCTCAGAATGCTTCTGTGTAGTTTTTATGTGAAGATATTTC

                *     *      *    **                       *   **       *    
     298791 CTTTCCCACCGTAGGCCACAAAAGGCTCCAAATATCCACTTGCAGATACTATGAAAAGAGAGTTT
          1 CTTTTCCACCATAGGCCTCAAAGCGCTCCAAATATCCACTTGCAGATTCTACAAAAAGAGTGTTT

                        *        *               ***            *            
     298856 CAAAACTGCTCATTCAAAAGATAGGTTCAACTCTGTGGTTTGAATGCACACAGCACAAAGAAGTT
         66 CAAAACTGCTCAATCAAAAGAAAGGTTCAACTCTGTGAGATGAATGCACACATCACAAAGAAGTT

              *       **                   **   *    
     298921 TCACAGAATGTGTCTGTGTAGTTTTTATGTGCGGATGTTTC
        131 TCTCAGAATGCTTCTGTGTAGTTTTTATGTGAAGATATTTC

                         *        ****   *   *               *    *          
     298962 CTTTTCCACCATATGCCT-AAATATTTCCCAATTTCCACTTGCAGATTCCACAAGAAGAGTGTTT
          1 CTTTTCCACCATAGGCCTCAAAGCGCTCCAAATATCCACTTGCAGATTCTACAAAAAGAGTGTTT

                      **       *       *           *            *      ***   
     299026 CAAAACTGCTGTATC-AAATAAA-GTTGAACTCTGTGAGGTGAATGCACACAGCACAAAATGGTT
         66 CAAAACTGCTCAATCAAAAGAAAGGTTCAACTCTGTGAGATGAATGCACACATCACAAAGAAGTT

                          **   *        *       *    
     299089 TCTCAGAATGCTTCCTTGTTGTTTTTATATGAAGATGTTTC
        131 TCTCAGAATGCTTCTGTGTAGTTTTTATGTGAAGATATTTC

                  *  *             *   *     *                               
     299130 CTTTTCAACAATAGGCCTCAAAGTGCTTCAAATGTCCACTTGCAGATTCTACAAAAAGAGTGTTT
          1 CTTTTCCACCATAGGCCTCAAAGCGCTCCAAATATCCACTTGCAGATTCTACAAAAAGAGTGTTT

                                        *      *  *  *                       
     299195 CAAAACTGCTCAATCAAAAGAAAGGTTCGACTCTGGGAAATTAATGCACACATCACAAAGAAGTT
         66 CAAAACTGCTCAATCAAAAGAAAGGTTCAACTCTGTGAGATGAATGCACACATCACAAAGAAGTT

                                     *        *      
     299260 TCTC---A-GCTTCTGTGTAGTTTTCATGTGAAGTTATTTC
        131 TCTCAGAATGCTTCTGTGTAGTTTTTATGTGAAGATATTTC

                     *       *     *            *    *         **       *    
     299297 CTTTTCCACAATAGGCCGCAAAGGGCTCCAAATATCAACTTACAGATTCTAGGAAAAGAGAGTTT
          1 CTTTTCCACCATAGGCCTCAAAGCGCTCCAAATATCCACTTGCAGATTCTACAAAAAGAGTGTTT

                       * **      *     *                                     
     299362 CAAAACTGCTCTACGAAAAGATAGGTTGAACTCTGTGAGATGAATGCACACATCACAAAGAAGTT
         66 CAAAACTGCTCAATCAAAAGAAAGGTTCAACTCTGTGAGATGAATGCACACATCACAAAGAAGTT

                       *               * *     *     
     299427 TCTCAGAATGCATCTGTGTAGTTTTTACGGGAAGACATTTC
        131 TCTCAGAATGCTTCTGTGTAGTTTTTATGTGAAGATATTTC

                        ***  *     **      *  *                  *     ***   
     299468 CTTTTCCACCATCTTCCACAAAGGTCTCCAAGTAACCACTTGCAGATTCTACAGAAAGACACTTT
          1 CTTTTCCACCATAGGCCTCAAAGCGCTCCAAATATCCACTTGCAGATTCTACAAAAAGAGTGTTT

            *          *         ***      *      ***                      *  
     299533 AAAAACTGCTCTATCAAAAGATCAGTTCAAGTCTGTGGTTTGAATGCACACATCACAAAGAATTT
         66 CAAAACTGCTCAATCAAAAGAAAGGTTCAACTCTGTGAGATGAATGCACACATCACAAAGAAGTT

                                     *  *            
     299598 TCTCAGAATGCTTCTGTGTAGTTTTCATATGAAGATATTTC
        131 TCTCAGAATGCTTCTGTGTAGTTTTTATGTGAAGATATTTC

                                    *                                   **   
     299639 CTTTTCCACCATAGGCCTCAAAGCACTCCAAATATCCACTTGCAGATTCTACAAAAAGAGATTTT
          1 CTTTTCCACCATAGGCCTCAAAGCGCTCCAAATATCCACTTGCAGATTCTACAAAAAGAGTGTTT

                   **                           *  *          *        *     
     299704 CAAAACTAGTCAATCAAAAGAAAGGTTCAACTCTGTCAGTTGAATGCACATATCACAAACAAGTT
         66 CAAAACTGCTCAATCAAAAGAAAGGTTCAACTCTGTGAGATGAATGCACACATCACAAAGAAGTT

                *      *                             
     299769 TCTCGGAATGCGTCTGTGTAGTTTTTATGTGAAGATATTTC
        131 TCTCAGAATGCTTCTGTGTAGTTTTTATGTGAAGATATTTC

               *     * *           *     *                  *    *           
     299810 CTTCTCCACAACAGGCCTCAAAGTGCTCCGAATATCCACTTGCAGATTTTAC-TAAAGAGTGTTT
          1 CTTTTCCACCATAGGCCTCAAAGCGCTCCAAATATCCACTTGCAGATTCTACAAAAAGAGTGTTT

             *               *  *   *     *        *    *               *    
     299874 CCAAACTGCTCAATCAAGAGGAAGTTTCAAGTCTGTGAGCTGAACGCACACATCACAAAGTAGTT
         66 CAAAACTGCTCAATCAAAAGAAAGGTTCAACTCTGTGAGATGAATGCACACATCACAAAGAAGTT

               *    *                           *    
     299939 TCTGAGAAGGCTTCTGTGTAGTTTTTATGTGAAGATGTTTC
        131 TCTCAGAATGCTTCTGTGTAGTTTTTATGTGAAGATATTTC

                            **     *                                    *  * 
     299980 CTTTTCCACCATAGGCTGCAAAGGGCTCCAAATATCCACTTGCAGATTCTACAAAAAGAGAGTCT
          1 CTTTTCCACCATAGGCCTCAAAGCGCTCCAAATATCCACTTGCAGATTCTACAAAAAGAGTGTTT

                 *     *         *          *     *                     *    
     300045 CAAAAGTGCTCTATCAAAAGATAGGTTCAACTATGTGATATGAATGCACACATCACAAAGTAGTT
         66 CAAAACTGCTCAATCAAAAGAAAGGTTCAACTCTGTGAGATGAATGCACACATCACAAAGAAGTT

                                          *          
     300110 TCTCAGAATGCTTCTGTGTAGTTTTTATGTAAAGATATTTC
        131 TCTCAGAATGCTTCTGTGTAGTTTTTATGTGAAGATATTTC

                                    *                                        
     300151 CTTTTCCACCATAGGCCTCAAAGCACTCCAAATATCCACTTGCAGATTCTACAAAAAGAGATG-T
          1 CTTTTCCACCATAGGCCTCAAAGCGCTCCAAATATCCACTTGCAGATTCTACAAAAAGAG-TGTT

                    ** *                   *     *  *    * *   *        *    
     300215 TCAAAACTATTTAATCAAAAGAAAGGTTCAAATCTGTCAGTTGAAGGTACATATCACAAACAAGT
         65 TCAAAACTGCTCAATCAAAAGAAAGGTTCAACTCTGTGAGATGAATGCACACATCACAAAGAAGT

              * **                                    
     300280 TTATTGGAATGCTTCTGTGTAGTTTTTATGTGAAGATATTTC
        130 TTCTCAGAATGCTTCTGTGTAGTTTTTATGTGAAGATATTTC

                     * *         * *                        **  *     *      
     300322 CTTTTCCACAACAGGCCTCAAGGTGCTCCAAATATCCACTTGCAGATTTCACTAAAAGTGTGTTT
          1 CTTTTCCACCATAGGCCTCAAAGCGCTCCAAATATCCACTTGCAGATTCTACAAAAAGAGTGTTT

             *  *            *  *   *     *        *             *           
     300387 CCAAGCTGCTCAATCAAGAGGAAGTTTCAAGTCTGTGAGGTGAATGCACACATTACAAAGAAGTT
         66 CAAAACTGCTCAATCAAAAGAAAGGTTCAACTCTGTGAGATGAATGCACACATCACAAAGAAGTT

            *  *                                     
     300452 ACTGAGAATGCTTCTGTGTAGTTTTTATGTGAAGATATTTC
        131 TCTCAGAATGCTTCTGTGTAGTTTTTATGTGAAGATATTTC

                      **               *                                *    
     300493 CTTTTCCACCGCAGGCCTCAAAGCGCTGCAAATATCCACTTGCAGATTCTACAAAAAGAGAGTTT
          1 CTTTTCCACCATAGGCCTCAAAGCGCTCCAAATATCCACTTGCAGATTCTACAAAAAGAGTGTTT

                      **         *   *         *   *     **            *     
     300558 CAAAACTGCTGTATCAAAAGATAGGGTCAACTCTGCGAGTTGAATAAACACATCACAAATAAGTT
         66 CAAAACTGCTCAATCAAAAGAAAGGTTCAACTCTGTGAGATGAATGCACACATCACAAAGAAGTT

               **   *        *               *       
     300623 TCTGGGAACGCTTCTGTATAG-TTTTATGTGAATATATTTC
        131 TCTCAGAATGCTTCTGTGTAGTTTTTATGTGAAGATATTTC

                         *          *                   *   *  *   * *   *   
     300663 CTTTTCCACCATATGCCTCAAAGCACTCCAAATATCCACTTGCACATTATAGAAACATAGTCTTT
          1 CTTTTCCACCATAGGCCTCAAAGCGCTCCAAATATCCACTTGCAGATTCTACAAAAAGAGTGTTT

                   **                        *         *             *       
     300728 CAAAACTTGTCAATC-AAAGAAAGGTTCAACTCCGTGAGATGAGTGCACACATCACAGAGAAGTT
         66 CAAAACTGCTCAATCAAAAGAAAGGTTCAACTCTGTGAGATGAATGCACACATCACAAAGAAGTT

                *     *                            * 
     300792 TCTCGGAATGTTTCTGTGTAGTTTTTATGTGAAGATATTGC
        131 TCTCAGAATGCTTCTGTGTAGTTTTTATGTGAAGATATTTC

                     *               *            *          *       *   *   
     300833 CTTTTCCACAATAGGCCTCAAAGCGTTCCAAATATCCAATTGCAGATTCCACAAAAAAAGTTTTT
          1 CTTTTCCACCATAGGCCTCAAAGCGCTCCAAATATCCACTTGCAGATTCTACAAAAAGAGTGTTT

            *                 *  *  *  *             * *         **    *     
     300898 TAAAACTGCTCAATCAAATGATAGATTAAACTCTGTGAGATTAGTGCACACATGTCAAAAAAGTT
         66 CAAAACTGCTCAATCAAAAGAAAGGTTCAACTCTGTGAGATGAATGCACACATCACAAAGAAGTT

                                *      * *           
     300963 TCTCAGAATGCTTCTGTGTACTTTTTAGGGGAAGATATTTC
        131 TCTCAGAATGCTTCTGTGTAGTTTTTATGTGAAGATATTTC

                        *    *     **         *    *           **  * *  *    
     301004 CTTTTCCACCATCGGCCACAAAGGACTCCAAATAACCACATGCAGATTCTAGTAACACAGAGTTT
          1 CTTTTCCACCATAGGCCTCAAAGCGCTCCAAATATCCACTTGCAGATTCTACAAAAAGAGTGTTT

                       *         * *           *   * * *    **    ***        
     301069 CAAAACTGCTCTATCAAAAGATAAGTTCAACTCTGAGAGTTTAGTGCAACCATCGTGAAGAAGTT
         66 CAAAACTGCTCAATCAAAAGAAAGGTTCAACTCTGTGAGATGAATGCACACATCACAAAGAAGTT

                            *     *                  
     301134 TCTCAGAATGCTTCTGAGTAGTGTTTATGTGAAGATATTTC
        131 TCTCAGAATGCTTCTGTGTAGTTTTTATGTGAAGATATTTC

                          *   *     *                      *           **    
     301175 CTTTTCCACCATAGACCTGAAAGCCCTCCAAATATCCACTTGCAGATCCTACAAAAAGAAAGTTT
          1 CTTTTCCACCATAGGCCTCAAAGCGCTCCAAATATCCACTTGCAGATTCTACAAAAAGAGTGTTT

             *         **     *  *  *   *        **      *                   
     301240 CGAAA-TGCTCTCTCAAACGATAGTTTCGACTCTGTGGTATGAATACACACATCACAAAGAAGTT
         66 CAAAACTGCTCAATCAAAAGAAAGGTTCAACTCTGTGAGATGAATGCACACATCACAAAGAAGTT

                                       **            
     301304 TCTCAGAATGCTTCTGTGTAGTTTTTAAATGAAGATATTTC
        131 TCTCAGAATGCTTCTGTGTAGTTTTTATGTGAAGATATTTC

            *                       *          *     *                       
     301345 TTTTTCCACCATAGGCCTCAAAGCACTCCAAATATGCACTTCCAGATTCTACAAAAAGAGTGTTT
          1 CTTTTCCACCATAGGCCTCAAAGCGCTCCAAATATCCACTTGCAGATTCTACAAAAAGAGTGTTT

              *                 *       * *         **   *         *  * *    
     301410 CAGAACTGCTCAATCAAAAGGAAGGTTCCAGTC--TGAGACAAATACACACATCAAAAGGTAGTT
         66 CAAAACTGCTCAATCAAAAGAAAGGTTCAACTCTGTGAGATGAATGCACACATCACAAAGAAGTT

                                                    *
     301473 TCTCAGAATGCTTCTGTGTAGTTTTTATGTGAAGATATTTT
        131 TCTCAGAATGCTTCTGTGTAGTTTTTATGTGAAGATATTTC

             *               *    **    *     *      *  **   *          *    
     301514 CCTTTCCACCATAGGCCACAAATGGCTCTAAATACCCACTTACATTTTCCACAAAAAGAGAGTTT
          1 CTTTTCCACCATAGGCCTCAAAGCGCTCCAAATATCCACTTGCAGATTCTACAAAAAGAGTGTTT

                       * *    * *      *   *         *                       
     301579 CAAAACTGCTCTACCAAAGGTAA-GTTTAACGCTGTGAG-TTAA-G-A-ACATCACAAAGAAGTT
         66 CAAAACTGCTCAATCAAAAGAAAGGTTCAACTCTGTGAGATGAATGCACACATCACAAAGAAGTT

                                   *   *  *          
     301639 TCTCAGAATGCTTCTGTGTAGTTCTTACGTAAAGATATTTC
        131 TCTCAGAATGCTTCTGTGTAGTTTTTATGTGAAGATATTTC

                 *   *      ***    *                  *              **      
     301680 CTTTTACACAATAGGCAGAAAAGTGCTCCAAATATCCACTTGAAGATTCTACAAAAACCGTGTTT
          1 CTTTTCCACCATAGGCCTCAAAGCGCTCCAAATATCCACTTGCAGATTCTACAAAAAGAGTGTTT

                     **                                          *      *    
     301745 CAAAACTGCCGAATCAAAAGAAAGGTTCAACTCTGTGAGATGAATGCACACATAACAAAGGAGTT
         66 CAAAACTGCTCAATCAAAAGAAAGGTTCAACTCTGTGAGATGAATGCACACATCACAAAGAAGTT

                                 *      *      *    *
     301810 TCTCAGAATGCTTCTGTGTAGCTTTTATATGAAGACATTTA
        131 TCTCAGAATGCTTCTGTGTAGTTTTTATGTGAAGATATTTC

            *        * *            *   ***                     **        *  
     301851 GTTTTCCACAACAGGCCTCAAAGCTCTCTCCATATCCACTTGCAGATTCTACCGAAAGAGTGCTT
          1 CTTTTCCACCATAGGCCTCAAAGCGCTCCAAATATCCACTTGCAGATTCTACAAAAAGAGTGTTT

             *                   * **     *        *        *     **         
     301916 CCAAACTGCTCAATCAAAAGAGACATTCAAATCTGTGAGGTGAATGCAGACATCGTAAAGAAGTT
         66 CAAAACTGCTCAATCAAAAGAAAGGTTCAACTCTGTGAGATGAATGCACACATCACAAAGAAGTT

                                *     *       *    **
     301981 TCTCAGAATGCTTCTGTGTATTTTTTGTGTGAAGTTATTCG
        131 TCTCAGAATGCTTCTGTGTAGTTTTTATGTGAAGATATTTC

            *    *             *     *  *            **                 *    
     302022 TTTTTGCACCATAGGCCTCCAAGCGTTCTAAATATCCACTTCTAGATTCTACAAAAAGAGAGTTT
          1 CTTTTCCACCATAGGCCTCAAAGCGCTCCAAATATCCACTTGCAGATTCTACAAAAAGAGTGTTT

                   *         *            *        *    *   *                
     302087 CAAAACTACTC-A--AACA-AAAGGTTCAATTCTGTGAGTTGAAAGCAAACATCACAAAGAAGTT
         66 CAAAACTGCTCAATCAAAAGAAAGGTTCAACTCTGTGAGATGAATGCACACATCACAAAGAAGTT

                       *             *               
     302148 TCTCAGAATGCGTCTGTGTAGTTTTGATGTGAAGATATTTC
        131 TCTCAGAATGCTTCTGTGTAGTTTTTATGTGAAGATATTTC

                     **   ****     *                  *                      
     302189 CTTTT-CACAGTAGAATGCAAAGGGCTCCAAATATCCACTTGGAGATTCTACAAAAAGA--GTTT
          1 CTTTTCCACCATAGGCCTCAAAGCGCTCCAAATATCCACTTGCAGATTCTACAAAAAGAGTGTTT

                  *    **     *  *     *     ***   *     *               *   
     302251 CAAAACCGCTCTGTCAAATGATAGGTTGAACTCCCGGAGGTGAATACACACATCACAAAGAGGTT
         66 CAAAACTGCTCAATCAAAAGAAAGGTTCAACTCTGTGAGATGAATGCACACATCACAAAGAAGTT

                  *                       *  *       
     302316 TCTCAGCATGCTTCTGTGTAGTTTTTATGTAAACATATTTC
        131 TCTCAGAATGCTTCTGTGTAGTTTTTATGTGAAGATATTTC

             *    * *              *           *      * *       *   *    *   
     302357 CGTTTCTATCATAGGCCTCAAAGTGCTCCAAATATTCACTTGTACATTCTACCAAACGAGTATTT
          1 CTTTTCCACCATAGGCCTCAAAGCGCTCCAAATATCCACTTGCAGATTCTACAAAAAGAGTGTTT

                              * *         ** *    *        *            *    
     302422 CAAAACTGCTCAATCAAATGGAAGGTTCAAAACCGTGACATGAATGCCCACATCACAAAGTAGTT
         66 CAAAACTGCTCAATCAAAAGAAAGGTTCAACTCTGTGAGATGAATGCACACATCACAAAGAAGTT

                                                     
     302487 TCTCAGAATGCTTCTGTGTAGTTTTTATGTGAAGATATTTC
        131 TCTCAGAATGCTTCTGTGTAGTTTTTATGTGAAGATATTTC

                     * *    *      *    *       * *   **      *              
     302528 CTTTTCCACAACA-GCGTGCAAAACGCTTCAAATATGCCCTTAGAGATTCCACAAAAAGAGTGTT
          1 CTTTTCCACCATAGGCCT-CAAAGCGCTCCAAATATCCACTTGCAGATTCTACAAAAAGAGTGTT

              *     *               *  *                                  ** 
     302592 TCCAAACTACTCAAATCAAAA-AATGATTTCAACTCTGTGAGATGAATGCACACATCACAAACTA
         65 TCAAAACTGCTC-AATCAAAAGAAAG-GTTCAACTCTGTGAGATGAATGCACACATCACAAAGAA

                         *     ** *   * *   *       *   
     302656 GTTTCTCAGAATGTTTCTGCCTGGTTCTCATGCGAAGATAGTTC
        128 GTTTCTCAGAATGCTTCTGTGTAGTTTTTATGTGAAGATATTTC

                 *           *   * **              *                  * *    
     302700 CTTTTTCACCATAGGCCGCAATGTACTCCAAATATCCACCTGCAGATTCTAC-AAAAGTGAGTTT
          1 CTTTTCCACCATAGGCCTCAAAGCGCTCCAAATATCCACTTGCAGATTCTACAAAAAGAGTGTTT

                       *         ***    **         *        *      *       * 
     302764 CAAAACTGCTCTATCAAAAGATCAGTTCGTCTCTGTGAGTTGAATGCATACATCAAAAAGAAGCT
         66 CAAAACTGCTCAATCAAAAGAAAGGTTCAACTCTGTGAGATGAATGCACACATCACAAAGAAGTT

                 *             *      **         *   
     302829 TCTCAAAATGCTTCTGTGTGGTTTTTCGGTGAAGATAGTTC
        131 TCTCAGAATGCTTCTGTGTAGTTTTTATGTGAAGATATTTC

            *     *        *      * *                 *        *        *    
     302870 TTTTTCTACCATAGGTCTCAAACCACTCCAAATATCCACTTGTAGATTCTATAAAAAGGAATG-T
          1 CTTTTCCACCATAGGCCTCAAAGCGCTCCAAATATCCACTTGCAGATTCTACAAAAA-GAGTGTT

                  *        *    *    *      * *         *      *             
     302934 TCAAAATTGCTCAATAAAAATAAAGTTTCAACACCGTGAGATGAGTGCACAAATCACAAAGAAGT
         65 TCAAAACTGCTCAATCAAAAGAAAGGTTCAACTCTGTGAGATGAATGCACACATCACAAAGAAGT

                  *          *         *          *   
     302999 TTCTCAAAATGCTTCTGGGTAGTTTTTCTGTGAAGATAGTTC
        130 TTCTCAGAATGCTTCTGTGTAGTTTTTATGTGAAGATATTTC

                  *     *    *     *          *                         *    
     303041 CTTTTCTACCATGGGCCACAAAGGGCTCCAAATACCCACTTGCAGATTCTACAAAAAGAGAGTTT
          1 CTTTTCCACCATAGGCCTCAAAGCGCTCCAAATATCCACTTGCAGATTCTACAAAAAGAGTGTTT

              *        *      ** * *        *    * *    **  * *           *  
     303106 CACAACTGCTCTATCAAACAATATGTTCAACTTTGTGGGTTGAACACAAATATCAC-AAGAATTT
         66 CAAAACTGCTCAATCAAAAGAAAGGTTCAACTCTGTGAGATGAATGCACACATCACAAAGAAGTT

                **                             *     
     303170 TCTCCCAATGCTTCTGTGTAGTTTTTATGTGAAGACATTTC
        131 TCTCAGAATGCTTCTGTGTAGTTTTTATGTGAAGATATTTC

            *   *  *      *  *     *                 *  *      *       *  *  
     303211 TTTTCCCTCCATAGTCCACAAAGTGCTCCAAATATCCACTTACATATTCTAGAAAAAGATTGCTT
          1 CTTTTCCACCATAGGCCTCAAAGCGCTCCAAATATCCACTTGCAGATTCTACAAAAAGAGTGTTT

            **       *    *               * * *                              
     303276 GGAAACTGCACAATGAAAAGAAAGGTTCAAATATATGAGATGAATGCACACATCACAAAGAAGTT
         66 CAAAACTGCTCAATCAAAAGAAAGGTTCAACTCTGTGAGATGAATGCACACATCACAAAGAAGTT

                                 *                    
     303341 TCTCAGAAT-CTCTCTGTGTAATTTTTATGTGAAGATATTTC
        131 TCTCAGAATGCT-TCTGTGTAGTTTTTATGTGAAGATATTTC

                *     *    *  *   *                        *      *    *     
     303382 CTTTCCCACCTTAGGTCTTAAAACGCTCCAAATATCCACTTGCAGATACTACAAGAAGATTGTTT
          1 CTTTTCCACCATAGGCCTCAAAGCGCTCCAAATATCCACTTGCAGATTCTACAAAAAGAGTGTTT

                     *    *        *      *     **                           
     303447 CAAAACTGCACAA-AAAAAGAAATGTTCAATTCTGTTTGATGAATGCACACATCACAAAGAAGTT
         66 CAAAACTGCTCAATCAAAAGAAAGGTTCAACTCTGTGAGATGAATGCACACATCACAAAGAAGTT

                           *                         
     303511 TCTCAGAATGCTTCTCTGTAGTTTTTATGTGAAGATATTTC
        131 TCTCAGAATGCTTCTGTGTAGTTTTTATGTGAAGATATTTC

                     *             *                 *         **      * *   
     303552 CTTTTCCACAATAGGCCTCAAAGGGCTCCAAATATCCACTTCCAGATTCTATGAAAAGAATATTT
          1 CTTTTCCACCATAGGCCTCAAAGCGCTCCAAATATCCACTTGCAGATTCTACAAAAAGAGTGTTT

             *              * *    *                                      *  
     303617 CCAAACTGCTCAATCATAGGAAATGTTCAACTCTGTGAGATGAATGCACACATCAC-AAGAAATT
         66 CAAAACTGCTCAATCAAAAGAAAGGTTCAACTCTGTGAGATGAATGCACACATCACAAAGAAGTT

                     *    *      *       *       *   
     303681 TCTCAGAATCCTTCAGTGTAGGTTTTATGAGAAGATAATTC
        131 TCTCAGAATGCTTCTGTGTAGTTTTTATGTGAAGATATTTC

                     *    **        *   *                           *    *   
     303722 CTTTTCCACAATAGTTCTCAAAGCACTCAAAATATCCACTTGCAGATTCTACAAAAGGAGTATTT
          1 CTTTTCCACCATAGGCCTCAAAGCGCTCCAAATATCCACTTGCAGATTCTACAAAAAGAGTGTTT

                                                          *                  
     303787 CAAAACTGCTCAATCAAAAGAAAGGTTCAACTCTGTGAGATGAATGGACACATCACAAAGAAGTT
         66 CAAAACTGCTCAATCAAAAGAAAGGTTCAACTCTGTGAGATGAATGCACACATCACAAAGAAGTT

                                  *   *              
     303852 TCTCAGAATGCTTCTGTGTAGTATTTTTGTGAAGATATTT-
        131 TCTCAGAATGCTTCTGTGTAGTTTTTATGTGAAGATATTTC

                          *  * * * * ***             *            *     * *  
     303892 CTTTTCCACCATAGACCGCCAGGGGACACAAATATCCACTTTCAGATTCTACAACAAGAGAGGTT
          1 CTTTTCCACCATAGGCCTCAAAGCGCTCCAAATATCCACTTGCAGATTCTACAAAAAGAGTGTTT

                   *   *     *   ** *       *      *                      * *
     303957 CAAAACTACTCGATCAAGAGATGGTTTCAACTATGTGAGTTGAATGCACACATCACAAAGAACTA
         66 CAAAACTGCTCAATCAAAAGAAAGGTTCAACTCTGTGAGATGAATGCACACATCACAAAGAAGTT

             *  *    *                               
     304022 TGTCGGAATTCTTCTGTGTAGTTTTTATGTGAAGATATTTC
        131 TCTCAGAATGCTTCTGTGTAGTTTTTATGTGAAGATATTTC

                     *    * *      * *    *                  *               
     304063 CTTTTCCACAATAGACGTCAAAGTGATCCAGATATCCACTTGCAGATTCCACAAAAAGAGTGTTT
          1 CTTTTCCACCATAGGCCTCAAAGCGCTCCAAATATCCACTTGCAGATTCTACAAAAAGAGTGTTT

                 *   *   *                  **                     *  *      
     304128 CAAAAGTGCACAACCAAAAGAAAGGTTCAACTAGGTGAGATGAATGCACACATCAGAAGGAAGTT
         66 CAAAACTGCTCAATCAAAAGAAAGGTTCAACTCTGTGAGATGAATGCACACATCACAAAGAAGTT

                            **   *     * *       *   
     304193 TCTCAGAATGCTTCTGCATAGCTTTTAAGGGAAGATACTTC
        131 TCTCAGAATGCTTCTGTGTAGTTTTTATGTGAAGATATTTC

                    *               *            * *  *    * *               
     304234 CTTTTCCAACATAGGCCTCAAAGCACTCCAAATATCCTCCTGGAGATACCACAAAAAGAGTGTTT
          1 CTTTTCCACCATAGGCCTCAAAGCGCTCCAAATATCCACTTGCAGATTCTACAAAAAGAGTGTTT

            **                      *  *                 *       *           
     304299 GCAAACTGCTCAATCAAAAGAAAGATTTAACTCTGTGAGATGAATCCACACATGACAAAGAAGTT
         66 CAAAACTGCTCAATCAAAAGAAAGGTTCAACTCTGTGAGATGAATGCACACATCACAAAGAAGTT

                                                     
     304364 TCTCAGAATGCTTCTGTGTAGTTTTTATGTGAAGATATTTC
        131 TCTCAGAATGCTTCTGTGTAGTTTTTATGTGAAGATATTTC

                     *   * * *    **           *                *     *      
     304405 CTTTTCCACAATAAGACCCAAAAGGCTCCAAATATTCACTTGCAGATTCTAAAAAAAACAGTGTT
          1 CTTTTCCACCATAGGCCTCAAAGCGCTCCAAATATCCACTTGCAGATTCT-ACAAAAAGAGTGTT

                                  *                  *      *        **      
     304470 TCAAAACTGCTCAATCAAAAGATA-GTTCAACTCTGTGAGAAGAATGCTCACATCACTGAGAAGT
         65 TCAAAACTGCTCAATCAAAAGAAAGGTTCAACTCTGTGAGATGAATGCACACATCACAAAGAAGT

                                         *            
     304534 TTCTCAGAATGCTTCTGTGTAGTTTTTATATGAAGATATTTC
        130 TTCTCAGAATGCTTCTGTGTAGTTTTTATGTGAAGATATTTC

                *     *      *    **                       *   **       *    
     304576 CTTTCCCACCGTAGGCCACAAAAGGCTCCAAATATCCACTTGCAGATACTATGAAAAGAGAGTTT
          1 CTTTTCCACCATAGGCCTCAAAGCGCTCCAAATATCCACTTGCAGATTCTACAAAAAGAGTGTTT

                        *        *               ***            *            
     304641 CAAAACTGCTCATTCAAAAGATAGGTTCAACTCTGTGGTTTGAATGCACACAGCACAAAGAAGTT
         66 CAAAACTGCTCAATCAAAAGAAAGGTTCAACTCTGTGAGATGAATGCACACATCACAAAGAAGTT

              *       **                   **   *    
     304706 TCACAGAATGTGTCTGTGTAGTTTTTATGTGCGGATGTTTC
        131 TCTCAGAATGCTTCTGTGTAGTTTTTATGTGAAGATATTTC

                         *        ****   *   *               *    *          
     304747 CTTTTCCACCATATGCCT-AAATATTTCCCAATTTCCACTTGCAGATTCCACAAGAAGAGTGTTT
          1 CTTTTCCACCATAGGCCTCAAAGCGCTCCAAATATCCACTTGCAGATTCTACAAAAAGAGTGTTT

                      **       *       *           *            *      ***   
     304811 CAAAACTGCTGTATC-AAATAAA-GTTGAACTCTGTGAGGTGAATGCACACAGCACAAAATGGTT
         66 CAAAACTGCTCAATCAAAAGAAAGGTTCAACTCTGTGAGATGAATGCACACATCACAAAGAAGTT

                          **   *        *       *    
     304874 TCTCAGAATGCTTCCTTGTTGTTTTTATATGAAGATGTTTC
        131 TCTCAGAATGCTTCTGTGTAGTTTTTATGTGAAGATATTTC

                  *  *             *   *     *                               
     304915 CTTTTCAACAATAGGCCTCAAAGTGCTTCAAATGTCCACTTGCAGATTCTACAAAAAGAGTGTTT
          1 CTTTTCCACCATAGGCCTCAAAGCGCTCCAAATATCCACTTGCAGATTCTACAAAAAGAGTGTTT

                                        *      *  *  *                       
     304980 CAAAACTGCTCAATCAAAAGAAAGGTTCGACTCTGGGAAATTAATGCACACATCACAAAGAAGTT
         66 CAAAACTGCTCAATCAAAAGAAAGGTTCAACTCTGTGAGATGAATGCACACATCACAAAGAAGTT

                                     *        *      
     305045 TCTC---A-GCTTCTGTGTAGTTTTCATGTGAAGTTATTTC
        131 TCTCAGAATGCTTCTGTGTAGTTTTTATGTGAAGATATTTC

                     *       *     *            *    *         **       *    
     305082 CTTTTCCACAATAGGCCGCAAAGGGCTCCAAATATCAACTTACAGATTCTAGGAAAAGAGAGTTT
          1 CTTTTCCACCATAGGCCTCAAAGCGCTCCAAATATCCACTTGCAGATTCTACAAAAAGAGTGTTT

                       * **      *     *                                     
     305147 CAAAACTGCTCTACGAAAAGATAGGTTGAACTCTGTGAGATGAATGCACACATCACAAAGAAGTT
         66 CAAAACTGCTCAATCAAAAGAAAGGTTCAACTCTGTGAGATGAATGCACACATCACAAAGAAGTT

                       *               * *           
     305212 TCTCAGAATGCATCTGTGTAGTTTTTACGGGAAGATATTTC
        131 TCTCAGAATGCTTCTGTGTAGTTTTTATGTGAAGATATTTC

                        ***  *     **      *  *           *      *     ***   
     305253 CTTTTCCACCATCTTCCACAAAGGTCTCCAAGTAACCACTTGCAGACTCTACAGAAAGACACTTT
          1 CTTTTCCACCATAGGCCTCAAAGCGCTCCAAATATCCACTTGCAGATTCTACAAAAAGAGTGTTT

            *          *         ***      *      ***                      *  
     305318 AAAAACTGCTCTATCAAAAGATCAGTTCAAGTCTGTGGTTTGAATGCACACATCACAAAGAATTT
         66 CAAAACTGCTCAATCAAAAGAAAGGTTCAACTCTGTGAGATGAATGCACACATCACAAAGAAGTT

                                     *  *            
     305383 TCTCAGAATGCTTCTGTGTAGTTTTCATATGAAGATATTTC
        131 TCTCAGAATGCTTCTGTGTAGTTTTTATGTGAAGATATTTC

                                    *                                   **   
     305424 CTTTTCCACCATAGGCCTCAAAGCACTCCAAATATCCACTTGCAGATTCTACAAAAAGAGATTTT
          1 CTTTTCCACCATAGGCCTCAAAGCGCTCCAAATATCCACTTGCAGATTCTACAAAAAGAGTGTTT

                   **                           *  *          *        *     
     305489 CAAAACTAGTCAATCAAAAGAAAGGTTCAACTCTGTCAGTTGAATGCACATATCACAAACAAGTT
         66 CAAAACTGCTCAATCAAAAGAAAGGTTCAACTCTGTGAGATGAATGCACACATCACAAAGAAGTT

                *      *                             
     305554 TCTCGGAATGCGTCTGTGTAGTTTTTATGTGAAGATATTTC
        131 TCTCAGAATGCTTCTGTGTAGTTTTTATGTGAAGATATTTC

               *     * *           *     *                  *    *           
     305595 CTTCTCCACAACAGGCCTCAAAGTGCTCCGAATATCCACTTGCAGATTTTAC-TAAAGAGTGTTT
          1 CTTTTCCACCATAGGCCTCAAAGCGCTCCAAATATCCACTTGCAGATTCTACAAAAAGAGTGTTT

             *               *  *   *     *        *    *               *    
     305659 CCAAACTGCTCAATCAAGAGGAAGTTTCAAGTCTGTGAGCTGAACGCACACATCACAAAGTAGTT
         66 CAAAACTGCTCAATCAAAAGAAAGGTTCAACTCTGTGAGATGAATGCACACATCACAAAGAAGTT

               *                                *    
     305724 TCTGAGAATGCTTCTGTGTAGTTTTTATGTGAAGATGTTTC
        131 TCTCAGAATGCTTCTGTGTAGTTTTTATGTGAAGATATTTC

                            **     *                                    *  * 
     305765 CTTTTCCACCATAGGCTGCAAAGGGCTCCAAATATCCACTTGCAGATTCTACAAAAAGAGAGTCT
          1 CTTTTCCACCATAGGCCTCAAAGCGCTCCAAATATCCACTTGCAGATTCTACAAAAAGAGTGTTT

                 *     *         *          *     *                     *    
     305830 CAAAAGTGCTCTATCAAAAGATAGGTTCAACTATGTGATATGAATGCACACATCACAAAGTAGTT
         66 CAAAACTGCTCAATCAAAAGAAAGGTTCAACTCTGTGAGATGAATGCACACATCACAAAGAAGTT

                                          *          
     305895 TCTCAGAATGCTTCTGTGTAGTTTTTATGTAAAGATATTTC
        131 TCTCAGAATGCTTCTGTGTAGTTTTTATGTGAAGATATTTC

                                    *                                   **   
     305936 CTTTTCCACCATAGGCCTCAAAGCACTCCAAATATCCACTTGCAGATTCTACAAAAAGAGATTTT
          1 CTTTTCCACCATAGGCCTCAAAGCGCTCCAAATATCCACTTGCAGATTCTACAAAAAGAGTGTTT

                   ** *                   *     *  *    * *   *        *     
     306001 CAAAACTATTTAATCAAAAGAAAGGTTCAAATCTGTCAGTTGAAGGTACATATCACAAACAAGTT
         66 CAAAACTGCTCAATCAAAAGAAAGGTTCAACTCTGTGAGATGAATGCACACATCACAAAGAAGTT

             * **                                    
     306066 TATTGGAATGCTTCTGTGTAGTTTTTATGTGAAGATATTTC
        131 TCTCAGAATGCTTCTGTGTAGTTTTTATGTGAAGATATTTC

                     * *         * *                        **  *     *      
     306107 CTTTTCCACAACAGGCCTCAAGGTGCTCCAAATATCCACTTGCAGATTTCACTAAAAGTGTGTTT
          1 CTTTTCCACCATAGGCCTCAAAGCGCTCCAAATATCCACTTGCAGATTCTACAAAAAGAGTGTTT

             *  *            *  *   *     *        *             *           
     306172 CCAAGCTGCTCAATCAAGAGGAAGTTTCAAGTCTGTGAGGTGAATGCACACATTACAAAGAAGTT
         66 CAAAACTGCTCAATCAAAAGAAAGGTTCAACTCTGTGAGATGAATGCACACATCACAAAGAAGTT

            *  *                                     
     306237 ACTGAGAATGCTTCTGTGTAGTTTTTATGTGAAGATATTTC
        131 TCTCAGAATGCTTCTGTGTAGTTTTTATGTGAAGATATTTC

                      **               *                                *    
     306278 CTTTTCCACCGCAGGCCTCAAAGCGCTGCAAATATCCACTTGCAGATTCTACAAAAAGAGAGTTT
          1 CTTTTCCACCATAGGCCTCAAAGCGCTCCAAATATCCACTTGCAGATTCTACAAAAAGAGTGTTT

                      **         *   *         *   *     **            *     
     306343 CAAAACTGCTGTATCAAAAGATAGGGTCAACTCTGCGAGTTGAATAAACACATCACAAATAAGTT
         66 CAAAACTGCTCAATCAAAAGAAAGGTTCAACTCTGTGAGATGAATGCACACATCACAAAGAAGTT

               **   *        *               *       
     306408 TCTGGGAACGCTTCTGTATAG-TTTTATGTGAATATATTTC
        131 TCTCAGAATGCTTCTGTGTAGTTTTTATGTGAAGATATTTC

                         *          *                   *   *  *   * *   *   
     306448 CTTTTCCACCATATGCCTCAAAGCACTCCAAATATCCACTTGCACATTATAGAAACATAGTCTTT
          1 CTTTTCCACCATAGGCCTCAAAGCGCTCCAAATATCCACTTGCAGATTCTACAAAAAGAGTGTTT

                   **                        *         *             *       
     306513 CAAAACTTGTCAATC-AAAGAAAGGTTCAACTCCGTGAGATGAGTGCACACATCACAGAGAAGTT
         66 CAAAACTGCTCAATCAAAAGAAAGGTTCAACTCTGTGAGATGAATGCACACATCACAAAGAAGTT

                *     *                            * 
     306577 TCTCGGAATGTTTCTGTGTAGTTTTTATGTGAAGATATTGC
        131 TCTCAGAATGCTTCTGTGTAGTTTTTATGTGAAGATATTTC

                     *               *            *          *       *   *   
     306618 CTTTTCCACAATAGGCCTCAAAGCGTTCCAAATATCCAATTGCAGATTCCACAAAAAAAGTTTTT
          1 CTTTTCCACCATAGGCCTCAAAGCGCTCCAAATATCCACTTGCAGATTCTACAAAAAGAGTGTTT

            *                 *  *  *  *             * *         **    *     
     306683 TAAAACTGCTCAATCAAATGATAGATTAAACTCTGTGAGATTAGTGCACACATGTCAAAAAAGTT
         66 CAAAACTGCTCAATCAAAAGAAAGGTTCAACTCTGTGAGATGAATGCACACATCACAAAGAAGTT

                                *      * *           
     306748 TCTCAGAATGCTTCTGTGTACTTTTTAGGGGAAGATATTTC
        131 TCTCAGAATGCTTCTGTGTAGTTTTTATGTGAAGATATTTC

                        *    *     **         *    *           **  * *  *    
     306789 CTTTTCCACCATCGGCCACAAAGGACTCCAAATAACCACATGCAGATTCTAGTAACACAGAGTTT
          1 CTTTTCCACCATAGGCCTCAAAGCGCTCCAAATATCCACTTGCAGATTCTACAAAAAGAGTGTTT

                       *         * *           *   * * *    **    ***        
     306854 CAAAACTGCTCTATCAAAAGATAAGTTCAACTCTGAGAGTTTAGTGCAACCATCGTGAAGAAGTT
         66 CAAAACTGCTCAATCAAAAGAAAGGTTCAACTCTGTGAGATGAATGCACACATCACAAAGAAGTT

                            *     *                  
     306919 TCTCAGAATGCTTCTGAGTAGTGTTTATGTGAAGATATTTC
        131 TCTCAGAATGCTTCTGTGTAGTTTTTATGTGAAGATATTTC

                              *     *                      *           **    
     306960 CTTTTCCACCATAGGCCTGAAAGCCCTCCAAATATCCACTTGCAGATCCTACAAAAAGAAAGTTT
          1 CTTTTCCACCATAGGCCTCAAAGCGCTCCAAATATCCACTTGCAGATTCTACAAAAAGAGTGTTT

             *         **     *  *  *   *        **      *                   
     307025 CGAAA-TGCTCTCTCAAACGATAGTTTCGACTCTGTGGTATGAATACACACATCACAAAGAAGTT
         66 CAAAACTGCTCAATCAAAAGAAAGGTTCAACTCTGTGAGATGAATGCACACATCACAAAGAAGTT

                                       **            
     307089 TCTCAGAATGCTTCTGTGTAGTTTTTAAATGAAGATATTTC
        131 TCTCAGAATGCTTCTGTGTAGTTTTTATGTGAAGATATTTC

            *                       *          *     *                       
     307130 TTTTTCCACCATAGGCCTCAAAGCACTCCAAATATGCACTTCCAGATTCTACAAAAAGAGTGTTT
          1 CTTTTCCACCATAGGCCTCAAAGCGCTCCAAATATCCACTTGCAGATTCTACAAAAAGAGTGTTT

              *                 *       * *         **   *         *  * *    
     307195 CAGAACTGCTCAATCAAAAGGAAGGTTCCAGTC--TGAGACAAATACACACATCAAAAGGTAGTT
         66 CAAAACTGCTCAATCAAAAGAAAGGTTCAACTCTGTGAGATGAATGCACACATCACAAAGAAGTT

                                                    *
     307258 TCTCAGAATGCTTCTGTGTAGTTTTTATGTGAAGATATTTT
        131 TCTCAGAATGCTTCTGTGTAGTTTTTATGTGAAGATATTTC

             *               *    **    *     *      *  **   *          *    
     307299 CCTTTCCACCATAGGCCACAAATGGCTCTAAATACCCACTTACATTTTCCACAAAAAGAGAGTTT
          1 CTTTTCCACCATAGGCCTCAAAGCGCTCCAAATATCCACTTGCAGATTCTACAAAAAGAGTGTTT

                       * *    * *      *   *         *                       
     307364 CAAAACTGCTCTACCAAAGGTAA-GTTTAACGCTGTGAG-TTAA-G-A-ACATCACAAAGAAGTT
         66 CAAAACTGCTCAATCAAAAGAAAGGTTCAACTCTGTGAGATGAATGCACACATCACAAAGAAGTT

                                   *   *  *          
     307424 TCTCAGAATGCTTCTGTGTAGTTCTTACGTAAAGATATTTC
        131 TCTCAGAATGCTTCTGTGTAGTTTTTATGTGAAGATATTTC

                 *   *      ***    *                  *              **      
     307465 CTTTTACACAATAGGCAGAAAAGTGCTCCAAATATCCACTTGAAGATTCTACAAAAACCGTGTTT
          1 CTTTTCCACCATAGGCCTCAAAGCGCTCCAAATATCCACTTGCAGATTCTACAAAAAGAGTGTTT

                     **                                          *      *    
     307530 CAAAACTGCCGAATCAAAAGAAAGGTTCAACTCTGTGAGATGAATGCACACATAACAAAGGAGTT
         66 CAAAACTGCTCAATCAAAAGAAAGGTTCAACTCTGTGAGATGAATGCACACATCACAAAGAAGTT

                                 *      *      *    *
     307595 TCTCAGAATGCTTCTGTGTAGCTTTTATATGAAGACATTTA
        131 TCTCAGAATGCTTCTGTGTAGTTTTTATGTGAAGATATTTC

            *        * *            *   ***                     **        *  
     307636 GTTTTCCACAACAGGCCTCAAAGCTCTCTCCATATCCACTTGCAGATTCTACCGAAAGAGTGCTT
          1 CTTTTCCACCATAGGCCTCAAAGCGCTCCAAATATCCACTTGCAGATTCTACAAAAAGAGTGTTT

             *                   * **     *        *        *     **         
     307701 CCAAACTGCTCAATCAAAAGAGACATTCAAATCTGTGAGGTGAATGCAGACATCGTAAAGAAGTT
         66 CAAAACTGCTCAATCAAAAGAAAGGTTCAACTCTGTGAGATGAATGCACACATCACAAAGAAGTT

                                *     *       *    **
     307766 TCTCAGAATGCTTCTGTGTATTTTTTGTGTGAAGTTATTCG
        131 TCTCAGAATGCTTCTGTGTAGTTTTTATGTGAAGATATTTC

            *    *             *     *  *            **                 *    
     307807 TTTTTGCACCATAGGCCTCCAAGCGTTCTAAATATCCACTTCTAGATTCTACAAAAAGAGAGTTT
          1 CTTTTCCACCATAGGCCTCAAAGCGCTCCAAATATCCACTTGCAGATTCTACAAAAAGAGTGTTT

                   *         *            *        *    *   *                
     307872 CAAAACTACTC-A--AACA-AAAGGTTCAATTCTGTGAGTTGAAAGCAAACATCACAAAGAAGTT
         66 CAAAACTGCTCAATCAAAAGAAAGGTTCAACTCTGTGAGATGAATGCACACATCACAAAGAAGTT

                       *             *               
     307933 TCTCAGAATGCGTCTGTGTAGTTTTGATGTGAAGATATTTC
        131 TCTCAGAATGCTTCTGTGTAGTTTTTATGTGAAGATATTTC

                     **   ****     *                  *                      
     307974 CTTTT-CACAGTAGAATGCAAAGGGCTCCAAATATCCACTTGGAGATTCTACAAAAAGA--GTTT
          1 CTTTTCCACCATAGGCCTCAAAGCGCTCCAAATATCCACTTGCAGATTCTACAAAAAGAGTGTTT

                  *    **     *  *     *     ***   *     *               *   
     308036 CAAAACCGCTCTGTCAAATGATAGGTTGAACTCCCGGAGGTGAATACACACATCACAAAGAGGTT
         66 CAAAACTGCTCAATCAAAAGAAAGGTTCAACTCTGTGAGATGAATGCACACATCACAAAGAAGTT

                  *                       *  *       
     308101 TCTCAGCATGCTTCTGTGTAGTTTTTATGTAAACATATTTC
        131 TCTCAGAATGCTTCTGTGTAGTTTTTATGTGAAGATATTTC

             *    * *              *           *      * *       *   *    *   
     308142 CGTTTCTATCATAGGCCTCAAAGTGCTCCAAATATTCACTTGTACATTCTACCAAACGAGTATTT
          1 CTTTTCCACCATAGGCCTCAAAGCGCTCCAAATATCCACTTGCAGATTCTACAAAAAGAGTGTTT

                              * *         **      *        *            *    
     308207 CAAAACTGCTCAATCAAATGGAAGGTTCAAAACTGTGACATGAATGCCCACATCACAAAGTAGTT
         66 CAAAACTGCTCAATCAAAAGAAAGGTTCAACTCTGTGAGATGAATGCACACATCACAAAGAAGTT

                                                     
     308272 TCTCAGAATGCTTCTGTGTAGTTTTTATGTGAAGATATTTC
        131 TCTCAGAATGCTTCTGTGTAGTTTTTATGTGAAGATATTTC

                     * *    *      *    *       * *   **      *              
     308313 CTTTTCCACAACA-GCGTGCAAAACGCTTCAAATATGCCCTTAGAGATTCCACAAAAAGAGTGTT
          1 CTTTTCCACCATAGGCCT-CAAAGCGCTCCAAATATCCACTTGCAGATTCTACAAAAAGAGTGTT

              *     *               *  *                                  ** 
     308377 TCCAAACTACTCAAATCAAAA-AATGATTTCAACTCTGTGAGATGAATGCACACATCACAAACTA
         65 TCAAAACTGCTC-AATCAAAAGAAAG-GTTCAACTCTGTGAGATGAATGCACACATCACAAAGAA

                         *     ** *   * *   *       *   
     308441 GTTTCTCAGAATGTTTCTGCCTGGTTCTCATGCGAAGATAGTTC
        128 GTTTCTCAGAATGCTTCTGTGTAGTTTTTATGTGAAGATATTTC

                 *        *  *   * **              *                  * *    
     308485 CTTTTTCACCATAGACCGCAATGTACTCCAAATATCCACCTGCAGATTCTAC-AAAAGTGAGTTT
          1 CTTTTCCACCATAGGCCTCAAAGCGCTCCAAATATCCACTTGCAGATTCTACAAAAAGAGTGTTT

                       *         ***    **         *        *      *       * 
     308549 CAAAACTGCTCTATCAAAAGATCAGTTCGTCTCTGTGAGTTGAATGCATACATCAAAAAGAAGCT
         66 CAAAACTGCTCAATCAAAAGAAAGGTTCAACTCTGTGAGATGAATGCACACATCACAAAGAAGTT

                 *             *      **         *   
     308614 TCTCAAAATGCTTCTGTGTGGTTTTTCGGTGAAGATAGTTC
        131 TCTCAGAATGCTTCTGTGTAGTTTTTATGTGAAGATATTTC

            *     *        *      * *                 *        *        *    
     308655 TTTTTCTACCATAGGTCTCAAACCACTCCAAATATCCACTTGTAGATTCTATAAAAAGGAATG-T
          1 CTTTTCCACCATAGGCCTCAAAGCGCTCCAAATATCCACTTGCAGATTCTACAAAAA-GAGTGTT

                  *        *    *    *      * *         *      *             
     308719 TCAAAATTGCTCAATAAAAATAAAGTTTCAACACCGTGAGATGAGTGCACAAATCACAAAGAAGT
         65 TCAAAACTGCTCAATCAAAAGAAAGGTTCAACTCTGTGAGATGAATGCACACATCACAAAGAAGT

                  *          *         *          *   
     308784 TTCTCAAAATGCTTCTGGGTAGTTTTTCTGTGAAGATAGTTC
        130 TTCTCAGAATGCTTCTGTGTAGTTTTTATGTGAAGATATTTC

                  *     *    *     *          *                         *    
     308826 CTTTTCTACCATGGGCCACAAAGGGCTCCAAATACCCACTTGCAGATTCTACAAAAAGAGAGTTT
          1 CTTTTCCACCATAGGCCTCAAAGCGCTCCAAATATCCACTTGCAGATTCTACAAAAAGAGTGTTT

              *        *      ** * *        *    * *    **  * *           *  
     308891 CACAACTGCTCTATCAAACAATATGTTCAACTTTGTGGGTTGAACACAAATATCAC-AAGAATTT
         66 CAAAACTGCTCAATCAAAAGAAAGGTTCAACTCTGTGAGATGAATGCACACATCACAAAGAAGTT

                **                             *     
     308955 TCTCCCAATGCTTCTGTGTAGTTTTTATGTGAAGACATTTC
        131 TCTCAGAATGCTTCTGTGTAGTTTTTATGTGAAGATATTTC

            *   *  *      *  *     *                 *  *      *       *  *  
     308996 TTTTCCCTCCATAGTCCACAAAGTGCTCCAAATATCCACTTACATATTCTAGAAAAAGATTGCTT
          1 CTTTTCCACCATAGGCCTCAAAGCGCTCCAAATATCCACTTGCAGATTCTACAAAAAGAGTGTTT

            **       *    *               * * *                              
     309061 GGAAACTGCACAATGAAAAGAAAGGTTCAAATATATGAGATGAATGCACACATCACAAAGAAGTT
         66 CAAAACTGCTCAATCAAAAGAAAGGTTCAACTCTGTGAGATGAATGCACACATCACAAAGAAGTT

                                 *                    
     309126 TCTCAGAAT-CTCTCTGTGTAATTTTTATGTGAAGATATTTC
        131 TCTCAGAATGCT-TCTGTGTAGTTTTTATGTGAAGATATTTC

                *     *    *  *   *                        *      *    *  *  
     309167 CTTTCCCACCTTAGGTCTTAAAACGCTCCAAATATCCACTTGCAGATACTACAAGAAGATTGCTT
          1 CTTTTCCACCATAGGCCTCAAAGCGCTCCAAATATCCACTTGCAGATTCTACAAAAAGAGTGTTT

                     *    *        *      *     **                           
     309232 CAAAACTGCACAA-AAAAAGAAATGTTCAATTCTGTTTGATGAATGCACACATCACAAAGAAGTT
         66 CAAAACTGCTCAATCAAAAGAAAGGTTCAACTCTGTGAGATGAATGCACACATCACAAAGAAGTT

                           *                         
     309296 TCTCAGAATGCTTCTCTGTAGTTTTTATGTGAAGATATTTC
        131 TCTCAGAATGCTTCTGTGTAGTTTTTATGTGAAGATATTTC

                     *             *                 *         **      * *   
     309337 CTTTTCCACAATAGGCCTCAAAGGGCTCCAAATATCCACTTCCAGATTCTATGAAAAGAATATTT
          1 CTTTTCCACCATAGGCCTCAAAGCGCTCCAAATATCCACTTGCAGATTCTACAAAAAGAGTGTTT

             *              * *    *                                      *  
     309402 CCAAACTGCTCAATCATAGGAAATGTTCAACTCTGTGAGATGAATGCACACATCAC-AAGAAATT
         66 CAAAACTGCTCAATCAAAAGAAAGGTTCAACTCTGTGAGATGAATGCACACATCACAAAGAAGTT

                     *    *      *       *       *   
     309466 TCTCAGAATCCTTCAGTGTAGGTTTTATGAGAAGATAATTC
        131 TCTCAGAATGCTTCTGTGTAGTTTTTATGTGAAGATATTTC

                     *    **        *   *                           *    *   
     309507 CTTTTCCACAATAGTTCTCAAAGCACTCAAAATATCCACTTGCAGATTCTACAAAAGGAGTATTT
          1 CTTTTCCACCATAGGCCTCAAAGCGCTCCAAATATCCACTTGCAGATTCTACAAAAAGAGTGTTT

                                                          *                  
     309572 CAAAACTGCTCAATCAAAAGAAAGGTTCAACTCTGTGAGATGAATGGACACATCACAAAGAAGTT
         66 CAAAACTGCTCAATCAAAAGAAAGGTTCAACTCTGTGAGATGAATGCACACATCACAAAGAAGTT

                                  *   *              
     309637 TCTCAGAATGCTTCTGTGTAGTATTTTTGTGAAGATATTT-
        131 TCTCAGAATGCTTCTGTGTAGTTTTTATGTGAAGATATTTC

                          *  * * * * ***             *            *     * *  
     309677 CTTTTCCACCATAGACCGCCAGGGGACACAAATATCCACTTTCAGATTCTACAACAAGAGAGGTT
          1 CTTTTCCACCATAGGCCTCAAAGCGCTCCAAATATCCACTTGCAGATTCTACAAAAAGAGTGTTT

                   *   *     *   ** *       *      *                      * *
     309742 CAAAACTACTCGATCAAGAGATGGTTTCAACTATGTGAGTTGAATGCACACATCACAAAGAACTA
         66 CAAAACTGCTCAATCAAAAGAAAGGTTCAACTCTGTGAGATGAATGCACACATCACAAAGAAGTT

             *  *    *                               
     309807 TGTCGGAATTCTTCTGTGTAGTTTTTATGTGAAGATATTTC
        131 TCTCAGAATGCTTCTGTGTAGTTTTTATGTGAAGATATTTC

                     *    * *      * *    *                  *               
     309848 CTTTTCCACAATAGACGTCAAAGTGATCCAGATATCCACTTGCAGATTCCACAAAAAGAGTGTTT
          1 CTTTTCCACCATAGGCCTCAAAGCGCTCCAAATATCCACTTGCAGATTCTACAAAAAGAGTGTTT

                 *   *   *                  **                     *  *      
     309913 CAAAAGTGCACAACCAAAAGAAAGGTTCAACTAGGTGAGATGAATGCACACATCAGAAGGAAGTT
         66 CAAAACTGCTCAATCAAAAGAAAGGTTCAACTCTGTGAGATGAATGCACACATCACAAAGAAGTT

                            **   *     * *       *   
     309978 TCTCAGAATGCTTCTGCATAGCTTTTAAGGGAAGATACTTC
        131 TCTCAGAATGCTTCTGTGTAGTTTTTATGTGAAGATATTTC

                    *               *            * *  *    * *               
     310019 CTTTTCCAACATAGGCCTCAAAGCACTCCAAATATCCTCCTGGAGATCCCACAAAAAGAGTGTTT
          1 CTTTTCCACCATAGGCCTCAAAGCGCTCCAAATATCCACTTGCAGATTCTACAAAAAGAGTGTTT

            **                      *  *                 *                   
     310084 GCAAACTGCTCAATCAAAAGAAAGATTTAACTCTGTGAGATGAATCCACACATCACAAAGAAGTT
         66 CAAAACTGCTCAATCAAAAGAAAGGTTCAACTCTGTGAGATGAATGCACACATCACAAAGAAGTT

                                       **            
     310149 TCTCAGAATGCTTCTGTGTAGTTTTTAATTGAAGATATTTC
        131 TCTCAGAATGCTTCTGTGTAGTTTTTATGTGAAGATATTTC

            *        *                  *            *  *                    
     310190 TTTTTCCACAATAGGCCTCAAAGCGCTCAAAATATCCACTTTCACATTCTACAAAAAGAGTGTTT
          1 CTTTTCCACCATAGGCCTCAAAGCGCTCCAAATATCCACTTGCAGATTCTACAAAAAGAGTGTTT

                     *           *                 *       *    *  *     *   
     310255 CAAAACTGCACAATCAAAAGATA-GTTCAACTCTGTGAGTTGAATGCGCACAACAAAAAGATGTT
         66 CAAAACTGCTCAATCAAAAGAAAGGTTCAACTCTGTGAGATGAATGCACACATCACAAAGAAGTT

                *     **                              
     310319 TCTCGGAATTATTTCTGTGTAGTTTTTATGTGAAGATATTTC
        131 TCTCAGAA-TGCTTCTGTGTAGTTTTTATGTGAAGATATTTC

                     *  *          *                                     *  *
     310361 CTTTTCCACAATGGGCCTCAAAGTGCTCCAAATATCCACTTGCAGATTCTACAAAAAGAGTTTTC
          1 CTTTTCCACCATAGGCCTCAAAGCGCTCCAAATATCCACTTGCAGATTCTACAAAAAGAGTGTTT

                       *            *    *                    * *           *
     310426 CAAAACTGCTCCATCAAAAGAAAGTTTCACCTCTGTGAGATGAATGCACATACCACAAAGAAGTG
         66 CAAAACTGCTCAATCAAAAGAAAGGTTCAACTCTGTGAGATGAATGCACACATCACAAAGAAGTT

                                                     
     310491 TCTCAGAATGCTTCTGTGTAGTTTTTATGTGAAGATATTTC
        131 TCTCAGAATGCTTCTGTGTAGTTTTTATGTGAAGATATTTC

                     *   *   *    **           *               *     *       
     310532 CTTTTCCACAATAAGCCCCAAAAGGCTCCAAATATTCACTTGCAGATTCTAAAAAAACAGTGTTT
          1 CTTTTCCACCATAGGCCTCAAAGCGCTCCAAATATCCACTTGCAGATTCTACAAAAAGAGTGTTT

                                                    *      *        **       
     310597 CAAAACTGCTCAATCAAAAGAAAGGTTCAACTCTGTGAGAAGAATGCTCACATCACTGAGAAGTT
         66 CAAAACTGCTCAATCAAAAGAAAGGTTCAACTCTGTGAGATGAATGCACACATCACAAAGAAGTT

                                        *            
     310662 TCTCAGAATGCTTCTGTGTAGTTTTTATATGAAGATATTTC
        131 TCTCAGAATGCTTCTGTGTAGTTTTTATGTGAAGATATTTC

                *     *      *    **                       *   **       *    
     310703 CTTTCCCACCGTAGGCCACAAAAGGCTCCAAATATCCACTTGCAGATACTATGAAAAGAGAGTTT
          1 CTTTTCCACCATAGGCCTCAAAGCGCTCCAAATATCCACTTGCAGATTCTACAAAAAGAGTGTTT

                        *        *               ***            *            
     310768 CAAAACTGCTCATTCAAAAGATAGGTTCAACTCTGTGGTTTGAATGCACACAGCACAAAGAAGTT
         66 CAAAACTGCTCAATCAAAAGAAAGGTTCAACTCTGTGAGATGAATGCACACATCACAAAGAAGTT

              *       **                   **   *    
     310833 TCACAGAATGTGTCTGTGTAGTTTTTATGTGCGGATGTTTC
        131 TCTCAGAATGCTTCTGTGTAGTTTTTATGTGAAGATATTTC

                         *        ****   *   *                    *          
     310874 CTTTTCCACCATATGCCT-AAATATTTCCCAATTTCCACTTGCAGATTCTACAAGAAGAGTGTTT
          1 CTTTTCCACCATAGGCCTCAAAGCGCTCCAAATATCCACTTGCAGATTCTACAAAAAGAGTGTTT

                      **       *       *           *            *      ***   
     310938 CAAAACTGCTGTATC-AAATAAA-GTTGAACTCTGTGAGGTGAATGCACACAGCACAAAATGGTT
         66 CAAAACTGCTCAATCAAAAGAAAGGTTCAACTCTGTGAGATGAATGCACACATCACAAAGAAGTT

                          **   *        *       *    
     311001 TCTCAGAATGCTTCCTTGTTGTTTTTATATGAAGATGTTTC
        131 TCTCAGAATGCTTCTGTGTAGTTTTTATGTGAAGATATTTC

                  *  *             *   *     *                               
     311042 CTTTTCAACAATAGGCCTCAAAGTGCTTCAAATGTCCACTTGCAGATTCTACAAAAAGAGTGTTT
          1 CTTTTCCACCATAGGCCTCAAAGCGCTCCAAATATCCACTTGCAGATTCTACAAAAAGAGTGTTT

                                        *      *  *  *                       
     311107 CAAAACTGCTCAATCAAAAGAAAGGTTCGACTCTGGGAAATTAATGCACACATCACAAAGAAGTT
         66 CAAAACTGCTCAATCAAAAGAAAGGTTCAACTCTGTGAGATGAATGCACACATCACAAAGAAGTT

                                     *        *      
     311172 TCTC---A-GCTTCTGTGTAGTTTTCATGTGAAGTTATTTC
        131 TCTCAGAATGCTTCTGTGTAGTTTTTATGTGAAGATATTTC

                     *       *     *            *    *         **       *    
     311209 CTTTTCCACAATAGGCCGCAAAGGGCTCCAAATATCAACTTACAGATTCTAGGAAAAGAGAGTTT
          1 CTTTTCCACCATAGGCCTCAAAGCGCTCCAAATATCCACTTGCAGATTCTACAAAAAGAGTGTTT

                       * **      *     *                                     
     311274 CAAAACTGCTCTACGAAAAGATAGGTTGAACTCTGTGAGATGAATGCACACATCACAAAGAAGTT
         66 CAAAACTGCTCAATCAAAAGAAAGGTTCAACTCTGTGAGATGAATGCACACATCACAAAGAAGTT

                       *               * *           
     311339 TCTCAGAATGCATCTGTGTAGTTTTTACGGGAAGATATTTC
        131 TCTCAGAATGCTTCTGTGTAGTTTTTATGTGAAGATATTTC

                        ***  *     **      *  *                  *     ***   
     311380 CTTTTCCACCATCTTCCACAAAGGTCTCCAAGTAACCACTTGCAGATTCTACAGAAAGACACTTT
          1 CTTTTCCACCATAGGCCTCAAAGCGCTCCAAATATCCACTTGCAGATTCTACAAAAAGAGTGTTT

            *          *         ***      *        *                       * 
     311445 AAAAACTGCTCTATCAAAAGATCAGTTCAAGTCTGTG-GTTGGAATGCACACATCACAAAGAATT
         66 CAAAACTGCTCAATCAAAAGAAAGGTTCAACTCTGTGAGAT-GAATGCACACATCACAAAGAAGT

                                      *  *           *
     311509 TTCTCAGAATGCTTCTGTGTAGTTTTCATATGAAGATATTTT
        130 TTCTCAGAATGCTTCTGTGTAGTTTTTATGTGAAGATATTTC

                                   *                                         
     311551 CTTTTCCACCATAGGCCTCAAAGTGCTCCAAATATCCACTTGCAGATTCTACAAAAAGAGTGTTT
          1 CTTTTCCACCATAGGCCTCAAAGCGCTCCAAATATCCACTTGCAGATTCTACAAAAAGAGTGTTT

                                      *       * **       *   **  *       *   
     311616 CAAAACTGCTCAATCAAAAGAAAGGTACAACTCTTTTTGATGAATTCACTAATAACAAAGACGTT
         66 CAAAACTGCTCAATCAAAAGAAAGGTTCAACTCTGTGAGATGAATGCACACATCACAAAGAAGTT

                 *         *                    *    
     311681 TCTCACAATGCTTCTCTGTAGTTTTTATGTGAAGATCTTTC
        131 TCTCAGAATGCTTCTGTGTAGTTTTTATGTGAAGATATTTC

                  *         *       *  *          * *                        
     311722 CTTTTCTACCATAGGCATCAAAGCACTACAAATATCCAATAGCAGATTCTACAAAAAGAGTGTTT
          1 CTTTTCCACCATAGGCCTCAAAGCGCTCCAAATATCCACTTGCAGATTCTACAAAAAGAGTGTTT

                    *         *    *       *                *    *           
     311787 CAAAACTGTTCAATCAAATGAAACGTTCAACCCTGTGAGATGAATGCAGACATTA-AAAGATAGT
         66 CAAAACTGCTCAATCAAAAGAAAGGTTCAACTCTGTGAGATGAATGCACACATCACAAAGA-AGT

                  **   *     *                **      
     311851 TTCTCATTATGGTTCTGGGTAGTTTTTATGTGAACTTATTTC
        130 TTCTCAGAATGCTTCTGTGTAGTTTTTATGTGAAGATATTTC

                    **       **    *       *                              *  
     311893 CTTTTCCAAAATAGGCCGGAAAGTGCTCCAAGTATCCACTTGCAGATTCTACAAAAAGA--GATT
          1 CTTTTCCACCATAGGCCTCAAAGCGCTCCAAATATCCACTTGCAGATTCTACAAAAAGAGTGTTT

                   *   *         *     *     *       *      *         *      
     311956 CAAAACTCCTCCATCAAAAGATAGGTTGAACTCCGTGAGATTAATGCATACATCACAACGAAGTT
         66 CAAAACTGCTCAATCAAAAGAAAGGTTCAACTCTGTGAGATGAATGCACACATCACAAAGAAGTT

                   **     **             *           
     312021 TCTCAGAGGGCTTCATTGTAG-TTTTATGGGAAGATATTTC
        131 TCTCAGAATGCTTCTGTGTAGTTTTTATGTGAAGATATTTC

                     *   * *       ***         *                    *   *    
     312061 CTTTTCCACTATATGTCTCAAAGATATCCAAATATACACTTGCAGATTCTACAAAATGAGAGTTT
          1 CTTTTCCACCATAGGCCTCAAAGCGCTCCAAATATCCACTTGCAGATTCTACAAAAAGAGTGTTT

                       **      *          *  ***    *                        
     312126 CAAAACTGCTCTGTCAAAAAAAGAGGTTCAGCTGCATGAGTTGAATGCACACATCACAAAGAAGT
         66 CAAAACTGCTCAATCAAAAGAA-AGGTTCAACTCTGTGAGATGAATGCACACATCACAAAGAAGT

                                 *         *         *
     312191 TTCTCAGAATGCTTCTGTGTATTTTTTATGTTAAGATATTTT
        130 TTCTCAGAATGCTTCTGTGTAGTTTTTATGTGAAGATATTTC

                            **     *                                    **   
     312233 CTTTTCCACCATAGGCTGCAAAGGGCTCCAAATATCCACTTGCAGATTCTACAAAAAGAGATTTT
          1 CTTTTCCACCATAGGCCTCAAAGCGCTCCAAATATCCACTTGCAGATTCTACAAAAAGAGTGTTT

                                     *                          **      *    
     312298 CAAAACTGCTCAATCAAAAGAAAGGCTCAACTCTGTGAGATGAATGCACACAAAACAAAGGAGTT
         66 CAAAACTGCTCAATCAAAAGAAAGGTTCAACTCTGTGAGATGAATGCACACATCACAAAGAAGTT

                                        *         *   
     312363 TCTGCA-AATGCTTCTGTGTAGTTTTTAAGTGAAGATACTTC
        131 TCT-CAGAATGCTTCTGTGTAGTTTTTATGTGAAGATATTTC

                     *     *   *    *             **  **                     
     312404 CTTTTCCACAATAGGTCTCGAAGCCCTCCAAATATCCAAATGTGGATTCTACAAAAAGAGTGTTT
          1 CTTTTCCACCATAGGCCTCAAAGCGCTCCAAATATCCACTTGCAGATTCTACAAAAAGAGTGTTT

                              * *   *         **  *      *     * *           
     312469 CAAAACTGCTCAATCAAATGGAAGCTTCAACTCTAAGAAATGAATACACACCTGACAAAGAAGTT
         66 CAAAACTGCTCAATCAAAAGAAAGGTTCAACTCTGTGAGATGAATGCACACATCACAAAGAAGTT

                      *  *            ** *    ***   *
     312534 TCTCAGAATGATTTTGTGTAGTTTTTTGGGGAAGGCGTTTT
        131 TCTCAGAATGCTTCTGTGTAGTTTTTATGTGAAGATATTTC

             *          *  * *     **         *    *             *  *   *    
     312575 CCTTTCCACCATCGGTCACAAAGGCCTCCAAATAACCACATGCAGATTCTACACAAGGAGAGTTT
          1 CTTTTCCACCATAGGCCTCAAAGCGCTCCAAATATCCACTTGCAGATTCTACAAAAAGAGTGTTT

                       **        *       *     *   *                 * *     
     312640 CAAAACTGCTCTGTCAAAAGATAGGTTCAGCTCTGCGAGTTGAATGCACACATCACAGACAAGTT
         66 CAAAACTGCTCAATCAAAAGAAAGGTTCAACTCTGTGAGATGAATGCACACATCACAAAGAAGTT

                                     *               
     312705 TCTCAGAATGCTTCTGTGTAGTTTTCATGTGAAGATATTTC
        131 TCTCAGAATGCTTCTGTGTAGTTTTTATGTGAAGATATTTC

                     **      *      *                     **             *   
     312746 CTTTTCCACTTTAGGCCACAAAGCACTCCAAATATCCACTTGCAGAAACTACAAAAAGAGTCTTT
          1 CTTTTCCACCATAGGCCTCAAAGCGCTCCAAATATCCACTTGCAGATTCTACAAAAAGAGTGTTT

                         *         *                                    *    
     312811 CAAAACTGCTCAAGCAAAAGAAAAGTTCAACTCTGTGAGATGAATGCACACATCACAAAGGAGTT
         66 CAAAACTGCTCAATCAAAAGAAAGGTTCAACTCTGTGAGATGAATGCACACATCACAAAGAAGTT

               *                                     
     312876 TCTGAGAATGCTTCTGTGTAGTTTTTATGTGAA-ATATTTC
        131 TCTCAGAATGCTTCTGTGTAGTTTTTATGTGAAGATATTTC

                            *      *           *      * *   *  *   *   * *   
     312916 CTTTTCCACCATAGGCTTCAAAGTGCTCCAAATATTCACTTGTAAATTATAAAAACAGAATTTTT
          1 CTTTTCCACCATAGGCCTCAAAGCGCTCCAAATATCCACTTGCAGATTCTACAAAAAGAGTGTTT

                 *      * *       * *      *                         *       
     312981 CAAAAATGCTCAGTTAAAAGAATGTTTCAACACTGTGAGATGAATGCACACATCACAGA-AAGTT
         66 CAAAACTGCTCAATCAAAAGAAAGGTTCAACTCTGTGAGATGAATGCACACATCACAAAGAAGTT

               **              *            *        
     313045 TCTGGGAATGCTTCTGTGTGGTTTTTATGTGAGGATATTTC
        131 TCTCAGAATGCTTCTGTGTAGTTTTTATGTGAAGATATTTC

                          * ** *   *                                    *    
     313086 CTTTTCCACCATAGACTACCAAGGGCTCCAAATATCCACTTGCAGATTCTACAAAAAGAGAGTTT
          1 CTTTTCCACCATAGGCCTCAAAGCGCTCCAAATATCCACTTGCAGATTCTACAAAAAGAGTGTTT

             *         *         *        * *     *                     *    
     313151 CGAAA-TGCTCTATCAAAAGATAGGTTCAAATATGTGATATGAATGCACACATCACAAAGTAGTT
         66 CAAAACTGCTCAATCAAAAGAAAGGTTCAACTCTGTGAGATGAATGCACACATCACAAAGAAGTT

                          *               *          
     313215 TCTCAGAATGCTTCCGTGTAGTTTTTATGTAAAGATATTTC
        131 TCTCAGAATGCTTCTGTGTAGTTTTTATGTGAAGATATTTC

                                    *                                   **   
     313256 CTTTTCCACCATAGGCCTCAAAGCACTCCAAATATCCACTTGCAGATTCTACAAAAAGAGATTTT
          1 CTTTTCCACCATAGGCCTCAAAGCGCTCCAAATATCCACTTGCAGATTCTACAAAAAGAGTGTTT

             *     **                           *  *          *        *     
     313321 CCAAACTAGTCAATCAAAAGAAAGGTTCAACTCTGTCAGTTGAATGCACATATCACAAACAAGTT
         66 CAAAACTGCTCAATCAAAAGAAAGGTTCAACTCTGTGAGATGAATGCACACATCACAAAGAAGTT

                *      *                             
     313386 TCTCGGAATGCGTCTGTGTAGTTTTTATGTGAAGATATTTC
        131 TCTCAGAATGCTTCTGTGTAGTTTTTATGTGAAGATATTTC

               *     * *           *     *                  *    *           
     313427 CTTCTCCACAACAGGCCTCAAAGTGCTCCGAATATCCACTTGCAGATTTTAC-TAAAGAGTGTTT
          1 CTTTTCCACCATAGGCCTCAAAGCGCTCCAAATATCCACTTGCAGATTCTACAAAAAGAGTGTTT

             *               *  *   *     *        *    *               *    
     313491 CCAAACTGCTCAATCAAGAGGAAGTTTCAAGTCTGTGAGCTGAACGCACACATCACAAAGTAGTT
         66 CAAAACTGCTCAATCAAAAGAAAGGTTCAACTCTGTGAGATGAATGCACACATCACAAAGAAGTT

               *                                *    
     313556 TCTGAGAATGCTTCTGTGTAGTTTTTATGTGAAGATGTTTC
        131 TCTCAGAATGCTTCTGTGTAGTTTTTATGTGAAGATATTTC

                            **     *                                    *    
     313597 CTTTTCCACCATAGGCTGCAAAGGGCTCCAAATATCCACTTGCAGATTCTACAAAAAGAGAGTTT
          1 CTTTTCCACCATAGGCCTCAAAGCGCTCCAAATATCCACTTGCAGATTCTACAAAAAGAGTGTTT

                 *     *         *          *     *                     *    
     313662 CAAAAGTGCTCTATCAAAAGATAGGTTCAACTATGTGATATGAATGCACACATCACAAAGTAGTT
         66 CAAAACTGCTCAATCAAAAGAAAGGTTCAACTCTGTGAGATGAATGCACACATCACAAAGAAGTT

                                          *          
     313727 TCTCAGAATGCTTCTGTGTAGTTTTTATGTAAAGATATTTC
        131 TCTCAGAATGCTTCTGTGTAGTTTTTATGTGAAGATATTTC

                                    *                                        
     313768 CTTTTCCACCATAGGCCTCAAAGCACTCCAAATATCCACTTGCAGATTCTACAAAAAGAGATG-T
          1 CTTTTCCACCATAGGCCTCAAAGCGCTCCAAATATCCACTTGCAGATTCTACAAAAAGAG-TGTT

                    ** *                   *     *  *    * *   *        *    
     313832 TCAAAACTATTTAATCAAAAGAAAGGTTCAAATCTGTCAGTTGAAGGTACATATCACAAACAAGT
         65 TCAAAACTGCTCAATCAAAAGAAAGGTTCAACTCTGTGAGATGAATGCACACATCACAAAGAAGT

              * **                                    
     313897 TTATTGGAATGCTTCTGTGTAGTTTTTATGTGAAGATATTTC
        130 TTCTCAGAATGCTTCTGTGTAGTTTTTATGTGAAGATATTTC

                     * *         * *                        **  *     *      
     313939 CTTTTCCACAACAGGCCTCAAGGTGCTCCAAATATCCACTTGCAGATTTCACTAAAAGTGTGTTT
          1 CTTTTCCACCATAGGCCTCAAAGCGCTCCAAATATCCACTTGCAGATTCTACAAAAAGAGTGTTT

             *               *  *   *     *        *             *           
     314004 CCAAACTGCTCAATCAAGAGGAAGTTTCAAGTCTGTGAGGTGAATGCACACATTACAAAGAAGTT
         66 CAAAACTGCTCAATCAAAAGAAAGGTTCAACTCTGTGAGATGAATGCACACATCACAAAGAAGTT

            *  *                                     
     314069 ACTGAGAATGCTTCTGTGTAGTTTTTATGTGAAGATATTTC
        131 TCTCAGAATGCTTCTGTGTAGTTTTTATGTGAAGATATTTC

                      **               *                                *    
     314110 CTTTTCCACCGCAGGCCTCAAAGCGCTGCAAATATCCACTTGCAGATTCTACAAAAAGAGAGTTT
          1 CTTTTCCACCATAGGCCTCAAAGCGCTCCAAATATCCACTTGCAGATTCTACAAAAAGAGTGTTT

                      **         *   *         *   *     **            *     
     314175 CAAAACTGCTGTATCAAAAGATAGGGTCAACTCTGCGAGTTGAATAAACACATCACAAATAAGTT
         66 CAAAACTGCTCAATCAAAAGAAAGGTTCAACTCTGTGAGATGAATGCACACATCACAAAGAAGTT

               **   *        *               *       
     314240 TCTGGGAACGCTTCTGTATAG-TTTTATGTGAATATATTTC
        131 TCTCAGAATGCTTCTGTGTAGTTTTTATGTGAAGATATTTC

                         *          *                   *   *  *   * *   *   
     314280 CTTTTCCACCATATGCCTCAAAGCACTCCAAATATCCACTTGCACATTATAGAAACATAGTCTTT
          1 CTTTTCCACCATAGGCCTCAAAGCGCTCCAAATATCCACTTGCAGATTCTACAAAAAGAGTGTTT

                   **                        *         *             *       
     314345 CAAAACTTGTCAATC-AAAGAAAGGTTCAACTCCGTGAGATGAGTGCACACATCACAGAGAAGTT
         66 CAAAACTGCTCAATCAAAAGAAAGGTTCAACTCTGTGAGATGAATGCACACATCACAAAGAAGTT

                *     *                            * 
     314409 TCTCGGAATGTTTCTGTGTAGTTTTTATGTGAAGATATTGC
        131 TCTCAGAATGCTTCTGTGTAGTTTTTATGTGAAGATATTTC

                     *               *            *          *       *   *   
     314450 CTTTTCCACAATAGGCCTCAAAGCGTTCCAAATATCCAATTGCAGATTCCACAAAAAAAGTTTTT
          1 CTTTTCCACCATAGGCCTCAAAGCGCTCCAAATATCCACTTGCAGATTCTACAAAAAGAGTGTTT

            *                 *  *  *  *             * *         **    *     
     314515 TAAAACTGCTCAATCAAATGATAGATTAAACTCTGTGAGATTAGTGCACACATGTCAAAAAAGTT
         66 CAAAACTGCTCAATCAAAAGAAAGGTTCAACTCTGTGAGATGAATGCACACATCACAAAGAAGTT

                                *      * *           
     314580 TCTCAGAATGCTTCTGTGTACTTTTTAGGGGAAGATATTTC
        131 TCTCAGAATGCTTCTGTGTAGTTTTTATGTGAAGATATTTC

                        *  * *     **         *    *           **  * *  *    
     314621 CTTTTCCACCATCGGTCACAAAGGACTCCAAATAACCACATGCAGATTCTAGTAACACAGAGTTT
          1 CTTTTCCACCATAGGCCTCAAAGCGCTCCAAATATCCACTTGCAGATTCTACAAAAAGAGTGTTT

                       *         * *           *   * * *    **    ***        
     314686 CAAAACTGCTCTATCAAAAGATAAGTTCAACTCTGAGAGTTTAGTGCAACCATCGTGAAGAAGTT
         66 CAAAACTGCTCAATCAAAAGAAAGGTTCAACTCTGTGAGATGAATGCACACATCACAAAGAAGTT

                            *     *                  
     314751 TCTCAGAATGCTTCTGAGTAGTGTTTATGTGAAGATATTTC
        131 TCTCAGAATGCTTCTGTGTAGTTTTTATGTGAAGATATTTC

                              *     *                      *           **    
     314792 CTTTTCCACCATAGGCCTGAAAGCCCTCCAAATATCCACTTGCAGATCCTACAAAAAGAAAGTTT
          1 CTTTTCCACCATAGGCCTCAAAGCGCTCCAAATATCCACTTGCAGATTCTACAAAAAGAGTGTTT

             *         **     *  *  *   *        **      *                   
     314857 CGAAA-TGCTCTCTCAAACGATAGTTTCGACTCTGTGGTATGAATACACACATCACAAAGAAGTT
         66 CAAAACTGCTCAATCAAAAGAAAGGTTCAACTCTGTGAGATGAATGCACACATCACAAAGAAGTT

                                       **            
     314921 TCTCAGAATGCTTCTGTGTAGTTTTTAAATGAAGATATTTC
        131 TCTCAGAATGCTTCTGTGTAGTTTTTATGTGAAGATATTTC

            *                       *          *     *                       
     314962 TTTTTCCACCATAGGCCTCAAAGCACTCCAAATATGCACTTCCAGATTCTACAAAAAGAGTGTTT
          1 CTTTTCCACCATAGGCCTCAAAGCGCTCCAAATATCCACTTGCAGATTCTACAAAAAGAGTGTTT

              *          *      *       * *         **   *         *  * * *  
     315027 CAGAACTGCTCAAACAAAAGGAAGGTTCCAGTC--TGAGACAAATACACACATCAAAAGGTAATT
         66 CAAAACTGCTCAATCAAAAGAAAGGTTCAACTCTGTGAGATGAATGCACACATCACAAAGAAGTT

                                                    *
     315090 TCTCAGAATGCTTCTGTGTAGTTTTTATGTGAAGATATTTT
        131 TCTCAGAATGCTTCTGTGTAGTTTTTATGTGAAGATATTTC

             *               *    **    *     *      *  **   *   *      *    
     315131 CCTTTCCACCATAGGCCACAAATGGCTCTAAATACCCACTTACATTTTCCACAGAAAGAGAGTTT
          1 CTTTTCCACCATAGGCCTCAAAGCGCTCCAAATATCCACTTGCAGATTCTACAAAAAGAGTGTTT

                       * *    * *      *   *         *                       
     315196 CAAAACTGCTCTACCAAAGGTAA-GTTTAACGCTGTGAG-TTAA-G-A-ACATCACAAAGAAGTT
         66 CAAAACTGCTCAATCAAAAGAAAGGTTCAACTCTGTGAGATGAATGCACACATCACAAAGAAGTT

                                   *      *          
     315256 TCTCAGAATGCTTCTGTGTAGTTCTTATGTAAAGATATTTC
        131 TCTCAGAATGCTTCTGTGTAGTTTTTATGTGAAGATATTTC

                 *   *      ***    *                  *              **      
     315297 CTTTTACACAATAGGCAGAAAAGTGCTCCAAATATCCACTTGAAGATTCTACAAAAACCGTGTTT
          1 CTTTTCCACCATAGGCCTCAAAGCGCTCCAAATATCCACTTGCAGATTCTACAAAAAGAGTGTTT

                     **             *                            *      *    
     315362 CAAAACTGCCGAATCAAAAGAAAGTTTCAACTCTGTGAGATGAATGCACACATAACAAAGGAGTT
         66 CAAAACTGCTCAATCAAAAGAAAGGTTCAACTCTGTGAGATGAATGCACACATCACAAAGAAGTT

                                 *      *           *
     315427 TCTCAGAATGCTTCTGTGTAGCTTTTATATGAAGATATTTA
        131 TCTCAGAATGCTTCTGTGTAGTTTTTATGTGAAGATATTTC

            *        * *            *   ***                     **        *  
     315468 GTTTTCCACAACAGGCCTCAAAGCTCTCTCCATATCCACTTGCAGATTCTACCGAAAGAGTGCTT
          1 CTTTTCCACCATAGGCCTCAAAGCGCTCCAAATATCCACTTGCAGATTCTACAAAAAGAGTGTTT

             *                   * **     *        *        *     **         
     315533 CCAAACTGCTCAATCAAAAGAGACATTCAAATCTGTGAGGTGAATGCAGACATCGTAAAGAAGTT
         66 CAAAACTGCTCAATCAAAAGAAAGGTTCAACTCTGTGAGATGAATGCACACATCACAAAGAAGTT

                                *     *       *    **
     315598 TCTCAGAATGCTTCTGTGTATTTTTTGTGTGAAGTTATTCG
        131 TCTCAGAATGCTTCTGTGTAGTTTTTATGTGAAGATATTTC

            *    *             *     *  *            **                 *    
     315639 TTTTTGCACCATAGGCCTCCAAGCGTTCTAAATATCCACTTCTAGATTCTACAAAAAGAGAGTTT
          1 CTTTTCCACCATAGGCCTCAAAGCGCTCCAAATATCCACTTGCAGATTCTACAAAAAGAGTGTTT

                   *         *            *        *    *   *                
     315704 CAAAACTACTC-A--AACA-AAAGGTTCAATTCTGTGAGTTGAAAGCAAACATCACAAAGAAGTT
         66 CAAAACTGCTCAATCAAAAGAAAGGTTCAACTCTGTGAGATGAATGCACACATCACAAAGAAGTT

                       *                             
     315765 TCTCAGAATGCGTCTGTGTAGTTTTTATGTGAAGATATTTC
        131 TCTCAGAATGCTTCTGTGTAGTTTTTATGTGAAGATATTTC

                     *    ****     *                  *                      
     315806 CTTTT-CACAATAGAATGCAAAGGGCTCCAAATATCCACTTGGAGATTCTACAAAAAGA--GTTT
          1 CTTTTCCACCATAGGCCTCAAAGCGCTCCAAATATCCACTTGCAGATTCTACAAAAAGAGTGTTT

                  *    **     *  *     *     ***   *     *               *   
     315868 CAAAACCGCTCTGTCAAATGATAGGTTGAACTCCCGGAGGTGAATACACACATCACAAAGAGGTT
         66 CAAAACTGCTCAATCAAAAGAAAGGTTCAACTCTGTGAGATGAATGCACACATCACAAAGAAGTT

                  *                       *  *       
     315933 TCTCAGCATGCTTCTGTGTAGTTTTTATGTAAACATATTTC
        131 TCTCAGAATGCTTCTGTGTAGTTTTTATGTGAAGATATTTC

             *    * *              *           *      * *       *   *    *   
     315974 CGTTTCTATCATAGGCCTCAAAGTGCTCCAAATATTCACTTGTACATTCTACCAAACGAGTATTT
          1 CTTTTCCACCATAGGCCTCAAAGCGCTCCAAATATCCACTTGCAGATTCTACAAAAAGAGTGTTT

                              * *         **      *        *            *    
     316039 CAAAACTGCTCAATCAAATGGAAGGTTCAAAACTGTGACATGAATGCCCACATCACAAAGTAGTT
         66 CAAAACTGCTCAATCAAAAGAAAGGTTCAACTCTGTGAGATGAATGCACACATCACAAAGAAGTT

                                                     
     316104 TCTCAGAATGCTTCTGTGTAGTTTTTATGTGAAGATATTTC
        131 TCTCAGAATGCTTCTGTGTAGTTTTTATGTGAAGATATTTC

                     *     ***          **  **    *     * **           *     
     316145 CTTTTCCACAATAGGTAACAAAGCGCTCTGAAGTTCCAATTGCACACCCTACAAAAAGAATGTTT
          1 CTTTTCCACCATAGGCCTCAAAGCGCTCCAAATATCCACTTGCAGATTCTACAAAAAGAGTGTTT

                              *        **                                    
     316210 CAAAACTGCTCAATCAAATGAAAGGTTACACTCTGTGAGATGAATGCACACATCACAAAGAAGTT
         66 CAAAACTGCTCAATCAAAAGAAAGGTTCAACTCTGTGAGATGAATGCACACATCACAAAGAAGTT

                                       * *   * *     
     316275 TCTCAGAATGCTTCTGTGTAGTTTTTAGGGGAACACATTTC
        131 TCTCAGAATGCTTCTGTGTAGTTTTTATGTGAAGATATTTC

                        *    *    **          **   **     *             *    
     316316 CTTTTCCACCATTGGCCACAAAAGGCTCCAAATAATCACACGCAGAATCTACAAAAAGAGAGTTT
          1 CTTTTCCACCATAGGCCTCAAAGCGCTCCAAATATCCACTTGCAGATTCTACAAAAAGAGTGTTT

                      **  *      *            **   *                         
     316381 CAAAACTGCTGTATGAAAAGATAGGTTCAACTCTACGAGTTGAATGCACACATCACAAAGAAGTT
         66 CAAAACTGCTCAATCAAAAGAAAGGTTCAACTCTGTGAGATGAATGCACACATCACAAAGAAGTT

               *     **  *                 *    *    
     316446 TCTTAGAATTTTTTTGTGTAGTTTTTATGTGGAGATGTTTC
        131 TCTCAGAATGCTTCTGTGTAGTTTTTATGTGAAGATATTTC

            *   *        *          **        *  *             **            
     316487 ATTTCCCACCATATGCCTCAAAGCATTCCAAATAACCCCTTGCAGATTCTATGAAAAGAGTGTTT
          1 CTTTTCCACCATAGGCCTCAAAGCGCTCCAAATATCCACTTGCAGATTCTACAAAAAGAGTGTTT

                 * **   *          *               *       *       *     *   
     316552 CAAAAGTTTTCAGTCAAAAGAAAAGTTCAACTCTGTGAGTTGAATGCCCACATCAGAAAGAGGTT
         66 CAAAACTGCTCAATCAAAAGAAAGGTTCAACTCTGTGAGATGAATGCACACATCACAAAGAAGTT

                 *   *  * *  *                     * 
     316617 TCTCATAATTCTCCCGTATAGTTTTTATGTGAAGATATTGC
        131 TCTCAGAATGCTTCTGTGTAGTTTTTATGTGAAGATATTTC

             *       * *  *   **   *    *   *                           *    
     316658 CATTTCCACAACAGACCTGCAAGGGCTCAAAAAATCCACTTGCAGATTCTACAAAAAGAGAGTTT
          1 CTTTTCCACCATAGGCCTCAAAGCGCTCCAAATATCCACTTGCAGATTCTACAAAAAGAGTGTTT

     316723 AGGCCGGGCA


Statistics
Matches: 254444,  Mismatches: 59851, Indels: 4512
        0.80            0.19        0.01

Matches are distributed among these distances:
 164  702  0.00
 166  8376  0.03
 167  12443  0.05
 168  9166  0.04
 169  15683  0.06
 170  65587  0.26
 171  134448  0.53
 172  7934  0.03
 173  105  0.00

ACGTcount: A:0.33, C:0.20, G:0.17, T:0.30


Consensus pattern (171 bp):   
CTTTTCCACCATAGGCCTCAAAGCGCTCCAAATATCCACTTGCAGATTCTACAAAAAGAGTGTTT
CAAAACTGCTCAATCAAAAGAAAGGTTCAACTCTGTGAGATGAATGCACACATCACAAAGAAGTT
TCTCAGAATGCTTCTGTGTAGTTTTTATGTGAAGATATTTC

Found at i:3144 original size:26 final size:25

Alignment explanation  
  

    Indices: 3115--3163  Score: 61
    Period size: 26  Copynumber: 1.9  Consensus size: 25

       3105 ACTTGCAGAT

                           *          
       3115 TCTACAAAAAGAGAGTTTCGAAATGC
          1 TCTACAAAAAGAGAGGTTC-AAATGC

                **      *          
       3141 TCTATCAAAAGATAGGTTCAAAT
          1 TCTACAAAAAGAGAGGTTCAAAT

       3164 ATGTGATATG


Statistics
Matches: 19,  Mismatches: 4, Indels: 1
        0.79            0.17        0.04

Matches are distributed among these distances:
  25    4  0.21
  26   15  0.79

ACGTcount: A:0.43, C:0.14, G:0.16, T:0.27


Consensus pattern (25 bp):   
TCTACAAAAAGAGAGGTTCAAATGC

Found at i:313162 original size:26 final size:25

Alignment explanation  
  

    Indices: 313133--313181  Score: 61
    Period size: 26  Copynumber: 1.9  Consensus size: 25

     313123 ACTTGCAGAT

                           *          
     313133 TCTACAAAAAGAGAGTTTCGAAATGC
          1 TCTACAAAAAGAGAGGTTC-AAATGC

                **      *          
     313159 TCTATCAAAAGATAGGTTCAAAT
          1 TCTACAAAAAGAGAGGTTCAAAT

     313182 ATGTGATATG


Statistics
Matches: 19,  Mismatches: 4, Indels: 1
        0.79            0.17        0.04

Matches are distributed among these distances:
  25    4  0.21
  26   15  0.79

ACGTcount: A:0.43, C:0.14, G:0.16, T:0.27


Consensus pattern (25 bp):   
TCTACAAAAAGAGAGGTTCAAATGC
Done.
```
