# Supplementary material for: Tigerfish designs oligonucleotide-based in situ hybridization probes targeting intervals of highly repetitive DNA at the scale of genomes
Source: Nat Commun. 2024 Feb 3;15:1027. doi: 10.1038/s41467-024-45385-x (PMC10838309; doi:10.1038/s41467-024-45385-x)
Supplement: Supplementary file 10 — Source Data [file 41467_2024_45385_MOESM10_ESM.gz › Tigerfish_full_data/Figure_S1_HOR_TRF_Analysis/TRF/trf_output/chr2_8_21_Y_hor.fa.s2.2.5.7.80.10.50.2000.1.txt.html]

   chr2_8_21_Y_hor.fa.s2.2.5.7.80.10.50.2000.txt.html    
Tandem Repeats Finder Program written by:

                 Gary Benson
      Program in Bioinformatics
          Boston University

Version 4.09

Sequence: chr8:44214155-46350589

Parameters: 2 5 7 80 10 50 2000

Pmatch=0.80,Pindel=0.10
tuple sizes 0,4,5,7
tuple distances 0, 29, 159, 2000

Length: 2136434
ACGTcount: A:0.30, C:0.17, G:0.22, T:0.31


Found at i:1282 original size:171 final size:171

   Alignment explanation   
    Indices: 949--9753  Score: 8047
    Period size: 171  Copynumber: 51.7  Consensus size: 171

        939 GGTAATTTAC

                 *  *     *                       *      * *                *
        949 TGAACTTTTCTTTTGATAGAGCAGTTTTGAAACACTCTGTTTGTAAAGTCTGCAAGTGGATATTA
          1 TGAACCTTCCTTTTCATAGAGCAGTTTTGAAACACTCTTTTTGTAGAATCTGCAAGTGGATATTT

                ***           ***         *          * *     *     *    *    
       1014 GGAGGATTTTGAGGCCTTCTTTGGAAAAGGGAATATCTTCACAGAAAAATTAGACCGAAGTATTC
         66 GGAGAGCTTTGAGGCCTTTGGTGGAAAAGGAAATATCTTCATATAAAAACTAGACAGAAGCATTC

              *                  *                 * 
       1079 TCTGAAACTTCTTTGTGATGTGTGCATTCAACTCACAGATT
        131 TCAGAAACTTCTTTGTGATGTTTGCATTCAACTCACAGAGT

                          *                                * *               
       1120 TGAACCTTCCTTTTGATAGAGCAGTTTTGAAACACTCTTTTTGTAGATTTTGCAAGTGGATATTT
          1 TGAACCTTCCTTTTCATAGAGCAGTTTTGAAACACTCTTTTTGTAGAATCTGCAAGTGGATATTT

            *   *            *    *      *                      *      * *   
       1185 AGAGCGCTTTGAGGCCTATGGTAGAAAAGTAAATATCTTCATATAAAAACTACACAGAATCCTTC
         66 GGAGAGCTTTGAGGCCTTTGGTGGAAAAGGAAATATCTTCATATAAAAACTAGACAGAAGCATTC

                   * *      *  * *   *               
       1250 TCAGAAAGTACTTTGTTATATGTGCGTTCAACTCACAGAGT
        131 TCAGAAACTTCTTTGTGATGTTTGCATTCAACTCACAGAGT

             *      *     *                       *      * *          *      
       1291 TTAACCTTTCTTTTGATAGAGCAGTTTTGAAACACTCTGTTTGTAAAGTCTGCAAGTGAATATTT
          1 TGAACCTTCCTTTTCATAGAGCAGTTTTGAAACACTCTTTTTGTAGAATCTGCAAGTGGATATTT

                **         *  ***      *  *           * *    *    **    *    
       1356 GGAGCACTTTGAGGCTTTCTTTGGAAACGGGAATATCTTCA-CTTAAAAGTAGATGGAAGTATTC
         66 GGAGAGCTTTGAGGCCTTTGGTGGAAAAGGAAATATCTTCATATAAAAACTAGACAGAAGCATTC

                                 *    * ** *         
       1420 TCAGAAACTTCTTTGTGATGTCTGCACTTTATTCACAGAGT
        131 TCAGAAACTTCTTTGTGATGTTTGCATTCAACTCACAGAGT

                         ***    *               *            *           *   
       1461 TGAACCTTCCTTTCTTTAGAACAGTTTTGAAACACTATTTTTGTAGAATTTGCAAGTGGATGTTT
          1 TGAACCTTCCTTTTCATAGAGCAGTTTTGAAACACTCTTTTTGTAGAATCTGCAAGTGGATATTT

            ** *       *     *    *         *          *      * *            
       1526 ACAAAGCTTTGGGGCCTGTGGTAGAAAAGGAAGTATCTTCATAGAAAAACAACACAGAAGCATTC
         66 GGAGAGCTTTGAGGCCTTTGGTGGAAAAGGAAATATCTTCATATAAAAACTAGACAGAAGCATTC

                     *                              *
       1591 TCAGAAACTACTTTGTGATGTTTGCATTCAACTCACAGAGG
        131 TCAGAAACTTCTTTGTGATGTTTGCATTCAACTCACAGAGT

             *  **       **                *   *          *                  
       1632 TAAATATTCCTTTCGATAGAGCAGTTTTGAACCACCCTTTTTGTAGGATCTGCAAGTGGATATTT
          1 TGAACCTTCCTTTTCATAGAGCAGTTTTGAAACACTCTTTTTGTAGAATCTGCAAGTGGATATTT

               *          **  * *      *  *   *        *    **               
       1697 GGATAGCTTTGAGGATTTCGTTGGAAACGGGAATGTCTTCATAGAAAATTTAGACAGAAGCATTC
         66 GGAGAGCTTTGAGGCCTTTGGTGGAAAAGGAAATATCTTCATATAAAAACTAGACAGAAGCATTC

                  *   **         *  ***  *   *       
       1762 TCAGAACCTTGATTGTGATGTGTGTTCTCCACTAACAGAGT
        131 TCAGAAACTTCTTTGTGATGTTTGCATTCAACTCACAGAGT

                    *     * **  * *           *       *         *            
       1803 TGAACCTTTCTTTTGACGGAACTGTTTTGAAACATTCTTTTTATAGAATCTGGAAGTGGATATTT
          1 TGAACCTTCCTTTTCATAGAGCAGTTTTGAAACACTCTTTTTGTAGAATCTGCAAGTGGATATTT

               *          **  * *      *  *                 *     *          
       1868 GGAAAGCTTTGAGGATTTCGTTGGAAACGGGAATATCTTCATATAAAATCTAGAAAGAAGCATTC
         66 GGAGAGCTTTGAGGCCTTTGGTGGAAAAGGAAATATCTTCATATAAAAACTAGACAGAAGCATTC

             *      *    * *       *       *         
       1933 TAAGAAACATCTTAGGGATGTTTACATTCAAGTCACAGAGT
        131 TCAGAAACTTCTTTGTGATGTTTGCATTCAACTCACAGAGT

                 *    *     *       *         *    * **  **     *       *    
       1974 TGAACATTCCCTTTCACAGAGCAGGTTTGAAACAATCTTCTCATACTATCTGGAAGTGGACATTT
          1 TGAACCTTCCTTTTCATAGAGCAGTTTTGAAACACTCTTTTTGTAGAATCTGCAAGTGGATATTT

            *   ** *   *     *  *  *                ** *                     
       2039 TGAGCTCCTTGGGGCCTATGCTGAAAAAGGAAATATCTTCCGACAAAAACTAGACAGAAGCATTC
         66 GGAGAGCTTTGAGGCCTTTGGTGGAAAAGGAAATATCTTCATATAAAAACTAGACAGAAGCATTC

            *     * * *          *    *      **    * 
       2104 GCAGAATCATGTTTGTGATGTGTGCACTCAACTGTCAGAAT
        131 TCAGAAACTTCTTTGTGATGTTTGCATTCAACTCACAGAGT

                    **   ** *      *                              *          
       2145 TGAACCTTTGTTTGGACAGAGCACTTTTGAAACACTCTTTTTGTAGAATCTGCAGGTGGATATTT
          1 TGAACCTTCCTTTTCATAGAGCAGTTTTGAAACACTCTTTTTGTAGAATCTGCAAGTGGATATTT

              **          **  * *      *  *   *      * *    *          *   * 
       2210 GGCTAGCTTTGAGGATTTCGTTGGAAACGGTAATGTCTTCAAAGAAAATCTAGACAGAAACATCC
         66 GGAGAGCTTTGAGGCCTTTGGTGGAAAAGGAAATATCTTCATATAAAAACTAGACAGAAGCATTC

                    *    *            *    *         
       2275 TCAGAAACATCTTCGTGATGTTTGCAATCAAGTCACAGAGT
        131 TCAGAAACTTCTTTGTGATGTTTGCATTCAACTCACAGAGT

                      *             *  *                  *     *       *    
       2316 TGAACCTTCCGTTTCATAGAGCAGGTTGGAAACACTCTTTTTGTAGTATCTGGAAGTGGACATTT
          1 TGAACCTTCCTTTTCATAGAGCAGTTTTGAAACACTCTTTTTGTAGAATCTGCAAGTGGATATTT

                *     *      *     *                **        * * *      **  
       2381 GGAGCGCTTTCAGGCCTATGGTGAAAAAGGAAATATCTTCCCATAAAAACGACATAGAAGCTATC
         66 GGAGAGCTTTGAGGCCTTTGGTGGAAAAGGAAATATCTTCATATAAAAACTAGACAGAAGCATTC

                *     *   *     ** ** *      *    *  
       2446 TCAGGAACTTGTTTATGATGCATCTAATCAACTAACAGTGT
        131 TCAGAAACTTCTTTGTGATGTTTGCATTCAACTCACAGAGT

                    ** ** * *                         * *                    
       2487 TGAACCTTTGTACTGACAGAGCAG-TTTGAAACACTCTTTTTTTGGAATCTGCAAGTGGATATTT
          1 TGAACCTTCCTTTTCATAGAGCAGTTTTGAAACACTCTTTTTGTAGAATCTGCAAGTGGATATTT

               **         **  * *      *   *   *   *       ***  *    *     * 
       2551 GGATCGCTTTGAGGATTTCGTTGGAAACGG-GAT-GC--AATATAAATCGTACACAGCAGCATAC
         66 GGAGAGCTTTGAGGCCTTTGGTGGAAAAGGAAATATCTTCATATAAAAACTAGACAGAAGCATTC

                   * *     **  *   *       *         
       2612 TCAGAAAATACTTTGCCATATTTCCATTCAAGTCACAGAGT
        131 TCAGAAACTTCTTTGTGATGTTTGCATTCAACTCACAGAGT

            *    *    **            *  *                  *     *       *    
       2653 GGAACATTCCCATTCATAGAGCAGGTTGGAAACACTCTTTTTGTAGTATCTGGAAGTGGACATTT
          1 TGAACCTTCCTTTTCATAGAGCAGTTTTGAAACACTCTTTTTGTAGAATCTGCAAGTGGATATTT

                *     ****   *     *                **        *              
       2718 GGAGCGCTTTCTTACCTGTGGTGAAAAAGGAAATATCTTCCCATAAAAACAAGACAGAAGCATTC
         66 GGAGAGCTTTGAGGCCTTTGGTGGAAAAGGAAATATCTTCATATAAAAACTAGACAGAAGCATTC

                      *          *  ***      *  *  * 
       2783 TCAGAAACTTTTTTGTGATGTGTGTCCTCAACTAACGGACT
        131 TCAGAAACTTCTTTGTGATGTTTGCATTCAACTCACAGAGT

                    *        **  * *  *  *             *   *      *          
       2824 TGAACCTTTCTTTTCATGCAGTACTTCTGGAACACTCTTTTTGAAGATTCTGCATGTGGATATTT
          1 TGAACCTTCCTTTTCATAGAGCAGTTTTGAAACACTCTTTTTGTAGAATCTGCAAGTGGATATTT

               **          *   *  * *   *   **    *          *       *       
       2889 GGATGGCTTTGAGG-ATTTCGTTGTAAACG--GGAT-TACATATAAAAAGTAGACAGCAGCA-T-
         66 GGAGAGCTTTGAGGCCTTTGGTGGAAAAGGAAATATCTTCATATAAAAACTAGACAGAAGCATTC

                             *   *         *       * 
       2948 TCAGAAACTTCTTTGTGGTGTCTGCATTCAAGTCACAGAAT
        131 TCAGAAACTTCTTTGTGATGTTTGCATTCAACTCACAGAGT

                 * *  * **            *    *     **       *    **            
       2989 TGAACATCCCCTCACATAGAGCAGTTGTGAAGCACTCCATTTGTAGTATCTCGAAGTGGATATTT
          1 TGAACCTTCCTTTTCATAGAGCAGTTTTGAAACACTCTTTTTGTAGAATCTGCAAGTGGATATTT

                *      **    *  *         *          **  *  ** *   *       * 
       3054 GGAGGGCTTTGTAGCCTAT-CTGGAAAAAGAAAATATCTTCCCATGAATGCGAGATAGAAGCAAT
         66 GGAGAGCTTTGAGGCCTTTGGTGG-AAAAGGAAATATCTTCATATAAAAACTAGACAGAAGCATT

                       *   *  *   * ** *      *  * * *
       3118 CTCAGAAACTTGTTTATGCTGTATCTACTCAACTAACTGTGC
        130 CTCAGAAACTTCTTTGTGATGTTTGCATTCAACTCACAGAGT

                    *  *  *               *        *  * *                  * 
       3160 TGAACCTTTCTATTGATAGAGCAGTTTTGAGACACTCTTCTTTTGGAATCTGCAAGTGGATATAT
          1 TGAACCTTCCTTTTCATAGAGCAGTTTTGAAACACTCTTTTTGTAGAATCTGCAAGTGGATATTT

               *  *       **  * *      *     *    *          *       *  *    
       3225 GGATAGATTTGAGGATTTCGTTGGAAACGG---GAT-TACATATAAAAAGTAGACAGCAGTATTC
         66 GGAGAGCTTTGAGGCCTTTGGTGGAAAAGGAAATATCTTCATATAAAAACTAGACAGAAGCATTC

                                       *  *   *      
       3286 TCAGAAACTTCTTTGTGATGTTTGCATCCAGCTCTCAGAGT
        131 TCAGAAACTTCTTTGTGATGTTTGCATTCAACTCACAGAGT

                 *    *          *  *        *        *   **    *   *  ** *  
       3327 TGAACATTCCCTTTCATAGAGTAGGTTTGAAACCCTCTTTTTATAGTGTCTGGAAGCGGGCAATT
          1 TGAACCTTCCTTTTCATAGAGCAGTTTTGAAACACTCTTTTTGTAGAATCTGCAAGTGGATATTT

                *     *      *  *  *              * **   *                   
       3392 GGAGCGCTTTCAGGCCTATGCTGAAAAAGG-AA-AT-TACCCATAGAAACTAGACAGAAGCATTC
         66 GGAGAGCTTTGAGGCCTTTGGTGGAAAAGGAAATATCTTCATATAAAAACTAGACAGAAGCATTC

             *    * ***          *  * *      * *     
       3454 TGAGAATCACGTTTGTGATGTGTGTACTCAACTAAGAGAGT
        131 TCAGAAACTTCTTTGTGATGTTTGCATTCAACTCACAGAGT

                  * *     *   *  *          *   *                            
       3495 TGAACCATTCTTTTGATACAGTAGTTTTGAAAAACTGTTTTTGTAGAATCTGCAAGTGGATATTT
          1 TGAACCTTCCTTTTCATAGAGCAGTTTTGAAACACTCTTTTTGTAGAATCTGCAAGTGGATATTT

               ***      *     ***      *   *  **        *   **  *       *    
       3560 GGACGTCTTTGATGCCTTCATTGGAAACGG-TATTCCTTCATATGAAAGATAAACAGAAGAATTC
         66 GGAGAGCTTTGAGGCCTTTGGTGGAAAAGGAAATATCTTCATATAAAAACTAGACAGAAGCATTC

              *                  *       *     *     
       3624 TCCGAAACTTCTTTGTGATGTGTGCATTCTACTCAAAGAGT
        131 TCAGAAACTTCTTTGTGATGTTTGCATTCAACTCACAGAGT

                 *       **   *  *      *             *      * *             
       3665 TGAACATTCCTTTCTATATAGAAGTTTTTAAACACTCTTTTTCTAGAATTTCCAAGTGGATATTT
          1 TGAACCTTCCTTTTCATAGAGCAGTTTTGAAACACTCTTTTTGTAGAATCTGCAAGTGGATATTT

            * * *            *  * *     *     *              *    * *  *     
       3730 AGTGCGCTTTGAGGCCTATGTTAGAAAATGAAATGTCTTCATATAAAAAATAGAGATAATCATTC
         66 GGAGAGCTTTGAGGCCTTTGGTGGAAAAGGAAATATCTTCATATAAAAACTAGACAGAAGCATTC

                    ***        * *   * *            *
       3795 TCAGAAACAATTTTGTGATATGTGCGTGCAACTCACAGAGC
        131 TCAGAAACTTCTTTGTGATGTTTGCATTCAACTCACAGAGT

             *      *     *  * ** * *           *            *        *      
       3836 TTAA-CTTTCTTTTGATGGGTCTGATTTGAAACACTTTTTTTGTAGAATTTGCAAGTGTATATTT
          1 TGAACCTTCCTTTTCATAGAGCAGTTTTGAAACACTCTTTTTGTAGAATCTGCAAGTGGATATTT

            *** **    *  *   *    *        *  *      *                       
       3900 ATTGCTCTTTAAGACCTGTGGTAGAAAAGGATATCTCTTCACATAAAAACTAGACAGAAGCATTC
         66 GGAGAGCTTTGAGGCCTTTGGTGGAAAAGGAAATATCTTCATATAAAAACTAGACAGAAGCATTC

                 *  ** *        *                    
       3965 TCAGAGACCACATTGTGATGCTTGCATTCAACTCACAGAGT
        131 TCAGAAACTTCTTTGTGATGTTTGCATTCAACTCACAGAGT

                 *     *  * *                 *           **     *           
       4006 TGAACATTCCTCTTGAGAGAGCAGTTTTGAAACAGTCTTTTTGTAGGCTCTGCTAGTGGATATTT
          1 TGAACCTTCCTTTTCATAGAGCAGTTTTGAAACACTCTTTTTGTAGAATCTGCAAGTGGATATTT

               ***     * *    * *              *       * *    *    *     **  
       4071 -GACCTCTTTGTGACCTTCGTTGGAAACAGG--ATTTCTTCATTTGAAAATTAGAAAGAAGATTT
         66 GGAGAGCTTTGAGGCCTTTGGTGGAAA-AGGAAATATCTTCATATAAAAACTAGACAGAAGCATT

                                  *      * **         
       4133 CTCAGAAACTTCTTTGTGATGTGTGCATTTATTTCACAGAGT
        130 CTCAGAAACTTCTTTGTGATGTTTGCATTCAACTCACAGAGT

                **  *    **      *                           * *      *      
       4175 TGAATTTTGCTTTCAATAGAGTAGTTTTGAAACACTCTTTTTGTAGAATTTCCAAGTGAATATTT
          1 TGAACCTTCCTTTTCATAGAGCAGTTTTGAAACACTCTTTTTGTAGAATCTGCAAGTGGATATTT

            *   * *   *      **** *                     **      *      *     
       4240 AGAGTGGTTTAAGGCCTAACCTAGAAAAGGAAATATCTTCATATGGAAACTACACAGAATCATTC
         66 GGAGAGCTTTGAGGCCTTTGGTGGAAAAGGAAATATCTTCATATAAAAACTAGACAGAAGCATTC

                     *   **      *   *         *     
       4305 TCAGAAACTACTTCATGATGTGTGCGTTCAACTCAAAGAGT
        131 TCAGAAACTTCTTTGTGATGTTTGCATTCAACTCACAGAGT

             *      *    **  * *                          *                  
       4346 TTAACCTTTCTTTGGATGGGGCAGTTTTGAAACACTCTTTTTGTAGTATCTGCAAGTGGATATTT
          1 TGAACCTTCCTTTTCATAGAGCAGTTTTGAAACACTCTTTTTGTAGAATCTGCAAGTGGATATTT

            *  ***     *      * *      *  *   *      **      *         **    
       4411 TGACCTCTTTGTGGCCTTCGTTGGAAACGGGAATTTCTTCACTTAAAAAGTAGACAGAAAAATTC
         66 GGAGAGCTTTGAGGCCTTTGGTGGAAAAGGAAATATCTTCATATAAAAACTAGACAGAAGCATTC

                         *       * ***     *         
       4476 TCAGAAACTTCTTCGTGATGTGTATGTTCAATTCACAGAGT
        131 TCAGAAACTTCTTTGTGATGTTTGCATTCAACTCACAGAGT

                         **                           * * *  * *   *         
       4517 TGAACCTTCCTTTCGATAGAGCAGTTTTGAAACACTCTTTTTATTGTATTTTCAACTGGATATTT
          1 TGAACCTTCCTTTTCATAGAGCAGTTTTGAAACACTCTTTTTGTAGAATCTGCAAGTGGATATTT

            *   * *   *      *    *        *              *            *     
       4582 AGAGTGGTTTAAGGCCTATGGTAGAAAAGGATATATCTTCATATAACAACTAGACAGAATCATTC
         66 GGAGAGCTTTGAGGCCTTTGGTGGAAAAGGAAATATCTTCATATAAAAACTAGACAGAAGCATTC

                     *         * *   *               
       4647 TCAGAAACTACTTTGTGATCTCTGCGTTCAACTCACAGAGT
        131 TCAGAAACTTCTTTGTGATGTTTGCATTCAACTCACAGAGT

             *   *  *     *                    ***           *     *  *     *
       4688 TTAACATTTCTTTTGATAGAGCAGTTTTGAAACACCGCTTTTGTAGAATTTGCAATTGTATATTG
          1 TGAACCTTCCTTTTCATAGAGCAGTTTTGAAACACTCTTTTTGTAGAATCTGCAAGTGGATATTT

            **  **   *  *    * ** *     *            *       *               
       4753 ACAGCACTTAGACGCCTATTTTAGAAAACGAAATATCTTCACATAAAAATTAG--A-AA-CATTC
         66 GGAGAGCTTTGAGGCCTTTGGTGGAAAAGGAAATATCTTCATATAAAAACTAGACAGAAGCATTC

                     * *   *               **   * *  
       4814 TCAGAAACTACATTGGGATGTTTGCATTCAATGCACTGTGT
        131 TCAGAAACTTCTTTGTGATGTTTGCATTCAACTCACAGAGT

                 *    **  ** **               * *     *                      
       4855 TGAACATTCCCCTTGGTGTAGCAGTTTTGAAACATTTTTTTTCTAGAATCTGCAAGTGGATATTT
          1 TGAACCTTCCTTTTCATAGAGCAGTTTTGAAACACTCTTTTTGTAGAATCTGCAAGTGGATATTT

            *  ***       *    * *      **  *  *                        ** *  
       4920 AGACCTCTTTGAGACCTTCGTTGGAAACTG-GATTTCTTCATATAAAAACTAGACAGAAAGACTC
         66 GGAGAGCTTTGAGGCCTTTGGTGGAAAAGGAAATATCTTCATATAAAAACTAGACAGAAGCATTC

                          *      *                   
       4984 TCAGAAACTTCTTTTTGATGTGTGCATTCAACTCACAGAGT
        131 TCAGAAACTTCTTTGTGATGTTTGCATTCAACTCACAGAGT

                *        **                 *          *     * *             
       5025 TGAAACTTCCTTTCGATAGAGCAGTTTTGAAAAACTCTTTTTGCAGAATTTCCAAGTGGATATTT
          1 TGAACCTTCCTTTTCATAGAGCAGTTTTGAAACACTCTTTTTGTAGAATCTGCAAGTGGATATTT

            *  **  *    *    *    *                          *  *      * *   
       5090 AGATCGCCTTGAAGCCTATGGTAGAAAAGGAAATATCTTCATATAAAAATTATACAGAAACTTTC
         66 GGAGAGCTTTGAGGCCTTTGGTGGAAAAGGAAATATCTTCATATAAAAACTAGACAGAAGCATTC

                     ***       * *   *     *    *  * 
       5155 TCAGAAACTAGATTGTGATTTGTGCTTTCAATTCACTGAAT
        131 TCAGAAACTTCTTTGTGATGTTTGCATTCAACTCACAGAGT

             *      *     **                    * *      * *          *      
       5196 TTAACCTTTCTTTTGGTAGAGCAGTTTTGAAACACTATGTTTGTAAAGTCTGCAAGTGTATATTT
          1 TGAACCTTCCTTTTCATAGAGCAGTTTTGAAACACTCTTTTTGTAGAATCTGCAAGTGGATATTT

                **    * *     ***      *              *       *  *       *   
       5261 GGAGCACTTTAAAGCCTTCTTTGGAATCAGG-AATATCTTCACATAAAAAGTAAACAGAAGTATT
         66 GGAGAGCTTTGAGGCCTTTGGTGGAA-AAGGAAATATCTTCATATAAAAACTAGACAGAAGCATT

                              *   *    *      *       
       5325 CTCAGAAACTTCTTTGTGTTGTCTGCACTCAACTAACAGAGT
        130 CTCAGAAACTTCTTTGTGATGTTTGCATTCAACTCACAGAGT

             *            *                      *         * *   *          *
       5367 TTAACCTTCCTTTTGATAGAGCAGTTTTGAAACACTCATTTTGTAGAGTTTGCGAGTGGATATTA
          1 TGAACCTTCCTTTTCATAGAGCAGTTTTGAAACACTCTTTTTGTAGAATCTGCAAGTGGATATTT

            *   * *   **     *    *                     *    *  *     *      
       5432 AGAGCGTTTTTTGGCCTATGGTAGAAAAGGAAATATCTTCATATTAAAAATACACAGAGGCATTC
         66 GGAGAGCTTTGAGGCCTTTGGTGGAAAAGGAAATATCTTCATATAAAAACTAGACAGAAGCATTC

                     *                  *            
       5497 TCAGAAACTACTTTGTGATGTTTGCATTAAACTCACAGAGT
        131 TCAGAAACTTCTTTGTGATGTTTGCATTCAACTCACAGAGT

              *  *  *    **      *       *                 *   *             
       5538 TGTACATTACTTTCGATAGAGTAGTTTTGTAACACTCTTTTTGTAGACTCTACAAGTGGATATTT
          1 TGAACCTTCCTTTTCATAGAGCAGTTTTGAAACACTCTTTTTGTAGAATCTGCAAGTGGATATTT

               ***            * *      *  * * *                         *    
       5603 GGACCTCTTTGAGGCCTTCGTTGGAAACGGGACTTTCTTCATATAAAAACTAGACAGAAGAATTC
         66 GGAGAGCTTTGAGGCCTTTGGTGGAAAAGGAAATATCTTCATATAAAAACTAGACAGAAGCATTC

                      *   *      *         *         
       5668 TCAGAAACTTGTTTATGATGTGTGCATTCAATTCACAGAGT
        131 TCAGAAACTTCTTTGTGATGTTTGCATTCAACTCACAGAGT

                 *  *    **                *                 * *             
       5709 TGAACTTTTCTTTCAATAGAGCAGTTTTGAATCACTCTTTTTGTAGAATTTCCAAGTGGATATTT
          1 TGAACCTTCCTTTTCATAGAGCAGTTTTGAAACACTCTTTTTGTAGAATCTGCAAGTGGATATTT

            *   ***      *   *    *                                    *     
       5774 AGAGCTGTTTGAGACCTATGGTAGAAAAGGAAATATCTTCATATAAAAACTAGACAGAATCATTC
         66 GGAGAGCTTTGAGGCCTTTGGTGGAAAAGGAAATATCTTCATATAAAAACTAGACAGAAGCATTC

                     *           *               *   
       5839 TCAGAAACTACTTTGTGATGTGTGCATTCAACTCACATAGT
        131 TCAGAAACTTCTTTGTGATGTTTGCATTCAACTCACAGAGT

             *   *  *     *                    ***           *        *     *
       5880 TTAACATTTCTTTTGATAGAGCAGTTTTGAAACACCACTTTTGTAGAATTTGCAAGTGTATATTC
          1 TGAACCTTCCTTTTCATAGAGCAGTTTTGAAACACTCTTTTTGTAGAATCTGCAAGTGGATATTT

            *   *            *    *     *            *   **   *  *           
       5945 AGAGCGCTTTGAGGCCTATGGTAGAAAATGAAATATCTTCACATACCAACGAGGCAGAAGCATTC
         66 GGAGAGCTTTGAGGCCTTTGGTGGAAAAGGAAATATCTTCATATAAAAACTAGACAGAAGCATTC

                     *  *                    *       
       6010 TCAGAAACTACTGTGTGATGTTTGCATTCAACTGACAGAGT
        131 TCAGAAACTTCTTTGTGATGTTTGCATTCAACTCACAGAGT

              *  *     *  *  *               *    *                          
       6051 TGGACATTCCTCTTGATGGAGCAGTTTTGAAACTCTC-CTTTGTAGAATCTGCAAGTGGATATTT
          1 TGAACCTTCCTTTTCATAGAGCAGTTTTGAAACACTCTTTTTGTAGAATCTGCAAGTGGATATTT

               ***            ***      *  *   *        **                * * 
       6115 GGAACTCTTTGAGGCCTTCATTGGAAACGGGAATTTCTTCACTTGAAAAAC-AGACAGAAGAAAT
         66 GGAGAGCTTTGAGGCCTTTGGTGGAAAAGGAAATATCTTCA-TATAAAAACTAGACAGAAGCATT

               *         *     ** ***                 
       6179 CTCTGAAACTT-TCTGTGACATGGACATTCAACTCACAGAGT
        130 CTCAGAAACTTCTTTGTGATGTTTGCATTCAACTCACAGAGT

                *   *     *                                  * *             
       6220 TGAATCTTTCTTTTGATAGAGCAGTTTTGAAACACTCTTTTTGTAGAATTTCCAAGTGGATATTT
          1 TGAACCTTCCTTTTCATAGAGCAGTTTTGAAACACTCTTTTTGTAGAATCTGCAAGTGGATATTT

            * * *   *   *    *    *                    *        *            
       6285 AGTGCGCTCTGAAGCCTGTGGTAGAAAAGGAAATATCTTCATAGAAAAACTACACAGAAGCATTC
         66 GGAGAGCTTTGAGGCCTTTGGTGGAAAAGGAAATATCTTCATATAAAAACTAGACAGAAGCATTC

             *   *   *       *                       
       6350 TGAGATACTACTTTGTGTTGTTTGCATTCAACTCACAGAGT
        131 TCAGAAACTTCTTTGTGATGTTTGCATTCAACTCACAGAGT

                 *        *              *                             *    *
       6391 TGAACATTCCTTTTGATAGAGCAGTTTTGTAACACTCTTTTTGTAGAATCTGCAAGTGGTTATTA
          1 TGAACCTTCCTTTTCATAGAGCAGTTTTGAAACACTCTTTTTGTAGAATCTGCAAGTGGATATTT

               ***   *        ***      *   *  *     *        *          *    
       6456 GGACTTCTTAGAGGCCTTCTTTGGAAACGG-GATTTCTTCCTATAAAAAGTAGACAGAAGAATTC
         66 GGAGAGCTTTGAGGCCTTTGGTGGAAAAGGAAATATCTTCATATAAAAACTAGACAGAAGCATTC

            *                    *                   
       6520 CCAGAAACTTCTTTGTGATGTGTGCATTCAACTCACAGAGT
        131 TCAGAAACTTCTTTGTGATGTTTGCATTCAACTCACAGAGT

                *        **   *                 *      *  *  * *      *      
       6561 TGAAACTTCCTTTCAATACAGCAGTTTTGAAACACTATTTTTGAAGTATTTTCAAGTGAATATTT
          1 TGAACCTTCCTTTTCATAGAGCAGTTTTGAAACACTCTTTTTGTAGAATCTGCAAGTGGATATTT

            * * *  *    *    *    **      *   *      *   *   *         * *   
       6626 AGGGCGCCTTGAAGCCTATGGTAAAAAAGGGAATGTCTTCACATAGAAATTAGACAGAACCTTTC
         66 GGAGAGCTTTGAGGCCTTTGGTGGAAAAGGAAATATCTTCATATAAAAACTAGACAGAAGCATTC

                     **          *   *  *       *    
       6691 TCAGAAACTAGTTTGTGATGTGTGCTTTAAACTCACTGAGT
        131 TCAGAAACTTCTTTGTGATGTTTGCATTCAACTCACAGAGT

             *      *     **  *                          * *                 
       6732 TTAACCTTTCTTTTGTTAAAGCAGTTTTGAAACACTC----TGTAAAGTCTGCAAGTGGATATTT
          1 TGAACCTTCCTTTTCATAGAGCAGTTTTGAAACACTCTTTTTGTAGAATCTGCAAGTGGATATTT

               **     * *     ***  *   *  *          *       *  *       *    
       6793 GGAATGCTTTAATGCCTTCTTTGTAAAGGGGAATATCTTCACAT-AAAAGTAAACAGAAGTATTC
         66 GGAGAGCTTTGAGGCCTTTGGTGGAAAAGGAAATATCTTCATATAAAAACTAGACAGAAGCATTC

                     *           * *  *      *    *  
       6857 TCAGAAACTCCTTTGTGATGTCTTCACTCAACTAACAGGGT
        131 TCAGAAACTTCTTTGTGATGTTTGCATTCAACTCACAGAGT

                      *   *    *                             *   *         * 
       6898 TGAACCTTCCCTTTGATAGTGCAGTTTTGAAACACTCTTTTTGTAGAATTTGCGAGTGGATATAT
          1 TGAACCTTCCTTTTCATAGAGCAGTTTTGAAACACTCTTTTTGTAGAATCTGCAAGTGGATATTT

            *   * *    *     *  * *                     *       *     *      
       6963 AGAGCGTTTTGGGGCCTATGCTAGAAAAGGAAATATCTTCATATTAAAACTACACAGAGGCATTC
         66 GGAGAGCTTTGAGGCCTTTGGTGGAAAAGGAAATATCTTCATATAAAAACTAGACAGAAGCATTC

                     *   **                          
       7028 TCAGAAACTACTTGTTGATGTTTGCATTCAACTCACAGAGT
        131 TCAGAAACTTCTTTGTGATGTTTGCATTCAACTCACAGAGT

                 *        **       *                  *   *  *               
       7069 TGAACATTCCTTTTGGTAGAGCACTTTTGAAACACTCTTTTTATAGGATATGCAAGTGGATATTT
          1 TGAACCTTCCTTTTCATAGAGCAGTTTTGAAACACTCTTTTTGTAGAATCTGCAAGTGGATATTT

            *  ****           ***      *   *  *       *       *   *     *    
       7134 TGACCTATTTGAGGCCTTCCTTGGAAACGG-GATTTCTTCATTTAAAAACGAGATAGAAGAATTC
         66 GGAGAGCTTTGAGGCCTTTGGTGGAAAAGGAAATATCTTCATATAAAAACTAGACAGAAGCATTC

            *       *        *   *        ***        
       7198 ACAGAAACCTCTTTGTGGTGTGTGCATTCAGTACACAGAGT
        131 TCAGAAACTTCTTTGTGATGTTTGCATTCAACTCACAGAGT

                         **                           ** *   * *             
       7239 TGAACCTTCCTTTCGATAGAGCAGTTTTG-AA-ACTCTTTTTACATAATTTCCAAGTGGATATTT
          1 TGAACCTTCCTTTTCATAGAGCAGTTTTGAAACACTCTTTTTGTAGAATCTGCAAGTGGATATTT

            *   *         *  *    **    *                     *        *     
       7302 AGAGCGCTTTGAGGTCTATGGTTAAAAACGAAATATCTTCATATAAAAACCAGACAGAATCATTC
         66 GGAGAGCTTTGAGGCCTTTGGTGGAAAAGGAAATATCTTCATATAAAAACTAGACAGAAGCATTC

                     *           *  **               
       7367 TCAGAAACTACTTTGTGATGTGTGTGTTCAACTCACAGAGT
        131 TCAGAAACTTCTTTGTGATGTTTGCATTCAACTCACAGAGT

             *      *        *   * *         *               * **            
       7408 TTAACCTTTCTTTTCATGGAGAAATTTTGAAACCCTCTTTTTGTAGAATTTTGAAGTGGATATTT
          1 TGAACCTTCCTTTTCATAGAGCAGTTTTGAAACACTCTTTTTGTAGAATCTGCAAGTGGATATTT

                ** *    *    *    *                  *          *            
       7473 GGAGCACGTTGAAGCCTATGGTAGAAAAGGAAATATCTTCACATAAAAACTAAACAGAAGCATTC
         66 GGAGAGCTTTGAGGCCTTTGGTGGAAAAGGAAATATCTTCATATAAAAACTAGACAGAAGCATTC

                     *  *                            
       7538 TCAGAAACTACTGTGTGATGTTTGCATTCAACTCACAGAGT
        131 TCAGAAACTTCTTTGTGATGTTTGCATTCAACTCACAGAGT

                 *     *  *  *                          *                    
       7579 TGAACATTCCTCTTGATGGAGCAGTTTTGAAACACTCTTTTTGTGGAATCTGCAAGTGGATATTT
          1 TGAACCTTCCTTTTCATAGAGCAGTTTTGAAACACTCTTTTTGTAGAATCTGCAAGTGGATATTT

             *  **            ***      *   ** *     *   *   *           *    
       7644 GAAGCTCTTTGAGGCCTTCATTGGAAACGG-GCTTTCTTCTTATGAAAGCTAGACAGAAGAATTC
         66 GGAGAGCTTTGAGGCCTTTGGTGGAAAAGGAAATATCTTCATATAAAAACTAGACAGAAGCATTC

                    *        * * *         *         
       7708 TCAGAAACATCTTTGTGTTCTATGCATTCAATTCACAGAGT
        131 TCAGAAACTTCTTTGTGATGTTTGCATTCAACTCACAGAGT

                *       ***                            **    * *             
       7749 TGAATCTTCCTTCCAATAGAGCAGTTTTGAAACACTCTTTTTGAGGAATTTCCAAGTGGATATTT
          1 TGAACCTTCCTTTTCATAGAGCAGTTTTGAAACACTCTTTTTGTAGAATCTGCAAGTGGATATTT

            *   * *               *  *   *  *     *        *           **    
       7814 AGAGTGGTTTGAGGCCTTTGGTAGATAAGAAATTATCTACATATAAACACTAGACAGAATAATTC
         66 GGAGAGCTTTGAGGCCTTTGGTGGAAAAGGAAATATCTTCATATAAAAACTAGACAGAAGCATTC

                     *  *        *   *     *         
       7879 TCAGAAACTACTCTGTGATGTGTGCGTTCAAATCACAGAGT
        131 TCAGAAACTTCTTTGTGATGTTTGCATTCAACTCACAGAGT

             *                   *               *            * *            
       7920 TTAACCTT-CTTTTCAATAGAACAGTTTTGAAACACTTTTTTTGTAGAATTTCCAAGTGGATATT
          1 TGAACCTTCCTTTTC-ATAGAGCAGTTTTGAAACACTCTTTTTGTAGAATCTGCAAGTGGATATT

             * * **      *    *  * *         *          *      * *    *      
       7984 TAGTGCCCTTTGAAGCCTATGTTAGAAAAGGAACTATCTTCATAGAAAAACAACACAGGAGCATT
         65 TGGAGAGCTTTGAGGCCTTTGGTGGAAAAGGAAATATCTTCATATAAAAACTAGACAGAAGCATT

                     ***          *      *  *         
       8049 CTCAGAAACCAGTTTGTGATGTGTGCATTGAATTCACAGAGT
        130 CTCAGAAACTTCTTTGTGATGTTTGCATTCAACTCACAGAGT

             *      *      *  *   *                 *     **  *        *     
       8091 TTAACCTTTCTTTTGAATGGAGAAGTTTTGAAACACTCTTCTTGTATGATTTGCAAGTGTATATT
          1 TGAACCTTCCTTTT-CATAGAGCAGTTTTGAAACACTCTTTTTGTAGAATCTGCAAGTGGATATT

             * * *      **    *   **    **            *       *    **        
       8156 TAGGGCGCTTTGTAGCCTGTGGCAGAAAGTGAAATATCTTCACATAAAAAGTAGAGTGAAGCATT
         65 TGGAGAGCTTTGAGGCCTTTGGTGGAAAAGGAAATATCTTCATATAAAAACTAGACAGAAGCATT

                      **                             *
       8221 CTCAGAAACTAATTTGTGATGTTTGCATTCAACTCACAGAGC
        130 CTCAGAAACTTCTTTGTGATGTTTGCATTCAACTCACAGAGT

                 **   *   *                *   *           *        *   *    
       8263 TGAACAAT-CATTTGATAGAGCAGTTTTGAATCACCCTTTTTGTAGATTCTGCAAGCGGACATTT
          1 TGAACCTTCCTTTTCATAGAGCAGTTTTGAAACACTCTTTTTGTAGAATCTGCAAGTGGATATTT

               **   *         * *  *   *  **        **       **         *    
       8327 GGACCGCTGTGAGGCCTTCGTTGAAAACGGGCATATCTTCTCATAAAAATCAGACAGAAGGATTC
         66 GGAGAGCTTTGAGGCCTTTGGTGGAAAAGGAAATATCTTCATATAAAAACTAGACAGAAGCATTC

            *            *       *           *     * 
       8392 CCAGAAACTTCTTAGTGATGTGTGCATTCAACTTACAGATT
        131 TCAGAAACTTCTTTGTGATGTTTGCATTCAACTCACAGAGT

             *   *  *     *    *                    *        *   *         * 
       8433 TTAACTTTTCTTTTGATAGGGCAGTTTTGAAACACTCTTTCTGTAGAATTTGCTAGTGGATATGT
          1 TGAACCTTCCTTTTCATAGAGCAGTTTTGAAACACTCTTTTTGTAGAATCTGCAAGTGGATATTT

             *****         * * **                    *   *   *    **   *     
       8498 GCCTCCCTTTGAGGCTTATTTTGGAAAAGGAAATATCTTCACATAGAAAGTAGAAGGAATCATTC
         66 GGAGAGCTTTGAGGCCTTTGGTGGAAAAGGAAATATCTTCATATAAAAACTAGACAGAAGCATTC

                     *    *      *  **               
       8563 TCAGAAACTACTTTTTGATGTGTGACTTCAACTCACAGAGT
        131 TCAGAAACTTCTTTGTGATGTTTGCATTCAACTCACAGAGT

                          *        *           *                         *   
       8604 TGAACCTTCCTTTTGATAGAGCATTTTTGAAACACACTTTTTGTAGAATCTGCAAGTGGATTTTT
          1 TGAACCTTCCTTTTCATAGAGCAGTTTTGAAACACTCTTTTTGTAGAATCTGCAAGTGGATATTT

                *            **** *    ** **    *    * * *   *          *    
       8669 GGAGGGCTTTGAGGCCTACTTTTGAAACTGGTATATTTTCACAGAGAAAGTAGACAGAAGTATTC
         66 GGAGAGCTTTGAGGCCTTTGGTGGAAAAGGAAATATCTTCATATAAAAACTAGACAGAAGCATTC

                                 *              *   *
       8734 TCAGAAACTTCTTTGTGATGTCTGCATTCAACTCACTGAGC
        131 TCAGAAACTTCTTTGTGATGTTTGCATTCAACTCACAGAGT

                   *     **               *      *           * *             
       8775 TGAACCTGCCTTTGGATAGAGCAGTTTTG-TACACTCATTTTGTAGAATTTCCAAGTGGATATTT
          1 TGAACCTTCCTTTTCATAGAGCAGTTTTGAAACACTCTTTTTGTAGAATCTGCAAGTGGATATTT

            *   *   **** *   *    *   *     *   *            *               
       8839 AGAGTGCTGCATGTCCTATGGTAGAATAGGAAGTATTTTCATA-AAAAAGTAGACAGAAGCATTC
         66 GGAGAGCTTTGAGGCCTTTGGTGGAAAAGGAAATATCTTCATATAAAAACTAGACAGAAGCATTC

             *       *             *         *       
       8903 TAAGAAACTACTTTGTGATGTTTCCATTCAACTAACAGAGT
        131 TCAGAAACTTCTTTGTGATGTTTGCATTCAACTCACAGAGT

                 *        *      *                      *               *    
       8944 TGAACATTCCTTTTGATAGAGAAGTTTTGAAACACTCTTTTTGTGGAATCTGCAAGTGGACATTT
          1 TGAACCTTCCTTTTCATAGAGCAGTTTTGAAACACTCTTTTTGTAGAATCTGCAAGTGGATATTT

            *  **        *    * *      *  * *        *        *   *          
       9009 AGACCGCTTTGAGTCCTTGGTTGGAAACGGGATTATCTTCACATAAAAACCAGAGAGAAGCATTC
         66 GGAGAGCTTTGAGGCCTTTGGTGGAAAAGGAAATATCTTCATATAAAAACTAGACAGAAGCATTC

                           *     *                  *
       9074 TCAGAAACTTCTTTGCGATGTGTGCATTCAACTCACAGAGG
        131 TCAGAAACTTCTTTGTGATGTTTGCATTCAACTCACAGAGT

                **  *     *   *         **                     *             
       9115 TGAATATTTCTTTTGATATAGCAGTTTTCTAACACTCTTTTTGTAGAATCTTCAAGTGGATATTT
          1 TGAACCTTCCTTTTCATAGAGCAGTTTTGAAACACTCTTTTTGTAGAATCTGCAAGTGGATATTT

            ******          **  *          **        *       *    **  **     
       9180 TATTCCCTTTGAGGCCCATGTTGGAAAAGGAGTTATCTTCACATAAAAATTAGAATGATACATTC
         66 GGAGAGCTTTGAGGCCTTTGGTGGAAAAGGAAATATCTTCATATAAAAACTAGACAGAAGCATTC

               *           *         *  *              * 
       9245 TCATAAACTTCTTTGAGATAGATAAGTGAATTCAACTCACAGACT
        131 TCAGAAACTTCTTTGTGAT-G-T--TTGCATTCAACTCACAGAGT

                    *     * * *              *    *      *** *          *    
       9290 TGAACCTTTCTTTTGACACAGCAGTTTTGAAACGCTCTGTTTGTAACGTTTGCAAGTGGACATTT
          1 TGAACCTTCCTTTTCATAGAGCAGTTTTGAAACACTCTTTTTGTAGAATCTGCAAGTGGATATTT

             *  *             ***      *  *         **       *      *   *    
       9355 GTAGTGCTTTGAGGCCTTCTTTGGAAAGGGGAATATCTTCCCATAAAAAGTAGACATAAGTATTC
         66 GGAGAGCTTTGAGGCCTTTGGTGGAAAAGGAAATATCTTCATATAAAAACTAGACAGAAGCATTC

              *                  *    *             *
       9420 TCTGAAACTTCTTTGTGATGTCTGCACTCAACTCACAGAGA
        131 TCAGAAACTTCTTTGTGATGTTTGCATTCAACTCACAGAGT

             *        *   *                                * *          *  * 
       9461 TCAACCTTCCCTTTGATAGAGCAGTTTTGAAACACTCTTTTTGTAGAGTTTGCAAGTGGAGATAT
          1 TGAACCTTCCTTTTCATAGAGCAGTTTTGAAACACTCTTTTTGTAGAATCTGCAAGTGGATATTT

            *   * *    *     *    *             *      *        *  *       * 
       9526 AGAGCGTTTTGGGGCCTATGGTAGAAAAGGAAATATATTCATAGAAAAACTACACGGAAGCATGC
         66 GGAGAGCTTTGAGGCCTTTGGTGGAAAAGGAAATATCTTCATATAAAAACTAGACAGAAGCATTC

                     *                               
       9591 TCAGAAACTGCTTTGTGATGTTTGCATTCAACTCACAGAGT
        131 TCAGAAACTTCTTTGTGATGTTTGCATTCAACTCACAGAGT

                **        *                   *           *           *      
       9632 TGAATATTCCTTTTGATAGAGCAGTTTTGAAACATTCTTTTTGTAGGATCTGCAAGTGAATATTT
          1 TGAACCTTCCTTTTCATAGAGCAGTTTTGAAACACTCTTTTTGTAGAATCTGCAAGTGGATATTT

               ***  **    *   * *      **             **              
       9697 GGACCTCTACGAGGTCTTCGTTGGATACGGGAAAT-TCTTCACTTAAAAACTAGACAG
         66 GGAGAGCTTTGAGGCCTTTGGTGGA-AAAGGAAATATCTTCATATAAAAACTAGACAG

       9754 GGCCGGGAAC


Statistics
Matches: 6492,  Mismatches: 2086, Indels: 112
        0.75            0.24        0.01

Matches are distributed among these distances:
 165   88  0.01
 166  156  0.02
 167  328  0.05
 168  175  0.03
 169  353  0.05
 170  1334  0.21
 171  3780  0.58
 172  148  0.02
 173    2  0.00
 174    1  0.00
 175  127  0.02

ACGTcount: A:0.30, C:0.17, G:0.20, T:0.34


Consensus pattern (171 bp):   
TGAACCTTCCTTTTCATAGAGCAGTTTTGAAACACTCTTTTTGTAGAATCTGCAAGTGGATATTT
GGAGAGCTTTGAGGCCTTTGGTGGAAAAGGAAATATCTTCATATAAAAACTAGACAGAAGCATTC
TCAGAAACTTCTTTGTGATGTTTGCATTCAACTCACAGAGT

Found at i:17488 original size:149 final size:149

   Alignment explanation   
    Indices: 17206--17504  Score: 416
    Period size: 149  Copynumber: 2.0  Consensus size: 149

      17196 CGGGATTTCT

                *             *                 * *                          
      17206 TCATGTAAAAACTAGACAGAAAAATTATCAGAAACTTCTTTGTGATATGTGCATTCAACTCACAG
          1 TCATATAAAAACTAGACACAAAAATTATCAGAAACTACATTGTGATATGTGCATTCAACTCACAG

                               *               **         *                * 
      17271 AATTTAACCTTCCTTTCGATAGAGCAGTTTTGAAATCCTCTTTTTGTAGAACTTCCAAGTGGATA
         66 AATTTAACCTTCCTTTCGAAAGAGCAGTTTTGAAACACTCTTTTTGCAGAACTTCCAAGTGGACA

                 *          *  
      17336 TTTAGTGTGCTTTGAGTCC
        131 TTTAGAGTGCTTTGAGGCC

                                 **   * *        *        *                  
      17355 TCATATAAAAACTAGACACAATGATTCTTAGAAACTAGATTGTGATGTGTGCATTCAACTCACAG
          1 TCATATAAAAACTAGACACAAAAATTATCAGAAACTACATTGTGATATGTGCATTCAACTCACAG

             *         **   *   *                        *     *  *      *   
      17420 ATTTTAACCTTTGTTTTGAAGGAGCAGTTTTGAAACACTCTTTTTTCAGAATTTGCAAGTGTACA
         66 AATTTAACCTTCCTTTCGAAAGAGCAGTTTTGAAACACTCTTTTTGCAGAACTTCCAAGTGGACA

                               
      17485 TTTAGAGTGCTTTGAGGCC
        131 TTTAGAGTGCTTTGAGGCC

             
      17504 T
          1 T

      17505 ATGGTAGAAA


Statistics
Matches: 124,  Mismatches: 26, Indels: 0
        0.83            0.17        0.00

Matches are distributed among these distances:
 149  124  1.00

ACGTcount: A:0.31, C:0.16, G:0.17, T:0.36


Consensus pattern (149 bp):   
TCATATAAAAACTAGACACAAAAATTATCAGAAACTACATTGTGATATGTGCATTCAACTCACAG
AATTTAACCTTCCTTTCGAAAGAGCAGTTTTGAAACACTCTTTTTGCAGAACTTCCAAGTGGACA
TTTAGAGTGCTTTGAGGCC

Found at i:20512 original size:153 final size:153

   Alignment explanation   
    Indices: 20225--21039  Score: 509
    Period size: 169  Copynumber: 5.0  Consensus size: 153

      20215 AGAGCAGTTT

                     *            *               *    **          ** * *    
      20225 TGAAACACTGTTTTTGTAGAATATGCAAGTGGATATTTGGAACTCTTTGAGGCCTTCGTTGGAAA
          1 TGAAACACTCTTTTTGTAGAATTTGCAAGTGGATATTTAGAACGTTTTGAGGCCTAAGGTAGAAA

             *       *      **        *           *           *         *    
      20290 CGGGAATT-GCTTCAATTAAAAACTAGACAGAAGAATTTTCAGAAACTACCTTGTGATGTCTGCA
         66 -AGGAATTACCTTCAAACAAAAACTACACAGAAGAATTCTCAGAAACTACTTTGTGATGTTTGCA

            *     *     *           
      20354 CTCAACGCACAGTGTTCAACCTTC
        130 TTCAACTCACAGAGTTCAACCTTC

                                                     *       *      *        
      20378 TGAAACACTCTTTTTGTAGAATTTGCAAGTGGATATTTAGAGCGTTTTGGGGCCTATGGTAGAAA
          1 TGAAACACTCTTTTTGTAGAATTTGCAAGTGGATATTTAGAACGTTTTGAGGCCTAAGGTAGAAA

                          **       *         *     *     * *                 
      20443 AGGAATTACCTTCACCCAAAAACCACACAGAAGCATTCTGAGAAAATGCTTTGTGATGTTTGCAT
         66 AGGAATTACCTTCAAACAAAAACTACACAGAAGAATTCTCAGAAACTACTTTGTGATGTTTGCAT

                        *  *          *
      20508 TCAACTCACAGATTTTAACTTTTCTTT
        131 TCAACTCACAGAGTTCAAC----CTTC

                             *  *  *            *                *           
      20535 TGATGGCGCAGTTTTGAAACACTCTTTGTAGAATTTTCAAGTGGATATTTAGATCGTTTTGAGGC
          1 TGA------A------ACACTCTTTTTGTAGAATTTGCAAGTGGATATTTAGAACGTTTTGAGGC

            *        *       *  *    ** *                *                   
      20600 GTAAGGTAGGAAAGGAAATATCTTCTTAGAAAAACTACACAGAAGGATTCTCAGAAACTACTTTG
         54 CTAAGGTAGAAAAGGAATTACCTTCAAACAAAAACTACACAGAAGAATTCTCAGAAACTACTTTG

                *  *     *                   *    *
      20665 TGATATTGGCATTTAACTCACAGAGTTGAACATTCCTTT
        119 TGATGTTTGCATTCAACTCACAGAGTT---CA-ACCTTC

                                *             *               * *  *   *     
      20704 TGATAGAGCAGTTGTGAAACACTTTTTGTAGAATCTGCAAGTGGATA-TTGGGACATCTCTGAGG
          1 TGA-A-A-C---------ACTCTTTTTGTAGAATTTGCAAGTGGATATTTAGAACGT-TTTGAGG

               **** *     *       *     * *        *                     *   
      20768 CCTTCATTGGAAACGGGAATT-TCTTCATATAAAAACTAGACAGAAGAATTCTCAGAAACTTCTT
         53 CCTAAGGTAGAAA-AGGAATTACCTTCAAACAAAAACTACACAGAAGAATTCTCAGAAACTACTT

                    *         *          *            
      20832 TGTGATGTGTGCATTCAATTCACAGAGTTGAACCTTCCTTTC
        117 TGTGATGTTTGCATTCAACTCACAGAGTTCAA----CC-TTC

                                     *           *                * **  *    
      20874 AATAGAGCAGTTTTGAAACACTCTTCTTGTAGAATTTCCAAGTGGATATTTAGAGCAGTTAGAGG
          1 --T---G-A-------AACACTCTTTTTGTAGAATTTGCAAGTGGATATTTAGAACGTTTTGAGG

                *    *        * **       *      * *       *             *    
      20939 CCTATGGTACAAAAGGAAATGTCTTCAAATAAAAACAAGACAGAAGCATTCTCAGAAACTTCTTT
         53 CCTAAGGTAGAAAAGGAATTACCTTCAAACAAAAACTACACAGAAGAATTCTCAGAAACTACTTT

                   *                    *       
      21004 GTGATGTGTGCATTCAACTCACAGAGTTGAACCTTC
        118 GTGATGTTTGCATTCAACTCACAGAGTTCAACCTTC

      21040 GTTTCGATAG


Statistics
Matches: 498,  Mismatches: 112, Indels: 91
        0.71            0.16        0.13

Matches are distributed among these distances:
 152    6  0.01
 153  112  0.22
 157    6  0.01
 159    2  0.00
 163    1  0.00
 164    1  0.00
 166    4  0.01
 167    2  0.00
 168    5  0.01
 169  226  0.45
 170   12  0.02
 171  108  0.22
 172    7  0.01
 173    1  0.00
 175    1  0.00
 176    1  0.00
 180    1  0.00
 181    1  0.00
 182    1  0.00

ACGTcount: A:0.32, C:0.17, G:0.20, T:0.32


Consensus pattern (153 bp):   
TGAAACACTCTTTTTGTAGAATTTGCAAGTGGATATTTAGAACGTTTTGAGGCCTAAGGTAGAAA
AGGAATTACCTTCAAACAAAAACTACACAGAAGAATTCTCAGAAACTACTTTGTGATGTTTGCAT
TCAACTCACAGAGTTCAACCTTC

Found at i:21949 original size:511 final size:511

   Alignment explanation   
    Indices: 20378--22357  Score: 2260
    Period size: 511  Copynumber: 3.9  Consensus size: 511

      20368 TTCAACCTTC

                                                        *    *               
      20378 TGAAACACTCTTTTTGTAGAATTTGCAAGTGGATATTTAGAGCGTTTTGGGGCCTATGGTAGAAA
          1 TGAAACACTCTTTTTGTAGAATTTGCAAGTGGATATTTAGAGCGCTTTGAGGCCTATGGTAGAAA

                 *  *      **      *               *     * *           *     
      20443 AGGAATTACCTTCACCCAAAAACCACACAGAAGCATTCTGAGAAAATGCTTTGTGATGTTTGCAT
         66 AGGAAATATCTTCACATAAAAACTACACAGAAGCATTCTCAGAAACTTCTTTGTGATGTGTGCAT

                        *  *   *  *        * *                    *  *   * * 
      20508 TCAACTCACAGATTTTAACTTTTCTTTTGATGGCGCAGTTTTGAAACACTCTTTGTAGAATTTTC
        131 TCAACTCACAGAGTTGAACATTCCTTTTGATAGAGCAGTTTTGAAACACTCTTTTTATAATCTGC

                              *        *   *  *                  *           
      20573 AAGTGGATATTTAGATCGTTTTGAGGCGTAAGGTAGGAAA-GGAAATATCTTCTTAGAAAAACTA
        196 AAGTGGATATTTAGATCGCTTTGAGGCCT-ACGTTGGAAACGGAAATATCTTCATAGAAAAACTA

                    *                       *  *     *                 *     
      20637 CACAGAAGGATTCTCAGAAACTACTTTGTGATATTGGCATTTAACTCACAGAGTTGAACATTCCT
        260 CACAGAAGAATTCTCAGAAACTACTTTGTGATGTTTGCATTCAACTCACAGAGTTGAACGTTCCT

                      *    *                                     **   **  *  
      20702 TTTGATAGAGCAGTTGTGAAACACT-TTT-TGTAGAATCTGCAAGTGGATATTGGGACATCTCTG
        325 TTTGATAGAGAAGTTTTGAAACACTCTTTGTGTAGAATCTGCAAGTGGATATTTAGACCGCTTTG

                  *    *    **     *                        **               
      20765 AGGCCTTCATTGGAAACGGGAATTTCTTCATATAAAAACTAGACAGAAGAATTCTCAGAAACTTC
        390 AGGCCTACATTAGAAAACGGAATATCTTCATATAAAAACTAGACAGAAACATTCTCAGAAACTTC

                *               *              *  *    **            
      20830 TTTGTGATGTGTGCATTCAATTCACAGAGTTGAACCTTCCTTTCAATAGAGCAGTTT
        455 TTTGGGATGTGTGCATTCAACTCACAGAGTTGAACATTTCTTTTGATAGAGCAGTTT

                        *           *                  **  *             *   
      20887 TGAAACACTCTTCTTGTAGAATTTCCAAGTGGATATTTAGAGCAGTTAGAGGCCTATGGTACAAA
          1 TGAAACACTCTTTTTGTAGAATTTGCAAGTGGATATTTAGAGCGCTTTGAGGCCTATGGTAGAAA

                   *      *        * *                                       
      20952 AGGAAATGTCTTCAAATAAAAACAAGACAGAAGCATTCTCAGAAACTTCTTTGTGATGTGTGCAT
         66 AGGAAATATCTTCACATAAAAACTACACAGAAGCATTCTCAGAAACTTCTTTGTGATGTGTGCAT

                               *   *   *                     *               
      21017 TCAACTCACAGAGTTGAACCTTCGTTTCGATAGAGCAGTTTTGAAACACGCTTTTTATCGAAT-T
        131 TCAACTCACAGAGTTGAACATTCCTTTTGATAGAGCAGTTTTGAAACACTCTTTTTAT--AATCT

            * *               *              * * *    *       *              
      21081 CCCAGCTGGATATTTAGAGCGCTTTGAGGCCTATGGTAGAAAAGGAAATACCTTCATAGAAAAAC
        194 GCAAG-TGGATATTTAGATCGCTTTGAGGCCTACGTTGGAAACGGAAATATCTTCATAGAAAAAC

                     ***             *       *                *              
      21146 TACACAGAATCGTTCTCAGAAACTAATTTGTGAGGTTTGCATTCAACTCATAGAGTTGAACGTTC
        258 TACACAGAAGAATTCTCAGAAACTACTTTGTGATGTTTGCATTCAACTCACAGAGTTGAACGTTC

                 *                *          *         *                     
      21211 C-TTTCATAGAGAAGTTTTGAATCACTTTTTTGTTGTTGTTGTTGAATCTGCAAGTGGATATTTA
        323 CTTTTGATAGAGAAGTTTTGAAACAC-----T-CT-TTG-TGTAGAATCTGCAAGTGGATATTTA

                * *         *    *    *   **        *     *  *        *      
      21275 GACCACGTTGAGGCCTTCATTGGAAACCGGTTTATCTTCACATAAATACCAGACAGAAGCATTCT
        380 GACCGCTTTGAGGCCTACATTAGAAAACGGAATATCTTCATATAAAAACTAGACAGAAACATTCT

                 *                                 *    **          *        
      21340 CAGAACCTTCTTTGGGATGTGTGCATTCAACTCACAGAGGTGAAATTTTCTTTTGAAAGAGCAGT
        445 CAGAAACTTCTTTGGGATGTGTGCATTCAACTCACAGAGTTGAACATTTCTTTTGATAGAGCAGT

            * 
      21405 CT
        510 TT

                     *            *                  ** *           *  * *   
      21407 TGAAACACTATTTTTGTAGAATCTGCAAGTGGATACTTTA-TTCCCTTTGAGGCCTTTGTTGGAA
          1 TGAAACACTCTTTTTGTAGAATTTGCAAGTGGATA-TTTAGAGCGCTTTGAGGCCTATGGTAGAA

                  *                *  * *    *       ** *       * *    *     
      21471 AAGGAATTATCTTCACATAAAAATTAGAAAGAAACATTCTCGTATACTTCTTGGAGATGAGTGCA
         65 AAGGAAATATCTTCACATAAAAACTACACAGAAGCATTCTCAGAAACTTCTTTGTGATGTGTGCA

                               *                   *             *           
      21536 TTCAACTCACAGAGTTGAAGATTCCTTTTGATAGAGCAGATTTGAAACACTCTGTTTATAAGTCT
        130 TTCAACTCACAGAGTTGAACATTCCTTTTGATAGAGCAGTTTTGAAACACTCTTTTTATAA-TCT

                          * * *         *  * *          *          ***     * 
      21601 GCAAGTGGATATTTGGTTGGCTTTGAGGTCTTCTTTGGAAACGGTAATATCTTCACCTAAAAAGT
        194 GCAAGTGGATATTTAGATCGCTTTGAGGCCTACGTTGGAAACGGAAATATCTTCATAGAAAAACT

             *     * *             *           *    *     *         *    *   
      21666 AGACAGAGGTATTCTCAGAAACTTC-TTGTGATGTCTGCACTCAACCCACAGAGTTCAACGGTCC
        259 ACACAGAAGAATTCTCAGAAACTACTTTGTGATGTTTGCATTCAACTCACAGAGTTGAACGTTCC

                        * *                        *      *     *     *      
      21730 TTTTGATAGAGAGGATTTGAAACACTCTTTGTGTAGAATTTGCAAGAGGATAATTAGAGCGCTTT
        324 TTTTGATAGAGAAGTTTTGAAACACTCTTTGTGTAGAATCTGCAAGTGGATATTTAGACCGCTTT

                    **                       *     *  *                     *
      21795 GAGGCCTATGTTAGAAAACGGAATATCTTCATAGAAAAATTACACAGAAACATTCTCAGAAACTA
        389 GAGGCCTACATTAGAAAACGGAATATCTTCATATAAAAACTAGACAGAAACATTCTCAGAAACTT

            *          *  *          **     **    *                * *
      21860 TTTTGGGATGTTTGTATTCAACTCAAGGAGTTATACATATCTTTTGATAGAGCAGATG
        454 CTTTGGGATGTGTGCATTCAACTCACAGAGTTGAACATTTCTTTTGATAGAGCAGTTT

                                    *                       * *     *   *    
      21918 TGAAACACTCTTTTTGTAGAATTTTCAAGTGGATATTTAGAGCGCTTTTATGCCTACGGTGGAAA
          1 TGAAACACTCTTTTTGTAGAATTTGCAAGTGGATATTTAGAGCGCTTTGAGGCCTATGGTAGAAA

                          * *     *                        *     *     *     
      21983 AGGAAATATCTTCATAGAAAAATTACACAGAAGCATTCTCAGAAACTACTTTGGGATGTTTGCAT
         66 AGGAAATATCTTCACATAAAAACTACACAGAAGCATTCTCAGAAACTTCTTTGTGATGTGTGCAT

                     *                   *              *               *    
      22048 TCAACTCACGGAGTTGAACATTCCTTTTTCATAGAGCAGTTTTGTAACACTCTTTTTGCAGAATC
        131 TCAACTCACAGAGTTGAACATTCC-TTTTGATAGAGCAGTTTTGAAACACTCTTTTT--ATAATC

                           *  * *       *   *             *  *        *      
      22113 TGCAAGTGGATATTTGGACCTCTTTGAGTCCTTCGTTGGAAACGG-GATTTCTTCATATAAAAAC
        193 TGCAAGTGGATATTTAGATCGCTTTGAGGCCTACGTTGGAAACGGAAATATCTTCATAGAAAAAC

              *  **                 *        *  *               ** *     *   
      22177 TAGACCAAAGAATTCTCAGAAACTTCTTTGTGAAGTGTGCATTCAACTCACAAGGATGAACCTTC
        258 TACACAGAAGAATTCTCAGAAACTACTTTGTGATGTTTGCATTCAACTCACAGAGTTGAACGTTC

                **      *      *           *        * *                *     
      22242 CTTTCAATAGAGCAGTTTTTAAACACTCTTTTTGTAGAATTTCCAAGTGGATATTTAGAGCGCTT
        323 CTTTTGATAGAGAAGTTTTGAAACACTCTTTGTGTAGAATCTGCAAGTGGATATTTAGACCGCTT

               * *   ***       * *     *                       
      22307 TGAAGTCTATGGTAGAAAAGGAAATATTTTCATATAAAAACTAGACAGAAA
        388 TGAGGCCTACATTAGAAAACGGAATATCTTCATATAAAAACTAGACAGAAA

      22358 GGCTGGTTCA


Statistics
Matches: 1181,  Mismatches: 267, Indels: 42
        0.79            0.18        0.03

Matches are distributed among these distances:
 509  158  0.13
 510   34  0.03
 511  343  0.29
 512   67  0.06
 513  166  0.14
 514    4  0.00
 515    1  0.00
 517    1  0.00
 518   36  0.03
 519   82  0.07
 520  285  0.24
 521    4  0.00

ACGTcount: A:0.31, C:0.16, G:0.20, T:0.33


Consensus pattern (511 bp):   
TGAAACACTCTTTTTGTAGAATTTGCAAGTGGATATTTAGAGCGCTTTGAGGCCTATGGTAGAAA
AGGAAATATCTTCACATAAAAACTACACAGAAGCATTCTCAGAAACTTCTTTGTGATGTGTGCAT
TCAACTCACAGAGTTGAACATTCCTTTTGATAGAGCAGTTTTGAAACACTCTTTTTATAATCTGC
AAGTGGATATTTAGATCGCTTTGAGGCCTACGTTGGAAACGGAAATATCTTCATAGAAAAACTAC
ACAGAAGAATTCTCAGAAACTACTTTGTGATGTTTGCATTCAACTCACAGAGTTGAACGTTCCTT
TTGATAGAGAAGTTTTGAAACACTCTTTGTGTAGAATCTGCAAGTGGATATTTAGACCGCTTTGA
GGCCTACATTAGAAAACGGAATATCTTCATATAAAAACTAGACAGAAACATTCTCAGAAACTTCT
TTGGGATGTGTGCATTCAACTCACAGAGTTGAACATTTCTTTTGATAGAGCAGTTT

Found at i:24666 original size:12 final size:12

   Alignment explanation   
    Indices: 24649--24686  Score: 55
    Period size: 12  Copynumber: 3.2  Consensus size: 12

      24639 CTTAAAGTAT

                        
      24649 AATAAAAAAAAA
          1 AATAAAAAAAAA

                      * 
      24661 AATAAAAAAACA
          1 AATAAAAAAAAA

              *   *     
      24673 AACAAACAAAAA
          1 AATAAAAAAAAA

              
      24685 AA
          1 AA

      24687 CCAGACAGAA


Statistics
Matches: 22,  Mismatches: 4, Indels: 0
        0.85            0.15        0.00

Matches are distributed among these distances:
  12   22  1.00

ACGTcount: A:0.87, C:0.08, G:0.00, T:0.05


Consensus pattern (12 bp):   
AATAAAAAAAAA

Found at i:24669 original size:8 final size:8

   Alignment explanation   
    Indices: 24652--24687  Score: 51
    Period size: 8  Copynumber: 4.5  Consensus size: 8

      24642 AAAGTATAAT

               *    
      24652 AAAAAAAA
          1 AAACAAAA

               *    
      24660 AAATAAAA
          1 AAACAAAA

                   *
      24668 AAACAAAC
          1 AAACAAAA

                    
      24676 AAACAAAA
          1 AAACAAAA

                
      24684 AAAC
          1 AAAC

      24688 CAGACAGAAT


Statistics
Matches: 24,  Mismatches: 4, Indels: 0
        0.86            0.14        0.00

Matches are distributed among these distances:
   8   24  1.00

ACGTcount: A:0.86, C:0.11, G:0.00, T:0.03


Consensus pattern (8 bp):   
AAACAAAA

Found at i:24975 original size:171 final size:171

   Alignment explanation   
    Indices: 24682--2125761  Score: 1651403
    Period size: 171  Copynumber: 12362.8  Consensus size: 171

      24672 AAACAAACAA

                  *        *             **           *  ***               * 
      24682 AAAAACCAGACAGAATCATTCTCAGAAACTCCTTTGTGATGTGTGGGTTCAACTCACAGAGTTTA
          1 AAAAACTAGACAGAAGCATTCTCAGAAACATCTTTGTGATGTTTGCACTCAACTCACAGAGTTGA

              *       **     **         *      *      * *    *     *       **
      24747 ACATTTCTTTCGATAGAATAGTTTTGAAGCACTCTGTTTGTAAAGTCTGCAAGTGAATATTTGAC
         66 ACCTTTCTTTTCATAGAGCAGTTTTGAAACACTCTTTTTGTAGAATCTGGAAGTGGATATTTGGA

              *        **   *       *  *          *  
      24812 GCACTTTGAGGCCTTCTTTGGAAAAGGTAATATCTTCACAT
        131 GCGCTTTGAGGATTTCGTTGGAAACGGAAATATCTTCATAT

                 *          *            *          * *       *     *   *    
      24853 AAAAAGTAGACAGAAGTATTCTCAGAAACTTCTTTGTGATCTCTGCACTCCACTCAGAGATTTGA
          1 AAAAACTAGACAGAAGCATTCTCAGAAACATCTTTGTGATGTTTGCACTCAACTCACAGAGTTGA

             *   *     *                     *         *  *  *     *      *  
      24918 AACTTCCTTTTGATAGAGCAGTTTTGAAACACTATTTTTGTAGGATTTGAAAGTGAATATTTAGA
         66 ACCTTTCTTTTCATAGAGCAGTTTTGAAACACTCTTTTTGTAGAATCTGGAAGTGGATATTTGGA

               *       ** *          * **     *     **
      24983 GCGTTTTGGAGCCTAT-GTTGGAAAAGATAATATATTCATTC
        131 GCGCTTT-GAGGATTTCGTTGGAAACGGAAATATCTTCATAT

                    *                      **              *                 
      25024 AAAAACTACACAGAAGCATTCTCAGAAAC-TACTT-TGATGTTTGCATTCAACTCACAGAGTTGA
          1 AAAAACTAGACAGAAGCATTCTCAGAAACATCTTTGTGATGTTTGCACTCAACTCACAGAGTTGA

              *  *     *              *      * *             *            *  
      25087 ACATTCCTTTTGATAGAGCAGTTTTGTAACACTTTCTTTGTAGAATCTGCAAGTGGATATTTTGA
         66 ACCTTTCTTTTCATAGAGCAGTTTTGAAACACTCTTTTTGTAGAATCTGGAAGTGGATATTTGGA

            * *  *     **              *   *   * *   
      25152 CCTCTCTGAGGCCTTCGTTGGAAACGGGAATTTCTACGTAT
        131 GCGCTTTGAGGATTTCGTTGGAAACGGAAATATCTTCATAT

                            *            *            *    *    *            
      25193 AAAAACTAGACAGAAGAATTCTCAGAAACTTCTTTGTGATGTGTGCATTCAATTCACAGAGTTGA
          1 AAAAACTAGACAGAAGCATTCTCAGAAACATCTTTGTGATGTTTGCACTCAACTCACAGAGTTGA

                **    **           *             *        * **           **  
      25258 ACCTGACTTTCGATAGAGCAGTTCTGAAACACTCTTTCTGTAGAATTTCCAAGTGGATATTCAGA
         66 ACCTTTCTTTTCATAGAGCAGTTTTGAAACACTCTTTTTGTAGAATCTGGAAGTGGATATTTGGA

             * *      *** ** * *    *                
      25323 GTGGTTTGAGTCCTATGGTAGAAAAGGAAATATCTTCATAT
        131 GCGCTTTGAGGATTTCGTTGGAAACGGAAATATCTTCATAT

                 *      ** *    *        **           *    *            *    
      25364 AAAAATTAGACATTATCATTTTCAGAAACTACTTTGTGATGTGTGCATTCAACTCACAGATTTGA
          1 AAAAACTAGACAGAAGCATTCTCAGAAACATCTTTGTGATGTTTGCACTCAACTCACAGAGTTGA

                 *     *                                  *  *     *      *  
      25429 ACCTTCCTTTTGATAGAGCAGTTTTGAAACACTCTTTTTGTAGAATTTGCAAGTGAATATTTAGA
         66 ACCTTTCTTTTCATAGAGCAGTTTTGAAACACTCTTTTTGTAGAATCTGGAAGTGGATATTTGGA

             *         ** * *   *    *               *
      25494 GGGCTTTGATGCCTATTG-TAGAAAAGGAAATATCTTCATAG
        131 GCGCTTTGA-GGATTTCGTTGGAAACGGAAATATCTTCATAT

              *     *      *            ****           *   *                 
      25535 AATAACTACACAGAAACATTCTCAGAAAGTAATTTGTGATGTTAGCATTCAACTCACAGAGTTGA
          1 AAAAACTAGACAGAAGCATTCTCAGAAACATCTTTGTGATGTTTGCACTCAACTCACAGAGTTGA

                 *     *         *              *      ****   *              
      25600 ACCTTCCTTTTGATAGAGCATTTTTTGAAACACTCTCTTTGTAATGGCTGCAAGTGGATATTTGG
         66 ACCTTTCTTTTCATAGAGCA-GTTTTGAAACACTCTTTTTGTAGAATCTGGAAGTGGATATTTGG

               **       **   *   *   * **          ** 
      25665 AGCAATTTGAGGTCTTCTTTGTAAATGAGAATATCTTCACGT
        130 AGCGCTTTGAGGATTTCGTTGGAAACGGAAATATCTTCATAT

                 *          *     *      **                            * *   
      25707 AAAAAGTAGACAGAAGTATTCTGAGAAACTACTTTGTGATGTTTGCACTCAACTCACAGTGCTGA
          1 AAAAACTAGACAGAAGCATTCTCAGAAACATCTTTGTGATGTTTGCACTCAACTCACAGAGTTGA

             *   *     *                *    *            * **     *   *  *  
      25772 AACTTCCTTTTGATAGAGCAGTTTTGAACCACTATTTTTGTAGAATTTCCAAGTGCATAGTTAGA
         66 ACCTTTCTTTTCATAGAGCAGTTTTGAAACACTCTTTTTGTAGAATCTGGAAGTGGATATTTGGA

                       ** ** * *    *               *
      25837 GCGCTTTGAGGCCTATGGTAGAAAAGGAAATATCTTCATAG
        131 GCGCTTTGAGGATTTCGTTGGAAACGGAAATATCTTCATAT

                    *                    **                *        *        
      25878 AAAAACTACACAGAAGCATTCTCAGAAACTACTTTGTGATGTTTGCATTCAACTCAAAGAGTTGA
          1 AAAAACTAGACAGAAGCATTCTCAGAAACATCTTTGTGATGTTTGCACTCAACTCACAGAGTTGA

             **  *    **    *         *                      *               
      25943 AGATTCCTTTCGATAGGGCAGTTTTGTAACACTCTTTTTGTAGAATCTGCAAGTGGATATTTGGA
         66 ACCTTTCTTTTCATAGAGCAGTTTTGAAACACTCTTTTTGTAGAATCTGGAAGTGGATATTTGGA

            * **    *   *           *   *  *         
      26008 CCTTTTTGGGGACTTCGTTGGAAATGG-GATTTCTTCATAT
        131 GCGCTTTGAGGATTTCGTTGGAAACGGAAATATCTTCATAT

                    *       *            *            *    *            *    
      26048 AAAAACTATACAGAAGAATTCTCAGAAACTTCTTTGTGATGTGTGCATTCAACTCACAGATTTGA
          1 AAAAACTAGACAGAAGCATTCTCAGAAACATCTTTGTGATGTTTGCACTCAACTCACAGAGTTGA

              *    *               *                    *  * ***          ***
      26113 A-ATTTCCTTTCGATAGAGCAGTATTGAAACACTCTTTTTGTAGCATTTCCGAGTGGATATTAAC
         66 ACCTTTCTTTTC-ATAGAGCAGTTTTGAAACACTCTTTTTGTAGAATCTGGAAGTGGATATTTGG

                      * ** **   *    ***     *        
      26177 AGCGCTTTGAAGCCTGAGTTAGAAAAAAAAATAACTTCATAT
        130 AGCGCTTTGAGGATTTCGTTGGAAACGGAAATATCTTCATAT

                           *             **           *   **     *   * **  * 
      26219 AAAAACTAGACAGAATCATTCTCAGAAACTACTTTGTGATGTGTGCGTTCAACACACGGCATTTA
          1 AAAAACTAGACAGAAGCATTCTCAGAAACATCTTTGTGATGTTTGCACTCAACTCACAGAGTTGA

                       *   *      *   *    *         *    *  * *          *  
      26284 ACCTTTCTTTTGATAAAGCAGTATTGCAACATTCTTTTTGTGGAATTTGCAGGTGGATATTTAGA
         66 ACCTTTCTTTTCATAGAGCAGTTTTGAAACACTCTTTTTGTAGAATCTGGAAGTGGATATTTGGA

                  *    **   *  * * *      *   *       
      26349 GCGCTTGGAGTCCTTTGGTAGAATAC-GAAGTATTTTCATA-
        131 GCGCTTTGAG-GATTTCGTTGGAAACGGAAATATCTTCATAT

                 * * *                                    * *     * *  ** *  
      26389 AAAAAATGGCCAGAAGCATTCTCAGAAACTAT-TTTGTGATGTTTGTATTCAACACGCAAGGCTG
          1 AAAAACTAGACAGAAGCATTCTCAGAAAC-ATCTTTGTGATGTTTGCACTCAACTCACAGAGTTG

                * *     *      *                  *   *       *            * 
      26453 AACCATCCTTTTGATAGAGGAGTTTTGAAACACTCTTTGTGTGGAATCTGCAAGTGGATATTTTG
         65 AACCTTTCTTTTCATAGAGCAGTTTTGAAACACTCTTTTTGTAGAATCTGGAAGTGGATATTTGG

            ** *      * ** **        *       *     *  
      26518 TTCCCTTTGAAGCCTAAGTTGGAAAAGGAAATACCTTCACAT
        130 AGCGCTTTGAGGATTTCGTTGGAAACGGAAATATCTTCATAT

                      *    *        *    *           **    *       * *       
      26560 AAAAACTAGAAAGAAACATTCTCATAAACTTCTTTGTGATGAGTGCATTCAACTCTCGGAGTTGA
          1 AAAAACTAGACAGAAGCATTCTCAGAAACATCTTTGTGATGTTTGCACTCAACTCACAGAGTTGA

                 *     *                              * *    *               
      26625 ACCTTCCTTTTGATAGAGCAGTTTTGAAACA--C--TTTGTAAAGTCTGCAAGTGGATATTTGGA
         66 ACCTTTCTTTTCATAGAGCAGTTTTGAAACACTCTTTTTGTAGAATCTGGAAGTGGATATTTGGA

             *     *   **   *       *  * *  *     *  
      26686 GTGCTTTAAGGCCTTCTTTGGAAATGGGAGTACCTTCACAT
        131 GCGCTTTGAGGATTTCGTTGGAAACGGAAATATCTTCATAT

                 *    *     *   *        *            *         *       *    
      26727 AAAAAGTAGAGAGAAGTATTTTCAGAAACTTCTTTGTGATGTCTGCACTCAAATCACAGATTTGA
          1 AAAAACTAGACAGAAGCATTCTCAGAAACATCTTTGTGATGTTTGCACTCAACTCACAGAGTTGA

             *   *     *                 *   **         * *  *  *      *     
      26792 AACTTCCTTTTGATAGAGCAGTTTTGAAAAACTGCTTTTGTAGATTTTGCAATTGGATAATTGGA
         66 ACCTTTCTTTTCATAGAGCAGTTTTGAAACACTCTTTTTGTAGAATCTGGAAGTGGATATTTGGA

                       ** ** * **   *   *     *     *
      26857 GCGCTTTGAGGCCTATGGTATAAAAGGATATATCATCATAG
        131 GCGCTTTGAGGATTTCGTTGGAAACGGAAATATCTTCATAT

                    *  *    *            *                 *         * *   * 
      26898 AAAAACTACACGGAAGTATTCTCAGAAACTTCTTTGTGATGTTTGCATTCAACTCACTGTGTTAA
          1 AAAAACTAGACAGAAGCATTCTCAGAAACATCTTTGTGATGTTTGCACTCAACTCACAGAGTTGA

              *  *     *           *  *    *   *             *               
      26963 ACATTCCTTTTTATAGAGCAGTTATGCAACAGTCTGTTTGTAGAATCTGCAAGTGGATATTTGGA
         66 ACCTTTCTTTTCATAGAGCAGTTTTGAAACACTCTTTTTGTAGAATCTGGAAGTGGATATTTGGA

            * *        **           *  *   *      ** 
      27028 CCTCTTTGAGGCCTTCGTTGGAAATGGGAATTTCTTCACTT
        131 GCGCTTTGAGGATTTCGTTGGAAACGGAAATATCTTCATAT

                         *  * *            *   *      *    *                 
      27069 AAAAACTAGACAGGAGAAATCTCAGAAAC-TTTTTCTGATGTGTGCATTCAACTCACAGAGTTGA
          1 AAAAACTAGACAGAAGCATTCTCAGAAACATCTTTGTGATGTTTGCACTCAACTCACAGAGTTGA

            *    *     *             *                    * **            *  
      27133 GCCTTCCTTTTGATAGAGCAGTTTTTAAACACTCTTTTT-TAGAATTTCCAAGTGGATATTTAGA
         66 ACCTTTCTTTTCATAGAGCAGTTTTGAAACACTCTTTTTGTAGAATCTGGAAGTGGATATTTGGA

                       ** * *   *    **            * *
      27197 GCGCTTTGAAGCCTATTG-TAGAAAAAGAAATATCTTCAGAG
        131 GCGCTTTG-AGGATTTCGTTGGAAACGGAAATATCTTCATAT

                    *        *                              *         * *   *
      27238 AAAAACTACACAGAAGCCTTCTCAGAAA-ATACTTTGTGATGTTTGCATTCAACTCACTGTGTTA
          1 AAAAACTAGACAGAAGCATTCTCAGAAACAT-CTTTGTGATGTTTGCACTCAACTCACAGAGTTG

               *  *     *           *  *    *   *             *              
      27302 AACATTCCTTTTTATAGAGCAGTTATGCAACAGTCTGTTTGTAGAATCTGCAAGTGGATATTTGG
         65 AACCTTTCTTTTCATAGAGCAGTTTTGAAACACTCTTTTTGTAGAATCTGGAAGTGGATATTTGG

             * *        **           *   *  *         
      27367 ACCTCTTTGAGGCCTTCGTTGGAAATGG-GATTTCTTCATAT
        130 AGCGCTTTGAGGATTTCGTTGGAAACGGAAATATCTTCATAT

                            *   *        *         *  ***  *             *   
      27408 AAAAACTAGACAGAAGAATTATCAGAAACTTCTTTGTGAAGTGCACATTCAACTCACAGAGATGA
          1 AAAAACTAGACAGAAGCATTCTCAGAAACATCTTTGTGATGTTTGCACTCAACTCACAGAGTTGA

             *    **  **                          *       * **            *  
      27473 AAC-TTGATTCGATAGAGCAGTTTTGAAACACTCTTTTGGTAGAATTTCCAAGTGGATATTTAGA
         66 ACCTTTCTTTTCATAGAGCAGTTTTGAAACACTCTTTTTGTAGAATCTGGAAGTGGATATTTGGA

               *     * ** ** * **   *                
      27537 GCGTTTTGAAGCCTATGGTACAAAAGGAAATATCTTCATAT
        131 GCGCTTTGAGGATTTCGTTGGAAACGGAAATATCTTCATAT

                           **          * **           *  ***           *   * 
      27578 AAAAACTAGACAGAATTATTCTCAGAAGCTACTTTGTGATGTGTGTGTTCAACTCACAGGGTTTA
          1 AAAAACTAGACAGAAGCATTCTCAGAAACATCTTTGTGATGTTTGCACTCAACTCACAGAGTTGA

                       *                                  *  *     *      *  
      27643 ACCTTTCTTTTGATAGAGCAGTTTTGAAACACTCTTTTTGTAGAATTTGCAAGTGTATATTTAGA
         66 ACCTTTCTTTTCATAGAGCAGTTTTGAAACACTCTTTTTGTAGAATCTGGAAGTGGATATTTGGA

            **         ** ** * *    *             *  
      27708 TTGCTTTGAGGCCTATGATAGAAAAGGAAATATCTTCACAT
        131 GCGCTTTGAGGATTTCGTTGGAAACGGAAATATCTTCATAT

                      *                  **        *    *  *   *        *    
      27749 AAAAACTAGAGAGAAGCATTCTCAGAAACTACTTTGTGAAGTTTCCATTCACCTCACAGACTTGA
          1 AAAAACTAGACAGAAGCATTCTCAGAAACATCTTTGTGATGTTTGCACTCAACTCACAGAGTTGA

               *   *   *                  * *        *       *               
      27814 A-CGTTCCTTTGATAGAGCAGTTTTGAAACGCCCTTTTTGTGGAATCTGCAAGTGGATATTTGGA
         66 ACCTTTCTTTTCATAGAGCAGTTTTGAAACACTCTTTTTGTAGAATCTGGAAGTGGATATTTGGA

            *          **   *  *       **    *    *  
      27878 CCGCTTTGAGGCCTTCTTTAGAAACGGGTATATGTTCACAT
        131 GCGCTTTGAGGATTTCGTTGGAAACGGAAATATCTTCATAT

                 **   *  *  *            * *          *    *           *     
      27919 AAAAAGCAGAAAGGAGGATTCTCAGAAACTTTTTTGTGATGTGTGCATTCAACTCACAGTGTTGA
          1 AAAAACTAGACAGAAGCATTCTCAGAAACATCTTTGTGATGTTTGCACTCAACTCACAGAGTTGA

                *      *                          *   *      *          *   *
      27984 ACCTGTCTTTTGATAGAGCAGTTTTGAAACACTCTTTTGGTAAAATCTGCAAGTGGATATGTGGC
         66 ACCTTTCTTTTCATAGAGCAGTTTTGAAACACTCTTTTTGTAGAATCTGGAAGTGGATATTTGGA

            * *        ** * ** *    *             ** 
      28049 TCCCTTTGAGGCCTACTATTGAAAAGGAAATATCTTCACGT
        131 GCGCTTTGAGGATTTCGTTGGAAACGGAAATATCTTCATAT

                      *  * *     *       *     *      *   **                 
      28090 AAAAACTAGAAAGTATCATTCCCAGAAACTTCTTTTTGATGTGTGCTTTCAACTCACAGAGTTGA
          1 AAAAACTAGACAGAAGCATTCTCAGAAACATCTTTGTGATGTTTGCACTCAACTCACAGAGTTGA

             *   *     *          *                          *               
      28155 AACTTCCTTTTGATAGAGCAGTATTGAAACACTCTTTTTGTAGAATCTGCAAGTGGATATTTGGA
         66 ACCTTTCTTTTCATAGAGCAGTTTTGAAACACTCTTTTTGTAGAATCTGGAAGTGGATATTTGGA

            *          **   *          **         *  
      28220 TCGCTTTGAGGCCTTCTTTGGAAACGGGTATATCTTCACAT
        131 GCGCTTTGAGGATTTCGTTGGAAACGGAAATATCTTCATAT

             *   *          *          * *       *    *    *                 
      28261 AGAAAGTAGACAGAAGTATTCTCAGAAGCTTCTTTGTTATGTCTGCATTCAACTCACAGAGTTGA
          1 AAAAACTAGACAGAAGCATTCTCAGAAACATCTTTGTGATGTTTGCACTCAACTCACAGAGTTGA

                 *  *  *     ** *       *       *            *            *  
      28326 ACCTTCCTATTGATAGAAGATTTTTGAAGCACTCTTGTTGTAGAATCTGCAAGTGGATATTTTGA
         66 ACCTTTCTTTTCATAGAGCAGTTTTGAAACACTCTTTTTGTAGAATCTGGAAGTGGATATTTGGA

            ***         *            * *   *      ** 
      28391 CATCTTTGAGGACTTCGTTGGAAACTGGAATTTCTTCACTT
        131 GCGCTTTGAGGATTTCGTTGGAAACGGAAATATCTTCATAT

             *  *  *  *     * *                       *    *    *          * 
      28432 ACAACCTTGAGAGAAGAAATCTCAGAAACAT-TTTGTGATGTGTGCATTCAAATCACAGAGTTTA
          1 AAAAACTAGACAGAAGCATTCTCAGAAACATCTTTGTGATGTTTGCACTCAACTCACAGAGTTGA

             *   *     **                   *    *       *   *       * *  ** 
      28496 AACTTCCTTTTGGTAGAGCAGTTTTGAAACACACTTTCTGTAGAAACTGCAAGTGGAAAATTACA
         66 ACCTTTCTTTTCATAGAGCAGTTTTGAAACACTCTTTTTGTAGAATCTGGAAGTGGATATTTGGA

             *        *****  * *    * *      *    *  
      28561 GTGCTTTGAGACCCACGGTAGAAAAGAAAATATGTTCACAT
        131 GCGCTTTGAGGATTTCGTTGGAAACGGAAATATCTTCATAT

                    *                    **       *        *                 
      28602 AAAAACTACACAGAAGCATTCTCAGAAACTACTTTGTGTTGTTTGCATTCAACTCACAGAGTTGA
          1 AAAAACTAGACAGAAGCATTCTCAGAAACATCTTTGTGATGTTTGCACTCAACTCACAGAGTTGA

              *  **    *  *     *                            * *             
      28667 ACATTCGTTTTGATTGAGCACTTTTGAAACACTCTTTTTGTAGAATCTGCAGGTGGATATTTGGA
         66 ACCTTTCTTTTCATAGAGCAGTTTTGAAACACTCTTTTTGTAGAATCTGGAAGTGGATATTTGGA

            * *        **   *          *   *      ** 
      28732 CCTCTTTGAGGCATTCATTGGAAACGGGAATTTCTTCACTT
        131 GCGCTTTGAGGATTTCGTTGGAAACGGAAATATCTTCATAT

               **           * *   *        *   *      *    *       *  **    *
      28773 AAATGCTAGACAGAAGAAATCTGAGAAA-ATTTTTTTGATGTCTGCATTCAACTCCCATTGTTGT
          1 AAAAACTAGACAGAAGCATTCTCAGAAACATCTTTGTGATGTTTGCACTCAACTCACAGAGTTGA

              *  *     *          *   *  *                    *              
      28837 ACATTCCTTTTGATAGAGCAGTATTGTAAGACTCTTTTTGTAGAATCTGCCAAGTGGATATTTGG
         66 ACCTTTCTTTTCATAGAGCAGTTTTGAAACACTCTTTTTGTAGAATCTG-GAAGTGGATATTTGG

             * *  *     **   *          *   *         
      28902 ACCTCTCTGAGGCCTTCATTGGAAACGGGAATTTCTTCATAT
        130 AGCGCTTTGAGGATTTCGTTGGAAACGGAAATATCTTCATAT

                            *                         *    * ** *      **    
      28944 AAAAACTAGACAGAAGAATTCTCAGAAACATCTTTGTGATGTGTGCATTACATTCACAGTATTGA
          1 AAAAACTAGACAGAAGCATTCTCAGAAACATCTTTGTGATGTTTGCACTCAACTCACAGAGTTGA

                      **                  *               * **            *  
      29009 ACC-TTCTTTCGATAGAGCAGTTTTGAAACTCTCTTTTTGTAGAATTTACAAGTGGATATTTAGA
         66 ACCTTTCTTTTCATAGAGCAGTTTTGAAACACTCTTTTTGTAGAATCTGGAAGTGGATATTTGGA

              **       ** ** * *    *                
      29073 GCAGTTTGAGGCCTATGGTAGAAAAGGAAATATCTTCATAT
        131 GCGCTTTGAGGATTTCGTTGGAAACGGAAATATCTTCATAT

                   *       **             *        *  *  ***           *   * 
      29114 AAAAACTGGACAGAAATATTCTCAGAAACAACTTTGTGAGGTGTGTGTTCAACTCACAGTGTTTA
          1 AAAAACTAGACAGAAGCATTCTCAGAAACATCTTTGTGATGTTTGCACTCAACTCACAGAGTTGA

              *  *     *                   *        * ** **  *  *  *      *  
      29179 A-ATTACTTTTGATAGAGCAGTTTTGAAACAATCTTTTTGAACTACTTGCAAATGTATATTTAGA
         66 ACCTTTCTTTTCATAGAGCAGTTTTGAAACACTCTTTTTGTAGAATCTGGAAGTGGATATTTGGA

                       ** **   *    *     *       *  
      29243 GCGCTTTGAGGCCTATGTTAGAAAAGGAAACATCTTCACAT
        131 GCGCTTTGAGGATTTCGTTGGAAACGGAAATATCTTCATAT

                           *             **                *             * * 
      29284 AAAAACTAGACAGAAACATTCTCAGAAACTACTTTGTGATGTTTGCATTCAACTCACAGAGGTAA
          1 AAAAACTAGACAGAAGCATTCTCAGAAACATCTTTGTGATGTTTGCACTCAACTCACAGAGTTGA

             **  *    **                *     *              *               
      29349 ATATTCCTTTCAATAGAGCAGTTTTGAACCAC-CCTTTTGTAGAATCTGCAAGTGGATATTTGGA
         66 ACCTTTCTTTTCATAGAGCAGTTTTGAAACACTCTTTTTGTAGAATCTGGAAGTGGATATTTGGA

            **              *          *   *        *
      29413 TAGCTTTGAGGATTTCATTGGAAACGGGAATGTCTTCATAG
        131 GCGCTTTGAGGATTTCGTTGGAAACGGAAATATCTTCATAT

                **                     * * **         *  **   *   *          
      29454 AAAATTTAGACAGAAGCATTCTCAGAACCTTGATTGTGATGTGTGTTCTCCACTAACAGAGTTGA
          1 AAAAACTAGACAGAAGCATTCTCAGAAACATCTTTGTGATGTTTGCACTCAACTCACAGAGTTGA

                       * *   * *           *       *                         
      29519 ACCTTTCTTTTGACAGAACTGTTTTGAAACATTCTTTTTATAGAATCTGGAAGTGGATATTTGGA
         66 ACCTTTCTTTTCATAGAGCAGTTTTGAAACACTCTTTTTGTAGAATCTGGAAGTGGATATTTGGA

            **                         *          *  
      29584 AAGCTTTGAGGATTTCGTTGGAAACGGGAATATCTTCAAAT
        131 GCGCTTTGAGGATTTCGTTGGAAACGGAAATATCTTCATAT

                *    *            *           * *       *  *    *            
      29625 AAAATCTAGCCAGAAGCATTCTAAGAAACATCTTAGGGATGTTTACATTCAAGTCACAGAGTTGA
          1 AAAAACTAGACAGAAGCATTCTCAGAAACATCTTTGTGATGTTTGCACTCAACTCACAGAGTTGA

              *  * *     *       *         *    * *   **             *    *  
      29690 ACATTCCCTTTCACAGAGCAGGTTTGAAACAATCTTCTCGTACTATCTGGAAGTGGACATTTTGA
         66 ACCTTTCTTTTCATAGAGCAGTTTTGAAACACTCTTTTTGTAGAATCTGGAAGTGGATATTTGGA

              * *   *  *  ** *  *   *            ** *
      29755 GCTCCTTGGGGCTTATGCTGAAAAAGGAAATATCTTCCGAC
        131 GCGCTTTGAGGATTTCGTTGGAAACGGAAATATCTTCATAT

              *   *              *     *  **          *           **    *    
      29796 AAGAACCAGACAGAAGCATTCGCAGAATCACGTTTGTGATGTGTGCACTCAACTGTCAGAATTGA
          1 AAAAACTAGACAGAAGCATTCTCAGAAACATCTTTGTGATGTTTGCACTCAACTCACAGAGTTGA

                  *   ** *      *                            * *            *
      29861 ACCTTTGTTTGGACAGAGCACTTTTGAAACACTCTTTTTGTAGAATCTGCAGGTGGATATTTGGC
         66 ACCTTTCTTTTCATAGAGCAGTTTTGAAACACTCTTTTTGTAGAATCTGGAAGTGGATATTTGGA

            **                         *   *      * *
      29926 TAGCTTTGAGGATTTCGTTGGAAACGGTAATGTCTTCAAAG
        131 GCGCTTTGAGGATTTCGTTGGAAACGGAAATATCTTCATAT

                *          *   *          *   *            *    *            
      29967 AAAATCTAGACAGAAACATCCTCAGAAACACCTTCGTGATGTTTGCAATCAAGTCACAGAGTTGA
          1 AAAAACTAGACAGAAGCATTCTCAGAAACATCTTTGTGATGTTTGCACTCAACTCACAGAGTTGA

                 * *             *  *                  *             *  *    
      30032 ACCTTCCGTTTCATAGAGCAGGTTGGAAACACTCTTTTTGTAGTATCTGGAAGTGGACATCTGGA
         66 ACCTTTCTTTTCATAGAGCAGTTTTGAAACACTCTTTTTGTAGAATCTGGAAGTGGATATTTGGA

                   *   ** ** *  *   *       *    **  
      30097 GCGCTTTCAGGCCTATGGTGAAAAAGGAAATAGCTTCCCAT
        131 GCGCTTTGAGGATTTCGTTGGAAACGGAAATATCTTCATAT

                  * * *      **      *   * *   *     ** ** *      *    *     
      30138 AAAAACGACATAGAAGCTATCTCAGGAACTTGTTTATGATGCATCTAATCAACTAACAGTGTTGA
          1 AAAAACTAGACAGAAGCATTCTCAGAAACATCTTTGTGATGTTTGCACTCAACTCACAGAGTTGA

                  * ** * *                    *    * *       *               
      30203 ACCTTTGTACTGACAGAGCAG-TTTGAAACACTCATTTTTTGGAATCTGCAAGTGGATATTTGGA
         66 ACCTTTCTTTTCATAGAGCAGTTTTGAAACACTCTTTTTGTAGAATCTGGAAGTGGATATTTGGA

                                        *   *   *    
      30267 GCGCTTTGAGGATTTCGTTGGAAACGG-GAT-GC--AATAT
        131 GCGCTTTGAGGATTTCGTTGGAAACGGAAATATCTTCATAT

                **  *    *     *                 **  *   *  *    *         * 
      30304 AAAACGTACACAGCAGCATACTCAGAAA-ATACTTTGCCATATTTCCATTCAAGTCACAGAGTGG
          1 AAAAACTAGACAGAAGCATTCTCAGAAACAT-CTTTGTGATGTTTGCACTCAACTCACAGAGTTG

               *  * **      *     *                  *  *             *      
      30368 AACATTCCCATTCATAAAGCAGGTTTGAAACACTCTTTTTGGAGTATCTGGAAGTGGACATTTGG
         65 AACCTTTCTTTTCATAGAGCAGTTTTGAAACACTCTTTTTGTAGAATCTGGAAGTGGATATTTGG

                    ** * * ** *  *   *            **  
      30433 AGCGCTTTCTGAACTATGGTGAAAAAGGAAATATCTTCCAAT
        130 AGCGCTTTGAGGATTTCGTTGGAAACGGAAATATCTTCATAT

            *     *               *      * *    *   * *  **       *  *  *    
      30475 GAAAACAAGACAGAAGCATTCTGAGAAACTTATTTGCGATATGTGTCCTCAACTAACGGACTTGA
          1 AAAAACTAGACAGAAGCATTCTCAGAAACATCTTTGTGATGTTTGCACTCAACTCACAGAGTTGA

                   *      **  * ** *  *             *   *    * *             
      30540 ACCTTTCGTTTCATGCAGTACCTCTGGAACACTCTTTTTGAAGATTCTGCATGTGGATATTTGGA
         66 ACCTTTCTTTTCATAGAGCAGTTTTGAAACACTCTTTTTGTAGAATCTGGAAGTGGATATTTGGA

            **                            **   *     
      30605 TAGCTTTGAGGATTTCGTTGGAAACGG---GCT-TACATAT
        131 GCGCTTTGAGGATTTCGTTGGAAACGGAAATATCTTCATAT

                 *       *               *        *   *    *    *       *    
      30642 AAAAATTAGACAGCAGCATTCTCAGAAACTTCTTTGTGGTGTCTGCATTCAAGTCACAGAATTGA
          1 AAAAACTAGACAGAAGCATTCTCAGAAACATCTTTGTGATGTTTGCACTCAACTCACAGAGTTGA

             ** ** * **            *  * *      * *     *    *        *       
      30707 AAATCCCCTCACATAGAGCAGTTGTGCAGCACTCTATCTGTAGTATCTCGAAGTGGACATTTGGA
         66 ACCTTTCTTTTCATAGAGCAGTTTTGAAACACTCTTTTTGTAGAATCTGGAAGTGGATATTTGGA

             *         ** *    * *   *            **  
      30772 GGGCTTTGTAGCCTATC-TGGAAAAAGGAAATATCTTCCCAT
        131 GCGCTTTG-AGGATTTCGTTGGAAACGGAAATATCTTCATAT

            *  ** *   *     * *            *   *  *   * **        *  * * *   
      30813 GAATGCGAGATAGAAGTAATCTCAGAAACATGTTTATGCTGTATCTACTCAACTAACTGTGCTGA
          1 AAAAACTAGACAGAAGCATTCTCAGAAACATCTTTGTGATGTTTGCACTCAACTCACAGAGTTGA

              *     *  * *          *  *        *  * *       *               
      30878 ACATTTCTATTGAAAGAGCAGTTTCGAGACACTCTTCTTTTGGAATCTGCAAGTGGATATTTGGA
         66 ACCTTTCTTTTCATAGAGCAGTTTTGAAACACTCTTTTTGTAGAATCTGGAAGTGGATATTTGGA

            ** *                        *     *      
      30943 TAGATTTGAGGATTTCGTTGGAAACGG-GAT-T-AT-ATAT
        131 GCGCTTTGAGGATTTCGTTGGAAACGGAAATATCTTCATAT

                 *       *               *                 **  *   *         
      30980 AAAAAGTAGACAGCAGCATTCTCAGAAACTTCTTTGTGATGTTTGCATCCAGCTCTCAGAGTTGA
          1 AAAAACTAGACAGAAGCATTCTCAGAAACATCTTTGTGATGTTTGCACTCAACTCACAGAGTTGA

              *  * *          *  *  *     *        *   **        *  **       
      31045 ACATTCCCTTTCATAGAGTAGGTTGGAAACCCTCTTTTTATAGTGTCTGGAAGCGGGCATTTGGA
         66 ACCTTTCTTTTCATAGAGCAGTTTTGAAACACTCTTTTTGTAGAATCTGGAAGTGGATATTTGGA

                   *   ** ** *  *   *      *   * *   
      31110 GCGCTTTCAGGCCTATGCTGAAAAAGGAAATGTCTACCTAT
        131 GCGCTTTGAGGATTTCGTTGGAAACGGAAATATCTTCATAT

             *                    *    *               ** *        *    *    
      31151 AGAAACTAGACAGAAGCATTCTGAGAATC-TCGTTTGTGATGTGGGTACTCAACTAACAGTGTTG
          1 AAAAACTAGACAGAAGCATTCTCAGAAACATC-TTTGTGATGTTTGCACTCAACTCACAGAGTTG

             *  *    *  *   *            *   *                *              
      31215 ATCCATTCTATTGATACAGCAGTTTTGAACCACCCTTTTTGTAGAATCTGCAAGTGGATATTTGG
         65 AACCTTTCTTTTCATAGAGCAGTTTTGAAACACTCTTTTTGTAGAATCTGGAAGTGGATATTTGG

             **   *                     *   *        *
      31280 ATAGCTGTGAGGATTTCGTTGGAAACGGGAATGTCTTCATAG
        130 AGCGCTTTGAGGATTTCGTTGGAAACGGAAATATCTTCATAT

                **                     * * **         *  **   *   *          
      31322 AAAATTTAGACAGAAGCATTCTCAGAACCTTGATTGTGATGTGTGTTCTCCACTAACAGAGTTGA
          1 AAAAACTAGACAGAAGCATTCTCAGAAACATCTTTGTGATGTTTGCACTCAACTCACAGAGTTGA

                       * *   * *           *       *                     *   
      31387 ACCTTTCTTTTGACAGAACTGTTTTGAAACATTCTTTTTATAGAATCTGGAAGTGGATATTGGGA
         66 ACCTTTCTTTTCATAGAGCAGTTTTGAAACACTCTTTTTGTAGAATCTGGAAGTGGATATTTGGA

            **                         *          *  
      31452 AAGCTTTGAGGATTTCGTTGGAAACGGGAATATCTTCAAAT
        131 GCGCTTTGAGGATTTCGTTGGAAACGGAAATATCTTCATAT

            *   *    *         *  *           * *       *  *    *            
      31493 CAAATCTAGCCAGAAGCATGCTAAGAAACATCTTAGGGATGTTTACATTCAAGTCACAGAGTTGA
          1 AAAAACTAGACAGAAGCATTCTCAGAAACATCTTTGTGATGTTTGCACTCAACTCACAGAGTTGA

              *  * *     *       *         *    * *   **             *    *  
      31558 ACATTCCCTTTCACAGAGCAGGTTTGAAACAATCTTCTCGTACTATCTGGAAGTGGACATTTTGA
         66 ACCTTTCTTTTCATAGAGCAGTTTTGAAACACTCTTTTTGTAGAATCTGGAAGTGGATATTTGGA

              * *   *  ** ** *  *   *            ** *
      31623 GCTCCTTGGGGCCTATGCTGAAAAAGGAAATATCTTCCAAC
        131 GCGCTTTGAGGATTTCGTTGGAAACGGAAATATCTTCATAT

                                 *     *  **          *           **    *    
      31664 AAAAACTAGACAGAAGCATTCGCAGAATCACGTTTGTGATGTGTGCACTCAACTGTCAGAATTGA
          1 AAAAACTAGACAGAAGCATTCTCAGAAACATCTTTGTGATGTTTGCACTCAACTCACAGAGTTGA

                 **   ** *      *                            * *            *
      31729 ACCTTGGTTTGGACAGAGCACTTTTGAAACACTCTTTTTGTAGAATCTGCAGGTGGATATTTGGC
         66 ACCTTTCTTTTCATAGAGCAGTTTTGAAACACTCTTTTTGTAGAATCTGGAAGTGGATATTTGGA

            **                         *   *      * *
      31794 TAGCTTTGAGGATTTCGTTGGAAACGGTAATGTCTTCAAAG
        131 GCGCTTTGAGGATTTCGTTGGAAACGGAAATATCTTCATAT

                *          *   *          *   *            *    *            
      31835 AAAATCTAGACAGAAACATCCTCAGAAACACCTTCGTGATGTTTGCAATCAAGTCACAGAGTTGA
          1 AAAAACTAGACAGAAGCATTCTCAGAAACATCTTTGTGATGTTTGCACTCAACTCACAGAGTTGA

                 * *             *  *                  *             *  *    
      31900 ACCTTCCGTTTCATAGAGCAGGTTGGAAACACTCTTTTTGTAGTATCTGGAAGTGGACATCTGGA
         66 ACCTTTCTTTTCATAGAGCAGTTTTGAAACACTCTTTTTGTAGAATCTGGAAGTGGATATTTGGA

                   *   ** ** ** *   *       *    **  
      31965 GCGCTTTCAGGCCTATGGAGAAAAAGGAAATAGCTTCCCAT
        131 GCGCTTTGAGGATTTCGTTGGAAACGGAAATATCTTCATAT

                  * * *      **      *   * *   *     ** ** *      *    *     
      32006 AAAAACGACATAGAAGCTATCTCAGGAACTTGTTTATGATGCATCTAATCAACTAACAGTGTTGA
          1 AAAAACTAGACAGAAGCATTCTCAGAAACATCTTTGTGATGTTTGCACTCAACTCACAGAGTTGA

                  * ** * *                         * *       *    *          
      32071 ACCTTTGTACTGACAGAGCAG-TTTGAAACACTCTTTTTTTGGAATCTGCAAGTAGATATTTGGA
         66 ACCTTTCTTTTCATAGAGCAGTTTTGAAACACTCTTTTTGTAGAATCTGGAAGTGGATATTTGGA

            *                           *   *   *    
      32135 TCGCTTTGAGGATTTCGTTGGAAACGG-GAT-GC--AATAT
        131 GCGCTTTGAGGATTTCGTTGGAAACGGAAATATCTTCATAT

                **  * *  *     *                 **  *   *  *    *         * 
      32172 AAAACGTACAAAGCAGCATACTCAGAAA-ATACTTTGCCATATTTCCATTCAAGTCACAGAGTGG
          1 AAAAACTAGACAGAAGCATTCTCAGAAACAT-CTTTGTGATGTTTGCACTCAACTCACAGAGTTG

               *  * **            *                  *  *             *      
      32236 AACATTCCCATTCATAGAGCAGGTTTGAAACACTCTTTTTGGAGTATCTGGAAGTGGACATTTGG
         65 AACCTTTCTTTTCATAGAGCAGTTTTGAAACACTCTTTTTGTAGAATCTGGAAGTGGATATTTGG

                    ** * * ** *  *   *            **  
      32301 AGCGCTTTCTGAACTATGGTGAAAAAGGAAATATCTTCCAAT
        130 AGCGCTTTGAGGATTTCGTTGGAAACGGAAATATCTTCATAT

            *     *               *      * *         **  **       *  *  * *  
      32343 GAAAACAAGACAGAAGCATTCTGAGAAACTTATTTGTGATGCGTGTCCTCAACTAACGGACTCGA
          1 AAAAACTAGACAGAAGCATTCTCAGAAACATCTTTGTGATGTTTGCACTCAACTCACAGAGTTGA

                   *      **  * *  *  *         *   *   *    * * *           
      32408 ACCTTTCGTTTCATGCAGTACTTCTGGAACACTCTTGTTGAAGATTCTGCATGCGGATATTTGGA
         66 ACCTTTCTTTTCATAGAGCAGTTTTGAAACACTCTTTTTGTAGAATCTGGAAGTGGATATTTGGA

            **                            **   *     
      32473 TAGCTTTGAGGATTTCGTTGGAAACGG---GCT-TACATAT
        131 GCGCTTTGAGGATTTCGTTGGAAACGGAAATATCTTCATAT

                 *       *               *        *   *         *       *    
      32510 AAAAATTAGACAGCAGCATTCTCAGAAACTTCTTTGTGGTGTCTGCACTCAAGTCACAGAATTGA
          1 AAAAACTAGACAGAAGCATTCTCAGAAACATCTTTGTGATGTTTGCACTCAACTCACAGAGTTGA

              * ** * **            *  * *      *       *    *        *       
      32575 ACATCCCCTCACATAGAGCAGTTGTGCAGCACTCTATTTGTAGTATCTCGAAGTGGACATTTGGA
         66 ACCTTTCTTTTCATAGAGCAGTTTTGAAACACTCTTTTTGTAGAATCTGGAAGTGGATATTTGGA

             *           *    **  * *   *            **  
      32640 GGGCTTTGTAGCCTATCTATGTAGAAAAAGGAAATATCTTCCCAT
        131 GCGCTTTG-AG--GAT-TTCGTTGGAAACGGAAATATCTTCATAT

            *  ** *   *     * *            *   *  *   * **        *  * * *   
      32685 GAATGCGAGATAGAAGTAATCTCAGAAACATGTTTATGCTGTATCTACTCAACTAACTGTGCTGA
          1 AAAAACTAGACAGAAGCATTCTCAGAAACATCTTTGTGATGTTTGCACTCAACTCACAGAGTTGA

              *     *  *               *        *  * *       *              *
      32750 ACATTTCTATTGATAGAGCAGTTTTGAGACACTCTTCTTTTGGAATCTGCAAGTGGATATTTGGC
         66 ACCTTTCTTTTCATAGAGCAGTTTTGAAACACTCTTTTTGTAGAATCTGGAAGTGGATATTTGGA

            ** *                        *     *      
      32815 TAGATTTGAGGATTTCGTTGGAAACGG-GAT-T-AT-ATAT
        131 GCGCTTTGAGGATTTCGTTGGAAACGGAAATATCTTCATAT

                 *       *               *                 **  *   *         
      32852 AAAAAGTAGACAGCAGCATTCTCAGAAACTTCTTTGTGATGTTTGCATCCAGCTCTCAGAGTTGA
          1 AAAAACTAGACAGAAGCATTCTCAGAAACATCTTTGTGATGTTTGCACTCAACTCACAGAGTTGA

             **  * *          *  *        * *          **        *  **       
      32917 ATATTCCCTTTCATAGAGTAGGTTTGAAACCCCCTTTTTGTAGTGTCTGGAAGCGGGCATTTGGA
         66 ACCTTTCTTTTCATAGAGCAGTTTTGAAACACTCTTTTTGTAGAATCTGGAAGTGGATATTTGGA

                   *   ** ** *  *   *          * *   
      32982 GCGCTTTCAGGCCTATGCTGAAAAAGGAAATATCTACCTAT
        131 GCGCTTTGAGGATTTCGTTGGAAACGGAAATATCTTCATAT

             *                    *    *               ** *        *    *    
      33023 AGAAACTAGACAGAAGCATTCTGAGAATC-TCGTTTGTGATGTGGGTACTCAACTAACAGTGTTG
          1 AAAAACTAGACAGAAGCATTCTCAGAAACATC-TTTGTGATGTTTGCACTCAACTCACAGAGTTG

             *  *       *   *            *   *                *              
      33087 ATCCATTCTTTTGATACAGCAGTTTTGAACCACCCTTTTTGTAGAATCTGCAAGTGGATATTTGG
         65 AACCTTTCTTTTCATAGAGCAGTTTTGAAACACTCTTTTTGTAGAATCTGGAAGTGGATATTTGG

             **   *                     *   *        *
      33152 ATAGCTGTGAGGATTTCGTTGGAAACGGGAATGTCTTCATAG
        130 AGCGCTTTGAGGATTTCGTTGGAAACGGAAATATCTTCATAT

                **                     * * **         *  **   *   *          
      33194 AAAATTTAGACAGAAGCATTCTCAGAACCTTGATTGTGATGTGTGTTCTCCACTAACAGAGTTGA
          1 AAAAACTAGACAGAAGCATTCTCAGAAACATCTTTGTGATGTTTGCACTCAACTCACAGAGTTGA

                       * *   * *           *       *                         
      33259 ACCTTTCTTTTGACAGAACTGTTTTGAAACATTCTTTTTATAGAATCTGGAAGTGGATATTTGGA
         66 ACCTTTCTTTTCATAGAGCAGTTTTGAAACACTCTTTTTGTAGAATCTGGAAGTGGATATTTGGA

            **                         *          *  
      33324 AAGCTTTGAGGATTTCGTTGGAAACGGGAATATCTTCAAAT
        131 GCGCTTTGAGGATTTCGTTGGAAACGGAAATATCTTCATAT

                *    *            *           * *       *  *    *            
      33365 AAAATCTAGCCAGAAGCATTCTAAGAAACATCTTAGGGATGTTTACATTCAAGTCACAGAGTTGA
          1 AAAAACTAGACAGAAGCATTCTCAGAAACATCTTTGTGATGTTTGCACTCAACTCACAGAGTTGA

              *  * *     *      **         *    * *   **             *    *  
      33430 ACATTCCCTTTCACAGAGCACGTTTGAAACAATCTTCTCGTACTATCTGGAAGTGGACATTTTGA
         66 ACCTTTCTTTTCATAGAGCAGTTTTGAAACACTCTTTTTGTAGAATCTGGAAGTGGATATTTGGA

              * *   *  ** ** *  *   *            ** *
      33495 GCTCCTTGGGGCCTATGCTGAAAAAGGAAATATCTTCCGAC
        131 GCGCTTTGAGGATTTCGTTGGAAACGGAAATATCTTCATAT

                                 *     *  **          *           **    *    
      33536 AAAAACTAGACAGAAGCATTCGCAGAATCACGTTTGTGATGTGTGCACTCAACTGTCAGAATTGA
          1 AAAAACTAGACAGAAGCATTCTCAGAAACATCTTTGTGATGTTTGCACTCAACTCACAGAGTTGA

                 **   ** *      *                            * *            *
      33601 ACCTTGGTTTGGACAGAGCACTTTTGAAACACTCTTTTTGTAGAATCTGCAGGTGGATATTTGGC
         66 ACCTTTCTTTTCATAGAGCAGTTTTGAAACACTCTTTTTGTAGAATCTGGAAGTGGATATTTGGA

            **                         *   *      * *
      33666 TAGCTTTGAGGATTTCGTTGGAAACGGTAATGTCTTCAAAG
        131 GCGCTTTGAGGATTTCGTTGGAAACGGAAATATCTTCATAT

                *          *   *          *   *            *    *            
      33707 AAAATCTAGACAGAAACATCCTCAGAAACACCTTCGTGATGTTTGCAATCAAGTCACAGAGTTGA
          1 AAAAACTAGACAGAAGCATTCTCAGAAACATCTTTGTGATGTTTGCACTCAACTCACAGAGTTGA

                 * *             *  *         *        *             *       
      33772 ACCTTCCGTTTCATAGAGCAGGTTGGAAACACTCATTTTGTAGTATCTGGAAGTGGACATTTGGA
         66 ACCTTTCTTTTCATAGAGCAGTTTTGAAACACTCTTTTTGTAGAATCTGGAAGTGGATATTTGGA

                   *   ** ** *  *   *       *    **  
      33837 GCGCTTTCAGGCCTATGGTGTAAAAGGAAATAGCTTCCCAT
        131 GCGCTTTGAGGATTTCGTTGGAAACGGAAATATCTTCATAT

                * * * *      **      *   * *   *     ** ** *      *    *     
      33878 AAAAGCGACATAGAAGCTATCTCAGGAACTTGTTTATGATGCATCTAATCAACTAACAGTGTTGA
          1 AAAAACTAGACAGAAGCATTCTCAGAAACATCTTTGTGATGTTTGCACTCAACTCACAGAGTTGA

                  * ** * *                         * *       *             * 
      33943 ACCTTTGTACTGACAGAGCAG-TTTGAAACACTCTTTTTTTGGAATCTGCAAGTGGATATTTGTA
         66 ACCTTTCTTTTCATAGAGCAGTTTTGAAACACTCTTTTTGTAGAATCTGGAAGTGGATATTTGGA

            * *                      *  *   *   *    
      34007 TCACTTTGAGGATTTCGTTGGAAACAG-GAT-GC--AATAT
        131 GCGCTTTGAGGATTTCGTTGGAAACGGAAATATCTTCATAT

                **  *    *     *                 **  *   *  *    *         * 
      34044 AAAACTTACACAGCAGCATACTCAGAAA-ATACTTTGCCATATTTCCATTCAAGTCACAGAGTGG
          1 AAAAACTAGACAGAAGCATTCTCAGAAACAT-CTTTGTGATGTTTGCACTCAACTCACAGAGTTG

               *  * **            *                  *  *             *      
      34108 AACATTCCCATTCATAGAGCAGGTTTGAAACACTCTTTTTGGAGTATCTGGAAGTGGACATTTGG
         65 AACCTTTCTTTTCATAGAGCAGTTTTGAAACACTCTTTTTGTAGAATCTGGAAGTGGATATTTGG

                    ** * * ** *  *   *            **  
      34173 AGCGCTTTCTGAACTATGGTGAAAAAGGAAATATCTTCCAAT
        130 AGCGCTTTGAGGATTTCGTTGGAAACGGAAATATCTTCATAT

            *     *               *      * *         **  **       *  *  * *  
      34215 GAAAACAAGACAGAAGCATTCTGAGAAACTTATTTGTGATGCGTGTCCTCAACTAACGGACTCGA
          1 AAAAACTAGACAGAAGCATTCTCAGAAACATCTTTGTGATGTTTGCACTCAACTCACAGAGTTGA

                   *      **  * *  *  *             *   *    * * *           
      34280 ACCTTTCGTTTCATGCAGTACTTCTGGAACACTCTTTTTGAAGATTCTGCATGCGGATATTTGGA
         66 ACCTTTCTTTTCATAGAGCAGTTTTGAAACACTCTTTTTGTAGAATCTGGAAGTGGATATTTGGA

            **                            **   * *   
      34345 TAGCTTTGAGGATTTCGTTGGAAACGG---GCT-TACGTAT
        131 GCGCTTTGAGGATTTCGTTGGAAACGGAAATATCTTCATAT

                 *       *               *        *   *    *    *       **   
      34382 AAAAATTAGACAGCAGCATTCTCAGAAACTTCTTTGTGGTGTCTGCATTCAAGTCACAGAACTGA
          1 AAAAACTAGACAGAAGCATTCTCAGAAACATCTTTGTGATGTTTGCACTCAACTCACAGAGTTGA

              * ** * **            *  * *      *       *    *        *       
      34447 ACATCCCCTCACATAGAGCAGTTGTGCAGCACTCTATTTGTAGTATCTCGAAGTGGACATTTGGA
         66 ACCTTTCTTTTCATAGAGCAGTTTTGAAACACTCTTTTTGTAGAATCTGGAAGTGGATATTTGGA

             *         ** *    * *   *            **  
      34512 GGGCTTTGTAGCCTATC-TGGAAAAAGGAAATATCTTCCCAT
        131 GCGCTTTG-AGGATTTCGTTGGAAACGGAAATATCTTCATAT

            *  ** *   *     * *            *   *  *   * ** *      * ** * *   
      34553 GAATGCGAGATAGAAGTAATCTCAGAAACATGTTTATGCTGTATCTATTCAACTAAGTGTGCTGA
          1 AAAAACTAGACAGAAGCATTCTCAGAAACATCTTTGTGATGTTTGCACTCAACTCACAGAGTTGA

              *     *  *               *        *  * *       *              *
      34618 ACATTTCTATTAATAGAGCAGTTTTGAGACACTCTTCTTTTCGAATCTGCAAGTGGATATTTGGC
         66 ACCTTTCTTTTCATAGAGCAGTTTTGAAACACTCTTTTTGTAGAATCTGGAAGTGGATATTTGGA

            ** *                        *     *      
      34683 TAGATTTGAGGATTTCGTTGGAAACGG-GAT-T-AT-ATAT
        131 GCGCTTTGAGGATTTCGTTGGAAACGGAAATATCTTCATAT

                 *       *               *                 **  **  *         
      34720 AAAAAGTAGACAGCAGCATTCTCAGAAACTTCTTTGTGATGTTTGCATCCAGGTCCCAGAGTTGA
          1 AAAAACTAGACAGAAGCATTCTCAGAAACATCTTTGTGATGTTTGCACTCAACTCACAGAGTTGA

              *  * *          *  *        * *      *   **        *  **       
      34785 ACATTCCGTTTCATAGAGTAGGTTTGAAACCCCCTTTTTATAGTGTCTGGAAGCGGGCATTTGGA
         66 ACCTTTCTTTTCATAGAGCAGTTTTGAAACACTCTTTTTGTAGAATCTGGAAGTGGATATTTGGA

             *     *   ** ** *  *   *          * *  *
      34850 GTGCTTTCAGGCCTATGCTGAAAAAGGAAATATCTACCTAC
        131 GCGCTTTGAGGATTTCGTTGGAAACGGAAATATCTTCATAT

             *                    *    *  **          ** *        *    *     
      34891 AGAAACTAGACAGAAGCATTCTGAGAATCACGTTTGTGATGTGGGTACTCAACTAACAGTGTTGA
          1 AAAAACTAGACAGAAGCATTCTCAGAAACATCTTTGTGATGTTTGCACTCAACTCACAGAGTTGA

            ** *       *   *            *   *                *               
      34956 TTCATTCTTTTGATACAGCAGTTTTGAACCACCCTTTTTGTAGAATCTGCAAGTGGATATTTGGA
         66 ACCTTTCTTTTCATAGAGCAGTTTTGAAACACTCTTTTTGTAGAATCTGGAAGTGGATATTTGGA

            **   *               *     *   *        *
      35021 TAGCTGTGAGGA-TTCGTTGGGAACGGGAATTTCTTCATAG
        131 GCGCTTTGAGGATTTCGTTGGAAACGGAAATATCTTCATAT

                **                     * ****         *  **   *   *          
      35061 AAAATTTAGACAGAAGCATTCTCAGAACCTGGATTGTGATGTGTGTTCTCCACTAACAGAGTTGA
          1 AAAAACTAGACAGAAGCATTCTCAGAAACATCTTTGTGATGTTTGCACTCAACTCACAGAGTTGA

                       * *   * *           *       *                         
      35126 ACCTTTCTTTTGACAGAACTGTTTTGAAACATTCTTTTTATAGAATCTGGAAGTGGATATTTGGA
         66 ACCTTTCTTTTCATAGAGCAGTTTTGAAACACTCTTTTTGTAGAATCTGGAAGTGGATATTTGGA

            **                         *          *  
      35191 AAGCTTTGAGGATTTCGTTGGAAACGGGAATATCTTCAAAT
        131 GCGCTTTGAGGATTTCGTTGGAAACGGAAATATCTTCATAT

                *    *            *           * *     * *  *    *            
      35232 AAAATCTAGCCAGAAGCATTCTAAGAAACATCTTAGGGATGTGTACATTCAAGTCACAGAGTTGA
          1 AAAAACTAGACAGAAGCATTCTCAGAAACATCTTTGTGATGTTTGCACTCAACTCACAGAGTTGA

              *   * *    **       *         *    * *   **      *      *    * 
      35297 ACATTCCCCTTTCTCAGAGCAGGTTTGAAACAATCTTCTCGTACTATCTGGCAGTGGACATTTTG
         66 ACCTT-TCTTTTCATAGAGCAGTTTTGAAACACTCTTTTTGTAGAATCTGGAAGTGGATATTTGG

               * *   *  ** ** *  *   *            ** *
      35362 AGCTCCTTGGGGCCTATGCTGAAAAAGGAAATATCTTCCGAC
        130 AGCGCTTTGAGGATTTCGTTGGAAACGGAAATATCTTCATAT

                                 *     *  **          *           **    *    
      35404 AAAAACTAGACAGAAGCATTCGCAGAATCACGTTTGTGATGTGTGCACTCAACTGTCAGAATTGA
          1 AAAAACTAGACAGAAGCATTCTCAGAAACATCTTTGTGATGTTTGCACTCAACTCACAGAGTTGA

                  *   ** *      *                            * *            *
      35469 ACCTTTGTTTGGACAGAGCACTTTTGAAACACTCTTTTTGTAGAATCTGCAGGTGGATATTTGGC
         66 ACCTTTCTTTTCATAGAGCAGTTTTGAAACACTCTTTTTGTAGAATCTGGAAGTGGATATTTGGA

            **                         *   *      * *
      35534 TAGCTTTGAGGATTTCGTTGGAAACGGTAATGTCTTCAAAG
        131 GCGCTTTGAGGATTTCGTTGGAAACGGAAATATCTTCATAT

                *                         *   *            *    *            
      35575 AAAATCTAGACAGAAGCATTCTCAGAAACACCTTCGTGATGTTTGCAATCAAGTCACAGAGTTGA
          1 AAAAACTAGACAGAAGCATTCTCAGAAACATCTTTGTGATGTTTGCACTCAACTCACAGAGTTGA

                 * *             * **         *        *             *       
      35640 ACCTTCCGTTTCATAGAGCAGGTAGGAAACACTCATTTTGTAGTATCTGGAAGTGGACATTTGGA
         66 ACCTTTCTTTTCATAGAGCAGTTTTGAAACACTCTTTTTGTAGAATCTGGAAGTGGATATTTGGA

                   *   ** ** *  *   *            **  
      35705 GCGCTTTCAGGCCTATGGTGTAAAAGGAAATATCTTCCCAT
        131 GCGCTTTGAGGATTTCGTTGGAAACGGAAATATCTTCATAT

                * * * *      **      *   * *   *     ** ** *      *    *     
      35746 AAAAGCGACATAGAAGCTATCTCAGGAACTTGTTTATGATGCATCTAATCAACTAACAGTGTTGA
          1 AAAAACTAGACAGAAGCATTCTCAGAAACATCTTTGTGATGTTTGCACTCAACTCACAGAGTTGA

                  * ** * *                         * *       *               
      35811 ACCTTTGTACTGACAGAGCAG-TTTGAAACACTCTTTTTTTGGAATCTGCAAGTGGATATTTGGA
         66 ACCTTTCTTTTCATAGAGCAGTTTTGAAACACTCTTTTTGTAGAATCTGGAAGTGGATATTTGGA

            *                           *   *   *    
      35875 TCGCTTTGAGGATTTCGTTGGAAACGG-GAT-GC--AATAT
        131 GCGCTTTGAGGATTTCGTTGGAAACGGAAATATCTTCATAT

                **  **   *     *                 **  *   *  *    *         * 
      35912 AAAACGTACTCAGCAGCATACTCAGAAA-ATACTTTGCCATATTTCCATTCAAGTCACAGAGTGG
          1 AAAAACTAGACAGAAGCATTCTCAGAAACAT-CTTTGTGATGTTTGCACTCAACTCACAGAGTTG

               *  * **            *  *               *  *             *      
      35976 AACATTCCCATTCATAGAGCAGGTTGGAAACACTCTTTTTGGAGTATCTGGAAGTGGACATTTGG
         65 AACCTTTCTTTTCATAGAGCAGTTTTGAAACACTCTTTTTGTAGAATCTGGAAGTGGATATTTGG

                    ** * * ** *  *   *        *   **  
      36041 AGCGCTTTCTGAACTATGGTGAAAAAGGAAATATGTTCCAAT
        130 AGCGCTTTGAGGATTTCGTTGGAAACGGAAATATCTTCATAT

            *     *               *      * *         **  **       *  ** * ** 
      36083 GAAAACAAGACAGAAGCATTCTGAGAAACTTATTTGTGATGCGTGTCCTCAACTAACGTACTCAA
          1 AAAAACTAGACAGAAGCATTCTCAGAAACATCTTTGTGATGTTTGCACTCAACTCACAGAGTTGA

                   *      **  * *  *  *             *   *    * * *           
      36148 ACCTTTCGTTTCATGCAGTACTTCTGGAACACTCTTTTTGAAGATTCTGCATGCGGATATTTGGA
         66 ACCTTTCTTTTCATAGAGCAGTTTTGAAACACTCTTTTTGTAGAATCTGGAAGTGGATATTTGGA

            **                    *     **    *      
      36213 TAGCTTTGAGGATTTCGTTGGATACGG-GCT-T-AT-ATAT
        131 GCGCTTTGAGGATTTCGTTGGAAACGGAAATATCTTCATAT

                 *       *               *        *   *    *    *       *    
      36250 AAAAATTAGACAGCAGCATTCTCAGAAACTTCTTTGTGGTGTCTGCATTCAAGTCACAGAATTGA
          1 AAAAACTAGACAGAAGCATTCTCAGAAACATCTTTGTGATGTTTGCACTCAACTCACAGAGTTGA

              * ** * **            *  * *      *       *    *        *       
      36315 ACATCCCCTCACATAGAGCAGTTGTGCAGCACTCTATTTGTAGTATCTCGAAGTGGACATTTGGA
         66 ACCTTTCTTTTCATAGAGCAGTTTTGAAACACTCTTTTTGTAGAATCTGGAAGTGGATATTTGGA

             *         ** *    * *   *  *         **  
      36380 GGGCTTTGTAGCCTATC-TGGAAAAAGGGAATATCTTCCCAT
        131 GCGCTTTG-AGGATTTCGTTGGAAACGGAAATATCTTCATAT

            *  ** *   *     * *           **   *  *   * **        *  * * *   
      36421 GAATGCGAGATAGAAGTAATCTCAGAAACAGGTTTATGCTGTATCTACTCAACTAACTGTGCTGA
          1 AAAAACTAGACAGAAGCATTCTCAGAAACATCTTTGTGATGTTTGCACTCAACTCACAGAGTTGA

              *     *  *               *        *  * *       *               
      36486 ACATTTCTATTGATAGAGCAGTTTTGAGACACTCTTCTTTTGGAATCTGCAAGTGGATATTTGGA
         66 ACCTTTCTTTTCATAGAGCAGTTTTGAAACACTCTTTTTGTAGAATCTGGAAGTGGATATTTGGA

            ** *           *            *     *      
      36551 TAGATTTGAGGATTTAGTTGGAAACGG-GAT-T-AT-ATAT
        131 GCGCTTTGAGGATTTCGTTGGAAACGGAAATATCTTCATAT

            *    *       *               *                 **  *   *         
      36588 CAAAAGTAGACAGCAGCATTCTCAGAAACTTCTTTGTGATGTTTGCATCCAGCTCTCAGAGTTGA
          1 AAAAACTAGACAGAAGCATTCTCAGAAACATCTTTGTGATGTTTGCACTCAACTCACAGAGTTGA

              *  * *          *  *        * *      *   **        *  **       
      36653 ACATTCCCTTTCATAGAGTAGGTTTGAAACCCCCTTTTTATAGTGTCTGGAAGCGGGCATTTGGA
         66 ACCTTTCTTTTCATAGAGCAGTTTTGAAACACTCTTTTTGTAGAATCTGGAAGTGGATATTTGGA

                   *   ** ** *  *   *          * *  *
      36718 GCGCTTTCAGGCCTATGCTGAAAAAGGAAATATCTACCTAC
        131 GCGCTTTGAGGATTTCGTTGGAAACGGAAATATCTTCATAT

             *                    *    *  **          ** **       *    *     
      36759 AGAAACTAGACAGAAGCATTCTGAGAATCACGTTTGTGATGTGGGTCCTCAACTAACAGTGTTGA
          1 AAAAACTAGACAGAAGCATTCTCAGAAACATCTTTGTGATGTTTGCACTCAACTCACAGAGTTGA

            ** *       *   *            *   *                *               
      36824 TTCATTCTTTTGATACAGCAGTTTTGAACCACACTTTTTGTAGAATCTGCAAGTGGATATTTGGA
         66 ACCTTTCTTTTCATAGAGCAGTTTTGAAACACTCTTTTTGTAGAATCTGGAAGTGGATATTTGGA

            **   *                     *   *        *
      36889 TAGCTGTGAGGATTTCGTTGGAAACGGGAATGTCTTCATAG
        131 GCGCTTTGAGGATTTCGTTGGAAACGGAAATATCTTCATAT

                **                     * ****         *  **   *   *          
      36930 AAAATTTAGACAGAAGCATTCTCAGAACCTGGATTGTGATGTGTGTTCTCCACTAACAGAGTTGA
          1 AAAAACTAGACAGAAGCATTCTCAGAAACATCTTTGTGATGTTTGCACTCAACTCACAGAGTTGA

                       * *   * *           *       *                         
      36995 ACCTTTCTTTTGACAGAACTGTTTTGAAACATTCTTTTTATAGAATCTGGAAGTGGATATTTGGA
         66 ACCTTTCTTTTCATAGAGCAGTTTTGAAACACTCTTTTTGTAGAATCTGGAAGTGGATATTTGGA

            **                         *          *  
      37060 AAGCTTTGAGGATTTCGTTGGAAACGGGAATATCTTCAAAT
        131 GCGCTTTGAGGATTTCGTTGGAAACGGAAATATCTTCATAT

                *    *            *           * *     * *  *    *            
      37101 AAAATCTAGCCAGAAGCATTCTAAGAAACATCTTAGGGATGTGTACATTCAAGTCACAGAGTTGA
          1 AAAAACTAGACAGAAGCATTCTCAGAAACATCTTTGTGATGTTTGCACTCAACTCACAGAGTTGA

              *   * *    **       *         *    * *   **      *      *    * 
      37166 ACATTCCCCTTTCTCAGAGCAGGTTTGAAACAATCTTCTCGTACTATCTGGCAGTGGACATTTTG
         66 ACCTT-TCTTTTCATAGAGCAGTTTTGAAACACTCTTTTTGTAGAATCTGGAAGTGGATATTTGG

               * *   *  ** ** *  *   *            ** *
      37231 AGCTCCTTGGGGCCTATGCTGAAAAAGGAAATATCTTCCGAC
        130 AGCGCTTTGAGGATTTCGTTGGAAACGGAAATATCTTCATAT

                                 *     *  **          *  *        **    *    
      37273 AAAAACTAGACAGAAGCATTCGCAGAATCACGTTTGTGATGTGTGAACTCAACTGTCAGAATTGA
          1 AAAAACTAGACAGAAGCATTCTCAGAAACATCTTTGTGATGTTTGCACTCAACTCACAGAGTTGA

                 **   ** *      *                            * *            *
      37338 ACCTTGGTTTGGACAGAGCACTTTTGAAACACTCTTTTTGTAGAATCTGCAGGTGGATATTTGGC
         66 ACCTTTCTTTTCATAGAGCAGTTTTGAAACACTCTTTTTGTAGAATCTGGAAGTGGATATTTGGA

            **                         *   *      * *
      37403 TAGCTTTGAGGATTTCGTTGGAAACGGTAATGTCTTCAAAG
        131 GCGCTTTGAGGATTTCGTTGGAAACGGAAATATCTTCATAT

                *          *   *          *   *            *    *            
      37444 AAAATCTAGACAGAAACATCCTCAGAAACACCTTCGTGATGTTTGCAATCAAGTCACAGAGTTGA
          1 AAAAACTAGACAGAAGCATTCTCAGAAACATCTTTGTGATGTTTGCACTCAACTCACAGAGTTGA

                 * *             *  *       * *        *             *       
      37509 ACCTTCCGTTTCATAGAGCAGGTTGGAAACACGCATTTTGTAGTATCTGGAAGTGGACATTTGGA
         66 ACCTTTCTTTTCATAGAGCAGTTTTGAAACACTCTTTTTGTAGAATCTGGAAGTGGATATTTGGA

                   *   ** ** *  *   *            **  
      37574 GCGCTTTCAGGCCTATGGTGTAAAAGGAAATATCTTCCCAT
        131 GCGCTTTGAGGATTTCGTTGGAAACGGAAATATCTTCATAT

                * * * *      **      *   * *   *     ** ** *      *    *     
      37615 AAAAGCGACATAGAAGCTATCTCAGGAACTTGTTTATGATGCATCTAATCAACTAACAGTGTTGA
          1 AAAAACTAGACAGAAGCATTCTCAGAAACATCTTTGTGATGTTTGCACTCAACTCACAGAGTTGA

                  * ** * *                         * *       *               
      37680 ACCTTTGTACTGACAGAGCAG-TTTGAAACACTCTTTTTTTGGAATCTGCAAGTGGATATTTGGA
         66 ACCTTTCTTTTCATAGAGCAGTTTTGAAACACTCTTTTTGTAGAATCTGGAAGTGGATATTTGGA

            *                  *        *   *   *    
      37744 TCGCTTTGAGGATTTCGTTAGAAACGG-GAT-GC--AATAT
        131 GCGCTTTGAGGATTTCGTTGGAAACGGAAATATCTTCATAT

                **  **   *     *                 **  *   *  *    *         * 
      37781 AAAACGTACTCAGCAGCATACTCAGAAA-ATACTTTGCCATATTTCCATTCAAGTCACAGAGTGG
          1 AAAAACTAGACAGAAGCATTCTCAGAAACAT-CTTTGTGATGTTTGCACTCAACTCACAGAGTTG

               *  * **            *                  *  *             *      
      37845 AACATTCCCATTCATAGAGCAGGTTTGAAACACTCTTTTTGGAGTATCTGGAAGTGGACATTTGG
         65 AACCTTTCTTTTCATAGAGCAGTTTTGAAACACTCTTTTTGTAGAATCTGGAAGTGGATATTTGG

                    ** * * ** *  *   *        *   **  
      37910 AGCGCTTTCTGAACTATGGTGAAAAAGGAAATATGTTCCAAT
        130 AGCGCTTTGAGGATTTCGTTGGAAACGGAAATATCTTCATAT

            *     *               *      * *         **  **       *  ** * ** 
      37952 GAAAACAAGACAGAAGCATTCTGAGAAACTTATTTGTGATGCGTGTCCTCAACTAACGTACTCAA
          1 AAAAACTAGACAGAAGCATTCTCAGAAACATCTTTGTGATGTTTGCACTCAACTCACAGAGTTGA

                   *      **  * *  *  *             *   *    * * *           
      38017 ACCTTTCGTTTCATGCAGTACTTCTGGAACACTCTTTTTGAAGATTCTGCATGCGGATATTTGGA
         66 ACCTTTCTTTTCATAGAGCAGTTTTGAAACACTCTTTTTGTAGAATCTGGAAGTGGATATTTGGA

            **                    *     **    *      
      38082 TAGCTTTGAGGATTTCGTTGGATACGG-GCT-T-AT-ATAT
        131 GCGCTTTGAGGATTTCGTTGGAAACGGAAATATCTTCATAT

                 *       *               *        *   *    *    *       *    
      38119 AAAAATTAGACAGCAGCATTCTCAGAAACTTCTTTGTGGTGTCTGCATTCAAGTCACAGAATTGA
          1 AAAAACTAGACAGAAGCATTCTCAGAAACATCTTTGTGATGTTTGCACTCAACTCACAGAGTTGA

              * ** * **            *  * *      *       *    *        *       
      38184 ACATCCCCTCACATAGAGCAGTTGTGCAGCACTCTATTTGTAGTATCTCGAAGTGGACATTTGGA
         66 ACCTTTCTTTTCATAGAGCAGTTTTGAAACACTCTTTTTGTAGAATCTGGAAGTGGATATTTGGA

             *         ** *    * *   *  *         **  
      38249 GGGCTTTGTAGCCTATC-TGGAAAAAGGGAATATCTTCCCAT
        131 GCGCTTTG-AGGATTTCGTTGGAAACGGAAATATCTTCATAT

            *  ** *   *     * *           **   *  *   * **        *  * * *   
      38290 GAATGCGAGATAGAAGTAATCTCAGAAACAGGTTTATGCTGTATCTACTCAACTAACTGTGCTGA
          1 AAAAACTAGACAGAAGCATTCTCAGAAACATCTTTGTGATGTTTGCACTCAACTCACAGAGTTGA

              *     *  *               *        *  * *       *              *
      38355 ACATTTCTATTGATAGAGCAGTTTTGAGACACTCTTCTTTTGGAATCTGCAAGTGGATATTTGGC
         66 ACCTTTCTTTTCATAGAGCAGTTTTGAAACACTCTTTTTGTAGAATCTGGAAGTGGATATTTGGA

            ** *                        *     *      
      38420 TAGATTTGAGGATTTCGTTGGAAACGG-GAT-T-AT-ATAT
        131 GCGCTTTGAGGATTTCGTTGGAAACGGAAATATCTTCATAT

            *    *       *               *                 **  *   *         
      38457 CAAAAGTAGACAGCAGCATTCTCAGAAACTTCTTTGTGATGTTTGCATCCAGCTCTCAGAGTTGA
          1 AAAAACTAGACAGAAGCATTCTCAGAAACATCTTTGTGATGTTTGCACTCAACTCACAGAGTTGA

              *  * *          *  *        * *      *   **        *  **       
      38522 ACATTCCCTTTCATAGAGTAGGTTTGAAACCCCCTTTTTATAGTGTCTGGAAGCGGGCATTTGGA
         66 ACCTTTCTTTTCATAGAGCAGTTTTGAAACACTCTTTTTGTAGAATCTGGAAGTGGATATTTGGA

                   *   ** ** *  *   *          * *  *
      38587 GCGCTTTCAGGCCTATGCTGAAAAAGGAAATATCTACCTAC
        131 GCGCTTTGAGGATTTCGTTGGAAACGGAAATATCTTCATAT

             *                    *    *  **          ** **       *    *     
      38628 AGAAACTAGACAGAAGCATTCTGAGAATCACGTTTGTGATGTGGGTCCTCAACTAACAGTGTTGA
          1 AAAAACTAGACAGAAGCATTCTCAGAAACATCTTTGTGATGTTTGCACTCAACTCACAGAGTTGA

            *  *       *   *            *   *                *               
      38693 TCCATTCTTTTGATACAGCAGTTTTGAACCACACTTTTTGTAGAATCTGCAAGTGGATATTTGGA
         66 ACCTTTCTTTTCATAGAGCAGTTTTGAAACACTCTTTTTGTAGAATCTGGAAGTGGATATTTGGA

            **   *                     *   *        *
      38758 TAGCTGTGAGGATTTCGTTGGAAACGGGAATGTCTTCATAG
        131 GCGCTTTGAGGATTTCGTTGGAAACGGAAATATCTTCATAT

                **                     * ****         *  **   *   *          
      38799 AAAATTTAGACAGAAGCATTCTCAGAACCTGGATTGTGATGTGTGTTCTCCACTAACAGAGTTGA
          1 AAAAACTAGACAGAAGCATTCTCAGAAACATCTTTGTGATGTTTGCACTCAACTCACAGAGTTGA

                 *    ** *   * *           *       *                         
      38864 ACCTTCCTTTGGACAGAACTGTTTTGAAACATTCTTTTTATAGAATCTGGAAGTGGATATTTGGA
         66 ACCTTTCTTTTCATAGAGCAGTTTTGAAACACTCTTTTTGTAGAATCTGGAAGTGGATATTTGGA

            **                         *          *  
      38929 AAGCTTTGAGGATTTCGTTGGAAACGGGAATATCTTCAAAT
        131 GCGCTTTGAGGATTTCGTTGGAAACGGAAATATCTTCATAT

            *   *    *            *           * *     * *  *    *            
      38970 CAAATCTAGCCAGAAGCATTCTAAGAAACATCTTAGGGATGTGTACATTCAAGTCACAGAGTTGA
          1 AAAAACTAGACAGAAGCATTCTCAGAAACATCTTTGTGATGTTTGCACTCAACTCACAGAGTTGA

              *   * *    **       *         *    * *   **      *      *    * 
      39035 ACATTCCCCTTTCTCAGAGCAGGTTTGAAACAATCTTCTCGTACTATCTGGCAGTGGACATTTTG
         66 ACCTT-TCTTTTCATAGAGCAGTTTTGAAACACTCTTTTTGTAGAATCTGGAAGTGGATATTTGG

               * *   *  ** ** *  *   *        *   ** *
      39100 AGCTCCTTGGGGCCTATGCTGAAAAAGGAAATATATTCCGAC
        130 AGCGCTTTGAGGATTTCGTTGGAAACGGAAATATCTTCATAT

                      *   *      *     *  **          *           **    *    
      39142 AAAAACTAGAGAGAGGCATTCGCAGAATCACGTTTGTGATGTGTGCACTCAACTGTCAGAATTGA
          1 AAAAACTAGACAGAAGCATTCTCAGAAACATCTTTGTGATGTTTGCACTCAACTCACAGAGTTGA

                 **   ** *      *                            * *            *
      39207 ACCTTGGTTTGGACAGAGCACTTTTGAAACACTCTTTTTGTAGAATCTGCAGGTGGATATTTGGC
         66 ACCTTTCTTTTCATAGAGCAGTTTTGAAACACTCTTTTTGTAGAATCTGGAAGTGGATATTTGGA

            **                         *   *      * *
      39272 TAGCTTTGAGGATTTCGTTGGAAACGGTAATGTCTTCAAAG
        131 GCGCTTTGAGGATTTCGTTGGAAACGGAAATATCTTCATAT

                *          *   *   *      *   *            *    *            
      39313 AAAATCTAGACAGAAACATCCTCTGAAACACCTTCGTGATGTTTGCAATCAAGTCACAGAGTTGA
          1 AAAAACTAGACAGAAGCATTCTCAGAAACATCTTTGTGATGTTTGCACTCAACTCACAGAGTTGA

                 * *      *      *            *        *             *       
      39378 ACCTTCCGTTTCATGGAGCAGGTTTGAAACACTCATTTTGTAGTATCTGGAAGTGGACATTTGGA
         66 ACCTTTCTTTTCATAGAGCAGTTTTGAAACACTCTTTTTGTAGAATCTGGAAGTGGATATTTGGA

                   *   ** ** *  *   *            **  
      39443 GCGCTTTCAGGCCTATGGTGTAAAAGGAAATATCTTCCCAT
        131 GCGCTTTGAGGATTTCGTTGGAAACGGAAATATCTTCATAT

                * * * *      **      *   * *   *     ** ** *      *    *     
      39484 AAAAGCGACATAGAAGCTATCTCAGGAACTTGTTTATGATGCATCTAATCAACTAACAGTGTTGA
          1 AAAAACTAGACAGAAGCATTCTCAGAAACATCTTTGTGATGTTTGCACTCAACTCACAGAGTTGA

                  * ** * *                         * *       *               
      39549 ACCTTTGTACTGACAGAGCAG-TTTGAAACACTCTTTTTTTGGAATCTGCAAGTGGATATTTGGA
         66 ACCTTTCTTTTCATAGAGCAGTTTTGAAACACTCTTTTTGTAGAATCTGGAAGTGGATATTTGGA

            *                           *   *   *    
      39613 TCGCTTTGAGGATTTCGTTGGAAACGG-GAT-GC--AATAT
        131 GCGCTTTGAGGATTTCGTTGGAAACGGAAATATCTTCATAT

            *   **  *    *     *                 **  *   *  *    *         * 
      39650 CAAACGTACACAGCAGCATACTCAGAAA-ATACTTTGCCATATTTCCATTCAAGTCACAGAGTGG
          1 AAAAACTAGACAGAAGCATTCTCAGAAACAT-CTTTGTGATGTTTGCACTCAACTCACAGAGTTG

               *  * **            *                  *  *             *      
      39714 AACATTCCCATTCATAGAGCAGGTTTGAAACACTCTTTTTGGAGTATCTGGAAGTGGACATTTGG
         65 AACCTTTCTTTTCATAGAGCAGTTTTGAAACACTCTTTTTGTAGAATCTGGAAGTGGATATTTGG

                    ** * * ** *  *   *            **  
      39779 AGCGCTTTCTGAACTATGGTGAAAAAGGAAATATCTTCCAAT
        130 AGCGCTTTGAGGATTTCGTTGGAAACGGAAATATCTTCATAT

            *     *               *      * *         **  **       *  ** * ** 
      39821 GAAAACAAGACAGAAGCATTCTGAGAAACTTATTTGTGATGCGTGTCCTCAACTAACGTACTCAA
          1 AAAAACTAGACAGAAGCATTCTCAGAAACATCTTTGTGATGTTTGCACTCAACTCACAGAGTTGA

                   *      **  * *  *  *             *   *    * * *           
      39886 ACCTTTCGTTTCATGCAGTACTTCTGGAACACTCTTTTTGAAGATTCTGCATGCGGATATTTGGA
         66 ACCTTTCTTTTCATAGAGCAGTTTTGAAACACTCTTTTTGTAGAATCTGGAAGTGGATATTTGGA

            ***               *           **   *     
      39951 TACCTTTGAGGATTTCGTGGGAAACGG---GCT-TACATAT
        131 GCGCTTTGAGGATTTCGTTGGAAACGGAAATATCTTCATAT

                 *       *               *        *   *    *    *     * *    
      39988 AAAAATTAGACAGCAGCATTCTCAGAAACTTCTTTGTGGTGTCTGCATTCAAGTCACATAATTGA
          1 AAAAACTAGACAGAAGCATTCTCAGAAACATCTTTGTGATGTTTGCACTCAACTCACAGAGTTGA

              * ** * **            *  * *      *       *    *        *       
      40053 ACATCCCCTCACATAGAGCAGTTGTGCAGCACTCTATTTGTAGTATCTCGAAGTGGACATTTGGA
         66 ACCTTTCTTTTCATAGAGCAGTTTTGAAACACTCTTTTTGTAGAATCTGGAAGTGGATATTTGGA

             *         ** *    * *   *            **  
      40118 GGGCTTTGTAGCCTATC-TGGAAAAAGGAAATATCTTCCCAT
        131 GCGCTTTG-AGGATTTCGTTGGAAACGGAAATATCTTCATAT

            *  ** *   *     * *            *   *  *   * **        *  * * *   
      40159 GAATGCGAGATAGAAGTAATCTCAGAAACATGTTTATGCTGTATCTACTCAACTAACTGTGCTGA
          1 AAAAACTAGACAGAAGCATTCTCAGAAACATCTTTGTGATGTTTGCACTCAACTCACAGAGTTGA

              *     *  *               *        *  * *       *              *
      40224 ACATTTCTATTGATAGAGCAGTTTTGAGACACTCTTCTTTTGGAATCTGCAAGTGGATATTTGGC
         66 ACCTTTCTTTTCATAGAGCAGTTTTGAAACACTCTTTTTGTAGAATCTGGAAGTGGATATTTGGA

            ** *                        *     *      
      40289 TAGATTTGAGGATTTCGTTGGAAACGG-GAT-T-AT-ATAT
        131 GCGCTTTGAGGATTTCGTTGGAAACGGAAATATCTTCATAT

                 *       *               *                 **  *   *         
      40326 AAAAAGTAGACAGCAGCATTCTCAGAAACTTCTTTGTGATGTTTGCATCCAGCTCTCAGAGTTGA
          1 AAAAACTAGACAGAAGCATTCTCAGAAACATCTTTGTGATGTTTGCACTCAACTCACAGAGTTGA

              *  * *          *  *        * *      *   ***       *  **       
      40391 ACATTCCCTTTCATAGAGTAGATTTGAAACCCCCTTTTTATAGTGACTGGAAGCGGGCATTTGGA
         66 ACCTTTCTTTTCATAGAGCAGTTTTGAAACACTCTTTTTGTAGAATCTGGAAGTGGATATTTGGA

              *    *   ** ** *  *   *          * *  *
      40456 GCCCTTTCAGGCCTATGCTGAAAAAGGAAATATCTACCTAC
        131 GCGCTTTGAGGATTTCGTTGGAAACGGAAATATCTTCATAT

             *                    *    *  **          ** *        *    *     
      40497 AGAAACTAGACAGAAGCATTCTGAGAATCACGTTTGTGATGTGGGTACTCAACTAACAGTGTTGA
          1 AAAAACTAGACAGAAGCATTCTCAGAAACATCTTTGTGATGTTTGCACTCAACTCACAGAGTTGA

            *  *       *   *            *   *                *               
      40562 TCCATTCTTTTGATACAGCAGTTTTGAACCACCCTTTTTGTAGAATCTGCAA-TGGATATTTGGA
         66 ACCTTTCTTTTCATAGAGCAGTTTTGAAACACTCTTTTTGTAGAATCTGGAAGTGGATATTTGGA

            **   *               *     *   *        *
      40626 TAGCTGTGAGGA-TTCGTTGGGAACGGGAATTTCTTCATAG
        131 GCGCTTTGAGGATTTCGTTGGAAACGGAAATATCTTCATAT

                **                     * ****         *  **   *   *          
      40666 AAAATTTAGACAGAAGCATTCTCAGAACCTGGATTGTGATGTGTGTTCTCCACTAACAGAGTTGA
          1 AAAAACTAGACAGAAGCATTCTCAGAAACATCTTTGTGATGTTTGCACTCAACTCACAGAGTTGA

                      ** *   * *           *       *                         
      40731 ACCTTTCTTTCGACAGAACTGTTTTGAAACATTCTTTTTATAGAATCTGGAAGTGGATATTTGGA
         66 ACCTTTCTTTTCATAGAGCAGTTTTGAAACACTCTTTTTGTAGAATCTGGAAGTGGATATTTGGA

            **                         *          *  
      40796 AAGCTTTGAGGATTTCGTTGGAAACGGGAATATCTTCAAAT
        131 GCGCTTTGAGGATTTCGTTGGAAACGGAAATATCTTCATAT

                *    *            *           * *       *  *    *            
      40837 AAAATCTAGCCAGAAGCATTCTAAGAAACATCTTAGGGATGTTTACATTCAAGTCACAGAGTTGA
          1 AAAAACTAGACAGAAGCATTCTCAGAAACATCTTTGTGATGTTTGCACTCAACTCACAGAGTTGA

              *   * *    **       *         *    * *   **      *      *    * 
      40902 ACATTCCCCTTTCTCAGAGCAGGTTTGAAACAATCTTCTCGTACTATCTGGCAGTGGACATTTTG
         66 ACCTT-TCTTTTCATAGAGCAGTTTTGAAACACTCTTTTTGTAGAATCTGGAAGTGGATATTTGG

               * *   *  ** ** *  **  *            ** *
      40967 AGCTCCTTGGGGCCTATGCTGATAAAGGAAATATCTTCCGAC
        130 AGCGCTTTGAGGATTTCGTTGGAAACGGAAATATCTTCATAT

                                 *     *  **          *           **    *    
      41009 AAAAACTAGACAGAAGCATTCGCAGAATCACGTTTGTGATGTGTGCACTCAACTGTCAGAATTGA
          1 AAAAACTAGACAGAAGCATTCTCAGAAACATCTTTGTGATGTTTGCACTCAACTCACAGAGTTGA

                  *   ** *      *                      *     * *            *
      41074 ACCTTTGTTTGGACAGAGCACTTTTGAAACACTCTTTTTGTAGGATCTGCAGGTGGATATTTGGC
         66 ACCTTTCTTTTCATAGAGCAGTTTTGAAACACTCTTTTTGTAGAATCTGGAAGTGGATATTTGGA

            **                         *   *      * *
      41139 TAGCTTTGAGGATTTCGTTGGAAACGGTAATGTCTTCAAAG
        131 GCGCTTTGAGGATTTCGTTGGAAACGGAAATATCTTCATAT

                *          *   *          *   *            *    *            
      41180 AAAATCTAGACAGAAACATCCTCAGAAACACCTTCGTGATGTTTGCAATCAAGTCACAGAGTTGA
          1 AAAAACTAGACAGAAGCATTCTCAGAAACATCTTTGTGATGTTTGCACTCAACTCACAGAGTTGA

                 * *             *  *         *        *             *       
      41245 ACCTTCCGTTTCATAGAGCAGGTTGGAAACACTCATTTTGTAGTATCTGGAAGTGGACATTTGGA
         66 ACCTTTCTTTTCATAGAGCAGTTTTGAAACACTCTTTTTGTAGAATCTGGAAGTGGATATTTGGA

                   *   ** ** *  *   *            **  
      41310 GCGCTTTCAGGCCTATGGTGTAAAAGGAAATATCTTCCCAT
        131 GCGCTTTGAGGATTTCGTTGGAAACGGAAATATCTTCATAT

                * * * *      **      *   * *   *     ** *  *      *    *     
      41351 AAAAGCGACATAGAAGCTATCTCAGGAACTTGTTTATGATGCCTCCAATCAACTAACAGTGTTGA
          1 AAAAACTAGACAGAAGCATTCTCAGAAACATCTTTGTGATGTTTGCACTCAACTCACAGAGTTGA

                  * ** * *                         * *       *               
      41416 ACCTTTGTACTGACAGAGCAG-TTTGAAACACTCTTTTTTTGGAATCTGCAAGTGGATATTTGGA
         66 ACCTTTCTTTTCATAGAGCAGTTTTGAAACACTCTTTTTGTAGAATCTGGAAGTGGATATTTGGA

            *                           *   *   *    
      41480 TCGCTTTGAGGATTTCGTTGGAAACGG-GAT-GC--AATAT
        131 GCGCTTTGAGGATTTCGTTGGAAACGGAAATATCTTCATAT

                **  *    *     *   *        *    **  *   *  *    *         * 
      41517 AAAACGTACACAGCAGCATACTCCGAAA-ATATTTTGCCATATTTCCATTCAAGTCACAGAGTGG
          1 AAAAACTAGACAGAAGCATTCTCAGAAACAT-CTTTGTGATGTTTGCACTCAACTCACAGAGTTG

               *  * **            *                  *  *             *      
      41581 AACATTCCCATTCATAGAGCAGGTTTGAAACACTCTTTTTGGAGTATCTGGAAGTGGACATTTGG
         65 AACCTTTCTTTTCATAGAGCAGTTTTGAAACACTCTTTTTGTAGAATCTGGAAGTGGATATTTGG

                    ** * * ** *  *   *        *   **  
      41646 AGCGCTTTCTGAACTATGGTGAAAAGGGAAATATGTTCCAAT
        130 AGCGCTTTGAGGATTTCGTTGGAAACGGAAATATCTTCATAT

            *     *               *      * *         **  **       *  *  * *  
      41688 GAAAACAAGACAGAAGCATTCTGAGAAACTTATTTGTGATGCGTGTCCTCAACTAACGGACTCGA
          1 AAAAACTAGACAGAAGCATTCTCAGAAACATCTTTGTGATGTTTGCACTCAACTCACAGAGTTGA

             *    **      **  * *  *  *             *   *    * * *          *
      41753 AGCTTTGGTTTCATGCAGTACTTCTGGAACACTCTTTTTGAAGATTCTGCATGCGGATATTTGGT
         66 ACCTTTCTTTTCATAGAGCAGTTTTGAAACACTCTTTTTGTAGAATCTGGAAGTGGATATTTGGA

            **                            **   *     
      41818 TAGCTTTGAGGATTTCGTTGGAAACGG---GCT-TACATAT
        131 GCGCTTTGAGGATTTCGTTGGAAACGGAAATATCTTCATAT

                 *       *               *   *    *   *    *    *       *    
      41855 AAAAATTAGACAGCAGCATTCTCAGAAACTTCTATGTGGTGTCTGCATTCAAGTCACAGAATTGA
          1 AAAAACTAGACAGAAGCATTCTCAGAAACATCTTTGTGATGTTTGCACTCAACTCACAGAGTTGA

              * ** * **            *  * *      *       *    *        *       
      41920 ACATCCCCTCACATAGAGCAGTTGTGCAGCACTCTATTTGTAGTATCTCGAAGTGGACATTTGGA
         66 ACCTTTCTTTTCATAGAGCAGTTTTGAAACACTCTTTTTGTAGAATCTGGAAGTGGATATTTGGA

             *         ** *    * *   *            **  
      41985 GGGCTTTGTAGCCTATC-TGGAAAAAGGAAATATCTTCCCAT
        131 GCGCTTTG-AGGATTTCGTTGGAAACGGAAATATCTTCATAT

            *  ** *   *     * *            *   *  *   * **        *  * * *   
      42026 GAATGCGAGATAGAAGTAATCTCAGAAACATGTTTATGCTGTATCTACTCAACTAACTGTGCTGA
          1 AAAAACTAGACAGAAGCATTCTCAGAAACATCTTTGTGATGTTTGCACTCAACTCACAGAGTTGA

              *     *  *               *        *  * *       *              *
      42091 ACATTTCTATTGATAGAGCAGTTTTGAGACACTCTTCTTTTGGAATCTGCAAGTGGATATTTGGC
         66 ACCTTTCTTTTCATAGAGCAGTTTTGAAACACTCTTTTTGTAGAATCTGGAAGTGGATATTTGGA

            ** *                        *     *      
      42156 TAGATTTGAGGATTTCGTTGGAAACGG-GAT-T-AT-ATAT
        131 GCGCTTTGAGGATTTCGTTGGAAACGGAAATATCTTCATAT

            *    *       *               *                 **  *   *         
      42193 CAAAAGTAGACAGCAGCATTCTCAGAAACTTCTTTGTGATGTTTGCATCCAGCTCTCAGAGTTGA
          1 AAAAACTAGACAGAAGCATTCTCAGAAACATCTTTGTGATGTTTGCACTCAACTCACAGAGTTGA

              *  * *          *  *        * *      *   **        *  **       
      42258 ACATTCCCTTTCATAGAGTAGGTTTGAAACCCCCTTTTTATAGTGTCTGGAAGCGGGCATTTGGA
         66 ACCTTTCTTTTCATAGAGCAGTTTTGAAACACTCTTTTTGTAGAATCTGGAAGTGGATATTTGGA

                   *   ** ** *  *   *          * *  *
      42323 GCGCTTTCAGGCCTATGCTGAAAAAGGAAATATCTACCTAC
        131 GCGCTTTGAGGATTTCGTTGGAAACGGAAATATCTTCATAT

             *                    *    *  **          ** *        *    *     
      42364 AGAAACTAGACAGAAGCATTCTGAGAATCACGTTTGTGATGTGGGTACTCAACTAACAGTGTTGA
          1 AAAAACTAGACAGAAGCATTCTCAGAAACATCTTTGTGATGTTTGCACTCAACTCACAGAGTTGA

            *  *       *   *            *   **               *               
      42429 TCCATTCTTTTGATACAGCAGTTTTGAACCACCTTTTTTGTAGAATCTGCAAGTGGATATTTGGA
         66 ACCTTTCTTTTCATAGAGCAGTTTTGAAACACTCTTTTTGTAGAATCTGGAAGTGGATATTTGGA

            **   *                     *   *        *
      42494 TAGCTGTGAGGATTTCGTTGGAAACGGGAATGTCTTCATAG
        131 GCGCTTTGAGGATTTCGTTGGAAACGGAAATATCTTCATAT

                **                     * ****         *  **   *   *          
      42535 AAAATTTAGACAGAAGCATTCTCAGAACCTGGATTGTGATGTGTGTTCTCCACTAACAGAGTTGA
          1 AAAAACTAGACAGAAGCATTCTCAGAAACATCTTTGTGATGTTTGCACTCAACTCACAGAGTTGA

                      ** *   * *           *       *               *         
      42600 ACCTTTCTTTGGACAGAACTGTTTTGAAACATTCTTTTTATAGAATCTGGAAGTGTATATTTGGA
         66 ACCTTTCTTTTCATAGAGCAGTTTTGAAACACTCTTTTTGTAGAATCTGGAAGTGGATATTTGGA

            **                         *          *  
      42665 AAGCTTTGAGGATTTCGTTGGAAACGGGAATATCTTCAAAT
        131 GCGCTTTGAGGATTTCGTTGGAAACGGAAATATCTTCATAT

                *    *            *           * *     * *  *    *            
      42706 AAAATCTAGCCAGAAGCATTCTAAGAAACATCTTAGGGATGTGTACATTCAAGTCACAGAGTTGA
          1 AAAAACTAGACAGAAGCATTCTCAGAAACATCTTTGTGATGTTTGCACTCAACTCACAGAGTTGA

              *   * *    **       *         *    * *   **      *      *    * 
      42771 ACATTCCCCTTTCTCAGAGCAGGTTTGAAACAATCTTCTCGTACTATCTGGCAGTGGACATTTTG
         66 ACCTT-TCTTTTCATAGAGCAGTTTTGAAACACTCTTTTTGTAGAATCTGGAAGTGGATATTTGG

               * *   *  ** ** *  *   *            ** *
      42836 AGCTCCTTGGGGCCTATGCTGAAAAAGGAAATATCTTCCGAC
        130 AGCGCTTTGAGGATTTCGTTGGAAACGGAAATATCTTCATAT

                                 *     *  **          *           **    *    
      42878 AAAAACTAGACAGAAGCATTCGCAGAATCACGTTTGTGATGTGTGCACTCAACTGTCAGAATTGA
          1 AAAAACTAGACAGAAGCATTCTCAGAAACATCTTTGTGATGTTTGCACTCAACTCACAGAGTTGA

                  *   ** *      *                      *     * *            *
      42943 ACCTTTGTTTGGACAGAGCACTTTTGAAACACTCTTTTTGTAGGATCTGCAGGTGGATATTTGGC
         66 ACCTTTCTTTTCATAGAGCAGTTTTGAAACACTCTTTTTGTAGAATCTGGAAGTGGATATTTGGA

            **                         *   *      * *
      43008 TAGCTTTGAGGATTTCGTTGGAAACGGTAATGTCTTCAAAG
        131 GCGCTTTGAGGATTTCGTTGGAAACGGAAATATCTTCATAT

                *          *   *          *   *            *    *            
      43049 AAAATCTAGACAGAAACATCCTCAGAAACACCTTCGTGATGTTTGCAATCAAGTCACAGAGTTGA
          1 AAAAACTAGACAGAAGCATTCTCAGAAACATCTTTGTGATGTTTGCACTCAACTCACAGAGTTGA

                 * *             *  *         *        *             *       
      43114 ACCTTCCGTTTCATAGAGCAGGTTGGAAACACTCATTTTGTAGTATCTGGAAGTGGACATTTGGA
         66 ACCTTTCTTTTCATAGAGCAGTTTTGAAACACTCTTTTTGTAGAATCTGGAAGTGGATATTTGGA

                   *   ** ** *  *   *            **  
      43179 GCGCTTTCAGGCCTATGGTGTAAAAGGAAATATCTTCCCAT
        131 GCGCTTTGAGGATTTCGTTGGAAACGGAAATATCTTCATAT

                * * * *      **      *   * *   *     ** ** *      *    *     
      43220 AAAAGCGACATAGAAGCTATCTCAGGAACTTGTTTATGATGCCTCTAATCAACTAACAGTGTTGA
          1 AAAAACTAGACAGAAGCATTCTCAGAAACATCTTTGTGATGTTTGCACTCAACTCACAGAGTTGA

                  * ** * *                         * *       *               
      43285 ACCTTTGTACTGACAGAGCAG-TTTGAAACACTCTTTTTTTGGAATCTGCAAGTGGATATTTGGA
         66 ACCTTTCTTTTCATAGAGCAGTTTTGAAACACTCTTTTTGTAGAATCTGGAAGTGGATATTTGGA

            *                           *   *   *    
      43349 TCGCTTTGAGGATTTCGTTGGAAACGG-GAT-GC--AATAT
        131 GCGCTTTGAGGATTTCGTTGGAAACGGAAATATCTTCATAT

                **  *    *     *                 **  *   *  *    *         * 
      43386 AAAACGTACACAGCAGCATACTCAGAAA-ATACTTTGCCATATTTCCATTCAAGTCACAGAGTGG
          1 AAAAACTAGACAGAAGCATTCTCAGAAACAT-CTTTGTGATGTTTGCACTCAACTCACAGAGTTG

               *  * **            *  *               *  *             *      
      43450 AACATTCCCATTCATAGAGCAGGTTGGAAACACTCTTTTTGGAGTATCTGGAAGTGGACATTTGG
         65 AACCTTTCTTTTCATAGAGCAGTTTTGAAACACTCTTTTTGTAGAATCTGGAAGTGGATATTTGG

                    ** * * ** *  *   *        *   **  
      43515 AGCGCTTTCTGAACTATGGTGAAAAGGGAAATATGTTCCAAT
        130 AGCGCTTTGAGGATTTCGTTGGAAACGGAAATATCTTCATAT

            *     *               *      * *         **  **       *  *  * *  
      43557 GAAAACAAGACAGAAGCATTCTGAGAAACTTATTTGTGATGCGTGTCCTCAACTAACGGACTCGA
          1 AAAAACTAGACAGAAGCATTCTCAGAAACATCTTTGTGATGTTTGCACTCAACTCACAGAGTTGA

             *     *      **  * *  *  *             *   *    * * *          *
      43622 AGCTTTCGTTTCATGCAGTACTTCTGGAACACTCTTTTTGAAGATTCTGCATGCGGATATTTGGT
         66 ACCTTTCTTTTCATAGAGCAGTTTTGAAACACTCTTTTTGTAGAATCTGGAAGTGGATATTTGGA

            ***                           **   *     
      43687 TATCTTTGAGGATTTCGTTGGAAACGG---GCT-TACATAT
        131 GCGCTTTGAGGATTTCGTTGGAAACGGAAATATCTTCATAT

                 *       *               *        *   *   **    *       *    
      43724 AAAAATTAGACAGCAGCATTCTCAGAAACTTCTTTGTGGTGTCTGCTTTCAAGTCACAGAATTGA
          1 AAAAACTAGACAGAAGCATTCTCAGAAACATCTTTGTGATGTTTGCACTCAACTCACAGAGTTGA

              * ** * **            *  * *      *       *    *        *       
      43789 ACATCCCCTCACATAGAGCAGTTGTGCAGCACTCTATTTGTAGTATCTCGAAGTGGACATTTGGA
         66 ACCTTTCTTTTCATAGAGCAGTTTTGAAACACTCTTTTTGTAGAATCTGGAAGTGGATATTTGGA

             *         ** *    * *   *            **  
      43854 GGGCTTTGTAGCCTATC-TGGAAAAAGGAAATATCTTCCCAT
        131 GCGCTTTG-AGGATTTCGTTGGAAACGGAAATATCTTCATAT

            *  ** *   *     * *            *   *  *   * **        *  * * *   
      43895 GAATGCGAGATAGAAGTAATCTCAGAAACATGTTTATGCTGTATCTACTCAACTAACTGTGCTGA
          1 AAAAACTAGACAGAAGCATTCTCAGAAACATCTTTGTGATGTTTGCACTCAACTCACAGAGTTGA

              *     *  *               *        *  * *       *              *
      43960 ACATTTCTATTGATAGAGCAGTTTTGAGACACTCTTCTTTTGGAATCTGCAAGTGGATATTTGGC
         66 ACCTTTCTTTTCATAGAGCAGTTTTGAAACACTCTTTTTGTAGAATCTGGAAGTGGATATTTGGA

            ** *                        *     *      
      44025 TAGATTTGAGGATTTCGTTGGAAACGG-GAT-T-AT-ATAT
        131 GCGCTTTGAGGATTTCGTTGGAAACGGAAATATCTTCATAT

            *    *       *               *                 **  *   *         
      44062 CAAAAGTAGACAGCAGCATTCTCAGAAACTTCTTTGTGATGTTTGCATCCAGCTCTCAGAGTTGA
          1 AAAAACTAGACAGAAGCATTCTCAGAAACATCTTTGTGATGTTTGCACTCAACTCACAGAGTTGA

              *  * *          *  *        * *      *   **           **       
      44127 ACATTCCCTTTCATAGAGTAGGTTTGAAACCCCCTTTTTATAGTGTCTGGAAGTGGGCATTTGGA
         66 ACCTTTCTTTTCATAGAGCAGTTTTGAAACACTCTTTTTGTAGAATCTGGAAGTGGATATTTGGA

                   *   ** ** *  *   *          * *  *
      44192 GCGCTTTCAGGCCTATGCTGAAAAAGGAAATATCTACCTAC
        131 GCGCTTTGAGGATTTCGTTGGAAACGGAAATATCTTCATAT

             *                    *    *  **          ** *        *    *     
      44233 AGAAACTAGACAGAAGCATTCTGAGAATCACGTTTGTGATGTGGGTACTCAACTAACAGTGTTGA
          1 AAAAACTAGACAGAAGCATTCTCAGAAACATCTTTGTGATGTTTGCACTCAACTCACAGAGTTGA

            *  *       *   *            *   *                *               
      44298 TCCATTCTTTTGATACAGCAGTTTTGAACCACCCTTTTTGTAGAATCTGCAAGTGGATATTTGGA
         66 ACCTTTCTTTTCATAGAGCAGTTTTGAAACACTCTTTTTGTAGAATCTGGAAGTGGATATTTGGA

            **   *                     *   *        *
      44363 TAGCTGTGAGGATTTCGTTGGAAACGGGAATGTCTTCATAG
        131 GCGCTTTGAGGATTTCGTTGGAAACGGAAATATCTTCATAT

                **                     * ****     *   *  **   *   *          
      44404 AAAATTTAGACAGAAGCATTCTCAGAACCTGGATTGTGGTGTGTGTTCTCCACTAACAGAGTTGA
          1 AAAAACTAGACAGAAGCATTCTCAGAAACATCTTTGTGATGTTTGCACTCAACTCACAGAGTTGA

                       * *   * *           *       *                         
      44469 ACCTTTCTTTTGACAGAACTGTTTTGAAACATTCTTTTTATAGAATCTGGAAGTGGATATTTGGA
         66 ACCTTTCTTTTCATAGAGCAGTTTTGAAACACTCTTTTTGTAGAATCTGGAAGTGGATATTTGGA

            **                 *       *          *  
      44534 AAGCTTTGAGGATTTCGTTAGAAACGGGAATATCTTCAAAT
        131 GCGCTTTGAGGATTTCGTTGGAAACGGAAATATCTTCATAT

                *    *            *           * *     * *  *    *            
      44575 AAAATCTAGCCAGAAGCATTCTAAGAAACATCTTAGGGATGTGTACATTCAAGTCACAGAGTTGA
          1 AAAAACTAGACAGAAGCATTCTCAGAAACATCTTTGTGATGTTTGCACTCAACTCACAGAGTTGA

              *   * *    **       *         *    * *   **      *      *    * 
      44640 ACATTCCCCTTTCTCAGAGCAGGTTTGAAACAATCTTCTCGTACTATCTGGCAGTGGACATTTTG
         66 ACCTT-TCTTTTCATAGAGCAGTTTTGAAACACTCTTTTTGTAGAATCTGGAAGTGGATATTTGG

               * *   *  ** ** *  *   *            ** *
      44705 AGCTCCTTGGGGCCTATGCTGAAAAAGGAAATATCTTCCGAC
        130 AGCGCTTTGAGGATTTCGTTGGAAACGGAAATATCTTCATAT

                                 *     *  **          *           **    *    
      44747 AAAAACTAGACAGAAGCATTCGCAGAATCACGTTTGTGATGTGTGCACTCAACTGTCAGAATTGA
          1 AAAAACTAGACAGAAGCATTCTCAGAAACATCTTTGTGATGTTTGCACTCAACTCACAGAGTTGA

                  *   ** *      *                      *     * *            *
      44812 ACCTTTGTTTGGACAGAGCACTTTTGAAACACTCTTTTTGTAGGATCTGCAGGTGGATATTTGGC
         66 ACCTTTCTTTTCATAGAGCAGTTTTGAAACACTCTTTTTGTAGAATCTGGAAGTGGATATTTGGA

            **                         *   *      * *
      44877 TAGCTTTGAGGATTTCGTTGGAAACGGTAATGTCTTCAAAG
        131 GCGCTTTGAGGATTTCGTTGGAAACGGAAATATCTTCATAT

                *          *   *          *   *            *    *            
      44918 AAAATCTAGACAGAAACATCCTCAGAAACACCTTCGTGATGTTTGCAATCAAGTCACAGAGTTGA
          1 AAAAACTAGACAGAAGCATTCTCAGAAACATCTTTGTGATGTTTGCACTCAACTCACAGAGTTGA

                 * *             *  *         *        *             *       
      44983 ACCTTCCGTTTCATAGAGCAGGTTGGAAACACTCATTTTGTAGTATCTGGAAGTGGACATTTGGA
         66 ACCTTTCTTTTCATAGAGCAGTTTTGAAACACTCTTTTTGTAGAATCTGGAAGTGGATATTTGGA

                   *   ** ** *  *   *            **  
      45048 GCGCTTTCAGGCCTATGGTGTAAAAGGAAATATCTTCCCAT
        131 GCGCTTTGAGGATTTCGTTGGAAACGGAAATATCTTCATAT

                * * * *      **      *   * *   *     ** ** *      *    *     
      45089 AAAAGCGACATAGAAGCTATCTCAGGAACTTGTTTATGATGCCTCTAATCAACTAACAGTGTTGA
          1 AAAAACTAGACAGAAGCATTCTCAGAAACATCTTTGTGATGTTTGCACTCAACTCACAGAGTTGA

                  * ** * *                         * *       *               
      45154 ACCTTTGTACTGACAGAGCAG-TTTGAAACACTCTTTTTTTGGAATCTGCAAGTGGATATTTGGA
         66 ACCTTTCTTTTCATAGAGCAGTTTTGAAACACTCTTTTTGTAGAATCTGGAAGTGGATATTTGGA

            *                           *   *   *    
      45218 TCGCTTTGAGGATTTCGTTGGAAACGG-GAT-GC--AATAT
        131 GCGCTTTGAGGATTTCGTTGGAAACGGAAATATCTTCATAT

                **  *    *     *                 **  *   *  *    *         * 
      45255 AAAACGTACACAGCAGCATACTCAGAAA-ATACTTTGCCATATTTCCATTCAAGTCACAGAGTGG
          1 AAAAACTAGACAGAAGCATTCTCAGAAACAT-CTTTGTGATGTTTGCACTCAACTCACAGAGTTG

               *  * **            *  *               *  *             *      
      45319 AACATTCCCATTCATAGAGCAGGTTGGAAACACTCTTTTTGGAGTATCTGGAAGTGGACATTTGG
         65 AACCTTTCTTTTCATAGAGCAGTTTTGAAACACTCTTTTTGTAGAATCTGGAAGTGGATATTTGG

                    ** * * ** *  *   *        *   **  
      45384 AGCGCTTTCTGAACTATGGTGAAAAGGGAAATATGTTCCAAT
        130 AGCGCTTTGAGGATTTCGTTGGAAACGGAAATATCTTCATAT

            *     *               *      * *         **  **       *  *  * *  
      45426 GAAAACAAGACAGAAGCATTCTGAGAAACTTATTTGTGATGCGTGTCCTCAACTAACGGACTCGA
          1 AAAAACTAGACAGAAGCATTCTCAGAAACATCTTTGTGATGTTTGCACTCAACTCACAGAGTTGA

             *     *      **  * *  *  *             *   *    * * *          *
      45491 AGCTTTCGTTTCATGCAGTACTTCTGGAACACTCTTTTTGAAGATTCTGCATGCGGATATTTGGT
         66 ACCTTTCTTTTCATAGAGCAGTTTTGAAACACTCTTTTTGTAGAATCTGGAAGTGGATATTTGGA

            **                            **   *     
      45556 TAGCTTTGAGGATTTCGTTGGAAACGG---GCT-TACATAT
        131 GCGCTTTGAGGATTTCGTTGGAAACGGAAATATCTTCATAT

                 *       *               *        *   *   **    *       *    
      45593 AAAAATTAGACAGCAGCATTCTCAGAAACTTCTTTGTGGTGTCTGCTTTCAAGTCACAGAATTGA
          1 AAAAACTAGACAGAAGCATTCTCAGAAACATCTTTGTGATGTTTGCACTCAACTCACAGAGTTGA

              * ** * **            *  * *      *       *    *        *       
      45658 ACATCCCCTCACATAGAGCAGTTGTGCAGCACTCTATTTGTAGTATCTCGAAGTGGACATTTGGA
         66 ACCTTTCTTTTCATAGAGCAGTTTTGAAACACTCTTTTTGTAGAATCTGGAAGTGGATATTTGGA

             *         ** *    * *   *            **  
      45723 GGGCTTTGTAGCCTATC-TGGAAAAAGGAAATATCTTCCCAT
        131 GCGCTTTG-AGGATTTCGTTGGAAACGGAAATATCTTCATAT

            *  ** *   *     * *            *   *  *   * **        *  * * *   
      45764 GAATGCGAGATAGAAGTAATCTCAGAAACATGTTTATGCTGTATCTACTCAACTAACTGTGCTGA
          1 AAAAACTAGACAGAAGCATTCTCAGAAACATCTTTGTGATGTTTGCACTCAACTCACAGAGTTGA

              *     *  *               *        *  * *       *              *
      45829 ACATTTCTATTGATAGAGCAGTTTTGAGACACTCTTCTTTTGGAATCTGCAAGTGGATATTTGGC
         66 ACCTTTCTTTTCATAGAGCAGTTTTGAAACACTCTTTTTGTAGAATCTGGAAGTGGATATTTGGA

            ** *                        *     *      
      45894 TAGATTTGAGGATTTCGTTGGAAACGG-GAT-T-AT-ATAT
        131 GCGCTTTGAGGATTTCGTTGGAAACGGAAATATCTTCATAT

            *    *       *               *                 **  *   *         
      45931 CAAAAGTAGACAGCAGCATTCTCAGAAACTTCTTTGTGATGTTTGCATCCAGCTCTCAGAGTTGA
          1 AAAAACTAGACAGAAGCATTCTCAGAAACATCTTTGTGATGTTTGCACTCAACTCACAGAGTTGA

              *  * *          *  *        * *      *   **           **       
      45996 ACATTCCCTTTCATAGAGTAGGTTTGAAACCCCCTTTTTATAGTGTCTGGAAGTGGGCATTTGGA
         66 ACCTTTCTTTTCATAGAGCAGTTTTGAAACACTCTTTTTGTAGAATCTGGAAGTGGATATTTGGA

                   *   ** ** *  *   *          * *  *
      46061 GCGCTTTCAGGCCTATGCTGAAAAAGGAAATATCTACCTAC
        131 GCGCTTTGAGGATTTCGTTGGAAACGGAAATATCTTCATAT

             *                    *    *  **          ** *        *    *     
      46102 AGAAACTAGACAGAAGCATTCTGAGAATCACGTTTGTGATGTGGGTACTCAACTAACAGTGTTGA
          1 AAAAACTAGACAGAAGCATTCTCAGAAACATCTTTGTGATGTTTGCACTCAACTCACAGAGTTGA

            *  *       *   *            *   **               *               
      46167 TCCATTCTTTTGATACAGCAGTTTTGAACCACCTTTTTTGTAGAATCTGCAAGTGGATATTTGGA
         66 ACCTTTCTTTTCATAGAGCAGTTTTGAAACACTCTTTTTGTAGAATCTGGAAGTGGATATTTGGA

            **   *                     *   *        *
      46232 TAGCTGTGAGGATTTCGTTGGAAACGGGAATGTCTTCATAG
        131 GCGCTTTGAGGATTTCGTTGGAAACGGAAATATCTTCATAT

                **                     * ****     *   *  **   *   *          
      46273 AAAATTTAGACAGAAGCATTCTCAGAACCTGGATTGTGGTGTGTGTTCTCCACTAACAGAGTTGA
          1 AAAAACTAGACAGAAGCATTCTCAGAAACATCTTTGTGATGTTTGCACTCAACTCACAGAGTTGA

                       * *   * *           *       *                         
      46338 ACCTTTCTTTTGACAGAACTGTTTTGAAACATTCTTTTTATAGAATCTGGAAGTGGATATTTGGA
         66 ACCTTTCTTTTCATAGAGCAGTTTTGAAACACTCTTTTTGTAGAATCTGGAAGTGGATATTTGGA

            **                 *       *          *  
      46403 AAGCTTTGAGGATTTCGTTAGAAACGGGAATATCTTCAAAT
        131 GCGCTTTGAGGATTTCGTTGGAAACGGAAATATCTTCATAT

                *    *            *           * *     * *  *    *            
      46444 AAAATCTAGCCAGAAGCATTCTAAGAAACATCTTAGGGATGTGTACATTCAAGTCACAGAGTTGA
          1 AAAAACTAGACAGAAGCATTCTCAGAAACATCTTTGTGATGTTTGCACTCAACTCACAGAGTTGA

              *   * *    **       *         *    * *   **      *      *    * 
      46509 ACATTCCCCTTTCTCAGAGCAGGTTTGAAACAATCTTCTCGTACTATCTGGCAGTGGACATTTTG
         66 ACCTT-TCTTTTCATAGAGCAGTTTTGAAACACTCTTTTTGTAGAATCTGGAAGTGGATATTTGG

               * *   *  ** ** *  *   *            ** *
      46574 AGCTCCTTGGGGCCTATGCTGAAAAAGGAAATATCTTCCGAC
        130 AGCGCTTTGAGGATTTCGTTGGAAACGGAAATATCTTCATAT

                                 *     *  **          * *         **    *    
      46616 AAAAACTAGACAGAAGCATTCGCAGAATCACGTTTGTGATGTGTTCACTCAACTGTCAGAATTGA
          1 AAAAACTAGACAGAAGCATTCTCAGAAACATCTTTGTGATGTTTGCACTCAACTCACAGAGTTGA

                  *   ** *      *                      *     * *            *
      46681 ACCTTTGTTTGGACAGAGCACTTTTGAAACACTCTTTTTGTAGGATCTGCAGGTGGATATTTGGC
         66 ACCTTTCTTTTCATAGAGCAGTTTTGAAACACTCTTTTTGTAGAATCTGGAAGTGGATATTTGGA

            **                         *   *      * *
      46746 TAGCTTTGAGGATTTCGTTGGAAACGGTAATGTCTTCAAAG
        131 GCGCTTTGAGGATTTCGTTGGAAACGGAAATATCTTCATAT

                *          *   *          *   *            *    *            
      46787 AAAATCTAGACAGAAACATCCTCAGAAACACCTTCGTGATGTTTGCAATCAAGTCACAGAGTTGA
          1 AAAAACTAGACAGAAGCATTCTCAGAAACATCTTTGTGATGTTTGCACTCAACTCACAGAGTTGA

                 * *             *  *         *        *             *       
      46852 ACCTTCCGTTTCATAGAGCAGGTTGGAAACACTCATTTTGTAGTATCTGGAAGTGGACATTTGGA
         66 ACCTTTCTTTTCATAGAGCAGTTTTGAAACACTCTTTTTGTAGAATCTGGAAGTGGATATTTGGA

                   *   ** ** *  *   *            **  
      46917 GCGCTTTCAGGCCTATGGTGTAAAAGGAAATATCTTCCCAT
        131 GCGCTTTGAGGATTTCGTTGGAAACGGAAATATCTTCATAT

                * * * *      **      *   * *   *     ** ** *      *    *     
      46958 AAAAGCGACATAGAAGCTATCTCAGGAACTTGTTTATGATGCCTCTAATCAACTAACAGTGTTGA
          1 AAAAACTAGACAGAAGCATTCTCAGAAACATCTTTGTGATGTTTGCACTCAACTCACAGAGTTGA

                  * ** * *                         * *       *               
      47023 ACCTTTGTACTGACAGAGCAG-TTTGAAACACTCTTTTTTTGGAATCTGCAAGTGGATATTTGGA
         66 ACCTTTCTTTTCATAGAGCAGTTTTGAAACACTCTTTTTGTAGAATCTGGAAGTGGATATTTGGA

            *                           *   *   *    
      47087 TCGCTTTGAGGATTTCGTTGGAAACGG-GAT-GC--AATAT
        131 GCGCTTTGAGGATTTCGTTGGAAACGGAAATATCTTCATAT

                **  *    *     *                 **  *   *  *    *         * 
      47124 AAAACGTACACAGCAGCATACTCAGAAA-ATACTTTGCCATATTTCCATTCAAGTCACAGAGTGG
          1 AAAAACTAGACAGAAGCATTCTCAGAAACAT-CTTTGTGATGTTTGCACTCAACTCACAGAGTTG

               *  * **            *  *               *  *             *      
      47188 AACATTCCCATTCATAGAGCAGGTTGGAAACACTCTTTTTGGAGTATCTGGAAGTGGACATTTGG
         65 AACCTTTCTTTTCATAGAGCAGTTTTGAAACACTCTTTTTGTAGAATCTGGAAGTGGATATTTGG

                    ** * * ** *  *   *        *   **  
      47253 AGCGCTTTCTGAACTATGGTGAAAAGGGAAATATGTTCCAAT
        130 AGCGCTTTGAGGATTTCGTTGGAAACGGAAATATCTTCATAT

            *     *               *      * *         **  **       *  *  * *  
      47295 GAAAACAAGACAGAAGCATTCTGAGAAACTTATTTGTGATGCGTGTCCTCAACTAACGGACTCGA
          1 AAAAACTAGACAGAAGCATTCTCAGAAACATCTTTGTGATGTTTGCACTCAACTCACAGAGTTGA

             *    **      **  * *  *  *             *   *    * * *          *
      47360 AGCTTTGGTTTCATGCAGTACTTCTGGAACACTCTTTTTGAAGATTCTGCATGCGGATATTTGGT
         66 ACCTTTCTTTTCATAGAGCAGTTTTGAAACACTCTTTTTGTAGAATCTGGAAGTGGATATTTGGA

            **                            **   *     
      47425 TAGCTTTGAGGATTTCGTTGGAAACGG---GCT-TACATAT
        131 GCGCTTTGAGGATTTCGTTGGAAACGGAAATATCTTCATAT

                 *       *               *        *   *    *    *       *    
      47462 AAAAATTAGACAGCAGCATTCTCAGAAACTTCTTTGTGGTGTCTGCATTCAAGTCACAGAATTGA
          1 AAAAACTAGACAGAAGCATTCTCAGAAACATCTTTGTGATGTTTGCACTCAACTCACAGAGTTGA

              * ** * **            *  * *      *       *    *        *       
      47527 ACATCCCCTCACATAGAGCAGTTGTGCAGCACTCTATTTGTAGTATCTCGAAGTGGACATTTGGA
         66 ACCTTTCTTTTCATAGAGCAGTTTTGAAACACTCTTTTTGTAGAATCTGGAAGTGGATATTTGGA

             *         ** *    * *   *            **  
      47592 GGGCTTTGTAGCCTATC-TGGAAAAAGGAAATATCTTCCCAT
        131 GCGCTTTG-AGGATTTCGTTGGAAACGGAAATATCTTCATAT

            *  ** *   *     * *            *   *  *   * **        *  * * *   
      47633 GAATGCGAGATAGAAGTAATCTCAGAAACATGTTTATGCTGTATCTACTCAACTAACTGTGCTGA
          1 AAAAACTAGACAGAAGCATTCTCAGAAACATCTTTGTGATGTTTGCACTCAACTCACAGAGTTGA

              *     *  *               *        *  * *       **              
      47698 ACATTTCTATTGATAGAGCAGTTTTGAGACACTCTTCTTTTGGAATCTGCTAGTGGATATTTGGA
         66 ACCTTTCTTTTCATAGAGCAGTTTTGAAACACTCTTTTTGTAGAATCTGGAAGTGGATATTTGGA

            ** *            *           *     *      
      47763 TAGATTTGAGGATTTCATTGGAAACGG-GAT-T-AT-ATAT
        131 GCGCTTTGAGGATTTCGTTGGAAACGGAAATATCTTCATAT

                 *       *               *                 **  *   *         
      47800 AAAAAGTAGACAGCAGCATTCTCAGAAACTTCTTTGTGATGTTTGCATCCAGCTCTCAGAGTTGA
          1 AAAAACTAGACAGAAGCATTCTCAGAAACATCTTTGTGATGTTTGCACTCAACTCACAGAGTTGA

              *  * *     *    *  *        * *      *   **        *  **       
      47865 ACATTCCCTTTCACAGAGTAGGTTTGAAACCCCCTTTTTATAGTGTCTGGAAGCGGGCATTTGGA
         66 ACCTTTCTTTTCATAGAGCAGTTTTGAAACACTCTTTTTGTAGAATCTGGAAGTGGATATTTGGA

            **     *   ** ** *  *   *          * *  *
      47930 ATGCTTTCAGGCCTATGCTGAAAAAGGAAATATCTACCTAC
        131 GCGCTTTGAGGATTTCGTTGGAAACGGAAATATCTTCATAT

             *                    *    *  **          ** *        *    *     
      47971 AGAAACTAGACAGAAGCATTCTGAGAATCACGTTTGTGATGTGGGTACTCAACTAACAGTGTTGA
          1 AAAAACTAGACAGAAGCATTCTCAGAAACATCTTTGTGATGTTTGCACTCAACTCACAGAGTTGA

            *  *       *   *            *   *       *        *               
      48036 TCCATTCTTTTGATACAGCAGTTTTGAACCACCCTTTTTGAAGAATCTGCAAGTGGATATTTGGA
         66 ACCTTTCTTTTCATAGAGCAGTTTTGAAACACTCTTTTTGTAGAATCTGGAAGTGGATATTTGGA

            **   *                     *   *        *
      48101 TAGCTGTGAGGATTTCGTTGGAAACGGGAATGTCTTCATAG
        131 GCGCTTTGAGGATTTCGTTGGAAACGGAAATATCTTCATAT

                **                     * ****         *  **   *   *          
      48142 AAAATTTAGACAGAAGCATTCTCAGAACCTGGATTGTGATGTGTGTTCTCCACTAACAGAGTTGA
          1 AAAAACTAGACAGAAGCATTCTCAGAAACATCTTTGTGATGTTTGCACTCAACTCACAGAGTTGA

                      ** *   * *           *       *                         
      48207 ACCTTTCTTTGGACAGAACTGTTTTGAAACATTCTTTTTATAGAATCTGGAAGTGGATATTTGGA
         66 ACCTTTCTTTTCATAGAGCAGTTTTGAAACACTCTTTTTGTAGAATCTGGAAGTGGATATTTGGA

            **     *                   *          *  
      48272 AAGCTTTTAGGATTTCGTTGGAAACGGGAATATCTTCAAAT
        131 GCGCTTTGAGGATTTCGTTGGAAACGGAAATATCTTCATAT

                *    *            *           * *     * *  *    *            
      48313 AAAATCTAGCCAGAAGCATTCTAAGAAACATCTTAGGGATGTGTACATTCAAGTCACAGAGTTGA
          1 AAAAACTAGACAGAAGCATTCTCAGAAACATCTTTGTGATGTTTGCACTCAACTCACAGAGTTGA

              *   * *    **       *         *    * *   **             *    * 
      48378 ACATTCCCCTTTCTCAGAGCAGGTTTGAAACAATCTTCTCGTACTATCTGGAAGTGGACATTTTG
         66 ACCTT-TCTTTTCATAGAGCAGTTTTGAAACACTCTTTTTGTAGAATCTGGAAGTGGATATTTGG

               * *   *  ** ** *  *   *            ** *
      48443 AGCTCCTTGGGGCCTATGCTGAAAAAGGAAATATCTTCCGAC
        130 AGCGCTTTGAGGATTTCGTTGGAAACGGAAATATCTTCATAT

                                 *     *  **          *           **    *    
      48485 AAAAACTAGACAGAAGCATTCGCAGAATCACGTTTGTGATGTGTGCACTCAACTGTCAGAATTGA
          1 AAAAACTAGACAGAAGCATTCTCAGAAACATCTTTGTGATGTTTGCACTCAACTCACAGAGTTGA

                  *   ** *      *                      *     * *            *
      48550 ACCTTTGTTTGGACAGAGCACTTTTGAAACACTCTTTTTGTAGCATCTGCAGGTGGATATTTGGC
         66 ACCTTTCTTTTCATAGAGCAGTTTTGAAACACTCTTTTTGTAGAATCTGGAAGTGGATATTTGGA

            **                         *   *      * *
      48615 TAGCTTTGAGGATTTCGTTGGAAACGGTAATGTCTTCAAAG
        131 GCGCTTTGAGGATTTCGTTGGAAACGGAAATATCTTCATAT

                *          *   *          *   *            *    *            
      48656 AAAATCTAGACAGAAACATCCTCAGAAACACCTTCGTGATGTTTGCAATCAAGTCACAGAGTTGA
          1 AAAAACTAGACAGAAGCATTCTCAGAAACATCTTTGTGATGTTTGCACTCAACTCACAGAGTTGA

                 * *             *  *         *      * *     *       *       
      48721 ACCTTCCGTTTCATAGAGCAGGTTGGAAACACTCATTTTGTGGTATCTGCAAGTGGACATTTGGA
         66 ACCTTTCTTTTCATAGAGCAGTTTTGAAACACTCTTTTTGTAGAATCTGGAAGTGGATATTTGGA

                   *   ** ** *  *   *   *    *   **  
      48786 GCGCTTTCAGGCCTATGGTGTAAAAGGATATATGTTCCCAT
        131 GCGCTTTGAGGATTTCGTTGGAAACGGAAATATCTTCATAT

                * * * *      **      *   * *   *     ** ** *      *    *     
      48827 AAAAGCGACATAGAAGCTATCTCAGGAACTTGTTTATGATGCATCTAATCAACTAACAGTGTTGA
          1 AAAAACTAGACAGAAGCATTCTCAGAAACATCTTTGTGATGTTTGCACTCAACTCACAGAGTTGA

                  * ** * *                         * *       *               
      48892 ACCTTTGTACTGACAGAGCAG-TTTGAAACACTCTTTTTTTGGAATCTGCAAGTGGATATTTGGA
         66 ACCTTTCTTTTCATAGAGCAGTTTTGAAACACTCTTTTTGTAGAATCTGGAAGTGGATATTTGGA

            *                           *   *   *    
      48956 TCGCTTTGAGGATTTCGTTGGAAACGG-GAT-GC--AATAT
        131 GCGCTTTGAGGATTTCGTTGGAAACGGAAATATCTTCATAT

                **  *    *     *                 **  *   *  *    *         * 
      48993 AAAACGTACACAGCAGCATACTCAGAAA-ATACTTTGCCATATTTCCATTCAAGTCACAGAGTGG
          1 AAAAACTAGACAGAAGCATTCTCAGAAACAT-CTTTGTGATGTTTGCACTCAACTCACAGAGTTG

               *  * **            *                  *  *             *      
      49057 AACATTCCCATTCATAGAGCAGGTTTGAAACACTCTTTTTGGAGTATCTGGAAGTGGACATTTGG
         65 AACCTTTCTTTTCATAGAGCAGTTTTGAAACACTCTTTTTGTAGAATCTGGAAGTGGATATTTGG

                    ** * * ** *  *   *        *   **  
      49122 AGCGCTTTCTGAACTATGGTGAAAAAGGAAATATGTTCCAAT
        130 AGCGCTTTGAGGATTTCGTTGGAAACGGAAATATCTTCATAT

            *     *               *      * *         **  **       *  *  * *  
      49164 GAAAACAAGACAGAAGCATTCTGAGAAACTTATTTGTGATGCGTGTCCTCAACTAACGGACTCGA
          1 AAAAACTAGACAGAAGCATTCTCAGAAACATCTTTGTGATGTTTGCACTCAACTCACAGAGTTGA

             *    **      **  * *  *  *             *   *    * * *           
      49229 AGCTTTGGTTTCATGCAGTACTTCTGGAACACTCTTTTTGAAGATTCTGCATGCGGATATTTGGA
         66 ACCTTTCTTTTCATAGAGCAGTTTTGAAACACTCTTTTTGTAGAATCTGGAAGTGGATATTTGGA

            **                            **   *     
      49294 TAGCTTTGAGGATTTCGTTGGAAACGG---GCT-TACATAT
        131 GCGCTTTGAGGATTTCGTTGGAAACGGAAATATCTTCATAT

                 *       *               *        *   *    *    *       **   
      49331 AAAAATTAGACAGCAGCATTCTCAGAAACTTCTTTGTGGTGTCTGCATTCAAGTCACAGAACTGA
          1 AAAAACTAGACAGAAGCATTCTCAGAAACATCTTTGTGATGTTTGCACTCAACTCACAGAGTTGA

              * ** * **            *  * *      *       *    *        *       
      49396 ACATCCCCTCACATAGAGCAGTTGTGCAGCACTCTATTTGTAGTATCTCGAAGTGGACATTTGGA
         66 ACCTTTCTTTTCATAGAGCAGTTTTGAAACACTCTTTTTGTAGAATCTGGAAGTGGATATTTGGA

             *         ** *    * *   *            **  
      49461 GGGCTTTGTAGCCTATC-TGGAAAAAGGAAATATCTTCCCAT
        131 GCGCTTTG-AGGATTTCGTTGGAAACGGAAATATCTTCATAT

            *  ** *   *     * *            *   *  *   * **  *     * ** * *   
      49502 GAATGCGAGATAGAAGTAATCTCAGAAACATGTTTATGCTGTATCTACCCAACTAAGTGTGCTGA
          1 AAAAACTAGACAGAAGCATTCTCAGAAACATCTTTGTGATGTTTGCACTCAACTCACAGAGTTGA

              *     *  *               *        *  * *       *              *
      49567 ACATTTCTATTAATAGAGCAGTTTTGAGACACTCTTCTTTTCGAATCTGCAAGTGGATATTTGGC
         66 ACCTTTCTTTTCATAGAGCAGTTTTGAAACACTCTTTTTGTAGAATCTGGAAGTGGATATTTGGA

            ** *                        *     *      
      49632 TAGATTTGAGGATTTCGTTGGAAACGG-GAT-T-AT-ATAT
        131 GCGCTTTGAGGATTTCGTTGGAAACGGAAATATCTTCATAT

                 **      *               *                 **  *   *         
      49669 AAAAAGCAGACAGCAGCATTCTCAGAAACTTCTTTGTGATGTTTGCATCCAGCTCTCAGAGTTGA
          1 AAAAACTAGACAGAAGCATTCTCAGAAACATCTTTGTGATGTTTGCACTCAACTCACAGAGTTGA

              *  * *          *  *        * *      *   **        *  **       
      49734 ACATTCCGTTTCATAGAGTAGGTTTGAAACCCCCTTTTTATAGTGTCTGGAAGCGGGCATTTGGA
         66 ACCTTTCTTTTCATAGAGCAGTTTTGAAACACTCTTTTTGTAGAATCTGGAAGTGGATATTTGGA

                   *   ** ** *  *   *          * *  *
      49799 GCGCTTTCAGGCCTATGCTGAAAAAGGAAATATCTACCTAC
        131 GCGCTTTGAGGATTTCGTTGGAAACGGAAATATCTTCATAT

             *                    *    *  **          ** *        *    *     
      49840 AGAAACTAGACAGAAGCATTCTGAGAATCACGTTTGTGATGTGGGTACTCAACTAACAGTGTTGA
          1 AAAAACTAGACAGAAGCATTCTCAGAAACATCTTTGTGATGTTTGCACTCAACTCACAGAGTTGA

            *  *       *   *            *   *                *               
      49905 TCCATTCTTTTGATACAGCAGTTTTGAACCACCCTTTTTGTAGAATCTGCAAGTGGATATTTGGA
         66 ACCTTTCTTTTCATAGAGCAGTTTTGAAACACTCTTTTTGTAGAATCTGGAAGTGGATATTTGGA

            **   *               *     *   *        *
      49970 TAGCTGTGAGGA-TTCGTTGGGAACGGGAATTTCTTCATAG
        131 GCGCTTTGAGGATTTCGTTGGAAACGGAAATATCTTCATAT

                **                     * ****         *  **   *   *          
      50010 AAAATTTAGACAGAAGCATTCTCAGAACCTGGATTGTGATGTGTGTTCTCCACTAACAGAGTTGA
          1 AAAAACTAGACAGAAGCATTCTCAGAAACATCTTTGTGATGTTTGCACTCAACTCACAGAGTTGA

                       * *   * *           *       *                         
      50075 ACCTTTCTTTTGACAGAACTGTTTTGAAACATTCTTTTTATAGAATCTGGAAGTGGATATTTGGA
         66 ACCTTTCTTTTCATAGAGCAGTTTTGAAACACTCTTTTTGTAGAATCTGGAAGTGGATATTTGGA

            **                         *          *  
      50140 AAGCTTTGAGGATTTCGTTGGAAACGGGAATATCTTCAAAT
        131 GCGCTTTGAGGATTTCGTTGGAAACGGAAATATCTTCATAT

                *    *            *           * *     * *  *    *            
      50181 AAAATCTAGCCAGAAGCATTCTAAGAAACATCTTAGGGATGTGTACATTCAAGTCACAGAGTTGA
          1 AAAAACTAGACAGAAGCATTCTCAGAAACATCTTTGTGATGTTTGCACTCAACTCACAGAGTTGA

              *   * *    **       *         *    * *   **      *      *    * 
      50246 ACATTCCCCTTTCTCAGAGCAGGTTTGAAACAATCTTCTCGTACTATCTGGCAGTGGACATTTTG
         66 ACCTT-TCTTTTCATAGAGCAGTTTTGAAACACTCTTTTTGTAGAATCTGGAAGTGGATATTTGG

               * *   *  ** ** *  *   *            ** *
      50311 AGCTCCTTGGGGCCTATGCTGAAAAAGGAAATATCTTCCGAC
        130 AGCGCTTTGAGGATTTCGTTGGAAACGGAAATATCTTCATAT

                                 *     *  **          *           **    *    
      50353 AAAAACTAGACAGAAGCATTCGCAGAATCACGTTTGTGATGTGTGCACTCAACTGTCAGAATTGA
          1 AAAAACTAGACAGAAGCATTCTCAGAAACATCTTTGTGATGTTTGCACTCAACTCACAGAGTTGA

                  *   ** *      *                            * *            *
      50418 ACCTTTGTTTGGACAGAGCACTTTTGAAACACTCTTTTTGTAGAATCTGCAGGTGGATATTTGGC
         66 ACCTTTCTTTTCATAGAGCAGTTTTGAAACACTCTTTTTGTAGAATCTGGAAGTGGATATTTGGA

            **                         *   *      * *
      50483 TAGCTTTGAGGATTTCGTTGGAAACGGTAATGTCTTCAAAG
        131 GCGCTTTGAGGATTTCGTTGGAAACGGAAATATCTTCATAT

                *          *   *          *   *            *    *            
      50524 AAAATCTAGACAGAAACATCCTCAGAAACACCTTCGTGATGTTTGCAATCAAGTCACAGAGTTGA
          1 AAAAACTAGACAGAAGCATTCTCAGAAACATCTTTGTGATGTTTGCACTCAACTCACAGAGTTGA

                 * *             *  *         *        *             *       
      50589 ACCTTCCGTTTCATAGAGCAGGTTGGAAACACTCATTTTGTAGTATCTGGAAGTGGACATTTGGA
         66 ACCTTTCTTTTCATAGAGCAGTTTTGAAACACTCTTTTTGTAGAATCTGGAAGTGGATATTTGGA

                   *   ** ** *  *   *            **  
      50654 GCGCTTTCAGGCCTATGGTGTAAAAGGAAATATCTTCCCAT
        131 GCGCTTTGAGGATTTCGTTGGAAACGGAAATATCTTCATAT

                * * * *      **      *   * *   *     ** ** *      *    *     
      50695 AAAAGCGACATAGAAGCTATCTCAGGAACTTGTTTATGATGCATCTAATCAACTAACAGTGTTGA
          1 AAAAACTAGACAGAAGCATTCTCAGAAACATCTTTGTGATGTTTGCACTCAACTCACAGAGTTGA

                  * ** * *                         * *       *               
      50760 ACCTTTGTACTGACAGAGCAG-TTTGAAACACTCTTTTTTTGGAATCTGCAAGTGGATATTTGGA
         66 ACCTTTCTTTTCATAGAGCAGTTTTGAAACACTCTTTTTGTAGAATCTGGAAGTGGATATTTGGA

            *                        *  *   *   *    
      50824 TCGCTTTGAGGATTTCGTTGGAAACTG-GAT-GC--AATAT
        131 GCGCTTTGAGGATTTCGTTGGAAACGGAAATATCTTCATAT

                **  *    *     *                 **  *   *  *    *         * 
      50861 AAAACGTACACAGCAGCATACTCAGAAA-ATACTTTGCCATATTTCCATTCAAGTCACAGAGTGG
          1 AAAAACTAGACAGAAGCATTCTCAGAAACAT-CTTTGTGATGTTTGCACTCAACTCACAGAGTTG

               *  * **            *                 **  *             *      
      50925 AACATTCCCATTCATAGAGCAGGTTTGAAACACTCTTTTTAGAGTATCTGGAAGTGGACATTTGG
         65 AACCTTTCTTTTCATAGAGCAGTTTTGAAACACTCTTTTTGTAGAATCTGGAAGTGGATATTTGG

                    ** * * ** *  *   *            **  
      50990 AGCGCTTTCTGAACTATGGTGAAAAAGGAAATATCTTCCAAT
        130 AGCGCTTTGAGGATTTCGTTGGAAACGGAAATATCTTCATAT

            *     *               *      * *         **  **       *  *  * *  
      51032 GAAAACAAGACAGAAGCATTCTGAGAAACTTATTTGTGATGCGTGTCCTCAACTAACGGACTCGA
          1 AAAAACTAGACAGAAGCATTCTCAGAAACATCTTTGTGATGTTTGCACTCAACTCACAGAGTTGA

                   *      **  * *  *  *             *   *    * * *           
      51097 ACCTTTCGTTTCATGCAGTACTTCTGGAACACTCTTTTTGAAGATTCTGCATGCGGATATTTGGA
         66 ACCTTTCTTTTCATAGAGCAGTTTTGAAACACTCTTTTTGTAGAATCTGGAAGTGGATATTTGGA

            **   *                        **   *     
      51162 TAGCTGTGAGGATTTCGTTGGAAACGG---GCT-TACATAT
        131 GCGCTTTGAGGATTTCGTTGGAAACGGAAATATCTTCATAT

                 *       *               *        *   *    *    *       *    
      51199 AAAAATTAGACAGCAGCATTCTCAGAAACTTCTTTGTGGTGTCTGCATTCAAGTCACAGAATTGA
          1 AAAAACTAGACAGAAGCATTCTCAGAAACATCTTTGTGATGTTTGCACTCAACTCACAGAGTTGA

              * ** * **            *  * *      *       *    *        *       
      51264 ACATCCCCTCACATAGAGCAGTTGTGCAGCACTCTATTTGTAGTATCTCGAAGTGGACATTTGGA
         66 ACCTTTCTTTTCATAGAGCAGTTTTGAAACACTCTTTTTGTAGAATCTGGAAGTGGATATTTGGA

             *         ** *    * *   *            **  
      51329 GGGCTTTGTAGCCTATC-TGGAAAAAGGAAATATCTTCCCAT
        131 GCGCTTTG-AGGATTTCGTTGGAAACGGAAATATCTTCATAT

            *  ** *   *     * *            *   *  *   * **   *    *  * * *   
      51370 GAATGCGAGATAGAAGTAATCTCAGAAACATGTTTATGCTGTATCTACTAAACTAACTGTGCTGA
          1 AAAAACTAGACAGAAGCATTCTCAGAAACATCTTTGTGATGTTTGCACTCAACTCACAGAGTTGA

              *     *  *               *        *  * *       *               
      51435 ACATTTCTATTGATAGAGCAGTTTTGAGACACTCTTCTTTTGGAATCTGCAAGTGGATATTTGGA
         66 ACCTTTCTTTTCATAGAGCAGTTTTGAAACACTCTTTTTGTAGAATCTGGAAGTGGATATTTGGA

            ** *                         **    *     
      51500 TAGATTTGAGGATTTCGTTGGAAAC-G--TGAT-TACATAT
        131 GCGCTTTGAGGATTTCGTTGGAAACGGAAATATCTTCATAT

                ***      *               *                 **  *   *         
      51537 AAAATGCAGACAGCAGCATTCTCAGAAACTTCTTTGTGATGTTTGCATCCAGCTCTCAGAGTTGA
          1 AAAAACTAGACAGAAGCATTCTCAGAAACATCTTTGTGATGTTTGCACTCAACTCACAGAGTTGA

              *  * *          *  *        * *      *   **        *  **       
      51602 ACATTCCCTTTCATAGAGTAGGTTTGAAACCCCCTTTTTATAGTGTCTGGAAGCGGGCATTTGGA
         66 ACCTTTCTTTTCATAGAGCAGTTTTGAAACACTCTTTTTGTAGAATCTGGAAGTGGATATTTGGA

                   *   ** ** *  *   *          * *  *
      51667 GCGCTTTCAGGCCTATGCTGAAAAAGGAAATATCTACCTAC
        131 GCGCTTTGAGGATTTCGTTGGAAACGGAAATATCTTCATAT

             *                    *    *  **          ** *        *    *     
      51708 AGAAACTAGACAGAAGCATTCTGAGAATCACGTTTGTGATGTGGGTACTCAACTAACAGTGTTGA
          1 AAAAACTAGACAGAAGCATTCTCAGAAACATCTTTGTGATGTTTGCACTCAACTCACAGAGTTGA

            *  *       *   *        *   *   *                *               
      51773 TCCATTCTTTTGATACAGCAGTTTAGAACCACCCTTTTTGTAGAATCTGCAAGTGGATATTTGGA
         66 ACCTTTCTTTTCATAGAGCAGTTTTGAAACACTCTTTTTGTAGAATCTGGAAGTGGATATTTGGA

            **   *                     *   *        *
      51838 TAGCTGTGAGGATTTCGTTGGAAACGGGAATGTCTTCATAG
        131 GCGCTTTGAGGATTTCGTTGGAAACGGAAATATCTTCATAT

                **                     * ****         *  **   *   *          
      51879 AAAATTTAGACAGAAGCATTCTCAGAACCTGGATTGTGATGTGTGTTCTCCACTAACAGAGTTGA
          1 AAAAACTAGACAGAAGCATTCTCAGAAACATCTTTGTGATGTTTGCACTCAACTCACAGAGTTGA

                       * *   * *           *       *                         
      51944 ACCTTTCTTTTGACAGAACTGTTTTGAAACATTCTTTTTATAGAATCTGGAAGTGGATATTTGGA
         66 ACCTTTCTTTTCATAGAGCAGTTTTGAAACACTCTTTTTGTAGAATCTGGAAGTGGATATTTGGA

            **                         *          *  
      52009 AAGCTTTGAGGATTTCGTTGGAAACGGGAATATCTTCAAAT
        131 GCGCTTTGAGGATTTCGTTGGAAACGGAAATATCTTCATAT

                *    *            *           * *       *  *    *            
      52050 AAAATCTAGCCAGAAGCATTCTAAGAAACATCTTAGGGATGTTTACATTCAAGTCACAGAGTTGA
          1 AAAAACTAGACAGAAGCATTCTCAGAAACATCTTTGTGATGTTTGCACTCAACTCACAGAGTTGA

              *   * *    **       *         *    * *   **      *      *    * 
      52115 ACATTCCCCTTTCTCAGAGCAGGTTTGAAACAATCTTCTCGTACTATCTGGCAGTGGACATTTTG
         66 ACCTT-TCTTTTCATAGAGCAGTTTTGAAACACTCTTTTTGTAGAATCTGGAAGTGGATATTTGG

               * *   *  ** ** *  *   *            ** *
      52180 AGCTCCTTGGGGCCTATGCTGAAAAAGGAAATATCTTCCGAC
        130 AGCGCTTTGAGGATTTCGTTGGAAACGGAAATATCTTCATAT

                                **     *  **          *           **    *    
      52222 AAAAACTAGACAGAAGCATTTGCAGAATCACGTTTGTGATGTGTGCACTCAACTGTCAGAATTGA
          1 AAAAACTAGACAGAAGCATTCTCAGAAACATCTTTGTGATGTTTGCACTCAACTCACAGAGTTGA

                  *   ** *      *      *          *    *     * *            *
      52287 ACCTTTGTTTGGACAGAGCACTTTTGAGACACTCTTTTCGTAGGATCTGCAGGTGGATATTTGGC
         66 ACCTTTCTTTTCATAGAGCAGTTTTGAAACACTCTTTTTGTAGAATCTGGAAGTGGATATTTGGA

            **                         *   *      * *
      52352 TAGCTTTGAGGATTTCGTTGGAAACGGTAATGTCTTCAAAG
        131 GCGCTTTGAGGATTTCGTTGGAAACGGAAATATCTTCATAT

                *          *   *          *   *            *    *            
      52393 AAAATCTAGACAGAAACATCCTCAGAAACACCTTCGTGATGTTTGCAATCAAGTCACAGAGTTGA
          1 AAAAACTAGACAGAAGCATTCTCAGAAACATCTTTGTGATGTTTGCACTCAACTCACAGAGTTGA

                 * *             *  *         *        *             *       
      52458 ACCTTCCGTTTCATAGAGCAGGTTGGAAACACTCATTTTGTAGTATCTGGAAGTGGACATTTGGA
         66 ACCTTTCTTTTCATAGAGCAGTTTTGAAACACTCTTTTTGTAGAATCTGGAAGTGGATATTTGGA

                   *   ** ** *  *   *            **  
      52523 GCGCTTTCAGGCCTATGGTGTAAAAGGAAATATCTTCCCAT
        131 GCGCTTTGAGGATTTCGTTGGAAACGGAAATATCTTCATAT

                * * * *      **      *   * *   *     ** ** *      *    *     
      52564 AAAAGCGACATAGAAGCTATCTCAGGAACTTGTTTATGATGCCTCTAATCAACTAACAGTGTTGA
          1 AAAAACTAGACAGAAGCATTCTCAGAAACATCTTTGTGATGTTTGCACTCAACTCACAGAGTTGA

                  * ** * *                         * *       *               
      52629 ACCTTTGTACTGACAGAGCAG-TTTGAAACACTCTTTTTTTGGAATCTGCAAGTGGATATTTGGA
         66 ACCTTTCTTTTCATAGAGCAGTTTTGAAACACTCTTTTTGTAGAATCTGGAAGTGGATATTTGGA

            *                           *   *   *    
      52693 TCGCTTTGAGGATTTCGTTGGAAACGG-GAT-GC--AATAT
        131 GCGCTTTGAGGATTTCGTTGGAAACGGAAATATCTTCATAT

                **  *    *     *                 **  *   *  *    *         * 
      52730 AAAACGTACACAGCAGCATACTCAGAAA-ATACTTTGCCATATTTCCATTCAAGTCACAGAGTGG
          1 AAAAACTAGACAGAAGCATTCTCAGAAACAT-CTTTGTGATGTTTGCACTCAACTCACAGAGTTG

               *  * **            *  *               *  *             *      
      52794 AACATTCCCATTCATAGAGCAGGTTGGAAACACTCTTTTTGGAGTATCTGGAAGTGGACATTTGG
         65 AACCTTTCTTTTCATAGAGCAGTTTTGAAACACTCTTTTTGTAGAATCTGGAAGTGGATATTTGG

                    ** * * ** *  *   *        *   **  
      52859 AGCGCTTTCTGAACTATGGTGAAAAGGGAAATATGTTCCAAT
        130 AGCGCTTTGAGGATTTCGTTGGAAACGGAAATATCTTCATAT

            *     *               *      * *         **  **       *  *  * *  
      52901 GAAAACAAGACAGAAGCATTCTGAGAAACTTATTTGTGATGCGTGTCCTCAACTAACGGACTCGA
          1 AAAAACTAGACAGAAGCATTCTCAGAAACATCTTTGTGATGTTTGCACTCAACTCACAGAGTTGA

             *    **      **  * *  *  *             *   *    * * *          *
      52966 AGCTTTGGTTTCATGCAGTACTTCTGGAACACTCTTTTTGAAGATTCTGCATGCGGATATTTGGT
         66 ACCTTTCTTTTCATAGAGCAGTTTTGAAACACTCTTTTTGTAGAATCTGGAAGTGGATATTTGGA

            **                            **   *     
      53031 TAGCTTTGAGGATTTCGTTGGAAACGG---GCT-TACATAT
        131 GCGCTTTGAGGATTTCGTTGGAAACGGAAATATCTTCATAT

                 *       *               *        *   *    *    *       *    
      53068 AAAAATTAGACAGCAGCATTCTCAGAAACTTCTTTGTGGTGTCTGCATTCAAGTCACAGAATTGA
          1 AAAAACTAGACAGAAGCATTCTCAGAAACATCTTTGTGATGTTTGCACTCAACTCACAGAGTTGA

              * ** * **            *  * *      *       *    *        *       
      53133 ACATCCCCTCACATAGAGCAGTTGTGCAGCACTCTATTTGTAGTATCTCGAAGTGGACATTTGGA
         66 ACCTTTCTTTTCATAGAGCAGTTTTGAAACACTCTTTTTGTAGAATCTGGAAGTGGATATTTGGA

             *         ** *    * *   *            **  
      53198 GGGCTTTGTAGCCTATC-TGGAAAAAGGAAATATCTTCCCAT
        131 GCGCTTTG-AGGATTTCGTTGGAAACGGAAATATCTTCATAT

            *  ** *   *     * *            *   *  *   * **        *  * * *   
      53239 GAATGCGAGATAGAAGTAATCTCAGAAACATGTTTATGCTGTATCTACTCAACTAACTGTGCTGA
          1 AAAAACTAGACAGAAGCATTCTCAGAAACATCTTTGTGATGTTTGCACTCAACTCACAGAGTTGA

              *     *  *               *        *  * *       **              
      53304 ACATTTCTATTGATAGAGCAGTTTTGAGACACTCTTCTTTTGGAATCTGCTAGTGGATATTTGGA
         66 ACCTTTCTTTTCATAGAGCAGTTTTGAAACACTCTTTTTGTAGAATCTGGAAGTGGATATTTGGA

            ** *            *           *     *      
      53369 TAGATTTGAGGATTTCATTGGAAACGG-GAT-T-AT-ATAT
        131 GCGCTTTGAGGATTTCGTTGGAAACGGAAATATCTTCATAT

                 *       *               *                 **  *   *         
      53406 AAAAAGTAGACAGCAGCATTCTCAGAAACTTCTTTGTGATGTTTGCATCCAGCTCTCAGAGTTGA
          1 AAAAACTAGACAGAAGCATTCTCAGAAACATCTTTGTGATGTTTGCACTCAACTCACAGAGTTGA

              *  * *     *    *  *        * *      *   **        *  **       
      53471 ACATTCCCTTTCACAGAGTAGGTTTGAAACCCCCTTTTTATAGTGTCTGGAAGCGGGCATTTGGA
         66 ACCTTTCTTTTCATAGAGCAGTTTTGAAACACTCTTTTTGTAGAATCTGGAAGTGGATATTTGGA

            **     *   ** ** *  *   *          * *  *
      53536 ATGCTTTCAGGCCTATGCTGAAAAAGGAAATATCTACCTAC
        131 GCGCTTTGAGGATTTCGTTGGAAACGGAAATATCTTCATAT

             *                    *    *  **          ** *        *    *     
      53577 AGAAACTAGACAGAAGCATTCTGAGAATCACGTTTGTGATGTGGGTACTCAACTAACAGTGTTGA
          1 AAAAACTAGACAGAAGCATTCTCAGAAACATCTTTGTGATGTTTGCACTCAACTCACAGAGTTGA

            *  *       *   *            *   *       *        *               
      53642 TCCATTCTTTTGATACAGCAGTTTTGAACCACCCTTTTTGAAGAATCTGCAAGTGGATATTTGGA
         66 ACCTTTCTTTTCATAGAGCAGTTTTGAAACACTCTTTTTGTAGAATCTGGAAGTGGATATTTGGA

            **   *                     *   *        *
      53707 TAGCTGTGAGGATTTCGTTGGAAACGGGAATGTCTTCATAG
        131 GCGCTTTGAGGATTTCGTTGGAAACGGAAATATCTTCATAT

                **                     * ****         *  **   *   *          
      53748 AAAATTTAGACAGAAGCATTCTCAGAACCTGGATTGTGATGTGTGTTCTCCACTAACAGAGTTGA
          1 AAAAACTAGACAGAAGCATTCTCAGAAACATCTTTGTGATGTTTGCACTCAACTCACAGAGTTGA

                       * *   * *           *       *                         
      53813 ACCTTTCTTTTGACAGAACTGTTTTGAAACATTCTTTTTATAGAATCTGGAAGTGGATATTTGGA
         66 ACCTTTCTTTTCATAGAGCAGTTTTGAAACACTCTTTTTGTAGAATCTGGAAGTGGATATTTGGA

            **     *                   *          *  
      53878 AAGCTTTTAGGATTTCGTTGGAAACGGGAATATCTTCAAAT
        131 GCGCTTTGAGGATTTCGTTGGAAACGGAAATATCTTCATAT

                *    *            *           * *     * *  *    *            
      53919 AAAATCTAGCCAGAAGCATTCTAAGAAACATCTTAGGGATGTGTACATTCAAGTCACAGAGTTGA
          1 AAAAACTAGACAGAAGCATTCTCAGAAACATCTTTGTGATGTTTGCACTCAACTCACAGAGTTGA

              *   * *    **       *         *    * *   **             *    * 
      53984 ACATTCCCCTTTCTCAGAGCAGGTTTGAAACAATCTTCTCGTACTATCTGGAAGTGGACATTTTG
         66 ACCTT-TCTTTTCATAGAGCAGTTTTGAAACACTCTTTTTGTAGAATCTGGAAGTGGATATTTGG

               * *   *  ** ** *  *   *            ** *
      54049 AGCTCCTTGGGGCCTATGCTGAAAAAGGAAATATCTTCCGAC
        130 AGCGCTTTGAGGATTTCGTTGGAAACGGAAATATCTTCATAT

                                 *     *  **          *           **    *    
      54091 AAAAACTAGACAGAAGCATTCGCAGAATCACGTTTGTGATGTGTGCACTCAACTGTCAGAATTGA
          1 AAAAACTAGACAGAAGCATTCTCAGAAACATCTTTGTGATGTTTGCACTCAACTCACAGAGTTGA

                  *   ** *      *                      *     * *            *
      54156 ACCTTTGTTTGGACAGAGCACTTTTGAAACACTCTTTTTGTAGCATCTGCAGGTGGATATTTGGC
         66 ACCTTTCTTTTCATAGAGCAGTTTTGAAACACTCTTTTTGTAGAATCTGGAAGTGGATATTTGGA

            **                         *   *      * *
      54221 TAGCTTTGAGGATTTCGTTGGAAACGGTAATGTCTTCAAAG
        131 GCGCTTTGAGGATTTCGTTGGAAACGGAAATATCTTCATAT

                *          *   *          *   *            *    *            
      54262 AAAATCTAGACAGAAACATCCTCAGAAACACCTTCGTGATGTTTGCAATCAAGTCACAGAGTTGA
          1 AAAAACTAGACAGAAGCATTCTCAGAAACATCTTTGTGATGTTTGCACTCAACTCACAGAGTTGA

                 * *             *  *         *      * *     *       *       
      54327 ACCTTCCGTTTCATAGAGCAGGTTGGAAACACTCATTTTGTGGTATCTGCAAGTGGACATTTGGA
         66 ACCTTTCTTTTCATAGAGCAGTTTTGAAACACTCTTTTTGTAGAATCTGGAAGTGGATATTTGGA

                   *   ** ** *  *   *   *    *   **  
      54392 GCGCTTTCAGGCCTATGGTGTAAAAGGATATATGTTCCCAT
        131 GCGCTTTGAGGATTTCGTTGGAAACGGAAATATCTTCATAT

                * * * *      **      *   * *   *     ** ** *      *    *     
      54433 AAAAGCGACATAGAAGCTATCTCAGGAACTTGTTTATGATGCATCTAATCAACTAACAGTGTTGA
          1 AAAAACTAGACAGAAGCATTCTCAGAAACATCTTTGTGATGTTTGCACTCAACTCACAGAGTTGA

                  * ** * *                         * *       *               
      54498 ACCTTTGTACTGACAGAGCAG-TTTGAAACACTCTTTTTTTGGAATCTGCAAGTGGATATTTGGA
         66 ACCTTTCTTTTCATAGAGCAGTTTTGAAACACTCTTTTTGTAGAATCTGGAAGTGGATATTTGGA

            *                           *   *   *    
      54562 TCGCTTTGAGGATTTCGTTGGAAACGG-GAT-GC--AATAT
        131 GCGCTTTGAGGATTTCGTTGGAAACGGAAATATCTTCATAT

                **  *    *     *                 **  *   *  *    *         * 
      54599 AAAACGTACACAGCAGCATACTCAGAAA-ATACTTTGCCATATTTCCATTCAAGTCACAGAGTGG
          1 AAAAACTAGACAGAAGCATTCTCAGAAACAT-CTTTGTGATGTTTGCACTCAACTCACAGAGTTG

               *  * **            *                  *  *             *      
      54663 AACATTCCCATTCATAGAGCAGGTTTGAAACACTCTTTTTGGAGTATCTGGAAGTGGACATTTGG
         65 AACCTTTCTTTTCATAGAGCAGTTTTGAAACACTCTTTTTGTAGAATCTGGAAGTGGATATTTGG

                    ** * * ** *  *   *        *   **  
      54728 AGCGCTTTCTGAACTATGGTGAAAAAGGAAATATGTTCCAAT
        130 AGCGCTTTGAGGATTTCGTTGGAAACGGAAATATCTTCATAT

            *     *               *      * *         **  **       *  *  * *  
      54770 GAAAACAAGACAGAAGCATTCTGAGAAACTTATTTGTGATGCGTGTCCTCAACTAACGGACTCGA
          1 AAAAACTAGACAGAAGCATTCTCAGAAACATCTTTGTGATGTTTGCACTCAACTCACAGAGTTGA

             *    **      **  * *  *  *             *   *    * * *           
      54835 AGCTTTGGTTTCATGCAGTACTTCTGGAACACTCTTTTTGAAGATTCTGCATGCGGATATTTGGA
         66 ACCTTTCTTTTCATAGAGCAGTTTTGAAACACTCTTTTTGTAGAATCTGGAAGTGGATATTTGGA

            **                            **   *     
      54900 TAGCTTTGAGGATTTCGTTGGAAACGG---GCT-TACATAT
        131 GCGCTTTGAGGATTTCGTTGGAAACGGAAATATCTTCATAT

                 *       *               *        *   *    *    *       **   
      54937 AAAAATTAGACAGCAGCATTCTCAGAAACTTCTTTGTGGTGTCTGCATTCAAGTCACAGAACTGA
          1 AAAAACTAGACAGAAGCATTCTCAGAAACATCTTTGTGATGTTTGCACTCAACTCACAGAGTTGA

              * ** * **            *  * *      *       *    *        *       
      55002 ACATCCCCTCACATAGAGCAGTTGTGCAGCACTCTATTTGTAGTATCTCGAAGTGGACATTTGGA
         66 ACCTTTCTTTTCATAGAGCAGTTTTGAAACACTCTTTTTGTAGAATCTGGAAGTGGATATTTGGA

             *         ** *    * *   *            **  
      55067 GGGCTTTGTAGCCTATC-TGGAAAAAGGAAATATCTTCCCAT
        131 GCGCTTTG-AGGATTTCGTTGGAAACGGAAATATCTTCATAT

            *  ** *   *     * *            *   *  *   * **  *     * ** * *   
      55108 GAATGCGAGATAGAAGTAATCTCAGAAACATGTTTATGCTGTATCTACCCAACTAAGTGTGCTGA
          1 AAAAACTAGACAGAAGCATTCTCAGAAACATCTTTGTGATGTTTGCACTCAACTCACAGAGTTGA

              *     *  *               *        *  * *       *              *
      55173 ACATTTCTATTAATAGAGCAGTTTTGAGACACTCTTCTTTTCGAATCTGCAAGTGGATATTTGGC
         66 ACCTTTCTTTTCATAGAGCAGTTTTGAAACACTCTTTTTGTAGAATCTGGAAGTGGATATTTGGA

            ** *                        *     *      
      55238 TAGATTTGAGGATTTCGTTGGAAACGG-GAT-T-AT-ATAT
        131 GCGCTTTGAGGATTTCGTTGGAAACGGAAATATCTTCATAT

                 **      *               *                 **  *   *         
      55275 AAAAAGCAGACAGCAGCATTCTCAGAAACTTCTTTGTGATGTTTGCATCCAGCTCTCAGAGTTGA
          1 AAAAACTAGACAGAAGCATTCTCAGAAACATCTTTGTGATGTTTGCACTCAACTCACAGAGTTGA

              *  * *          *  *        * *      *   **        *  **       
      55340 ACATTCCGTTTCATAGAGTAGGTTTGAAACCCCCTTTTTATAGTGTCTGGAAGCGGGCATTTGGA
         66 ACCTTTCTTTTCATAGAGCAGTTTTGAAACACTCTTTTTGTAGAATCTGGAAGTGGATATTTGGA

                   *   ** ** *  *   *          * *  *
      55405 GCGCTTTCAGGCCTATGCTGAAAAAGGAAATATCTACCTAC
        131 GCGCTTTGAGGATTTCGTTGGAAACGGAAATATCTTCATAT

             *                    *    *  **          ** *        *    *     
      55446 AGAAACTAGACAGAAGCATTCTGAGAATCACGTTTGTGATGTGGGTACTCAACTAACAGTGTTGA
          1 AAAAACTAGACAGAAGCATTCTCAGAAACATCTTTGTGATGTTTGCACTCAACTCACAGAGTTGA

            *  *       *   *            *   *                *               
      55511 TCCATTCTTTTGATACAGCAGTTTTGAACCACCCTTTTTGTAGAATCTGCAAGTGGATATTTGGA
         66 ACCTTTCTTTTCATAGAGCAGTTTTGAAACACTCTTTTTGTAGAATCTGGAAGTGGATATTTGGA

            **   *               *     *   *        *
      55576 TAGCTGTGAGGA-TTCGTTGGGAACGGGAATTTCTTCATAG
        131 GCGCTTTGAGGATTTCGTTGGAAACGGAAATATCTTCATAT

                **                     * ****         *  **   *   *          
      55616 AAAATTTAGACAGAAGCATTCTCAGAACCTGGATTGTGATGTGTGTTCTCCACTAACAGAGTTGA
          1 AAAAACTAGACAGAAGCATTCTCAGAAACATCTTTGTGATGTTTGCACTCAACTCACAGAGTTGA

                       * *   * *           *       *                         
      55681 ACCTTTCTTTTGACAGAACTGTTTTGAAACATTCTTTTTATAGAATCTGGAAGTGGATATTTGGA
         66 ACCTTTCTTTTCATAGAGCAGTTTTGAAACACTCTTTTTGTAGAATCTGGAAGTGGATATTTGGA

            **                         *          *  
      55746 AAGCTTTGAGGATTTCGTTGGAAACGGGAATATCTTCAAAT
        131 GCGCTTTGAGGATTTCGTTGGAAACGGAAATATCTTCATAT

                *    *            *           * *     * *  *    *            
      55787 AAAATCTAGCCAGAAGCATTCTAAGAAACATCTTAGGGATGTGTACATTCAAGTCACAGAGTTGA
          1 AAAAACTAGACAGAAGCATTCTCAGAAACATCTTTGTGATGTTTGCACTCAACTCACAGAGTTGA

              *   * *    **       *         *    * *   **      *      *    * 
      55852 ACATTCCCCTTTCTCAGAGCAGGTTTGAAACAATCTTCTCGTACTATCTGGCAGTGGACATTTTG
         66 ACCTT-TCTTTTCATAGAGCAGTTTTGAAACACTCTTTTTGTAGAATCTGGAAGTGGATATTTGG

               * *   *  ** ** *  *   *            ** *
      55917 AGCTCCTTGGGGCCTATGCTGAAAAAGGAAATATCTTCCGAC
        130 AGCGCTTTGAGGATTTCGTTGGAAACGGAAATATCTTCATAT

                                 *     *  **          *           **    *    
      55959 AAAAACTAGACAGAAGCATTCGCAGAATCACGTTTGTGATGTGTGCACTCAACTGTCAGAATTGA
          1 AAAAACTAGACAGAAGCATTCTCAGAAACATCTTTGTGATGTTTGCACTCAACTCACAGAGTTGA

                  *   ** *      *                            * *            *
      56024 ACCTTTGTTTGGACAGAGCACTTTTGAAACACTCTTTTTGTAGAATCTGCAGGTGGATATTTGGC
         66 ACCTTTCTTTTCATAGAGCAGTTTTGAAACACTCTTTTTGTAGAATCTGGAAGTGGATATTTGGA

            **                         *   *      * *
      56089 TAGCTTTGAGGATTTCGTTGGAAACGGTAATGTCTTCAAAG
        131 GCGCTTTGAGGATTTCGTTGGAAACGGAAATATCTTCATAT

                *          *   *          *   *            *    *            
      56130 AAAATCTAGACAGAAACATCCTCAGAAACACCTTCGTGATGTTTGCAATCAAGTCACAGAGTTGA
          1 AAAAACTAGACAGAAGCATTCTCAGAAACATCTTTGTGATGTTTGCACTCAACTCACAGAGTTGA

                 * *             *  *         *        *             *       
      56195 ACCTTCCGTTTCATAGAGCAGGTTGGAAACACTCATTTTGTAGTATCTGGAAGTGGACATTTGGA
         66 ACCTTTCTTTTCATAGAGCAGTTTTGAAACACTCTTTTTGTAGAATCTGGAAGTGGATATTTGGA

                   *   ** ** *  *   *            **  
      56260 GCGCTTTCAGGCCTATGGTGTAAAAGGAAATATCTTCCCAT
        131 GCGCTTTGAGGATTTCGTTGGAAACGGAAATATCTTCATAT

                * * * *      **      *   * *   *     ** ** *      *    *     
      56301 AAAAGCGACATAGAAGCTATCTCAGGAACTTGTTTATGATGCATCTAATCAACTAACAGTGTTGA
          1 AAAAACTAGACAGAAGCATTCTCAGAAACATCTTTGTGATGTTTGCACTCAACTCACAGAGTTGA

                  * ** * *                         * *       *               
      56366 ACCTTTGTACTGACAGAGCAG-TTTGAAACACTCTTTTTTTGGAATCTGCAAGTGGATATTTGGA
         66 ACCTTTCTTTTCATAGAGCAGTTTTGAAACACTCTTTTTGTAGAATCTGGAAGTGGATATTTGGA

            *                        *  *   *   *    
      56430 TCGCTTTGAGGATTTCGTTGGAAACTG-GAT-GC--AATAT
        131 GCGCTTTGAGGATTTCGTTGGAAACGGAAATATCTTCATAT

                **  *    *     *                 **  *   *  *    *         * 
      56467 AAAACGTACACAGCAGCATACTCAGAAA-ATACTTTGCCATATTTCCATTCAAGTCACAGAGTGG
          1 AAAAACTAGACAGAAGCATTCTCAGAAACAT-CTTTGTGATGTTTGCACTCAACTCACAGAGTTG

               *  * **            *                 **  *             *      
      56531 AACATTCCCATTCATAGAGCAGGTTTGAAACACTCTTTTTAGAGTATCTGGAAGTGGACATTTGG
         65 AACCTTTCTTTTCATAGAGCAGTTTTGAAACACTCTTTTTGTAGAATCTGGAAGTGGATATTTGG

                    ** * * ** *  *   *            **  
      56596 AGCGCTTTCTGAACTATGGTGAAAAAGGAAATATCTTCCAAT
        130 AGCGCTTTGAGGATTTCGTTGGAAACGGAAATATCTTCATAT

            *     *               *      * *         **  **       *  *  * *  
      56638 GAAAACAAGACAGAAGCATTCTGAGAAACTTATTTGTGATGCGTGTCCTCAACTAACGGACTCGA
          1 AAAAACTAGACAGAAGCATTCTCAGAAACATCTTTGTGATGTTTGCACTCAACTCACAGAGTTGA

                   *      **  * *  *  *             *   *    * * *           
      56703 ACCTTTCGTTTCATGCAGTACTTCTGGAACACTCTTTTTGAAGATTCTGCATGCGGATATTTGGA
         66 ACCTTTCTTTTCATAGAGCAGTTTTGAAACACTCTTTTTGTAGAATCTGGAAGTGGATATTTGGA

            **   *                        **   *     
      56768 TAGCTGTGAGGATTTCGTTGGAAACGG---GCT-TACATAT
        131 GCGCTTTGAGGATTTCGTTGGAAACGGAAATATCTTCATAT

                 *       *               *        *   *    *    *       *    
      56805 AAAAATTAGACAGCAGCATTCTCAGAAACTTCTTTGTGGTGTCTGCATTCAAGTCACAGAATTGA
          1 AAAAACTAGACAGAAGCATTCTCAGAAACATCTTTGTGATGTTTGCACTCAACTCACAGAGTTGA

              * ** * **            *  * *      *       *    *        *       
      56870 ACATCCCCTCACATAGAGCAGTTGTGCAGCACTCTATTTGTAGTATCTCGAAGTGGACATTTGGA
         66 ACCTTTCTTTTCATAGAGCAGTTTTGAAACACTCTTTTTGTAGAATCTGGAAGTGGATATTTGGA

             *         ** *    * *   *            **  
      56935 GGGCTTTGTAGCCTATC-TGGAAAAAGGAAATATCTTCCCAT
        131 GCGCTTTG-AGGATTTCGTTGGAAACGGAAATATCTTCATAT

            *  ** *   *     * *            *   *  *   * **   *    *  * * *   
      56976 GAATGCGAGATAGAAGTAATCTCAGAAACATGTTTATGCTGTATCTACTAAACTAACTGTGCTGA
          1 AAAAACTAGACAGAAGCATTCTCAGAAACATCTTTGTGATGTTTGCACTCAACTCACAGAGTTGA

              *     *  *               *        *  * *       *               
      57041 ACATTTCTATTGATAGAGCAGTTTTGAGACACTCTTCTTTTGGAATCTGCAAGTGGATATTTGGA
         66 ACCTTTCTTTTCATAGAGCAGTTTTGAAACACTCTTTTTGTAGAATCTGGAAGTGGATATTTGGA

            ** *                         **    *     
      57106 TAGATTTGAGGATTTCGTTGGAAAC-G--TGAT-TACATAT
        131 GCGCTTTGAGGATTTCGTTGGAAACGGAAATATCTTCATAT

                ***      *               *                 **  *   *         
      57143 AAAATGCAGACAGCAGCATTCTCAGAAACTTCTTTGTGATGTTTGCATCCAGCTCTCAGAGTTGA
          1 AAAAACTAGACAGAAGCATTCTCAGAAACATCTTTGTGATGTTTGCACTCAACTCACAGAGTTGA

              *  * *          *  *        * *      *   **        *  **       
      57208 ACATTCCCTTTCATAGAGTAGGTTTGAAACCCCCTTTTTATAGTGTCTGGAAGCGGGCATTTGGA
         66 ACCTTTCTTTTCATAGAGCAGTTTTGAAACACTCTTTTTGTAGAATCTGGAAGTGGATATTTGGA

                   *   ** ** *  *   *          * *  *
      57273 GCGCTTTCAGGCCTATGCTGAAAAAGGAAATATCTACCTAC
        131 GCGCTTTGAGGATTTCGTTGGAAACGGAAATATCTTCATAT

             *                    *    *  **          ** *        *    *     
      57314 AGAAACTAGACAGAAGCATTCTGAGAATCACGTTTGTGATGTGGGTACTCAACTAACAGTGTTGA
          1 AAAAACTAGACAGAAGCATTCTCAGAAACATCTTTGTGATGTTTGCACTCAACTCACAGAGTTGA

            *  *       *   *        *   *   *                *               
      57379 TCCATTCTTTTGATACAGCAGTTTAGAACCACCCTTTTTGTAGAATCTGCAAGTGGATATTTGGA
         66 ACCTTTCTTTTCATAGAGCAGTTTTGAAACACTCTTTTTGTAGAATCTGGAAGTGGATATTTGGA

            **   *                     *   *        *
      57444 TAGCTGTGAGGATTTCGTTGGAAACGGGAATGTCTTCATAG
        131 GCGCTTTGAGGATTTCGTTGGAAACGGAAATATCTTCATAT

                **                     * ****         *  **   *   *          
      57485 AAAATTTAGACAGAAGCATTCTCAGAACCTGGATTGTGATGTGTGTTCTCCACTAACAGAGTTGA
          1 AAAAACTAGACAGAAGCATTCTCAGAAACATCTTTGTGATGTTTGCACTCAACTCACAGAGTTGA

                       * *   * *           *       *                         
      57550 ACCTTTCTTTTGACAGAACTGTTTTGAAACATTCTTTTTATAGAATCTGGAAGTGGATATTTGGA
         66 ACCTTTCTTTTCATAGAGCAGTTTTGAAACACTCTTTTTGTAGAATCTGGAAGTGGATATTTGGA

            **                         *          *  
      57615 AAGCTTTGAGGATTTCGTTGGAAACGGGAATATCTTCAAAT
        131 GCGCTTTGAGGATTTCGTTGGAAACGGAAATATCTTCATAT

                *    *            *           * *       *  *    *            
      57656 AAAATCTAGCCAGAAGCATTCTAAGAAACATCTTAGGGATGTTTACATTCAAGTCACAGAGTTGA
          1 AAAAACTAGACAGAAGCATTCTCAGAAACATCTTTGTGATGTTTGCACTCAACTCACAGAGTTGA

              *   * *    **       *         *    * *   **      *      *    * 
      57721 ACATTCCCCTTTCTCAGAGCAGGTTTGAAACAATCTTCTCGTACTATCTGGCAGTGGACATTTTG
         66 ACCTT-TCTTTTCATAGAGCAGTTTTGAAACACTCTTTTTGTAGAATCTGGAAGTGGATATTTGG

               * *   *  ** ** *  *   *            ** *
      57786 AGCTCCTTGGGGCCTATGCTGAAAAAGGAAATATCTTCCGAC
        130 AGCGCTTTGAGGATTTCGTTGGAAACGGAAATATCTTCATAT

                                **     *  **          *           **    *    
      57828 AAAAACTAGACAGAAGCATTTGCAGAATCACGTTTGTGATGTGTGCACTCAACTGTCAGAATTGA
          1 AAAAACTAGACAGAAGCATTCTCAGAAACATCTTTGTGATGTTTGCACTCAACTCACAGAGTTGA

                  *   ** *      *      *          *    *     * *            *
      57893 ACCTTTGTTTGGACAGAGCACTTTTGAGACACTCTTTTCGTAGGATCTGCAGGTGGATATTTGGC
         66 ACCTTTCTTTTCATAGAGCAGTTTTGAAACACTCTTTTTGTAGAATCTGGAAGTGGATATTTGGA

            **                         *   *      * *
      57958 TAGCTTTGAGGATTTCGTTGGAAACGGTAATGTCTTCAAAG
        131 GCGCTTTGAGGATTTCGTTGGAAACGGAAATATCTTCATAT

                *          *   *          *   *            *    *            
      57999 AAAATCTAGACAGAAACATCCTCAGAAACACCTTCGTGATGTTTGCAATCAAGTCACAGAGTTGA
          1 AAAAACTAGACAGAAGCATTCTCAGAAACATCTTTGTGATGTTTGCACTCAACTCACAGAGTTGA

                 * *             *  *         *        *             *       
      58064 ACCTTCCGTTTCATAGAGCAGGTTGGAAACACTCATTTTGTAGTATCTGGAAGTGGACATTTGGA
         66 ACCTTTCTTTTCATAGAGCAGTTTTGAAACACTCTTTTTGTAGAATCTGGAAGTGGATATTTGGA

                   *   ** ** *  *   *  *         **  
      58129 GCGCTTTCAGGCCTATGGTGTAAAAGGCAATATCTTCCCAT
        131 GCGCTTTGAGGATTTCGTTGGAAACGGAAATATCTTCATAT

                * * * *      **      *   * *   *     ** ** *      *    *     
      58170 AAAAGCGACATAGAAGCTATCTCAGGAACTTGTTTATGATGCCTCTAATCAACTAACAGTGTTGA
          1 AAAAACTAGACAGAAGCATTCTCAGAAACATCTTTGTGATGTTTGCACTCAACTCACAGAGTTGA

                  * ** * *                         * *       *               
      58235 ACCTTTGTACTGACAGAGCAG-TTTGAAACACTCTTTTTTTGGAATCTGCAAGTGGATATTTGGA
         66 ACCTTTCTTTTCATAGAGCAGTTTTGAAACACTCTTTTTGTAGAATCTGGAAGTGGATATTTGGA

            *                           *   *   *    
      58299 TCGCTTTGAGGATTTCGTTGGAAACGG-GAT-GC--AATAT
        131 GCGCTTTGAGGATTTCGTTGGAAACGGAAATATCTTCATAT

                **  *    *     *                 **  *   *  * *  *         * 
      58336 AAAACGTACACAGCAGCATACTCAGAAA-ATACTTTGCCATATTTCCATTAAAGTCACAGAGTGG
          1 AAAAACTAGACAGAAGCATTCTCAGAAACAT-CTTTGTGATGTTTGCACTCAACTCACAGAGTTG

               *  * **            *                  *  *             *      
      58400 AACATTCCCATTCATAGAGCAGGTTTGAAACACTCTTTTTGGAGTATCTGGAAGTGGACATTTGG
         65 AACCTTTCTTTTCATAGAGCAGTTTTGAAACACTCTTTTTGTAGAATCTGGAAGTGGATATTTGG

                    ** * * ** *  *   *            **  
      58465 AGCGCTTTCTGAACTATGGTGTAAAAGGAAATATCTTCCAAT
        130 AGCGCTTTGAGGATTTCGTTGGAAACGGAAATATCTTCATAT

            *     *               *      * *         **  **       *  *  * *  
      58507 GAAAACAAGACAGAAGCATTCTGAGAAACTTATTTGTGATGCGTGTCCTCAACTAACGGACTCGA
          1 AAAAACTAGACAGAAGCATTCTCAGAAACATCTTTGTGATGTTTGCACTCAACTCACAGAGTTGA

             *     *      **  * *  *  *             *   *    * * *          *
      58572 AGCTTTCGTTTCATGCAGTACTTCTGGAACACTCTTTTTGAAGATTCTGCATGCGGATATTTGGT
         66 ACCTTTCTTTTCATAGAGCAGTTTTGAAACACTCTTTTTGTAGAATCTGGAAGTGGATATTTGGA

            **  *                         **   *     
      58637 TAGCATTGAGGATTTCGTTGGAAACGG---GCT-TACATAT
        131 GCGCTTTGAGGATTTCGTTGGAAACGGAAATATCTTCATAT

                 *       *               *        *   *    *    *       *    
      58674 AAAAATTAGACAGCAGCATTCTCAGAAACTTCTTTGTGGTGTCTGCATTCAAGTCACAGAATTGA
          1 AAAAACTAGACAGAAGCATTCTCAGAAACATCTTTGTGATGTTTGCACTCAACTCACAGAGTTGA

              * ** * **            *  * *      *   *   *    *        *       
      58739 ACATCCCCTCACATAGAGCAGTTGTGCAGCACTCTATTTTTAGTATCTCGAAGTGGACATTTGGA
         66 ACCTTTCTTTTCATAGAGCAGTTTTGAAACACTCTTTTTGTAGAATCTGGAAGTGGATATTTGGA

             *         ** *    * *   *            **  
      58804 GGGCTTTGTAGCCTATC-TGGAAAAAGGAAATATCTTCCCAT
        131 GCGCTTTG-AGGATTTCGTTGGAAACGGAAATATCTTCATAT

            *  ** *   *     * *            *   *  *   * **        *  * * *   
      58845 GAATGCGAGATAGAAGTAATCTCAGAAACATGTTTATGCTGTATCTACTCAACTAACTGTGCTGA
          1 AAAAACTAGACAGAAGCATTCTCAGAAACATCTTTGTGATGTTTGCACTCAACTCACAGAGTTGA

              *     *  *               *        *  * *       *              *
      58910 ACATTTCTATTGATAGAGCAGTTTTGAGACACTCTTCTTTTGGAATCTGCAAGTGGATATTTGGC
         66 ACCTTTCTTTTCATAGAGCAGTTTTGAAACACTCTTTTTGTAGAATCTGGAAGTGGATATTTGGA

            ** *                        *     *      
      58975 TAGATTTGAGGATTTCGTTGGAAACGG-GAT-T-AT-ATAT
        131 GCGCTTTGAGGATTTCGTTGGAAACGGAAATATCTTCATAT

                 *       *               *                 **  *   *         
      59012 AAAAAGTAGACAGCAGCATTCTCAGAAACTTCTTTGTGATGTTTGCATCCAGCTCTCAGAGTTGA
          1 AAAAACTAGACAGAAGCATTCTCAGAAACATCTTTGTGATGTTTGCACTCAACTCACAGAGTTGA

              *  * *          *  **       * *      *   **        *  ***    * 
      59077 ACATTCCCTTTCATAGAGTAGGCTTGAAACCCCCTTTTTATAGTGTCTGGAAGCGGGCTTTTGTA
         66 ACCTTTCTTTTCATAGAGCAGTTTTGAAACACTCTTTTTGTAGAATCTGGAAGTGGATATTTGGA

                   *   ** ** *  *   *          * *  *
      59142 GCGCTTTCAGGCCTGTGCTGAAAAAGGAAATATCTACCTAC
        131 GCGCTTTGAGGATTTCGTTGGAAACGGAAATATCTTCATAT

             *                    *    *  **          ** *        *    *     
      59183 AGAAACTAGACAGAAGCATTCTGAGAATCACGTTTGTGATGTGGGTACTCAACTAACAGTGTTGA
          1 AAAAACTAGACAGAAGCATTCTCAGAAACATCTTTGTGATGTTTGCACTCAACTCACAGAGTTGA

            *  *       *   *        *   *   *                *               
      59248 TCCATTCTTTTGATACAGCAGTTTAGAACCACCCTTTTTGTAGAATCTGCAAGTGGATATTTGGA
         66 ACCTTTCTTTTCATAGAGCAGTTTTGAAACACTCTTTTTGTAGAATCTGGAAGTGGATATTTGGA

            **   *                     *   *        *
      59313 TAGCTGTGAGGATTTCGTTGGAAACGGGAATGTCTTCATAG
        131 GCGCTTTGAGGATTTCGTTGGAAACGGAAATATCTTCATAT

                **                     * ****         *  **   *   *          
      59354 AAAATTTAGACAGAAGCATTCTCAGAACCTGGATTGTGATGTGTGTTCTCCACTAACAGAGTTGA
          1 AAAAACTAGACAGAAGCATTCTCAGAAACATCTTTGTGATGTTTGCACTCAACTCACAGAGTTGA

                       * *   * *           *       *                         
      59419 ACCTTTCTTTTGACAGAACTGTTTTGAAACATTCTTTTTATAGAATCTGGAAGTGGATATTTGGA
         66 ACCTTTCTTTTCATAGAGCAGTTTTGAAACACTCTTTTTGTAGAATCTGGAAGTGGATATTTGGA

            **                         *          *  
      59484 AAGCTTTGAGGATTTCGTTGGAAACGGGAATATCTTCAAAT
        131 GCGCTTTGAGGATTTCGTTGGAAACGGAAATATCTTCATAT

                *    *            *           * *     * *  *    *            
      59525 AAAATCTAGCCAGAAGCATTCTAAGAAACATCTTAGGGATGTGTACATTCAAGTCACAGAGTTGA
          1 AAAAACTAGACAGAAGCATTCTCAGAAACATCTTTGTGATGTTTGCACTCAACTCACAGAGTTGA

              *   * *    **       *         *    * *   **      *      *    * 
      59590 ACATTCCCCTTTCTCAGAGCAGGTTTGAAACAATCTTCTCGTACTATCTGGCAGTGGACATTTTG
         66 ACCTT-TCTTTTCATAGAGCAGTTTTGAAACACTCTTTTTGTAGAATCTGGAAGTGGATATTTGG

               * *   *  ** ** *  *   *           *** *
      59655 AGCTCCTTGGGGCCTATGCTGAAAAAGGAAATATCTTTCGAC
        130 AGCGCTTTGAGGATTTCGTTGGAAACGGAAATATCTTCATAT

                                 *     *  **          *           **    *    
      59697 AAAAACTAGACAGAAGCATTCGCAGAATCACGTTTGTGATGTGTGCACTCAACTGTCAGAATTGA
          1 AAAAACTAGACAGAAGCATTCTCAGAAACATCTTTGTGATGTTTGCACTCAACTCACAGAGTTGA

                  *   ** *      *                      *     * *            *
      59762 ACCTTTGTTTGGACAGAGCACTTTTGAAACACTCTTTTTGTAGGATCTGCAGGTGGATATTTGGC
         66 ACCTTTCTTTTCATAGAGCAGTTTTGAAACACTCTTTTTGTAGAATCTGGAAGTGGATATTTGGA

            ***                        *   *      * *
      59827 TACCTTTGAGGATTTCGTTGGAAACGGTAATGTCTTCAAAG
        131 GCGCTTTGAGGATTTCGTTGGAAACGGAAATATCTTCATAT

                *          *   *          *   *            *    *            
      59868 AAAATCTAGACAGAAACATCCTCAGAAACAACTTCGTGATGTTTGCAATCAAGTCACAGAGTTGA
          1 AAAAACTAGACAGAAGCATTCTCAGAAACATCTTTGTGATGTTTGCACTCAACTCACAGAGTTGA

                 * *             *  *         *        *             *       
      59933 ACCTTCCGTTTCATAGAGCAGGTTGGAAACACTCATTTTGTAGTATCTGGAAGTGGACATTTGGA
         66 ACCTTTCTTTTCATAGAGCAGTTTTGAAACACTCTTTTTGTAGAATCTGGAAGTGGATATTTGGA

                   *   ** ** *  *   *            **  
      59998 GCGCTTTCAGGCCTATGGTGTAAAAGGAAATATCTTCCCAT
        131 GCGCTTTGAGGATTTCGTTGGAAACGGAAATATCTTCATAT

                * * ***      **   *  *   * *   *     ** ** *      *    *     
      60039 AAAAGCGACTTAGAAGCTATCTAAGGAACTTGTTTATGATGCATCTAATCAACTAACAGTGTTGA
          1 AAAAACTAGACAGAAGCATTCTCAGAAACATCTTTGTGATGTTTGCACTCAACTCACAGAGTTGA

                  * ** * *                         * *       *               
      60104 ACCTTTGTACTGACAGAGCAG-TTTGAAACACTCTTTTTTTGGAATCTGCAAGTGGATATTTGGA
         66 ACCTTTCTTTTCATAGAGCAGTTTTGAAACACTCTTTTTGTAGAATCTGGAAGTGGATATTTGGA

            *                        *  *   *   *    
      60168 TCGCTTTGAGGATTTCGTTGGAAACTG-GAT-GC--AATAT
        131 GCGCTTTGAGGATTTCGTTGGAAACGGAAATATCTTCATAT

                **  *    *     *                 **  *   *  *    *         * 
      60205 AAAACGTACACAGCAGCATACTCAGAAA-ATACTTTGCCATATTTCCATTCAAGTCACAGAGTGG
          1 AAAAACTAGACAGAAGCATTCTCAGAAACAT-CTTTGTGATGTTTGCACTCAACTCACAGAGTTG

               *  * **            *                  *  **            *      
      60269 AACATTCCCATTCATAGAGCAGGTTTGAAACACTCTTTTTGGAGTCTCTGGAAGTGGACATTTGG
         65 AACCTTTCTTTTCATAGAGCAGTTTTGAAACACTCTTTTTGTAGAATCTGGAAGTGGATATTTGG

                    ** * * ** *  *   *            **  
      60334 AGCGCTTTCTGAACTATGGTGAAAAAGGAAATATCTTCCAAT
        130 AGCGCTTTGAGGATTTCGTTGGAAACGGAAATATCTTCATAT

            *     *               *      * *         **  **       *  *  * *  
      60376 GAAAACAAGACAGAAGCATTCTGAGAAACTTATTTGTGATGCGTGTCCTCAACTAACGGACTCGA
          1 AAAAACTAGACAGAAGCATTCTCAGAAACATCTTTGTGATGTTTGCACTCAACTCACAGAGTTGA

             *     *      **  * *  *  *             *   *    * * *          *
      60441 AGCTTTCGTTTCATGCAGTACTTCTGGAACACTCTTTTTGAAGATTCTGCATGCGGATATTTGGT
         66 ACCTTTCTTTTCATAGAGCAGTTTTGAAACACTCTTTTTGTAGAATCTGGAAGTGGATATTTGGA

            **                            **   *     
      60506 TAGCTTTGAGGATTTCGTTGGAAACGG---GCT-TACATAT
        131 GCGCTTTGAGGATTTCGTTGGAAACGGAAATATCTTCATAT

                 *       *               *        *   *    *    *       * *  
      60543 AAAAATTAGACAGGAGCATTCTCAGAAACTTCTTTGTGGTGTCTGCATTCAAGTCACAGAATCGA
          1 AAAAACTAGACAGAAGCATTCTCAGAAACATCTTTGTGATGTTTGCACTCAACTCACAGAGTTGA

              * ** * **            * ** *      *       *    *        *       
      60608 ACATCCCCTCACATAGAGCAGTTGTACAGCACTCTATTTGTAGTATCTCGAAGTGGACATTTGGA
         66 ACCTTTCTTTTCATAGAGCAGTTTTGAAACACTCTTTTTGTAGAATCTGGAAGTGGATATTTGGA

             *         ** *    * *   *            **  
      60673 GGGCTTTGTAGCCTATC-TGGAAAAAGGAAATATCTTCCCAT
        131 GCGCTTTG-AGGATTTCGTTGGAAACGGAAATATCTTCATAT

            *  ** *   *     * *            *   *  *   * **        *  * * *   
      60714 GAATGCGAGATAGAAGTAATCTCAGAAACATGTTTATGCTGTATCTACTCAACTAACTGTGCTGA
          1 AAAAACTAGACAGAAGCATTCTCAGAAACATCTTTGTGATGTTTGCACTCAACTCACAGAGTTGA

              *     *  *               *        *  * *       *              *
      60779 ACATTTCTATTGATAGAGCAGTTTTGAGACACTCTTCTTTTGGAATCTGCAAGTGGATATTTGGC
         66 ACCTTTCTTTTCATAGAGCAGTTTTGAAACACTCTTTTTGTAGAATCTGGAAGTGGATATTTGGA

            ** *                        *     *      
      60844 TAGATTTGAGGATTTCGTTGGAAACGG-GAT-T-AT-ATAT
        131 GCGCTTTGAGGATTTCGTTGGAAACGGAAATATCTTCATAT

            *   **       *               *                 **  *   *         
      60881 CAAAGGTAGACAGCAGCATTCTCAGAAACTTCTTTGTGATGTTTGCATCCAGCTCTCAGAGTTGA
          1 AAAAACTAGACAGAAGCATTCTCAGAAACATCTTTGTGATGTTTGCACTCAACTCACAGAGTTGA

              *  * *     *    *  *        * *      *   **        *  **       
      60946 ACATTCCCTTTCACAGAGTAGGTTTGAAACCCCCTTTTTATAGTGTCTGGAAGCGGGCATTTGGA
         66 ACCTTTCTTTTCATAGAGCAGTTTTGAAACACTCTTTTTGTAGAATCTGGAAGTGGATATTTGGA

            *      *   ** ** *  *   *          * * **
      61011 ACGCTTTCAGGCCTATGCTGAAAAAGGAAATATCTACCTTC
        131 GCGCTTTGAGGATTTCGTTGGAAACGGAAATATCTTCATAT

             *      *             *    *  **          ** *        *    *     
      61052 AGAAACTACACAGAAGCATTCTGAGAATCACGTTTGTGATGTGGGTACTCAACTAACAGTGTTGA
          1 AAAAACTAGACAGAAGCATTCTCAGAAACATCTTTGTGATGTTTGCACTCAACTCACAGAGTTGA

            *  *       *   *            *   *                *   *           
      61117 TCCATTCTTTTGATACAGCAGTTTTGAACCACCCTTTTTGTAGAATCTGCAAGAGGATATTTGGA
         66 ACCTTTCTTTTCATAGAGCAGTTTTGAAACACTCTTTTTGTAGAATCTGGAAGTGGATATTTGGA

            **   *         *           *   *        *
      61182 TAGCTGTGAGGATTTAGTTGGAAACGGGAATGTCTTCATAG
        131 GCGCTTTGAGGATTTCGTTGGAAACGGAAATATCTTCATAT

                **                     * ****         *  **   *   *          
      61223 AAAATTTAGACAGAAGCATTCTCAGAACCTGGATTGTGATGTGTGTTCTCCACTAACAGAGTTGA
          1 AAAAACTAGACAGAAGCATTCTCAGAAACATCTTTGTGATGTTTGCACTCAACTCACAGAGTTGA

                       * *   * *           *     * *                         
      61288 ACCTTTCTTTTGACAGAACTGTTTTGAAACATTCTTTGTATAGAATCTGGAAGTGGATATTTGGA
         66 ACCTTTCTTTTCATAGAGCAGTTTTGAAACACTCTTTTTGTAGAATCTGGAAGTGGATATTTGGA

            **          *              *   *        *
      61353 AAGCTTTGAGGAATTCGTTGGAAACGGGAATGTCTTCATAG
        131 GCGCTTTGAGGATTTCGTTGGAAACGGAAATATCTTCATAT

                **                     * ****         *  *   ** *  ****   *  
      61394 AAAATTTAGACAGAAGCATTCTCAGAACCTGGATTGTGATGTGTGTA-TAGAATCTGGAAGTGGA
          1 AAAAACTAGACAGAAGCATTCTCAGAAACATCTTTGTGATGTTTGCACTCAACTCACAGAGTTGA

            ***  ****** * *   * *                  *    ****  *       **   * 
      61458 TATTTGGAAAGCTTTGAGGATTTCGTTGGAAACGGGAATATCTTCAAATAAAATCTAGCCAGAAG
         66 ACCTTTCTTTTCATAGAGCAGTT--TT-GAAAC----A-CTCTTTTTGTAGAATCT-GGAAG-TG

            *   * ** * *  *   *             * * ***  * **  ** ** 
      61523 CATTCTAAGAAACATCTTAG-GGA--T-G-TGTACATTCAAGTCAC-AGAGTT
        121 GA-TATTTGGAGC-GCTTTGAGGATTTCGTTGGAAACGGAAATATCTTCATAT

            *        *    ** *         *  **       **  *      *     *   **   
      61570 GAAAA-T--TC---CCCTTTCTCAG-AGCAGGTTTGAAACAATCTTCTCGTACT-ATCT-GGA-A
          1 AAAAACTAGACAGAAGCATTCTCAGAAACATCTTTG---TGATGTT-T-GCACTCAACTCACAGA

              *    **  **** * * * *  ** **     * ***** * * ***** ***    *  * 
      61625 GTGGACATTTTGAGCTCCTTGGGGCTATGCTGAAAAAGGAAATATCTTCCGACAAAAAGTAGACA
         61 GTTGA-ACCTTTCTTTTCATAGAGCAGTTTTGAAACACTCTTTTTGTAGAATCTGGAAGTGGATA

                *    *  *    *  **         ***   * *   *       *  
      61690 ---GAAGC-ATTCGCAGAATCACGTTTGTGATGTGTGCACTCAACTGTCAGAAT
        125 TTTGGAGCGCTTTG-AGGATTTCG-TTG-GAAACG-GAAAT-ATCT-TCA-TAT

            **  *   *   *   *       *  **                    *       *   *   
      61740 TGAACCTTTGTTTCGACAGAGCA-CCTATGAAACACTCTTT-T--TG-TAG-A---ATCT-GCAG
          1 AAAAAC-TAG--AC-AGA-AGCATTCTCAGAAACA-TCTTTGTGATGTTTGCACTCAACTCACAG

               *  ***  *** ** * *   * *                * * *  ***        **  
      61795 -GTGGATATTTGGCTAGCTTTGAGGATTTCGTTGGAAACGGTAATGTCTTCAAAGAAAATCAAGA
         60 AGTTGAACCTTTCTTTTCATAGAGCAGTT--TT-GAAAC---ACTCTTTTTGTAG--AATCTGGA

                ***   * ** * * * *  ***   *      *  *       *   *  *   
      61859 CAGAAACATCCTCAGAAACACCTTCGTGATGT--TTGCAATC---AA-GTC-ACAGAGT
        117 -AG-TGGAT-ATTTGGAGCGCTTTGAGGATTTCGTTGGAAACGGAAATATCTTCATA-T

            **  *    *   *  *       ** **         *  *  **  *    ***  *  *   
      61911 TGAACCTTCCGTTTCATAGAGCA-GGTTGGAAACACTCATTTTGTAGTAT-C-TGGAAGTGGACA
          1 AAAAAC-T-AG--ACAGA-AGCATTCTCAGAAACA-TCTTTGTGATGTTTGCACTCAACT-CACA

             ** *  *       ****    * * *  ****    *   *  ****  *  * ***      
      61973 -TTTGGAGCGCTTTCAGGCCTATGGTGTA-AAAGGAAATA-TGTTCCCATAAAAACGACATAGAA
         59 GAGTTGAAC-CTTTCTTTTC-ATAGAGCAGTTTTGAAACACTCTTTTTGTAGAATCTGGA-AG--

             **    **   * ***  * *     *    ****   **   * *   ** ** 
      62035 TCTATATCAGGAACTTGTTTATGATGCATC-TAATCAACTAACAGTGT-TGAACCT
        119 TGGATATTTGGAGCGCTTTGAGGAT--TTCGTTGGAAACGGA-AATATCTTCATAT

            ****              *    *                    * *        *  ** * * 
      62089 TTGTACT-GACAG-AGCAGT-T-TGAAACACTCTTT-T--T-TTGGAATCTGCAAGT-GGATATT
          1 AAAAACTAGACAGAAGCATTCTCAGAAACA-TCTTTGTGATGTTTGCA-CT-CAACTCACAGAGT

              *    **     *  *  *****  *     ****  *** *  *  ** **     * *   
      62145 TGGA--TCGC-TTTGA-GGATTTCGTTGGAAACGGGATGCAATATAAAACGTACACAGCAGCATA
         63 TGAACCTTTCTTTTCATAGAGCAGTTTTGAAACACTCT-TTTTGTAGAATCTGGA-AG-TGGATA

             ***  * ***      ***     **  **  *** * * ***** *  
      62206 CCCAGAAAATACTTTGCCATATTTCCAT-TCAAGTCACAGAGTGGAACAT
        125 -TTTG-GAGCGCTTTG-AGGATTTCGTTGGAAACGGAAATATCTTCATAT

            ****   ** *        ** **            *  **  *    ***  *  *    ** *
      62255 TCCCA-TTCATAG-AGCA-GGTTGGAAACACTCTTTTTGGAGTAT-C-TGGAAGTGGACA-TTTG
          1 AAAAACTAGACAGAAGCATTCTCAGAAACA-TCTTTGTGATGTTTGCACTCAACT-CACAGAGTT

              *        ***    * *   *****    *      ****        **    ***  * 
      62314 GAGCGCTTTCTGAACTATGGTG-AAAAAGGAAATA-TCTTCCAAT-GAATACAAGACACAAG-CA
         64 GAAC-CTTTCTTTTC-ATAGAGCAGTTTTGAAACACTCTTTTTGTAGAAT-CTGGA-AGTGGATA

              **    **     **      *       ** **  *  * **** **
      62375 TTCTGAGAAACTTATTTGTGA-TGCG-T-G-TCCTCAACTAACGGACTCG
        125 TTTGGAG-CGCTT-TGAG-GATTTCGTTGGAAACGGAAATATCTTCATAT

              **** * **** *     *    * *          *   * *       ** **  * * * 
      62421 AAGCTTTCGTTTCATGCAGTACTTCTGGAACACTCTTTTTGAAGATT---CTGCATGCAGATATT
          1 AAAAACTAGACAGAAGCA-TTC-TCAGAAACA-TCTTTGTGATGTTTGCACTCAACTCACAGAGT

                ** **    **** * *      *     ***    ** *  *  ** *      * *   
      62483 TG--GTTAGCTTTGAGGATATC-G-TTGGAAACGGGCTTACATATAAAAATTAGACAGCAGCATT
         63 TGAACCTTTCTTTTCATAGAGCAGTTTTGAAACACTCTT-TTTGTAGAATCTGGA-AG-TGGA-T

            * ** * *  *     *    *    *****  *** * *  *  *   *
      62544 CTCAGAAACTTCTTTGTGG-TGTCTGCATTCAAGTCACAGA-ATTGA-AC
        124 ATTTGGAGC-GCTTTGAGGATTTC-GTTGGAAACGGAAATATCTTCATAT

             ****   *   *         *      *         * *    *   *  *  *  *    *
      62591 ATCCCCT-CACATAGAGCAGTTGTGCAG-CAC-TCTATTTTTA-GTAT-CTC-GAAGTGGACA-T
          1 AAAAACTAGACAGA-AGCA-TTCT-CAGAAACATCT-TTGTGATGTTTGCACTCAACT-CACAGA

            * *   **     * ** *   ** * *****    *        **  *    * **       
      62649 TTGGAGGGCTTT-GTAGCCTATCTGGATAAAGGAAATA-TCTTCCCATGAATGCGAGATGGAAGT
         61 GTTGA-ACCTTTCTTTTCATA-GAGCAGTTTTGAAACACTCTT--TTTGTA-G-AATCTGGAAGT

             *  * ** * * ***  * * * * *** **      *  * *    ** *  
      62712 -AATCTCAGAAACATGTTTATGCTGTATCTACACAAC-TAACTGTGCTGAACAT
        120 GGATATTTGGAGCGCTTTGAGGATTTCGTTGGA-AACGGAAATAT-CTTCATAT

            ****   *  *          * *   *          *  *** *     *  *    * **  
      62764 TTCTA-TTGATAG-AGCAGTTTTGAGACAC-TCTTCTTTTGGAATCTGCAAGTGGATATTTGGCT
          1 AAAAACTAGACAGAAGCA-TTCTCAGAAACATC-T-TTGTGATGTTTGC-ACT-CA-A-CTCAC-

               *    ***    *  ** * ** * *       *     *  ***** *    *    *   
      62826 AGATTTGAGGATTTCGTTGGAAACGGGATTATATATCAAA-AGT-AGACAGCAGCATTCTCAGAA
         58 AGAGTTGAACCTTTCTTTTCATAGAGCAGT-T-T-TGAAACACTCTTTTTGTAG-AATCT-GGAA

              *** *        *         ** **    **   ***    *    * *   
      62889 --ACTTCTTTGTGA-TG-TTTGCATCCAGCTC-TCAG-AGTTG-AACAT-TCCGT-T
        118 GTGGATATTTG-GAGCGCTTTG-A-GGATTTCGTTGGAAACGGAAATATCTTCATAT

              **         *   *           ** *  * *     *   **** * ***   ** * 
      62937 --TCA-TAG--AGTAG-GTT-T--GAAACCCCCTTTTTATAGTGT-CTGGAAGCGGGCA-TTTGG
          1 AAAAACTAGACAGAAGCATTCTCAGAAACATCTTTGTGAT-GTTTGCACTCAACTCACAGAGTTG

             *       ***      *     *****    *     *** **    *  *      * *   
      62991 AGCGCTTTCAGGTC-T-GTGCTGAAAAAGGAAATA-TCTACCTACAGAAACTAGACAGAAGCAT-
         65 AAC-CTTTCTTTTCATAGAGC--AGTTTTGAAACACTCTTTTTGTAGAATCTGGA-AG-TGGATA

             *      * * *  ***     **     **      * ***             **
      63052 TCTGAGAATCACGTTTTTGATGTGGGTACTCAACTAACAGTGTTGATCCATTCTATTG
        125 TTTG-G-AGCGCTTTGAGGAT-TTCGT--TGGA--AACGGAAAT-AT-C-TTC-ATAT

             * * **   ***   *    * * ** * **      **      *    *     *  ***  
      63110 ATACAGCAGTTTTGAACCACCCTTTTTGTAGAATCTGCAAGTGGATATTTGGA-T-AGCTGTGAG
          1 AAAAACTAG-ACAGAAGCA--TTCTCAGAAACATCT--TTGT-GATGTTTGCACTCAACTCACA-

                             *** * *         * *   *  * * ** *  *  *    *    
      63173 GATTTCGTTGGAAACGGGAATGTCTTCATAGA-AAATTTAGACAGAAGCATTCT-CAGAACCTGG
         59 GA----GTT-G-AAC--CTTTCTTTTCATAGAGCAGTTTTGAAACACTCTTTTTGTAGAATCTGG

              *       * *       ** *   **    ***    *                     *  
      63236 ATTGTGATGTGTGTTCTCCACTAACAGAGTTGAACCTTTCTTTGGACAGAACTGTTTTGAAACAT
        116 A-AGTG--G-ATATT-T---GGAGC-GCTTTGAGGATTTCGTTGG--A-AAC-G----GAAATA-

                **   
      63301 TCTTTTTAT
        163 TCTTCATAT

             *  *    **   * *  **  * *    *      *      * *****   **** *  *  
      63310 AGAATCT-GTAAG-TGGATAGT-TGGAA-AGCTTTGAGGAT-TTCGTTGGAAACGGGAATATCTT
          1 AAAAACTAGACAGAAGCATTCTCAGAAACATCTTTG-TGATGTTTGCACTCAACTCACAGA-GTT

            *    ****   ***  *      *   **           ***    * * ** **     ** 
      63370 CAAGTAAAATCTAGCCAGA-AGCATTCTAAGAAACA-TCTTAGGGATGTGTACATTCAAGTCACA
         64 GAA--CCTTTCTTTTCATAGAGCAGT-TTTGAAACACTCTTTTTG-T-AGAATCTGGAAGT-GGA

            * *       * *   ****      **   * *         *      *** 
      63433 GAGTT-GA--ACATT-CCCCTTTC-TCAGAGCAGGTTTGAAACAATCTTCTCGT
        123 TATTTGGAGCGCTTTGAGGATTTCGTTGGA-AACG---GAAA-TATCTTCATAT

             ** *    *   **         *   *    *   * *** *   ****  *** * *  *  
      63482 ACTATCT-GGCAGTGGACATT-T-TG-AGC-TCCTTGGGGCCTATGCTGAAAAAGGAAATATCTT
          1 AAAAACTAGACAGAAG-CATTCTCAGAAACATCTTTGTGATGTTTGCACTCAACTCACAGA-GTT

                ******  ***  *      *  ***   *     *   **    * *     *  *   *
      63542 CCGACAAAAACTAGACAGA-AGCATTCGCAGAATCACGTTTGTGATGTGTGCA-CT-CAACTGTC
         64 --GAACCTTTCTTTTCATAGAGCAGT-TTTGAAACAC-TCT-TTTTGT-AGAATCTGGAAGTG-G

             * *     *              * ***   *     *    *      ** * 
      63604 AGAATT-GAAC-CTTT---G-TTTGGACAGAGCACTTTTGAAACACTCTTTTTGT
        122 ATATTTGGAGCGCTTTGAGGATTTCGTTGGA-AAC---GGAAATA-TCTTCATAT

             ** *        ** *   * **  *** *      *      * *****    ** ** **  
      63653 AGGATCT-G-CAGGTGGA-TATTTG-GCTAGCTTTGAGGAT-TTCGTTGGAAACGGTAATGTCTT
          1 AAAAACTAGACAGAAGCATTCTCAGAAACATCTTTG-TGATGTTTGCACTCAAC-TCACAGAGTT

            *  ****** * ** *    *   ** **           * **    *              **
      63713 CAAAGAAAATCTAGACAGAAACATCCTCAGAAACACCTTCGTGATGTTTGCAATC---AAGTCAC
         64 GAACCTTTCTTTTCATAG-AGCA-GTTTTGAAACA-C-TCTTTTTG-TAG-AATCTGGAAGT-GG

             * *   * *   *     *  *  ****         ****  * ** ** * 
      63775 AGAGTTGAACCTTCCGTTTCATAGAGCAGGTTGGAAACACTCATTTTGTAGTGT
        122 ATATTTGGAGC--GC-TTTGA-GGATTTCGTTGGAAACGGAAATATCTTCATAT

             * *  * *        *   ** *    ****           * **  *    * ** *  **
      63829 CTAGAAGTGGACATTTGGAGCGCTTTCAGGCCTATGCTGTAAAAGGAAATATCTTCCCATAAAAG
          1 -AAAAACTAGACA---GAAGCATTCTCAGAAACAT-CT-T----TGTGATGT-TTGCACTCAACT

              * *   *   *  *         *  *  *        **         ****   * * * *
      63894 C-GACA-TAGAAGCTATC---TCA-GGAACTTGTTTATGATGC-CTCTAATCAACTAACAGTGTT
         55 CACAGAGTTGAACCTTTCTTTTCATAGAGC-AGTTT-TGAAACACTCT--TTTTGT-AGAATCTG

                * ***     ***    **      **       **      * *  *    * **    
      63952 GAACCTTTGTACTGACAGAGCAGTTTGAAACACTCTTTTTTTGGAATCTG-CA-A-GTGGATAT
        115 GAA-GTGGATA-T-TTGGAGCGCTTTG-AGGA---TTTCGTTGGAAACGGAAATATCTTCATAT

            ****   *  ***  * **     **               * *** *  ***     *      
      64013 TTGGA-TCGCTTTGAGGATTTCGTTGGAAACGGGATGCAATATAAAACGTACACAGCAGCATACT
          1 AAAAACTAG-ACAGAAGCATTC-TCAGAAAC---AT-C--TTTGTGATGTTTGCA-C-TCA-ACT

                 * *  *    * **    **        **    **  *    *   **  **       
      64077 CAGAAAATACTTTGCCATATTTCCATTCA-AGTCACAG-AGTGGAACATTCCCATTCATAGAGCA
         55 C---ACAGA-GTTG-AACCTTTCTTTTCATAG-AGCAGTTTTGAAACACT-CTTTTTGTAGA--A

               *  *  ***** *        **    ** *        *  *  **** *   *   ** *
      64140 GGTTTGAAACACTCTTTTTGGAGTATCTGGAAGTGGACATTTGGAGTGCTTTCTGAACTATGGTG
        111 --TCTGGAAGTGGATATTTGGAG-CGCT--TTGAGG--ATTTCG-TTGGAAACGGAAATATCTTC

             * *
      64205 AAAA
        168 ATAT

            ***  *      *        *   ***     *      **   * * *     *      ***
      64209 GGGAAATATGTTCCAATGAAAACA-AGACAGAAGCATTCTGAGAAAGTTATTTGTGATGCGTGTC
          1 AAAAACTA-G--AC-A-G-AAGCATTCTCAGAAACA-TCTTTG--TGATGTTTG-CA--C-TCAA

                *** ***  ** *****     * **              *             *   *  
      64273 CTCAACTAACGGACTCGAAGCTTTCGTTTCATGCAGTACTTCTGGAACACTCTTTTTGAAGATTC
         53 CTCACAGAGTTGAACCTTTCTTTTC-ATAGA-GCAGT--TT-TGAAACACTCTTTTTGTAGAATC

              * * *          ***              *             **   *     
      64338 TGCATGCGGATATTTGGTTAGCTTTGAGGATTTCATTGGAAACGG---GCT-TACATAT
        113 TGGAAGTGGATATTTGGAGCGCTTTGAGGATTTCGTTGGAAACGGAAATATCTTCATAT

                 *       *               *        *   *    *    *       *    
      64393 AAAAATTAGACAGCAGCATTCTCAGAAACTTCTTTGTGGTGTCTGCATTCAAGTCACAGAATTGA
          1 AAAAACTAGACAGAAGCATTCTCAGAAACATCTTTGTGATGTTTGCACTCAACTCACAGAGTTGA

              * ** * **            *    *      *       *    *        *       
      64458 ACATCCCCTCACATAGAGCAGTTGTGAAGCACTCTATTTGTAGTATCTCGAAGTGGACATTTGGA
         66 ACCTTTCTTTTCATAGAGCAGTTTTGAAACACTCTTTTTGTAGAATCTGGAAGTGGATATTTGGA

             *         ** *    * *   *           ***  
      64523 GGGCTTTGTAGCCTATC-TGGAAAAAGGAAATATCTTTCCAT
        131 GCGCTTTG-AGGATTTCGTTGGAAACGGAAATATCTTCATAT

            *  ** *   *     * *            *   *  *   * **        *  * * *   
      64564 GAATGCGAGATAGAAGTAATCTCAGAAACATGTTTATGCTGTATCTACTCAACTAACTGTGCTGA
          1 AAAAACTAGACAGAAGCATTCTCAGAAACATCTTTGTGATGTTTGCACTCAACTCACAGAGTTGA

              *     *  *               *        *  * *       *              *
      64629 ACATTTCTATTGATAGAGCAGTTTTGAGACACTCTTCTTTTGGAATCTGCAAGTGGATATTTGGC
         66 ACCTTTCTTTTCATAGAGCAGTTTTGAAACACTCTTTTTGTAGAATCTGGAAGTGGATATTTGGA

            ** *                        *     *      
      64694 TAGATTTGAGGATTTCGTTGGAAACGG-GAT-T-AT-ATAT
        131 GCGCTTTGAGGATTTCGTTGGAAACGGAAATATCTTCATAT

            *   **       *               *                 **  *   *         
      64731 CAAAGGTAGACAGCAGCATTCTCAGAAACTTCTTTGTGATGTTTGCATCCAGCTCTCAGAGTTGA
          1 AAAAACTAGACAGAAGCATTCTCAGAAACATCTTTGTGATGTTTGCACTCAACTCACAGAGTTGA

              *  * *          *  *        *        *   **        *  **       
      64796 ACATTCCCTTTCATAGAGTAGGTTTGAAACCCTCTTTTTATAGTGTCTGGAAGCGGGCATTTGGA
         66 ACCTTTCTTTTCATAGAGCAGTTTTGAAACACTCTTTTTGTAGAATCTGGAAGTGGATATTTGGA

                   *   ** ** *  *   *          * *  *
      64861 GCGCTTTCAGGCCTATGCTGAAAAAGGAAATATCTACCTAC
        131 GCGCTTTGAGGATTTCGTTGGAAACGGAAATATCTTCATAT

             *                    *    *  **          ** *        *    *     
      64902 AGAAACTAGACAGAAGCATTCTGAGAATCACGTTTGTGATGTGGGTACTCAACTAACAGTGTTGA
          1 AAAAACTAGACAGAAGCATTCTCAGAAACATCTTTGTGATGTTTGCACTCAACTCACAGAGTTGA

            *  *       **  *            *   *                *               
      64967 TCCATTCTTTTGGTACAGCAGTTTTGAACCACCCTTTTTGTAGAATCTGCAAGTGGATATTTGGA
         66 ACCTTTCTTTTCATAGAGCAGTTTTGAAACACTCTTTTTGTAGAATCTGGAAGTGGATATTTGGA

            **   *                     *   *        *
      65032 TAGCTGTGAGGATTTCGTTGGAAACGGGAATGTCTTCATAG
        131 GCGCTTTGAGGATTTCGTTGGAAACGGAAATATCTTCATAT

                **                     * ****         *  **   *   *          
      65073 AAAATTTAGACAGAAGCATTCTCAGAACCTGGATTGTGATGTGTGTTCTCCACTAACAGAGTTGA
          1 AAAAACTAGACAGAAGCATTCTCAGAAACATCTTTGTGATGTTTGCACTCAACTCACAGAGTTGA

                      ** *   * *           *       *                         
      65138 ACCTTTCTTTGGACAGAACTGTTTTGAAACATTCTTTTTATAGAATCTGGAAGTGGATATTTGGA
         66 ACCTTTCTTTTCATAGAGCAGTTTTGAAACACTCTTTTTGTAGAATCTGGAAGTGGATATTTGGA

            **          *              *          *  
      65203 AAGCTTTGAGGAATTCGTTGGAAACGGGAATATCTTCAAAT
        131 GCGCTTTGAGGATTTCGTTGGAAACGGAAATATCTTCATAT

                *    *            *           * *       *  *    *            
      65244 AAAATCTAGCCAGAAGCATTCTAAGAAACATCTTAGGGATGTTTACATTCAAGTCACAGAGTTGA
          1 AAAAACTAGACAGAAGCATTCTCAGAAACATCTTTGTGATGTTTGCACTCAACTCACAGAGTTGA

              *   * *    **       *         *    * *   **      *      *    * 
      65309 ACATTCCCCTTTCTCAGAGCAGGTTTGAAACAATCTTCTCGTACTATCTGGCAGTGGACATTTTG
         66 ACCTT-TCTTTTCATAGAGCAGTTTTGAAACACTCTTTTTGTAGAATCTGGAAGTGGATATTTGG

               * *   *  ** ** *  *   *            ** *
      65374 AGCTCCTTGGGGCCTATGCTGAAAAAGGAAATATCTTCCGAC
        130 AGCGCTTTGAGGATTTCGTTGGAAACGGAAATATCTTCATAT

                                 *     *  **          *           **    *    
      65416 AAAAACTAGACAGAAGCATTCGCAGAATCACGTTTGTGATGTGTGCACTCAACTGTCAGAATTGA
          1 AAAAACTAGACAGAAGCATTCTCAGAAACATCTTTGTGATGTTTGCACTCAACTCACAGAGTTGA

                  *   ** *      *                            * *            *
      65481 ACCTTTGTTTGGACAGAGCACTTTTGAAACACTCTTTTTGTAGAATCTGCAGGTGGATATTTGGC
         66 ACCTTTCTTTTCATAGAGCAGTTTTGAAACACTCTTTTTGTAGAATCTGGAAGTGGATATTTGGA

            **                         *   *      * *
      65546 TAGCTTTGAGGATTTCGTTGGAAACGGTAATGTCTTCAAAG
        131 GCGCTTTGAGGATTTCGTTGGAAACGGAAATATCTTCATAT

                *          *   *          *   *            *    *            
      65587 AAAATCTAGACAGAAACATCCTCAGAAACACCTTCGTGATGTTTGCAATCAAGTCACAGAGTTGA
          1 AAAAACTAGACAGAAGCATTCTCAGAAACATCTTTGTGATGTTTGCACTCAACTCACAGAGTTGA

                 * *             *  *         *        *             *       
      65652 ACCTTCCGTTTCATAGAGCAGGTTGGAAACACTCATTTTGTAGTATCTGGAAGTGGACATTTGGA
         66 ACCTTTCTTTTCATAGAGCAGTTTTGAAACACTCTTTTTGTAGAATCTGGAAGTGGATATTTGGA

                   *   ** ** *  *   *            **  
      65717 GCGCTTTCAGGCCTATGGTGTAAAAGGAAATATCTTCCCAT
        131 GCGCTTTGAGGATTTCGTTGGAAACGGAAATATCTTCATAT

                * * * *      **      *   * *   *     **  * *      *    *     
      65758 AAAAGCGACATAGAAGCTATCTCAGGAACTTGTTTATGATGCATGTAATCAACTAACAGTGTTGA
          1 AAAAACTAGACAGAAGCATTCTCAGAAACATCTTTGTGATGTTTGCACTCAACTCACAGAGTTGA

                  * ** * *                         * *       *               
      65823 ACCTTTGTACTGACAGAGCAG-TTTGAAACACTCTTTTTTTGGAATCTGCAAGTGGATATTTGGA
         66 ACCTTTCTTTTCATAGAGCAGTTTTGAAACACTCTTTTTGTAGAATCTGGAAGTGGATATTTGGA

            *                           *   *   *    
      65887 TCGCTTTGAGGATTTCGTTGGAAACGG-GAT-GC--AATAT
        131 GCGCTTTGAGGATTTCGTTGGAAACGGAAATATCTTCATAT

                **  *    *     *                 **  *   *  *    *         * 
      65924 AAAACGTACACAGCAGCATACTCAGAAA-ATACTTTGCCATATTTCCATTCAAGTCACAGAGTGG
          1 AAAAACTAGACAGAAGCATTCTCAGAAACAT-CTTTGTGATGTTTGCACTCAACTCACAGAGTTG

               *  * **            *                  *  **            *      
      65988 AACATTCCCATTCATAGAGCAGGTTTGAAACACTCTTTTTGGAGTCTCTGGAAGTGGACATTTGG
         65 AACCTTTCTTTTCATAGAGCAGTTTTGAAACACTCTTTTTGTAGAATCTGGAAGTGGATATTTGG

                   *** * * ** *  *   *            **  
      66053 AGCGCTTACTGAACTATGGTGAAAAAGGAAATATCTTCCAAT
        130 AGCGCTTTGAGGATTTCGTTGGAAACGGAAATATCTTCATAT

            *     *               *     ** *         **  **       *  *  * ** 
      66095 GAAAACAAGACAGAAGCATTCTGAGAAAATTATTTGTGATGCGTGTCCTCAACTAACGGACTCAA
          1 AAAAACTAGACAGAAGCATTCTCAGAAACATCTTTGTGATGTTTGCACTCAACTCACAGAGTTGA

                   *      **  * *  *  *             *   *    * * *          *
      66160 ACCTTTCGTTTCATGCAGTACTTCTGGAACACTCTTTTTGAAGATTCTGCACGCGGATATTTGGT
         66 ACCTTTCTTTTCATAGAGCAGTTTTGAAACACTCTTTTTGTAGAATCTGGAAGTGGATATTTGGA

            **                            **   *     
      66225 TAGCTTTGAGGATTTCGTTGGAAACGG---GCT-TACATAT
        131 GCGCTTTGAGGATTTCGTTGGAAACGGAAATATCTTCATAT

                 *       *               **       *   *   **    *   *   *    
      66262 AAAAATTAGACAGCAGCATTCTCAGAAACTACTTTGTGGTGTCTGCTTTCAAGTCAGAGAATTGA
          1 AAAAACTAGACAGAAGCATTCTCAGAAACATCTTTGTGATGTTTGCACTCAACTCACAGAGTTGA

              * ** * **  *         *  * *      *       *    *        *       
      66327 ACATCCCCTCACACAGAGCAGTTGTGCAGCACTCTATTTGTAGTATCTCGAAGTGGACATTTGGA
         66 ACCTTTCTTTTCATAGAGCAGTTTTGAAACACTCTTTTTGTAGAATCTGGAAGTGGATATTTGGA

             *         *     *         *            **  
      66392 GGGCTTT---GTTTTCTATCTGGAAAAAGGAAATATCTTCCCAT
        131 GCGCTTTGAGGATTTC-GT-TGG-AAACGGAAATATCTTCATAT

            *  ** * * *     * *            *   *  *   * **        * ** * *   
      66433 GAATGCGACATAGAAGTAATCTCAGAAACATGTTTATGCTGTATCTACTCAACTAAGTGTGCTGA
          1 AAAAACTAGACAGAAGCATTCTCAGAAACATCTTTGTGATGTTTGCACTCAACTCACAGAGTTGA

              *     *  *               *        ** * *       *               
      66498 ACATTTCTATTGATAGAGCAGTTTTGAGACACTCTTCATTTGGAATCTGCAAGTGGATATTTGGA
         66 ACCTTTCTTTTCATAGAGCAGTTTTGAAACACTCTTTTTGTAGAATCTGGAAGTGGATATTTGGA

            ** *            *           *     *   *  
      66563 TAGATTTGAGGATTTCTTTGGAAACGG-GAT-T-AT-ACAT
        131 GCGCTTTGAGGATTTCGTTGGAAACGGAAATATCTTCATAT

                 *       *               *        *   *    *    *       *    
      66600 AAAAAGTAGACAGCAGCATTCTCAGAAACTTCTTTGTGGTGTCTGCATTCAAGTCACAGAATTGA
          1 AAAAACTAGACAGAAGCATTCTCAGAAACATCTTTGTGATGTTTGCACTCAACTCACAGAGTTGA

              * ** * **            *  * *      *       *    *  *     *       
      66665 ACATCCCCTCACATAGAGCAGTTGTGCAGCACTCTATTTGTAGTATCTCGATGTGGACATTTGGA
         66 ACCTTTCTTTTCATAGAGCAGTTTTGAAACACTCTTTTTGTAGAATCTGGAAGTGGATATTTGGA

             *         ** *    * *   *            **  
      66730 GGGCTTTGTAGCCTATC-TGGAAAAAGGAAATATCTTCCCAT
        131 GCGCTTTG-AGGATTTCGTTGGAAACGGAAATATCTTCATAT

            *  ** *   *     * *            *   *  *   * **        *  * * *   
      66771 GAATGCGAGATAGAAGTAATCTCAGAAACATGTTTATGCTGTATCTACTCAACTAACTGTGCTGA
          1 AAAAACTAGACAGAAGCATTCTCAGAAACATCTTTGTGATGTTTGCACTCAACTCACAGAGTTGA

              *     *  *               *        *  * *       *               
      66836 ACATTTCTATTGATAGAGCAGTTTTGAGACACTCTTCTTTTGGAATCTGCAAGTGGATATTTGGA
         66 ACCTTTCTTTTCATAGAGCAGTTTTGAAACACTCTTTTTGTAGAATCTGGAAGTGGATATTTGGA

            ** *                        *     *      
      66901 TAGATTTGAGGATTTCGTTGGAAACGG-GAT-T-AT-ATAT
        131 GCGCTTTGAGGATTTCGTTGGAAACGGAAATATCTTCATAT

            *    *       *               *                 **  *   * *       
      66938 GAAAAGTAGACAGCAGCATTCTCAGAAACTTCTTTGTGATGTTTGCATCCAGCTCTCCGAGTTGA
          1 AAAAACTAGACAGAAGCATTCTCAGAAACATCTTTGTGATGTTTGCACTCAACTCACAGAGTTGA

               *               *  *        * *      *   **        *  **      
      67003 A-CATTCTCTTTCATAGAGTAGGTTTGAAACCCCCTTTTTATAGTGTCTGGAAGCGGGCATTTGG
         66 ACCTTTCT-TTTCATAGAGCAGTTTTGAAACACTCTTTTTGTAGAATCTGGAAGTGGATATTTGG

                    *   ** ** *  *   *          * *  *
      67067 AGCGCTTTCAGGCCTATGCTGAAAAAGGAAATATCTACCTAC
        130 AGCGCTTTGAGGATTTCGTTGGAAACGGAAATATCTTCATAT

             *         *      *   *    *  **          ** *        *    *     
      67109 AGAAACTAGACTGAAGCAATCTGAGAATCACGTTTGTGATGTGGGTACTCAACTAACAGTGTTGA
          1 AAAAACTAGACAGAAGCATTCTCAGAAACATCTTTGTGATGTTTGCACTCAACTCACAGAGTTGA

            *  *       *   *            *   *                *               
      67174 TCCATTCTTTTGATACAGCAGTTTTGAACCACCCTTTTTGTAGAATCTGCAAGTGGATATTTGGA
         66 ACCTTTCTTTTCATAGAGCAGTTTTGAAACACTCTTTTTGTAGAATCTGGAAGTGGATATTTGGA

            **   *  *                  *   *        *
      67239 TAGCTGTGGGGATTTCGTTGGAAACGGGAATGTCTTCATAG
        131 GCGCTTTGAGGATTTCGTTGGAAACGGAAATATCTTCATAT

                **                     * * **         *  **   *   *        * 
      67280 AAAATTTAGACAGAAGCATTCTCAGAACCTTGATTGTGATGTGTGTTCTCCACTAACAGAGTTCA
          1 AAAAACTAGACAGAAGCATTCTCAGAAACATCTTTGTGATGTTTGCACTCAACTCACAGAGTTGA

                       * *   * *           *       *                         
      67345 ACCTTTCTTTTGACAGAACTGTTTTGAAACATTCTTTTTATAGAATCTGGAAGTGGATATTTGGA
         66 ACCTTTCTTTTCATAGAGCAGTTTTGAAACACTCTTTTTGTAGAATCTGGAAGTGGATATTTGGA

            **              *         **          *  
      67410 AAGCTTTGAGGATTTCATTGGAAACGAGAATATCTTCAAAT
        131 GCGCTTTGAGGATTTCGTTGGAAACGGAAATATCTTCATAT

                *    *            *           * *       *  *    *            
      67451 AAAATCTAGCCAGAAGCATTCTAAGAAACATCTTAGGGATGTTTACATTCAAGTCACAGAGTTGA
          1 AAAAACTAGACAGAAGCATTCTCAGAAACATCTTTGTGATGTTTGCACTCAACTCACAGAGTTGA

              *  * *     *       *         *    * *   **      *      *    *  
      67516 ACATTCCCTTTCACAGAGCAGGTTTGAAACAATCTTCTCGTACTATCTGGCAGTGGACATTTTGA
         66 ACCTTTCTTTTCATAGAGCAGTTTTGAAACACTCTTTTTGTAGAATCTGGAAGTGGATATTTGGA

              *     *  ** ** *  *   *            ** *
      67581 GCTCTTTGGGGTCTATGCTGAAAAAGGAAATATCTTCCAAC
        131 GCGCTTTGAGGATTTCGTTGGAAACGGAAATATCTTCATAT

                                 *     *  **          *           **    *    
      67622 AAAAACTAGACAGAAGCATTCGCAGAATCACGTTTGTGATGTGTGCACTCAACTGTCAGAATTGA
          1 AAAAACTAGACAGAAGCATTCTCAGAAACATCTTTGTGATGTTTGCACTCAACTCACAGAGTTGA

                  *   ** *      **                           * *            *
      67687 ACCTTTGTTTGGACAGAGCACATTTGAAACACTCTTTTTGTAGAATCTGCAGGTGGATATTTGGC
         66 ACCTTTCTTTTCATAGAGCAGTTTTGAAACACTCTTTTTGTAGAATCTGGAAGTGGATATTTGGA

            **         *               *   *      * *
      67752 TAGCTTTGAGGTTTTCGTTGGAAACGGTAATGTCTTCAAAG
        131 GCGCTTTGAGGATTTCGTTGGAAACGGAAATATCTTCATAT

                *          *   *          *   *           **    *            
      67793 AAAATCTAGACAGAAACATCCTCAGAAACAACTTCGTGATGTTTGCCATCAAGTCACAGAGTTGA
          1 AAAAACTAGACAGAAGCATTCTCAGAAACATCTTTGTGATGTTTGCACTCAACTCACAGAGTTGA

                 * *             *  *                  *             *       
      67858 ACCTTCCGTTTCATAGAGCAGGTTGGAAACACTCTTTTTGTAGTATCTGGAAGTGGACATTTGGA
         66 ACCTTTCTTTTCATAGAGCAGTTTTGAAACACTCTTTTTGTAGAATCTGGAAGTGGATATTTGGA

                   *   ** ** *  *   *            **  
      67923 GCGCTTTCAGGCCTATGGTGTAAAAGGAAATATCTTCCCAT
        131 GCGCTTTGAGGATTTCGTTGGAAACGGAAATATCTTCATAT

                * * * *    * **      *     *   *      * ** *      *    *     
      67964 AAAAGCGACATAGAACCTATCTCAGGAACATGTTTATGATGTATCTAATCAACTAACAGTGTTGA
          1 AAAAACTAGACAGAAGCATTCTCAGAAACATCTTTGTGATGTTTGCACTCAACTCACAGAGTTGA

                  * ** * *                                    *              
      68029 ACCTTTGTACTGACAGAGCAG-TTTGAAACACTCTTTTT-TATGAATCTGCAAGTGGATATTTGG
         66 ACCTTTCTTTTCATAGAGCAGTTTTGAAACACTCTTTTTGTA-GAATCTGGAAGTGGATATTTGG

             *                           **  *   *    
      68092 ATCGCTTTGAGGATTTCGTTGGAAACGG-GGT-GC--AATAT
        130 AGCGCTTTGAGGATTTCGTTGGAAACGGAAATATCTTCATAT

                **  *    *     *                 **  *   *  *    *         * 
      68130 AAAACGTACACAGCAGCATACTCAGAAA-ATACTTTGCCATATTTCCATTCAAGTCACAGAGTGG
          1 AAAAACTAGACAGAAGCATTCTCAGAAACAT-CTTTGTGATGTTTGCACTCAACTCACAGAGTTG

               *  * **            *                  *  **            *      
      68194 AACATTCCCATTCATAGAGCAGGTTTGAAACACTCTTTTTGGAGTGTCTGGAAGTGGACATTTGG
         65 AACCTTTCTTTTCATAGAGCAGTTTTGAAACACTCTTTTTGTAGAATCTGGAAGTGGATATTTGG

                  * ** * * ** *  *   *            **  
      68259 AGCGCTATCTGAACTATGGTGAAAAAGGAAATATCTTCCAAT
        130 AGCGCTTTGAGGATTTCGTTGGAAACGGAAATATCTTCATAT

            *     *               *      * **        **  **       *  *  * *  
      68301 GAAAACAAGACAGAAGCATTCTGAGAAACTTACTTGTGATGCGTGTCCTCAACTAACGGACTCGA
          1 AAAAACTAGACAGAAGCATTCTCAGAAACATCTTTGTGATGTTTGCACTCAACTCACAGAGTTGA

                   **     **  * *  *  *             *   *    * * *           
      68366 ACCTTTCGATTCATGCAGTACTTCTGGAACACTCTTTTTGAAGATTCTGCATGCGGATATTTGGA
         66 ACCTTTCTTTTCATAGAGCAGTTTTGAAACACTCTTTTTGTAGAATCTGGAAGTGGATATTTGGA

            **                 *    *     **   *     
      68431 TAGCTTTGAGGATTTCGTTTGAAATGG---GCT-TACATAT
        131 GCGCTTTGAGGATTTCGTTGGAAACGGAAATATCTTCATAT

                 *       *               *        *   *    *    *       *    
      68468 AAAAATTAGACAGCAGCATTCTCAGAAACTTCTTTGTGGTGTCTGCATTCAAGTCACAGAATTGA
          1 AAAAACTAGACAGAAGCATTCTCAGAAACATCTTTGTGATGTTTGCACTCAACTCACAGAGTTGA

              * *  * **            *  * *      *       *    *    *   *       
      68533 ACATCTCCTCACATAGAGCAGTTGTGCAGCACTCTATTTGTAGTATCTCGAAGAGGACATTTGGA
         66 ACCTTTCTTTTCATAGAGCAGTTTTGAAACACTCTTTTTGTAGAATCTGGAAGTGGATATTTGGA

             ** *      ** *    * *   *            **  
      68598 GGTCATTGTAGCCTATC-TGGAAAAAGGAAATATCTTCCCAT
        131 GCGCTTTG-AGGATTTCGTTGGAAACGGAAATATCTTCATAT

            *  ** *   *     * *            *   *  *   * **        *  * * *   
      68639 GAATGCGAGATAGAAGTAATCTCAGAAACATGTTTATGCTGTATCTACTCAACTAACTGTGCTGA
          1 AAAAACTAGACAGAAGCATTCTCAGAAACATCTTTGTGATGTTTGCACTCAACTCACAGAGTTGA

              *     *  *               *        *  * *       *               
      68704 ACATTTCTATTGATAGAGCAGTTTTGAGACACTCTTCTTTTGGAATCTGCAAGTGGATATTTGGA
         66 ACCTTTCTTTTCATAGAGCAGTTTTGAAACACTCTTTTTGTAGAATCTGGAAGTGGATATTTGGA

            ** *                        *     *      
      68769 TAGATTTGAGGATTTCGTTGGAAACGG-GAT-T-AT-ATAT
        131 GCGCTTTGAGGATTTCGTTGGAAACGGAAATATCTTCATAT

                 *       *               *                 **  *   *         
      68806 AAAAAGTAGACAGCAGCATTCTCAG-AACTTCTTTGTGATGTTTGCATCCAGCTCTCAGAGTTGA
          1 AAAAACTAGACAGAAGCATTCTCAGAAACATCTTTGTGATGTTTGCACTCAACTCACAGAGTTGA

              *  * *       *  *  *                  *   **        *  **      
      68870 ACATTCCCTTTCATATAGTAGGTTTGAAACCAC-CTTTTTATAGTGTCTGGAAGCGGGCATTTGG
         66 ACCTTTCTTTTCATAGAGCAGTTTTGAAA-CACTCTTTTTGTAGAATCTGGAAGTGGATATTTGG

                    *   ** ** *  *   *          * *  *
      68934 AGCGCTTTCAGGCCTATGCTGAAAAAGGAAATATCTACCTAC
        130 AGCGCTTTGAGGATTTCGTTGGAAACGGAAATATCTTCATAT

             *                    *    *  **          ** *        *    *     
      68976 AGAAACTAGACAGAAGCATTCTGAGAATCACGTTTGTGATGTGGGTACTCAACTAACAGTGTTGA
          1 AAAAACTAGACAGAAGCATTCTCAGAAACATCTTTGTGATGTTTGCACTCAACTCACAGAGTTGA

            *  *       *   *            *   *                *               
      69041 TCCATTCTTTTGATACAGCAGTTTTGAACCACCCTTTTTGTAGAATCTGCAAGTGGATATTTGGA
         66 ACCTTTCTTTTCATAGAGCAGTTTTGAAACACTCTTTTTGTAGAATCTGGAAGTGGATATTTGGA

            **   *                     *   *        *
      69106 TAGCTGTGAGGATTTCGTTGGAAACGGGAATGTCTTCATAG
        131 GCGCTTTGAGGATTTCGTTGGAAACGGAAATATCTTCATAT

                **                     * * **         *  **       *          
      69147 AAAATTTAGACAGAAGCATTCTCAGAACCTTGATTGTGATGTGTGTTCTCAACTAACAGAGTTGA
          1 AAAAACTAGACAGAAGCATTCTCAGAAACATCTTTGTGATGTTTGCACTCAACTCACAGAGTTGA

                      ** *   * *           *       *                         
      69212 ACCTTTCTTTGGACAGAACTGTTTTGAAACATTCTTTTTATAGAATCTGGAAGTGGATATTTGGA
         66 ACCTTTCTTTTCATAGAGCAGTTTTGAAACACTCTTTTTGTAGAATCTGGAAGTGGATATTTGGA

            **                         *          *  
      69277 AAGCTTTGAGGATTTCGTTGGAAACGGGAATATCTTCAAAT
        131 GCGCTTTGAGGATTTCGTTGGAAACGGAAATATCTTCATAT

                *    *            *           * *       *  *    *            
      69318 AAAATCTAGCCAGAAGCATTCTAAGAAACATCTTAGGGATGTTTACATTCAAGTCACAGAGTTGA
          1 AAAAACTAGACAGAAGCATTCTCAGAAACATCTTTGTGATGTTTGCACTCAACTCACAGAGTTGA

              *   * *    **       *         *    * *   **      *      *    * 
      69383 ACATTCCCCTTTCTCAGAGCAGGTTTGAAACAATCTTCTCGTACTATCTGGCAGTGGACATTTTG
         66 ACCTT-TCTTTTCATAGAGCAGTTTTGAAACACTCTTTTTGTAGAATCTGGAAGTGGATATTTGG

               * *   *  ** ** *  *   *            ** *
      69448 AGCTCCTTGGGGCCTATGCTGAAAAAGGAAATATCTTCCGAC
        130 AGCGCTTTGAGGATTTCGTTGGAAACGGAAATATCTTCATAT

                                **     *  **          *           **    *    
      69490 AAAAACTAGACAGAAGCATTTGCAGAATCACGTTTGTGATGTGTGCACTCAACTGTCAGAATTGA
          1 AAAAACTAGACAGAAGCATTCTCAGAAACATCTTTGTGATGTTTGCACTCAACTCACAGAGTTGA

                 **   ** *      *                            * *            *
      69555 ACCTTGGTTTGGACAGAGCACTTTTGAAACACTCTTTTTGTAGAATCTGCAGGTGGATATTTGGC
         66 ACCTTTCTTTTCATAGAGCAGTTTTGAAACACTCTTTTTGTAGAATCTGGAAGTGGATATTTGGA

            ***                        *   *      * *
      69620 TACCTTTGAGGATTTCGTTGGAAACGGTAATGTCTTCAAAG
        131 GCGCTTTGAGGATTTCGTTGGAAACGGAAATATCTTCATAT

                *          *   **         *   *            *    *            
      69661 AAAATCTAGACAGAAACATCGTCAGAAACACCTTCGTGATGTTTGCAATCAAGTCACAGAGTTGA
          1 AAAAACTAGACAGAAGCATTCTCAGAAACATCTTTGTGATGTTTGCACTCAACTCACAGAGTTGA

                 * *             *  *         *        *             *       
      69726 ACCTTCCGTTTCATAGAGCAGGTTGGAAACACTCATTTTGTAGTATCTGGAAGTGGACATTTGGA
         66 ACCTTTCTTTTCATAGAGCAGTTTTGAAACACTCTTTTTGTAGAATCTGGAAGTGGATATTTGGA

                   *   ** ** *  *   *            **  
      69791 GCGCTTTCAGGCCTATGGTGTAAAAGGAAATATCTTCCCAT
        131 GCGCTTTGAGGATTTCGTTGGAAACGGAAATATCTTCATAT

                * * * *      **      *   * *   *     ** ** *      *    *     
      69832 AAAAGCGACATAGAAGCTATCTCAGGAACTTGTTTATGATGCATCTAATCAACTAACAGTGTTGA
          1 AAAAACTAGACAGAAGCATTCTCAGAAACATCTTTGTGATGTTTGCACTCAACTCACAGAGTTGA

                  * ** * *                         * *       *               
      69897 ACCTTTGTACTGACAGAGCAG-TTTGAAACACTCTTTTTTTGGAATCTGCAAGTGGATATTTGGA
         66 ACCTTTCTTTTCATAGAGCAGTTTTGAAACACTCTTTTTGTAGAATCTGGAAGTGGATATTTGGA

            *                           *   *   *    
      69961 TCGCTTTGAGGATTTCGTTGGAAACGG-GAT-GC--AATAT
        131 GCGCTTTGAGGATTTCGTTGGAAACGGAAATATCTTCATAT

                **  *    *     *                 **  *   *  *    *         * 
      69998 AAAACGTACACAGCAGCATACTCAGAAA-ATACTTTGCCATATTTCCATTCAAGTCACAGAGTGG
          1 AAAAACTAGACAGAAGCATTCTCAGAAACAT-CTTTGTGATGTTTGCACTCAACTCACAGAGTTG

               *  * **            *           *      * ***            *      
      70062 AACATTCCCATTCATAGAGCAGGTTTGAAACACTTTTTTTGGAATGTCTGGAAGTGGACATTTGG
         65 AACCTTTCTTTTCATAGAGCAGTTTTGAAACACTCTTTTTGTAGAATCTGGAAGTGGATATTTGG

                    *  * * ** *  *   *            **  
      70127 AGCGCTTTCAGAACTATGGTGAAAAAGGAAATATCTTCCAAT
        130 AGCGCTTTGAGGATTTCGTTGGAAACGGAAATATCTTCATAT

            *     *               *      * *         **  **       *  *  * *  
      70169 GAAAACAAGACAGAAGCATTCTGAGAAACTTATTTGTGATGCGTGTCCTCAACTAACGGACTCGA
          1 AAAAACTAGACAGAAGCATTCTCAGAAACATCTTTGTGATGTTTGCACTCAACTCACAGAGTTGA

                   *      **  * *  *  *             *   *    * * *          *
      70234 ACCTTTCGTTTCATGCAGTACTTCTGGAACACTCTTTTTGAAGATTCTGCATGCGGATATTTGGT
         66 ACCTTTCTTTTCATAGAGCAGTTTTGAAACACTCTTTTTGTAGAATCTGGAAGTGGATATTTGGA

            **                            **   *   * 
      70299 TAGCTTTGAGGATTTCGTTGGAAACGG---GCT-TACATGT
        131 GCGCTTTGAGGATTTCGTTGGAAACGGAAATATCTTCATAT

                 *       *               *        *   *    *    *       *    
      70336 AAAAATTAGACAGCAGCATTCTCAGAAACTTCTTTGTGGTGTCTGCATTCAAGTCACAGAATTGA
          1 AAAAACTAGACAGAAGCATTCTCAGAAACATCTTTGTGATGTTTGCACTCAACTCACAGAGTTGA

              * ** * **            *  * *      *       *    *        *       
      70401 ACATCCCCTCACATAGAGCAGTTGTGCAGCACTCTATTTGTAGTATCTCGAAGTGGACATTTGGA
         66 ACCTTTCTTTTCATAGAGCAGTTTTGAAACACTCTTTTTGTAGAATCTGGAAGTGGATATTTGGA

             *         ** *    * *   *            **  
      70466 GGGCTTTGTAGCCTATC-TGGAAAAAGGAAATATCTTCCCAT
        131 GCGCTTTG-AGGATTTCGTTGGAAACGGAAATATCTTCATAT

            *  ** *   *     * *            *   *  *   * **        *  * * *   
      70507 GAATGCGAGATAGAAGTAATCTCAGAAACATGTTTATGCTGTATCTACTCAACTAACTGTGCTGA
          1 AAAAACTAGACAGAAGCATTCTCAGAAACATCTTTGTGATGTTTGCACTCAACTCACAGAGTTGA

              *     *  *               *        *  * *       *               
      70572 ACATTTCTATTGATAGAGCAGTTTTGAGACACTCTTCTTTTGGAATCTGCAAGTGGATATTTGGA
         66 ACCTTTCTTTTCATAGAGCAGTTTTGAAACACTCTTTTTGTAGAATCTGGAAGTGGATATTTGGA

            ** *                        *     *      
      70637 TAGATTTGAGGATTTCGTTGGAAACGG-GAT-T-AT-ATAT
        131 GCGCTTTGAGGATTTCGTTGGAAACGGAAATATCTTCATAT

            *    *       *               *                 **  *   *         
      70674 CAAAAGTAGACAGCAGCATTCTCAGAAACTTCTTTGTGATGTTTGCATCCAGCTCTCAGAGTTGA
          1 AAAAACTAGACAGAAGCATTCTCAGAAACATCTTTGTGATGTTTGCACTCAACTCACAGAGTTGA

              *  * *          *  *        * *      *   **        *  **       
      70739 ACATTCCCTTTCATAGAGTAGGTTTGAAACCCCCTTTTTATAGTGTCTGGAAGCGGGCATTTGGA
         66 ACCTTTCTTTTCATAGAGCAGTTTTGAAACACTCTTTTTGTAGAATCTGGAAGTGGATATTTGGA

                   *   ** ** *  *   *          * *  *
      70804 GCGCTTTCAGGCCTATGCTGAAAAAGGAAATATCTACCTAC
        131 GCGCTTTGAGGATTTCGTTGGAAACGGAAATATCTTCATAT

             *                    *    *  **          ** *        *    *     
      70845 AGAAACTAGACAGAAGCATTCTGAGAATCACGTTTGTGATGTGGGTACTCAACTAACAGTGTTGA
          1 AAAAACTAGACAGAAGCATTCTCAGAAACATCTTTGTGATGTTTGCACTCAACTCACAGAGTTGA

            *  *       *   *            *   *     *          *               
      70910 TCCATTCTTTTGATACAGCAGTTTTGAACCACCCTTTTCGTAGAATCTGCAAGTGGATATTTGGA
         66 ACCTTTCTTTTCATAGAGCAGTTTTGAAACACTCTTTTTGTAGAATCTGGAAGTGGATATTTGGA

            **   *                     *   *        *
      70975 TAGCTGTGAGGATTTCGTTGGAAACGGGAATGTCTTCATAG
        131 GCGCTTTGAGGATTTCGTTGGAAACGGAAATATCTTCATAT

                **                     * ****         *  **   *   *          
      71016 AAAATTTAGACAGAAGCATTCTCAGAACCTGGATTGTGATGTGTGTTCTCCACTAACAGAGTTGA
          1 AAAAACTAGACAGAAGCATTCTCAGAAACATCTTTGTGATGTTTGCACTCAACTCACAGAGTTGA

                      ** *   * *           *       *               *         
      71081 ACCTTTCTTTGGACAGAACTGTTTTGAAACATTCTTTTTATAGAATCTGGAAGTGTATATTTGGA
         66 ACCTTTCTTTTCATAGAGCAGTTTTGAAACACTCTTTTTGTAGAATCTGGAAGTGGATATTTGGA

            **                         *          *  
      71146 AAGCTTTGAGGATTTCGTTGGAAACGGGAATATCTTCAAAT
        131 GCGCTTTGAGGATTTCGTTGGAAACGGAAATATCTTCATAT

            *   *    *            *           * *       *  *    *            
      71187 CAAATCTAGCCAGAAGCATTCTAAGAAACATCTTAGGGATGTTTACATTCAAGTCACAGAGTTGA
          1 AAAAACTAGACAGAAGCATTCTCAGAAACATCTTTGTGATGTTTGCACTCAACTCACAGAGTTGA

              *   * *    **       *         *    * *   **      *      *    * 
      71252 ACATTCCCCTTTCTCAGAGCAGGTTTGAAACAATCTTCTCGTACTATCTGGCAGTGGACATTTTG
         66 ACCTT-TCTTTTCATAGAGCAGTTTTGAAACACTCTTTTTGTAGAATCTGGAAGTGGATATTTGG

               * *   *  ** ** *  *   *            ** *
      71317 AGCTCCTTGGGGCCTATGCTGAAAAAGGAAATATCTTCCGAC
        130 AGCGCTTTGAGGATTTCGTTGGAAACGGAAATATCTTCATAT

                                 *     *  **          *           **    *    
      71359 AAAAACTAGACAGAAGCATTCGCAGAATCACGTTTGTGATGTGTGCACTCAACTGTCAGAATTGA
          1 AAAAACTAGACAGAAGCATTCTCAGAAACATCTTTGTGATGTTTGCACTCAACTCACAGAGTTGA

                 **   ** *      *                            * *            *
      71424 ACCTTGGTTTGGACAGAGCACTTTTGAAACACTCTTTTTGTAGAATCTGCAGGTGGATATTTGGC
         66 ACCTTTCTTTTCATAGAGCAGTTTTGAAACACTCTTTTTGTAGAATCTGGAAGTGGATATTTGGA

            **                         *   *      * *
      71489 TAGCTTTGAGGATTTCGTTGGAAACGGTAATGTCTTCAAAG
        131 GCGCTTTGAGGATTTCGTTGGAAACGGAAATATCTTCATAT

                *          *   *          *   *            *    *            
      71530 AAAATCTAGACAGAAACATCCTCAGAAACACCTTCGTGATGTTTGCAATCAAGTCACAGAGTTGA
          1 AAAAACTAGACAGAAGCATTCTCAGAAACATCTTTGTGATGTTTGCACTCAACTCACAGAGTTGA

                 * *             *  *         *        *             *       
      71595 ACCTTCCGTTTCATAGAGCAGGTTGGAAACACTCATTTTGTAGTATCTGGAAGTGGACATTTGGA
         66 ACCTTTCTTTTCATAGAGCAGTTTTGAAACACTCTTTTTGTAGAATCTGGAAGTGGATATTTGGA

                   *   ** ** *  *   *            **  
      71660 GCGCTTTCAGGCCTATGGTGTAAAAGGAAATATCTTCCCAT
        131 GCGCTTTGAGGATTTCGTTGGAAACGGAAATATCTTCATAT

                * * * *      **      *   * *   *     ** ** *      *    *     
      71701 AAAAGCGACATAGAAGCTATCTCAGGAACTTGTTTATGATGCATCTAATCAACTAACAGTGTTGA
          1 AAAAACTAGACAGAAGCATTCTCAGAAACATCTTTGTGATGTTTGCACTCAACTCACAGAGTTGA

                  * ** * *        *                * *       *               
      71766 A-CTTTGTACTGACAGAGCAG-GTTGAAACACTCTTTTTTTGGAATCTGCAAGTGGATATTTGGA
         66 ACCTTTCTTTTCATAGAGCAGTTTTGAAACACTCTTTTTGTAGAATCTGGAAGTGGATATTTGGA

            *                           *   *   *    
      71829 TCGCTTTGAGGATTTCGTTGGAAACGG-GAT-GC--AATAT
        131 GCGCTTTGAGGATTTCGTTGGAAACGGAAATATCTTCATAT

                **  *    *     *                 **  *   *  *    *      *  * 
      71866 AAAACGTACACAGCAGCATACTCAGAAA-ATACTTTGCCATATTTCCATTCAAGTCACAGTGTGG
          1 AAAAACTAGACAGAAGCATTCTCAGAAACAT-CTTTGTGATGTTTGCACTCAACTCACAGAGTTG

               *  * **            *           *      *  **            *      
      71930 AACATTCCCATTCATAGAGCAGGTTTGAAACACTTTTTTTGGAGTGTCTGGAAGTGGACATTTGG
         65 AACCTTTCTTTTCATAGAGCAGTTTTGAAACACTCTTTTTGTAGAATCTGGAAGTGGATATTTGG

                    *  * * ** *  *   *            **  
      71995 AGCGCTTTCAGAACTATGGTGAAAAAGGAAATATCTTCCAAT
        130 AGCGCTTTGAGGATTTCGTTGGAAACGGAAATATCTTCATAT

            *     *               *      * *         **  **       *  *  * *  
      72037 GAAAACAAGACAGAAGCATTCTGAGAAACTTATTTGTGATGCGTGTCCTCAACTAACGGACTCGA
          1 AAAAACTAGACAGAAGCATTCTCAGAAACATCTTTGTGATGTTTGCACTCAACTCACAGAGTTGA

                   *      **  * *  *  *             *   *    * * *          *
      72102 ACCTTTCGTTTCATGCAGTACTTCTGGAACACTCTTTTTGAAGATTCTGCATGCGGATATTTGGT
         66 ACCTTTCTTTTCATAGAGCAGTTTTGAAACACTCTTTTTGTAGAATCTGGAAGTGGATATTTGGA

            **                            **   *     
      72167 TAGCTTTGAGGATTTCGTTGGAAACGG---GCT-TACATAT
        131 GCGCTTTGAGGATTTCGTTGGAAACGGAAATATCTTCATAT

                 *       *               *        *   *    *    *       *    
      72204 AAAAATTAGACAGCAGCATTCTCAGAAACTTCTTTGTGGTGTCTGCATTCAAGTCACAGAATTGA
          1 AAAAACTAGACAGAAGCATTCTCAGAAACATCTTTGTGATGTTTGCACTCAACTCACAGAGTTGA

              * ** * **            *  * *      *       *    *    *   *       
      72269 ACATCCCCTCACATAGAGCAGTTGTGCAGCACTCTATTTGTAGTATCTCGAAGAGGACATTTGGA
         66 ACCTTTCTTTTCATAGAGCAGTTTTGAAACACTCTTTTTGTAGAATCTGGAAGTGGATATTTGGA

             *         ** *  * * *   *            **  
      72334 GGGCTTTGTAGCCTAT-ATGGAAAAAGGAAATATCTTCCCAT
        131 GCGCTTTG-AGGATTTCGTTGGAAACGGAAATATCTTCATAT

            *  ** *   *     * *            *   *  *   * **        *  * * *   
      72375 GAATGCGAGATAGAAGTAATCTCAGAAACATGTTTATGCTGTATCTACTCAACTAACTGTGCTGA
          1 AAAAACTAGACAGAAGCATTCTCAGAAACATCTTTGTGATGTTTGCACTCAACTCACAGAGTTGA

              *     *  *               *        *  * *       *               
      72440 ACATTTCTATTGATAGAGCAGTTTTGAGACACTCTTCTTTTGGAATCTGCAAGTGGATATTTGGA
         66 ACCTTTCTTTTCATAGAGCAGTTTTGAAACACTCTTTTTGTAGAATCTGGAAGTGGATATTTGGA

            ** *                        *     *      
      72505 TAGATTTGAGGATTTCGTTGGAAACGG-GAT-T-AT-ATAT
        131 GCGCTTTGAGGATTTCGTTGGAAACGGAAATATCTTCATAT

            *    *       *               *                 **  *   *         
      72542 CAAAAGTAGACAGCAGCATTCTCAGAAACTTCTTTGTGATGTTTGCATCCAGCTCTCAGAGTTGA
          1 AAAAACTAGACAGAAGCATTCTCAGAAACATCTTTGTGATGTTTGCACTCAACTCACAGAGTTGA

              *  * *          *  *        * *      *   **        *  **       
      72607 ACATTCCCTTTCATAGAGTAGGTTTGAAACCCCCTTTTTATAGTGTCTGGAAGCGGGCATTTGGA
         66 ACCTTTCTTTTCATAGAGCAGTTTTGAAACACTCTTTTTGTAGAATCTGGAAGTGGATATTTGGA

                   *   ** ** *  *   *          * *  *
      72672 GCGCTTTCAGGCCTATGCTGAAAAAGGAAATATCTACCTAC
        131 GCGCTTTGAGGATTTCGTTGGAAACGGAAATATCTTCATAT

             *                    *    *  **          ** *        *    *     
      72713 AGAAACTAGACAGAAGCATTCTGAGAATCACGTTTGTGATGTGGGTACTCAACTAACAGTGTTGA
          1 AAAAACTAGACAGAAGCATTCTCAGAAACATCTTTGTGATGTTTGCACTCAACTCACAGAGTTGA

            *  *       *   *            *   *                *               
      72778 TCCATTCTTTTGATACAGCAGTTTTGAACCACCCTTTTTGTAGAATCTGCAAGTGGATATTTGGA
         66 ACCTTTCTTTTCATAGAGCAGTTTTGAAACACTCTTTTTGTAGAATCTGGAAGTGGATATTTGGA

            **   *                     *   *        *
      72843 TAGCTGTGAGGATTTCGTTGGAAACGGGAATGTCTTCATAG
        131 GCGCTTTGAGGATTTCGTTGGAAACGGAAATATCTTCATAT

                **                     * ****         *  **   *   *          
      72884 AAAATTTAGACAGAAGCATTCTCAGAACCTGGATTGTGATGTGTGTTCTCCACTAACAGAGTTGA
          1 AAAAACTAGACAGAAGCATTCTCAGAAACATCTTTGTGATGTTTGCACTCAACTCACAGAGTTGA

                      ** *   * *           * *     *                         
      72949 ACCTTTCTTTGGACAGAACTGTTTTGAAACATTGTTTTTATAGAATCTGGAAGTGGATATTTGGA
         66 ACCTTTCTTTTCATAGAGCAGTTTTGAAACACTCTTTTTGTAGAATCTGGAAGTGGATATTTGGA

            **                         *          *  
      73014 AAGCTTTGAGGATTTCGTTGGAAACGGGAATATCTTCAAAT
        131 GCGCTTTGAGGATTTCGTTGGAAACGGAAATATCTTCATAT

                *    *            *           * *       *  *    *            
      73055 AAAATCTAGCCAGAAGCATTCTAAGAAACATCTTAGGGATGTTTACATTCAAGTCACAGAGTTGA
          1 AAAAACTAGACAGAAGCATTCTCAGAAACATCTTTGTGATGTTTGCACTCAACTCACAGAGTTGA

              *   * *    **       *         *    * *   **      *      *    * 
      73120 ACATTCCCCTTTCTCAGAGCAGGTTTGAAACAATCTTCTCGTACTATCTGGCAGTGGACATTTTG
         66 ACCTT-TCTTTTCATAGAGCAGTTTTGAAACACTCTTTTTGTAGAATCTGGAAGTGGATATTTGG

               * *   *  ** ** *  *   *            ** *
      73185 AGCTCCTTGGGGCCTATGCTGAAAAAGGAAATATCTTCCGAC
        130 AGCGCTTTGAGGATTTCGTTGGAAACGGAAATATCTTCATAT

                                 *     *  **          *           **    *    
      73227 AAAAACTAGACAGAAGCATTCGCAGAATCACGTTTGTGATGTGTGCACTCAACTGTCAGAATTGA
          1 AAAAACTAGACAGAAGCATTCTCAGAAACATCTTTGTGATGTTTGCACTCAACTCACAGAGTTGA

                 **   ** *      *          *                 * *            *
      73292 ACCTTGGTTTGGACAGAGCACTTTTGAAACAATCTTTTTGTAGAATCTGCAGGTGGATATTTGGC
         66 ACCTTTCTTTTCATAGAGCAGTTTTGAAACACTCTTTTTGTAGAATCTGGAAGTGGATATTTGGA

            **                         *   *      * *
      73357 TAGCTTTGAGGATTTCGTTGGAAACGGTAATGTCTTCAAAG
        131 GCGCTTTGAGGATTTCGTTGGAAACGGAAATATCTTCATAT

                *          *   *          *   *            *    *            
      73398 AAAATCTAGACAGAAACATCCTCAGAAACACCTTCGTGATGTTTGCAATCAAGTCACAGAGTTGA
          1 AAAAACTAGACAGAAGCATTCTCAGAAACATCTTTGTGATGTTTGCACTCAACTCACAGAGTTGA

                 * *             *  *         *        *             *       
      73463 ACCTTCCGTTTCATAGAGCAGGTTGGAAACACTCATTTTGTAGTATCTGGAAGTGGACATTTGGA
         66 ACCTTTCTTTTCATAGAGCAGTTTTGAAACACTCTTTTTGTAGAATCTGGAAGTGGATATTTGGA

                   *   ** ** *  *   *            **  
      73528 GCGCTTTCAGGCCTATGGTGTAAAAGGAAATATCTTCCCAT
        131 GCGCTTTGAGGATTTCGTTGGAAACGGAAATATCTTCATAT

                * * * *      **      *   * *   *     ** ** *      *    *     
      73569 AAAAGCGACATAGAAGCTATCTCAGGAACTTGTTTATGATGCATCTAATCAACTAACAGTGTTGA
          1 AAAAACTAGACAGAAGCATTCTCAGAAACATCTTTGTGATGTTTGCACTCAACTCACAGAGTTGA

                  * ** * *                         * *       *               
      73634 ACCTTTGTACTGACAGAGCAG-TTTGAAACACTCTTTTTTTGGAATCTGCAAGTGGATATTTGGA
         66 ACCTTTCTTTTCATAGAGCAGTTTTGAAACACTCTTTTTGTAGAATCTGGAAGTGGATATTTGGA

            *                           *   *   *    
      73698 TCGCTTTGAGGATTTCGTTGGAAACGG-GAT-GC--AATAT
        131 GCGCTTTGAGGATTTCGTTGGAAACGGAAATATCTTCATAT

                **  *    *     *                 **  *   *  *    *         * 
      73735 AAAACGTACACAGCAGCATACTCAGAAA-ATACTTTGCCATATTTCCATTCAAGTCACAGAGTGG
          1 AAAAACTAGACAGAAGCATTCTCAGAAACAT-CTTTGTGATGTTTGCACTCAACTCACAGAGTTG

               *  * **            *           *      *  **            *      
      73799 AACATTCCCATTCATAGAGCAGGTTTGAAACACTTTTTTTGGAGTGTCTGGAAGTGGACATTTGG
         65 AACCTTTCTTTTCATAGAGCAGTTTTGAAACACTCTTTTTGTAGAATCTGGAAGTGGATATTTGG

                    *  * * ** *  *   *            **  
      73864 AGCGCTTTCAGAACTATGGTGAAAAAGGAAATATCTTCCAAT
        130 AGCGCTTTGAGGATTTCGTTGGAAACGGAAATATCTTCATAT

            *     *               *      * *         **  **       *  *  * *  
      73906 GAAAACAAGACAGAAGCATTCTGAGAAACTTATTTGTGATGCGTGTCCTCAACTAACGGACTCGA
          1 AAAAACTAGACAGAAGCATTCTCAGAAACATCTTTGTGATGTTTGCACTCAACTCACAGAGTTGA

                   *     ***  * *  *  *             *   *    * * *           
      73971 ACCTTTCGTTTCACGCAGTACTTCTGGAACACTCTTTTTGAAGATTCTGCATGCGGATATTTGGA
         66 ACCTTTCTTTTCATAGAGCAGTTTTGAAACACTCTTTTTGTAGAATCTGGAAGTGGATATTTGGA

            **                            **   *     
      74036 TAGCTTTGAGGATTTCGTTGGAAACGG---GCT-TACATAT
        131 GCGCTTTGAGGATTTCGTTGGAAACGGAAATATCTTCATAT

                 *       *               *        *   *    *    *       *    
      74073 AAAAATTAGACAGCAGCATTCTCAGAAACTTCTTTGTGGTGTCTGCATTCAAGTCACAGAATTGA
          1 AAAAACTAGACAGAAGCATTCTCAGAAACATCTTTGTGATGTTTGCACTCAACTCACAGAGTTGA

              * ** * **   *        *  * *      *       *    *    *   *       
      74138 ACATCCCCTCACATTGAGCAGTTGTGCAGCACTCTATTTGTAGTATCTCGAAGAGGACATTTGGA
         66 ACCTTTCTTTTCATAGAGCAGTTTTGAAACACTCTTTTTGTAGAATCTGGAAGTGGATATTTGGA

             *         ** *  * * *   *            **  
      74203 GGGCTTTGTAGCCTAT-ATGGAAAAAGGAAATATCTTCCCAT
        131 GCGCTTTG-AGGATTTCGTTGGAAACGGAAATATCTTCATAT

            *   * *   *     * *            *   *  *   * **        *  * * *   
      74244 GAAAGCGAGATAGAAGTAATCTCAGAAACATGTTTATGCTGTATCTACTCAACTAACTGTGCTGA
          1 AAAAACTAGACAGAAGCATTCTCAGAAACATCTTTGTGATGTTTGCACTCAACTCACAGAGTTGA

              *     *  *               *        *  * *       *               
      74309 ACATTTCTATTGATAGAGCAGTTTTGAGACACTCTTCTTTTGGAATCTGCAAGTGGATATTTGGA
         66 ACCTTTCTTTTCATAGAGCAGTTTTGAAACACTCTTTTTGTAGAATCTGGAAGTGGATATTTGGA

            ** *                        *     *      
      74374 TAGATTTGAGGATTTCGTTGGAAACGG-GAT-T-AT-ATAT
        131 GCGCTTTGAGGATTTCGTTGGAAACGGAAATATCTTCATAT

                 *       *               *                 **  *   *         
      74411 AAAAAGTAGACAGCAGCATTCTCA-AAACTTCTTTGTGATGTTTGCATCCAGCTCTCAGAGTTGA
          1 AAAAACTAGACAGAAGCATTCTCAGAAACATCTTTGTGATGTTTGCACTCAACTCACAGAGTTGA

              *  * *          *  *        * *   *  *   **        *  **   *   
      74475 ACATTCCCTTTCATAGAGTAGGTTTGAAACCCCCTTCTTATAGTGTCTGGAAGCGGGCATTAGGA
         66 ACCTTTCTTTTCATAGAGCAGTTTTGAAACACTCTTTTTGTAGAATCTGGAAGTGGATATTTGGA

            *      *   ** ** *  *   *          * *  *
      74540 ACGCTTTCAGGCCTATGCTGAAAAAGGAAATATCTACCTAC
        131 GCGCTTTGAGGATTTCGTTGGAAACGGAAATATCTTCATAT

             *                    *    *  **          ** *        *    *     
      74581 AGAAACTAGACAGAAGCATTCTGAGAATCACGTTTGTGATGTGGGTACTCAACTAACAGTGTTGA
          1 AAAAACTAGACAGAAGCATTCTCAGAAACATCTTTGTGATGTTTGCACTCAACTCACAGAGTTGA

            *  *       *   *            *   *                *               
      74646 TCCATTCTTTTGATACAGCAGTTTTGAACCACCCTTTTTGTAGAATCTGCAAGTGGATATTTGGA
         66 ACCTTTCTTTTCATAGAGCAGTTTTGAAACACTCTTTTTGTAGAATCTGGAAGTGGATATTTGGA

            **   *                     *   *        *
      74711 TAGCTGTGAGGATTTCGTTGGAAACGGGAATGTCTTCATAG
        131 GCGCTTTGAGGATTTCGTTGGAAACGGAAATATCTTCATAT

                **                     * ****         *  **   *   *          
      74752 AAAATTTAGACAGAAGCATTCTCAGAACCTGGATTGTGATGTGTGTTCTCCACTAACAGAGTTGA
          1 AAAAACTAGACAGAAGCATTCTCAGAAACATCTTTGTGATGTTTGCACTCAACTCACAGAGTTGA

                      ** *   * *           *       *                         
      74817 ACCTTTCTTTGGACAGAACTGTTTTGAAACATTCTTTTTATAGAATCTGGAAGTGGATATTTGGA
         66 ACCTTTCTTTTCATAGAGCAGTTTTGAAACACTCTTTTTGTAGAATCTGGAAGTGGATATTTGGA

            **                         *          *  
      74882 AAGCTTTGAGGATTTCGTTGGAAACGGGAATATCTTCAAAT
        131 GCGCTTTGAGGATTTCGTTGGAAACGGAAATATCTTCATAT

                *    *            *           * *       *  *    *            
      74923 AAAATCTAGCCAGAAGCATTCTAAGAAACATCTTAGGGATGTTTACATTCAAGTCACAGAGTTGA
          1 AAAAACTAGACAGAAGCATTCTCAGAAACATCTTTGTGATGTTTGCACTCAACTCACAGAGTTGA

              *   * *    **       *         *    * *   **      *      *    * 
      74988 ACATTCCCCTTTCTCAGAGCAGGTTTGAAACAATCTTCTCGTACTATCTGGCAGTGGACATTTTG
         66 ACCTT-TCTTTTCATAGAGCAGTTTTGAAACACTCTTTTTGTAGAATCTGGAAGTGGATATTTGG

               * *   *  ** ** *  *   *            ** *
      75053 AGCTCCTTGGGGCCTATGCTGAAAAAGGAAATATCTTCCGAC
        130 AGCGCTTTGAGGATTTCGTTGGAAACGGAAATATCTTCATAT

                                 *     *  **          *           **    *    
      75095 AAAAACTAGACAGAAGCATTCGCAGAATCACGTTTGTGATGTGTGCACTCAACTGTCAGAATTGA
          1 AAAAACTAGACAGAAGCATTCTCAGAAACATCTTTGTGATGTTTGCACTCAACTCACAGAGTTGA

                 **   ** *      *                            * *            *
      75160 ACCTTGGTTTGGACAGAGCACTTTTGAAACACTCTTTTTGTAGAATCTGCAGGTGGATATTTGGC
         66 ACCTTTCTTTTCATAGAGCAGTTTTGAAACACTCTTTTTGTAGAATCTGGAAGTGGATATTTGGA

            **                         *   *      * *
      75225 TAGCTTTGAGGATTTCGTTGGAAACGGTAATGTCTTCAAAG
        131 GCGCTTTGAGGATTTCGTTGGAAACGGAAATATCTTCATAT

                *          *   *          *   *            *    *            
      75266 AAAATCTAGACAGAAACATCCTCAGAAACACCTTCGTGATGTTTGCAATCAAGTCACAGAGTTGA
          1 AAAAACTAGACAGAAGCATTCTCAGAAACATCTTTGTGATGTTTGCACTCAACTCACAGAGTTGA

                 * *             *  *         *        *             *       
      75331 ACCTTCCGTTTCATAGAGCAGGTTGGAAACACTCATTTTGTAGTATCTGGAAGTGGACATTTGGA
         66 ACCTTTCTTTTCATAGAGCAGTTTTGAAACACTCTTTTTGTAGAATCTGGAAGTGGATATTTGGA

                   *   ** ** *  *   *            **  
      75396 GCGCTTTCAGGCCTATGGTGTAAAAGGAAATATCTTCCCAT
        131 GCGCTTTGAGGATTTCGTTGGAAACGGAAATATCTTCATAT

                * * * *      **      *   * *   *     ** ** *      *    *     
      75437 AAAAGCGACATAGAAGCTATCTCAGGAACTTGTTTATGATGCATCTAATCAACTAACAGTGTTGA
          1 AAAAACTAGACAGAAGCATTCTCAGAAACATCTTTGTGATGTTTGCACTCAACTCACAGAGTTGA

                  * ** * *                         * *       *               
      75502 ACCTTTGTACTGACAGAGCAG-TTTGAAACACTCTTTTTTTGGAATCTGCAAGTGGATATTTGGA
         66 ACCTTTCTTTTCATAGAGCAGTTTTGAAACACTCTTTTTGTAGAATCTGGAAGTGGATATTTGGA

            *                           *   *   *    
      75566 TCGCTTTGAGGATTTCGTTGGAAACGG-GAT-GC--AATAT
        131 GCGCTTTGAGGATTTCGTTGGAAACGGAAATATCTTCATAT

                **  *    *     *                 **  *   *  *    *         * 
      75603 AAAACGTACACAGCAGCATACTCAGAAA-ATACTTTGCCATATTTCCATTCAAGTCACAGAGTGG
          1 AAAAACTAGACAGAAGCATTCTCAGAAACAT-CTTTGTGATGTTTGCACTCAACTCACAGAGTTG

               *  * **            *           *      *  **            *      
      75667 AACATTCCCATTCATAGAGCAGGTTTGAAACACTTTTTTTGGAGTGTCTGGAAGTGGACATTTGG
         65 AACCTTTCTTTTCATAGAGCAGTTTTGAAACACTCTTTTTGTAGAATCTGGAAGTGGATATTTGG

                    *  * * ** *  *   *            **  
      75732 AGCGCTTTCAGAACTATGGTGAAAAAGGAAATATCTTCCAAT
        130 AGCGCTTTGAGGATTTCGTTGGAAACGGAAATATCTTCATAT

            *     *               *      * *         **  **       *  *  * *  
      75774 GAAAACAAGACAGAAGCATTCTGAGAAACTTATTTGTGATGCGTGTCCTCAACTAACGGACTCGA
          1 AAAAACTAGACAGAAGCATTCTCAGAAACATCTTTGTGATGTTTGCACTCAACTCACAGAGTTGA

                   *      **  * *  *  *             *   *    * * *          *
      75839 ACCTTTCGTTTCATGCAGTACTTCTGGAACACTCTTTTTGAAGATTCTGCATGCGGATATTTGGT
         66 ACCTTTCTTTTCATAGAGCAGTTTTGAAACACTCTTTTTGTAGAATCTGGAAGTGGATATTTGGA

            **                            **   *     
      75904 TAGCTTTGAGGATTTCGTTGGAAACGG---GCT-TACATAT
        131 GCGCTTTGAGGATTTCGTTGGAAACGGAAATATCTTCATAT

                 *       *               *        *   *    *    *       *    
      75941 AAAAATTAGACAGCAGCATTCTCAGAAACTTCTTTGTGGTGTCTGCATTCAAGTCACAGAATTGA
          1 AAAAACTAGACAGAAGCATTCTCAGAAACATCTTTGTGATGTTTGCACTCAACTCACAGAGTTGA

              * ** * **            *  * *      *       *    *        *       
      76006 ACATCCCCTCACATAGAGCAGTTGTGCAGCACTCTATTTGTAGTATCTCGAAGTGGACATTTGGA
         66 ACCTTTCTTTTCATAGAGCAGTTTTGAAACACTCTTTTTGTAGAATCTGGAAGTGGATATTTGGA

             *         ****           *            **  
      76071 GGGCTTTGTAGCCCATC--TGGATAAAGGAAATATCTTCCCAT
        131 GCGCTTTG-AGGATTTCGTTGGA-AACGGAAATATCTTCATAT

            *  ** *   *     * *            *   *  *   * **        *  * * *   
      76112 GAATGCGAGATAGAAGTAATCTCAGAAACATGTTTATGCTGTATCTACTCAACTAACTGTGCTGA
          1 AAAAACTAGACAGAAGCATTCTCAGAAACATCTTTGTGATGTTTGCACTCAACTCACAGAGTTGA

              *     *  *               *        *  * *       *               
      76177 ACATTTCTATTGATAGAGCAGTTTTGAGACACTCTTCTTTTGGAATCTGCAAGTGGATATTTGGA
         66 ACCTTTCTTTTCATAGAGCAGTTTTGAAACACTCTTTTTGTAGAATCTGGAAGTGGATATTTGGA

            ** *                        *     *      
      76242 TAGATTTGAGGATTTCGTTGGAAACGG-GAT-T-AT-ATAT
        131 GCGCTTTGAGGATTTCGTTGGAAACGGAAATATCTTCATAT

            *    *       *               *                 **  *   *         
      76279 CAAAAGTAGACAGCAGCATTCTCAGAAACTTCTTTGTGATGTTTGCATCCAGCTCTCAGAGTTGA
          1 AAAAACTAGACAGAAGCATTCTCAGAAACATCTTTGTGATGTTTGCACTCAACTCACAGAGTTGA

              *  * *          *  *        *        *   **        *  **       
      76344 ACATTCCCTTTCATAGAGTAGGTTTGAAACCCTCTTTTTATAGTGTCTGGAAGCGGGCATTTGGA
         66 ACCTTTCTTTTCATAGAGCAGTTTTGAAACACTCTTTTTGTAGAATCTGGAAGTGGATATTTGGA

                   *   ** ** *  *   *          * *  *
      76409 GCGCTTTCAGGCCTATGCTGAAAAAGGAAATATCTACCTAC
        131 GCGCTTTGAGGATTTCGTTGGAAACGGAAATATCTTCATAT

             *                    *    *  **          ** *        *    *     
      76450 AGAAACTAGACAGAAGCATTCTGAGAATCACGTTTGTGATGTGGGTACTCAACTAACAGTGTTGA
          1 AAAAACTAGACAGAAGCATTCTCAGAAACATCTTTGTGATGTTTGCACTCAACTCACAGAGTTGA

            *  *       *   *            *   *                *               
      76515 TCCATTCTTTTGATACAGCAGTTTTGAACCACCCTTTTTGTAGAATCTGCAAGTGGATATTTGGA
         66 ACCTTTCTTTTCATAGAGCAGTTTTGAAACACTCTTTTTGTAGAATCTGGAAGTGGATATTTGGA

            **   *                     *   *        *
      76580 TAGCTGTGAGGATTTCGTTGGAAACGGGAATGTCTTCATAG
        131 GCGCTTTGAGGATTTCGTTGGAAACGGAAATATCTTCATAT

                **                     * ****         *  **   *   *          
      76621 AAAATTTAGACAGAAGCATTCTCAGAACCTGGATTGTGATGTGTGTTCTCCACTAACAGAGTTGA
          1 AAAAACTAGACAGAAGCATTCTCAGAAACATCTTTGTGATGTTTGCACTCAACTCACAGAGTTGA

                      ** *   * *           *       *                         
      76686 ACCTTTCTTTGGACAGAACTGTTTTGAAACATTCTTTTTATAGAATCTGGAAGTGGATATTTGGA
         66 ACCTTTCTTTTCATAGAGCAGTTTTGAAACACTCTTTTTGTAGAATCTGGAAGTGGATATTTGGA

            **                         *          *  
      76751 AAGCTTTGAGGATTTCGTTGGAAACGGGAATATCTTCAAAT
        131 GCGCTTTGAGGATTTCGTTGGAAACGGAAATATCTTCATAT

                *    *            *           * *       *  *    *            
      76792 AAAATCTAGCCAGAAGCATTCTAAGAAACATCTTAGGGATGTTTACATTCAAGTCACAGAGTTGA
          1 AAAAACTAGACAGAAGCATTCTCAGAAACATCTTTGTGATGTTTGCACTCAACTCACAGAGTTGA

              *   * *    **       *         *    * *   **      *      *    * 
      76857 ACATTCCCCTTTCTCAGAGCAGGTTTGAAACAATCTTCTCGTACTATCTGGCAGTGGACATTTTG
         66 ACCTT-TCTTTTCATAGAGCAGTTTTGAAACACTCTTTTTGTAGAATCTGGAAGTGGATATTTGG

               * *   *  ** ** *  *   *            ** *
      76922 AGCTCCTTGGGGCCTATGCTGAAAAAGGAAATATCTTCCGAC
        130 AGCGCTTTGAGGATTTCGTTGGAAACGGAAATATCTTCATAT

                                 *     *  **          *           **    *    
      76964 AAAAACTAGACAGAAGCATTCGCAGAATCACGTTTGTGATGTGTGCACTCAACTGTCAGAATTGA
          1 AAAAACTAGACAGAAGCATTCTCAGAAACATCTTTGTGATGTTTGCACTCAACTCACAGAGTTGA

                 **   ** *      *                            * *            *
      77029 ACCTTGGTTTGGACAGAGCACTTTTGAAACACTCTTTTTGTAGAATCTGCAGGTGGATATTTGGC
         66 ACCTTTCTTTTCATAGAGCAGTTTTGAAACACTCTTTTTGTAGAATCTGGAAGTGGATATTTGGA

            **                         *   *      * *
      77094 TAGCTTTGAGGATTTCGTTGGAAACGGTAATGTCTTCAAAG
        131 GCGCTTTGAGGATTTCGTTGGAAACGGAAATATCTTCATAT

                *          *   *          *   *            *    *            
      77135 AAAATCTAGACAGAAACATCCTCAGAAACACCTTCGTGATGTTTGCAATCAAGTCACAGAGTTGA
          1 AAAAACTAGACAGAAGCATTCTCAGAAACATCTTTGTGATGTTTGCACTCAACTCACAGAGTTGA

                 * **       *    *  *         *        *             *       
      77200 ACCTTCCGATTCATAGTGCAGGTTGGAAACACTCATTTTGTAGTATCTGGAAGTGGACATTTGGA
         66 ACCTTTCTTTTCATAGAGCAGTTTTGAAACACTCTTTTTGTAGAATCTGGAAGTGGATATTTGGA

                   *   ** ** *  *   *            **  
      77265 GCGCTTTCAGGCCTATGGTGTAAAAGGAAATATCTTCCCAT
        131 GCGCTTTGAGGATTTCGTTGGAAACGGAAATATCTTCATAT

                * * * *      **      *   * *   *     ** ** *      *    *     
      77306 AAAAGCGACATAGAAGCTATCTCAGGAACTTGTTTATGATGCATCTAATCAACTAACAGTGTTGA
          1 AAAAACTAGACAGAAGCATTCTCAGAAACATCTTTGTGATGTTTGCACTCAACTCACAGAGTTGA

                  * ** * *                         * *       *               
      77371 ACCTTTGTACTGACAGAGCAG-TTTGAAACACTCTTTTTTTGGAATCTGCAAGTGGATATTTGGA
         66 ACCTTTCTTTTCATAGAGCAGTTTTGAAACACTCTTTTTGTAGAATCTGGAAGTGGATATTTGGA

            **  *                       *   *   *    
      77435 TGGCCTTGAGGATTTCGTTGGAAACGG-GAT-GC--AATAT
        131 GCGCTTTGAGGATTTCGTTGGAAACGGAAATATCTTCATAT

                **  *    *     *                 **  *   *  *    *         * 
      77472 AAAACGTACACAGCAGCATACTCAGAAA-ATACTTTGCCATATTTCCATTCAAGTCACAGAGTGG
          1 AAAAACTAGACAGAAGCATTCTCAGAAACAT-CTTTGTGATGTTTGCACTCAACTCACAGAGTTG

               *  * **            *           *      *  **            *      
      77536 AACATTCCCATTCATAGAGCAGGTTTGAAACACTTTTTTTGGAGTGTCTGGAAGTGGACATTTGG
         65 AACCTTTCTTTTCATAGAGCAGTTTTGAAACACTCTTTTTGTAGAATCTGGAAGTGGATATTTGG

                    *  * * ** *  *   *            **  
      77601 AGCGCTTTCAGAACTATGGTGAAAAAGGAAATATCTTCCAAT
        130 AGCGCTTTGAGGATTTCGTTGGAAACGGAAATATCTTCATAT

            *     *               *      * *         **  **       *  *  * *  
      77643 GAAAACAAGACAGAAGCATTCTGAGAAACTTATTTGTGATGCGTGTCCTCAACTAACGGACTCGA
          1 AAAAACTAGACAGAAGCATTCTCAGAAACATCTTTGTGATGTTTGCACTCAACTCACAGAGTTGA

                   *      **  * *  *  *             *   *    * * *          *
      77708 ACCTTTCGTTTCATGCAGTACTTCTGGAACACTCTTTTTGAAGATTCTGCATGCGGATATTTGGT
         66 ACCTTTCTTTTCATAGAGCAGTTTTGAAACACTCTTTTTGTAGAATCTGGAAGTGGATATTTGGA

            **                            **   *     
      77773 TAGCTTTGAGGATTTCGTTGGAAACGG---GCT-TACATAT
        131 GCGCTTTGAGGATTTCGTTGGAAACGGAAATATCTTCATAT

                 *       *               *        *   *    *    *       *    
      77810 AAAAATTAGACAGCAGCATTCTCAGAAACTTCTTTGTGGTGTCTGCATTCAAGTCACAGAATTGA
          1 AAAAACTAGACAGAAGCATTCTCAGAAACATCTTTGTGATGTTTGCACTCAACTCACAGAGTTGA

              * ** * **            *  * *      *       *    *        *       
      77875 ACATCCCCTCACATAGAGCAGTTGTGCAGCACTCTATTTGTAGTATCTCGAAGTGGACATTTGGA
         66 ACCTTTCTTTTCATAGAGCAGTTTTGAAACACTCTTTTTGTAGAATCTGGAAGTGGATATTTGGA

             *         ****           *            **  
      77940 GGGCTTTGTAGCCCATC--TGGATAAAGGAAATATCTTCCCAT
        131 GCGCTTTG-AGGATTTCGTTGGA-AACGGAAATATCTTCATAT

            *  ** *   *     * *            *   *  *   * **        *  * * *   
      77981 GAATGCGAGATAGAAGTAATCTCAGAAACATGTTTATGCTGTATCTACTCAACTAACTGTGCTGA
          1 AAAAACTAGACAGAAGCATTCTCAGAAACATCTTTGTGATGTTTGCACTCAACTCACAGAGTTGA

              *     *  *               *        *  * *       *              *
      78046 ACATTTCTATTGATAGAGCAGTTTTGAGACACTCTTCTTTTGGAATCTGCAAGTGGATATTTGGC
         66 ACCTTTCTTTTCATAGAGCAGTTTTGAAACACTCTTTTTGTAGAATCTGGAAGTGGATATTTGGA

            ** *                        *     *      
      78111 TAGATTTGAGGATTTCGTTGGAAACGG-GAT-T-AT-ATAT
        131 GCGCTTTGAGGATTTCGTTGGAAACGGAAATATCTTCATAT

            *    *       *               *                 **  *   *         
      78148 CAAAAGTAGACAGCAGCATTCTCAGAAACTTCTTTGTGATGTTTGCATCCAGCTCTCAGAGTTGA
          1 AAAAACTAGACAGAAGCATTCTCAGAAACATCTTTGTGATGTTTGCACTCAACTCACAGAGTTGA

              *  * *          *  *        *        *   **        *  **  *    
      78213 ACATTCCCTTTCATAGAGTAGGTTTGAAACCCTCTTTTTATAGTGTCTGGAAGCGGGCATGTGGA
         66 ACCTTTCTTTTCATAGAGCAGTTTTGAAACACTCTTTTTGTAGAATCTGGAAGTGGATATTTGGA

                   *   ** ** *  *   *          * *  *
      78278 GCGCTTTCAGGCCTATGCTGAAAAAGGAAATATCTACCTAC
        131 GCGCTTTGAGGATTTCGTTGGAAACGGAAATATCTTCATAT

             *                    *    *  **          ** **       *    *     
      78319 AGAAACTAGACAGAAGCATTCTGAGAATCACGTTTGTGATGTGGGTCCTCAACTAACAGTGTTGA
          1 AAAAACTAGACAGAAGCATTCTCAGAAACATCTTTGTGATGTTTGCACTCAACTCACAGAGTTGA

            *  *       *   *            *   *                *               
      78384 TCCATTCTTTTGATACAGCAGTTTTGAACCACCCTTTTTGTAGAATCTGCAAGTGGATATTTGGA
         66 ACCTTTCTTTTCATAGAGCAGTTTTGAAACACTCTTTTTGTAGAATCTGGAAGTGGATATTTGGA

            **   *                     *   *        *
      78449 TAGCTGTGAGGATTTCGTTGGAAACGGGAATGTCTTCATAG
        131 GCGCTTTGAGGATTTCGTTGGAAACGGAAATATCTTCATAT

                **                     * ****         *  **   *   *          
      78490 AAAATTTAGACAGAAGCATTCTCAGAACCTGGATTGTGATGTGTGTTCTCCACTAACAGAGTTGA
          1 AAAAACTAGACAGAAGCATTCTCAGAAACATCTTTGTGATGTTTGCACTCAACTCACAGAGTTGA

                      ** *   * *           *       *                   *     
      78555 ACCTTTCTTTGGACAGAACTGTTTTGAAACATTCTTTTTATAGAATCTGGAAGTGGATAGTTGGA
         66 ACCTTTCTTTTCATAGAGCAGTTTTGAAACACTCTTTTTGTAGAATCTGGAAGTGGATATTTGGA

            **                         *          *  
      78620 AAGCTTTGAGGATTTCGTTGGAAACGGGAATATCTTCAAAT
        131 GCGCTTTGAGGATTTCGTTGGAAACGGAAATATCTTCATAT

                *    *            *           * *       *  *    *            
      78661 AAAATCTAGCCAGAAGCATTCTAAGAAACATCTTAGGGATGTTTACATTCAAGTCACAGAGTTGA
          1 AAAAACTAGACAGAAGCATTCTCAGAAACATCTTTGTGATGTTTGCACTCAACTCACAGAGTTGA

              *   * *    **       *         *    * *   **      *      *    * 
      78726 ACATTCCCCTTTCTCAGAGCAGGTTTGAAACAATCTTCTCGTACTATCTGGCAGTGGACATTTTG
         66 ACCTT-TCTTTTCATAGAGCAGTTTTGAAACACTCTTTTTGTAGAATCTGGAAGTGGATATTTGG

               * *   *  ** ** *  *   *            ** *
      78791 AGCTCCTTGGGGCCTATGCTGAAAAAGGAAATATCTTCCGAC
        130 AGCGCTTTGAGGATTTCGTTGGAAACGGAAATATCTTCATAT

                                 *     *  **          *           **    *    
      78833 AAAAACTAGACAGAAGCATTCGCAGAATCACGTTTGTGATGTGTGCACTCAACTGTCAGAATTGA
          1 AAAAACTAGACAGAAGCATTCTCAGAAACATCTTTGTGATGTTTGCACTCAACTCACAGAGTTGA

                 **   ** *      *                            * *            *
      78898 ACCTTGGTTTGGACAGAGCACTTTTGAAACACTCTTTTTGTAGAATCTGCAGGTGGATATTTGGC
         66 ACCTTTCTTTTCATAGAGCAGTTTTGAAACACTCTTTTTGTAGAATCTGGAAGTGGATATTTGGA

            **                         *   *      * *
      78963 TAGCTTTGAGGATTTCGTTGGAAACGGTAATGTCTTCAAAG
        131 GCGCTTTGAGGATTTCGTTGGAAACGGAAATATCTTCATAT

                *          *   *          *   *            *    *            
      79004 AAAATCTAGACAGAAACATCCTCAGAAACACCTTCGTGATGTTTGCAATCAATTCACAGAGTTGA
          1 AAAAACTAGACAGAAGCATTCTCAGAAACATCTTTGTGATGTTTGCACTCAACTCACAGAGTTGA

                 * *        *    *  *         *        *             *       
      79069 ACCTTCCGTTTCATAGTGCAGGTTGGAAACACTCATTTTGTAGTATCTGGAAGTGGACATTTGGA
         66 ACCTTTCTTTTCATAGAGCAGTTTTGAAACACTCTTTTTGTAGAATCTGGAAGTGGATATTTGGA

                   *   ** ** *  *   *            **  
      79134 GCGCTTTCAGGCCTATGGTGTAAAAGGAAATATCTTCCCAT
        131 GCGCTTTGAGGATTTCGTTGGAAACGGAAATATCTTCATAT

                * * * *      **      *   * *   *     ** ** *      *    *     
      79175 AAAAGCGACATAGAAGCTATCTCAGGAACTTGTTTATGATGCATCTAATCAACTAACAGTGTTGA
          1 AAAAACTAGACAGAAGCATTCTCAGAAACATCTTTGTGATGTTTGCACTCAACTCACAGAGTTGA

                  * ** * *                         * *       *               
      79240 ACCTTTGTACTGACAGAGCAG-TTTGAAACACTCTTTTTTTGGAATCTGCAAGTGGATATTTGGA
         66 ACCTTTCTTTTCATAGAGCAGTTTTGAAACACTCTTTTTGTAGAATCTGGAAGTGGATATTTGGA

            *                           *   *   *    
      79304 TCGCTTTGAGGATTTCGTTGGAAACGG-GAT-GC--AATAT
        131 GCGCTTTGAGGATTTCGTTGGAAACGGAAATATCTTCATAT

                **  *    *    **                 **  *   *  *    *         * 
      79341 AAAACGTACACAGCAGCACACTCAGAAA-ATACTTTGCCATATTTCCATTCAAGTCACAGAGTGG
          1 AAAAACTAGACAGAAGCATTCTCAGAAACAT-CTTTGTGATGTTTGCACTCAACTCACAGAGTTG

               *  * **            *                  *  *            **      
      79405 AACATTCCCATTCATAGAGCAGGTTTGAAACACTCTTTTTGGAGTATCTGGAAGTGGGCATTTGG
         65 AACCTTTCTTTTCATAGAGCAGTTTTGAAACACTCTTTTTGTAGAATCTGGAAGTGGATATTTGG

              *     ** * * ** *  *   *            **  
      79470 AGAGCTTTCTGAACTATGGTGAAAAAGGAAATATCTTCCAAT
        130 AGCGCTTTGAGGATTTCGTTGGAAACGGAAATATCTTCATAT

            *     *               *      * *         **  **       * **  * *  
      79512 GAAAACAAGACAGAAGCATTCTGAGAAACTTATTTGTGATGCGTGTCCTCAACTAAAGGACTCGA
          1 AAAAACTAGACAGAAGCATTCTCAGAAACATCTTTGTGATGTTTGCACTCAACTCACAGAGTTGA

                   *      **  * *  *  *             *   *    * * *          *
      79577 ACCTTTCGTTTCATGCAGTACTTCTGGAACACTCTTTTTGAAGATTCTGCATGCGGATATTTGGT
         66 ACCTTTCTTTTCATAGAGCAGTTTTGAAACACTCTTTTTGTAGAATCTGGAAGTGGATATTTGGA

            **   *                        **   *     
      79642 TAGCTGTGAGGATTTCGTTGGAAAC-G--AGCT-TACATAT
        131 GCGCTTTGAGGATTTCGTTGGAAACGGAAATATCTTCATAT

                 *       *               *        *   *    *    *       *    
      79679 AAAAATTAGACAGCAGCATTCTCAGAAACTTCTTTGTGGTGTCTGCATTCAAGTCACAGAATTGA
          1 AAAAACTAGACAGAAGCATTCTCAGAAACATCTTTGTGATGTTTGCACTCAACTCACAGAGTTGA

              * ** * **            *  * *      *       *    *        *       
      79744 ACATCCCCTCACATAGAGCAGTTGTGCAGCACTCTATTTGTAGTATCTCGAAGTGGACATTTGGA
         66 ACCTTTCTTTTCATAGAGCAGTTTTGAAACACTCTTTTTGTAGAATCTGGAAGTGGATATTTGGA

             *         ** *    * *   *            **  
      79809 GGGCTTTGTAGCCTATC-TGGAAAAAGGAAATATCTTCCCAT
        131 GCGCTTTG-AGGATTTCGTTGGAAACGGAAATATCTTCATAT

            *  ** *   *     * *            *   *  *   * **        *  * * *   
      79850 GAATGCGAGATAGAAGTAATCTCAGAAACATGTTTATGCTGTATCTACTCAACTAACTGTGCTGA
          1 AAAAACTAGACAGAAGCATTCTCAGAAACATCTTTGTGATGTTTGCACTCAACTCACAGAGTTGA

              *     *  *               *        *  * *       *              *
      79915 ACATTTCTATTGATAGAGCAGTTTTGAGACACTCTTCTTTTGGAATCTGCAAGTGGATATTTGGC
         66 ACCTTTCTTTTCATAGAGCAGTTTTGAAACACTCTTTTTGTAGAATCTGGAAGTGGATATTTGGA

            ** *                        *     *      
      79980 TAGATTTGAGGATTTCGTTGGAAACGG-GAT-T-AT-ATAT
        131 GCGCTTTGAGGATTTCGTTGGAAACGGAAATATCTTCATAT

                 *       *               *                 **  *   *         
      80017 AAAAAGTAGACAGCAGCATTCTCAGAAACTTCTTTGTGATGTTTGCATCCAGCTCTCAGAGTTGA
          1 AAAAACTAGACAGAAGCATTCTCAGAAACATCTTTGTGATGTTTGCACTCAACTCACAGAGTTGA

              *  * *          *  *        * *      *   **        *  **     * 
      80082 ACATTCCGTTTCATAGAGTAGGTTTGAAACCCCCTTTTTATAGTGTCTGGAAGCGGGCATTTGCA
         66 ACCTTTCTTTTCATAGAGCAGTTTTGAAACACTCTTTTTGTAGAATCTGGAAGTGGATATTTGGA

             *         ** ** *  *   *          * *  *
      80147 GTGCTTTGAGGCCTAAGCTGAAAAAGGAAATATCTACCTAC
        131 GCGCTTTGAGGATTTCGTTGGAAACGGAAATATCTTCATAT

             *                    *    *  **          ** *        *    *     
      80188 AGAAACTAGACAGAAGCATTCTGAGAATCACGTTTGTGATGTGGGTACTCAACTAACAGTGTTGA
          1 AAAAACTAGACAGAAGCATTCTCAGAAACATCTTTGTGATGTTTGCACTCAACTCACAGAGTTGA

            *  *       *   *            *   *                *               
      80253 TCCATTCTTTTGATACAGCAGTTTTGAACCACCCTTTTTGTAGAATCTGCAAGTGGATATTTGGA
         66 ACCTTTCTTTTCATAGAGCAGTTTTGAAACACTCTTTTTGTAGAATCTGGAAGTGGATATTTGGA

            **   *                     *   *        *
      80318 TAGCTGTGAGGATTTCGTTGGAAACGGGAATGTCTTCATAG
        131 GCGCTTTGAGGATTTCGTTGGAAACGGAAATATCTTCATAT

                **                     * ****         *  **   *   *          
      80359 AAAATTTAGACAGAAGCATTCTCAGAACCTGGATTGTGATGTGTGTTCTCCACTAACAGAGTTGA
          1 AAAAACTAGACAGAAGCATTCTCAGAAACATCTTTGTGATGTTTGCACTCAACTCACAGAGTTGA

                       * *   * *           *       *                         
      80424 ACCTTTCTTTTGACAGAACTGTTTTGAAACATTCTTTTTATAGAATCTGGAAGTGGATATTTGGA
         66 ACCTTTCTTTTCATAGAGCAGTTTTGAAACACTCTTTTTGTAGAATCTGGAAGTGGATATTTGGA

            **                         *          *  
      80489 AAGCTTTGAGGATTTCGTTGGAAACGGGAATATCTTCAAAT
        131 GCGCTTTGAGGATTTCGTTGGAAACGGAAATATCTTCATAT

                *    *            *           * *     * *  *    *            
      80530 AAAATCTAGCCAGAAGCATTCTAAGAAACATCTTAGGGATGTGTACATTCAAGTCACAGAGTTGA
          1 AAAAACTAGACAGAAGCATTCTCAGAAACATCTTTGTGATGTTTGCACTCAACTCACAGAGTTGA

              *   * *    **       *         *    * *   **      *      *    * 
      80595 ACATTCCCCTTTCTCAGAGCAGGTTTGAAACAATCTTCTCGTACTATCTGGCAGTGGACATTTTG
         66 ACCTT-TCTTTTCATAGAGCAGTTTTGAAACACTCTTTTTGTAGAATCTGGAAGTGGATATTTGG

               * *   *  ** ** *  *   *            ** *
      80660 AGCTCCTTGGGGCCTATGCTGAAAAAGGAAATATCTTCCGAC
        130 AGCGCTTTGAGGATTTCGTTGGAAACGGAAATATCTTCATAT

                                 *     *  **          * *       * **    *    
      80702 AAAAACTAGACAGAAGCATTCGCAGAATCACGTTTGTGATGTGTTCACTCAATTGTCAGAATTGA
          1 AAAAACTAGACAGAAGCATTCTCAGAAACATCTTTGTGATGTTTGCACTCAACTCACAGAGTTGA

                  *   ** *  *   *      *          * *  *     * *            *
      80767 ACCTTTGTTTGGACAGGGCACTTTTGAGACACTCTTTTCGCAGGATCTGCAGGTGGATATTTGGC
         66 ACCTTTCTTTTCATAGAGCAGTTTTGAAACACTCTTTTTGTAGAATCTGGAAGTGGATATTTGGA

            **                         *   *      * *
      80832 TAGCTTTGAGGATTTCGTTGGAAACGGTAATGTCTTCAAAG
        131 GCGCTTTGAGGATTTCGTTGGAAACGGAAATATCTTCATAT

                *          *   *          *   *            *    *            
      80873 AAAATCTAGACAGAAACATCCTCAGAAACACCTTCGTGATGTTTGCAATCAAGTCACAGAGTTGA
          1 AAAAACTAGACAGAAGCATTCTCAGAAACATCTTTGTGATGTTTGCACTCAACTCACAGAGTTGA

                 * *             *  *         *        *             *       
      80938 ACCTTCCGTTTCATAGAGCAGGTTGGAAACACTCATTTTGTAGTATCTGGAAGTGGACATTTGGA
         66 ACCTTTCTTTTCATAGAGCAGTTTTGAAACACTCTTTTTGTAGAATCTGGAAGTGGATATTTGGA

                   *   ** ** *  *   *            **  
      81003 GCGCTTTCAGGCCTATGGTGTAAAAGGAAATATCTTCCCAT
        131 GCGCTTTGAGGATTTCGTTGGAAACGGAAATATCTTCATAT

                * * * *      **      *   * *   *     ** ** *      *    *     
      81044 AAAAGCGACATAGAAGCTATCTCAGGAACTTGTTTATGATGCCTCTAATCAACTAACAGTGTTGA
          1 AAAAACTAGACAGAAGCATTCTCAGAAACATCTTTGTGATGTTTGCACTCAACTCACAGAGTTGA

                  * ** * *                         * *       *               
      81109 ACCTTTGTACTGACAGAGCAG-TTTGAAACACTCTTTTTTTGGAATCTGCAAGTGGATATTTGGA
         66 ACCTTTCTTTTCATAGAGCAGTTTTGAAACACTCTTTTTGTAGAATCTGGAAGTGGATATTTGGA

            *                           *   *   *    
      81173 TCGCTTTGAGGATTTCGTTGGAAACGG-GAT-GC--AATAT
        131 GCGCTTTGAGGATTTCGTTGGAAACGGAAATATCTTCATAT

                **  *    *     * *               **  *   *  *    *         * 
      81210 AAAACGTACACAGCAGCATACCCAGAAA-ATACTTTGCCATATTTCCATTCAAGTCACAGAGTGG
          1 AAAAACTAGACAGAAGCATTCTCAGAAACAT-CTTTGTGATGTTTGCACTCAACTCACAGAGTTG

               *  * **            *  *               *  *             *      
      81274 AACATTCCCATTCATAGAGCAGGTTGGAAACACTCTTTTTGGAGTATCTGGAAGTGGACATTTGG
         65 AACCTTTCTTTTCATAGAGCAGTTTTGAAACACTCTTTTTGTAGAATCTGGAAGTGGATATTTGG

                    ** * * ** *  *   *            **  
      81339 AGCGCTTTCTGAACTATGGTGAAAAAGGAAATATCTTCCAAT
        130 AGCGCTTTGAGGATTTCGTTGGAAACGGAAATATCTTCATAT

            *  *  *     *         *      * *         **  **      **  *  * *  
      81381 GAATACAAGACACAAGCATTCTGAGAAACTTATTTGTGATGCGTGTCCTCAACAAACGGACTCGA
          1 AAAAACTAGACAGAAGCATTCTCAGAAACATCTTTGTGATGTTTGCACTCAACTCACAGAGTTGA

             *     *      **  * *  *  *             *   *    * * **         *
      81446 AGCTTTCGTTTCATGCAGTACTTCTGGAACACTCTTTTTGAAGATTCTGCATGCAGATATTTGGT
         66 ACCTTTCTTTTCATAGAGCAGTTTTGAAACACTCTTTTTGTAGAATCTGGAAGTGGATATTTGGA

            **           *                **   *     
      81511 TAGCTTTGAGGATATCGTTGGAAACGG---GCT-TACATAT
        131 GCGCTTTGAGGATTTCGTTGGAAACGGAAATATCTTCATAT

                 *       *               *        *   *    *    *       *    
      81548 AAAAATTAGACAGCAGCATTCTCAGAAACTTCTTTGTGGTGTCTGCATTCAAGTCACAGAATTGA
          1 AAAAACTAGACAGAAGCATTCTCAGAAACATCTTTGTGATGTTTGCACTCAACTCACAGAGTTGA

              * ** * **            *  * *      *   *   *    *        *       
      81613 ACATCCCCTCACATAGAGCAGTTGTGCAGCACTCTATTTTTAGTATCTCGAAGTGGACATTTGGA
         66 ACCTTTCTTTTCATAGAGCAGTTTTGAAACACTCTTTTTGTAGAATCTGGAAGTGGATATTTGGA

             *         ** *           *            **  
      81678 GGGCTTTGTAGCCTATC--TGGATAAAGGAAATATCTTCCCAT
        131 GCGCTTTG-AGGATTTCGTTGGA-AACGGAAATATCTTCATAT

            *  ** *   **    * *            *   *  *   * **        *  * * *   
      81719 GAATGCGAGATGGAAGTAATCTCAGAAACATGTTTATGCTGTATCTACTCAACTAACTGTGCTGA
          1 AAAAACTAGACAGAAGCATTCTCAGAAACATCTTTGTGATGTTTGCACTCAACTCACAGAGTTGA

              *     *  *               *        *  * *       *              *
      81784 ACATTTCTATTGATAGAGCAGTTTTGAGACACTCTTCTTTTGGAATCTGCAAGTGGATATTTGGC
         66 ACCTTTCTTTTCATAGAGCAGTTTTGAAACACTCTTTTTGTAGAATCTGGAAGTGGATATTTGGA

            ** *                        *     *      
      81849 TAGATTTGAGGATTTCGTTGGAAACGG-GAT-T-AT-ATAT
        131 GCGCTTTGAGGATTTCGTTGGAAACGGAAATATCTTCATAT

                 *       *               *                 **  *   *         
      81886 AAAAAGTAGACAGCAGCATTCTCAGAAACTTCTTTGTGATGTTTGCATCCAGCTCTCAGAGTTGA
          1 AAAAACTAGACAGAAGCATTCTCAGAAACATCTTTGTGATGTTTGCACTCAACTCACAGAGTTGA

              *  * *          *  *        * *      *   **        *  **       
      81951 ACATTCCCTTTCATAGAGTAGGTTTGAAACCCCCTTTTTATAGTGTCTGGAAGCGGGCATTTGGA
         66 ACCTTTCTTTTCATAGAGCAGTTTTGAAACACTCTTTTTGTAGAATCTGGAAGTGGATATTTGGA

                   *   ** ** *  *   *          * *  *
      82016 GCGCTTTCAGGTCTGTGCTGAAAAAGGAAATATCTACCTAC
        131 GCGCTTTGAGGATTTCGTTGGAAACGGAAATATCTTCATAT

             *                    *    *  **   *      ** *        *    *     
      82057 AGAAACTAGACAGAAGCATTCTGAGAATCACGTTTTTGATGTGGGTACTCAACTAACAGTGTTGA
          1 AAAAACTAGACAGAAGCATTCTCAGAAACATCTTTGTGATGTTTGCACTCAACTCACAGAGTTGA

            *  *    *  *   *            *   *                *               
      82122 TCCATTCTATTGATACAGCAGTTTTGAACCACCCTTTTTGTAGAATCTGCAAGTGGATATTTGGA
         66 ACCTTTCTTTTCATAGAGCAGTTTTGAAACACTCTTTTTGTAGAATCTGGAAGTGGATATTTGGA

            **   *                     *   *        *
      82187 TAGCTGTGAGGATTTCGTTGGAAACGGGAATGTCTTCATAG
        131 GCGCTTTGAGGATTTCGTTGGAAACGGAAATATCTTCATAT

                **                     * ****         *  **   *   *          
      82228 AAAATTTAGACAGAAGCATTCTCAGAACCTGGATTGTGATGTGTGTTCTCCACTAACAGAGTTGA
          1 AAAAACTAGACAGAAGCATTCTCAGAAACATCTTTGTGATGTTTGCACTCAACTCACAGAGTTGA

                       * *   * *           *       *         *         *     
      82293 ACCTTTCTTTTGACAGAACTGTTTTGAAACATTCTTTTTATAGAATCTGTAAGTGGATAGTTGGA
         66 ACCTTTCTTTTCATAGAGCAGTTTTGAAACACTCTTTTTGTAGAATCTGGAAGTGGATATTTGGA

            **                         *          ** 
      82358 AAGCTTTGAGGATTTCGTTGGAAACGGGAATATCTTCAAGT
        131 GCGCTTTGAGGATTTCGTTGGAAACGGAAATATCTTCATAT

                *    *            *           * *     * *  *    *            
      82399 AAAATCTAGCCAGAAGCATTCTAAGAAACATCTTAGGGATGTGTACATTCAAGTCACAGAGTTGA
          1 AAAAACTAGACAGAAGCATTCTCAGAAACATCTTTGTGATGTTTGCACTCAACTCACAGAGTTGA

              *   * *    **       *         *    * *   **      *      *    * 
      82464 ACATTCCCCTTTCTCAGAGCAGGTTTGAAACAATCTTCTCGTACTATCTGGCAGTGGACATTTTG
         66 ACCTT-TCTTTTCATAGAGCAGTTTTGAAACACTCTTTTTGTAGAATCTGGAAGTGGATATTTGG

               * *   *  ** ** *  *   *            ** *
      82529 AGCTCCTTGGGGCCTATGCTGAAAAAGGAAATATCTTCCGAC
        130 AGCGCTTTGAGGATTTCGTTGGAAACGGAAATATCTTCATAT

                                 *     *  **          *           **    *    
      82571 AAAAACTAGACAGAAGCATTCACAGAATCACGTTTGTGATGTGTGCACTCAACTGTCAGAATTGA
          1 AAAAACTAGACAGAAGCATTCTCAGAAACATCTTTGTGATGTTTGCACTCAACTCACAGAGTTGA

                  *   ** *      *                            * *            *
      82636 ACCTTTGTTTGGACAGAGCACTTTTGAAACACTCTTTTTGTAGAATCTGCAGGTGGATATTTGGC
         66 ACCTTTCTTTTCATAGAGCAGTTTTGAAACACTCTTTTTGTAGAATCTGGAAGTGGATATTTGGA

            **                         *   *      * *
      82701 TAGCTTTGAGGATTTCGTTGGAAACGGTAATGTCTTCAAAG
        131 GCGCTTTGAGGATTTCGTTGGAAACGGAAATATCTTCATAT

                *          *   *          *   *            *    *            
      82742 AAAATCTAGACAGAAACATCCTCAGAAACACCTTCGTGATGTTTGCAATCAAGTCACAGAGTTGA
          1 AAAAACTAGACAGAAGCATTCTCAGAAACATCTTTGTGATGTTTGCACTCAACTCACAGAGTTGA

                 * *             *  *         *        **   *        *       
      82807 ACCTTCCGTTTCATAGAGCAGGTTGGAAACACTCATTTTGTAGTGTCTAGAAGTGGACATTTGGA
         66 ACCTTTCTTTTCATAGAGCAGTTTTGAAACACTCTTTTTGTAGAATCTGGAAGTGGATATTTGGA

                   *   ** ** *  *   *            **  
      82872 GCGCTTTCAGGCCTATGGTGTAAAAGGAAATATCTTCCCAT
        131 GCGCTTTGAGGATTTCGTTGGAAACGGAAATATCTTCATAT

                * * * *      **      *   * *   *     ** ** *      *    *     
      82913 AAAAGCGACATAGAAGCTATCTCAGGAACTTGTTTATGATGCCTCTAATCAACTAACAGTGTTGA
          1 AAAAACTAGACAGAAGCATTCTCAGAAACATCTTTGTGATGTTTGCACTCAACTCACAGAGTTGA

                  * ** * *                         * *       *               
      82978 ACCTTTGTACTGACAGAGCAG-TTTGAAACACTCTTTTTTTGGAATCTGCAAGTGGATATTTGGA
         66 ACCTTTCTTTTCATAGAGCAGTTTTGAAACACTCTTTTTGTAGAATCTGGAAGTGGATATTTGGA

            *                           *   *   *    
      83042 TCGCTTTGAGGATTTCGTTGGAAACGG-GAT-GC--AATAT
        131 GCGCTTTGAGGATTTCGTTGGAAACGGAAATATCTTCATAT

                **  *    *     *                 **  *   *  * *  *         * 
      83079 AAAACGTACACAGCAGCATACTCAGAAA-ATACTTTGCCATATTTCCATTAAAGTCACAGAGTGG
          1 AAAAACTAGACAGAAGCATTCTCAGAAACAT-CTTTGTGATGTTTGCACTCAACTCACAGAGTTG

               *  * **            *                  *  *             *      
      83143 AACATTCCCATTCATAGAGCAGGTTTGAAACACTCTTTTTGGAGTATCTGGAAGTGGACATTTGG
         65 AACCTTTCTTTTCATAGAGCAGTTTTGAAACACTCTTTTTGTAGAATCTGGAAGTGGATATTTGG

              *     ** * * ** *  *   *        *   **  
      83208 AGTGCTTTCTGAACTATGGTGAAAAGGGAAATATGTTCCAAT
        130 AGCGCTTTGAGGATTTCGTTGGAAACGGAAATATCTTCATAT

            *     *               *      * *         **  **       *  *  * *  
      83250 GAAAACAAGACAGAAGCATTCTGAGAAACTTATTTGTGATGCGTGTCCTCAACTAACGGACTCGA
          1 AAAAACTAGACAGAAGCATTCTCAGAAACATCTTTGTGATGTTTGCACTCAACTCACAGAGTTGA

                   *      **  * *  *  *             *   *    * * *          *
      83315 ACCTTTCGTTTCATGCAGTACTTCTGGAACACTCTTTTTGAAGATTCTGCATGCGGATATTTGGT
         66 ACCTTTCTTTTCATAGAGCAGTTTTGAAACACTCTTTTTGTAGAATCTGGAAGTGGATATTTGGA

            **                            **   *     
      83380 TAGCTTTGAGGATTTCGTTGGAAACGG---GCT-TACATAT
        131 GCGCTTTGAGGATTTCGTTGGAAACGGAAATATCTTCATAT

                 *       *               *        *   *    *    *       *    
      83417 AAAAATTAGACAGCAGCATTCTCAGAAACTTCTTTGTGGTGTCTGCATTCAAGTCACAGAATTGA
          1 AAAAACTAGACAGAAGCATTCTCAGAAACATCTTTGTGATGTTTGCACTCAACTCACAGAGTTGA

              * ** * **            *    *      *       *    *        *       
      83482 ACATCCCCTCACATAGAGCAGTTGTGAAGCACTCTATTTGTAGTATCTCGAAGTGGACATTTGGA
         66 ACCTTTCTTTTCATAGAGCAGTTTTGAAACACTCTTTTTGTAGAATCTGGAAGTGGATATTTGGA

             *         ** *    * *   *           ***  
      83547 GGGCTTTGTAGCCTATC-TGGAAAAAGGAAATATCTTTCCAT
        131 GCGCTTTG-AGGATTTCGTTGGAAACGGAAATATCTTCATAT

            *  ** *   *     * *            *   *  *   * **        *  * * *   
      83588 GAATGCGAGATAGAAGTAATCTCAGAAACATGTTTATGCTGTATCTACTCAACTAACTGTGCTGA
          1 AAAAACTAGACAGAAGCATTCTCAGAAACATCTTTGTGATGTTTGCACTCAACTCACAGAGTTGA

              *     *  *               *        *  * *       *              *
      83653 ACATTTCTATTGATAGAGCAGTTTTGAGACACTCTTCTTTTGGAATCTGCAAGTGGATATTTGGC
         66 ACCTTTCTTTTCATAGAGCAGTTTTGAAACACTCTTTTTGTAGAATCTGGAAGTGGATATTTGGA

            ** *                        *     *      
      83718 TAGATTTGAGGATTTCGTTGGAAACGG-GAT-T-AT-ATAT
        131 GCGCTTTGAGGATTTCGTTGGAAACGGAAATATCTTCATAT

            *   **       *               *                 **  *   *         
      83755 CAAAGGTAGACAGCAGCATTCTCAGAAACTTCTTTGTGATGTTTGCATCCAGCTCTCAGAGTTGA
          1 AAAAACTAGACAGAAGCATTCTCAGAAACATCTTTGTGATGTTTGCACTCAACTCACAGAGTTGA

              *  * *          *  *        *        *   **        *  **       
      83820 ACATTCCCTTTCATAGAGTAGGTTTGAAACCCTCTTTTTATAGTGTCTGGAAGCGGGCATTTGGA
         66 ACCTTTCTTTTCATAGAGCAGTTTTGAAACACTCTTTTTGTAGAATCTGGAAGTGGATATTTGGA

                   *   ** ** *  *   *          * *  *
      83885 GCGCTTTCAGGCCTATGCTGAAAAAGGAAATATCTACCTAC
        131 GCGCTTTGAGGATTTCGTTGGAAACGGAAATATCTTCATAT

             *                    *    *  **          ** *        *    *     
      83926 AGAAACTAGACAGAAGCATTCTGAGAATCACGTTTGTGATGTGGGTACTCAACTAACAGTGTTGA
          1 AAAAACTAGACAGAAGCATTCTCAGAAACATCTTTGTGATGTTTGCACTCAACTCACAGAGTTGA

            *  *       *   *            *   *                *               
      83991 TCCATTCTTTTGATACAGCAGTTTTGAACCACCCTTTTTGTAGAATCTGCAAGTGGATATTTGGA
         66 ACCTTTCTTTTCATAGAGCAGTTTTGAAACACTCTTTTTGTAGAATCTGGAAGTGGATATTTGGA

            **   *                     *   *        *
      84056 TAGCTGTGAGGATTTCGTTGGAAACGGGAATGTCTTCATAG
        131 GCGCTTTGAGGATTTCGTTGGAAACGGAAATATCTTCATAT

                **                     * ****         *  **   *   *          
      84097 AAAATTTAGACAGAAGCATTCTCAGAACCTGGATTGTGATGTGTGTTCTCCACTAACAGAGTTGA
          1 AAAAACTAGACAGAAGCATTCTCAGAAACATCTTTGTGATGTTTGCACTCAACTCACAGAGTTGA

                       * *   * *           *       *                         
      84162 ACCTTTCTTTTGACAGAACTGTTTTGAAACATTCTTTTTATAGAATCTGGAAGTGGATATTTGGA
         66 ACCTTTCTTTTCATAGAGCAGTTTTGAAACACTCTTTTTGTAGAATCTGGAAGTGGATATTTGGA

            **          *              *          *  
      84227 AAGCTTTGAGGAATTCGTTGGAAACGGGAATATCTTCAAAT
        131 GCGCTTTGAGGATTTCGTTGGAAACGGAAATATCTTCATAT

                *    *            *           * *       *  *    *            
      84268 AAAATCTAGCCAGAAGCATTCTAAGAAACATCTTAGGGATGTTTACATTCAAGTCACAGAGTTGA
          1 AAAAACTAGACAGAAGCATTCTCAGAAACATCTTTGTGATGTTTGCACTCAACTCACAGAGTTGA

              *   * *    **       *         *    * *   **      *      *    * 
      84333 ACATTCCCCTTTCTCAGAGCAGGTTTGAAACAATCTTCTCGTACTATCTGGCAGTGGACATTTTG
         66 ACCTT-TCTTTTCATAGAGCAGTTTTGAAACACTCTTTTTGTAGAATCTGGAAGTGGATATTTGG

               * *   *  ** ** *  *   *            ** *
      84398 AGCTCCTTGGGGCCTATGCTGAAAAAGGAAATATCTTCTGAC
        130 AGCGCTTTGAGGATTTCGTTGGAAACGGAAATATCTTCATAT

                                 *     *  **          *           **    *    
      84440 AAAAACTAGACAGAAGCATTCGCAGAATCACGTTTGTGATGTGTGCACTCAACTGTCAGAATTGA
          1 AAAAACTAGACAGAAGCATTCTCAGAAACATCTTTGTGATGTTTGCACTCAACTCACAGAGTTGA

                  *   ** *      *                            * *            *
      84505 ACCTTTGTTTGGACAGAGCACTTTTGAAACACTCTTTTTGTAGAATCTGCAGGTGGATATTTGGC
         66 ACCTTTCTTTTCATAGAGCAGTTTTGAAACACTCTTTTTGTAGAATCTGGAAGTGGATATTTGGA

            ***                        *   *      * *
      84570 TACCTTTGAGGATTTCGTTGGAAACGGTAATGTCTTCAAAG
        131 GCGCTTTGAGGATTTCGTTGGAAACGGAAATATCTTCATAT

                *          *   *          *   *            *    *            
      84611 AAAATCTAGACAGAAACATCCTCAGAAACAACTTCGTGATGTTTGCAATCAAGTCACAGAGTTGA
          1 AAAAACTAGACAGAAGCATTCTCAGAAACATCTTTGTGATGTTTGCACTCAACTCACAGAGTTGA

                 * *             *  *         *        *             *       
      84676 ACCTTCCGTTTCATAGAGCAGGTTGGAAACACTCATTTTGTAGTATCTGGAAGTGGACATTTGGA
         66 ACCTTTCTTTTCATAGAGCAGTTTTGAAACACTCTTTTTGTAGAATCTGGAAGTGGATATTTGGA

                   *   ** ** *  *   *            **  
      84741 GCGCTTTCAGGCCTATGGTGTAAAAGGAAATATCTTCCCAT
        131 GCGCTTTGAGGATTTCGTTGGAAACGGAAATATCTTCATAT

                * * ***      **   *  *   * *   *     ** ** *      *    *     
      84782 AAAAGCGACTTAGAAGCTATCTAAGGAACTTGTTTATGATGCATCTAATCAACTAACAGTGTTGA
          1 AAAAACTAGACAGAAGCATTCTCAGAAACATCTTTGTGATGTTTGCACTCAACTCACAGAGTTGA

                  * ** * *                         * *       *               
      84847 ACCTTTGTACTGACAGAGCAG-TTTGAAACACTCTTTTTTTGGAATCTGCAAGTGGATATTTGGA
         66 ACCTTTCTTTTCATAGAGCAGTTTTGAAACACTCTTTTTGTAGAATCTGGAAGTGGATATTTGGA

            *                           *   *   *    
      84911 TCGCTTTGAGGATTTCGTTGGAAACGG-GAT-GC--AATAT
        131 GCGCTTTGAGGATTTCGTTGGAAACGGAAATATCTTCATAT

                **  *    *     *                 **  *   *  *    *         * 
      84948 AAAACGTACACAGCAGCATACTCAGAAA-ATACTTTGCCATATTTCCATTCAAGTCACAGAGTGG
          1 AAAAACTAGACAGAAGCATTCTCAGAAACAT-CTTTGTGATGTTTGCACTCAACTCACAGAGTTG

               *  * **            *                  *  **            *      
      85012 AACATTCCCATTCATAGAGCAGGTTTGAAACACTCTTTTTGGAGTCTCTGGAAGTGGACATTTGG
         65 AACCTTTCTTTTCATAGAGCAGTTTTGAAACACTCTTTTTGTAGAATCTGGAAGTGGATATTTGG

                    ** * * ** *  *   *            **  
      85077 AGCGCTTTCTGAACTATGGTGAAAAAGGAAATATCTTCCAAT
        130 AGCGCTTTGAGGATTTCGTTGGAAACGGAAATATCTTCATAT

            *     *               *      * *         **  **       *  *  * *  
      85119 GAAAACAAGACAGAAGCATTCTGAGAAACTTATTTGTGATGCGTGTCCTCAACTAACGGACTCGA
          1 AAAAACTAGACAGAAGCATTCTCAGAAACATCTTTGTGATGTTTGCACTCAACTCACAGAGTTGA

             *     *      **  * *  *  *             *   *    * * *          *
      85184 AGCTTTCGTTTCATGCAGTACTTCTGGAACACTCTTTTTGAAGATTCTGCATGCGGATATTTGGT
         66 ACCTTTCTTTTCATAGAGCAGTTTTGAAACACTCTTTTTGTAGAATCTGGAAGTGGATATTTGGA

            **                            **   *     
      85249 TAGCTTTGAGGATTTCGTTGGAAACGG---GCT-TACATAT
        131 GCGCTTTGAGGATTTCGTTGGAAACGGAAATATCTTCATAT

                 *       *               *        *   *    *    *       *    
      85286 AAAAATTAGACAGGAGCATTCTCAGAAACTTCTTTGTGGTGTCTGCATTCAAGTCACAGAATTGA
          1 AAAAACTAGACAGAAGCATTCTCAGAAACATCTTTGTGATGTTTGCACTCAACTCACAGAGTTGA

              * ** * **            * ** *      *       *    *        *       
      85351 ACATCCCCTCACATAGAGCAGTTGTACAGCACTCTATTTGTAGTATCTCGAAGTGGACATTTGGA
         66 ACCTTTCTTTTCATAGAGCAGTTTTGAAACACTCTTTTTGTAGAATCTGGAAGTGGATATTTGGA

             *         ** *    * *   *            **  
      85416 GGGCTTTGTAGCCTATC-TGGAAAAAGGAAATATCTTCCCAT
        131 GCGCTTTG-AGGATTTCGTTGGAAACGGAAATATCTTCATAT

            *  ** *   *     * *            *   *  *   * **        *  * * *   
      85457 GAATGCGAGATAGAAGTAATCTCAGAAACATGTTTATGCTGTATCTACTCAACTAACTGTGCTGA
          1 AAAAACTAGACAGAAGCATTCTCAGAAACATCTTTGTGATGTTTGCACTCAACTCACAGAGTTGA

              *     *  *               *        *  * *       *              *
      85522 ACATTTCTATTGATAGAGCAGTTTTGAGACACTCTTCTTTTGGAATCTGCAAGTGGATATTTGGC
         66 ACCTTTCTTTTCATAGAGCAGTTTTGAAACACTCTTTTTGTAGAATCTGGAAGTGGATATTTGGA

            ** *                        *     *      
      85587 TAGATTTGAGGATTTCGTTGGAAACGG-GAT-T-AT-ATAT
        131 GCGCTTTGAGGATTTCGTTGGAAACGGAAATATCTTCATAT

            *   **       *               *                 **  *   *         
      85624 CAAAGGTAGACAGCAGCATTCTCAGAAACTTCTTTGTGATGTTTGCATCCAGCTCTCAGAGTTGA
          1 AAAAACTAGACAGAAGCATTCTCAGAAACATCTTTGTGATGTTTGCACTCAACTCACAGAGTTGA

              *  * *     *    *  *        * *      *   **        *  **       
      85689 ACATTCCCTTTCACAGAGTAGGTTTGAAACCCCCTTTTTATAGTGTCTGGAAGCGGGCATTTGGA
         66 ACCTTTCTTTTCATAGAGCAGTTTTGAAACACTCTTTTTGTAGAATCTGGAAGTGGATATTTGGA

            *      *   ** ** *  *   *          * * **
      85754 ACGCTTTCAGGCCTATGCTGAAAAAGGAAATATCTACCTTC
        131 GCGCTTTGAGGATTTCGTTGGAAACGGAAATATCTTCATAT

             *               *    *    *  **          ** *        *    *     
      85795 AGAAACTAGACAGAAGCGTTCTGAGAATCACGTTTGTGATGTGGGTACTCAACTAACAGTGTTGA
          1 AAAAACTAGACAGAAGCATTCTCAGAAACATCTTTGTGATGTTTGCACTCAACTCACAGAGTTGA

            *  *       *   *            *   *                *   *           
      85860 TCCATTCTTTTGATACAGCAGTTTTGAACCACCCTTTTTGTAGAATCTGCAAGCGGATATTTGGA
         66 ACCTTTCTTTTCATAGAGCAGTTTTGAAACACTCTTTTTGTAGAATCTGGAAGTGGATATTTGGA

            **   *         *           *   *        *
      85925 TAGCTGTGAGGATTTAGTTGGAAACGGGAATGTCTTCATAG
        131 GCGCTTTGAGGATTTCGTTGGAAACGGAAATATCTTCATAT

                **                     * ****         *  **   *   *          
      85966 AAAATTTAGACAGAAGCATTCTCAGAACCTGGATTGTGATGTGTGTTCTCCACTAACAGAGTTGA
          1 AAAAACTAGACAGAAGCATTCTCAGAAACATCTTTGTGATGTTTGCACTCAACTCACAGAGTTGA

                       * *   * *           *     * *                         
      86031 ACCTTTCTTTTGACAGAACTGTTTTGAAACATTCTTTGTATAGAATCTGGAAGTGGATATTTGGA
         66 ACCTTTCTTTTCATAGAGCAGTTTTGAAACACTCTTTTTGTAGAATCTGGAAGTGGATATTTGGA

            **          *              *   *        *
      86096 AAGCTTTGAGGAATTCGTTGGAAACGGGAATGTCTTCATAG
        131 GCGCTTTGAGGATTTCGTTGGAAACGGAAATATCTTCATAT

                **                     * ****         *  *   ** *  ****   *  
      86137 AAAATTTAGACAGAAGCATTCTCAGAACCTGGATTGTGATGTGTGTA-TAGAATCTGGAAGTGGA
          1 AAAAACTAGACAGAAGCATTCTCAGAAACATCTTTGTGATGTTTGCACTCAACTCACAGAGTTGA

            ***  ****** * *   * *                  *    ****  *       **   * 
      86201 TATTTGGAAAGCTTTGAGGATTTCGTTGGAAACGGGAATATCTTCAAATAAAATCTAGCCAGAAG
         66 ACCTTTCTTTTCATAGAGCAGTT--TT-GAAAC----A-CTCTTTTTGTAGAATCT-GGAAG-TG

            *   * ** * *  *   *            *    *  *** * * ** ** *  
      86266 CATTCTAAGAAACATCTTAG-GGATGTGTACATT--CAAGTCACAGAGTTGAAAAT
        121 GA-TATTTGGAGC-GCTTTGAGGAT-T-T-CGTTGGAAACGGAAATATCTTCATAT

            ****** ***         ** **                ** * *  *     ****    ** 
      86319 TCCCCTTTCTCAG-AGCA-GGTTTGAAACAATCTTCTCGTACTATCTGGA---AGTGGACA-TTT
          1 AAAAACTAGACAGAAGCATTCTCAGAAAC-ATCTT-T-GTGATGTTTGCACTCAACTCACAGAGT

               *   *   **** *    *** *****    *      ***** *  ** *     **  * 
      86378 TGAGCTCCTT-GGGGCCTATGCTGAAAAAGGAAATA-TCTTCCGACAAAAAGTAGACAGAAG-CA
         63 TGAAC-CTTTCTTTTCATA-GAGCAGTTTTGAAACACTCTTTTTGTAGAATCTGGA-AGTGGATA

              *      * *       *   *      *       * ** *  *  * ***
      86440 TTCGCAGAATCACGTTTG-TGATGT-G-TGCACTCAACTGTCAGA-ATTGAACC
        125 TTTG--G-AGCGC-TTTGAGGATTTCGTTGGA---AACGGAAATATCTTCATAT

            ****** *           *  **                    *       *   *      * 
      86490 TTGGTTTGGACAG-AGCA-CCTATGAAACACTCTTT-T--TG-TAG-A---ATCT-GCAG-GTGG
          1 AAAAACTAGACAGAAGCATTCTCAGAAACA-TCTTTGTGATGTTTGCACTCAACTCACAGAGTTG

             ***  *** ** * *   * *                * * *  ***        **      *
      86543 ATATTTGGCTAGCTTTGAGGATTTCGTTGGAAACGGTAATGTCTTCAAAGAAAATCAAGACAGAA
         65 AACCTTTCTTTTCATAGAGCAGTT--TT-GAAAC---ACTCTTTTTGTAG--AATCTGGA-AG-T

            **   * ** * * * *  ***   *      *  *       *   *  *   
      86608 ACATCCTCAGAAACACCTTCGTGATGT--TTGCAATC---AA-GTC-ACAGAGT
        120 GGAT-ATTTGGAGCGCTTTGAGGATTTCGTTGGAAACGGAAATATCTTCATA-T

            **  *    *   *  *       ** **         *  *  **  *    ***  *  *   
      86655 TGAACCTTCCGTTTCATAGAGCA-GGTTGGAAACACTCATTTTGTAGTAT-C-TGGAAGTGGACA
          1 AAAAAC-T-AG--ACAGA-AGCATTCTCAGAAACA-TCTTTGTGATGTTTGCACTCAACT-CACA

             ** *  *       ****    * * *  ****    *   *  ****  *  * ***      
      86717 -TTTGGAGCGCTTTCAGGCCTATGGTGTA-AAAGGAAATA-TGTTCCCATAAAAACGACATAGAA
         59 GAGTTGAAC-CTTTCTTTTC-ATAGAGCAGTTTTGAAACACTCTTTTTGTAGAATCTGGA-AG--

             **    **   * ***  * *     *    ****   **   * *   ** ** 
      86779 TCTATATCAGGAACTTGTTTATGATGCATC-TAATCAACTAACAGTGT-TGAACCT
        119 TGGATATTTGGAGCGCTTTGAGGAT--TTCGTTGGAAACGGA-AATATCTTCATAT

            ****              *    *                    * *        *  ** * * 
      86833 TTGTACT-GACAG-AGCAGT-T-TGAAACACTCTTT-T--T-TTGGAATCTGCAAGT-GGATATT
          1 AAAAACTAGACAGAAGCATTCTCAGAAACA-TCTTTGTGATGTTTGCA-CT-CAACTCACAGAGT

              *    **     *  *  *****  *     ****  *** *  *  ** **     * *   
      86889 TGGA--TCGC-TTTGA-GGATTTCGTTGGAAACGGGATGCAATATAAAACGTACACAGCAGCATA
         63 TGAACCTTTCTTTTCATAGAGCAGTTTTGAAACACTCT-TTTTGTAGAATCTGGA-AG-TGGATA

              **  * ***      ***     **  **  *** * * ***** *  
      86950 CTCAGAAAATACTTTGCCATATTTCCAT-TCAAGTCACAGAGTGGAACAT
        125 -TTTG-GAGCGCTTTG-AGGATTTCGTTGGAAACGGAAATATCTTCATAT

            ****   ** *        ** **            *  **  *    ***  *  *    ** *
      86999 TCCCA-TTCATAG-AGCA-GGTTTGAAACACTCTTTTTGGAGTAT-C-TGGAAGTGGACA-TTTG
          1 AAAAACTAGACAGAAGCATTCTCAGAAACA-TCTTTGTGATGTTTGCACTCAACT-CACAGAGTT

              *        ***    * *   *****    *      **** **  * **      * *   
      87058 GAGCGCTTTCTGAACTATGGTG-AAAAAGGAAATA-TCTTCCAATGAAAACAAGACAGAAGCAT-
         64 GAAC-CTTTCTTTTC-ATAGAGCAGTTTTGAAACACTCTTTTTGTAGAATCTGGA-AG-TGGATA

             *      *   **        *  *  *  ****        *** *     *   
      87120 TCTGAGAAACTTATTTGTGATGCATGTCCTCAACTAACGGACTCGAACCTTTCGT-T
        125 TTTG-G-AGC--GCTT-TGA-GGATTTCGT-TGGAAACGGA--AATATC-TTCATAT

              **         *           *          *   *            **    *  * *
      87176 --TCA-T-G-CAGTA-C-TTCT--GGAACACTCTTTTTGAAGATTCTGCATGCGGATATTTGGAT
          1 AAAAACTAGACAGAAGCATTCTCAGAAACA-TCTTTGTGATG-TT-TGCA--CTCA-A-CT-CAC

              **    ***    *  ** *   *     ** *       * ****** *    *    *   
      87232 AGCTTTGAGGATTTCGTTGGAAACGGGCTTACATAT-AAA-AATTAGACAGCAGCATTCTCAGAA
         58 AGAGTTGAACCTTTCTTTTCATA-GAGC--AGTTTTGAAACACTCTTTTTGTAG-AATCT-GGAA

            ***** * *   *      *  * *   **        *    * **** *   ***     * 
      87295 ACTTCTTTGTGGTGTCTGCATTCAAG-TCACAGAATTGAACATCCCCTCACATTGGGCATTTGT
        118 GTGGATATTTGGAG-C-GCTTTGAGGATTTC-G--TTGGA-A-ACGGAAATA-TCTTCA--TAT

             * **   **  ** *  *           **       * * ***    *   ** *   * * 
      87358 GCAGCACTCTATTTGTAGTA-TCTCGATGTGGACAT-TTGGAGGGCTTTGTAGCCTATCTGGAAA
          1 -AAAAACTAGA-CAGAAGCATTCTC-A-G-AAACATCTTTGTGATGTTTGCA-CTCAACT-CACA

            *        * *    **     **      * *   ** *     ** * **  * ** *    
      87421 AAG--GAA-ATATCTTCCCATGAATGCGAG-ATAGAAGTAATC--TCAGAAACATGTTTATGCTG
         59 GAGTTGAACCTTTCTTTTCAT-AGAGC-AGTTTTGAAACACTCTTTTTGTAGAATCTGGAAG-TG

            *  *  * **  ***         **      *    *   *         * *       * * 
      87480 TATCTACTCAACTAACTGTGCTGAACATTTCTATTGATAGAGCAGTTTTGAGACACTCTTCTTTT
        121 GATAT-TTGGA-GCGCT-T--TGAGGATTTC-GTTG-GA-AAC-G----GAAATA-TCTTCATAT

            **  *       *** *  ** ** ** *          * *  * ** **   *  * **  **
      87545 GGAATCT-G-CAAGTGGATATTTGGCTAGA---TT-TGAGGATTTCGTTGGA--AACGGGATTAT
          1 AAAAACTAGACAGAAGCATTCTCAGAAACATCTTTGTGATGTTTGCACTCAACTCACAGAGTTGA

             **  ***** ** *       *   **      *             * *   ** ***  * *
      87602 ATATCAAAAGTAGACAGCAGCATTCTCAGAAAC-TTCTTTGTGATGTTTGCATCCAGCTCT-CAG
         66 ACCT-TTCTTTTCATAG-AGCAGT-TTTGAAACACTCTTT-T--TG-TAGAATCTGGAAGTGGAT

             *     *  * *    *  *  * **   *      ****           
      87665 AGTTGAACATTCCCTTTCATAGAGTAGGTTTGAAAC-CCCCT-T-TT--TAT
        124 ATTTG--GA-GCGCTTTGA-GGATTTCGTTGGAAACGGAAATATCTTCATAT

             ****  *      **          * *  *  * ** *** *      *  ***** * ** *
      87712 AGTGTCTGGA-AGCGGGCATT-T--GGAGC-GCTCTCAGGCCTATG--CTGAAAAAGGAAATATC
          1 AAAAACTAGACAG-AAGCATTCTCAGAAACATCTTTGTGATGTTTGCACTCAACTCACAGAGTTG

            *    *******   * *  *      *            *   **   * ***   *  *  * 
      87770 TACCTACAGAAACTAGACAGAAGCATTCTGAGAATCACGTTTGTGATGTGGGTACT-CAACT-AA
         65 AACCTTTCTTTTC-ATAGAGCAG--TTTTGA-AA-CAC-TCT-TTTTGTAGAATCTGGAAGTGGA

            *   * *  * **    ***     **      *   * ** ** * ** ***  
      87833 CAGTGTTGATCCATTCTTTTGATACAGCAGTTTTGAACCACACTTTTTGTAGAAT
        123 TA-TTTGGAGCGCTT-TGAGGAT--TTC-G-TTGGAAACGGAAATATCTTCATAT

             ***  * *  * *** *   *  **   * **  *      ***** *  *  ** ** * * *
      87888 CTGCAAGTGGATATTTGGATAGCTGTGAGGATTTCGTTG-GAAACGGGAATGTCTTCATAGAAAA
          1 -AAAAACTAGACAGAAGCAT-TCTCAGA-AACATCTTTGTGATGTTTGCA-CTCAACTCACAGAG

              *     ****    *   *   * * **     **   * * * *                  
      87952 TTTAGACAGAAGCATTCTCAGA-ACCTGGATTG--TGA-TGTGTGTATAGAATCTGGAAGTGGAT
         62 TTGA-AC-CTTTC-TTTTCATAGAGCAGTTTTGAAACACTCTTTTTGTAGAATCTGGAAGTGGAT

                   ** *                       *          *  
      88013 ATTTGGAAAGTTTTGAGGATTTCGTTGGAAACGGGAATATCTTCAAAT
        124 ATTTGGAGCGCTTTGAGGATTTCGTTGGAAACGGAAATATCTTCATAT

                *    *            *           * *     * *  *    *            
      88061 AAAATCTAGCCAGAAGCATTCTAAGAAACATCTTAGGGATGTGTACATTCAAGTCACAGAGTTGA
          1 AAAAACTAGACAGAAGCATTCTCAGAAACATCTTTGTGATGTTTGCACTCAACTCACAGAGTTGA

              *   * *    **       *         *    * *   **             *    * 
      88126 ACATTCCCCTTTCTCAGAGCAGGTTTGAAACAATCTTCTCGTACTATCTGGAAGTGGACATTTTG
         66 ACCTT-TCTTTTCATAGAGCAGTTTTGAAACACTCTTTTTGTAGAATCTGGAAGTGGATATTTGG

               *     *  ** ** *  *   *            ** *
      88191 AGCTCTTTGGGGCCTATGCTGAAAAAGGAAATATCTTCCGAC
        130 AGCGCTTTGAGGATTTCGTTGGAAACGGAAATATCTTCATAT

                 *               *     *  **          *           **    *    
      88233 AAAAAGTAGACAGAAGCATTCGCAGAATCACGTTTGTGATGTGTGCACTCAACTGTCAGAATTGA
          1 AAAAACTAGACAGAAGCATTCTCAGAAACATCTTTGTGATGTTTGCACTCAACTCACAGAGTTGA

                  *   ** *      **                           * *            *
      88298 ACCTTTGTTTGGACAGAGCACCTTTGAAACACTCTTTTTGTAGAATCTGCAGGTGGATATTTGGC
         66 ACCTTTCTTTTCATAGAGCAGTTTTGAAACACTCTTTTTGTAGAATCTGGAAGTGGATATTTGGA

            **                         *   *      * *
      88363 TAGCTTTGAGGATTTCGTTGGAAACGGTAATGTCTTCAAAG
        131 GCGCTTTGAGGATTTCGTTGGAAACGGAAATATCTTCATAT

                *          *   *          *   *            *    *            
      88404 AAAATCTAGACAGAAACATCCTCAGAAACACCTTCGTGATGTTTGCAATCAAGTCACAGAGTTGA
          1 AAAAACTAGACAGAAGCATTCTCAGAAACATCTTTGTGATGTTTGCACTCAACTCACAGAGTTGA

                 * *             *  *         *        *             *       
      88469 ACCTTCCGTTTCATAGAGCAGGTTGGAAACACTCATTTTGTAGTATCTGGAAGTGGACATTTGGA
         66 ACCTTTCTTTTCATAGAGCAGTTTTGAAACACTCTTTTTGTAGAATCTGGAAGTGGATATTTGGA

                   *   ** ** *  *   *        *   **  
      88534 GCGCTTTCAGGCCTATGGTGTAAAAGGAAATATGTTCCCAT
        131 GCGCTTTGAGGATTTCGTTGGAAACGGAAATATCTTCATAT

                  * * *      **      *   * *   *     ** ** *      *    *     
      88575 AAAAACGACATAGAAGCTATCTCAGGAACTTGTTTATGATGCATCTAATCAACTAACAGTGTTGA
          1 AAAAACTAGACAGAAGCATTCTCAGAAACATCTTTGTGATGTTTGCACTCAACTCACAGAGTTGA

                  * ** * *                         * *       *               
      88640 ACCTTTGTACTGACAGAGCAG-TTTGAAACACTCTTTTTTTGGAATCTGCAAGTGGATATTTGGA
         66 ACCTTTCTTTTCATAGAGCAGTTTTGAAACACTCTTTTTGTAGAATCTGGAAGTGGATATTTGGA

            *                           *   *   *    
      88704 TCGCTTTGAGGATTTCGTTGGAAACGG-GAT-GC--AATAT
        131 GCGCTTTGAGGATTTCGTTGGAAACGGAAATATCTTCATAT

                **  *    *     *                 **  *   *  *    *         * 
      88741 AAAACGTACACAGCAGCATACTCAGAAA-ATACTTTGCCATATTTCCATTCAAGTCACAGAGTGG
          1 AAAAACTAGACAGAAGCATTCTCAGAAACAT-CTTTGTGATGTTTGCACTCAACTCACAGAGTTG

               *  * **            *                  *  *             *      
      88805 AACATTCCCATTCATAGAGCAGGTTTGAAACACTCTTTTTGGAGTATCTGGAAGTGGACATTTGG
         65 AACCTTTCTTTTCATAGAGCAGTTTTGAAACACTCTTTTTGTAGAATCTGGAAGTGGATATTTGG

                    ** * * ** *  *   *            **  
      88870 AGCGCTTTCTGAACTATGGTGAAAAAGGAAATATCTTCCAAT
        130 AGCGCTTTGAGGATTTCGTTGGAAACGGAAATATCTTCATAT

            *     *               *      * *         **  **       *  *  * *  
      88912 GAAAACAAGACAGAAGCATTCTGAGAAACTTATTTGTGATGCGTGTCCTCAACTAACGGACTCGA
          1 AAAAACTAGACAGAAGCATTCTCAGAAACATCTTTGTGATGTTTGCACTCAACTCACAGAGTTGA

                   *      **  * *  *  *             *   *    * * *           
      88977 ACCTTTCGTTTCATGCAGTACTTCTGGAACACTCTTTTTGAAGATTCTGCATGCGGATATTTGGA
         66 ACCTTTCTTTTCATAGAGCAGTTTTGAAACACTCTTTTTGTAGAATCTGGAAGTGGATATTTGGA

            ** * *                        **   *     
      89042 TAGGTGTGAGGATTTCGTTGGAAACGG---GCT-TACATAT
        131 GCGCTTTGAGGATTTCGTTGGAAACGGAAATATCTTCATAT

                 *       *               *        *   *    *    *       *    
      89079 AAAAATTAGACAGCAGCATTCTCAGAAACTTCTTTGTGGTGTCTGCATTCAAGTCACAGAATTGA
          1 AAAAACTAGACAGAAGCATTCTCAGAAACATCTTTGTGATGTTTGCACTCAACTCACAGAGTTGA

              * ** * **            *  * *      *       *    *        *       
      89144 ACATCCCCTCACATAGAGCAGTTGTGCAGCACTCTATTTGTAGTATCTCGAAGTGGACATTTGGA
         66 ACCTTTCTTTTCATAGAGCAGTTTTGAAACACTCTTTTTGTAGAATCTGGAAGTGGATATTTGGA

             *         ** *    * *   *            **  
      89209 GGGCTTTGTAGCCTATC-TGGAAAAAGGAAATATCTTCCCAT
        131 GCGCTTTG-AGGATTTCGTTGGAAACGGAAATATCTTCATAT

            *  ** *   *     * *            *   *  *   * **        *  * * *   
      89250 GAATGCGAGATAGAAGTAATCTCAGAAACATGTTTATGCTGTATCTACTCAACTAACTGTGCTGA
          1 AAAAACTAGACAGAAGCATTCTCAGAAACATCTTTGTGATGTTTGCACTCAACTCACAGAGTTGA

              *     *  *               *        *  * *       *              *
      89315 ACATTTCTATTGATAGAGCAGTTTTGAGACACTCTTCTTTTGGAATCTGCAAGTGGATATTTGGC
         66 ACCTTTCTTTTCATAGAGCAGTTTTGAAACACTCTTTTTGTAGAATCTGGAAGTGGATATTTGGA

            ** *                        *     *      
      89380 TAGATTTGAGGATTTCGTTGGAAACGG-GAT-T-AT-ATAT
        131 GCGCTTTGAGGATTTCGTTGGAAACGGAAATATCTTCATAT

            *   **       *               *                 **  *   *         
      89417 CAAACGTAGACAGCAGCATTCTCAGAAACTTCTTTGTGATGTTTGCATCCAGCTCTCAGAGTTGA
          1 AAAAACTAGACAGAAGCATTCTCAGAAACATCTTTGTGATGTTTGCACTCAACTCACAGAGTTGA

              *  * *          *  *        * *      *   **        *  **       
      89482 ACATTCCCTTTCATAGAGTAGGTTTGAAACCCCCTTTTTATAGTGTCTGGAAGCGGGCATTTGGA
         66 ACCTTTCTTTTCATAGAGCAGTTTTGAAACACTCTTTTTGTAGAATCTGGAAGTGGATATTTGGA

                   *   ** ** *  *   *          * *  *
      89547 GCGCTTTCAGGCCTATGCTGAAAAAGGAAATATCTACCTAC
        131 GCGCTTTGAGGATTTCGTTGGAAACGGAAATATCTTCATAT

             *                    *    *  **          ** *        *    *     
      89588 AGAAACTAGACAGAAGCATTCTGAGAATCACGTTTGTGATGTGGGTACTCAACTAACAGTGTTGA
          1 AAAAACTAGACAGAAGCATTCTCAGAAACATCTTTGTGATGTTTGCACTCAACTCACAGAGTTGA

            *  *       *   *            *   *                *               
      89653 TCCATTCTTTTGATACAGCAGTTTTGAACCACCCTTTTTGTAGAATCTGCAAGTGGATATTTGGA
         66 ACCTTTCTTTTCATAGAGCAGTTTTGAAACACTCTTTTTGTAGAATCTGGAAGTGGATATTTGGA

            **   *                     *   *        *
      89718 TAGCTGTGAGGATTTCGTTGGAAACGGGAATGTCTTCATAG
        131 GCGCTTTGAGGATTTCGTTGGAAACGGAAATATCTTCATAT

                **                     * ****         *  **   *   *          
      89759 AAAATTTAGACAGAAGCATTCTCAGAACCTGGATTGTGATGTGTGTTCTCCACTAACAGAGTTGA
          1 AAAAACTAGACAGAAGCATTCTCAGAAACATCTTTGTGATGTTTGCACTCAACTCACAGAGTTGA

                       * *   * *           *       **                        
      89824 ACCTTTCTTTTGACAGAACTGTTTTGAAACATTCTTTTTAGAGAATCTGGAAGTGGATATTTGGA
         66 ACCTTTCTTTTCATAGAGCAGTTTTGAAACACTCTTTTTGTAGAATCTGGAAGTGGATATTTGGA

            **                         *          *  
      89889 AAGCTTTGAGGATTTCGTTGGAAACGGGAATATCTTCAAAT
        131 GCGCTTTGAGGATTTCGTTGGAAACGGAAATATCTTCATAT

                *    *            *           * *       *  *                 
      89930 AAAATCTAGCCAGAAGCATTCTAAGAAACATCTTAGGGATGTTTACATTCAACTCACAGAGTTGA
          1 AAAAACTAGACAGAAGCATTCTCAGAAACATCTTTGTGATGTTTGCACTCAACTCACAGAGTTGA

              *   * *    **       *         *    * *   **      *      *    * 
      89995 ACATTCCCCTTTCTCAGAGCAGGTTTGAAACAATCTTCTCGTACTATCTGGCAGTGGACATTTTG
         66 ACCTT-TCTTTTCATAGAGCAGTTTTGAAACACTCTTTTTGTAGAATCTGGAAGTGGATATTTGG

               * *   *  ** ** *  *   *            ** *
      90060 AGCTCCTTGGGGCCTATGCTGAAAAAGGAAATATCTTCCGAC
        130 AGCGCTTTGAGGATTTCGTTGGAAACGGAAATATCTTCATAT

                                 *     *   *          *           **    *    
      90102 AAAAACTAGACAGAAGCATTCGCAGAATCATGTTTGTGATGTGTGCACTCAACTGTCAGAATTGA
          1 AAAAACTAGACAGAAGCATTCTCAGAAACATCTTTGTGATGTTTGCACTCAACTCACAGAGTTGA

                  *   ** *      *        *             *     * *     *      *
      90167 ACCTTTGTTTGGACAGAGCACTTTTGAAAGACTCTTTTTGTAGGATCTGCAGGTGGACATTTGGC
         66 ACCTTTCTTTTCATAGAGCAGTTTTGAAACACTCTTTTTGTAGAATCTGGAAGTGGATATTTGGA

            **              *          *  **      * *
      90232 TAGCTTTGAGGATTTCATTGGAAACGGTAAAGTCTTCAAAG
        131 GCGCTTTGAGGATTTCGTTGGAAACGGAAATATCTTCATAT

                *          *   *          *   *            *    *            
      90273 AAAATCTAGACAGAAACATCCTCAGAAACACCTTCGTGATGTTTGCAATCAAGTCACAGAGTTGA
          1 AAAAACTAGACAGAAGCATTCTCAGAAACATCTTTGTGATGTTTGCACTCAACTCACAGAGTTGA

                 * *             *  *         *        *             *       
      90338 ACCTTCCGTTTCATAGAGCAGGTTGGAAACACTCATTTTGTAGTATCTGGAAGTGGACATTTGGA
         66 ACCTTTCTTTTCATAGAGCAGTTTTGAAACACTCTTTTTGTAGAATCTGGAAGTGGATATTTGGA

                   *   ** ** *  *   *            **  
      90403 GCGCTTTCAGGCCTATGGTGTAAAAGGAAATATCTTCCCAT
        131 GCGCTTTGAGGATTTCGTTGGAAACGGAAATATCTTCATAT

                * * * *      **      *   * *   *     **  * *      *    *     
      90444 AAAAGCGACATAGAAGCTATCTCAGGAACTTGTTTATGATGCATGTAATCAACTAACAGTGTTGA
          1 AAAAACTAGACAGAAGCATTCTCAGAAACATCTTTGTGATGTTTGCACTCAACTCACAGAGTTGA

                  * ** * *                         * *       *               
      90509 ACCTTTGTACTGACAGAGCAG-TTTGAAACACTCTTTTTTTGGAATCTGCAAGTGGATATTTGGA
         66 ACCTTTCTTTTCATAGAGCAGTTTTGAAACACTCTTTTTGTAGAATCTGGAAGTGGATATTTGGA

            *                           *   *   *    
      90573 TCGCTTTGAGGATTTCGTTGGAAACGG-GAT-GC--AATAT
        131 GCGCTTTGAGGATTTCGTTGGAAACGGAAATATCTTCATAT

                **  *    *     *                 **  *   *  *    *         * 
      90610 AAAACGTACACAGCAGCATACTCAGAAA-ATACTTTGCCATATTTCCATTCAAGTCACAGAGTGG
          1 AAAAACTAGACAGAAGCATTCTCAGAAACAT-CTTTGTGATGTTTGCACTCAACTCACAGAGTTG

               *  * **            *                  *  **            *      
      90674 AACATTCCCATTCATAGAGCAGGTTTGAAACACTCTTTTTGGAGTCTCTGGAAGTGGACATTTGG
         65 AACCTTTCTTTTCATAGAGCAGTTTTGAAACACTCTTTTTGTAGAATCTGGAAGTGGATATTTGG

                   *** * * ** *  *   *            **  
      90739 AGCGCTTACTGAACTATGGTGAAAAAGGAAATATCTTCCAAT
        130 AGCGCTTTGAGGATTTCGTTGGAAACGGAAATATCTTCATAT

            *     *               *     ** *         **  **       *  *  * ** 
      90781 GAAAACAAGACAGAAGCATTCTGAGAAAATTATTTGTGATGCGTGTCCTCAACTAACGGACTCAA
          1 AAAAACTAGACAGAAGCATTCTCAGAAACATCTTTGTGATGTTTGCACTCAACTCACAGAGTTGA

                   *      **  * *  *  *             *   *    * * *          *
      90846 ACCTTTCGTTTCATGCAGTACTTCTGGAACACTCTTTTTGAAGATTCTGCATGCGGATATTTGGT
         66 ACCTTTCTTTTCATAGAGCAGTTTTGAAACACTCTTTTTGTAGAATCTGGAAGTGGATATTTGGA

            **                            **   *     
      90911 TAGCTTTGAGGATTTCGTTGGAAACGG---GCT-TACATAT
        131 GCGCTTTGAGGATTTCGTTGGAAACGGAAATATCTTCATAT

                 *       *               **       *   *   **    *   *   *    
      90948 AAAAATTAGACAGCAGCATTCTCAGAAACTACTTTGTGGTGTCTGCTTTCAAGTCAGAGAATTGA
          1 AAAAACTAGACAGAAGCATTCTCAGAAACATCTTTGTGATGTTTGCACTCAACTCACAGAGTTGA

              * ** * **  *         *  * *      *       *    *        *       
      91013 ACATCCCCTCACACAGAGCAGTTGTGCAGCACTCTATTTGTAGTATCTCGAAGTGGACATTTGGA
         66 ACCTTTCTTTTCATAGAGCAGTTTTGAAACACTCTTTTTGTAGAATCTGGAAGTGGATATTTGGA

             *         *     *         *            **  
      91078 GGGCTTT---GTTTTCTATCTGGAAAAAGGAAATATCTTCCCAT
        131 GCGCTTTGAGGATTTC-GT-TGG-AAACGGAAATATCTTCATAT

            *  ** * * *     * *            *   *  *   * **        * ** * *   
      91119 GAATGCGACATAGAAGTAATCTCAGAAACATGTTTATGCTGTATCTACTCAACTAAGTGTGCTGA
          1 AAAAACTAGACAGAAGCATTCTCAGAAACATCTTTGTGATGTTTGCACTCAACTCACAGAGTTGA

              *     *  *               *        ** * *       *               
      91184 ACATTTCTATTGATAGAGCAGTTTTGAGACACTCTTCATTTGGAATCTGCAAGTGGATATTTGGA
         66 ACCTTTCTTTTCATAGAGCAGTTTTGAAACACTCTTTTTGTAGAATCTGGAAGTGGATATTTGGA

            ** *            *           *     *   *  
      91249 TAGATTTGAGGATTTCTTTGGAAACGG-GAT-T-AT-ACAT
        131 GCGCTTTGAGGATTTCGTTGGAAACGGAAATATCTTCATAT

                 *       *               *        *   *    *    *       *    
      91286 AAAAAGTAGACAGCAGCATTCTCAGAAACTTCTTTGTGGTGTCTGCATTCAAGTCACAGAATTGA
          1 AAAAACTAGACAGAAGCATTCTCAGAAACATCTTTGTGATGTTTGCACTCAACTCACAGAGTTGA

              * ** * **            *  * *      *       *    *  *     *       
      91351 ACATCCCCTCACATAGAGCAGTTGTGCAGCACTCTATTTGTAGTATCTCGATGTGGACATTTGGA
         66 ACCTTTCTTTTCATAGAGCAGTTTTGAAACACTCTTTTTGTAGAATCTGGAAGTGGATATTTGGA

             *         ** *    * *   *            **  
      91416 GGGCTTTGTAGCCTATC-TGGAAAAAGGAAATATCTTCCCAT
        131 GCGCTTTG-AGGATTTCGTTGGAAACGGAAATATCTTCATAT

            *  ** *   *     * *            *   *  *   * **        *  * * *   
      91457 GAATGCGAGATAGAAGTAATCTCAGAAACATGTTTATGCTGTATCTACTCAACTAACTGTGCTGA
          1 AAAAACTAGACAGAAGCATTCTCAGAAACATCTTTGTGATGTTTGCACTCAACTCACAGAGTTGA

             **     *  *               *        *  * *       *               
      91522 AGATTTCTATTGATAGAGCAGTTTTGAGACACTCTTCTTTTGGAATCTGCAAGTGGATATTTGGA
         66 ACCTTTCTTTTCATAGAGCAGTTTTGAAACACTCTTTTTGTAGAATCTGGAAGTGGATATTTGGA

            ** *                        *     *      
      91587 TAGATTTGAGGATTTCGTTGGAAACGG-GAT-T-AT-ATAT
        131 GCGCTTTGAGGATTTCGTTGGAAACGGAAATATCTTCATAT

            *    *       *               *                 **  *   * *       
      91624 GAAAAGTAGACAGCAGCATTCTCAGAAACTTCTTTGTGATGTTTGCATCCAGCTCTCCGAGTTGA
          1 AAAAACTAGACAGAAGCATTCTCAGAAACATCTTTGTGATGTTTGCACTCAACTCACAGAGTTGA

               *               *  *        * *      *   **        *  **      
      91689 A-CATTCTCTTTCATAGAGTAGGTTTGAAACCCCCTTTTTATAGTGTCTGGAAGCGGGCATTTGG
         66 ACCTTTCT-TTTCATAGAGCAGTTTTGAAACACTCTTTTTGTAGAATCTGGAAGTGGATATTTGG

                    *   ** ** *  *   *          * *  *
      91753 AGCGCTTTCAGGCCTATGCTGAAAAAGGAAATATCTACCTAC
        130 AGCGCTTTGAGGATTTCGTTGGAAACGGAAATATCTTCATAT

             *         *      *   *    *  **          ** *        *    *     
      91795 AGAAACTAGACTGAAGCAATCTGAGAATCACGTTTGTGATGTGGGTACTCAACTAACAGTGTTGA
          1 AAAAACTAGACAGAAGCATTCTCAGAAACATCTTTGTGATGTTTGCACTCAACTCACAGAGTTGA

            *  *       *   *            *   *                *               
      91860 TCCATTCTTTTGATACAGCAGTTTTGAACCACCCTTTTTGTAGAATCTGCAAGTGGATATTTGGA
         66 ACCTTTCTTTTCATAGAGCAGTTTTGAAACACTCTTTTTGTAGAATCTGGAAGTGGATATTTGGA

            **   *  *                  *   *        *
      91925 TAGCTGTGGGGATTTCGTTGGAAACGGGAATGTCTTCATAG
        131 GCGCTTTGAGGATTTCGTTGGAAACGGAAATATCTTCATAT

                **                     * * **         *  **   *   *        * 
      91966 AAAATTTAGACAGAAGCATTCTCAGAACCTTGATTGTGATGTGTGTTCTCCACTAACAGAGTTCA
          1 AAAAACTAGACAGAAGCATTCTCAGAAACATCTTTGTGATGTTTGCACTCAACTCACAGAGTTGA

                       * *   * *           *       *                         
      92031 ACCTTTCTTTTGACAGAACTGTTTTGAAACATTCTTTTTATAGAATCTGGAAGTGGATATTTGGA
         66 ACCTTTCTTTTCATAGAGCAGTTTTGAAACACTCTTTTTGTAGAATCTGGAAGTGGATATTTGGA

            **              *         **          *  
      92096 AAGCTTTGAGGATTTCATTGGAAACGAGAATATCTTCAAAT
        131 GCGCTTTGAGGATTTCGTTGGAAACGGAAATATCTTCATAT

                *    *            *           * *       *  *    *            
      92137 AAAATCTAGCCAGAAGCATTCTAAGAAACATCTTAGGGATGTTTACATTCAAGTCACAGAGTTGA
          1 AAAAACTAGACAGAAGCATTCTCAGAAACATCTTTGTGATGTTTGCACTCAACTCACAGAGTTGA

              *  * *     *       *         *    * *   **      *      *    *  
      92202 ACATTCCCTTTCACAGAGCAGGTTTGAAACAATCTTCTCGTACTATCTGGCAGTGGACATTTTGA
         66 ACCTTTCTTTTCATAGAGCAGTTTTGAAACACTCTTTTTGTAGAATCTGGAAGTGGATATTTGGA

              *     *  ** **    *   *            ** *
      92267 GCTCTTTGGGGTCTATGTTGAAAAAGGAAATATCTTCCAAC
        131 GCGCTTTGAGGATTTCGTTGGAAACGGAAATATCTTCATAT

                                 *     *  **          *           **    *    
      92308 AAAAACTAGACAGAAGCATTCGCAGAATCACGTTTGTGATGTGTGCACTCAACTGTCAGAATTGA
          1 AAAAACTAGACAGAAGCATTCTCAGAAACATCTTTGTGATGTTTGCACTCAACTCACAGAGTTGA

                  *   ** *      **                           * *            *
      92373 ACCTTTGTTTGGACAGAGCACATTTGAAACACTCTTTTTGTAGAATCTGCAGGTGGATATTTGGC
         66 ACCTTTCTTTTCATAGAGCAGTTTTGAAACACTCTTTTTGTAGAATCTGGAAGTGGATATTTGGA

            **         *               *   *      * *
      92438 TAGCTTTGAGGTTTTCGTTGGAAACGGTAATGTCTTCAAAG
        131 GCGCTTTGAGGATTTCGTTGGAAACGGAAATATCTTCATAT

                *          *   *          *   *            *    *            
      92479 AAAATCTAGACAGAAACATCCTCAGAAACACCTTCGTGATGTTTGCAATCAAGTCACAGAGTTGA
          1 AAAAACTAGACAGAAGCATTCTCAGAAACATCTTTGTGATGTTTGCACTCAACTCACAGAGTTGA

                 * *             *  *         *        *             *       
      92544 ACCTTCCGTTTCATAGAGCAGGTTGGAAACACTCATTTTGTAGTATCTGGAAGTGGACATTTGGA
         66 ACCTTTCTTTTCATAGAGCAGTTTTGAAACACTCTTTTTGTAGAATCTGGAAGTGGATATTTGGA

                   *   ** ** *  *   *            **  
      92609 GCGCTTTCAGGCCTATGGTGTAAAAGGAAATATCTTCCCAT
        131 GCGCTTTGAGGATTTCGTTGGAAACGGAAATATCTTCATAT

                * * * *      **      *   * *   *     ** ** *      *    *     
      92650 AAAAGCGACATAGAAGCTATCTCAGGAACTTGTTTATGATGCATCTAATCAACTAACAGTGTTGA
          1 AAAAACTAGACAGAAGCATTCTCAGAAACATCTTTGTGATGTTTGCACTCAACTCACAGAGTTGA

              *   * ** * *                         * *       *               
      92715 ACATTTGTACTGACAGAGCAG-TTTGAAACACTCTTTTTTTGGAATCTGCAAGTGGATATTTGGA
         66 ACCTTTCTTTTCATAGAGCAGTTTTGAAACACTCTTTTTGTAGAATCTGGAAGTGGATATTTGGA

            *                           *   *   *    
      92779 TCGCTTTGAGGATTTCGTTGGAAACGG-GAT-GC--AATAT
        131 GCGCTTTGAGGATTTCGTTGGAAACGGAAATATCTTCATAT

                **  *    *     *                 **  *   *  *    *         * 
      92816 AAAACGTACACAGCAGCATACTCAGAAA-ATACTTTGCCATATTTCCATTCAAGTCACAGAGTGG
          1 AAAAACTAGACAGAAGCATTCTCAGAAACAT-CTTTGTGATGTTTGCACTCAACTCACAGAGTTG

               *  * **            *           *      *  **            *      
      92880 AACATTCCCATTCATAGAGCAGGTTTGAAACACTTTTTTTGGAGTGTCTGGAAGTGGACATTTGG
         65 AACCTTTCTTTTCATAGAGCAGTTTTGAAACACTCTTTTTGTAGAATCTGGAAGTGGATATTTGG

                    *  * * ** *  *   *            **  
      92945 AGCGCTTTCAGAACTATGGTGAAAAAGGAAATATCTTCCAAT
        130 AGCGCTTTGAGGATTTCGTTGGAAACGGAAATATCTTCATAT

            *     *               *      * *         **  **       *  *  * *  
      92987 GAAAACAAGACAGAAGCATTCTGAGAAACTTATTTGTGATGCGTGTCCTCAACTAACGGACTCGA
          1 AAAAACTAGACAGAAGCATTCTCAGAAACATCTTTGTGATGTTTGCACTCAACTCACAGAGTTGA

                   *     ***  * *  *  *             *   *    * * *           
      93052 ACCTTTCGTTTCACGCAGTACTTCTGGAACACTCTTTTTGAAGATTCTGCATGCGGATATTTGGA
         66 ACCTTTCTTTTCATAGAGCAGTTTTGAAACACTCTTTTTGTAGAATCTGGAAGTGGATATTTGGA

            **                            **   *     
      93117 TAGCTTTGAGGATTTCGTTGGAAACGG---GCT-TACATAT
        131 GCGCTTTGAGGATTTCGTTGGAAACGGAAATATCTTCATAT

                 *       *               *        *   *    *    *       *    
      93154 AAAAATTAGACAGCAGCATTCTCAGAAACTTCTTTGTGGTGTCTGCATTCAAGTCACAGAATTGA
          1 AAAAACTAGACAGAAGCATTCTCAGAAACATCTTTGTGATGTTTGCACTCAACTCACAGAGTTGA

              * ** * **   *        *  * *      *       *    *    *   *       
      93219 ACATCCCCTCACATTGAGCAGTTGTGCAGCACTCTATTTGTAGTATCTCGAAGAGGACATTTGGA
         66 ACCTTTCTTTTCATAGAGCAGTTTTGAAACACTCTTTTTGTAGAATCTGGAAGTGGATATTTGGA

             *         ** *  * * *   *            **  
      93284 GGGCTTTGTAGCCTAT-ATGGAAAAAGGAAATATCTTCCCAT
        131 GCGCTTTG-AGGATTTCGTTGGAAACGGAAATATCTTCATAT

            *   * *   *     * *            *   *  *   * **        *  * * *   
      93325 GAAAGCGAGATAGAAGTAATCTCAGAAACATGTTTATGCTGTATCTACTCAACTAACTGTGCTGA
          1 AAAAACTAGACAGAAGCATTCTCAGAAACATCTTTGTGATGTTTGCACTCAACTCACAGAGTTGA

              *     *  *               *        *  * *       *               
      93390 ACATTTCTATTGATAGAGCAGTTTTGAGACACTCTTCTTTTGGAATCTGCAAGTGGATATTTGGA
         66 ACCTTTCTTTTCATAGAGCAGTTTTGAAACACTCTTTTTGTAGAATCTGGAAGTGGATATTTGGA

            ** *                        *     *      
      93455 TAGATTTGAGGATTTCGTTGGAAACGG-GAT-T-AT-ATAT
        131 GCGCTTTGAGGATTTCGTTGGAAACGGAAATATCTTCATAT

                 *       *               *                 **  *   *         
      93492 AAAAAGTAGACAGCAGCATTCTCA-AAACTTCTTTGTGATGTTTGCATCCAGCTCTCAGAGTTGA
          1 AAAAACTAGACAGAAGCATTCTCAGAAACATCTTTGTGATGTTTGCACTCAACTCACAGAGTTGA

              *  * *          *  *        * *      *   **        *  **   *   
      93556 ACATTCCCTTTCATAGAGTAGGTTTGAAACCCCCTTTTTATAGTGTCTGGAAGCGGGCATTAGGA
         66 ACCTTTCTTTTCATAGAGCAGTTTTGAAACACTCTTTTTGTAGAATCTGGAAGTGGATATTTGGA

            *      *   ** ** *  *   *          * *  *
      93621 ACGCTTTCAGGCCTATGCTGAAAAAGGAAATATCTACCTAC
        131 GCGCTTTGAGGATTTCGTTGGAAACGGAAATATCTTCATAT

             *                    *    *  **          ** *        *    *     
      93662 AGAAACTAGACAGAAGCATTCTGAGAATCACGTTTGTGATGTGGGTACTCAACTAACAGTGTTGA
          1 AAAAACTAGACAGAAGCATTCTCAGAAACATCTTTGTGATGTTTGCACTCAACTCACAGAGTTGA

            *  *       *   *            *   *                *               
      93727 TCCATTCTTTTGATACAGCAGTTTTGAACCACCCTTTTTGTAGAATCTGCAAGTGGATATTTGGA
         66 ACCTTTCTTTTCATAGAGCAGTTTTGAAACACTCTTTTTGTAGAATCTGGAAGTGGATATTTGGA

            **   *                     *   *        *
      93792 TAGCTGTGAGGATTTCGTTGGAAACGGGAATGTCTTCATAG
        131 GCGCTTTGAGGATTTCGTTGGAAACGGAAATATCTTCATAT

                **                     * ****         *  **   *   *          
      93833 AAAATTTAGACAGAAGCATTCTCAGAACCTGGATTGTGATGTGTGTTCTCCACTAACAGAGTTGA
          1 AAAAACTAGACAGAAGCATTCTCAGAAACATCTTTGTGATGTTTGCACTCAACTCACAGAGTTGA

                      ** *   * *           *       *                         
      93898 ACCTTTCTTTGGACAGAACTGTTTTGAAACATTCTTTTTATAGAATCTGGAAGTGGATATTTGGA
         66 ACCTTTCTTTTCATAGAGCAGTTTTGAAACACTCTTTTTGTAGAATCTGGAAGTGGATATTTGGA

            **                         *          *  
      93963 AAGCTTTGAGGATTTCGTTGGAAACGGGAATATCTTCAAAT
        131 GCGCTTTGAGGATTTCGTTGGAAACGGAAATATCTTCATAT

                *    *            *           * *       *  *    *            
      94004 AAAATCTAGCCAGAAGCATTCTAAGAAACATCTTAGGGATGTTTACATTCAAGTCACAGAGTTGA
          1 AAAAACTAGACAGAAGCATTCTCAGAAACATCTTTGTGATGTTTGCACTCAACTCACAGAGTTGA

              *   * *    **       *         *    * *   **      *      *    * 
      94069 ACATTCCCCTTTCTCAGAGCAGGTTTGAAACAATCTTCTCGTACTATCTGGCAGTGGACATTTTG
         66 ACCTT-TCTTTTCATAGAGCAGTTTTGAAACACTCTTTTTGTAGAATCTGGAAGTGGATATTTGG

               * *   *  ** ** *  *   *            ** *
      94134 AGCTCCTTGGGGCCTATGCTGAAAAAGGAAATATCTTCCGAC
        130 AGCGCTTTGAGGATTTCGTTGGAAACGGAAATATCTTCATAT

                                 *     *  **          *           **    *    
      94176 AAAAACTAGACAGAAGCATTCGCAGAATCACGTTTGTGATGTGTGCACTCAACTGTCAGAATTGA
          1 AAAAACTAGACAGAAGCATTCTCAGAAACATCTTTGTGATGTTTGCACTCAACTCACAGAGTTGA

                 **   ** *      *                            * *            *
      94241 ACCTTGGTTTGGACAGAGCACTTTTGAAACACTCTTTTTGTAGAATCTGCAGGTGGATATTTGGC
         66 ACCTTTCTTTTCATAGAGCAGTTTTGAAACACTCTTTTTGTAGAATCTGGAAGTGGATATTTGGA

            **                         *   *      * *
      94306 TAGCTTTGAGGATTTCGTTGGAAACGGTAATGTCTTCAAAG
        131 GCGCTTTGAGGATTTCGTTGGAAACGGAAATATCTTCATAT

                *          *   *          *   *            *    *            
      94347 AAAATCTAGACAGAAACATCCTCAGAAACACCTTCGTGATGTTTGCAATCAAGTCACAGAGTTGA
          1 AAAAACTAGACAGAAGCATTCTCAGAAACATCTTTGTGATGTTTGCACTCAACTCACAGAGTTGA

                 * *             *  *         *        *             *       
      94412 ACCTTCCGTTTCATAGAGCAGGTTGGAAACACTCATTTTGTAGTATCTGGAAGTGGACATTTGGA
         66 ACCTTTCTTTTCATAGAGCAGTTTTGAAACACTCTTTTTGTAGAATCTGGAAGTGGATATTTGGA

                   *   ** ** *  *   *            **  
      94477 GCGCTTTCAGGCCTATGGTGTAAAAGGAAATATCTTCCCAT
        131 GCGCTTTGAGGATTTCGTTGGAAACGGAAATATCTTCATAT

                * * * *      **      *   * *   *     ** ** *      *    *     
      94518 AAAAGCGACATAGAAGCTATCTCAGGAACTTGTTTATGATGCATCTAATCAACTAACAGTGTTGA
          1 AAAAACTAGACAGAAGCATTCTCAGAAACATCTTTGTGATGTTTGCACTCAACTCACAGAGTTGA

                  * ** * *                         * *       *               
      94583 ACCTTTGTACTGACAGAGCAG-TTTGAAACACTCTTTTTTTGGAATCTGCAAGTGGATATTTGGA
         66 ACCTTTCTTTTCATAGAGCAGTTTTGAAACACTCTTTTTGTAGAATCTGGAAGTGGATATTTGGA

            *                           *   *   *    
      94647 TCGCTTTGAGGATTTCGTTGGAAACGG-GAT-GC--AATAT
        131 GCGCTTTGAGGATTTCGTTGGAAACGGAAATATCTTCATAT

                **  *    *     *                 **  *   *  **   *         * 
      94684 AAAACGTACACAGCAGCATACTCAGAAA-ATACTTTGCCATATTTCCATCCAAGTCACAGAGTGG
          1 AAAAACTAGACAGAAGCATTCTCAGAAACAT-CTTTGTGATGTTTGCACTCAACTCACAGAGTTG

               *  * **            *           *      *  **            *      
      94748 AACATTCCCATTCATAGAGCAGGTTTGAAACACTTTTTTTGGAGTGTCTGGAAGTGGACATTTGG
         65 AACCTTTCTTTTCATAGAGCAGTTTTGAAACACTCTTTTTGTAGAATCTGGAAGTGGATATTTGG

                    *  * * ** *  *   *            **  
      94813 AGCGCTTTCAGAACTATGGTGAAAAAGGAAATATCTTCCAAT
        130 AGCGCTTTGAGGATTTCGTTGGAAACGGAAATATCTTCATAT

            *     *               *      * *         **  **       *  *  * *  
      94855 GAAAACAAGACAGAAGCATTCTGAGAAACTTATTTGTGATGCGTGTCCTCAACTAACGGACTCGA
          1 AAAAACTAGACAGAAGCATTCTCAGAAACATCTTTGTGATGTTTGCACTCAACTCACAGAGTTGA

                   *      **  * *  *  *             *   *    * * *          *
      94920 ACCTTTCGTTTCATGCAGTACTTCTGGAACACTCTTTTTGAAGATTCTGCATGCGGATATTTGGT
         66 ACCTTTCTTTTCATAGAGCAGTTTTGAAACACTCTTTTTGTAGAATCTGGAAGTGGATATTTGGA

            **                            **   *     
      94985 TAGCTTTGAGGATTTCGTTGGAAACGG---GCT-TACATAT
        131 GCGCTTTGAGGATTTCGTTGGAAACGGAAATATCTTCATAT

                 *       *               *        *   *    *    *       *    
      95022 AAAAATTAGACAGCAGCATTCTCAGAAACTTCTTTGTGGTGTCTGCATTCAAGTCACAGAATTGA
          1 AAAAACTAGACAGAAGCATTCTCAGAAACATCTTTGTGATGTTTGCACTCAACTCACAGAGTTGA

              * ** * **            *  * *      *       *    *        *       
      95087 ACATCCCCTCACATAGAGCAGTTGTGCAGCACTCTATTTGTAGTATCTCGAAGTGGACATTTGGA
         66 ACCTTTCTTTTCATAGAGCAGTTTTGAAACACTCTTTTTGTAGAATCTGGAAGTGGATATTTGGA

             *         ****           *            **  
      95152 GGGCTTTGTAGCCCATC--TGGATAAAGGAAATATCTTCCCAT
        131 GCGCTTTG-AGGATTTCGTTGGA-AACGGAAATATCTTCATAT

            *  ** *   *     * *            *   *  *   * **        *  * * *   
      95193 GAATGCGAGATAGAAGTAATCTCAGAAACATGTTTATGCTGTATCTACTCAACTAACTGTGCTGA
          1 AAAAACTAGACAGAAGCATTCTCAGAAACATCTTTGTGATGTTTGCACTCAACTCACAGAGTTGA

              *     *  *               *        *  * *       *               
      95258 ACATTTCTATTGATAGAGCAGTTTTGAGACACTCTTCTTTTGGAATCTGCAAGTGGATATTTGGA
         66 ACCTTTCTTTTCATAGAGCAGTTTTGAAACACTCTTTTTGTAGAATCTGGAAGTGGATATTTGGA

            ** *                        *     *      
      95323 TAGATTTGAGGATTTCGTTGGAAACGG-GAT-T-AT-ATAT
        131 GCGCTTTGAGGATTTCGTTGGAAACGGAAATATCTTCATAT

                 *       *               *                 **  *   *         
      95360 AAAAAGTAGACAGCAGCATTCTCAGAAACTTCTTTGTGATGTTTGCATCCAGCTCTCAGAGTTGA
          1 AAAAACTAGACAGAAGCATTCTCAGAAACATCTTTGTGATGTTTGCACTCAACTCACAGAGTTGA

              *  * *          *  *        *        *   **        *  **       
      95425 ACATTCCCTTTCATAGAGTAGGTTTGAAACCCTCTTTTTATAGTGTCTGGAAGCGGGCATTTGGA
         66 ACCTTTCTTTTCATAGAGCAGTTTTGAAACACTCTTTTTGTAGAATCTGGAAGTGGATATTTGGA

                   *   ** ** *  *   *          * *  *
      95490 GCGCTTTCAGGCCTATGCTGAAAAAGGAAATATCTACCTAC
        131 GCGCTTTGAGGATTTCGTTGGAAACGGAAATATCTTCATAT

             *                    *    *  **          ** *        *    *     
      95531 AGAAACTAGACAGAAGCATTCTGAGAATCACGTTTGTGATGTGGGTACTCAACTAACAGTGTTGA
          1 AAAAACTAGACAGAAGCATTCTCAGAAACATCTTTGTGATGTTTGCACTCAACTCACAGAGTTGA

            *  *       *   *            *   *                *               
      95596 TCCATTCTTTTGATACAGCAGTTTTGAACCACCCTTTTTGTAGAATCTGCAAGTGGATATTTGGA
         66 ACCTTTCTTTTCATAGAGCAGTTTTGAAACACTCTTTTTGTAGAATCTGGAAGTGGATATTTGGA

            **   *                     *   *        *
      95661 TAGCTGTGAGGATTTCGTTGGAAACGGGAATGTCTTCATAG
        131 GCGCTTTGAGGATTTCGTTGGAAACGGAAATATCTTCATAT

                **                     * ****         *  **   *   *          
      95702 AAAATTTAGACAGAAGCATTCTCAGAACCTGGATTGTGATGTGTGTTCTCCACTAACAGAGTTGA
          1 AAAAACTAGACAGAAGCATTCTCAGAAACATCTTTGTGATGTTTGCACTCAACTCACAGAGTTGA

                      ** *   * *           *       *                         
      95767 ACCTTTCTTTGGACAGAACTGTTTTGAAACATTCTTTTTATAGAATCTGGAAGTGGATATTTGGA
         66 ACCTTTCTTTTCATAGAGCAGTTTTGAAACACTCTTTTTGTAGAATCTGGAAGTGGATATTTGGA

            **                         *          *  
      95832 AAGCTTTGAGGATTTCGTTGGAAACGGGAATATCTTCAAAT
        131 GCGCTTTGAGGATTTCGTTGGAAACGGAAATATCTTCATAT

                *    *            *           * *       *  *    *            
      95873 AAAATCTAGCCAGAAGCATTCTAAGAAACATCTTAGGGATGTTTACATTCAAGTCACAGAGTTGA
          1 AAAAACTAGACAGAAGCATTCTCAGAAACATCTTTGTGATGTTTGCACTCAACTCACAGAGTTGA

              *   * *    **       *         *    * *   **      *      *    * 
      95938 ACATTCCCCTTTCTCAGAGCAGGTTTGAAACAATCTTCTCGTACTATCTGGCAGTGGACATTTTG
         66 ACCTT-TCTTTTCATAGAGCAGTTTTGAAACACTCTTTTTGTAGAATCTGGAAGTGGATATTTGG

               * *   *  ** ** *  *   *            ** *
      96003 AGCTCCTTGGGGCCTATGCTGAAAAAGGAAATATCTTCCGAC
        130 AGCGCTTTGAGGATTTCGTTGGAAACGGAAATATCTTCATAT

                                 *     *  **          *           **    *    
      96045 AAAAACTAGACAGAAGCATTCGCAGAATCACGTTTGTGATGTGTGCACTCAACTGTCAGAATTGA
          1 AAAAACTAGACAGAAGCATTCTCAGAAACATCTTTGTGATGTTTGCACTCAACTCACAGAGTTGA

                 **   ** *      *                            * *            *
      96110 ACCTTGGTTTGGACAGAGCACTTTTGAAACACTCTTTTTGTAGAATCTGCAGGTGGATATTTGGC
         66 ACCTTTCTTTTCATAGAGCAGTTTTGAAACACTCTTTTTGTAGAATCTGGAAGTGGATATTTGGA

            **                         *   *      * *
      96175 TAGCTTTGAGGATTTCGTTGGAAACGGTAATGTCTTCAAAG
        131 GCGCTTTGAGGATTTCGTTGGAAACGGAAATATCTTCATAT

                *          *   *          *   *            *    *            
      96216 AAAATCTAGACAGAAACATCCTCAGAAACACCTTCGTGATGTTTGCAATCAAGTCACAGAGTTGA
          1 AAAAACTAGACAGAAGCATTCTCAGAAACATCTTTGTGATGTTTGCACTCAACTCACAGAGTTGA

                 * *        *    *  *         *        *             *       
      96281 ACCTTCCGTTTCATAGTGCAGGTTGGAAACACTCATTTTGTAGTATCTGGAAGTGGACATTTGGA
         66 ACCTTTCTTTTCATAGAGCAGTTTTGAAACACTCTTTTTGTAGAATCTGGAAGTGGATATTTGGA

                   *   ** ** *  *   *            **  
      96346 GCGCTTTCAGGCCTATGGTGTAAAAGGAAATATCTTCCCAT
        131 GCGCTTTGAGGATTTCGTTGGAAACGGAAATATCTTCATAT

                * * * *      **      *   * *   *     ** ** *      *    *     
      96387 AAAAGCGACATAGAAGCTATCTCAGGAACTTGTTTATGATGCATCTAATCAACTAACAGTGTTGA
          1 AAAAACTAGACAGAAGCATTCTCAGAAACATCTTTGTGATGTTTGCACTCAACTCACAGAGTTGA

                  * ** * *                         * *       *               
      96452 ACCTTTGTACTGACAGAGCAG-TTTGAAACACTCTTTTTTTGGAATCTGCAAGTGGATATTTGGA
         66 ACCTTTCTTTTCATAGAGCAGTTTTGAAACACTCTTTTTGTAGAATCTGGAAGTGGATATTTGGA

            **  *                       *   *   *    
      96516 TGGCCTTGAGGATTTCGTTGGAAACGG-GAT-GC--AATAT
        131 GCGCTTTGAGGATTTCGTTGGAAACGGAAATATCTTCATAT

                **  *    *     *                 **  *   *  *    *         * 
      96553 AAAACGTACACAGCAGCATACTCAGAAA-ATACTTTGCCATATTTCCATTCAAGTCACAGAGTGG
          1 AAAAACTAGACAGAAGCATTCTCAGAAACAT-CTTTGTGATGTTTGCACTCAACTCACAGAGTTG

               *  * **            *           *      *  **            *      
      96617 AACATTCCCATTCATAGAGCAGGTTTGAAACACTTTTTTTGGAGTGTCTGGAAGTGGACATTTGG
         65 AACCTTTCTTTTCATAGAGCAGTTTTGAAACACTCTTTTTGTAGAATCTGGAAGTGGATATTTGG

                    *  * * ** *  *   *            **  
      96682 AGCGCTTTCAGAACTATGGTGAAAAAGGAAATATCTTCCAAT
        130 AGCGCTTTGAGGATTTCGTTGGAAACGGAAATATCTTCATAT

            *     *               *      * *         **  **       *  *  * *  
      96724 GAAAACAAGACAGAAGCATTCTGAGAAACTTATTTGTGATGCGTGTCCTCAACTAACGGACTCGA
          1 AAAAACTAGACAGAAGCATTCTCAGAAACATCTTTGTGATGTTTGCACTCAACTCACAGAGTTGA

                   *      **  * *  *  *      *      *   *    * * *          *
      96789 ACCTTTCGTTTCATGCAGTACTTCTGGAACACTGTTTTTGAAGATTCTGCATGCGGATATTTGGT
         66 ACCTTTCTTTTCATAGAGCAGTTTTGAAACACTCTTTTTGTAGAATCTGGAAGTGGATATTTGGA

            **                            **   *     
      96854 TAGCTTTGAGGATTTCGTTGGAAACGG---GCT-TACATAT
        131 GCGCTTTGAGGATTTCGTTGGAAACGGAAATATCTTCATAT

                 *       *               *        *   *    *    *       *    
      96891 AAAAATTAGACAGCAGCATTCTCAGAAACTTCTTTGTGGTGTCTGCATTCAAGTCACAGAATTGA
          1 AAAAACTAGACAGAAGCATTCTCAGAAACATCTTTGTGATGTTTGCACTCAACTCACAGAGTTGA

              * ** * **            *  * *      *       *    *        *       
      96956 ACATCCCCTCACATAGAGCAGTTGTGCAGCACTCTATTTGTAGTATCTCGAAGTGGACATTTGGA
         66 ACCTTTCTTTTCATAGAGCAGTTTTGAAACACTCTTTTTGTAGAATCTGGAAGTGGATATTTGGA

             *         ****           *            **  
      97021 GGGCTTTGTAGCCCATC--TGGATAAAGGAAATATCTTCCCAT
        131 GCGCTTTG-AGGATTTCGTTGGA-AACGGAAATATCTTCATAT

            *  ** *   *     * *            *   *  *   * **        *  * * *   
      97062 GAATGCGAGATAGAAGTAATCTCAGAAACATGTTTATGCTGTATCTACTCAACTAACTGTGCTGA
          1 AAAAACTAGACAGAAGCATTCTCAGAAACATCTTTGTGATGTTTGCACTCAACTCACAGAGTTGA

              *     *  *               *        *  * *       *              *
      97127 ACATTTCTATTGATAGAGCAGTTTTGAGACACTCTTCTTTTGGAATCTGCAAGTGGATATTTGGC
         66 ACCTTTCTTTTCATAGAGCAGTTTTGAAACACTCTTTTTGTAGAATCTGGAAGTGGATATTTGGA

            ** *                        *     *      
      97192 TAGATTTGAGGATTTCGTTGGAAACGG-GAT-T-AT-ATAT
        131 GCGCTTTGAGGATTTCGTTGGAAACGGAAATATCTTCATAT

                 *       *               *                 **  *   *         
      97229 AAAAAGTAGACAGCAGCATTCTCAGAAACTTCTTTGTGATGTTTGCATCCAGCTCTCAGAGTTGA
          1 AAAAACTAGACAGAAGCATTCTCAGAAACATCTTTGTGATGTTTGCACTCAACTCACAGAGTTGA

              *  * *          *  *        * *      *   **           **       
      97294 ACATTCCCTTTCATAGAGTAGGTTTGAAACCCCCTTTTTATAGTGTCTGGAAGTGGGCATTTGGA
         66 ACCTTTCTTTTCATAGAGCAGTTTTGAAACACTCTTTTTGTAGAATCTGGAAGTGGATATTTGGA

                   *   ** ** *  *   *  *       * *  *
      97359 GCGCTTTCAGGCCTATGCTGAAAAAGGGAATATCTACCTAC
        131 GCGCTTTGAGGATTTCGTTGGAAACGGAAATATCTTCATAT

             *                    *    *  **          ** *        *    *     
      97400 AGAAACTAGACAGAAGCATTCTGAGAATCACGTTTGTGATGTGGGTACTCAACTAACAGTGTTGA
          1 AAAAACTAGACAGAAGCATTCTCAGAAACATCTTTGTGATGTTTGCACTCAACTCACAGAGTTGA

            *  *       *   *            *   *                *               
      97465 TCCATTCTTTTGATACAGCAGTTTTGAACCACCCTTTTTGTAGAATCTGCAAGTGGATATTTGGA
         66 ACCTTTCTTTTCATAGAGCAGTTTTGAAACACTCTTTTTGTAGAATCTGGAAGTGGATATTTGGA

            **   *                     *   *        *
      97530 TAGCTGTGAGGATTTCGTTGGAAACGGGAATGTCTTCATAG
        131 GCGCTTTGAGGATTTCGTTGGAAACGGAAATATCTTCATAT

                **                     * ****         ** **   *   *          
      97571 AAAATTTAGACAGAAGCATTCTCAGAACCTGGATTGTGATGTGAGTTCTCCACTAACAGAGTTGA
          1 AAAAACTAGACAGAAGCATTCTCAGAAACATCTTTGTGATGTTTGCACTCAACTCACAGAGTTGA

                      ** *   * *           *       *                         
      97636 ACCTTTCTTTGGACAGAACTGTTTTGAAACATTCTTTTTATAGAATCTGGAAGTGGATATTTGGA
         66 ACCTTTCTTTTCATAGAGCAGTTTTGAAACACTCTTTTTGTAGAATCTGGAAGTGGATATTTGGA

            **                         *          *  
      97701 AAGCTTTGAGGATTTCGTTGGAAACGGGAATATCTTCAAAT
        131 GCGCTTTGAGGATTTCGTTGGAAACGGAAATATCTTCATAT

                *    *            *           * *       *  *    *            
      97742 AAAATCTAGCCAGAAGCATTCTAAGAAACATCTTAGGGATGTTTACATTCAAGTCACAGAGTTGA
          1 AAAAACTAGACAGAAGCATTCTCAGAAACATCTTTGTGATGTTTGCACTCAACTCACAGAGTTGA

              *   * *    **       *         *    * *   **      *      *    * 
      97807 ACATTCCCCTTTCTCAGAGCAGGTTTGAAACAATCTTCTCGTACTATCTGGCAGTGGACATTTTG
         66 ACCTT-TCTTTTCATAGAGCAGTTTTGAAACACTCTTTTTGTAGAATCTGGAAGTGGATATTTGG

               * *   *  ** ** *  *   *            ** *
      97872 AGCTCCTTGGGGCCTATGCTGAAAAAGGAAATATCTTCCGAC
        130 AGCGCTTTGAGGATTTCGTTGGAAACGGAAATATCTTCATAT

                                 *     *  **          *           **    *    
      97914 AAAAACTAGACAGAAGCATTCGCAGAATCACGTTTGTGATGTGTGCACTCAACTGTCAGAATTGA
          1 AAAAACTAGACAGAAGCATTCTCAGAAACATCTTTGTGATGTTTGCACTCAACTCACAGAGTTGA

                 **   ** *      *                            * *            *
      97979 ACCTTGGTTTGGACAGAGCACTTTTGAAACACTCTTTTTGTAGAATCTGCAGGTGGATATTTGGC
         66 ACCTTTCTTTTCATAGAGCAGTTTTGAAACACTCTTTTTGTAGAATCTGGAAGTGGATATTTGGA

            **                         *   *      * *
      98044 TAGCTTTGAGGATTTCGTTGGAAACGGTAATGTCTTCAAAG
        131 GCGCTTTGAGGATTTCGTTGGAAACGGAAATATCTTCATAT

                *          *   *          *   *            *    *            
      98085 AAAATCTAGACAGAAACATCCTCAGAAACACCTTCGTGATGTTTGCAATCAAGTCACAGAGTTGA
          1 AAAAACTAGACAGAAGCATTCTCAGAAACATCTTTGTGATGTTTGCACTCAACTCACAGAGTTGA

                 * *             *  *         *        *        *    *       
      98150 ACCTTCCGTTTCATAGAGCAGGTTGGAAACACTCATTTTGTAGTATCTGGAATTGGACATTTGGA
         66 ACCTTTCTTTTCATAGAGCAGTTTTGAAACACTCTTTTTGTAGAATCTGGAAGTGGATATTTGGA

               *   *   ** ** *  *   *            **  
      98215 GCGATTTCAGGCCTATGGTGTAAAAGGAAATATCTTCCCAT
        131 GCGCTTTGAGGATTTCGTTGGAAACGGAAATATCTTCATAT

                * * * *      **      *   * *   *     ** ** *      *    *     
      98256 AAAAGCGACATAGAAGCTATCTCAGGAACTTGTTTATGATGCATCTAATCAACTAACAGTGTTGA
          1 AAAAACTAGACAGAAGCATTCTCAGAAACATCTTTGTGATGTTTGCACTCAACTCACAGAGTTGA

                  * ** * *                         * *       *               
      98321 ACCTTTGTACTGACAGAGCAG-TTTGAAACACTCTTTTTTTGGAATCTGCAAGTGGATATTTGGA
         66 ACCTTTCTTTTCATAGAGCAGTTTTGAAACACTCTTTTTGTAGAATCTGGAAGTGGATATTTGGA

            *        *                  *   *   *    
      98385 TCGCTTTGATGATTTCGTTGGAAACGG-GAT-GC--AATAT
        131 GCGCTTTGAGGATTTCGTTGGAAACGGAAATATCTTCATAT

                **  *    *     *                 **  *   *  *    *         * 
      98422 AAAACGTACACAGCAGCATACTCAGAAA-ATACTTTGCCATATTTCCATTCAAGTCACAGAGTGG
          1 AAAAACTAGACAGAAGCATTCTCAGAAACAT-CTTTGTGATGTTTGCACTCAACTCACAGAGTTG

               *  * **            *           *      *  **            *      
      98486 AACATTCCCATTCATAGAGCAGGTTTGAAACACTTTTTTTGGAGTGTCTGGAAGTGGACATTTGG
         65 AACCTTTCTTTTCATAGAGCAGTTTTGAAACACTCTTTTTGTAGAATCTGGAAGTGGATATTTGG

                    *  * * ** *  *   *            **  
      98551 AGCGCTTTCAGAACTATGGTGAAAAAGGAAATATCTTCCAAT
        130 AGCGCTTTGAGGATTTCGTTGGAAACGGAAATATCTTCATAT

            *     *               *      * *         **  **       *  *  * *  
      98593 GAAAACAAGACAGAAGCATTCTGAGAAACTTATTTGTGATGCGTGTCCTCAACTTACGGACTCGA
          1 AAAAACTAGACAGAAGCATTCTCAGAAACATCTTTGTGATGTTTGCACTCAACTCACAGAGTTGA

                   *      **  * *  *  *             *   *    * * *          *
      98658 ACCTTTCGTTTCATGCAGTACTTCTGGAACACTCTTTTTGAAGATTCTGCATGCGGATATTTGGT
         66 ACCTTTCTTTTCATAGAGCAGTTTTGAAACACTCTTTTTGTAGAATCTGGAAGTGGATATTTGGA

            **                            **   *     
      98723 TAGCTTTGAGGATTTCGTTGGAAACGG---GCT-TACATAT
        131 GCGCTTTGAGGATTTCGTTGGAAACGGAAATATCTTCATAT

                 *       *               *        *   *    *    *       *    
      98760 AAAAATTAGACAGCAGCATTCTCAGAAACTTCTTTGTGGTGTCTGCATTCAAGTCACAGAATTGA
          1 AAAAACTAGACAGAAGCATTCTCAGAAACATCTTTGTGATGTTTGCACTCAACTCACAGAGTTGA

              * ** * **            *  * *      *       *    *        *       
      98825 ACATCCCCTCACATAGAGCAGTTGTGCAGCACTCTATTTGTAGTATCTCGAAGTGGACATTTGGA
         66 ACCTTTCTTTTCATAGAGCAGTTTTGAAACACTCTTTTTGTAGAATCTGGAAGTGGATATTTGGA

             *         ****    * *   *            **  
      98890 GGGCTTTGTAGCCAATC-TGGAAAAAGGAAATATCTTCCCAT
        131 GCGCTTTG-AGGATTTCGTTGGAAACGGAAATATCTTCATAT

            *   * *   *     * *            *   *  *   * **        *  * * *   
      98931 GAAAGCCAGATAGAAGTAATCTCAGAAACATGTTTATGCTGTATCTACTCAACTAACTGTGCTGA
          1 AAAAACTAGACAGAAGCATTCTCAGAAACATCTTTGTGATGTTTGCACTCAACTCACAGAGTTGA

              *     *  *               *        *  * *       *               
      98996 ACATTTCTATTGATAGAGCAGTTTTGAGACACTCTTCTTTTGGAATCTGCAAGTGGATATTTGGA
         66 ACCTTTCTTTTCATAGAGCAGTTTTGAAACACTCTTTTTGTAGAATCTGGAAGTGGATATTTGGA

            ** *                        *     *      
      99061 TAGATTTGAGGATTTCGTTGGAAACGG-GAT-T-AT-ATAT
        131 GCGCTTTGAGGATTTCGTTGGAAACGGAAATATCTTCATAT

                 *       *               *                 **  *   *         
      99098 AAAAAGTAGACAGCAGCATTCTCAGAAACTTCTTTGTGATGTTTGCATCCAGCTCTCAGAGTTGA
          1 AAAAACTAGACAGAAGCATTCTCAGAAACATCTTTGTGATGTTTGCACTCAACTCACAGAGTTGA

              *  * *          *  *        * *      *  ***        *  **       
      99163 ACATTCCCTTTCATAGAGTAGGTTTGAAACCCCCTTTTTATACTGTCTGGAAGCGGGCATTTGGA
         66 ACCTTTCTTTTCATAGAGCAGTTTTGAAACACTCTTTTTGTAGAATCTGGAAGTGGATATTTGGA

                   *   ** ** *  *   *    *     * *  *
      99228 GCGCTTTCAGGCCTATGCTGAAAAAGGAATTATCTACCTAC
        131 GCGCTTTGAGGATTTCGTTGGAAACGGAAATATCTTCATAT

             *                    *    *  **          ** *        *    *     
      99269 AGAAACTAGACAGAAGCATTCTGAGAATCACGTTTGTGATGTGGGTACTCAACTAACAGTGTTGA
          1 AAAAACTAGACAGAAGCATTCTCAGAAACATCTTTGTGATGTTTGCACTCAACTCACAGAGTTGA

            *  *       *   *            *   *                *               
      99334 TCCATTCTTTTGATACAGCAGTTTTGAACCACCCTTTTTGTAGAATCTGCAAGTGGATATTTGGA
         66 ACCTTTCTTTTCATAGAGCAGTTTTGAAACACTCTTTTTGTAGAATCTGGAAGTGGATATTTGGA

            **   *          *          *   *        *
      99399 TAGCTGTGAGGATTTCATTGGAAACGGGAATGTCTTCATAG
        131 GCGCTTTGAGGATTTCGTTGGAAACGGAAATATCTTCATAT

                **                     * ****         *  **   *   *          
      99440 AAAATTTAGACAGAAGCATTCTCAGAACCTGGATTGTGATGTGTGTTCTCCACTAACAGAGTTGA
          1 AAAAACTAGACAGAAGCATTCTCAGAAACATCTTTGTGATGTTTGCACTCAACTCACAGAGTTGA

                      ** *   * * *         *  *    *                         
      99505 ACCTTTCTTTGGACAGAACTGATTTGAAACATTCATTTTATAGAATCTGGAAGTGGATATTTGGA
         66 ACCTTTCTTTTCATAGAGCAGTTTTGAAACACTCTTTTTGTAGAATCTGGAAGTGGATATTTGGA

            ** *               *    *  *          *  
      99570 AAGTTTTGAGGATTTCGTTAGAAATGGGAATATCTTCAAAT
        131 GCGCTTTGAGGATTTCGTTGGAAACGGAAATATCTTCATAT

                *    *            *           * *       *  *    *            
      99611 AAAATCTAGCCAGAAGCATTCTAAGAAACATCTTAGGGATGTTTACATTCAAGTCACAGAGTTGA
          1 AAAAACTAGACAGAAGCATTCTCAGAAACATCTTTGTGATGTTTGCACTCAACTCACAGAGTTGA

              *   * *    **       *         *    * *   **      *      *    * 
      99676 ACATTCCCCTTTCTCAGAGCAGGTTTGAAACAATCTTCTCGTACTATCTGGCAGTGGACATTTTG
         66 ACCTT-TCTTTTCATAGAGCAGTTTTGAAACACTCTTTTTGTAGAATCTGGAAGTGGATATTTGG

               * *   *  ** ** *  *   *            ** *
      99741 AGCTCCTTGGGGCCTATGCTGAAAAAGGAAATATCTTCCGAC
        130 AGCGCTTTGAGGATTTCGTTGGAAACGGAAATATCTTCATAT

                                 *     *  **          *           **    *    
      99783 AAAAACTAGACAGAAGCATTCGCAGAATCACGTTTGTGATGTGTGCACTCAACTGTCAGAATTGA
          1 AAAAACTAGACAGAAGCATTCTCAGAAACATCTTTGTGATGTTTGCACTCAACTCACAGAGTTGA

                 **   ** *      *                            * *            *
      99848 ACCTTGGTTTGGACAGAGCACTTTTGAAACACTCTTTTTGTAGAATCTGCAGGTGGATATTTGGC
         66 ACCTTTCTTTTCATAGAGCAGTTTTGAAACACTCTTTTTGTAGAATCTGGAAGTGGATATTTGGA

            **                         *   *      * *
      99913 TAGCTTTGAGGATTTCGTTGGAAACGGTAATGTCTTCAAAG
        131 GCGCTTTGAGGATTTCGTTGGAAACGGAAATATCTTCATAT

                *          *   *          *   *            *    *            
      99954 AAAATCTAGACAGAAACATCCTCAGAAACACCTTCGTGATGTTTGCAATCAAGTCACAGAGTTGA
          1 AAAAACTAGACAGAAGCATTCTCAGAAACATCTTTGTGATGTTTGCACTCAACTCACAGAGTTGA

                 * *             *  *         *        *             *       
     100019 ACCTTCCGTTTCATAGAGCAGGTTGGAAACACTCATTTTGTAGTATCTGGAAGTGGACATTTGGA
         66 ACCTTTCTTTTCATAGAGCAGTTTTGAAACACTCTTTTTGTAGAATCTGGAAGTGGATATTTGGA

                   *   ** ** *  *   *            **  
     100084 GCGCTTTCAGGCCTATGGTGTAAAAGGAAATATCTTCCCAT
        131 GCGCTTTGAGGATTTCGTTGGAAACGGAAATATCTTCATAT

                * * * *      **      *   * *   *     ** ** *      *    *     
     100125 AAAAGCGACATAGAAGCTATCTCAGGAACTTGTTTATGATGCATCTAATCAACTAACAGTGTTGA
          1 AAAAACTAGACAGAAGCATTCTCAGAAACATCTTTGTGATGTTTGCACTCAACTCACAGAGTTGA

             *    * ** * *                         * *       *               
     100190 AACTTTGTACTGACAGAGCAG-TTTGAAACACTCTTTTTTTGGAATCTGCAAGTGGATATTTGGA
         66 ACCTTTCTTTTCATAGAGCAGTTTTGAAACACTCTTTTTGTAGAATCTGGAAGTGGATATTTGGA

            *                           *   *   *    
     100254 TCGCTTTGAGGATTTCGTTGGAAACGG-GAT-GC--AATAT
        131 GCGCTTTGAGGATTTCGTTGGAAACGGAAATATCTTCATAT

                **  *    *     *                 **  *   *  *    *         * 
     100291 AAAACGTACACAGCAGCATACTCAGAAA-ATACTTTGCCATATTTCCATTCAAGTCACAGAGTGG
          1 AAAAACTAGACAGAAGCATTCTCAGAAACAT-CTTTGTGATGTTTGCACTCAACTCACAGAGTTG

               *  * **            *           *      *  **            *      
     100355 AACATTCCCATTCATAGAGCAGGTTTGAAACACTTTTTTTGGAGTGTCTGGAAGTGGACATTTGG
         65 AACCTTTCTTTTCATAGAGCAGTTTTGAAACACTCTTTTTGTAGAATCTGGAAGTGGATATTTGG

                    *  * * ** *  *   *            **  
     100420 AGCGCTTTCAGAACTATGGTGAAAAAGGAAATATCTTCCAAT
        130 AGCGCTTTGAGGATTTCGTTGGAAACGGAAATATCTTCATAT

            *     *               *      * *         ** ***       *  *  * *  
     100462 GAAAACAAGACAGAAGCATTCTGAGAAACTTATTTGTGATGCGTATCCTCAACTAACGGACTCGA
          1 AAAAACTAGACAGAAGCATTCTCAGAAACATCTTTGTGATGTTTGCACTCAACTCACAGAGTTGA

                   *      **  * *  *  *             *   *    * * *          *
     100527 ACCTTTCGTTTCATGCAGTACTTCTGGAACACTCTTTTTGAAGATTCTGCATGCGGATATTTGGT
         66 ACCTTTCTTTTCATAGAGCAGTTTTGAAACACTCTTTTTGTAGAATCTGGAAGTGGATATTTGGA

            **                            **   *   * 
     100592 TAGCTTTGAGGATTTCGTTGGAAACGG---GCT-TACATGT
        131 GCGCTTTGAGGATTTCGTTGGAAACGGAAATATCTTCATAT

                 *       *               *        *   *    *    *       *    
     100629 AAAAATTAGACAGCAGCATTCTCAGAAACTTCTTTGTGGTGTCTGCATTCAAGTCACAGAATTGA
          1 AAAAACTAGACAGAAGCATTCTCAGAAACATCTTTGTGATGTTTGCACTCAACTCACAGAGTTGA

              * ** * **            *  * *      *       *    *        *       
     100694 ACATCCCCTCACATAGAGCAGTTGTGCAGCACTCTATTTGTAGTATCTCGAAGTGGACATTTGGA
         66 ACCTTTCTTTTCATAGAGCAGTTTTGAAACACTCTTTTTGTAGAATCTGGAAGTGGATATTTGGA

             *         ** *    * *   *            **  
     100759 GGGCTTTGTAGCCTATC-TGGAAAAAGGAAATATCTTCCCAT
        131 GCGCTTTG-AGGATTTCGTTGGAAACGGAAATATCTTCATAT

            *  ** *   *     * *            *   *  *   * **        *  * * *   
     100800 GAATGCGAGATAGAAGTAATCTCAGAAACATGTTTATGCTGTATCTACTCAACTAACTGTGCTGA
          1 AAAAACTAGACAGAAGCATTCTCAGAAACATCTTTGTGATGTTTGCACTCAACTCACAGAGTTGA

              *     *  *               *        *  * *       *               
     100865 ACATTTCTATTGATAGAGCAGTTTTGAGACACTCTTCTTTTGGAATCTGCAAGTGGATATTTGGA
         66 ACCTTTCTTTTCATAGAGCAGTTTTGAAACACTCTTTTTGTAGAATCTGGAAGTGGATATTTGGA

            ** *                        *     *      
     100930 TAGATTTGAGGATTTCGTTGGAAACGG-GAT-T-AT-ATAT
        131 GCGCTTTGAGGATTTCGTTGGAAACGGAAATATCTTCATAT

                 *       *               *                 **  *   *         
     100967 AAAAAGTAGACAGCAGCATTCTCAGAAACTTCTTTGTGATGTTTGCATCCAGCTCTCAGAGTTGA
          1 AAAAACTAGACAGAAGCATTCTCAGAAACATCTTTGTGATGTTTGCACTCAACTCACAGAGTTGA

              *  * *          *  *        * *      *   **           **       
     101032 ACATTCCCTTTCATAGAGTAGGTTTGAAACCCCCTTTTTATAGTGTCTGGAAGTGGGCATTTGGA
         66 ACCTTTCTTTTCATAGAGCAGTTTTGAAACACTCTTTTTGTAGAATCTGGAAGTGGATATTTGGA

                   *   ** ** *  *   *          * *  *
     101097 GCGCTTTCAGGCCTATGCTGAAAAAGGAAATATCTACCTAC
        131 GCGCTTTGAGGATTTCGTTGGAAACGGAAATATCTTCATAT

             *                    *    *  **          ** *        *    *     
     101138 AGAAACTAGACAGAAGCATTCTGAGAATCACGTTTGTGATGTGGGTACTCAACTAACAGTGTTGA
          1 AAAAACTAGACAGAAGCATTCTCAGAAACATCTTTGTGATGTTTGCACTCAACTCACAGAGTTGA

            *  *       *   *            *   *                *               
     101203 TCCATTCTTTTGATACAGCAGTTTTGAACCACCCTTTTTGTAGAATCTGCAAGTGGATATTTGGA
         66 ACCTTTCTTTTCATAGAGCAGTTTTGAAACACTCTTTTTGTAGAATCTGGAAGTGGATATTTGGA

            **   *                     *   *        *
     101268 TAGCTGTGAGGATTTCGTTGGAAACGGGAATGTCTTCATAG
        131 GCGCTTTGAGGATTTCGTTGGAAACGGAAATATCTTCATAT

                **                     * ****         ** **   *   *          
     101309 AAAATTTAGACAGAAGCATTCTCAGAACCTGGATTGTGATGTGAGTTCTCCACTAACAGAGTTGA
          1 AAAAACTAGACAGAAGCATTCTCAGAAACATCTTTGTGATGTTTGCACTCAACTCACAGAGTTGA

                      ** *   * *           *       *                         
     101374 ACCTTTCTTTGGACAGAACTGTTTTGAAACATTCTTTTTATAGAATCTGGAAGTGGATATTTGGA
         66 ACCTTTCTTTTCATAGAGCAGTTTTGAAACACTCTTTTTGTAGAATCTGGAAGTGGATATTTGGA

            **                         *          *  
     101439 AAGCTTTGAGGATTTCGTTGGAAACGGGAATATCTTCAAAT
        131 GCGCTTTGAGGATTTCGTTGGAAACGGAAATATCTTCATAT

                *    *            *           * *       *  *    *            
     101480 AAAATCTAGCCAGAAGCATTCTAAGAAACATCTTAGGGATGTTTACATTCAAGTCACAGAGTTGA
          1 AAAAACTAGACAGAAGCATTCTCAGAAACATCTTTGTGATGTTTGCACTCAACTCACAGAGTTGA

              *   * *    **       *         *    * *   **      *      *    * 
     101545 ACATTCCCCTTTCTCAGAGCAGGTTTGAAACAATCTTCTCGTACTATCTGGCAGTGGACATTTTG
         66 ACCTT-TCTTTTCATAGAGCAGTTTTGAAACACTCTTTTTGTAGAATCTGGAAGTGGATATTTGG

               * *   *  ** ** *  *   *            ** *
     101610 AGCTCCTTGGGGCCTATGCTGAAAAAGGAAATATCTTCCGAC
        130 AGCGCTTTGAGGATTTCGTTGGAAACGGAAATATCTTCATAT

                                 *     *  **          *           **    *    
     101652 AAAAACTAGACAGAAGCATTCGCAGAATCACGTTTGTGATGTGTGCACTCAACTGTCAGAATTGA
          1 AAAAACTAGACAGAAGCATTCTCAGAAACATCTTTGTGATGTTTGCACTCAACTCACAGAGTTGA

                 **   ** *      *                            * *            *
     101717 ACCTTGGTTTGGACAGAGCACTTTTGAAACACTCTTTTTGTAGAATCTGCAGGTGGATATTTGGC
         66 ACCTTTCTTTTCATAGAGCAGTTTTGAAACACTCTTTTTGTAGAATCTGGAAGTGGATATTTGGA

            **                         *   *      ***
     101782 TAGCTTTGAGGATTTCGTTGGAAACGGTAATGTCTTCAACG
        131 GCGCTTTGAGGATTTCGTTGGAAACGGAAATATCTTCATAT

                *          *   *          *   *            *    *            
     101823 AAAATCTAGACAGAAACATCCTCAGAAACACCTTCGTGATGTTTGCAATCAAGTCACAGAGTTGA
          1 AAAAACTAGACAGAAGCATTCTCAGAAACATCTTTGTGATGTTTGCACTCAACTCACAGAGTTGA

                 * *             *  *         *        *        *    *       
     101888 ACCTTCCGTTTCATAGAGCAGGTTGGAAACACTCATTTTGTAGTATCTGGAATTGGACATTTGGA
         66 ACCTTTCTTTTCATAGAGCAGTTTTGAAACACTCTTTTTGTAGAATCTGGAAGTGGATATTTGGA

               *   *   ** ** *  *   *            **  
     101953 GCGATTTCAGGCCTATGGTGTAAAAGGAAATATCTTCCCAT
        131 GCGCTTTGAGGATTTCGTTGGAAACGGAAATATCTTCATAT

                * * * *      **      *   * *   *     ** ** *      *    *     
     101994 AAAAGCGACATAGAAGCTATCTCAGGAACTTGTTTATGATGCATCTAATCAACTAACAGTGTTGA
          1 AAAAACTAGACAGAAGCATTCTCAGAAACATCTTTGTGATGTTTGCACTCAACTCACAGAGTTGA

             *    * ** * *                         * *       *               
     102059 AACTTTGTACTGACAGAGCAG-TTTGAAACACTCTTTTTTTGGAATCTGCAAGTGGATATTTGGA
         66 ACCTTTCTTTTCATAGAGCAGTTTTGAAACACTCTTTTTGTAGAATCTGGAAGTGGATATTTGGA

            *                           *   *   *    
     102123 TCGCTTTGAGGATTTCGTTGGAAACGG-GAT-GC--AATAT
        131 GCGCTTTGAGGATTTCGTTGGAAACGGAAATATCTTCATAT

                **  *    *     *                 **  *   *  *    *         * 
     102160 AAAACGTACACAGCAGCATACTCAGAAA-ATACTTTGCCATATTTCCATTCAAGTCACAGAGTGG
          1 AAAAACTAGACAGAAGCATTCTCAGAAACAT-CTTTGTGATGTTTGCACTCAACTCACAGAGTTG

               *  * **            *           *      *  **            *      
     102224 AACATTCCCATTCATAGAGCAGGTTTGAAACACTTTTTTTGGAGTGTCTGGAAGTGGACATTTGG
         65 AACCTTTCTTTTCATAGAGCAGTTTTGAAACACTCTTTTTGTAGAATCTGGAAGTGGATATTTGG

                    *  * * ** *  *   *            **  
     102289 AGCGCTTTCAGAACTATGGTGAAAAAGGAAATATCTTCCAAT
        130 AGCGCTTTGAGGATTTCGTTGGAAACGGAAATATCTTCATAT

            *     *               *      * *         **  **       *  *  * *  
     102331 GAAAACAAGACAGAAGCATTCTGAGAAACTTATTTGTGATGCGTGTCCTCAACTAACGGACTCGA
          1 AAAAACTAGACAGAAGCATTCTCAGAAACATCTTTGTGATGTTTGCACTCAACTCACAGAGTTGA

                   *      **  * *  *  *             *   *    * * *          *
     102396 ACCTTTCGTTTCATGCAGTACTTCTGGAACACTCTTTTTGAAGATTCTGCATGCGGATATTTGGT
         66 ACCTTTCTTTTCATAGAGCAGTTTTGAAACACTCTTTTTGTAGAATCTGGAAGTGGATATTTGGA

            **                            **   *     
     102461 TAGCTTTGAGGATTTCGTTGGAAACGG---GCT-TACATAT
        131 GCGCTTTGAGGATTTCGTTGGAAACGGAAATATCTTCATAT

                 *       *               *        *   *    *    *       *    
     102498 AAAAATTAGACAGCAGCATTCTCAGAAACTTCTTTGTGGTGTCTGCATTCAAGTCACAGAATTGA
          1 AAAAACTAGACAGAAGCATTCTCAGAAACATCTTTGTGATGTTTGCACTCAACTCACAGAGTTGA

              * ** * **            *  * *      *       *    *        *       
     102563 ACATCCCCTCACATAGAGCAGTTGTGCAGCACTCTATTTGTAGTATCTCGAAGTGGACATTTGGA
         66 ACCTTTCTTTTCATAGAGCAGTTTTGAAACACTCTTTTTGTAGAATCTGGAAGTGGATATTTGGA

             *         ** *    * *   *            **  
     102628 GGGCTTTGTAGCCTATC-TGGAAAAAGGAAATATCTTCCCAT
        131 GCGCTTTG-AGGATTTCGTTGGAAACGGAAATATCTTCATAT

            *   * *   *     * *            *   *  *   * **        *  * * *   
     102669 GAAAGCCAGATAGAAGTAATCTCAGAAACATGTTTATGCTGTATCTACTCAACTAACTGTGCTGA
          1 AAAAACTAGACAGAAGCATTCTCAGAAACATCTTTGTGATGTTTGCACTCAACTCACAGAGTTGA

              *     *  *               *        *  * *       *               
     102734 ACATTTCTATTGATAGAGCAGTTTTGAGACACTCTTCTTTTGGAATCTGCAAGTGGATATTTGGA
         66 ACCTTTCTTTTCATAGAGCAGTTTTGAAACACTCTTTTTGTAGAATCTGGAAGTGGATATTTGGA

            ** *                        *     *      
     102799 TAGATTTGAGGATTTCGTTGGAAACGG-GAT-T-AT-ATAT
        131 GCGCTTTGAGGATTTCGTTGGAAACGGAAATATCTTCATAT

                 *       *               *                 **  *   *         
     102836 AAAAAGTAGACAGCAGCATTCTCAGAAACTTCTTTGTGATGTTTGCATCCAGCTCTCAGAGTTGA
          1 AAAAACTAGACAGAAGCATTCTCAGAAACATCTTTGTGATGTTTGCACTCAACTCACAGAGTTGA

              *  * *          *  *        * *      *  ***        *  **       
     102901 ACATTCCCTTTCATAGAGTAGGTTTGAAACCCCCTTTTTATACTGTCTGGAAGCGGGCATTTGGA
         66 ACCTTTCTTTTCATAGAGCAGTTTTGAAACACTCTTTTTGTAGAATCTGGAAGTGGATATTTGGA

                   *   ** ** *  *   *    *     * *  *
     102966 GCGCTTTCAGGCCTATGCTGAAAAAGGAATTATCTACCTAC
        131 GCGCTTTGAGGATTTCGTTGGAAACGGAAATATCTTCATAT

             *                    *    *  **          ** *        *    *     
     103007 AGAAACTAGACAGAAGCATTCTGAGAATCACGTTTGTGATGTGGGTACTCAACTAACAGTGTTGA
          1 AAAAACTAGACAGAAGCATTCTCAGAAACATCTTTGTGATGTTTGCACTCAACTCACAGAGTTGA

            *  *       *   *            *   *                *               
     103072 TCCATTCTTTTGATACAGCAGTTTTGAACCACCCTTTTTGTAGAATCTGCAAGTGGATATTTGGA
         66 ACCTTTCTTTTCATAGAGCAGTTTTGAAACACTCTTTTTGTAGAATCTGGAAGTGGATATTTGGA

            **   *                     *   *        *
     103137 TAGCTGTGAGGATTTCGTTGGAAACGGGAATGTCTTCATAG
        131 GCGCTTTGAGGATTTCGTTGGAAACGGAAATATCTTCATAT

                **                     * ****         ** **   *   *          
     103178 AAAATTTAGACAGAAGCATTCTCAGAACCTGGATTGTGATGTGAGTTCTCCACTAACAGAGTTGA
          1 AAAAACTAGACAGAAGCATTCTCAGAAACATCTTTGTGATGTTTGCACTCAACTCACAGAGTTGA

                      ** *   * * *         *       *                         
     103243 ACCTTTCTTTGGACAGAACTGATTTGAAACATTCTTTTTATAGAATCTGGAAGTGGATATTTGGA
         66 ACCTTTCTTTTCATAGAGCAGTTTTGAAACACTCTTTTTGTAGAATCTGGAAGTGGATATTTGGA

            ** *                       *          *  
     103308 AAGTTTTGAGGATTTCGTTGGAAACGGGAATATCTTCAAAT
        131 GCGCTTTGAGGATTTCGTTGGAAACGGAAATATCTTCATAT

                *    *            *           * *       *  *    *            
     103349 AAAATCTAGCCAGAAGCATTCTAAGAAACATCTTAGGGATGTTTACATTCAAGTCACAGAGTTGA
          1 AAAAACTAGACAGAAGCATTCTCAGAAACATCTTTGTGATGTTTGCACTCAACTCACAGAGTTGA

              *   * *    **       *         *    * *   **      *      *    * 
     103414 ACATTCCCCTTTCTCAGAGCAGGTTTGAAACAATCTTCTCGTACTATCTGGCAGTGGACATTTTG
         66 ACCTT-TCTTTTCATAGAGCAGTTTTGAAACACTCTTTTTGTAGAATCTGGAAGTGGATATTTGG

             * * *   *  ** ** *  *   *            ** *
     103479 AACTCCTTGGGGCCTATGCTGAAAAAGGAAATATCTTCCGAC
        130 AGCGCTTTGAGGATTTCGTTGGAAACGGAAATATCTTCATAT

                                 *     *   *          *           **    *    
     103521 AAAAACTAGACAGAAGCATTCGCAGAATCATGTTTGTGATGTGTGCACTCAACTGTCAGAATTGA
          1 AAAAACTAGACAGAAGCATTCTCAGAAACATCTTTGTGATGTTTGCACTCAACTCACAGAGTTGA

                 **   ** *      *                            * *          * *
     103586 ACCTTGGTTTGGACAGAGCACTTTTGAAACACTCTTTTTGTAGAATCTGCAGGTGGATATTTCGC
         66 ACCTTTCTTTTCATAGAGCAGTTTTGAAACACTCTTTTTGTAGAATCTGGAAGTGGATATTTGGA

            **                         *   *      * *
     103651 TAGCTTTGAGGATTTCGTTGGAAACGGTAATGTCTTCAAAG
        131 GCGCTTTGAGGATTTCGTTGGAAACGGAAATATCTTCATAT

                *          *   *          *   *            *    *            
     103692 AAAATCTAGACAGAAACATCCTCAGAAACACCTTCGTGATGTTTGCAATCAAGTCACAGAGTTGA
          1 AAAAACTAGACAGAAGCATTCTCAGAAACATCTTTGTGATGTTTGCACTCAACTCACAGAGTTGA

                 * *             *  *         *        *             *       
     103757 ACCTTCCGTTTCATAGAGCAGGTTGGAAACACTCATTTTGTAGTATCTGGAAGTGGACATTTGGA
         66 ACCTTTCTTTTCATAGAGCAGTTTTGAAACACTCTTTTTGTAGAATCTGGAAGTGGATATTTGGA

                   *   ** ** *  *   *            **  
     103822 GCGCTTTCAGGCCTATGGTGTAAAAGGAAATATCTTCCCAT
        131 GCGCTTTGAGGATTTCGTTGGAAACGGAAATATCTTCATAT

                * * * *      **      *   * *   *     ** ** *      *    *     
     103863 AAAAGCGACATAGAAGCTATCTCAGGAACTTGTTTATGATGCATCTAATCAACTAACAGTGTTGA
          1 AAAAACTAGACAGAAGCATTCTCAGAAACATCTTTGTGATGTTTGCACTCAACTCACAGAGTTGA

                  * ** * *                         * *       *               
     103928 ACCTTTGTACTGACAGAGCAG-TTTGAAACACTCTTTTTTTGGAATCTGCAAGTGGATATTTGGA
         66 ACCTTTCTTTTCATAGAGCAGTTTTGAAACACTCTTTTTGTAGAATCTGGAAGTGGATATTTGGA

            *                           *   *   *    
     103992 TCGCTTTGAGGATTTCGTTGGAAACGG-GAT-GC--AATAT
        131 GCGCTTTGAGGATTTCGTTGGAAACGGAAATATCTTCATAT

                **  *    *     *                 **  *   *  *    **        * 
     104029 AAAACGTACACAGCAGCATACTCAGAAA-ATACTTTGCCATATTTCCATTCAAGACACAGAGTGG
          1 AAAAACTAGACAGAAGCATTCTCAGAAACAT-CTTTGTGATGTTTGCACTCAACTCACAGAGTTG

               *  * **            *           *      *  **            *      
     104093 AACATTCCCATTCATAGAGCAGGTTTGAAACACTTTTTTTGGAGTGTCTGGAAGTGGACATTTGG
         65 AACCTTTCTTTTCATAGAGCAGTTTTGAAACACTCTTTTTGTAGAATCTGGAAGTGGATATTTGG

                    *  * * ** *  *   *            **  
     104158 AGCGCTTTCAGAACTATGGTGAAAAAGGAAATATCTTCCAAT
        130 AGCGCTTTGAGGATTTCGTTGGAAACGGAAATATCTTCATAT

            *     *               *      * *         **  **       *  *  * *  
     104200 GAAAACAAGACAGAAGCATTCTGAGAAACTTATTTGTGATGCGTGTCCTCAACTAACGGACTCGA
          1 AAAAACTAGACAGAAGCATTCTCAGAAACATCTTTGTGATGTTTGCACTCAACTCACAGAGTTGA

                   *      **  * *  *  *             *   *    * * *           
     104265 ACCTTTCGTTTCATGCAGTACTTCTGGAACACTCTTTTTGAAGATTCTGCATGCGGATATTTGGA
         66 ACCTTTCTTTTCATAGAGCAGTTTTGAAACACTCTTTTTGTAGAATCTGGAAGTGGATATTTGGA

            **                            **   *     
     104330 TAGCTTTGAGGATTTCGTTGGAAACGG---GCT-TACATAT
        131 GCGCTTTGAGGATTTCGTTGGAAACGGAAATATCTTCATAT

                 *       *               *        *   *    *    *       *    
     104367 AAAAATTAGACAGCAGCATTCTCAGAAACTTCTTTGTGGTGTCTGCATTCAAGTCACAGAATTGA
          1 AAAAACTAGACAGAAGCATTCTCAGAAACATCTTTGTGATGTTTGCACTCAACTCACAGAGTTGA

              * ** * **            *  * *      *       *    *        *       
     104432 ACATCCCCTCACATAGAGCAGTTGTGCAGCACTCTATTTGTAGTATCTCGAAGTGGACATTTGGA
         66 ACCTTTCTTTTCATAGAGCAGTTTTGAAACACTCTTTTTGTAGAATCTGGAAGTGGATATTTGGA

             *         ** *    * *   *            **  
     104497 GGGCTTTGTAGCCTATC-TGGAAAAAGGAAATATCTTCCCAT
        131 GCGCTTTG-AGGATTTCGTTGGAAACGGAAATATCTTCATAT

            *   * *   *     * *            *   *  *   * **        *  * * *   
     104538 GAAAGCCAGATAGAAGTAATCTCAGAAACATGTTTATGCTGTATCTACTCAACTAACTGTGCTGA
          1 AAAAACTAGACAGAAGCATTCTCAGAAACATCTTTGTGATGTTTGCACTCAACTCACAGAGTTGA

              *     *  *               *        *             *              
     104603 ACATTTCTATTGATAGAGCAGTTTTGAGACACTCTTCTT-TAGGAATCTGCAAGTGGATATTTGG
         66 ACCTTTCTTTTCATAGAGCAGTTTTGAAACACTCTTTTTGTA-GAATCTGGAAGTGGATATTTGG

             ** *                        *     *      
     104667 ATAGATTTGAGGATTTCGTTGGAAACGG-GAT-T-AT-ATAT
        130 AGCGCTTTGAGGATTTCGTTGGAAACGGAAATATCTTCATAT

                 *       *               *                 **  *   *         
     104705 AAAAAGTAGACAGCAGCATTCTCAGAAACTTCTTTGTGATGTTTGCATCCAGCTCTCAGAGTTGA
          1 AAAAACTAGACAGAAGCATTCTCAGAAACATCTTTGTGATGTTTGCACTCAACTCACAGAGTTGA

              *  * *          *  *        * *      *  ***        *  **       
     104770 ACATTCCCTTTCATAGAGTAGGTTTGAAACCCCCTTTTTATACTGTCTGGAAGCGGGCATTTGGA
         66 ACCTTTCTTTTCATAGAGCAGTTTTGAAACACTCTTTTTGTAGAATCTGGAAGTGGATATTTGGA

                   *   ** ** *  *   *    *     * *  *
     104835 GCGCTTTCAGGCCTATGCTGAAAAAGGAATTATCTACCTAC
        131 GCGCTTTGAGGATTTCGTTGGAAACGGAAATATCTTCATAT

             *                    *    *  **          ** *        *    *     
     104876 AGAAACTAGACAGAAGCATTCTGAGAATCACGTTTGTGATGTGGGTACTCAACTAACAGTGTTGA
          1 AAAAACTAGACAGAAGCATTCTCAGAAACATCTTTGTGATGTTTGCACTCAACTCACAGAGTTGA

            *  *           *         *  *   *             *  *               
     104941 TCCATTCTTTTCATACAGCAGTTTTTAACCACACTTTTTGTAGAATGTGCAAGTGGATATTTGGA
         66 ACCTTTCTTTTCATAGAGCAGTTTTGAAACACTCTTTTTGTAGAATCTGGAAGTGGATATTTGGA

            **   *                     *   *        *
     105006 TAGCTGTGAGGATTTCGTTGGAAACGGGAATGTCTTCATAG
        131 GCGCTTTGAGGATTTCGTTGGAAACGGAAATATCTTCATAT

                **       *             * ****         *  **   *   *          
     105047 AAAATTTAGACAGGAGCATTCTCAGAACCTGGATTGTGATGTGTGTTCTCCACTAACAGAGTTGA
          1 AAAAACTAGACAGAAGCATTCTCAGAAACATCTTTGTGATGTTTGCACTCAACTCACAGAGTTGA

                       * *   * *           *       **               *        
     105112 ACCTTTCTTTTGACAGAACTGTTTTGAAACATTCTTTTTAGAGAATCTGGAAGTGGTTATTTGGA
         66 ACCTTTCTTTTCATAGAGCAGTTTTGAAACACTCTTTTTGTAGAATCTGGAAGTGGATATTTGGA

            **                       * *          *  
     105177 AAGCTTTGAGGATTTCGTTGGAAACAGGAATATCTTCAAAT
        131 GCGCTTTGAGGATTTCGTTGGAAACGGAAATATCTTCATAT

                *    *            *           * *       *  *    *            
     105218 AAAATCTAGCCAGAAGCATTCTAAGAAACATCTTAGGGATGTTTACATTCAAGTCACAGAGTTGA
          1 AAAAACTAGACAGAAGCATTCTCAGAAACATCTTTGTGATGTTTGCACTCAACTCACAGAGTTGA

              *   * *    **       *         *    * *   **      *      *    * 
     105283 ACATTCCCCTTTCTCAGAGCAGGTTTGAAACAATCTTCTCGTACTATCTGGCAGTGGACATTTTG
         66 ACCTT-TCTTTTCATAGAGCAGTTTTGAAACACTCTTTTTGTAGAATCTGGAAGTGGATATTTGG

               * *   ** ** ** *  *   *            ** *
     105348 AGCTCCTTGGCGCCTATGCTGAAAAAGGAAATATCTTCTGAC
        130 AGCGCTTTGAGGATTTCGTTGGAAACGGAAATATCTTCATAT

                                 *     *  **          *           **    *    
     105390 AAAAACTAGACAGAAGCATTCGCAGAATCACGTTTGTGATGTGTGCACTCAACTGTCAGAATTGA
          1 AAAAACTAGACAGAAGCATTCTCAGAAACATCTTTGTGATGTTTGCACTCAACTCACAGAGTTGA

                  *   ** *      *              *             * *            *
     105455 ACCTTTGTTTGGACAGAGCACTTTTG-AA-ACTCTCTTTGTAGAATCTGCAGGTGGATATTTGGC
         66 ACCTTTCTTTTCATAGAGCAGTTTTGAAACACTCTTTTTGTAGAATCTGGAAGTGGATATTTGGA

            **              *          *   *     ** *
     105518 TAGCTTTGAGGATTTCTTTGGAAACGGTAATGTCTTCGAAG
        131 GCGCTTTGAGGATTTCGTTGGAAACGGAAATATCTTCATAT

                *          *   *          *   *            *    *            
     105559 AAAATCTAGACAGAAACATCCTCAGAAACACCTTCGTGATGTTTGCAATCAAGTCACAGAGTTGA
          1 AAAAACTAGACAGAAGCATTCTCAGAAACATCTTTGTGATGTTTGCACTCAACTCACAGAGTTGA

                 * *             *  *           *      *             *       
     105624 ACCTTCCGTTTCATAGAGCAGGTTGGAAACACTCTTATTGTAGTATCTGGAAGTGGACATTTGGA
         66 ACCTTTCTTTTCATAGAGCAGTTTTGAAACACTCTTTTTGTAGAATCTGGAAGTGGATATTTGGA

                   *   ** ** *  *   *            **  
     105689 GCGCTTTCAGGCCTATGGTGTAAAAGGAAATATCTTCCCAT
        131 GCGCTTTGAGGATTTCGTTGGAAACGGAAATATCTTCATAT

                * * * *      **      *   * *   *     ** ** *      *    *     
     105730 AAAAGCGACATAGAAGCTATCTCAGGAACTTGTTTATGATGCATCTAATCAACTAACAGTGTTGA
          1 AAAAACTAGACAGAAGCATTCTCAGAAACATCTTTGTGATGTTTGCACTCAACTCACAGAGTTGA

                  * ** * *         *               * *       *               
     105795 ACCTTTGTACTGACAGAGCAG-TATGAAACACTCTTTTTTTGGAATCTGCAAGTGGATATTTGGA
         66 ACCTTTCTTTTCATAGAGCAGTTTTGAAACACTCTTTTTGTAGAATCTGGAAGTGGATATTTGGA

            *                           *   *   *    
     105859 TCGCTTTGAGGATTTCGTTGGAAACGG-GAT-GC--AATAT
        131 GCGCTTTGAGGATTTCGTTGGAAACGGAAATATCTTCATAT

                **  *    *     *                 **  *   *  *    *         * 
     105896 AAAACGTACACAGCAGCATACTCAGAAA-ATACTTTGCCATATTTCCATTCAAGTCACAGAGTGG
          1 AAAAACTAGACAGAAGCATTCTCAGAAACAT-CTTTGTGATGTTTGCACTCAACTCACAGAGTTG

               *  * **            *                  *  *             *      
     105960 AACATTCCCATTCATAGAGCAGGTTTGAAACACTCTTTTTGGAGTATCTGGAAGTGGACATTTGG
         65 AACCTTTCTTTTCATAGAGCAGTTTTGAAACACTCTTTTTGTAGAATCTGGAAGTGGATATTTGG

                    ** * * ** *  *   *            **  
     106025 AGCGCTTTCTGAACTATGGTGAAAAAGGAAATATCTTCCAAT
        130 AGCGCTTTGAGGATTTCGTTGGAAACGGAAATATCTTCATAT

            *     *               *      * *         **  **       *  *  * *  
     106067 GAAAACAAGACAGAAGCATTCTGAGAAACTTATTTGTGATGCGTGTCCTCAACTAACGGACTCGA
          1 AAAAACTAGACAGAAGCATTCTCAGAAACATCTTTGTGATGTTTGCACTCAACTCACAGAGTTGA

                   *      **  * *  *  *             *   *    * * *           
     106132 ACCTTTCGTTTCATGCAGTACTTCTGGAACACTCTTTTTGAAGATTCTGCATGCGGATATTTGGA
         66 ACCTTTCTTTTCATAGAGCAGTTTTGAAACACTCTTTTTGTAGAATCTGGAAGTGGATATTTGGA

            **                            **   *     
     106197 TAGCTTTGAGGATTTCGTTGGAAACGG---GCT-TACATAT
        131 GCGCTTTGAGGATTTCGTTGGAAACGGAAATATCTTCATAT

                 *       *               *        *   *    *    *       *    
     106234 AAAAATTAGACAGCAGCATTCTCAGAAACTTCTTTGTGGTGTCTGCATTCAAGTCACAGAATTGA
          1 AAAAACTAGACAGAAGCATTCTCAGAAACATCTTTGTGATGTTTGCACTCAACTCACAGAGTTGA

              * ** * **            *  * *      *       *    *        *       
     106299 ACATCCCCTCACATAGAGCAGTTGTGCAGCACTCTCTTTGTAGTATCTCGAAGTGGACATTTGGA
         66 ACCTTTCTTTTCATAGAGCAGTTTTGAAACACTCTTTTTGTAGAATCTGGAAGTGGATATTTGGA

             *         ** *    * *   *            **  
     106364 GGGCTTTGTAGCCTATC-TGGAAAAAGGAAATATCTTCCCAT
        131 GCGCTTTG-AGGATTTCGTTGGAAACGGAAATATCTTCATAT

            *  ** *   *     * *            *   *  *   * **        *  * * *   
     106405 GAATGCGAGATAGAAGTAATCTCAGAAACATGTTTATGCTGTATCTACTCAACTAACTGTGCTGA
          1 AAAAACTAGACAGAAGCATTCTCAGAAACATCTTTGTGATGTTTGCACTCAACTCACAGAGTTGA

              *     *  *               *        *  * *       *               
     106470 ACATTTCTATTGATAGAGCAGTTTTGAGACACTCTTCTTTTGGAATCTGCAAGTGGATATTTGGA
         66 ACCTTTCTTTTCATAGAGCAGTTTTGAAACACTCTTTTTGTAGAATCTGGAAGTGGATATTTGGA

            ** *                        *     *      
     106535 TAGATTTGAGGATTTCGTTGGAAACGG-GAT-T-AT-ATAT
        131 GCGCTTTGAGGATTTCGTTGGAAACGGAAATATCTTCATAT

                 *       *               *                 **  *   *         
     106572 AAAAAGTAGACAGCAGCATTCTCAGAAACTTCTTTGTGATGTTTGCATCCAGCTCTCAGAGTTGA
          1 AAAAACTAGACAGAAGCATTCTCAGAAACATCTTTGTGATGTTTGCACTCAACTCACAGAGTTGA

              *  * *         **  *        * *      *   **        *  **  *    
     106637 ACATTCCCTTTCATAGATTAGGTTTGAAACCCCCTTTTTATAGTGTCTGGAAGCGGGCATGTGGA
         66 ACCTTTCTTTTCATAGAGCAGTTTTGAAACACTCTTTTTGTAGAATCTGGAAGTGGATATTTGGA

                   *   ** ** *  *   *          * *  *
     106702 GCGCTTTCAGGCCTATGCTGAAAAAGGAAATATCTACCTAC
        131 GCGCTTTGAGGATTTCGTTGGAAACGGAAATATCTTCATAT

             *                    *    *  **          ** *        *    *     
     106743 AGAAACTAGACAGAAGCATTCTGAGAATCACGTTTGTGATGTGGGTACTCAACTAACAGTGTTGA
          1 AAAAACTAGACAGAAGCATTCTCAGAAACATCTTTGTGATGTTTGCACTCAACTCACAGAGTTGA

            *  *       *   *   *        *   *                *               
     106808 TCCATTCTTTTGATACAGCGGTTTTGAACCACACTTTTTGTAGAATCTGCAAGTGGATATTTGGA
         66 ACCTTTCTTTTCATAGAGCAGTTTTGAAACACTCTTTTTGTAGAATCTGGAAGTGGATATTTGGA

            **   *                     *   *  *     *
     106873 TAGCTGTGAGGATTTCGTTGGAAACGGGAATGTCCTCATAG
        131 GCGCTTTGAGGATTTCGTTGGAAACGGAAATATCTTCATAT

                **                     * * **         *  **   *   *          
     106914 AAAATTTAGACAGAAGCATTCTCAGAACCTTGATTGTGATGTGTGTTCTCCACTAACAGAGTTGA
          1 AAAAACTAGACAGAAGCATTCTCAGAAACATCTTTGTGATGTTTGCACTCAACTCACAGAGTTGA

                       * *   * *   *       *       *                         
     106979 ACCTTTCTTTTGACAGAACTGTTCTGAAACATTCTTTTTATAGAATCTGGAAGTGGATATTTGGA
         66 ACCTTTCTTTTCATAGAGCAGTTTTGAAACACTCTTTTTGTAGAATCTGGAAGTGGATATTTGGA

            **                         *          *  
     107044 AAGCTTTGAGGATTTCGTTGGAAACGGGAATATCTTCAAAT
        131 GCGCTTTGAGGATTTCGTTGGAAACGGAAATATCTTCATAT

                *    *            *           * *       *  *    *            
     107085 AAAATCTAGCCAGAAGCATTCTAAGAAACATCTTAGGGATGTTTACATTCAAGTCACAGAGTTGA
          1 AAAAACTAGACAGAAGCATTCTCAGAAACATCTTTGTGATGTTTGCACTCAACTCACAGAGTTGA

              *  * *     *       *         *    * *   **      *      *    *  
     107150 ACATTCCCTTTCACAGAGCAGGTTTGAAACAATCTTCTCGTACTATCTGGCAGTGGACATTTTGA
         66 ACCTTTCTTTTCATAGAGCAGTTTTGAAACACTCTTTTTGTAGAATCTGGAAGTGGATATTTGGA

              * *   *  ** ** *  *   *            ** *
     107215 GCTCCTTGGGGCCTATGCTGAAAAAGGAAATATCTTCCGAC
        131 GCGCTTTGAGGATTTCGTTGGAAACGGAAATATCTTCATAT

                                 *     *  **          *           **    *    
     107256 AAAAACTAGACAGAAGCATTCGCAGAATCACGTTTGTGATGTGTGCACTCAACTGTCAGAATTGA
          1 AAAAACTAGACAGAAGCATTCTCAGAAACATCTTTGTGATGTTTGCACTCAACTCACAGAGTTGA

                  *   ** *      *                            * *            *
     107321 ACCTTTGTTTGGACAGAGCACTTTTGAAACACTCTTTTTGTAGAATCTGCAGGTGGATATTTGGC
         66 ACCTTTCTTTTCATAGAGCAGTTTTGAAACACTCTTTTTGTAGAATCTGGAAGTGGATATTTGGA

            **                         *   *      * *
     107386 TAGCTTTGAGGATTTCGTTGGAAACGGTAATGTCTTCAAAG
        131 GCGCTTTGAGGATTTCGTTGGAAACGGAAATATCTTCATAT

                *          *   *          *   *            *    *            
     107427 AAAATCTAGACAGAAACATCCTCAGAAACACCTTCGTGATGTTTGCAATCAAGTCACAGAGTTGA
          1 AAAAACTAGACAGAAGCATTCTCAGAAACATCTTTGTGATGTTTGCACTCAACTCACAGAGTTGA

                 * *             *  *                  *             *       
     107492 ACCTTCCGTTTCATAGAGCAGGTTGGAAACACTCTTTTTGTAGTATCTGGAAGTGGACATTTGGA
         66 ACCTTTCTTTTCATAGAGCAGTTTTGAAACACTCTTTTTGTAGAATCTGGAAGTGGATATTTGGA

                   *   ** ** *  *   *            **  
     107557 GCGCTTTCAGGCCTATGGTGAAAAAGGAAATATCTTCCCAT
        131 GCGCTTTGAGGATTTCGTTGGAAACGGAAATATCTTCATAT

                  * * *      **      *   * *   *     ** ** *      *    *     
     107598 AAAAACGACATAGAAGCTATCTCAGGAACTTGTTTATGATGCATCTAATCAACTAACAGTGTTGA
          1 AAAAACTAGACAGAAGCATTCTCAGAAACATCTTTGTGATGTTTGCACTCAACTCACAGAGTTGA

                  * ** * *                         * *       *               
     107663 ACCTTTGTACTGACAGAGCAG-TTTGAAACACTCTTTTTTTGGAATCTGCAAGTGGATATTTGGA
         66 ACCTTTCTTTTCATAGAGCAGTTTTGAAACACTCTTTTTGTAGAATCTGGAAGTGGATATTTGGA

            *                           *   *   *    
     107727 TCGCTTTGAGGATTTCGTTGGAAACGG-GAT-GC--AATAT
        131 GCGCTTTGAGGATTTCGTTGGAAACGGAAATATCTTCATAT

                **  *    *     *                 **  *   *  *    *         * 
     107764 AAAACGTACACAGCAGCATACTCAGAAA-ATACTTTGCCATATTTCCATTCAAGTCACAGAGTGG
          1 AAAAACTAGACAGAAGCATTCTCAGAAACAT-CTTTGTGATGTTTGCACTCAACTCACAGAGTTG

               *  * **            *                  *  *             *      
     107828 AACATTCCCATTCATAGAGCAGGTTTGAAACACTCTTTTTGGAGTATCTGGAAGTGGACATTTGG
         65 AACCTTTCTTTTCATAGAGCAGTTTTGAAACACTCTTTTTGTAGAATCTGGAAGTGGATATTTGG

                    ** * * ** *  *   *            **  
     107893 AGCGCTTTCTGAACTATGGTGAAAAAGGAAATATCTTCCAAT
        130 AGCGCTTTGAGGATTTCGTTGGAAACGGAAATATCTTCATAT

            *     *               *      * *          *  **      **  *  *    
     107935 GAAAACAAGACAGAAGCATTCTGAGAAACTTATTTGTGATGTGTGTCCTCAACAAACGGACTTGA
          1 AAAAACTAGACAGAAGCATTCTCAGAAACATCTTTGTGATGTTTGCACTCAACTCACAGAGTTGA

                   *      **  * *  *  *             *   *    * * *           
     108000 ACCTTTCGTTTCATGCAGTACTTCTGGAACACTCTTTTTGAAGATTCTGCATGCGGATATTTGGA
         66 ACCTTTCTTTTCATAGAGCAGTTTTGAAACACTCTTTTTGTAGAATCTGGAAGTGGATATTTGGA

            **                            **   *   * 
     108065 TAGCTTTGAGGATTTCGTTGGAAACGG---GCT-TACATGT
        131 GCGCTTTGAGGATTTCGTTGGAAACGGAAATATCTTCATAT

                 *       *               *        *   *    *    *       *    
     108102 AAAAATTAGACAGCAGCATTCTCAGAAACTTCTTTGTGGTGTCTGCATTCAAGTCACAGAATTGA
          1 AAAAACTAGACAGAAGCATTCTCAGAAACATCTTTGTGATGTTTGCACTCAACTCACAGAGTTGA

              * ** * **            *  * *      *       *             *       
     108167 ACATCCCCTCACATAGAGCAGTTGTGCAGCACTCTATTTGTAGTATCTGGAAGTGGACATTTGGA
         66 ACCTTTCTTTTCATAGAGCAGTTTTGAAACACTCTTTTTGTAGAATCTGGAAGTGGATATTTGGA

             *         ** *    * *   *            **  
     108232 GGGCTTTGTAGCCTATC-TGGAAAAAGGAAATATCTTCCCAT
        131 GCGCTTTG-AGGATTTCGTTGGAAACGGAAATATCTTCATAT

            *  ** *   *     * *            *   *  *   * **        *  * * *   
     108273 GAATGCGAGATAGAAGTAATCTCAGAAACATGTTTATGCTGTATCTACTCAACTAACTGTGCTGA
          1 AAAAACTAGACAGAAGCATTCTCAGAAACATCTTTGTGATGTTTGCACTCAACTCACAGAGTTGA

              *     *  *               *        *  * *       *               
     108338 ACATTTCTATTGATAGAGCAGTTTTGAGACACTCTTCTTTTGGAATCTGCAAGTGGATATTTGGA
         66 ACCTTTCTTTTCATAGAGCAGTTTTGAAACACTCTTTTTGTAGAATCTGGAAGTGGATATTTGGA

            ** *                        *     *      
     108403 TAGATTTGAGGATTTCGTTGGAAACGG-GAT-T-AT-ATAT
        131 GCGCTTTGAGGATTTCGTTGGAAACGGAAATATCTTCATAT

            *    *       *               *                 **  *   *         
     108440 CAAAAGTAGACAGCAGCATTCTCAGAAACTTCTTTGTGATGTTTGCATCCAGCTCTCAGAGTTGA
          1 AAAAACTAGACAGAAGCATTCTCAGAAACATCTTTGTGATGTTTGCACTCAACTCACAGAGTTGA

              *  * *          *  *        *        *   **        *  **       
     108505 ACATTCCCTTTCATAGAGTAGGTTTGAAACCCTCTTTTTATAGTGTCTGGAAGCGGGCATTTGGA
         66 ACCTTTCTTTTCATAGAGCAGTTTTGAAACACTCTTTTTGTAGAATCTGGAAGTGGATATTTGGA

                   *   ** ** *  *   *          * *   
     108570 GCGCTTTCAGGCCTATGCTGAAAAAGGAAATATCTACCTAT
        131 GCGCTTTGAGGATTTCGTTGGAAACGGAAATATCTTCATAT

             *                    *    *  **          ** *        *    *     
     108611 AGAAACTAGACAGAAGCATTCTGAGAATCACGTTTGTGATGTGGGTACTCAACTAACAGTGTTGA
          1 AAAAACTAGACAGAAGCATTCTCAGAAACATCTTTGTGATGTTTGCACTCAACTCACAGAGTTGA

            *  *       *   *            *   *                *               
     108676 TCCATTCTTTTGATACAGCAGTTTTGAACCACACTTTTTGTAGAATCTGCAAGTGGATATTTGGA
         66 ACCTTTCTTTTCATAGAGCAGTTTTGAAACACTCTTTTTGTAGAATCTGGAAGTGGATATTTGGA

            **   *                     *   *        *
     108741 TAGCTGTGAGGATTTCGTTGGAAACGGGAATGTCTTCATAG
        131 GCGCTTTGAGGATTTCGTTGGAAACGGAAATATCTTCATAT

                **                     * * **         *  **   *   *          
     108782 AAAATTTAGACAGAAGCATTCTCAGAACCTTGATTGTGATGTGTGTTCTCCACTAACAGAGTTGA
          1 AAAAACTAGACAGAAGCATTCTCAGAAACATCTTTGTGATGTTTGCACTCAACTCACAGAGTTGA

                       * *   * *   *       *       *                         
     108847 ACCTTTCTTTTGACAGAACTGTTCTGAAACATTCTTTTTATAGAATCTGGAAGTGGATATTTGGA
         66 ACCTTTCTTTTCATAGAGCAGTTTTGAAACACTCTTTTTGTAGAATCTGGAAGTGGATATTTGGA

            **                         *          *  
     108912 AAGCTTTGAGGATTTCGTTGGAAACGGGAATATCTTCAAAT
        131 GCGCTTTGAGGATTTCGTTGGAAACGGAAATATCTTCATAT

                *    *            *           * *       *  *    *            
     108953 AAAATCTAGCCAGAAGCATTCTAAGAAACATCTTAGGGATGTTTACATTCAAGTCACAGAGTTGA
          1 AAAAACTAGACAGAAGCATTCTCAGAAACATCTTTGTGATGTTTGCACTCAACTCACAGAGTTGA

              *  * *     *       *         *    * *   **      *      *    *  
     109018 ACATTCCCTTTCACAGAGCAGGTTTGAAACAATCTTCTCGTACTATCTGGCAGTGGACATTTTGA
         66 ACCTTTCTTTTCATAGAGCAGTTTTGAAACACTCTTTTTGTAGAATCTGGAAGTGGATATTTGGA

              * *   *  ** ** *  *   *            ** *
     109083 GCTCCTTGGGGCCTATGCTGAAAAAGGAAATATCTTCCGAC
        131 GCGCTTTGAGGATTTCGTTGGAAACGGAAATATCTTCATAT

                                 *     *  **          *           **    *    
     109124 AAAAACTAGACAGAAGCATTCGCAGAATCACGTTTGTGATGTGTGCACTCAACTGTCAGAATTGA
          1 AAAAACTAGACAGAAGCATTCTCAGAAACATCTTTGTGATGTTTGCACTCAACTCACAGAGTTGA

                 **   ** *      *                            * *            *
     109189 ACCTTGGTTTGGACAGAGCACTTTTGAAACACTCTTTTTGTAGAATCTGCAGGTGGATATTTGGC
         66 ACCTTTCTTTTCATAGAGCAGTTTTGAAACACTCTTTTTGTAGAATCTGGAAGTGGATATTTGGA

            **                         *   *      * *
     109254 TAGCTTTGAGGATTTCGTTGGAAACGGTAATGTCTTCAAAG
        131 GCGCTTTGAGGATTTCGTTGGAAACGGAAATATCTTCATAT

                *                         *   *            *    *            
     109295 AAAATCTAGACAGAAGCATTCTCAGAAACACCTTCGTGATGTTTGCAATCAAGTCACAGAGTTGA
          1 AAAAACTAGACAGAAGCATTCTCAGAAACATCTTTGTGATGTTTGCACTCAACTCACAGAGTTGA

                 * *      *      *  *         *        *        *    *       
     109360 ACCTTCCGTTTCATGGAGCAGGTTGGAAACACTCATTTTGTAGTATCTGGAATTGGACATTTGGA
         66 ACCTTTCTTTTCATAGAGCAGTTTTGAAACACTCTTTTTGTAGAATCTGGAAGTGGATATTTGGA

               *   *   ** ** *  *   *            **  
     109425 GCGATTTCAGGCCTATGGTGTAAAAGGAAATATCTTCCCAT
        131 GCGCTTTGAGGATTTCGTTGGAAACGGAAATATCTTCATAT

                * * * *      **      *   * *   *     ** ** *      *    *     
     109466 AAAAGCGACATAGAAGCTATCTCAGGAACTTGTTTATGATGCATCTAATCAACTAACAGTGTTGA
          1 AAAAACTAGACAGAAGCATTCTCAGAAACATCTTTGTGATGTTTGCACTCAACTCACAGAGTTGA

             *    * ** * *                         * *       *               
     109531 AACTTTGTACTGACAGAGCAG-TTTGAAACACTCTTTTTTTGGAATCTGCAAGTGGATATTTGGA
         66 ACCTTTCTTTTCATAGAGCAGTTTTGAAACACTCTTTTTGTAGAATCTGGAAGTGGATATTTGGA

            *                           *   *   *    
     109595 TCGCTTTGAGGATTTCGTTGGAAACGG-GAT-GC--AATAT
        131 GCGCTTTGAGGATTTCGTTGGAAACGGAAATATCTTCATAT

                **  *    *     *                 **  *   *  *    *         * 
     109632 AAAACGTACACAGCAGCATACTCAGAAA-ATACTTTGCCATATTTCCATTCAAGTCACAGAGTGG
          1 AAAAACTAGACAGAAGCATTCTCAGAAACAT-CTTTGTGATGTTTGCACTCAACTCACAGAGTTG

               *  * **            *           *      *  **            *      
     109696 AACATTCCCATTCATAGAGCAGGTTTGAAACACTTTTTTTGGAGTGTCTGGAAGTGGACATTTGG
         65 AACCTTTCTTTTCATAGAGCAGTTTTGAAACACTCTTTTTGTAGAATCTGGAAGTGGATATTTGG

                    *  * * ** *  *   *            **  
     109761 AGCGCTTTAAGAACTATGGTGAAAAAGGAAATATCTTCCAAT
        130 AGCGCTTTGAGGATTTCGTTGGAAACGGAAATATCTTCATAT

            *     *               *      * *         **  **       *  *  * *  
     109803 GAAAACAAGACAGAAGCATTCTGAGAAACTTATTTGTGATGCGTGTCCTCAACTAACGGACTCGA
          1 AAAAACTAGACAGAAGCATTCTCAGAAACATCTTTGTGATGTTTGCACTCAACTCACAGAGTTGA

                   *      **  * *  *  *             *   *    * * *          *
     109868 ACCTTTCGTTTCATGCAGTACTTCTGGAACACTCTTTTTGAAGATTCTGCATGCGGATATTTGGT
         66 ACCTTTCTTTTCATAGAGCAGTTTTGAAACACTCTTTTTGTAGAATCTGGAAGTGGATATTTGGA

            **                            **   *     
     109933 TAGCTTTGAGGATTTCGTTGGAAACGG---GCT-TACATAT
        131 GCGCTTTGAGGATTTCGTTGGAAACGGAAATATCTTCATAT

                 *       *               *        *   *    *    *       *    
     109970 AAAAATTAGACAGCAGCATTCTCAGAAACTTCTTTGTGGTGTCTGCATTCAAGTCACAGAATTGA
          1 AAAAACTAGACAGAAGCATTCTCAGAAACATCTTTGTGATGTTTGCACTCAACTCACAGAGTTGA

              * ** * **            *  * *      *       *    *        *       
     110035 ACATCCCCTCACATAGAGCAGTTGTGCAGCACTCTATTTGTAGTATCTCGAAGTGGACATTTGGA
         66 ACCTTTCTTTTCATAGAGCAGTTTTGAAACACTCTTTTTGTAGAATCTGGAAGTGGATATTTGGA

             *         ** *    * *   *            **  
     110100 GGGCTTTGTAGCCTATC-TGGAAAAAGGAAATATCTTCCCAT
        131 GCGCTTTG-AGGATTTCGTTGGAAACGGAAATATCTTCATAT

            *   * *   *     * *            *   *  *   * **        *  * * *   
     110141 GAAAGCCAGATAGAAGTAATCTCAGAAACATGTTTATGCTGTATCTACTCAACTAACTGTGCTGA
          1 AAAAACTAGACAGAAGCATTCTCAGAAACATCTTTGTGATGTTTGCACTCAACTCACAGAGTTGA

              *     *  *               *        *  * *       *               
     110206 ACATTTCTATTGATAGAGCAGTTTTGAGACACTCTTCTTTTGGAATCTGCAAGTGGATATTTGGA
         66 ACCTTTCTTTTCATAGAGCAGTTTTGAAACACTCTTTTTGTAGAATCTGGAAGTGGATATTTGGA

            ** *                        *     *      
     110271 TAGATTTGAGGATTTCGTTGGAAACGG-GAT-T-AT-ATAT
        131 GCGCTTTGAGGATTTCGTTGGAAACGGAAATATCTTCATAT

                 *       *               *                 **  *   *         
     110308 AAAAAGTAGACAGCAGCATTCTCAGAAACTTCTTTGTGATGTTTGCATCCAGCTCTCAGAGTTGA
          1 AAAAACTAGACAGAAGCATTCTCAGAAACATCTTTGTGATGTTTGCACTCAACTCACAGAGTTGA

              *  * *          *  *        * *      *  ***        *  **       
     110373 ACATTCCCTTTCATAGAGTAGGTTTGAAACCCCCTTTTTATACTGTCTGGAAGCGGGCATTTGGA
         66 ACCTTTCTTTTCATAGAGCAGTTTTGAAACACTCTTTTTGTAGAATCTGGAAGTGGATATTTGGA

                   *   ** ** *  *   *    *     * *  *
     110438 GCGCTTTCAGGCCTATGCTGAAAAAGGAATTATCTACCTAC
        131 GCGCTTTGAGGATTTCGTTGGAAACGGAAATATCTTCATAT

             *                    *    *  **          ** *        *    *     
     110479 AGAAACTAGACAGAAGCATTCTGAGAATCACGTTTGTGATGTGGGTACTCAACTAACAGTGTTGA
          1 AAAAACTAGACAGAAGCATTCTCAGAAACATCTTTGTGATGTTTGCACTCAACTCACAGAGTTGA

            *  *       *   *            *   *                *               
     110544 TCCATTCTTTTGATACAGCAGTTTTGAACCACCCTTTTTGTAGAATCTGCAAGTGGATATTTGGA
         66 ACCTTTCTTTTCATAGAGCAGTTTTGAAACACTCTTTTTGTAGAATCTGGAAGTGGATATTTGGA

            **   *                     *   *        *
     110609 TAGCTGTGAGGATTTCGTTGGAAACGGGAATGTCTTCATAG
        131 GCGCTTTGAGGATTTCGTTGGAAACGGAAATATCTTCATAT

                **                     * ****         ** **   *   *          
     110650 AAAATTTAGACAGAAGCATTCTCAGAACCTGGATTGTGATGTGAGTTCTCCACTAACAGAGTTGA
          1 AAAAACTAGACAGAAGCATTCTCAGAAACATCTTTGTGATGTTTGCACTCAACTCACAGAGTTGA

                      ** *   * * *         *       *                         
     110715 ACCTTTCTTTGGACAGAACTGATTTGAAACATTCTTTTTATAGAATCTGGAAGTGGATATTTGGA
         66 ACCTTTCTTTTCATAGAGCAGTTTTGAAACACTCTTTTTGTAGAATCTGGAAGTGGATATTTGGA

            ** *                    *  *          *  
     110780 AAGTTTTGAGGATTTCGTTGGAAATGGGAATATCTTCAAAT
        131 GCGCTTTGAGGATTTCGTTGGAAACGGAAATATCTTCATAT

                *    *            *           * *       *  *    *            
     110821 AAAATCTAGCCAGAAGCATTCTAAGAAACATCTTAGGGATGTTTACATTCAAGTCACAGAGTTGA
          1 AAAAACTAGACAGAAGCATTCTCAGAAACATCTTTGTGATGTTTGCACTCAACTCACAGAGTTGA

              *   * *    **       *         *    * *   **      *      *    * 
     110886 ACATTCCCCTTTCTCAGAGCAGGTTTGAAACAATCTTCTCGTACTATCTGGCAGTGGACATTTTG
         66 ACCTT-TCTTTTCATAGAGCAGTTTTGAAACACTCTTTTTGTAGAATCTGGAAGTGGATATTTGG

               * *   *  ** ** *  *   *            ** *
     110951 AGCTCCTTGGGGCCTATGCTGAAAAAGGAAATATCTTCCGAC
        130 AGCGCTTTGAGGATTTCGTTGGAAACGGAAATATCTTCATAT

                                 *     *   *          *           **    *    
     110993 AAAAACTAGACAGAAGCATTCGCAGAATCATGTTTGTGATGTGTGCACTCAACTGTCAGAATTGA
          1 AAAAACTAGACAGAAGCATTCTCAGAAACATCTTTGTGATGTTTGCACTCAACTCACAGAGTTGA

                 **   ** *      *                            * *          * *
     111058 ACCTTGGTTTGGACAGAGCACTTTTGAAACACTCTTTTTGTAGAATCTGCAGGTGGATATTTCGC
         66 ACCTTTCTTTTCATAGAGCAGTTTTGAAACACTCTTTTTGTAGAATCTGGAAGTGGATATTTGGA

            **                         *   *      * *
     111123 TAGCTTTGAGGATTTCGTTGGAAACGGTAATGTCTTCAAAG
        131 GCGCTTTGAGGATTTCGTTGGAAACGGAAATATCTTCATAT

                *          *   *          *   *            *    *            
     111164 AAAATCTAGACAGAAACATCCTCAGAAACACCTTCGTGATGTTTGCAATCAAGTCACAGAGTTGA
          1 AAAAACTAGACAGAAGCATTCTCAGAAACATCTTTGTGATGTTTGCACTCAACTCACAGAGTTGA

                 * *             *  *         *        *             *       
     111229 ACCTTCCGTTTCATAGAGCAGGTTGGAAACACTCATTTTGTAGTATCTGGAAGTGGACATTTGGA
         66 ACCTTTCTTTTCATAGAGCAGTTTTGAAACACTCTTTTTGTAGAATCTGGAAGTGGATATTTGGA

                   *   ** ** *  *   *            **  
     111294 GCGCTTTCAGGCCTATGGTGTAAAAGGAAATATCTTCCCAT
        131 GCGCTTTGAGGATTTCGTTGGAAACGGAAATATCTTCATAT

                * * * *      **      *   * *   *     ** ** *      *    *     
     111335 AAAAGCGACATAGAAGCTATCTCAGGAACTTGTTTATGATGCATCTAATCAACTAACAGTGTTGA
          1 AAAAACTAGACAGAAGCATTCTCAGAAACATCTTTGTGATGTTTGCACTCAACTCACAGAGTTGA

                  * ** * *                         * *       *               
     111400 ACCTTTGTACTGACAGAGCAG-TTTGAAACACTCTTTTTTTGGAATCTGCAAGTGGATATTTGGA
         66 ACCTTTCTTTTCATAGAGCAGTTTTGAAACACTCTTTTTGTAGAATCTGGAAGTGGATATTTGGA

            *                           *   *   *    
     111464 TCGCTTTGAGGATTTCGTTGGAAACGG-GAT-GC--AATAT
        131 GCGCTTTGAGGATTTCGTTGGAAACGGAAATATCTTCATAT

                **  *    *     *                 **  *   *  *    **        * 
     111501 AAAACGTACACAGCAGCATACTCAGAAA-ATACTTTGCCATATTTCCATTCAAGACACAGAGTGG
          1 AAAAACTAGACAGAAGCATTCTCAGAAACAT-CTTTGTGATGTTTGCACTCAACTCACAGAGTTG

               *  * **            *           *      *  **            *      
     111565 AACATTCCCATTCATAGAGCAGGTTTGAAACACTTTTTTTGGAGTGTCTGGAAGTGGACATTTGG
         65 AACCTTTCTTTTCATAGAGCAGTTTTGAAACACTCTTTTTGTAGAATCTGGAAGTGGATATTTGG

                    *  * * ** *  *   *            **  
     111630 AGCGCTTTCAGAACTATGGTGAAAAAGGAAATATCTTCCAAT
        130 AGCGCTTTGAGGATTTCGTTGGAAACGGAAATATCTTCATAT

            *     *               *      * *         **  **       *  *  * *  
     111672 GAAAACAAGACAGAAGCATTCTGAGAAACTTATTTGTGATGCGTGTCCTCAACTAACGGACTCGA
          1 AAAAACTAGACAGAAGCATTCTCAGAAACATCTTTGTGATGTTTGCACTCAACTCACAGAGTTGA

                   *      **  * *  *  *             *   *    * * *           
     111737 ACCTTTCGTTTCATGCAGTACTTCTGGAACACTCTTTTTGAAGATTCTGCATGCGGATATTTGGA
         66 ACCTTTCTTTTCATAGAGCAGTTTTGAAACACTCTTTTTGTAGAATCTGGAAGTGGATATTTGGA

            **                            **   *     
     111802 TAGCTTTGAGGATTTCGTTGGAAACGG---GCT-TACATAT
        131 GCGCTTTGAGGATTTCGTTGGAAACGGAAATATCTTCATAT

                 *       *               *        *   *    *    *       *    
     111839 AAAAATTAGACAGCAGCATTCTCAGAAACTTCTTTGTGGTGTCTGCATTCAAGTCACAGAATTGA
          1 AAAAACTAGACAGAAGCATTCTCAGAAACATCTTTGTGATGTTTGCACTCAACTCACAGAGTTGA

              * ** * **            *  * *      *       *    *        *       
     111904 ACATCCCCTCACATAGAGCAGTTGTGCAGCACTCTATTTGTAGTATCTCGAAGTGGACATTTGGA
         66 ACCTTTCTTTTCATAGAGCAGTTTTGAAACACTCTTTTTGTAGAATCTGGAAGTGGATATTTGGA

             *         ** *    * *   *            **  
     111969 GGGCTTTGTAGCCTATC-TGGAAAAAGGAAATATCTTCCCAT
        131 GCGCTTTG-AGGATTTCGTTGGAAACGGAAATATCTTCATAT

            *  ** *   *     * *            *   *  *   * **        *  * * *   
     112010 GAATGCGAGATAGAAGTAATCTCAGAAACATGTTTATGCTGTATCTACTCAACTAACTGTGCTGA
          1 AAAAACTAGACAGAAGCATTCTCAGAAACATCTTTGTGATGTTTGCACTCAACTCACAGAGTTGA

              *     *  *               *        *  * *       *               
     112075 ACATTTCTATTGATAGAGCAGTTTTGAGACACTCTTCTTTTGGAATCTGCAAGTGGATATTTGGA
         66 ACCTTTCTTTTCATAGAGCAGTTTTGAAACACTCTTTTTGTAGAATCTGGAAGTGGATATTTGGA

            ** *                        *     *      
     112140 TAGATTTGAGGATTTCGTTGGAAACGG-GAT-T-AT-ATAT
        131 GCGCTTTGAGGATTTCGTTGGAAACGGAAATATCTTCATAT

                 *       *               *                 **  *   *         
     112177 AAAAAGTAGACAGCAGCATTCTCAGAAACTTCTTTGTGATGTTTGCATCCAGCTCTCAGAGTTGA
          1 AAAAACTAGACAGAAGCATTCTCAGAAACATCTTTGTGATGTTTGCACTCAACTCACAGAGTTGA

              *  * *          *  *        * *      *   **        *  **       
     112242 ACATTCCCTTTCATAGAGTAGGTTTGAAACCCCCTTTTTATAGTGTCTGGAAGCGGGCATTTGGA
         66 ACCTTTCTTTTCATAGAGCAGTTTTGAAACACTCTTTTTGTAGAATCTGGAAGTGGATATTTGGA

                   *   ** ** *  *   *          * *  *
     112307 GCGCTTTCAGGCCTATGCTGAAAAAGGAAATATCTACCTAC
        131 GCGCTTTGAGGATTTCGTTGGAAACGGAAATATCTTCATAT

             *                    *    *  **          ** *        *    *     
     112348 AGAAACTAGACAGAAGCATTCTGAGAATCACGTTTGTGATGTGGGTACTCAACTAACAGTGTTGA
          1 AAAAACTAGACAGAAGCATTCTCAGAAACATCTTTGTGATGTTTGCACTCAACTCACAGAGTTGA

            *  *           *            *   *             *  *    *          
     112413 TCCATTCTTTTCATACAGCAGTTTTGAACCACACTTTTTGTAGAATGTGCAAGTAGATATTTGGA
         66 ACCTTTCTTTTCATAGAGCAGTTTTGAAACACTCTTTTTGTAGAATCTGGAAGTGGATATTTGGA

            **   *                     *   *        *
     112478 TAGCTGTGAGGATTTCGTTGGAAACGGGAATGTCTTCATAG
        131 GCGCTTTGAGGATTTCGTTGGAAACGGAAATATCTTCATAT

                **       *             * ****         *  **   *   *          
     112519 AAAATTTAGACAGGAGCATTCTCAGAACCTGGATTGTGATGTGTGTTCTCCACTAACAGAGTTGA
          1 AAAAACTAGACAGAAGCATTCTCAGAAACATCTTTGTGATGTTTGCACTCAACTCACAGAGTTGA

                       * *   * *           *       *                         
     112584 ACCTTTCTTTTGACAGAACTGTTTTGAAACATTCTTTTTATAGAATCTGGAAGTGGATATTTGGA
         66 ACCTTTCTTTTCATAGAGCAGTTTTGAAACACTCTTTTTGTAGAATCTGGAAGTGGATATTTGGA

            **                         *          *  
     112649 AAGCTTTGAGGATTTCGTTGGAAACGGGAATATCTTCAAAT
        131 GCGCTTTGAGGATTTCGTTGGAAACGGAAATATCTTCATAT

                *   **            *           * *       *  *    *            
     112690 AAAATCTACCCAGAAGCATTCTAAGAAACATCTTAGGGATGTTTACATTCAAGTCACAGAGTTGA
          1 AAAAACTAGACAGAAGCATTCTCAGAAACATCTTTGTGATGTTTGCACTCAACTCACAGAGTTGA

              *   * *    **       *         *    * *   **      *      *    * 
     112755 ACATTCCCCTTTCTCAGAGCAGGTTTGAAACAATCTTCTCGTACTATCTGGCAGTGGACATTTTG
         66 ACCTT-TCTTTTCATAGAGCAGTTTTGAAACACTCTTTTTGTAGAATCTGGAAGTGGATATTTGG

               * *   ** ** ** *  *   *            ** *
     112820 AGCTCCTTGGCGCCTATGCTGAAAAAGGAAATATCTTCCGAC
        130 AGCGCTTTGAGGATTTCGTTGGAAACGGAAATATCTTCATAT

                                 *     *  **          *           **    *    
     112862 AAAAACTAGACAGAAGCATTCGCAGAATCACGTTTGTGATGTGTGCACTCAACTGTCAGAATTGA
          1 AAAAACTAGACAGAAGCATTCTCAGAAACATCTTTGTGATGTTTGCACTCAACTCACAGAGTTGA

                  *   ** *      *              *             * *  *         *
     112927 ACCTTTGTTTGGACAGAGCACTTTTG-AA-ACTCTCTTTGTAGAATCTGCAGGTTGATATTTGGC
         66 ACCTTTCTTTTCATAGAGCAGTTTTGAAACACTCTTTTTGTAGAATCTGGAAGTGGATATTTGGA

            **              *          *   *     ** *
     112990 TAGCTTTGAGGATTTCTTTGGAAACGGTAATGTCTTCGAAG
        131 GCGCTTTGAGGATTTCGTTGGAAACGGAAATATCTTCATAT

                *          *   *          *   *            *    *            
     113031 AAAATCTAGACAGAAACATCCTCAGAAACACCTTCGTGATGTTTGCAATCAAGTCACAGAGTTGA
          1 AAAAACTAGACAGAAGCATTCTCAGAAACATCTTTGTGATGTTTGCACTCAACTCACAGAGTTGA

                 * *             *  *           *      *             *       
     113096 ACCTTCCGTTTCATAGAGCAGGTTGGAAACACTCTTATTGTAGTATCTGGAAGTGGACATTTGGA
         66 ACCTTTCTTTTCATAGAGCAGTTTTGAAACACTCTTTTTGTAGAATCTGGAAGTGGATATTTGGA

                   *   ** ** *  *   *            **  
     113161 GCGCTTTCAGGCCTATGGTGTAAAAGGAAATATCTTCCCAT
        131 GCGCTTTGAGGATTTCGTTGGAAACGGAAATATCTTCATAT

                * * * *      **      *   * *   *     ** ** *      *    *     
     113202 AAAAGCGACATAGAAGCTATCTCAGGAACTTGTTTATGATGCATCTAATCAACTAACAGTGTTGA
          1 AAAAACTAGACAGAAGCATTCTCAGAAACATCTTTGTGATGTTTGCACTCAACTCACAGAGTTGA

                  * ** * *         *               * *       *               
     113267 ACCTTTGTACTGACAGAGCAG-TATGAAACACTCTTTTTTTGGAATCTGCAAGTGGATATTTGGA
         66 ACCTTTCTTTTCATAGAGCAGTTTTGAAACACTCTTTTTGTAGAATCTGGAAGTGGATATTTGGA

            *                           *   *   *    
     113331 TCGCTTTGAGGATTTCGTTGGAAACGG-GAT-GC--AATAT
        131 GCGCTTTGAGGATTTCGTTGGAAACGGAAATATCTTCATAT

                **  *    *     *                 **  *   *  *    *         * 
     113368 AAAACGTACACAGCAGCATACTCAGAAA-ATACTTTGCCATATTTCCATTCAAGTCACAGAGTGG
          1 AAAAACTAGACAGAAGCATTCTCAGAAACAT-CTTTGTGATGTTTGCACTCAACTCACAGAGTTG

               *  * **            *                  *  *             *      
     113432 AACATTCCCATTCATAGAGCAGGTTTGAAACACTCTTTTTGGAGTATCTGGAAGTGGACATTTGG
         65 AACCTTTCTTTTCATAGAGCAGTTTTGAAACACTCTTTTTGTAGAATCTGGAAGTGGATATTTGG

                    ** * * ** *  *   *            **  
     113497 AGCGCTTTCTGAACTATGGTGAAAAAGGAAATATCTTCCAAT
        130 AGCGCTTTGAGGATTTCGTTGGAAACGGAAATATCTTCATAT

            *     *               *      * *         **  **       *  *  * *  
     113539 GAAAACAAGACAGAAGCATTCTGAGAAACTTATTTGTGATGCGTGTCCTCAACTAACGGACTCGA
          1 AAAAACTAGACAGAAGCATTCTCAGAAACATCTTTGTGATGTTTGCACTCAACTCACAGAGTTGA

                   *      **  * *  *  *             *   *    * * *           
     113604 ACCTTTCGTTTCATGCAGTACTTCTGGAACACTCTTTTTGAAGATTCTGCATGCGGATATTTGGA
         66 ACCTTTCTTTTCATAGAGCAGTTTTGAAACACTCTTTTTGTAGAATCTGGAAGTGGATATTTGGA

            **                            **   *     
     113669 TAGCTTTGAGGATTTCGTTGGAAACGG---GCT-TACATAT
        131 GCGCTTTGAGGATTTCGTTGGAAACGGAAATATCTTCATAT

                 *       *               *        *   *    *    *       *    
     113706 AAAAATTAGACAGCAGCATTCTCAGAAACTTCTTTGTGGTGTCTGCATTCAAGTCACAGAATTGA
          1 AAAAACTAGACAGAAGCATTCTCAGAAACATCTTTGTGATGTTTGCACTCAACTCACAGAGTTGA

              * ** * **            *  * *      *       *    *        *       
     113771 ACATCCCCTCACATAGAGCAGTTGTGCAGCACTCTCTTTGTAGTATCTCGAAGTGGACATTTGGA
         66 ACCTTTCTTTTCATAGAGCAGTTTTGAAACACTCTTTTTGTAGAATCTGGAAGTGGATATTTGGA

             *         ** *    * *   *            **  
     113836 GGGCTTTGTAGCCTATC-TGGAAAAAGGAAATATCTTCCCAT
        131 GCGCTTTG-AGGATTTCGTTGGAAACGGAAATATCTTCATAT

            *  ** *   *     * *            *   *  *   * **        *  * * *   
     113877 GAATGCGAGATAGAAGTAATCTCAGAAACATGTTTATGCTGTATCTACTCAACTAACTGTGCTGA
          1 AAAAACTAGACAGAAGCATTCTCAGAAACATCTTTGTGATGTTTGCACTCAACTCACAGAGTTGA

              *     *  *               *        *  * *       *               
     113942 ACATTTCTATTGATAGAGCAGTTTTGAGACACTCTTCTTTTGGAATCTGCAAGTGGATATTTGGA
         66 ACCTTTCTTTTCATAGAGCAGTTTTGAAACACTCTTTTTGTAGAATCTGGAAGTGGATATTTGGA

            ** *                        *     *      
     114007 TAGATTTGAGGATTTCGTTGGAAACGG-GAT-T-AT-ATAT
        131 GCGCTTTGAGGATTTCGTTGGAAACGGAAATATCTTCATAT

                 *       *               *                 **  *   *         
     114044 AAAAAGTAGACAGCAGCATTCTCAGAAACTTCTTTGTGATGTTTGCATCCAGCTCTCAGAGTTGA
          1 AAAAACTAGACAGAAGCATTCTCAGAAACATCTTTGTGATGTTTGCACTCAACTCACAGAGTTGA

              *  * *          *  *        *        *   **        *  **       
     114109 ACATTCCCTTTCATAGAGTAGGTTTGAAACCCTCTTTTTATAGTGTCTGGAAGCGGGCATTTGGA
         66 ACCTTTCTTTTCATAGAGCAGTTTTGAAACACTCTTTTTGTAGAATCTGGAAGTGGATATTTGGA

                   *   ** ** *  *   *          * *   
     114174 GCGCTTTCAGGCCTATGCTGAAAAAGGAAATATCTACCTAT
        131 GCGCTTTGAGGATTTCGTTGGAAACGGAAATATCTTCATAT

             *                    *    *  **          ** *        *    *     
     114215 AGAAACTAGACAGAAGCATTCTGAGAATCACGTTTGTGATGTGGGTACTCAACTAACAGTGTTGA
          1 AAAAACTAGACAGAAGCATTCTCAGAAACATCTTTGTGATGTTTGCACTCAACTCACAGAGTTGA

            *  *       *   *   *        *                    *               
     114280 TCCATTCTTTTGATACAGCGGTTTTGAACCACTCTTTTTGTAGAATCTGCAAGTGGATATTTGGA
         66 ACCTTTCTTTTCATAGAGCAGTTTTGAAACACTCTTTTTGTAGAATCTGGAAGTGGATATTTGGA

            **   *                     *   *        *
     114345 TAGCTGTGAGGATTTCGTTGGAAACGGGAATGTCTTCATAG
        131 GCGCTTTGAGGATTTCGTTGGAAACGGAAATATCTTCATAT

                **                     * ****         ** **   *   *          
     114386 AAAATTTAGACAGAAGCATTCTCAGAACCTGGATTGTGATGTGAGTTCTCCACTAACAGAGTTGA
          1 AAAAACTAGACAGAAGCATTCTCAGAAACATCTTTGTGATGTTTGCACTCAACTCACAGAGTTGA

                      ** *   * *           *       *                         
     114451 ACCTTTCTTTGGACAGAACTGTTTTGAAACATTCTTTTTATAGAATCTGGAAGTGGATATTTGGA
         66 ACCTTTCTTTTCATAGAGCAGTTTTGAAACACTCTTTTTGTAGAATCTGGAAGTGGATATTTGGA

            **                         *          *  
     114516 AAGCTTTGAGGATTTCGTTGGAAACGGGAATATCTTCAAAT
        131 GCGCTTTGAGGATTTCGTTGGAAACGGAAATATCTTCATAT

                *    *            *           * *       *  *    *            
     114557 AAAATCTAGCCAGAAGCATTCTAAGAAACATCTTAGGGATGTTTACATTCAAGTCACAGAGTTGA
          1 AAAAACTAGACAGAAGCATTCTCAGAAACATCTTTGTGATGTTTGCACTCAACTCACAGAGTTGA

              *   * *    **       *         *    * *   **      *      *    * 
     114622 ACATTCCCCTTTCTCAGAGCAGGTTTGAAACAATCTTCTCGTACTATCTGGCAGTGGACATTTTG
         66 ACCTT-TCTTTTCATAGAGCAGTTTTGAAACACTCTTTTTGTAGAATCTGGAAGTGGATATTTGG

               * *   *  ** ** *  *   *            ** *
     114687 AGCTCCTTGGGGCCTATGCTGAAAAAGGAAATATCTTCCGAC
        130 AGCGCTTTGAGGATTTCGTTGGAAACGGAAATATCTTCATAT

                                 *     *  **          *           **    *    
     114729 AAAAACTAGACAGAAGCATTCGCAGAATCACGTTTGTGATGTGTGCACTCAACTGTCAGAATTGA
          1 AAAAACTAGACAGAAGCATTCTCAGAAACATCTTTGTGATGTTTGCACTCAACTCACAGAGTTGA

                 **   ** *      *                            * *            *
     114794 ACCTTGGTTTGGACAGAGCACTTTTGAAACACTCTTTTTGTAGAATCTGCAGGTGGATATTTGGC
         66 ACCTTTCTTTTCATAGAGCAGTTTTGAAACACTCTTTTTGTAGAATCTGGAAGTGGATATTTGGA

            **                         *   *      * *
     114859 TAGCTTTGAGGATTTCGTTGGAAACGGTAATGTCTTCAAAG
        131 GCGCTTTGAGGATTTCGTTGGAAACGGAAATATCTTCATAT

                *          *   *          *   *            *    *            
     114900 AAAATCTAGACAGAAACATCCTCAGAAACACCTTCGTGATGTTTGCAATCAAGTCACAGAGTTGA
          1 AAAAACTAGACAGAAGCATTCTCAGAAACATCTTTGTGATGTTTGCACTCAACTCACAGAGTTGA

                 * *             *  *         *        *        *    *       
     114965 ACCTTCCGTTTCATAGAGCAGGTTGGAAACACTCATTTTGTAGTATCTGGAATTGGACATTTGGA
         66 ACCTTTCTTTTCATAGAGCAGTTTTGAAACACTCTTTTTGTAGAATCTGGAAGTGGATATTTGGA

               *   *   ** ** *  *   *            **  
     115030 GCGATTTCAGGCCTATGGTGTAAAAGGAAATATCTTCCCAT
        131 GCGCTTTGAGGATTTCGTTGGAAACGGAAATATCTTCATAT

                * * * **     **      *   * *   *     ** ** *      *    *     
     115071 AAAAGCGACATTGAAGCTATCTCAGGAACTTGTTTATGATGCATCTAATCAACTAACAGTGTTGA
          1 AAAAACTAGACAGAAGCATTCTCAGAAACATCTTTGTGATGTTTGCACTCAACTCACAGAGTTGA

             *    * ** * *                         * *       *               
     115136 AACTTTGTACTGACAGAGCAG-TTTGAAACACTCTTTTTTTGGAATCTGCAAGTGGATATTTGGA
         66 ACCTTTCTTTTCATAGAGCAGTTTTGAAACACTCTTTTTGTAGAATCTGGAAGTGGATATTTGGA

            *                           *   *   *    
     115200 TCGCTTTGAGGATTTCGTTGGAAACGG-GAT-GC--AATAT
        131 GCGCTTTGAGGATTTCGTTGGAAACGGAAATATCTTCATAT

                **  *    *     *                 **  *   *  *    *         * 
     115237 AAAACGTACACAGCAGCATACTCAGAAA-ATACTTTGCCATATTTCCATTCAAGTCACAGAGTGG
          1 AAAAACTAGACAGAAGCATTCTCAGAAACAT-CTTTGTGATGTTTGCACTCAACTCACAGAGTTG

               *  * **            *           *      *  **            *      
     115301 AACATTCCCATTCATAGAGCAGGTTTGAAACACTTTTTTTGGAGTGTCTGGAAGTGGACATTTGG
         65 AACCTTTCTTTTCATAGAGCAGTTTTGAAACACTCTTTTTGTAGAATCTGGAAGTGGATATTTGG

                    *  * * ** *  *   *            **  
     115366 AGCGCTTTCAGAACTATGGTGAAAAAGGAAATATCTTCCAAT
        130 AGCGCTTTGAGGATTTCGTTGGAAACGGAAATATCTTCATAT

            *     *               *      * *         **  **       *  *  * *  
     115408 GAAAACAAGACAGAAGCATTCTGAGAAACTTATTTGTGATGCGTGTCCTCAACTAACGGACTCGA
          1 AAAAACTAGACAGAAGCATTCTCAGAAACATCTTTGTGATGTTTGCACTCAACTCACAGAGTTGA

                   *      **  * *  *  *             *   *    * * *          *
     115473 ACCTTTCGTTTCATGCAGTACTTCTGGAACACTCTTTTTGAAGATTCTGCATGCGGATATTTGGT
         66 ACCTTTCTTTTCATAGAGCAGTTTTGAAACACTCTTTTTGTAGAATCTGGAAGTGGATATTTGGA

            **                            **   *     
     115538 TAGCTTTGAGGATTTCGTTGGAAACGG---GCT-TACATAT
        131 GCGCTTTGAGGATTTCGTTGGAAACGGAAATATCTTCATAT

                 *       *               *        *   *    *    *       *    
     115575 AAAAATTAGACAGCAGCATTCTCAGAAACTTCTTTGTGGTGTCTGCATTCAAGTCACAGAATTGA
          1 AAAAACTAGACAGAAGCATTCTCAGAAACATCTTTGTGATGTTTGCACTCAACTCACAGAGTTGA

              * ** * **            *  * *      *       *    *        *       
     115640 ACATCCCCTCACATAGAGCAGTTGTGCAGCACTCTATTTGTAGTATCTCGAAGTGGACATTTGGA
         66 ACCTTTCTTTTCATAGAGCAGTTTTGAAACACTCTTTTTGTAGAATCTGGAAGTGGATATTTGGA

             *         ** *    * *   *            **  
     115705 GGGCTTTGTAGCCTATC-TGGAAAAAGGAAATATCTTCCCAT
        131 GCGCTTTG-AGGATTTCGTTGGAAACGGAAATATCTTCATAT

            *   * *   *     * *            *   *  *   * **        *  * * *   
     115746 GAAAGCCAGATAGAAGTAATCTCAGAAACATGTTTATGCTGTATCTACTCAACTAACTGTGCTGA
          1 AAAAACTAGACAGAAGCATTCTCAGAAACATCTTTGTGATGTTTGCACTCAACTCACAGAGTTGA

              *     *  *               *        *             *              
     115811 ACATTTCTATTGATAGAGCAGTTTTGAGACACTCTTCTT-TAGGAATCTGCAAGTGGATATTTGG
         66 ACCTTTCTTTTCATAGAGCAGTTTTGAAACACTCTTTTTGTA-GAATCTGGAAGTGGATATTTGG

             ** *                    *   *     *      
     115875 ATAGATTTGAGGATTTCGTTGGAAAAGG-GAT-T-AT-ATAT
        130 AGCGCTTTGAGGATTTCGTTGGAAACGGAAATATCTTCATAT

                 *       *               *                 **  *   *         
     115913 AAAAAGTAGACAGCAGCATTCTCAGAAACTTCTTTGTGATGTTTGCATCCAGCTCTCAGAGTTGA
          1 AAAAACTAGACAGAAGCATTCTCAGAAACATCTTTGTGATGTTTGCACTCAACTCACAGAGTTGA

              *  * *          *  *        * *      *  ***        *  **       
     115978 ACATTCCCTTTCATAGAGTAGGTTTGAAACCCCCTTTTTATACTGTCTGGAAGCGGGCATTTGGA
         66 ACCTTTCTTTTCATAGAGCAGTTTTGAAACACTCTTTTTGTAGAATCTGGAAGTGGATATTTGGA

                   *   ** ** *  *   *    *     * *  *
     116043 GCGCTTTCAGGCCTATGCTGAAAAAGGAATTATCTACCTAC
        131 GCGCTTTGAGGATTTCGTTGGAAACGGAAATATCTTCATAT

             *                    *    *  **          ** *        *    *     
     116084 AGAAACTAGACAGAAGCATTCTGAGAATCACGTTTGTGATGTGGGTACTCAACTAACAGTGTTGA
          1 AAAAACTAGACAGAAGCATTCTCAGAAACATCTTTGTGATGTTTGCACTCAACTCACAGAGTTGA

            *  *       *   *            *   *                *               
     116149 TCCATTCTTTTGATACAGCAGTTTTGAACCACCCTTTTTGTAGAATCTGCAAGTGGATATTTGGA
         66 ACCTTTCTTTTCATAGAGCAGTTTTGAAACACTCTTTTTGTAGAATCTGGAAGTGGATATTTGGA

            **   *                     *   *        *
     116214 TAGCTGTGAGGATTTCGTTGGAAACGGGAATGTCTTCATAG
        131 GCGCTTTGAGGATTTCGTTGGAAACGGAAATATCTTCATAT

                **                     * ****         ** **   *   *          
     116255 AAAATTTAGACAGAAGCATTCTCAGAACCTGGATTGTGATGTGAGTTCTCCACTAACAGAGTTGA
          1 AAAAACTAGACAGAAGCATTCTCAGAAACATCTTTGTGATGTTTGCACTCAACTCACAGAGTTGA

                      ** *   * * *         *       *                         
     116320 ACCTTTCTTTGGACAGAACTGATTTGAAACATTCTTTTTATAGAATCTGGAAGTGGATATTTGGA
         66 ACCTTTCTTTTCATAGAGCAGTTTTGAAACACTCTTTTTGTAGAATCTGGAAGTGGATATTTGGA

            ** *                    *  *          *  
     116385 AAGTTTTGAGGATTTCGTTGGAAATGGGAATATCTTCAAAT
        131 GCGCTTTGAGGATTTCGTTGGAAACGGAAATATCTTCATAT

                *    *            *           * *       *  *    *            
     116426 AAAATCTAGCCAGAAGCATTCTAAGAAACATCTTAGGGATGTTTACATTCAAGTCACAGAGTTGA
          1 AAAAACTAGACAGAAGCATTCTCAGAAACATCTTTGTGATGTTTGCACTCAACTCACAGAGTTGA

              *   * *    **       *         *  * * *   **      *      *    * 
     116491 ACATTCCCCTTTCTCAGAGCAGGTTTGAAACAATCATCTCGTACTATCTGGCAGTGGACATTTTG
         66 ACCTT-TCTTTTCATAGAGCAGTTTTGAAACACTCTTTTTGTAGAATCTGGAAGTGGATATTTGG

               * *   *  ** ** *  *   *            ** *
     116556 AGCTCCTTGCGGCCTATGCTGAAAAAGGAAATATCTTCCGAC
        130 AGCGCTTTGAGGATTTCGTTGGAAACGGAAATATCTTCATAT

                                 *     *  **          *           **    *    
     116598 AAAAACTAGACAGAAGCATTCGCAGAATCACGTTTGTGATGTGTGCACTCAACTGTCAGAATTGA
          1 AAAAACTAGACAGAAGCATTCTCAGAAACATCTTTGTGATGTTTGCACTCAACTCACAGAGTTGA

                 **   ** *      *                            * *            *
     116663 ACCTTGGTTTGGACAGAGCACTTTTGAAACACTCTTTTTGTAGAATCTGCAGGTGGATATTTGGC
         66 ACCTTTCTTTTCATAGAGCAGTTTTGAAACACTCTTTTTGTAGAATCTGGAAGTGGATATTTGGA

            **                         *   *      * *
     116728 TAGCTTTGAGGATTTCGTTGGAAACGGTAATGTCTTCAAAG
        131 GCGCTTTGAGGATTTCGTTGGAAACGGAAATATCTTCATAT

                *          *   *          *   *            *    *            
     116769 AAAATCTAGACAGAAACATCCTCAGAAACACCTTCGTGATGTTTGCAATCAAGTCACAGAGTTGA
          1 AAAAACTAGACAGAAGCATTCTCAGAAACATCTTTGTGATGTTTGCACTCAACTCACAGAGTTGA

                 * *             *  *         *        *             *       
     116834 ACCTTCCGTTTCATAGAGCAGGTTGGAAACACTCATTTTGTAGTATCTGGAAGTGGACATTTGGA
         66 ACCTTTCTTTTCATAGAGCAGTTTTGAAACACTCTTTTTGTAGAATCTGGAAGTGGATATTTGGA

                   *   ** ** *  *   *            **  
     116899 GCGCTTTCAGGCCTATGGTGTAAAAGGAAATATCTTCCCAT
        131 GCGCTTTGAGGATTTCGTTGGAAACGGAAATATCTTCATAT

                * * * *      **      *   * *   *     ** ** *      *    *     
     116940 AAAAGCGACATAGAAGCTATCTCAGGAACTTGTTTATGATGCATCTAATCAACTAACAGTGTTGA
          1 AAAAACTAGACAGAAGCATTCTCAGAAACATCTTTGTGATGTTTGCACTCAACTCACAGAGTTGA

                  * ** * *                         * *       *               
     117005 ACCTTTGTACTGACAGAGCAG-TTTGAAACACTCTTTTTTTGGAATCTGCAAGTGGATATTTGGA
         66 ACCTTTCTTTTCATAGAGCAGTTTTGAAACACTCTTTTTGTAGAATCTGGAAGTGGATATTTGGA

            *                           *   *   *    
     117069 TCGCTTTGAGGATTTCGTTGGAAACGG-GAT-GC--AATAT
        131 GCGCTTTGAGGATTTCGTTGGAAACGGAAATATCTTCATAT

                **  *    *     *                 **  *   *  *    *         * 
     117106 AAAACGTACACAGCAGCATACTCAGAAA-ATACTTTGCCATATTTCCATTCAAGTCACAGAGTGG
          1 AAAAACTAGACAGAAGCATTCTCAGAAACAT-CTTTGTGATGTTTGCACTCAACTCACAGAGTTG

               *  * **            *           *      *  **            *      
     117170 AACATTCCCATTCATAGAGCAGGTTTGAAACACTTTTTTTGGAGTGTCTGGAAGTGGACATTTGG
         65 AACCTTTCTTTTCATAGAGCAGTTTTGAAACACTCTTTTTGTAGAATCTGGAAGTGGATATTTGG

                    *  * * ** *  *   *            **  
     117235 AGCGCTTTCAGAACTATGGTGAAAAAGGAAATATCTTCCAAT
        130 AGCGCTTTGAGGATTTCGTTGGAAACGGAAATATCTTCATAT

            *     *               *      * *         **  **       *  *  * *  
     117277 GAAAACAAGACAGAAGCATTCTGAGAAACTTATTTGTGATGCGTGTCCTCAACTAACGGACTCGA
          1 AAAAACTAGACAGAAGCATTCTCAGAAACATCTTTGTGATGTTTGCACTCAACTCACAGAGTTGA

                   *      **  * *  *  *             *   *    * * *           
     117342 ACCTTTCGTTTCATGCAGTACTTCTGGAACACTCTTTTTGAAGATTCTGCATGCGGATATTTGGA
         66 ACCTTTCTTTTCATAGAGCAGTTTTGAAACACTCTTTTTGTAGAATCTGGAAGTGGATATTTGGA

            **                            **   *     
     117407 TAGCTTTGAGGATTTCGTTGGAAACGG---GCT-TACATAT
        131 GCGCTTTGAGGATTTCGTTGGAAACGGAAATATCTTCATAT

                 *       *               *        *   *    *    *       *    
     117444 AAAAATTAGACAGCAGCATTCTCAGAAACTTCTTTGTGGTGTCTGCATTCAAGTCACAGAATTGA
          1 AAAAACTAGACAGAAGCATTCTCAGAAACATCTTTGTGATGTTTGCACTCAACTCACAGAGTTGA

              * ** * **            *  * *      *       *    *        *       
     117509 ACATCCCCTCACATAGAGCAGTTGTGCAGCACTCTATTTGTAGTATCTCGAAGTGGACATTTGGA
         66 ACCTTTCTTTTCATAGAGCAGTTTTGAAACACTCTTTTTGTAGAATCTGGAAGTGGATATTTGGA

             *         ** *    * *   *            **  
     117574 GGGCTTTGTAGCCTATC-TGGAAAAAGGAAATATCTTCCCAT
        131 GCGCTTTG-AGGATTTCGTTGGAAACGGAAATATCTTCATAT

            *  ** *   *     * *            *   *  *   * **        *  * * *   
     117615 GAATGCGAGATAGAAGTAATCTCAGAAACATGTTTATGCTGTATCTACTCAACTAACTGTGCTGA
          1 AAAAACTAGACAGAAGCATTCTCAGAAACATCTTTGTGATGTTTGCACTCAACTCACAGAGTTGA

              *     *  *               *        *  * *       *               
     117680 ACATTTCTATTGATAGAGCAGTTTTGAGACACTCTTCTTTTGGAATCTGCAAGTGGATATTTGGA
         66 ACCTTTCTTTTCATAGAGCAGTTTTGAAACACTCTTTTTGTAGAATCTGGAAGTGGATATTTGGA

            ** *                        *     *      
     117745 TAGATTTGAGGATTTCGTTGGAAACGG-GAT-T-AT-ATAT
        131 GCGCTTTGAGGATTTCGTTGGAAACGGAAATATCTTCATAT

            *    *       *               *                 **  *   *         
     117782 CAAAAGTAGACAGCAGCATTCTCAGAAACTTCTTTGTGATGTTTGCATCCAGCTCTCAGAGTTGA
          1 AAAAACTAGACAGAAGCATTCTCAGAAACATCTTTGTGATGTTTGCACTCAACTCACAGAGTTGA

              *  * *          *  *        * *      *   **        *  **       
     117847 ACATTCCCTTTCATAGAGTAGGTTTGAAACCCCCTTTTTATAGTGTCTGGAAGCGGGCATTTGGA
         66 ACCTTTCTTTTCATAGAGCAGTTTTGAAACACTCTTTTTGTAGAATCTGGAAGTGGATATTTGGA

                   *   ** ** *  *   *          * *  *
     117912 GCGCTTTCAGGCCTATGCTGAAAAAGGAAATATCTACCTAC
        131 GCGCTTTGAGGATTTCGTTGGAAACGGAAATATCTTCATAT

             *                    *    *  **          ** *        *    *     
     117953 AGAAACTAGACAGAAGCATTCTGAGAATCACGTTTGTGATGTGGGTACTCAACTAACAGTGTTGA
          1 AAAAACTAGACAGAAGCATTCTCAGAAACATCTTTGTGATGTTTGCACTCAACTCACAGAGTTGA

            *  *           *            *   *             *  *               
     118018 TCCATTCTTTTCATACAGCAGTTTTGAACCACACTTTTTGTAGAATGTGCAAGTGGATATTTGGA
         66 ACCTTTCTTTTCATAGAGCAGTTTTGAAACACTCTTTTTGTAGAATCTGGAAGTGGATATTTGGA

            **   *                     *   *        *
     118083 TAGCTGTGAGGATTTCGTTGGAAACGGGAATGTCTTCATAG
        131 GCGCTTTGAGGATTTCGTTGGAAACGGAAATATCTTCATAT

                **       *             * ****         *  **   *   *          
     118124 AAAATTTAGACAGGAGCATTCTCAGAACCTGGATTGTGATGTGTGTTCTCCACTAACAGAGTTGA
          1 AAAAACTAGACAGAAGCATTCTCAGAAACATCTTTGTGATGTTTGCACTCAACTCACAGAGTTGA

                       * *   * *           *       **               *        
     118189 ACCTTTCTTTTGACAGAACTGTTTTGAAACATTCTTTTTAGAGAATCTGGAAGTGGTTATTTGGA
         66 ACCTTTCTTTTCATAGAGCAGTTTTGAAACACTCTTTTTGTAGAATCTGGAAGTGGATATTTGGA

            **                         *          *  
     118254 AAGCTTTGAGGATTTCGTTGGAAACGGGAATATCTTCAAAT
        131 GCGCTTTGAGGATTTCGTTGGAAACGGAAATATCTTCATAT

                *    *            *           * *       *  *    *            
     118295 AAAATCTAGCCAGAAGCATTCTAAGAAACATCTTAGGGATGTTTACATTCAAGTCACAGAGTTGA
          1 AAAAACTAGACAGAAGCATTCTCAGAAACATCTTTGTGATGTTTGCACTCAACTCACAGAGTTGA

              *   * *    **       *         *    * *   **      *      *    * 
     118360 ACATTCCCCTTTCTCAGAGCAGGTTTGAAACAATCTTCTCGTACTATCTGGCAGTGGACATTTTG
         66 ACCTT-TCTTTTCATAGAGCAGTTTTGAAACACTCTTTTTGTAGAATCTGGAAGTGGATATTTGG

               * *   ** ** ** *  *   *            ** *
     118425 AGCTCCTTGGCGCCTATGCTGAAAAAGGAAATATCTTCTGAC
        130 AGCGCTTTGAGGATTTCGTTGGAAACGGAAATATCTTCATAT

                                 *     *  **          *           **    *    
     118467 AAAAACTAGACAGAAGCATTCGCAGAATCACGTTTGTGATGTGTGCACTCAACTGTCAGAATTGA
          1 AAAAACTAGACAGAAGCATTCTCAGAAACATCTTTGTGATGTTTGCACTCAACTCACAGAGTTGA

                  *   ** *      *              *             * *            *
     118532 ACCTTTGTTTGGACAGAGCACTTTTG-AA-ACTCTCTTTGTAGAATCTGCAGGTGGATATTTGGC
         66 ACCTTTCTTTTCATAGAGCAGTTTTGAAACACTCTTTTTGTAGAATCTGGAAGTGGATATTTGGA

            **              *          *   *     ** *
     118595 TAGCTTTGAGGATTTCTTTGGAAACGGTAATGTCTTCGAAG
        131 GCGCTTTGAGGATTTCGTTGGAAACGGAAATATCTTCATAT

                *          *   *          *   *            *    *            
     118636 AAAATCTAGACAGAAACATCCTCAGAAACACCTTCGTGATGTTTGCAATCAAGTCACAGAGTTGA
          1 AAAAACTAGACAGAAGCATTCTCAGAAACATCTTTGTGATGTTTGCACTCAACTCACAGAGTTGA

                 * *             *  *           *      *             *       
     118701 ACCTTCCGTTTCATAGAGCAGGTTGGAAACACTCTTATTGTAGTATCTGGAAGTGGACATTTGGA
         66 ACCTTTCTTTTCATAGAGCAGTTTTGAAACACTCTTTTTGTAGAATCTGGAAGTGGATATTTGGA

                   *   ** ** *  *   *            **  
     118766 GCGCTTTCAGGCCTATGGTGTAAAAGGAAATATCTTCCCAT
        131 GCGCTTTGAGGATTTCGTTGGAAACGGAAATATCTTCATAT

                * * * *      **      *   * *   *     ** ** *      *    *     
     118807 AAAAGCGACATAGAAGCTATCTCAGGAACTTGTTTATGATGCATCTAATCAACTAACAGTGTTGA
          1 AAAAACTAGACAGAAGCATTCTCAGAAACATCTTTGTGATGTTTGCACTCAACTCACAGAGTTGA

                  * ** * *         *               * *       *               
     118872 ACCTTTGTACTGACAGAGCAG-TATGAAACACTCTTTTTTTGGAATCTGCAAGTGGATATTTGGA
         66 ACCTTTCTTTTCATAGAGCAGTTTTGAAACACTCTTTTTGTAGAATCTGGAAGTGGATATTTGGA

            *                           *   *   *    
     118936 TCGCTTTGAGGATTTCGTTGGAAACGG-GAT-GC--AATAT
        131 GCGCTTTGAGGATTTCGTTGGAAACGGAAATATCTTCATAT

                **  *    *     *                 **  *   *  *    *         * 
     118973 AAAACGTACACAGCAGCATACTCAGAAA-ATACTTTGCCATATTTCCATTCAAGTCACAGAGTGG
          1 AAAAACTAGACAGAAGCATTCTCAGAAACAT-CTTTGTGATGTTTGCACTCAACTCACAGAGTTG

               *  * **            *                  *  *             *      
     119037 AACATTCCCATTCATAGAGCAGGTTTGAAACACTCTTTTTGGAGTATCTGGAAGTGGACATTTGG
         65 AACCTTTCTTTTCATAGAGCAGTTTTGAAACACTCTTTTTGTAGAATCTGGAAGTGGATATTTGG

                    ** * * ** *  *   *            **  
     119102 AGCGCTTTCTGAACTATGGTGAAAAAGGAAATATCTTCCAAT
        130 AGCGCTTTGAGGATTTCGTTGGAAACGGAAATATCTTCATAT

            *     *               *      * *         **  **       *  *  * *  
     119144 GAAAACAAGACAGAAGCATTCTGAGAAACTTATTTGTGATGCGTGTCCTCAACTAACGGACTCGA
          1 AAAAACTAGACAGAAGCATTCTCAGAAACATCTTTGTGATGTTTGCACTCAACTCACAGAGTTGA

                   *      **  * *  *  *             *   *    * * *           
     119209 ACCTTTCGTTTCATGCAGTACTTCTGGAACACTCTTTTTGAAGATTCTGCATGCGGATATTTGGA
         66 ACCTTTCTTTTCATAGAGCAGTTTTGAAACACTCTTTTTGTAGAATCTGGAAGTGGATATTTGGA

            **                            **   *     
     119274 TAGCTTTGAGGATTTCGTTGGAAACGG---GCT-TACATAT
        131 GCGCTTTGAGGATTTCGTTGGAAACGGAAATATCTTCATAT

                 *       *               *        *   *    *    *       *    
     119311 AAAAATTAGACAGCAGCATTCTCAGAAACTTCTTTGTGGTGTCTGCATTCAAGTCACAGAATTGA
          1 AAAAACTAGACAGAAGCATTCTCAGAAACATCTTTGTGATGTTTGCACTCAACTCACAGAGTTGA

              * ** * **            *  * *      *       *    *        *       
     119376 ACATCCCCTCACATAGAGCAGTTGTGCAGCACTCTCTTTGTAGTATCTCGAAGTGGACATTTGGA
         66 ACCTTTCTTTTCATAGAGCAGTTTTGAAACACTCTTTTTGTAGAATCTGGAAGTGGATATTTGGA

             *         ** *    * *   *            **  
     119441 GGGCTTTGTAGCCTATC-TGGAAAAAGGAAATATCTTCCCAT
        131 GCGCTTTG-AGGATTTCGTTGGAAACGGAAATATCTTCATAT

            *  ** *   *     * *            *   *  *   * **        *  * * *   
     119482 GAATGCGAGATAGAAGTAATCTCAGAAACATGTTTATGCTGTATCTACTCAACTAACTGTGCTGA
          1 AAAAACTAGACAGAAGCATTCTCAGAAACATCTTTGTGATGTTTGCACTCAACTCACAGAGTTGA

              *     *  *               *        *  * *       *               
     119547 ACATTTCTATTGATAGAGCAGTTTTGAGACACTCTTCTTTTGGAATCTGCAAGTGGATATTTGGA
         66 ACCTTTCTTTTCATAGAGCAGTTTTGAAACACTCTTTTTGTAGAATCTGGAAGTGGATATTTGGA

            ** *                        *     *      
     119612 TAGATTTGAGGATTTCGTTGGAAACGG-GAT-T-AT-ATAT
        131 GCGCTTTGAGGATTTCGTTGGAAACGGAAATATCTTCATAT

                 *       *               *                 **  *   *         
     119649 AAAAAGTAGACAGCAGCATTCTCAGAAACTTCTTTGTGATGTTTGCATCCAGCTCTCAGAGTTGA
          1 AAAAACTAGACAGAAGCATTCTCAGAAACATCTTTGTGATGTTTGCACTCAACTCACAGAGTTGA

              *  * *         **  *        * *      *   **        *  **  *    
     119714 ACATTCCCTTTCATAGATTAGGTTTGAAACCCCCTTTTTATAGTGTCTGGAAGCGGGCATGTGGA
         66 ACCTTTCTTTTCATAGAGCAGTTTTGAAACACTCTTTTTGTAGAATCTGGAAGTGGATATTTGGA

                   *   ** ** *  *   *          * *  *
     119779 GCGCTTTCAGGCCTATGCTGAAAAAGGAAATATCTACCTAC
        131 GCGCTTTGAGGATTTCGTTGGAAACGGAAATATCTTCATAT

             *                    *    *  **          ** *        *    *     
     119820 AGAAACTAGACAGAAGCATTCTGAGAATCACGTTTGTGATGTGGGTACTCAACTAACAGTGTTGA
          1 AAAAACTAGACAGAAGCATTCTCAGAAACATCTTTGTGATGTTTGCACTCAACTCACAGAGTTGA

            *  *       *   *   *        *   *                *               
     119885 TCCATTCTTTTGATACAGCGGTTTTGAACCACACTTTTTGTAGAATCTGCAAGTGGATATTTGGA
         66 ACCTTTCTTTTCATAGAGCAGTTTTGAAACACTCTTTTTGTAGAATCTGGAAGTGGATATTTGGA

            **   *                     *   *  *     *
     119950 TAGCTGTGAGGATTTCGTTGGAAACGGGAATGTCCTCATAG
        131 GCGCTTTGAGGATTTCGTTGGAAACGGAAATATCTTCATAT

                **                     * * **         *  **   *   *          
     119991 AAAATTTAGACAGAAGCATTCTCAGAACCTTGATTGTGATGTGTGTTCTCCACTAACAGAGTTGA
          1 AAAAACTAGACAGAAGCATTCTCAGAAACATCTTTGTGATGTTTGCACTCAACTCACAGAGTTGA

                       * *   * *   *       *       *                         
     120056 ACCTTTCTTTTGACAGAACTGTTCTGAAACATTCTTTTTATAGAATCTGGAAGTGGATATTTGGA
         66 ACCTTTCTTTTCATAGAGCAGTTTTGAAACACTCTTTTTGTAGAATCTGGAAGTGGATATTTGGA

            **                         *          *  
     120121 AAGCTTTGAGGATTTCGTTGGAAACGGGAATATCTTCAAAT
        131 GCGCTTTGAGGATTTCGTTGGAAACGGAAATATCTTCATAT

                *    *            *           * *       *  *    *            
     120162 AAAATCTAGCCAGAAGCATTCTAAGAAACATCTTAGGGATGTTTACATTCAAGTCACAGAGTTGA
          1 AAAAACTAGACAGAAGCATTCTCAGAAACATCTTTGTGATGTTTGCACTCAACTCACAGAGTTGA

              *  * *     *       *         *    * *   **      *      *    *  
     120227 ACATTCCCTTTCACAGAGCAGGTTTGAAACAATCTTCTCGTACTATCTGGCAGTGGACATTTTGA
         66 ACCTTTCTTTTCATAGAGCAGTTTTGAAACACTCTTTTTGTAGAATCTGGAAGTGGATATTTGGA

              * *   *  ** ** *  *   *            ** *
     120292 GCTCCTTGGGGCCTATGCTGAAAAAGGAAATATCTTCCGAC
        131 GCGCTTTGAGGATTTCGTTGGAAACGGAAATATCTTCATAT

                                 *     *  **          *           **    *    
     120333 AAAAACTAGACAGAAGCATTCGCAGAATCACGTTTGTGATGTGTGCACTCAACTGTCAGAATTGA
          1 AAAAACTAGACAGAAGCATTCTCAGAAACATCTTTGTGATGTTTGCACTCAACTCACAGAGTTGA

                 **   ** *      *                            * *            *
     120398 ACCTTGGTTTGGACAGAGCACTTTTGAAACACTCTTTTTGTAGAATCTGCAGGTGGATATTTGGC
         66 ACCTTTCTTTTCATAGAGCAGTTTTGAAACACTCTTTTTGTAGAATCTGGAAGTGGATATTTGGA

            **                         *   *      * *
     120463 TAGCTTTGAGGATTTCGTTGGAAACGGTAATGTCTTCAAAG
        131 GCGCTTTGAGGATTTCGTTGGAAACGGAAATATCTTCATAT

                *                         *   *            *    *            
     120504 AAAATCTAGACAGAAGCATTCTCAGAAACACCTTCGTGATGTTTGCAATCAAGTCACAGAGTTGA
          1 AAAAACTAGACAGAAGCATTCTCAGAAACATCTTTGTGATGTTTGCACTCAACTCACAGAGTTGA

                 * *             *  *                  *             *       
     120569 ACCTTCCGTTTCATAGAGCAGGTTGGAAACACTCTTTTTGTAGTATCTGGAAGTGGACATTTGGA
         66 ACCTTTCTTTTCATAGAGCAGTTTTGAAACACTCTTTTTGTAGAATCTGGAAGTGGATATTTGGA

                   *   ** ** *  *   *            **  
     120634 GCGCTTTCAGGCCTATGGTGAAAAAGGAAATATCTTCCCAT
        131 GCGCTTTGAGGATTTCGTTGGAAACGGAAATATCTTCATAT

                  * * *      **      *   * *   *     ** ** *      *    *     
     120675 AAAAACGACATAGAAGCTATCTCAGGAACTTGTTTATGATGCATCTAATCAACTAACAGTGTTGA
          1 AAAAACTAGACAGAAGCATTCTCAGAAACATCTTTGTGATGTTTGCACTCAACTCACAGAGTTGA

                  * ** * *                         * *       *           *   
     120740 ACCTTTGTACTGACAGAGCAG-TTTGAAACACTCTTTTTTTGGAATCTGCAAGTGGATATTAGGA
         66 ACCTTTCTTTTCATAGAGCAGTTTTGAAACACTCTTTTTGTAGAATCTGGAAGTGGATATTTGGA

            *                           *   *   *    
     120804 TCGCTTTGAGGATTTCGTTGGAAACGG-GAT-GC--AATAT
        131 GCGCTTTGAGGATTTCGTTGGAAACGGAAATATCTTCATAT

                **  *    *     *                 **  *   *  *    *         * 
     120841 AAAACGTACACAGCAGCATACTCAGAAA-ATACTTTGCCATATTTCCATTCAAGTCACAGAGTGG
          1 AAAAACTAGACAGAAGCATTCTCAGAAACAT-CTTTGTGATGTTTGCACTCAACTCACAGAGTTG

               *  * **            *  *               *  *             *      
     120905 AACATTCCCATTCATAGAGCAGGTTGGAAACACTCTTTTTGGAGTATCTGGAAGTGGACATTTGG
         65 AACCTTTCTTTTCATAGAGCAGTTTTGAAACACTCTTTTTGTAGAATCTGGAAGTGGATATTTGG

                    ** * * ** *  *   *            **  
     120970 AGCGCTTTCTGAACTATGGTGAAAAAGGAAATATCTTCCAAT
        130 AGCGCTTTGAGGATTTCGTTGGAAACGGAAATATCTTCATAT

            *     *               *      * *          *  **      **  *  *    
     121012 GAAAACAAGACAGAAGCATTCTGAGAAACTTATTTGTGATGTGTGTCCTCAACAAACGGACTTGA
          1 AAAAACTAGACAGAAGCATTCTCAGAAACATCTTTGTGATGTTTGCACTCAACTCACAGAGTTGA

                   *      **  * *  *  *             *   *    * * *           
     121077 ACCTTTCGTTTCATGCAGTACTTCTGGAACACTCTTTTTGAAGATTCTGCATGCGGATATTTGGA
         66 ACCTTTCTTTTCATAGAGCAGTTTTGAAACACTCTTTTTGTAGAATCTGGAAGTGGATATTTGGA

            **                            **   *   * 
     121142 TAGCTTTGAGGATTTCGTTGGAAACGG---GCT-TACATGT
        131 GCGCTTTGAGGATTTCGTTGGAAACGGAAATATCTTCATAT

                 *       *               *        *   *    *    *       *    
     121179 AAAAATTAGACAGCAGCATTCTCAGAAACTTCTTTGTGGTGTCTGCATTCAAGTCACAGAATTGA
          1 AAAAACTAGACAGAAGCATTCTCAGAAACATCTTTGTGATGTTTGCACTCAACTCACAGAGTTGA

              * ** * **            *  * *      *       *    *        *       
     121244 ACATCCCCTCACATAGAGCAGTTGTGCAGCACTCTATTTGTAGTATCTCGAAGTGGACATTTGGA
         66 ACCTTTCTTTTCATAGAGCAGTTTTGAAACACTCTTTTTGTAGAATCTGGAAGTGGATATTTGGA

             *         ** *    * *   *            **  
     121309 GGGCTTTGTAGCCTATC-TGGAAAAAGGAAATATCTTCCCAT
        131 GCGCTTTG-AGGATTTCGTTGGAAACGGAAATATCTTCATAT

            *  ** *   *     * *            *   *  *   * **        *  * * *   
     121350 GAATGCGAGATAGAAGTAATCTCAGAAACATGTTTATGCTGTATCTACTCAACTAACTGTGCTGA
          1 AAAAACTAGACAGAAGCATTCTCAGAAACATCTTTGTGATGTTTGCACTCAACTCACAGAGTTGA

              *     *  *               *        *  * *       *               
     121415 ACATTTCTATTGATAGAGCAGTTTTGAGACACTCTTCTTTTGGAATCTGCAAGTGGATATTTGGA
         66 ACCTTTCTTTTCATAGAGCAGTTTTGAAACACTCTTTTTGTAGAATCTGGAAGTGGATATTTGGA

            ** *                        *     *      
     121480 TAGATTTGAGGATTTCGTTGGAAACGG-GAT-T-AT-ATAT
        131 GCGCTTTGAGGATTTCGTTGGAAACGGAAATATCTTCATAT

                 *       *               *                 **  *   *         
     121517 AAAAAGTAGACAGCAGCATTCTCAGAAACTTCTTTGTGATGTTTGCATCCAGCTCTCAGAGTTGA
          1 AAAAACTAGACAGAAGCATTCTCAGAAACATCTTTGTGATGTTTGCACTCAACTCACAGAGTTGA

              *  * *          *  *        *   *    *   **        *  **       
     121582 ACATTCCCTTTCATAGAGTAGGTTTGAAACCCTCCTTTTATAGTGTCTGGAAGCGGGCATTTGGA
         66 ACCTTTCTTTTCATAGAGCAGTTTTGAAACACTCTTTTTGTAGAATCTGGAAGTGGATATTTGGA

                   *   ** ** *  *   *          * *   
     121647 GCGCTTTCAGGCCTATGCTGAAAAAGGAAATATCTACCTAT
        131 GCGCTTTGAGGATTTCGTTGGAAACGGAAATATCTTCATAT

             *                    *    *  **          ** *        *    *     
     121688 AGAAACTAGACAGAAGCATTCTGAGAATCACGTTTGTGATGTGGGTACTCAACTAACAGTGTTGA
          1 AAAAACTAGACAGAAGCATTCTCAGAAACATCTTTGTGATGTTTGCACTCAACTCACAGAGTTGA

            *  *       *   *            *   *                *               
     121753 TCCATTCTTTTGATACAGCAGTTTTGAACCACACTTTTTGTAGAATCTGCAAGTGGATATTTGGA
         66 ACCTTTCTTTTCATAGAGCAGTTTTGAAACACTCTTTTTGTAGAATCTGGAAGTGGATATTTGGA

            **   *                     *   *        *
     121818 TAGCTGTGAGGATTTCGTTGGAAACGGGAATGTCTTCATAG
        131 GCGCTTTGAGGATTTCGTTGGAAACGGAAATATCTTCATAT

                **                 *   * * **         *  **   *   *          
     121859 AAAATTTAGACAGAAGCATTCTCTGAACCTTGATTGTGATGTGTGTTCTCCACTAACAGAGTTGA
          1 AAAAACTAGACAGAAGCATTCTCAGAAACATCTTTGTGATGTTTGCACTCAACTCACAGAGTTGA

                       * *   * *   *       *       *                         
     121924 ACCTTTCTTTTGACAGAACTGTTCTGAAACATTCTTTTTATAGAATCTGGAAGTGGATATTTGGA
         66 ACCTTTCTTTTCATAGAGCAGTTTTGAAACACTCTTTTTGTAGAATCTGGAAGTGGATATTTGGA

            **                         *          *  
     121989 AAGCTTTGAGGATTTCGTTGGAAACGGGAATATCTTCAAAT
        131 GCGCTTTGAGGATTTCGTTGGAAACGGAAATATCTTCATAT

                *    *            *           * *       *  *    *          * 
     122030 AAAATCTAGCCAGAAGCATTCTAAGAAACATCTTAGGGATGTTTACATTCAAGTCACAGAGTTCA
          1 AAAAACTAGACAGAAGCATTCTCAGAAACATCTTTGTGATGTTTGCACTCAACTCACAGAGTTGA

              *  * *     *       *         *    * *   **      *      *    *  
     122095 ACATTCCCTTTCACAGAGCAGGTTTGAAACAATCTTCTCGTACTATCTGGCAGTGGACATTTTGA
         66 ACCTTTCTTTTCATAGAGCAGTTTTGAAACACTCTTTTTGTAGAATCTGGAAGTGGATATTTGGA

              * *   *  ** ** *  *   *            ** *
     122160 GCTCCTTGGGGCCTATGCTGAAAAAGGAAATATCTTCCGAC
        131 GCGCTTTGAGGATTTCGTTGGAAACGGAAATATCTTCATAT

                                 *     *  **          *           **  * *    
     122201 AAAAACTAGACAGAAGCATTCGCAGAATCACGTTTGTGATGTGTGCACTCAACTGTCACAATTGA
          1 AAAAACTAGACAGAAGCATTCTCAGAAACATCTTTGTGATGTTTGCACTCAACTCACAGAGTTGA

                 **   ** *      *                            * *            *
     122266 ACCTTGGTTTGGACAGAGCACTTTTGAAACACTCTTTTTGTAGAATCTGCAGGTGGATATTTGGC
         66 ACCTTTCTTTTCATAGAGCAGTTTTGAAACACTCTTTTTGTAGAATCTGGAAGTGGATATTTGGA

            **                         *   *      * *
     122331 TAGCTTTGAGGATTTCGTTGGAAACGGTAATGTCTTCAAAG
        131 GCGCTTTGAGGATTTCGTTGGAAACGGAAATATCTTCATAT

                *                         *   *            *    *            
     122372 AAAATCTAGACAGAAGCATTCTCAGAAACACCTTCGTGATGTTTGCAATCAAGTCACAGAGTTGA
          1 AAAAACTAGACAGAAGCATTCTCAGAAACATCTTTGTGATGTTTGCACTCAACTCACAGAGTTGA

                 * *             *  *                  *             *       
     122437 ACCTTCCGTTTCATAGAGCAGGTTGGAAACACTCTTTTTGTAGTATCTGGAAGTGGACATTTGGA
         66 ACCTTTCTTTTCATAGAGCAGTTTTGAAACACTCTTTTTGTAGAATCTGGAAGTGGATATTTGGA

             *         ** *    * *   *            **  
     122502 GGGCTTTGTAGCCTATC-TGGAAAAAGGAAATATCTTCCCAT
        131 GCGCTTTG-AGGATTTCGTTGGAAACGGAAATATCTTCATAT

            *  ** *   *     * *            *   *  *   * **        *  * * *   
     122543 GAATGCGAGATAGAAGTAATCTCAGAAACATGTTTATGCTGTATCTACTCAACTAACTGTGCTGA
          1 AAAAACTAGACAGAAGCATTCTCAGAAACATCTTTGTGATGTTTGCACTCAACTCACAGAGTTGA

              *     *  *               *        *  * *       *               
     122608 ACATTTCTATTGATAGAGCAGTTTTGAGACACTCTTCTTTTGGAATCTGCAAGTGGATATTTGGA
         66 ACCTTTCTTTTCATAGAGCAGTTTTGAAACACTCTTTTTGTAGAATCTGGAAGTGGATATTTGGA

            ** *                        *     *      
     122673 TAGATTTGAGGATTTCGTTGGAAACGG-GAT-T-AT-ATAT
        131 GCGCTTTGAGGATTTCGTTGGAAACGGAAATATCTTCATAT

                 *       *               *                 **  *   *         
     122710 AAAAAGTAGACAGCAGCATTCTCAGAAACTTCTTTGTGATGTTTGCATCCAGCTCTCAGAGTTGA
          1 AAAAACTAGACAGAAGCATTCTCAGAAACATCTTTGTGATGTTTGCACTCAACTCACAGAGTTGA

            * *  * *          *  *        *        *   **        *  **       
     122775 GCATTCCCTTTCATAGAGTAGGTTTGAAACCCTCTTTTTATAGTGTCTGGAAGCGGGCATTTGGA
         66 ACCTTTCTTTTCATAGAGCAGTTTTGAAACACTCTTTTTGTAGAATCTGGAAGTGGATATTTGGA

                   *    * * **  **  **          * *  *
     122840 GCGCTTTCAGGCCTATGCTTAAAATAGGAAATATCTACCTAC
        131 GCGCTTTGAGG-ATTTCGTTGGAAACGGAAATATCTTCATAT

             *                    *    *  **          ** *        *   **     
     122882 AGAAACTAGACAGAAGCATTCTGAGAATCACGTTTGTGATGTGGGTACTCAACTAACAATGTTGA
          1 AAAAACTAGACAGAAGCATTCTCAGAAACATCTTTGTGATGTTTGCACTCAACTCACAGAGTTGA

            *  *       *   *            *   *                *   *           
     122947 TCCATTCTTTTGATACAGCAGTTTTGAACCACACTTTTTGTAGAATCTGCAAGAGGATATTTGGA
         66 ACCTTTCTTTTCATAGAGCAGTTTTGAAACACTCTTTTTGTAGAATCTGGAAGTGGATATTTGGA

            **   *                     *   *      * *
     123012 TAGCTGTGAGGATTTCGTTGGAAACGGGAATGTCTTCAAAG
        131 GCGCTTTGAGGATTTCGTTGGAAACGGAAATATCTTCATAT

                *                         *   *            *    *            
     123053 AAAATCTAGACAGAAGCATTCTCAGAAACACCTTCGTGATGTTTGCAATCAAGTCACAGAGTTGA
          1 AAAAACTAGACAGAAGCATTCTCAGAAACATCTTTGTGATGTTTGCACTCAACTCACAGAGTTGA

                 * *             *  *           *      *             *       
     123118 ACCTTCCGTTTCATAGAGCAGGTTGGAAACACTCTTATTGTAGTATCTGGAAGTGGACATTTGGA
         66 ACCTTTCTTTTCATAGAGCAGTTTTGAAACACTCTTTTTGTAGAATCTGGAAGTGGATATTTGGA

                   *   ** ** *  *   *            **  
     123183 GCGCTTTCAGGCCTATGGTGAAAAAGGAAATATCTTCCCAT
        131 GCGCTTTGAGGATTTCGTTGGAAACGGAAATATCTTCATAT

                  * * *      **      *   * *   *     ** ** *      *    *     
     123224 AAAAACGACATAGAAGCTATCTCAGGAACTTGTTTATGATGCATCTAATCAACTAACAGTGTTGA
          1 AAAAACTAGACAGAAGCATTCTCAGAAACATCTTTGTGATGTTTGCACTCAACTCACAGAGTTGA

                  * ** * *                         * *       *               
     123289 ACCTTTGTACTGACAGAGCAG-TTTGAAACACTCTTTTTTTGGAATCTGCAAGTGGATATTTGGA
         66 ACCTTTCTTTTCATAGAGCAGTTTTGAAACACTCTTTTTGTAGAATCTGGAAGTGGATATTTGGA

            *                           *   *   *    
     123353 TCGCTTTGAGGATTTCGTTGGAAACGG-GAT-GC--AATAT
        131 GCGCTTTGAGGATTTCGTTGGAAACGGAAATATCTTCATAT

                **  *    *     *                 **  *   *  *    *         * 
     123390 AAAACGTACACAGCAGCATACTCAGAAA-ATACTTTGCCATATTTCCATTCAAGTCACAGAGTGG
          1 AAAAACTAGACAGAAGCATTCTCAGAAACAT-CTTTGTGATGTTTGCACTCAACTCACAGAGTTG

               *  * **            *  *               *  *             *      
     123454 AACATTCCCATTCATAGAGCAGGTTGGAAACACTCTTTTTGGAGTATCTGGAAGTGGACATTTGG
         65 AACCTTTCTTTTCATAGAGCAGTTTTGAAACACTCTTTTTGTAGAATCTGGAAGTGGATATTTGG

                    ** * * ** *  *   *            **  
     123519 AGCGCTTTCTGAACTGTGGTGAAAAAGGAAATATCTTCCAAT
        130 AGCGCTTTGAGGATTTCGTTGGAAACGGAAATATCTTCATAT

            *     *               *      * *          *  **      **  *  *    
     123561 GAAAACAAGACAGAAGCATTCTGAGAAACTTATTTGTGATGTGTGTCCTCAACAAACGGACTTGA
          1 AAAAACTAGACAGAAGCATTCTCAGAAACATCTTTGTGATGTTTGCACTCAACTCACAGAGTTGA

                   *      **  * *  *  *             *   *    * * *           
     123626 ACCTTTCGTTTCATGCAGTACTTCTGGAACACTCTTTTTGAAGATTCTGCATGCGGATATTTGGA
         66 ACCTTTCTTTTCATAGAGCAGTTTTGAAACACTCTTTTTGTAGAATCTGGAAGTGGATATTTGGA

            **                            **   *   * 
     123691 TAGCTTTGAGGATTTCGTTGGAAACGG---GCT-TACATGT
        131 GCGCTTTGAGGATTTCGTTGGAAACGGAAATATCTTCATAT

                 *       *               *        *   *    *    *       *    
     123728 AAAAATTAGACAGCAGCATTCTCAGAAACTTCTTTGTGGTGTCTGCATTCAAGTCACAGAATTGA
          1 AAAAACTAGACAGAAGCATTCTCAGAAACATCTTTGTGATGTTTGCACTCAACTCACAGAGTTGA

              * ** * **            *  * *      *       *             *       
     123793 ACATCCCCTCACATAGAGCAGTTGTGCAGCACTCTATTTGTAGTATCTGGAAGTGGACATTTGGA
         66 ACCTTTCTTTTCATAGAGCAGTTTTGAAACACTCTTTTTGTAGAATCTGGAAGTGGATATTTGGA

             *         ** *    * *   *            **  
     123858 GGGCTTTGTAGCCTATC-TGGAAAAAGGAAATATCTTCCCAT
        131 GCGCTTTG-AGGATTTCGTTGGAAACGGAAATATCTTCATAT

            *  ** *   *     * *            *   *  *   * **        *  * * *   
     123899 GAATGCGAGATAGAAGTAATCTCAGAAACATGTTTATGCTGTATCTACTCAACTAACTGTGCTGA
          1 AAAAACTAGACAGAAGCATTCTCAGAAACATCTTTGTGATGTTTGCACTCAACTCACAGAGTTGA

              *     *  *               *        *  * *       *               
     123964 ACATTTCTATTGATAGAGCAGTTTTGAGACACTCTTCTTTTGGAATCTGCAAGTGGATATTTGGA
         66 ACCTTTCTTTTCATAGAGCAGTTTTGAAACACTCTTTTTGTAGAATCTGGAAGTGGATATTTGGA

            ** *                        *     *      
     124029 TAGATTTGAGGATTTCGTTGGAAACGG-GAT-T-AT-ATAT
        131 GCGCTTTGAGGATTTCGTTGGAAACGGAAATATCTTCATAT

            *    *       *               *                 **  *   *         
     124066 CAAAAGTAGACAGCAGCATTCTCAGAAACTTCTTTGTGATGTTTGCATCCAGCTCTCAGAGTTGA
          1 AAAAACTAGACAGAAGCATTCTCAGAAACATCTTTGTGATGTTTGCACTCAACTCACAGAGTTGA

              *  * *          *  *        *        *   **        *  **       
     124131 ACATTCCCTTTCATAGAGTAGGTTTGAAACCCTCTTTTTATAGTGTCTGGAAGCGGGCATTTGGA
         66 ACCTTTCTTTTCATAGAGCAGTTTTGAAACACTCTTTTTGTAGAATCTGGAAGTGGATATTTGGA

                   *   ** ** *  *   *          * *   
     124196 GCGCTTTCAGGCCTATGCTGAAAAAGGAAATATCTACCTAT
        131 GCGCTTTGAGGATTTCGTTGGAAACGGAAATATCTTCATAT

             *                    *    *  **          ** *        *    *     
     124237 AGAAACTAGACAGAAGCATTCTGAGAATCACGTTTGTGATGTGGGTACTCAACTAACAGTGTTGA
          1 AAAAACTAGACAGAAGCATTCTCAGAAACATCTTTGTGATGTTTGCACTCAACTCACAGAGTTGA

            *  *       *   *            *   *                *               
     124302 TCCATTCTTTTGATACAGCAGTTTTGAACCACACTTTTTGTAGAATCTGCAAGTGGATATTTGGA
         66 ACCTTTCTTTTCATAGAGCAGTTTTGAAACACTCTTTTTGTAGAATCTGGAAGTGGATATTTGGA

            **   *                     *   *        *
     124367 TAGCTGTGAGGATTTCGTTGGAAACGGGAATGTCTTCATAG
        131 GCGCTTTGAGGATTTCGTTGGAAACGGAAATATCTTCATAT

                **                     *** **         *  **   *   *          
     124408 AAAATTTAGACAGAAGCATTCTCAGAACTTTGATTGTGATGTGTGTTCTCCACTAACAGAGTTGA
          1 AAAAACTAGACAGAAGCATTCTCAGAAACATCTTTGTGATGTTTGCACTCAACTCACAGAGTTGA

                       * *   * *   *       *       *                         
     124473 ACCTTTCTTTTGACAGAACTGTTCTGAAACATTCTTTTTATAGAATCTGGAAGTGGATATTTGGA
         66 ACCTTTCTTTTCATAGAGCAGTTTTGAAACACTCTTTTTGTAGAATCTGGAAGTGGATATTTGGA

            **                         *          *  
     124538 AAGCTTTGAGGATTTCGTTGGAAACGGGAATATCTTCAAAT
        131 GCGCTTTGAGGATTTCGTTGGAAACGGAAATATCTTCATAT

                *    *            *           * *       *  *    *            
     124579 AAAATCTAGCCAGAAGCATTCTAAGAAACATCTTAGGGATGTTTACATTCAAGTCACAGAGTTGA
          1 AAAAACTAGACAGAAGCATTCTCAGAAACATCTTTGTGATGTTTGCACTCAACTCACAGAGTTGA

              *  * *     *       *         *    * *   **      *      *    *  
     124644 ACATTACCTTTCACAGAGCAGGTTTGAAACAATCTTCTCGTACTATCTGGCAGTGGACATTTTGA
         66 ACCTTTCTTTTCATAGAGCAGTTTTGAAACACTCTTTTTGTAGAATCTGGAAGTGGATATTTGGA

              *     *  ** ** *  *   *            ** *
     124709 GCTCTTTGGGGCCTATGCTGAAAAAGGAAATATCTTCCGAC
        131 GCGCTTTGAGGATTTCGTTGGAAACGGAAATATCTTCATAT

                                 *     *  **          *           **  * *    
     124750 AAAAACTAGACAGAAGCATTCGCAGAATCACGTTTGTGATGTGTGCACTCAACTGTCACAATTGA
          1 AAAAACTAGACAGAAGCATTCTCAGAAACATCTTTGTGATGTTTGCACTCAACTCACAGAGTTGA

                 **   ** *      *                            * *            *
     124815 ACCTTGGTTTGGACAGAGCACTTTTGAAACACTCTTTTTGTAGAATCTGCAGGTGGATATTTGGC
         66 ACCTTTCTTTTCATAGAGCAGTTTTGAAACACTCTTTTTGTAGAATCTGGAAGTGGATATTTGGA

            **                         *   *      * *
     124880 TAGCTTTGAGGATTTCGTTGGAAACGGTAATGTCTTCAAAG
        131 GCGCTTTGAGGATTTCGTTGGAAACGGAAATATCTTCATAT

                *                         *   *            *    *            
     124921 AAAATCTAGACAGAAGCATTCTCAGAAACACCTTCGTGATGTTTGCAATCAAGTCACAGAGTTGA
          1 AAAAACTAGACAGAAGCATTCTCAGAAACATCTTTGTGATGTTTGCACTCAACTCACAGAGTTGA

                 * *             *  *                  *             *       
     124986 ACCTTCCGTTTCATAGAGCAGGTTGGAAACACTCTTTTTGTAGTATCTGGAAGTGGACATTTGGA
         66 ACCTTTCTTTTCATAGAGCAGTTTTGAAACACTCTTTTTGTAGAATCTGGAAGTGGATATTTGGA

             *         ** *    * *   *            **  
     125051 GGGCTTTGTAGCCTATC-TGGAAAAAGGAAATATCTTCCCAT
        131 GCGCTTTG-AGGATTTCGTTGGAAACGGAAATATCTTCATAT

            *  ** *   *     * *            *   *  *   * **        *  * * *   
     125092 GAATGCGAGATAGAAGTAATCTCAGAAACATGTTTATGCTGTATCTACTCAACTAACTGTGCTGA
          1 AAAAACTAGACAGAAGCATTCTCAGAAACATCTTTGTGATGTTTGCACTCAACTCACAGAGTTGA

              *     *  *               *        *  * *       *               
     125157 ACATTTCTATTGATAGAGCAGTTTTGAGACACTCTTCTTTTGGAATCTGCAAGTGGATATTTGGA
         66 ACCTTTCTTTTCATAGAGCAGTTTTGAAACACTCTTTTTGTAGAATCTGGAAGTGGATATTTGGA

            ** *                        *     *      
     125222 TAGATTTGAGGATTTCGTTGGAAACGG-GAT-T-AT-ATAT
        131 GCGCTTTGAGGATTTCGTTGGAAACGGAAATATCTTCATAT

            *    *       *               *                 **  *   *         
     125259 CAAAAGTAGACAGCAGCATTCTCAGAAACTTCTTTGTGATGTTTGCATCCAGCTCTCAGAGTTGA
          1 AAAAACTAGACAGAAGCATTCTCAGAAACATCTTTGTGATGTTTGCACTCAACTCACAGAGTTGA

              *  * *          *  *        *        *   **        *  **       
     125324 ACATTCCCTTTCATAGAGTAGGTTTGAAACCCTCTTTTTATAGTGTCTGGAAGCGGGCATTTGGA
         66 ACCTTTCTTTTCATAGAGCAGTTTTGAAACACTCTTTTTGTAGAATCTGGAAGTGGATATTTGGA

                   *   ** ** *  *   *          * *   
     125389 GCGCTTTCAGGCCTATGCTGAAAAAGGAAATATCTACCTAT
        131 GCGCTTTGAGGATTTCGTTGGAAACGGAAATATCTTCATAT

             *                    *    *  **          ** *        *    *     
     125430 AGAAACTAGACAGAAGCATTCTGAGAATCACGTTTGTGATGTGGGTACTCAACTAACAGTGTTGA
          1 AAAAACTAGACAGAAGCATTCTCAGAAACATCTTTGTGATGTTTGCACTCAACTCACAGAGTTGA

            *  *       *   *            *   *                *               
     125495 TCCATTCTTTTGATACAGCAGTTTTGAACCACACTTTTTGTAGAATCTGCAAGTGGATATTTGGA
         66 ACCTTTCTTTTCATAGAGCAGTTTTGAAACACTCTTTTTGTAGAATCTGGAAGTGGATATTTGGA

            **   *                     *   *        *
     125560 TAGCTGTGAGGATTTCGTTGGAAACGGGAATGTCTTCATAG
        131 GCGCTTTGAGGATTTCGTTGGAAACGGAAATATCTTCATAT

                **                     * * **         *  **   *   *          
     125601 AAAATTTAGACAGAAGCATTCTCAGAACCTTGATTGTGATGTGTGTTCTCCACTAACAGAGTTGA
          1 AAAAACTAGACAGAAGCATTCTCAGAAACATCTTTGTGATGTTTGCACTCAACTCACAGAGTTGA

                       * *   * *   *       *       *                         
     125666 ACCTTTCTTTTGACAGAACTGTTCTGAAACATTCTTTTTATAGAATCTGGAAGTGGATATTTGGA
         66 ACCTTTCTTTTCATAGAGCAGTTTTGAAACACTCTTTTTGTAGAATCTGGAAGTGGATATTTGGA

            **              *          *          *  
     125731 AAGCTTTGAGGATTTCATTGGAAACGGGAATATCTTCAAAT
        131 GCGCTTTGAGGATTTCGTTGGAAACGGAAATATCTTCATAT

                *    *            *           * *       *  *    *            
     125772 AAAATCTAGCCAGAAGCATTCTAAGAAACATCTTAGGGATGTTTACATTCAAGTCACAGAGTTGA
          1 AAAAACTAGACAGAAGCATTCTCAGAAACATCTTTGTGATGTTTGCACTCAACTCACAGAGTTGA

              *  * *     *       *         *    * *   **      *      *    *  
     125837 ACATTCCCTTTCACAGAGCAGGTTTGAAACAATCTTCTCGTACTATCTGGCAGTGGACATTTTGA
         66 ACCTTTCTTTTCATAGAGCAGTTTTGAAACACTCTTTTTGTAGAATCTGGAAGTGGATATTTGGA

              * *   *  ** ** *  *   *            ** *
     125902 GCTCCTTGTGGCCTATGCTGAAAAAGGAAATATCTTCCGAC
        131 GCGCTTTGAGGATTTCGTTGGAAACGGAAATATCTTCATAT

                                 *     *  **          *           **    *    
     125943 AAAAACTAGACAGAAGCATTCGCAGAATCACGTTTGTGATGTGTGCACTCAACTGTCAGAATTGA
          1 AAAAACTAGACAGAAGCATTCTCAGAAACATCTTTGTGATGTTTGCACTCAACTCACAGAGTTGA

                 **   ** *      *                            * *            *
     126008 ACCTTGGTTTGGACAGAGCACTTTTGAAACACTCTTTTTGTAGAATCTGCAGGTGGATATTTGGC
         66 ACCTTTCTTTTCATAGAGCAGTTTTGAAACACTCTTTTTGTAGAATCTGGAAGTGGATATTTGGA

            **                         *   *      * *
     126073 TAGCTTTGAGGATTTCGTTGGAAACGGTAATGTCTTCACAG
        131 GCGCTTTGAGGATTTCGTTGGAAACGGAAATATCTTCATAT

                *                         *   *            *    *            
     126114 AAAATCTAGACAGAAGCATTCTCAGAAACACCTTCGTGATGTTTGCAATCAAGTCACAGAGTTGA
          1 AAAAACTAGACAGAAGCATTCTCAGAAACATCTTTGTGATGTTTGCACTCAACTCACAGAGTTGA

                 * *             *  *                  *             *       
     126179 ACCTTCCGTTTCATAGAGCAGGTTGGAAACACTCTTTTTGTAGTATCTGGAAGTGGACATTTGGA
         66 ACCTTTCTTTTCATAGAGCAGTTTTGAAACACTCTTTTTGTAGAATCTGGAAGTGGATATTTGGA

             *     *   ** ** *  *   *            **  
     126244 GAGCTTTCAGGCCTATGGTGAAAAAGGAAATATCTTCCCAT
        131 GCGCTTTGAGGATTTCGTTGGAAACGGAAATATCTTCATAT

                  * * *    * **      *   * *   *     ** ** *      *    *     
     126285 AAAAACGACATAGAATCTATCTCAGGAACTTGTTTATGATGCATCTAATCAACTAACAGTGTTGA
          1 AAAAACTAGACAGAAGCATTCTCAGAAACATCTTTGTGATGTTTGCACTCAACTCACAGAGTTGA

                  * ** * *                         * *       *               
     126350 ACCTTTGTACTGACAGAGCAG-TTTGAAACACTCTTTTTTTGGAATCTGCAAGTGGATATTTGGA
         66 ACCTTTCTTTTCATAGAGCAGTTTTGAAACACTCTTTTTGTAGAATCTGGAAGTGGATATTTGGA

            *                           *   *   *    
     126414 TCGCTTTGAGGATTTCGTTGGAAACGG-GAT-GC--AATAT
        131 GCGCTTTGAGGATTTCGTTGGAAACGGAAATATCTTCATAT

                **  *    *     *                 **  *   *  *    *         * 
     126451 AAAACGTACACAGCAGCATACTCAGAAA-ATACTTTGCCATATTTCCATTCAAGTCACAGAGTGG
          1 AAAAACTAGACAGAAGCATTCTCAGAAACAT-CTTTGTGATGTTTGCACTCAACTCACAGAGTTG

               *  * **            *     *            *  *             *      
     126515 AACATTCCCATTCATAGAGCAGGTTTGATACACTCTTTTTGGAGTATCTGGAAGTGGACATTTGG
         65 AACCTTTCTTTTCATAGAGCAGTTTTGAAACACTCTTTTTGTAGAATCTGGAAGTGGATATTTGG

                    ** * * ** *  *   *            **  
     126580 AGCGCTTTCTGAACTAAGGTGAAAAAGGAAATATCTTCCAAT
        130 AGCGCTTTGAGGATTTCGTTGGAAACGGAAATATCTTCATAT

            *     *               *      * *          *  **      **  *  *    
     126622 GAAAACAAGACAGAAGCATTCTGAGAAACTTATTTGTGATGTGTGTCCTCAACAAACGGACTTGA
          1 AAAAACTAGACAGAAGCATTCTCAGAAACATCTTTGTGATGTTTGCACTCAACTCACAGAGTTGA

                   *      **  * *  *  *             *   *    * * *           
     126687 ACCTTTCGTTTCATGCAGTACTTCTGGAACACTCTTTTTGAAGATTCTGCATGCGGATATTTGGA
         66 ACCTTTCTTTTCATAGAGCAGTTTTGAAACACTCTTTTTGTAGAATCTGGAAGTGGATATTTGGA

            **                            **   *   * 
     126752 TAGCTTTGAGGATTTCGTTGGAAACGG---GCT-TACATGT
        131 GCGCTTTGAGGATTTCGTTGGAAACGGAAATATCTTCATAT

                 *       *               *        *   *    *    *       *    
     126789 AAAAATTAGACAGCAGCATTCTCAGAAACTTCTTTGTGGTGTCTGCATTCAAGTCACAGAATTGA
          1 AAAAACTAGACAGAAGCATTCTCAGAAACATCTTTGTGATGTTTGCACTCAACTCACAGAGTTGA

             ** ** * **            *  * *      *       *             *       
     126854 AATTCCCCTCACATAGAGCAGTTGTGCAGCACTCTATTTGTAGTATCTGGAAGTGGACATTTGGA
         66 ACCTTTCTTTTCATAGAGCAGTTTTGAAACACTCTTTTTGTAGAATCTGGAAGTGGATATTTGGA

             *         ** *    * *   *            **  
     126919 GGGCTTTGTAGCCTATC-TGGAAAAAGGAAATATCTTCCCAT
        131 GCGCTTTG-AGGATTTCGTTGGAAACGGAAATATCTTCATAT

            *  ** *   *     * *            *   *  *   * **        *  * * *   
     126960 GAATGCGAGATAGAAGTAATCTCAGAAACATGTTTATGCTGTATCTACTCAACTAACTGTGCTGA
          1 AAAAACTAGACAGAAGCATTCTCAGAAACATCTTTGTGATGTTTGCACTCAACTCACAGAGTTGA

              *     *  *               *        *  * *       *               
     127025 ACATTTCTATTGATAGAGCAGTTTTGAGACACTCTTCTTTTGGAATCTGCAAGTGGATATTTGGA
         66 ACCTTTCTTTTCATAGAGCAGTTTTGAAACACTCTTTTTGTAGAATCTGGAAGTGGATATTTGGA

            ** *                        *     *      
     127090 TAGATTTGAGGATTTCGTTGGAAACGG-GAT-T-AT-ATAT
        131 GCGCTTTGAGGATTTCGTTGGAAACGGAAATATCTTCATAT

                 *       *               *                 **  *   *         
     127127 AAAAAGTAGACAGCAGCATTCTCAGAAACTTCTTTGTGATGTTTGCATCCAGCTCTCAGAGTTGA
          1 AAAAACTAGACAGAAGCATTCTCAGAAACATCTTTGTGATGTTTGCACTCAACTCACAGAGTTGA

            * *  * *          *  *        *        *   **        *  **       
     127192 GCATTCCCTTTCATAGAGTAGGTTTGAAACCCTCTTTTTATAGTGTCTGGAAGCGGGCATTTGGA
         66 ACCTTTCTTTTCATAGAGCAGTTTTGAAACACTCTTTTTGTAGAATCTGGAAGTGGATATTTGGA

                   *    * * **  **  **          * *  *
     127257 GCGCTTTCAGGCCTATGCTTAAAATAGGAAATATCTACCTAC
        131 GCGCTTTGAGG-ATTTCGTTGGAAACGGAAATATCTTCATAT

             *                    *    *  **          ** *        *    *     
     127299 AGAAACTAGACAGAAGCATTCTGAGAATCACGTTTGTGATGTGGGTACTCAACTAACAGTGTTGA
          1 AAAAACTAGACAGAAGCATTCTCAGAAACATCTTTGTGATGTTTGCACTCAACTCACAGAGTTGA

            *  *       *   *            *   *                *   *           
     127364 TCCATTCTTTTGATACAGCAGTTTTGAACCACACTTTTTGTAGAATCTGCAAGAGGATATTTGGA
         66 ACCTTTCTTTTCATAGAGCAGTTTTGAAACACTCTTTTTGTAGAATCTGGAAGTGGATATTTGGA

            **   *                     *   *     ** *
     127429 TAGCTGTGAGGATTTCGTTGGAAACGGGAATGTCTTCTAAG
        131 GCGCTTTGAGGATTTCGTTGGAAACGGAAATATCTTCATAT

                *                         *   *            *    *            
     127470 AAAATCTAGACAGAAGCATTCTCAGAAACACCTTCGTGATGTTTGCAATCAAGTCACAGAGTTGA
          1 AAAAACTAGACAGAAGCATTCTCAGAAACATCTTTGTGATGTTTGCACTCAACTCACAGAGTTGA

                 * *             *  *           *      *             *  *    
     127535 ACCTTCCGTTTCATAGAGCAGGTTGGAAACACTCTTATTGTAGTATCTGGAAGTGGACATCTGGA
         66 ACCTTTCTTTTCATAGAGCAGTTTTGAAACACTCTTTTTGTAGAATCTGGAAGTGGATATTTGGA

                   *   ** ** *  *   *            **  
     127600 GCGCTTTCAGGCCTATGGTGAAAAAGGAAATATCTTCCCAT
        131 GCGCTTTGAGGATTTCGTTGGAAACGGAAATATCTTCATAT

                  * * *      **      *   * *  **     ** ** *      *    *     
     127641 AAAAACGACATAGAAGCTATCTCAGGAACTTGTTGATGATGCATCTAATCAACTAACAGTGTTGA
          1 AAAAACTAGACAGAAGCATTCTCAGAAACATCTTTGTGATGTTTGCACTCAACTCACAGAGTTGA

                  * ** * *                         * *       *               
     127706 ACCTTTGTACTGACAGAGCAG-TTTGAAACACTCTTTTTTTGGAATCTGCAAGTGGATATTTGGA
         66 ACCTTTCTTTTCATAGAGCAGTTTTGAAACACTCTTTTTGTAGAATCTGGAAGTGGATATTTGGA

            *                           *   *   *    
     127770 TCGCTTTGAGGATTTCGTTGGAAACGG-GAT-GC--AATAT
        131 GCGCTTTGAGGATTTCGTTGGAAACGGAAATATCTTCATAT

                **  *    *     *                 **  *   *  *    *         * 
     127807 AAAACGTACACAGCAGCATACTCAGAAA-ATACTTTGCCATATTTCCATTCAAGTCACAGAGTGG
          1 AAAAACTAGACAGAAGCATTCTCAGAAACAT-CTTTGTGATGTTTGCACTCAACTCACAGAGTTG

               *  * **            *  *               *  *             *      
     127871 AACATTCCCATTCATAGAGCAGGTTGGAAACACTCTTTTTGGAGTATCTGGAAGTGGACATTTGG
         65 AACCTTTCTTTTCATAGAGCAGTTTTGAAACACTCTTTTTGTAGAATCTGGAAGTGGATATTTGG

                    ** * * ** *  *   *            **  
     127936 AGCGCTTTCTGAACTATGGTGAAAAAGGAAATATCTTCCAAT
        130 AGCGCTTTGAGGATTTCGTTGGAAACGGAAATATCTTCATAT

            *     *               *      * *          *  **      **  *  *    
     127978 GAAAACAAGACAGAAGCATTCTGAGAAACTTATTTGTGATGTGTGTCCTCAACAAACGGACTTGA
          1 AAAAACTAGACAGAAGCATTCTCAGAAACATCTTTGTGATGTTTGCACTCAACTCACAGAGTTGA

                   *      **  * *  *  *             *   *    * * *           
     128043 ACCTTTCGTTTCATGCAGTACTTCTGGAACACTCTTTTTGAAGATTCTGCATGCGGATATTTGGA
         66 ACCTTTCTTTTCATAGAGCAGTTTTGAAACACTCTTTTTGTAGAATCTGGAAGTGGATATTTGGA

            **                            **   *   * 
     128108 TAGCTTTGAGGATTTCGTTGGAAACGG---GCT-TACATGT
        131 GCGCTTTGAGGATTTCGTTGGAAACGGAAATATCTTCATAT

                 *       *               **       *   *    *    *       *    
     128145 AAAAATTAGACAGCAGCATTCTCAGAAACTACTTTGTGGTGTCTGCATTCAAGTCACAGAATTGA
          1 AAAAACTAGACAGAAGCATTCTCAGAAACATCTTTGTGATGTTTGCACTCAACTCACAGAGTTGA

              * ** * **            *  * *      *       *    *        *       
     128210 ACTTCCCCTCACATAGAGCAGTTGTGCAGCACTCTATTTGTAGTATCTCGAAGTGGACATTTGGA
         66 ACCTTTCTTTTCATAGAGCAGTTTTGAAACACTCTTTTTGTAGAATCTGGAAGTGGATATTTGGA

             *         ** *    * *   *            **  
     128275 GGGCTTTGTAGCCTATC-TGGAAAAAGGAAATATCTTCCCAT
        131 GCGCTTTG-AGGATTTCGTTGGAAACGGAAATATCTTCATAT

            *  ** *   *     * *            *   *  *   * **        *  * * *   
     128316 GAATGCGAGATAGAAGTAATCTCAGAAACATGTTTATGCTGTATCTACTCAACTAACTGTGCTGA
          1 AAAAACTAGACAGAAGCATTCTCAGAAACATCTTTGTGATGTTTGCACTCAACTCACAGAGTTGA

              *     *  *               *        *  * *       *               
     128381 ACATTTCTATTGATAGAGCAGTTTTGAGACACTCTTCTTTTGGAATCTGCAAGTGGATATTTGGA
         66 ACCTTTCTTTTCATAGAGCAGTTTTGAAACACTCTTTTTGTAGAATCTGGAAGTGGATATTTGGA

            ** *                        *     *      
     128446 TAGATTTGAGGATTTCGTTGGAAACGG-GAT-T-AT-ATAT
        131 GCGCTTTGAGGATTTCGTTGGAAACGGAAATATCTTCATAT

                 *       *               *                 **  *   *    *    
     128483 AAAAAGTAGACAGCAGCATTCTCAGAAACTTCTTTGTGATGTTTGCATCCAGCTCTCAGATTTGA
          1 AAAAACTAGACAGAAGCATTCTCAGAAACATCTTTGTGATGTTTGCACTCAACTCACAGAGTTGA

              *  * *          *  *        *        *   **        *  **       
     128548 ACATTCCCTTTCATAGAGTAGGTTTGAAACCCTCTTTTTATAGTGTCTGGAAGCGGGCATTTGGA
         66 ACCTTTCTTTTCATAGAGCAGTTTTGAAACACTCTTTTTGTAGAATCTGGAAGTGGATATTTGGA

                   *    * * **  **  **          * *  *
     128613 GCGCTTTCAGGCCTATGCTTAAAATAGGAAATATCTACCTAC
        131 GCGCTTTGAGG-ATTTCGTTGGAAACGGAAATATCTTCATAT

             *                    *    *   *          ** *        *    *     
     128655 AGAAACTAGACAGAAGCATTCTGAGAATCATGTTTGTGATGTGGGTACTCAACTAACAGTGTTGA
          1 AAAAACTAGACAGAAGCATTCTCAGAAACATCTTTGTGATGTTTGCACTCAACTCACAGAGTTGA

            *  *       *   *            *   *                *   *           
     128720 TCCATTCTTTTGATACAGCAGTTTTGAACCACACTTTTTGTAGAATCTGCAAGAGGATATTTGGA
         66 ACCTTTCTTTTCATAGAGCAGTTTTGAAACACTCTTTTTGTAGAATCTGGAAGTGGATATTTGGA

            **   *                     *   *      * *
     128785 TAGCTGTGAGGATTTCGTTGGAAACGGGAATGTCTTCAAAG
        131 GCGCTTTGAGGATTTCGTTGGAAACGGAAATATCTTCATAT

                *                         *   *            *    *            
     128826 AAAATCTAGACAGAAGCATTCTCAGAAACACCTTCGTGATGTTTGCAATCAAGTCACAGAGTTGA
          1 AAAAACTAGACAGAAGCATTCTCAGAAACATCTTTGTGATGTTTGCACTCAACTCACAGAGTTGA

                 * *             *  *                  *             *       
     128891 ACCTTCCGTTTCATAGAGCAGGTTGGAAACACTCTTTTTGTAGTATCTGGAAGTGGACATTTGGA
         66 ACCTTTCTTTTCATAGAGCAGTTTTGAAACACTCTTTTTGTAGAATCTGGAAGTGGATATTTGGA

                   *   ** ** *  *   *            **  
     128956 GCGCTTTCAGGCCTATGGTGAAAAAGGAAATATCTTCCCAT
        131 GCGCTTTGAGGATTTCGTTGGAAACGGAAATATCTTCATAT

                  * * *      **      *   * *   *     ** ** *      *    *     
     128997 AAAAACGACATAGAAGCTATCTCAGGAACTTGTTTATGATGCATCTAATCAACTAACAGTGTTGA
          1 AAAAACTAGACAGAAGCATTCTCAGAAACATCTTTGTGATGTTTGCACTCAACTCACAGAGTTGA

                  * ** * *                         * *       *               
     129062 ACCTTTGTACTGACAGAGCAG-TTTGAAACACTCTTTTTTTGGAATCTGCAAGTGGATATTTGGA
         66 ACCTTTCTTTTCATAGAGCAGTTTTGAAACACTCTTTTTGTAGAATCTGGAAGTGGATATTTGGA

            *                           *   *   *    
     129126 TCGCTTTGAGGATTTCGTTGGAAACGG-GAT-GC--AATAT
        131 GCGCTTTGAGGATTTCGTTGGAAACGGAAATATCTTCATAT

                **  *    *     *                 **  *   *  *    *         * 
     129163 AAAACGTACACAGCAGCATACTCAGAAA-ATACTTTGCCATATTTCCATTCAAGTCACAGAGTGG
          1 AAAAACTAGACAGAAGCATTCTCAGAAACAT-CTTTGTGATGTTTGCACTCAACTCACAGAGTTG

               *  * **            *                  *  *             *      
     129227 AACATTCCCATTCATAGAGCAGGTTTGAAACACTCTTTTTGGAGTATCTGGAAGTGGACATTTGG
         65 AACCTTTCTTTTCATAGAGCAGTTTTGAAACACTCTTTTTGTAGAATCTGGAAGTGGATATTTGG

                    ** * * ** *  *   *            **  
     129292 AGCGCTTTCTGAACTATGGTGAAAAAGGAAATATCTTCCAAT
        130 AGCGCTTTGAGGATTTCGTTGGAAACGGAAATATCTTCATAT

            *     * *             *      * *          *  **      **  *  *    
     129334 GAAAACAACACAGAAGCATTCTGAGAAACTTATTTGTGATGTGTGTCCTCAACAAACGGACTTGA
          1 AAAAACTAGACAGAAGCATTCTCAGAAACATCTTTGTGATGTTTGCACTCAACTCACAGAGTTGA

                   *      **  * *  *  *             *   *    * * *           
     129399 ACCTTTCGTTTCATGCAGTACTTCTGGAACACTCTTTTTGAAGATTCTGCATGCGGATATTTGGA
         66 ACCTTTCTTTTCATAGAGCAGTTTTGAAACACTCTTTTTGTAGAATCTGGAAGTGGATATTTGGA

            **                            **   *   * 
     129464 TAGCTTTGAGGATTTCGTTGGAAACGG---GCT-TACATGT
        131 GCGCTTTGAGGATTTCGTTGGAAACGGAAATATCTTCATAT

                 *       *               *        *   *    *    *       *    
     129501 AAAAATTAGACAGCAGCATTCTCAGAAACTTCTTTGTGGTGTCTGCATTCAAGTCACAGAATTGA
          1 AAAAACTAGACAGAAGCATTCTCAGAAACATCTTTGTGATGTTTGCACTCAACTCACAGAGTTGA

              * ** * **            *  * *      *       *    *        *       
     129566 ACTTCCCCTCACATAGAGCAGTTGTGCAGCACTCTATTTGTAGTATCTCGAAGTGGACATTTGGA
         66 ACCTTTCTTTTCATAGAGCAGTTTTGAAACACTCTTTTTGTAGAATCTGGAAGTGGATATTTGGA

             *         ** *    * *   *            **  
     129631 GGGCTTTGTAGCCTATC-TGGAAAAAGGAAATATCTTCCCAT
        131 GCGCTTTG-AGGATTTCGTTGGAAACGGAAATATCTTCATAT

            *  ** *   *     * *            *   *  *   * **        *  * * *   
     129672 GAATGCGAGATAGAAGTAATCTCAGAAACATGTTTATGCTGTATCTACTCAACTAACTGTGCTGA
          1 AAAAACTAGACAGAAGCATTCTCAGAAACATCTTTGTGATGTTTGCACTCAACTCACAGAGTTGA

              *     *  *               *        *  * *       *               
     129737 ACATTTCTATTGATAGAGCAGTTTTGAGACACTCTTCTTTTGGAATCTGCAAGTGGATATTTGGA
         66 ACCTTTCTTTTCATAGAGCAGTTTTGAAACACTCTTTTTGTAGAATCTGGAAGTGGATATTTGGA

            ** *                        *     *      
     129802 TAGATTTGAGGATTTCGTTGGAAACGG-GAT-T-AT-ATAT
        131 GCGCTTTGAGGATTTCGTTGGAAACGGAAATATCTTCATAT

                 *       *               *                 **  *   *         
     129839 AAAAAGTAGACAGCAGCATTCTCAGAAACTTCTTTGTGATGTTTGCATCCAGCTCTCAGAGTTGA
          1 AAAAACTAGACAGAAGCATTCTCAGAAACATCTTTGTGATGTTTGCACTCAACTCACAGAGTTGA

              *  * *          *  *        *        *   **        *  **       
     129904 ACATTCCCTTTCATAGAGTAGGTTTGAAACCCTCTTTTTATAGTGTCTGGAAGCGGGCATTTGGA
         66 ACCTTTCTTTTCATAGAGCAGTTTTGAAACACTCTTTTTGTAGAATCTGGAAGTGGATATTTGGA

                   *   ** ** *  *   *          * *   
     129969 GCGCTTTCAGGCCTATGCTGAAAAAGGAAATATCTACCTAT
        131 GCGCTTTGAGGATTTCGTTGGAAACGGAAATATCTTCATAT

             *                    *    *  **          ** *        *    *     
     130010 AGAAACTAGACAGAAGCATTCTGAGAATCACGTTTGTGATGTGGGTACTCAACTAACAGTGTTGA
          1 AAAAACTAGACAGAAGCATTCTCAGAAACATCTTTGTGATGTTTGCACTCAACTCACAGAGTTGA

            *  *       *   *            *   *                *               
     130075 TCCATTCTTTTGATACAGCAGTTTTGAACCACACTTTTTGTAGAATCTGCAAGTGGATATTTGGA
         66 ACCTTTCTTTTCATAGAGCAGTTTTGAAACACTCTTTTTGTAGAATCTGGAAGTGGATATTTGGA

            **   *                     *   *        *
     130140 TAGCTGTGAGGATTTCGTTGGAAACGGGAATGTCTTCATAG
        131 GCGCTTTGAGGATTTCGTTGGAAACGGAAATATCTTCATAT

                **                     * * **         *  **   *   *          
     130181 AAAATTTAGACAGAAGCATTCTCAGAACCTTGATTGTGATGTGTGTTCTCCACTAACAGAGTTGA
          1 AAAAACTAGACAGAAGCATTCTCAGAAACATCTTTGTGATGTTTGCACTCAACTCACAGAGTTGA

                       * *   * *   *       *       *                         
     130246 ACCTTTCTTTTGACAGAACTGTTCTGAAACATTCTTTTTATAGAATCTGGAAGTGGATATTTGGA
         66 ACCTTTCTTTTCATAGAGCAGTTTTGAAACACTCTTTTTGTAGAATCTGGAAGTGGATATTTGGA

            **                         *          *  
     130311 AAGCTTTGAGGATTTCGTTGGAAACGGGAATATCTTCAAAT
        131 GCGCTTTGAGGATTTCGTTGGAAACGGAAATATCTTCATAT

                *    *            *           * *       *  *    *            
     130352 AAAATCTAGCCAGAAGCATTCTAAGAAACATCTTAGGGATGTTTACATTCAAGTCACAGAGTTGA
          1 AAAAACTAGACAGAAGCATTCTCAGAAACATCTTTGTGATGTTTGCACTCAACTCACAGAGTTGA

              *  * *     *       *         *    * *   **      *      *    *  
     130417 ACATTCCCTTTCACAGAGCAGGTTTGAAACAATCTTCTCGTACTATCTGGCAGTGGACATTTTGA
         66 ACCTTTCTTTTCATAGAGCAGTTTTGAAACACTCTTTTTGTAGAATCTGGAAGTGGATATTTGGA

              * *   *  ** ** *  *   *            ** *
     130482 GCTCCTTGGGGCCTATGCTGAAAAAGGAAATATCTTCCGAC
        131 GCGCTTTGAGGATTTCGTTGGAAACGGAAATATCTTCATAT

                                 *     *  **          *           **    *    
     130523 AAAAACTAGACAGAAGCATTCGCAGAATCACGTTTGTGATGTGTGCACTCAACTGTCAGAATTGA
          1 AAAAACTAGACAGAAGCATTCTCAGAAACATCTTTGTGATGTTTGCACTCAACTCACAGAGTTGA

                 **   ** *      *                            * *            *
     130588 ACCTTGGTTTGGACAGAGCACTTTTGAAACACTCTTTTTGTAGAATCTGCAGGTGGATATTTGGC
         66 ACCTTTCTTTTCATAGAGCAGTTTTGAAACACTCTTTTTGTAGAATCTGGAAGTGGATATTTGGA

            **                         *   *      * *
     130653 TAGCTTTGAGGATTTCGTTGGAAACGGTAATGTCTTCAAAG
        131 GCGCTTTGAGGATTTCGTTGGAAACGGAAATATCTTCATAT

                *                         *   *            *    *            
     130694 AAAATCTAGACAGAAGCATTCTCAGAAACAGCTTCGTGATGTTTGCAATCAAGTCACAGAGTTGA
          1 AAAAACTAGACAGAAGCATTCTCAGAAACATCTTTGTGATGTTTGCACTCAACTCACAGAGTTGA

                 * *             *  *                  *             *       
     130759 ACCTTCCGTTTCATAGAGCAGGTTGGAAACACTCTTTTTGTAGTATCTGGAAGTGGACATTTGGA
         66 ACCTTTCTTTTCATAGAGCAGTTTTGAAACACTCTTTTTGTAGAATCTGGAAGTGGATATTTGGA

             *         ** *    * *   *            **  
     130824 GGGCTTTGTAGCCTATC-TGGAAAAAGGAAATATCTTCCCAT
        131 GCGCTTTG-AGGATTTCGTTGGAAACGGAAATATCTTCATAT

            *  ** *   *     * *            *   *  *   * **        *  * * *   
     130865 GAATGCGAGATAGAAGTAATCTCAGAAACATGTTTATGCTGTATCTACTCAACTAACTGTGCTGA
          1 AAAAACTAGACAGAAGCATTCTCAGAAACATCTTTGTGATGTTTGCACTCAACTCACAGAGTTGA

              *     *  *               *        *  * *       *             * 
     130930 ACATTTCTATTGATAGAGCAGTTTTGAGACACTCTTCTTTTGGAATCTGCAAGTGGATATTTGCA
         66 ACCTTTCTTTTCATAGAGCAGTTTTGAAACACTCTTTTTGTAGAATCTGGAAGTGGATATTTGGA

            ** *            *           *     *      
     130995 TAGATTTGAGGATTTCTTTGGAAACGG-GAT-T-AT-ATAT
        131 GCGCTTTGAGGATTTCGTTGGAAACGGAAATATCTTCATAT

                 *       *               *                 **  *   *         
     131032 AAAAAGTAGACAGCAGCATTCTCAGAAACTTCTTTGTGATGTTTGCATCCAGCTCTCAGAGTTGA
          1 AAAAACTAGACAGAAGCATTCTCAGAAACATCTTTGTGATGTTTGCACTCAACTCACAGAGTTGA

            * *  * *          *  *        *        *   **        *  **       
     131097 GCATTCCCTTTCATAGAGTAGGTTTGAAACCCTCTTTTTATAGTGTCTGGAAGCGGGCATTTGGA
         66 ACCTTTCTTTTCATAGAGCAGTTTTGAAACACTCTTTTTGTAGAATCTGGAAGTGGATATTTGGA

                   *    * * **  **  **          * *  *
     131162 GCGCTTTCAGGCCTATGCTTAAAATAGGAAATATCTACCTAC
        131 GCGCTTTGAGG-ATTTCGTTGGAAACGGAAATATCTTCATAT

             *                    *    *  **          ** *        *    *     
     131204 AGAAACTAGACAGAAGCATTCTGAGAATCACGTTTGTGATGTGGGTACTCAACTAACAGTGTTGA
          1 AAAAACTAGACAGAAGCATTCTCAGAAACATCTTTGTGATGTTTGCACTCAACTCACAGAGTTGA

            *  *       *   *            *   *                *   *           
     131269 TCCATTCTTTTGATACAGCAGTTTTGAACCACACTTTTTGTAGAATCTGCAAGAGGATATTTGGA
         66 ACCTTTCTTTTCATAGAGCAGTTTTGAAACACTCTTTTTGTAGAATCTGGAAGTGGATATTTGGA

            **   *                     *   *      * *
     131334 TAGCTGTGAGGATTTCGTTGGAAACGGGAATGTCTTCAAAG
        131 GCGCTTTGAGGATTTCGTTGGAAACGGAAATATCTTCATAT

                *                         *   *            *    *            
     131375 AAAATCTAGACAGAAGCATTCTCAGAAACACCTTCGTGATGTTTGCAATCAAGTCACAGAGTTGA
          1 AAAAACTAGACAGAAGCATTCTCAGAAACATCTTTGTGATGTTTGCACTCAACTCACAGAGTTGA

                 * *             *  *           *      *             *       
     131440 ACCTTCCGTTTCATAGAGCAGGTTGGAAACACTCTTATTGTAGTATCTGGAAGTGGACATTTGGA
         66 ACCTTTCTTTTCATAGAGCAGTTTTGAAACACTCTTTTTGTAGAATCTGGAAGTGGATATTTGGA

                   *   ** ** *  *   *            **  
     131505 GCGCTTTCAGGCCTATGGTGAAAAAGGAAATATCTTCCCAT
        131 GCGCTTTGAGGATTTCGTTGGAAACGGAAATATCTTCATAT

                  * * *      **      *   * *   *     ** ** *      *    *     
     131546 AAAAACGACATAGAAGCTATCTCAGGAACTTGTTTATGATGCATCTAATCAACTAACAGTGTTGA
          1 AAAAACTAGACAGAAGCATTCTCAGAAACATCTTTGTGATGTTTGCACTCAACTCACAGAGTTGA

                  * ** * *                         * *       *               
     131611 ACCTTTGTACTGACAGAGCAG-TTTGAAACACTCTTTTTTTGGAATCTGCAAGTGGATATTTGGA
         66 ACCTTTCTTTTCATAGAGCAGTTTTGAAACACTCTTTTTGTAGAATCTGGAAGTGGATATTTGGA

            *                           *   *   *    
     131675 TCGCTTTGAGGATTTCGTTGGAAACGG-GAT-GC--AATAT
        131 GCGCTTTGAGGATTTCGTTGGAAACGGAAATATCTTCATAT

                **  *    *     *                 **  *   *  *    *         * 
     131712 AAAACGTACACAGCAGCATACTCAGAAA-ATACTTTGCCATATTTCCATTCAAGTCACAGAGTGG
          1 AAAAACTAGACAGAAGCATTCTCAGAAACAT-CTTTGTGATGTTTGCACTCAACTCACAGAGTTG

               *  * **            *                  *  *             *      
     131776 AACATTCCCATTCATAGAGCAGGTTTGAAACACTCTTTTTGGAGTATCTGGAAGTGGACATTTGG
         65 AACCTTTCTTTTCATAGAGCAGTTTTGAAACACTCTTTTTGTAGAATCTGGAAGTGGATATTTGG

                    ** * * ** *      *            **  
     131841 AGCGCTTTCTGAACTATGGTGGAAAAGGAAATATCTTCCAAT
        130 AGCGCTTTGAGGATTTCGTTGGAAACGGAAATATCTTCATAT

            *     *               *      * *          *  **       *  *  *    
     131883 GAAAACAAGACAGAAGCATTCTGAGAAACTTATTTGTGATGTGTGTCCTCAACTAACGGACTTGA
          1 AAAAACTAGACAGAAGCATTCTCAGAAACATCTTTGTGATGTTTGCACTCAACTCACAGAGTTGA

                   *      **  * *  *  *             *   *    * * *           
     131948 ACCTTTCGTTTCATGCAGTACTTCTGGAACACTCTTTTTGAAGATTCTGCATGCGGATATTTGGA
         66 ACCTTTCTTTTCATAGAGCAGTTTTGAAACACTCTTTTTGTAGAATCTGGAAGTGGATATTTGGA

            **                            **   *   * 
     132013 TAGCTTTGAGGATTTCGTTGGAAACGG---GCT-TACATGT
        131 GCGCTTTGAGGATTTCGTTGGAAACGGAAATATCTTCATAT

                 *       *               *        *   *    *    *       *    
     132050 AAAAATTAGACAGCAGCATTCTCAGAAACTTCTTTGTGGTGTCTGCATTCAAGTCACAGAATTGA
          1 AAAAACTAGACAGAAGCATTCTCAGAAACATCTTTGTGATGTTTGCACTCAACTCACAGAGTTGA

              * ** * **            *  * *      *       *    *        *       
     132115 ACTTCCCCTCACATAGAGCAGTTGTGCAGCACTCTATTTGTAGTATCTCGAAGTGGACATTTGGA
         66 ACCTTTCTTTTCATAGAGCAGTTTTGAAACACTCTTTTTGTAGAATCTGGAAGTGGATATTTGGA

             *         ** *    * *   *            **  
     132180 GGGCTTTGTAGCCTATC-TGGAAAAAGGAAATATCTTCCCAT
        131 GCGCTTTG-AGGATTTCGTTGGAAACGGAAATATCTTCATAT

            *  ** *   *     * *            *   *  *   * **        *  * * *   
     132221 GAATGCGAGATAGAAGTAATCTCAGAAACATGTTTATGCTGTATCTACTCAACTAACTGTGCTGA
          1 AAAAACTAGACAGAAGCATTCTCAGAAACATCTTTGTGATGTTTGCACTCAACTCACAGAGTTGA

              *     *  *               *        *             *              
     132286 ACATTTCTATTGATAGAGCAGTTTTGAGACACTCTTCTT-TAGGAATCTGCAAGTGGATATTTGG
         66 ACCTTTCTTTTCATAGAGCAGTTTTGAAACACTCTTTTTGTA-GAATCTGGAAGTGGATATTTGG

             ** *                        *     *      
     132350 ATAGATTTGAGGATTTCGTTGGAAACGG-GAT-T-AT-ATAT
        130 AGCGCTTTGAGGATTTCGTTGGAAACGGAAATATCTTCATAT

            *    *       *               *                 **  *   *         
     132388 CAAAAGTAGACAGCAGCATTCTCAGAAACTTCTTTGTGATGTTTGCATCCAGCTCTCAGAGTTGA
          1 AAAAACTAGACAGAAGCATTCTCAGAAACATCTTTGTGATGTTTGCACTCAACTCACAGAGTTGA

              *  * *          *  *        *        *   **        *  **       
     132453 ACATTCCCTTTCATAGAGTAGGTTTGAAACCCTCTTTTTATAGTGTCTGGAAGCGGGCATTTGGA
         66 ACCTTTCTTTTCATAGAGCAGTTTTGAAACACTCTTTTTGTAGAATCTGGAAGTGGATATTTGGA

                   *   ** ** *  *   *          * *   
     132518 GCGCTTTCAGGCCTATGCTGAAAAAGGAAATATCTACCTAT
        131 GCGCTTTGAGGATTTCGTTGGAAACGGAAATATCTTCATAT

             *                    *    *  **          ** *        *    *     
     132559 AGAAACTAGACAGAAGCATTCTGAGAATCACGTTTGTGATGTGGGTACTCAACTAACAGTGTTGA
          1 AAAAACTAGACAGAAGCATTCTCAGAAACATCTTTGTGATGTTTGCACTCAACTCACAGAGTTGA

            *  *       *   *            *   *                *               
     132624 TCCATTCTTTTGATACAGCAGTTTTGAACCACACTTTTTGTAGAATCTGCAAGTGGATATTTGGA
         66 ACCTTTCTTTTCATAGAGCAGTTTTGAAACACTCTTTTTGTAGAATCTGGAAGTGGATATTTGGA

            **   *          *          *   *        *
     132689 TAGCTGTGAGGATTTCCTTGGAAACGGGAATGTCTTCATAG
        131 GCGCTTTGAGGATTTCGTTGGAAACGGAAATATCTTCATAT

                **                     * * **         *  **   *   *          
     132730 AAAATTTAGACAGAAGCATTCTCAGAACCTTGATTGTGATGTGTGTTCTCCACTAACAGAGTTGA
          1 AAAAACTAGACAGAAGCATTCTCAGAAACATCTTTGTGATGTTTGCACTCAACTCACAGAGTTGA

                       * *   * *   *       *       *                         
     132795 ACCTTTCTTTTGACAGAACTGTTCTGAAACATTCTTTTTATAGAATCTGGAAGTGGATATTTGGA
         66 ACCTTTCTTTTCATAGAGCAGTTTTGAAACACTCTTTTTGTAGAATCTGGAAGTGGATATTTGGA

            **      *                  *          *  
     132860 AAGCTTTGCGGATTTCGTTGGAAACGGGAATATCTTCAAAT
        131 GCGCTTTGAGGATTTCGTTGGAAACGGAAATATCTTCATAT

                *    *            *           * *       *  *    *            
     132901 AAAATCTAGCCAGAAGCATTCTAAGAAACATCTTAGGGATGTTTACATTCAAGTCACAGAGTTGA
          1 AAAAACTAGACAGAAGCATTCTCAGAAACATCTTTGTGATGTTTGCACTCAACTCACAGAGTTGA

              *  * *     **      *         *    * *   **      *      *    *  
     132966 ACATTCCATTTCACGGAGCAGGTTTGAAACAATCTTCTCGTACTATCTGGCAGTGGACATTTTGA
         66 ACCTTTCTTTTCATAGAGCAGTTTTGAAACACTCTTTTTGTAGAATCTGGAAGTGGATATTTGGA

              *     *  ** ** *  *   *            ** *
     133031 GCTCTTTGGGGCCTATGCTGAAAAAGGAAATATCTTCCGAC
        131 GCGCTTTGAGGATTTCGTTGGAAACGGAAATATCTTCATAT

                                 *     *  **          *           **    *    
     133072 AAAAACTAGACAGAAGCATTCGCAGAATCACGTTTGTGATGTGTGCACTCAACTGTCAGAATTGA
          1 AAAAACTAGACAGAAGCATTCTCAGAAACATCTTTGTGATGTTTGCACTCAACTCACAGAGTTGA

                 **   ** *      *                            * *            *
     133137 ACCTTGGTTTGGAGAGAGCACTTTTGAAACACTCTTTTTGTAGAATCTGCAGGTGGATATTTGGC
         66 ACCTTTCTTTTCATAGAGCAGTTTTGAAACACTCTTTTTGTAGAATCTGGAAGTGGATATTTGGA

            **                         *   *      * *
     133202 TAGCTTTGAGGATTTCGTTGGAAACGGTAATGTCTTCAAAG
        131 GCGCTTTGAGGATTTCGTTGGAAACGGAAATATCTTCATAT

                *                         *   *            *    *            
     133243 AAAATCTAGACAGAAGCATTCTCAGAAACACCTTCGTGATGTTTGCAATCAAGTCACAGAGTTGA
          1 AAAAACTAGACAGAAGCATTCTCAGAAACATCTTTGTGATGTTTGCACTCAACTCACAGAGTTGA

                 * *             *  *                  *             *       
     133308 ACCTTCCGTTTCATAGAGCAGGTTGGAAACACTCTTTTTGTAGTATCTGGAAGTGGACATTTGGA
         66 ACCTTTCTTTTCATAGAGCAGTTTTGAAACACTCTTTTTGTAGAATCTGGAAGTGGATATTTGGA

             *         ** *    * *   *            **  
     133373 GGGCTTTGTAGCCTATC-TGGAAAAAGGAAATATCTTCCCAT
        131 GCGCTTTG-AGGATTTCGTTGGAAACGGAAATATCTTCATAT

            *  ** *   *     * *            *   *  *   * **        *  * * *   
     133414 GAATGCGAGATAGAAGTAATCTCAGAAACATGTTTATGCTGTATCTACTCAACTAACTGTGCTGA
          1 AAAAACTAGACAGAAGCATTCTCAGAAACATCTTTGTGATGTTTGCACTCAACTCACAGAGTTGA

              *     *  *               *        *  * *       *               
     133479 ACATTTCTATTGATAGAGCAGTTTTGAGACACTCTTCTTTTGGAATCTGCAAGTGGATATTTGGA
         66 ACCTTTCTTTTCATAGAGCAGTTTTGAAACACTCTTTTTGTAGAATCTGGAAGTGGATATTTGGA

            ** *                        *     *      
     133544 TAGATTTGAGGATTTCGTTGGAAACGG-GAT-T-AT-ATAT
        131 GCGCTTTGAGGATTTCGTTGGAAACGGAAATATCTTCATAT

                 *       *               *                 **  *   *         
     133581 AAAAAGTAGACAGCAGCATTCTCAGAAACTTCTTTGTGATGTTTGCATCCAGCTCTCAGAGTTGA
          1 AAAAACTAGACAGAAGCATTCTCAGAAACATCTTTGTGATGTTTGCACTCAACTCACAGAGTTGA

              *  * *          *  *        *        *   **        *  **       
     133646 ACATTCCCTTTCATAGAGTAGGTTTGAAACCCTCTTTTTATAGTGTCTGGAAGCGGGCATTTGGA
         66 ACCTTTCTTTTCATAGAGCAGTTTTGAAACACTCTTTTTGTAGAATCTGGAAGTGGATATTTGGA

                   *   ** ** *  *   *          * *   
     133711 GCGCTTTCAGGCCTATGCTGAAAAAGGAAATATCTACCTAT
        131 GCGCTTTGAGGATTTCGTTGGAAACGGAAATATCTTCATAT

             *                    *    *  **          ** *        *    *     
     133752 AGAAACTAGACAGAAGCATTCTGAGAATCACGTTTGTGATGTGGGTACTCAACTAACAGTGTTGA
          1 AAAAACTAGACAGAAGCATTCTCAGAAACATCTTTGTGATGTTTGCACTCAACTCACAGAGTTGA

            *  *       *   *            *   *                *               
     133817 TCCATTCTTTTGATACAGCAGTTTTGAACCACACTTTTTGTAGAATCTGCAAGTGGATATTTGGA
         66 ACCTTTCTTTTCATAGAGCAGTTTTGAAACACTCTTTTTGTAGAATCTGGAAGTGGATATTTGGA

            **   *                     *   *        *
     133882 TAGCTGTGAGGATTTCGTTGGAAACGGGAATGTCTTCATAG
        131 GCGCTTTGAGGATTTCGTTGGAAACGGAAATATCTTCATAT

                **                     * * **         *  **   *   *          
     133923 AAAATTTAGACAGAAGCATTCTCAGAACCTTGATTGTGATGTGTGTTCTCCACTAACAGAGTTGA
          1 AAAAACTAGACAGAAGCATTCTCAGAAACATCTTTGTGATGTTTGCACTCAACTCACAGAGTTGA

                       * *   * *   *       *       *                         
     133988 ACCTTTCTTTTGACAGAACTGTTCTGAAACATTCTTTTTATAGAATCTGGAAGTGGATATTTGGA
         66 ACCTTTCTTTTCATAGAGCAGTTTTGAAACACTCTTTTTGTAGAATCTGGAAGTGGATATTTGGA

            **                         *          *  
     134053 AAGCTTTGAGGATTTCGTTGGAAACGGGAATATCTTCAAAT
        131 GCGCTTTGAGGATTTCGTTGGAAACGGAAATATCTTCATAT

                *    *            *           * *       *  *    *            
     134094 AAAATCTAGCCAGAAGCATTCTAAGAAACATCTTAGGGATGTTTACATTCAAGTCACAGAGTTGA
          1 AAAAACTAGACAGAAGCATTCTCAGAAACATCTTTGTGATGTTTGCACTCAACTCACAGAGTTGA

              *  * *     *       *         *    * *   **      *      *    *  
     134159 ACATTCCCTTTCACAGAGCAGGTTTGAAACAATCTTCTCGTACTATCTGGCAGTGGACATTTTGA
         66 ACCTTTCTTTTCATAGAGCAGTTTTGAAACACTCTTTTTGTAGAATCTGGAAGTGGATATTTGGA

             ** *   *  ** ** *  *   *            ** *
     134224 GTTCCTTGGGGCCTATGCTGAAAAAGGAAATATCTTCCGAC
        131 GCGCTTTGAGGATTTCGTTGGAAACGGAAATATCTTCATAT

                                 *     *  **          *           **    *    
     134265 AAAAACTAGACAGAAGCATTCGCAGAATCACGTTTGTGATGTGTGCACTCAACTGTCAGAATTGA
          1 AAAAACTAGACAGAAGCATTCTCAGAAACATCTTTGTGATGTTTGCACTCAACTCACAGAGTTGA

                 **   ** *      *                            * *            *
     134330 ACCTTGGTTTGGACAGAGCACTTTTGAAACACTCTTTTTGTAGAATCTGCAGGTGGATATTTGGC
         66 ACCTTTCTTTTCATAGAGCAGTTTTGAAACACTCTTTTTGTAGAATCTGGAAGTGGATATTTGGA

            **                         *   *      * *
     134395 TAGCTTTGAGGATTTCGTTGGAAACGGTAATGTCTTCAAAG
        131 GCGCTTTGAGGATTTCGTTGGAAACGGAAATATCTTCATAT

                *                         *   *            *    *            
     134436 AAAATCTAGACAGAAGCATTCTCAGAAACACCTTCGTGATGTTTGCAATCAAGTCACAGAGTTGA
          1 AAAAACTAGACAGAAGCATTCTCAGAAACATCTTTGTGATGTTTGCACTCAACTCACAGAGTTGA

                 * *             *  *                  *             *       
     134501 ACCTTCCGTTTCATAGAGCAGGTTGGAAACACTCTTTTTGTAGTATCTGGAAGTGGACATTTGGA
         66 ACCTTTCTTTTCATAGAGCAGTTTTGAAACACTCTTTTTGTAGAATCTGGAAGTGGATATTTGGA

             *         ** *    * *   *            **  
     134566 GGGCTTTGTAGCCTATC-TGGAAAAAGGAAATATCTTCCCAT
        131 GCGCTTTG-AGGATTTCGTTGGAAACGGAAATATCTTCATAT

            *  ** *   *     * *            *   *  *   * **        *  * * *   
     134607 GAATGCGAGATAGAAGTAATCTCAGAAACATGTTTATGCTGTATCTACTCAACTAACTGTGCTGA
          1 AAAAACTAGACAGAAGCATTCTCAGAAACATCTTTGTGATGTTTGCACTCAACTCACAGAGTTGA

              *     *  *               *        *  * *       *               
     134672 ACATTTCTATTGATAGAGCAGTTTTGAGACACTCTTCTTTTGGAATCTGCAAGTGGATATTTGGA
         66 ACCTTTCTTTTCATAGAGCAGTTTTGAAACACTCTTTTTGTAGAATCTGGAAGTGGATATTTGGA

            ** *                        *     *      
     134737 TAGATTTGAGGATTTCGTTGGAAACGG-GAT-T-AT-ATAT
        131 GCGCTTTGAGGATTTCGTTGGAAACGGAAATATCTTCATAT

                 *       *               *                 **  *   *         
     134774 -AAAAGTAGACAGCAGCATTCTCAGAAACTTCTTTGTGATGTTTGCATCCAGCTCTCAGAGTTGA
          1 AAAAACTAGACAGAAGCATTCTCAGAAACATCTTTGTGATGTTTGCACTCAACTCACAGAGTTGA

            * *  * *          *  *        *        *   **        *  **       
     134838 GCATTCCCTTTCATAGAGTAGGTTTGAAACCCTCTTTTTATAGTGTCTGGAAGCGGGCATTTGGA
         66 ACCTTTCTTTTCATAGAGCAGTTTTGAAACACTCTTTTTGTAGAATCTGGAAGTGGATATTTGGA

                   *    * * **  **  **          * *  *
     134903 GCGCTTTCAGGCCTATGCTTAAAATAGGAAATATCTACCTAC
        131 GCGCTTTGAGG-ATTTCGTTGGAAACGGAAATATCTTCATAT

             *                    *    *  **          ** *        *    *     
     134945 AGAAACTAGACAGAAGCATTCTGAGAATCACGTTTGTGATGTGGGTACTCAACTAACAGTGTTGA
          1 AAAAACTAGACAGAAGCATTCTCAGAAACATCTTTGTGATGTTTGCACTCAACTCACAGAGTTGA

            *  *       *   *            *   *                *   *           
     135010 TCCATTCTTTTGATACAGCAGTTTTGAACCACACTTTTTGTAGAATCTGCAAGAGGATATTTGGA
         66 ACCTTTCTTTTCATAGAGCAGTTTTGAAACACTCTTTTTGTAGAATCTGGAAGTGGATATTTGGA

            **   *                     *   *      * *
     135075 TAGCTGTGAGGATTTCGTTGGAAACGGGAATGTCTTCAAAG
        131 GCGCTTTGAGGATTTCGTTGGAAACGGAAATATCTTCATAT

                *                         *   *            *    *            
     135116 AAAATCTAGACAGAAGCATTCTCAGAAACACCTTCGTGATGTTTGCAATCAAGTCACAGAGTTGA
          1 AAAAACTAGACAGAAGCATTCTCAGAAACATCTTTGTGATGTTTGCACTCAACTCACAGAGTTGA

                 * *             *  *           *      *             *       
     135181 ACCTTCCGTTTCATAGAGCAGGTTGGAAACACTCTTATTGTAGTATCTGGAAGTGGACATTTGGA
         66 ACCTTTCTTTTCATAGAGCAGTTTTGAAACACTCTTTTTGTAGAATCTGGAAGTGGATATTTGGA

                   *   ** ** *  *   *            **  
     135246 GCGCTTTCAGGCCTATGGTGAAAAAGGAAATATCTTCCCAT
        131 GCGCTTTGAGGATTTCGTTGGAAACGGAAATATCTTCATAT

                  * * *      **      *   * *   *     ** ** *      *    *     
     135287 AAAAACGACATAGAAGCTATCTCAGGAACTTGTTTATGATGCATCTAATCAACTAACAGTGTTGA
          1 AAAAACTAGACAGAAGCATTCTCAGAAACATCTTTGTGATGTTTGCACTCAACTCACAGAGTTGA

                  * ** * *                           *       *               
     135352 ACCTTTGTACTGACAGAGCAG-TTTGAAACACTCTTTTT-TGGAATCTGCAAGTGGATATTTGGA
         66 ACCTTTCTTTTCATAGAGCAGTTTTGAAACACTCTTTTTGTAGAATCTGGAAGTGGATATTTGGA

            *                           *   *   *    
     135415 TCGCTTTGAGGATTTCGTTGGAAACGG-GAT-GC--AATAT
        131 GCGCTTTGAGGATTTCGTTGGAAACGGAAATATCTTCATAT

                **  *    *     *                 **  *   *  *    *         * 
     135452 AAAACGTACACAGCAGCATACTCAGAAA-ATACTTTGCCATATTTCCATTCAAGTCACAGAGTGG
          1 AAAAACTAGACAGAAGCATTCTCAGAAACAT-CTTTGTGATGTTTGCACTCAACTCACAGAGTTG

               *  * **            *  *               *  *             *      
     135516 AACATTCCCATTCATAGAGCAGGTTGGAAACACTCTTTTTGGAGTATCTGGAAGTGGACATTTGG
         65 AACCTTTCTTTTCATAGAGCAGTTTTGAAACACTCTTTTTGTAGAATCTGGAAGTGGATATTTGG

                    ** * * ** *      *            **  
     135581 AGCGCTTTCTGAACTATGGTGGAAAAGGAAATATCTTCCAAT
        130 AGCGCTTTGAGGATTTCGTTGGAAACGGAAATATCTTCATAT

            *     *               *      * *          *  **      **  *  *    
     135623 GAAAACAAGACAGAAGCATTCTGAGAAACTTATTTGTGATGTGTGTCCTCAACAAACGGACTTGA
          1 AAAAACTAGACAGAAGCATTCTCAGAAACATCTTTGTGATGTTTGCACTCAACTCACAGAGTTGA

                   *      **  * *  *  *             *   *    * * *           
     135688 ACCTTTCGTTTCATGCAGTACTTCTGGAACACTCTTTTTGAAGATTCTGCATGCGGATATTTGGA
         66 ACCTTTCTTTTCATAGAGCAGTTTTGAAACACTCTTTTTGTAGAATCTGGAAGTGGATATTTGGA

            **                            **   *   * 
     135753 TAGCTTTGAGGATTTCGTTGGAAACGG---GCT-TACATGT
        131 GCGCTTTGAGGATTTCGTTGGAAACGGAAATATCTTCATAT

                 *       *               *        *   *    *    *       *    
     135790 AAAAATTAGACAGCAGCATTCTCAGAAACTTCTTTGTGGTGTCTGCATTCAAGTCACAGAATTGA
          1 AAAAACTAGACAGAAGCATTCTCAGAAACATCTTTGTGATGTTTGCACTCAACTCACAGAGTTGA

              * ** * **            *  * *      *       *    *        *       
     135855 ACTTCCCCTCACATAGAGCAGTTGTGCAGCACTCTATTTGTAGTATCTCGAAGTGGACATTTGGA
         66 ACCTTTCTTTTCATAGAGCAGTTTTGAAACACTCTTTTTGTAGAATCTGGAAGTGGATATTTGGA

             *         ** *    * *   *            **  
     135920 GGGCTTTGTAGCCTATC-TGGAAAAAGGAAATATCTTCCCAT
        131 GCGCTTTG-AGGATTTCGTTGGAAACGGAAATATCTTCATAT

            *  ** *   *     * *            *   *  *   * **        *  * * *   
     135961 GAATGCGAGATAGAAGTAATCTCAGAAACATGTTTATGCTGTATCTACTCAACTAACTGTGCTGA
          1 AAAAACTAGACAGAAGCATTCTCAGAAACATCTTTGTGATGTTTGCACTCAACTCACAGAGTTGA

              *     *  *               *        *             *              
     136026 ACATTTCTATTGATAGAGCAGTTTTGAGACACTCTTCTT-TAGGAATCTGCAAGTGGATATTTGG
         66 ACCTTTCTTTTCATAGAGCAGTTTTGAAACACTCTTTTTGTA-GAATCTGGAAGTGGATATTTGG

             ** *                        *     *      
     136090 ATAGATTTGAGGATTTCGTTGGAAACGG-GAT-T-AT-ATAT
        130 AGCGCTTTGAGGATTTCGTTGGAAACGGAAATATCTTCATAT

            *    *       *               *                 **  *   *        *
     136128 CAAAAGTAGACAGCAGCATTCTCAGAAACTTCTTTGTGATGTTTGCATCCAGCTCTCAGAGTTGG
          1 AAAAACTAGACAGAAGCATTCTCAGAAACATCTTTGTGATGTTTGCACTCAACTCACAGAGTTGA

              *  * *          *  *        *        *   **        *  **       
     136193 ACATTCCCTTTCATAGAGTAGGTTTGAAACCCTCTTTTTATAGTGTCTGGAAGCGGGCATTTGGA
         66 ACCTTTCTTTTCATAGAGCAGTTTTGAAACACTCTTTTTGTAGAATCTGGAAGTGGATATTTGGA

                   *   ** ** *  *   *          * *   
     136258 GCGCTTTCAGGCCTATGCTGAAAAAGGAAATATCTACCTAT
        131 GCGCTTTGAGGATTTCGTTGGAAACGGAAATATCTTCATAT

             *                    *    *  **          ** *        *    *     
     136299 AGAAACTAGACAGAAGCATTCTGAGAATCACGTTTGTGATGTGGGTACTCAACTAACAGTGTTGA
          1 AAAAACTAGACAGAAGCATTCTCAGAAACATCTTTGTGATGTTTGCACTCAACTCACAGAGTTGA

            *  *       *   *            *   *                *               
     136364 TCCATTCTTTTGATACAGCAGTTTTGAACCACACTTTTTGTAGAATCTGCAAGTGGATATTTGGA
         66 ACCTTTCTTTTCATAGAGCAGTTTTGAAACACTCTTTTTGTAGAATCTGGAAGTGGATATTTGGA

            **   *                     *   *        *
     136429 TAGCTGTGAGGATTTCGTTGGAAACGGGAATGTCTTCATAG
        131 GCGCTTTGAGGATTTCGTTGGAAACGGAAATATCTTCATAT

                **                     *   **         *  **   *   *          
     136470 AAAATTTAGACAGAAGCATTCTCAGAACCATGATTGTGATGTGTGTTCTCCACTAACAGAGTTGA
          1 AAAAACTAGACAGAAGCATTCTCAGAAACATCTTTGTGATGTTTGCACTCAACTCACAGAGTTGA

                       * *   * *   *       *       *                         
     136535 ACCTTTCTTTTGACAGAACTGTTCTGAAACATTCTTTTTATAGAATCTGGAAGTGGATATTTGGA
         66 ACCTTTCTTTTCATAGAGCAGTTTTGAAACACTCTTTTTGTAGAATCTGGAAGTGGATATTTGGA

            **      *                  *          *  
     136600 AAGCTTTGCGGATTTCGTTGGAAACGGGAATATCTTCAAAT
        131 GCGCTTTGAGGATTTCGTTGGAAACGGAAATATCTTCATAT

                *    *            *           * *       *  *    *            
     136641 AAAATCTAGCCAGAAGCATTCTAAGAAACATCTTAGGGATGTTTACATTCAAGTCACAGAGTTGA
          1 AAAAACTAGACAGAAGCATTCTCAGAAACATCTTTGTGATGTTTGCACTCAACTCACAGAGTTGA

              *  * *     **      *         *    * *   **      *      *    *  
     136706 ACATTCCCTTTCACGGAGCAGGTTTGAAACAATCTTCTCGTACTATCTGGCAGTGGACATTTTGA
         66 ACCTTTCTTTTCATAGAGCAGTTTTGAAACACTCTTTTTGTAGAATCTGGAAGTGGATATTTGGA

              *     *  ** ** *  *   *            ** *
     136771 GCTCTTTGGGGCCTATGCTGAAAAAGGAAATATCTTCCGAC
        131 GCGCTTTGAGGATTTCGTTGGAAACGGAAATATCTTCATAT

                                 *     *  **          *           **    *    
     136812 AAAAACTAGACAGAAGCATTCGCAGAATCACGTTTGTGATGTGTGCACTCAACTGTCAGAATTGA
          1 AAAAACTAGACAGAAGCATTCTCAGAAACATCTTTGTGATGTTTGCACTCAACTCACAGAGTTGA

                 **   ** *      *                            * *            *
     136877 ACCTTGGTTTGGAGAGAGCACTTTTGAAACACTCTTTTTGTAGAATCTGCAGGTGGATATTTGGC
         66 ACCTTTCTTTTCATAGAGCAGTTTTGAAACACTCTTTTTGTAGAATCTGGAAGTGGATATTTGGA

            **                         *   *      * *
     136942 TAGCTTTGAGGATTTCGTTGGAAACGGTAATGTCTTCAAAG
        131 GCGCTTTGAGGATTTCGTTGGAAACGGAAATATCTTCATAT

                *                         *   *            *    *            
     136983 AAAATCTAGACAGAAGCATTCTCAGAAACACCTTCGTGATGTTTGCAATCAAGTCACAGAGTTGA
          1 AAAAACTAGACAGAAGCATTCTCAGAAACATCTTTGTGATGTTTGCACTCAACTCACAGAGTTGA

                 * *             *  *                  *             *       
     137048 ACCTTCCGTTTCATAGAGCAGGTTGGAAACACTCTTTTTGTAGTATCTGGAAGTGGACATTTGGA
         66 ACCTTTCTTTTCATAGAGCAGTTTTGAAACACTCTTTTTGTAGAATCTGGAAGTGGATATTTGGA

             *         ** *    * *   *            **  
     137113 GGGCTTTGTAGCCTATC-TGGAAAAAGGAAATATCTTCCCAT
        131 GCGCTTTG-AGGATTTCGTTGGAAACGGAAATATCTTCATAT

            *  ** *   *     * *            *   *  *   * **        *  * * *   
     137154 GAATGCGAGATAGAAGTAATCTCAGAAACATGTTTATGCTGTATCTACTCAACTAACTGTGCTGA
          1 AAAAACTAGACAGAAGCATTCTCAGAAACATCTTTGTGATGTTTGCACTCAACTCACAGAGTTGA

              *     *  *               *        *  * *       *               
     137219 ACATTTCTATTGATAGAGCAGTTTTGAGACACTCTTCTTTTGGAATCTGCAAGTGGATATTTGGA
         66 ACCTTTCTTTTCATAGAGCAGTTTTGAAACACTCTTTTTGTAGAATCTGGAAGTGGATATTTGGA

            ** *                        *     *      
     137284 TAGATTTGAGGATTTCGTTGGAAACGG-GAT-T-AT-ATAT
        131 GCGCTTTGAGGATTTCGTTGGAAACGGAAATATCTTCATAT

            *    *       *               *                 **  *   *         
     137321 CAAAAGTAGACAGCAGCATTCTCAGAAACTTCTTTGTGATGTTTGCATCCAGCTCTCAGAGTTGA
          1 AAAAACTAGACAGAAGCATTCTCAGAAACATCTTTGTGATGTTTGCACTCAACTCACAGAGTTGA

              *  * *          *  *        *        *   **        *  **       
     137386 ACATTCCCTTTCATAGAGTAGGTTTGAAACCCTCTTTTTATAGTGTCTGGAAGCGGGCATTTGGA
         66 ACCTTTCTTTTCATAGAGCAGTTTTGAAACACTCTTTTTGTAGAATCTGGAAGTGGATATTTGGA

                   *   ** ** *  *   *          * *   
     137451 GCGCTTTCAGGCCTATGCTGAAAAAGGAAATATCTACCTAT
        131 GCGCTTTGAGGATTTCGTTGGAAACGGAAATATCTTCATAT

             *                    *    *  **          ** *        *    *     
     137492 AGAAACTAGACAGAAGCATTCTGAGAATCACGTTTGTGATGTGGGTACTCAACTAACAGTGTTGA
          1 AAAAACTAGACAGAAGCATTCTCAGAAACATCTTTGTGATGTTTGCACTCAACTCACAGAGTTGA

            *  *       *   *            *   *                *               
     137557 TCCATTCTTTTGATACAGCAGTTTTGAACCACACTTTTTGTAGAATCTGCAAGTGGATATTTGGA
         66 ACCTTTCTTTTCATAGAGCAGTTTTGAAACACTCTTTTTGTAGAATCTGGAAGTGGATATTTGGA

            **   *                     *   *        *
     137622 TAGCTGTGAGGATTTCGTTGGAAACGGGAATGTCTTCATAG
        131 GCGCTTTGAGGATTTCGTTGGAAACGGAAATATCTTCATAT

                **                     * * **         *  **   *   *          
     137663 AAAATTTAGACAGAAGCATTCTCAGAACCTTGATTGTGATGTGTGTTCTCCACTAACAGAGTTGA
          1 AAAAACTAGACAGAAGCATTCTCAGAAACATCTTTGTGATGTTTGCACTCAACTCACAGAGTTGA

                       * *   * *   *       *       *                         
     137728 ACCTTTCTTTTGACAGAACTGTTCTGAAACATTCTTTTTATAGAATCTGGAAGTGGATATTTGGA
         66 ACCTTTCTTTTCATAGAGCAGTTTTGAAACACTCTTTTTGTAGAATCTGGAAGTGGATATTTGGA

            **                         *          *  
     137793 AAGCTTTGAGGATTTCGTTGGAAACGGGAATATCTTCAAAT
        131 GCGCTTTGAGGATTTCGTTGGAAACGGAAATATCTTCATAT

                *    *            *           * *       *  *    *            
     137834 AAAATCTAGCCAGAAGCATTCTAAGAAACATCTTAGGGATGTTTACATTCAAGTCACAGAGTTGA
          1 AAAAACTAGACAGAAGCATTCTCAGAAACATCTTTGTGATGTTTGCACTCAACTCACAGAGTTGA

              *  * *     *       *         *    * *   **      *      *    *  
     137899 ACATTCCCTTTCACAGAGCAGGTTTGAAACAATCTTCTCGTACTATCTGGCAGTGGACATTTTGA
         66 ACCTTTCTTTTCATAGAGCAGTTTTGAAACACTCTTTTTGTAGAATCTGGAAGTGGATATTTGGA

              * *   *  ** ** *  *   *            ** *
     137964 GCTCCTTGGGGCCTATGCTGAAAAAGGAAATATCTTCCGAC
        131 GCGCTTTGAGGATTTCGTTGGAAACGGAAATATCTTCATAT

                                 *     *  **          *           **    *    
     138005 AAAAACTAGACAGAAGCATTCGCAGAATCACGTTTGTGATGTGTGCACTCAACTGTCAGAATTGA
          1 AAAAACTAGACAGAAGCATTCTCAGAAACATCTTTGTGATGTTTGCACTCAACTCACAGAGTTGA

                 **   ** *      *                            * *            *
     138070 ACCTTGGTTTGGACAGAGCACTTTTGAAACACTCTTTTTGTAGAATCTGCAGGTGGATATTTGGC
         66 ACCTTTCTTTTCATAGAGCAGTTTTGAAACACTCTTTTTGTAGAATCTGGAAGTGGATATTTGGA

            **                         *   *      * *
     138135 TAGCTTTGAGGATTTCGTTGGAAACGGTAATGTCTTCAAAG
        131 GCGCTTTGAGGATTTCGTTGGAAACGGAAATATCTTCATAT

                *                         *   *            *    *            
     138176 AAAATCTAGACAGAAGCATTCTCAGAAACAGCTTCGTGATGTTTGCAATCAAGTCACAGAGTTGA
          1 AAAAACTAGACAGAAGCATTCTCAGAAACATCTTTGTGATGTTTGCACTCAACTCACAGAGTTGA

                 * *             *  *                  *             *       
     138241 ACCTTCCGTTTCATAGAGCAGGTTGGAAACACTCTTTTTGTAGTATCTGGAAGTGGACATTTGGA
         66 ACCTTTCTTTTCATAGAGCAGTTTTGAAACACTCTTTTTGTAGAATCTGGAAGTGGATATTTGGA

             *         ** *    * *   *            **  
     138306 GGGCTTTGTAGCCTATC-TGGAAAAAGGAAATATCTTCCCAT
        131 GCGCTTTG-AGGATTTCGTTGGAAACGGAAATATCTTCATAT

            *  ** *   *     * *            *   *  *   * **        *  * * *   
     138347 GAATGCGAGATAGAAGTAATCTCAGAAACATGTTTATGCTGTATCTACTCAACTAACTGTGCTGA
          1 AAAAACTAGACAGAAGCATTCTCAGAAACATCTTTGTGATGTTTGCACTCAACTCACAGAGTTGA

              *     *  *               *        *  * *       *             * 
     138412 ACATTTCTATTGATAGAGCAGTTTTGAGACACTCTTCTTTTGGAATCTGCAAGTGGATATTTGCA
         66 ACCTTTCTTTTCATAGAGCAGTTTTGAAACACTCTTTTTGTAGAATCTGGAAGTGGATATTTGGA

            ** *            *           *     *      
     138477 TAGATTTGAGGATTTCTTTGGAAACGG-GAT-T-AT-ATAT
        131 GCGCTTTGAGGATTTCGTTGGAAACGGAAATATCTTCATAT

                 *       *               *                 **  *   *         
     138514 AAAAAGTAGACAGCAGCATTCTCAGAAACTTCTTTGTGATGTTTGCATCCAGCTCTCAGAGTTGA
          1 AAAAACTAGACAGAAGCATTCTCAGAAACATCTTTGTGATGTTTGCACTCAACTCACAGAGTTGA

            * *  * *          *  *        *        *   **        *  **       
     138579 GCATTCCCTTTCATAGAGTAGGTTTGAAACCCTCTTTTTATAGTGTCTGGAAGCGGGCATTTGGA
         66 ACCTTTCTTTTCATAGAGCAGTTTTGAAACACTCTTTTTGTAGAATCTGGAAGTGGATATTTGGA

                   *    * * **  **  **          * *  *
     138644 GCGCTTTCAGGCCTATGCTTAAAATAGGAAATATCTACCTAC
        131 GCGCTTTGAGG-ATTTCGTTGGAAACGGAAATATCTTCATAT

             *                    *    *  **          ** *        *    *     
     138686 AGAAACTAGACAGAAGCATTCTGAGAATCACGTTTGTGATGTGGGTACTCAACTAACAGTGTTGA
          1 AAAAACTAGACAGAAGCATTCTCAGAAACATCTTTGTGATGTTTGCACTCAACTCACAGAGTTGA

            *  *       *   *            *   *                *   *           
     138751 TCCATTCTTTTGATACAGCAGTTTTGAACCACACTTTTTGTAGAATCTGCAAGAGGATATTTGGA
         66 ACCTTTCTTTTCATAGAGCAGTTTTGAAACACTCTTTTTGTAGAATCTGGAAGTGGATATTTGGA

            **   *                     *   *      * *
     138816 TAGCTGTGAGGATTTCGTTGGAAACGGGAATGTCTTCAAAG
        131 GCGCTTTGAGGATTTCGTTGGAAACGGAAATATCTTCATAT

                *                         *   *            *    *            
     138857 AAAATCTAGACAGAAGCATTCTCAGAAACACCTTCGTGATGTTTGCAATCAAGTCACAGAGTTGA
          1 AAAAACTAGACAGAAGCATTCTCAGAAACATCTTTGTGATGTTTGCACTCAACTCACAGAGTTGA

                 * *             *  *           *      *             *       
     138922 ACCTTCCGTTTCATAGAGCAGGTTGGAAACACTCTTATTGTAGTATCTGGAAGTGGACATTTGGA
         66 ACCTTTCTTTTCATAGAGCAGTTTTGAAACACTCTTTTTGTAGAATCTGGAAGTGGATATTTGGA

                   *   ** ** *  *   *            **  
     138987 GCGCTTTCAGGCCTATGGTGAAAAAGGAAATATCTTCCCAT
        131 GCGCTTTGAGGATTTCGTTGGAAACGGAAATATCTTCATAT

                  * * *      **      *   * *   *     ** ** *      *    *     
     139028 AAAAACGACATAGAAGCTATCTCAGGAACTTGTTTATGATGCATCTAATCAACTAACAGTGTTGA
          1 AAAAACTAGACAGAAGCATTCTCAGAAACATCTTTGTGATGTTTGCACTCAACTCACAGAGTTGA

                  * ** * *                         * *       *               
     139093 ACCTTTGTACTGACAGAGCAG-TTTGAAACACTCTTTTTTTGGAATCTGCAAGTGGATATTTGGA
         66 ACCTTTCTTTTCATAGAGCAGTTTTGAAACACTCTTTTTGTAGAATCTGGAAGTGGATATTTGGA

            *                           *   *   *    
     139157 TCGCTTTGAGGATTTCGTTGGAAACGG-GAT-GC--AATAT
        131 GCGCTTTGAGGATTTCGTTGGAAACGGAAATATCTTCATAT

                **  *    *     *                 **  *   *  *    *         * 
     139194 AAAACGTACACAGCAGCATACTCAGAAA-ATACTTTGCCATATTTCCATTCAAGTCACAGAGTGG
          1 AAAAACTAGACAGAAGCATTCTCAGAAACAT-CTTTGTGATGTTTGCACTCAACTCACAGAGTTG

               *  * **            *                  *  *             *      
     139258 AACATTCCCATTCATAGAGCAGGTTTGAAACACTCTTTTTGGAGTATCTGGAAGTGGACATTTGG
         65 AACCTTTCTTTTCATAGAGCAGTTTTGAAACACTCTTTTTGTAGAATCTGGAAGTGGATATTTGG

                    ** * * ** *  *   *            **  
     139323 AGCGCTTTCTGAACTATGGTGAAAAAGGAAATATCTTCCAAT
        130 AGCGCTTTGAGGATTTCGTTGGAAACGGAAATATCTTCATAT

            *     * *             *      * *          *  **      **  *  *    
     139365 GAAAACAACACAGAAGCATTCTGAGAAACTTATTTGTGATGTGTGTCCTCAACAAACGGACTTGA
          1 AAAAACTAGACAGAAGCATTCTCAGAAACATCTTTGTGATGTTTGCACTCAACTCACAGAGTTGA

                   *      **  * *  *  *             *   *    * * *           
     139430 ACCTTTCGTTTCATGCAGTACTTCTGGAACACTCTTTTTGAAGATTCTGCATGCGGATATTTGGA
         66 ACCTTTCTTTTCATAGAGCAGTTTTGAAACACTCTTTTTGTAGAATCTGGAAGTGGATATTTGGA

            **                            **   *   * 
     139495 TAGCTTTGAGGATTTCGTTGGAAACGG---GCT-TACATGT
        131 GCGCTTTGAGGATTTCGTTGGAAACGGAAATATCTTCATAT

                 *       *               *   *    *   *    *    *       *    
     139532 AAAAATTAGACAGCAGCATTCTCAGAAACTTCTGTGTGGTGTCTGCATTCAAGTCACAGAATTGA
          1 AAAAACTAGACAGAAGCATTCTCAGAAACATCTTTGTGATGTTTGCACTCAACTCACAGAGTTGA

              * ** * **            *  * *      *       *    *        *       
     139597 ACTTCCCCTCACATAGAGCAGTTGTGCAGCACTCTATTTGTAGTATCTCGAAGTGGACATTTGGA
         66 ACCTTTCTTTTCATAGAGCAGTTTTGAAACACTCTTTTTGTAGAATCTGGAAGTGGATATTTGGA

             *         ** *    * *   *            **  
     139662 GGGCTTTGTAGCCTATC-TGGAAAAAGGAAATATCTTCCCAT
        131 GCGCTTTG-AGGATTTCGTTGGAAACGGAAATATCTTCATAT

            *  ** *   *     * *            *   *  *   * **        *  * * *   
     139703 GAATGCGAGATAGAAGTAATCTCAGAAACATGTTTATGCTGTATCTACTCAACTAACTGTGCTGA
          1 AAAAACTAGACAGAAGCATTCTCAGAAACATCTTTGTGATGTTTGCACTCAACTCACAGAGTTGA

              *     *  *               *        *  * *       *               
     139768 ACATTTCTATTGATAGAGCAGTTTTGAGACACTCTTCTTTTGGAATCTGCAAGTGGATATTTGGA
         66 ACCTTTCTTTTCATAGAGCAGTTTTGAAACACTCTTTTTGTAGAATCTGGAAGTGGATATTTGGA

            ** *                        *     *      
     139833 TAGATTTGAGGATTTCGTTGGAAACGG-GAT-T-AT-ATAT
        131 GCGCTTTGAGGATTTCGTTGGAAACGGAAATATCTTCATAT

                 *       *               *                 **  *   *         
     139870 AAAAAGTAGACAGCAGCATTCTCAGAAACTTCTTTGTGATGTTTGCATCCAGCTCTCAGAGTTGA
          1 AAAAACTAGACAGAAGCATTCTCAGAAACATCTTTGTGATGTTTGCACTCAACTCACAGAGTTGA

              *  * *          *  *        *        *   **        *  **       
     139935 ACATTCCCTTTCATAGAGTAGGTTTGAAACCCTCTTTTTATAGTGTCTGGAAGCGGGCATTTGGA
         66 ACCTTTCTTTTCATAGAGCAGTTTTGAAACACTCTTTTTGTAGAATCTGGAAGTGGATATTTGGA

                   *   ** ** *  *   *          * *   
     140000 GCGCTTTCAGGCCTATGCTGAAAAAGGAAATATCTACCTAT
        131 GCGCTTTGAGGATTTCGTTGGAAACGGAAATATCTTCATAT

             *                    *    *  **          ** *        *    *     
     140041 AGAAACTAGACAGAAGCATTCTGAGAATCACGTTTGTGATGTGGGTACTCAACTAACAGTGTTGA
          1 AAAAACTAGACAGAAGCATTCTCAGAAACATCTTTGTGATGTTTGCACTCAACTCACAGAGTTGA

            *  *       *   *            *   *                *               
     140106 TCCATTCTTTTGATACAGCAGTTTTGAACCACACTTTTTGTAGAATCTGCAAGTGGATATTTGGA
         66 ACCTTTCTTTTCATAGAGCAGTTTTGAAACACTCTTTTTGTAGAATCTGGAAGTGGATATTTGGA

            **   *                     *   *        *
     140171 TAGCTGTGAGGATTTCGTTGGAAACGGGAATGTCTTCATAG
        131 GCGCTTTGAGGATTTCGTTGGAAACGGAAATATCTTCATAT

                **                     * * **         *  **   *   *          
     140212 AAAATTTAGACAGAAGCATTCTCAGAACCTTGATTGTGATGTGTGTTCTCCACTAACAGAGTTGA
          1 AAAAACTAGACAGAAGCATTCTCAGAAACATCTTTGTGATGTTTGCACTCAACTCACAGAGTTGA

                       * *   * *   *       *       *                         
     140277 ACCTTTCTTTTGACAGAACTGTTCTGAAACATTCTTTTTATAGAATCTGGAAGTGGATATTTGGA
         66 ACCTTTCTTTTCATAGAGCAGTTTTGAAACACTCTTTTTGTAGAATCTGGAAGTGGATATTTGGA

            **                         *          *  
     140342 AAGCTTTGAGGATTTCGTTGGAAACGGGAATATCTTCAAAT
        131 GCGCTTTGAGGATTTCGTTGGAAACGGAAATATCTTCATAT

                *    *            *           * *       *  *    *            
     140383 AAAATCTAGCCAGAAGCATTCTAAGAAACATCTTAGGGATGTTTACATTCAAGTCACAGAGTTGA
          1 AAAAACTAGACAGAAGCATTCTCAGAAACATCTTTGTGATGTTTGCACTCAACTCACAGAGTTGA

              *  * *     *       *         *    * *   **      *      *    *  
     140448 ACATTCCCTTTCACAGAGCAGGTTTGAAACAATCTTCTCGTACTATCTGGCAGTGGACATTTTGA
         66 ACCTTTCTTTTCATAGAGCAGTTTTGAAACACTCTTTTTGTAGAATCTGGAAGTGGATATTTGGA

              * *   *  ** ** *  *   *            ** *
     140513 GCTCCTTGGGGCCTATGCTGAAAAAGGAAATATCTTCCGAC
        131 GCGCTTTGAGGATTTCGTTGGAAACGGAAATATCTTCATAT

                                 *     *  **          *           **    *    
     140554 AAAAACTAGACAGAAGCATTCGCAGAATCACGTTTGTGATGTGTGCACTCAACTGTCAGAATTGA
          1 AAAAACTAGACAGAAGCATTCTCAGAAACATCTTTGTGATGTTTGCACTCAACTCACAGAGTTGA

                  *   ** *      *                            * *            *
     140619 ACCTTTGTTTGGACAGAGCACTTTTGAAACACTCTTTTTGTAGAATCTGCAGGTGGATATTTGGC
         66 ACCTTTCTTTTCATAGAGCAGTTTTGAAACACTCTTTTTGTAGAATCTGGAAGTGGATATTTGGA

            **                         *   *      * *
     140684 TAGCTTTGAGGATTTCGTTGGAAACGGTAATGTCTTCAAAG
        131 GCGCTTTGAGGATTTCGTTGGAAACGGAAATATCTTCATAT

                *                         * * *            *    *            
     140725 AAAATCTAGACAGAAGCATTCTCAGAAACAGCGTCGTGATGTTTGCAATCAAGTCACAGAGTTGA
          1 AAAAACTAGACAGAAGCATTCTCAGAAACATCTTTGTGATGTTTGCACTCAACTCACAGAGTTGA

                 * *             *  *                  *             *       
     140790 ACCTTCCGTTTCATAGAGCAGGTTGGAAACACTCTTTTTGTAGTATCTGGAAGTGGACATTTGGA
         66 ACCTTTCTTTTCATAGAGCAGTTTTGAAACACTCTTTTTGTAGAATCTGGAAGTGGATATTTGGA

             *         ** *    * *   *            **  
     140855 GGGCTTTGTAGCCTATC-TGGAAAAAGGAAATATCTTCCCAT
        131 GCGCTTTG-AGGATTTCGTTGGAAACGGAAATATCTTCATAT

            *  ** *   *     * *            *   *  *   * **        *  * * *   
     140896 GAATGCGAGATAGAAGTAATCTCAGAAACATGTTTATGCTGTATCTACTCAACTAACTGTGCTGA
          1 AAAAACTAGACAGAAGCATTCTCAGAAACATCTTTGTGATGTTTGCACTCAACTCACAGAGTTGA

              *     *  *               *        *  * *       *               
     140961 ACATTTCTATTGATAGAGCAGTTTTGAGACACTCTTCTTTTGGAATCTGCAAGTGGATATTTGGA
         66 ACCTTTCTTTTCATAGAGCAGTTTTGAAACACTCTTTTTGTAGAATCTGGAAGTGGATATTTGGA

            ** *                        *     *      
     141026 TAGATTTGAGGATTTCGTTGGAAACGG-GAT-T-AT-ATAT
        131 GCGCTTTGAGGATTTCGTTGGAAACGGAAATATCTTCATAT

                 *       *               *                 **  *   *         
     141063 AAAAAGTAGACAGCAGCATTCTCAGAAACTTCTTTGTGATGTTTGCATCCAGCTCTCAGAGTTGA
          1 AAAAACTAGACAGAAGCATTCTCAGAAACATCTTTGTGATGTTTGCACTCAACTCACAGAGTTGA

            * *  * *          *  *        *        *   **        *  **       
     141128 GCATTCCCTTTCATAGAGTAGGTTTGAAACCCTCTTTTTATAGTGTCTGGAAGCGGGCATTTGGA
         66 ACCTTTCTTTTCATAGAGCAGTTTTGAAACACTCTTTTTGTAGAATCTGGAAGTGGATATTTGGA

                   *    * * **  **  **          * *  *
     141193 GCGCTTTCAGGCCTATGCTTAAAATAGGAAATATCTACCTAC
        131 GCGCTTTGAGG-ATTTCGTTGGAAACGGAAATATCTTCATAT

             *                    *    *  **          ** *        *    *     
     141235 AGAAACTAGACAGAAGCATTCTGAGAATCACGTTTGTGATGTGGGTACTCAACTAACAGTGTTGA
          1 AAAAACTAGACAGAAGCATTCTCAGAAACATCTTTGTGATGTTTGCACTCAACTCACAGAGTTGA

            *  *       *   *            *   *                *   *           
     141300 TCCATTCTTTTGATACAGCAGTTTTGAACCACACTTTTTGTAGAATCTGCAAGAGGATATTTGGA
         66 ACCTTTCTTTTCATAGAGCAGTTTTGAAACACTCTTTTTGTAGAATCTGGAAGTGGATATTTGGA

            **   *                     *   *      * *
     141365 TAGCTGTGAGGATTTCGTTGGAAACGGGAATGTCTTCAAAG
        131 GCGCTTTGAGGATTTCGTTGGAAACGGAAATATCTTCATAT

                *                         *   *            *    *            
     141406 AAAATCTAGACAGAAGCATTCTCAGAAACACCTTCGTGATGTTTGCAATCAAGTCACAGAGTTGA
          1 AAAAACTAGACAGAAGCATTCTCAGAAACATCTTTGTGATGTTTGCACTCAACTCACAGAGTTGA

                 * *             *  *           *      *             *       
     141471 ACCTTCCGTTTCATAGAGCAGGTTGGAAACACTCTTATTGTAGTATCTGGAAGTGGACATTTGGA
         66 ACCTTTCTTTTCATAGAGCAGTTTTGAAACACTCTTTTTGTAGAATCTGGAAGTGGATATTTGGA

                   *   ** ** *  *   *            **  
     141536 GCGCTTTCAGGCCTATGGTGAAAAAGGAAATATCTTCCCAT
        131 GCGCTTTGAGGATTTCGTTGGAAACGGAAATATCTTCATAT

                  * * *      **      *   * *   *     ** ** *      *    *     
     141577 AAAAACGACATAGAAGCTATCTCAGGAACTTGTTTATGATGCATCTAATCAACTAACAGTGTTGA
          1 AAAAACTAGACAGAAGCATTCTCAGAAACATCTTTGTGATGTTTGCACTCAACTCACAGAGTTGA

                  * ** * *                           *       *               
     141642 ACCTTTGTACTGACAGAGCAG-TTTGAAACACTCTTTTT-TGGAATCTGCAAGTGGATATTTGGA
         66 ACCTTTCTTTTCATAGAGCAGTTTTGAAACACTCTTTTTGTAGAATCTGGAAGTGGATATTTGGA

            *                           *   *   *    
     141705 TCGCTTTGAGGATTTCGTTGGAAACGG-GAT-GC--AATAT
        131 GCGCTTTGAGGATTTCGTTGGAAACGGAAATATCTTCATAT

                **  *    *     *                 **  *   *  *    *         * 
     141742 AAAACGTACACAGCAGCATACTCAGAAA-ATACTTTGCCATATTTCCATTCAAGTCACAGAGTGG
          1 AAAAACTAGACAGAAGCATTCTCAGAAACAT-CTTTGTGATGTTTGCACTCAACTCACAGAGTTG

               *  * **            *  *       *       *  *             *      
     141806 AACATTCCCATTCATAGAGCAGGTTGGAAACACACTTTTTGGAGTATCTGGAAGTGGACATTTGG
         65 AACCTTTCTTTTCATAGAGCAGTTTTGAAACACTCTTTTTGTAGAATCTGGAAGTGGATATTTGG

                    ** * * ** *      *            **  
     141871 AGCGCTTTCTGAACTATGGTGGAAAAGGAAATATCTTCCAAT
        130 AGCGCTTTGAGGATTTCGTTGGAAACGGAAATATCTTCATAT

            *     *               *      * *          *  **       *  *  *    
     141913 GAAAACAAGACAGAAGCATTCTGAGAAACTTATTTGTGATGTGTGTCCTCAACTAACGGACTTGA
          1 AAAAACTAGACAGAAGCATTCTCAGAAACATCTTTGTGATGTTTGCACTCAACTCACAGAGTTGA

                   *      **  * *  *  *             *   *    * * *           
     141978 ACCTTTCGTTTCATGCAGTACTTCTGGAACACTCTTTTTGAAGATTCTGCATGCGGATATTTGGA
         66 ACCTTTCTTTTCATAGAGCAGTTTTGAAACACTCTTTTTGTAGAATCTGGAAGTGGATATTTGGA

            **                            **   *   * 
     142043 TAGCTTTGAGGATTTCGTTGGAAACGG---GCT-TACATGT
        131 GCGCTTTGAGGATTTCGTTGGAAACGGAAATATCTTCATAT

                 *       *               *        *   *    *    *       *    
     142080 AAAAATTAGACAGCAGCATTCTCAGAAACTTCTTTGTGGTGTCTGCATTCAAGTCACAGAATTGA
          1 AAAAACTAGACAGAAGCATTCTCAGAAACATCTTTGTGATGTTTGCACTCAACTCACAGAGTTGA

              * ** * **            *  * *   *  *       *    *        *       
     142145 ACTTCCCCTCACATAGAGCAGTTGTGCAGCACACTATTTGTAGTATCTCGAAGTGGACATTTGGA
         66 ACCTTTCTTTTCATAGAGCAGTTTTGAAACACTCTTTTTGTAGAATCTGGAAGTGGATATTTGGA

             *         ** *    * *   *            **  
     142210 GGGCTTTGTAGCCTATC-TGGAAAAAGGAAATATCTTCCCAT
        131 GCGCTTTG-AGGATTTCGTTGGAAACGGAAATATCTTCATAT

            *  ** *   *     * *            *   *  *   * **        *  * * *   
     142251 GAATGCGAGATAGAAGTAATCTCAGAAACATGTTTATGCTGTATCTACTCAACTAACTGTGCTGA
          1 AAAAACTAGACAGAAGCATTCTCAGAAACATCTTTGTGATGTTTGCACTCAACTCACAGAGTTGA

              *     *  *               *        *             *              
     142316 ACATTTCTATTGATAGAGCAGTTTTGAGACACTCTTCTT-TAGGAATCTGCAAGTGGATATTTGG
         66 ACCTTTCTTTTCATAGAGCAGTTTTGAAACACTCTTTTTGTA-GAATCTGGAAGTGGATATTTGG

             ** *                        *     *      
     142380 ATAGATTTGAGGATTTCGTTGGAAACGG-GAT-T-AT-ATAT
        130 AGCGCTTTGAGGATTTCGTTGGAAACGGAAATATCTTCATAT

            *    *       *               *                 **  *   *         
     142418 CAAAAGTAGACAGCAGCATTCTCAGAAACTTCTTTGTGATGTTTGCATCCAGCTCTCAGAGTTGA
          1 AAAAACTAGACAGAAGCATTCTCAGAAACATCTTTGTGATGTTTGCACTCAACTCACAGAGTTGA

              *  * *          *  *        *        *   **        *  **       
     142483 ACATTCCCTTTCATAGAGTAGGTTTGAAACCCTCTTTTTATAGTGTCTGGAAGCGGGCATTTGGA
         66 ACCTTTCTTTTCATAGAGCAGTTTTGAAACACTCTTTTTGTAGAATCTGGAAGTGGATATTTGGA

                   *   ** ** *  *   *          * *   
     142548 GCGCTTTCAGGCCTATGCTGAAAAAGGAAATATCTACCTAT
        131 GCGCTTTGAGGATTTCGTTGGAAACGGAAATATCTTCATAT

             *                    *    *  **          ** *        *    *     
     142589 AGAAACTAGACAGAAGCATTCTGAGAATCACGTTTGTGATGTGGGTACTCAACTAACAGTGTTGA
          1 AAAAACTAGACAGAAGCATTCTCAGAAACATCTTTGTGATGTTTGCACTCAACTCACAGAGTTGA

            *  *       *   *            *   *                *               
     142654 TCCATTCTTTTGATACAGCAGTTTTGAACCACACTTTTTGTAGAATCTGCAAGTGGATATTTGGA
         66 ACCTTTCTTTTCATAGAGCAGTTTTGAAACACTCTTTTTGTAGAATCTGGAAGTGGATATTTGGA

            **   *          *          *   *        *
     142719 TAGCTGTGAGGATTTCCTTGGAAACGGGAATGTCTTCATAG
        131 GCGCTTTGAGGATTTCGTTGGAAACGGAAATATCTTCATAT

                **                     * * **         *  **   *   *          
     142760 AAAATTTAGACAGAAGCATTCTCAGAACCTTGATTGTGATGTGTGTTCTCCACTAACAGAGTTGA
          1 AAAAACTAGACAGAAGCATTCTCAGAAACATCTTTGTGATGTTTGCACTCAACTCACAGAGTTGA

                       * *   * *   *       *       *                         
     142825 ACCTTTCTTTTGACAGAACTGTTCTGAAACATTCTTTTTATAGAATCTGGAAGTGGATATTTGGA
         66 ACCTTTCTTTTCATAGAGCAGTTTTGAAACACTCTTTTTGTAGAATCTGGAAGTGGATATTTGGA

            **      *                  *          *  
     142890 AAGCTTTGCGGATTTCGTTGGAAACGGGAATATCTTCAAAT
        131 GCGCTTTGAGGATTTCGTTGGAAACGGAAATATCTTCATAT

            *   *    *            *           * *       *  *    *            
     142931 CAAATCTAGCCAGAAGCATTCTAAGAAACATCTTAGGGATGTTTACATTCAAGTCACAGAGTTGA
          1 AAAAACTAGACAGAAGCATTCTCAGAAACATCTTTGTGATGTTTGCACTCAACTCACAGAGTTGA

              *  * *     **      *         *    * *   **      *      *    *  
     142996 ACATTCCATTTCACGGAGCAGGTTTGAAACAATCTTCTCGTACTATCTGGCAGTGGACATTTTGA
         66 ACCTTTCTTTTCATAGAGCAGTTTTGAAACACTCTTTTTGTAGAATCTGGAAGTGGATATTTGGA

              *     *  ** ** *  *   *            ** *
     143061 GCTCTTTGGGGCCTATGCTGAAAAAGGAAATATCTTCCGAC
        131 GCGCTTTGAGGATTTCGTTGGAAACGGAAATATCTTCATAT

                                 *     *  **          *           **    *    
     143102 AAAAACTAGACAGAAGCATTCGCAGAATCACGTTTGTGATGTGTGCACTCAACTGTCAGAATTGA
          1 AAAAACTAGACAGAAGCATTCTCAGAAACATCTTTGTGATGTTTGCACTCAACTCACAGAGTTGA

                 **   ** *      *                            * *            *
     143167 ACCTTGGTTTGGAGAGAGCACTTTTGAAACACTCTTTTTGTAGAATCTGCAGGTGGATATTTGGC
         66 ACCTTTCTTTTCATAGAGCAGTTTTGAAACACTCTTTTTGTAGAATCTGGAAGTGGATATTTGGA

            **                         *   *      * *
     143232 TAGCTTTGAGGATTTCGTTGGAAACGGTAATGTCTTCAAAG
        131 GCGCTTTGAGGATTTCGTTGGAAACGGAAATATCTTCATAT

                *                         *   *            *    *            
     143273 AAAATCTAGACAGAAGCATTCTCAGAAACACCTTCGTGATGTTTGCAATCAAGTCACAGAGTTGA
          1 AAAAACTAGACAGAAGCATTCTCAGAAACATCTTTGTGATGTTTGCACTCAACTCACAGAGTTGA

                 * *             *  *                  *             *       
     143338 ACCTTCCGTTTCATAGAGCAGGTTGGAAACACTCTTTTTGTAGTATCTGGAAGTGGACATTTGGA
         66 ACCTTTCTTTTCATAGAGCAGTTTTGAAACACTCTTTTTGTAGAATCTGGAAGTGGATATTTGGA

             *         ** *    * *   *            **  
     143403 GGGCTTTGTAGCCTATC-TGGAAAAAGGAAATATCTTCCCAT
        131 GCGCTTTG-AGGATTTCGTTGGAAACGGAAATATCTTCATAT

            *  ** *   *     * *            *   *  *   * **        *  * * *   
     143444 GAATGCGAGATAGAAGTAATCTCAGAAACATGTTTATGCTGTATCTACTCAACTAACTGTGCTGA
          1 AAAAACTAGACAGAAGCATTCTCAGAAACATCTTTGTGATGTTTGCACTCAACTCACAGAGTTGA

              *     *  *               *        *  * *       *               
     143509 ACATTTCTATTGATAGAGCAGTTTTGAGACACTCTTCTTTTGGAATCTGCAAGTGGATATTTGGA
         66 ACCTTTCTTTTCATAGAGCAGTTTTGAAACACTCTTTTTGTAGAATCTGGAAGTGGATATTTGGA

            ** *                        *     *      
     143574 TAGATTTGAGGATTTCGTTGGAAACGG-GAT-T-AT-ATAT
        131 GCGCTTTGAGGATTTCGTTGGAAACGGAAATATCTTCATAT

                 *       *               *                 **  *   *         
     143611 AAAAAGTAGACAGCAGCATTCTCAGAAACTTCTTTGTGATGTTTGCATCCAGCTCTCAGAGTTGA
          1 AAAAACTAGACAGAAGCATTCTCAGAAACATCTTTGTGATGTTTGCACTCAACTCACAGAGTTGA

              *  * *          *  *        *        *   **        *  **       
     143676 ACATTCCCTTTCATAGAGTAGGTTTGAAACCCTCTTTTTATAGTGTCTGGAAGCGGGCATTTGGA
         66 ACCTTTCTTTTCATAGAGCAGTTTTGAAACACTCTTTTTGTAGAATCTGGAAGTGGATATTTGGA

                   *   ** ** *  *   *          * *   
     143741 GCGCTTTCAGGCCTATGCTGAAAAAGGAAATATCTACCTAT
        131 GCGCTTTGAGGATTTCGTTGGAAACGGAAATATCTTCATAT

             *                    *    *  **          ** *        *    *     
     143782 AGAAACTAGACAGAAGCATTCTGAGAATCACGTTTGTGATGTGGGTACTCAACTAACAGTGTTGA
          1 AAAAACTAGACAGAAGCATTCTCAGAAACATCTTTGTGATGTTTGCACTCAACTCACAGAGTTGA

            *  *       *   *            *   *                *               
     143847 TCCATTCTTTTGATACAGCAGTTTTGAACCACACTTTTTGTAGAATCTGCAAGTGGATATTTGGA
         66 ACCTTTCTTTTCATAGAGCAGTTTTGAAACACTCTTTTTGTAGAATCTGGAAGTGGATATTTGGA

            **   *                     *   *        *
     143912 TAGCTGTGAGGATTTCGTTGGAAACGGGAATGTCTTCATAG
        131 GCGCTTTGAGGATTTCGTTGGAAACGGAAATATCTTCATAT

                **                     * * **         *  **   *   *          
     143953 AAAATTTAGACAGAAGCATTCTCAGAACCTTGATTGTGATGTGTGTTCTCCACTAACAGAGTTGA
          1 AAAAACTAGACAGAAGCATTCTCAGAAACATCTTTGTGATGTTTGCACTCAACTCACAGAGTTGA

                       * *   * *   *       *       *                         
     144018 ACCTTTCTTTTGACAGAACTGTTCTGAAACATTCTTTTTATAGAATCTGGAAGTGGATATTTGGA
         66 ACCTTTCTTTTCATAGAGCAGTTTTGAAACACTCTTTTTGTAGAATCTGGAAGTGGATATTTGGA

            **                         *          *  
     144083 AAGCTTTGAGGATTTCGTTGGAAACGGGAATATCTTCAAAT
        131 GCGCTTTGAGGATTTCGTTGGAAACGGAAATATCTTCATAT

                *    *            *           * *       *  *    *            
     144124 AAAATCTAGCCAGAAGCATTCTAAGAAACATCTTAGGGATGTTTACATTCAAGTCACAGAGTTGA
          1 AAAAACTAGACAGAAGCATTCTCAGAAACATCTTTGTGATGTTTGCACTCAACTCACAGAGTTGA

              *  * *     *       *       * *    * *   **      *      *    *  
     144189 ACATTCCCTTTCACAGAGCAGGTTTGAAAAAATCTTCTCGTACTATCTGGCAGTGGACATTTTGA
         66 ACCTTTCTTTTCATAGAGCAGTTTTGAAACACTCTTTTTGTAGAATCTGGAAGTGGATATTTGGA

             ** *   *  ** ** *  *   *            ** *
     144254 GTTCCTTGGGGCCTATGCTGAAAAAGGAAATATCTTCCGAC
        131 GCGCTTTGAGGATTTCGTTGGAAACGGAAATATCTTCATAT

                                 *     *  **          *           **    *    
     144295 AAAAACTAGACAGAAGCATTCGCAGAATCACGTTTGTGATGTGTGCACTCAACTGTCAGAATTGA
          1 AAAAACTAGACAGAAGCATTCTCAGAAACATCTTTGTGATGTTTGCACTCAACTCACAGAGTTGA

                 **   ** *      *                            * *            *
     144360 ACCTTGGTTTGGACAGAGCACTTTTGAAACACTCTTTTTGTAGAATCTGCAGGTGGATATTTGGC
         66 ACCTTTCTTTTCATAGAGCAGTTTTGAAACACTCTTTTTGTAGAATCTGGAAGTGGATATTTGGA

            **                         *   *      * *
     144425 TAGCTTTGAGGATTTCGTTGGAAACGGTAATGTCTTCAAAG
        131 GCGCTTTGAGGATTTCGTTGGAAACGGAAATATCTTCATAT

                *                         * * *            *    *            
     144466 AAAATCTAGACAGAAGCATTCTCAGAAACAGCGTCGTGATGTTTGCAATCAAGTCACAGAGTTGA
          1 AAAAACTAGACAGAAGCATTCTCAGAAACATCTTTGTGATGTTTGCACTCAACTCACAGAGTTGA

                 * *             *  *                  *             *       
     144531 ACCTTCCGTTTCATAGAGCAGGTTGGAAACACTCTTTTTGTAGTATCTGGAAGTGGACATTTGGA
         66 ACCTTTCTTTTCATAGAGCAGTTTTGAAACACTCTTTTTGTAGAATCTGGAAGTGGATATTTGGA

             *         ** *    * *   *            **  
     144596 GGGCTTTGTAGCCTATC-TGGAAAAAGGAAATATCTTCCCAT
        131 GCGCTTTG-AGGATTTCGTTGGAAACGGAAATATCTTCATAT

            *  ** *   *     * *            *   *  *   * **        *  * * *   
     144637 GAATGCGAGATAGAAGTAATCTCAGAAACATGTTTATGCTGTATCTACTCAACTAACTGTGCTGA
          1 AAAAACTAGACAGAAGCATTCTCAGAAACATCTTTGTGATGTTTGCACTCAACTCACAGAGTTGA

              *     *  *               *        *  * *       *               
     144702 ACATTTCTATTGATAGAGCAGTTTTGAGACACTCTTCTTTTGGAATCTGCAAGTGGATATTTGGA
         66 ACCTTTCTTTTCATAGAGCAGTTTTGAAACACTCTTTTTGTAGAATCTGGAAGTGGATATTTGGA

            ** *                        *     *      
     144767 TAGATTTGAGGATTTCGTTGGAAACGG-GAT-T-AT-ATAT
        131 GCGCTTTGAGGATTTCGTTGGAAACGGAAATATCTTCATAT

                 *       *               *                 **  *   *         
     144804 AAAAAGTAGACAGCAGCATTCTCAGAAACTTCTTTGTGATGTTTGCATCCAGCTCTCAGAGTTGA
          1 AAAAACTAGACAGAAGCATTCTCAGAAACATCTTTGTGATGTTTGCACTCAACTCACAGAGTTGA

            * *  * *          *  *        *        *   **        *  **       
     144869 GCATTCCCTTTCATAGAGTAGGTTTGAAACCCTCTTTTTATAGTGTCTGGAAGCGGGCATTTGGA
         66 ACCTTTCTTTTCATAGAGCAGTTTTGAAACACTCTTTTTGTAGAATCTGGAAGTGGATATTTGGA

                   *    * * **  **  **          * *  *
     144934 GCGCTTTCAGGCCTATGCTTAAAATAGGAAATATCTACCTAC
        131 GCGCTTTGAGG-ATTTCGTTGGAAACGGAAATATCTTCATAT

             *                    *    *  **          ** *        *    *     
     144976 AGAAACTAGACAGAAGCATTCTGAGAATCACGTTTGTGATGTGGGTACTCAACTAACAGTGTTGA
          1 AAAAACTAGACAGAAGCATTCTCAGAAACATCTTTGTGATGTTTGCACTCAACTCACAGAGTTGA

            *  *       *   *            *   *                *   *           
     145041 TCCATTCTTTTGATACAGCAGTTTTGAACCACACTTTTTGTAGAATCTGCAAGAGGATATTTGGA
         66 ACCTTTCTTTTCATAGAGCAGTTTTGAAACACTCTTTTTGTAGAATCTGGAAGTGGATATTTGGA

            **   *                     *   *      * *
     145106 TAGCTGTGAGGATTTCGTTGGAAACGGGAATGTCTTCAAAG
        131 GCGCTTTGAGGATTTCGTTGGAAACGGAAATATCTTCATAT

                *                         *   *            *    *            
     145147 AAAATCTAGACAGAAGCATTCTCAGAAACACCTTCGTGATGTTTGCAATCAAGTCACAGAGTTGA
          1 AAAAACTAGACAGAAGCATTCTCAGAAACATCTTTGTGATGTTTGCACTCAACTCACAGAGTTGA

                 * *             *  *           *      *             *       
     145212 ACCTTCCGTTTCATAGAGCAGGTTGGAAACACTCTTATTGTAGTATCTGGAAGTGGACATTTGGA
         66 ACCTTTCTTTTCATAGAGCAGTTTTGAAACACTCTTTTTGTAGAATCTGGAAGTGGATATTTGGA

                   *   ** ** *  *   *            **  
     145277 GCGCTTTCAGGCCTATGGTGAAAAAGGAAATATCTTCCCAT
        131 GCGCTTTGAGGATTTCGTTGGAAACGGAAATATCTTCATAT

                  * * *      **      *   * *   *     ** ** *      *    *     
     145318 AAAAACGACATAGAAGCTATCTCAGGAACTTGTTTATGATGCATCTAATCAACTAACAGTGTTGA
          1 AAAAACTAGACAGAAGCATTCTCAGAAACATCTTTGTGATGTTTGCACTCAACTCACAGAGTTGA

                  * ** * *                           *       *               
     145383 ACCTTTGTACTGACAGAGCAG-TTTGAAACACTCTTTTT-TGGAATCTGCAAGTGGATATTTGGA
         66 ACCTTTCTTTTCATAGAGCAGTTTTGAAACACTCTTTTTGTAGAATCTGGAAGTGGATATTTGGA

            *                           *   *   *    
     145446 TCGCTTTGAGGATTTCGTTGGAAACGG-GAT-GC--AATAT
        131 GCGCTTTGAGGATTTCGTTGGAAACGGAAATATCTTCATAT

                **  *    *     *                 **  *   *  *    *         * 
     145483 AAAACGTACACAGCAGCATACTCAGAAA-ATACTTTGCCATATTTCCATTCAAGTCACAGAGTGG
          1 AAAAACTAGACAGAAGCATTCTCAGAAACAT-CTTTGTGATGTTTGCACTCAACTCACAGAGTTG

               *  * **            *  *               *  *             *      
     145547 AACATTCCCATTCATAGAGCAGGTTGGAAACACTCTTTTTGGAGTATCTGGAAGTGGACATTTGG
         65 AACCTTTCTTTTCATAGAGCAGTTTTGAAACACTCTTTTTGTAGAATCTGGAAGTGGATATTTGG

                    ** * * ** *      *            **  
     145612 AGCGCTTTCTGAACTATGGTGGAAAAGGAAATATCTTCCAAT
        130 AGCGCTTTGAGGATTTCGTTGGAAACGGAAATATCTTCATAT

            *     *               *      * *          *  **      **  *  *    
     145654 GAAAACAAGACAGAAGCATTCTGAGAAACTTATTTGTGATGTGTGTCCTCAACAAACGGACTTGA
          1 AAAAACTAGACAGAAGCATTCTCAGAAACATCTTTGTGATGTTTGCACTCAACTCACAGAGTTGA

                   *      **  * *  *  *             *   *    * * *           
     145719 ACCTTTCGTTTCATGCAGTACTTCTGGAACACTCTTTTTGAAGATTCTGCATGCGGATATTTGGA
         66 ACCTTTCTTTTCATAGAGCAGTTTTGAAACACTCTTTTTGTAGAATCTGGAAGTGGATATTTGGA

            **                            **   *   * 
     145784 TAGCTTTGAGGATTTCGTTGGAAACGG---GCT-TACATGT
        131 GCGCTTTGAGGATTTCGTTGGAAACGGAAATATCTTCATAT

                 *       *               *        *   *    *    *       *    
     145821 AAAAATTAGACAGCAGCATTCTCAGAAACTTCTTTGTGGTGTCTGCATTCAAGTCACAGAATTGA
          1 AAAAACTAGACAGAAGCATTCTCAGAAACATCTTTGTGATGTTTGCACTCAACTCACAGAGTTGA

              * ** * **            *  * *      *       *    *        *       
     145886 ACTTCCCCTCACATAGAGCAGTTGTGCAGCACTCTATTTGTAGTATCTCGAAGTGGACATTTGGA
         66 ACCTTTCTTTTCATAGAGCAGTTTTGAAACACTCTTTTTGTAGAATCTGGAAGTGGATATTTGGA

             *         ** *    * *   *            **  
     145951 GGGCTTTGTAGCCTATC-TGGAAAAAGGAAATATCTTCCCAT
        131 GCGCTTTG-AGGATTTCGTTGGAAACGGAAATATCTTCATAT

            *  ** *   *     * *            *   *  *   * **        ** * * *   
     145992 GAATGCGAGATAGAAGTAATCTCAGAAACATGTTTATGCTGTATCTACTCAACTACCTGTGCTGA
          1 AAAAACTAGACAGAAGCATTCTCAGAAACATCTTTGTGATGTTTGCACTCAACTCACAGAGTTGA

              *     *  *               *        *             *              
     146057 ACATTTCTATTGATAGAGCAGTTTTGAGACACTCTTCTT-TAGGAATCTGCAAGTGGATATTTGG
         66 ACCTTTCTTTTCATAGAGCAGTTTTGAAACACTCTTTTTGTA-GAATCTGGAAGTGGATATTTGG

             ** *                        *     *      
     146121 ATAGATTTGAGGATTTCGTTGGAAACGG-GAT-T-AT-ATAT
        130 AGCGCTTTGAGGATTTCGTTGGAAACGGAAATATCTTCATAT

                 *       *               *                 **  *   *        *
     146159 AAAAAGTAGACAGCAGCATTCTCAGAAACTTCTTTGTGATGTTTGCATCCAGCTCTCAGAGTTGG
          1 AAAAACTAGACAGAAGCATTCTCAGAAACATCTTTGTGATGTTTGCACTCAACTCACAGAGTTGA

              *  * *          *  *        *        *   **        *  **       
     146224 ACATTCCCTTTCATAGAGTAGGTTTGAAACCCTCTTTTTATAGTGTCTGGAAGCGGGCATTTGGA
         66 ACCTTTCTTTTCATAGAGCAGTTTTGAAACACTCTTTTTGTAGAATCTGGAAGTGGATATTTGGA

                   *   ** ** *  *   *          * *   
     146289 GCGCTTTCAGGCCTATGCTGAAAAAGGAAATATCTACCTAT
        131 GCGCTTTGAGGATTTCGTTGGAAACGGAAATATCTTCATAT

             *                    *    *  **          ** *        *    *     
     146330 AGAAACTAGACAGAAGCATTCTGAGAATCACGTTTGTGATGTGGGTACTCAACTAACAGTGTTGA
          1 AAAAACTAGACAGAAGCATTCTCAGAAACATCTTTGTGATGTTTGCACTCAACTCACAGAGTTGA

            *  *       *   *            *   *                *               
     146395 TCCATTCTTTTGATACAGCAGTTTTGAACCACACTTTTTGTAGAATCTGCAAGTGGATATTTGGA
         66 ACCTTTCTTTTCATAGAGCAGTTTTGAAACACTCTTTTTGTAGAATCTGGAAGTGGATATTTGGA

            **   *                     *   *        *
     146460 TAGCTGTGAGGATTTCGTTGGAAACGGGAATGTCTTCATAG
        131 GCGCTTTGAGGATTTCGTTGGAAACGGAAATATCTTCATAT

                **                     * * **         *  **   *   *          
     146501 AAAATTTAGACAGAAGCATTCTCAGAACCTTGATTGTGATGTGTGTTCTCCACTAACAGAGTTGA
          1 AAAAACTAGACAGAAGCATTCTCAGAAACATCTTTGTGATGTTTGCACTCAACTCACAGAGTTGA

                       * *   * *   *       *       *                         
     146566 ACCTTTCTTTTGACAGAACTGTTCTGAAACATTCTTTTTATAGAATCTGGAAGTGGATATTTGGA
         66 ACCTTTCTTTTCATAGAGCAGTTTTGAAACACTCTTTTTGTAGAATCTGGAAGTGGATATTTGGA

            **      *                  *          *  
     146631 AAGCTTTGCGGATTTCGTTGGAAACGGGAATATCTTCAAAT
        131 GCGCTTTGAGGATTTCGTTGGAAACGGAAATATCTTCATAT

                *    *            *           * *       *  *    *            
     146672 AAAATCTAGCCAGAAGCATTCTAAGAAACATCTTAGGGATGTTTACATTCAAGTCACAGAGTTGA
          1 AAAAACTAGACAGAAGCATTCTCAGAAACATCTTTGTGATGTTTGCACTCAACTCACAGAGTTGA

              *  * *     **      *         *    * *   **      *      *    *  
     146737 ACATTCCCTTTCACGGAGCAGGTTTGAAACAATCTTCTCGTACTATCTGGCAGTGGACATTTTGA
         66 ACCTTTCTTTTCATAGAGCAGTTTTGAAACACTCTTTTTGTAGAATCTGGAAGTGGATATTTGGA

              *     *  ** ** *  *   *            ** *
     146802 GCTCTTTGGGGCCTATGCTGAAAAAGGAAATATCTTCCGAC
        131 GCGCTTTGAGGATTTCGTTGGAAACGGAAATATCTTCATAT

                                 *     *  **          *           **    *    
     146843 AAAAACTAGACAGAAGCATTCGCAGAATCACGTTTGTGATGTGTGCACTCAACTGTCAGAATTGA
          1 AAAAACTAGACAGAAGCATTCTCAGAAACATCTTTGTGATGTTTGCACTCAACTCACAGAGTTGA

                 **   ** *      *                            * *            *
     146908 ACCTTGGTTTGGAGAGAGCACTTTTGAAACACTCTTTTTGTAGAATCTGCAGGTGGATATTTGGC
         66 ACCTTTCTTTTCATAGAGCAGTTTTGAAACACTCTTTTTGTAGAATCTGGAAGTGGATATTTGGA

            **                         *   *      * *
     146973 TAGCTTTGAGGATTTCGTTGGAAACGGTAATGTCTTCAAAG
        131 GCGCTTTGAGGATTTCGTTGGAAACGGAAATATCTTCATAT

                *                         *   *            *    *            
     147014 AAAATCTAGACAGAAGCATTCTCAGAAACACCTTCGTGATGTTTGCAATCAAGTCACAGAGTTGA
          1 AAAAACTAGACAGAAGCATTCTCAGAAACATCTTTGTGATGTTTGCACTCAACTCACAGAGTTGA

                 * *             *  *                  *             *       
     147079 ACCTTCCGTTTCATAGAGCAGGTTGGAAACACTCTTTTTGTAGTATCTGGAAGTGGACATTTGGA
         66 ACCTTTCTTTTCATAGAGCAGTTTTGAAACACTCTTTTTGTAGAATCTGGAAGTGGATATTTGGA

             *         ** *    * *   *            **  
     147144 GGGCTTTGTAGCCTATC-TGGAAAAAGGAAATATCTTCCCAT
        131 GCGCTTTG-AGGATTTCGTTGGAAACGGAAATATCTTCATAT

            *  ** *   *     * *            *   *  *   * **        *  * * *   
     147185 GAATGCGAGATAGAAGTAATCTCAGAAACATGTTTATGCTGTATCTACTCAACTAACTGTGCTGA
          1 AAAAACTAGACAGAAGCATTCTCAGAAACATCTTTGTGATGTTTGCACTCAACTCACAGAGTTGA

              *     *  *               *        *  * *       *               
     147250 ACATTTCTATTGATAGAGCAGTTTTGAGACACTCTTCTTTTGGAATCTGCAAGTGGATATTTGGA
         66 ACCTTTCTTTTCATAGAGCAGTTTTGAAACACTCTTTTTGTAGAATCTGGAAGTGGATATTTGGA

            ** *                        *     *      
     147315 TAGATTTGAGGATTTCGTTGGAAACGG-GAT-T-AT-ATAT
        131 GCGCTTTGAGGATTTCGTTGGAAACGGAAATATCTTCATAT

                 *       *               *                 **  *   *         
     147352 AAAAAGTAGACAGCAGCATTCTCAGAAACTTCTTTGTGATGTTTGCATCCAGCTCTCAGAGTTGA
          1 AAAAACTAGACAGAAGCATTCTCAGAAACATCTTTGTGATGTTTGCACTCAACTCACAGAGTTGA

              *  * *          *  *        *        *   **        *  **       
     147417 ACATTCCCTTTCATAGAGTAGGTTTGAAACCCTCTTTTTATAGTGTCTGGAAGCGGGCATTTGGA
         66 ACCTTTCTTTTCATAGAGCAGTTTTGAAACACTCTTTTTGTAGAATCTGGAAGTGGATATTTGGA

                   *   ** ** *  *   *          * *   
     147482 GCGCTTTCAGGCCTATGCTGAAAAAGGAAATATCTACCTAT
        131 GCGCTTTGAGGATTTCGTTGGAAACGGAAATATCTTCATAT

             *                    *    *  **          ** *        *    *     
     147523 AGAAACTAGACAGAAGCATTCTGAGAATCACGTTTGTGATGTGGGTACTCAACTAACAGTGTTGA
          1 AAAAACTAGACAGAAGCATTCTCAGAAACATCTTTGTGATGTTTGCACTCAACTCACAGAGTTGA

            *  *       *   *            *   *                *               
     147588 TCCATTCTTTTGATACAGCAGTTTTGAACCACACTTTTTGTAGAATCTGCAAGTGGATATTTGGA
         66 ACCTTTCTTTTCATAGAGCAGTTTTGAAACACTCTTTTTGTAGAATCTGGAAGTGGATATTTGGA

            **   *                     *   *        *
     147653 TAGCTGTGAGGATTTCGTTGGAAACGGGAATGTCTTCATAG
        131 GCGCTTTGAGGATTTCGTTGGAAACGGAAATATCTTCATAT

                **                     * * **         *  **   *   *          
     147694 AAAATTTAGACAGAAGCATTCTCAGAACCTTGATTGTGATGTGTGTTCTCCACTAACAGAGTTGA
          1 AAAAACTAGACAGAAGCATTCTCAGAAACATCTTTGTGATGTTTGCACTCAACTCACAGAGTTGA

                       * *   ***   *       *       *                         
     147759 ACCTTTCTTTTGACAGAAATGTTCTGAAACATTCTTTTTATAGAATCTGGAAGTGGATATTTGGA
         66 ACCTTTCTTTTCATAGAGCAGTTTTGAAACACTCTTTTTGTAGAATCTGGAAGTGGATATTTGGA

            **                        **          *  
     147824 AAGCTTTGAGGATTTCGTTGGAAACGAGAATATCTTCAAAT
        131 GCGCTTTGAGGATTTCGTTGGAAACGGAAATATCTTCATAT

                *    *            *           * *       *  *    *            
     147865 AAAATCTAGCCAGAAGCATTCTAAGAAACATCTTAGGGATGTTTACATTCAAGTCACAGAGTTGA
          1 AAAAACTAGACAGAAGCATTCTCAGAAACATCTTTGTGATGTTTGCACTCAACTCACAGAGTTGA

              *  * *     *       *         *    * *   **      *      *    *  
     147930 ACATTCCCTTTCACAGAGCAGGTTTGAAACAATCTTCTCGTACTATCTGGCAGTGGACATTTTGA
         66 ACCTTTCTTTTCATAGAGCAGTTTTGAAACACTCTTTTTGTAGAATCTGGAAGTGGATATTTGGA

              * *   *  ** ** *  *   *            ** *
     147995 GCTCCTTGGGGCCTATGCTGAAAAAGGAAATATCTTCCGAC
        131 GCGCTTTGAGGATTTCGTTGGAAACGGAAATATCTTCATAT

                                 *     *  **          *           **    *    
     148036 AAAAACTAGACAGAAGCATTCGCAGAATCACGTTTGTGATGTGTGCACTCAACTGTCAGAATTGA
          1 AAAAACTAGACAGAAGCATTCTCAGAAACATCTTTGTGATGTTTGCACTCAACTCACAGAGTTGA

                 **   ** *      *                            * *            *
     148101 ACCTTGGTTTGGACAGAGCACTTTTGAAACACTCTTTTTGTAGAATCTGCAGGTGGATATTTGGC
         66 ACCTTTCTTTTCATAGAGCAGTTTTGAAACACTCTTTTTGTAGAATCTGGAAGTGGATATTTGGA

            **                         *   *      * *
     148166 TAGCTTTGAGGATTTCGTTGGAAACGGTAATGTCTTCAAAG
        131 GCGCTTTGAGGATTTCGTTGGAAACGGAAATATCTTCATAT

                *                         *   **           *    *            
     148207 AAAATCTAGACAGAAGCATTCTCAGAAACACCTTCATGATGTTTGCAATCAAGTCACAGAGTTGA
          1 AAAAACTAGACAGAAGCATTCTCAGAAACATCTTTGTGATGTTTGCACTCAACTCACAGAGTTGA

                 * *             *  *                  *             *       
     148272 ACCTTCCGTTTCATAGAGCAGGTTGGAAACACTCTTTTTGTAGTATCTGGAAGTGGACATTTGGA
         66 ACCTTTCTTTTCATAGAGCAGTTTTGAAACACTCTTTTTGTAGAATCTGGAAGTGGATATTTGGA

             *         ** *    * *   *        *   **  
     148337 GGGCTTTGTAGCCTATC-TGGAAAAAGGAAATATATTCCCAT
        131 GCGCTTTG-AGGATTTCGTTGGAAACGGAAATATCTTCATAT

            *  ** *   *     * *            *   *  *   * **        *  * * *   
     148378 GAATGCGAGATAGAAGTAATCTCAGAAACATGTTTATGCTGTATCTACTCAACTAACTGTGCTGA
          1 AAAAACTAGACAGAAGCATTCTCAGAAACATCTTTGTGATGTTTGCACTCAACTCACAGAGTTGA

              *     *  *               *        *  * *       *               
     148443 ACATTTCTATTGATAGAGCAGTTTTGAGACACTCTTCTTTTGGAATCTGCAAGTGGATATTTGGA
         66 ACCTTTCTTTTCATAGAGCAGTTTTGAAACACTCTTTTTGTAGAATCTGGAAGTGGATATTTGGA

            ** *            *           *     *      
     148508 TAGATTTGAGGATTTCTTTGGAAACGG-GAT-T-AT-ATAT
        131 GCGCTTTGAGGATTTCGTTGGAAACGGAAATATCTTCATAT

                 *       *               *                 **  **  *         
     148545 AAAAAGTAGACAGCAGCATTCTCAGAAACTTCTTTGTGATGTTTGCATCCAGTTCTCAGAGTTGA
          1 AAAAACTAGACAGAAGCATTCTCAGAAACATCTTTGTGATGTTTGCACTCAACTCACAGAGTTGA

              *  * *          *  *        *        *   **        *  **       
     148610 ACATTCCCTTTCATAGAGTAGGTTTGAAACCCTCTTTTTATAGTGTCTGGAAGCGGGCATTTGGA
         66 ACCTTTCTTTTCATAGAGCAGTTTTGAAACACTCTTTTTGTAGAATCTGGAAGTGGATATTTGGA

                   *    * * **  **  **          * *  *
     148675 GCGCTTTCAGGCCTATGCTTAAAATAGGAAATATCTACCTAC
        131 GCGCTTTGAGG-ATTTCGTTGGAAACGGAAATATCTTCATAT

             *                    *    *  **          ** *        *    *     
     148717 AGAAACTAGACAGAAGCATTCTGAGAATCACGTTTGTGATGTGGGTACTCAACTAACAGTGTTGA
          1 AAAAACTAGACAGAAGCATTCTCAGAAACATCTTTGTGATGTTTGCACTCAACTCACAGAGTTGA

            *  *       *   *            *   *                *   *           
     148782 TCCATTCTTTTGATACAGCAGTTTTGAACCACACTTTTTGTAGAATCTGCAAGAGGATATTTGGA
         66 ACCTTTCTTTTCATAGAGCAGTTTTGAAACACTCTTTTTGTAGAATCTGGAAGTGGATATTTGGA

            **   *                     *   *      * *
     148847 AAGCTGTGAGGATTTCGTTGGAAACGGGAATGTCTTCAAAG
        131 GCGCTTTGAGGATTTCGTTGGAAACGGAAATATCTTCATAT

                *                         *   *            *    *            
     148888 AAAATCTAGACAGAAGCATTCTCAGAAACACCTTCGTGATGTTTGCAATCAAGTCACAGAGTTGA
          1 AAAAACTAGACAGAAGCATTCTCAGAAACATCTTTGTGATGTTTGCACTCAACTCACAGAGTTGA

                 * *             *  *                  ** *          *       
     148953 ACCTTCCGTTTCATAGAGCAGGTTGGAAACACTCTTTTTGTAGTTTGTGGAAGTGGACATTTGGA
         66 ACCTTTCTTTTCATAGAGCAGTTTTGAAACACTCTTTTTGTAGAATCTGGAAGTGGATATTTGGA

                   *   ** ** *  *   *            **  
     149018 GCGCTTTCAGGCCTATGGTGAAAAAGGAAATATCTTCCCAT
        131 GCGCTTTGAGGATTTCGTTGGAAACGGAAATATCTTCATAT

                  * * *      **      *   * *   *     ** ** *      *    *     
     149059 AAAAACGACATAGAAGCTATCTCAGGAACTTGTTTATGATGCATCTAATCAACTAACAGTGTTGA
          1 AAAAACTAGACAGAAGCATTCTCAGAAACATCTTTGTGATGTTTGCACTCAACTCACAGAGTTGA

                  * ** * *                         * *       *               
     149124 ACCTTTGTACTGACAGAGCAG-TTTGAAACACTCTTTTTTTGGAATCTGCAAGTGGATATTTGGA
         66 ACCTTTCTTTTCATAGAGCAGTTTTGAAACACTCTTTTTGTAGAATCTGGAAGTGGATATTTGGA

            *                           *   *   *    
     149188 TCGCTTTGAGGATTTCGTTGGAAACGG-GAT-GC--AATAT
        131 GCGCTTTGAGGATTTCGTTGGAAACGGAAATATCTTCATAT

                **  *    *     *                 **  *   *  *    *         * 
     149225 AAAACGTACACAGCAGCATACTCAGAAA-ATACTTTGCCATATTTCCATTCAAGTCACAGAGTGG
          1 AAAAACTAGACAGAAGCATTCTCAGAAACAT-CTTTGTGATGTTTGCACTCAACTCACAGAGTTG

               *  * **            *                  *  *             *      
     149289 AACATTCCCATTCATAGAGCAGGTTTGAAACACTCTTTTTGGAGTATCTGGAAGTGGACATTTGG
         65 AACCTTTCTTTTCATAGAGCAGTTTTGAAACACTCTTTTTGTAGAATCTGGAAGTGGATATTTGG

                    ** * * ** *  *   *            **  
     149354 AGCGCTTTCTGAACTATGGTGAAAAAGGAAATATCTTCCAAT
        130 AGCGCTTTGAGGATTTCGTTGGAAACGGAAATATCTTCATAT

            *     *               *      * *          *  **      **  *  *    
     149396 GAAAACAAGACAGAAGCATTCTGAGAAACTTATTTGTGATGTGTGTCCTCAACAAACGGACTTGA
          1 AAAAACTAGACAGAAGCATTCTCAGAAACATCTTTGTGATGTTTGCACTCAACTCACAGAGTTGA

                   *      **  * *  *  *             *   *    * * *           
     149461 ACCTTTCGTTTCATGCAGTACTTCTGGAACACTCTTTTTGAAGATTCTGCATGCGGATATTTGGA
         66 ACCTTTCTTTTCATAGAGCAGTTTTGAAACACTCTTTTTGTAGAATCTGGAAGTGGATATTTGGA

            **                            **   *   * 
     149526 TAGCTTTGAGGATTTCGTTGGAAACGG---GCT-TACATGT
        131 GCGCTTTGAGGATTTCGTTGGAAACGGAAATATCTTCATAT

                 *       *               *        *   *    *    *       *    
     149563 AAAAATTAGACAGCAGCATTCTCAGAAACTTCTTTGTGGTGTCTGCATTCAAGTCACAGAATTGA
          1 AAAAACTAGACAGAAGCATTCTCAGAAACATCTTTGTGATGTTTGCACTCAACTCACAGAGTTGA

              * ** * **            *  * *      *       *    *        *       
     149628 ACTTCCCCTCACATAGAGCAGTTGTGCAGCACTCTATTTGTAGTATCTCGAAGTGGACATTTGGA
         66 ACCTTTCTTTTCATAGAGCAGTTTTGAAACACTCTTTTTGTAGAATCTGGAAGTGGATATTTGGA

             *      ** ** *          *            **  
     149693 GGGCTTTGTAGCCTAC-TTGGAAAAAGGAAATATCTTCCCAT
        131 GCGCTTTGAGGATTTCGTTGG-AAACGGAAATATCTTCATAT

            *  ** *   *     * *            *   *  *   * **        *  * * *   
     149734 GAATGCGAGATAGAAGTAATCTCAGAAACATGTTTATGCTGTATCTACTCAACTAACTGTGCTGA
          1 AAAAACTAGACAGAAGCATTCTCAGAAACATCTTTGTGATGTTTGCACTCAACTCACAGAGTTGA

              *     *  *               *        *  * **      *               
     149799 ACATTTCTATTGATAGAGCAGTTTTGAGACACTCTTCTTTTGAAATCTGCAAGTGGATATTTGGA
         66 ACCTTTCTTTTCATAGAGCAGTTTTGAAACACTCTTTTTGTAGAATCTGGAAGTGGATATTTGGA

            ** *                        *     *      
     149864 TAGATTTGAGGATTTCGTTGGAAACGG-GAT-T-AT-ATAT
        131 GCGCTTTGAGGATTTCGTTGGAAACGGAAATATCTTCATAT

                 *       *               *                 **  *   *         
     149901 AAAAAGTAGACAGCAGCATTCTCAGAAACTTCTTTGTGATGTTTGCATCCAGCTCTCAGAGTTGA
          1 AAAAACTAGACAGAAGCATTCTCAGAAACATCTTTGTGATGTTTGCACTCAACTCACAGAGTTGA

              *  * *          *  *        *        *   **   *    *  **       
     149966 ACATTCCCTTTCATAGAGTAGGTTTGAAACCCTCTTTTTATAGTGTCTAGAAGCGGGCATTTGGA
         66 ACCTTTCTTTTCATAGAGCAGTTTTGAAACACTCTTTTTGTAGAATCTGGAAGTGGATATTTGGA

                   *    * * **  **  **          * *  *
     150031 GCGCTTTCAGGCCTATGCTTAAAATAGGAAATATCTACCTAC
        131 GCGCTTTGAGG-ATTTCGTTGGAAACGGAAATATCTTCATAT

             *                    *    *  **          ** *        *    *     
     150073 AGAAACTAGACAGAAGCATTCTGAGAATCACGTTTGTGATGTGGGTACTCAACTAACAGTGTTGA
          1 AAAAACTAGACAGAAGCATTCTCAGAAACATCTTTGTGATGTTTGCACTCAACTCACAGAGTTGA

            *  *       *   *            *   *                *   *           
     150138 TCCATTCTTTTGATACAGCAGTTTTGAACCACACTTTTTGTAGAATCTGCAAGAGGATATTTGGA
         66 ACCTTTCTTTTCATAGAGCAGTTTTGAAACACTCTTTTTGTAGAATCTGGAAGTGGATATTTGGA

            **   *                     *   *      * *
     150203 TAGCTGTGAGGATTTCGTTGGAAACGGGAATGTCTTCAAAG
        131 GCGCTTTGAGGATTTCGTTGGAAACGGAAATATCTTCATAT

                *                         *   *            *    *            
     150244 AAAATCTAGACAGAAGCATTCTCAGAAACACCTTCGTGATGTTTGCAATCAAGTCACAGAGTTGA
          1 AAAAACTAGACAGAAGCATTCTCAGAAACATCTTTGTGATGTTTGCACTCAACTCACAGAGTTGA

                 * *             *  *           *      *             *       
     150309 ACCTTCCGTTTCATAGAGCAGGTTGGAAACACTCTTATTGTAGTATCTGGAAGTGGACATTTGGA
         66 ACCTTTCTTTTCATAGAGCAGTTTTGAAACACTCTTTTTGTAGAATCTGGAAGTGGATATTTGGA

                   *   ** ** *  *   *            **  
     150374 GCGCTTTCAGGCCTATGGTGAAAAAGGAAATATCTTCCCAT
        131 GCGCTTTGAGGATTTCGTTGGAAACGGAAATATCTTCATAT

                  * * *      ***     *   * *   *     ** ** *      *    *     
     150415 AAAAACGACATAGAAGCTACCTCAGGAACTTGTTTATGATGCATCTAATCAACTAACAGTGTTGA
          1 AAAAACTAGACAGAAGCATTCTCAGAAACATCTTTGTGATGTTTGCACTCAACTCACAGAGTTGA

                  * ** * *                         * *       *               
     150480 ACCTTTGTACTGACAGAGCAG-TTTGAAACACTCTTTTTTTGGAATCTGCAAGTGGATATTTGGA
         66 ACCTTTCTTTTCATAGAGCAGTTTTGAAACACTCTTTTTGTAGAATCTGGAAGTGGATATTTGGA

            * *                         *   *   *    
     150544 TCACTTTGAGGATTTCGTTGGAAACGG-GAT-GC--AATAT
        131 GCGCTTTGAGGATTTCGTTGGAAACGGAAATATCTTCATAT

                **       *     *                 **  *   *  *    *         * 
     150581 AAAACGTAGACAGCAGCATACTCAGAAA-ATACTTTGCCATATTTCCATTCAAGTCACAGAGTGG
          1 AAAAACTAGACAGAAGCATTCTCAGAAACAT-CTTTGTGATGTTTGCACTCAACTCACAGAGTTG

               *  * **            *  *               *  *             *      
     150645 AACATTCCCATTCATAGAGCAGGTTGGAAACACTCTTTTTGGAGTATCTGGAAGTGGACATTTGG
         65 AACCTTTCTTTTCATAGAGCAGTTTTGAAACACTCTTTTTGTAGAATCTGGAAGTGGATATTTGG

                    ** * * ** *  *   *            **  
     150710 AGCGCTTTCTGAACTATGGTGAAAAAGGAAATATCTTCCAAT
        130 AGCGCTTTGAGGATTTCGTTGGAAACGGAAATATCTTCATAT

            *     *               *      * *          *  **      **  *  *    
     150752 GAAAACAAGACAGAAGCATTCTGAGAAACTTATTTGTGATGTGTGTCCTCAACAAACGGACTTGA
          1 AAAAACTAGACAGAAGCATTCTCAGAAACATCTTTGTGATGTTTGCACTCAACTCACAGAGTTGA

                   *      **  * *  *  *             *   *    * * *           
     150817 ACCTTTCGTTTCATGCAGTACTTCTGGAACACTCTTTTTGAAGATTCTGCATGCGGATATTTGGA
         66 ACCTTTCTTTTCATAGAGCAGTTTTGAAACACTCTTTTTGTAGAATCTGGAAGTGGATATTTGGA

            **                            **   *   * 
     150882 TAGCTTTGAGGATTTCGTTGGAAACGG---GCT-TACATGT
        131 GCGCTTTGAGGATTTCGTTGGAAACGGAAATATCTTCATAT

                 *       *               *        *   *    *    *       *    
     150919 AAAAATTAGACAGCAGCATTCTCAGAAACTTCTTTGTGGTGTCTGCATTCAAGTCACAGAATTGA
          1 AAAAACTAGACAGAAGCATTCTCAGAAACATCTTTGTGATGTTTGCACTCAACTCACAGAGTTGA

              * ** * **           **  * *      *       *    *        *       
     150984 ACTTCCCCTCACATAGAGCAGTAGTGCAGCACTCTATTTGTAGTATCTCGAAGTGGACATTTGGA
         66 ACCTTTCTTTTCATAGAGCAGTTTTGAAACACTCTTTTTGTAGAATCTGGAAGTGGATATTTGGA

             *         ** *    * *   *            **  
     151049 GGGCTTTGTAGCCTATC-TGGAAAAAGGAAATATCTTCCCAT
        131 GCGCTTTG-AGGATTTCGTTGGAAACGGAAATATCTTCATAT

            *  ** *   *     * *            *   *  *   * **        *  * * *   
     151090 GAATGCGAGATAGAAGTAATCTCAGAAACATGTTTATGCTGTATCTACTCAACTAACTGTGCTGA
          1 AAAAACTAGACAGAAGCATTCTCAGAAACATCTTTGTGATGTTTGCACTCAACTCACAGAGTTGA

              *     *  *               *        *  * *       *               
     151155 ACATTTCTATTGATAGAGCAGTTTTGAGACACTCTTCTTTTGGAATCTGCAAGTGGATATTTGGA
         66 ACCTTTCTTTTCATAGAGCAGTTTTGAAACACTCTTTTTGTAGAATCTGGAAGTGGATATTTGGA

            ** *                        *     *      
     151220 TAGATTTGAGGATTTCGTTGGAAACGG-GAT-T-AT-ATAT
        131 GCGCTTTGAGGATTTCGTTGGAAACGGAAATATCTTCATAT

            *    *       *               *                 **  *   *         
     151257 CAAAAGTAGACAGCAGCATTCTCAGAAACTTCTTTGTGATGTTTGCATCCAGCTCTCAGAGTTGA
          1 AAAAACTAGACAGAAGCATTCTCAGAAACATCTTTGTGATGTTTGCACTCAACTCACAGAGTTGA

              *  * *          *  *        *        *   **        *  **       
     151322 ACATTCCCTTTCATAGAGTAGGTTTGAAACCCTCTTTTTCTAGTGTCTGGAAGCGGGCATTTGGA
         66 ACCTTTCTTTTCATAGAGCAGTTTTGAAACACTCTTTTTGTAGAATCTGGAAGTGGATATTTGGA

                   *   ** ** *  *   *          * *   
     151387 GCGCTTTCAGGCCTATGCTGAAAAAGGAAATATCTACCTAT
        131 GCGCTTTGAGGATTTCGTTGGAAACGGAAATATCTTCATAT

             *                    *    *  **          ** *        *    *     
     151428 AGAAACTAGACAGAAGCATTCTGAGAATCACGTTTGTGATGTGGGTACTCAACTAACAGTGTTGA
          1 AAAAACTAGACAGAAGCATTCTCAGAAACATCTTTGTGATGTTTGCACTCAACTCACAGAGTTGA

            *  *       *   *            *   *                *               
     151493 TCCATTCTTTTGATACAGCAGTTTTGAACCACACTTTTTGTAGAATCTGCAAGTGGATATTTGGA
         66 ACCTTTCTTTTCATAGAGCAGTTTTGAAACACTCTTTTTGTAGAATCTGGAAGTGGATATTTGGA

            **   *                     *   *        *
     151558 TAGCTGTGAGGATTTCGTTGGAAACGGGAATGTCTTCATAG
        131 GCGCTTTGAGGATTTCGTTGGAAACGGAAATATCTTCATAT

                **                     * * **         *  **   *   *          
     151599 AAAATTTAGACAGAAGCATTCTCAGAACCTTGATTGTGATGTGTGTTCTCCACTAACAGAGTTGA
          1 AAAAACTAGACAGAAGCATTCTCAGAAACATCTTTGTGATGTTTGCACTCAACTCACAGAGTTGA

                       * *   * *   *       *       *                         
     151664 ACCTTTCTTTTGACAGAACTGTTCTGAAACATTCTTTTTATAGAATCTGGAAGTGGATATTTGGA
         66 ACCTTTCTTTTCATAGAGCAGTTTTGAAACACTCTTTTTGTAGAATCTGGAAGTGGATATTTGGA

            **      *                  *          *  
     151729 AAGCTTTGCGGATTTCGTTGGAAACGGGAATATCTTCAAAT
        131 GCGCTTTGAGGATTTCGTTGGAAACGGAAATATCTTCATAT

                *    *            *           * *       *  *    *            
     151770 AAAATCTAGCCAGAAGCATTCTAAGAAACATCTTAGGGATGTTTACATTCAAGTCACAGAGTTGA
          1 AAAAACTAGACAGAAGCATTCTCAGAAACATCTTTGTGATGTTTGCACTCAACTCACAGAGTTGA

              *  * *     **      *         *    * *   **      *      *    *  
     151835 ACATTCCCTTTCACGGAGCAGGTTTGAAACAATCTTCTCGTACTATCTGGCAGTGGACATTTTGA
         66 ACCTTTCTTTTCATAGAGCAGTTTTGAAACACTCTTTTTGTAGAATCTGGAAGTGGATATTTGGA

              *     *  ** ** *  *   *            ** *
     151900 GCTCTTTGGGGCCTATGCTGAAAAAGGAAATATCTTCCGAC
        131 GCGCTTTGAGGATTTCGTTGGAAACGGAAATATCTTCATAT

                                 *     *  **          *           **    *    
     151941 AAAAACTAGACAGAAGCATTCGCAGAATCACGTTTGTGATGTGTGCACTCAACTGTCAGAATTGA
          1 AAAAACTAGACAGAAGCATTCTCAGAAACATCTTTGTGATGTTTGCACTCAACTCACAGAGTTGA

                 **   ** *      *                            * *            *
     152006 ACCTTGGTTTGGAGAGAGCACTTTTGAAACACTCTTTTTGTAGAATCTGCAGGTGGATATTTGGC
         66 ACCTTTCTTTTCATAGAGCAGTTTTGAAACACTCTTTTTGTAGAATCTGGAAGTGGATATTTGGA

            **                         *   *      * *
     152071 TAGCTTTGAGGATTTCGTTGGAAACGGTAATGTCTTCAAAG
        131 GCGCTTTGAGGATTTCGTTGGAAACGGAAATATCTTCATAT

                *                         *   *            *    *            
     152112 AAAATCTAGACAGAAGCATTCTCAGAAACACCTTCGTGATGTTTGCAATCAAGTCACAGAGTTGA
          1 AAAAACTAGACAGAAGCATTCTCAGAAACATCTTTGTGATGTTTGCACTCAACTCACAGAGTTGA

                 * *             *  *                  *             *       
     152177 ACCTTCCGTTTCATAGAGCAGGTTGGAAACACTCTTTTTGTAGTATCTGGAAGTGGACATTTGGA
         66 ACCTTTCTTTTCATAGAGCAGTTTTGAAACACTCTTTTTGTAGAATCTGGAAGTGGATATTTGGA

             *         ** *    * *   *            **  
     152242 GGGCTTTGTAGCCTATC-TGGAAAAAGGAAATATCTTCCCAT
        131 GCGCTTTG-AGGATTTCGTTGGAAACGGAAATATCTTCATAT

            *  ** *   *     * *            *   *  *   * **        *  * * *   
     152283 GAATGCGAGATAGAAGTAATCTCAGAAACATGTTTATGCTGTATCTACTCAACTAACTGTGCTGA
          1 AAAAACTAGACAGAAGCATTCTCAGAAACATCTTTGTGATGTTTGCACTCAACTCACAGAGTTGA

              *     *  *               *        *  * *       *               
     152348 ACATTTCTATTGATAGAGCAGTTTTGAGACACTCTTCTTTTGGAATCTGCAAGTGGATATTTGGA
         66 ACCTTTCTTTTCATAGAGCAGTTTTGAAACACTCTTTTTGTAGAATCTGGAAGTGGATATTTGGA

            ** *                        *     *      
     152413 TAGATTTGAGGATTTCGTTGGAAACGG-GAT-T-AT-ATAT
        131 GCGCTTTGAGGATTTCGTTGGAAACGGAAATATCTTCATAT

                 *       *               *                 **  *   *         
     152450 AAAAAGTAGACAGCAGCATTCTCAGAAACTTCTTTGTGATGTTTGCATCCAGCTCTCAGAGTTGA
          1 AAAAACTAGACAGAAGCATTCTCAGAAACATCTTTGTGATGTTTGCACTCAACTCACAGAGTTGA

              *  * *          *  *        *        *   **        *  **       
     152515 ACATTCCCTTTCATAGAGTAGGTTTGAAACCCTCTTTTTATAGTGTCTGGAAGCGGGCATTTGGA
         66 ACCTTTCTTTTCATAGAGCAGTTTTGAAACACTCTTTTTGTAGAATCTGGAAGTGGATATTTGGA

             *     *   ** ** *  *   *          * *   
     152580 GTGCTTTCAGGCCTATGCTGAAAAAGGAAATATCTACCTAT
        131 GCGCTTTGAGGATTTCGTTGGAAACGGAAATATCTTCATAT

             *                    *    *  **          ** *        *    *     
     152621 AGAAACTAGACAGAAGCATTCTGAGAATCACGTTTGTGATGTGGGTACTCAACTAACAGTGTTGA
          1 AAAAACTAGACAGAAGCATTCTCAGAAACATCTTTGTGATGTTTGCACTCAACTCACAGAGTTGA

            *  *       *   *            *   *                *               
     152686 TCCATTCTTTTGATACAGCAGTTTTGAACCACACTTTTTGTAGAATCTGCAAGTGGATATTTGGA
         66 ACCTTTCTTTTCATAGAGCAGTTTTGAAACACTCTTTTTGTAGAATCTGGAAGTGGATATTTGGA

            **   *                     *   *        *
     152751 TAGCTGTGAGGATTTCGTTGGAAACGGGAATGTCTTCATAG
        131 GCGCTTTGAGGATTTCGTTGGAAACGGAAATATCTTCATAT

                **                     * * **         *  **   *   *          
     152792 AAAATTTAGACAGAAGCATTCTCAGAACCTTGATTGTGATGTGTGTTCTCCACTAACAGAGTTGA
          1 AAAAACTAGACAGAAGCATTCTCAGAAACATCTTTGTGATGTTTGCACTCAACTCACAGAGTTGA

                       * *   ***   *       *       *                         
     152857 ACCTTTCTTTTGACAGAAATGTTCTGAAACATTCTTTTTATAGAATCTGGAAGTGGATATTTGGA
         66 ACCTTTCTTTTCATAGAGCAGTTTTGAAACACTCTTTTTGTAGAATCTGGAAGTGGATATTTGGA

            **                        **          *  
     152922 AAGCTTTGAGGATTTCGTTGGAAACGAGAATATCTTCAAAT
        131 GCGCTTTGAGGATTTCGTTGGAAACGGAAATATCTTCATAT

                *    *            *           * *       *  *    *            
     152963 AAAATCTAGCCAGAAGCATTCTAAGAAACATCTTAGGGATGTTTACATTCAAGTCACAGAGTTGA
          1 AAAAACTAGACAGAAGCATTCTCAGAAACATCTTTGTGATGTTTGCACTCAACTCACAGAGTTGA

              *  * *     *       *         *    * *   **      *      *    *  
     153028 ACATTCCCTTTCACAGAGCAGGTTTGAAACAATCTTCTCGTACTATCTGGCAGTGGACATTTTGA
         66 ACCTTTCTTTTCATAGAGCAGTTTTGAAACACTCTTTTTGTAGAATCTGGAAGTGGATATTTGGA

              * *   *  ** ** *  *   *            ** *
     153093 GCTCCTTGGGGCCTATGCTGAAAAAGGAAATATCTTCCGAC
        131 GCGCTTTGAGGATTTCGTTGGAAACGGAAATATCTTCATAT

                                 *     *  **          *           **    *    
     153134 AAAAACTAGACAGAAGCATTCGCAGAATCACGTTTGTGATGTGTGCACTCAACTGTCAGAATTGA
          1 AAAAACTAGACAGAAGCATTCTCAGAAACATCTTTGTGATGTTTGCACTCAACTCACAGAGTTGA

                 **   ** *      *                            * *            *
     153199 ACCTTGGTTTGGACAGAGCACTTTTGAAACACTCTTTTTGTAGAATCTGCAGGTGGATATTTGGC
         66 ACCTTTCTTTTCATAGAGCAGTTTTGAAACACTCTTTTTGTAGAATCTGGAAGTGGATATTTGGA

            **                         *   *      * *
     153264 TAGCTTTGAGGATTTCGTTGGAAACGGTAATGTCTTCAAAG
        131 GCGCTTTGAGGATTTCGTTGGAAACGGAAATATCTTCATAT

                *                         *   **           *    *            
     153305 AAAATCTAGACAGAAGCATTCTCAGAAACACCTTCATGATGTTTGCAATCAAGTCACAGAGTTGA
          1 AAAAACTAGACAGAAGCATTCTCAGAAACATCTTTGTGATGTTTGCACTCAACTCACAGAGTTGA

                 * *             *  *                  *             *       
     153370 ACCTTCCGTTTCATAGAGCAGGTTGGAAACACTCTTTTTGTAGTATCTGGAAGTGGACATTTGGA
         66 ACCTTTCTTTTCATAGAGCAGTTTTGAAACACTCTTTTTGTAGAATCTGGAAGTGGATATTTGGA

             *         ** *    * *   *        *   **  
     153435 GGGCTTTGTAGCCTATC-TGGAAAAAGGAAATATATTCCCAT
        131 GCGCTTTG-AGGATTTCGTTGGAAACGGAAATATCTTCATAT

            *  ** *   *     * *            *   *  *   * **        *  * * *   
     153476 GAATGCGAGATAGAAGTAATCTCAGAAACATGTTTATGCTGTATCTACTCAACTAACTGTGCTGA
          1 AAAAACTAGACAGAAGCATTCTCAGAAACATCTTTGTGATGTTTGCACTCAACTCACAGAGTTGA

              *     *  *               *        *  * *       *               
     153541 ACATTTCTATTGATAGAGCAGTTTTGAGACACTCTTCTTTTGGAATCTGCAAGTGGATATTTGGA
         66 ACCTTTCTTTTCATAGAGCAGTTTTGAAACACTCTTTTTGTAGAATCTGGAAGTGGATATTTGGA

            ** *            *           *     *      
     153606 TAGATTTGAGGATTTCTTTGGAAACGG-GAT-T-AT-ATAT
        131 GCGCTTTGAGGATTTCGTTGGAAACGGAAATATCTTCATAT

                 *       *               *                 **  **  *         
     153643 AAAAAGTAGACAGCAGCATTCTCAGAAACTTCTTTGTGATGTTTGCATCCAGTTCTCAGAGTTGA
          1 AAAAACTAGACAGAAGCATTCTCAGAAACATCTTTGTGATGTTTGCACTCAACTCACAGAGTTGA

              *  * *          *  *        *        *   **        *  **       
     153708 ACATTCCCTTTCATAGAGTAGGTTTGAAACCCTCTTTTTATAGTGTCTGGAAGCGGGCATTTGGA
         66 ACCTTTCTTTTCATAGAGCAGTTTTGAAACACTCTTTTTGTAGAATCTGGAAGTGGATATTTGGA

                   *    * * **  **  **          * *  *
     153773 GCGCTTTCAGGCCTATGCTTAAAATAGGAAATATCTACCTAC
        131 GCGCTTTGAGG-ATTTCGTTGGAAACGGAAATATCTTCATAT

             *                    *    *  **          ** *        *    *     
     153815 AGAAACTAGACAGAAGCATTCTGAGAATCACGTTTGTGATGTGGGTACTCAACTAACAGTGTTGA
          1 AAAAACTAGACAGAAGCATTCTCAGAAACATCTTTGTGATGTTTGCACTCAACTCACAGAGTTGA

            *  *       *   *            *   *                *   *           
     153880 TCCATTCTTTTGATACAGCAGTTTTGAACCACACTTTTTGTAGAATCTGCAAGAGGATATTTGGA
         66 ACCTTTCTTTTCATAGAGCAGTTTTGAAACACTCTTTTTGTAGAATCTGGAAGTGGATATTTGGA

            **   *                     *   *      * *
     153945 AAGCTGTGAGGATTTCGTTGGAAACGGGAATGTCTTCAAAG
        131 GCGCTTTGAGGATTTCGTTGGAAACGGAAATATCTTCATAT

                *                         *   *            *    *            
     153986 AAAATCTAGACAGAAGCATTCTCAGAAACACCTTCGTGATGTTTGCAATCAAGTCACAGAGTTGA
          1 AAAAACTAGACAGAAGCATTCTCAGAAACATCTTTGTGATGTTTGCACTCAACTCACAGAGTTGA

                 * *             *  *                  ** *          *       
     154051 ACCTTCCGTTTCATAGAGCAGGTTGGAAACACTCTTTTTGTAGTTTGTGGAAGTGGACATTTGGA
         66 ACCTTTCTTTTCATAGAGCAGTTTTGAAACACTCTTTTTGTAGAATCTGGAAGTGGATATTTGGA

                       ** ** *  *   *            **  
     154116 GCGCTTTGAGGCCTATGGTGAAAAAGGAAATATCTTCCCAT
        131 GCGCTTTGAGGATTTCGTTGGAAACGGAAATATCTTCATAT

                  * * *      **      *   * *   *     ** ** *      *    *     
     154157 AAAAACGACATAGAAGCTATCTCAGGAACTTGTTTATGATGCATCTAATCAACTAACAGTGTTGA
          1 AAAAACTAGACAGAAGCATTCTCAGAAACATCTTTGTGATGTTTGCACTCAACTCACAGAGTTGA

                  * ** * *                         * *       *               
     154222 ACCTTTGTACTGACAGAGCAG-TTTGAAACACTCTTTTTTTGGAATCTGCAAGTGGATATTTGGA
         66 ACCTTTCTTTTCATAGAGCAGTTTTGAAACACTCTTTTTGTAGAATCTGGAAGTGGATATTTGGA

            *                           *   *   *    
     154286 TCGCTTTGAGGATTTCGTTGGAAACGG-GAT-GC--AATAT
        131 GCGCTTTGAGGATTTCGTTGGAAACGGAAATATCTTCATAT

                **  *    *     *                 **  *   *  *    *         * 
     154323 AAAACGTACACAGCAGCATACTCAGAAA-ATACTTTGCCATATTTCCATTCAAGTCACAGAGTGG
          1 AAAAACTAGACAGAAGCATTCTCAGAAACAT-CTTTGTGATGTTTGCACTCAACTCACAGAGTTG

               *  * **            *                  *  *             *      
     154387 AACATTCCCATTCATAGAGCAGGTTTGAAACACTCTTTTTGGAGTATCTGGAAGTGGACATTTGG
         65 AACCTTTCTTTTCATAGAGCAGTTTTGAAACACTCTTTTTGTAGAATCTGGAAGTGGATATTTGG

                    ** * * ** *  *   *            **  
     154452 AGCGCTTTCTGAACTATGGTGAAAAAGGAAATATCTTCCAAT
        130 AGCGCTTTGAGGATTTCGTTGGAAACGGAAATATCTTCATAT

            *     *               *      * *          *  **      **  *  *    
     154494 GAAAACAAGACAGAAGCATTCTGAGAAACTTATTTGTGATGTGTGTCCTCAACAAACGGACTTGA
          1 AAAAACTAGACAGAAGCATTCTCAGAAACATCTTTGTGATGTTTGCACTCAACTCACAGAGTTGA

                   *      **  * *  *  *             *   *    * * *           
     154559 ACCTTTCGTTTCATGCAGTACTTCTGGAACACTCTTTTTGAAGATTCTGCATGCGGATATTTGGA
         66 ACCTTTCTTTTCATAGAGCAGTTTTGAAACACTCTTTTTGTAGAATCTGGAAGTGGATATTTGGA

            **                            **   *   * 
     154624 TAGCTTTGAGGATTTCGTTGGAAACGG---GCT-TACATGT
        131 GCGCTTTGAGGATTTCGTTGGAAACGGAAATATCTTCATAT

                 *       *               *        *   *    *    *       *    
     154661 AAAAATTAGACAGCAGCATTCTCAGAAACTTCTTTGTGGTGTCTGCATTCAAGTCACAGAATTGA
          1 AAAAACTAGACAGAAGCATTCTCAGAAACATCTTTGTGATGTTTGCACTCAACTCACAGAGTTGA

              * ** * **            *  * *      *       *    *        *       
     154726 ACTTCCCCTCACATAGAGCAGTTGTGCAGCACTCTATTTGTAGTATCTCGAAGTGGACATTTGGA
         66 ACCTTTCTTTTCATAGAGCAGTTTTGAAACACTCTTTTTGTAGAATCTGGAAGTGGATATTTGGA

             *      ** ** *          *            **  
     154791 GGGCTTTGTAGCCTAC-TTGGAAAAAGGAAATATCTTCCCAT
        131 GCGCTTTGAGGATTTCGTTGG-AAACGGAAATATCTTCATAT

            *  ** *   *     * *            *   *  *   * **        *  * * *   
     154832 GAATGCGAGATAGAAGTAATCTCAGAAACATGTTTATGCTGTATCTACTCAACTAACTGTGCTGA
          1 AAAAACTAGACAGAAGCATTCTCAGAAACATCTTTGTGATGTTTGCACTCAACTCACAGAGTTGA

              *     *  *               *        *  * **      *               
     154897 ACATTTCTATTGATAGAGCAGTTTTGAGACACTCTTCTTTTGAAATCTGCAAGTGGATATTTGGA
         66 ACCTTTCTTTTCATAGAGCAGTTTTGAAACACTCTTTTTGTAGAATCTGGAAGTGGATATTTGGA

            ** *                        *     *      
     154962 TAGATTTGAGGATTTCGTTGGAAACGG-GAT-T-AT-ATAT
        131 GCGCTTTGAGGATTTCGTTGGAAACGGAAATATCTTCATAT

                 *       *               *                 **  *   *         
     154999 AAAAAGTAGACAGCAGCATTCTCAGAAACTTCTTTGTGATGTTTGCATCCAGCTCTCAGAGTTGA
          1 AAAAACTAGACAGAAGCATTCTCAGAAACATCTTTGTGATGTTTGCACTCAACTCACAGAGTTGA

              *  * *          *  *        *        *   **   *    *  **       
     155064 ACATTCCCTTTCATAGAGTAGGTTTGAAACCCTCTTTTTATAGTGTCTAGAAGCGGGCATTTGGA
         66 ACCTTTCTTTTCATAGAGCAGTTTTGAAACACTCTTTTTGTAGAATCTGGAAGTGGATATTTGGA

                   *    * * **  **  **          * *  *
     155129 GCGCTTTCAGGCCTATGCTTAAAATAGGAAATATCTACCTAC
        131 GCGCTTTGAGG-ATTTCGTTGGAAACGGAAATATCTTCATAT

             *                    *    *  **          ** *        *    *     
     155171 AGAAACTAGACAGAAGCATTCTGAGAATCACGTTTGTGATGTGGGTACTCAACTAACAGTGTTGA
          1 AAAAACTAGACAGAAGCATTCTCAGAAACATCTTTGTGATGTTTGCACTCAACTCACAGAGTTGA

            *  *       *   *            *   *                *   *           
     155236 TCCATTCTTTTGATACAGCAGTTTTGAACCACACTTTTTGTAGAATCTGCAAGAGGATATTTGGA
         66 ACCTTTCTTTTCATAGAGCAGTTTTGAAACACTCTTTTTGTAGAATCTGGAAGTGGATATTTGGA

            **   *                     *   *      * *
     155301 TAGCTGTGAGGATTTCGTTGGAAACGGGAATGTCTTCAAAG
        131 GCGCTTTGAGGATTTCGTTGGAAACGGAAATATCTTCATAT

                *                         *   *            *    *            
     155342 AAAATCTAGACAGAAGCATTCTCAGAAACACCTTCGTGATGTTTGCAATCAAGTCACAGAGTTGA
          1 AAAAACTAGACAGAAGCATTCTCAGAAACATCTTTGTGATGTTTGCACTCAACTCACAGAGTTGA

                 * *             *  *           *      *             *       
     155407 ACCTTCCGTTTCATAGAGCAGGTTGGAAACACTCTTATTGTAGTATCTGGAAGTGGACATTTGGA
         66 ACCTTTCTTTTCATAGAGCAGTTTTGAAACACTCTTTTTGTAGAATCTGGAAGTGGATATTTGGA

                   *   ** ** *  *   *            **  
     155472 GCGCTTTCAGGCCTATGGTGAAAAAGGAAATATCTTCCCAT
        131 GCGCTTTGAGGATTTCGTTGGAAACGGAAATATCTTCATAT

                  * * *      ***     *   * *   *     ** ** *      *    *     
     155513 AAAAACGACATAGAAGCTACCTCAGGAACTTGTTTATGATGCATCTAATCAACTAACAGTGTTGA
          1 AAAAACTAGACAGAAGCATTCTCAGAAACATCTTTGTGATGTTTGCACTCAACTCACAGAGTTGA

                  * ** * *                         * *       *               
     155578 ACCTTTGTACTGACAGAGCAG-TTTGAAACACTCTTTTTTTGGAATCTGCAAGTGGATATTTGGA
         66 ACCTTTCTTTTCATAGAGCAGTTTTGAAACACTCTTTTTGTAGAATCTGGAAGTGGATATTTGGA

            * *                         *   *   *    
     155642 TCACTTTGAGGATTTCGTTGGAAACGG-GAT-GC--AATAT
        131 GCGCTTTGAGGATTTCGTTGGAAACGGAAATATCTTCATAT

                **       *     *                 **  *   *  *    *         * 
     155679 AAAACGTAGACAGCAGCATACTCAGAAA-ATACTTTGCCATATTTCCATTCAAGTCACAGAGTGG
          1 AAAAACTAGACAGAAGCATTCTCAGAAACAT-CTTTGTGATGTTTGCACTCAACTCACAGAGTTG

               *  * **            *  *               *  *             *      
     155743 AACATTCCCATTCATAGAGCAGGTTGGAAACACTCTTTTTGGAGTATCTGGAAGTGGACATTTGG
         65 AACCTTTCTTTTCATAGAGCAGTTTTGAAACACTCTTTTTGTAGAATCTGGAAGTGGATATTTGG

                    ** * * ** *  *   *            **  
     155808 AGCGCTTTCTGAACTATGGTGAAAAAGGAAATATCTTCCAAT
        130 AGCGCTTTGAGGATTTCGTTGGAAACGGAAATATCTTCATAT

            *     *               *      * *          *  **       *  *  *    
     155850 GAAAACAAGACAGAAGCATTCTGAGAAACTTATTTGTGATGTGTGTCCTCAACTAACGGACTTGA
          1 AAAAACTAGACAGAAGCATTCTCAGAAACATCTTTGTGATGTTTGCACTCAACTCACAGAGTTGA

                   *      **  * *  *  *             *   *    * * *           
     155915 ACCTTTCGTTTCATGCAGTACTTCTGGAACACTCTTTTTGAAGATTCTGCATGCGGATATTTGGA
         66 ACCTTTCTTTTCATAGAGCAGTTTTGAAACACTCTTTTTGTAGAATCTGGAAGTGGATATTTGGA

            **                            **   *   * 
     155980 TAGCTTTGAGGATTTCGTTGGAAACGG---GCT-TACATGT
        131 GCGCTTTGAGGATTTCGTTGGAAACGGAAATATCTTCATAT

                 *       *               *        *   *    *    *       *    
     156017 AAAAATTAGACAGCAGCATTCTCAGAAACTTCTTTGTGGTGTCTGCATTCAAGTCACAGAATTGA
          1 AAAAACTAGACAGAAGCATTCTCAGAAACATCTTTGTGATGTTTGCACTCAACTCACAGAGTTGA

              * ** * **            *  * *      *       *             *       
     156082 ACTTCCCCTCACATAGAGCAGTTGTGCAGCACTCTATTTGTAGTATCTGGAAGTGGACATTTGGA
         66 ACCTTTCTTTTCATAGAGCAGTTTTGAAACACTCTTTTTGTAGAATCTGGAAGTGGATATTTGGA

             *         ** *    * *   *            **  
     156147 GGGCTTTGTAGCCTATC-TGGAAAAAGGAAATATCTTCCCAT
        131 GCGCTTTG-AGGATTTCGTTGGAAACGGAAATATCTTCATAT

            *  ** *   *     * *            *   *  *   * **        *  * * *   
     156188 GAATGCGAGATAGAAGTAATCTCAGAAACATGTTTATGCTGTATCTACTCAACTAACTGTGCTGA
          1 AAAAACTAGACAGAAGCATTCTCAGAAACATCTTTGTGATGTTTGCACTCAACTCACAGAGTTGA

              *     *  *               *        *  * *       *               
     156253 ACATTTCTATTGATAGAGCAGTTTTGAGACACTCTTCTTTTGGAATCTGCAAGTGGATATTTGGA
         66 ACCTTTCTTTTCATAGAGCAGTTTTGAAACACTCTTTTTGTAGAATCTGGAAGTGGATATTTGGA

            ** *                        *     *      
     156318 TAGATTTGAGGATTTCGTTGGAAACGG-GAT-T-AT-ATAT
        131 GCGCTTTGAGGATTTCGTTGGAAACGGAAATATCTTCATAT

                 *       *               *                 **  *   *         
     156355 AAAAAGTAGACAGCAGCATTCTCAGAAACTTCTTTGTGATGTTTGCATCCAGCTCTCAGAGTTGA
          1 AAAAACTAGACAGAAGCATTCTCAGAAACATCTTTGTGATGTTTGCACTCAACTCACAGAGTTGA

              *  * *          *  *        *        *   **        *  **       
     156420 ACATTCCCTTTCATAGAGTAGGTTTGAAACCCTCTTTTTATAGTGTCTGGAAGCGGGCATTTGGA
         66 ACCTTTCTTTTCATAGAGCAGTTTTGAAACACTCTTTTTGTAGAATCTGGAAGTGGATATTTGGA

                   *   ** ** *  *   *          * *   
     156485 GCGCTTTCAGGCCTATGCTGAAAAAGGAAATATCTACCTAT
        131 GCGCTTTGAGGATTTCGTTGGAAACGGAAATATCTTCATAT

             *                    *    *  **          ** *        *    *     
     156526 AGAAACTAGACAGAAGCATTCTGAGAATCACGTTTGTGATGTGGGTACTCAACTAACAGTGTTGA
          1 AAAAACTAGACAGAAGCATTCTCAGAAACATCTTTGTGATGTTTGCACTCAACTCACAGAGTTGA

            *  *       *   *            *   *                *               
     156591 TCCATTCTTTTGATACAGCAGTTTTGAACCACACTTTTTGTAGAATCTGCAAGTGGATATTTGGA
         66 ACCTTTCTTTTCATAGAGCAGTTTTGAAACACTCTTTTTGTAGAATCTGGAAGTGGATATTTGGA

            **   *         *           *   *        *
     156656 TAGCTGTGAGGATTTTGTTGGAAACGGGAATGTCTTCATAG
        131 GCGCTTTGAGGATTTCGTTGGAAACGGAAATATCTTCATAT

                **                     * * **         *  **   *   *          
     156697 AAAATTTAGACAGAAGCATTCTCAGAACCTTGATTGTGATGTGTGTTCTCCACTAACAGAGTTGA
          1 AAAAACTAGACAGAAGCATTCTCAGAAACATCTTTGTGATGTTTGCACTCAACTCACAGAGTTGA

                       * *   * *   *       *       *                         
     156762 ACCTTTCTTTTGACAGAACTGTTCTGAAACATTCTTTTTATAGAATCTGGAAGTGGATATTTGGA
         66 ACCTTTCTTTTCATAGAGCAGTTTTGAAACACTCTTTTTGTAGAATCTGGAAGTGGATATTTGGA

            **                         *          *  
     156827 AAGCTTTGAGGATTTCGTTGGAAACGGGAATATCTTCAAAT
        131 GCGCTTTGAGGATTTCGTTGGAAACGGAAATATCTTCATAT

                *    *            *           * *       *  *    *            
     156868 AAAATCTAGCCAGAAGCATTCTAAGAAACATCTTAGGGATGTTTACATTCAAGTCACAGAGTTGA
          1 AAAAACTAGACAGAAGCATTCTCAGAAACATCTTTGTGATGTTTGCACTCAACTCACAGAGTTGA

              *  * *     *       *         *    * *   **      *      *    *  
     156933 ACATTCCCTTTCACAGAGCAGGTTTGAAACAATCTTCTCGTACTATCTGGCAGTGGACATTTTGA
         66 ACCTTTCTTTTCATAGAGCAGTTTTGAAACACTCTTTTTGTAGAATCTGGAAGTGGATATTTGGA

              * *   *  ** ** *  *   *            ** *
     156998 GCTCCTTGGGGCCTATGCTGAAAAAGGAAATATCTTCCGAC
        131 GCGCTTTGAGGATTTCGTTGGAAACGGAAATATCTTCATAT

                                 *     *  **          *           **    *    
     157039 AAAAACTAGACAGAAGCATTCGCAGAATCACGTTTGTGATGTGTGCACTCAACTGTCAGAATTGA
          1 AAAAACTAGACAGAAGCATTCTCAGAAACATCTTTGTGATGTTTGCACTCAACTCACAGAGTTGA

                 **   ** *      *                            * *            *
     157104 ACCTTGGTTTGGACAGAGCACTTTTGAAACACTCTTTTTGTAGAATCTGCAGGTGGATATTTGGC
         66 ACCTTTCTTTTCATAGAGCAGTTTTGAAACACTCTTTTTGTAGAATCTGGAAGTGGATATTTGGA

            **                         *   *      * *
     157169 TAGCTTTGAGGATTTCGTTGGAAACGGTAATGTCTTCAAAG
        131 GCGCTTTGAGGATTTCGTTGGAAACGGAAATATCTTCATAT

                *                         *   *            *    *            
     157210 AAAATCTAGACAGAAGCATTCTCAGAAACACCTTCGTGATGTTTGCAATCAAGTCACAGAGTTGA
          1 AAAAACTAGACAGAAGCATTCTCAGAAACATCTTTGTGATGTTTGCACTCAACTCACAGAGTTGA

                 * *             *  *                  *             *       
     157275 ACCTTCCGTTTCATAGAGCAGGTTGGAAACACTCTTTTTGTAGTATCTGGAAGTGGACATTTGGA
         66 ACCTTTCTTTTCATAGAGCAGTTTTGAAACACTCTTTTTGTAGAATCTGGAAGTGGATATTTGGA

             *         ** *    * *   *            **  
     157340 GGGCTTTGTAGCCTATC-TGGAAAAAGGAAATATCTTCCCAT
        131 GCGCTTTG-AGGATTTCGTTGGAAACGGAAATATCTTCATAT

            *  ** *   *     * *            *   *  *   * **        *  * * *   
     157381 GAATGCGAGATAGAAGTAATCTCAGAAACATGTTTATGCTGTATCTACTCAACTAACTGTGCTGA
          1 AAAAACTAGACAGAAGCATTCTCAGAAACATCTTTGTGATGTTTGCACTCAACTCACAGAGTTGA

              *     *  *               *        *  * *       *               
     157446 ACATTTCTATTGATAGAGCAGTTTTGAGACACTCTTCTTTTGGAATCTGCAAGTGGATATTTGGA
         66 ACCTTTCTTTTCATAGAGCAGTTTTGAAACACTCTTTTTGTAGAATCTGGAAGTGGATATTTGGA

            ** *                        *     *      
     157511 TAGATTTGAGGATTTCGTTGGAAACGG-GAT-T-AT-ATAT
        131 GCGCTTTGAGGATTTCGTTGGAAACGGAAATATCTTCATAT

                 *       *               *                 **  *   *         
     157548 AAAAAGTAGACAGCAGCATTCTCAGAAACTTCTTTGTGATGTTTGCATCCAGCTCTCAGAGTTGA
          1 AAAAACTAGACAGAAGCATTCTCAGAAACATCTTTGTGATGTTTGCACTCAACTCACAGAGTTGA

            * *  * *          *  *        *        *   **        *  **       
     157613 GCATTCCCTTTCATAGAGTAGGTTTGAAACCCTCTTTTTATAGTGTCTGGAAGCGGGCATTTGGA
         66 ACCTTTCTTTTCATAGAGCAGTTTTGAAACACTCTTTTTGTAGAATCTGGAAGTGGATATTTGGA

                   *    * * **  **  **          * *  *
     157678 GCGCTTTCAGGCCTATGCTTAAAATAGGAAATATCTACCTAC
        131 GCGCTTTGAGG-ATTTCGTTGGAAACGGAAATATCTTCATAT

             *                    *    *  **          ** *        *    *     
     157720 AGAAACTAGACAGAAGCATTCTGAGAATCACGTTTGTGATGTGGGTACTCAACTAACAGTGTTGA
          1 AAAAACTAGACAGAAGCATTCTCAGAAACATCTTTGTGATGTTTGCACTCAACTCACAGAGTTGA

            *  *       *   *            *   *                *   *           
     157785 TCCATTCTTTTGATACAGCAGTTTTGAACCACACTTTTTGTAGAATCTGCAAGAGGATATTTGGA
         66 ACCTTTCTTTTCATAGAGCAGTTTTGAAACACTCTTTTTGTAGAATCTGGAAGTGGATATTTGGA

            **   *                     *   *      * *
     157850 TAGCTGTGAGGATTTCGTTGGAAACGGGAATGTCTTCAAAG
        131 GCGCTTTGAGGATTTCGTTGGAAACGGAAATATCTTCATAT

                *                         *   *            *    *            
     157891 AAAATCTAGACAGAAGCATTCTCAGAAACACCTTCGTGATGTTTGCAATCAAGTCACAGAGTTGA
          1 AAAAACTAGACAGAAGCATTCTCAGAAACATCTTTGTGATGTTTGCACTCAACTCACAGAGTTGA

                 * *             *  *                  *             *       
     157956 ACCTTCCGTTTCATAGAGCAGGTTGGAAACACTCTTTTTGTAGTATCTGGAAGTGGACATTTGGA
         66 ACCTTTCTTTTCATAGAGCAGTTTTGAAACACTCTTTTTGTAGAATCTGGAAGTGGATATTTGGA

                   *   ** ** *  *   *            **  
     158021 GCGCTTTCAGGCCTATGGTGAAAAAGGAAATATCTTCCCAT
        131 GCGCTTTGAGGATTTCGTTGGAAACGGAAATATCTTCATAT

                  * * *      **      *   * *   *     ** ** *      * *  *     
     158062 AAAAACGACATAGAAGCTATCTCAGGAACTTGTTTATGATGCATCTAATCAACTAAGAGTGTTGA
          1 AAAAACTAGACAGAAGCATTCTCAGAAACATCTTTGTGATGTTTGCACTCAACTCACAGAGTTGA

              *   * ** * *                         * *       *               
     158127 ACATTTGTACTGACAGAGCAG-TTTGAAACACTCTTTTTTTGGAATCTGCAAGTGGATATTTGGA
         66 ACCTTTCTTTTCATAGAGCAGTTTTGAAACACTCTTTTTGTAGAATCTGGAAGTGGATATTTGGA

            *                           *   *   *    
     158191 TCGCTTTGAGGATTTCGTTGGAAACGG-GAT-GC--AATAT
        131 GCGCTTTGAGGATTTCGTTGGAAACGGAAATATCTTCATAT

                **  *    *     *                 **  *   *  *    *         * 
     158228 AAAACGTACACAGCAGCATACTCAGAAA-ATACTTTGCCATATTTCCATTCAAGTCACAGAGTGG
          1 AAAAACTAGACAGAAGCATTCTCAGAAACAT-CTTTGTGATGTTTGCACTCAACTCACAGAGTTG

               *  * **            *                  *  *             *      
     158292 AACATTCCCATTCATAGAGCAGGTTTGAAACACTCTTTTTGGAGTATCTGGAAGTGGACATTTGG
         65 AACCTTTCTTTTCATAGAGCAGTTTTGAAACACTCTTTTTGTAGAATCTGGAAGTGGATATTTGG

                    ** * * ** *  *   *            **  
     158357 AGCGCTTTCTGAACTATGGTGAAAAAGGAAATATCTTCCAAT
        130 AGCGCTTTGAGGATTTCGTTGGAAACGGAAATATCTTCATAT

            *     *               *      * *          *  **      **  *  *    
     158399 GAAAACAAGACAGAAGCATTCTGAGAAACTTATTTGTGATGTGTGTCCTCAACAAACGGACTTGA
          1 AAAAACTAGACAGAAGCATTCTCAGAAACATCTTTGTGATGTTTGCACTCAACTCACAGAGTTGA

                   *      **  * *  *  *             *   *    * * *           
     158464 ACCTTTCGTTTCATGCAGTACTTCTGGAACACTCTTTTTGAAGATTCTGCATGCGGATATTTGGA
         66 ACCTTTCTTTTCATAGAGCAGTTTTGAAACACTCTTTTTGTAGAATCTGGAAGTGGATATTTGGA

            **                            **   *   * 
     158529 TAGCTTTGAGGATTTCGTTGGAAACGG---GCT-TACATGT
        131 GCGCTTTGAGGATTTCGTTGGAAACGGAAATATCTTCATAT

                 *       *               *        *   *    *    *       *    
     158566 AAAAATTAGACAGCAGCATTCTCAGAAACTTCTTTGTGGTGTCTGCATTCAAGTCACAGAATTGA
          1 AAAAACTAGACAGAAGCATTCTCAGAAACATCTTTGTGATGTTTGCACTCAACTCACAGAGTTGA

              * ** * **            *  * *      *       *    *        *       
     158631 ACTTCCCCTCACATAGAGCAGTTGTGCAGCACTCTATTTGTAGTATCTCGAAGTGGACATTTGGA
         66 ACCTTTCTTTTCATAGAGCAGTTTTGAAACACTCTTTTTGTAGAATCTGGAAGTGGATATTTGGA

             *         ** *    * *   *            **  
     158696 GGGCTTTGTAGCCTATC-TGGAAAAAGGAAATATCTTCCCAT
        131 GCGCTTTG-AGGATTTCGTTGGAAACGGAAATATCTTCATAT

            *  ** *   *     * *            *   *  **  * **        *  * * *   
     158737 GAATGCGAGATAGAAGTAATCTCAGAAACATGTTTATGCAGTATCTACTCAACTAACTGTGCTGA
          1 AAAAACTAGACAGAAGCATTCTCAGAAACATCTTTGTGATGTTTGCACTCAACTCACAGAGTTGA

              *     *  *               *        *  * **      *               
     158802 ACATTTCTATTGATAGAGCAGTTTTGAGACACTCTTCTTTTGAAATCTGCAAGTGGATATTTGGA
         66 ACCTTTCTTTTCATAGAGCAGTTTTGAAACACTCTTTTTGTAGAATCTGGAAGTGGATATTTGGA

            ** *                        *     *      
     158867 AAGATTTGAGGATTTCGTTGGAAACGG-GAT-T-AT-ATAT
        131 GCGCTTTGAGGATTTCGTTGGAAACGGAAATATCTTCATAT

                 *       *               *                 **  *   *         
     158904 AAAAAGTAGACAGCAGCATTCTCAGAAACTTCTTTGTGATGTTTGCATCCAGCTCTCAGAGTTGA
          1 AAAAACTAGACAGAAGCATTCTCAGAAACATCTTTGTGATGTTTGCACTCAACTCACAGAGTTGA

              *  * *          *  *        *        *   **   *    *  **       
     158969 ACATTCCCTTTCATAGAGTAGGTTTGAAACCCTCTTTTTATAGTGTCTAGAAGCGGGCATTTGGA
         66 ACCTTTCTTTTCATAGAGCAGTTTTGAAACACTCTTTTTGTAGAATCTGGAAGTGGATATTTGGA

                   *    * * **  **  **          * *  *
     159034 GCGCTTTCAGGCCTATGCTTAAAATAGGAAATATCTACCTAC
        131 GCGCTTTGAGG-ATTTCGTTGGAAACGGAAATATCTTCATAT

             *                    *    *  **          ** *        *    *     
     159076 AGAAACTAGACAGAAGCATTCTGAGAATCACGTTTGTGATGTGGGTACTCAACTAACAGTGTTGA
          1 AAAAACTAGACAGAAGCATTCTCAGAAACATCTTTGTGATGTTTGCACTCAACTCACAGAGTTGA

            *  *       *   *            *   *                *   *           
     159141 TCCATTCTTTTGATACAGCAGTTTTGAACCACACTTTTTGTAGAATCTGCAAGAGGATATTTGGA
         66 ACCTTTCTTTTCATAGAGCAGTTTTGAAACACTCTTTTTGTAGAATCTGGAAGTGGATATTTGGA

            **   *                     *   *      * *
     159206 TAGCTGTGAGGATTTCGTTGGAAACGGGAATGTCTTCAAAG
        131 GCGCTTTGAGGATTTCGTTGGAAACGGAAATATCTTCATAT

                *                         *   *            *    *            
     159247 AAAATCTAGACAGAAGCATTCTCAGAAACACCTTCGTGATGTTTGCAATCAAGTCACAGAGTTGA
          1 AAAAACTAGACAGAAGCATTCTCAGAAACATCTTTGTGATGTTTGCACTCAACTCACAGAGTTGA

                 * *             *  *                  *             *       
     159312 ACCTTCCGTTTCATAGAGCAGGTTGGAAACACTCTTTTTGTAGTATCTGGAAGTGGACATTTGGA
         66 ACCTTTCTTTTCATAGAGCAGTTTTGAAACACTCTTTTTGTAGAATCTGGAAGTGGATATTTGGA

                   *   ** ** *  *   *            **  
     159377 GCGCTTTCAGGCCTATGGTGAAAAAGGAAATATCTTCCCAT
        131 GCGCTTTGAGGATTTCGTTGGAAACGGAAATATCTTCATAT

                  * * *      **      *   * *   *     ** ** *      *    *     
     159418 AAAAACGACATAGAAGCTATCTCAGGAACTTGTTTATGATGCATCTAATCAACTAACAGTGTTGA
          1 AAAAACTAGACAGAAGCATTCTCAGAAACATCTTTGTGATGTTTGCACTCAACTCACAGAGTTGA

                  * ** * *                         * *       *               
     159483 ACCTTTGTACTGACAGAGCAG-TTTGAAACACTCTTTTTTTGGAATCTGCAAGTGGATATTTGGA
         66 ACCTTTCTTTTCATAGAGCAGTTTTGAAACACTCTTTTTGTAGAATCTGGAAGTGGATATTTGGA

            *                           *   *   *    
     159547 TCGCTTTGAGGATTTCGTTGGAAACGG-GAT-GC--AATAT
        131 GCGCTTTGAGGATTTCGTTGGAAACGGAAATATCTTCATAT

                **  *    *     *                 **  *   *  *    *         * 
     159584 AAAACGTACACAGCAGCATACTCAGAAA-ATACTTTGCCATATTTCCATTCAAGTCACAGAGTGG
          1 AAAAACTAGACAGAAGCATTCTCAGAAACAT-CTTTGTGATGTTTGCACTCAACTCACAGAGTTG

               *  * **            *                  *  *             *      
     159648 AACATTCCCATTCATAGAGCAGGTTTGAAACACTCTTTTTGGAGTATCTGGAAGTGGACATTTGG
         65 AACCTTTCTTTTCATAGAGCAGTTTTGAAACACTCTTTTTGTAGAATCTGGAAGTGGATATTTGG

                    ** * * ** *  *   *            **  
     159713 AGCGCTTTCTGAACTATGGTGAAAAAGGAAATATCTTCCAAT
        130 AGCGCTTTGAGGATTTCGTTGGAAACGGAAATATCTTCATAT

            *     *               *      * *          *  **      **  *  *    
     159755 GAAAACAAGACAGAAGCATTCTGAGAAACTTATTTGTGATGTGTGTCCTCAACAAACGGACTTGA
          1 AAAAACTAGACAGAAGCATTCTCAGAAACATCTTTGTGATGTTTGCACTCAACTCACAGAGTTGA

                   *      **  * *  *  *             *   *    * * *           
     159820 ACCTTTCGTTTCATGCAGTACTTCTGGAACACTCTTTTTGAAGATTCTGCATGCGGATATTTGGA
         66 ACCTTTCTTTTCATAGAGCAGTTTTGAAACACTCTTTTTGTAGAATCTGGAAGTGGATATTTGGA

            **                            **   *   * 
     159885 TAGCTTTGAGGATTTCGTTGGAAACGG---GCT-TACATGT
        131 GCGCTTTGAGGATTTCGTTGGAAACGGAAATATCTTCATAT

                 *       *               *        *   *    *    *       *    
     159922 AAAAATTAGACAGCAGCATTCTCAGAAACTTCTTTGTGGTGTCTGCATTCAAGTCACAGAATTGA
          1 AAAAACTAGACAGAAGCATTCTCAGAAACATCTTTGTGATGTTTGCACTCAACTCACAGAGTTGA

              * ** * **            *  * *              *             *       
     159987 ACATCCCCTCACATAGAGCAGTTGTGCAGCACTCTTTTTGTAGTATCTGGAAGTGGACATTTGGA
         66 ACCTTTCTTTTCATAGAGCAGTTTTGAAACACTCTTTTTGTAGAATCTGGAAGTGGATATTTGGA

             *         ** *    * *   *            **  
     160052 GGGCTTTGTAGCCTATC-TGGAAAAAGGAAATATCTTCCCAT
        131 GCGCTTTG-AGGATTTCGTTGGAAACGGAAATATCTTCATAT

            *  ** *   *     * *            *   *  *   * **        *  * * *   
     160093 GAATGCGAGATAGAAGTAATCTCAGAAACATGTTTATGCTGTATCTACTCAACTAACTGTGCTGA
          1 AAAAACTAGACAGAAGCATTCTCAGAAACATCTTTGTGATGTTTGCACTCAACTCACAGAGTTGA

              *     *  *               *        *  * *       *               
     160158 ACATTTCTATTGATAGAGCAGTTTTGAGACACTCTTCTTTTGGAATCTGCAAGTGGATATTTGGA
         66 ACCTTTCTTTTCATAGAGCAGTTTTGAAACACTCTTTTTGTAGAATCTGGAAGTGGATATTTGGA

            ** *                        *     *      
     160223 TAGATTTGAGGATTTCGTTGGAAACGG-GAT-T-AT-ATAT
        131 GCGCTTTGAGGATTTCGTTGGAAACGGAAATATCTTCATAT

                 *       *               *                 **  *   *         
     160260 AAAAAGTAGACAGCAGCATTCTCAGAAACTTCTTTGTGATGTTTGCATCCAGCTCTCAGAGTTGA
          1 AAAAACTAGACAGAAGCATTCTCAGAAACATCTTTGTGATGTTTGCACTCAACTCACAGAGTTGA

              *  * *          *  *        *        *   **        *  **       
     160325 ACATTCCCTTTCATAGAGTAGGTTTGAAACCCTCTTTTTATAGTGTCTGGAAGCGGGCATTTGGA
         66 ACCTTTCTTTTCATAGAGCAGTTTTGAAACACTCTTTTTGTAGAATCTGGAAGTGGATATTTGGA

                   *   ** ** *  *   *          * *   
     160390 GCGCTTTCAGGCCTATGCTGAAAAAGGAAATATCTACCTAT
        131 GCGCTTTGAGGATTTCGTTGGAAACGGAAATATCTTCATAT

             *                    *    *  **          ** *        *    *     
     160431 AGAAACTAGACAGAAGCATTCTGAGAATCACGTTTGTGATGTGAGTACTCAACTAACAGTGTTGA
          1 AAAAACTAGACAGAAGCATTCTCAGAAACATCTTTGTGATGTTTGCACTCAACTCACAGAGTTGA

            *  *       *   *            *   *                *               
     160496 TCCATTCTTTTGATACAGCAGTTTTGAACCACACTTTTTGTAGAATCTGCAAGTGGATATTTGGA
         66 ACCTTTCTTTTCATAGAGCAGTTTTGAAACACTCTTTTTGTAGAATCTGGAAGTGGATATTTGGA

            **   *                     *   *        *
     160561 TAGCTGTGAGGATTTCGTTGGAAACGGGAATGTCTTCATAG
        131 GCGCTTTGAGGATTTCGTTGGAAACGGAAATATCTTCATAT

                **                     * * **         *  **   *   *          
     160602 AAAATTTAGACAGAAGCATTCTCAGAACCTTGATTGTGATGTGTGTTCTCCACTAACAGAGTTGA
          1 AAAAACTAGACAGAAGCATTCTCAGAAACATCTTTGTGATGTTTGCACTCAACTCACAGAGTTGA

                       * *   * *   *       *       *                         
     160667 ACCTTTCTTTTGACAGAACTGTTCTGAAACATTCTTTTTATAGAATCTGGAAGTGGATATTTGGA
         66 ACCTTTCTTTTCATAGAGCAGTTTTGAAACACTCTTTTTGTAGAATCTGGAAGTGGATATTTGGA

            **                         *          *  
     160732 AAGCTTTGAGGATTTCGTTGGAAACGGGAATATCTTCAAAT
        131 GCGCTTTGAGGATTTCGTTGGAAACGGAAATATCTTCATAT

                *    *            *           * *       *  *    *            
     160773 AAAATCTAGCCAGAAGCATTCTAAGAAACATCTTAGGGATGTTTACATTCAAGTCACAGAGTTGA
          1 AAAAACTAGACAGAAGCATTCTCAGAAACATCTTTGTGATGTTTGCACTCAACTCACAGAGTTGA

              *  * *     *       *         *    * *   **      *      *    *  
     160838 ACATTCCCTTTCACAGAGCAGGTTTGAAACAATCTTCTCGTACTATCTGGCAGTGGACATTTTGA
         66 ACCTTTCTTTTCATAGAGCAGTTTTGAAACACTCTTTTTGTAGAATCTGGAAGTGGATATTTGGA

              * *   *  ** ** *  *   *            ** *
     160903 GCTCCTTGGGGCCTATGCTGAAAAAGGAAATATCTTCCGAC
        131 GCGCTTTGAGGATTTCGTTGGAAACGGAAATATCTTCATAT

                                 *     *  **          *           **    *    
     160944 AAAAACTAGACAGAAGCATTCGCAGAATCACGTTTGTGATGTGTGCACTCAACTGTCAGAATTGA
          1 AAAAACTAGACAGAAGCATTCTCAGAAACATCTTTGTGATGTTTGCACTCAACTCACAGAGTTGA

                 **   ** *      *                            * *            *
     161009 ACCTTGGTTTGGACAGAGCACTTTTGAAACACTCTTTTTGTAGAATCTGCAGGTGGATATTTGGC
         66 ACCTTTCTTTTCATAGAGCAGTTTTGAAACACTCTTTTTGTAGAATCTGGAAGTGGATATTTGGA

            **                         *   *      * *
     161074 TAGCTTTGAGGATTTCGTTGGAAACGGTAATGTCTTCAAAG
        131 GCGCTTTGAGGATTTCGTTGGAAACGGAAATATCTTCATAT

                *                         *   *            *    *            
     161115 AAAATCTAGACAGAAGCATTCTCAGAAACACCTTCGTGATGTTTGCAATCAAGTCACAGAGTTGA
          1 AAAAACTAGACAGAAGCATTCTCAGAAACATCTTTGTGATGTTTGCACTCAACTCACAGAGTTGA

                 * *             *  *                  *             *       
     161180 ACCTTCCGTTTCATAGAGCAGGTTGGAAACACTCTTTTTGTAGTATCTGGAAGTGGACATTTGGA
         66 ACCTTTCTTTTCATAGAGCAGTTTTGAAACACTCTTTTTGTAGAATCTGGAAGTGGATATTTGGA

                   *   ** ** *  *   *            **  
     161245 GCGCTTTCAGGCCTATGGTGAAAAAGGAAATATCTTCCCAT
        131 GCGCTTTGAGGATTTCGTTGGAAACGGAAATATCTTCATAT

                  * * *      **      *   * *   *     ** ** *      *    *     
     161286 AAAAACGACATAGAAGCTATCTCAGGAACTTGTTTATGATGCATCTAATCAACTAACAGTGTTGA
          1 AAAAACTAGACAGAAGCATTCTCAGAAACATCTTTGTGATGTTTGCACTCAACTCACAGAGTTGA

                  * ** * *                         * *       *               
     161351 ACCTTTGTACTGACAGAGCAG-TTTGAAACACTCTTTTTTTGGAATCTGCAAGTGGATATTTGGA
         66 ACCTTTCTTTTCATAGAGCAGTTTTGAAACACTCTTTTTGTAGAATCTGGAAGTGGATATTTGGA

            *                           *   *   *    
     161415 TCGCTTTGAGGATTTCGTTGGAAACGG-GAT-GC--AATAT
        131 GCGCTTTGAGGATTTCGTTGGAAACGGAAATATCTTCATAT

                **  *    *     *                 **  *   *  *    *         * 
     161452 AAAACGTACACAGCAGCATACTCAGAAA-ATACTTTGCCATATTTCCATTCAAGTCACAGAGTGG
          1 AAAAACTAGACAGAAGCATTCTCAGAAACAT-CTTTGTGATGTTTGCACTCAACTCACAGAGTTG

               *  * **            *                  *  *             **     
     161516 AACATTCCCATTCATAGAGCAGGTTTGAAACACTCTTTTTGGAGTATCTGGAAGTGGACTTTTGG
         65 AACCTTTCTTTTCATAGAGCAGTTTTGAAACACTCTTTTTGTAGAATCTGGAAGTGGATATTTGG

                    ** * * ** *  *   *    *       **  
     161581 AGCGCTTTCTGAACTATGGTGAAAAAGGAAGTATCTTCCAAT
        130 AGCGCTTTGAGGATTTCGTTGGAAACGGAAATATCTTCATAT

            *     *          *  * *      * *      *   *  **      **  *  *    
     161623 GAAAACAAGACAGAAGCTTTATGAGAAACTTATTTGTGGTGTGTGTCCTCAACAAACGGACTTGA
          1 AAAAACTAGACAGAAGCATTCTCAGAAACATCTTTGTGATGTTTGCACTCAACTCACAGAGTTGA

                   *      **  * *  *  *             *   *    * * *           
     161688 ACCTTTCGTTTCATGCAGTACTTCTGGAACACTCTTTTTGAAGATTCTGCATGCGGATATTTGGA
         66 ACCTTTCTTTTCATAGAGCAGTTTTGAAACACTCTTTTTGTAGAATCTGGAAGTGGATATTTGGA

            **              *             **   *  ** 
     161753 TAGCTTTGAGGATTTCATTGGAAACGG---GCT-TACAAGT
        131 GCGCTTTGAGGATTTCGTTGGAAACGGAAATATCTTCATAT

                 *       *               *        *   *    *    *       *    
     161790 AAAAATTAGACAGCAGCATTCTCAGAAACTTCTTTGTGGTGTCTGCATTCAAGTCACAGAATTGA
          1 AAAAACTAGACAGAAGCATTCTCAGAAACATCTTTGTGATGTTTGCACTCAACTCACAGAGTTGA

              * ** * **            *  * *      *       *             *       
     161855 ACATCCCCTCACATAGAGCAGTTGTGCAGCACTCTATTTGTAGTATCTGGAAGTGGACATTTGGA
         66 ACCTTTCTTTTCATAGAGCAGTTTTGAAACACTCTTTTTGTAGAATCTGGAAGTGGATATTTGGA

             *         ** *    * *   *            **  
     161920 GGGCTTTGTAGCCTATC-TGGAAAAAGGAAATATCTTCCCAT
        131 GCGCTTTG-AGGATTTCGTTGGAAACGGAAATATCTTCATAT

            *  ** *   *     * * *          *   *  *   * **        *  * * *   
     161961 GAATGCGAGATAGAAGTAATGTCAGAAACATGTTTATGCTGTATCTACTCAACTAACTGTGCTGA
          1 AAAAACTAGACAGAAGCATTCTCAGAAACATCTTTGTGATGTTTGCACTCAACTCACAGAGTTGA

              *     *  *               *        *  * *       *               
     162026 ACATTTCTATTGATAGAGCAGTTTTGAGACACTCTTCTTTTGGAATCTGCAAGTGGATATTTGGA
         66 ACCTTTCTTTTCATAGAGCAGTTTTGAAACACTCTTTTTGTAGAATCTGGAAGTGGATATTTGGA

            ** *                        *     *      
     162091 TAGATTTGAGGATTTCGTTGGAAACGG-GAT-T-AT-ATAT
        131 GCGCTTTGAGGATTTCGTTGGAAACGGAAATATCTTCATAT

                 *       *               *                 **  *   *         
     162128 AAAAAGTAGACAGCAGCATTCTCAGAAACTTCTTTGTGATGTTTGCATCCAGCTCTCAGAGTTGA
          1 AAAAACTAGACAGAAGCATTCTCAGAAACATCTTTGTGATGTTTGCACTCAACTCACAGAGTTGA

              *  * *          *  *        *        *   **        *  ***      
     162193 ACATTCCCTTTCATAGAGTAGGTTTGAAACCCTCTTTTTATAGTGTCTGGAAGCGGGCGTTTGGA
         66 ACCTTTCTTTTCATAGAGCAGTTTTGAAACACTCTTTTTGTAGAATCTGGAAGTGGATATTTGGA

                   *   ** ** *  *   *          * *   
     162258 GCGCTTTCAGGCCTATGCTGAAAAAGGAAATATCTACCTAT
        131 GCGCTTTGAGGATTTCGTTGGAAACGGAAATATCTTCATAT

             *                    *    *  **          ** *        *    *     
     162299 AGAAACTAGACAGAAGCATTCTGAGAATCACGTTTGTGATGTGGGTACTCAACTAACAGTGTTGA
          1 AAAAACTAGACAGAAGCATTCTCAGAAACATCTTTGTGATGTTTGCACTCAACTCACAGAGTTGA

            *  *       *   *            *   *                *               
     162364 TCCATTCTTTTGATACAGCAGTTTTGAACCACACTTTTTGTAGAATCTGCAAGTGGATATTTGGA
         66 ACCTTTCTTTTCATAGAGCAGTTTTGAAACACTCTTTTTGTAGAATCTGGAAGTGGATATTTGGA

            **   *                     *   *        *
     162429 TAGCTGTGAGGATTTCGTTGGAAACGGGAATGTCTTCATAG
        131 GCGCTTTGAGGATTTCGTTGGAAACGGAAATATCTTCATAT

                **                     * * **         *  **   *   *          
     162470 AAAATTTAGACAGAAGCATTCTCAGAACCTTGATTGTGATGTGTGTTCTCCACTAACAGAGTTGA
          1 AAAAACTAGACAGAAGCATTCTCAGAAACATCTTTGTGATGTTTGCACTCAACTCACAGAGTTGA

                       * *   * *   *       *       *                         
     162535 ACCTTTCTTTTGACAGAACTGTTCTGAAACATTCTTTTTATAGAATCTGGAAGTGGATATTTGGA
         66 ACCTTTCTTTTCATAGAGCAGTTTTGAAACACTCTTTTTGTAGAATCTGGAAGTGGATATTTGGA

            **                         *          *  
     162600 AAGCTTTGAGGATTTCGTTGGAAACGGGAATATCTTCAAAT
        131 GCGCTTTGAGGATTTCGTTGGAAACGGAAATATCTTCATAT

                *    *       *    *           * *       *  *    *            
     162641 AAAATCTAGCCAGAAGCTTTCTAAGAAACATCTTAGGGATGTTTACATTCAAGTCACAGAGTTGA
          1 AAAAACTAGACAGAAGCATTCTCAGAAACATCTTTGTGATGTTTGCACTCAACTCACAGAGTTGA

              *  * *     *       *         *    * *   **      *      *    *  
     162706 ACATTCCCTTTCACAGAGCAGGTTTGAAACAATCTTCTCGTACTATCTGGCAGTGGACATTTTGA
         66 ACCTTTCTTTTCATAGAGCAGTTTTGAAACACTCTTTTTGTAGAATCTGGAAGTGGATATTTGGA

              * *   *  ** ** *  *   *            ** *
     162771 GCTCCTTGGGGCCTATGCTGAAAAAGGAAATATCTTCCGAC
        131 GCGCTTTGAGGATTTCGTTGGAAACGGAAATATCTTCATAT

                                 *     *  **          *           **    *    
     162812 AAAAACTAGACAGAAGCATTCGCAGAATCACGTTTGTGATGTGTGCACTCAACTGTCAGAATTGA
          1 AAAAACTAGACAGAAGCATTCTCAGAAACATCTTTGTGATGTTTGCACTCAACTCACAGAGTTGA

                 **   ** *      *                            * *            *
     162877 ACCTTGGTTTGGACAGAGCACTTTTGAAACACTCTTTTTGTAGAATCTGCAGGTGGATATTTGGC
         66 ACCTTTCTTTTCATAGAGCAGTTTTGAAACACTCTTTTTGTAGAATCTGGAAGTGGATATTTGGA

            **                         *   *      * *
     162942 TAGCTTTGAGGATTTCGTTGGAAACGGTAATGTCTTCAAAG
        131 GCGCTTTGAGGATTTCGTTGGAAACGGAAATATCTTCATAT

                *                         *   *            *    *            
     162983 AAAATCTAGACAGAAGCATTCTCAGAAACACCTTCGTGATGTTTGCAATCAAGTCACAGAGTTGA
          1 AAAAACTAGACAGAAGCATTCTCAGAAACATCTTTGTGATGTTTGCACTCAACTCACAGAGTTGA

                 * *             *  *                  *             *       
     163048 ACCTTCCGTTTCATAGAGCAGGTTGGAAACACTCTTTTTGTAGTATCTGGAAGTGGACATTTGGA
         66 ACCTTTCTTTTCATAGAGCAGTTTTGAAACACTCTTTTTGTAGAATCTGGAAGTGGATATTTGGA

                   *   ** ** *  *   *            **  
     163113 GCGCTTTCAGGCCTATGGTGAAAAAGGAAATATCTTCCCAT
        131 GCGCTTTGAGGATTTCGTTGGAAACGGAAATATCTTCATAT

                  * * *      **      *   * *   *     ** ** *      *    *     
     163154 AAAAACGACATAGAAGCTATCTCAGGAACTTGTTTATGATGCATCTAATCAACTAACAGTGTTGA
          1 AAAAACTAGACAGAAGCATTCTCAGAAACATCTTTGTGATGTTTGCACTCAACTCACAGAGTTGA

                  * ** * *                         * *       *               
     163219 ACCTTTGTACTGACAGAGCAG-TTTGAAACACTCTTTTTTTGGAATCTGCAAGTGGATATTTGGA
         66 ACCTTTCTTTTCATAGAGCAGTTTTGAAACACTCTTTTTGTAGAATCTGGAAGTGGATATTTGGA

            *                           *   *   *    
     163283 TCGCTTTGAGGATTTCGTTGGAAACGG-GAT-GC--AATAT
        131 GCGCTTTGAGGATTTCGTTGGAAACGGAAATATCTTCATAT

                **  *    *     *                 **  *   *  *    *         * 
     163320 AAAACGTACACAGCAGCATACTCAGAAA-ATACTTTGCCATATTTCCATTCAAGTCACAGAGTGG
          1 AAAAACTAGACAGAAGCATTCTCAGAAACAT-CTTTGTGATGTTTGCACTCAACTCACAGAGTTG

               *  * **            *                  *  *             *      
     163384 AACATTCCCATTCATAGAGCAGGTTTGAAACACTCTTTTTGGAGTATCTGGAAGTGGACATTTGG
         65 AACCTTTCTTTTCATAGAGCAGTTTTGAAACACTCTTTTTGTAGAATCTGGAAGTGGATATTTGG

                    ** * * ** *  *   *            **  
     163449 AGCGCTTTCTGAACTATGGTGAAAAAGGAAATATCTTCCAAT
        130 AGCGCTTTGAGGATTTCGTTGGAAACGGAAATATCTTCATAT

            *     *               *      * *          *  **      **  *  *    
     163491 GAAAACAAGACAGAAGCATTCTGAGAAACTTATTTGTGATGTGTGTCCTCAACAAACGGACTTGA
          1 AAAAACTAGACAGAAGCATTCTCAGAAACATCTTTGTGATGTTTGCACTCAACTCACAGAGTTGA

                   *      **  * *  *  *             *   *    * * *           
     163556 ACCTTTCGTTTCATGCAGTACTTCTGGAACACTCTTTTTGAAGATTCTGCATGCGGATATTTGGA
         66 ACCTTTCTTTTCATAGAGCAGTTTTGAAACACTCTTTTTGTAGAATCTGGAAGTGGATATTTGGA

            **                          **         * 
     163621 TAGCTTTGAGGATTTCGTTGGAAACGG-GCT-TC--CATGT
        131 GCGCTTTGAGGATTTCGTTGGAAACGGAAATATCTTCATAT

                 *       *               *        *   *    *    *       *    
     163658 AAAAATTAGACAGCAGCATTCTCAGAAACTTCTTTGTGGTGTCTGCATTCAAGTCACAGAATTGA
          1 AAAAACTAGACAGAAGCATTCTCAGAAACATCTTTGTGATGTTTGCACTCAACTCACAGAGTTGA

              * ** * ***           *  * *              *             *       
     163723 ACATCCCCTCATATAGAGCAGTTGTGCAGCACTCTTTTTGTAGTATCTGGAAGTGGACATTTGGA
         66 ACCTTTCTTTTCATAGAGCAGTTTTGAAACACTCTTTTTGTAGAATCTGGAAGTGGATATTTGGA

             *         ** *    * *   *            **  
     163788 GGGCTTTGTAGCCTATC-TGGAAAAAGGAAATATCTTCCCAT
        131 GCGCTTTG-AGGATTTCGTTGGAAACGGAAATATCTTCATAT

            *  ** *   *     * *            *   *  *   * **        *  * * *   
     163829 GAATGCGAGATAGAAGTAATCTCAGAAACATGTTTATGCTGTATCTACTCAACTAACTGTGCTGA
          1 AAAAACTAGACAGAAGCATTCTCAGAAACATCTTTGTGATGTTTGCACTCAACTCACAGAGTTGA

              *     *  *               *        *  * *       *   *           
     163894 ACATTTCTATTGATAGAGCAGTTTTGAGACACTCTTCTTTTGGAATCTGCAAGCGGATATTTGGA
         66 ACCTTTCTTTTCATAGAGCAGTTTTGAAACACTCTTTTTGTAGAATCTGGAAGTGGATATTTGGA

            ** *                        *     *      
     163959 TAGATTTGAGGATTTCGTTGGAAACGG-GAT-T-AT-ATAT
        131 GCGCTTTGAGGATTTCGTTGGAAACGGAAATATCTTCATAT

                 *       *               *                 **  *   *         
     163996 AAAAAGTAGACAGCAGCATTCTCAGAAACTTCTTTGTGATGTTTGCATCCAGCTCTCAGAGTTGA
          1 AAAAACTAGACAGAAGCATTCTCAGAAACATCTTTGTGATGTTTGCACTCAACTCACAGAGTTGA

              *  * *          *  *        *        *   **        *  **       
     164061 ACATTCCCTTTCATAGAGTAGGTTTGAAACCCTCTTTTTATAGTGTCTGGAAGCGGGCATTTGGA
         66 ACCTTTCTTTTCATAGAGCAGTTTTGAAACACTCTTTTTGTAGAATCTGGAAGTGGATATTTGGA

                   *   ** ** *  *   *          * *   
     164126 GCGCTTTCAGGCCTATGCTGAAAAAGGAAATATCTACCTAT
        131 GCGCTTTGAGGATTTCGTTGGAAACGGAAATATCTTCATAT

             *                    *    *  **          ** *        *    *     
     164167 AGAAACTAGACAGAAGCATTCTGAGAATCACGTTTGTGATGTGAGTACTCAACTAACAGTGTTGA
          1 AAAAACTAGACAGAAGCATTCTCAGAAACATCTTTGTGATGTTTGCACTCAACTCACAGAGTTGA

            *  *       *   *            *   *                *               
     164232 TCCATTCTTTTGATACAGCAGTTTTGAACCACACTTTTTGTAGAATCTGCAAGTGGATATTTGGA
         66 ACCTTTCTTTTCATAGAGCAGTTTTGAAACACTCTTTTTGTAGAATCTGGAAGTGGATATTTGGA

            **   *                     *   *        *
     164297 TAGCTGTGAGGATTTCGTTGGAAACGGGAATGTCTTCATAG
        131 GCGCTTTGAGGATTTCGTTGGAAACGGAAATATCTTCATAT

                **                     * * **         *  **   *   *          
     164338 AAAATTTAGACAGAAGCATTCTCAGAACCTTGATTGTGATGTGTGTTCTCCACTAACAGAGTTGA
          1 AAAAACTAGACAGAAGCATTCTCAGAAACATCTTTGTGATGTTTGCACTCAACTCACAGAGTTGA

                       * *   * *   *       *       *                         
     164403 ACCTTTCTTTTGACAGAACTGTTCTGAAACATTCTTTTTATAGAATCTGGAAGTGGATATTTGGA
         66 ACCTTTCTTTTCATAGAGCAGTTTTGAAACACTCTTTTTGTAGAATCTGGAAGTGGATATTTGGA

            **                         *          *  
     164468 AAGCTTTGAGGATTTCGTTGGAAACGGGAATATCTTCAAAT
        131 GCGCTTTGAGGATTTCGTTGGAAACGGAAATATCTTCATAT

                *    *            *           * *       *  *    *            
     164509 AAAATCTAGCCAGAAGCATTCTAAGAAACATCTTAGGGATGTTTACATTCAAGTCACAGAGTTGA
          1 AAAAACTAGACAGAAGCATTCTCAGAAACATCTTTGTGATGTTTGCACTCAACTCACAGAGTTGA

              *  * *     *       *         *    * *   **      *      *    *  
     164574 ACATTCCCTTTCACAGAGCAGGTTTGAAACAATCTTCTCGTACTATCTGGCAGTGGACATTTTGA
         66 ACCTTTCTTTTCATAGAGCAGTTTTGAAACACTCTTTTTGTAGAATCTGGAAGTGGATATTTGGA

              * *   *  ** ** *  *   *            ** *
     164639 GCTCCTTGGGGCCTATGCTGAAAAAGGAAATATCTTCCGAC
        131 GCGCTTTGAGGATTTCGTTGGAAACGGAAATATCTTCATAT

                                 *     *  **          *           **    *    
     164680 AAAAACTAGACAGAAGCATTCGCAGAATCACGTTTGTGATGTGTGCACTCAACTGTCAGAATTGA
          1 AAAAACTAGACAGAAGCATTCTCAGAAACATCTTTGTGATGTTTGCACTCAACTCACAGAGTTGA

                 **   ** *      *                            * *            *
     164745 ACCTTGGTTTGGACAGAGCACTTTTGAAACACTCTTTTTGTAGAATCTGCAGGTGGATATTTGGC
         66 ACCTTTCTTTTCATAGAGCAGTTTTGAAACACTCTTTTTGTAGAATCTGGAAGTGGATATTTGGA

            **   *                     *   *      * *
     164810 TAGCTGTGAGGATTTCGTTGGAAACGGGAATGTCTTCAAAG
        131 GCGCTTTGAGGATTTCGTTGGAAACGGAAATATCTTCATAT

                *                         *   *            *    *            
     164851 AAAATCTAGACAGAAGCATTCTCAGAAACACCTTCGTGATGTTTGCAATCAAGTCACAGAGTTGA
          1 AAAAACTAGACAGAAGCATTCTCAGAAACATCTTTGTGATGTTTGCACTCAACTCACAGAGTTGA

                 * *             *  *                  *             *       
     164916 ACCTTCCGTTTCATAGAGCAGGTTGGAAACACTCTTTTTGTAGTATCTGGAAGTGGACATTTGGA
         66 ACCTTTCTTTTCATAGAGCAGTTTTGAAACACTCTTTTTGTAGAATCTGGAAGTGGATATTTGGA

                   *   ** ** *  *   *            **  
     164981 GCGCTTTCAGGCCTATGGTGAAAAAGGAAATATCTTCCCAT
        131 GCGCTTTGAGGATTTCGTTGGAAACGGAAATATCTTCATAT

                  * * *      **      *   * *   *     ** ** *      *    *     
     165022 AAAAACGACATAGAAGCTATCTCAGGAACTTGTTTATGATGCATCTAATCAACTAACAGTGTTGA
          1 AAAAACTAGACAGAAGCATTCTCAGAAACATCTTTGTGATGTTTGCACTCAACTCACAGAGTTGA

                  * ** * *                         * *       *               
     165087 ACCTTTGTACTGACAGAGCAG-TTTGAAACACTCTTTTTTTGGAATCTGCAAGTGGATATTTGGA
         66 ACCTTTCTTTTCATAGAGCAGTTTTGAAACACTCTTTTTGTAGAATCTGGAAGTGGATATTTGGA

            *                           *   *   *    
     165151 TCGCTTTGAGGATTTCGTTGGAAACGG-GAT-GC--AATAT
        131 GCGCTTTGAGGATTTCGTTGGAAACGGAAATATCTTCATAT

                **  *    *     *                 **  *   *  *    *         * 
     165188 AAAACGTACACAGCAGCATACTCAGAAA-ATACTTTGCCATATTTCCATTCAAGTCACAGAGTGG
          1 AAAAACTAGACAGAAGCATTCTCAGAAACAT-CTTTGTGATGTTTGCACTCAACTCACAGAGTTG

               *  * **            *                  *  *             *      
     165252 AACATTCCCATTCATAGAGCAGGTTTGAAACACTCTTTTTGGAGTATCTGGAAGTGGACATTTGG
         65 AACCTTTCTTTTCATAGAGCAGTTTTGAAACACTCTTTTTGTAGAATCTGGAAGTGGATATTTGG

                    ** * * ** *  *   *            **  
     165317 AGCGCTTTCTGAACTATGGTGAAAAAGGAAATATCTTCCAAT
        130 AGCGCTTTGAGGATTTCGTTGGAAACGGAAATATCTTCATAT

            *     *               *      * *          *  **      **  *  *    
     165359 GAAAACAAGACAGAAGCATTCTGAGAAACTTATTTGTGATGTGTGTCCTCAACAAACGGACTTGA
          1 AAAAACTAGACAGAAGCATTCTCAGAAACATCTTTGTGATGTTTGCACTCAACTCACAGAGTTGA

                   *      **  * *  *  *             *   *    * * *           
     165424 ACCTTTCGTTTCATGCAGTACTTCTGGAACACTCTTTTTGAAGATTCTGCATGCGGATATTTGGA
         66 ACCTTTCTTTTCATAGAGCAGTTTTGAAACACTCTTTTTGTAGAATCTGGAAGTGGATATTTGGA

            **                            **   *   * 
     165489 TAGCTTTGAGGATTTCGTTGGAAACGG---GCT-TACATGT
        131 GCGCTTTGAGGATTTCGTTGGAAACGGAAATATCTTCATAT

                 *       *               *        *   *    *    *       *    
     165526 AAAAATTAGACAGCAGCATTCTCAGAAACTTCTTTGTGGTGTCTGCATTCAAGTCACAGAATTGA
          1 AAAAACTAGACAGAAGCATTCTCAGAAACATCTTTGTGATGTTTGCACTCAACTCACAGAGTTGA

              * ** * **            *  * *              *             *       
     165591 ACATCCCCTCACATAGAGCAGTTGTGCAGCACTCTTTTTGTAGTATCTGGAAGTGGACATTTGGA
         66 ACCTTTCTTTTCATAGAGCAGTTTTGAAACACTCTTTTTGTAGAATCTGGAAGTGGATATTTGGA

             *         ** *    * *   *            **  
     165656 GGGCTTTGTAGCCTATC-TGGAAAAAGGAAATATCTTCCCAT
        131 GCGCTTTG-AGGATTTCGTTGGAAACGGAAATATCTTCATAT

            *  ** *   *     * *            *   *  *   * **        *  * * *   
     165697 GAATGCGAGATAGAAGTAATCTCAGAAACATGTTTATGCTGTATCTACTCAACTAACTGTGCTGA
          1 AAAAACTAGACAGAAGCATTCTCAGAAACATCTTTGTGATGTTTGCACTCAACTCACAGAGTTGA

              *     *  *               *        *  * *       *               
     165762 ACATTTCTATTGATAGAGCAGTTTTGAGACACTCTTCTTTTGGAATCTGCAAGTGGATATTTGGA
         66 ACCTTTCTTTTCATAGAGCAGTTTTGAAACACTCTTTTTGTAGAATCTGGAAGTGGATATTTGGA

            ** *                        *     *      
     165827 TAGATTTGAGGATTTCGTTGGAAACGG-GAT-T-AT-ATAT
        131 GCGCTTTGAGGATTTCGTTGGAAACGGAAATATCTTCATAT

                 *       *               *                 **  *   *         
     165864 AAAAAGTAGACAGCAGCATTCTCAGAAACTTCTTTGTGATGTTTGCATCCAGCTCTCAGAGTTGA
          1 AAAAACTAGACAGAAGCATTCTCAGAAACATCTTTGTGATGTTTGCACTCAACTCACAGAGTTGA

              *  * *          *  *        *        *   **        *  **       
     165929 ACATTCCCTTTCATAGAGTAGGTTTGAAACCCTCTTTTTATAGTGTCTGGAAGCGGGCATTTGGA
         66 ACCTTTCTTTTCATAGAGCAGTTTTGAAACACTCTTTTTGTAGAATCTGGAAGTGGATATTTGGA

                   *   ** ** *  *   *          * *   
     165994 GCGCTTTCAGGCCTATGCTGAAAAAGGAAATATCTACCTAT
        131 GCGCTTTGAGGATTTCGTTGGAAACGGAAATATCTTCATAT

             *                    *    *  **          ** *        *    *     
     166035 AGAAACTAGACAGAAGCATTCTGAGAATCACGTTTGTGATGTGAGTACTCAACTAACAGTGTTGA
          1 AAAAACTAGACAGAAGCATTCTCAGAAACATCTTTGTGATGTTTGCACTCAACTCACAGAGTTGA

            *  *       *   *            *   *                *               
     166100 TCCATTCTTTTGATACAGCAGTTTTGAACCACACTTTTTGTAGAATCTGCAAGTGGATATTTGGA
         66 ACCTTTCTTTTCATAGAGCAGTTTTGAAACACTCTTTTTGTAGAATCTGGAAGTGGATATTTGGA

            **   *                     *   *        *
     166165 TAGCTGTGAGGATTTCGTTGGAAACGGGAATGTCTTCATAG
        131 GCGCTTTGAGGATTTCGTTGGAAACGGAAATATCTTCATAT

                **                     * * **         *  **   *   *          
     166206 AAAATTTAGACAGAAGCATTCTCAGAACCTTGATTGTGATGTGTGTTCTCCACTAACAGAGTTGA
          1 AAAAACTAGACAGAAGCATTCTCAGAAACATCTTTGTGATGTTTGCACTCAACTCACAGAGTTGA

                       * *   * *   *       *       *                         
     166271 ACCTTTCTTTTGACAGAACTGTTCTGAAACATTCTTTTTATAGAATCTGGAAGTGGATATTTGGA
         66 ACCTTTCTTTTCATAGAGCAGTTTTGAAACACTCTTTTTGTAGAATCTGGAAGTGGATATTTGGA

            **                         *          *  
     166336 AAGCTTTGAGGATTTCGTTGGAAACGGGAATATCTTCAAAT
        131 GCGCTTTGAGGATTTCGTTGGAAACGGAAATATCTTCATAT

                *    *            *           * *       *  *    *            
     166377 AAAATCTAGCCAGAAGCATTCTAAGAAACATCTTAGGGATGTTTACATTCAAGTCACAGAGTTGA
          1 AAAAACTAGACAGAAGCATTCTCAGAAACATCTTTGTGATGTTTGCACTCAACTCACAGAGTTGA

              *  * *     *       *         *    * *   **      *      *    *  
     166442 ACATTCCCTTTCACAGAGCAGGTTTGAAACAATCTTCTCGTACTATCTGGCAGTGGACATTTTGA
         66 ACCTTTCTTTTCATAGAGCAGTTTTGAAACACTCTTTTTGTAGAATCTGGAAGTGGATATTTGGA

              * *   *  ** ** *  *   *            ** *
     166507 GCTCCTTGGGGCCTATGCTGAAAAAGGAAATATCTTCCGAC
        131 GCGCTTTGAGGATTTCGTTGGAAACGGAAATATCTTCATAT

                                 *     *  **          *           **    *    
     166548 AAAAACTAGACAGAAGCATTCGCAGAATCACGTTTGTGATGTGTGCACTCAACTGTCAGAATTGA
          1 AAAAACTAGACAGAAGCATTCTCAGAAACATCTTTGTGATGTTTGCACTCAACTCACAGAGTTGA

                 **   ** *      *                            * *            *
     166613 ACCTTGGTTTGGACAGAGCACTTTTGAAACACTCTTTTTGTAGAATCTGCAGGTGGATATTTGGC
         66 ACCTTTCTTTTCATAGAGCAGTTTTGAAACACTCTTTTTGTAGAATCTGGAAGTGGATATTTGGA

            **                         *   *      * *
     166678 TAGCTTTGAGGATTTCGTTGGAAACGGTAATGTCTTCAAAG
        131 GCGCTTTGAGGATTTCGTTGGAAACGGAAATATCTTCATAT

                *                         *   *            *    *            
     166719 AAAATCTAGACAGAAGCATTCTCAGAAACACCTTCGTGATGTTTGCAATCAAGTCACAGAGTTGA
          1 AAAAACTAGACAGAAGCATTCTCAGAAACATCTTTGTGATGTTTGCACTCAACTCACAGAGTTGA

                 * *             *  *                  *             *       
     166784 ACCTTCCGTTTCATAGAGCAGGTTGGAAACACTCTTTTTGTAGTATCTGGAAGTGGACATTTGGA
         66 ACCTTTCTTTTCATAGAGCAGTTTTGAAACACTCTTTTTGTAGAATCTGGAAGTGGATATTTGGA

                   *   ** ** *  *   *            **  
     166849 GCGCTTTCAGGCCTATGGTGAAAAAGGAAATATCTTCCCAT
        131 GCGCTTTGAGGATTTCGTTGGAAACGGAAATATCTTCATAT

                  * * *      **      *   * *   *     ** ** *      *    *     
     166890 AAAAACGACATAGAAGCTATCTCAGGAACTTGTTTATGATGCATCTAATCAACTAACAGTGTTGA
          1 AAAAACTAGACAGAAGCATTCTCAGAAACATCTTTGTGATGTTTGCACTCAACTCACAGAGTTGA

                  * ** * *                         * *       *               
     166955 ACCTTTGTACTGACAGAGCAG-TTTGAAACACTCTTTTTTTGGAATCTGCAAGTGGATATTTGGA
         66 ACCTTTCTTTTCATAGAGCAGTTTTGAAACACTCTTTTTGTAGAATCTGGAAGTGGATATTTGGA

            *                           *   *   *    
     167019 TCGCTTTGAGGATTTCGTTGGAAACGG-GAT-GC--AATAT
        131 GCGCTTTGAGGATTTCGTTGGAAACGGAAATATCTTCATAT

                **  *    *     *                 **  *   *  *    *         * 
     167056 AAAACGTACACAGCAGCATACTCAGAAA-ATACTTTGCCATATTTCCATTCAAGTCACAGAGTGG
          1 AAAAACTAGACAGAAGCATTCTCAGAAACAT-CTTTGTGATGTTTGCACTCAACTCACAGAGTTG

               *  * **            *                  *  *             **     
     167120 AACATTCCCATTCATAGAGCAGGTTTGAAACACTCTTTTTGGAGTATCTGGAAGTGGACTTTTGG
         65 AACCTTTCTTTTCATAGAGCAGTTTTGAAACACTCTTTTTGTAGAATCTGGAAGTGGATATTTGG

                    ** * * ** *  *   *    *       **  
     167185 AGCGCTTTCTGAACTATGGTGAAAAAGGAAGTATCTTCCAAT
        130 AGCGCTTTGAGGATTTCGTTGGAAACGGAAATATCTTCATAT

            *     *          *  * *      * *      *   *  **      **  *  *    
     167227 GAAAACAAGACAGAAGCTTTATGAGAAACTTATTTGTGGTGTGTGTCCTCAACAAACGGACTTGA
          1 AAAAACTAGACAGAAGCATTCTCAGAAACATCTTTGTGATGTTTGCACTCAACTCACAGAGTTGA

                   *      **  * *  *  *             *   *    * * *           
     167292 ACCTTTCGTTTCATGCAGTACTTCTGGAACACTCTTTTTGAAGATTCTGCATGCGGATATTTGGA
         66 ACCTTTCTTTTCATAGAGCAGTTTTGAAACACTCTTTTTGTAGAATCTGGAAGTGGATATTTGGA

            **              *             **   *  ** 
     167357 TAGCTTTGAGGATTTCATTGGAAACGG---GCT-TACAAGT
        131 GCGCTTTGAGGATTTCGTTGGAAACGGAAATATCTTCATAT

                 *       *               *        *   *    *    *       *    
     167394 AAAAATTAGACAGCAGCATTCTCAGAAACTTCTTTGTGGTGTCTGCATTCAAGTCACAGAATTGA
          1 AAAAACTAGACAGAAGCATTCTCAGAAACATCTTTGTGATGTTTGCACTCAACTCACAGAGTTGA

              * ** * **            *  * *      *       *             *       
     167459 ACATCCCCTCACATAGAGCAGTTGTGCAGCACTCTATTTGTAGTATCTGGAAGTGGACATTTGGA
         66 ACCTTTCTTTTCATAGAGCAGTTTTGAAACACTCTTTTTGTAGAATCTGGAAGTGGATATTTGGA

             *         ** *    * *   *            **  
     167524 GGGCTTTGTAGCCTATC-TGGAAAAAGGAAATATCTTCCCAT
        131 GCGCTTTG-AGGATTTCGTTGGAAACGGAAATATCTTCATAT

            *  ** *   *     * *            *   *  *   * **        *  * * *   
     167565 GAATGCGAGATAGAAGTAATCTCAGAAACATGTTTATGCTGTATCTACTCAACTAACTGTGCTGA
          1 AAAAACTAGACAGAAGCATTCTCAGAAACATCTTTGTGATGTTTGCACTCAACTCACAGAGTTGA

              *     *  *               *        *  * *       *               
     167630 ACATTTCTATTGATAGAGCAGTTTTGAGACACTCTTCTTTTGGAATCTGCAAGTGGATATTTGGA
         66 ACCTTTCTTTTCATAGAGCAGTTTTGAAACACTCTTTTTGTAGAATCTGGAAGTGGATATTTGGA

            ** *                        *     *      
     167695 TAGATTTGAGGATTTCGTTGGAAACGG-GAT-T-AT-ATAT
        131 GCGCTTTGAGGATTTCGTTGGAAACGGAAATATCTTCATAT

                 *       *               *                 **  *   *         
     167732 AAAAAGTAGACAGCAGCATTCTCAGAAACTTCTTTGTGATGTTTGCATCCAGCTCTCAGAGTTGA
          1 AAAAACTAGACAGAAGCATTCTCAGAAACATCTTTGTGATGTTTGCACTCAACTCACAGAGTTGA

              *  * *          *  *        *        *   **        *  ***      
     167797 ACATTCCCTTTCATAGAGTAGGTTTGAAACCCTCTTTTTATAGTGTCTGGAAGCGGGCGTTTGGA
         66 ACCTTTCTTTTCATAGAGCAGTTTTGAAACACTCTTTTTGTAGAATCTGGAAGTGGATATTTGGA

                   *   ** ** *  *   *          * *   
     167862 GCGCTTTCAGGCCTATGCTGAAAAAGGAAATATCTACCTAT
        131 GCGCTTTGAGGATTTCGTTGGAAACGGAAATATCTTCATAT

             *                    *    *  **          ** *        *    *     
     167903 AGAAACTAGACAGAAGCATTCTGAGAATCACGTTTGTGATGTGGGTACTCAACTAACAGTGTTGA
          1 AAAAACTAGACAGAAGCATTCTCAGAAACATCTTTGTGATGTTTGCACTCAACTCACAGAGTTGA

            *  *       *   *            *   *                *               
     167968 TCCATTCTTTTGATACAGCAGTTTTGAACCACACTTTTTGTAGAATCTGCAAGTGGATATTTGGA
         66 ACCTTTCTTTTCATAGAGCAGTTTTGAAACACTCTTTTTGTAGAATCTGGAAGTGGATATTTGGA

            **   *                     *   *        *
     168033 TAGCTGTGAGGATTTCGTTGGAAACGGGAATGTCTTCATAG
        131 GCGCTTTGAGGATTTCGTTGGAAACGGAAATATCTTCATAT

                **                     * * **         *  **   *   *          
     168074 AAAATTTAGACAGAAGCATTCTCAGAACCTTGATTGTGATGTGTGTTCTCCACTAACAGAGTTGA
          1 AAAAACTAGACAGAAGCATTCTCAGAAACATCTTTGTGATGTTTGCACTCAACTCACAGAGTTGA

                       * *   * *   *       *       *                         
     168139 ACCTTTCTTTTGACAGAACTGTTCTGAAACATTCTTTTTATAGAATCTGGAAGTGGATATTTGGA
         66 ACCTTTCTTTTCATAGAGCAGTTTTGAAACACTCTTTTTGTAGAATCTGGAAGTGGATATTTGGA

            **                         *          *  
     168204 AAGCTTTGAGGATTTCGTTGGAAACGGGAATATCTTCAAAT
        131 GCGCTTTGAGGATTTCGTTGGAAACGGAAATATCTTCATAT

                *    *       *    *           * *       *  *    *            
     168245 AAAATCTAGCCAGAAGCTTTCTAAGAAACATCTTAGGGATGTTTACATTCAAGTCACAGAGTTGA
          1 AAAAACTAGACAGAAGCATTCTCAGAAACATCTTTGTGATGTTTGCACTCAACTCACAGAGTTGA

              *  * *     *       *         *    * *   **      *      *    *  
     168310 ACATTCCCTTTCACAGAGCAGGTTTGAAACAATCTTCTCGTACTATCTGGCAGTGGACATTTTGA
         66 ACCTTTCTTTTCATAGAGCAGTTTTGAAACACTCTTTTTGTAGAATCTGGAAGTGGATATTTGGA

              * *   *  ** ** *  *   *            ** *
     168375 GCTCCTTGGGGCCTATGCTGAAAAAGGAAATATCTTCCGAC
        131 GCGCTTTGAGGATTTCGTTGGAAACGGAAATATCTTCATAT

                                 *     *  **          *           **    *    
     168416 AAAAACTAGACAGAAGCATTCGCAGAATCACGTTTGTGATGTGTGCACTCAACTGTCAGAATTGA
          1 AAAAACTAGACAGAAGCATTCTCAGAAACATCTTTGTGATGTTTGCACTCAACTCACAGAGTTGA

                 **   ** *      *                            * *            *
     168481 ACCTTGGTTTGGACAGAGCACTTTTGAAACACTCTTTTTGTAGAATCTGCAGGTGGATATTTGGC
         66 ACCTTTCTTTTCATAGAGCAGTTTTGAAACACTCTTTTTGTAGAATCTGGAAGTGGATATTTGGA

            **                         *   *      * *
     168546 TAGCTTTGAGGATTTCGTTGGAAACGGTAATGTCTTCAAAG
        131 GCGCTTTGAGGATTTCGTTGGAAACGGAAATATCTTCATAT

                *                         *   *            *    *            
     168587 AAAATCTAGACAGAAGCATTCTCAGAAACACCTTCGTGATGTTTGCAATCAAGTCACAGAGTTGA
          1 AAAAACTAGACAGAAGCATTCTCAGAAACATCTTTGTGATGTTTGCACTCAACTCACAGAGTTGA

                 * *             *  *                  *             *       
     168652 ACCTTCCGTTTCATAGAGCAGGTTGGAAACACTCTTTTTGTAGTATCTGGAAGTGGACATTTGGA
         66 ACCTTTCTTTTCATAGAGCAGTTTTGAAACACTCTTTTTGTAGAATCTGGAAGTGGATATTTGGA

                   *   ** ** *  *   *            **  
     168717 GCGCTTTCAGGCCTATGGTGAAAAAGGAAATATCTTCCCAT
        131 GCGCTTTGAGGATTTCGTTGGAAACGGAAATATCTTCATAT

                  * * *      **      *   * *   *     ** ** *      *    *     
     168758 AAAAACGACATAGAAGCTATCTCAGGAACTTGTTTATGATGCATCTAATCAACTAACAGTGTTGA
          1 AAAAACTAGACAGAAGCATTCTCAGAAACATCTTTGTGATGTTTGCACTCAACTCACAGAGTTGA

                  * ** * *                         * *       *               
     168823 ACCTTTGTACTGACAGAGCAG-TTTGAAACACTCTTTTTTTGGAATCTGCAAGTGGATATTTGGA
         66 ACCTTTCTTTTCATAGAGCAGTTTTGAAACACTCTTTTTGTAGAATCTGGAAGTGGATATTTGGA

            *                           *   *   *    
     168887 TCGCTTTGAGGATTTCGTTGGAAACGG-GAT-GC--AATAT
        131 GCGCTTTGAGGATTTCGTTGGAAACGGAAATATCTTCATAT

                **  *    *     *                 **  *   *  *    *         * 
     168924 AAAACGTACACAGCAGCATACTCAGAAA-ATACTTTGCCATATTTCCATTCAAGTCACAGAGTGG
          1 AAAAACTAGACAGAAGCATTCTCAGAAACAT-CTTTGTGATGTTTGCACTCAACTCACAGAGTTG

               *  * **            *                  *  *             *      
     168988 AACATTCCCATTCATAGAGCAGGTTTGAAACACTCTTTTTGGAGTATCTGGAAGTGGACATTTGG
         65 AACCTTTCTTTTCATAGAGCAGTTTTGAAACACTCTTTTTGTAGAATCTGGAAGTGGATATTTGG

                    ** * * ** *  *   *            **  
     169053 AGCGCTTTCTGAACTATGGTGAAAAAGGAAATATCTTCCAAT
        130 AGCGCTTTGAGGATTTCGTTGGAAACGGAAATATCTTCATAT

            *     *               *      * *          *  **      **  *  *    
     169095 GAAAACAAGACAGAAGCATTCTGAGAAACTTATTTGTGATGTGTGTCCTCAACAAACGGACTTGA
          1 AAAAACTAGACAGAAGCATTCTCAGAAACATCTTTGTGATGTTTGCACTCAACTCACAGAGTTGA

                   *      **  * *  *  *             *   *    * * *           
     169160 ACCTTTCGTTTCATGCAGTACTTCTGGAACACTCTTTTTGAAGATTCTGCATGCGGATATTTGGA
         66 ACCTTTCTTTTCATAGAGCAGTTTTGAAACACTCTTTTTGTAGAATCTGGAAGTGGATATTTGGA

            **                          **         * 
     169225 TAGCTTTGAGGATTTCGTTGGAAACGG-GCT-TC--CATGT
        131 GCGCTTTGAGGATTTCGTTGGAAACGGAAATATCTTCATAT

                 *       *               *        *   *    *    *       *    
     169262 AAAAATTAGACAGCAGCATTCTCAGAAACTTCTTTGTGGTGTCTGCATTCAAGTCACAGAATTGA
          1 AAAAACTAGACAGAAGCATTCTCAGAAACATCTTTGTGATGTTTGCACTCAACTCACAGAGTTGA

              * ** * ***           *  * *              *             *       
     169327 ACATCCCCTCATATAGAGCAGTTGTGCAGCACTCTTTTTGTAGTATCTGGAAGTGGACATTTGGA
         66 ACCTTTCTTTTCATAGAGCAGTTTTGAAACACTCTTTTTGTAGAATCTGGAAGTGGATATTTGGA

             *         ** *    * *   *            **  
     169392 GGGCTTTGTAGCCTATC-TGGAAAAAGGAAATATCTTCCCAT
        131 GCGCTTTG-AGGATTTCGTTGGAAACGGAAATATCTTCATAT

            *  ** *   *     * *            *   *  *   * **        *  * * *   
     169433 GAATGCGAGATAGAAGTAATCTCAGAAACATGTTTATGCTGTATCTACTCAACTAACTGTGCTGA
          1 AAAAACTAGACAGAAGCATTCTCAGAAACATCTTTGTGATGTTTGCACTCAACTCACAGAGTTGA

              *     *  *               *        *  * *       *   *           
     169498 ACATTTCTATTGATAGAGCAGTTTTGAGACACTCTTCTTTTGGAATCTGCAAGCGGATATTTGGA
         66 ACCTTTCTTTTCATAGAGCAGTTTTGAAACACTCTTTTTGTAGAATCTGGAAGTGGATATTTGGA

            ** *                        *     *      
     169563 TAGATTTGAGGATTTCGTTGGAAACGG-GAT-T-AT-ATAT
        131 GCGCTTTGAGGATTTCGTTGGAAACGGAAATATCTTCATAT

                 *       *               *                 **  *   *         
     169600 AAAAAGTAGACAGCAGCATTCTCAGAAACTTCTTTGTGATGTTTGCATCCAGCTCTCAGAGTTGA
          1 AAAAACTAGACAGAAGCATTCTCAGAAACATCTTTGTGATGTTTGCACTCAACTCACAGAGTTGA

              *  * *          *  *        *        *   **        *  **       
     169665 ACATTCCCTTTCATAGAGTAGGTTTGAAACCCTCTTTTTATAGTGTCTGGAAGCGGGCATTTGGA
         66 ACCTTTCTTTTCATAGAGCAGTTTTGAAACACTCTTTTTGTAGAATCTGGAAGTGGATATTTGGA

                   *   ** ** *  *   *          * *   
     169730 GCGCTTTCAGGCCTATGCTGAAAAAGGAAATATCTACCTAT
        131 GCGCTTTGAGGATTTCGTTGGAAACGGAAATATCTTCATAT

             *                    *    *  **          ** *        *    *     
     169771 AGAAACTAGACAGAAGCATTCTGAGAATCACGTTTGTGATGTGAGTACTCAACTAACAGTGTTGA
          1 AAAAACTAGACAGAAGCATTCTCAGAAACATCTTTGTGATGTTTGCACTCAACTCACAGAGTTGA

            *  *       *   *            *   *                *               
     169836 TCCATTCTTTTGATACAGCAGTTTTGAACCACACTTTTTGTAGAATCTGCAAGTGGATATTTGGA
         66 ACCTTTCTTTTCATAGAGCAGTTTTGAAACACTCTTTTTGTAGAATCTGGAAGTGGATATTTGGA

            **   *                     *   *        *
     169901 TAGCTGTGAGGATTTCGTTGGAAACGGGAATGTCTTCATAG
        131 GCGCTTTGAGGATTTCGTTGGAAACGGAAATATCTTCATAT

                **                     * * **         *  **   *   *          
     169942 AAAATTTAGACAGAAGCATTCTCAGAACCTTGATTGTGATGTGTGTTCTCCACTAACAGAGTTGA
          1 AAAAACTAGACAGAAGCATTCTCAGAAACATCTTTGTGATGTTTGCACTCAACTCACAGAGTTGA

                       * *   * *   *       *       *                         
     170007 ACCTTTCTTTTGACAGAACTGTTCTGAAACATTCTTTTTATAGAATCTGGAAGTGGATATTTGGA
         66 ACCTTTCTTTTCATAGAGCAGTTTTGAAACACTCTTTTTGTAGAATCTGGAAGTGGATATTTGGA

            **                         *          *  
     170072 AAGCTTTGAGGATTTCGTTGGAAACGGGAATATCTTCAAAT
        131 GCGCTTTGAGGATTTCGTTGGAAACGGAAATATCTTCATAT

                *    *            *           * *       *  *    *            
     170113 AAAATCTAGCCAGAAGCATTCTAAGAAACATCTTAGGGATGTTTACATTCAAGTCACAGAGTTGA
          1 AAAAACTAGACAGAAGCATTCTCAGAAACATCTTTGTGATGTTTGCACTCAACTCACAGAGTTGA

              *  * *     *       *         *    * *   **      *      *    *  
     170178 ACATTCCCTTTCACAGAGCAGGTTTGAAACAATCTTCTCGTACTATCTGGCAGTGGACATTTTGA
         66 ACCTTTCTTTTCATAGAGCAGTTTTGAAACACTCTTTTTGTAGAATCTGGAAGTGGATATTTGGA

              * *   *  ** ** *  *   *            ** *
     170243 GCTCCTTGGGGCCTATGCTGAAAAAGGAAATATCTTCCGAC
        131 GCGCTTTGAGGATTTCGTTGGAAACGGAAATATCTTCATAT

                                 *     *  **          *           **    *    
     170284 AAAAACTAGACAGAAGCATTCGCAGAATCACGTTTGTGATGTGTGCACTCAACTGTCAGAATTGA
          1 AAAAACTAGACAGAAGCATTCTCAGAAACATCTTTGTGATGTTTGCACTCAACTCACAGAGTTGA

                 **   ** *      *                            * *            *
     170349 ACCTTGGTTTGGACAGAGCACTTTTGAAACACTCTTTTTGTAGAATCTGCAGGTGGATATTTGGC
         66 ACCTTTCTTTTCATAGAGCAGTTTTGAAACACTCTTTTTGTAGAATCTGGAAGTGGATATTTGGA

            **                         *   *      * *
     170414 TAGCTTTGAGGATTTCGTTGGAAACGGTAATGTCTTCAAAG
        131 GCGCTTTGAGGATTTCGTTGGAAACGGAAATATCTTCATAT

                *                         *   *            *    *            
     170455 AAAATCTAGACAGAAGCATTCTCAGAAACACCTTCGTGATGTTTGCAATCAAGTCACAGAGTTGA
          1 AAAAACTAGACAGAAGCATTCTCAGAAACATCTTTGTGATGTTTGCACTCAACTCACAGAGTTGA

                 * *             *  *           *      *             *       
     170520 ACCTTCCGTTTCATAGAGCAGGTTGGAAACACTCTTATTGTAGTATCTGGAAGTGGACATTTGGA
         66 ACCTTTCTTTTCATAGAGCAGTTTTGAAACACTCTTTTTGTAGAATCTGGAAGTGGATATTTGGA

                   *   ** ** *  *   *            **  
     170585 GCGCTTTCAGGCCTATGGTGAAAAAGGAAATATCTTCCCAT
        131 GCGCTTTGAGGATTTCGTTGGAAACGGAAATATCTTCATAT

                  * * *      **      *   * *   *     ** ** *      *    *     
     170626 AAAAACGACATAGAAGCTATCTCAGGAACTTGTTTATGATGCATCTAATCAACTAACAGTGTTGA
          1 AAAAACTAGACAGAAGCATTCTCAGAAACATCTTTGTGATGTTTGCACTCAACTCACAGAGTTGA

                  * ** * *                         * *       *               
     170691 ACCTTTGTACTGACAGAGCAG-TTTGAAACACTCTTTTTTTGGAATCTGCAAGTGGATATTTGGA
         66 ACCTTTCTTTTCATAGAGCAGTTTTGAAACACTCTTTTTGTAGAATCTGGAAGTGGATATTTGGA

            *                           *   *   *    
     170755 TCGCTTTGAGGATTTCGTTGGAAACGG-GAT-GC--AATAT
        131 GCGCTTTGAGGATTTCGTTGGAAACGGAAATATCTTCATAT

                **  *    *     *                 **  *   *  *    *         * 
     170792 AAAACGTACACAGCAGCATACTCAGAAA-ATACTTTGCCATATTTCCATTCAAGTCACAGAGTGG
          1 AAAAACTAGACAGAAGCATTCTCAGAAACAT-CTTTGTGATGTTTGCACTCAACTCACAGAGTTG

               *  * **            *  *               *  *             *      
     170856 AACATTCCCATTCATAGAGCAGGTTGGAAACACTCTTTTTGGAGTATCTGGAAGTGGACATTTGG
         65 AACCTTTCTTTTCATAGAGCAGTTTTGAAACACTCTTTTTGTAGAATCTGGAAGTGGATATTTGG

                    ** * * ** *  *   *            **  
     170921 AGCGCTTTCTGAACTATGGTGAAAAAGGAAATATCTTCCAAT
        130 AGCGCTTTGAGGATTTCGTTGGAAACGGAAATATCTTCATAT

            *     *               *      * *          *  **      **  *  *    
     170963 GAAAACAAGACAGAAGCATTCTGAGAAACTTATTTGTGATGTGTGTCCTCAACAAACGGACTTGA
          1 AAAAACTAGACAGAAGCATTCTCAGAAACATCTTTGTGATGTTTGCACTCAACTCACAGAGTTGA

                   *      **  * *  *  *             *   *    * * *           
     171028 ACCTTTCGTTTCATGCAGTACTTCTGGAACACTCTTTTTGAAGATTCTGCATGCGGATATTTGGA
         66 ACCTTTCTTTTCATAGAGCAGTTTTGAAACACTCTTTTTGTAGAATCTGGAAGTGGATATTTGGA

            **                  *         **   *   * 
     171093 TAGCTTTGAGGATTTCGTTGAAAACGG---GCT-TACATGT
        131 GCGCTTTGAGGATTTCGTTGGAAACGGAAATATCTTCATAT

                 *       *               *        *   *    *    *       *    
     171130 AAAAATTAGACAGCAGCATTCTCAGAAACTTCTTTGTGGTGTCTGCATTCAAGTCACAGAATTGA
          1 AAAAACTAGACAGAAGCATTCTCAGAAACATCTTTGTGATGTTTGCACTCAACTCACAGAGTTGA

              * ** * **            *  * *      *       *    *        *       
     171195 ACTTCCCCTCACATAGAGCAGTTGTGCAGCACTCTATTTGTAGTATCTCGAAGTGGACATTTGGA
         66 ACCTTTCTTTTCATAGAGCAGTTTTGAAACACTCTTTTTGTAGAATCTGGAAGTGGATATTTGGA

             *         ** *    * *   *            **  
     171260 GGGCTTTGTAGCCTATC-TGGAAAAAGGAAATATCTTCCCAT
        131 GCGCTTTG-AGGATTTCGTTGGAAACGGAAATATCTTCATAT

            *  ** *   *     * *            *   *  *   * **        *  * * *   
     171301 GAATGCGAGATAGAAGTAATCTCAGAAACATGTTTATGCTGTATCTACTCAACTAACTGTGCTGA
          1 AAAAACTAGACAGAAGCATTCTCAGAAACATCTTTGTGATGTTTGCACTCAACTCACAGAGTTGA

              *     *  *               *        *  * *       *               
     171366 ACATTTCTATTGATAGAGCAGTTTTGAGACACTCTTCTTTTGGAATCTGCAAGTGGATATTTGGA
         66 ACCTTTCTTTTCATAGAGCAGTTTTGAAACACTCTTTTTGTAGAATCTGGAAGTGGATATTTGGA

            ** *                        *     *      
     171431 TAGATTTGAGGATTTCGTTGGAAACGG-GAT-T-AT-ATAT
        131 GCGCTTTGAGGATTTCGTTGGAAACGGAAATATCTTCATAT

                 *       *               *                 **  *   *         
     171468 AAAAAGTAGACAGCAGCATTCTCAGAAACTTCTTTGTGATGTTTGCATCCAGCTCTCAGAGTTGA
          1 AAAAACTAGACAGAAGCATTCTCAGAAACATCTTTGTGATGTTTGCACTCAACTCACAGAGTTGA

              *  * *          *  *        *        *   **        *  **       
     171533 ACATTCCCTTTCATAGAGTAGGTTTGAAACCCTCTTTTTATAGTGTCTGGAAGCGGGCATTTGGA
         66 ACCTTTCTTTTCATAGAGCAGTTTTGAAACACTCTTTTTGTAGAATCTGGAAGTGGATATTTGGA

                   *   ** ** *  *   *          * *   
     171598 GCGCTTTCAGGCCTATGCTGAAAAAGGAAATATCTACCTAT
        131 GCGCTTTGAGGATTTCGTTGGAAACGGAAATATCTTCATAT

             *                    *    *  **          ** *        *    *     
     171639 AGAAACTAGACAGAAGCATTCTGAGAATCACGTTTGTGATGTGGGTACTCAACTAACAGTGTTGA
          1 AAAAACTAGACAGAAGCATTCTCAGAAACATCTTTGTGATGTTTGCACTCAACTCACAGAGTTGA

            *  *       *   *            *   *                *               
     171704 TCCATTCTTTTGATACAGCAGTTTTGAACCACACTTTTTGTAGAATCTGCAAGTGGATATTTGGA
         66 ACCTTTCTTTTCATAGAGCAGTTTTGAAACACTCTTTTTGTAGAATCTGGAAGTGGATATTTGGA

            **   *                     *   *        *
     171769 TAGCTGTGAGGATTTCGTTGGAAACGGGAATGTCTTCATAG
        131 GCGCTTTGAGGATTTCGTTGGAAACGGAAATATCTTCATAT

                **                     * ****         *  **   *   *          
     171810 AAAATTTAGACAGAAGCATTCTCAGAACCTGGATTGTGATGTGTGTTCTCCACTAACAGAGTTGA
          1 AAAAACTAGACAGAAGCATTCTCAGAAACATCTTTGTGATGTTTGCACTCAACTCACAGAGTTGA

                       * *   * *   *       *       *         *               
     171875 ACCTTTCTTTTGACAGAACTGTTCTGAAACATTCTTTTTATAGAATCTGCAAGTGGATATTTGGA
         66 ACCTTTCTTTTCATAGAGCAGTTTTGAAACACTCTTTTTGTAGAATCTGGAAGTGGATATTTGGA

            **                         *          *  
     171940 AAGCTTTGAGGATTTCGTTGGAAACGGGAATATCTTCAAAT
        131 GCGCTTTGAGGATTTCGTTGGAAACGGAAATATCTTCATAT

                *    *            *           * *       *  *    *            
     171981 AAAATCTAGCCAGAAGCATTCTAAGAAACATCTTAGGGATGTTTACATTCAAGTCACAGAGTTGA
          1 AAAAACTAGACAGAAGCATTCTCAGAAACATCTTTGTGATGTTTGCACTCAACTCACAGAGTTGA

              *  * *     *       *         *    * *   **      *      *    *  
     172046 ACATTCCCTTTCACAGAGCAGGTTTGAAACAATCTTCTCGTACTATCTGGCAGTGGACATTTTGA
         66 ACCTTTCTTTTCATAGAGCAGTTTTGAAACACTCTTTTTGTAGAATCTGGAAGTGGATATTTGGA

              * *   *  ** ** *  *   *            ** *
     172111 GCTCCTTGGGGCCTATGCTGAAAAAGGAAATATCTTCCGAC
        131 GCGCTTTGAGGATTTCGTTGGAAACGGAAATATCTTCATAT

                                 *     *  **          *           **    *    
     172152 AAAAACTAGACAGAAGCATTCGCAGAATCACGTTTGTGATGTGTGCACTCAACTGTCAGAATTGA
          1 AAAAACTAGACAGAAGCATTCTCAGAAACATCTTTGTGATGTTTGCACTCAACTCACAGAGTTGA

                 **   ** *      *                            * *            *
     172217 ACCTTGGTTTGGACAGAGCACTTTTGAAACACTCTTTTTGTAGAATCTGCAGGTGGATATTTGGC
         66 ACCTTTCTTTTCATAGAGCAGTTTTGAAACACTCTTTTTGTAGAATCTGGAAGTGGATATTTGGA

            **              *          *   *      * *
     172282 TAGCTTTGAGGATTTCATTGGAAACGGTAATGTCTTCAAAG
        131 GCGCTTTGAGGATTTCGTTGGAAACGGAAATATCTTCATAT

                *                         *   *            *    *          * 
     172323 AAAATCTAGACAGAAGCATTCTCAGAAACAACTTCGTGATGTTTGCAATCAAGTCACAGAGTTAA
          1 AAAAACTAGACAGAAGCATTCTCAGAAACATCTTTGTGATGTTTGCACTCAACTCACAGAGTTGA

                 * *             *  *                  *             *       
     172388 ACCTTCCGTTTCATAGAGCAGGTTGGAAACACTCTTTTTGTAGTATCTGGAAGTGGACATTTGGA
         66 ACCTTTCTTTTCATAGAGCAGTTTTGAAACACTCTTTTTGTAGAATCTGGAAGTGGATATTTGGA

             *         ** *    * *   *            **  
     172453 GGGCTTTGTAGCCTATC-TGGAAAAAGGAAATATCTTCCCAT
        131 GCGCTTTG-AGGATTTCGTTGGAAACGGAAATATCTTCATAT

            *  ** *   *     * *            *   *  *   * **        *  * * *   
     172494 GAATGCGAGATAGAAGTAATCTCAGAAACATGTTTATGCTGTATCTACTCAACTAACTGTGCTGA
          1 AAAAACTAGACAGAAGCATTCTCAGAAACATCTTTGTGATGTTTGCACTCAACTCACAGAGTTGA

              *     *  *               *        *  * *       *               
     172559 ACATTTCTATTGATAGAGCAGTTTTGAGACACTCTTCTTTTGGAATCTGCAAGTGGATATTTGGA
         66 ACCTTTCTTTTCATAGAGCAGTTTTGAAACACTCTTTTTGTAGAATCTGGAAGTGGATATTTGGA

            ** *                        *     *      
     172624 TAGATTTGAGGATTTCGTTGGAAACGG-GAT-T-AT-ATAT
        131 GCGCTTTGAGGATTTCGTTGGAAACGGAAATATCTTCATAT

                 *       *               *                 **  *   *         
     172661 AAAAAGTAGACAGCAGCATTCTCAGAAACTTCTTTGTGATGTTTGCATCCAGCTCTCAGAGTTGA
          1 AAAAACTAGACAGAAGCATTCTCAGAAACATCTTTGTGATGTTTGCACTCAACTCACAGAGTTGA

              *  * *          *  *        *        *   **        *  **       
     172726 ACATTCCCTTTCATAGAGTAGGTTTGAAACCCTCTTTTTATAGTGTCTGGAAGCGGGCATTTGGA
         66 ACCTTTCTTTTCATAGAGCAGTTTTGAAACACTCTTTTTGTAGAATCTGGAAGTGGATATTTGGA

                   *    * * **  **  **          * *  *
     172791 GCGCTTTCAGGCCTATGCTTAAAATAGGAAATATCTACCTAC
        131 GCGCTTTGAGG-ATTTCGTTGGAAACGGAAATATCTTCATAT

             *                    *    *  **          ** *        *    *     
     172833 AGAAACTAGACAGAAGCATTCTGAGAATCACGTTTGTGATGTGGGTACTCAACTAACAGTGTTGA
          1 AAAAACTAGACAGAAGCATTCTCAGAAACATCTTTGTGATGTTTGCACTCAACTCACAGAGTTGA

            *  *       *   *            *   *                *   *           
     172898 TCCATTCTTTTGATACAGCAGTTTTGAACCACACTTTTTGTAGAATCTGCAAGAGGATATTTGGA
         66 ACCTTTCTTTTCATAGAGCAGTTTTGAAACACTCTTTTTGTAGAATCTGGAAGTGGATATTTGGA

            **   *                     *   *      * *
     172963 TAGCTGTGAGGATTTCGTTGGAAACGGGAATGTCTTCAAAG
        131 GCGCTTTGAGGATTTCGTTGGAAACGGAAATATCTTCATAT

                *                         *   *            *    *            
     173004 AAAATCTAGACAGAAGCATTCTCAGAAACACCTTCGTGATGTTTGCAATCAAGTCACAGAGTTGA
          1 AAAAACTAGACAGAAGCATTCTCAGAAACATCTTTGTGATGTTTGCACTCAACTCACAGAGTTGA

                   *             *  *           *      *             *       
     173069 ACCTTTCGTTTCATAGAGCAGGTTGGAAACACTCTTATTGTAGTATCTGGAAGTGGACATTTGGA
         66 ACCTTTCTTTTCATAGAGCAGTTTTGAAACACTCTTTTTGTAGAATCTGGAAGTGGATATTTGGA

                   *   ** ** *  *   *            **  
     173134 GCGCTTTCAGGCCTATGGTGAAAAAGGAAATATCTTCCCAT
        131 GCGCTTTGAGGATTTCGTTGGAAACGGAAATATCTTCATAT

                  * * *      **      *   * *   *     ** ** *      *    *     
     173175 AAAAACGACATAGAAGCTATCTCAGGAACTTGTTTATGATGCATCTAATCAACTAACAGTGTTGA
          1 AAAAACTAGACAGAAGCATTCTCAGAAACATCTTTGTGATGTTTGCACTCAACTCACAGAGTTGA

                  * ** * *                         * *       *               
     173240 ACCTTTGTACTGACAGAGCAG-TTTGAAACACTCTTTTTTTGGAATCTGCAAGTGGATATTTGGA
         66 ACCTTTCTTTTCATAGAGCAGTTTTGAAACACTCTTTTTGTAGAATCTGGAAGTGGATATTTGGA

            *                           *   *   *    
     173304 TCGCTTTGAGGATTTCGTTGGAAACGG-GAT-GC--AATAT
        131 GCGCTTTGAGGATTTCGTTGGAAACGGAAATATCTTCATAT

                **  *    *     *                 **  *   *  *    *         * 
     173341 AAAACGTACACAGCAGCATACTCAGAAA-ATACTTTGCCATATTTCCATTCAAGTCACAGAGTGG
          1 AAAAACTAGACAGAAGCATTCTCAGAAACAT-CTTTGTGATGTTTGCACTCAACTCACAGAGTTG

               *  * **            *  *               *  *             *      
     173405 AACATTCCCATTCATAGAGCAGGTTGGAAACACTCTTTTTGGAGTATCTGGAAGTGGACATTTGG
         65 AACCTTTCTTTTCATAGAGCAGTTTTGAAACACTCTTTTTGTAGAATCTGGAAGTGGATATTTGG

                    ** * * ** *  *   *            **  
     173470 AGCGCTTTCTGAACTATGGTGAAAAAGGAAATATCTTCCAAT
        130 AGCGCTTTGAGGATTTCGTTGGAAACGGAAATATCTTCATAT

            *     *               *      * *          *  **      **  *  *    
     173512 GAAAACAAGACAGAAGCATTCTGAGAAACTTATTTGTGATGTGTGTCCTCAACAAACGGACTTGA
          1 AAAAACTAGACAGAAGCATTCTCAGAAACATCTTTGTGATGTTTGCACTCAACTCACAGAGTTGA

                   *      **  * *  *  *             *   *    * * *           
     173577 ACCTTTCGTTTCATGCAGTACTTCTGGAACACTCTTTTTGAAGATTCTGCATGCGGATATTTGGA
         66 ACCTTTCTTTTCATAGAGCAGTTTTGAAACACTCTTTTTGTAGAATCTGGAAGTGGATATTTGGA

            **                            **   *   * 
     173642 TAGCTTTGAGGATTTCGTTGGAAACGG---GCT-TACATGT
        131 GCGCTTTGAGGATTTCGTTGGAAACGGAAATATCTTCATAT

                 *       *               *        *   *    *    *       *    
     173679 AAAAATTAGACAGCAGCATTCTCAGAAACTTCTTTGTGGTGTCTGCATTCAAGTCACAGAATTGA
          1 AAAAACTAGACAGAAGCATTCTCAGAAACATCTTTGTGATGTTTGCACTCAACTCACAGAGTTGA

              * ** * **            *  * *      *       *    *        *       
     173744 ACTTCCCCTCACATAGAGCAGTTGTGCAGCACTCTATTTGTAGTATCTCGAAGTGGACATTTGGA
         66 ACCTTTCTTTTCATAGAGCAGTTTTGAAACACTCTTTTTGTAGAATCTGGAAGTGGATATTTGGA

             *         ** *    * *   *            **  
     173809 GGGCTTTGTAGCCTATC-TGGAAAAAGGAAATATCTTCCCAT
        131 GCGCTTTG-AGGATTTCGTTGGAAACGGAAATATCTTCATAT

            *  ** *   *     * *            *   *  *   * **        *  * * *   
     173850 GAATGCGAGATAGAAGTAATCTCAGAAACATGTTTATGCTGTATCTACTCAACTAACTGTGCTGA
          1 AAAAACTAGACAGAAGCATTCTCAGAAACATCTTTGTGATGTTTGCACTCAACTCACAGAGTTGA

              *     *  *               *        *  * *       *               
     173915 ACATTTCTATTGATAGAGCAGTTTTGAGACACTCTTCTTTTGGAATCTGCAAGTGGATATTTGGA
         66 ACCTTTCTTTTCATAGAGCAGTTTTGAAACACTCTTTTTGTAGAATCTGGAAGTGGATATTTGGA

            ** *                        *     *      
     173980 TAGATTTGAGGATTTCGTTGGAAACGG-GAT-T-AT-ATAT
        131 GCGCTTTGAGGATTTCGTTGGAAACGGAAATATCTTCATAT

                 *       *               *                 **  *   *         
     174017 AAAAAGTAGACAGCAGCATTCTCAGAAACTTCTTTGTGATGTTTGCATCCAGCTCTCAGAGTTGA
          1 AAAAACTAGACAGAAGCATTCTCAGAAACATCTTTGTGATGTTTGCACTCAACTCACAGAGTTGA

              *  * *          *  *        *        *   **        *  **       
     174082 ACATTCCCTTTCATAGAGTAGGTTTGAAACCCTCTTTTTATAGTGTCTGGAAGCGGGCATTTGGA
         66 ACCTTTCTTTTCATAGAGCAGTTTTGAAACACTCTTTTTGTAGAATCTGGAAGTGGATATTTGGA

                   *   ** ** *  *   *          * *   
     174147 GCGCTTTCAGGCCTATGCTGAAAAAGGAAATATCTACCTAT
        131 GCGCTTTGAGGATTTCGTTGGAAACGGAAATATCTTCATAT

             *                    *    *  **          ** *        **   *     
     174188 AGAAACTAGACAGAAGCATTCTGAGAATCACGTTTGTGATGTGGGTACTCAACTAGCAGTGTTGA
          1 AAAAACTAGACAGAAGCATTCTCAGAAACATCTTTGTGATGTTTGCACTCAACTCACAGAGTTGA

            *  *       *   *            *   *                *               
     174253 TCCATTCTTTTGATACAGCAGTTTTGAACCACACTTTTTGTAGAATCTGCAAGTGGATATTTGGA
         66 ACCTTTCTTTTCATAGAGCAGTTTTGAAACACTCTTTTTGTAGAATCTGGAAGTGGATATTTGGA

            **   *                     *   *        *
     174318 TAGCTGTGAGGATTTCGTTGGAAACGGGAATGTCTTCATAG
        131 GCGCTTTGAGGATTTCGTTGGAAACGGAAATATCTTCATAT

                **                     * * **         *  **   *   *          
     174359 AAAATTTAGACAGAAGCATTCTCAGAACCTTGATTGTGATGTGTGTTCTCCACTAACAGAGTTGA
          1 AAAAACTAGACAGAAGCATTCTCAGAAACATCTTTGTGATGTTTGCACTCAACTCACAGAGTTGA

                       * *   * *   *       *       *                      *  
     174424 ACCTTTCTTTTGACAGAACTGTTCTGAAACATTCTTTTTATAGAATCTGGAAGTGGATATTTTGA
         66 ACCTTTCTTTTCATAGAGCAGTTTTGAAACACTCTTTTTGTAGAATCTGGAAGTGGATATTTGGA

            **              *          *          *  
     174489 AAGCTTTGAGGATTTCATTGGAAACGGGAATATCTTCAAAT
        131 GCGCTTTGAGGATTTCGTTGGAAACGGAAATATCTTCATAT

                *    *            *           * *       *  *    *          * 
     174530 AAAATCTAGCCAGAAGCATTCTAAGAAACATCTTAGGGATGTTTACATTCAAGTCACAGAGTTCA
          1 AAAAACTAGACAGAAGCATTCTCAGAAACATCTTTGTGATGTTTGCACTCAACTCACAGAGTTGA

              *  * *     *       *         *    * *   **      *      *    *  
     174595 ACATTCCCTTTCACAGAGCAGGTTTGAAACAATCTTCTCGTACTATCTGGCAGTGGACATTTTGA
         66 ACCTTTCTTTTCATAGAGCAGTTTTGAAACACTCTTTTTGTAGAATCTGGAAGTGGATATTTGGA

              * *   *  ** ** *  *   *            ** *
     174660 GCTCCTTGGGGCCTATGCTGAAAAAGGAAATATCTTCCGAC
        131 GCGCTTTGAGGATTTCGTTGGAAACGGAAATATCTTCATAT

                                 *     *  **          *           **    *    
     174701 AAAAACTAGACAGAAGCATTCGCAGAATCACGTTTGTGATGTGTGCACTCAACTGTCAGAATTGA
          1 AAAAACTAGACAGAAGCATTCTCAGAAACATCTTTGTGATGTTTGCACTCAACTCACAGAGTTGA

                 **   ** *      *                            * *            *
     174766 ACCTTGGTTTAGACAGAGCACTTTTGAAACACTCTTTTTGTAGAATCTGCAGGTGGATATTTGGC
         66 ACCTTTCTTTTCATAGAGCAGTTTTGAAACACTCTTTTTGTAGAATCTGGAAGTGGATATTTGGA

            **                         *   *      * *
     174831 TAGCTTTGAGGATTTCGTTGGAAACGGTAATGTCTTCAAAG
        131 GCGCTTTGAGGATTTCGTTGGAAACGGAAATATCTTCATAT

                *                         *   *            *    *            
     174872 AAAATCTAGACAGAAGCATTCTCAGAAACACCTTCGTGATGTTTGCAATCAAGTCACAGAGTTGA
          1 AAAAACTAGACAGAAGCATTCTCAGAAACATCTTTGTGATGTTTGCACTCAACTCACAGAGTTGA

                 * *             *  *           *      *             *       
     174937 ACCTTCCGTTTCATAGAGCAGGTTGGAAACACTCTTATTGTAGTATCTGGAAGTGGACATTTGGA
         66 ACCTTTCTTTTCATAGAGCAGTTTTGAAACACTCTTTTTGTAGAATCTGGAAGTGGATATTTGGA

                   *   ** ** *  *   *            **  
     175002 GCGCTTTCAGGCCTATGGTGAAAAAGGAAATATCTTCCCAT
        131 GCGCTTTGAGGATTTCGTTGGAAACGGAAATATCTTCATAT

                  * * *      **      *   * *   *     ** ** *      *    *   * 
     175043 AAAAACGACATAGAAGCTATCTCAGGAACTTGTTTATGATGCATCTAATCAACTAACAGTGTTCA
          1 AAAAACTAGACAGAAGCATTCTCAGAAACATCTTTGTGATGTTTGCACTCAACTCACAGAGTTGA

                  * ** * *                         * *       *               
     175108 ACCTTTGTACTGACAGAGCAG-TTTGAAACACTCTTTTTTTGGAATCTGCAAGTGGATATTTGGA
         66 ACCTTTCTTTTCATAGAGCAGTTTTGAAACACTCTTTTTGTAGAATCTGGAAGTGGATATTTGGA

            *                           *   *   *    
     175172 TCGCTTTGAGGATTTCGTTGGAAACGG-GAT-GC--AATAT
        131 GCGCTTTGAGGATTTCGTTGGAAACGGAAATATCTTCATAT

                **  *    *     *                 **  *   *  *    *         * 
     175209 AAAACGTACACAGCAGCATACTCAGAAA-ATACTTTGCCATATTTCCATTCAAGTCACAGAGTGG
          1 AAAAACTAGACAGAAGCATTCTCAGAAACAT-CTTTGTGATGTTTGCACTCAACTCACAGAGTTG

               *  * **            *  *               *  *             *      
     175273 AACATTCCCATTCATAGAGCAGGTTGGAAACACTCTTTTTGGAGTATCTGGAAGTGGACATTTGG
         65 AACCTTTCTTTTCATAGAGCAGTTTTGAAACACTCTTTTTGTAGAATCTGGAAGTGGATATTTGG

                    ** * * ** *  *   *            **  
     175338 AGCGCTTTCTGAACTATGGTGAAAAAGGAAATATCTTCCAAT
        130 AGCGCTTTGAGGATTTCGTTGGAAACGGAAATATCTTCATAT

            *     * *             *      * *          *  **      **  *  *    
     175380 GAAAACAACACAGAAGCATTCTGAGAAACTTATTTGTGATGTGTGTCCTCAACAAACGGACTTGA
          1 AAAAACTAGACAGAAGCATTCTCAGAAACATCTTTGTGATGTTTGCACTCAACTCACAGAGTTGA

                   *      **  * *  *  *             *   *    * * *           
     175445 ACCTTTCGTTTCATGCAGTACTTCTGGAACACTCTTTTTGAAGATTCTGCATGCGGATATTTGGA
         66 ACCTTTCTTTTCATAGAGCAGTTTTGAAACACTCTTTTTGTAGAATCTGGAAGTGGATATTTGGA

            **                            **   *   * 
     175510 TAGCTTTGAGGATTTCGTTGGAAACGG---GCT-TACATGT
        131 GCGCTTTGAGGATTTCGTTGGAAACGGAAATATCTTCATAT

                 *       *               **       *   *    *    *       *    
     175547 AAAAATTAGACAGCAGCATTCTCAGAAACTACTTTGTGGTGTCTGCATTCAAGTCACAGAATTGA
          1 AAAAACTAGACAGAAGCATTCTCAGAAACATCTTTGTGATGTTTGCACTCAACTCACAGAGTTGA

              * ** * **            *  * *      *       *    *        *    *  
     175612 ACTTCCCCTCACATAGAGCAGTTGTGCAGCACTCTATTTGTAGTATCTCGAAGTGGACATTTAGA
         66 ACCTTTCTTTTCATAGAGCAGTTTTGAAACACTCTTTTTGTAGAATCTGGAAGTGGATATTTGGA

             *         ** *    * *   *            **  
     175677 GGGCTTTGTAGCCTATC-TGGAAAAAGGAAATATCTTCCCAT
        131 GCGCTTTG-AGGATTTCGTTGGAAACGGAAATATCTTCATAT

            *  ** *   *     * *            *   *  *   * **        *  * * *   
     175718 GAATGCGAGATAGAAGTAATCTCAGAAACATGTTTATGCTGTATCTACTCAACTAACTGTGCTGA
          1 AAAAACTAGACAGAAGCATTCTCAGAAACATCTTTGTGATGTTTGCACTCAACTCACAGAGTTGA

              *     *  *               *        *  * *       *               
     175783 ACATTTCTATTGATAGAGCAGTTTTGAGACACTCTTCTTTTGGAATCTGCAAGTGGATATTTGGA
         66 ACCTTTCTTTTCATAGAGCAGTTTTGAAACACTCTTTTTGTAGAATCTGGAAGTGGATATTTGGA

            ** *                        *     *      
     175848 TAGATTTGAGGATTTCGTTGGAAACGG-GAT-T-AT-ATAT
        131 GCGCTTTGAGGATTTCGTTGGAAACGGAAATATCTTCATAT

            *    *       *               *                 **  *   *         
     175885 CAAAAGTAGACAGCAGCATTCTCAGAAACTTCTTTGTGATGTTTGCATCCAGCTCTCAGAGTTGA
          1 AAAAACTAGACAGAAGCATTCTCAGAAACATCTTTGTGATGTTTGCACTCAACTCACAGAGTTGA

              *  * *          *  *        *        *   **        *  **       
     175950 ACATTCCCTTTCATAGAGTAGGTTTGAAACCCTCTTTTTATAGTGTCTGGAAGCGGGCATTTGGA
         66 ACCTTTCTTTTCATAGAGCAGTTTTGAAACACTCTTTTTGTAGAATCTGGAAGTGGATATTTGGA

                *  *    * * **  **  **          * *  *
     176015 GCGCATTCAGGCCTATGCTTAAAATAGGAAATATCTACCTAC
        131 GCGCTTTGAGG-ATTTCGTTGGAAACGGAAATATCTTCATAT

             *                    *    *  **          ** *        *    *     
     176057 AGAAACTAGACAGAAGCATTCTGAGAATCACGTTTGTGATGTGGGTACTCAACTAACAGTGTTGA
          1 AAAAACTAGACAGAAGCATTCTCAGAAACATCTTTGTGATGTTTGCACTCAACTCACAGAGTTGA

            *  *       *   *            *   *                *   *           
     176122 TCCATTCTTTTGATACAGCAGTTTTGAACCACACTTTTTGTAGAATCTGCAAGAGGATATTTGGA
         66 ACCTTTCTTTTCATAGAGCAGTTTTGAAACACTCTTTTTGTAGAATCTGGAAGTGGATATTTGGA

            **   *                     *   *      * *
     176187 TAGCTGTGAGGATTTCGTTGGAAACGGGAATGTCTTCAAAG
        131 GCGCTTTGAGGATTTCGTTGGAAACGGAAATATCTTCATAT

                *                         *   *            *    *            
     176228 AAAATCTAGACAGAAGCATTCTCAGAAACACCTTCGTGATGTTTGCAATCAAGTCACAGAGTTGA
          1 AAAAACTAGACAGAAGCATTCTCAGAAACATCTTTGTGATGTTTGCACTCAACTCACAGAGTTGA

                 * *             *  *           *      *             *       
     176293 ACCTTCCGTTTCATAGAGCAGGTTGGAAACACTCTTATTGTAGTATCTGGAAGTGGACATTTGGA
         66 ACCTTTCTTTTCATAGAGCAGTTTTGAAACACTCTTTTTGTAGAATCTGGAAGTGGATATTTGGA

                   *   ** ** *  *   *            **  
     176358 GCGCTTTCAGGCCTATGGTGAAAAAGGAAATATCTTCCCAT
        131 GCGCTTTGAGGATTTCGTTGGAAACGGAAATATCTTCATAT

                  * * *      **      *   * *   *     ** ** *      *    *     
     176399 AAAAACGACATAGAAGCTATCTCAGGAACTTGTTTATGATGCATCTAATCAACTAACAGTGTTGA
          1 AAAAACTAGACAGAAGCATTCTCAGAAACATCTTTGTGATGTTTGCACTCAACTCACAGAGTTGA

                  * ** * *                         * *       *               
     176464 ACCTTTGTACTGACAGAGCAG-TTTGAAACACTCTTTTTTTGGAATCTGCAAGTGGATATTTGGA
         66 ACCTTTCTTTTCATAGAGCAGTTTTGAAACACTCTTTTTGTAGAATCTGGAAGTGGATATTTGGA

            *                           *   *   *    
     176528 TCGCTTTGAGGATTTCGTTGGAAACGG-GAT-GC--AATAT
        131 GCGCTTTGAGGATTTCGTTGGAAACGGAAATATCTTCATAT

                **  *    *     *                 **  *   *  *    *         * 
     176565 AAAACGTACACAGCAGCATACTCAGAAA-ATACTTTGCCATATTTCCATTCAAGTCACAGAGTGG
          1 AAAAACTAGACAGAAGCATTCTCAGAAACAT-CTTTGTGATGTTTGCACTCAACTCACAGAGTTG

               *  * **            *                  *  *             *      
     176629 AACATTCCCATTCATAGAGCAGGTTTGAAACACTCTTTTTGGAGTATCTGGAAGTGGACATTTGG
         65 AACCTTTCTTTTCATAGAGCAGTTTTGAAACACTCTTTTTGTAGAATCTGGAAGTGGATATTTGG

                    ** * * ** *  *   *            **  
     176694 AGCGCTTTCTGAACTATGGTGAAAAAGGAAATATCTTCCAAT
        130 AGCGCTTTGAGGATTTCGTTGGAAACGGAAATATCTTCATAT

            *     * *             *      * *          *  **      **  *  *    
     176736 GAAAACAACACAGAAGCATTCTGAGAAACTTATTTGTGATGTGTGTCCTCAACAAACGGACTTGA
          1 AAAAACTAGACAGAAGCATTCTCAGAAACATCTTTGTGATGTTTGCACTCAACTCACAGAGTTGA

                   *      **  * *  *  *             *   *    * * *           
     176801 ACCTTTCGTTTCATGCAGTACTTCTGGAACACTCTTTTTGAAGATTCTGCATGCGGATATTTGGA
         66 ACCTTTCTTTTCATAGAGCAGTTTTGAAACACTCTTTTTGTAGAATCTGGAAGTGGATATTTGGA

            **                      *     **   *   * 
     176866 TAGCTTTGAGGATTTCGTTGGAAAAGG---GCT-TACATGT
        131 GCGCTTTGAGGATTTCGTTGGAAACGGAAATATCTTCATAT

                 *       *               *        *   *    *    *       *    
     176903 AAAAATTAGACAGCAGCATTCTCAGAAACTTCTTTGTGGTGTCTGCATTCAAGTCACAGAATTGA
          1 AAAAACTAGACAGAAGCATTCTCAGAAACATCTTTGTGATGTTTGCACTCAACTCACAGAGTTGA

              * ** * **            *  * *      *       *    *        *       
     176968 ACTTCCCCTCACATAGAGCAGTTGTGCAGCACTCTATTTGTAGTATCTCGAAGTGGACATTTGGA
         66 ACCTTTCTTTTCATAGAGCAGTTTTGAAACACTCTTTTTGTAGAATCTGGAAGTGGATATTTGGA

             *         ** *    * *   *            **  
     177033 GGGCTTTGTAGCCTATC-TGGAAAAAGGAAATATCTTCCCAT
        131 GCGCTTTG-AGGATTTCGTTGGAAACGGAAATATCTTCATAT

            *  ** *   *     * *            *   *  *   * **        *  * * *   
     177074 GAATGCGAGATAGAAGTAATCTCAGAAACATGTTTATGCTGTATCTACTCAACTAACTGTGCTGA
          1 AAAAACTAGACAGAAGCATTCTCAGAAACATCTTTGTGATGTTTGCACTCAACTCACAGAGTTGA

              *     *  *               *        *  * *       *               
     177139 ACATTTCTATTGATAGAGCAGTTTTGAGACACTCTTCTTTTGGAATCTGCAAGTGGATATTTGGA
         66 ACCTTTCTTTTCATAGAGCAGTTTTGAAACACTCTTTTTGTAGAATCTGGAAGTGGATATTTGGA

            ** *                        *     *      
     177204 TAGATTTGAGGATTTCGTTGGAAACGG-GAT-T-AT-ATAT
        131 GCGCTTTGAGGATTTCGTTGGAAACGGAAATATCTTCATAT

                 *       *               *                 **  *   *         
     177241 AAAAAGTAGACAGCAGCATTCTCAGAAACTTCTTTGTGATGTTTGCATCCAGCTCTCAGAGTTGA
          1 AAAAACTAGACAGAAGCATTCTCAGAAACATCTTTGTGATGTTTGCACTCAACTCACAGAGTTGA

              *  * *          *  *        *        *   **        *  **       
     177306 ACATTCCCTTTCATAGAGTAGGTTTGAAACCCTCTTTTTATAGTGTCTGGAAGCGGGCATTTGGA
         66 ACCTTTCTTTTCATAGAGCAGTTTTGAAACACTCTTTTTGTAGAATCTGGAAGTGGATATTTGGA

                   *   ** ** *  *   *          * *   
     177371 GCGCTTTCAGGCCTATGCTGAAAAAGGAAATATCTACCTAT
        131 GCGCTTTGAGGATTTCGTTGGAAACGGAAATATCTTCATAT

             *                    *    *  **          ** *        *    *     
     177412 AGAAACTAGACAGAAGCATTCTGAGAATCACGTTTGTGATGTGGGTACTCAACTAACAGTGTTGA
          1 AAAAACTAGACAGAAGCATTCTCAGAAACATCTTTGTGATGTTTGCACTCAACTCACAGAGTTGA

            *  *       *   *            *   *                *               
     177477 TCCATTCTTTTGATACAGCAGTTTTGAACCACACTTTTTGTAGAATCTGCAAGTGGATATTTGGA
         66 ACCTTTCTTTTCATAGAGCAGTTTTGAAACACTCTTTTTGTAGAATCTGGAAGTGGATATTTGGA

            **   *                     *   *        *
     177542 TAGCTGTGAGGATTTCGTTGGAAACGGGAATGTCTTCATAG
        131 GCGCTTTGAGGATTTCGTTGGAAACGGAAATATCTTCATAT

                **                     * * **         *  **   *   *          
     177583 AAAATTTAGACAGAAGCATTCTCAGAACCTTGATTGTGATGTGTGTTCTCCACTAACAGAGTTGA
          1 AAAAACTAGACAGAAGCATTCTCAGAAACATCTTTGTGATGTTTGCACTCAACTCACAGAGTTGA

                       * *   * *   *       *       *                         
     177648 ACCTTTCTTTTGACAGAACTGTTCTGAAACATTCTTTTTATAGAATCTGGAAGTGGATATTTGGA
         66 ACCTTTCTTTTCATAGAGCAGTTTTGAAACACTCTTTTTGTAGAATCTGGAAGTGGATATTTGGA

            **                         *          *  
     177713 AAGCTTTGAGGATTTCGTTGGAAACGGGAATATCTTCAAAT
        131 GCGCTTTGAGGATTTCGTTGGAAACGGAAATATCTTCATAT

                *    *            *          ** *       *  *    *            
     177754 AAAATCTAGCCAGAAGCATTCTAAGAAACATCTCAGGGATGTTTACATTCAAGTCACAGAGTTGA
          1 AAAAACTAGACAGAAGCATTCTCAGAAACATCTTTGTGATGTTTGCACTCAACTCACAGAGTTGA

              *  * *     *       *         *    * *   **      *      *    *  
     177819 ACATTCCCTTTCACAGAGCAGGTTTGAAACAATCTTCTCGTACTATCTGGCAGTGGACATTTTGA
         66 ACCTTTCTTTTCATAGAGCAGTTTTGAAACACTCTTTTTGTAGAATCTGGAAGTGGATATTTGGA

              * *   *  ** ** *  *   *            ** *
     177884 GCTCCTTGGGGCCTATGCTGAAAAAGGAAATATCTTCCGAC
        131 GCGCTTTGAGGATTTCGTTGGAAACGGAAATATCTTCATAT

                                 *     *  **          *           **    *    
     177925 AAAAACTAGACAGAAGCATTCGCAGAATCACGTTTGTGATGTGTGCACTCAACTGTCAGAATTGA
          1 AAAAACTAGACAGAAGCATTCTCAGAAACATCTTTGTGATGTTTGCACTCAACTCACAGAGTTGA

                 **   ** *      *                     *      * *            *
     177990 ACCTTGGTTTGGACAGAGCACTTTTGAAACACTCTTTTTGTACAATCTGCAGGTGGATATTTGGC
         66 ACCTTTCTTTTCATAGAGCAGTTTTGAAACACTCTTTTTGTAGAATCTGGAAGTGGATATTTGGA

            **                         *   *      * *
     178055 TAGCTTTGAGGATTTCGTTGGAAACGGTAATGTCTTCAAAG
        131 GCGCTTTGAGGATTTCGTTGGAAACGGAAATATCTTCATAT

                *                         * * *            *    *            
     178096 AAAATCTAGACAGAAGCATTCTCAGAAACAGCGTCGTGATGTTTGCAATCAAGTCACAGAGTTGA
          1 AAAAACTAGACAGAAGCATTCTCAGAAACATCTTTGTGATGTTTGCACTCAACTCACAGAGTTGA

                 * *             *  *                  *             *       
     178161 ACCTTCCGTTTCATAGAGCAGGTTGGAAACACTCTTTTTGTAGTATCTGGAAGTGGACATTTGGA
         66 ACCTTTCTTTTCATAGAGCAGTTTTGAAACACTCTTTTTGTAGAATCTGGAAGTGGATATTTGGA

             *         ** *    * *   *            **  
     178226 GGGCTTTGTAGCCTATC-TGGAAAAAGGAAATATCTTCCCAT
        131 GCGCTTTG-AGGATTTCGTTGGAAACGGAAATATCTTCATAT

            *  ** *   *     * *            *   *  *   * **        *  * * *   
     178267 GAATGCGAGATAGAAGTAATCTCAGAAACATGTTTATGCTGTATCTACTCAACTAACTGTGCTGA
          1 AAAAACTAGACAGAAGCATTCTCAGAAACATCTTTGTGATGTTTGCACTCAACTCACAGAGTTGA

              *     *  *               *        *  * *       *               
     178332 ACATTTCTATTGATAGAGCAGTTTTGAGACACTCTTCTTTTGGAATCTGCAAGTGGATATTTGGA
         66 ACCTTTCTTTTCATAGAGCAGTTTTGAAACACTCTTTTTGTAGAATCTGGAAGTGGATATTTGGA

            ** *                    *   *     *      
     178397 TAGATTTGAGGATTTCGTTGGAAATGG-GAT-T-AT-ATAT
        131 GCGCTTTGAGGATTTCGTTGGAAACGGAAATATCTTCATAT

                 *       *               *                 **  *   *         
     178434 AAAAAGTAGACAGCAGCATTCTCAGAAACTTCTTTGTGATGTTTGCATCCAGCTCTCAGAGTTGA
          1 AAAAACTAGACAGAAGCATTCTCAGAAACATCTTTGTGATGTTTGCACTCAACTCACAGAGTTGA

            * *  * *          *  *        *        *   **        *  **       
     178499 GCATTCCCTTTCATAGAGTAGGTTTGAAACCCTCTTTTTATAGTGTCTGGAAGCGGGCATTTGGA
         66 ACCTTTCTTTTCATAGAGCAGTTTTGAAACACTCTTTTTGTAGAATCTGGAAGTGGATATTTGGA

                   *    * * **  **  **          * *  *
     178564 GCGCTTTCAGGCCTATGCTTAAAATAGGAAATATCTACCTAC
        131 GCGCTTTGAGG-ATTTCGTTGGAAACGGAAATATCTTCATAT

             *                    *    *  **          ** *        *    *     
     178606 AGAAACTAGACAGAAGCATTCTGAGAATCACGTTTGTGATGTGGGTACTCAACTAACAGTGTTGA
          1 AAAAACTAGACAGAAGCATTCTCAGAAACATCTTTGTGATGTTTGCACTCAACTCACAGAGTTGA

            *  *       *   *            *   *                *   *           
     178671 TCCATTCTTTTGATACAGCAGTTTTGAACCACACTTTTTGTAGAATCTGCAAGAGGATATTTGGA
         66 ACCTTTCTTTTCATAGAGCAGTTTTGAAACACTCTTTTTGTAGAATCTGGAAGTGGATATTTGGA

            **   *                     *   *      * *
     178736 TAGCTGTGAGGATTTCGTTGGAAACGGGAATGTCTTCAAAG
        131 GCGCTTTGAGGATTTCGTTGGAAACGGAAATATCTTCATAT

                *                         *   *            *    *            
     178777 AAAATCTAGACAGAAGCATTCTCAGAAACACCTTCGTGATGTTTGCAATCAAGTCACAGAGTTGA
          1 AAAAACTAGACAGAAGCATTCTCAGAAACATCTTTGTGATGTTTGCACTCAACTCACAGAGTTGA

                 * *             *  *           *      *             *       
     178842 ACCTTCCGTTTCATAGAGCAGGTTGGAAACACTCTTATTGTAGTATCTGGAAGTGGACATTTGGA
         66 ACCTTTCTTTTCATAGAGCAGTTTTGAAACACTCTTTTTGTAGAATCTGGAAGTGGATATTTGGA

                   *   ** ** *  *   *            **  
     178907 GCGCTTTCAGGCCTATGGTGAAAAAGGAAATATCTTCCCAT
        131 GCGCTTTGAGGATTTCGTTGGAAACGGAAATATCTTCATAT

                  * * *      **      *   * *   *     ** ** *      *    *     
     178948 AAAAACGACATAGAAGCTATCTCAGGAACTTGTTTATGATGCATCTAATCAACTAACAGTGTTGA
          1 AAAAACTAGACAGAAGCATTCTCAGAAACATCTTTGTGATGTTTGCACTCAACTCACAGAGTTGA

                  * ** * *                         * *       *               
     179013 ACCTTTGTACTGACAGAGCAG-TTTGAAACACTCTTTTTTTGGAATCTGCAAGTGGATATTTGGA
         66 ACCTTTCTTTTCATAGAGCAGTTTTGAAACACTCTTTTTGTAGAATCTGGAAGTGGATATTTGGA

            *                           *   *   *    
     179077 TCGCTTTGAGGATTTCGTTGGAAACGG-GAT-GC--AATAT
        131 GCGCTTTGAGGATTTCGTTGGAAACGGAAATATCTTCATAT

                **  *    *     *                 **  *   *  *    *         * 
     179114 AAAACGTACACAGCAGCATACTCAGAAA-ATACTTTGCCATATTTCCATTCAAGTCACAGAGTGG
          1 AAAAACTAGACAGAAGCATTCTCAGAAACAT-CTTTGTGATGTTTGCACTCAACTCACAGAGTTG

               *  * **            *                  *  *             *      
     179178 AACATTCCCATTCATAGAGCAGGTTTGAAACACTCTTTTTGGAGTATCTGGAAGTGGACATTTGG
         65 AACCTTTCTTTTCATAGAGCAGTTTTGAAACACTCTTTTTGTAGAATCTGGAAGTGGATATTTGG

                    ** * * ** *  *   *            **  
     179243 AGCGCTTTCTGAACTATGGTGAAAAAGGAAATATCTTCCAAT
        130 AGCGCTTTGAGGATTTCGTTGGAAACGGAAATATCTTCATAT

            *     * *             *      * *          *  **      **  *  *    
     179285 GAAAACAACACAGAAGCATTCTGAGAAACTTATTTGTGATGTGTGTCCTCAACAAACGGACTTGA
          1 AAAAACTAGACAGAAGCATTCTCAGAAACATCTTTGTGATGTTTGCACTCAACTCACAGAGTTGA

                   *      **  * *  *  *             *   *    * * *           
     179350 ACCTTTCGTTTCATGCAGTACTTCTGGAACACTCTTTTTGAAGATTCTGCATGCGGATATTTGGA
         66 ACCTTTCTTTTCATAGAGCAGTTTTGAAACACTCTTTTTGTAGAATCTGGAAGTGGATATTTGGA

            **                      *     **   *   * 
     179415 TAGCTTTGAGGATTTCGTTGGAAAAGG---GCT-TACATGT
        131 GCGCTTTGAGGATTTCGTTGGAAACGGAAATATCTTCATAT

                 *       *               *        *   *    *    *       *    
     179452 AAAAATTAGACAGCAGCATTCTCAGAAACTTCTTTGTGGTGTCTGCATTCAAGTCACAGAATTGA
          1 AAAAACTAGACAGAAGCATTCTCAGAAACATCTTTGTGATGTTTGCACTCAACTCACAGAGTTGA

              * ** * **            *  * *      *       *    *        *       
     179517 ACTTCCCCTCACATAGAGCAGTTGTGCAGCACTCTATTTGTAGTATCTCGAAGTGGACATTTGGA
         66 ACCTTTCTTTTCATAGAGCAGTTTTGAAACACTCTTTTTGTAGAATCTGGAAGTGGATATTTGGA

             *         ** *    * *   *            **  
     179582 GGGCTTTGTAGCCTATC-TGGAAAAAGGAAATATCTTCCCAT
        131 GCGCTTTG-AGGATTTCGTTGGAAACGGAAATATCTTCATAT

            *  ** *   *     * *            *   *  *   * **        *  * * *   
     179623 GAATGCGAGATAGAAGTAATCTCAGAAACATGTTTATGCTGTATCTACTCAACTAACTGTGCTGA
          1 AAAAACTAGACAGAAGCATTCTCAGAAACATCTTTGTGATGTTTGCACTCAACTCACAGAGTTGA

              *     *  *               *        *  * *       *               
     179688 ACATTTCTATTGATAGAGCAGTTTTGAGACACTCTTCTTTTGGAATCTGCAAGTGGATATTTGGA
         66 ACCTTTCTTTTCATAGAGCAGTTTTGAAACACTCTTTTTGTAGAATCTGGAAGTGGATATTTGGA

            ** *                        *     *      
     179753 TAGATTTGAGGATTTCGTTGGAAACGG-GAT-T-AT-ATAT
        131 GCGCTTTGAGGATTTCGTTGGAAACGGAAATATCTTCATAT

                 *       *               *                 **  *   *         
     179790 AAAAAGTAGACAGCAGCATTCTCAGAAACTTCTTTGTGATGTTTGCATCCAGCTCTCAGAGTTGA
          1 AAAAACTAGACAGAAGCATTCTCAGAAACATCTTTGTGATGTTTGCACTCAACTCACAGAGTTGA

              *  * *          *  *        *        *   **        *  **       
     179855 ACATTCCCTTTCATAGAGTAGGTTTGAAACCCTCTTTTTATAGTGTCTGGAAGCGGGCATTTGGA
         66 ACCTTTCTTTTCATAGAGCAGTTTTGAAACACTCTTTTTGTAGAATCTGGAAGTGGATATTTGGA

                   *   ** ** *  *   *          * *   
     179920 GCGCTTTCAGGCCTATGCTGAAAAAGGAAATATCTACCTAT
        131 GCGCTTTGAGGATTTCGTTGGAAACGGAAATATCTTCATAT

             *                    *    *  **          ** *        *    *     
     179961 AGAAACTAGACAGAAGCATTCTGAGAATCACGTTTGTGATGTGGGTACTCAACTAACAGTGTTGA
          1 AAAAACTAGACAGAAGCATTCTCAGAAACATCTTTGTGATGTTTGCACTCAACTCACAGAGTTGA

            *  *       *   *            *   *                *               
     180026 TCCATTCTTTTGATACAGCAGTTTTGAACCACACTTTTTGTAGAATCTGCAAGTGGATATTTGGA
         66 ACCTTTCTTTTCATAGAGCAGTTTTGAAACACTCTTTTTGTAGAATCTGGAAGTGGATATTTGGA

            **   *                     *   *        *
     180091 TAGCTGTGAGGATTTCGTTGGAAACGGGAATGTCTTCATAG
        131 GCGCTTTGAGGATTTCGTTGGAAACGGAAATATCTTCATAT

                **                     * * **         *  **   *   *          
     180132 AAAATTTAGACAGAAGCATTCTCAGAACCTTGATTGTGATGTGTGTTCTCCACTAACAGAGTTGA
          1 AAAAACTAGACAGAAGCATTCTCAGAAACATCTTTGTGATGTTTGCACTCAACTCACAGAGTTGA

                       * *   * *   *       *       *                         
     180197 ACCTTTCTTTTGACAGAACTGTTCTGAAACATTCTTTTTATAGAATCTGGAAGTGGATATTTGGA
         66 ACCTTTCTTTTCATAGAGCAGTTTTGAAACACTCTTTTTGTAGAATCTGGAAGTGGATATTTGGA

            **                         *          *  
     180262 AAGCTTTGAGGATTTCGTTGGAAACGGGAATATCTTCAAAT
        131 GCGCTTTGAGGATTTCGTTGGAAACGGAAATATCTTCATAT

                *    *            *          ** *       *  *    *            
     180303 AAAATCTAGCCAGAAGCATTCTAAGAAACATCTCAGGGATGTTTACATTCAAGTCACAGAGTTGA
          1 AAAAACTAGACAGAAGCATTCTCAGAAACATCTTTGTGATGTTTGCACTCAACTCACAGAGTTGA

              *  * *     *       *         *    * *   **      *      *    *  
     180368 ACATTCCCTTTCACAGAGCAGGTTTGAAACAATCTTCTCGTACTATCTGGCAGTGGACATTTTGA
         66 ACCTTTCTTTTCATAGAGCAGTTTTGAAACACTCTTTTTGTAGAATCTGGAAGTGGATATTTGGA

              * *   *  ** ** *  *   *            ** *
     180433 GCTCCTTGGGGCCTATGCTGAAAAAGGAAATATCTTCCGAC
        131 GCGCTTTGAGGATTTCGTTGGAAACGGAAATATCTTCATAT

                                 *     *  **          *           **    *    
     180474 AAAAACTAGACAGAAGCATTCGCAGAATCACGTTTGTGATGTGTGCACTCAACTGTCAGAATTGA
          1 AAAAACTAGACAGAAGCATTCTCAGAAACATCTTTGTGATGTTTGCACTCAACTCACAGAGTTGA

                 **   ** *      *                     *      * *            *
     180539 ACCTTGGTTTGGACAGAGCACTTTTGAAACACTCTTTTTGTACAATCTGCAGGTGGATATTTGGC
         66 ACCTTTCTTTTCATAGAGCAGTTTTGAAACACTCTTTTTGTAGAATCTGGAAGTGGATATTTGGA

            **                         *   *      * *
     180604 TAGCTTTGAGGATTTCGTTGGAAACGGTAATGTCTTCAAAG
        131 GCGCTTTGAGGATTTCGTTGGAAACGGAAATATCTTCATAT

                *                         * * *            *    *            
     180645 AAAATCTAGACAGAAGCATTCTCAGAAACAGCGTCGTGATGTTTGCAATCAAGTCACAGAGTTGA
          1 AAAAACTAGACAGAAGCATTCTCAGAAACATCTTTGTGATGTTTGCACTCAACTCACAGAGTTGA

                 * *             *  *                  *             *       
     180710 ACCTTCCGTTTCATAGAGCAGGTTGGAAACACTCTTTTTGTAGTATCTGGAAGTGGACATTTGGA
         66 ACCTTTCTTTTCATAGAGCAGTTTTGAAACACTCTTTTTGTAGAATCTGGAAGTGGATATTTGGA

             *         ** *    * *   *            **  
     180775 GGGCTTTGTAGCCTATC-TGGAAAAAGGAAATATCTTCCCAT
        131 GCGCTTTG-AGGATTTCGTTGGAAACGGAAATATCTTCATAT

            *  ** *   *     * *            *   *  *   * **        *  * * *   
     180816 GAATGCGAGATAGAAGTAATCTCAGAAACATGTTTATGCTGTATCTACTCAACTAACTGTGCTGA
          1 AAAAACTAGACAGAAGCATTCTCAGAAACATCTTTGTGATGTTTGCACTCAACTCACAGAGTTGA

              *     *  *               *        *  * *       *               
     180881 ACATTTCTATTGATAGAGCAGTTTTGAGACACTCTTCTTTTGGAATCTGCAAGTGGATATTTGGA
         66 ACCTTTCTTTTCATAGAGCAGTTTTGAAACACTCTTTTTGTAGAATCTGGAAGTGGATATTTGGA

            ** *                    *   *     *      
     180946 TAGATTTGAGGATTTCGTTGGAAATGG-GAT-T-AT-ATAT
        131 GCGCTTTGAGGATTTCGTTGGAAACGGAAATATCTTCATAT

                 *       *               *                 **  *   *         
     180983 AAAAAGTAGACAGCAGCATTCTCAGAAACTTCTTTGTGATGTTTGCATCCAGCTCTCAGAGTTGA
          1 AAAAACTAGACAGAAGCATTCTCAGAAACATCTTTGTGATGTTTGCACTCAACTCACAGAGTTGA

            * *  * *          *  *        *        *   **        *  **       
     181048 GCATTCCCTTTCATAGAGTAGGTTTGAAACCCTCTTTTTATAGTGTCTGGAAGCGGGCATTTGGA
         66 ACCTTTCTTTTCATAGAGCAGTTTTGAAACACTCTTTTTGTAGAATCTGGAAGTGGATATTTGGA

                   *    * * **  **  **          * *  *
     181113 GCGCTTTCAGGCCTATGCTTAAAATAGGAAATATCTACCTAC
        131 GCGCTTTGAGG-ATTTCGTTGGAAACGGAAATATCTTCATAT

             *                    *    *  **          ** *        *    *     
     181155 AGAAACTAGACAGAAGCATTCTGAGAATCACGTTTGTGATGTGGGTACTCAACTAACAGTGTTGA
          1 AAAAACTAGACAGAAGCATTCTCAGAAACATCTTTGTGATGTTTGCACTCAACTCACAGAGTTGA

            *  *       *   *            *   *                *   *           
     181220 TCCATTCTTTTGATACAGCAGTTTTGAACCACACTTTTTGTAGAATCTGCAAGAGGATATTTGGA
         66 ACCTTTCTTTTCATAGAGCAGTTTTGAAACACTCTTTTTGTAGAATCTGGAAGTGGATATTTGGA

            **   *                     *   *      * *
     181285 TAGCTGTGAGGATTTCGTTGGAAACGGGAATGTCTTCAAAG
        131 GCGCTTTGAGGATTTCGTTGGAAACGGAAATATCTTCATAT

                *                         *   *            *    *            
     181326 AAAATCTAGACAGAAGCATTCTCAGAAACACCTTCGTGATGTTTGCAATCAAGTCACAGAGTTGA
          1 AAAAACTAGACAGAAGCATTCTCAGAAACATCTTTGTGATGTTTGCACTCAACTCACAGAGTTGA

                 * *             *  *           *      *             *       
     181391 ACCTTCCGTTTCATAGAGCAGGTTGGAAACACTCTTATTGTAGTATCTGGAAGTGGACATTTGGA
         66 ACCTTTCTTTTCATAGAGCAGTTTTGAAACACTCTTTTTGTAGAATCTGGAAGTGGATATTTGGA

                   *   ** ** *  *   *            **  
     181456 GCGCTTTCAGGCCTATGGTGAAAAAGGAAATATCTTCCCAT
        131 GCGCTTTGAGGATTTCGTTGGAAACGGAAATATCTTCATAT

                  * * *      **      *   * *   *     ** ** *      *    *     
     181497 AAAAACGACATAGAAGCTATCTCAGGAACTTGTTTATGATGCATCTAATCAACTAACAGTGTTGA
          1 AAAAACTAGACAGAAGCATTCTCAGAAACATCTTTGTGATGTTTGCACTCAACTCACAGAGTTGA

                  * ** * *                           *       *               
     181562 ACCTTTGTACTGACAGAGCAG-TTTGAAACACTCTTTTT-TGGAATCTGCAAGTGGATATTTGGA
         66 ACCTTTCTTTTCATAGAGCAGTTTTGAAACACTCTTTTTGTAGAATCTGGAAGTGGATATTTGGA

            *                           *   *   *    
     181625 TCGCTTTGAGGATTTCGTTGGAAACGG-GAT-GC--AATAT
        131 GCGCTTTGAGGATTTCGTTGGAAACGGAAATATCTTCATAT

                **  *    *     *        ***     **  *   *  *    *         *  
     181662 AAAACGTACACAGCAGCATACTCAGAAAGTACTTTGCCATATTTCCATTCAAGTCACAGAGTGGA
          1 AAAAACTAGACAGAAGCATTCTCAGAAACATCTTTGTGATGTTTGCACTCAACTCACAGAGTTGA

              *  * **            *  *               *  *             *       
     181727 ACATTCCCATTCATAGAGCAGGTTGGAAACACTCTTTTTGGAGTATCTGGAAGTGGACATTTGGA
         66 ACCTTTCTTTTCATAGAGCAGTTTTGAAACACTCTTTTTGTAGAATCTGGAAGTGGATATTTGGA

                   ** * * ** *      *            **  
     181792 GCGCTTTCTGAACTATGGTGGAAAAGGAAATATCTTCCAAT
        131 GCGCTTTGAGGATTTCGTTGGAAACGGAAATATCTTCATAT

            *     *               *      * *          *  **      **  *  *    
     181833 GAAAACAAGACAGAAGCATTCTGAGAAACTTATTTGTGATGTGTGTCCTCAACAAACGGACTTGA
          1 AAAAACTAGACAGAAGCATTCTCAGAAACATCTTTGTGATGTTTGCACTCAACTCACAGAGTTGA

                   *      **  * *  *  *             *   *    * * *           
     181898 ACCTTTCGTTTCATGCAGTACTTCTGGAACACTCTTTTTGAAGATTCTGCATGCGGATATTTGGA
         66 ACCTTTCTTTTCATAGAGCAGTTTTGAAACACTCTTTTTGTAGAATCTGGAAGTGGATATTTGGA

            **                            **   *   * 
     181963 TAGCTTTGAGGATTTCGTTGGAAACGG---GCT-TACATGT
        131 GCGCTTTGAGGATTTCGTTGGAAACGGAAATATCTTCATAT

                 *       *         *     *        *   *    *    *       *    
     182000 AAAAATTAGACAGCAGCATTCTCTGAAACTTCTTTGTGGTGTCTGCATTCAAGTCACAGAATTGA
          1 AAAAACTAGACAGAAGCATTCTCAGAAACATCTTTGTGATGTTTGCACTCAACTCACAGAGTTGA

              * ** * **            *  * *      *       *    *        *       
     182065 ACTTCCCCTCACATAGAGCAGTTGTGCAGCACTCTATTTGTAGTATCTCGAAGTGGACATTTGGA
         66 ACCTTTCTTTTCATAGAGCAGTTTTGAAACACTCTTTTTGTAGAATCTGGAAGTGGATATTTGGA

             *         ** *    * *   *            **  
     182130 GGGCTTTGTAGCCTATC-TGGAAAAAGGAAATATCTTCCCAT
        131 GCGCTTTG-AGGATTTCGTTGGAAACGGAAATATCTTCATAT

            *  ** *   *     * *            *   *  *   * **        *  * * *   
     182171 GAATGCGAGATAGAAGTAATCTCAGAAACATGTTTATGCTGTATCTACTCAACTAACTGTGCTGA
          1 AAAAACTAGACAGAAGCATTCTCAGAAACATCTTTGTGATGTTTGCACTCAACTCACAGAGTTGA

              *     *  *               *        *  * *       *               
     182236 ACATTTCTATTGATAGAGCAGTTTTGAGACACTCTTCTTTTGGAATCTGCAAGTGGATATTTGGA
         66 ACCTTTCTTTTCATAGAGCAGTTTTGAAACACTCTTTTTGTAGAATCTGGAAGTGGATATTTGGA

            ** *                        *     *      
     182301 TAGATTTGAGGATTTCGTTGGAAACGG-GAT-T-AT-ATAT
        131 GCGCTTTGAGGATTTCGTTGGAAACGGAAATATCTTCATAT

                 *       *               *                 **  *   *         
     182338 AAAAAGTAGACAGCAGCATTCTCAGAAACTTCTTTGTGATGTTTGCATCCAGCTCTCAGAGTTGA
          1 AAAAACTAGACAGAAGCATTCTCAGAAACATCTTTGTGATGTTTGCACTCAACTCACAGAGTTGA

              *  * *          *  *        *        *   **        *  **       
     182403 ACATTCCCTTTCATAGAGTAGGTTTGAAACCCTCTTTTTATAGTGTCTGGAAGCGGGCATTTGGA
         66 ACCTTTCTTTTCATAGAGCAGTTTTGAAACACTCTTTTTGTAGAATCTGGAAGTGGATATTTGGA

                   *   ** ** *  *   *          * *   
     182468 GCGCTTTCAGGCCTATGCTGAAAAAGGAAATATCTACCTAT
        131 GCGCTTTGAGGATTTCGTTGGAAACGGAAATATCTTCATAT

             *                    *    *  **          ** *        *    *     
     182509 AGAAACTAGACAGAAGCATTCTGAGAATCACGTTTGTGATGTGGGTACTCAACTAACAGTGTTGA
          1 AAAAACTAGACAGAAGCATTCTCAGAAACATCTTTGTGATGTTTGCACTCAACTCACAGAGTTGA

            *  *       *   *            *   *                *               
     182574 TCCATTCTTTTGATACAGCAGTTTTGAACCACACTTTTTGTAGAATCTGCAAGTGGATATTTGGA
         66 ACCTTTCTTTTCATAGAGCAGTTTTGAAACACTCTTTTTGTAGAATCTGGAAGTGGATATTTGGA

            **   *          *  *       *   *        *
     182639 TAGCTGTGAGGATTTCCTTCGAAACGGGAATGTCTTCATAG
        131 GCGCTTTGAGGATTTCGTTGGAAACGGAAATATCTTCATAT

                **                     * * **         *  **   *   *          
     182680 AAAATTTAGACAGAAGCATTCTCAGAACCTTGATTGTGATGTGTGTTCTCCACTAACAGAGTTGA
          1 AAAAACTAGACAGAAGCATTCTCAGAAACATCTTTGTGATGTTTGCACTCAACTCACAGAGTTGA

                       * *   * *   *       *       *                         
     182745 ACCTTTCTTTTGACAGAACTGTTCTGAAACATTCTTTTTATAGAATCTGGAAGTGGATATTTGGA
         66 ACCTTTCTTTTCATAGAGCAGTTTTGAAACACTCTTTTTGTAGAATCTGGAAGTGGATATTTGGA

            **      *                  *          *  
     182810 AAGCTTTGCGGATTTCGTTGGAAACGGGAATATCTTCAAAT
        131 GCGCTTTGAGGATTTCGTTGGAAACGGAAATATCTTCATAT

                *    *            *           * *       *  *    *            
     182851 AAAATCTAGCCAGAAGCATTCTAAGAAACATCTTAGGGATGTTTACATTCAAGTCACAGAGTTGA
          1 AAAAACTAGACAGAAGCATTCTCAGAAACATCTTTGTGATGTTTGCACTCAACTCACAGAGTTGA

              *  * *     **      *         *    * *   **      *      *    *  
     182916 ACATTCCCTTTCACGGAGCAGGTTTGAAACAATCTTCTCGTACTATCTGGCAGTGGACATTTTGA
         66 ACCTTTCTTTTCATAGAGCAGTTTTGAAACACTCTTTTTGTAGAATCTGGAAGTGGATATTTGGA

              *     *  ** ** *  *   *            ** *
     182981 GCTCTTTGGGGCCTATGCTGAAAAAGGAAATATCTTCCGAC
        131 GCGCTTTGAGGATTTCGTTGGAAACGGAAATATCTTCATAT

                                 *     *  **          *           **    *    
     183022 AAAAACTAGACAGAAGCATTCGCAGAATCACGTTTGTGATGTGTGCACTCAACTGTCAGAATTGA
          1 AAAAACTAGACAGAAGCATTCTCAGAAACATCTTTGTGATGTTTGCACTCAACTCACAGAGTTGA

                 **   ** *      *                            * *            *
     183087 ACCTTGGTTTGGAGAGAGCACTTTTGAAACACTCTTTTTGTAGAATCTGCAGGTGGATATTTGGC
         66 ACCTTTCTTTTCATAGAGCAGTTTTGAAACACTCTTTTTGTAGAATCTGGAAGTGGATATTTGGA

            **                         *   *      * *
     183152 TAGCTTTGAGGATTTCGTTGGAAACGGTAATGTCTTCAAAG
        131 GCGCTTTGAGGATTTCGTTGGAAACGGAAATATCTTCATAT

                *                         *   *            *    *            
     183193 AAAATCTAGACAGAAGCATTCTCAGAAACACCTTCGTGATGTTTGCAATCAAGTCACAGAGTTGA
          1 AAAAACTAGACAGAAGCATTCTCAGAAACATCTTTGTGATGTTTGCACTCAACTCACAGAGTTGA

                 * *             *  *                  *             *       
     183258 ACCTTCCGTTTCATAGAGCAGGTTGGAAACACTCTTTTTGTAGTATCTGGAAGTGGACATTTGGA
         66 ACCTTTCTTTTCATAGAGCAGTTTTGAAACACTCTTTTTGTAGAATCTGGAAGTGGATATTTGGA

             *         ** *    * *   *            **  
     183323 GGGCTTTGTAGCCTATC-TGGAAAAAGGAAATATCTTCCCAT
        131 GCGCTTTG-AGGATTTCGTTGGAAACGGAAATATCTTCATAT

            *  ** *   *     * *            *   *  *   * **        *  * * *   
     183364 GAATGCGAGATAGAAGTAATCTCAGAAACATGTTTATGCTGTATCTACTCAACTAACTGTGCTGA
          1 AAAAACTAGACAGAAGCATTCTCAGAAACATCTTTGTGATGTTTGCACTCAACTCACAGAGTTGA

              *     *  *               *        *  * *       *               
     183429 ACATTTCTATTGATAGAGCAGTTTTGAGACACTCTTCTTTTGGAATCTGCAAGTGGATATTTGGA
         66 ACCTTTCTTTTCATAGAGCAGTTTTGAAACACTCTTTTTGTAGAATCTGGAAGTGGATATTTGGA

            ** *                        *     *      
     183494 TAGATTTGAGGATTTCGTTGGAAACGG-GAT-T-AT-ATAT
        131 GCGCTTTGAGGATTTCGTTGGAAACGGAAATATCTTCATAT

                 *       *               *                 **  *   *         
     183531 AAAAAGTAGACAGCAGCATTCTCAGAAACTTCTTTGTGATGTTTGCATCCAGCTCTCAGAGTTGA
          1 AAAAACTAGACAGAAGCATTCTCAGAAACATCTTTGTGATGTTTGCACTCAACTCACAGAGTTGA

              *  * *          *  *        *        *   **        *  **       
     183596 ACATTCCCTTTCATAGAGTAGGTTTGAAACCCTCTTTTTATAGTGTCTGGAAGCGGGCATTTGGA
         66 ACCTTTCTTTTCATAGAGCAGTTTTGAAACACTCTTTTTGTAGAATCTGGAAGTGGATATTTGGA

                   *   ** ** *  *   *          * *   
     183661 GCGCTTTCAGGCCTATGCTGAAAAAGGAAATATCTACCTAT
        131 GCGCTTTGAGGATTTCGTTGGAAACGGAAATATCTTCATAT

             *                    *    *  **          ** *        *    *     
     183702 AGAAACTAGACAGAAGCATTCTGAGAATCACGTTTGTGATGTGGGTACTCAACTAACAGTGTTGA
          1 AAAAACTAGACAGAAGCATTCTCAGAAACATCTTTGTGATGTTTGCACTCAACTCACAGAGTTGA

            *  *       *   *            *   *                *               
     183767 TCCATTCTTTTGATACAGCAGTTTTGAACCACACTTTTTGTAGAATCTGCAAGTGGATATTTGGA
         66 ACCTTTCTTTTCATAGAGCAGTTTTGAAACACTCTTTTTGTAGAATCTGGAAGTGGATATTTGGA

            **   *                     *   *        *
     183832 TAGCTGTGAGGATTTCGTTGGAAACGGGAATGTCTTCATAG
        131 GCGCTTTGAGGATTTCGTTGGAAACGGAAATATCTTCATAT

                **                     * * **         *  **   *   *          
     183873 AAAATTTAGACAGAAGCATTCTCAGAACCTTGATTGTGATGTGTGTTCTCCACTAACAGAGTTGA
          1 AAAAACTAGACAGAAGCATTCTCAGAAACATCTTTGTGATGTTTGCACTCAACTCACAGAGTTGA

                       * *   * *   *       *       *         *               
     183938 ACCTTTCTTTTGACAGAACTGTTCTGAAACATTCTTTTTATAGAATCTGCAAGTGGATATTTGGA
         66 ACCTTTCTTTTCATAGAGCAGTTTTGAAACACTCTTTTTGTAGAATCTGGAAGTGGATATTTGGA

            **                         *          *  
     184003 AAGCTTTGAGGATTTCGTTGGAAACGGGAATATCTTCAAAT
        131 GCGCTTTGAGGATTTCGTTGGAAACGGAAATATCTTCATAT

                *    *            *           * *       *  *    *            
     184044 AAAATCTAGCCAGAAGCATTCTAAGAAACATCTTAGGGATGTTTACATTCAAGTCACAGAGTTGA
          1 AAAAACTAGACAGAAGCATTCTCAGAAACATCTTTGTGATGTTTGCACTCAACTCACAGAGTTGA

              *  * *     *       *         *    * *   **      *      *    *  
     184109 ACATTCCCTTTCACAGAGCAGGTTTGAAACAATCTTCTCGTACTATCTGGCAGTGGACATTTTGA
         66 ACCTTTCTTTTCATAGAGCAGTTTTGAAACACTCTTTTTGTAGAATCTGGAAGTGGATATTTGGA

              * *   *  ** ** *  *   *            ** *
     184174 GCTCCTTGGGGCCTATGCTGAAAAAGGAAATATCTTCCGAC
        131 GCGCTTTGAGGATTTCGTTGGAAACGGAAATATCTTCATAT

                                 *     *  **          *           **    *    
     184215 AAAAACTAGACAGAAGCATTCGCAGAATCACGTTTGTGATGTGTGCACTCAACTGTCAGAATTGA
          1 AAAAACTAGACAGAAGCATTCTCAGAAACATCTTTGTGATGTTTGCACTCAACTCACAGAGTTGA

                 **   ** *      *                            * *            *
     184280 ACCTTGGTTTGGACAGAGCACTTTTGAAACACTCTTTTTGTAGAATCTGCAGGTGGATATTTGGC
         66 ACCTTTCTTTTCATAGAGCAGTTTTGAAACACTCTTTTTGTAGAATCTGGAAGTGGATATTTGGA

            **                         *   *      * *
     184345 TAGCTTTGAGGATTTCGTTGGAAACGGTAATGTCTTCAAAG
        131 GCGCTTTGAGGATTTCGTTGGAAACGGAAATATCTTCATAT

                *                         *   *            *    *          * 
     184386 AAAATCTAGACAGAAGCATTCTCAGAAACAACTTCGTGATGTTTGCAATCAAGTCACAGAGTTAA
          1 AAAAACTAGACAGAAGCATTCTCAGAAACATCTTTGTGATGTTTGCACTCAACTCACAGAGTTGA

                 * *             *  *                  *             *       
     184451 ACCTTCCGTTTCATAGAGCAGGTTGGAAACACTCTTTTTGTAGTATCTGGAAGTGGACATTTGGA
         66 ACCTTTCTTTTCATAGAGCAGTTTTGAAACACTCTTTTTGTAGAATCTGGAAGTGGATATTTGGA

             *         ** *    * *   *            **  
     184516 GGGCTTTGTAGCCTATC-TGGAAAAAGGAAATATCTTCCCAT
        131 GCGCTTTG-AGGATTTCGTTGGAAACGGAAATATCTTCATAT

            *  ** *   *     * *            *   *  *   * **        *  * * *   
     184557 GAATGCGAGATAGAAGTAATCTCAGAAACATGTTTATGCTGTATCTACTCAACTAACTGTGCTGA
          1 AAAAACTAGACAGAAGCATTCTCAGAAACATCTTTGTGATGTTTGCACTCAACTCACAGAGTTGA

              *     *  *               *        *  * *       *               
     184622 ACATTTCTATTGATAGAGCAGTTTTGAGACACTCTTCTTTTGGAATCTGCAAGTGGATATTTGGA
         66 ACCTTTCTTTTCATAGAGCAGTTTTGAAACACTCTTTTTGTAGAATCTGGAAGTGGATATTTGGA

            ** *                        *     *      
     184687 TAGATTTGAGGATTTCGTTGGAAACGG-GAT-T-AT-ATAT
        131 GCGCTTTGAGGATTTCGTTGGAAACGGAAATATCTTCATAT

                 *       *               *                 **  *   *         
     184724 AAAAAGTAGACAGCAGCATTCTCAGAAACTTCTTTGTGATGTTTGCATCCAGCTCTCAGAGTTGA
          1 AAAAACTAGACAGAAGCATTCTCAGAAACATCTTTGTGATGTTTGCACTCAACTCACAGAGTTGA

              *  * *          *  *        *        *   **        *  **       
     184789 ACATTCCCTTTCATAGAGTAGGTTTGAAACCCTCTTTTTATAGTGTCTGGAAGCGGGCATTTGGA
         66 ACCTTTCTTTTCATAGAGCAGTTTTGAAACACTCTTTTTGTAGAATCTGGAAGTGGATATTTGGA

                   *   ** ** *  *   *          * *   
     184854 GCGCTTTCAGGCCTATGCTGAAAAAGGAAATATCTACCTAT
        131 GCGCTTTGAGGATTTCGTTGGAAACGGAAATATCTTCATAT

             *                    *    *  **          ** *        *    *     
     184895 AGAAACTAGACAGAAGCATTCTGAGAATCACGTTTGTGATGTGGGTACTCAACTAACAGTGTTGA
          1 AAAAACTAGACAGAAGCATTCTCAGAAACATCTTTGTGATGTTTGCACTCAACTCACAGAGTTGA

            *  *       *   *            *   *                *               
     184960 TCCATTCTTTTGATACAGCAGTTTTGAACCACACTTTTTGTAGAATCTGCAAGTGGATATTTGGA
         66 ACCTTTCTTTTCATAGAGCAGTTTTGAAACACTCTTTTTGTAGAATCTGGAAGTGGATATTTGGA

            **   *                     *   *        *
     185025 TAGCTGTGAGGATTTCGTTGGAAACGGGAATGTCTTCATAG
        131 GCGCTTTGAGGATTTCGTTGGAAACGGAAATATCTTCATAT

                **                     * * **         *  **   *   *          
     185066 AAAACTTAGACAGAAGCATTCTCAGAACCTTGATTGTGATGTGTGTTCTCCACTAACAGAGTTGA
          1 AAAAACTAGACAGAAGCATTCTCAGAAACATCTTTGTGATGTTTGCACTCAACTCACAGAGTTGA

                       * *   * *   *       *       *                         
     185131 ACCTTTCTTTTGACAGAACTGTTCTGAAACATTCTTTTTATAGAATCTGGAAGTGGATATTTGGA
         66 ACCTTTCTTTTCATAGAGCAGTTTTGAAACACTCTTTTTGTAGAATCTGGAAGTGGATATTTGGA

            **                         *          *  
     185196 AAGCTTTGAGGATTTCGTTGGAAACGGGAATATCTTCAAAT
        131 GCGCTTTGAGGATTTCGTTGGAAACGGAAATATCTTCATAT

                *    *            *          ** *       *  *    *            
     185237 AAAATCTAGCCAGAAGCATTCTAAGAAACATCTCAGGGATGTTTACATTCAAGTCACAGAGTTGA
          1 AAAAACTAGACAGAAGCATTCTCAGAAACATCTTTGTGATGTTTGCACTCAACTCACAGAGTTGA

              *  * *     *       *         *    * *   **      *      *    *  
     185302 ACATTCCCTTTCACAGAGCAGGTTTGAAACAATCTTCTCGTACTATCTGGCAGTGGACATTTTGA
         66 ACCTTTCTTTTCATAGAGCAGTTTTGAAACACTCTTTTTGTAGAATCTGGAAGTGGATATTTGGA

              * *   *  ** ** *  *   *            ** *
     185367 GCTCCTTGGGGCCTATGCTGAAAAAGGAAATATCTTCCGAC
        131 GCGCTTTGAGGATTTCGTTGGAAACGGAAATATCTTCATAT

                                 *     *  **          *           **    *    
     185408 AAAAACTAGACAGAAGCATTCGCAGAATCACGTTTGTGATGTGTGCACTCAACTGTCAGAATTGA
          1 AAAAACTAGACAGAAGCATTCTCAGAAACATCTTTGTGATGTTTGCACTCAACTCACAGAGTTGA

                 **   ** *      *                            * *            *
     185473 ACCTTGGTTTAGACAGAGCACTTTTGAAACACTCTTTTTGTAGAATCTGCAGGTGGATATTTGGC
         66 ACCTTTCTTTTCATAGAGCAGTTTTGAAACACTCTTTTTGTAGAATCTGGAAGTGGATATTTGGA

            **                         *   *      * *
     185538 TAGCTTTGAGGATTTCGTTGGAAACGGTAATGTCTTCAAAG
        131 GCGCTTTGAGGATTTCGTTGGAAACGGAAATATCTTCATAT

                *                         * * *            *    *            
     185579 AAAATCTAGACAGAAGCATTCTCAGAAACAGCGTCGTGATGTTTGCAATCAAGTCACAGAGTTGA
          1 AAAAACTAGACAGAAGCATTCTCAGAAACATCTTTGTGATGTTTGCACTCAACTCACAGAGTTGA

                 * *             *  *                  *             *       
     185644 ACCTTCCGTTTCATAGAGCAGGTTGGAAACACTCTTTTTGTAGTATCTGGAAGTGGACATTTGGA
         66 ACCTTTCTTTTCATAGAGCAGTTTTGAAACACTCTTTTTGTAGAATCTGGAAGTGGATATTTGGA

             *         ** *    * *   *            **  
     185709 GGGCTTTGTAGCCTATC-TGGAAAAAGGAAATATCTTCCCAT
        131 GCGCTTTG-AGGATTTCGTTGGAAACGGAAATATCTTCATAT

            *  ** *   *     * *            *   *  *   * **        *  * * *   
     185750 GAATGCGAGATAGAAGTAATCTCAGAAACATGTTTATGCTGTATCTACTCAACTAACTGTGCTGA
          1 AAAAACTAGACAGAAGCATTCTCAGAAACATCTTTGTGATGTTTGCACTCAACTCACAGAGTTGA

              *     *  *               *        *  * *       *               
     185815 ACATTTCTATTGATAGAGCAGTTTTGAGACACTCTTCTTTTGGAATCTGCAAGTGGATATTTGGA
         66 ACCTTTCTTTTCATAGAGCAGTTTTGAAACACTCTTTTTGTAGAATCTGGAAGTGGATATTTGGA

            ** *                    *   *     *      
     185880 TAGATTTGAGGATTTCGTTGGAAATGG-GAT-T-AT-ATAT
        131 GCGCTTTGAGGATTTCGTTGGAAACGGAAATATCTTCATAT

                 *       *               *                 **  *   *         
     185917 AAAAAGTAGACAGCAGCATTCTCAGAAACTTCTTTGTGATGTTTGCATCCAGCTCTCAGAGTTGA
          1 AAAAACTAGACAGAAGCATTCTCAGAAACATCTTTGTGATGTTTGCACTCAACTCACAGAGTTGA

            * *  * *          *  *        *        *   **        *  **       
     185982 GCATTCCCTTTCATAGAGTAGGTTTGAAACCCTCTTTTTATAGTGTCTGGAAGCGGGCATTTGGA
         66 ACCTTTCTTTTCATAGAGCAGTTTTGAAACACTCTTTTTGTAGAATCTGGAAGTGGATATTTGGA

                   *    * * **  **  **          *    *
     186047 GCGCTTTCAGGCCTATGCTTAAAATAGGAAATATCTACATAC
        131 GCGCTTTGAGG-ATTTCGTTGGAAACGGAAATATCTTCATAT

             *                    *    *  **          ** *        *    *     
     186089 AGAAACTAGACAGAAGCATTCTGAGAATCACGTTTGTGATGTGGGTACTCAACTAACAGTGTTGA
          1 AAAAACTAGACAGAAGCATTCTCAGAAACATCTTTGTGATGTTTGCACTCAACTCACAGAGTTGA

            *  *       *   *            *   *                *   *           
     186154 TCCATTCTTTTGATACAGCAGTTTTGAACCACACTTTTTGTAGAATCTGCAAGAGGATATTTGGA
         66 ACCTTTCTTTTCATAGAGCAGTTTTGAAACACTCTTTTTGTAGAATCTGGAAGTGGATATTTGGA

            **   *                     *   *      * *
     186219 TAGCTGTGAGGATTTCGTTGGAAACGGGAATGTCTTCAAAG
        131 GCGCTTTGAGGATTTCGTTGGAAACGGAAATATCTTCATAT

                *                         *   *            *    *            
     186260 AAAATCTAGACAGAAGCATTCTCAGAAACACCTTCGTGATGTTTGCAATCAAGTCACAGAGTTGA
          1 AAAAACTAGACAGAAGCATTCTCAGAAACATCTTTGTGATGTTTGCACTCAACTCACAGAGTTGA

                 * *             *  *           *      *             *       
     186325 ACCTTCCGTTTCATAGAGCAGGTTGGAAACACTCTTATTGTAGTATCTGGAAGTGGACATTTGGA
         66 ACCTTTCTTTTCATAGAGCAGTTTTGAAACACTCTTTTTGTAGAATCTGGAAGTGGATATTTGGA

                   *   ** ** *  *   *            **  
     186390 GCGCTTTCAGGCCTATGGTGAAAAAGGAAATATCTTCCCAT
        131 GCGCTTTGAGGATTTCGTTGGAAACGGAAATATCTTCATAT

                  * * *      **      *   * *   *     ** ** *      *    *     
     186431 AAAAACGACATAGAAGCTATCTCAGGAACTTGTTTATGATGCATCTAATCAACTAACAGTGTTGA
          1 AAAAACTAGACAGAAGCATTCTCAGAAACATCTTTGTGATGTTTGCACTCAACTCACAGAGTTGA

                  * ** * *                           *       *               
     186496 ACCTTTGTACTGACAGAGCAG-TTTGAAACACTCTTTTT-TGGAATCTGCAAGTGGATATTTGGA
         66 ACCTTTCTTTTCATAGAGCAGTTTTGAAACACTCTTTTTGTAGAATCTGGAAGTGGATATTTGGA

            *                           *   *   *    
     186559 TCGCTTTGAGGATTTCGTTGGAAACGG-GAT-GC--AATAT
        131 GCGCTTTGAGGATTTCGTTGGAAACGGAAATATCTTCATAT

                **  *    *     *        ***     **  *   *  *    *         *  
     186596 AAAACGTACACAGCAGCATACTCAGAAAGTACTTTGCCATATTTCCATTCAAGTCACAGAGTGGA
          1 AAAAACTAGACAGAAGCATTCTCAGAAACATCTTTGTGATGTTTGCACTCAACTCACAGAGTTGA

              *  * **            *  *               *  *             *       
     186661 ACATTCCCATTCATAGAGCAGGTTGGAAACACTCTTTTTGGAGTATCTGGAAGTGGACATTTGGA
         66 ACCTTTCTTTTCATAGAGCAGTTTTGAAACACTCTTTTTGTAGAATCTGGAAGTGGATATTTGGA

                   ** * * ** *      *            **  
     186726 GCGCTTTCTGAACTATGGTGGAAAAGGAAATATCTTCCAAT
        131 GCGCTTTGAGGATTTCGTTGGAAACGGAAATATCTTCATAT

            *     *               *      * *          *  **      **  *  *    
     186767 GAAAACAAGACAGAAGCATTCTGAGAAACTTATTTGTGATGTGTGTCCTCAACAAACGGACTTGA
          1 AAAAACTAGACAGAAGCATTCTCAGAAACATCTTTGTGATGTTTGCACTCAACTCACAGAGTTGA

                   *      **  * *  *  *             *   *    * * *           
     186832 ACCTTTCGTTTCATGCAGTACTTCTGGAACACTCTTTTTGAAGATTCTGCATGCGGATATTTGGA
         66 ACCTTTCTTTTCATAGAGCAGTTTTGAAACACTCTTTTTGTAGAATCTGGAAGTGGATATTTGGA

            **                            **   *   * 
     186897 TAGCTTTGAGGATTTCGTTGGAAACGG---GCT-TACATGT
        131 GCGCTTTGAGGATTTCGTTGGAAACGGAAATATCTTCATAT

                 *       *               *        *   *    *    *       *    
     186934 AAAAATTAGACAGCAGCATTCTCAGAAACTTCTTTGTGGTGTCTGCATTCAAGTCACAGAATTGA
          1 AAAAACTAGACAGAAGCATTCTCAGAAACATCTTTGTGATGTTTGCACTCAACTCACAGAGTTGA

              * ** * **            *  * *      *       *    *        *       
     186999 ACTTCCCCTCACATAGAGCAGTTGTGCAGCACTCTATTTGTAGTATCTCGAAGTGGACATTTGGA
         66 ACCTTTCTTTTCATAGAGCAGTTTTGAAACACTCTTTTTGTAGAATCTGGAAGTGGATATTTGGA

             *         ** *    * *   *            **  
     187064 GGGCTTTGTAGCCTATC-TGGAAAAAGGAAATATCTTCCCAT
        131 GCGCTTTG-AGGATTTCGTTGGAAACGGAAATATCTTCATAT

            *  ** *   *     * *            *   *  *   * **        *  * * *   
     187105 GAATGCGAGATAGAAGTAATCTCAGAAACATGTTTATGCTGTATCTACTCAACTAACTGTGCTGA
          1 AAAAACTAGACAGAAGCATTCTCAGAAACATCTTTGTGATGTTTGCACTCAACTCACAGAGTTGA

              *     *  *               *        *  * *       *               
     187170 ACATTTCTATTGATAGAGCAGTTTTGAGACACTCTTCTTTTGGAATCTGCAAGTGGATATTTGGA
         66 ACCTTTCTTTTCATAGAGCAGTTTTGAAACACTCTTTTTGTAGAATCTGGAAGTGGATATTTGGA

            ** *                        *     *      
     187235 TAGATTTGAGGATTTCGTTGGAAACGG-GAT-T-AT-ATAT
        131 GCGCTTTGAGGATTTCGTTGGAAACGGAAATATCTTCATAT

            *    *       *               *                 **  *   *         
     187272 CAAAAGTAGACAGCAGCATTCTCAGAAACTTCTTTGTGATGTTTGCATCCAGCTCTCAGAGTTGA
          1 AAAAACTAGACAGAAGCATTCTCAGAAACATCTTTGTGATGTTTGCACTCAACTCACAGAGTTGA

              *  * *          *  *        *        *   **        *  **       
     187337 ACATTCCCTTTCATAGAGTAGGTTTGAAACCCTCTTTTTATAGTGTCTGGAAGCGGGCATTTGGA
         66 ACCTTTCTTTTCATAGAGCAGTTTTGAAACACTCTTTTTGTAGAATCTGGAAGTGGATATTTGGA

                   *   ** ** *  *   *          * *   
     187402 GCGCTTTCAGGCCTATGCTGAAAAAGGAAATATCTACCTAT
        131 GCGCTTTGAGGATTTCGTTGGAAACGGAAATATCTTCATAT

             *                    *    *  **          ** *        *    *     
     187443 AGAAACTAGACAGAAGCATTCTGAGAATCACGTTTGTGATGTGGGTACTCAACTAACAGTGTTGA
          1 AAAAACTAGACAGAAGCATTCTCAGAAACATCTTTGTGATGTTTGCACTCAACTCACAGAGTTGA

            *  *       *   *            *   *                *               
     187508 TCCATTCTTTTGATACAGCAGTTTTGAACCACACTTTTTGTAGAATCTGCAAGTGGATATTTGGA
         66 ACCTTTCTTTTCATAGAGCAGTTTTGAAACACTCTTTTTGTAGAATCTGGAAGTGGATATTTGGA

            **   *          *  *       *   *        *
     187573 TAGCTGTGAGGATTTCCTTCGAAACGGGAATGTCTTCATAG
        131 GCGCTTTGAGGATTTCGTTGGAAACGGAAATATCTTCATAT

                **                     * * **         *  **   *   *          
     187614 AAAATTTAGACAGAAGCATTCTCAGAACCTTGATTGTGATGTGTGTTCTCCACTAACAGAGTTGA
          1 AAAAACTAGACAGAAGCATTCTCAGAAACATCTTTGTGATGTTTGCACTCAACTCACAGAGTTGA

                       * *   * *   *       *       *                         
     187679 ACCTTTCTTTTGACAGAACTGTTCTGAAACATTCTTTTTATAGAATCTGGAAGTGGATATTTGGA
         66 ACCTTTCTTTTCATAGAGCAGTTTTGAAACACTCTTTTTGTAGAATCTGGAAGTGGATATTTGGA

            **      *                  *          *  
     187744 AAGCTTTGCGGATTTCGTTGGAAACGGGAATATCTTCAAAT
        131 GCGCTTTGAGGATTTCGTTGGAAACGGAAATATCTTCATAT

                *    *            *           * *       *  *    *            
     187785 AAAATCTAGCCAGAAGCATTCTAAGAAACATCTTAGGGATGTTTACATTCAAGTCACAGAGTTGA
          1 AAAAACTAGACAGAAGCATTCTCAGAAACATCTTTGTGATGTTTGCACTCAACTCACAGAGTTGA

              *  * *     **      *         *    * *   **      *      *    *  
     187850 ACATTCCCTTTCACGGAGCAGGTTTGAAACAATCTTCTCGTACTATCTGGCAGTGGACATTTTGA
         66 ACCTTTCTTTTCATAGAGCAGTTTTGAAACACTCTTTTTGTAGAATCTGGAAGTGGATATTTGGA

              *     *  ** ** *  *   *            ** *
     187915 GCTCTTTGGGGCCTATGCTGAAAAAGGAAATATCTTCCGAC
        131 GCGCTTTGAGGATTTCGTTGGAAACGGAAATATCTTCATAT

                                 *     *  **          *           **    *    
     187956 AAAAACTAGACAGAAGCATTCGCAGAATCACGTTTGTGATGTGTGCACTCAACTGTCAGAATTGA
          1 AAAAACTAGACAGAAGCATTCTCAGAAACATCTTTGTGATGTTTGCACTCAACTCACAGAGTTGA

                 **   ** *      *                            * *            *
     188021 ACCTTGGTTTGGAGAGAGCACTTTTGAAACACTCTTTTTGTAGAATCTGCAGGTGGATATTTGGC
         66 ACCTTTCTTTTCATAGAGCAGTTTTGAAACACTCTTTTTGTAGAATCTGGAAGTGGATATTTGGA

            **                         *   *      * *
     188086 TAGCTTTGAGGATTTCGTTGGAAACGGTAATGTCTTCAAAG
        131 GCGCTTTGAGGATTTCGTTGGAAACGGAAATATCTTCATAT

                *                         *   *            *    *            
     188127 AAAATCTAGACAGAAGCATTCTCAGAAACACCTTCGTGATGTTTGCAATCAAGTCACAGAGTTGA
          1 AAAAACTAGACAGAAGCATTCTCAGAAACATCTTTGTGATGTTTGCACTCAACTCACAGAGTTGA

                 * *             *  *                  *             *       
     188192 ACCTTCCGTTTCATAGAGCAGGTTGGAAACACTCTTTTTGTAGTATCTGGAAGTGGACATTTGGA
         66 ACCTTTCTTTTCATAGAGCAGTTTTGAAACACTCTTTTTGTAGAATCTGGAAGTGGATATTTGGA

             *         ** *    * *   *            **  
     188257 GGGCTTTGTAGCCTATC-TGGAAAAAGGAAATATCTTCCCAT
        131 GCGCTTTG-AGGATTTCGTTGGAAACGGAAATATCTTCATAT

            *  ** *   *     * *            *   *  *   * **        *  * * *   
     188298 GAATGCGAGATAGAAGTAATCTCAGAAACATGTTTATGCTGTATCTACTCAACTAACTGTGCTGA
          1 AAAAACTAGACAGAAGCATTCTCAGAAACATCTTTGTGATGTTTGCACTCAACTCACAGAGTTGA

              *     *  *               *        *  * *       *               
     188363 ACATTTCTATTGATAGAGCAGTTTTGAGACACTCTTCTTTTGGAATCTGCAAGTGGATATTTGGA
         66 ACCTTTCTTTTCATAGAGCAGTTTTGAAACACTCTTTTTGTAGAATCTGGAAGTGGATATTTGGA

            ** *                        *     *      
     188428 TAGATTTGAGGATTTCGTTGGAAACGG-GAT-T-AT-ATAT
        131 GCGCTTTGAGGATTTCGTTGGAAACGGAAATATCTTCATAT

                 *       *               *                 **  *   *         
     188465 AAAAAGTAGACAGCAGCATTCTCAGAAACTTCTTTGTGATGTTTGCATCCAGCTCTCAGAGTTGA
          1 AAAAACTAGACAGAAGCATTCTCAGAAACATCTTTGTGATGTTTGCACTCAACTCACAGAGTTGA

              *  * *          *  *        *        *   **        *  **       
     188530 ACATTCCCTTTCATAGAGTAGGTTTGAAACCCTCTTTTTATAGTGTCTGGAAGCGGGCATTTGGA
         66 ACCTTTCTTTTCATAGAGCAGTTTTGAAACACTCTTTTTGTAGAATCTGGAAGTGGATATTTGGA

                   *   ** ** *  *   *          * *   
     188595 GCGCTTTCAGGCCTATGCTGAAAAAGGAAATATCTACCTAT
        131 GCGCTTTGAGGATTTCGTTGGAAACGGAAATATCTTCATAT

             *                    *    *  **          ** *        *    *     
     188636 AGAAACTAGACAGAAGCATTCTGAGAATCACGTTTGTGATGTGGGTACTCAACTAACAGTGTTGA
          1 AAAAACTAGACAGAAGCATTCTCAGAAACATCTTTGTGATGTTTGCACTCAACTCACAGAGTTGA

            *  *       *   *            *   *                *               
     188701 TCCATTCTTTTGATACAGCAGTTTTGAACCACACTTTTTGTAGAATCTGCAAGTGGATATTTGGA
         66 ACCTTTCTTTTCATAGAGCAGTTTTGAAACACTCTTTTTGTAGAATCTGGAAGTGGATATTTGGA

            **   *                     *   *        *
     188766 TAGCTGTGAGGATTTCGTTGGAAACGGGAATGTCTTCATAG
        131 GCGCTTTGAGGATTTCGTTGGAAACGGAAATATCTTCATAT

                **                     * * **         *  **   *   *          
     188807 AAAATTTAGACAGAAGCATTCTCAGAACCTTGATTGTGATGTGTGTTCTCCACTAACAGAGTTGA
          1 AAAAACTAGACAGAAGCATTCTCAGAAACATCTTTGTGATGTTTGCACTCAACTCACAGAGTTGA

                       * *   * *   *       *       *         *               
     188872 ACCTTTCTTTTGACAGAACTGTTCTGAAACATTCTTTTTATAGAATCTGCAAGTGGATATTTGGA
         66 ACCTTTCTTTTCATAGAGCAGTTTTGAAACACTCTTTTTGTAGAATCTGGAAGTGGATATTTGGA

            **                         *          *  
     188937 AAGCTTTGAGGATTTCGTTGGAAACGGGAATATCTTCAAAT
        131 GCGCTTTGAGGATTTCGTTGGAAACGGAAATATCTTCATAT

                *    *            *           * *       *  *    *            
     188978 AAAATCTAGCCAGAAGCATTCTAAGAAACATCTTAGGGATGTTTACATTCAAGTCACAGAGTTGA
          1 AAAAACTAGACAGAAGCATTCTCAGAAACATCTTTGTGATGTTTGCACTCAACTCACAGAGTTGA

              *  * *     *       *         *    * *   **      *      *    *  
     189043 ACATTCCCTTTCACAGAGCAGGTTTGAAACAATCTTCTCGTACTATCTGGCAGTGGACATTTTGA
         66 ACCTTTCTTTTCATAGAGCAGTTTTGAAACACTCTTTTTGTAGAATCTGGAAGTGGATATTTGGA

              * *   *  ** ** *  *   *            ** *
     189108 GCTCCTTGGGGCCTATGCTGAAAAAGGAAATATCTTCCGAC
        131 GCGCTTTGAGGATTTCGTTGGAAACGGAAATATCTTCATAT

                                 *     *  **          *           **    *    
     189149 AAAAACTAGACAGAAGCATTCGCAGAATCACGTTTGTGATGTGTGCACTCAACTGTCAGAATTGA
          1 AAAAACTAGACAGAAGCATTCTCAGAAACATCTTTGTGATGTTTGCACTCAACTCACAGAGTTGA

                 **   ** *      *                            * *            *
     189214 ACCTTGGTTTGGACAGAGCACTTTTGAAACACTCTTTTTGTAGAATCTGCAGGTGGATATTTGGC
         66 ACCTTTCTTTTCATAGAGCAGTTTTGAAACACTCTTTTTGTAGAATCTGGAAGTGGATATTTGGA

            **                         *   *      * *
     189279 TAGCTTTGAGGATTTCGTTGGAAACGGTAATGTCTTCAAAG
        131 GCGCTTTGAGGATTTCGTTGGAAACGGAAATATCTTCATAT

                *                         *   *            *    *          * 
     189320 AAAATCTAGACAGAAGCATTCTCAGAAACAACTTCGTGATGTTTGCAATCAAGTCACAGAGTTAA
          1 AAAAACTAGACAGAAGCATTCTCAGAAACATCTTTGTGATGTTTGCACTCAACTCACAGAGTTGA

                 * *             *  *                  *             *       
     189385 ACCTTCCGTTTCATAGAGCAGGTTGGAAACACTCTTTTTGTAGTATCTGGAAGTGGACATTTGGA
         66 ACCTTTCTTTTCATAGAGCAGTTTTGAAACACTCTTTTTGTAGAATCTGGAAGTGGATATTTGGA

             *         ** *    * *   *            **  
     189450 GGGCTTTGTAGCCTATC-TGGAAAAAGGAAATATCTTCCCAT
        131 GCGCTTTG-AGGATTTCGTTGGAAACGGAAATATCTTCATAT

            *  ** *   *     * *            *   *  *   * **        *  * * *   
     189491 GAATGCGAGATAGAAGTAATCTCAGAAACATGTTTATGCTGTATCTACTCAACTAACTGTGCTGA
          1 AAAAACTAGACAGAAGCATTCTCAGAAACATCTTTGTGATGTTTGCACTCAACTCACAGAGTTGA

              *     *  *               *        *  * *       *               
     189556 ACATTTCTATTGATAGAGCAGTTTTGAGACACTCTTCTTTTGGAATCTGCAAGTGGATATTTGGA
         66 ACCTTTCTTTTCATAGAGCAGTTTTGAAACACTCTTTTTGTAGAATCTGGAAGTGGATATTTGGA

            ** *                        *     *      
     189621 TAGATTTGAGGATTTCGTTGGAAACGG-GAT-T-AT-ATAT
        131 GCGCTTTGAGGATTTCGTTGGAAACGGAAATATCTTCATAT

                 *       *               *                 **  *   *         
     189658 AAAAAGTAGACAGCAGCATTCTCAGAAACTTCTTTGTGATGTTTGCATCCAGCTCTCAGAGTTGA
          1 AAAAACTAGACAGAAGCATTCTCAGAAACATCTTTGTGATGTTTGCACTCAACTCACAGAGTTGA

              *  * *          *  *        *        *   **        *  **       
     189723 ACATTCCCTTTCATAGAGTAGGTTTGAAACCCTCTTTTTATAGTGTCTGGAAGCGGGCATTTGGA
         66 ACCTTTCTTTTCATAGAGCAGTTTTGAAACACTCTTTTTGTAGAATCTGGAAGTGGATATTTGGA

                   *    * * **  **  **          * *  *
     189788 GCGCTTTCAGGCCTATGCTTAAAATAGGAAATATCTACCTAC
        131 GCGCTTTGAGG-ATTTCGTTGGAAACGGAAATATCTTCATAT

             *                    *    *  **          ** *        *    *     
     189830 AGAAACTAGACAGAAGCATTCTGAGAATCACGTTTGTGATGTGGGTACTCAACTAACAGTGTTGA
          1 AAAAACTAGACAGAAGCATTCTCAGAAACATCTTTGTGATGTTTGCACTCAACTCACAGAGTTGA

            *  *       *   *            *   *                *   *           
     189895 TCCATTCTTTTGATACAGCAGTTTTGAACCACACTTTTTGTAGAATCTGCAAGAGGATATTTGGA
         66 ACCTTTCTTTTCATAGAGCAGTTTTGAAACACTCTTTTTGTAGAATCTGGAAGTGGATATTTGGA

            **   *                     *   *      * *
     189960 TAGCTGTGAGGATTTCGTTGGAAACGGGAATGTCTTCAAAG
        131 GCGCTTTGAGGATTTCGTTGGAAACGGAAATATCTTCATAT

                *                         *   *            *    *            
     190001 AAAATCTAGACAGAAGCATTCTCAGAAACACCTTCGTGATGTTTGCAATCAAGTCACAGAGTTGA
          1 AAAAACTAGACAGAAGCATTCTCAGAAACATCTTTGTGATGTTTGCACTCAACTCACAGAGTTGA

                   *             *  *           *      *             *       
     190066 ACCTTTCGTTTCATAGAGCAGGTTGGAAACACTCTTATTGTAGTATCTGGAAGTGGACATTTGGA
         66 ACCTTTCTTTTCATAGAGCAGTTTTGAAACACTCTTTTTGTAGAATCTGGAAGTGGATATTTGGA

                   *   ** ** *  *   *            **  
     190131 GCGCTTTCAGGCCTATGGTGAAAAAGGAAATATCTTCCCAT
        131 GCGCTTTGAGGATTTCGTTGGAAACGGAAATATCTTCATAT

                  * * *      **      *   * *   *     ** ** *      *    *     
     190172 AAAAACGACATAGAAGCTATCTCAGGAACTTGTTTATGATGCATCTAATCAACTAACAGTGTTGA
          1 AAAAACTAGACAGAAGCATTCTCAGAAACATCTTTGTGATGTTTGCACTCAACTCACAGAGTTGA

                  * ** * *                         * *       *               
     190237 ACCTTTGTACTGACAGAGCAG-TTTGAAACACTCTTTTTTTGGAATCTGCAAGTGGATATTTGGA
         66 ACCTTTCTTTTCATAGAGCAGTTTTGAAACACTCTTTTTGTAGAATCTGGAAGTGGATATTTGGA

            *                           *   *   *    
     190301 TCGCTTTGAGGATTTCGTTGGAAACGG-GAT-GC--AATAT
        131 GCGCTTTGAGGATTTCGTTGGAAACGGAAATATCTTCATAT

                **  *    *     *                 **  *   *  *    *         * 
     190338 AAAACGTACACAGCAGCATACTCAGAAA-ATACTTTGCCATATTTCCATTCAAGTCACAGAGTGG
          1 AAAAACTAGACAGAAGCATTCTCAGAAACAT-CTTTGTGATGTTTGCACTCAACTCACAGAGTTG

               *  * **            *                  *  *             *      
     190402 AACATTCCCATTCATAGAGCAGGTTTGAAACACTCTTTTTGGAGTATCTGGAAGTGGACATTTGG
         65 AACCTTTCTTTTCATAGAGCAGTTTTGAAACACTCTTTTTGTAGAATCTGGAAGTGGATATTTGG

                    ** * * ** *  *   *            **  
     190467 AGCGCTTTCTGAACTATGGTGAAAAAGGAAATATCTTCCAAT
        130 AGCGCTTTGAGGATTTCGTTGGAAACGGAAATATCTTCATAT

            *     *               *      * *          *  **       *  *  *    
     190509 GAAAACAAGACAGAAGCATTCTGAGAAACTTATTTGTGATGTGTGTCCTCAACTAACGGACTTGA
          1 AAAAACTAGACAGAAGCATTCTCAGAAACATCTTTGTGATGTTTGCACTCAACTCACAGAGTTGA

                   *      **  * *  *  *             *   *    * * *           
     190574 ACCTTTCGTTTCATGCAGTACTTCTGGAACACTCTTTTTGAAGATTCTGCATGCGGATATTTGGA
         66 ACCTTTCTTTTCATAGAGCAGTTTTGAAACACTCTTTTTGTAGAATCTGGAAGTGGATATTTGGA

            **                            **   *   * 
     190639 TTGCTTTGAGGATTTCGTTGGAAACGG---GCT-TACATGT
        131 GCGCTTTGAGGATTTCGTTGGAAACGGAAATATCTTCATAT

                 *       *               *        *   *    *    *       *    
     190676 AAAAATTAGACAGCAGCATTCTCAGAAACTTCTTTGTGCTGTCTGCATTCAAGTCACAGAATTGA
          1 AAAAACTAGACAGAAGCATTCTCAGAAACATCTTTGTGATGTTTGCACTCAACTCACAGAGTTGA

              * ** * **            *  * *      *       *    *        *       
     190741 ACTTCCCCTCACATAGAGCAGTTGTGCAGCACTCTCTTTGTAGTATCTCGAAGTGGACATTTGGA
         66 ACCTTTCTTTTCATAGAGCAGTTTTGAAACACTCTTTTTGTAGAATCTGGAAGTGGATATTTGGA

             *         ** *    * *   *            **  
     190806 GGGCTTTGTAGCCTATC-TGGAAAAAGGAAATATCTTCCCAT
        131 GCGCTTTG-AGGATTTCGTTGGAAACGGAAATATCTTCATAT

            *  ** *   *     * *            *   *  *   * **        *  * * *   
     190847 GAATGCGAGATAGAAGTAATCTCAGAAACATGTTTATGCTGTATCTACTCAACTAACTGTGCTGA
          1 AAAAACTAGACAGAAGCATTCTCAGAAACATCTTTGTGATGTTTGCACTCAACTCACAGAGTTGA

              *     *  *               *        *  * *       *               
     190912 ACATTTCTATTGATAGAGCAGTTTTGAGACACTCTTCTTTTGGAATCTGCAAGTGGATATTTGGA
         66 ACCTTTCTTTTCATAGAGCAGTTTTGAAACACTCTTTTTGTAGAATCTGGAAGTGGATATTTGGA

            ** *                        *     *      
     190977 TAGATTTGAGGATTTCGTTGGAAACGG-GAT-T-AT-ATAT
        131 GCGCTTTGAGGATTTCGTTGGAAACGGAAATATCTTCATAT

                 *       *               *                 **  *   *         
     191014 AAAAAGTAGACAGCAGCATTCTCAGAAACTTCTTTGTGATGTTTGCATCCAGCTCTCAGAGTTGA
          1 AAAAACTAGACAGAAGCATTCTCAGAAACATCTTTGTGATGTTTGCACTCAACTCACAGAGTTGA

              *  * *          *  *        *        *   **        *  **       
     191079 ACATTCCCTTTCATAGAGTAGGTTTGAAACCCTCTTTTTATAGTGTCTGGAAGCGGGCATTTGGA
         66 ACCTTTCTTTTCATAGAGCAGTTTTGAAACACTCTTTTTGTAGAATCTGGAAGTGGATATTTGGA

                   *    * * **  **  **          * *  *
     191144 GCGCTTTCAGGCCTATGCTTAAAATAGGAAATATCTACCTAC
        131 GCGCTTTGAGG-ATTTCGTTGGAAACGGAAATATCTTCATAT

             *                    *    *  **          ** *        *    *     
     191186 AGAAACTAGACAGAAGCATTCTGAGAATCACGTTTGTGATGTGGGTACTCAACTAACAGTGTTGA
          1 AAAAACTAGACAGAAGCATTCTCAGAAACATCTTTGTGATGTTTGCACTCAACTCACAGAGTTGA

            *  *       *   *            *   *                *   *           
     191251 TCCATTCTTTTGATACAGCAGTTTTGAACCACACTTTTTGTAGAATCTGCAAGAGGATATTTGGA
         66 ACCTTTCTTTTCATAGAGCAGTTTTGAAACACTCTTTTTGTAGAATCTGGAAGTGGATATTTGGA

            **   *                     *   *      * *
     191316 TAGCTGTGAGGATTTCGTTGGAAACGGGAATGTCTTCAAAG
        131 GCGCTTTGAGGATTTCGTTGGAAACGGAAATATCTTCATAT

                *                         *   *            *    *            
     191357 AAAATCTAGACAGAAGCATTCTCAGAAACACCTTCGTGATGTTTGCAATCAAGTCACAGAGTTGA
          1 AAAAACTAGACAGAAGCATTCTCAGAAACATCTTTGTGATGTTTGCACTCAACTCACAGAGTTGA

                   *             *  *           *      *             *       
     191422 ACCTTTCGTTTCATAGAGCAGGTTGGAAACACTCTTATTGTAGTATCTGGAAGTGGACATTTGGA
         66 ACCTTTCTTTTCATAGAGCAGTTTTGAAACACTCTTTTTGTAGAATCTGGAAGTGGATATTTGGA

                   *   ** ** *  *   *            **  
     191487 GCGCTTTCAGGCCTATGGTGAAAAAGGAAATATCTTCCCAT
        131 GCGCTTTGAGGATTTCGTTGGAAACGGAAATATCTTCATAT

                  * * *      **      *   * *   *     ** ** *      *    *     
     191528 AAAAACGACATAGAAGCTATCTCAGGAACTTGTTTATGATGCATCTAATCAACTAACAGTGTTGA
          1 AAAAACTAGACAGAAGCATTCTCAGAAACATCTTTGTGATGTTTGCACTCAACTCACAGAGTTGA

                  * ** * *                         * *       *               
     191593 ACCTTTGTACTGACAGAGCAG-TTTGAAACACTCTTTTTTTGGAATCTGCAAGTGGATATTTGGA
         66 ACCTTTCTTTTCATAGAGCAGTTTTGAAACACTCTTTTTGTAGAATCTGGAAGTGGATATTTGGA

            *                           *   *   *    
     191657 TCGCTTTGAGGATTTCGTTGGAAACGG-GAT-GC--AATAT
        131 GCGCTTTGAGGATTTCGTTGGAAACGGAAATATCTTCATAT

                **  *    *     *                 **  *   *  *    *         * 
     191694 AAAACGTACACAGCAGCATACTCAGAAA-ATACTTTGCCATATTTCCATTCAAGTCACAGAGTGG
          1 AAAAACTAGACAGAAGCATTCTCAGAAACAT-CTTTGTGATGTTTGCACTCAACTCACAGAGTTG

               *  * **            *                  *  *             *      
     191758 AACATTCCCATTCATAGAGCAGGTTTGAAACACTCTTTTTGGAGTATCTGGAAGTGGACATTTGG
         65 AACCTTTCTTTTCATAGAGCAGTTTTGAAACACTCTTTTTGTAGAATCTGGAAGTGGATATTTGG

                    ** * * ** *  *   *            **  
     191823 AGCGCTTTCTGAACTATGGTGAAAAAGGAAATATCTTCCAAT
        130 AGCGCTTTGAGGATTTCGTTGGAAACGGAAATATCTTCATAT

            *     *               *      * *          *  **       *  *  *    
     191865 GAAAACAAGACAGAAGCATTCTGAGAAACTTATTTGTGATGTGTGTCCTCAACTAACGGACTTGA
          1 AAAAACTAGACAGAAGCATTCTCAGAAACATCTTTGTGATGTTTGCACTCAACTCACAGAGTTGA

                   *      **  * *  *  *             *   *    * * *           
     191930 ACCTTTCGTTTCATGCAGTACTTCTGGAACACTCTTTTTGAAGATTCTGCATGCGGATATTTGGA
         66 ACCTTTCTTTTCATAGAGCAGTTTTGAAACACTCTTTTTGTAGAATCTGGAAGTGGATATTTGGA

            **                            **   *   * 
     191995 TTGCTTTGAGGATTTCGTTGGAAACGG---GCT-TACATGT
        131 GCGCTTTGAGGATTTCGTTGGAAACGGAAATATCTTCATAT

                 *       *               *        *   *    *    *       *    
     192032 AAAAATTAGACAGCAGCATTCTCAGAAACTTCTTTGTGCTGTCTGCATTCAAGTCACAGAATTGA
          1 AAAAACTAGACAGAAGCATTCTCAGAAACATCTTTGTGATGTTTGCACTCAACTCACAGAGTTGA

              * ** * **            *  * *      *       *             *       
     192097 ACTTCCCCTCACATAGAGCAGTTGTGCAGCACTCTATTTGTAGTATCTGGAAGTGGACATTTGGA
         66 ACCTTTCTTTTCATAGAGCAGTTTTGAAACACTCTTTTTGTAGAATCTGGAAGTGGATATTTGGA

             *         ** *    * *   *            **  
     192162 GGGCTTTGTAGCCTATC-TGGAAAAAGGAAATATCTTCCCAT
        131 GCGCTTTG-AGGATTTCGTTGGAAACGGAAATATCTTCATAT

            *  ** *   *     * *            *   *  *   * **        *  * * *   
     192203 GAATGCGAGATAGAAGTAATCTCAGAAACATGTTTATGCTGTATCTACTCAACTAACTGTGCTGA
          1 AAAAACTAGACAGAAGCATTCTCAGAAACATCTTTGTGATGTTTGCACTCAACTCACAGAGTTGA

              *     *  *               *        *  * *       *               
     192268 ACATTTCTATTGATAGAGCAGTTTTGAGACACTCTTCTTTTGGAATCTGCAAGTGGATATTTGGA
         66 ACCTTTCTTTTCATAGAGCAGTTTTGAAACACTCTTTTTGTAGAATCTGGAAGTGGATATTTGGA

            ** *                        *     *      
     192333 TAGATTTGAGGATTTCGTTGGAAACGG-GAT-T-AT-ATAT
        131 GCGCTTTGAGGATTTCGTTGGAAACGGAAATATCTTCATAT

                 *       *               *                 **  *   *         
     192370 AAAAAGTAGACAGCAGCATTCTCAGAAACTTCTTTGTGATGTTTGCATCCAGCTCTCAGAGTTGA
          1 AAAAACTAGACAGAAGCATTCTCAGAAACATCTTTGTGATGTTTGCACTCAACTCACAGAGTTGA

              *  * *          *  *        *        *   **        *   *       
     192435 ACATTCCCTTTCATAGAGTAGGTTTGAAACCCTCTTTTTATAGTGTCTGGAAGCGGACATTTGGA
         66 ACCTTTCTTTTCATAGAGCAGTTTTGAAACACTCTTTTTGTAGAATCTGGAAGTGGATATTTGGA

             *     *   ** ** *  *   *          * *   
     192500 GAGCTTTCAGGCCTATGCTGAAAAAGGAAATATCTACCTAT
        131 GCGCTTTGAGGATTTCGTTGGAAACGGAAATATCTTCATAT

             *                    *    *  **   *      ** *        *    *     
     192541 AGAAACTAGACAGAAGCATTCTGAGAATCACGTTTCTGATGTGGGTACTCAACTAACAGTGTTGA
          1 AAAAACTAGACAGAAGCATTCTCAGAAACATCTTTGTGATGTTTGCACTCAACTCACAGAGTTGA

            *  *       *   *            *   *             *  *               
     192606 TCCATTCTTTTGATACAGCAGTTTTGAACCACACTTTTTGTAGAATGTGCAAGTGGATATTTGGA
         66 ACCTTTCTTTTCATAGAGCAGTTTTGAAACACTCTTTTTGTAGAATCTGGAAGTGGATATTTGGA

            **   *                     *   *        *
     192671 TAGCTGTGAGGATTTCGTTGGAAACGGGAATGTCTTCATAG
        131 GCGCTTTGAGGATTTCGTTGGAAACGGAAATATCTTCATAT

                **                     * * **         *  **   *   *          
     192712 AAAATTTAGACAGAAGCATTCTCAGAACCTTGATTGTGATGTGTGTTCTCCACTAACAGAGTTGA
          1 AAAAACTAGACAGAAGCATTCTCAGAAACATCTTTGTGATGTTTGCACTCAACTCACAGAGTTGA

                       * *   * *   *       *       *                         
     192777 ACCTTTCTTTTGACAGAACTGTTCTGAAACATTCTTTTTATAGAATCTGGAAGTGGATATTTGGA
         66 ACCTTTCTTTTCATAGAGCAGTTTTGAAACACTCTTTTTGTAGAATCTGGAAGTGGATATTTGGA

            **              *          *          *  
     192842 AAGCTTTGAGGATTTCATTGGAAACGGGAATATCTTCAAAT
        131 GCGCTTTGAGGATTTCGTTGGAAACGGAAATATCTTCATAT

                *    *            *           * *       *  *    *            
     192883 AAAATCTAGCCAGAAGCATTCTAAGAAACATCTTAGGGATGTTTACATTCAAGTCACAGAGTTGA
          1 AAAAACTAGACAGAAGCATTCTCAGAAACATCTTTGTGATGTTTGCACTCAACTCACAGAGTTGA

              *  * *     *       *         *    * *   **      *      *    *  
     192948 ACATTCCCTTTCACAGAGCAGGTTTGAAACAATCTTCTCGTACTATCTGGCAGTGGACATTTTGA
         66 ACCTTTCTTTTCATAGAGCAGTTTTGAAACACTCTTTTTGTAGAATCTGGAAGTGGATATTTGGA

               *        ** ** *  *   *            ** *
     193013 GCTCCTTTG-GGCCTATGCTGAAAAAGGAAATATCTTCCGAC
        131 GC-GCTTTGAGGATTTCGTTGGAAACGGAAATATCTTCATAT

                                 *     *  **          *           **    *    
     193054 AAAAACTAGACAGAAGCATTCGCAGAATCACGTTTGTGATGTGTGCACTCAACTGTCAGAATTGA
          1 AAAAACTAGACAGAAGCATTCTCAGAAACATCTTTGTGATGTTTGCACTCAACTCACAGAGTTGA

                 **   ** *      *                      *     * *            *
     193119 ACCTTGGTTTGGACAGAGCACTTTTGAAACACTCTTTTTGTAGTATCTGCAGGTGGATATTTGGC
         66 ACCTTTCTTTTCATAGAGCAGTTTTGAAACACTCTTTTTGTAGAATCTGGAAGTGGATATTTGGA

            **   *          *          *   *      * *
     193184 TAGCTCTGAGGATTTCATTGGAAACGGTAATGTCTTCAAAG
        131 GCGCTTTGAGGATTTCGTTGGAAACGGAAATATCTTCATAT

                *                         *   *            *    *            
     193225 AAAATCTAGACAGAAGCATTCTCAGAAACACCTTCGTGATGTTTGCAATCAAGTCACAGAGTTGA
          1 AAAAACTAGACAGAAGCATTCTCAGAAACATCTTTGTGATGTTTGCACTCAACTCACAGAGTTGA

                 * *             *  *                  *             *       
     193290 ACCTTCCGTTTCATAGAGCAGGTTGGAAACACTCTTTTTGTAGTATCTGGAAGTGGACATTTGGA
         66 ACCTTTCTTTTCATAGAGCAGTTTTGAAACACTCTTTTTGTAGAATCTGGAAGTGGATATTTGGA

                   *   ** ** *  *   *            **  
     193355 GCGCTTTCAGGCCTATGGTGAAAAAGGAAATATCTTCCCAT
        131 GCGCTTTGAGGATTTCGTTGGAAACGGAAATATCTTCATAT

                  * * *      **      *   * *   *     ** ** *      *    *     
     193396 AAAAACGACATAGAAGCTATCTCAGGAACTTGTTTATGATGCATCTAATCAACTAACAGTGTTGA
          1 AAAAACTAGACAGAAGCATTCTCAGAAACATCTTTGTGATGTTTGCACTCAACTCACAGAGTTGA

                  * ** * *                         * *       *               
     193461 ACCTTTGTACTGACAGAGCAG-TTTGAAACACTCTTTTTTTGGAATCTGCAAGTGGATATTTGGA
         66 ACCTTTCTTTTCATAGAGCAGTTTTGAAACACTCTTTTTGTAGAATCTGGAAGTGGATATTTGGA

            *                           *   *   *    
     193525 TCGCTTTGAGGATTTCGTTGGAAACGG-GAT-GC--AATAT
        131 GCGCTTTGAGGATTTCGTTGGAAACGGAAATATCTTCATAT

                **  *    *     *                 **  *   *  *    *         * 
     193562 AAAACGTACACAGCAGCATACTCAGAAA-ATACTTTGCCATATTTCCATTCAAGTCACAGAGTGG
          1 AAAAACTAGACAGAAGCATTCTCAGAAACAT-CTTTGTGATGTTTGCACTCAACTCACAGAGTTG

               *  * **            *  *               *  *             *      
     193626 AACATTCCCATTCATAGAGCAGGTTGGAAACACTCTTTTTGGAGTATCTGGAAGTGGACATTTGG
         65 AACCTTTCTTTTCATAGAGCAGTTTTGAAACACTCTTTTTGTAGAATCTGGAAGTGGATATTTGG

                    ** * * ** *  *   *            **  
     193691 AGCGCTTTCTGAACTATGGTGAAAAAGGAAATATCTTCCAAT
        130 AGCGCTTTGAGGATTTCGTTGGAAACGGAAATATCTTCATAT

            *     *               *      * *          *  **      **  *  *    
     193733 GAAAACAAGACAGAAGCATTCTGAGAAACTTATTTGTGATGTGTGTCCTCAACAAACGGACTTGA
          1 AAAAACTAGACAGAAGCATTCTCAGAAACATCTTTGTGATGTTTGCACTCAACTCACAGAGTTGA

                   *      **  * *  *  *             *   *    * * *           
     193798 ACCTTTCGTTTCATGCAGTACTTCTGGAACACTCTTTTTGAAGATTCTGCATGCGGATATTTGGA
         66 ACCTTTCTTTTCATAGAGCAGTTTTGAAACACTCTTTTTGTAGAATCTGGAAGTGGATATTTGGA

            **                            **   *   * 
     193863 TAGCTTTGAGGATTTCGTTGGAAACGG---GCT-TACATGT
        131 GCGCTTTGAGGATTTCGTTGGAAACGGAAATATCTTCATAT

                 *       *               **       *   *    *    *       *    
     193900 AAAAATTAGACAGCAGCATTCTCAGAAACTACTTTGTGGTGTCTGCATTCAAGTCACAGAATTGA
          1 AAAAACTAGACAGAAGCATTCTCAGAAACATCTTTGTGATGTTTGCACTCAACTCACAGAGTTGA

              * ** * **            *  * *      *       *             *       
     193965 ACTTCCCCTCACATAGAGCAGTTGTGCAGCACTCTATTTGTAGTATCTGGAAGTGGACATTTGGA
         66 ACCTTTCTTTTCATAGAGCAGTTTTGAAACACTCTTTTTGTAGAATCTGGAAGTGGATATTTGGA

             *         ** *    * *   *            **  
     194030 GGGCTTTGTAGCCTATC-TGGAAAAAGGAAATATCTTCCCAT
        131 GCGCTTTG-AGGATTTCGTTGGAAACGGAAATATCTTCATAT

            *  ** *   *     * *            *   *  *   * **        *  * * *   
     194071 GAATGCGAGATAGAAGTAATCTCAGAAACATGTTTATGCTGTATCTACTCAACTAACTGTGCTGA
          1 AAAAACTAGACAGAAGCATTCTCAGAAACATCTTTGTGATGTTTGCACTCAACTCACAGAGTTGA

              *     *  *               *        *  * *       *               
     194136 ACATTTCTATTGATAGAGCAGTTTTGAGACACTCTTCTTTTGGAATCTGCAAGTGGATATTTGGA
         66 ACCTTTCTTTTCATAGAGCAGTTTTGAAACACTCTTTTTGTAGAATCTGGAAGTGGATATTTGGA

            ** *                        *     *      
     194201 TAGATTTGAGGATTTCGTTGGAAACGG-GAT-T-AT-ATAT
        131 GCGCTTTGAGGATTTCGTTGGAAACGGAAATATCTTCATAT

            *    *       *               *                 **  *   *         
     194238 CAAAAGTAGACAGCAGCATTCTCAGAAACTTCTTTGTGATGTTTGCATCCAGCTCTCAGAGTTGA
          1 AAAAACTAGACAGAAGCATTCTCAGAAACATCTTTGTGATGTTTGCACTCAACTCACAGAGTTGA

              *  * *          *  *        *        *   **        *  **       
     194303 ACATTCCCTTTCATAGAGTAGGTTTGAAACCCTCTTTTTATAGTGTCTGGAAGCGGGCATTTGGA
         66 ACCTTTCTTTTCATAGAGCAGTTTTGAAACACTCTTTTTGTAGAATCTGGAAGTGGATATTTGGA

                   *    * * **  **  **          * *  *
     194368 GCGCTTTCAGGCCTATGCTTAAAATAGGAAATATCTACCTAC
        131 GCGCTTTGAGG-ATTTCGTTGGAAACGGAAATATCTTCATAT

             *                    *    *   *          ** *        *    *     
     194410 AGAAACTAGACAGAAGCATTCTGAGAATCATGTTTGTGATGTGGGTACTCAACTAACAGTGTTGA
          1 AAAAACTAGACAGAAGCATTCTCAGAAACATCTTTGTGATGTTTGCACTCAACTCACAGAGTTGA

            *  *       *   *            *   *                *   *           
     194475 TCCATTCTTTTGATACAGCAGTTTTGAACCACACTTTTTGTAGAATCTGCAAGAGGATATTTGGA
         66 ACCTTTCTTTTCATAGAGCAGTTTTGAAACACTCTTTTTGTAGAATCTGGAAGTGGATATTTGGA

            **   *                     *   *      * *
     194540 TAGCTGTGAGGATTTCGTTGGAAACGGGAATGTCTTCAAAG
        131 GCGCTTTGAGGATTTCGTTGGAAACGGAAATATCTTCATAT

                *                         *   *            *    *            
     194581 AAAATCTAGACAGAAGCATTCTCAGAAACACCTTCGTGATGTTTGCAATCAAGTCACAGAGTTGA
          1 AAAAACTAGACAGAAGCATTCTCAGAAACATCTTTGTGATGTTTGCACTCAACTCACAGAGTTGA

                 * *             *  *           *      *             *       
     194646 ACCTTCCGTTTCATAGAGCAGGTTGGAAACACTCTTATTGTAGTATCTGGAAGTGGACATTTGGA
         66 ACCTTTCTTTTCATAGAGCAGTTTTGAAACACTCTTTTTGTAGAATCTGGAAGTGGATATTTGGA

                   *   ** ** *  *   *            **  
     194711 GCGCTTTCAGGCCTATGGTGAAAAAGGAAATATCTTCCCAT
        131 GCGCTTTGAGGATTTCGTTGGAAACGGAAATATCTTCATAT

                  * * *      **      *   * *   *     ** ** *      *    *     
     194752 AAAAACGACATAGAAGCTATCTCAGGAACTTGTTTATGATGCATCTAATCAACTAACAGTGTTGA
          1 AAAAACTAGACAGAAGCATTCTCAGAAACATCTTTGTGATGTTTGCACTCAACTCACAGAGTTGA

                  * ** * *                         * *       *               
     194817 ACCTTTGTACTGACAGAGCAG-TTTGAAACACTCTTTTTTTGGAATCTGCAAGTGGATATTTGGA
         66 ACCTTTCTTTTCATAGAGCAGTTTTGAAACACTCTTTTTGTAGAATCTGGAAGTGGATATTTGGA

            *                           *   *   *    
     194881 TCGCTTTGAGGATTTCGTTGGAAACGG-GAT-GC--AATAT
        131 GCGCTTTGAGGATTTCGTTGGAAACGGAAATATCTTCATAT

                **  *    *     *                 **  *   *  *    *         * 
     194918 AAAACGTACACAGCAGCATACTCAGAAA-ATACTTTGCCATATTTCCATTCAAGTCACAGAGTGG
          1 AAAAACTAGACAGAAGCATTCTCAGAAACAT-CTTTGTGATGTTTGCACTCAACTCACAGAGTTG

               *  * **            *                  *  *             *      
     194982 AACATTCCCATTCATAGAGCAGGTTTGAAACACTCTTTTTGGAGTATCTGGAAGTGGACATTTGG
         65 AACCTTTCTTTTCATAGAGCAGTTTTGAAACACTCTTTTTGTAGAATCTGGAAGTGGATATTTGG

                    ** * * ** *  *   *            **  
     195047 AGCGCTTTCTGAACTATGGTGAAAAAGGAAATATCTTCCAAT
        130 AGCGCTTTGAGGATTTCGTTGGAAACGGAAATATCTTCATAT

            *     * *             *      * *          *  **      **  *  *    
     195089 GAAAACAACACAGAAGCATTCTGAGAAACTTATTTGTGATGTGTGTCCTCAACAAACGGACTTGA
          1 AAAAACTAGACAGAAGCATTCTCAGAAACATCTTTGTGATGTTTGCACTCAACTCACAGAGTTGA

                   *      **  * *  *  *             *   *    * * *           
     195154 ACCTTTCGTTTCATGCAGTACTTCTGGAACACTCTTTTTGAAGATTCTGCATGCGGATATTTGGA
         66 ACCTTTCTTTTCATAGAGCAGTTTTGAAACACTCTTTTTGTAGAATCTGGAAGTGGATATTTGGA

            **                            **   *   * 
     195219 TAGCTTTGAGGATTTCGTTGGAAACGG---GCT-TACATGT
        131 GCGCTTTGAGGATTTCGTTGGAAACGGAAATATCTTCATAT

                 *       *               *        *   *    *    *       *    
     195256 AAAAATTAGACAGCAGCATTCTCAGAAACTTCTTTGTGGTGTCTGCATTCAAGTCACAGAATTGA
          1 AAAAACTAGACAGAAGCATTCTCAGAAACATCTTTGTGATGTTTGCACTCAACTCACAGAGTTGA

              * ** * **            *  * *      *       *             *       
     195321 ACTTCCCCTCACATAGAGCAGTTGTGCAGCACTCTATTTGTAGTATCTGGAAGTGGACATTTGGA
         66 ACCTTTCTTTTCATAGAGCAGTTTTGAAACACTCTTTTTGTAGAATCTGGAAGTGGATATTTGGA

             *         ** *    * *   *            **  
     195386 GGGCTTTGTAGCCTATC-TGGAAAAAGGAAATATCTTCCCAT
        131 GCGCTTTG-AGGATTTCGTTGGAAACGGAAATATCTTCATAT

            *  ** *   *     * *            *   *  *   * **        *  * * *   
     195427 GAATGCGAGATAGAAGTAATCTCAGAAACATGTTTATGCTGTATCTACTCAACTAACTGTGCTGA
          1 AAAAACTAGACAGAAGCATTCTCAGAAACATCTTTGTGATGTTTGCACTCAACTCACAGAGTTGA

              *     *  *               *        *  * *       *               
     195492 ACATTTCTATTGATAGAGCAGTTTTGAGACACTCTTCTTTTGGAATCTGCAAGTGGATATTTGGA
         66 ACCTTTCTTTTCATAGAGCAGTTTTGAAACACTCTTTTTGTAGAATCTGGAAGTGGATATTTGGA

            ** *                        *     *      
     195557 TAGATTTGAGGATTTCGTTGGAAACGG-GAT-T-AT-ATAT
        131 GCGCTTTGAGGATTTCGTTGGAAACGGAAATATCTTCATAT

            *    *       *               *                 **  *   *         
     195594 CAAAAGTAGACAGCAGCATTCTCAGAAACTTCTTTGTGATGTTTGCATCCAGCTCTCAGAGTTGA
          1 AAAAACTAGACAGAAGCATTCTCAGAAACATCTTTGTGATGTTTGCACTCAACTCACAGAGTTGA

              *  * *          *  *        *        *   **        *  **       
     195659 ACATTCCCTTTCATAGAGTAGGTTTGAAACCCTCTTTTTATAGTGTCTGGAAGCGGGCATTTGGA
         66 ACCTTTCTTTTCATAGAGCAGTTTTGAAACACTCTTTTTGTAGAATCTGGAAGTGGATATTTGGA

                   *   ** ** *  *   *          * *   
     195724 GCGCTTTCAGGCCTATGCTGAAAAAGGAAATATCTACCTAT
        131 GCGCTTTGAGGATTTCGTTGGAAACGGAAATATCTTCATAT

             *                    *    *  **          ** *        *    *     
     195765 AGAAACTAGACAGAAGCATTCTGAGAATCACGTTTGTGATGTGGGTACTCAACTAACAGTGTTGA
          1 AAAAACTAGACAGAAGCATTCTCAGAAACATCTTTGTGATGTTTGCACTCAACTCACAGAGTTGA

            *  *       *   *            *   *                *               
     195830 TCCATTCTTTTGATACAGCAGTTTTGAACCACACTTTTTGTAGAATCTGCAAGTGGATATTTGGA
         66 ACCTTTCTTTTCATAGAGCAGTTTTGAAACACTCTTTTTGTAGAATCTGGAAGTGGATATTTGGA

            **   *                     *   *        *
     195895 TAGCTGTGAGGATTTCGTTGGAAACGGGAATGTCTTCATAG
        131 GCGCTTTGAGGATTTCGTTGGAAACGGAAATATCTTCATAT

                **                     * * **         *  **   *   *          
     195936 AAAATTTAGACAGAAGCATTCTCAGAACCTTGATTGTGATGTGTGTTCTCCACTAACAGAGTTGA
          1 AAAAACTAGACAGAAGCATTCTCAGAAACATCTTTGTGATGTTTGCACTCAACTCACAGAGTTGA

                       * *   * *   *       *       *   *                     
     196001 ACCTTTCTTTTGACAGAACTGTTCTGAAACATTCTTTTTATAGTATCTGGAAGTGGATATTTGGA
         66 ACCTTTCTTTTCATAGAGCAGTTTTGAAACACTCTTTTTGTAGAATCTGGAAGTGGATATTTGGA

            **                         *          *  
     196066 AAGCTTTGAGGATTTCGTTGGAAACGGGAATATCTTCAAAT
        131 GCGCTTTGAGGATTTCGTTGGAAACGGAAATATCTTCATAT

                *    *            *          ** *       *  *    *            
     196107 AAAATCTAGCCAGAAGCATTCTAAGAAACATCTCAGGGATGTTTACATTCAAGTCACAGAGTTGA
          1 AAAAACTAGACAGAAGCATTCTCAGAAACATCTTTGTGATGTTTGCACTCAACTCACAGAGTTGA

              *  * *     *       *         *    * *   **      *      *    *  
     196172 ACATTCCCTTTCACAGAGCAGGTTTGAAACAATCTTCTCGTACTATCTGGCAGTGGACATTTTGA
         66 ACCTTTCTTTTCATAGAGCAGTTTTGAAACACTCTTTTTGTAGAATCTGGAAGTGGATATTTGGA

              * *   *  ** ** *  *   *            ** *
     196237 GCTCCTTGGGGCCTATGCTGAAAAAGGAAATATCTTCCGAC
        131 GCGCTTTGAGGATTTCGTTGGAAACGGAAATATCTTCATAT

                                 *     *  **          *           **    **   
     196278 AAAAACTAGACAGAAGCATTCGCAGAATCACGTTTGTGATGTGTGCACTCAACTGTCAGAACTGA
          1 AAAAACTAGACAGAAGCATTCTCAGAAACATCTTTGTGATGTTTGCACTCAACTCACAGAGTTGA

                 **   ** *      *                            * *            *
     196343 ACCTTGGTTTGGACAGAGCACTTTTGAAACACTCTTTTTGTAGAATCTGCAGGTGGATATTTGGC
         66 ACCTTTCTTTTCATAGAGCAGTTTTGAAACACTCTTTTTGTAGAATCTGGAAGTGGATATTTGGA

            **                         *   *      * *
     196408 TAGCTTTGAGGATTTCGTTGGAAACGGTAATGTCTTCAAAG
        131 GCGCTTTGAGGATTTCGTTGGAAACGGAAATATCTTCATAT

                *                         * * *            *    *            
     196449 AAAATCTAGACAGAAGCATTCTCAGAAACAGCGTCGTGATGTTTGCAATCAAGTCACAGAGTTGA
          1 AAAAACTAGACAGAAGCATTCTCAGAAACATCTTTGTGATGTTTGCACTCAACTCACAGAGTTGA

                 * *             *  *                  *             *       
     196514 ACCTTCCGTTTCATAGAGCAGGTTGGAAACACTCTTTTTGTAGTATCTGGAAGTGGACATTTGGA
         66 ACCTTTCTTTTCATAGAGCAGTTTTGAAACACTCTTTTTGTAGAATCTGGAAGTGGATATTTGGA

             *         ** *    * *   *            **  
     196579 GGGCTTTGTAGCCTATC-TGGAAAAAGGAAATATCTTCCCAT
        131 GCGCTTTG-AGGATTTCGTTGGAAACGGAAATATCTTCATAT

            *  ** *   *     * *            *   *  *   * **        *  * * *   
     196620 GAATGCGAGATAGAAGTAATCTCAGAAACATGTTTATGCTGTATCTACTCAACTAACTGTGCTGA
          1 AAAAACTAGACAGAAGCATTCTCAGAAACATCTTTGTGATGTTTGCACTCAACTCACAGAGTTGA

              *     *  *               *        *  * *       *               
     196685 ACATTTCTATTGATAGAGCAGTTTTGAGACACTCTTCTTTTGGAATCTGCAAGTGGATATTTGGA
         66 ACCTTTCTTTTCATAGAGCAGTTTTGAAACACTCTTTTTGTAGAATCTGGAAGTGGATATTTGGA

            ** *                        *     *      
     196750 TAGATTTGAGGATTTCGTTGGAAACGG-GAT-T-AT-ATAT
        131 GCGCTTTGAGGATTTCGTTGGAAACGGAAATATCTTCATAT

                 *       *               *                 **  *   *         
     196787 AAAAAGTAGACAGCAGCATTCTCAGAAACTTCTTTGTGATGTTTGCATCCAGCTCTCAGAGTTGA
          1 AAAAACTAGACAGAAGCATTCTCAGAAACATCTTTGTGATGTTTGCACTCAACTCACAGAGTTGA

            * *  * *          *  *        *        *   **        *  **       
     196852 GCATTCCCTTTCATAGAGTAGGTTTGAAACCCTCTTTTTATAGTGTCTGGAAGCGGGCATTTGGA
         66 ACCTTTCTTTTCATAGAGCAGTTTTGAAACACTCTTTTTGTAGAATCTGGAAGTGGATATTTGGA

                   *    *        **  **          * *  *
     196917 GCGCTTTCAGGCCTATTC-TTAAAATAGGAAATATCTACCTAC
        131 GCGCTTTGAGG-AT-TTCGTTGGAAACGGAAATATCTTCATAT

             *                    *    *  **          ** *        *    *     
     196959 AGAAACTAGACAGAAGCATTCTGAGAATCACGTTTGTGATGTGGGTACTCAACTAACAGTGTTGA
          1 AAAAACTAGACAGAAGCATTCTCAGAAACATCTTTGTGATGTTTGCACTCAACTCACAGAGTTGA

            *  *       *   *            *   *                *   *           
     197024 TCCATTCTTTTGATACAGCAGTTTTGAACCACACTTTTTGTAGAATCTGCAAGAGGATATTTGGA
         66 ACCTTTCTTTTCATAGAGCAGTTTTGAAACACTCTTTTTGTAGAATCTGGAAGTGGATATTTGGA

            **   *                     *   *      * *
     197089 TAGCTGTGAGGATTTCGTTGGAAACGGGAATGTCTTCAAAG
        131 GCGCTTTGAGGATTTCGTTGGAAACGGAAATATCTTCATAT

                *                         *   *            *    *            
     197130 AAAATCTAGACAGAAGCATTCTCAGAAACACCTTCGTGATGTTTGCAATCAAGTCACAGAGTTGA
          1 AAAAACTAGACAGAAGCATTCTCAGAAACATCTTTGTGATGTTTGCACTCAACTCACAGAGTTGA

                 * *             *  *                  *             *       
     197195 ACCTTCCGTTTCATAGAGCAGGTTGGAAACACTCTTTTTGTAGTATCTGGAAGTGGACATTTGGA
         66 ACCTTTCTTTTCATAGAGCAGTTTTGAAACACTCTTTTTGTAGAATCTGGAAGTGGATATTTGGA

                   *   ** ** *  *   *            **  
     197260 GCGCTTTCAGGCCTATGGTGAAAAAGGAAATATCTTCCCAT
        131 GCGCTTTGAGGATTTCGTTGGAAACGGAAATATCTTCATAT

                  * * *      **      *   * *   *     ** ** *      *    *     
     197301 AAAAACGACATAGAAGCTATCTCAGGAACTTGTTTATGATGCATCTAATCAACTAACAGTGTTGA
          1 AAAAACTAGACAGAAGCATTCTCAGAAACATCTTTGTGATGTTTGCACTCAACTCACAGAGTTGA

                  * ** * *                         * *       *               
     197366 ACCTTTGTACTGACAGAGCAG-TTTGAAACACTCTTTTTTTGGAATCTGCAAGTGGATATTTGGA
         66 ACCTTTCTTTTCATAGAGCAGTTTTGAAACACTCTTTTTGTAGAATCTGGAAGTGGATATTTGGA

            *                           *   *   *    
     197430 TCGCTTTGAGGATTTCGTTGGAAACGG-GAT-GC--AATAT
        131 GCGCTTTGAGGATTTCGTTGGAAACGGAAATATCTTCATAT

                **  *    *     *                 **  *   *  *    *         * 
     197467 AAAACGTACACAGCAGCATACTCAGAAA-ATACTTTGCCATATTTCCATTCAAGTCACAGAGTGG
          1 AAAAACTAGACAGAAGCATTCTCAGAAACAT-CTTTGTGATGTTTGCACTCAACTCACAGAGTTG

               *  * **            *                  *  *             *      
     197531 AACATTCCCATTCATAGAGCAGGTTTGAAACACTCTTTTTGGAGTATCTGGAAGTGGACATTTGG
         65 AACCTTTCTTTTCATAGAGCAGTTTTGAAACACTCTTTTTGTAGAATCTGGAAGTGGATATTTGG

                    ** * * ** *  *   *            **  
     197596 AGCGCTTTCTGAACTATGGTGAAAAAGGAAATATCTTCCAAT
        130 AGCGCTTTGAGGATTTCGTTGGAAACGGAAATATCTTCATAT

            *     * *             *      * *          *  **      **  *  *    
     197638 GAAAACAACACAGAAGCATTCTGAGAAACTTATTTGTGATGTGTGTCCTCAACAAACGGACTTGA
          1 AAAAACTAGACAGAAGCATTCTCAGAAACATCTTTGTGATGTTTGCACTCAACTCACAGAGTTGA

                   *      **  * *  *  *             *   *    * * *           
     197703 ACCTTTCGTTTCATGCAGTACTTCTGGAACACTCTTTTTGAAGATTCTGCATGCGGATATTTGGA
         66 ACCTTTCTTTTCATAGAGCAGTTTTGAAACACTCTTTTTGTAGAATCTGGAAGTGGATATTTGGA

            **                            **   *   * 
     197768 TAGCTTTGAGGATTTCGTTGGAAACGG---GCT-TACATGT
        131 GCGCTTTGAGGATTTCGTTGGAAACGGAAATATCTTCATAT

                 *       *               *        *   *    *    *       *    
     197805 AAAAATTAGACAGCAGCATTCTCAGAAACTTCTTTGTGGTGTCTGCATTCAAGTCACAGAATTGA
          1 AAAAACTAGACAGAAGCATTCTCAGAAACATCTTTGTGATGTTTGCACTCAACTCACAGAGTTGA

              * ** * **            *  * *      *       *             *       
     197870 ACTTCCCCTCACATAGAGCAGTTGTGCAGCACTCTATTTGTAGTATCTGGAAGTGGACATTTGGA
         66 ACCTTTCTTTTCATAGAGCAGTTTTGAAACACTCTTTTTGTAGAATCTGGAAGTGGATATTTGGA

             *         ** *    * *   *            **  
     197935 GGGCTTTGTAGCCTATC-TGGAAAAAGGAAATATCTTCCCAT
        131 GCGCTTTG-AGGATTTCGTTGGAAACGGAAATATCTTCATAT

            *  ** *   *     * *            *   *  *   * **        *  * * *   
     197976 GAATGCGAGATAGAAGTAATCTCAGAAACATGTTTATGCTGTATCTACTCAACTAACTGTGCTGA
          1 AAAAACTAGACAGAAGCATTCTCAGAAACATCTTTGTGATGTTTGCACTCAACTCACAGAGTTGA

              *     *  *               *        *  * *       *        *      
     198041 ACATTTCTATTGATAGAGCAGTTTTGAGACACTCTTCTTTTGGAATCTGCAAGTGGATTTTTGGA
         66 ACCTTTCTTTTCATAGAGCAGTTTTGAAACACTCTTTTTGTAGAATCTGGAAGTGGATATTTGGA

            ** *                        *     *      
     198106 TAGATTTGAGGATTTCGTTGGAAACGG-GAT-T-AT-ATAT
        131 GCGCTTTGAGGATTTCGTTGGAAACGGAAATATCTTCATAT

            *    *       *               *                 **  *   *         
     198143 CAAAAGTAGACAGCAGCATTCTCAGAAACTTCTTTGTGATGTTTGCATCCAGCTCTCAGAGTTGA
          1 AAAAACTAGACAGAAGCATTCTCAGAAACATCTTTGTGATGTTTGCACTCAACTCACAGAGTTGA

              *  * *          *  *        *        *   **        *  **       
     198208 ACATTCCCTTTCATAGAGTAGGTTTGAAACCCTCTTTTTATAGTGTCTGGAAGCGGGCATTTGGA
         66 ACCTTTCTTTTCATAGAGCAGTTTTGAAACACTCTTTTTGTAGAATCTGGAAGTGGATATTTGGA

                   *   ** ** * **   *          * *   
     198273 GCGCTTTCAGGCCTATGCTAAAAAAGGAAATATCTACCTAT
        131 GCGCTTTGAGGATTTCGTTGGAAACGGAAATATCTTCATAT

             *                    *    *  **          ** *        *    *     
     198314 AGAAACTAGACAGAAGCATTCTGAGAATCACGTTTGTGATGTGGGTACTCAACTAACAGTGTTGA
          1 AAAAACTAGACAGAAGCATTCTCAGAAACATCTTTGTGATGTTTGCACTCAACTCACAGAGTTGA

            *  *       *   *            *   *                *               
     198379 TCCATTCTTTTGATACAGCAGTTTTGAACCACACTTTTTGTAGAATCTGCAAGTGGATATTTGGA
         66 ACCTTTCTTTTCATAGAGCAGTTTTGAAACACTCTTTTTGTAGAATCTGGAAGTGGATATTTGGA

            **   *                     *   *        *
     198444 TAGCTGTGAGGATTTCGTTGGAAACGGGAATGTCTTCATAG
        131 GCGCTTTGAGGATTTCGTTGGAAACGGAAATATCTTCATAT

                **                     * * **         *  **   *   *          
     198485 AAAATTTAGACAGAAGCATTCTCAGAACCTTGATTGTGATGTGTGTTCTCCACTAACAGAGTTGA
          1 AAAAACTAGACAGAAGCATTCTCAGAAACATCTTTGTGATGTTTGCACTCAACTCACAGAGTTGA

                       * *   * *   *       *       *   *                     
     198550 ACCTTTCTTTTGACAGAACTGTTCTGAAACATTCTTTTTATAGTATCTGGAAGTGGATATTTGGA
         66 ACCTTTCTTTTCATAGAGCAGTTTTGAAACACTCTTTTTGTAGAATCTGGAAGTGGATATTTGGA

            **                         *          *  
     198615 AAGCTTTGAGGATTTCGTTGGAAACGGGAATATCTTCAAAT
        131 GCGCTTTGAGGATTTCGTTGGAAACGGAAATATCTTCATAT

                *    *            *          ** *       *  *    *            
     198656 AAAATCTAGCCAGAAGCATTCTAAGAAACATCTCAGGGATGTTTACATTCAAGTCACAGAGTTGA
          1 AAAAACTAGACAGAAGCATTCTCAGAAACATCTTTGTGATGTTTGCACTCAACTCACAGAGTTGA

              *  * *     **      *         *    * *   **      *      *    *  
     198721 ACATTCCCTTTCACGGAGCAGGTTTGAAACAATCTTCTCGTACTATCTGGCAGTGGACATTTTGA
         66 ACCTTTCTTTTCATAGAGCAGTTTTGAAACACTCTTTTTGTAGAATCTGGAAGTGGATATTTGGA

              * *   *  ** ** *  *   *            ** *
     198786 GCTCCTTGGGGCCTATGCTGAAAAAGGAAATATCTTCCGAC
        131 GCGCTTTGAGGATTTCGTTGGAAACGGAAATATCTTCATAT

                                 *     *  **          *           **    **   
     198827 AAAAACTAGACAGAAGCATTCGCAGAATCACGTTTGTGATGTGTGCACTCAACTGTCAGAACTGA
          1 AAAAACTAGACAGAAGCATTCTCAGAAACATCTTTGTGATGTTTGCACTCAACTCACAGAGTTGA

                 **   ** *      *                            * *            *
     198892 ACCTTGGTTTGGACAGAGCACTTTTGAAACACTCTTTTTGTAGAATCTGCAGGTGGATATTTGGC
         66 ACCTTTCTTTTCATAGAGCAGTTTTGAAACACTCTTTTTGTAGAATCTGGAAGTGGATATTTGGA

            **                         *   *      * *
     198957 TAGCTTTGAGGATTTCGTTGGAAACGGTAATGTCTTCAAAG
        131 GCGCTTTGAGGATTTCGTTGGAAACGGAAATATCTTCATAT

                *                         * * *            *    *            
     198998 AAAATCTAGACAGAAGCATTCTCAGAAACAGCGTCGTGATGTTTGCAATCAAGTCACAGAGTTGA
          1 AAAAACTAGACAGAAGCATTCTCAGAAACATCTTTGTGATGTTTGCACTCAACTCACAGAGTTGA

                 * *             *  *                  *             *       
     199063 ACCTTCCGTTTCATAGAGCAGGTTGGAAACACTCTTTTTGTAGTATCTGGAAGTGGACATTTGGA
         66 ACCTTTCTTTTCATAGAGCAGTTTTGAAACACTCTTTTTGTAGAATCTGGAAGTGGATATTTGGA

             *         ** *    * *   *            **  
     199128 GGGCTTTGTAGCCTATC-TGGAAAAAGGAAATATCTTCCCAT
        131 GCGCTTTG-AGGATTTCGTTGGAAACGGAAATATCTTCATAT

            *  ** *   *     * *            *   *  *   * **        *  * * *   
     199169 GAATGCGAGATAGAAGTAATCTCAGAAACATGTTTATGCTGTATCTACTCAACTAACTGTGCTGA
          1 AAAAACTAGACAGAAGCATTCTCAGAAACATCTTTGTGATGTTTGCACTCAACTCACAGAGTTGA

              *     *  *               *        *  * *       *               
     199234 ACATTTCTATTGATAGAGCAGTTTTGAGACACTCTTCTTTTGGAATCTGCAAGTGGATATTTGGA
         66 ACCTTTCTTTTCATAGAGCAGTTTTGAAACACTCTTTTTGTAGAATCTGGAAGTGGATATTTGGA

            ** *                        *     *      
     199299 TAGATTTGAGGATTTCGTTGGAAACGG-GAT-T-AT-ATAT
        131 GCGCTTTGAGGATTTCGTTGGAAACGGAAATATCTTCATAT

                 *       *               *                 **  *   *         
     199336 AAAAAGTAGACAGCAGCATTCTCAGAAACTTCTTTGTGATGTTTGCATCCAGCTCTCAGAGTTGA
          1 AAAAACTAGACAGAAGCATTCTCAGAAACATCTTTGTGATGTTTGCACTCAACTCACAGAGTTGA

            * *  * *          *  *        *        *   **        *  **       
     199401 GCATTCCCTTTCATAGAGTAGGTTTGAAACCCTCTTTTTATAGTGTCTGGAAGCGGGCATTTGGA
         66 ACCTTTCTTTTCATAGAGCAGTTTTGAAACACTCTTTTTGTAGAATCTGGAAGTGGATATTTGGA

                   *    *        **  **          * *  *
     199466 GCGCTTTCAGGCCTATTC-TTAAAATAGGAAATATCTACCTAC
        131 GCGCTTTGAGG-AT-TTCGTTGGAAACGGAAATATCTTCATAT

             *                    *    *  **          ** *        *    *     
     199508 AGAAACTAGACAGAAGCATTCTGAGAATCACGTTTGTGATGTGGGTACTCAACTAACAGTGTTGA
          1 AAAAACTAGACAGAAGCATTCTCAGAAACATCTTTGTGATGTTTGCACTCAACTCACAGAGTTGA

            *  *       *   *            *   *                *   *           
     199573 TCCATTCTTTTGATACAGCAGTTTTGAACCACACTTTTTGTAGAATCTGCAAGAGGATATTTGGA
         66 ACCTTTCTTTTCATAGAGCAGTTTTGAAACACTCTTTTTGTAGAATCTGGAAGTGGATATTTGGA

            **   *                     *   *  *   * *
     199638 TAGCTGTGAGGATTTCGTTGGAAACGGGAATGTCCTCAAAG
        131 GCGCTTTGAGGATTTCGTTGGAAACGGAAATATCTTCATAT

                *                         *   *            *    *            
     199679 AAAATCTAGACAGAAGCATTCTCAGAAACACCTTCGTGATGTTTGCAATCAAGTCACAGAGTTGA
          1 AAAAACTAGACAGAAGCATTCTCAGAAACATCTTTGTGATGTTTGCACTCAACTCACAGAGTTGA

                 * *             *  *           *      *             *       
     199744 ACCTTCCGTTTCATAGAGCAGGTTGGAAACACTCTTATTGTAGTATCTGGAAGTGGACATTTGGA
         66 ACCTTTCTTTTCATAGAGCAGTTTTGAAACACTCTTTTTGTAGAATCTGGAAGTGGATATTTGGA

                   *   ** ** *  *   *            **  
     199809 GCGCTTTCAGGCCTATGGTGAAAAAGGAAATATCTTCCCAT
        131 GCGCTTTGAGGATTTCGTTGGAAACGGAAATATCTTCATAT

                  * * *      **      *   * *   *     ** ** *      *    *     
     199850 AAAAACGACATAGAAGCTATCTCAGGAACTTGTTTATGATGCATCTAATCAACTAACAGTGTTGA
          1 AAAAACTAGACAGAAGCATTCTCAGAAACATCTTTGTGATGTTTGCACTCAACTCACAGAGTTGA

                  * ** * *                           *       *               
     199915 ACCTTTGTACTGACAGAGCAG-TTTGAAACACTCTTTTT-TGGAATCTGCAAGTGGATATTTGGA
         66 ACCTTTCTTTTCATAGAGCAGTTTTGAAACACTCTTTTTGTAGAATCTGGAAGTGGATATTTGGA

            *                           *   *   *    
     199978 TCGCTTTGAGGATTTCGTTGGAAACGG-GAT-GC--AATAT
        131 GCGCTTTGAGGATTTCGTTGGAAACGGAAATATCTTCATAT

                **  *    *     *                 **  *   *  *    *         * 
     200015 AAAACGTACACAGCAGCATACTCAGAAA-ATACTTTGCCATATTTCCATTCAAGTCACAGAGTGG
          1 AAAAACTAGACAGAAGCATTCTCAGAAACAT-CTTTGTGATGTTTGCACTCAACTCACAGAGTTG

               *  * **            *  *               *  *             *      
     200079 AACATTCCCATTCATAGAGCAGGTTGGAAACACTCTTTTTGGAGTATCTGGAAGTGGACATTTGG
         65 AACCTTTCTTTTCATAGAGCAGTTTTGAAACACTCTTTTTGTAGAATCTGGAAGTGGATATTTGG

                    ** * * ** *      *            **  
     200144 AGCGCTTTCTGAACTATGGTGGAAAAGGAAATATCTTCCAAT
        130 AGCGCTTTGAGGATTTCGTTGGAAACGGAAATATCTTCATAT

            *     *               *      * *          *  **      **  *  *    
     200186 GAAAACAAGACAGAAGCATTCTGAGAAACTTATTTGTGATGTGTGTCCTCAACAAACGGACTTGA
          1 AAAAACTAGACAGAAGCATTCTCAGAAACATCTTTGTGATGTTTGCACTCAACTCACAGAGTTGA

                   *      **  * *  *  *             *   *    * * *           
     200251 ACCTTTCGTTTCATGCAGTACTTCTGGAACACTCTTTTTGAAGATTCTGCATGCGGATATTTGGA
         66 ACCTTTCTTTTCATAGAGCAGTTTTGAAACACTCTTTTTGTAGAATCTGGAAGTGGATATTTGGA

            **                            **   *   * 
     200316 TAGCTTTGAGGATTTCGTTGGAAACGG---GCT-TACATGT
        131 GCGCTTTGAGGATTTCGTTGGAAACGGAAATATCTTCATAT

                 *       *               *        *   *    *    *       *    
     200353 AAAAATTAGACAGCAGCATTCTCAGAAACTTCTTTGTGGTGTCTGCATTCAAGTCACAGAATTGA
          1 AAAAACTAGACAGAAGCATTCTCAGAAACATCTTTGTGATGTTTGCACTCAACTCACAGAGTTGA

              * ** * **            *  * *      *       *    *        *       
     200418 ACTTCCCCTCACATAGAGCAGTTGTGCAGCACTCTATTTGTAGTATCTCGAAGTGGACATTTGGA
         66 ACCTTTCTTTTCATAGAGCAGTTTTGAAACACTCTTTTTGTAGAATCTGGAAGTGGATATTTGGA

             *         ** *    * *   *            **  
     200483 GGGCTTTGTAGCCTATC-TGGAAAAAGGAAATATCTTCCCAT
        131 GCGCTTTG-AGGATTTCGTTGGAAACGGAAATATCTTCATAT

            *  ** *   *     * *            *   *  *   * **        *  * * *   
     200524 GAATGCGAGATAGAAGTAATCTCAGAAACATGTTTATGCTGTATCTACTCAACTAACTGTGCTGA
          1 AAAAACTAGACAGAAGCATTCTCAGAAACATCTTTGTGATGTTTGCACTCAACTCACAGAGTTGA

              *     *  *               *        *  * *       *               
     200589 ACATTTCTATTGATAGAGCAGTTTTGAGACACTCTTCTTTTGGAATCTGCAAGTGGATATTTGGA
         66 ACCTTTCTTTTCATAGAGCAGTTTTGAAACACTCTTTTTGTAGAATCTGGAAGTGGATATTTGGA

            ** *                        *     *      
     200654 TAGATTTGAGGATTTCGTTGGAAACGG-GAT-T-AT-ATAT
        131 GCGCTTTGAGGATTTCGTTGGAAACGGAAATATCTTCATAT

                 *       *               *                 **  *   *         
     200691 AAAAAGTAGACAGCAGCATTCTCAGAAACTTCTTTGTGATGTTTGCATCCAGCTCTCAGAGTTGA
          1 AAAAACTAGACAGAAGCATTCTCAGAAACATCTTTGTGATGTTTGCACTCAACTCACAGAGTTGA

              *  * *          *  *        *        *   **        *  **       
     200756 ACATTCCCTTTCATAGAGTAGGTTTGAAACCCTCTTTTTATAGTGTCTGGAAGCGGGCATTTGGA
         66 ACCTTTCTTTTCATAGAGCAGTTTTGAAACACTCTTTTTGTAGAATCTGGAAGTGGATATTTGGA

                   *   ** ** *  *   *          * *   
     200821 GCGCTTTCAGGCCTATGCTGAAAAAGGAAATATCTACCTAT
        131 GCGCTTTGAGGATTTCGTTGGAAACGGAAATATCTTCATAT

             *                    *    *  **          ** *        *    *     
     200862 AGAAACTAGACAGAAGCATTCTGAGAATCACGTTTGTGATGTGGGTACTCAACTAACAGTGTTGA
          1 AAAAACTAGACAGAAGCATTCTCAGAAACATCTTTGTGATGTTTGCACTCAACTCACAGAGTTGA

            *  *       *   *            *   *                *               
     200927 TCCATTCTTTTGATACAGCAGTTTTGAACCACACTTTTTGTAGAATCTGCAAGTGGATATTTGGA
         66 ACCTTTCTTTTCATAGAGCAGTTTTGAAACACTCTTTTTGTAGAATCTGGAAGTGGATATTTGGA

            **   *          *  *       *   *        *
     200992 TAGCTGTGAGGATTTCCTTCGAAACGGGAATGTCTTCATAG
        131 GCGCTTTGAGGATTTCGTTGGAAACGGAAATATCTTCATAT

                **                     * * **         *  **   *   *          
     201033 AAAATTTAGACAGAAGCATTCTCAGAACCTTGATTGTGATGTGTGTTCTCTACTAACAGAGTTGA
          1 AAAAACTAGACAGAAGCATTCTCAGAAACATCTTTGTGATGTTTGCACTCAACTCACAGAGTTGA

                       * *   ***   *       *       *                         
     201098 ACCTTTCTTTTGACAGAAATGTTCTGAAACATTCTTTTTATAGAATCTGGAAGTGGATATTTGGA
         66 ACCTTTCTTTTCATAGAGCAGTTTTGAAACACTCTTTTTGTAGAATCTGGAAGTGGATATTTGGA

            **                         *          *  
     201163 AAGCTTTGAGGATTTCGTTGGAAACGGGAATATCTTCAAAT
        131 GCGCTTTGAGGATTTCGTTGGAAACGGAAATATCTTCATAT

                *    *            *           * *       *  *    *            
     201204 AAAATCTAGCCAGAAGCATTCTAAGAAACATCTTAGGGATGTTTACATTCAAGTCACAGAGTTGA
          1 AAAAACTAGACAGAAGCATTCTCAGAAACATCTTTGTGATGTTTGCACTCAACTCACAGAGTTGA

              *  * *     *       *         *    * *   **      *      *    *  
     201269 ACATTCCCTTTCACAGAGCAGGTTTGAAACAATCTTCTCGTACTATCTGGCAGTGGACATTTTGA
         66 ACCTTTCTTTTCATAGAGCAGTTTTGAAACACTCTTTTTGTAGAATCTGGAAGTGGATATTTGGA

              * *   *  ** ** *  *   *            ** *
     201334 GCTCCTTGGGGCCTATGCTGAAAAAGGAAATATCTTCCGAC
        131 GCGCTTTGAGGATTTCGTTGGAAACGGAAATATCTTCATAT

                                 *     *  **          *           **    *    
     201375 AAAAACTAGACAGAAGCATTCGCAGAATCACGTTTGTGATGTGTGCACTCAACTGTCAGAATTGA
          1 AAAAACTAGACAGAAGCATTCTCAGAAACATCTTTGTGATGTTTGCACTCAACTCACAGAGTTGA

                 **   ** *      *                            * *            *
     201440 ACCTTGGTTTGGACAGAGCACTTTTGAAACACTCTTTTTGTAGAATCTGCAGGTGGATATTTGGC
         66 ACCTTTCTTTTCATAGAGCAGTTTTGAAACACTCTTTTTGTAGAATCTGGAAGTGGATATTTGGA

            **                         *   *      * *
     201505 TAGCTTTGAGGATTTCGTTGGAAACGGTAATGTCTTCAAAG
        131 GCGCTTTGAGGATTTCGTTGGAAACGGAAATATCTTCATAT

                *                         *   **           *    *            
     201546 AAAATCTAGACAGAAGCATTCTCAGAAACACCTTCATGATGTTTGCAATCAAGTCACAGAGTTGA
          1 AAAAACTAGACAGAAGCATTCTCAGAAACATCTTTGTGATGTTTGCACTCAACTCACAGAGTTGA

                 * *             *  *                  *             *       
     201611 ACCTTCCGTTTCATAGAGCAGGTTGGAAACACTCTTTTTGTAGTATCTGGAAGTGGACATTTGGA
         66 ACCTTTCTTTTCATAGAGCAGTTTTGAAACACTCTTTTTGTAGAATCTGGAAGTGGATATTTGGA

             *         ** *    * *   *        *   **  
     201676 GGGCTTTGTAGCCTATC-TGGAAAAAGGAAATATATTCCCAT
        131 GCGCTTTG-AGGATTTCGTTGGAAACGGAAATATCTTCATAT

            *  ** *   *     * *            *   *  *   * **        *  * * *   
     201717 GAATGCGAGATAGAAGTAATCTCAGAAACATGTTTATGCTGTATCTACTCAACTAACTGTGCTGA
          1 AAAAACTAGACAGAAGCATTCTCAGAAACATCTTTGTGATGTTTGCACTCAACTCACAGAGTTGA

              *     *  *               *        *  * *       *               
     201782 ACATTTCTATTGATAGAGCAGTTTTGAGACACTCTTCTTTTGGAATCTGCAAGTGGATATTTGGA
         66 ACCTTTCTTTTCATAGAGCAGTTTTGAAACACTCTTTTTGTAGAATCTGGAAGTGGATATTTGGA

            ** *                        *     *      
     201847 TAGATTTGAGGATTTCGTTGGAAACGG-GAT-T-AT-ATAT
        131 GCGCTTTGAGGATTTCGTTGGAAACGGAAATATCTTCATAT

                 *       *               *                 **  **  *         
     201884 AAAAAGTAGACAGCAGCATTCTCAGAAACTTCTTTGTGATGTTTGCATCCAGTTCTCAGAGTTGA
          1 AAAAACTAGACAGAAGCATTCTCAGAAACATCTTTGTGATGTTTGCACTCAACTCACAGAGTTGA

              *  * *          *  *        *        *   **        *  **       
     201949 ACATTCCCTTTCATAGAGTAGGTTTGAAACCCTCTTTTTATAGTGTCTGGAAGCGGGCATTTGGA
         66 ACCTTTCTTTTCATAGAGCAGTTTTGAAACACTCTTTTTGTAGAATCTGGAAGTGGATATTTGGA

                   *    * * **  **  **          * *  *
     202014 GCGCTTTCAGGCCTATGCTTAAAATAGGAAATATCTACCTAC
        131 GCGCTTTGAGG-ATTTCGTTGGAAACGGAAATATCTTCATAT

             *                    *    *  **          ** *        *    *     
     202056 AGAAACTAGACAGAAGCATTCTGAGAATCACGTTTGTGATGTGGGTACTCAACTAACAGTGTTGA
          1 AAAAACTAGACAGAAGCATTCTCAGAAACATCTTTGTGATGTTTGCACTCAACTCACAGAGTTGA

            *  *       *   *            *   *                *   *           
     202121 TCCATTCTTTTGATACAGCAGTTTTGAACCACACTTTTTGTAGAATCTGCAAGAGGATATTTGGA
         66 ACCTTTCTTTTCATAGAGCAGTTTTGAAACACTCTTTTTGTAGAATCTGGAAGTGGATATTTGGA

            **   *                     *   *      * *
     202186 TAGCTGTGAGGATTTCGTTGGAAACGGGAATGTCTTCAAAG
        131 GCGCTTTGAGGATTTCGTTGGAAACGGAAATATCTTCATAT

                *                         *   *            *    *            
     202227 AAAATCTAGACAGAAGCATTCTCAGAAACACCTTCGTGATGTTTGCAATCAAGTCACAGAGTTGA
          1 AAAAACTAGACAGAAGCATTCTCAGAAACATCTTTGTGATGTTTGCACTCAACTCACAGAGTTGA

                 * *             *  *                  ** *          *       
     202292 ACCTTCCGTTTCATAGAGCAGGTTGGAAACACTCTTTTTGTAGTTTGTGGAAGTGGACATTTGGA
         66 ACCTTTCTTTTCATAGAGCAGTTTTGAAACACTCTTTTTGTAGAATCTGGAAGTGGATATTTGGA

                   *   ** ** *  *   *            **  
     202357 GCGCTTTCAGGCCTATGGTGAAAAAGGAAATATCTTCCCAT
        131 GCGCTTTGAGGATTTCGTTGGAAACGGAAATATCTTCATAT

                  * * *      **      *   * *   *     ** ** *      *    *     
     202398 AAAAACGACATAGAAGCTATCTCAGGAACTTGTTTATGATGCATCTAATCAACTAACAGTGTTGA
          1 AAAAACTAGACAGAAGCATTCTCAGAAACATCTTTGTGATGTTTGCACTCAACTCACAGAGTTGA

                  * ** * *                         * *       *               
     202463 ACCTTTGTACTGACAGAGCAG-TTTGAAACACTCTTTTTTTGGAATCTGCAAGTGGATATTTGGA
         66 ACCTTTCTTTTCATAGAGCAGTTTTGAAACACTCTTTTTGTAGAATCTGGAAGTGGATATTTGGA

            *                           *   *   *    
     202527 TCGCTTTGAGGATTTCGTTGGAAACGG-GAT-GC--AATAT
        131 GCGCTTTGAGGATTTCGTTGGAAACGGAAATATCTTCATAT

                **  *    *     *                 **  *   *  *    *         * 
     202564 AAAACGTACACAGCAGCATACTCAGAAA-ATACTTTGCCATATTTCCATTCAAGTCACAGAGTGG
          1 AAAAACTAGACAGAAGCATTCTCAGAAACAT-CTTTGTGATGTTTGCACTCAACTCACAGAGTTG

               *  * **            *                  *  *             *      
     202628 AACATTCCCATTCATAGAGCAGGTTTGAAACACTCTTTTTGGAGTATCTGGAAGTGGACATTTGG
         65 AACCTTTCTTTTCATAGAGCAGTTTTGAAACACTCTTTTTGTAGAATCTGGAAGTGGATATTTGG

                    ** * * ** *  *   *            **  
     202693 AGCGCTTTCTGAACTATGGTGAAAAAGGAAATATCTTCCAAT
        130 AGCGCTTTGAGGATTTCGTTGGAAACGGAAATATCTTCATAT

            *     *               *      * *          *  **      **  *  *    
     202735 GAAAACAAGACAGAAGCATTCTGAGAAACTTATTTGTGATGTGTGTCCTCAACAAACGGACTTGA
          1 AAAAACTAGACAGAAGCATTCTCAGAAACATCTTTGTGATGTTTGCACTCAACTCACAGAGTTGA

                   *      **  * *  *  *             *   *    * * *           
     202800 ACCTTTCGTTTCATGCAGTACTTCTGGAACACTCTTTTTGAAGATTCTGCATGCGGATATTTGGA
         66 ACCTTTCTTTTCATAGAGCAGTTTTGAAACACTCTTTTTGTAGAATCTGGAAGTGGATATTTGGA

            **              *             **   *   * 
     202865 TAGCTTTGAGGATTTCATTGGAAACGG---GCT-TACATGT
        131 GCGCTTTGAGGATTTCGTTGGAAACGGAAATATCTTCATAT

                 *       *               *        *   *    *    *       *    
     202902 AAAAATTAGACAGCAGCATTCTCAGAAACTTCTTTGTGGTGTCTGCATTCAAGTCACAGAATTGA
          1 AAAAACTAGACAGAAGCATTCTCAGAAACATCTTTGTGATGTTTGCACTCAACTCACAGAGTTGA

              * ** * **            *  * *      *       *    *        *       
     202967 ACTTCCCCTCACATAGAGCAGTTGTGCAGCACTCTATTTGTAGTATCTCGAAGTGGACATTTGGA
         66 ACCTTTCTTTTCATAGAGCAGTTTTGAAACACTCTTTTTGTAGAATCTGGAAGTGGATATTTGGA

             *         ** *    * *   *            **  
     203032 GGGCTTTGTAGCCTATC-TGGAAAAAGGAAATATCTTCCCAT
        131 GCGCTTTG-AGGATTTCGTTGGAAACGGAAATATCTTCATAT

            *  ** *   *     * *            *   *  *   * **        *  * * *   
     203073 GAATGCGAGATAGAAGTAATCTCAGAAACATGTTTATGCTGTATCTACTCAACTAACTGTGCTGA
          1 AAAAACTAGACAGAAGCATTCTCAGAAACATCTTTGTGATGTTTGCACTCAACTCACAGAGTTGA

              *     *  *               *        *  * **      *               
     203138 ACATTTCTATTGATAGAGCAGTTTTGAGACACTCTTCTTTTGAAATCTGCAAGTGGATATTTGGA
         66 ACCTTTCTTTTCATAGAGCAGTTTTGAAACACTCTTTTTGTAGAATCTGGAAGTGGATATTTGGA

            ** *                        *     *      
     203203 TAGATTTGAGGATTTCGTTGGAAACGG-GAT-T-AT-ATAT
        131 GCGCTTTGAGGATTTCGTTGGAAACGGAAATATCTTCATAT

                 *       *               *                 **  *   *         
     203240 AAAAAGTAGACAGCAGCATTCTCAGAAACTTCTTTGTGATGTTTGCATCCAGCTCTCAGAGTTGA
          1 AAAAACTAGACAGAAGCATTCTCAGAAACATCTTTGTGATGTTTGCACTCAACTCACAGAGTTGA

              *  * *          *  *        *        *   **   *    *  **       
     203305 ACATTCCCTTTCATAGAGTAGGTTTGAAACCCTCTTTTTATAGTGTCTAGAAGCGGGCATTTGGA
         66 ACCTTTCTTTTCATAGAGCAGTTTTGAAACACTCTTTTTGTAGAATCTGGAAGTGGATATTTGGA

                   *    * * **  **  **          * *  *
     203370 GCGCTTTCAGGCCTATGCTTAAAATAGGAAATATCTACCTAC
        131 GCGCTTTGAGG-ATTTCGTTGGAAACGGAAATATCTTCATAT

             *                    *    *  **          ** *        *    *     
     203412 AGAAACTAGACAGAAGCATTCTGAGAATCACGTTTGTGATGTGGGTACTCAACTAACAGTGTTGA
          1 AAAAACTAGACAGAAGCATTCTCAGAAACATCTTTGTGATGTTTGCACTCAACTCACAGAGTTGA

            *  *       *   *            *   *                *   *           
     203477 TCCATTCTTTTGATACAGCAGTTTTGAACCACACTTTTTGTAGAATCTGCAAGAGGATATTTGGA
         66 ACCTTTCTTTTCATAGAGCAGTTTTGAAACACTCTTTTTGTAGAATCTGGAAGTGGATATTTGGA

            **   *                     *   *      * *
     203542 TAGCTGTGAGGATTTCGTTGGAAACGGGAATGTCTTCAAAG
        131 GCGCTTTGAGGATTTCGTTGGAAACGGAAATATCTTCATAT

                *                         *   *            *  * *            
     203583 AAAATCTAGACAGAAGCATTCTCAGAAACACCTTCGTGATGTTTGCAATCCAGTCACAGAGTTGA
          1 AAAAACTAGACAGAAGCATTCTCAGAAACATCTTTGTGATGTTTGCACTCAACTCACAGAGTTGA

                 * *             *  *           *      *             *       
     203648 ACCTTCCGTTTCATAGAGCAGGTTGGAAACACTCTTATTGTAGTATCTGGAAGTGGACATTTGGA
         66 ACCTTTCTTTTCATAGAGCAGTTTTGAAACACTCTTTTTGTAGAATCTGGAAGTGGATATTTGGA

                   *   ** ** *  *   *            **  
     203713 GCGCTTTCAGGCCTATGGTGAAAAAGGAAATATCTTCCCAT
        131 GCGCTTTGAGGATTTCGTTGGAAACGGAAATATCTTCATAT

                  * * *      ***     *   * *   *     ** ** *      *    *     
     203754 AAAAACGACATAGAAGCTACCTCAGGAACTTGTTTATGATGCATCTAATCAACTAACAGTGTTGA
          1 AAAAACTAGACAGAAGCATTCTCAGAAACATCTTTGTGATGTTTGCACTCAACTCACAGAGTTGA

                  * ** * *                         * *       *               
     203819 ACCTTTGTACTGACAGAGCAG-TTTGAAACACTCTTTTTTTGGAATCTGCAAGTGGATATTTGGA
         66 ACCTTTCTTTTCATAGAGCAGTTTTGAAACACTCTTTTTGTAGAATCTGGAAGTGGATATTTGGA

            * *                         *   *   *    
     203883 TCACTTTGAGGATTTCGTTGGAAACGG-GAT-GC--AATAT
        131 GCGCTTTGAGGATTTCGTTGGAAACGGAAATATCTTCATAT

                **  *    *     *                 **  *   *  *    *         * 
     203920 AAAACGTACACAGCAGCATACTCAGAAA-ATACTTTGCCATATTTCCATTCAAGTCACAGAGTGG
          1 AAAAACTAGACAGAAGCATTCTCAGAAACAT-CTTTGTGATGTTTGCACTCAACTCACAGAGTTG

               *  * **            *  *               *  *             *      
     203984 AACATTCCCATTCATAGAGCAGGTTGGAAACACTCTTTTTGGAGTATCTGGAAGTGGACATTTGG
         65 AACCTTTCTTTTCATAGAGCAGTTTTGAAACACTCTTTTTGTAGAATCTGGAAGTGGATATTTGG

                    ** * * ** *  *   *            **  
     204049 AGCGCTTTCTGAACTATGGTGAAAAAGGAAATATCTTCCAAT
        130 AGCGCTTTGAGGATTTCGTTGGAAACGGAAATATCTTCATAT

            *     *               *      * *          *  **      **  *  *    
     204091 GAAAACAAGACAGAAGCATTCTGAGAAACTTATTTGTGATGTGTGTCCTCAACAAACGGACTTGA
          1 AAAAACTAGACAGAAGCATTCTCAGAAACATCTTTGTGATGTTTGCACTCAACTCACAGAGTTGA

                   *      **  * *  *  *             *   *    * * *           
     204156 ACCTTTCGTTTCATGCAGTACTTCTGGAACACTCTTTTTGAAGATTCTGCATGCGGATATTTGGA
         66 ACCTTTCTTTTCATAGAGCAGTTTTGAAACACTCTTTTTGTAGAATCTGGAAGTGGATATTTGGA

            **                            **   *   * 
     204221 TAGCTTTGAGGATTTCGTTGGAAACGG---GCT-TACATGT
        131 GCGCTTTGAGGATTTCGTTGGAAACGGAAATATCTTCATAT

                 *       *               *        *   *    *    *       *    
     204258 AAAAATTAGACAGCAGCATTCTCAGAAACTTCTTTGTGGTGTCTGCATTCAAGTCACAGAATTGA
          1 AAAAACTAGACAGAAGCATTCTCAGAAACATCTTTGTGATGTTTGCACTCAACTCACAGAGTTGA

              * ** * **            *  * *      *       *    *        *       
     204323 ACTTCCCCTCACATAGAGCAGTTGTGCAGCACTCTATTTGTAGTATCTCGAAGTGGACATTTGGA
         66 ACCTTTCTTTTCATAGAGCAGTTTTGAAACACTCTTTTTGTAGAATCTGGAAGTGGATATTTGGA

             *         ** *    * *   *            **  
     204388 GGGCTTTGTAGCCTATC-TGGAAAAAGGAAATATCTTCCCAT
        131 GCGCTTTG-AGGATTTCGTTGGAAACGGAAATATCTTCATAT

            *  ** *   *     * *            *   *  *   * **        *  * * *   
     204429 GAATGCGAGATAGAAGTAATCTCAGAAACATGTTTATGCTGTATCTACTCAACTAACTGTGCTGA
          1 AAAAACTAGACAGAAGCATTCTCAGAAACATCTTTGTGATGTTTGCACTCAACTCACAGAGTTGA

              *     *  *               *        *  * *       *               
     204494 ACATTTCTATTGATAGAGCAGTTTTGAGACACTCTTCTTTTGGAATCTGCAAGTGGATATTTGGA
         66 ACCTTTCTTTTCATAGAGCAGTTTTGAAACACTCTTTTTGTAGAATCTGGAAGTGGATATTTGGA

            ** *                        *     *      
     204559 TAGATTTGAGGATTTCGTTGGAAACGG-GAT-T-AT-ATAT
        131 GCGCTTTGAGGATTTCGTTGGAAACGGAAATATCTTCATAT

                 *       *               *                 **  *   *         
     204596 AAAAAGTAGACAGCAGCATTCTCAGAAACTTCTTTGTGATGTTTGCATCCAGCTCTCAGAGTTGA
          1 AAAAACTAGACAGAAGCATTCTCAGAAACATCTTTGTGATGTTTGCACTCAACTCACAGAGTTGA

              *  * *          *  *        *        *   **        *  **       
     204661 ACATTCCCTTTCATAGAGTAGGTTTGAAACCCTCTTTTTATAGTGTCTGGAAGCGGGCATTTGGA
         66 ACCTTTCTTTTCATAGAGCAGTTTTGAAACACTCTTTTTGTAGAATCTGGAAGTGGATATTTGGA

                   *   ** ** *  *   *          * *   
     204726 GCGCTTTCAGGCCTATGCTGAAAAAGGAAATATCTACCTAT
        131 GCGCTTTGAGGATTTCGTTGGAAACGGAAATATCTTCATAT

             *                    *    *  **          ** *        *    *     
     204767 AGAAACTAGACAGAAGCATTCTGAGAATCACGTTTGTGATGTGGGTACTCAACTAACAGTGTTGA
          1 AAAAACTAGACAGAAGCATTCTCAGAAACATCTTTGTGATGTTTGCACTCAACTCACAGAGTTGA

            *  *       *   *            *   *                *               
     204832 TCCATTCTTTTGATACAGCAGTTTTGAACCACACTTTTTGTAGAATCTGCAAGTGGATATTTGGA
         66 ACCTTTCTTTTCATAGAGCAGTTTTGAAACACTCTTTTTGTAGAATCTGGAAGTGGATATTTGGA

            **   *                     *   *        *
     204897 TAGCTGTGAGGATTTCGTTGGAAACGGGAATGTCTTCATAG
        131 GCGCTTTGAGGATTTCGTTGGAAACGGAAATATCTTCATAT

                **                     * * **         *  **   *   *          
     204938 AAAATTTAGACAGAAGCATTCTCAGAACCTTGATTGTGATGTGTGTTCTCCACTAACAGAGTTGA
          1 AAAAACTAGACAGAAGCATTCTCAGAAACATCTTTGTGATGTTTGCACTCAACTCACAGAGTTGA

                       * *   * *   *       *       *                         
     205003 ACCTTTCTTTTGACAGAACTGTTCTGAAACATTCTTTTTATAGAATCTGGAAGTGGATATTTGGA
         66 ACCTTTCTTTTCATAGAGCAGTTTTGAAACACTCTTTTTGTAGAATCTGGAAGTGGATATTTGGA

            **              *          *          *  
     205068 AAGCTTTGAGGATTTCATTGGAAACGGGAATATCTTCAAAT
        131 GCGCTTTGAGGATTTCGTTGGAAACGGAAATATCTTCATAT

                *    *            *         * * *     * ** *    *            
     205109 AAAATCTAGCCAGAAGCATTCTAAGAAACATCGTAGGGATGTATAGATTCAAGTCACAGAGTTGA
          1 AAAAACTAGACAGAAGCATTCTCAGAAACATCTTTGTGATGTTTGCACTCAACTCACAGAGTTGA

              *  * *     *       *         *    * *   **      *      *    *  
     205174 ACATTCCCTTTCACAGAGCAGGTTTGAAACAATCTTCTCGTACTATCTGGCAGTGGACATTTTGA
         66 ACCTTTCTTTTCATAGAGCAGTTTTGAAACACTCTTTTTGTAGAATCTGGAAGTGGATATTTGGA

              * *   *  ** ** *  *   *            ** *
     205239 GCTCCTTGGGGCCTATGCTGAAAAAGGAAATATCTTCCGAC
        131 GCGCTTTGAGGATTTCGTTGGAAACGGAAATATCTTCATAT

                                 *     *  **          *           **    *    
     205280 AAAAACTAGACAGAAGCATTCGCAGAATCACGTTTGTGATGTGTGCACTCAACTGTCAGAATTGA
          1 AAAAACTAGACAGAAGCATTCTCAGAAACATCTTTGTGATGTTTGCACTCAACTCACAGAGTTGA

                 **   ** *      *                            * *            *
     205345 ACCTTGGTTTGGACAGAGCACTTTTGAAACACTCTTTTTGTAGAATCTGCAGGTGGATATTTGGC
         66 ACCTTTCTTTTCATAGAGCAGTTTTGAAACACTCTTTTTGTAGAATCTGGAAGTGGATATTTGGA

            **                         *   *      * *
     205410 TAGCTTTGAGGATTTCGTTGGAAACGGTAATGTCTTCAAAG
        131 GCGCTTTGAGGATTTCGTTGGAAACGGAAATATCTTCATAT

                *                         *   *            *    *            
     205451 AAAATCTAGACAGAAGCATTCTCAGAAACACCTTCGTGATGTTTGCAATCAAGTCACAGAGTTGA
          1 AAAAACTAGACAGAAGCATTCTCAGAAACATCTTTGTGATGTTTGCACTCAACTCACAGAGTTGA

                 * *             *  *           *      *             *       
     205516 ACCTTCCGTTTCATAGAGCAGGTTGGAAACACTCTTATTGTAGTATCTGGAAGTGGACATTTGGA
         66 ACCTTTCTTTTCATAGAGCAGTTTTGAAACACTCTTTTTGTAGAATCTGGAAGTGGATATTTGGA

                   *   ** ** *  *   *            **  
     205581 GCGCTTTCAGGCCTATGGTGAAAAAGGAAATATCTTCCCAT
        131 GCGCTTTGAGGATTTCGTTGGAAACGGAAATATCTTCATAT

                  * * *      **      *   * *   *     ** ** *      *    *     
     205622 AAAAACGACATAGAAGCTATCTCAGGAACTTGTTTATGATGCATCTAATCAACTAACAGTGTTGA
          1 AAAAACTAGACAGAAGCATTCTCAGAAACATCTTTGTGATGTTTGCACTCAACTCACAGAGTTGA

                  * ** * *                         * *       *               
     205687 ACCTTTGTACTGACAGAGCAG-TTTGAAACACTCTTTTTTTGGAATCTGCAAGTGGATATTTGGA
         66 ACCTTTCTTTTCATAGAGCAGTTTTGAAACACTCTTTTTGTAGAATCTGGAAGTGGATATTTGGA

            *                           *   *   *    
     205751 TCGCTTTGAGGATTTCGTTGGAAACGG-GAT-GC--AATAT
        131 GCGCTTTGAGGATTTCGTTGGAAACGGAAATATCTTCATAT

                **  *    *     *                 **  *   *  *    *         * 
     205788 AAAACGTACACAGCAGCATACTCAGAAA-ATACTTTGCCATATTTCCATTCAAGTCACAGAGTGG
          1 AAAAACTAGACAGAAGCATTCTCAGAAACAT-CTTTGTGATGTTTGCACTCAACTCACAGAGTTG

               *  * **            *                  *  *             *      
     205852 AACATTCCCATTCATAGAGCAGGTTTGAAACACTCTTTTTGGAGTATCTGGAAGTGGACATTTGG
         65 AACCTTTCTTTTCATAGAGCAGTTTTGAAACACTCTTTTTGTAGAATCTGGAAGTGGATATTTGG

                    ** * * ** *  *   *            **  
     205917 AGCGCTTTCTGAACTATGGTGAAAAAGGAAATATCTTCCAAT
        130 AGCGCTTTGAGGATTTCGTTGGAAACGGAAATATCTTCATAT

            *     *               *      * *          *  **      **  *  *    
     205959 GAAAACAAGACAGAAGCATTCTGAGAAACTTATTTGTGATGTGTGTCCTCAACAAACGGACTTGA
          1 AAAAACTAGACAGAAGCATTCTCAGAAACATCTTTGTGATGTTTGCACTCAACTCACAGAGTTGA

                   *      **  * *  *  *             *   *    * * *           
     206024 ACCTTTCGTTTCATGCAGTACTTCTGGAACACTCTTTTTGAAGATTCTGCATGCGGATATTTGGA
         66 ACCTTTCTTTTCATAGAGCAGTTTTGAAACACTCTTTTTGTAGAATCTGGAAGTGGATATTTGGA

            **                            **   *   * 
     206089 TAGCTTTGAGGATTTCGTTGGAAACGG---GCT-TACATGT
        131 GCGCTTTGAGGATTTCGTTGGAAACGGAAATATCTTCATAT

                 *       *               *        *   *    *    *       *    
     206126 AAAAATTAGACAGCAGCATTCTCAGAAACTTCTTTGTGGTGTCTGCATTCAAGTCACAGAATTGA
          1 AAAAACTAGACAGAAGCATTCTCAGAAACATCTTTGTGATGTTTGCACTCAACTCACAGAGTTGA

              * ** * **            *  * *      *       *             *       
     206191 ACATCCCCTCACATAGAGCAGTTGTGCAGCACTCTATTTGTAGTATCTGGAAGTGGACATTTGGA
         66 ACCTTTCTTTTCATAGAGCAGTTTTGAAACACTCTTTTTGTAGAATCTGGAAGTGGATATTTGGA

             *         ** *    ***   *            **  
     206256 GGGCTTTGTAGCCTATC-TGCAAAAAGGAAATATCTTCCCAT
        131 GCGCTTTG-AGGATTTCGTTGGAAACGGAAATATCTTCATAT

            *  ** *   *     * *            *   *  *   * **        *  * * *   
     206297 GAATGCGAGATAGAAGTAATCTCAGAAACATGTTTATGCTGTATCTACTCAACTAACTGTGCTGA
          1 AAAAACTAGACAGAAGCATTCTCAGAAACATCTTTGTGATGTTTGCACTCAACTCACAGAGTTGA

              *     *  *               *        *  * *       *               
     206362 ACATTTCTATTGATAGAGCAGTTTTGAGACACTCTTCTTTTGGAATCTGCAAGTGGATATTTGGA
         66 ACCTTTCTTTTCATAGAGCAGTTTTGAAACACTCTTTTTGTAGAATCTGGAAGTGGATATTTGGA

            ** *                 *      *     *      
     206427 TAGATTTGAGGATTTCGTTGGCAACGG-GAT-T-AT-ATAT
        131 GCGCTTTGAGGATTTCGTTGGAAACGGAAATATCTTCATAT

                 *       *               *                 **  *   *         
     206464 AAAAAGTAGACAGCAGCATTCTCAGAAACTTCTTTGTGATGTTTGCATCCAGCTCTCAGAGTTGA
          1 AAAAACTAGACAGAAGCATTCTCAGAAACATCTTTGTGATGTTTGCACTCAACTCACAGAGTTGA

              *  * *          *  *        *        *   **        *  **       
     206529 ACATTCCCTTTCATAGAGTAGGTTTGAAACCCTCTTTTTATAGTGTCTGGAAGCGGGCATTTGGA
         66 ACCTTTCTTTTCATAGAGCAGTTTTGAAACACTCTTTTTGTAGAATCTGGAAGTGGATATTTGGA

                   *   ** ** *  *   *          * *   
     206594 GCGCTTTCAGGCCTATGCTGAAAAAGGAAATATCTACCTAT
        131 GCGCTTTGAGGATTTCGTTGGAAACGGAAATATCTTCATAT

             *                    *    *  **          ** *        *    *     
     206635 AGAAACTAGACAGAAGCATTCTGAGAATCACGTTTGTGATGTGGGTACTCAACTAACAGTGTTGA
          1 AAAAACTAGACAGAAGCATTCTCAGAAACATCTTTGTGATGTTTGCACTCAACTCACAGAGTTGA

            *  *       *   *            *   *                * *             
     206700 TCCATTCTTTTGATACAGCAGTTTTGAACCACACTTTTTGTAGAATCTGCAGGTGGATATTTGGA
         66 ACCTTTCTTTTCATAGAGCAGTTTTGAAACACTCTTTTTGTAGAATCTGGAAGTGGATATTTGGA

            **   *                     *   *        *
     206765 TAGCTGTGAGGATTTCGTTGGAAACGGGAATGTCTTCATAG
        131 GCGCTTTGAGGATTTCGTTGGAAACGGAAATATCTTCATAT

                **    *                * * *          *  **   *   * *        
     206806 AAAATTTAGATAGAAGCATTCTCAGAACCTTGTTTGTGATGTGTGTTCTCCACTAAGAGAGTTGA
          1 AAAAACTAGACAGAAGCATTCTCAGAAACATCTTTGTGATGTTTGCACTCAACTCACAGAGTTGA

                       * *   * *   *       *       *                         
     206871 ACCTTTCTTTTGACAGAACTGTTCTGAAACATTCTTTTTATAGAATCTGGAAGTGGATATTTGGA
         66 ACCTTTCTTTTCATAGAGCAGTTTTGAAACACTCTTTTTGTAGAATCTGGAAGTGGATATTTGGA

            **                         *          *  
     206936 AAGCTTTGAGGATTTCGTTGGAAACGGGAATATCTTCAAAT
        131 GCGCTTTGAGGATTTCGTTGGAAACGGAAATATCTTCATAT

                *    *            *           * *       *  *    *            
     206977 AAAATCTAGCCAGAAGCATTCTAAGAAACATCTTAGGGATGTTTACATTCAAGTCACAGAGTTGA
          1 AAAAACTAGACAGAAGCATTCTCAGAAACATCTTTGTGATGTTTGCACTCAACTCACAGAGTTGA

              *  * *     *       *         *    * *   **      *      *    *  
     207042 ACATTCCCTTTCACAGAGCAGGTTTGAAACAATCTTCTCGTACTATCTGGCAGTGGACATTTTGA
         66 ACCTTTCTTTTCATAGAGCAGTTTTGAAACACTCTTTTTGTAGAATCTGGAAGTGGATATTTGGA

              * *   *  ** ** *  *   *            ** *
     207107 GCTCCTTGGGGCCTATGCTGAAAAAGGAAATATCTTCCGAC
        131 GCGCTTTGAGGATTTCGTTGGAAACGGAAATATCTTCATAT

                                 *     *  **          *           **    *    
     207148 AAAAACTAGACAGAAGCATTCGCAGAATCACGTTTGTGATGTGTGCACTCAACTGTCAGAATTGA
          1 AAAAACTAGACAGAAGCATTCTCAGAAACATCTTTGTGATGTTTGCACTCAACTCACAGAGTTGA

                 **   ** *      *                            * *            *
     207213 ACCTTGGTTTGGACAGAGCACTTTTGAAACACTCTTTTTGTAGAATCTGCAGGTGGATATTTGGC
         66 ACCTTTCTTTTCATAGAGCAGTTTTGAAACACTCTTTTTGTAGAATCTGGAAGTGGATATTTGGA

            **                         *   *      * *
     207278 TAGCTTTGAGGATTTCGTTGGAAACGGTAATGTCTTCAAAG
        131 GCGCTTTGAGGATTTCGTTGGAAACGGAAATATCTTCATAT

                *                         *   *            *    *            
     207319 AAAATCTAGACAGAAGCATTCTCAGAAACACCTTCGTGATGTTTGCAATCAAGTCACAGAGTTGA
          1 AAAAACTAGACAGAAGCATTCTCAGAAACATCTTTGTGATGTTTGCACTCAACTCACAGAGTTGA

                 * *             *  *           *      *             *       
     207384 ACCTTCCGTTTCATAGAGCAGGTTGGAAACACTCTTATTGTAGTATCTGGAAGTGGACATTTGGA
         66 ACCTTTCTTTTCATAGAGCAGTTTTGAAACACTCTTTTTGTAGAATCTGGAAGTGGATATTTGGA

                   *   ** ** *  *   *            **  
     207449 GCGCTTTCAGGCCTATGGTGAAAAAGGAAATATCTTCCCAT
        131 GCGCTTTGAGGATTTCGTTGGAAACGGAAATATCTTCATAT

                  * * *      **      *   * *   *     ** ** *      *    *     
     207490 AAAAACGACATAGAAGCTATCTCAGGAACTTGTTTATGATGCATCTAATCAACTAACAGTGTTGA
          1 AAAAACTAGACAGAAGCATTCTCAGAAACATCTTTGTGATGTTTGCACTCAACTCACAGAGTTGA

                  * ** * **                        * *       *               
     207555 ACCTTTGTACTGACGGAGCAG-TTTGAAACACTCTTTTTTTGGAATCTGCAAGTGGATATTTGGA
         66 ACCTTTCTTTTCATAGAGCAGTTTTGAAACACTCTTTTTGTAGAATCTGGAAGTGGATATTTGGA

            *                           *   *   *    
     207619 TCGCTTTGAGGATTTCGTTGGAAACGG-GAT-GC--AATAT
        131 GCGCTTTGAGGATTTCGTTGGAAACGGAAATATCTTCATAT

                **  *    *     *                 **  *   *  *    *         * 
     207656 AAAACGTACACAGCAGCATACTCAGAAA-ATACTTTGCCATATTTCCATTCAAGTCACAGAGTGG
          1 AAAAACTAGACAGAAGCATTCTCAGAAACAT-CTTTGTGATGTTTGCACTCAACTCACAGAGTTG

               *  * **            *                  *  *             *      
     207720 AACATTCCCATTCATAGAGCAGGTTTGAAACACTCTTTTTGGAGTATCTGGAAGTGGACATTTGG
         65 AACCTTTCTTTTCATAGAGCAGTTTTGAAACACTCTTTTTGTAGAATCTGGAAGTGGATATTTGG

                    ** * * ** *  *   *            **  
     207785 AGCGCTTTCTGAACTATGGTGAAAAAGGAAATATCTTCCAAT
        130 AGCGCTTTGAGGATTTCGTTGGAAACGGAAATATCTTCATAT

            *     *               *      * *        * *  **      **  *  *    
     207827 GAAAACAAGACAGAAGCATTCTGAGAAACTTATTTGTGATATGTGTCCTCAACAAACGGACTTGA
          1 AAAAACTAGACAGAAGCATTCTCAGAAACATCTTTGTGATGTTTGCACTCAACTCACAGAGTTGA

                   *      **  * *  *  *             *   *    * * *           
     207892 ACCTTTCGTTTCATGCAGTACTTCTGGAACACTCTTTTTGAAGATTCTGCATGCGGATATTTGGA
         66 ACCTTTCTTTTCATAGAGCAGTTTTGAAACACTCTTTTTGTAGAATCTGGAAGTGGATATTTGGA

            **                            **   *   * 
     207957 TAGCTTTGAGGATTTCGTTGGAAACGG---GCT-TACATGT
        131 GCGCTTTGAGGATTTCGTTGGAAACGGAAATATCTTCATAT

                 *       *               *        *   *    *    *       *    
     207994 AAAAATTAGACAGCAGCATTCTCAGAAACTTCTTTGTGGTGTCTGCATTCAAGTCACAGAATTGA
          1 AAAAACTAGACAGAAGCATTCTCAGAAACATCTTTGTGATGTTTGCACTCAACTCACAGAGTTGA

              * ** * **            *  * *      *       *             *       
     208059 ACATCCCCTCACATAGAGCAGTTGTGCAGCACTCTATTTGTAGTATCTGGAAGTGGACATTTGGA
         66 ACCTTTCTTTTCATAGAGCAGTTTTGAAACACTCTTTTTGTAGAATCTGGAAGTGGATATTTGGA

             *         ** *    * *   *            **  
     208124 GGGCTTTGTAGCCTATC-TGGAAAAAGGAAATATCTTCCCAT
        131 GCGCTTTG-AGGATTTCGTTGGAAACGGAAATATCTTCATAT

            *  ** *   *     * *            *   *  *   * **        *  * * *   
     208165 GAATGCGAGATAGAAGTAATCTCAGAAACATGTTTATGCTGTATCTACTCAACTAACTGTGCTGA
          1 AAAAACTAGACAGAAGCATTCTCAGAAACATCTTTGTGATGTTTGCACTCAACTCACAGAGTTGA

              *     *  *               *        *  * *       *               
     208230 ACATTTCTATTGATAGAGCAGTTTTGAGACACTCTTCTTTTGGAATCTGCAAGTGGATATTTGGA
         66 ACCTTTCTTTTCATAGAGCAGTTTTGAAACACTCTTTTTGTAGAATCTGGAAGTGGATATTTGGA

            ** *                        *     *      
     208295 TAGATTTGAGGATTTCGTTGGAAACGG-GAT-T-AT-ATAT
        131 GCGCTTTGAGGATTTCGTTGGAAACGGAAATATCTTCATAT

                 *       *               *                 **  *   *         
     208332 AAAAAGTAGACAGCAGCATTCTCAGAAACTTCTTTGTGATGTTTGCATCCAGCTCTCAGAGTTGA
          1 AAAAACTAGACAGAAGCATTCTCAGAAACATCTTTGTGATGTTTGCACTCAACTCACAGAGTTGA

              *  * *          *  *        *        *   **        *  **       
     208397 ACATTCCCTTTCATAGAGTAGGTTTGAAACCCTCTTTTTATAGTGTCTGGAAGCGGGCATTTGGA
         66 ACCTTTCTTTTCATAGAGCAGTTTTGAAACACTCTTTTTGTAGAATCTGGAAGTGGATATTTGGA

                   *   ** ** *  *   *          * *   
     208462 GCGCTTTCAGGCCTATGCTGAAAAAGGAAATATCTACCTAT
        131 GCGCTTTGAGGATTTCGTTGGAAACGGAAATATCTTCATAT

             *                    *    *  **          ** *        *    *     
     208503 AGAAACTAGACAGAAGCATTCTGAGAATCACGTTTGTGATGTGGGTACTCAACTAACAGTGTTGA
          1 AAAAACTAGACAGAAGCATTCTCAGAAACATCTTTGTGATGTTTGCACTCAACTCACAGAGTTGA

            *  *       *   *            *   *                *               
     208568 TCCATTCTTTTGATACAGCAGTTTTGAACCACACTTTTTGTAGAATCTGCAAGTGGATATTTGGA
         66 ACCTTTCTTTTCATAGAGCAGTTTTGAAACACTCTTTTTGTAGAATCTGGAAGTGGATATTTGGA

            **   *                  *  *   *        *
     208633 TAGCTGTGAGGATTTCGTTGGAAATGGGAATGTCTTCATAG
        131 GCGCTTTGAGGATTTCGTTGGAAACGGAAATATCTTCATAT

                **                     * * **         *  **   *   *          
     208674 AAAATTTAGACAGAAGCATTCTCAGAACCTTGATTGTGATGTGTGTTCTCCACTAACAGAGTTGA
          1 AAAAACTAGACAGAAGCATTCTCAGAAACATCTTTGTGATGTTTGCACTCAACTCACAGAGTTGA

                       * *   * *   *       *       *                         
     208739 ACCTTTCTTTTGACAGAACTGTTCTGAAACATTCTTTTTATAGAATCTGGAAGTGGATATTTGGA
         66 ACCTTTCTTTTCATAGAGCAGTTTTGAAACACTCTTTTTGTAGAATCTGGAAGTGGATATTTGGA

            **                         *          *  
     208804 AAGCTTTGAGGATTTCGTTGGAAACGGGAATATCTTCAAAT
        131 GCGCTTTGAGGATTTCGTTGGAAACGGAAATATCTTCATAT

                *    *            *           * *       *  *    *            
     208845 AAAATCTAGCCAGAAGCATTCTAAGAAACATCTTAGGGATGTTTACATTCAAGTCACAGAGTTGA
          1 AAAAACTAGACAGAAGCATTCTCAGAAACATCTTTGTGATGTTTGCACTCAACTCACAGAGTTGA

              *  * *     *       *         *    * *   **      *      *    *  
     208910 ACATTCCCTTTCACAGAGCAGGTTTGAAACAATCTTCTCGTACTATCTGGCAGTGGACATTTTGA
         66 ACCTTTCTTTTCATAGAGCAGTTTTGAAACACTCTTTTTGTAGAATCTGGAAGTGGATATTTGGA

              * *   *  ** ** *  *   *            ** *
     208975 GCTCCTTGGGGCCTATGCTGAAAAAGGAAATATCTTCCGAC
        131 GCGCTTTGAGGATTTCGTTGGAAACGGAAATATCTTCATAT

                                 *     *  **          *           **    *    
     209016 AAAAACTAGACAGAAGCATTCGCAGAATCACGTTTGTGATGTGTGCACTCAACTGTCAGAATTGA
          1 AAAAACTAGACAGAAGCATTCTCAGAAACATCTTTGTGATGTTTGCACTCAACTCACAGAGTTGA

                 **   ** *      *                            * *            *
     209081 ACCTTGGTTTGGACAGAGCACTTTTGAAACACTCTTTTTGTAGAATCTGCAGGTGGATATTTGGC
         66 ACCTTTCTTTTCATAGAGCAGTTTTGAAACACTCTTTTTGTAGAATCTGGAAGTGGATATTTGGA

            **                         *   *      * *
     209146 TAGCTTTGAGGATTTCGTTGGAAACGGTAATGTCTTCAAAG
        131 GCGCTTTGAGGATTTCGTTGGAAACGGAAATATCTTCATAT

                *                         *   *            *    *            
     209187 AAAATCTAGACAGAAGCATTCTCAGAAACACCTTCGTGATGTTTGCAATCAAGTCACAGAGTTGA
          1 AAAAACTAGACAGAAGCATTCTCAGAAACATCTTTGTGATGTTTGCACTCAACTCACAGAGTTGA

                 * *             ** *                  *             *       
     209252 ACCTTCCGTTTCATAGAGCAGGATGGAAACACTCTTTTTGTAGTATCTGGAAGTGGACATTTGGA
         66 ACCTTTCTTTTCATAGAGCAGTTTTGAAACACTCTTTTTGTAGAATCTGGAAGTGGATATTTGGA

             *         ** *    * *   *            **  
     209317 GGGCTTTGTAGCCTATC-TGGAAAAAGGAAATATCTTCCCAT
        131 GCGCTTTG-AGGATTTCGTTGGAAACGGAAATATCTTCATAT

            *  ** *   *     * *            *   *  *   * **        *  * * *   
     209358 GAATGCGAGATAGAAGTAATCTCAGAAACATGTTTATGCTGTATCTACTCAACTAACTGTGCTGA
          1 AAAAACTAGACAGAAGCATTCTCAGAAACATCTTTGTGATGTTTGCACTCAACTCACAGAGTTGA

              *     *  *               *        *  * *       *               
     209423 ACATTTCTATTGATAGAGCAGTTTTGAGACACTCTTCTTTTGGAATCTGCAAGTGGATATTTGGA
         66 ACCTTTCTTTTCATAGAGCAGTTTTGAAACACTCTTTTTGTAGAATCTGGAAGTGGATATTTGGA

            ** *                        *     *      
     209488 TAGATTTGAGGATTTCGTTGGAAACGG-GAT-T-AT-ATAT
        131 GCGCTTTGAGGATTTCGTTGGAAACGGAAATATCTTCATAT

                 *       *               *                 **  *   *         
     209525 AAAAAGTAGACAGCAGCATTCTCAGAAACTTCTTTGTGATGTTTGCATCCAGCTCTCAGAGTTGA
          1 AAAAACTAGACAGAAGCATTCTCAGAAACATCTTTGTGATGTTTGCACTCAACTCACAGAGTTGA

              *  * *          *  *        *        *   **        *  **       
     209590 ACATTCCCTTTCATAGAGTAGGTTTGAAACCCTCTTTTTATAGTGTCTGGAAGCGGGCATTTGGA
         66 ACCTTTCTTTTCATAGAGCAGTTTTGAAACACTCTTTTTGTAGAATCTGGAAGTGGATATTTGGA

                   *   ** ** *  *   *          * *   
     209655 GCGCTTTCAGGCCTATGCTGAAAAAGGAAATATCTACCTAT
        131 GCGCTTTGAGGATTTCGTTGGAAACGGAAATATCTTCATAT

             *                    *    *  **          ** *        *    *     
     209696 AGAAACTAGACAGAAGCATTCTGAGAATCACGTTTGTGATGTGGGTACTCAACTAACAGTGTTGA
          1 AAAAACTAGACAGAAGCATTCTCAGAAACATCTTTGTGATGTTTGCACTCAACTCACAGAGTTGA

            *  *       *   *            *   *                *               
     209761 TCCATTCTTTTGATACAGCAGTTTTGAACCACACTTTTTGTAGAATCTGCAAGTGGATATTTGGA
         66 ACCTTTCTTTTCATAGAGCAGTTTTGAAACACTCTTTTTGTAGAATCTGGAAGTGGATATTTGGA

            **   *                     *   *        *
     209826 TAGCTGTGAGGATTTCGTTGGAAACGGGAATGTCTTCATAG
        131 GCGCTTTGAGGATTTCGTTGGAAACGGAAATATCTTCATAT

                **                     * * **         *  **   *   *          
     209867 AAAATTTAGACAGAAGCATTCTCAGAACCTTGATTGTGATGTGTGTTCTCCACTAACAGAGTTGA
          1 AAAAACTAGACAGAAGCATTCTCAGAAACATCTTTGTGATGTTTGCACTCAACTCACAGAGTTGA

                       * *   * *   *       *       *                         
     209932 ACCTTTCTTTTGACAGAACTGTTCTGAAACATTCTTTTTATAGAATCTGGAAGTGGATATTTGGA
         66 ACCTTTCTTTTCATAGAGCAGTTTTGAAACACTCTTTTTGTAGAATCTGGAAGTGGATATTTGGA

            **                         *          *  
     209997 AAGCTTTGAGGATTTCGTTGGAAACGGGAATATCTTCAAAT
        131 GCGCTTTGAGGATTTCGTTGGAAACGGAAATATCTTCATAT

                *    *            *           * *       *  *    *            
     210038 AAAATCTAGCCAGAAGCATTCTAAGAAACATCTTAGGGATGTTTACATTCAAGTCACAGAGTTGA
          1 AAAAACTAGACAGAAGCATTCTCAGAAACATCTTTGTGATGTTTGCACTCAACTCACAGAGTTGA

              *  * *     *       *         *    * *   **      *      *    *  
     210103 ACATTCCCTTTCACAGAGCAGGTTTGAAACAATCTTCTCGTACTATCTGGCAGTGGACATTTTGA
         66 ACCTTTCTTTTCATAGAGCAGTTTTGAAACACTCTTTTTGTAGAATCTGGAAGTGGATATTTGGA

              * *   *  ** ** *  *   *            ** *
     210168 GCTCCTTGGGGCCTATGCTGAAAAAGGAAATATCTTCCGAC
        131 GCGCTTTGAGGATTTCGTTGGAAACGGAAATATCTTCATAT

                                 *     *  **          *           **    *    
     210209 AAAAACTAGACAGAAGCATTCGCAGAATCACGTTTGTGATGTGTGCACTCAACTGTCAGAATTGA
          1 AAAAACTAGACAGAAGCATTCTCAGAAACATCTTTGTGATGTTTGCACTCAACTCACAGAGTTGA

                 **   ** *      *                            * *            *
     210274 ACCTTGGTTTGGACAGAGCACTTTTGAAACACTCTTTTTGTAGAATCTGCAGGTGGATATTTGGC
         66 ACCTTTCTTTTCATAGAGCAGTTTTGAAACACTCTTTTTGTAGAATCTGGAAGTGGATATTTGGA

            **                         *   *      * *
     210339 TAGCTTTGAGGATTTCGTTGGAAACGGTAATGTCTTCAAAG
        131 GCGCTTTGAGGATTTCGTTGGAAACGGAAATATCTTCATAT

                *                         *   *            *    *            
     210380 AAAATCTAGACAGAAGCATTCTCAGAAACACCTTCGTGATGTTTGCAATCAAGTCACAGAGTTGA
          1 AAAAACTAGACAGAAGCATTCTCAGAAACATCTTTGTGATGTTTGCACTCAACTCACAGAGTTGA

                 * *             ** *                  *             *       
     210445 ACCTTCCGTTTCATAGAGCAGGATGGAAACACTCTTTTTGTAGTATCTGGAAGTGGACATTTGGA
         66 ACCTTTCTTTTCATAGAGCAGTTTTGAAACACTCTTTTTGTAGAATCTGGAAGTGGATATTTGGA

             *         ** *    * *   *            **  
     210510 GGGCTTTGTAGCCTATC-TGGAAAAAGGAAATATCTTCCCAT
        131 GCGCTTTG-AGGATTTCGTTGGAAACGGAAATATCTTCATAT

            *  ** *   *     * *            *   *  *   * **        *  * * *   
     210551 GAATGCGAGATAGAAGTAATCTCAGAAACATGTTTATGCTGTATCTACTCAACTAACTGTGCTGA
          1 AAAAACTAGACAGAAGCATTCTCAGAAACATCTTTGTGATGTTTGCACTCAACTCACAGAGTTGA

              *     *  *               *        *  * *       *               
     210616 ACATTTCTATTGATAGAGCAGTTTTGAGACACTCTTCTTTTGGAATCTGCAAGTGGATATTTGGA
         66 ACCTTTCTTTTCATAGAGCAGTTTTGAAACACTCTTTTTGTAGAATCTGGAAGTGGATATTTGGA

            ** *                        *     *      
     210681 TAGATTTGAGGATTTCGTTGGAAACGG-GAT-T-AT-ATAT
        131 GCGCTTTGAGGATTTCGTTGGAAACGGAAATATCTTCATAT

                 *       *               *                 **  *   *         
     210718 AAAAAGTAGACAGCAGCATTCTCAGAAACTTCTTTGTGATGTTTGCATCCAGCTCTCAGAGTTGA
          1 AAAAACTAGACAGAAGCATTCTCAGAAACATCTTTGTGATGTTTGCACTCAACTCACAGAGTTGA

              *  * *          *  *        *        *   **        *  **       
     210783 ACATTCCCTTTCATAGAGTAGGTTTGAAACCCTCTTTTTATAGTGTCTGGAAGCGGGCATTTGGA
         66 ACCTTTCTTTTCATAGAGCAGTTTTGAAACACTCTTTTTGTAGAATCTGGAAGTGGATATTTGGA

                   *   ** ** *  *   *          * *   
     210848 GCGCTTTCAGGCCTATGCTGAAAAAGGAAATATCTACCTAT
        131 GCGCTTTGAGGATTTCGTTGGAAACGGAAATATCTTCATAT

             *                    *    *  **          ** *        *    *     
     210889 AGAAACTAGACAGAAGCATTCTGAGAATCACGTTTGTGATGTGGGTACTCAACTAACAGTGTTGA
          1 AAAAACTAGACAGAAGCATTCTCAGAAACATCTTTGTGATGTTTGCACTCAACTCACAGAGTTGA

            *  *       *   *            *   *                *               
     210954 TCCATTCTTTTGATACAGCAGTTTTGAACCACACTTTTTGTAGAATCTGCAAGTGGATATTTGGA
         66 ACCTTTCTTTTCATAGAGCAGTTTTGAAACACTCTTTTTGTAGAATCTGGAAGTGGATATTTGGA

            **   *                     *   *        *
     211019 TAGCTGTGAGGATTTCGTTGGAAACGGGAATGTCTTCATAG
        131 GCGCTTTGAGGATTTCGTTGGAAACGGAAATATCTTCATAT

                **                     * * **         *  **   *   *          
     211060 AAAATTTAGACAGAAGCATTCTCAGAACCTTGATTGTGATGTGTGTTCTCCACTAACAGAGTTGA
          1 AAAAACTAGACAGAAGCATTCTCAGAAACATCTTTGTGATGTTTGCACTCAACTCACAGAGTTGA

                       * *   * *   *       *       *                         
     211125 ACCTTTCTTTTGACAGAACTGTTCTGAAACATTCTTTTTATAGAATCTGGAAGTGGATATTTGGA
         66 ACCTTTCTTTTCATAGAGCAGTTTTGAAACACTCTTTTTGTAGAATCTGGAAGTGGATATTTGGA

            **                         *          *  
     211190 AAGCTTTGAGGATTTCGTTGGAAACGGGAATATCTTCAAAT
        131 GCGCTTTGAGGATTTCGTTGGAAACGGAAATATCTTCATAT

                *    *            *           * *       *  *    *            
     211231 AAAATCTAGCCAGAAGCATTCTAAGAAACATCTTAGGGATGTTTACATTCAAGTCACAGAGTTGA
          1 AAAAACTAGACAGAAGCATTCTCAGAAACATCTTTGTGATGTTTGCACTCAACTCACAGAGTTGA

              *  * *     *       *         *    * *   **      *      *    *  
     211296 ACATTCCCTTTCACAGAGCAGGTTTGAAACAATCTTCTCGTACTATCTGGCAGTGGACATTTTGA
         66 ACCTTTCTTTTCATAGAGCAGTTTTGAAACACTCTTTTTGTAGAATCTGGAAGTGGATATTTGGA

              * *   *  ** ** *  *   *            ** *
     211361 GCTCCTTGGGGCCTATGCTGAAAAAGGAAATATCTTCCGAC
        131 GCGCTTTGAGGATTTCGTTGGAAACGGAAATATCTTCATAT

                                 *     *  **          *           **    *    
     211402 AAAAACTAGACAGAAGCATTCGCAGAATCACGTTTGTGATGTGTGCACTCAACTGTCAGAATTGA
          1 AAAAACTAGACAGAAGCATTCTCAGAAACATCTTTGTGATGTTTGCACTCAACTCACAGAGTTGA

                 **   ** *      *                            * *            *
     211467 ACCTTGGTTTGGACAGAGCACTTTTGAAACACTCTTTTTGTAGAATCTGCAGGTGGATATTTGGC
         66 ACCTTTCTTTTCATAGAGCAGTTTTGAAACACTCTTTTTGTAGAATCTGGAAGTGGATATTTGGA

            **                         *   *      * *
     211532 TAGCTTTGAGGATTTCGTTGGAAACGGTAATGTCTTCAAAG
        131 GCGCTTTGAGGATTTCGTTGGAAACGGAAATATCTTCATAT

                *                         *   *            *    *            
     211573 AAAATCTAGACAGAAGCATTCTCAGAAACACCTTCGTGATGTTTGCAATCAAGTCACAGAGTTGA
          1 AAAAACTAGACAGAAGCATTCTCAGAAACATCTTTGTGATGTTTGCACTCAACTCACAGAGTTGA

                 * *             *  *                  *             *       
     211638 ACCTTCCGTTTCATAGAGCAGGTTGGAAACACTCTTTTTGTAGTATCTGGAAGTGGACATTTGGA
         66 ACCTTTCTTTTCATAGAGCAGTTTTGAAACACTCTTTTTGTAGAATCTGGAAGTGGATATTTGGA

             *         ** *    * *   *            **  
     211703 GGGCTTTGTAGCCTATC-TGGAAAAAGGAAATATCTTCCCAT
        131 GCGCTTTG-AGGATTTCGTTGGAAACGGAAATATCTTCATAT

            *  ** *   *     * *            *   *  *   * **        *  * * *   
     211744 GAATGCGAGATAGAAGTAATCTCAGAAACATGTTTATGCTGTATCTACTCAACTAACTGTGCTGA
          1 AAAAACTAGACAGAAGCATTCTCAGAAACATCTTTGTGATGTTTGCACTCAACTCACAGAGTTGA

              *     *  *               *        *  * *       *               
     211809 ACATTTCTATTGATAGAGCAGTTTTGAGACACTCTTCTTTTGGAATCTGCAAGTGGATATTTGGA
         66 ACCTTTCTTTTCATAGAGCAGTTTTGAAACACTCTTTTTGTAGAATCTGGAAGTGGATATTTGGA

            ** *                        *     *      
     211874 TAGATTTGAGGATTTCGTTGGAAACGG-GAT-T-AT-ATAT
        131 GCGCTTTGAGGATTTCGTTGGAAACGGAAATATCTTCATAT

                 *       *               *                 **  *   *         
     211911 AAAAAGTAGACAGCAGCATTCTCAGAAACTTCTTTGTGATGTTTGCATCCAGCTCTCAGAGTTGA
          1 AAAAACTAGACAGAAGCATTCTCAGAAACATCTTTGTGATGTTTGCACTCAACTCACAGAGTTGA

            * *  * *          *  *        *        *   **        *  **       
     211976 GCATTCCCTTTCATAGAGTAGGTTTGAAACCCTCTTTTTATAGTGTCTGGAAGCGGGCATTTGGA
         66 ACCTTTCTTTTCATAGAGCAGTTTTGAAACACTCTTTTTGTAGAATCTGGAAGTGGATATTTGGA

                   *    * * **  **  **          * *  *
     212041 GCGCTTTCAGGCCTATGCTTAAAATAGGAAATATCTACCTAC
        131 GCGCTTTGAGG-ATTTCGTTGGAAACGGAAATATCTTCATAT

             *                    *    *   *          ** *        *    *     
     212083 AGAAACTAGACAGAAGCATTCTGAGAATCATGTTTGTGATGTGGGTACTCAACTAACAGTGTTGA
          1 AAAAACTAGACAGAAGCATTCTCAGAAACATCTTTGTGATGTTTGCACTCAACTCACAGAGTTGA

            *  *       *   *            *   *                *   *           
     212148 TCCATTCTTTTGATACAGCAGTTTTGAACCACACTTTTTGTAGAATCTGCAAGAGGATATTTGGA
         66 ACCTTTCTTTTCATAGAGCAGTTTTGAAACACTCTTTTTGTAGAATCTGGAAGTGGATATTTGGA

            **   *                     *   *      * *
     212213 TAGCTGTGAGGATTTCGTTGGAAACGGGAATGTCTTCAAAG
        131 GCGCTTTGAGGATTTCGTTGGAAACGGAAATATCTTCATAT

                *                         *   *            *    *            
     212254 AAAATCTAGACAGAAGCATTCTCAGAAACACCTTCGTGATGTTTGCAATCAAGTCACAGAGTTGA
          1 AAAAACTAGACAGAAGCATTCTCAGAAACATCTTTGTGATGTTTGCACTCAACTCACAGAGTTGA

                 * *             *  *           *      *             *       
     212319 ACCTTCCGTTTCATAGAGCAGGTTGGAAACACTCTTATTGTAGTATCTGGAAGTGGACATTTGGA
         66 ACCTTTCTTTTCATAGAGCAGTTTTGAAACACTCTTTTTGTAGAATCTGGAAGTGGATATTTGGA

                   *   ** ** *  *   *            **  
     212384 GCGCTTTCAGGCCTATGGTGAAAAAGGAAATATCTTCCCAT
        131 GCGCTTTGAGGATTTCGTTGGAAACGGAAATATCTTCATAT

                  * * *      **      *   * *   *     ** ** *      *    *     
     212425 AAAAACGACATAGAAGCTATCTCAGGAACTTGTTTATGATGCATCTAATCAACTAACAGTGTTGA
          1 AAAAACTAGACAGAAGCATTCTCAGAAACATCTTTGTGATGTTTGCACTCAACTCACAGAGTTGA

                  * ** * *                         * *       *               
     212490 ACCTTTGTACTGACAGAGCAG-TTTGAAACACTCTTTTTTTGGAATCTGCAAGTGGATATTTGGA
         66 ACCTTTCTTTTCATAGAGCAGTTTTGAAACACTCTTTTTGTAGAATCTGGAAGTGGATATTTGGA

            *                           *   *   *    
     212554 TCGCTTTGAGGATTTCGTTGGAAACGG-GAT-GC--AATAT
        131 GCGCTTTGAGGATTTCGTTGGAAACGGAAATATCTTCATAT

                **  *    *     *                 **  *   *  *    *         * 
     212591 AAAACGTACACAGCAGCATACTCAGAAA-ATACTTTGCCATATTTCCATTCAAGTCACAGAGTGG
          1 AAAAACTAGACAGAAGCATTCTCAGAAACAT-CTTTGTGATGTTTGCACTCAACTCACAGAGTTG

               *  * **            *                  *  *             *      
     212655 AACATTCCCATTCATAGAGCAGGTTTGAAACACTCTTTTTGGAGTATCTGGAAGTGGACATTTGG
         65 AACCTTTCTTTTCATAGAGCAGTTTTGAAACACTCTTTTTGTAGAATCTGGAAGTGGATATTTGG

                    ** * * ** *  *   *            **  
     212720 AGCGCTTTCTGAACTATGGTGAAAAAGGAAATATCTTCCAAT
        130 AGCGCTTTGAGGATTTCGTTGGAAACGGAAATATCTTCATAT

            *     * *             *      * *          *  **      **  *  *    
     212762 GAAAACAACACAGAAGCATTCTGAGAAACTTATTTGTGATGTGTGTCCTCAACAAACGGACTTGA
          1 AAAAACTAGACAGAAGCATTCTCAGAAACATCTTTGTGATGTTTGCACTCAACTCACAGAGTTGA

                   *      **  * *  *  *             *   *    * * *           
     212827 ACCTTTCGTTTCATGCAGTACTTCTGGAACACTCTTTTTGAAGATTCTGCATGCGGATATTTGGA
         66 ACCTTTCTTTTCATAGAGCAGTTTTGAAACACTCTTTTTGTAGAATCTGGAAGTGGATATTTGGA

            **                            **   *   * 
     212892 TAGCTTTGAGGATTTCGTTGGAAACGG---GCT-TACATGT
        131 GCGCTTTGAGGATTTCGTTGGAAACGGAAATATCTTCATAT

                 *       *               *        *   *    *    *       *    
     212929 AAAAATTAGACAGCAGCATTCTCAGAAACTTCTTTGTGGTGTCTGCATTCAAGTCACAGAATTGA
          1 AAAAACTAGACAGAAGCATTCTCAGAAACATCTTTGTGATGTTTGCACTCAACTCACAGAGTTGA

              * ** * **            *  * *      *       *             *       
     212994 ACTTCCCCTCACATAGAGCAGTTGTGCAGCACTCTATTTGTAGTATCTGGAAGTGGACATTTGGA
         66 ACCTTTCTTTTCATAGAGCAGTTTTGAAACACTCTTTTTGTAGAATCTGGAAGTGGATATTTGGA

             *         ** *    * *   *            **  
     213059 GGGCTTTGTAGCCTATC-TGGAAAAAGGAAATATCTTCCCAT
        131 GCGCTTTG-AGGATTTCGTTGGAAACGGAAATATCTTCATAT

            *  ** *   *     * *            *   *  *   * **        *  * * *   
     213100 GAATGCGAGATAGAAGTAATCTCAGAAACATGTTTATGCTGTATCTACTCAACTAACTGTGCTGA
          1 AAAAACTAGACAGAAGCATTCTCAGAAACATCTTTGTGATGTTTGCACTCAACTCACAGAGTTGA

              *     *  *               *        *  * *       *               
     213165 ACATTTCTATTGATAGAGCAGTTTTGAGACACTCTTCTTTTGGAATCTGCAAGTGGATATTTGGA
         66 ACCTTTCTTTTCATAGAGCAGTTTTGAAACACTCTTTTTGTAGAATCTGGAAGTGGATATTTGGA

            ** *                        *     *      
     213230 TAGATTTGAGGATTTCGTTGGAAACGG-GAT-T-AT-ATAT
        131 GCGCTTTGAGGATTTCGTTGGAAACGGAAATATCTTCATAT

            *    *       *               *                 **  *   *         
     213267 CAAAAGTAGACAGCAGCATTCTCAGAAACTTCTTTGTGATGTTTGCATCCAGCTCTCAGAGTTGA
          1 AAAAACTAGACAGAAGCATTCTCAGAAACATCTTTGTGATGTTTGCACTCAACTCACAGAGTTGA

              *  * *          *  *        *        *   **        *  **       
     213332 ACATTCCCTTTCATAGAGTAGGTTTGAAACCCTCTTTTTATAGTGTCTGGAAGCGGGCATTTGGA
         66 ACCTTTCTTTTCATAGAGCAGTTTTGAAACACTCTTTTTGTAGAATCTGGAAGTGGATATTTGGA

                   *   ** ** *  *   *          * *   
     213397 GCGCTTTCAGGCCTATGCTGAAAAAGGAAATATCTACCTAT
        131 GCGCTTTGAGGATTTCGTTGGAAACGGAAATATCTTCATAT

             *                    *    *  **          ** *        *    *     
     213438 AGAAACTAGACAGAAGCATTCTGAGAATCACGTTTGTGATGTGGGTACTCAACTAACAGTGTTGA
          1 AAAAACTAGACAGAAGCATTCTCAGAAACATCTTTGTGATGTTTGCACTCAACTCACAGAGTTGA

            *  *       *   *            *   *                *               
     213503 TCCATTCTTTTGATACAGCAGTTTTGAACCACACTTTTTGTAGAATCTGCAAGTGGATATTTGGA
         66 ACCTTTCTTTTCATAGAGCAGTTTTGAAACACTCTTTTTGTAGAATCTGGAAGTGGATATTTGGA

            **   *                     *   *        *
     213568 TAGCTGTGAGGATTTCGTTGGAAACGGGAATGTCTTCATAG
        131 GCGCTTTGAGGATTTCGTTGGAAACGGAAATATCTTCATAT

                **                     * * **         *  **   *   *          
     213609 AAAATTTAGACAGAAGCATTCTCAGAACCTTGATTGTGATGTGTGTTCTCCACTAACAGAGTTGA
          1 AAAAACTAGACAGAAGCATTCTCAGAAACATCTTTGTGATGTTTGCACTCAACTCACAGAGTTGA

                       * *   * *   *       *       *                         
     213674 ACCTTTCTTTTGACAGAACTGTTCTGAAACATTCTTTTTATAGAATCTGGAAGTGGATATTTGGA
         66 ACCTTTCTTTTCATAGAGCAGTTTTGAAACACTCTTTTTGTAGAATCTGGAAGTGGATATTTGGA

            **                         *          *  
     213739 AAGCTTTGAGGATTTCGTTGGAAACGGGAATATCTTCAAAT
        131 GCGCTTTGAGGATTTCGTTGGAAACGGAAATATCTTCATAT

                *    *            *          ** *       *  *    *            
     213780 AAAATCTAGCCAGAAGCATTCTAAGAAACATCTCAGGGATGTTTACATTCAAGTCACAGAGTTGA
          1 AAAAACTAGACAGAAGCATTCTCAGAAACATCTTTGTGATGTTTGCACTCAACTCACAGAGTTGA

              *  * *     *       *         *    * *   **      *      *    *  
     213845 ACATTCCCTTTCACAGAGCAGGTTTGAAACAATCTTCTCGTACTATCTGGCAGTGGACATTTTGA
         66 ACCTTTCTTTTCATAGAGCAGTTTTGAAACACTCTTTTTGTAGAATCTGGAAGTGGATATTTGGA

              * *   *  ** ** *  *   *            ** *
     213910 GCTCCTTGGGGCCTATGCTGAAAAAGGAAATATCTTCCGAC
        131 GCGCTTTGAGGATTTCGTTGGAAACGGAAATATCTTCATAT

                                 *     *  **          *           **    **   
     213951 AAAAACTAGACAGAAGCATTCGCAGAATCACGTTTGTGATGTGTGCACTCAACTGTCAGAACTGA
          1 AAAAACTAGACAGAAGCATTCTCAGAAACATCTTTGTGATGTTTGCACTCAACTCACAGAGTTGA

                 **   ** *      *                            * *            *
     214016 ACCTTGGTTTGGACAGAGCACTTTTGAAACACTCTTTTTGTAGAATCTGCAGGTGGATATTTGGC
         66 ACCTTTCTTTTCATAGAGCAGTTTTGAAACACTCTTTTTGTAGAATCTGGAAGTGGATATTTGGA

            **                         *   *      * *
     214081 TAGCTTTGAGGATTTCGTTGGAAACGGTAATGTCTTCAAAG
        131 GCGCTTTGAGGATTTCGTTGGAAACGGAAATATCTTCATAT

                *                         * * *            *    *            
     214122 AAAATCTAGACAGAAGCATTCTCAGAAACAGCGTCGTGATGTTTGCAATCAAGTCACAGAGTTGA
          1 AAAAACTAGACAGAAGCATTCTCAGAAACATCTTTGTGATGTTTGCACTCAACTCACAGAGTTGA

                 * *             *  *                  *             *       
     214187 ACCTTCCGTTTCATAGAGCAGGTTGGAAACACTCTTTTTGTAGTATCTGGAAGTGGACATTTGGA
         66 ACCTTTCTTTTCATAGAGCAGTTTTGAAACACTCTTTTTGTAGAATCTGGAAGTGGATATTTGGA

             *         ** *    * *   *            **  
     214252 GGGCTTTGTAGCCTATC-TGGAAAAAGGAAATATCTTCCCAT
        131 GCGCTTTG-AGGATTTCGTTGGAAACGGAAATATCTTCATAT

            *  ** *   *     * *            *   *  *   * **        *  * * *   
     214293 GAATGCGAGATAGAAGTAATCTCAGAAACATGTTTATGCTGTATCTACTCAACTAACTGTGCTGA
          1 AAAAACTAGACAGAAGCATTCTCAGAAACATCTTTGTGATGTTTGCACTCAACTCACAGAGTTGA

              *     *  *               *        *  * *       *               
     214358 ACATTTCTATTGATAGAGCAGTTTTGAGACACTCTTCTTTTGGAATCTGCAAGTGGATATTTGGA
         66 ACCTTTCTTTTCATAGAGCAGTTTTGAAACACTCTTTTTGTAGAATCTGGAAGTGGATATTTGGA

            ** *                        *     *      
     214423 TAGATTTGAGGATTTCGTTGGAAACGG-GAT-T-AT-ATAT
        131 GCGCTTTGAGGATTTCGTTGGAAACGGAAATATCTTCATAT

                 *       *               *                 **  *   *         
     214460 AAAAAGTAGACAGCAGCATTCTCAGAAACTTCTTTGTGATGTTTGCATCCAGCTCTCAGAGTTGA
          1 AAAAACTAGACAGAAGCATTCTCAGAAACATCTTTGTGATGTTTGCACTCAACTCACAGAGTTGA

            * *  * *          *  *        *        *   **        *  **       
     214525 GCATTCCCTTTCATAGAGTAGGTTTGAAACCCTCTTTTTATAGTGTCTGGAAGCGGGCATTTGGA
         66 ACCTTTCTTTTCATAGAGCAGTTTTGAAACACTCTTTTTGTAGAATCTGGAAGTGGATATTTGGA

                   *    *        **  **          * *  *
     214590 GCGCTTTCAGGCCTATTC-TTAAAATAGGAAATATCTACCTAC
        131 GCGCTTTGAGG-AT-TTCGTTGGAAACGGAAATATCTTCATAT

             *                    *    *  **          ** *        *    *     
     214632 AGAAACTAGACAGAAGCATTCTGAGAATCACGTTTGTGATGTGGGTACTCAACTAACAGTGTTGA
          1 AAAAACTAGACAGAAGCATTCTCAGAAACATCTTTGTGATGTTTGCACTCAACTCACAGAGTTGA

            *  *       *   *            *   *                *   *           
     214697 TCCATTCTTTTGATACAGCAGTTTTGAACCACACTTTTTGTAGAATCTGCAAGAGGATATTTGGA
         66 ACCTTTCTTTTCATAGAGCAGTTTTGAAACACTCTTTTTGTAGAATCTGGAAGTGGATATTTGGA

            **   *                     *   *  *   * *
     214762 TAGCTGTGAGGATTTCGTTGGAAACGGGAATGTCCTCAAAG
        131 GCGCTTTGAGGATTTCGTTGGAAACGGAAATATCTTCATAT

                *                         *   *            *    *            
     214803 AAAATCTAGACAGAAGCATTCTCAGAAACACCTTCGTGATGTTTGCAATCAAGTCACAGAGTTGA
          1 AAAAACTAGACAGAAGCATTCTCAGAAACATCTTTGTGATGTTTGCACTCAACTCACAGAGTTGA

                 * *             *  *           *      *             *       
     214868 ACCTTCCGTTTCATAGAGCAGGTTGGAAACACTCTTATTGTAGTATCTGGAAGTGGACATTTGGA
         66 ACCTTTCTTTTCATAGAGCAGTTTTGAAACACTCTTTTTGTAGAATCTGGAAGTGGATATTTGGA

                   *   ** ** *  *   *            **  
     214933 GCGCTTTCAGGCCTATGGTGAAAAAGGAAATATCTTCCCAT
        131 GCGCTTTGAGGATTTCGTTGGAAACGGAAATATCTTCATAT

                  * * *      **      *   * *   *     ** ** *      *    *     
     214974 AAAAACGACATAGAAGCTATCTCAGGAACTTGTTTATGATGCATCTAATCAACTAACAGTGTTGA
          1 AAAAACTAGACAGAAGCATTCTCAGAAACATCTTTGTGATGTTTGCACTCAACTCACAGAGTTGA

                  * ** * *        *                  *       *               
     215039 ACCTTTGTACTGACAGAGCAG-CTTGAAACACTCTTTTT-TGGAATCTGCAAGTGGATATTTGGA
         66 ACCTTTCTTTTCATAGAGCAGTTTTGAAACACTCTTTTTGTAGAATCTGGAAGTGGATATTTGGA

            *                           *   *   *    
     215102 TCGCTTTGAGGATTTCGTTGGAAACGG-GAT-GC--AATAT
        131 GCGCTTTGAGGATTTCGTTGGAAACGGAAATATCTTCATAT

                **  *    *     *                 **  *   *  *    *         * 
     215139 AAAACGTACACAGCAGCATACTCAGAAA-ATACTTTGCCATATTTCCATTCAAGTCACAGAGTGG
          1 AAAAACTAGACAGAAGCATTCTCAGAAACAT-CTTTGTGATGTTTGCACTCAACTCACAGAGTTG

               *  * **            *  *               *  *             *      
     215203 AACATTCCCATTCATAGAGCAGGTTGGAAACACTCTTTTTGGAGTATCTGGAAGTGGACATTTGG
         65 AACCTTTCTTTTCATAGAGCAGTTTTGAAACACTCTTTTTGTAGAATCTGGAAGTGGATATTTGG

                    ** * * ** *      *            **  
     215268 AGCGCTTTCTGAACTATGGTGGAAAAGGAAATATCTTCCAAT
        130 AGCGCTTTGAGGATTTCGTTGGAAACGGAAATATCTTCATAT

            *     *               *      * *          *  **      **  *  *    
     215310 GAAAACAAGACAGAAGCATTCTGAGAAACTTATTTGTGATGTGTGTCCTCAACAAACGGACTTGA
          1 AAAAACTAGACAGAAGCATTCTCAGAAACATCTTTGTGATGTTTGCACTCAACTCACAGAGTTGA

                   *      **  * *  *  *             *   *    * * *           
     215375 ACCTTTCGTTTCATGCAGTACTTCTGGAACACTCTTTTTGAAGATTCTGCATGCGGATATTTGGA
         66 ACCTTTCTTTTCATAGAGCAGTTTTGAAACACTCTTTTTGTAGAATCTGGAAGTGGATATTTGGA

            **        *                   **   *   * 
     215440 TAGCTTTGAGAATTTCGTTGGAAACGG---GCT-TACATGT
        131 GCGCTTTGAGGATTTCGTTGGAAACGGAAATATCTTCATAT

                 *       *               *        *   *    *    *       *    
     215477 AAAAATTAGACAGCAGCATTCTCAGAAACTTCTTTGTGGTGTCTGCATTCAAGTCACAGAATTGA
          1 AAAAACTAGACAGAAGCATTCTCAGAAACATCTTTGTGATGTTTGCACTCAACTCACAGAGTTGA

              * ** * **            *  * *      *       *    *        *       
     215542 ACTTCCCCTCACATAGAGCAGTTGTGCAGCACTCTATTTGTAGTATCTCGAAGTGGACATTTGGA
         66 ACCTTTCTTTTCATAGAGCAGTTTTGAAACACTCTTTTTGTAGAATCTGGAAGTGGATATTTGGA

             *         ** *    * *   *            **  
     215607 GGGCTTTGTAGCCTATC-TGGAAAAAGGAAATATCTTCCCAT
        131 GCGCTTTG-AGGATTTCGTTGGAAACGGAAATATCTTCATAT

            *  ** *   *     * *            *   *  *   * **        *  * * *   
     215648 GAATGCGAGATAGAAGTAATCTCAGAAACATGTTTATGCTGTATCTACTCAACTAACTGTGCTGA
          1 AAAAACTAGACAGAAGCATTCTCAGAAACATCTTTGTGATGTTTGCACTCAACTCACAGAGTTGA

              *     *  *               *        *  * *       *               
     215713 ACATTTCTATTGATAGAGCAGTTTTGAGACACTCTTCTTTTGGAATCTGCAAGTGGATATTTGGA
         66 ACCTTTCTTTTCATAGAGCAGTTTTGAAACACTCTTTTTGTAGAATCTGGAAGTGGATATTTGGA

            ** *                        *     *      
     215778 TAGATTTGAGGATTTCGTTGGAAACGG-GAT-T-AT-ATAT
        131 GCGCTTTGAGGATTTCGTTGGAAACGGAAATATCTTCATAT

                 *       *               *                 **  *   *         
     215815 AAAAAGTAGACAGCAGCATTCTCAGAAACTTCTTTGTGATGTTTGCATCCAGCTCTCAGAGTTGA
          1 AAAAACTAGACAGAAGCATTCTCAGAAACATCTTTGTGATGTTTGCACTCAACTCACAGAGTTGA

            * *  * *          *  *        *        *   **        *  **       
     215880 GCATTCCCTTTCATAGAGTAGGTTTGAAACCCTCTTTTTATAGTGTCTGGAAGCGGGCATTTGGA
         66 ACCTTTCTTTTCATAGAGCAGTTTTGAAACACTCTTTTTGTAGAATCTGGAAGTGGATATTTGGA

                   *    * * **  **  **          * *  *
     215945 GCGCTTTCAGGCCTATGCTTAAAATAGGAAATATCTACCTAC
        131 GCGCTTTGAGG-ATTTCGTTGGAAACGGAAATATCTTCATAT

             *                    *    *   *          ** *        *    *     
     215987 AGAAACTAGACAGAAGCATTCTGAGAATCATGTTTGTGATGTGGGTACTCAACTAACAGTGTTGA
          1 AAAAACTAGACAGAAGCATTCTCAGAAACATCTTTGTGATGTTTGCACTCAACTCACAGAGTTGA

            *  *       *  **            *   *                *   *           
     216052 TCCATTCTTTTGATTCAGCAGTTTTGAACCACACTTTTTGTAGAATCTGCAAGAGGATATTTGGA
         66 ACCTTTCTTTTCATAGAGCAGTTTTGAAACACTCTTTTTGTAGAATCTGGAAGTGGATATTTGGA

            **   *                     *   *        *
     216117 TAGCTGTGAGGATTTCGTTGGAAACGGGAATGTCTTCATAG
        131 GCGCTTTGAGGATTTCGTTGGAAACGGAAATATCTTCATAT

                **    *                * * *          *  **   *   * *        
     216158 AAAATTTAGATAGAAGCATTCTCAGAACCTTGTTTGTGATGTGTGTTCTCCACTAAGAGAGTTGA
          1 AAAAACTAGACAGAAGCATTCTCAGAAACATCTTTGTGATGTTTGCACTCAACTCACAGAGTTGA

                       * *   * *   *       *       *                         
     216223 ACCTTTCTTTTGACAGAACTGTTCTGAAACATTCTTTTTATAGAATCTGGAAGTGGATATTTGGA
         66 ACCTTTCTTTTCATAGAGCAGTTTTGAAACACTCTTTTTGTAGAATCTGGAAGTGGATATTTGGA

            **                         *          *  
     216288 AAGCTTTGAGGATTTCGTTGGAAACGGGAATATCTTCAAAT
        131 GCGCTTTGAGGATTTCGTTGGAAACGGAAATATCTTCATAT

                *    *            *           * *       *  *    *            
     216329 AAAATCTAGCCAGAAGCATTCTAAGAAACATCTTAGGGATGTTTACATTCAAGTCACAGAGTTGA
          1 AAAAACTAGACAGAAGCATTCTCAGAAACATCTTTGTGATGTTTGCACTCAACTCACAGAGTTGA

              *  * *     *       *         *    * *   **      *      *    *  
     216394 ACATTCCCTTTCACAGAGCAGGTTTGAAACAATCTTCTCGTACTATCTGGCAGTGGACATTTTGA
         66 ACCTTTCTTTTCATAGAGCAGTTTTGAAACACTCTTTTTGTAGAATCTGGAAGTGGATATTTGGA

              * *   *  ** ** *  *   *            ** *
     216459 GCTCCTTGGGGCCTATGCTGAAAAAGGAAATATCTTCCGAC
        131 GCGCTTTGAGGATTTCGTTGGAAACGGAAATATCTTCATAT

                                 *     *  **          *           **    *    
     216500 AAAAACTAGACAGAAGCATTCGCAGAATCACGTTTGTGATGTGTGCACTCAACTGTCAGAATTGA
          1 AAAAACTAGACAGAAGCATTCTCAGAAACATCTTTGTGATGTTTGCACTCAACTCACAGAGTTGA

                 **   ** *      *                            * *            *
     216565 ACCTTGGTTTGGACAGAGCACTTTTGAAACACTCTTTTTGTAGAATCTGCAGGTGGATATTTGGC
         66 ACCTTTCTTTTCATAGAGCAGTTTTGAAACACTCTTTTTGTAGAATCTGGAAGTGGATATTTGGA

            **                         *   *      * *
     216630 TAGCTTTGAGGATTTCGTTGGAAACGGTAATGTCTTCAAAG
        131 GCGCTTTGAGGATTTCGTTGGAAACGGAAATATCTTCATAT

                *                         *   *            *    *            
     216671 AAAATCTAGACAGAAGCATTCTCAGAAACACCTTCGTGATGTTTGCAATCAAGTCACAGAGTTGA
          1 AAAAACTAGACAGAAGCATTCTCAGAAACATCTTTGTGATGTTTGCACTCAACTCACAGAGTTGA

                 * *             *  *           *      *             *       
     216736 ACCTTCCGTTTCATAGAGCAGGTTGGAAACACTCTTATTGTAGTATCTGGAAGTGGACATTTGGA
         66 ACCTTTCTTTTCATAGAGCAGTTTTGAAACACTCTTTTTGTAGAATCTGGAAGTGGATATTTGGA

                   *   ** ** *  *   *            **  
     216801 GCGCTTTCAGGCCTATGGTGAAAAAGGAAATATCTTCCCAT
        131 GCGCTTTGAGGATTTCGTTGGAAACGGAAATATCTTCATAT

                  * * *      **      *   * *   *     ** ** *      *    *     
     216842 AAAAACGACATAGAAGCTATCTCAGGAACTTGTTTATGATGCATCTAATCAACTAACAGTGTTGA
          1 AAAAACTAGACAGAAGCATTCTCAGAAACATCTTTGTGATGTTTGCACTCAACTCACAGAGTTGA

                  * ** * *                         * *       *               
     216907 ACCTTTGTACTGACAGAGCAG-TTTGAAACACTCTTTTTTTGGAATCTGCAAGTGGATATTTGGA
         66 ACCTTTCTTTTCATAGAGCAGTTTTGAAACACTCTTTTTGTAGAATCTGGAAGTGGATATTTGGA

            *                           *   *   *    
     216971 TCGCTTTGAGGATTTCGTTGGAAACGG-GAT-GC--AATAT
        131 GCGCTTTGAGGATTTCGTTGGAAACGGAAATATCTTCATAT

                **  *    *     *                 **  *   *  *    *         * 
     217008 AAAACGTACACAGCAGCATACTCAGAAA-ATACTTTGCCATATTTCCATTCAAGTCACAGAGTGG
          1 AAAAACTAGACAGAAGCATTCTCAGAAACAT-CTTTGTGATGTTTGCACTCAACTCACAGAGTTG

               *  * **            *                  *  *             *      
     217072 AACATTCCCATTCATAGAGCAGGTTTGAAACACTCTTTTTGGAGTATCTGGAAGTGGACATTTGG
         65 AACCTTTCTTTTCATAGAGCAGTTTTGAAACACTCTTTTTGTAGAATCTGGAAGTGGATATTTGG

                    ** * * ** *  *   *            **  
     217137 AGCGCTTTCTGAACTATGGTGAAAAAGGAAATATCTTCCAAT
        130 AGCGCTTTGAGGATTTCGTTGGAAACGGAAATATCTTCATAT

            *     *               *      * *        * *  **      **  *  *    
     217179 GAAAACAAGACAGAAGCATTCTGAGAAACTTATTTGTGATATGTGTCCTCAACAAACGGACTTGA
          1 AAAAACTAGACAGAAGCATTCTCAGAAACATCTTTGTGATGTTTGCACTCAACTCACAGAGTTGA

                   *      **  * *  *  *             *   *    * * *           
     217244 ACCTTTCGTTTCATGCAGTACTTCTGGAACACTCTTTTTGAAGATTCTGCATGCGGATATTTGGA
         66 ACCTTTCTTTTCATAGAGCAGTTTTGAAACACTCTTTTTGTAGAATCTGGAAGTGGATATTTGGA

            **                            **   *   * 
     217309 TAGCTTTGAGGATTTCGTTGGAAACGG---GCT-TACATGT
        131 GCGCTTTGAGGATTTCGTTGGAAACGGAAATATCTTCATAT

                 *       *               *        *   *    *    *       *    
     217346 AAAAATTAGACAGCAGCATTCTCAGAAACTTCTTTGTGGTGTCTGCATTCAAGTCACAGAATTGA
          1 AAAAACTAGACAGAAGCATTCTCAGAAACATCTTTGTGATGTTTGCACTCAACTCACAGAGTTGA

              * ** * **            *  * *      *       *             *       
     217411 ACATCCCCTCACATAGAGCAGTTGTGCAGCACTCTATTTGTAGTATCTGGAAGTGGACATTTGGA
         66 ACCTTTCTTTTCATAGAGCAGTTTTGAAACACTCTTTTTGTAGAATCTGGAAGTGGATATTTGGA

             *         ** *    * *   *            **  
     217476 GGGCTTTGTAGCCTATC-TGGAAAAAGGAAATATCTTCCCAT
        131 GCGCTTTG-AGGATTTCGTTGGAAACGGAAATATCTTCATAT

            *  ** *   *     * *            *   *  *   * **        *  * * *   
     217517 GAATGCGAGATAGAAGTAATCTCAGAAACATGTTTATGCTGTATCTACTCAACTAACTGTGCTGA
          1 AAAAACTAGACAGAAGCATTCTCAGAAACATCTTTGTGATGTTTGCACTCAACTCACAGAGTTGA

              *     *  *               *        *  * *       *               
     217582 ACATTTCTATTGATAGAGCAGTTTTGAGACACTCTTCTTTTGGAATCTGCAAGTGGATATTTGGA
         66 ACCTTTCTTTTCATAGAGCAGTTTTGAAACACTCTTTTTGTAGAATCTGGAAGTGGATATTTGGA

            ** *                        *     *      
     217647 TAGATTTGAGGATTTCGTTGGAAACGG-GAT-T-AT-ATAT
        131 GCGCTTTGAGGATTTCGTTGGAAACGGAAATATCTTCATAT

                 *       *               *                 **  *   *         
     217684 AAAAAGTAGACAGCAGCATTCTCAGAAACTTCTTTGTGATGTTTGCATCCAGCTCTCAGAGTTGA
          1 AAAAACTAGACAGAAGCATTCTCAGAAACATCTTTGTGATGTTTGCACTCAACTCACAGAGTTGA

              *  * *          *  *        *        *   **        *  **       
     217749 ACATTCCCTTTCATAGAGTAGGTTTGAAACCCTCTTTTTATAGTGTCTGGAAGCGGGCATTTGGA
         66 ACCTTTCTTTTCATAGAGCAGTTTTGAAACACTCTTTTTGTAGAATCTGGAAGTGGATATTTGGA

                   *   ** ** *  *   *          * *   
     217814 GCGCTTTCAGGCCTATGCTGAAAAAGGAAATATCTACCTAT
        131 GCGCTTTGAGGATTTCGTTGGAAACGGAAATATCTTCATAT

             *                    *    *  **          ** *        *    *     
     217855 AGAAACTAGACAGAAGCATTCTGAGAATCACGTTTGTGATGTGGGTACTCAACTAACAGTGTTGA
          1 AAAAACTAGACAGAAGCATTCTCAGAAACATCTTTGTGATGTTTGCACTCAACTCACAGAGTTGA

            *  *       *   *            *   *                *               
     217920 TCCATTCTTTTGATACAGCAGTTTTGAACCACACTTTTTGTAGAATCTGCAAGTGGATATTTGGA
         66 ACCTTTCTTTTCATAGAGCAGTTTTGAAACACTCTTTTTGTAGAATCTGGAAGTGGATATTTGGA

            **   *                     *   *        *
     217985 TAGCTGTGAGGATTTCGTTGGAAACGGGAATGTCTTCATAG
        131 GCGCTTTGAGGATTTCGTTGGAAACGGAAATATCTTCATAT

                **                     * * **         *  **   *   *          
     218026 AAAATTTAGACAGAAGCATTCTCAGAACCTTGATTGTGATGTGTGTTCTCCACTAACAGAGTTGA
          1 AAAAACTAGACAGAAGCATTCTCAGAAACATCTTTGTGATGTTTGCACTCAACTCACAGAGTTGA

                       * *   * *   *       *       *                         
     218091 ACCTTTCTTTTGACAGAACTGTTCTGAAACATTCTTTTTATAGAATCTGGAAGTGGATATTTGGA
         66 ACCTTTCTTTTCATAGAGCAGTTTTGAAACACTCTTTTTGTAGAATCTGGAAGTGGATATTTGGA

            **                         *          *  
     218156 AAGCTTTGAGGATTTCGTTGGAAACGGGAATATCTTCAAAT
        131 GCGCTTTGAGGATTTCGTTGGAAACGGAAATATCTTCATAT

            *   *    *            *     *     * *       *  *    *            
     218197 CAAATCTAGCCAGAAGCATTCTAAGAAATATCTTAGGGATGTTTACATTCAAGTCACAGAGTTGA
          1 AAAAACTAGACAGAAGCATTCTCAGAAACATCTTTGTGATGTTTGCACTCAACTCACAGAGTTGA

              *  * *     *       *         *    * *   **      *      *    *  
     218262 ACATTCCCTTTCACAGAGCAGGTTTGAAACAATCTTCTCGTACTATCTGGCAGTGGACATTTTGA
         66 ACCTTTCTTTTCATAGAGCAGTTTTGAAACACTCTTTTTGTAGAATCTGGAAGTGGATATTTGGA

              * *   *  ** ** *  *   *            ** *
     218327 GCTCCTTGGGGCCTATGCTGAAAAAGGAAATATCTTCCGAC
        131 GCGCTTTGAGGATTTCGTTGGAAACGGAAATATCTTCATAT

                                 *     *  **          *           **    *    
     218368 AAAAACTAGACAGAAGCATTCGCAGAATCACGTTTGTGATGTGTGCACTCAACTGTCAGAATTGA
          1 AAAAACTAGACAGAAGCATTCTCAGAAACATCTTTGTGATGTTTGCACTCAACTCACAGAGTTGA

                 **   ** *      *                            * *            *
     218433 ACCTTGGTTTGGACAGAGCACTTTTGAAACACTCTTTTTGTAGAATCTGCAGGTGGATATTTGGC
         66 ACCTTTCTTTTCATAGAGCAGTTTTGAAACACTCTTTTTGTAGAATCTGGAAGTGGATATTTGGA

            **                         *   *      * *
     218498 TAGCTTTGAGGATTTCGTTGGAAACGGTAATGTCTTCAAAG
        131 GCGCTTTGAGGATTTCGTTGGAAACGGAAATATCTTCATAT

                *                         *   *            *    *            
     218539 AAAATCTAGACAGAAGCATTCTCAGAAACACCTTCGTGATGTTTGCAATCAAGTCACAGAGTTGA
          1 AAAAACTAGACAGAAGCATTCTCAGAAACATCTTTGTGATGTTTGCACTCAACTCACAGAGTTGA

                 * *             *  *           *      *             *       
     218604 ACCTTCCGTTTCATAGAGCAGGTTGGAAACACTCTTATTGTAGTATCTGGAAGTGGACATTTGGA
         66 ACCTTTCTTTTCATAGAGCAGTTTTGAAACACTCTTTTTGTAGAATCTGGAAGTGGATATTTGGA

                   *   ** ** *  *   *            **  
     218669 GCGCTTTCAGGCCTATGGTGAAAAAGGAAATATCTTCCCAT
        131 GCGCTTTGAGGATTTCGTTGGAAACGGAAATATCTTCATAT

                  * * *      **      *   * *   *     ** ** *      *    *     
     218710 AAAAACGACATAGAAGCTATCTCAGGAACTTGTTTATGATGCATCTAATCAACTAACAGTGTTGA
          1 AAAAACTAGACAGAAGCATTCTCAGAAACATCTTTGTGATGTTTGCACTCAACTCACAGAGTTGA

                  * ** * *                         * *       *               
     218775 ACCTTTGTACTGACAGAGCAG-TTTGAAACACTCTTTTTTTGGAATCTGCAAGTGGATATTTGGA
         66 ACCTTTCTTTTCATAGAGCAGTTTTGAAACACTCTTTTTGTAGAATCTGGAAGTGGATATTTGGA

            *                           *   *   *    
     218839 TCGCTTTGAGGATTTCGTTGGAAACGG-GAT-GC--AATAT
        131 GCGCTTTGAGGATTTCGTTGGAAACGGAAATATCTTCATAT

                **  *    *     *                 **  *   *  *    *         * 
     218876 AAAACGTACACAGCAGCATACTCAGAAA-ATACTTTGCCATATTTCCATTCAAGTCACAGAGTGG
          1 AAAAACTAGACAGAAGCATTCTCAGAAACAT-CTTTGTGATGTTTGCACTCAACTCACAGAGTTG

               *  * **            *  *               *  *             *      
     218940 AACATTCCCATTCATAGAGCAGGTTGGAAACACTCTTTTTGGAGTATCTGGAAGTGGACATTTGG
         65 AACCTTTCTTTTCATAGAGCAGTTTTGAAACACTCTTTTTGTAGAATCTGGAAGTGGATATTTGG

                    ** * * ** *  *   *            **  
     219005 AGCGCTTTCTGAACTATGGTGAAAAAGGAAATATCTTCCAAT
        130 AGCGCTTTGAGGATTTCGTTGGAAACGGAAATATCTTCATAT

            *     *               *      * *          *  **      **  *  *    
     219047 GAAAACAAGACAGAAGCATTCTGAGAAACTTATTTGTGATGTGTGTCCTCAACAAACGGACTTGA
          1 AAAAACTAGACAGAAGCATTCTCAGAAACATCTTTGTGATGTTTGCACTCAACTCACAGAGTTGA

                   *      **  * *  *  *             *   *    * * *           
     219112 ACCTTTCGTTTCATGCAGTACTTCTGGAACACTCTTTTTGAAGATTCTGCATGCGGATATTTGGA
         66 ACCTTTCTTTTCATAGAGCAGTTTTGAAACACTCTTTTTGTAGAATCTGGAAGTGGATATTTGGA

            **                  *         **   *   * 
     219177 TAGCTTTGAGGATTTCGTTGAAAACGG---GCT-TACATGT
        131 GCGCTTTGAGGATTTCGTTGGAAACGGAAATATCTTCATAT

                 *       *               *        *   *    *    *       *    
     219214 AAAAATTAGACAGCAGCATTCTCAGAAACTTCTTTGTGGTGTCTGCATTCAAGTCACAGAATTGA
          1 AAAAACTAGACAGAAGCATTCTCAGAAACATCTTTGTGATGTTTGCACTCAACTCACAGAGTTGA

              * ** * **            *  * *      *       *             *       
     219279 ACTTCCCCTCACATAGAGCAGTTGTGCAGCACTCTATTTGTAGTATCTGGAAGTGGACATTTGGA
         66 ACCTTTCTTTTCATAGAGCAGTTTTGAAACACTCTTTTTGTAGAATCTGGAAGTGGATATTTGGA

             *         ** *    * *   *            **  
     219344 GGGCTTTGTAGCCTATC-TGGAAAAAGGAAATATCTTCCCAT
        131 GCGCTTTG-AGGATTTCGTTGGAAACGGAAATATCTTCATAT

            *  ** *   *     * *            *   *  *   * **        *  * * *   
     219385 GAATGCGAGATAGAAGTAATCTCAGAAACATGTTTATGCTGTATCTACTCAACTAACTGTGCTGA
          1 AAAAACTAGACAGAAGCATTCTCAGAAACATCTTTGTGATGTTTGCACTCAACTCACAGAGTTGA

              *     *  *               *        *  * *       *               
     219450 ACATTTCTATTGATAGAGCAGTTTTGAGACACTCTTCTTTTGGAATCTGCAAGTGGATATTTGGA
         66 ACCTTTCTTTTCATAGAGCAGTTTTGAAACACTCTTTTTGTAGAATCTGGAAGTGGATATTTGGA

            ** *                        *     *      
     219515 TAGATTTGAGGATTTCGTTGGAAACGG-GAT-T-AT-ATAT
        131 GCGCTTTGAGGATTTCGTTGGAAACGGAAATATCTTCATAT

                 *       *               *                 **  *   *         
     219552 AAAAAGTAGACAGCAGCATTCTCAGAAACTTCTTTGTGATGTTTGCATCCAGCTCTCAGAGTTGA
          1 AAAAACTAGACAGAAGCATTCTCAGAAACATCTTTGTGATGTTTGCACTCAACTCACAGAGTTGA

              *  * *          *  *        *        *   **        *  **       
     219617 ACATTCCCTTTCATAGAGTAGGTTTGAAACCCTCTTTTTATAGTGTCTGGAAGCGGGCATTTGGA
         66 ACCTTTCTTTTCATAGAGCAGTTTTGAAACACTCTTTTTGTAGAATCTGGAAGTGGATATTTGGA

                   *   ** ** *  *   *          * *   
     219682 GCGCTTTCAGGCCTATGCTGAAAAAGGAAATATCTACCTAT
        131 GCGCTTTGAGGATTTCGTTGGAAACGGAAATATCTTCATAT

             *                    *    *  **          ** *        *    *     
     219723 AGAAACTAGACAGAAGCATTCTGAGAATCACGTTTGTGATGTGGGTACTCAACTAACAGTGTTGA
          1 AAAAACTAGACAGAAGCATTCTCAGAAACATCTTTGTGATGTTTGCACTCAACTCACAGAGTTGA

            *  *       *   *            *   *                *               
     219788 TCCATTCTTTTGATACAGCAGTTTTGAACCACACTTTTTGTAGAATCTGCAAGTGGATATTTGGA
         66 ACCTTTCTTTTCATAGAGCAGTTTTGAAACACTCTTTTTGTAGAATCTGGAAGTGGATATTTGGA

            **   *                     *   *        *
     219853 TAGCTGTGAGGATTTCGTTGGAAACGGGAATGTCTTCATAG
        131 GCGCTTTGAGGATTTCGTTGGAAACGGAAATATCTTCATAT

                **                     * * **         *  **   *   *          
     219894 AAAATTTAGACAGAAGCATTCTCAGAACCTTGATTGTGATGTGTGTTCTCCACTAACAGAGTTGA
          1 AAAAACTAGACAGAAGCATTCTCAGAAACATCTTTGTGATGTTTGCACTCAACTCACAGAGTTGA

                       * *   * *   *       *       *                         
     219959 ACCTTTCTTTTGACAGAACTGTTCTGAAACATTCTTTTTATAGAATCTGGAAGTGGATATTTGGA
         66 ACCTTTCTTTTCATAGAGCAGTTTTGAAACACTCTTTTTGTAGAATCTGGAAGTGGATATTTGGA

            **                         *          *  
     220024 AAGCTTTGAGGATTTCGTTGGAAACGGGAATATCTTCAAAT
        131 GCGCTTTGAGGATTTCGTTGGAAACGGAAATATCTTCATAT

                *    *            *           * *       *  *    *            
     220065 AAAATCTAGCCAGAAGCATTCTAAGAAACATCTTAGGGATGTTTACATTCAAGTCACAGAGTTGA
          1 AAAAACTAGACAGAAGCATTCTCAGAAACATCTTTGTGATGTTTGCACTCAACTCACAGAGTTGA

              *  * *     *       *         *    * *   **      *      *    *  
     220130 ACATTCCCTTTCACAGAGCAGGTTTGAAACAATCTTCTCGTACTATCTGGCAGTGGACATTTTGA
         66 ACCTTTCTTTTCATAGAGCAGTTTTGAAACACTCTTTTTGTAGAATCTGGAAGTGGATATTTGGA

              * *   *  ** ** *  *   *            ** *
     220195 GCTCCTTGGGGCCTATGCTGAAAAAGGAAATATCTTCCGAC
        131 GCGCTTTGAGGATTTCGTTGGAAACGGAAATATCTTCATAT

                                 *     *  **          *           **    *    
     220236 AAAAACTAGACAGAAGCATTCGCAGAATCACGTTTGTGATGTGTGCACTCAACTGTCAGAATTGA
          1 AAAAACTAGACAGAAGCATTCTCAGAAACATCTTTGTGATGTTTGCACTCAACTCACAGAGTTGA

                 **   ** *      *                            * *            *
     220301 ACCTTGGTTTGGACAGAGCACTTTTGAAACACTCTTTTTGTAGAATCTGCAGGTGGATATTTGGC
         66 ACCTTTCTTTTCATAGAGCAGTTTTGAAACACTCTTTTTGTAGAATCTGGAAGTGGATATTTGGA

            **                         *   *      * *
     220366 TAGCTTTGAGGATTTCGTTGGAAACGGTAATGTCTTCAAAG
        131 GCGCTTTGAGGATTTCGTTGGAAACGGAAATATCTTCATAT

                *                         *   *            *    *          * 
     220407 AAAATCTAGACAGAAGCATTCTCAGAAACAACTTCGTGATGTTTGCAATCAAGTCACAGAGTTAA
          1 AAAAACTAGACAGAAGCATTCTCAGAAACATCTTTGTGATGTTTGCACTCAACTCACAGAGTTGA

                 * *             *  *       *          *             *       
     220472 ACCTTCCGTTTCATAGAGCAGGTTGGAAACACACTTTTTGTAGTATCTGGAAGTGGACATTTGGA
         66 ACCTTTCTTTTCATAGAGCAGTTTTGAAACACTCTTTTTGTAGAATCTGGAAGTGGATATTTGGA

             *         ** *    * *   *            **  
     220537 GGGCTTTGTAGCCTATC-TGGAAAAAGGAAATATCTTCCCAT
        131 GCGCTTTG-AGGATTTCGTTGGAAACGGAAATATCTTCATAT

            *  ** *   *     * *            *   *  *   * **        *  * * *   
     220578 GAATGCGAGATAGAAGTAATCTCAGAAACATGTTTATGCTGTATCTACTCAACTAACTGTGCTGA
          1 AAAAACTAGACAGAAGCATTCTCAGAAACATCTTTGTGATGTTTGCACTCAACTCACAGAGTTGA

              *     *  *               *        *  * *       *               
     220643 ACATTTCTATTGATAGAGCAGTTTTGAGACACTCTTCTTTTGGAATCTGCAAGTGGATATTTGGA
         66 ACCTTTCTTTTCATAGAGCAGTTTTGAAACACTCTTTTTGTAGAATCTGGAAGTGGATATTTGGA

            ** *                        *     *      
     220708 TAGATTTGAGGATTTCGTTGGAAACGG-GAT-T-AT-ATAT
        131 GCGCTTTGAGGATTTCGTTGGAAACGGAAATATCTTCATAT

                 *       *               *                 **  *   *         
     220745 AAAAAGTAGACAGCAGCATTCTCAGAAACTTCTTTGTGATGTTTGCATCCAGCTCTCAGAGTTGA
          1 AAAAACTAGACAGAAGCATTCTCAGAAACATCTTTGTGATGTTTGCACTCAACTCACAGAGTTGA

              *  * *          *  *        *        *   **        *  **       
     220810 ACATTCCCTTTCATAGAGTAGGTTTGAAACCCTCTTTTTATAGTGTCTGGAAGCGGGCATTTGGA
         66 ACCTTTCTTTTCATAGAGCAGTTTTGAAACACTCTTTTTGTAGAATCTGGAAGTGGATATTTGGA

                   *    * * **  **  **          * *  *
     220875 GCGCTTTCAGGCCTATGCTTAAAATAGGAAATATCTACCTAC
        131 GCGCTTTGAGG-ATTTCGTTGGAAACGGAAATATCTTCATAT

             *                    *    *  **          ** *        *    *     
     220917 AGAAACTAGACAGAAGCATTCTGAGAATCACGTTTGTGATGTGGGTACTCAACTAACAGTGTTGA
          1 AAAAACTAGACAGAAGCATTCTCAGAAACATCTTTGTGATGTTTGCACTCAACTCACAGAGTTGA

            *  *       *   *            *   *                *   *           
     220982 TCCATTCTTTTGATACAGCAGTTTTGAACCACACTTTTTGTAGAATCTGCAAGAGGATATTTGGA
         66 ACCTTTCTTTTCATAGAGCAGTTTTGAAACACTCTTTTTGTAGAATCTGGAAGTGGATATTTGGA

            **   *                     *   *      * *
     221047 TAGCTGTGAGGATTTCGTTGGAAACGGGAATGTCTTCAAAG
        131 GCGCTTTGAGGATTTCGTTGGAAACGGAAATATCTTCATAT

                *                         *   *            *    *            
     221088 AAAATCTAGACAGAAGCATTCTCAGAAACACCTTCGTGATGTTTGCAATCAAGTCACAGAGTTGA
          1 AAAAACTAGACAGAAGCATTCTCAGAAACATCTTTGTGATGTTTGCACTCAACTCACAGAGTTGA

                   *             *  *           *      *             *       
     221153 ACCTTTCGTTTCATAGAGCAGGTTGGAAACACTCTTATTGTAGTATCTGGAAGTGGACATTTGGA
         66 ACCTTTCTTTTCATAGAGCAGTTTTGAAACACTCTTTTTGTAGAATCTGGAAGTGGATATTTGGA

                   *   ** ** *  *   *            **  
     221218 GCGCTTTCAGGCCTATGGTGAAAAAGGAAATATCTTCCCAT
        131 GCGCTTTGAGGATTTCGTTGGAAACGGAAATATCTTCATAT

                  * * *      **      *   * *   *     ** ** *      *    *     
     221259 AAAAACGACATAGAAGCTATCTCAGGAACTTGTTTATGATGCATCTAATCAACTAACAGTGTTGA
          1 AAAAACTAGACAGAAGCATTCTCAGAAACATCTTTGTGATGTTTGCACTCAACTCACAGAGTTGA

                  * ** * *                         * *       *               
     221324 ACCTTTGTACTGACAGAGCAG-TTTGAAACACTCTTTTTTTGGAATCTGCAAGTGGATATTTGGA
         66 ACCTTTCTTTTCATAGAGCAGTTTTGAAACACTCTTTTTGTAGAATCTGGAAGTGGATATTTGGA

            *                           *   *   *    
     221388 TCGCTTTGAGGATTTCGTTGGAAACGG-GAT-GC--AATAT
        131 GCGCTTTGAGGATTTCGTTGGAAACGGAAATATCTTCATAT

                **  *    *     *                 **  *   *  *    *         * 
     221425 AAAACGTACACAGCAGCATACTCAGAAA-ATACTTTGCCATATTTCCATTCAAGTCACAGAGTGG
          1 AAAAACTAGACAGAAGCATTCTCAGAAACAT-CTTTGTGATGTTTGCACTCAACTCACAGAGTTG

               *  * **            *                  *  *             *      
     221489 AACATTCCCATTCATAGAGCAGGTTTGAAACACTCTTTTTGGAGTATCTGGAAGTGGACATTTGG
         65 AACCTTTCTTTTCATAGAGCAGTTTTGAAACACTCTTTTTGTAGAATCTGGAAGTGGATATTTGG

                    ** * * ** *  *   *            **  
     221554 AGCGCTTTCTGAACTATGGTGAAAAAGGAAATATCTTCCAAT
        130 AGCGCTTTGAGGATTTCGTTGGAAACGGAAATATCTTCATAT

            *     *               *      * *          *  **       *  *  *    
     221596 GAAAACAAGACAGAAGCATTCTGAGAAACTTATTTGTGATGTGTGTCCTCAACTAACGGACTTGA
          1 AAAAACTAGACAGAAGCATTCTCAGAAACATCTTTGTGATGTTTGCACTCAACTCACAGAGTTGA

                   *      **  * *  *  *             *   *    * * *           
     221661 ACCTTTCGTTTCATGCAGTACTTCTGGAACACTCTTTTTGAAGATTCTGCATGCGGATATTTGGA
         66 ACCTTTCTTTTCATAGAGCAGTTTTGAAACACTCTTTTTGTAGAATCTGGAAGTGGATATTTGGA

            **                            **   *   * 
     221726 TTGCTTTGAGGATTTCGTTGGAAACGG---GCT-TACATGT
        131 GCGCTTTGAGGATTTCGTTGGAAACGGAAATATCTTCATAT

                 *       *               *        *   *    *    *       *    
     221763 AAAAATTAGACAGCAGCATTCTCAGAAACTTCTTTGTGGTGTCTGCATTCAAGTCACAGAATTGA
          1 AAAAACTAGACAGAAGCATTCTCAGAAACATCTTTGTGATGTTTGCACTCAACTCACAGAGTTGA

              * ** * **            *  * *      *       *             *       
     221828 ACTTCCCCTCACATAGAGCAGTTGTGCAGCACTCTATTTGTAGTATCTGGAAGTGGACATTTGGA
         66 ACCTTTCTTTTCATAGAGCAGTTTTGAAACACTCTTTTTGTAGAATCTGGAAGTGGATATTTGGA

             *         ** *    * *   *            **  
     221893 GGGCTTTGTAGCCTATC-TGGAAAAAGGAAATATCTTCCCAT
        131 GCGCTTTG-AGGATTTCGTTGGAAACGGAAATATCTTCATAT

            *  ** *   *     * *            *   *  *   * **        *  * * *   
     221934 GAATGCGAGATAGAAGTAATCTCAGAAACATGTTTATGCTGTATCTACTCAACTAACTGTGCTGA
          1 AAAAACTAGACAGAAGCATTCTCAGAAACATCTTTGTGATGTTTGCACTCAACTCACAGAGTTGA

              *     *  *               *        *  * *       *               
     221999 ACATTTCTATTGATAGAGCAGTTTTGAGACACTCTTCTTTTGGAATCTGCAAGTGGATATTTGGA
         66 ACCTTTCTTTTCATAGAGCAGTTTTGAAACACTCTTTTTGTAGAATCTGGAAGTGGATATTTGGA

            ** *                        *     *      
     222064 TAGATTTGAGGATTTCGTTGGAAACGG-GAT-T-AT-ATAT
        131 GCGCTTTGAGGATTTCGTTGGAAACGGAAATATCTTCATAT

                 *       *               *                 **  *   *         
     222101 AAAAAGTAGACAGCAGCATTCTCAGAAACTTCTTTGTGATGTTTGCATCCAGCTCTCAGAGTTGA
          1 AAAAACTAGACAGAAGCATTCTCAGAAACATCTTTGTGATGTTTGCACTCAACTCACAGAGTTGA

              *  * *          *  *        *        *   **        *  **       
     222166 ACATTCCCTTTCATAGAGTAGGTTTGAAACCCTCTTTTTATAGTGTCTGGAAGCGGGCATTTGGA
         66 ACCTTTCTTTTCATAGAGCAGTTTTGAAACACTCTTTTTGTAGAATCTGGAAGTGGATATTTGGA

                   *   ** ** *  *   *          * *   
     222231 GCGCTTTCAGGCCTATGCTGAAAAAGGAAATATCTACCTAT
        131 GCGCTTTGAGGATTTCGTTGGAAACGGAAATATCTTCATAT

             *                    *    *  **          ** *        **   *     
     222272 AGAAACTAGACAGAAGCATTCTGAGAATCACGTTTGTGATGTGGGTACTCAACTAGCAGTGTTGA
          1 AAAAACTAGACAGAAGCATTCTCAGAAACATCTTTGTGATGTTTGCACTCAACTCACAGAGTTGA

            *  *       *   *            *   *                *               
     222337 TCCATTCTTTTGATACAGCAGTTTTGAACCACACTTTTTGTAGAATCTGCAAGTGGATATTTGGA
         66 ACCTTTCTTTTCATAGAGCAGTTTTGAAACACTCTTTTTGTAGAATCTGGAAGTGGATATTTGGA

            **   *                     *   *        *
     222402 TAGCTGTGAGGATTTCGTTGGAAACGGGAATGTCTTCATAG
        131 GCGCTTTGAGGATTTCGTTGGAAACGGAAATATCTTCATAT

                **                     * * **         *  **   *   *          
     222443 AAAATTTAGACAGAAGCATTCTCAGAACCTTGATTGTGATGTGTGTTCTCCACTAACAGAGTTGA
          1 AAAAACTAGACAGAAGCATTCTCAGAAACATCTTTGTGATGTTTGCACTCAACTCACAGAGTTGA

                       * *   * *   *       *       *                      *  
     222508 ACCTTTCTTTTGACAGAACTGTTCTGAAACATTCTTTTTATAGAATCTGGAAGTGGATATTTTGA
         66 ACCTTTCTTTTCATAGAGCAGTTTTGAAACACTCTTTTTGTAGAATCTGGAAGTGGATATTTGGA

            **              *          *          *  
     222573 AAGCTTTGAGGATTTCATTGGAAACGGGAATATCTTCAAAT
        131 GCGCTTTGAGGATTTCGTTGGAAACGGAAATATCTTCATAT

                *    *            *           * *       *  *    *            
     222614 AAAATCTAGCCAGAAGCATTCTAAGAAACATCTTAGGGATGTTTACATTCAAGTCACAGAGTTGA
          1 AAAAACTAGACAGAAGCATTCTCAGAAACATCTTTGTGATGTTTGCACTCAACTCACAGAGTTGA

              *  * *     *       *         *    * *   **      *      *    *  
     222679 ACATTCCCTTTCACAGAGCAGGTTTGAAACAATCTTCTCGTACTATCTGGCAGTGGACATTTTGA
         66 ACCTTTCTTTTCATAGAGCAGTTTTGAAACACTCTTTTTGTAGAATCTGGAAGTGGATATTTGGA

              * *   *  ** ** *  *   *            ** *
     222744 GCTCCTTGGGGCCTATGCTGAAAAAGGAAATATCTTCCGAC
        131 GCGCTTTGAGGATTTCGTTGGAAACGGAAATATCTTCATAT

                                 *     *  **          *           **    *    
     222785 AAAAACTAGACAGAAGCATTCGCAGAATCACGTTTGTGATGTGTGCACTCAACTGTCAGAATTGA
          1 AAAAACTAGACAGAAGCATTCTCAGAAACATCTTTGTGATGTTTGCACTCAACTCACAGAGTTGA

                 **   ** *      *                            * *            *
     222850 ACCTTGGTTTAGACAGAGCACTTTTGAAACACTCTTTTTGTAGAATCTGCAGGTGGATATTTGGC
         66 ACCTTTCTTTTCATAGAGCAGTTTTGAAACACTCTTTTTGTAGAATCTGGAAGTGGATATTTGGA

            **                         *   *      * *
     222915 TAGCTTTGAGGATTTCGTTGGAAACGGTAATGTCTTCAAAG
        131 GCGCTTTGAGGATTTCGTTGGAAACGGAAATATCTTCATAT

                *                         *   *            *    *            
     222956 AAAATCTAGACAGAAGCATTCTCAGAAACACCTTCGTGATGTTTGCAATCAAGTCACAGAGTTGA
          1 AAAAACTAGACAGAAGCATTCTCAGAAACATCTTTGTGATGTTTGCACTCAACTCACAGAGTTGA

                 * *             *  *           *      *             *       
     223021 ACCTTCCGTTTCATAGAGCAGGTTGGAAACACTCTTATTGTAGTATCTGGAAGTGGACATTTGGA
         66 ACCTTTCTTTTCATAGAGCAGTTTTGAAACACTCTTTTTGTAGAATCTGGAAGTGGATATTTGGA

                   *   ** ** *  *   *            **  
     223086 GCGCTTTCAGGCCTATGGTGAAAAAGGAAATATCTTCCCAT
        131 GCGCTTTGAGGATTTCGTTGGAAACGGAAATATCTTCATAT

                  * * *      **      *   * *   *     ** ** *      *    *     
     223127 AAAAACGACATAGAAGCTATCTCAGGAACTTGTTTATGATGCATCTAATCAACTAACAGTGTTGA
          1 AAAAACTAGACAGAAGCATTCTCAGAAACATCTTTGTGATGTTTGCACTCAACTCACAGAGTTGA

                  * ** * *                         * *       *               
     223192 ACCTTTGTACTGACAGAGCAG-TTTGAAACACTCTTTTTTTGGAATCTGCAAGTGGATATTTGGA
         66 ACCTTTCTTTTCATAGAGCAGTTTTGAAACACTCTTTTTGTAGAATCTGGAAGTGGATATTTGGA

            *                           *   *   *    
     223256 TCGCTTTGAGGATTTCGTTGGAAACGG-GAT-GC--AATAT
        131 GCGCTTTGAGGATTTCGTTGGAAACGGAAATATCTTCATAT

                **  *    *     *                 **  *   *  *    *         * 
     223293 AAAACGTACACAGCAGCATACTCAGAAA-ATACTTTGCCATATTTCCATTCAAGTCACAGAGTGG
          1 AAAAACTAGACAGAAGCATTCTCAGAAACAT-CTTTGTGATGTTTGCACTCAACTCACAGAGTTG

               *  * **            *  *               *  *             *      
     223357 AACATTCCCATTCATAGAGCAGGTTGGAAACACTCTTTTTGGAGTATCTGGAAGTGGACATTTGG
         65 AACCTTTCTTTTCATAGAGCAGTTTTGAAACACTCTTTTTGTAGAATCTGGAAGTGGATATTTGG

                    ** * * ** *  *   *            **  
     223422 AGCGCTTTCTGAACTATGGTGAAAAAGGAAATATCTTCCAAT
        130 AGCGCTTTGAGGATTTCGTTGGAAACGGAAATATCTTCATAT

            *     *               *      * *          *  **      **  *  *    
     223464 GAAAACAAGACAGAAGCATTCTGAGAAACTTATTTGTGATGTGTGTCCTCAACAAACGGACTTGA
          1 AAAAACTAGACAGAAGCATTCTCAGAAACATCTTTGTGATGTTTGCACTCAACTCACAGAGTTGA

                   *      **  * *  *  *             *   *    * ***           
     223529 ACCTTTCGTTTCATGCAGTACTTCTGGAACACTCTTTTTGAAGATTCTGCATTCGGATATTTGGA
         66 ACCTTTCTTTTCATAGAGCAGTTTTGAAACACTCTTTTTGTAGAATCTGGAAGTGGATATTTGGA

            **                            **   *   * 
     223594 TAGCTTTGAGGATTTCGTTGGAAACGG---GCT-TACATGT
        131 GCGCTTTGAGGATTTCGTTGGAAACGGAAATATCTTCATAT

              *  *       *               *        *   *    *    *       *    
     223631 AAGAATTAGACAGCAGCATTCTCAGAAACTTCTTTGTGGTGTCTGCATTCAAGTCACAGAATTGA
          1 AAAAACTAGACAGAAGCATTCTCAGAAACATCTTTGTGATGTTTGCACTCAACTCACAGAGTTGA

              * ** * **            *  * *      *       *             *       
     223696 ACTTCCCCTCACATAGAGCAGTTGTGCAGCACTCTATTTGTAGTATCTGGAAGTGGACATTTGGA
         66 ACCTTTCTTTTCATAGAGCAGTTTTGAAACACTCTTTTTGTAGAATCTGGAAGTGGATATTTGGA

             *         ** *    * *   *            **  
     223761 GGGCTTTGTAGCCTATC-TGGAAAAAGGAAATATCTTCCCAT
        131 GCGCTTTG-AGGATTTCGTTGGAAACGGAAATATCTTCATAT

            *  ** *   *     * *            *   *  *   * **        *  * * *   
     223802 GAATGCGAGATAGAAGTAATCTCAGAAACATGTTTATGCTGTATCTACTCAACTAACTGTGCTGA
          1 AAAAACTAGACAGAAGCATTCTCAGAAACATCTTTGTGATGTTTGCACTCAACTCACAGAGTTGA

              *     *  *               *        *  * *       *               
     223867 ACATTTCTATTGATAGAGCAGTTTTGAGACACTCTTCTTTTGGAATCTGCAAGTGGATATTTGGA
         66 ACCTTTCTTTTCATAGAGCAGTTTTGAAACACTCTTTTTGTAGAATCTGGAAGTGGATATTTGGA

            ** *                        *     *      
     223932 TAGATTTGAGGATTTCGTTGGAAACGG-GAT-T-AT-ATAT
        131 GCGCTTTGAGGATTTCGTTGGAAACGGAAATATCTTCATAT

                 *       *               *                 **  *   *         
     223969 AAAAAGTAGACAGCAGCATTCTCAGAAACTTCTTTGTGATGTTTGCATCCAGCTCTCAGAGTTGA
          1 AAAAACTAGACAGAAGCATTCTCAGAAACATCTTTGTGATGTTTGCACTCAACTCACAGAGTTGA

              *  * *          *  *        *        *   **        *  **       
     224034 ACATTCCCTTTCATAGAGTAGGTTTGAAACCCTCTTTTTATAGTGTCTGGAAGCGGGCATTTGGA
         66 ACCTTTCTTTTCATAGAGCAGTTTTGAAACACTCTTTTTGTAGAATCTGGAAGTGGATATTTGGA

             *     *   ** ** *  *   *          * *   
     224099 GAGCTTTCAGGCCTATGCTGAAAAAGGAAATATCTACCTAT
        131 GCGCTTTGAGGATTTCGTTGGAAACGGAAATATCTTCATAT

             *                    *    *  **   *      ** *        *    *     
     224140 AGAAACTAGACAGAAGCATTCTGAGAATCACGTTTCTGATGTGGGTACTCAACTAACAGTGTTGA
          1 AAAAACTAGACAGAAGCATTCTCAGAAACATCTTTGTGATGTTTGCACTCAACTCACAGAGTTGA

            *  *       *   *            *   *             *  *               
     224205 TCCATTCTTTTGATACAGCAGTTTTGAACCACACTTTTTGTAGAATGTGCAAGTGGATATTTGGA
         66 ACCTTTCTTTTCATAGAGCAGTTTTGAAACACTCTTTTTGTAGAATCTGGAAGTGGATATTTGGA

            **   *                     *   *        *
     224270 TAGCTGTGAGGATTTCGTTGGAAACGGGAATGTCTTCATAG
        131 GCGCTTTGAGGATTTCGTTGGAAACGGAAATATCTTCATAT

                **                     * * **         *  **   *   *          
     224311 AAAATTTAGACAGAAGCATTCTCAGAACCTTGATTGTGATGTGTGTTCTCCACTAACAGAGTTGA
          1 AAAAACTAGACAGAAGCATTCTCAGAAACATCTTTGTGATGTTTGCACTCAACTCACAGAGTTGA

                       * *   * *   *       *       *                         
     224376 ACCTTTCTTTTGACAGAACTGTTCTGAAACATTCTTTTTATAGAATCTGGAAGTGGATATTTGGA
         66 ACCTTTCTTTTCATAGAGCAGTTTTGAAACACTCTTTTTGTAGAATCTGGAAGTGGATATTTGGA

            **              *          *          *  
     224441 AAGCTTTGAGGATTTCATTGGAAACGGGAATATCTTCAAAT
        131 GCGCTTTGAGGATTTCGTTGGAAACGGAAATATCTTCATAT

                *    *            *           * *       *  *    *          * 
     224482 AAAATCTAGCCAGAAGCATTCTAAGAAACATCTTAGGGATGTTTACATTCAAGTCACAGAGTTCA
          1 AAAAACTAGACAGAAGCATTCTCAGAAACATCTTTGTGATGTTTGCACTCAACTCACAGAGTTGA

              *  * *     *       *         *    * *   **      *      *    *  
     224547 ACATTCCCTTTCACAGAGCAGGTTTGAAACAATCTTCTCGTACTATCTGGCAGTGGACATTTTGA
         66 ACCTTTCTTTTCATAGAGCAGTTTTGAAACACTCTTTTTGTAGAATCTGGAAGTGGATATTTGGA

              * *   *  ** ** *  *   *            ** *
     224612 GCTCCTTGGGGCCTATGCTGAAAAAGGAAATATCTTCCGAC
        131 GCGCTTTGAGGATTTCGTTGGAAACGGAAATATCTTCATAT

                                 *     *  **          *           **    *    
     224653 AAAAACTAGACAGAAGCATTCGCAGAATCACGTTTGTGATGTGTGCACTCAACTGTCAGAATTGA
          1 AAAAACTAGACAGAAGCATTCTCAGAAACATCTTTGTGATGTTTGCACTCAACTCACAGAGTTGA

                 **   ** *      *                            * *            *
     224718 ACCTTGGTTTAGACAGAGCACTTTTGAAACACTCTTTTTGTAGAATCTGCAGGTGGATATTTGGC
         66 ACCTTTCTTTTCATAGAGCAGTTTTGAAACACTCTTTTTGTAGAATCTGGAAGTGGATATTTGGA

            **                         *   *      * *
     224783 TAGCTTTGAGGATTTCGTTGGAAACGGTAATGTCTTCAAAG
        131 GCGCTTTGAGGATTTCGTTGGAAACGGAAATATCTTCATAT

                *                         *   *            *    *            
     224824 AAAATCTAGACAGAAGCATTCTCAGAAACACCTTCGTGATGTTTGCAATCAAGTCACAGAGTTGA
          1 AAAAACTAGACAGAAGCATTCTCAGAAACATCTTTGTGATGTTTGCACTCAACTCACAGAGTTGA

                 * *             *  *           *      *             *       
     224889 ACCTTCCGTTTCATAGAGCAGGTTGGAAACACTCTTATTGTAGTATCTGGAAGTGGACATTTGGA
         66 ACCTTTCTTTTCATAGAGCAGTTTTGAAACACTCTTTTTGTAGAATCTGGAAGTGGATATTTGGA

                   *   ** ** *  *   *            **  
     224954 GCGCTTTCAGGCCTATGGTGAAAAAGGAAATATCTTCCCAT
        131 GCGCTTTGAGGATTTCGTTGGAAACGGAAATATCTTCATAT

                  * * *      **      *   * *   *     ** ** *      *    *     
     224995 AAAAACGACATAGAAGCTATCTCAGGAACTTGTTTATGATGCATCTAATCAACTAACAGTGTTGA
          1 AAAAACTAGACAGAAGCATTCTCAGAAACATCTTTGTGATGTTTGCACTCAACTCACAGAGTTGA

                  * ** * *                         * *       *               
     225060 ACCTTTGTACTGACAGAGCAG-TTTGAAACACTCTTTTTTTGGAATCTGCAAGTGGATATTTGGA
         66 ACCTTTCTTTTCATAGAGCAGTTTTGAAACACTCTTTTTGTAGAATCTGGAAGTGGATATTTGGA

            *                           *   *   *    
     225124 TCGCTTTGAGGATTTCGTTGGAAACGG-GAT-GC--AATAT
        131 GCGCTTTGAGGATTTCGTTGGAAACGGAAATATCTTCATAT

                **  *    *     *                 **  *   *  *    *         * 
     225161 AAAACGTACACAGCAGCATACTCAGAAA-ATACTTTGCCATATTTCCATTCAAGTCACAGAGTGG
          1 AAAAACTAGACAGAAGCATTCTCAGAAACAT-CTTTGTGATGTTTGCACTCAACTCACAGAGTTG

               *  * **            *                  *  *             *      
     225225 AACATTCCCATTCATAGAGCAGGTTTGAAACACTCTTTTTGGAGTATCTGGAAGTGGACATTTGG
         65 AACCTTTCTTTTCATAGAGCAGTTTTGAAACACTCTTTTTGTAGAATCTGGAAGTGGATATTTGG

                    ** * * ** *  *   *            **  
     225290 AGCGCTTTCTGAACTATGGTGAAAAAGGAAATATCTTCCAAT
        130 AGCGCTTTGAGGATTTCGTTGGAAACGGAAATATCTTCATAT

            *     *               *      * *          *  **      **  *  *    
     225332 GAAAACAAGACAGAAGCATTCTGAGAAACTTATTTGTGATGTGTGTCCTCAACAAACGGACTTGA
          1 AAAAACTAGACAGAAGCATTCTCAGAAACATCTTTGTGATGTTTGCACTCAACTCACAGAGTTGA

                   *      **  * *  *  *             *   *    * ***           
     225397 ACCTTTCGTTTCATGCAGTACTTCTGGAACACTCTTTTTGAAGATTCTGCATTCGGATATTTGGA
         66 ACCTTTCTTTTCATAGAGCAGTTTTGAAACACTCTTTTTGTAGAATCTGGAAGTGGATATTTGGA

            **                            **   *   * 
     225462 TAGCTTTGAGGATTTCGTTGGAAACGG---GCT-TACATGT
        131 GCGCTTTGAGGATTTCGTTGGAAACGGAAATATCTTCATAT

                 *       *               *        *   *    *    *       *    
     225499 AAAAATTAGACAGCAGCATTCTCAGAAACTTCTTTGTGGTGTCTGCATTCAAGTCACAGAATTGA
          1 AAAAACTAGACAGAAGCATTCTCAGAAACATCTTTGTGATGTTTGCACTCAACTCACAGAGTTGA

              * ** * **            *  * *      *       *             *       
     225564 ACTTCCCCTCACATAGAGCAGTTGTGCAGCACTCTATTTGTAGTATCTGGAAGTGGACATTTGGA
         66 ACCTTTCTTTTCATAGAGCAGTTTTGAAACACTCTTTTTGTAGAATCTGGAAGTGGATATTTGGA

             *         ** *    * *   *            **  
     225629 GGGCTTTGTAGCCTATC-TGGAAAAAGGAAATATCTTCCCAT
        131 GCGCTTTG-AGGATTTCGTTGGAAACGGAAATATCTTCATAT

            *  ** *   *     * *            *   *  *   * **        *  * * *   
     225670 GAATGCGAGATAGAAGTAATCTCAGAAACATGTTTATGCTGTATCTACTCAACTAACTGTGCTGA
          1 AAAAACTAGACAGAAGCATTCTCAGAAACATCTTTGTGATGTTTGCACTCAACTCACAGAGTTGA

              *     *  *               *        *  * *       *               
     225735 ACATTTCTATTGATAGAGCAGTTTTGAGACACTCTTCTTTTGGAATCTGCAAGTGGATATTTGGA
         66 ACCTTTCTTTTCATAGAGCAGTTTTGAAACACTCTTTTTGTAGAATCTGGAAGTGGATATTTGGA

            ** *                        *     *      
     225800 TAGATTTGAGGATTTCGTTGGAAACGG-GAT-T-AT-ATAT
        131 GCGCTTTGAGGATTTCGTTGGAAACGGAAATATCTTCATAT

                 *       *               *                 **  *   *         
     225837 AAAAAGTAGACAGCAGCATTCTCAGAAACTTCTTTGTGATGTTTGCATCCAGCTCTCAGAGTTGA
          1 AAAAACTAGACAGAAGCATTCTCAGAAACATCTTTGTGATGTTTGCACTCAACTCACAGAGTTGA

              *  * *          *  *        *        *   **        *  **       
     225902 ACATTCCCTTTCATAGAGTAGGTTTGAAACCCTCTTTTTATAGTGTCTGGAAGCGGGCATTTGGA
         66 ACCTTTCTTTTCATAGAGCAGTTTTGAAACACTCTTTTTGTAGAATCTGGAAGTGGATATTTGGA

             *     *   ** ** *  *   *          * *   
     225967 GAGCTTTCAGGCCTATGCTGAAAAAGGAAATATCTACCTAT
        131 GCGCTTTGAGGATTTCGTTGGAAACGGAAATATCTTCATAT

             *                    *    *  **   *      ** *        *    *     
     226008 AGAAACTAGACAGAAGCATTCTGAGAATCACGTTTCTGATGTGGGTACTCAACTAACAGTGTTGA
          1 AAAAACTAGACAGAAGCATTCTCAGAAACATCTTTGTGATGTTTGCACTCAACTCACAGAGTTGA

            *  *       *   *            *   *             *  *               
     226073 TCCATTCTTTTGATACAGCAGTTTTGAACCACACTTTTTGTAGAATGTGCAAGTGGATATTTGGA
         66 ACCTTTCTTTTCATAGAGCAGTTTTGAAACACTCTTTTTGTAGAATCTGGAAGTGGATATTTGGA

            **   *                   * *   *        *
     226138 TAGCTGTGAGGATTTCGTTGGAAACAGGAATGTCTTCATAG
        131 GCGCTTTGAGGATTTCGTTGGAAACGGAAATATCTTCATAT

                **                     * * **         *  **   *   *          
     226179 AAAATTTAGACAGAAGCATTCTCAGAACCTTGATTGTGATGTGTGTTCTCCACTAACAGAGTTGA
          1 AAAAACTAGACAGAAGCATTCTCAGAAACATCTTTGTGATGTTTGCACTCAACTCACAGAGTTGA

                       * *   * *   *       *       *                         
     226244 ACCTTTCTTTTGACAGAACTGTTCTGAAACATTCTTTTTATAGAATCTGGAAGTGGATATTTGGA
         66 ACCTTTCTTTTCATAGAGCAGTTTTGAAACACTCTTTTTGTAGAATCTGGAAGTGGATATTTGGA

            **              *          *          *  
     226309 AAGCTTTGAGGATTTCATTGGAAACGGGAATATCTTCAAAT
        131 GCGCTTTGAGGATTTCGTTGGAAACGGAAATATCTTCATAT

            *   *    *            *           * *       *  *    *            
     226350 CAAATCTAGCCAGAAGCATTCTAAGAAACATCTTAGGGATGTTTACATTCAAGTCACAGAGTTGA
          1 AAAAACTAGACAGAAGCATTCTCAGAAACATCTTTGTGATGTTTGCACTCAACTCACAGAGTTGA

              *  * *     *       *         *    * *   **      *      *    *  
     226415 ACATTCCCTTTCACAGAGCAGGTTTGAAACAATCTTCTCGTACTATCTGGCAGTGGACATTTTGA
         66 ACCTTTCTTTTCATAGAGCAGTTTTGAAACACTCTTTTTGTAGAATCTGGAAGTGGATATTTGGA

               *        ** ** *  *   *            ** *
     226480 GCTACTTTG-GGCCTATGCTGAAAAAGGAAATATCTTCCGAC
        131 GC-GCTTTGAGGATTTCGTTGGAAACGGAAATATCTTCATAT

                                 *     *  **          *           **    *    
     226521 AAAAACTAGACAGAAGCATTCGCAGAATCACGTTTGTGATGTGTGCACTCAACTGTCAGAATTGA
          1 AAAAACTAGACAGAAGCATTCTCAGAAACATCTTTGTGATGTTTGCACTCAACTCACAGAGTTGA

                 **   ** *      *                            * *            *
     226586 ACCTTGGTTTGGACAGAGCACTTTTGAAACACTCTTTTTGTAGAATCTGCAGGTGGATATTTGGC
         66 ACCTTTCTTTTCATAGAGCAGTTTTGAAACACTCTTTTTGTAGAATCTGGAAGTGGATATTTGGA

            **   *          *          *   *      * *
     226651 TAGCTCTGAGGATTTCATTGGAAACGGTAATGTCTTCAAAG
        131 GCGCTTTGAGGATTTCGTTGGAAACGGAAATATCTTCATAT

                *                         *   *            *    *            
     226692 AAAATCTAGACAGAAGCATTCTCAGAAACACCTTCGTGATGTTTGCAATCAAGTCACAGAGTTGA
          1 AAAAACTAGACAGAAGCATTCTCAGAAACATCTTTGTGATGTTTGCACTCAACTCACAGAGTTGA

                 * *             *  *                  *             *       
     226757 ACCTTCCGTTTCATAGAGCAGGTTGGAAACACTCTTTTTGTAGTATCTGGAAGTGGACATTTGGA
         66 ACCTTTCTTTTCATAGAGCAGTTTTGAAACACTCTTTTTGTAGAATCTGGAAGTGGATATTTGGA

                   *   ** ** *  *   *            **  
     226822 GCGCTTTCAGGCCTATGGTGAAAAAGGAAATATCTTCCCAT
        131 GCGCTTTGAGGATTTCGTTGGAAACGGAAATATCTTCATAT

                  * * *      **      *   * *   *     ** ** *      *    *     
     226863 AAAAACGACATAGAAGCTATCTCAGGAACTTGTTTATGATGCATCTAATCAACTAACAGTGTTGA
          1 AAAAACTAGACAGAAGCATTCTCAGAAACATCTTTGTGATGTTTGCACTCAACTCACAGAGTTGA

                  * ** * *                         * *       *               
     226928 ACCTTTGTACTGACAGAGCAG-TTTGAAACACTCTTTTTTTGGAATCTGCAAGTGGATATTTGGA
         66 ACCTTTCTTTTCATAGAGCAGTTTTGAAACACTCTTTTTGTAGAATCTGGAAGTGGATATTTGGA

            *                           *   *   *    
     226992 TCGCTTTGAGGATTTCGTTGGAAACGG-GAT-GC--AATAT
        131 GCGCTTTGAGGATTTCGTTGGAAACGGAAATATCTTCATAT

                **  *    *     *                 **  *   *  * *  *         * 
     227029 AAAACGTACACAGCAGCATACTCAGAAA-ATACTTTGCCATATTTCCATTAAAGTCACAGAGTGG
          1 AAAAACTAGACAGAAGCATTCTCAGAAACAT-CTTTGTGATGTTTGCACTCAACTCACAGAGTTG

               *  * **            *  *               *  *             *      
     227093 AACATTCCCATTCATAGAGCAGGTTGGAAACACTCTTTTTGGAGTATCTGGAAGTGGACATTTGG
         65 AACCTTTCTTTTCATAGAGCAGTTTTGAAACACTCTTTTTGTAGAATCTGGAAGTGGATATTTGG

                    ** * * ** *  *   *            **  
     227158 AGCGCTTTCTGAACTATGGTGAAAAAGGAAATATCTTCCAAT
        130 AGCGCTTTGAGGATTTCGTTGGAAACGGAAATATCTTCATAT

            *     *               *      * *          *  **      **  *  *    
     227200 GAAAACAAGACAGAAGCATTCTGAGAAACTTATTTGTGATGTGTGTCCTCAACAAACGGACTTGA
          1 AAAAACTAGACAGAAGCATTCTCAGAAACATCTTTGTGATGTTTGCACTCAACTCACAGAGTTGA

                   *      **  * *  *  *             *   *    * * *           
     227265 ACCTTTCGTTTCATGCAGTACTTCTGGAACACTCTTTTTGAAGATTCTGCATGCGGATATTTGGA
         66 ACCTTTCTTTTCATAGAGCAGTTTTGAAACACTCTTTTTGTAGAATCTGGAAGTGGATATTTGGA

            **                            **   *   * 
     227330 TAGCTTTGAGGATTTCGTTGGAAACGG---GCT-TACATGT
        131 GCGCTTTGAGGATTTCGTTGGAAACGGAAATATCTTCATAT

                 *       *               *        *   *    *    *       *    
     227367 AAAAATTAGACAGCAGCATTCTCAGAAACTTCTTTGTGGTGTCTGCATTCAAGTCACAGAATTGA
          1 AAAAACTAGACAGAAGCATTCTCAGAAACATCTTTGTGATGTTTGCACTCAACTCACAGAGTTGA

              * ** * **            *  * *      *       *    *        *       
     227432 ACTTCCCCTCACATAGAGCAGTTGTGCAGCACTCTATTTGTAGTATCTCGAAGTGGACATTTGGA
         66 ACCTTTCTTTTCATAGAGCAGTTTTGAAACACTCTTTTTGTAGAATCTGGAAGTGGATATTTGGA

             *         ** *    ***   *            **  
     227497 GGGCTTTGTAGCCTATC-TGCAAAAAGGAAATATCTTCCCAT
        131 GCGCTTTG-AGGATTTCGTTGGAAACGGAAATATCTTCATAT

            *  ** *   *     * *            *   *  *   * **        *  * * *   
     227538 GAATGCGAGATAGAAGTAATCTCAGAAACATGTTTATGCTGTATCTACTCAACTAACTGTGCTGA
          1 AAAAACTAGACAGAAGCATTCTCAGAAACATCTTTGTGATGTTTGCACTCAACTCACAGAGTTGA

              *     *  *               *        *  * *       *               
     227603 ACATTTCTATTGATAGAGCAGTTTTGAGACACTCTTCTTTTGGAATCTGCAAGTGGATATTTGGA
         66 ACCTTTCTTTTCATAGAGCAGTTTTGAAACACTCTTTTTGTAGAATCTGGAAGTGGATATTTGGA

            ** *               *        *     *      
     227668 TAGATTTGAGGATTTCGTTAGAAACGG-GAT-T-AT-ATAT
        131 GCGCTTTGAGGATTTCGTTGGAAACGGAAATATCTTCATAT

                 *       *               *                 **  *   *         
     227705 AAAAAGTAGACAGCAGCATTCTCAGAAACTTCTTTGTGATGTTTGCATCCAGCTCTCAGAGTTGA
          1 AAAAACTAGACAGAAGCATTCTCAGAAACATCTTTGTGATGTTTGCACTCAACTCACAGAGTTGA

              *  * *          *  *        *        *   **        *  **       
     227770 ACATTCCCTTTCATAGAGTAGGTTTGAAACCCTCTTTTTATAGTGTCTGGAAGCGGGCATTTGGA
         66 ACCTTTCTTTTCATAGAGCAGTTTTGAAACACTCTTTTTGTAGAATCTGGAAGTGGATATTTGGA

                   *   ** ** *  *   *          * *   
     227835 GCGCTTTCAGGCCTATGCTGAAAAAGGAAATATCTACCTAT
        131 GCGCTTTGAGGATTTCGTTGGAAACGGAAATATCTTCATAT

             *                    *    *  **          ** *        *    *     
     227876 AGAAACTAGACAGAAGCATTCTGAGAATCACGTTTGTGATGTGGGTACTCAACTAACAGTGTTGA
          1 AAAAACTAGACAGAAGCATTCTCAGAAACATCTTTGTGATGTTTGCACTCAACTCACAGAGTTGA

            *  *       *   *            *   *                *               
     227941 TCCATTCTTTTGATACAGCAGTTTTGAACCACACTTTTTGTAGAATCTGCAAGTGGATATTTGGA
         66 ACCTTTCTTTTCATAGAGCAGTTTTGAAACACTCTTTTTGTAGAATCTGGAAGTGGATATTTGGA

            **   *                     *   *        *
     228006 TAGCTGTGAGGATTTCGTTGGAAACGGGAATGTCTTCATAG
        131 GCGCTTTGAGGATTTCGTTGGAAACGGAAATATCTTCATAT

                **                     * * **         *  **   *   *          
     228047 AAAATTTAGACAGAAGCATTCTCAGAACCTTGATTGTGATGTGTGTTCTCCACTAACAGAGTTGA
          1 AAAAACTAGACAGAAGCATTCTCAGAAACATCTTTGTGATGTTTGCACTCAACTCACAGAGTTGA

                       * *   * *   *       *       *                         
     228112 ACCTTTCTTTTGACAGAACTGTTCTGAAACATTCTTTTTATAGAATCTGGAAGTGGATATTTGGA
         66 ACCTTTCTTTTCATAGAGCAGTTTTGAAACACTCTTTTTGTAGAATCTGGAAGTGGATATTTGGA

            **                         *          *  
     228177 AAGCTTTGAGGATTTCGTTGGAAACGGGAATATCTTCAAAT
        131 GCGCTTTGAGGATTTCGTTGGAAACGGAAATATCTTCATAT

                *    *            *           * *       *  *    *            
     228218 AAAATCTAGCCAGAAGCATTCTAAGAAACATCTTAGGGATGTTTACATTCAAGTCACAGAGTTGA
          1 AAAAACTAGACAGAAGCATTCTCAGAAACATCTTTGTGATGTTTGCACTCAACTCACAGAGTTGA

              *  * *     *       *         *    * *   **      *      *    *  
     228283 ACATTCCCTTTCACAGAGCAGGTTTGAAACAATCTTCTCGTACTATCTGGCAGTGGACATTTTGA
         66 ACCTTTCTTTTCATAGAGCAGTTTTGAAACACTCTTTTTGTAGAATCTGGAAGTGGATATTTGGA

              * *   *  ** ** *  *   *            ** *
     228348 GCTCCTTGGGGCCTATGCTGAAAAAGGAAATATCTTCCGAC
        131 GCGCTTTGAGGATTTCGTTGGAAACGGAAATATCTTCATAT

                                 *     *  **          *           **    *    
     228389 AAAAACTAGACAGAAGCATTCGCAGAATCACGTTTGTGATGTGTGCACTCAACTGTCAGAATTGA
          1 AAAAACTAGACAGAAGCATTCTCAGAAACATCTTTGTGATGTTTGCACTCAACTCACAGAGTTGA

                 **   ** *      *                            * *             
     228454 ACCTTGGTTTGGACAGAGCACTTTTGAAACACTCTTTTTGTAGAATCTGCAGGTGGATATTTGG-
         66 ACCTTTCTTTTCATAGAGCAGTTTTGAAACACTCTTTTTGTAGAATCTGGAAGTGGATATTTGGA

            ***                        *   *      * *
     228518 CTACTTTGAGGATTTCGTTGGAAACGGTAATGTCTTCAAAG
        131 GCGCTTTGAGGATTTCGTTGGAAACGGAAATATCTTCATAT

                *                         *   *            *    *            
     228559 AAAATCTAGACAGAAGCATTCTCAGAAACACCTTCGTGATGTTTGCAATCAAGTCACAGAGTTGA
          1 AAAAACTAGACAGAAGCATTCTCAGAAACATCTTTGTGATGTTTGCACTCAACTCACAGAGTTGA

                 * *             *  *                  *             *       
     228624 ACCTTCCGTTTCATAGAGCAGGTTGGAAACACTCTTTTTGTAGTATCTGGAAGTGGACATTTGGA
         66 ACCTTTCTTTTCATAGAGCAGTTTTGAAACACTCTTTTTGTAGAATCTGGAAGTGGATATTTGGA

             *         ** *    * *   *            **  
     228689 GGGCTTTGTAGCCTATC-TGGAAAAAGGAAATATCTTCCCAT
        131 GCGCTTTG-AGGATTTCGTTGGAAACGGAAATATCTTCATAT

            *  ** *   *     * *            *   *  *   * **        *  * * *   
     228730 GAATGCGAGATAGAAGTAATCTCAGAAACATGTTTATGCTGTATCTACTCAACTAACTGTGCTGA
          1 AAAAACTAGACAGAAGCATTCTCAGAAACATCTTTGTGATGTTTGCACTCAACTCACAGAGTTGA

              *     *  *               *        *  * *       *               
     228795 ACATTTCTATTGATAGAGCAGTTTTGAGACACTCTTCTTTTGGAATCTGCAAGTGGATATTTGGA
         66 ACCTTTCTTTTCATAGAGCAGTTTTGAAACACTCTTTTTGTAGAATCTGGAAGTGGATATTTGGA

            ** *                        *     *      
     228860 TAGATTTGAGGATTTCGTTGGAAACGG-GAT-T-AT-ATAT
        131 GCGCTTTGAGGATTTCGTTGGAAACGGAAATATCTTCATAT

                 *       *               *                 **  *   *         
     228897 AAAAAGTAGACAGCAGCATTCTCAGAAACTTCTTTGTGATGTTTGCATCCAGCTCTCAGAGTTGA
          1 AAAAACTAGACAGAAGCATTCTCAGAAACATCTTTGTGATGTTTGCACTCAACTCACAGAGTTGA

            * *  * *          *  *        *        *   **        *  **       
     228962 GCATTCCCTTTCATAGAGTAGGTTTGAAACCCTCTTTTTATAGTGTCTGGAAGCGGGCATTTGGA
         66 ACCTTTCTTTTCATAGAGCAGTTTTGAAACACTCTTTTTGTAGAATCTGGAAGTGGATATTTGGA

                   *    * * **  **  **          * *  *
     229027 GCGCTTTCAGGTCTATGCTTAAAATAGGAAATATCTACCTAC
        131 GCGCTTTGAGG-ATTTCGTTGGAAACGGAAATATCTTCATAT

             *                    *    *  **          ** *        *    *     
     229069 AGAAACTAGACAGAAGCATTCTGAGAATCACGTTTGTGATGTGGGTACTCAACTAACAGTGTTGA
          1 AAAAACTAGACAGAAGCATTCTCAGAAACATCTTTGTGATGTTTGCACTCAACTCACAGAGTTGA

            *  *       *   *            *   *                *   *           
     229134 TCCATTCTTTTGATACAGCAGTTTTGAACCACACTTTTTGTAGAATCTGCAAGAGGATATTTGGA
         66 ACCTTTCTTTTCATAGAGCAGTTTTGAAACACTCTTTTTGTAGAATCTGGAAGTGGATATTTGGA

            **   *                     *   *      * *
     229199 TAGCTGTGAGGATTTCGTTGGAAACGGGAATGTCTTCAAAG
        131 GCGCTTTGAGGATTTCGTTGGAAACGGAAATATCTTCATAT

                *                   *     *   *            *    *            
     229240 AAAATCTAGACAGAAGCATTCTCAAAAACACCTTCGTGATGTTTGCAATCAAGTCACAGAGTTGA
          1 AAAAACTAGACAGAAGCATTCTCAGAAACATCTTTGTGATGTTTGCACTCAACTCACAGAGTTGA

                 * *             *  *           *      * *           *       
     229305 ACCTTCCGTTTCATAGAGCAGGTTGGAAACACTCTTATTGTAGTACCTGGAAGTGGACATTTGGA
         66 ACCTTTCTTTTCATAGAGCAGTTTTGAAACACTCTTTTTGTAGAATCTGGAAGTGGATATTTGGA

                   *   ** ** *  *   *            **  
     229370 GCGCTTTCAGGCCTATGGTGAAAAAGGAAATATCTTCCCAT
        131 GCGCTTTGAGGATTTCGTTGGAAACGGAAATATCTTCATAT

                  * * *      **      *   * *   *     ** ** *      *    *     
     229411 AAAAACGACATAGAAGCTATCTCAGGAACTTGTTTATGATGCATCTAATCAACTAACAGTGTTGA
          1 AAAAACTAGACAGAAGCATTCTCAGAAACATCTTTGTGATGTTTGCACTCAACTCACAGAGTTGA

                  * ** * *                         * *       *               
     229476 ACCTTTGTACTGACAGAGCAG-TTTGAAACACTCTTTTTTTGGAATCTGCAAGTGGATATTTGGA
         66 ACCTTTCTTTTCATAGAGCAGTTTTGAAACACTCTTTTTGTAGAATCTGGAAGTGGATATTTGGA

            *                           *   *   *    
     229540 TCGCTTTGAGGATTTCGTTGGAAACGG-GAT-GC--AATAT
        131 GCGCTTTGAGGATTTCGTTGGAAACGGAAATATCTTCATAT

                **  *    *     *                 **  *   *  *    *         * 
     229577 AAAACGTACACAGCAGCATACTCAGAAA-ATACTTTGCCATATTTCCATTCAAGTCACAGAGTGG
          1 AAAAACTAGACAGAAGCATTCTCAGAAACAT-CTTTGTGATGTTTGCACTCAACTCACAGAGTTG

               *  * **            *                  *  *             *      
     229641 AACATTCCCATTCATAGAGCAGGTTTGAAACACTCTTTTTGGAGTATCTGGAAGTGGACATTTGG
         65 AACCTTTCTTTTCATAGAGCAGTTTTGAAACACTCTTTTTGTAGAATCTGGAAGTGGATATTTGG

                    ** * * ** *  *   *            **  
     229706 AGCGCTTTCTGAACTATGGTGAAAAAGGAAATATCTTCCAAT
        130 AGCGCTTTGAGGATTTCGTTGGAAACGGAAATATCTTCATAT

            *     *               *      * *          *  **      **  *  *    
     229748 GAAAACAAGACAGAAGCATTCTGAGAAACTTATTTGTGATGTGTGTCCTCAACAAACGGACTTGA
          1 AAAAACTAGACAGAAGCATTCTCAGAAACATCTTTGTGATGTTTGCACTCAACTCACAGAGTTGA

                   *      **  * *  *  *             *         * * *          
     229813 ACCTTTCGTTTCATGCAGTACTTCTGGAACACTCTTTTTGAAGATAT-TGCATGCGGATATTTGG
         66 ACCTTTCTTTTCATAGAGCAGTTTTGAAACACTCTTTTTGTAGA-ATCTGGAAGTGGATATTTGG

             **                            **   *   * 
     229877 ATAGCTTTGAGGATTTCGTTGGAAACGG---GCT-TACATGT
        130 AGCGCTTTGAGGATTTCGTTGGAAACGGAAATATCTTCATAT

                 *       *               *        *   *    *    *       *    
     229915 AAAAATTAGACAGCAGCATTCTCAGAAACTTCTTTGTGGTGTCTGCATTCAAGTCACAGAATTGA
          1 AAAAACTAGACAGAAGCATTCTCAGAAACATCTTTGTGATGTTTGCACTCAACTCACAGAGTTGA

              * ** * **            *  * *      *       *             *       
     229980 ACTTCCCCTCACATAGAGCAGTTGTGCAGCACTCTATTTGTAGTATCTGGAAGTGGACATTTGGA
         66 ACCTTTCTTTTCATAGAGCAGTTTTGAAACACTCTTTTTGTAGAATCTGGAAGTGGATATTTGGA

             *         ** *    * *   *            **  
     230045 GGGCTTTGTAGCCTATC-TGGAAAAAGGAAATATCTTCCCAT
        131 GCGCTTTG-AGGATTTCGTTGGAAACGGAAATATCTTCATAT

            *  ** *   *     * *            *   *  *   * **        *  * * *   
     230086 GAATGCGAGATAGAAGTAATCTCAGAAACATGTTTATGCTGTATCTACTCAACTAACTGTGCTGA
          1 AAAAACTAGACAGAAGCATTCTCAGAAACATCTTTGTGATGTTTGCACTCAACTCACAGAGTTGA

              *     *  *               *        *  * *       *               
     230151 ACATTTCTATTGATAGAGCAGTTTTGAGACACTCTTCTTTTGGAATCTGCAAGTGGATATTTGGA
         66 ACCTTTCTTTTCATAGAGCAGTTTTGAAACACTCTTTTTGTAGAATCTGGAAGTGGATATTTGGA

            ** *                        *     *      
     230216 TAGATTTGAGGATTTCGTTGGAAACGG-GAT-T-AT-ATAT
        131 GCGCTTTGAGGATTTCGTTGGAAACGGAAATATCTTCATAT

                 *       *               *                 **  *   *         
     230253 AAAAAGTAGACAGCAGCATTCTCAGAAACTTCTTTGTGATGTTTGCATCCAGCTCTCAGAGTTGA
          1 AAAAACTAGACAGAAGCATTCTCAGAAACATCTTTGTGATGTTTGCACTCAACTCACAGAGTTGA

              *  * *          *  *        *        *   **        *  **       
     230318 ACATTCCCTTTCATAGAGTAGGTTTGAAACCCTCTTTTTATAGTGTCTGGAAGCGGGCATTTGGA
         66 ACCTTTCTTTTCATAGAGCAGTTTTGAAACACTCTTTTTGTAGAATCTGGAAGTGGATATTTGGA

                   *    * * **  **  **          * *  *
     230383 GCGCTTTCAGGCCTATGCTTAAAATAGGAAATATCTACCTAC
        131 GCGCTTTGAGG-ATTTCGTTGGAAACGGAAATATCTTCATAT

             *                    *    *  **          ** *        *    *     
     230425 AGAAACTAGACAGAAGCATTCTGAGAATCACGTTTGTGATGTGGGTACTCAACTAACAGTGTTGA
          1 AAAAACTAGACAGAAGCATTCTCAGAAACATCTTTGTGATGTTTGCACTCAACTCACAGAGTTGA

            *  *           *            *   *                *   *           
     230490 TCCATTCTTTTCATACAGCAGTTTTGAACCACACTTTTTGTAGAATCTGCAAGAGGATATTTGGA
         66 ACCTTTCTTTTCATAGAGCAGTTTTGAAACACTCTTTTTGTAGAATCTGGAAGTGGATATTTGGA

            **   *                     *   *      * *
     230555 TAGCTGTGAGGATTTCGTTGGAAACGGGAATGTCTTCAAAG
        131 GCGCTTTGAGGATTTCGTTGGAAACGGAAATATCTTCATAT

                *                         *   *            *    *            
     230596 AAAATCTAGACAGAAGCATTCTCAGAAACACCTTCGTGATGTTTGCAATCAAGTCACAGAGTTGA
          1 AAAAACTAGACAGAAGCATTCTCAGAAACATCTTTGTGATGTTTGCACTCAACTCACAGAGTTGA

                 * *             *  *           *      *             *       
     230661 ACCTTCCGTTTCATAGAGCAGGTTGGAAACACTCTTATTGTAGTATCTGGAAGTGGACATTTGGA
         66 ACCTTTCTTTTCATAGAGCAGTTTTGAAACACTCTTTTTGTAGAATCTGGAAGTGGATATTTGGA

                   *   ** ** *  *   *            **  
     230726 GCGCTTTCAGGCCTATGGTGAAAAAGGAAATATCTTCCCAT
        131 GCGCTTTGAGGATTTCGTTGGAAACGGAAATATCTTCATAT

                  * * *      **      *   * *   *     ** ** *      *    *     
     230767 AAAAACGACATAGAAGCTATCTCAGGAACTTGTTTATGATGCATCTAATCAACTAACAGTGTTGA
          1 AAAAACTAGACAGAAGCATTCTCAGAAACATCTTTGTGATGTTTGCACTCAACTCACAGAGTTGA

                  * ** * *                         * *       *               
     230832 ACCTTTGTACTGACAGAGCAG-TTTGAAACACTCTTTTTTTGGAATCTGCAAGTGGATATTTGGA
         66 ACCTTTCTTTTCATAGAGCAGTTTTGAAACACTCTTTTTGTAGAATCTGGAAGTGGATATTTGGA

            *                           *   *   *    
     230896 TCGCTTTGAGGATTTCGTTGGAAACGG-GAT-GC--AATAT
        131 GCGCTTTGAGGATTTCGTTGGAAACGGAAATATCTTCATAT

                **  *    *     *                 **  *   *  *    *         * 
     230933 AAAACGTACACAGCAGCATACTCAGAAA-ATACTTTGCCATATTTCCATTCAAGTCACAGAGTGG
          1 AAAAACTAGACAGAAGCATTCTCAGAAACAT-CTTTGTGATGTTTGCACTCAACTCACAGAGTTG

               *  * **            *                  *  *             *      
     230997 AACATTCCCATTCATAGAGCAGGTTTGAAACACTCTTTTTGGAGTATCTGGAAGTGGACATTTGG
         65 AACCTTTCTTTTCATAGAGCAGTTTTGAAACACTCTTTTTGTAGAATCTGGAAGTGGATATTTGG

                    ** * * ** *  *   *            **  
     231062 AGCGCTTTCTGAACTATGGTGAAAAAGGAAATATCTTCCAAT
        130 AGCGCTTTGAGGATTTCGTTGGAAACGGAAATATCTTCATAT

            *     *               *      * *          *  **      **  *  *    
     231104 GAAAACAAGACAGAAGCATTCTGAGAAACTTATTTGTGATGTGTGTCCTCAACAAACGGACTTGA
          1 AAAAACTAGACAGAAGCATTCTCAGAAACATCTTTGTGATGTTTGCACTCAACTCACAGAGTTGA

                   *      **  * *  *  *             *   *    * * *    *    * 
     231169 ACCTTTCGTTTCATGCAGTACTTCTGGAACACTCTTTTTGAAGATTCTGCATGCGGATGTTTGCA
         66 ACCTTTCTTTTCATAGAGCAGTTTTGAAACACTCTTTTTGTAGAATCTGGAAGTGGATATTTGGA

            **                            **   *     
     231234 TAGCTTTGAGGATTTCGTTGGAAACGG---GCT-TACATAT
        131 GCGCTTTGAGGATTTCGTTGGAAACGGAAATATCTTCATAT

                 *       *  *            *        *   *    *    *       *    
     231271 AAAAATTAGACAGCAGAATTCTCAGAAACTTCTTTGTGGTGTCTGCATTCAAGTCACAGAATTGA
          1 AAAAACTAGACAGAAGCATTCTCAGAAACATCTTTGTGATGTTTGCACTCAACTCACAGAGTTGA

              * ** * **            *  * * *    *       *             *       
     231336 ACTTCCCCTCACATAGAGCAGTTGTGCAGCTCTCTATTTGTAGTATCTGGAAGTGGACATTTGGA
         66 ACCTTTCTTTTCATAGAGCAGTTTTGAAACACTCTTTTTGTAGAATCTGGAAGTGGATATTTGGA

             *         ** *    * *   *            **  
     231401 GGGCTTTGTAGCCTATC-TGGAAAAAGGAAATATCTTCCCAT
        131 GCGCTTTG-AGGATTTCGTTGGAAACGGAAATATCTTCATAT

            *  ** *   *     * *            *   *  *   * **        *  * * *   
     231442 GAATGCGAGATAGAAGTAATCTCAGAAACATGTTTATGCTGTATCTACTCAACTAACTGTGCTGA
          1 AAAAACTAGACAGAAGCATTCTCAGAAACATCTTTGTGATGTTTGCACTCAACTCACAGAGTTGA

              *     *  *               *        *  * *       *               
     231507 ACATTTCTATTGATAGAGCAGTTTTGAGACACTCTTCTTTTGGAATCTGCAAGTGGATATTTGGA
         66 ACCTTTCTTTTCATAGAGCAGTTTTGAAACACTCTTTTTGTAGAATCTGGAAGTGGATATTTGGA

            ** *                        *     *      
     231572 TAGATTTGAGGATTTCGTTGGAAACGG-GAT-T-AT-ATAT
        131 GCGCTTTGAGGATTTCGTTGGAAACGGAAATATCTTCATAT

                 *       *               *                 **  *   *         
     231609 AAAAAGTAGACAGCAGCATTCTCAGAAACTTCTTTGTGATGTTTGCATCCAGCTCTCAGAGTTGA
          1 AAAAACTAGACAGAAGCATTCTCAGAAACATCTTTGTGATGTTTGCACTCAACTCACAGAGTTGA

              *  * *          *  *        *        *   **        *  **       
     231674 ACATTCCCTTTCATAGAGTAGGTTTGAAACCCTCTTTTTATAGTGTCTGGAAGCGGGCATTTGGA
         66 ACCTTTCTTTTCATAGAGCAGTTTTGAAACACTCTTTTTGTAGAATCTGGAAGTGGATATTTGGA

                   *    * * **  **  **          * *  *
     231739 GCGCTTTCAGGCCTATGCTTAAAATAGGAAATATCTACCTAC
        131 GCGCTTTGAGG-ATTTCGTTGGAAACGGAAATATCTTCATAT

             *                    *    *  **          ** *        *    *     
     231781 AGAAACTAGACAGAAGCATTCTGAGAATCACGTTTGTGATGTGGGTACTCAACTAACAGTGTTGA
          1 AAAAACTAGACAGAAGCATTCTCAGAAACATCTTTGTGATGTTTGCACTCAACTCACAGAGTTGA

            *  *       *   *            *   *                *   *           
     231846 TCCATTCTTTTGATACAGCAGTTTTGAACCACACTTTTTGTAGAATCTGCAAGAGGATATTTGGA
         66 ACCTTTCTTTTCATAGAGCAGTTTTGAAACACTCTTTTTGTAGAATCTGGAAGTGGATATTTGGA

            **   *                     *   *      * *
     231911 TAGCTGTGAGGATTTCGTTGGAAACGGGAATGTCTTCAAAG
        131 GCGCTTTGAGGATTTCGTTGGAAACGGAAATATCTTCATAT

                *                         *   *            *    *            
     231952 AAAATCTAGACAGAAGCATTCTCAGAAACACCTTCGTGATGTTTGCAATCAAGTCACAGAGTTGA
          1 AAAAACTAGACAGAAGCATTCTCAGAAACATCTTTGTGATGTTTGCACTCAACTCACAGAGTTGA

                 * *             *  *           *      *             *       
     232017 ACCTTCCGTTTCATAGAGCAGGTTGGAAACACTCTTATTGTAGTATCTGGAAGTGGACATTTGGA
         66 ACCTTTCTTTTCATAGAGCAGTTTTGAAACACTCTTTTTGTAGAATCTGGAAGTGGATATTTGGA

                   *   ** ** *  *   *            **  
     232082 GCGCTTTCAGGCCTATGGTGAAAAAGGAAATATCTTCCCAT
        131 GCGCTTTGAGGATTTCGTTGGAAACGGAAATATCTTCATAT

                  * * *      **      *   * *   *     ** ** *      *    *     
     232123 AAAAACGACATAGAAGCTATCTCAGGAACTTGTTTATGATGCATCTAATCAACTAACAGTGTTGA
          1 AAAAACTAGACAGAAGCATTCTCAGAAACATCTTTGTGATGTTTGCACTCAACTCACAGAGTTGA

                  * ** * *                         * *       *               
     232188 ACCTTTGTACTGACAGAGCAG-TTTGAAACACTCTTTTTTTGGAATCTGCAAGTGGATATTTGGA
         66 ACCTTTCTTTTCATAGAGCAGTTTTGAAACACTCTTTTTGTAGAATCTGGAAGTGGATATTTGGA

            *                           *   *   *    
     232252 TCGCTTTGAGGATTTCGTTGGAAACGG-GAT-GC--AATAT
        131 GCGCTTTGAGGATTTCGTTGGAAACGGAAATATCTTCATAT

                **  *    *     *                 **  *   *  *    *         * 
     232289 AAAACGTACACAGCAGCATACTCAGAAA-ATACTTTGCCATATTTCCATTCAAGTCACAGAGTGG
          1 AAAAACTAGACAGAAGCATTCTCAGAAACAT-CTTTGTGATGTTTGCACTCAACTCACAGAGTTG

               *  * **            *  *               *  *             *      
     232353 AACATTCCCATTCATAGAGCAGGTTGGAAACACTCTTTTTGGAGTATCTGGAAGTGGACATTTGG
         65 AACCTTTCTTTTCATAGAGCAGTTTTGAAACACTCTTTTTGTAGAATCTGGAAGTGGATATTTGG

                    ** * * ** *  *   *            **  
     232418 AGCGCTTTCTGAACTATGGTGAAAAAGGAAATATCTTCCAAT
        130 AGCGCTTTGAGGATTTCGTTGGAAACGGAAATATCTTCATAT

            *     *               *      * *          *  **      **  *  *    
     232460 GAAAACAAGACAGAAGCATTCTGAGAAACTTATTTGTGATGTGTGTCCTCAACAAACGGACTTGA
          1 AAAAACTAGACAGAAGCATTCTCAGAAACATCTTTGTGATGTTTGCACTCAACTCACAGAGTTGA

                   *      **  * *  *  *             *   *    * * *           
     232525 ACCTTTCGTTTCATGCAGTACTTCTGGAACACTCTTTTTGAAGATTCTGCATGCGGATATTTGGA
         66 ACCTTTCTTTTCATAGAGCAGTTTTGAAACACTCTTTTTGTAGAATCTGGAAGTGGATATTTGGA

            **                            **   *   * 
     232590 TAGCTTTGAGGATTTCGTTGGAAACGG---GCT-TACATGT
        131 GCGCTTTGAGGATTTCGTTGGAAACGGAAATATCTTCATAT

                 *       *  *            *        *   *    *    *       *    
     232627 AAAAATTAGACAGCAGAATTCTCAGAAACTTCTTTGTGGTGTCTGCATTCAAGTCACAGAATTGA
          1 AAAAACTAGACAGAAGCATTCTCAGAAACATCTTTGTGATGTTTGCACTCAACTCACAGAGTTGA

              * ** * **            *  * *      *       *             *       
     232692 ACTTCCCCTCACATAGAGCAGTTGTGCAGCACTCTATTTGTAGTATCTGGAAGTGGACATTTGGA
         66 ACCTTTCTTTTCATAGAGCAGTTTTGAAACACTCTTTTTGTAGAATCTGGAAGTGGATATTTGGA

             *         ** *    * *   *            **  
     232757 GGGCTTTGTAGCCTATC-TGGAAAAAGGAAATATCTTCCCAT
        131 GCGCTTTG-AGGATTTCGTTGGAAACGGAAATATCTTCATAT

            *  ** *   *     * *            *   *  *   * **        *  * * *   
     232798 GAATGCGAGATAGAAGTAATCTCAGAAACATGTTTATGCTGTATCTACTCAACTAACTGTGCTGA
          1 AAAAACTAGACAGAAGCATTCTCAGAAACATCTTTGTGATGTTTGCACTCAACTCACAGAGTTGA

              *     *  *               *        *  * *       *               
     232863 ACATTTCTATTGATAGAGCAGTTTTGAGACACTCTTCTTTTGGAATCTGCAAGTGGATATTTGGA
         66 ACCTTTCTTTTCATAGAGCAGTTTTGAAACACTCTTTTTGTAGAATCTGGAAGTGGATATTTGGA

            ** *                        *     *      
     232928 TAGATTTGAGGATTTCGTTGGAAACGG-GAT-T-AT-ATAT
        131 GCGCTTTGAGGATTTCGTTGGAAACGGAAATATCTTCATAT

                 *       *               *                 **  *   *         
     232965 AAAAAGTAGACAGCAGCATTCTCAGAAACTTCTTTGTGATGTTTGCATCCAGCTCTCAGAGTTGA
          1 AAAAACTAGACAGAAGCATTCTCAGAAACATCTTTGTGATGTTTGCACTCAACTCACAGAGTTGA

              *  * *          *  *        *        *   **        *  **       
     233030 ACATTCCCTTTCATAGAGTAGGTTTGAAACCCTCTTTTTATAGTGTCTGGAAGCGGGCATTTGGA
         66 ACCTTTCTTTTCATAGAGCAGTTTTGAAACACTCTTTTTGTAGAATCTGGAAGTGGATATTTGGA

                   *    * * **  **  **          * *  *
     233095 GCGCTTTCAGGTCTATGCTTAAAATAGGAAATATCTACCTAC
        131 GCGCTTTGAGG-ATTTCGTTGGAAACGGAAATATCTTCATAT

             *                    *    *  **          ** *        *    *     
     233137 AGAAACTAGACAGAAGCATTCTGAGAATCACGTTTGTGATGTGGGTACTCAACTAACAGTGTTGA
          1 AAAAACTAGACAGAAGCATTCTCAGAAACATCTTTGTGATGTTTGCACTCAACTCACAGAGTTGA

            *  *       *   *            *   *                *   *           
     233202 TCCATTCTTTTGATACAGCAGTTTTGAACCACACTTTTTGTAGAATCTGCAAGAGGATATTTGGA
         66 ACCTTTCTTTTCATAGAGCAGTTTTGAAACACTCTTTTTGTAGAATCTGGAAGTGGATATTTGGA

            **   *                     *   *      * *
     233267 TAGCTGTGAGGATTTCGTTGGAAACGGGAATGTCTTCAAAG
        131 GCGCTTTGAGGATTTCGTTGGAAACGGAAATATCTTCATAT

                *                         *   *            *    *            
     233308 AAAATCTAGACAGAAGCATTCTCAGAAACACCTTCGTGATGTTTGCAATCAAGTCACAGAGTTGA
          1 AAAAACTAGACAGAAGCATTCTCAGAAACATCTTTGTGATGTTTGCACTCAACTCACAGAGTTGA

                 * *             *  *           *      *             *       
     233373 ACCTTCCGTTTCATAGAGCAGGTTGGAAACACTCTTATTGTAGTATCTGGAAGTGGACATTTGGA
         66 ACCTTTCTTTTCATAGAGCAGTTTTGAAACACTCTTTTTGTAGAATCTGGAAGTGGATATTTGGA

                   *   ** ** *  *   *            **  
     233438 GCGCTTTCAGGCCTATGGTGAAAAAGGAAATATCTTCCCAT
        131 GCGCTTTGAGGATTTCGTTGGAAACGGAAATATCTTCATAT

                  * * *      **      *   * *   *     ** ** *      *    *     
     233479 AAAAACGACATAGAAGCTATCTCAGGAACTTGTTTATGATGCATCTAATCAACTAACAGTGTTGA
          1 AAAAACTAGACAGAAGCATTCTCAGAAACATCTTTGTGATGTTTGCACTCAACTCACAGAGTTGA

                  * ** * *                         * *       *               
     233544 ACCTTTGTACTGACAGAGCAG-TTTGAAACACTCTTTTTTTGGAATCTGCAAGTGGATATTTGGA
         66 ACCTTTCTTTTCATAGAGCAGTTTTGAAACACTCTTTTTGTAGAATCTGGAAGTGGATATTTGGA

            *                           *   *   *    
     233608 TCGCTTTGAGGATTTCGTTGGAAACGG-GAT-GC--AATAT
        131 GCGCTTTGAGGATTTCGTTGGAAACGGAAATATCTTCATAT

                **  *    *     *                 **  *   *  *    *         * 
     233645 AAAACGTACACAGCAGCATACTCAGAAA-ATACTTTGCCATATTTCCATTCAAGTCACAGAGTGG
          1 AAAAACTAGACAGAAGCATTCTCAGAAACAT-CTTTGTGATGTTTGCACTCAACTCACAGAGTTG

               *  * **            *  *               *  *             *      
     233709 AACATTCCCATTCATAGAGCAGGTTGGAAACACTCTTTTTGGAGTATCTGGAAGTGGACATTTGG
         65 AACCTTTCTTTTCATAGAGCAGTTTTGAAACACTCTTTTTGTAGAATCTGGAAGTGGATATTTGG

                    ** * * ** *  *   *            **  
     233774 AGCGCTTTCTGAACTATGGTGAAAAAGGAAATATCTTCCAAT
        130 AGCGCTTTGAGGATTTCGTTGGAAACGGAAATATCTTCATAT

            *     *               *      * *          *  **      **  *  *    
     233816 GAAAACAAGACAGAAGCATTCTGAGAAACTTATTTGTGATGTGTGTCCTCAACAAACGGACTTGA
          1 AAAAACTAGACAGAAGCATTCTCAGAAACATCTTTGTGATGTTTGCACTCAACTCACAGAGTTGA

                   *      **  * *  *  *             *         * * *          
     233881 ACCTTTCGTTTCATGCAGTACTTCTGGAACACTCTTTTTGAAGATAT-TGCATGCGGATATTTGG
         66 ACCTTTCTTTTCATAGAGCAGTTTTGAAACACTCTTTTTGTAGA-ATCTGGAAGTGGATATTTGG

             **                            **   *   * 
     233945 ATAGCTTTGAGGATTTCGTTGGAAACGG---GCT-TACATGT
        130 AGCGCTTTGAGGATTTCGTTGGAAACGGAAATATCTTCATAT

                 *       *               *        *   *    *    *       *    
     233983 AAAAATTAGACAGCAGCATTCTCAGAAACTTCTTTGTGGTGTCTGCATTCAAGTCACAGAATTGA
          1 AAAAACTAGACAGAAGCATTCTCAGAAACATCTTTGTGATGTTTGCACTCAACTCACAGAGTTGA

              * ** * **            *  * *      *       *             *       
     234048 ACTTCCCCTCACATAGAGCAGTTGTGCAGCACTCTATTTGTAGTATCTGGAAGTGGACATTTGGA
         66 ACCTTTCTTTTCATAGAGCAGTTTTGAAACACTCTTTTTGTAGAATCTGGAAGTGGATATTTGGA

             *         ** *    * *   *            **  
     234113 GGGCTTTGTAGCCTATC-TGGAAAAAGGAAATATCTTCCCAT
        131 GCGCTTTG-AGGATTTCGTTGGAAACGGAAATATCTTCATAT

            *  ** *   *     * *            *   *  *   * **        *  * * *   
     234154 GAATGCGAGATAGAAGTAATCTCAGAAACATGTTTATGCTGTATCTACTCAACTAACTGTGCTGA
          1 AAAAACTAGACAGAAGCATTCTCAGAAACATCTTTGTGATGTTTGCACTCAACTCACAGAGTTGA

              *     *  *               *        *  * *       *               
     234219 ACATTTCTATTGATAGAGCAGTTTTGAGACACTCTTCTTTTGGAATCTGCAAGTGGATATTTGGA
         66 ACCTTTCTTTTCATAGAGCAGTTTTGAAACACTCTTTTTGTAGAATCTGGAAGTGGATATTTGGA

            ** *                        *     *      
     234284 TAGATTTGAGGATTTCGTTGGAAACGG-GAT-T-AT-ATAT
        131 GCGCTTTGAGGATTTCGTTGGAAACGGAAATATCTTCATAT

                 *       *               *                 **  *   *         
     234321 AAAAAGTAGACAGCAGCATTCTCAGAAACTTCTTTGTGATGTTTGCATCCAGCTCTCAGAGTTGA
          1 AAAAACTAGACAGAAGCATTCTCAGAAACATCTTTGTGATGTTTGCACTCAACTCACAGAGTTGA

              *  * *          *  *        *        *   **        *  **       
     234386 ACATTCCCTTTCATAGAGTAGGTTTGAAACCCTCTTTTTATAGTGTCTGGAAGCGGGCATTTGGA
         66 ACCTTTCTTTTCATAGAGCAGTTTTGAAACACTCTTTTTGTAGAATCTGGAAGTGGATATTTGGA

                   *    * * **  **  **          * *  *
     234451 GCGCTTTCAGGCCTATGCTTAAAATAGGAAATATCTACCTAC
        131 GCGCTTTGAGG-ATTTCGTTGGAAACGGAAATATCTTCATAT

             *                    *    *  **          ** *        *    *     
     234493 AGAAACTAGACAGAAGCATTCTGAGAATCACGTTTGTGATGTGGGTACTCAACTAACAGTGTTGA
          1 AAAAACTAGACAGAAGCATTCTCAGAAACATCTTTGTGATGTTTGCACTCAACTCACAGAGTTGA

            *  *           *            *   *                *   *           
     234558 TCCATTCTTTTCATACAGCAGTTTTGAACCACACTTTTTGTAGAATCTGCAAGAGGATATTTGGA
         66 ACCTTTCTTTTCATAGAGCAGTTTTGAAACACTCTTTTTGTAGAATCTGGAAGTGGATATTTGGA

            **   *                     *   *      * *
     234623 TAGCTGTGAGGATTTCGTTGGAAACGGGAATGTCTTCAAAG
        131 GCGCTTTGAGGATTTCGTTGGAAACGGAAATATCTTCATAT

                *                         *   *            *    *            
     234664 AAAATCTAGACAGAAGCATTCTCAGAAACACCTTCGTGATGTTTGCAATCAAGTCACAGAGTTGA
          1 AAAAACTAGACAGAAGCATTCTCAGAAACATCTTTGTGATGTTTGCACTCAACTCACAGAGTTGA

                 * *             *  *           *      *             *       
     234729 ACCTTCCGTTTCATAGAGCAGGTTGGAAACACTCTTATTGTAGTATCTGGAAGTGGACATTTGGA
         66 ACCTTTCTTTTCATAGAGCAGTTTTGAAACACTCTTTTTGTAGAATCTGGAAGTGGATATTTGGA

                   *   ** ** *  *   *            **  
     234794 GCGCTTTCAGGCCTATGGTGAAAAAGGAAATATCTTCCCAT
        131 GCGCTTTGAGGATTTCGTTGGAAACGGAAATATCTTCATAT

                  * * *      **      *   * *   *     ** ** *      *    *     
     234835 AAAAACGACATAGAAGCTATCTCAGGAACTTGTTTATGATGCATCTAATCAACTAACAGTGTTGA
          1 AAAAACTAGACAGAAGCATTCTCAGAAACATCTTTGTGATGTTTGCACTCAACTCACAGAGTTGA

                  * ** * *                         * *       *               
     234900 ACCTTTGTACTGACAGAGCAG-TTTGAAACACTCTTTTTTTGGAATCTGCAAGTGGATATTTGGA
         66 ACCTTTCTTTTCATAGAGCAGTTTTGAAACACTCTTTTTGTAGAATCTGGAAGTGGATATTTGGA

            *                           *   *   *    
     234964 TCGCTTTGAGGATTTCGTTGGAAACGG-GAT-GC--AATAT
        131 GCGCTTTGAGGATTTCGTTGGAAACGGAAATATCTTCATAT

                **  *    *     *                 **  *   *  *    *         * 
     235001 AAAACGTACACAGCAGCATACTCAGAAA-ATACTTTGCCATATTTCCATTCAAGTCACAGAGTGG
          1 AAAAACTAGACAGAAGCATTCTCAGAAACAT-CTTTGTGATGTTTGCACTCAACTCACAGAGTTG

               *  * **            *                  *  *             *      
     235065 AACATTCCCATTCATAGAGCAGGTTTGAAACACTCTTTTTGGAGTATCTGGAAGTGGACATTTGG
         65 AACCTTTCTTTTCATAGAGCAGTTTTGAAACACTCTTTTTGTAGAATCTGGAAGTGGATATTTGG

                    ** * * ** *  *   *            **  
     235130 AGCGCTTTCTGAACTATGGTGAAAAAGGAAATATCTTCCAAT
        130 AGCGCTTTGAGGATTTCGTTGGAAACGGAAATATCTTCATAT

            *     *               *      * *          *  **      **  *  *    
     235172 GAAAACAAGACAGAAGCATTCTGAGAAACTTATTTGTGATGTGTGTCCTCAACAAACGGACTTGA
          1 AAAAACTAGACAGAAGCATTCTCAGAAACATCTTTGTGATGTTTGCACTCAACTCACAGAGTTGA

                   *      **  * *  *  *             *   *    * * *         * 
     235237 ACCTTTCGTTTCATGCAGTACTTCTGGAACACTCTTTTTGAAGATTCTGCATGCGGATATTTGCA
         66 ACCTTTCTTTTCATAGAGCAGTTTTGAAACACTCTTTTTGTAGAATCTGGAAGTGGATATTTGGA

            **                            **   *   * 
     235302 TAGCTTTGAGGATTTCGTTGGAAACGG---GCT-TACATGT
        131 GCGCTTTGAGGATTTCGTTGGAAACGGAAATATCTTCATAT

                 *       *  *            *        *   *    *    *       *    
     235339 AAAAATTAGACAGCAGAATTCTCAGAAACTTCTTTGTGGTGTCTGCATTCAAGTCACAGAATTGA
          1 AAAAACTAGACAGAAGCATTCTCAGAAACATCTTTGTGATGTTTGCACTCAACTCACAGAGTTGA

              * ** * **            *  * *      *       *             *       
     235404 ACTTCCCCTCACATAGAGCAGTTGTGCAGCACTCTATTTGTAGTATCTGGAAGTGGACATTTGGA
         66 ACCTTTCTTTTCATAGAGCAGTTTTGAAACACTCTTTTTGTAGAATCTGGAAGTGGATATTTGGA

             *         ** *    * *   *            **  
     235469 GGGCTTTGTAGCCTATC-TGGAAAAAGGAAATATCTTCCCAT
        131 GCGCTTTG-AGGATTTCGTTGGAAACGGAAATATCTTCATAT

            *  ** *   *     * *            *   *  *   * **        *  * * *   
     235510 GAATGCGAGATAGAAGTAATCTCAGAAACATGTTTATGCTGTATCTACTCAACTAACTGTGCTGA
          1 AAAAACTAGACAGAAGCATTCTCAGAAACATCTTTGTGATGTTTGCACTCAACTCACAGAGTTGA

              *     *  *               *        *  * *       *               
     235575 ACATTTCTATTGATAGAGCAGTTTTGAGACACTCTTCTTTTGGAATCTGCAAGTGGATATTTGGA
         66 ACCTTTCTTTTCATAGAGCAGTTTTGAAACACTCTTTTTGTAGAATCTGGAAGTGGATATTTGGA

            ** *           *            *     *      
     235640 TAGATTTGAGGATTTTGTTGGAAACGG-GAT-T-AT-ATAT
        131 GCGCTTTGAGGATTTCGTTGGAAACGGAAATATCTTCATAT

                 *       *               *                 **  *   *         
     235677 AAAAAGTAGACAGCAGCATTCTCAGAAACTTCTTTGTGATGTTTGCATCCAGCTCTCAGAGTTGA
          1 AAAAACTAGACAGAAGCATTCTCAGAAACATCTTTGTGATGTTTGCACTCAACTCACAGAGTTGA

              *  * *          *  *        *        *   **        *  **       
     235742 ACATTCCCTTTCATAGAGTAGGTTTGAAACCCTCTTTTTATAGTGTCTGGAAGCGGGCATTTGGA
         66 ACCTTTCTTTTCATAGAGCAGTTTTGAAACACTCTTTTTGTAGAATCTGGAAGTGGATATTTGGA

                   *    * * **  **  **          * *  *
     235807 GCGCTTTCAGGTCTATGCTTAAAATAGGAAATATCTACCTAC
        131 GCGCTTTGAGG-ATTTCGTTGGAAACGGAAATATCTTCATAT

             *                    *    *  **          ** *        *    *     
     235849 AGAAACTAGACAGAAGCATTCTGAGAATCACGTTTGTGATGTGGGTACTCAACTAACAGTGTTGA
          1 AAAAACTAGACAGAAGCATTCTCAGAAACATCTTTGTGATGTTTGCACTCAACTCACAGAGTTGA

            *  *       *   *            *   *                *   *           
     235914 TCCATTCTTTTGATACAGCAGTTTTGAACCACACTTTTTGTAGAATCTGCAAGAGGATATTTGGA
         66 ACCTTTCTTTTCATAGAGCAGTTTTGAAACACTCTTTTTGTAGAATCTGGAAGTGGATATTTGGA

            **   *                     *   *      * *
     235979 TAGCTGTGAGGATTTCGTTGGAAACGGGAATGTCTTCAAAG
        131 GCGCTTTGAGGATTTCGTTGGAAACGGAAATATCTTCATAT

                *                         *   *            *    *            
     236020 AAAATCTAGACAGAAGCATTCTCAGAAACACCTTCGTGATGTTTGCAATCAAGTCACAGAGTTGA
          1 AAAAACTAGACAGAAGCATTCTCAGAAACATCTTTGTGATGTTTGCACTCAACTCACAGAGTTGA

                 * *             *  *           *      *             *       
     236085 ACCTTCCGTTTCATAGAGCAGGTTGGAAACACTCTTATTGTAGTATCTGGAAGTGGACATTTGGA
         66 ACCTTTCTTTTCATAGAGCAGTTTTGAAACACTCTTTTTGTAGAATCTGGAAGTGGATATTTGGA

                   *   ** ** *  *   *            **  
     236150 GCGCTTTCAGGCCTATGGTGAAAAAGGAAATATCTTCCCAT
        131 GCGCTTTGAGGATTTCGTTGGAAACGGAAATATCTTCATAT

                  * * *      **      *   * *   *     ** ** *      *    *     
     236191 AAAAACGACATAGAAGCTATCTCAGGAACTTGTTTATGATGCATCTAATCAACTAACAGTGTTGA
          1 AAAAACTAGACAGAAGCATTCTCAGAAACATCTTTGTGATGTTTGCACTCAACTCACAGAGTTGA

                  * ** * *                         * *       *               
     236256 ACCTTTGTACTGACAGAGCAG-TTTGAAACACTCTTTTTTTGGAATCTGCAAGTGGATATTTGGA
         66 ACCTTTCTTTTCATAGAGCAGTTTTGAAACACTCTTTTTGTAGAATCTGGAAGTGGATATTTGGA

            *                           *   *   *    
     236320 TCGCTTTGAGGATTTCGTTGGAAACGG-GAT-GC--AATAT
        131 GCGCTTTGAGGATTTCGTTGGAAACGGAAATATCTTCATAT

                **  *    *     *                 **  *   *  *    *         * 
     236357 AAAACGTACACAGCAGCATACTCAGAAA-ATACTTTGCCATATTTCCATTCAAGTCACAGAGTGG
          1 AAAAACTAGACAGAAGCATTCTCAGAAACAT-CTTTGTGATGTTTGCACTCAACTCACAGAGTTG

               *  * **            *                  *  *             *      
     236421 AACATTCCCATTCATAGAGCAGGTTTGAAACACTCTTTTTGGAGTATCTGGAAGTGGACATTTGG
         65 AACCTTTCTTTTCATAGAGCAGTTTTGAAACACTCTTTTTGTAGAATCTGGAAGTGGATATTTGG

                    ** * * ** *  *   *            **  
     236486 AGCGCTTTCTGAACTATGGTGAAAAAGGAAATATCTTCCAAT
        130 AGCGCTTTGAGGATTTCGTTGGAAACGGAAATATCTTCATAT

            *     *               *      * *          *  **      **  *  *    
     236528 GAAAACAAGACAGAAGCATTCTGAGAAACTTATTTGTGATGTGTGTCCTCAACAAACGGACTTGA
          1 AAAAACTAGACAGAAGCATTCTCAGAAACATCTTTGTGATGTTTGCACTCAACTCACAGAGTTGA

                   *      **  * *  *  *             *         * * *          
     236593 ACCTTTCGTTTCATGCAGTACTTCTGGAACACTCTTTTTGAAGATAT-TGCATGCGGATATTTGG
         66 ACCTTTCTTTTCATAGAGCAGTTTTGAAACACTCTTTTTGTAGA-ATCTGGAAGTGGATATTTGG

             **                            **   *   * 
     236657 ATAGCTTTGAGGATTTCGTTGGAAACGG---GCT-TACATGT
        130 AGCGCTTTGAGGATTTCGTTGGAAACGGAAATATCTTCATAT

                 *       *               *        *   *    *    *       *    
     236695 AAAAATTAGACAGCAGCATTCTCAGAAACTTCTTTGTGGTGTCTGCATTCAAGTCACAGAATTGA
          1 AAAAACTAGACAGAAGCATTCTCAGAAACATCTTTGTGATGTTTGCACTCAACTCACAGAGTTGA

              * ** * **            *  * *      *       *             *       
     236760 ACTTCCCCTCACATAGAGCAGTTGTGCAGCACTCTATTTGTAGTATCTGGAAGTGGACATTTGGA
         66 ACCTTTCTTTTCATAGAGCAGTTTTGAAACACTCTTTTTGTAGAATCTGGAAGTGGATATTTGGA

             *         ** *    * *   *            **  
     236825 GGGCTTTGTAGCCTATC-TGGAAAAAGGAAATATCTTCCCAT
        131 GCGCTTTG-AGGATTTCGTTGGAAACGGAAATATCTTCATAT

            *  ** *   *     * *            *   *  *   * **        *  * * *   
     236866 GAATGCGAGATAGAAGTAATCTCAGAAACATGTTTATGCTGTATCTACTCAACTAACTGTGCTGA
          1 AAAAACTAGACAGAAGCATTCTCAGAAACATCTTTGTGATGTTTGCACTCAACTCACAGAGTTGA

              *     *  *               *        *  * *       *               
     236931 ACATTTCTATTGATAGAGCAGTTTTGAGACACTCTTCTTTTGGAATCTGCAAGTGGATATTTGGA
         66 ACCTTTCTTTTCATAGAGCAGTTTTGAAACACTCTTTTTGTAGAATCTGGAAGTGGATATTTGGA

            ** *                        *     *      
     236996 TAGATTTGAGGATTTCGTTGGAAACGG-GAT-T-AT-ATAT
        131 GCGCTTTGAGGATTTCGTTGGAAACGGAAATATCTTCATAT

                 *       *               *                 **  *   *         
     237033 AAAAAGTAGACAGCAGCATTCTCAGAAACTTCTTTGTGATGTTTGCATCCAGCTCTCAGAGTTGA
          1 AAAAACTAGACAGAAGCATTCTCAGAAACATCTTTGTGATGTTTGCACTCAACTCACAGAGTTGA

              *  * *          *  *        *        *   **        *  **       
     237098 ACATTCCCTTTCATAGAGTAGGTTTGAAACCCTCTTTTTATAGTGTCTGGAAGCGGGCATTTGGA
         66 ACCTTTCTTTTCATAGAGCAGTTTTGAAACACTCTTTTTGTAGAATCTGGAAGTGGATATTTGGA

                   *    * * **  **  **          * *  *
     237163 GCGCTTTCAGGCCTATGCTTAAAATAGGAAATATCTACCTAC
        131 GCGCTTTGAGG-ATTTCGTTGGAAACGGAAATATCTTCATAT

             *                    *    *  **          ** *        *    *     
     237205 AGAAACTAGACAGAAGCATTCTGAGAATCACGTTTGTGATGTGGGTACTCAACTAACAGTGTTGA
          1 AAAAACTAGACAGAAGCATTCTCAGAAACATCTTTGTGATGTTTGCACTCAACTCACAGAGTTGA

            *  *       *   *            *   *                *   *           
     237270 TCCATTCTTTTGATACAGCAGTTTTGAACCACACTTTTTGTAGAATCTGCAAGAGGATATTTGGA
         66 ACCTTTCTTTTCATAGAGCAGTTTTGAAACACTCTTTTTGTAGAATCTGGAAGTGGATATTTGGA

            **   *                     *  **      * *
     237335 TAGCTGTGAGGATTTCGTTGGAAACGGGAAAGTCTTCAAAG
        131 GCGCTTTGAGGATTTCGTTGGAAACGGAAATATCTTCATAT

                *                         *   *            *    *            
     237376 AAAATCTAGACAGAAGCATTCTCAGAAACACCTTCGTGATGTTTGCAATCAAGTCACAGAGTTGA
          1 AAAAACTAGACAGAAGCATTCTCAGAAACATCTTTGTGATGTTTGCACTCAACTCACAGAGTTGA

                 * *             *  *           *      *             *       
     237441 ACCTTCCGTTTCATAGAGCAGGTTGGAAACACTCTTATTGTAGTATCTGGAAGTGGACATTTGGA
         66 ACCTTTCTTTTCATAGAGCAGTTTTGAAACACTCTTTTTGTAGAATCTGGAAGTGGATATTTGGA

                   *   ** ** *  *   *            **  
     237506 GCGCTTTCAGGCCTATGGTGAAAAAGGAAATATCTTCCCAT
        131 GCGCTTTGAGGATTTCGTTGGAAACGGAAATATCTTCATAT

                  * * *      **      *   * *   *     ** ** *      *    *     
     237547 AAAAACGACATAGAAGCTATCTCAGGAACTTGTTTATGATGCATCTAATCAACTAACAGTGTTGA
          1 AAAAACTAGACAGAAGCATTCTCAGAAACATCTTTGTGATGTTTGCACTCAACTCACAGAGTTGA

                  * ** * *                         * *       *               
     237612 ACCTTTGTACTGACAGAGCAG-TTTGAAACACTCTTTTTTTGGAATCTGCAAGTGGATATTTGGA
         66 ACCTTTCTTTTCATAGAGCAGTTTTGAAACACTCTTTTTGTAGAATCTGGAAGTGGATATTTGGA

            *                           *   *   *    
     237676 TCGCTTTGAGGATTTCGTTGGAAACGG-GAT-GC--AATAT
        131 GCGCTTTGAGGATTTCGTTGGAAACGGAAATATCTTCATAT

                **  *    *     *                 **  *   *  *    *         * 
     237713 AAAACGTACACAGCAGCATACTCAGAAA-ATACTTTGCCATATTTCCATTCAAGTCACAGAGTGG
          1 AAAAACTAGACAGAAGCATTCTCAGAAACAT-CTTTGTGATGTTTGCACTCAACTCACAGAGTTG

               *  * **            *                  *  *             *      
     237777 AACATTCCCATTCATAGAGCAGGTTTGAAACACTCTTTTTGGAGTATCTGGAAGTGGACATTTGG
         65 AACCTTTCTTTTCATAGAGCAGTTTTGAAACACTCTTTTTGTAGAATCTGGAAGTGGATATTTGG

                    ** * * ** *  *   *            **  
     237842 AGCGCTTTCTGAACTATGGTGAAAAAGGAAATATCTTCCAAT
        130 AGCGCTTTGAGGATTTCGTTGGAAACGGAAATATCTTCATAT

            *     *               *      * *          *  **      **  *  *    
     237884 GAAAACAAGACAGAAGCATTCTGAGAAACTTATTTGTGATGTGTGTCCTCAACAAACGGACTTGA
          1 AAAAACTAGACAGAAGCATTCTCAGAAACATCTTTGTGATGTTTGCACTCAACTCACAGAGTTGA

                   *      **  * *  *  *             *   *    * * *           
     237949 ACCTTTCGTTTCATGCAGTACTTCTGGAACACTCTTTTTGAAGATTCTGCATGCGGATATTTGGA
         66 ACCTTTCTTTTCATAGAGCAGTTTTGAAACACTCTTTTTGTAGAATCTGGAAGTGGATATTTGGA

            **                            **   *   * 
     238014 TAGCTTTGAGGATTTCGTTGGAAACGG---GCT-TACATGT
        131 GCGCTTTGAGGATTTCGTTGGAAACGGAAATATCTTCATAT

                 *       *               * *      *   *    *    *       *    
     238051 AAAAATTAGACAGCAGCATTCTCAGAAACTTTTTTGTGGTGTCTGCATTCAAGTCACAGAATTGA
          1 AAAAACTAGACAGAAGCATTCTCAGAAACATCTTTGTGATGTTTGCACTCAACTCACAGAGTTGA

              * ** * **            *  * *      *       *             *       
     238116 ACTTCCCCTCACATAGAGCAGTTGTGCAGCACTCTATTTGTAGTATCTGGAAGTGGACATTTGGA
         66 ACCTTTCTTTTCATAGAGCAGTTTTGAAACACTCTTTTTGTAGAATCTGGAAGTGGATATTTGGA

             *         ** *          *            **  
     238181 GGGCTTTGTAGCCTATC--TGGAAAAGGAAATATCTTCCCAT
        131 GCGCTTTG-AGGATTTCGTTGGAAACGGAAATATCTTCATAT

            *  ** *   *     * *            *  **  *   * **        *  * * *   
     238221 GAATGCGAGATAGAAGTAATCTCAGAAACATGTTCATGCTGTATCTACTCAACTAACTGTGCTGA
          1 AAAAACTAGACAGAAGCATTCTCAGAAACATCTTTGTGATGTTTGCACTCAACTCACAGAGTTGA

              *     *  *               *        *  * *       *               
     238286 ACATTTCTATTGATAGAGCAGTTTTGAGACACTCTTCTTTTGGAATCTGCAAGTGGATATTTGGA
         66 ACCTTTCTTTTCATAGAGCAGTTTTGAAACACTCTTTTTGTAGAATCTGGAAGTGGATATTTGGA

            ** *                        *     *      
     238351 TAGATTTGAGGATTTCGTTGGAAACGG-GAT-T-AT-ATAT
        131 GCGCTTTGAGGATTTCGTTGGAAACGGAAATATCTTCATAT

                 *       *               *                 **  *   *         
     238388 AAAAAGTAGACAGCAGCATTCTCAGAAACTTCTTTGTGATGTTTGCATCCAGCTCTCAGAGTTGA
          1 AAAAACTAGACAGAAGCATTCTCAGAAACATCTTTGTGATGTTTGCACTCAACTCACAGAGTTGA

              *  * *          *  *        *        *   **        *  **       
     238453 ACATTCCCTTTCATAGAGTAGGTTTGAAACCCTCTTTTTATAGTGTCTGGAAGCGGGCATTTGGA
         66 ACCTTTCTTTTCATAGAGCAGTTTTGAAACACTCTTTTTGTAGAATCTGGAAGTGGATATTTGGA

                   *    * * **  **  **          * *  *
     238518 GCGCTTTCAGGCCTATGCTTAAAATAGGAAATATCTACCTAC
        131 GCGCTTTGAGG-ATTTCGTTGGAAACGGAAATATCTTCATAT

             *                    *    *  **          ** *        *    *     
     238560 AGAAACTAGACAGAAGCATTCTGAGAATCACGTTTGTGATGTGGGTACTCAACTAACAGTGTTGA
          1 AAAAACTAGACAGAAGCATTCTCAGAAACATCTTTGTGATGTTTGCACTCAACTCACAGAGTTGA

            *  *       *   *            *   *                *   *           
     238625 TCCATTCTTTTGATACAGCAGTTTTGAACCACACTTTTTGTAGAATCTGCAAGAGGATATTTGGA
         66 ACCTTTCTTTTCATAGAGCAGTTTTGAAACACTCTTTTTGTAGAATCTGGAAGTGGATATTTGGA

            **   *                     *  **      * *
     238690 TAGCTGTGAGGATTTCGTTGGAAACGGGAAAGTCTTCAAAG
        131 GCGCTTTGAGGATTTCGTTGGAAACGGAAATATCTTCATAT

                *                         *   *            *    *            
     238731 AAAATCTAGACAGAAGCATTCTCAGAAACACCTTCGTGATGTTTGCAATCAAGTCACAGAGTTGA
          1 AAAAACTAGACAGAAGCATTCTCAGAAACATCTTTGTGATGTTTGCACTCAACTCACAGAGTTGA

                 * *             *  *           *      *             *       
     238796 ACCTTCCGTTTCATAGAGCAGGTTGGAAACACTCTTATTGTAGTATCTGGAAGTGGACATTTGGA
         66 ACCTTTCTTTTCATAGAGCAGTTTTGAAACACTCTTTTTGTAGAATCTGGAAGTGGATATTTGGA

                   *   ** ** *  *   *            **  
     238861 GCGCTTTCAGGCCTATGGTGAAAAAGGAAATATCTTCCCAT
        131 GCGCTTTGAGGATTTCGTTGGAAACGGAAATATCTTCATAT

                  * * *      **      *   * *   *     ** ** *      *    *     
     238902 AAAAACGACATAGAAGCTATCTCAGGAACTTGTTTATGATGCATCTAATCAACTAACAGTGTTGA
          1 AAAAACTAGACAGAAGCATTCTCAGAAACATCTTTGTGATGTTTGCACTCAACTCACAGAGTTGA

                  * ** * *                         * *       *               
     238967 ACCTTTGTACTGACAGAGCAG-TTTGAAACACTCTTTTTTTGGAATCTGCAAGTGGATATTTGGA
         66 ACCTTTCTTTTCATAGAGCAGTTTTGAAACACTCTTTTTGTAGAATCTGGAAGTGGATATTTGGA

            *                           *   *   *    
     239031 TCGCTTTGAGGATTTCGTTGGAAACGG-GAT-GC--AATAT
        131 GCGCTTTGAGGATTTCGTTGGAAACGGAAATATCTTCATAT

                **  *    *     *                 **  *   *  *    *         * 
     239068 AAAACGTACACAGCAGCATACTCAGAAA-ATACTTTGCCATATTTCCATTCAAGTCACAGAGTGG
          1 AAAAACTAGACAGAAGCATTCTCAGAAACAT-CTTTGTGATGTTTGCACTCAACTCACAGAGTTG

               *  * **            *                  *  *             *      
     239132 AACATTCCCATTCATAGAGCAGGTTTGAAACACTCTTTTTGGAGTATCTGGAAGTGGACATTTGG
         65 AACCTTTCTTTTCATAGAGCAGTTTTGAAACACTCTTTTTGTAGAATCTGGAAGTGGATATTTGG

                    ** * * ** *  *   *            **  
     239197 AGCGCTTTCTGAACTATGGTGAAAAAGGAAATATCTTCCAAT
        130 AGCGCTTTGAGGATTTCGTTGGAAACGGAAATATCTTCATAT

            *     *               *      * *          *  **      **  *  *    
     239239 GAAAACAAGACAGAAGCATTCTGAGAAACTTATTTGTGATGTGTGTCCTCAACAAACGGACTTGA
          1 AAAAACTAGACAGAAGCATTCTCAGAAACATCTTTGTGATGTTTGCACTCAACTCACAGAGTTGA

                   *      **  * *  *  *             *   *    * * *           
     239304 ACCTTTCGTTTCATGCAGTACTTCTGGAACACTCTTTTTGAAGATTCTGCATGCGGATATTTGGA
         66 ACCTTTCTTTTCATAGAGCAGTTTTGAAACACTCTTTTTGTAGAATCTGGAAGTGGATATTTGGA

            **                            **   *   * 
     239369 TAGCTTTGAGGATTTCGTTGGAAACGG---GCT-TACATGT
        131 GCGCTTTGAGGATTTCGTTGGAAACGGAAATATCTTCATAT

                 *       *               * *      *   *    *    *       *    
     239406 AAAAATTAGACAGCAGCATTCTCAGAAACTTTTTTGTGGTGTCTGCATTCAAGTCACAGAATTGA
          1 AAAAACTAGACAGAAGCATTCTCAGAAACATCTTTGTGATGTTTGCACTCAACTCACAGAGTTGA

              * ** * **            *  * *      *       *             *       
     239471 ACTTCCCCTCACATAGAGCAGTTGTGCAGCACTCTATTTGTAGTATCTGGAAGTGGACATTTGGA
         66 ACCTTTCTTTTCATAGAGCAGTTTTGAAACACTCTTTTTGTAGAATCTGGAAGTGGATATTTGGA

             *         ** *          *            **  
     239536 GGGCTTTGTAGCCTATC--TGGAAAAGGAAATATCTTCCCAT
        131 GCGCTTTG-AGGATTTCGTTGGAAACGGAAATATCTTCATAT

            *  ** *   *     * *            *  **  *   * **        *  * * *   
     239576 GAATGCGAGATAGAAGTAATCTCAGAAACATGTTCATGCTGTATCTACTCAACTAACTGTGCTGA
          1 AAAAACTAGACAGAAGCATTCTCAGAAACATCTTTGTGATGTTTGCACTCAACTCACAGAGTTGA

              *     *  *               *        *  * *       *               
     239641 ACATTTCTATTGATAGAGCAGTTTTGAGACACTCTTCTTTTGGAATCTGCAAGTGGATATTTGGA
         66 ACCTTTCTTTTCATAGAGCAGTTTTGAAACACTCTTTTTGTAGAATCTGGAAGTGGATATTTGGA

            ** *                        *     *      
     239706 TAGATTTGAGGATTTCGTTGGAAACGG-GAT-T-AT-ATAT
        131 GCGCTTTGAGGATTTCGTTGGAAACGGAAATATCTTCATAT

                 *       *               *                 **  *   *         
     239743 AAAAAGTAGACAGCAGCATTCTCAGAAACTTCTTTGTGATGTTTGCATCCAGCTCTCAGAGTTGA
          1 AAAAACTAGACAGAAGCATTCTCAGAAACATCTTTGTGATGTTTGCACTCAACTCACAGAGTTGA

              *  * *          *  *        *        *   **        *  **       
     239808 ACATTCCCTTTCATAGAGTAGGTTTGAAACCCTCTTTTTATAGTGTCTGGAAGCGGGCATTTGGA
         66 ACCTTTCTTTTCATAGAGCAGTTTTGAAACACTCTTTTTGTAGAATCTGGAAGTGGATATTTGGA

                   *    * * **  **  **          * *  *
     239873 GCGCTTTCAGGCCTATGCTTAAAATAGGAAATATCTACCTAC
        131 GCGCTTTGAGG-ATTTCGTTGGAAACGGAAATATCTTCATAT

             *                    *    *  **          ** *        *    *     
     239915 AGAAACTAGACAGAAGCATTCTGAGAATCACGTTTGTGATGTGGGTACTCAACTAACAGTGTTGA
          1 AAAAACTAGACAGAAGCATTCTCAGAAACATCTTTGTGATGTTTGCACTCAACTCACAGAGTTGA

            *  *       *   *            *   *                *   *           
     239980 TCCATTCTTTTGATACAGCAGTTTTGAACCACACTTTTTGTAGAATCTGCAAGAGGATATTTGGA
         66 ACCTTTCTTTTCATAGAGCAGTTTTGAAACACTCTTTTTGTAGAATCTGGAAGTGGATATTTGGA

            **   *                     *   *      * *
     240045 TAGCTGTGAGGATTTCGTTGGAAACGGGAATGTCTTCAAAG
        131 GCGCTTTGAGGATTTCGTTGGAAACGGAAATATCTTCATAT

                *                         *   *            *   **            
     240086 AAAATCTAGACAGAAGCATTCTCAGAAACACCTTCGTGATGTTTGCAATCATGTCACAGAGTTGA
          1 AAAAACTAGACAGAAGCATTCTCAGAAACATCTTTGTGATGTTTGCACTCAACTCACAGAGTTGA

                 * *             *  *           *      *             *       
     240151 ACCTTCCGTTTCATAGAGCAGGTTGGAAACACTCTTATTGTAGTATCTGGAAGTGGACATTTGGA
         66 ACCTTTCTTTTCATAGAGCAGTTTTGAAACACTCTTTTTGTAGAATCTGGAAGTGGATATTTGGA

                   *   ** ** *  *   *            **  
     240216 GCGCTTTCAGGCCTATGGTGAAAAAGGAAATATCTTCCCAT
        131 GCGCTTTGAGGATTTCGTTGGAAACGGAAATATCTTCATAT

                  * * *      **      *   * *   *     ** ** *      *    *     
     240257 AAAAACGACATAGAAGCTATCTCAGGAACTTGTTTATGATGCATCTAATCAACTAACAGTGTTGA
          1 AAAAACTAGACAGAAGCATTCTCAGAAACATCTTTGTGATGTTTGCACTCAACTCACAGAGTTGA

                  * ** * *                         * *       *               
     240322 ACCTTTGTACTGACAGAGCAG-TTTGAAACACTCTTTTTTTGGAATCTGCAAGTGGATATTTGGA
         66 ACCTTTCTTTTCATAGAGCAGTTTTGAAACACTCTTTTTGTAGAATCTGGAAGTGGATATTTGGA

            *                           *   *   *    
     240386 TCGCTTTGAGGATTTCGTTGGAAACGG-GAT-GC--AATAT
        131 GCGCTTTGAGGATTTCGTTGGAAACGGAAATATCTTCATAT

                **  *    *     *                 **  *   *  *    *         * 
     240423 AAAACGTACACAGCAGCATACTCAGAAA-ATACTTTGCCATATTTCCATTCAAGTCACAGAGTGG
          1 AAAAACTAGACAGAAGCATTCTCAGAAACAT-CTTTGTGATGTTTGCACTCAACTCACAGAGTTG

               *  * **            *                  *  *             *      
     240487 AACATTCCCATTCATAGAGCAGGTTTGAAACACTCTTTTTGGAGTATCTGGAAGTGGACATTTGG
         65 AACCTTTCTTTTCATAGAGCAGTTTTGAAACACTCTTTTTGTAGAATCTGGAAGTGGATATTTGG

                    ** * * ** *  *   *            **  
     240552 AGCGCTTTCTGAACTATGGTGAAAAAGGAAATATCTTCCAAT
        130 AGCGCTTTGAGGATTTCGTTGGAAACGGAAATATCTTCATAT

            *     *               *      * *          *  **      **  *  *    
     240594 GAAAACAAGACAGAAGCATTCTGAGAAACTTATTTGTGATGTGTGTCCTCAACAAACGGACTTGA
          1 AAAAACTAGACAGAAGCATTCTCAGAAACATCTTTGTGATGTTTGCACTCAACTCACAGAGTTGA

                   *      **  * *  *  *             *   *    * * *           
     240659 ACCTTTCGTTTCATGCAGTACTTCTGGAACACTCTTTTTGAAGATTCTGCATGCGGATATTTGGA
         66 ACCTTTCTTTTCATAGAGCAGTTTTGAAACACTCTTTTTGTAGAATCTGGAAGTGGATATTTGGA

            **                            **   *   * 
     240724 TAGCTTTGAGGATTTCGTTGGAAACGG---GCT-TACATGT
        131 GCGCTTTGAGGATTTCGTTGGAAACGGAAATATCTTCATAT

                 *       *  *            *        *   *    *    *       *    
     240761 AAAAATTAGACAGCAGAATTCTCAGAAACTTCTTTGTGGTGTCTGCATTCAAGTCACAGAATTGA
          1 AAAAACTAGACAGAAGCATTCTCAGAAACATCTTTGTGATGTTTGCACTCAACTCACAGAGTTGA

              * ** * **            *  * *      *       *             *       
     240826 ACTTCCCCTCACATAGAGCAGTTGTGCAGCACTCTATTTGTAGTATCTGGAAGTGGACATTTGGA
         66 ACCTTTCTTTTCATAGAGCAGTTTTGAAACACTCTTTTTGTAGAATCTGGAAGTGGATATTTGGA

             *         ** *    * *   *            **  
     240891 GGGCTTTGTAGCCTATC-TGGAAAAAGGAAATATCTTCCCAT
        131 GCGCTTTG-AGGATTTCGTTGGAAACGGAAATATCTTCATAT

            *  ** *   *     * *            *   *  *   * **        *  * * *   
     240932 GAATGCGAGATAGAAGTAATCTCAGAAACATGTTTATGCTGTATCTACTCAACTAACTGTGCTGA
          1 AAAAACTAGACAGAAGCATTCTCAGAAACATCTTTGTGATGTTTGCACTCAACTCACAGAGTTGA

              *     *  *               *        *  * *       *               
     240997 ACATTTCTATTGATAGAGCAGTTTTGAGACACTCTTCTTTTGGAATCTGCAAGTGGATATTTGGA
         66 ACCTTTCTTTTCATAGAGCAGTTTTGAAACACTCTTTTTGTAGAATCTGGAAGTGGATATTTGGA

            ** *                        *     *      
     241062 TAGATTTGAGGATTTCGTTGGAAACGG-GAT-T-AT-ATAT
        131 GCGCTTTGAGGATTTCGTTGGAAACGGAAATATCTTCATAT

                 *       *               *          *      **  *   *         
     241099 AAAAAGTAGACAGCAGCATTCTCAGAAACTTCTTTGTGATATTTGCATCCAGCTCTCAGAGTTGA
          1 AAAAACTAGACAGAAGCATTCTCAGAAACATCTTTGTGATGTTTGCACTCAACTCACAGAGTTGA

              *  * *          *  *        *        *   **        *  **       
     241164 ACATTCCCTTTCATAGAGTAGGTTTGAAACCCTCTTTTTATAGTGTCTGGAAGCGGGCATTTGGA
         66 ACCTTTCTTTTCATAGAGCAGTTTTGAAACACTCTTTTTGTAGAATCTGGAAGTGGATATTTGGA

                   *    * * **  **  **          * *  *
     241229 GCGCTTTCAGGTCTATGCTTAAAATAGGAAATATCTACCTAC
        131 GCGCTTTGAGG-ATTTCGTTGGAAACGGAAATATCTTCATAT

             *                    *    *  **          ** *      * *    *     
     241271 AGAAACTAGACAGAAGCATTCTGAGAATCACGTTTGTGATGTGGGTACTCAAGTAACAGTGTTGA
          1 AAAAACTAGACAGAAGCATTCTCAGAAACATCTTTGTGATGTTTGCACTCAACTCACAGAGTTGA

            *  *       *   *            *   *                *   *           
     241336 TCCATTCTTTTGATACAGCAGTTTTGAACCACACTTTTTGTAGAATCTGCAAGAGGATATTTGGA
         66 ACCTTTCTTTTCATAGAGCAGTTTTGAAACACTCTTTTTGTAGAATCTGGAAGTGGATATTTGGA

            **   *                     *   *      * *
     241401 TAGCTGTGAGGATTTCGTTGGAAACGGGAATGTCTTCAAAG
        131 GCGCTTTGAGGATTTCGTTGGAAACGGAAATATCTTCATAT

                *                         *   *            *    *            
     241442 AAAATCTAGACAGAAGCATTCTCAGAAACACCTTCGTGATGTTTGCAATCAAGTCACAGAGTTGA
          1 AAAAACTAGACAGAAGCATTCTCAGAAACATCTTTGTGATGTTTGCACTCAACTCACAGAGTTGA

                 * *             *  *           *      *             *       
     241507 ACCTTCCGTTTCATAGAGCAGGTTGGAAACACTCTTATTGTAGTATCTGGAAGTGGACATTTGGA
         66 ACCTTTCTTTTCATAGAGCAGTTTTGAAACACTCTTTTTGTAGAATCTGGAAGTGGATATTTGGA

                   *   ** ** *  *   *            **  
     241572 GCGCTTTCAGGCCTATGGTGAAAAAGGAAATATCTTCCCAT
        131 GCGCTTTGAGGATTTCGTTGGAAACGGAAATATCTTCATAT

                  * * *      **      *   * *   *     ** ** *      *    *     
     241613 AAAAACGACATAGAAGCTATCTCAGGAACTTGTTTATGATGCATCTAATCAACTAACAGTGTTGA
          1 AAAAACTAGACAGAAGCATTCTCAGAAACATCTTTGTGATGTTTGCACTCAACTCACAGAGTTGA

                  * ** * *                         * *       *               
     241678 ACCTTTGTACTGACAGAGCAG-TTTGAAACACTCTTTTTTTGGAATCTGCAAGTGGATATTTGGA
         66 ACCTTTCTTTTCATAGAGCAGTTTTGAAACACTCTTTTTGTAGAATCTGGAAGTGGATATTTGGA

            *                           *   *   *    
     241742 TCGCTTTGAGGATTTCGTTGGAAACGG-GAT-GC--AATAT
        131 GCGCTTTGAGGATTTCGTTGGAAACGGAAATATCTTCATAT

                **  *    *     *                 **  *   *  *    *         * 
     241779 AAAACGTACACAGCAGCATACTCAGAAA-ATACTTTGCCATATTTCCATTCAAGTCACAGAGTGG
          1 AAAAACTAGACAGAAGCATTCTCAGAAACAT-CTTTGTGATGTTTGCACTCAACTCACAGAGTTG

               *  * **            *                  *  *             *      
     241843 AACATTCCCATTCATAGAGCAGGTTTGAAACACTCTTTTTGGAGTATCTGGAAGTGGACATTTGG
         65 AACCTTTCTTTTCATAGAGCAGTTTTGAAACACTCTTTTTGTAGAATCTGGAAGTGGATATTTGG

                    ** * * ** *  *   *            **  
     241908 AGCGCTTTCTGAACTATGGTGAAAAAGGAAATATCTTCCAAT
        130 AGCGCTTTGAGGATTTCGTTGGAAACGGAAATATCTTCATAT

            *     *               *      * *          *  **      **  *  *    
     241950 GAAAACAAGACAGAAGCATTCTGAGAAACTTATTTGTGATGTGTGTCCTCAACAAACGGACTTGA
          1 AAAAACTAGACAGAAGCATTCTCAGAAACATCTTTGTGATGTTTGCACTCAACTCACAGAGTTGA

                   *      **  * *  *  *             *         * * *          
     242015 ACCTTTCGTTTCATGCAGTACTTCTGGAACACTCTTTTTGAAGATAT-TGCATGCGGATATTTGG
         66 ACCTTTCTTTTCATAGAGCAGTTTTGAAACACTCTTTTTGTAGA-ATCTGGAAGTGGATATTTGG

             **                            **   *   * 
     242079 ATAGCTTTGAGGATTTCGTTGGAAACGG---GCT-TACATGT
        130 AGCGCTTTGAGGATTTCGTTGGAAACGGAAATATCTTCATAT

                 *       *               *        *   *    *    *       *    
     242117 AAAAATTAGACAGCAGCATTCTCAGAAACTTCTTTGTGGTGTCTGCATTCAAGTCACAGAATTGA
          1 AAAAACTAGACAGAAGCATTCTCAGAAACATCTTTGTGATGTTTGCACTCAACTCACAGAGTTGA

              * ** * **            *  * *      *       *             *       
     242182 ACTTCCCCTCACATAGAGCAGTTGTGCAGCACTCTATTTGTAGTATCTGGAAGTGGACATTTGGA
         66 ACCTTTCTTTTCATAGAGCAGTTTTGAAACACTCTTTTTGTAGAATCTGGAAGTGGATATTTGGA

             *         ** *    * *   *            **  
     242247 GGGCTTTGTAGCCTATC-TGGAAAAAGGAAATATCTTCCCAT
        131 GCGCTTTG-AGGATTTCGTTGGAAACGGAAATATCTTCATAT

            *  ** *   *     * *            *   *  **  * **        *  * * *   
     242288 GAATGCGAGATAGAAGTAATCTCAGAAACATGTTTATGCCGTATCTACTCAACTAACTGTGCTGA
          1 AAAAACTAGACAGAAGCATTCTCAGAAACATCTTTGTGATGTTTGCACTCAACTCACAGAGTTGA

              *     *  *               *            * **** * ***** *** *   **
     242353 ACATTTCTATTGATAGAGCAGTTTTGAGACACTCTTCTTTTTTTTTTTTTTTTTTTTTTTTTTTT
         66 ACCTTTCTTTTCATAGAGCAGTTTTGAAACACTCTT-TTTGTAGAATCTGGAAGTGGATATTTGG

            *****   *****   **  *  *      * *   *   * 
     242418 TTTTTTTTTTTTTTTTTTTTTGAGACGG-AGTCTC-GC-TCT
        130 AGCGCTTTGAGGATTTCGTTGGAAACGGAAATATCTTCATAT

            ***** *  *  *   *   * ** * * *     *** *    ***  *   **     * *  
     242457 GTCGCCCAGGCTGGAGTGCAGTGGCGGGATC-TC-GGCTCACTGCAAGCTC-CGCCTC-CCGGGT
          1 AAAAACTAGAC-AGA-AGCATTCTCAGAAACATCTTTGTGA-TGTTTGCACTCAACTCACAGAGT

             *  *  *     * * *  *     * ***  **    ***** **   *** * ** *    *
     242518 TCACGCCATTCTCCTGCCT-CAGC--CTCCCAAGTAGCTGGGACTACAGGCGCCCGCCACT--AC
         63 TGA-ACCTTTCT-TTTCATAGAGCAGTTTTGAAACA-CTCTTTTTGTA-GAATCTGGAAGTGGAT

            ****  *****   ** *     *** *  *     ** *  ** *   
     242578 GCCCGGCTAATTTTTTGTATTTTTAGTAGAGACGG-GGTTTCACCGT-T
        124 ATTTGGAGCGCTTTGAGGA-TTTCGTTGGAAACGGAAATATCTTCATAT

            ** ** **  ** *** **     ***       *        ***       **   *  *   
     242625 TTAGCCGGGATGGTCTCGATCTC-CTGAC--C-TCGTGATCCGCCCGC-CTCGGCCTCCCAAAGT
          1 AAAAACTAGACAGAAGCATTCTCAGAAACATCTTTGTGAT--GTTTGCACTC-AACTCACAGAGT

                       *****  ***  ** *  ******  *        *  * *       *     
     242685 GCTGGGATTACAGGCGTGAGCCACCGCGCCCGGCCGAGACACTCTTCTTTTGGAATCTGCAAGTG
         63 --T--GA--ACCTTTCT-TTTCATAGAGCAGTTTTGAAACACTCTTTTTGTAGAATCTGGAAGTG

                      ** *                        *     *      
     242750 GATATTTGGATAGATTTGAGGATTTCGTTGGAAACGG-GAT-T-AT-ATAT
        121 GATATTTGGAGCGCTTTGAGGATTTCGTTGGAAACGGAAATATCTTCATAT

                 *       *               *                 **  *   *         
     242797 AAAAAGTAGACAGCAGCATTCTCAGAAACTTCTTTGTGATGTTTGCATCCAGCTCTCAGAGTTGA
          1 AAAAACTAGACAGAAGCATTCTCAGAAACATCTTTGTGATGTTTGCACTCAACTCACAGAGTTGA

              *  * *          *  *        *        *   **        *  **       
     242862 ACATTCCCTTTCATAGAGTAGGTTTGAAACCCTCTTTTTATAGTGTCTGGAAGCGGGCATTTGGA
         66 ACCTTTCTTTTCATAGAGCAGTTTTGAAACACTCTTTTTGTAGAATCTGGAAGTGGATATTTGGA

                   *    * * **  **  **          * *  *
     242927 GCGCTTTCAGGCCTATGCTTAAAATAGGAAATATCTACCTAC
        131 GCGCTTTGAGG-ATTTCGTTGGAAACGGAAATATCTTCATAT

             *                    *    *  **          ** *        *    *     
     242969 AGAAACTAGACAGAAGCATTCTGAGAATCACGTTTGTGATGTGGGTACTCAACTAACAGTGTTGA
          1 AAAAACTAGACAGAAGCATTCTCAGAAACATCTTTGTGATGTTTGCACTCAACTCACAGAGTTGA

            *  *       *   *            *   *                *   *           
     243034 TCCATTCTTTTGATACAGCAGTTTTGAACCACACTTTTTGTAGAATCTGCAAGAGGATATTTGGA
         66 ACCTTTCTTTTCATAGAGCAGTTTTGAAACACTCTTTTTGTAGAATCTGGAAGTGGATATTTGGA

            **   *                     *  **      * *
     243099 TAGCTGTGAGGATTTCGTTGGAAACGGGAAAGTCTTCAAAG
        131 GCGCTTTGAGGATTTCGTTGGAAACGGAAATATCTTCATAT

                *                         *   *            *    *            
     243140 AAAATCTAGACAGAAGCATTCTCAGAAACACCTTCGTGATGTTTGCAATCAAGTCACAGAGTTGA
          1 AAAAACTAGACAGAAGCATTCTCAGAAACATCTTTGTGATGTTTGCACTCAACTCACAGAGTTGA

                 * *             *  *           *      *             *       
     243205 ACCTTCCGTTTCATAGAGCAGGTTGGAAACACTCTTATTGTAGTATCTGGAAGTGGACATTTGGA
         66 ACCTTTCTTTTCATAGAGCAGTTTTGAAACACTCTTTTTGTAGAATCTGGAAGTGGATATTTGGA

                   *   ** ** *  *   *            **  
     243270 GCGCTTTCAGGCCTATGGTGAAAAAGGAAATATCTTCCCAT
        131 GCGCTTTGAGGATTTCGTTGGAAACGGAAATATCTTCATAT

                  * * *      **      *   * *   *     ** ** *      *    *     
     243311 AAAAACGACATAGAAGCTATCTCAGGAACTTGTTTATGATGCATCTAATCAACTAACAGTGTTGA
          1 AAAAACTAGACAGAAGCATTCTCAGAAACATCTTTGTGATGTTTGCACTCAACTCACAGAGTTGA

                  * ** * *                         * *       *               
     243376 ACCTTTGTACTGACAGAGCAG-TTTGAAACACTCTTTTTTTGGAATCTGCAAGTGGATATTTGGA
         66 ACCTTTCTTTTCATAGAGCAGTTTTGAAACACTCTTTTTGTAGAATCTGGAAGTGGATATTTGGA

            *                           *   *   *    
     243440 TCGCTTTGAGGATTTCGTTGGAAACGG-GAT-GC--AATAT
        131 GCGCTTTGAGGATTTCGTTGGAAACGGAAATATCTTCATAT

                **  *    *     *                 **  *   *  *    *         * 
     243477 AAAACGTACACAGCAGCATACTCAGAAA-ATACTTTGCCATATTTCCATTCAAGTCACAGAGTGG
          1 AAAAACTAGACAGAAGCATTCTCAGAAACAT-CTTTGTGATGTTTGCACTCAACTCACAGAGTTG

               *  * **            *                  *  *             *      
     243541 AACATTCCCATTCATAGAGCAGGTTTGAAACACTCTTTTTGGAGTATCTGGAAGTGGACATTTGG
         65 AACCTTTCTTTTCATAGAGCAGTTTTGAAACACTCTTTTTGTAGAATCTGGAAGTGGATATTTGG

                    ** * * ** *  *   *            **  
     243606 AGCGCTTTCTGAACTATGGTGAAAAAGGAAATATCTTCCAAT
        130 AGCGCTTTGAGGATTTCGTTGGAAACGGAAATATCTTCATAT

            *     *               *      * *          *  **      **  *  *    
     243648 GAAAACAAGACAGAAGCATTCTGAGAAACTTATTTGTGATGTGTGTCCTCAACAAACGGACTTGA
          1 AAAAACTAGACAGAAGCATTCTCAGAAACATCTTTGTGATGTTTGCACTCAACTCACAGAGTTGA

                   *      **  * *  *  *             *   *    * * *           
     243713 ACCTTTCGTTTCATGCAGTACTTCTGGAACACTCTTTTTGAAGATTCTGCATGCGGATATTTGGA
         66 ACCTTTCTTTTCATAGAGCAGTTTTGAAACACTCTTTTTGTAGAATCTGGAAGTGGATATTTGGA

            **                            **   *   * 
     243778 TAGCTTTGAGGATTTCGTTGGAAACGG---GCT-TACATGT
        131 GCGCTTTGAGGATTTCGTTGGAAACGGAAATATCTTCATAT

                 *       *               * *      *   *    *    *       *    
     243815 AAAAATTAGACAGCAGCATTCTCAGAAACTTTTTTGTGGTGTCTGCATTCAAGTCACAGAATTGA
          1 AAAAACTAGACAGAAGCATTCTCAGAAACATCTTTGTGATGTTTGCACTCAACTCACAGAGTTGA

              * ** * **            *  * *      *       *             *       
     243880 ACTTCCCCTCACATAGAGCAGTTGTGCAGCACTCTATTTGTAGTATCTGGAAGTGGACATTTGGA
         66 ACCTTTCTTTTCATAGAGCAGTTTTGAAACACTCTTTTTGTAGAATCTGGAAGTGGATATTTGGA

             *         ** *          *            **  
     243945 GGGCTTTGTAGCCTATC--TGGAAAAGGAAATATCTTCCCAT
        131 GCGCTTTG-AGGATTTCGTTGGAAACGGAAATATCTTCATAT

            *  ** *   *     * *            *  **  *   * **        *  * * *   
     243985 GAATGCGAGATAGAAGTAATCTCAGAAACATGTTCATGCTGTATCTACTCAACTAACTGTGCTGA
          1 AAAAACTAGACAGAAGCATTCTCAGAAACATCTTTGTGATGTTTGCACTCAACTCACAGAGTTGA

              *     *  *               *        *  * *       *               
     244050 ACATTTCTATTGATAGAGCAGTTTTGAGACACTCTTCTTTTGGAATCTGCAAGTGGATATTTGGA
         66 ACCTTTCTTTTCATAGAGCAGTTTTGAAACACTCTTTTTGTAGAATCTGGAAGTGGATATTTGGA

            ** *                        *     *      
     244115 TAGATTTGAGGATTTCGTTGGAAACGG-GAT-T-AT-ATAT
        131 GCGCTTTGAGGATTTCGTTGGAAACGGAAATATCTTCATAT

                 *       *               *                 **  *   *         
     244152 AAAAAGTAGACAGCAGCATTCTCAGAAACTTCTTTGTGATGTTTGCATCCAGCTCTCAGAGTTGA
          1 AAAAACTAGACAGAAGCATTCTCAGAAACATCTTTGTGATGTTTGCACTCAACTCACAGAGTTGA

              *  * *          *  *        *        *   **        *  **       
     244217 ACATTCCCTTTCATAGAGTAGGTTTGAAACCCTCTTTTTATAGTGTCTGGAAGCGGGCATTTGGA
         66 ACCTTTCTTTTCATAGAGCAGTTTTGAAACACTCTTTTTGTAGAATCTGGAAGTGGATATTTGGA

                   *    * * **  **  **          * *  *
     244282 GCGCTTTCAGGCCTATGCTTAAAATAGGAAATATCTACCTAC
        131 GCGCTTTGAGG-ATTTCGTTGGAAACGGAAATATCTTCATAT

             *                    *    *  **          ** *        *    *     
     244324 AGAAACTAGACAGAAGCATTCTGAGAATCACGTTTGTGATGTGGGTACTCAACTAACAGTGTTGA
          1 AAAAACTAGACAGAAGCATTCTCAGAAACATCTTTGTGATGTTTGCACTCAACTCACAGAGTTGA

            *  *       *   *            *   *                *   *           
     244389 TCCATTCTTTTGATACAGCAGTTTTGAACCACACTTTTTGTAGAATCTGCAAGAGGATATTTGGA
         66 ACCTTTCTTTTCATAGAGCAGTTTTGAAACACTCTTTTTGTAGAATCTGGAAGTGGATATTTGGA

            **   *                     *   *      * *
     244454 TAGCTGTGAGGATTTCGTTGGAAACGGGAATGTCTTCAAAG
        131 GCGCTTTGAGGATTTCGTTGGAAACGGAAATATCTTCATAT

                *                         *   *            *   **            
     244495 AAAATCTAGACAGAAGCATTCTCAGAAACACCTTCGTGATGTTTGCAATCATGTCACAGAGTTGA
          1 AAAAACTAGACAGAAGCATTCTCAGAAACATCTTTGTGATGTTTGCACTCAACTCACAGAGTTGA

                 * *             *  *           *      *             *       
     244560 ACCTTCCGTTTCATAGAGCAGGTTGGAAACACTCTTATTGTAGTATCTGGAAGTGGACATTTGGA
         66 ACCTTTCTTTTCATAGAGCAGTTTTGAAACACTCTTTTTGTAGAATCTGGAAGTGGATATTTGGA

                   *   ** ** *  *   *            **  
     244625 GCGCTTTCAGGCCTATGGTGAAAAAGGAAATATCTTCCCAT
        131 GCGCTTTGAGGATTTCGTTGGAAACGGAAATATCTTCATAT

                  * * *      **      *   * *   *     ** ** *      *    *     
     244666 AAAAACGACATAGAAGCTATCTCAGGAACTTGTTTATGATGCATCTAATCAACTAACAGTGTTGA
          1 AAAAACTAGACAGAAGCATTCTCAGAAACATCTTTGTGATGTTTGCACTCAACTCACAGAGTTGA

                  * ** * *                         * *       *               
     244731 ACCTTTGTACTGACAGAGCAG-TTTGAAACACTCTTTTTTTGGAATCTGCAAGTGGATATTTGGA
         66 ACCTTTCTTTTCATAGAGCAGTTTTGAAACACTCTTTTTGTAGAATCTGGAAGTGGATATTTGGA

            *                           *   *   *    
     244795 TCGCTTTGAGGATTTCGTTGGAAACGG-GAT-GC--AATAT
        131 GCGCTTTGAGGATTTCGTTGGAAACGGAAATATCTTCATAT

                **  *    *     *                 **  *   *  *    *         * 
     244832 AAAACGTACACAGCAGCATACTCAGAAA-ATACTTTGCCATATTTCCATTCAAGTCACAGAGTGG
          1 AAAAACTAGACAGAAGCATTCTCAGAAACAT-CTTTGTGATGTTTGCACTCAACTCACAGAGTTG

               *  * **            *                  *  *             *      
     244896 AACATTCCCATTCATAGAGCAGGTTTGAAACACTCTTTTTGGAGTATCTGGAAGTGGACATTTGG
         65 AACCTTTCTTTTCATAGAGCAGTTTTGAAACACTCTTTTTGTAGAATCTGGAAGTGGATATTTGG

                    ** * * ** *  *   *            **  
     244961 AGCGCTTTCTGAACTATGGTGAAAAAGGAAATATCTTCCAAT
        130 AGCGCTTTGAGGATTTCGTTGGAAACGGAAATATCTTCATAT

            *     *               *      * *          *  **      **  *  *    
     245003 GAAAACAAGACAGAAGCATTCTGAGAAACTTATTTGTGATGTGTGTCCTCAACAAACGGACTTGA
          1 AAAAACTAGACAGAAGCATTCTCAGAAACATCTTTGTGATGTTTGCACTCAACTCACAGAGTTGA

                   *      **  * *  *  *             *   *    * * *           
     245068 ACCTTTCGTTTCATGCAGTACTTCTGGAACACTCTTTTTGAAGATTCTGCATGCGGATATTTGGA
         66 ACCTTTCTTTTCATAGAGCAGTTTTGAAACACTCTTTTTGTAGAATCTGGAAGTGGATATTTGGA

            **                            **   *   * 
     245133 TAGCTTTGAGGATTTCGTTGGAAACGG---GCT-TACATGT
        131 GCGCTTTGAGGATTTCGTTGGAAACGGAAATATCTTCATAT

                 *       *  *            *        *   *    *    *       *    
     245170 AAAAATTAGACAGCAGAATTCTCAGAAACTTCTTTGTGGTGTCTGCATTCAAGTCACAGAATTGA
          1 AAAAACTAGACAGAAGCATTCTCAGAAACATCTTTGTGATGTTTGCACTCAACTCACAGAGTTGA

              * ** * **            *  * *      *       *             *       
     245235 ACTTCCCCTCACATAGAGCAGTTGTGCAGCACTCTATTTGTAGTATCTGGAAGTGGACATTTGGA
         66 ACCTTTCTTTTCATAGAGCAGTTTTGAAACACTCTTTTTGTAGAATCTGGAAGTGGATATTTGGA

             *         ** *    * *   *            **  
     245300 GGGCTTTGTAGCCTATC-TGGAAAAAGGAAATATCTTCCCAT
        131 GCGCTTTG-AGGATTTCGTTGGAAACGGAAATATCTTCATAT

            *  ** *   *     * *            *   *  *   * **        *  * * *   
     245341 GAATGCGAGATAGAAGTAATCTCAGAAACATGTTTATGCTGTATCTACTCAACTAACTGTGCTGA
          1 AAAAACTAGACAGAAGCATTCTCAGAAACATCTTTGTGATGTTTGCACTCAACTCACAGAGTTGA

              *     *  *               *        *  * *       *               
     245406 ACATTTCTATTGATAGAGCAGTTTTGAGACACTCTTCTTTTGGAATCTGCAAGTGGATATTTGGA
         66 ACCTTTCTTTTCATAGAGCAGTTTTGAAACACTCTTTTTGTAGAATCTGGAAGTGGATATTTGGA

            ** *                        *     *      
     245471 TAGATTTGAGGATTTCGTTGGAAACGG-GAT-T-AT-ATAT
        131 GCGCTTTGAGGATTTCGTTGGAAACGGAAATATCTTCATAT

                 *       *               *          *      **  *   *         
     245508 AAAAAGTAGACAGCAGCATTCTCAGAAACTTCTTTGTGATATTTGCATCCAGCTCTCAGAGTTGA
          1 AAAAACTAGACAGAAGCATTCTCAGAAACATCTTTGTGATGTTTGCACTCAACTCACAGAGTTGA

              *  * *          *  *        *        *   **        *  **       
     245573 ACATTCCCTTTCATAGAGTAGGTTTGAAACCCTCTTTTTATAGTGTCTGGAAGCGGGCATTTGGA
         66 ACCTTTCTTTTCATAGAGCAGTTTTGAAACACTCTTTTTGTAGAATCTGGAAGTGGATATTTGGA

                   *    * * **  **  **          * *  *
     245638 GCGCTTTCAGGTCTATGCTTAAAATAGGAAATATCTACCTAC
        131 GCGCTTTGAGG-ATTTCGTTGGAAACGGAAATATCTTCATAT

             *                    *    *  **          ** *        *    *     
     245680 AGAAACTAGACAGAAGCATTCTGAGAATCACGTTTGTGATGTGGGTACTCAACTAACAGTGTTGA
          1 AAAAACTAGACAGAAGCATTCTCAGAAACATCTTTGTGATGTTTGCACTCAACTCACAGAGTTGA

            *  *       *   *            *   *                *   *           
     245745 TCCATTCTTTTGATACAGCAGTTTTGAACCACACTTTTTGTAGAATCTGCAAGAGGATATTTGGA
         66 ACCTTTCTTTTCATAGAGCAGTTTTGAAACACTCTTTTTGTAGAATCTGGAAGTGGATATTTGGA

            **   *                     *   *      * *
     245810 TAGCTGTGAGGATTTCGTTGGAAACGGGAATGTCTTCAAAG
        131 GCGCTTTGAGGATTTCGTTGGAAACGGAAATATCTTCATAT

                *                         *   *            *    *            
     245851 AAAATCTAGACAGAAGCATTCTCAGAAACACCTTCGTGATGTTTGCAATCAAGTCACAGAGTTGA
          1 AAAAACTAGACAGAAGCATTCTCAGAAACATCTTTGTGATGTTTGCACTCAACTCACAGAGTTGA

                 * *             *  *           *      *             *       
     245916 ACCTTCCGTTTCATAGAGCAGGTTGGAAACACTCTTATTGTAGTATCTGGAAGTGGACATTTGGA
         66 ACCTTTCTTTTCATAGAGCAGTTTTGAAACACTCTTTTTGTAGAATCTGGAAGTGGATATTTGGA

                   *   ** ** *  *   *            **  
     245981 GCGCTTTCAGGCCTATGGTGAAAAAGGAAATATCTTCCCAT
        131 GCGCTTTGAGGATTTCGTTGGAAACGGAAATATCTTCATAT

                  * * *      **      *   * *   *     ** ** *      *    *     
     246022 AAAAACGACATAGAAGCTATCTCAGGAACTTGTTTATGATGCATCTAATCAACTAACAGTGTTGA
          1 AAAAACTAGACAGAAGCATTCTCAGAAACATCTTTGTGATGTTTGCACTCAACTCACAGAGTTGA

                  * ** * *                         * *       *               
     246087 ACCTTTGTACTGACAGAGCAG-TTTGAAACACTCTTTTTTTGGAATCTGCAAGTGGATATTTGGA
         66 ACCTTTCTTTTCATAGAGCAGTTTTGAAACACTCTTTTTGTAGAATCTGGAAGTGGATATTTGGA

            *                           *   *   *    
     246151 TCGCTTTGAGGATTTCGTTGGAAACGG-GAT-GC--AATAT
        131 GCGCTTTGAGGATTTCGTTGGAAACGGAAATATCTTCATAT

                **  *    *     *                 **  *   *  *    *         * 
     246188 AAAACGTACACAGCAGCATACTCAGAAA-ATACTTTGCCATATTTCCATTCAAGTCACAGAGTGG
          1 AAAAACTAGACAGAAGCATTCTCAGAAACAT-CTTTGTGATGTTTGCACTCAACTCACAGAGTTG

               *  * **            *                  *  *             *      
     246252 AACATTCCCATTCATAGAGCAGGTTTGAAACACTCTTTTTGGAGTATCTGGAAGTGGACATTTGG
         65 AACCTTTCTTTTCATAGAGCAGTTTTGAAACACTCTTTTTGTAGAATCTGGAAGTGGATATTTGG

                    ** * * ** *  *   *            **  
     246317 AGCGCTTTCTGAACTATGGTGAAAAAGGAAATATCTTCCAAT
        130 AGCGCTTTGAGGATTTCGTTGGAAACGGAAATATCTTCATAT

            *     *               *      * *          *  **      **  *  *    
     246359 GAAAACAAGACAGAAGCATTCTGAGAAACTTATTTGTGATGTGTGTCCTCAACAAACGGACTTGA
          1 AAAAACTAGACAGAAGCATTCTCAGAAACATCTTTGTGATGTTTGCACTCAACTCACAGAGTTGA

                   *      **  * *  *  *             *         * * *          
     246424 ACCTTTCGTTTCATGCAGTACTTCTGGAACACTCTTTTTGAAGATAT-TGCATGCGGATATTTGG
         66 ACCTTTCTTTTCATAGAGCAGTTTTGAAACACTCTTTTTGTAGA-ATCTGGAAGTGGATATTTGG

             **                            **   *   * 
     246488 ATAGCTTTGAGGATTTCGTTGGAAACGG---GCT-TACATGT
        130 AGCGCTTTGAGGATTTCGTTGGAAACGGAAATATCTTCATAT

                 *       *               *        *   *    *    *       *    
     246526 AAAAATTAGACAGCAGCATTCTCAGAAACTTCTTTGTGGTGTCTGCATTCAAGTCACAGAATTGA
          1 AAAAACTAGACAGAAGCATTCTCAGAAACATCTTTGTGATGTTTGCACTCAACTCACAGAGTTGA

              * ** * **            *  * *      *       *             *       
     246591 ACTTCCCCTCACATAGAGCAGTTGTGCAGCACTCTATTTGTAGTATCTGGAAGTGGACATTTGGA
         66 ACCTTTCTTTTCATAGAGCAGTTTTGAAACACTCTTTTTGTAGAATCTGGAAGTGGATATTTGGA

             *         ** *    * *   *            **  
     246656 GGGCTTTGTAGCCTATC-TGGAAAAAGGAAATATCTTCCCAT
        131 GCGCTTTG-AGGATTTCGTTGGAAACGGAAATATCTTCATAT

            *  ** *   *     * *            *   *  **  * **        *  * * *   
     246697 GAATGCGAGATAGAAGTAATCTCAGAAACATGTTTATGCCGTATCTACTCAACTAACTGTGCTGA
          1 AAAAACTAGACAGAAGCATTCTCAGAAACATCTTTGTGATGTTTGCACTCAACTCACAGAGTTGA

              *     *  *                    *            * **** * ***** *** *
     246762 ACATTTCTATTGATAGAGCAGTTTTGAGACACTCTTCTTTTTTTTTTTTTTTTTTTTTTTTTTTT
         66 ACCTTTCTTTTCATAGAGCAGTTTTGA-A-ACAC-TC---TTTTTGTAGAATCTGGAAGTGGATA

               *******   *****   **  *  *      * *   *   * 
     246827 TTTTTTTTTTTTTTTTTTTTTTTTTTGAGACGG-AGTCTC-GC-TCT
        125 TTTGGAGCGCTTTGAGGATTTCGTTGGAAACGGAAATATCTTCATAT

            ***** *  *  *   *   * ** * * *     *** *    ***  *   **     * *  
     246871 GTCGCCCAGGCTGGAGTGCAGTGGCGGGATC-TC-GGCTCACTGCAAGCTC-CGCCTC-CCGGGT
          1 AAAAACTAGAC-AGA-AGCATTCTCAGAAACATCTTTGTGA-TGTTTGCACTCAACTCACAGAGT

             *  *  *     * * *  *     * ***  **    ***** **   *** * ** *    *
     246932 TCACGCCATTCTCCTGCCT-CAGC--CTCCCAAGTAGCTGGGACTACAGGCGCCCGCCACT--AC
         63 TGA-ACCTTTCT-TTTCATAGAGCAGTTTTGAAACA-CTCTTTTTGTA-GAATCTGGAAGTGGAT

            ****  *****   ** *     *** *  *     ** *  ** *   
     246992 GCCCGGCTAATTTTTTGTATTTTTAGTAGAGACGG-GGTTTCACCGT-T
        124 ATTTGGAGCGCTTTGAGGA-TTTCGTTGGAAACGGAAATATCTTCATAT

            ** ** **  ** *** **     ***       *        ***       **   *  *   
     247039 TTAGCCGGGATGGTCTCGATCTC-CTGAC--C-TCGTGATCCGCCCGC-CTCGGCCTCCCAAAGT
          1 AAAAACTAGACAGAAGCATTCTCAGAAACATCTTTGTGAT--GTTTGCACTC-AACTCACAGAGT

                       *****  ***  ** *  ******  *        *  * *       *     
     247099 GCTGGGATTACAGGCGTGAGCCACCGCGCCCGGCCGAGACACTCTTCTTTTGGAATCTGCAAGTG
         63 --T--GA--ACCTTTCT-TTTCATAGAGCAGTTTTGAAACACTCTTTTTGTAGAATCTGGAAGTG

                      ** *                        *     *      
     247164 GATATTTGGATAGATTTGAGGATTTCGTTGGAAACGG-GAT-T-AT-ATAT
        121 GATATTTGGAGCGCTTTGAGGATTTCGTTGGAAACGGAAATATCTTCATAT

                 *       *               *                 **  *   *         
     247211 AAAAAGTAGACAGCAGCATTCTCAGAAACTTCTTTGTGATGTTTGCATCCAGCTCTCAGAGTTGA
          1 AAAAACTAGACAGAAGCATTCTCAGAAACATCTTTGTGATGTTTGCACTCAACTCACAGAGTTGA

              *  * *          *  *        *        *   **        *  **       
     247276 ACATTCCCTTTCATAGAGTAGGTTTGAAACCCTCTTTTTATAGTGTCTGGAAGCGGGCATTTGGA
         66 ACCTTTCTTTTCATAGAGCAGTTTTGAAACACTCTTTTTGTAGAATCTGGAAGTGGATATTTGGA

                   *    * * **  **  **          * *  *
     247341 GCGCTTTCAGGCCTATGCTTAAAATAGGAAATATCTACCTAC
        131 GCGCTTTGAGG-ATTTCGTTGGAAACGGAAATATCTTCATAT

             *                    *    *  **          ** *        *    *     
     247383 AGAAACTAGACAGAAGCATTCTGAGAATCACGTTTGTGATGTGGGTACTCAACTAACAGTGTTGA
          1 AAAAACTAGACAGAAGCATTCTCAGAAACATCTTTGTGATGTTTGCACTCAACTCACAGAGTTGA

            *  *       *   *            *   *                *   *           
     247448 TCCATTCTTTTGATACAGCAGTTTTGAACCACACTTTTTGTAGAATCTGCAAGAGGATATTTGGA
         66 ACCTTTCTTTTCATAGAGCAGTTTTGAAACACTCTTTTTGTAGAATCTGGAAGTGGATATTTGGA

            **   *                     *  **      * *
     247513 TAGCTGTGAGGATTTCGTTGGAAACGGGAAAGTCTTCAAAG
        131 GCGCTTTGAGGATTTCGTTGGAAACGGAAATATCTTCATAT

                *                         *   *            *    *            
     247554 AAAATCTAGACAGAAGCATTCTCAGAAACACCTTCGTGATGTTTGCAATCAAGTCACAGAGTTGA
          1 AAAAACTAGACAGAAGCATTCTCAGAAACATCTTTGTGATGTTTGCACTCAACTCACAGAGTTGA

                 * *             *  *           *      *             *       
     247619 ACCTTCCGTTTCATAGAGCAGGTTGGAAACACTCTTATTGTAGTATCTGGAAGTGGACATTTGGA
         66 ACCTTTCTTTTCATAGAGCAGTTTTGAAACACTCTTTTTGTAGAATCTGGAAGTGGATATTTGGA

                   *   ** ** *  *   *            **  
     247684 GCGCTTTCAGGCCTATGGTGAAAAAGGAAATATCTTCCCAT
        131 GCGCTTTGAGGATTTCGTTGGAAACGGAAATATCTTCATAT

                  * * *      **      *   * *   *     ** ** *      *    *     
     247725 AAAAACGACATAGAAGCTATCTCAGGAACTTGTTTATGATGCATCTAATCAACTAACAGTGTTGA
          1 AAAAACTAGACAGAAGCATTCTCAGAAACATCTTTGTGATGTTTGCACTCAACTCACAGAGTTGA

                  * ** * *                         * *       *               
     247790 ACCTTTGTACTGACAGAGCAG-TTTGAAACACTCTTTTTTTGGAATCTGCAAGTGGATATTTGGA
         66 ACCTTTCTTTTCATAGAGCAGTTTTGAAACACTCTTTTTGTAGAATCTGGAAGTGGATATTTGGA

            *                           *   *   *    
     247854 TCGCTTTGAGGATTTCGTTGGAAACGG-GAT-GC--AATAT
        131 GCGCTTTGAGGATTTCGTTGGAAACGGAAATATCTTCATAT

                **  *    *     *                 **  *   *  *    *         * 
     247891 AAAACGTACACAGCAGCATACTCAGAAA-ATACTTTGCCATATTTCCATTCAAGTCACAGAGTGG
          1 AAAAACTAGACAGAAGCATTCTCAGAAACAT-CTTTGTGATGTTTGCACTCAACTCACAGAGTTG

               *  * **            *                  *  *             *      
     247955 AACATTCCCATTCATAGAGCAGGTTTGAAACACTCTTTTTGGAGTATCTGGAAGTGGACATTTGG
         65 AACCTTTCTTTTCATAGAGCAGTTTTGAAACACTCTTTTTGTAGAATCTGGAAGTGGATATTTGG

                    ** * * ** *  *   *            **  
     248020 AGCGCTTTCTGAACTATGGTGAAAAAGGAAATATCTTCCAAT
        130 AGCGCTTTGAGGATTTCGTTGGAAACGGAAATATCTTCATAT

            *     *               *      * *          *  **      **  *  *    
     248062 GAAAACAAGACAGAAGCATTCTGAGAAACTTATTTGTGATGTGTGTCCTCAACAAACGGACTTGA
          1 AAAAACTAGACAGAAGCATTCTCAGAAACATCTTTGTGATGTTTGCACTCAACTCACAGAGTTGA

                   *      **  * *  *  *             *   *    * * *           
     248127 ACCTTTCGTTTCATGCAGTACTTCTGGAACACTCTTTTTGAAGATTCTGCATGCGGATATTTGGA
         66 ACCTTTCTTTTCATAGAGCAGTTTTGAAACACTCTTTTTGTAGAATCTGGAAGTGGATATTTGGA

            **                          **    *    * 
     248192 TAGCTTTGAGGATTTCGTTGGAAACGG-GCT-T-AT-ATGT
        131 GCGCTTTGAGGATTTCGTTGGAAACGGAAATATCTTCATAT

                 *       *               * *      *   *    *    *       *    
     248229 AAAAATTAGACAGCAGCATTCTCAGAAACTTTTTTGTGGTGTCTGCATTCAAGTCACAGAATTGA
          1 AAAAACTAGACAGAAGCATTCTCAGAAACATCTTTGTGATGTTTGCACTCAACTCACAGAGTTGA

              * ** * **  *         *  * *      *       *             *       
     248294 ACTTCCCCTCACAAAGAGCAGTTGTGCAGCACTCTATTTGTAGTATCTGGAAGTGGACATTTGGA
         66 ACCTTTCTTTTCATAGAGCAGTTTTGAAACACTCTTTTTGTAGAATCTGGAAGTGGATATTTGGA

             *         ** *          *            **  
     248359 GGGCTTTGTAGCCTATC--TGGAAAAGGAAATATCTTCCCAT
        131 GCGCTTTG-AGGATTTCGTTGGAAACGGAAATATCTTCATAT

            *  ** *   *     * *            *  **  *   * **        *  * * *   
     248399 GAATGCGAGATAGAAGTAATCTCAGAAACATGTTCATGCTGTATCTACTCAACTAACTGTGCTGA
          1 AAAAACTAGACAGAAGCATTCTCAGAAACATCTTTGTGATGTTTGCACTCAACTCACAGAGTTGA

              *     *  *               *        *  * *       *               
     248464 ACATTTCTATTGATAGAGCAGTTTTGAGACACTCTTCTTTTGGAATCTGCAAGTGGATATTTGGA
         66 ACCTTTCTTTTCATAGAGCAGTTTTGAAACACTCTTTTTGTAGAATCTGGAAGTGGATATTTGGA

            ** *                        *     *      
     248529 TAGATTTGAGGATTTCGTTGGAAACGG-GAT-T-AT-ATAT
        131 GCGCTTTGAGGATTTCGTTGGAAACGGAAATATCTTCATAT

                 *       *               *                 **  *   *         
     248566 AAAAAGTAGACAGCAGCATTCTCAGAAACTTCTTTGTGATGTTTGCATCCAGCTCTCAGAGTTGA
          1 AAAAACTAGACAGAAGCATTCTCAGAAACATCTTTGTGATGTTTGCACTCAACTCACAGAGTTGA

              *  * *          *  *        *        *   **        *  **       
     248631 ACATTCCCTTTCATAGAGTAGGTTTGAAACCCTCTTTTTATAGTGTCTGGAAGCGGGCATTTGGA
         66 ACCTTTCTTTTCATAGAGCAGTTTTGAAACACTCTTTTTGTAGAATCTGGAAGTGGATATTTGGA

                   *    * * **  **  **          * *  *
     248696 GCGCTTTCAGGCCTATGCTTAAAATAGGAAATATCTACCTAC
        131 GCGCTTTGAGG-ATTTCGTTGGAAACGGAAATATCTTCATAT

             *                    *    *  **          ** *        *    *     
     248738 AGAAACTAGACAGAAGCATTCTGAGAATCACGTTTGTGATGTGGGTACTCAACTAACAGTGTTGA
          1 AAAAACTAGACAGAAGCATTCTCAGAAACATCTTTGTGATGTTTGCACTCAACTCACAGAGTTGA

            *  *       *   *            *   *                *   *           
     248803 TCCATTCTTTTGATACAGCAGTTTTGAACCACACTTTTTGTAGAATCTGCAAGAGGATATTTGGA
         66 ACCTTTCTTTTCATAGAGCAGTTTTGAAACACTCTTTTTGTAGAATCTGGAAGTGGATATTTGGA

            **   *                     *  **      * *
     248868 TAGCTGTGAGGATTTCGTTGGAAACGGGAAAGTCTTCAAAG
        131 GCGCTTTGAGGATTTCGTTGGAAACGGAAATATCTTCATAT

                *                         *   *            *    *            
     248909 AAAATCTAGACAGAAGCATTCTCAGAAACACCTTCGTGATGTTTGCAATCAAGTCACAGAGTTGA
          1 AAAAACTAGACAGAAGCATTCTCAGAAACATCTTTGTGATGTTTGCACTCAACTCACAGAGTTGA

                 * *             *  *           *      *             *       
     248974 ACCTTCCGTTTCATAGAGCAGGTTGGAAACACTCTTATTGTAGTATCTGGAAGTGGACATTTGGA
         66 ACCTTTCTTTTCATAGAGCAGTTTTGAAACACTCTTTTTGTAGAATCTGGAAGTGGATATTTGGA

                   *   ** ** *  *   *            **  
     249039 GCGCTTTCAGGCCTATGGTGAAAAAGGAAATATCTTCCCAT
        131 GCGCTTTGAGGATTTCGTTGGAAACGGAAATATCTTCATAT

                  * * *      **      *   * *   *     ** ** *      *    *     
     249080 AAAAACGACATAGAAGCTATCTCAGGAACTTGTTTATGATGCATCTAATCAACTAACAGTGTTGA
          1 AAAAACTAGACAGAAGCATTCTCAGAAACATCTTTGTGATGTTTGCACTCAACTCACAGAGTTGA

                  * ** * *                         * *       *               
     249145 ACCTTTGTACTGACAGAGCAG-TTTGAAACACTCTTTTTTTGGAATCTGCAAGTGGATATTTGGA
         66 ACCTTTCTTTTCATAGAGCAGTTTTGAAACACTCTTTTTGTAGAATCTGGAAGTGGATATTTGGA

            *                           *   *   *    
     249209 TCGCTTTGAGGATTTCGTTGGAAACGG-GAT-GC--AATAT
        131 GCGCTTTGAGGATTTCGTTGGAAACGGAAATATCTTCATAT

                **  *    *     *                 **  *   *  *    *         * 
     249246 AAAACGTACACAGCAGCATACTCAGAAA-ATACTTTGCCATATTTCCATTCAAGTCACAGAGTGG
          1 AAAAACTAGACAGAAGCATTCTCAGAAACAT-CTTTGTGATGTTTGCACTCAACTCACAGAGTTG

               *  * **            *                  *  *             *      
     249310 AACATTCCCATTCATAGAGCAGGTTTGAAACACTCTTTTTGGAGTATCTGGAAGTGGACATTTGG
         65 AACCTTTCTTTTCATAGAGCAGTTTTGAAACACTCTTTTTGTAGAATCTGGAAGTGGATATTTGG

                    ** * * ** *  *   *            **  
     249375 AGCGCTTTCTGAACTATGGTGAAAAAGGAAATATCTTCCAAT
        130 AGCGCTTTGAGGATTTCGTTGGAAACGGAAATATCTTCATAT

            *     *               *      * *          *  **      **  *  *    
     249417 GAAAACAAGACAGAAGCATTCTGAGAAACTTATTTGTGATGTGTGTCCTCAACAAACGGACTTGA
          1 AAAAACTAGACAGAAGCATTCTCAGAAACATCTTTGTGATGTTTGCACTCAACTCACAGAGTTGA

                   *      **  * *  *  *             *   *    * * *           
     249482 ACCTTTCGTTTCATGCAGTACTTCTGGAACACTCTTTTTGAAGATTCTGCATGCGGATATTTGGA
         66 ACCTTTCTTTTCATAGAGCAGTTTTGAAACACTCTTTTTGTAGAATCTGGAAGTGGATATTTGGA

            **                          **    *    * 
     249547 TAGCTTTGAGGATTTCGTTGGAAACGG-GCT-T-AT-ATGT
        131 GCGCTTTGAGGATTTCGTTGGAAACGGAAATATCTTCATAT

                 *       *               * *      *   *    *    *       *    
     249584 AAAAATTAGACAGCAGCATTCTCAGAAACTTTTTTGTGGTGTCTGCATTCAAGTCACAGAATTGA
          1 AAAAACTAGACAGAAGCATTCTCAGAAACATCTTTGTGATGTTTGCACTCAACTCACAGAGTTGA

              * ** * **  *         *  * *      *       *             *       
     249649 ACTTCCCCTCACAAAGAGCAGTTGTGCAGCACTCTATTTGTAGTATCTGGAAGTGGACATTTGGA
         66 ACCTTTCTTTTCATAGAGCAGTTTTGAAACACTCTTTTTGTAGAATCTGGAAGTGGATATTTGGA

             *         ** *          *            **  
     249714 GGGCTTTGTAGCCTATC--TGGAAAAGGAAATATCTTCCCAT
        131 GCGCTTTG-AGGATTTCGTTGGAAACGGAAATATCTTCATAT

            *  ** *   *     * *            *  **  *   * **        *  * * *   
     249754 GAATGCGAGATAGAAGTAATCTCAGAAACATGTTCATGCTGTATCTACTCAACTAACTGTGCTGA
          1 AAAAACTAGACAGAAGCATTCTCAGAAACATCTTTGTGATGTTTGCACTCAACTCACAGAGTTGA

              *     *  *               *        *  * *       *               
     249819 ACATTTCTATTGATAGAGCAGTTTTGAGACACTCTTCTTTTGGAATCTGCAAGTGGATATTTGGA
         66 ACCTTTCTTTTCATAGAGCAGTTTTGAAACACTCTTTTTGTAGAATCTGGAAGTGGATATTTGGA

            ** *                        *     *      
     249884 TAGATTTGAGGATTTCGTTGGAAACGG-GAT-T-AT-ATAT
        131 GCGCTTTGAGGATTTCGTTGGAAACGGAAATATCTTCATAT

                 *       *               *                 **  *   *         
     249921 AAAAAGTAGACAGCAGCATTCTCAGAAACTTCTTTGTGATGTTTGCATCCAGCTCTCAGAGTTGA
          1 AAAAACTAGACAGAAGCATTCTCAGAAACATCTTTGTGATGTTTGCACTCAACTCACAGAGTTGA

              *  * *          *  *        *        *   **        *  **       
     249986 ACATTCCCTTTCATAGAGTAGGTTTGAAACCCTCTTTTTATAGTGTCTGGAAGCGGGCATTTGGA
         66 ACCTTTCTTTTCATAGAGCAGTTTTGAAACACTCTTTTTGTAGAATCTGGAAGTGGATATTTGGA

                   *    * * **  **  **          * *  *
     250051 GCGCTTTCAGGCCTATGCTTAAAATAGGAAATATCTACCTAC
        131 GCGCTTTGAGG-ATTTCGTTGGAAACGGAAATATCTTCATAT

             *                    *    *  **          ** *        *    *     
     250093 AGAAACTAGACAGAAGCATTCTGAGAATCACGTTTGTGATGTGGGTACTCAACTAACAGTGTTGA
          1 AAAAACTAGACAGAAGCATTCTCAGAAACATCTTTGTGATGTTTGCACTCAACTCACAGAGTTGA

            *  *       *   *            *   *                *   *           
     250158 TCCATTCTTTTGATACAGCAGTTTTGAACCACACTTTTTGTAGAATCTGCAAGAGGATATTTGGA
         66 ACCTTTCTTTTCATAGAGCAGTTTTGAAACACTCTTTTTGTAGAATCTGGAAGTGGATATTTGGA

            **   *                     *   *      * *
     250223 TAGCTGTGAGGATTTCGTTGGAAACGGGAATGTCTTCAAAG
        131 GCGCTTTGAGGATTTCGTTGGAAACGGAAATATCTTCATAT

                *                         *   *            *   **            
     250264 AAAATCTAGACAGAAGCATTCTCAGAAACACCTTCGTGATGTTTGCAATCATGTCACAGAGTTGA
          1 AAAAACTAGACAGAAGCATTCTCAGAAACATCTTTGTGATGTTTGCACTCAACTCACAGAGTTGA

                 * *             *  *           *      *             *       
     250329 ACCTTCCGTTTCATAGAGCAGGTTGGAAACACTCTTATTGTAGTATCTGGAAGTGGACATTTGGA
         66 ACCTTTCTTTTCATAGAGCAGTTTTGAAACACTCTTTTTGTAGAATCTGGAAGTGGATATTTGGA

                   *   ** ** *  *   *            **  
     250394 GCGCTTTCAGGCCTATGGTGAAAAAGGAAATATCTTCCCAT
        131 GCGCTTTGAGGATTTCGTTGGAAACGGAAATATCTTCATAT

                  * * *      **      *   * *   *     ** ** *      *    *     
     250435 AAAAACGACATAGAAGCTATCTCAGGAACTTGTTTATGATGCATCTAATCAACTAACAGTGTTGA
          1 AAAAACTAGACAGAAGCATTCTCAGAAACATCTTTGTGATGTTTGCACTCAACTCACAGAGTTGA

                  * ** * *                         * *       *               
     250500 ACCTTTGTACTGACAGAGCAG-TTTGAAACACTCTTTTTTTGGAATCTGCAAGTGGATATTTGGA
         66 ACCTTTCTTTTCATAGAGCAGTTTTGAAACACTCTTTTTGTAGAATCTGGAAGTGGATATTTGGA

            *                           *   *   *    
     250564 TCGCTTTGAGGATTTCGTTGGAAACGG-GAT-GC--AATAT
        131 GCGCTTTGAGGATTTCGTTGGAAACGGAAATATCTTCATAT

                **  *    *     *                 **  *   *  *    *         * 
     250601 AAAACGTACACAGCAGCATACTCAGAAA-ATACTTTGCCATATTTCCATTCAAGTCACAGAGTGG
          1 AAAAACTAGACAGAAGCATTCTCAGAAACAT-CTTTGTGATGTTTGCACTCAACTCACAGAGTTG

               *  * **            *                  *  *             *      
     250665 AACATTCCCATTCATAGAGCAGGTTTGAAACACTCTTTTTGGAGTATCTGGAAGTGGACATTTGG
         65 AACCTTTCTTTTCATAGAGCAGTTTTGAAACACTCTTTTTGTAGAATCTGGAAGTGGATATTTGG

                    ** * * ** *  *   *            **  
     250730 AGCGCTTTCTGAACTATGGTGAAAAAGGAAATATCTTCCAAT
        130 AGCGCTTTGAGGATTTCGTTGGAAACGGAAATATCTTCATAT

            *     *               *      * *          *  **      **  *  *    
     250772 GAAAACAAGACAGAAGCATTCTGAGAAACTTATTTGTGATGTGTGTCCTCAACAAACGGACTTGA
          1 AAAAACTAGACAGAAGCATTCTCAGAAACATCTTTGTGATGTTTGCACTCAACTCACAGAGTTGA

                   *      **  * *  *  *             *   *    * * *           
     250837 ACCTTTCGTTTCATGCAGTACTTCTGGAACACTCTTTTTGAAGATTCTGCATGCGGATATTTGGA
         66 ACCTTTCTTTTCATAGAGCAGTTTTGAAACACTCTTTTTGTAGAATCTGGAAGTGGATATTTGGA

            **                            **   *   * 
     250902 TAGCTTTGAGGATTTCGTTGGAAACGG---GCT-TACATGT
        131 GCGCTTTGAGGATTTCGTTGGAAACGGAAATATCTTCATAT

                 *       *               *        *   *    *    *       *    
     250939 AAAAATTAGACAGCAGCATTCTCAGAAACTTCTTTGTGGTGTCTGCATTCAAGTCACAGAATTGA
          1 AAAAACTAGACAGAAGCATTCTCAGAAACATCTTTGTGATGTTTGCACTCAACTCACAGAGTTGA

              * ** * **            *  * *      *       *             *       
     251004 ACTTCCCCTCACATAGAGCAGTTGTGCAGCACTCTATTTGTAGTATCTGGAAGTGGACATTTGGA
         66 ACCTTTCTTTTCATAGAGCAGTTTTGAAACACTCTTTTTGTAGAATCTGGAAGTGGATATTTGGA

             *         ** *    * *   *            **  
     251069 GGGCTTTGTAGCCTATC-TGGAAAAAGGAAATATCTTCCCAT
        131 GCGCTTTG-AGGATTTCGTTGGAAACGGAAATATCTTCATAT

            *  ** *   *     * *            *   *  *   * **        *  * * *   
     251110 GAATGCGAGATAGAAGTAATCTCAGAAACATGTTTATGCTGTATCTACTCAACTAACTGTGCTGA
          1 AAAAACTAGACAGAAGCATTCTCAGAAACATCTTTGTGATGTTTGCACTCAACTCACAGAGTTGA

              *     *  *               *        *  * *       *               
     251175 ACATTTCTATTGATAGAGCAGTTTTGAGACACTCTTCTTTTGGAATCTGCAAGTGGATATTTGGA
         66 ACCTTTCTTTTCATAGAGCAGTTTTGAAACACTCTTTTTGTAGAATCTGGAAGTGGATATTTGGA

            ** *                        *     *      
     251240 TAGATTTGAGGATTTCGTTGGAAACGG-GAT-T-AT-ATAT
        131 GCGCTTTGAGGATTTCGTTGGAAACGGAAATATCTTCATAT

                 *       *               *                 **  *   *         
     251277 AAAAAGTAGACAGCAGCATTCTCAGAAACTTCTTTGTGATGTTTGCATCCAGCTCTCAGAGTTGA
          1 AAAAACTAGACAGAAGCATTCTCAGAAACATCTTTGTGATGTTTGCACTCAACTCACAGAGTTGA

              *  * *          *  *        *        *   **        *  **       
     251342 ACATTCCCTTTCATAGAGTAGGTTTGAAACCCTCTTTTTATAGTGTCTGGAAGCGGGCATTTGGA
         66 ACCTTTCTTTTCATAGAGCAGTTTTGAAACACTCTTTTTGTAGAATCTGGAAGTGGATATTTGGA

                   *    * * **  **  **          * *  *
     251407 GCGCTTTCAGGCCTATGCTTAAAATAGGAAATATCTACCTAC
        131 GCGCTTTGAGG-ATTTCGTTGGAAACGGAAATATCTTCATAT

             *                    *    *  **          ** *        *    *     
     251449 AGAAACTAGACAGAAGCATTCTGAGAATCACGTTTGTGATGTGGGTACTCAACTAACAGTGTTGA
          1 AAAAACTAGACAGAAGCATTCTCAGAAACATCTTTGTGATGTTTGCACTCAACTCACAGAGTTGA

            *  *       *   *            *   *                *   *           
     251514 TCCATTCTTTTGATACAGCAGTTTTGAACCACACTTTTTGTAGAATCTGCAAGAGGATATTTGGA
         66 ACCTTTCTTTTCATAGAGCAGTTTTGAAACACTCTTTTTGTAGAATCTGGAAGTGGATATTTGGA

            **   *                     *   *      * *
     251579 TAGCTGTGAGGATTTCGTTGGAAACGGGAATGTCTTCAAAG
        131 GCGCTTTGAGGATTTCGTTGGAAACGGAAATATCTTCATAT

                *                         *   *            *    *            
     251620 AAAATCTAGACAGAAGCATTCTCAGAAACACCTTCGTGATGTTTGCAATCAAGTCACAGAGTTGA
          1 AAAAACTAGACAGAAGCATTCTCAGAAACATCTTTGTGATGTTTGCACTCAACTCACAGAGTTGA

                 * *             *  *           *      *             *       
     251685 ACCTTCCGTTTCATAGAGCAGGTTGGAAACACTCTTATTGTAGTATCTGGAAGTGGACATTTGGA
         66 ACCTTTCTTTTCATAGAGCAGTTTTGAAACACTCTTTTTGTAGAATCTGGAAGTGGATATTTGGA

                   *   ** ** *  *   *            **  
     251750 GCGCTTTCAGGCCTATGGTGAAAAAGGAAATATCTTCCCAT
        131 GCGCTTTGAGGATTTCGTTGGAAACGGAAATATCTTCATAT

                  * * *      **      *   * *   *     ** ** *      *    *     
     251791 AAAAACGACATAGAAGCTATCTCAGGAACTTGTTTATGATGCATCTAATCAACTAACAGTGTTGA
          1 AAAAACTAGACAGAAGCATTCTCAGAAACATCTTTGTGATGTTTGCACTCAACTCACAGAGTTGA

                  * ** * *                         * *       *               
     251856 ACCTTTGTACTGACAGAGCAG-TTTGAAACACTCTTTTTTTGGAATCTGCAAGTGGATATTTGGA
         66 ACCTTTCTTTTCATAGAGCAGTTTTGAAACACTCTTTTTGTAGAATCTGGAAGTGGATATTTGGA

            *                           *   *   *    
     251920 TCGCTTTGAGGATTTCGTTGGAAACGG-GAT-GC--AATAT
        131 GCGCTTTGAGGATTTCGTTGGAAACGGAAATATCTTCATAT

                **  *    *     *                 **  *   *  *    *         * 
     251957 AAAACGTACACAGCAGCATACTCAGAAA-ATACTTTGCCATATTTCCATTCAAGTCACAGAGTGG
          1 AAAAACTAGACAGAAGCATTCTCAGAAACAT-CTTTGTGATGTTTGCACTCAACTCACAGAGTTG

               *  * **            *                  *  *             *      
     252021 AACATTCCCATTCATAGAGCAGGTTTGAAACACTCTTTTTGGAGTATCTGGAAGTGGACATTTGG
         65 AACCTTTCTTTTCATAGAGCAGTTTTGAAACACTCTTTTTGTAGAATCTGGAAGTGGATATTTGG

                    ** * * ** *  *   *            **  
     252086 AGCGCTTTCTGAACTATGGTGAAAAAGGAAATATCTTCCAAT
        130 AGCGCTTTGAGGATTTCGTTGGAAACGGAAATATCTTCATAT

            *     *               *      * *          *  **      **  *  *    
     252128 GAAAACAAGACAGAAGCATTCTGAGAAACTTATTTGTGATGTGTGTCCTCAACAAACGGACTTGA
          1 AAAAACTAGACAGAAGCATTCTCAGAAACATCTTTGTGATGTTTGCACTCAACTCACAGAGTTGA

                   *      **  * *  *  *             *         * * *          
     252193 ACCTTTCGTTTCATGCAGTACTTCTGGAACACTCTTTTTGAAGATAT-TGCATGCGGATATTTGG
         66 ACCTTTCTTTTCATAGAGCAGTTTTGAAACACTCTTTTTGTAGA-ATCTGGAAGTGGATATTTGG

             **                           *     *   * 
     252257 ATAGCTTTGAGGATTTCGTTGGAAACGG--GT-T-TACATGT
        130 AGCGCTTTGAGGATTTCGTTGGAAACGGAAATATCTTCATAT

                 *       *               *        **  *    *    *       *    
     252295 AAAAATTAGACAGCAGCATTCTCAGAAACTTCTTTGTGGAGTCTGCATTCAAGTCACAGAATTGA
          1 AAAAACTAGACAGAAGCATTCTCAGAAACATCTTTGTGATGTTTGCACTCAACTCACAGAGTTGA

              * ** * **            *  * *      *       *             *       
     252360 ACTTCCCCTCACATAGAGCAGTTGTGCAGCACTCTATTTGTAGTATCTGGAAGTGGACATTTGGA
         66 ACCTTTCTTTTCATAGAGCAGTTTTGAAACACTCTTTTTGTAGAATCTGGAAGTGGATATTTGGA

             *         ** *    * *   *            **  
     252425 GGGCTTTGTAGCCTATC-TGGAAAAAGGAAATATCTTCCCAT
        131 GCGCTTTG-AGGATTTCGTTGGAAACGGAAATATCTTCATAT

            *  ** *   *     * *            *   *  *   * **        *  * * *   
     252466 GAATGCGAGATAGAAGTAATCTCAGAAACATGTTTATGCTGTATCTACTCAACTAACTGTGCTGA
          1 AAAAACTAGACAGAAGCATTCTCAGAAACATCTTTGTGATGTTTGCACTCAACTCACAGAGTTGA

              *     *  *               *        *  * *       *               
     252531 ACATTTCTATTGATAGAGCAGTTTTGAGACACTCTTCTTTTGGAATCTGCAAGTGGATATTTGGA
         66 ACCTTTCTTTTCATAGAGCAGTTTTGAAACACTCTTTTTGTAGAATCTGGAAGTGGATATTTGGA

            ** *                        *     *      
     252596 TAGATTTGAGGATTTCGTTGGAAACGG-GAT-T-AT-ATAT
        131 GCGCTTTGAGGATTTCGTTGGAAACGGAAATATCTTCATAT

                 *       *               *                 **  *   *         
     252633 AAAAAGTAGACAGCAGCATTCTCAGAAACTTCTTTGTGATGTTTGCATCCAGCTCTCAGAGTTGA
          1 AAAAACTAGACAGAAGCATTCTCAGAAACATCTTTGTGATGTTTGCACTCAACTCACAGAGTTGA

              *  * *          *  *        *        *   **        *  **       
     252698 ACATTCCCTTTCATAGAGTAGGTTTGAAACCCTCTTTTTATAGTGTCTGGAAGCGGGCATTTGGA
         66 ACCTTTCTTTTCATAGAGCAGTTTTGAAACACTCTTTTTGTAGAATCTGGAAGTGGATATTTGGA

                   *    * * **  **  **          * *  *
     252763 GCGCTTTCAGGCCTATGCTTAAAATAGGAAATATCTACCTAC
        131 GCGCTTTGAGG-ATTTCGTTGGAAACGGAAATATCTTCATAT

             *                    *    *  **          ** *        *    *     
     252805 AGAAACTAGACAGAAGCATTCTGAGAATCACGTTTGTGATGTGGGTACTCAACTAACAGTGTTGA
          1 AAAAACTAGACAGAAGCATTCTCAGAAACATCTTTGTGATGTTTGCACTCAACTCACAGAGTTGA

            *  *       *   *            *   *                *   *           
     252870 TCCATTCTTTTGATACAGCAGTTTTGAACCACACTTTTTGTAGAATCTGCAAGAGGATATTTGGA
         66 ACCTTTCTTTTCATAGAGCAGTTTTGAAACACTCTTTTTGTAGAATCTGGAAGTGGATATTTGGA

            **   *                     *   *      * *
     252935 TAGCTGTGAGGATTTCGTTGGAAACGGGAATGTCTTCAAAG
        131 GCGCTTTGAGGATTTCGTTGGAAACGGAAATATCTTCATAT

                *                         *   *            *    *            
     252976 AAAATCTAGACAGAAGCATTCTCAGAAACACCTTCGTGATGTTTGCAATCAAGTCACAGAGTTGA
          1 AAAAACTAGACAGAAGCATTCTCAGAAACATCTTTGTGATGTTTGCACTCAACTCACAGAGTTGA

                 * *             *  *           *      *             *       
     253041 ACCTTCCGTTTCATAGAGCAGGTTGGAAACACTCTTATTGTAGTATCTGGAAGTGGACATTTGGA
         66 ACCTTTCTTTTCATAGAGCAGTTTTGAAACACTCTTTTTGTAGAATCTGGAAGTGGATATTTGGA

                   *   ** ** *  *   *            **  
     253106 GCGCTTTCAGGCCTATGGTGAAAAAGGAAATATCTTCCCAT
        131 GCGCTTTGAGGATTTCGTTGGAAACGGAAATATCTTCATAT

                  * * *      **      *   * *   *     ** ** *      *    *     
     253147 AAAAACGACATAGAAGCTATCTCAGGAACTTGTTTATGATGCATCTAATCAACTAACAGTGTTGA
          1 AAAAACTAGACAGAAGCATTCTCAGAAACATCTTTGTGATGTTTGCACTCAACTCACAGAGTTGA

                  * ** * *                         * *       *               
     253212 ACCTTTGTACTGACAGAGCAG-TTTGAAACACTCTTTTTTTGGAATCTGCAAGTGGATATTTGGA
         66 ACCTTTCTTTTCATAGAGCAGTTTTGAAACACTCTTTTTGTAGAATCTGGAAGTGGATATTTGGA

            *                           *   *   *    
     253276 TCGCTTTGAGGATTTCGTTGGAAACGG-GAT-GC--AATAT
        131 GCGCTTTGAGGATTTCGTTGGAAACGGAAATATCTTCATAT

                **  *    *     *                 **  *   *  *    *         * 
     253313 AAAACGTACACAGCAGCATACTCAGAAA-ATACTTTGCCATATTTCCATTCAAGTCACAGAGTGG
          1 AAAAACTAGACAGAAGCATTCTCAGAAACAT-CTTTGTGATGTTTGCACTCAACTCACAGAGTTG

               *  * **            *                  *  *             *      
     253377 AACATTCCCATTCATAGAGCAGGTTTGAAACACTCTTTTTGGAGTATCTGGAAGTGGACATTTGG
         65 AACCTTTCTTTTCATAGAGCAGTTTTGAAACACTCTTTTTGTAGAATCTGGAAGTGGATATTTGG

                    ** * * ** *  *   *            **  
     253442 AGCGCTTTCTGAACTATGGTGAAAAAGGAAATATCTTCCAAT
        130 AGCGCTTTGAGGATTTCGTTGGAAACGGAAATATCTTCATAT

            *     *               *      * *          *  **      **  *  *    
     253484 GAAAACAAGACAGAAGCATTCTGAGAAACTTATTTGTGATGTGTGTCCTCAACAAACGGACTTGA
          1 AAAAACTAGACAGAAGCATTCTCAGAAACATCTTTGTGATGTTTGCACTCAACTCACAGAGTTGA

                   *      **  * *  *  *             *   *    * * *           
     253549 ACCTTTCGTTTCATGCAGTACTTCTGGAACACTCTTTTTGAAGATTCTGCATGCGGATATTTGGA
         66 ACCTTTCTTTTCATAGAGCAGTTTTGAAACACTCTTTTTGTAGAATCTGGAAGTGGATATTTGGA

            **                            **   *   * 
     253614 TAGCTTTGAGGATTTCGTTGGAAACGG---GCT-TACATGT
        131 GCGCTTTGAGGATTTCGTTGGAAACGGAAATATCTTCATAT

                 *       *               *        *   *    *    *       *    
     253651 AAAAATTAGACAGCAGCATTCTCAGAAACTTCTTTGTGGTGTCTGCATTCAAGTCACAGAATTGA
          1 AAAAACTAGACAGAAGCATTCTCAGAAACATCTTTGTGATGTTTGCACTCAACTCACAGAGTTGA

              * ** * **            *  * *      *       *             *       
     253716 ACTTCCCCTCACATAGAGCAGTTGTGCAGCACTCTATTTGTAGTATCTGGAAGTGGACATTTGGA
         66 ACCTTTCTTTTCATAGAGCAGTTTTGAAACACTCTTTTTGTAGAATCTGGAAGTGGATATTTGGA

             *         ** *    * *   *            **  
     253781 GGGCTTTGTAGCCTATC-TGGAAAAAGGAAATATCTTCCCAT
        131 GCGCTTTG-AGGATTTCGTTGGAAACGGAAATATCTTCATAT

            *  ** *   *     * *            *   *  *   * **        *  * * *   
     253822 GAATGCGAGATAGAAGTAATCTCAGAAACATGTTTATGCTGTATCTACTCAACTAACTGTGCTGA
          1 AAAAACTAGACAGAAGCATTCTCAGAAACATCTTTGTGATGTTTGCACTCAACTCACAGAGTTGA

              *     *  *               *        *  * *       *               
     253887 ACATTTCTATTGATAGAGCAGTTTTGAGACACTCTTCTTTTGGAATCTGCAAGTGGATATTTGGA
         66 ACCTTTCTTTTCATAGAGCAGTTTTGAAACACTCTTTTTGTAGAATCTGGAAGTGGATATTTGGA

            ** *                        *     *      
     253952 TAGATTTGAGGATTTCGTTGGAAACGG-GAT-T-AT-ATAT
        131 GCGCTTTGAGGATTTCGTTGGAAACGGAAATATCTTCATAT

                 *       *            *  *                 **  *   *         
     253989 AAAAAGTAGACAGCAGCATTCTCAGACACTTCTTTGTGATGTTTGCATCCAGCTCTCAGAGTTGA
          1 AAAAACTAGACAGAAGCATTCTCAGAAACATCTTTGTGATGTTTGCACTCAACTCACAGAGTTGA

              *  * *          *  *        *        *   **        *  **       
     254054 ACATTCCCTTTCATAGAGTAGGTTTGAAACCCTCTTTTTATAGTGTCTGGAAGCGGGCATTTGGA
         66 ACCTTTCTTTTCATAGAGCAGTTTTGAAACACTCTTTTTGTAGAATCTGGAAGTGGATATTTGGA

                   *    * * **  **  **          * *  *
     254119 GCGCTTTCAGGCCTATGCTTAAAATAGGAAATATCTACCTAC
        131 GCGCTTTGAGG-ATTTCGTTGGAAACGGAAATATCTTCATAT

             *                    *    *  **          ** *        *    *     
     254161 AGAAACTAGACAGAAGCATTCTGAGAATCACGTTTGTGATGTGGGTACTCAACTAACAGTGTTGA
          1 AAAAACTAGACAGAAGCATTCTCAGAAACATCTTTGTGATGTTTGCACTCAACTCACAGAGTTGA

            *  *       *   *            *   *                *   *           
     254226 TCCATTCTTTTGATACAGCAGTTTTGAACCACACTTTTTGTAGAATCTGCAAGAGGATATTTGGA
         66 ACCTTTCTTTTCATAGAGCAGTTTTGAAACACTCTTTTTGTAGAATCTGGAAGTGGATATTTGGA

            **   *                     *   *      * *
     254291 TAGCTGTGAGGATTTCGTTGGAAACGGGAATGTCTTCAAAG
        131 GCGCTTTGAGGATTTCGTTGGAAACGGAAATATCTTCATAT

                *                         *   *            *    *            
     254332 AAAATCTAGACAGAAGCATTCTCAGAAACACCTTCGTGATGTTTGCAATCAAGTCACAGAGTTGA
          1 AAAAACTAGACAGAAGCATTCTCAGAAACATCTTTGTGATGTTTGCACTCAACTCACAGAGTTGA

                 * *             *  *           *      *             *       
     254397 ACCTTCCGTTTCATAGAGCAGGTTGGAAACACTCTTATTGTAGTATCTGGAAGTGGACATTTGGA
         66 ACCTTTCTTTTCATAGAGCAGTTTTGAAACACTCTTTTTGTAGAATCTGGAAGTGGATATTTGGA

                   *   ** ** *  *   *            **  
     254462 GCGCTTTCAGGCCTATGGTGAAAAAGGAAATATCTTCCCAT
        131 GCGCTTTGAGGATTTCGTTGGAAACGGAAATATCTTCATAT

                  * * *      **      *   * *   *     ** ** *      *    *     
     254503 AAAAACGACATAGAAGCTATCTCAGGAACTTGTTTATGATGCATCTAATCAACTAACAGTGTTGA
          1 AAAAACTAGACAGAAGCATTCTCAGAAACATCTTTGTGATGTTTGCACTCAACTCACAGAGTTGA

                  * ** * *                         * *       *               
     254568 ACCTTTGTACTGACAGAGCAG-TTTGAAACACTCTTTTTTTGGAATCTGCAAGTGGATATTTGGA
         66 ACCTTTCTTTTCATAGAGCAGTTTTGAAACACTCTTTTTGTAGAATCTGGAAGTGGATATTTGGA

            *                           *   *   *    
     254632 TCGCTTTGAGGATTTCGTTGGAAACGG-GAT-GC--AATAT
        131 GCGCTTTGAGGATTTCGTTGGAAACGGAAATATCTTCATAT

                **  *    *     *                 **  *   *  *    *         * 
     254669 AAAACGTACACAGCAGCATACTCAGAAA-ATACTTTGCCATATTTCCATTCAAGTCACAGAGTGG
          1 AAAAACTAGACAGAAGCATTCTCAGAAACAT-CTTTGTGATGTTTGCACTCAACTCACAGAGTTG

               *  * **            *  *               *  *             *      
     254733 AACATTCCCATTCATAGAGCAGGTTGGAAACACTCTTTTTGGAGTATCTGGAAGTGGACATTTGG
         65 AACCTTTCTTTTCATAGAGCAGTTTTGAAACACTCTTTTTGTAGAATCTGGAAGTGGATATTTGG

                    ** * * ** *  *   *            **  
     254798 AGCGCTTTCTGAACTATGGTGAAAAAGGAAATATCTTCCAAT
        130 AGCGCTTTGAGGATTTCGTTGGAAACGGAAATATCTTCATAT

            *     *               *      * *          *  **      **  *  *    
     254840 GAAAACAAGACAGAAGCATTCTGAGAAACTTATTTGTGATGTGTGTCCTCAACAAACGGACTTGA
          1 AAAAACTAGACAGAAGCATTCTCAGAAACATCTTTGTGATGTTTGCACTCAACTCACAGAGTTGA

                   *      **  * *  *  *             *   *    * * *           
     254905 ACCTTTCGTTTCATGCAGTACTTCTGGAACACTCTTTTTGAAGATTCTGCATGCGGATATTTGGA
         66 ACCTTTCTTTTCATAGAGCAGTTTTGAAACACTCTTTTTGTAGAATCTGGAAGTGGATATTTGGA

            **                            **   *   * 
     254970 TAGCTTTGAGGATTTCGTTGGAAACGG---GCT-TACATGT
        131 GCGCTTTGAGGATTTCGTTGGAAACGGAAATATCTTCATAT

                 *       *               *        *   *    *    *       *    
     255007 AAAAATTAGACAGCAGCATTCTCAGAAACTTCTTTGTGGTGTCTGCATTCAAGTCACAGAATTGA
          1 AAAAACTAGACAGAAGCATTCTCAGAAACATCTTTGTGATGTTTGCACTCAACTCACAGAGTTGA

              * ** * **            *  * *      *       *             *       
     255072 ACTTCCCCTCACATAGAGCAGTTGTGCAGCACTCTATTTGTAGTATCTGGAAGTGGACATTTGGA
         66 ACCTTTCTTTTCATAGAGCAGTTTTGAAACACTCTTTTTGTAGAATCTGGAAGTGGATATTTGGA

             *         ** *    * *   *            **  
     255137 GGGCTTTGTAGCCTAT-GTGGAAAAAGGAAATATCTTCCCAT
        131 GCGCTTTG-AGGATTTCGTTGGAAACGGAAATATCTTCATAT

            *  ** *   *     * *            *   *  *   * **        *  * * *   
     255178 GAATGCGAGATAGAAGTAATCTCAGAAACATGTTTATGCTGTATCTACTCAACTAACTGTGCTGA
          1 AAAAACTAGACAGAAGCATTCTCAGAAACATCTTTGTGATGTTTGCACTCAACTCACAGAGTTGA

              *     *  *               *        *  * *       *               
     255243 ACATTTCTATTGATAGAGCAGTTTTGAGACACTCTTCTTTTGGAATCTGCAAGTGGATATTTGGA
         66 ACCTTTCTTTTCATAGAGCAGTTTTGAAACACTCTTTTTGTAGAATCTGGAAGTGGATATTTGGA

            ** *                        *     *      
     255308 TAGATTTGAGGATTTCGTTGGAAACGG-GAT-T-AT-ATAT
        131 GCGCTTTGAGGATTTCGTTGGAAACGGAAATATCTTCATAT

                 *       *            *  *                 **  *   *         
     255345 AAAAAGTAGACAGCAGCATTCTCAGACACTTCTTTGTGATGTTTGCATCCAGCTCTCAGAGTTGA
          1 AAAAACTAGACAGAAGCATTCTCAGAAACATCTTTGTGATGTTTGCACTCAACTCACAGAGTTGA

              *  * *          *  *        *        *   **        *  **       
     255410 ACATTCCCTTTCATAGAGTAGGTTTGAAACCCTCTTTTTATAGTGTCTGGAAGCGGGCATTTGGA
         66 ACCTTTCTTTTCATAGAGCAGTTTTGAAACACTCTTTTTGTAGAATCTGGAAGTGGATATTTGGA

                   *    * * **  **  **          * *  *
     255475 GCGCTTTCAGGCCTATGCTTAAAATAGGAAATATCTACCTAC
        131 GCGCTTTGAGG-ATTTCGTTGGAAACGGAAATATCTTCATAT

             *                    *    *  **          ** *        *    *     
     255517 AGAAACTAGACAGAAGCATTCTGAGAATCACGTTTGTGATGTGGGTACTCAACTAACAGTGTTGA
          1 AAAAACTAGACAGAAGCATTCTCAGAAACATCTTTGTGATGTTTGCACTCAACTCACAGAGTTGA

            *  *       *   *            *   *                *   *           
     255582 TCCATTCTTTTGATACAGCAGTTTTGAACCACACTTTTTGTAGAATCTGCAAGAGGATATTTGGA
         66 ACCTTTCTTTTCATAGAGCAGTTTTGAAACACTCTTTTTGTAGAATCTGGAAGTGGATATTTGGA

            **   *                     *   *      * *
     255647 TAGCTGTGAGGATTTCGTTGGAAACGGGAATGTCTTCAAAG
        131 GCGCTTTGAGGATTTCGTTGGAAACGGAAATATCTTCATAT

                *                         *   *            *    *            
     255688 AAAATCTAGACAGAAGCATTCTCAGAAACACCTTCGTGATGTTTGCAATCAAGTCACAGAGTTGA
          1 AAAAACTAGACAGAAGCATTCTCAGAAACATCTTTGTGATGTTTGCACTCAACTCACAGAGTTGA

                 * *             *  *           *      *             *       
     255753 ACCTTCCGTTTCATAGAGCAGGTTGGAAACACTCTTATTGTAGTATCTGGAAGTGGACATTTGGA
         66 ACCTTTCTTTTCATAGAGCAGTTTTGAAACACTCTTTTTGTAGAATCTGGAAGTGGATATTTGGA

                   *   ** ** *  *   *            **  
     255818 GCGCTTTCAGGCCTATGGTGAAAAAGGAAATATCTTCCCAT
        131 GCGCTTTGAGGATTTCGTTGGAAACGGAAATATCTTCATAT

                  * * *      **      *   * *   *     ** ** *      *    *     
     255859 AAAAACGACATAGAAGCTATCTCAGGAACTTGTTTATGATGCATCTAATCAACTAACAGTGTTGA
          1 AAAAACTAGACAGAAGCATTCTCAGAAACATCTTTGTGATGTTTGCACTCAACTCACAGAGTTGA

                  * ** * *                         * *       *               
     255924 ACCTTTGTACTGACAGAGCAG-TTTGAAACACTCTTTTTTTGGAATCTGCAAGTGGATATTTGGA
         66 ACCTTTCTTTTCATAGAGCAGTTTTGAAACACTCTTTTTGTAGAATCTGGAAGTGGATATTTGGA

            *                           *   *   *    
     255988 TCGCTTTGAGGATTTCGTTGGAAACGG-GAT-GC--AATAT
        131 GCGCTTTGAGGATTTCGTTGGAAACGGAAATATCTTCATAT

                **  *    *     *                 **  *   *  *    *         * 
     256025 AAAACGTACACAGCAGCATACTCAGAAA-ATACTTTGCCATATTTCCATTCAAGTCACAGAGTGG
          1 AAAAACTAGACAGAAGCATTCTCAGAAACAT-CTTTGTGATGTTTGCACTCAACTCACAGAGTTG

               *  * **            *                  *  *             *      
     256089 AACATTCCCATTCATAGAGCAGGTTTGAAACACTCTTTTTGGAGTATCTGGAAGTGGACATTTGG
         65 AACCTTTCTTTTCATAGAGCAGTTTTGAAACACTCTTTTTGTAGAATCTGGAAGTGGATATTTGG

                    ** * * ** *  *   *            **  
     256154 AGCGCTTTCTGAACTATGGTGAAAAAGGAAATATCTTCCAAT
        130 AGCGCTTTGAGGATTTCGTTGGAAACGGAAATATCTTCATAT

            *     *               *      * *          *  **      **  *  *    
     256196 GAAAACAAGACAGAAGCATTCTGAGAAACTTATTTGTGATGTGTGTCCTCAACAAACGGACTTGA
          1 AAAAACTAGACAGAAGCATTCTCAGAAACATCTTTGTGATGTTTGCACTCAACTCACAGAGTTGA

                   *      **  * *  *  *             *   *    * * *           
     256261 ACCTTTCGTTTCATGCAGTACTTCTGGAACACTCTTTTTGAAGATTCTGCATGCGGATATTTGGA
         66 ACCTTTCTTTTCATAGAGCAGTTTTGAAACACTCTTTTTGTAGAATCTGGAAGTGGATATTTGGA

            **                            **   *   * 
     256326 TAGCTTTGAGGATTTCGTTGGAAACGG---GCT-TACATGT
        131 GCGCTTTGAGGATTTCGTTGGAAACGGAAATATCTTCATAT

                 *       *               *        *   *    *    *       *    
     256363 AAAAATTAGACAGCAGCATTCTCAGAAACTTCTTTGTGGTGTCTGCATTCAAGTCACAGAATTGA
          1 AAAAACTAGACAGAAGCATTCTCAGAAACATCTTTGTGATGTTTGCACTCAACTCACAGAGTTGA

              * ** * **            *  * *      *       *             *       
     256428 ACTTCCCCTCACATAGAGCAGTTGTGCAGCACTCTATTTGTAGTATCTGGAAGTGGACATTTGGA
         66 ACCTTTCTTTTCATAGAGCAGTTTTGAAACACTCTTTTTGTAGAATCTGGAAGTGGATATTTGGA

             *         ** *    * *   *            **  
     256493 GGGCTTTGTAGCCTATC-TGGAAAAAGGAAATATCTTCCCAT
        131 GCGCTTTG-AGGATTTCGTTGGAAACGGAAATATCTTCATAT

            *  ** *   *     * *            *   *  *   * **        *  * * *   
     256534 GAATGCGAGATAGAAGTAATCTCAGAAACATGTTTATGCTGTATCTACTCAACTAACTGTGCTGA
          1 AAAAACTAGACAGAAGCATTCTCAGAAACATCTTTGTGATGTTTGCACTCAACTCACAGAGTTGA

              *     *  *               *        *  * *       *               
     256599 ACATTTCTATTGATAGAGCAGTTTTGAGACACTCTTCTTTTGGAATCTGCAAGTGGATATTTGGA
         66 ACCTTTCTTTTCATAGAGCAGTTTTGAAACACTCTTTTTGTAGAATCTGGAAGTGGATATTTGGA

            ** *                        *     *      
     256664 TAGATTTGAGGATTTCGTTGGAAACGG-GAT-T-AT-ATAT
        131 GCGCTTTGAGGATTTCGTTGGAAACGGAAATATCTTCATAT

                 *       *               *                 **  *   *         
     256701 AAAAAGTAGACAGCAGCATTCTCAGAAACTTCTTTGTGATGTTTGCATCCAGCTCTCAGAGTTGA
          1 AAAAACTAGACAGAAGCATTCTCAGAAACATCTTTGTGATGTTTGCACTCAACTCACAGAGTTGA

              *  * *          *  *        *        *   **        *  **       
     256766 ACATTCCCTTTCATAGAGTAGGTTTGAAACCCTCTTTTTATAGTGTCTGGAAGCGGGCATTTGGA
         66 ACCTTTCTTTTCATAGAGCAGTTTTGAAACACTCTTTTTGTAGAATCTGGAAGTGGATATTTGGA

                   *    * * **  **  **          * *  *
     256831 GCGCTTTCAGGCCTATGCTTAAAATAGGAAATATCTACCTAC
        131 GCGCTTTGAGG-ATTTCGTTGGAAACGGAAATATCTTCATAT

             *                    *    *  **          ** *        *    *     
     256873 AGAAACTAGACAGAAGCATTCTGAGAATCACGTTTGTGATGTGGGTACTCAACTAACAGTGTTGA
          1 AAAAACTAGACAGAAGCATTCTCAGAAACATCTTTGTGATGTTTGCACTCAACTCACAGAGTTGA

            *  *       *   *            *   *                *   *           
     256938 TCCATTCTTTTGATACAGCAGTTTTGAACCACACTTTTTGTAGAATCTGCAAGAGGATATTTGGA
         66 ACCTTTCTTTTCATAGAGCAGTTTTGAAACACTCTTTTTGTAGAATCTGGAAGTGGATATTTGGA

            **   *                     *   *      * *
     257003 TAGCTGTGAGGATTTCGTTGGAAACGGGAATGTCTTCAAAG
        131 GCGCTTTGAGGATTTCGTTGGAAACGGAAATATCTTCATAT

                *                         *   *            *    *            
     257044 AAAATCTAGACAGAAGCATTCTCAGAAACACCTTCGTGATGTTTGCAATCAAGTCACAGAGTTGA
          1 AAAAACTAGACAGAAGCATTCTCAGAAACATCTTTGTGATGTTTGCACTCAACTCACAGAGTTGA

                 * *             *  *           *      *             *       
     257109 ACCTTCCGTTTCATAGAGCAGGTTGGAAACACTCTTATTGTAGTATCTGGAAGTGGACATTTGGA
         66 ACCTTTCTTTTCATAGAGCAGTTTTGAAACACTCTTTTTGTAGAATCTGGAAGTGGATATTTGGA

                   *   ** ** *  *   *            **  
     257174 GCGCTTTCAGGCCTATGGTGAAAAAGGAAATATCTTCCCAT
        131 GCGCTTTGAGGATTTCGTTGGAAACGGAAATATCTTCATAT

                  * * *      **      *   * *   *     ** ** *      *    *     
     257215 AAAAACGACATAGAAGCTATCTCAGGAACTTGTTTATGATGCATCTAATCAACTAACAGTGTTGA
          1 AAAAACTAGACAGAAGCATTCTCAGAAACATCTTTGTGATGTTTGCACTCAACTCACAGAGTTGA

                  * ** * *                         * *       *               
     257280 ACCTTTGTACTGACAGAGCAG-TTTGAAACACTCTTTTTTTGGAATCTGCAAGTGGATATTTGGA
         66 ACCTTTCTTTTCATAGAGCAGTTTTGAAACACTCTTTTTGTAGAATCTGGAAGTGGATATTTGGA

            *                           *   *   *    
     257344 TCGCTTTGAGGATTTCGTTGGAAACGG-GAT-GC--AATAT
        131 GCGCTTTGAGGATTTCGTTGGAAACGGAAATATCTTCATAT

                **  *    *     *                 **  *   *  *    *         * 
     257381 AAAACGTACACAGCAGCATACTCAGAAA-ATACTTTGCCATATTTCCATTCAAGTCACAGAGTGG
          1 AAAAACTAGACAGAAGCATTCTCAGAAACAT-CTTTGTGATGTTTGCACTCAACTCACAGAGTTG

               *  * **            *                  *  *             *      
     257445 AACATTCCCATTCATAGAGCAGGTTTGAAACACTCTTTTTGGAGTATCTGGAAGTGGACATTTGG
         65 AACCTTTCTTTTCATAGAGCAGTTTTGAAACACTCTTTTTGTAGAATCTGGAAGTGGATATTTGG

                    ** * * ** *  *   *            **  
     257510 AGCGCTTTCTGAACTATGGTGAAAAAGGAAATATCTTCCAAT
        130 AGCGCTTTGAGGATTTCGTTGGAAACGGAAATATCTTCATAT

            *     *               *      * *          *  **      **  *  *    
     257552 GAAAACAAGACAGAAGCATTCTGAGAAACTTATTTGTGATGTGTGTCCTCAACAAACGGACTTGA
          1 AAAAACTAGACAGAAGCATTCTCAGAAACATCTTTGTGATGTTTGCACTCAACTCACAGAGTTGA

                   *      **  * *  *  *             *   *    * * *           
     257617 ACCTTTCGTTTCATGCAGTACTTCTGGAACACTCTTTTTGAAGATTCTGCATGCGGATATTTGGA
         66 ACCTTTCTTTTCATAGAGCAGTTTTGAAACACTCTTTTTGTAGAATCTGGAAGTGGATATTTGGA

            **                            **   *   * 
     257682 TAGCTTTGAGGATTTCGTTGGAAACGG---GCT-TACATGT
        131 GCGCTTTGAGGATTTCGTTGGAAACGGAAATATCTTCATAT

                 *       *               * *      *   *    *    *       *    
     257719 AAAAATTAGACAGCAGCATTCTCAGAAACTTTTTTGTGGTGTCTGCATTCAAGTCACAGAATTGA
          1 AAAAACTAGACAGAAGCATTCTCAGAAACATCTTTGTGATGTTTGCACTCAACTCACAGAGTTGA

              * ** * **            *  * *  *   *       *             *       
     257784 ACTTCCCCTCACATAGAGCAGTTGTGCAGCAATCTATTTGTAGTATCTGGAAGTGGACATTTGGA
         66 ACCTTTCTTTTCATAGAGCAGTTTTGAAACACTCTTTTTGTAGAATCTGGAAGTGGATATTTGGA

             *         ** *          *            **  
     257849 GGGCTTTGTAGCCTATC--TGGAAAAGGAAATATCTTCCCAT
        131 GCGCTTTG-AGGATTTCGTTGGAAACGGAAATATCTTCATAT

            *  ** *   *     * *            *  **  *   * **        *  * * *   
     257889 GAATGCGAGATAGAAGTAATCTCAGAAACATGTTCATGCTGTATCTACTCAACTAACTGTGCTGA
          1 AAAAACTAGACAGAAGCATTCTCAGAAACATCTTTGTGATGTTTGCACTCAACTCACAGAGTTGA

              *     *  *               *        *  * *       *               
     257954 ACATTTCTATTGATAGAGCAGTTTTGAGACACTCTTCTTTTGGAATCTGCAAGTGGATATTTGGA
         66 ACCTTTCTTTTCATAGAGCAGTTTTGAAACACTCTTTTTGTAGAATCTGGAAGTGGATATTTGGA

            ** *                        *     *      
     258019 TAGATTTGAGGATTTCGTTGGAAACGG-GAT-T-AT-ATAT
        131 GCGCTTTGAGGATTTCGTTGGAAACGGAAATATCTTCATAT

                 *       *               *                 **  *   *         
     258056 AAAAAGTAGACAGCAGCATTCTCAGAAACTTCTTTGTGATGTTTGCATCCAGCTCTCAGAGTTGA
          1 AAAAACTAGACAGAAGCATTCTCAGAAACATCTTTGTGATGTTTGCACTCAACTCACAGAGTTGA

              *  * *          *  *        *        *   **        *  **       
     258121 ACATTCCCTTTCATAGAGTAGGTTTGAAACCCTCTTTTTATAGTGTCTGGAAGCGGGCATTTGGA
         66 ACCTTTCTTTTCATAGAGCAGTTTTGAAACACTCTTTTTGTAGAATCTGGAAGTGGATATTTGGA

                   *    * * **  **  **          * *  *
     258186 GCGCTTTCAGGCCTATGCTTAAAATAGGAAATATCTACCTAC
        131 GCGCTTTGAGG-ATTTCGTTGGAAACGGAAATATCTTCATAT

             *                    *    *  **          ** *        *    *     
     258228 AGAAACTAGACAGAAGCATTCTGAGAATCACGTTTGTGATGTGGGTACTCAACTAACAGTGTTGA
          1 AAAAACTAGACAGAAGCATTCTCAGAAACATCTTTGTGATGTTTGCACTCAACTCACAGAGTTGA

            *  *       *   *            *   *                *   *           
     258293 TCCATTCTTTTGATACAGCAGTTTTGAACCACACTTTTTGTAGAATCTGCAAGAGGATATTTGGA
         66 ACCTTTCTTTTCATAGAGCAGTTTTGAAACACTCTTTTTGTAGAATCTGGAAGTGGATATTTGGA

            **   *                     *   *      * *
     258358 TAGCTGTGAGGATTTCGTTGGAAACGGGAATGTCTTCAAAG
        131 GCGCTTTGAGGATTTCGTTGGAAACGGAAATATCTTCATAT

                *                         *   *            *   **            
     258399 AAAATCTAGACAGAAGCATTCTCAGAAACACCTTCGTGATGTTTGCAATCATGTCACAGAGTTGA
          1 AAAAACTAGACAGAAGCATTCTCAGAAACATCTTTGTGATGTTTGCACTCAACTCACAGAGTTGA

                 * *             *  *           *      *             *       
     258464 ACCTTCCGTTTCATAGAGCAGGTTGGAAACACTCTTATTGTAGTATCTGGAAGTGGACATTTGGA
         66 ACCTTTCTTTTCATAGAGCAGTTTTGAAACACTCTTTTTGTAGAATCTGGAAGTGGATATTTGGA

                   *   ** ** *  *   *            **  
     258529 GCGCTTTCAGGCCTATGGTGAAAAAGGAAATATCTTCCCAT
        131 GCGCTTTGAGGATTTCGTTGGAAACGGAAATATCTTCATAT

                  * * *      **      *   * *   *     ** ** *      *    *     
     258570 AAAAACGACATAGAAGCTATCTCAGGAACTTGTTTATGATGCATCTAATCAACTAACAGTGTTGA
          1 AAAAACTAGACAGAAGCATTCTCAGAAACATCTTTGTGATGTTTGCACTCAACTCACAGAGTTGA

                  * ** * *                         * *       *               
     258635 ACCTTTGTACTGACAGAGCAG-TTTGAAACACTCTTTTTTTGGAATCTGCAAGTGGATATTTGGA
         66 ACCTTTCTTTTCATAGAGCAGTTTTGAAACACTCTTTTTGTAGAATCTGGAAGTGGATATTTGGA

            *                           *   *   *    
     258699 TCGCTTTGAGGATTTCGTTGGAAACGG-GAT-GC--AATAT
        131 GCGCTTTGAGGATTTCGTTGGAAACGGAAATATCTTCATAT

                **  *    *     *                 **  *   *  *    *         * 
     258736 AAAACGTACACAGCAGCATACTCAGAAA-ATACTTTGCCATATTTCCATTCAAGTCACAGAGTGG
          1 AAAAACTAGACAGAAGCATTCTCAGAAACAT-CTTTGTGATGTTTGCACTCAACTCACAGAGTTG

               *  * **            *                  *  *             *      
     258800 AACATTCCCATTCATAGAGCAGGTTTGAAACACTCTTTTTGGAGTATCTGGAAGTGGACATTTGG
         65 AACCTTTCTTTTCATAGAGCAGTTTTGAAACACTCTTTTTGTAGAATCTGGAAGTGGATATTTGG

                    ** * * ** *  *   *            **  
     258865 AGCGCTTTCTGAACTATGGTGAAAAAGGAAATATCTTCCAAT
        130 AGCGCTTTGAGGATTTCGTTGGAAACGGAAATATCTTCATAT

            *     *               *      * *          *  **      **  *  *    
     258907 GAAAACAAGACAGAAGCATTCTGAGAAACTTATTTGTGATGTGTGTCCTCAACAAACGGACTTGA
          1 AAAAACTAGACAGAAGCATTCTCAGAAACATCTTTGTGATGTTTGCACTCAACTCACAGAGTTGA

                   *      **  * *  *  *             *   *    * * *           
     258972 ACCTTTCGTTTCATGCAGTACTTCTGGAACACTCTTTTTGAAGATTCTGCATGCGGATATTTGGA
         66 ACCTTTCTTTTCATAGAGCAGTTTTGAAACACTCTTTTTGTAGAATCTGGAAGTGGATATTTGGA

            **                            **   *   * 
     259037 TAGCTTTGAGGATTTCGTTGGAAACGG---GCT-TACATGT
        131 GCGCTTTGAGGATTTCGTTGGAAACGGAAATATCTTCATAT

                 *       *               *        *   *    *    *       *    
     259074 AAAAATTAGACAGCAGCATTCTCAGAAACTTCTTTGTGGTGTCTGCATTCAAGTCACAGAATTGA
          1 AAAAACTAGACAGAAGCATTCTCAGAAACATCTTTGTGATGTTTGCACTCAACTCACAGAGTTGA

              * ** * **            *  * *      *       *             *       
     259139 ACTTCCCCTCACATAGAGCAGTTGTGCAGCACTCTATTTGTAGTATCTGGAAGTGGACATTTGGA
         66 ACCTTTCTTTTCATAGAGCAGTTTTGAAACACTCTTTTTGTAGAATCTGGAAGTGGATATTTGGA

             *         ** *    * *   *            **  
     259204 GGGCTTTGTAGCCTATC-TGGAAAAAGGAAATATCTTCCCAT
        131 GCGCTTTG-AGGATTTCGTTGGAAACGGAAATATCTTCATAT

            *  ** *   *     * *            *   *  *   * **        *  * * *   
     259245 GAATGCGAGATAGAAGTAATCTCAGAAACATGTTTATGCTGTATCTACTCAACTAACTGTGCTGA
          1 AAAAACTAGACAGAAGCATTCTCAGAAACATCTTTGTGATGTTTGCACTCAACTCACAGAGTTGA

              *     *  *               *        *  * *       *               
     259310 ACATTTCTATTGATAGAGCAGTTTTGAGACACTCTTCTTTTGGAATCTGCAAGTGGATATTTGGA
         66 ACCTTTCTTTTCATAGAGCAGTTTTGAAACACTCTTTTTGTAGAATCTGGAAGTGGATATTTGGA

            ** *                        *     *      
     259375 TAGATTTGAGGATTTCGTTGGAAACGG-GAT-T-AT-ATAT
        131 GCGCTTTGAGGATTTCGTTGGAAACGGAAATATCTTCATAT

                 *       *               *                 **  *   *         
     259412 AAAAAGTAGACAGCAGCATTCTCAGAAACTTCTTTGTGATGTTTGCATCCAGCTCTCAGAGTTGA
          1 AAAAACTAGACAGAAGCATTCTCAGAAACATCTTTGTGATGTTTGCACTCAACTCACAGAGTTGA

              *  * *          *  *        *        *   **        *  **       
     259477 ACATTCCCTTTCATAGAGTAGGTTTGAAACCCTCTTTTTATAGTGTCTGGAAGCGGGCATTTGGA
         66 ACCTTTCTTTTCATAGAGCAGTTTTGAAACACTCTTTTTGTAGAATCTGGAAGTGGATATTTGGA

                   *    * * **  **  **          * *  *
     259542 GCGCTTTCAGGCCTATGCTTAAAATAGGAAATATCTACCTAC
        131 GCGCTTTGAGG-ATTTCGTTGGAAACGGAAATATCTTCATAT

             *                    *    *  **          ** *        *    *     
     259584 AGAAACTAGACAGAAGCATTCTGAGAATCACGTTTGTGATGTGGGTACTCAACTAACAGTGTTGA
          1 AAAAACTAGACAGAAGCATTCTCAGAAACATCTTTGTGATGTTTGCACTCAACTCACAGAGTTGA

            *  *       *   *  *         *   **               *   *           
     259649 TCCATTCTTTTGATACAGAAGTTTTGAACCACAGTTTTTGTAGAATCTGCAAGAGGATATTTGGA
         66 ACCTTTCTTTTCATAGAGCAGTTTTGAAACACTCTTTTTGTAGAATCTGGAAGTGGATATTTGGA

            **   *                     *   *      * *
     259714 TAGCTGTGAGGATTTCGTTGGAAACGGGAATGTCTTCAAAG
        131 GCGCTTTGAGGATTTCGTTGGAAACGGAAATATCTTCATAT

                *                         *   *            *    *            
     259755 AAAATCTAGACAGAAGCATTCTCAGAAACACCTTCGTGATGTTTGCAATCAAGTCACAGAGTTGA
          1 AAAAACTAGACAGAAGCATTCTCAGAAACATCTTTGTGATGTTTGCACTCAACTCACAGAGTTGA

                 * *             *  *           *      *             *       
     259820 ACCTTCCGTTTCATAGAGCAGGTTGGAAACACTCTTATTGTAGTATCTGGAAGTGGACATTTGGA
         66 ACCTTTCTTTTCATAGAGCAGTTTTGAAACACTCTTTTTGTAGAATCTGGAAGTGGATATTTGGA

                   *   ** ** *  *   *            **  
     259885 GCGCTTTCAGGCCTATGGTGAAAAAGGAAATATCTTCCCAT
        131 GCGCTTTGAGGATTTCGTTGGAAACGGAAATATCTTCATAT

                  * * *      **      *   * *   *     ** ** *      *    *     
     259926 AAAAACGACATAGAAGCTATCTCAGGAACTTGTTTATGATGCATCTAATCAACTAACAGTGTTGA
          1 AAAAACTAGACAGAAGCATTCTCAGAAACATCTTTGTGATGTTTGCACTCAACTCACAGAGTTGA

                  * ** * *                         * *       *               
     259991 ACCTTTGTACTGACAGAGCAG-TTTGAAACACTCTTTTTTTGGAATCTGCAAGTGGATATTTGGA
         66 ACCTTTCTTTTCATAGAGCAGTTTTGAAACACTCTTTTTGTAGAATCTGGAAGTGGATATTTGGA

            *                           *   *   *    
     260055 TCGCTTTGAGGATTTCGTTGGAAACGG-GAT-GC--AATAT
        131 GCGCTTTGAGGATTTCGTTGGAAACGGAAATATCTTCATAT

                **  *    *     *                 **  *   *  *    *         * 
     260092 AAAACGTACACAGCAGCATACTCAGAAA-ATACTTTGCCATATTTCCATTCAAGTCACAGAGTGG
          1 AAAAACTAGACAGAAGCATTCTCAGAAACAT-CTTTGTGATGTTTGCACTCAACTCACAGAGTTG

               *  * **            *                  *  *             *      
     260156 AACATTCCCATTCATAGAGCAGGTTTGAAACACTCTTTTTGGAGTATCTGGAAGTGGACATTTGG
         65 AACCTTTCTTTTCATAGAGCAGTTTTGAAACACTCTTTTTGTAGAATCTGGAAGTGGATATTTGG

                    ** * * ** *  *   *            **  
     260221 AGCGCTTTCTGAACTATGGTGAAAAAGGAAATATCTTCCAAT
        130 AGCGCTTTGAGGATTTCGTTGGAAACGGAAATATCTTCATAT

            *     *               *      * *          *  **      **  *  *    
     260263 GAAAACAAGACAGAAGCATTCTGAGAAACTTATTTGTGATGTGTGTCCTCAACAAACGGACTTGA
          1 AAAAACTAGACAGAAGCATTCTCAGAAACATCTTTGTGATGTTTGCACTCAACTCACAGAGTTGA

                   *      **  * *  *  *             *         * * *          
     260328 ACCTTTCGTTTCATGCAGTACTTCTGGAACACTCTTTTTGAAGATAT-TGCATGCGGATATTTGG
         66 ACCTTTCTTTTCATAGAGCAGTTTTGAAACACTCTTTTTGTAGA-ATCTGGAAGTGGATATTTGG

             **                           *     *   * 
     260392 ATAGCTTTGAGGATTTCGTTGGAAACGG--GT-T-TACATGT
        130 AGCGCTTTGAGGATTTCGTTGGAAACGGAAATATCTTCATAT

                 *       *               *        *   *    *    *       *    
     260430 AAAAATTAGACAGCAGCATTCTCAGAAACTTCTTTGTGGTGTCTGCATTCAAGTCACAGAATTGA
          1 AAAAACTAGACAGAAGCATTCTCAGAAACATCTTTGTGATGTTTGCACTCAACTCACAGAGTTGA

              * ** * **            *  * *      *       *             *       
     260495 ACTTCCCCTCACATAGAGCAGTTGTGCAGCACTCTATTTGTAGTATCTGGAAGTGGACATTTGGA
         66 ACCTTTCTTTTCATAGAGCAGTTTTGAAACACTCTTTTTGTAGAATCTGGAAGTGGATATTTGGA

             *         ** *    * *   *            **  
     260560 GGGCTTTGTAGCCTATC-TGGAAAAAGGAAATATCTTCCCAT
        131 GCGCTTTG-AGGATTTCGTTGGAAACGGAAATATCTTCATAT

            *  ** *   *     * *            *   *  *   * **        *  * * *   
     260601 GAATGCGAGATAGAAGTAATCTCAGAAACATGTTTATGCTGTATCTACTCAACTAACTGTGCTGA
          1 AAAAACTAGACAGAAGCATTCTCAGAAACATCTTTGTGATGTTTGCACTCAACTCACAGAGTTGA

              *     *  *               *        *  * *       *               
     260666 ACATTTCTATTGATAGAGCAGTTTTGAGACACTCTTCTTTTGGAATCTGCAAGTGGATATTTGGA
         66 ACCTTTCTTTTCATAGAGCAGTTTTGAAACACTCTTTTTGTAGAATCTGGAAGTGGATATTTGGA

            ** *                        *     *      
     260731 TAGATTTGAGGATTTCGTTGGAAACGG-GAT-T-AT-ATAT
        131 GCGCTTTGAGGATTTCGTTGGAAACGGAAATATCTTCATAT

                 *       *               *                 **  *   *         
     260768 AAAAAGTAGACAGCAGCATTCTCAGAAACTTCTTTGTGATGTTTGCATCCAGCTCTCAGAGTTGA
          1 AAAAACTAGACAGAAGCATTCTCAGAAACATCTTTGTGATGTTTGCACTCAACTCACAGAGTTGA

              *  * *          *  *        *        *   **        *  **       
     260833 ACATTCCCTTTCATAGAGTAGGTTTGAAACCCTCTTTTTATAGTGTCTGGAAGCGGGCATTTGGA
         66 ACCTTTCTTTTCATAGAGCAGTTTTGAAACACTCTTTTTGTAGAATCTGGAAGTGGATATTTGGA

                   *    * * **  **  **          * *  *
     260898 GCGCTTTCAGGCCTATGCTTAAAATAGGAAATATCTACCTAC
        131 GCGCTTTGAGG-ATTTCGTTGGAAACGGAAATATCTTCATAT

             *                    *    *  **          ** *        *    *     
     260940 AGAAACTAGACAGAAGCATTCTGAGAATCACGTTTGTGATGTGGGTACTCAACTAACAGTGTTGA
          1 AAAAACTAGACAGAAGCATTCTCAGAAACATCTTTGTGATGTTTGCACTCAACTCACAGAGTTGA

            *  *       *   *            *   *                *   *           
     261005 TCCATTCTTTTGATACAGCAGTTTTGAACCACACTTTTTGTAGAATCTGCAAGAGGATATTTGGA
         66 ACCTTTCTTTTCATAGAGCAGTTTTGAAACACTCTTTTTGTAGAATCTGGAAGTGGATATTTGGA

            **   *                     *   *      * *
     261070 TAGCTGTGAGGATTTCGTTGGAAACGGGAATGTCTTCAAAG
        131 GCGCTTTGAGGATTTCGTTGGAAACGGAAATATCTTCATAT

                *                         *   *            *    *            
     261111 AAAATCTAGACAGAAGCATTCTCAGAAACACCTTCGTGATGTTTGCAATCAAGTCACAGAGTTGA
          1 AAAAACTAGACAGAAGCATTCTCAGAAACATCTTTGTGATGTTTGCACTCAACTCACAGAGTTGA

                 * *             *  *           *      *             *       
     261176 ACCTTCCGTTTCATAGAGCAGGTTGGAAACACTCTTATTGTAGTATCTGGAAGTGGACATTTGGA
         66 ACCTTTCTTTTCATAGAGCAGTTTTGAAACACTCTTTTTGTAGAATCTGGAAGTGGATATTTGGA

                   *   ** ** *  *   *            **  
     261241 GCGCTTTCAGGCCTATGGTGAAAAAGGAAATATCTTCCCAT
        131 GCGCTTTGAGGATTTCGTTGGAAACGGAAATATCTTCATAT

                  * * *      **      *   * *   *     ** ** *      *    *     
     261282 AAAAACGACATAGAAGCTATCTCAGGAACTTGTTTATGATGCATCTAATCAACTAACAGTGTTGA
          1 AAAAACTAGACAGAAGCATTCTCAGAAACATCTTTGTGATGTTTGCACTCAACTCACAGAGTTGA

                  * ** * *                         * *       *               
     261347 ACCTTTGTACTGACAGAGCAG-TTTGAAACACTCTTTTTTTGGAATCTGCAAGTGGATATTTGGA
         66 ACCTTTCTTTTCATAGAGCAGTTTTGAAACACTCTTTTTGTAGAATCTGGAAGTGGATATTTGGA

            *                           *   *   *    
     261411 TCGCTTTGAGGATTTCGTTGGAAACGG-GAT-GC--AATAT
        131 GCGCTTTGAGGATTTCGTTGGAAACGGAAATATCTTCATAT

                **  *    *     *                 **  *   *  *    *         * 
     261448 AAAACGTACACAGCAGCATACTCAGAAA-ATACTTTGCCATATTTCCATTCAAGTCACAGAGTGG
          1 AAAAACTAGACAGAAGCATTCTCAGAAACAT-CTTTGTGATGTTTGCACTCAACTCACAGAGTTG

               *  * **            *                  *  *             *      
     261512 AACATTCCCATTCATAGAGCAGGTTTGAAACACTCTTTTTGGAGTATCTGGAAGTGGACATTTGG
         65 AACCTTTCTTTTCATAGAGCAGTTTTGAAACACTCTTTTTGTAGAATCTGGAAGTGGATATTTGG

                    ** * * ** *  *   *            **  
     261577 AGCGCTTTCTGAACTATGGTGAAAAAGGAAATATCTTCCAAT
        130 AGCGCTTTGAGGATTTCGTTGGAAACGGAAATATCTTCATAT

            *     *               *      * *          *  **      **  *  *    
     261619 GAAAACAAGACAGAAGCATTCTGAGAAACTTATTTGTGATGTGTGTCCTCAACAAACGGACTTGA
          1 AAAAACTAGACAGAAGCATTCTCAGAAACATCTTTGTGATGTTTGCACTCAACTCACAGAGTTGA

                   *      **  * *  *  *             *   *    * * *           
     261684 ACCTTTCGTTTCATGCAGTACTTCTGGAACACTCTTTTTGAAGATTCTGCATGCGGATATTTGGA
         66 ACCTTTCTTTTCATAGAGCAGTTTTGAAACACTCTTTTTGTAGAATCTGGAAGTGGATATTTGGA

            **                            **   *   * 
     261749 TAGCTTTGAGGATTTCGTTGGAAACGG---GCT-TACATGT
        131 GCGCTTTGAGGATTTCGTTGGAAACGGAAATATCTTCATAT

                 *       *               *        *   *    *    *       *    
     261786 AAAAATTAGACAGCAGCATTCTCAGAAACTTCTTTGTGGTGTCTGCATTCAAGTCACAGAATTGA
          1 AAAAACTAGACAGAAGCATTCTCAGAAACATCTTTGTGATGTTTGCACTCAACTCACAGAGTTGA

              * ** * **            *  * *      *       *             *       
     261851 ACTTCCCCTCACATAGAGCAGTTGTGCAGCACTCTATTTGTAGTATCTGGAAGTGGACATTTGGA
         66 ACCTTTCTTTTCATAGAGCAGTTTTGAAACACTCTTTTTGTAGAATCTGGAAGTGGATATTTGGA

             *         ** *    * *   *            **  
     261916 GGGCTTTGTAGCCTATC-TGGAAAAAGGAAATATCTTCCCAT
        131 GCGCTTTG-AGGATTTCGTTGGAAACGGAAATATCTTCATAT

            *  ** *   *     * *            *   *  *   * **        *  * * *   
     261957 GAATGCGAGATAGAAGTAATCTCAGAAACATGTTTATGCTGTATCTACTCAACTAACTGTGCTGA
          1 AAAAACTAGACAGAAGCATTCTCAGAAACATCTTTGTGATGTTTGCACTCAACTCACAGAGTTGA

              *     *  *               *        *  * *       *               
     262022 ACATTTCTATTGATAGAGCAGTTTTGAGACACTCTTCTTTTGGAATCTGCAAGTGGATATTTGGA
         66 ACCTTTCTTTTCATAGAGCAGTTTTGAAACACTCTTTTTGTAGAATCTGGAAGTGGATATTTGGA

            ** *                        *     *      
     262087 TAGATTTGAGGATTTCGTTGGAAACGG-GAT-T-AT-ATAT
        131 GCGCTTTGAGGATTTCGTTGGAAACGGAAATATCTTCATAT

                 *       *               *                 **  *   *         
     262124 AAAAAGTAGACAGCAGCATTCTCAGAAACTTCTTTGTGATGTTTGCATCCAGCTCTCAGAGTTGA
          1 AAAAACTAGACAGAAGCATTCTCAGAAACATCTTTGTGATGTTTGCACTCAACTCACAGAGTTGA

              *  * *          *  *        *        *   **        *  **       
     262189 ACATTCCCTTTCATAGAGTAGGTTTGAAACCCTCTTTTTATAGTGTCTGGAAGCGGGCATTTGGA
         66 ACCTTTCTTTTCATAGAGCAGTTTTGAAACACTCTTTTTGTAGAATCTGGAAGTGGATATTTGGA

                   *   ** ** *  *   *          * *   
     262254 GCGCTTTCAGGCCTATGCTGAAAAAGGAAATATCTACCTAT
        131 GCGCTTTGAGGATTTCGTTGGAAACGGAAATATCTTCATAT

             *                    *    *  **          ** *        *    *     
     262295 AGAAACTAGACAGAAGCATTCTGAGAATCACGTTTGTGATGTGGGTACTCAACTAACAGTGTTGA
          1 AAAAACTAGACAGAAGCATTCTCAGAAACATCTTTGTGATGTTTGCACTCAACTCACAGAGTTGA

            *  *       *   *            *   *                *               
     262360 TCCATTCTTTTGATACAGCAGTTTTGAACCACACTTTTTGTAGAATCTGCAAGTGGATATTTGGA
         66 ACCTTTCTTTTCATAGAGCAGTTTTGAAACACTCTTTTTGTAGAATCTGGAAGTGGATATTTGGA

            **   *                     *   *        *
     262425 TAGCTGTGAGGATTTCGTTGGAAACGGGAATGTCTTCATAG
        131 GCGCTTTGAGGATTTCGTTGGAAACGGAAATATCTTCATAT

                **                     * * **         *  **   *   *          
     262466 AAAATTTAGACAGAAGCATTCTCAGAACCTTGATTGTGATGTGTGTTCTCCACTAACAGAGTTGA
          1 AAAAACTAGACAGAAGCATTCTCAGAAACATCTTTGTGATGTTTGCACTCAACTCACAGAGTTGA

                       * *   * *   *       *       *                         
     262531 ACCTTTCTTTTGACAGAACTGTTCTGAAACATTCTTTTTATAGAATCTGGAAGTGGATATTTGGA
         66 ACCTTTCTTTTCATAGAGCAGTTTTGAAACACTCTTTTTGTAGAATCTGGAAGTGGATATTTGGA

            **                         *          *  
     262596 AAGCTTTGAGGATTTCGTTGGAAACGGGAATATCTTCAAAT
        131 GCGCTTTGAGGATTTCGTTGGAAACGGAAATATCTTCATAT

            *   *    *            *           * *       *  *    *            
     262637 CAAATCTAGCCAGAAGCATTCTAAGAAACATCTTAGGGATGTTTACATTCAAGTCACAGAGTTGA
          1 AAAAACTAGACAGAAGCATTCTCAGAAACATCTTTGTGATGTTTGCACTCAACTCACAGAGTTGA

              *  * *     *       *         *    * *   **      *      *    *  
     262702 ACATTCCCTTTCACAGAGCAGGTTTGAAACAATCTTCTCGTACTATCTGGCAGTGGACATTTTGA
         66 ACCTTTCTTTTCATAGAGCAGTTTTGAAACACTCTTTTTGTAGAATCTGGAAGTGGATATTTGGA

              * *   *  ** ** *  *   *            ** *
     262767 GCTCCTTGGGGCCTATGCTGAAAAAGGAAATATCTTCCGAC
        131 GCGCTTTGAGGATTTCGTTGGAAACGGAAATATCTTCATAT

                                 *     *  **          *           **    *    
     262808 AAAAACTAGACAGAAGCATTCGCAGAATCACGTTTGTGATGTGTGCACTCAACTGTCAGAATTGA
          1 AAAAACTAGACAGAAGCATTCTCAGAAACATCTTTGTGATGTTTGCACTCAACTCACAGAGTTGA

                 **   ** *      *                            * *            *
     262873 ACCTTGGTTTGGACAGAGCACTTTTGAAACACTCTTTTTGTAGAATCTGCAGGTGGATATTTGGC
         66 ACCTTTCTTTTCATAGAGCAGTTTTGAAACACTCTTTTTGTAGAATCTGGAAGTGGATATTTGGA

            **                         *   *      * *
     262938 TAGCTTTGAGGATTTCGTTGGAAACGGTAATGTCTTCAAAG
        131 GCGCTTTGAGGATTTCGTTGGAAACGGAAATATCTTCATAT

                *                         *   *            *    *            
     262979 AAAATCTAGACAGAAGCATTCTCAGAAACACCTTCGTGATGTTTGCAATCAAGTCACAGAGTTGA
          1 AAAAACTAGACAGAAGCATTCTCAGAAACATCTTTGTGATGTTTGCACTCAACTCACAGAGTTGA

                 * *             *  *       *          *    *        *       
     263044 ACCTTCCGTTTCATAGAGCAGGTTGGAAACACACTTTTTGTAGTATCTAGAAGTGGACATTTGGA
         66 ACCTTTCTTTTCATAGAGCAGTTTTGAAACACTCTTTTTGTAGAATCTGGAAGTGGATATTTGGA

                   *   ** ** *  *   *            **  
     263109 GCGCTTTCAGGCCTATGGTGAAAAAGGAAATATCTTCCCAT
        131 GCGCTTTGAGGATTTCGTTGGAAACGGAAATATCTTCATAT

                  * * *      **      *   * * * *     ** ** *      *    *     
     263150 AAAAACGACATAGAAGCTATCTCAGGAACTTGTCTATGATGCATCTAATCAACTAACAGTGTTGA
          1 AAAAACTAGACAGAAGCATTCTCAGAAACATCTTTGTGATGTTTGCACTCAACTCACAGAGTTGA

                  * ** * *   *                     * *       *               
     263215 ACCTTTGTACTGACAGAACAG-TTTGAAACACTCTTTTTTTGGAATCTGCAAGTGGATATTTGGA
         66 ACCTTTCTTTTCATAGAGCAGTTTTGAAACACTCTTTTTGTAGAATCTGGAAGTGGATATTTGGA

            *                           *   *   *    
     263279 TCGCTTTGAGGATTTCGTTGGAAACGG-GAT-GC--AATAT
        131 GCGCTTTGAGGATTTCGTTGGAAACGGAAATATCTTCATAT

                **  *    *     *                 **  *   *  *    *         * 
     263316 AAAACGTACACAGCAGCATACTCAGAAA-ATACTTTGCCATATTTCCATTCAAGTCACAGAGTGG
          1 AAAAACTAGACAGAAGCATTCTCAGAAACAT-CTTTGTGATGTTTGCACTCAACTCACAGAGTTG

               *  * **            *  *               *  *             *      
     263380 AACATTCCCATTCATAGAGCAGGTTGGAAACACTCTTTTTGGAGTATCTGGAAGTGGACATTTGG
         65 AACCTTTCTTTTCATAGAGCAGTTTTGAAACACTCTTTTTGTAGAATCTGGAAGTGGATATTTGG

                    ** * * ** *  *   *            **  
     263445 AGCGCTTTCTGAACTATGGTGAAAAAGGAAATATCTTCCAAT
        130 AGCGCTTTGAGGATTTCGTTGGAAACGGAAATATCTTCATAT

            *     *               *      * *          *  **      **  *  *    
     263487 GAAAACAAGACAGAAGCATTCTGAGAAACTTATTTGTGATGTCTGTCCTCAACAAACGGACTTGA
          1 AAAAACTAGACAGAAGCATTCTCAGAAACATCTTTGTGATGTTTGCACTCAACTCACAGAGTTGA

                   *      **  * *  *  *             *   *    * * *           
     263552 ACCTTTCGTTTCATGCAGTACTTCTGGAACACTCTTTTTGAAGATTCTGCATGCGGATATTTGGA
         66 ACCTTTCTTTTCATAGAGCAGTTTTGAAACACTCTTTTTGTAGAATCTGGAAGTGGATATTTGGA

            **                            **   *   * 
     263617 TAGCTTTGAGGATTTCGTTGGAAACGG---GCT-TACATGT
        131 GCGCTTTGAGGATTTCGTTGGAAACGGAAATATCTTCATAT

                 *       *               *        *   *    *    *       *    
     263654 AAAAATTAGACAGCAGCATTCTCAGAAACTTCTTTGTGGTGTCTGCATTCAAGTCACAGAATTGA
          1 AAAAACTAGACAGAAGCATTCTCAGAAACATCTTTGTGATGTTTGCACTCAACTCACAGAGTTGA

              * ** * **            *  * *      *       *   *         *       
     263719 ACTTCCCCTCACATAGAGCAGTTGTGCAGCACTCTATTTGTAGTATCGGGAAGTGGACATTTGGA
         66 ACCTTTCTTTTCATAGAGCAGTTTTGAAACACTCTTTTTGTAGAATCTGGAAGTGGATATTTGGA

             *         ** *    * *   *            **  
     263784 GGGCTTTGTAGCCTATC-TGGAAAAAGGAAATATCTTCCCAT
        131 GCGCTTTG-AGGATTTCGTTGGAAACGGAAATATCTTCATAT

            *  ** *   *     * *            *   *  *   * **        *  * * *   
     263825 GAATGCGAGATAGAAGTAATCTCAGAAACATGTTTATGCTGTATCTACTCAACTAACTGTGCTGA
          1 AAAAACTAGACAGAAGCATTCTCAGAAACATCTTTGTGATGTTTGCACTCAACTCACAGAGTTGA

              *     *  *               *        *  * *       *               
     263890 ACATTTCTATTGATAGAGCAGTTTTGAGACACTCTTCTTTTGGAATCTGCAAGTGGATATTTGGA
         66 ACCTTTCTTTTCATAGAGCAGTTTTGAAACACTCTTTTTGTAGAATCTGGAAGTGGATATTTGGA

            ** *                        *     *      
     263955 TAGATTTGAGGATTTCGTTGGAAACGG-GAT-T-AT-ATAT
        131 GCGCTTTGAGGATTTCGTTGGAAACGGAAATATCTTCATAT

                 *       *               *                 **  *   *         
     263992 AAAAAGTAGACAGCAGCATTCTCAGAAACTTCTTTGTGATGTTTGCATCCAGCTCTCAGAGTTGA
          1 AAAAACTAGACAGAAGCATTCTCAGAAACATCTTTGTGATGTTTGCACTCAACTCACAGAGTTGA

              *  * *          *  *        *        *   **        *  **       
     264057 ACATTCCCTTTCATAGAGTAGGTTTGAAACCCTCTTTTTATAGTGTCTGGAAGCGGGCATTTGGA
         66 ACCTTTCTTTTCATAGAGCAGTTTTGAAACACTCTTTTTGTAGAATCTGGAAGTGGATATTTGGA

                   *    * * **  **  **          * *  *
     264122 GCGCTTTCAGGCCTATGCTTAAAATAGGAAATATCTACCTAC
        131 GCGCTTTGAGG-ATTTCGTTGGAAACGGAAATATCTTCATAT

             *                    *    *  **          ** *        *    *     
     264164 AGAAACTAGACAGAAGCATTCTGAGAATCACGTTTGTGATGTGGGTACTCAACTAACAGTGTTGA
          1 AAAAACTAGACAGAAGCATTCTCAGAAACATCTTTGTGATGTTTGCACTCAACTCACAGAGTTGA

            *  *       *   *            *   *                *   *           
     264229 TCCATTCTTTTGATACAGCAGTTTTGAACCACACTTTTTGTAGAATCTGCAAGAGGATATTTGGA
         66 ACCTTTCTTTTCATAGAGCAGTTTTGAAACACTCTTTTTGTAGAATCTGGAAGTGGATATTTGGA

            **   *                     *   *      * *
     264294 TAGCTGTGAGGATTTCGTTGGAAACGGGAATGTCTTCAAAG
        131 GCGCTTTGAGGATTTCGTTGGAAACGGAAATATCTTCATAT

                *                         *   *            *    *            
     264335 AAAATCTAGACAGAAGCATTCTCAGAAACACCTTCGTGATGTTTGCAATCAAGTCACAGAGTTGA
          1 AAAAACTAGACAGAAGCATTCTCAGAAACATCTTTGTGATGTTTGCACTCAACTCACAGAGTTGA

                 * *             *  *           *      *             *       
     264400 ACCTTCCGTTTCATAGAGCAGGTTGGAAACACTCTTATTGTAGTATCTGGAAGTGGACATTTGGA
         66 ACCTTTCTTTTCATAGAGCAGTTTTGAAACACTCTTTTTGTAGAATCTGGAAGTGGATATTTGGA

                   *   ** ** *  *   *            **  
     264465 GCGCTTTCAGGCCTATGGTGAAAAAGGAAATATCTTCCCAT
        131 GCGCTTTGAGGATTTCGTTGGAAACGGAAATATCTTCATAT

                  * * *      **      *   * *   *     ** ** *      *    *     
     264506 AAAAACGACATAGAAGCTATCTCAGGAACTTGTTTATGATGCATCTAATCAACTAACAGTGTTGA
          1 AAAAACTAGACAGAAGCATTCTCAGAAACATCTTTGTGATGTTTGCACTCAACTCACAGAGTTGA

                  * ** * *                         * *       *               
     264571 ACCTTTGTACTGACAGAGCAG-TTTGAAACACTCTTTTTTTGGAATCTGCAAGTGGATATTTGGA
         66 ACCTTTCTTTTCATAGAGCAGTTTTGAAACACTCTTTTTGTAGAATCTGGAAGTGGATATTTGGA

            *                           *   *   *    
     264635 TCGCTTTGAGGATTTCGTTGGAAACGG-GAT-GC--AATAT
        131 GCGCTTTGAGGATTTCGTTGGAAACGGAAATATCTTCATAT

                **  *    *     *                 **  *   *  *    *         * 
     264672 AAAACGTACACAGCAGCATACTCAGAAA-ATACTTTGCCATATTTCCATTCAAGTCACAGAGTGG
          1 AAAAACTAGACAGAAGCATTCTCAGAAACAT-CTTTGTGATGTTTGCACTCAACTCACAGAGTTG

               *  * **            *                  *  *             *      
     264736 AACATTCCCATTCATAGAGCAGGTTTGAAACACTCTTTTTGGAGTATCTGGAAGTGGACATTTGG
         65 AACCTTTCTTTTCATAGAGCAGTTTTGAAACACTCTTTTTGTAGAATCTGGAAGTGGATATTTGG

                    ** * * ** *  *   *            **  
     264801 AGCGCTTTCTGAACTATGGTGAAAAAGGAAATATCTTCCAAT
        130 AGCGCTTTGAGGATTTCGTTGGAAACGGAAATATCTTCATAT

            *     *               *      * *          *  **      **  *  *    
     264843 GAAAACAAGACAGAAGCATTCTGAGAAACTTATTTGTGATGTGTGTCCTCAACAAACGGACTTGA
          1 AAAAACTAGACAGAAGCATTCTCAGAAACATCTTTGTGATGTTTGCACTCAACTCACAGAGTTGA

                   *      **  * *  *  *             *   *    * * *           
     264908 ACCTTTCGTTTCATGCAGTACTTCTGGAACACTCTTTTTGAAGATTCTGCATGCGGATATTTGGA
         66 ACCTTTCTTTTCATAGAGCAGTTTTGAAACACTCTTTTTGTAGAATCTGGAAGTGGATATTTGGA

            **                            **   *   * 
     264973 TAGCTTTGAGGATTTCGTTGGAAACGG---GCT-TACATGT
        131 GCGCTTTGAGGATTTCGTTGGAAACGGAAATATCTTCATAT

                 *       *               *        *   *    *    *       *    
     265010 AAAAATTAGACAGCAGCATTCTCAGAAACTTCTTTGTGGTGTCTGCATTCAAGTCACAGAATTGA
          1 AAAAACTAGACAGAAGCATTCTCAGAAACATCTTTGTGATGTTTGCACTCAACTCACAGAGTTGA

              * ** * **            *  * *      *       *             *       
     265075 ACTTCCCCTCACATAGAGCAGTTGTGCAGCACTCTATTTGTAGTATCTGGAAGTGGACATTTGGA
         66 ACCTTTCTTTTCATAGAGCAGTTTTGAAACACTCTTTTTGTAGAATCTGGAAGTGGATATTTGGA

             *         ** *    * *   *            **  
     265140 GGGCTTTGTAGCCTATC-TGGAAAAAGGAAATATCTTCCCAT
        131 GCGCTTTG-AGGATTTCGTTGGAAACGGAAATATCTTCATAT

            *  ** *   *     * *            *   *  *   * **        *  * * *   
     265181 GAATGCGAGATAGAAGTAATCTCAGAAACATGTTTATGCTGTATCTACTCAACTAACTGTGCTGA
          1 AAAAACTAGACAGAAGCATTCTCAGAAACATCTTTGTGATGTTTGCACTCAACTCACAGAGTTGA

              *     *  *               *        *  * *       *               
     265246 ACATTTCTATTGATAGAGCAGTTTTGAGACACTCTTCTTTTGGAATCTGCAAGTGGATATTTGGA
         66 ACCTTTCTTTTCATAGAGCAGTTTTGAAACACTCTTTTTGTAGAATCTGGAAGTGGATATTTGGA

            ** *                        *     *      
     265311 TAGATTTGAGGATTTCGTTGGAAACGG-GAT-T-AT-ATAT
        131 GCGCTTTGAGGATTTCGTTGGAAACGGAAATATCTTCATAT

                 *       *            *  *                 **  *   *         
     265348 AAAAAGTAGACAGCAGCATTCTCAGACACTTCTTTGTGATGTTTGCATCCAGCTCTCAGAGTTGA
          1 AAAAACTAGACAGAAGCATTCTCAGAAACATCTTTGTGATGTTTGCACTCAACTCACAGAGTTGA

              *  * *          *  *        *        *   **        *  **       
     265413 ACATTCCCTTTCATAGAGTAGGTTTGAAACCCTCTTTTTATAGTGTCTGGAAGCGGGCATTTGGA
         66 ACCTTTCTTTTCATAGAGCAGTTTTGAAACACTCTTTTTGTAGAATCTGGAAGTGGATATTTGGA

                   *    * * **  **  **          * *  *
     265478 GCGCTTTCAGGCCTATGCTTAAAATAGGAAATATCTACCTAC
        131 GCGCTTTGAGG-ATTTCGTTGGAAACGGAAATATCTTCATAT

             *                    *    *  **          ** *        *    *     
     265520 AGAAACTAGACAGAAGCATTCTGAGAATCACGTTTGTGATGTGGGTACTCAACTAACAGTGTTGA
          1 AAAAACTAGACAGAAGCATTCTCAGAAACATCTTTGTGATGTTTGCACTCAACTCACAGAGTTGA

            *  *       *   *            *   *                *   *           
     265585 TCCATTCTTTTGATACAGCAGTTTTGAACCACACTTTTTGTAGAATCTGCAAGAGGATATTTGGA
         66 ACCTTTCTTTTCATAGAGCAGTTTTGAAACACTCTTTTTGTAGAATCTGGAAGTGGATATTTGGA

            **   *                     *   *      * *
     265650 TAGCTGTGAGGATTTCGTTGGAAACGGGAATGTCTTCAAAG
        131 GCGCTTTGAGGATTTCGTTGGAAACGGAAATATCTTCATAT

                *                         *   *            *    *            
     265691 AAAATCTAGACAGAAGCATTCTCAGAAACACCTTCGTGATGTTTGCAATCAAGTCACAGAGTTGA
          1 AAAAACTAGACAGAAGCATTCTCAGAAACATCTTTGTGATGTTTGCACTCAACTCACAGAGTTGA

                 * *             *  *           *      *             *       
     265756 ACCTTCCGTTTCATAGAGCAGGTTGGAAACACTCTTATTGTAGTATCTGGAAGTGGACATTTGGA
         66 ACCTTTCTTTTCATAGAGCAGTTTTGAAACACTCTTTTTGTAGAATCTGGAAGTGGATATTTGGA

                   *   ** ** *  *   *            **  
     265821 GCGCTTTCAGGCCTATGGTGAAAAAGGAAATATCTTCCCAT
        131 GCGCTTTGAGGATTTCGTTGGAAACGGAAATATCTTCATAT

                  * * *      **      *   * *   *     ** ** *      *    *     
     265862 AAAAACGACATAGAAGCTATCTCAGGAACTTGTTTATGATGCATCTAATCAACTAACAGTGTTGA
          1 AAAAACTAGACAGAAGCATTCTCAGAAACATCTTTGTGATGTTTGCACTCAACTCACAGAGTTGA

                  * ** * *                         * *       *               
     265927 ACCTTTGTACTGACAGAGCAG-TTTGAAACACTCTTTTTTTGGAATCTGCAAGTGGATATTTGGA
         66 ACCTTTCTTTTCATAGAGCAGTTTTGAAACACTCTTTTTGTAGAATCTGGAAGTGGATATTTGGA

            *                           *   *   *    
     265991 TCGCTTTGAGGATTTCGTTGGAAACGG-GAT-GC--AATAT
        131 GCGCTTTGAGGATTTCGTTGGAAACGGAAATATCTTCATAT

                **  *    *     *                 **  *   *  *    *         * 
     266028 AAAACGTACACAGCAGCATACTCAGAAA-ATACTTTGCCATATTTCCATTCAAGTCACAGAGTGG
          1 AAAAACTAGACAGAAGCATTCTCAGAAACAT-CTTTGTGATGTTTGCACTCAACTCACAGAGTTG

               *  * **            *  *               *  *             *      
     266092 AACATTCCCATTCATAGAGCAGGTTGGAAACACTCTTTTTGGAGTATCTGGAAGTGGACATTTGG
         65 AACCTTTCTTTTCATAGAGCAGTTTTGAAACACTCTTTTTGTAGAATCTGGAAGTGGATATTTGG

                    ** * * ** *  *   *            **  
     266157 AGCGCTTTCTGAACTATGGTGAAAAAGGAAATATCTTCCAAT
        130 AGCGCTTTGAGGATTTCGTTGGAAACGGAAATATCTTCATAT

            *     *               *      * *          *  **      **  *  *    
     266199 GAAAACAAGACAGAAGCATTCTGAGAAACTTATTTGTGATGTGTGTCCTCAACAAACGGACTTGA
          1 AAAAACTAGACAGAAGCATTCTCAGAAACATCTTTGTGATGTTTGCACTCAACTCACAGAGTTGA

                   *      **  * *  *  *             *   *    * * *           
     266264 ACCTTTCGTTTCATGCAGTACTTCTGGAACACTCTTTTTGAAGATTCTGCATGCGGATATTTGGA
         66 ACCTTTCTTTTCATAGAGCAGTTTTGAAACACTCTTTTTGTAGAATCTGGAAGTGGATATTTGGA

            **                            **   *   * 
     266329 TAGCTTTGAGGATTTCGTTGGAAACGG---GCT-TACATGT
        131 GCGCTTTGAGGATTTCGTTGGAAACGGAAATATCTTCATAT

                 *       *               *        *   *    *    *       *    
     266366 AAAAATTAGACAGCAGCATTCTCAGAAACTTCTTTGTGGTGTCTGCATTCAAGTCACAGAATTGA
          1 AAAAACTAGACAGAAGCATTCTCAGAAACATCTTTGTGATGTTTGCACTCAACTCACAGAGTTGA

              * ** * **            *  * *      *       *             *       
     266431 ACTTCCCCTCACATAGAGCAGTTGTGCAGCACTCTATTTGTAGTATCTGGAAGTGGACATTTGGA
         66 ACCTTTCTTTTCATAGAGCAGTTTTGAAACACTCTTTTTGTAGAATCTGGAAGTGGATATTTGGA

             *         ** *    * *   *            **  
     266496 GGGCTTTGTAGCCTAT-GTGGAAAAAGGAAATATCTTCCCAT
        131 GCGCTTTG-AGGATTTCGTTGGAAACGGAAATATCTTCATAT

            *  ** *   *     * *            *   *  *   * **        *  * * *   
     266537 GAATGCGAGATAGAAGTAATCTCAGAAACATGTTTATGCTGTATCTACTCAACTAACTGTGCTGA
          1 AAAAACTAGACAGAAGCATTCTCAGAAACATCTTTGTGATGTTTGCACTCAACTCACAGAGTTGA

              *     *  *               *        *  * *       *               
     266602 ACATTTCTATTGATAGAGCAGTTTTGAGACACTCTTCTTTTGGAATCTGCAAGTGGATATTTGGA
         66 ACCTTTCTTTTCATAGAGCAGTTTTGAAACACTCTTTTTGTAGAATCTGGAAGTGGATATTTGGA

            ** *                        *     *      
     266667 TAGATTTGAGGATTTCGTTGGAAACGG-GAT-T-AT-ATAT
        131 GCGCTTTGAGGATTTCGTTGGAAACGGAAATATCTTCATAT

                 *       *            *  *                 **  *   *         
     266704 AAAAAGTAGACAGCAGCATTCTCAGACACTTCTTTGTGATGTTTGCATCCAGCTCTCAGAGTTGA
          1 AAAAACTAGACAGAAGCATTCTCAGAAACATCTTTGTGATGTTTGCACTCAACTCACAGAGTTGA

              *  * *          *  *        *        *   **        *  **       
     266769 ACATTCCCTTTCATAGAGTAGGTTTGAAACCCTCTTTTTATAGTGTCTGGAAGCGGGCATTTGGA
         66 ACCTTTCTTTTCATAGAGCAGTTTTGAAACACTCTTTTTGTAGAATCTGGAAGTGGATATTTGGA

                   *    * * **  **  **          * *  *
     266834 GCGCTTTCAGGCCTATGCTTAAAATAGGAAATATCTACCTAC
        131 GCGCTTTGAGG-ATTTCGTTGGAAACGGAAATATCTTCATAT

             *                    *    *  **          ** *        *    *     
     266876 AGAAACTAGACAGAAGCATTCTGAGAATCACGTTTGTGATGTGGGTACTCAACTAACAGTGTTGA
          1 AAAAACTAGACAGAAGCATTCTCAGAAACATCTTTGTGATGTTTGCACTCAACTCACAGAGTTGA

            *  *       *   *            *   *                *   *           
     266941 TCCATTCTTTTGATACAGCAGTTTTGAACCACACTTTTTGTAGAATCTGCAAGAGGATATTTGGA
         66 ACCTTTCTTTTCATAGAGCAGTTTTGAAACACTCTTTTTGTAGAATCTGGAAGTGGATATTTGGA

            **   *                     *   *      * *
     267006 TAGCTGTGAGGATTTCGTTGGAAACGGGAATGTCTTCAAAG
        131 GCGCTTTGAGGATTTCGTTGGAAACGGAAATATCTTCATAT

                *                         *   *            *    *            
     267047 AAAATCTAGACAGAAGCATTCTCAGAAACACCTTCGTGATGTTTGCAATCAAGTCACAGAGTTGA
          1 AAAAACTAGACAGAAGCATTCTCAGAAACATCTTTGTGATGTTTGCACTCAACTCACAGAGTTGA

                 * *             *  *           *      *             *       
     267112 ACCTTCCGTTTCATAGAGCAGGTTGGAAACACTCTTATTGTAGTATCTGGAAGTGGACATTTGGA
         66 ACCTTTCTTTTCATAGAGCAGTTTTGAAACACTCTTTTTGTAGAATCTGGAAGTGGATATTTGGA

                   *   ** ** *  *   *            **  
     267177 GCGCTTTCAGGCCTATGGTGAAAAAGGAAATATCTTCCCAT
        131 GCGCTTTGAGGATTTCGTTGGAAACGGAAATATCTTCATAT

                  * * *      **      *   * *   *     ** ** *      *    *     
     267218 AAAAACGACATAGAAGCTATCTCAGGAACTTGTTTATGATGCATCTAATCAACTAACAGTGTTGA
          1 AAAAACTAGACAGAAGCATTCTCAGAAACATCTTTGTGATGTTTGCACTCAACTCACAGAGTTGA

                  * ** * *                         * *       *               
     267283 ACCTTTGTACTGACAGAGCAG-TTTGAAACACTCTTTTTTTGGAATCTGCAAGTGGATATTTGGA
         66 ACCTTTCTTTTCATAGAGCAGTTTTGAAACACTCTTTTTGTAGAATCTGGAAGTGGATATTTGGA

            *                           *   *   *    
     267347 TCGCTTTGAGGATTTCGTTGGAAACGG-GAT-GC--AATAT
        131 GCGCTTTGAGGATTTCGTTGGAAACGGAAATATCTTCATAT

                **  *    *     *                 **  *   *  *    *         * 
     267384 AAAACGTACACAGCAGCATACTCAGAAA-ATACTTTGCCATATTTCCATTCAAGTCACAGAGTGG
          1 AAAAACTAGACAGAAGCATTCTCAGAAACAT-CTTTGTGATGTTTGCACTCAACTCACAGAGTTG

               *  * **            *                  *  *             *      
     267448 AACATTCCCATTCATAGAGCAGGTTTGAAACACTCTTTTTGGAGTATCTGGAAGTGGACATTTGG
         65 AACCTTTCTTTTCATAGAGCAGTTTTGAAACACTCTTTTTGTAGAATCTGGAAGTGGATATTTGG

                    ** * * ** *  *   *            **  
     267513 AGCGCTTTCTGAACTATGGTGAAAAAGGAAATATCTTCCAAT
        130 AGCGCTTTGAGGATTTCGTTGGAAACGGAAATATCTTCATAT

            *     *               *      * *          *  **      **  *  *    
     267555 GAAAACAAGACAGAAGCATTCTGAGAAACTTATTTGTGATGTGTGTCCTCAACAAACGGACTTGA
          1 AAAAACTAGACAGAAGCATTCTCAGAAACATCTTTGTGATGTTTGCACTCAACTCACAGAGTTGA

                   *      **  * *  *  *             *   *    * * *           
     267620 ACCTTTCGTTTCATGCAGTACTTCTGGAACACTCTTTTTGAAGATTCTGCATGCGGATATTTGGA
         66 ACCTTTCTTTTCATAGAGCAGTTTTGAAACACTCTTTTTGTAGAATCTGGAAGTGGATATTTGGA

            **                            **   *   * 
     267685 TAGCTTTGAGGATTTCGTTGGAAACGG---GCT-TACATGT
        131 GCGCTTTGAGGATTTCGTTGGAAACGGAAATATCTTCATAT

                 *       *               *        *   *    *    *       *    
     267722 AAAAATTAGACAGCAGCATTCTCAGAAACTTCTTTGTGGTGTCTGCATTCAAGTCACAGAATTGA
          1 AAAAACTAGACAGAAGCATTCTCAGAAACATCTTTGTGATGTTTGCACTCAACTCACAGAGTTGA

              * ** * **            *  * *      *       *             *       
     267787 ACTTCCCCTCACATAGAGCAGTTGTGCAGCACTCTATTTGTAGTATCTGGAAGTGGACATTTGGA
         66 ACCTTTCTTTTCATAGAGCAGTTTTGAAACACTCTTTTTGTAGAATCTGGAAGTGGATATTTGGA

             *         ** *    * *   *            **  
     267852 GGGCTTTGTAGCCTATC-TGGAAAAAGGAAATATCTTCCCAT
        131 GCGCTTTG-AGGATTTCGTTGGAAACGGAAATATCTTCATAT

            *  ** *   *     * *            *   *  *   * **        *  * * *   
     267893 GAATGCGAGATAGAAGTAATCTCAGAAACATGTTTATGCTGTATCTACTCAACTAACTGTGCTGA
          1 AAAAACTAGACAGAAGCATTCTCAGAAACATCTTTGTGATGTTTGCACTCAACTCACAGAGTTGA

              *     *  *               *        *  * *       *               
     267958 ACATTTCTATTGATAGAGCAGTTTTGAGACACTCTTCTTTTGGAATCTGCAAGTGGATATTTGGA
         66 ACCTTTCTTTTCATAGAGCAGTTTTGAAACACTCTTTTTGTAGAATCTGGAAGTGGATATTTGGA

            ** *                        *     *      
     268023 TAGATTTGAGGATTTCGTTGGAAACGG-GAT-T-AT-ATAT
        131 GCGCTTTGAGGATTTCGTTGGAAACGGAAATATCTTCATAT

                 *       *               *                 **  *   *         
     268060 AAAAAGTAGACAGCAGCATTCTCAGAAACTTCTTTGTGATGTTTGCATCCAGCTCTCAGAGTTGA
          1 AAAAACTAGACAGAAGCATTCTCAGAAACATCTTTGTGATGTTTGCACTCAACTCACAGAGTTGA

              *  * *          *  *        *        *   **        *  **       
     268125 ACATTCCCTTTCATAGAGTAGGTTTGAAACCCTCTTTTTATAGTGTCTGGAAGCGGGCATTTGGA
         66 ACCTTTCTTTTCATAGAGCAGTTTTGAAACACTCTTTTTGTAGAATCTGGAAGTGGATATTTGGA

                   *    * * **  **  **          * *  *
     268190 GCGCTTTCAGGCCTATGCTTAAAATAGGAAATATCTACCTAC
        131 GCGCTTTGAGG-ATTTCGTTGGAAACGGAAATATCTTCATAT

             *                    *    *  **          ** *        *    *     
     268232 AGAAACTAGACAGAAGCATTCTGAGAATCACGTTTGTGATGTGGGTACTCAACTAACAGTGTTGA
          1 AAAAACTAGACAGAAGCATTCTCAGAAACATCTTTGTGATGTTTGCACTCAACTCACAGAGTTGA

            *  *       *   *            *   *                *   *           
     268297 TCCATTCTTTTGATACAGCAGTTTTGAACCACACTTTTTGTAGAATCTGCAAGAGGATATTTGGA
         66 ACCTTTCTTTTCATAGAGCAGTTTTGAAACACTCTTTTTGTAGAATCTGGAAGTGGATATTTGGA

            **   *                     *   *      * *
     268362 TAGCTGTGAGGATTTCGTTGGAAACGGGAATGTCTTCAAAG
        131 GCGCTTTGAGGATTTCGTTGGAAACGGAAATATCTTCATAT

                *                         *   *            *    *            
     268403 AAAATCTAGACAGAAGCATTCTCAGAAACACCTTCGTGATGTTTGCAATCAAGTCACAGAGTTGA
          1 AAAAACTAGACAGAAGCATTCTCAGAAACATCTTTGTGATGTTTGCACTCAACTCACAGAGTTGA

                 * *             *  *           *      *             *       
     268468 ACCTTCCGTTTCATAGAGCAGGTTGGAAACACTCTTATTGTAGTATCTGGAAGTGGACATTTGGA
         66 ACCTTTCTTTTCATAGAGCAGTTTTGAAACACTCTTTTTGTAGAATCTGGAAGTGGATATTTGGA

                   *   ** ** *  *   *            **  
     268533 GCGCTTTCAGGCCTATGGTGAAAAAGGAAATATCTTCCCAT
        131 GCGCTTTGAGGATTTCGTTGGAAACGGAAATATCTTCATAT

                  * * *      **      *   * *   *     ** ** *      *    *     
     268574 AAAAACGACATAGAAGCTATCTCAGGAACTTGTTTATGATGCATCTAATCAACTAACAGTGTTGA
          1 AAAAACTAGACAGAAGCATTCTCAGAAACATCTTTGTGATGTTTGCACTCAACTCACAGAGTTGA

                  * ** * *                         * *       *               
     268639 ACCTTTGTACTGACAGAGCAG-TTTGAAACACTCTTTTTTTGGAATCTGCAAGTGGATATTTGGA
         66 ACCTTTCTTTTCATAGAGCAGTTTTGAAACACTCTTTTTGTAGAATCTGGAAGTGGATATTTGGA

            *                           *   *   *    
     268703 TCGCTTTGAGGATTTCGTTGGAAACGG-GAT-GC--AATAT
        131 GCGCTTTGAGGATTTCGTTGGAAACGGAAATATCTTCATAT

                **  *    *     *                 **  *   *  *    *         * 
     268740 AAAACGTACACAGCAGCATACTCAGAAA-ATACTTTGCCATATTTCCATTCAAGTCACAGAGTGG
          1 AAAAACTAGACAGAAGCATTCTCAGAAACAT-CTTTGTGATGTTTGCACTCAACTCACAGAGTTG

               *  * **            *                  *  *             *      
     268804 AACATTCCCATTCATAGAGCAGGTTTGAAACACTCTTTTTGGAGTATCTGGAAGTGGACATTTGG
         65 AACCTTTCTTTTCATAGAGCAGTTTTGAAACACTCTTTTTGTAGAATCTGGAAGTGGATATTTGG

                    ** * * ** *  *   *            **  
     268869 AGCGCTTTCTGAACTATGGTGAAAAAGGAAATATCTTCCAAT
        130 AGCGCTTTGAGGATTTCGTTGGAAACGGAAATATCTTCATAT

            *     *               *      * *          *  **      **  *  *    
     268911 GAAAACAAGACAGAAGCATTCTGAGAAACTTATTTGTGATGTGTGTCCTCAACAAACGGACTTGA
          1 AAAAACTAGACAGAAGCATTCTCAGAAACATCTTTGTGATGTTTGCACTCAACTCACAGAGTTGA

                   *      **  * *  *  *             *   *    * * *           
     268976 ACCTTTCGTTTCATGCAGTACTTCTGGAACACTCTTTTTGAAGATTCTGCATGCGGATATTTGGA
         66 ACCTTTCTTTTCATAGAGCAGTTTTGAAACACTCTTTTTGTAGAATCTGGAAGTGGATATTTGGA

            **                            **   *   * 
     269041 TAGCTTTGAGGATTTCGTTGGAAACGG---GCT-TACATGT
        131 GCGCTTTGAGGATTTCGTTGGAAACGGAAATATCTTCATAT

                 *       *               * *      *   *    *    *       *    
     269078 AAAAATTAGACAGCAGCATTCTCAGAAACTTTTTTGTGGTGTCTGCATTCAAGTCACAGAATTGA
          1 AAAAACTAGACAGAAGCATTCTCAGAAACATCTTTGTGATGTTTGCACTCAACTCACAGAGTTGA

              * ** * **            *  * *  *   *       *             *       
     269143 ACTTCCCCTCACATAGAGCAGTTGTGCAGCAATCTATTTGTAGTATCTGGAAGTGGACATTTGGA
         66 ACCTTTCTTTTCATAGAGCAGTTTTGAAACACTCTTTTTGTAGAATCTGGAAGTGGATATTTGGA

             *         ** *          *            **  
     269208 GGGCTTTGTAGCCTATC--TGGAAAAGGAAATATCTTCCCAT
        131 GCGCTTTG-AGGATTTCGTTGGAAACGGAAATATCTTCATAT

            *  ** *   *     * *            *  **  *   * **        *  * * *   
     269248 GAATGCGAGATAGAAGTAATCTCAGAAACATGTTCATGCTGTATCTACTCAACTAACTGTGCTGA
          1 AAAAACTAGACAGAAGCATTCTCAGAAACATCTTTGTGATGTTTGCACTCAACTCACAGAGTTGA

              *     *  *               *        *  * *       *               
     269313 ACATTTCTATTGATAGAGCAGTTTTGAGACACTCTTCTTTTGGAATCTGCAAGTGGATATTTGGA
         66 ACCTTTCTTTTCATAGAGCAGTTTTGAAACACTCTTTTTGTAGAATCTGGAAGTGGATATTTGGA

            ** *                        *     *      
     269378 TAGATTTGAGGATTTCGTTGGAAACGG-GAT-T-AT-ATAT
        131 GCGCTTTGAGGATTTCGTTGGAAACGGAAATATCTTCATAT

                 *       *               *                 **  *   *         
     269415 AAAAAGTAGACAGCAGCATTCTCAGAAACTTCTTTGTGATGTTTGCATCCAGCTCTCAGAGTTGA
          1 AAAAACTAGACAGAAGCATTCTCAGAAACATCTTTGTGATGTTTGCACTCAACTCACAGAGTTGA

              *  * *          *  *        *        *   **        *  **       
     269480 ACATTCCCTTTCATAGAGTAGGTTTGAAACCCTCTTTTTATAGTGTCTGGAAGCGGGCATTTGGA
         66 ACCTTTCTTTTCATAGAGCAGTTTTGAAACACTCTTTTTGTAGAATCTGGAAGTGGATATTTGGA

                   *    * * **  **  **          * *  *
     269545 GCGCTTTCAGGCCTATGCTTAAAATAGGAAATATCTACCTAC
        131 GCGCTTTGAGG-ATTTCGTTGGAAACGGAAATATCTTCATAT

             *                    *    *  **          ** *        *    *     
     269587 AGAAACTAGACAGAAGCATTCTGAGAATCACGTTTGTGATGTGGGTACTCAACTAACAGTGTTGA
          1 AAAAACTAGACAGAAGCATTCTCAGAAACATCTTTGTGATGTTTGCACTCAACTCACAGAGTTGA

            *  *       *   *            *   *                *   *           
     269652 TCCATTCTTTTGATACAGCAGTTTTGAACCACACTTTTTGTAGAATCTGCAAGAGGATATTTGGA
         66 ACCTTTCTTTTCATAGAGCAGTTTTGAAACACTCTTTTTGTAGAATCTGGAAGTGGATATTTGGA

            **   *                     *   *      * *
     269717 TAGCTGTGAGGATTTCGTTGGAAACGGGAATGTCTTCAAAG
        131 GCGCTTTGAGGATTTCGTTGGAAACGGAAATATCTTCATAT

                *                         *   *            *   **            
     269758 AAAATCTAGACAGAAGCATTCTCAGAAACACCTTCGTGATGTTTGCAATCATGTCACAGAGTTGA
          1 AAAAACTAGACAGAAGCATTCTCAGAAACATCTTTGTGATGTTTGCACTCAACTCACAGAGTTGA

                 * *             *  *           *      *             *       
     269823 ACCTTCCGTTTCATAGAGCAGGTTGGAAACACTCTTATTGTAGTATCTGGAAGTGGACATTTGGA
         66 ACCTTTCTTTTCATAGAGCAGTTTTGAAACACTCTTTTTGTAGAATCTGGAAGTGGATATTTGGA

                   *   ** ** *  *   *            **  
     269888 GCGCTTTCAGGCCTATGGTGAAAAAGGAAATATCTTCCCAT
        131 GCGCTTTGAGGATTTCGTTGGAAACGGAAATATCTTCATAT

                  * * *      **      *   * *   *     ** ** *      *    *     
     269929 AAAAACGACATAGAAGCTATCTCAGGAACTTGTTTATGATGCATCTAATCAACTAACAGTGTTGA
          1 AAAAACTAGACAGAAGCATTCTCAGAAACATCTTTGTGATGTTTGCACTCAACTCACAGAGTTGA

                  * ** * *                         * *       *               
     269994 ACCTTTGTACTGACAGAGCAG-TTTGAAACACTCTTTTTTTGGAATCTGCAAGTGGATATTTGGA
         66 ACCTTTCTTTTCATAGAGCAGTTTTGAAACACTCTTTTTGTAGAATCTGGAAGTGGATATTTGGA

            *                           *   *   *    
     270058 TCGCTTTGAGGATTTCGTTGGAAACGG-GAT-GC--AATAT
        131 GCGCTTTGAGGATTTCGTTGGAAACGGAAATATCTTCATAT

                **  *    *     *                 **  *   *  *    *         * 
     270095 AAAACGTACACAGCAGCATACTCAGAAA-ATACTTTGCCATATTTCCATTCAAGTCACAGAGTGG
          1 AAAAACTAGACAGAAGCATTCTCAGAAACAT-CTTTGTGATGTTTGCACTCAACTCACAGAGTTG

               *  * **            *                  *  *             *      
     270159 AACATTCCCATTCATAGAGCAGGTTTGAAACACTCTTTTTGGAGTATCTGGAAGTGGACATTTGG
         65 AACCTTTCTTTTCATAGAGCAGTTTTGAAACACTCTTTTTGTAGAATCTGGAAGTGGATATTTGG

                    ** * * ** *  *   *            **  
     270224 AGCGCTTTCTGAACTATGGTGAAAAAGGAAATATCTTCCAAT
        130 AGCGCTTTGAGGATTTCGTTGGAAACGGAAATATCTTCATAT

            *     *               *      * *          *  **      **  *  *    
     270266 GAAAACAAGACAGAAGCATTCTGAGAAACTTATTTGTGATGTGTGTCCTCAACAAACGGACTTGA
          1 AAAAACTAGACAGAAGCATTCTCAGAAACATCTTTGTGATGTTTGCACTCAACTCACAGAGTTGA

                   *      **  * *  *  *             *   *    * * *           
     270331 ACCTTTCGTTTCATGCAGTACTTCTGGAACACTCTTTTTGAAGATTCTGCATGCGGATATTTGGA
         66 ACCTTTCTTTTCATAGAGCAGTTTTGAAACACTCTTTTTGTAGAATCTGGAAGTGGATATTTGGA

            **                            **   *   * 
     270396 TAGCTTTGAGGATTTCGTTGGAAACGG---GCT-TACATGT
        131 GCGCTTTGAGGATTTCGTTGGAAACGGAAATATCTTCATAT

                 *       *               *        *   *    *    *       *    
     270433 AAAAATTAGACAGCAGCATTCTCAGAAACTTCTTTGTGGTGTCTGCATTCAAGTCACAGAATTGA
          1 AAAAACTAGACAGAAGCATTCTCAGAAACATCTTTGTGATGTTTGCACTCAACTCACAGAGTTGA

              * ** * **            *  * *      *       *             *       
     270498 ACTTCCCCTCACATAGAGCAGTTGTGCAGCACTCTATTTGTAGTATCTGGAAGTGGACATTTGGA
         66 ACCTTTCTTTTCATAGAGCAGTTTTGAAACACTCTTTTTGTAGAATCTGGAAGTGGATATTTGGA

             *         ** *    * *   *            **  
     270563 GGGCTTTGTAGCCTATC-TGGAAAAAGGAAATATCTTCCCAT
        131 GCGCTTTG-AGGATTTCGTTGGAAACGGAAATATCTTCATAT

            *  ** *   *     * *            *   *  *   * **        *  * * *   
     270604 GAATGCGAGATAGAAGTAATCTCAGAAACATGTTTATGCTGTATCTACTCAACTAACTGTGCTGA
          1 AAAAACTAGACAGAAGCATTCTCAGAAACATCTTTGTGATGTTTGCACTCAACTCACAGAGTTGA

              *     *  *               *        *  * *       *               
     270669 ACATTTCTATTGATAGAGCAGTTTTGAGACACTCTTCTTTTGGAATCTGCAAGTGGATATTTGGA
         66 ACCTTTCTTTTCATAGAGCAGTTTTGAAACACTCTTTTTGTAGAATCTGGAAGTGGATATTTGGA

            ** *                        *     *      
     270734 TAGATTTGAGGATTTCGTTGGAAACGG-GAT-T-AT-ATAT
        131 GCGCTTTGAGGATTTCGTTGGAAACGGAAATATCTTCATAT

                 *       *               *                 **  *   *         
     270771 AAAAAGTAGACAGCAGCATTCTCAGAAACTTCTTTGTGATGTTTGCATCCAGCTCTCAGAGTTGA
          1 AAAAACTAGACAGAAGCATTCTCAGAAACATCTTTGTGATGTTTGCACTCAACTCACAGAGTTGA

              *  * *          *  *        *        *   **        *  **       
     270836 ACATTCCCTTTCATAGAGTAGGTTTGAAACCCTCTTTTTATAGTGTCTGGAAGCGGGCATTTGGA
         66 ACCTTTCTTTTCATAGAGCAGTTTTGAAACACTCTTTTTGTAGAATCTGGAAGTGGATATTTGGA

                   *    * * **  **  **          * *  *
     270901 GCGCTTTCAGGCCTATGCTTAAAATAGGAAATATCTACCTAC
        131 GCGCTTTGAGG-ATTTCGTTGGAAACGGAAATATCTTCATAT

             *                    *    *  **          ** *        *    *     
     270943 AGAAACTAGACAGAAGCATTCTGAGAATCACGTTTGTGATGTGGGTACTCAACTAACAGTGTTGA
          1 AAAAACTAGACAGAAGCATTCTCAGAAACATCTTTGTGATGTTTGCACTCAACTCACAGAGTTGA

            *  *       *   *  *         *   **               *   *           
     271008 TCCATTCTTTTGATACAGAAGTTTTGAACCACAGTTTTTGTAGAATCTGCAAGAGGATATTTGGA
         66 ACCTTTCTTTTCATAGAGCAGTTTTGAAACACTCTTTTTGTAGAATCTGGAAGTGGATATTTGGA

            **   *                     *   *      * *
     271073 TAGCTGTGAGGATTTCGTTGGAAACGGGAATGTCTTCAAAG
        131 GCGCTTTGAGGATTTCGTTGGAAACGGAAATATCTTCATAT

                *                         *   *            *    *            
     271114 AAAATCTAGACAGAAGCATTCTCAGAAACACCTTCGTGATGTTTGCAATCAAGTCACAGAGTTGA
          1 AAAAACTAGACAGAAGCATTCTCAGAAACATCTTTGTGATGTTTGCACTCAACTCACAGAGTTGA

                 * *             *  *           *      *             *       
     271179 ACCTTCCGTTTCATAGAGCAGGTTGGAAACACTCTTATTGTAGTATCTGGAAGTGGACATTTGGA
         66 ACCTTTCTTTTCATAGAGCAGTTTTGAAACACTCTTTTTGTAGAATCTGGAAGTGGATATTTGGA

                   *   ** ** *  *   *            **  
     271244 GCGCTTTCAGGCCTATGGTGAAAAAGGAAATATCTTCCCAT
        131 GCGCTTTGAGGATTTCGTTGGAAACGGAAATATCTTCATAT

                  * * *      **      *   * *   *     ** ** *      *    *     
     271285 AAAAACGACATAGAAGCTATCTCAGGAACTTGTTTATGATGCATCTAATCAACTAACAGTGTTGA
          1 AAAAACTAGACAGAAGCATTCTCAGAAACATCTTTGTGATGTTTGCACTCAACTCACAGAGTTGA

                  * ** * *                         * *       *               
     271350 ACCTTTGTACTGACAGAGCAG-TTTGAAACACTCTTTTTTTGGAATCTGCAAGTGGATATTTGGA
         66 ACCTTTCTTTTCATAGAGCAGTTTTGAAACACTCTTTTTGTAGAATCTGGAAGTGGATATTTGGA

            *                           *   *   *    
     271414 TCGCTTTGAGGATTTCGTTGGAAACGG-GAT-GC--AATAT
        131 GCGCTTTGAGGATTTCGTTGGAAACGGAAATATCTTCATAT

                **  *    *     *                 **  *   *  *    *         * 
     271451 AAAACGTACACAGCAGCATACTCAGAAA-ATACTTTGCCATATTTCCATTCAAGTCACAGAGTGG
          1 AAAAACTAGACAGAAGCATTCTCAGAAACAT-CTTTGTGATGTTTGCACTCAACTCACAGAGTTG

               *  * **            *                  *  *             *      
     271515 AACATTCCCATTCATAGAGCAGGTTTGAAACACTCTTTTTGGAGTATCTGGAAGTGGACATTTGG
         65 AACCTTTCTTTTCATAGAGCAGTTTTGAAACACTCTTTTTGTAGAATCTGGAAGTGGATATTTGG

                    ** * * ** *  *   *            **  
     271580 AGCGCTTTCTGAACTATGGTGAAAAAGGAAATATCTTCCAAT
        130 AGCGCTTTGAGGATTTCGTTGGAAACGGAAATATCTTCATAT

            *     *               *      * *          *  **      **  *  *    
     271622 GAAAACAAGACAGAAGCATTCTGAGAAACTTATTTGTGATGTGTGTCCTCAACAAACGGACTTGA
          1 AAAAACTAGACAGAAGCATTCTCAGAAACATCTTTGTGATGTTTGCACTCAACTCACAGAGTTGA

                   *      **  * *  *  *             *         * * *          
     271687 ACCTTTCGTTTCATGCAGTACTTCTGGAACACTCTTTTTGAAGATAT-TGCATGCGGATATTTGG
         66 ACCTTTCTTTTCATAGAGCAGTTTTGAAACACTCTTTTTGTAGA-ATCTGGAAGTGGATATTTGG

             **                           *     *   * 
     271751 ATAGCTTTGAGGATTTCGTTGGAAACGG--GT-T-TACATGT
        130 AGCGCTTTGAGGATTTCGTTGGAAACGGAAATATCTTCATAT

                 *       *               *        *   *    *    *       *    
     271789 AAAAATTAGACAGCAGCATTCTCAGAAACTTCTTTGTGGTGTCTGCATTCAAGTCACAGAATTGA
          1 AAAAACTAGACAGAAGCATTCTCAGAAACATCTTTGTGATGTTTGCACTCAACTCACAGAGTTGA

              * ** * **            *  * *      *       *             *       
     271854 ACTTCCCCTCACATAGAGCAGTTGTGCAGCACTCTATTTGTAGTATCTGGAAGTGGACATTTGGA
         66 ACCTTTCTTTTCATAGAGCAGTTTTGAAACACTCTTTTTGTAGAATCTGGAAGTGGATATTTGGA

             *         ** *    * *   *            **  
     271919 GGGCTTTGTAGCCTATC-TGGAAAAAGGAAATATCTTCCCAT
        131 GCGCTTTG-AGGATTTCGTTGGAAACGGAAATATCTTCATAT

            *  ** *   *     * *            *   *  *   * **        *  * * *   
     271960 GAATGCGAGATAGAAGTAATCTCAGAAACATGTTTATGCTGTATCTACTCAACTAACTGTGCTGA
          1 AAAAACTAGACAGAAGCATTCTCAGAAACATCTTTGTGATGTTTGCACTCAACTCACAGAGTTGA

              *     *  *               *        *  * *       *               
     272025 ACATTTCTATTGATAGAGCAGTTTTGAGACACTCTTCTTTTGGAATCTGCAAGTGGATATTTGGA
         66 ACCTTTCTTTTCATAGAGCAGTTTTGAAACACTCTTTTTGTAGAATCTGGAAGTGGATATTTGGA

            ** *                        *     *      
     272090 TAGATTTGAGGATTTCGTTGGAAACGG-GAT-T-AT-ATAT
        131 GCGCTTTGAGGATTTCGTTGGAAACGGAAATATCTTCATAT

                 *       *               *                 **  *   *         
     272127 AAAAAGTAGACAGCAGCATTCTCAGAAACTTCTTTGTGATGTTTGCATCCAGCTCTCAGAGTTGA
          1 AAAAACTAGACAGAAGCATTCTCAGAAACATCTTTGTGATGTTTGCACTCAACTCACAGAGTTGA

              *  * *          *  *        *        *   **        *  **       
     272192 ACATTCCCTTTCATAGAGTAGGTTTGAAACCCTCTTTTTATAGTGTCTGGAAGCGGGCATTTGGA
         66 ACCTTTCTTTTCATAGAGCAGTTTTGAAACACTCTTTTTGTAGAATCTGGAAGTGGATATTTGGA

                   *    * * **  **  **          * *  *
     272257 GCGCTTTCAGGCCTATGCTTAAAATAGGAAATATCTACCTAC
        131 GCGCTTTGAGG-ATTTCGTTGGAAACGGAAATATCTTCATAT

             *                    *    *  **          ** *        *    *     
     272299 AGAAACTAGACAGAAGCATTCTGAGAATCACGTTTGTGATGTGGGTACTCAACTAACAGTGTTGA
          1 AAAAACTAGACAGAAGCATTCTCAGAAACATCTTTGTGATGTTTGCACTCAACTCACAGAGTTGA

            *  *       *   *            *   *                *   *           
     272364 TCCATTCTTTTGATACAGCAGTTTTGAACCACACTTTTTGTAGAATCTGCAAGAGGATATTTGGA
         66 ACCTTTCTTTTCATAGAGCAGTTTTGAAACACTCTTTTTGTAGAATCTGGAAGTGGATATTTGGA

            **   *                     *   *      * *
     272429 TAGCTGTGAGGATTTCGTTGGAAACGGGAATGTCTTCAAAG
        131 GCGCTTTGAGGATTTCGTTGGAAACGGAAATATCTTCATAT

                *                         *   *            *    *            
     272470 AAAATCTAGACAGAAGCATTCTCAGAAACACCTTCGTGATGTTTGCAATCAAGTCACAGAGTTGA
          1 AAAAACTAGACAGAAGCATTCTCAGAAACATCTTTGTGATGTTTGCACTCAACTCACAGAGTTGA

                 * *             *  *           *      *             *       
     272535 ACCTTCCGTTTCATAGAGCAGGTTGGAAACACTCTTATTGTAGTATCTGGAAGTGGACATTTGGA
         66 ACCTTTCTTTTCATAGAGCAGTTTTGAAACACTCTTTTTGTAGAATCTGGAAGTGGATATTTGGA

                   *   ** ** *  *   *            **  
     272600 GCGCTTTCAGGCCTATGGTGAAAAAGGAAATATCTTCCCAT
        131 GCGCTTTGAGGATTTCGTTGGAAACGGAAATATCTTCATAT

                  * * *      **      *   * *   *     ** ** *      *    *     
     272641 AAAAACGACATAGAAGCTATCTCAGGAACTTGTTTATGATGCATCTAATCAACTAACAGTGTTGA
          1 AAAAACTAGACAGAAGCATTCTCAGAAACATCTTTGTGATGTTTGCACTCAACTCACAGAGTTGA

                  * ** * *                         * *       *               
     272706 ACCTTTGTACTGACAGAGCAG-TTTGAAACACTCTTTTTTTGGAATCTGCAAGTGGATATTTGGA
         66 ACCTTTCTTTTCATAGAGCAGTTTTGAAACACTCTTTTTGTAGAATCTGGAAGTGGATATTTGGA

            *                           *   *   *    
     272770 TCGCTTTGAGGATTTCGTTGGAAACGG-GAT-GC--AATAT
        131 GCGCTTTGAGGATTTCGTTGGAAACGGAAATATCTTCATAT

                **  *    *     *                 **  *   *  *    *         * 
     272807 AAAACGTACACAGCAGCATACTCAGAAA-ATACTTTGCCATATTTCCATTCAAGTCACAGAGTGG
          1 AAAAACTAGACAGAAGCATTCTCAGAAACAT-CTTTGTGATGTTTGCACTCAACTCACAGAGTTG

               *  * **            *                  *  *             *      
     272871 AACATTCCCATTCATAGAGCAGGTTTGAAACACTCTTTTTGGAGTATCTGGAAGTGGACATTTGG
         65 AACCTTTCTTTTCATAGAGCAGTTTTGAAACACTCTTTTTGTAGAATCTGGAAGTGGATATTTGG

                    ** * * ** *  *   *            **  
     272936 AGCGCTTTCTGAACTATGGTGAAAAAGGAAATATCTTCCAAT
        130 AGCGCTTTGAGGATTTCGTTGGAAACGGAAATATCTTCATAT

            *     *               *      * *          *  **      **  *  *    
     272978 GAAAACAAGACAGAAGCATTCTGAGAAACTTATTTGTGATGTGTGTCCTCAACAAACGGACTTGA
          1 AAAAACTAGACAGAAGCATTCTCAGAAACATCTTTGTGATGTTTGCACTCAACTCACAGAGTTGA

                   *      **  * *  *  *             *   *    * * *           
     273043 ACCTTTCGTTTCATGCAGTACTTCTGGAACACTCTTTTTGAAGATTCTGCATGCGGATATTTGGA
         66 ACCTTTCTTTTCATAGAGCAGTTTTGAAACACTCTTTTTGTAGAATCTGGAAGTGGATATTTGGA

            **                            **   *   * 
     273108 TAGCTTTGAGGATTTCGTTGGAAACGG---GCT-TACATGT
        131 GCGCTTTGAGGATTTCGTTGGAAACGGAAATATCTTCATAT

                 *       *               *        *   *    *    *       *    
     273145 AAAAATTAGACAGCAGCATTCTCAGAAACTTCTTTGTGGTGTCTGCATTCAAGTCACAGAATTGA
          1 AAAAACTAGACAGAAGCATTCTCAGAAACATCTTTGTGATGTTTGCACTCAACTCACAGAGTTGA

              * ** * **            *  * *      *       *             *       
     273210 ACTTCCCCTCACATAGAGCAGTTGTGCAGCACTCTATTTGTAGTATCTGGAAGTGGACATTTGGA
         66 ACCTTTCTTTTCATAGAGCAGTTTTGAAACACTCTTTTTGTAGAATCTGGAAGTGGATATTTGGA

             *         ** *    * *   *            **  
     273275 GGGCTTTGTAGCCTATC-TGGAAAAAGGAAATATCTTCCCAT
        131 GCGCTTTG-AGGATTTCGTTGGAAACGGAAATATCTTCATAT

            *  ** *   *     * *            *   *  *   * **        *  * * *   
     273316 GAATGCGAGATAGAAGTAATCTCAGAAACATGTTTATGCTGTATCTACTCAACTAACTGTGCTGA
          1 AAAAACTAGACAGAAGCATTCTCAGAAACATCTTTGTGATGTTTGCACTCAACTCACAGAGTTGA

              *     *  *               *        *  * *       *               
     273381 ACATTTCTATTGATAGAGCAGTTTTGAGACACTCTTCTTTTGGAATCTGCAAGTGGATATTTGGA
         66 ACCTTTCTTTTCATAGAGCAGTTTTGAAACACTCTTTTTGTAGAATCTGGAAGTGGATATTTGGA

            ** *                        *     *      
     273446 TAGATTTGAGGATTTCGTTGGAAACGG-GAT-T-AT-ATAT
        131 GCGCTTTGAGGATTTCGTTGGAAACGGAAATATCTTCATAT

                 *       *               *                 **  *   *         
     273483 AAAAAGTAGACAGCAGCATTCTCAGAAACTTCTTTGTGATGTTTGCATCCAGCTCTCAGAGTTGA
          1 AAAAACTAGACAGAAGCATTCTCAGAAACATCTTTGTGATGTTTGCACTCAACTCACAGAGTTGA

              *  * *          *  *        *        *   **        *  **       
     273548 ACATTCCCTTTCATAGAGTAGGTTTGAAACCCTCTTTTTATAGTGTCTGGAAGCGGGCATTTGGA
         66 ACCTTTCTTTTCATAGAGCAGTTTTGAAACACTCTTTTTGTAGAATCTGGAAGTGGATATTTGGA

                   *   ** ** *  *   *          * *   
     273613 GCGCTTTCAGGCCTATGCTGAAAAAGGAAATATCTACCTAT
        131 GCGCTTTGAGGATTTCGTTGGAAACGGAAATATCTTCATAT

             *                    *    *  **          ** *        *    *     
     273654 AGAAACTAGACAGAAGCATTCTGAGAATCACGTTTGTGATGTGGGTACTCAACTAACAGTGTTGA
          1 AAAAACTAGACAGAAGCATTCTCAGAAACATCTTTGTGATGTTTGCACTCAACTCACAGAGTTGA

            *  *       *   *            *   *                *               
     273719 TCCATTCTTTTGATACAGCAGTTTTGAACCACACTTTTTGTAGAATCTGCAAGTGGATATTTGGA
         66 ACCTTTCTTTTCATAGAGCAGTTTTGAAACACTCTTTTTGTAGAATCTGGAAGTGGATATTTGGA

            **   *                     *   *        *
     273784 TAGCTGTGAGGATTTCGTTGGAAACGGGAATGTCTTCATAG
        131 GCGCTTTGAGGATTTCGTTGGAAACGGAAATATCTTCATAT

                **                     * * **         *  **   *   *          
     273825 AAAATTTAGACAGAAGCATTCTCAGAACCTTGATTGTGATGTGTGTTCTCCACTAACAGAGTTGA
          1 AAAAACTAGACAGAAGCATTCTCAGAAACATCTTTGTGATGTTTGCACTCAACTCACAGAGTTGA

                       * *   * *   *       *       *                         
     273890 ACCTTTCTTTTGACAGAACTGTTCTGAAACATTCTTTTTATAGAATCTGGAAGTGGATATTTGGA
         66 ACCTTTCTTTTCATAGAGCAGTTTTGAAACACTCTTTTTGTAGAATCTGGAAGTGGATATTTGGA

            **                         *          *  
     273955 AAGCTTTGAGGATTTCGTTGGAAACGGGAATATCTTCAAAT
        131 GCGCTTTGAGGATTTCGTTGGAAACGGAAATATCTTCATAT

            *   *    *            *           * *       *  *    *            
     273996 CAAATCTAGCCAGAAGCATTCTAAGAAACATCTTAGGGATGTTTACATTCAAGTCACAGAGTTGA
          1 AAAAACTAGACAGAAGCATTCTCAGAAACATCTTTGTGATGTTTGCACTCAACTCACAGAGTTGA

              *  * *     *       *         *    * *   **      *      *    *  
     274061 ACATTCCCTTTCACAGAGCAGGTTTGAAACAATCTTCTCGTACTATCTGGCAGTGGACATTTTGA
         66 ACCTTTCTTTTCATAGAGCAGTTTTGAAACACTCTTTTTGTAGAATCTGGAAGTGGATATTTGGA

              * *   *  ** ** *  *   *            ** *
     274126 GCTCCTTGGGGCCTATGCTGAAAAAGGAAATATCTTCCGAC
        131 GCGCTTTGAGGATTTCGTTGGAAACGGAAATATCTTCATAT

                                 *     *  **          *           **    *    
     274167 AAAAACTAGACAGAAGCATTCGCAGAATCACGTTTGTGATGTGTGCACTCAACTGTCAGAATTGA
          1 AAAAACTAGACAGAAGCATTCTCAGAAACATCTTTGTGATGTTTGCACTCAACTCACAGAGTTGA

                 **   ** *      *                            * *            *
     274232 ACCTTGGTTTGGACAGAGCACTTTTGAAACACTCTTTTTGTAGAATCTGCAGGTGGATATTTGGC
         66 ACCTTTCTTTTCATAGAGCAGTTTTGAAACACTCTTTTTGTAGAATCTGGAAGTGGATATTTGGA

            **                         *   *      * *
     274297 TAGCTTTGAGGATTTCGTTGGAAACGGTAATGTCTTCAAAG
        131 GCGCTTTGAGGATTTCGTTGGAAACGGAAATATCTTCATAT

                *                         *   *            *    *            
     274338 AAAATCTAGACAGAAGCATTCTCAGAAACACCTTCGTGATGTTTGCAATCAAGTCACAGAGTTGA
          1 AAAAACTAGACAGAAGCATTCTCAGAAACATCTTTGTGATGTTTGCACTCAACTCACAGAGTTGA

                 * *             *  *       *          *    *        *       
     274403 ACCTTCCGTTTCATAGAGCAGGTTGGAAACACACTTTTTGTAGTATCTAGAAGTGGACATTTGGA
         66 ACCTTTCTTTTCATAGAGCAGTTTTGAAACACTCTTTTTGTAGAATCTGGAAGTGGATATTTGGA

                   *   ** ** *  *   *            **  
     274468 GCGCTTTCAGGCCTATGGTGAAAAAGGAAATATCTTCCCAT
        131 GCGCTTTGAGGATTTCGTTGGAAACGGAAATATCTTCATAT

                  * * *      **      *   * * * *     ** ** *      *    *     
     274509 AAAAACGACATAGAAGCTATCTCAGGAACTTGTCTATGATGCATCTAATCAACTAACAGTGTTGA
          1 AAAAACTAGACAGAAGCATTCTCAGAAACATCTTTGTGATGTTTGCACTCAACTCACAGAGTTGA

                  * ** * *   *                     * *       *               
     274574 ACCTTTGTACTGACAGAACAG-TTTGAAACACTCTTTTTTTGGAATCTGCAAGTGGATATTTGGA
         66 ACCTTTCTTTTCATAGAGCAGTTTTGAAACACTCTTTTTGTAGAATCTGGAAGTGGATATTTGGA

            *                           *   *   *    
     274638 TCGCTTTGAGGATTTCGTTGGAAACGG-GAT-GC--AATAT
        131 GCGCTTTGAGGATTTCGTTGGAAACGGAAATATCTTCATAT

                **  *    *     *                 **  *   *  *    *         * 
     274675 AAAACGTACACAGCAGCATACTCAGAAA-ATACTTTGCCATATTTCCATTCAAGTCACAGAGTGG
          1 AAAAACTAGACAGAAGCATTCTCAGAAACAT-CTTTGTGATGTTTGCACTCAACTCACAGAGTTG

               *  * **            *  *               *  *             *      
     274739 AACATTCCCATTCATAGAGCAGGTTGGAAACACTCTTTTTGGAGTATCTGGAAGTGGACATTTGG
         65 AACCTTTCTTTTCATAGAGCAGTTTTGAAACACTCTTTTTGTAGAATCTGGAAGTGGATATTTGG

                    ** * * ** *  *   *            **  
     274804 AGCGCTTTCTGAACTATGGTGAAAAAGGAAATATCTTCCAAT
        130 AGCGCTTTGAGGATTTCGTTGGAAACGGAAATATCTTCATAT

            *     *               *      * *          *  **      **  *  *    
     274846 GAAAACAAGACAGAAGCATTCTGAGAAACTTATTTGTGATGTGTGTCCTCAACAAACGGACTTGA
          1 AAAAACTAGACAGAAGCATTCTCAGAAACATCTTTGTGATGTTTGCACTCAACTCACAGAGTTGA

                   *      **  * *  *  *             *   *    * * *           
     274911 ACCTTTCGTTTCATGCAGTACTTCTGGAACACTCTTTTTGAAGATTCTGCATGCGGATATTTGGA
         66 ACCTTTCTTTTCATAGAGCAGTTTTGAAACACTCTTTTTGTAGAATCTGGAAGTGGATATTTGGA

            **                            **   *   * 
     274976 TAGCTTTGAGGATTTCGTTGGAAACGG---GCT-TACATGT
        131 GCGCTTTGAGGATTTCGTTGGAAACGGAAATATCTTCATAT

                 *       *               *        *   *    *    *       *    
     275013 AAAAATTAGACAGCAGCATTCTCAGAAACTTCTTTGTGGTGTCTGCATTCAAGTCACAGAATTGA
          1 AAAAACTAGACAGAAGCATTCTCAGAAACATCTTTGTGATGTTTGCACTCAACTCACAGAGTTGA

              * ** * **            *  * *      *       *   *         *       
     275078 ACTTCCCCTCACATAGAGCAGTTGTGCAGCACTCTATTTGTAGTATCGGGAAGTGGACATTTGGA
         66 ACCTTTCTTTTCATAGAGCAGTTTTGAAACACTCTTTTTGTAGAATCTGGAAGTGGATATTTGGA

             *         ** *    * *   *            **  
     275143 GGGCTTTGTAGCCTATC-TGGAAAAAGGAAATATCTTCCCAT
        131 GCGCTTTG-AGGATTTCGTTGGAAACGGAAATATCTTCATAT

            *  ** *   *     * *            *   *  *   * **        *  * * *   
     275184 GAATGCGAGATAGAAGTAATCTCAGAAACATGTTTATGCTGTATCTACTCAACTAACTGTGCTGA
          1 AAAAACTAGACAGAAGCATTCTCAGAAACATCTTTGTGATGTTTGCACTCAACTCACAGAGTTGA

              *     *  *               *        *  * *       *               
     275249 ACATTTCTATTGATAGAGCAGTTTTGAGACACTCTTCTTTTGGAATCTGCAAGTGGATATTTGGA
         66 ACCTTTCTTTTCATAGAGCAGTTTTGAAACACTCTTTTTGTAGAATCTGGAAGTGGATATTTGGA

            ** *                        *     *      
     275314 TAGATTTGAGGATTTCGTTGGAAACGG-GAT-T-AT-ATAT
        131 GCGCTTTGAGGATTTCGTTGGAAACGGAAATATCTTCATAT

                 *       *               *                 **  *   *         
     275351 AAAAAGTAGACAGCAGCATTCTCAGAAACTTCTTTGTGATGTTTGCATCCAGCTCTCAGAGTTGA
          1 AAAAACTAGACAGAAGCATTCTCAGAAACATCTTTGTGATGTTTGCACTCAACTCACAGAGTTGA

              *  * *          *  *        *        *    *        *  **       
     275416 ACATTCCCTTTCATAGAGTAGGTTTGAAACCCTCTTTTTATAGAGTCTGGAAGCGGGCATTTGGA
         66 ACCTTTCTTTTCATAGAGCAGTTTTGAAACACTCTTTTTGTAGAATCTGGAAGTGGATATTTGGA

                   *   ** ** *  *   *          * *   
     275481 GCGCTTTCAGGCCTATGCTGAAAAAGGAAATATCTACCTAT
        131 GCGCTTTGAGGATTTCGTTGGAAACGGAAATATCTTCATAT

             *                    *    *  **          ** *        *    *     
     275522 AGAAACTAGACAGAAGCATTCTGAGAATCACGTTTGTGATGTGGGTACTCAACTAACAGTGTTGA
          1 AAAAACTAGACAGAAGCATTCTCAGAAACATCTTTGTGATGTTTGCACTCAACTCACAGAGTTGA

            *  *       *   *            *   *                *               
     275587 TCCATTCTTTTGATACAGCAGTTTTGAACCACACTTTTTGTAGAATCTGCAAGTGGATATTTGGA
         66 ACCTTTCTTTTCATAGAGCAGTTTTGAAACACTCTTTTTGTAGAATCTGGAAGTGGATATTTGGA

            **   *                     *   *        *
     275652 TAGCTGTGAGGATTTCGTTGGAAACGGGAATGTCTTCATAG
        131 GCGCTTTGAGGATTTCGTTGGAAACGGAAATATCTTCATAT

                **                     * * **         *  **   *   *          
     275693 AAAATTTAGACAGAAGCATTCTCAGAACCTTGATTGTGATGTGTGTTCTCCACTAACAGAGTTGA
          1 AAAAACTAGACAGAAGCATTCTCAGAAACATCTTTGTGATGTTTGCACTCAACTCACAGAGTTGA

                       * *   * *   *       *       *                         
     275758 ACCTTTCTTTTGACAGAACTGTTCTGAAACATTCTTTTTATAGAATCTGGAAGTGGATATTTGGA
         66 ACCTTTCTTTTCATAGAGCAGTTTTGAAACACTCTTTTTGTAGAATCTGGAAGTGGATATTTGGA

            **                         *          *  
     275823 AAGCTTTGAGGATTTCGTTGGAAACGGGAATATCTTCAAAT
        131 GCGCTTTGAGGATTTCGTTGGAAACGGAAATATCTTCATAT

            *   *    *            *           * *       *  *    *            
     275864 CAAATCTAGCCAGAAGCATTCTAAGAAACATCTTAGGGATGTTTACATTCAAGTCACAGAGTTGA
          1 AAAAACTAGACAGAAGCATTCTCAGAAACATCTTTGTGATGTTTGCACTCAACTCACAGAGTTGA

              *  * *     *       *         *    * *   **      *      *    *  
     275929 ACATTCCCTTTCACAGAGCAGGTTTGAAACAATCTTCTCGTACTATCTGGCAGTGGACATTTTGA
         66 ACCTTTCTTTTCATAGAGCAGTTTTGAAACACTCTTTTTGTAGAATCTGGAAGTGGATATTTGGA

              * *   *  ** ** *  *   *            ** *
     275994 GCTCCTTGGGGCCTATGCTGAAAAAGGAAATATCTTCCGAC
        131 GCGCTTTGAGGATTTCGTTGGAAACGGAAATATCTTCATAT

                                 *     *  **          *           **    *    
     276035 AAAAACTAGACAGAAGCATTCGCAGAATCACGTTTGTGATGTGTGCACTCAACTGTCAGAATTGA
          1 AAAAACTAGACAGAAGCATTCTCAGAAACATCTTTGTGATGTTTGCACTCAACTCACAGAGTTGA

                 **   ** *      *                            * *            *
     276100 ACCTTGGTTTGGACAGAGCACTTTTGAAACACTCTTTTTGTAGAATCTGCAGGTGGATATTTGGC
         66 ACCTTTCTTTTCATAGAGCAGTTTTGAAACACTCTTTTTGTAGAATCTGGAAGTGGATATTTGGA

            **                         *   *      * *
     276165 TAGCTTTGAGGATTTCGTTGGAAACGGTAATGTCTTCAAAG
        131 GCGCTTTGAGGATTTCGTTGGAAACGGAAATATCTTCATAT

                *                         *   *            *    *            
     276206 AAAATCTAGACAGAAGCATTCTCAGAAACACCTTCGTGATGTTTGCAATCAAGTCACAGAGTTGA
          1 AAAAACTAGACAGAAGCATTCTCAGAAACATCTTTGTGATGTTTGCACTCAACTCACAGAGTTGA

                 * *             *  *       *          *    *        *       
     276271 ACCTTCCGTTTCATAGAGCAGGTTGGAAACACACTTTTTGTAGTATCTAGAAGTGGACATTTGGA
         66 ACCTTTCTTTTCATAGAGCAGTTTTGAAACACTCTTTTTGTAGAATCTGGAAGTGGATATTTGGA

                   *   ** ** *  *   *            **  
     276336 GCGCTTTCAGGCCTATGGTGAAAAAGGAAATATCTTCCCAT
        131 GCGCTTTGAGGATTTCGTTGGAAACGGAAATATCTTCATAT

                  * * *      **      *   * * * *     ** ** *      *    *     
     276377 AAAAACGACATAGAAGCTATCTCAGGAACTTGTCTATGATGCATCTAATCAACTAACAGTGTTGA
          1 AAAAACTAGACAGAAGCATTCTCAGAAACATCTTTGTGATGTTTGCACTCAACTCACAGAGTTGA

                  * ** * *   *                     * *       *               
     276442 ACCTTTGTACTGACAGAACAG-TTTGAAACACTCTTTTTTTGGAATCTGCAAGTGGATATTTGGA
         66 ACCTTTCTTTTCATAGAGCAGTTTTGAAACACTCTTTTTGTAGAATCTGGAAGTGGATATTTGGA

            *                           *   *   *    
     276506 TCGCTTTGAGGATTTCGTTGGAAACGG-GAT-GC--AATAT
        131 GCGCTTTGAGGATTTCGTTGGAAACGGAAATATCTTCATAT

                **  *    *     *                 **  *   *  *    *         * 
     276543 AAAACGTACACAGCAGCATACTCAGAAA-ATACTTTGCCATATTTCCATTCAAGTCACAGAGTGG
          1 AAAAACTAGACAGAAGCATTCTCAGAAACAT-CTTTGTGATGTTTGCACTCAACTCACAGAGTTG

               *  * **            *  *               *  *             *      
     276607 AACATTCCCATTCATAGAGCAGGTTGGAAACACTCTTTTTGGAGTATCTGGAAGTGGACATTTGG
         65 AACCTTTCTTTTCATAGAGCAGTTTTGAAACACTCTTTTTGTAGAATCTGGAAGTGGATATTTGG

                    ** * * ** *  *   *            **  
     276672 AGCGCTTTCTGAACTATGGTGAAAAAGGAAATATCTTCCAAT
        130 AGCGCTTTGAGGATTTCGTTGGAAACGGAAATATCTTCATAT

            *     *               *      * *          *  **      **  *  *    
     276714 GAAAACAAGACAGAAGCATTCTGAGAAACTTATTTGTGATGTCTGTCCTCAACAAACGGACTTGA
          1 AAAAACTAGACAGAAGCATTCTCAGAAACATCTTTGTGATGTTTGCACTCAACTCACAGAGTTGA

                   *      **  * *  *  *             *   *    * * *           
     276779 ACCTTTCGTTTCATGCAGTACTTCTGGAACACTCTTTTTGAAGATTCTGCATGCGGATATTTGGA
         66 ACCTTTCTTTTCATAGAGCAGTTTTGAAACACTCTTTTTGTAGAATCTGGAAGTGGATATTTGGA

            **                            **   *   * 
     276844 TAGCTTTGAGGATTTCGTTGGAAACGG---GCT-TACATGT
        131 GCGCTTTGAGGATTTCGTTGGAAACGGAAATATCTTCATAT

                 *       *               *        *   *    *    *       *    
     276881 AAAAATTAGACAGCAGCATTCTCAGAAACTTCTTTGTGGTGTCTGCATTCAAGTCACAGAATTGA
          1 AAAAACTAGACAGAAGCATTCTCAGAAACATCTTTGTGATGTTTGCACTCAACTCACAGAGTTGA

              * ** * **            *  * *      *       *             *       
     276946 ACTTCCCCTCACATAGAGCAGTTGTGCAGCACTCTATTTGTAGTATCTGGAAGTGGACATTTGGA
         66 ACCTTTCTTTTCATAGAGCAGTTTTGAAACACTCTTTTTGTAGAATCTGGAAGTGGATATTTGGA

             *         ** *    * *   *            **  
     277011 GGGCTTTGTAGCCTATC-TGGAAAAAGGAAATATCTTCCCAT
        131 GCGCTTTG-AGGATTTCGTTGGAAACGGAAATATCTTCATAT

            *  ** *   *     * *            *   *  *   * **        *  * * *   
     277052 GAATGCGAGATAGAAGTAATCTCAGAAACATGTTTATGCTGTATCTACTCAACTAACTGTGCTGA
          1 AAAAACTAGACAGAAGCATTCTCAGAAACATCTTTGTGATGTTTGCACTCAACTCACAGAGTTGA

              *     *  *               *        *  * *       *               
     277117 ACATTTCTATTGATAGAGCAGTTTTGAGACACTCTTCTTTTGGAATCTGCAAGTGGATATTTGGA
         66 ACCTTTCTTTTCATAGAGCAGTTTTGAAACACTCTTTTTGTAGAATCTGGAAGTGGATATTTGGA

            ** *                        *     *      
     277182 TAGATTTGAGGATTTCGTTGGAAACGG-GAT-T-AT-ATAT
        131 GCGCTTTGAGGATTTCGTTGGAAACGGAAATATCTTCATAT

                 *       *               *                 **  *   *         
     277219 AAAAAGTAGACAGCAGCATTCTCAGAAACTTCTTTGTGATGTTTGCATCCAGCTCTCAGAGTTGA
          1 AAAAACTAGACAGAAGCATTCTCAGAAACATCTTTGTGATGTTTGCACTCAACTCACAGAGTTGA

              *  * *          *  *        *        *   **        *  **       
     277284 ACATTCCCTTTCATAGAGTAGGTTTGAAACCCTCTTTTTATAGTGTCTGGAAGCGGGCATTTGGA
         66 ACCTTTCTTTTCATAGAGCAGTTTTGAAACACTCTTTTTGTAGAATCTGGAAGTGGATATTTGGA

                   *   ** ** *  *   *          * *   
     277349 GCGCTTTCAGGCCTATGCTGAAAAAGGAAATATCTACCTAT
        131 GCGCTTTGAGGATTTCGTTGGAAACGGAAATATCTTCATAT

             *                    *    *  **          ** *        *    *     
     277390 AGAAACTAGACAGAAGCATTCTGAGAATCACGTTTGTGATGTGGGTACTCAACTAACAGTGTTGA
          1 AAAAACTAGACAGAAGCATTCTCAGAAACATCTTTGTGATGTTTGCACTCAACTCACAGAGTTGA

            *  *       *   *            *   *                *               
     277455 TCCATTCTTTTGATACAGCAGTTTTGAACCACACTTTTTGTAGAATCTGCAAGTGGATATTTGGA
         66 ACCTTTCTTTTCATAGAGCAGTTTTGAAACACTCTTTTTGTAGAATCTGGAAGTGGATATTTGGA

            **   *                     *   *        *
     277520 TAGCTGTGAGGATTTCGTTGGAAACGGGAATGTCTTCATAG
        131 GCGCTTTGAGGATTTCGTTGGAAACGGAAATATCTTCATAT

                **                     * * **         *  **   *   *          
     277561 AAAATTTAGACAGAAGCATTCTCAGAACCTTGATTGTGATGTGTGTTCTCCACTAACAGAGTTGA
          1 AAAAACTAGACAGAAGCATTCTCAGAAACATCTTTGTGATGTTTGCACTCAACTCACAGAGTTGA

                       * *   * *   *       *       *                         
     277626 ACCTTTCTTTTGACAGAACTGTTCTGAAACATTCTTTTTATAGAATCTGGAAGTGGATATTTGGA
         66 ACCTTTCTTTTCATAGAGCAGTTTTGAAACACTCTTTTTGTAGAATCTGGAAGTGGATATTTGGA

            **                         *          *  
     277691 AAGCTTTGAGGATTTCGTTGGAAACGGGAATATCTTCAAAT
        131 GCGCTTTGAGGATTTCGTTGGAAACGGAAATATCTTCATAT

                *    *            *           * *       *  *    *            
     277732 AAAATCTAGCCAGAAGCATTCTAAGAAACATCTTAGGGATGTTTACATTCAAGTCACAGAGTTGA
          1 AAAAACTAGACAGAAGCATTCTCAGAAACATCTTTGTGATGTTTGCACTCAACTCACAGAGTTGA

              *  * *     *       *         *    * *   **      *      *    *  
     277797 ACATTCCCTTTCACAGAGCAGGTTTGAAACAATCTTCTCGTACTATCTGGCAGTGGACATTTTGA
         66 ACCTTTCTTTTCATAGAGCAGTTTTGAAACACTCTTTTTGTAGAATCTGGAAGTGGATATTTGGA

              * *   *  ** ** *  *   **           ** *
     277862 GCTCCTTGGGGCCTATGCTGAAAAACGAAATATCTTCCGAC
        131 GCGCTTTGAGGATTTCGTTGGAAACGGAAATATCTTCATAT

                                 *     *  **          *           **    *    
     277903 AAAAACTAGACAGAAGCATTCGCAGAATCACGTTTGTGATGTGTGCACTCAACTGTCAGAATTGA
          1 AAAAACTAGACAGAAGCATTCTCAGAAACATCTTTGTGATGTTTGCACTCAACTCACAGAGTTGA

                 **   ** *      *                            * *            *
     277968 ACCTTGGTTTGGACAGAGCAATTTTGAAACACTCTTTTTGTAGAATCTGCAGGTGGATATTTGGC
         66 ACCTTTCTTTTCATAGAGCAGTTTTGAAACACTCTTTTTGTAGAATCTGGAAGTGGATATTTGGA

            **                         *   *      * *
     278033 TAGCTTTGAGGATTTCGTTGGAAACGGTAATGTCTTCAAAG
        131 GCGCTTTGAGGATTTCGTTGGAAACGGAAATATCTTCATAT

                *                         *   *            *    *            
     278074 AAAATCTAGACAGAAGCATTCTCAGAAACACCTTCGTGATGTTTGCAATCAAGTCACAGAGTTGA
          1 AAAAACTAGACAGAAGCATTCTCAGAAACATCTTTGTGATGTTTGCACTCAACTCACAGAGTTGA

                 * *             *  *                  *             *       
     278139 ACCTTCCGTTTCATAGAGCAGGTTGGAAACACTCTTTTTGTAGTATCTGGAAGTGGACATTTGGA
         66 ACCTTTCTTTTCATAGAGCAGTTTTGAAACACTCTTTTTGTAGAATCTGGAAGTGGATATTTGGA

             *     ** * * ** *  *   *        *   **  
     278204 GGGCTTTCTGAACTATGGTGAAAAGGGAAATATGTTCCAAT
        131 GCGCTTTGAGGATTTCGTTGGAAACGGAAATATCTTCATAT

            *     *               *      * *         **  **       *  *  * *  
     278245 GAAAACAAGACAGAAGCATTCTGAGAAACTTATTTGTGATGCGTGTCCTCAACTAACGGACTCGA
          1 AAAAACTAGACAGAAGCATTCTCAGAAACATCTTTGTGATGTTTGCACTCAACTCACAGAGTTGA

             *     *      **  * *  *  *             *   *    * * *           
     278310 AGCTTTCGTTTCATGCAGTACTTCTGGAACACTCTTTTTGAAGATTCTGCATGCGGATATTTGGA
         66 ACCTTTCTTTTCATAGAGCAGTTTTGAAACACTCTTTTTGTAGAATCTGGAAGTGGATATTTGGA

            **                            **   *   * 
     278375 TAGCTTTGAGGATTTCGTTGGAAACGG---GCT-TACATGT
        131 GCGCTTTGAGGATTTCGTTGGAAACGGAAATATCTTCATAT

                 *       *  *            *        *   *    *    *       *    
     278412 AAAAATTAGACAGCAGAATTCTCAGAAACTTCTTTGTGGTGTCTGCATTCAAGTCACAGAATTGA
          1 AAAAACTAGACAGAAGCATTCTCAGAAACATCTTTGTGATGTTTGCACTCAACTCACAGAGTTGA

              * ** * **            *  * *      *       *             *       
     278477 ACATCCCCTCACATAGAGCAGTTGTGCAGCACTCTATTTGTAGTATCTGGAAGTGGACATTTGGA
         66 ACCTTTCTTTTCATAGAGCAGTTTTGAAACACTCTTTTTGTAGAATCTGGAAGTGGATATTTGGA

             *  *      ** *    * *   *            **  
     278542 GGGCATTGTAGCCTATC-TGGAAAAAGGAAATATCTTCCCAT
        131 GCGCTTTG-AGGATTTCGTTGGAAACGGAAATATCTTCATAT

            *  ** *   *     * *            *   *  *   * **        *  * * *   
     278583 GAATGCGAGATAGAAGTAATCTCAGAAACATGTTTATGCTGTATCTACTCAACTAACTGTGCTGA
          1 AAAAACTAGACAGAAGCATTCTCAGAAACATCTTTGTGATGTTTGCACTCAACTCACAGAGTTGA

              *     *  *               *        *  * *       *               
     278648 ACATTTCTATTGATAGAGCAGTTTTGAGACACTCTTCTTTTGGAATCTGCAAGTGGATATTTGGA
         66 ACCTTTCTTTTCATAGAGCAGTTTTGAAACACTCTTTTTGTAGAATCTGGAAGTGGATATTTGGA

             * *                    *   *     *      
     278713 GAGATTTGAGGATTTCGTTGGAAATGG-GAT-T-AT-ATAT
        131 GCGCTTTGAGGATTTCGTTGGAAACGGAAATATCTTCATAT

                 *       *               *                 **  *   *         
     278750 AAAAAGTAGACAGCAGCATTCTCAGAAACTTCTTTGTGATGTTTGCATCCAGCTCTCAGAGTTGA
          1 AAAAACTAGACAGAAGCATTCTCAGAAACATCTTTGTGATGTTTGCACTCAACTCACAGAGTTGA

              *  * *          *  *        *        *   **        *  **       
     278815 ACATTCCCTTTCATAGAGTAGGTTTGAAACCCTCTTTTTATAGTGTCTGGAAGCGGGCATTTGGA
         66 ACCTTTCTTTTCATAGAGCAGTTTTGAAACACTCTTTTTGTAGAATCTGGAAGTGGATATTTGGA

                   *    * * **  **  **          * *  *
     278880 GCGCTTTCAGGCCTATGCTTAAAATAGGAAATATCTACCTAC
        131 GCGCTTTGAGG-ATTTCGTTGGAAACGGAAATATCTTCATAT

             *                    *    *  **          ** *        *    *     
     278922 AGAAACTAGACAGAAGCATTCTGAGAATCACGTTTGTGATGTGGGTACTCAACTAACAGTGTTGA
          1 AAAAACTAGACAGAAGCATTCTCAGAAACATCTTTGTGATGTTTGCACTCAACTCACAGAGTTGA

            *  *       *   *            *   *                *   *           
     278987 TCCATTCTTTTGATACAGCAGTTTTGAACCACACTTTTTGTAGAATCTGCAAGAGGATATTTGGA
         66 ACCTTTCTTTTCATAGAGCAGTTTTGAAACACTCTTTTTGTAGAATCTGGAAGTGGATATTTGGA

            **   *                     *   *      * *
     279052 TAGCTGTGAGGATTTCGTTGGAAACGGGAATGTCTTCAAAG
        131 GCGCTTTGAGGATTTCGTTGGAAACGGAAATATCTTCATAT

                *    *            *           * *       *  *    *            
     279093 AAAATCTAGCCAGAAGCATTCTAAGAAACATCTTAGGGATGTTTACATTCAAGTCACAGAGTTGA
          1 AAAAACTAGACAGAAGCATTCTCAGAAACATCTTTGTGATGTTTGCACTCAACTCACAGAGTTGA

              *  * *     *       *         *    * *   **      *      *    *  
     279158 ACATTCCCTTTCACAGAGCAGGTTTGAAACAATCTTCTCGTACTATCTGGCAGTGGACATTTTGA
         66 ACCTTTCTTTTCATAGAGCAGTTTTGAAACACTCTTTTTGTAGAATCTGGAAGTGGATATTTGGA

              * *   *  ** ** *  *   *            ** *
     279223 GCTCCTTGGGGCCTATGCTGAAAAAGGAAATATCTTCCGAC
        131 GCGCTTTGAGGATTTCGTTGGAAACGGAAATATCTTCATAT

            *                    *     *  **          *           **    *    
     279264 GAAAACTAGACAGAAGCATTCGCAGAATCACGTTTGTGATGTGTGCACTCAACTGTCAGAATTGA
          1 AAAAACTAGACAGAAGCATTCTCAGAAACATCTTTGTGATGTTTGCACTCAACTCACAGAGTTGA

                 **   ** *      *                            * *            *
     279329 ACCTTGGTTTGGACAGAGCACTTTTGAAACACTCTTTTTGTAGAATCTGCAGGTGGATATTTGGC
         66 ACCTTTCTTTTCATAGAGCAGTTTTGAAACACTCTTTTTGTAGAATCTGGAAGTGGATATTTGGA

            **                         *   *      * *
     279394 TAGCTTTGAGGATTTCGTTGGAAACGGTAATGTCTTCAAAG
        131 GCGCTTTGAGGATTTCGTTGGAAACGGAAATATCTTCATAT

                *                         *   *            *    *            
     279435 AAAATCTAGACAGAAGCATTCTCAGAAACACCTTCGTGATGTTTGCAATCAAGTCACAGAGTTGA
          1 AAAAACTAGACAGAAGCATTCTCAGAAACATCTTTGTGATGTTTGCACTCAACTCACAGAGTTGA

                 * *             *  *                  *             *       
     279500 ACCTTCCGTTTCATAGAGCAGGTTGGAAACACTCTTTTTGTAGTATCTGGAAGTGGACATTTGGA
         66 ACCTTTCTTTTCATAGAGCAGTTTTGAAACACTCTTTTTGTAGAATCTGGAAGTGGATATTTGGA

             *         ** *    * *   *            **  
     279565 GGGCTTTGTAGCCTATC-TGGAAAAAGGAAATATCTTCCCAT
        131 GCGCTTTG-AGGATTTCGTTGGAAACGGAAATATCTTCATAT

            *  ** *   *     * *            *   *  *   * **        *  * * *   
     279606 GAATGCGAGATAGAAGTAATCTCAGAAACATGTTTATGCTGTATCTACTCAACTAACTGTGCTGA
          1 AAAAACTAGACAGAAGCATTCTCAGAAACATCTTTGTGATGTTTGCACTCAACTCACAGAGTTGA

              *     *  *               *        *  * *       *               
     279671 ACATTTCTATTGATAGAGCAGTTTTGAGACACTCTTCTTTTGGAATCTGCAAGTGGATATTTGGA
         66 ACCTTTCTTTTCATAGAGCAGTTTTGAAACACTCTTTTTGTAGAATCTGGAAGTGGATATTTGGA

            ** *                        *     *      
     279736 TAGATTTGAGGATTTCGTTGGAAACGG-GAT-T-AT-ATAT
        131 GCGCTTTGAGGATTTCGTTGGAAACGGAAATATCTTCATAT

                 *       *               *      *          **  *   *         
     279773 AAAAAGTAGACAGCAGCATTCTCAGAAACTTCTTTGCGATGTTTGCATCCAGCTCTCAGAGTTGA
          1 AAAAACTAGACAGAAGCATTCTCAGAAACATCTTTGTGATGTTTGCACTCAACTCACAGAGTTGA

              *  * *          *  *        *        *   **        *  **       
     279838 ACATTCCCTTTCATAGAGTAGGTTTGAAACCCTCTTTTTATAGTGTCTGGAAGCGGGCATTTGGA
         66 ACCTTTCTTTTCATAGAGCAGTTTTGAAACACTCTTTTTGTAGAATCTGGAAGTGGATATTTGGA

             *     *   ** ** *  *   *          * *   
     279903 GTGCTTTCAGGCCTATGCTGAAAAAGGAAATATCTACCTAT
        131 GCGCTTTGAGGATTTCGTTGGAAACGGAAATATCTTCATAT

             *                    *    *  **          ** *        *    *     
     279944 AGAAACTAGACAGAAGCATTCTGAGAATCACGTTTGTGATGTGGGTACTCAACTAACAGTGTTGA
          1 AAAAACTAGACAGAAGCATTCTCAGAAACATCTTTGTGATGTTTGCACTCAACTCACAGAGTTGA

            *  *       *   *            *   *        *       *               
     280009 TCCATTCTTTTGATACAGCAGTTTTGAACCACACTTTTTGTGGAATCTGCAAGTGGATATTTGGA
         66 ACCTTTCTTTTCATAGAGCAGTTTTGAAACACTCTTTTTGTAGAATCTGGAAGTGGATATTTGGA

            **   *                     *   *        *
     280074 TAGCTGTGAGGATTTCGTTGGAAACGGGAATGTCTTCATAG
        131 GCGCTTTGAGGATTTCGTTGGAAACGGAAATATCTTCATAT

                **                     * * **         *  **   *   *          
     280115 AAAATTTAGACAGAAGCATTCTCAGAACCTTGATTGTGATGTGTGTTCTCCACTAACAGAGTTGA
          1 AAAAACTAGACAGAAGCATTCTCAGAAACATCTTTGTGATGTTTGCACTCAACTCACAGAGTTGA

                       * *   * *   *       *       *                         
     280180 ACCTTTCTTTTGACAGAACTGTTCTGAAACATTCTTTTTATAGAATCTGGAAGTGGATATTTGGA
         66 ACCTTTCTTTTCATAGAGCAGTTTTGAAACACTCTTTTTGTAGAATCTGGAAGTGGATATTTGGA

            **                         *          *  
     280245 AAGCTTTGAGGATTTCGTTGGAAACGGGAATATCTTCAAAT
        131 GCGCTTTGAGGATTTCGTTGGAAACGGAAATATCTTCATAT

                *    *            *           * *       *  *    *            
     280286 AAAATCTAGCCAGAAGCATTCTAAGAAACATCTTAGGGATGTTTACATTCAAGTCACAGAGTTGA
          1 AAAAACTAGACAGAAGCATTCTCAGAAACATCTTTGTGATGTTTGCACTCAACTCACAGAGTTGA

              *  * *     *       *         *    * *   **      *      *    *  
     280351 ACATTCCCTTTCACAGAGCAGGTTTGAAACAATCTTCTCGTACTATCTGGCAGTGGACATTTTGA
         66 ACCTTTCTTTTCATAGAGCAGTTTTGAAACACTCTTTTTGTAGAATCTGGAAGTGGATATTTGGA

              * *   *  ** ** *  *   *            ** *
     280416 GCTCCTTGGGGCCTATGCTGAAAAAGGAAATATCTTCCGAC
        131 GCGCTTTGAGGATTTCGTTGGAAACGGAAATATCTTCATAT

                                 *     *  **          *           **    *    
     280457 AAAAACTAGACAGAAGCATTCGCAGAATCACGTTTGTGATGTGTGCACTCAACTGTCAGAATTGA
          1 AAAAACTAGACAGAAGCATTCTCAGAAACATCTTTGTGATGTTTGCACTCAACTCACAGAGTTGA

                 **   ** *      *                            * *            *
     280522 ACCTTGGTTTGGACAGAGCACTTTTGAAACACTCTTTTTGTAGAATCTGCAGGTGGATATTTGGC
         66 ACCTTTCTTTTCATAGAGCAGTTTTGAAACACTCTTTTTGTAGAATCTGGAAGTGGATATTTGGA

            **                         *   *      * *
     280587 TAGCTTTGAGGATTTCGTTGGAAACGGTAATGTCTTCAAAG
        131 GCGCTTTGAGGATTTCGTTGGAAACGGAAATATCTTCATAT

                *                         *   *            *    *            
     280628 AAAATCTAGACAGAAGCATTCTCAGAAACACCTTCGTGATGTTTGCAATCAAGTCACAGAGTTGA
          1 AAAAACTAGACAGAAGCATTCTCAGAAACATCTTTGTGATGTTTGCACTCAACTCACAGAGTTGA

                 * *             *  *                  *             *       
     280693 ACCTTCCGTTTCATAGAGCAGGTTGGAAACACTCTTTTTGTAGTATCTGGAAGTGGACATTTGGA
         66 ACCTTTCTTTTCATAGAGCAGTTTTGAAACACTCTTTTTGTAGAATCTGGAAGTGGATATTTGGA

             *         ** *    * *   *            **  
     280758 GGGCTTTGTAGCCTATC-TGGAAAAAGGAAATATCTTCCCAT
        131 GCGCTTTG-AGGATTTCGTTGGAAACGGAAATATCTTCATAT

            *  ** *   *     * *            *   *  *   * **        *  * * *   
     280799 GAATGCGAGATAGAAGTAATCTCAGAAACATGTTTATGCTGTATCTACTCAACTAACTGTGCTGA
          1 AAAAACTAGACAGAAGCATTCTCAGAAACATCTTTGTGATGTTTGCACTCAACTCACAGAGTTGA

              *     *  *               *        *  * *       *               
     280864 ACATTTCTATTGATAGAGCAGTTTTGAGACACTCTTCTTTTGGAATCTGCAAGTGGATATTTGGA
         66 ACCTTTCTTTTCATAGAGCAGTTTTGAAACACTCTTTTTGTAGAATCTGGAAGTGGATATTTGGA

            ** *                        *     *      
     280929 TAGATTTGAGGATTTCGTTGGAAACGG-GAT-T-AT-ATAT
        131 GCGCTTTGAGGATTTCGTTGGAAACGGAAATATCTTCATAT

                 *       *               *    *            **  *   *         
     280966 AAAAAGTAGACAGCAGCATTCTCAGAAACTTCTTGGTGATGTTTGCATCCAGCTCTCAGAGTTGA
          1 AAAAACTAGACAGAAGCATTCTCAGAAACATCTTTGTGATGTTTGCACTCAACTCACAGAGTTGA

              *  * *          *  *        *        *   **        *  **       
     281031 ACATTCCCTTTCATAGAGTAGGTTTGAAACCCTCTTTTTATAGTGTCTGGAAGCGGGCATTTGGA
         66 ACCTTTCTTTTCATAGAGCAGTTTTGAAACACTCTTTTTGTAGAATCTGGAAGTGGATATTTGGA

                   *   ** ** *  *   *          * *   
     281096 GCGCTTTCAGGCCTATGCTGAAAAAGGAAATATCTACCTAT
        131 GCGCTTTGAGGATTTCGTTGGAAACGGAAATATCTTCATAT

             *                    *    *  **          ** *        *    *     
     281137 AGAAACTAGACAGAAGCATTCTGAGAATCACGTTTGTGATGTGGGTACTCAACTAACAGTGTTGA
          1 AAAAACTAGACAGAAGCATTCTCAGAAACATCTTTGTGATGTTTGCACTCAACTCACAGAGTTGA

            *  *       *   *            *   *                *               
     281202 TCCATTCTTTTGATACAGCAGTTTTGAACCACACTTTTTGTAGAATCTGCAAGTGGATATTTGGA
         66 ACCTTTCTTTTCATAGAGCAGTTTTGAAACACTCTTTTTGTAGAATCTGGAAGTGGATATTTGGA

            **   *                     *   *        *
     281267 TAGCTGTGAGGATTTCGTTGGAAACGGGAATGTCTTCATAG
        131 GCGCTTTGAGGATTTCGTTGGAAACGGAAATATCTTCATAT

                **                     * * **         *  **   *   *          
     281308 AAAATGTAGACAGAAGCATTCTCAGAACCTTGATTGTGATGTGTGTTCTCCACTAACAGAGTTGA
          1 AAAAACTAGACAGAAGCATTCTCAGAAACATCTTTGTGATGTTTGCACTCAACTCACAGAGTTGA

                       * *   * *   *       *       *                         
     281373 ACCTTTCTTTTGACAGAACTGTTCTGAAACATTCTTTTTATAGAATCTGGAAGTGGATATTTGGA
         66 ACCTTTCTTTTCATAGAGCAGTTTTGAAACACTCTTTTTGTAGAATCTGGAAGTGGATATTTGGA

            **                         *          *  
     281438 AAGCTTTGAGGATTTCGTTGGAAACGGGAATATCTTCAAAT
        131 GCGCTTTGAGGATTTCGTTGGAAACGGAAATATCTTCATAT

            *   *    *            *           * *       *  *    *            
     281479 CAAATCTAGCCAGAAGCATTCTAAGAAACATCTTAGGGATGTTTACATTCAAGTCACAGAGTTGA
          1 AAAAACTAGACAGAAGCATTCTCAGAAACATCTTTGTGATGTTTGCACTCAACTCACAGAGTTGA

              *  * *     *       *         *    * *   **      *      *    *  
     281544 ACATTCCCTTTCACAGAGCAGGTTTGAAACAATCTTCTCGTACTATCTGGCAGTGGACATTTTGA
         66 ACCTTTCTTTTCATAGAGCAGTTTTGAAACACTCTTTTTGTAGAATCTGGAAGTGGATATTTGGA

              * *   *  ** ** *  *   *            ** *
     281609 GCTCCTTGGGGCCTATGCTGAAAAAGGAAATATCTTCCGAC
        131 GCGCTTTGAGGATTTCGTTGGAAACGGAAATATCTTCATAT

                                 *     *  **          *           **    *    
     281650 AAAAACTAGACAGAAGCATTCGCAGAATCACGTTTGTGATGTATGCACTCAACTGTCAGAATTGA
          1 AAAAACTAGACAGAAGCATTCTCAGAAACATCTTTGTGATGTTTGCACTCAACTCACAGAGTTGA

                 **   ** *      *                            * *            *
     281715 ACCTTGGTTTGGACAGAGCACTTTTGAAACACTCTTTTTGTAGAATCTGCAGGTGGATATTTGGC
         66 ACCTTTCTTTTCATAGAGCAGTTTTGAAACACTCTTTTTGTAGAATCTGGAAGTGGATATTTGGA

            **                         *   *      * *
     281780 TAGCTTTGAGGATTTCGTTGGAAACGGTAATGTCTTCAAAG
        131 GCGCTTTGAGGATTTCGTTGGAAACGGAAATATCTTCATAT

                *                         *   *            *    **           
     281821 AAAATCTAGACAGAAGCATTCTCAGAAACACCTTCGTGATGTTTGCAATCAAGACACAGAGTTGA
          1 AAAAACTAGACAGAAGCATTCTCAGAAACATCTTTGTGATGTTTGCACTCAACTCACAGAGTTGA

                 * *             *  *                  *             *       
     281886 ACCTTCCGTTTCATAGAGCAGGTTGGAAACACTCTTTTTGTAGTATCTGGAAGTGGACATTTGGA
         66 ACCTTTCTTTTCATAGAGCAGTTTTGAAACACTCTTTTTGTAGAATCTGGAAGTGGATATTTGGA

             *         ** *     **   *            **  
     281951 GGGCTTTGTAGCCTATC--TAAAAAAGGAAATATCTTCCCAT
        131 GCGCTTTG-AGGATTTCGTTGGAAACGGAAATATCTTCATAT

            *  ** *   *     * *            *   *  *   * **        *  * * *   
     281991 GAATGCGAGATAGAAGTAATCTCAGAAACATGTTTATGCTGTATCTACTCAACTAACTGTGCTGA
          1 AAAAACTAGACAGAAGCATTCTCAGAAACATCTTTGTGATGTTTGCACTCAACTCACAGAGTTGA

              *     *  *               *        *  * *       *               
     282056 ACATTTCTATTGATAGAGCAGTTTTGAGACACTCTTCTTTTGGAATCTGCAAGTGGATATTTGGA
         66 ACCTTTCTTTTCATAGAGCAGTTTTGAAACACTCTTTTTGTAGAATCTGGAAGTGGATATTTGGA

            ** *                        *     *      
     282121 TAGATTTGAGGATTTCGTTGGAAACGG-GAT-T-AT-ATAT
        131 GCGCTTTGAGGATTTCGTTGGAAACGGAAATATCTTCATAT

                 *       *               *                 **  *   *         
     282158 AAAAAGTAGACAGCAGCATTCTCAGAAACTTCTTTGTGATGTTTGCATCCAGCTCTCAGAGTTGA
          1 AAAAACTAGACAGAAGCATTCTCAGAAACATCTTTGTGATGTTTGCACTCAACTCACAGAGTTGA

              *  * *          *  *        *        *   **    *   *  **       
     282223 ACATTCCCTTTCATAGAGTAGGTTTGAAACCCTCTTTTTATAGTGTCTGCAAGCGGGCATTTGGA
         66 ACCTTTCTTTTCATAGAGCAGTTTTGAAACACTCTTTTTGTAGAATCTGGAAGTGGATATTTGGA

                   *    * * **  **  **          * *  *
     282288 GCGCTTTCAGGCCTATGCTTAAAATAGGAAATATCTACCTAC
        131 GCGCTTTGAGG-ATTTCGTTGGAAACGGAAATATCTTCATAT

             *                    *    *  **          ** *        *    *     
     282330 AGAAACTAGACAGAAGCATTCTGAGAATCACGTTTGTGATGTGGGTACTCAACTAACAGTGTTGA
          1 AAAAACTAGACAGAAGCATTCTCAGAAACATCTTTGTGATGTTTGCACTCAACTCACAGAGTTGA

            *  *       *   *            *   *                *   *           
     282395 TCCATTCTTTTGATACAGCAGTTTTGAACCACACTTTTTGTAGAATCTGCAAGAGGATATTTGGA
         66 ACCTTTCTTTTCATAGAGCAGTTTTGAAACACTCTTTTTGTAGAATCTGGAAGTGGATATTTGGA

            **   *                     *   *      * *
     282460 TAGCTGTGAGGATTTCGTTGGAAACGGGAATGTCTTCAAAG
        131 GCGCTTTGAGGATTTCGTTGGAAACGGAAATATCTTCATAT

                *                         * * *            *    *            
     282501 AAAATCTAGACAGAAGCATTCTCAGAAACACCATCGTGATGTTTGCAATCAAGTCACAGAGTTGA
          1 AAAAACTAGACAGAAGCATTCTCAGAAACATCTTTGTGATGTTTGCACTCAACTCACAGAGTTGA

                 * *             *  *           *      *             *       
     282566 ACCTTCCGTTTCATAGAGCAGGTTGGAAACACTCTTATTGTAGTATCTGGAAGTGGACATTTGGA
         66 ACCTTTCTTTTCATAGAGCAGTTTTGAAACACTCTTTTTGTAGAATCTGGAAGTGGATATTTGGA

                   *   ** ** *  *   *            **  
     282631 GCGCTTTCAGGCCTATGGTGAAAAAGGAAATATCTTCCCAT
        131 GCGCTTTGAGGATTTCGTTGGAAACGGAAATATCTTCATAT

                  * * *      **      *   * *   *     ** ** *      *    *     
     282672 AAAAACGACATAGAAGCTATCTCAGGAACTTGTTTATGATGCATCTAATCAACTAACAGTGTTGA
          1 AAAAACTAGACAGAAGCATTCTCAGAAACATCTTTGTGATGTTTGCACTCAACTCACAGAGTTGA

                  * ** * *                         * *       *               
     282737 ACCTTTGTACTGACAGAGCAG-TTTGAAACACTCTTTTTTTGGAATCTGCAAGTGGATATTTGGA
         66 ACCTTTCTTTTCATAGAGCAGTTTTGAAACACTCTTTTTGTAGAATCTGGAAGTGGATATTTGGA

            *                           *   *   *    
     282801 TCGCTTTGAGGATTTCGTTGGAAACGG-GAT-GC--AATAT
        131 GCGCTTTGAGGATTTCGTTGGAAACGGAAATATCTTCATAT

                **  *    *     *                 **  *   *  *    *         * 
     282838 AAAACGTACACAGCAGCATACTCAGAAA-ATACTTTGCCATATTTCCATTCAAGTCACAGAGTGG
          1 AAAAACTAGACAGAAGCATTCTCAGAAACAT-CTTTGTGATGTTTGCACTCAACTCACAGAGTTG

              **  * **            *                  *  *             *      
     282902 AATATTCCCATTCATAGAGCAGGTTTGAAACACTCTTTTTGGAGTATCTGGAAGTGGACATTTGG
         65 AACCTTTCTTTTCATAGAGCAGTTTTGAAACACTCTTTTTGTAGAATCTGGAAGTGGATATTTGG

                    ** * * ** *  *   *            **  
     282967 AGCGCTTTCTGAACTATGGTGAAAAAGGAAATATCTTCCAAT
        130 AGCGCTTTGAGGATTTCGTTGGAAACGGAAATATCTTCATAT

            *     *               *      * *          *  **      **  *  *    
     283009 GAAAACAAGACAGAAGCATTCTGAGAAACTTATTTGTGATGTGTGTCCTCAACAAACGGACTTGA
          1 AAAAACTAGACAGAAGCATTCTCAGAAACATCTTTGTGATGTTTGCACTCAACTCACAGAGTTGA

                   *      **  * *  *  *             *   *    * * *           
     283074 ACCTTTCGTTTCATGCAGTACTTCTGGAACACTCTTTTTGAAGATTCTGCATGCGGATATTTGGA
         66 ACCTTTCTTTTCATAGAGCAGTTTTGAAACACTCTTTTTGTAGAATCTGGAAGTGGATATTTGGA

            **                            **   *   * 
     283139 TAGCTTTGAGGATTTCGTTGGAAACGG---GCT-TACATGT
        131 GCGCTTTGAGGATTTCGTTGGAAACGGAAATATCTTCATAT

                 *       *               *        *   *    *    *       *    
     283176 AAAAATTAGACAGCAGCATTCTCAGAAACTTCTTTGTGGTGTCTGCATTCAAGTCACAGAATTGA
          1 AAAAACTAGACAGAAGCATTCTCAGAAACATCTTTGTGATGTTTGCACTCAACTCACAGAGTTGA

              * ** * **            *  * *      *       *             *       
     283241 ACTTCCCCTCACATAGAGCAGTTGTGCAGCACTCTATTTGTAGTATCTGGAAGTGGACATTTGGA
         66 ACCTTTCTTTTCATAGAGCAGTTTTGAAACACTCTTTTTGTAGAATCTGGAAGTGGATATTTGGA

             *         ** *    * *   *            **  
     283306 GGGCTTTGTAGCCTATC-TGGAAAAAGGAAATATCTTCCCAT
        131 GCGCTTTG-AGGATTTCGTTGGAAACGGAAATATCTTCATAT

            *  ** *   *     * *            *   *  *   * **        *  * * *   
     283347 GAATGCGAGATAGAAGTAATCTCAGAAACATGTTTATGCTGTATCTACTCAACTAACTGTGCTGA
          1 AAAAACTAGACAGAAGCATTCTCAGAAACATCTTTGTGATGTTTGCACTCAACTCACAGAGTTGA

              *     *  *               *        *  * *       *               
     283412 ACATTTCTATTGATAGAGCAGTTTTGAGACACTCTTCTTTTGGAATCTGCAAGTGGATATTTGGA
         66 ACCTTTCTTTTCATAGAGCAGTTTTGAAACACTCTTTTTGTAGAATCTGGAAGTGGATATTTGGA

            ** *                        *     *      
     283477 TAGATTTGAGGATTTCGTTGGAAACGG-GAT-T-AT-ATAT
        131 GCGCTTTGAGGATTTCGTTGGAAACGGAAATATCTTCATAT

                 *       *               *                 **  *   *         
     283514 AAAAAGTAGACAGCAGCATTCTCAGAAACTTCTTTGTGATGTTTGCATCCAGCTCTCAGAGTTGA
          1 AAAAACTAGACAGAAGCATTCTCAGAAACATCTTTGTGATGTTTGCACTCAACTCACAGAGTTGA

              *  * *          *  *        *        *   **        *  **       
     283579 ACATTCCCTTTCATAGAGTAGGTTTGAAACCCTCTTTTTATAGTGTCTGGAAGCGGGCATTTGGA
         66 ACCTTTCTTTTCATAGAGCAGTTTTGAAACACTCTTTTTGTAGAATCTGGAAGTGGATATTTGGA

                   *   ** ** *  *   *          * *   
     283644 GCGCTTTCAGGCCTATGCTGAAAAAGGAAATATCTACCTAT
        131 GCGCTTTGAGGATTTCGTTGGAAACGGAAATATCTTCATAT

             *                    *    *  **          ** *        *    *     
     283685 AGAAACTAGACAGAAGCATTCTGAGAATCACGTTTGTGATGTGGGTACTCAACTAACAGTGTTGA
          1 AAAAACTAGACAGAAGCATTCTCAGAAACATCTTTGTGATGTTTGCACTCAACTCACAGAGTTGA

            *  *       *   *            *   *                *               
     283750 TCCATTCTTTTGATACAGCAGTTTTGAACCACACTTTTTGTAGAATCTGCAAGTGGATATTTGGA
         66 ACCTTTCTTTTCATAGAGCAGTTTTGAAACACTCTTTTTGTAGAATCTGGAAGTGGATATTTGGA

            **   *                     *   *        *
     283815 TAGCTGTGAGGATTTCGTTGGAAACGGGAATGTCTTCATAG
        131 GCGCTTTGAGGATTTCGTTGGAAACGGAAATATCTTCATAT

                **                     * * **         *  **   *   *          
     283856 AAAATTTAGACAGAAGCATTCTCAGAACCTTGATTGTGATGTGTGTTCTCCACTAACAGAGTTGA
          1 AAAAACTAGACAGAAGCATTCTCAGAAACATCTTTGTGATGTTTGCACTCAACTCACAGAGTTGA

                       * *   * *   *       *       *                         
     283921 ACCTTTCTTTTGACAGAACTGTTCTGAAACATTCTTTTTATAGAATCTGGAAGTGGATATTTGGA
         66 ACCTTTCTTTTCATAGAGCAGTTTTGAAACACTCTTTTTGTAGAATCTGGAAGTGGATATTTGGA

            **                         *          *  
     283986 AAGCTTTGAGGATTTCGTTGGAAACGGGAATATCTTCAAAT
        131 GCGCTTTGAGGATTTCGTTGGAAACGGAAATATCTTCATAT

                *    *            *           * *       *  *    *            
     284027 AAAATCTAGCCAGAAGCATTCTAAGAAACATCTTAGGGATGTTTACATTCAAGTCACAGAGTTGA
          1 AAAAACTAGACAGAAGCATTCTCAGAAACATCTTTGTGATGTTTGCACTCAACTCACAGAGTTGA

              *  * *     *       *         *    * *   **      *      *    *  
     284092 ACATTCCCTTTCACAGAGCAGGTTTGAAACAATCTTCTCGTACTATCTGGCAGTGGACATTTTGA
         66 ACCTTTCTTTTCATAGAGCAGTTTTGAAACACTCTTTTTGTAGAATCTGGAAGTGGATATTTGGA

              * *   *  ** ** *  *   *            ** *
     284157 GCTCCTTGGGGCCTATGCTGAAAAAGGAAATATCTTCCGAC
        131 GCGCTTTGAGGATTTCGTTGGAAACGGAAATATCTTCATAT

                                 *     *  **          *           **    *    
     284198 AAAAACTAGACAGAAGCATTCGCAGAATCACGTTTGTGATGTGTGCACTCAACTGTCAGAATTGA
          1 AAAAACTAGACAGAAGCATTCTCAGAAACATCTTTGTGATGTTTGCACTCAACTCACAGAGTTGA

                 **   ** *      *                            * *            *
     284263 ACCTTGGTTTGGACAGAGCACTTTTGAAACACTCTTTTTGTAGAATCTGCAGGTGGATATTTGGC
         66 ACCTTTCTTTTCATAGAGCAGTTTTGAAACACTCTTTTTGTAGAATCTGGAAGTGGATATTTGGA

            ***                        *   *      * *
     284328 TACCTTTGAGGATTTCGTTGGAAACGGTAATGTCTTCAAAG
        131 GCGCTTTGAGGATTTCGTTGGAAACGGAAATATCTTCATAT

                *                         *   *            *    *            
     284369 AAAATCTAGACAGAAGCATTCTCAGAAACACCTTCGTGATGTTTGCAATCAAGTCACAGAGTTGA
          1 AAAAACTAGACAGAAGCATTCTCAGAAACATCTTTGTGATGTTTGCACTCAACTCACAGAGTTGA

                 * *             *  *                  *             *       
     284434 ACCTTCCGTTTCATAGAGCAGGTTGGAAACACTCTTTTTGTAGTATCTGGAAGTGGACATTTGGA
         66 ACCTTTCTTTTCATAGAGCAGTTTTGAAACACTCTTTTTGTAGAATCTGGAAGTGGATATTTGGA

             *         ** *    * *   *            **  
     284499 GGGCTTTGTAGCCTATC-TGGAAAAAGGAAATATCTTCCCAT
        131 GCGCTTTG-AGGATTTCGTTGGAAACGGAAATATCTTCATAT

            *  ** *   *     * *            *   *  *   * **        *  * * *   
     284540 GAATGCGAGATAGAAGTAATCTCAGAAACATGTTTATGCTGTATCTACTCAACTAACTGTGCTGA
          1 AAAAACTAGACAGAAGCATTCTCAGAAACATCTTTGTGATGTTTGCACTCAACTCACAGAGTTGA

              *     *  *               *        *  * *       *               
     284605 ACATTTCTATTGATAGAGCAGTTTTGAGACACTCTTCTTTTGGAATCTGCAAGTGGATATTTGGA
         66 ACCTTTCTTTTCATAGAGCAGTTTTGAAACACTCTTTTTGTAGAATCTGGAAGTGGATATTTGGA

            ** *                        *     *      
     284670 TAGATTTGAGGATTTCGTTGGAAACGG-GAT-T-AT-ATAT
        131 GCGCTTTGAGGATTTCGTTGGAAACGGAAATATCTTCATAT

                 *       *               *                 **  *   *         
     284707 AAAAAGTAGACAGCAGCATTCTCAGAAACTTCTTTGTGATGTTTGCATCCAGCTCTCAGAGTTGA
          1 AAAAACTAGACAGAAGCATTCTCAGAAACATCTTTGTGATGTTTGCACTCAACTCACAGAGTTGA

              *  * *          *  *        *        *   **        *  **       
     284772 ACATTCCCTTTCATAGAGTAGGTTTGAAACCCTCTTTTTATAGTGTCTGGAAGCGGGCATTTGGA
         66 ACCTTTCTTTTCATAGAGCAGTTTTGAAACACTCTTTTTGTAGAATCTGGAAGTGGATATTTGGA

                   *    * * **  **  **          * *  *
     284837 GCGCTTTCAGGGCTATGCTTAAAATAGGAAATATCTACCTAC
        131 GCGCTTTGA-GGATTTCGTTGGAAACGGAAATATCTTCATAT

             *                    *    *  **          ** *        *    *     
     284879 AGAAACTAGACAGAAGCA-TCTGAGAATCACGTTTGTGATGTGGGTACTCAACTAACAGTGTTGA
          1 AAAAACTAGACAGAAGCATTCTCAGAAACATCTTTGTGATGTTTGCACTCAACTCACAGAGTTGA

            *  *       *   *            *   *                *   *           
     284943 TCCATTCTTTTGATACAGCAGTTTTGAACCACACTTTTTGTAGAATCTGCAAGAGGATATTTGGA
         66 ACCTTTCTTTTCATAGAGCAGTTTTGAAACACTCTTTTTGTAGAATCTGGAAGTGGATATTTGGA

            **   *                     *   *      * *
     285008 TAGCTGTGAGGATTTCGTTGGAAACGGGAATGTCTTCAAAG
        131 GCGCTTTGAGGATTTCGTTGGAAACGGAAATATCTTCATAT

                *                         *   *            *    *            
     285049 AAAATCTAGACAGAAGCATTCTCAGAAACACCTTCGTGATGTTTGCAATCAAGTCACAGAGTTGA
          1 AAAAACTAGACAGAAGCATTCTCAGAAACATCTTTGTGATGTTTGCACTCAACTCACAGAGTTGA

                 * *             *  *           *      *             *       
     285114 ACCTTCCGTTTCATAGAGCAGGTTGGAAACACTCTTATTGTAGTATCTGGAAGTGGACATTTGGA
         66 ACCTTTCTTTTCATAGAGCAGTTTTGAAACACTCTTTTTGTAGAATCTGGAAGTGGATATTTGGA

                   *   ** ** *  *   *            **  
     285179 GCGCTTTCAGGCCTATGGTGAAAAAGGAAATATCTTCCCAT
        131 GCGCTTTGAGGATTTCGTTGGAAACGGAAATATCTTCATAT

                  * * *      **      *   * *   *     ** ** *      *    *  *  
     285220 AAAAACGACATAGAAGCTATCTCAGGAACTTGTTTATGATGCATCTAATCAACTAACAGTGTAGA
          1 AAAAACTAGACAGAAGCATTCTCAGAAACATCTTTGTGATGTTTGCACTCAACTCACAGAGTTGA

                  * ** * *                         * *       *               
     285285 ACCTTTGTACTGACAGAGCAG-TTTGAAACACTCTTTTTTTGGAATCTGCAAGTGGATATTTGGA
         66 ACCTTTCTTTTCATAGAGCAGTTTTGAAACACTCTTTTTGTAGAATCTGGAAGTGGATATTTGGA

            *                             *   ***    
     285349 TCGCTTTGAGGATTTCGTTGGAAACGG---GAT-GGAATAT
        131 GCGCTTTGAGGATTTCGTTGGAAACGGAAATATCTTCATAT

                **  *    *     *                 **  *   *  *    *         * 
     285386 AAAACGTACACAGCAGCATACTCAGAAA-ATACTTTGCCATATTTCCATTCAAGTCACAGAGTGG
          1 AAAAACTAGACAGAAGCATTCTCAGAAACAT-CTTTGTGATGTTTGCACTCAACTCACAGAGTTG

               *  * **            *                  *  *             *      
     285450 AACATTCCCATTCATAGAGCAGGTTTGAAACACTCTTTTTGGAGTATCTGGAAGTGGACATTTGG
         65 AACCTTTCTTTTCATAGAGCAGTTTTGAAACACTCTTTTTGTAGAATCTGGAAGTGGATATTTGG

                    ** * * ** *  *   *            **  
     285515 AGCGCTTTCTGAACTATGGTGAAAAAGGAAATATCTTCCAAT
        130 AGCGCTTTGAGGATTTCGTTGGAAACGGAAATATCTTCATAT

            *     *               *      * *          *  **      **  *  *    
     285557 GAAAACAAGACAGAAGCATTCTGAGAAACTTATTTGTGATGTGTGTCCTCAACAAACGGACTTGA
          1 AAAAACTAGACAGAAGCATTCTCAGAAACATCTTTGTGATGTTTGCACTCAACTCACAGAGTTGA

                   *      **  * *  *  *             *   *    * * *           
     285622 ACCTTTCGTTTCATGCAGTACTTCTGGAACACTCTTTTTGAAGATTCTGCATGCGGATATTTGGA
         66 ACCTTTCTTTTCATAGAGCAGTTTTGAAACACTCTTTTTGTAGAATCTGGAAGTGGATATTTGGA

            **                            **   *   * 
     285687 TTGCTTTGAGGATTTCGTTGGAAACGG---GCT-TACATGT
        131 GCGCTTTGAGGATTTCGTTGGAAACGGAAATATCTTCATAT

                 *       *               *        *   *    *    *       *    
     285724 AAAAATTAGACAGCAGCATTCTCAGAAACTTCTTTGTGGTGTCTGCATTCAAGTCACAGAATTGA
          1 AAAAACTAGACAGAAGCATTCTCAGAAACATCTTTGTGATGTTTGCACTCAACTCACAGAGTTGA

              * ** * **            *  * *      *       *             *       
     285789 ACTTCCCCTCACATAGAGCAGTTGTGCAGCACTCTATTTGTAGTATCTGGAAGTGGACATTTGGA
         66 ACCTTTCTTTTCATAGAGCAGTTTTGAAACACTCTTTTTGTAGAATCTGGAAGTGGATATTTGGA

             *         ** *    * *   *            **  
     285854 GGGCTTTGTAGCCTATC-TGGAAAAAGGAAATATCTTCCCAT
        131 GCGCTTTG-AGGATTTCGTTGGAAACGGAAATATCTTCATAT

            *  ** *   *     * *            *   *  *   * **        *  * * *   
     285895 GAATGCGAGATAGAAGTAATCTCAGAAACATGTTTATGCTGTATCTACTCAACTAACTGTGCTGA
          1 AAAAACTAGACAGAAGCATTCTCAGAAACATCTTTGTGATGTTTGCACTCAACTCACAGAGTTGA

              *     *  *               *        *  * *       *               
     285960 ACATTTCTGTTGATAGAGCAGTTTTGAGACACTCTTCTTTTGGAATCTGCAAGTGGATATTTGGA
         66 ACCTTTCTTTTCATAGAGCAGTTTTGAAACACTCTTTTTGTAGAATCTGGAAGTGGATATTTGGA

            ** *                        *     *      
     286025 TAGATTTGAGGATTTCGTTGGAAACGG-GAT-T-AT-ATAT
        131 GCGCTTTGAGGATTTCGTTGGAAACGGAAATATCTTCATAT

                 *       *               *                 **  *   *         
     286062 AAAAAGTAGACAGCAGCATTCTCAGAAACTTCTTTGTGATGTTTGCATCCAGCTCTCAGAGTTGA
          1 AAAAACTAGACAGAAGCATTCTCAGAAACATCTTTGTGATGTTTGCACTCAACTCACAGAGTTGA

              *  * *          *  *        *        *   **        *  **       
     286127 ACATTCCCTTTCATAGAGTAGGTTTGAAACCCTCTTTTTATAGTGTCTGGAAGCGGGCATTTGGA
         66 ACCTTTCTTTTCATAGAGCAGTTTTGAAACACTCTTTTTGTAGAATCTGGAAGTGGATATTTGGA

                   *    * * **  **  **          * *  *
     286192 GCGCTTTCAGGCCTATGCTTAAAATAGGAAATATCTACCTAC
        131 GCGCTTTGAGG-ATTTCGTTGGAAACGGAAATATCTTCATAT

             *                    *    *  **          ** *        *    *     
     286234 AGAAACTAGACAGAAGCATTCTGAGAATCACGTTTGTGATGTGGGTACTCAACTAACAGTGTTGA
          1 AAAAACTAGACAGAAGCATTCTCAGAAACATCTTTGTGATGTTTGCACTCAACTCACAGAGTTGA

            *  *       *   *            *   *                *               
     286299 TCCATTCTTTTGATACAGCAGTTTTGAACCACACTTTTTGTAGAATCTGCAAGTGGATATTTGGA
         66 ACCTTTCTTTTCATAGAGCAGTTTTGAAACACTCTTTTTGTAGAATCTGGAAGTGGATATTTGGA

            **   *                     *   *      * *
     286364 TAGCTGTGAGGATTTCGTTGGAAACGGGAATGTCTTCAAAG
        131 GCGCTTTGAGGATTTCGTTGGAAACGGAAATATCTTCATAT

                *                         *   *            *    *            
     286405 AAAATCTAGACAGAAGCATTCTCAGAAACACCTTCGTGATGTTTGCAATCAAGTCACAGAGTTGA
          1 AAAAACTAGACAGAAGCATTCTCAGAAACATCTTTGTGATGTTTGCACTCAACTCACAGAGTTGA

                 * *             *  *           *      *             *       
     286470 ACCTTCCGTTTCATAGAGCAGGTTGGAAACACTCTTATTGTAGTATCTGGAAGTGGACATTTGGA
         66 ACCTTTCTTTTCATAGAGCAGTTTTGAAACACTCTTTTTGTAGAATCTGGAAGTGGATATTTGGA

                   *   ** ** *  *   *            **  
     286535 GCGCTTTCAGGCCTATGGTGAAAAAGGAAATATCTTCCCAT
        131 GCGCTTTGAGGATTTCGTTGGAAACGGAAATATCTTCATAT

                  * * *      **      *   * *   *     ** ** *      *    *     
     286576 AAAAACGACATAGAAGCTATCTCAGGAACTTGTTTATGATGCATCTAATCAACTAACAGTGTTGA
          1 AAAAACTAGACAGAAGCATTCTCAGAAACATCTTTGTGATGTTTGCACTCAACTCACAGAGTTGA

                  * ** * *                         * *       *               
     286641 ACCTTTGTACTGACAGAGCAG-TTTGAAACACTCTTTTTTTGGAATCTGCAAGTGGATATTTGGA
         66 ACCTTTCTTTTCATAGAGCAGTTTTGAAACACTCTTTTTGTAGAATCTGGAAGTGGATATTTGGA

            *                             *   ***    
     286705 TCGCTTTGAGGATTTCGTTGGAAACGG---GAT-GGAATAT
        131 GCGCTTTGAGGATTTCGTTGGAAACGGAAATATCTTCATAT

                **  *    *     *                 **  *   *  *    *         * 
     286742 AAAACGTACACAGCAGCATACTCAGAAA-ATACTTTGCCATATTTCCATTCAAGTCACAGAGTGG
          1 AAAAACTAGACAGAAGCATTCTCAGAAACAT-CTTTGTGATGTTTGCACTCAACTCACAGAGTTG

               *  * **            *                  *  *             *      
     286806 AACATTCCCATTCATAGAGCAGGTTTGAAACACTCTTTTTGGAGTATCTGGAAGTGGACATTTGG
         65 AACCTTTCTTTTCATAGAGCAGTTTTGAAACACTCTTTTTGTAGAATCTGGAAGTGGATATTTGG

                    ** * * ** *  *   *            **  
     286871 AGCGCTTTCTGAACTATGGTGAAAAAGGAAATATCTTCCAAT
        130 AGCGCTTTGAGGATTTCGTTGGAAACGGAAATATCTTCATAT

            *     *               *      * *          *  **      **  *  *    
     286913 GAAAACAAGACAGAAGCATTCTGAGAAACTTATTTGTGATGTGTGTCCTCAACAAACGGACTTGA
          1 AAAAACTAGACAGAAGCATTCTCAGAAACATCTTTGTGATGTTTGCACTCAACTCACAGAGTTGA

                   *      **  * *  *  *             *   *    * * *           
     286978 ACCTTTCGTTTCATGCAGTACTTCTGGAACACTCTTTTTGAAGATTCTGCATGCGGATATTTGGA
         66 ACCTTTCTTTTCATAGAGCAGTTTTGAAACACTCTTTTTGTAGAATCTGGAAGTGGATATTTGGA

            **                            **   *   * 
     287043 TAGCTTTGAGGATTTCGTTGGAAACGG---GCT-TACATGT
        131 GCGCTTTGAGGATTTCGTTGGAAACGGAAATATCTTCATAT

                 *       *               *        *   *    *    *       *    
     287080 AAAAATTAGACAGCAGCATTCTCAGAAACTTCTTTGTGGTGTCTGCATTCAAGTCACAGAATTGA
          1 AAAAACTAGACAGAAGCATTCTCAGAAACATCTTTGTGATGTTTGCACTCAACTCACAGAGTTGA

              * ** * **            *  * *      *       *             *       
     287145 ACTTCCCCTCACATAGAGCAGTTGTGCAGCACTCTATTTGTAGTATCTGGAAGTGGACATTTGGA
         66 ACCTTTCTTTTCATAGAGCAGTTTTGAAACACTCTTTTTGTAGAATCTGGAAGTGGATATTTGGA

             *         ** *    * *   *            **  
     287210 GGGCTTTGTAGCCTATC-TGGAAAAAGGAAATATCTTCCCAT
        131 GCGCTTTG-AGGATTTCGTTGGAAACGGAAATATCTTCATAT

            *  ** *   *     * *            *   *  *   * **        *  * * *   
     287251 GAATGCGAGATAGAAGTAATCTCAGAAACATGTTTATGCTGTATCTACTCAACTAACTGTGCTGA
          1 AAAAACTAGACAGAAGCATTCTCAGAAACATCTTTGTGATGTTTGCACTCAACTCACAGAGTTGA

              *     *  *               *        *  * *       *               
     287316 ACATTTCTATTGATAGAGCAGTTTTGAGACACTCTTCTTTTGGAATCTGCAAGTGGATATTTGGA
         66 ACCTTTCTTTTCATAGAGCAGTTTTGAAACACTCTTTTTGTAGAATCTGGAAGTGGATATTTGGA

            ** *                        *     *      
     287381 TAGATTTGAGGATTTCGTTGGAAACGG-GAT-T-AT-ATAT
        131 GCGCTTTGAGGATTTCGTTGGAAACGGAAATATCTTCATAT

                 *       *               *                 **  *   *         
     287418 AAAAAGTAGACAGCAGCATTCTCAGAAACTTCTTTGTGATGTTTGCATCCAGCTCTCAGAGTTGA
          1 AAAAACTAGACAGAAGCATTCTCAGAAACATCTTTGTGATGTTTGCACTCAACTCACAGAGTTGA

              *  * *          *  *        *        *   **        *  **       
     287483 ACATTCCCTTTCATAGAGTAGGTTTGAAACCCTCTTTTTATAGTGTCTGGAAGCGGGCATTTGGA
         66 ACCTTTCTTTTCATAGAGCAGTTTTGAAACACTCTTTTTGTAGAATCTGGAAGTGGATATTTGGA

                   *   ** ** *  *   *          * *   
     287548 GCGCTTTCAGGCCTATGCTGAAAAAGGAAATATCTACCTAT
        131 GCGCTTTGAGGATTTCGTTGGAAACGGAAATATCTTCATAT

             *                    *    *  **          ** *        *    *     
     287589 AGAAACTAGACAGAAGCATTCTGAGAATCACGTTTGTGATGTGGGTACTCAACTAACAGTGTTGA
          1 AAAAACTAGACAGAAGCATTCTCAGAAACATCTTTGTGATGTTTGCACTCAACTCACAGAGTTGA

            *  *       *   *            *   *                *               
     287654 TCCATTCTTTTGATACAGCAGTTTTGAACCACACTTTTTGTAGAATCTGCAAGTGGATATTTGGA
         66 ACCTTTCTTTTCATAGAGCAGTTTTGAAACACTCTTTTTGTAGAATCTGGAAGTGGATATTTGGA

            **   *                     *   *      * *
     287719 TAGCTGTGAGGATTTCGTTGGAAACGGGAATGTCTTCAAAG
        131 GCGCTTTGAGGATTTCGTTGGAAACGGAAATATCTTCATAT

                *     *                * * **         *  **   *   *          
     287760 AAAATCTAGAGAGAAGCATTCTCAGAACCTTGATTGTGATGTGTGTTCTCCACTAACAGAGTTGA
          1 AAAAACTAGACAGAAGCATTCTCAGAAACATCTTTGTGATGTTTGCACTCAACTCACAGAGTTGA

                       * *   * *   *       *       *                         
     287825 ACCTTTCTTTTGACAGAACTGTTCTGAAACATTCTTTTTATAGAATCTGGAAGTGGATATTTGGA
         66 ACCTTTCTTTTCATAGAGCAGTTTTGAAACACTCTTTTTGTAGAATCTGGAAGTGGATATTTGGA

            **                         *          *  
     287890 AAGCTTTGAGGATTTCGTTGGAAACGGGAATATCTTCAAAT
        131 GCGCTTTGAGGATTTCGTTGGAAACGGAAATATCTTCATAT

                *    *            *           * *       *  *    *            
     287931 AAAATCTAGCCAGAAGCATTCTAAGAAACATCTTAGGGATGTTTACATTCAAGTCACAGAGTTGA
          1 AAAAACTAGACAGAAGCATTCTCAGAAACATCTTTGTGATGTTTGCACTCAACTCACAGAGTTGA

              *  * *     *       *         *    * *   **      *      *    *  
     287996 ACATTCCCTTTCACAGAGCAGGTTTGAAACAATCTTCTCGTACTATCTGGCAGTGGACATTTTGA
         66 ACCTTTCTTTTCATAGAGCAGTTTTGAAACACTCTTTTTGTAGAATCTGGAAGTGGATATTTGGA

              * *   *  ** ** *  *   *            ** *
     288061 GCTCCTTGGGGCCTATGCTGAAAAAGGAAATATCTTCCGAC
        131 GCGCTTTGAGGATTTCGTTGGAAACGGAAATATCTTCATAT

                                 *     *  **          *           **    *    
     288102 AAAAACTAGACAGAAGCATTCGCAGAATCACGTTTGTGATGTGTGCACTCAACTGTCAGAATTGA
          1 AAAAACTAGACAGAAGCATTCTCAGAAACATCTTTGTGATGTTTGCACTCAACTCACAGAGTTGA

                 **   ** *      *                            * *            *
     288167 ACCTTGGTTTGGACAGAGCACTTTTGAAACACTCTTTTTGTAGAATCTGCAGGTGGATATTTGGC
         66 ACCTTTCTTTTCATAGAGCAGTTTTGAAACACTCTTTTTGTAGAATCTGGAAGTGGATATTTGGA

            **                         *   *      * *
     288232 TAGCTTTGAGGATTTCGTTGGAAACGGTAATGTCTTCAAAG
        131 GCGCTTTGAGGATTTCGTTGGAAACGGAAATATCTTCATAT

                *                         *   *            *    *            
     288273 AAAATCTAGACAGAAGCATTCTCAGAAACACCTTCGTGATGTTTGCAATCAAGTCACAGAGTTGA
          1 AAAAACTAGACAGAAGCATTCTCAGAAACATCTTTGTGATGTTTGCACTCAACTCACAGAGTTGA

                 * *             *  *                  *             *       
     288338 ACCTTCCGTTTCATAGAGCAGGTTGGAAACACTCTTTTTGTAGTATCTGGAAGTGGACATTTGGA
         66 ACCTTTCTTTTCATAGAGCAGTTTTGAAACACTCTTTTTGTAGAATCTGGAAGTGGATATTTGGA

             *         ** *    * *   *            **  
     288403 GGGCTTTGTAGCCTATC-TGGAAAAAGGAAATATCTTCCCAT
        131 GCGCTTTG-AGGATTTCGTTGGAAACGGAAATATCTTCATAT

            *  ** *   *     * *            *   *  *   * **        *  * * *   
     288444 GAATGCGAGATAGAAGTAATCTCAGAAACATGTTTATGCTGTATCTACTCAACTAACTGTGCTGA
          1 AAAAACTAGACAGAAGCATTCTCAGAAACATCTTTGTGATGTTTGCACTCAACTCACAGAGTTGA

              *     *  *               *        *  * *       *               
     288509 ACATTTCTATTGATAGAGCAGTTTTGAGACACTCTTCTTTTGGAATCTGCAAGTGGATATTTGGA
         66 ACCTTTCTTTTCATAGAGCAGTTTTGAAACACTCTTTTTGTAGAATCTGGAAGTGGATATTTGGA

             * *                        *     *      
     288574 GAGATTTGAGGATTTCGTTGGAAACGG-GAT-T-AT-ATAT
        131 GCGCTTTGAGGATTTCGTTGGAAACGGAAATATCTTCATAT

                 *       *               *                 **  *   *         
     288611 AAAAAGTAGACAGCAGCATTCTCAGAAACTTCTTTGTGATGTTTGCATCCAGCTCTCAGAGTTGA
          1 AAAAACTAGACAGAAGCATTCTCAGAAACATCTTTGTGATGTTTGCACTCAACTCACAGAGTTGA

              *  * *          *  *        * *      *   **        *  **       
     288676 ACATTCCCTTTCATAGAGTAGGTTTGAAACCCCCTTTTTATAGTGTCTGGAAGCGGGCATTTGGA
         66 ACCTTTCTTTTCATAGAGCAGTTTTGAAACACTCTTTTTGTAGAATCTGGAAGTGGATATTTGGA

                   *    * * **  **  **          * *  *
     288741 GCGCTTTCAGGCCTATGCTTAAAATAGGAAATATCTACCTAC
        131 GCGCTTTGAGG-ATTTCGTTGGAAACGGAAATATCTTCATAT

             *                    *    *  **          ** *        *    *     
     288783 AGAAACTAGACAGAAGCATTCTGAGAATCACGTTTGTGATGTGGGTACTCAACTAACAGTGTTGA
          1 AAAAACTAGACAGAAGCATTCTCAGAAACATCTTTGTGATGTTTGCACTCAACTCACAGAGTTGA

            *  *       *   *    *       *   *                *   *           
     288848 TCCATTCTTTTGATACAGCACTTTTGAACCACACTTTTTGTAGAATCTGCAAGAGGATATTTGGA
         66 ACCTTTCTTTTCATAGAGCAGTTTTGAAACACTCTTTTTGTAGAATCTGGAAGTGGATATTTGGA

            **   *                     *   *      * *
     288913 TAGCTGTGAGGATTTCGTTGGAAACGGGAATGTCTTCAAAG
        131 GCGCTTTGAGGATTTCGTTGGAAACGGAAATATCTTCATAT

                *                         *   *            *    *            
     288954 AAAATCTAGACAGAAGCATTCTCAGAAACACCTTCGTGATGTTTGCAATCAAGTCACAGAGTTGA
          1 AAAAACTAGACAGAAGCATTCTCAGAAACATCTTTGTGATGTTTGCACTCAACTCACAGAGTTGA

                 * *             *  *           *      *             *       
     289019 ACCTTCCGTTTCATAGAGCAGGTTGGAAACACTCTTATTGTAGTATCTGGAAGTGGACATTTGGA
         66 ACCTTTCTTTTCATAGAGCAGTTTTGAAACACTCTTTTTGTAGAATCTGGAAGTGGATATTTGGA

                   *   ** ** *  *   *            **  
     289084 GCGCTTTCAGGCCTATGGTGAAAAAGGAAATATCTTCCCAT
        131 GCGCTTTGAGGATTTCGTTGGAAACGGAAATATCTTCATAT

                  * * *      **      *   * *   *     ** ** *      *    *     
     289125 AAAAACGACATAGAAGCTATCTCAGGAACTTGTTTATGATGCATCTAATCAACTAACAGTGTTGA
          1 AAAAACTAGACAGAAGCATTCTCAGAAACATCTTTGTGATGTTTGCACTCAACTCACAGAGTTGA

                  * ** * *                         * *       *               
     289190 ACCTTTGTACTGACAGAGCAG-TTTGAAACACTCTTTTTTTGGAATCTGCAAGTGGATATTTGGA
         66 ACCTTTCTTTTCATAGAGCAGTTTTGAAACACTCTTTTTGTAGAATCTGGAAGTGGATATTTGGA

            *                           *   *   *    
     289254 TCGCTTTGAGGATTTCGTTGGAAACGG-GAT-GC--AATAT
        131 GCGCTTTGAGGATTTCGTTGGAAACGGAAATATCTTCATAT

                **  *    *     *                 **  *   *  *    *         * 
     289291 AAAACGTACACAGCAGCATACTCAGAAA-ATACTTTGCCATATTTCCATTCAAGTCACAGAGTGG
          1 AAAAACTAGACAGAAGCATTCTCAGAAACAT-CTTTGTGATGTTTGCACTCAACTCACAGAGTTG

               *  * **            *                  *  *             *      
     289355 AACATTCCCATTCATAGAGCAGGTTTGAAACACTCTTTTTGGAGTATCTGGAAGTGGACATTTGG
         65 AACCTTTCTTTTCATAGAGCAGTTTTGAAACACTCTTTTTGTAGAATCTGGAAGTGGATATTTGG

                    ** * * ** *  *   *            **  
     289420 AGCGCTTTCTGAACTATGGTGAAAAAGGAAATATCTTCCAAT
        130 AGCGCTTTGAGGATTTCGTTGGAAACGGAAATATCTTCATAT

            *     *               *      * *          *  **      **  *  *    
     289462 GAAAACAAGACAGAAGCATTCTGAGAAACTTATTTGTGATGTGTGTCCTCAACAAACGGACTTGA
          1 AAAAACTAGACAGAAGCATTCTCAGAAACATCTTTGTGATGTTTGCACTCAACTCACAGAGTTGA

                   *      **  * *  *  *             *   *    * * *           
     289527 ACCTTTCGTTTCATGCAGTACTTCTGGAACACTCTTTTTGAAGATTCTGCATGCGGATATTTGGA
         66 ACCTTTCTTTTCATAGAGCAGTTTTGAAACACTCTTTTTGTAGAATCTGGAAGTGGATATTTGGA

            **                            **   *   * 
     289592 TAGCTTTGAGGATTTCGTTGGAAACGG---GCT-TACATGT
        131 GCGCTTTGAGGATTTCGTTGGAAACGGAAATATCTTCATAT

                 *       *               *        *   *    *    *       *    
     289629 AAAAATTAGACAGCAGCATTCTCAGAAACTTCTTTGTGGTGTCTGCATTCAAGTCACAGAATTGA
          1 AAAAACTAGACAGAAGCATTCTCAGAAACATCTTTGTGATGTTTGCACTCAACTCACAGAGTTGA

              * ** * **            *  * *      *       *    *        *       
     289694 ACTTCCCCTCACATAGAGCAGTTGTGCAGCACTCTATTTGTAGTATCTCGAAGTGGACATTTGGA
         66 ACCTTTCTTTTCATAGAGCAGTTTTGAAACACTCTTTTTGTAGAATCTGGAAGTGGATATTTGGA

             *         ** *    * *   *            **  
     289759 GGGCTTTGTAGCCTATC-TGGAAAAAGGAAATATCTTCCCAT
        131 GCGCTTTG-AGGATTTCGTTGGAAACGGAAATATCTTCATAT

            *  ** *   *     * *            *   *  *   * **        *  * * *   
     289800 GAATGCGAGATAGAAGTAATCTCAGAAACATGTTTATGCTGTATCTACTCAACTAACTGTGCTGA
          1 AAAAACTAGACAGAAGCATTCTCAGAAACATCTTTGTGATGTTTGCACTCAACTCACAGAGTTGA

              *     *  *               *        *  * *       *               
     289865 ACATTTCTATTGATAGAGCAGTTTTGAGACACTCTTCTTTTGGAATCTGCAAGTGGATATTTGGA
         66 ACCTTTCTTTTCATAGAGCAGTTTTGAAACACTCTTTTTGTAGAATCTGGAAGTGGATATTTGGA

            ** *                        *     *      
     289930 TAGATTTGAGGATTTCGTTGGAAACGG-GAT-T-AT-ATAT
        131 GCGCTTTGAGGATTTCGTTGGAAACGGAAATATCTTCATAT

                 *       *               *                 **  *   *         
     289967 AAAAAGTAGACAGCAGCATTCTCAGAAACTTCTTTGTGATGTTTGCATCCAGCTCTCAGAGTTGA
          1 AAAAACTAGACAGAAGCATTCTCAGAAACATCTTTGTGATGTTTGCACTCAACTCACAGAGTTGA

              *  * *          *  *        *        *   **        *  **       
     290032 ACATTCCCTTTCATAGAGTAGGTTTGAAACCCTCTTTTTATAGTGTCTGGAAGCGGGCATTTGGA
         66 ACCTTTCTTTTCATAGAGCAGTTTTGAAACACTCTTTTTGTAGAATCTGGAAGTGGATATTTGGA

                   *    * * **  **  **          * *  *
     290097 GCGCTTTCAGGCCTATGCTTAAAATAGGAAATATCTACCTAC
        131 GCGCTTTGAGG-ATTTCGTTGGAAACGGAAATATCTTCATAT

             *                    *    *  **          ** *        *    *     
     290139 AGAAACTAGACAGAAGCATTCTGAGAATCACGTTTGTGATGTGGGTACTCAACTAACAGTGTTGA
          1 AAAAACTAGACAGAAGCATTCTCAGAAACATCTTTGTGATGTTTGCACTCAACTCACAGAGTTGA

            *  *       *   *            *   *                *   *           
     290204 TCCATTCTTTTGATACAGCAGTTTTGAACCACACTTTTTGTAGAATCTGCAAGAGGATATTTGGA
         66 ACCTTTCTTTTCATAGAGCAGTTTTGAAACACTCTTTTTGTAGAATCTGGAAGTGGATATTTGGA

            **   *                     *   *      * *
     290269 TAGCTGTGAGGATTTCGTTGGAAACGGGAATGTCTTCAAAG
        131 GCGCTTTGAGGATTTCGTTGGAAACGGAAATATCTTCATAT

                *                         *   *            *    *            
     290310 AAAATCTAGACAGAAGCATTCTCAGAAACACCTTCGTGATGTTTGCAATCAAGTCACAGAGTTGA
          1 AAAAACTAGACAGAAGCATTCTCAGAAACATCTTTGTGATGTTTGCACTCAACTCACAGAGTTGA

                 * *             *  *           *      *             *       
     290375 ACCTTCCGTTTCATAGAGCAGGTTGGAAACACTCTTATTGTAGTATCTGGAAGTGGACATTTGGA
         66 ACCTTTCTTTTCATAGAGCAGTTTTGAAACACTCTTTTTGTAGAATCTGGAAGTGGATATTTGGA

                   *   ** ** *  *   *            **  
     290440 GCGCTTTCAGGCCTATGGTGAAAAAGGAAATATCTTCCCAT
        131 GCGCTTTGAGGATTTCGTTGGAAACGGAAATATCTTCATAT

                  * * *      **      *   * *   *     ** ** *      *    *     
     290481 AAAAACGACATAGAAGCTATCTCAGGAACTTGTTTATGATGCATCTAATCAACTAACAGTGTTGA
          1 AAAAACTAGACAGAAGCATTCTCAGAAACATCTTTGTGATGTTTGCACTCAACTCACAGAGTTGA

                  * ** * *                         * *       *               
     290546 ACCTTTGTACTGACAGAGCAG-TTTGAAACACTCTTTTTTTGGAATCTGCAAGTGGATATTTGGA
         66 ACCTTTCTTTTCATAGAGCAGTTTTGAAACACTCTTTTTGTAGAATCTGGAAGTGGATATTTGGA

            *                           *   *   *    
     290610 TCGCTTTGAGGATTTCGTTGGAAACGG-GAT-GC--AATAT
        131 GCGCTTTGAGGATTTCGTTGGAAACGGAAATATCTTCATAT

                **  *    *     *                 **  *   *  *    *         * 
     290647 AAAACGTACACAGCAGCATACTCAGAAA-ATACTTTGCCATATTTCCATTCAAGTCACAGAGTGG
          1 AAAAACTAGACAGAAGCATTCTCAGAAACAT-CTTTGTGATGTTTGCACTCAACTCACAGAGTTG

               *  * **            *  *               *  *             *      
     290711 AACATTCCCATTCATAGAGCAGGTTGGAAACACTCTTTTTGGAGTATCTGGAAGTGGACATTTGG
         65 AACCTTTCTTTTCATAGAGCAGTTTTGAAACACTCTTTTTGTAGAATCTGGAAGTGGATATTTGG

                    ** * * ** *  *   *            **  
     290776 AGCGCTTTCTGAACTATGGTGAAAAAGGAAATATCTTCCAAT
        130 AGCGCTTTGAGGATTTCGTTGGAAACGGAAATATCTTCATAT

            *     *               *      * *          *  **      **  *  *    
     290818 GAAAACAAGACAGAAGCATTCTGAGAAACTTATTTGTGATGTGTGTCCTCAACAAACGGACTTGA
          1 AAAAACTAGACAGAAGCATTCTCAGAAACATCTTTGTGATGTTTGCACTCAACTCACAGAGTTGA

                   *      **  * *  *  *             *   *    * * *           
     290883 ACCTTTCGTTTCATGCAGTACTTCTGGAACACTCTTTTTGAAGATTCTGCATGCGGATATTTGGA
         66 ACCTTTCTTTTCATAGAGCAGTTTTGAAACACTCTTTTTGTAGAATCTGGAAGTGGATATTTGGA

            **                            **   *   * 
     290948 TAGCTTTGAGGATTTCGTTGGAAACGG---GCT-TACATGT
        131 GCGCTTTGAGGATTTCGTTGGAAACGGAAATATCTTCATAT

                 *       *               *        *   *    *    *       *    
     290985 AAAAATTAGACAGCAGCATTCTCAGAAACTTCTTTGTGGTGTCTGCATTCAAGTCACAGAATTGA
          1 AAAAACTAGACAGAAGCATTCTCAGAAACATCTTTGTGATGTTTGCACTCAACTCACAGAGTTGA

              * ** * **            *  * *      *       *    *        *       
     291050 ACTTCCCCTCACATAGAGCAGTTGTGCAGCACTCTATTTGTAGTATCTCGAAGTGGACATTTGGA
         66 ACCTTTCTTTTCATAGAGCAGTTTTGAAACACTCTTTTTGTAGAATCTGGAAGTGGATATTTGGA

             *         ** *    * *   *            **  
     291115 GGGCTTTGTAGCCTATC-TGGAAAAAGGAAATATCTTCCCAT
        131 GCGCTTTG-AGGATTTCGTTGGAAACGGAAATATCTTCATAT

            *  ** *   *     * *            *   *  *   * **        *  * * *   
     291156 GAATGCGAGATAGAAGTAATCTCAGAAACATGTTTATGCTGTATCTACTCAACTAACTGTGCTGA
          1 AAAAACTAGACAGAAGCATTCTCAGAAACATCTTTGTGATGTTTGCACTCAACTCACAGAGTTGA

              *     *  *               *        *  * *       *               
     291221 ACATTTCTATTGATAGAGCAGTTTTGAGACACTCTTCTTTTGGAATCTGCAAGTGGATATTTGGA
         66 ACCTTTCTTTTCATAGAGCAGTTTTGAAACACTCTTTTTGTAGAATCTGGAAGTGGATATTTGGA

            ** *                        *     *      
     291286 TAGATTTGAGGATTTCGTTGGAAACGG-GAT-T-AT-ATAT
        131 GCGCTTTGAGGATTTCGTTGGAAACGGAAATATCTTCATAT

            *    *       *               *                 **  *   *         
     291323 CAAAAGTAGACAGCAGCATTCTCAGAAACTTCTTTGTGATGTTTGCATCCAGCTCTCAGAGTTGA
          1 AAAAACTAGACAGAAGCATTCTCAGAAACATCTTTGTGATGTTTGCACTCAACTCACAGAGTTGA

              *  * *          *  *        *        *   **        *  **       
     291388 ACATTCCCTTTCATAGAGTAGGTTTGAAACCCTCTTTTTATAGTGTCTGGAAGCGGGCATTTGGA
         66 ACCTTTCTTTTCATAGAGCAGTTTTGAAACACTCTTTTTGTAGAATCTGGAAGTGGATATTTGGA

                   *   ** ** *  *   *          * *   
     291453 GCGCTTTCAGGCCTATGCTGAAAAAGGAAATATCTACCTAT
        131 GCGCTTTGAGGATTTCGTTGGAAACGGAAATATCTTCATAT

             *                    *    *  **          ** *        *    *     
     291494 AGAAACTAGACAGAAGCATTCTGAGAATCACGTTTGTGATGTGGGTACTCAACTAACAGTGTTGA
          1 AAAAACTAGACAGAAGCATTCTCAGAAACATCTTTGTGATGTTTGCACTCAACTCACAGAGTTGA

            *  *       *   *            *   *                *               
     291559 TCCATTCTTTTGATACAGCAGTTTTGAACCACACTTTTTGTAGAATCTGCAAGTGGATATTTGGA
         66 ACCTTTCTTTTCATAGAGCAGTTTTGAAACACTCTTTTTGTAGAATCTGGAAGTGGATATTTGGA

            **   *                     *   *        *
     291624 TAGCTGTGAGGATTTCGTTGGAAACGGGAATGTCTTCATAG
        131 GCGCTTTGAGGATTTCGTTGGAAACGGAAATATCTTCATAT

                **                     * * **         *  **   *   *          
     291665 AAAATGTAGACAGAAGCATTCTCAGAACCTTAATTGTGATGTGTGTTCTCCACTAACAGAGTTGA
          1 AAAAACTAGACAGAAGCATTCTCAGAAACATCTTTGTGATGTTTGCACTCAACTCACAGAGTTGA

              *        * *   * *   *       *       *    *        *           
     291730 ACTTTTCTTTTGACAGAACTGTTCTGAAACATTCTTTTTATAGAGTCTGGAAGAGGATATTTGGA
         66 ACCTTTCTTTTCATAGAGCAGTTTTGAAACACTCTTTTTGTAGAATCTGGAAGTGGATATTTGGA

            **                         *          *  
     291795 AAGCTTTGAGGATTTCGTTGGAAACGGGAATATCTTCAAAT
        131 GCGCTTTGAGGATTTCGTTGGAAACGGAAATATCTTCATAT

                *    *            *           * *       *  *    *            
     291836 AAAATCTAGCCAGAAGCATTCTAAGAAACATCTTAGGGATGTTTACATTCAAGTCACAGAGTTGA
          1 AAAAACTAGACAGAAGCATTCTCAGAAACATCTTTGTGATGTTTGCACTCAACTCACAGAGTTGA

              *  * *     *       *         *    * *   **      *      *    *  
     291901 ACATTCCCTTTCACAGAGCAGGTTTGAAACAATCTTCTCGTACTATCTGGCAGTGGACATTTTGA
         66 ACCTTTCTTTTCATAGAGCAGTTTTGAAACACTCTTTTTGTAGAATCTGGAAGTGGATATTTGGA

              * *   *  ** ** *  *   *            ** *
     291966 GCTCCTTGGGGCCTATGCTGAAAAAGGAAATATCTTCCGAC
        131 GCGCTTTGAGGATTTCGTTGGAAACGGAAATATCTTCATAT

                                 *     *  **          *           **    *    
     292007 AAAAACTAGACAGAAGCATTCGCAGAATCACGTTTGTGATGTGTGCACTCAACTGTCAGAATTGA
          1 AAAAACTAGACAGAAGCATTCTCAGAAACATCTTTGTGATGTTTGCACTCAACTCACAGAGTTGA

                 **   ** *      *                            * *            *
     292072 ACCTTGGTTTGGACAGAGCACTTTTGAAACACTCTTTTTGTAGAATCTGCAGGTGGATATTTGGC
         66 ACCTTTCTTTTCATAGAGCAGTTTTGAAACACTCTTTTTGTAGAATCTGGAAGTGGATATTTGGA

            ** *                       *   *      * *
     292137 TAGATTTGAGGATTTCGTTGGAAACGGTAATGTCTTCAAAG
        131 GCGCTTTGAGGATTTCGTTGGAAACGGAAATATCTTCATAT

                *                         *   *            *    *            
     292178 AAAATCTAGACAGAAGCATTCTCAGAAACACCTTCGTGATGTTTGCAATCAAGTCACAGAGTTGA
          1 AAAAACTAGACAGAAGCATTCTCAGAAACATCTTTGTGATGTTTGCACTCAACTCACAGAGTTGA

                 * *             *  *                  *             *       
     292243 ACCTTCCGTTTCATAGAGCAGGTTGGAAACACTCTTTTTGTAGTATCTGGAAGTGGACATTTGGA
         66 ACCTTTCTTTTCATAGAGCAGTTTTGAAACACTCTTTTTGTAGAATCTGGAAGTGGATATTTGGA

                   *   ** ** *  *   *            **  
     292308 GCGCTTTCAGGCCTATGGTGAAAAAGGAAATATCTTCCCAT
        131 GCGCTTTGAGGATTTCGTTGGAAACGGAAATATCTTCATAT

                  * * *      **      *   * *   *     ** ** *      *    *     
     292349 AAAAACGACATAGAAGCTATCTCAGGAACTTGTTTATGATGCATCTAATCAACTAACAGTGTTGA
          1 AAAAACTAGACAGAAGCATTCTCAGAAACATCTTTGTGATGTTTGCACTCAACTCACAGAGTTGA

                  * ** * *                         * *       *               
     292414 ACCTTTGTACTGACAGAGCAG-TTTGAAACACTCTTTTTTTGGAATCTGCAAGTGGATATTTGGA
         66 ACCTTTCTTTTCATAGAGCAGTTTTGAAACACTCTTTTTGTAGAATCTGGAAGTGGATATTTGGA

            *                           *   *   *    
     292478 TCGCTTTGAGGATTTCGTTGGAAACGG-GAT-GC--AATAT
        131 GCGCTTTGAGGATTTCGTTGGAAACGGAAATATCTTCATAT

                **  *    *     *                 **  *   *  *    *         * 
     292515 AAAACGTACACAGCAGCATACTCAGAAA-ATACTTTGCCATATTTCCATTCAAGTCACAGAGTGG
          1 AAAAACTAGACAGAAGCATTCTCAGAAACAT-CTTTGTGATGTTTGCACTCAACTCACAGAGTTG

               *  * **            *  *               *  *             *      
     292579 AACATTCCCATTCATAGAGCAGGTTGGAAACACTCTTTTTGGAGTATCTGGAAGTGGACATTTGG
         65 AACCTTTCTTTTCATAGAGCAGTTTTGAAACACTCTTTTTGTAGAATCTGGAAGTGGATATTTGG

                    ** * * ** *  *   *            **  
     292644 AGCGCTTTCTGAACTATGGTGAAAAAGGAAATATCTTCCAAT
        130 AGCGCTTTGAGGATTTCGTTGGAAACGGAAATATCTTCATAT

            *     *               *      * *          *  **      **  * **    
     292686 GAAAACAAGACAGAAGCATTCTGAGAAACTTATTTGTGATGTGTGTCCTCAACAAACGGTCTTGA
          1 AAAAACTAGACAGAAGCATTCTCAGAAACATCTTTGTGATGTTTGCACTCAACTCACAGAGTTGA

                   *      **  * *  *  *             *   *    * * *           
     292751 ACCTTTCGTTTCATGCAGTACTTCTGGAACACTCTTTTTGAAGATTCTGCATGCGGATATTTGGA
         66 ACCTTTCTTTTCATAGAGCAGTTTTGAAACACTCTTTTTGTAGAATCTGGAAGTGGATATTTGGA

            **                            **   *   * 
     292816 TAGCTTTGAGGATTTCGTTGGAAACGG---GCT-TACATGT
        131 GCGCTTTGAGGATTTCGTTGGAAACGGAAATATCTTCATAT

                 *       *  *            *        *   *    *    *       *    
     292853 AAAAATTAGACAGCAGAATTCTCAGAAACTTCTTTGTGGTGTCTGCATTCAAGTCACAGAATTGA
          1 AAAAACTAGACAGAAGCATTCTCAGAAACATCTTTGTGATGTTTGCACTCAACTCACAGAGTTGA

              * ** * **            *  * *      *       *    **       *       
     292918 ACTTCACCTCACATAGAGCAGTTGTGCAGCACTCTATTTGTAGTATCTCAAAGTGGACATTTTGG
         66 ACCTTTCTTTTCATAGAGCAGTTTTGAAACACTCTTTTTGTAGAATCTGGAAGTGGATA-TTTGG

              *         ** *    * *   *            **  
     292983 AGGGCTTTGTAGCCTATC-TGGAAAAAGGAAATATCTTCCCAT
        130 AGCGCTTTG-AGGATTTCGTTGGAAACGGAAATATCTTCATAT

            *  ** *   *     * *            *   *  *   * **        *  * * *   
     293025 GAATGCGAGATAGAAGTAATCTCAGAAACATGTTTATGCTGTATCTACTCAACTAACTGTGCTGA
          1 AAAAACTAGACAGAAGCATTCTCAGAAACATCTTTGTGATGTTTGCACTCAACTCACAGAGTTGA

              *     *  *               *        *  * *       *               
     293090 ACATTTCTATTGATAGAGCAGTTTTGAGACACTCTTCTTTTGGAATCTGCAAGTGGATATTTGGA
         66 ACCTTTCTTTTCATAGAGCAGTTTTGAAACACTCTTTTTGTAGAATCTGGAAGTGGATATTTGGA

            ** *                        *     *      
     293155 TAGATTTGAGGATTTCGTTGGAAACGG-GAT-T-AT-ATAT
        131 GCGCTTTGAGGATTTCGTTGGAAACGGAAATATCTTCATAT

                 *       *               *                 **  *   *         
     293192 AAAAAGTAGACAGCAGCATTCTCAGAAACTTCTTTGTGATGTTTGCATCCAGCTCTCAGAGTTGA
          1 AAAAACTAGACAGAAGCATTCTCAGAAACATCTTTGTGATGTTTGCACTCAACTCACAGAGTTGA

              *  * *          *  *        *        *   **        *  **       
     293257 ACATTCCCTTTCATAGAGTAGGTTTGAAACCCTCTTTTTATAGTGTCTGGAAGCGGGCATTTGGA
         66 ACCTTTCTTTTCATAGAGCAGTTTTGAAACACTCTTTTTGTAGAATCTGGAAGTGGATATTTGGA

                   *   ** ** *  *   *          * *   
     293322 GCGCTTTCAGGCCTATGCTGAAAAAGGAAATATCTACCTAT
        131 GCGCTTTGAGGATTTCGTTGGAAACGGAAATATCTTCATAT

             *                    *    *  **          ** *        *    *     
     293363 ACAAACTAGACAGAAGCATTCTGAGAATCACGTTTGTGATGTGGGTACTCAACTAACAGTGTTGA
          1 AAAAACTAGACAGAAGCATTCTCAGAAACATCTTTGTGATGTTTGCACTCAACTCACAGAGTTGA

            *  *       *   *            *   **               *               
     293428 TCCATTCTTTTGATACAGCAGTTTTGAACCACATTTTTTGTAGAATCTGCAAGTGGATATTTGGA
         66 ACCTTTCTTTTCATAGAGCAGTTTTGAAACACTCTTTTTGTAGAATCTGGAAGTGGATATTTGGA

            **   *                     *   *        *
     293493 TAGCTGTGAGGATTTCGTTGGAAACGGGAATGTCTTCATAG
        131 GCGCTTTGAGGATTTCGTTGGAAACGGAAATATCTTCATAT

                **                     * * **         *  **   *   *          
     293534 AAAATTTAGACAGAAGCATTCTCAGAACCTTGATTGTGATGTGTGTTCTCCACTAACAGAGTTGA
          1 AAAAACTAGACAGAAGCATTCTCAGAAACATCTTTGTGATGTTTGCACTCAACTCACAGAGTTGA

                       * *   * *   *       *       *                         
     293599 ACCTTTCTTTTGACAGAACTGTTCTGAAACATTCTTTTTATAGAATCTGGAAGTGGATATTTGGA
         66 ACCTTTCTTTTCATAGAGCAGTTTTGAAACACTCTTTTTGTAGAATCTGGAAGTGGATATTTGGA

            **              *          *          *  
     293664 AAGCTTTGAGGATTTCATTGGAAACGGGAATATCTTCAAAT
        131 GCGCTTTGAGGATTTCGTTGGAAACGGAAATATCTTCATAT

                *    *            *           * *       *  *    *            
     293705 AAAATCTAGCCAGAAGCATTCTAAGAAACATCTTAGGGATGTTTACATTCAAGTCACAGAGTTGA
          1 AAAAACTAGACAGAAGCATTCTCAGAAACATCTTTGTGATGTTTGCACTCAACTCACAGAGTTGA

              *  * *     *       *         *    ***   **      *      *   **  
     293770 ACATTCCCTTTCACAGAGCAGGTTTGAAACAATCTTCCCGTACTATCTGGCAGTGGACATTATGA
         66 ACCTTTCTTTTCATAGAGCAGTTTTGAAACACTCTTTTTGTAGAATCTGGAAGTGGATATTTGGA

              * *   *  ** ** *  *   *            ** *
     293835 GCTCCTTGGGGCCTATGCTGAAAAAGGAAATATCTTCCGAC
        131 GCGCTTTGAGGATTTCGTTGGAAACGGAAATATCTTCATAT

                                 *     *  **          *           **    *    
     293876 AAAAACTAGACAGAAGCATTCGCAGAATCACGTTTGTGATGTGTGCACTCAACTGTCAGAATTGA
          1 AAAAACTAGACAGAAGCATTCTCAGAAACATCTTTGTGATGTTTGCACTCAACTCACAGAGTTGA

                 **   ** *      *                            * *            *
     293941 ACCTTGGTTTGGACAGAGCACTTTTGAAACACTCTTTTTGTAGAATCTGCAGGTGGATATTTGGC
         66 ACCTTTCTTTTCATAGAGCAGTTTTGAAACACTCTTTTTGTAGAATCTGGAAGTGGATATTTGGA

            **                         *   *      * *
     294006 TAGCTTTGAGGATTTCGTTGGAAACGGTAATGTCTTCAAAG
        131 GCGCTTTGAGGATTTCGTTGGAAACGGAAATATCTTCATAT

                *                         *   *            *    *            
     294047 AAAATCTAGACAGAAGCATTCTCAGAAACACCTTCGTGATGTTTGCAATCAAGTCACAGAGTTGA
          1 AAAAACTAGACAGAAGCATTCTCAGAAACATCTTTGTGATGTTTGCACTCAACTCACAGAGTTGA

                 * *             *  *                  *             *       
     294112 ACCTTCCGTTTCATAGAGCAGGTTGGAAACACTCTTTTTGTAGTATCTGGAAGTGGACATTTGGA
         66 ACCTTTCTTTTCATAGAGCAGTTTTGAAACACTCTTTTTGTAGAATCTGGAAGTGGATATTTGGA

             *         ** *    * *   *            **  
     294177 GGGCTTTGTAGCCTATC-TGGAAAAAGGAAATATCTTCCCAT
        131 GCGCTTTG-AGGATTTCGTTGGAAACGGAAATATCTTCATAT

            *  ** *   *     * *            *   *  *   * **        *  * * *   
     294218 GAATGCGAGATAGAAGTAATCTCAGAAACATGTTTATGCTGTATCTACTCAACTAACTGTGCTGA
          1 AAAAACTAGACAGAAGCATTCTCAGAAACATCTTTGTGATGTTTGCACTCAACTCACAGAGTTGA

              *     *  *               *        *  * *       *               
     294283 ACATTTCTATTGATAGAGCAGTTTTGAGACACTCTTCTTTTGGAATCTGCAAGTGGATATTTGGA
         66 ACCTTTCTTTTCATAGAGCAGTTTTGAAACACTCTTTTTGTAGAATCTGGAAGTGGATATTTGGA

             * *                        *     *      
     294348 GAGATTTGAGGATTTCGTTGGAAACGG-GAT-T-AT-ATAT
        131 GCGCTTTGAGGATTTCGTTGGAAACGGAAATATCTTCATAT

                 *       *               *                 **  *   *         
     294385 AAAAAGTAGACAGCAGCATTCTCAGAAACTTCTTTGTGATGTTTGCATCCAGCTCTCAGAGTTGA
          1 AAAAACTAGACAGAAGCATTCTCAGAAACATCTTTGTGATGTTTGCACTCAACTCACAGAGTTGA

              *  * *          *  *        *        *   **    *   *  **       
     294450 ACATTCCCTTTCATAGAGTAGGTTTGAAACCCTCTTTTTATAGTGTCTGCAAGCGGGCATTTGGA
         66 ACCTTTCTTTTCATAGAGCAGTTTTGAAACACTCTTTTTGTAGAATCTGGAAGTGGATATTTGGA

                *  *    * * **  **  **          * *  *
     294515 GCGCATTCAGGCCTGTGCTTAAAATAGGAAATATCTACCTAC
        131 GCGCTTTGAGG-ATTTCGTTGGAAACGGAAATATCTTCATAT

             *                    *    *  **          ** *        *    *     
     294557 AGAAACTAGACAGAAGCATTCTGAGAATCACGTTTGTGATGTGGGTACTCAACTAACAGTGTTGA
          1 AAAAACTAGACAGAAGCATTCTCAGAAACATCTTTGTGATGTTTGCACTCAACTCACAGAGTTGA

            *  *       *   *            *   *                *   *           
     294622 TCCATTCTTTTGATACAGCAGTTTTGAACCACACTTTTTGTAGAATCTGCAAGAGGATATTTGGA
         66 ACCTTTCTTTTCATAGAGCAGTTTTGAAACACTCTTTTTGTAGAATCTGGAAGTGGATATTTGGA

            **   *                     *   *      * *
     294687 TAGCTGTGAGGATTTCGTTGGAAACGGGAATGTCTTCAAAG
        131 GCGCTTTGAGGATTTCGTTGGAAACGGAAATATCTTCATAT

                *                         *   *            *    *            
     294728 AAAATCTAGACAGAAGCATTCTCAGAAACACCTTCGTGATGTTTGCAATCAAGTCACAGAGTTGA
          1 AAAAACTAGACAGAAGCATTCTCAGAAACATCTTTGTGATGTTTGCACTCAACTCACAGAGTTGA

                 * *             *  *           *      *             *       
     294793 ACCTTCCGTTTCATAGAGCAGGTTGGAAACACTCTTATTGTAGTATCTGGAAGTGGACATTTGGA
         66 ACCTTTCTTTTCATAGAGCAGTTTTGAAACACTCTTTTTGTAGAATCTGGAAGTGGATATTTGGA

                   *   ** ** *  *   *            **  
     294858 GCGCTTTCAGGCCTATGGTGAAAAAGGAAATATCTTCCCAT
        131 GCGCTTTGAGGATTTCGTTGGAAACGGAAATATCTTCATAT

                  * * *      **      *   * *   *     ** ** *      *    *     
     294899 AAAAACGACATAGAAGCTATCTCAGGAACTTGTTTATGATGCATCTAATCAACTAACAGTGTTGA
          1 AAAAACTAGACAGAAGCATTCTCAGAAACATCTTTGTGATGTTTGCACTCAACTCACAGAGTTGA

                  * ** * *                         * *       *               
     294964 ACCTTTGTACTGACAGAGCAG-TTTGAAACACTCTTTTTTTGGAATCTGCAAGTGGATATTTGGA
         66 ACCTTTCTTTTCATAGAGCAGTTTTGAAACACTCTTTTTGTAGAATCTGGAAGTGGATATTTGGA

            *                           *   *   *    
     295028 TCGCTTTGAGGATTTCGTTGGAAACGG-GAT-GC--AATAT
        131 GCGCTTTGAGGATTTCGTTGGAAACGGAAATATCTTCATAT

                **  *    *     *                 **  *   *  *    *         * 
     295065 AAAACGTACACAGCAGCATACTCAGAAA-ATACTTTGCCATATTTCCATTCAAGTCACAGAGTGG
          1 AAAAACTAGACAGAAGCATTCTCAGAAACAT-CTTTGTGATGTTTGCACTCAACTCACAGAGTTG

               *  * **            *                  *  *             *      
     295129 AACATTCCCATTCATAGAGCAGGTTTGAAACACTCTTTTTGGAGTATCTGGAAGTGGACATTTGG
         65 AACCTTTCTTTTCATAGAGCAGTTTTGAAACACTCTTTTTGTAGAATCTGGAAGTGGATATTTGG

                    ** * * ** *  *   *            **  
     295194 AGCGCTTTCTGAACTATGGTGAAAAAGGAAATATCTTCCAAT
        130 AGCGCTTTGAGGATTTCGTTGGAAACGGAAATATCTTCATAT

            *     *               *      * *          *  **      **  *  *    
     295236 GAAAACAAGACAGAAGCATTCTGAGAAACTTATTTGTGATGTGTGTCCTCAACAAACGGACTTGA
          1 AAAAACTAGACAGAAGCATTCTCAGAAACATCTTTGTGATGTTTGCACTCAACTCACAGAGTTGA

                   *      **  * *  *  *             *   *    * * *           
     295301 ACCTTTCGTTTCATGCAGTACTTCTGGAACACTCTTTTTGAAGATTCTGCATGCGGATATTTGGA
         66 ACCTTTCTTTTCATAGAGCAGTTTTGAAACACTCTTTTTGTAGAATCTGGAAGTGGATATTTGGA

            **                            **   *   * 
     295366 TAGCTTTGAGGATTTCGTTGGAAACGG---GCT-TACATGT
        131 GCGCTTTGAGGATTTCGTTGGAAACGGAAATATCTTCATAT

                 *       *               *        *   *    *    *       *    
     295403 AAAAATTAGACAGCAGCATTCTCAGAAACTTCTTTGTGGTGTCTGCATTCAAGTCACAGAATTGA
          1 AAAAACTAGACAGAAGCATTCTCAGAAACATCTTTGTGATGTTTGCACTCAACTCACAGAGTTGA

              * ** * **            *  * *      *       *             *       
     295468 ACTTCCCCTCACATAGAGCAGTTGTGCAGCACTCTATTTGTAGTATCTGGAAGTGGACATTTGGA
         66 ACCTTTCTTTTCATAGAGCAGTTTTGAAACACTCTTTTTGTAGAATCTGGAAGTGGATATTTGGA

             *         ** *    * *   *            **  
     295533 GGGCTTTGTAGCCTATC-TGGAAAAAGGAAATATCTTCCCAT
        131 GCGCTTTG-AGGATTTCGTTGGAAACGGAAATATCTTCATAT

            *  ** *   *     * *            *   *  *   * **        *  * * *   
     295574 GAATGCGAGATAGAAGTAATCTCAGAAACATGTTTATGCTGTATCTACTCAACTAACTGTGCTGA
          1 AAAAACTAGACAGAAGCATTCTCAGAAACATCTTTGTGATGTTTGCACTCAACTCACAGAGTTGA

              *     *  *               *        *  * *       *               
     295639 ACATTTCTATTGATAGAGCAGTTTTGAGACACTCTTCTTTTGGAATCTGCAAGTGGATATTTGGA
         66 ACCTTTCTTTTCATAGAGCAGTTTTGAAACACTCTTTTTGTAGAATCTGGAAGTGGATATTTGGA

            ** *                        *     *      
     295704 TAGATTTGAGGATTTCGTTGGAAACGG-GAT-T-AT-ATAT
        131 GCGCTTTGAGGATTTCGTTGGAAACGGAAATATCTTCATAT

                 *       *               *                 **  *   *         
     295741 AAAAAGTAGACAGCAGCATTCTCAGAAACTTCTTTGTGATGTTTGCATCCAGCTCTCAGAGTTGA
          1 AAAAACTAGACAGAAGCATTCTCAGAAACATCTTTGTGATGTTTGCACTCAACTCACAGAGTTGA

              *  * *          *  *        *        *   **        *  **       
     295806 ACATTCCCTTTCATAGAGTAGGTTTGAAACCCTCTTTTTATAGTGTCTGGAAGCGGGCATTTGGA
         66 ACCTTTCTTTTCATAGAGCAGTTTTGAAACACTCTTTTTGTAGAATCTGGAAGTGGATATTTGGA

                   *   ** ** *  *   *          * *   
     295871 GCGCTTTCAGGCCTATGCTGAAAAAGGAAATATCTACCTAT
        131 GCGCTTTGAGGATTTCGTTGGAAACGGAAATATCTTCATAT

             *                    *    *  **          ** *        *    *     
     295912 AGAAACTAGACAGAAGCATTCTGAGAATCACGTTTGTGATGTGGGTACTCAACTAACAGTGTTGA
          1 AAAAACTAGACAGAAGCATTCTCAGAAACATCTTTGTGATGTTTGCACTCAACTCACAGAGTTGA

            *  *       *   *            *   *                *               
     295977 TCCATTCTTTTGATACAGCAGTTTTGAACCACACTTTTTGTAGAATCTGCAAGTGGATATTTGGA
         66 ACCTTTCTTTTCATAGAGCAGTTTTGAAACACTCTTTTTGTAGAATCTGGAAGTGGATATTTGGA

            **   *                     *   *        *
     296042 TAGCTGTGAGGATTTCGTTGGAAACGGGAATGTCTTCATAG
        131 GCGCTTTGAGGATTTCGTTGGAAACGGAAATATCTTCATAT

                **                     * * **         *  **   *   *          
     296083 AAAATGTAGACAGAAGCATTCTCAGAACCTTGATTGTGATGTGTGTTCTCCACTAACAGAGTTGA
          1 AAAAACTAGACAGAAGCATTCTCAGAAACATCTTTGTGATGTTTGCACTCAACTCACAGAGTTGA

                       * *   * *   *       *       *                         
     296148 ACCTTTCTTTTGACAGAACTGTTCTGAAACATTCTTTTTATAGAATCTGGAAGTGGATATTTGGA
         66 ACCTTTCTTTTCATAGAGCAGTTTTGAAACACTCTTTTTGTAGAATCTGGAAGTGGATATTTGGA

            **                         *          *  
     296213 AAGCTTTGAGGATTTCGTTGGAAACGGGAATATCTTCAAAT
        131 GCGCTTTGAGGATTTCGTTGGAAACGGAAATATCTTCATAT

                *    *            *           * *       *  *    *            
     296254 AAAATCTAGCCAGAAGCATTCTAAGAAACATCTTAGGGATGTTTACATTCAAGTCACAGAGTTGA
          1 AAAAACTAGACAGAAGCATTCTCAGAAACATCTTTGTGATGTTTGCACTCAACTCACAGAGTTGA

              *  * *     *       *         *    * *   **      *      *    *  
     296319 ACATTCCCTTTCACAGAGCAGGTTTGAAACAATCTTCTCGTACTATCTGGCAGTGGACATTTTGA
         66 ACCTTTCTTTTCATAGAGCAGTTTTGAAACACTCTTTTTGTAGAATCTGGAAGTGGATATTTGGA

              * *   *  ** ** *  *   *            ** *
     296384 GCTCCTTGGGGCCTATGCTGAAAAAGGAAATATCTTCCGAC
        131 GCGCTTTGAGGATTTCGTTGGAAACGGAAATATCTTCATAT

                                 *     *  **          *           **    *    
     296425 AAAAACTAGACAGAAGCATTCGCAGAATCACGTTTGTGATGTGTGCACTCAACTGTCAGAATTGA
          1 AAAAACTAGACAGAAGCATTCTCAGAAACATCTTTGTGATGTTTGCACTCAACTCACAGAGTTGA

                 **   ** *      *                            * *            *
     296490 ACCTTGGTTTGGACAGAGCACTTTTGAAACACTCTTTTTGTAGAATCTGCAGGTGGATATTTGGC
         66 ACCTTTCTTTTCATAGAGCAGTTTTGAAACACTCTTTTTGTAGAATCTGGAAGTGGATATTTGGA

            **                         *   *      * *
     296555 TAGCTTTGAGGATTTCGTTGGAAACGGGAATGTCTTCAAAG
        131 GCGCTTTGAGGATTTCGTTGGAAACGGAAATATCTTCATAT

                *                         *   *            *    *            
     296596 AAAATCTAGACAGAAGCATTCTCAGAAACACCTTCGTGATGTTTGCAATCAAGTCACAGAGTTGA
          1 AAAAACTAGACAGAAGCATTCTCAGAAACATCTTTGTGATGTTTGCACTCAACTCACAGAGTTGA

                 * *             *  *           *      *             *       
     296661 ACCTTCCGTTTCATAGAGCAGGTTGGAAACACTCTTATTGTAGTATCTGGAAGTGGACATTTGGA
         66 ACCTTTCTTTTCATAGAGCAGTTTTGAAACACTCTTTTTGTAGAATCTGGAAGTGGATATTTGGA

                   *   ** ** *  *   *            **  
     296726 GCGCTTTCAGGCCTATGGTGAAAAAGGAAATATCTTCCCAT
        131 GCGCTTTGAGGATTTCGTTGGAAACGGAAATATCTTCATAT

                  * * *      **      *   * *   *     ** ** *      *    *     
     296767 AAAAACGACATAGAAGCTATCTCAGGAACTTGTTTATGATGCATCTAATCAACTAACAGTGTTGA
          1 AAAAACTAGACAGAAGCATTCTCAGAAACATCTTTGTGATGTTTGCACTCAACTCACAGAGTTGA

                  * ** * *                         * *       *               
     296832 ACCTTTGTACTGACAGAGCAG-TTTGAAACACTCTTTTTTTGGAATCTGCAAGTGGATATTTGGA
         66 ACCTTTCTTTTCATAGAGCAGTTTTGAAACACTCTTTTTGTAGAATCTGGAAGTGGATATTTGGA

            *                           *   *   *    
     296896 TCGCTTTGAGGATTTCGTTGGAAACGG-GAT-GC--AATAT
        131 GCGCTTTGAGGATTTCGTTGGAAACGGAAATATCTTCATAT

                **  *    *     *                 **  *   *  *    *         * 
     296933 AAAACGTACACAGCAGCATACTCAGAAA-ATACTTTGCCATATTTCCATTCAAGTCACAGAGTGG
          1 AAAAACTAGACAGAAGCATTCTCAGAAACAT-CTTTGTGATGTTTGCACTCAACTCACAGAGTTG

               *  * **            *              *   *  *             *      
     296997 AACATTCCCATTCATAGAGCAGGTTTGAAACACTCTTCTTGGAGTATCTGGAAGTGGACATTTGG
         65 AACCTTTCTTTTCATAGAGCAGTTTTGAAACACTCTTTTTGTAGAATCTGGAAGTGGATATTTGG

                    ** * * ** *  *   *            **  
     297062 AGCGCTTTCTGAACTATGGTGAAAAAGGAAATATCTTCCAAT
        130 AGCGCTTTGAGGATTTCGTTGGAAACGGAAATATCTTCATAT

            *     *               *      * *          *  **      **  *  *    
     297104 GAAAACAAGACAGAAGCATTCTGAGAAACTTATTTGTGATGTGTGTCCTCAACAAACGGACTTGA
          1 AAAAACTAGACAGAAGCATTCTCAGAAACATCTTTGTGATGTTTGCACTCAACTCACAGAGTTGA

                   *      **  * *  *  *             *   *    * * *           
     297169 ACCTTTCGTTTCATGCAGTACTTCTGGAACACTCTTTTTGAAGATTCTGCATGCGGATATTTGGA
         66 ACCTTTCTTTTCATAGAGCAGTTTTGAAACACTCTTTTTGTAGAATCTGGAAGTGGATATTTGGA

            **                            **   *   * 
     297234 TAGCTTTGAGGATTTCGTTGGAAACGG---GCT-TACATGT
        131 GCGCTTTGAGGATTTCGTTGGAAACGGAAATATCTTCATAT

                 *       *               *        *   *    *    *       *    
     297271 AAAAATTAGACAGCAGCATTCTCAGAAACTTCTTTGTGGTGTCTGCATTCAAGTCACAGAATTGA
          1 AAAAACTAGACAGAAGCATTCTCAGAAACATCTTTGTGATGTTTGCACTCAACTCACAGAGTTGA

              * ** * **            *  * *      *       *             *       
     297336 ACATCCCCTCACATAGAGCAGTTGTGCAGCACTCTATTTGTAGTATCTGGAAGTGGACATTTGGA
         66 ACCTTTCTTTTCATAGAGCAGTTTTGAAACACTCTTTTTGTAGAATCTGGAAGTGGATATTTGGA

             *         ** *    * *   *            **  
     297401 GGGCTTTGTAGCCTATC-TGGAAAAAGGAAATATCTTCCCAT
        131 GCGCTTTG-AGGATTTCGTTGGAAACGGAAATATCTTCATAT

            *  ** *   *     * *            *   *  *   * **        *  * * *   
     297442 GAATGCGAGATAGAAGTAATCTCAGAAACATGTTTATGCTGTATCTACTCAACTAACTGTGCTGA
          1 AAAAACTAGACAGAAGCATTCTCAGAAACATCTTTGTGATGTTTGCACTCAACTCACAGAGTTGA

              *     *  *               *        *  * *       *           *   
     297507 ACATTTCTATTGATAGAGCAGTTTTGAGACACTCTTCTTTTGGAATCTGCAAGTGGATATTAGGA
         66 ACCTTTCTTTTCATAGAGCAGTTTTGAAACACTCTTTTTGTAGAATCTGGAAGTGGATATTTGGA

            ** *                        *     *      
     297572 TAGATTTGAGGATTTCGTTGGAAACGG-GAT-T-AT-ATAT
        131 GCGCTTTGAGGATTTCGTTGGAAACGGAAATATCTTCATAT

            *    *       *               *                 **  *   *         
     297609 CAAAAGTAGACAGCAGCATTCTCAGAAACTTCTTTGTGATGTTTGCATCCAGCTCTCAGAGTTGA
          1 AAAAACTAGACAGAAGCATTCTCAGAAACATCTTTGTGATGTTTGCACTCAACTCACAGAGTTGA

              *  * *          *  *        *        *   **        *  **       
     297674 ACATTCCCTTTCATAGAGTAGGTTTGAAACCCTCTTTTTATAGTGTCTGGAAGCGGGCATTTGGA
         66 ACCTTTCTTTTCATAGAGCAGTTTTGAAACACTCTTTTTGTAGAATCTGGAAGTGGATATTTGGA

                   *   ** ** *  *   *          * *   
     297739 GCGCTTTCAGGCCTATGCTGAAAAAGGAAATATCTACCTAT
        131 GCGCTTTGAGGATTTCGTTGGAAACGGAAATATCTTCATAT

             *                    *    *  **          ** *        *    *     
     297780 AGAAACTAGACAGAAGCATTCTGAGAATCACGTTTGTGATGTGGGTACTCAACTAACAGTGTTGA
          1 AAAAACTAGACAGAAGCATTCTCAGAAACATCTTTGTGATGTTTGCACTCAACTCACAGAGTTGA

            *  *       *   *            *   **               *               
     297845 TCCATTCTTTTGATACAGCAGTTTTGAACCACATTTTTTGTAGAATCTGCAAGTGGATATTTGGA
         66 ACCTTTCTTTTCATAGAGCAGTTTTGAAACACTCTTTTTGTAGAATCTGGAAGTGGATATTTGGA

            **   *          *          *   *        *
     297910 TAGCTGTGAGGATTTCCTTGGAAACGGGAATGTCTTCATAG
        131 GCGCTTTGAGGATTTCGTTGGAAACGGAAATATCTTCATAT

                **              *      *** **         *  **   *   *          
     297951 AAAATTTAGACAGAAGCATTTTCAGAACTTTGATTGTGATGTGTGTTCTCCACTAACAGAGTTGA
          1 AAAAACTAGACAGAAGCATTCTCAGAAACATCTTTGTGATGTTTGCACTCAACTCACAGAGTTGA

                       * *   * *   *       *       *                         
     298016 ACCTTTCTTTTGACAGAACTGTTCTGAAACATTCTTTTTATAGAATCTGGAAGTGGATATTTGGA
         66 ACCTTTCTTTTCATAGAGCAGTTTTGAAACACTCTTTTTGTAGAATCTGGAAGTGGATATTTGGA

            **                         *          *  
     298081 AAGCTTTGAGGATTTCGTTGGAAACGGGAATATCTTCAAAT
        131 GCGCTTTGAGGATTTCGTTGGAAACGGAAATATCTTCATAT

            *   *    *            *           * *       *  *    *            
     298122 CAAATCTAGCCAGAAGCATTCTAAGAAACATCTTAGGGATGTTTACATTCAAGTCACAGAGTTGA
          1 AAAAACTAGACAGAAGCATTCTCAGAAACATCTTTGTGATGTTTGCACTCAACTCACAGAGTTGA

              *  * *     *       *         *    * *   **      *      *    *  
     298187 ACATTCCCTTTCACAGAGCAGGTTTGAAACAATCTTCTCGTACTATCTGGCAGTGGACATTTTGA
         66 ACCTTTCTTTTCATAGAGCAGTTTTGAAACACTCTTTTTGTAGAATCTGGAAGTGGATATTTGGA

              * *   *  ** ** *  *   *            ** *
     298252 GCTCCTTGGGGCCTATGCTGAAAAAGGAAATATCTTCCGAC
        131 GCGCTTTGAGGATTTCGTTGGAAACGGAAATATCTTCATAT

                                 *     *  **          *           **    *    
     298293 AAAAACTAGACAGAAGCATTCGCAGAATCACGTTTGTGATGTGTGCACTCAACTGTCAGAATTGA
          1 AAAAACTAGACAGAAGCATTCTCAGAAACATCTTTGTGATGTTTGCACTCAACTCACAGAGTTGA

                 **   ** *      *                            * *            *
     298358 ACCTTGGTTTGGACAGAGCACTTTTGAAACACTCTTTTTGTAGAATCTGCAGGTGGATATTTGGC
         66 ACCTTTCTTTTCATAGAGCAGTTTTGAAACACTCTTTTTGTAGAATCTGGAAGTGGATATTTGGA

            **                         *   *      * *
     298423 TAGCTTTGAGGATTTCGTTGGAAACGGTAATGTCTTCAAAG
        131 GCGCTTTGAGGATTTCGTTGGAAACGGAAATATCTTCATAT

                *                         *   *            *    *            
     298464 AAAATCTAGACAGAAGCATTCTCAGAAACACCTTCGTGATGTTTGCAATCAAGTCACAGAGTTGA
          1 AAAAACTAGACAGAAGCATTCTCAGAAACATCTTTGTGATGTTTGCACTCAACTCACAGAGTTGA

                 * *             *  *                  *             *       
     298529 ACCTTCCGTTTCATAGAGCAGGTTGGAAACACTCTTTTTGTAGTATCTGGAAGTGGACATTTGGA
         66 ACCTTTCTTTTCATAGAGCAGTTTTGAAACACTCTTTTTGTAGAATCTGGAAGTGGATATTTGGA

             *         ** *    * *   *            **  
     298594 GGGCTTTGTAGCCTATC-TGGAAAAAGGAAATATCTTCCCAT
        131 GCGCTTTG-AGGATTTCGTTGGAAACGGAAATATCTTCATAT

            *  ** *   *     * *            *   *  *   * **        *  * * *   
     298635 GAATGCGAGATAGAAGTAATCTCAGAAACATGTTTATGCTGTATCTACTCAACTAACTGTGCTGA
          1 AAAAACTAGACAGAAGCATTCTCAGAAACATCTTTGTGATGTTTGCACTCAACTCACAGAGTTGA

              *     *  *               *        *  * *       *               
     298700 ACATTTCTATTGATAGAGCAGTTTTGAGACACTCTTCTTTTGGAATCTGCAAGTGGATATTTGGA
         66 ACCTTTCTTTTCATAGAGCAGTTTTGAAACACTCTTTTTGTAGAATCTGGAAGTGGATATTTGGA

            ** *                        *     *      
     298765 TAGATTTGAGGATTTCGTTGGAAACGG-GAT-T-AT-ATAT
        131 GCGCTTTGAGGATTTCGTTGGAAACGGAAATATCTTCATAT

                 *       *               *                 **  *   *         
     298802 AAAAAGTAGACAGCAGCATTCTCAGAAACTTCTTTGTGATGTTTGCATCCAGCTCTCAGAGTTGA
          1 AAAAACTAGACAGAAGCATTCTCAGAAACATCTTTGTGATGTTTGCACTCAACTCACAGAGTTGA

              *  * *          *  *        *        *   **        *  **       
     298867 ACATTCCCTTTCATAGAGTAGGTTTGAAACCCTCTTTTTATAGTGTCTGGAAGCGGGCATTTGGA
         66 ACCTTTCTTTTCATAGAGCAGTTTTGAAACACTCTTTTTGTAGAATCTGGAAGTGGATATTTGGA

                   *    * * **  **  **          * *  *
     298932 GCGCTTTCAGGCCTATGCTTAAAATAGGAAATATCTACCTAC
        131 GCGCTTTGAGG-ATTTCGTTGGAAACGGAAATATCTTCATAT

             *                    *    *  **          ** *        *    *     
     298974 AGAAACTAGACAGAAGCATTCTGAGAATCACGTTTGTGATGTGGGTACTCAACTAACAGTGTTGA
          1 AAAAACTAGACAGAAGCATTCTCAGAAACATCTTTGTGATGTTTGCACTCAACTCACAGAGTTGA

            *  *       *   *            *   *                *               
     299039 TCCATTCTTTTGATACAGCAGTTTTGAACCACACTTTTTGTAGAATCTGCAAGTGGATATTTGGA
         66 ACCTTTCTTTTCATAGAGCAGTTTTGAAACACTCTTTTTGTAGAATCTGGAAGTGGATATTTGGA

            **   *                     *   *      * *
     299104 TAGCTGTGAGGATTTCGTTGGAAACGGGAATGTCTTCAAAG
        131 GCGCTTTGAGGATTTCGTTGGAAACGGAAATATCTTCATAT

                *                         *   *            *    *            
     299145 AAAATCTAGACAGAAGCATTCTCAGAAACACCTTCGTGATGTTTGCAATCAAGTCACAGAGTTGA
          1 AAAAACTAGACAGAAGCATTCTCAGAAACATCTTTGTGATGTTTGCACTCAACTCACAGAGTTGA

                 * *             *  *           *      *             *       
     299210 ACCTTCCGTTTCATAGAGCAGGTTGGAAACACTCTTATTGTAGTATCTGGAAGTGGACATTTGGA
         66 ACCTTTCTTTTCATAGAGCAGTTTTGAAACACTCTTTTTGTAGAATCTGGAAGTGGATATTTGGA

                   *   ** ** *  *   *            **  
     299275 GCGCTTTCAGGCCTATGGTGAAAAAGGAAATATCTTCCCAT
        131 GCGCTTTGAGGATTTCGTTGGAAACGGAAATATCTTCATAT

                  * * *      **      *   * *   *     ** ** *      *    *     
     299316 AAAAACGACATAGAAGCTATCTCAGGAACTTGTTTATGATGCATCTAATCAACTAACAGTGTTGA
          1 AAAAACTAGACAGAAGCATTCTCAGAAACATCTTTGTGATGTTTGCACTCAACTCACAGAGTTGA

                  * ** * *                         * *       *               
     299381 ACCTTTGTACTGACAGAGCAG-TTTGAAACACTCTTTTTTTGGAATCTGCAAGTGGATATTTGGA
         66 ACCTTTCTTTTCATAGAGCAGTTTTGAAACACTCTTTTTGTAGAATCTGGAAGTGGATATTTGGA

            *                           *   *   *    
     299445 TCGCTTTGAGGATTTCGTTGGAAACGG-GAT-GC--AATAT
        131 GCGCTTTGAGGATTTCGTTGGAAACGGAAATATCTTCATAT

                **  *    *     *                 **  *   *  *    *         * 
     299482 AAAACGTACACAGCAGCATACTCAGAAA-ATACTTTGCCATATTTCCATTCAAGTCACAGAGTGG
          1 AAAAACTAGACAGAAGCATTCTCAGAAACAT-CTTTGTGATGTTTGCACTCAACTCACAGAGTTG

               *  * **            *                  *  *             *      
     299546 AACATTCCCATTCATAGAGCAGGTTTGAAACACTCTTTTTGGAGTATCTGGAAGTGGACATTTGG
         65 AACCTTTCTTTTCATAGAGCAGTTTTGAAACACTCTTTTTGTAGAATCTGGAAGTGGATATTTGG

                    ** * * ** *  *   *            **  
     299611 AGCGCTTTCTGAACTATGGTGAAAAAGGAAATATCTTCCAAT
        130 AGCGCTTTGAGGATTTCGTTGGAAACGGAAATATCTTCATAT

            *     * *             *      * *          *  **      **  *  *    
     299653 GAAAACAACACAGAAGCATTCTGAGAAACTTATTTGTGATGTGTGTCCTCAACAAACGGACTTGA
          1 AAAAACTAGACAGAAGCATTCTCAGAAACATCTTTGTGATGTTTGCACTCAACTCACAGAGTTGA

                   *      **  * *  *  *             *   *    * * *           
     299718 ACCTTTCGTTTCATGCAGTACTTCTGGAACACTCTTTTTGAAGATTCTGCATGCGGATATTTGGA
         66 ACCTTTCTTTTCATAGAGCAGTTTTGAAACACTCTTTTTGTAGAATCTGGAAGTGGATATTTGGA

            **                            **   *   * 
     299783 TAGCTTTGAGGATTTCGTTGGAAACGG---GCT-TACATGT
        131 GCGCTTTGAGGATTTCGTTGGAAACGGAAATATCTTCATAT

                 *       *               *        *   *    *    *       *    
     299820 AAAAATTAGACAGCAGCATTCTCAGAAACTTCTTTGTGGTGTCTGCATTCAAGTCACAGAATTGA
          1 AAAAACTAGACAGAAGCATTCTCAGAAACATCTTTGTGATGTTTGCACTCAACTCACAGAGTTGA

              * ** * **            *  * *      *       *    *        *       
     299885 ACTTCCCCTCACATAGAGCAGTTGTGCAGCACTCTATTTGTAGTATCTCGAAGTGGACATTTGGA
         66 ACCTTTCTTTTCATAGAGCAGTTTTGAAACACTCTTTTTGTAGAATCTGGAAGTGGATATTTGGA

             *         ** *    * *   *            **  
     299950 GGGCTTTGTAGCCTATC-TGGAAAAAGGAAATATCTTCCCAT
        131 GCGCTTTG-AGGATTTCGTTGGAAACGGAAATATCTTCATAT

            *  ** *   *     * *            *   *  *   * **        *  * * *   
     299991 GAATGCGAGATAGAAGTAATCTCAGAAACATGTTTATGCTGTATCTACTCAACTAACTGTGCTGA
          1 AAAAACTAGACAGAAGCATTCTCAGAAACATCTTTGTGATGTTTGCACTCAACTCACAGAGTTGA

              *     *  *               *        *  * *       *               
     300056 ACATTTCTATTGATAGAGCAGTTTTGAGACACTCTTCTTTTGGAATCTGCAAGTGGATATTTGGA
         66 ACCTTTCTTTTCATAGAGCAGTTTTGAAACACTCTTTTTGTAGAATCTGGAAGTGGATATTTGGA

            ** *                        *     *      
     300121 TAGATTTGAGGATTTCGTTGGAAACGG-GAT-T-AT-ATAT
        131 GCGCTTTGAGGATTTCGTTGGAAACGGAAATATCTTCATAT

                 *       *               *                 **  *   *         
     300158 AAAAAGTAGACAGCAGCATTCTCAGAAACTTCTTTGTGATGTTTGCATCCAGCTCTCAGAGTTGA
          1 AAAAACTAGACAGAAGCATTCTCAGAAACATCTTTGTGATGTTTGCACTCAACTCACAGAGTTGA

              *  * *          *  *        *        *   **        *  **       
     300223 ACATTCCCTTTCATAGAGTAGGTTTGAAACCCTCTTTTTATAGTGTCTGGAAGCGGGCATTTGGA
         66 ACCTTTCTTTTCATAGAGCAGTTTTGAAACACTCTTTTTGTAGAATCTGGAAGTGGATATTTGGA

                   *   ** ** *  *   *          * *   
     300288 GCGCTTTCAGGCCTATGCTGAAAAAGGAAATATCTACCTAT
        131 GCGCTTTGAGGATTTCGTTGGAAACGGAAATATCTTCATAT

             *                    *    *  **          ** *        *    *     
     300329 AGAAACTAGACAGAAGCATTCTGAGAATCACGTTTGTGATGTGGGTACTCAACTAACAGTGTTGA
          1 AAAAACTAGACAGAAGCATTCTCAGAAACATCTTTGTGATGTTTGCACTCAACTCACAGAGTTGA

            *  *       *   *            *   *                *               
     300394 TCCATTCTTTTGATACAGCAGTTTTGAACCACACTTTTTGTAGAATCTGCAAGTGGATATTTGGA
         66 ACCTTTCTTTTCATAGAGCAGTTTTGAAACACTCTTTTTGTAGAATCTGGAAGTGGATATTTGGA

            **   *           *         *   *        *
     300459 TAGCTGTGAGGATTTCGGTGGAAACGGGAATGTCTTCATAG
        131 GCGCTTTGAGGATTTCGTTGGAAACGGAAATATCTTCATAT

                **                     * * **         *  **   *   *          
     300500 AAAATGTAGACAGAAGCATTCTCAGAACCTTGATTGTGATGTGTGTTCTCCACTAACAGAGTTGA
          1 AAAAACTAGACAGAAGCATTCTCAGAAACATCTTTGTGATGTTTGCACTCAACTCACAGAGTTGA

                       * *   * *   *       *       *                         
     300565 ACCTTTCTTTTGACAGAACTGTTCTGAAACATTCTTTTTATAGAATCTGGAAGTGGATATTTGGA
         66 ACCTTTCTTTTCATAGAGCAGTTTTGAAACACTCTTTTTGTAGAATCTGGAAGTGGATATTTGGA

            **                         *          *  
     300630 AAGCTTTGAGGATTTCGTTGGAAACGGGAATATCTTCAAAT
        131 GCGCTTTGAGGATTTCGTTGGAAACGGAAATATCTTCATAT

                *    *            *           * *       *  *    *            
     300671 AAAATCTAGCCAGAAGCATTCTAAGAAACATCTTAGGGATGTTTACATTCAAGTCACAGAGTTGA
          1 AAAAACTAGACAGAAGCATTCTCAGAAACATCTTTGTGATGTTTGCACTCAACTCACAGAGTTGA

              *  * *     *       *         *    * *   **      *      *    *  
     300736 ACATTCCCTTTCACAGAGCAGGTTTGAAACAATCTTCTCGTACTATCTGGCAGTGGACATTTTGA
         66 ACCTTTCTTTTCATAGAGCAGTTTTGAAACACTCTTTTTGTAGAATCTGGAAGTGGATATTTGGA

              * *   *  ** ** *  *   *            ** *
     300801 GCTCCTTGGGGCCTATGCTGAAAAAGGAAATATCTTCCGAC
        131 GCGCTTTGAGGATTTCGTTGGAAACGGAAATATCTTCATAT

                                 *     *  **          *           **    *    
     300842 AAAAACTAGACAGAAGCATTCGCAGAATCACGTTTGTGATGTGTGCACTCAACTGTCAGAATTGA
          1 AAAAACTAGACAGAAGCATTCTCAGAAACATCTTTGTGATGTTTGCACTCAACTCACAGAGTTGA

                 **   ** *      *                            * *            *
     300907 ACCTTGGTTTGGACAGAGCACTTTTGAAACACTCTTTTTGTAGAATCTGCAGGTGGATATTTGGC
         66 ACCTTTCTTTTCATAGAGCAGTTTTGAAACACTCTTTTTGTAGAATCTGGAAGTGGATATTTGGA

            **                         *   *      * *
     300972 TAGCTTTGAGGATTTCGTTGGAAACGGGAATGTCTTCAAAG
        131 GCGCTTTGAGGATTTCGTTGGAAACGGAAATATCTTCATAT

                *                         *   *            *    *            
     301013 AAAATCTAGACAGAAGCATTCTCAGAAACACCTTCGTGATGTTTGCAATCAAGTCACAGAGTTGA
          1 AAAAACTAGACAGAAGCATTCTCAGAAACATCTTTGTGATGTTTGCACTCAACTCACAGAGTTGA

                 * *             *  *                  *             *       
     301078 ACCTTCCGTTTCATAGAGCAGGTTGGAAACACTCTTTTTGTAGTATCTGGAAGTGGACATTTGGA
         66 ACCTTTCTTTTCATAGAGCAGTTTTGAAACACTCTTTTTGTAGAATCTGGAAGTGGATATTTGGA

                   *   ** ** *  *   *            **  
     301143 GCGCTTTCAGGCCTATGGTGAAAAAGGAAATATCTTCCCAT
        131 GCGCTTTGAGGATTTCGTTGGAAACGGAAATATCTTCATAT

                  * * *      **      *   * *   *     ** ** *      *    *     
     301184 AAAAACGACATAGAAGCTATCTCAGGAACTTGTTTATGATGCATCTAATCAACTAACAGTGTTGA
          1 AAAAACTAGACAGAAGCATTCTCAGAAACATCTTTGTGATGTTTGCACTCAACTCACAGAGTTGA

                  * ** * *                         * *       *               
     301249 ACCTTTGTACTGACAGAGCAG-TTTGAAACACTCTTTTTTTGGAATCTGCAAGTGGATATTTGGA
         66 ACCTTTCTTTTCATAGAGCAGTTTTGAAACACTCTTTTTGTAGAATCTGGAAGTGGATATTTGGA

            *                           *   *   *    
     301313 TCGCTTTGAGGATTTCGTTGGAAACGG-GAT-GC--AATAT
        131 GCGCTTTGAGGATTTCGTTGGAAACGGAAATATCTTCATAT

                **  *    *     *                 **  *   *  *    *         * 
     301350 AAAACGTACACAGCAGCATACTCAGAAA-ATACTTTGCCATATTTCCATTCAAGTCACAGAGTGG
          1 AAAAACTAGACAGAAGCATTCTCAGAAACAT-CTTTGTGATGTTTGCACTCAACTCACAGAGTTG

               *  * **            *  *               *  *             *      
     301414 AACATTCCCATTCATAGAGCAGGTTGGAAACACTCTTTTTGGAGTATCTGGAAGTGGACATTTGG
         65 AACCTTTCTTTTCATAGAGCAGTTTTGAAACACTCTTTTTGTAGAATCTGGAAGTGGATATTTGG

                    ** * * ** *  *   *            **  
     301479 AGCGCTTTCTGAACTATGGTGAAAAAGGAAATATCTTCCAAT
        130 AGCGCTTTGAGGATTTCGTTGGAAACGGAAATATCTTCATAT

            *     *               *      * *          *  **      **  *  *    
     301521 GAAAACAAGACAGAAGCATTCTGAGAAACTTATTTGTGATGTGTGTCCTCAACAAACGGACTTGA
          1 AAAAACTAGACAGAAGCATTCTCAGAAACATCTTTGTGATGTTTGCACTCAACTCACAGAGTTGA

                   *      **  * *  *  *             *   *    * * *           
     301586 ACCTTTCGTTTCATGCAGTACTTCTGGAACACTCTTTTTGAAGATTCTGCATGCGGATATTTGGA
         66 ACCTTTCTTTTCATAGAGCAGTTTTGAAACACTCTTTTTGTAGAATCTGGAAGTGGATATTTGGA

            **                            **   *   * 
     301651 TAGCTTTGAGGATTTCGTTGGAAACGG---GCT-TACATGT
        131 GCGCTTTGAGGATTTCGTTGGAAACGGAAATATCTTCATAT

                 *       *               *        *   *    *    *       *    
     301688 AAAAATTAGACAGCAGCATTCTCAGAAACTTCTTTGTGGTGTCTGCATTCAAGTCACAGAATTGA
          1 AAAAACTAGACAGAAGCATTCTCAGAAACATCTTTGTGATGTTTGCACTCAACTCACAGAGTTGA

              * ** * **            *  * *      *       *    *        *       
     301753 ACTTCCCCTCACATAGAGCAGTTGTGCAGCACTCTATTTGTAGTATCTCGAAGTGGACATTTGGA
         66 ACCTTTCTTTTCATAGAGCAGTTTTGAAACACTCTTTTTGTAGAATCTGGAAGTGGATATTTGGA

             *         ** *    * *   *            **  
     301818 GGGCTTTGTAGCCTATC-TGGAAAAAGGAAATATCTTCCCAT
        131 GCGCTTTG-AGGATTTCGTTGGAAACGGAAATATCTTCATAT

            *  ** *   *     * *            *   *  *   * **        *  * * *   
     301859 GAATGCGAGATAGAAGTAATCTCAGAAACATGTTTATGCTGTATCTACTCAACTAACTGTGCTGA
          1 AAAAACTAGACAGAAGCATTCTCAGAAACATCTTTGTGATGTTTGCACTCAACTCACAGAGTTGA

              *     *  *               *        *  * *       *           *   
     301924 ACATTTCTATTGATAGAGCAGTTTTGAGACACTCTTCTTTTGGAATCTGCAAGTGGATATTAGGA
         66 ACCTTTCTTTTCATAGAGCAGTTTTGAAACACTCTTTTTGTAGAATCTGGAAGTGGATATTTGGA

            ** *                        *     *      
     301989 TAGATTTGAGGATTTCGTTGGAAACGG-GAT-T-AT-ATAT
        131 GCGCTTTGAGGATTTCGTTGGAAACGGAAATATCTTCATAT

            *    *       *               *                 **  *   *         
     302026 CAAAAGTAGACAGCAGCATTCTCAGAAACTTCTTTGTGATGTTTGCATCCAGCTCTCAGAGTTGA
          1 AAAAACTAGACAGAAGCATTCTCAGAAACATCTTTGTGATGTTTGCACTCAACTCACAGAGTTGA

              *  * *          *  *        *        *   **        *  **       
     302091 ACATTCCCTTTCATAGAGTAGGTTTGAAACCCTCTTTTTATAGTGTCTGGAAGCGGGCATTTGGA
         66 ACCTTTCTTTTCATAGAGCAGTTTTGAAACACTCTTTTTGTAGAATCTGGAAGTGGATATTTGGA

                   *   ** ** *  *   *          * *   
     302156 GCGCTTTCAGGCCTATGCTGAAAAAGGAAATATCTACCTAT
        131 GCGCTTTGAGGATTTCGTTGGAAACGGAAATATCTTCATAT

             *                    *    *  **          ** *        *    *     
     302197 AGAAACTAGACAGAAGCATTCTGAGAATCACGTTTGTGATGTGGGTACTCAACTAACAGTGTTGA
          1 AAAAACTAGACAGAAGCATTCTCAGAAACATCTTTGTGATGTTTGCACTCAACTCACAGAGTTGA

            *  *       *   *            *   **               *               
     302262 TCCATTCTTTTGATACAGCAGTTTTGAACCACATTTTTTGTAGAATCTGCAAGTGGATATTTGGA
         66 ACCTTTCTTTTCATAGAGCAGTTTTGAAACACTCTTTTTGTAGAATCTGGAAGTGGATATTTGGA

            **   *                     *   *        *
     302327 TAGCTGTGAGGATTTCGTTGGAAACGGGAATGTCTTCATAG
        131 GCGCTTTGAGGATTTCGTTGGAAACGGAAATATCTTCATAT

                **              *      *** **         *  **   *   *          
     302368 AAAATTTAGACAGAAGCATTTTCAGAACTTTGATTGTGATGTGTGTTCTCCACTAACAGAGTTGA
          1 AAAAACTAGACAGAAGCATTCTCAGAAACATCTTTGTGATGTTTGCACTCAACTCACAGAGTTGA

                       * *   * *   *       *       *                         
     302433 ACCTTTCTTTTGACAGAACTGTTCTGAAACATTCTTTTTATAGAATCTGGAAGTGGATATTTGGA
         66 ACCTTTCTTTTCATAGAGCAGTTTTGAAACACTCTTTTTGTAGAATCTGGAAGTGGATATTTGGA

            **                         *          *  
     302498 AAGCTTTGAGGATTTCGTTGGAAACGGGAATATCTTCAAAT
        131 GCGCTTTGAGGATTTCGTTGGAAACGGAAATATCTTCATAT

            *   *    *            *           * *       *  *    *            
     302539 CAAATCTAGCCAGAAGCATTCTAAGAAACATCTTAGGGATGTTTACATTCAAGTCACAGAGTTGA
          1 AAAAACTAGACAGAAGCATTCTCAGAAACATCTTTGTGATGTTTGCACTCAACTCACAGAGTTGA

              *  * *     *       *         *    * *   **      *      *    *  
     302604 ACATTCCCTTTCACAGAGCAGGTTTGAAACAATCTTCTCGTACTATCTGGCAGTGGACATTTTGA
         66 ACCTTTCTTTTCATAGAGCAGTTTTGAAACACTCTTTTTGTAGAATCTGGAAGTGGATATTTGGA

              * *   *  ** ** *  *   *            ** *
     302669 GCTCCTTGGGGCCTATGCTGAAAAAGGAAATATCTTCCGAC
        131 GCGCTTTGAGGATTTCGTTGGAAACGGAAATATCTTCATAT

                                 *     *  **          *           **    *    
     302710 AAAAACTAGACAGAAGCATTCGCAGAATCACGTTTGTGATGTGTGCACTCAACTGTCAGAATTGA
          1 AAAAACTAGACAGAAGCATTCTCAGAAACATCTTTGTGATGTTTGCACTCAACTCACAGAGTTGA

                 **   ** *      *                            * *            *
     302775 ACCTTGGTTTGGACAGAGCACTTTTGAAACACTCTTTTTGTAGAATCTGCAGGTGGATATTTGGC
         66 ACCTTTCTTTTCATAGAGCAGTTTTGAAACACTCTTTTTGTAGAATCTGGAAGTGGATATTTGGA

            **                         *   *      * *
     302840 TAGCTTTGAGGATTTCGTTGGAAACGGTAATGTCTTCAAAG
        131 GCGCTTTGAGGATTTCGTTGGAAACGGAAATATCTTCATAT

                *                         *   *            *    *            
     302881 AAAATCTAGACAGAAGCATTCTCAGAAACACCTTCGTGATGTTTGCAATCAAGTCACAGAGTTGA
          1 AAAAACTAGACAGAAGCATTCTCAGAAACATCTTTGTGATGTTTGCACTCAACTCACAGAGTTGA

                 * *             *  *                  *             *       
     302946 ACCTTCCGTTTCATAGAGCAGGTTGGAAACACTCTTTTTGTAGTATCTGGAAGTGGACATTTGGA
         66 ACCTTTCTTTTCATAGAGCAGTTTTGAAACACTCTTTTTGTAGAATCTGGAAGTGGATATTTGGA

             *         ** *    * *   *            **  
     303011 GGGCTTTGTAGCCTAT-GTGGAAAAAGGAAATATCTTCCCAT
        131 GCGCTTTG-AGGATTTCGTTGGAAACGGAAATATCTTCATAT

            *  ** *   *     * *            *   *  *   * **        *  * * *   
     303052 GAATGCGAGATAGAAGTAATCTCAGAAACATGTTTATGCTGTATCTACTCAACTAACTGTGCTGA
          1 AAAAACTAGACAGAAGCATTCTCAGAAACATCTTTGTGATGTTTGCACTCAACTCACAGAGTTGA

              *     *  *               *        *  * *       *               
     303117 ACATTTCTATTGATAGAGCAGTTTTGAGACACTCTTCTTTTGGAATCTGCAAGTGGATATTTGGA
         66 ACCTTTCTTTTCATAGAGCAGTTTTGAAACACTCTTTTTGTAGAATCTGGAAGTGGATATTTGGA

            ** *                        *     *      
     303182 TAGATTTGAGGATTTCGTTGGAAACGG-GAT-T-AT-ATAT
        131 GCGCTTTGAGGATTTCGTTGGAAACGGAAATATCTTCATAT

                 *       *               *                 **  *   *         
     303219 AAAAAGTAGACAGCAGCATTCTCAGAAACTTCTTTGTGATGTTTGCATCCAGCTCTCAGAGTTGA
          1 AAAAACTAGACAGAAGCATTCTCAGAAACATCTTTGTGATGTTTGCACTCAACTCACAGAGTTGA

              *  * *          *  *        *        *   **        *  **       
     303284 ACATTCCCTTTCATAGAGTAGGTTTGAAACCCTCTTTTTATAGTGTCTGGAAGCGGGCATTTGGA
         66 ACCTTTCTTTTCATAGAGCAGTTTTGAAACACTCTTTTTGTAGAATCTGGAAGTGGATATTTGGA

                   *    * * **  **  **          * *  *
     303349 GCGCTTTCAGGCCTATGCTTAAAATAGGAAATATCTACCTAC
        131 GCGCTTTGAGG-ATTTCGTTGGAAACGGAAATATCTTCATAT

             *                    *    *  **          ** *        *    *     
     303391 AGAAACTAGACAGAAGCATTCTGAGAATCACGTTTGTGATGTGGGTACTCAACTAACAGTGTTGA
          1 AAAAACTAGACAGAAGCATTCTCAGAAACATCTTTGTGATGTTTGCACTCAACTCACAGAGTTGA

            *  *       *   *            *   *                *               
     303456 TCCATTCTTTTGATACAGCAGTTTTGAACCACACTTTTTGTAGAATCTGCAAGTGGATATTTGGA
         66 ACCTTTCTTTTCATAGAGCAGTTTTGAAACACTCTTTTTGTAGAATCTGGAAGTGGATATTTGGA

            **   *                     *   *      * *
     303521 TAGCTGTGAGGATTTCGTTGGAAACGGGAATGTCTTCAAAG
        131 GCGCTTTGAGGATTTCGTTGGAAACGGAAATATCTTCATAT

                *                         *   *            *    *            
     303562 AAAATCTAGACAGAAGCATTCTCAGAAACACCTTCGTGATGTTTGCAATCAAGTCACAGAGTTGA
          1 AAAAACTAGACAGAAGCATTCTCAGAAACATCTTTGTGATGTTTGCACTCAACTCACAGAGTTGA

                 * *             *  *           *      *             *       
     303627 ACCTTCCGTTTCATAGAGCAGGTTGGAAACACTCTTATTGTAGTATCTGGAAGTGGACATTTGGA
         66 ACCTTTCTTTTCATAGAGCAGTTTTGAAACACTCTTTTTGTAGAATCTGGAAGTGGATATTTGGA

                   *   ** ** *  *   *            **  
     303692 GCGCTTTCAGGCCTATGGTGAAAAAGGAAATATCTTCCCAT
        131 GCGCTTTGAGGATTTCGTTGGAAACGGAAATATCTTCATAT

                  * * *      **      *   * *   *     ** ** *      *    *     
     303733 AAAAACGACATAGAAGCTATCTCAGGAACTTGTTTATGATGCATCTAATCAACTAACAGTGTTGA
          1 AAAAACTAGACAGAAGCATTCTCAGAAACATCTTTGTGATGTTTGCACTCAACTCACAGAGTTGA

                  * ** * *                         * *       *               
     303798 ACCTTTGTACTGACAGAGCAG-TTTGAAACACTCTTTTTTTGGAATCTGCAAGTGGATATTTGGA
         66 ACCTTTCTTTTCATAGAGCAGTTTTGAAACACTCTTTTTGTAGAATCTGGAAGTGGATATTTGGA

            *                           *   *   *    
     303862 TCGCTTTGAGGATTTCGTTGGAAACGG-GAT-GC--AATAT
        131 GCGCTTTGAGGATTTCGTTGGAAACGGAAATATCTTCATAT

                **  *    *     *                 **  *   *  *    *         * 
     303899 AAAACGTACACAGCAGCATACTCAGAAA-ATACTTTGCCATATTTCCATTCAAGTCACAGAGTGG
          1 AAAAACTAGACAGAAGCATTCTCAGAAACAT-CTTTGTGATGTTTGCACTCAACTCACAGAGTTG

               *  * **            *                  *  *             *      
     303963 AACATTCCCATTCATAGAGCAGGTTTGAAACACTCTTTTTGGAGTATCTGGAAGTGGACATTTGG
         65 AACCTTTCTTTTCATAGAGCAGTTTTGAAACACTCTTTTTGTAGAATCTGGAAGTGGATATTTGG

                    ** * * ** *  *   *            **  
     304028 AGCGCTTTCTGAACTATGGTGAAAAAGGAAATATCTTCCAAT
        130 AGCGCTTTGAGGATTTCGTTGGAAACGGAAATATCTTCATAT

            *     *               *      * *          *  **      **  *  *    
     304070 GAAAACAAGACAGAAGCATTCTGAGAAACTTATTTGTGATGTGTGTCCTCAACAAACGGACTTGA
          1 AAAAACTAGACAGAAGCATTCTCAGAAACATCTTTGTGATGTTTGCACTCAACTCACAGAGTTGA

                   *      **  * *  *  *             *   *    * * *           
     304135 ACCTTTCGTTTCATGCAGTACTTCTGGAACACTCTTTTTGAAGATTCTGCATGCGGATATTTGGA
         66 ACCTTTCTTTTCATAGAGCAGTTTTGAAACACTCTTTTTGTAGAATCTGGAAGTGGATATTTGGA

            **                            **   *   * 
     304200 TAGCTTTGAGGATTTCGTTGGAAACGG---GCT-TACATGT
        131 GCGCTTTGAGGATTTCGTTGGAAACGGAAATATCTTCATAT

                 *       *               *        *   *    *    *       *    
     304237 AAAAATTAGACAGCAGCATTCTCAGAAACTTCTTTGTGGTGTCTGCATTCAAGTCACAGAATTGA
          1 AAAAACTAGACAGAAGCATTCTCAGAAACATCTTTGTGATGTTTGCACTCAACTCACAGAGTTGA

              * ** * **            *  * *      *       *    *        *       
     304302 ACTTCCCCTCACATAGAGCAGTTGTGCAGCACTCTATTTGTAGTATCTCGAAGTGGACATTTGGA
         66 ACCTTTCTTTTCATAGAGCAGTTTTGAAACACTCTTTTTGTAGAATCTGGAAGTGGATATTTGGA

             *         ** *    * *   *            **  
     304367 GGGCTTTGTAGCCTATC-TGGAAAAAGGAAATATCTTCCCAT
        131 GCGCTTTG-AGGATTTCGTTGGAAACGGAAATATCTTCATAT

            *  ** *   *     * *            *   *  *   * **        *  * * *   
     304408 GAATGCGAGATAGAAGTAATCTCAGAAACATGTTTATGCTGTATCTACTCAACTAACTGTGCTGA
          1 AAAAACTAGACAGAAGCATTCTCAGAAACATCTTTGTGATGTTTGCACTCAACTCACAGAGTTGA

              *     *  *               *        *  * *       *               
     304473 ACATTTCTATTGATAGAGCAGTTTTGAGACACTCTTCTTTTGGAATCTGCAAGTGGATATTTGGA
         66 ACCTTTCTTTTCATAGAGCAGTTTTGAAACACTCTTTTTGTAGAATCTGGAAGTGGATATTTGGA

            ** *                        *     *      
     304538 TAGATTTGAGGATTTCGTTGGAAACGG-GAT-T-AT-ATAT
        131 GCGCTTTGAGGATTTCGTTGGAAACGGAAATATCTTCATAT

                 *       *               *                 **  *   *         
     304575 AAAAAGTAGACAGCAGCATTCTCAGAAACTTCTTTGTGATGTTTGCATCCAGCTCTCAGAGTTGA
          1 AAAAACTAGACAGAAGCATTCTCAGAAACATCTTTGTGATGTTTGCACTCAACTCACAGAGTTGA

              *  * *          *  *        *        *   **        *  **       
     304640 ACATTCCCTTTCATAGAGTAGGTTTGAAACCCTCTTTTTATAGTGTCTGGAAGCGGGCATTTGGA
         66 ACCTTTCTTTTCATAGAGCAGTTTTGAAACACTCTTTTTGTAGAATCTGGAAGTGGATATTTGGA

                   *   ** ** *  *   *          * *   
     304705 GCGCTTTCAGGCCTATGCTGAAAAAGGAAATATCTACCTAT
        131 GCGCTTTGAGGATTTCGTTGGAAACGGAAATATCTTCATAT

             *                    *    *  **          ** *        *    *     
     304746 AGAAACTAGACAGAAGCATTCTGAGAATCACGTTTGTGATGTGGGTACTCAACTAACAGTGTTGA
          1 AAAAACTAGACAGAAGCATTCTCAGAAACATCTTTGTGATGTTTGCACTCAACTCACAGAGTTGA

            *  *       *   *            *   *                *               
     304811 TCCATTCTTTTGATACAGCAGTTTTGAACCACACTTTTTGTAGAATCTGCAAGTGGATATTTGGA
         66 ACCTTTCTTTTCATAGAGCAGTTTTGAAACACTCTTTTTGTAGAATCTGGAAGTGGATATTTGGA

            **   *                     *   *        *
     304876 TAGCTGTGAGGATTTCGTTGGAAACGGGAATGTCTTCATAG
        131 GCGCTTTGAGGATTTCGTTGGAAACGGAAATATCTTCATAT

                **                     * * **         *  **   *   *          
     304917 AAAATGTAGACAGAAGCATTCTCAGAACCTTGATTGTGATGTGTGTTCTCCACTAACAGAGTTGA
          1 AAAAACTAGACAGAAGCATTCTCAGAAACATCTTTGTGATGTTTGCACTCAACTCACAGAGTTGA

                       * *   * *   *       *       *                         
     304982 ACCTTTCTTTTGACAGAACTGTTCTGAAACATTCTTTTTATAGAATCTGGAAGTGGATATTTGGA
         66 ACCTTTCTTTTCATAGAGCAGTTTTGAAACACTCTTTTTGTAGAATCTGGAAGTGGATATTTGGA

            **                         *          *  
     305047 AAGCTTTGAGGATTTCGTTGGAAACGGGAATATCTTCAAAT
        131 GCGCTTTGAGGATTTCGTTGGAAACGGAAATATCTTCATAT

                *    *            *           * *       *  *    *            
     305088 AAAATCTAGCCAGAAGCATTCTAAGAAACATCTTAGGGATGTTTACATTCAAGTCACAGAGTTGA
          1 AAAAACTAGACAGAAGCATTCTCAGAAACATCTTTGTGATGTTTGCACTCAACTCACAGAGTTGA

              *  * *     *       *         *    * *   **      *      *    *  
     305153 ACATTCCCTTTCACAGAGCAGGTTTGAAACAATCTTCTCGTACTATCTGGCAGTGGACATTTTGA
         66 ACCTTTCTTTTCATAGAGCAGTTTTGAAACACTCTTTTTGTAGAATCTGGAAGTGGATATTTGGA

              * *   *  ** ** *  *   *            ** *
     305218 GCTCCTTGGGGCCTATGCTGAAAAAGGAAATATCTTCCGAA
        131 GCGCTTTGAGGATTTCGTTGGAAACGGAAATATCTTCATAT

                                 *     *  **          *           **    *    
     305259 AAAAACTAGACAGAAGCATTCGCAGAATCACGTTTGTGATGTGTGCACTCAACTGTCAGAATTGA
          1 AAAAACTAGACAGAAGCATTCTCAGAAACATCTTTGTGATGTTTGCACTCAACTCACAGAGTTGA

                 **   ** *      *                            * *            *
     305324 ACCTTGGTTTGGACAGAGCACTTTTGAAACACTCTTTTTGTAGAATCTGCAGGTGGATATTTGGC
         66 ACCTTTCTTTTCATAGAGCAGTTTTGAAACACTCTTTTTGTAGAATCTGGAAGTGGATATTTGGA

            **                         *   *      * *
     305389 TAGCTTTGAGGATTTCGTTGGAAACGGGAATGTCTTCAAAG
        131 GCGCTTTGAGGATTTCGTTGGAAACGGAAATATCTTCATAT

                *                         *   *            *    *            
     305430 AAAATCTAGACAGAAGCATTCTCAGAAACACCTTCGTGATGTTTGCAATCAAGTCACAGAGTTGA
          1 AAAAACTAGACAGAAGCATTCTCAGAAACATCTTTGTGATGTTTGCACTCAACTCACAGAGTTGA

                 * *             *  *           *      *             *       
     305495 ACCTTCCGTTTCATAGAGCAGGTTGGAAACACTCTTATTGTAGTATCTGGAAGTGGACATTTGGA
         66 ACCTTTCTTTTCATAGAGCAGTTTTGAAACACTCTTTTTGTAGAATCTGGAAGTGGATATTTGGA

                   *   ** ** *  *   *            **  
     305560 GCGCTTTCAGGCCTATGGTGAAAAAGGAAATATCTTCCCAT
        131 GCGCTTTGAGGATTTCGTTGGAAACGGAAATATCTTCATAT

                  * * *      **      *   * *   *     ** ** *      *    *     
     305601 AAAAACGACATAGAAGCTATCTCAGGAACTTGTTTATGATGCATCTAATCAACTAACAGTGTTGA
          1 AAAAACTAGACAGAAGCATTCTCAGAAACATCTTTGTGATGTTTGCACTCAACTCACAGAGTTGA

                  * ** * *                         * *       *               
     305666 ACCTTTGTACTGACAGAGCAG-TTTGAAACACTCTTTTTTTGGAATCTGCAAGTGGATATTTGGA
         66 ACCTTTCTTTTCATAGAGCAGTTTTGAAACACTCTTTTTGTAGAATCTGGAAGTGGATATTTGGA

            *                           *   *   *    
     305730 TCGCTTTGAGGATTTCGTTGGAAACGG-GAT-GC--AATAT
        131 GCGCTTTGAGGATTTCGTTGGAAACGGAAATATCTTCATAT

                **  *    *     *                 **  *   *  *    *         * 
     305767 AAAACGTACACAGCAGCATACTCAGAAA-ATACTTTGCCATATTTCCATTCAAGTCACAGAGTGG
          1 AAAAACTAGACAGAAGCATTCTCAGAAACAT-CTTTGTGATGTTTGCACTCAACTCACAGAGTTG

               *  * **            ** *               *  *             *      
     305831 AACATTCCCATTCATAGAGCAGGCTGGAAACACTCTTTTTGGAGTATCTGGAAGTGGACATTTGG
         65 AACCTTTCTTTTCATAGAGCAGTTTTGAAACACTCTTTTTGTAGAATCTGGAAGTGGATATTTGG

                    ** * * ** *  *   *            **  
     305896 AGCGCTTTCTGAACTATGGTGAAAAAGGAAATATCTTCCAAT
        130 AGCGCTTTGAGGATTTCGTTGGAAACGGAAATATCTTCATAT

            *     *               * *    * *          *  **      **  *  *    
     305938 GAAAACAAGACAGAAGCATTCTGAAAAACTTATTTGTGATGTGTGTCCTCAACAAACGGACTTGA
          1 AAAAACTAGACAGAAGCATTCTCAGAAACATCTTTGTGATGTTTGCACTCAACTCACAGAGTTGA

                   *      **  * *  *  *             *   *    * * *           
     306003 ACCTTTCGTTTCATGCAGTACTTCTGGAACACTCTTTTTGAAGATTCTGCATGCGGATATTTGGA
         66 ACCTTTCTTTTCATAGAGCAGTTTTGAAACACTCTTTTTGTAGAATCTGGAAGTGGATATTTGGA

            **                            **   *   * 
     306068 TAGCTTTGAGGATTTCGTTGGAAACGG---GCT-TACATGT
        131 GCGCTTTGAGGATTTCGTTGGAAACGGAAATATCTTCATAT

                 *       *               *        *   *    *    *       *    
     306105 AAAAATTAGACAGCAGCATTCTCAGAAACTTCTTTGTGGTGTCTGCATTCAAGTCACAGAATTGA
          1 AAAAACTAGACAGAAGCATTCTCAGAAACATCTTTGTGATGTTTGCACTCAACTCACAGAGTTGA

              * ** * **            *  * *      *       *    *        *       
     306170 ACTTCCCCTCACATAGAGCAGTTGTGCAGCACTCTATTTGTAGTATCTCGAAGTGGACATTTGGA
         66 ACCTTTCTTTTCATAGAGCAGTTTTGAAACACTCTTTTTGTAGAATCTGGAAGTGGATATTTGGA

             *         ** *    * *   *            **  
     306235 GGGCTTTGTAGCCTATC-TGGAAAAAGGAAATATCTTCCCAT
        131 GCGCTTTG-AGGATTTCGTTGGAAACGGAAATATCTTCATAT

            *  ** *   *     * *            *   *  *   * **        *  * * *   
     306276 GAATGCGAGATAGAAGTAATCTCAGAAACATGTTTATGCTGTATCTACTCAACTAACTGTGCTGA
          1 AAAAACTAGACAGAAGCATTCTCAGAAACATCTTTGTGATGTTTGCACTCAACTCACAGAGTTGA

              *     *  *               *        *  * *       *           *   
     306341 ACATTTCTATTGATAGAGCAGTTTTGAGACACTCTTCTTTTGGAATCTGCAAGTGGATATTAGGA
         66 ACCTTTCTTTTCATAGAGCAGTTTTGAAACACTCTTTTTGTAGAATCTGGAAGTGGATATTTGGA

            ** *                        *     *      
     306406 TAGATTTGAGGATTTCGTTGGAAACGG-GAT-T-AT-ATAT
        131 GCGCTTTGAGGATTTCGTTGGAAACGGAAATATCTTCATAT

                 *       *               *                 **  *   *         
     306443 AAAAAGTAGACAGCAGCATTCTCAGAAACTTCTTTGTGATGTTTGCATCCAGCTCTCAGAGTTGA
          1 AAAAACTAGACAGAAGCATTCTCAGAAACATCTTTGTGATGTTTGCACTCAACTCACAGAGTTGA

              *  * *          *  *        *        *   **        *  **       
     306508 ACATTCCCTTTCATAGAGTAGGTTTGAAACCCTCTTTTTATAGTGTCTGGAAGCGGGCATTTGGA
         66 ACCTTTCTTTTCATAGAGCAGTTTTGAAACACTCTTTTTGTAGAATCTGGAAGTGGATATTTGGA

                   *   ** ** *  *   *          * *   
     306573 GCGCTTTCAGGCCTATGCTGAAAAAGGAAATATCTACCTAT
        131 GCGCTTTGAGGATTTCGTTGGAAACGGAAATATCTTCATAT

             *                    *    ** **   *      ** *        *    *     
     306614 AGAAACTAGACAGAAGCATTCTGAGAATGACGTTTTTGATGTGGGTACTCAACTAACAGTGTTGA
          1 AAAAACTAGACAGAAGCATTCTCAGAAACATCTTTGTGATGTTTGCACTCAACTCACAGAGTTGA

            *  *       *   *            *   **               *               
     306679 TCCATTCTTTTGATACAGCAGTTTTGAACCACATTTTTTGTAGAATCTGCAAGTGGATATTTGGA
         66 ACCTTTCTTTTCATAGAGCAGTTTTGAAACACTCTTTTTGTAGAATCTGGAAGTGGATATTTGGA

            **   *                     *   *        *
     306744 TAGCTGTGAGGATTTCGTTGGAAACGGGAATGTCTTCATAG
        131 GCGCTTTGAGGATTTCGTTGGAAACGGAAATATCTTCATAT

                **              *      *** **         *  **   *   *          
     306785 AAAATTTAGACAGAAGCATTTTCAGAACTTTGATTGTGATGTGTGTTCTCCACTAACAGAGTTGA
          1 AAAAACTAGACAGAAGCATTCTCAGAAACATCTTTGTGATGTTTGCACTCAACTCACAGAGTTGA

                       * *   * *   *       *                                 
     306850 ACCTTTCTTTTGACAGAACTGTTCTGAAACATTCTTTTTGTAGAATCTGGAAGTGGATATTTGGA
         66 ACCTTTCTTTTCATAGAGCAGTTTTGAAACACTCTTTTTGTAGAATCTGGAAGTGGATATTTGGA

            **                         *          *  
     306915 AAGCTTTGAGGATTTCGTTGGAAACGGGAATATCTTCAAAT
        131 GCGCTTTGAGGATTTCGTTGGAAACGGAAATATCTTCATAT

            *   *    *            *           * *       *  *    *            
     306956 CAAATCTAGCCAGAAGCATTCTAAGAAACATCTTAGGGATGTTTACATTCAAGTCACAGAGTTGA
          1 AAAAACTAGACAGAAGCATTCTCAGAAACATCTTTGTGATGTTTGCACTCAACTCACAGAGTTGA

              *  * *     *       *         *    * *   **      *      *    *  
     307021 ACATTCCCTTTCACAGAGCAGGTTTGAAACAATCTTCTCGTACTATCTGGCAGTGGACATTTTGA
         66 ACCTTTCTTTTCATAGAGCAGTTTTGAAACACTCTTTTTGTAGAATCTGGAAGTGGATATTTGGA

              * *   *  ** ** *  *   *            ** *
     307086 GCTCCTTGGGGCCTATGCTGAAAAAGGAAATATCTTCCGAC
        131 GCGCTTTGAGGATTTCGTTGGAAACGGAAATATCTTCATAT

                                 *     *  **       *  *           **    *    
     307127 AAAAACTAGACAGAAGCATTCGCAGAATCACGTTTGTGACGTGTGCACTCAACTGTCAGAATTGA
          1 AAAAACTAGACAGAAGCATTCTCAGAAACATCTTTGTGATGTTTGCACTCAACTCACAGAGTTGA

                 **   ** *      *                            * *            *
     307192 ACCTTGGTTTGGACAGAGCACTTTTGAAACACTCTTTTTGTAGAATCTGCAGGTGGATATTTGGC
         66 ACCTTTCTTTTCATAGAGCAGTTTTGAAACACTCTTTTTGTAGAATCTGGAAGTGGATATTTGGA

            **                         *   *      * *
     307257 TAGCTTTGAGGATTTCGTTGGAAACGGTAATGTCTTCAAAG
        131 GCGCTTTGAGGATTTCGTTGGAAACGGAAATATCTTCATAT

                *                         *   *            *    *            
     307298 AAAATCTAGACAGAAGCATTCTCAGAAACACCTTCGTGATGTTTGCAATCAAGTCACAGAGTTGA
          1 AAAAACTAGACAGAAGCATTCTCAGAAACATCTTTGTGATGTTTGCACTCAACTCACAGAGTTGA

                 * *             *  *                  *             *       
     307363 ACCTTCCGTTTCATAGAGCAGGTTGGAAACACTCTTTTTGTAGTATCTGGAAGTGGACATTTGGA
         66 ACCTTTCTTTTCATAGAGCAGTTTTGAAACACTCTTTTTGTAGAATCTGGAAGTGGATATTTGGA

             *         ** *    * *   *            **  
     307428 GGGCTTTGTAGCCTAT-GTGGAAAAAGGAAATATCTTCCCAT
        131 GCGCTTTG-AGGATTTCGTTGGAAACGGAAATATCTTCATAT

            *  ** *   *     * *            *   *  *   * **        *  * * *   
     307469 GAATGCGAGATAGAAGTAATCTCAGAAACATGTTTATGCTGTATCTACTCAACTAACTGTGCTGA
          1 AAAAACTAGACAGAAGCATTCTCAGAAACATCTTTGTGATGTTTGCACTCAACTCACAGAGTTGA

              *     *  *               *        *  * *       *               
     307534 ACATTTCTATTGATAGAGCAGTTTTGAGACACTCTTCTTTTGGAATCTGCAAGTGGATATTTGGA
         66 ACCTTTCTTTTCATAGAGCAGTTTTGAAACACTCTTTTTGTAGAATCTGGAAGTGGATATTTGGA

            ** *                        *     *      
     307599 TAGATTTGAGGATTTCGTTGGAAACGG-GAT-T-AT-ATAT
        131 GCGCTTTGAGGATTTCGTTGGAAACGGAAATATCTTCATAT

                 *       *               *                 **  *   *         
     307636 AAAAAGTAGACAGCAGCATTCTCAGAAACTTCTTTGTGATGTTTGCATCCAGCTCTCAGAGTTGA
          1 AAAAACTAGACAGAAGCATTCTCAGAAACATCTTTGTGATGTTTGCACTCAACTCACAGAGTTGA

              *  * *          *  *        *        *   **        *  **       
     307701 ACATTCCCTTTCATAGAGTAGGTTTGAAACCCTCTTTTTATAGTGTCTGGAAGCGGGCATTTGGA
         66 ACCTTTCTTTTCATAGAGCAGTTTTGAAACACTCTTTTTGTAGAATCTGGAAGTGGATATTTGGA

                   *    * * **  **  **          * *  *
     307766 GCGCTTTCAGGCCTATGCTTAAAATAGGAAATATCTACCTAC
        131 GCGCTTTGAGG-ATTTCGTTGGAAACGGAAATATCTTCATAT

             *                    *    *  **          ** *        *    *     
     307808 AGAAACTAGACAGAAGCATTCTGAGAATCACGTTTGTGATGTGGGTACTCAACTAACAGTGTTGA
          1 AAAAACTAGACAGAAGCATTCTCAGAAACATCTTTGTGATGTTTGCACTCAACTCACAGAGTTGA

            *  *       *   *            *   *                *               
     307873 TCCATTCTTTTGATACAGCAGTTTTGAACCACACTTTTTGTAGAATCTGCAAGTGGATATTTGGA
         66 ACCTTTCTTTTCATAGAGCAGTTTTGAAACACTCTTTTTGTAGAATCTGGAAGTGGATATTTGGA

            **   *                     *   *      * *
     307938 TAGCTGTGAGGATTTCGTTGGAAACGGGAATGTCTTCAAAG
        131 GCGCTTTGAGGATTTCGTTGGAAACGGAAATATCTTCATAT

                *                         *   *            *    *            
     307979 AAAATCTAGACAGAAGCATTCTCAGAAACACCTTCGTGATGTTTGCAATCAAGTCACAGAGTTGA
          1 AAAAACTAGACAGAAGCATTCTCAGAAACATCTTTGTGATGTTTGCACTCAACTCACAGAGTTGA

                 * *             *  *           *      *             *       
     308044 ACCTTCCGTTTCATAGAGCAGGTTGGAAACACTCTTATTGTAGTATCTGGAAGTGGACATTTGGA
         66 ACCTTTCTTTTCATAGAGCAGTTTTGAAACACTCTTTTTGTAGAATCTGGAAGTGGATATTTGGA

                   *   ** ** *  *   *            **  
     308109 GCGCTTTCAGGCCTATGGTGAAAAAGGAAATATCTTCCCAT
        131 GCGCTTTGAGGATTTCGTTGGAAACGGAAATATCTTCATAT

                  * * *      **      *   * *   *     ** ** *      *    *     
     308150 AAAAACGACATAGAAGCTATCTCAGGAACTTGTTTATGATGCATCTAATCAACTAACAGTGTTGA
          1 AAAAACTAGACAGAAGCATTCTCAGAAACATCTTTGTGATGTTTGCACTCAACTCACAGAGTTGA

                  * ** * *                         * *       *               
     308215 ACCTTTGTACTGACAGAGCAG-TTTGAAACACTCTTTTTTTGGAATCTGCAAGTGGATATTTGGA
         66 ACCTTTCTTTTCATAGAGCAGTTTTGAAACACTCTTTTTGTAGAATCTGGAAGTGGATATTTGGA

            *                           *   *   *    
     308279 TCGCTTTGAGGATTTCGTTGGAAACGG-GAT-GC--AATAT
        131 GCGCTTTGAGGATTTCGTTGGAAACGGAAATATCTTCATAT

                **  *    *     *                 **  *   *  *    *         * 
     308316 AAAACGTACACAGCAGCATACTCAGAAA-ATACTTTGCCATATTTCCATTCAAGTCACAGAGTGG
          1 AAAAACTAGACAGAAGCATTCTCAGAAACAT-CTTTGTGATGTTTGCACTCAACTCACAGAGTTG

               *  * **            *                  *  *             *      
     308380 AACATTCCCATTCATAGAGCAGGTTTGAAACACTCTTTTTGGAGTATCTGGAAGTGGACATTTGG
         65 AACCTTTCTTTTCATAGAGCAGTTTTGAAACACTCTTTTTGTAGAATCTGGAAGTGGATATTTGG

                    ** * * ** *  *   *            **  
     308445 AGCGCTTTCTGAACTATGGTGAAAAAGGAAATATCTTCCAAT
        130 AGCGCTTTGAGGATTTCGTTGGAAACGGAAATATCTTCATAT

            *     *               *      * *          *  **      **  *  *    
     308487 GAAAACAAGACAGAAGCATTCTGAGAAACTTATTTGTGATGTGTGTCCTCAACAAACGGACTTGA
          1 AAAAACTAGACAGAAGCATTCTCAGAAACATCTTTGTGATGTTTGCACTCAACTCACAGAGTTGA

                   *      **  * *  *  *             *   *    * * *           
     308552 ACCTTTCGTTTCATGCAGTACTTCTGGAACACTCTTTTTGAAGATTCTGCATGCGGATATTTGGA
         66 ACCTTTCTTTTCATAGAGCAGTTTTGAAACACTCTTTTTGTAGAATCTGGAAGTGGATATTTGGA

            **                            **   *   * 
     308617 TAGCTTTGAGGATTTCGTTGGAAACGG---GCT-TACATGT
        131 GCGCTTTGAGGATTTCGTTGGAAACGGAAATATCTTCATAT

                 *       *               *        *   *    *    *       *    
     308654 AAAAATTAGACAGCAGCATTCTCAGAAACTTCTTTGTGGTGTCTGCATTCAAGTCACAGAATTGA
          1 AAAAACTAGACAGAAGCATTCTCAGAAACATCTTTGTGATGTTTGCACTCAACTCACAGAGTTGA

              * ** * **            *  * *      *       *    *        *       
     308719 ACTTCCCCTCACATAGAGCAGTTGTGCAGCACTCTATTTGTAGTATCTCGAAGTGGACATTTGGA
         66 ACCTTTCTTTTCATAGAGCAGTTTTGAAACACTCTTTTTGTAGAATCTGGAAGTGGATATTTGGA

             *         ** *    * *   *            **  
     308784 GGGCTTTGTAGCCTATC-TGGAAAAAGGAAATATCTTCCCAT
        131 GCGCTTTG-AGGATTTCGTTGGAAACGGAAATATCTTCATAT

            *  ** *   *     * *            *   *  *   * **        *  * * *   
     308825 GAATGCGAGATAGAAGTAATCTCAGAAACATGTTTATGCTGTATCTACTCAACTAACTGTGCTGA
          1 AAAAACTAGACAGAAGCATTCTCAGAAACATCTTTGTGATGTTTGCACTCAACTCACAGAGTTGA

              *     *  *               *        *  * *       *               
     308890 ACATTTCTATTGATAGAGCAGTTTTGAGACACTCTTCTTTTGGAATCTGCAAGTGGATATTTGGA
         66 ACCTTTCTTTTCATAGAGCAGTTTTGAAACACTCTTTTTGTAGAATCTGGAAGTGGATATTTGGA

            ** *                        *     *      
     308955 TAGATTTGAGGATTTCGTTGGAAACGG-GAT-T-AT-ATAT
        131 GCGCTTTGAGGATTTCGTTGGAAACGGAAATATCTTCATAT

                 *       *               *                 **  *   *         
     308992 AAAAAGTAGACAGCAGCATTCTCAGAAACTTCTTTGTGATGTTTGCATCCAGCTCTCAGAGTTGA
          1 AAAAACTAGACAGAAGCATTCTCAGAAACATCTTTGTGATGTTTGCACTCAACTCACAGAGTTGA

              *  * *          *  *        *        *   **        *  **       
     309057 ACATTCCCTTTCATAGAGTAGGTTTGAAACCCTCTTTTTATAGTGTCTGGAAGCGGGCATTTGGA
         66 ACCTTTCTTTTCATAGAGCAGTTTTGAAACACTCTTTTTGTAGAATCTGGAAGTGGATATTTGGA

             *     *    * * **  **  **          * *  *
     309122 GTGCTTTCAGGCCTATGCTTAAAATAGGAAATATCTACTTAC
        131 GCGCTTTGAGG-ATTTCGTTGGAAACGGAAATATCTTCATAT

             *                    *    *  **          ** *        *    *     
     309164 AGAAACTAGACAGAAGCATTCTGAGAATCACGTTTGTGATGTGGGTACTCAACTAACAGTGTTGA
          1 AAAAACTAGACAGAAGCATTCTCAGAAACATCTTTGTGATGTTTGCACTCAACTCACAGAGTTGA

            *  *       *   *            *   *                *   *           
     309229 TCCATTCTTTTGATACAGCAGTTTTGAACCACACTTTTTGTAGAATCTGCAAGAGGATATTTGGA
         66 ACCTTTCTTTTCATAGAGCAGTTTTGAAACACTCTTTTTGTAGAATCTGGAAGTGGATATTTGGA

            **   *                     *   *      * *
     309294 TAGCTGTGAGGATTTCGTTGGAAACGGGAATGTCTTCAAAG
        131 GCGCTTTGAGGATTTCGTTGGAAACGGAAATATCTTCATAT

                *                         *   *            *    *            
     309335 AAAATCTAGACAGAAGCATTCTCAGAAACACCTTCGTGATGTTTGCAATCAAGTCACAGAGTTGA
          1 AAAAACTAGACAGAAGCATTCTCAGAAACATCTTTGTGATGTTTGCACTCAACTCACAGAGTTGA

                 * *             *  *           *      *             *       
     309400 ACCTTCCGTTTCATAGAGCAGGTTGGAAACACTCTTATTGTAGTATCTGGAAGTGGACATTTGGA
         66 ACCTTTCTTTTCATAGAGCAGTTTTGAAACACTCTTTTTGTAGAATCTGGAAGTGGATATTTGGA

                   *   ** ** *  *   *            **  
     309465 GCGCTTTCAGGCCTATGGTGAAAAAGGAAATATCTTCCCAT
        131 GCGCTTTGAGGATTTCGTTGGAAACGGAAATATCTTCATAT

                  * * *      **      *   * *   *     ** ** *      *    *     
     309506 AAAAACGACATAGAAGCTATCTCAGGAACTTGTTTATGATGCATCTAATCAACTAACAGTGTTGA
          1 AAAAACTAGACAGAAGCATTCTCAGAAACATCTTTGTGATGTTTGCACTCAACTCACAGAGTTGA

                  * ** * *                         * *       *               
     309571 ACCTTTGTACTGACAGAGCAG-TTTGAAACACTCTTTTTTTGGAATCTGCAAGTGGATATTTGGA
         66 ACCTTTCTTTTCATAGAGCAGTTTTGAAACACTCTTTTTGTAGAATCTGGAAGTGGATATTTGGA

            *                           *   *   *    
     309635 TCGCTTTGAGGATTTCGTTGGAAACGG-GAT-GC--AATAT
        131 GCGCTTTGAGGATTTCGTTGGAAACGGAAATATCTTCATAT

                **  *    *     *                 **  *   *  *    *         * 
     309672 AAAACGTACACAGCAGCATACTCAGAAA-ATACTTTGCCATATTTCCATTCAAGTCACAGAGTGG
          1 AAAAACTAGACAGAAGCATTCTCAGAAACAT-CTTTGTGATGTTTGCACTCAACTCACAGAGTTG

               *  * **            *                  *  *             *      
     309736 AACATTCCCATTCATAGAGCAGGTTTGAAACACTCTTTTTGGAGTATCTGGAAGTGGACATTTGG
         65 AACCTTTCTTTTCATAGAGCAGTTTTGAAACACTCTTTTTGTAGAATCTGGAAGTGGATATTTGG

                    ** * * ** *  *   *            **  
     309801 AGCGCTTTCTGAACTATGGTGAAAAAGGAAATATCTTCCAAT
        130 AGCGCTTTGAGGATTTCGTTGGAAACGGAAATATCTTCATAT

            *     * *             *      * *          *  **      **  *  *    
     309843 GAAAACAACACAGAAGCATTCTGAGAAACTTATTTGTGATGTGTGTCCTCAACAAACGGACTTGA
          1 AAAAACTAGACAGAAGCATTCTCAGAAACATCTTTGTGATGTTTGCACTCAACTCACAGAGTTGA

                   *      **  * *  *  *             *   *    * * *           
     309908 ACCTTTCGTTTCATGCAGTACTTCTGGAACACTCTTTTTGAAGATTCTGCATGCGGATATTTGGA
         66 ACCTTTCTTTTCATAGAGCAGTTTTGAAACACTCTTTTTGTAGAATCTGGAAGTGGATATTTGGA

            **                            **   *   * 
     309973 TAGCTTTGAGGATTTCGTTGGAAACGG---GCT-TACATGT
        131 GCGCTTTGAGGATTTCGTTGGAAACGGAAATATCTTCATAT

                 *       *               *        *   *    *    *       *    
     310010 AAAAATTAGACAGCAGCATTCTCAGAAACTTCTTTGTGGTGTCTGCATTCAAGTCACAGAATTGA
          1 AAAAACTAGACAGAAGCATTCTCAGAAACATCTTTGTGATGTTTGCACTCAACTCACAGAGTTGA

              * ** * **            *  * *      *       *             *       
     310075 ACTTCCCCTCACATAGAGCAGTTGTGCAGCACTCTATTTGTAGTATCTGGAAGTGGACATTTGGA
         66 ACCTTTCTTTTCATAGAGCAGTTTTGAAACACTCTTTTTGTAGAATCTGGAAGTGGATATTTGGA

             *         ** *    * *   *            **  
     310140 GGGCTTTGTAGCCTATC-TGGAAAAAGGAAATATCTTCCCAT
        131 GCGCTTTG-AGGATTTCGTTGGAAACGGAAATATCTTCATAT

            *  ** *   *  *  * *            *   *  *   * **        *  * * *   
     310181 GAATGCGAGATAGTAGTAATCTCAGAAACATGTTTATGCTGTATCTACTCAACTAACTGTGCTGA
          1 AAAAACTAGACAGAAGCATTCTCAGAAACATCTTTGTGATGTTTGCACTCAACTCACAGAGTTGA

              *     *  *               *        *  * *       *               
     310246 ACATTTCTATTGATAGAGCAGTTTTGAGACACTCTTCTTTTGGAATCTGCAAGTGGATATTTGGA
         66 ACCTTTCTTTTCATAGAGCAGTTTTGAAACACTCTTTTTGTAGAATCTGGAAGTGGATATTTGGA

            ** *                        *     *      
     310311 TAGATTTGAGGATTTCGTTGGAAACGG-GAT-T-AT-ATAT
        131 GCGCTTTGAGGATTTCGTTGGAAACGGAAATATCTTCATAT

                 *       *               *                 **  *   *         
     310348 AAAAAGTAGACAGCAGCATTCTCAGAAACTTCTTTGTGATGTTTGCATCCAGCTCTCAGAGTTGA
          1 AAAAACTAGACAGAAGCATTCTCAGAAACATCTTTGTGATGTTTGCACTCAACTCACAGAGTTGA

              *  * *          *  *        *        *   **        *  **       
     310413 ACATTCCCTTTCATAGAGTAGGTTTGAAACCCTCTTTTTATAGTGTCTGGAAGCGGGCATTTGGA
         66 ACCTTTCTTTTCATAGAGCAGTTTTGAAACACTCTTTTTGTAGAATCTGGAAGTGGATATTTGGA

                   *   ** ** *  *   *          * *   
     310478 GCGCTTTCAGGCCTATGCTGAAAAAGGAAATATCTACCTAT
        131 GCGCTTTGAGGATTTCGTTGGAAACGGAAATATCTTCATAT

             *                    *    *  **          ** *        *    *     
     310519 AGAAACTAGACAGAAGCATTCTGAGAATCACGTTTGTGATGTGGGTACTCAACTAACAGTGTTGA
          1 AAAAACTAGACAGAAGCATTCTCAGAAACATCTTTGTGATGTTTGCACTCAACTCACAGAGTTGA

            *  *       *   *            *   *                *               
     310584 TCCATTCTTTTGATACAGCAGTTTTGAACCACACTTTTTGTAGAATCTGCAAGTGGATATTTGGA
         66 ACCTTTCTTTTCATAGAGCAGTTTTGAAACACTCTTTTTGTAGAATCTGGAAGTGGATATTTGGA

            **   *                     *   *        *
     310649 TAGCTGTGAGGATTTCGTTGGAAACGGGAATGTCTTCATAG
        131 GCGCTTTGAGGATTTCGTTGGAAACGGAAATATCTTCATAT

                **                *    * * **         *  **   *   *          
     310690 AAAATTTAGACAGAAGCATTCTTAGAACCTTGATTGTGATGTGTGTTCTCCACTAACAGAGTTGA
          1 AAAAACTAGACAGAAGCATTCTCAGAAACATCTTTGTGATGTTTGCACTCAACTCACAGAGTTGA

                       * *   * *   *       *       *                         
     310755 ACCTTTCTTTTGACAGAACTGTTCTGAAACATTCTTTTTATAGAATCTGGAAGTGGATATTTGGA
         66 ACCTTTCTTTTCATAGAGCAGTTTTGAAACACTCTTTTTGTAGAATCTGGAAGTGGATATTTGGA

            **                         *          *  
     310820 AAGCTTTGAGGATTTCGTTGGAAACGGGAATATCTTCAAAT
        131 GCGCTTTGAGGATTTCGTTGGAAACGGAAATATCTTCATAT

                *    *            *           * *       *  *    *            
     310861 AAAATCTAGCCAGAAGCATTCTAAGAAACATCTTAGGGATGTTTACATTCAAGTCACAGAGTTGA
          1 AAAAACTAGACAGAAGCATTCTCAGAAACATCTTTGTGATGTTTGCACTCAACTCACAGAGTTGA

              *  * *     *       *         *    * *   **      *      *    *  
     310926 ACATTCCCTTTCACAGAGCAGGTTTGAAACAATCTTCTCGTACTATCTGGCAGTGGACATTTTGA
         66 ACCTTTCTTTTCATAGAGCAGTTTTGAAACACTCTTTTTGTAGAATCTGGAAGTGGATATTTGGA

              * *   *  ** ** *  *   *            ** *
     310991 GCTCCTTGGGGCCTATGCTGAAAAAGGAAATATCTTCCGAC
        131 GCGCTTTGAGGATTTCGTTGGAAACGGAAATATCTTCATAT

                                 *     *  **          *           **    *    
     311032 AAAAACTAGACAGAAGCATTCGCAGAATCACGTTTGTGATGTGTGCACTCAACTGTCAGAATTGA
          1 AAAAACTAGACAGAAGCATTCTCAGAAACATCTTTGTGATGTTTGCACTCAACTCACAGAGTTGA

                 **   ** *      *                            * *            *
     311097 ACCTTGGTTTGGACAGAGCACTTTTGAAACACTCTTTTTGTAGAATCTGCAGGTGGATATTTGGC
         66 ACCTTTCTTTTCATAGAGCAGTTTTGAAACACTCTTTTTGTAGAATCTGGAAGTGGATATTTGGA

            **                         *   *      * *
     311162 TAGCTTTGAGGATTTCGTTGGAAACGGTAATGTCTTCAAAG
        131 GCGCTTTGAGGATTTCGTTGGAAACGGAAATATCTTCATAT

                *                         *   *            *    *            
     311203 AAAATCTAGACAGAAGCATTCTCAGAAACACCTTCGTGATGTTTGCAATCAAGTCACAGAGTTGA
          1 AAAAACTAGACAGAAGCATTCTCAGAAACATCTTTGTGATGTTTGCACTCAACTCACAGAGTTGA

                 * *             *  *                  *             *       
     311268 ACCTTCCGTTTCATAGAGCAGGTTGGAAACACTCTTTTTGTAGTATCTGGAAGTGGACATTTGGA
         66 ACCTTTCTTTTCATAGAGCAGTTTTGAAACACTCTTTTTGTAGAATCTGGAAGTGGATATTTGGA

                   *   ** ** *  *   *            **  
     311333 GCGCTTTCAGGCCTATGGTGAAAAAGGAAATATCTTCCCAT
        131 GCGCTTTGAGGATTTCGTTGGAAACGGAAATATCTTCATAT

                  * * *      **      *   * *   *     ** ** *      *    *     
     311374 AAAAACGACATAGAAGCTATCTCAGGAACTTGTTTATGATGCATCTAATCAACTAACAGTGTTGA
          1 AAAAACTAGACAGAAGCATTCTCAGAAACATCTTTGTGATGTTTGCACTCAACTCACAGAGTTGA

                  * ** * *                         * *       *               
     311439 ACCTTTGTACTGACAGAGCAG-TTTGAAACACTCTTTTTTTGGAATCTGCAAGTGGATATTTGGA
         66 ACCTTTCTTTTCATAGAGCAGTTTTGAAACACTCTTTTTGTAGAATCTGGAAGTGGATATTTGGA

            *                           *   *   *    
     311503 TCGCTTTGAGGATTTCGTTGGAAACGG-GAT-GC--AATAT
        131 GCGCTTTGAGGATTTCGTTGGAAACGGAAATATCTTCATAT

                **  *    *     *                 **  *   *  *    *         * 
     311540 AAAACGTACACAGCAGCATACTCAGAAA-ATACTTTGCCATATTTCCATTCAAGTCACAGAGTGG
          1 AAAAACTAGACAGAAGCATTCTCAGAAACAT-CTTTGTGATGTTTGCACTCAACTCACAGAGTTG

               *  * **            *                  *  *             *      
     311604 AACATTCCCATTCATAGAGCAGGTTTGAAACACTCTTTTTGGAGTATCTGGAAGTGGACATTTGG
         65 AACCTTTCTTTTCATAGAGCAGTTTTGAAACACTCTTTTTGTAGAATCTGGAAGTGGATATTTGG

                    ** * * ** *  *   *            **  
     311669 AGCGCTTTCTGAACTATGGTGAAAAAGGAAATATCTTCCAAT
        130 AGCGCTTTGAGGATTTCGTTGGAAACGGAAATATCTTCATAT

            *     *               *      * *          *  **      **  *  *    
     311711 GAAAACAAGACAGAAGCATTCTGAGAAACTTATTTGTGATGTGTGTCCTCAACAAACGGACTTGA
          1 AAAAACTAGACAGAAGCATTCTCAGAAACATCTTTGTGATGTTTGCACTCAACTCACAGAGTTGA

                   *      **  * *  *  *             *   *    * * *           
     311776 ACCTTTCGTTTCATGCAGTACTTCTGGAACACTCTTTTTGAAGATTCTGCATGCGGATATTTGGA
         66 ACCTTTCTTTTCATAGAGCAGTTTTGAAACACTCTTTTTGTAGAATCTGGAAGTGGATATTTGGA

            **                            **   *   * 
     311841 TAGCTTTGAGGATTTCGTTGGAAACGG---GCT-TACATGT
        131 GCGCTTTGAGGATTTCGTTGGAAACGGAAATATCTTCATAT

                 *       *               *        *   *    *    *       *    
     311878 AAAAATTAGACAGCAGCATTCTCAGAAACTTCTTTGTGGTGTCTGCATTCAAGTCACAGAATTGA
          1 AAAAACTAGACAGAAGCATTCTCAGAAACATCTTTGTGATGTTTGCACTCAACTCACAGAGTTGA

              * ** * **            *  * *      *       *             *       
     311943 ACTTCCCCTCACATAGAGCAGTTGTGCAGCACTCTATTTGTAGTATCTGGAAGTGGACATTTGGA
         66 ACCTTTCTTTTCATAGAGCAGTTTTGAAACACTCTTTTTGTAGAATCTGGAAGTGGATATTTGGA

             *         ** *    * *   *            **  
     312008 GGGCTTTGTAGCCTATC-TGGAAAAAGGAAATATCTTCCCAT
        131 GCGCTTTG-AGGATTTCGTTGGAAACGGAAATATCTTCATAT

            *  ** *   *     * *            *   *  *   * **        *  * * *   
     312049 GAATGCGAGATAGAAGTAATCTCAGAAACATGTTTATGCTGTATCTACTCAACTAACTGTGCTGA
          1 AAAAACTAGACAGAAGCATTCTCAGAAACATCTTTGTGATGTTTGCACTCAACTCACAGAGTTGA

              *     *  *               *        *  * *       *               
     312114 ACATTTCTATTGATAGAGCAGTTTTGAGACACTCTTCTTTTGGAATCTGCAAGTGGATATTTGGA
         66 ACCTTTCTTTTCATAGAGCAGTTTTGAAACACTCTTTTTGTAGAATCTGGAAGTGGATATTTGGA

            ** *                        *     *      
     312179 TAGATTTGAGGATTTCGTTGGAAACGG-GAT-T-AT-ATAT
        131 GCGCTTTGAGGATTTCGTTGGAAACGGAAATATCTTCATAT

            *    *       *               *                 **  *   *         
     312216 CAAAAGTAGACAGCAGCATTCTCAGAAACTTCTTTGTGATGTTTGCATCCAGCTCTCAGAGTTGA
          1 AAAAACTAGACAGAAGCATTCTCAGAAACATCTTTGTGATGTTTGCACTCAACTCACAGAGTTGA

              *  * *          *  *        *        *   **        *  **       
     312281 ACATTCCCTTTCATAGAGTAGGTTTGAAACCCTCTTTTTATAGTGTCTGGAAGCGGGCATTTGGA
         66 ACCTTTCTTTTCATAGAGCAGTTTTGAAACACTCTTTTTGTAGAATCTGGAAGTGGATATTTGGA

                   *   ** ** *  *   *          * *   
     312346 GCGCTTTCAGGCCTATGCTGAAAAAGGAAATATCTACCTAT
        131 GCGCTTTGAGGATTTCGTTGGAAACGGAAATATCTTCATAT

             *                    *    *  **          ** *        *    *     
     312387 AGAAACTAGACAGAAGCATTCTGAGAATCACGTTTGTGATGTGGGTACTCAACTAACAGTGTTGA
          1 AAAAACTAGACAGAAGCATTCTCAGAAACATCTTTGTGATGTTTGCACTCAACTCACAGAGTTGA

            *  *       *   *            *   *                *               
     312452 TCCATTCTTTTGATACAGCAGTTTTGAACCACACTTTTTGTAGAATCTGCAAGTGGATATTTGGA
         66 ACCTTTCTTTTCATAGAGCAGTTTTGAAACACTCTTTTTGTAGAATCTGGAAGTGGATATTTGGA

            **   *                     *   *        *
     312517 TAGCTGTGAGGATTTCGTTGGAAACGGGAATGTCTTCATAG
        131 GCGCTTTGAGGATTTCGTTGGAAACGGAAATATCTTCATAT

                **                     * * **         *  **   *   *          
     312558 AAAATTTAGACAGAAGCATTCTCAGAACCTTGATTGTGATGTGTGTTCTCCACTAACAGAGTTGA
          1 AAAAACTAGACAGAAGCATTCTCAGAAACATCTTTGTGATGTTTGCACTCAACTCACAGAGTTGA

                       * *   * *   *       *       *                         
     312623 ACCTTTCTTTTGACAGAACTGTTCTGAAACATTCTTTTTATAGAATCTGGAAGTGGATATTTGGA
         66 ACCTTTCTTTTCATAGAGCAGTTTTGAAACACTCTTTTTGTAGAATCTGGAAGTGGATATTTGGA

            **                         *          *  
     312688 AAGCTTTGAGGATTTCGTTGGAAACGGGAATATCTTCAAAT
        131 GCGCTTTGAGGATTTCGTTGGAAACGGAAATATCTTCATAT

                *    *            *           * *       *  *    *            
     312729 AAAATCTAGCCAGAAGCATTCTAAGAAACATCTTAGGGATGTTTACATTCAAGTCACAGAGTTGA
          1 AAAAACTAGACAGAAGCATTCTCAGAAACATCTTTGTGATGTTTGCACTCAACTCACAGAGTTGA

              *  * *     *       *         *    * *   **      *      *    *  
     312794 ACATTCCCTTTCACAGAGCAGGTTTGAAACAATCTTCTCGTACTATCTGGCAGTGGACATTTTGA
         66 ACCTTTCTTTTCATAGAGCAGTTTTGAAACACTCTTTTTGTAGAATCTGGAAGTGGATATTTGGA

              * *   *  ** ** *  *   *            ** *
     312859 GCTCCTTGGGGCCTATGCTGAAAAAGGAAATATCTTCCGAC
        131 GCGCTTTGAGGATTTCGTTGGAAACGGAAATATCTTCATAT

                                 *     *  **          *           **    *    
     312900 AAAAACTAGACAGAAGCATTCGCAGAATCACGTTTGTGATGTGTGCACTCAACTGTCAGAATTGA
          1 AAAAACTAGACAGAAGCATTCTCAGAAACATCTTTGTGATGTTTGCACTCAACTCACAGAGTTGA

                 **   ** *      *                            * *            *
     312965 ACCTTGGTTTGGACAGAGCACTTTTGAAACACTCTTTTTGTAGAATCTGCAGGTGGATATTTGGC
         66 ACCTTTCTTTTCATAGAGCAGTTTTGAAACACTCTTTTTGTAGAATCTGGAAGTGGATATTTGGA

            **                         *   *      * *
     313030 TAGCTTTGAGGATTTCGTTGGAAACGGTAATGTCTTCAAAG
        131 GCGCTTTGAGGATTTCGTTGGAAACGGAAATATCTTCATAT

                *                         *   *            *    *            
     313071 AAAATCTAGACAGAAGCATTCTCAGAAACACCTTCGTGATGTTTGCAATCAAGTCACAGAGTTGA
          1 AAAAACTAGACAGAAGCATTCTCAGAAACATCTTTGTGATGTTTGCACTCAACTCACAGAGTTGA

                 * *             *  *                  *             *       
     313136 ACCTTCCGTTTCATAGAGCAGGTTGGAAACACTCTTTTTGTAGTATCTGGAAGTGGACATTTGGA
         66 ACCTTTCTTTTCATAGAGCAGTTTTGAAACACTCTTTTTGTAGAATCTGGAAGTGGATATTTGGA

             *         ** *    * *   *            **  
     313201 GGGCTTTGTAGCCTATC-TGGAAAAAGGAAATATCTTCCCAT
        131 GCGCTTTG-AGGATTTCGTTGGAAACGGAAATATCTTCATAT

            *  ** *   *     * *            *   *  *   * **        *  * * *   
     313242 GAATGCGAGATAGAAGTAATCTCAGAAACATGTTTATGCTGTATCTACTCAACTAACTGTGCTGA
          1 AAAAACTAGACAGAAGCATTCTCAGAAACATCTTTGTGATGTTTGCACTCAACTCACAGAGTTGA

              *     *  *               *        *  * *       *               
     313307 ACATTTCTATTGATAGAGCAGTTTTGAGACACTCTTCTTTTGGAATCTGCAAGTGGATATTTGGA
         66 ACCTTTCTTTTCATAGAGCAGTTTTGAAACACTCTTTTTGTAGAATCTGGAAGTGGATATTTGGA

            ** *                        **    *      
     313372 TAGATTTGAGGATTTCGTTGGAAACGG-GGT-T-AT-ATAT
        131 GCGCTTTGAGGATTTCGTTGGAAACGGAAATATCTTCATAT

                 *       *               *                 **  *   *         
     313409 AAAAAGTAGACAGCAGCATTCTCAGAAACTTCTTTGTGATGTTTGCATCCAGCTCTCAGAGTTGA
          1 AAAAACTAGACAGAAGCATTCTCAGAAACATCTTTGTGATGTTTGCACTCAACTCACAGAGTTGA

              *  * *          *  *        *        *   **        *  **       
     313474 ACATTCCCTTTCATAGAGTAGGTTTGAAACCCTCTTTTTATAGTGTCTGGAAGCGGGCATTTGGA
         66 ACCTTTCTTTTCATAGAGCAGTTTTGAAACACTCTTTTTGTAGAATCTGGAAGTGGATATTTGGA

                   *    * * **  **  **          * *  *
     313539 GCGCTTTCAGGCCTATGCTTAAAATAGGAAATATCTACCTAC
        131 GCGCTTTGAGG-ATTTCGTTGGAAACGGAAATATCTTCATAT

             *                    *    *  **          ** *        *    *     
     313581 AGAAACTAGACAGAAGCATTCTGAGAATCACGTTTGTGATGTGGGTACTCAACTAACAGTGTTGA
          1 AAAAACTAGACAGAAGCATTCTCAGAAACATCTTTGTGATGTTTGCACTCAACTCACAGAGTTGA

            *  *       *   *            *   *                *   *           
     313646 TCCATTCTTTTGATACAGCAGTTTTGAACCACACTTTTTGTAGAATCTGCAAGAGGATATTTGGA
         66 ACCTTTCTTTTCATAGAGCAGTTTTGAAACACTCTTTTTGTAGAATCTGGAAGTGGATATTTGGA

            **   *                     *   *      * *
     313711 TAGCTGTGAGGATTTCGTTGGAAACGGGAATGTCTTCAAAG
        131 GCGCTTTGAGGATTTCGTTGGAAACGGAAATATCTTCATAT

                *                         *   *            *    *            
     313752 AAAATCTAGACAGAAGCATTCTCAGAAACACCTTCGTGATGTTTGCAATCAAGTCACAGAGTTGA
          1 AAAAACTAGACAGAAGCATTCTCAGAAACATCTTTGTGATGTTTGCACTCAACTCACAGAGTTGA

                 * *             *  *           *      *             *       
     313817 ACCTTCCGTTTCATAGAGCAGGTTGGAAACACTCTTATTGTAGTATCTGGAAGTGGACATTTGGA
         66 ACCTTTCTTTTCATAGAGCAGTTTTGAAACACTCTTTTTGTAGAATCTGGAAGTGGATATTTGGA

                   *   ** ** *  *   *            **  
     313882 GCGCTTTCAGGCCTATGGTGAAAAAGGAAATATCTTCCCAT
        131 GCGCTTTGAGGATTTCGTTGGAAACGGAAATATCTTCATAT

                  * * *      **      *   * *   *     ** ** *      *    *     
     313923 AAAAACGACATAGAAGCTATCTCAGGAACTTGTTTATGATGCATCTAATCAACTAACAGTGTTGA
          1 AAAAACTAGACAGAAGCATTCTCAGAAACATCTTTGTGATGTTTGCACTCAACTCACAGAGTTGA

                  * ** * *                         * *       *               
     313988 ACCTTTGTACTGACAGAGCAG-TTTGAAACACTCTTTTTTTGGAATCTGCAAGTGGATATTTGGA
         66 ACCTTTCTTTTCATAGAGCAGTTTTGAAACACTCTTTTTGTAGAATCTGGAAGTGGATATTTGGA

            *                           *   *   *    
     314052 TCGCTTTGAGGATTTCGTTGGAAACGG-GAT-GC--AATAT
        131 GCGCTTTGAGGATTTCGTTGGAAACGGAAATATCTTCATAT

                **  *    *     *                 **  *   *  *    *         * 
     314089 AAAACGTACACAGCAGCATACTCAGAAA-ATACTTTGCCATATTTCCATTCAAGTCACAGAGTGG
          1 AAAAACTAGACAGAAGCATTCTCAGAAACAT-CTTTGTGATGTTTGCACTCAACTCACAGAGTTG

               *  * **            *                  *  *             *      
     314153 AACATTCCCATTCATAGAGCAGGTTTGAAACACTCTTTTTGGAGTATCTGGAAGTGGACATTTGG
         65 AACCTTTCTTTTCATAGAGCAGTTTTGAAACACTCTTTTTGTAGAATCTGGAAGTGGATATTTGG

                    ** * * ** *  *   *            **  
     314218 AGCGCTTTCTGAACTATGGTGAAAAAGGAAATATCTTCCAAT
        130 AGCGCTTTGAGGATTTCGTTGGAAACGGAAATATCTTCATAT

            *     *               *      * *          *  **      **  *  *    
     314260 GAAAACAAGACAGAAGCATTCTGAGAAACTTATTTGTGATGTGTGTCCTCAACAAACGGACTTGA
          1 AAAAACTAGACAGAAGCATTCTCAGAAACATCTTTGTGATGTTTGCACTCAACTCACAGAGTTGA

                   *      **  * *  *  *             *   *    * * *           
     314325 ACCTTTCGTTTCATGCAGTACTTCTGGAACACTCTTTTTGAAGATTCTGCATGCGGATATTTGGA
         66 ACCTTTCTTTTCATAGAGCAGTTTTGAAACACTCTTTTTGTAGAATCTGGAAGTGGATATTTGGA

            **                            **   *   * 
     314390 TTGCTTTGAGGATTTCGTTGGAAACGG---GCT-TACATGT
        131 GCGCTTTGAGGATTTCGTTGGAAACGGAAATATCTTCATAT

                 *       *               *        *   *    *    *       *    
     314427 AAAAATTAGACAGCAGCATTCTCAGAAACTTCTTTGTGGTGTCTGCATTCAAGTCACAGAATTGA
          1 AAAAACTAGACAGAAGCATTCTCAGAAACATCTTTGTGATGTTTGCACTCAACTCACAGAGTTGA

              * ** * **            *  * *      *       *             *       
     314492 ACTTCCCCTCACATAGAGCAGTTGTGCAGCACTCTATTTGTAGTATCTGGAAGTGGACATTTGGA
         66 ACCTTTCTTTTCATAGAGCAGTTTTGAAACACTCTTTTTGTAGAATCTGGAAGTGGATATTTGGA

             *         ** *    * *   *            **  
     314557 GGGCTTTGTAGCCTATC-TGGAAAAAGGAAATATCTTCCCAT
        131 GCGCTTTG-AGGATTTCGTTGGAAACGGAAATATCTTCATAT

            *  ** *   *     * *            *   *  *   * **        *  * * *   
     314598 GAATGCGAGATAGAAGTAATCTCAGAAACATGTTTATGCTGTATCTACTCAACTAACTGTGCTGA
          1 AAAAACTAGACAGAAGCATTCTCAGAAACATCTTTGTGATGTTTGCACTCAACTCACAGAGTTGA

              *     *  *               *        *  * *       *               
     314663 ACATTTCTATTGATAGAGCAGTTTTGAGACACTCTTCTTTTGGAATCTGCAAGTGGATATTTGGA
         66 ACCTTTCTTTTCATAGAGCAGTTTTGAAACACTCTTTTTGTAGAATCTGGAAGTGGATATTTGGA

            ** *                        *     *      
     314728 TAGATTTGAGGATTTCGTTGGAAACGG-GAT-T-AT-ATAT
        131 GCGCTTTGAGGATTTCGTTGGAAACGGAAATATCTTCATAT

                 *       *               *                 **  *   *         
     314765 AAAAAGTAGACAGCAGCATTCTCAGAAACTTCTTTGTGATGTTTGCATCCAGCTCTCAGAGTTGA
          1 AAAAACTAGACAGAAGCATTCTCAGAAACATCTTTGTGATGTTTGCACTCAACTCACAGAGTTGA

              *  * *          *  *        *        *   **        *  **       
     314830 ACATTCCCTTTCATAGAGTAGGTTTGAAACCCTCTTTTTATAGTGTCTGGAAGCGGGCATTTGGA
         66 ACCTTTCTTTTCATAGAGCAGTTTTGAAACACTCTTTTTGTAGAATCTGGAAGTGGATATTTGGA

                   *    * * **  **  **          * *  *
     314895 GCGCTTTCAGGCCTATGCTTAAAATAGGAAATATCTACCTAC
        131 GCGCTTTGAGG-ATTTCGTTGGAAACGGAAATATCTTCATAT

             *                    *    *  **          ** *        *    *     
     314937 AGAAACTAGACAGAAGCATTCTGAGAATCACGTTTGTGATGTGGGTACTCAACTAACAGTGTTGA
          1 AAAAACTAGACAGAAGCATTCTCAGAAACATCTTTGTGATGTTTGCACTCAACTCACAGAGTTGA

            *  *       *   *            *   *                *   *           
     315002 TCCATTCTTTTGATACAGCAGTTTTGAACCACACTTTTTGTAGAATCTGCAAGAGGATATTTGGA
         66 ACCTTTCTTTTCATAGAGCAGTTTTGAAACACTCTTTTTGTAGAATCTGGAAGTGGATATTTGGA

            **   *                     *   *      * *
     315067 TAGCTGTGAGGATTTCGTTGGAAACGGGAATGTCTTCAAAG
        131 GCGCTTTGAGGATTTCGTTGGAAACGGAAATATCTTCATAT

                *                         *   *            *    *            
     315108 AAAATCTAGACAGAAGCATTCTCAGAAACACCTTCGTGATGTTTGCAATCAAGTCACAGAGTTGA
          1 AAAAACTAGACAGAAGCATTCTCAGAAACATCTTTGTGATGTTTGCACTCAACTCACAGAGTTGA

                 * *             *  *           *      *             *       
     315173 ACCTTCCGTTTCATAGAGCAGGTTGGAAACACTCTTATTGTAGTATCTGGAAGTGGACATTTGGA
         66 ACCTTTCTTTTCATAGAGCAGTTTTGAAACACTCTTTTTGTAGAATCTGGAAGTGGATATTTGGA

                   *   ** ** *  *   *            **  
     315238 GCGCTTTCAGGCCTATGGTGAAAAAGGAAATATCTTCCCAT
        131 GCGCTTTGAGGATTTCGTTGGAAACGGAAATATCTTCATAT

                  * * *      **      *   * *   *     ** ** *      *    *     
     315279 AAAAACGACATAGAAGCTATCTCAGGAACTTGTTTATGATGCATCTAATCAACTAACAGTGTTGA
          1 AAAAACTAGACAGAAGCATTCTCAGAAACATCTTTGTGATGTTTGCACTCAACTCACAGAGTTGA

                  * ** * *                         * *       *               
     315344 ACCTTTGTACTGACAGAGCAG-TTTGAAACACTCTTTTTTTGGAATCTGCAAGTGGATATTTGGA
         66 ACCTTTCTTTTCATAGAGCAGTTTTGAAACACTCTTTTTGTAGAATCTGGAAGTGGATATTTGGA

            *                           *   *   *    
     315408 TCGCTTTGAGGATTTCGTTGGAAACGG-GAT-GC--AATAT
        131 GCGCTTTGAGGATTTCGTTGGAAACGGAAATATCTTCATAT

                **  *    *     *                 **  *   *  *    *         * 
     315445 AAAACGTACACAGCAGCATACTCAGAAA-ATACTTTGCCATATTTCCATTCAAGTCACAGAGTGG
          1 AAAAACTAGACAGAAGCATTCTCAGAAACAT-CTTTGTGATGTTTGCACTCAACTCACAGAGTTG

               *  * **            *                  *  *             *      
     315509 AACATTCCCATTCATAGAGCAGGTTTGAAACACTCTTTTTGGAGTATCTGGAAGTGGACATTTGG
         65 AACCTTTCTTTTCATAGAGCAGTTTTGAAACACTCTTTTTGTAGAATCTGGAAGTGGATATTTGG

                    ** * * ** *  *   *            **  
     315574 AGCGCTTTCTGAACTATGGTGAAAAAGGAAATATCTTCCAAT
        130 AGCGCTTTGAGGATTTCGTTGGAAACGGAAATATCTTCATAT

            *     * *             *      * *          *  **      **  *  *    
     315616 GAAAACAACACAGAAGCATTCTGAGAAACTTATTTGTGATGTGTGTCCTCAACAAACGGACTTGA
          1 AAAAACTAGACAGAAGCATTCTCAGAAACATCTTTGTGATGTTTGCACTCAACTCACAGAGTTGA

                   *      **  * *  *  *             *   *    * ***           
     315681 ACCTTTCGTTTCATGCAGTACTTCTGGAACACTCTTTTTGAAGATTCTGCATTCGGATATTTGGA
         66 ACCTTTCTTTTCATAGAGCAGTTTTGAAACACTCTTTTTGTAGAATCTGGAAGTGGATATTTGGA

            **                            **   *   * 
     315746 TAGCTTTGAGGATTTCGTTGGAAACGG---GCT-TACATGT
        131 GCGCTTTGAGGATTTCGTTGGAAACGGAAATATCTTCATAT

                 *       *               *        *   *    *    *       *    
     315783 AAAAATTAGACAGCAGCATTCTCAGAAACTTCTTTGTGGTGTCTGCATTCAAGTCACAGAATTGA
          1 AAAAACTAGACAGAAGCATTCTCAGAAACATCTTTGTGATGTTTGCACTCAACTCACAGAGTTGA

              * ** * **            *  * *      *       *    *        *       
     315848 ACTTCCCCTCACATAGAGCAGTTGTGCAGCACTCTATTTGTAGTATCTCGAAGTGGACATTTGGA
         66 ACCTTTCTTTTCATAGAGCAGTTTTGAAACACTCTTTTTGTAGAATCTGGAAGTGGATATTTGGA

             *         ** *    * *   *            **  
     315913 GGGCTTTGTAGCCTATC-TGGAAAAAGGAAATATCTTCCCAT
        131 GCGCTTTG-AGGATTTCGTTGGAAACGGAAATATCTTCATAT

            *  ** *   *     * *            *   *  *   * **        *  * * *   
     315954 GAATGCGAGATAGAAGTAATCTCAGAAACATGTTTATGCTGTATCTACTCAACTAACTGTGCTGA
          1 AAAAACTAGACAGAAGCATTCTCAGAAACATCTTTGTGATGTTTGCACTCAACTCACAGAGTTGA

              *     *  *               *        *  * *       *               
     316019 ACATTTCTATTGATAGAGCAGTTTTGAGACACTCTTCTTTTGGAATCTGCAAGTGGATATTTGGA
         66 ACCTTTCTTTTCATAGAGCAGTTTTGAAACACTCTTTTTGTAGAATCTGGAAGTGGATATTTGGA

            ** *                        *     *      
     316084 TAGATTTGAGGATTTCGTTGGAAACGG-GAT-T-AT-ATAT
        131 GCGCTTTGAGGATTTCGTTGGAAACGGAAATATCTTCATAT

                 *       *               *                 **  *   *         
     316121 AAAAAGTAGACAGCAGCATTCTCAGAAACTTCTTTGTGATGTTTGCATCCAGCTCTCAGAGTTGA
          1 AAAAACTAGACAGAAGCATTCTCAGAAACATCTTTGTGATGTTTGCACTCAACTCACAGAGTTGA

              *  * *          *  *        *        *   **        *  **       
     316186 ACATTCCCTTTCATAGAGTAGGTTTGAAACCCTCTTTTTATAGTGTCTGGAAGCGGGCATTTGGA
         66 ACCTTTCTTTTCATAGAGCAGTTTTGAAACACTCTTTTTGTAGAATCTGGAAGTGGATATTTGGA

                   *    * * **  **  **          * *  *
     316251 GCGCTTTCAGGCCTATGCTTAAAATAGGAAATATCTACCTAC
        131 GCGCTTTGAGG-ATTTCGTTGGAAACGGAAATATCTTCATAT

             *                    *    *  **          ** *        *    *     
     316293 AGAAACTAGACAGAAGCATTCTGAGAATCACGTTTGTGATGTGGGTACTCAACTAACAGTGTTGA
          1 AAAAACTAGACAGAAGCATTCTCAGAAACATCTTTGTGATGTTTGCACTCAACTCACAGAGTTGA

            *  *       *   *            *   *                *   *           
     316358 TCCATTCTTTTGATACAGCAGTTTTGAACCACACTTTTTGTAGAATCTGCAAGAGGATATTTGGA
         66 ACCTTTCTTTTCATAGAGCAGTTTTGAAACACTCTTTTTGTAGAATCTGGAAGTGGATATTTGGA

            **   *                     *   *      * *
     316423 TAGCTGTGAGGATTTCGTTGGAAACGGGAATGTCTTCAAAG
        131 GCGCTTTGAGGATTTCGTTGGAAACGGAAATATCTTCATAT

                *                         *   *            *    *            
     316464 AAAATCTAGACAGAAGCATTCTCAGAAACACCTTCGTGATGTTTGCAATCAAGTCACAGAGTTGA
          1 AAAAACTAGACAGAAGCATTCTCAGAAACATCTTTGTGATGTTTGCACTCAACTCACAGAGTTGA

                 * *             *  *           *      *             *       
     316529 ACCTTCCGTTTCATAGAGCAGGTTGGAAACACTCTTATTGTAGTATCTGGAAGTGGACATTTGGA
         66 ACCTTTCTTTTCATAGAGCAGTTTTGAAACACTCTTTTTGTAGAATCTGGAAGTGGATATTTGGA

                   *   ** ** *  *   *            **  
     316594 GCGCTTTCAGGCCTATGGTGAAAAAGGAAATATCTTCCCAT
        131 GCGCTTTGAGGATTTCGTTGGAAACGGAAATATCTTCATAT

                  * * *      **      *   * *   *     ** ** *      *    *     
     316635 AAAAACGACATAGAAGCTATCTCAGGAACTTGTTTATGATGCATCTAATCAACTAACAGTGTTGA
          1 AAAAACTAGACAGAAGCATTCTCAGAAACATCTTTGTGATGTTTGCACTCAACTCACAGAGTTGA

                  * ** * *                         * *       *               
     316700 ACCTTTGTACTGACAGAGCAG-TTTGAAACACTCTTTTTTTGGAATCTGAAAGTGGATATTTGGA
         66 ACCTTTCTTTTCATAGAGCAGTTTTGAAACACTCTTTTTGTAGAATCTGGAAGTGGATATTTGGA

            *                           *   *   *    
     316764 TCGCTTTGAGGATTTCGTTGGAAACGG-GAT-GC--AATAT
        131 GCGCTTTGAGGATTTCGTTGGAAACGGAAATATCTTCATAT

                **  *    *     *                 **  *   *  *    *         * 
     316801 AAAACGTACACAGCAGCATACTCAGAAA-ATACTTTGCCATATTTCCATTCAAGTCACAGAGTGG
          1 AAAAACTAGACAGAAGCATTCTCAGAAACAT-CTTTGTGATGTTTGCACTCAACTCACAGAGTTG

               *  * **            *                  *  *             *      
     316865 AACATTCCCATTCATAGAGCAGGTTTGAAACACTCTTTTTGGAGTATCTGGAAGTGGACATTTGG
         65 AACCTTTCTTTTCATAGAGCAGTTTTGAAACACTCTTTTTGTAGAATCTGGAAGTGGATATTTGG

                    ** * * ** *  *   *            **  
     316930 AGCGCTTTCTGAACTATGGTGAAAAAGGAAATATCTTCCAAT
        130 AGCGCTTTGAGGATTTCGTTGGAAACGGAAATATCTTCATAT

            *     *             * *      * *          *  **      **  *  *    
     316972 GAAAACAAGACAGAAGCATTGTGAGAAACTTATTTGTGATGTGTGTCCTCAACAAACGGACTTGA
          1 AAAAACTAGACAGAAGCATTCTCAGAAACATCTTTGTGATGTTTGCACTCAACTCACAGAGTTGA

                   *      **  * *  *  *             *   *    * * *           
     317037 ACCTTTCGTTTCATGCAGTACTTCTGGAACACTCTTTTTGAAGATTCTGCATGCGGATATTTGGA
         66 ACCTTTCTTTTCATAGAGCAGTTTTGAAACACTCTTTTTGTAGAATCTGGAAGTGGATATTTGGA

            **                            **   *   * 
     317102 TAGCTTTGAGGATTTCGTTGGAAACGG---GCT-TACATGT
        131 GCGCTTTGAGGATTTCGTTGGAAACGGAAATATCTTCATAT

                 *       *               *        *   *    **   *       *    
     317139 AAAAATTAGACAGCAGCATTCTCAGAAACTTCTTTGTGGTGTCTGCATCCAAGTCACAGAATTGA
          1 AAAAACTAGACAGAAGCATTCTCAGAAACATCTTTGTGATGTTTGCACTCAACTCACAGAGTTGA

              * ** * **            *  * *      *       *             *       
     317204 ACTTCCCCTCACATAGAGCAGTTGTGCAGCACTCTATTTGTAGTATCTGGAAGTGGACATTTGGA
         66 ACCTTTCTTTTCATAGAGCAGTTTTGAAACACTCTTTTTGTAGAATCTGGAAGTGGATATTTGGA

             *         ** *    * *   *            **  
     317269 GGGCTTTGTAGCCTATC-TGGAAAAAGGAAATATCTTCCCAT
        131 GCGCTTTG-AGGATTTCGTTGGAAACGGAAATATCTTCATAT

            *  ** *   *     * *            *   *  *   * **        *  * * *   
     317310 GAATGCGAGATAGAAGTAATCTCAGAAACATGTTTATGCTGTATCTACTCAACTAACTGTGCTGA
          1 AAAAACTAGACAGAAGCATTCTCAGAAACATCTTTGTGATGTTTGCACTCAACTCACAGAGTTGA

              *     *  *               *        *  * *       *               
     317375 ACATTTCTATTGATAGAGCAGTTTTGAGACACTCTTCTTTTGGAATCTGCAAGTGGATATTTGGA
         66 ACCTTTCTTTTCATAGAGCAGTTTTGAAACACTCTTTTTGTAGAATCTGGAAGTGGATATTTGGA

            ** *                        *     *      
     317440 TAGATTTGAGGATTTCGTTGGAAACGG-GAT-T-AT-ATAT
        131 GCGCTTTGAGGATTTCGTTGGAAACGGAAATATCTTCATAT

                 *       *               *                 **  *   *         
     317477 AAAAAGTAGACAGCAGCATTCTCAGAAACTTCTTTGTGATGTTTGCATCCAGCTCTCAGAGTTGA
          1 AAAAACTAGACAGAAGCATTCTCAGAAACATCTTTGTGATGTTTGCACTCAACTCACAGAGTTGA

              *  * *          *  *        *        *   **        *  **       
     317542 ACATTCCCTTTCATAGAGTAGGTTTGAAACCCTCTTTTTATAGTGTCTGGAAGCGGGCATTTGGA
         66 ACCTTTCTTTTCATAGAGCAGTTTTGAAACACTCTTTTTGTAGAATCTGGAAGTGGATATTTGGA

                   *    * * **  **  **          * *  *
     317607 GCGCTTTCAGGCCTATGCTTAAAATAGGAAATATCTACCTAC
        131 GCGCTTTGAGG-ATTTCGTTGGAAACGGAAATATCTTCATAT

             *                    *    *  **          ** *        *    *     
     317649 AGAAACTAGACAGAAGCATTCTGAGAATCACGTTTGTGATGTGGGTACTCAACTAACAGTGTTGA
          1 AAAAACTAGACAGAAGCATTCTCAGAAACATCTTTGTGATGTTTGCACTCAACTCACAGAGTTGA

            *  *       *   *            *   *    *           *   *           
     317714 TCCATTCTTTTGATACAGCAGTTTTGAACCACACTTTCTGTAGAATCTGCAAGAGGATATTTGGA
         66 ACCTTTCTTTTCATAGAGCAGTTTTGAAACACTCTTTTTGTAGAATCTGGAAGTGGATATTTGGA

            **   *                     *   *      * *
     317779 TAGCTGTGAGGATTTCGTTGGAAACGGGAATGTCTTCAAAG
        131 GCGCTTTGAGGATTTCGTTGGAAACGGAAATATCTTCATAT

                *                         *   *            *    *            
     317820 AAAATCTAGACAGAAGCATTCTCAGAAACACCTTCGTGATGTTTGCAATCAAGTCACAGAGTTGA
          1 AAAAACTAGACAGAAGCATTCTCAGAAACATCTTTGTGATGTTTGCACTCAACTCACAGAGTTGA

                 * *             *  *           *      *             *       
     317885 ACCTTCCGTTTCATAGAGCAGGTTGGAAACACTCTTATTGTAGTATCTGGAAGTGGACATTTGGA
         66 ACCTTTCTTTTCATAGAGCAGTTTTGAAACACTCTTTTTGTAGAATCTGGAAGTGGATATTTGGA

                   *   ** ** *  *   *            **  
     317950 GCGCTTTCAGGCCTATGGTGAAAAAGGAAATATCTTCCCAT
        131 GCGCTTTGAGGATTTCGTTGGAAACGGAAATATCTTCATAT

                  * * *      **      *   * *   *     ** ** *      *    *     
     317991 AAAAACGACATAGAAGCTATCTCAGGAACTTGTTTATGATGCATCTAATCAACTAACAGTGTTGA
          1 AAAAACTAGACAGAAGCATTCTCAGAAACATCTTTGTGATGTTTGCACTCAACTCACAGAGTTGA

                  * ** * *                         * *       *               
     318056 ACCTTTGTACTGACAGAGCAG-TTTGAAACACTCTTTTTTTGGAATCTGCAAGTGGATATTTGGA
         66 ACCTTTCTTTTCATAGAGCAGTTTTGAAACACTCTTTTTGTAGAATCTGGAAGTGGATATTTGGA

            *                           *   *   *    
     318120 TCGCTTTGAGGATTTCGTTGGAAACGG-GAT-GC--AATAT
        131 GCGCTTTGAGGATTTCGTTGGAAACGGAAATATCTTCATAT

                **  *    *     *                 **  *   *  *    *         * 
     318157 AAAACGTACACAGCAGCATACTCAGAAA-ATACTTTGCCATATTTCCATTCAAGTCACAGAGTGG
          1 AAAAACTAGACAGAAGCATTCTCAGAAACAT-CTTTGTGATGTTTGCACTCAACTCACAGAGTTG

               *  * **            *                  *  *             *      
     318221 AACATTCCCATTCATAGAGCAGGTTTGAAACACTCTTTTTGGAGTATCTGGAAGTGGACATTTGG
         65 AACCTTTCTTTTCATAGAGCAGTTTTGAAACACTCTTTTTGTAGAATCTGGAAGTGGATATTTGG

                    ** * * ** *  *   *            **  
     318286 AGCGCTTTCTGAACTATGGTGAAAAAGGAAATATCTTCCAAT
        130 AGCGCTTTGAGGATTTCGTTGGAAACGGAAATATCTTCATAT

            *     *               *      * *          *  **      **  *  *    
     318328 GAAAACAAGACAGAAGCATTCTGAGAAACTTATTTGTGATGTGTGTCCTCAACAAACGGACTTGA
          1 AAAAACTAGACAGAAGCATTCTCAGAAACATCTTTGTGATGTTTGCACTCAACTCACAGAGTTGA

                   *      **  * *  *  *             *   *    * ***           
     318393 ACCTTTCGTTTCATGCAGTACTTCTGGAACACTCTTTTTGAAGATTCTGCATTCGGATATTTGGA
         66 ACCTTTCTTTTCATAGAGCAGTTTTGAAACACTCTTTTTGTAGAATCTGGAAGTGGATATTTGGA

            **                            **   *   * 
     318458 TAGCTTTGAGGATTTCGTTGGAAACGG---GCT-TACATGT
        131 GCGCTTTGAGGATTTCGTTGGAAACGGAAATATCTTCATAT

                 *       *               *        *   *    *    *       *    
     318495 AAAAATTAGACAGCAGCATTCTCAGAAACTTCTTTGTGGTGTCTGCATTCAAGTCACAGAATTGA
          1 AAAAACTAGACAGAAGCATTCTCAGAAACATCTTTGTGATGTTTGCACTCAACTCACAGAGTTGA

              * ** * **            *  * *      *       *    *        *       
     318560 ACTTCCCCTCACATAGAGCAGTTGTGCAGCACTCTATTTGTAGTATCTCGAAGTGGACATTTGGA
         66 ACCTTTCTTTTCATAGAGCAGTTTTGAAACACTCTTTTTGTAGAATCTGGAAGTGGATATTTGGA

             *         ** *    * *   *            **  
     318625 GGGCTTTGTAGCCTATC-TGGAAAAAGGAAATATCTTCCCAT
        131 GCGCTTTG-AGGATTTCGTTGGAAACGGAAATATCTTCATAT

            *  ** *   *     * *            *   *  *   * **        *  * * *   
     318666 GAATGCGAGATAGAAGTAATCTCAGAAACATGTTTATGCTGTATCTACTCAACTAACTGTGCTGA
          1 AAAAACTAGACAGAAGCATTCTCAGAAACATCTTTGTGATGTTTGCACTCAACTCACAGAGTTGA

              *     *  *               *        *  * *       *               
     318731 ACATTTCTATTGATAGAGCAGTTTTGAGACACTCTTCTTTTGGAATCTGCAAGTGGATATTTGGA
         66 ACCTTTCTTTTCATAGAGCAGTTTTGAAACACTCTTTTTGTAGAATCTGGAAGTGGATATTTGGA

            ** *                        *     *      
     318796 TAGATTTGAGGATTTCGTTGGAAACGG-GAT-T-AT-ATAT
        131 GCGCTTTGAGGATTTCGTTGGAAACGGAAATATCTTCATAT

                 *       *               *                 **  *   *         
     318833 AAAAAGTAGACAGCAGCATTCTCAGAAACTTCTTTGTGATGTTTGCATCCAGCTCTCAGAGTTGA
          1 AAAAACTAGACAGAAGCATTCTCAGAAACATCTTTGTGATGTTTGCACTCAACTCACAGAGTTGA

              *  * *          *  *        *        *   **        *  **       
     318898 ACATTCCCTTTCATAGAGTAGGTTTGAAACCCTCTTTTTATAGTGTCTGGAAGCGGGCATTTGGA
         66 ACCTTTCTTTTCATAGAGCAGTTTTGAAACACTCTTTTTGTAGAATCTGGAAGTGGATATTTGGA

                   *    * * **  **  **          * *  *
     318963 GCGCTTTCAGGCCTATGCTTAAAATAGGAAATATCTACCTAC
        131 GCGCTTTGAGG-ATTTCGTTGGAAACGGAAATATCTTCATAT

             *                    *    *  **          ** *        *    *     
     319005 AGAAACTAGACAGAAGCATTCTGAGAATCACGTTTGTGATGTGGGTACTCAACTAACAGTGTTGA
          1 AAAAACTAGACAGAAGCATTCTCAGAAACATCTTTGTGATGTTTGCACTCAACTCACAGAGTTGA

            *  *       *   *            *   *                *   *           
     319070 TCCATTCTTTTGATACAGCAGTTTTGAACCACACTTTTTGTAGAATCTGCAAGAGGATATTTGGA
         66 ACCTTTCTTTTCATAGAGCAGTTTTGAAACACTCTTTTTGTAGAATCTGGAAGTGGATATTTGGA

            **   *                     *   *      * *
     319135 TAGCTGTGAGGATTTCGTTGGAAACGGGAATGTCTTCAAAG
        131 GCGCTTTGAGGATTTCGTTGGAAACGGAAATATCTTCATAT

                *                         *   *            *    *            
     319176 AAAATCTAGACAGAAGCATTCTCAGAAACACCTTCGTGATGTTTGCAATCAAGTCACAGAGTTGA
          1 AAAAACTAGACAGAAGCATTCTCAGAAACATCTTTGTGATGTTTGCACTCAACTCACAGAGTTGA

                 * *             *  *           *      *             *       
     319241 ACCTTCCGTTTCATAGAGCAGGTTGGAAACACTCTTATTGTAGTATCTGGAAGTGGACATTTGGA
         66 ACCTTTCTTTTCATAGAGCAGTTTTGAAACACTCTTTTTGTAGAATCTGGAAGTGGATATTTGGA

                   *   ** ** *  *   *            **  
     319306 GCGCTTTCAGGCCTATGGTGAAAAAGGAAATATCTTCCCAT
        131 GCGCTTTGAGGATTTCGTTGGAAACGGAAATATCTTCATAT

                  * * *      **      *   * *   *     ** ** *      *    *     
     319347 AAAAACGACATAGAAGCTATCTCAGGAACTTGTTTATGATGCATCTAATCAACTAACAGTGTTGA
          1 AAAAACTAGACAGAAGCATTCTCAGAAACATCTTTGTGATGTTTGCACTCAACTCACAGAGTTGA

                  * ** * *                         * *       *               
     319412 ACCTTTGTACTGACAGAGCAG-TTTGAAACACTCTTTTTTTGGAATCTGCAAGTGGATATTTGGA
         66 ACCTTTCTTTTCATAGAGCAGTTTTGAAACACTCTTTTTGTAGAATCTGGAAGTGGATATTTGGA

            *                           *   *   *    
     319476 TCGCTTTGAGGATTTCGTTGGAAACGG-GAT-GC--AATAT
        131 GCGCTTTGAGGATTTCGTTGGAAACGGAAATATCTTCATAT

                **  *    *     *                 **  *   *  *    *         * 
     319513 AAAACGTACACAGCAGCATACTCAGAAA-ATACTTTGCCATATTTCCATTCAAGTCACAGAGTGG
          1 AAAAACTAGACAGAAGCATTCTCAGAAACAT-CTTTGTGATGTTTGCACTCAACTCACAGAGTTG

               *  * **            *                  *  *             *      
     319577 AACATTCCCATTCATAGAGCAGGTTTGAAACACTCTTTTTGGAGTATCTGGAAGTGGACATTTGG
         65 AACCTTTCTTTTCATAGAGCAGTTTTGAAACACTCTTTTTGTAGAATCTGGAAGTGGATATTTGG

                    ** * * ** *  *   *            **  
     319642 AGCGCTTTCTGAACTATGGTGAAAAAGGAAATATCTTCCAAT
        130 AGCGCTTTGAGGATTTCGTTGGAAACGGAAATATCTTCATAT

            *     *               *      * *          *  **      **  *  *    
     319684 GAAAACAAGACAGAAGCATTCTGAGAAACTTATTTGTGATGTGTGTCCTCAACAAACGGACTTGA
          1 AAAAACTAGACAGAAGCATTCTCAGAAACATCTTTGTGATGTTTGCACTCAACTCACAGAGTTGA

                   *      **  * *  *  *             *   *    * * *           
     319749 ACCTTTCGTTTCATGCAGTACTTCTGGAACACTCTTTTTGAAGATTCTGCATGCGGATATTTGGA
         66 ACCTTTCTTTTCATAGAGCAGTTTTGAAACACTCTTTTTGTAGAATCTGGAAGTGGATATTTGGA

            **                            **   *   * 
     319814 TAGCTTTGAGGATTTCGTTGGAAACGG---GCT-TACATGT
        131 GCGCTTTGAGGATTTCGTTGGAAACGGAAATATCTTCATAT

                 *       *               *        *   *    *    *       *    
     319851 AAAAATTAGACAGCAGCATTCTCAGAAACTTCTTTGTGGTGTCTGCATTCAAGTCACAGAATTGA
          1 AAAAACTAGACAGAAGCATTCTCAGAAACATCTTTGTGATGTTTGCACTCAACTCACAGAGTTGA

              * ** * **            *  * *      *       *             *       
     319916 ACTTCCCCTCACATAGAGCAGTTGTGCAGCACTCTATTTGTAGTATCTGGAAGTGGACATTTGGA
         66 ACCTTTCTTTTCATAGAGCAGTTTTGAAACACTCTTTTTGTAGAATCTGGAAGTGGATATTTGGA

             *         ** *    * *   *            **  
     319981 GGGCTTTGTAGCCTATC-TGGAAAAAGGAAATATCTTCCCAT
        131 GCGCTTTG-AGGATTTCGTTGGAAACGGAAATATCTTCATAT

            *  ** *   *     * *            *   *  *   * **        *  * * *   
     320022 GAATGCGAGATAGAAGTAATCTCAGAAACATGTTTATGCTGTATCTACTCAACTAACTGTGCTGA
          1 AAAAACTAGACAGAAGCATTCTCAGAAACATCTTTGTGATGTTTGCACTCAACTCACAGAGTTGA

              *     *  *               *        *  * *       *               
     320087 ACATTTCTATTGATAGAGCAGTTTTGAGACACTCTTCTTTTGGAATCTGCAAGTGGATATTTGGA
         66 ACCTTTCTTTTCATAGAGCAGTTTTGAAACACTCTTTTTGTAGAATCTGGAAGTGGATATTTGGA

            ** *                        *     *      
     320152 TAGATTTGAGGATTTCGTTGGAAACGG-GAT-T-AT-ATAT
        131 GCGCTTTGAGGATTTCGTTGGAAACGGAAATATCTTCATAT

                 *       *               *                 **  *   *         
     320189 AAAAAGTAGACAGCAGCATTCTCAGAAACTTCTTTGTGATGTTTGCATCCAGCTCTCAGAGTTGA
          1 AAAAACTAGACAGAAGCATTCTCAGAAACATCTTTGTGATGTTTGCACTCAACTCACAGAGTTGA

              *  * *          *  *        *        *   **        *  **       
     320254 ACATTCCCTTTCATAGAGTAGGTTTGAAACCCTCTTTTTATAGTGTCTGGAAGCGGGCATTTGGA
         66 ACCTTTCTTTTCATAGAGCAGTTTTGAAACACTCTTTTTGTAGAATCTGGAAGTGGATATTTGGA

                   *    * * **  **  **          * *  *
     320319 GCGCTTTCAGGCCTATGCTTAAAATAGGAAATATCTACCTAC
        131 GCGCTTTGAGG-ATTTCGTTGGAAACGGAAATATCTTCATAT

             *                    *    *  **          ** *        *    *     
     320361 AGAAACTAGACAGAAGCATTCTGAGAATCACGTTTGTGATGTGGGTACTCAACTAACAGTGTTGA
          1 AAAAACTAGACAGAAGCATTCTCAGAAACATCTTTGTGATGTTTGCACTCAACTCACAGAGTTGA

            *  *       *   *            *   *                *   *           
     320426 TCCATTCTTTTGATACAGCAGTTTTGAACCACACTTTTTGTAGAATCTGCAAGAGGATATTTGGA
         66 ACCTTTCTTTTCATAGAGCAGTTTTGAAACACTCTTTTTGTAGAATCTGGAAGTGGATATTTGGA

            **   *                     *   *      * *
     320491 TAGCTGTGAGGATTTCGTTGGAAACGGGAATGTCTTCAAAG
        131 GCGCTTTGAGGATTTCGTTGGAAACGGAAATATCTTCATAT

                *                         *   *            *    *            
     320532 AAAATCTAGACAGAAGCATTCTCAGAAACACCTTCGTGATGTTTGCAATCAAGTCACAGAGTTGA
          1 AAAAACTAGACAGAAGCATTCTCAGAAACATCTTTGTGATGTTTGCACTCAACTCACAGAGTTGA

                 * *             *  *           *      *             *       
     320597 ACCTTCCGTTTCATAGAGCAGGTTGGAAACACTCTTATTGTAGTATCTGGAAGTGGACATTTGGA
         66 ACCTTTCTTTTCATAGAGCAGTTTTGAAACACTCTTTTTGTAGAATCTGGAAGTGGATATTTGGA

                   *   ** ** *  *   *            **  
     320662 GCGCTTTCAGGCCTATGGTGAAAAAGGAAATATCTTCCCAT
        131 GCGCTTTGAGGATTTCGTTGGAAACGGAAATATCTTCATAT

                  * * *      **      *   * *   *     ** ** *      *    *     
     320703 AAAAACGACATAGAAGCTATCTCAGGAACTTGTTTATGATGCATCTAATCAACTAACAGTGTTGA
          1 AAAAACTAGACAGAAGCATTCTCAGAAACATCTTTGTGATGTTTGCACTCAACTCACAGAGTTGA

                  * ** * *                         * *       *               
     320768 ACCTTTGTACTGACAGAGCAG-TTTGAAACACTCTTTTTTTGGAATCTGCAAGTGGATATTTGGA
         66 ACCTTTCTTTTCATAGAGCAGTTTTGAAACACTCTTTTTGTAGAATCTGGAAGTGGATATTTGGA

            *                           *   *   *    
     320832 TCGCTTTGAGGATTTCGTTGGAAACGG-GAT-GC--AATAT
        131 GCGCTTTGAGGATTTCGTTGGAAACGGAAATATCTTCATAT

                **  *    *     *                 **  *   *  *    *         * 
     320869 AAAACGTACACAGCAGCATACTCAGAAA-ATACTTTGCCATATTTCCATTCAAGTCACAGAGTGG
          1 AAAAACTAGACAGAAGCATTCTCAGAAACAT-CTTTGTGATGTTTGCACTCAACTCACAGAGTTG

               *  * **            *                  *  *             *      
     320933 AACATTCCCATTCATAGAGCAGGTTTGAAACACTCTTTTTGGAGTATCTGGAAGTGGACATTTGG
         65 AACCTTTCTTTTCATAGAGCAGTTTTGAAACACTCTTTTTGTAGAATCTGGAAGTGGATATTTGG

                    ** * * ** *  *   *            **  
     320998 AGCGCTTTCTGAACTATGGTGAAAAAGGAAATATCTTCCAAT
        130 AGCGCTTTGAGGATTTCGTTGGAAACGGAAATATCTTCATAT

            *     *               *      * *          *  **      **  *  *    
     321040 GAAAACAAGACAGAAGCATTCTGAGAAACTTATTTGTGATGTGTGTCCTCAACAAACGGACTTGA
          1 AAAAACTAGACAGAAGCATTCTCAGAAACATCTTTGTGATGTTTGCACTCAACTCACAGAGTTGA

                   *      **  * *  *  *             *   *    * * *           
     321105 ACCTTTCGTTTCATGCAGTACTTCTGGAACACTCTTTTTGAAGATTCTGCATGCGGATATTTGGA
         66 ACCTTTCTTTTCATAGAGCAGTTTTGAAACACTCTTTTTGTAGAATCTGGAAGTGGATATTTGGA

            **                            **   *   * 
     321170 TAGCTTTGAGGATTTCGTTGGAAACGG---GCT-TACATGT
        131 GCGCTTTGAGGATTTCGTTGGAAACGGAAATATCTTCATAT

                 *       *               *        *   *    *    *       *    
     321207 AAAAATTAGACAGCAGCATTCTCAGAAACTTCTTTGTGGTGTCTGCATTCAAGTCACAGAATTGA
          1 AAAAACTAGACAGAAGCATTCTCAGAAACATCTTTGTGATGTTTGCACTCAACTCACAGAGTTGA

              * ** * **            *  * *      *       *    *        *       
     321272 ACTTCCCCTCACATAGAGCAGTTGTGCAGCACTCTATTTGTAGTATCTCGAAGTGGACATTTGGA
         66 ACCTTTCTTTTCATAGAGCAGTTTTGAAACACTCTTTTTGTAGAATCTGGAAGTGGATATTTGGA

             *         ** *    * *   *            **  
     321337 GGGCTTTGTAGCCTATC-TGGAAAAAGGAAATATCTTCCCAT
        131 GCGCTTTG-AGGATTTCGTTGGAAACGGAAATATCTTCATAT

            *  ** *   *     * *            *   *  *   * **        *  * * *   
     321378 GAATGCGAGATAGAAGTAATCTCAGAAACATGTTTATGCTGTATCTACTCAACTAACTGTGCTGA
          1 AAAAACTAGACAGAAGCATTCTCAGAAACATCTTTGTGATGTTTGCACTCAACTCACAGAGTTGA

              *     *  *               *        *  * *       *               
     321443 ACATTTCTATTGATAGAGCAGTTTTGAGACACTCTTCTTTTGGAATCTGCAAGTGGATATTTGGA
         66 ACCTTTCTTTTCATAGAGCAGTTTTGAAACACTCTTTTTGTAGAATCTGGAAGTGGATATTTGGA

            ** *                        *     *      
     321508 TAGATTTGAGGATTTCGTTGGAAACGG-GAT-T-AT-ATAT
        131 GCGCTTTGAGGATTTCGTTGGAAACGGAAATATCTTCATAT

            *    *       *               *                 **  *   *         
     321545 CAAAAGTAGACAGCAGCATTCTCAGAAACTTCTTTGTGATGTTTGCATCCAGCTCTCAGAGTTGA
          1 AAAAACTAGACAGAAGCATTCTCAGAAACATCTTTGTGATGTTTGCACTCAACTCACAGAGTTGA

              *  * *          *  *        *        *   **        *  **       
     321610 ACATTCCCTTTCATAGAGTAGGTTTGAAACCCTCTTTTTATAGTGTCTGGAAGCGGGCATTTGGA
         66 ACCTTTCTTTTCATAGAGCAGTTTTGAAACACTCTTTTTGTAGAATCTGGAAGTGGATATTTGGA

                   *   ** ** *  *   *          * *   
     321675 GCGCTTTCAGGCCTATGCTGAAAAAGGAAATATCTACCTAT
        131 GCGCTTTGAGGATTTCGTTGGAAACGGAAATATCTTCATAT

             *                    *    *  **          ** *        *    *     
     321716 AGAAACTAGACAGAAGCATTCTGAGAATCACGTTTGTGATGTGGGTACTCAACTAACAGTGTTGA
          1 AAAAACTAGACAGAAGCATTCTCAGAAACATCTTTGTGATGTTTGCACTCAACTCACAGAGTTGA

            *  *       *   *            *   *                *               
     321781 TCCATTCTTTTGATACAGCAGTTTTGAACCACACTTTTTGTAGAATCTGCAAGTGGATATTTGGA
         66 ACCTTTCTTTTCATAGAGCAGTTTTGAAACACTCTTTTTGTAGAATCTGGAAGTGGATATTTGGA

            **   *                     *   *        *
     321846 TAGCTGTGAGGATTTCGTTGGAAACGGGAATGTCTTCATAG
        131 GCGCTTTGAGGATTTCGTTGGAAACGGAAATATCTTCATAT

                **                     * * **         *  **   *   *          
     321887 AAAATTTAGACAGAAGCATTCTCAGAACCTTGATTGTGATGTGTGTTCTCCACTAACAGAGTTGA
          1 AAAAACTAGACAGAAGCATTCTCAGAAACATCTTTGTGATGTTTGCACTCAACTCACAGAGTTGA

                       * *   * *   *       *       *                         
     321952 ACCTTTCTTTTGACAGAACTGTTCTGAAACATTCTTTTTATAGAATCTGGAAGTGGATATTTGGA
         66 ACCTTTCTTTTCATAGAGCAGTTTTGAAACACTCTTTTTGTAGAATCTGGAAGTGGATATTTGGA

            **                         *          *  
     322017 AAGCTTTGAGGATTTCGTTGGAAACGGGAATATCTTCAAAT
        131 GCGCTTTGAGGATTTCGTTGGAAACGGAAATATCTTCATAT

                *    *            *           * *       *  *    *            
     322058 AAAATCTAGCCAGAAGCATTCTAAGAAACATCTTAGGGATGTTTACATTCAAGTCACAGAGTTGA
          1 AAAAACTAGACAGAAGCATTCTCAGAAACATCTTTGTGATGTTTGCACTCAACTCACAGAGTTGA

              *  * *     *       *         *    * *   **      *      *    *  
     322123 ACATTCCCTTTCACAGAGCAGGTTTGAAACAATCTTCTCGTACTATCTGGCAGTGGACATTTTGA
         66 ACCTTTCTTTTCATAGAGCAGTTTTGAAACACTCTTTTTGTAGAATCTGGAAGTGGATATTTGGA

              * **  *  ** ** *  *   *            ** *
     322188 GCTCCCTGGGGCCTATGCTGAAAAAGGAAATATCTTCCGAC
        131 GCGCTTTGAGGATTTCGTTGGAAACGGAAATATCTTCATAT

                                 *     *  **          *           **    *    
     322229 AAAAACTAGACAGAAGCATTCGCAGAATCACGTTTGTGATGTGTGCACTCAACTGTCAGAATTGA
          1 AAAAACTAGACAGAAGCATTCTCAGAAACATCTTTGTGATGTTTGCACTCAACTCACAGAGTTGA

                 **   ** *      *                            * *            *
     322294 ACCTTGGTTTGGACAGAGCACTTTTGAAACACTCTTTTTGTAGAATCTGCAGGTGGATATTTGGC
         66 ACCTTTCTTTTCATAGAGCAGTTTTGAAACACTCTTTTTGTAGAATCTGGAAGTGGATATTTGGA

            **                         *   *      * *
     322359 TAGCTTTGAGGATTTCGTTGGAAACGGTAATGTCTTCAAAG
        131 GCGCTTTGAGGATTTCGTTGGAAACGGAAATATCTTCATAT

                *                         *   *            *    *            
     322400 AAAATCTAGACAGAAGCATTCTCAGAAACACCTTCGTGATGTTTGCAATCAAGTCACAGAGTTGA
          1 AAAAACTAGACAGAAGCATTCTCAGAAACATCTTTGTGATGTTTGCACTCAACTCACAGAGTTGA

                 * *             *  *                  *             *       
     322465 ACCTTCCGTTTCATAGAGCAGGTTGGAAACACTCTTTTTGTAGTATCTGGAAGTGGACATTTGGA
         66 ACCTTTCTTTTCATAGAGCAGTTTTGAAACACTCTTTTTGTAGAATCTGGAAGTGGATATTTGGA

                   *   ** ** *  *   *            **  
     322530 GCGCTTTCAGGCCTATGGTGAAAAAGGAAATATCTTCCCAT
        131 GCGCTTTGAGGATTTCGTTGGAAACGGAAATATCTTCATAT

                  * * *      **      *   * *   *     ** ** *      *    *     
     322571 AAAAACGACATAGAAGCTATCTCAGGAACTTGTTTATGATGCATCTAATCAACTAACAGTGTTGA
          1 AAAAACTAGACAGAAGCATTCTCAGAAACATCTTTGTGATGTTTGCACTCAACTCACAGAGTTGA

                  * ** * *                         * *       *               
     322636 ACCTTTGTACTGACAGAGCAG-TTTGAAACACTCTTTTTTTGGAATCTGCAAGTGGATATTTGGA
         66 ACCTTTCTTTTCATAGAGCAGTTTTGAAACACTCTTTTTGTAGAATCTGGAAGTGGATATTTGGA

            *                           *   *   *    
     322700 TCGCTTTGAGGATTTCGTTGGAAACGG-GAT-GC--AATAT
        131 GCGCTTTGAGGATTTCGTTGGAAACGGAAATATCTTCATAT

                **  *    *     *                 **  *   *  *    *         * 
     322737 AAAACGTACACAGCAGCATACTCAGAAA-ATACTTTGCCATATTTCCATTCAAGTCACAGAGTGG
          1 AAAAACTAGACAGAAGCATTCTCAGAAACAT-CTTTGTGATGTTTGCACTCAACTCACAGAGTTG

               *  * **          * *  *               *  *             *     *
     322801 AACATTCCCATTCATAGAGCGGGTTGGAAACACTCTTTTTGGAGTATCTGGAAGTGGACATTTGC
         65 AACCTTTCTTTTCATAGAGCAGTTTTGAAACACTCTTTTTGTAGAATCTGGAAGTGGATATTTGG

                    ** * * ** *  *   *            **  
     322866 AGCGCTTTCTGAACTATGGTGAAAAAGGAAATATCTTCCAAT
        130 AGCGCTTTGAGGATTTCGTTGGAAACGGAAATATCTTCATAT

            *     *               *      * *          *  **      **  * **    
     322908 GAAAACAAGACAGAAGCATTCTGAGAAACTTATTTGTGATGTGTGTCCTCAACAAACGGTCTTGA
          1 AAAAACTAGACAGAAGCATTCTCAGAAACATCTTTGTGATGTTTGCACTCAACTCACAGAGTTGA

                   *      **  * *  *  *             *   *    * * *           
     322973 ACCTTTCGTTTCATGCAGTACTTCTGGAACACTCTTTTTGAAGATTCTGCATGCGGATATTTGGA
         66 ACCTTTCTTTTCATAGAGCAGTTTTGAAACACTCTTTTTGTAGAATCTGGAAGTGGATATTTGGA

            **                            **   *   * 
     323038 TAGCTTTGAGGATTTCGTTGGAAACGG---GCT-TACATGT
        131 GCGCTTTGAGGATTTCGTTGGAAACGGAAATATCTTCATAT

                 *       *               *        *   *    *    *       *    
     323075 AAAAATTAGACAGCAGCATTCTCAGAAACTTCTTTGTGGTGTCTGCATTCAAGTCACAGAATTGA
          1 AAAAACTAGACAGAAGCATTCTCAGAAACATCTTTGTGATGTTTGCACTCAACTCACAGAGTTGA

              * ** * **            *  * *      *       *    *        *       
     323140 ACTTCCCCTCACATAGAGCAGTTGTGCAGCACTCTATTTGTAGTATCTCGAAGTGGACATTTGGA
         66 ACCTTTCTTTTCATAGAGCAGTTTTGAAACACTCTTTTTGTAGAATCTGGAAGTGGATATTTGGA

             *         ** *    * *   *            **  
     323205 GGGCTTTGTAGCCTATC-TGGAAAAAGGAAATATCTTCCCAT
        131 GCGCTTTG-AGGATTTCGTTGGAAACGGAAATATCTTCATAT

            *  ** *   *     * *            *   *  *   * **        *  * * *   
     323246 GAATGCGAGATAGAAGTAATCTCAGAAACATGTTTATGCTGTATCTACTCAACTAACTGTGCTGA
          1 AAAAACTAGACAGAAGCATTCTCAGAAACATCTTTGTGATGTTTGCACTCAACTCACAGAGTTGA

              *     *  *               *        *  * *       *               
     323311 ACATTTCTATTGATAGAGCAGTTTTGAGACACTCTTCTTTTGGAATCTGCAAGTGGATATTTGGA
         66 ACCTTTCTTTTCATAGAGCAGTTTTGAAACACTCTTTTTGTAGAATCTGGAAGTGGATATTTGGA

            ** *                        *     *      
     323376 TAGATTTGAGGATTTCGTTGGAAACGG-GAT-T-AT-ATAT
        131 GCGCTTTGAGGATTTCGTTGGAAACGGAAATATCTTCATAT

                 *       *               *                 **  *   *         
     323413 AAAAAGTAGACAGCAGCATTCTCAGAAACTTCTTTGTGATGTTTGCATCCAGCTCTCAGAGTTGA
          1 AAAAACTAGACAGAAGCATTCTCAGAAACATCTTTGTGATGTTTGCACTCAACTCACAGAGTTGA

              *  * *          *  *        *        *   **    *   *  **       
     323478 ACATTCCCTTTCATAGAGTAGGTTTGAAACCCTCTTTTTATAGTGTCTGCAAGCGGGCATTTGGA
         66 ACCTTTCTTTTCATAGAGCAGTTTTGAAACACTCTTTTTGTAGAATCTGGAAGTGGATATTTGGA

                   *    * * **  **  **          * *  *
     323543 GCGCTTTCAGGCCTATGCTTAAAATAGGAAATATCTACCTAC
        131 GCGCTTTGAGG-ATTTCGTTGGAAACGGAAATATCTTCATAT

             *                    *    *  **          ** *        *    *     
     323585 AGAAACTAGACAGAAGCATTCTGAGAATCACGTTTGTGATGTGGGTACTCAACTAACAGTGTTGA
          1 AAAAACTAGACAGAAGCATTCTCAGAAACATCTTTGTGATGTTTGCACTCAACTCACAGAGTTGA

            *  *       *   *            *   *                *   *           
     323650 TCCATTCTTTTGATACAGCAGTTTTGAACCACACTTTTTGTAGAATCTGCAAGAGGATATTTGGA
         66 ACCTTTCTTTTCATAGAGCAGTTTTGAAACACTCTTTTTGTAGAATCTGGAAGTGGATATTTGGA

            **   *                     *   *      * *
     323715 TAGCTGTGAGGATTTCGTTGGAAACGGGAATGTCTTCAAAG
        131 GCGCTTTGAGGATTTCGTTGGAAACGGAAATATCTTCATAT

                *                         *   *            *    *            
     323756 AAAATCTAGACAGAAGCATTCTCAGAAACACCTTCGTGATGTTTGCAATCAAGTCACAGAGTTGA
          1 AAAAACTAGACAGAAGCATTCTCAGAAACATCTTTGTGATGTTTGCACTCAACTCACAGAGTTGA

                 * *             *  *           *      *             *       
     323821 ACCTTCCGTTTCATAGAGCAGGTTGGAAACACTCTTATTGTAGTATCTGGAAGTGGACATTTGGA
         66 ACCTTTCTTTTCATAGAGCAGTTTTGAAACACTCTTTTTGTAGAATCTGGAAGTGGATATTTGGA

                   *   ** ** *  *   *            **  
     323886 GCGCTTTCAGGCCTATGGTGAAAAAGGAAATATCTTCCCAT
        131 GCGCTTTGAGGATTTCGTTGGAAACGGAAATATCTTCATAT

                  * * *      **      *   * *   *     ** ** *      *    *     
     323927 AAAAACGACATAGAAGCTATCTCAGGAACTTGTTTATGATGCATCTAATCAACTAACAGTGTTGA
          1 AAAAACTAGACAGAAGCATTCTCAGAAACATCTTTGTGATGTTTGCACTCAACTCACAGAGTTGA

                  * ** * *                         * *       *               
     323992 ACCTTTGTACTGACAGAGCAG-TTTGAAACACTCTTTTTTTGGAATCTGCAAGTGGATATTTGGA
         66 ACCTTTCTTTTCATAGAGCAGTTTTGAAACACTCTTTTTGTAGAATCTGGAAGTGGATATTTGGA

            *                           *   *   *    
     324056 TCGCTTTGAGGATTTCGTTGGAAACGG-GAT-GC--AATAT
        131 GCGCTTTGAGGATTTCGTTGGAAACGGAAATATCTTCATAT

                **  *    *     *                 **  *   *  *    *         * 
     324093 AAAACGTACACAGCAGCATACTCAGAAA-ATACTTTGCCATATTTCCATTCAAGTCACAGAGTGG
          1 AAAAACTAGACAGAAGCATTCTCAGAAACAT-CTTTGTGATGTTTGCACTCAACTCACAGAGTTG

               *  * **            *                  *  *             *      
     324157 AACATTCCCATTCATAGAGCAGGTTTGAAACACTCTTTTTGGAGTATCTGGAAGTGGACATTTGG
         65 AACCTTTCTTTTCATAGAGCAGTTTTGAAACACTCTTTTTGTAGAATCTGGAAGTGGATATTTGG

                    ** * * ** *  *   *            **  
     324222 AGCGCTTTCTGAACTATGGTGAAAAAGGAAATATCTTCCAAT
        130 AGCGCTTTGAGGATTTCGTTGGAAACGGAAATATCTTCATAT

            *     *               *      * *          *  **      **  *  *    
     324264 GAAAACAAGACAGAAGCATTCTGAGAAACTTATTTGTGATGTGTGTCCTCAACAAACGGACTTGA
          1 AAAAACTAGACAGAAGCATTCTCAGAAACATCTTTGTGATGTTTGCACTCAACTCACAGAGTTGA

                   *      **  * *  *  *             *   *    * * *           
     324329 ACCTTTCGTTTCATGCAGTACTTCTGGAACACTCTTTTTGAAGATTCTGCATGCGGATATTTGGA
         66 ACCTTTCTTTTCATAGAGCAGTTTTGAAACACTCTTTTTGTAGAATCTGGAAGTGGATATTTGGA

            **                            **   *   * 
     324394 TAGCTTTGAGGATTTCGTTGGAAACGG---GCT-TACATGT
        131 GCGCTTTGAGGATTTCGTTGGAAACGGAAATATCTTCATAT

                 *       *               *        *   *    *    *       *    
     324431 AAAAATTAGACAGCAGCATTCTCAGAAACTTCTTTGTGGTGTCTGCATTCAAGTCACAGAATTGA
          1 AAAAACTAGACAGAAGCATTCTCAGAAACATCTTTGTGATGTTTGCACTCAACTCACAGAGTTGA

              * ** * **            *  * *      *       *             *       
     324496 ACTTCCCCTCACATAGAGCAGTTGTGCAGCACTCTATTTGTAGTATCTGGAAGTGGACATTTGGA
         66 ACCTTTCTTTTCATAGAGCAGTTTTGAAACACTCTTTTTGTAGAATCTGGAAGTGGATATTTGGA

             *         ** *    * *   *            **  
     324561 GGGCTTTGTAGCCTATC-TGGAAAAAGGAAATATCTTCCCAT
        131 GCGCTTTG-AGGATTTCGTTGGAAACGGAAATATCTTCATAT

            *  ** *   *     * *            *   *  *   * **        *  * * *   
     324602 GAATGCGAGATAGAAGTAATCTCAGAAACATGTTTATGCTGTATCTACTCAACTAACTGTGCTGA
          1 AAAAACTAGACAGAAGCATTCTCAGAAACATCTTTGTGATGTTTGCACTCAACTCACAGAGTTGA

              *     *  *               *        *  * *       *               
     324667 ACATTTCTATTGATAGAGCAGTTTTGAGACACTCTTCTTTTGGAATCTGCAAGTGGATATTTGGA
         66 ACCTTTCTTTTCATAGAGCAGTTTTGAAACACTCTTTTTGTAGAATCTGGAAGTGGATATTTGGA

            ** *                        *     *      
     324732 TAGATTTGAGGATTTCGTTGGAAACGG-GAT-T-AT-ATAT
        131 GCGCTTTGAGGATTTCGTTGGAAACGGAAATATCTTCATAT

                 *       *               *                 **  *   *         
     324769 AAAAAGTAGACAGCAGCATTCTCAGAAACTTCTTTGTGATGTTTGCATCCAGCTCTCAGAGTTGA
          1 AAAAACTAGACAGAAGCATTCTCAGAAACATCTTTGTGATGTTTGCACTCAACTCACAGAGTTGA

              *  * *          *  *        *        *   **        *  **       
     324834 ACATTCCCTTTCATAGAGTAGGTTTGAAACCCTCTTTTTATAGTGTCTGGAAGCGGGCATTTGGA
         66 ACCTTTCTTTTCATAGAGCAGTTTTGAAACACTCTTTTTGTAGAATCTGGAAGTGGATATTTGGA

                   *   ** ** *  *   *          * *   
     324899 GCGCTTTCAGGCCTATGCTGAAAAAGGAAATATCTACCTAT
        131 GCGCTTTGAGGATTTCGTTGGAAACGGAAATATCTTCATAT

             *                    *    *  **          ** *        *    *     
     324940 AGAAACTAGACAGAAGCATTCTGAGAATCACGTTTGTGATGTGGGTACTCAACTAACAGTGTTGA
          1 AAAAACTAGACAGAAGCATTCTCAGAAACATCTTTGTGATGTTTGCACTCAACTCACAGAGTTGA

            *  *       *   *            *   *                *               
     325005 TCCATTCTTTTGATACAGCAGTTTTGAACCACACTTTTTGTAGAATCTGCAAGTGGATATTTGGA
         66 ACCTTTCTTTTCATAGAGCAGTTTTGAAACACTCTTTTTGTAGAATCTGGAAGTGGATATTTGGA

            **   *                     *   *        *
     325070 TAGCTGTGAGGATTTCGTTGGAAACGGGAATGTCTTCATAG
        131 GCGCTTTGAGGATTTCGTTGGAAACGGAAATATCTTCATAT

                **                     * * **         *  **   *   *          
     325111 AAAATTTAGACAGAAGCATTCTCAGAACCTTGATTGTGATGTGTGTTCTCCACTAACAGAGTTGA
          1 AAAAACTAGACAGAAGCATTCTCAGAAACATCTTTGTGATGTTTGCACTCAACTCACAGAGTTGA

                       * *   * *   *       *       *                         
     325176 ACCTTTCTTTTGACAGAACTGTTCTGAAACATTCTTTTTATAGAATCTGGAAGTGGATATTTGGA
         66 ACCTTTCTTTTCATAGAGCAGTTTTGAAACACTCTTTTTGTAGAATCTGGAAGTGGATATTTGGA

            **                         *          *  
     325241 AAGCTTTGAGGATTTCGTTGGAAACGGGAATATCTTCAAAT
        131 GCGCTTTGAGGATTTCGTTGGAAACGGAAATATCTTCATAT

                *    *            *           * *       *  *    *            
     325282 AAAATCTAGCCAGAAGCATTCTAAGAAACATCTTAGGGATGTTTACATTCAAGTCACAGAGTTGA
          1 AAAAACTAGACAGAAGCATTCTCAGAAACATCTTTGTGATGTTTGCACTCAACTCACAGAGTTGA

              *  * *     *       *         *    * *   **      *      *    *  
     325347 ACATTCCCTTTCACAGAGCAGGTTTGAAACAATCTTCTCGTACTATCTGGCAGTGGACATTTTGA
         66 ACCTTTCTTTTCATAGAGCAGTTTTGAAACACTCTTTTTGTAGAATCTGGAAGTGGATATTTGGA

              * **  *  ** ** *  *   *            ** *
     325412 GCTCCCTGGGGCCTATGCTGAAAAAGGAAATATCTTCCGAC
        131 GCGCTTTGAGGATTTCGTTGGAAACGGAAATATCTTCATAT

                                 *     *  **          *           **    *    
     325453 AAAAACTAGACAGAAGCATTCGCAGAATCACGTTTGTGATGTGTGCACTCAACTGTCAGAATTGA
          1 AAAAACTAGACAGAAGCATTCTCAGAAACATCTTTGTGATGTTTGCACTCAACTCACAGAGTTGA

                 **   ** *      *                            * *            *
     325518 ACCTTGGTTTGGACAGAGCACTTTTGAAACACTCTTTTTGTAGAATCTGCAGGTGGATATTTGGC
         66 ACCTTTCTTTTCATAGAGCAGTTTTGAAACACTCTTTTTGTAGAATCTGGAAGTGGATATTTGGA

            **                         *   *      * *
     325583 TAGCTTTGAGGATTTCGTTGGAAACGGTAATGTCTTCAAAG
        131 GCGCTTTGAGGATTTCGTTGGAAACGGAAATATCTTCATAT

                *                         *   *            *    *            
     325624 AAAATCTAGACAGAAGCATTCTCAGAAACACCTTCGTGATGTTTGCAATCAAGTCACAGAGTTGA
          1 AAAAACTAGACAGAAGCATTCTCAGAAACATCTTTGTGATGTTTGCACTCAACTCACAGAGTTGA

                 * *             *  *                  *             *       
     325689 ACCTTCCGTTTCATAGAGCAGGTTGGAAACACTCTTTTTGTAGTATCTGGAAGTGGACATTTGGA
         66 ACCTTTCTTTTCATAGAGCAGTTTTGAAACACTCTTTTTGTAGAATCTGGAAGTGGATATTTGGA

                   *   ** ** *  *   *            **  
     325754 GCGCTTTCAGGCCTATGGTGAAAAAGGAAATATCTTCCCAT
        131 GCGCTTTGAGGATTTCGTTGGAAACGGAAATATCTTCATAT

                  * * *      **      *   * *   *     ** ** *      *    *     
     325795 AAAAACGACATAGAAGCTATCTCAGGAACTTGTTTATGATGCATCTAATCAACTAACAGTGTTGA
          1 AAAAACTAGACAGAAGCATTCTCAGAAACATCTTTGTGATGTTTGCACTCAACTCACAGAGTTGA

                  * ** * *                         * *       *               
     325860 ACCTTTGTACTGACAGAGCAG-TTTGAAACACTCTTTTTTTGGAATCTGCAAGTGGATATTTGGA
         66 ACCTTTCTTTTCATAGAGCAGTTTTGAAACACTCTTTTTGTAGAATCTGGAAGTGGATATTTGGA

            *                           *   *   *    
     325924 TCGCTTTGAGGATTTCGTTGGAAACGG-GAT-GC--AATAT
        131 GCGCTTTGAGGATTTCGTTGGAAACGGAAATATCTTCATAT

                **  *    *     *                 **  *   *  *    *        ** 
     325961 AAAACGTACACAGCAGCATACTCAGAAA-ATACTTTGCCATATTTCCATTCAAGTCACAGAGCGG
          1 AAAAACTAGACAGAAGCATTCTCAGAAACAT-CTTTGTGATGTTTGCACTCAACTCACAGAGTTG

               *  * **          * *  *               *  *             *      
     326025 AACATTCCCATTCATAGAGCGGGTTGGAAACACTCTTTTTGGAGTATCTGGAAGTGGACATTTGG
         65 AACCTTTCTTTTCATAGAGCAGTTTTGAAACACTCTTTTTGTAGAATCTGGAAGTGGATATTTGG

                    ** * * ** *  *   *            **  
     326090 AGCGCTTTCTGAACTATGGTGAAAAAGGAAATATCTTCCAAT
        130 AGCGCTTTGAGGATTTCGTTGGAAACGGAAATATCTTCATAT

            *     *               *      * *          *  **      **  * **    
     326132 GAAAACAAGACAGAAGCATTCTGAGAAACTTATTTGTGATGTGTGTCCTCAACAAACGGTCTTGA
          1 AAAAACTAGACAGAAGCATTCTCAGAAACATCTTTGTGATGTTTGCACTCAACTCACAGAGTTGA

                   *      **  * *  *  *             *   *    * * *           
     326197 ACCTTTCGTTTCATGCAGTACTTCTGGAACACTCTTTTTGAAGATTCTGCATGCGGATATTTGGA
         66 ACCTTTCTTTTCATAGAGCAGTTTTGAAACACTCTTTTTGTAGAATCTGGAAGTGGATATTTGGA

            **                            **   *   * 
     326262 TAGCTTTGAGGATTTCGTTGGAAACGG---GCT-TACATGT
        131 GCGCTTTGAGGATTTCGTTGGAAACGGAAATATCTTCATAT

                 *       *               *        *   *    *    *       *    
     326299 AAAAATTAGACAGCAGCATTCTCAGAAACTTCTTTGTGGTGTCTGCATTCAAGTCACAGAATTGA
          1 AAAAACTAGACAGAAGCATTCTCAGAAACATCTTTGTGATGTTTGCACTCAACTCACAGAGTTGA

              * ** * **            *  * *      *       *    *        *       
     326364 ACTTCCCCTCACATAGAGCAGTTGTGCAGCACTCTATTTGTAGTATCTCGAAGTGGACATTTGGA
         66 ACCTTTCTTTTCATAGAGCAGTTTTGAAACACTCTTTTTGTAGAATCTGGAAGTGGATATTTGGA

             *         ** *    * *   *            **  
     326429 GGGCTTTGTAGCCTATC-TGGAAAAAGGAAATATCTTCCCAT
        131 GCGCTTTG-AGGATTTCGTTGGAAACGGAAATATCTTCATAT

            *  ** *   *     * *            *   *  *   * **        *  * * *   
     326470 GAATGCGAGATAGAAGTAATCTCAGAAACATGTTTATGCTGTATCTACTCAACTAACTGTGCTGA
          1 AAAAACTAGACAGAAGCATTCTCAGAAACATCTTTGTGATGTTTGCACTCAACTCACAGAGTTGA

              *     *  *               *        *  * *     * *               
     326535 ACATTTCTATTGATAGAGCAGTTTTGAGACACTCTTCTTTTGGAATCCGCAAGTGGATATTTGGA
         66 ACCTTTCTTTTCATAGAGCAGTTTTGAAACACTCTTTTTGTAGAATCTGGAAGTGGATATTTGGA

             * *                        *     *      
     326600 GAGATTTGAGGATTTCGTTGGAAACGG-GAT-T-AT-ATAT
        131 GCGCTTTGAGGATTTCGTTGGAAACGGAAATATCTTCATAT

                 *       *               *                 **  *   *         
     326637 AAAAAGTAGACAGCAGCATTCTCAGAAACTTCTTTGTGATGTTTGCATCCAGCTCTCAGAGTTGA
          1 AAAAACTAGACAGAAGCATTCTCAGAAACATCTTTGTGATGTTTGCACTCAACTCACAGAGTTGA

              *  * *          *  *        *        *   **    *   *  **       
     326702 ACATTCCCTTTCATAGAGTAGGTTTGAAACCCTCTTTTTATAGTGTCTGCAAGCGGGCATTTGGA
         66 ACCTTTCTTTTCATAGAGCAGTTTTGAAACACTCTTTTTGTAGAATCTGGAAGTGGATATTTGGA

                   *    * * **  **  **          * *  *
     326767 GCGCTTTCAGGCCTATGCTTAAAATAGGAAATATCTACCTAC
        131 GCGCTTTGAGG-ATTTCGTTGGAAACGGAAATATCTTCATAT

             *                    *    *  **          ** *        *    *     
     326809 AGAAACTAGACAGAAGCATTCTGAGAATCACGTTTGTGATGTGGGTACTCAACTAACAGTGTTGA
          1 AAAAACTAGACAGAAGCATTCTCAGAAACATCTTTGTGATGTTTGCACTCAACTCACAGAGTTGA

            *  *       *   *            *   *                *   *           
     326874 TCCATTCTTTTGATACAGCAGTTTTGAACCACACTTTTTGTAGAATCTGCAAGAGGATATTTGGA
         66 ACCTTTCTTTTCATAGAGCAGTTTTGAAACACTCTTTTTGTAGAATCTGGAAGTGGATATTTGGA

            **   *                     *   *      * *
     326939 TAGCTGTGAGGATTTCGTTGGAAACGGGAATGTCTTCAAAG
        131 GCGCTTTGAGGATTTCGTTGGAAACGGAAATATCTTCATAT

                *                         *   *            *    *            
     326980 AAAATCTAGACAGAAGCATTCTCAGAAACACCTTCGTGATGTTTGCAATCAAGTCACAGAGTTGA
          1 AAAAACTAGACAGAAGCATTCTCAGAAACATCTTTGTGATGTTTGCACTCAACTCACAGAGTTGA

                 * *             *  *           *      *             *       
     327045 ACCTTCCGTTTCATAGAGCAGGTTGGAAACACTCTTATTGTAGTATCTGGAAGTGGACATTT-GA
         66 ACCTTTCTTTTCATAGAGCAGTTTTGAAACACTCTTTTTGTAGAATCTGGAAGTGGATATTTGGA

                   *   ** ** *  *   *            **  
     327109 GCGCTTTCAGGCCTATGGTGAAAAAGGAAATATCTTCCCAT
        131 GCGCTTTGAGGATTTCGTTGGAAACGGAAATATCTTCATAT

                  * * *      **      *   * *   *     ** ** *      *    *     
     327150 AAAAACGACATAGAAGCTATCTCAGGAACTTGTTTATGATGCATCTAATCAACTAACAGTGTTGA
          1 AAAAACTAGACAGAAGCATTCTCAGAAACATCTTTGTGATGTTTGCACTCAACTCACAGAGTTGA

                  * ** * *                         * *       *               
     327215 ACCTTTGTACTGACAGAGCAG-TTTGAAACACTCTTTTTTTGGAATCTGCAAGTGGATATTTGGA
         66 ACCTTTCTTTTCATAGAGCAGTTTTGAAACACTCTTTTTGTAGAATCTGGAAGTGGATATTTGGA

            *                           *   *   *    
     327279 TCGCTTTGAGGATTTCGTTGGAAACGG-GAT-GC--AATAT
        131 GCGCTTTGAGGATTTCGTTGGAAACGGAAATATCTTCATAT

                **  *    *     *                 **  *   *  *    *         * 
     327316 AAAACGTACACAGCAGCATACTCAGAAA-ATACTTTGCCATATTTCCATTCAAGTCACAGAGTGG
          1 AAAAACTAGACAGAAGCATTCTCAGAAACAT-CTTTGTGATGTTTGCACTCAACTCACAGAGTTG

               *  * **            *                  *  *             *      
     327380 AACATTCCCATTCATAGAGCAGGTTTGAAACACTCTTTTTGGAGTATCTGGAAGTGGACATTTGG
         65 AACCTTTCTTTTCATAGAGCAGTTTTGAAACACTCTTTTTGTAGAATCTGGAAGTGGATATTTGG

                    ** * * ** *  *   *            **  
     327445 AGCGCTTTCTGAACTATGGTGAAAAAGGAAATATCTTCCAAT
        130 AGCGCTTTGAGGATTTCGTTGGAAACGGAAATATCTTCATAT

            *     *               *      * *          *  **      **  *  *    
     327487 GAAAACAAGACAGAAGCATTCTGAGAAACTTATTTGTGATGTGTGTCCTCAACAAACGGACTTGA
          1 AAAAACTAGACAGAAGCATTCTCAGAAACATCTTTGTGATGTTTGCACTCAACTCACAGAGTTGA

                   *      **  * *  *  *             *   *    * * *           
     327552 ACCTTTCGTTTCATGCAGTACTTCTGGAACACTCTTTTTGAAGATTCTGCATGCGGATATTTGGA
         66 ACCTTTCTTTTCATAGAGCAGTTTTGAAACACTCTTTTTGTAGAATCTGGAAGTGGATATTTGGA

            **                            **   *   * 
     327617 TAGCTTTGAGGATTTCGTTGGAAACGG---GCT-TACATGT
        131 GCGCTTTGAGGATTTCGTTGGAAACGGAAATATCTTCATAT

                 *       *               *        *   *    *    *       *    
     327654 AAAAATTAGACAGCAGCATTCTCAGAAACTTCTTTGTGGTGTCTGCATTCAAGTCACAGAATTGA
          1 AAAAACTAGACAGAAGCATTCTCAGAAACATCTTTGTGATGTTTGCACTCAACTCACAGAGTTGA

              * ** * **            *  * *      *       *             *       
     327719 ACTTCCCCTCACATAGAGCAGTTGTGCAGCACTCTATTTGTAGTATCTGGAAGTGGACATTTGGA
         66 ACCTTTCTTTTCATAGAGCAGTTTTGAAACACTCTTTTTGTAGAATCTGGAAGTGGATATTTGGA

             *         ** *    * *   *            **  
     327784 GGGCTTTGTAGCCTATC-TGGAAAAAGGAAATATCTTCCCAT
        131 GCGCTTTG-AGGATTTCGTTGGAAACGGAAATATCTTCATAT

            *  ** *   *     * *            *   *  *   * **        *  * * *   
     327825 GAATGCGAGATAGAAGTAATCTCAGAAACATGTTTATGCTGTATCTACTCAACTAACTGTGCTGA
          1 AAAAACTAGACAGAAGCATTCTCAGAAACATCTTTGTGATGTTTGCACTCAACTCACAGAGTTGA

              *     *  *               *        *  * *       *               
     327890 ACATTTCTATTGATAGAGCAGTTTTGAGACACTCTTCTTTTGGAATCTGCAAGTGGATATTTGGA
         66 ACCTTTCTTTTCATAGAGCAGTTTTGAAACACTCTTTTTGTAGAATCTGGAAGTGGATATTTGGA

            ** *                        *     *      
     327955 TAGATTTGAGGATTTCGTTGGAAACGG-GAT-T-AT-ATAT
        131 GCGCTTTGAGGATTTCGTTGGAAACGGAAATATCTTCATAT

                 *       *               *                 **  *   *         
     327992 AAAAAGTAGACAGCAGCATTCTCAGAAACTTCTTTGTGATGTTTGCATCCAGCTCTCAGAGTTGA
          1 AAAAACTAGACAGAAGCATTCTCAGAAACATCTTTGTGATGTTTGCACTCAACTCACAGAGTTGA

              *  * *          *  *        *        *   **    *   *  **       
     328057 ACATTCCCTTTCATAGAGTAGGTTTGAAACCCTCTTTTTATAGTGTCTGCAAGCGGGCATTTGGA
         66 ACCTTTCTTTTCATAGAGCAGTTTTGAAACACTCTTTTTGTAGAATCTGGAAGTGGATATTTGGA

                   *    * * **  **  **          * *  *
     328122 GCGCTTTCAGGCCTATGCTTAAAATAGGAAATATCTACCTAC
        131 GCGCTTTGAGG-ATTTCGTTGGAAACGGAAATATCTTCATAT

             *                    *    *  **          ** *        *    *     
     328164 AGAAACTAGACAGAAGCATTCTGAGAATCACGTTTGTGATGTGGGTACTCAACTAACAGTGTTGA
          1 AAAAACTAGACAGAAGCATTCTCAGAAACATCTTTGTGATGTTTGCACTCAACTCACAGAGTTGA

            *  *       *   *            *   *                *   *           
     328229 TCCATTCTTTTGATACAGCAGTTTTGAACCACACTTTTTGTAGAATCTGCAAGAGGATATTTGGA
         66 ACCTTTCTTTTCATAGAGCAGTTTTGAAACACTCTTTTTGTAGAATCTGGAAGTGGATATTTGGA

            **   *                     *   *      * *
     328294 TAGCTGTGAGGATTTCGTTGGAAACGGGAATGTCTTCAAAG
        131 GCGCTTTGAGGATTTCGTTGGAAACGGAAATATCTTCATAT

                *                         *   *            *    *            
     328335 AAAATCTAGACAGAAGCATTCTCAGAAACACCTTCGTGATGTTTGCAATCAAGTCACAGAGTTGA
          1 AAAAACTAGACAGAAGCATTCTCAGAAACATCTTTGTGATGTTTGCACTCAACTCACAGAGTTGA

                 * *             *  *           *      *             *       
     328400 ACCTTCCGTTTCATAGAGCAGGTTGGAAACACTCTTATTGTAGTATCTGGAAGTGGACATTTGGA
         66 ACCTTTCTTTTCATAGAGCAGTTTTGAAACACTCTTTTTGTAGAATCTGGAAGTGGATATTTGGA

                   *   ** ** *  *   *            **  
     328465 GCGCTTTCAGGCCTATGGTGAAAAAGGAAATATCTTCCCAT
        131 GCGCTTTGAGGATTTCGTTGGAAACGGAAATATCTTCATAT

                  * * *      **      *   * *   *     ** ** *      *    *     
     328506 AAAAACGACATAGAAGCTATCTCAGGAACTTGTTTATGATGCATCTAATCAACTAACAGTGTTGA
          1 AAAAACTAGACAGAAGCATTCTCAGAAACATCTTTGTGATGTTTGCACTCAACTCACAGAGTTGA

                  * ** * *                         * *       *               
     328571 ACCTTTGTACTGACAGAGCAG-TTTGAAACACTCTTTTTTTGGAATCTGCAAGTGGATATTTGGA
         66 ACCTTTCTTTTCATAGAGCAGTTTTGAAACACTCTTTTTGTAGAATCTGGAAGTGGATATTTGGA

            *                           *   *   *    
     328635 TCGCTTTGAGGATTTCGTTGGAAACGG-GAT-GC--AATAT
        131 GCGCTTTGAGGATTTCGTTGGAAACGGAAATATCTTCATAT

                **  *    *     **                **  *   *  *    *         * 
     328672 AAAACGTACACAGCAGCATAGTCAGAAA-ATACTTTGCCATATTTCCATTCAAGTCACAGAGTGG
          1 AAAAACTAGACAGAAGCATTCTCAGAAACAT-CTTTGTGATGTTTGCACTCAACTCACAGAGTTG

               *  * **            *  *               *  *             *      
     328736 AACATTCCCATTCATAGAGCAGGTTGGAAACACTCTTTTTGGAGTATCTGGAAGTGGACATTTGG
         65 AACCTTTCTTTTCATAGAGCAGTTTTGAAACACTCTTTTTGTAGAATCTGGAAGTGGATATTTGG

                    ** * * ** *  *   *            **  
     328801 AGCGCTTTCTGAACTATGGTGAAAAAGGAAATATCTTCCAAT
        130 AGCGCTTTGAGGATTTCGTTGGAAACGGAAATATCTTCATAT

            *     *               *      * *          *  **      **  *  *    
     328843 GAAAACAAGACAGAAGCATTCTGAGAAACTTATTTGTGATGTGTGTCCTCAACAAACGGACTTGA
          1 AAAAACTAGACAGAAGCATTCTCAGAAACATCTTTGTGATGTTTGCACTCAACTCACAGAGTTGA

                   *      **  * *  *  *             *   *    * * *           
     328908 ACCTTTCGTTTCATGCAGTACTTCTGGAACACTCTTTTTGAAGATTCTGCATGCGGATATTTGGA
         66 ACCTTTCTTTTCATAGAGCAGTTTTGAAACACTCTTTTTGTAGAATCTGGAAGTGGATATTTGGA

            **                            **   *   * 
     328973 TAGCTTTGAGGATTTCGTTGGAAACGG---GCT-TACATGT
        131 GCGCTTTGAGGATTTCGTTGGAAACGGAAATATCTTCATAT

                 *       *               *        *   *    *    *       *    
     329010 AAAAATTAGACAGCAGCATTCTCAGAAACTTCTTTGTGGTGTCTGCATTCAAGTCACAGAATTGA
          1 AAAAACTAGACAGAAGCATTCTCAGAAACATCTTTGTGATGTTTGCACTCAACTCACAGAGTTGA

              * ** * **            *  * *      *       *             *       
     329075 ACTTCCCCTCACATAGAGCAGTTGTGCAGCACTCTATTTGTAGTATCTGGAAGTGGACATTTGGA
         66 ACCTTTCTTTTCATAGAGCAGTTTTGAAACACTCTTTTTGTAGAATCTGGAAGTGGATATTTGGA

             *         ** *    * *   *            **  
     329140 GGGCTTTGTAGCCTATC-TGGAAAAAGGAAATATCTTCCCAT
        131 GCGCTTTG-AGGATTTCGTTGGAAACGGAAATATCTTCATAT

            *  ** *   *     * *            *   *  *   * **        *  * * *   
     329181 GAATGCGAGATAGAAGTAATCTCAGAAACATGTTTATGCTGTATCTACTCAACTAACTGTGCTGA
          1 AAAAACTAGACAGAAGCATTCTCAGAAACATCTTTGTGATGTTTGCACTCAACTCACAGAGTTGA

              *     *  *               *        *  * *       *               
     329246 ACATTTCTATTGATAGAGCAGTTTTGAGACACTCTTCTTTTGGAATCTGCAAGTGGATATTTGGA
         66 ACCTTTCTTTTCATAGAGCAGTTTTGAAACACTCTTTTTGTAGAATCTGGAAGTGGATATTTGGA

            ** *                        *     *      
     329311 TAGATTTGAGGATTTCGTTGGAAACGG-GAT-T-AT-ATAT
        131 GCGCTTTGAGGATTTCGTTGGAAACGGAAATATCTTCATAT

            *    *       *               *                 **  *   *         
     329348 CAAAAGTAGACAGCAGCATTCTCAGAAACTTCTTTGTGATGTTTGCATCCAGCTCTCAGAGTTGA
          1 AAAAACTAGACAGAAGCATTCTCAGAAACATCTTTGTGATGTTTGCACTCAACTCACAGAGTTGA

              *  * *          *  *        *        *   **           **       
     329413 ACATTCCCTTTCATAGAGTAGGTTTGAAACCCTCTTTTTATAGTGTCTGGAAGTGGTCATTTGGA
         66 ACCTTTCTTTTCATAGAGCAGTTTTGAAACACTCTTTTTGTAGAATCTGGAAGTGGATATTTGGA

                   *   ** ** *  *   *          * *   
     329478 GCGCTTTCAGGCCTATGCTGAAAAAGGAAATATCTACCTAT
        131 GCGCTTTGAGGATTTCGTTGGAAACGGAAATATCTTCATAT

             *                    *    *  **          ** *        *    *     
     329519 AGAAACTAGACAGAAGCATTCTGAGAATCACGTTTGTGATGTGGGTACTCAACTAACAGTGTTGA
          1 AAAAACTAGACAGAAGCATTCTCAGAAACATCTTTGTGATGTTTGCACTCAACTCACAGAGTTGA

            *  *       *   *            *   *                *               
     329584 TCCATTCTTTTGATACAGCAGTTTTGAACCACACTTTTTGTAGAATCTGCAAGTGGATATTTGGA
         66 ACCTTTCTTTTCATAGAGCAGTTTTGAAACACTCTTTTTGTAGAATCTGGAAGTGGATATTTGGA

            **   *                     *   *        *
     329649 TAGCTGTGAGGATTTCGTTGGAAACGGGAATGTCTTCATAG
        131 GCGCTTTGAGGATTTCGTTGGAAACGGAAATATCTTCATAT

                **                     * * **         *  **   *   *          
     329690 AAAATTTAGACAGAAGCATTCTCAGAACCTTGATTGTGATGTGTGTTCTCCACTAACAGAGTTGA
          1 AAAAACTAGACAGAAGCATTCTCAGAAACATCTTTGTGATGTTTGCACTCAACTCACAGAGTTGA

                       * *   * *   *       *       *                         
     329755 ACCTTTCTTTTGACAGAACTGTTCTGAAACATTCTTTTTATAGAATCTGGAAGTGGATATTTGGA
         66 ACCTTTCTTTTCATAGAGCAGTTTTGAAACACTCTTTTTGTAGAATCTGGAAGTGGATATTTGGA

            **              *          *          *  
     329820 AAGCTTTGAGGATTTCATTGGAAACGGGAATATCTTCAAAT
        131 GCGCTTTGAGGATTTCGTTGGAAACGGAAATATCTTCATAT

            *   *    *            *           * *       *  *    *            
     329861 CAAATCTAGCCAGAAGCATTCTAAGAAACATCTTAGGGATGTTTACATTCAAGTCACAGAGTTGA
          1 AAAAACTAGACAGAAGCATTCTCAGAAACATCTTTGTGATGTTTGCACTCAACTCACAGAGTTGA

              *  * *     *       *         *    * *   **      *      *    *  
     329926 ACATTCCCTTTCACAGAGCAGGTTTGAAACAATCTTCTCGTACTATCTGGCAGTGGACATTTTGA
         66 ACCTTTCTTTTCATAGAGCAGTTTTGAAACACTCTTTTTGTAGAATCTGGAAGTGGATATTTGGA

               *        *  ** *  *   *            ** *
     329991 GCTCCTTTG-GGCTTATGGTGAAAAAGGAAATATCTTCCGAC
        131 GC-GCTTTGAGGATTTCGTTGGAAACGGAAATATCTTCATAT

                                 *     *  **          *           **    *    
     330032 AAAAACTAGACAGAAGCATTCGCAGAATCACGTTTGTGATGTGTGCACTCAACTGTCAGAATTGA
          1 AAAAACTAGACAGAAGCATTCTCAGAAACATCTTTGTGATGTTTGCACTCAACTCACAGAGTTGA

                 **   ** *      *                            * *            *
     330097 ACCTTGGTTTGGACAGAGCACTTTTGAAACACTCTTTTTGTAGAATCTGCAGGTGGATATTTGGC
         66 ACCTTTCTTTTCATAGAGCAGTTTTGAAACACTCTTTTTGTAGAATCTGGAAGTGGATATTTGGA

            **   *                     *   *      * *
     330162 TAGCTCTGAGGATTTCGTTGGAAACGGTAATGTCTTCAAAG
        131 GCGCTTTGAGGATTTCGTTGGAAACGGAAATATCTTCATAT

                *                         *   *            *    *            
     330203 AAAATCTAGACAGAAGCATTCTCAGAAACACCTTCGTGATGTTTGCAATCAAGTCACAGAGTTGA
          1 AAAAACTAGACAGAAGCATTCTCAGAAACATCTTTGTGATGTTTGCACTCAACTCACAGAGTTGA

                 * *             *  *                  *             *       
     330268 ACCTTCCGTTTCATAGAGCAGGTTGGAAACACTCTTTTTGTAGTATCTGGAAGTGGACATTTGGA
         66 ACCTTTCTTTTCATAGAGCAGTTTTGAAACACTCTTTTTGTAGAATCTGGAAGTGGATATTTGGA

                   *   ** ** *  *   *            **  
     330333 GCGCTTTCAGGCCTATGGTGAAAAAGGAAATATCTTCCCAT
        131 GCGCTTTGAGGATTTCGTTGGAAACGGAAATATCTTCATAT

                  * * *      **      *   * *   *     ** ** *      *    *     
     330374 AAAAACGACATAGAAGCTATCTCAGGAACTTGTTTATGATGCATCTAATCAACTAACAGTGTTGA
          1 AAAAACTAGACAGAAGCATTCTCAGAAACATCTTTGTGATGTTTGCACTCAACTCACAGAGTTGA

                  * ** * *                         * *       *               
     330439 ACCTTTGTACTGACAGAGCAG-TTTGAAACACTCTTTTTTTGGAATCTGCAAGTGGATATTTGGA
         66 ACCTTTCTTTTCATAGAGCAGTTTTGAAACACTCTTTTTGTAGAATCTGGAAGTGGATATTTGGA

            *                           *   *   *    
     330503 TCGCTTTGAGGATTTCGTTGGAAACGG-GAT-GC--AATAT
        131 GCGCTTTGAGGATTTCGTTGGAAACGGAAATATCTTCATAT

                **  *    *     *                 **  *   *  *    *         * 
     330540 AAAACGTACACAGCAGCATACTCAGAAA-ATACTTTGCCATATTTCCATTCAAGTCACAGAGTGG
          1 AAAAACTAGACAGAAGCATTCTCAGAAACAT-CTTTGTGATGTTTGCACTCAACTCACAGAGTTG

               *  * **            *  *               *  *             *      
     330604 AACATTCCCATTCATAGAGCAGGTTGGAAACACTCTTTTTGGAGTATCTGGAAGTGGACATTTGG
         65 AACCTTTCTTTTCATAGAGCAGTTTTGAAACACTCTTTTTGTAGAATCTGGAAGTGGATATTTGG

                    ** * * ** *  *   *            **  
     330669 AGCGCTTTCTGAACTATGGTGAAAAAGGAAATATCTTCCAAT
        130 AGCGCTTTGAGGATTTCGTTGGAAACGGAAATATCTTCATAT

            *     *               *      * *          *  **      **  *  *    
     330711 GAAAACAAGACAGAAGCATTCTGAGAAACTTATTTGTGATGTGTGTCCTCAACAAACGGACTTGA
          1 AAAAACTAGACAGAAGCATTCTCAGAAACATCTTTGTGATGTTTGCACTCAACTCACAGAGTTGA

                   *      **  * *  *  *             *   *    * * *           
     330776 ACCTTTCGTTTCATGCAGTACTTCTGGAACACTCTTTTTGAAGATTCTGCATGCGGATATTTGGA
         66 ACCTTTCTTTTCATAGAGCAGTTTTGAAACACTCTTTTTGTAGAATCTGGAAGTGGATATTTGGA

            **                            **   *   * 
     330841 TAGCTTTGAGGATTTCGTTGGAAACGG---GCT-TACATGT
        131 GCGCTTTGAGGATTTCGTTGGAAACGGAAATATCTTCATAT

                 *       *               *        *   *    *    *       *    
     330878 AAAAATTAGACAGCAGCATTCTCAGAAACTTCTTTGTGGTGTCTGCATTCAAGTCACAGAATTGA
          1 AAAAACTAGACAGAAGCATTCTCAGAAACATCTTTGTGATGTTTGCACTCAACTCACAGAGTTGA

              * ** * **            *  * *      *       *    *        *       
     330943 ACTTCCCCTCACATAGAGCAGTTGTGCAGCACTCTATTTGTAGTATCTCGAAGTGGACATTTGGA
         66 ACCTTTCTTTTCATAGAGCAGTTTTGAAACACTCTTTTTGTAGAATCTGGAAGTGGATATTTGGA

             *         ** *    * *   *            **  
     331008 GGGCTTTGTAGCCTATC-TGGAAAAAGGAAATATCTTCCCAT
        131 GCGCTTTG-AGGATTTCGTTGGAAACGGAAATATCTTCATAT

            *  ** *   *     * *            *   *  *   * **        *  * * *   
     331049 GAATGCGAGATAGAAGTAATCTCAGAAACATGTTTATGCTGTATCTACTCAACTAACTGTGCTGA
          1 AAAAACTAGACAGAAGCATTCTCAGAAACATCTTTGTGATGTTTGCACTCAACTCACAGAGTTGA

              *     *  *               *        *  * *     * *               
     331114 ACATTTCTATTGATAGAGCAGTTTTGAGACACTCTTCTTTTGGAATCCGCAAGTGGATATTTGGA
         66 ACCTTTCTTTTCATAGAGCAGTTTTGAAACACTCTTTTTGTAGAATCTGGAAGTGGATATTTGGA

             * *                        *     *      
     331179 GAGATTTGAGGATTTCGTTGGAAACGG-GAT-T-AT-ATAT
        131 GCGCTTTGAGGATTTCGTTGGAAACGGAAATATCTTCATAT

                **       *               *                 **  *   *         
     331216 AAAAGGTAGACAGCAGCATTCTCAGAAACTTCTTTGTGATGTTTGCATCCAGCTCTCAGAGTTGA
          1 AAAAACTAGACAGAAGCATTCTCAGAAACATCTTTGTGATGTTTGCACTCAACTCACAGAGTTGA

              *  * *          *  *        *        *   **    *   *  **       
     331281 ACATTCCCTTTCATAGAGTAGGTTTGAAACCCTCTTTTTATAGTGTCTGCAAGCGGGCATTTGGA
         66 ACCTTTCTTTTCATAGAGCAGTTTTGAAACACTCTTTTTGTAGAATCTGGAAGTGGATATTTGGA

                   *    * * **  **  **          * *  *
     331346 GCGCTTTCAGGCCTATGCTTAAAATAGGAAATATCTACCTAC
        131 GCGCTTTGAGG-ATTTCGTTGGAAACGGAAATATCTTCATAT

             *                    *    *  **          ** *        *    *     
     331388 AGAAACTAGACAGAAGCATTCTGAGAATCACGTTTGTGATGTGGGTACTCAACTAACAGTGTTGA
          1 AAAAACTAGACAGAAGCATTCTCAGAAACATCTTTGTGATGTTTGCACTCAACTCACAGAGTTGA

            *  *       *   *            *   *                *   *           
     331453 TCCATTCTTTTGATACAGCAGTTTTGAACCACACTTTTTGTAGAATCTGCAAGAGGATATTTGGA
         66 ACCTTTCTTTTCATAGAGCAGTTTTGAAACACTCTTTTTGTAGAATCTGGAAGTGGATATTTGGA

            **   *                     *   *      * *
     331518 TAGCTGTGAGGATTTCGTTGGAAACGGGAATGTCTTCAAAG
        131 GCGCTTTGAGGATTTCGTTGGAAACGGAAATATCTTCATAT

                *                         *   *            *    *            
     331559 AAAATCTAGACAGAAGCATTCTCAGAAACACCTTCGTGATGTTTGCAATCAAGTCACAGAGTTGA
          1 AAAAACTAGACAGAAGCATTCTCAGAAACATCTTTGTGATGTTTGCACTCAACTCACAGAGTTGA

                 * *             *  *           *      *             *       
     331624 ACCTTCCGTTTCATAGAGCAGGTTGGAAACACTCTTATTGTAGTATCTGGAAGTGGACATTT-GA
         66 ACCTTTCTTTTCATAGAGCAGTTTTGAAACACTCTTTTTGTAGAATCTGGAAGTGGATATTTGGA

                   *   ** ** *  *   *            **  
     331688 GCGCTTTCAGGCCTATGGTGAAAAAGGAAATATCTTCCCAT
        131 GCGCTTTGAGGATTTCGTTGGAAACGGAAATATCTTCATAT

                  * * *      **      *   * *   *     ** ** *      *    *     
     331729 AAAAACGACATAGAAGCTATCTCAGGAACTTGTTTATGATGCATCTAATCAACTAACAGTGTTGA
          1 AAAAACTAGACAGAAGCATTCTCAGAAACATCTTTGTGATGTTTGCACTCAACTCACAGAGTTGA

                  * ** * *                         * *       *               
     331794 ACCTTTGTACTGACAGAGCAG-TTTGAAACACTCTTTTTTTGGAATCTGCAAGTGGATATTTGGA
         66 ACCTTTCTTTTCATAGAGCAGTTTTGAAACACTCTTTTTGTAGAATCTGGAAGTGGATATTTGGA

            *                           *   *   *    
     331858 TCGCTTTGAGGATTTCGTTGGAAACGG-GAT-GC--AATAT
        131 GCGCTTTGAGGATTTCGTTGGAAACGGAAATATCTTCATAT

                **  *    *     *                 **  *   *  *    *         * 
     331895 AAAACGTACACAGCAGCATACTCAGAAA-ATACTTTGCCATATTTCCATTCAAGTCACAGAGTGG
          1 AAAAACTAGACAGAAGCATTCTCAGAAACAT-CTTTGTGATGTTTGCACTCAACTCACAGAGTTG

               *  * **            *                  *  *             *      
     331959 AACATTCCCATTCATAGAGCAGGTTTGAAACACTCTTTTTGGAGTATCTGGAAGTGGACATTTGG
         65 AACCTTTCTTTTCATAGAGCAGTTTTGAAACACTCTTTTTGTAGAATCTGGAAGTGGATATTTGG

                    ** * * ** *  *   *            **  
     332024 AGCGCTTTCTGAACTATGGTGAAAAAGGAAATATCTTCCAAT
        130 AGCGCTTTGAGGATTTCGTTGGAAACGGAAATATCTTCATAT

            *     *               *      * *          *  **      **  *  *    
     332066 GAAAACAAGACAGAAGCATTCTGAGAAACTTATTTGTGATGTGTGTCCTCAACAAACGGACTTGA
          1 AAAAACTAGACAGAAGCATTCTCAGAAACATCTTTGTGATGTTTGCACTCAACTCACAGAGTTGA

                   *      **  * *  *  *             *   *    * * *           
     332131 ACCTTTCGTTTCATGCAGTACTTCTGGAACACTCTTTTTGAAGATTCTGCATGCGGATATTTGGA
         66 ACCTTTCTTTTCATAGAGCAGTTTTGAAACACTCTTTTTGTAGAATCTGGAAGTGGATATTTGGA

            **                            **   *   * 
     332196 TAGCTTTGAGGATTTCGTTGGAAACGG---GCT-TACATGT
        131 GCGCTTTGAGGATTTCGTTGGAAACGGAAATATCTTCATAT

                 *       *               *        *   *    *    *       *    
     332233 AAAAATTAGACAGCAGCATTCTCAGAAACTTCTTTGTGGTGTCTGCATTCAAGTCACAGAATTGA
          1 AAAAACTAGACAGAAGCATTCTCAGAAACATCTTTGTGATGTTTGCACTCAACTCACAGAGTTGA

              * ** * **            *  * *      *       *             *       
     332298 ACTTCCCCTCACATAGAGCAGTTGTGCAGCACTCTATTTGTAGTATCTGGAAGTGGACATTTGGA
         66 ACCTTTCTTTTCATAGAGCAGTTTTGAAACACTCTTTTTGTAGAATCTGGAAGTGGATATTTGGA

             *         ** *    * *   *            **  
     332363 GGGCTTTGTAGCCTATC-TGGAAAAAGGAAATATCTTCCCAT
        131 GCGCTTTG-AGGATTTCGTTGGAAACGGAAATATCTTCATAT

            *  ** *   *     * *            *   *  *   * **        *  * * *   
     332404 GAATGCGAGATAGAAGTAATCTCAGAAACATGTTTATGCTGTATCTACTCAACTAACTGTGCTGA
          1 AAAAACTAGACAGAAGCATTCTCAGAAACATCTTTGTGATGTTTGCACTCAACTCACAGAGTTGA

              *     *  *               *        *  * *       *               
     332469 ACATTTCTATTGATAGAGCAGTTTTGAGACACTCTTATTTTGGAATCTGCAAGTGGATATTTGGA
         66 ACCTTTCTTTTCATAGAGCAGTTTTGAAACACTCTTTTTGTAGAATCTGGAAGTGGATATTTGGA

            ** *                        *     *      
     332534 TAGATTTGAGGATTTCGTTGGAAACGG-GAT-T-AT-ATAT
        131 GCGCTTTGAGGATTTCGTTGGAAACGGAAATATCTTCATAT

            *    *       *               *                 **  *   *         
     332571 CAAAAGTAGACAGCAGCATTCTCAGAAACTTCTTTGTGATGTTTGCATCCAGCTCTCAGAGTTGA
          1 AAAAACTAGACAGAAGCATTCTCAGAAACATCTTTGTGATGTTTGCACTCAACTCACAGAGTTGA

              *  * *          *  *        *        *   **        *  **       
     332636 ACATTCCCTTTCATAGAGTAGGTTTGAAACCCTCTTTTTATAGTGTCTGGAAGCGGGCATTTGGA
         66 ACCTTTCTTTTCATAGAGCAGTTTTGAAACACTCTTTTTGTAGAATCTGGAAGTGGATATTTGGA

                   *    * * **  **  **          * *  *
     332701 GCGCTTTCAGGCCTATGCTTAAAATAGGAAATATCTACCTAC
        131 GCGCTTTGAGG-ATTTCGTTGGAAACGGAAATATCTTCATAT

             *                    *    *  **          ** *        *    *     
     332743 AGAAACTAGACAGAAGCATTCTGAGAATCACGTTTGTGATGTGGGTACTCAACTAACAGTGTTGA
          1 AAAAACTAGACAGAAGCATTCTCAGAAACATCTTTGTGATGTTTGCACTCAACTCACAGAGTTGA

            *  *       *   *            *   *                *   *           
     332808 TCCATTCTTTTGATACAGCAGTTTTGAACCACACTTTTTGTAGAATCTGCAAGAGGATATTTGGA
         66 ACCTTTCTTTTCATAGAGCAGTTTTGAAACACTCTTTTTGTAGAATCTGGAAGTGGATATTTGGA

            **   *                     *   *      * *
     332873 TAGCTGTGAGGATTTCGTTGGAAACGGGAATGTCTTCAAAG
        131 GCGCTTTGAGGATTTCGTTGGAAACGGAAATATCTTCATAT

                *                         *   *            *    *            
     332914 AAAATCTAGACAGAAGCATTCTCAGAAACACCTTCGTGATGTTTGCAATCAAGTCACAGAGTTGA
          1 AAAAACTAGACAGAAGCATTCTCAGAAACATCTTTGTGATGTTTGCACTCAACTCACAGAGTTGA

                 * *             *  *           *      *             *       
     332979 ACCTTCCGTTTCATAGAGCAGGTTGGAAACACTCTTATTGTAGTATCTGGAAGTGGACATTTGGA
         66 ACCTTTCTTTTCATAGAGCAGTTTTGAAACACTCTTTTTGTAGAATCTGGAAGTGGATATTTGGA

                   *   ** ** *  *   *            **  
     333044 GCGCTTTCAGGCCTATGGTGAAAAAGGAAATATCTTCCCAT
        131 GCGCTTTGAGGATTTCGTTGGAAACGGAAATATCTTCATAT

                  * * *      **      *   * *   *     ** ** *      *    *     
     333085 AAAAACGACATAGAAGCTATCTCAGGAACTTGTTTATGATGCATCTAATCAACTAACAGTGTTGA
          1 AAAAACTAGACAGAAGCATTCTCAGAAACATCTTTGTGATGTTTGCACTCAACTCACAGAGTTGA

                  * ** * *                         * *       *               
     333150 ACCTTTGTACTGACAGAGCAG-TTTGAAACACTCTTTTTTTGGAATCTGCAAGTGGATATTTGGA
         66 ACCTTTCTTTTCATAGAGCAGTTTTGAAACACTCTTTTTGTAGAATCTGGAAGTGGATATTTGGA

            *                           *   *   *    
     333214 TCGCTTTGAGGATTTCGTTGGAAACGG-GAT-GC--AATAT
        131 GCGCTTTGAGGATTTCGTTGGAAACGGAAATATCTTCATAT

                **  *    *     *                 **  *   *  *    *         * 
     333251 AAAACGTACACAGCAGCATACTCAGAAA-ATACTTTGCCATATTTCCATTCAAGTCACAGAGTGG
          1 AAAAACTAGACAGAAGCATTCTCAGAAACAT-CTTTGTGATGTTTGCACTCAACTCACAGAGTTG

               *  * **            *                  *  *             *      
     333315 AACATTCCCATTCATAGAGCAGGTTTGAAACACTCTTTTTGGAGTATCTGGAAGTGGACATTTGG
         65 AACCTTTCTTTTCATAGAGCAGTTTTGAAACACTCTTTTTGTAGAATCTGGAAGTGGATATTTGG

                    ** * * ** *  *   *            **  
     333380 AGCGCTTTCTGAACTATGGTGAAAAAGGAAATATCTTCCAAT
        130 AGCGCTTTGAGGATTTCGTTGGAAACGGAAATATCTTCATAT

            *     *               *      * *          *  **      **  *  *    
     333422 GAAAACAAGACAGAAGCATTCTGAGAAACTTATTTGTGATGTGTGTCCTCAACAAACGGACTTGA
          1 AAAAACTAGACAGAAGCATTCTCAGAAACATCTTTGTGATGTTTGCACTCAACTCACAGAGTTGA

                   *      **  * *  *  *             *   *    * * *           
     333487 ACCTTTCGTTTCATGCAGTACTTCTGGAACACTCTTTTTGAAGATTCTGCATGCGGATATTTGGA
         66 ACCTTTCTTTTCATAGAGCAGTTTTGAAACACTCTTTTTGTAGAATCTGGAAGTGGATATTTGGA

            **                            **   *   * 
     333552 TAGCTTTGAGGATTTCGTTGGAAACGG---GCT-TACATGT
        131 GCGCTTTGAGGATTTCGTTGGAAACGGAAATATCTTCATAT

                 *       *               *        *   *    *    *       *    
     333589 AAAAATTAGACAGCAGCATTCTCAGAAACTTCTTTGTGGTGTCTGCATTCAAGTCACAGAATTGA
          1 AAAAACTAGACAGAAGCATTCTCAGAAACATCTTTGTGATGTTTGCACTCAACTCACAGAGTTGA

              * ** * **            *  * *      *       *             *       
     333654 ACTTCCCCTCACATAGAGCAGTTGTGCAGCACTCTATTTGTAGTATCTGGAAGTGGACATTTGGA
         66 ACCTTTCTTTTCATAGAGCAGTTTTGAAACACTCTTTTTGTAGAATCTGGAAGTGGATATTTGGA

             *         ** *    * *   *            **  
     333719 GGGCTTTGTAGCCTATC-TGGAAAAAGGAAATATCTTCCCAT
        131 GCGCTTTG-AGGATTTCGTTGGAAACGGAAATATCTTCATAT

            *  ** *   *     * *            *   *  *   * **        *  * * *   
     333760 GAATGCGAGATAGAAGTAATCTCAGAAACATGTTTATGCTGTATCTACTCAACTAACTGTGCTGA
          1 AAAAACTAGACAGAAGCATTCTCAGAAACATCTTTGTGATGTTTGCACTCAACTCACAGAGTTGA

              *     *  *               *        *  * *       *               
     333825 ACATTTCTATTGATAGAGCAGTTTTGAGACACTCTTCTTTTGGAATCTGCAAGTGGATATTTGGA
         66 ACCTTTCTTTTCATAGAGCAGTTTTGAAACACTCTTTTTGTAGAATCTGGAAGTGGATATTTGGA

            ** *                        *     *      
     333890 TAGATTTGAGGATTTCGTTGGAAACGG-GAT-T-AT-ATAT
        131 GCGCTTTGAGGATTTCGTTGGAAACGGAAATATCTTCATAT

            *    *       *               *                 **  *   *         
     333927 CAAAAGTAGACAGCAGCATTCTCAGAAACTTCTTTGTGATGTTTGCATCCAGCTCTCAGAGTTGA
          1 AAAAACTAGACAGAAGCATTCTCAGAAACATCTTTGTGATGTTTGCACTCAACTCACAGAGTTGA

              *  * *          *  *        *        *   **           **       
     333992 ACATTCCCTTTCATAGAGTAGGTTTGAAACCCTCTTTTTATAGTGTCTGGAAGTGGTCATTTGGA
         66 ACCTTTCTTTTCATAGAGCAGTTTTGAAACACTCTTTTTGTAGAATCTGGAAGTGGATATTTGGA

                   *   ** ** *  *   *          * *   
     334057 GCGCTTTCAGGCCTATGCTGAAAAAGGAAATATCTACCTAT
        131 GCGCTTTGAGGATTTCGTTGGAAACGGAAATATCTTCATAT

             *                    *    *  **          ** *        *    *     
     334098 AGAAACTAGACAGAAGCATTCTGAGAATCACGTTTGTGATGTGGGTACTCAACTAACAGTGTTGA
          1 AAAAACTAGACAGAAGCATTCTCAGAAACATCTTTGTGATGTTTGCACTCAACTCACAGAGTTGA

            *  *       *   *            *   *                *               
     334163 TCCATTCTTTTGATACAGCAGTTTTGAACCACACTTTTTGTAGAATCTGCAAGTGGATATTTGGA
         66 ACCTTTCTTTTCATAGAGCAGTTTTGAAACACTCTTTTTGTAGAATCTGGAAGTGGATATTTGGA

            **   *                     *   *        *
     334228 TAGCTGTGAGGATTTCGTTGGAAACGGGAATGTCTTCATAG
        131 GCGCTTTGAGGATTTCGTTGGAAACGGAAATATCTTCATAT

                **                     * * **         *  **   *   *          
     334269 AAAATTTAGACAGAAGCATTCTCAGAACCTTGATTGTGATGTGTGTTCTCCACTAACAGAGTTGA
          1 AAAAACTAGACAGAAGCATTCTCAGAAACATCTTTGTGATGTTTGCACTCAACTCACAGAGTTGA

                       * *   * *   *       *       *                         
     334334 ACCTTTCTTTTGACAGAACTGTTCTGAAACATTCTTTTTATAGAATCTGGAAGTGGATATTTGGA
         66 ACCTTTCTTTTCATAGAGCAGTTTTGAAACACTCTTTTTGTAGAATCTGGAAGTGGATATTTGGA

            **              *          *          *  
     334399 AAGCTTTGAGGATTTCATTGGAAACGGGAATATCTTCAAAT
        131 GCGCTTTGAGGATTTCGTTGGAAACGGAAATATCTTCATAT

            *   *    *            *           * *       *  *    *            
     334440 CAAATCTAGCCAGAAGCATTCTAAGAAACATCTTAGGGATGTTTACATTCAAGTCACAGAGTTGA
          1 AAAAACTAGACAGAAGCATTCTCAGAAACATCTTTGTGATGTTTGCACTCAACTCACAGAGTTGA

              *  * *     *       *         *    * *   **      *      *    *  
     334505 ACATTCCCTTTCACAGAGCAGGTTTGAAACAATCTTCTCGTACTATCTGGCAGTGGACATTTTGA
         66 ACCTTTCTTTTCATAGAGCAGTTTTGAAACACTCTTTTTGTAGAATCTGGAAGTGGATATTTGGA

               *        ** ** *  *   *            ** *
     334570 GCTCCTTTG-GGCCTATGGTGAAAAAGGAAATATCTTCCGAC
        131 GC-GCTTTGAGGATTTCGTTGGAAACGGAAATATCTTCATAT

                                 *     *  **          *           **    *    
     334611 AAAAACTAGACAGAAGCATTCGCAGAATCACGTTTGTGATGTGTGCACTCAACTGTCAGAATTGA
          1 AAAAACTAGACAGAAGCATTCTCAGAAACATCTTTGTGATGTTTGCACTCAACTCACAGAGTTGA

                 **   ** *      *                            * *            *
     334676 ACCTTGGTTTGGACAGAGCACTTTTGAAACACTCTTTTTGTAGAATCTGCAGGTGGATATTTGGC
         66 ACCTTTCTTTTCATAGAGCAGTTTTGAAACACTCTTTTTGTAGAATCTGGAAGTGGATATTTGGA

            **   *                     *   *      * *
     334741 TAGCTCTGAGGATTTCGTTGGAAACGGTAATGTCTTCAAAG
        131 GCGCTTTGAGGATTTCGTTGGAAACGGAAATATCTTCATAT

                *                         *   *            *    *            
     334782 AAAATCTAGACAGAAGCATTCTCAGAAACACCTTCGTGATGTTTGCAATCAAGTCACAGAGTTGA
          1 AAAAACTAGACAGAAGCATTCTCAGAAACATCTTTGTGATGTTTGCACTCAACTCACAGAGTTGA

                 * *             *  *                  *             *       
     334847 ACCTTCCGTTTCATAGAGCAGGTTGGAAACACTCTTTTTGTAGTATCTGGAAGTGGACATTTGGA
         66 ACCTTTCTTTTCATAGAGCAGTTTTGAAACACTCTTTTTGTAGAATCTGGAAGTGGATATTTGGA

                   *   ** ** *  *   *            **  
     334912 GCGCTTTCAGGCCTATGGTGAAAAAGGAAATATCTTCCCAT
        131 GCGCTTTGAGGATTTCGTTGGAAACGGAAATATCTTCATAT

                  * * *      **      *   * *   *     ** ** *      *    *     
     334953 AAAAACGACATAGAAGCTATCTCAGGAACTTGTTTATGATGCATCTAATCAACTAACAGTGTTGA
          1 AAAAACTAGACAGAAGCATTCTCAGAAACATCTTTGTGATGTTTGCACTCAACTCACAGAGTTGA

                  * ** * *                         * *       *               
     335018 ACCTTTGTACTGACAGAGCAG-TTTGAAACACTCTTTTTTTGGAATCTGCAAGTGGATATTTGGA
         66 ACCTTTCTTTTCATAGAGCAGTTTTGAAACACTCTTTTTGTAGAATCTGGAAGTGGATATTTGGA

            *                           *   *   *    
     335082 TCGCTTTGAGGATTTCGTTGGAAACGG-GAT-GC--AATAT
        131 GCGCTTTGAGGATTTCGTTGGAAACGGAAATATCTTCATAT

                **  *    *     *                 **  *   *  *    *         * 
     335119 AAAACGTACACAGCAGCATACTCAGAAA-ATACTTTGCCATATTTCCATTCAAGTCACAGAGTGG
          1 AAAAACTAGACAGAAGCATTCTCAGAAACAT-CTTTGTGATGTTTGCACTCAACTCACAGAGTTG

               *  * **            *  *               *  *             *      
     335183 AACATTCCCATTCATAGAGCAGGTTGGAAACACTCTTTTTGGAGTATCTGGAAGTGGACATTTGG
         65 AACCTTTCTTTTCATAGAGCAGTTTTGAAACACTCTTTTTGTAGAATCTGGAAGTGGATATTTGG

                    ** * * ** *  *   *            **  
     335248 AGCGCTTTCTGAACTATGGTGAAAAAGGAAATATCTTCCAAT
        130 AGCGCTTTGAGGATTTCGTTGGAAACGGAAATATCTTCATAT

            *     *               *      * *          *  **      **  *  *    
     335290 GAAAACAAGACAGAAGCATTCTGAGAAACTTATTTGTGATGTGTGTCCTCAACAAACGGACTTGA
          1 AAAAACTAGACAGAAGCATTCTCAGAAACATCTTTGTGATGTTTGCACTCAACTCACAGAGTTGA

                   *      **  * *  *  *             *   *    * * *           
     335355 ACCTTTCGTTTCATGCAGTACTTCTGGAACACTCTTTTTGAAGATTCTGCATGCGGATATTTGGA
         66 ACCTTTCTTTTCATAGAGCAGTTTTGAAACACTCTTTTTGTAGAATCTGGAAGTGGATATTTGGA

            **                            **   *   * 
     335420 TAGCTTTGAGGATTTCGTTGGAAACGG---GCT-TACATGT
        131 GCGCTTTGAGGATTTCGTTGGAAACGGAAATATCTTCATAT

                 *       *               *        *   *    *    *       *    
     335457 AAAAATTAGACAGCAGCATTCTCAGAAACTTCTTTGTGGTGTCTGCATTCAAGTCACAGAATTGA
          1 AAAAACTAGACAGAAGCATTCTCAGAAACATCTTTGTGATGTTTGCACTCAACTCACAGAGTTGA

              * ** * **            *  * *      *       *    *        *       
     335522 ACATCCCCTCACATAGAGCAGTTGTGCAGCACTCTATTTGTAGTATCTCGAAGTGGACATTTGGA
         66 ACCTTTCTTTTCATAGAGCAGTTTTGAAACACTCTTTTTGTAGAATCTGGAAGTGGATATTTGGA

             *         ** *    * *   *            **  
     335587 GGGCTTTGTAGCCTATC-TGGAAAAAGGAAATATCTTCCCAT
        131 GCGCTTTG-AGGATTTCGTTGGAAACGGAAATATCTTCATAT

            *  ** *   *     * *            *   *  *   * **        *  * * *   
     335628 GAATGCGAGATAGAAGTAATCTCAGAAACATGTTTATGCTGTATCTACTCAACTAACTGTGCTGA
          1 AAAAACTAGACAGAAGCATTCTCAGAAACATCTTTGTGATGTTTGCACTCAACTCACAGAGTTGA

              *     *  *               *        *  * *       *               
     335693 ACATTTCTATTGATAGAGCAGTTTTGAGACACTCTTCTTTTGGAATCTGCAAGTGGATATTTGGA
         66 ACCTTTCTTTTCATAGAGCAGTTTTGAAACACTCTTTTTGTAGAATCTGGAAGTGGATATTTGGA

            ** *                        *     *      
     335758 TAGATTTGAGGATTTCGTTGGAAACGG-GAT-T-AT-ATAT
        131 GCGCTTTGAGGATTTCGTTGGAAACGGAAATATCTTCATAT

                 *       *               *                 **  *   *         
     335795 AAAAAGTAGACAGCAGCATTCTCAGAAACTTCTTTGTGATGTTTGCATCCAGCTCTCAGAGTTGA
          1 AAAAACTAGACAGAAGCATTCTCAGAAACATCTTTGTGATGTTTGCACTCAACTCACAGAGTTGA

              *  * *          *  *        *        *   **        *  **       
     335860 ACATTCCCTTTCATAGAGTAGGTTTGAAACCCTCTTTTTATAGTGTCTGGAAGCGGGCATTTGGA
         66 ACCTTTCTTTTCATAGAGCAGTTTTGAAACACTCTTTTTGTAGAATCTGGAAGTGGATATTTGGA

                   *   ** ** *  *   *          * *   
     335925 GCGCTTTCAGGCCTATGCTGAAAAAGGAAATATCTACCTAT
        131 GCGCTTTGAGGATTTCGTTGGAAACGGAAATATCTTCATAT

             *                    *    *  **          ** *        *    *     
     335966 AGAAACTAGACAGAAGCATTCTGAGAATCACGTTTGTGATGTGGGTACTCAACTAACAGTGTTGA
          1 AAAAACTAGACAGAAGCATTCTCAGAAACATCTTTGTGATGTTTGCACTCAACTCACAGAGTTGA

            *  *       *   *            *   *                *               
     336031 TCCATTCTTTTGATACAGCAGTTTTGAACCACACTTTTTGTAGAATCTGCAAGTGGATATTTGGA
         66 ACCTTTCTTTTCATAGAGCAGTTTTGAAACACTCTTTTTGTAGAATCTGGAAGTGGATATTTGGA

            **   *                     *   *        *
     336096 TAGCTGTGAGGATTTCGTTGGAAACGGGAATGTCTTCATAG
        131 GCGCTTTGAGGATTTCGTTGGAAACGGAAATATCTTCATAT

                **                     * * **         *  **   *   *          
     336137 AAAATTTAGACAGAAGCATTCTCAGAACCTTGATTGTGATGTGTGTTCTCCACTAACAGAGTTGA
          1 AAAAACTAGACAGAAGCATTCTCAGAAACATCTTTGTGATGTTTGCACTCAACTCACAGAGTTGA

                       * *   * *   *       *       *                         
     336202 ACCTTTCTTTTGACAGAACTGTTCTGAAACATTCTTTTTATAGAATCTGGAAGTGGATATTTGGA
         66 ACCTTTCTTTTCATAGAGCAGTTTTGAAACACTCTTTTTGTAGAATCTGGAAGTGGATATTTGGA

            **                         *          *  
     336267 AAGCTTTGAGGATTTCGTTGGAAACGGGAATATCTTCAAAT
        131 GCGCTTTGAGGATTTCGTTGGAAACGGAAATATCTTCATAT

                *    *            *           * *       *  *    *            
     336308 AAAATCTAGCCAGAAGCATTCTAAGAAACATCTTAGGGATGTTTACATTCAAGTCACAGAGTTGA
          1 AAAAACTAGACAGAAGCATTCTCAGAAACATCTTTGTGATGTTTGCACTCAACTCACAGAGTTGA

              *  * *     *       *         *    * *   **      *      *    *  
     336373 ACATTCCCTTTCACAGAGCAGGTTTGAAACAATCTTCTCGTACTATCTGGCAGTGGACATTTTGA
         66 ACCTTTCTTTTCATAGAGCAGTTTTGAAACACTCTTTTTGTAGAATCTGGAAGTGGATATTTGGA

              * *   *  ** ** *  *   *            ** *
     336438 GCTCCTTGGGGCCTATGCTGAAAAAGGAAATATCTTCCGAC
        131 GCGCTTTGAGGATTTCGTTGGAAACGGAAATATCTTCATAT

                                 *     *  **          *           **    *    
     336479 AAAAACTAGACAGAAGCATTCGCAGAATCACGTTTGTGATGTGTGCACTCAACTGTCAGAATTGA
          1 AAAAACTAGACAGAAGCATTCTCAGAAACATCTTTGTGATGTTTGCACTCAACTCACAGAGTTGA

                 **   ** *      *                            * *            *
     336544 ACCTTGGTTTGGACAGAGCACTTTTGAAACACTCTTTTTGTAGAATCTGCAGGTGGATATTTGGC
         66 ACCTTTCTTTTCATAGAGCAGTTTTGAAACACTCTTTTTGTAGAATCTGGAAGTGGATATTTGGA

            **                         *   *      * *
     336609 TAGCTTTGAGGATTTCGTTGGAAACGGTAATGTCTTCAAAG
        131 GCGCTTTGAGGATTTCGTTGGAAACGGAAATATCTTCATAT

                *                         *   *            *    *            
     336650 AAAATCTAGACAGAAGCATTCTCAGAAACACCTTCGTGATGTTTGCAATCAAGTCACAGAGTTGA
          1 AAAAACTAGACAGAAGCATTCTCAGAAACATCTTTGTGATGTTTGCACTCAACTCACAGAGTTGA

                 * *             *  *                  *             *       
     336715 ACCTTCCGTTTCATAGAGCAGGTTGGAAACACTCTTTTTGTAGTATCTGGAAGTGGACATTTGGA
         66 ACCTTTCTTTTCATAGAGCAGTTTTGAAACACTCTTTTTGTAGAATCTGGAAGTGGATATTTGGA

             *         ** *    * *   *            **  
     336780 GGGCTTTGTAGCCTATC-TGGAAAAAGGAAATATCTTCCCAT
        131 GCGCTTTG-AGGATTTCGTTGGAAACGGAAATATCTTCATAT

            *  ** *   *     * *            *   *  *   * **        *  * * *   
     336821 GAATGCGAGATAGAAGTAATCTCAGAAACATGTTTATGCTGTATCTACTCAACTAACTGTGCTGA
          1 AAAAACTAGACAGAAGCATTCTCAGAAACATCTTTGTGATGTTTGCACTCAACTCACAGAGTTGA

              *     *  *               *        *  * *       *               
     336886 ACATTTCTATTGATAGAGCAGTTTTGAGACACTCTTCTTTTGGAATCTGCAAGTGGATATTTGGA
         66 ACCTTTCTTTTCATAGAGCAGTTTTGAAACACTCTTTTTGTAGAATCTGGAAGTGGATATTTGGA

            ** *                        *     *      
     336951 TAGATTTGAGGATTTCGTTGGAAACGG-GAT-T-AT-ATAT
        131 GCGCTTTGAGGATTTCGTTGGAAACGGAAATATCTTCATAT

                 *       *               *                 **  *   *         
     336988 AAAAAGTAGACAGCAGCATTCTCAGAAACTTCTTTGTGATGTTTGCATCCAGCTCTCAGAGTTGA
          1 AAAAACTAGACAGAAGCATTCTCAGAAACATCTTTGTGATGTTTGCACTCAACTCACAGAGTTGA

              *  * *          *  *        *        *   **        ** **       
     337053 ACATTCCCTTTCATAGAGTAGGTTTGAAACCCTCTTTTTATAGTGTCTGGAAGCAGGCATTTGGA
         66 ACCTTTCTTTTCATAGAGCAGTTTTGAAACACTCTTTTTGTAGAATCTGGAAGTGGATATTTGGA

                   *    * * **  **   *          * *  *
     337118 GCGCTTTCAGGCCTATGCTTAAAAAAGGAAATATCTACCTAC
        131 GCGCTTTGAGG-ATTTCGTTGGAAACGGAAATATCTTCATAT

             *                    *    *  **          ** *        *    *     
     337160 AGAAACTAGACAGAAGCATTCTGAGAATCACGTTTGTGATGTGGGTACTCAACTAACAGTGTTGA
          1 AAAAACTAGACAGAAGCATTCTCAGAAACATCTTTGTGATGTTTGCACTCAACTCACAGAGTTGA

            *  *       *   *            *   *                *   *           
     337225 TCCATTCTTTTGATACAGCAGTTTTGAACCACACTTTTTGTAGAATCTGCAAGAGGATATTTGGA
         66 ACCTTTCTTTTCATAGAGCAGTTTTGAAACACTCTTTTTGTAGAATCTGGAAGTGGATATTTGGA

            **   *                     *   *      * *
     337290 TAGCTGTGAGGATTTCGTTGGAAACGGGAATGTCTTCAAAG
        131 GCGCTTTGAGGATTTCGTTGGAAACGGAAATATCTTCATAT

                *                         *   *            *    *            
     337331 AAAATCTAGACAGAAGCATTCTCAGAAACACCTTCGTGATGTTTGCAATCAAGTCACAGAGTTGA
          1 AAAAACTAGACAGAAGCATTCTCAGAAACATCTTTGTGATGTTTGCACTCAACTCACAGAGTTGA

                 * *             *  *           *      *             *       
     337396 ACCTTCCGTTTCATAGAGCAGGTTGGAAACACTCTTATTGTAGTATCTGGAAGTGGACATTTGGA
         66 ACCTTTCTTTTCATAGAGCAGTTTTGAAACACTCTTTTTGTAGAATCTGGAAGTGGATATTTGGA

                   *   ** ** *  *   *            **  
     337461 GCGCTTTCAGGCCTATGGTGAAAAAGGAAATATCTTCCCAT
        131 GCGCTTTGAGGATTTCGTTGGAAACGGAAATATCTTCATAT

                  * * *      **      *   * *   *     ** ** *      *    *     
     337502 AAAAACGACATAGAAGCTATCTCAGGAACTTGTTTATGATGCATCTAATCAACTAACAGTGTTGA
          1 AAAAACTAGACAGAAGCATTCTCAGAAACATCTTTGTGATGTTTGCACTCAACTCACAGAGTTGA

                  * ** * *                         * *       *               
     337567 ACCTTTGTACTGACAGAGCAG-TTTGAAACACTCTTTTTTTGGAATCTGCAAGTGGATATTTGGA
         66 ACCTTTCTTTTCATAGAGCAGTTTTGAAACACTCTTTTTGTAGAATCTGGAAGTGGATATTTGGA

            *                           *   *   *    
     337631 TCGCTTTGAGGATTTCGTTGGAAACGG-GAT-GC--AATAT
        131 GCGCTTTGAGGATTTCGTTGGAAACGGAAATATCTTCATAT

                **  *    *     *                 **  *   *  *    *         * 
     337668 AAAACGTACACAGCAGCATACTCAGAAA-ATACTTTGCCATATTTCCATTCAAGTCACAGAGTGG
          1 AAAAACTAGACAGAAGCATTCTCAGAAACAT-CTTTGTGATGTTTGCACTCAACTCACAGAGTTG

               *  * **       *    *  *               *  *             *      
     337732 AACATTCCCATTCATAGCGCAGGTTGGAAACACTCTTTTTGGAGTATCTGGAAGTGGACATTTGG
         65 AACCTTTCTTTTCATAGAGCAGTTTTGAAACACTCTTTTTGTAGAATCTGGAAGTGGATATTTGG

                    ** * * ** *  *   *            **  
     337797 AGCGCTTTCTGAACTATGGTGAAAAAGGAAATATCTTCCAAT
        130 AGCGCTTTGAGGATTTCGTTGGAAACGGAAATATCTTCATAT

            *     *               *      * *          *  **      **  *  *    
     337839 GAAAACAAGACAGAAGCATTCTGAGAAACTTATTTGTGATGTGTGTCCTCAACAAACGGACTTGA
          1 AAAAACTAGACAGAAGCATTCTCAGAAACATCTTTGTGATGTTTGCACTCAACTCACAGAGTTGA

                   *      **  * *  *  *             *   *    * * *           
     337904 ACCTTTCGTTTCATGCAGTACTTCTGGAACACTCTTTTTGAAGATTCTGCATGCGGATATTTGGA
         66 ACCTTTCTTTTCATAGAGCAGTTTTGAAACACTCTTTTTGTAGAATCTGGAAGTGGATATTTGGA

            **                            **   *   * 
     337969 TTGCTTTGAGGATTTCGTTGGAAACGG---GCT-TACATGT
        131 GCGCTTTGAGGATTTCGTTGGAAACGGAAATATCTTCATAT

                 *       *               *        *   *    *    *       *    
     338006 AAAAATTAGACAGCAGCATTCTCAGAAACTTCTTTGTGGTGTCTGCATTCAAGTCACAGAATTGA
          1 AAAAACTAGACAGAAGCATTCTCAGAAACATCTTTGTGATGTTTGCACTCAACTCACAGAGTTGA

              * ** * **            *  * *      *       *             *   *   
     338071 ACTTCCCCTCACATAGAGCAGTTGTGCAGCACTCTATTTGTAGTATCTGGAAGTGGACATTCGGA
         66 ACCTTTCTTTTCATAGAGCAGTTTTGAAACACTCTTTTTGTAGAATCTGGAAGTGGATATTTGGA

             *         ** *    * *   *            **  
     338136 GGGCTTTGTAGCCTATC-TGGAAAAAGGAAATATCTTCCCAT
        131 GCGCTTTG-AGGATTTCGTTGGAAACGGAAATATCTTCATAT

            *  ** *   *     * *            *   *  *   * **        *  *** *   
     338177 GAATGCGAGATAGAAGTAATCTCAGAAACATGTTTATGCTGTATCTACTCAACTAACTTTGCTGA
          1 AAAAACTAGACAGAAGCATTCTCAGAAACATCTTTGTGATGTTTGCACTCAACTCACAGAGTTGA

              *     *  *               *        *  * *       *               
     338242 ACATTTCTATTGATAGAGCAGTTTTGAGACACTCTTCTTTTGGAATCTGCAAGTGGATATTTGGA
         66 ACCTTTCTTTTCATAGAGCAGTTTTGAAACACTCTTTTTGTAGAATCTGGAAGTGGATATTTGGA

            ** *                        *     *      
     338307 TAGATTTGAGGATTTCGTTGGAAACGG-GAT-T-AT-ATAT
        131 GCGCTTTGAGGATTTCGTTGGAAACGGAAATATCTTCATAT

            *    *       *               *                 **  *   *         
     338344 CAAAAGTAGACAGCAGCATTCTCAGAAACTTCTTTGTGATGTTTGCATCCAGCTCTCAGAGTTGA
          1 AAAAACTAGACAGAAGCATTCTCAGAAACATCTTTGTGATGTTTGCACTCAACTCACAGAGTTGA

              *  * *          *  *        *        *   **        *  **       
     338409 ACATTCCCTTTCATAGAGTAGGTTTGAAACCCTCTTTTTATAGTGTCTGGAAGCGGGCATTTGGA
         66 ACCTTTCTTTTCATAGAGCAGTTTTGAAACACTCTTTTTGTAGAATCTGGAAGTGGATATTTGGA

                   *   ** ** *  *   *          * *   
     338474 GCGCTTTCAGGCCTATGCTGAAAAAGGAAATATCTACCTAT
        131 GCGCTTTGAGGATTTCGTTGGAAACGGAAATATCTTCATAT

             *                    *    *  **          ** *        *    *     
     338515 AGAAACTAGACAGAAGCATTCTGAGAATCACGTTTGTGATGTGGGTACTCAACTAACAGTGTTGA
          1 AAAAACTAGACAGAAGCATTCTCAGAAACATCTTTGTGATGTTTGCACTCAACTCACAGAGTTGA

            *  *       *   *            *   *                *               
     338580 TCCATTCTTTTGATACAGCAGTTTTGAACCACACTTTTTGTAGAATCTGCAAGTGGATATTTGGA
         66 ACCTTTCTTTTCATAGAGCAGTTTTGAAACACTCTTTTTGTAGAATCTGGAAGTGGATATTTGGA

            **   *                     *   *        *
     338645 TAGCTGTGAGGATTTCGTTGGAAACGGGAATGTCTTCATAG
        131 GCGCTTTGAGGATTTCGTTGGAAACGGAAATATCTTCATAT

                **                     * * **         *  **   *   *          
     338686 AAAATTTAGACAGAAGCATTCTCAGAACCTTGATTGTGATGTGTGTTCTCCACTAACAGAGTTGA
          1 AAAAACTAGACAGAAGCATTCTCAGAAACATCTTTGTGATGTTTGCACTCAACTCACAGAGTTGA

                       * *   * *   *       *       *                         
     338751 ACCTTTCTTTTGACAGAACTGTTCTGAAACATTCTTTTTATAGAATCTGGAAGTGGATATTTGGA
         66 ACCTTTCTTTTCATAGAGCAGTTTTGAAACACTCTTTTTGTAGAATCTGGAAGTGGATATTTGGA

            **              *          *          *  
     338816 AAGCTTTGAGGATTTCATTGGAAACGGGAATATCTTCAAAT
        131 GCGCTTTGAGGATTTCGTTGGAAACGGAAATATCTTCATAT

            *   *    *            *           * *       *  *    *            
     338857 CAAATCTAGCCAGAAGCATTCTAAGAAACATCTTAGGGATGTTTACATTCAAGTCACAGAGTTGA
          1 AAAAACTAGACAGAAGCATTCTCAGAAACATCTTTGTGATGTTTGCACTCAACTCACAGAGTTGA

              *  * *     *       *         *    * *   **      *      *    *  
     338922 ACATTCCCTTTCACAGAGCAGGTTTGAAACAATCTTCTCGTACTATCTGGCAGTGGACATTTTGA
         66 ACCTTTCTTTTCATAGAGCAGTTTTGAAACACTCTTTTTGTAGAATCTGGAAGTGGATATTTGGA

               *        ** ** *  *   *            ** *
     338987 GCTCCTTTG-GGCCTATGGTGAAAAAGGAAATATCTTCCGAC
        131 GC-GCTTTGAGGATTTCGTTGGAAACGGAAATATCTTCATAT

                                 *     *  **          *           **    *    
     339028 AAAAACTAGACAGAAGCATTCGCAGAATCACGTTTGTGATGTGTGCACTCAACTGTCAGAATTGA
          1 AAAAACTAGACAGAAGCATTCTCAGAAACATCTTTGTGATGTTTGCACTCAACTCACAGAGTTGA

                 **   ** *      *                            * *            *
     339093 ACCTTGGTTTGGACAGAGCACTTTTGAAACACTCTTTTTGTAGAATCTGCAGGTGGATATTTGGC
         66 ACCTTTCTTTTCATAGAGCAGTTTTGAAACACTCTTTTTGTAGAATCTGGAAGTGGATATTTGGA

            **   *                     *   *      * *
     339158 TAGCTCTGAGGATTTCGTTGGAAACGGTAATGTCTTCAAAG
        131 GCGCTTTGAGGATTTCGTTGGAAACGGAAATATCTTCATAT

                *                         *   *            *    *            
     339199 AAAATCTAGACAGAAGCATTCTCAGAAACACCTTCGTGATGTTTGCAATCAAGTCACAGAGTTGA
          1 AAAAACTAGACAGAAGCATTCTCAGAAACATCTTTGTGATGTTTGCACTCAACTCACAGAGTTGA

                 * *             *  *                  *             *       
     339264 ACCTTCCGTTTCATAGAGCAGGTTGGAAACACTCTTTTTGTAGTATCTGGAAGTGGACATTTGGA
         66 ACCTTTCTTTTCATAGAGCAGTTTTGAAACACTCTTTTTGTAGAATCTGGAAGTGGATATTTGGA

             *         ** *    * *   *            **  
     339329 GGGCTTTGTAGCCTATC-TGGAAAAAGGAAATATCTTCCCAT
        131 GCGCTTTG-AGGATTTCGTTGGAAACGGAAATATCTTCATAT

            *  ** *   *     * *            *   *  *   * **        *  * * *   
     339370 GAATGCGAGATAGAAGTAATCTCAGAAACATGTTTATGCTGTATCTACTCAACTAACTGTGCTGA
          1 AAAAACTAGACAGAAGCATTCTCAGAAACATCTTTGTGATGTTTGCACTCAACTCACAGAGTTGA

              *     *  *               *        *  * *       *               
     339435 ACATTTCTATTGATAGAGCAGTTTTGAGACACTCTTCTTTTGGAATCTGCAAGTGGATATTTGGA
         66 ACCTTTCTTTTCATAGAGCAGTTTTGAAACACTCTTTTTGTAGAATCTGGAAGTGGATATTTGGA

            ** *                        *     *      
     339500 TAGATTTGAGGATTTCGTTGGAAACGG-GAT-T-AT-ATAT
        131 GCGCTTTGAGGATTTCGTTGGAAACGGAAATATCTTCATAT

                 *       *               *                 **  *   *         
     339537 AAAAAGTAGACAGCAGCATTCTCAGAAACTTCTTTGTGATGTTTGCATCCAGCTCTCAGAGTTGA
          1 AAAAACTAGACAGAAGCATTCTCAGAAACATCTTTGTGATGTTTGCACTCAACTCACAGAGTTGA

              *  * *          *  *        *        *   **        *  **       
     339602 ACATTCCCTTTCATAGAGTAGGTTTGAAACCCTCTTTTTATAGTGTCTGGAAGCGGGCATTTGGA
         66 ACCTTTCTTTTCATAGAGCAGTTTTGAAACACTCTTTTTGTAGAATCTGGAAGTGGATATTTGGA

                   *    * * **  **  **          * *  *
     339667 GCGCTTTCAGGCCTATGCTTAAAATAGGAAATATCTACCTAC
        131 GCGCTTTGAGG-ATTTCGTTGGAAACGGAAATATCTTCATAT

             *                    *    *  **          ** *        *    *     
     339709 AGAAACTAGACAGAAGCATTCTGAGAATCACGTTTGTGATGTGGGTACTCAACTAACAGTGTTGA
          1 AAAAACTAGACAGAAGCATTCTCAGAAACATCTTTGTGATGTTTGCACTCAACTCACAGAGTTGA

            *  *       *   *            *   *                *   *           
     339774 TCCATTCTTTTGATACAGCAGTTTTGAACCACACTTTTTGTAGAATCTGCAAGAGGATATTTGGA
         66 ACCTTTCTTTTCATAGAGCAGTTTTGAAACACTCTTTTTGTAGAATCTGGAAGTGGATATTTGGA

            **   *                     *   *      * *
     339839 TAGCTGTGAGGATTTCGTTGGAAACGGGAATGTCTTCAAAG
        131 GCGCTTTGAGGATTTCGTTGGAAACGGAAATATCTTCATAT

                *                         *   *            *    *            
     339880 AAAATCTAGACAGAAGCATTCTCAGAAACACCTTCGTGATGTTTGCAATCAAGTCACAGAGTTGA
          1 AAAAACTAGACAGAAGCATTCTCAGAAACATCTTTGTGATGTTTGCACTCAACTCACAGAGTTGA

                 * *             *  *           *      *             *       
     339945 ACCTTCCGTTTCATAGAGCAGGTTGGAAACACTCTTATTGTAGTATCTGGAAGTGGACATTTGGA
         66 ACCTTTCTTTTCATAGAGCAGTTTTGAAACACTCTTTTTGTAGAATCTGGAAGTGGATATTTGGA

                   *   ** ** *  *   *            **  
     340010 GCGCTTTCAGGCCTATGGTGAAAAAGGAAATATCTTCCCAT
        131 GCGCTTTGAGGATTTCGTTGGAAACGGAAATATCTTCATAT

                  * * *      **      *   * *   *     ** ** *      *    *     
     340051 AAAAACGACATAGAAGCTATCTCAGGAACTTGTTTATGATGCATCTAATCAACTAACAGTGTTGA
          1 AAAAACTAGACAGAAGCATTCTCAGAAACATCTTTGTGATGTTTGCACTCAACTCACAGAGTTGA

                  * ** * *                         * *       *               
     340116 ACCTTTGTACTGACAGAGCAG-TTTGAAACACTCTTTTTTTGGAATCTGCAAGTGGATATTTGGA
         66 ACCTTTCTTTTCATAGAGCAGTTTTGAAACACTCTTTTTGTAGAATCTGGAAGTGGATATTTGGA

            *                           *   *   *    
     340180 TCGCTTTGAGGATTTCGTTGGAAACGG-GAT-GC--AATAT
        131 GCGCTTTGAGGATTTCGTTGGAAACGGAAATATCTTCATAT

                **  *    *     *                 **  *   *  *    *         * 
     340217 AAAACGTACACAGCAGCATACTCAGAAA-ATACTTTGCCATATTTCCATTCAAGTCACAGAGTGG
          1 AAAAACTAGACAGAAGCATTCTCAGAAACAT-CTTTGTGATGTTTGCACTCAACTCACAGAGTTG

               *  * **            *  *               *  *             *      
     340281 AACATTCCCATTCATAGAGCAGGTTGGAAACACTCTTTTTGGAGTATCTGGAAGTGGACATTTGG
         65 AACCTTTCTTTTCATAGAGCAGTTTTGAAACACTCTTTTTGTAGAATCTGGAAGTGGATATTTGG

                    ** * * ** *  *   *            **  
     340346 AGCGCTTTCTGAACTATGGTGAAAAAGGAAATATCTTCCAAT
        130 AGCGCTTTGAGGATTTCGTTGGAAACGGAAATATCTTCATAT

            *     *               *      * *          *  **      **  *  *    
     340388 GAAAACAAGACAGAAGCATTCTGAGAAACTTATTTGTGATGTGTGTCCTCAACAAACGGACTTGA
          1 AAAAACTAGACAGAAGCATTCTCAGAAACATCTTTGTGATGTTTGCACTCAACTCACAGAGTTGA

                   *      **  * *  *  *             *   *    * * *           
     340453 ACCTTTCGTTTCATGCAGTACTTCTGGAACACTCTTTTTGAAGATTCTGCATGCGGATATTTGGA
         66 ACCTTTCTTTTCATAGAGCAGTTTTGAAACACTCTTTTTGTAGAATCTGGAAGTGGATATTTGGA

            **                            **   *   * 
     340518 TAGCTTTGAGGATTTCGTTGGAAACGG---GCT-TACATGT
        131 GCGCTTTGAGGATTTCGTTGGAAACGGAAATATCTTCATAT

                 *       *  *            *        *   *    *    *       *    
     340555 AAAAATTAGACAGCAGAATTCTCAGAAACTTCTTTGTGGTGTCTGCATTCAAGTCACAGAATTGA
          1 AAAAACTAGACAGAAGCATTCTCAGAAACATCTTTGTGATGTTTGCACTCAACTCACAGAGTTGA

              * ** * ***           *  ***      *       *             *       
     340620 ACTTCCCCTCATATAGAGCAGTTGTGCTGCACTCTATTTGTAGTATCTGGAAGTGGACATTTGGA
         66 ACCTTTCTTTTCATAGAGCAGTTTTGAAACACTCTTTTTGTAGAATCTGGAAGTGGATATTTGGA

             *         ** *    * *   *            **  
     340685 GGGCTTTGTAGCCTATC-TGGAAAAAGGAAATATCTTCCCAT
        131 GCGCTTTG-AGGATTTCGTTGGAAACGGAAATATCTTCATAT

            *  ** *   *     * *            *   *  *   * **        *  * * *   
     340726 GAATGCGAGATAGAAGTAATCTCAGAAACATGTTTATGCTGTATCTACTCAACTAACTGTGCTGA
          1 AAAAACTAGACAGAAGCATTCTCAGAAACATCTTTGTGATGTTTGCACTCAACTCACAGAGTTGA

              *     *  *               *        *  * *       *               
     340791 ACATTTCTATTGATAGAGCAGTTTTGAGACACTCTTCTTTTGGAATCTGCAAGTGGATATTTGGA
         66 ACCTTTCTTTTCATAGAGCAGTTTTGAAACACTCTTTTTGTAGAATCTGGAAGTGGATATTTGGA

            ** *                        *     *      
     340856 TAGATTTGAGGATTTCGTTGGAAACGG-GAT-T-AT-ATAT
        131 GCGCTTTGAGGATTTCGTTGGAAACGGAAATATCTTCATAT

                 *       *               *                 **  *   *         
     340893 AAAAAGTAGACAGCAGCATTCTCAGAAACTTCTTTGTGATGTTTGCATCCAGCTCTCAGAGTTGA
          1 AAAAACTAGACAGAAGCATTCTCAGAAACATCTTTGTGATGTTTGCACTCAACTCACAGAGTTGA

              *  * *          *  *        *        *   **        *  **       
     340958 ACATTCCCTTTCATAGAGTAGGTTTGAAACCCTCTTTTTATAGTGTCTGGAAGCGGGCATTTGGA
         66 ACCTTTCTTTTCATAGAGCAGTTTTGAAACACTCTTTTTGTAGAATCTGGAAGTGGATATTTGGA

                   *    * * **  **  **          * *  *
     341023 GCGCTTTCAGGCCTATGCTTAAAATAGGAAATATCTACCTAC
        131 GCGCTTTGAGG-ATTTCGTTGGAAACGGAAATATCTTCATAT

             *                    *    *  **          ** *        *    *     
     341065 AGAAACTAGACAGAAGCATTCTGAGAATCACGTTTGTGATGTGGGTACTCAACTAACAGTGTTGA
          1 AAAAACTAGACAGAAGCATTCTCAGAAACATCTTTGTGATGTTTGCACTCAACTCACAGAGTTGA

            *  *       *   *            *   *                *   *           
     341130 TCCATTCTTTTGATACAGCAGTTTTGAACCACACTTTTTGTAGAATCTGCAAGAGGATATTTGGA
         66 ACCTTTCTTTTCATAGAGCAGTTTTGAAACACTCTTTTTGTAGAATCTGGAAGTGGATATTTGGA

            **   *                     *   *      * *
     341195 TAGCTGTGAGGATTTCGTTGGAAACGGGAATGTCTTCAAAG
        131 GCGCTTTGAGGATTTCGTTGGAAACGGAAATATCTTCATAT

                *                         *   *            *    *            
     341236 AAAATCTAGACAGAAGCATTCTCAGAAACACCTTCGTGATGTTTGCAATCAAGTCACAGAGTTGA
          1 AAAAACTAGACAGAAGCATTCTCAGAAACATCTTTGTGATGTTTGCACTCAACTCACAGAGTTGA

                 * *             *  *           *      *             *       
     341301 ACCTTCCGTTTCATAGAGCAGGTTGGAAACACTCTTATTGTAGTATCTGGAAGTGGACATTTGGA
         66 ACCTTTCTTTTCATAGAGCAGTTTTGAAACACTCTTTTTGTAGAATCTGGAAGTGGATATTTGGA

                   *   ** ** *  *   *            **  
     341366 GCGCTTTCAGGCCTATGGTGAAAAAGGAAATATCTTCCCAT
        131 GCGCTTTGAGGATTTCGTTGGAAACGGAAATATCTTCATAT

                  * * *      **      *   * *         ** ** *      *    *     
     341407 AAAAACGACATAGAAGCTATCTCAGGAACTTGTTTGTGATGCATCTAATCAACTAACAGTGTTGA
          1 AAAAACTAGACAGAAGCATTCTCAGAAACATCTTTGTGATGTTTGCACTCAACTCACAGAGTTGA

                  * ** * *                         * *       *               
     341472 ACCTTTGTACTGACAGAGCAG-TTTGAAACACTCTTTTTTTGGAATCTGCAAGTGGATATTTGGA
         66 ACCTTTCTTTTCATAGAGCAGTTTTGAAACACTCTTTTTGTAGAATCTGGAAGTGGATATTTGGA

            *                           *   *   *    
     341536 TCGCTTTGAGGATTTCGTTGGAAACGG-GAT-GC--AATAT
        131 GCGCTTTGAGGATTTCGTTGGAAACGGAAATATCTTCATAT

                **  *    **    *                 **  *   *  *    *         * 
     341573 AAAACGTACACAGCGGCATACTCAGAAA-ATACTTTGCCATATTTCCATTCAAGTCACAGAGTGG
          1 AAAAACTAGACAGAAGCATTCTCAGAAACAT-CTTTGTGATGTTTGCACTCAACTCACAGAGTTG

               *  * **     *      *                  *  *             *      
     341637 AACATTCCCATTCATGGAGCAGGTTTGAAACACTCTTTTTGGAGTATCTGGAAGTGGACATTTGG
         65 AACCTTTCTTTTCATAGAGCAGTTTTGAAACACTCTTTTTGTAGAATCTGGAAGTGGATATTTGG

                    ** * * ** *  *   *            **  
     341702 AGCGCTTTCTGAACTATGGTGAAAAAGGAAATATCTTCCAAT
        130 AGCGCTTTGAGGATTTCGTTGGAAACGGAAATATCTTCATAT

            *     *               *      * *          *  **      **  *  *    
     341744 GAAAACAAGACAGAAGCATTCTGAGAAACTTATTTGTGATGTGTGTCCTCAACAAACGGACTTGA
          1 AAAAACTAGACAGAAGCATTCTCAGAAACATCTTTGTGATGTTTGCACTCAACTCACAGAGTTGA

                   *      **  * *  *  *             *   *    * * *           
     341809 ACCTTTCGTTTCATGCAGTACTTCTGGAACACTCTTTTTGAAGATTCTGCATGCGGATATTTGGA
         66 ACCTTTCTTTTCATAGAGCAGTTTTGAAACACTCTTTTTGTAGAATCTGGAAGTGGATATTTGGA

            **                            **   *   * 
     341874 TTGCTTTGAGGATTTCGTTGGAAACGG---GCT-TACATGT
        131 GCGCTTTGAGGATTTCGTTGGAAACGGAAATATCTTCATAT

                 *       *               *        *   *    *    *       *    
     341911 AAAAATTAGACAGCAGCATTCTCAGAAACTTCTTTGTGGTGTCTGCATTCAAGTCACAGAATTGA
          1 AAAAACTAGACAGAAGCATTCTCAGAAACATCTTTGTGATGTTTGCACTCAACTCACAGAGTTGA

              * ** * **            *  * *      *       *             *       
     341976 ACTTCACCTCACATAGAGCAGTTGTGCAGCACTCTATTTGTAGTATCTGGAAGTGGACATTTGGA
         66 ACCTTTCTTTTCATAGAGCAGTTTTGAAACACTCTTTTTGTAGAATCTGGAAGTGGATATTTGGA

             *         ** *    * *   *            **  
     342041 GGGCTTTGTAGCCTATC-TGGAAAAAGGAAATATCTTCCCAT
        131 GCGCTTTG-AGGATTTCGTTGGAAACGGAAATATCTTCATAT

            *  ** *   *  *  * *            *   *  *   * **        *  * * *   
     342082 GAATGCGAGATAGTAGTAATCTCAGAAACATGTTTATGCTGTATCTACTCAACTAACTGTGCTGA
          1 AAAAACTAGACAGAAGCATTCTCAGAAACATCTTTGTGATGTTTGCACTCAACTCACAGAGTTGA

              *     *  *               *        *  * *       *               
     342147 ACATTTCTATTGATAGAGCAGTTTTGAGACACTCTTCTTTTGGAATCTGCAAGTGGATATTTGGA
         66 ACCTTTCTTTTCATAGAGCAGTTTTGAAACACTCTTTTTGTAGAATCTGGAAGTGGATATTTGGA

            ** *                        *     *      
     342212 TAGATTTGAGGATTTCGTTGGAAACGG-GAT-T-AT-ATAT
        131 GCGCTTTGAGGATTTCGTTGGAAACGGAAATATCTTCATAT

                 *       *               *                 **  *   *         
     342249 AAAAAGTAGACAGCAGCATTCTCAGAAACTTCTTTGTGATGTTTGCATCCAGCTCTCAGAGTTGA
          1 AAAAACTAGACAGAAGCATTCTCAGAAACATCTTTGTGATGTTTGCACTCAACTCACAGAGTTGA

              *  * *          *  *        *        *   **        *  **       
     342314 ACATTCCCTTTCATAGAGTAGGTTTGAAACCCTCTTTTTATAGTGTCTGGAAGCGGGCATTTGGA
         66 ACCTTTCTTTTCATAGAGCAGTTTTGAAACACTCTTTTTGTAGAATCTGGAAGTGGATATTTGGA

                   *   ** ** *  *   *          * *   
     342379 GCGCTTTCAGGCCTATGCTGAAAAAGGAAATATCTACCTAT
        131 GCGCTTTGAGGATTTCGTTGGAAACGGAAATATCTTCATAT

             *                    *    *  **          ** *        *    *     
     342420 AGAAACTAGACAGAAGCATTCTGAGAATCACGTTTGTGATGTGGGTACTCAACTAACAGTGTTGA
          1 AAAAACTAGACAGAAGCATTCTCAGAAACATCTTTGTGATGTTTGCACTCAACTCACAGAGTTGA

            *  *       *   *            *   *                *               
     342485 TCCATTCTTTTGATACAGCAGTTTTGAACCACACTTTTTGTAGAATCTGCAAGTGGATATTTGGA
         66 ACCTTTCTTTTCATAGAGCAGTTTTGAAACACTCTTTTTGTAGAATCTGGAAGTGGATATTTGGA

            **   *                     *   *        *
     342550 TAGCTGTGAGGATTTCGTTGGAAACGGGAATGTCTTCATAG
        131 GCGCTTTGAGGATTTCGTTGGAAACGGAAATATCTTCATAT

                **                     * * **         *  **   *   *          
     342591 AAAATTTAGACAGAAGCATTCTCAGAACCTTGATTGTGATGTGTGTTCTCCACTAACAGAGTTGA
          1 AAAAACTAGACAGAAGCATTCTCAGAAACATCTTTGTGATGTTTGCACTCAACTCACAGAGTTGA

                       * *   * *   *       *       *                         
     342656 ACCTTTCTTTTGACAGAACTGTTCTGAAACATTCTTTTTATAGAATCTGGAAGTGGATATTTGGA
         66 ACCTTTCTTTTCATAGAGCAGTTTTGAAACACTCTTTTTGTAGAATCTGGAAGTGGATATTTGGA

            **                         *          *  
     342721 AAGCTTTGAGGATTTCGTTGGAAACGGGAATATCTTCAAAT
        131 GCGCTTTGAGGATTTCGTTGGAAACGGAAATATCTTCATAT

            *   *    *            *           * *       *  *    *            
     342762 CAAATCTAGCCAGAAGCATTCTAAGAAACATCTTAGGGATGTTTACATTCAAGTCACAGAGTTGA
          1 AAAAACTAGACAGAAGCATTCTCAGAAACATCTTTGTGATGTTTGCACTCAACTCACAGAGTTGA

              *  * *     *       *         *    * *   **      *      *    *  
     342827 ACATTCCCTTTCACAGAGCAGGTTTGAAACAATCTTCTCGTACTATCTGGCAGTGGACATTTTGA
         66 ACCTTTCTTTTCATAGAGCAGTTTTGAAACACTCTTTTTGTAGAATCTGGAAGTGGATATTTGGA

              * *   *  ** ** *  *   *            ** *
     342892 GCTCCTTGGGGCCTATGCTGAAAAAGGAAATATCTTCCGAC
        131 GCGCTTTGAGGATTTCGTTGGAAACGGAAATATCTTCATAT

                                 *     *  **          *           **    *    
     342933 AAAAACTAGACAGAAGCATTCGCAGAATCACGTTTGTGATGTGTGCACTCAACTGTCAGAATTGA
          1 AAAAACTAGACAGAAGCATTCTCAGAAACATCTTTGTGATGTTTGCACTCAACTCACAGAGTTGA

                 **   ** *      *                            * *            *
     342998 ACCTTGGTTTGGACAGAGCACTTTTGAAACACTCTTTTTGTAGAATCTGCAGGTGGATATTTGGC
         66 ACCTTTCTTTTCATAGAGCAGTTTTGAAACACTCTTTTTGTAGAATCTGGAAGTGGATATTTGGA

            **                         *   *      * *
     343063 TAGCTTTGAGGATTTCGTTGGAAACGGTAATGTCTTCAAAG
        131 GCGCTTTGAGGATTTCGTTGGAAACGGAAATATCTTCATAT

                *                         *   *            *    *            
     343104 AAAATCTAGACAGAAGCATTCTCAGAAACACCTTCGTGATGTTTGCAATCAAGTCACAGAGTTGA
          1 AAAAACTAGACAGAAGCATTCTCAGAAACATCTTTGTGATGTTTGCACTCAACTCACAGAGTTGA

                 * *             *  *                  *             *       
     343169 ACCTTCCGTTTCATAGAGCAGGTTGGAAACACTCTTTTTGTAGTATCTGGAAGTGGACATTTGGA
         66 ACCTTTCTTTTCATAGAGCAGTTTTGAAACACTCTTTTTGTAGAATCTGGAAGTGGATATTTGGA

             *         ** *    * *   *            **  
     343234 GGGCTTTGTAGCCTATC-TGGAAAAAGGAAATATCTTCCCAT
        131 GCGCTTTG-AGGATTTCGTTGGAAACGGAAATATCTTCATAT

            *  ** *   *     * *            *   *  *   * **        *  * ***   
     343275 GAATGCGAGATAGAAGTAATCTCAGAAACATGTTTATGCTGTATCTACTCAACTAACTGTCCTGA
          1 AAAAACTAGACAGAAGCATTCTCAGAAACATCTTTGTGATGTTTGCACTCAACTCACAGAGTTGA

              *     *  *               *        *  * *       *               
     343340 ACATTTCTATTGATAGAGCAGTTTTGAGACACTCTTCTTTTGGAATCTGCAAGTGGATATTTGGA
         66 ACCTTTCTTTTCATAGAGCAGTTTTGAAACACTCTTTTTGTAGAATCTGGAAGTGGATATTTGGA

            ** *                        *     *      
     343405 TAGATTTGAGGATTTCGTTGGAAACGG-GAT-T-AT-ATAT
        131 GCGCTTTGAGGATTTCGTTGGAAACGGAAATATCTTCATAT

                 *       *               *                 **  *   *         
     343442 AAAAAGTAGACAGCAGCATTCTCAGAAACTTCTTTGTGATGTTTGCATCCAGCTCTCAGAGTTGA
          1 AAAAACTAGACAGAAGCATTCTCAGAAACATCTTTGTGATGTTTGCACTCAACTCACAGAGTTGA

              *  * *          *  *        *        *   **        ** **       
     343507 ACATTCCCTTTCATAGAGTAGGTTTGAAACCCTCTTTTTATAGTGTCTGGAAGCAGGCATTTGGA
         66 ACCTTTCTTTTCATAGAGCAGTTTTGAAACACTCTTTTTGTAGAATCTGGAAGTGGATATTTGGA

               *   *    * * **  **  **          * *  *
     343572 GCGTTTTCAGGCCTATGCTTAAAATAGGAAATATCTACCTAC
        131 GCGCTTTGAGG-ATTTCGTTGGAAACGGAAATATCTTCATAT

             *                    *    *  **          ** *        *    *     
     343614 AGAAACTAGACAGAAGCATTCTGAGAATCACGTTTGTGATGTGGGTACTCAACTAACAGTGTTGA
          1 AAAAACTAGACAGAAGCATTCTCAGAAACATCTTTGTGATGTTTGCACTCAACTCACAGAGTTGA

            *  *       *   *            *   *                *   *           
     343679 TCCATTCTTTTGATACAGCAGTTTTGAACCACACTTTTTGTAGAATCTGCAAGAGGATATTTGGA
         66 ACCTTTCTTTTCATAGAGCAGTTTTGAAACACTCTTTTTGTAGAATCTGGAAGTGGATATTTGGA

            **   *                     *   *      * *
     343744 TAGCTGTGAGGATTTCGTTGGAAACGGGAATGTCTTCAAAG
        131 GCGCTTTGAGGATTTCGTTGGAAACGGAAATATCTTCATAT

                *                         *   *            *    *            
     343785 AAAATCTAGACAGAAGCATTCTCAGAAACACCTTCGTGATGTTTGCAATCAAGTCACAGAGTTGA
          1 AAAAACTAGACAGAAGCATTCTCAGAAACATCTTTGTGATGTTTGCACTCAACTCACAGAGTTGA

                 * *             *  *           *      *             *       
     343850 ACCTTCCGTTTCATAGAGCAGGTTGGAAACACTCTTATTGTAGTATCTGGAAGTGGACATTTGGA
         66 ACCTTTCTTTTCATAGAGCAGTTTTGAAACACTCTTTTTGTAGAATCTGGAAGTGGATATTTGGA

                   *   ** ** *  *   *            **  
     343915 GCGCTTTCAGGCCTATGGTGAAAAAGGAAATATCTTCCCAT
        131 GCGCTTTGAGGATTTCGTTGGAAACGGAAATATCTTCATAT

                  * * *      **      *   * *   *     ** ** *      *    *     
     343956 AAAAACGACATAGAAGCTATCTCAGGAACTTGTTTATGATGCATCTAATCAACTAACAGTGTTGA
          1 AAAAACTAGACAGAAGCATTCTCAGAAACATCTTTGTGATGTTTGCACTCAACTCACAGAGTTGA

                  * ** * *                         * *       *               
     344021 ACCTTTGTACTGACAGAGCAG-TTTGAAACACTCTTTTTTTGGAATCTGCAAGTGGATATTTGGA
         66 ACCTTTCTTTTCATAGAGCAGTTTTGAAACACTCTTTTTGTAGAATCTGGAAGTGGATATTTGGA

            *                           *   *   *    
     344085 TCGCTTTGAGGATTTCGTTGGAAACGG-GAT-GC--AATAT
        131 GCGCTTTGAGGATTTCGTTGGAAACGGAAATATCTTCATAT

                **  *    *     *                 **  *   *  *    *         * 
     344122 AAAACGTACACAGCAGCATACTCAGAAA-ATACTTTGCCATATTTCCATTCAAGTCACAGAGTGG
          1 AAAAACTAGACAGAAGCATTCTCAGAAACAT-CTTTGTGATGTTTGCACTCAACTCACAGAGTTG

               *  * **            *  *               *  *             *      
     344186 AACATTCCCATTCATAGAGCAGGTTGGAAACACTCTTTTTGGAGTATCTGGAAGTGGACATTTGG
         65 AACCTTTCTTTTCATAGAGCAGTTTTGAAACACTCTTTTTGTAGAATCTGGAAGTGGATATTTGG

                    ** * * ** *  *   *            **  
     344251 AGCGCTTTCTGAACTATGGTGAAAAAGGAAATATCTTCCAAT
        130 AGCGCTTTGAGGATTTCGTTGGAAACGGAAATATCTTCATAT

            *     *               *      * *          *  **      **  *  *    
     344293 GAAAACAAGACAGAAGCATTCTGAGAAACTTATTTGTGATGTGTGTCCTCAACAAACGGACTTGA
          1 AAAAACTAGACAGAAGCATTCTCAGAAACATCTTTGTGATGTTTGCACTCAACTCACAGAGTTGA

                   *      **  * *  *  *             *   *    * * *           
     344358 ACCTTTCGTTTCATGCAGTACTTCTGGAACACTCTTTTTGAAGATTCTGCATGCGGATATTTGGA
         66 ACCTTTCTTTTCATAGAGCAGTTTTGAAACACTCTTTTTGTAGAATCTGGAAGTGGATATTTGGA

            **                            **   *   * 
     344423 TAGCTTTGAGGATTTCGTTGGAAACGG---GCT-TACATGT
        131 GCGCTTTGAGGATTTCGTTGGAAACGGAAATATCTTCATAT

                 *       *               *        *   *    *    *       *    
     344460 AAAAATTAGACAGCAGCATTCTCAGAAACTTCTTTGTGGTGTCTGCATTCAAGTCACAGAATTGA
          1 AAAAACTAGACAGAAGCATTCTCAGAAACATCTTTGTGATGTTTGCACTCAACTCACAGAGTTGA

              * ** * **            *  * *      *       *             *       
     344525 ACTTCCCCTCACATAGAGCAGTTGTGCAGCACTCTATTTGTAGTATCTGGAAGTGGACATTTGGA
         66 ACCTTTCTTTTCATAGAGCAGTTTTGAAACACTCTTTTTGTAGAATCTGGAAGTGGATATTTGGA

             *         ** *    * *   *            **  
     344590 GGGCTTTGTAGCCTATC-TGGAAAAAGGAAATATCTTCCCAT
        131 GCGCTTTG-AGGATTTCGTTGGAAACGGAAATATCTTCATAT

            *  ** *   *     * *            *   *  *   * **        *  * * *   
     344631 GAATGCGAGATAGAAGTAATCTCAGAAACATGTTTATGCTGTATCTACTCAACTAACTGTGCTGA
          1 AAAAACTAGACAGAAGCATTCTCAGAAACATCTTTGTGATGTTTGCACTCAACTCACAGAGTTGA

              *     *  *               *        *  * *       *               
     344696 ACATTTCTATTGATAGAGCAGTTTTGAGACACTCTTCTTTTGGAATCTGCAAGTGGATATTTGGA
         66 ACCTTTCTTTTCATAGAGCAGTTTTGAAACACTCTTTTTGTAGAATCTGGAAGTGGATATTTGGA

            ** *            *           *     *      
     344761 TAGATTTGAGGATTTCATTGGAAACGG-GAT-T-AT-ATAT
        131 GCGCTTTGAGGATTTCGTTGGAAACGGAAATATCTTCATAT

                 *       *               *                 **  *   *         
     344798 AAAAAGTAGACAGCAGCATTCTCAGAAACTTCTTTGTGATGTTTGCATCCAGCTCTCAGAGTTGA
          1 AAAAACTAGACAGAAGCATTCTCAGAAACATCTTTGTGATGTTTGCACTCAACTCACAGAGTTGA

              *  * *          *  *        *        *   **        *  **       
     344863 ACATTCCCTTTCATAGAGTAGGTTTGAAACCCTCTTTTTATAGTGTCTGGAAGCGGGCATTTGGA
         66 ACCTTTCTTTTCATAGAGCAGTTTTGAAACACTCTTTTTGTAGAATCTGGAAGTGGATATTTGGA

                   *   ** ** *  *   *          * *   
     344928 GCGCTTTCAGGCCTATGCTGAAAAAGGAAATATCTACCTAT
        131 GCGCTTTGAGGATTTCGTTGGAAACGGAAATATCTTCATAT

             *                    *    *  **          ** *        *    *     
     344969 AGAAACTAGACAGAAGCATTCTGAGAATCACGTTTGTGATGTGGGTACTCAACTAACAGTGTTGA
          1 AAAAACTAGACAGAAGCATTCTCAGAAACATCTTTGTGATGTTTGCACTCAACTCACAGAGTTGA

            *  *       *   *            *   *                *               
     345034 TCCATTCTTTTGATACAGCAGTTTTGAACCACACTTTTTGTAGAATCTGCAAGTGGATATTTGGA
         66 ACCTTTCTTTTCATAGAGCAGTTTTGAAACACTCTTTTTGTAGAATCTGGAAGTGGATATTTGGA

            **   *           *         *   *        *
     345099 TAGCTGTGAGGATTTCGGTGGAAACGGGAATGTCTTCATAG
        131 GCGCTTTGAGGATTTCGTTGGAAACGGAAATATCTTCATAT

                **                     * * **         *  **   *   *          
     345140 AAAATTTAGACAGAAGCATTCTCAGAACCTTGATTGTGATGTGTGTTCTCCACTAACAGAGTTGA
          1 AAAAACTAGACAGAAGCATTCTCAGAAACATCTTTGTGATGTTTGCACTCAACTCACAGAGTTGA

                       * *   * *   *       *       *                         
     345205 ACCTTTCTTTTGACAGAACTGTTCTGAAACATTCTTTTTATAGAATCTGGAAGTGGATATTTGGA
         66 ACCTTTCTTTTCATAGAGCAGTTTTGAAACACTCTTTTTGTAGAATCTGGAAGTGGATATTTGGA

            **                         *          *  
     345270 AAGCTTTGAGGATTTCGTTGGAAACGGGAATATCTTCAAAT
        131 GCGCTTTGAGGATTTCGTTGGAAACGGAAATATCTTCATAT

                *    *            *           * *    *  *  *    *            
     345311 AAAATCTAGCCAGAAGCATTCTAAGAAACATCTTAGGGATGATTACATTCAAGTCACAGAGTTGA
          1 AAAAACTAGACAGAAGCATTCTCAGAAACATCTTTGTGATGTTTGCACTCAACTCACAGAGTTGA

              *  * *     *       *         *    * *   **      *      *    *  
     345376 ACATTCCCTTTCACAGAGCAGGTTTGAAACAATCTTCTCGTACTATCTGGCAGTGGACATTTTGA
         66 ACCTTTCTTTTCATAGAGCAGTTTTGAAACACTCTTTTTGTAGAATCTGGAAGTGGATATTTGGA

              * *   *  ** ** *  *   *            ** *
     345441 GCTCCTTGGGGCCTATGCTGAAAAAGGAAATATCTTCCGAC
        131 GCGCTTTGAGGATTTCGTTGGAAACGGAAATATCTTCATAT

                                 *     *  **          *           **    *    
     345482 AAAAACTAGACAGAAGCATTCGCAGAATCACGTTTGTGATGTGTGCACTCAACTGTCAGAATTGA
          1 AAAAACTAGACAGAAGCATTCTCAGAAACATCTTTGTGATGTTTGCACTCAACTCACAGAGTTGA

                 **   ** *      *                            * *            *
     345547 ACCTTGGTTTGGACAGAGCACTTTTGAAACACTCTTTTTGTAGAATCTGCAGGTGGATATTTGGC
         66 ACCTTTCTTTTCATAGAGCAGTTTTGAAACACTCTTTTTGTAGAATCTGGAAGTGGATATTTGGA

            **                         *   *      * *
     345612 TAGCTTTGAGGATTTCGTTGGAAACGGTAATGTCTTCAAAG
        131 GCGCTTTGAGGATTTCGTTGGAAACGGAAATATCTTCATAT

                *                         *   *            *    *            
     345653 AAAATCTAGACAGAAGCATTCTCAGAAACACCTTCGTGATGTTTGCAATCAAGTCACAGAGTTGA
          1 AAAAACTAGACAGAAGCATTCTCAGAAACATCTTTGTGATGTTTGCACTCAACTCACAGAGTTGA

                 * *             *  *                  *             *       
     345718 ACCTTCCGTTTCATAGAGCAGGTTGGAAACACTCTTTTTGTAGTATCTGGAAGTGGACATTTGGA
         66 ACCTTTCTTTTCATAGAGCAGTTTTGAAACACTCTTTTTGTAGAATCTGGAAGTGGATATTTGGA

             *         ** *    * *   *            **  
     345783 GGGCTTTGTAGCCTATC-TGGAAAAAGGAAATATCTTCCCAT
        131 GCGCTTTG-AGGATTTCGTTGGAAACGGAAATATCTTCATAT

            *  ** *   *     * *            *   *  *   * **        *  * * *   
     345824 GAATGCGAGATAGAAGTAATCTCAGAAACATGTTTATGCTGTATCTACTCAACTAACTGTGCTGA
          1 AAAAACTAGACAGAAGCATTCTCAGAAACATCTTTGTGATGTTTGCACTCAACTCACAGAGTTGA

              *     *  *               *        *  * *       *               
     345889 ACATTTCTATTGATAGAGCAGTTTTGAGACACTCTTCTTTTGGAATCTGCAAGTGGATATTTGGA
         66 ACCTTTCTTTTCATAGAGCAGTTTTGAAACACTCTTTTTGTAGAATCTGGAAGTGGATATTTGGA

            ** *                        *     *      
     345954 TAGATTTGAGGATTTCGTTGGAAACGG-GAT-T-AT-ATAT
        131 GCGCTTTGAGGATTTCGTTGGAAACGGAAATATCTTCATAT

                 *       *               *                 **  *   *         
     345991 AAAAAGTAGACAGCAGCATTCTCAGAAACTTCTTTGTGATGTTTGCATCCAGCTCTCAGAGTTGA
          1 AAAAACTAGACAGAAGCATTCTCAGAAACATCTTTGTGATGTTTGCACTCAACTCACAGAGTTGA

              *  * *          *  *        *        *   **        *  **       
     346056 ACATTCCCTTTCATAGAGTAGGTTTGAAACCCTCTTTTTATAGTGTCTGGAAGCGGGCATTTGGA
         66 ACCTTTCTTTTCATAGAGCAGTTTTGAAACACTCTTTTTGTAGAATCTGGAAGTGGATATTTGGA

                   *    * * **  **  **          * *  *
     346121 GCGCTTTCAGGCCTATGCTTAAAATAGGAAATATCTACCTAC
        131 GCGCTTTGAGG-ATTTCGTTGGAAACGGAAATATCTTCATAT

             *                    *    *  **          ** *        *    *     
     346163 AGAAACTAGACAGAAGCATTCTGAGAATCACGTTTGTGATGTGGGTACTCAACTAACAGTGTTGA
          1 AAAAACTAGACAGAAGCATTCTCAGAAACATCTTTGTGATGTTTGCACTCAACTCACAGAGTTGA

            *  *       *   *            *   *                *   *           
     346228 TCCATTCTTTTGATACAGCAGTTTTGAACCACACTTTTTGTAGAATCTGCAAGAGGATATTTGGA
         66 ACCTTTCTTTTCATAGAGCAGTTTTGAAACACTCTTTTTGTAGAATCTGGAAGTGGATATTTGGA

            **   *                     *   *      * *
     346293 TAGCTGTGAGGATTTCGTTGGAAACGGGAATGTCTTCAAAG
        131 GCGCTTTGAGGATTTCGTTGGAAACGGAAATATCTTCATAT

                *                         *   *            *    *            
     346334 AAAATCTAGACAGAAGCATTCTCAGAAACACCTTCGTGATGTTTGCAATCAAGTCACAGAGTTGA
          1 AAAAACTAGACAGAAGCATTCTCAGAAACATCTTTGTGATGTTTGCACTCAACTCACAGAGTTGA

                 * *             *  *           *      *             *       
     346399 ACCTTCCGTTTCATAGAGCAGGTTGGAAACACTCTTATTGTAGTATCTGGAAGTGGACATTTGGA
         66 ACCTTTCTTTTCATAGAGCAGTTTTGAAACACTCTTTTTGTAGAATCTGGAAGTGGATATTTGGA

                   *   ** ** *  *   *            **  
     346464 GCGCTTTCAGGCCTATGGTGAAAAAGGAAATATCTTCCCAT
        131 GCGCTTTGAGGATTTCGTTGGAAACGGAAATATCTTCATAT

                  * * *      **      *   * *   *     ** ** *      *    *     
     346505 AAAAACGACATAGAAGCTATCTCAGGAACTTGTTTATGATGCATCTAATCAACTAACAGTGTTGA
          1 AAAAACTAGACAGAAGCATTCTCAGAAACATCTTTGTGATGTTTGCACTCAACTCACAGAGTTGA

                  * ** * *                         * *       *               
     346570 ACCTTTGTACTGACAGAGCAG-TTTGAAACACTCTTTTTTTGGAATCTGCAAGTGGATATTTGGA
         66 ACCTTTCTTTTCATAGAGCAGTTTTGAAACACTCTTTTTGTAGAATCTGGAAGTGGATATTTGGA

            *                           *   *   *    
     346634 TCGCTTTGAGGATTTCGTTGGAAACGG-GAT-GC--AATAT
        131 GCGCTTTGAGGATTTCGTTGGAAACGGAAATATCTTCATAT

                **  *    *     *                 **  *   *  *    *         * 
     346671 AAAACGTACACAGCAGCATACTCAGAAA-ATACTTTGCCATATTTCCATTCAAGTCACAGAGTGG
          1 AAAAACTAGACAGAAGCATTCTCAGAAACAT-CTTTGTGATGTTTGCACTCAACTCACAGAGTTG

               *  * **            *                  *  *             *      
     346735 AACATTCCCATTCATAGAGCAGGTTTGAAACACTCTTTTTGGAGTATCTGGAAGTGGACATTTGG
         65 AACCTTTCTTTTCATAGAGCAGTTTTGAAACACTCTTTTTGTAGAATCTGGAAGTGGATATTTGG

                    ** * * ** *  *   *            **  
     346800 AGCGCTTTCTGAACTATGGTGAAAAAGGAAATATCTTCCAAT
        130 AGCGCTTTGAGGATTTCGTTGGAAACGGAAATATCTTCATAT

            *     *               *      * *          *  **      **  *  *    
     346842 GAAAACAAGACAGAAGCATTCTGAGAAACTTATTTGTGATGTGTGTCCTCAACAAACGGACTTGA
          1 AAAAACTAGACAGAAGCATTCTCAGAAACATCTTTGTGATGTTTGCACTCAACTCACAGAGTTGA

                   *      **  * *  *  *             *   *    * * *           
     346907 ACCTTTCGTTTCATGCAGTACTTCTGGAACACTCTTTTTGAAGATTCTGCATGCGGATATTTGGA
         66 ACCTTTCTTTTCATAGAGCAGTTTTGAAACACTCTTTTTGTAGAATCTGGAAGTGGATATTTGGA

            **                            **   *   * 
     346972 TTGCTTTGAGGATTTCGTTGGAAACGG---GCT-TACATGT
        131 GCGCTTTGAGGATTTCGTTGGAAACGGAAATATCTTCATAT

                 *       *               *        *   *    *     *       *   
     347009 AAAAATTAGACAGCAGCATTCTCAGAAACTTCTTTGTGGTGTCTGCATTCAAAGTCACAGAATTG
          1 AAAAACTAGACAGAAGCATTCTCAGAAACATCTTTGTGATGTTTGCACTC-AACTCACAGAGTTG

               * ** * **            *  * *      *       *             *      
     347074 AACATCCCCTCACATAGAGCAGTTGTGCAGCACTCTATTTGTAGTATCTGGAAGTGGACATTTGG
         65 AACCTTTCTTTTCATAGAGCAGTTTTGAAACACTCTTTTTGTAGAATCTGGAAGTGGATATTTGG

              * *       ** *    * *   *            **  
     347139 AGGGTTTTGTAGCCTATC-TGGAAAAAGGAAATATCTTCCCAT
        130 AGCGCTTTG-AGGATTTCGTTGGAAACGGAAATATCTTCATAT

            *  ** *   *     * *            *   *  *   * **        *  * * *   
     347181 GAATGCGAGATAGAAGTAATCTCAGAAACATGTTTATGCTGTATCTACTCAACTAACTGTGCTGA
          1 AAAAACTAGACAGAAGCATTCTCAGAAACATCTTTGTGATGTTTGCACTCAACTCACAGAGTTGA

              *     *  *               *        *  * *       *               
     347246 ACATTTCTATTGATAGAGCAGTTTTGAGACACTCTTCTTTTGGAATCTGCAAGTGGATATTTGGA
         66 ACCTTTCTTTTCATAGAGCAGTTTTGAAACACTCTTTTTGTAGAATCTGGAAGTGGATATTTGGA

            ** *                        *     *      
     347311 TAGATTTGAGGATTTCGTTGGAAACGG-GAT-T-AT-ATAT
        131 GCGCTTTGAGGATTTCGTTGGAAACGGAAATATCTTCATAT

                 *       *               *                 **  *   *         
     347348 AAAAAGTAGACAGCAGCATTCTCAGAAACTTCTTTGTGATGTTTGCATCCAGCTCTCAGAGTTGA
          1 AAAAACTAGACAGAAGCATTCTCAGAAACATCTTTGTGATGTTTGCACTCAACTCACAGAGTTGA

              *  * *          *  *        *        *   **        *  **       
     347413 ACATTCCCTTTCATAGAGTAGGTTTGAAACCCTCTTTTTATAGTGTCTGGAAGCGGGCATTTGGA
         66 ACCTTTCTTTTCATAGAGCAGTTTTGAAACACTCTTTTTGTAGAATCTGGAAGTGGATATTTGGA

                   *   ** ** *  *   *          * *   
     347478 GCGCTTTCAGGCCTATGCTGAAAAAGGAAATATCTACCTAT
        131 GCGCTTTGAGGATTTCGTTGGAAACGGAAATATCTTCATAT

             *                    *    *  **          ** *        *    *     
     347519 AGAAACTAGACAGAAGCATTCTGAGAATCACGTTTGTGATGTGGGTACTCAACTAACAGTGTTGA
          1 AAAAACTAGACAGAAGCATTCTCAGAAACATCTTTGTGATGTTTGCACTCAACTCACAGAGTTGA

            *  *       *   *            *   *                *               
     347584 TCCATTCTTTTGATACAGCAGTTTTGAACCACACTTTTTGTAGAATCTGCAAGTGGATATTTGGA
         66 ACCTTTCTTTTCATAGAGCAGTTTTGAAACACTCTTTTTGTAGAATCTGGAAGTGGATATTTGGA

            **   *                     *   *        *
     347649 TAGCTGTGAGGATTTCGTTGGAAACGGGAATGTCTTCATAG
        131 GCGCTTTGAGGATTTCGTTGGAAACGGAAATATCTTCATAT

                **                     * * **         *  **   *   *          
     347690 AAAATTTAGACAGAAGCATTCTCAGAACCTTGATTGTGATGTGTGTTCTCCACTAACAGAGTTGA
          1 AAAAACTAGACAGAAGCATTCTCAGAAACATCTTTGTGATGTTTGCACTCAACTCACAGAGTTGA

                       * *   * *   *       *       *                         
     347755 ACCTTTCTTTTGACAGAACTGTTCTGAAACATTCTTTTTATAGAATCTGGAAGTGGATATTTGGA
         66 ACCTTTCTTTTCATAGAGCAGTTTTGAAACACTCTTTTTGTAGAATCTGGAAGTGGATATTTGGA

            **                         *          *  
     347820 AAGCTTTGAGGATTTCGTTGGAAACGGGAATATCTTCAAAT
        131 GCGCTTTGAGGATTTCGTTGGAAACGGAAATATCTTCATAT

                *    *            *           * *       *  *    *            
     347861 AAAATCTAGCCAGAAGCATTCTAAGAAACATCTTAGGGATGTTTACATTCAAGTCACAGAGTTGA
          1 AAAAACTAGACAGAAGCATTCTCAGAAACATCTTTGTGATGTTTGCACTCAACTCACAGAGTTGA

              *  * *     *       *         *    * *   **      *      *    *  
     347926 ACATTCCCTTTCACAGAGCAGGTTTGAAACAATCTTCTCGTACTATCTGGCAGTGGACATTTTGA
         66 ACCTTTCTTTTCATAGAGCAGTTTTGAAACACTCTTTTTGTAGAATCTGGAAGTGGATATTTGGA

              * *   *  ** ** *  *   *            ** *
     347991 GCTCCTTGGGGCCTATGCTGAAAAAGGAAATATCTTCCGAC
        131 GCGCTTTGAGGATTTCGTTGGAAACGGAAATATCTTCATAT

                                 *     *  **          *           **    *    
     348032 AAAAACTAGACAGAAGCATTCGCAGAATCACGTTTGTGATGTGTGCACTCAACTGTCAGAATTGA
          1 AAAAACTAGACAGAAGCATTCTCAGAAACATCTTTGTGATGTTTGCACTCAACTCACAGAGTTGA

                 **   ** *      *                            * *            *
     348097 ACCTTGGTTTGGACAGAGCACTTTTGAAACACTCTTTTTGTAGAATCTGCAGGTGGATATTTGGC
         66 ACCTTTCTTTTCATAGAGCAGTTTTGAAACACTCTTTTTGTAGAATCTGGAAGTGGATATTTGGA

            **                         *   *      * *
     348162 TAGCTTTGAGGATTTCGTTGGAAACGGTAATGTCTTCAAAG
        131 GCGCTTTGAGGATTTCGTTGGAAACGGAAATATCTTCATAT

                *      *                  *   *            *    *            
     348203 AAAATCTAGACGGAAGCATTCTCAGAAACACCTTCGTGATGTTTGCAATCAAGTCACAGAGTTGA
          1 AAAAACTAGACAGAAGCATTCTCAGAAACATCTTTGTGATGTTTGCACTCAACTCACAGAGTTGA

                 * *             *  *                  *             *       
     348268 ACCTTCCGTTTCATAGAGCAGGTTGGAAACACTCTTTTTGTAGTATCTGGAAGTGGACATTTGGA
         66 ACCTTTCTTTTCATAGAGCAGTTTTGAAACACTCTTTTTGTAGAATCTGGAAGTGGATATTTGGA

             *         ** *    * *   *            **  
     348333 GGGCTTTGTAGCCTATC-TGGAAAAAGGAAATATCTTCCCAT
        131 GCGCTTTG-AGGATTTCGTTGGAAACGGAAATATCTTCATAT

            *  ** *   *     * *            *   *  *   * **        *  * * *   
     348374 GAATGCGAGATAGAAGTAATCTCAGAAACATGTTTATGCTGTATCTACTCAACTAACTGTGCTGA
          1 AAAAACTAGACAGAAGCATTCTCAGAAACATCTTTGTGATGTTTGCACTCAACTCACAGAGTTGA

              *     *  *               *        *  * *       *               
     348439 ACATTTCTATTTATAGAGCAGTTTTGAGACACTCTTCTTTTGGAATCTGCAAGTGGATATTTGGA
         66 ACCTTTCTTTTCATAGAGCAGTTTTGAAACACTCTTTTTGTAGAATCTGGAAGTGGATATTTGGA

            ** *                        *     *      
     348504 TAGATTTGAGGATTTCGTTGGAAACGG-GAT-T-AT-ATAT
        131 GCGCTTTGAGGATTTCGTTGGAAACGGAAATATCTTCATAT

                 *       *               *                 **  *   *         
     348541 AAAAAGTAGACAGCAGCATTCTCAGAAACTTCTTTGTGATGTTTGCATCCAGCTCTCAGAGTTGA
          1 AAAAACTAGACAGAAGCATTCTCAGAAACATCTTTGTGATGTTTGCACTCAACTCACAGAGTTGA

              *  * *          *  *        *        *   **        *  **       
     348606 ACATTCCCTTTCATAGAGTAGGTTTGAAACCCTCTTTTTATAGTGTCTGGAAGCGGGCATTTGGA
         66 ACCTTTCTTTTCATAGAGCAGTTTTGAAACACTCTTTTTGTAGAATCTGGAAGTGGATATTTGGA

                   *    * * **  **  **          * *  *
     348671 GCGCTTTCAGGCCTATGCTTAAAATAGGAAATATCTACCTAC
        131 GCGCTTTGAGG-ATTTCGTTGGAAACGGAAATATCTTCATAT

             *               *    *    *  **          ** *        *    *     
     348713 AGAAACTAGACAGAAGCCTTCTGAGAATCACGTTTGTGATGTGGGTACTCAACTAACAGTGTTGA
          1 AAAAACTAGACAGAAGCATTCTCAGAAACATCTTTGTGATGTTTGCACTCAACTCACAGAGTTGA

            *  *       *   *            *   *                *   *           
     348778 TCCATTCTTTTGATACAGCAGTTTTGAACCACACTTTTTGTAGAATCTGCAAGAGGATATTTGGA
         66 ACCTTTCTTTTCATAGAGCAGTTTTGAAACACTCTTTTTGTAGAATCTGGAAGTGGATATTTGGA

            **   *                     *   *      * *
     348843 TAGCTGTGAGGATTTCGTTGGAAACGGGAATGTCTTCAAAG
        131 GCGCTTTGAGGATTTCGTTGGAAACGGAAATATCTTCATAT

                *                         *   *            *    *            
     348884 AAAATCTAGACAGAAGCATTCTCAGAAACACCTTCGTGATGTTTGCAATCAAGTCACAGAGTTGA
          1 AAAAACTAGACAGAAGCATTCTCAGAAACATCTTTGTGATGTTTGCACTCAACTCACAGAGTTGA

                 * *             *  *                  *             *       
     348949 ACCTTCCGTTTCATAGAGCAGGTTGGAAACACTCTTTTTGTAGTATCTGGAAGTGGACATTTGGA
         66 ACCTTTCTTTTCATAGAGCAGTTTTGAAACACTCTTTTTGTAGAATCTGGAAGTGGATATTTGGA

                   *   ** ** *  *   *            **  
     349014 GCGCTTTCAGGCCTATGGTGAAAAAGGAAATATCTTCCCAT
        131 GCGCTTTGAGGATTTCGTTGGAAACGGAAATATCTTCATAT

                  * * *      **      *   * *   *     ** ** *      *    *     
     349055 AAAAACGACATAGAAGCTATCTCAGGAACTTGTTTATGATGCATCTAATCAACTAACAGTGTTGA
          1 AAAAACTAGACAGAAGCATTCTCAGAAACATCTTTGTGATGTTTGCACTCAACTCACAGAGTTGA

                  * ** * *                         * *       *               
     349120 ACCTTTGTACTGACAGAGCAG-TTTGAAACACTCTTTTTTTGGAATCTGCAAGTGGATATTTGGA
         66 ACCTTTCTTTTCATAGAGCAGTTTTGAAACACTCTTTTTGTAGAATCTGGAAGTGGATATTTGGA

            *                           *   *   *    
     349184 TCGCTTTGAGGATTTCGTTGGAAACGG-GAT-GC--AATAT
        131 GCGCTTTGAGGATTTCGTTGGAAACGGAAATATCTTCATAT

                **  *    *     *                 **  *   *  *    *         * 
     349221 AAAACGTACACAGCAGCATACTCAGAAA-ATACTTTGCCATATTTCCATTCAAGTCACAGAGTGG
          1 AAAAACTAGACAGAAGCATTCTCAGAAACAT-CTTTGTGATGTTTGCACTCAACTCACAGAGTTG

               *  * **            *  *               *  *             *      
     349285 AACATTCCCATTCATAGAGCAGGTTGGAAACACTCTTTTTGGAGTATCTGGAAGTGGACATTTGG
         65 AACCTTTCTTTTCATAGAGCAGTTTTGAAACACTCTTTTTGTAGAATCTGGAAGTGGATATTTGG

                    ** * * ** *  *   *            **  
     349350 AGCGCTTTCTGAACTATGGTGAAAAAGGAAATATCTTCCAAT
        130 AGCGCTTTGAGGATTTCGTTGGAAACGGAAATATCTTCATAT

            *     *               *      * *          *  **      **  *  *    
     349392 GAAAACAAGACAGAAGCATTCTGAGAAACTTATTTGTGATGTGTGTCCTCAACAAACGGACTTGA
          1 AAAAACTAGACAGAAGCATTCTCAGAAACATCTTTGTGATGTTTGCACTCAACTCACAGAGTTGA

                   *      **  * *  *  *             *   *    * * *           
     349457 ACCTTTCGTTTCATGCAGTACTTCTGGAACACTCTTTTTGAAGATTCTGCATGCGGATATTTGGA
         66 ACCTTTCTTTTCATAGAGCAGTTTTGAAACACTCTTTTTGTAGAATCTGGAAGTGGATATTTGGA

            **                            **   *   * 
     349522 TTGCTTTGAGGATTTCGTTGGAAACGG---GCT-TACATGT
        131 GCGCTTTGAGGATTTCGTTGGAAACGGAAATATCTTCATAT

                 *       *               *        *   *    *    *       *    
     349559 AAAAATTAGACAGCAGCATTCTCAGAAACTTCTTTGTGGTGTCTGCATTCAAGTCACAGAATTGA
          1 AAAAACTAGACAGAAGCATTCTCAGAAACATCTTTGTGATGTTTGCACTCAACTCACAGAGTTGA

              * ** * **            *  * *      *       *             *       
     349624 ACATCCCCTCACATAGAGCAGTTGTGCAGCACTCTATTTGTAGTATCTGGAAGTGGACATTTGGA
         66 ACCTTTCTTTTCATAGAGCAGTTTTGAAACACTCTTTTTGTAGAATCTGGAAGTGGATATTTGGA

             *         ** *    * *   *            **  
     349689 GGGCTTTGTAGCCTATC-TGGAAAAAGGAAATATCTTCCCAT
        131 GCGCTTTG-AGGATTTCGTTGGAAACGGAAATATCTTCATAT

            *  ** *   *  *  * *            *   *  *   * **        *  * * *   
     349730 GAATGCGAGATAGTAGTAATCTCAGAAACATGTTTATGCTGTATCTACTCAACTAACTGTGCTGA
          1 AAAAACTAGACAGAAGCATTCTCAGAAACATCTTTGTGATGTTTGCACTCAACTCACAGAGTTGA

              *     *  *               *        *  * *       *               
     349795 ACATTTCTATTGATAGAGCAGTTTTGAGACACTCTTCTTTTGGAATCTGCAAGTGGATATTTGGA
         66 ACCTTTCTTTTCATAGAGCAGTTTTGAAACACTCTTTTTGTAGAATCTGGAAGTGGATATTTGGA

            ** *                        *     *      
     349860 TAGATTTGAGGATTTCGTTGGAAACGG-GAT-T-AT-ATAT
        131 GCGCTTTGAGGATTTCGTTGGAAACGGAAATATCTTCATAT

                 *       *               *                 **  *   *         
     349897 AAAAAGTAGACAGCAGCATTCTCAGAAACTTCTTTGTGATGTTTGCATCCAGCTCTCAGAGTTGA
          1 AAAAACTAGACAGAAGCATTCTCAGAAACATCTTTGTGATGTTTGCACTCAACTCACAGAGTTGA

              *  * *          *  *        *        *   **        *  **       
     349962 ACATTCCCTTTCATAGAGTAGGTTTGAAACCCTCTTTTTATAGTGTCTGGAAGCGGGCATTTGGA
         66 ACCTTTCTTTTCATAGAGCAGTTTTGAAACACTCTTTTTGTAGAATCTGGAAGTGGATATTTGGA

                   *   ** ** *  *   *          * *   
     350027 GCGCTTTCAGGCCTATGCTGAAAAAGGAAATATCTACCTAT
        131 GCGCTTTGAGGATTTCGTTGGAAACGGAAATATCTTCATAT

             *                    *    *  **          ** *        *    *     
     350068 AGAAACTAGACAGAAGCATTCTGAGAATCACGTTTGTGATGTGGGTACTCAACTAACAGTGTTGA
          1 AAAAACTAGACAGAAGCATTCTCAGAAACATCTTTGTGATGTTTGCACTCAACTCACAGAGTTGA

            *  *       *   *            *   *                *               
     350133 TCCATTCTTTTGATACAGCAGTTTTGAACCACACTTTTTGTAGAATCTGCAAGTGGATATTTGGA
         66 ACCTTTCTTTTCATAGAGCAGTTTTGAAACACTCTTTTTGTAGAATCTGGAAGTGGATATTTGGA

            **   *           *         *   *        *
     350198 TAGCTGTGAGGATTTCGGTGGAAACGGGAATGTCTTCATAG
        131 GCGCTTTGAGGATTTCGTTGGAAACGGAAATATCTTCATAT

                **                     * * **         *  **   *   *          
     350239 AAAATTTAGACAGAAGCATTCTCAGAACCTTGATTGTGATGTGTGTTCTCCACTAACAGAGTTGA
          1 AAAAACTAGACAGAAGCATTCTCAGAAACATCTTTGTGATGTTTGCACTCAACTCACAGAGTTGA

                       * *   * *   *       *       *                         
     350304 ACCTTTCTTTTGACAGAACTGTTCTGAAACATTCTTTTTATAGAATCTGGAAGTGGATATTTGGA
         66 ACCTTTCTTTTCATAGAGCAGTTTTGAAACACTCTTTTTGTAGAATCTGGAAGTGGATATTTGGA

            **                         *          *  
     350369 AAGCTTTGAGGATTTCGTTGGAAACGGGAATATCTTCAAAT
        131 GCGCTTTGAGGATTTCGTTGGAAACGGAAATATCTTCATAT

                *    *            *           * *       *  *    *            
     350410 AAAATCTAGCCAGAAGCATTCTAAGAAACATCTTAGGGATGTTTACATTCAAGTCACAGAGTTGA
          1 AAAAACTAGACAGAAGCATTCTCAGAAACATCTTTGTGATGTTTGCACTCAACTCACAGAGTTGA

              *  * *     *       *         *    * *   **      *      *    *  
     350475 ACATTCCCTTTCACAGAGCAGGTTTGAAACAATCTTCTCGTACTATCTGGCAGTGGACATTTTGA
         66 ACCTTTCTTTTCATAGAGCAGTTTTGAAACACTCTTTTTGTAGAATCTGGAAGTGGATATTTGGA

              * *   *  ** ** *  *   *            ** *
     350540 GCTCCTTGGGGCCTATGCTGAAAAAGGAAATATCTTCCGAC
        131 GCGCTTTGAGGATTTCGTTGGAAACGGAAATATCTTCATAT

                                 *     *  **          *           **    *    
     350581 AAAAACTAGACAGAAGCATTCGCAGAATCACGTTTGTGATGTGTGCACTCAACTGTCAGAATTGA
          1 AAAAACTAGACAGAAGCATTCTCAGAAACATCTTTGTGATGTTTGCACTCAACTCACAGAGTTGA

                 **   ** *      *                            * *            *
     350646 ACCTTGGTTTGGACAGAGCACTTTTGAAACACTCTTTTTGTAGAATCTGCAGGTGGATATTTGGC
         66 ACCTTTCTTTTCATAGAGCAGTTTTGAAACACTCTTTTTGTAGAATCTGGAAGTGGATATTTGGA

            **                         *   *      * *
     350711 TAGCTTTGAGGATTTCGTTGGAAACGGGAATGTCTTCAAAG
        131 GCGCTTTGAGGATTTCGTTGGAAACGGAAATATCTTCATAT

                *                         *   *            *    *            
     350752 AAAATCTAGACAGAAGCATTCTCAGAAACACCTTCGTGATGTTTGCAATCAAGTCACAGAGTTGA
          1 AAAAACTAGACAGAAGCATTCTCAGAAACATCTTTGTGATGTTTGCACTCAACTCACAGAGTTGA

                 * *             *  *                  *             *       
     350817 ACCTTCCGTTTCATAGAGCAGGTTGGAAACACTCTTTTTGTAGTATCTGGAAGTGGACATTTGGA
         66 ACCTTTCTTTTCATAGAGCAGTTTTGAAACACTCTTTTTGTAGAATCTGGAAGTGGATATTTGGA

                   *   ** ** *  *   *            **  
     350882 GCGCTTTCAGGCCTATGGTGAAAAAGGAAATATCTTCCCAT
        131 GCGCTTTGAGGATTTCGTTGGAAACGGAAATATCTTCATAT

                  * * *      **      *   * *   *     ** ** *      *    *     
     350923 AAAAACGACATAGAAGCTATCTCAGGAACTTGTTTATGATGCATCTAATCAACTAACAGTGTTGA
          1 AAAAACTAGACAGAAGCATTCTCAGAAACATCTTTGTGATGTTTGCACTCAACTCACAGAGTTGA

                  * ** * *                         * *       *               
     350988 ACCTTTGTACTGACAGAGCAG-TTTGAAACACTCTTTTTTTGGAATCTGCAAGTGGATATTTGGA
         66 ACCTTTCTTTTCATAGAGCAGTTTTGAAACACTCTTTTTGTAGAATCTGGAAGTGGATATTTGGA

            *                           *   *   *    
     351052 TCGCTTTGAGGATTTCGTTGGAAACGG-GAT-GC--AATAT
        131 GCGCTTTGAGGATTTCGTTGGAAACGGAAATATCTTCATAT

                **  *    *     *                 **  *   *  *    *         * 
     351089 AAAACGTACACAGCAGCATACTCAGAAA-ATACTTTGCCATATTTCCATTCAAGTCACAGAGTGG
          1 AAAAACTAGACAGAAGCATTCTCAGAAACAT-CTTTGTGATGTTTGCACTCAACTCACAGAGTTG

               *  * **            *  *               *  *             *      
     351153 AACATTCCCATTCATAGAGCAGGTTGGAAACACTCTTTTTGGAGTATCTGGAAGTGGACATTTGG
         65 AACCTTTCTTTTCATAGAGCAGTTTTGAAACACTCTTTTTGTAGAATCTGGAAGTGGATATTTGG

                    ** * * ** *  *   *            **  
     351218 AGCGCTTTCTGAACTATGGTGAAAAAGGAAATATCTTCCAAT
        130 AGCGCTTTGAGGATTTCGTTGGAAACGGAAATATCTTCATAT

            *     *               *      * *          *  **      **  *  *    
     351260 GAAAACAAGACAGAAGCATTCTGAGAAACTTATTTGTGATGTGTGTCCTCAACAAACGGACTTGA
          1 AAAAACTAGACAGAAGCATTCTCAGAAACATCTTTGTGATGTTTGCACTCAACTCACAGAGTTGA

                   *      **  * *  *  *             *   *    * * *           
     351325 ACCTTTCGTTTCATGCAGTACTTCTGGAACACTCTTTTTGAAGATTCTGCATGCGGATATTTGGA
         66 ACCTTTCTTTTCATAGAGCAGTTTTGAAACACTCTTTTTGTAGAATCTGGAAGTGGATATTTGGA

            **                            **   *   * 
     351390 TAGCTTTGAGGATTTCGTTGGAAACGG---GCT-TACATGT
        131 GCGCTTTGAGGATTTCGTTGGAAACGGAAATATCTTCATAT

                 *       *               *        *   *    *    *       *    
     351427 AAAAATTAGACAGCAGCATTCTCAGAAACTTCTTTGTGGTGTCTGCATTCAAGTCACAGAATTGA
          1 AAAAACTAGACAGAAGCATTCTCAGAAACATCTTTGTGATGTTTGCACTCAACTCACAGAGTTGA

              * ** * **            *  * *      *       *             *       
     351492 ACTTCCCCTCACATAGAGCAGTTGTGCAGCACTCTATTTGTAGTATCTGGAAGTGGACATTTGGA
         66 ACCTTTCTTTTCATAGAGCAGTTTTGAAACACTCTTTTTGTAGAATCTGGAAGTGGATATTTGGA

             *         ** *    * *   *            **  
     351557 GGGCTTTGTAGCCTATC-TGGAAAAAGGAAATATCTTCCCAT
        131 GCGCTTTG-AGGATTTCGTTGGAAACGGAAATATCTTCATAT

            *  ** *   *     * *            *   *  *   * **        *  * * *   
     351598 GAATGCGAGATAGAAGTAATCTCAGAAACATGTTTATGCTGTATCTACTCAACTAACTGTGCTGA
          1 AAAAACTAGACAGAAGCATTCTCAGAAACATCTTTGTGATGTTTGCACTCAACTCACAGAGTTGA

              *     *  *               *        *  * *       *               
     351663 ACATTTCTATTGATAGAGCAGTTTTGAGACACTCTTCTTTTGGAATCTGCAAGTGGATATTTGGA
         66 ACCTTTCTTTTCATAGAGCAGTTTTGAAACACTCTTTTTGTAGAATCTGGAAGTGGATATTTGGA

            ** *                        *     *      
     351728 TAGATTTGAGGATTTCGTTGGAAACGG-GAT-T-AT-ATAT
        131 GCGCTTTGAGGATTTCGTTGGAAACGGAAATATCTTCATAT

                 *       *               *              *  **  *   *         
     351765 AAAAAGTAGACAGCAGCATTCTCAGAAACTTCTTTGTGATGTTTCCATCCAGCTCTCAGAGTTGA
          1 AAAAACTAGACAGAAGCATTCTCAGAAACATCTTTGTGATGTTTGCACTCAACTCACAGAGTTGA

              *  * *          *  *        *        *   **        *  **       
     351830 ACATTCCCTTTCATAGAGTAGGTTTGAAACCCTCTTTTTATAGTGTCTGGAAGCGGGCATTTGGA
         66 ACCTTTCTTTTCATAGAGCAGTTTTGAAACACTCTTTTTGTAGAATCTGGAAGTGGATATTTGGA

                   *   ** ** *  *   *          * *   
     351895 GCGCTTTCAGGCCTATGCTGAAAAAGGAAATATCTACCTAT
        131 GCGCTTTGAGGATTTCGTTGGAAACGGAAATATCTTCATAT

             *                    *    *  **          ** *        *    *     
     351936 AGAAACTAGACAGAAGCATTCTGAGAATCACGTTTGTGATGTGGGTACTCAACTAACAGTGTTGA
          1 AAAAACTAGACAGAAGCATTCTCAGAAACATCTTTGTGATGTTTGCACTCAACTCACAGAGTTGA

            *  *       *   *            *   *                                
     352001 TCCATTCTTTTGATACAGCAGTTTTGAACCACACTTTTTGTAGAATCTGGAAGTGGATATTTGGA
         66 ACCTTTCTTTTCATAGAGCAGTTTTGAAACACTCTTTTTGTAGAATCTGGAAGTGGATATTTGGA

            **                         *   *        *
     352066 AAGCTTTGAGGATTTCGTTGGAAACGGGAATGTCTTCATAG
        131 GCGCTTTGAGGATTTCGTTGGAAACGGAAATATCTTCATAT

                **     *               * * **         *  **   *   *          
     352107 AAAATTTAGACGGAAGCATTCTCAGAACCTTGATTGTGATGTGTGTTCTCCACTAACAGAGTTGA
          1 AAAAACTAGACAGAAGCATTCTCAGAAACATCTTTGTGATGTTTGCACTCAACTCACAGAGTTGA

                       * *   * *   *       *       *                         
     352172 ACCTTTCTTTTGACAGAACTGTTCTGAAACATTCTTTTTATAGAATCTGGAAGTGGATATTTGGA
         66 ACCTTTCTTTTCATAGAGCAGTTTTGAAACACTCTTTTTGTAGAATCTGGAAGTGGATATTTGGA

            **                         *          *  
     352237 AAGCTTTGAGGATTTCGTTGGAAACGGGAATATCTTCAAAT
        131 GCGCTTTGAGGATTTCGTTGGAAACGGAAATATCTTCATAT

                *    *            *           * *       *  *    *            
     352278 AAAATCTAGCCAGAAGCATTCTAAGAAACATCTTAGGGATGTTTACATTCAAGTCACAGAGTTGA
          1 AAAAACTAGACAGAAGCATTCTCAGAAACATCTTTGTGATGTTTGCACTCAACTCACAGAGTTGA

              *  * *     *       *         *    * *   **      *      *    *  
     352343 ACATTCCCTTTCACAGAGCAGGTTTGAAACAATCTTCTCGTACTATCTGGCAGTGGACATTTTGA
         66 ACCTTTCTTTTCATAGAGCAGTTTTGAAACACTCTTTTTGTAGAATCTGGAAGTGGATATTTGGA

              * *   *  ** ** *  *   *            ** *
     352408 GCTCCTTGGGGCCTATGCTGAAAAAGGAAATATCTTCCGAC
        131 GCGCTTTGAGGATTTCGTTGGAAACGGAAATATCTTCATAT

                                 *     *  **          *           **    *    
     352449 AAAAACTAGACAGAAGCATTCGCAGAATCACGTTTGTGATGTGTGCACTCAACTGTCAGAATTGA
          1 AAAAACTAGACAGAAGCATTCTCAGAAACATCTTTGTGATGTTTGCACTCAACTCACAGAGTTGA

                 **   ** *      *                            * *            *
     352514 ACCTTGGTTTGGACAGAGCACTTTTGAAACACTCTTTTTGTAGAATCTGCAGGTGGATATTTGGC
         66 ACCTTTCTTTTCATAGAGCAGTTTTGAAACACTCTTTTTGTAGAATCTGGAAGTGGATATTTGGA

            **                         *   *      * *
     352579 TAGCTTTGAGGATTTCGTTGGAAACGGTAATGTCTTCAAAG
        131 GCGCTTTGAGGATTTCGTTGGAAACGGAAATATCTTCATAT

                *                         *   *            *    *            
     352620 AAAATCTAGACAGAAGCATTCTCAGAAACACCTTCGTGATGTTTGCAATCAAGTCACAGAGTTGA
          1 AAAAACTAGACAGAAGCATTCTCAGAAACATCTTTGTGATGTTTGCACTCAACTCACAGAGTTGA

                 * *             *  *                  *             *       
     352685 ACCTTCCGTTTCATAGAGCAGGTTGGAAACACTCTTTTTGTAGTATCTGGAAGTGGACATTTGGA
         66 ACCTTTCTTTTCATAGAGCAGTTTTGAAACACTCTTTTTGTAGAATCTGGAAGTGGATATTTGGA

             *         ** *    * *   *            **  
     352750 GGGCTTTGTAGCCTATC-TGGAAAAAGGAAATATCTTCCCAT
        131 GCGCTTTG-AGGATTTCGTTGGAAACGGAAATATCTTCATAT

            *  ** *   *     * *            *   *  *   * **        *  * * *   
     352791 GAATGCGAGATAGAAGTAATCTCAGAAACATGTTTATGCTGTATCTACTCAACTAACTGTGCTGA
          1 AAAAACTAGACAGAAGCATTCTCAGAAACATCTTTGTGATGTTTGCACTCAACTCACAGAGTTGA

              *     *  *               *        *  * *       *               
     352856 ACATTTCTATTGATAGAGCAGTTTTGAGACACTCTTCTTTTGGAATCTGCAAGTGGATATTTGGA
         66 ACCTTTCTTTTCATAGAGCAGTTTTGAAACACTCTTTTTGTAGAATCTGGAAGTGGATATTTGGA

            ** *                        *     *      
     352921 TAGATTTGAGGATTTCGTTGGAAACGG-GAT-T-AT-ATAT
        131 GCGCTTTGAGGATTTCGTTGGAAACGGAAATATCTTCATAT

                 *       *               *                 **  *   *         
     352958 AAAAAGTAGACAGCAGCATTCTCAGAAACTTCTTTGTGATGTTTGCATCCAGCTCTCAGAGTTGA
          1 AAAAACTAGACAGAAGCATTCTCAGAAACATCTTTGTGATGTTTGCACTCAACTCACAGAGTTGA

              *  * *          *  *        *        *   **        *  **       
     353023 ACATTCCCTTTCATAGAGTAGGTTTGAAACCCTCTTTTTATAGTGTCTGGAAGCGGGCATTTGGA
         66 ACCTTTCTTTTCATAGAGCAGTTTTGAAACACTCTTTTTGTAGAATCTGGAAGTGGATATTTGGA

                   *    * * **  **  **          * *  *
     353088 GCGCTTTCAGGCCTATGCTTAAAATAGGAAATATCTACCTAC
        131 GCGCTTTGAGG-ATTTCGTTGGAAACGGAAATATCTTCATAT

             *                    *    *  **          ** *        *    *     
     353130 AGAAACTAGACAGAAGCATTCTGAGAATCACGTTTGTGATGTGGGTACTCAACTAACAGTGTTGA
          1 AAAAACTAGACAGAAGCATTCTCAGAAACATCTTTGTGATGTTTGCACTCAACTCACAGAGTTGA

            *  *       *   *            *   *                *   *           
     353195 TCCATTCTTTTGATACAGCAGTTTTGAACCACACTTTTTGTAGAATCTGCAAGAGGATATTTGGA
         66 ACCTTTCTTTTCATAGAGCAGTTTTGAAACACTCTTTTTGTAGAATCTGGAAGTGGATATTTGGA

            **   *                     *   *      * *
     353260 TAGCTGTGAGGATTTCGTTGGAAACGGGAATGTCTTCAAAG
        131 GCGCTTTGAGGATTTCGTTGGAAACGGAAATATCTTCATAT

                *                         *   *            *    *            
     353301 AAAATCTAGACAGAAGCATTCTCAGAAACACCTTCGTGATGTTTGCAATCAAGTCACAGAGTTGA
          1 AAAAACTAGACAGAAGCATTCTCAGAAACATCTTTGTGATGTTTGCACTCAACTCACAGAGTTGA

                 * *             *  *           *      *             *       
     353366 ACCTTCCGTTTCATAGAGCAGGTTGGAAACACTCTTATTGTAGTATCTGGAAGTGGACATTTGGA
         66 ACCTTTCTTTTCATAGAGCAGTTTTGAAACACTCTTTTTGTAGAATCTGGAAGTGGATATTTGGA

                   *      *   *  *   *            **  
     353431 GCGCTTTCAGGCATAT-GGTGAAAAAGGAAATATCTTCCCAT
        131 GCGCTTTGAGG-ATTTCGTTGGAAACGGAAATATCTTCATAT

                  * * *      **      *   * *   *     ** ** *      *    *     
     353472 AAAAACGACATAGAAGCTATCTCAGGAACTTGTTTATGATGCATCTAATCAACTAACAGTGTTGA
          1 AAAAACTAGACAGAAGCATTCTCAGAAACATCTTTGTGATGTTTGCACTCAACTCACAGAGTTGA

                  * ** * *                         * *       *               
     353537 ACCTTTGTACTGACAGAGCAG-TTTGAAACACTCTTTTTTTGGAATCTGCAAGTGGATATTTGGA
         66 ACCTTTCTTTTCATAGAGCAGTTTTGAAACACTCTTTTTGTAGAATCTGGAAGTGGATATTTGGA

            *                           *   *   *    
     353601 TCGCTTTGAGGATTTCGTTGGAAACGG-GAT-GC--AATAT
        131 GCGCTTTGAGGATTTCGTTGGAAACGGAAATATCTTCATAT

                **  *    *     *                 **  *   *  *    *         * 
     353638 AAAACGTACACAGCAGCATACTCAGAAA-ATACTTTGCCATATTTCCATTCAAGTCACAGAGTGG
          1 AAAAACTAGACAGAAGCATTCTCAGAAACAT-CTTTGTGATGTTTGCACTCAACTCACAGAGTTG

               *  * **            *  *               *  *             *      
     353702 AACATTCCCATTCATAGAGCAGGTTGGAAACACTCTTTTTGGAGTATCTGGAAGTGGACATTTGG
         65 AACCTTTCTTTTCATAGAGCAGTTTTGAAACACTCTTTTTGTAGAATCTGGAAGTGGATATTTGG

                    ** * * ** *  *   *            **  
     353767 AGCGCTTTCTGAACTATGGTGAAAAAGGAAATATCTTCCAAT
        130 AGCGCTTTGAGGATTTCGTTGGAAACGGAAATATCTTCATAT

            *     *               *      * *          *  **      **  *  *    
     353809 GAAAACAAGACAGAAGCATTCTGAGAAACTTATTTGTGATGTGTGTCCTCAACAAACGGACTTGA
          1 AAAAACTAGACAGAAGCATTCTCAGAAACATCTTTGTGATGTTTGCACTCAACTCACAGAGTTGA

                   *      **  * *  *  *             *   *    * * *           
     353874 ACCTTTCGTTTCATGCAGTACTTCTGGAACACTCTTTTTGAAGATTCTGCATGCGGATATTTGGA
         66 ACCTTTCTTTTCATAGAGCAGTTTTGAAACACTCTTTTTGTAGAATCTGGAAGTGGATATTTGGA

            **                            **   *   * 
     353939 TTGCTTTGAGGATTTCGTTGGAAACGG---GCT-TACATGT
        131 GCGCTTTGAGGATTTCGTTGGAAACGGAAATATCTTCATAT

                 *       *               *        *   *    *    *       *    
     353976 AAAAATTAGACAGCAGCATTCTCAGAAACTTCTTTGTGGTGTCTGCATTCAAGTCACAGAATTGA
          1 AAAAACTAGACAGAAGCATTCTCAGAAACATCTTTGTGATGTTTGCACTCAACTCACAGAGTTGA

              * ** * **            *  * *      *       *             *       
     354041 ACTTCCCCTCACATAGAGCAGTTGTGCAGCACTCTATTTGTAGTATCTGGAAGTGGACATTTGGA
         66 ACCTTTCTTTTCATAGAGCAGTTTTGAAACACTCTTTTTGTAGAATCTGGAAGTGGATATTTGGA

             *         ** *    * *   *            **  
     354106 GGGCTTTGTAGCCTATC-TGGAAAAAGGAAATATCTTCCCAT
        131 GCGCTTTG-AGGATTTCGTTGGAAACGGAAATATCTTCATAT

            *  ** *   *     * *            *   *  *   * **        *  * * *   
     354147 GAATGCGAGATAGAAGTAATCTCAGAAACATGTTTATGCTGTATCTACTCAACTAACTGTGCTGA
          1 AAAAACTAGACAGAAGCATTCTCAGAAACATCTTTGTGATGTTTGCACTCAACTCACAGAGTTGA

              *     *  *               *        *  * *       *               
     354212 ACATTTCTATTGATAGAGCAGTTTTGAGACACTCTTCTTTTGGAATCTGCAAGTGGATATTTGGA
         66 ACCTTTCTTTTCATAGAGCAGTTTTGAAACACTCTTTTTGTAGAATCTGGAAGTGGATATTTGGA

            ** *                        *     *      
     354277 TAGATTTGAGGATTTCGTTGGAAACGG-GAT-T-AT-ATAT
        131 GCGCTTTGAGGATTTCGTTGGAAACGGAAATATCTTCATAT

                 *       *               *                 **  *   *         
     354314 AAAAAGTAGACAGCAGCATTCTCAGAAACTTCTTTGTGATGTTTGCATCCAGCTCTCAGAGTTGA
          1 AAAAACTAGACAGAAGCATTCTCAGAAACATCTTTGTGATGTTTGCACTCAACTCACAGAGTTGA

              *  * *          *  *        *        *   **        *  **       
     354379 ACATTCCCTTTCATAGAGTAGGTTTGAAACCCTCTTTTTATAGTGTCTGGAAGCGGGCATTTGGA
         66 ACCTTTCTTTTCATAGAGCAGTTTTGAAACACTCTTTTTGTAGAATCTGGAAGTGGATATTTGGA

                   *   ** ** *  *   *          * *   
     354444 GCGCTTTCAGGCCTATGCTGAAAAAGGAAATATCTACCTAT
        131 GCGCTTTGAGGATTTCGTTGGAAACGGAAATATCTTCATAT

             *                    *    *  **          ** *        *    *     
     354485 AGAAACTAGACAGAAGCATTCTGAGAATCACGTTTGTGATGTGGGTACTCAACTAACAGTGTTGA
          1 AAAAACTAGACAGAAGCATTCTCAGAAACATCTTTGTGATGTTTGCACTCAACTCACAGAGTTGA

            *  *       *   *            *   *                *               
     354550 TCCATTCTTTTGATACAGCAGTTTTGAACCACACTTTTTGTAGAATCTGCAAGTGGATATTTGGA
         66 ACCTTTCTTTTCATAGAGCAGTTTTGAAACACTCTTTTTGTAGAATCTGGAAGTGGATATTTGGA

            **   *                     *   *        *
     354615 TAGCTGTGAGGATTTCGTTGGAAACGGGAATGTCTTCATAG
        131 GCGCTTTGAGGATTTCGTTGGAAACGGAAATATCTTCATAT

                **                     * * **         *  **   *   *          
     354656 AAAATTTAGACAGAAGCATTCTCAGAACCTTGATTGTGATGTGTGTTCTCCACTAACAGAGTTGA
          1 AAAAACTAGACAGAAGCATTCTCAGAAACATCTTTGTGATGTTTGCACTCAACTCACAGAGTTGA

                       * *   * *   *       *       *                         
     354721 ACCTTTCTTTTGACAGAACTGTTCTGAAACATTCTTTTTATAGAATCTGGAAGTGGATATTTGGA
         66 ACCTTTCTTTTCATAGAGCAGTTTTGAAACACTCTTTTTGTAGAATCTGGAAGTGGATATTTGGA

            **                         *          *  
     354786 AAGCTTTGAGGATTTCGTTGGAAACGGGAATATCTTCAAAT
        131 GCGCTTTGAGGATTTCGTTGGAAACGGAAATATCTTCATAT

            *   *    *            *           * *       *  *    *            
     354827 GAAATCTAGCCAGAAGCATTCTAAGAAACATCTTAGGGATGTTTACATTCAAGTCACAGAGTTGA
          1 AAAAACTAGACAGAAGCATTCTCAGAAACATCTTTGTGATGTTTGCACTCAACTCACAGAGTTGA

              *  * *     *       *         *    * *   **      *      *    *  
     354892 ACATTCCCTTTCACAGAGCAGGTTTGAAACAATCTTCTCGTACTATCTGGCAGTGGACATTTTGA
         66 ACCTTTCTTTTCATAGAGCAGTTTTGAAACACTCTTTTTGTAGAATCTGGAAGTGGATATTTGGA

              * *   *  ** ** *  *   *            ** *
     354957 GCTCCTTGGGGCCTATGCTGAAAAAGGAAATATCTTCCGAC
        131 GCGCTTTGAGGATTTCGTTGGAAACGGAAATATCTTCATAT

                                 *     *  **          *           **    *    
     354998 AAAAACTAGACAGAAGCATTCGCAGAATCACGTTTGTGATGTGTGCACTCAACTGTCAGAATTGA
          1 AAAAACTAGACAGAAGCATTCTCAGAAACATCTTTGTGATGTTTGCACTCAACTCACAGAGTTGA

                 **   ** *      *                            * *            *
     355063 ACCTTGGTTTGGACAGAGCACTTTTGAAACACTCTTTTTGTAGAATCTGCAGGTGGATATTTGGC
         66 ACCTTTCTTTTCATAGAGCAGTTTTGAAACACTCTTTTTGTAGAATCTGGAAGTGGATATTTGGA

            **                         *   *      * *
     355128 TAGCTTTGAGGATTTCGTTGGAAACGGTAATGTCTTCAAAG
        131 GCGCTTTGAGGATTTCGTTGGAAACGGAAATATCTTCATAT

                *      *                  *   *            *    *            
     355169 AAAATCTAGACGGAAGCATTCTCAGAAACACCTTCGTGATGTTTGCAATCAAGTCACAGAGTTGA
          1 AAAAACTAGACAGAAGCATTCTCAGAAACATCTTTGTGATGTTTGCACTCAACTCACAGAGTTGA

                 * *             *  *                  *             *       
     355234 ACCTTCCGTTTCATAGAGCAGGTTGGAAACACTCTTTTTGTAGTATCTGGAAGTGGACATTTGGA
         66 ACCTTTCTTTTCATAGAGCAGTTTTGAAACACTCTTTTTGTAGAATCTGGAAGTGGATATTTGGA

             *         ** *    * *   *            **  
     355299 GGGCTTTGTAGCCTATC-TGGAAAAAGGAAATATCTTCCCAT
        131 GCGCTTTG-AGGATTTCGTTGGAAACGGAAATATCTTCATAT

            *  ** *   *     * *            *   *  *   * **        *  * * *   
     355340 GAATGCGAGATAGAAGTAATCTCAGAAACATGTTTATGCTGTATCTACTCAACTAACTGTGCTGA
          1 AAAAACTAGACAGAAGCATTCTCAGAAACATCTTTGTGATGTTTGCACTCAACTCACAGAGTTGA

              *     *  *               *        *  * *       *               
     355405 ACATTTCTATTGATAGAGCAGTTTTGAGACACTCTTCTTTTGGAATCTGCAAGTGGATATTTGGA
         66 ACCTTTCTTTTCATAGAGCAGTTTTGAAACACTCTTTTTGTAGAATCTGGAAGTGGATATTTGGA

            ** *                        *     *      
     355470 TAGATTTGAGGATTTCGTTGGAAACGG-GAT-T-AT-ATAT
        131 GCGCTTTGAGGATTTCGTTGGAAACGGAAATATCTTCATAT

                 *       *               *                 **  *   *         
     355507 AAAAAGTAGACAGCAGCATTCTCAGAAACTTCTTTGTGATGTTTGCATCCAGCTCTCAGAGTTGA
          1 AAAAACTAGACAGAAGCATTCTCAGAAACATCTTTGTGATGTTTGCACTCAACTCACAGAGTTGA

              *  * *          *  *        *        *   **        *  **       
     355572 ACATTCCCTTTCATAGAGTAGGTTTGAAACCCTCTTTTTATAGTGTCTGGAAGCGGGCATTTGGA
         66 ACCTTTCTTTTCATAGAGCAGTTTTGAAACACTCTTTTTGTAGAATCTGGAAGTGGATATTTGGA

                   *    * * **  **  **          * *  *
     355637 GCGCTTTCAGGCCTATGCTTAAAATAGGAAATATCTACCTAC
        131 GCGCTTTGAGG-ATTTCGTTGGAAACGGAAATATCTTCATAT

             *               *    *    *  **          ** *        *    *     
     355679 AGAAACTAGACAGAAGCCTTCTGAGAATCACGTTTGTGATGTGGGTACTCAACTAACAGTGTTGA
          1 AAAAACTAGACAGAAGCATTCTCAGAAACATCTTTGTGATGTTTGCACTCAACTCACAGAGTTGA

            *  *       *   *            *   *                *   *           
     355744 TCCATTCTTTTGATACAGCAGTTTTGAACCACACTTTTTGTAGAATCTGCAAGAGGATATTTGGA
         66 ACCTTTCTTTTCATAGAGCAGTTTTGAAACACTCTTTTTGTAGAATCTGGAAGTGGATATTTGGA

            **   *                     *   *      * *
     355809 TAGCTGTGAGGATTTCGTTGGAAACGGGAATGTCTTCAAAG
        131 GCGCTTTGAGGATTTCGTTGGAAACGGAAATATCTTCATAT

                *                         *   *            *    *            
     355850 AAAATCTAGACAGAAGCATTCTCAGAAACACCTTCGTGATGTTTGCAATCAAGTCACAGAGTTGA
          1 AAAAACTAGACAGAAGCATTCTCAGAAACATCTTTGTGATGTTTGCACTCAACTCACAGAGTTGA

                 * *             *  *                  *             *       
     355915 ACCTTCCGTTTCATAGAGCAGGTTGGAAACACTCTTTTTGTAGTATCTGGAAGTGGACATTTGGA
         66 ACCTTTCTTTTCATAGAGCAGTTTTGAAACACTCTTTTTGTAGAATCTGGAAGTGGATATTTGGA

                   *   ** ** *  *   *            **  
     355980 GCGCTTTCAGGCCTATGGTGAAAAAGGAAATATCTTCCCAT
        131 GCGCTTTGAGGATTTCGTTGGAAACGGAAATATCTTCATAT

                  * * *      **      *   * *   *     ** ** *      *    *     
     356021 AAAAACGACATAGAAGCTATCTCAGGAACTTGTTTATGATGCATCTAATCAACTAACAGTGTTGA
          1 AAAAACTAGACAGAAGCATTCTCAGAAACATCTTTGTGATGTTTGCACTCAACTCACAGAGTTGA

                  * ** * *                         * *       *               
     356086 ACCTTTGTACTGACAGAGCAG-TTTGAAACACTCTTTTTTTGGAATCTGCAAGTGGATATTTGGA
         66 ACCTTTCTTTTCATAGAGCAGTTTTGAAACACTCTTTTTGTAGAATCTGGAAGTGGATATTTGGA

            *                           *   *   *    
     356150 TCGCTTTGAGGATTTCGTTGGAAACGG-GAT-GC--AATAT
        131 GCGCTTTGAGGATTTCGTTGGAAACGGAAATATCTTCATAT

                **  *    *     *                 **  *   *  *    *         * 
     356187 AAAACGTACACAGCAGCATACTCAGAAA-ATACTTTGCCATATTTCCATTCAAGTCACAGAGTGG
          1 AAAAACTAGACAGAAGCATTCTCAGAAACAT-CTTTGTGATGTTTGCACTCAACTCACAGAGTTG

               *  * **            *  *               *  *             *      
     356251 AACATTCCCATTCATAGAGCAGGTTGGAAACACTCTTTTTGGAGTATCTGGAAGTGGACATTTGG
         65 AACCTTTCTTTTCATAGAGCAGTTTTGAAACACTCTTTTTGTAGAATCTGGAAGTGGATATTTGG

                    ** * * ** *  *   *            **  
     356316 AGCGCTTTCTGAACTATGGTGAAAAAGGAAATATCTTCCAAT
        130 AGCGCTTTGAGGATTTCGTTGGAAACGGAAATATCTTCATAT

            *     *               *      * *          *  **      **  *  *    
     356358 GAAAACAAGACAGAAGCATTCTGAGAAACTTATTTGTGATGTGTGTCCTCAACAAACGGACTTGA
          1 AAAAACTAGACAGAAGCATTCTCAGAAACATCTTTGTGATGTTTGCACTCAACTCACAGAGTTGA

                   *      **  * *  *  *             *   *    * * *           
     356423 ACCTTTCGTTTCATGCAGTACTTCTGGAACACTCTTTTTGAAGATTCTGCATGCGGATATTTGGA
         66 ACCTTTCTTTTCATAGAGCAGTTTTGAAACACTCTTTTTGTAGAATCTGGAAGTGGATATTTGGA

            **                            **   *   * 
     356488 TTGCTTTGAGGATTTCGTTGGAAACGG---GCT-TACATGT
        131 GCGCTTTGAGGATTTCGTTGGAAACGGAAATATCTTCATAT

                 *       *               *        *   *    *    *       *    
     356525 AAAAATTAGACAGCAGCATTCTCAGAAACTTCTTTGTGGTGTCTGCATTCAAGTCACAGAATTGA
          1 AAAAACTAGACAGAAGCATTCTCAGAAACATCTTTGTGATGTTTGCACTCAACTCACAGAGTTGA

              * ** * **            *  * *      *       *             *       
     356590 ACTTCCCCTCACATAGAGCAGTTGTGCAGCACTCTATTTGTAGTATCTGGAAGTGGACATTTGGA
         66 ACCTTTCTTTTCATAGAGCAGTTTTGAAACACTCTTTTTGTAGAATCTGGAAGTGGATATTTGGA

             *         ** *    * *   *            **  
     356655 GGGCTTTGTAGCCTATC-TGGAAAAAGGAAATATCTTCCCAT
        131 GCGCTTTG-AGGATTTCGTTGGAAACGGAAATATCTTCATAT

            *  ** *   *  *  * *            *   *  *   * **        *  * * *   
     356696 GAATGCGAGATAGTAGTAATCTCAGAAACATGTTTATGCTGTATCTACTCAACTAACTGTGCTGA
          1 AAAAACTAGACAGAAGCATTCTCAGAAACATCTTTGTGATGTTTGCACTCAACTCACAGAGTTGA

              *     *  *               *        *  * *       *               
     356761 ACATTTCTATTGATAGAGCAGTTTTGAGACACTCTTCTTTTGGAATCTGCAAGTGGATATTTGGA
         66 ACCTTTCTTTTCATAGAGCAGTTTTGAAACACTCTTTTTGTAGAATCTGGAAGTGGATATTTGGA

            ** *                        *     *      
     356826 TAGATTTGAGGATTTCGTTGGAAACGG-GAT-T-AT-ATAT
        131 GCGCTTTGAGGATTTCGTTGGAAACGGAAATATCTTCATAT

                 *       *               *                 **  *   *         
     356863 AAAAAGTAGACAGCAGCATTCTCAGAAACTTCTTTGTGATGTTTGCATCCAGCTCTCAGAGTTGA
          1 AAAAACTAGACAGAAGCATTCTCAGAAACATCTTTGTGATGTTTGCACTCAACTCACAGAGTTGA

              *  * *          *  *        *        *   **        *  **       
     356928 ACATTCCCTTTCATAGAGTAGGTTTGAAACCCTCTTTTTATAGTGTCTGGAAGCGGGCATTTGGA
         66 ACCTTTCTTTTCATAGAGCAGTTTTGAAACACTCTTTTTGTAGAATCTGGAAGTGGATATTTGGA

                   *   ** ** *  *   *          * *   
     356993 GCGCTTTCAGGCCTATGCTGAAAAAGGAAATATCTACCTAT
        131 GCGCTTTGAGGATTTCGTTGGAAACGGAAATATCTTCATAT

             *                    *    *  **          ** *        *    *     
     357034 AGAAACTAGACAGAAGCATTCTGAGAATCACGTTTGTGATGTGGGTACTCAACTAACAGTGTTGA
          1 AAAAACTAGACAGAAGCATTCTCAGAAACATCTTTGTGATGTTTGCACTCAACTCACAGAGTTGA

            *  *       *   *            *   *                *               
     357099 TCCATTCTTTTGATACAGCAGTTTTGAACCACACTTTTTGTAGAATCTGCAAGTGGATATTTGGA
         66 ACCTTTCTTTTCATAGAGCAGTTTTGAAACACTCTTTTTGTAGAATCTGGAAGTGGATATTTGGA

            **   *                     *   *        *
     357164 TAGCTGTGAGGATTTCGTTGGAAACGGGAATGTCTTCATAG
        131 GCGCTTTGAGGATTTCGTTGGAAACGGAAATATCTTCATAT

                **                     * * **         *  **   *   *          
     357205 AAAATTTAGACAGAAGCATTCTCAGAACCTTGATTGTGATGTGTGTTCTCCACTAACAGAGTTGA
          1 AAAAACTAGACAGAAGCATTCTCAGAAACATCTTTGTGATGTTTGCACTCAACTCACAGAGTTGA

                       * *   * *   *       *       *                         
     357270 ACCTTTCTTTTGACAGAACTGTTCTGAAACATTCTTTTTATAGAATCTGGAAGTGGATATTTGGA
         66 ACCTTTCTTTTCATAGAGCAGTTTTGAAACACTCTTTTTGTAGAATCTGGAAGTGGATATTTGGA

            **                         *          *  
     357335 AAGCTTTGAGGATTTCGTTGGAAACGGGAATATCTTCAAAT
        131 GCGCTTTGAGGATTTCGTTGGAAACGGAAATATCTTCATAT

            *   *    *            *           * *       *  *    *            
     357376 CAAATCTAGCCAGAAGCATTCTAAGAAACATCTTAGGGATGTTTACATTCAAGTCACAGAGTTGA
          1 AAAAACTAGACAGAAGCATTCTCAGAAACATCTTTGTGATGTTTGCACTCAACTCACAGAGTTGA

              *  * *     *       *         *    * *   **      *      *    *  
     357441 ACATTCCCTTTCACAGAGCAGGTTTGAAACAATCTTCTCGTACTATCTGGCAGTGGACATTTTGA
         66 ACCTTTCTTTTCATAGAGCAGTTTTGAAACACTCTTTTTGTAGAATCTGGAAGTGGATATTTGGA

              * *   *  ** ** *  *   *            ** *
     357506 GCTCCTTGGGGCCTATGCTGAAAAAGGAAATATCTTCCGAC
        131 GCGCTTTGAGGATTTCGTTGGAAACGGAAATATCTTCATAT

                                 *     *  **          *           **    *    
     357547 AAAAACTAGACAGAAGCATTCGCAGAATCACGTTTGTGATGTGTGCACTCAACTGTCAGAATTGA
          1 AAAAACTAGACAGAAGCATTCTCAGAAACATCTTTGTGATGTTTGCACTCAACTCACAGAGTTGA

                 **   ** *      *                            * *            *
     357612 ACCTTGGTTTGGACAGAGCACTTTTGAAACACTCTTTTTGTAGAATCTGCAGGTGGATATTTGGC
         66 ACCTTTCTTTTCATAGAGCAGTTTTGAAACACTCTTTTTGTAGAATCTGGAAGTGGATATTTGGA

            **                         *   *      * *
     357677 TAGCTTTGAGGATTTCGTTGGAAACGGGAATGTCTTCAAAG
        131 GCGCTTTGAGGATTTCGTTGGAAACGGAAATATCTTCATAT

                *                         *   *            *    *            
     357718 AAAATCTAGACAGAAGCATTCTCAGAAACACCTTCGTGATGTTTGCAATCAAGTCACAGAGTTGA
          1 AAAAACTAGACAGAAGCATTCTCAGAAACATCTTTGTGATGTTTGCACTCAACTCACAGAGTTGA

                 * *             *  *                  *             *       
     357783 ACCTTCCGTTTCATAGAGCAGGTTGGAAACACTCTTTTTGTAGTATCTGGAAGTGGACATTTGGA
         66 ACCTTTCTTTTCATAGAGCAGTTTTGAAACACTCTTTTTGTAGAATCTGGAAGTGGATATTTGGA

                   *   ** ** *  *   *            **  
     357848 GCGCTTTCAGGCCTATGGTGAAAAAGGAAATATCTTCCCAT
        131 GCGCTTTGAGGATTTCGTTGGAAACGGAAATATCTTCATAT

                  * * *      **      *   * *   *     ** ** *      *    *     
     357889 AAAAACGACATAGAAGCTATCTCAGGAACTTGTTTATGATGCATCTAATCAACTAACAGTGTTGA
          1 AAAAACTAGACAGAAGCATTCTCAGAAACATCTTTGTGATGTTTGCACTCAACTCACAGAGTTGA

                  * ** * *                         * *       *               
     357954 ACCTTTGTACTGACAGAGCAG-TTTGAAACACTCTTTTTTTGGAATCTGCAAGTGGATATTTGGA
         66 ACCTTTCTTTTCATAGAGCAGTTTTGAAACACTCTTTTTGTAGAATCTGGAAGTGGATATTTGGA

            *                           *   *   *    
     358018 TCGCTTTGAGGATTTCGTTGGAAACGG-GAT-GC--AATAT
        131 GCGCTTTGAGGATTTCGTTGGAAACGGAAATATCTTCATAT

                **  *    *     *                 **  *   *  *    *         * 
     358055 AAAACGTACACAGCAGCATACTCAGAAA-ATACTTTGCCATATTTCCATTCAAGTCACAGAGTGG
          1 AAAAACTAGACAGAAGCATTCTCAGAAACAT-CTTTGTGATGTTTGCACTCAACTCACAGAGTTG

               *  * **            *  *               *  *             *      
     358119 AACATTCCCATTCATAGAGCAGGTTGGAAACACTCTTTTTGGAGTATCTGGAAGTGGACATTTGG
         65 AACCTTTCTTTTCATAGAGCAGTTTTGAAACACTCTTTTTGTAGAATCTGGAAGTGGATATTTGG

                    ** * * ** *  *   *            **  
     358184 AGCGCTTTCTGAACTATGGTGAAAAAGGAAATATCTTCCAAT
        130 AGCGCTTTGAGGATTTCGTTGGAAACGGAAATATCTTCATAT

            *     *               *      * *          *  **      **  *  *    
     358226 GAAAACAAGACAGAAGCATTCTGAGAAACTTATTTGTGATGTGTGTCCTCAACAAACGGACTTGA
          1 AAAAACTAGACAGAAGCATTCTCAGAAACATCTTTGTGATGTTTGCACTCAACTCACAGAGTTGA

                   *      **  * *  *  *             *   *    * * *           
     358291 ACCTTTCGTTTCATGCAGTACTTCTGGAACACTCTTTTTGAAGATTCTGCATGCGGATATTTGGA
         66 ACCTTTCTTTTCATAGAGCAGTTTTGAAACACTCTTTTTGTAGAATCTGGAAGTGGATATTTGGA

            **                            **   *   * 
     358356 TAGCTTTGAGGATTTCGTTGGAAACGG---GCT-TACATGT
        131 GCGCTTTGAGGATTTCGTTGGAAACGGAAATATCTTCATAT

                 *       *               *        *   *    *    *       *    
     358393 AAAAATTAGACAGCAGCATTCTCAGAAACTTCTTTGTGGTGTCTGCATTCAAGTCACAGAATTGA
          1 AAAAACTAGACAGAAGCATTCTCAGAAACATCTTTGTGATGTTTGCACTCAACTCACAGAGTTGA

              * ** * **            *  * *      *       *             *       
     358458 ACTTCCCCTCACATAGAGCAGTTGTGCAGCACTCTATTTGTAGTATCTGGAAGTGGACATTTGGA
         66 ACCTTTCTTTTCATAGAGCAGTTTTGAAACACTCTTTTTGTAGAATCTGGAAGTGGATATTTGGA

             *         ** *    * *   *            **  
     358523 GGGCTTTGTAGCCTATC-TGGAAAAAGGAAATATCTTCCCAT
        131 GCGCTTTG-AGGATTTCGTTGGAAACGGAAATATCTTCATAT

            *  ** *   *     * *            *   *  *   * **        *  * * *   
     358564 GAATGCGAGATAGAAGTAATCTCAGAAACATGTTTATGCTGTATCTACTCAACTAACTGTGCTGA
          1 AAAAACTAGACAGAAGCATTCTCAGAAACATCTTTGTGATGTTTGCACTCAACTCACAGAGTTGA

              *     *  *               *        *  * *       *               
     358629 ACATTTCTATTGATAGAGCAGTTTTGAGACACTCTTCTTTTGGAATCTGCAAGTGGATATTTGGA
         66 ACCTTTCTTTTCATAGAGCAGTTTTGAAACACTCTTTTTGTAGAATCTGGAAGTGGATATTTGGA

            ** *                        *     *      
     358694 TAGATTTGAGGATTTCGTTGGAAACGG-GAT-T-AT-ATAT
        131 GCGCTTTGAGGATTTCGTTGGAAACGGAAATATCTTCATAT

                 *       *               *                 **  *   *         
     358731 AAAAAGTAGACAGCAGCATTCTCAGAAACTTCTTTGTGATGTTTGCATCCAGCTCTCAGAGTTGA
          1 AAAAACTAGACAGAAGCATTCTCAGAAACATCTTTGTGATGTTTGCACTCAACTCACAGAGTTGA

              *  * *          *  *        *        *   **        *  **       
     358796 ACATTCCCTTTCATAGAGTAGGTTTGAAACCCTCTTTTTATAGTGTCTGGAAGCGGGCATTTGGA
         66 ACCTTTCTTTTCATAGAGCAGTTTTGAAACACTCTTTTTGTAGAATCTGGAAGTGGATATTTGGA

                   *   ** ** *  *   *          * *   
     358861 GCGCTTTCAGGCCTATGCTGAAAAAGGAAATATCTACCTAT
        131 GCGCTTTGAGGATTTCGTTGGAAACGGAAATATCTTCATAT

             *                    *    *  **          ** *        *    *     
     358902 AGAAACTAGACAGAAGCATTCTGAGAATCACGTTTGTGATGTGGGTACTCAACTAACAGTGTTGA
          1 AAAAACTAGACAGAAGCATTCTCAGAAACATCTTTGTGATGTTTGCACTCAACTCACAGAGTTGA

            *  *       *   *            *   *                *               
     358967 TCCATTCTTTTGATACAGCAGTTTTGAACCACACTTTTTGTAGAATCTGCAAGTGGATATTTGGA
         66 ACCTTTCTTTTCATAGAGCAGTTTTGAAACACTCTTTTTGTAGAATCTGGAAGTGGATATTTGGA

            **   *                     *   *        *
     359032 TAGCTGTGAGGATTTCGTTGGAAACGGGAATGTCTTCATAG
        131 GCGCTTTGAGGATTTCGTTGGAAACGGAAATATCTTCATAT

                **                     * * **         *  **   *   *          
     359073 AAAATTTAGACAGAAGCATTCTCAGAACCTTGATTGTGATGTGTGTTCTCCACTAACAGAGTTGA
          1 AAAAACTAGACAGAAGCATTCTCAGAAACATCTTTGTGATGTTTGCACTCAACTCACAGAGTTGA

                       * *   * *   *       *       *                         
     359138 ACCTTTCTTTTGACAGAACTGTTCTGAAACATTCTTTTTATAGAATCTGGAAGTGGATATTTGGA
         66 ACCTTTCTTTTCATAGAGCAGTTTTGAAACACTCTTTTTGTAGAATCTGGAAGTGGATATTTGGA

            **                         *          *  
     359203 AAGCTTTGAGGATTTCGTTGGAAACGGGAATATCTTCAAAT
        131 GCGCTTTGAGGATTTCGTTGGAAACGGAAATATCTTCATAT

                *    *            *           * *       *  *    *            
     359244 AAAATCTAGCCAGAAGCATTCTAAGAAACATCTTAGGGATGTTTACATTCAAGTCACAGAGTTGA
          1 AAAAACTAGACAGAAGCATTCTCAGAAACATCTTTGTGATGTTTGCACTCAACTCACAGAGTTGA

              *  * *     *       *         *    * *   **      *      *    *  
     359309 ACATTCCCTTTCACAGAGCAGGTTTGAAACAATCTTCTCGTACTATCTGGCAGTGGACATTTTGA
         66 ACCTTTCTTTTCATAGAGCAGTTTTGAAACACTCTTTTTGTAGAATCTGGAAGTGGATATTTGGA

              * *   *  ** ** *  *   *            ** *
     359374 GCTCCTTGGGGCCTATGCTGAAAAAGGAAATATCTTCCGAC
        131 GCGCTTTGAGGATTTCGTTGGAAACGGAAATATCTTCATAT

                                 *     *  **          *           **    *    
     359415 AAAAACTAGACAGAAGCATTCGCAGAATCACGTTTGTGATGTGTGCACTCAACTGTCAGAATTGA
          1 AAAAACTAGACAGAAGCATTCTCAGAAACATCTTTGTGATGTTTGCACTCAACTCACAGAGTTGA

                 **   ** *      *                            * *            *
     359480 ACCTTGGTTTGGACAGAGCACTTTTGAAACACTCTTTTTGTAGAATCTGCAGGTGGATATTTGGC
         66 ACCTTTCTTTTCATAGAGCAGTTTTGAAACACTCTTTTTGTAGAATCTGGAAGTGGATATTTGGA

            **                         *   *      * *
     359545 TAGCTTTGAGGATTTCGTTGGAAACGGGAATGTCTTCAAAG
        131 GCGCTTTGAGGATTTCGTTGGAAACGGAAATATCTTCATAT

                *                         *   *            *    *            
     359586 AAAATCTAGACAGAAGCATTCTCAGAAACACCTTCGTGATGTTTGCAATCAAGTCACAGAGTTGA
          1 AAAAACTAGACAGAAGCATTCTCAGAAACATCTTTGTGATGTTTGCACTCAACTCACAGAGTTGA

                 * *             *  *                  *             *       
     359651 ACCTTCCGTTTCATAGAGCAGGTTGGAAACACTCTTTTTGTAGTATCTGGAAGTGGACATTTGGA
         66 ACCTTTCTTTTCATAGAGCAGTTTTGAAACACTCTTTTTGTAGAATCTGGAAGTGGATATTTGGA

                   *   ** ** *  *   *            **  
     359716 GCGCTTTCAGGCCTATGGTGAAAAAGGAAATATCTTCCCAT
        131 GCGCTTTGAGGATTTCGTTGGAAACGGAAATATCTTCATAT

                  * * *      **      *   * *   *     ** ** *      *    *     
     359757 AAAAACGACATAGAAGCTATCTCAGGAACTTGTTTATGATGCATCTAATCAACTAACAGTGTTGA
          1 AAAAACTAGACAGAAGCATTCTCAGAAACATCTTTGTGATGTTTGCACTCAACTCACAGAGTTGA

                  * ** * *                         * *       *               
     359822 ACCTTTGTACTGACAGAGCAG-TTTGAAACACTCTTTTTTTGGAATCTGCAAGTGGATATTTGGA
         66 ACCTTTCTTTTCATAGAGCAGTTTTGAAACACTCTTTTTGTAGAATCTGGAAGTGGATATTTGGA

            *                           *   *   *    
     359886 TCGCTTTGAGGATTTCGTTGGAAACGG-GAT-GC--AATAT
        131 GCGCTTTGAGGATTTCGTTGGAAACGGAAATATCTTCATAT

                **  *    *     *                 **  *   *  *    *         * 
     359923 AAAACGTACACAGCAGCATACTCAGAAA-ATACTTTGCCATATTTCCATTCAAGTCACAGAGTGG
          1 AAAAACTAGACAGAAGCATTCTCAGAAACAT-CTTTGTGATGTTTGCACTCAACTCACAGAGTTG

               *  * **            *  *               *  *             *      
     359987 AACATTCCCATTCATAGAGCAGGTTGGAAACACTCTTTTTGGAGTATCTGGAAGTGGACATTTGG
         65 AACCTTTCTTTTCATAGAGCAGTTTTGAAACACTCTTTTTGTAGAATCTGGAAGTGGATATTTGG

                    ** * * ** *  *   *            **  
     360052 AGCGCTTTCTGAACTATGGTGAAAAAGGAAATATCTTCCAAT
        130 AGCGCTTTGAGGATTTCGTTGGAAACGGAAATATCTTCATAT

            *     *               *      * *          *  **      **  *  *    
     360094 GAAAACAAGACAGAAGCATTCTGAGAAACTTATTTGTGATGTGTGTCCTCAACAAACGGACTTGA
          1 AAAAACTAGACAGAAGCATTCTCAGAAACATCTTTGTGATGTTTGCACTCAACTCACAGAGTTGA

                   *      **  * *  *  *             *   *    * * *           
     360159 ACCTTTCGTTTCATGCAGTACTTCTGGAACACTCTTTTTGAAGATTCTGCATGCGGATATTTGGA
         66 ACCTTTCTTTTCATAGAGCAGTTTTGAAACACTCTTTTTGTAGAATCTGGAAGTGGATATTTGGA

            **                            **   *   * 
     360224 TAGCTTTGAGGATTTCGTTGGAAACGG---GCT-TACATGT
        131 GCGCTTTGAGGATTTCGTTGGAAACGGAAATATCTTCATAT

                 *       *               *        *   *    *    *       *    
     360261 AAAAATTAGACAGCAGCATTCTCAGAAACTTCTTTGTGGTGTCTGCATTCAAGTCACAGAATTGA
          1 AAAAACTAGACAGAAGCATTCTCAGAAACATCTTTGTGATGTTTGCACTCAACTCACAGAGTTGA

              * ** * **            *  * *      *       *             *       
     360326 ACTTCCCCTCACATAGAGCAGTTGTGCAGCACTCTATTTGTAGTATCTGGAAGTGGACATTTGGA
         66 ACCTTTCTTTTCATAGAGCAGTTTTGAAACACTCTTTTTGTAGAATCTGGAAGTGGATATTTGGA

             *         ** *    * *   *            **  
     360391 GGGCTTTGTAGCCTATC-TGGAAAAAGGAAATATCTTCCCAT
        131 GCGCTTTG-AGGATTTCGTTGGAAACGGAAATATCTTCATAT

            *  ** *   *     * *            *   *  *   * **        *  * * *   
     360432 GAATGCGAGATAGAAGTAATCTCAGAAACATGTTTATGCTGTATCTACTCAACTAACTGTGCTGA
          1 AAAAACTAGACAGAAGCATTCTCAGAAACATCTTTGTGATGTTTGCACTCAACTCACAGAGTTGA

              *     *  *               *        *  * *       *               
     360497 ACATTTCTATTGATAGAGCAGTTTTGAGACACTCTTCTTTTGGAATCTGCAAGTGGATATTTGGA
         66 ACCTTTCTTTTCATAGAGCAGTTTTGAAACACTCTTTTTGTAGAATCTGGAAGTGGATATTTGGA

            ** *                        *     *      
     360562 TAGATTTGAGGATTTCGTTGGAAACGG-GAT-T-AT-ATAT
        131 GCGCTTTGAGGATTTCGTTGGAAACGGAAATATCTTCATAT

                 *       *               *                 **  *   *         
     360599 AAAAAGTAGACAGCAGCATTCTCAGAAACTTCTTTGTGATGTTTGCATCCAGCTCTCAGAGTTGA
          1 AAAAACTAGACAGAAGCATTCTCAGAAACATCTTTGTGATGTTTGCACTCAACTCACAGAGTTGA

              *  * *          *  *        *        *   **        *  **       
     360664 ACATTCCCTTTCATAGAGTAGGTTTGAAACCCTCTTTTTATAGTGTCTGGAAGCGGGCATTTGGA
         66 ACCTTTCTTTTCATAGAGCAGTTTTGAAACACTCTTTTTGTAGAATCTGGAAGTGGATATTTGGA

                   *   ** ** *  *   *          * *   
     360729 GCGCTTTCAGGCCTATGCTGAAAAAGGAAATATCTACCTAT
        131 GCGCTTTGAGGATTTCGTTGGAAACGGAAATATCTTCATAT

             *                    *    *  **          ** *        *    *     
     360770 AGAAACTAGACAGAAGCATTCTGAGAATCACGTTTGTGATGTGGGTACTCAACTAACAGTGTTGA
          1 AAAAACTAGACAGAAGCATTCTCAGAAACATCTTTGTGATGTTTGCACTCAACTCACAGAGTTGA

            *  *       *   *            *   *                *               
     360835 TCCATTCTTTTGATACAGCAGTTTTGAACCACACTTTTTGTAGAATCTGCAAGTGGATATTTGGA
         66 ACCTTTCTTTTCATAGAGCAGTTTTGAAACACTCTTTTTGTAGAATCTGGAAGTGGATATTTGGA

            **   *                     *   *        *
     360900 TAGCTGTGAGGATTTCGTTGGAAACGGGAATGTCTTCATAG
        131 GCGCTTTGAGGATTTCGTTGGAAACGGAAATATCTTCATAT

                **                     * * **         *  **   *   *          
     360941 AAAATTTAGACAGAAGCATTCTCAGAACCTTGATTGTGATGTGTGTTCTCCACTAACAGAGTTGA
          1 AAAAACTAGACAGAAGCATTCTCAGAAACATCTTTGTGATGTTTGCACTCAACTCACAGAGTTGA

                     * * *   * *   *       *       *                         
     361006 ACCTTTCTTCTGACAGAACTGTTCTGAAACATTCTTTTTATAGAATCTGGAAGTGGATATTTGGA
         66 ACCTTTCTTTTCATAGAGCAGTTTTGAAACACTCTTTTTGTAGAATCTGGAAGTGGATATTTGGA

            **                         *          *  
     361071 AAGCTTTGAGGATTTCGTTGGAAACGGGAATATCTTCAAAT
        131 GCGCTTTGAGGATTTCGTTGGAAACGGAAATATCTTCATAT

                *    *            *           * *   *   *  *    *            
     361112 AAAATCTAGCCAGAAGCATTCTAAGAAACATCTTAGGGATATTTACATTCAAGTCACAGAGTTGA
          1 AAAAACTAGACAGAAGCATTCTCAGAAACATCTTTGTGATGTTTGCACTCAACTCACAGAGTTGA

              *  * *     *       *         *    * *   **      *      *    *  
     361177 ACATTCCCTTTCACAGAGCAGGTTTGAAACAATCTTCTCGTACTATCTGGCAGTGGACATTTTGA
         66 ACCTTTCTTTTCATAGAGCAGTTTTGAAACACTCTTTTTGTAGAATCTGGAAGTGGATATTTGGA

              * *   *  ** ** *  *   *            ** *
     361242 GCTCCTTGGGGCCTATGCTGAAAAAGGAAATATCTTCCGAC
        131 GCGCTTTGAGGATTTCGTTGGAAACGGAAATATCTTCATAT

                                 *     *  **          *           **    *    
     361283 AAAAACTAGACAGAAGCATTCGCAGAATCACGTTTGTGATGTGTGCACTCAACTGTCAGAATTGA
          1 AAAAACTAGACAGAAGCATTCTCAGAAACATCTTTGTGATGTTTGCACTCAACTCACAGAGTTGA

                 **   ** *      *                            * *            *
     361348 ACCTTGGTTTGGACAGAGCACTTTTGAAACACTCTTTTTGTAGAATCTGCAGGTGGATATTTGGC
         66 ACCTTTCTTTTCATAGAGCAGTTTTGAAACACTCTTTTTGTAGAATCTGGAAGTGGATATTTGGA

            **                         *   *      * *
     361413 TAGCTTTGAGGATTTCGTTGGAAACGGTAATGTCTTCAAAG
        131 GCGCTTTGAGGATTTCGTTGGAAACGGAAATATCTTCATAT

                *                         *   *            *    *            
     361454 AAAATCTAGACAGAAGCATTCTCAGAAACACCTTCGTGATGTTTGCAATCAAGTCACAGAGTTGA
          1 AAAAACTAGACAGAAGCATTCTCAGAAACATCTTTGTGATGTTTGCACTCAACTCACAGAGTTGA

                 * *             *  *                  *             *       
     361519 ACCTTCCGTTTCATAGAGCAGGTTGGAAACACTCTTTTTGTAGTATCTGGAAGTGGACATTTGGA
         66 ACCTTTCTTTTCATAGAGCAGTTTTGAAACACTCTTTTTGTAGAATCTGGAAGTGGATATTTGGA

             *         ** *    * *   *            **  
     361584 GGGCTTTGTAGCCTATC-TGGAAAAAGGAAATATCTTCCCAT
        131 GCGCTTTG-AGGATTTCGTTGGAAACGGAAATATCTTCATAT

            *  ** *   *     * *            *   *  *   * **        *  * * *   
     361625 GAATGCGAGATAGAAGTAATCTCAGAAACATGTTTATGCTGTATCTACTCAACTAACTGTGCTGA
          1 AAAAACTAGACAGAAGCATTCTCAGAAACATCTTTGTGATGTTTGCACTCAACTCACAGAGTTGA

              *     *  *               *        *  * *       *               
     361690 ACATTTCTATTGATAGAGCAGTTTTGAGACACTCTTCTTTTGGAATCTGCAAGTGGATATTTGGA
         66 ACCTTTCTTTTCATAGAGCAGTTTTGAAACACTCTTTTTGTAGAATCTGGAAGTGGATATTTGGA

            ** *                        *     *      
     361755 TAGATTTGAGGATTTCGTTGGAAACGG-GAT-T-AT-ATAT
        131 GCGCTTTGAGGATTTCGTTGGAAACGGAAATATCTTCATAT

                 *       *               *                 **  *   *         
     361792 AAAAAGTAGACAGCAGCATTCTCAGAAACTTCTTTGTGATGTTTGCATCCAGCTCTCAGAGTTGA
          1 AAAAACTAGACAGAAGCATTCTCAGAAACATCTTTGTGATGTTTGCACTCAACTCACAGAGTTGA

              *  * *          *  *        *        *   **        *  **       
     361857 ACATTCCCTTTCATAGAGTAGGTTTGAAACCCTCTTTTTATAGTGTCTGGAAGCGGGCATTTGGA
         66 ACCTTTCTTTTCATAGAGCAGTTTTGAAACACTCTTTTTGTAGAATCTGGAAGTGGATATTTGGA

                   *    * * **  **  **          * *  *
     361922 GCGCTTTCAGGCCTATGCTTAAAATAGGAAATATCTACCTAC
        131 GCGCTTTGAGG-ATTTCGTTGGAAACGGAAATATCTTCATAT

             *                    *    *  **          ** *        *    *     
     361964 AGAAACTAGACAGAAGCATTCTGAGAATCACGTTTGTGATGTGGGTACTCAACTAACAGTGTTGA
          1 AAAAACTAGACAGAAGCATTCTCAGAAACATCTTTGTGATGTTTGCACTCAACTCACAGAGTTGA

            *  *       *   *            *   *                *   *           
     362029 TCCATTCTTTTGATACAGCAGTTTTGAACCACACTTTTTGTAGAATCTGCAAGAGGATATTTGGA
         66 ACCTTTCTTTTCATAGAGCAGTTTTGAAACACTCTTTTTGTAGAATCTGGAAGTGGATATTTGGA

            **   *                     *   *      * *
     362094 TAGCTGTGAGGATTTCGTTGGAAACGGGAATGTCTTCAAAG
        131 GCGCTTTGAGGATTTCGTTGGAAACGGAAATATCTTCATAT

                *                         *   *            *    *            
     362135 AAAATCTAGACAGAAGCATTCTCAGAAACACCTTCGTGATGTTTGCAATCAAGTCACAGAGTTGA
          1 AAAAACTAGACAGAAGCATTCTCAGAAACATCTTTGTGATGTTTGCACTCAACTCACAGAGTTGA

                 * *             *  *           *      *             *       
     362200 ACCTTCCGTTTCATAGAGCAGGTTGGAAACACTCTTATTGTAGTATCTGGAAGTGGACATTTGGA
         66 ACCTTTCTTTTCATAGAGCAGTTTTGAAACACTCTTTTTGTAGAATCTGGAAGTGGATATTTGGA

                   *   ** ** *  *   *            **  
     362265 GCGCTTTCAGGCCTATGGTGAAAAAGGAAATATCTTCCCAT
        131 GCGCTTTGAGGATTTCGTTGGAAACGGAAATATCTTCATAT

                  * * *      **      *   * *   *     ** ** *      *    *     
     362306 AAAAACGACATAGAAGCTATCTCAGGAACTTGTTTATGATGCATCTAATCAACTAACAGTGTTGA
          1 AAAAACTAGACAGAAGCATTCTCAGAAACATCTTTGTGATGTTTGCACTCAACTCACAGAGTTGA

                  * ** * *                         * *       *               
     362371 ACCTTTGTACTGACAGAGCAG-TTTGAAACACTCTTTTTTTGGAATCTGCAAGTGGATATTTGGA
         66 ACCTTTCTTTTCATAGAGCAGTTTTGAAACACTCTTTTTGTAGAATCTGGAAGTGGATATTTGGA

            *                           *   *   *    
     362435 TCGCTTTGAGGATTTCGTTGGAAACGG-GAT-GC--AATAT
        131 GCGCTTTGAGGATTTCGTTGGAAACGGAAATATCTTCATAT

                **  *    *     *                 **  *   *  *    *         * 
     362472 AAAACGTACACAGCAGCATACTCAGAAA-ATACTTTGCCATATTTCCATTCAAGTCACAGAGTGG
          1 AAAAACTAGACAGAAGCATTCTCAGAAACAT-CTTTGTGATGTTTGCACTCAACTCACAGAGTTG

               *  * **            *  *               *  *             *      
     362536 AACATTCCCATTCATAGAGCAGGTTGGAAACACTCTTTTTGGAGTATCTGGAAGTGGACATTTGG
         65 AACCTTTCTTTTCATAGAGCAGTTTTGAAACACTCTTTTTGTAGAATCTGGAAGTGGATATTTGG

                    ** * * ** *  *   *            **  
     362601 AGCGCTTTCTGAACTATGGTGAAAAAGGAAATATCTTCCAAT
        130 AGCGCTTTGAGGATTTCGTTGGAAACGGAAATATCTTCATAT

            *     *               *      * *          *  **      **  *  *    
     362643 GAAAACAAGACAGAAGCATTCTGAGAAACTTATTTGTGATGTGTGTCCTCAACAAACGGACTTGA
          1 AAAAACTAGACAGAAGCATTCTCAGAAACATCTTTGTGATGTTTGCACTCAACTCACAGAGTTGA

                   *      **  * *  *  *             *   *    * * *           
     362708 ACCTTTCGTTTCATGCAGTACTTCTGGAACACTCTTTTTGAAGATTCTGCATGCGGATATTTGGA
         66 ACCTTTCTTTTCATAGAGCAGTTTTGAAACACTCTTTTTGTAGAATCTGGAAGTGGATATTTGGA

            **                            **   *   * 
     362773 TAGCTTTGAGGATTTCGTTGGAAACGG---GCT-TACATGT
        131 GCGCTTTGAGGATTTCGTTGGAAACGGAAATATCTTCATAT

                 *       *               *        *   *    *    *       *    
     362810 AAAAATTAGACAGCAGCATTCTCAGAAACTTCTTTGTGGTGTCTGCATTCAAGTCACAGAATTGA
          1 AAAAACTAGACAGAAGCATTCTCAGAAACATCTTTGTGATGTTTGCACTCAACTCACAGAGTTGA

              * ** * **            *  * *      *       *             *       
     362875 ACTTCCCCTCACATAGAGCAGTTGTGCAGCACTCTATTTGTAGTATCTGGAAGTGGACATTTGGA
         66 ACCTTTCTTTTCATAGAGCAGTTTTGAAACACTCTTTTTGTAGAATCTGGAAGTGGATATTTGGA

             *         ** *    * *   *            **  
     362940 GGGCTTTGTAGCCTATC-TGGAAAAAGGAAATATCTTCCCAT
        131 GCGCTTTG-AGGATTTCGTTGGAAACGGAAATATCTTCATAT

            *  ** *   *     * *            *   *  *   * **        *  * * *   
     362981 GAATGCGAGATAGAAGTAATCTCAGAAACATGTTTATGCTGTATCTACTCAACTAACTGTGCTGA
          1 AAAAACTAGACAGAAGCATTCTCAGAAACATCTTTGTGATGTTTGCACTCAACTCACAGAGTTGA

              *     *  *               *        *  * *       *               
     363046 ACATTTCTATTGATAGAGCAGTTTTGAGACACTCTTCTTTTGGAATCTGCAAGTGGATATTTGGA
         66 ACCTTTCTTTTCATAGAGCAGTTTTGAAACACTCTTTTTGTAGAATCTGGAAGTGGATATTTGGA

            ** *                        *     *      
     363111 TAGATTTGAGGATTTCGTTGGAAACGG-GAT-T-AT-ATAT
        131 GCGCTTTGAGGATTTCGTTGGAAACGGAAATATCTTCATAT

                 *       *               *                 **  *   *         
     363148 AAAAAGTAGACAGCAGCATTCTCAGAAACTTCTTTGTGATGTTTGCATCCAGCTCTCAGAGTTGA
          1 AAAAACTAGACAGAAGCATTCTCAGAAACATCTTTGTGATGTTTGCACTCAACTCACAGAGTTGA

              *  * *          *  *        *        *   **        *  **       
     363213 ACATTCCCTTTCATAGAGTAGGTTTGAAACCCTCTTTTTATAGTGTCTGGAAGCGGGCATTTGGA
         66 ACCTTTCTTTTCATAGAGCAGTTTTGAAACACTCTTTTTGTAGAATCTGGAAGTGGATATTTGGA

                   *   ** ** *  *   *          * *   
     363278 GCGCTTTCAGGCCTATGCTGAAAAAGGAAATATCTACCTAT
        131 GCGCTTTGAGGATTTCGTTGGAAACGGAAATATCTTCATAT

             *                    *    *  **          ** *        *    *     
     363319 AGAAACTAGACAGAAGCATTCTGAGAATCACGTTTGTGATGTGGGTACTCAACTAACAGTGTTGA
          1 AAAAACTAGACAGAAGCATTCTCAGAAACATCTTTGTGATGTTTGCACTCAACTCACAGAGTTGA

            *  *       *   *            *   *                *               
     363384 TCCATTCTTTTGATACAGCAGTTTTGAACCACACTTTTTGTAGAATCTGCAAGTGGATATTTGGA
         66 ACCTTTCTTTTCATAGAGCAGTTTTGAAACACTCTTTTTGTAGAATCTGGAAGTGGATATTTGGA

            **   *                     *   *        *
     363449 TAGCTGTGAGGATTTCGTTGGAAACGGGAATGTCTTCATAG
        131 GCGCTTTGAGGATTTCGTTGGAAACGGAAATATCTTCATAT

                **                     * * **         *  **   *   *          
     363490 AAAATTTAGACAGAAGCATTCTCAGAACCTTGATTGTGATGTGTGTTCTCCACTAACAGAGTTGA
          1 AAAAACTAGACAGAAGCATTCTCAGAAACATCTTTGTGATGTTTGCACTCAACTCACAGAGTTGA

                       * *   * *   *       *       *                         
     363555 ACCTTTCTTTTGACAGAACTGTTCTGAAACATTCTTTTTATAGAATCTGGAAGTGGATATTTGGA
         66 ACCTTTCTTTTCATAGAGCAGTTTTGAAACACTCTTTTTGTAGAATCTGGAAGTGGATATTTGGA

            **                         *          *  
     363620 AAGCTTTGAGGATTTCGTTGGAAACGGGAATATCTTCAAAT
        131 GCGCTTTGAGGATTTCGTTGGAAACGGAAATATCTTCATAT

                *    *            *           * *       *  *    *            
     363661 AAAATCTAGCCAGAAGCATTCTAAGAAACATCTTAGGGATGTTTACATTCAAGTCACAGAGTTGA
          1 AAAAACTAGACAGAAGCATTCTCAGAAACATCTTTGTGATGTTTGCACTCAACTCACAGAGTTGA

              *  * *     *       *         *    * *   **      *      *    *  
     363726 ACATTCCCTTTCACAGAGCAGGTTTGAAACAATCTTCTCGTACTATCTGGCAGTGGACATTTTGA
         66 ACCTTTCTTTTCATAGAGCAGTTTTGAAACACTCTTTTTGTAGAATCTGGAAGTGGATATTTGGA

              * *   *  ** ** *  *   *            ** *
     363791 GCTCCTTGGGGCCTATGCTGAAAAAGGAAATATCTTCCGAC
        131 GCGCTTTGAGGATTTCGTTGGAAACGGAAATATCTTCATAT

                                 *     *  **          *           **    *    
     363832 AAAAACTAGACAGAAGCATTCGCAGAATCACGTTTGTGATGTGTGCACTCAACTGTCAGAATTGA
          1 AAAAACTAGACAGAAGCATTCTCAGAAACATCTTTGTGATGTTTGCACTCAACTCACAGAGTTGA

                 **   ** *      *                            * *            *
     363897 ACCTTGGTTTGGACAGAGCACTTTTGAAACACTCTTTTTGTAGAATCTGCAGGTGGATATTTGGC
         66 ACCTTTCTTTTCATAGAGCAGTTTTGAAACACTCTTTTTGTAGAATCTGGAAGTGGATATTTGGA

            **                         *   *      * *
     363962 TAGCTTTGAGGATTTCGTTGGAAACGGTAATGTCTTCAAAG
        131 GCGCTTTGAGGATTTCGTTGGAAACGGAAATATCTTCATAT

                *                         *   *            *    *            
     364003 AAAATCTAGACAGAAGCATTCTCAGAAACACCTTCGTGATGTTTGCAATCAAGTCACAGAGTTGA
          1 AAAAACTAGACAGAAGCATTCTCAGAAACATCTTTGTGATGTTTGCACTCAACTCACAGAGTTGA

                 * *             *  *                  *             *       
     364068 ACCTTCCGTTTCATAGAGCAGGTTGGAAACACTCTTTTTGTAGTATCTGGAAGTGGACATTTGGA
         66 ACCTTTCTTTTCATAGAGCAGTTTTGAAACACTCTTTTTGTAGAATCTGGAAGTGGATATTTGGA

             *         ** *    * *   *            **  
     364133 GGGCTTTGTAGCCTATC-TGGAAAAAGGAAATATCTTCCCAT
        131 GCGCTTTG-AGGATTTCGTTGGAAACGGAAATATCTTCATAT

            *  ** *   *     * *            *   *  *   * **        *  * * *   
     364174 GAATGCGAGATAGAAGTAATCTCAGAAACATGTTTATGCTGTATCTACTCAACTAACTGTGCTGA
          1 AAAAACTAGACAGAAGCATTCTCAGAAACATCTTTGTGATGTTTGCACTCAACTCACAGAGTTGA

              *     *  *               *        *  * *       *               
     364239 ACATTTCTATTGATAGAGCAGTTTTGAGACACTCTTCTTTTGGAATCTGCAAGTGGATATTTGGA
         66 ACCTTTCTTTTCATAGAGCAGTTTTGAAACACTCTTTTTGTAGAATCTGGAAGTGGATATTTGGA

            ** *                        *     *      
     364304 TAGATTTGAGGATTTCGTTGGAAACGG-GAT-T-AT-ATAT
        131 GCGCTTTGAGGATTTCGTTGGAAACGGAAATATCTTCATAT

                 *       *               *                 **  *   *         
     364341 AAAAAGTAGACAGCAGCATTCTCAGAAACTTCTTTGTGATGTTTGCATCCAGCTCTCAGAGTTGA
          1 AAAAACTAGACAGAAGCATTCTCAGAAACATCTTTGTGATGTTTGCACTCAACTCACAGAGTTGA

              *  * *          *  *        *        *   **        *  **       
     364406 ACATTCCCTTTCATAGAGTAGGTTTGAAACCCTCTTTTTATAGTGTCTGGAAGCGGGCATTTGGA
         66 ACCTTTCTTTTCATAGAGCAGTTTTGAAACACTCTTTTTGTAGAATCTGGAAGTGGATATTTGGA

                   *    * * **  **  **          * *  *
     364471 GCGCTTTCAGGCCTATGCTTAAAATAGGAAATATCTACCTAC
        131 GCGCTTTGAGG-ATTTCGTTGGAAACGGAAATATCTTCATAT

             *                    *    *  **          ** *        *    *     
     364513 AGAAACTAGACAGAAGCATTCTGAGAATCACGTTTGTGATGTGGGTACTCAACTAACAGTGTTGA
          1 AAAAACTAGACAGAAGCATTCTCAGAAACATCTTTGTGATGTTTGCACTCAACTCACAGAGTTGA

            *  *       *   *            *   *                *   *           
     364578 TCCATTCTTTTGATACAGCAGTTTTGAACCACACTTTTTGTAGAATCTGCAAGAGGATATTTGGA
         66 ACCTTTCTTTTCATAGAGCAGTTTTGAAACACTCTTTTTGTAGAATCTGGAAGTGGATATTTGGA

            **   *                     *   *      * *
     364643 TAGCTGTGAGGATTTCGTTGGAAACGGGAATGTCTTCAAAG
        131 GCGCTTTGAGGATTTCGTTGGAAACGGAAATATCTTCATAT

                *                         *   *            *    *            
     364684 AAAATCTAGACAGAAGCATTCTCAGAAACACCTTCGTGATGTTTGCAATCAAGTCACAGAGTTGA
          1 AAAAACTAGACAGAAGCATTCTCAGAAACATCTTTGTGATGTTTGCACTCAACTCACAGAGTTGA

                 * *             *  *           *      *             *       
     364749 ACCTTCCGTTTCATAGAGCAGGTTGGAAACACTCTTATTGTAGTATCTGGAAGTGGACATTTGGA
         66 ACCTTTCTTTTCATAGAGCAGTTTTGAAACACTCTTTTTGTAGAATCTGGAAGTGGATATTTGGA

                   *   ** ** *  *   *            **  
     364814 GCGCTTTCAGGCCTATGGTGAAAAAGGAAATATCTTCCCAT
        131 GCGCTTTGAGGATTTCGTTGGAAACGGAAATATCTTCATAT

                  * * *      **      *   * *   *     ** ** *      *    *     
     364855 AAAAACGACATAGAAGCTATCTCAGGAACTTGTTTATGATGCATCTAATCAACTAACAGTGTTGA
          1 AAAAACTAGACAGAAGCATTCTCAGAAACATCTTTGTGATGTTTGCACTCAACTCACAGAGTTGA

                  * ** * *                         * *       *               
     364920 ACCTTTGTACTGACAGAGCAG-TTTGAAACACTCTTTTTTTGGAATCTGCAAGTGGATATTTGGA
         66 ACCTTTCTTTTCATAGAGCAGTTTTGAAACACTCTTTTTGTAGAATCTGGAAGTGGATATTTGGA

            *                           *   *   *    
     364984 TCGCTTTGAGGATTTCGTTGGAAACGG-GAT-GC--AATAT
        131 GCGCTTTGAGGATTTCGTTGGAAACGGAAATATCTTCATAT

                **  *    *     *                 **  *   *  *    *         * 
     365021 AAAACGTACACAGCAGCATACTCAGAAA-ATACTTTGCCATATTTCCATTCAAGTCACAGAGTGG
          1 AAAAACTAGACAGAAGCATTCTCAGAAACAT-CTTTGTGATGTTTGCACTCAACTCACAGAGTTG

               *  * **            *                  *  *             *      
     365085 AACATTCCCATTCATAGAGCAGGTTTGAAACACTCTTTTTGGAGTATCTGGAAGTGGACATTTGG
         65 AACCTTTCTTTTCATAGAGCAGTTTTGAAACACTCTTTTTGTAGAATCTGGAAGTGGATATTTGG

                    ** * * ** *  *   *            **  
     365150 AGCGCTTTCTGAACTATGGTGAAAAAGGAAATATCTTCCAAT
        130 AGCGCTTTGAGGATTTCGTTGGAAACGGAAATATCTTCATAT

            *     *               *      * *          *  **      **  *  *    
     365192 GAAAACAAGACAGAAGCATTCTGAGAAACTTATTTGTGATGTGTGTCCTCAACAAACGGACTTGA
          1 AAAAACTAGACAGAAGCATTCTCAGAAACATCTTTGTGATGTTTGCACTCAACTCACAGAGTTGA

                   *      **  * *  *  *             *   *    * * *           
     365257 ACCTTTCGTTTCATGCAGTACTTCTGGAACACTCTTTTTGAAGATTCTGCATGCGGATATTTGGA
         66 ACCTTTCTTTTCATAGAGCAGTTTTGAAACACTCTTTTTGTAGAATCTGGAAGTGGATATTTGGA

            **                            **   *   * 
     365322 TTGCTTTGAGGATTTCGTTGGAAACGG---GCT-TACATGT
        131 GCGCTTTGAGGATTTCGTTGGAAACGGAAATATCTTCATAT

                 *       *               *        *   *    *    *       *    
     365359 AAAAATTAGACAGCAGCATTCTCAGAAACTTCTTTGTGGTGTCTGCATTCAAGTCACAGAATTGA
          1 AAAAACTAGACAGAAGCATTCTCAGAAACATCTTTGTGATGTTTGCACTCAACTCACAGAGTTGA

              * ** * **            *  * *      *       *             *       
     365424 ACTTCCCCTCACATAGAGCAGTTGTGCAGCACTCTATTTGTAGTATCTGGAAGTGGACATTTGGA
         66 ACCTTTCTTTTCATAGAGCAGTTTTGAAACACTCTTTTTGTAGAATCTGGAAGTGGATATTTGGA

             *         ** *    * *   *            **  
     365489 GGGCTTTGTAGCCTATC-TGGAAAAAGGAAATATCTTCCCAT
        131 GCGCTTTG-AGGATTTCGTTGGAAACGGAAATATCTTCATAT

            *  ** *   *     * *            *   *  *   * **        *  * * *   
     365530 GAATGCGAGATAGAAGTAATCTCAGAAACATGTTTATGCTGTATCTACTCAACTAACTGTGCTGA
          1 AAAAACTAGACAGAAGCATTCTCAGAAACATCTTTGTGATGTTTGCACTCAACTCACAGAGTTGA

              *     *  *               *        *  * *       *               
     365595 ACATTTCTATTGATAGAGCAGTTTTGAGACACTCTTCTTTTGGAATCTGCAAGTGGATATTTGGA
         66 ACCTTTCTTTTCATAGAGCAGTTTTGAAACACTCTTTTTGTAGAATCTGGAAGTGGATATTTGGA

            ** *                        *     *      
     365660 TAGATTTGAGGATTTCGTTGGAAACGG-GAT-T-AT-ATAT
        131 GCGCTTTGAGGATTTCGTTGGAAACGGAAATATCTTCATAT

                 *       *               *                 **  *   *         
     365697 AAAAAGTAGACAGCAGCATTCTCAGAAACTTCTTTGTGATGTTTGCATCCAGCTCTCAGAGTTGA
          1 AAAAACTAGACAGAAGCATTCTCAGAAACATCTTTGTGATGTTTGCACTCAACTCACAGAGTTGA

              *  * *          *  *        *        *   **        *  **       
     365762 ACATTCCCTTTCATAGAGTAGGTTTGAAACCCTCTTTTTATAGTGTCTGGAAGCGGGCATTTGGA
         66 ACCTTTCTTTTCATAGAGCAGTTTTGAAACACTCTTTTTGTAGAATCTGGAAGTGGATATTTGGA

                   *   ** ** *  *   *          * *   
     365827 GCGCTTTCAGGCCTATGCTGAAAAAGGAAATATCTACCTAT
        131 GCGCTTTGAGGATTTCGTTGGAAACGGAAATATCTTCATAT

             *                    *    *  **          ** *        *    *     
     365868 AGAAACTAGACAGAAGCATTCTGAGAATCACGTTTGTGATGTGGGTACTCAACTAACAGTGTTGA
          1 AAAAACTAGACAGAAGCATTCTCAGAAACATCTTTGTGATGTTTGCACTCAACTCACAGAGTTGA

            *  *       *   *            *   *                *               
     365933 TCCATTCTTTTGATACAGCAGTTTTGAACCACACTTTTTGTAGAATCTGCAAGTGGATATTTGGA
         66 ACCTTTCTTTTCATAGAGCAGTTTTGAAACACTCTTTTTGTAGAATCTGGAAGTGGATATTTGGA

            **   *                     *   *        *
     365998 TAGCTGTGAGGATTTCGTTGGAAACGGGAATGTCTTCATAG
        131 GCGCTTTGAGGATTTCGTTGGAAACGGAAATATCTTCATAT

                **                     * * **         *  **   *   *          
     366039 AAAATTTAGACAGAAGCATTCTCAGAACCTTGATTGTGATGTGTGTTCTCCACTAACAGAGTTGA
          1 AAAAACTAGACAGAAGCATTCTCAGAAACATCTTTGTGATGTTTGCACTCAACTCACAGAGTTGA

                       * *   * *   *       *       *                         
     366104 ACCTTTCTTTTGACAGAACTGTTCTGAAACATTCTTTTTATAGAATCTGGAAGTGGATATTTGGA
         66 ACCTTTCTTTTCATAGAGCAGTTTTGAAACACTCTTTTTGTAGAATCTGGAAGTGGATATTTGGA

            **                         *          *  
     366169 AAGCTTTGAGGATTTCGTTGGAAACGGGAATATCTTCAAAT
        131 GCGCTTTGAGGATTTCGTTGGAAACGGAAATATCTTCATAT

                *    *            *           * *       *  *    *            
     366210 AAAATCTAGCCAGAAGCATTCTAAGAAACATCTTAGGGATGTTTACATTCAAGTCACAGAGTTGA
          1 AAAAACTAGACAGAAGCATTCTCAGAAACATCTTTGTGATGTTTGCACTCAACTCACAGAGTTGA

              *  * *     *       *         *    * *   **      *      *    *  
     366275 ACATTCCCTTTCACAGAGCAGGTTTGAAACAATCTTCTCGTACTATCTGGCAGTGGACATTTTGA
         66 ACCTTTCTTTTCATAGAGCAGTTTTGAAACACTCTTTTTGTAGAATCTGGAAGTGGATATTTGGA

              * *   *  ** ** *  *   *            ** *
     366340 GCTCCTTGGGGCCTATGCTGAAAAAGGAAATATCTTCCGAA
        131 GCGCTTTGAGGATTTCGTTGGAAACGGAAATATCTTCATAT

                                 *     *  **          *           **    *    
     366381 AAAAACTAGACAGAAGCATTCGCAGAATCACGTTTGTGATGTGTGCACTCAACTGTCAGAATTGA
          1 AAAAACTAGACAGAAGCATTCTCAGAAACATCTTTGTGATGTTTGCACTCAACTCACAGAGTTGA

                 **   ** *      *                            * *            *
     366446 ACCTTGGTTTGGACAGAGCACTTTTGAAACACTCTTTTTGTAGAATCTGCAGGTGGATATTTGGC
         66 ACCTTTCTTTTCATAGAGCAGTTTTGAAACACTCTTTTTGTAGAATCTGGAAGTGGATATTTGGA

            **                         *   *      * *
     366511 TAGCTTTGAGGATTTCGTTGGAAACGGTAATGTCTTCAAAG
        131 GCGCTTTGAGGATTTCGTTGGAAACGGAAATATCTTCATAT

                *      *                  *   *            *    *            
     366552 AAAATCTAGACGGAAGCATTCTCAGAAACACCTTCGTGATGTTTGCAATCAAGTCACAGAGTTGA
          1 AAAAACTAGACAGAAGCATTCTCAGAAACATCTTTGTGATGTTTGCACTCAACTCACAGAGTTGA

                 * *             *  *                  *             *       
     366617 ACCTTCCGTTTCATAGAGCAGGTTGGAAACACTCTTTTTGTAGTATCTGGAAGTGGACATTTGGA
         66 ACCTTTCTTTTCATAGAGCAGTTTTGAAACACTCTTTTTGTAGAATCTGGAAGTGGATATTTGGA

             *         ** *    * *   *            **  
     366682 GGGCTTTGTAGCCTATC-TGGAAAAAGGAAATATCTTCCCAT
        131 GCGCTTTG-AGGATTTCGTTGGAAACGGAAATATCTTCATAT

            *  ** *   *     * *            *   *  *   * **        *  * * *   
     366723 GAATGCGAGATAGAAGTAATCTCAGAAACATGTTTATGCTGTATCTACTCAACTAACTGTGCTGA
          1 AAAAACTAGACAGAAGCATTCTCAGAAACATCTTTGTGATGTTTGCACTCAACTCACAGAGTTGA

              *     *  *               *        *  * *       *               
     366788 ACATTTCTATTGATAGAGCAGTTTTGAGACACTCTTCTTTTGGAATCTGCAAGTGGATATTTGGA
         66 ACCTTTCTTTTCATAGAGCAGTTTTGAAACACTCTTTTTGTAGAATCTGGAAGTGGATATTTGGA

            ** *                        *     *      
     366853 TAGATTTGAGGATTTCGTTGGAAACGG-GAT-T-AT-ATAT
        131 GCGCTTTGAGGATTTCGTTGGAAACGGAAATATCTTCATAT

                 *       *               *                 **  *   *         
     366890 AAAAAGTAGACAGCAGCATTCTCAGAAACTTCTTTGTGATGTTTGCATCCAGCTCTCAGAGTTGA
          1 AAAAACTAGACAGAAGCATTCTCAGAAACATCTTTGTGATGTTTGCACTCAACTCACAGAGTTGA

              *  * *          *  *        *        *   **        *  **       
     366955 ACATTCCCTTTCATAGAGTAGGTTTGAAACCCTCTTTTTATAGTGTCTGGAAGCGGGCATTTGGA
         66 ACCTTTCTTTTCATAGAGCAGTTTTGAAACACTCTTTTTGTAGAATCTGGAAGTGGATATTTGGA

                   *    * * **  **  **          * *  *
     367020 GCGCTTTCAGGCCTATGCTTAAAATAGGAAATATCTACCTAC
        131 GCGCTTTGAGG-ATTTCGTTGGAAACGGAAATATCTTCATAT

             *               *    *    *  **          ** *        *    *     
     367062 AGAAACTAGACAGAAGCCTTCTGAGAATCACGTTTGTGATGTGGGTACTCAACTAACAGTGTTGA
          1 AAAAACTAGACAGAAGCATTCTCAGAAACATCTTTGTGATGTTTGCACTCAACTCACAGAGTTGA

            *  *       *   *            *   *                *   *           
     367127 TCCATTCTTTTGATACAGCAGTTTTGAACCACACTTTTTGTAGAATCTGCAAGAGGATATTTGGA
         66 ACCTTTCTTTTCATAGAGCAGTTTTGAAACACTCTTTTTGTAGAATCTGGAAGTGGATATTTGGA

            **   *                     *   *      * *
     367192 TAGCTGTGAGGATTTCGTTGGAAACGGGAATGTCTTCAAAG
        131 GCGCTTTGAGGATTTCGTTGGAAACGGAAATATCTTCATAT

                *                         *   *            *    *            
     367233 AAAATCTAGACAGAAGCATTCTCAGAAACACCTTCGTGATGTTTGCAATCAAGTCACAGAGTTGA
          1 AAAAACTAGACAGAAGCATTCTCAGAAACATCTTTGTGATGTTTGCACTCAACTCACAGAGTTGA

                 * *             *  *                  *             *       
     367298 ACCTTCCGTTTCATAGAGCAGGTTGGAAACACTCTTTTTGTAGTATCTGGAAGTGGACATTTGGA
         66 ACCTTTCTTTTCATAGAGCAGTTTTGAAACACTCTTTTTGTAGAATCTGGAAGTGGATATTTGGA

                   *   ** ** *  *   *            **  
     367363 GCGCTTTCAGGCCTATGGTGAAAAAGGAAATATCTTCCCAT
        131 GCGCTTTGAGGATTTCGTTGGAAACGGAAATATCTTCATAT

                  * * *      **      *   * *   *     ** ** *      *    *     
     367404 AAAAACGACATAGAAGCTATCTCAGGAACTTGTTTATGATGCATCTAATCAACTAACAGTGTTGA
          1 AAAAACTAGACAGAAGCATTCTCAGAAACATCTTTGTGATGTTTGCACTCAACTCACAGAGTTGA

                  * ** * *                         * *       *               
     367469 ACCTTTGTACTGACAGAGCAG-TTTGAAACACTCTTTTTTTGGAATCTGCAAGTGGATATTTGGA
         66 ACCTTTCTTTTCATAGAGCAGTTTTGAAACACTCTTTTTGTAGAATCTGGAAGTGGATATTTGGA

            *                           *   *   *    
     367533 TCGCTTTGAGGATTTCGTTGGAAACGG-GAT-GC--AATAT
        131 GCGCTTTGAGGATTTCGTTGGAAACGGAAATATCTTCATAT

                **  *    *     *                 **  *   *  *    *         * 
     367570 AAAACGTACACAGCAGCATACTCAGAAA-ATACTTTGCCATATTTCCATTCAAGTCACAGAGTGG
          1 AAAAACTAGACAGAAGCATTCTCAGAAACAT-CTTTGTGATGTTTGCACTCAACTCACAGAGTTG

              **  * **            *  *               *  *             *      
     367634 AAGATTCCCATTCATAGAGCAGGTTGGAAACACTCTTTTTGGAGTATCTGGAAGTGGACATTTGG
         65 AACCTTTCTTTTCATAGAGCAGTTTTGAAACACTCTTTTTGTAGAATCTGGAAGTGGATATTTGG

                    ** * * ** *  *   *            **  
     367699 AGCGCTTTCTGAACTATGGTGAAAAAGGAAATATCTTCCAAT
        130 AGCGCTTTGAGGATTTCGTTGGAAACGGAAATATCTTCATAT

            *     *               *      * *          *  **      **  *  *    
     367741 GAAAACAAGACAGAAGCATTCTGAGAAACTTATTTGTGATGTGTGTCCTCAACAAACGGACTTGA
          1 AAAAACTAGACAGAAGCATTCTCAGAAACATCTTTGTGATGTTTGCACTCAACTCACAGAGTTGA

                   *      **  * *  *  *             *   *    * * *           
     367806 ACCTTTCGTTTCATGCAGTACTTCTGGAACACTCTTTTTGAAGATTCTGCATGCGGATATTTGGA
         66 ACCTTTCTTTTCATAGAGCAGTTTTGAAACACTCTTTTTGTAGAATCTGGAAGTGGATATTTGGA

            **                            **   *   * 
     367871 TAGCTTTGAGGATTTCGTTGGAAACGG---GCT-TACATGT
        131 GCGCTTTGAGGATTTCGTTGGAAACGGAAATATCTTCATAT

                 *       *               *        *   *    *    *       *    
     367908 AAAAATTAGACAGCAGCATTCTCAGAAACTTCTTTGTGGTGTCTGCATTCAAGTCACAGAATTGA
          1 AAAAACTAGACAGAAGCATTCTCAGAAACATCTTTGTGATGTTTGCACTCAACTCACAGAGTTGA

              * ** * **            *  * *      *       *             *       
     367973 ACTTCCCCTCACATAGAGCAGTTGTGCAGCACTCTATTTGTAGTATCTGGAAGTGGACATTTGGA
         66 ACCTTTCTTTTCATAGAGCAGTTTTGAAACACTCTTTTTGTAGAATCTGGAAGTGGATATTTGGA

             *         ** *    * *   *            **  
     368038 GGGCTTTGTAGCCTATC-TGGAAAAAGGAAATATCTTCCCAT
        131 GCGCTTTG-AGGATTTCGTTGGAAACGGAAATATCTTCATAT

            *  ** *   *     * *            *   *  *   * **        *  * * *   
     368079 GAATGCGAGATAGAAGTAATCTCAGAAACATGTTTATGCTGTATCTACTCAACTAACTGTGCTGA
          1 AAAAACTAGACAGAAGCATTCTCAGAAACATCTTTGTGATGTTTGCACTCAACTCACAGAGTTGA

              *     *  *               *        *  * *       *               
     368144 ACATTTCTATTGATAGAGCAGTTTTGAGACACTCTTCTTTTGGAATCTGCAAGTGGATATTTGGA
         66 ACCTTTCTTTTCATAGAGCAGTTTTGAAACACTCTTTTTGTAGAATCTGGAAGTGGATATTTGGA

            ** *                        *     *      
     368209 TAGATTTGAGGATTTCGTTGGAAACGG-GAT-T-AT-ATAT
        131 GCGCTTTGAGGATTTCGTTGGAAACGGAAATATCTTCATAT

                 *       *               *                 **  *   *         
     368246 AAAAAGTAGACAGCAGCATTCTCAGAAACTTCTTTGTGATGTTTGCATCCAGCTCTCAGAGTTGA
          1 AAAAACTAGACAGAAGCATTCTCAGAAACATCTTTGTGATGTTTGCACTCAACTCACAGAGTTGA

              *  * *          *  *        *        *   **        *  **       
     368311 ACATTCCCTTTCATAGAGTAGGTTTGAAACCCTCTTTTTATAGTGTCTGGAAGCGGGCATTTGGA
         66 ACCTTTCTTTTCATAGAGCAGTTTTGAAACACTCTTTTTGTAGAATCTGGAAGTGGATATTTGGA

                   *   ** ** *  *   *          * *   
     368376 GCGCTTTCAGGCCTATGCTGAAAAAGGAAATATCTACCTAT
        131 GCGCTTTGAGGATTTCGTTGGAAACGGAAATATCTTCATAT

             *                    *    *  **          ** *        *    *     
     368417 AGAAACTAGACAGAAGCATTCTGAGAATCACGTTTGTGATGTGGGTACTCAACTAACAGTGTTGA
          1 AAAAACTAGACAGAAGCATTCTCAGAAACATCTTTGTGATGTTTGCACTCAACTCACAGAGTTGA

            *  *       *   *            *   *                *               
     368482 TCCATTCTTTTGATACAGCAGTTTTGAACCACACTTTTTGTAGAATCTGCAAGTGGATATTTGGA
         66 ACCTTTCTTTTCATAGAGCAGTTTTGAAACACTCTTTTTGTAGAATCTGGAAGTGGATATTTGGA

            **   *                     *   *        *
     368547 TAGCTGTGAGGATTTCGTTGGAAACGGGAATGTCTTCATAG
        131 GCGCTTTGAGGATTTCGTTGGAAACGGAAATATCTTCATAT

                **                     * * **         *  **   *   *          
     368588 AAAATTTAGACAGAAGCATTCTCAGAACCTTGATTGTGATGTGTGTTCTCCACTAACAGAGTTGA
          1 AAAAACTAGACAGAAGCATTCTCAGAAACATCTTTGTGATGTTTGCACTCAACTCACAGAGTTGA

                       * *   * *   *       *       *                         
     368653 ACCTTTCTTTTGACAGAACTGTTCTGAAACATTCTTTTTATAGAATCTGGAAGTGGATATTTGGA
         66 ACCTTTCTTTTCATAGAGCAGTTTTGAAACACTCTTTTTGTAGAATCTGGAAGTGGATATTTGGA

            **                         *          *  
     368718 AAGCTTTGAGGATTTCGTTGGAAACGGGAATATCTTCAAAT
        131 GCGCTTTGAGGATTTCGTTGGAAACGGAAATATCTTCATAT

            *   *    *            *           * *       *  *    *            
     368759 CAAATCTAGCCAGAAGCATTCTAAGAAACATCTTAGGGATGTTTACATTCAAGTCACAGAGTTGA
          1 AAAAACTAGACAGAAGCATTCTCAGAAACATCTTTGTGATGTTTGCACTCAACTCACAGAGTTGA

              *  * *     *       *         *    * *   **      *      *    *  
     368824 ACATTCCCTTTCACAGAGCAGGTTTGAAACAATCTTCTCGTACTATCTGGCAGTGGACATTTTGA
         66 ACCTTTCTTTTCATAGAGCAGTTTTGAAACACTCTTTTTGTAGAATCTGGAAGTGGATATTTGGA

              * *   *  ** ** *  *   *            ** *
     368889 GCTCCTTGGGGCCTATGCTGAAAAAGGAAATATCTTCCGAC
        131 GCGCTTTGAGGATTTCGTTGGAAACGGAAATATCTTCATAT

                                 *     *  **          *           **    *    
     368930 AAAAACTAGACAGAAGCATTCGCAGAATCACGTTTGTGATGTGTGCACTCAACTGTCAGAATTGA
          1 AAAAACTAGACAGAAGCATTCTCAGAAACATCTTTGTGATGTTTGCACTCAACTCACAGAGTTGA

                 **   ** *      *                            * *            *
     368995 ACCTTGGTTTGGACAGAGCACTTTTGAAACACTCTTTTTGTAGAATCTGCAGGTGGATATTTGGC
         66 ACCTTTCTTTTCATAGAGCAGTTTTGAAACACTCTTTTTGTAGAATCTGGAAGTGGATATTTGGA

            **                         *   *      * *
     369060 TAGCTTTGAGGATTTCGTTGGAAACGGTAATGTCTTCAAAG
        131 GCGCTTTGAGGATTTCGTTGGAAACGGAAATATCTTCATAT

                *                         *   *            *    *            
     369101 AAAATCTAGACAGAAGCATTCTCAGAAACACCTTCGTGATGTTTGCAATCAAGTCACAGAGTTGA
          1 AAAAACTAGACAGAAGCATTCTCAGAAACATCTTTGTGATGTTTGCACTCAACTCACAGAGTTGA

                 * *             *  *                  *             *       
     369166 ACCTTCCGTTTCATAGAGCAGGTTGGAAACACTCTTTTTGTAGTATCTGGAAGTGGACATTTGGA
         66 ACCTTTCTTTTCATAGAGCAGTTTTGAAACACTCTTTTTGTAGAATCTGGAAGTGGATATTTGGA

             *         ** *    * *   *            **  
     369231 GGGCTTTGTAGCCTATC-TGGAAAAAGGAAATATCTTCCCAT
        131 GCGCTTTG-AGGATTTCGTTGGAAACGGAAATATCTTCATAT

            *  ** *   *     * *            *   *  *   * **        *  * * *   
     369272 GAATGCGAGATAGAAGTAATCTCAGAAACATGTTTATGCTGTATCTACTCAACTAACTGTGCTGA
          1 AAAAACTAGACAGAAGCATTCTCAGAAACATCTTTGTGATGTTTGCACTCAACTCACAGAGTTGA

              *     *  *               *        *  * *       *               
     369337 ACATTTCTATTGATAGAGCAGTTTTGAGACACTCTTCTTTTGGAATCTGCAAGTGGATATTTGGA
         66 ACCTTTCTTTTCATAGAGCAGTTTTGAAACACTCTTTTTGTAGAATCTGGAAGTGGATATTTGGA

            ** *                        *     *      
     369402 TAGATTTGAGGATTTCGTTGGAAACGG-GAT-T-AT-ATAT
        131 GCGCTTTGAGGATTTCGTTGGAAACGGAAATATCTTCATAT

                 *       *               *                 **  *   *         
     369439 AAAAAGTAGACAGCAGCATTCTCAGAAACTTCTTTGTGATGTTTGCATCCAGCTCTCAGAGTTGA
          1 AAAAACTAGACAGAAGCATTCTCAGAAACATCTTTGTGATGTTTGCACTCAACTCACAGAGTTGA

              *  * *          *  *        *        *   **        *  **       
     369504 ACATTCCCTTTCATAGAGTAGGTTTGAAACCCTCTTTTTATAGTGTCTGGAAGCGGGCATTTGGA
         66 ACCTTTCTTTTCATAGAGCAGTTTTGAAACACTCTTTTTGTAGAATCTGGAAGTGGATATTTGGA

                   *    * * **  **  **          * *  *
     369569 GCGCTTTCAGGCCTATGCTTAAAATAGGAAATATCTACCTAC
        131 GCGCTTTGAGG-ATTTCGTTGGAAACGGAAATATCTTCATAT

             *               *    *    *  **          ** *        *    *     
     369611 AGAAACTAGACAGAAGCCTTCTGAGAATCACGTTTGTGATGTGGGTACTCAACTAACAGTGTTGA
          1 AAAAACTAGACAGAAGCATTCTCAGAAACATCTTTGTGATGTTTGCACTCAACTCACAGAGTTGA

            *  *       *   *            *   *                *   *           
     369676 TCCATTCTTTTGATACAGCAGTTTTGAACCACACTTTTTGTAGAATCTGCAAGAGGATATTTGGA
         66 ACCTTTCTTTTCATAGAGCAGTTTTGAAACACTCTTTTTGTAGAATCTGGAAGTGGATATTTGGA

            **   *                     *   *      * *
     369741 TAGCTGTGAGGATTTCGTTGGAAACGGGAATGTCTTCAAAG
        131 GCGCTTTGAGGATTTCGTTGGAAACGGAAATATCTTCATAT

                *                         *   *            *    *            
     369782 AAAATCTAGACAGAAGCATTCTCAGAAACACCTTCGTGATGTTTGCAATCAAGTCACAGAGTTGA
          1 AAAAACTAGACAGAAGCATTCTCAGAAACATCTTTGTGATGTTTGCACTCAACTCACAGAGTTGA

                 * *             *  *                  *             *       
     369847 ACCTTCCGTTTCATAGAGCAGGTTGGAAACACTCTTTTTGTAGTATCTGGAAGTGGACATTTGGA
         66 ACCTTTCTTTTCATAGAGCAGTTTTGAAACACTCTTTTTGTAGAATCTGGAAGTGGATATTTGGA

                   *   ** ** *  *   *            **  
     369912 GCGCTTTCAGGCCTATGGTGAAAAAGGAAATATCTTCCCAT
        131 GCGCTTTGAGGATTTCGTTGGAAACGGAAATATCTTCATAT

                  * * *      **      *   * *   *     ** ** *      *    *     
     369953 AAAAACGACATAGAAGCTATCTCAGGAACTTGTTTATGATGCATCTAATCAACTAACAGTGTTGA
          1 AAAAACTAGACAGAAGCATTCTCAGAAACATCTTTGTGATGTTTGCACTCAACTCACAGAGTTGA

                  * ** * *                         * *       *               
     370018 ACCTTTGTACTGACAGAGCAG-TTTGAAACACTCTTTTTTTGGAATCTGCAAGTGGATATTTGGA
         66 ACCTTTCTTTTCATAGAGCAGTTTTGAAACACTCTTTTTGTAGAATCTGGAAGTGGATATTTGGA

            *                           *   *   *    
     370082 TCGCTTTGAGGATTTCGTTGGAAACGG-GAT-GC--AATAT
        131 GCGCTTTGAGGATTTCGTTGGAAACGGAAATATCTTCATAT

                **  *    *     *                 **  *   *  *    *         * 
     370119 AAAACGTACACAGCAGCATACTCAGAAA-ATACTTTGCCATATTTCCATTCAAGTCACAGAGTGG
          1 AAAAACTAGACAGAAGCATTCTCAGAAACAT-CTTTGTGATGTTTGCACTCAACTCACAGAGTTG

               *  * **            *  *               *  *             *      
     370183 AACATTCCCATTCATAGAGCAGGTTGGAAACACTCTTTTTGGAGTATCTGGAAGTGGACATTTGG
         65 AACCTTTCTTTTCATAGAGCAGTTTTGAAACACTCTTTTTGTAGAATCTGGAAGTGGATATTTGG

                    ** * * ** *  *   *            **  
     370248 AGCGCTTTCTGAACTATGGTGAAAAAGGAAATATCTTCCAAT
        130 AGCGCTTTGAGGATTTCGTTGGAAACGGAAATATCTTCATAT

            *     *               *      * *          *  **      **  *  *    
     370290 GAAAACAAGACAGAAGCATTCTGAGAAACTTATTTGTGATGTGTGTCCTCAACAAACGGACTTGA
          1 AAAAACTAGACAGAAGCATTCTCAGAAACATCTTTGTGATGTTTGCACTCAACTCACAGAGTTGA

                   *      **  * *  *  *             *   *    * * *           
     370355 ACCTTTCGTTTCATGCAGTACTTCTGGAACACTCTTTTTGAAGATTCTGCATGCGGATATTTGGA
         66 ACCTTTCTTTTCATAGAGCAGTTTTGAAACACTCTTTTTGTAGAATCTGGAAGTGGATATTTGGA

            **                            **   *   * 
     370420 TAGCTTTGAGGATTTCGTTGGAAACGG---GCT-TACATGT
        131 GCGCTTTGAGGATTTCGTTGGAAACGGAAATATCTTCATAT

                 *       *               *        *   *    *    *       *    
     370457 AAAAATTAGACAGCAGCATTCTCAGAAACTTCTTTGTGGTGTCTGCATTCAAGTCACAGAATTGA
          1 AAAAACTAGACAGAAGCATTCTCAGAAACATCTTTGTGATGTTTGCACTCAACTCACAGAGTTGA

              * ** * **            *  * *      *       *             *       
     370522 ACTTCCCCTCACATAGAGCAGTTGTGCAGCACTCTATTTGTAGTATCTGGAAGTGGACATTTGGA
         66 ACCTTTCTTTTCATAGAGCAGTTTTGAAACACTCTTTTTGTAGAATCTGGAAGTGGATATTTGGA

             *         ** *    * *   *            **  
     370587 GGGCTTTGTAGCCTATC-TGGAAAAAGGAAATATCTTCCCAT
        131 GCGCTTTG-AGGATTTCGTTGGAAACGGAAATATCTTCATAT

            *  ** *   *     * *            *   *  *   * **        *  * * *   
     370628 GAATGCGAGATAGAAGTAATCTCAGAAACATGTTTATGCTGTATCTACTCAACTAACTGTGCTGA
          1 AAAAACTAGACAGAAGCATTCTCAGAAACATCTTTGTGATGTTTGCACTCAACTCACAGAGTTGA

              *     *  *               *        *  * *       *               
     370693 ACATTTCTATTGATAGAGCAGTTTTGAGACACTCTTCTTTTGGAATCTGCAAGTGGATATTTGGA
         66 ACCTTTCTTTTCATAGAGCAGTTTTGAAACACTCTTTTTGTAGAATCTGGAAGTGGATATTTGGA

            ** *                        *     *      
     370758 TAGATTTGAGGATTTCGTTGGAAACGG-GAT-T-AT-ATAT
        131 GCGCTTTGAGGATTTCGTTGGAAACGGAAATATCTTCATAT

                 *       *               *                 **  *   *         
     370795 AAAAAGTAGACAGCAGCATTCTCAGAAACTTCTTTGTGATGTTTGCATCCAGCTCTCAGAGTTGA
          1 AAAAACTAGACAGAAGCATTCTCAGAAACATCTTTGTGATGTTTGCACTCAACTCACAGAGTTGA

              *  * *          *  *        *        *   **        *  **       
     370860 ACATTCCCTTTCATAGAGTAGGTTTGAAACCCTCTTTTTATAGTGTCTGGAAGCGGGCATTTGGA
         66 ACCTTTCTTTTCATAGAGCAGTTTTGAAACACTCTTTTTGTAGAATCTGGAAGTGGATATTTGGA

                   *   ** ** *  *   *          * *   
     370925 GCGCTTTCAGGCCTATGCTGAAAAAGGAAATATCTACCTAT
        131 GCGCTTTGAGGATTTCGTTGGAAACGGAAATATCTTCATAT

             *                    *    *  **          ** *        *    *     
     370966 AGAAACTAGACAGAAGCATTCTGAGAATCACGTTTGTGATGTGGGTACTCAACTAACAGTGTTGA
          1 AAAAACTAGACAGAAGCATTCTCAGAAACATCTTTGTGATGTTTGCACTCAACTCACAGAGTTGA

            *  *       *   *            *   *                *               
     371031 TCCATTCTTTTGATACAGCAGTTTTGAACCACACTTTTTGTAGAATCTGCAAGTGGATATTTGGA
         66 ACCTTTCTTTTCATAGAGCAGTTTTGAAACACTCTTTTTGTAGAATCTGGAAGTGGATATTTGGA

            **   *                     *   *        *
     371096 TAGCTGTGAGGATTTCGTTGGAAACGGGAATGTCTTCATAG
        131 GCGCTTTGAGGATTTCGTTGGAAACGGAAATATCTTCATAT

                **                     * * **         *  **   *   *          
     371137 AAAATTTAGACAGAAGCATTCTCAGAACCTTGATTGTGATGTGTGTTCTCCACTAACAGAGTTGA
          1 AAAAACTAGACAGAAGCATTCTCAGAAACATCTTTGTGATGTTTGCACTCAACTCACAGAGTTGA

                       * *   * *   *       *       *                         
     371202 ACCTTTCTTTTGACAGAACTGTTCTGAAACATTCTTTTTATAGAATCTGGAAGTGGATATTTGGA
         66 ACCTTTCTTTTCATAGAGCAGTTTTGAAACACTCTTTTTGTAGAATCTGGAAGTGGATATTTGGA

            **                         *          *  
     371267 AAGCTTTGAGGATTTCGTTGGAAACGGGAATATCTTCAAAT
        131 GCGCTTTGAGGATTTCGTTGGAAACGGAAATATCTTCATAT

            *   *    *            *           * *       *  *    *            
     371308 CAAATCTAGCCAGAAGCATTCTAAGAAACATCTTAGGGATGTTTACATTCAAGTCACAGAGTTGA
          1 AAAAACTAGACAGAAGCATTCTCAGAAACATCTTTGTGATGTTTGCACTCAACTCACAGAGTTGA

              *  * *     *       *         *    * *   **      *      *    *  
     371373 ACATTCCCTTTCACAGAGCAGGTTTGAAACAATCTTCTCGTACTATCTGGCAGTGGACATTTTGA
         66 ACCTTTCTTTTCATAGAGCAGTTTTGAAACACTCTTTTTGTAGAATCTGGAAGTGGATATTTGGA

              * *   *  ** ** *  *   *            ** *
     371438 GCTCCTTGGGGCCTATGCTGAAAAAGGAAATATCTTCCGAC
        131 GCGCTTTGAGGATTTCGTTGGAAACGGAAATATCTTCATAT

                       *         *     *  **          *           **    *    
     371479 AAAAACTAGACGGAAGCATTCGCAGAATCACGTTTGTGATGTGTGCACTCAACTGTCAGAATTGA
          1 AAAAACTAGACAGAAGCATTCTCAGAAACATCTTTGTGATGTTTGCACTCAACTCACAGAGTTGA

                 **   ** *      *                            * *            *
     371544 ACCTTGGTTTGGACAGAGCACTTTTGAAACACTCTTTTTGTAGAATCTGCAGGTGGATATTTGGC
         66 ACCTTTCTTTTCATAGAGCAGTTTTGAAACACTCTTTTTGTAGAATCTGGAAGTGGATATTTGGA

            **                         *   *      * *
     371609 TAGCTTTGAGGATTTCGTTGGAAACGGTAATGTCTTCAAAG
        131 GCGCTTTGAGGATTTCGTTGGAAACGGAAATATCTTCATAT

                *                         *   *            *    *            
     371650 AAAATCTAGACAGAAGCATTCTCAGAAACACCTTCGTGATGTTTGCAATCAAGTCACAGAGTTGA
          1 AAAAACTAGACAGAAGCATTCTCAGAAACATCTTTGTGATGTTTGCACTCAACTCACAGAGTTGA

                 * *             *  *                  *             *       
     371715 ACCTTCCGTTTCATAGAGCAGGTTGGAAACACTCTTTTTGTAGTATCTGGAAGTGGACATTTGGA
         66 ACCTTTCTTTTCATAGAGCAGTTTTGAAACACTCTTTTTGTAGAATCTGGAAGTGGATATTTGGA

             *         ** *    * *   *            **  
     371780 GGGCTTTGTAGCCTATC-TGGAAAAAGGAAATATCTTCCCAT
        131 GCGCTTTG-AGGATTTCGTTGGAAACGGAAATATCTTCATAT

            *  ** *   *     * *            *   *  *   * **        *  * * *   
     371821 GAATGCGAGATAGAAGTAATCTCAGAAACATGTTTATGCTGTATCTACTCAACTAACTGTGCTGA
          1 AAAAACTAGACAGAAGCATTCTCAGAAACATCTTTGTGATGTTTGCACTCAACTCACAGAGTTGA

              *     *  *               *        *  * *       *               
     371886 ACATTTCTATTGATAGAGCAGTTTTGAGACACTCTTCTTTTGGAATCTGCAAGTGGATATTTGGA
         66 ACCTTTCTTTTCATAGAGCAGTTTTGAAACACTCTTTTTGTAGAATCTGGAAGTGGATATTTGGA

            ** *                        *     *      
     371951 TAGATTTGAGGATTTCGTTGGAAACGG-GAT-T-AT-ATAT
        131 GCGCTTTGAGGATTTCGTTGGAAACGGAAATATCTTCATAT

                 *       *               *                 **  *   *         
     371988 AAAAAGTAGACAGCAGCATTCTCAGAAACTTCTTTGTGATGTTTGCATCCAGCTCTCAGAGTTGA
          1 AAAAACTAGACAGAAGCATTCTCAGAAACATCTTTGTGATGTTTGCACTCAACTCACAGAGTTGA

              *  * *          *  *        *        *   **        *  **       
     372053 ACATTCCCTTTCATAGAGTAGGTTTGAAACCCTCTTTTTATAGTGTCTGGAAGCGGGCATTTGGA
         66 ACCTTTCTTTTCATAGAGCAGTTTTGAAACACTCTTTTTGTAGAATCTGGAAGTGGATATTTGGA

                   *    * * **  **  **          * *  *
     372118 GCGCTTTCAGGCCTATGCTTAAAATAGGAAATATCTACCTAC
        131 GCGCTTTGAGG-ATTTCGTTGGAAACGGAAATATCTTCATAT

             *                    *    *  **          ** *        *    *     
     372160 AGAAACTAGACAGAAGCATTCTGAGAATCACGTTTGTGATGTGGGTACTCAACTAACAGTGTTGA
          1 AAAAACTAGACAGAAGCATTCTCAGAAACATCTTTGTGATGTTTGCACTCAACTCACAGAGTTGA

            *  *       *   *            *   *                *   *           
     372225 TCCATTCTTTTGATACAGCAGTTTTGAACCACACTTTTTGTAGAATCTGCAAGAGGATATTTGGA
         66 ACCTTTCTTTTCATAGAGCAGTTTTGAAACACTCTTTTTGTAGAATCTGGAAGTGGATATTTGGA

            **   *                     *   *      * *
     372290 TAGCTGTGAGGATTTCGTTGGAAACGGGAATGTCTTCAAAG
        131 GCGCTTTGAGGATTTCGTTGGAAACGGAAATATCTTCATAT

                *                         *   *            *    *            
     372331 AAAATCTAGACAGAAGCATTCTCAGAAACACCTTCGTGATGTTTGCAATCAAGTCACAGAGTTGA
          1 AAAAACTAGACAGAAGCATTCTCAGAAACATCTTTGTGATGTTTGCACTCAACTCACAGAGTTGA

                 * *             *  *           *      *             *       
     372396 ACCTTCCGTTTCATAGAGCAGGTTGGAAACACTCTTATTGTAGTATCTGGAAGTGGACATTTGGA
         66 ACCTTTCTTTTCATAGAGCAGTTTTGAAACACTCTTTTTGTAGAATCTGGAAGTGGATATTTGGA

                   *   ** ** *  *   *            **  
     372461 GCGCTTTCAGGCCTATGGTGAAAAAGGAAATATCTTCCCAT
        131 GCGCTTTGAGGATTTCGTTGGAAACGGAAATATCTTCATAT

                  * * *      **      *   * *   *     ** ** *      *    *     
     372502 AAAAACGACATAGAAGCTATCTCAGGAACTTGTTTATGATGCATCTAATCAACTAACAGTGTTGA
          1 AAAAACTAGACAGAAGCATTCTCAGAAACATCTTTGTGATGTTTGCACTCAACTCACAGAGTTGA

                  * ** * *                         * *       *               
     372567 ACCTTTGTACTGACAGAGCAG-TTTGAAACACTCTTTTTTTGGAATCTGCAAGTGGATATTTGGA
         66 ACCTTTCTTTTCATAGAGCAGTTTTGAAACACTCTTTTTGTAGAATCTGGAAGTGGATATTTGGA

            *                           *   *   *    
     372631 TCGCTTTGAGGATTTCGTTGGAAACGG-GAT-GC--AATAT
        131 GCGCTTTGAGGATTTCGTTGGAAACGGAAATATCTTCATAT

                **  *    *     *                 **  *   *  *    *         * 
     372668 AAAACGTACACAGCAGCATACTCAGAAA-ATACTTTGCCATATTTCCATTCAAGTCACAGAGTGG
          1 AAAAACTAGACAGAAGCATTCTCAGAAACAT-CTTTGTGATGTTTGCACTCAACTCACAGAGTTG

               *  * **            *                  *  *             *      
     372732 AACATTCCCATTCATAGAGCAGGTTTGAAACACTCTTTTTGGAGTATCTGGAAGTGGACATTTGG
         65 AACCTTTCTTTTCATAGAGCAGTTTTGAAACACTCTTTTTGTAGAATCTGGAAGTGGATATTTGG

                    ** * * ** *  *   *            **  
     372797 AGCGCTTTCTGAACTATGGTGAAAAAGGAAATATCTTCCAAT
        130 AGCGCTTTGAGGATTTCGTTGGAAACGGAAATATCTTCATAT

            *     *               *      * *          *  **      **  *  *    
     372839 GAAAACAAGACAGAAGCATTCTGAGAAACTTATTTGTGATGTGTGTCCTCAACAAACGGACTTGA
          1 AAAAACTAGACAGAAGCATTCTCAGAAACATCTTTGTGATGTTTGCACTCAACTCACAGAGTTGA

                   *      **  * *  *  *             *   *    * * *           
     372904 ACCTTTCGTTTCATGCAGTACTTCTGGAACACTCTTTTTGAAGATTCTGCATGCGGATATTTGGA
         66 ACCTTTCTTTTCATAGAGCAGTTTTGAAACACTCTTTTTGTAGAATCTGGAAGTGGATATTTGGA

            **                            **   *   * 
     372969 TTGCTTTGAGGATTTCGTTGGAAACGG---GCT-TACATGT
        131 GCGCTTTGAGGATTTCGTTGGAAACGGAAATATCTTCATAT

                 *       *               *        *   *    *    *       *    
     373006 AAAAATTAGACAGCAGCATTCTCAGAAACTTCTTTGTGGTGTCTGCATTCAAGTCACAGAATTGA
          1 AAAAACTAGACAGAAGCATTCTCAGAAACATCTTTGTGATGTTTGCACTCAACTCACAGAGTTGA

              * ** * **            *  * *      *       *             *       
     373071 ACTTCCCCTCACATAGAGCAGTTGTGCAGCACTCTATTTGTAGTATCTGGAAGTGGACATTTGGA
         66 ACCTTTCTTTTCATAGAGCAGTTTTGAAACACTCTTTTTGTAGAATCTGGAAGTGGATATTTGGA

             *         ** *    * *   *            ** *
     373136 GGGCTTTGTAGCCTATC-TGGAAAAAGGAAATATCTTCCCAA
        131 GCGCTTTG-AGGATTTCGTTGGAAACGGAAATATCTTCATAT

            *  ** *   *     * *            *   *  *   * **        *  * * *   
     373177 GAATGCGAGATAGAAGTAATCTCAGAAACATGTTTATGCTGTATCTACTCAACTAACTGTGCTGA
          1 AAAAACTAGACAGAAGCATTCTCAGAAACATCTTTGTGATGTTTGCACTCAACTCACAGAGTTGA

              *     *  *               *        *  * *       *               
     373242 ACATTTCTATTGATAGAGCAGTTTTGAGACACTCTTCTTTTGGAATCTGCAAGTGGATATTTGGA
         66 ACCTTTCTTTTCATAGAGCAGTTTTGAAACACTCTTTTTGTAGAATCTGGAAGTGGATATTTGGA

            ** *                        *     *      
     373307 TAGATTTGAGGATTTCGTTGGAAACGG-GAT-T-AT-ATAT
        131 GCGCTTTGAGGATTTCGTTGGAAACGGAAATATCTTCATAT

                 *       *               *                 **  *   *         
     373344 AAAAAGTAGACAGCAGCATTCTCAGAAACTTCTTTGTGATGTTTGCATCCAGCTCTCAGAGTTGA
          1 AAAAACTAGACAGAAGCATTCTCAGAAACATCTTTGTGATGTTTGCACTCAACTCACAGAGTTGA

              *  * *          *  *        *        *   **        *  **       
     373409 ACATTCCCTTTCATAGAGTAGGTTTGAAACCCTCTTTTTATAGTGTCTGGAAGCGGGCATTTGGA
         66 ACCTTTCTTTTCATAGAGCAGTTTTGAAACACTCTTTTTGTAGAATCTGGAAGTGGATATTTGGA

                   *   ** ** *  *   *          * *   
     373474 GCGCTTTCAGGCCTATGCTGAAAAAGGAAATATCTACCTAT
        131 GCGCTTTGAGGATTTCGTTGGAAACGGAAATATCTTCATAT

             *                    *    *  **          ** *        *    *     
     373515 AGAAACTAGACAGAAGCATTCTAAGAATCACGTTTGTGATGTGGGTACTCAACTAACAGTGTTGA
          1 AAAAACTAGACAGAAGCATTCTCAGAAACATCTTTGTGATGTTTGCACTCAACTCACAGAGTTGA

            *  *       *   *            *   *                *               
     373580 TCCATTCTTTTGATACAGCAGTTTTGAACCACACTTTTTGTAGAATCTGCAAGTGGATATTTGGA
         66 ACCTTTCTTTTCATAGAGCAGTTTTGAAACACTCTTTTTGTAGAATCTGGAAGTGGATATTTGGA

            **   *                     *   *        *
     373645 TAGCTGTGAGGATTTCGTTGGAAACGGGAATGTCTTCATAG
        131 GCGCTTTGAGGATTTCGTTGGAAACGGAAATATCTTCATAT

                **                     * * **         *  **   *   *          
     373686 AAAATTTAGACAGAAGCATTCTCAGAACCTTGATTGTGATGTGTGTTCTCCACTAACAGAGTTGA
          1 AAAAACTAGACAGAAGCATTCTCAGAAACATCTTTGTGATGTTTGCACTCAACTCACAGAGTTGA

                       * *   * *   *       *       *                         
     373751 ACCTTTCTTTTGACAGAACTGTTCTGAAACATTCTTTTTATAGAATCTGGAAGTGGATATTTGGA
         66 ACCTTTCTTTTCATAGAGCAGTTTTGAAACACTCTTTTTGTAGAATCTGGAAGTGGATATTTGGA

            **                         *          *  
     373816 AAGCTTTGAGGATTTCGTTGGAAACGGGAATATCTTCAAAT
        131 GCGCTTTGAGGATTTCGTTGGAAACGGAAATATCTTCATAT

                *    *            *           * *       *  *    *            
     373857 AAAATCTAGCCAGAAGCATTCTAAGAAACATCTTAGGGATGTTTACATTCAAGTCACAGAGTTGA
          1 AAAAACTAGACAGAAGCATTCTCAGAAACATCTTTGTGATGTTTGCACTCAACTCACAGAGTTGA

              *  * *     *       *         *    * *   **      *      *    *  
     373922 ACATTCCCTTTCACAGAGCAGGTTTGAAACAATCTTCTCGTACTATCTGGCAGTGGACATTTTGA
         66 ACCTTTCTTTTCATAGAGCAGTTTTGAAACACTCTTTTTGTAGAATCTGGAAGTGGATATTTGGA

              * *   *  ** ** *  *   *            ** *
     373987 GCTCCTTGGGGCCTATGCTGAAAAAGGAAATATCTTCCGAC
        131 GCGCTTTGAGGATTTCGTTGGAAACGGAAATATCTTCATAT

                                 *     *  **          *           **    *    
     374028 AAAAACTAGACAGAAGCATTCGCAGAATCACGTTTGTGATGTGTGCACTCAACTGTCAGAATTGA
          1 AAAAACTAGACAGAAGCATTCTCAGAAACATCTTTGTGATGTTTGCACTCAACTCACAGAGTTGA

                 **   ** *      *                            * *            *
     374093 ACCTTGGTTTGGACAGAGCACTTTTGAAACACTCTTTTTGTAGAATCTGCAGGTGGATATTTGGC
         66 ACCTTTCTTTTCATAGAGCAGTTTTGAAACACTCTTTTTGTAGAATCTGGAAGTGGATATTTGGA

            **                         *   *      * *
     374158 TAGCTTTGAGGATTTCGTTGGAAACGGTAATGTCTTCAAAG
        131 GCGCTTTGAGGATTTCGTTGGAAACGGAAATATCTTCATAT

                *      *                  *   *            *    *            
     374199 AAAATCTAGACGGAAGCATTCTCAGAAACACCTTCGTGATGTTTGCAATCAAGTCACAGAGTTGA
          1 AAAAACTAGACAGAAGCATTCTCAGAAACATCTTTGTGATGTTTGCACTCAACTCACAGAGTTGA

                 * *             *  *                  *             *       
     374264 ACCTTCCGTTTCATAGAGCAGGTTGGAAACACTCTTTTTGTAGTATCTGGAAGTGGACATTTGGA
         66 ACCTTTCTTTTCATAGAGCAGTTTTGAAACACTCTTTTTGTAGAATCTGGAAGTGGATATTTGGA

             *         ** *    * *   *            **  
     374329 GGGCTTTGTAGCCTATC-TGGAAAAAGGAAATATCTTCCCAT
        131 GCGCTTTG-AGGATTTCGTTGGAAACGGAAATATCTTCATAT

            *  ** *   *     * *            *   *  *   * **        *  * * *   
     374370 GAATGCGAGATAGAAGTAATCTCAGAAACATGTTTATGCTGTATCTACTCAACTAACTGTGCTGA
          1 AAAAACTAGACAGAAGCATTCTCAGAAACATCTTTGTGATGTTTGCACTCAACTCACAGAGTTGA

              *     *  *               *        *  * *       *               
     374435 ACATTTCTATTGATAGAGCAGTTTTGAGACACTCTTCTTTTGGAATCTGCAAGTGGATATTTGGA
         66 ACCTTTCTTTTCATAGAGCAGTTTTGAAACACTCTTTTTGTAGAATCTGGAAGTGGATATTTGGA

            ** *                        *     *      
     374500 TAGATTTGAGGATTTCGTTGGAAACGG-GAT-T-AT-ATAT
        131 GCGCTTTGAGGATTTCGTTGGAAACGGAAATATCTTCATAT

                 *       *               *                 **  *   *         
     374537 AAAAAGTAGACAGCAGCATTCTCAGAAACTTCTTTGTGATGTTTGCATCCAGCTCTCAGAGTTGA
          1 AAAAACTAGACAGAAGCATTCTCAGAAACATCTTTGTGATGTTTGCACTCAACTCACAGAGTTGA

              *  * *          *  *        *        *   **        *  **       
     374602 ACATTCCCTTTCATAGAGTAGGTTTGAAACCCTCTTTTTATAGTGTCTGGAAGCGGGCATTTGGA
         66 ACCTTTCTTTTCATAGAGCAGTTTTGAAACACTCTTTTTGTAGAATCTGGAAGTGGATATTTGGA

                   *    * * **  **  **          * *  *
     374667 GCGCTTTCAGGCCTATGCTTAAAATAGGAAATATCTACCTAC
        131 GCGCTTTGAGG-ATTTCGTTGGAAACGGAAATATCTTCATAT

             *               *    *    *  **          ** *        *    *     
     374709 AGAAACTAGACAGAAGCCTTCTGAGAATCACGTTTGTGATGTGGGTACTCAACTAACAGTGTTGA
          1 AAAAACTAGACAGAAGCATTCTCAGAAACATCTTTGTGATGTTTGCACTCAACTCACAGAGTTGA

            *  *       *   *            *   *                *   *           
     374774 TCCATTCTTTTGATACAGCAGTTTTGAACCACACTTTTTGTAGAATCTGCAAGAGGATATTTGGA
         66 ACCTTTCTTTTCATAGAGCAGTTTTGAAACACTCTTTTTGTAGAATCTGGAAGTGGATATTTGGA

            **   *                     *   *      * *
     374839 TAGCTGTGAGGATTTCGTTGGAAACGGGAATGTCTTCAAAG
        131 GCGCTTTGAGGATTTCGTTGGAAACGGAAATATCTTCATAT

                *                         *   *            *    *            
     374880 AAAATCTAGACAGAAGCATTCTCAGAAACACCTTCGTGATGTTTGCAATCAAGTCACAGAGTTGA
          1 AAAAACTAGACAGAAGCATTCTCAGAAACATCTTTGTGATGTTTGCACTCAACTCACAGAGTTGA

                 * *             *  *                  *             *       
     374945 ACCTTCCGTTTCATAGAGCAGGTTGGAAACACTCTTTTTGTAGTATCTGGAAGTGGACATTTGGA
         66 ACCTTTCTTTTCATAGAGCAGTTTTGAAACACTCTTTTTGTAGAATCTGGAAGTGGATATTTGGA

                   *   ** ** *  *   *            **  
     375010 GCGCTTTCAGGCCTATGGTGAAAAAGGAAATATCTTCCCAT
        131 GCGCTTTGAGGATTTCGTTGGAAACGGAAATATCTTCATAT

                  * * *      **      *   * *   *     ** ** *      *    *     
     375051 AAAAACGACATAGAAGCTATCTCAGGAACTTGTTTATGATGCATCTAATCAACTAACAGTGTTGA
          1 AAAAACTAGACAGAAGCATTCTCAGAAACATCTTTGTGATGTTTGCACTCAACTCACAGAGTTGA

                  * ** * *                         * *       *               
     375116 ACCTTTGTACTGACAGAGCAG-TTTGAAACACTCTTTTTTTGGAATCTGCAAGTGGATATTTGGA
         66 ACCTTTCTTTTCATAGAGCAGTTTTGAAACACTCTTTTTGTAGAATCTGGAAGTGGATATTTGGA

            *                           *   *   *    
     375180 TCGCTTTGAGGATTTCGTTGGAAACGG-GAT-GC--AATAT
        131 GCGCTTTGAGGATTTCGTTGGAAACGGAAATATCTTCATAT

                **  *    *     *                 **  *   *  *    *         * 
     375217 AAAACGTACACAGCAGCATACTCAGAAA-ATACTTTGCCATATTTCCATTCAAGTCACAGAGTGG
          1 AAAAACTAGACAGAAGCATTCTCAGAAACAT-CTTTGTGATGTTTGCACTCAACTCACAGAGTTG

               *  * **            *  *               *  *             *      
     375281 AACATTCCCATTCATAGAGCAGGTTGGAAACACTCTTTTTGGAGTATCTGGAAGTGGACATTTGG
         65 AACCTTTCTTTTCATAGAGCAGTTTTGAAACACTCTTTTTGTAGAATCTGGAAGTGGATATTTGG

                    ** * * ** *  *   *            **  
     375346 AGCGCTTTCTGAACTATGGTGAAAAAGGAAATATCTTCCAAT
        130 AGCGCTTTGAGGATTTCGTTGGAAACGGAAATATCTTCATAT

            *     *               *      * *          *  **      **  *  *    
     375388 GAAAACAAGACAGAAGCATTCTGAGAAACTTATTTGTGATGTGTGTCCTCAACAAACGGACTTGA
          1 AAAAACTAGACAGAAGCATTCTCAGAAACATCTTTGTGATGTTTGCACTCAACTCACAGAGTTGA

                   *      **  * *  *  *             *   *    * * *           
     375453 ACCTTTCGTTTCATGCAGTACTTCTGGAACACTCTTTTTGAAGATTCTGCATGCGGATATTTGGA
         66 ACCTTTCTTTTCATAGAGCAGTTTTGAAACACTCTTTTTGTAGAATCTGGAAGTGGATATTTGGA

            **                            **   *   * 
     375518 TAGCTTTGAGGATTTCGTTGGAAACGG---GCT-TACATGT
        131 GCGCTTTGAGGATTTCGTTGGAAACGGAAATATCTTCATAT

                 *       *               *        *   *    *    *       *    
     375555 AAAAATTAGACAGCAGCATTCTCAGAAACTTCTTTGTGGTGTCTGCATTCAAGTCACAGAATTGA
          1 AAAAACTAGACAGAAGCATTCTCAGAAACATCTTTGTGATGTTTGCACTCAACTCACAGAGTTGA

              * ** * **            *  * *      *       *    *        *       
     375620 ACATCCCCTCACATAGAGCAGTTGTGCAGCACTCTATTTGTAGTATCTCGAAGTGGACATTTGGA
         66 ACCTTTCTTTTCATAGAGCAGTTTTGAAACACTCTTTTTGTAGAATCTGGAAGTGGATATTTGGA

             *         ** *    * *   *            **  
     375685 GGGCTTTGTAGCCTATC-TGGAAAAAGGAAATATCTTCCCAT
        131 GCGCTTTG-AGGATTTCGTTGGAAACGGAAATATCTTCATAT

            *  ** *   *     * *            *   *  *   * **        *  * * *   
     375726 GAATGCGAGATAGAAGTAATCTCAGAAACATGTTTATGCTGTATCTACTCAACTAACTGTGCTGA
          1 AAAAACTAGACAGAAGCATTCTCAGAAACATCTTTGTGATGTTTGCACTCAACTCACAGAGTTGA

              *     *  *               *        *  * *       *               
     375791 ACATTTCTATTGATAGAGCAGTTTTGAGACACTCTTCTTTTGGAATCTGCAAGTGGATATTTGGA
         66 ACCTTTCTTTTCATAGAGCAGTTTTGAAACACTCTTTTTGTAGAATCTGGAAGTGGATATTTGGA

            ** *                        *     *      
     375856 TAGATTTGAGGATTTCGTTGGAAACGG-GAT-T-AT-ATAT
        131 GCGCTTTGAGGATTTCGTTGGAAACGGAAATATCTTCATAT

                 *       *               *                 **  *   *         
     375893 AAAAAGTAGACAGCAGCATTCTCAGAAACTTCTTTGTGATGTTTGCATCCAGCTCTCAGAGTTGA
          1 AAAAACTAGACAGAAGCATTCTCAGAAACATCTTTGTGATGTTTGCACTCAACTCACAGAGTTGA

              *  * *          *  *        *        *   **        *  **       
     375958 ACATTCCCTTTCATAGAGTAGGTTTGAAACCCTCTTTTTATAGTGTCTGGAAGCGGGCATTTGGA
         66 ACCTTTCTTTTCATAGAGCAGTTTTGAAACACTCTTTTTGTAGAATCTGGAAGTGGATATTTGGA

                   *   ** ** *  *   *          * *   
     376023 GCGCTTTCAGGCCTATGCTGAAAAAGGAAATATCTACCTAT
        131 GCGCTTTGAGGATTTCGTTGGAAACGGAAATATCTTCATAT

             *                    *    *  **          ** *        *    *     
     376064 AGAAACTAGACAGAAGCATTCTGAGAATCACGTTTGTGATGTGGGTACTCAACTAACAGTGTTGA
          1 AAAAACTAGACAGAAGCATTCTCAGAAACATCTTTGTGATGTTTGCACTCAACTCACAGAGTTGA

            *  *       *   *            *   *                *               
     376129 TCCATTCTTTTGATACAGCAGTTTTGAACCACACTTTTTGTAGAATCTGCAAGTGGATATTTGGA
         66 ACCTTTCTTTTCATAGAGCAGTTTTGAAACACTCTTTTTGTAGAATCTGGAAGTGGATATTTGGA

            **   *                     *   *        *
     376194 TAGCTGTGAGGATTTCGTTGGAAACGGGAATGTCTTCATAG
        131 GCGCTTTGAGGATTTCGTTGGAAACGGAAATATCTTCATAT

                **                     * * **         *  **   *   *          
     376235 AAAATTTAGACAGAAGCATTCTCAGAACCTTGATTGTGATGTGTGTTCTCCACTAACAGAGTTGA
          1 AAAAACTAGACAGAAGCATTCTCAGAAACATCTTTGTGATGTTTGCACTCAACTCACAGAGTTGA

                       * *   * *   *       *       *                         
     376300 ACCTTTCTTTTGACAGAACTGTTCTGAAACATTCTTTTTATAGAATCTGGAAGTGGATATTTGGA
         66 ACCTTTCTTTTCATAGAGCAGTTTTGAAACACTCTTTTTGTAGAATCTGGAAGTGGATATTTGGA

            **                         *          *  
     376365 AAGCTTTGAGGATTTCGTTGGAAACGGGAATATCTTCAAAT
        131 GCGCTTTGAGGATTTCGTTGGAAACGGAAATATCTTCATAT

            *   *    *            *           * *       *  *    *            
     376406 CAAATCTAGCCAGAAGCATTCTAAGAAACATCTTAGGGATGTTTACATTCAAGTCACAGAGTTGA
          1 AAAAACTAGACAGAAGCATTCTCAGAAACATCTTTGTGATGTTTGCACTCAACTCACAGAGTTGA

              *  * *     *       *         *    * *   **      *      *    *  
     376471 ACATTCCCTTTCACAGAGCAGGTTTGAAACAATCTTCTCGTACTATCTGGCAGTGGACATTTTGA
         66 ACCTTTCTTTTCATAGAGCAGTTTTGAAACACTCTTTTTGTAGAATCTGGAAGTGGATATTTGGA

              * *   *  ** ** *  *   *            ** *
     376536 GCTCCTTGGGGCCTATGCTGAAAAAGGAAATATCTTCCGAC
        131 GCGCTTTGAGGATTTCGTTGGAAACGGAAATATCTTCATAT

                                 *     *  **          *           **    *    
     376577 AAAAACTAGACAGAAGCATTCGCAGAATCACGTTTGTGATGTGTGCACTCAACTGTCAGAATTGA
          1 AAAAACTAGACAGAAGCATTCTCAGAAACATCTTTGTGATGTTTGCACTCAACTCACAGAGTTGA

                 **   ** *      *                            * *            *
     376642 ACCTTGGTTTGGACAGAGCACTTTTGAAACACTCTTTTTGTAGAATCTGCAGGTGGATATTTGGC
         66 ACCTTTCTTTTCATAGAGCAGTTTTGAAACACTCTTTTTGTAGAATCTGGAAGTGGATATTTGGA

            **                         *   *      * *
     376707 TAGCTTTGAGGATTTCGTTGGAAACGGTAATGTCTTCAAAG
        131 GCGCTTTGAGGATTTCGTTGGAAACGGAAATATCTTCATAT

                *                         *   *            *    *            
     376748 AAAATCTAGACAGAAGCATTCTCAGAAACACCTTCGTGATGTTTGCAATCAAGTCACAGAGTTGA
          1 AAAAACTAGACAGAAGCATTCTCAGAAACATCTTTGTGATGTTTGCACTCAACTCACAGAGTTGA

                 * *             *  *                  *             *       
     376813 ACCTTCCGTTTCATAGAGCAGGTTGGAAACACTCTTTTTGTAGTATCTGGAAGTGGACATTTGGA
         66 ACCTTTCTTTTCATAGAGCAGTTTTGAAACACTCTTTTTGTAGAATCTGGAAGTGGATATTTGGA

             *         ** *    * *   *            **  
     376878 GGGCTTTGTAGCCTATC-TGGAAAATGGAAATATCTTCCCAT
        131 GCGCTTTG-AGGATTTCGTTGGAAACGGAAATATCTTCATAT

            *  ** *   *     * *            *   *  *   * **        *  * * *   
     376919 GAATGCGAGATAGAAGTAATCTCAGAAACATGTTTATGCTGTATCTACTCAACTAACTGTGCTGA
          1 AAAAACTAGACAGAAGCATTCTCAGAAACATCTTTGTGATGTTTGCACTCAACTCACAGAGTTGA

              *     *  *               *        *  * *       *               
     376984 ACATTTCTATTGATAGAGCAGTTTTGAGACACTCTTCTTTTGGAATCTGCAAGTGGATATTTGGA
         66 ACCTTTCTTTTCATAGAGCAGTTTTGAAACACTCTTTTTGTAGAATCTGGAAGTGGATATTTGGA

            ** *                        *     *      
     377049 TAGATTTGAGGATTTCGTTGGAAACGG-GAT-T-AT-ATAT
        131 GCGCTTTGAGGATTTCGTTGGAAACGGAAATATCTTCATAT

                 *       *               *      *          **  *   *         
     377086 AAAAAGTAGACAGCAGCATTCTCAGAAACTTCTTTGAGATGTTTGCATCCAGCTCTCAGAGTTGA
          1 AAAAACTAGACAGAAGCATTCTCAGAAACATCTTTGTGATGTTTGCACTCAACTCACAGAGTTGA

              *  * *          *  *        *        *   **        *  **       
     377151 ACATTCCCTTTCATAGAGTAGGTTTGAAACCCTCTTTTTATAGTGTCTGGAAGCGGGCATTTGGA
         66 ACCTTTCTTTTCATAGAGCAGTTTTGAAACACTCTTTTTGTAGAATCTGGAAGTGGATATTTGGA

                   *    * * **  **  **          * *  *
     377216 GCGCTTTCAGGCCTATGCTTAAAATAGGAAATATCTACCTAC
        131 GCGCTTTGAGG-ATTTCGTTGGAAACGGAAATATCTTCATAT

             *                    *    *  **          ** *        *    *     
     377258 AGAAACTAGACAGAAGCATTCTGAGAATCACGTTTGTGATGTGGGTACTCAACTAACAGTGTTGA
          1 AAAAACTAGACAGAAGCATTCTCAGAAACATCTTTGTGATGTTTGCACTCAACTCACAGAGTTGA

            *  *       *   *            *   *                *   *           
     377323 TCCATTCTTTTGATACAGCAGTTTTGAACCACACTTTTTGTAGAATCTGCAAGAGGATATTTGGA
         66 ACCTTTCTTTTCATAGAGCAGTTTTGAAACACTCTTTTTGTAGAATCTGGAAGTGGATATTTGGA

            **   *                     *   *      * *
     377388 TAGCTGTGAGGATTTCGTTGGAAACGGGAATGTCTTCAAAG
        131 GCGCTTTGAGGATTTCGTTGGAAACGGAAATATCTTCATAT

                *                         *   *            *    *            
     377429 AAAATCTAGACAGAAGCATTCTCAGAAACACCTTCGTGATGTTTGCAATCAAGTCACAGAGTTGA
          1 AAAAACTAGACAGAAGCATTCTCAGAAACATCTTTGTGATGTTTGCACTCAACTCACAGAGTTGA

                 * *             *  *           *      *             *       
     377494 ACCTTCCGTTTCATAGAGCAGGTTGGAAACACTCTTATTGTAGTATCTGGAAGTGGACATTTGGA
         66 ACCTTTCTTTTCATAGAGCAGTTTTGAAACACTCTTTTTGTAGAATCTGGAAGTGGATATTTGGA

                   *   ** ** *  *   *            **  
     377559 GCGCTTTCAGGCCTATGGTGAAAAAGGAAATATCTTCCCAT
        131 GCGCTTTGAGGATTTCGTTGGAAACGGAAATATCTTCATAT

                  * * *      **      *   * *   *     ** ** *      *    *     
     377600 AAAAACGACATAGAAGCTATCTCAGGAACTTGTTTATGATGCATCTAATCAACTAACAGTGTTGA
          1 AAAAACTAGACAGAAGCATTCTCAGAAACATCTTTGTGATGTTTGCACTCAACTCACAGAGTTGA

                  * ** * *                         * *       *               
     377665 ACCTTTGTACTGACAGAGCAG-TTTGAAACACTCTTTTTTTGGAATCTGCAAGTGGATATTTGGA
         66 ACCTTTCTTTTCATAGAGCAGTTTTGAAACACTCTTTTTGTAGAATCTGGAAGTGGATATTTGGA

            *                           *   *   *    
     377729 TCGCTTTGAGGATTTCGTTGGAAACGG-GAT-GC--AATAT
        131 GCGCTTTGAGGATTTCGTTGGAAACGGAAATATCTTCATAT

                **  *    *     *                 **  *   *  *    *         * 
     377766 AAAACGTACACAGCAGCATACTCAGAAA-ATACTTTGCCATATTTCCATTCAAGTCACAGAGTGG
          1 AAAAACTAGACAGAAGCATTCTCAGAAACAT-CTTTGTGATGTTTGCACTCAACTCACAGAGTTG

               *  * **            *  *               *  *             *      
     377830 AACATTCCCATTCATAGAGCAGGTTGGAAACACTCTTTTTGGAGTATCTGGAAGTGGACATTTGG
         65 AACCTTTCTTTTCATAGAGCAGTTTTGAAACACTCTTTTTGTAGAATCTGGAAGTGGATATTTGG

                    ** * * ** *  *   *            **  
     377895 AGCGCTTTCTGAACTATGGTGAAAAAGGAAATATCTTCCAAT
        130 AGCGCTTTGAGGATTTCGTTGGAAACGGAAATATCTTCATAT

            *     *               *      * *          *  **      **  *  *    
     377937 GAAAACAAGACAGAAGCATTCTGAGAAACTTATTTGTGATGTGTGTCCTCAACAAACGGACTTGA
          1 AAAAACTAGACAGAAGCATTCTCAGAAACATCTTTGTGATGTTTGCACTCAACTCACAGAGTTGA

                   *      **  * *  *  *             *   *    * * *           
     378002 ACCTTTCGTTTCATGCAGTACTTCTGGAACACTCTTTTTGAAGATTCTGCATGCGGATATTTGGA
         66 ACCTTTCTTTTCATAGAGCAGTTTTGAAACACTCTTTTTGTAGAATCTGGAAGTGGATATTTGGA

            **                      *     **   *   * 
     378067 TAGCTTTGAGGATTTCGTTGGAAATGG---GCT-TACATGT
        131 GCGCTTTGAGGATTTCGTTGGAAACGGAAATATCTTCATAT

                 *       *  *            *        *   *    *    *       *    
     378104 AAAAATTAGACAGCAGAATTCTCAGAAACTTCTTTGTGGTGTCTGCATTCAAGTCACAGAATTGA
          1 AAAAACTAGACAGAAGCATTCTCAGAAACATCTTTGTGATGTTTGCACTCAACTCACAGAGTTGA

              * ** * ***           *  * *      *       *    *        *       
     378169 ACTTCCCCTCATATAGAGCAGTTGTGCAGCACTCTATTTGTAGTATCTCGAAGTGGACATTTGGA
         66 ACCTTTCTTTTCATAGAGCAGTTTTGAAACACTCTTTTTGTAGAATCTGGAAGTGGATATTTGGA

             *         ** *    * *   *            **  
     378234 GGGCTTTGTAGCCTATC-TGGAAAAAGGAAATATCTTCCCAT
        131 GCGCTTTG-AGGATTTCGTTGGAAACGGAAATATCTTCATAT

            *  ** *   *     * *            *   *  *   * **        *  * * *   
     378275 GAATGCGAGATAGAAGTAATCTCAGAAACATGTTTATGCTGTATCTACTCAACTAACTGTGCTGA
          1 AAAAACTAGACAGAAGCATTCTCAGAAACATCTTTGTGATGTTTGCACTCAACTCACAGAGTTGA

              *     *  *               *        *  * *       *               
     378340 ACATTTCTATTGATAGAGCAGTTTTGAGACACTCTTCTTTTGGAATCTGCAAGTGGATATTTGGA
         66 ACCTTTCTTTTCATAGAGCAGTTTTGAAACACTCTTTTTGTAGAATCTGGAAGTGGATATTTGGA

            ** *                        *     *      
     378405 TAGATTTGAGGATTTCGTTGGAAACGG-GAT-T-AT-ATAT
        131 GCGCTTTGAGGATTTCGTTGGAAACGGAAATATCTTCATAT

                 *       *               *                 **  *   *         
     378442 AAAAAGTAGACAGCAGCATTCTCAGAAACTTCTTTGTGATGTTTGCATCCAGCTCTCAGAGTTGA
          1 AAAAACTAGACAGAAGCATTCTCAGAAACATCTTTGTGATGTTTGCACTCAACTCACAGAGTTGA

              *  * *          *  *        *        *   **        *  **       
     378507 ACATTCCCTTTCATAGAGTAGGTTTGAAACCCTCTTTTTATAGTGTCTGGAAGCGGGCATTTGGA
         66 ACCTTTCTTTTCATAGAGCAGTTTTGAAACACTCTTTTTGTAGAATCTGGAAGTGGATATTTGGA

                   *    * * **  **  **          * *  *
     378572 GCGCTTTCAGGCCTATGCTTAAAATAGGAAATATCTACCTAC
        131 GCGCTTTGAGG-ATTTCGTTGGAAACGGAAATATCTTCATAT

             *                    *    *  **          ** *        *    *     
     378614 AGAAACTAGACAGAAGCATTCTGAGAATCACGTTTGTGATGTGGGTACTCAACTAACAGTGTTGA
          1 AAAAACTAGACAGAAGCATTCTCAGAAACATCTTTGTGATGTTTGCACTCAACTCACAGAGTTGA

            *  *       *   *            *   *                *   *           
     378679 TCCATTCTTTTGATACAGCAGTTTTGAACCACACTTTTTGTAGAATCTGCAAGAGGATATTTGGA
         66 ACCTTTCTTTTCATAGAGCAGTTTTGAAACACTCTTTTTGTAGAATCTGGAAGTGGATATTTGGA

            **   *                     *   *      * *
     378744 TAGCTGTGAGGATTTCGTTGGAAACGGGAATGTCTTCAAAG
        131 GCGCTTTGAGGATTTCGTTGGAAACGGAAATATCTTCATAT

                *                         *   *            *    *            
     378785 AAAATCTAGACAGAAGCATTCTCAGAAACACCTTCGTGATGTTTGCAATCAAGTCACAGAGTTGA
          1 AAAAACTAGACAGAAGCATTCTCAGAAACATCTTTGTGATGTTTGCACTCAACTCACAGAGTTGA

                 * *             *  *           *      *             *       
     378850 ACCTTCCGTTTCATAGAGCAGGTTGGAAACACTCTTATTGTAGTATCTGGAAGTGGACATTTGGA
         66 ACCTTTCTTTTCATAGAGCAGTTTTGAAACACTCTTTTTGTAGAATCTGGAAGTGGATATTTGGA

                   *   ** ** *  *   *            **  
     378915 GCGCTTTCAGGCCTATGGTGAAAAAGGAAATATCTTCCCAT
        131 GCGCTTTGAGGATTTCGTTGGAAACGGAAATATCTTCATAT

                  * * *      **      *   * *         ** ** *      *    *     
     378956 AAAAACGACATAGAAGCTATCTCAGGAACTTGTTTGTGATGCATCTAATCAACTAACAGTGTTGA
          1 AAAAACTAGACAGAAGCATTCTCAGAAACATCTTTGTGATGTTTGCACTCAACTCACAGAGTTGA

                  * ** * *                         * *       *               
     379021 ACCTTTGTACTGACAGAGCAG-TTTGAAACACTCTTTTTTTGGAATCTGCAAGTGGATATTTGGA
         66 ACCTTTCTTTTCATAGAGCAGTTTTGAAACACTCTTTTTGTAGAATCTGGAAGTGGATATTTGGA

            *                           *   *   *    
     379085 TCGCTTTGAGGATTTCGTTGGAAACGG-GAT-GC--AATAT
        131 GCGCTTTGAGGATTTCGTTGGAAACGGAAATATCTTCATAT

                **  *    **    *                 **  *   *  *    *         * 
     379122 AAAACGTACACAGCGGCATACTCAGAAA-ATACTTTGCCATATTTCCATTCAAGTCACAGAGTGG
          1 AAAAACTAGACAGAAGCATTCTCAGAAACAT-CTTTGTGATGTTTGCACTCAACTCACAGAGTTG

               *  * **     *      *                  *  *             *      
     379186 AACATTCCCATTCATGGAGCAGGTTTGAAACACTCTTTTTGGAGTATCTGGAAGTGGACATTTGG
         65 AACCTTTCTTTTCATAGAGCAGTTTTGAAACACTCTTTTTGTAGAATCTGGAAGTGGATATTTGG

                    ** * * ** *  *   *            **  
     379251 AGCGCTTTCTGAACTATGGTGAAAAAGGAAATATCTTCCAAT
        130 AGCGCTTTGAGGATTTCGTTGGAAACGGAAATATCTTCATAT

            *     *               *      * *          *  **      **  *  *    
     379293 GAAAACAAGACAGAAGCATTCTGAGAAACTTATTTGTGATGTGTGTCCTCAACAAACGGACTTGA
          1 AAAAACTAGACAGAAGCATTCTCAGAAACATCTTTGTGATGTTTGCACTCAACTCACAGAGTTGA

                   *      **  * *  *  *             *   *    * * *           
     379358 ACCTTTCGTTTCATGCAGTACTTCTGGAACACTCTTTTTGAAGATTCTGCATGCGGATATTTGGA
         66 ACCTTTCTTTTCATAGAGCAGTTTTGAAACACTCTTTTTGTAGAATCTGGAAGTGGATATTTGGA

            **                            **   *   * 
     379423 TTGCTTTGAGGATTTCGTTGGAAACGG---GCT-TACATGT
        131 GCGCTTTGAGGATTTCGTTGGAAACGGAAATATCTTCATAT

                 *       *               *        *   *    *    *       *    
     379460 AAAAATTAGACAGCAGCATTCTCAGAAACTTCTTTGTGGTGTCTGCATTCAAGTCACAGAATTGA
          1 AAAAACTAGACAGAAGCATTCTCAGAAACATCTTTGTGATGTTTGCACTCAACTCACAGAGTTGA

              * ** * **            *  * *      *       *             *       
     379525 ACTTCCCCTCACATAGAGCAGTTGTGCAGCACTCTATTTGTAGTATCTGGAAGTGGACATTTGGA
         66 ACCTTTCTTTTCATAGAGCAGTTTTGAAACACTCTTTTTGTAGAATCTGGAAGTGGATATTTGGA

             *         ** *    * *   *            **  
     379590 GGGCTTTGTAGCCTATC-TGGAAAAAGGAAATATCTTCCCAT
        131 GCGCTTTG-AGGATTTCGTTGGAAACGGAAATATCTTCATAT

            *  ** *   *  *  * *            *   *  *   * **        *  * * *   
     379631 GAATGCGAGATAGTAGTAATCTCAGAAACATGTTTATGCTGTATCTACTCAACTAACTGTGCTGA
          1 AAAAACTAGACAGAAGCATTCTCAGAAACATCTTTGTGATGTTTGCACTCAACTCACAGAGTTGA

              *     *  *               *        *  * *       *               
     379696 ACATTTCTATTGATAGAGCAGTTTTGAGACACTCTTCTTTTGGAATCTGCAAGTGGATATTTGGA
         66 ACCTTTCTTTTCATAGAGCAGTTTTGAAACACTCTTTTTGTAGAATCTGGAAGTGGATATTTGGA

            ** *                        *     *      
     379761 TAGATTTGAGGATTTCGTTGGAAACGG-GAT-T-AT-ATAT
        131 GCGCTTTGAGGATTTCGTTGGAAACGGAAATATCTTCATAT

                 *       *               *                 **  *   *         
     379798 AAAAAGTAGACAGCAGCATTCTCAGAAACTTCTTTGTGATGTTTGCATCCAGCTCTCAGAGTTGA
          1 AAAAACTAGACAGAAGCATTCTCAGAAACATCTTTGTGATGTTTGCACTCAACTCACAGAGTTGA

              *  * *          *  *        *        *   **        *  **       
     379863 ACATTCCCTTTCATAGAGTAGGTTTGAAACCCTCTTTTTATAGTGTCTGGAAGCGGGCATTTGGA
         66 ACCTTTCTTTTCATAGAGCAGTTTTGAAACACTCTTTTTGTAGAATCTGGAAGTGGATATTTGGA

                   *   ** ** *  *   *          * *   
     379928 GCGCTTTCAGGCCTATGCTGAAAAAGGAAATATCTACCTAT
        131 GCGCTTTGAGGATTTCGTTGGAAACGGAAATATCTTCATAT

             *                    *    *  **          ** *        *    *     
     379969 AGAAACTAGACAGAAGCATTCTGAGAATCACGTTTGTGATGTGGGTACTCAACTAACAGTGTTGA
          1 AAAAACTAGACAGAAGCATTCTCAGAAACATCTTTGTGATGTTTGCACTCAACTCACAGAGTTGA

            *  *       *   *            *   *                *               
     380034 TCCATTCTTTTGATACAGCAGTTTTGAACCACACTTTTTGTAGAATCTGCAAGTGGATATTTGGA
         66 ACCTTTCTTTTCATAGAGCAGTTTTGAAACACTCTTTTTGTAGAATCTGGAAGTGGATATTTGGA

            **   *                     *   *        *
     380099 TAGCTGTGAGGATTTCGTTGGAAACGGGAATGTCTTCATAG
        131 GCGCTTTGAGGATTTCGTTGGAAACGGAAATATCTTCATAT

                **                     * * **         *  **   *   *          
     380140 AAAATTTAGACAGAAGCATTCTCAGAACCTTGATTGTGATGTGTGTTCTCCACTAACAGAGTTGA
          1 AAAAACTAGACAGAAGCATTCTCAGAAACATCTTTGTGATGTTTGCACTCAACTCACAGAGTTGA

                       * *   * *   *       *       *                         
     380205 ACCTTTCTTTTGACAGAACTGTTCTGAAACATTCTTTTTATAGAATCTGGAAGTGGATATTTGGA
         66 ACCTTTCTTTTCATAGAGCAGTTTTGAAACACTCTTTTTGTAGAATCTGGAAGTGGATATTTGGA

            **                         *          *  
     380270 AAGCTTTGAGGATTTCGTTGGAAACGGGAATATCTTCAAAT
        131 GCGCTTTGAGGATTTCGTTGGAAACGGAAATATCTTCATAT

            *   *    *            *           * *       *  *    *            
     380311 CAAATCTAGCCAGAAGCATTCTAAGAAACATCTTAGGGATGTTTACATTCAAGTCACAGAGTTGA
          1 AAAAACTAGACAGAAGCATTCTCAGAAACATCTTTGTGATGTTTGCACTCAACTCACAGAGTTGA

              *  * *     *       *         *    * *   **      *      *    *  
     380376 ACATTCCCTTTCACAGAGCAGGTTTGAAACAATCTTCTCGTACTATCTGGCAGTGGACATTTTGA
         66 ACCTTTCTTTTCATAGAGCAGTTTTGAAACACTCTTTTTGTAGAATCTGGAAGTGGATATTTGGA

              * *   *  ** ** *  *   *            ** *
     380441 GCTCCTTGGGGCCTATGCTGAAAAAGGAAATATCTTCCGAC
        131 GCGCTTTGAGGATTTCGTTGGAAACGGAAATATCTTCATAT

                                 *     *  **          *           **    *    
     380482 AAAAACTAGACAGAAGCATTCGCAGAATCACGTTTGTGATGTGTGCACTCAACTGTCAGAATTGA
          1 AAAAACTAGACAGAAGCATTCTCAGAAACATCTTTGTGATGTTTGCACTCAACTCACAGAGTTGA

                 **   ** *      *                            * *            *
     380547 ACCTTGGTTTGGACAGAGCACTTTTGAAACACTCTTTTTGTAGAATCTGCAGGTGGATATTTGGC
         66 ACCTTTCTTTTCATAGAGCAGTTTTGAAACACTCTTTTTGTAGAATCTGGAAGTGGATATTTGGA

            **                         *   *      * *
     380612 TAGCTTTGAGGATTTCGTTGGAAACGGGAATGTCTTCAAAG
        131 GCGCTTTGAGGATTTCGTTGGAAACGGAAATATCTTCATAT

                *                         *   *            *    *            
     380653 AAAATCTAGACAGAAGCATTCTCAGAAACACCTTCGTGATGTTTGCAATCAAGTCACAGAGTTGA
          1 AAAAACTAGACAGAAGCATTCTCAGAAACATCTTTGTGATGTTTGCACTCAACTCACAGAGTTGA

                 * *             *  *                  *             *       
     380718 ACCTTCCGTTTCATAGAGCAGGTTGGAAACACTCTTTTTGTAGTATCTGGAAGTGGACATTTGGA
         66 ACCTTTCTTTTCATAGAGCAGTTTTGAAACACTCTTTTTGTAGAATCTGGAAGTGGATATTTGGA

             *         ** *    * *   *            **  
     380783 GGGCTTTGTAGCCTATC-TGGAAAAAGGAAATATCTTCCCAT
        131 GCGCTTTG-AGGATTTCGTTGGAAACGGAAATATCTTCATAT

            *  ** *   *     * *            **  *  *   * **        *  * * *   
     380824 GAATGCGAGATAGAAGTAATCTCAGAAACATGCTTATGCTGTATCTACTCAACTAACTGTGCTGA
          1 AAAAACTAGACAGAAGCATTCTCAGAAACATCTTTGTGATGTTTGCACTCAACTCACAGAGTTGA

              *     *  *               *        *  * *       *               
     380889 ACATTTCTATTGATAGAGCAGTTTTGAGACACTCTTCTTTTGGAATCTGCAAGTGGATATTTGGA
         66 ACCTTTCTTTTCATAGAGCAGTTTTGAAACACTCTTTTTGTAGAATCTGGAAGTGGATATTTGGA

             * *                        *     *      
     380954 GAGATTTGAGGATTTCGTTGGAAACGG-GAT-T-AT-ATAT
        131 GCGCTTTGAGGATTTCGTTGGAAACGGAAATATCTTCATAT

                 *       *               *                 **  *   *         
     380991 AAAAAGTAGACAGCAGCATTCTCAGAAACTTCTTTGTGATGTTTGCATCCAGCTCTCAGAGTTGA
          1 AAAAACTAGACAGAAGCATTCTCAGAAACATCTTTGTGATGTTTGCACTCAACTCACAGAGTTGA

              *  * *          *  *        *        *   **        *  **       
     381056 ACATTCCCTTTCATAGAGTAGGTTTGAAACCCTCTTTTTATAGTGTCTGGAAGCGGGCATTTGGA
         66 ACCTTTCTTTTCATAGAGCAGTTTTGAAACACTCTTTTTGTAGAATCTGGAAGTGGATATTTGGA

                   *    * * **  **  **          * *  *
     381121 GCGCTTTCAGGCCTATGCTTAAAATAGGAAATATCTACCTAC
        131 GCGCTTTGAGG-ATTTCGTTGGAAACGGAAATATCTTCATAT

             *                    *    *  **          ** *        *    *     
     381163 AGAAACTAGACAGAAGCATTCTGAGAATCACGTTTGTGATGTGGGTACTCAACTAACAGTGTTGA
          1 AAAAACTAGACAGAAGCATTCTCAGAAACATCTTTGTGATGTTTGCACTCAACTCACAGAGTTGA

            *  *       *   *            *   *                *   *           
     381228 TCCATTCTTTTGATACAGCAGTTTTGAACCACACTTTTTGTAGAATCTGCAAGAGGATATTTGGA
         66 ACCTTTCTTTTCATAGAGCAGTTTTGAAACACTCTTTTTGTAGAATCTGGAAGTGGATATTTGGA

            **   *                     *   *      * *
     381293 TAGCTGTGAGGATTTCGTTGGAAACGGGAATGTCTTCAAAG
        131 GCGCTTTGAGGATTTCGTTGGAAACGGAAATATCTTCATAT

                *                         *   *            *    *            
     381334 AAAATCTAGACAGAAGCATTCTCAGAAACACCTTCGTGATGTTTGCAATCAAGTCACAGAGTTGA
          1 AAAAACTAGACAGAAGCATTCTCAGAAACATCTTTGTGATGTTTGCACTCAACTCACAGAGTTGA

                 * *             *  *           *      *             *       
     381399 ACCTTCCGTTTCATAGAGCAGGTTGGAAACACTCTTATTGTAGTATCTGGAAGTGGACATTTGGA
         66 ACCTTTCTTTTCATAGAGCAGTTTTGAAACACTCTTTTTGTAGAATCTGGAAGTGGATATTTGGA

                   *   ** ** *  *   *            **  
     381464 GCGCTTTCAGGCCTATGGTGAAAAAGGAAATATCTTCCCAT
        131 GCGCTTTGAGGATTTCGTTGGAAACGGAAATATCTTCATAT

                  * * *      **      *   * *   *     ** ** *      *    *     
     381505 AAAAACGACATAGAAGCTATCTCAGGAACTTGTTTATGATGCATCTAATCAACTAACAGTGTTGA
          1 AAAAACTAGACAGAAGCATTCTCAGAAACATCTTTGTGATGTTTGCACTCAACTCACAGAGTTGA

                  * ** * *                         * *       *               
     381570 ACCTTTGTACTGACAGAGCAG-TTTGAAACACTCTTTTTTTGGAATCTGCAAGTGGATATTTGGA
         66 ACCTTTCTTTTCATAGAGCAGTTTTGAAACACTCTTTTTGTAGAATCTGGAAGTGGATATTTGGA

            *                           *   *   *    
     381634 TCGCTTTGAGGATTTCGTTGGAAACGG-GAT-GC--AATA-
        131 GCGCTTTGAGGATTTCGTTGGAAACGGAAATATCTTCATAT

                     *    *     *                 **  *   *  *    *         *
     381670 AAAAACGTACACAGCAGCATACTCAGAAA-ATACTTTGCCATATTTCCATTCAAGTCACAGAGTG
          1 AAAAAC-TAGACAGAAGCATTCTCAGAAACAT-CTTTGTGATGTTTGCACTCAACTCACAGAGTT

                *  * **            *                  *  *             *     
     381734 GAACATTCCCATTCATAGAGCAGGTTTGAAACACTCTTTTTGGAGTATCTGGAAGTGGACATTTG
         64 GAACCTTTCTTTTCATAGAGCAGTTTTGAAACACTCTTTTTGTAGAATCTGGAAGTGGATATTTG

                     ** * * ** *  *   *            **  
     381799 GAGCGCTTTCTGAACTATGGTGAAAAAGGAAATATCTTCCAAT
        129 GAGCGCTTTGAGGATTTCGTTGGAAACGGAAATATCTTCATAT

            *     *               *      * *          *  **      **  *  *    
     381842 GAAAACAAGACAGAAGCATTCTGAGAAACTTATTTGTGATGTGTGTCCTCAACAAACGGACTTGA
          1 AAAAACTAGACAGAAGCATTCTCAGAAACATCTTTGTGATGTTTGCACTCAACTCACAGAGTTGA

                   *      **  * *  *  *             *   *    * * *           
     381907 ACCTTTCGTTTCATGCAGTACTTCTGGAACACTCTTTTTGAAGATTCTGCATGCGGATATTTGGA
         66 ACCTTTCTTTTCATAGAGCAGTTTTGAAACACTCTTTTTGTAGAATCTGGAAGTGGATATTTGGA

            **                            **   *   * 
     381972 TAGCTTTGAGGATTTCGTTGGAAACGG---GCT-TACATGT
        131 GCGCTTTGAGGATTTCGTTGGAAACGGAAATATCTTCATAT

                 *       *               *     *  *   *    *    *       *    
     382009 AAAAATTAGACAGCAGCATTCTCAGAAACTTCTTTCTGGTGTCTGCATTCAAGTCACAGAATTGA
          1 AAAAACTAGACAGAAGCATTCTCAGAAACATCTTTGTGATGTTTGCACTCAACTCACAGAGTTGA

              * ** * **            *  * *      *       *    *      * *       
     382074 ACTTCCCCTCACATAGAGCAGTTGTGCAGCACTCTATTTGTAGTATCTCGAAGTGCACATTTGGA
         66 ACCTTTCTTTTCATAGAGCAGTTTTGAAACACTCTTTTTGTAGAATCTGGAAGTGGATATTTGGA

             *         ** *    * *   *            **  
     382139 GGGCTTTGTAGCCTATC-TGGAAAAAGGAAATATCTTCCCAT
        131 GCGCTTTG-AGGATTTCGTTGGAAACGGAAATATCTTCATAT

            *  ** *   *     * *            *   *  *   * **        *  * * *   
     382180 GAATGCGAGATAGAAGTAATCTCAGAAACATGTTTATGCTGTATCTACTCAACTAACTGTGCTGA
          1 AAAAACTAGACAGAAGCATTCTCAGAAACATCTTTGTGATGTTTGCACTCAACTCACAGAGTTGA

              *     *  *               *        *  * *       *               
     382245 ACATTTCTATTGATAGAGCAGTTTTGAGACACTCTTCTTTTGGAATCTGCAAGTGGATATTTGGA
         66 ACCTTTCTTTTCATAGAGCAGTTTTGAAACACTCTTTTTGTAGAATCTGGAAGTGGATATTTGGA

            ** *                        *     *      
     382310 TAGATTTGAGGATTTCGTTGGAAACGG-GAT-T-AT-ATAT
        131 GCGCTTTGAGGATTTCGTTGGAAACGGAAATATCTTCATAT

            *    **      *               *                 **  *   *         
     382347 CAAAAGCAGACAGCAGCATTCTCAGAAACTTCTTTGTGATGTTTGCATCCAGCTCTCAGAGTTGA
          1 AAAAACTAGACAGAAGCATTCTCAGAAACATCTTTGTGATGTTTGCACTCAACTCACAGAGTTGA

              *  * *          *  *        *        *   **        *  **       
     382412 ACATTCCCTTTCATAGAGTAGGTTTGAAACCCTCTTTTTATAGTGTCTGGAAGCGGGCATTTGGA
         66 ACCTTTCTTTTCATAGAGCAGTTTTGAAACACTCTTTTTGTAGAATCTGGAAGTGGATATTTGGA

                   *   ** ** *  *   *          * *   
     382477 GCGCTTTCAGGCCTATGCTGAAAAAGGAAATATCTACCTAT
        131 GCGCTTTGAGGATTTCGTTGGAAACGGAAATATCTTCATAT

             *                    *    *  **          ** *        *    *     
     382518 AGAAACTAGACAGAAGCATTCTGAGAATCACGTTTGTGATGTGGGTACTCAACTAACAGTGTTGA
          1 AAAAACTAGACAGAAGCATTCTCAGAAACATCTTTGTGATGTTTGCACTCAACTCACAGAGTTGA

            *  *       *   *            *   *                *               
     382583 TCCATTCTTTTGATACAGCAGTTTTGAACCACACTTTTTGTAGAATCTGCAAGTGGATATTTGGA
         66 ACCTTTCTTTTCATAGAGCAGTTTTGAAACACTCTTTTTGTAGAATCTGGAAGTGGATATTTGGA

            **   *          *          *   *        *
     382648 TAGCTGTGAGGATTTCCTTGGAAACGGGAATGTCTTCATAG
        131 GCGCTTTGAGGATTTCGTTGGAAACGGAAATATCTTCATAT

                **                     * * **         *  **   *   *          
     382689 AAAATTTAGACAGAAGCATTCTCAGAACCTTGATTGTGATGTGTGTTCTCCACTAACAGAGTTGA
          1 AAAAACTAGACAGAAGCATTCTCAGAAACATCTTTGTGATGTTTGCACTCAACTCACAGAGTTGA

                       * *   * *   *       *       *                         
     382754 ACCTTTCTTTTGACAGAACTGTTCTGAAACATTCTTTTTATAGAATCTGGAAGTGGATATTTGGA
         66 ACCTTTCTTTTCATAGAGCAGTTTTGAAACACTCTTTTTGTAGAATCTGGAAGTGGATATTTGGA

            **                         *          *  
     382819 AAGCTTTGAGGATTTCGTTGGAAACGGGAATATCTTCAAAT
        131 GCGCTTTGAGGATTTCGTTGGAAACGGAAATATCTTCATAT

                *    *            *           * *       *  *    *            
     382860 AAAATCTAGCCAGAAGCATTCTAAGAAACATCTTAGGGATGTTTACATTCAAGTCACAGAGTTGA
          1 AAAAACTAGACAGAAGCATTCTCAGAAACATCTTTGTGATGTTTGCACTCAACTCACAGAGTTGA

              *  * *     *       *         *    * *   **      *      *    *  
     382925 ACATTCCCTTTCACAGAGCAGGTTTGAAACAATCTTCTCGTACTATCTGGCAGTGGACATTTTGA
         66 ACCTTTCTTTTCATAGAGCAGTTTTGAAACACTCTTTTTGTAGAATCTGGAAGTGGATATTTGGA

              * *   *  ** ** *  *   *            ** *
     382990 GCTCCTTGGGGCCTATGCTGAAAAAGGAAATATCTTCCGAC
        131 GCGCTTTGAGGATTTCGTTGGAAACGGAAATATCTTCATAT

                                 *     *  **          *      *    **    *    
     383031 AAAAACTAGACAGAAGCATTCGCAGAATCACGTTTGTGATGTGTGCACTAAACTGTCAGAATTGA
          1 AAAAACTAGACAGAAGCATTCTCAGAAACATCTTTGTGATGTTTGCACTCAACTCACAGAGTTGA

                 **   ** *      *                            * *            *
     383096 ACCTTGGTTTGGACAGAGCACTTTTGAAACACTCTTTTTGTAGAATCTGCAGGTGGATATTTGGC
         66 ACCTTTCTTTTCATAGAGCAGTTTTGAAACACTCTTTTTGTAGAATCTGGAAGTGGATATTTGGA

            **                         *   *      * *
     383161 TAGCTTTGAGGATTTCGTTGGAAACGGTAATGTCTTCAAAG
        131 GCGCTTTGAGGATTTCGTTGGAAACGGAAATATCTTCATAT

                *                         *   *            *    *            
     383202 AAAATCTAGACAGAAGCATTCTCAGAAACACCTTCGTGATGTTTGCAATCAAGTCACAGAGTTGA
          1 AAAAACTAGACAGAAGCATTCTCAGAAACATCTTTGTGATGTTTGCACTCAACTCACAGAGTTGA

                 * *       *     *  *                  *             *       
     383267 ACCTTCCGTTTCATACAGCAGGTTGGAAACACTCTTTTTGTAGTATCTGGAAGTGGACATTTGGA
         66 ACCTTTCTTTTCATAGAGCAGTTTTGAAACACTCTTTTTGTAGAATCTGGAAGTGGATATTTGGA

                   *   ** ** *  *   *            **  
     383332 GCGCTTTAAGGCCTATGGTGAAAAAGGAAATATCTTCCCAT
        131 GCGCTTTGAGGATTTCGTTGGAAACGGAAATATCTTCATAT

                  * * *      **      *   * *   *     ** ** *      *    *     
     383373 AAAAACGACATAGAAGCTATCTCAGGAACTTGTTTATGATGCATCTAATCAACTAACAGTGTTGA
          1 AAAAACTAGACAGAAGCATTCTCAGAAACATCTTTGTGATGTTTGCACTCAACTCACAGAGTTGA

                  * ** * *                         * *       *               
     383438 ACCTTTGTACTGACAGAGCAG-TTTGAAACACTCTTTTTTTGGAATCTGCAAGTGGATATTTGGA
         66 ACCTTTCTTTTCATAGAGCAGTTTTGAAACACTCTTTTTGTAGAATCTGGAAGTGGATATTTGGA

            *                           *   *   *    
     383502 TCGCTTTGAGGATTTCGTTGGAAACGG-GAT-GC--AATAT
        131 GCGCTTTGAGGATTTCGTTGGAAACGGAAATATCTTCATAT

                **  *    *     *                 **  *   *  *    *         * 
     383539 AAAACGTACACAGCAGCATACTCAGAAA-ATACTTTGCCATATTTCCATTCAAGTCACAGAGTGG
          1 AAAAACTAGACAGAAGCATTCTCAGAAACAT-CTTTGTGATGTTTGCACTCAACTCACAGAGTTG

               *  * **            *          *       *  *             *      
     383603 AACATTCCCATTCATAGAGCAGGTTTGAAACACACTTTTTGGAGTATCTGGAAGTGGACATTTGG
         65 AACCTTTCTTTTCATAGAGCAGTTTTGAAACACTCTTTTTGTAGAATCTGGAAGTGGATATTTGG

                    ** * * ** *  *   *            **  
     383668 AGCGCTTTCTGAACTATGGTGAAAAAGGAAATATCTTCCAAT
        130 AGCGCTTTGAGGATTTCGTTGGAAACGGAAATATCTTCATAT

            *     * *             *      * *          *  **      **  *  *    
     383710 GAAAACAACACAGAAGCATTCTGAGAAACTTATTTGTGATGTGTGTCCTCAACAAACGGACTTGA
          1 AAAAACTAGACAGAAGCATTCTCAGAAACATCTTTGTGATGTTTGCACTCAACTCACAGAGTTGA

                   *      **  * *  *  *             *   *    * * *           
     383775 ACCTTTCGTTTCATGCAGTACTTCTGGAACACTCTTTTTGAAGATTCTGCATGCGGATATTTGGA
         66 ACCTTTCTTTTCATAGAGCAGTTTTGAAACACTCTTTTTGTAGAATCTGGAAGTGGATATTTGGA

            **                            **   *   * 
     383840 TAGCTTTGAGGATTTCGTTGGAAACGG---GCT-TACATGT
        131 GCGCTTTGAGGATTTCGTTGGAAACGGAAATATCTTCATAT

                 *       *               *        *   *    *    *       *    
     383877 AAAAATTAGACAGCAGCATTCTCAGAAACTTCTTTGTGGTGTCTGCATTCAAGTCACAGAATTGA
          1 AAAAACTAGACAGAAGCATTCTCAGAAACATCTTTGTGATGTTTGCACTCAACTCACAGAGTTGA

              * ** * **            *  * *      *       *    *        *       
     383942 ACTTCCCCTCACATAGAGCAGTTGTGCAGCACTCTATTTGTAGTATCTCGAAGTGGACATTTGGA
         66 ACCTTTCTTTTCATAGAGCAGTTTTGAAACACTCTTTTTGTAGAATCTGGAAGTGGATATTTGGA

             *         ** *    * *   *            **  
     384007 GGGCTTTGTAGCCTATC-TGGAAAAAGGAAATATCTTCCCAT
        131 GCGCTTTG-AGGATTTCGTTGGAAACGGAAATATCTTCATAT

            *  ** *   *     * *            *   *  *   * **        *  * * *   
     384048 GAATGCGAGATAGAAGTAATCTCAGAAACATGTTTATGCTGTATCTACTCAACTAACTGTGCTGA
          1 AAAAACTAGACAGAAGCATTCTCAGAAACATCTTTGTGATGTTTGCACTCAACTCACAGAGTTGA

              *     *  *               *        *  * *       *               
     384113 ACATTTCTATTGATAGAGCAGTTTTGAGACACTCTTCTTTTGGAATCTGCAAGTGGATATTTGGA
         66 ACCTTTCTTTTCATAGAGCAGTTTTGAAACACTCTTTTTGTAGAATCTGGAAGTGGATATTTGGA

            ** *                        *     *      
     384178 TAGATTTGAGGATTTCGTTGGAAACGG-GAT-T-AT-ATAT
        131 GCGCTTTGAGGATTTCGTTGGAAACGGAAATATCTTCATAT

                 *       *               *                 **  *   *         
     384215 AAAAAGTAGACAGCAGCATTCTCAGAAACTTCTTTGTGATGTTTGCATCCAGCTCTCAGAGTTGA
          1 AAAAACTAGACAGAAGCATTCTCAGAAACATCTTTGTGATGTTTGCACTCAACTCACAGAGTTGA

              *  * *          *  *        *        *   **        *  **       
     384280 ACATTCCCTTTCATAGAGTAGGTTTGAAACCCTCTTTTTATAGTGTCTGGAAGCGGGCATTTGGA
         66 ACCTTTCTTTTCATAGAGCAGTTTTGAAACACTCTTTTTGTAGAATCTGGAAGTGGATATTTGGA

                   *   ** ** *  *   *          * *   
     384345 GCGCTTTCAGGCCTATGCTGAAAAAGGAAATATCTACCTAT
        131 GCGCTTTGAGGATTTCGTTGGAAACGGAAATATCTTCATAT

             *                    *    *  **          ** *        *    *     
     384386 AGAAACTAGACAGAAGCATTCTGAGAATCACGTTTGTGATGTGGGTACTCAACTAACAGTGTTGA
          1 AAAAACTAGACAGAAGCATTCTCAGAAACATCTTTGTGATGTTTGCACTCAACTCACAGAGTTGA

            *  *       *   *            *   *                *               
     384451 TCCATTCTTTTGATACAGCAGTTTTGAACCACACTTTTTGTAGAATCTGCAAGTGGATATTTGGA
         66 ACCTTTCTTTTCATAGAGCAGTTTTGAAACACTCTTTTTGTAGAATCTGGAAGTGGATATTTGGA

            **   *                  *  *   *        *
     384516 TAGCTGTGAGGATTTCGTTGGAAATGGGAATGTCTTCATAG
        131 GCGCTTTGAGGATTTCGTTGGAAACGGAAATATCTTCATAT

                **                     * * **         *  **   *   *          
     384557 AAAATTTAGACAGAAGCATTCTCAGAACCTTGATTGTGATGTGTGTTCTCCACTAACAGAGTTGA
          1 AAAAACTAGACAGAAGCATTCTCAGAAACATCTTTGTGATGTTTGCACTCAACTCACAGAGTTGA

                      ** * * * *   *       *       *                         
     384622 ACCTTTCTTTAGACACAACTGTTCTGAAACATTCTTTTTATAGAATCTGGAAGTGGATATTTGGA
         66 ACCTTTCTTTTCATAGAGCAGTTTTGAAACACTCTTTTTGTAGAATCTGGAAGTGGATATTTGGA

            **                         *          *  
     384687 AAGCTTTGAGGATTTCGTTGGAAACGGGAATATCTTCAAAT
        131 GCGCTTTGAGGATTTCGTTGGAAACGGAAATATCTTCATAT

                *    *            *           * *       *  *    *            
     384728 AAAATCTAGCCAGAAGCATTCTAAGAAACATCTTAGGGATGTTTACATTCAAGTCACAGAGTTGA
          1 AAAAACTAGACAGAAGCATTCTCAGAAACATCTTTGTGATGTTTGCACTCAACTCACAGAGTTGA

              *  * *     *       *         *    * *   **      *      *    *  
     384793 ACATTCCCTTTCACAGAGCAGGTTTGAAACAATCTTCTCGTACTATCTGGCAGTGGACATTTTGA
         66 ACCTTTCTTTTCATAGAGCAGTTTTGAAACACTCTTTTTGTAGAATCTGGAAGTGGATATTTGGA

              * *   *  ** ** *  *   *            ** *
     384858 GCTCCTTGGGGCCTATGCTGAAAAAGGAAATATCTTCCGAC
        131 GCGCTTTGAGGATTTCGTTGGAAACGGAAATATCTTCATAT

                                 *     *  **          *           **    *    
     384899 AAAAACTAGACAGAAGCATTCGCAGAATCACGTTTGTGATGTGTGCACTCAACTGTCAGAATTGA
          1 AAAAACTAGACAGAAGCATTCTCAGAAACATCTTTGTGATGTTTGCACTCAACTCACAGAGTTGA

                 **   ** *      *                            * *            *
     384964 ACCTTGGTTTGGACAGAGCACTTTTGAAACACTCTTTTTGTAGAATCTGCAGGTGGATATTTGGC
         66 ACCTTTCTTTTCATAGAGCAGTTTTGAAACACTCTTTTTGTAGAATCTGGAAGTGGATATTTGGA

            **                         *   *      * *
     385029 TAGCTTTGAGGATTTCGTTGGAAACGGTAATGTCTTCAAAG
        131 GCGCTTTGAGGATTTCGTTGGAAACGGAAATATCTTCATAT

                *                         *   *            *    *            
     385070 AAAATCTAGACAGAAGCATTCTCAGAAACACCTTCGTGATGTTTGCAATCAAGTCACAGAGTTGA
          1 AAAAACTAGACAGAAGCATTCTCAGAAACATCTTTGTGATGTTTGCACTCAACTCACAGAGTTGA

                 * *             *  *       *          *             *       
     385135 ACCTTCCGTTTCATAGAGCAGGTTGGAAACACACTTTTTGTAGTATCTGGAAGTGGACATTTGGA
         66 ACCTTTCTTTTCATAGAGCAGTTTTGAAACACTCTTTTTGTAGAATCTGGAAGTGGATATTTGGA

             *         ** *    * *   *            **  
     385200 GGGCTTTGTAGCCTATC-TGGAAAAAGGAAATATCTTCCCAT
        131 GCGCTTTG-AGGATTTCGTTGGAAACGGAAATATCTTCATAT

            *  ** *   *     * *            *   *  *   * **        *  * * *   
     385241 GAATGCGAGATAGAAGTAATCTCAGAAACATGTTTATGCTGTATCTACTCAACTAACTGTGCTGA
          1 AAAAACTAGACAGAAGCATTCTCAGAAACATCTTTGTGATGTTTGCACTCAACTCACAGAGTTGA

              *     *  *               *        *  * *       *           *   
     385306 ACATTTCTATTGATAGAGCAGTTTTGAGACACTCTTCTTTTGGAATCTGCAAGTGGATATTAGGA
         66 ACCTTTCTTTTCATAGAGCAGTTTTGAAACACTCTTTTTGTAGAATCTGGAAGTGGATATTTGGA

            ** *                        *     *      
     385371 TAGATTTGAGGATTTCGTTGGAAACGG-GAT-T-AT-ATAT
        131 GCGCTTTGAGGATTTCGTTGGAAACGGAAATATCTTCATAT

                 *       *               *                 **  *   *         
     385408 AAAAAGTAGACAGCAGCATTCTCAGAAACTTCTTTGTGATGTTTGCATCCAGCTCTCAGAGTTGA
          1 AAAAACTAGACAGAAGCATTCTCAGAAACATCTTTGTGATGTTTGCACTCAACTCACAGAGTTGA

            * *  * *          *  *        *        *   **        *  **       
     385473 GCATTCCCTTTCATAGAGTAGGTTTGAAACCCTCTTTTTATAGTGTCTGGAAGCGGGCATTTGGA
         66 ACCTTTCTTTTCATAGAGCAGTTTTGAAACACTCTTTTTGTAGAATCTGGAAGTGGATATTTGGA

                   *    * * **  **  **          * *  *
     385538 GCGCTTTCAGGCCTATGCTTAAAATAGGAAATATCTACCTAC
        131 GCGCTTTGAGG-ATTTCGTTGGAAACGGAAATATCTTCATAT

             *                    *    *  **          ** *        *    *     
     385580 AGAAACTAGACAGAAGCATTCTGAGAATCACGTTTGTGATGTGGGTACTCAACTAACAGTGTTGA
          1 AAAAACTAGACAGAAGCATTCTCAGAAACATCTTTGTGATGTTTGCACTCAACTCACAGAGTTGA

            *  *       *   *            *   *                *   *           
     385645 TCCATTCTTTTGATACAGCAGTTTTGAACCACACTTTTTGTAGAATCTGCAAGAGGATATTTGGA
         66 ACCTTTCTTTTCATAGAGCAGTTTTGAAACACTCTTTTTGTAGAATCTGGAAGTGGATATTTGGA

            **   *                     *   *      * *
     385710 TAGCTGTGAGGATTTCGTTGGAAACGGGAATGTCTTCAAAG
        131 GCGCTTTGAGGATTTCGTTGGAAACGGAAATATCTTCATAT

                *                         *   *            *    *            
     385751 AAAATCTAGACAGAAGCATTCTCAGAAACACCTTCGTGATGTTTGCAATCAAGTCACAGAGTTGA
          1 AAAAACTAGACAGAAGCATTCTCAGAAACATCTTTGTGATGTTTGCACTCAACTCACAGAGTTGA

                 * *             *  *           *      *             *       
     385816 ACCTTCCGTTTCATAGAGCAGGTTGGAAACACTCTTATTGTAGTATCTGGAAGTGGACATTTGGA
         66 ACCTTTCTTTTCATAGAGCAGTTTTGAAACACTCTTTTTGTAGAATCTGGAAGTGGATATTTGGA

                   *   ** ** *  *   *            **  
     385881 GCGCTTTCAGGCCTATGGTGAAAAAGGAAATATCTTCCCAT
        131 GCGCTTTGAGGATTTCGTTGGAAACGGAAATATCTTCATAT

                  * * *      **      *   * *   *     ** ** *      *    *     
     385922 AAAAACGACATAGAAGCTATCTCAGGAACTTGTTTATGATGCATCTAATCAACTAACAGTGTTGA
          1 AAAAACTAGACAGAAGCATTCTCAGAAACATCTTTGTGATGTTTGCACTCAACTCACAGAGTTGA

                  * ** * *                         * *       *               
     385987 ACCTTTGTACTGACAGAGCAG-TTTGAAACACTCTTTTTTTGGAATCTGCAAGTGGATATTTGGA
         66 ACCTTTCTTTTCATAGAGCAGTTTTGAAACACTCTTTTTGTAGAATCTGGAAGTGGATATTTGGA

            *                           *   *   *    
     386051 TCGCTTTGAGGATTTCGTTGGAAACGG-GAT-GC--AATAT
        131 GCGCTTTGAGGATTTCGTTGGAAACGGAAATATCTTCATAT

                **  *    *     *                 **  *   *  *    *         * 
     386088 AAAACGTACACAGCAGCATACTCAGAAA-ATACTTTGCCATATTTCCATTCAAGTCACAGAGTGG
          1 AAAAACTAGACAGAAGCATTCTCAGAAACAT-CTTTGTGATGTTTGCACTCAACTCACAGAGTTG

               *  * **            *  *               *  *             *      
     386152 AACATTCCCATTCATAGAGCAGGTTGGAAACACTCTTTTTGGAGTATCTGGAAGTGGACATTTGG
         65 AACCTTTCTTTTCATAGAGCAGTTTTGAAACACTCTTTTTGTAGAATCTGGAAGTGGATATTTGG

                    ** * * ** *  *   *            **  
     386217 AGCGCTTTCTGAACTATGGTGAAAAAGGAAATATCTTCCAAT
        130 AGCGCTTTGAGGATTTCGTTGGAAACGGAAATATCTTCATAT

            *     *               *      * *          *  **      **  *  *    
     386259 GAAAACAAGACAGAAGCATTCTGAGAAACTTATTTGTGATGTGTGTCCTCAACAAACGGACTTGA
          1 AAAAACTAGACAGAAGCATTCTCAGAAACATCTTTGTGATGTTTGCACTCAACTCACAGAGTTGA

                   *      **  * *  *  *             *   *    * * *           
     386324 ACCTTTCGTTTCATGCAGTACTTCTGGAACACTCTTTTTGAAGATTCTGCATGCGGATATTTGGA
         66 ACCTTTCTTTTCATAGAGCAGTTTTGAAACACTCTTTTTGTAGAATCTGGAAGTGGATATTTGGA

            **                            **   *   * 
     386389 TAGCTTTGAGGATTTCGTTGGAAACGG---GCT-TACATGT
        131 GCGCTTTGAGGATTTCGTTGGAAACGGAAATATCTTCATAT

                 *       *               *        *   *    *    *       *    
     386426 AAAAATTAGACAGCAGCATTCTCAGAAACTTCTTTGTGGTGTCTGCATTCAAGTCACAGAATTGA
          1 AAAAACTAGACAGAAGCATTCTCAGAAACATCTTTGTGATGTTTGCACTCAACTCACAGAGTTGA

              * ** * **            *  * *      *       *             *       
     386491 ACTTCCCCTCACATAGAGCAGTTGTGCAGCACTCTATTTGTAGTATCTGGAAGTGGACATTTGGA
         66 ACCTTTCTTTTCATAGAGCAGTTTTGAAACACTCTTTTTGTAGAATCTGGAAGTGGATATTTGGA

             *         ** *    * *   *            **  
     386556 GGGCTTTGTAGCCTATC-TGGAAAAAGGAAATATCTTCCCAT
        131 GCGCTTTG-AGGATTTCGTTGGAAACGGAAATATCTTCATAT

            *  ** *   *     * *            *   *  *   * **        *  * * *   
     386597 GAATGCGAGATAGAAGTAATCTCAGAAACATGTTTATGCTGTATCTACTCAACTAACTGTGCTGA
          1 AAAAACTAGACAGAAGCATTCTCAGAAACATCTTTGTGATGTTTGCACTCAACTCACAGAGTTGA

              *     *  *               *        *  * *       *               
     386662 ACATTTCTATTGATAGAGCAGTTTTGAGACACTCTTCTTTTGGAATCTGCAAGTGGATATTTGGA
         66 ACCTTTCTTTTCATAGAGCAGTTTTGAAACACTCTTTTTGTAGAATCTGGAAGTGGATATTTGGA

            ** *                        *     *      
     386727 TAGATTTGAGGATTTCGTTGGAAACGG-GAT-T-AT-ATAT
        131 GCGCTTTGAGGATTTCGTTGGAAACGGAAATATCTTCATAT

                 *       *               *                 **  *   *         
     386764 AAAAAGTAGACAGCAGCATTCTCAGAAACTTCTTTGTGATGTTTGCATCCAGCTCTCAGAGTTGA
          1 AAAAACTAGACAGAAGCATTCTCAGAAACATCTTTGTGATGTTTGCACTCAACTCACAGAGTTGA

              *  * *          *  *        *        *   **        *  **       
     386829 ACATTCCCTTTCATAGAGTAGGTTTGAAACCCTCTTTTTATAGTGTCTGGAAGCGGGCATTTGGA
         66 ACCTTTCTTTTCATAGAGCAGTTTTGAAACACTCTTTTTGTAGAATCTGGAAGTGGATATTTGGA

                   *   ** ** *  *   *          * *   
     386894 GCGCTTTCAGGCCTATGCTGAAAAAGGAAATATCTACGTAT
        131 GCGCTTTGAGGATTTCGTTGGAAACGGAAATATCTTCATAT

             *                    *    *  **          ** *        *    *     
     386935 AGAAACTAGACAGAAGCATTCTGAGAATCACGTTTGTGATGTGGGTACTCAACTAACAGTGTTGA
          1 AAAAACTAGACAGAAGCATTCTCAGAAACATCTTTGTGATGTTTGCACTCAACTCACAGAGTTGA

            *  *       *   *            *   *                *   *           
     387000 TCCATTCTTTTGATACAGCAGTTTTGAACCACACTTTTTGTAGAATCTGCAAGAGGATATTTGGA
         66 ACCTTTCTTTTCATAGAGCAGTTTTGAAACACTCTTTTTGTAGAATCTGGAAGTGGATATTTGGA

            **   *                     *   *        *
     387065 TAGCTGTGAGGATTTCGTTGGAAACGGGAATGTCTTCATAG
        131 GCGCTTTGAGGATTTCGTTGGAAACGGAAATATCTTCATAT

                **                     * * **         *  **   *   *          
     387106 AAAATTTAGACAGAAGCATTCTCAGAACCTTGATTGTGATGTGTGTTCTCCACTAACAGAGTTGA
          1 AAAAACTAGACAGAAGCATTCTCAGAAACATCTTTGTGATGTTTGCACTCAACTCACAGAGTTGA

                       * *   * *   *       *       *                         
     387171 ACCTTTCTTTTGACAGAACTGTTCTGAAACATTCTTTTTATAGAATCTGGAAGTGGATATTTGGA
         66 ACCTTTCTTTTCATAGAGCAGTTTTGAAACACTCTTTTTGTAGAATCTGGAAGTGGATATTTGGA

            **                         *          *  
     387236 AAGCTTTGAGGATTTCGTTGGAAACGGGAATATCTTCAAAT
        131 GCGCTTTGAGGATTTCGTTGGAAACGGAAATATCTTCATAT

                *    *            *           * *       *  *    *            
     387277 AAAATCTAGCCAGAAGCATTCTAAGAAACATCTTAGGGATGTTTACATTCAAGTCACAGAGTTGA
          1 AAAAACTAGACAGAAGCATTCTCAGAAACATCTTTGTGATGTTTGCACTCAACTCACAGAGTTGA

              *  * *     *       *         *    * *   **      *      *    *  
     387342 ACATTCCCTTTCACAGAGCAGGTTTGAAACAATCTTCTCGTACTATCTGGCAGTGGACATTTTGA
         66 ACCTTTCTTTTCATAGAGCAGTTTTGAAACACTCTTTTTGTAGAATCTGGAAGTGGATATTTGGA

              * *   *  ** ** *  *   *            ** *
     387407 GCTCCTTGGGGCCTATGCTGAAAAAGGAAATATCTTCCGAC
        131 GCGCTTTGAGGATTTCGTTGGAAACGGAAATATCTTCATAT

                                 *     *  **          *           **    *    
     387448 AAAAACTAGACAGAAGCATTCGCAGAATCACGTTTGTGATGTGTGCACTCAACTGTCAGAATTGA
          1 AAAAACTAGACAGAAGCATTCTCAGAAACATCTTTGTGATGTTTGCACTCAACTCACAGAGTTGA

                 **   ** *      *                            * *            *
     387513 ACCTTGGTTTGGACAGAGCACTTTTGAAACACTCTTTTTGTAGAATCTGCAGGTGGATATTTGGC
         66 ACCTTTCTTTTCATAGAGCAGTTTTGAAACACTCTTTTTGTAGAATCTGGAAGTGGATATTTGGA

            **                         *   *      * *
     387578 TAGCTTTGAGGATTTCGTTGGAAACGGGAATGTCTTCAAAG
        131 GCGCTTTGAGGATTTCGTTGGAAACGGAAATATCTTCATAT

                *                         *   *            *    *            
     387619 AAAATCTAGACAGAAGCATTCTCAGAAACACCTTCGTGATGTTTGCAATCAAGTCACAGAGTTGA
          1 AAAAACTAGACAGAAGCATTCTCAGAAACATCTTTGTGATGTTTGCACTCAACTCACAGAGTTGA

                 * *             *  *                  *             *       
     387684 ACCTTCCGTTTCATAGAGCAGGTTGGAAACACTCTTTTTGTAGTATCTGGAAGTGGACATTTGGA
         66 ACCTTTCTTTTCATAGAGCAGTTTTGAAACACTCTTTTTGTAGAATCTGGAAGTGGATATTTGGA

             *         ** *    * *   *            **  
     387749 GGGCTTTGTAGCCTATC-TGGAAAAAGGAAATATCTTCCCAT
        131 GCGCTTTG-AGGATTTCGTTGGAAACGGAAATATCTTCATAT

            *  ** *   *     * *            *   *  *   * **        *  * * *   
     387790 GAATGCGAGATAGAAGTAATCTCAGAAACATGTTTATGCTGTATCTACTCAACTAACTGTGCTGA
          1 AAAAACTAGACAGAAGCATTCTCAGAAACATCTTTGTGATGTTTGCACTCAACTCACAGAGTTGA

              *     *  *               *        *  * *       *               
     387855 ACATTTCTATTGATAGAGCAGTTTTGAGACACTCTTCTTTTGGAATCTGCAAGTGGATATTTGGA
         66 ACCTTTCTTTTCATAGAGCAGTTTTGAAACACTCTTTTTGTAGAATCTGGAAGTGGATATTTGGA

            ** *            *           *     *      
     387920 TAGATTTGAGGATTTCTTTGGAAACGG-GAT-T-AT-ATAT
        131 GCGCTTTGAGGATTTCGTTGGAAACGGAAATATCTTCATAT

                 *       *               *                 **  *   *         
     387957 AAAAAGTAGACAGCAGCATTCTCAGAAACTTCTTTGTGATGTTTGCATCCAGCTCTCAGAGTTGA
          1 AAAAACTAGACAGAAGCATTCTCAGAAACATCTTTGTGATGTTTGCACTCAACTCACAGAGTTGA

            * *  * *   *      *  *        *        *   **        *  **       
     388022 GCATTCCCTTTTATAGAGTAGGTTTGAAACCCTCTTTTTATAGTGTCTGGAAGCGGGCATTTGGA
         66 ACCTTTCTTTTCATAGAGCAGTTTTGAAACACTCTTTTTGTAGAATCTGGAAGTGGATATTTGGA

                   *    * * **  **  **          * *  *
     388087 GCGCTTTCAGGCCTATGCTTAAAATAGGAAATATCTACCTAC
        131 GCGCTTTGAGG-ATTTCGTTGGAAACGGAAATATCTTCATAT

             *                    *    *  **          ** *        *    *     
     388129 AGAAACTAGACAGAAGCATTCTGAGAATCACGTTTGTGATGTGGGTACTCAACTAACAGTGTTGA
          1 AAAAACTAGACAGAAGCATTCTCAGAAACATCTTTGTGATGTTTGCACTCAACTCACAGAGTTGA

            *  *       *   *            *   *                *   *           
     388194 TCCATTCTTTTGATACAGCAGTTTTGAACCACACTTTTTGTAGAATCTGCAAGAGGATATTTGGA
         66 ACCTTTCTTTTCATAGAGCAGTTTTGAAACACTCTTTTTGTAGAATCTGGAAGTGGATATTTGGA

            **   *                  *  *   *      * *
     388259 TAGCTGTGAGGATTTCGTTGGAAAGGGGAATGTCTTCAAAG
        131 GCGCTTTGAGGATTTCGTTGGAAACGGAAATATCTTCATAT

                *                         *   *            *    *            
     388300 AAAATCTAGACAGAAGCATTCTCAGAAACACCTTCGTGATGTTTGCAATCAAGTCACAGAGTTGA
          1 AAAAACTAGACAGAAGCATTCTCAGAAACATCTTTGTGATGTTTGCACTCAACTCACAGAGTTGA

                 * *             *  *           *      *             *       
     388365 ACCTTCCGTTTCATAGAGCAGGTTGGAAACACTCTTATTGTAGTATCTGGAAGTGGACATTTGGA
         66 ACCTTTCTTTTCATAGAGCAGTTTTGAAACACTCTTTTTGTAGAATCTGGAAGTGGATATTTGGA

                   *   ** ** *  *   *            **  
     388430 GCGCTTTCAGGCCTATGGTGAAAAAGGAAATATCTTCCCAT
        131 GCGCTTTGAGGATTTCGTTGGAAACGGAAATATCTTCATAT

                  * * *      **      *   * *   *     ** ** *      *    *     
     388471 AAAAACGACATAGAAGCTATCTCAGGAACTTGTTTATGATGCATCTAATCAACTAACAGTGTTGA
          1 AAAAACTAGACAGAAGCATTCTCAGAAACATCTTTGTGATGTTTGCACTCAACTCACAGAGTTGA

                  * ** * *                         * *       *               
     388536 ACCTTTGTACTGACAGAGCAG-TTTGAAACACTCTTTTTTTGGAATCTGCAAGTGGATATTTGGA
         66 ACCTTTCTTTTCATAGAGCAGTTTTGAAACACTCTTTTTGTAGAATCTGGAAGTGGATATTTGGA

            *                           *   *   *    
     388600 TCGCTTTGAGGATTTCGTTGGAAACGG-GAT-GC--AATAT
        131 GCGCTTTGAGGATTTCGTTGGAAACGGAAATATCTTCATAT

                **  *    *     *                 **  *   *  *    *         * 
     388637 AAAACGTACACAGCAGCATACTCAGAAA-ATACTTTGCCATATTTCCATTCAAGTCACAGAGTGG
          1 AAAAACTAGACAGAAGCATTCTCAGAAACAT-CTTTGTGATGTTTGCACTCAACTCACAGAGTTG

               *  * **            *  *               *  *             *      
     388701 AACATTCCCATTCATAGAGCAGGTTGGAAACACTCTTTTTGGAGTATCTGGAAGTGGACATTTGG
         65 AACCTTTCTTTTCATAGAGCAGTTTTGAAACACTCTTTTTGTAGAATCTGGAAGTGGATATTTGG

                    ** * * ** *  *   *            **  
     388766 AGCGCTTTCTGAACTATGGTGAAAAAGGAAATATCTTCCAAT
        130 AGCGCTTTGAGGATTTCGTTGGAAACGGAAATATCTTCATAT

            *     *               *      * *          *  **      **  *  *    
     388808 GAAAACAAGACAGAAGCATTCTGAGAAACTTATTTGTGATGTGTGTCCTCAACAAACGGACTTGA
          1 AAAAACTAGACAGAAGCATTCTCAGAAACATCTTTGTGATGTTTGCACTCAACTCACAGAGTTGA

                   *      **  * *  *  *             *   *    * * *           
     388873 ACCTTTCGTTTCATGCAGTACTTCTGGAACACTCTTTTTGAAGATTCTGCATGCGGATATTTGGA
         66 ACCTTTCTTTTCATAGAGCAGTTTTGAAACACTCTTTTTGTAGAATCTGGAAGTGGATATTTGGA

            **                            **   *   * 
     388938 TAGCTTTGAGGATTTCGTTGGAAACGG---GCT-TACATGT
        131 GCGCTTTGAGGATTTCGTTGGAAACGGAAATATCTTCATAT

                 *       *               *        *   *    *    *       *    
     388975 AAAAATTAGACAGCAGCATTCTCAGAAACTTCTTTGTGGTGTCTGCATTCAAGTCACAGAATTGA
          1 AAAAACTAGACAGAAGCATTCTCAGAAACATCTTTGTGATGTTTGCACTCAACTCACAGAGTTGA

              * ** * **            *  * *      *       *             *       
     389040 ACTTCCCCTCACATAGAGCAGTTGTGCAGCACTCTATTTGTAGTATCTGGAAGTGGACATTTGGA
         66 ACCTTTCTTTTCATAGAGCAGTTTTGAAACACTCTTTTTGTAGAATCTGGAAGTGGATATTTGGA

             *         ** *    * *   *            **  
     389105 GGGCTTTGTAGCCTATC-TGGAAAAAGGAAATATCTTCCCAT
        131 GCGCTTTG-AGGATTTCGTTGGAAACGGAAATATCTTCATAT

            *  ** *   *     * *            *   *  *   * **        *  * * *   
     389146 GAATGCGAGATAGAAGTAATCTCAGAAACATGTTTATGCTGTATCTACTCAACTAACTGTGCTGA
          1 AAAAACTAGACAGAAGCATTCTCAGAAACATCTTTGTGATGTTTGCACTCAACTCACAGAGTTGA

              *     *  *               *        *  * *       *            *  
     389211 ACATTTCTATTGATAGAGCAGTTTTGAGACACTCTTCTTTTGGAATCTGCAAGTGGATATTTCGA
         66 ACCTTTCTTTTCATAGAGCAGTTTTGAAACACTCTTTTTGTAGAATCTGGAAGTGGATATTTGGA

            ** *                        *     *      
     389276 TAGATTTGAGGATTTCGTTGGAAACGG-GAT-T-AT-ATAT
        131 GCGCTTTGAGGATTTCGTTGGAAACGGAAATATCTTCATAT

                 *       *               *             *   **  *   *         
     389313 AAAAAGTAGACAGCAGCATTCTCAGAAACTTCTTTGTGATGTTGGCATCCAGCTCTCAGAGTTGA
          1 AAAAACTAGACAGAAGCATTCTCAGAAACATCTTTGTGATGTTTGCACTCAACTCACAGAGTTGA

              *  * *          *  *        *        *   **        *  **       
     389378 ACATTCCCTTTCATAGAGTAGGTTTGAAACCCTCTTTTTATAGTGTCTGGAAGCGGGCATTTGGA
         66 ACCTTTCTTTTCATAGAGCAGTTTTGAAACACTCTTTTTGTAGAATCTGGAAGTGGATATTTGGA

               *   *   ** ** ** *   *          * *   
     389443 GCGATTTCAGGCCTATGCGGAAAAAGGAAATATCTACCTAT
        131 GCGCTTTGAGGATTTCGTTGGAAACGGAAATATCTTCATAT

             *                    *    *  **          ** *        *    *     
     389484 AGAAACTAGACAGAAGCATTCTGAGAATCACGTTTGTGATGTGGGTACTCAACTAACAGTGTTGA
          1 AAAAACTAGACAGAAGCATTCTCAGAAACATCTTTGTGATGTTTGCACTCAACTCACAGAGTTGA

            *  *       *   * *          *   *                *               
     389549 TCCATTCTTTTGATACACCAGTTTTGAACCACACTTTTTGTAGAATCTGCAAGTGGATATTTGGA
         66 ACCTTTCTTTTCATAGAGCAGTTTTGAAACACTCTTTTTGTAGAATCTGGAAGTGGATATTTGGA

            **   *                     *   *        *
     389614 TAGCTGTGAGGATTTCGTTGGAAACGGGAATGTCTTCATAG
        131 GCGCTTTGAGGATTTCGTTGGAAACGGAAATATCTTCATAT

                **         *           * * **         *  **   *   *          
     389655 AAAATTTAGACAGAAACATTCTCAGAACCTTGATTGTGATGTGTGTTCTCCACTAACAGAGTTGA
          1 AAAAACTAGACAGAAGCATTCTCAGAAACATCTTTGTGATGTTTGCACTCAACTCACAGAGTTGA

                       * *   * *   *       *       *                         
     389720 ACCTTTCTTTTGACAGAACTGTTCTGAAACATTCTTTTTATAGAATCTGGAAGTGGATATTTGGA
         66 ACCTTTCTTTTCATAGAGCAGTTTTGAAACACTCTTTTTGTAGAATCTGGAAGTGGATATTTGGA

            **                         *          *  
     389785 AAGCTTTGAGGATTTCGTTGGAAACGGGAATATCTTCAAAT
        131 GCGCTTTGAGGATTTCGTTGGAAACGGAAATATCTTCATAT

            *   *    *            *           * *       *  *    *            
     389826 CAAATCTAGCCAGAAGCATTCTAAGAAACATCTTAGGGATGTTTACATTCAAGTCACAGAGTTGA
          1 AAAAACTAGACAGAAGCATTCTCAGAAACATCTTTGTGATGTTTGCACTCAACTCACAGAGTTGA

              *  * *     *       *      *  *    * *   **      *      *    *  
     389891 ACATTCCCTTTCACAGAGCAGGTTTGAACCAATCTTCTCGTACTATCTGGCAGTGGACATTTTGA
         66 ACCTTTCTTTTCATAGAGCAGTTTTGAAACACTCTTTTTGTAGAATCTGGAAGTGGATATTTGGA

              * *   *  ** ** *  *   *            ** *
     389956 GCTCCTTGGGGCCTATGCTGAAAAAGGAAATATCTTCCGAC
        131 GCGCTTTGAGGATTTCGTTGGAAACGGAAATATCTTCATAT

                                 *     *  **          *           **    *    
     389997 AAAAACTAGACAGAAGCATTCGCAGAATCACGTTTGTGATGTGTGCACTCAACTGTCAGAATTGA
          1 AAAAACTAGACAGAAGCATTCTCAGAAACATCTTTGTGATGTTTGCACTCAACTCACAGAGTTGA

                 **   ** *      *              *             * *            *
     390062 ACCTTGGTTTGGACAGAGCACTTTTGAAACACTCTCTTTGTAGAATCTGCAGGTGGATATTTGGC
         66 ACCTTTCTTTTCATAGAGCAGTTTTGAAACACTCTTTTTGTAGAATCTGGAAGTGGATATTTGGA

            **                         *   *      * *
     390127 TAGCTTTGAGGATTTCGTTGGAAACGGTAATGTCTTCAAAG
        131 GCGCTTTGAGGATTTCGTTGGAAACGGAAATATCTTCATAT

                *                         *   *     *      *    *            
     390168 AAAATCTAGACAGAAGCATTCTCAGAAACACCTTCGTGATATTTGCAATCAAGTCACAGAGTTGA
          1 AAAAACTAGACAGAAGCATTCTCAGAAACATCTTTGTGATGTTTGCACTCAACTCACAGAGTTGA

                 * *             *  *                  *             *       
     390233 ACCTTCCGTTTCATAGAGCAGGTTGGAAACACTCTTTTTGTAGTATCTGGAAGTGGACATTTGGA
         66 ACCTTTCTTTTCATAGAGCAGTTTTGAAACACTCTTTTTGTAGAATCTGGAAGTGGATATTTGGA

             *         ** *    * *   *            **  
     390298 GGGCTTTGTAGCCTATC-TGGAAAAAGGAAATATCTTCCCAT
        131 GCGCTTTG-AGGATTTCGTTGGAAACGGAAATATCTTCATAT

            *  ** *   *     * *            *   *  *   * **        *  * * *   
     390339 GAATGCGAGATAGAAGTAATCTCAGAAACATGTTTATGCTGTATCTACTCAACTAACTGTGCTGA
          1 AAAAACTAGACAGAAGCATTCTCAGAAACATCTTTGTGATGTTTGCACTCAACTCACAGAGTTGA

              *     *  *               *        *  * *       *               
     390404 ACATTTCTATTGATAGAGCAGTTTTGAGACACTCTTCTTTTGGAATCTGCAAGTGGATATTTGGA
         66 ACCTTTCTTTTCATAGAGCAGTTTTGAAACACTCTTTTTGTAGAATCTGGAAGTGGATATTTGGA

            ** *                        *     *     *
     390469 TAGATTTGAGGATTTCGTTGGAAACGG-GAT-T-AT-ATAC
        131 GCGCTTTGAGGATTTCGTTGGAAACGGAAATATCTTCATAT

                 *       *               *                 **  *   *         
     390506 AAAAAGTAGACAGCAGCATTCTCAGAAACTTCTTTGTGATGTTTGCATCCAGCTCTCAGAGTTGA
          1 AAAAACTAGACAGAAGCATTCTCAGAAACATCTTTGTGATGTTTGCACTCAACTCACAGAGTTGA

            * *  * *          *  *        *        *   **        *  **       
     390571 GCATTCCCTTTCATAGAGTAGGTTTGAAACCCTCTTTTTATAGTGTCTGGAAGCGGGCATTTGGA
         66 ACCTTTCTTTTCATAGAGCAGTTTTGAAACACTCTTTTTGTAGAATCTGGAAGTGGATATTTGGA

                   *    * * **  **  **          * *  *
     390636 GCGCTTTCAGGCCTATGCTTAAAATAGGAAATATCTACCTAC
        131 GCGCTTTGAGG-ATTTCGTTGGAAACGGAAATATCTTCATAT

             *                    *    *  **          ** *        *    *     
     390678 AGAAACTAGACAGAAGCATTCTGAGAATCACGTTTGTGATGTGGGTACTCAACTAACAGTGTTGA
          1 AAAAACTAGACAGAAGCATTCTCAGAAACATCTTTGTGATGTTTGCACTCAACTCACAGAGTTGA

            *  *       *   *            *   *                *   *           
     390743 TCCATTCTTTTGATACAGCAGTTTTGAACCACACTTTTTGTAGAATCTGCAAGAGGATATTTGGA
         66 ACCTTTCTTTTCATAGAGCAGTTTTGAAACACTCTTTTTGTAGAATCTGGAAGTGGATATTTGGA

            **   *                     *   *      * *
     390808 TAGCTGTGAGGATTTCGTTGGAAACGGGAATGTCTTCAAAG
        131 GCGCTTTGAGGATTTCGTTGGAAACGGAAATATCTTCATAT

                *                         *   *            *    *            
     390849 AAAATCTAGACAGAAGCATTCTCAGAAACACCTTCGTGATGTTTGCAATCAAGTCACAGAGTTGA
          1 AAAAACTAGACAGAAGCATTCTCAGAAACATCTTTGTGATGTTTGCACTCAACTCACAGAGTTGA

                 * *             *  *           *      *             *       
     390914 ACCTTCCGTTTCATAGAGCAGGTTGGAAACACTCTTATTGTAGTATCTGGAAGTGGACATTTGGA
         66 ACCTTTCTTTTCATAGAGCAGTTTTGAAACACTCTTTTTGTAGAATCTGGAAGTGGATATTTGGA

                   *   ** ** *  *   *            **  
     390979 GCGCTTTCAGGCCTATGGTGAAAAAGGAAATATCTTCCCAT
        131 GCGCTTTGAGGATTTCGTTGGAAACGGAAATATCTTCATAT

                  * * *      **      *   * *   *     ** ** *      *    *     
     391020 AAAAACGACATAGAAGCTATCTCAGGAACTTGTTTATGATGCATCTAATCAACTAACAGTGTTGA
          1 AAAAACTAGACAGAAGCATTCTCAGAAACATCTTTGTGATGTTTGCACTCAACTCACAGAGTTGA

                  * ** * *                         * *       *               
     391085 ACCTTTGTACTGACAGAGCAG-TTTGAAACACTCTTTTTTTGGAATCTGCAAGTGGATATTTGGA
         66 ACCTTTCTTTTCATAGAGCAGTTTTGAAACACTCTTTTTGTAGAATCTGGAAGTGGATATTTGGA

            *                           *   *   *    
     391149 TCGCTTTGAGGATTTCGTTGGAAACGG-GAT-GC--AATA-
        131 GCGCTTTGAGGATTTCGTTGGAAACGGAAATATCTTCATAT

                     *    *     *                 **  *   *  *    *         *
     391185 AAAAACGTACACAGCAGCATACTCAGAAA-ATACTTTGCCATATTTCCATTCAAGTCACAGAGTG
          1 AAAAAC-TAGACAGAAGCATTCTCAGAAACAT-CTTTGTGATGTTTGCACTCAACTCACAGAGTT

                *  * **            *                  *  *             *     
     391249 GAACATTCCCATTCATAGAGCAGGTTTGAAACACTCTTTTTGGAGTATCTGGAAGTGGACATTTG
         64 GAACCTTTCTTTTCATAGAGCAGTTTTGAAACACTCTTTTTGTAGAATCTGGAAGTGGATATTTG

                     ** * * ** *  *   *            **  
     391314 GAGCGCTTTCTGAACTATGGTGAAAAAGGAAATATCTTCCAAT
        129 GAGCGCTTTGAGGATTTCGTTGGAAACGGAAATATCTTCATAT

            *     *               *      * *          *  **      **  *  *    
     391357 GAAAACAAGACAGAAGCATTCTGAGAAACTTATTTGTGATGTGTGTCCTCAACAAACGGACTTGA
          1 AAAAACTAGACAGAAGCATTCTCAGAAACATCTTTGTGATGTTTGCACTCAACTCACAGAGTTGA

                   *      **  * *  *  *             *   *    * * *           
     391422 ACCTTTCGTTTCATGCAGTACTTCTGGAACACTCTTTTTGAAGATTCTGCATGCGGATATTTGGA
         66 ACCTTTCTTTTCATAGAGCAGTTTTGAAACACTCTTTTTGTAGAATCTGGAAGTGGATATTTGGA

            **                            **   *   * 
     391487 TAGCTTTGAGGATTTCGTTGGAAACGG---GCT-TACATGT
        131 GCGCTTTGAGGATTTCGTTGGAAACGGAAATATCTTCATAT

                 *       *               *        *   *    *    *       *    
     391524 AAAAATTAGACAGCAGCATTCTCAGAAACTTCTTTGTGGTGTCTGCATTCAAGTCACAGAATTGA
          1 AAAAACTAGACAGAAGCATTCTCAGAAACATCTTTGTGATGTTTGCACTCAACTCACAGAGTTGA

              * ** * **            *  * *      *       *    *        *       
     391589 ACTTCCCCTCACATAGAGCAGTTGTGCAGCACTCTATTTGTAGTATCTCGAAGTGGACATTTGGA
         66 ACCTTTCTTTTCATAGAGCAGTTTTGAAACACTCTTTTTGTAGAATCTGGAAGTGGATATTTGGA

             *         ** *    * *   *            **  
     391654 GGGCTTTGTAGCCTATC-TGGAAAAAGGAAATATCTTCCCAT
        131 GCGCTTTG-AGGATTTCGTTGGAAACGGAAATATCTTCATAT

            *  ** *   *     * *            *   *  *   * **        *  * * *   
     391695 GAATGCGAGATAGAAGTAATCTCAGAAACATGTTTATGCTGTATCTACTCAACTAACTGTGCTGA
          1 AAAAACTAGACAGAAGCATTCTCAGAAACATCTTTGTGATGTTTGCACTCAACTCACAGAGTTGA

              *     *  *               *        *  * *       *               
     391760 ACATTTCTATTGATAGAGCAGTTTTGAGACACTCTTCTTTTGGAATCTGCAAGTGGATATTTGGA
         66 ACCTTTCTTTTCATAGAGCAGTTTTGAAACACTCTTTTTGTAGAATCTGGAAGTGGATATTTGGA

            ** *                        *     *      
     391825 TAGATTTGAGGATTTCGTTGGAAACGG-GAT-T-AT-ATAT
        131 GCGCTTTGAGGATTTCGTTGGAAACGGAAATATCTTCATAT

                 *       *               *                 **  *   *         
     391862 AAAAAGTAGACAGCAGCATTCTCAGAAACTTCTTTGTGATGTTTGCATCCAGCTCTCAGAGTTGA
          1 AAAAACTAGACAGAAGCATTCTCAGAAACATCTTTGTGATGTTTGCACTCAACTCACAGAGTTGA

              *  * *          *  *        *        *   **        *  **       
     391927 ACATTCCCTTTCATAGAGTAGGTTTGAAACCCTCTTTTTATAGTGTCTGGAAGCGGGCATTTGGA
         66 ACCTTTCTTTTCATAGAGCAGTTTTGAAACACTCTTTTTGTAGAATCTGGAAGTGGATATTTGGA

                   *    * * **  **  **          * *   
     391992 GCGCTTTCAGGCCTATGCTTAAAATAGGAAATATCTACCTAT
        131 GCGCTTTGAGG-ATTTCGTTGGAAACGGAAATATCTTCATAT

             *                    *    *  **          ** *        *    *     
     392034 AGAAACTAGACAGAAGCATTCTGAGAATCACGTTTGTGATGTGGGTACTCAACTAACAGTGTTGA
          1 AAAAACTAGACAGAAGCATTCTCAGAAACATCTTTGTGATGTTTGCACTCAACTCACAGAGTTGA

            *  *       *   *            *   *                *               
     392099 TCCATTCTTTTGATACAGCAGTTTTGAACCACACTTTTTGTAGAATCTGCAAGTGGATATTTGGA
         66 ACCTTTCTTTTCATAGAGCAGTTTTGAAACACTCTTTTTGTAGAATCTGGAAGTGGATATTTGGA

            **   *                     *   *        *
     392164 TAGCTGTGAGGATTTCGTTGGAAACGGGAATGTCTTCATAG
        131 GCGCTTTGAGGATTTCGTTGGAAACGGAAATATCTTCATAT

                **                   * * * **         *  **   *   *          
     392205 AAAATTTAGACAGAAGCATTCTCAGGACCTTGATTGTGATGTGTGTTCTCCACTAACAGAGTTGA
          1 AAAAACTAGACAGAAGCATTCTCAGAAACATCTTTGTGATGTTTGCACTCAACTCACAGAGTTGA

                       * *   * *   *       *       *                         
     392270 ACCTTTCTTTTGACAGAACTGTTCTGAAACATTCTTTTTATAGAATCTGGAAGTGGATATTTGGA
         66 ACCTTTCTTTTCATAGAGCAGTTTTGAAACACTCTTTTTGTAGAATCTGGAAGTGGATATTTGGA

            **                         *          *  
     392335 AAGCTTTGAGGATTTCGTTGGAAACGGGAATATCTTCAAAT
        131 GCGCTTTGAGGATTTCGTTGGAAACGGAAATATCTTCATAT

            *   *    *            *           * *       *  *    *            
     392376 CAAATCTAGCCAGAAGCATTCTAAGAAACATCTTAGGGATGTTTACATTCAAGTCACAGAGTTGA
          1 AAAAACTAGACAGAAGCATTCTCAGAAACATCTTTGTGATGTTTGCACTCAACTCACAGAGTTGA

              *  * *     *       *         *    * *   **      *      *    *  
     392441 ACATTCCCTTTCACAGAGCAGGTTTGAAACAATCTTCTCGTACTATCTGGCAGTGGACATTTTGA
         66 ACCTTTCTTTTCATAGAGCAGTTTTGAAACACTCTTTTTGTAGAATCTGGAAGTGGATATTTGGA

              * *   *  ** ** *  *   *            ** *
     392506 GCTCCTTGGGGCCTATGCTGAAAAAGGAAATATCTTCCGAC
        131 GCGCTTTGAGGATTTCGTTGGAAACGGAAATATCTTCATAT

                                 *     *  **          *           **    *    
     392547 AAAAACTAGACAGAAGCATTCGCAGAATCACGTTTGTGATGTGTGCACTCAACTGTCAGAATTGA
          1 AAAAACTAGACAGAAGCATTCTCAGAAACATCTTTGTGATGTTTGCACTCAACTCACAGAGTTGA

                 **   ** *      *                            * *            *
     392612 ACCTTGGTTTGGACAGAGCACTTTTGAAACACTCTTTTTGTAGAATCTGCAGGTGGATATTTGGC
         66 ACCTTTCTTTTCATAGAGCAGTTTTGAAACACTCTTTTTGTAGAATCTGGAAGTGGATATTTGGA

            **                         *   *      * *
     392677 TAGCTTTGAGGATTTCGTTGGAAACGGTAATGTCTTCAAAG
        131 GCGCTTTGAGGATTTCGTTGGAAACGGAAATATCTTCATAT

                *                         *   *            *    *            
     392718 AAAATCTAGACAGAAGCATTCTCAGAAACAACTTCGTGATGTTTGCAATCAAGTCACAGAGTTGA
          1 AAAAACTAGACAGAAGCATTCTCAGAAACATCTTTGTGATGTTTGCACTCAACTCACAGAGTTGA

                 * *             *  *                  *             *       
     392783 ACCTTCCGTTTCATAGAGCAGGTTGGAAACACTCTTTTTGTAGTATCTGGAAGTGGACATTTGGA
         66 ACCTTTCTTTTCATAGAGCAGTTTTGAAACACTCTTTTTGTAGAATCTGGAAGTGGATATTTGGA

             *         ** *    * *   *            **  
     392848 GGGCTTTGTAGCCTATC-TGGAAAAAGGAAATATCTTCCCAT
        131 GCGCTTTG-AGGATTTCGTTGGAAACGGAAATATCTTCATAT

            *  ** *   *     * *            *   *  *   * **        *  * * *   
     392889 GAATGCGAGATAGAAGTAATCTCAGAAACATGTTTATGCTGTATCTACTCAACTAACTGTGCTGA
          1 AAAAACTAGACAGAAGCATTCTCAGAAACATCTTTGTGATGTTTGCACTCAACTCACAGAGTTGA

              *     *  *               *        *  * *       *               
     392954 ACATTTCTATTGATAGAGCAGTTTTGAGACACTCTTCTTTTGGAATCTGCAAGTGGATATTTGGA
         66 ACCTTTCTTTTCATAGAGCAGTTTTGAAACACTCTTTTTGTAGAATCTGGAAGTGGATATTTGGA

            ** *                        *     *      
     393019 TAGATTTGAGGATTTCGTTGGAAACGG-GAT-T-AT-ATAT
        131 GCGCTTTGAGGATTTCGTTGGAAACGGAAATATCTTCATAT

                 *       *               *                 **  *   *  *      
     393056 AAAAAGTAGACAGCAGCATTCTCAGAAACTTCTTTGTGATGTTTGCATCCAGCTCTCACAGTTGA
          1 AAAAACTAGACAGAAGCATTCTCAGAAACATCTTTGTGATGTTTGCACTCAACTCACAGAGTTGA

            * *  * *          *  *        *        *   **        *  **       
     393121 GCATTCCCTTTCATAGAGTAGGTTTGAAACCCTCTTTTTATAGTGTCTGGAAGCGGGCATTTGGA
         66 ACCTTTCTTTTCATAGAGCAGTTTTGAAACACTCTTTTTGTAGAATCTGGAAGTGGATATTTGGA

                   *    * * **  **  **          * *  *
     393186 GCGCTTTCAGGCCTATGCTTAAAATAGGAAATATCTACCTAC
        131 GCGCTTTGAGG-ATTTCGTTGGAAACGGAAATATCTTCATAT

             *                    *    *  **          ** *        *    *     
     393228 AGAAACTAGACAGAAGCATTCTGAGAATCACGTTTGTGATGTGGGTACTCAACTAACAGTGTTGA
          1 AAAAACTAGACAGAAGCATTCTCAGAAACATCTTTGTGATGTTTGCACTCAACTCACAGAGTTGA

            *  *       *   *            *   *                *               
     393293 TCCATTCTTTTGATACAGCAGTTTTGAACCACACTTTTTGTAGAATCTGCAAGTGGATATTTGGA
         66 ACCTTTCTTTTCATAGAGCAGTTTTGAAACACTCTTTTTGTAGAATCTGGAAGTGGATATTTGGA

            **   *                     *   *      * *
     393358 TAGCTGTGAGGATTTCGTTGGAAACGGTAATGTCTTCAAAG
        131 GCGCTTTGAGGATTTCGTTGGAAACGGAAATATCTTCATAT

                *                         *   *            *    *            
     393399 AAAATCTAGACAGAAGCATTCTCAGAAACACCTTCGTGATGTTTGCAATCAAGTCAC--AGTTGA
          1 AAAAACTAGACAGAAGCATTCTCAGAAACATCTTTGTGATGTTTGCACTCAACTCACAGAGTTGA

                 * *             *  *           *      *             *       
     393462 ACCTTCCGTTTCATAGAGCAGGTTGGAAACACTCTTATTGTAGTATCTGGAAGTGGACATTTGGA
         66 ACCTTTCTTTTCATAGAGCAGTTTTGAAACACTCTTTTTGTAGAATCTGGAAGTGGATATTTGGA

                   *   ** ** *  *   *            **  
     393527 GCGCTTTCAGGCCTATGGTGAAAAAGGAAATATCTTCCCAT
        131 GCGCTTTGAGGATTTCGTTGGAAACGGAAATATCTTCATAT

                  * * *      **      *   * *   *     ** ** *      *    *     
     393568 AAAAACGACATAGAAGCTATCTCAGGAACTTGTTTATGATGCATCTAATCAACTAACAGTGTTGA
          1 AAAAACTAGACAGAAGCATTCTCAGAAACATCTTTGTGATGTTTGCACTCAACTCACAGAGTTGA

                  * ** * *                         * *       *               
     393633 ACCTTTGTACTGACAGAGCAG-TTTGAAACACTCTTTTTTTGGAATCTGCAAGTGGATATTTGGA
         66 ACCTTTCTTTTCATAGAGCAGTTTTGAAACACTCTTTTTGTAGAATCTGGAAGTGGATATTTGGA

            *                           *   *   *    
     393697 TCGCTTTGAGGATTTCGTTGGAAACGG-GAT-GC--AATAT
        131 GCGCTTTGAGGATTTCGTTGGAAACGGAAATATCTTCATAT

                **  *    *     *                 **  *   *  *    *         * 
     393734 AAAACGTACACAGCAGCATACTCAGAAA-ATACTTTGCCATATTTCCATTCAAGTCACAGAGTGG
          1 AAAAACTAGACAGAAGCATTCTCAGAAACAT-CTTTGTGATGTTTGCACTCAACTCACAGAGTTG

               *  * **            *  *               *  *             *      
     393798 AACATTCCCATTCATAGAGCAGGTTGGAAACACTCTTTTTGGAGTATCTGGAAGTGGACATTTGG
         65 AACCTTTCTTTTCATAGAGCAGTTTTGAAACACTCTTTTTGTAGAATCTGGAAGTGGATATTTGG

                    ** * * ** *  *   *            **  
     393863 AGCGCTTTCTGAACTATGGTGAAAAAGGAAATATCTTCCAAT
        130 AGCGCTTTGAGGATTTCGTTGGAAACGGAAATATCTTCATAT

            *     *               * *    * *          *  **      **  *  *    
     393905 GAAAACAAGACAGAAGCATTCTGAAAAACTTATTTGTGATGTGTGTCCTCAACAAACGGACTTGA
          1 AAAAACTAGACAGAAGCATTCTCAGAAACATCTTTGTGATGTTTGCACTCAACTCACAGAGTTGA

                   *      **  * *  *  *             *   *    * * *           
     393970 ACCTTTCGTTTCATGCAGTACTTCTGGAACACTCTTTTTGAAGATTCTGCATGCGGATATTTGGA
         66 ACCTTTCTTTTCATAGAGCAGTTTTGAAACACTCTTTTTGTAGAATCTGGAAGTGGATATTTGGA

            **                            **   *   * 
     394035 TAGCTTTGAGGATTTCGTTGGAAACGG---GCT-TACATGT
        131 GCGCTTTGAGGATTTCGTTGGAAACGGAAATATCTTCATAT

                 *       *               *        *   *    *    *       *    
     394072 AAAAATTAGACAGCAGCATTCTCAGAAACTTCTTTGTGGTGTCTGCATTCAAGTCACAGAATTGA
          1 AAAAACTAGACAGAAGCATTCTCAGAAACATCTTTGTGATGTTTGCACTCAACTCACAGAGTTGA

              * ** * **            *  * *      *       *             *       
     394137 ACTTCCCCTCACATAGAGCAGTTGTGCAGCACTCTATTTGTAGTATCTGGAAGTGGACATTTGGA
         66 ACCTTTCTTTTCATAGAGCAGTTTTGAAACACTCTTTTTGTAGAATCTGGAAGTGGATATTTGGA

             *         ** *    * *   *            **  
     394202 GGGCTTTGTAGCCTATC-TGGAAAAAGGAAATATCTTCCCAT
        131 GCGCTTTG-AGGATTTCGTTGGAAACGGAAATATCTTCATAT

            *  ** *   *     * *            *   *  *   * **        *  * * *   
     394243 GAATGCGAGATAGAAGTAATCTCAGAAACATGTTTATGCTGTATCTACTCAACTAACTGTGCTGA
          1 AAAAACTAGACAGAAGCATTCTCAGAAACATCTTTGTGATGTTTGCACTCAACTCACAGAGTTGA

              *     *  *               *        *  * *       *               
     394308 ACATTTCTATTGATAGAGCAGTTTTGAGACACTCTTCTTTTGGAATCTGCAAGTGGATATTTGGA
         66 ACCTTTCTTTTCATAGAGCAGTTTTGAAACACTCTTTTTGTAGAATCTGGAAGTGGATATTTGGA

            ** *                        *     *      
     394373 TAGATTTGAGGATTTCGTTGGAAACGG-GAT-T-AT-ATAT
        131 GCGCTTTGAGGATTTCGTTGGAAACGGAAATATCTTCATAT

                 *       *               *                 **  *   *         
     394410 AAAAAGTAGACAGCAGCATTCTCAGAAACTTCTTTGTGATGTTTGCATCCAGCTCTCAGAGTTGA
          1 AAAAACTAGACAGAAGCATTCTCAGAAACATCTTTGTGATGTTTGCACTCAACTCACAGAGTTGA

              *  * *          *  *        *        *   **        *  **       
     394475 ACATTCCCTTTCATAGAGTAGGTTTGAAACCCTCTTTTTATAGTGTCTGGAAGCGGGCATTTGGA
         66 ACCTTTCTTTTCATAGAGCAGTTTTGAAACACTCTTTTTGTAGAATCTGGAAGTGGATATTTGGA

                   *    * * **  **  **          * *   
     394540 GCGCTTTCAGGCCTATGCTTAAAATAGGAAATATCTACCTAT
        131 GCGCTTTGAGG-ATTTCGTTGGAAACGGAAATATCTTCATAT

             *                    *    *  **          ** *        *    *     
     394582 AGAAACTAGACAGAAGCATTCTGAGAATCACGTTTGTGATGTGGGTACTCAACTAACAGTGTTGA
          1 AAAAACTAGACAGAAGCATTCTCAGAAACATCTTTGTGATGTTTGCACTCAACTCACAGAGTTGA

            *  *       *   *            *   *                *               
     394647 TCCATTCTTTTGATACAGCAGTTTTGAACCACACTTTTTGTAGAATCTGCAAGTGGATATTTGGA
         66 ACCTTTCTTTTCATAGAGCAGTTTTGAAACACTCTTTTTGTAGAATCTGGAAGTGGATATTTGGA

            **   *                     *   *        *
     394712 TAGCTGTGAGGATTTCGTTGGAAACGGGAATGTCTTCATAG
        131 GCGCTTTGAGGATTTCGTTGGAAACGGAAATATCTTCATAT

                **                     * * **         *  **   *   *          
     394753 AAAATTTAGACAGAAGCATTCTCAGAACCTTGATTGTGATGTGTGTTCTCCACTAACAGAGTTGA
          1 AAAAACTAGACAGAAGCATTCTCAGAAACATCTTTGTGATGTTTGCACTCAACTCACAGAGTTGA

                       * *   * *   *       *       *                         
     394818 ACCTTTCTTTTGACAGAACTGTTCTGAAACATTCTTTTTATAGAATCTGGAAGTGGATATTTGGA
         66 ACCTTTCTTTTCATAGAGCAGTTTTGAAACACTCTTTTTGTAGAATCTGGAAGTGGATATTTGGA

            **                         *          *  
     394883 AAGCTTTGAGGATTTCGTTGGAAACGGGAATATCTTCAAAT
        131 GCGCTTTGAGGATTTCGTTGGAAACGGAAATATCTTCATAT

                *    *            *           * *       *  *    *            
     394924 AAAATCTAGCCAGAAGCATTCTAAGAAACATCTTAGGGATGTTTACATTCAAGTCACAGAGTTGA
          1 AAAAACTAGACAGAAGCATTCTCAGAAACATCTTTGTGATGTTTGCACTCAACTCACAGAGTTGA

              *  * *     *       *         *    * *   **      *      *    *  
     394989 ACATTCCCTTTCACAGAGCAGGTTTGAAACAATCTTCTCGTACTATCTGGCAGTGGACATTTTGA
         66 ACCTTTCTTTTCATAGAGCAGTTTTGAAACACTCTTTTTGTAGAATCTGGAAGTGGATATTTGGA

              * *   *  ** ** *  *   *            ** *
     395054 GCTCCTTGGGGCCTATGCTGAAAAAGGAAATATCTTCCGAC
        131 GCGCTTTGAGGATTTCGTTGGAAACGGAAATATCTTCATAT

                                 *     *  **          *           **    *    
     395095 AAAAACTAGACAGAAGCATTCGCAGAATCACGTTTGTGATGTGTGCACTCAACTGTCAGAATTGA
          1 AAAAACTAGACAGAAGCATTCTCAGAAACATCTTTGTGATGTTTGCACTCAACTCACAGAGTTGA

                 **   ** *      *                            * *            *
     395160 ACCTTGGTTTGGACAGAGCACTTTTGAAACACTCTTTTTGTAGAATCTGCAGGTGGATATTTGGC
         66 ACCTTTCTTTTCATAGAGCAGTTTTGAAACACTCTTTTTGTAGAATCTGGAAGTGGATATTTGGA

            **                         *   *      * *
     395225 TAGCTTTGAGGATTTCGTTGGAAACGGTAATGTCTTCAAAG
        131 GCGCTTTGAGGATTTCGTTGGAAACGGAAATATCTTCATAT

                *                         *   *            *    *            
     395266 AAAATCTAGACAGAAGCATTCTCAGAAACAACTTCGTGATGTTTGCAATCAAGTCACAGAGTTGA
          1 AAAAACTAGACAGAAGCATTCTCAGAAACATCTTTGTGATGTTTGCACTCAACTCACAGAGTTGA

                 * *             *  *                  *             *       
     395331 ACCTTCCGTTTCATAGAGCAGGTTGGAAACACTCTTTTTGTAGTATCTGGAAGTGGACATTTGGA
         66 ACCTTTCTTTTCATAGAGCAGTTTTGAAACACTCTTTTTGTAGAATCTGGAAGTGGATATTTGGA

             *         ** *    * *   *            **  
     395396 GGGCTTTGTAGCCTATC-TGGAAAAAGGAAATATCTTCCCAT
        131 GCGCTTTG-AGGATTTCGTTGGAAACGGAAATATCTTCATAT

            *  ** *   *     * *            *   *  *   * **        *  * * *   
     395437 GAATGCGAGATAGAAGTAATCTCAGAAACATGTTTATGCTGTATCTACTCAACTAACTGTGCTGA
          1 AAAAACTAGACAGAAGCATTCTCAGAAACATCTTTGTGATGTTTGCACTCAACTCACAGAGTTGA

              *     *  *     *         *        *  * *       *               
     395502 ACATTTCTATTGATAGAACAGTTTTGAGACACTCTTCTTTTGGAATCTGCAAGTGGATATTTGGA
         66 ACCTTTCTTTTCATAGAGCAGTTTTGAAACACTCTTTTTGTAGAATCTGGAAGTGGATATTTGGA

            ** *                        *     *      
     395567 TAGATTTGAGGATTTCGTTGGAAACGG-GAT-T-AT-ATAT
        131 GCGCTTTGAGGATTTCGTTGGAAACGGAAATATCTTCATAT

                 *       *               *                 **  *   *         
     395604 AAAAAGTAGACAGCAGCATTCTCAGAAACTTCTTTGTGATGTTTGCATCCAGCTCTCAGAGTTGA
          1 AAAAACTAGACAGAAGCATTCTCAGAAACATCTTTGTGATGTTTGCACTCAACTCACAGAGTTGA

            * *  * *          *  *        *        *   **        *  **       
     395669 GCATTCCCTTTCATAGAGTAGGTTTGAAACCCTCTTTTTATAGTGTCTGGAAGCGGGCATTTGGA
         66 ACCTTTCTTTTCATAGAGCAGTTTTGAAACACTCTTTTTGTAGAATCTGGAAGTGGATATTTGGA

                   *    * * **  **  **          * *  *
     395734 GCGCTTTCAGGCCTATGCTTAAAATAGGAAATATCTACCTAC
        131 GCGCTTTGAGG-ATTTCGTTGGAAACGGAAATATCTTCATAT

             *                    *    *  **          ** *        *    *     
     395776 AGAAACTAGACAGAAGCATTCTGAGAATCACGTTTGTGATGTGGGTACTCAACTAACAGTGTTGA
          1 AAAAACTAGACAGAAGCATTCTCAGAAACATCTTTGTGATGTTTGCACTCAACTCACAGAGTTGA

            *  *       *   *            *   *       *        *               
     395841 TCCATTCTTTTGATACAGCAGTTTTGAACCACACTTTTTGGAGAATCTGCAAGTGGATATTTGGA
         66 ACCTTTCTTTTCATAGAGCAGTTTTGAAACACTCTTTTTGTAGAATCTGGAAGTGGATATTTGGA

            **   *                     *   *      * *
     395906 TAGCTGTGAGGATTTCGTTGGAAACGGTAATGTCTTCAAAG
        131 GCGCTTTGAGGATTTCGTTGGAAACGGAAATATCTTCATAT

                *                         *   *            *    *            
     395947 AAAATCTAGACAGAAGCATTCTCAGAAACACCTTCGTGATGTTTGCAATCAAGTCACAGAGTTGA
          1 AAAAACTAGACAGAAGCATTCTCAGAAACATCTTTGTGATGTTTGCACTCAACTCACAGAGTTGA

                 * *             *  *           *      *             *       
     396012 ACCTTCCGTTTCATAGAGCAGGTTGGAAACACTCTTATTGTAGTATCTGGAAGTGGACATTTGGA
         66 ACCTTTCTTTTCATAGAGCAGTTTTGAAACACTCTTTTTGTAGAATCTGGAAGTGGATATTTGGA

                   *   ** ** *  *   *            **  
     396077 GCGCTTTCAGGCCTATGGTGAAAAAGGAAATATCTTCCCAT
        131 GCGCTTTGAGGATTTCGTTGGAAACGGAAATATCTTCATAT

                  * * *      **      *   * *   *     ** ** *      *    *     
     396118 AAAAACGACATAGAAGCTATCTCAGGAACTTGTTTATGATGCATCTAATCAACTAACAGTGTTGA
          1 AAAAACTAGACAGAAGCATTCTCAGAAACATCTTTGTGATGTTTGCACTCAACTCACAGAGTTGA

                  * ** * *                         * *       *               
     396183 ACCTTTGTACTGACAGAGCAG-TTTGAAACACTCTTTTTTTGGAATCTGCAAGTGGATATTTGGA
         66 ACCTTTCTTTTCATAGAGCAGTTTTGAAACACTCTTTTTGTAGAATCTGGAAGTGGATATTTGGA

            *                           *   *   *    
     396247 TCGCTTTGAGGATTTCGTTGGAAACGG-GAT-GC--AATAT
        131 GCGCTTTGAGGATTTCGTTGGAAACGGAAATATCTTCATAT

                **  *    *     *                 **  *   *  *    *         * 
     396284 AAAACGTACACAGCAGCATACTCAGAAA-ATACTTTGCCATATTTCCATTCAAGTCACAGAGTGG
          1 AAAAACTAGACAGAAGCATTCTCAGAAACAT-CTTTGTGATGTTTGCACTCAACTCACAGAGTTG

               *  * **            *  *               *  *             *      
     396348 AACATTCCCATTCATAGAGCAGGTTGGAAACACTCTTTTTGGAGTATCTGGAAGTGGACATTTGG
         65 AACCTTTCTTTTCATAGAGCAGTTTTGAAACACTCTTTTTGTAGAATCTGGAAGTGGATATTTGG

                    ** * * ** *  *   *            **  
     396413 AGCGCTTTCTGAACTATGGTGAAAAAGGAAATATCTTCCAAT
        130 AGCGCTTTGAGGATTTCGTTGGAAACGGAAATATCTTCATAT

            *     *               *      * *          *  **      **  *  *    
     396455 GAAAACAAGACAGAAGCATTCTGAGAAACTTATTTGTGATGTGTGTCCTCAACAAACGGACTTGA
          1 AAAAACTAGACAGAAGCATTCTCAGAAACATCTTTGTGATGTTTGCACTCAACTCACAGAGTTGA

                   *      **  * *  *  *             *   *    * * *           
     396520 ACCTTTCGTTTCATGCAGTACTTCTGGAACACTCTTTTTGAAGATTCTGCATGCGGATATTTGGA
         66 ACCTTTCTTTTCATAGAGCAGTTTTGAAACACTCTTTTTGTAGAATCTGGAAGTGGATATTTGGA

            **                            **   *   * 
     396585 TAGCTTTGAGGATTTCGTTGGAAACGG---GCT-TACATGT
        131 GCGCTTTGAGGATTTCGTTGGAAACGGAAATATCTTCATAT

                 *       *               *        *   *    *    *       *    
     396622 AAAAATTAGACAGCAGCATTCTCAGAAACTTCTTTGTGGTGTCTGCATTCAAGTCACAGAATTGA
          1 AAAAACTAGACAGAAGCATTCTCAGAAACATCTTTGTGATGTTTGCACTCAACTCACAGAGTTGA

              * ** * **            *  * *      *       *             *       
     396687 ACTTCCCCTCACATAGAGCAGTTGTGCAGCACTCTATTTGTAGTATCTGGAAGTGGACATTTGGA
         66 ACCTTTCTTTTCATAGAGCAGTTTTGAAACACTCTTTTTGTAGAATCTGGAAGTGGATATTTGGA

             *         ** *    * *   *            **  
     396752 GGGCTTTGTAGCCTATC-TGGAAAAAGGAAATATCTTCCCAT
        131 GCGCTTTG-AGGATTTCGTTGGAAACGGAAATATCTTCATAT

            *  ** *   *     * *            *   *  *   * **        *  * * *   
     396793 GAATGCGAGATAGAAGTAATCTCAGAAACATGTTTATGCTGTATCTACTCAACTAACTGTGCTGA
          1 AAAAACTAGACAGAAGCATTCTCAGAAACATCTTTGTGATGTTTGCACTCAACTCACAGAGTTGA

              *     *  *               *        *  * *       *               
     396858 ACATTTCTATTGATAGAGCAGTTTTGAGACACTCTTCTTTTGGAATCTGCAAGTGGATATTTGGA
         66 ACCTTTCTTTTCATAGAGCAGTTTTGAAACACTCTTTTTGTAGAATCTGGAAGTGGATATTTGGA

            ** *                        *     *     *
     396923 TAGATTTGAGGATTTCGTTGGAAACGG-GAT-T-AT-ATAC
        131 GCGCTTTGAGGATTTCGTTGGAAACGGAAATATCTTCATAT

                 *       *               *                 **  *   *         
     396960 AAAAAGTAGACAGCAGCATTCTCAGAAACTTCTTTGTGATGTTTGCATCCAGCTCTCAGAGTTGA
          1 AAAAACTAGACAGAAGCATTCTCAGAAACATCTTTGTGATGTTTGCACTCAACTCACAGAGTTGA

            * *  * *          *  *        *        *   **        *  **       
     397025 GCATTCCCTTTCATAGAGTAGGTTTGAAACCCTCTTTTTATAGTGTCTGGAAGCGGGCATTTGGA
         66 ACCTTTCTTTTCATAGAGCAGTTTTGAAACACTCTTTTTGTAGAATCTGGAAGTGGATATTTGGA

                   *    * * **  **  **          * *  *
     397090 GCGCTTTCAGGCCTATGCTTAAAATAGGAAATATCTACCTAC
        131 GCGCTTTGAGG-ATTTCGTTGGAAACGGAAATATCTTCATAT

             *                    *    *  **          ** *        *    *     
     397132 AGAAACTAGACAGAAGCATTCTGAGAATCACGTTTGTGATGTGGGTACTCAACTAACAGTGTTGA
          1 AAAAACTAGACAGAAGCATTCTCAGAAACATCTTTGTGATGTTTGCACTCAACTCACAGAGTTGA

            *  *       *   *            *   *                *   *           
     397197 TCCATTCTTTTGATACAGCAGTTTTGAACCACACTTTTTGTAGAATCTGCAAGAGGATATTTGGA
         66 ACCTTTCTTTTCATAGAGCAGTTTTGAAACACTCTTTTTGTAGAATCTGGAAGTGGATATTTGGA

            **   *                     *   *      * *
     397262 TAGCTGTGAGGATTTCGTTGGAAACGGGAATGTCTTCAAAG
        131 GCGCTTTGAGGATTTCGTTGGAAACGGAAATATCTTCATAT

                *                         *   *            *    *            
     397303 AAAATCTAGACAGAAGCATTCTCAGAAACACCTTCGTGATGTTTGCAATCAAGTCACAGAGTTGA
          1 AAAAACTAGACAGAAGCATTCTCAGAAACATCTTTGTGATGTTTGCACTCAACTCACAGAGTTGA

                 * *             *  *           *      *             *       
     397368 ACCTTCCGTTTCATAGAGCAGGTTGGAAACACTCTTATTGTAGTATCTGGAAGTGGACATTTGGA
         66 ACCTTTCTTTTCATAGAGCAGTTTTGAAACACTCTTTTTGTAGAATCTGGAAGTGGATATTTGGA

                   *   ** ** *  *   *            **  
     397433 GCGCTTTCAGGCCTATGGTGAAAAAGGAAATATCTTCCCAT
        131 GCGCTTTGAGGATTTCGTTGGAAACGGAAATATCTTCATAT

                  * * *      **      *   * *   *     ** ** *      *    *     
     397474 AAAAACGACATAGAAGCTATCTCAGGAACTTGTTTATGATGCATCTAATCAACTAACAGTGTTGA
          1 AAAAACTAGACAGAAGCATTCTCAGAAACATCTTTGTGATGTTTGCACTCAACTCACAGAGTTGA

                  * ** * *                         * *       *               
     397539 ACCTTTGTACTGACAGAGCAG-TTTGAAACACTCTTTTTTTGGAATCTGCAAGTGGATATTTGGA
         66 ACCTTTCTTTTCATAGAGCAGTTTTGAAACACTCTTTTTGTAGAATCTGGAAGTGGATATTTGGA

            *                           *   *   *    
     397603 TCGCTTTGAGGATTTCGTTGGAAACGG-GAT-GC--AATA-
        131 GCGCTTTGAGGATTTCGTTGGAAACGGAAATATCTTCATAT

                     *    *     *                 **  *   *  *    *         *
     397639 AAAAACGTACACAGCAGCATACTCAGAAA-ATACTTTGCCATATTTCCATTCAAGTCACAGAGTG
          1 AAAAAC-TAGACAGAAGCATTCTCAGAAACAT-CTTTGTGATGTTTGCACTCAACTCACAGAGTT

                *  * **            *                  *  *             *     
     397703 GAACATTCCCATTCATAGAGCAGGTTTGAAACACTCTTTTTGGAGTATCTGGAAGTGGACATTTG
         64 GAACCTTTCTTTTCATAGAGCAGTTTTGAAACACTCTTTTTGTAGAATCTGGAAGTGGATATTTG

                     ** * * ** *  *   *            **  
     397768 GAGCGCTTTCTGAACTATGGTGAAAAAGGAAATATCTTCCAAT
        129 GAGCGCTTTGAGGATTTCGTTGGAAACGGAAATATCTTCATAT

            *     *               *      * *          *  **      **  *  *    
     397811 GAAAACAAGACAGAAGCATTCTGAGAAACTTATTTGTGATGTGTGTCCTCAACAAACGGACTTGA
          1 AAAAACTAGACAGAAGCATTCTCAGAAACATCTTTGTGATGTTTGCACTCAACTCACAGAGTTGA

                   *      **  * *  *  *             *   *    * * *           
     397876 ACCTTTCGTTTCATGCAGTACTTCTGGAACACTCTTTTTGAAGATTCTGCATGCGGATATTTGGA
         66 ACCTTTCTTTTCATAGAGCAGTTTTGAAACACTCTTTTTGTAGAATCTGGAAGTGGATATTTGGA

            **                            **   *   * 
     397941 TAGCTTTGAGGATTTCGTTGGAAACGG---GCT-TACATGT
        131 GCGCTTTGAGGATTTCGTTGGAAACGGAAATATCTTCATAT

                 *       *               *        *   *    *    *       *    
     397978 AAAAATTAGACAGCAGCATTCTCAGAAACTTCTTTGTGGTGTCTGCATTCAAGTCACAGAATTGA
          1 AAAAACTAGACAGAAGCATTCTCAGAAACATCTTTGTGATGTTTGCACTCAACTCACAGAGTTGA

              * ** * **            *  * *      *       *    *        *       
     398043 ACTTCCCCTCACATAGAGCAGTTGTGCAGCACTCTATTTGTAGTATCTCGAAGTGGACATTTGGA
         66 ACCTTTCTTTTCATAGAGCAGTTTTGAAACACTCTTTTTGTAGAATCTGGAAGTGGATATTTGGA

             *         ** *    * *   *            **  
     398108 GGGCTTTGTAGCCTATC-TGGAAAAAGGAAATATCTTCCCAT
        131 GCGCTTTG-AGGATTTCGTTGGAAACGGAAATATCTTCATAT

            *  ** *   *     * *            *   *  *   * **        *  * * *   
     398149 GAATGCGAGATAGAAGTAATCTCAGAAACATGTTTATGCTGTATCTACTCAACTAACTGTGCTGA
          1 AAAAACTAGACAGAAGCATTCTCAGAAACATCTTTGTGATGTTTGCACTCAACTCACAGAGTTGA

              *     *  *               *        *  * *       *               
     398214 ACATTTCTATTGATAGAGCAGTTTTGAGACACTCTTCTTTTGGAATCTGCAAGTGGATATTTGGA
         66 ACCTTTCTTTTCATAGAGCAGTTTTGAAACACTCTTTTTGTAGAATCTGGAAGTGGATATTTGGA

            ** *                        *     *      
     398279 TAGATTTGAGGATTTCGTTGGAAACGG-GAT-T-AT-ATAT
        131 GCGCTTTGAGGATTTCGTTGGAAACGGAAATATCTTCATAT

                 *       *               *                 **  *   *         
     398316 AAAAAGTAGACAGCAGCATTCTCAGAAACTTCTTTGTGATGTTTGCATCCAGCTCTCAGAGTTGA
          1 AAAAACTAGACAGAAGCATTCTCAGAAACATCTTTGTGATGTTTGCACTCAACTCACAGAGTTGA

              *  * *          *  *        *        *   **        *  **       
     398381 ACATTCCCTTTCATAGAGTAGGTTTGAAACCCTCTTTTTATAGTGTCTGGAAGCGGGCATTTGGA
         66 ACCTTTCTTTTCATAGAGCAGTTTTGAAACACTCTTTTTGTAGAATCTGGAAGTGGATATTTGGA

                   *    * * **  **  **          * *   
     398446 GCGCTTTCAGGCCTATGCTTAAAATAGGAAATATCTACCTAT
        131 GCGCTTTGAGG-ATTTCGTTGGAAACGGAAATATCTTCATAT

             *                    *    *  **          ** *        *    *     
     398488 AGAAACTAGACAGAAGCATTCTGAGAATCACGTTTGTGATGTGGGTACTCAACTAACAGTGTTGA
          1 AAAAACTAGACAGAAGCATTCTCAGAAACATCTTTGTGATGTTTGCACTCAACTCACAGAGTTGA

            *  *       *   *            *   *                *               
     398553 TCCATTCTTTTGATACAGCAGTTTTGAACCACACTTTTTGTAGAATCTGCAAGTGGATATTTGGA
         66 ACCTTTCTTTTCATAGAGCAGTTTTGAAACACTCTTTTTGTAGAATCTGGAAGTGGATATTTGGA

            **   *                     *   *        *
     398618 TAGCTGTGAGGATTTCGTTGGAAACGGGAATGTCTTCATAG
        131 GCGCTTTGAGGATTTCGTTGGAAACGGAAATATCTTCATAT

                **                     * * **         *  **   *   *          
     398659 AAAATTTAGACAGAAGCATTCTCAGAACCTTGATTGTGATGTGTGTTCTCCACTAACAGAGTTGA
          1 AAAAACTAGACAGAAGCATTCTCAGAAACATCTTTGTGATGTTTGCACTCAACTCACAGAGTTGA

                       * *   * *   *       *       *                         
     398724 ACCTTTCTTTTGACAGAACTGTTCTGAAACATTCTTTTTATAGAATCTGGAAGTGGATATTTGGA
         66 ACCTTTCTTTTCATAGAGCAGTTTTGAAACACTCTTTTTGTAGAATCTGGAAGTGGATATTTGGA

            **                         *          *  
     398789 AAGCTTTGAGGATTTCGTTGGAAACGGGAATATCTTCAAAT
        131 GCGCTTTGAGGATTTCGTTGGAAACGGAAATATCTTCATAT

            *   *    *            *           * *       *  *    *            
     398830 CAAATCTAGCCAGAAGCATTCTAAGAAACATCTTAGGGATGTTTACATTCAAGTCACAGAGTTGA
          1 AAAAACTAGACAGAAGCATTCTCAGAAACATCTTTGTGATGTTTGCACTCAACTCACAGAGTTGA

              *  * *     *       *         *    * *   **      *      *    *  
     398895 ACATTCCCTTTCACAGAGCAGGTTTGAAACAATCTTCTCGTACTATCTGGCAGTGGACATTTTGA
         66 ACCTTTCTTTTCATAGAGCAGTTTTGAAACACTCTTTTTGTAGAATCTGGAAGTGGATATTTGGA

              * *   *  ** ** *  *   *            ** *
     398960 GCTCCTTGGGGCCTATGCTGAAAAAGGAAATATCTTCCGAC
        131 GCGCTTTGAGGATTTCGTTGGAAACGGAAATATCTTCATAT

                                 *     *  **          *           **    *    
     399001 AAAAACTAGACAGAAGCATTCGCAGAATCACGTTTGTGATGTGTGCACTCAACTGTCAGAATTGA
          1 AAAAACTAGACAGAAGCATTCTCAGAAACATCTTTGTGATGTTTGCACTCAACTCACAGAGTTGA

                 **   ** *      *                            * *            *
     399066 ACCTTGGTTTGGACAGAGCACTTTTGAAACACTCTTTTTGTAGAATCTGCAGGTGGATATTTGGC
         66 ACCTTTCTTTTCATAGAGCAGTTTTGAAACACTCTTTTTGTAGAATCTGGAAGTGGATATTTGGA

            **                         *   *      * *
     399131 TAGCTTTGAGGATTTCGTTGGAAACGGTAATGTCTTCAAAG
        131 GCGCTTTGAGGATTTCGTTGGAAACGGAAATATCTTCATAT

                *                         *   *            *    *            
     399172 AAAATCTAGACAGAAGCATTCTCAGAAACAACTTCGTGATGTTTGCAATCAAGTCACAGAGTTGA
          1 AAAAACTAGACAGAAGCATTCTCAGAAACATCTTTGTGATGTTTGCACTCAACTCACAGAGTTGA

                 * *             *  *                  *             *       
     399237 ACCTTCCGTTTCATAGAGCAGGTTGGAAACACTCTTTTTGTAGTATCTGGAAGTGGACATTTGGA
         66 ACCTTTCTTTTCATAGAGCAGTTTTGAAACACTCTTTTTGTAGAATCTGGAAGTGGATATTTGGA

             *         ** *    * *   *            **  
     399302 GGGCTTTGTAGCCTATC-TGGAAAAAGGAAATATCTTCCCAT
        131 GCGCTTTG-AGGATTTCGTTGGAAACGGAAATATCTTCATAT

            *  ** *   *     * *            *   *  *   * **        *  * * *   
     399343 GAATGCGAGATAGAAGTAATCTCAGAAACATGTTTATGCTGTATCTACTCAACTAACTGTGCTGA
          1 AAAAACTAGACAGAAGCATTCTCAGAAACATCTTTGTGATGTTTGCACTCAACTCACAGAGTTGA

              *     *  *               *        *  * *       *               
     399408 ACATTTCTATTGATAGAGCAGTTTTGAGACACTCTTCTTTTGGAATCTGCAAGTGGATATTTGGA
         66 ACCTTTCTTTTCATAGAGCAGTTTTGAAACACTCTTTTTGTAGAATCTGGAAGTGGATATTTGGA

            ** *                        *     *      
     399473 TAGATTTGAGGATTTCGTTGGAAACGG-GAT-T-AT-ATAT
        131 GCGCTTTGAGGATTTCGTTGGAAACGGAAATATCTTCATAT

                 *       *               *                 **  *   *         
     399510 AAAAAGTAGACAGCAGCATTCTCAGAAACTTCTTTGTGATGTTTGCATCCAGCTCTCAGAGTTGA
          1 AAAAACTAGACAGAAGCATTCTCAGAAACATCTTTGTGATGTTTGCACTCAACTCACAGAGTTGA

            * *  * *          *  *        *        *   **        *  **       
     399575 GCATTCCCTTTCATAGAGTAGGTTTGAAACCCTCTTTTTATAGTGTCTGGAAGCGGGCATTTGGA
         66 ACCTTTCTTTTCATAGAGCAGTTTTGAAACACTCTTTTTGTAGAATCTGGAAGTGGATATTTGGA

                   *    * * **  **  **          * *  *
     399640 GCGCTTTCAGGCCTATGCTTAAAATAGGAAATATCTACCTAC
        131 GCGCTTTGAGG-ATTTCGTTGGAAACGGAAATATCTTCATAT

             *                    *    *  **          ** *        *    *     
     399682 AGAAACTAGACAGAAGCATTCTGAGAATCACGTTTGTGATGTGGGTACTCAACTAACAGTGTTGA
          1 AAAAACTAGACAGAAGCATTCTCAGAAACATCTTTGTGATGTTTGCACTCAACTCACAGAGTTGA

            *  *       *   *            *   *                *               
     399747 TCCATTCTTTTGATACAGCAGTTTTGAACCACACTTTTTGTAGAATCTGCAAGTGGATATTTGGA
         66 ACCTTTCTTTTCATAGAGCAGTTTTGAAACACTCTTTTTGTAGAATCTGGAAGTGGATATTTGGA

            **   *                     *   *      * *
     399812 TAGCTGTGAGGATTTCGTTGGAAACGGTAATGTCTTCAAAG
        131 GCGCTTTGAGGATTTCGTTGGAAACGGAAATATCTTCATAT

                *                         *   *            *    *            
     399853 AAAATCTAGACAGAAGCATTCTCAGAAACACCTTCGTGATGTTTGCAATCAAGTCACAGAGTTGA
          1 AAAAACTAGACAGAAGCATTCTCAGAAACATCTTTGTGATGTTTGCACTCAACTCACAGAGTTGA

                 * *             *  *           *      *             *       
     399918 ACCTTCCGTTTCATAGAGCAGGTTGGAAACACTCTTATTGTAGTATCTGGAAGTGGACATTTGGA
         66 ACCTTTCTTTTCATAGAGCAGTTTTGAAACACTCTTTTTGTAGAATCTGGAAGTGGATATTTGGA

                   *   ** ** *  *   *            **  
     399983 GCGCTTTCAGGCCTATGGTGAAAAAGGAAATATCTTCCCAT
        131 GCGCTTTGAGGATTTCGTTGGAAACGGAAATATCTTCATAT

                  * * *      **      *   * *   *     ** ** *      *    *     
     400024 AAAAACGACATAGAAGCTATCTCAGGAACTTGTTTATGATGCATCTAATCAACTAACAGTGTTGA
          1 AAAAACTAGACAGAAGCATTCTCAGAAACATCTTTGTGATGTTTGCACTCAACTCACAGAGTTGA

                  * ** * *                         * *       *               
     400089 ACCTTTGTACTGACAGAGCAG-TTTGAAACACTCTTTTTTTGGAATCTGCAAGTGGATATTTGGA
         66 ACCTTTCTTTTCATAGAGCAGTTTTGAAACACTCTTTTTGTAGAATCTGGAAGTGGATATTTGGA

            *                           *   *   *    
     400153 TCGCTTTGAGGATTTCGTTGGAAACGG-GAT-GC--AATAT
        131 GCGCTTTGAGGATTTCGTTGGAAACGGAAATATCTTCATAT

                **  *    *     *                 **  *   *  *    *         * 
     400190 AAAACGTACACAGCAGCATACTCAGAAA-ATACTTTGCCATATTTCCATTCAAGTCACAGAGTGG
          1 AAAAACTAGACAGAAGCATTCTCAGAAACAT-CTTTGTGATGTTTGCACTCAACTCACAGAGTTG

               *  * **            *  *               *  *             *      
     400254 AACATTCCCATTCATAGAGCAGGTTGGAAACACTCTTTTTGGAGTATCTGGAAGTGGACATTTGG
         65 AACCTTTCTTTTCATAGAGCAGTTTTGAAACACTCTTTTTGTAGAATCTGGAAGTGGATATTTGG

                    ** * * ** *  *   *            **  
     400319 AGCGCTTTCTGAACTATGGTGAAAAAGGAAATATCTTCCAAT
        130 AGCGCTTTGAGGATTTCGTTGGAAACGGAAATATCTTCATAT

            *     *            *  * *    * *          *  **      **  *  *    
     400361 GAAAACAAGACAGAAGCATCCTGAAAAACTTATTTGTGATGTGTGTCCTCAACAAACGGACTTGA
          1 AAAAACTAGACAGAAGCATTCTCAGAAACATCTTTGTGATGTTTGCACTCAACTCACAGAGTTGA

                   *      **  * *  *  *             *   *    * * *           
     400426 ACCTTTCGTTTCATGCAGTACTTCTGGAACACTCTTTTTGAAGATTCTGCATGCGGATATTTGGA
         66 ACCTTTCTTTTCATAGAGCAGTTTTGAAACACTCTTTTTGTAGAATCTGGAAGTGGATATTTGGA

            **                            **   *   * 
     400491 TAGCTTTGAGGATTTCGTTGGAAACGG---GCT-TACATGT
        131 GCGCTTTGAGGATTTCGTTGGAAACGGAAATATCTTCATAT

                 *       *               *        *   *    *    *       *    
     400528 AAAAATTAGACAGCAGCATTCTCAGAAACTTCTTTGTGGTGTCTGCATTCAAGTCACAGAATTGA
          1 AAAAACTAGACAGAAGCATTCTCAGAAACATCTTTGTGATGTTTGCACTCAACTCACAGAGTTGA

              * ** * **            *  * *      *       *             *       
     400593 ACTTCCCCTCACATAGAGCAGTTGTGCAGCACTCTATTTGTAGTATCTGGAAGTGGACATTTGGA
         66 ACCTTTCTTTTCATAGAGCAGTTTTGAAACACTCTTTTTGTAGAATCTGGAAGTGGATATTTGGA

             *         ** *    * *   *            **  
     400658 GGGCTTTGTAGCCTATC-TGGAAAAAGGAAATATCTTCCCAT
        131 GCGCTTTG-AGGATTTCGTTGGAAACGGAAATATCTTCATAT

            *  ** *   *     * *            *   *  *   * **        *  * * *   
     400699 GAATGCGAGATAGAAGTAATCTCAGAAACATGTTTATGCTGTATCTACTCAACTAACTGTGCTGA
          1 AAAAACTAGACAGAAGCATTCTCAGAAACATCTTTGTGATGTTTGCACTCAACTCACAGAGTTGA

              *     *  *               *        *  * *       *               
     400764 ACATTTCTATTGATAGAGCAGTTTTGAGACACTCTTCTTTTGGAATCTGCAAGTGGATATTTGGA
         66 ACCTTTCTTTTCATAGAGCAGTTTTGAAACACTCTTTTTGTAGAATCTGGAAGTGGATATTTGGA

            ** *                        *     *      
     400829 TAGATTTGAGGATTTCGTTGGAAACGG-GAT-T-AT-ATAT
        131 GCGCTTTGAGGATTTCGTTGGAAACGGAAATATCTTCATAT

                 *       *               *                 **  *   *         
     400866 AAAAAGTAGACAGCAGCATTCTCAGAAACTTCTTTGTGATGTTTGCATCCAGCTCTCAGAGTTGA
          1 AAAAACTAGACAGAAGCATTCTCAGAAACATCTTTGTGATGTTTGCACTCAACTCACAGAGTTGA

              *  * *          *  *        *        *   **        *  **       
     400931 ACATTCCCTTTCATAGAGTAGGTTTGAAACCCTCTTTTTATAGTGTCTGGAAGCGGGCATTTGGA
         66 ACCTTTCTTTTCATAGAGCAGTTTTGAAACACTCTTTTTGTAGAATCTGGAAGTGGATATTTGGA

                   *    * * **  **  **          * *   
     400996 GCGCTTTCAGGCCTATGCTTAAAATAGGAAATATCTACCTAT
        131 GCGCTTTGAGG-ATTTCGTTGGAAACGGAAATATCTTCATAT

             *                    *    *  **          ** *        *    *     
     401038 AGAAACTAGACAGAAGCATTCTGAGAATCACGTTTGTGATGTGGGTACTCAACTAACAGTGTTGA
          1 AAAAACTAGACAGAAGCATTCTCAGAAACATCTTTGTGATGTTTGCACTCAACTCACAGAGTTGA

            *  *       *   *            *   *                *               
     401103 TCCATTCTTTTGATACAGCAGTTTTGAACCACACTTTTTGTAGAATCTGCAAGTGGATATTTGGA
         66 ACCTTTCTTTTCATAGAGCAGTTTTGAAACACTCTTTTTGTAGAATCTGGAAGTGGATATTTGGA

            **   *                     *   *        *
     401168 TAGCTGTGAGGATTTCGTTGGAAACGGGAATGTCTTCATAG
        131 GCGCTTTGAGGATTTCGTTGGAAACGGAAATATCTTCATAT

                **                     * * **         *  **   *   *          
     401209 AAAATTTAGACAGAAGCATTCTCAGAACCTTGATTGTGATGTGTGTTCTCCACTAACAGAGTTGA
          1 AAAAACTAGACAGAAGCATTCTCAGAAACATCTTTGTGATGTTTGCACTCAACTCACAGAGTTGA

                       * *   * *   *       *       *                         
     401274 ACCTTTCTTTTGACAGAACTGTTCTGAAACATTCTTTTTATAGAATCTGGAAGTGGATATTTGGA
         66 ACCTTTCTTTTCATAGAGCAGTTTTGAAACACTCTTTTTGTAGAATCTGGAAGTGGATATTTGGA

            **                         *          *  
     401339 AAGCTTTGAGGATTTCGTTGGAAACGGGAATATCTTCAAAT
        131 GCGCTTTGAGGATTTCGTTGGAAACGGAAATATCTTCATAT

            *   *    *            *           * *       *  *    *            
     401380 CAAATCTAGCCAGAAGCATTCTAAGAAACATCTTAGGGATGTTTACATTCAAGTCACAGAGTTGA
          1 AAAAACTAGACAGAAGCATTCTCAGAAACATCTTTGTGATGTTTGCACTCAACTCACAGAGTTGA

              *  * *     *       *         *    * *   **      *      *    *  
     401445 ACATTCCCTTTCACAGAGCAGGTTTGAAACAATCTTCTCGTACTATCTGGCAGTGGACATTTTGA
         66 ACCTTTCTTTTCATAGAGCAGTTTTGAAACACTCTTTTTGTAGAATCTGGAAGTGGATATTTGGA

              * *   *  ** ** *  *   *            ** *
     401510 GCTCCTTGGGGCCTATGCTGAAAAAGGAAATATCTTCCGAC
        131 GCGCTTTGAGGATTTCGTTGGAAACGGAAATATCTTCATAT

                                 *     *  **          *           **    *    
     401551 AAAAACTAGACAGAAGCATTCGCAGAATCACGTTTGTGATGTGTGCACTCAACTGTCAGAATTGA
          1 AAAAACTAGACAGAAGCATTCTCAGAAACATCTTTGTGATGTTTGCACTCAACTCACAGAGTTGA

                 **   ** *      *                            * *            *
     401616 ACCTTGGTTTGGACAGAGCACTTTTGAAACACTCTTTTTGTAGAATCTGCAGGTGGATATTTGGC
         66 ACCTTTCTTTTCATAGAGCAGTTTTGAAACACTCTTTTTGTAGAATCTGGAAGTGGATATTTGGA

            **                         *   *      * *
     401681 TAGCTTTGAGGATTTCGTTGGAAACGGTAATGTCTTCAAAG
        131 GCGCTTTGAGGATTTCGTTGGAAACGGAAATATCTTCATAT

                *                         *   *            *    *            
     401722 AAAATCTAGACAGAAGCATTCTCAGAAACAACTTCGTGATGTTTGCAATCAAGTCACAGAGTTGA
          1 AAAAACTAGACAGAAGCATTCTCAGAAACATCTTTGTGATGTTTGCACTCAACTCACAGAGTTGA

                 * *             *  *                  *             *       
     401787 ACCTTCCGTTTCATAGAGCAGGTTGGAAACACTCTTTTTGTAGTATCTGGAAGTGGACATTTGGA
         66 ACCTTTCTTTTCATAGAGCAGTTTTGAAACACTCTTTTTGTAGAATCTGGAAGTGGATATTTGGA

             *         ** *    * *   *            **  
     401852 GGGCTTTGTAGCCTATC-TGGAAAAAGGAAATATCTTCCCAT
        131 GCGCTTTG-AGGATTTCGTTGGAAACGGAAATATCTTCATAT

            *  ** *   *     * *            *   *  *   * **        *  * * *   
     401893 GAATGCGAGATAGAAGTAATCTCAGAAACATGTTTATGCTGTATCTACTCAACTAACTGTGCTGA
          1 AAAAACTAGACAGAAGCATTCTCAGAAACATCTTTGTGATGTTTGCACTCAACTCACAGAGTTGA

              *     *  *     *         *        *  * *       *               
     401958 ACATTTCTATTGATAGAACAGTTTTGAGACACTCTTCTTTTGGAATCTGCAAGTGGATATTTGGA
         66 ACCTTTCTTTTCATAGAGCAGTTTTGAAACACTCTTTTTGTAGAATCTGGAAGTGGATATTTGGA

            ** *                        *     *      
     402023 TAGATTTGAGGATTTCGTTGGAAACGG-GAT-T-AT-ATAT
        131 GCGCTTTGAGGATTTCGTTGGAAACGGAAATATCTTCATAT

                 *       *               *                 **  *   *         
     402060 AAAAAGTAGACAGCAGCATTCTCAGAAACTTCTTTGTGATGTTTGCATCCAGCTCTCAGAGTTGA
          1 AAAAACTAGACAGAAGCATTCTCAGAAACATCTTTGTGATGTTTGCACTCAACTCACAGAGTTGA

            * *  * *          *  *        *        *   **        *  **       
     402125 GCATTCCCTTTCATAGAGTAGGTTTGAAACCCTCTTTTTATAGTGTCTGGAAGCGGGCATTTGGA
         66 ACCTTTCTTTTCATAGAGCAGTTTTGAAACACTCTTTTTGTAGAATCTGGAAGTGGATATTTGGA

                   *    * * **  **  **          * *  *
     402190 GCGCTTTCAGGCCTATGCTTAAAATAGGAAATATCTACCTAC
        131 GCGCTTTGAGG-ATTTCGTTGGAAACGGAAATATCTTCATAT

             *                    *    *  **          ** *        *    *     
     402232 AGAAACTAGACAGAAGCATTCTGAGAATCACGTTTGTGATGTGGGTACTCAACTAACAGTGTTGA
          1 AAAAACTAGACAGAAGCATTCTCAGAAACATCTTTGTGATGTTTGCACTCAACTCACAGAGTTGA

            *  *       *   *            *   *                *               
     402297 TCCATTCTTTTGATACAGCAGTTTTGAACCACACTTTTTGTAGAATCTGCAAGTGGATATTTGGA
         66 ACCTTTCTTTTCATAGAGCAGTTTTGAAACACTCTTTTTGTAGAATCTGGAAGTGGATATTTGGA

            **   *                     *   *      * *
     402362 TAGCTGTGAGGATTTCGTTGGAAACGGTAATGTCTTCAAAG
        131 GCGCTTTGAGGATTTCGTTGGAAACGGAAATATCTTCATAT

                *                         *   *            *    *            
     402403 AAAATCTAGACAGAAGCATTCTCAGAAACACCTTCGTGATGTTTGCAATCAAGTCACAGAGTTGA
          1 AAAAACTAGACAGAAGCATTCTCAGAAACATCTTTGTGATGTTTGCACTCAACTCACAGAGTTGA

                 * *             *  *           *      *             *       
     402468 ACCTTCCGTTTCATAGAGCAGGTTGGAAACACTCTTATTGTAGTATCTGGAAGTGGACATTTGGA
         66 ACCTTTCTTTTCATAGAGCAGTTTTGAAACACTCTTTTTGTAGAATCTGGAAGTGGATATTTGGA

                   *   ** ** *  *   *            **  
     402533 GCGCTTTCAGGCCTATGGTGAAAAAGGAAATATCTTCCCAT
        131 GCGCTTTGAGGATTTCGTTGGAAACGGAAATATCTTCATAT

                  * * *      **      *   * *   *     ** ** *      *    *     
     402574 AAAAACGACATAGAAGCTATCTCAGGAACTTGTTTATGATGCATCTAATCAACTAACAGTGTTGA
          1 AAAAACTAGACAGAAGCATTCTCAGAAACATCTTTGTGATGTTTGCACTCAACTCACAGAGTTGA

                  * ** * *                         * *       *               
     402639 ACCTTTGTACTGACAGAGCAG-TTTGAAACACTCTTTTTTTGGAATCTGCAAGTGGATATTTGGA
         66 ACCTTTCTTTTCATAGAGCAGTTTTGAAACACTCTTTTTGTAGAATCTGGAAGTGGATATTTGGA

            *                           *   *   *    
     402703 TCGCTTTGAGGATTTCGTTGGAAACGG-GAT-GC--AATAT
        131 GCGCTTTGAGGATTTCGTTGGAAACGGAAATATCTTCATAT

                **  *    *     *                 **  *   *  *    *         * 
     402740 AAAACGTACACAGCAGCATACTCAGAAA-ATACTTTGCCATATTTCCATTCAAGTCACAGAGTGG
          1 AAAAACTAGACAGAAGCATTCTCAGAAACAT-CTTTGTGATGTTTGCACTCAACTCACAGAGTTG

               *  * **            *  *               *  *             *      
     402804 AACATTCCCATTCATAGAGCAGGTTGGAAACACTCTTTTTGGAGTATCTGGAAGTGGACATTTGG
         65 AACCTTTCTTTTCATAGAGCAGTTTTGAAACACTCTTTTTGTAGAATCTGGAAGTGGATATTTGG

                    ** * * ** *  *   *            **  
     402869 AGCGCTTTCTGAACTATGGTGAAAAAGGAAATATCTTCCAAT
        130 AGCGCTTTGAGGATTTCGTTGGAAACGGAAATATCTTCATAT

            *     *               *      * *          *  **      **  *  *    
     402911 GAAAACAAGACAGAAGCATTCTGAGAAACTTATTTGTGATGTGTGTCCTCAACAAACGGACTTGA
          1 AAAAACTAGACAGAAGCATTCTCAGAAACATCTTTGTGATGTTTGCACTCAACTCACAGAGTTGA

                   *      **  * *  *  *             *   *    * * *           
     402976 ACCTTTCGTTTCATGCAGTACTTCTGGAACACTCTTTTTGAAGATTCTGCATGCGGATATTTGGA
         66 ACCTTTCTTTTCATAGAGCAGTTTTGAAACACTCTTTTTGTAGAATCTGGAAGTGGATATTTGGA

            **                            **   *   * 
     403041 TAGCTTTGAGGATTTCGTTGGAAACGG---GCT-TACATGT
        131 GCGCTTTGAGGATTTCGTTGGAAACGGAAATATCTTCATAT

                 *       *               *        *   *    *    *       *    
     403078 AAAAATTAGACAGCAGCATTCTCAGAAACTTCTTTGTGGTGTCTGCATTCAAGTCACAGAATTGA
          1 AAAAACTAGACAGAAGCATTCTCAGAAACATCTTTGTGATGTTTGCACTCAACTCACAGAGTTGA

              * ** * **            *  * *      *       *             *       
     403143 ACTTCCCCTCACATAGAGCAGTTGTGCAGCACTCTATTTGTAGTATCTGGAAGTGGACATTTGGA
         66 ACCTTTCTTTTCATAGAGCAGTTTTGAAACACTCTTTTTGTAGAATCTGGAAGTGGATATTTGGA

             *         ** *    * *   *            **  
     403208 GGGCTTTGTAGCCTATC-TGGAAAAAGGAAATATCTTCCCAT
        131 GCGCTTTG-AGGATTTCGTTGGAAACGGAAATATCTTCATAT

            *  ** *   *     * *            *   *  * * * **        *  * * *   
     403249 GAATGCGAGATAGAAGTAATCTCAGAAACATGTTTATGCTCTATCTACTCAACTAACTGTGCTGA
          1 AAAAACTAGACAGAAGCATTCTCAGAAACATCTTTGTGATGTTTGCACTCAACTCACAGAGTTGA

              *     *  *               *        *  * *       *               
     403314 ACATTTCTATTGATAGAGCAGTTTTGAGACACTCTTCTTTTGGAATCTGCAAGTGGATATTTGGA
         66 ACCTTTCTTTTCATAGAGCAGTTTTGAAACACTCTTTTTGTAGAATCTGGAAGTGGATATTTGGA

            ** *                        *     *      
     403379 TAGATTTGAGGATTTCGTTGGAAACGG-GAT-T-AT-ATAT
        131 GCGCTTTGAGGATTTCGTTGGAAACGGAAATATCTTCATAT

                 *       *               *                 **  *   *         
     403416 AAAAAGTAGACAGCAGCATTCTCAGAAACTTCTTTGTGATGTTTGCATCCAGCTCTCAGAGTTGA
          1 AAAAACTAGACAGAAGCATTCTCAGAAACATCTTTGTGATGTTTGCACTCAACTCACAGAGTTGA

              *  * *          *  *        *        *   **        *  **       
     403481 ACATTCCCTTTCATAGAGTAGGTTTGAAACCCTCTTTTTATAGTGTCTGGAAGCGGGCATTTGGA
         66 ACCTTTCTTTTCATAGAGCAGTTTTGAAACACTCTTTTTGTAGAATCTGGAAGTGGATATTTGGA

                   *    * * **  **  **          * *  *
     403546 GCGCTTTCAGGCCTATGCTTAAAATAGGAAATATCTACCTAC
        131 GCGCTTTGAGG-ATTTCGTTGGAAACGGAAATATCTTCATAT

             *                    *    *  **          ** *        *    *     
     403588 AGAAACTAGACAGAAGCATTCTGAGAATCACGTTTGTGATGTGGGTACTCAACTAACAGTGTTGA
          1 AAAAACTAGACAGAAGCATTCTCAGAAACATCTTTGTGATGTTTGCACTCAACTCACAGAGTTGA

            *  *       *   *            *   *                *   *           
     403653 TCCATTCTTTTGATACAGCAGTTTTGAACCACACTTTTTGTAGAATCTGCAAGAGGATATTTGGA
         66 ACCTTTCTTTTCATAGAGCAGTTTTGAAACACTCTTTTTGTAGAATCTGGAAGTGGATATTTGGA

            **   *                     *   *      * *
     403718 TAGCTGTGAGGATTTCGTTGGAAACGGGAATGTCTTCAAAG
        131 GCGCTTTGAGGATTTCGTTGGAAACGGAAATATCTTCATAT

                *                         *   *            *    *            
     403759 AAAATCTAGACAGAAGCATTCTCAGAAACACCTTCGTGATGTTTGCAATCAAGTCACAGAGTTGA
          1 AAAAACTAGACAGAAGCATTCTCAGAAACATCTTTGTGATGTTTGCACTCAACTCACAGAGTTGA

                 * *             *  *           *      *             *       
     403824 ACCTTCCGTTTCATAGAGCAGGTTGGAAACACTCTTATTGTAGTATCTGGAAGTGGACATTTGGA
         66 ACCTTTCTTTTCATAGAGCAGTTTTGAAACACTCTTTTTGTAGAATCTGGAAGTGGATATTTGGA

                   *   ** ** *  *   *            **  
     403889 GCGCTTTCAGGCCTATGGTGAAAAAGGAAATATCTTCCCAT
        131 GCGCTTTGAGGATTTCGTTGGAAACGGAAATATCTTCATAT

                  * * *      **      *   * *   *     ** ** *      *    *     
     403930 AAAAACGACATAGAAGCTATCTCAGGAACTTGTTTATGATGCATCTAATCAACTAACAGTGTTGA
          1 AAAAACTAGACAGAAGCATTCTCAGAAACATCTTTGTGATGTTTGCACTCAACTCACAGAGTTGA

                  * ** * *                         * *       *               
     403995 ACCTTTGTACTGACAGAGCAG-TTTGAAACACTCTTTTTTTGGAATCTGCAAGTGGATATTTGGA
         66 ACCTTTCTTTTCATAGAGCAGTTTTGAAACACTCTTTTTGTAGAATCTGGAAGTGGATATTTGGA

            *                           *   *   *    
     404059 TCGCTTTGAGGATTTCGTTGGAAACGG-GAT-GC--AATAT
        131 GCGCTTTGAGGATTTCGTTGGAAACGGAAATATCTTCATAT

                **  *    *     *                 **  *   *  *    *         * 
     404096 AAAACGTACACAGCAGCATACTCAGAAA-ATACTTTGCCATATTTCCATTCAAGTCACAGAGTGG
          1 AAAAACTAGACAGAAGCATTCTCAGAAACAT-CTTTGTGATGTTTGCACTCAACTCACAGAGTTG

               *  * **            *          *       *  *             *      
     404160 AACATTCCCATTCATAGAGCAGGTTTGAAACACACTTTTTGGAGTATCTGGAAGTGGACATTTGG
         65 AACCTTTCTTTTCATAGAGCAGTTTTGAAACACTCTTTTTGTAGAATCTGGAAGTGGATATTTGG

                    ** * * ** *  *   *            **  
     404225 AGCGCTTTCTGAACTATGGTGAAAAAGGAAATATCTTCCAAT
        130 AGCGCTTTGAGGATTTCGTTGGAAACGGAAATATCTTCATAT

            *     *        *      *      * *          *  **      **  *  *    
     404267 GAAAACAAGACAGAAACATTCTGAGAAACTTATTTGTGATGTGTGTCCTCAACAAACGGACTTGA
          1 AAAAACTAGACAGAAGCATTCTCAGAAACATCTTTGTGATGTTTGCACTCAACTCACAGAGTTGA

                   *      **  * *  *  *             *   *    * * *           
     404332 ACCTTTCGTTTCATGCAGTACTTCTGGAACACTCTTTTTGAAGATTCTGCATGCGGATATTTGGA
         66 ACCTTTCTTTTCATAGAGCAGTTTTGAAACACTCTTTTTGTAGAATCTGGAAGTGGATATTTGGA

            **                            **   *   * 
     404397 TAGCTTTGAGGATTTCGTTGGAAACGG---GCT-TACATGT
        131 GCGCTTTGAGGATTTCGTTGGAAACGGAAATATCTTCATAT

                 *       * *             *        *   *    *    *       *    
     404434 AAAAATTAGACAGCAACATTCTCAGAAACTTCTTTGTGGTGTCTGCATTCAAGTCACAGAATTGA
          1 AAAAACTAGACAGAAGCATTCTCAGAAACATCTTTGTGATGTTTGCACTCAACTCACAGAGTTGA

              * ** * **            *  * *      *       *    *        *       
     404499 ACTTCCCCTCACATAGAGCAGTTGTGCAGCACTCTATTTGTAGTATCTCGAAGTGGACATTTGGA
         66 ACCTTTCTTTTCATAGAGCAGTTTTGAAACACTCTTTTTGTAGAATCTGGAAGTGGATATTTGGA

             *         ** *    * *   *            **  
     404564 GGGCTTTGTAGCCTATC-TGGAAAAAGGAAATATCTTCCCAT
        131 GCGCTTTG-AGGATTTCGTTGGAAACGGAAATATCTTCATAT

            *  ** *   *     * *            *   *  *   * **        *  * * *   
     404605 GAATGCGAGATAGAAGTAATCTCAGAAACATGTTTATGCTGTATCTACTCAACTAACTGTGCTGA
          1 AAAAACTAGACAGAAGCATTCTCAGAAACATCTTTGTGATGTTTGCACTCAACTCACAGAGTTGA

              *     *  *               *        *  * *       *               
     404670 ACATTTCTATTGATAGAGCAGTTTTGAGACACTCTTCTTTTGGAATCTGCAAGTGGATATTTGGA
         66 ACCTTTCTTTTCATAGAGCAGTTTTGAAACACTCTTTTTGTAGAATCTGGAAGTGGATATTTGGA

            ** *                        *     *      
     404735 TAGATTTGAGGATTTCGTTGGAAACGG-GAT-T-AT-ATAT
        131 GCGCTTTGAGGATTTCGTTGGAAACGGAAATATCTTCATAT

            *    *       *               *                 **  *   *         
     404772 CAAAAGTAGACAGCAGCATTCTCAGAAACTTCTTTGTGATGTTTGCATCCAGCTCTCAGAGTTGA
          1 AAAAACTAGACAGAAGCATTCTCAGAAACATCTTTGTGATGTTTGCACTCAACTCACAGAGTTGA

              *  * *          *  *        *        *   **        *  **       
     404837 ACATTCCCTTTCATAGAGTAGGTTTGAAACCCTCTTTTTATAGTGTCTGGAAGCGGGCATTTGGA
         66 ACCTTTCTTTTCATAGAGCAGTTTTGAAACACTCTTTTTGTAGAATCTGGAAGTGGATATTTGGA

                   *    * * **  **  **          * *   
     404902 GCGCTTTCAGGCCTATGCTTAAAATAGGAAATATCTACCTAT
        131 GCGCTTTGAGG-ATTTCGTTGGAAACGGAAATATCTTCATAT

             *                    *    *  **          ** *        *    *     
     404944 AGAAACTAGACAGAAGCATTCTGAGAATCACGTTTGTGATGTGGGTACTCAACTAACAGTGTTGA
          1 AAAAACTAGACAGAAGCATTCTCAGAAACATCTTTGTGATGTTTGCACTCAACTCACAGAGTTGA

            *  *       *   *            *   *                *               
     405009 TCCATTCTTTTGATACAGCAGTTTTGAACCACACTTTTTGTAGAATCTGCAAGTGGATATTTGGA
         66 ACCTTTCTTTTCATAGAGCAGTTTTGAAACACTCTTTTTGTAGAATCTGGAAGTGGATATTTGGA

            **   *                     *   *        *
     405074 TAGCTGTGAGGATTTCGTTGGAAACGGGAATGTCTTCATAG
        131 GCGCTTTGAGGATTTCGTTGGAAACGGAAATATCTTCATAT

                **                     * * **         *  **   *   *          
     405115 AAAATTTAGACAGAAGCATTCTCAGAACCTTGATTGTGATGTGTGTTCTCCACTAACAGAGTTGA
          1 AAAAACTAGACAGAAGCATTCTCAGAAACATCTTTGTGATGTTTGCACTCAACTCACAGAGTTGA

                       * *   * *   *       *       *                         
     405180 ACCTTTCTTTTGACAGAACTGTTCTGAAACATTCTTTTTATAGAATCTGGAAGTGGATATTTGGA
         66 ACCTTTCTTTTCATAGAGCAGTTTTGAAACACTCTTTTTGTAGAATCTGGAAGTGGATATTTGGA

            **                         *          *  
     405245 AAGCTTTGAGGATTTCGTTGGAAACGGGAATATCTTCAAAT
        131 GCGCTTTGAGGATTTCGTTGGAAACGGAAATATCTTCATAT

            *   *    *            *           * *       *  *    *            
     405286 CAAATCTAGCCAGAAGCATTCTAAGAAACATCTTAGGGATGTTTACATTCAAGTCACAGAGTTGA
          1 AAAAACTAGACAGAAGCATTCTCAGAAACATCTTTGTGATGTTTGCACTCAACTCACAGAGTTGA

              *  * *     *       *         *    * *   **      *      *    *  
     405351 ACATTCCCTTTCACAGAGCAGGTTTGAAACAATCTTCTCGTACTATCTGGCAGTGGACATTTTGA
         66 ACCTTTCTTTTCATAGAGCAGTTTTGAAACACTCTTTTTGTAGAATCTGGAAGTGGATATTTGGA

              * *   *  ** ** *  *   *            ** *
     405416 GCTCCTTGGGGCCTATGCTGAAAAAGGAAATATCTTCCGAC
        131 GCGCTTTGAGGATTTCGTTGGAAACGGAAATATCTTCATAT

                                 *     *  **          *           **    *    
     405457 AAAAACTAGACAGAAGCATTCGCAGAATCACGTTTGTGATGTGTGCACTCAACTGTCAGAATTGA
          1 AAAAACTAGACAGAAGCATTCTCAGAAACATCTTTGTGATGTTTGCACTCAACTCACAGAGTTGA

                 **   ** *      *              *             * *            *
     405522 ACCTTGGTTTGGACAGAGCACTTTTGAAACACTCTCTTTGTAGAATCTGCAGGTGGATATTTGGC
         66 ACCTTTCTTTTCATAGAGCAGTTTTGAAACACTCTTTTTGTAGAATCTGGAAGTGGATATTTGGA

            **                         *   *      * *
     405587 TAGCTTTGAGGATTTCGTTGGAAACGGTAATGTCTTCAAAG
        131 GCGCTTTGAGGATTTCGTTGGAAACGGAAATATCTTCATAT

                *                         *   *     *      *    *            
     405628 AAAATCTAGACAGAAGCATTCTCAGAAACACCTTCGTGATATTTGCAATCAAGTCACAGAGTTGA
          1 AAAAACTAGACAGAAGCATTCTCAGAAACATCTTTGTGATGTTTGCACTCAACTCACAGAGTTGA

                 * *             *  *                  *             *       
     405693 ACCTTCCGTTTCATAGAGCAGGTTGGAAACACTCTTTTTGTAGTATCTGGAAGTGGACATTTGGA
         66 ACCTTTCTTTTCATAGAGCAGTTTTGAAACACTCTTTTTGTAGAATCTGGAAGTGGATATTTGGA

             *         ** *    * *   *            **  
     405758 GGGCTTTGTAGCCTATC-TGGAAAAAGGAAATATCTTCCCAT
        131 GCGCTTTG-AGGATTTCGTTGGAAACGGAAATATCTTCATAT

            *  ** *   *     * *            *   *  *   * **        *  * * *   
     405799 GAATGCGAGATAGAAGTAATCTCAGAAACATGTTTATGCTGTATCTACTCAACTAACTGTGCTGA
          1 AAAAACTAGACAGAAGCATTCTCAGAAACATCTTTGTGATGTTTGCACTCAACTCACAGAGTTGA

              *     *  *               *        *  * *       *               
     405864 ACATTTCTATTGATAGAGCAGTTTTGAGACACTCTTCTTTTGGAATCTGCAAGTGGATATTTGGA
         66 ACCTTTCTTTTCATAGAGCAGTTTTGAAACACTCTTTTTGTAGAATCTGGAAGTGGATATTTGGA

            ** *                        *     *      
     405929 TAGATTTGAGGATTTCGTTGGAAACGG-GAT-T-AT-ATAT
        131 GCGCTTTGAGGATTTCGTTGGAAACGGAAATATCTTCATAT

                 *       *               *                 **  *   *         
     405966 AAAAAGTAGACAGCAGCATTCTCAGAAACTTCTTTGTGATGTTTGCATCCAGCTCTCAGAGTTGA
          1 AAAAACTAGACAGAAGCATTCTCAGAAACATCTTTGTGATGTTTGCACTCAACTCACAGAGTTGA

            * ** * *          *  *        *        *   **        *  **       
     406031 GCAATCCCTTTCATAGAGTAGGTTTGAAACCCTCTTTTTATAGTGTCTGGAAGCGGGCATTTGGA
         66 ACCTTTCTTTTCATAGAGCAGTTTTGAAACACTCTTTTTGTAGAATCTGGAAGTGGATATTTGGA

                   *    * * **  **  **          * *  *
     406096 GCGCTTTCAGGCCTATGCTTAAAATAGGAAATATCTACCTAC
        131 GCGCTTTGAGG-ATTTCGTTGGAAACGGAAATATCTTCATAT

             *                    *    *  **          ** *        *    *     
     406138 AGAAACTAGACAGAAGCATTCTGAGAATCACGTTTGTGATGTGGGTACTCAACTAACAGTGTTGA
          1 AAAAACTAGACAGAAGCATTCTCAGAAACATCTTTGTGATGTTTGCACTCAACTCACAGAGTTGA

            *  *       *   *            *   *                *   *           
     406203 TCCATTCTTTTGATACAGCAGTTTTGAACCACACTTTTTGTAGAATCTGCAAGAGGATATTTGGA
         66 ACCTTTCTTTTCATAGAGCAGTTTTGAAACACTCTTTTTGTAGAATCTGGAAGTGGATATTTGGA

            **   *                     *   *      * *
     406268 TAGCTGTGAGGATTTCGTTGGAAACGGGAATGTCTTCAAAG
        131 GCGCTTTGAGGATTTCGTTGGAAACGGAAATATCTTCATAT

                *                         *   *            *    *            
     406309 AAAATCTAGACAGAAGCATTCTCAGAAACACCTTCGTGATGTTTGCAATCAAGTCACAGAGTTGA
          1 AAAAACTAGACAGAAGCATTCTCAGAAACATCTTTGTGATGTTTGCACTCAACTCACAGAGTTGA

                 * *             *  *           *      *             *       
     406374 ACCTTCCGTTTCATAGAGCAGGTTGGAAACACTCTTATTGTAGTATCTGGAAGTGGACATTTGGA
         66 ACCTTTCTTTTCATAGAGCAGTTTTGAAACACTCTTTTTGTAGAATCTGGAAGTGGATATTTGGA

                   *   ** ** *  *   *            **  
     406439 GCGCTTTCAGGCCTATGGTGAAAAAGGAAATATCTTCCCAT
        131 GCGCTTTGAGGATTTCGTTGGAAACGGAAATATCTTCATAT

                  * * *      **      *   * *   *     ** ** *      *    *     
     406480 AAAAACGACATAGAAGCTATCTCAGGAACTTGTTTATGATGCATCTAATCAACTAACAGTGTTGA
          1 AAAAACTAGACAGAAGCATTCTCAGAAACATCTTTGTGATGTTTGCACTCAACTCACAGAGTTGA

                  * ** * *                         * *       *               
     406545 ACCTTTGTACTGACAGAGCAG-TTTGAAACACTCTTTTTTTGGAATCTGCAAGTGGATATTTGGA
         66 ACCTTTCTTTTCATAGAGCAGTTTTGAAACACTCTTTTTGTAGAATCTGGAAGTGGATATTTGGA

            *                           *   *   *    
     406609 TCGCTTTGAGGATTTCGTTGGAAACGG-GAT-GC--AATA-
        131 GCGCTTTGAGGATTTCGTTGGAAACGGAAATATCTTCATAT

                     *    *     *                 **  *   *  *    *         *
     406645 AAAAACGTACACAGCAGCATACTCAGAAA-ATACTTTGCCATATTTCCATTCAAGTCACAGAGTG
          1 AAAAAC-TAGACAGAAGCATTCTCAGAAACAT-CTTTGTGATGTTTGCACTCAACTCACAGAGTT

                *  * **            *                  *  *             *     
     406709 GAACATTCCCATTCATAGAGCAGGTTTGAAACACTCTTTTTGGAGTATCTGGAAGTGGACATTTG
         64 GAACCTTTCTTTTCATAGAGCAGTTTTGAAACACTCTTTTTGTAGAATCTGGAAGTGGATATTTG

                     ** * * ** *  *   *            **  
     406774 GAGCGCTTTCTGAACTATGGTGAAAAAGGAAATATCTTCCAAT
        129 GAGCGCTTTGAGGATTTCGTTGGAAACGGAAATATCTTCATAT

            *     *               *      * *          *  **      **  *  *    
     406817 GAAAACAAGACAGAAGCATTCTGAGAAACTTATTTGTGATGTGTGTCCTCAACAAACGGACTTGA
          1 AAAAACTAGACAGAAGCATTCTCAGAAACATCTTTGTGATGTTTGCACTCAACTCACAGAGTTGA

                   *      **  * *  *  *             *   *    * * *           
     406882 ACCTTTCGTTTCATGCAGTACTTCTGGAACACTCTTTTTGAAGATTCTGCATGCGGATATTTGGA
         66 ACCTTTCTTTTCATAGAGCAGTTTTGAAACACTCTTTTTGTAGAATCTGGAAGTGGATATTTGGA

            **                            **   *   * 
     406947 TAGCTTTGAGGATTTCGTTGGAAACGG---GCT-TACATGT
        131 GCGCTTTGAGGATTTCGTTGGAAACGGAAATATCTTCATAT

                 *       *               *        *   *    *    *       *    
     406984 AAAAATTAGACAGCAGCATTCTCAGAAACTTCTTTGTGGTGTCTGCATTCAAGTCACAGAATTGA
          1 AAAAACTAGACAGAAGCATTCTCAGAAACATCTTTGTGATGTTTGCACTCAACTCACAGAGTTGA

              * ** * **            *  * *      *       *             *       
     407049 ACTTCCCCTCACATAGAGCAGTTGTGCAGCACTCTATTTGTAGTATCTGGAAGTGGACATTTGGA
         66 ACCTTTCTTTTCATAGAGCAGTTTTGAAACACTCTTTTTGTAGAATCTGGAAGTGGATATTTGGA

             *         ** *    * *   *            **  
     407114 GGGCTTTGTAGCCTATC-TGGAAAAAGGAAATATCTTCCCAT
        131 GCGCTTTG-AGGATTTCGTTGGAAACGGAAATATCTTCATAT

            *  ** *   *     * *            *   *  *   * **        *  * * *   
     407155 GAATGCGAGATAGAAGTAATCTCAGAAACATGTTTATGCTGTATCTACTCAACTAACTGTGCTGA
          1 AAAAACTAGACAGAAGCATTCTCAGAAACATCTTTGTGATGTTTGCACTCAACTCACAGAGTTGA

              *     *  *               *        *  * *       *               
     407220 ACATTTCTATTGATAGAGCAGTTTTGAGACACTCTTCTTTTGGAATCTGCAAGTGGATATTTGGA
         66 ACCTTTCTTTTCATAGAGCAGTTTTGAAACACTCTTTTTGTAGAATCTGGAAGTGGATATTTGGA

            ** *                        *     *      
     407285 TAGATTTGAGGATTTCGTTGGAAACGG-GAT-T-AT-ATAT
        131 GCGCTTTGAGGATTTCGTTGGAAACGGAAATATCTTCATAT

                 *       *               *                 **  *   *         
     407322 AAAAAGTAGACAGCAGCATTCTCAGAAACTTCTTTGTGATGTTTGCATCCAGCTCTCAGAGTTGA
          1 AAAAACTAGACAGAAGCATTCTCAGAAACATCTTTGTGATGTTTGCACTCAACTCACAGAGTTGA

              *  * *          *  *        *        *   **        *  **       
     407387 ACATTCCCTTTCATAGAGTAGGTTTGAAACCCTCTTTTTATAGTGTCTGGAAGCGGGCATTTGGA
         66 ACCTTTCTTTTCATAGAGCAGTTTTGAAACACTCTTTTTGTAGAATCTGGAAGTGGATATTTGGA

                   *    * * **  **  **          * *   
     407452 GCGCTTTCAGGCCTATGCTTAAAATAGGAAATATCTACCTAT
        131 GCGCTTTGAGG-ATTTCGTTGGAAACGGAAATATCTTCATAT

             *                    *    *  **          ** *        *    *     
     407494 AGAAACTAGACAGAAGCATTCTGAGAATCACGTTTGTGATGTGGGTACTCAACTAACAGTGTTGA
          1 AAAAACTAGACAGAAGCATTCTCAGAAACATCTTTGTGATGTTTGCACTCAACTCACAGAGTTGA

            *  *       *   *            *   *                *               
     407559 TCCATTCTTTTGATACAGCAGTTTTGAACCACACTTTTTGTAGAATCTGCAAGTGGATATTTGGA
         66 ACCTTTCTTTTCATAGAGCAGTTTTGAAACACTCTTTTTGTAGAATCTGGAAGTGGATATTTGGA

            **   *                     *   *        *
     407624 TAGCTGTGAGGATTTCGTTGGAAACGGGAATGTCTTCATAG
        131 GCGCTTTGAGGATTTCGTTGGAAACGGAAATATCTTCATAT

                **                     * * **         *  **   *   *          
     407665 AAAATTTAGACAGAAGCATTCTCAGAACCTTGATTGTGATGTGTGTTCTCCACTAACAGAGTTGA
          1 AAAAACTAGACAGAAGCATTCTCAGAAACATCTTTGTGATGTTTGCACTCAACTCACAGAGTTGA

                       * *   * *   *       *       *                         
     407730 ACCTTTCTTTTGACAGAACTGTTCTGAAACATTCTTTTTATAGAATCTGGAAGTGGATATTTGGA
         66 ACCTTTCTTTTCATAGAGCAGTTTTGAAACACTCTTTTTGTAGAATCTGGAAGTGGATATTTGGA

            **                         *          *  
     407795 AAGCTTTGAGGATTTCGTTGGAAACGGGAATATCTTCAAAT
        131 GCGCTTTGAGGATTTCGTTGGAAACGGAAATATCTTCATAT

                *    *            *           * *       *  *    *            
     407836 AAAATCTAGCCAGAAGCATTCTAAGAAACATCTTAGGGATGTTTACATTCAAGTCACAGAGTTGA
          1 AAAAACTAGACAGAAGCATTCTCAGAAACATCTTTGTGATGTTTGCACTCAACTCACAGAGTTGA

              *  * *     *       *         *    * *   **      *      *    *  
     407901 ACATTCCCTTTCACAGAGCAGGTTTGAAACAATCTTCTCGTACTATCTGGCAGTGGACATTTTGA
         66 ACCTTTCTTTTCATAGAGCAGTTTTGAAACACTCTTTTTGTAGAATCTGGAAGTGGATATTTGGA

              * *   *  ** ** *  *   *            ** *
     407966 GCTCCTTGGGGCCTATGCTGAAAAAGGAAATATCTTCCGAC
        131 GCGCTTTGAGGATTTCGTTGGAAACGGAAATATCTTCATAT

                                 *     *  **          *           **    *    
     408007 AAAAACTAGACAGAAGCATTCGCAGAATCACGTTTGTGATGTGTGCACTCAACTGTCAGAATTGA
          1 AAAAACTAGACAGAAGCATTCTCAGAAACATCTTTGTGATGTTTGCACTCAACTCACAGAGTTGA

                 **   ** *      *                            * *            *
     408072 ACCTTGGTTTGGAGAGAGCACTTTTGAAACACTCTTTTTGTAGAATCTGCAGGTGGATATTTGGC
         66 ACCTTTCTTTTCATAGAGCAGTTTTGAAACACTCTTTTTGTAGAATCTGGAAGTGGATATTTGGA

            **                         *   *      * *
     408137 TAGCTTTGAGGATTTCGTTGGAAACGGTAATGTCTTCAAAG
        131 GCGCTTTGAGGATTTCGTTGGAAACGGAAATATCTTCATAT

                *                         *   *            *    *            
     408178 AAAATCTAGACAGAAGCATTCTCAGAAACACCTTCGTGATGTTTGCAATCAAGTCACAGAGTTGA
          1 AAAAACTAGACAGAAGCATTCTCAGAAACATCTTTGTGATGTTTGCACTCAACTCACAGAGTTGA

                 * *             *  *                  *             *       
     408243 ACCTTCCGTTTCATAGAGCAGGTTGGAAACACTCTTTTTGTAGTATCTGGAAGTGGACATTTGGA
         66 ACCTTTCTTTTCATAGAGCAGTTTTGAAACACTCTTTTTGTAGAATCTGGAAGTGGATATTTGGA

             *         ** *    * *   *            **  
     408308 GGGCTTTGTAGCCTATC-TGGAAAAAGGAAATATCTTCCCAT
        131 GCGCTTTG-AGGATTTCGTTGGAAACGGAAATATCTTCATAT

            *  ** *   *     * *            *   *  *   * **        *  * * *   
     408349 GAATGCGAGATAGAAGTAATCTCAGAAACATGTTTATGCTGTATCTACTCAACTAACTGTGCTGA
          1 AAAAACTAGACAGAAGCATTCTCAGAAACATCTTTGTGATGTTTGCACTCAACTCACAGAGTTGA

              *     *  *               *        *  * *       *               
     408414 ACATTTCTATTGATAGAGCAGTTTTGAGACACTCTTCTTTTGGAATCTGCAAGTGGATATTTGGA
         66 ACCTTTCTTTTCATAGAGCAGTTTTGAAACACTCTTTTTGTAGAATCTGGAAGTGGATATTTGGA

             * *                        *     *      
     408479 GAGATTTGAGGATTTCGTTGGAAACGG-GAT-T-AT-ATAT
        131 GCGCTTTGAGGATTTCGTTGGAAACGGAAATATCTTCATAT

                 *       *               *                 **  *   *         
     408516 AAAAAGTAGACAGCAGCATTCTCAGAAACTTCTTTGTGATGTTTGCATCCAGCTCTCAGAGTTGA
          1 AAAAACTAGACAGAAGCATTCTCAGAAACATCTTTGTGATGTTTGCACTCAACTCACAGAGTTGA

              *  * *          *  *        *        *   **        *  **       
     408581 ACATTCCCTTTCATAGAGTAGGTTTGAAACCCTCTTTTTATAGTGTCTGGAAGCGGGCATTTGGA
         66 ACCTTTCTTTTCATAGAGCAGTTTTGAAACACTCTTTTTGTAGAATCTGGAAGTGGATATTTGGA

                   *    * * **  **  **          * *  *
     408646 GCGCTTTCAGGCCTATGCTTAAAATAGGAAATATCTACCTAC
        131 GCGCTTTGAGG-ATTTCGTTGGAAACGGAAATATCTTCATAT

             *                    *    *  **          ** *        *    *     
     408688 AGAAACTAGACAGAAGCATTCTGAGAATCACGTTTGTGATGTGGGTACTCAACTAACAGTGTTGA
          1 AAAAACTAGACAGAAGCATTCTCAGAAACATCTTTGTGATGTTTGCACTCAACTCACAGAGTTGA

            *  *       *   *            *   *                *   *           
     408753 TCCATTCTTTTGATACAGCAGTTTTGAACCACACTTTTTGTAGAATCTGCAAGAGGATATTTGGA
         66 ACCTTTCTTTTCATAGAGCAGTTTTGAAACACTCTTTTTGTAGAATCTGGAAGTGGATATTTGGA

            **   *                     *   *      * *
     408818 TAGCTGTGAGGATTTCGTTGGAAACGGGAATGTCTTCAAAG
        131 GCGCTTTGAGGATTTCGTTGGAAACGGAAATATCTTCATAT

                *                         *   *            *    *            
     408859 AAAATCTAGACAGAAGCATTCTCAGAAACACCTTCGTGATGTTTGCAATCAAGTCACAGAGTTGA
          1 AAAAACTAGACAGAAGCATTCTCAGAAACATCTTTGTGATGTTTGCACTCAACTCACAGAGTTGA

                 * *             *  *           *      *             *       
     408924 ACCTTCCGTTTCATAGAGCAGGTTGGAAACACTCTTATTGTAGTATCTGGAAGTGGACATTTGGA
         66 ACCTTTCTTTTCATAGAGCAGTTTTGAAACACTCTTTTTGTAGAATCTGGAAGTGGATATTTGGA

                   *   ** ** *  *   *            **  
     408989 GCGCTTTCAGGCCTATGGTGAAAAAGGAAATATCTTCCCAT
        131 GCGCTTTGAGGATTTCGTTGGAAACGGAAATATCTTCATAT

                  * * *      **      *   * *   *     ** ** *      *    *     
     409030 AAAAACGACATAGAAGCTATCTCAGGAACTTGTTTATGATGCATCTAATCAACTAACAGTGTTGA
          1 AAAAACTAGACAGAAGCATTCTCAGAAACATCTTTGTGATGTTTGCACTCAACTCACAGAGTTGA

                  * ** * *                         * *       *               
     409095 ACCTTTGTACTGACAGAGCAG-TTTGAAACACTCTTTTTTTGGAATCTGCAAGTGGATATTTGGA
         66 ACCTTTCTTTTCATAGAGCAGTTTTGAAACACTCTTTTTGTAGAATCTGGAAGTGGATATTTGGA

            *                           *   *   *    
     409159 TCGCTTTGAGGATTTCGTTGGAAACGG-GAT-GC--AATAT
        131 GCGCTTTGAGGATTTCGTTGGAAACGGAAATATCTTCATAT

                **  *    *     *                 **  *   *  *    *         * 
     409196 AAAACGTACACAGCAGCATACTCAGAAA-ATACTTTGCCATATTTCCATTCAAGTCACAGAGTGG
          1 AAAAACTAGACAGAAGCATTCTCAGAAACAT-CTTTGTGATGTTTGCACTCAACTCACAGAGTTG

               *  * **            *  *               *  *             *      
     409260 AACATTCCCATTCATAGAGCAGGTTGGAAACACTCTTTTTGGAGTATCTGGAAGTGGACATTTGG
         65 AACCTTTCTTTTCATAGAGCAGTTTTGAAACACTCTTTTTGTAGAATCTGGAAGTGGATATTTGG

                    ** * * ** *  *   *            **  
     409325 AGCGCTTTCTGAACTATGGTGAAAAAGGAAATATCTTCCAAT
        130 AGCGCTTTGAGGATTTCGTTGGAAACGGAAATATCTTCATAT

            *     *               *      * *          *  **      **  *  *    
     409367 GAAAACAAGACAGAAGCATTCTGAGAAACTTATTTGTGATGTGTGTCCTCAACAAACGGACTTGA
          1 AAAAACTAGACAGAAGCATTCTCAGAAACATCTTTGTGATGTTTGCACTCAACTCACAGAGTTGA

                   *      **  * *  *  *             *   *    * * *           
     409432 ACCTTTCGTTTCATGCAGTACTTCTGGAACACTCTTTTTGAAGATTCTGCATGCGGATATTTGGA
         66 ACCTTTCTTTTCATAGAGCAGTTTTGAAACACTCTTTTTGTAGAATCTGGAAGTGGATATTTGGA

            **                            **   *   * 
     409497 TAGCTTTGAGGATTTCGTTGGAAACGG---GCT-TACATGT
        131 GCGCTTTGAGGATTTCGTTGGAAACGGAAATATCTTCATAT

                 *       *               *        *   *    *    *       *    
     409534 AAAAATTAGACAGCAGCATTCTCAGAAACTTCTTTGTGGTGTCTGCATTCAAGTCACAGAATTGA
          1 AAAAACTAGACAGAAGCATTCTCAGAAACATCTTTGTGATGTTTGCACTCAACTCACAGAGTTGA

              * ** * **            *  * *      *       *             *       
     409599 ACTTCCCCTCACATAGAGCAGTTGTGCAGCACTCTATTTGTAGTATCTGGAAGTGGACATTTGGA
         66 ACCTTTCTTTTCATAGAGCAGTTTTGAAACACTCTTTTTGTAGAATCTGGAAGTGGATATTTGGA

             *         ** *    * *   *            **  
     409664 GGGCTTTGTAGCCTAT-GTGGAAAAAGGAAATATCTTCCCAT
        131 GCGCTTTG-AGGATTTCGTTGGAAACGGAAATATCTTCATAT

            *  ** *   *     * *            *   *  *   ****        *  * * *   
     409705 GAATGCGAGATAGAAGTAATCTCAGAAACATGTTTATGCTGTACCTACTCAACTAACTGTGCTGA
          1 AAAAACTAGACAGAAGCATTCTCAGAAACATCTTTGTGATGTTTGCACTCAACTCACAGAGTTGA

              *     *  *               *        *  * *       *               
     409770 ACATTTCTATTGATAGAGCAGTTTTGAGACACTCTTCTTTTGGAATCTGCAAGTGGATATTTGGA
         66 ACCTTTCTTTTCATAGAGCAGTTTTGAAACACTCTTTTTGTAGAATCTGGAAGTGGATATTTGGA

            ** *                        *     *      
     409835 TAGATTTGAGGATTTCGTTGGAAACGG-GAT-T-AT-ATAT
        131 GCGCTTTGAGGATTTCGTTGGAAACGGAAATATCTTCATAT

            *    *       *               *                 **  *   *         
     409872 CAAAAGTAGACAGCAGCATTCTCAGAAACTTCTTTGTGATGTTTGCATCCAGCTCTCAGAGTTGA
          1 AAAAACTAGACAGAAGCATTCTCAGAAACATCTTTGTGATGTTTGCACTCAACTCACAGAGTTGA

              *  * *          *  *        *        *   **        *  **       
     409937 ACATTCCCTTTCATAGAGTAGGTTTGAAACCCTCTTTTTATAGTGTCTGGAAGCGGGCATTTGGA
         66 ACCTTTCTTTTCATAGAGCAGTTTTGAAACACTCTTTTTGTAGAATCTGGAAGTGGATATTTGGA

                   *   ** ** *  *   *          * *   
     410002 GCGCTTTCAGGCCTATGCTGAAAAAGGAAATATCTACGTAT
        131 GCGCTTTGAGGATTTCGTTGGAAACGGAAATATCTTCATAT

             *                    *    *  **          ** *        *    *     
     410043 AGAAACTAGACAGAAGCATTCTGAGAATCACGTTTGTGATGTGGGTACTCAACTAACAGTGTTGA
          1 AAAAACTAGACAGAAGCATTCTCAGAAACATCTTTGTGATGTTTGCACTCAACTCACAGAGTTGA

            *  *       *   *            *   *                *               
     410108 TCCATTCTTTTGATACAGCAGTTTTGAACCACACTTTTTGTAGAATCTGCAAGTGGATATTTGGA
         66 ACCTTTCTTTTCATAGAGCAGTTTTGAAACACTCTTTTTGTAGAATCTGGAAGTGGATATTTGGA

            **   *                     *   *        *
     410173 TAGCTGTGAGGATTTCGTTGGAAACGGGAATGTCTTCATAG
        131 GCGCTTTGAGGATTTCGTTGGAAACGGAAATATCTTCATAT

                **                     * * **         *  **   *   *          
     410214 AAAATTTAGACAGAAGCATTCTCAGAACCTTGATTGTGATGTGTGTTCTCCACTAACAGAGTTGA
          1 AAAAACTAGACAGAAGCATTCTCAGAAACATCTTTGTGATGTTTGCACTCAACTCACAGAGTTGA

                       * *   * *   *       *       *                         
     410279 ACCTTTCTTTTGACAGAACTGTTCTGAAACATTCTTTTTATAGAATCTGGAAGTGGATATTTGGA
         66 ACCTTTCTTTTCATAGAGCAGTTTTGAAACACTCTTTTTGTAGAATCTGGAAGTGGATATTTGGA

            **                         *          *  
     410344 AAGCTTTGAGGATTTCGTTGGAAACGGGAATATCTTCAAAT
        131 GCGCTTTGAGGATTTCGTTGGAAACGGAAATATCTTCATAT

                *    *            *           * *       *  *    *      *     
     410385 AAAATCTAGCCAGAAGCATTCTAAGAAACATCTTAGGGATGTTTACATTCAAGTCACAGTGTTGA
          1 AAAAACTAGACAGAAGCATTCTCAGAAACATCTTTGTGATGTTTGCACTCAACTCACAGAGTTGA

              *  * *     *       *         *    * *   **      *      *    *  
     410450 ACATTCCCTTTCACAGAGCAGGTTTGAAACAATCTTCTCGTACTATCTGGCAGTGGACATTTTGA
         66 ACCTTTCTTTTCATAGAGCAGTTTTGAAACACTCTTTTTGTAGAATCTGGAAGTGGATATTTGGA

              * *   *  ** ** *  *   *            ** *
     410515 GCTCCTTGGGGCCTATGCTGAAAAAGGAAATATCTTCCGAC
        131 GCGCTTTGAGGATTTCGTTGGAAACGGAAATATCTTCATAT

                                 *     *  **          *           **    *    
     410556 AAAAACTAGACAGAAGCATTCGCAGAATCACGTTTGTGATGTGTGCACTCAACTGTCAGAATTGA
          1 AAAAACTAGACAGAAGCATTCTCAGAAACATCTTTGTGATGTTTGCACTCAACTCACAGAGTTGA

                 **   ** *      *                            * *            *
     410621 ACCTTGGTTTGGACAGAGCACTTTTGAAACACTCTTTTTGTAGAATCTGCAGGTGGATATTTGGC
         66 ACCTTTCTTTTCATAGAGCAGTTTTGAAACACTCTTTTTGTAGAATCTGGAAGTGGATATTTGGA

            **                         *   *      * *
     410686 TAGCTTTGAGGATTTCGTTGGAAACGGTAATGTCTTCAAAG
        131 GCGCTTTGAGGATTTCGTTGGAAACGGAAATATCTTCATAT

                *                         *   *            *    *            
     410727 AAAATCTAGACAGAAGCATTCTCAGAAACACCTTCGTGATGTTTGCAATCAAGTCACAGAGTTGA
          1 AAAAACTAGACAGAAGCATTCTCAGAAACATCTTTGTGATGTTTGCACTCAACTCACAGAGTTGA

                 * *             *  *                  *             *       
     410792 ACCTTCCGTTTCATAGAGCAGGTTGGAAACACTCTTTTTGTAGTATCTGGAAGTGGACATTTGGA
         66 ACCTTTCTTTTCATAGAGCAGTTTTGAAACACTCTTTTTGTAGAATCTGGAAGTGGATATTTGGA

             *         ** *    * *   *            **  
     410857 GGGCTTTGTAGCCTATC-TGGAAAAAGGAAATATCTTCCCAT
        131 GCGCTTTG-AGGATTTCGTTGGAAACGGAAATATCTTCATAT

            *  ** *   *     * *            *   *  *   * **        *  * * *   
     410898 GAATGCGAGATAGAAGTAATCTCAGAAACATGTTTATGCTGTATCTACTCAACTAACTGTGCTGA
          1 AAAAACTAGACAGAAGCATTCTCAGAAACATCTTTGTGATGTTTGCACTCAACTCACAGAGTTGA

              *     *  *               *        *  * *       *               
     410963 ACATTTCTATTGATAGAGCAGTTTTGAGACACTCTTCTTTTGGAATCTGCAAGTGGATATTTGGA
         66 ACCTTTCTTTTCATAGAGCAGTTTTGAAACACTCTTTTTGTAGAATCTGGAAGTGGATATTTGGA

            ** *                        *     *      
     411028 TAGATTTGAGGATTTCGTTGGAAACGG-GAT-T-AT-ATAT
        131 GCGCTTTGAGGATTTCGTTGGAAACGGAAATATCTTCATAT

                 *       *               *                 **  *   *         
     411065 AAAAAGTAGACAGCAGCATTCTCAGAAACTTCTTTGTGATGTTTGCATCCAGCTCTCAGAGTTGA
          1 AAAAACTAGACAGAAGCATTCTCAGAAACATCTTTGTGATGTTTGCACTCAACTCACAGAGTTGA

            * *  * *   *      *  *        *        *   **        *  **       
     411130 GCATTCCCTTTTATAGAGTAGGTTTGAAACCCTCTTTTTATAGTGTCTGGAAGCGGGCATTTGGA
         66 ACCTTTCTTTTCATAGAGCAGTTTTGAAACACTCTTTTTGTAGAATCTGGAAGTGGATATTTGGA

                   *    * * **  **  **          * *  *
     411195 GCGCTTTCAGGCCTATGCTTAAAATAGGAAATATCTACCTAC
        131 GCGCTTTGAGG-ATTTCGTTGGAAACGGAAATATCTTCATAT

             *                    *    *  **          ** *        *    *     
     411237 AGAAACTAGACAGAAGCATTCTGAGAATCACGTTTGTGATGTGGGTACTCAACTAACAGTGTTGA
          1 AAAAACTAGACAGAAGCATTCTCAGAAACATCTTTGTGATGTTTGCACTCAACTCACAGAGTTGA

            *  *       *   *            *   *                *   *           
     411302 TCCATTCTTTTGATACAGCAGTTTTGAACCACACTTTTTGTAGAATCTGCAAGAGGATATTTGGA
         66 ACCTTTCTTTTCATAGAGCAGTTTTGAAACACTCTTTTTGTAGAATCTGGAAGTGGATATTTGGA

            **   *                  *  *   *      * *
     411367 TAGCTGTGAGGATTTCGTTGGAAAGGGGAATGTCTTCAAAG
        131 GCGCTTTGAGGATTTCGTTGGAAACGGAAATATCTTCATAT

                *                         *   *            *    *            
     411408 AAAATCTAGACAGAAGCATTCTCAGAAACACCTTCGTGATGTTTGCAATCAAGTCACAGAGTTGA
          1 AAAAACTAGACAGAAGCATTCTCAGAAACATCTTTGTGATGTTTGCACTCAACTCACAGAGTTGA

                 * *             *  *           *      *             *       
     411473 ACCTTCCGTTTCATAGAGCAGGTTGGAAACACTCTTATTGTAGTATCTGGAAGTGGACATTTGGA
         66 ACCTTTCTTTTCATAGAGCAGTTTTGAAACACTCTTTTTGTAGAATCTGGAAGTGGATATTTGGA

                   *   ** ** *  *   *            **  
     411538 GCGCTTTCAGGCCTATGGTGAAAAAGGAAATATCTTCCCAT
        131 GCGCTTTGAGGATTTCGTTGGAAACGGAAATATCTTCATAT

                  * * *      **      *   * *   *     ** ** *      *    *     
     411579 AAAAACGACATAGAAGCTATCTCAGGAACTTGTTTATGATGCATCTAATCAACTAACAGTGTTGA
          1 AAAAACTAGACAGAAGCATTCTCAGAAACATCTTTGTGATGTTTGCACTCAACTCACAGAGTTGA

                  * ** * *                         * *       *               
     411644 ACCTTTGTACTGACAGAGCAG-TTTGAAACACTCTTTTTTTGGAATCTGCAAGTGGATATTTGGA
         66 ACCTTTCTTTTCATAGAGCAGTTTTGAAACACTCTTTTTGTAGAATCTGGAAGTGGATATTTGGA

            *                           *   *   *    
     411708 TCGCTTTGAGGATTTCGTTGGAAACGG-GAT-GC--AATAT
        131 GCGCTTTGAGGATTTCGTTGGAAACGGAAATATCTTCATAT

                **  *    *     *                 **  *   *  *    *         * 
     411745 AAAACGTACACAGCAGCATACTCAGAAA-ATACTTTGCCATATTTCCATTCAAGTCACAGAGTGG
          1 AAAAACTAGACAGAAGCATTCTCAGAAACAT-CTTTGTGATGTTTGCACTCAACTCACAGAGTTG

               *  * **            *  *               *  *             *      
     411809 AACATTCCCATTCATAGAGCAGGTTGGAAACACTCTTTTTGGAGTATCTGGAAGTGGACATTTGG
         65 AACCTTTCTTTTCATAGAGCAGTTTTGAAACACTCTTTTTGTAGAATCTGGAAGTGGATATTTGG

                    ** * * ** *  *   *            **  
     411874 AGCGCTTTCTGAACTATGGTGAAAAAGGAAATATCTTCCAAT
        130 AGCGCTTTGAGGATTTCGTTGGAAACGGAAATATCTTCATAT

            *     *               *      * *          *  **      **  *  *    
     411916 GAAAACAAGACAGAAGCATTCTGAGAAACTTATTTGTGATGTGTGTCCTCAACAAACGGACTTGA
          1 AAAAACTAGACAGAAGCATTCTCAGAAACATCTTTGTGATGTTTGCACTCAACTCACAGAGTTGA

                   *      **  * *  *  *             *   *    * * *           
     411981 ACCTTTCGTTTCATGCAGTACTTCTGGAACACTCTTTTTGAAGATTCTGCATGCGGATATTTGGA
         66 ACCTTTCTTTTCATAGAGCAGTTTTGAAACACTCTTTTTGTAGAATCTGGAAGTGGATATTTGGA

            **                            **   *   * 
     412046 TAGCTTTGAGGATTTCGTTGGAAACGG---GCT-TACATGT
        131 GCGCTTTGAGGATTTCGTTGGAAACGGAAATATCTTCATAT

                 *       *               *        *   *    *    *       *    
     412083 AAAAATTAGACAGCAGCATTCTCAGAAACTTCTTTGTGGTGTCTGCATTCAAGTCACAGAATTGA
          1 AAAAACTAGACAGAAGCATTCTCAGAAACATCTTTGTGATGTTTGCACTCAACTCACAGAGTTGA

              * ** * **            *  * *      *       *             *       
     412148 ACTTCCCCTCACATAGAGCAGTTGTGCAGCACTCTATTTGTAGTATCTGGAAGTGGACATTTGGA
         66 ACCTTTCTTTTCATAGAGCAGTTTTGAAACACTCTTTTTGTAGAATCTGGAAGTGGATATTTGGA

             *         ** *    * *   *            **  
     412213 GGGCTTTGTAGCCTATC-TGGAAAAAGGAAATATCTTCCCAT
        131 GCGCTTTG-AGGATTTCGTTGGAAACGGAAATATCTTCATAT

            *  ** *   *     * *            *   *  *   * **        *  * * *   
     412254 GAATGCGAGATAGAAGTAATCTCAGAAACATGTTTATGCTGTATCTACTCAACTAACTGTGCTGA
          1 AAAAACTAGACAGAAGCATTCTCAGAAACATCTTTGTGATGTTTGCACTCAACTCACAGAGTTGA

              *     *  *               *        *  * *       *               
     412319 ACATTTCTATTGATAGAGCAGTTTTGAGACACTCTTCTTTTGGAATCTGCAAGTGGATATTTGGA
         66 ACCTTTCTTTTCATAGAGCAGTTTTGAAACACTCTTTTTGTAGAATCTGGAAGTGGATATTTGGA

            ** *                        *     *      
     412384 TAGATTTGAGGATTTCGTTGGAAACGG-GAT-T-AT-ATAT
        131 GCGCTTTGAGGATTTCGTTGGAAACGGAAATATCTTCATAT

                 *       *               *                 **  *   *         
     412421 AAAAAGTAGACAGCAGCATTCTCAGAAACTTCTTTGTGATGTTTGCATCCAGCTCTCAGAGTTGA
          1 AAAAACTAGACAGAAGCATTCTCAGAAACATCTTTGTGATGTTTGCACTCAACTCACAGAGTTGA

            * *  * *   *      *  *        *        *   **        *  **       
     412486 GCATTCCCTTTTATAGAGTAGGTTTGAAACCCTCTTTTTATAGTGTCTGGAAGCGGGCATTTGGA
         66 ACCTTTCTTTTCATAGAGCAGTTTTGAAACACTCTTTTTGTAGAATCTGGAAGTGGATATTTGGA

                   *    * * **  **  **          * *  *
     412551 GCGCTTTCAGGCCTATGCTTAAAATAGGAAATATCTACCTAC
        131 GCGCTTTGAGG-ATTTCGTTGGAAACGGAAATATCTTCATAT

             *                    *    *  **          ** *        *    *     
     412593 AGAAACTAGACAGAAGCATTCTGAGAATCACGTTTGTGATGTGGGTACTCAACTAACAGTGTTGA
          1 AAAAACTAGACAGAAGCATTCTCAGAAACATCTTTGTGATGTTTGCACTCAACTCACAGAGTTGA

            *  *       *   *            *   *                *   *           
     412658 TCCATTCTTTTGATACAGCAGTTTTGAACCACACTTTTTGTAGAATCTGCAAGAGGATATTTGGA
         66 ACCTTTCTTTTCATAGAGCAGTTTTGAAACACTCTTTTTGTAGAATCTGGAAGTGGATATTTGGA

            **   *                  *  *   *      * *
     412723 TAGCTGTGAGGATTTCGTTGGAAAGGGGAATGTCTTCAAAG
        131 GCGCTTTGAGGATTTCGTTGGAAACGGAAATATCTTCATAT

                *                         *   *            *    *            
     412764 AAAATCTAGACAGAAGCATTCTCAGAAACACCTTCGTGATGTTTGCAATCAAGTCACAGAGTTGA
          1 AAAAACTAGACAGAAGCATTCTCAGAAACATCTTTGTGATGTTTGCACTCAACTCACAGAGTTGA

                 * *             *  *           *      *             *       
     412829 ACCTTCCGTTTCATAGAGCAGGTTGGAAACACTCTTATTGTAGTATCTGGAAGTGGACATTTGGA
         66 ACCTTTCTTTTCATAGAGCAGTTTTGAAACACTCTTTTTGTAGAATCTGGAAGTGGATATTTGGA

                   *   ** ** *  *   *            **  
     412894 GCGCTTTCAGGCCTATGGTGAAAAAGGAAATATCTTCCCAT
        131 GCGCTTTGAGGATTTCGTTGGAAACGGAAATATCTTCATAT

                  * * *      **      *   * *   *     ** ** *      *    *     
     412935 AAAAACGACATAGAAGCTATCTCAGGAACTTGTTTATGATGCATCTAATCAACTAACAGTGTTGA
          1 AAAAACTAGACAGAAGCATTCTCAGAAACATCTTTGTGATGTTTGCACTCAACTCACAGAGTTGA

                  * ** * *                         * *       *               
     413000 ACCTTTGTACTGACAGAGCAG-TTTGAAACACTCTTTTTTTGGAATCTGCAAGTGGATATTTGGA
         66 ACCTTTCTTTTCATAGAGCAGTTTTGAAACACTCTTTTTGTAGAATCTGGAAGTGGATATTTGGA

            *                           *   *   *    
     413064 TCGCTTTGAGGATTTCGTTGGAAACGG-GAT-GC--AATAT
        131 GCGCTTTGAGGATTTCGTTGGAAACGGAAATATCTTCATAT

                **  *    *     *                 **  *   *  *    *         * 
     413101 AAAACGTACACAGCAGCATACTCAGAAA-ATACTTTGCCATATTTCCATTCAAGTCACAGAGTGG
          1 AAAAACTAGACAGAAGCATTCTCAGAAACAT-CTTTGTGATGTTTGCACTCAACTCACAGAGTTG

               *  * **            *  *               *  *             *      
     413165 AACATTCCCATTCATAGAGCAGGTTGGAAACACTCTTTTTGGAGTATCTGGAAGTGGACATTTGG
         65 AACCTTTCTTTTCATAGAGCAGTTTTGAAACACTCTTTTTGTAGAATCTGGAAGTGGATATTTGG

                    ** * * ** *  *   *            **  
     413230 AGCGCTTTCTGAACTATGGTGAAAAAGGAAATATCTTCCAAT
        130 AGCGCTTTGAGGATTTCGTTGGAAACGGAAATATCTTCATAT

            *     *               *      * *          *  **      **  *  *    
     413272 GAAAACAAGACAGAAGCATTCTGAGAAACTTATTTGTGATGTGTGTCCTCAACAAACGGACTTGA
          1 AAAAACTAGACAGAAGCATTCTCAGAAACATCTTTGTGATGTTTGCACTCAACTCACAGAGTTGA

                   *      **  * *  *  *             *   *    * * *           
     413337 ACCTTTCGTTTCATGCAGTACTTCTGGAACACTCTTTTTGAAGATTCTGCATGCGGATATTTGGA
         66 ACCTTTCTTTTCATAGAGCAGTTTTGAAACACTCTTTTTGTAGAATCTGGAAGTGGATATTTGGA

            **                            **   *   * 
     413402 TAGCTTTGAGGATTTCGTTGGAAACGG---GCT-TACATGT
        131 GCGCTTTGAGGATTTCGTTGGAAACGGAAATATCTTCATAT

                 *       *               *        *   *    *    *       *    
     413439 AAAAATTAGACAGCAGCATTCTCAGAAACTTCTTTGTGGTGTCTGCATTCAAGTCACAGAATTGA
          1 AAAAACTAGACAGAAGCATTCTCAGAAACATCTTTGTGATGTTTGCACTCAACTCACAGAGTTGA

              * ** * **            *  * *      *       *             *       
     413504 ACTTCCCCTCACATAGAGCAGTTGTGCAGCACTCTATTTGTAGTATCTGGAAGTGGACATTTGGA
         66 ACCTTTCTTTTCATAGAGCAGTTTTGAAACACTCTTTTTGTAGAATCTGGAAGTGGATATTTGGA

             *         ** *    * *   *            **  
     413569 GGGCTTTGTAGCCTATC-TGGAAAAAGGAAATATCTTCCCAT
        131 GCGCTTTG-AGGATTTCGTTGGAAACGGAAATATCTTCATAT

            *  ** *   *     * *            *   *  *   * **        *  * * *   
     413610 GAATGCGAGATAGAAGTAATCTCAGAAACATGTTTATGCTGTATCTACTCAACTAACTGTGCTGA
          1 AAAAACTAGACAGAAGCATTCTCAGAAACATCTTTGTGATGTTTGCACTCAACTCACAGAGTTGA

              *     *  *               *        *  * *       *               
     413675 ACATTTCTATTGATAGAGCAGTTTTGAGACACTCTTCTTTTGGAATCTGCAAGTGGATATTTGGA
         66 ACCTTTCTTTTCATAGAGCAGTTTTGAAACACTCTTTTTGTAGAATCTGGAAGTGGATATTTGGA

            ** *                        *     *      
     413740 TAGATTTGAGGATTTCGTTGGAAACGG-GAT-T-AT-ATAT
        131 GCGCTTTGAGGATTTCGTTGGAAACGGAAATATCTTCATAT

                 *       *               *                 **  *   *         
     413777 AAAAAGTAGACAGCAGCATTCTCAGAAACTTCTTTGTGATGTTTGCATCCAGCTCTCAGAGTTGA
          1 AAAAACTAGACAGAAGCATTCTCAGAAACATCTTTGTGATGTTTGCACTCAACTCACAGAGTTGA

              *  * *          *  *        *        *   **        *  **       
     413842 ACATTCCCTTTCATAGAGTAGGTTTGAAACCCTCTTTTTATAGTGTCTGGAAGCGGGCATTTGGA
         66 ACCTTTCTTTTCATAGAGCAGTTTTGAAACACTCTTTTTGTAGAATCTGGAAGTGGATATTTGGA

               *   *   ** ** ** *   *          * *   
     413907 GCGATTTCAGGCCTATGCGGAAAAAGGAAATATCTACCTAT
        131 GCGCTTTGAGGATTTCGTTGGAAACGGAAATATCTTCATAT

             *                    *    *  **          ** *        *    *     
     413948 AGAAACTAGACAGAAGCATTCTGAGAATCACGTTTGTGATGTGGGTACTCAACTAACAGTGTTGA
          1 AAAAACTAGACAGAAGCATTCTCAGAAACATCTTTGTGATGTTTGCACTCAACTCACAGAGTTGA

            *  *       *   *            *   *                *               
     414013 TCCATTCTTTTGATACAGCAGTTTTGAACCACACTTTTTGTAGAATCTGCAAGTGGATATTTGGA
         66 ACCTTTCTTTTCATAGAGCAGTTTTGAAACACTCTTTTTGTAGAATCTGGAAGTGGATATTTGGA

            **   *          *          *   *        *
     414078 TAGCTGTGAGGATTTCTTTGGAAACGGGAATGTCTTCATAG
        131 GCGCTTTGAGGATTTCGTTGGAAACGGAAATATCTTCATAT

                **         *           * * **         *  **   *   *          
     414119 AAAATTTAGACAGAAACATTCTCAGAACCTTGATTGTGATGTGTGTTCTCCACTAACAGAGTTGA
          1 AAAAACTAGACAGAAGCATTCTCAGAAACATCTTTGTGATGTTTGCACTCAACTCACAGAGTTGA

                       * *   * *   *       *       *                         
     414184 ACCTTTCTTTTGACAGAACTGTTCTGAAACATTCTTTTTATAGAATCTGGAAGTGGATATTTGGA
         66 ACCTTTCTTTTCATAGAGCAGTTTTGAAACACTCTTTTTGTAGAATCTGGAAGTGGATATTTGGA

            **                         *          *  
     414249 AAGCTTTGAGGATTTCGTTGGAAACGGGAATATCTTCAAAT
        131 GCGCTTTGAGGATTTCGTTGGAAACGGAAATATCTTCATAT

            *   *    *            *           * *       *  *    *            
     414290 CAAATCTAGCCAGAAGCATTCTAAGAAACATCTTAGGGATGTTTACATTCAAGTCACAGAGTTGA
          1 AAAAACTAGACAGAAGCATTCTCAGAAACATCTTTGTGATGTTTGCACTCAACTCACAGAGTTGA

              *  * *     *       *         *    * *   **      *      *    *  
     414355 ACATTCCCTTTCACAGAGCAGGTTTGAAACAATCTTCTCGTACTATCTGGCAGTGGACATTTTGA
         66 ACCTTTCTTTTCATAGAGCAGTTTTGAAACACTCTTTTTGTAGAATCTGGAAGTGGATATTTGGA

              * *   *  ** ** *  *   *            ** *
     414420 GCTCCTTGGGGCCTATGCTGAAAAAGGAAATATCTTCCGAC
        131 GCGCTTTGAGGATTTCGTTGGAAACGGAAATATCTTCATAT

                                 *     *  **          *           **    *    
     414461 AAAAACTAGACAGAAGCATTCGCAGAATCACGTTTGTGATGTGTGCACTCAACTGTCAGAATTGA
          1 AAAAACTAGACAGAAGCATTCTCAGAAACATCTTTGTGATGTTTGCACTCAACTCACAGAGTTGA

                 **   ** *      *              *             * *            *
     414526 ACCTTGGTTTGGACAGAGCACTTTTGAAACACTCTCTTTGTAGAATCTGCAGGTGGATATTTGGC
         66 ACCTTTCTTTTCATAGAGCAGTTTTGAAACACTCTTTTTGTAGAATCTGGAAGTGGATATTTGGA

            **                         *   *      * *
     414591 TAGCTTTGAGGATTTCGTTGGAAACGGTAATGTCTTCAAAG
        131 GCGCTTTGAGGATTTCGTTGGAAACGGAAATATCTTCATAT

                *                         *   *     *      *    *            
     414632 AAAATCTAGACAGAAGCATTCTCAGAAACACCTTCGTGATATTTGCAATCAAGTCACAGAGTTGA
          1 AAAAACTAGACAGAAGCATTCTCAGAAACATCTTTGTGATGTTTGCACTCAACTCACAGAGTTGA

                 * *             *  *                  *             *       
     414697 ACCTTCCGTTTCATAGAGCAGGTTGGAAACACTCTTTTTGTAGTATCTGGAAGTGGACATTTGGA
         66 ACCTTTCTTTTCATAGAGCAGTTTTGAAACACTCTTTTTGTAGAATCTGGAAGTGGATATTTGGA

             *         ** *    * *   *            **  
     414762 GGGCTTTGTAGCCTATC-TGGAAAAAGGAAATATCTTCCCAT
        131 GCGCTTTG-AGGATTTCGTTGGAAACGGAAATATCTTCATAT

            *  ** *   *     * *            *   *  *   * **        *  * * *   
     414803 GAATGCGAGATAGAAGTAATCTCAGAAACATGTTTATGCTGTATCTACTCAACTAACTGTGCTGA
          1 AAAAACTAGACAGAAGCATTCTCAGAAACATCTTTGTGATGTTTGCACTCAACTCACAGAGTTGA

              *     *  *               *        *  * *       *               
     414868 ACATTTCTATTGATAGAGCAGTTTTGAGACACTCTTCTTTTGGAATCTGCAAGTGGATATTTGGA
         66 ACCTTTCTTTTCATAGAGCAGTTTTGAAACACTCTTTTTGTAGAATCTGGAAGTGGATATTTGGA

            ** *                        *     *      
     414933 TAGATTTGAGGATTTCGTTGGAAACGG-GAT-T-AT-ATAT
        131 GCGCTTTGAGGATTTCGTTGGAAACGGAAATATCTTCATAT

            *    *       *               *                 **  * * *         
     414970 CAAAAGTAGACAGCAGCATTCTCAGAAACTTCTTTGTGATGTTTGCATCCAGCACTCAGAGTTGA
          1 AAAAACTAGACAGAAGCATTCTCAGAAACATCTTTGTGATGTTTGCACTCAACTCACAGAGTTGA

            * *  * *          *  *        *        *   **        *  **       
     415035 GCATTCCCTTTCATAGAGTAGGTTTGAAACCCTCTTTTTATAGTGTCTGGAAGCGGGCATTTGGA
         66 ACCTTTCTTTTCATAGAGCAGTTTTGAAACACTCTTTTTGTAGAATCTGGAAGTGGATATTTGGA

                   *    * * **  **  **          * *  *
     415100 GCGCTTTCAGGCCTATGCTTAAAATAGGAAATATCTACCTAC
        131 GCGCTTTGAGG-ATTTCGTTGGAAACGGAAATATCTTCATAT

             *                    *    *  **          ** *        *    *     
     415142 AGAAACTAGACAGAAGCATTCTGAGAATCACGTTTGTGATGTGGGTACTCAACTAACAGTGTTGA
          1 AAAAACTAGACAGAAGCATTCTCAGAAACATCTTTGTGATGTTTGCACTCAACTCACAGAGTTGA

            *  *       *   *            *   *                *   *           
     415207 TCCATTCTTTTGATACAGCAGTTTTGAACCACACTTTTTGTAGAATCTGCAAGAGGATATTTGGA
         66 ACCTTTCTTTTCATAGAGCAGTTTTGAAACACTCTTTTTGTAGAATCTGGAAGTGGATATTTGGA

            **   *                     *   *      * *
     415272 TAGCTGTGAGGATTTCGTTGGAAACGGGAATGTCTTCAAAG
        131 GCGCTTTGAGGATTTCGTTGGAAACGGAAATATCTTCATAT

                *                         *   *            *    *            
     415313 AAAATCTAGACAGAAGCATTCTCAGAAACACCTTCGTGATGTTTGCAATCAAGTCACAGAGTTGA
          1 AAAAACTAGACAGAAGCATTCTCAGAAACATCTTTGTGATGTTTGCACTCAACTCACAGAGTTGA

                 * *             *  *           *      *             *       
     415378 ACCTTCCGTTTCATAGAGCAGGTTGGAAACACTCTTATTGTAGTATCTGGAAGTGGACATTTGGA
         66 ACCTTTCTTTTCATAGAGCAGTTTTGAAACACTCTTTTTGTAGAATCTGGAAGTGGATATTTGGA

                   *   ** ** *  *   *            **  
     415443 GCGCTTTCAGGCCTATGGTGAAAAAGGAAATATCTTCCCAT
        131 GCGCTTTGAGGATTTCGTTGGAAACGGAAATATCTTCATAT

                  * * *      **      *   * *   *     ** ** *      *    *     
     415484 AAAAACGACATAGAAGCTATCTCAGGAACTTGTTTATGATGCATCTAATCAACTAACAGTGTTGA
          1 AAAAACTAGACAGAAGCATTCTCAGAAACATCTTTGTGATGTTTGCACTCAACTCACAGAGTTGA

                  * ** * *                         * *       *               
     415549 ACCTTTGTACTGACAGAGCAG-TTTGAAACACTCTTTTTTTGGAATCTGCAAGTGGATATTTGGA
         66 ACCTTTCTTTTCATAGAGCAGTTTTGAAACACTCTTTTTGTAGAATCTGGAAGTGGATATTTGGA

            *                           *   *   *    
     415613 TCGCTTTGAGGATTTCGTTGGAAACGG-GAT-GC--AATA-
        131 GCGCTTTGAGGATTTCGTTGGAAACGGAAATATCTTCATAT

                     *    *     *                 **  *   *  *    *         *
     415649 AAAAACGTAAACAGCAGCATACTCAGAAA-ATACTTTGCCATATTTCCATTCAAGTCACAGAGTG
          1 AAAAAC-TAGACAGAAGCATTCTCAGAAACAT-CTTTGTGATGTTTGCACTCAACTCACAGAGTT

                *  * **            *                  *  *             *     
     415713 GAACATTCCCATTCATAGAGCAGGTTTGAAACACTCTTTTTGGAGTATCTGGAAGTGGACATTTG
         64 GAACCTTTCTTTTCATAGAGCAGTTTTGAAACACTCTTTTTGTAGAATCTGGAAGTGGATATTTG

                     ** * * ** *  *   *            **  
     415778 GAGCGCTTTCTGAACTATGGTGAAAAAGGAAATATCTTCCAAT
        129 GAGCGCTTTGAGGATTTCGTTGGAAACGGAAATATCTTCATAT

            *     *               *      * *          *  **      **  *  *    
     415821 GAAAACAAGACAGAAGCATTCTGAGAAACTTATTTGTGATGTGTGTCCTCAACAAACGGACTTGA
          1 AAAAACTAGACAGAAGCATTCTCAGAAACATCTTTGTGATGTTTGCACTCAACTCACAGAGTTGA

                   *      **  * *  *  *             *   *    * * *           
     415886 ACCTTTCGTTTCATGCAGTACTTCTGGAACACTCTTTTTGAAGATTCTGCATGCGGATATTTGGA
         66 ACCTTTCTTTTCATAGAGCAGTTTTGAAACACTCTTTTTGTAGAATCTGGAAGTGGATATTTGGA

            **                            **   *   * 
     415951 TAGCTTTGAGGATTTCGTTGGAAACGG---GCT-TACATGT
        131 GCGCTTTGAGGATTTCGTTGGAAACGGAAATATCTTCATAT

                 *       *               *        *   *    *    *       *    
     415988 AAAAATTAGACAGCAGCATTCTCAGAAACTTCTTTGTGGTGTCTGCATTCAAGTCACAGAATTGA
          1 AAAAACTAGACAGAAGCATTCTCAGAAACATCTTTGTGATGTTTGCACTCAACTCACAGAGTTGA

              * ** * **            *  * *      *       *    *    *   *       
     416053 ACTTCCCCTCACATAGAGCAGTTGTGCAGCACTCTATTTGTAGTATCTCGAAGGGGACATTTGGA
         66 ACCTTTCTTTTCATAGAGCAGTTTTGAAACACTCTTTTTGTAGAATCTGGAAGTGGATATTTGGA

             *         ** *    * *   *            **  
     416118 GGGCTTTGTAGCCTATC-TGGAAAAAGGAAATATCTTCCCAT
        131 GCGCTTTG-AGGATTTCGTTGGAAACGGAAATATCTTCATAT

            *  ** *   *     * *            *   *  *   * **        *  * * *   
     416159 GAATGCGAGATAGAAGTAATCTCAGAAACATGTTTATGCTGTATCTACTCAACTAACTGTGCTGA
          1 AAAAACTAGACAGAAGCATTCTCAGAAACATCTTTGTGATGTTTGCACTCAACTCACAGAGTTGA

              *     *  *               *        *  * *       *               
     416224 ACATTTCTATTGATAGAGCAGTTTTGAGACACTCTTCTTTTGGAATCTGCAAGTGGATATTTGGA
         66 ACCTTTCTTTTCATAGAGCAGTTTTGAAACACTCTTTTTGTAGAATCTGGAAGTGGATATTTGGA

            ** *                        *     *      
     416289 TAGATTTGAGGATTTCGTTGGAAACGG-GAT-T-AT-ATAT
        131 GCGCTTTGAGGATTTCGTTGGAAACGGAAATATCTTCATAT

                 *       *               *                 **  *   *         
     416326 AAAAAGTAGACAGCAGCATTCTCAGAAACTTCTTTGTGATGTTTGCATCCAGCTCTCAGAGTTGA
          1 AAAAACTAGACAGAAGCATTCTCAGAAACATCTTTGTGATGTTTGCACTCAACTCACAGAGTTGA

              *  * *          *  *        *        *   **        *  **       
     416391 ACATTCCCTTTCATAGAGTAGGTTTGAAACCCTCTTTTTATAGTGTCTGGAAGCGGGCATTTGGA
         66 ACCTTTCTTTTCATAGAGCAGTTTTGAAACACTCTTTTTGTAGAATCTGGAAGTGGATATTTGGA

                   *    * * **  **  **          * *   
     416456 GCGCTTTCAGGCCTATGCTTAAAATAGGAAATATCTACCTAT
        131 GCGCTTTGAGG-ATTTCGTTGGAAACGGAAATATCTTCATAT

             *                    *    *  **          ** *        *    *     
     416498 AGAAACTAGACAGAAGCATTCTGAGAATCACGTTTGTGATGTGGGTACTCAACTAACAGTGTTGA
          1 AAAAACTAGACAGAAGCATTCTCAGAAACATCTTTGTGATGTTTGCACTCAACTCACAGAGTTGA

            *  *       *   *            *   *                *               
     416563 TCCATTCTTTTGATACAGCAGTTTTGAACCACACTTTTTGTAGAATCTGCAAGTGGATATTTGGA
         66 ACCTTTCTTTTCATAGAGCAGTTTTGAAACACTCTTTTTGTAGAATCTGGAAGTGGATATTTGGA

            **   *                     *   *        *
     416628 TAGCTGTGAGGATTTCGTTGGAAACGGGAATGTCTTCATAG
        131 GCGCTTTGAGGATTTCGTTGGAAACGGAAATATCTTCATAT

                **                     * * **         *  **   *   *          
     416669 AAAATTTAGACAGAAGCATTCTCAGAACCTTGATTGTGATGTGTGTTCTCCACTAACAGAGTTGA
          1 AAAAACTAGACAGAAGCATTCTCAGAAACATCTTTGTGATGTTTGCACTCAACTCACAGAGTTGA

                       * *   * *   *       *       *                         
     416734 ACCTTTCTTTTGACAGAACTGTTCTGAAACATTCTTTTTATAGAATCTGGAAGTGGATATTTGGA
         66 ACCTTTCTTTTCATAGAGCAGTTTTGAAACACTCTTTTTGTAGAATCTGGAAGTGGATATTTGGA

            **                         *          *  
     416799 AAGCTTTGAGGATTTCGTTGGAAACGGGAATATCTTCAAAT
        131 GCGCTTTGAGGATTTCGTTGGAAACGGAAATATCTTCATAT

            *   *    *            *           * *       *  *    *            
     416840 CAAATCTAGCCAGAAGCATTCTAAGAAACATCTTAGGGATGTTTACATTCAAGTCACAGAGTTGA
          1 AAAAACTAGACAGAAGCATTCTCAGAAACATCTTTGTGATGTTTGCACTCAACTCACAGAGTTGA

              *  * *     *       *         *    * *   **      *      *    *  
     416905 ACATTCCCTTTCACAGAGCAGGTTTGAAACAATCTTCTCGTACTATCTGGCAGTGGACATTTTGA
         66 ACCTTTCTTTTCATAGAGCAGTTTTGAAACACTCTTTTTGTAGAATCTGGAAGTGGATATTTGGA

              * *   *  ** ** *  *   *            ** *
     416970 GCTCCTTGGGGCCTATGCTGAAAAAGGAAATATCTTCCGAC
        131 GCGCTTTGAGGATTTCGTTGGAAACGGAAATATCTTCATAT

                                 *     *  **          *           **    *    
     417011 AAAAACTAGACAGAAGCATTCGCAGAATCACGTTTGTGATGTGTGCACTCAACTGTCAGAATTGA
          1 AAAAACTAGACAGAAGCATTCTCAGAAACATCTTTGTGATGTTTGCACTCAACTCACAGAGTTGA

                 **   ** *      *                            * *            *
     417076 ACCTTGGTTTGGACAGAGCACTTTTGAAACACTCTTTTTGTAGAATCTGCAGGTGGATATTTGGC
         66 ACCTTTCTTTTCATAGAGCAGTTTTGAAACACTCTTTTTGTAGAATCTGGAAGTGGATATTTGGA

            **                         *   *      * *
     417141 TAGCTTTGAGGATTTCGTTGGAAACGGTAATGTCTTCAAAG
        131 GCGCTTTGAGGATTTCGTTGGAAACGGAAATATCTTCATAT

                *                         *   *            *    *            
     417182 AAAATCTAGACAGAAGCATTCTCAGAAACAACTTCGTGATGTTTGCAATCAAGTCACAGAGTTGA
          1 AAAAACTAGACAGAAGCATTCTCAGAAACATCTTTGTGATGTTTGCACTCAACTCACAGAGTTGA

                 * *             *  *                  *             *       
     417247 ACCTTCCGTTTCATAGAGCAGGTTGGAAACACTCTTTTTGTAGTATCTGGAAGTGGACATTTGGA
         66 ACCTTTCTTTTCATAGAGCAGTTTTGAAACACTCTTTTTGTAGAATCTGGAAGTGGATATTTGGA

             *         ** *    * *   *            **  
     417312 GGGCTTTGTAGCCTATC-TGGAAAAAGGAAATATCTTCCCAT
        131 GCGCTTTG-AGGATTTCGTTGGAAACGGAAATATCTTCATAT

            *  ** *   *     * *            *   *  *   * **        *  * * *   
     417353 GAATGCGAGATAGAAGTAATCTCAGAAACATGTTTATGCTGTATCTACTCAACTAACTGTGCTGA
          1 AAAAACTAGACAGAAGCATTCTCAGAAACATCTTTGTGATGTTTGCACTCAACTCACAGAGTTGA

              *     *  *               *        *  * *       *               
     417418 ACATTTCTATTGATAGAGCAGTTTTGAGACACTCTTCTTTTGGAATCTGCAAGTGGATATTTGGA
         66 ACCTTTCTTTTCATAGAGCAGTTTTGAAACACTCTTTTTGTAGAATCTGGAAGTGGATATTTGGA

            ** *                        *     *      
     417483 TAGATTTGAGGATTTCGTTGGAAACGG-GAT-T-AT-ATAT
        131 GCGCTTTGAGGATTTCGTTGGAAACGGAAATATCTTCATAT

                 *       *               *                 **  *   *         
     417520 AAAAAGTAGACAGCAGCATTCTCAGAAACTTCTTTGTGATGTTTGCATCCAGCTCTCAGAGTTGA
          1 AAAAACTAGACAGAAGCATTCTCAGAAACATCTTTGTGATGTTTGCACTCAACTCACAGAGTTGA

            * *  * *          *  *        *        *   **        *  **       
     417585 GCATTCCCTTTCATAGAGTAGGTTTGAAACCCTCTTTTTATAGTGTCTGGAAGCGGGCATTTGGA
         66 ACCTTTCTTTTCATAGAGCAGTTTTGAAACACTCTTTTTGTAGAATCTGGAAGTGGATATTTGGA

                   *    * * **  **  **          * *  *
     417650 GCGCTTTCAGGCCTATGCTTAAAATAGGAAATATCTACCTAC
        131 GCGCTTTGAGG-ATTTCGTTGGAAACGGAAATATCTTCATAT

             *                    *    *  **          ** *        *    *     
     417692 AGAAACTAGACAGAAGCATTCTGAGAATCACGTTTGTGATGTGGGTACTCAACTAACAGTGTTGA
          1 AAAAACTAGACAGAAGCATTCTCAGAAACATCTTTGTGATGTTTGCACTCAACTCACAGAGTTGA

            *  *       *   *            *   *                *               
     417757 TCCATTCTTTTGATACAGCAGTTTTGAACCACACTTTTTGTAGAATCTGCAAGTGGATATTTGGA
         66 ACCTTTCTTTTCATAGAGCAGTTTTGAAACACTCTTTTTGTAGAATCTGGAAGTGGATATTTGGA

            **   *                     *   *      * *
     417822 TAGCTGTGAGGATTTCGTTGGAAACGGGAATGTCTTCAAAG
        131 GCGCTTTGAGGATTTCGTTGGAAACGGAAATATCTTCATAT

                *                         *   *            *    *            
     417863 AAAATCTAGACAGAAGCATTCTCAGAAACACCTTCGTGATGTTTGCAATCAAGTCACAGAGTTGA
          1 AAAAACTAGACAGAAGCATTCTCAGAAACATCTTTGTGATGTTTGCACTCAACTCACAGAGTTGA

                 * *             *  *           *      *             *       
     417928 ACCTTCCGTTTCATAGAGCAGGTTGGAAACACTCTTATTGTAGTATCTGGAAGTGGACATTTGGA
         66 ACCTTTCTTTTCATAGAGCAGTTTTGAAACACTCTTTTTGTAGAATCTGGAAGTGGATATTTGGA

                   *   ** ** *  *   *            **  
     417993 GCGCTTTCAGGCCTATGGTGAAAAAGGAAATATCTTCCCAT
        131 GCGCTTTGAGGATTTCGTTGGAAACGGAAATATCTTCATAT

                  * * *      **      *   * *   *     ** ** *      *    *     
     418034 AAAAACGACATAGAAGCTATCTCAGGAACTTGTTTATGATGCATCTAATCAACTAACAGTGTTGA
          1 AAAAACTAGACAGAAGCATTCTCAGAAACATCTTTGTGATGTTTGCACTCAACTCACAGAGTTGA

                  * ** * *                         * *       *               
     418099 ACCTTTGTACTGACAGAGCAG-TTTGAAACACTCTTTTTTTGGAATCTGCAAGTGGATATTTGGA
         66 ACCTTTCTTTTCATAGAGCAGTTTTGAAACACTCTTTTTGTAGAATCTGGAAGTGGATATTTGGA

            *                           *   *   *    
     418163 TCGCTTTGAGGATTTCGTTGGAAACGG-GAT-GC--AATAT
        131 GCGCTTTGAGGATTTCGTTGGAAACGGAAATATCTTCATAT

               ***  *    *     *                 **  *   *  *    *         * 
     418200 AAATCGTACACAGCAGCATACTCAGAAA-ATACTTTGCCATATTTCCATTCAAGTCACAGAGTGG
          1 AAAAACTAGACAGAAGCATTCTCAGAAACAT-CTTTGTGATGTTTGCACTCAACTCACAGAGTTG

               *  * **            *  *               *  *             *      
     418264 AACATTCCCATTCATAGAGCAGGTTGGAAACACTCTTTTTGGAGTATCTGGAAGTGGACATTTGG
         65 AACCTTTCTTTTCATAGAGCAGTTTTGAAACACTCTTTTTGTAGAATCTGGAAGTGGATATTTGG

                    ** * * ** *  *   *            **  
     418329 AGCGCTTTCTGAACTATGGTGAAAAAGGAAATATCTTCCAAT
        130 AGCGCTTTGAGGATTTCGTTGGAAACGGAAATATCTTCATAT

            *     *               *      * *          *  **      **  *  *    
     418371 GAAAACAAGACAGAAGCATTCTGAGAAACTTATTTGTGATGTGTGTCCTCAACAAACGGACTTGA
          1 AAAAACTAGACAGAAGCATTCTCAGAAACATCTTTGTGATGTTTGCACTCAACTCACAGAGTTGA

                   *      **  * *  *  *             *   *    * * *           
     418436 ACCTTTCGTTTCATGCAGTACTTCTGGAACACTCTTTTTGAAGATTCTGCATGCGGATATTTGGA
         66 ACCTTTCTTTTCATAGAGCAGTTTTGAAACACTCTTTTTGTAGAATCTGGAAGTGGATATTTGGA

            **                            **   *   * 
     418501 TAGCTTTGAGGATTTCGTTGGAAACGG---GCT-TACATGT
        131 GCGCTTTGAGGATTTCGTTGGAAACGGAAATATCTTCATAT

                 *       *               *        *   *    *    *       *    
     418538 AAAAATTAGACAGCAGCATTCTCAGAAACTTCTTTGTGGTGTCTGCATTCAAGTCACAGAATTGA
          1 AAAAACTAGACAGAAGCATTCTCAGAAACATCTTTGTGATGTTTGCACTCAACTCACAGAGTTGA

              * ** * **            *  * *      *       *             *       
     418603 ACTTCCCCTCACATAGAGCAGTTGTGCAGCACTCTATTTGTAGTATCTGGAAGTGGACATTTGGA
         66 ACCTTTCTTTTCATAGAGCAGTTTTGAAACACTCTTTTTGTAGAATCTGGAAGTGGATATTTGGA

             *         ** *    * *   *            **  
     418668 GGGCTTTGTAGCCTATC-TGGAAAAAGGAAATATCTTCCCAT
        131 GCGCTTTG-AGGATTTCGTTGGAAACGGAAATATCTTCATAT

            *  ** *   *     * *            *   *  *   * **        *  * * *   
     418709 GAATGCGAGATAGAAGTAATCTCAGAAACATGTTTATGCTGTATCTACTCAACTAACTGTGCTGA
          1 AAAAACTAGACAGAAGCATTCTCAGAAACATCTTTGTGATGTTTGCACTCAACTCACAGAGTTGA

              *     *  *               *        *  * *       *               
     418774 ACATTTCTATTGATAGAGCAGTTTTGAGACACTCTTCTTTTGGAATCTGCAAGTGGATATTTGGA
         66 ACCTTTCTTTTCATAGAGCAGTTTTGAAACACTCTTTTTGTAGAATCTGGAAGTGGATATTTGGA

            ** *                        *     *      
     418839 TAGATTTGAGGATTTCGTTGGAAACGG-GAT-T-AT-ATAT
        131 GCGCTTTGAGGATTTCGTTGGAAACGGAAATATCTTCATAT

                 *       *               *                 **  *   *         
     418876 AAAAAGTAGACAGCAGCATTCTCAGAAACTTCTTTGTGATGTTTGCATCCAGCTCTCAGAGTTGA
          1 AAAAACTAGACAGAAGCATTCTCAGAAACATCTTTGTGATGTTTGCACTCAACTCACAGAGTTGA

            * *  * *          *  *        *        *   **        *  **       
     418941 GCATTCCCTTTCATAGAGTAGGTTTGAAACCCTCTTTTTATAGTGTCTGGAAGCGGGCATTTGGA
         66 ACCTTTCTTTTCATAGAGCAGTTTTGAAACACTCTTTTTGTAGAATCTGGAAGTGGATATTTGGA

                   *    * * **  **  **          * *  *
     419006 GCGCTTTCAGGCCTATGCTTAAAATAGGAAATATCTACCTAC
        131 GCGCTTTGAGG-ATTTCGTTGGAAACGGAAATATCTTCATAT

             *                    *    *  **          ** *        *    *     
     419048 AGAAACTAGACAGAAGCATTCTGAGAATCACGTTTGTGATGTGGGTACTCAACTAACAGTGTTGA
          1 AAAAACTAGACAGAAGCATTCTCAGAAACATCTTTGTGATGTTTGCACTCAACTCACAGAGTTGA

            *  *       *   *            *   *                *               
     419113 TCCATTCTTTTGATACAGCAGTTTTGAACCACACTTTTTGTAGAATCTGCAAGTGGATATTTGGA
         66 ACCTTTCTTTTCATAGAGCAGTTTTGAAACACTCTTTTTGTAGAATCTGGAAGTGGATATTTGGA

            **   *                     *   *      * *
     419178 TAGCTGTGAGGATTTCGTTGGAAACGGGAATGTCTTCAAAG
        131 GCGCTTTGAGGATTTCGTTGGAAACGGAAATATCTTCATAT

                *                         *   *            *    *            
     419219 AAAATCTAGACAGAAGCATTCTCAGAAACACCTTCGTGATGTTTGCAATCAAGTCACAGAGTTGA
          1 AAAAACTAGACAGAAGCATTCTCAGAAACATCTTTGTGATGTTTGCACTCAACTCACAGAGTTGA

                 * *             *  *           *      *             *       
     419284 ACCTTCCGTTTCATAGAGCAGGTTGGAAACACTCTTATTGTAGTATCTGGAAGTGGACATTTGGA
         66 ACCTTTCTTTTCATAGAGCAGTTTTGAAACACTCTTTTTGTAGAATCTGGAAGTGGATATTTGGA

                   *   ** ** *  *   *            **  
     419349 GCGCTTTCAGGCCTATGGTGAAAAAGGAAATATCTTCCCAT
        131 GCGCTTTGAGGATTTCGTTGGAAACGGAAATATCTTCATAT

                  * * *      **      *   * *   *     ** ** *      *    *     
     419390 AAAAACGACATAGAAGCTATCTCAGGAACTTGTTTATGATGCATCTAATCAACTAACAGTGTTGA
          1 AAAAACTAGACAGAAGCATTCTCAGAAACATCTTTGTGATGTTTGCACTCAACTCACAGAGTTGA

                  * ** * *                         * *       *               
     419455 ACCTTTGTACTGACAGAGCAG-TTTGAAACACTCTTTTTTTGGAATCTGCAAGTGGATATTTGGA
         66 ACCTTTCTTTTCATAGAGCAGTTTTGAAACACTCTTTTTGTAGAATCTGGAAGTGGATATTTGGA

            *                           *   *   *    
     419519 TCGCTTTGAGGATTTCGTTGGAAACGG-GAT-GC--AATAT
        131 GCGCTTTGAGGATTTCGTTGGAAACGGAAATATCTTCATAT

                **  *    *     *                 **  *   *  *    *         * 
     419556 AAAACGTACACAGCAGCATACTCAGAAA-ATACTTTGCCATATTTCCATTCAAGTCACAGAGTGG
          1 AAAAACTAGACAGAAGCATTCTCAGAAACAT-CTTTGTGATGTTTGCACTCAACTCACAGAGTTG

               *  * **            *  *               *  *             *      
     419620 AACATTCCCATTCATAGAGCAGGTTGGAAACACTCTTTTTGGAGTATCTGGAAGTGGACATTTGG
         65 AACCTTTCTTTTCATAGAGCAGTTTTGAAACACTCTTTTTGTAGAATCTGGAAGTGGATATTTGG

                    ** * * ** *  *   *            **  
     419685 AGCGCTTTCTGAACTATGGTGAAAAAGGAAATATCTTCCAAT
        130 AGCGCTTTGAGGATTTCGTTGGAAACGGAAATATCTTCATAT

            *     *               *      * *          *  **      **  *  *    
     419727 GAAAACAAGACAGAAGCATTCTGAGAAACTTATTTGTGATGTGTGTCCTCAACAAACGGACTTGA
          1 AAAAACTAGACAGAAGCATTCTCAGAAACATCTTTGTGATGTTTGCACTCAACTCACAGAGTTGA

                   *      **  * *  *  *             *   *    * * *           
     419792 ACCTTTCGTTTCATGCAGTACTTCTGGAACACTCTTTTTGAAGATTCTGCATGCGGATATTTGGA
         66 ACCTTTCTTTTCATAGAGCAGTTTTGAAACACTCTTTTTGTAGAATCTGGAAGTGGATATTTGGA

            **                            **   *   * 
     419857 TAGCTTTGAGGATTTCGTTGGAAACGG---GCT-TACATGT
        131 GCGCTTTGAGGATTTCGTTGGAAACGGAAATATCTTCATAT

                 *       *               *        *   *    *    *       *    
     419894 AAAAATTAGACAGCAGCATTCTCAGAAACTTCTTTGTGGTGTCTGCATTCAAGTCACAGAATTGA
          1 AAAAACTAGACAGAAGCATTCTCAGAAACATCTTTGTGATGTTTGCACTCAACTCACAGAGTTGA

              * ** * **            *  * *      *       *             *       
     419959 ACTTCCCCTCACATAGAGCAGTTGTGCAGCACTCTATTTGTAGTATCTGGAAGTGGACATTTGGA
         66 ACCTTTCTTTTCATAGAGCAGTTTTGAAACACTCTTTTTGTAGAATCTGGAAGTGGATATTTGGA

             *         ** *    * *   *            **  
     420024 GGGCTTTGTAGCCTATC-TGGAAAAAGGAAATATCTTCCCAT
        131 GCGCTTTG-AGGATTTCGTTGGAAACGGAAATATCTTCATAT

            *  ** *   *     * *            *   *  *   * **        *  * * *   
     420065 GAATGCGAGATAGAAGTAATCTCAGAAACATGTTTATGCTGTATCTACTCAACTAACTGTGCTGA
          1 AAAAACTAGACAGAAGCATTCTCAGAAACATCTTTGTGATGTTTGCACTCAACTCACAGAGTTGA

              *     *  *               *        *  * *       *               
     420130 ACATTTCTATTGATAGAGCAGTTTTGAGACACTCTTCTTTTGGAATCTGCAAGTGGATATTTGGA
         66 ACCTTTCTTTTCATAGAGCAGTTTTGAAACACTCTTTTTGTAGAATCTGGAAGTGGATATTTGGA

            ** *                        *     *      
     420195 TAGATTTGAGGATTTCGTTGGAAACGG-GAT-T-AT-ATAT
        131 GCGCTTTGAGGATTTCGTTGGAAACGGAAATATCTTCATAT

                 *       *               *                 **  *   *         
     420232 AAAAAGTAGACAGCAGCATTCTCAGAAACTTCTTTGTGATGTTTGCATCCAGCTCTCAGAGTTGA
          1 AAAAACTAGACAGAAGCATTCTCAGAAACATCTTTGTGATGTTTGCACTCAACTCACAGAGTTGA

              *  * *          *  *        *        *   **        *  **       
     420297 ACATTCCCTTTCATAGAGTAGGTTTGAAACCCTCTTTTTATAGTGTCTGGAAGCGGGCATTTGGA
         66 ACCTTTCTTTTCATAGAGCAGTTTTGAAACACTCTTTTTGTAGAATCTGGAAGTGGATATTTGGA

                   *    * * **  **  **          * *  *
     420362 GCGCTTTCAGGCCTATGCTTAAAATAGGAAATATCTACCTAC
        131 GCGCTTTGAGG-ATTTCGTTGGAAACGGAAATATCTTCATAT

             *                    *    *  **          ** *        *    *     
     420404 AGAAACTAGACAGAAGCATTCTGAGAATCACGTTTGTGATGTGGGTACTCAACTAACAGTGTTGA
          1 AAAAACTAGACAGAAGCATTCTCAGAAACATCTTTGTGATGTTTGCACTCAACTCACAGAGTTGA

            *  *       *   *            *   *                *   *           
     420469 TCCATTCTTTTGATACAGCAGTTTTGAACCACACTTTTTGTAGAATCTGCAAGAGGATATTTGGA
         66 ACCTTTCTTTTCATAGAGCAGTTTTGAAACACTCTTTTTGTAGAATCTGGAAGTGGATATTTGGA

            **   *                     *   *      * *
     420534 TAGCTGTGAGGATTTCGTTGGAAACGGGAATGTCTTCAAAG
        131 GCGCTTTGAGGATTTCGTTGGAAACGGAAATATCTTCATAT

                *                         *   *            *    *            
     420575 AAAATCTAGACAGAAGCATTCTCAGAAACACCTTCGTGATGTTTGCAATCAAGTCACAGAGTTGA
          1 AAAAACTAGACAGAAGCATTCTCAGAAACATCTTTGTGATGTTTGCACTCAACTCACAGAGTTGA

                 * *             *  *           *      *             *       
     420640 ACCTTCCGTTTCATAGAGCAGGTTGGAAACACTCTTATTGTAGTATCTGGAAGTGGACATTTGGA
         66 ACCTTTCTTTTCATAGAGCAGTTTTGAAACACTCTTTTTGTAGAATCTGGAAGTGGATATTTGGA

                   *   ** ** *  *   *            **  
     420705 GCGCTTTCAGGCCTATGGTGAAAAAGGAAATATCTTCCCAT
        131 GCGCTTTGAGGATTTCGTTGGAAACGGAAATATCTTCATAT

                  * * *      **      *   * *   *     ** ** *      *    *     
     420746 AAAAACGACATAGAAGCTATCTCAGGAACTTGTTTATGATGCATCTAATCAACTAACAGTGTTGA
          1 AAAAACTAGACAGAAGCATTCTCAGAAACATCTTTGTGATGTTTGCACTCAACTCACAGAGTTGA

                  * ** * *                         * *       *               
     420811 ACCTTTGTACTGACAGAGCAG-TTTGAAACACTCTTTTTTTGGAATCTGCAAGTGGATATTTGGA
         66 ACCTTTCTTTTCATAGAGCAGTTTTGAAACACTCTTTTTGTAGAATCTGGAAGTGGATATTTGGA

            *                           *   *   *    
     420875 TCGCTTTGAGGATTTCGTTGGAAACGG-GAT-GC--AATAT
        131 GCGCTTTGAGGATTTCGTTGGAAACGGAAATATCTTCATAT

                **  *    *     *                 **  *   *  *    *         * 
     420912 AAAACGTACACAGCAGCATACTCAGAAA-ATACTTTGCCATATTTCCATTCAAGTCACAGAGTGG
          1 AAAAACTAGACAGAAGCATTCTCAGAAACAT-CTTTGTGATGTTTGCACTCAACTCACAGAGTTG

               *  * **            *          *       *  *             *      
     420976 AACATTCCCATTCATAGAGCAGGTTTGAAACACACTTTTTGGAGTATCTGGAAGTGGACATTTGG
         65 AACCTTTCTTTTCATAGAGCAGTTTTGAAACACTCTTTTTGTAGAATCTGGAAGTGGATATTTGG

                    ** * * ** *  *   *            **  
     421041 AGCGCTTTCTGAACTATGGTGAAAAAGGAAATATCTTCCAAT
        130 AGCGCTTTGAGGATTTCGTTGGAAACGGAAATATCTTCATAT

            *     *               *      * *          *  **      **  *  *    
     421083 GAAAACAAGACAGAAGCATTCTGAGAAACTTATTTGTGATGTGTGTCCTCAACAAACGGACTTGA
          1 AAAAACTAGACAGAAGCATTCTCAGAAACATCTTTGTGATGTTTGCACTCAACTCACAGAGTTGA

                   *      **  * *  *  *             *   *    * * *           
     421148 ACCTTTCGTTTCATGCAGTACTTCTGGAACACTCTTTTTGAAGATTCTGCATGCGGATATTTGGA
         66 ACCTTTCTTTTCATAGAGCAGTTTTGAAACACTCTTTTTGTAGAATCTGGAAGTGGATATTTGGA

            **                            **   *   * 
     421213 TAGCTTTGAGGATTTCGTTGGAAACGG---GCT-TACATGT
        131 GCGCTTTGAGGATTTCGTTGGAAACGGAAATATCTTCATAT

                 *       *               *        *   *    *    *       *    
     421250 AAAAATTAGACAGCAGCATTCTCAGAAACTTCTTTGTGGTGTCTGCATTCAAGTCACAGAATTGA
          1 AAAAACTAGACAGAAGCATTCTCAGAAACATCTTTGTGATGTTTGCACTCAACTCACAGAGTTGA

              * ** * **            *  * *      *       *    *        *       
     421315 ACTTCCCCTCACATAGAGCAGTTGTGCAGCACTCTATTTGTAGTATCTCGAAGTGGACATTTGGA
         66 ACCTTTCTTTTCATAGAGCAGTTTTGAAACACTCTTTTTGTAGAATCTGGAAGTGGATATTTGGA

             *         ** *    * *   *            **  
     421380 GGGCTTTGTAGCCTATC-TGGAAAAAGGAAATATCTTCCCAT
        131 GCGCTTTG-AGGATTTCGTTGGAAACGGAAATATCTTCATAT

            *  ** *   *     * *            *   *  *   * **        *  * * *   
     421421 GAATGCGAGATAGAAGTAATCTCAGAAACATGTTTATGCTGTATCTACTCAACTAACTGTGCTGA
          1 AAAAACTAGACAGAAGCATTCTCAGAAACATCTTTGTGATGTTTGCACTCAACTCACAGAGTTGA

              *     *  *               *        *  * *       *               
     421486 ACATTTCTATTGATAGAGCAGTTTTGAGACACTCTTCTTTTGGAATCTGCAAGTGGATATTTGGA
         66 ACCTTTCTTTTCATAGAGCAGTTTTGAAACACTCTTTTTGTAGAATCTGGAAGTGGATATTTGGA

            ** *                        *     *      
     421551 TAGATTTGAGGATTTCGTTGGAAACGG-GAT-T-AT-ATAT
        131 GCGCTTTGAGGATTTCGTTGGAAACGGAAATATCTTCATAT

            *    *       *               *                 **  *   *         
     421588 CAAAAGTAGACAGCAGCATTCTCAGAAACTTCTTTGTGATGTTTGCATCCAGCTCTCAGAGTTGA
          1 AAAAACTAGACAGAAGCATTCTCAGAAACATCTTTGTGATGTTTGCACTCAACTCACAGAGTTGA

              *  * *          *  *        *        *   **        *  **       
     421653 ACATTCCCTTTCATAGAGTAGGTTTGAAACCCTCTTTTTATAGTGTCTGGAAGCGGGCATTTGGA
         66 ACCTTTCTTTTCATAGAGCAGTTTTGAAACACTCTTTTTGTAGAATCTGGAAGTGGATATTTGGA

                   *    * * **  **  **          * *   
     421718 GCGCTTTCAGGCCTATGCTTAAAATAGGAAATATCTACCTAT
        131 GCGCTTTGAGG-ATTTCGTTGGAAACGGAAATATCTTCATAT

             *                    *    *  **          ** *        *    *     
     421760 AGAAACTAGACAGAAGCATTCTGAGAATCACGTTTGTGATGTGGGTACTCAACTAACAGTGTTGA
          1 AAAAACTAGACAGAAGCATTCTCAGAAACATCTTTGTGATGTTTGCACTCAACTCACAGAGTTGA

            *  *       *   *            *   *                *               
     421825 TCCATTCTTTTGATACAGCAGTTTTGAACCACACTTTTTGTAGAATCTGCAAGTGGATATTTGGA
         66 ACCTTTCTTTTCATAGAGCAGTTTTGAAACACTCTTTTTGTAGAATCTGGAAGTGGATATTTGGA

            **   *                     *   *        *
     421890 TAGCTGTGAGGATTTCGTTGGAAACGGGAATGTCTTCATAG
        131 GCGCTTTGAGGATTTCGTTGGAAACGGAAATATCTTCATAT

                **                     * * **         *  **   *   *          
     421931 AAAATTTAGACAGAAGCATTCTCAGAACCTTGATTGTGATGTGTGTTCTCCACTAACAGAGTTGA
          1 AAAAACTAGACAGAAGCATTCTCAGAAACATCTTTGTGATGTTTGCACTCAACTCACAGAGTTGA

                       * *   * *   *       *       *                         
     421996 ACCTTTCTTTTGACAGAACTGTTCTGAAACATTCTTTTTATAGAATCTGGAAGTGGATATTTGGA
         66 ACCTTTCTTTTCATAGAGCAGTTTTGAAACACTCTTTTTGTAGAATCTGGAAGTGGATATTTGGA

            **                         *          *  
     422061 AAGCTTTGAGGATTTCGTTGGAAACGGGAATATCTTCAAAT
        131 GCGCTTTGAGGATTTCGTTGGAAACGGAAATATCTTCATAT

            *   *    *            *           * *       *  *    *            
     422102 CAAATCTAGCCAGAAGCATTCTAAGAAACATCTTAGGGATGTTTACATTCAAGTCACAGAGTTGA
          1 AAAAACTAGACAGAAGCATTCTCAGAAACATCTTTGTGATGTTTGCACTCAACTCACAGAGTTGA

              *  * *     *       *         *    * *   **      *      *    *  
     422167 ACATTCCCTTTCACAGAGCAGGTTTGAAACAATCTTCTCGTACTATCTGGCAGTGGACATTTTGA
         66 ACCTTTCTTTTCATAGAGCAGTTTTGAAACACTCTTTTTGTAGAATCTGGAAGTGGATATTTGGA

              * *   *  ** ** *  *   *            ** *
     422232 GCTCCTTGGGGCCTATGCTGAAAAAGGAAATATCTTCCGAC
        131 GCGCTTTGAGGATTTCGTTGGAAACGGAAATATCTTCATAT

                                 *     *  **          *           **    *    
     422273 AAAAACTAGACAGAAGCATTCGCAGAATCACGTTTGTGATGTGTGCACTCAACTGTCAGAATTGA
          1 AAAAACTAGACAGAAGCATTCTCAGAAACATCTTTGTGATGTTTGCACTCAACTCACAGAGTTGA

                 **   ** *      *                   *        * *            *
     422338 ACCTTGGTTTGGACAGAGCACTTTTGAAACACTCTTTTTGCAGAATCTGCAGGTGGATATTTGGC
         66 ACCTTTCTTTTCATAGAGCAGTTTTGAAACACTCTTTTTGTAGAATCTGGAAGTGGATATTTGGA

            **                         *   *      * *
     422403 TAGCTTTGAGGATTTCGTTGGAAACGGTAATGTCTTCAAAG
        131 GCGCTTTGAGGATTTCGTTGGAAACGGAAATATCTTCATAT

                *                         *   *            *    *            
     422444 AAAATCTAGACAGAAGCATTCTCAGAAACAACTTCGTGATGTTTGCAATCAAGTCACAGAGTTGA
          1 AAAAACTAGACAGAAGCATTCTCAGAAACATCTTTGTGATGTTTGCACTCAACTCACAGAGTTGA

                 * *             *  *                  *             *       
     422509 ACCTTCCGTTTCATAGAGCAGGTTGGAAACACTCTTTTTGTAGTATCTGGAAGTGGACATTTGGA
         66 ACCTTTCTTTTCATAGAGCAGTTTTGAAACACTCTTTTTGTAGAATCTGGAAGTGGATATTTGGA

             *         ** *    * *   *            **  
     422574 GGGCTTTGTAGCCTATC-TGGAAAAAGGAAATATCTTCCCAT
        131 GCGCTTTG-AGGATTTCGTTGGAAACGGAAATATCTTCATAT

            *  ** *   *     * *            *   *  *   * **        *  * * *   
     422615 GAATGCGAGATAGAAGTAATCTCAGAAACATGTTTATGCTGTATCTACTCAACTAACTGTGCTGA
          1 AAAAACTAGACAGAAGCATTCTCAGAAACATCTTTGTGATGTTTGCACTCAACTCACAGAGTTGA

              *     *  *               *        *  * *       *               
     422680 ACATTTCTATTGATAGAGCAGTTTTGAGACACTCTTCTTTTGGAATCTGCAAGTGGATATTTGGA
         66 ACCTTTCTTTTCATAGAGCAGTTTTGAAACACTCTTTTTGTAGAATCTGGAAGTGGATATTTGGA

            ** *                       **     *      
     422745 TAGATTTGAGGATTTCGTTGGAAAC-GCGAT-T-AT-ATAT
        131 GCGCTTTGAGGATTTCGTTGGAAACGGAAATATCTTCATAT

                 *       *               *         *       **  *   *         
     422782 AAAAAGTAGACAGCAGCATTCTCAGAAACTTCTTTGTGACGTTTGCATCCAGCTCTCAGAGTTGA
          1 AAAAACTAGACAGAAGCATTCTCAGAAACATCTTTGTGATGTTTGCACTCAACTCACAGAGTTGA

            * *  * *          *  *        *        *   **        *  **       
     422847 GCATTCCCTTTCATAGAGTAGGTTTGAAACCCTCTTTTTATAGTGTCTGGAAGCGGGCATTTGGA
         66 ACCTTTCTTTTCATAGAGCAGTTTTGAAACACTCTTTTTGTAGAATCTGGAAGTGGATATTTGGA

                   *    * * **  **  **          * *  *
     422912 GCGCTTTCAGGCCTATGCTTAAAATAGGAAATATCTACCTAC
        131 GCGCTTTGAGG-ATTTCGTTGGAAACGGAAATATCTTCATAT

             *                    *    *  **          ** *        *    *     
     422954 AGAAACTAGACAGAAGCATTCTGAGAATCACGTTTGTGATGTGGGTACTCAACTAACAGTGTTGA
          1 AAAAACTAGACAGAAGCATTCTCAGAAACATCTTTGTGATGTTTGCACTCAACTCACAGAGTTGA

            *  *       *   *            *   *                *               
     423019 TCCATTCTTTTGATACAGCAGTTTTGAACCACACTTTTTGTAGAATCTGCAAGTGGATATTTGGA
         66 ACCTTTCTTTTCATAGAGCAGTTTTGAAACACTCTTTTTGTAGAATCTGGAAGTGGATATTTGGA

            **   *                     *   *      * *
     423084 TAGCTGTGAGGATTTCGTTGGAAACGGTAATGTCTTCAAAG
        131 GCGCTTTGAGGATTTCGTTGGAAACGGAAATATCTTCATAT

                *                         *   *            *    *            
     423125 AAAATCTAGACAGAAGCATTCTCAGAAACACCTTCGTGATGTTTGCAATCAAGTCACAGAGTTGA
          1 AAAAACTAGACAGAAGCATTCTCAGAAACATCTTTGTGATGTTTGCACTCAACTCACAGAGTTGA

                 * *             *  *           *      *             *       
     423190 ACCTTCCGTTTCATAGAGCAGGTTGGAAACACTCTTATTGTAGTATCTGGAAGTGGACATTTGGA
         66 ACCTTTCTTTTCATAGAGCAGTTTTGAAACACTCTTTTTGTAGAATCTGGAAGTGGATATTTGGA

                   *   ** ** *  *   *            **  
     423255 GCGCTTTCAGGCCTATGGTGAAAAAGGAAATATCTTCCCAT
        131 GCGCTTTGAGGATTTCGTTGGAAACGGAAATATCTTCATAT

                  * * *      **      *   * *   *     ** ** *      *    *     
     423296 AAAAACGACATAGAAGCTATCTCAGGAACTTGTTTATGATGCATCTAATCAACTAACAGTGTTGA
          1 AAAAACTAGACAGAAGCATTCTCAGAAACATCTTTGTGATGTTTGCACTCAACTCACAGAGTTGA

                  * ** * *                         * *       *               
     423361 ACCTTTGTACTGACAGAGCAG-TTTGAAACACTCTTTTTTTGGAATCTGCAAGTGGATATTTGGA
         66 ACCTTTCTTTTCATAGAGCAGTTTTGAAACACTCTTTTTGTAGAATCTGGAAGTGGATATTTGGA

            *                           *   *   *    
     423425 TCGCTTTGAGGATTTCGTTGGAAACGG-GAT-GC--AATAT
        131 GCGCTTTGAGGATTTCGTTGGAAACGGAAATATCTTCATAT

                **  *    *     *                 **  *   *  *    *         * 
     423462 AAAACGTACACAGCAGCATACTCAGAAA-ATACTTTGCCATATTTCCATTCAAGTCACAGAGTGG
          1 AAAAACTAGACAGAAGCATTCTCAGAAACAT-CTTTGTGATGTTTGCACTCAACTCACAGAGTTG

               *  * **            *  *               *  *             *      
     423526 AACATTCCCATTCATAGAGCAGGTTGGAAACACTCTTTTTGGAGTATCTGGAAGTGGACATTTGG
         65 AACCTTTCTTTTCATAGAGCAGTTTTGAAACACTCTTTTTGTAGAATCTGGAAGTGGATATTTGG

                    ** * * ** *  *   *            **  
     423591 AGCGCTTTCTGAACTATGGTGAAAAAGGAAATATCTTCCAAT
        130 AGCGCTTTGAGGATTTCGTTGGAAACGGAAATATCTTCATAT

            *     *               *      * *          *  **      **  *  *    
     423633 GAAAACAAGACAGAAGCATTCTGAGAAACTTATTTGTGATGTGTGTCCTCAACAAACGGACTTGA
          1 AAAAACTAGACAGAAGCATTCTCAGAAACATCTTTGTGATGTTTGCACTCAACTCACAGAGTTGA

                   *      **  * *  *  *             *   *    * * *           
     423698 ACCTTTCGTTTCATGCAGTACTTCTGGAACACTCTTTTTGAAGATTCTGCATGCGGATATTTGGA
         66 ACCTTTCTTTTCATAGAGCAGTTTTGAAACACTCTTTTTGTAGAATCTGGAAGTGGATATTTGGA

            **                            **   *   * 
     423763 TAGCTTTGAGGATTTCGTTGGAAACGG---GCT-TACATGT
        131 GCGCTTTGAGGATTTCGTTGGAAACGGAAATATCTTCATAT

                 *       *               *        *   *    *    *       *    
     423800 AAAAATTAGACAGCAGCATTCTCAGAAACTTCTTTGTGGTGTCTGCATTCAAGTCACAGAATTGA
          1 AAAAACTAGACAGAAGCATTCTCAGAAACATCTTTGTGATGTTTGCACTCAACTCACAGAGTTGA

              * ** * **            *  * *      *       *             *       
     423865 ACTTCCCCTCACATAGAGCAGTTGTGCAGCACTCTATTTGTAGTATCTGGAAGTGGACATTTGGA
         66 ACCTTTCTTTTCATAGAGCAGTTTTGAAACACTCTTTTTGTAGAATCTGGAAGTGGATATTTGGA

             *         ** *    * *   *            **  
     423930 GGGCTTTGTAGCCTATC-TGGAAAAAGGAAATATCTTCCCAT
        131 GCGCTTTG-AGGATTTCGTTGGAAACGGAAATATCTTCATAT

            *  ** *   *     * *            *   *  *   * **        *  * * *   
     423971 GAATGCGAGATAGAAGTAATCTCAGAAACATGTTTATGCTGTATCTACTCAACTAACTGTGCTGA
          1 AAAAACTAGACAGAAGCATTCTCAGAAACATCTTTGTGATGTTTGCACTCAACTCACAGAGTTGA

              *     *  *               *        *  * *       *               
     424036 ACATTTCTATTGATAGAGCAGTTTTGAGACACTCTTCTTTTGGAATCTGCAAGTGGATATTTGGA
         66 ACCTTTCTTTTCATAGAGCAGTTTTGAAACACTCTTTTTGTAGAATCTGGAAGTGGATATTTGGA

            ** *                        *     *      
     424101 TAGATTTGAGGATTTCGTTGGAAACGG-GAT-T-AT-ATAT
        131 GCGCTTTGAGGATTTCGTTGGAAACGGAAATATCTTCATAT

            *    *       *               *                 **  *   *         
     424138 CAAAAGTAGACAGCAGCATTCTCAGAAACTTCTTTGTGATGTTTGCATCCAGCTCTCAGAGTTGA
          1 AAAAACTAGACAGAAGCATTCTCAGAAACATCTTTGTGATGTTTGCACTCAACTCACAGAGTTGA

              *  * *          *  *        *        *   **        *  **       
     424203 ACATTCCCTTTCATAGAGTAGGTTTGAAACCCTCTTTTTATAGTGTCTGGAAGCGGGCATTTGGA
         66 ACCTTTCTTTTCATAGAGCAGTTTTGAAACACTCTTTTTGTAGAATCTGGAAGTGGATATTTGGA

                *  *    * * **  **  **          * *  *
     424268 GCGCATTCAGGCCTATGCTTAAAATAGGAAATATCTACCTAC
        131 GCGCTTTGAGG-ATTTCGTTGGAAACGGAAATATCTTCATAT

             *                    *    *  **          ** *        *    *     
     424310 AGAAACTAGACAGAAGCATTCTGAGAATCACGTTTGTGATGTGGGTACTCAACTAACAGTGTTGA
          1 AAAAACTAGACAGAAGCATTCTCAGAAACATCTTTGTGATGTTTGCACTCAACTCACAGAGTTGA

            *  *       *   *            *   *                *   *           
     424375 TCCATTCTTTTGATACAGCAGTTTTGAACCACACTTTTTGTAGAATCTGCAAGAGGATATTTGGA
         66 ACCTTTCTTTTCATAGAGCAGTTTTGAAACACTCTTTTTGTAGAATCTGGAAGTGGATATTTGGA

            **   *                     *   *      * *
     424440 TAGCTGTGAGGATTTCGTTGGAAACGGGAATGTCTTCAAAG
        131 GCGCTTTGAGGATTTCGTTGGAAACGGAAATATCTTCATAT

                *                         *   *            *    *            
     424481 AAAATCTAGACAGAAGCATTCTCAGAAACACCTTCGTGATGTTTGCAATCAAGTCACAGAGTTGA
          1 AAAAACTAGACAGAAGCATTCTCAGAAACATCTTTGTGATGTTTGCACTCAACTCACAGAGTTGA

                 * *             *  *           *      *             *       
     424546 ACCTTCCGTTTCATAGAGCAGGTTGGAAACACTCTTATTGTAGTATCTGGAAGTGGACATTTGGA
         66 ACCTTTCTTTTCATAGAGCAGTTTTGAAACACTCTTTTTGTAGAATCTGGAAGTGGATATTTGGA

                   *   ** ** *  *   *            **  
     424611 GCGCTTTCAGGCCTATGGTGAAAAAGGAAATATCTTCCCAT
        131 GCGCTTTGAGGATTTCGTTGGAAACGGAAATATCTTCATAT

                  * * *      **      *   * *   *     ** ** *      *    *     
     424652 AAAAACGACATAGAAGCTATCTCAGGAACTTGTTTATGATGCATCTAATCAACTAACAGTGTTGA
          1 AAAAACTAGACAGAAGCATTCTCAGAAACATCTTTGTGATGTTTGCACTCAACTCACAGAGTTGA

                  * ** * *                         * *       *               
     424717 ACCTTTGTACTGACAGAGCAG-TTTGAAACACTCTTTTTTTGGAATCTGCAAGTGGATATTTGGA
         66 ACCTTTCTTTTCATAGAGCAGTTTTGAAACACTCTTTTTGTAGAATCTGGAAGTGGATATTTGGA

            *                           *   *   *    
     424781 TCGCTTTGAGGATTTCGTTGGAAACGG-GAT-GC--AATAT
        131 GCGCTTTGAGGATTTCGTTGGAAACGGAAATATCTTCATAT

                **  *    *     *                 **  *   *  *    *   *     * 
     424818 AAAACGTACACAGCAGCATACTCAGAAA-ATACTTTGCCATATTTCCATTCAAGTCAGAGAGTGG
          1 AAAAACTAGACAGAAGCATTCTCAGAAACAT-CTTTGTGATGTTTGCACTCAACTCACAGAGTTG

               *  * **            *                  *  *             *      
     424882 AACATTCCCATTCATAGAGCAGGTTTGAAACACTCTTTTTGGAGTATCTGGAAGTGGACATTTGG
         65 AACCTTTCTTTTCATAGAGCAGTTTTGAAACACTCTTTTTGTAGAATCTGGAAGTGGATATTTGG

                    ** * * ** *  *   *            **  
     424947 AGCGCTTTCTGAACTATGGTGAAAAAGGAAATATCTTCCAAT
        130 AGCGCTTTGAGGATTTCGTTGGAAACGGAAATATCTTCATAT

            *     *               *      * *          *  **      **  *  *    
     424989 GAAAACAAGACAGAAGCATTCTGAGAAACTTATTTGTGATGTGTGTCCTCAACAAACGGACTTGA
          1 AAAAACTAGACAGAAGCATTCTCAGAAACATCTTTGTGATGTTTGCACTCAACTCACAGAGTTGA

                   *      **  * *  *  *             *   *    * * * *         
     425054 ACCTTTCGTTTCATGCAGTACTTCTGGAACACTCTTTTTGAAGATTCTGCATGCGTATATTTGGA
         66 ACCTTTCTTTTCATAGAGCAGTTTTGAAACACTCTTTTTGTAGAATCTGGAAGTGGATATTTGGA

            **                            **   *   * 
     425119 TAGCTTTGAGGATTTCGTTGGAAACGG---GCT-TACATGT
        131 GCGCTTTGAGGATTTCGTTGGAAACGGAAATATCTTCATAT

                 *       *      *        *        *   *    *    *       *    
     425156 AAAAATTAGACAGCAGCATTTTCAGAAACTTCTTTGTGGTGTCTGCATTCAAGTCACAGAATTGA
          1 AAAAACTAGACAGAAGCATTCTCAGAAACATCTTTGTGATGTTTGCACTCAACTCACAGAGTTGA

              * ** * **            *  * *      *       *             *       
     425221 ACTTCCCCTCACATAGAGCAGTTGTGCAGCACTCTATTTGTAGTATCTGGAAGTGGACATTTGGA
         66 ACCTTTCTTTTCATAGAGCAGTTTTGAAACACTCTTTTTGTAGAATCTGGAAGTGGATATTTGGA

             *         ** *    * *   *            **  
     425286 GGGCTTTGTAGCCTATC-TGGAAAAAGGAAATATCTTCCCAT
        131 GCGCTTTG-AGGATTTCGTTGGAAACGGAAATATCTTCATAT

            *  ** *   *     * *            *   *  *   * **        *  * * *   
     425327 GAATGCGAGATAGAAGTAATCTCAGAAACATGTTTATGCTGTATCTACTCAACTAACTGTGCTGA
          1 AAAAACTAGACAGAAGCATTCTCAGAAACATCTTTGTGATGTTTGCACTCAACTCACAGAGTTGA

              *     *  *               *        *  * *       *               
     425392 ACATTTCTATTGATAGAGCAGTTTTGAGACACTCTTCTTTTGGAATCTGCAAGTGGATATTTGGA
         66 ACCTTTCTTTTCATAGAGCAGTTTTGAAACACTCTTTTTGTAGAATCTGGAAGTGGATATTTGGA

            ** *                        *     *      
     425457 TAGATTTGAGGATTTCGTTGGAAACGG-GAT-T-AT-ATAT
        131 GCGCTTTGAGGATTTCGTTGGAAACGGAAATATCTTCATAT

            *    **      *               *                 **  *   *         
     425494 CAAAAGCAGACAGCAGCATTCTCAGAAACTTCTTTGTGATGTTTGCATCCAGCTCTCAGAGTTGA
          1 AAAAACTAGACAGAAGCATTCTCAGAAACATCTTTGTGATGTTTGCACTCAACTCACAGAGTTGA

              *  * *          *  *        *        *   **        *  **       
     425559 ACATTCCCTTTCATAGAGTAGGTTTGAAACCCTCTTTTTATAGTGTCTGGAAGCGGGCATTTGGA
         66 ACCTTTCTTTTCATAGAGCAGTTTTGAAACACTCTTTTTGTAGAATCTGGAAGTGGATATTTGGA

                   *    * * **  **  **          * *  *
     425624 GCGCTTTCAGGCCTATGCTTAAAATAGGAAATATCTACCTAC
        131 GCGCTTTGAGG-ATTTCGTTGGAAACGGAAATATCTTCATAT

             *                    *    *  **          ** *        *    *     
     425666 AGAAACTAGACAGAAGCATTCTGAGAATCACGTTTGTGATGTGGGTACTCAACTAACAGTGTTGA
          1 AAAAACTAGACAGAAGCATTCTCAGAAACATCTTTGTGATGTTTGCACTCAACTCACAGAGTTGA

            *  *       *   *            *   *                *   *           
     425731 TCCATTCTTTTGATACAGCAGTTTTGAACCACACTTTTTGTAGAATCTGCAAGAGGATATTTGGA
         66 ACCTTTCTTTTCATAGAGCAGTTTTGAAACACTCTTTTTGTAGAATCTGGAAGTGGATATTTGGA

            **   *                     *   *      * *
     425796 TAGCTGTGAGGATTTCGTTGGAAACGGGAATGTCTTCAAAG
        131 GCGCTTTGAGGATTTCGTTGGAAACGGAAATATCTTCATAT

                *                         *   *            *    *            
     425837 AAAATCTAGACAGAAGCATTCTCAGAAACACCTTCGTGATGTTTGCAATCAAGTCACAGAGTTGA
          1 AAAAACTAGACAGAAGCATTCTCAGAAACATCTTTGTGATGTTTGCACTCAACTCACAGAGTTGA

                 * *             *  *           *      *             *       
     425902 ACCTTCCGTTTCATAGAGCAGGTTGGAAACACTCTTATTGTAGTATCTGGAAGTGGACATTTGGA
         66 ACCTTTCTTTTCATAGAGCAGTTTTGAAACACTCTTTTTGTAGAATCTGGAAGTGGATATTTGGA

                   *   ** ** *  *   *            **  
     425967 GCGCTTTCAGGCCTATGGTGAAAAAGGAAATATCTTCCCAT
        131 GCGCTTTGAGGATTTCGTTGGAAACGGAAATATCTTCATAT

                  * * *      **      *   * *   *     ** ** *      *    *     
     426008 AAAAACGACATAGAAGCTATCTCAGGAACTTGTTTATGATGCATCTAATCAACTAACAGTGTTGA
          1 AAAAACTAGACAGAAGCATTCTCAGAAACATCTTTGTGATGTTTGCACTCAACTCACAGAGTTGA

                  * ** * *                         * *       *               
     426073 ACCTTTGTACTGACAGAGCAG-TTTGAAACACTCTTTTTTTGGAATCTGCAAGTGGATATTTGGA
         66 ACCTTTCTTTTCATAGAGCAGTTTTGAAACACTCTTTTTGTAGAATCTGGAAGTGGATATTTGGA

            *                          **   *   *    
     426137 TCGCTTTGAGGATTTCGTTGGAAAC-GCGAT-GC--AATAT
        131 GCGCTTTGAGGATTTCGTTGGAAACGGAAATATCTTCATAT

                **  *    *     *                 **  *   *  *    *         * 
     426174 AAAACGTACACAGCAGCATACTCAGAAA-ATACTTTGCCATATTTCCATTCAAGTCACAGAGTGG
          1 AAAAACTAGACAGAAGCATTCTCAGAAACAT-CTTTGTGATGTTTGCACTCAACTCACAGAGTTG

               *  * **            *          *       *  *             *      
     426238 AACATTCCCATTCATAGAGCAGGTTTGAAACACACTTTTTGGAGTATCTGGAAGTGGACATTTGG
         65 AACCTTTCTTTTCATAGAGCAGTTTTGAAACACTCTTTTTGTAGAATCTGGAAGTGGATATTTGG

                    ** * * ** *  *   *            **  
     426303 AGCGCTTTCTGAACTATGGTGAAAAAGGAAATATCTTCCAAT
        130 AGCGCTTTGAGGATTTCGTTGGAAACGGAAATATCTTCATAT

            *     *               *      * *          *  **      **  *  *    
     426345 GAAAACAAGACAGAAGCATTCTGAGAAACTTATTTGTGATGTGTGTCCTCAACAAACGGACTTGA
          1 AAAAACTAGACAGAAGCATTCTCAGAAACATCTTTGTGATGTTTGCACTCAACTCACAGAGTTGA

                   *      **  * *  *  *             *   *    * * *           
     426410 ACCTTTCGTTTCATGCAGTACTTCTGGAACACTCTTTTTGAAGATTCTGCATGCGGATATTTGGA
         66 ACCTTTCTTTTCATAGAGCAGTTTTGAAACACTCTTTTTGTAGAATCTGGAAGTGGATATTTGGA

            **                            **   *   * 
     426475 TAGCTTTGAGGATTTCGTTGGAAACGG---GCT-TACATGT
        131 GCGCTTTGAGGATTTCGTTGGAAACGGAAATATCTTCATAT

                 *       *               *        *   *    *    *       *    
     426512 AAAAATTAGACAGCAGCATTCTCAGAAACTTCTTTGTGGTGTCTGCATTCAAGTCACAGAATTGA
          1 AAAAACTAGACAGAAGCATTCTCAGAAACATCTTTGTGATGTTTGCACTCAACTCACAGAGTTGA

              * ** * **            *  * *      *       *    *        *       
     426577 ACTTCCCCTCACATAGAGCAGTTGTGCAGCACTCTATTTGTAGTATCTCGAAGTGGACATTTGGA
         66 ACCTTTCTTTTCATAGAGCAGTTTTGAAACACTCTTTTTGTAGAATCTGGAAGTGGATATTTGGA

             *         ** *    * *   *            **  
     426642 GGGCTTTGTAGCCTATC-TGGAAAAAGGAAATATCTTCCCAT
        131 GCGCTTTG-AGGATTTCGTTGGAAACGGAAATATCTTCATAT

            *  ** *   *     * *            *   *  *   * **        *  * * *   
     426683 GAATGCGAGATAGAAGTAATCTCAGAAACATGTTTATGCTGTATCTACTCAACTAACTGTGCTGA
          1 AAAAACTAGACAGAAGCATTCTCAGAAACATCTTTGTGATGTTTGCACTCAACTCACAGAGTTGA

              *     *  *               *        *  * *       *               
     426748 ACATTTCTATTGATAGAGCAGTTTTGAGACACTCTTCTTTTGGAATCTGCAAGTGGATATTTGGA
         66 ACCTTTCTTTTCATAGAGCAGTTTTGAAACACTCTTTTTGTAGAATCTGGAAGTGGATATTTGGA

            ** *                        *     *      
     426813 TAGATTTGAGGATTTCGTTGGAAACGG-GAT-T-AT-ATAT
        131 GCGCTTTGAGGATTTCGTTGGAAACGGAAATATCTTCATAT

            *    *       *               *                 **  *   *         
     426850 CAAAAGTAGACAGCAGCATTCTCAGAAACTTCTTTGTGATGTTTGCATCCAGCTCTCAGAGTTGA
          1 AAAAACTAGACAGAAGCATTCTCAGAAACATCTTTGTGATGTTTGCACTCAACTCACAGAGTTGA

              *  * *          *  *        *        *   **        *  **       
     426915 ACATTCCCTTTCATAGAGTAGGTTTGAAACCCTCTTTTTATAGTGTCTGGAAGCGGGCATTTGGA
         66 ACCTTTCTTTTCATAGAGCAGTTTTGAAACACTCTTTTTGTAGAATCTGGAAGTGGATATTTGGA

                   *    * * **  **  **          * *   
     426980 GCGCTTTCAGGCCTATGCTTAAAATAGGAAATATCTACCTAT
        131 GCGCTTTGAGG-ATTTCGTTGGAAACGGAAATATCTTCATAT

             *                    *    *  **          ** *        *    *     
     427022 AGAAACTAGACAGAAGCATTCTGAGAATCACGTTTGTGATGTGGGTACTCAACTAACAGTGTTGA
          1 AAAAACTAGACAGAAGCATTCTCAGAAACATCTTTGTGATGTTTGCACTCAACTCACAGAGTTGA

            *  *       *   *            *   *                *               
     427087 TCCATTCTTTTGATACAGCAGTTTTGAACCACACTTTTTGTAGAATCTGCAAGTGGATATTTGGA
         66 ACCTTTCTTTTCATAGAGCAGTTTTGAAACACTCTTTTTGTAGAATCTGGAAGTGGATATTTGGA

            **   *                     *   *        *
     427152 TAGCTGTGAGGATTTCGTTGGAAACGGGAATGTCTTCATAG
        131 GCGCTTTGAGGATTTCGTTGGAAACGGAAATATCTTCATAT

                **         *           * * **         *  **   *   *          
     427193 AAAATTTAGACAGAAACATTCTCAGAACCTTGATTGTGATGTGTGTTCTCCACTAACAGAGTTGA
          1 AAAAACTAGACAGAAGCATTCTCAGAAACATCTTTGTGATGTTTGCACTCAACTCACAGAGTTGA

                       * *   * *   *       *       *                         
     427258 ACCTTTCTTTTGACAGAACTGTTCTGAAACATTCTTTTTATAGAATCTGGAAGTGGATATTTGGA
         66 ACCTTTCTTTTCATAGAGCAGTTTTGAAACACTCTTTTTGTAGAATCTGGAAGTGGATATTTGGA

            **                         *          *  
     427323 AAGCTTTGAGGATTTCGTTGGAAACGGGAATATCTTCAAAT
        131 GCGCTTTGAGGATTTCGTTGGAAACGGAAATATCTTCATAT

            *   *    *            *           * *       *  *    *            
     427364 CAAATCTAGCCAGAAGCATTCTAAGAAACATCTTAGGGATGTTTACATTCAAGTCACAGAGTTGA
          1 AAAAACTAGACAGAAGCATTCTCAGAAACATCTTTGTGATGTTTGCACTCAACTCACAGAGTTGA

              *  * *     *       *         *    * *   **      *      *    *  
     427429 ACATTCCCTTTCACAGAGCAGGTTTGAAACAATCTTCTCGTACTATCTGGCAGTGGACATTTTGA
         66 ACCTTTCTTTTCATAGAGCAGTTTTGAAACACTCTTTTTGTAGAATCTGGAAGTGGATATTTGGA

              * *   *  ** ** *  *   *            ** *
     427494 GCTCCTTGGGGCCTATGCTGAAAAAGGAAATATCTTCCGAC
        131 GCGCTTTGAGGATTTCGTTGGAAACGGAAATATCTTCATAT

                                 *     *  **          *           **    *    
     427535 AAAAACTAGACAGAAGCATTCGCAGAATCACGTTTGTGATGTGTGCACTCAACTGTCAGAATTGA
          1 AAAAACTAGACAGAAGCATTCTCAGAAACATCTTTGTGATGTTTGCACTCAACTCACAGAGTTGA

                 ***  ** *      *              *             * *            *
     427600 ACCTTGGATTGGACAGAGCACTTTTGAAACACTCTCTTTGTAGAATCTGCAGGTGGATATTTGGC
         66 ACCTTTCTTTTCATAGAGCAGTTTTGAAACACTCTTTTTGTAGAATCTGGAAGTGGATATTTGGA

            **                         *   *      * *
     427665 TAGCTTTGAGGATTTCGTTGGAAACGGTAATGTCTTCAAAG
        131 GCGCTTTGAGGATTTCGTTGGAAACGGAAATATCTTCATAT

                *                         *   *     *      *    *            
     427706 AAAATCTAGACAGAAGCATTCTCAGAAACACCTTCGTGATATTTGCAATCAAGTCACAGAGTTGA
          1 AAAAACTAGACAGAAGCATTCTCAGAAACATCTTTGTGATGTTTGCACTCAACTCACAGAGTTGA

                 * *             *  *                  *             *       
     427771 ACCTTCCGTTTCATAGAGCAGGTTGGAAACACTCTTTTTGTAGTATCTGGAAGTGGACATTTGGA
         66 ACCTTTCTTTTCATAGAGCAGTTTTGAAACACTCTTTTTGTAGAATCTGGAAGTGGATATTTGGA

             *         ** *    * *   *            **  
     427836 GGGCTTTGTAGCCTATC-TGGAAAAAGGAAATATCTTCCCAT
        131 GCGCTTTG-AGGATTTCGTTGGAAACGGAAATATCTTCATAT

            *  ** *   *     * *            *   *  *   * **        *  * * *   
     427877 GAATGCGAGATAGAAGTAATCTCAGAAACATGTTTATGCTGTATCTACTCAACTAACTGTGCTGA
          1 AAAAACTAGACAGAAGCATTCTCAGAAACATCTTTGTGATGTTTGCACTCAACTCACAGAGTTGA

              *     *  *               *        *  * *       *               
     427942 ACATTTCTATTGATAGAGCAGTTTTGAGACACTCTTCTTTTGGAATCTGCAAGTGGATATTTGGA
         66 ACCTTTCTTTTCATAGAGCAGTTTTGAAACACTCTTTTTGTAGAATCTGGAAGTGGATATTTGGA

            ** *                        *     *      
     428007 TAGATTTGAGGATTTCGTTGGAAACGG-GAT-T-AT-ATAT
        131 GCGCTTTGAGGATTTCGTTGGAAACGGAAATATCTTCATAT

            *    *       *               *                 **  *   *         
     428044 CAAAAGTAGACAGCAGCATTCTCAGAAACTTCTTTGTGATGTTTGCATCCAGCTCTCAGAGTTGA
          1 AAAAACTAGACAGAAGCATTCTCAGAAACATCTTTGTGATGTTTGCACTCAACTCACAGAGTTGA

            * *  * *          *  *        *        *   **        ** **       
     428109 GCATTCCCTTTCATAGAGTAGGTTTGAAACCCTCTTTTTATAGTGTCTGGAAGCAGGCATTTGGA
         66 ACCTTTCTTTTCATAGAGCAGTTTTGAAACACTCTTTTTGTAGAATCTGGAAGTGGATATTTGGA

                   *    * * **  **  **          * *  *
     428174 GCGCTTTCAGGCCTATGCTTAAAATAGGAAATATCTACCTAC
        131 GCGCTTTGAGG-ATTTCGTTGGAAACGGAAATATCTTCATAT

             *                    *    *  **          ** *        *    *     
     428216 AGAAACTAGACAGAAGCATTCTGAGAATCACGTTTGTGATGTGGGTACTCAACTAACAGTGTTGA
          1 AAAAACTAGACAGAAGCATTCTCAGAAACATCTTTGTGATGTTTGCACTCAACTCACAGAGTTGA

            *  *       *   *            *   *                *   *           
     428281 TCCATTCTTTTGATACAGCAGTTTTGAACCACACTTTTTGTAGAATCTGCAAGAGGATATTTGGA
         66 ACCTTTCTTTTCATAGAGCAGTTTTGAAACACTCTTTTTGTAGAATCTGGAAGTGGATATTTGGA

            **   *                     *   *      * *
     428346 TAGCTGTGAGGATTTCGTTGGAAACGGGAATGTCTTCAAAG
        131 GCGCTTTGAGGATTTCGTTGGAAACGGAAATATCTTCATAT

                *                         *   *            *    *            
     428387 AAAATCTAGACAGAAGCATTCTCAGAAACACCTTCGTGATGTTTGCAATCAAGTCACAGAGTTGA
          1 AAAAACTAGACAGAAGCATTCTCAGAAACATCTTTGTGATGTTTGCACTCAACTCACAGAGTTGA

                 * *             *  *           *      *             *       
     428452 ACCTTCCGTTTCATAGAGCAGGTTGGAAACACTCTTATTGTAGTATCTGGAAGTGGACATTTGGA
         66 ACCTTTCTTTTCATAGAGCAGTTTTGAAACACTCTTTTTGTAGAATCTGGAAGTGGATATTTGGA

                   *   ** ** *  *   *            **  
     428517 GCGCTTTCAGGCCTATGGTGAAAAAGGAAATATCTTCCCAT
        131 GCGCTTTGAGGATTTCGTTGGAAACGGAAATATCTTCATAT

                  * * *      **      *   * *   *     ** ** *      *   **     
     428558 AAAAACGACATAGAAGCTATCTCAGGAACTTGTTTATGATGCATCTAATCAACTAACACTGTTGA
          1 AAAAACTAGACAGAAGCATTCTCAGAAACATCTTTGTGATGTTTGCACTCAACTCACAGAGTTGA

                  * ** * *                         * *       *               
     428623 ACCTTTGTACTGACAGAGCAG-TTTGAAACACTCTTTTTTTGGAATCTGCAAGTGGATATTTGGA
         66 ACCTTTCTTTTCATAGAGCAGTTTTGAAACACTCTTTTTGTAGAATCTGGAAGTGGATATTTGGA

            *                           *   *   *    
     428687 TCGCTTTGAGGATTTCGTTGGAAACGG-GAT-GC--AATAT
        131 GCGCTTTGAGGATTTCGTTGGAAACGGAAATATCTTCATAT

                **  *    *     *                 **  *   *  *    *         * 
     428724 AAAACGTACACAGCAGCATACTCAGAAA-ATACTTTGCCATATTTCCATTCAAGTCACAGAGTGG
          1 AAAAACTAGACAGAAGCATTCTCAGAAACAT-CTTTGTGATGTTTGCACTCAACTCACAGAGTTG

               *  * **            *                  *  *             *      
     428788 AACATTCCCATTCATAGAGCAGGTTTGAAACACTCTTTTTGGAGTATCTGGAAGTGGACATTTGG
         65 AACCTTTCTTTTCATAGAGCAGTTTTGAAACACTCTTTTTGTAGAATCTGGAAGTGGATATTTGG

                    ** * * ** *  *   *            **  
     428853 AGCGCTTTCTGAACTATGGTGAAAAAGGAAATATCTTCCAAT
        130 AGCGCTTTGAGGATTTCGTTGGAAACGGAAATATCTTCATAT

            *     *               *      * *          *  **      **  *  *    
     428895 GAAAACAAGACAGAAGCATTCTGAGAAACTTATTTGTGATGTGTGTCCTCAACAAACGGACTTGA
          1 AAAAACTAGACAGAAGCATTCTCAGAAACATCTTTGTGATGTTTGCACTCAACTCACAGAGTTGA

                   *      **  * *  *  *             *   *    * * *           
     428960 ACCTTTCGTTTCATGCAGTACTTCTGGAACACTCTTTTTGAAGATTCTGCATGCGGATATTTGGA
         66 ACCTTTCTTTTCATAGAGCAGTTTTGAAACACTCTTTTTGTAGAATCTGGAAGTGGATATTTGGA

            **                            **   *   * 
     429025 TAGCTTTGAGGATTTCGTTGGAAACGG---GCT-TACATGT
        131 GCGCTTTGAGGATTTCGTTGGAAACGGAAATATCTTCATAT

                 *       *               *        *   *    *    *       *    
     429062 AAAAATTAGACAGCAGCATTCTCAGAAACTTCTTTGTGGTGTCTGCATTCAAGTCACAGAATTGA
          1 AAAAACTAGACAGAAGCATTCTCAGAAACATCTTTGTGATGTTTGCACTCAACTCACAGAGTTGA

              * ** * **            *  * *      *       *    *        *       
     429127 ACTTCCCCTCACATAGAGCAGTTGTGCAGCACTCTATTTGTAGTATCTCGAAGTGGACATTTGGA
         66 ACCTTTCTTTTCATAGAGCAGTTTTGAAACACTCTTTTTGTAGAATCTGGAAGTGGATATTTGGA

             *         ** *    * *   *            **  
     429192 GGGCTTTGTAGCCTATC-TGGAAAAAGGAAATATCTTCCCAT
        131 GCGCTTTG-AGGATTTCGTTGGAAACGGAAATATCTTCATAT

            *  ** *   *     * *            *   *  *   * **        *  * * *   
     429233 GAATGCGAGATAGAAGTAATCTCAGAAACATGTTTATGCTGTATCTACTCAACTAACTGTGCTGA
          1 AAAAACTAGACAGAAGCATTCTCAGAAACATCTTTGTGATGTTTGCACTCAACTCACAGAGTTGA

              *     *  *               *        *  * *       *               
     429298 ACATTTCTATTGATAGAGCAGTTTTGAGACACTCTTCTTTTGGAATCTGCAAGTGGATATTTGGA
         66 ACCTTTCTTTTCATAGAGCAGTTTTGAAACACTCTTTTTGTAGAATCTGGAAGTGGATATTTGGA

            ** *                        *     *      
     429363 TAGATTTGAGGATTTCGTTGGAAACGG-GAT-T-AT-ATAT
        131 GCGCTTTGAGGATTTCGTTGGAAACGGAAATATCTTCATAT

            *    *       *               *                 **  *   *         
     429400 CAAAAGTAGACAGCAGCATTCTCAGAAACTTCTTTGTGATGTTTGCATCCAGCTCTCAGAGTTGA
          1 AAAAACTAGACAGAAGCATTCTCAGAAACATCTTTGTGATGTTTGCACTCAACTCACAGAGTTGA

              *  * *          *  *        *        *   **        *  **       
     429465 ACATTCCCTTTCATAGAGTAGGTTTGAAACCCTCTTTTTATAGTGTCTGGAAGCGGGCATTTGGA
         66 ACCTTTCTTTTCATAGAGCAGTTTTGAAACACTCTTTTTGTAGAATCTGGAAGTGGATATTTGGA

                   *    * * **  **  **          * *   
     429530 GCGCTTTCAGGCCTATGCTTAAAATAGGAAATATCTACCTAT
        131 GCGCTTTGAGG-ATTTCGTTGGAAACGGAAATATCTTCATAT

             *                    *    *  **          ** *        *    *     
     429572 AGAAACTAGACAGAAGCATTCTGAGAATCACGTTTGTGATGTGGGTACTCAACTAACAGTGTTGA
          1 AAAAACTAGACAGAAGCATTCTCAGAAACATCTTTGTGATGTTTGCACTCAACTCACAGAGTTGA

            *  *       *   *            *   *                *               
     429637 TCCATTCTTTTGATACAGCAGTTTTGAACCACACTTTTTGTAGAATCTGCAAGTGGATATTTGGA
         66 ACCTTTCTTTTCATAGAGCAGTTTTGAAACACTCTTTTTGTAGAATCTGGAAGTGGATATTTGGA

            **   *                     *   *        *
     429702 TAGCTGTGAGGATTTCGTTGGAAACGGGAATGTCTTCATAG
        131 GCGCTTTGAGGATTTCGTTGGAAACGGAAATATCTTCATAT

                **                     * * **         *  **   *   *          
     429743 AAAATTTAGACAGAAGCATTCTCAGAACCTTGATTGTGATGTGTGTTCTCCACTAACAGAGTTGA
          1 AAAAACTAGACAGAAGCATTCTCAGAAACATCTTTGTGATGTTTGCACTCAACTCACAGAGTTGA

                       * *   * *   *       *       *                         
     429808 ACCTTTCTTTTGACAGAACTGTTCTGAAACATTCTTTTTATAGAATCTGGAAGTGGATATTTGGA
         66 ACCTTTCTTTTCATAGAGCAGTTTTGAAACACTCTTTTTGTAGAATCTGGAAGTGGATATTTGGA

            **                         *          *  
     429873 AAGCTTTGAGGATTTCGTTGGAAACGGGAATATCTTCAAAT
        131 GCGCTTTGAGGATTTCGTTGGAAACGGAAATATCTTCATAT

            *   *    *            *           * *       *  *    *            
     429914 CAAATCTAGCCAGAAGCATTCTAAGAAACATCTTAGGGATGTTTACATTCAAGTCACAGAGTTGA
          1 AAAAACTAGACAGAAGCATTCTCAGAAACATCTTTGTGATGTTTGCACTCAACTCACAGAGTTGA

              *  * *     *       *         *    * *   **      *      *    *  
     429979 ACATTCCCTTTCACAGAGCAGGTTTGAAACAATCTTCTCGTACTATCTGGCAGTGGACATTTTGA
         66 ACCTTTCTTTTCATAGAGCAGTTTTGAAACACTCTTTTTGTAGAATCTGGAAGTGGATATTTGGA

              * *   *  ** ** *  *   *            ** *
     430044 GCTCCTTGGGGCCTATGCTGAAAAAGGAAATATCTTCCGAC
        131 GCGCTTTGAGGATTTCGTTGGAAACGGAAATATCTTCATAT

                                 *     *  **          *           **    *    
     430085 AAAAACTAGACAGAAGCATTCGCAGAATCACGTTTGTGATGTGTGCACTCAACTGTCAGAATTGA
          1 AAAAACTAGACAGAAGCATTCTCAGAAACATCTTTGTGATGTTTGCACTCAACTCACAGAGTTGA

                 **   ** *      *                            * *            *
     430150 ACCTTGGTTTGGACAGAGCACTTTTGAAACACTCTTTTTGTAGAATCTGCAGGTGGATATTTGGC
         66 ACCTTTCTTTTCATAGAGCAGTTTTGAAACACTCTTTTTGTAGAATCTGGAAGTGGATATTTGGA

            **                      ** *   *      * *
     430215 TAGCTTTGAGGATTTCGTTGGAAAAAGTAATGTCTTCAAAG
        131 GCGCTTTGAGGATTTCGTTGGAAACGGAAATATCTTCATAT

                *                         *   *            *    *            
     430256 AAAATCTAGACAGAAGCATTCTCAGAAACAACTTCGTGATGTTTGCAATCAAGTCACAGAGTTGA
          1 AAAAACTAGACAGAAGCATTCTCAGAAACATCTTTGTGATGTTTGCACTCAACTCACAGAGTTGA

                 * *             *  *                  *             *       
     430321 ACCTTCCGTTTCATAGAGCAGGTTGGAAACACTCTTTTTGTAGTATCTGGAAGTGGACATTTGGA
         66 ACCTTTCTTTTCATAGAGCAGTTTTGAAACACTCTTTTTGTAGAATCTGGAAGTGGATATTTGGA

             *         ** *    * *   *            **  
     430386 GGGCTTTGTAGCCTATC-TGGAAAAAGGAAATATCTTCCCAT
        131 GCGCTTTG-AGGATTTCGTTGGAAACGGAAATATCTTCATAT

            *  ** *   *     * *                *  *   * **        *  * * *   
     430427 GAATGCGAGATAGAAGTAATCTCAGAAACATCTTTATGCTGTATCTACTCAACTAACTGTGCTGA
          1 AAAAACTAGACAGAAGCATTCTCAGAAACATCTTTGTGATGTTTGCACTCAACTCACAGAGTTGA

              *     *  *               *        *  * *       *               
     430492 ACATTTCTATTGATAGAGCAGTTTTGAGACACTCTTCTTTTGGAATCTGCAAGTGGATATTTGGA
         66 ACCTTTCTTTTCATAGAGCAGTTTTGAAACACTCTTTTTGTAGAATCTGGAAGTGGATATTTGGA

            ** *                        *     *      
     430557 TAGATTTGAGGATTTCGTTGGAAACGG-GAT-T-AT-ATAT
        131 GCGCTTTGAGGATTTCGTTGGAAACGGAAATATCTTCATAT

                 *       *               *                 **  *   *         
     430594 AAAAAGTAGACAGCAGCATTCTCAGAAACTTCTTTGTGATGTTTGCATCCAGCTCTCAGAGTTGA
          1 AAAAACTAGACAGAAGCATTCTCAGAAACATCTTTGTGATGTTTGCACTCAACTCACAGAGTTGA

            * *  * *          *  *        *        *   **        *  **       
     430659 GCATTCCCTTTCATAGAGTAGGTTTGAAACCCTCTTTTTATAGTGTCTGGAAGCGGGCATTTGGA
         66 ACCTTTCTTTTCATAGAGCAGTTTTGAAACACTCTTTTTGTAGAATCTGGAAGTGGATATTTGGA

                   *    * * **  **  **          * *  *
     430724 GCGCTTTCAGGCCTATGCTTAAAATAGGAAATATCTACCTAC
        131 GCGCTTTGAGG-ATTTCGTTGGAAACGGAAATATCTTCATAT

             *                    *    *  **          ** *        *    *     
     430766 AGAAACTAGACAGAAGCATTCTGAGAATCACGTTTGTGATGTGGGTACTCAACTAACAGTGTTGA
          1 AAAAACTAGACAGAAGCATTCTCAGAAACATCTTTGTGATGTTTGCACTCAACTCACAGAGTTGA

            *  *       *   *            *   *                *               
     430831 TCCATTCTTTTGATACAGCAGTTTTGAACCACACTTTTTGTAGAATCTGCAAGTGGATATTTGGA
         66 ACCTTTCTTTTCATAGAGCAGTTTTGAAACACTCTTTTTGTAGAATCTGGAAGTGGATATTTGGA

            **   *                     *   *      * *
     430896 TAGCTGTGAGGATTTCGTTGGAAACGGTAATGTCTTCAAAG
        131 GCGCTTTGAGGATTTCGTTGGAAACGGAAATATCTTCATAT

                *                         *   *            *    *            
     430937 AAAATCTAGACAGAAGCATTCTCAGAAACACCTTCGTGATGTTTGCAATCAAGTCACAGAGTTGA
          1 AAAAACTAGACAGAAGCATTCTCAGAAACATCTTTGTGATGTTTGCACTCAACTCACAGAGTTGA

                 * *             *  *           *      *             *       
     431002 ACCTTCCGTTTCATAGAGCAGGTTGGAAACACTCTTATTGTAGTATCTGGAAGTGGACATTTGGA
         66 ACCTTTCTTTTCATAGAGCAGTTTTGAAACACTCTTTTTGTAGAATCTGGAAGTGGATATTTGGA

                   *   ** ** *  *   *            **  
     431067 GCGCTTTCAGGCCTATGGTGAAAAAGGAAATATCTTCCCAT
        131 GCGCTTTGAGGATTTCGTTGGAAACGGAAATATCTTCATAT

                  * * *      **      *   * *   *     ** ** *      *    *     
     431108 AAAAACGACATAGAAGCTATCTCAGGAACTTGTTTATGATGCATCTAATCAACTAACAGTGTTGA
          1 AAAAACTAGACAGAAGCATTCTCAGAAACATCTTTGTGATGTTTGCACTCAACTCACAGAGTTGA

                  * ** * *                         * *       *               
     431173 ACCTTTGTACTGACAGAGCAG-TTTGAAACACTCTTTTTTTGGAATCTGCAAGTGGATATTTGGA
         66 ACCTTTCTTTTCATAGAGCAGTTTTGAAACACTCTTTTTGTAGAATCTGGAAGTGGATATTTGGA

            *                           *   *   *    
     431237 TCGCTTTGAGGATTTCGTTGGAAACGG-GAT-GC--AATAT
        131 GCGCTTTGAGGATTTCGTTGGAAACGGAAATATCTTCATAT

                **  *    *     *                 **  *   *  *    *         * 
     431274 AAAACGTACACAGCAGCATACTCAGAAA-ATACTTTGCCATATTTCCATTCAAGTCACAGAGTGG
          1 AAAAACTAGACAGAAGCATTCTCAGAAACAT-CTTTGTGATGTTTGCACTCAACTCACAGAGTTG

               *  * **            *  *               *  *             *      
     431338 AACATTCCCATTCATAGAGCAGGTTGGAAACACTCTTTTTGGAGTATCTGGAAGTGGACATTTGG
         65 AACCTTTCTTTTCATAGAGCAGTTTTGAAACACTCTTTTTGTAGAATCTGGAAGTGGATATTTGG

                    ** * * ** *  *   *            **  
     431403 AGCGCTTTCTGAACTATGGTGAAAAAGGAAATATCTTCCAAT
        130 AGCGCTTTGAGGATTTCGTTGGAAACGGAAATATCTTCATAT

            *     *               *      * *          *  **      **  *  *    
     431445 GAAAACAAGACAGAAGCATTCTGAGAAACTTATTTGTGATGTGTGTCCTCAACAAACGGACTTGA
          1 AAAAACTAGACAGAAGCATTCTCAGAAACATCTTTGTGATGTTTGCACTCAACTCACAGAGTTGA

                   *      **  * *  *  *             *   *    * * *           
     431510 ACCTTTCGTTTCATGCAGTACTTCTGGAACACTCTTTTTGAAGATTCTGCATGCGGATATTTGGA
         66 ACCTTTCTTTTCATAGAGCAGTTTTGAAACACTCTTTTTGTAGAATCTGGAAGTGGATATTTGGA

            **                            **   *   * 
     431575 TAGCTTTGAGGATTTCGTTGGAAACGG---GCT-TACATGT
        131 GCGCTTTGAGGATTTCGTTGGAAACGGAAATATCTTCATAT

                 *       *               *        *   *    *    *       *    
     431612 AAAAATTAGACAGCAGCATTCTCAGAAACTTCTTTGTGGTGTCTGCATTCAAGTCACAGAATTGA
          1 AAAAACTAGACAGAAGCATTCTCAGAAACATCTTTGTGATGTTTGCACTCAACTCACAGAGTTGA

              * ** * **            *  * *      *       *             *       
     431677 ACTTCCCCTCACATAGAGCAGTTGTGCAGCACTCTATTTGTAGTATCTGGAAGTGGACATTTGGA
         66 ACCTTTCTTTTCATAGAGCAGTTTTGAAACACTCTTTTTGTAGAATCTGGAAGTGGATATTTGGA

             *         ** *    * *   *            **  
     431742 GGGCTTTGTAGCCTATC-TGGAAAAAGGAAATATCTTCCCAT
        131 GCGCTTTG-AGGATTTCGTTGGAAACGGAAATATCTTCATAT

            *  ** *   *     * *            *   *  *   * **        *  * * *   
     431783 GAATGCGAGATAGAAGTAATCTCAGAAACATGTTTATGCTGTATCTACTCAACTAACTGTGCTGA
          1 AAAAACTAGACAGAAGCATTCTCAGAAACATCTTTGTGATGTTTGCACTCAACTCACAGAGTTGA

              *     *  *               *        *  * *       *               
     431848 ACATTTCTATTGATAGAGCAGTTTTGAGACACTCTTCTTTTGGAATCTGCAAGTGGATATTTGGA
         66 ACCTTTCTTTTCATAGAGCAGTTTTGAAACACTCTTTTTGTAGAATCTGGAAGTGGATATTTGGA

            ** *                        *     *      
     431913 TAGATTTGAGGATTTCGTTGGAAACGG-GAT-T-AT-ATAT
        131 GCGCTTTGAGGATTTCGTTGGAAACGGAAATATCTTCATAT

            *    *       *               *                 **  *   *         
     431950 CAAAAGTAGACAGCAGCATTCTCAGAAACTTCTTTGTGATGTTTGCATCCAGCTCTCAGAGTTGA
          1 AAAAACTAGACAGAAGCATTCTCAGAAACATCTTTGTGATGTTTGCACTCAACTCACAGAGTTGA

              *  * *          *  *        *        *   **        *  **       
     432015 ACATTCCCTTTCATAGAGTAGGTTTGAAACCCTCTTTTTATAGTGTCTGGAAGCGGGCATTTGGA
         66 ACCTTTCTTTTCATAGAGCAGTTTTGAAACACTCTTTTTGTAGAATCTGGAAGTGGATATTTGGA

                *  *    * * **  **  **          * *  *
     432080 GCGCATTCAGGCCTATGCTTAAAATAGGAAATATCTACCTAC
        131 GCGCTTTGAGG-ATTTCGTTGGAAACGGAAATATCTTCATAT

             *                    *    *  **          ** *        *    *     
     432122 AGAAACTAGACAGAAGCATTCTGAGAATCACGTTTGTGATGTGGGTACTCAACTAACAGTGTTGA
          1 AAAAACTAGACAGAAGCATTCTCAGAAACATCTTTGTGATGTTTGCACTCAACTCACAGAGTTGA

            *  *       *   *            *   *                *   *           
     432187 TCCATTCTTTTGATACAGCAGTTTTGAACCACACTTTTTGTAGAATCTGCAAGAGGATATTTGGA
         66 ACCTTTCTTTTCATAGAGCAGTTTTGAAACACTCTTTTTGTAGAATCTGGAAGTGGATATTTGGA

            **   *                     *   *      * *
     432252 TAGCTGTGAGGATTTCGTTGGAAACGGGAATGTCTTCAAAG
        131 GCGCTTTGAGGATTTCGTTGGAAACGGAAATATCTTCATAT

                *                         *   *            *    *            
     432293 AAAATCTAGACAGAAGCATTCTCAGAAACACCTTCGTGATGTTTGCAATCAAGTCACAGAGTTGA
          1 AAAAACTAGACAGAAGCATTCTCAGAAACATCTTTGTGATGTTTGCACTCAACTCACAGAGTTGA

                 * *             *  *           *      *             *       
     432358 ACCTTCCGTTTCATAGAGCAGGTTGGAAACACTCTTATTGTAGTATCTGGAAGTGGACATTTGGA
         66 ACCTTTCTTTTCATAGAGCAGTTTTGAAACACTCTTTTTGTAGAATCTGGAAGTGGATATTTGGA

                   *   ** ** *  *   *            **  
     432423 GCGCTTTCAGGCCTATGGTGAAAAAGGAAATATCTTCCCAT
        131 GCGCTTTGAGGATTTCGTTGGAAACGGAAATATCTTCATAT

                  * * *      **      *   * *   *     ** ** *      *    *     
     432464 AAAAACGACATAGAAGCTATCTCAGGAACTTGTTTATGATGCATCTAATCAACTAACAGTGTTGA
          1 AAAAACTAGACAGAAGCATTCTCAGAAACATCTTTGTGATGTTTGCACTCAACTCACAGAGTTGA

                  * ** * *                         * *       *               
     432529 ACCTTTGTACTGACAGAGCAG-TTTGAAACACTCTTTTTTTGGAATCTGCAAGTGGATATTTGGA
         66 ACCTTTCTTTTCATAGAGCAGTTTTGAAACACTCTTTTTGTAGAATCTGGAAGTGGATATTTGGA

            *                           *   *   *    
     432593 TCGCTTTGAGGATTTCGTTGGAAACGG-GAT-GC--AATAT
        131 GCGCTTTGAGGATTTCGTTGGAAACGGAAATATCTTCATAT

                **  *    *     *                 **  *   *  *    *   *     * 
     432630 AAAACGTACACAGCAGCATACTCAGAAA-ATACTTTGCCATATTTCCATTCAAGTCAGAGAGTGG
          1 AAAAACTAGACAGAAGCATTCTCAGAAACAT-CTTTGTGATGTTTGCACTCAACTCACAGAGTTG

               *  * **            *                  *  *             *      
     432694 AACATTCCCATTCATAGAGCAGGTTTGAAACACTCTTTTTGGAGTATCTGGAAGTGGACATTTGG
         65 AACCTTTCTTTTCATAGAGCAGTTTTGAAACACTCTTTTTGTAGAATCTGGAAGTGGATATTTGG

                    ** * * ** *  *   *            **  
     432759 AGCGCTTTCTGAACTATGGTGAAAAAGGAAATATCTTCCAAT
        130 AGCGCTTTGAGGATTTCGTTGGAAACGGAAATATCTTCATAT

            *     *               *      * *          *  **      **  *  *    
     432801 GAAAACAAGACAGAAGCATTCTGAGAAACTTATTTGTGATGTGTGTCCTCAACAAACGGACTTGA
          1 AAAAACTAGACAGAAGCATTCTCAGAAACATCTTTGTGATGTTTGCACTCAACTCACAGAGTTGA

                   *      **  * *  *  *             *   *    * * *           
     432866 ACCTTTCGTTTCATGCAGTACTTCTGGAACACTCTTTTTGAAGATTCTGCATGCGGATATTTGGA
         66 ACCTTTCTTTTCATAGAGCAGTTTTGAAACACTCTTTTTGTAGAATCTGGAAGTGGATATTTGGA

            **                            **   *   * 
     432931 TAGCTTTGAGGATTTCGTTGGAAACGG---GCT-TACATGT
        131 GCGCTTTGAGGATTTCGTTGGAAACGGAAATATCTTCATAT

                 *       *               *        *   *    *    *       *    
     432968 AAAAATTAGACAGCAGCATTCTCAGAAACTTCTTTGTGGTGTCTGCATTCAAGTCACAGAATTGA
          1 AAAAACTAGACAGAAGCATTCTCAGAAACATCTTTGTGATGTTTGCACTCAACTCACAGAGTTGA

              * ** * **            *  * *      *       *    *        *       
     433033 ACTTCCCCTCACATAGAGCAGTTGTGCAGCACTCTATTTGTAGTATCTCGAAGTGGACATTTGGA
         66 ACCTTTCTTTTCATAGAGCAGTTTTGAAACACTCTTTTTGTAGAATCTGGAAGTGGATATTTGGA

             *         ** *    * *   *            **  
     433098 GGGCTTTGTAGCCTATC-TGGAAAAAGGAAATATCTTCCCAT
        131 GCGCTTTG-AGGATTTCGTTGGAAACGGAAATATCTTCATAT

            *  ** *   *     * *            *   *  *   * **        *  * * *   
     433139 GAATGCGAGATAGAAGTAATCTCAGAAACATGTTTATGCTGTATCTACTCAACTAACTGTGCTGA
          1 AAAAACTAGACAGAAGCATTCTCAGAAACATCTTTGTGATGTTTGCACTCAACTCACAGAGTTGA

              *     *  *               *        *  * *       *               
     433204 ACATTTCTATTGATAGAGCAGTTTTGAGACACTCTTCTTTTGGAATCTGCAAGTGGATATTTGGA
         66 ACCTTTCTTTTCATAGAGCAGTTTTGAAACACTCTTTTTGTAGAATCTGGAAGTGGATATTTGGA

            ** *                        *     *      
     433269 TAGATTTGAGGATTTCGTTGGAAACGG-GAT-T-AT-ATAT
        131 GCGCTTTGAGGATTTCGTTGGAAACGGAAATATCTTCATAT

            *    *       *               *                 **  *   *         
     433306 CAAAAGTAGACAGCAGCATTCTCAGAAACTTCTTTGTGATGTTTGCATCCAGCTCTCAGAGTTGA
          1 AAAAACTAGACAGAAGCATTCTCAGAAACATCTTTGTGATGTTTGCACTCAACTCACAGAGTTGA

              *  * *          *  *        *        *   **        *  **       
     433371 ACATTCCCTTTCATAGAGTAGGTTTGAAACCCTCTTTTTATAGTGTCTGGAAGCGGGCATTTGGA
         66 ACCTTTCTTTTCATAGAGCAGTTTTGAAACACTCTTTTTGTAGAATCTGGAAGTGGATATTTGGA

                   *    * * **  **  **          * *  *
     433436 GCGCTTTCAGGCCTATGCTTAAAATAGGAAATATCTACCTAC
        131 GCGCTTTGAGG-ATTTCGTTGGAAACGGAAATATCTTCATAT

             *                    *    *  **          ** *        *    *     
     433478 AGAAACTAGACAGAAGCATTCTGAGAATCACGTTTGTGATGTGGGTACTCAACTAACAGTGTTGA
          1 AAAAACTAGACAGAAGCATTCTCAGAAACATCTTTGTGATGTTTGCACTCAACTCACAGAGTTGA

            *  *       *   *            *   *                *   *           
     433543 TCCATTCTTTTGATACAGCAGTTTTGAACCACACTTTTTGTAGAATCTGCAAGAGGATATTTGGA
         66 ACCTTTCTTTTCATAGAGCAGTTTTGAAACACTCTTTTTGTAGAATCTGGAAGTGGATATTTGGA

            **   *                     *   *      * *
     433608 TAGCTGTGAGGATTTCGTTGGAAACGGGAATGTCTTCAAAG
        131 GCGCTTTGAGGATTTCGTTGGAAACGGAAATATCTTCATAT

                *                         *   *            *    *            
     433649 AAAATCTAGACAGAAGCATTCTCAGAAACACCTTCGTGATGTTTGCAATCAAGTCACAGAGTTGA
          1 AAAAACTAGACAGAAGCATTCTCAGAAACATCTTTGTGATGTTTGCACTCAACTCACAGAGTTGA

                 * *             *  *           *      *             *       
     433714 ACCTTCCGTTTCATAGAGCAGGTTGGAAACACTCTTATTGTAGTATCTGGAAGTGGACATTTGGA
         66 ACCTTTCTTTTCATAGAGCAGTTTTGAAACACTCTTTTTGTAGAATCTGGAAGTGGATATTTGGA

                   *   ** ** *  *   *            **  
     433779 GCGCTTTCAGGCCTATGGTGAAAAAGGAAATATCTTCCCAT
        131 GCGCTTTGAGGATTTCGTTGGAAACGGAAATATCTTCATAT

                  * * *      **      *   * *   *     ** ** *      *    *     
     433820 AAAAACGACATAGAAGCTATCTCAGGAACTTGTTTATGATGCATCTAATCAACTAACAGTGTTGA
          1 AAAAACTAGACAGAAGCATTCTCAGAAACATCTTTGTGATGTTTGCACTCAACTCACAGAGTTGA

                  * ** * *                         * *       *               
     433885 ACCTTTGTACTGACAGAGCAG-TTTGAAACACTCTTTTTTTGGAATCTGCAAGTGGATATTTGGA
         66 ACCTTTCTTTTCATAGAGCAGTTTTGAAACACTCTTTTTGTAGAATCTGGAAGTGGATATTTGGA

            *                           *   *   *    
     433949 TCGCTTTGAGGATTTCGTTGGAAACGG-GAT-GC--AATAT
        131 GCGCTTTGAGGATTTCGTTGGAAACGGAAATATCTTCATAT

                **  *    *     *                 **  *   *  *    *         * 
     433986 AAAACGTACACAGCAGCATACTCAGAAA-ATACTTTGCCATATTTCCATTCAAGTCACAGAGTGG
          1 AAAAACTAGACAGAAGCATTCTCAGAAACAT-CTTTGTGATGTTTGCACTCAACTCACAGAGTTG

               *  * **            *          *       *  *             *      
     434050 AACATTCCCATTCATAGAGCAGGTTTGAAACACACTTTTTGGAGTATCTGGAAGTGGACATTTGG
         65 AACCTTTCTTTTCATAGAGCAGTTTTGAAACACTCTTTTTGTAGAATCTGGAAGTGGATATTTGG

                    ** * * ** *  *   *            **  
     434115 AGCGCTTTCTGAACTATGGTGAAAAAGGAAATATCTTCCAAT
        130 AGCGCTTTGAGGATTTCGTTGGAAACGGAAATATCTTCATAT

            *     *               *      * *          *  **    * **  *  *    
     434157 GAAAACAAGACAGAAGCATTCTGAGAAACTTATTTGTGATGTGTGTCCTCACCAAACGGACTTGA
          1 AAAAACTAGACAGAAGCATTCTCAGAAACATCTTTGTGATGTTTGCACTCAACTCACAGAGTTGA

                   *      **  * *  *  *             *   *    * * *           
     434222 ACCTTTCGTTTCATGCAGTACTTCTGGAACACTCTTTTTGAAGATTCTGCATGCGGATATTTGGA
         66 ACCTTTCTTTTCATAGAGCAGTTTTGAAACACTCTTTTTGTAGAATCTGGAAGTGGATATTTGGA

            **                            **   *   * 
     434287 TAGCTTTGAGGATTTCGTTGGAAACGG---GCT-TACATGT
        131 GCGCTTTGAGGATTTCGTTGGAAACGGAAATATCTTCATAT

                 *       *               *        *   *    *    *       *    
     434324 AAAAATTAGACAGCAGCATTCTCAGAAACTTCTTTGTGGTGTCTGCATTCAAGTCACAGAATTGA
          1 AAAAACTAGACAGAAGCATTCTCAGAAACATCTTTGTGATGTTTGCACTCAACTCACAGAGTTGA

              * ** * **            *  * *      *       *    *        *       
     434389 ACTTCCCCTCACATAGAGCAGTTGTGCAGCACTCTATTTGTAGTATCTCGAAGTGGACATTTGGA
         66 ACCTTTCTTTTCATAGAGCAGTTTTGAAACACTCTTTTTGTAGAATCTGGAAGTGGATATTTGGA

             *         ** *    * *   *            **  
     434454 GGGCTTTGTAGCCTATC-TGGAAAAAGGAAATATCTTCCCAT
        131 GCGCTTTG-AGGATTTCGTTGGAAACGGAAATATCTTCATAT

            *  ** *   *     * *            *   *  *   * **        *  * * *   
     434495 GAATGCGAGATAGAAGTAATCTCAGAAACATGTTTATGCTGTATCTACTCAACTAACTGTGCTGA
          1 AAAAACTAGACAGAAGCATTCTCAGAAACATCTTTGTGATGTTTGCACTCAACTCACAGAGTTGA

              *     *  *               *        *  * *       *               
     434560 ACATTTCTATTGATAGAGCAGTTTTGAGACACTCTTCTTTTGGAATCTGCAAGTGGATATTTGGA
         66 ACCTTTCTTTTCATAGAGCAGTTTTGAAACACTCTTTTTGTAGAATCTGGAAGTGGATATTTGGA

            ** *                        *     *      
     434625 TAGATTTGAGGATTTCGTTGGAAACGG-GAT-T-AT-ATAT
        131 GCGCTTTGAGGATTTCGTTGGAAACGGAAATATCTTCATAT

                 *       *               *                 **  *   *         
     434662 AAAAAGTAGACAGCAGCATTCTCAGAAACTTCTTTGTGATGTTTGCATCCAGCTCTCAGAGTTGA
          1 AAAAACTAGACAGAAGCATTCTCAGAAACATCTTTGTGATGTTTGCACTCAACTCACAGAGTTGA

              *  * *          *  *        *        *   **    *   *  **       
     434727 ACATTCCCTTTCATAGAGTAGGTTTGAAACCCTCTTTTTATAGTGTCTGCAAGCGGGCATTTGGA
         66 ACCTTTCTTTTCATAGAGCAGTTTTGAAACACTCTTTTTGTAGAATCTGGAAGTGGATATTTGGA

                   *    * * **  **  **          * *  *
     434792 GCGCTTTCAGGCCTATGCTTAAAATAGGAAATATCTACCTAC
        131 GCGCTTTGAGG-ATTTCGTTGGAAACGGAAATATCTTCATAT

             *                    *    *  **          ** *        *    *     
     434834 AGAAACTAGACAGAAGCATTCTGAGAATCACGTTTGTGATGTGGGTACTCAACTAACAGTGTTGA
          1 AAAAACTAGACAGAAGCATTCTCAGAAACATCTTTGTGATGTTTGCACTCAACTCACAGAGTTGA

            *  *       *   *            *   *                *   *           
     434899 TCCATTCTTTTGATACAGCAGTTTTGAACCACACTTTTTGTAGAATCTGCAAGAGGATATTTGGA
         66 ACCTTTCTTTTCATAGAGCAGTTTTGAAACACTCTTTTTGTAGAATCTGGAAGTGGATATTTGGA

            **   *                     *   *      * *
     434964 TAGCTGTGAGGATTTCGTTGGAAACGGGAATGTCTTCAAAG
        131 GCGCTTTGAGGATTTCGTTGGAAACGGAAATATCTTCATAT

                *                         *   *            *    *            
     435005 AAAATCTAGACAGAAGCATTCTCAGAAACACCTTCGTGATGTTTGCAATCAAGTCACAGAGTTGA
          1 AAAAACTAGACAGAAGCATTCTCAGAAACATCTTTGTGATGTTTGCACTCAACTCACAGAGTTGA

                 * *             *  *           *      *             *       
     435070 ACCTTCCGTTTCATAGAGCAGGTTGGAAACACTCTTATTGTAGTATCTGGAAGTGGACATTTGGA
         66 ACCTTTCTTTTCATAGAGCAGTTTTGAAACACTCTTTTTGTAGAATCTGGAAGTGGATATTTGGA

                   *   ** ** *  *   *            **  
     435135 GCGCTTTCAGGCCTATGGTGAAAAAGGAAATATCTTCCCAT
        131 GCGCTTTGAGGATTTCGTTGGAAACGGAAATATCTTCATAT

                  * * *      **      *   * *   *     ** ** *      *    *     
     435176 AAAAACGACATAGAAGCTATCTCAGGAACTTGTTTATGATGCATCTAATCAACTAACAGTGTTGA
          1 AAAAACTAGACAGAAGCATTCTCAGAAACATCTTTGTGATGTTTGCACTCAACTCACAGAGTTGA

                  * ** * *                         * *       *               
     435241 ACCTTTGTACTGACAGAGCAG-TTTGAAACACTCTTTTTTTGGAATCTGCAAGTGGATATTTGGA
         66 ACCTTTCTTTTCATAGAGCAGTTTTGAAACACTCTTTTTGTAGAATCTGGAAGTGGATATTTGGA

            *                           *   *   *    
     435305 TCGCTTTGAGGATTTCGTTGGAAACGG-GAT-GC--AATAT
        131 GCGCTTTGAGGATTTCGTTGGAAACGGAAATATCTTCATAT

                **  *    *     *                 **  *   *  *    *         * 
     435342 AAAACGTACACAGCAGCATACTCAGAAA-ATACTTTGCCATATTTCCATTCAAGTCACAGAGTGG
          1 AAAAACTAGACAGAAGCATTCTCAGAAACAT-CTTTGTGATGTTTGCACTCAACTCACAGAGTTG

               *  * **            *                  *  *             *      
     435406 AACATTCCCATTCATAGAGCAGGTTTGAAACACTCTTTTTGGAGTATCTGGAAGTGGACATTTGG
         65 AACCTTTCTTTTCATAGAGCAGTTTTGAAACACTCTTTTTGTAGAATCTGGAAGTGGATATTTGG

                    ** * * ** *  *   *            **  
     435471 AGCGCTTTCTGAACTATGGTGAAAAAGGAAATATCTTCCAAT
        130 AGCGCTTTGAGGATTTCGTTGGAAACGGAAATATCTTCATAT

            *     *               *                    *  **      **  *  *   
     435513 GAAAACAAGACAGAAGCATTCTGAGAAAC-TCATTTGTGATGTGTGTCCTCAACAAACGGACTTG
          1 AAAAACTAGACAGAAGCATTCTCAGAAACATC-TTTGTGATGTTTGCACTCAACTCACAGAGTTG

                    *      **  * *  *  *             *   *    * * *          
     435577 AACCTTTCGTTTCATGCAGTACTTCTGGAACACTCTTTTTGAAGATTCTGCATGCGGATATTTGG
         65 AACCTTTCTTTTCATAGAGCAGTTTTGAAACACTCTTTTTGTAGAATCTGGAAGTGGATATTTGG

             **                            **   *   * 
     435642 ATAGCTTTGAGGATTTCGTTGGAAACGG---GCT-TACATGT
        130 AGCGCTTTGAGGATTTCGTTGGAAACGGAAATATCTTCATAT

                 *       *               *        *   *    *    *       *    
     435680 AAAAATTAGACAGCAGCATTCTCAGAAACTTCTTTGTGGTGTCTGCATTCAAGTCACAGAATTGA
          1 AAAAACTAGACAGAAGCATTCTCAGAAACATCTTTGTGATGTTTGCACTCAACTCACAGAGTTGA

              * ** * **            *  * *      *       *             *       
     435745 ACTTCCCCTCACATAGAGCAGTTGTGCAGCACTCTATTTGTAGTATCTGGAAGTGGACATTTGGA
         66 ACCTTTCTTTTCATAGAGCAGTTTTGAAACACTCTTTTTGTAGAATCTGGAAGTGGATATTTGGA

             *         ** *    * *   *            **  
     435810 GGGCTTTGTAGCCTAT-GTGGAAAAAGGAAATATCTTCCCAT
        131 GCGCTTTG-AGGATTTCGTTGGAAACGGAAATATCTTCATAT

            *  ** *   *     * *            *   *  *   * **        *  * * *   
     435851 GAATGCGAGATAGAAGTAATCTCAGAAACATGTTTATGCTGTATCTACTCAACTAACTGTGCTGA
          1 AAAAACTAGACAGAAGCATTCTCAGAAACATCTTTGTGATGTTTGCACTCAACTCACAGAGTTGA

              *     *  *               *        *  * *       *               
     435916 ACATTTCTATTGATAGAGCAGTTTTGAGACACTCTTCTTTTGGAATCTGCAAGTGGATATTTGGA
         66 ACCTTTCTTTTCATAGAGCAGTTTTGAAACACTCTTTTTGTAGAATCTGGAAGTGGATATTTGGA

            ** *                        *     *      
     435981 TAGATTTGAGGATTTCGTTGGAAACGG-GAT-T-AT-ATAT
        131 GCGCTTTGAGGATTTCGTTGGAAACGGAAATATCTTCATAT

            *    *       *               *                 **  *   *         
     436018 CAAAAGTAGACAGCAGCATTCTCAGAAACTTCTTTGTGATGTTTGCATCCAGCTCTCAGAGTTGA
          1 AAAAACTAGACAGAAGCATTCTCAGAAACATCTTTGTGATGTTTGCACTCAACTCACAGAGTTGA

              *  * *          *  *        *        *   **        *  **       
     436083 ACATTCCCTTTCATAGAGTAGGTTTGAAACCCTCTTTTTATAGTGTCTGGAAGCGGGCATTTGGA
         66 ACCTTTCTTTTCATAGAGCAGTTTTGAAACACTCTTTTTGTAGAATCTGGAAGTGGATATTTGGA

                   *    * * **  **  **          * *  *
     436148 GCGCTTTCAGGCCTATGCTTAAAATAGGAAATATCTACCTAC
        131 GCGCTTTGAGG-ATTTCGTTGGAAACGGAAATATCTTCATAT

             *                    *    *  **          ** *        *    *     
     436190 AGAAACTAGACAGAAGCATTCTGAGAATCACGTTTGTGATGTGGGTACTCAACTAACAGTGTTGA
          1 AAAAACTAGACAGAAGCATTCTCAGAAACATCTTTGTGATGTTTGCACTCAACTCACAGAGTTGA

            *  *       *   *            *   *                *   *           
     436255 TCCATTCTTTTGATACAGCAGTTTTGAACCACACTTTTTGTAGAATCTGCAAGAGGATATTTGGA
         66 ACCTTTCTTTTCATAGAGCAGTTTTGAAACACTCTTTTTGTAGAATCTGGAAGTGGATATTTGGA

            **   *                     *   *      * *
     436320 TAGCTGTGAGGATTTCGTTGGAAACGGGAATGTCTTCAAAG
        131 GCGCTTTGAGGATTTCGTTGGAAACGGAAATATCTTCATAT

                *                         *   *            *    *            
     436361 AAAATCTAGACAGAAGCATTCTCAGAAACACCTTCGTGATGTTTGCAATCAAGTCACAGAGTTGA
          1 AAAAACTAGACAGAAGCATTCTCAGAAACATCTTTGTGATGTTTGCACTCAACTCACAGAGTTGA

                 * *             *  *           *      *             *       
     436426 ACCTTCCGTTTCATAGAGCAGGTTGGAAACACTCTTATTGTAGTATCTGGAAGTGGACATTTGGA
         66 ACCTTTCTTTTCATAGAGCAGTTTTGAAACACTCTTTTTGTAGAATCTGGAAGTGGATATTTGGA

                   *   ** ** *  *   *            **  
     436491 GCGCTTTCAGGCCTATGGTGAAAAAGGAAATATCTTCCCAT
        131 GCGCTTTGAGGATTTCGTTGGAAACGGAAATATCTTCATAT

                  * * *      **      *   * *   *     ** ** *      *    *     
     436532 AAAAACGACATAGAAGCTATCTCAGGAACTTGTTTATGATGCATCTAATCAACTAACAGTGTTGA
          1 AAAAACTAGACAGAAGCATTCTCAGAAACATCTTTGTGATGTTTGCACTCAACTCACAGAGTTGA

                  * ** * *                         * *       *               
     436597 ACCTTTGTACTGACAGAGCAG-TTTGAAACACTCTTTTTTTGGAATCTGCAAGTGGATATTTGGA
         66 ACCTTTCTTTTCATAGAGCAGTTTTGAAACACTCTTTTTGTAGAATCTGGAAGTGGATATTTGGA

            *                           *   *   *    
     436661 TCGCTTTGAGGATTTCGTTGGAAACGG-GAT-GC--AATAT
        131 GCGCTTTGAGGATTTCGTTGGAAACGGAAATATCTTCATAT

                **  *    *     *                 **  *   *  *    *         * 
     436698 AAAACGTACACAGCAGCATACTCAGAAA-ATACTTTGCCATATTTCCATTCAAGTCACAGAGTGG
          1 AAAAACTAGACAGAAGCATTCTCAGAAACAT-CTTTGTGATGTTTGCACTCAACTCACAGAGTTG

               *  * **            *                  *  *             *      
     436762 AACATTCCCATTCATAGAGCAGGTTTGAAACACTCTTTTTGGAGTATCTGGAAGTGGACATTTGG
         65 AACCTTTCTTTTCATAGAGCAGTTTTGAAACACTCTTTTTGTAGAATCTGGAAGTGGATATTTGG

                    ** * * ** *  *   *            **  
     436827 AGCGCTTTCTGAACTATGGTGAAAAAGGAAATATCTTCCAAT
        130 AGCGCTTTGAGGATTTCGTTGGAAACGGAAATATCTTCATAT

            *     *               *      * *          *  **      **  *  *    
     436869 GAAAACAAGACAGAAGCATTCTGAGAAACTTATTTGTGATGTGTGTCCTCAACAAACGGACTTGA
          1 AAAAACTAGACAGAAGCATTCTCAGAAACATCTTTGTGATGTTTGCACTCAACTCACAGAGTTGA

                   *      **  * *  *  *             *   *    * * *           
     436934 ACCTTTCGTTTCATGCAGTACTTCTGGAACACTCTTTTTGAAGATTCTGCATGCGGATATTTGGA
         66 ACCTTTCTTTTCATAGAGCAGTTTTGAAACACTCTTTTTGTAGAATCTGGAAGTGGATATTTGGA

            **                            **   *   * 
     436999 TAGCTTTGAGGATTTCGTTGGAAACGG---GCT-TACATGT
        131 GCGCTTTGAGGATTTCGTTGGAAACGGAAATATCTTCATAT

                 *       *               *        *   *    *    *       *    
     437036 AAAAATTAGACAGCAGCATTCTCAGAAACTTCTTTGTGGTGTCTGCATTCAAGTCACAGAATTGA
          1 AAAAACTAGACAGAAGCATTCTCAGAAACATCTTTGTGATGTTTGCACTCAACTCACAGAGTTGA

              * ** * **            *  * *      *       *             *       
     437101 ACTTCCCCTCACATAGAGCAGTTGTGCAGCACTCTATTTGTAGTATCTGGAAGTGGACATTTGGA
         66 ACCTTTCTTTTCATAGAGCAGTTTTGAAACACTCTTTTTGTAGAATCTGGAAGTGGATATTTGGA

             *         ** *    * *   *            **  
     437166 GGGCTTTGTAGCCTATC-TGGAAAAAGGAAATATCTTCCCAT
        131 GCGCTTTG-AGGATTTCGTTGGAAACGGAAATATCTTCATAT

            *  ** *   *     * *            *   *  *   * **        *  * * *   
     437207 GAATGCGAGATAGAAGTAATCTCAGAAACATGTTTATGCTGTATCTACTCAACTAACTGTGCTGA
          1 AAAAACTAGACAGAAGCATTCTCAGAAACATCTTTGTGATGTTTGCACTCAACTCACAGAGTTGA

              *     *  *               *        *  * *       *               
     437272 ACATTTCTATTGATAGAGCAGTTTTGAGACACTCTTCTTTTGGAATCTGCAAGTGGATATTTGGA
         66 ACCTTTCTTTTCATAGAGCAGTTTTGAAACACTCTTTTTGTAGAATCTGGAAGTGGATATTTGGA

            ** *                        *     *      
     437337 TAGATTTGAGGATTTCGTTGGAAACGG-GAT-T-AT-ATAT
        131 GCGCTTTGAGGATTTCGTTGGAAACGGAAATATCTTCATAT

            *    *       *               *                 **  *   *         
     437374 CAAAAGTAGACAGCAGCATTCTCAGAAACTTCTTTGTGATGTTTGCATCCAGCTCTCAGAGTTGA
          1 AAAAACTAGACAGAAGCATTCTCAGAAACATCTTTGTGATGTTTGCACTCAACTCACAGAGTTGA

              *  * *          *  *        *        *   **        *  **       
     437439 ACATTCCCTTTCATAGAGTAGGTTTGAAACCCTCTTTTTATAGTGTCTGGAAGCGGGCATTTGGA
         66 ACCTTTCTTTTCATAGAGCAGTTTTGAAACACTCTTTTTGTAGAATCTGGAAGTGGATATTTGGA

                   *    * * **  **  **          * *  *
     437504 GCGCTTTCAGGCCTATGCTTAAAATAGGAAATATCTACCTAC
        131 GCGCTTTGAGG-ATTTCGTTGGAAACGGAAATATCTTCATAT

             *                    *    *  **          ** *        *    *     
     437546 AGAAACTAGACAGAAGCATTCTGAGAATCACGTTTGTGATGTGGGTACTCAACTAACAGTGTTGA
          1 AAAAACTAGACAGAAGCATTCTCAGAAACATCTTTGTGATGTTTGCACTCAACTCACAGAGTTGA

            *  *       *   *            *   *                *   *           
     437611 TCCATTCTTTTGATACAGCAGTTTTGAACCACACTTTTTGTAGAATCTGCAAGAGGATATTTGGA
         66 ACCTTTCTTTTCATAGAGCAGTTTTGAAACACTCTTTTTGTAGAATCTGGAAGTGGATATTTGGA

            **   *                     *   *      * *
     437676 TAGCTGTGAGGATTTCGTTGGAAACGGGAATGTCTTCAAAG
        131 GCGCTTTGAGGATTTCGTTGGAAACGGAAATATCTTCATAT

                *                         *   *            *    *            
     437717 AAAATCTAGACAGAAGCATTCTCAGAAACACCTTCGTGATGTTTGCAATCAAGTCACAGAGTTGA
          1 AAAAACTAGACAGAAGCATTCTCAGAAACATCTTTGTGATGTTTGCACTCAACTCACAGAGTTGA

                 * *             *  *           *      *             *       
     437782 ACCTTCCGTTTCATAGAGCAGGTTGGAAACACTCTTATTGTAGTATCTGGAAGTGGACATTTGGA
         66 ACCTTTCTTTTCATAGAGCAGTTTTGAAACACTCTTTTTGTAGAATCTGGAAGTGGATATTTGGA

                   *   ** ** *  *   *            **  
     437847 GCGCTTTCAGGCCTATGGTGAAAAAGGAAATATCTTCCCAT
        131 GCGCTTTGAGGATTTCGTTGGAAACGGAAATATCTTCATAT

                  * * *      **      *   * *   *     ** ** *      *    *     
     437888 AAAAACGACATAGAAGCTATCTCAGGAACTTGTTTATGATGCATCTAATCAACTAACAGTGTTGA
          1 AAAAACTAGACAGAAGCATTCTCAGAAACATCTTTGTGATGTTTGCACTCAACTCACAGAGTTGA

                  * ** * *                         * *       *               
     437953 ACCTTTGTACTGACAGAGCAG-TTTGAAACACTCTTTTTTTGGAATCTGCAAGTGGATATTTGGA
         66 ACCTTTCTTTTCATAGAGCAGTTTTGAAACACTCTTTTTGTAGAATCTGGAAGTGGATATTTGGA

            *                           *   *   *    
     438017 TCGCTTTGAGGATTTCGTTGGAAACGG-GAT-GC--AATAT
        131 GCGCTTTGAGGATTTCGTTGGAAACGGAAATATCTTCATAT

                **  *    *     *                 **  *   *  *    *   *     * 
     438054 AAAACGTACACAGCAGCATACTCAGAAA-ATACTTTGCCATATTTCCATTCAAGTCAGAGAGTGG
          1 AAAAACTAGACAGAAGCATTCTCAGAAACAT-CTTTGTGATGTTTGCACTCAACTCACAGAGTTG

               *  * **            *                  *  *             *      
     438118 AACATTCCCATTCATAGAGCAGGTTTGAAACACTCTTTTTGGAGTATCTGGAAGTGGACATTTGG
         65 AACCTTTCTTTTCATAGAGCAGTTTTGAAACACTCTTTTTGTAGAATCTGGAAGTGGATATTTGG

                    ** * * ** *  *   *            **  
     438183 AGCGCTTTCTGAACTATGGTGAAAAAGGAAATATCTTCCAAT
        130 AGCGCTTTGAGGATTTCGTTGGAAACGGAAATATCTTCATAT

            *     *               *      * *          *  **      **  *  *    
     438225 GAAAACAAGACAGAAGCATTCTGAGAAACTTATTTGTGATGTGTGTCCTCAACAAACGGACTTGA
          1 AAAAACTAGACAGAAGCATTCTCAGAAACATCTTTGTGATGTTTGCACTCAACTCACAGAGTTGA

                   *      **  * *  *  *             *   *    * * *           
     438290 ACCTTTCGTTTCATGCAGTACTTCTGGAACACTCTTTTTGAAGATTCTGCATGCGGATATTTGGA
         66 ACCTTTCTTTTCATAGAGCAGTTTTGAAACACTCTTTTTGTAGAATCTGGAAGTGGATATTTGGA

            **                      *     **   *   * 
     438355 TAGCTTTGAGGATTTCGTTGGAAAAGG---GCT-TACATGT
        131 GCGCTTTGAGGATTTCGTTGGAAACGGAAATATCTTCATAT

                 *       *               *        *   *    *    *       *    
     438392 AAAAATTAGACAGCAGCATTCTCAGAAACTTCTTTGTGGTGTCTGCATTCAAGTCACAGAATTGA
          1 AAAAACTAGACAGAAGCATTCTCAGAAACATCTTTGTGATGTTTGCACTCAACTCACAGAGTTGA

              * ** * **            *  * *      *       *             *       
     438457 ACTTCCCCTCACATAGAGCAGTTGTGCAGCACTCTATTTGTAGTATCTGGAAGTGGACATTTGGA
         66 ACCTTTCTTTTCATAGAGCAGTTTTGAAACACTCTTTTTGTAGAATCTGGAAGTGGATATTTGGA

             *         ** *    * *   *            **  
     438522 GGGCTTTGTAGCCTATC-TGGAAAAAGGAAATATCTTCCCAT
        131 GCGCTTTG-AGGATTTCGTTGGAAACGGAAATATCTTCATAT

            *  ** *   *     * *            *   *  *   * **        *  * * *   
     438563 GAATGCGAGATAGAAGTAATCTCAGAAACATGTTTATGCTGTATCTACTCAACTAACTGTGCTGA
          1 AAAAACTAGACAGAAGCATTCTCAGAAACATCTTTGTGATGTTTGCACTCAACTCACAGAGTTGA

              *     *  *               *        *  * *       *               
     438628 ACATTTCTATTGATAGAGCAGTTTTGAGACACTCTTCTTTTGGAATCTGCAAGTGGATATTTGGA
         66 ACCTTTCTTTTCATAGAGCAGTTTTGAAACACTCTTTTTGTAGAATCTGGAAGTGGATATTTGGA

            ** *                        *     *      
     438693 TAGATTTGAGGATTTCGTTGGAAACGG-GAT-T-AT-ATAT
        131 GCGCTTTGAGGATTTCGTTGGAAACGGAAATATCTTCATAT

            *    *       *               *                 **  *   *         
     438730 CAAAAGTAGACAGCAGCATTCTCAGAAACTTCTTTGTGATGTTTGCATCCAGCTCTCAGAGTTGA
          1 AAAAACTAGACAGAAGCATTCTCAGAAACATCTTTGTGATGTTTGCACTCAACTCACAGAGTTGA

              *  * *          *  *        *        *   **        *  **       
     438795 ACATTCCCTTTCATAGAGTAGGTTTGAAACCCTCTTTTTATAGTGTCTGGAAGCGGGCATTTGGA
         66 ACCTTTCTTTTCATAGAGCAGTTTTGAAACACTCTTTTTGTAGAATCTGGAAGTGGATATTTGGA

                   *    * * **  **  **          * *  *
     438860 GCGCTTTCAGGCCTATGCTTAAAATAGGAAATATCTACCTAC
        131 GCGCTTTGAGG-ATTTCGTTGGAAACGGAAATATCTTCATAT

             *                    *    *  **          ** *        *    *     
     438902 AGAAACTAGACAGAAGCATTCTGAGAATCACGTTTGTGATGTGGGTACTCAACTAACAGTGTTGA
          1 AAAAACTAGACAGAAGCATTCTCAGAAACATCTTTGTGATGTTTGCACTCAACTCACAGAGTTGA

            *  *       *   *            *   *                *   *           
     438967 TCCATTCTTTTGATACAGCAGTTTTGAACCACACTTTTTGTAGAATCTGCAAGAGGATATTTGGA
         66 ACCTTTCTTTTCATAGAGCAGTTTTGAAACACTCTTTTTGTAGAATCTGGAAGTGGATATTTGGA

            **   *                     *   *      * *
     439032 TAGCTGTGAGGATTTCGTTGGAAACGGGAATGTCTTCAAAG
        131 GCGCTTTGAGGATTTCGTTGGAAACGGAAATATCTTCATAT

                *                         *   *            *    *            
     439073 AAAATCTAGACAGAAGCATTCTCAGAAACACCTTCGTGATGTTTGCAATCAAGTCACAGAGTTGA
          1 AAAAACTAGACAGAAGCATTCTCAGAAACATCTTTGTGATGTTTGCACTCAACTCACAGAGTTGA

                 * *             *  *           *      *             *       
     439138 ACCTTCCGTTTCATAGAGCAGGTTGGAAACACTCTTATTGTAGTATCTGGAAGTGGACATTTGGA
         66 ACCTTTCTTTTCATAGAGCAGTTTTGAAACACTCTTTTTGTAGAATCTGGAAGTGGATATTTGGA

                   *   ** ** *  *   *            **  
     439203 GCGCTTTCAGGCCTATGGTGAAAAAGGAAATATCTTCCCAT
        131 GCGCTTTGAGGATTTCGTTGGAAACGGAAATATCTTCATAT

                  * * *      **      *   * *   *     ** ** *      *    *     
     439244 AAAAACGACATAGAAGCTATCTCAGGAACTTGTTTATGATGCATCTAATCAACTAACAGTGTTGA
          1 AAAAACTAGACAGAAGCATTCTCAGAAACATCTTTGTGATGTTTGCACTCAACTCACAGAGTTGA

                  * ** * *                         * *       *               
     439309 ACCTTTGTACTGACAGAGCAG-TTTGAAACACTCTTTTTTTGGAATCTGCAAGTGGATATTTGGA
         66 ACCTTTCTTTTCATAGAGCAGTTTTGAAACACTCTTTTTGTAGAATCTGGAAGTGGATATTTGGA

            *                           *   *   *    
     439373 TCGCTTTGAGGATTTCGTTGGAAACGG-GAT-GC--AATAT
        131 GCGCTTTGAGGATTTCGTTGGAAACGGAAATATCTTCATAT

                **  *    *     *                 **  *   *  *    *   *     * 
     439410 AAAACGTACACAGCAGCATACTCAGAAA-ATACTTTGCCATATTTCCATTCAAGTCAGAGAGTGG
          1 AAAAACTAGACAGAAGCATTCTCAGAAACAT-CTTTGTGATGTTTGCACTCAACTCACAGAGTTG

               *  * **            *                  *  *             *      
     439474 AACATTCCCATTCATAGAGCAGGTTTGAAACACTCTTTTTGGAGTATCTGGAAGTGGACATTTGG
         65 AACCTTTCTTTTCATAGAGCAGTTTTGAAACACTCTTTTTGTAGAATCTGGAAGTGGATATTTGG

                    ** * * ** *  *   *            **  
     439539 AGCGCTTTCTGAACTATGGTGAAAAAGGAAATATCTTCCAAT
        130 AGCGCTTTGAGGATTTCGTTGGAAACGGAAATATCTTCATAT

            *     *               *      * *          *  **      **  *  *    
     439581 GAAAACAAGACAGAAGCATTCTGAGAAACTTATTTGTGATGTGTGTCCTCAACAAACGGACTTGA
          1 AAAAACTAGACAGAAGCATTCTCAGAAACATCTTTGTGATGTTTGCACTCAACTCACAGAGTTGA

                   *      **  * *  *  *             *   *    * * *           
     439646 ACCTTTCGTTTCATGCAGTACTTCTGGAACACTCTTTTTGAAGATTCTGCATGCGGATATTTGGA
         66 ACCTTTCTTTTCATAGAGCAGTTTTGAAACACTCTTTTTGTAGAATCTGGAAGTGGATATTTGGA

            **                            **   *   * 
     439711 TAGCTTTGAGGATTTCGTTGGAAACGG---GCT-TACATGT
        131 GCGCTTTGAGGATTTCGTTGGAAACGGAAATATCTTCATAT

                 *       *               *        *   *    *    *       *    
     439748 AAAAATTAGACAGCAGCATTCTCAGAAACTTCTTTGTGGTGTCTGCATTCAAGTCACAGAATTGA
          1 AAAAACTAGACAGAAGCATTCTCAGAAACATCTTTGTGATGTTTGCACTCAACTCACAGAGTTGA

              * ** * **            *  * *      *       *    *        *       
     439813 ACTTCCCCTCACATAGAGCAGTTGTGCAGCACTCTATTTGTAGTATCTCGAAGTGGACATTTGGA
         66 ACCTTTCTTTTCATAGAGCAGTTTTGAAACACTCTTTTTGTAGAATCTGGAAGTGGATATTTGGA

             *         ** *    * *   *            **  
     439878 GGGCTTTGTAGCCTATC-TGGAAAAAGGAAATATCTTCCCAT
        131 GCGCTTTG-AGGATTTCGTTGGAAACGGAAATATCTTCATAT

            *  ** *   *     * *            *   *  *   * **        *  * * *   
     439919 GAATGCGAGATAGAAGTAATCTCAGAAACATGTTTATGCTGTATCTACTCAACTAACTGTGCTGA
          1 AAAAACTAGACAGAAGCATTCTCAGAAACATCTTTGTGATGTTTGCACTCAACTCACAGAGTTGA

              *     *  *               *        *  * *       *               
     439984 ACATTTCTATTGATAGAGCAGTTTTGAGACACTCTTCTTTTGGAATCTGCAAGTGGATATTTGGA
         66 ACCTTTCTTTTCATAGAGCAGTTTTGAAACACTCTTTTTGTAGAATCTGGAAGTGGATATTTGGA

            ** *                        *     *      
     440049 TAGATTTGAGGATTTCGTTGGAAACGG-GAT-T-AT-ATAT
        131 GCGCTTTGAGGATTTCGTTGGAAACGGAAATATCTTCATAT

            *    *       *               *                 **  *   *         
     440086 CAAAAGTAGACAGCAGCATTCTCAGAAACTTCTTTGTGATGTTTGCATCCAGCTCTCAGAGTTGA
          1 AAAAACTAGACAGAAGCATTCTCAGAAACATCTTTGTGATGTTTGCACTCAACTCACAGAGTTGA

              *  * *          *  *        *        *   **    *   *  **       
     440151 ACATTCCCTTTCATAGAGTAGGTTTGAAACCCTCTTTTTATAGTGTCTGCAAGCGGGCATTTGGA
         66 ACCTTTCTTTTCATAGAGCAGTTTTGAAACACTCTTTTTGTAGAATCTGGAAGTGGATATTTGGA

                   *    * * **  **  **          * *  *
     440216 GCGCTTTCAGGCCTATGCTTAAAATAGGAAATATCTACCTAC
        131 GCGCTTTGAGG-ATTTCGTTGGAAACGGAAATATCTTCATAT

             *                    *    *  **          ** *        *    *     
     440258 AGAAACTAGACAGAAGCATTCTGAGAATCACGTTTGTGATGTGGGTACTCAACTAACAGTGTTGA
          1 AAAAACTAGACAGAAGCATTCTCAGAAACATCTTTGTGATGTTTGCACTCAACTCACAGAGTTGA

            *  *       *   *            *   *                *   *           
     440323 TCCATTCTTTTGATACAGCAGTTTTGAACCACACTTTTTGTAGAATCTGCAAGAGGATATTTGGA
         66 ACCTTTCTTTTCATAGAGCAGTTTTGAAACACTCTTTTTGTAGAATCTGGAAGTGGATATTTGGA

            **   *                     *   *      * *
     440388 TAGCTGTGAGGATTTCGTTGGAAACGGGAATGTCTTCAAAG
        131 GCGCTTTGAGGATTTCGTTGGAAACGGAAATATCTTCATAT

                *                         *   *            *    *            
     440429 AAAATCTAGACAGAAGCATTCTCAGAAACACCTTCGTGATGTTTGCAATCAAGTCACAGAGTTGA
          1 AAAAACTAGACAGAAGCATTCTCAGAAACATCTTTGTGATGTTTGCACTCAACTCACAGAGTTGA

                 * *             *  *           *      *             *       
     440494 ACCTTCCGTTTCATAGAGCAGGTTGGAAACACTCTTATTGTAGTATCTGGAAGTGGACATTTGGA
         66 ACCTTTCTTTTCATAGAGCAGTTTTGAAACACTCTTTTTGTAGAATCTGGAAGTGGATATTTGGA

                   *   ** ** *  *   *            **  
     440559 GCGCTTTCAGGCCTATGGTGAAAAAGGAAATATCTTCCCAT
        131 GCGCTTTGAGGATTTCGTTGGAAACGGAAATATCTTCATAT

                  * * *      **      *   * *   *     ** ** *      *    *     
     440600 AAAAACGACATAGAAGCTATCTCAGGAACTTGTTTATGATGCATCTAATCAACTAACAGTGTTGA
          1 AAAAACTAGACAGAAGCATTCTCAGAAACATCTTTGTGATGTTTGCACTCAACTCACAGAGTTGA

                  * ** * *                         * *       *               
     440665 ACCTTTGTACTGACAGAGCAG-TTTGAAACACTCTTTTTTTGGAATCTGCAAGTGGATATTTGGA
         66 ACCTTTCTTTTCATAGAGCAGTTTTGAAACACTCTTTTTGTAGAATCTGGAAGTGGATATTTGGA

            *                           *   *   *    
     440729 TCGCTTTGAGGATTTCGTTGGAAACGG-GAT-GC--AATAT
        131 GCGCTTTGAGGATTTCGTTGGAAACGGAAATATCTTCATAT

                **  *    *     *                 **  *   *  *    *   *     * 
     440766 AAAACGTACACAGCAGCATACTCAGAAA-ATACTTTGCCATATTTCCATTCAAGTCAGAGAGTGG
          1 AAAAACTAGACAGAAGCATTCTCAGAAACAT-CTTTGTGATGTTTGCACTCAACTCACAGAGTTG

               *  * **            *                  *  *             *      
     440830 AACATTCCCATTCATAGAGCAGGTTTGAAACACTCTTTTTGGAGTATCTGGAAGTGGACATTTGG
         65 AACCTTTCTTTTCATAGAGCAGTTTTGAAACACTCTTTTTGTAGAATCTGGAAGTGGATATTTGG

                    ** * * ** *  *   *            **  
     440895 AGCGCTTTCTGAACTATGGTGAAAAAGGAAATATCTTCCAAT
        130 AGCGCTTTGAGGATTTCGTTGGAAACGGAAATATCTTCATAT

            *     *               *      * *          *  **      **  *  *    
     440937 GAAAACAAGACAGAAGCATTCTGAGAAACTTATTTGTGATGTGTGTCCTCAACAAACGGACTTGA
          1 AAAAACTAGACAGAAGCATTCTCAGAAACATCTTTGTGATGTTTGCACTCAACTCACAGAGTTGA

                   *      **  * *  *  *             *   *    * * *           
     441002 ACCTTTCGTTTCATGCAGTACTTCTGGAACACTCTTTTTGAAGATTCTGCATGCGGATATTTGGA
         66 ACCTTTCTTTTCATAGAGCAGTTTTGAAACACTCTTTTTGTAGAATCTGGAAGTGGATATTTGGA

            **                            **   *   * 
     441067 TAGCTTTGAGGATTTCGTTGGAAACGG---GCT-TACATGT
        131 GCGCTTTGAGGATTTCGTTGGAAACGGAAATATCTTCATAT

                 *       *               *        *   *    *    *       *    
     441104 AAAAATTAGACAGCAGCATTCTCAGAAACTTCTTTGTGGTGTCTGCATTCAAGTCACAGAATTGA
          1 AAAAACTAGACAGAAGCATTCTCAGAAACATCTTTGTGATGTTTGCACTCAACTCACAGAGTTGA

              * ** * **            *  * *      *       *             *       
     441169 ACTTCCCCTCACATAGAGCAGTTGTGCAGCACTCTATTTGTAGTATCTGGAAGTGGACATTTGGA
         66 ACCTTTCTTTTCATAGAGCAGTTTTGAAACACTCTTTTTGTAGAATCTGGAAGTGGATATTTGGA

             *         ** *    * *   *            **  
     441234 GGGCTTTGTAGCCTATC-TGGAAAAAGGAAATATCTTCCCAT
        131 GCGCTTTG-AGGATTTCGTTGGAAACGGAAATATCTTCATAT

            *  ** *   *     * *            *   *  *   * **        *  * * *   
     441275 GAATGCGAGATAGAAGTAATCTCAGAAACATGTTTATGCTGTATCTACTCAACTAACTGTGCTGA
          1 AAAAACTAGACAGAAGCATTCTCAGAAACATCTTTGTGATGTTTGCACTCAACTCACAGAGTTGA

              *     *  *               *        *  * *       *               
     441340 ACATTTCTATTGATAGAGCAGTTTTGAGACACTCTTCTTTTGGAATCTGCAAGTGGATATTTGGA
         66 ACCTTTCTTTTCATAGAGCAGTTTTGAAACACTCTTTTTGTAGAATCTGGAAGTGGATATTTGGA

            ** *                        *     *      
     441405 TAGATTTGAGGATTTCGTTGGAAACGG-GAT-T-AT-ATAT
        131 GCGCTTTGAGGATTTCGTTGGAAACGGAAATATCTTCATAT

                 *       *               *                 **  *   *         
     441442 AAAAAGTAGACAGCAGCATTCTCAGAAACTTCTTTGTGATGTTTGCATCCAGCTCTCAGAGTTGA
          1 AAAAACTAGACAGAAGCATTCTCAGAAACATCTTTGTGATGTTTGCACTCAACTCACAGAGTTGA

              *  * *          *  *        *        *   **    *   *  **       
     441507 ACATTCCCTTTCATAGAGTAGGTTTGAAACCCTCTTTTTATAGTGTCTGCAAGCGGGCATTTGGA
         66 ACCTTTCTTTTCATAGAGCAGTTTTGAAACACTCTTTTTGTAGAATCTGGAAGTGGATATTTGGA

                   *    * * **  **  **          * *  *
     441572 GCGCTTTCAGGCCTATGCTTAAAATAGGAAATATCTACCTAC
        131 GCGCTTTGAGG-ATTTCGTTGGAAACGGAAATATCTTCATAT

             *                    *    *  **          ** *        *    *     
     441614 AGAAACTAGACAGAAGCATTCTGAGAATCACGTTTGTGATGTGGGTACTCAACTAACAGTGTTGA
          1 AAAAACTAGACAGAAGCATTCTCAGAAACATCTTTGTGATGTTTGCACTCAACTCACAGAGTTGA

            *  *       *   *            *   *                *   *           
     441679 TCCATTCTTTTGATACAGCAGTTTTGAACCACACTTTTTGTAGAATCTGCAAGAGGATATTTGGA
         66 ACCTTTCTTTTCATAGAGCAGTTTTGAAACACTCTTTTTGTAGAATCTGGAAGTGGATATTTGGA

            **   *                     *   *      * *
     441744 TAGCTGTGAGGATTTCGTTGGAAACGGGAATGTCTTCAAAG
        131 GCGCTTTGAGGATTTCGTTGGAAACGGAAATATCTTCATAT

                *                         *   *            *    *            
     441785 AAAATCTAGACAGAAGCATTCTCAGAAACACCTTCGTGATGTTTGCAATCAAGTCACAGAGTTGA
          1 AAAAACTAGACAGAAGCATTCTCAGAAACATCTTTGTGATGTTTGCACTCAACTCACAGAGTTGA

                 * *             *  *           *      *             *       
     441850 ACCTTCCGTTTCATAGAGCAGGTTGGAAACACTCTTATTGTAGTATCTGGAAGTGGACATTTGGA
         66 ACCTTTCTTTTCATAGAGCAGTTTTGAAACACTCTTTTTGTAGAATCTGGAAGTGGATATTTGGA

                   *   ** ** *  *   *            **  
     441915 GCGCTTTCAGGCCTATGGTGAAAAAGGAAATATCTTCCCAT
        131 GCGCTTTGAGGATTTCGTTGGAAACGGAAATATCTTCATAT

                  * * *      **      *   * *   *     ** ** *      *    *     
     441956 AAAAACGACATAGAAGCTATCTCAGGAACTTGTTTATGATGCATCTAATCAACTAACAGTGTTGA
          1 AAAAACTAGACAGAAGCATTCTCAGAAACATCTTTGTGATGTTTGCACTCAACTCACAGAGTTGA

                  * ** * *                         * *       *               
     442021 ACCTTTGTACTGACAGAGCAG-TTTGAAACACTCTTTTTTTGGAATCTGCAAGTGGATATTTGGA
         66 ACCTTTCTTTTCATAGAGCAGTTTTGAAACACTCTTTTTGTAGAATCTGGAAGTGGATATTTGGA

            *                           *   *   *    
     442085 TCGCTTTGAGGATTTCGTTGGAAACGG-GAT-GC--AATAT
        131 GCGCTTTGAGGATTTCGTTGGAAACGGAAATATCTTCATAT

                **  *    *     *                 **  *   *  *    *         * 
     442122 AAAACGTACACAGCAGCATACTCAGAAA-ATACTTTGCCATATTTCCATTCAAGTCACAGAGTGG
          1 AAAAACTAGACAGAAGCATTCTCAGAAACAT-CTTTGTGATGTTTGCACTCAACTCACAGAGTTG

               *  * **            *                  *  *             *      
     442186 AACATTCCCATTCATAGAGCAGGTTTGAAACACTCTTTTTGGAGTATCTGGAAGTGGACATTTGG
         65 AACCTTTCTTTTCATAGAGCAGTTTTGAAACACTCTTTTTGTAGAATCTGGAAGTGGATATTTGG

                    ** * * ** *  *   *            **  
     442251 AGCGCTTTCTGAACTATGGTGAAAAAGGAAATATCTTCCAAT
        130 AGCGCTTTGAGGATTTCGTTGGAAACGGAAATATCTTCATAT

            *     *               *      * *          *  **      **  *  *    
     442293 GAAAACAAGACAGAAGCATTCTGAGAAACTTATTTGTGATGTGTGTCCTCAACAAACGGACTTGA
          1 AAAAACTAGACAGAAGCATTCTCAGAAACATCTTTGTGATGTTTGCACTCAACTCACAGAGTTGA

                   *      **  * *  *  *             *   *    * * *           
     442358 ACCTTTCGTTTCATGCAGTACTTCTGGAACACTCTTTTTGAAGATTCTGCATGCGGATATTTGGA
         66 ACCTTTCTTTTCATAGAGCAGTTTTGAAACACTCTTTTTGTAGAATCTGGAAGTGGATATTTGGA

            **                            **   *   * 
     442423 TAGCTTTGAGGATTTCGTTGGAAACGG---GCT-TACATGT
        131 GCGCTTTGAGGATTTCGTTGGAAACGGAAATATCTTCATAT

                 *       *               *        *   *    *    *       *    
     442460 AAAAATTAGACAGCAGCATTCTCAGAAACTTCTTTGTGGTGTCTGCATTCAAGTCACAGAATTGA
          1 AAAAACTAGACAGAAGCATTCTCAGAAACATCTTTGTGATGTTTGCACTCAACTCACAGAGTTGA

              * ** * **            *  * *      *       *             *       
     442525 ACTTCCCCTCACATAGAGCAGTTGTGCAGCACTCTATTTGTAGTATCTGGAAGTGGACATTTGGA
         66 ACCTTTCTTTTCATAGAGCAGTTTTGAAACACTCTTTTTGTAGAATCTGGAAGTGGATATTTGGA

             *         ** *    * *   *            **  
     442590 GGGCTTTGTAGCCTATC-TGGAAAAAGGAAATATCTTCCCAT
        131 GCGCTTTG-AGGATTTCGTTGGAAACGGAAATATCTTCATAT

            *  ** *   *     * *            *   *  *   * ***       *  * * *   
     442631 GAATGCGAGATAGAAGTAATCTCAGAAACATGTTTATGCTGTATCTTCTCAACTAACTGTGCTGA
          1 AAAAACTAGACAGAAGCATTCTCAGAAACATCTTTGTGATGTTTGCACTCAACTCACAGAGTTGA

              *     *  *               *        *  * *       *               
     442696 ACATTTCTATTGATAGAGCAGTTTTGAGACACTCTTCTTTTGGAATCTGCAAGTGGATATTTGGA
         66 ACCTTTCTTTTCATAGAGCAGTTTTGAAACACTCTTTTTGTAGAATCTGGAAGTGGATATTTGGA

            ** *                        *     *      
     442761 TAGATTTGAGGATTTCGTTGGAAACGG-GAT-T-AT-ATAT
        131 GCGCTTTGAGGATTTCGTTGGAAACGGAAATATCTTCATAT

                 *       *               *                 **  *   *         
     442798 AAAAAGTAGACAGCAGCATTCTCAGAAACTTCTTTGTGATGTTTGCATCCAGCTCTCAGAGTTGA
          1 AAAAACTAGACAGAAGCATTCTCAGAAACATCTTTGTGATGTTTGCACTCAACTCACAGAGTTGA

              *  * *          *  *        *        *   **        *  **       
     442863 ACATTCCCTTTCATAGAGTAGGTTTGAAACCCTCTTTTTATAGTGTCTGGAAGCGGGCATTTGGA
         66 ACCTTTCTTTTCATAGAGCAGTTTTGAAACACTCTTTTTGTAGAATCTGGAAGTGGATATTTGGA

                   *    * * **  **  **          * *  *
     442928 GCGCTTTCAGGCCTATGCTTAAAATAGGAAATATCTACCTAC
        131 GCGCTTTGAGG-ATTTCGTTGGAAACGGAAATATCTTCATAT

             *                    *    *  **          ** *        *    *     
     442970 AGAAACTAGACAGAAGCATTCTGAGAATCACGTTTGTGATGTGGGTACTCAACTAACAGTGTTGA
          1 AAAAACTAGACAGAAGCATTCTCAGAAACATCTTTGTGATGTTTGCACTCAACTCACAGAGTTGA

            *  *       *   *            *   *                *   *           
     443035 TCCATTCTTTTGATACAGCAGTTTTGAACCACACTTTTTGTAGAATCTGCAAGAGGATATTTGGA
         66 ACCTTTCTTTTCATAGAGCAGTTTTGAAACACTCTTTTTGTAGAATCTGGAAGTGGATATTTGGA

            **   *                     *   *      * *
     443100 TAGCTGTGAGGATTTCGTTGGAAACGGGAATGTCTTCAAAG
        131 GCGCTTTGAGGATTTCGTTGGAAACGGAAATATCTTCATAT

                *                         *   *            *    *            
     443141 AAAATCTAGACAGAAGCATTCTCAGAAACACCTTCGTGATGTTTGCAATCAAGTCACAGAGTTGA
          1 AAAAACTAGACAGAAGCATTCTCAGAAACATCTTTGTGATGTTTGCACTCAACTCACAGAGTTGA

                 * *             *  *           *      *             *       
     443206 ACCTTCCGTTTCATAGAGCAGGTTGGAAACACTCTTATTGTAGTATCTGGAAGTGGACATTTGGA
         66 ACCTTTCTTTTCATAGAGCAGTTTTGAAACACTCTTTTTGTAGAATCTGGAAGTGGATATTTGGA

                   *   ** ** *  *   *            **  
     443271 GCGCTTTCAGGCCTATGGTGAAAAAGGAAATATCTTCCCAT
        131 GCGCTTTGAGGATTTCGTTGGAAACGGAAATATCTTCATAT

                  * * *      **      *   * *   *     ** ** *      *    *     
     443312 AAAAACGACATAGAAGCTATCTCAGGAACTTGTTTATGATGCATCTAATCAACTAACAGTGTTGA
          1 AAAAACTAGACAGAAGCATTCTCAGAAACATCTTTGTGATGTTTGCACTCAACTCACAGAGTTGA

                  * ** * *                         * *       *               
     443377 ACCTTTGTACTGACAGAGCAG-TTTGAAACACTCTTTTTTTGGAATCTGCAAGTGGATATTTGGA
         66 ACCTTTCTTTTCATAGAGCAGTTTTGAAACACTCTTTTTGTAGAATCTGGAAGTGGATATTTGGA

            *                           *   *   *    
     443441 TCGCTTTGAGGATTTCGTTGGAAACGG-GAT-GC--AATAT
        131 GCGCTTTGAGGATTTCGTTGGAAACGGAAATATCTTCATAT

                **  *    *     *                 **  *   *  *    *        ** 
     443478 AAAACGTACACAGCAGCATACTCAGAAA-ATACTTTGCCATATTTCCATTCAAGTCACAGAGCGG
          1 AAAAACTAGACAGAAGCATTCTCAGAAACAT-CTTTGTGATGTTTGCACTCAACTCACAGAGTTG

               *  * **            *                  *  *             *      
     443542 AACATTCCCATTCATAGAGCAGGTTTGAAACACTCTTTTTGGAGTATCTGGAAGTGGACATTTGG
         65 AACCTTTCTTTTCATAGAGCAGTTTTGAAACACTCTTTTTGTAGAATCTGGAAGTGGATATTTGG

                    ** * * ** *  *   *            **  
     443607 AGCGCTTTCTGAACTATGGTGAAAAAGGAAATATCTTCCAAT
        130 AGCGCTTTGAGGATTTCGTTGGAAACGGAAATATCTTCATAT

            *     *               *      * *          *  **      **  *  *    
     443649 GAAAACAAGACAGAAGCATTCTGAGAAACTTATTTGTGATGTGTGTCCTCAACAAACGGACTTGA
          1 AAAAACTAGACAGAAGCATTCTCAGAAACATCTTTGTGATGTTTGCACTCAACTCACAGAGTTGA

                   *      **  * *  *  *             *   *    * * *           
     443714 ACCTTTCGTTTCATGCAGTACTTCTGGAACACTCTTTTTGAAGATTCTGCATGCGGATATTTGGA
         66 ACCTTTCTTTTCATAGAGCAGTTTTGAAACACTCTTTTTGTAGAATCTGGAAGTGGATATTTGGA

            **                            **   *   * 
     443779 TAGCTTTGAGGATTTCGTTGGAAACGG---GCT-TACATGT
        131 GCGCTTTGAGGATTTCGTTGGAAACGGAAATATCTTCATAT

                 *       *               *        *   *    *    *       *    
     443816 AAAAATTAGACAGCAGCATTCTCAGAAACTTCTTTGTGGTGTCTGCATTCAAGTCACAGAATTGA
          1 AAAAACTAGACAGAAGCATTCTCAGAAACATCTTTGTGATGTTTGCACTCAACTCACAGAGTTGA

              * ** * **            *  * *      *       *             *       
     443881 ACTTCCCCTCACATAGAGCAGTTGTGCAGCACTCTATTTGTAGTATCTGGAAGTGGACATTTGGA
         66 ACCTTTCTTTTCATAGAGCAGTTTTGAAACACTCTTTTTGTAGAATCTGGAAGTGGATATTTGGA

             *         ** *    * *   *            **  
     443946 GGGCTTTGTAGCCTATC-TGGAAAAAGGAAATATCTTCCCAT
        131 GCGCTTTG-AGGATTTCGTTGGAAACGGAAATATCTTCATAT

            *  ** *   *     * *            *   *  *   * **        *  * * *   
     443987 GAATGCGAGATAGAAGTAATCTCAGAAACATGTTTATGCTGTATCTACTCAACTAACTGTGCTGA
          1 AAAAACTAGACAGAAGCATTCTCAGAAACATCTTTGTGATGTTTGCACTCAACTCACAGAGTTGA

              *     *  *               *        *  * *       *               
     444052 ACATTTCTATTTATAGAGCAGTTTTGAGACACTCTTCTTTTGGAATCTGCAAGTGGATATTTGGA
         66 ACCTTTCTTTTCATAGAGCAGTTTTGAAACACTCTTTTTGTAGAATCTGGAAGTGGATATTTGGA

            ** *                        *     *      
     444117 TAGATTTGAGGATTTCGTTGGAAACGG-GAT-T-AT-ATAT
        131 GCGCTTTGAGGATTTCGTTGGAAACGGAAATATCTTCATAT

            *    *       *               *                 **  *   *         
     444154 CAAAAGTAGACAGCAGCATTCTCAGAAACTTCTTTGTGATGTTTGCATCCAGCTCTCAGAGTTGA
          1 AAAAACTAGACAGAAGCATTCTCAGAAACATCTTTGTGATGTTTGCACTCAACTCACAGAGTTGA

              *  * *          *  *        *        *   **    *   *  **       
     444219 ACATTCCCTTTCATAGAGTAGGTTTGAAACCCTCTTTTTATAGTGTCTGCAAGCGGGCATTTGGA
         66 ACCTTTCTTTTCATAGAGCAGTTTTGAAACACTCTTTTTGTAGAATCTGGAAGTGGATATTTGGA

                   *    * * **  **  **          * *  *
     444284 GCGCTTTCAGGCCTATGCTTAAAATAGGAAATATCTACCTAC
        131 GCGCTTTGAGG-ATTTCGTTGGAAACGGAAATATCTTCATAT

             *                    *    *  **          ** *        *    *     
     444326 AGAAACTAGACAGAAGCATTCTGAGAATCACGTTTGTGATGTGGGTACTCAACTAACAGTGTTGA
          1 AAAAACTAGACAGAAGCATTCTCAGAAACATCTTTGTGATGTTTGCACTCAACTCACAGAGTTGA

            *  *       *   *            *   *                *   *           
     444391 TCCATTCTTTTGATACAGCAGTTTTGAACCACACTTTTTGTAGAATCTGCAAGAGGATATTTGGA
         66 ACCTTTCTTTTCATAGAGCAGTTTTGAAACACTCTTTTTGTAGAATCTGGAAGTGGATATTTGGA

            **   *                     *   *      * *
     444456 TAGCTGTGAGGATTTCGTTGGAAACGGGAATGTCTTCAAAG
        131 GCGCTTTGAGGATTTCGTTGGAAACGGAAATATCTTCATAT

                *                         *   *            *    *            
     444497 AAAATCTAGACAGAAGCATTCTCAGAAACACCTTCGTGATGTTTGCAATCAAGTCACAGAGTTGA
          1 AAAAACTAGACAGAAGCATTCTCAGAAACATCTTTGTGATGTTTGCACTCAACTCACAGAGTTGA

                 * *             *  *           *      *             *       
     444562 ACCTTCCGTTTCATAGAGCAGGTTGGAAACACTCTTATTGTAGTATCTGGAAGTGGACATTTGGA
         66 ACCTTTCTTTTCATAGAGCAGTTTTGAAACACTCTTTTTGTAGAATCTGGAAGTGGATATTTGGA

                   *   ** ** *  *   *            **  
     444627 GCGCTTTCAGGCCTATGGTGAAAAAGGAAATATCTTCCCAT
        131 GCGCTTTGAGGATTTCGTTGGAAACGGAAATATCTTCATAT

                  * * *      **      *   * *   *     ** ** *      *    *     
     444668 AAAAACGACATAGAAGCTATCTCAGGAACTTGTTTATGATGCATCTAATCAACTAACAGTGTTGA
          1 AAAAACTAGACAGAAGCATTCTCAGAAACATCTTTGTGATGTTTGCACTCAACTCACAGAGTTGA

                  * ** * *                         * *       **              
     444733 ACCTTTGTACTGACAGAGCAG-TTTGAAACACTCTTTTTTTGGAATCTGCGAGTGGATATTTGGA
         66 ACCTTTCTTTTCATAGAGCAGTTTTGAAACACTCTTTTTGTAGAATCTGGAAGTGGATATTTGGA

            *                           *   *   *    
     444797 TCGCTTTGAGGATTTCGTTGGAAACGG-GAT-GC--AATAT
        131 GCGCTTTGAGGATTTCGTTGGAAACGGAAATATCTTCATAT

                **  *    *     *                 **  *   *  *    *         * 
     444834 AAAACGTACACAGCAGCATACTCAGAAA-ATACTTTGCCATATTTCCATTCAAGTCACAGAGTGG
          1 AAAAACTAGACAGAAGCATTCTCAGAAACAT-CTTTGTGATGTTTGCACTCAACTCACAGAGTTG

               *  * **            *                  *  *             *      
     444898 AACATTCCCATTCATAGAGCAGGTTTGAAACACTCTTTTTGGAGTATCTGGAAGTGGACATTTGG
         65 AACCTTTCTTTTCATAGAGCAGTTTTGAAACACTCTTTTTGTAGAATCTGGAAGTGGATATTTGG

                    ** * * ** *  *   *            **  
     444963 AGCGCTTTCTGAACTATGGTGAAAAAGGAAATATCTTCCAAT
        130 AGCGCTTTGAGGATTTCGTTGGAAACGGAAATATCTTCATAT

            *     *               *      * *          *  **      **  *  *    
     445005 GAAAACAAGACAGAAGCATTCTGAGAAACTTATTTGTGATGTGTGTCCTCAACAAACGGACTTGA
          1 AAAAACTAGACAGAAGCATTCTCAGAAACATCTTTGTGATGTTTGCACTCAACTCACAGAGTTGA

                   *      **  * *                    *   *    * * *          
     445070 ACCTTTCGTTTCATGCAGTACTTCTTG-AACACTCTTTTTGAAGATTCTGCATGCGGATATTTGG
         66 ACCTTTCTTTTCATAGAGCAGTT-TTGAAACACTCTTTTTGTAGAATCTGGAAGTGGATATTTGG

             **                            **   *   * 
     445134 ATAGCTTTGAGGATTTCGTTGGAAACGG---GCT-TACATGT
        130 AGCGCTTTGAGGATTTCGTTGGAAACGGAAATATCTTCATAT

                 *       *               *        *   *    *    *       *    
     445172 AAAAATTAGACAGCAGCATTCTCAGAAACTTCTTTGTGGTGTCTGCATTCAAGTCACAGAATTGA
          1 AAAAACTAGACAGAAGCATTCTCAGAAACATCTTTGTGATGTTTGCACTCAACTCACAGAGTTGA

              * ** * **            *  * *      *       *             *       
     445237 ACTTCCCCTCACATAGAGCAGTTGTGCAGCACTCTATTTGTAGTATCTGGAAGTGGACATTTGGA
         66 ACCTTTCTTTTCATAGAGCAGTTTTGAAACACTCTTTTTGTAGAATCTGGAAGTGGATATTTGGA

             *         ** *    * *   *            **  
     445302 GGGCTTTGTAGCCTATC-TGGAAAAAGGAAATATCTTCCCAT
        131 GCGCTTTG-AGGATTTCGTTGGAAACGGAAATATCTTCATAT

            *  ** *   *     * *            *   *  *   * **        *  * * *   
     445343 GAATGCGAGATAGAAGTAATCTCAGAAACATGTTTATGCTGTATCTACTCAACTAACTGTGCTGA
          1 AAAAACTAGACAGAAGCATTCTCAGAAACATCTTTGTGATGTTTGCACTCAACTCACAGAGTTGA

              *     *  *               *        *  * *       *               
     445408 ACATTTCTATTGATAGAGCAGTTTTGAGACACTCTTCTTTTGGAATCTGCAAGTGGATATTTGGA
         66 ACCTTTCTTTTCATAGAGCAGTTTTGAAACACTCTTTTTGTAGAATCTGGAAGTGGATATTTGGA

            ** *                        *     *      
     445473 TAGATTTGAGGATTTCGTTGGAAACGG-GAT-T-AT-ATAT
        131 GCGCTTTGAGGATTTCGTTGGAAACGGAAATATCTTCATAT

            *    *       *               *                 **  *   *         
     445510 CAAAAGTAGACAGCAGCATTCTCAGAAACTTCTTTGTGATGTTTGCATCCAGCTCTCAGAGTTGA
          1 AAAAACTAGACAGAAGCATTCTCAGAAACATCTTTGTGATGTTTGCACTCAACTCACAGAGTTGA

              *  * *          *  *        *        *   **    *   *  **       
     445575 ACATTCCCTTTCATAGAGTAGGTTTGAAACCCTCTTTTTATAGTGTCTGCAAGCGGGCATTTGGA
         66 ACCTTTCTTTTCATAGAGCAGTTTTGAAACACTCTTTTTGTAGAATCTGGAAGTGGATATTTGGA

                   *    * * **  **  **          * *  *
     445640 GCGCTTTCAGGCCTATGCTTAAAATAGGAAATATCTACCTAC
        131 GCGCTTTGAGG-ATTTCGTTGGAAACGGAAATATCTTCATAT

             *                    *    *  **          ** *        *    *     
     445682 AGAAACTAGACAGAAGCATTCTGAGAATCACGTTTGTGATGTGGGTACTCAACTAACAGTGTTGA
          1 AAAAACTAGACAGAAGCATTCTCAGAAACATCTTTGTGATGTTTGCACTCAACTCACAGAGTTGA

            *  *       *   *            *   *                *   *           
     445747 TCCATTCTTTTGATACAGCAGTTTTGAACCACACTTTTTGTAGAATCTGCAAGAGGATATTTGGA
         66 ACCTTTCTTTTCATAGAGCAGTTTTGAAACACTCTTTTTGTAGAATCTGGAAGTGGATATTTGGA

            **   *                     *   *      * *
     445812 TAGCTGTGAGGATTTCGTTGGAAACGGGAATGTCTTCAAAG
        131 GCGCTTTGAGGATTTCGTTGGAAACGGAAATATCTTCATAT

                *                         *   *            *    *            
     445853 AAAATCTAGACAGAAGCATTCTCAGAAACACCTTCGTGATGTTTGCAATCAAGTCACAGAGTTGA
          1 AAAAACTAGACAGAAGCATTCTCAGAAACATCTTTGTGATGTTTGCACTCAACTCACAGAGTTGA

                 * *             *  *           *      *             *       
     445918 ACCTTCCGTTTCATAGAGCAGGTTGGAAACACTCTTATTGTAGTATCTGGAAGTGGACATTTGGA
         66 ACCTTTCTTTTCATAGAGCAGTTTTGAAACACTCTTTTTGTAGAATCTGGAAGTGGATATTTGGA

                   *   ** ** *  *   *            **  
     445983 GCGCTTTCAGGCCTATGGTGAAAAAGGAAATATCTTCCCAT
        131 GCGCTTTGAGGATTTCGTTGGAAACGGAAATATCTTCATAT

                  * * *      **      *   * *   *     ** ** *      *    *     
     446024 AAAAACGACATAGAAGCTATCTCAGGAACTTGTTTATGATGCATCTAATCAACTAACAGTGTTGA
          1 AAAAACTAGACAGAAGCATTCTCAGAAACATCTTTGTGATGTTTGCACTCAACTCACAGAGTTGA

                  * ** * *                         * *       *               
     446089 ACCTTTGTACTGACAGAGCAG-TTTGAAACACTCTTTTTTTGGAATCTGCAAGTGGATATTTGGA
         66 ACCTTTCTTTTCATAGAGCAGTTTTGAAACACTCTTTTTGTAGAATCTGGAAGTGGATATTTGGA

            *                           *   *   *    
     446153 TCGCTTTGAGGATTTCGTTGGAAACGG-GAT-GC--AATAT
        131 GCGCTTTGAGGATTTCGTTGGAAACGGAAATATCTTCATAT

                **  *    *     *                 **  *   *  *    *         * 
     446190 AAAACGTACACAGCAGCATACTCAGAAA-ATACTTTGCCATATTTCCATTCAAGTCACAGAGTGG
          1 AAAAACTAGACAGAAGCATTCTCAGAAACAT-CTTTGTGATGTTTGCACTCAACTCACAGAGTTG

               *  * **            *                  *  *             *      
     446254 AACATTCCCATTCATAGAGCAGGTTTGAAACACTCTTTTTGGAGTATCTGGAAGTGGACATTTGG
         65 AACCTTTCTTTTCATAGAGCAGTTTTGAAACACTCTTTTTGTAGAATCTGGAAGTGGATATTTGG

                    ** * * ** *  *   *            **  
     446319 AGCGCTTTCTGAACTATGGTGAAAAAGGAAATATCTTCCAAT
        130 AGCGCTTTGAGGATTTCGTTGGAAACGGAAATATCTTCATAT

            *     *               *      * *          *  **      **  *  *    
     446361 GAAAACAAGACAGAAGCATTCTGAGAAACTTATTTGTGATGTGTGTCCTCAACAAACGGACTTGA
          1 AAAAACTAGACAGAAGCATTCTCAGAAACATCTTTGTGATGTTTGCACTCAACTCACAGAGTTGA

                   *      **  * *  *  *             *   *    * * *           
     446426 ACCTTTCGTTTCATGCAGTACTTCTGGAACACTCTTTTTGAAGATTCTGCATGCGGATATTTGGA
         66 ACCTTTCTTTTCATAGAGCAGTTTTGAAACACTCTTTTTGTAGAATCTGGAAGTGGATATTTGGA

            **                            **   *   * 
     446491 TAGCTTTGAGGATTTCGTTGGAAACGG---GCT-TACATGT
        131 GCGCTTTGAGGATTTCGTTGGAAACGGAAATATCTTCATAT

                 *       *               *        *   *    *    *       *    
     446528 AAAAATTAGACAGCAGCATTCTCAGAAACTTCTTTGTGGTGTCTGCATTCAAGTCACAGAATTGA
          1 AAAAACTAGACAGAAGCATTCTCAGAAACATCTTTGTGATGTTTGCACTCAACTCACAGAGTTGA

              * ** * **            *  * *      *       *             *       
     446593 ACTTCCCCTCACATAGAGCAGTTGTGCAGCACTCTATTTGTAGTATCTGGAAGTGGACATTTGGA
         66 ACCTTTCTTTTCATAGAGCAGTTTTGAAACACTCTTTTTGTAGAATCTGGAAGTGGATATTTGGA

             *         ** *    * *   *            **  
     446658 GGGCTTTGTAGCCTATC-TGGAAAAAGGAAATATCTTCCCAT
        131 GCGCTTTG-AGGATTTCGTTGGAAACGGAAATATCTTCATAT

            *  ** *   *     * *            *   *  *   * **        *  * * *   
     446699 GAATGCGAGATAGAAGTAATCTCAGAAACATGTTTATGCTGTATCTACTCAACTAACTGTGCTGA
          1 AAAAACTAGACAGAAGCATTCTCAGAAACATCTTTGTGATGTTTGCACTCAACTCACAGAGTTGA

              *     *  *               *        *  * *       *               
     446764 ACATTTCTATTGATAGAGCAGTTTTGAGACACTCTTCTTTTGGAATCTGCAAGTGGATATTTGGA
         66 ACCTTTCTTTTCATAGAGCAGTTTTGAAACACTCTTTTTGTAGAATCTGGAAGTGGATATTTGGA

            ** *                        *     *      
     446829 TAGATTTGAGGATTTCGTTGGAAACGG-GAT-T-AT-ATAT
        131 GCGCTTTGAGGATTTCGTTGGAAACGGAAATATCTTCATAT

                 *       *               *                 **  *   *         
     446866 AAAAAGTAGACAGCAGCATTCTCAGAAACTTCTTTGTGATGTTTGCATCCAGCTCTCAGAGTTGA
          1 AAAAACTAGACAGAAGCATTCTCAGAAACATCTTTGTGATGTTTGCACTCAACTCACAGAGTTGA

              *  * *          *  *        *        *   **        *  **       
     446931 ACATTCCCTTTCATAGAGTAGGTTTGAAACCCTCTTTTTATAGTGTCTGGAAGCGGGCATTTGGA
         66 ACCTTTCTTTTCATAGAGCAGTTTTGAAACACTCTTTTTGTAGAATCTGGAAGTGGATATTTGGA

                   *    * * **  **  **          * *  *
     446996 GCGCTTTCAGGCCTATGCTTAAAATAGGAAATATCTACCTAC
        131 GCGCTTTGAGG-ATTTCGTTGGAAACGGAAATATCTTCATAT

             *                    *    *  **          ** *        *    *     
     447038 AGAAACTAGACAGAAGCATTCTGAGAATCACGTTTGTGATGTGGGTACTCAACTAACAGTGTTGA
          1 AAAAACTAGACAGAAGCATTCTCAGAAACATCTTTGTGATGTTTGCACTCAACTCACAGAGTTGA

            *  *       *   *            *   *                *   *           
     447103 TCCATTCTTTTGATACAGCAGTTTTGAACCACACTTTTTGTAGAATCTGCAAGAGGATATTTGGA
         66 ACCTTTCTTTTCATAGAGCAGTTTTGAAACACTCTTTTTGTAGAATCTGGAAGTGGATATTTGGA

            **   *                     *   *      * *
     447168 TAGCTGTGAGGATTTCGTTGGAAACGGGAATGTCTTCAAAG
        131 GCGCTTTGAGGATTTCGTTGGAAACGGAAATATCTTCATAT

                *                         *   *            *    *            
     447209 AAAATCTAGACAGAAGCATTCTCAGAAACACCTTCGTGATGTTTGCAATCAAGTCACAGAGTTGA
          1 AAAAACTAGACAGAAGCATTCTCAGAAACATCTTTGTGATGTTTGCACTCAACTCACAGAGTTGA

                 * *             *  *           *      *             *       
     447274 ACCTTCCGTTTCATAGAGCAGGTTGGAAACACTCTTATTGTAGTATCTGGAAGTGGACATTTGGA
         66 ACCTTTCTTTTCATAGAGCAGTTTTGAAACACTCTTTTTGTAGAATCTGGAAGTGGATATTTGGA

                   *   ** ** *  *   *            **  
     447339 GCGCTTTCAGGCCTATGGTGAAAAAGGAAATATCTTCCCAT
        131 GCGCTTTGAGGATTTCGTTGGAAACGGAAATATCTTCATAT

                  * * *      **      *   * *   *     ** ** *      *    *     
     447380 AAAAACGACATAGAAGCTATCTCAGGAACTTGTTTATGATGCATCTAATCAACTAACAGTGTTGA
          1 AAAAACTAGACAGAAGCATTCTCAGAAACATCTTTGTGATGTTTGCACTCAACTCACAGAGTTGA

                  * ** * *    *                    * *       *               
     447445 ACCTTTGTACTGACAGAGGAG-TTTGAAACACTCTTTTTTTGGAATCTGCAAGTGGATATTTGGA
         66 ACCTTTCTTTTCATAGAGCAGTTTTGAAACACTCTTTTTGTAGAATCTGGAAGTGGATATTTGGA

            *                           *   *   *    
     447509 TCGCTTTGAGGATTTCGTTGGAAACGG-GAT-GC--AATAT
        131 GCGCTTTGAGGATTTCGTTGGAAACGGAAATATCTTCATAT

                **  *    *     *                 **  *   *  *    *         * 
     447546 AAAACGTACACAGCAGCATACTCAGAAA-ATACTTTGCCATATTTCCATTCAAGTCACAGAGTGG
          1 AAAAACTAGACAGAAGCATTCTCAGAAACAT-CTTTGTGATGTTTGCACTCAACTCACAGAGTTG

               *  * **            *                  *  *             *      
     447610 AACATTCCCATTCATAGAGCAGGTTTGAAACACTCTTTTTGGAGTATCTGGAAGTGGACATTTGG
         65 AACCTTTCTTTTCATAGAGCAGTTTTGAAACACTCTTTTTGTAGAATCTGGAAGTGGATATTTGG

                    ** * * ** *  *   *            **  
     447675 AGCGCTTTCTGAACTATGGTGAAAAAGGAAATATCTTCCAAT
        130 AGCGCTTTGAGGATTTCGTTGGAAACGGAAATATCTTCATAT

            *     *               *      * *          *  **      **  *  *    
     447717 GAAAACAAGACAGAAGCATTCTGAGAAACTTATTTGTGATGTGTGTCCTCAACAAACGGACTTGA
          1 AAAAACTAGACAGAAGCATTCTCAGAAACATCTTTGTGATGTTTGCACTCAACTCACAGAGTTGA

                   *      **  * *  *  *             *   *    * * *           
     447782 ACCTTTCGTTTCATGCAGTACTTCTGGAACACTCTTTTTGAAGATTCTGCATGCGGATATTTGGA
         66 ACCTTTCTTTTCATAGAGCAGTTTTGAAACACTCTTTTTGTAGAATCTGGAAGTGGATATTTGGA

            **                            **   *   * 
     447847 TAGCTTTGAGGATTTCGTTGGAAACGG---GCT-TACATGT
        131 GCGCTTTGAGGATTTCGTTGGAAACGGAAATATCTTCATAT

                 *       *               *        *   *    *    *       *    
     447884 AAAAATTAGACAGCAGCATTCTCAGAAACTTCTTTGTGGTGTCTGCATTCAAGTCACAGAATTGA
          1 AAAAACTAGACAGAAGCATTCTCAGAAACATCTTTGTGATGTTTGCACTCAACTCACAGAGTTGA

              * ** * **            *  * *      *       *             *       
     447949 ACTTCCCCTCACATAGAGCAGTTGTGCAGCACTCTATTTGTAGTATCTGGAAGTGGACATTTGGA
         66 ACCTTTCTTTTCATAGAGCAGTTTTGAAACACTCTTTTTGTAGAATCTGGAAGTGGATATTTGGA

             *         ** *    * *   *            **  
     448014 GGGCTTTGTAGCCTATC-TGGAAAAAGGAAATATCTTCCCAT
        131 GCGCTTTG-AGGATTTCGTTGGAAACGGAAATATCTTCATAT

            *  ** *   *     * *            *   *  *   * **        *  * * *   
     448055 GAATGCGAGATAGAAGTAATCTCAGAAACATGTTTATGCTGTATCTACTCAACTAACTGTGCTGA
          1 AAAAACTAGACAGAAGCATTCTCAGAAACATCTTTGTGATGTTTGCACTCAACTCACAGAGTTGA

              *     *  *               *        *  * *       *               
     448120 ACATTTCTATTGATAGAGCAGTTTTGAGACACTCTTCTTTTGGAATCTGCAAGTGGATATTTGGA
         66 ACCTTTCTTTTCATAGAGCAGTTTTGAAACACTCTTTTTGTAGAATCTGGAAGTGGATATTTGGA

            ** *                        *     *      
     448185 TAGATTTGAGGATTTCGTTGGAAACGG-GAT-T-AT-ATAT
        131 GCGCTTTGAGGATTTCGTTGGAAACGGAAATATCTTCATAT

            *    *       *               *                 **  *   *         
     448222 CAAAAGTAGACAGCAGCATTCTCAGAAACTTCTTTGTGATGTTTGCATCCAGCTCTCAGAGTTGA
          1 AAAAACTAGACAGAAGCATTCTCAGAAACATCTTTGTGATGTTTGCACTCAACTCACAGAGTTGA

              *  * *          *  *        *        *   **        *  **       
     448287 ACATTCCCTTTCATAGAGTAGGTTTGAAACCCTCTTTTTATAGTGTCTGGAAGCGGGCATTTGGA
         66 ACCTTTCTTTTCATAGAGCAGTTTTGAAACACTCTTTTTGTAGAATCTGGAAGTGGATATTTGGA

                   *    * * **  **  **          * *  *
     448352 GCGC-TTCAGGCCTATGCTTAAAATAGGAAATATCTACCTAC
        131 GCGCTTTGAGG-ATTTCGTTGGAAACGGAAATATCTTCATAT

             *                    *    *  **          ** *        *    *     
     448393 AGAAACTAGACAGAAGCATTCTGAGAATCACGTTTGTGATGTGGGTACTCAACTAACAGTGTTGA
          1 AAAAACTAGACAGAAGCATTCTCAGAAACATCTTTGTGATGTTTGCACTCAACTCACAGAGTTGA

            *  *       *   *            *   *                *   *           
     448458 TCCATTCTTTTGATACAGCAGTTTTGAACCACACTTTTTGTAGAATCTGCAAGAGGATATTTGGA
         66 ACCTTTCTTTTCATAGAGCAGTTTTGAAACACTCTTTTTGTAGAATCTGGAAGTGGATATTTGGA

            **   *                     *   *      * *
     448523 TAGCTGTGAGGATTTCGTTGGAAACGGGAATGTCTTCAAAG
        131 GCGCTTTGAGGATTTCGTTGGAAACGGAAATATCTTCATAT

                *                         *   *            *    *            
     448564 AAAATCTAGACAGAAGCATTCTCAGAAACACCTTCGTGATGTTTGCAATCAAGTCACAGAGTTGA
          1 AAAAACTAGACAGAAGCATTCTCAGAAACATCTTTGTGATGTTTGCACTCAACTCACAGAGTTGA

                 * *             *  *           *      *             *       
     448629 ACCTTCCGTTTCATAGAGCAGGTTGGAAACACTCTTATTGTAGTATCTGGAAGTGGACATTTGGA
         66 ACCTTTCTTTTCATAGAGCAGTTTTGAAACACTCTTTTTGTAGAATCTGGAAGTGGATATTTGGA

                   *   ** ** *  *   *            **  
     448694 GCGCTTTCAGGCCTATGGTGAAAAAGGAAATATCTTCCCAT
        131 GCGCTTTGAGGATTTCGTTGGAAACGGAAATATCTTCATAT

                  * * *      **      *   * *   *     ** ** *      *    *     
     448735 AAAAACGACATAGAAGCTATCTCAGGAACTTGTTTATGATGCATCTAATCAACTAACAGTGTTGA
          1 AAAAACTAGACAGAAGCATTCTCAGAAACATCTTTGTGATGTTTGCACTCAACTCACAGAGTTGA

                  * ** * *                         * *       *               
     448800 ACCTTTGTACTGACAGAGCAG-TTTGAAACACTCTTTTTTTGGAATCTGCAAGTGGATATTTGGA
         66 ACCTTTCTTTTCATAGAGCAGTTTTGAAACACTCTTTTTGTAGAATCTGGAAGTGGATATTTGGA

            *                           *   *   *    
     448864 TCGCTTTGAGGATTTCGTTGGAAACGG-GAT-GC--AATAT
        131 GCGCTTTGAGGATTTCGTTGGAAACGGAAATATCTTCATAT

                **  *    *     *                 **  *   *  *    *         * 
     448901 AAAACGTACACAGCAGCATACTCAGAAA-ATACTTTGCCATATTTCCATTCAAGTCACAGAGTGG
          1 AAAAACTAGACAGAAGCATTCTCAGAAACAT-CTTTGTGATGTTTGCACTCAACTCACAGAGTTG

               *  * **            *                  *  *             *      
     448965 AACATTCCCATTCATAGAGCAGGTTTGAAACACTCTTTTTGGAGTATCTGGAAGTGGACATTTGG
         65 AACCTTTCTTTTCATAGAGCAGTTTTGAAACACTCTTTTTGTAGAATCTGGAAGTGGATATTTGG

                    ** * * ** *  *   *            **  
     449030 AGCGCTTTCTGAACTATGGTGAAAAAGGAAATATCTTCCAAT
        130 AGCGCTTTGAGGATTTCGTTGGAAACGGAAATATCTTCATAT

            *     * *             *      * *          *  **      **  *  *    
     449072 GAAAACAACACAGAAGCATTCTGAGAAACTTATTTGTGATGTGTGTCCTCAACAAACGGACTTGA
          1 AAAAACTAGACAGAAGCATTCTCAGAAACATCTTTGTGATGTTTGCACTCAACTCACAGAGTTGA

                   *      **  * *  *  *             *   *    * * *           
     449137 ACCTTTCGTTTCATGCAGTACTTCTGGAACACTCTTTTTGAAGATTCTGCATGCGGATATTTGGA
         66 ACCTTTCTTTTCATAGAGCAGTTTTGAAACACTCTTTTTGTAGAATCTGGAAGTGGATATTTGGA

            **                            **   *   * 
     449202 TAGCTTTGAGGATTTCGTTGGAAACGG---GCT-TACATGT
        131 GCGCTTTGAGGATTTCGTTGGAAACGGAAATATCTTCATAT

                 *       *               *        *   *    *    *       *    
     449239 AAAAATTAGACAGCAGCATTCTCAGAAACTTCTTTGTGGTGTCTGCATTCAAGTCACAGAATTGA
          1 AAAAACTAGACAGAAGCATTCTCAGAAACATCTTTGTGATGTTTGCACTCAACTCACAGAGTTGA

              * ** * **            *  * *      *       *             *       
     449304 ACTTCCCCTCACATAGAGCAGTTGTGCAGCACTCTATTTGTAGTATCTGGAAGTGGACATTTGGA
         66 ACCTTTCTTTTCATAGAGCAGTTTTGAAACACTCTTTTTGTAGAATCTGGAAGTGGATATTTGGA

             *         ** *    * *   *            **  
     449369 GGGCTTTGTAGCCTATC-TGGAAAAAGGAAATATCTTCCCAT
        131 GCGCTTTG-AGGATTTCGTTGGAAACGGAAATATCTTCATAT

            *  ** *   *     * *            *   *  *   * **        *  * * *   
     449410 GAATGCGAGATAGAAGTAATCTCAGAAACATGTTTATGCTGTATCTACTCAACTAACTGTGCTGA
          1 AAAAACTAGACAGAAGCATTCTCAGAAACATCTTTGTGATGTTTGCACTCAACTCACAGAGTTGA

              *     *  *               *        *  * *       *               
     449475 ACATTTCTATTGATAGAGCAGTTTTGAGACACTCTTCTTTTGGAATCTGCAAGTGGATATTTGGA
         66 ACCTTTCTTTTCATAGAGCAGTTTTGAAACACTCTTTTTGTAGAATCTGGAAGTGGATATTTGGA

            ** *                        *     *      
     449540 TAGATTTGAGGATTTCGTTGGAAACGG-GAT-T-AT-ATAT
        131 GCGCTTTGAGGATTTCGTTGGAAACGGAAATATCTTCATAT

                 *       *               *                 **  *   *         
     449577 AAAAAGTAGACAGCAGCATTCTCAGAAACTTCTTTGTGATGTTTGCATCCAGCTCTCAGAGTTGA
          1 AAAAACTAGACAGAAGCATTCTCAGAAACATCTTTGTGATGTTTGCACTCAACTCACAGAGTTGA

              *  * *          *  *        *        *   **    *   *  **       
     449642 ACATTCCCTTTCATAGAGTAGGTTTGAAACCCTCTTTTTATAGTGTCTGCAAGCGGGCATTTGGA
         66 ACCTTTCTTTTCATAGAGCAGTTTTGAAACACTCTTTTTGTAGAATCTGGAAGTGGATATTTGGA

                   *    * * **  **  **          * *  *
     449707 GCGCTTTCAGGCCTATGCTTAAAATAGGAAATATCTACCTAC
        131 GCGCTTTGAGG-ATTTCGTTGGAAACGGAAATATCTTCATAT

             *                    *    *  **          ** *        *    *     
     449749 AGAAACTAGACAGAAGCATTCTGAGAATCACGTTTGTGATGTGGGTACTCAACTAACAGTGTTGA
          1 AAAAACTAGACAGAAGCATTCTCAGAAACATCTTTGTGATGTTTGCACTCAACTCACAGAGTTGA

            *  *       *   *            *   *                *   *           
     449814 TCCATTCTTTTGATACAGCAGTTTTGAACCACACTTTTTGTAGAATCTGCAAGAGGATATTTGGA
         66 ACCTTTCTTTTCATAGAGCAGTTTTGAAACACTCTTTTTGTAGAATCTGGAAGTGGATATTTGGA

            **   *                     *   *      * *
     449879 TAGCTGTGAGGATTTCGTTGGAAACGGGAATGTCTTCAAAG
        131 GCGCTTTGAGGATTTCGTTGGAAACGGAAATATCTTCATAT

                *                         *   *            *    *            
     449920 AAAATCTAGACAGAAGCATTCTCAGAAACACCTTCGTGATGTTTGCAATCAAGTCACAGAGTTGA
          1 AAAAACTAGACAGAAGCATTCTCAGAAACATCTTTGTGATGTTTGCACTCAACTCACAGAGTTGA

                 * *             *  *           *      *             *       
     449985 ACCTTCCGTTTCATAGAGCAGGTTGGAAACACTCTTATTGTAGTATCTGGAAGTGGACATTTGGA
         66 ACCTTTCTTTTCATAGAGCAGTTTTGAAACACTCTTTTTGTAGAATCTGGAAGTGGATATTTGGA

                   *   ** ** *  *   *            **  
     450050 GCGCTTTCAGGCCTATGGTGAAAAAGGAAATATCTTCCCAT
        131 GCGCTTTGAGGATTTCGTTGGAAACGGAAATATCTTCATAT

                  * * *      **      *   * *   *     ** ** *      *    *     
     450091 AAAAACGACATAGAAGCTATCTCAGGAACTTGTTTATGATGCATCTAATCAACTAACAGTGTTGA
          1 AAAAACTAGACAGAAGCATTCTCAGAAACATCTTTGTGATGTTTGCACTCAACTCACAGAGTTGA

                  * ** * *                         * *       *               
     450156 ACCTTTGTACTGACAGAGCAG-TTTGAAACACTCTTTTTTTGGAATCTGCAAGTGGATATTTGGA
         66 ACCTTTCTTTTCATAGAGCAGTTTTGAAACACTCTTTTTGTAGAATCTGGAAGTGGATATTTGGA

            *                           *   *   *    
     450220 TCGCTTTGAGGATTTCGTTGGAAACGG-GAT-GC--AATAT
        131 GCGCTTTGAGGATTTCGTTGGAAACGGAAATATCTTCATAT

                **  *    *     *                 **  *   *  *    *   *     * 
     450257 AAAACGTACACAGCAGCATACTCAGAAA-ATACTTTGCCATATTTCCATTCAAGTCAGAGAGTGG
          1 AAAAACTAGACAGAAGCATTCTCAGAAACAT-CTTTGTGATGTTTGCACTCAACTCACAGAGTTG

               *  * **            *                  *  *             *      
     450321 AACATTCCCATTCATAGAGCAGGTTTGAAACACTCTTTTTGGAGTATCTGGAAGTGGACATTTGG
         65 AACCTTTCTTTTCATAGAGCAGTTTTGAAACACTCTTTTTGTAGAATCTGGAAGTGGATATTTGG

                    ** * * ** *  *   *            **  
     450386 AGCGCTTTCTGAACTATGGTGAAAAAGGAAATATCTTCCAAT
        130 AGCGCTTTGAGGATTTCGTTGGAAACGGAAATATCTTCATAT

            *     *               *      * *          *  **      **  *  *    
     450428 GAAAACAAGACAGAAGCATTCTGAGAAACTTATTTGTGATGTGTGTCCTCAACAAACGGACTTGA
          1 AAAAACTAGACAGAAGCATTCTCAGAAACATCTTTGTGATGTTTGCACTCAACTCACAGAGTTGA

                   *      **  * *  *  *             *   *    * * *           
     450493 ACCTTTCGTTTCATGCAGTACTTCTGGAACACTCTTTTTGAAGATTCTGCATGCGGATATTTGGA
         66 ACCTTTCTTTTCATAGAGCAGTTTTGAAACACTCTTTTTGTAGAATCTGGAAGTGGATATTTGGA

            **                            **   *   * 
     450558 TAGCTTTGAGGATTTCGTTGGAAACGG---GCT-TACATGT
        131 GCGCTTTGAGGATTTCGTTGGAAACGGAAATATCTTCATAT

                 *       *               *        *   *    *    *       *    
     450595 AAAAATTAGACAGCAGCATTCTCAGAAACTTCTTTGTGGTGTCTGCATTCAAGTCACAGAATTGA
          1 AAAAACTAGACAGAAGCATTCTCAGAAACATCTTTGTGATGTTTGCACTCAACTCACAGAGTTGA

              * ** * **            *  * *      *       *             *       
     450660 ACTTCCCCTCACATAGAGCAGTTGTGCAGCACTCTATTTGTAGTATCTGGAAGTGGACATTTGGA
         66 ACCTTTCTTTTCATAGAGCAGTTTTGAAACACTCTTTTTGTAGAATCTGGAAGTGGATATTTGGA

             *         ** *    * *   *            **  
     450725 GGGCTTTGTAGCCTATC-TGGAAAAAGGAAATATCTTCCCAT
        131 GCGCTTTG-AGGATTTCGTTGGAAACGGAAATATCTTCATAT

            *  ** *   *     * *            *   *  *   * **        *  * * *   
     450766 GAATGCGAGATAGAAGTAATCTCAGAAACATGTTTATGCTGTATCTACTCAACTAACTGTGCTGA
          1 AAAAACTAGACAGAAGCATTCTCAGAAACATCTTTGTGATGTTTGCACTCAACTCACAGAGTTGA

              *     *  *             * *        *  * *       *               
     450831 ACATTTCTATTGATAGAGCAGTTTTCAGACACTCTTCTTTTGGAATCTGCAAGTGGATATTTGGA
         66 ACCTTTCTTTTCATAGAGCAGTTTTGAAACACTCTTTTTGTAGAATCTGGAAGTGGATATTTGGA

            ** *                        *     *      
     450896 TAGATTTGAGGATTTCGTTGGAAACGG-GAT-T-AT-ATAT
        131 GCGCTTTGAGGATTTCGTTGGAAACGGAAATATCTTCATAT

            *    *       *               *                 **  *   *         
     450933 CAAAAGTAGACAGCAGCATTCTCAGAAACTTCTTTGTGATGTTTGCATCCAGCTCTCAGAGTTGA
          1 AAAAACTAGACAGAAGCATTCTCAGAAACATCTTTGTGATGTTTGCACTCAACTCACAGAGTTGA

              *  * *          *  *        *        *   **        *  **       
     450998 ACATTCCCTTTCATAGAGTAGGTTTGAAACCCTCTTTTTATAGTGTCTGGAAGCGGGCATTTGGA
         66 ACCTTTCTTTTCATAGAGCAGTTTTGAAACACTCTTTTTGTAGAATCTGGAAGTGGATATTTGGA

                   *    * * **  **  **          * *  *
     451063 GCGCTTTCAGGCCTATGCTTAAAATAGGAAATATCTACCTAC
        131 GCGCTTTGAGG-ATTTCGTTGGAAACGGAAATATCTTCATAT

             *                    *    *  **          ** *        *    *     
     451105 AGAAACTAGACAGAAGCATTCTGAGAATCACGTTTGTGATGTGGGTACTCAACTAACAGTGTTGA
          1 AAAAACTAGACAGAAGCATTCTCAGAAACATCTTTGTGATGTTTGCACTCAACTCACAGAGTTGA

            *  *       *   *            *   *                *   *           
     451170 TCCATTCTTTTGATACAGCAGTTTTGAACCACACTTTTTGTAGAATCTGCAAGAGGATATTTGGA
         66 ACCTTTCTTTTCATAGAGCAGTTTTGAAACACTCTTTTTGTAGAATCTGGAAGTGGATATTTGGA

            **   *                     *   *      * *
     451235 TAGCTGTGAGGATTTCGTTGGAAACGGGAATGTCTTCAAAG
        131 GCGCTTTGAGGATTTCGTTGGAAACGGAAATATCTTCATAT

                *                         *   *            *    *            
     451276 AAAATCTAGACAGAAGCATTCTCAGAAACACCTTCGTGATGTTTGCAATCAAGTCACAGAGTTGA
          1 AAAAACTAGACAGAAGCATTCTCAGAAACATCTTTGTGATGTTTGCACTCAACTCACAGAGTTGA

                 * *             *  *           *      *             *       
     451341 ACCTTCCGTTTCATAGAGCAGGTTGGAAACACTCTTATTGTAGTATCTGGAAGTGGACATTTGGA
         66 ACCTTTCTTTTCATAGAGCAGTTTTGAAACACTCTTTTTGTAGAATCTGGAAGTGGATATTTGGA

                   *   ** ** *  *   *            **  
     451406 GCGCTTTCAGGCCTATGGTGAAAAAGGAAATATCTTCCCAT
        131 GCGCTTTGAGGATTTCGTTGGAAACGGAAATATCTTCATAT

                  * * *      **      *   * *   *     ** ** *      *    *     
     451447 AAAAACGACATAGAAGCTATCTCAGGAACTTGTTTATGATGCATCTAATCAACTAACAGTGTTGA
          1 AAAAACTAGACAGAAGCATTCTCAGAAACATCTTTGTGATGTTTGCACTCAACTCACAGAGTTGA

                  * ** * *                         * *       *               
     451512 ACCTTTGTACTGACAGAGCAG-TTTGAAACACTCTTTTTTTGGAATCTGCAAGTGGATATTTGGA
         66 ACCTTTCTTTTCATAGAGCAGTTTTGAAACACTCTTTTTGTAGAATCTGGAAGTGGATATTTGGA

            *                           *   *   *    
     451576 TCGCTTTGAGGATTTCGTTGGAAACGG-GAT-GC--AATAT
        131 GCGCTTTGAGGATTTCGTTGGAAACGGAAATATCTTCATAT

                **  *    *     *                 **  *   *  *    *         * 
     451613 AAAACGTACACAGCAGCATACTCAGAAA-ATACTTTGCCATATTTCCATTCAAGTCACAGAGTGG
          1 AAAAACTAGACAGAAGCATTCTCAGAAACAT-CTTTGTGATGTTTGCACTCAACTCACAGAGTTG

               *  * **            *                  *  *             *      
     451677 AACATTCCCATTCATAGAGCAGGTTTGAAACACTCTTTTTGGAGTATCTGGAAGTGGACATTTGG
         65 AACCTTTCTTTTCATAGAGCAGTTTTGAAACACTCTTTTTGTAGAATCTGGAAGTGGATATTTGG

                    ** * * ** *  *   *            **  
     451742 AGCGCTTTCTGAACTATGGTGAAAAAGGAAATATCTTCCAAT
        130 AGCGCTTTGAGGATTTCGTTGGAAACGGAAATATCTTCATAT

            *     *               *      * *          *  **      **  *  *    
     451784 GAAAACAAGACAGAAGCATTCTGAGAAACTTATTTGTGATGTGTGTCCTCAACAAACGGACTTGA
          1 AAAAACTAGACAGAAGCATTCTCAGAAACATCTTTGTGATGTTTGCACTCAACTCACAGAGTTGA

                   *      **  * *  *  *             *   *    * * *           
     451849 ACCTTTCGTTTCATGCAGTACTTCTGGAACACTCTTTTTGAAGATTCTGCATGCGGATATTTGGA
         66 ACCTTTCTTTTCATAGAGCAGTTTTGAAACACTCTTTTTGTAGAATCTGGAAGTGGATATTTGGA

            **                            **   *   * 
     451914 TAGCTTTGAGGATTTCGTTGGAAACGG---GCT-TACATGT
        131 GCGCTTTGAGGATTTCGTTGGAAACGGAAATATCTTCATAT

                 *       *               *        *   *    *    *       *    
     451951 AAAAATTAGACAGCAGCATTCTCAGAAACTTCTTTGTGGTGTCTGCATTCAAGTCACAGAATTGA
          1 AAAAACTAGACAGAAGCATTCTCAGAAACATCTTTGTGATGTTTGCACTCAACTCACAGAGTTGA

              * ** * **            *  * *      *       *             *       
     452016 ACTTCCCCTCACATAGAGCAGTTGTGCAGCACTCTATTTGTAGTATCTGGAAGTGGACATTTGGA
         66 ACCTTTCTTTTCATAGAGCAGTTTTGAAACACTCTTTTTGTAGAATCTGGAAGTGGATATTTGGA

             *         ** *    * *   *            **  
     452081 GGGCTTTGTAGCCTATC-TGGAAAAAGGAAATATCTTCCCAT
        131 GCGCTTTG-AGGATTTCGTTGGAAACGGAAATATCTTCATAT

            *  ** *   *     * *            *   *  *   * **        *  * * *   
     452122 GAATGCGAGATAGAAGTAATCTCAGAAACATGTTTATGCTGTATCTACTCAACTAACTGTGCTGA
          1 AAAAACTAGACAGAAGCATTCTCAGAAACATCTTTGTGATGTTTGCACTCAACTCACAGAGTTGA

              *     *  *               *        *  * *       *               
     452187 ACATTTCTATTGATAGAGCAGTTTTGAGACACTCTTCTTTTGGAATCTGCAAGTGGATATTTGGA
         66 ACCTTTCTTTTCATAGAGCAGTTTTGAAACACTCTTTTTGTAGAATCTGGAAGTGGATATTTGGA

            ** *                        *     *      
     452252 TAGATTTGAGGATTTCGTTGGAAACGG-GAT-T-AT-ATAT
        131 GCGCTTTGAGGATTTCGTTGGAAACGGAAATATCTTCATAT

                 *       *               *                 **  *   *         
     452289 AAAAAGTAGACAGCAGCATTCTCAGAAACTTCTTTGTGATGTTTGCATCCAGCTCTCAGAGTTGA
          1 AAAAACTAGACAGAAGCATTCTCAGAAACATCTTTGTGATGTTTGCACTCAACTCACAGAGTTGA

              *  * *          *  *        *        *   **    *   *  **       
     452354 ACATTCCCTTTCATAGAGTAGGTTTGAAACCCTCTTTTTATAGTGTCTGCAAGCGGGCATTTGGA
         66 ACCTTTCTTTTCATAGAGCAGTTTTGAAACACTCTTTTTGTAGAATCTGGAAGTGGATATTTGGA

                   *    * * **   *  **          * *  *
     452419 GCGCTTTCAGGCCTATGCTTGAAATAGGAAATATCTACCTAC
        131 GCGCTTTGAGG-ATTTCGTTGGAAACGGAAATATCTTCATAT

             *                    *    *  **          ** *        *    *     
     452461 AGAAACTAGACAGAAGCATTCTGAGAATCACGTTTGTGATGTGGGTACTCAACTAACAGTGTTGA
          1 AAAAACTAGACAGAAGCATTCTCAGAAACATCTTTGTGATGTTTGCACTCAACTCACAGAGTTGA

            *  *       *   *            *   *                *   *           
     452526 TCCATTCTTTTGATACAGCAGTTTTGAACCACACTTTTTGTAGAATCTGCAAGAGGATATTTGGA
         66 ACCTTTCTTTTCATAGAGCAGTTTTGAAACACTCTTTTTGTAGAATCTGGAAGTGGATATTTGGA

            **   *                     *   *      * *
     452591 TAGCTGTGAGGATTTCGTTGGAAACGGGAATGTCTTCAAAG
        131 GCGCTTTGAGGATTTCGTTGGAAACGGAAATATCTTCATAT

                *                         *   *            *    *            
     452632 AAAATCTAGACAGAAGCATTCTCAGAAACACCTTCGTGATGTTTGCAATCAAGTCACAGAGTTGA
          1 AAAAACTAGACAGAAGCATTCTCAGAAACATCTTTGTGATGTTTGCACTCAACTCACAGAGTTGA

                 * *             *  *           *      *             *       
     452697 ACCTTCCGTTTCATAGAGCAGGTTGGAAACACTCTTATTGTAGTATCTGGAAGTGGACATTTGGA
         66 ACCTTTCTTTTCATAGAGCAGTTTTGAAACACTCTTTTTGTAGAATCTGGAAGTGGATATTTGGA

                   *   ** ** *  *   *            **  
     452762 GCGCTTTCAGGCCTATGGTGAAAAAGGAAATATCTTCCCAT
        131 GCGCTTTGAGGATTTCGTTGGAAACGGAAATATCTTCATAT

                  * * *      **      *   * *   *     ** ** *      *    *     
     452803 AAAAACGACATAGAAGCTATCTCAGGAACTTGTTTATGATGCATCTAATCAACTAACAGTGTTGA
          1 AAAAACTAGACAGAAGCATTCTCAGAAACATCTTTGTGATGTTTGCACTCAACTCACAGAGTTGA

                  * ** * *                         * *       *               
     452868 ACCTTTGTACTGACAGAGCAG-TTTGAAACACTCTTTTTTTGGAATCTGCAAGTGGATATTTGGA
         66 ACCTTTCTTTTCATAGAGCAGTTTTGAAACACTCTTTTTGTAGAATCTGGAAGTGGATATTTGGA

            *                           *   *   *    
     452932 TCGCTTTGAGGATTTCGTTGGAAACGG-GAT-GC--AATAT
        131 GCGCTTTGAGGATTTCGTTGGAAACGGAAATATCTTCATAT

                **  *    *     *                 **  *   *  *    *         * 
     452969 AAAACGTACACAGCAGCATACTCAGAAA-ATACTTTGCCATATTTCCATTCAAGTCACAGAGTGG
          1 AAAAACTAGACAGAAGCATTCTCAGAAACAT-CTTTGTGATGTTTGCACTCAACTCACAGAGTTG

               *  * **            *  *               *  *             *      
     453033 AACATTCCCATTCATAGAGCAGGTTGGAAACACTCTTTTTGGAGTATCTGGAAGTGGACATTTGG
         65 AACCTTTCTTTTCATAGAGCAGTTTTGAAACACTCTTTTTGTAGAATCTGGAAGTGGATATTTGG

                    ** * * ** *  *   *            **  
     453098 AGCGCTTTCTGAACTATGGTGAAAAAGGAAATATCTTCCAAT
        130 AGCGCTTTGAGGATTTCGTTGGAAACGGAAATATCTTCATAT

            *     *               *      * *          *  **      **  *  *    
     453140 GAAAACAAGACAGAAGCATTCTGAGAAACTTATTTGTGATGTGTGTCCTCAACAAACGGACTTGA
          1 AAAAACTAGACAGAAGCATTCTCAGAAACATCTTTGTGATGTTTGCACTCAACTCACAGAGTTGA

                   *      **  * *  *  *             *   *    * * *           
     453205 ACCTTTCGTTTCATGCAGTACTTCTGGAACACTCTTTTTGAAGATTCTGCATGCGGATATTTGGA
         66 ACCTTTCTTTTCATAGAGCAGTTTTGAAACACTCTTTTTGTAGAATCTGGAAGTGGATATTTGGA

            **                            **   *   * 
     453270 TAGCTTTGAGGATTTCGTTGGAAACGG---GCT-TACATGT
        131 GCGCTTTGAGGATTTCGTTGGAAACGGAAATATCTTCATAT

                 *       *               *        *   *    *    *       *    
     453307 AAAAATTAGACAGCAGCATTCTCAGAAACTTCTTTGTGGTGTCTGCATTCAAGTCACAGAATTGA
          1 AAAAACTAGACAGAAGCATTCTCAGAAACATCTTTGTGATGTTTGCACTCAACTCACAGAGTTGA

              * ** * **            *  * *      *       *    *        *       
     453372 ACTTCCCCTCACATAGAGCAGTTGTGCAGCACTCTATTTGTAGTATCTCGAAGTGGACATTTGGA
         66 ACCTTTCTTTTCATAGAGCAGTTTTGAAACACTCTTTTTGTAGAATCTGGAAGTGGATATTTGGA

             *         ** *    * *   *            **  
     453437 GGGCTTTGTAGCCTATC-TGGAAAAAGGAAATATCTTCCCAT
        131 GCGCTTTG-AGGATTTCGTTGGAAACGGAAATATCTTCATAT

            *  ** *   *     * *            *   *  *   * **        *  * * *   
     453478 GAATGCGAGATAGAAGTAATCTCAGAAACATGTTTATGCTGTATCTACTCAACTAACTGTGCTGA
          1 AAAAACTAGACAGAAGCATTCTCAGAAACATCTTTGTGATGTTTGCACTCAACTCACAGAGTTGA

              *     *  *               *        *  * *       *               
     453543 ACATTTCTATTGATAGAGCAGTTTTGAGACACTCTTCTTTTGGAATCTGCAAGTGGATATTTGGA
         66 ACCTTTCTTTTCATAGAGCAGTTTTGAAACACTCTTTTTGTAGAATCTGGAAGTGGATATTTGGA

            ** *                        *     *      
     453608 TAGATTTGAGGATTTCGTTGGAAACGG-GAT-T-AT-ATAT
        131 GCGCTTTGAGGATTTCGTTGGAAACGGAAATATCTTCATAT

                 *       *               *                 **  *   *         
     453645 AAAAAGTAGACAGCAGCATTCTCAGAAACTTCTTTGTGATGTTTGCATCCAGCTCTCAGAGTTGA
          1 AAAAACTAGACAGAAGCATTCTCAGAAACATCTTTGTGATGTTTGCACTCAACTCACAGAGTTGA

              *  * *          *  *        *        *   **        *  **       
     453710 ACATTCCCTTTCATAGAGTAGGTTTGAAACCCTCTTTTTATAGTGTCTGGAAGCGGGCATTTGGA
         66 ACCTTTCTTTTCATAGAGCAGTTTTGAAACACTCTTTTTGTAGAATCTGGAAGTGGATATTTGGA

                   *    * * **  **  **          * *  *
     453775 GCGCTTTCAGGCCTATGCTTAAAATAGGAAATATCTACCTAC
        131 GCGCTTTGAGG-ATTTCGTTGGAAACGGAAATATCTTCATAT

             *                    *    *  **          ** *        *    *     
     453817 AGAAACTAGACAGAAGCATTCTGAGAATCACGTTTGTGATGTGGGTACTCAACTAACAGTGTTGA
          1 AAAAACTAGACAGAAGCATTCTCAGAAACATCTTTGTGATGTTTGCACTCAACTCACAGAGTTGA

            *  *       *   *            *   *                *               
     453882 TCCATTCTTTTGATACAGCAGTTTTGAACCACACTTTTTGTAGAATCTGCAAGTGGATATTTGGA
         66 ACCTTTCTTTTCATAGAGCAGTTTTGAAACACTCTTTTTGTAGAATCTGGAAGTGGATATTTGGA

            **   *                     *   *        *
     453947 TAGCTGTGAGGATTTCGTTGGAAACGGGAATGTCTTCATAG
        131 GCGCTTTGAGGATTTCGTTGGAAACGGAAATATCTTCATAT

                **                     * * **         *  **   *   *          
     453988 AAAATTTAGACAGAAGCATTCTCAGAACCTTGATTGTGATGTGTGTTCTCCACTAACAGAGTTGA
          1 AAAAACTAGACAGAAGCATTCTCAGAAACATCTTTGTGATGTTTGCACTCAACTCACAGAGTTGA

                       * *   * *   *       *       *                         
     454053 ACCTTTCTTTTGACAGAACTGTTCTGAAACATTCTTTTTATAGAATCTGGAAGTGGATATTTGGA
         66 ACCTTTCTTTTCATAGAGCAGTTTTGAAACACTCTTTTTGTAGAATCTGGAAGTGGATATTTGGA

            **                         *          *  
     454118 AAGCTTTGAGGATTTCGTTGGAAACGGGAATATCTTCAAAT
        131 GCGCTTTGAGGATTTCGTTGGAAACGGAAATATCTTCATAT

                *    *            *           * *       *  *    *            
     454159 AAAATCTAGCCAGAAGCATTCTAAGAAACATCTTAGGGATGTTTACATTCAAGTCACAGAGTTGA
          1 AAAAACTAGACAGAAGCATTCTCAGAAACATCTTTGTGATGTTTGCACTCAACTCACAGAGTTGA

              *  * *    **       *         *    * *   **      *      *    *  
     454224 ACATTCCCTTTCGCAGAGCAGGTTTGAAACAATCTTCTCGTACTATCTGGCAGTGGACATTTTGA
         66 ACCTTTCTTTTCATAGAGCAGTTTTGAAACACTCTTTTTGTAGAATCTGGAAGTGGATATTTGGA

              * *   *  ** ** *  *   *            ** *
     454289 GCTCCTTGGGGCCTATGCTGAAAAAGGAAATATCTTCGGAC
        131 GCGCTTTGAGGATTTCGTTGGAAACGGAAATATCTTCATAT

                                 *     *  **          *           **    *    
     454330 AAAAACTAGACAGAAGCATTCGCAGAATCACGTTTGTGATGTGTGCACTCAACTGTCAGAATTGA
          1 AAAAACTAGACAGAAGCATTCTCAGAAACATCTTTGTGATGTTTGCACTCAACTCACAGAGTTGA

                 **   ****      *                            * *            *
     454395 ACCTTGGTTTGGCCAGAGCACTTTTGAAACACTCTTTTTGTAGAATCTGCAGGTGGATATTTGGC
         66 ACCTTTCTTTTCATAGAGCAGTTTTGAAACACTCTTTTTGTAGAATCTGGAAGTGGATATTTGGA

            **                         *   *      * *
     454460 TAGCTTTGAGGATTTCGTTGGAAACGGTAATGTCTTCAAAG
        131 GCGCTTTGAGGATTTCGTTGGAAACGGAAATATCTTCATAT

                *                         *   *            *    *            
     454501 AAAATCTAGACAGAAGCATTCTCAGAAACACCTTCGTGATGTTTGCAATCAAGTCACAGAGTTGA
          1 AAAAACTAGACAGAAGCATTCTCAGAAACATCTTTGTGATGTTTGCACTCAACTCACAGAGTTGA

                 * *             *  *                  *             *       
     454566 ACCTTCCGTTTCATAGAGCAGGTTGGAAACACTCTTTTTGTAGTATCTGGAAGTGGACATTTGGA
         66 ACCTTTCTTTTCATAGAGCAGTTTTGAAACACTCTTTTTGTAGAATCTGGAAGTGGATATTTGGA

             *         ** *    * *   *            **  
     454631 GGGCTTTGTAGCCTATC-TGGAAAAAGGAAATATCTTCCCAT
        131 GCGCTTTG-AGGATTTCGTTGGAAACGGAAATATCTTCATAT

            *  ** *   *     * *            *   *  *   * **        *  * * *   
     454672 GAATGCGAGATAGAAGTAATCTCAGAAACATGTTTATGCTGTATCTACTCAACTAACTGTGCTGA
          1 AAAAACTAGACAGAAGCATTCTCAGAAACATCTTTGTGATGTTTGCACTCAACTCACAGAGTTGA

              *     *  *               *        ** * *       *               
     454737 ACATTTCTATTGATAGAGCAGTTTTGAGACACTCTTCCTTTGGAATCTGCAAGTGGATATTTGGA
         66 ACCTTTCTTTTCATAGAGCAGTTTTGAAACACTCTTTTTGTAGAATCTGGAAGTGGATATTTGGA

             * *                        *     *      
     454802 GAGATTTGAGGATTTCGTTGGAAACGG-GAT-T-AT-ATAT
        131 GCGCTTTGAGGATTTCGTTGGAAACGGAAATATCTTCATAT

                 *       *               *                 **  *   *         
     454839 AAAAAGTAGACAGCAGCATTCTCAGAAACTTCTTTGTGATGTTTGCATCCAGCTCTCAGAGTTGA
          1 AAAAACTAGACAGAAGCATTCTCAGAAACATCTTTGTGATGTTTGCACTCAACTCACAGAGTTGA

              *  * *          *  *    *   *        *   **        *  **       
     454904 ACATTCCCTTTCATAGAGTAGGTTTGCAACCCTCTTTTTATAGTGTCTGGAAGCGGGCATTTGGA
         66 ACCTTTCTTTTCATAGAGCAGTTTTGAAACACTCTTTTTGTAGAATCTGGAAGTGGATATTTGGA

                   *    * * **  **  **          * *  *
     454969 GCGCTTTCAGGCCTATGCTTAAAATAGGAAATATCTACCTAC
        131 GCGCTTTGAGG-ATTTCGTTGGAAACGGAAATATCTTCATAT

             *                    *    *               ** *        *    *    
     455011 AGAAACTAGACAGAAGCATTCTGAGAATC-TCGTTTGTGATGTGGGTACTCAACTAACAGTGTTG
          1 AAAAACTAGACAGAAGCATTCTCAGAAACATC-TTTGTGATGTTTGCACTCAACTCACAGAGTTG

             *  *       *   *            *   *                *   *          
     455075 ATCCATTCTTTTGATACAGCAGTTTTGAACCACACTTTTTGTAGAATCTGCAAGAGGATATTTGG
         65 AACCTTTCTTTTCATAGAGCAGTTTTGAAACACTCTTTTTGTAGAATCTGGAAGTGGATATTTGG

             **   *                     *   *      * *
     455140 ATAGCTGTGAGGATTTCGTTGGAAACGGGAATGTCTTCAAAG
        130 AGCGCTTTGAGGATTTCGTTGGAAACGGAAATATCTTCATAT

                *                         *   *            *    *            
     455182 AAAATCTAGACAGAAGCATTCTCAGAAACACCTTCGTGATGTTTGCAATCAAGTCACAGAGTTGA
          1 AAAAACTAGACAGAAGCATTCTCAGAAACATCTTTGTGATGTTTGCACTCAACTCACAGAGTTGA

                 * *             *  *           *      *             *       
     455247 ACCTTCCGTTTCATAGAGCAGGTTGGAAACACTCTTATTGTAGTATCTGGAAGTGGACATTTGGA
         66 ACCTTTCTTTTCATAGAGCAGTTTTGAAACACTCTTTTTGTAGAATCTGGAAGTGGATATTTGGA

                   *   ** ** *  *   *            **  
     455312 GCGCTTTCAGGCCTATGGTGAAAAAGGAAATATCTTCCCAT
        131 GCGCTTTGAGGATTTCGTTGGAAACGGAAATATCTTCATAT

                  * * *      **      *   * *   *   * ** ** *      *    *     
     455353 AAAAACGACATAGAAGCTATCTCAGGAACTTGTTTATGAGGCATCTAATCAACTAACAGTGTTGA
          1 AAAAACTAGACAGAAGCATTCTCAGAAACATCTTTGTGATGTTTGCACTCAACTCACAGAGTTGA

                  * ** * *                         * *       *               
     455418 ACCTTTGTACTGACAGAGCAG-TTTGAAACACTCTTTTTTTGGAATCTGCAAGTGGATATTTGGA
         66 ACCTTTCTTTTCATAGAGCAGTTTTGAAACACTCTTTTTGTAGAATCTGGAAGTGGATATTTGGA

            *                           *   *   *    
     455482 TCGCTTTGAGGATTTCGTTGGAAACGG-GAT-GC--AATAT
        131 GCGCTTTGAGGATTTCGTTGGAAACGGAAATATCTTCATAT

                **  *    *     *                 **  *   *  *    *         * 
     455519 AAAACGTACACAGCAGCATACTCAGAAA-ATACTTTGCCATATTTCCATTCAAGTCACAGAGTGG
          1 AAAAACTAGACAGAAGCATTCTCAGAAACAT-CTTTGTGATGTTTGCACTCAACTCACAGAGTTG

               *  * **            *  *               *  *             *      
     455583 AACATTCCCATTCATAGAGCAGGTTGGAAACACTCTTTTTGGAGTATCTGGAAGTGGACATTTGG
         65 AACCTTTCTTTTCATAGAGCAGTTTTGAAACACTCTTTTTGTAGAATCTGGAAGTGGATATTTGG

                    ** * * ** *  *   *            **  
     455648 AGCGCTTTCTGAACTATGGTGAAAAAGGAAATATCTTCCAAT
        130 AGCGCTTTGAGGATTTCGTTGGAAACGGAAATATCTTCATAT

            *     *               *      * *          *  **      **  *  *    
     455690 GAAAACAAGACAGAAGCATTCTGAGAAACTTATTTGTGATGTGTGTCCTCAACAAACGGACTTGA
          1 AAAAACTAGACAGAAGCATTCTCAGAAACATCTTTGTGATGTTTGCACTCAACTCACAGAGTTGA

                   *      **  * *  *  *             *   *    * * *           
     455755 ACCTTTCGTTTCATGCAGTACTTCTGGAACACTCTTTTTGAAGATTCTGCATGCGGATATTTGGA
         66 ACCTTTCTTTTCATAGAGCAGTTTTGAAACACTCTTTTTGTAGAATCTGGAAGTGGATATTTGGA

            **                            **   *   * 
     455820 TAGCTTTGAGGATTTCGTTGGAAACGG---GCT-TACATGT
        131 GCGCTTTGAGGATTTCGTTGGAAACGGAAATATCTTCATAT

                 *       *               *        *   *    *    *       *    
     455857 AAAAATTAGACAGCAGCATTCTCAGAAACTTCTTTGTGGTGTCTGCATTCAAGTCACAGAATTGA
          1 AAAAACTAGACAGAAGCATTCTCAGAAACATCTTTGTGATGTTTGCACTCAACTCACAGAGTTGA

              * ** * **            *  * *      *       *             *       
     455922 ACATCCCCTCACATAGAGCAGTTGTGCAGCACTCTATTTGTAGTATCTGGAAGTGGACATTTGGA
         66 ACCTTTCTTTTCATAGAGCAGTTTTGAAACACTCTTTTTGTAGAATCTGGAAGTGGATATTTGGA

             *         ** *    * *   *            **  
     455987 GGGCTTTGTAGCCTAT-GTGGAAAAAGGAAATATCTTCCCAT
        131 GCGCTTTG-AGGATTTCGTTGGAAACGGAAATATCTTCATAT

            *  ** *   *     * *            *   *  *   * **        *  * * *   
     456028 GAATGCGAGATAGAAGTAATCTCAGAAACATGTTTATGCTGTATCTACTCAACTAACTGTGCTGA
          1 AAAAACTAGACAGAAGCATTCTCAGAAACATCTTTGTGATGTTTGCACTCAACTCACAGAGTTGA

              *     *  *               *        *  * *       *               
     456093 ACATTTCTATTGATAGAGCAGTTTTGAGACACTCTTCTTTTGGAATCTGCAAGTGGATATTTGGA
         66 ACCTTTCTTTTCATAGAGCAGTTTTGAAACACTCTTTTTGTAGAATCTGGAAGTGGATATTTGGA

            ** *                        *     *      
     456158 TAGATTTGAGGATTTCGTTGGAAACGG-GAT-T-AT-ATAT
        131 GCGCTTTGAGGATTTCGTTGGAAACGGAAATATCTTCATAT

                 *       *               *                 **  *   *         
     456195 AAAAAGTAGACAGCAGCATTCTCAGAAACTTCTTTGTGATGTTTGCATCCAGCTCTCAGAGTTGA
          1 AAAAACTAGACAGAAGCATTCTCAGAAACATCTTTGTGATGTTTGCACTCAACTCACAGAGTTGA

              *  * *          *  *        *        *   **        *  **       
     456260 ACATTCCCTTTCATAGAGTAGGTTTGAAACCCTCTTTTTATAGTGTCTGGAAGCGGGCATTTGGA
         66 ACCTTTCTTTTCATAGAGCAGTTTTGAAACACTCTTTTTGTAGAATCTGGAAGTGGATATTTGGA

                   *   ** ** *  *   *          * *   
     456325 GCGCTTTCAGGCCTATGCTGAAAAAGGAAATATCTACCTAT
        131 GCGCTTTGAGGATTTCGTTGGAAACGGAAATATCTTCATAT

             *                    *    *  **          ** *        *    *     
     456366 AGAAACTAGACAGAAGCATTCTGAGAATCACGTTTGTGATGTGGGTACTCAACTAACAGTGTTGA
          1 AAAAACTAGACAGAAGCATTCTCAGAAACATCTTTGTGATGTTTGCACTCAACTCACAGAGTTGA

            *  *       *   *            *   *                *               
     456431 TCCATTCTTTTGATACAGCAGTTTTGAACCACACTTTTTGTAGAATCTGCAAGTGGATATTTGGA
         66 ACCTTTCTTTTCATAGAGCAGTTTTGAAACACTCTTTTTGTAGAATCTGGAAGTGGATATTTGGA

            **   *                     *   *        *
     456496 TAGCTGTGAGGATTTCGTTGGAAACGGGAATGTCTTCATAG
        131 GCGCTTTGAGGATTTCGTTGGAAACGGAAATATCTTCATAT

                **                     * * **         *  **   *   *     *    
     456537 AAAATTTAGACAGAAGCATTCTCAGAACCTTGATTGTGATGTGTGTTCTCCACTAACAGATTTGA
          1 AAAAACTAGACAGAAGCATTCTCAGAAACATCTTTGTGATGTTTGCACTCAACTCACAGAGTTGA

                       * *   * *   *       *       *                         
     456602 ACCTTTCTTTTGACAGAACTGTTCTGAAACATTCTTTTTATAGAATCTGGAAGTGGATATTTGGA
         66 ACCTTTCTTTTCATAGAGCAGTTTTGAAACACTCTTTTTGTAGAATCTGGAAGTGGATATTTGGA

            **              *          *          *  
     456667 AAGCTTTGAGGATTTCATTGGAAACGGGAATATCTTCAAAT
        131 GCGCTTTGAGGATTTCGTTGGAAACGGAAATATCTTCATAT

                *    *            *           * *       *  *    *            
     456708 AAAATCTAGCCAGAAGCATTCTAAGAAACATCTTAGGGATGTTTACATTCAAGTCACAGAGTTGA
          1 AAAAACTAGACAGAAGCATTCTCAGAAACATCTTTGTGATGTTTGCACTCAACTCACAGAGTTGA

              *  * *     *       *         *    * *   **      *      *    *  
     456773 ACATTCCCTTTCACAGAGCAGGTTTGAAACAATCTTCTCGTACTATCTGGCAGTGGACATTTTGA
         66 ACCTTTCTTTTCATAGAGCAGTTTTGAAACACTCTTTTTGTAGAATCTGGAAGTGGATATTTGGA

              * *   *  ** ** *  *   *            ** *
     456838 GCTCCTTGGGGCCTATGCTGAAAAAGGAAATATCTTCCGAC
        131 GCGCTTTGAGGATTTCGTTGGAAACGGAAATATCTTCATAT

                                 *     *  **          *           **    *    
     456879 AAAAACTAGACAGAAGCATTCGCAGAATCACGTTTGTGATGTGTGCACTCAACTGTCAGAATTGA
          1 AAAAACTAGACAGAAGCATTCTCAGAAACATCTTTGTGATGTTTGCACTCAACTCACAGAGTTGA

                 **   ** *      *                            * *            *
     456944 ACCTTGGTTTGGACAGAGCACTTTTGAAACACTCTTTTTGTAGAATCTGCAGGTGGATATTTGGC
         66 ACCTTTCTTTTCATAGAGCAGTTTTGAAACACTCTTTTTGTAGAATCTGGAAGTGGATATTTGGA

            **                         *   *      * *
     457009 TAGCTTTGAGGATTTCGTTGGAAACGGTAATGTCTTCAAAG
        131 GCGCTTTGAGGATTTCGTTGGAAACGGAAATATCTTCATAT

                *                         *   *            *    *            
     457050 AAAATCTAGACAGAAGCATTCTCAGAAACACCTTCGTGATGTTTGCAATCAAGTCACAGAGTTGA
          1 AAAAACTAGACAGAAGCATTCTCAGAAACATCTTTGTGATGTTTGCACTCAACTCACAGAGTTGA

                 * *             *  *                  *             *       
     457115 ACCTTCCGTTTCATAGAGCAGGTTGGAAACACTCTTTTTGTAGTATCTGGAAGTGGACATTTGGA
         66 ACCTTTCTTTTCATAGAGCAGTTTTGAAACACTCTTTTTGTAGAATCTGGAAGTGGATATTTGGA

             *         ** *    * *   *            **  
     457180 GGGCTTTGTAGCCTAT-GTGGAAAAAGGAAATATCTTCCCAT
        131 GCGCTTTG-AGGATTTCGTTGGAAACGGAAATATCTTCATAT

            *  ** *   *     * *            *   *  *   * **        *  * * *   
     457221 GAATGCGAGATAGAAGTAATCTCAGAAACATGTTTATGCTGTATCTACTCAACTAACTGTGCTGA
          1 AAAAACTAGACAGAAGCATTCTCAGAAACATCTTTGTGATGTTTGCACTCAACTCACAGAGTTGA

              *     *  *               *        *  * *       *               
     457286 ACATTTCTATTGATAGAGCAGTTTTGAGACACTCTTCTTTTGGAATCTGCAAGTGGATATTTGGA
         66 ACCTTTCTTTTCATAGAGCAGTTTTGAAACACTCTTTTTGTAGAATCTGGAAGTGGATATTTGGA

            ** *                        *     *      
     457351 TAGATTTGAGGATTTCGTTGGAAACGG-GAT-T-AT-ATAT
        131 GCGCTTTGAGGATTTCGTTGGAAACGGAAATATCTTCATAT

                 *       *               *                 **  *   *         
     457388 AAAAAGTAGACAGCAGCATTCTCAGAAACTTCTTTGTGATGTTTGCATCCAGCTCTCAGAGTTGA
          1 AAAAACTAGACAGAAGCATTCTCAGAAACATCTTTGTGATGTTTGCACTCAACTCACAGAGTTGA

              *  * *          *  *        *        *   **        *  **       
     457453 ACATTCCCTTTCATAGAGTAGGTTTGAAACCCTCTTTTTATAGTGTCTGGAAGCGGGCATTTGGA
         66 ACCTTTCTTTTCATAGAGCAGTTTTGAAACACTCTTTTTGTAGAATCTGGAAGTGGATATTTGGA

                   *   ** ** *  *   *          * *   
     457518 GCGCTTTCAGGCCTATGCTGAAAAAGGAAATATCTACCTAT
        131 GCGCTTTGAGGATTTCGTTGGAAACGGAAATATCTTCATAT

             *                    *    *  **          ** *        *    *     
     457559 AGAAACTAGACAGAAGCATTCTGAGAATCACGTTTGTGATGTGGGTACTCAACTAACAGTGTTGA
          1 AAAAACTAGACAGAAGCATTCTCAGAAACATCTTTGTGATGTTTGCACTCAACTCACAGAGTTGA

            *  *       *   *            *   *                *               
     457624 TCCATTCTTTTGATACAGCAGTTTTGAACCACACTTTTTGTAGAATCTGCAAGTGGATATTTGGA
         66 ACCTTTCTTTTCATAGAGCAGTTTTGAAACACTCTTTTTGTAGAATCTGGAAGTGGATATTTGGA

            **   *                     *   *        *
     457689 TAGCTGTGAGGATTTCGTTGGAAACGGGAATGTCTTCATAG
        131 GCGCTTTGAGGATTTCGTTGGAAACGGAAATATCTTCATAT

                **                     * * **         *  **   *   *          
     457730 AAAATTTAGACAGAAGCATTCTCAGAACCTTGATTGTGATGTGTGTTCTCCACTAACAGAGTTGA
          1 AAAAACTAGACAGAAGCATTCTCAGAAACATCTTTGTGATGTTTGCACTCAACTCACAGAGTTGA

                       * *   * *   *       *       *                         
     457795 ACCTTTCTTTTGACAGAACTGTTCTGAAACATTCTTTTTATAGAATCTGGAAGTGGATATTTGGA
         66 ACCTTTCTTTTCATAGAGCAGTTTTGAAACACTCTTTTTGTAGAATCTGGAAGTGGATATTTGGA

            **                         *          *  
     457860 AAGCTTTGAGGATTTCGTTGGAAACGGGAATATCTTCAAAT
        131 GCGCTTTGAGGATTTCGTTGGAAACGGAAATATCTTCATAT

                *    *            *           * *       *  *    *            
     457901 AAAATCTAGCCAGAAGCATTCTAAGAAACATCTTAGGGATGTTTACATTCAAGTCACAGAGTTGA
          1 AAAAACTAGACAGAAGCATTCTCAGAAACATCTTTGTGATGTTTGCACTCAACTCACAGAGTTGA

              *  * *     *       *         *    * *   **      *      *    *  
     457966 ACATTCCCTTTCACAGAGCAGGTTTGAAACAATCTTCTCGTACTATCTGGCAGTGGACATTTTGA
         66 ACCTTTCTTTTCATAGAGCAGTTTTGAAACACTCTTTTTGTAGAATCTGGAAGTGGATATTTGGA

              * *   *  ** ** *  *   *            ** *
     458031 GCTCCTTGGGGCCTATGCTGAAAAAGGAAATATCTTCCGAC
        131 GCGCTTTGAGGATTTCGTTGGAAACGGAAATATCTTCATAT

                                 *     *  **          *           **    *    
     458072 AAAAACTAGACAGAAGCATTCGCAGAATCACGTTTGTGATGTGTGCACTCAACTGTCAGAATTGA
          1 AAAAACTAGACAGAAGCATTCTCAGAAACATCTTTGTGATGTTTGCACTCAACTCACAGAGTTGA

                 **   ** *      *                            * *            *
     458137 ACCTTGGTTTGGACAGAGCACTTTTGAAACACTCTTTTTGTAGAATCTGCAGGTGGATATTTGGC
         66 ACCTTTCTTTTCATAGAGCAGTTTTGAAACACTCTTTTTGTAGAATCTGGAAGTGGATATTTGGA

            **                         *   *      * *
     458202 TAGCTTTGAGGATTTCGTTGGAAACGGTAATGTCTTCAAAG
        131 GCGCTTTGAGGATTTCGTTGGAAACGGAAATATCTTCATAT

                *                         *   *            *    *            
     458243 AAAATCTAGACAGAAGCATTCTCAGAAACACCTTCGTGATGTTTGCAATCAAGTCACAGAGTTGA
          1 AAAAACTAGACAGAAGCATTCTCAGAAACATCTTTGTGATGTTTGCACTCAACTCACAGAGTTGA

                 * *             *  *                  *             *       
     458308 ACCTTCCGTTTCATAGAGCAGGTTGGAAACACTCTTTTTGTAGTATCTGGAAGTGGACATTTGGA
         66 ACCTTTCTTTTCATAGAGCAGTTTTGAAACACTCTTTTTGTAGAATCTGGAAGTGGATATTTGGA

             *         ** *    * *   *            **  
     458373 GGGCTTTGTAGCCTATC-TGGAAAAAGGAAATATCTTCCCAT
        131 GCGCTTTG-AGGATTTCGTTGGAAACGGAAATATCTTCATAT

            *  ** *   *     * *            **  *  *   * **        *  * * *   
     458414 GAATGCGAGATAGAAGTAATCTCAGAAACATGCTTATGCTGTATCTACTCAACTAACTGTGCTGA
          1 AAAAACTAGACAGAAGCATTCTCAGAAACATCTTTGTGATGTTTGCACTCAACTCACAGAGTTGA

              *     *  *               *        *  * *       *               
     458479 ACATTTCTATTGATAGAGCAGTTTTGAGACACTCTTCTTTTGGAATCTGCAAGTGGATATTTGGA
         66 ACCTTTCTTTTCATAGAGCAGTTTTGAAACACTCTTTTTGTAGAATCTGGAAGTGGATATTTGGA

             * *                        *     *      
     458544 GAGATTTGAGGATTTCGTTGGAAACGG-GAT-T-AT-ATAT
        131 GCGCTTTGAGGATTTCGTTGGAAACGGAAATATCTTCATAT

                 *       *               *                 **  *   *         
     458581 AAAAAGTAGACAGCAGCATTCTCAGAAACTTCTTTGTGATGTTTGCATCCAGCTCTCAGAGTTGA
          1 AAAAACTAGACAGAAGCATTCTCAGAAACATCTTTGTGATGTTTGCACTCAACTCACAGAGTTGA

              *  * *          *  *        *        *   **    *   *  **       
     458646 ACATTCCCTTTCATAGAGTAGGTTTGAAACCCTCTTTTTATAGTGTCTGCAAGCGGGCATTTGGA
         66 ACCTTTCTTTTCATAGAGCAGTTTTGAAACACTCTTTTTGTAGAATCTGGAAGTGGATATTTGGA

                   *    * * **  **  **          * *  *
     458711 GCGCTTTCAGGCCTATGCTTAAAATAGGAAATATCTACCTAC
        131 GCGCTTTGAGG-ATTTCGTTGGAAACGGAAATATCTTCATAT

             *                    *    *  **          ** *        *    *     
     458753 AGAAACTAGACAGAAGCATTCTGAGAATCACGTTTGTGATGTGGGTACTCAACTAACAGTGTTGA
          1 AAAAACTAGACAGAAGCATTCTCAGAAACATCTTTGTGATGTTTGCACTCAACTCACAGAGTTGA

            *  *       *   *            *   *                *   *           
     458818 TCCATTCTTTTGATACAGCAGTTTTGAACCACACTTTTTGTAGAATCTGCAAGAGGATATTTGGA
         66 ACCTTTCTTTTCATAGAGCAGTTTTGAAACACTCTTTTTGTAGAATCTGGAAGTGGATATTTGGA

            **   *                     *   *      * *
     458883 TAGCTGTGAGGATTTCGTTGGAAACGGGAATGTCTTCAAAG
        131 GCGCTTTGAGGATTTCGTTGGAAACGGAAATATCTTCATAT

                *                         *   *            *    *            
     458924 AAAATCTAGACAGAAGCATTCTCAGAAACACCTTCGTGATGTTTGCAATCAAGTCACAGAGTTGA
          1 AAAAACTAGACAGAAGCATTCTCAGAAACATCTTTGTGATGTTTGCACTCAACTCACAGAGTTGA

                 * *             *  *           *      *             *       
     458989 ACCTTCCGTTTCATAGAGCAGGTTGGAAACACTCTTATTGTAGTATCTGGAAGTGGACATTTGGA
         66 ACCTTTCTTTTCATAGAGCAGTTTTGAAACACTCTTTTTGTAGAATCTGGAAGTGGATATTTGGA

                   *   ** ** *  *   *            **  
     459054 GCGCTTTCAGGCCTATGGTGAAAAAGGAAATATCTTCCCAT
        131 GCGCTTTGAGGATTTCGTTGGAAACGGAAATATCTTCATAT

                  * * *      **      *   * *   *     ** ** *      *    *     
     459095 AAAAACGACATAGAAGCTATCTCAGGAACTTGTTTATGATGCATCTAATCAACTAACAGTGTTGA
          1 AAAAACTAGACAGAAGCATTCTCAGAAACATCTTTGTGATGTTTGCACTCAACTCACAGAGTTGA

                  * ** * *                         * *       *               
     459160 ACCTTTGTACTGACAGAGCAG-TTTGAAACACTCTTTTTTTGGAATCTGCAAGTGGATATTTGGA
         66 ACCTTTCTTTTCATAGAGCAGTTTTGAAACACTCTTTTTGTAGAATCTGGAAGTGGATATTTGGA

            *                           *   *   *    
     459224 TCGCTTTGAGGATTTCGTTGGAAACGG-GAT-GC--AATA-
        131 GCGCTTTGAGGATTTCGTTGGAAACGGAAATATCTTCATAT

                     *    *     *                 **  *   *  *    *         *
     459260 AAAAACGTACACAGCAGCATACTCAGAAA-ATACTTTGCCATATTTCCATTCAAGTCACAGAGTG
          1 AAAAAC-TAGACAGAAGCATTCTCAGAAACAT-CTTTGTGATGTTTGCACTCAACTCACAGAGTT

                *  * **            *                  *  *             *     
     459324 GAACATTCCCATTCATAGAGCAGGTTTGAAACACTCTTTTTGGAGTATCTGGAAGTGGACATTTG
         64 GAACCTTTCTTTTCATAGAGCAGTTTTGAAACACTCTTTTTGTAGAATCTGGAAGTGGATATTTG

                     ** * * ** *  *   *            **  
     459389 GAGCGCTTTCTGAACTATGGTGAAAAAGGAAATATCTTCCAAT
        129 GAGCGCTTTGAGGATTTCGTTGGAAACGGAAATATCTTCATAT

            *     *               *      * *          *  **      **  *  *    
     459432 GAAAACAAGACAGAAGCATTCTGAGAAACTTATTTGTGATGTGTGTCCTCAACAAACGGACTTGA
          1 AAAAACTAGACAGAAGCATTCTCAGAAACATCTTTGTGATGTTTGCACTCAACTCACAGAGTTGA

                   *      **  * *  *  *             *   *    * * *           
     459497 ACCTTTCATTTCATGCAGTACTTCTGGAACACTCTTTTTGAAGATTCTGCATGCGGATATTTGGA
         66 ACCTTTCTTTTCATAGAGCAGTTTTGAAACACTCTTTTTGTAGAATCTGGAAGTGGATATTTGGA

            **                            **   *   * 
     459562 TAGCTTTGAGGATTTCGTTGGAAACGG---GCT-TACATGT
        131 GCGCTTTGAGGATTTCGTTGGAAACGGAAATATCTTCATAT

                 *       *               *        *   *    *    *       *    
     459599 AAAAATTAGACAGCAGCATTCTCAGAAACTTCTTTGTGGTGTCTGCATTCAAGTCACAGAATTGA
          1 AAAAACTAGACAGAAGCATTCTCAGAAACATCTTTGTGATGTTTGCACTCAACTCACAGAGTTGA

              * ** * **            *  * *      *       *    *      * *       
     459664 ACTTCCCCTCACATAGAGCAGTTGTGCAGCACTCTATTTGTAGTATCTCGAAGTGCACATTTGGA
         66 ACCTTTCTTTTCATAGAGCAGTTTTGAAACACTCTTTTTGTAGAATCTGGAAGTGGATATTTGGA

             *         ** *    * *   *            **  
     459729 GGGCTTTGTAGCCTATC-TGGAAAAAGGAAATATCTTCCCAT
        131 GCGCTTTG-AGGATTTCGTTGGAAACGGAAATATCTTCATAT

            *  ** *   *     * *            *   *  *   * **        *  * * *   
     459770 GAATGCGAGATAGAAGTAATCTCAGAAACATGTTTATGCTGTATCTACTCAACTAACTGTGCTGA
          1 AAAAACTAGACAGAAGCATTCTCAGAAACATCTTTGTGATGTTTGCACTCAACTCACAGAGTTGA

              *     *  *               *        *  * *       *               
     459835 ACATTTCTATTGATAGAGCAGTTTTGAGACACTCTTCTTTTGGAATCTGCAAGTGGATATTTGGA
         66 ACCTTTCTTTTCATAGAGCAGTTTTGAAACACTCTTTTTGTAGAATCTGGAAGTGGATATTTGGA

            ** *                        *     *      
     459900 TAGATTTGAGGATTTCGTTGGAAACGG-GAT-T-AT-ATAT
        131 GCGCTTTGAGGATTTCGTTGGAAACGGAAATATCTTCATAT

            *    **      *               *                 **  *   *         
     459937 CAAAAGCAGACAGCAGCATTCTCAGAAACTTCTTTGTGATGTTTGCATCCAGCTCTCAGAGTTGA
          1 AAAAACTAGACAGAAGCATTCTCAGAAACATCTTTGTGATGTTTGCACTCAACTCACAGAGTTGA

              *  * *          *  *        *        *   **        *  **       
     460002 ACATTCCCTTTCATAGAGTAGGTTTGAAACCCTCTTTTTATAGTGTCTGGAAGCGGGCATTTGGA
         66 ACCTTTCTTTTCATAGAGCAGTTTTGAAACACTCTTTTTGTAGAATCTGGAAGTGGATATTTGGA

                   *   ** ** *  *   *          * *   
     460067 GCGCTTTCAGGCCTATGCTGAAAAAGGAAATATCTACCTAT
        131 GCGCTTTGAGGATTTCGTTGGAAACGGAAATATCTTCATAT

             *                    *    *  **          ** *        *    *     
     460108 AGAAACTAGACAGAAGCATTCTGAGAATCACGTTTGTGATGTGGGTACTCAACTAACAGTGTTGA
          1 AAAAACTAGACAGAAGCATTCTCAGAAACATCTTTGTGATGTTTGCACTCAACTCACAGAGTTGA

            *  *       *   *            *   *                *               
     460173 TCCATTCTTTTGATACAGCAGTTTTGAACCACACTTTTTGTAGAATCTGCAAGTGGATATTTGGA
         66 ACCTTTCTTTTCATAGAGCAGTTTTGAAACACTCTTTTTGTAGAATCTGGAAGTGGATATTTGGA

            **   *          *          *   *        *
     460238 TAGCTGTGAGGATTTCCTTGGAAACGGGAATGTCTTCATAG
        131 GCGCTTTGAGGATTTCGTTGGAAACGGAAATATCTTCATAT

                **                     * * **         *  **   *   *          
     460279 AAAATTTAGACAGAAGCATTCTCAGAACCTTGATTGTGATGTGTGTTCTCCACTAACAGAGTTGA
          1 AAAAACTAGACAGAAGCATTCTCAGAAACATCTTTGTGATGTTTGCACTCAACTCACAGAGTTGA

                       * *   * *   *       *       *                         
     460344 ACCTTTCTTTTGACAGAACTGTTCTGAAACATTCTTTTTATAGAATCTGGAAGTGGATATTTGGA
         66 ACCTTTCTTTTCATAGAGCAGTTTTGAAACACTCTTTTTGTAGAATCTGGAAGTGGATATTTGGA

            **                         *          *  
     460409 AAGCTTTGAGGATTTCGTTGGAAACGGGAATATCTTCAAAT
        131 GCGCTTTGAGGATTTCGTTGGAAACGGAAATATCTTCATAT

                *    *            *           * *       *  *    *            
     460450 AAAATCTAGCCAGAAGCATTCTAAGAAACATCTTAGGGATGTTTACATTCAAGTCACAGAGTTGA
          1 AAAAACTAGACAGAAGCATTCTCAGAAACATCTTTGTGATGTTTGCACTCAACTCACAGAGTTGA

              *  * *     *       *         *    * *   **      *      *    *  
     460515 ACATTCCCTTTCACAGAGCAGGTTTGAAACAATCTTCTCGTACTATCTGGCAGTGGACATTTTGA
         66 ACCTTTCTTTTCATAGAGCAGTTTTGAAACACTCTTTTTGTAGAATCTGGAAGTGGATATTTGGA

              * *   *  ** ** *  *   *            ** *
     460580 GCTCCTTGGGGCCTATGCTGAAAAAGGAAATATCTTCCGAC
        131 GCGCTTTGAGGATTTCGTTGGAAACGGAAATATCTTCATAT

                                 *     *  **          *           **    *    
     460621 AAAAACTAGACAGAAGCATTCGCAGAATCACGTTTGTGATGTGTGCACTCAACTGTCAGAATTGA
          1 AAAAACTAGACAGAAGCATTCTCAGAAACATCTTTGTGATGTTTGCACTCAACTCACAGAGTTGA

                 **   ** *      *                            * *            *
     460686 ACCTTGGTTTGGACAGAGCACTTTTGAAACACTCTTTTTGTAGAATCTGCAGGTGGATATTTGGC
         66 ACCTTTCTTTTCATAGAGCAGTTTTGAAACACTCTTTTTGTAGAATCTGGAAGTGGATATTTGGA

            **                         *   *      * *
     460751 TAGCTTTGAGGATTTCGTTGGAAACGGTAATGTCTTCAAAG
        131 GCGCTTTGAGGATTTCGTTGGAAACGGAAATATCTTCATAT

                *                         *   *            *    *            
     460792 AAAATCTAGACAGAAGCATTCTCAGAAACACCTTCGTGATGTTTGCAATCAAGTCACAGAGTTGA
          1 AAAAACTAGACAGAAGCATTCTCAGAAACATCTTTGTGATGTTTGCACTCAACTCACAGAGTTGA

                 * *       *     *  *                  *             *       
     460857 ACCTTCCGTTTCATACAGCAGGTTGGAAACACTCTTTTTGTAGTATCTGGAAGTGGACATTTGGA
         66 ACCTTTCTTTTCATAGAGCAGTTTTGAAACACTCTTTTTGTAGAATCTGGAAGTGGATATTTGGA

                   *   ** ** *  *   *            **  
     460922 GCGCTTTAAGGCCTATGGTGAAAAAGGAAATATCTTCCCAT
        131 GCGCTTTGAGGATTTCGTTGGAAACGGAAATATCTTCATAT

                  * * *      **      *   * *   *     ** ** *      *    *     
     460963 AAAAACGACATAGAAGCTATCTCAGGAACTTGTTTATGATGCATCTAATCAACTAACAGTGTTGA
          1 AAAAACTAGACAGAAGCATTCTCAGAAACATCTTTGTGATGTTTGCACTCAACTCACAGAGTTGA

                  * ** * *                         * *       *               
     461028 ACCTTTGTACTGACAGAGCAG-TTTGAAACACTCTTTTTTTGGAATCTGCAAGTGGATATTTGGA
         66 ACCTTTCTTTTCATAGAGCAGTTTTGAAACACTCTTTTTGTAGAATCTGGAAGTGGATATTTGGA

            *                           *   *   *    
     461092 TCGCTTTGAGGATTTCGTTGGAAACGG-GAT-GC--AATAT
        131 GCGCTTTGAGGATTTCGTTGGAAACGGAAATATCTTCATAT

                **  *    *     *                 **  *   *  *    *         * 
     461129 AAAACGTACACAGCAGCATACTCAGAAA-ATACTTTGCCATATTTCCATTCAAGTCACAGAGTGG
          1 AAAAACTAGACAGAAGCATTCTCAGAAACAT-CTTTGTGATGTTTGCACTCAACTCACAGAGTTG

               *  * **            *          *       *  *             *      
     461193 AACATTCCCATTCATAGAGCAGGTTTGAAACACACTTTTTGGAGTATCTGGAAGTGGACATTTGG
         65 AACCTTTCTTTTCATAGAGCAGTTTTGAAACACTCTTTTTGTAGAATCTGGAAGTGGATATTTGG

                    ** * * ** *  *   *            **  
     461258 AGCGCTTTCTGAACTATGGTGAAAAAGGAAATATCTTCCAAT
        130 AGCGCTTTGAGGATTTCGTTGGAAACGGAAATATCTTCATAT

            *     * *             *      * *          *  **      **  *  *    
     461300 GAAAACAACACAGAAGCATTCTGAGAAACTTATTTGTGATGTGTGTCCTCAACAAACGGACTTGA
          1 AAAAACTAGACAGAAGCATTCTCAGAAACATCTTTGTGATGTTTGCACTCAACTCACAGAGTTGA

                   *      **  * *  *  *             *   *    * * *           
     461365 ACCTTTCGTTTCATGCAGTACTTCTGGAACACTCTTTTTGAAGATTCTGCATGCGGATATTTGGA
         66 ACCTTTCTTTTCATAGAGCAGTTTTGAAACACTCTTTTTGTAGAATCTGGAAGTGGATATTTGGA

            **                            **   *   * 
     461430 TAGCTTTGAGGATTTCGTTGGAAACGG---GCT-TACATGT
        131 GCGCTTTGAGGATTTCGTTGGAAACGGAAATATCTTCATAT

                 *       *               *        *   *    *    *       *    
     461467 AAAAATTAGACAGCAGCATTCTCAGAAACTTCTTTGTGGTGTCTGCATTCAAGTCACAGAATTGA
          1 AAAAACTAGACAGAAGCATTCTCAGAAACATCTTTGTGATGTTTGCACTCAACTCACAGAGTTGA

              * ** * **            *  * *      *       *             *       
     461532 ACTTCCCCTCACATAGAGCAGTTGTGCAGCACTCTATTTGTAGTATCTGGAAGTGGACATTTGGA
         66 ACCTTTCTTTTCATAGAGCAGTTTTGAAACACTCTTTTTGTAGAATCTGGAAGTGGATATTTGGA

             *         ** *    * *   *            **  
     461597 GGGCTTTGTAGCCTATC-TGGAAAAAGGAAATATCTTCCCAT
        131 GCGCTTTG-AGGATTTCGTTGGAAACGGAAATATCTTCATAT

            *  ** *   *     * *            *   *  *   * **        *  * * *   
     461638 GAATGCGAGATAGAAGTAATCTCAGAAACATGTTTATGCTGTATCTACTCAACTAACTGTGCTGA
          1 AAAAACTAGACAGAAGCATTCTCAGAAACATCTTTGTGATGTTTGCACTCAACTCACAGAGTTGA

              *     *  *               *        *  * *       *               
     461703 ACATTTCTATTGATAGAGCAGTTTTGAGACACTCTTCTTTTGGAATCTGCAAGTGGATATTTGGA
         66 ACCTTTCTTTTCATAGAGCAGTTTTGAAACACTCTTTTTGTAGAATCTGGAAGTGGATATTTGGA

            ** *                        *     *      
     461768 TAGATTTGAGGATTTCGTTGGAAACGG-GAT-T-AT-ATAT
        131 GCGCTTTGAGGATTTCGTTGGAAACGGAAATATCTTCATAT

            *    *       *               *                 **  *   *         
     461805 CAAAAGTAGACAGCAGCATTCTCAGAAACTTCTTTGTGATGTTTGCATCCAGCTCTCAGAGTTGA
          1 AAAAACTAGACAGAAGCATTCTCAGAAACATCTTTGTGATGTTTGCACTCAACTCACAGAGTTGA

              *  * *          *  *        *        *   **        *  **       
     461870 ACATTCCCTTTCATAGAGTAGGTTTGAAACCCTCTTTTTATAGTGTCTGGAAGCGGGCATTTGGA
         66 ACCTTTCTTTTCATAGAGCAGTTTTGAAACACTCTTTTTGTAGAATCTGGAAGTGGATATTTGGA

                   *   ** ** *  *   *          * *   
     461935 GCGCTTTCAGGCCTATGCTGAAAAAGGAAATATCTACCTAT
        131 GCGCTTTGAGGATTTCGTTGGAAACGGAAATATCTTCATAT

             *                    *    *  **          ** *        *    *     
     461976 AGAAACTAGACAGAAGCATTCTGAGAATCACGTTTGTGATGTGGGTACTCAACTAACAGTGTTGA
          1 AAAAACTAGACAGAAGCATTCTCAGAAACATCTTTGTGATGTTTGCACTCAACTCACAGAGTTGA

            *  *       *   *            *   *                *               
     462041 TCCATTCTTTTGATACAGCAGTTTTGAACCACACTTTTTGTAGAATCTGCAAGTGGATATTTGGA
         66 ACCTTTCTTTTCATAGAGCAGTTTTGAAACACTCTTTTTGTAGAATCTGGAAGTGGATATTTGGA

            **   *                     *   *        *
     462106 TAGCTGTGAGGATTTCGTTGGAAACGGGAATGTCTTCATAG
        131 GCGCTTTGAGGATTTCGTTGGAAACGGAAATATCTTCATAT

                **         *           * * **         *  **   *   *          
     462147 AAAATTTAGACAGAAACATTCTCAGAACCTTGATTGTGATGTGTGTTCTCCACTAACAGAGTTGA
          1 AAAAACTAGACAGAAGCATTCTCAGAAACATCTTTGTGATGTTTGCACTCAACTCACAGAGTTGA

                       * *   * *   *       *       *                         
     462212 ACCTTTCTTTTGACAGAACTGTTCTGAAACATTCTTTTTATAGAATCTGGAAGTGGATATTTGGA
         66 ACCTTTCTTTTCATAGAGCAGTTTTGAAACACTCTTTTTGTAGAATCTGGAAGTGGATATTTGGA

            **                         *          *  
     462277 AAGCTTTGAGGATTTCGTTGGAAACGGGAATATCTTCAAAT
        131 GCGCTTTGAGGATTTCGTTGGAAACGGAAATATCTTCATAT

                *    *            *           * *       *  *    *            
     462318 AAAATCTAGCCAGAAGCATTCTAAGAAACATCTTAGGGATGTTTACATTCAAGTCACAGAGTTGA
          1 AAAAACTAGACAGAAGCATTCTCAGAAACATCTTTGTGATGTTTGCACTCAACTCACAGAGTTGA

              *  * *     *       *         *    * *   **      *      *    *  
     462383 ACATTCCCTTTCACAGAGCAGGTTTGAAACAATCTTCTCGTACTATCTGGCAGTGGACATTTTGA
         66 ACCTTTCTTTTCATAGAGCAGTTTTGAAACACTCTTTTTGTAGAATCTGGAAGTGGATATTTGGA

              * *   *  ** ** *  *   *            ** *
     462448 GCTCCTTGGGGCCTATGCTGAAAAAGGAAATATCTTCCGAC
        131 GCGCTTTGAGGATTTCGTTGGAAACGGAAATATCTTCATAT

                                 *  *  *  **          *           **    *    
     462489 AAAAACTAGACAGAAGCATTCGCACAATCACGTTTGTGATGTGTGCACTCAACTGTCAGAATTGA
          1 AAAAACTAGACAGAAGCATTCTCAGAAACATCTTTGTGATGTTTGCACTCAACTCACAGAGTTGA

                 **   ** *      *              *             * *            *
     462554 ACCTTGGTTTGGACAGAGCACTTTTGAAACACTCTCTTTGTAGAATCTGCAGGTGGATATTTGGC
         66 ACCTTTCTTTTCATAGAGCAGTTTTGAAACACTCTTTTTGTAGAATCTGGAAGTGGATATTTGGA

            **                         *   *      * *
     462619 TAGCTTTGAGGATTTCGTTGGAAACGGTAATGTCTTCAAAG
        131 GCGCTTTGAGGATTTCGTTGGAAACGGAAATATCTTCATAT

                *            *            *   *     *      *    *            
     462660 AAAATCTAGACAGAAGCCTTCTCAGAAACACCTTCGTGATATTTGCAATCAAGTCACAGAGTTGA
          1 AAAAACTAGACAGAAGCATTCTCAGAAACATCTTTGTGATGTTTGCACTCAACTCACAGAGTTGA

                 * *             *  *                  *             *       
     462725 ACCTTCCGTTTCATAGAGCAGGTTGGAAACACTCTTTTTGTAGTATCTGGAAGTGGACATTTGGA
         66 ACCTTTCTTTTCATAGAGCAGTTTTGAAACACTCTTTTTGTAGAATCTGGAAGTGGATATTTGGA

             *         ** *    * *   *            **  
     462790 GGGCTTTGTAGCCTAT-GTGGAAAAAGGAAATATCTTCCCAT
        131 GCGCTTTG-AGGATTTCGTTGGAAACGGAAATATCTTCATAT

            *  ** *   *     * *            *   *  *   * **        *  * * *   
     462831 GAATGCGAGATAGAAGTAATCTCAGAAACATGTTTATGCTGTATCTACTCAACTAACTGTGCTGA
          1 AAAAACTAGACAGAAGCATTCTCAGAAACATCTTTGTGATGTTTGCACTCAACTCACAGAGTTGA

              *     *  *               *        *  * *       *               
     462896 ACATTTCTATTGATAGAGCAGTTTTGAGACACTCTTCTTTTGGAATCTGCAAGTGGATATTTGGA
         66 ACCTTTCTTTTCATAGAGCAGTTTTGAAACACTCTTTTTGTAGAATCTGGAAGTGGATATTTGGA

            ** *                        *     *      
     462961 TAGATTTGAGGATTTCGTTGGAAACGG-GAT-T-AT-ATAT
        131 GCGCTTTGAGGATTTCGTTGGAAACGGAAATATCTTCATAT

                 *       *               *                 **  *   *         
     462998 AAAAAGTAGACAGCAGCATTCTCAGAAACTTCTTTGTGATGTTTGCATCCAGCTCTCAGAGTTGA
          1 AAAAACTAGACAGAAGCATTCTCAGAAACATCTTTGTGATGTTTGCACTCAACTCACAGAGTTGA

            * *  * *          *  *        *        *   **        *  **       
     463063 GCATTCCCTTTCATAGAGTAGGTTTGAAACCCTCTTTTTATAGTGTCTGGAAGCGGGCATTTGGA
         66 ACCTTTCTTTTCATAGAGCAGTTTTGAAACACTCTTTTTGTAGAATCTGGAAGTGGATATTTGGA

                   *    * * **  **  **          * *  *
     463128 GCGCTTTCAGGCCTATGCTTAAAATAGGAAATATCTACCTAC
        131 GCGCTTTGAGG-ATTTCGTTGGAAACGGAAATATCTTCATAT

             *                    *    *  **          ** *        *    *     
     463170 AGAAACTAGACAGAAGCATTCTGAGAATCACGTTTGTGATGTGGGTACTCAACTAACAGTGTTGA
          1 AAAAACTAGACAGAAGCATTCTCAGAAACATCTTTGTGATGTTTGCACTCAACTCACAGAGTTGA

            *  *       *   *            *   *                *   *           
     463235 TCCATTCTTTTGATACAGCAGTTTTGAACCACACTTTTTGTAGAATCTGCAAGAGGATATTTGGA
         66 ACCTTTCTTTTCATAGAGCAGTTTTGAAACACTCTTTTTGTAGAATCTGGAAGTGGATATTTGGA

            **   *                     *   *      * *
     463300 TAGCTGTGAGGATTTCGTTGGAAACGGGAATGTCTTCAAAG
        131 GCGCTTTGAGGATTTCGTTGGAAACGGAAATATCTTCATAT

                *                         *   *            *    *            
     463341 AAAATCTAGACAGAAGCATTCTCAGAAACACCTTCGTGATGTTTGCAATCAAGTCACAGAGTTGA
          1 AAAAACTAGACAGAAGCATTCTCAGAAACATCTTTGTGATGTTTGCACTCAACTCACAGAGTTGA

                 * *             *  *           *      *             *       
     463406 ACCTTCCGTTTCATAGAGCAGGTTGGAAACACTCTTATTGTAGTATCTGGAAGTGGACATTTGGA
         66 ACCTTTCTTTTCATAGAGCAGTTTTGAAACACTCTTTTTGTAGAATCTGGAAGTGGATATTTGGA

                   *   ** ** *  *   *            **  
     463471 GCGCTTTCAGGCCTATGGTGAAAAAGGAAATATCTTCCCAT
        131 GCGCTTTGAGGATTTCGTTGGAAACGGAAATATCTTCATAT

                  * * *      **      *   * *   *     ** ** *      *    *     
     463512 AAAAACGACATAGAAGCTATCTCAGGAACTTGTTTATGATGCATCTAATCAACTAACAGTGTTGA
          1 AAAAACTAGACAGAAGCATTCTCAGAAACATCTTTGTGATGTTTGCACTCAACTCACAGAGTTGA

                  * ** * *                         * *       *               
     463577 ACCTTTGTACTGACAGAGCAG-TTTGAAACACTCTTTTTTTGGAATCTGCAAGTGGATATTTGGA
         66 ACCTTTCTTTTCATAGAGCAGTTTTGAAACACTCTTTTTGTAGAATCTGGAAGTGGATATTTGGA

            *                           *   *   *    
     463641 TCGCTTTGAGGATTTCGTTGGAAACGG-GAT-GC--AATA-
        131 GCGCTTTGAGGATTTCGTTGGAAACGGAAATATCTTCATAT

                     *    *     *                 **  *   *  *    *         *
     463677 AAAAACGTACACAGCAGCATACTCAGAAA-ATACTTTGCCATATTTCCATTCAAGTCACAGAGTG
          1 AAAAAC-TAGACAGAAGCATTCTCAGAAACAT-CTTTGTGATGTTTGCACTCAACTCACAGAGTT

                *  * **            *                  *  *             *     
     463741 GAACATTCCCATTCATAGAGCAGGTTTGAAACACTCTTTTTGGAGTATCTGGAAGTGGACATTTG
         64 GAACCTTTCTTTTCATAGAGCAGTTTTGAAACACTCTTTTTGTAGAATCTGGAAGTGGATATTTG

                     ** * * ** *  *   *            **  
     463806 GAGCGCTTTCTGAACTATGGTGAAAAAGGAAATATCTTCCAAT
        129 GAGCGCTTTGAGGATTTCGTTGGAAACGGAAATATCTTCATAT

            *     *               *      * *          *  **      **  *  *    
     463849 GAAAACAAGACAGAAGCATTCTGAGAAACTTATTTGTGATGTGTGTCCTCAACAAACGGACTTGA
          1 AAAAACTAGACAGAAGCATTCTCAGAAACATCTTTGTGATGTTTGCACTCAACTCACAGAGTTGA

                   *      **  * *  *  *             *   *    * * *           
     463914 ACCTTTCGTTTCATGCAGTACTTCTGGAACACTCTTTTTGAAGATTCTGCATGCGGATATTTGGA
         66 ACCTTTCTTTTCATAGAGCAGTTTTGAAACACTCTTTTTGTAGAATCTGGAAGTGGATATTTGGA

            **                            **   *   * 
     463979 TAGCTTTGAGGATTTCGTTGGAAACGG---GCT-TACATGT
        131 GCGCTTTGAGGATTTCGTTGGAAACGGAAATATCTTCATAT

                 *       *               *        *   *    *    *       *    
     464016 AAAAATTAGACAGCAGCATTCTCAGAAACTTCTTTGTGGTGTCTGCATTCAAGTCACAGAATTGA
          1 AAAAACTAGACAGAAGCATTCTCAGAAACATCTTTGTGATGTTTGCACTCAACTCACAGAGTTGA

              * ** * **            *  * *      *       *             *       
     464081 ACTTCCCCTCACATAGAGCAGTTGTGCAGCACTCTATTTGTAGTATCTGGAAGTGGACATTTGGA
         66 ACCTTTCTTTTCATAGAGCAGTTTTGAAACACTCTTTTTGTAGAATCTGGAAGTGGATATTTGGA

             *         ** *    * *   *            **  
     464146 GGGCTTTGTAGCCTATC-TGGAAAAAGGAAATATCTTCCCAT
        131 GCGCTTTG-AGGATTTCGTTGGAAACGGAAATATCTTCATAT

            *  ** *   *     * *            *   *  *   * **        *  * * *   
     464187 GAATGCGAGATAGAAGTAATCTCAGAAACATGTTTATGCTGTATCTACTCAACTAACTGTGCTGA
          1 AAAAACTAGACAGAAGCATTCTCAGAAACATCTTTGTGATGTTTGCACTCAACTCACAGAGTTGA

              *     *  *               *        *  * *       *               
     464252 ACATTTCTATTGATAGAGCAGTTTTGAGACACTCTTCTTTTGGAATCTGCAAGTGGATATTTGGA
         66 ACCTTTCTTTTCATAGAGCAGTTTTGAAACACTCTTTTTGTAGAATCTGGAAGTGGATATTTGGA

            ** *                        *     *      
     464317 TAGATTTGAGGATTTCGTTGGAAACGG-GAT-T-AT-ATAT
        131 GCGCTTTGAGGATTTCGTTGGAAACGGAAATATCTTCATAT

                 *       *               *                 **  *   *         
     464354 AAAAAGTAGACAGCAGCATTCTCAGAAACTTCTTTGTGATGTTTGCATCCAGCTCTCAGAGTTGA
          1 AAAAACTAGACAGAAGCATTCTCAGAAACATCTTTGTGATGTTTGCACTCAACTCACAGAGTTGA

              *  * *          *  *        *        *   **        *  **       
     464419 ACATTCCCTTTCATAGAGTAGGTTTGAAACCCTCTTTTTATAGTGTCTGGAAGCGGGCATTTGGA
         66 ACCTTTCTTTTCATAGAGCAGTTTTGAAACACTCTTTTTGTAGAATCTGGAAGTGGATATTTGGA

                   *   ** ** *  *   *          * *   
     464484 GCGCTTTCAGGCCTATGCTGAAAAAGGAAATATCTACCTAT
        131 GCGCTTTGAGGATTTCGTTGGAAACGGAAATATCTTCATAT

             *                    *    *  **          ** *        *    *     
     464525 AGAAACTAGACAGAAGCATTCTGAGAATCACGTTTGTGATGTGGGTACTCAACTAACAGTGTTGA
          1 AAAAACTAGACAGAAGCATTCTCAGAAACATCTTTGTGATGTTTGCACTCAACTCACAGAGTTGA

            *  *       *   *            *   *                *               
     464590 TCCATTCTTTTGATACAGCAGTTTTGAACCACACTTTTTGTAGAATCTGCAAGTGGATATTTGGA
         66 ACCTTTCTTTTCATAGAGCAGTTTTGAAACACTCTTTTTGTAGAATCTGGAAGTGGATATTTGGA

            **   *                     *   *        *
     464655 TAGCTGTGAGGATTTCGTTGGAAACGGGAATGTCTTCATAG
        131 GCGCTTTGAGGATTTCGTTGGAAACGGAAATATCTTCATAT

                **         *           * * **         *  **   *   *          
     464696 AAAATTTAGACAGAAACATTCTCAGAACCTTGATTGTGATGTGTGTTCTCCACTAACAGAGTTGA
          1 AAAAACTAGACAGAAGCATTCTCAGAAACATCTTTGTGATGTTTGCACTCAACTCACAGAGTTGA

                       * *   * *   *       *       *                         
     464761 ACCTTTCTTTTGACAGAACTGTTCTGAAACATTCTTTTTATAGAATCTGGAAGTGGATATTTGGA
         66 ACCTTTCTTTTCATAGAGCAGTTTTGAAACACTCTTTTTGTAGAATCTGGAAGTGGATATTTGGA

            **                         *          *  
     464826 AAGCTTTGAGGATTTCGTTGGAAACGGGAATATCTTCAAAT
        131 GCGCTTTGAGGATTTCGTTGGAAACGGAAATATCTTCATAT

            *   *    *            *           * *       *  *    *            
     464867 CAAATCTAGCCAGAAGCATTCTAAGAAACATCTTAGGGATGTTTACATTCAAGTCACAGAGTTGA
          1 AAAAACTAGACAGAAGCATTCTCAGAAACATCTTTGTGATGTTTGCACTCAACTCACAGAGTTGA

              *  * *     *       *         *    * *   **      *      *    *  
     464932 ACATTCCCTTTCACAGAGCAGGTTTGAAACAATCTTCTCGTACTATCTGGCAGTGGACATTTTGA
         66 ACCTTTCTTTTCATAGAGCAGTTTTGAAACACTCTTTTTGTAGAATCTGGAAGTGGATATTTGGA

              * *   *  ** ** *  *   *            ** *
     464997 GCTCCTTGGGGCCTATGCTGAAAAAGGAAATATCTTCCGAC
        131 GCGCTTTGAGGATTTCGTTGGAAACGGAAATATCTTCATAT

                                 *     *  **          *           **    *    
     465038 AAAAACTAGACAGAAGCATTCGCAGAATCACGTTTGTGATGTGTGCACTCAACTGTCAGAATTGA
          1 AAAAACTAGACAGAAGCATTCTCAGAAACATCTTTGTGATGTTTGCACTCAACTCACAGAGTTGA

                 **   ** *      *              *             * *            *
     465103 ACCTTGGTTTGGACAGAGCACTTTTGAAACACTCTCTTTGTAGAATCTGCAGGTGGATATTTGGC
         66 ACCTTTCTTTTCATAGAGCAGTTTTGAAACACTCTTTTTGTAGAATCTGGAAGTGGATATTTGGA

            **                         *   *      * *
     465168 TAGCTTTGAGGATTTCGTTGGAAACGGTAATGTCTTCAAAG
        131 GCGCTTTGAGGATTTCGTTGGAAACGGAAATATCTTCATAT

                *                         *   *     *      *    *            
     465209 AAAATCTAGACAGAAGCATTCTCAGAAACACCTTCGTGATATTTGCAATCAAGTCACAGAGTTGA
          1 AAAAACTAGACAGAAGCATTCTCAGAAACATCTTTGTGATGTTTGCACTCAACTCACAGAGTTGA

                 * *             *  *                 **             *       
     465274 ACCTTCCGTTTCATAGAGCAGGTTGGAAACACTCTTTTTGTATTATCTGGAAGTGGACATTTGGA
         66 ACCTTTCTTTTCATAGAGCAGTTTTGAAACACTCTTTTTGTAGAATCTGGAAGTGGATATTTGGA

             *         ** *    * *   *            **  
     465339 GGGCTTTGTAGCCTATC-TGGAAAAAGGAAATATCTTCCCAT
        131 GCGCTTTG-AGGATTTCGTTGGAAACGGAAATATCTTCATAT

            *  ** *   *     * *            *   *  *   * **        *  * * *   
     465380 GAATGCGAGATAGAAGTAATCTCAGAAACATGTTTATGCTGTATCTACTCAACTAACTGTGCTGA
          1 AAAAACTAGACAGAAGCATTCTCAGAAACATCTTTGTGATGTTTGCACTCAACTCACAGAGTTGA

              *     *  *               *        *  * *       *               
     465445 ACATTTCTATTGATAGAGCAGTTTTGAGACACTCTTCTTTTGGAATCTGCAAGTGGATATTTGGA
         66 ACCTTTCTTTTCATAGAGCAGTTTTGAAACACTCTTTTTGTAGAATCTGGAAGTGGATATTTGGA

            ** *                        *     *      
     465510 TAGATTTGAGGATTTCGTTGGAAACGG-GAT-T-AT-ATAT
        131 GCGCTTTGAGGATTTCGTTGGAAACGGAAATATCTTCATAT

                 *       *               *                 **  *   *         
     465547 AAAAAGTAGACAGCAGCATTCTCAGAAACTTCTTTGTGATGTTTGCATCCAGCTCTCAGAGTTGA
          1 AAAAACTAGACAGAAGCATTCTCAGAAACATCTTTGTGATGTTTGCACTCAACTCACAGAGTTGA

            * *  * *          *  *        *        *   **        *  **       
     465612 GCATTCCCTTTCATAGAGTAGGTTTGAAACCCTCTTTTTATAGTGTCTGGAAGCGGGCATTTGGA
         66 ACCTTTCTTTTCATAGAGCAGTTTTGAAACACTCTTTTTGTAGAATCTGGAAGTGGATATTTGGA

                   *    * * **  **  **          * *  *
     465677 GCGCTTTCAGGCCTATGCTTAAAATAGGAAATATCTACCTAC
        131 GCGCTTTGAGG-ATTTCGTTGGAAACGGAAATATCTTCATAT

             *                    *    *  **          ** *        *    *     
     465719 AGAAACTAGACAGAAGCATTCTGAGAATCACGTTTGTGATGTGGGTACTCAACTAACAGTGTTGA
          1 AAAAACTAGACAGAAGCATTCTCAGAAACATCTTTGTGATGTTTGCACTCAACTCACAGAGTTGA

            *  *       *   *            *   *                *   *           
     465784 TCCATTCTTTTGATACAGCAGTTTTGAACCACACTTTTTGTAGAATCTGCAAGAGGATATTTGGA
         66 ACCTTTCTTTTCATAGAGCAGTTTTGAAACACTCTTTTTGTAGAATCTGGAAGTGGATATTTGGA

            **   *                     *   *      * *
     465849 TAGCTGTGAGGATTTCGTTGGAAACGGGAATGTCTTCAAAG
        131 GCGCTTTGAGGATTTCGTTGGAAACGGAAATATCTTCATAT

                *                         *   *            *    *            
     465890 AAAATCTAGACAGAAGCATTCTCAGAAACACCTTCGTGATGTTTGCAATCAAGTCACAGAGTTGA
          1 AAAAACTAGACAGAAGCATTCTCAGAAACATCTTTGTGATGTTTGCACTCAACTCACAGAGTTGA

                 * *             *  *           *      *             *       
     465955 ACCTTCCGTTTCATAGAGCAGGTTGGAAACACTCTTATTGTAGTATCTGGAAGTGGACATTTGGA
         66 ACCTTTCTTTTCATAGAGCAGTTTTGAAACACTCTTTTTGTAGAATCTGGAAGTGGATATTTGGA

                   *   ** ** *  *   *            **  
     466020 GCGCTTTCAGGCCTATGGTGAAAAAGGAAATATCTTCCCAT
        131 GCGCTTTGAGGATTTCGTTGGAAACGGAAATATCTTCATAT

                  * * *      **      *   * *   *     ** ** *      *    *     
     466061 AAAAACGACATAGAAGCTATCTCAGGAACTTGTTTATGATGCATCTAATCAACTAACAGTGTTGA
          1 AAAAACTAGACAGAAGCATTCTCAGAAACATCTTTGTGATGTTTGCACTCAACTCACAGAGTTGA

                  * ** * *                         * *       *               
     466126 ACCTTTGTACTGACAGAGCAG-TTTGAAACACTCTTTTTTTGGAATCTGCAAGTGGATATTTGGA
         66 ACCTTTCTTTTCATAGAGCAGTTTTGAAACACTCTTTTTGTAGAATCTGGAAGTGGATATTTGGA

            *                           *   *   *    
     466190 TCGCTTTGAGGATTTCGTTGGAAACGG-GAT-GC--AATA-
        131 GCGCTTTGAGGATTTCGTTGGAAACGGAAATATCTTCATAT

                     *    *     *                 **  *   *  *    *         *
     466226 AAAAACGTACACAGCAGCATACTCAGAAA-ATACTTTGCCATATTTCCATTCAAGTCACAGAGTG
          1 AAAAAC-TAGACAGAAGCATTCTCAGAAACAT-CTTTGTGATGTTTGCACTCAACTCACAGAGTT

                *  * **            *                  *  *             *     
     466290 GAACATTCCCATTCATAGAGCAGGTTTGAAACACTCTTTTTGGAGTATCTGGAAGTGGACATTTG
         64 GAACCTTTCTTTTCATAGAGCAGTTTTGAAACACTCTTTTTGTAGAATCTGGAAGTGGATATTTG

                     ** * * ** *  *   *            **  
     466355 GAGCGCTTTCTGAACTATGGTGAAAAAGGAAATATCTTCCAAT
        129 GAGCGCTTTGAGGATTTCGTTGGAAACGGAAATATCTTCATAT

            *     *               *      * *          *  **      **  *  *    
     466398 GAAAACAAGACAGAAGCATTCTGAGAAACTTATTTGTGATGTGTGTCCTCAACAAACGGACTTGA
          1 AAAAACTAGACAGAAGCATTCTCAGAAACATCTTTGTGATGTTTGCACTCAACTCACAGAGTTGA

                   *      **  * *  *  *             *   *    * * *           
     466463 ACCTTTCGTTTCATGCAGTACTTCTGGAACACTCTTTTTGAAGATTCTGCATGCGGATATTTGGA
         66 ACCTTTCTTTTCATAGAGCAGTTTTGAAACACTCTTTTTGTAGAATCTGGAAGTGGATATTTGGA

            **                            **   *   * 
     466528 TAGCTTTGAGGATTTCGTTGGAAACGG---GCT-TACATGT
        131 GCGCTTTGAGGATTTCGTTGGAAACGGAAATATCTTCATAT

                 *       *               *        *   *    *    *       *    
     466565 AAAAATTAGACAGCAGCATTCTCAGAAACTTCTTTGTGGTGTCTGCATTCAAGTCACAGAATTGA
          1 AAAAACTAGACAGAAGCATTCTCAGAAACATCTTTGTGATGTTTGCACTCAACTCACAGAGTTGA

              * ** * **            *  * *      *       *    *        *       
     466630 ACTTCCCCTCACATAGAGCAGTTGTGCAGCACTCTATTTGTAGTATCTCGAAGTGGACATTTGGA
         66 ACCTTTCTTTTCATAGAGCAGTTTTGAAACACTCTTTTTGTAGAATCTGGAAGTGGATATTTGGA

             *         ** *    * *   *            **  
     466695 GGGCTTTGTAGCCTATC-TGGAAAAAGGAAATATCTTCCCAT
        131 GCGCTTTG-AGGATTTCGTTGGAAACGGAAATATCTTCATAT

            *  ** *   *     * *            *   *  *   * **        *  * * *   
     466736 GAATGCGAGATAGAAGTAATCTCAGAAACATGTTTATGCTGTATCTACTCAACTAACTGTGCTGA
          1 AAAAACTAGACAGAAGCATTCTCAGAAACATCTTTGTGATGTTTGCACTCAACTCACAGAGTTGA

              *     *  *               *        *  * *       *               
     466801 ACATTTCTATTGATAGAGCAGTTTTGAGACACTCTTCTTTTGGAATCTGCAAGTGGATATTTGGA
         66 ACCTTTCTTTTCATAGAGCAGTTTTGAAACACTCTTTTTGTAGAATCTGGAAGTGGATATTTGGA

            ** *                        *     *      
     466866 TAGATTTGAGGATTTCGTTGGAAACGG-GAT-T-AT-ATAT
        131 GCGCTTTGAGGATTTCGTTGGAAACGGAAATATCTTCATAT

            *    *       *               *                 **  *   *         
     466903 CAAAAGTAGACAGCAGCATTCTCAGAAACTTCTTTGTGATGTTTGCATCCAGCTCTCAGAGTTGA
          1 AAAAACTAGACAGAAGCATTCTCAGAAACATCTTTGTGATGTTTGCACTCAACTCACAGAGTTGA

              *  * *          *  *        *        *   **        *  **       
     466968 ACATTCCCTTTCATAGAGTAGGTTTGAAACCCTCTTTTTATAGTGTCTGGAAGCGGGCATTTGGA
         66 ACCTTTCTTTTCATAGAGCAGTTTTGAAACACTCTTTTTGTAGAATCTGGAAGTGGATATTTGGA

                   *    * *  *  **  **          * *   
     467033 GCGCTTTCAGGCCTATCCTTAAAATAGGAAATATCTACCTAT
        131 GCGCTTTGAGG-ATTTCGTTGGAAACGGAAATATCTTCATAT

             *                    *    *  **          ** *        *    *     
     467075 AGAAACTAGACAGAAGCATTCTGAGAATCACGTTTGTGATGTGGGTACTCAACTAACAGTGTTGA
          1 AAAAACTAGACAGAAGCATTCTCAGAAACATCTTTGTGATGTTTGCACTCAACTCACAGAGTTGA

            *  *       *   *            *   *                *               
     467140 TCCATTCTTTTGATACAGCAGTTTTGAACCACACTTTTTGTAGAATCTGCAAGTGGATATTTGGA
         66 ACCTTTCTTTTCATAGAGCAGTTTTGAAACACTCTTTTTGTAGAATCTGGAAGTGGATATTTGGA

            **   *                     *   *        *
     467205 TAGCTGTGAGGATTTCGTTGGAAACGGGAATGTCTTCATAG
        131 GCGCTTTGAGGATTTCGTTGGAAACGGAAATATCTTCATAT

                **                     * * **         *  **   *   *          
     467246 AAAATTTAGACAGAAGCATTCTCAGAACCTTGATTGTGATGTGTGTTCTCCACTAACAGAGTTGA
          1 AAAAACTAGACAGAAGCATTCTCAGAAACATCTTTGTGATGTTTGCACTCAACTCACAGAGTTGA

                       * *   * *   *       *       *                         
     467311 ACCTTTCTTTTGACAGAACTGTTCTGAAACATTCTTTTTATAGAATCTGGAAGTGGATATTTGGA
         66 ACCTTTCTTTTCATAGAGCAGTTTTGAAACACTCTTTTTGTAGAATCTGGAAGTGGATATTTGGA

            **                         *          *  
     467376 AAGCTTTGAGGATTTCGTTGGAAACGGGAATATCTTCAAAT
        131 GCGCTTTGAGGATTTCGTTGGAAACGGAAATATCTTCATAT

                *    *            *           * *       *  *    *            
     467417 AAAATCTAGCCAGAAGCATTCTAAGAAACATCTTAGGGATGTTTACATTCAAGTCACAGAGTTGA
          1 AAAAACTAGACAGAAGCATTCTCAGAAACATCTTTGTGATGTTTGCACTCAACTCACAGAGTTGA

              *  * *     *       *         *    * *   **      *      *    *  
     467482 ACATTCCCTTTCACAGAGCAGGTTTGAAACAATCTTCTCGTACTATCTGGCAGTGGACATTTTGA
         66 ACCTTTCTTTTCATAGAGCAGTTTTGAAACACTCTTTTTGTAGAATCTGGAAGTGGATATTTGGA

              * *   *  ** ** *  *   *            ** *
     467547 GCTCCTTGGGGCCTATGCTGAAAAAGGAAATATCTTCCGAC
        131 GCGCTTTGAGGATTTCGTTGGAAACGGAAATATCTTCATAT

                                 *     *  **          *           **    *    
     467588 AAAAACTAGACAGAAGCATTCGCAGAATCACGTTTGTGATGTGTGCACTCAACTGTCAGAATTGA
          1 AAAAACTAGACAGAAGCATTCTCAGAAACATCTTTGTGATGTTTGCACTCAACTCACAGAGTTGA

                 **   ** *      *                            * *            *
     467653 ACCTTGGTTTGGAGAGAGCACTTTTGAAACACTCTTTTTGTAGAATCTGCAGGTGGATATTTGGC
         66 ACCTTTCTTTTCATAGAGCAGTTTTGAAACACTCTTTTTGTAGAATCTGGAAGTGGATATTTGGA

            **                         *   *      * *
     467718 TAGCTTTGAGGATTTCGTTGGAAACGGTAATGTCTTCAAAG
        131 GCGCTTTGAGGATTTCGTTGGAAACGGAAATATCTTCATAT

                *                         *   *            *    *            
     467759 AAAATCTAGACAGAAGCATTCTCAGAAACAACTTCGTGATGTTTGCAATCAAGTCACAGAGTTGA
          1 AAAAACTAGACAGAAGCATTCTCAGAAACATCTTTGTGATGTTTGCACTCAACTCACAGAGTTGA

                 * *             *  *                  *             *       
     467824 ACCTTCCGTTTCATAGAGCAGGTTGGAAACACTCTTTTTGTAGTATCTGGAAGTGGACATTTGGA
         66 ACCTTTCTTTTCATAGAGCAGTTTTGAAACACTCTTTTTGTAGAATCTGGAAGTGGATATTTGGA

             *         ** *    * *   *            **  
     467889 GGGCTTTGTAGCCTATC-TGGAAAAAGGAAATATCTTCCCAT
        131 GCGCTTTG-AGGATTTCGTTGGAAACGGAAATATCTTCATAT

            *  ** *   *     * *            *   *  *   * **        *  * * *   
     467930 GAATGCGAGATAGAAGTAATCTCAGAAACATGTTTATGCTGTATCTACTCAACTAACTGTGCTGA
          1 AAAAACTAGACAGAAGCATTCTCAGAAACATCTTTGTGATGTTTGCACTCAACTCACAGAGTTGA

              *     *  *               *        *  * *       *               
     467995 ACATTTCTATTGATAGAGCAGTTTTGAGACACTCTTCTTTTGGAATCTGCAAGTGGATATTTGGA
         66 ACCTTTCTTTTCATAGAGCAGTTTTGAAACACTCTTTTTGTAGAATCTGGAAGTGGATATTTGGA

            ** *                        *     *      
     468060 TAGATTTGAGGATTTCGTTGGAAACGG-GAT-T-AT-ATAT
        131 GCGCTTTGAGGATTTCGTTGGAAACGGAAATATCTTCATAT

                 *       *               *                 **  *   *         
     468097 AAAAAGTAGACAGCAGCATTCTCAGAAACTTCTTTGTGATGTTTGCATCCAGCTCTCAGAGTTGA
          1 AAAAACTAGACAGAAGCATTCTCAGAAACATCTTTGTGATGTTTGCACTCAACTCACAGAGTTGA

              *  * *          *  *        *        *   **        *  **       
     468162 ACATTCCCTTTCATAGAGTAGGTTTGAAACCCTCTTTTTATAGTGTCTGGAAGCGGGCATTTGGA
         66 ACCTTTCTTTTCATAGAGCAGTTTTGAAACACTCTTTTTGTAGAATCTGGAAGTGGATATTTGGA

                *  *    * * **  **  **          * *  *
     468227 GCGCATTCAGGCCTATGCTTAAAATAGGAAATATCTACCTAC
        131 GCGCTTTGAGG-ATTTCGTTGGAAACGGAAATATCTTCATAT

             *                    *    *  **          ** *        *    *     
     468269 AGAAACTAGACAGAAGCATTCTGAGAATCACGTTTGTGATGTGGGTACTCAACTAACAGTGTTGA
          1 AAAAACTAGACAGAAGCATTCTCAGAAACATCTTTGTGATGTTTGCACTCAACTCACAGAGTTGA

            *  *       *   *            *   *                *   *           
     468334 TCCATTCTTTTGATACAGCAGTTTTGAACCACACTTTTTGTAGAATCTGCAAGAGGATATTTGGA
         66 ACCTTTCTTTTCATAGAGCAGTTTTGAAACACTCTTTTTGTAGAATCTGGAAGTGGATATTTGGA

            **   *                     *   *      * *
     468399 TAGCTGTGAGGATTTCGTTGGAAACGGGAATGTCTTCAAAG
        131 GCGCTTTGAGGATTTCGTTGGAAACGGAAATATCTTCATAT

                *                         *   *            *    *            
     468440 AAAATCTAGACAGAAGCATTCTCAGAAACACCTTCGTGATGTTTGCAATCAAGTCACAGAGTTGA
          1 AAAAACTAGACAGAAGCATTCTCAGAAACATCTTTGTGATGTTTGCACTCAACTCACAGAGTTGA

                 * *             *  *           *      *             *       
     468505 ACCTTCCGTTTCATAGAGCAGGTTGGAAACACTCTTATTGTAGTATCTGGAAGTGGACATTTGGA
         66 ACCTTTCTTTTCATAGAGCAGTTTTGAAACACTCTTTTTGTAGAATCTGGAAGTGGATATTTGGA

                   *   ** ** *  *   *            **  
     468570 GCGCTTTCAGGCCTATGGTGAAAAAGGAAATATCTTCCCAT
        131 GCGCTTTGAGGATTTCGTTGGAAACGGAAATATCTTCATAT

                  * * *      **      *   * *   *     ** ** *      *    *     
     468611 AAAAACGACATAGAAGCTATCTCAGGAACTTGTTTATGATGCATCTAATCAACTAACAGTGTTGA
          1 AAAAACTAGACAGAAGCATTCTCAGAAACATCTTTGTGATGTTTGCACTCAACTCACAGAGTTGA

                  * ** * *                         * *       *               
     468676 ACCTTTGTACTGAGAGAGCAG-TTTGAAACACTCTTTTTTTGGAATCTGCAAGTGGATATTTGGA
         66 ACCTTTCTTTTCATAGAGCAGTTTTGAAACACTCTTTTTGTAGAATCTGGAAGTGGATATTTGGA

            *                           *   *   *    
     468740 TCGCTTTGAGGATTTCGTTGGAAACGG-GAT-GC--AATAT
        131 GCGCTTTGAGGATTTCGTTGGAAACGGAAATATCTTCATAT

                **  *    *     *                 **  *   *  *    *         * 
     468777 AAAACGTACACAGCAGCATACTCAGAAA-ATACTTTGCCATATTTCCATTCAAGTCACAGAGTGG
          1 AAAAACTAGACAGAAGCATTCTCAGAAACAT-CTTTGTGATGTTTGCACTCAACTCACAGAGTTG

               *  * **            *  *               *  *             *      
     468841 AACATTCCCATTCATAGAGCAGGTTGGAAACACTCTTTTTGGAGTATCTGGAAGTGGACATTTGG
         65 AACCTTTCTTTTCATAGAGCAGTTTTGAAACACTCTTTTTGTAGAATCTGGAAGTGGATATTTGG

                    ** * * ** *  *   *            **  
     468906 AGCGCTTTCTGAACTATGGTGAAAAAGGAAATATCTTCCAAT
        130 AGCGCTTTGAGGATTTCGTTGGAAACGGAAATATCTTCATAT

            *     *               *      * *          *  **      **  *  *    
     468948 GAAAACAAGACAGAAGCATTCTGAGAAACTTATTTGTGATGTGTGTCCTCAACAAACGGACTTGA
          1 AAAAACTAGACAGAAGCATTCTCAGAAACATCTTTGTGATGTTTGCACTCAACTCACAGAGTTGA

                   *      **  * *  *  *             *   *    * * *           
     469013 ACCTTTCGTTTCATGCAGTACTTCTGGAACACTCTTTTTGAAGATTCTGCATGCGGATATTTGGA
         66 ACCTTTCTTTTCATAGAGCAGTTTTGAAACACTCTTTTTGTAGAATCTGGAAGTGGATATTTGGA

            **                            **   *   * 
     469078 TAGCTTTGAGGATTTCGTTGGAAACGG---GCT-TACATGT
        131 GCGCTTTGAGGATTTCGTTGGAAACGGAAATATCTTCATAT

                 *       *               *        *   *    *    *       *    
     469115 AAAAATTAGACAGCAGCATTCTCAGAAACTTCTTTGTGGTGTCTGCATTCAAGTCACAGAATTGA
          1 AAAAACTAGACAGAAGCATTCTCAGAAACATCTTTGTGATGTTTGCACTCAACTCACAGAGTTGA

              * ** * **            *  * *      *       *             *       
     469180 ACTTCCCCTCACATAGAGCAGTTGTGCAGCACTCTATTTGTAGTATCTGGAAGTGGACATTTGGA
         66 ACCTTTCTTTTCATAGAGCAGTTTTGAAACACTCTTTTTGTAGAATCTGGAAGTGGATATTTGGA

             *         ** *    * *   *            **  
     469245 GGGCTTTGTAGCCTATC-TGGAAAAAGGAAATATCTTCCCAT
        131 GCGCTTTG-AGGATTTCGTTGGAAACGGAAATATCTTCATAT

            *  ** *   *     * *            *   *  * * * **        *  * * *   
     469286 GAATGCGAGATAGAAGTAATCTCAGAAACATGTTTATGCTCTATCTACTCAACTAACTGTGCTGA
          1 AAAAACTAGACAGAAGCATTCTCAGAAACATCTTTGTGATGTTTGCACTCAACTCACAGAGTTGA

              *     *  *               *        *  * *       *               
     469351 ACATTTCTATTGATAGAGCAGTTTTGAGACACTCTTCTTTTGGAATCTGCAAGTGGATATTTGGA
         66 ACCTTTCTTTTCATAGAGCAGTTTTGAAACACTCTTTTTGTAGAATCTGGAAGTGGATATTTGGA

            ** *                        *     *      
     469416 TAGATTTGAGGATTTCGTTGGAAACGG-GAT-T-AT-ATAT
        131 GCGCTTTGAGGATTTCGTTGGAAACGGAAATATCTTCATAT

            *    **      *               *                 **  *   *         
     469453 CAAAAGCAGACAGCAGCATTCTCAGAAACTTCTTTGTGATGTTTGCATCCAGCTCTCAGAGTTGA
          1 AAAAACTAGACAGAAGCATTCTCAGAAACATCTTTGTGATGTTTGCACTCAACTCACAGAGTTGA

              *  * *          *  *        *        *   **        *  **       
     469518 ACATTCCCTTTCATAGAGTAGGTTTGAAACCCTCTTTTTATAGTGTCTGGAAGCGGGCATTTGGA
         66 ACCTTTCTTTTCATAGAGCAGTTTTGAAACACTCTTTTTGTAGAATCTGGAAGTGGATATTTGGA

                   *    * * **  **  **          * *  *
     469583 GCGCTTTCAGGCCTATGCTTAAAATAGGAAATATCTACCTAC
        131 GCGCTTTGAGG-ATTTCGTTGGAAACGGAAATATCTTCATAT

             *                    *    *  **          ** *        *    *     
     469625 AGAAACTAGACAGAAGCATTCTGAGAATCACGTTTGTGATGTGGGTACTCAACTAACAGTGTTGA
          1 AAAAACTAGACAGAAGCATTCTCAGAAACATCTTTGTGATGTTTGCACTCAACTCACAGAGTTGA

            *  *       *   *            *   *                *   *           
     469690 TCCATTCTTTTGATACAGCAGTTTTGAACCACACTTTTTGTAGAATCTGCAAGAGGATATTTGGA
         66 ACCTTTCTTTTCATAGAGCAGTTTTGAAACACTCTTTTTGTAGAATCTGGAAGTGGATATTTGGA

            **   *                     *   *      * *
     469755 TAGCTGTGAGGATTTCGTTGGAAACGGGAATGTCTTCAAAG
        131 GCGCTTTGAGGATTTCGTTGGAAACGGAAATATCTTCATAT

                *                         *   *            *    *            
     469796 AAAATCTAGACAGAAGCATTCTCAGAAACACCTTCGTGATGTTTGCAATCAAGTCACAGAGTTGA
          1 AAAAACTAGACAGAAGCATTCTCAGAAACATCTTTGTGATGTTTGCACTCAACTCACAGAGTTGA

                 * *             *  *           *      *             *       
     469861 ACCTTCCGTTTCATAGAGCAGGTTGGAAACACTCTTATTGTAGTATCTGGAAGTGGACATTTGGA
         66 ACCTTTCTTTTCATAGAGCAGTTTTGAAACACTCTTTTTGTAGAATCTGGAAGTGGATATTTGGA

                   *   ** ** *  *   *            **  
     469926 GCGCTTTCAGGCCTATGGTGAAAAAGGAAATATCTTCCCAT
        131 GCGCTTTGAGGATTTCGTTGGAAACGGAAATATCTTCATAT

                  * * *      **      *   * *   *     ** ** *      *    *     
     469967 AAAAACGACATAGAAGCTATCTCAGGAACTTGTTTATGATGCATCTAATCAACTAACAGTGTTGA
          1 AAAAACTAGACAGAAGCATTCTCAGAAACATCTTTGTGATGTTTGCACTCAACTCACAGAGTTGA

                  * ** * *                         * *       *               
     470032 ACCTTTGTACTGACAGAGCAG-TTTGAAACACTCTTTTTTTGGAATCTGCAAGTGGATATTTGGA
         66 ACCTTTCTTTTCATAGAGCAGTTTTGAAACACTCTTTTTGTAGAATCTGGAAGTGGATATTTGGA

            *                           *   *   *    
     470096 TCGCTTTGAGGATTTCGTTGGAAACGG-GAT-GC--AATAT
        131 GCGCTTTGAGGATTTCGTTGGAAACGGAAATATCTTCATAT

                **  *    *     *                 **  *   *  *    *         * 
     470133 AAAACGTACACAGCAGCATACTCAGAAA-ATACTTTGCCATATTTCCATTCAAGTCACAGAGTGG
          1 AAAAACTAGACAGAAGCATTCTCAGAAACAT-CTTTGTGATGTTTGCACTCAACTCACAGAGTTG

               *  * **            *  *               *  *             *      
     470197 AACATTCCCATTCATAGAGCAGGTTGGAAACACTCTTTTTGGAGTATCTGGAAGTGGACATTTGG
         65 AACCTTTCTTTTCATAGAGCAGTTTTGAAACACTCTTTTTGTAGAATCTGGAAGTGGATATTTGG

                    ** * * ** *  *   *            **  
     470262 AGCGCTTTCTGAACTATGGTGAAAAAGGAAATATCTTCCAAT
        130 AGCGCTTTGAGGATTTCGTTGGAAACGGAAATATCTTCATAT

            *     *               *      * *          *  **      **  *  *    
     470304 GAAAACAAGACAGAAGCATTCTGAGAAACTTATTTGTGATGTGTGTCCTCAACAAACGGACTTGA
          1 AAAAACTAGACAGAAGCATTCTCAGAAACATCTTTGTGATGTTTGCACTCAACTCACAGAGTTGA

                   *      **  * *  *  *             *   *    * * *           
     470369 ACCTTTCGTTTCATGCAGTACTTCTGGAACACTCTTTTTGAAGATTCTGCATGCGGATATTTGGA
         66 ACCTTTCTTTTCATAGAGCAGTTTTGAAACACTCTTTTTGTAGAATCTGGAAGTGGATATTTGGA

            **                            **   *   * 
     470434 TAGCTTTGAGGATTTCGTTGGAAACGG---GCT-TACATGT
        131 GCGCTTTGAGGATTTCGTTGGAAACGGAAATATCTTCATAT

                 *       *               *        *   *    *    *       *    
     470471 AAAAATTAGACAGCAGCATTCTCAGAAACTTCTTTGTGGTGTCTGCATTCAAGTCACAGAATTGA
          1 AAAAACTAGACAGAAGCATTCTCAGAAACATCTTTGTGATGTTTGCACTCAACTCACAGAGTTGA

              * ** * **            *  * *      *       *             *       
     470536 ACTTCCCCTCACATAGAGCAGTTGTGCAGCACTCTATTTGTAGTATCTGGAAGTGGACATTTGGA
         66 ACCTTTCTTTTCATAGAGCAGTTTTGAAACACTCTTTTTGTAGAATCTGGAAGTGGATATTTGGA

             *         ** *    * *   *            **  
     470601 GGGCTTTGTAGCCTATC-TGGAAAAAGGAAATATCTTCCCAT
        131 GCGCTTTG-AGGATTTCGTTGGAAACGGAAATATCTTCATAT

            *  ** *   *     * *            *   *  *   * **        *  * * *   
     470642 GAATGCGAGATAGAAGTAATCTCAGAAACATGTTTATGCTGTATCTACTCAACTAACTGTGCTGA
          1 AAAAACTAGACAGAAGCATTCTCAGAAACATCTTTGTGATGTTTGCACTCAACTCACAGAGTTGA

              *     *  *               *        *  * *       *               
     470707 ACATTTCTATTGATAGAGCAGTTTTGAGACACTCTTCTTTTGGAATCTGCAAGTGGATATTTGGA
         66 ACCTTTCTTTTCATAGAGCAGTTTTGAAACACTCTTTTTGTAGAATCTGGAAGTGGATATTTGGA

            ** *                        *     *      
     470772 TAGATTTGAGGATTTCGTTGGAAACGG-GAT-T-AT-ATAT
        131 GCGCTTTGAGGATTTCGTTGGAAACGGAAATATCTTCATAT

                 *       *               *                 **  *   *         
     470809 AAAAAGTAGACAGCAGCATTCTCAGAAACTTCTTTGTGATGTTTGCATCCAGCTCTCAGAGTTGA
          1 AAAAACTAGACAGAAGCATTCTCAGAAACATCTTTGTGATGTTTGCACTCAACTCACAGAGTTGA

              *  * *          *  *        *        *   **        *  **       
     470874 ACATTCCCTTTCATAGAGTAGGTTTGAAACCCTCTTTTTATAGTGTCTGGAAGCGGGCATTTGGA
         66 ACCTTTCTTTTCATAGAGCAGTTTTGAAACACTCTTTTTGTAGAATCTGGAAGTGGATATTTGGA

               *   *   ** ** ** *   *          * *   
     470939 GCGATTTCAGGCCTATGCGGAAAAAGGAAATATCTACCTAT
        131 GCGCTTTGAGGATTTCGTTGGAAACGGAAATATCTTCATAT

             *                    *    *  **          ** *        *    *     
     470980 AGAAACTAGACAGAAGCATTCTGAGAATCACGTTTGTGATGTGGGTACTCAACTAACAGTGTTGA
          1 AAAAACTAGACAGAAGCATTCTCAGAAACATCTTTGTGATGTTTGCACTCAACTCACAGAGTTGA

            *  *       *   *            *   *                *               
     471045 TCCATTCTTTTGATACAGCAGTTTTGAACCACACTTTTTGTAGAATCTGCAAGTGGATATTTGGA
         66 ACCTTTCTTTTCATAGAGCAGTTTTGAAACACTCTTTTTGTAGAATCTGGAAGTGGATATTTGGA

            **   *                     *   *        *
     471110 TAGCTGTGAGGATTTCGTTGGAAACGGGAATGTCTTCATAG
        131 GCGCTTTGAGGATTTCGTTGGAAACGGAAATATCTTCATAT

                **         *           * * **         *  **   *   *          
     471151 AAAATTTAGACAGAAACATTCTCAGAACCTTGATTGTGATGTGTGTTCTCCACTAACAGAGTTGA
          1 AAAAACTAGACAGAAGCATTCTCAGAAACATCTTTGTGATGTTTGCACTCAACTCACAGAGTTGA

                       * *   * *   *       *       *                         
     471216 ACCTTTCTTTTGACAGAACTGTTCTGAAACATTCTTTTTATAGAATCTGGAAGTGGATATTTGGA
         66 ACCTTTCTTTTCATAGAGCAGTTTTGAAACACTCTTTTTGTAGAATCTGGAAGTGGATATTTGGA

            **                         *          *  
     471281 AAGCTTTGAGGATTTCGTTGGAAACGGGAATATCTTCAAAT
        131 GCGCTTTGAGGATTTCGTTGGAAACGGAAATATCTTCATAT

                *    *            *           * *       *  *    *            
     471322 AAAATCTAGCCAGAAGCATTCTAAGAAACATCTTAGGGATGTTTACATTCAAGTCACAGAGTTGA
          1 AAAAACTAGACAGAAGCATTCTCAGAAACATCTTTGTGATGTTTGCACTCAACTCACAGAGTTGA

              *  * *     *       *         *    * *   **      *      *    *  
     471387 ACATTCCCTTTCACAGAGCAGGTTTGAAACAATCTTCTCGTACTATCTGGCAGTGGACATTTTGA
         66 ACCTTTCTTTTCATAGAGCAGTTTTGAAACACTCTTTTTGTAGAATCTGGAAGTGGATATTTGGA

              * *   *  ** ** *  *   *            ** *
     471452 GCTCCTTGGGGCCTATGCTGAAAAAGGAAATATCTTCCGAC
        131 GCGCTTTGAGGATTTCGTTGGAAACGGAAATATCTTCATAT

                                 *     *  **          *           **    *    
     471493 AAAAACTAGACAGAAGCATTCGCAGAATCACGTTTGTGATGTGTGCACTCAACTGTCAGAATTGA
          1 AAAAACTAGACAGAAGCATTCTCAGAAACATCTTTGTGATGTTTGCACTCAACTCACAGAGTTGA

                 **   ** *      *              *             * *            *
     471558 ACCTTGGTTTGGACAGAGCACTTTTGAAACACTCTCTTTGTAGAATCTGCAGGTGGATATTTGGC
         66 ACCTTTCTTTTCATAGAGCAGTTTTGAAACACTCTTTTTGTAGAATCTGGAAGTGGATATTTGGA

            **                         *   *      * *
     471623 TAGCTTTGAGGATTTCGTTGGAAACGGTAATGTCTTCAAAG
        131 GCGCTTTGAGGATTTCGTTGGAAACGGAAATATCTTCATAT

                *                         *   *     *      *    *            
     471664 AAAATCTAGACAGAAGCATTCTCAGAAACACCTTCGTGATATTTGCAATCAAGTCACAGAGTTGA
          1 AAAAACTAGACAGAAGCATTCTCAGAAACATCTTTGTGATGTTTGCACTCAACTCACAGAGTTGA

                 * *             *  *                  *             *       
     471729 ACCTTCCGTTTCATAGAGCAGGTTGGAAACACTCTTTTTGTAGTATCTGGAAGTGGACATTTGGA
         66 ACCTTTCTTTTCATAGAGCAGTTTTGAAACACTCTTTTTGTAGAATCTGGAAGTGGATATTTGGA

             *         ** *    * *   *            **  
     471794 GGGCTTTGTAGCCTATC-TGGAAAAAGGAAATATCTTCCCAT
        131 GCGCTTTG-AGGATTTCGTTGGAAACGGAAATATCTTCATAT

            *  ** *   *     * *            *   *  *   * **        *  * * *   
     471835 GAATGCGAGATAGAAGTAATCTCAGAAACATGTTTATGCTGTATCTACTCAACTAACTGTGCTGA
          1 AAAAACTAGACAGAAGCATTCTCAGAAACATCTTTGTGATGTTTGCACTCAACTCACAGAGTTGA

              *     *  *               *        *  * *       *               
     471900 ACATTTCTATTGATAGAGCAGTTTTGAGACACTCTTCTTTTGGAATCTGCAAGTGGATATTTGGA
         66 ACCTTTCTTTTCATAGAGCAGTTTTGAAACACTCTTTTTGTAGAATCTGGAAGTGGATATTTGGA

            ** *                        *     *      
     471965 TAGATTTGAGGATTTCGTTGGAAACGG-GAT-T-AT-ATAT
        131 GCGCTTTGAGGATTTCGTTGGAAACGGAAATATCTTCATAT

                 *       *               *                 **  *   *         
     472002 AAAAAGTAGACAGCAGCATTCTCAGAAACTTCTTTGTGATGTTTGCATCCAGCTCTCAGAGTTGA
          1 AAAAACTAGACAGAAGCATTCTCAGAAACATCTTTGTGATGTTTGCACTCAACTCACAGAGTTGA

            * *  * *          *  *        *        *   **        *  **       
     472067 GCATTCCCTTTCATAGAGTAGGTTTGAAACCCTCTTTTTATAGTGTCTGGAAGCGGGCATTTGGA
         66 ACCTTTCTTTTCATAGAGCAGTTTTGAAACACTCTTTTTGTAGAATCTGGAAGTGGATATTTGGA

                   *    * * **  **  **          * *  *
     472132 GCGCTTTCAGGCCTATGCTTAAAATAGGAAATATCTACCTAC
        131 GCGCTTTGAGG-ATTTCGTTGGAAACGGAAATATCTTCATAT

             *                    *    *  **          ** *        *    *     
     472174 AGAAACTAGACAGAAGCATTCTGAGAATCACGTTTGTGATGTGGGTACTCAACTAACAGTGTTGA
          1 AAAAACTAGACAGAAGCATTCTCAGAAACATCTTTGTGATGTTTGCACTCAACTCACAGAGTTGA

            *  *       *   *            *   *                *   *           
     472239 TCCATTCTTTTGATACAGCAGTTTTGAACCACACTTTTTGTAGAATCTGCAAGAGGATATTTGGA
         66 ACCTTTCTTTTCATAGAGCAGTTTTGAAACACTCTTTTTGTAGAATCTGGAAGTGGATATTTGGA

            **   *                     *   *      * *
     472304 TAGCTGTGAGGATTTCGTTGGAAACGGGAATGTCTTCAAAG
        131 GCGCTTTGAGGATTTCGTTGGAAACGGAAATATCTTCATAT

                *                         *   *            *    *            
     472345 AAAATCTAGACAGAAGCATTCTCAGAAACACCTTCGTGATGTTTGCAATCAAGTCACAGAGTTGA
          1 AAAAACTAGACAGAAGCATTCTCAGAAACATCTTTGTGATGTTTGCACTCAACTCACAGAGTTGA

                 * *             *  *           *      *             *       
     472410 ACCTTCCGTTTCATAGAGCAGGTTGGAAACACTCTTATTGTAGTATCTGGAAGTGGACATTTGGA
         66 ACCTTTCTTTTCATAGAGCAGTTTTGAAACACTCTTTTTGTAGAATCTGGAAGTGGATATTTGGA

                   *   ** ** *  *   *            **  
     472475 GCGCTTTCAGGCCTATGGTGAAAAAGGAAATATCTTCCCAT
        131 GCGCTTTGAGGATTTCGTTGGAAACGGAAATATCTTCATAT

                  * * *      **      *   * *   *     ** ** *      *    *     
     472516 AAAAACGACATAGAAGCTATCTCAGGAACTTGTTTATGATGCATCTAATCAACTAACAGTGTTGA
          1 AAAAACTAGACAGAAGCATTCTCAGAAACATCTTTGTGATGTTTGCACTCAACTCACAGAGTTGA

                  * ** * *                         * *       *               
     472581 ACCTTTGTACTGACAGAGCAG-TTTGAAACACTCTTTTTTTGGAATCTGCAAGTGGATATTTGGA
         66 ACCTTTCTTTTCATAGAGCAGTTTTGAAACACTCTTTTTGTAGAATCTGGAAGTGGATATTTGGA

            *                           *   *   *    
     472645 TCGCTTTGAGGATTTCGTTGGAAACGG-GAT-GC--AATAT
        131 GCGCTTTGAGGATTTCGTTGGAAACGGAAATATCTTCATAT

                **  *    *     *                 **  *   *  *    *         * 
     472682 AAAACGTACACAGCAGCATACTCAGAAA-ATACTTTGCCATATTTCCATTCAAGTCACAGAGTGG
          1 AAAAACTAGACAGAAGCATTCTCAGAAACAT-CTTTGTGATGTTTGCACTCAACTCACAGAGTTG

               *  * **            *  *               *  *             *      
     472746 AACATTCCCATTCATAGAGCAGGTTGGAAACACTCTTTTTGGAGTATCTGGAAGTGGACATTTGG
         65 AACCTTTCTTTTCATAGAGCAGTTTTGAAACACTCTTTTTGTAGAATCTGGAAGTGGATATTTGG

                    ** * * ** *  *   *            **  
     472811 AGCGCTTTCTGAACTATGGTGAAAAAGGAAATATCTTCCAAT
        130 AGCGCTTTGAGGATTTCGTTGGAAACGGAAATATCTTCATAT

            *     *               *      * *          *  **      **  *  *    
     472853 GAAAACAAGACAGAAGCATTCTGAGAAACTTATTTGTGATGTGTGTCCTCAACAAACGGACTTGA
          1 AAAAACTAGACAGAAGCATTCTCAGAAACATCTTTGTGATGTTTGCACTCAACTCACAGAGTTGA

                   *      **  * *  *  *             *   *    * * *           
     472918 ACCTTTCGTTTCATGCAGTACTTCTGGAACACTCTTTTTGAAGATTCTGCATGCGGATATTTGGA
         66 ACCTTTCTTTTCATAGAGCAGTTTTGAAACACTCTTTTTGTAGAATCTGGAAGTGGATATTTGGA

            **                            **   *   * 
     472983 TAGCTTTGAGGATTTCGTTGGAAACGG---GCT-TACATGT
        131 GCGCTTTGAGGATTTCGTTGGAAACGGAAATATCTTCATAT

                 *       *               *        *   *    *    *       *    
     473020 AAAAATTAGACAGCAGCATTCTCAGAAACTTCTTTGTGGTGTCTGCATTCAAGTCACAGAATTGA
          1 AAAAACTAGACAGAAGCATTCTCAGAAACATCTTTGTGATGTTTGCACTCAACTCACAGAGTTGA

              * ** * **            *  * *      *       *             *       
     473085 ACTTCCCCTCACATAGAGCAGTTGTGCAGCACTCTATTTGTAGTATCTGGAAGTGGACATTTGGA
         66 ACCTTTCTTTTCATAGAGCAGTTTTGAAACACTCTTTTTGTAGAATCTGGAAGTGGATATTTGGA

             *         ** *    * *   *            **  
     473150 GGGCTTTGTAGCCTATC-TGGAAAAAGGAAATATCTTCCCAT
        131 GCGCTTTG-AGGATTTCGTTGGAAACGGAAATATCTTCATAT

            *  ** *   *     * *            *   *  *   * **        *  * * *   
     473191 GAATGCGAGATAGAAGTAATCTCAGAAACATGTTTATGCTGTATCTACTCAACTAACTGTGCTGA
          1 AAAAACTAGACAGAAGCATTCTCAGAAACATCTTTGTGATGTTTGCACTCAACTCACAGAGTTGA

              *     *  *               *        *  * *       *               
     473256 ACATTTCTATTGATAGAGCAGTTTTGAGACACTCTTCTTTTGGAATCTGCAAGTGGATATTTGGA
         66 ACCTTTCTTTTCATAGAGCAGTTTTGAAACACTCTTTTTGTAGAATCTGGAAGTGGATATTTGGA

            ** *                        *     *      
     473321 TAGATTTGAGGATTTCGTTGGAAACGG-GAT-T-AT-ATAT
        131 GCGCTTTGAGGATTTCGTTGGAAACGGAAATATCTTCATAT

                 *       *               *                 **  *   *         
     473358 AAAAAGTAGACAGCAGCATTCTCAGAAACTTCTTTGTGATGTTTGCATCCAGCTCTCAGAGTTGA
          1 AAAAACTAGACAGAAGCATTCTCAGAAACATCTTTGTGATGTTTGCACTCAACTCACAGAGTTGA

              *  * *          *  *        *        *   **        *  **       
     473423 ACATTCCCTTTCATAGAGTAGGTTTGAAACCCTCTTTTTATAGTGTCTGGAAGCGGGCATTTGGA
         66 ACCTTTCTTTTCATAGAGCAGTTTTGAAACACTCTTTTTGTAGAATCTGGAAGTGGATATTTGGA

               *   *   ** ** ** *   *          * *   
     473488 GCGATTTCAGGCCTATGCGGAAAAAGGAAATATCTACCTAT
        131 GCGCTTTGAGGATTTCGTTGGAAACGGAAATATCTTCATAT

             *                    *    *  **          ** *        *    *     
     473529 AGAAACTAGACAGAAGCATTCTGAGAATCACGTTTGTGATGTGGGTACTCAACTAACAGTGTTGA
          1 AAAAACTAGACAGAAGCATTCTCAGAAACATCTTTGTGATGTTTGCACTCAACTCACAGAGTTGA

            *  *       *   *            *   *                *               
     473594 TCCATTCTTTTGATACAGCAGTTTTGAACCACACTTTTTGTAGAATCTGCAAGTGGATATTTGGA
         66 ACCTTTCTTTTCATAGAGCAGTTTTGAAACACTCTTTTTGTAGAATCTGGAAGTGGATATTTGGA

            **   *                     *   *        *
     473659 TAGCTGTGAGGATTTCGTTGGAAACGGGAATGTCTTCATAG
        131 GCGCTTTGAGGATTTCGTTGGAAACGGAAATATCTTCATAT

                **         *           * * **         *  **   *   *          
     473700 AAAATTTAGACAGAAACATTCTCAGAACCTTGATTGTGATGTGTGTTCTCCACTAACAGAGTTGA
          1 AAAAACTAGACAGAAGCATTCTCAGAAACATCTTTGTGATGTTTGCACTCAACTCACAGAGTTGA

                       * *   * *   *       *       *                         
     473765 ACCTTTCTTTTGACAGAACTGTTCTGAAACATTCTTTTTATAGAATCTGGAAGTGGATATTTGGA
         66 ACCTTTCTTTTCATAGAGCAGTTTTGAAACACTCTTTTTGTAGAATCTGGAAGTGGATATTTGGA

            **                         *          *  
     473830 AAGCTTTGAGGATTTCGTTGGAAACGGGAATATCTTCAAAT
        131 GCGCTTTGAGGATTTCGTTGGAAACGGAAATATCTTCATAT

                *    *            *           * *       *  *    *            
     473871 AAAATCTAGCCAGAAGCATTCTAAGAAACATCTTAGGGATGTTTACATTCAAGTCACAGAGTTGA
          1 AAAAACTAGACAGAAGCATTCTCAGAAACATCTTTGTGATGTTTGCACTCAACTCACAGAGTTGA

              *  * *     *       *         *    * *   **      *      *    *  
     473936 ACATTCCCTTTCACAGAGCAGGTTTGAAACAATCTTCTCGTACTATCTGGCAGTGGACATTTTGA
         66 ACCTTTCTTTTCATAGAGCAGTTTTGAAACACTCTTTTTGTAGAATCTGGAAGTGGATATTTGGA

              * *   *  ** ** *  *   *            ** *
     474001 GCTCCTTGGGGCCTATGCTGAAAAAGGAAATATCTTCCGAC
        131 GCGCTTTGAGGATTTCGTTGGAAACGGAAATATCTTCATAT

                                 *     *  **          *           **    *    
     474042 AAAAACTAGACAGAAGCATTCGCAGAATCACGTTTGTGATGTGTGCACTCAACTGTCAGAATTGA
          1 AAAAACTAGACAGAAGCATTCTCAGAAACATCTTTGTGATGTTTGCACTCAACTCACAGAGTTGA

                 **   ** *      *              *             * *            *
     474107 ACCTTGGTTTGGACAGAGCACTTTTGAAACACTCTCTTTGTAGAATCTGCAGGTGGATATTTGGC
         66 ACCTTTCTTTTCATAGAGCAGTTTTGAAACACTCTTTTTGTAGAATCTGGAAGTGGATATTTGGA

            **                         *   *      * *
     474172 TAGCTTTGAGGATTTCGTTGGAAACGGTAATGTCTTCAAAG
        131 GCGCTTTGAGGATTTCGTTGGAAACGGAAATATCTTCATAT

                *                         *   *     *      *    *            
     474213 AAAATCTAGACAGAAGCATTCTCAGAAACACCTTCGTGATATTTGCAATCAAGTCACAGAGTTGA
          1 AAAAACTAGACAGAAGCATTCTCAGAAACATCTTTGTGATGTTTGCACTCAACTCACAGAGTTGA

                 * *             *  *                  *             *       
     474278 ACCTTCCGTTTCATAGAGCAGGTTGGAAACACTCTTTTTGTAGTATCTGGAAGTGGACATTTGGA
         66 ACCTTTCTTTTCATAGAGCAGTTTTGAAACACTCTTTTTGTAGAATCTGGAAGTGGATATTTGGA

             *         ** *    * *   *            **  
     474343 GGGCTTTGTAGCCTATC-TGGAAAAAGGAAATATCTTCCCAT
        131 GCGCTTTG-AGGATTTCGTTGGAAACGGAAATATCTTCATAT

            *  ** *   *     * *            *   *  *   * **        *  * * *   
     474384 GAATGCGAGATAGAAGTAATCTCAGAAACATGTTTATGCTGTATCTACTCAACTAACTGTGCTGA
          1 AAAAACTAGACAGAAGCATTCTCAGAAACATCTTTGTGATGTTTGCACTCAACTCACAGAGTTGA

              *     *  *               *        *  * *       *               
     474449 ACATTTCTATTGATAGAGCAGTTTTGAGACACTCTTCTTTTGGAATCTGCAAGTGGATATTTGGA
         66 ACCTTTCTTTTCATAGAGCAGTTTTGAAACACTCTTTTTGTAGAATCTGGAAGTGGATATTTGGA

            ** *                        *     *      
     474514 TAGATTTGAGGATTTCGTTGGAAACGG-GAT-T-AT-ATAT
        131 GCGCTTTGAGGATTTCGTTGGAAACGGAAATATCTTCATAT

                 *       *               *                 **  *   *         
     474551 AAAAAGTAGACAGCAGCATTCTCAGAAACTTCTTTGTGATGTTTGCATCCAGCTCTCAGAGTTGA
          1 AAAAACTAGACAGAAGCATTCTCAGAAACATCTTTGTGATGTTTGCACTCAACTCACAGAGTTGA

            * *  * *          *  *        *        *   **        *  **       
     474616 GCATTCCCTTTCATAGAGTAGGTTTGAAACCCTCTTTTTATAGTGTCTGGAAGCGGGCATTTGGA
         66 ACCTTTCTTTTCATAGAGCAGTTTTGAAACACTCTTTTTGTAGAATCTGGAAGTGGATATTTGGA

                   *    * * **  **  **          * *  *
     474681 GCGCTTTCAGGCCTATGCTTAAAATAGGAAATATCTACCTAC
        131 GCGCTTTGAGG-ATTTCGTTGGAAACGGAAATATCTTCATAT

             *                    *    *  **          ** *        *    *     
     474723 AGAAACTAGACAGAAGCATTCTGAGAATCACGTTTGTGATGTGGGTACTCAACTAACAGTGTTGA
          1 AAAAACTAGACAGAAGCATTCTCAGAAACATCTTTGTGATGTTTGCACTCAACTCACAGAGTTGA

            *  *       *   *            *   *                *   *           
     474788 TCCATTCTTTTGATACAGCAGTTTTGAACCACACTTTTTGTAGAATCTGCAAGAGGATATTTGGA
         66 ACCTTTCTTTTCATAGAGCAGTTTTGAAACACTCTTTTTGTAGAATCTGGAAGTGGATATTTGGA

            **   *                     *   *      * *
     474853 TAGCTGTGAGGATTTCGTTGGAAACGGGAATGTCTTCAAAG
        131 GCGCTTTGAGGATTTCGTTGGAAACGGAAATATCTTCATAT

                *                         *   *            *    *            
     474894 AAAATCTAGACAGAAGCATTCTCAGAAACACCTTCGTGATGTTTGCAATCAAGTCACAGAGTTGA
          1 AAAAACTAGACAGAAGCATTCTCAGAAACATCTTTGTGATGTTTGCACTCAACTCACAGAGTTGA

                 * *             *  *           *      *             *       
     474959 ACCTTCCGTTTCATAGAGCAGGTTGGAAACACTCTTATTGTAGTATCTGGAAGTGGACATTTGGA
         66 ACCTTTCTTTTCATAGAGCAGTTTTGAAACACTCTTTTTGTAGAATCTGGAAGTGGATATTTGGA

                   *   ** ** *  *   *            **  
     475024 GCGCTTTCAGGCCTATGGTGAAAAAGGAAATATCTTCCCAT
        131 GCGCTTTGAGGATTTCGTTGGAAACGGAAATATCTTCATAT

                  * * *      **      *   * *   *     ** ** *      *    *     
     475065 AAAAACGACATAGAAGCTATCTCAGGAACTTGTTTATGATGCATCTAATCAACTAACAGTGTTGA
          1 AAAAACTAGACAGAAGCATTCTCAGAAACATCTTTGTGATGTTTGCACTCAACTCACAGAGTTGA

                  * ** * *                         * *       *               
     475130 ACCTTTGTACTGACAGAGCAG-TTTGAAACACTCTTTTTTTGGAATCTGCAAGTGGATATTTGGA
         66 ACCTTTCTTTTCATAGAGCAGTTTTGAAACACTCTTTTTGTAGAATCTGGAAGTGGATATTTGGA

            *                  *        *   *   *    
     475194 TCGCTTTGAGGATTTCGTTTGAAACGG-GAT-GC--AATA-
        131 GCGCTTTGAGGATTTCGTTGGAAACGGAAATATCTTCATAT

                     *    *     *                 **  *   *  *    *         *
     475230 AAAAACGTACACAGCAGCATACTCAGAAA-ATACTTTGCCATATTTCCATTCAAGTCACAGAGTG
          1 AAAAAC-TAGACAGAAGCATTCTCAGAAACAT-CTTTGTGATGTTTGCACTCAACTCACAGAGTT

                *  * **            *                  *  *             *     
     475294 GAACATTCCCATTCATAGAGCAGGTTTGAAACACTCTTTTTGGAGTATCTGGAAGTGGACATTTG
         64 GAACCTTTCTTTTCATAGAGCAGTTTTGAAACACTCTTTTTGTAGAATCTGGAAGTGGATATTTG

                     ** * * ** *  *   *           * *  
     475359 GAGCGCTTTCTGAACTATGGTGAAAAAGGAAATATCTTAAAAT
        129 GAGCGCTTTGAGGATTTCGTTGGAAACGGAAATATCTTCATAT

            *     *               *      * *          *  **      **  *  *    
     475402 GAAAACAAGACAGAAGCATTCTGAGAAACTTATTTGTGATGTGTGTCCTCAACAAACGGACTTGA
          1 AAAAACTAGACAGAAGCATTCTCAGAAACATCTTTGTGATGTTTGCACTCAACTCACAGAGTTGA

                   *      **  * *  *  *             *   *    * * *           
     475467 ACCTTTCGTTTCATGCAGTACTTCTGGAACACTCTTTTTGAAGATTCTGCATGCGGATATTTGGA
         66 ACCTTTCTTTTCATAGAGCAGTTTTGAAACACTCTTTTTGTAGAATCTGGAAGTGGATATTTGGA

            **                            **   *   * 
     475532 TAGCTTTGAGGATTTCGTTGGAAACGG---GCT-TACATGT
        131 GCGCTTTGAGGATTTCGTTGGAAACGGAAATATCTTCATAT

                 *       *               *        *   *    *    *       *    
     475569 AAAAATTAGACAGCAGCATTCTCAGAAACTTCTTTGTGGTGTCTGCATTCAAGTCACAGAATTGA
          1 AAAAACTAGACAGAAGCATTCTCAGAAACATCTTTGTGATGTTTGCACTCAACTCACAGAGTTGA

              * ** * **            *  * *      *       *    *        *       
     475634 ACTTCCCCTCACATAGAGCAGTTGTGCAGCACTCTATTTGTAGTATCTCGAAGTGGACATTTGGA
         66 ACCTTTCTTTTCATAGAGCAGTTTTGAAACACTCTTTTTGTAGAATCTGGAAGTGGATATTTGGA

             *         ** *    * *   *            **  
     475699 GGGCTTTGTAGCCTATC-TGGAAAAAGGAAATATCTTCCCAT
        131 GCGCTTTG-AGGATTTCGTTGGAAACGGAAATATCTTCATAT

            *  ** *   *     * *            *   *  *   * **        *  * * *   
     475740 GAATGCGAGATAGAAGTAATCTCAGAAACATGTTTATGCTGTATCTACTCAACTAACTGTGCTGA
          1 AAAAACTAGACAGAAGCATTCTCAGAAACATCTTTGTGATGTTTGCACTCAACTCACAGAGTTGA

              *     *  *               *        *  * *       *               
     475805 ACATTTCTATTGATAGAGCAGTTTTGAGACACTCTTCTTTTGGAATCTGCAAGTGGATATTTGGA
         66 ACCTTTCTTTTCATAGAGCAGTTTTGAAACACTCTTTTTGTAGAATCTGGAAGTGGATATTTGGA

            ** *                        *     *      
     475870 TAGATTTGAGGATTTCGTTGGAAACGG-GAT-T-AT-ATAT
        131 GCGCTTTGAGGATTTCGTTGGAAACGGAAATATCTTCATAT

                 *       *               *                 **  *   *         
     475907 AAAAAGTAGACAGCAGCATTCTCAGAAACTTCTTTGTGATGTTTGCATCCAGCTCTCAGAGTTGA
          1 AAAAACTAGACAGAAGCATTCTCAGAAACATCTTTGTGATGTTTGCACTCAACTCACAGAGTTGA

              *  * *          *  *        *        *   **        *  **       
     475972 ACATTCCCTTTCATAGAGTAGGTTTGAAACCCTCTTTTTATAGTGTCTGGAAGCGGGCATTTGGA
         66 ACCTTTCTTTTCATAGAGCAGTTTTGAAACACTCTTTTTGTAGAATCTGGAAGTGGATATTTGGA

                   *    * * **  **  **          * *   
     476037 GCGCTTTCAGGCCTATGCTTAAAATAGGAAATATCTACCTAT
        131 GCGCTTTGAGG-ATTTCGTTGGAAACGGAAATATCTTCATAT

             *                    *    *  **          ** *        *    *     
     476079 AGAAACTAGACAGAAGCATTCTGAGAATCACGTTTGTGATGTGGGTACTCAACTAACAGTGTTGA
          1 AAAAACTAGACAGAAGCATTCTCAGAAACATCTTTGTGATGTTTGCACTCAACTCACAGAGTTGA

            *  *       *   *            *   *                *               
     476144 TCCATTCTTTTGATACAGCAGTTTTGAACCACACTTTTTGTAGAATCTGCAAGTGGATATTTGGA
         66 ACCTTTCTTTTCATAGAGCAGTTTTGAAACACTCTTTTTGTAGAATCTGGAAGTGGATATTTGGA

            **   *                     *   *        *
     476209 TAGCTGTGAGGATTTCGTTGGAAACGGGAATGTCTTCATAG
        131 GCGCTTTGAGGATTTCGTTGGAAACGGAAATATCTTCATAT

                **                     * * **         *  **   *   *          
     476250 AAAATTTAGACAGAAGCATTCTCAGAACCTTGATTGTGATGTGTGTTCTCCACTAACAGAGTTGA
          1 AAAAACTAGACAGAAGCATTCTCAGAAACATCTTTGTGATGTTTGCACTCAACTCACAGAGTTGA

                       * *   * *   *       *       *                         
     476315 ACCTTTCTTTTGACAGAACTGTTCTGAAACATTCTTTTTATAGAATCTGGAAGTGGATATTTGGA
         66 ACCTTTCTTTTCATAGAGCAGTTTTGAAACACTCTTTTTGTAGAATCTGGAAGTGGATATTTGGA

            **                         *          *  
     476380 AAGCTTTGAGGATTTCGTTGGAAACGGGAATATCTTCAAAT
        131 GCGCTTTGAGGATTTCGTTGGAAACGGAAATATCTTCATAT

                *    *            *           * *       *  *    *            
     476421 AAAATCTAGCCAGAAGCATTCTAAGAAACATCTTAGGGATGTTTACATTCAAGTCACAGAGTTGA
          1 AAAAACTAGACAGAAGCATTCTCAGAAACATCTTTGTGATGTTTGCACTCAACTCACAGAGTTGA

              *  * *     *       *         *    * *   **      *      *    *  
     476486 ACATTCCCTTTCACAGAGCAGGTTTGAAACAATCTTCTCGTACTATCTGGCAGTGGACATTTTGA
         66 ACCTTTCTTTTCATAGAGCAGTTTTGAAACACTCTTTTTGTAGAATCTGGAAGTGGATATTTGGA

              * *   *  ** ** *  *   *            ** *
     476551 GCTCCTTGGGGCCTATGCTGAAAAAGGAAATATCTTCCGAC
        131 GCGCTTTGAGGATTTCGTTGGAAACGGAAATATCTTCATAT

                                 *     *  **          *           **    *    
     476592 AAAAACTAGACAGAAGCATTCGCAGAATCACGTTTGTGATGTGTGCACTCAACTGTCAGAATTGA
          1 AAAAACTAGACAGAAGCATTCTCAGAAACATCTTTGTGATGTTTGCACTCAACTCACAGAGTTGA

                 **   ** *      *                            * *            *
     476657 ACCTTGGTTTGGACAGAGCACTTTTGAAACACTCTTTTTGTAGAATCTGCAGGTGGATATTTGGC
         66 ACCTTTCTTTTCATAGAGCAGTTTTGAAACACTCTTTTTGTAGAATCTGGAAGTGGATATTTGGA

            **                         *   *      * *
     476722 TAGCTTTGAGGATTTCGTTGGAAACGGTAATGTCTTCAAAG
        131 GCGCTTTGAGGATTTCGTTGGAAACGGAAATATCTTCATAT

                *                         *   *            *    *            
     476763 AAAATCTAGACAGAAGCATTCTCAGAAACAACTTCGTGATGTTTGCAATCAAGTCACAGAGTTGA
          1 AAAAACTAGACAGAAGCATTCTCAGAAACATCTTTGTGATGTTTGCACTCAACTCACAGAGTTGA

                 * *             *  *                  *             *       
     476828 ACCTTCCGTTTCATAGAGCAGGTTGGAAACACTCTTTTTGTAGTATCTGGAAGTGGACATTTGGA
         66 ACCTTTCTTTTCATAGAGCAGTTTTGAAACACTCTTTTTGTAGAATCTGGAAGTGGATATTTGGA

             *         ** *    * *   *            **  
     476893 GGGCTTTGTAGCCTATC-TGGAAAAAGGAAATATCTTCCCAT
        131 GCGCTTTG-AGGATTTCGTTGGAAACGGAAATATCTTCATAT

            *  ** *   *     * *            *   *  *   * **        *  * * *   
     476934 GAATGCGAGATAGAAGTAATCTCAGAAACATGTTTATGCTGTATCTACTCAACTAACTGTGCTGA
          1 AAAAACTAGACAGAAGCATTCTCAGAAACATCTTTGTGATGTTTGCACTCAACTCACAGAGTTGA

              *     *  *               *        *  * *       *               
     476999 ACATTTCTATTGATAGAGCAGTTTTGAGACACTCTTCTTTTGGAATCTGCAAGTGGATATTTGGA
         66 ACCTTTCTTTTCATAGAGCAGTTTTGAAACACTCTTTTTGTAGAATCTGGAAGTGGATATTTGGA

            ** *                        *     *      
     477064 TAGATTTGAGGATTTCGTTGGAAACGG-GAT-T-AT-ATAT
        131 GCGCTTTGAGGATTTCGTTGGAAACGGAAATATCTTCATAT

                 *       *               *                 **  *   *         
     477101 AAAAAGTAGACAGCAGCATTCTCAGAAACTTCTTTGTGATGTTTGCATCCAGCTCTCAGAGTTGA
          1 AAAAACTAGACAGAAGCATTCTCAGAAACATCTTTGTGATGTTTGCACTCAACTCACAGAGTTGA

            * *  * *          *  *        *        *   **        *  **       
     477166 GCATTCCCTTTCATAGAGTAGGTTTGAAACCCTCTTTTTATAGTGTCTGGAAGCGGGCATTTGGA
         66 ACCTTTCTTTTCATAGAGCAGTTTTGAAACACTCTTTTTGTAGAATCTGGAAGTGGATATTTGGA

                   *    * * **  **  **          * *  *
     477231 GCGCTTTCAGGCCTATGCTTAAAATAGGAAATATCTACCTAC
        131 GCGCTTTGAGG-ATTTCGTTGGAAACGGAAATATCTTCATAT

             *                    *    *  **          ** *        *    *     
     477273 AGAAACTAGACAGAAGCATTCTGAGAATCACGTTTGTGATGTGGGTACTCAACTAACAGTGTTGA
          1 AAAAACTAGACAGAAGCATTCTCAGAAACATCTTTGTGATGTTTGCACTCAACTCACAGAGTTGA

            *  *       *   *            *   *                *               
     477338 TCCATTCTTTTGATACAGCAGTTTTGAACCACACTTTTTGTAGAATCTGCAAGTGGATATTTGGA
         66 ACCTTTCTTTTCATAGAGCAGTTTTGAAACACTCTTTTTGTAGAATCTGGAAGTGGATATTTGGA

             *   *                     *   *      * *
     477403 GAGCTGTGAGGATTTCGTTGGAAACGGGAATGTCTTCAAAG
        131 GCGCTTTGAGGATTTCGTTGGAAACGGAAATATCTTCATAT

                *                         *   *            *    *            
     477444 AAAATCTAGACAGAAGCATTCTCAGAAACACCTTCGTGATGTTTGCAATCAAGTCACAGAGTTGA
          1 AAAAACTAGACAGAAGCATTCTCAGAAACATCTTTGTGATGTTTGCACTCAACTCACAGAGTTGA

                 * *             *  *           *      *             *       
     477509 ACCTTCCGTTTCATAGAGCAGGTTGGAAACACTCTTATTGTAGTATCTGGAAGTGGACATTTGGA
         66 ACCTTTCTTTTCATAGAGCAGTTTTGAAACACTCTTTTTGTAGAATCTGGAAGTGGATATTTGGA

                   *   ** ** *  *   *            **  
     477574 GCGCTTTCAGGCCTATGGTGAAAAAGGAAATATCTTCCCAT
        131 GCGCTTTGAGGATTTCGTTGGAAACGGAAATATCTTCATAT

            *     * * *      **      *   * *   *     ** ** *      *    *     
     477615 GAAAACGACATAGAAGCTATCTCAGGAACTTGTTTATGATGCATCTAATCAACTAACAGCGTTGA
          1 AAAAACTAGACAGAAGCATTCTCAGAAACATCTTTGTGATGTTTGCACTCAACTCACAGAGTTGA

                  * ** * *                         * *       *               
     477680 ACCTTTGTACTGACAGAGCAG-TTTGAAACACTCTTTTTTTGGAATCTGCAAGTGGATATTTGGA
         66 ACCTTTCTTTTCATAGAGCAGTTTTGAAACACTCTTTTTGTAGAATCTGGAAGTGGATATTTGGA

            *                           *   *   *    
     477744 TCGCTTTGAGGATTTCGTTGGAAACGG-GAT-GC--AATAT
        131 GCGCTTTGAGGATTTCGTTGGAAACGGAAATATCTTCATAT

                **  *    *     *                 **  *   *  *    *         * 
     477781 AAAACGTACACAGCAGCATACTCAGAAA-ATACTTTGCCATATTTCCATTCAAGTCACAGAGTGG
          1 AAAAACTAGACAGAAGCATTCTCAGAAACAT-CTTTGTGATGTTTGCACTCAACTCACAGAGTTG

               *  * **            *  *               *  *             *      
     477845 AACATTCCCATTCATAGAGCAGGTTGGAAACACTCTTTTTGGAGTATCTGGAAGTGGACATTTGG
         65 AACCTTTCTTTTCATAGAGCAGTTTTGAAACACTCTTTTTGTAGAATCTGGAAGTGGATATTTGG

                    ** * * ** *  *   *            **  
     477910 AGCGCTTTCTGAACTATGGTGAAAAAGGAAATATCTTCCAAT
        130 AGCGCTTTGAGGATTTCGTTGGAAACGGAAATATCTTCATAT

            *     *               *      * *          *  **      **  *  *    
     477952 GAAAACAAGACAGAAGCATTCTGAGAAACTTATTTGTGATGTGTGTCCTCAACAAACGGACTTGA
          1 AAAAACTAGACAGAAGCATTCTCAGAAACATCTTTGTGATGTTTGCACTCAACTCACAGAGTTGA

                   *      **  * *  *  *             *   *    * * *           
     478017 ACCTTTCGTTTCATGCAGTACTTCTGGAACACTCTTTTTGAAGATTCTGCATGCGGATATTTGGA
         66 ACCTTTCTTTTCATAGAGCAGTTTTGAAACACTCTTTTTGTAGAATCTGGAAGTGGATATTTGGA

            **                            **   *   * 
     478082 TAGCTTTGAGGATTTCGTTGGAAACGG---GCT-TACATGT
        131 GCGCTTTGAGGATTTCGTTGGAAACGGAAATATCTTCATAT

                 *       *               *        *   *    *    *       *    
     478119 AAAAATTAGACAGCAGCATTCTCAGAAACTTCTTTGTGGTGTCTGCATTCAAGTCACAGAATTGA
          1 AAAAACTAGACAGAAGCATTCTCAGAAACATCTTTGTGATGTTTGCACTCAACTCACAGAGTTGA

              * ** * **            *  * *      *       *             *       
     478184 ACTTCCCCTCACATAGAGCAGTTGTGCAGCACTCTATTTGTAGTATCTGGAAGTGGACATTTGGA
         66 ACCTTTCTTTTCATAGAGCAGTTTTGAAACACTCTTTTTGTAGAATCTGGAAGTGGATATTTGGA

             *         ** *    * *   *            **  
     478249 GGGCTTTGTAGCCTATC-TGGAAAAAGGAAATATCTTCCCAT
        131 GCGCTTTG-AGGATTTCGTTGGAAACGGAAATATCTTCATAT

            *  ** *   *     * *            *   *  *   * **        *  * * *   
     478290 GAATGCGAGATAGAAGTAATCTCAGAAACATGTTTATGCTGTATCTACTCAACTAACTGTGCTGA
          1 AAAAACTAGACAGAAGCATTCTCAGAAACATCTTTGTGATGTTTGCACTCAACTCACAGAGTTGA

              *     *  *               *        *  * *       *               
     478355 ACATTTCTATTGATAGAGCAGTTTTGAGACACTCTTCTTTTGGAATCTGCAAGTGGATATTTGGA
         66 ACCTTTCTTTTCATAGAGCAGTTTTGAAACACTCTTTTTGTAGAATCTGGAAGTGGATATTTGGA

            ** *                        *     *   *  
     478420 TAGATTTGAGGATTTCGTTGGAAACGG-GAT-T-AT-AGAT
        131 GCGCTTTGAGGATTTCGTTGGAAACGGAAATATCTTCATAT

            *    *       *               *                 **  *   *         
     478457 CAAAAGTAGACAGCAGCATTCTCAGAAACTTCTTTGTGATGTTTGCATCCAGCTCTCAGAGTTGA
          1 AAAAACTAGACAGAAGCATTCTCAGAAACATCTTTGTGATGTTTGCACTCAACTCACAGAGTTGA

              *  * *          *  *        *        *   **        *  **       
     478522 ACATTCCCTTTCATAGAGTAGGTTTGAAACCCTCTTTTTATAGTGTCTGGAAGCGGGCATTTGGA
         66 ACCTTTCTTTTCATAGAGCAGTTTTGAAACACTCTTTTTGTAGAATCTGGAAGTGGATATTTGGA

                *  *    * * **  **  **          * *  *
     478587 GCGCATTCAGGCCTATGCTTAAAATAGGAAATATCTACCTAC
        131 GCGCTTTGAGG-ATTTCGTTGGAAACGGAAATATCTTCATAT

             *                    *    *  **          ** *        *    *     
     478629 AGAAACTAGACAGAAGCATTCTGAGAATCACGTTTGTGATGTGGGTACTCAACTAACAGTGTTGA
          1 AAAAACTAGACAGAAGCATTCTCAGAAACATCTTTGTGATGTTTGCACTCAACTCACAGAGTTGA

            *  *       *   *            *   *                *   *           
     478694 TCCATTCTTTTGATACAGCAGTTTTGAACCACACTTTTTGTAGAATCTGCAAGAGGATATTTGGA
         66 ACCTTTCTTTTCATAGAGCAGTTTTGAAACACTCTTTTTGTAGAATCTGGAAGTGGATATTTGGA

            **   *                     *   *      * *
     478759 TAGCTGTGAGGATTTCGTTGGAAACGGGAATGTCTTCAAAG
        131 GCGCTTTGAGGATTTCGTTGGAAACGGAAATATCTTCATAT

                *                         *   *            *    *            
     478800 AAAATCTAGACAGAAGCATTCTCAGAAACACCTTCGTGATGTTTGCAATCAAGTCACAGAGTTGA
          1 AAAAACTAGACAGAAGCATTCTCAGAAACATCTTTGTGATGTTTGCACTCAACTCACAGAGTTGA

                 * *             *  *           *      *             *       
     478865 ACCTTCCGTTTCATAGAGCAGGTTGGAAACACTCTTATTGTAGTATCTGGAAGTGGACATTTGGA
         66 ACCTTTCTTTTCATAGAGCAGTTTTGAAACACTCTTTTTGTAGAATCTGGAAGTGGATATTTGGA

                   *   ** ** *  *   *            **  
     478930 GCGCTTTCAGGCCTATGGTGAAAAAGGAAATATCTTCCCAT
        131 GCGCTTTGAGGATTTCGTTGGAAACGGAAATATCTTCATAT

                  * * *      **      *   * *   *     ** ** *      *    *     
     478971 AAAAACGACATAGAAGCTATCTCAGGAACTTGTTTATGATGCATCTAATCAACTAACAGTGTTGA
          1 AAAAACTAGACAGAAGCATTCTCAGAAACATCTTTGTGATGTTTGCACTCAACTCACAGAGTTGA

                  * ** * *                         * *       *               
     479036 ACCTTTGTACTGACAGAGCAG-TTTGAAACACTCTTTTTTTGGAATCTGCAAGTGGATATTTGGA
         66 ACCTTTCTTTTCATAGAGCAGTTTTGAAACACTCTTTTTGTAGAATCTGGAAGTGGATATTTGGA

            *                           *   *   *    
     479100 TCGCTTTGAGGATTTCGTTGGAAACGG-GAT-GC--AATAT
        131 GCGCTTTGAGGATTTCGTTGGAAACGGAAATATCTTCATAT

                **  *    *     *                 **  *   *  *    *         * 
     479137 AAAACGTACACAGCAGCATACTCAGAAA-ATACTTTGCCATATTTCCATTCAAGTCACAGAGTGG
          1 AAAAACTAGACAGAAGCATTCTCAGAAACAT-CTTTGTGATGTTTGCACTCAACTCACAGAGTTG

               *  * **            *  *               *  *             *      
     479201 AACATTCCCATTCATAGAGCAGGTTGGAAACACTCTTTTTGGAGTATCTGGAAGTGGACATTTGG
         65 AACCTTTCTTTTCATAGAGCAGTTTTGAAACACTCTTTTTGTAGAATCTGGAAGTGGATATTTGG

                    ** * * ** *  *   *            **  
     479266 AGCGCTTTCTGAACTATGGTGAAAAAGGAAATATCTTCCAAT
        130 AGCGCTTTGAGGATTTCGTTGGAAACGGAAATATCTTCATAT

            *     *               *      * *          *  **      **  *  *    
     479308 GAAAACAAGACAGAAGCATTCTGAGAAACTTATTTGTGATGTGTGTCCTCAACAAACGGACTTGA
          1 AAAAACTAGACAGAAGCATTCTCAGAAACATCTTTGTGATGTTTGCACTCAACTCACAGAGTTGA

                   *      **  * *  *  *             *   *    * * *           
     479373 ACCTTTCGTTTCATGCAGTACTTCTGGAACACTCTTTTTGAAGATTCTGCATGCGGATATTTGGA
         66 ACCTTTCTTTTCATAGAGCAGTTTTGAAACACTCTTTTTGTAGAATCTGGAAGTGGATATTTGGA

            **                            **   *   * 
     479438 TAGCTTTGAGGATTTCGTTGGAAACGG---GCT-TACATGT
        131 GCGCTTTGAGGATTTCGTTGGAAACGGAAATATCTTCATAT

                 *       *               *        *   *    *    *       *    
     479475 AAAAATTAGACAGCAGCATTCTCAGAAACTTCTTTGTGGTGTCTGCATTCAAGTCACAGAATTGA
          1 AAAAACTAGACAGAAGCATTCTCAGAAACATCTTTGTGATGTTTGCACTCAACTCACAGAGTTGA

              * ** * **            *  * *      *       *             *       
     479540 ACTTCCCCTCACATAGAGCAGTTGTGCAGCACTCTATTTGTAGTATCTGGAAGTGGACATTTGGA
         66 ACCTTTCTTTTCATAGAGCAGTTTTGAAACACTCTTTTTGTAGAATCTGGAAGTGGATATTTGGA

             *         ** *    * *   *            **  
     479605 GGGCTTTGTAGCCTATC-TGGAAAAAGGAAATATCTTCCCAT
        131 GCGCTTTG-AGGATTTCGTTGGAAACGGAAATATCTTCATAT

            *  ** *   *     * *            *   *  *   * **        *  * * *   
     479646 GAATGCGAGATAGAAGTAATCTCAGAAACATGTTTATGCTGTATCTACTCAACTAACTGTGCTGA
          1 AAAAACTAGACAGAAGCATTCTCAGAAACATCTTTGTGATGTTTGCACTCAACTCACAGAGTTGA

              *     *  *               *        *  * *       *               
     479711 ACATTTCTATTGATAGAGCAGTTTTGAGACACTCTTCTTTTGGAATCTGCAAGTGGATATTTGGA
         66 ACCTTTCTTTTCATAGAGCAGTTTTGAAACACTCTTTTTGTAGAATCTGGAAGTGGATATTTGGA

            ** *                        *     *   *  
     479776 TAGATTTGAGGATTTCGTTGGAAACGG-GAT-T-AT-AGAT
        131 GCGCTTTGAGGATTTCGTTGGAAACGGAAATATCTTCATAT

            *    *       *               *                 **  *   *         
     479813 CAAAAGTAGACAGCAGCATTCTCAGAAACTTCTTTGTGATGTTTGCATCCAGCTCTCAGAGTTGA
          1 AAAAACTAGACAGAAGCATTCTCAGAAACATCTTTGTGATGTTTGCACTCAACTCACAGAGTTGA

              *  * *          *  *        *        *   **        *  **       
     479878 ACATTCCCTTTCATAGAGTAGGTTTGAAACCCTCTTTTTATAGTGTCTGGAAGCGGGCATTTGGA
         66 ACCTTTCTTTTCATAGAGCAGTTTTGAAACACTCTTTTTGTAGAATCTGGAAGTGGATATTTGGA

                *  *    * * **  **  **          * *  *
     479943 GCGCATTCAGGCCTATGCTTAAAATAGGAAATATCTACCTAC
        131 GCGCTTTGAGG-ATTTCGTTGGAAACGGAAATATCTTCATAT

             *                    *    *  **          ** *        *    *     
     479985 AGAAACTAGACAGAAGCATTCTGAGAATCACGTTTGTGATGTGGGTACTCAACTAACAGTGTTGA
          1 AAAAACTAGACAGAAGCATTCTCAGAAACATCTTTGTGATGTTTGCACTCAACTCACAGAGTTGA

            *  *       *   *            *   *                *   *           
     480050 TCCATTCTTTTGATACAGCAGTTTTGAACCACACTTTTTGTAGAATCTGCAAGAGGATATTTGGA
         66 ACCTTTCTTTTCATAGAGCAGTTTTGAAACACTCTTTTTGTAGAATCTGGAAGTGGATATTTGGA

            **   *                     *   *      * *
     480115 TAGCTGTGAGGATTTCGTTGGAAACGGGAATGTCTTCAAAG
        131 GCGCTTTGAGGATTTCGTTGGAAACGGAAATATCTTCATAT

                *                         *   *            *    *            
     480156 AAAATCTAGACAGAAGCATTCTCAGAAACACCTTCGTGATGTTTGCAATCAAGTCACAGAGTTGA
          1 AAAAACTAGACAGAAGCATTCTCAGAAACATCTTTGTGATGTTTGCACTCAACTCACAGAGTTGA

                 * *             *  *           *      *             *       
     480221 ACCTTCCGTTTCATAGAGCAGGTTGGAAACACTCTTATTGTAGTATCTGGAAGTGGACATTTGGA
         66 ACCTTTCTTTTCATAGAGCAGTTTTGAAACACTCTTTTTGTAGAATCTGGAAGTGGATATTTGGA

                   *   ** ** *  *   *            **  
     480286 GCGCTTTCAGGCCTATGGTGAAAAAGGAAATATCTTCCCAT
        131 GCGCTTTGAGGATTTCGTTGGAAACGGAAATATCTTCATAT

                  * * *      **      *   * *   *     ** ** *      *    *     
     480327 AAAAACGACATAGAAGCTATCTCAGGAACTTGTTTATGATGCATCTAATCAACTAACAGTGTTGA
          1 AAAAACTAGACAGAAGCATTCTCAGAAACATCTTTGTGATGTTTGCACTCAACTCACAGAGTTGA

                  * ** * *                         * *       *               
     480392 ACCTTTGTACTGACAGAGCAG-TTTGAAACACTCTTTTTTTGGAATCTGCAAGTGGATATTTGGA
         66 ACCTTTCTTTTCATAGAGCAGTTTTGAAACACTCTTTTTGTAGAATCTGGAAGTGGATATTTGGA

            *                           *   *   *    
     480456 TCGCTTTGAGGATTTCGTTGGAAACGG-GAT-GC--AATAT
        131 GCGCTTTGAGGATTTCGTTGGAAACGGAAATATCTTCATAT

                **  *    *     *                 **  *   *  *    *         * 
     480493 AAAACGTACACAGCAGCATACTCAGAAA-ATACTTTGCCATATTTCCATTCAAGTCACAGAGTGG
          1 AAAAACTAGACAGAAGCATTCTCAGAAACAT-CTTTGTGATGTTTGCACTCAACTCACAGAGTTG

               *  * **            *                  *  *             *      
     480557 AACATTCCCATTCATAGAGCAGGTTTGAAACACTCTTTTTGGAGTATCTGGAAGTGGACATTTGG
         65 AACCTTTCTTTTCATAGAGCAGTTTTGAAACACTCTTTTTGTAGAATCTGGAAGTGGATATTTGG

                    ** * * ** *  *   *            **  
     480622 AGCGCTTTCTGAACTATGGTGAAAAAGGAAATATCTTCCAAT
        130 AGCGCTTTGAGGATTTCGTTGGAAACGGAAATATCTTCATAT

            *     *               *      * *          *  **      **  *  *    
     480664 GAAAACAAGACAGAAGCATTCTGAGAAACTTATTTGTGATGTGTGTCCTCAACAAACGGACTTGA
          1 AAAAACTAGACAGAAGCATTCTCAGAAACATCTTTGTGATGTTTGCACTCAACTCACAGAGTTGA

                   *      **  * *  *  *             *   *    * *             
     480729 ACCTTTCGTTTCATGCAGTACTTCTGGAACACTCTTTTTGAAGATTCTGCATGTGGATATTTGGA
         66 ACCTTTCTTTTCATAGAGCAGTTTTGAAACACTCTTTTTGTAGAATCTGGAAGTGGATATTTGGA

            **                            **   *   * 
     480794 TAGCTTTGAGGATTTCGTTGGAAACGG---GCT-TACATGT
        131 GCGCTTTGAGGATTTCGTTGGAAACGGAAATATCTTCATAT

                 *       *               *        *   *    *    *       *    
     480831 AAAAATTAGACAGCAGCATTCTCAGAAACTTCTTTGTGGTGTCTGCATTCAAGTCACAGAATTGA
          1 AAAAACTAGACAGAAGCATTCTCAGAAACATCTTTGTGATGTTTGCACTCAACTCACAGAGTTGA

              * ** * **            *  * *      *       *             *       
     480896 ACTTCCCCTCACATAGAGCAGTTGTGCAGCACTCTATTTGTAGTATCTGGAAGTGGACATTTGGA
         66 ACCTTTCTTTTCATAGAGCAGTTTTGAAACACTCTTTTTGTAGAATCTGGAAGTGGATATTTGGA

             *         ** *    * *   *            **  
     480961 GGGCTTTGTAGCCTATC-TGGAAAAAGGAAATATCTTCCCAT
        131 GCGCTTTG-AGGATTTCGTTGGAAACGGAAATATCTTCATAT

            *  ** *   *     * *            *   *  * * * **        *  * * *   
     481002 GAATGCGAGATAGAAGTAATCTCAGAAACATGTTTATGCTCTATCTACTCAACTAACTGTGCTGA
          1 AAAAACTAGACAGAAGCATTCTCAGAAACATCTTTGTGATGTTTGCACTCAACTCACAGAGTTGA

              *     *  *               *        *  * *       *               
     481067 ACATTTCTATTGATAGAGCAGTTTTGAGACACTCTTCTTTTGGAATCTGCAAGTGGATATTTGGA
         66 ACCTTTCTTTTCATAGAGCAGTTTTGAAACACTCTTTTTGTAGAATCTGGAAGTGGATATTTGGA

            ** *                       **     *      
     481132 TAGATTTGAGGATTTCGTTGGAAAC-GCGAT-T-AT-ATAT
        131 GCGCTTTGAGGATTTCGTTGGAAACGGAAATATCTTCATAT

                 *       *               *                 **  *   *         
     481169 AAAAAGTAGACAGCAGCATTCTCAGAAACTTCTTTGTGATGTTTGCATCCAGCTCTCAGAGTTGA
          1 AAAAACTAGACAGAAGCATTCTCAGAAACATCTTTGTGATGTTTGCACTCAACTCACAGAGTTGA

            * *  * *          *  *        *        *   **        *  **       
     481234 GCATTCCCTTTCATAGAGTAGGTTTGAAACCCTCTTTTTATAGTGTCTGGAAGCGGGCATTTGGA
         66 ACCTTTCTTTTCATAGAGCAGTTTTGAAACACTCTTTTTGTAGAATCTGGAAGTGGATATTTGGA

                   *    * * **  **  **          * *  *
     481299 GCGCTTTCAGGCCTATGCTTAAAATAGGAAATATCTACCTAC
        131 GCGCTTTGAGG-ATTTCGTTGGAAACGGAAATATCTTCATAT

             *                    *    *  **          ** *        *    *     
     481341 AGAAACTAGACAGAAGCATTCTGAGAATCACGTTTGTGATGTGGGTACTCAACTAACAGTGTTGA
          1 AAAAACTAGACAGAAGCATTCTCAGAAACATCTTTGTGATGTTTGCACTCAACTCACAGAGTTGA

            *  *       *   *            *   *                *               
     481406 TCCATTCTTTTGATACAGCAGTTTTGAACCACACTTTTTGTAGAATCTGCAAGTGGATATTTGGA
         66 ACCTTTCTTTTCATAGAGCAGTTTTGAAACACTCTTTTTGTAGAATCTGGAAGTGGATATTTGGA

            **   *   *                 *   *      * *
     481471 TAGCTGTGAAGATTTCGTTGGAAACGGGAATGTCTTCAAAG
        131 GCGCTTTGAGGATTTCGTTGGAAACGGAAATATCTTCATAT

                *                         *   *            *    *            
     481512 AAAATCTAGACAGAAGCATTCTCAGAAACACCTTCGTGATGTTTGCAATCAAGTCACAGAGTTGA
          1 AAAAACTAGACAGAAGCATTCTCAGAAACATCTTTGTGATGTTTGCACTCAACTCACAGAGTTGA

                 * *             *  *           *      *             *       
     481577 ACCTTCCGTTTCATAGAGCAGGTTGGAAACACTCTTATTGTAGTATCTGGAAGTGGACATTTGGA
         66 ACCTTTCTTTTCATAGAGCAGTTTTGAAACACTCTTTTTGTAGAATCTGGAAGTGGATATTTGGA

                   *   ** ** *  *   *            **  
     481642 GCGCTTTCAGGCCTATGGTGAAAAAGGAAATATCTTCCCAT
        131 GCGCTTTGAGGATTTCGTTGGAAACGGAAATATCTTCATAT

                  * * *      **      *   * *   *     ** ** *      *    *     
     481683 AAAAACGACATAGAAGCTATCTCAGGAACTTGTTTATGATGCATCTAATCAACTAACAGTGTTGA
          1 AAAAACTAGACAGAAGCATTCTCAGAAACATCTTTGTGATGTTTGCACTCAACTCACAGAGTTGA

                  * ** * *                         * *       *               
     481748 ACCTTTGTACTGACAGAGCAG-TTTGAAACACTCTTTTTTTGGAATCTGCAAGTGGATATTTGGA
         66 ACCTTTCTTTTCATAGAGCAGTTTTGAAACACTCTTTTTGTAGAATCTGGAAGTGGATATTTGGA

            *                           *   *   *    
     481812 TCGCTTTGAGGATTTCGTTGGAAACGG-GAT-GC--AATAT
        131 GCGCTTTGAGGATTTCGTTGGAAACGGAAATATCTTCATAT

                **  *    *     *                 **  *   *  *    *         * 
     481849 AAAACGTACACAGCAGCATACTCAGAAA-ATACTTTGCCATATTTCCATTCAAGTCACAGAGTGG
          1 AAAAACTAGACAGAAGCATTCTCAGAAACAT-CTTTGTGATGTTTGCACTCAACTCACAGAGTTG

               *  * **            *  *               *  *             *      
     481913 AACATTCCCATTCATAGAGCAGGTTGGAAACACTCTTTTTGGAGTATCTGGAAGTGGACATTTGG
         65 AACCTTTCTTTTCATAGAGCAGTTTTGAAACACTCTTTTTGTAGAATCTGGAAGTGGATATTTGG

                    ** * * ** *  *   *            **  
     481978 AGCGCTTTCTGAACTATGGTGAAAAAGGAAATATCTTCCAAT
        130 AGCGCTTTGAGGATTTCGTTGGAAACGGAAATATCTTCATAT

            *     *               *      * *          *  **      **  *  *    
     482020 GAAAACAAGACAGAAGCATTCTGAGAAACTTATTTGTGATGTGTGTCCTCAACAAACGGACTTGA
          1 AAAAACTAGACAGAAGCATTCTCAGAAACATCTTTGTGATGTTTGCACTCAACTCACAGAGTTGA

                   *      **  * *  *  *             *   *    * * *           
     482085 ACCTTTCGTTTCATGCAGTACTTCTGGAACACTCTTTTTGAAGATTCTGCATGCGGATATTTGGA
         66 ACCTTTCTTTTCATAGAGCAGTTTTGAAACACTCTTTTTGTAGAATCTGGAAGTGGATATTTGGA

            **                            **   *   * 
     482150 TAGCTTTGAGGATTTCGTTGGAAACGG---GCT-TACATGT
        131 GCGCTTTGAGGATTTCGTTGGAAACGGAAATATCTTCATAT

                 *       *               *        *   *    *    *       *    
     482187 AAAAATTAGACAGCAGCATTCTCAGAAACTTCTTTGTGGTGTCTGCATTCAAGTCACAGAATTGA
          1 AAAAACTAGACAGAAGCATTCTCAGAAACATCTTTGTGATGTTTGCACTCAACTCACAGAGTTGA

              * ** * **            *  * *      *       *             *       
     482252 ACTTCCCCTCACATAGAGCAGTTGTGCAGCACTCTATTTGTAGTATCTGGAAGTGGACATTTGGA
         66 ACCTTTCTTTTCATAGAGCAGTTTTGAAACACTCTTTTTGTAGAATCTGGAAGTGGATATTTGGA

             *         ** *    * *   *            **  
     482317 GGGCTTTGTAGCCTATC-TGGAAAAAGGAAATATCTTCCCAT
        131 GCGCTTTG-AGGATTTCGTTGGAAACGGAAATATCTTCATAT

            *  ** *   *     * *            *   *  *   * **        *  * * *   
     482358 GAATGCGAGATAGAAGTAATCTCAGAAACATGTTTATGCTGTATCTACTCAACTAACTGTGCTGA
          1 AAAAACTAGACAGAAGCATTCTCAGAAACATCTTTGTGATGTTTGCACTCAACTCACAGAGTTGA

              *     *  *               *        *  * *       *               
     482423 ACATTTCTATTGATAGAGCAGTTTTGAGACACTCTTCTTTTGGAATCTGCAAGTGGATATTTGGA
         66 ACCTTTCTTTTCATAGAGCAGTTTTGAAACACTCTTTTTGTAGAATCTGGAAGTGGATATTTGGA

            ** *                        *     *      
     482488 TAGATTTGAGGATTTCGTTGGAAACGG-GAT-T-AT-ATAT
        131 GCGCTTTGAGGATTTCGTTGGAAACGGAAATATCTTCATAT

                 *       *               *                 **  *   *         
     482525 AAAAAGTAGACAGCAGCATTCTCAGAAACTTCTTTGTGATGTTTGCATCCAGCTCTCAGAGTTGA
          1 AAAAACTAGACAGAAGCATTCTCAGAAACATCTTTGTGATGTTTGCACTCAACTCACAGAGTTGA

              *  * *          *  *        *        *   **        *  **       
     482590 ACATTCCCTTTCATAGAGTAGGTTTGAAACCCTCTTTTTATAGTGTCTGGAAGCGGGCATTTGGA
         66 ACCTTTCTTTTCATAGAGCAGTTTTGAAACACTCTTTTTGTAGAATCTGGAAGTGGATATTTGGA

                   *   ** ** *  *   *          * *   
     482655 GCGCTTTCAGGCCTATGCTGAAAAAGGAAATATCTACCTAT
        131 GCGCTTTGAGGATTTCGTTGGAAACGGAAATATCTTCATAT

             *                    *    *  **          ** *        *    *     
     482696 AGAAACTAGACAGAAGCATTCTGAGAATCACGTTTGTGATGTGGGTACTCAACTAACAGTGTTGA
          1 AAAAACTAGACAGAAGCATTCTCAGAAACATCTTTGTGATGTTTGCACTCAACTCACAGAGTTGA

            *  *       *   *            *   *                *               
     482761 TCCATTCTTTTGATACAGCAGTTTTGAACCACACTTTTTGTAGAATCTGCAAGTGGATATTTGGA
         66 ACCTTTCTTTTCATAGAGCAGTTTTGAAACACTCTTTTTGTAGAATCTGGAAGTGGATATTTGGA

            **   *                     *   *        *
     482826 TAGCTGTGAGGATTTCGTTGGAAACGGGAATGTCTTCATAG
        131 GCGCTTTGAGGATTTCGTTGGAAACGGAAATATCTTCATAT

                **                     * * **         *  **   *   *          
     482867 AAAATTTAGACAGAAGCATTCTCAGAACCTTGATTGTGATGTGTGTTCTCCACTAACAGAGTTGA
          1 AAAAACTAGACAGAAGCATTCTCAGAAACATCTTTGTGATGTTTGCACTCAACTCACAGAGTTGA

                       * *   * *   *       *                                 
     482932 ACCTTTCTTTTGACAGAACTGTTCTGAAACATTCTTTTTGTAGAATCTGGAAGTGGATATTTGGA
         66 ACCTTTCTTTTCATAGAGCAGTTTTGAAACACTCTTTTTGTAGAATCTGGAAGTGGATATTTGGA

            **                         *          *  
     482997 AAGCTTTGAGGATTTCGTTGGAAACGGGAATATCTTCAAAT
        131 GCGCTTTGAGGATTTCGTTGGAAACGGAAATATCTTCATAT

            *   *    *            *           * *       *  *    *            
     483038 CAAATCTAGCCAGAAGCATTCTAAGAAACATCTTAGGGATGTTTACATTCAAGTCACAGAGTTGA
          1 AAAAACTAGACAGAAGCATTCTCAGAAACATCTTTGTGATGTTTGCACTCAACTCACAGAGTTGA

              *  * *     *       *         *    * *   **      *      *    *  
     483103 ACATTCCCTTTCACAGAGCAGGTTTGAAACAATCTTCTCGTACTATCTGGCAGTGGACATTTTGA
         66 ACCTTTCTTTTCATAGAGCAGTTTTGAAACACTCTTTTTGTAGAATCTGGAAGTGGATATTTGGA

              * *   *  ** ** *  *   *            ** *
     483168 GCTCCTTGGGGCCTATGCTGAAAAAGGAAATATCTTCCGAC
        131 GCGCTTTGAGGATTTCGTTGGAAACGGAAATATCTTCATAT

                                 *     *  **          *           **    *    
     483209 AAAAACTAGACAGAAGCATTCGCAGAATCACGTTTGTGATGTGTGCACTCAACTGTCAGAATTGA
          1 AAAAACTAGACAGAAGCATTCTCAGAAACATCTTTGTGATGTTTGCACTCAACTCACAGAGTTGA

                 **   ** *      *                            * *            *
     483274 ACCTTGGTTTGGACAGAGCACTTTTGAAACACTCTTTTTGTAGAATCTGCAGGTGGATATTTGGC
         66 ACCTTTCTTTTCATAGAGCAGTTTTGAAACACTCTTTTTGTAGAATCTGGAAGTGGATATTTGGA

            **                         *   *      * *
     483339 TAGCTTTGAGGATTTCGTTGGAAACGGTAATGTCTTCAAAG
        131 GCGCTTTGAGGATTTCGTTGGAAACGGAAATATCTTCATAT

                *                         *   *            *    *            
     483380 AAAATCTAGACAGAAGCATTCTCAGAAACACCTTCGTGATGTTTGCAATCAAGTCACAGAGTTGA
          1 AAAAACTAGACAGAAGCATTCTCAGAAACATCTTTGTGATGTTTGCACTCAACTCACAGAGTTGA

                 * *             *  *                  *             *       
     483445 ACCTTCCGTTTCATAGAGCAGGTTGGAAACACTCTTTTTGTAGTATCTGGAAGTGGACATTTGGA
         66 ACCTTTCTTTTCATAGAGCAGTTTTGAAACACTCTTTTTGTAGAATCTGGAAGTGGATATTTGGA

                   *   ** ** *  *   *            **  
     483510 GCGCTTTCAGGCCTATGGTGAAAAAGGAAATATCTTCCCAT
        131 GCGCTTTGAGGATTTCGTTGGAAACGGAAATATCTTCATAT

                  * * *      **      *   * *   *     ** ** *      *    *     
     483551 AAAAACGACATAGAAGCTATCTCAGGAACTTGTTTATGATGCATCTAATCAACTAACAGTGTTGA
          1 AAAAACTAGACAGAAGCATTCTCAGAAACATCTTTGTGATGTTTGCACTCAACTCACAGAGTTGA

                  * ** * *                         * *       *               
     483616 ACCTTTGTACTGACAGAGCAG-TTTGAAACACTCTTTTTTTGGAATCTGCAAGTGGATATTTGGA
         66 ACCTTTCTTTTCATAGAGCAGTTTTGAAACACTCTTTTTGTAGAATCTGGAAGTGGATATTTGGA

            *                           *   *   *    
     483680 TCGCTTTGAGGATTTCGTTGGAAACGG-GAT-GC--AATAT
        131 GCGCTTTGAGGATTTCGTTGGAAACGGAAATATCTTCATAT

                **  *    *     *                 **  *   *  *    *         * 
     483717 AAAACGTACACAGCAGCATACTCAGAAA-ATACTTTGCCATATTTCCATTCAAGTCACAGAGTGG
          1 AAAAACTAGACAGAAGCATTCTCAGAAACAT-CTTTGTGATGTTTGCACTCAACTCACAGAGTTG

               *  * **            *                  *  *             *      
     483781 AACATTCCCATTCATAGAGCAGGTTTGAAACACTCTTTTTGGAGTATCTGGAAGTGGACATTTGG
         65 AACCTTTCTTTTCATAGAGCAGTTTTGAAACACTCTTTTTGTAGAATCTGGAAGTGGATATTTGG

                    ** * * ** *  *   *            **  
     483846 AGCGCTTTCTGAACTATGGTGAAAAAGGAAATATCTTCCAAT
        130 AGCGCTTTGAGGATTTCGTTGGAAACGGAAATATCTTCATAT

            *     *               *      * *          *  **      **  *  *    
     483888 GAAAACAAGACAGAAGCATTCTGAGAAACTTATTTGTGATGTGTGTCCTCAACAAACGGACTTGA
          1 AAAAACTAGACAGAAGCATTCTCAGAAACATCTTTGTGATGTTTGCACTCAACTCACAGAGTTGA

                   *      **  * *  *  *             *   *    * * *           
     483953 ACCTTTCGTTTCATGCAGTACTTCTGGAACACTCTTTTTGAAGATTCTGCATGCGGATATTTGGA
         66 ACCTTTCTTTTCATAGAGCAGTTTTGAAACACTCTTTTTGTAGAATCTGGAAGTGGATATTTGGA

            **           *                **   *   * 
     484018 TAGCTTTGAGGATCTCGTTGGAAACGG---GCT-TACATGT
        131 GCGCTTTGAGGATTTCGTTGGAAACGGAAATATCTTCATAT

                 *       *               *        *   *    *    *       *    
     484055 AAAAATTAGACAGCAGCATTCTCAGAAACTTCTTTGTGGTGTCTGCATTCAAGTCACAGAATTGA
          1 AAAAACTAGACAGAAGCATTCTCAGAAACATCTTTGTGATGTTTGCACTCAACTCACAGAGTTGA

              * ** * **            *  * *      *       *             *       
     484120 ACATCCCCTCACATAGAGCAGTTGTGCAGCACTCTATTTGTAGTATCTGGAAGTGGACATTTGGA
         66 ACCTTTCTTTTCATAGAGCAGTTTTGAAACACTCTTTTTGTAGAATCTGGAAGTGGATATTTGGA

             *         ** *    * *   *            **  
     484185 GGGCTTTGTAGCCTATC-TGGAAAAAGGAAATATCTTCCCAT
        131 GCGCTTTG-AGGATTTCGTTGGAAACGGAAATATCTTCATAT

            *  ** *   *     * *            *   *  *   * **        *  * * *   
     484226 GAATGCGAGATAGAAGTAATCTCAGAAACATGTTTATGCTGTATCTACTCAACTAACTGTGCTGA
          1 AAAAACTAGACAGAAGCATTCTCAGAAACATCTTTGTGATGTTTGCACTCAACTCACAGAGTTGA

              *     *  *               *        *  * *       *               
     484291 ACATTTCTATTGATAGAGCAGTTTTGAGACACTCTTCTTTTGGAATCTGCAAGTGGATATTTGGA
         66 ACCTTTCTTTTCATAGAGCAGTTTTGAAACACTCTTTTTGTAGAATCTGGAAGTGGATATTTGGA

            ** *                        *     *      
     484356 TAGATTTGAGGATTTCGTTGGAAACGG-GAT-T-AT-ATAT
        131 GCGCTTTGAGGATTTCGTTGGAAACGGAAATATCTTCATAT

                 *       *               *                 **  *   *         
     484393 AAAAAGTAGACAGCAGCATTCTCAGAAACTTCTTTGTGATGTTTGCATCCAGCTCTCAGAGTTGA
          1 AAAAACTAGACAGAAGCATTCTCAGAAACATCTTTGTGATGTTTGCACTCAACTCACAGAGTTGA

              *  * *          *  *        *        *   **           **       
     484458 ACATTCCCTTTCATAGAGTAGGTTTGAAACCCTCTTTTTATAGTGTCTGGAAGTGGGCATTTGGA
         66 ACCTTTCTTTTCATAGAGCAGTTTTGAAACACTCTTTTTGTAGAATCTGGAAGTGGATATTTGGA

                   *   ** ** *  *   *          * *   
     484523 GCGCTTTCAGGCCTATGCTGAAAAAGGAAATATCTACCTAT
        131 GCGCTTTGAGGATTTCGTTGGAAACGGAAATATCTTCATAT

             *                    *    *  **          ** *        *    *     
     484564 AGAAACTAGACAGAAGCATTCTGAGAATCACGTTTGTGATGTGGGTACTCAACTAACAGTGTTGA
          1 AAAAACTAGACAGAAGCATTCTCAGAAACATCTTTGTGATGTTTGCACTCAACTCACAGAGTTGA

            *  *       *   *  *         *   *                *               
     484629 TCCATTCTTTTGATACAGAAGTTTTGAACCACACTTTTTGTAGAATCTGCAAGTGGATATTTGGA
         66 ACCTTTCTTTTCATAGAGCAGTTTTGAAACACTCTTTTTGTAGAATCTGGAAGTGGATATTTGGA

            **   *                     *   *        *
     484694 TAGCTGTGAGGATTTCGTTGGAAACGGGAATGTCTTCATAG
        131 GCGCTTTGAGGATTTCGTTGGAAACGGAAATATCTTCATAT

                **                     * * **         *  **   *   *          
     484735 AAAATTTAGACAGAAGCATTCTCAGAACCTTGATTGTGATGTGTGTTCTCCACTAACAGAGTTGA
          1 AAAAACTAGACAGAAGCATTCTCAGAAACATCTTTGTGATGTTTGCACTCAACTCACAGAGTTGA

                       * *   * *   *       *       *                         
     484800 ACCTTTCTTTTGACAGAACTGTTCTGAAACATTCTTTTTATAGAATCTGGAAGTGGATATTTGGA
         66 ACCTTTCTTTTCATAGAGCAGTTTTGAAACACTCTTTTTGTAGAATCTGGAAGTGGATATTTGGA

            **                       * *          *  
     484865 AAGCTTTGAGGATTTCGTTGGAAACTGGAATATCTTCAAAT
        131 GCGCTTTGAGGATTTCGTTGGAAACGGAAATATCTTCATAT

            *   *    *            *           * *       *  *    *            
     484906 CAAATCTAGCCAGAAGCATTCTAAGAAACATCTTAGGGATGTTTACATTCAAGTCACAGAGTTGA
          1 AAAAACTAGACAGAAGCATTCTCAGAAACATCTTTGTGATGTTTGCACTCAACTCACAGAGTTGA

              *  * *     *       *         *    * *   **      *      *    *  
     484971 ACATTCCCTTTCACAGAGCAGGTTTGAAACAATCTTCTCGTACTATCTGGCAGTGGACATTTTGA
         66 ACCTTTCTTTTCATAGAGCAGTTTTGAAACACTCTTTTTGTAGAATCTGGAAGTGGATATTTGGA

              * *   *  ** ** *  *   *            ** *
     485036 GCTCCTTGGGGCCTATGCTGAAAAAGGAAATATCTTCCGAC
        131 GCGCTTTGAGGATTTCGTTGGAAACGGAAATATCTTCATAT

                                 *     *  **          *           **    *    
     485077 AAAAACTAGACAGAAGCATTCGCAGAATCACGTTTGTGATGTGTGCACTCAACTGTCAGAATTGA
          1 AAAAACTAGACAGAAGCATTCTCAGAAACATCTTTGTGATGTTTGCACTCAACTCACAGAGTTGA

                 **   ** *      *                            * *            *
     485142 ACCTTGGTTTGGACAGAGCACTTTTGAAACACTCTTTTTGTAGAATCTGCAGGTGGATATTTGGC
         66 ACCTTTCTTTTCATAGAGCAGTTTTGAAACACTCTTTTTGTAGAATCTGGAAGTGGATATTTGGA

            **                         *   *      * *
     485207 TAGCTTTGAGGATTTCGTTGGAAACGGTAATGTCTTCAAAG
        131 GCGCTTTGAGGATTTCGTTGGAAACGGAAATATCTTCATAT

                *                         *   *            *    *            
     485248 AAAATCTAGACAGAAGCATTCTCAGAAACACCTTCGTGATGTTTGCAATCAAGTCACAGAGTTGA
          1 AAAAACTAGACAGAAGCATTCTCAGAAACATCTTTGTGATGTTTGCACTCAACTCACAGAGTTGA

                 * *             *  *                  *             *       
     485313 ACCTTCCGTTTCATAGAGCAGGTTGGAAACACTCTTTTTGTAGTATCTGGAAGTGGACATTTGGA
         66 ACCTTTCTTTTCATAGAGCAGTTTTGAAACACTCTTTTTGTAGAATCTGGAAGTGGATATTTGGA

             *         ** *    * *   *            **  
     485378 GGGCTTTGTAGCCTATC-TGGAAAAAGGAAATATCTTCCCAT
        131 GCGCTTTG-AGGATTTCGTTGGAAACGGAAATATCTTCATAT

            *  ** *   *     * *            *   *  *   * **        *  * * *   
     485419 GAATGCGAGATAGAAGTAATCTCAGAAACATGTTTATGCTGTATCTACTCAACTAACTGTGCTGA
          1 AAAAACTAGACAGAAGCATTCTCAGAAACATCTTTGTGATGTTTGCACTCAACTCACAGAGTTGA

              *     *  *               *        *  * *       *               
     485484 ACATTTCTATTGATAGAGCAGTTTTGAGACACTCTTCTTTTGGAATCTGCAAGTGGATATTTGGA
         66 ACCTTTCTTTTCATAGAGCAGTTTTGAAACACTCTTTTTGTAGAATCTGGAAGTGGATATTTGGA

            ** *                        *     *      
     485549 TAGATTTGAGGATTTCGTTGGAAACGG-GAT-T-AT-ATAT
        131 GCGCTTTGAGGATTTCGTTGGAAACGGAAATATCTTCATAT

            *    *       *               *                 **  *   *         
     485586 CAAAAGTAGACAGCAGCATTCTCAGAAACTTCTTTGTGATGTTTGCATCCAGCTCTCAGAGTTGA
          1 AAAAACTAGACAGAAGCATTCTCAGAAACATCTTTGTGATGTTTGCACTCAACTCACAGAGTTGA

              *  * *          *  *        *        *   **        *  **       
     485651 ACATTCCCTTTCATAGAGTAGGTTTGAAACCCTCTTTTTATAGTGTCTGGAAGCGGGCATTTGGA
         66 ACCTTTCTTTTCATAGAGCAGTTTTGAAACACTCTTTTTGTAGAATCTGGAAGTGGATATTTGGA

                   *   ** ** *  *   *          * *   
     485716 GCGCTTTCAGGCCTATGCTGAAAAAGGAAATATCTACCTAT
        131 GCGCTTTGAGGATTTCGTTGGAAACGGAAATATCTTCATAT

             *                    *    *  **          ** *        *    *     
     485757 AGAAACTAGACAGAAGCATTCTGAGAATCACGTTTGTGATGTGGGTACTCAACTAACAGTGTTGA
          1 AAAAACTAGACAGAAGCATTCTCAGAAACATCTTTGTGATGTTTGCACTCAACTCACAGAGTTGA

            *  *       *   *            *   *                *               
     485822 TCCATTCTTTTGATACAGCAGTTTTGAACCACACTTTTTGTAGAATCTGCAAGTGGATATTTGGA
         66 ACCTTTCTTTTCATAGAGCAGTTTTGAAACACTCTTTTTGTAGAATCTGGAAGTGGATATTTGGA

            **   *                     *   *        *
     485887 TAGCTGTGAGGATTTCGTTGGAAACGGGAATGTCTTCATAG
        131 GCGCTTTGAGGATTTCGTTGGAAACGGAAATATCTTCATAT

                **                     * * **         *  **   *   *          
     485928 AAAATTTAGACAGAAGCATTCTCAGAACCTTGATTGTGATGTGTGTTCTCCACTAACAGAGTTGA
          1 AAAAACTAGACAGAAGCATTCTCAGAAACATCTTTGTGATGTTTGCACTCAACTCACAGAGTTGA

                       * *   * *   *       *       *                         
     485993 ACCTTTCTTTTGACAGAACTGTTCTGAAACATTCTTTTTATAGAATCTGGAAGTGGATATTTGGA
         66 ACCTTTCTTTTCATAGAGCAGTTTTGAAACACTCTTTTTGTAGAATCTGGAAGTGGATATTTGGA

            **                         *          *  
     486058 AAGCTTTGAGGATTTCGTTGGAAACGGGAATATCTTCAAAT
        131 GCGCTTTGAGGATTTCGTTGGAAACGGAAATATCTTCATAT

                *    *            *           * *       *  *    *            
     486099 AAAATCTAGCCAGAAGCATTCTAAGAAACATCTTAGGGATGTTTACATTCAAGTCACAGAGTTGA
          1 AAAAACTAGACAGAAGCATTCTCAGAAACATCTTTGTGATGTTTGCACTCAACTCACAGAGTTGA

              *  * *     *       *         *    * *   **      *      *    *  
     486164 ACATTCCCTTTCACAGAGCAGGTTTGAAACAATCTTCTCGTACTATCTGGCAGTGGACATTTTGA
         66 ACCTTTCTTTTCATAGAGCAGTTTTGAAACACTCTTTTTGTAGAATCTGGAAGTGGATATTTGGA

              * *   *  ** ** *  *   *            ** *
     486229 GCTCCTTGGGGCCTATGCTGAAAAAGGAAATATCTTCCGAC
        131 GCGCTTTGAGGATTTCGTTGGAAACGGAAATATCTTCATAT

                                 *     *  **          *           **    *    
     486270 AAAAACTAGACAGAAGCATTCGCAGAATCACGTTTGTGATGTGTGCACTCAACTGTCAGAATTGA
          1 AAAAACTAGACAGAAGCATTCTCAGAAACATCTTTGTGATGTTTGCACTCAACTCACAGAGTTGA

                 **   ** *      *                            * *            *
     486335 ACCTTGGTTTGGACAGAGCACTTTTGAAACACTCTTTTTGTAGAATCTGCAGGTGGATATTTGGC
         66 ACCTTTCTTTTCATAGAGCAGTTTTGAAACACTCTTTTTGTAGAATCTGGAAGTGGATATTTGGA

            **                         *   *      * *
     486400 TAGCTTTGAGGATTTCGTTGGAAACGGTAATGTCTTCAAAG
        131 GCGCTTTGAGGATTTCGTTGGAAACGGAAATATCTTCATAT

                *                         *   *            *    *            
     486441 AAAATCTAGACAGAAGCATTCTCAGAAACACCTTCGTGATGTTTGCAATCAAGTCACAGAGTTGA
          1 AAAAACTAGACAGAAGCATTCTCAGAAACATCTTTGTGATGTTTGCACTCAACTCACAGAGTTGA

                 * *             *  *                  *             *       
     486506 ACCTTCCGTTTCATAGAGCAGGTTGGAAACACTCTTTTTGTAGTATCTGGAAGTGGACATTTGGA
         66 ACCTTTCTTTTCATAGAGCAGTTTTGAAACACTCTTTTTGTAGAATCTGGAAGTGGATATTTGGA

             *         ** *    * *   *            **  
     486571 GGGCTTTGTAGCCTATC-TGGAAAAAGGAAATATCTTCCCAT
        131 GCGCTTTG-AGGATTTCGTTGGAAACGGAAATATCTTCATAT

            *  ** *   *     * *            *   *  *   * **        *  * * *   
     486612 GAATGCGAGATAGAAGTAATCTCAGAAACATGTTTATGCTGTATCTACTCAACTAACTGTGCTGA
          1 AAAAACTAGACAGAAGCATTCTCAGAAACATCTTTGTGATGTTTGCACTCAACTCACAGAGTTGA

              *     *  *               *        *  * *       *               
     486677 ACATTTCTATTGATAGAGCAGTTTTGAGACACTCTTCTTTTGGAATCTGCAAGTGGATATTTGGA
         66 ACCTTTCTTTTCATAGAGCAGTTTTGAAACACTCTTTTTGTAGAATCTGGAAGTGGATATTTGGA

            ** *                        *     *      
     486742 TAGATTTGAGGATTTCGTTGGAAACGG-GAT-T-AT-ATAT
        131 GCGCTTTGAGGATTTCGTTGGAAACGGAAATATCTTCATAT

            *    *       *               *                 **  *   *         
     486779 CAAAAGTAGACAGCAGCATTCTCAGAAACTTCTTTGTGATGTTTGCATCCAGCTCTCAGAGTTGA
          1 AAAAACTAGACAGAAGCATTCTCAGAAACATCTTTGTGATGTTTGCACTCAACTCACAGAGTTGA

              *  * *          *  *        *        *   **        *  **       
     486844 ACATTCCCTTTCATAGAGTAGGTTTGAAACCCTCTTTTTATAGTGTCTGGAAGCGGGCATTTGGA
         66 ACCTTTCTTTTCATAGAGCAGTTTTGAAACACTCTTTTTGTAGAATCTGGAAGTGGATATTTGGA

                   *   ** ** *  *   *          * *   
     486909 GCGCTTTCAGGCCTATGCTGAAAAAGGAAATATCTACCTAT
        131 GCGCTTTGAGGATTTCGTTGGAAACGGAAATATCTTCATAT

             *                    *    *  **          ** *        *    *     
     486950 AGAAACTAGACAGAAGCATTCTGAGAATCACGTTTGTGATGTGGGTACTCAACTAACAGTGTTGA
          1 AAAAACTAGACAGAAGCATTCTCAGAAACATCTTTGTGATGTTTGCACTCAACTCACAGAGTTGA

            *  *       *   *            *   *                *               
     487015 TCCATTCTTTTGATACAGCAGTTTTGAACCACACTTTTTGTAGAATCTGCAAGTGGATATTTGGA
         66 ACCTTTCTTTTCATAGAGCAGTTTTGAAACACTCTTTTTGTAGAATCTGGAAGTGGATATTTGGA

            **   *                     *   *        *
     487080 TAGCTGTGAGGATTTCGTTGGAAACGGGAATGTCTTCATAG
        131 GCGCTTTGAGGATTTCGTTGGAAACGGAAATATCTTCATAT

                **                     * * **         *  **   *   *          
     487121 AAAATTTAGACAGAAGCATTCTCAGAACCTTGATTGTGATGTGTGTTCTCCACTAACAGAGTTGA
          1 AAAAACTAGACAGAAGCATTCTCAGAAACATCTTTGTGATGTTTGCACTCAACTCACAGAGTTGA

                       * *   * *   *       *       *                         
     487186 ACCTTTCTTTTGACAGAACTGTTCTGAAACATTCTTTTTATAGAATCTGGAAGTGGATATTTGGA
         66 ACCTTTCTTTTCATAGAGCAGTTTTGAAACACTCTTTTTGTAGAATCTGGAAGTGGATATTTGGA

            **                         *          *  
     487251 AAGCTTTGAGGATTTCGTTGGAAACGGGAATATCTTCAAAT
        131 GCGCTTTGAGGATTTCGTTGGAAACGGAAATATCTTCATAT

                *    *            *           * *       *  *    *            
     487292 AAAATCTAGCCAGAAGCATTCTAAGAAACATCTTAGGGATGTTTACATTCAAGTCACAGAGTTGA
          1 AAAAACTAGACAGAAGCATTCTCAGAAACATCTTTGTGATGTTTGCACTCAACTCACAGAGTTGA

              *  * *     *       *         *    * *   **      *      *    *  
     487357 ACATTCCCTTTCACAGAGCAGGTTTGAAACAATCTTCTCGTACTATCTGGCAGTGGACATTTTGA
         66 ACCTTTCTTTTCATAGAGCAGTTTTGAAACACTCTTTTTGTAGAATCTGGAAGTGGATATTTGGA

              * *   *  ** ** *  *   *            ** *
     487422 GCTCCTTGGGGCCTATGCTGAAAAAGGAAATATCTTCCGAC
        131 GCGCTTTGAGGATTTCGTTGGAAACGGAAATATCTTCATAT

                                 *     *  **          *           **    *    
     487463 AAAAACTAGACAGAAGCATTCGCAGAATCACGTTTGTGATGTGTGCACTCAACTGTCAGAATTGA
          1 AAAAACTAGACAGAAGCATTCTCAGAAACATCTTTGTGATGTTTGCACTCAACTCACAGAGTTGA

                 **   ** *      *                            * *            *
     487528 ACCTTGGTTTGGACAGAGCACTTTTGAAACACTCTTTTTGTAGAATCTGCAGGTGGATATTTGGC
         66 ACCTTTCTTTTCATAGAGCAGTTTTGAAACACTCTTTTTGTAGAATCTGGAAGTGGATATTTGGA

            **                         *   *      * *
     487593 TAGCTTTGAGGATTTCGTTGGAAACGGTAATGTCTTCAAAG
        131 GCGCTTTGAGGATTTCGTTGGAAACGGAAATATCTTCATAT

                *                         *   *            *    *            
     487634 AAAATCTAGACAGAAGCATTCTCAGAAACACCTTCGTGATGTTTGCAATCAAGTCACAGAGTTGA
          1 AAAAACTAGACAGAAGCATTCTCAGAAACATCTTTGTGATGTTTGCACTCAACTCACAGAGTTGA

                 * *             *  *                  *             *       
     487699 ACCTTCCGTTTCATAGAGCAGGTTGGAAACACTCTTTTTGTAGTATCTGGAAGTGGACATTTGGA
         66 ACCTTTCTTTTCATAGAGCAGTTTTGAAACACTCTTTTTGTAGAATCTGGAAGTGGATATTTGGA

             *         ** *    * *   *            **  
     487764 GGGCTTTGTAGCCTATC-TGGAAAAAGGAAATATCTTCCCAT
        131 GCGCTTTG-AGGATTTCGTTGGAAACGGAAATATCTTCATAT

            *  ** *   *     * *            *   *  *   * **        *  * * *   
     487805 GAATGCGAGATAGAAGTAATCTCAGAAACATGTTTATGCTGTATCTACTCAACTAACTGTGCTGA
          1 AAAAACTAGACAGAAGCATTCTCAGAAACATCTTTGTGATGTTTGCACTCAACTCACAGAGTTGA

              *     *  *               *        *  * *       *               
     487870 ACATTTCTATTGATAGAGCAGTTTTGAGACACTCTTCTTTTGGAATCTGCAAGTGGATATTTGGA
         66 ACCTTTCTTTTCATAGAGCAGTTTTGAAACACTCTTTTTGTAGAATCTGGAAGTGGATATTTGGA

            ** *                        *     *      
     487935 TAGATTTGAGGATTTCGTTGGAAACGG-GAT-T-AT-ATAT
        131 GCGCTTTGAGGATTTCGTTGGAAACGGAAATATCTTCATAT

            *    *       *               *                 **  *   *         
     487972 CAAAAGTAGACAGCAGCATTCTCAGAAACTTCTTTGTGATGTTTGCATCCAGCTCTCAGAGTTGA
          1 AAAAACTAGACAGAAGCATTCTCAGAAACATCTTTGTGATGTTTGCACTCAACTCACAGAGTTGA

              *  * *          *  *        *        *   **        *  **       
     488037 ACATTCCCTTTCATAGAGTAGGTTTGAAACCCTCTTTTTATAGTGTCTGGAAGCGGGCATTTGGA
         66 ACCTTTCTTTTCATAGAGCAGTTTTGAAACACTCTTTTTGTAGAATCTGGAAGTGGATATTTGGA

                   *   ** ** *  *   *          * *   
     488102 GCGCTTTCAGGCCTATGCTGAAAAAGGAAATATCTACCTAT
        131 GCGCTTTGAGGATTTCGTTGGAAACGGAAATATCTTCATAT

             *                    *    *  **          ** *        *    *     
     488143 AGAAACTAGACAGAAGCATTCTGAGAATCACGTTTGTGATGTGGGTACTCAACTAACAGTGTTGA
          1 AAAAACTAGACAGAAGCATTCTCAGAAACATCTTTGTGATGTTTGCACTCAACTCACAGAGTTGA

            *  *       *   *            *   *                                
     488208 TCCATTCTTTTGATACAGCAGTTTTGAACCACACTTTTTGTAGAATCTGGAAGTGGATATTTGGA
         66 ACCTTTCTTTTCATAGAGCAGTTTTGAAACACTCTTTTTGTAGAATCTGGAAGTGGATATTTGGA

            **                         *          *  
     488273 AAGCTTTGAGGATTTCGTTGGAAACGGGAATATCTTCAAAT
        131 GCGCTTTGAGGATTTCGTTGGAAACGGAAATATCTTCATAT

                *    *            *           * *       *  *    *            
     488314 AAAATCTAGCCAGAAGCATTCTAAGAAACATCTTAGGGATGTTTACATTCAAGTCACAGAGTTGA
          1 AAAAACTAGACAGAAGCATTCTCAGAAACATCTTTGTGATGTTTGCACTCAACTCACAGAGTTGA

              *  * *     *       *         *    * *   **      *      *    *  
     488379 ACATTCCCTTTCACAGAGCAGGTTTGAAACAATCTTCTCGTACTATCTGGCAGTGGACATTTTGA
         66 ACCTTTCTTTTCATAGAGCAGTTTTGAAACACTCTTTTTGTAGAATCTGGAAGTGGATATTTGGA

              * *   *  ** ** *  *   *            ** *
     488444 GCTCCTTGGGGCCTATGCTGAAAAAGGAAATATCTTCCGAC
        131 GCGCTTTGAGGATTTCGTTGGAAACGGAAATATCTTCATAT

                                 *     *  **          *           **    *    
     488485 AAAAACTAGACAGAAGCATTCGCAGAATCACGTTTGTGATGTGTGCACTCAACTGTCAGAATTGA
          1 AAAAACTAGACAGAAGCATTCTCAGAAACATCTTTGTGATGTTTGCACTCAACTCACAGAGTTGA

                 **   ** *      *                            * *            *
     488550 ACCTTGGTTTGGACAGAGCACTTTTGAAACACTCTTTTTGTAGAATCTGCAGGTGGATATTTGGC
         66 ACCTTTCTTTTCATAGAGCAGTTTTGAAACACTCTTTTTGTAGAATCTGGAAGTGGATATTTGGA

            **                         *   *      * *
     488615 TAGCTTTGAGGATTTCGTTGGAAACGGTAATGTCTTCAAAG
        131 GCGCTTTGAGGATTTCGTTGGAAACGGAAATATCTTCATAT

                *           *             *   *            *    *            
     488656 AAAATCTAGACAGAAGAATTCTCAGAAACACCTTCGTGATGTTTGCAATCAAGTCACAGAGTTGA
          1 AAAAACTAGACAGAAGCATTCTCAGAAACATCTTTGTGATGTTTGCACTCAACTCACAGAGTTGA

                 * *             *  *                  *             *       
     488721 ACCTTCCGTTTCATAGAGCAGGTTGGAAACACTCTTTTTGTAGTATCTGGAAGTGGACATTTGGA
         66 ACCTTTCTTTTCATAGAGCAGTTTTGAAACACTCTTTTTGTAGAATCTGGAAGTGGATATTTGGA

             *         ** *    * *   *            **  
     488786 GGGCTTTGTAGCCTATC-TGGAAAAAGGAAATATCTTCCCAT
        131 GCGCTTTG-AGGATTTCGTTGGAAACGGAAATATCTTCATAT

            *  ** *   *     * *            *   *  *   * **        *  * * *   
     488827 GAATGCGAGATAGAAGTAATCTCAGAAACATGTTTATGCTGTATCTACTCAACTAACTGTGCTGA
          1 AAAAACTAGACAGAAGCATTCTCAGAAACATCTTTGTGATGTTTGCACTCAACTCACAGAGTTGA

              *     *  *               *        *  * *       *               
     488892 ACATTTCTATTGATAGAGCAGTTTTGAGACACTCTTCTTTTGGAATCTGCAAGTGGATATTTGGA
         66 ACCTTTCTTTTCATAGAGCAGTTTTGAAACACTCTTTTTGTAGAATCTGGAAGTGGATATTTGGA

            ** *                        *     *      
     488957 TAGATTTGAGGATTTCGTTGGAAACGG-GAT-T-AT-ATAT
        131 GCGCTTTGAGGATTTCGTTGGAAACGGAAATATCTTCATAT

                 *       *               *                 **  *   *         
     488994 AAAAAGTAGACAGCAGCATTCTCAGAAACTTCTTTGTGATGTTTGCATCCAGCTCTCAGAGTTGA
          1 AAAAACTAGACAGAAGCATTCTCAGAAACATCTTTGTGATGTTTGCACTCAACTCACAGAGTTGA

              *  * *          *  *        *        *   **        *  **       
     489059 ACATTCCCTTTCATAGAGTAGGTTTGAAACCCTCTTTTTATAGTGTCTGGAAGCGGGCATTTGGA
         66 ACCTTTCTTTTCATAGAGCAGTTTTGAAACACTCTTTTTGTAGAATCTGGAAGTGGATATTTGGA

                   *   ** ** *  *   *          * *   
     489124 GCGCTTTCAGGCCTATGCTGAAAAAGGAAATATCTACCTAT
        131 GCGCTTTGAGGATTTCGTTGGAAACGGAAATATCTTCATAT

             *                    *    *  **          ** *        *    *     
     489165 AGAAACTAGACAGAAGCATTCTGAGAATCACGTTTGTGATGTGGGTACTCAACTAACAGTGTTGA
          1 AAAAACTAGACAGAAGCATTCTCAGAAACATCTTTGTGATGTTTGCACTCAACTCACAGAGTTGA

            *  *       *   *            *   *                *               
     489230 TCCATTCTTTTGATACAGCAGTTTTGAACCACACTTTTTGTAGAATCTGCAAGTGGATATTTGGA
         66 ACCTTTCTTTTCATAGAGCAGTTTTGAAACACTCTTTTTGTAGAATCTGGAAGTGGATATTTGGA

            **   *                     *   *        *
     489295 TAGCTGTGAGGATTTCGTTGGAAACGGGAATGTCTTCATAG
        131 GCGCTTTGAGGATTTCGTTGGAAACGGAAATATCTTCATAT

                **                     * * **         *  **   *   *          
     489336 AAAATTTAGACAGAAGCATTCTCAGAACCTTGATTGTGATGTGTGTTCTCCACTAACAGAGTTGA
          1 AAAAACTAGACAGAAGCATTCTCAGAAACATCTTTGTGATGTTTGCACTCAACTCACAGAGTTGA

                       * *   * *   *       *                                 
     489401 ACCTTTCTTTTGACAGAACTGTTCTGAAACATTCTTTTTGTAGAATCTGGAAGTGGATATTTGGA
         66 ACCTTTCTTTTCATAGAGCAGTTTTGAAACACTCTTTTTGTAGAATCTGGAAGTGGATATTTGGA

            **                         *          *  
     489466 AAGCTTTGAGGATTTCGTTGGAAACGGGAATATCTTCAAAT
        131 GCGCTTTGAGGATTTCGTTGGAAACGGAAATATCTTCATAT

                *    *            *           * *       *  *    *            
     489507 AAAATCTAGCCAGAAGCATTCTAAGAAACATCTTAGGGATGTTTACATTCAAGTCACAGAGTTGA
          1 AAAAACTAGACAGAAGCATTCTCAGAAACATCTTTGTGATGTTTGCACTCAACTCACAGAGTTGA

              *  * *     *       *         *    * *   **      *      *    *  
     489572 ACATTCCCTTTCACAGAGCAGGTTTGAAACAATCTTCTCGTACTATCTGGCAGTGGACATTTTGA
         66 ACCTTTCTTTTCATAGAGCAGTTTTGAAACACTCTTTTTGTAGAATCTGGAAGTGGATATTTGGA

              * *   *  ** ** *  *   *            ** *
     489637 GCTCCTTGGGGCCTATGCTGAAAAAGGAAATATCTTCCGAC
        131 GCGCTTTGAGGATTTCGTTGGAAACGGAAATATCTTCATAT

                                 *     *  **          *           **    *    
     489678 AAAAACTAGACAGAAGCATTCGCAGAATCACGTTTGTGATGTGTGCA-TCAACTGTCAGAATTGA
          1 AAAAACTAGACAGAAGCATTCTCAGAAACATCTTTGTGATGTTTGCACTCAACTCACAGAGTTGA

                 **   ** *      *                            * *            *
     489742 ACCTTGGTTTGGACAGAGCACTTTTGAAACACTCTTTTTGTAGAATCTGCAGGTGGATATTTGGC
         66 ACCTTTCTTTTCATAGAGCAGTTTTGAAACACTCTTTTTGTAGAATCTGGAAGTGGATATTTGGA

            **                      *  *   *      * *
     489807 TAGCTTTGAGGATTTCGTTGGAAAGGGTAATGTCTTCAAAG
        131 GCGCTTTGAGGATTTCGTTGGAAACGGAAATATCTTCATAT

                *                         *   *            *    *            
     489848 AAAATCTAGACAGAAGCATTCTCAGAAACACCTTCGTGATGTTTGCAATCAAGTCACAGAGTTGA
          1 AAAAACTAGACAGAAGCATTCTCAGAAACATCTTTGTGATGTTTGCACTCAACTCACAGAGTTGA

                 * *             *  *                  *             *       
     489913 ACCTTCCGTTTCATAGAGCAGGTTGGAAACACTCTTTTTGTAGTATCTGGAAGTGGACATTTGGA
         66 ACCTTTCTTTTCATAGAGCAGTTTTGAAACACTCTTTTTGTAGAATCTGGAAGTGGATATTTGGA

             *         ** *    * *   *            **  
     489978 GGGCTTTGTAGCCTATC-TGGAAAAAGGAAATATCTTCCCAT
        131 GCGCTTTG-AGGATTTCGTTGGAAACGGAAATATCTTCATAT

            *  ** *   *     * *            *   *  *   * **        *  * * *   
     490019 GAATGCGAGATAGAAGTAATCTCAGAAACATGTTTATGCTGTATCTACTCAACTAACTGTGCTGA
          1 AAAAACTAGACAGAAGCATTCTCAGAAACATCTTTGTGATGTTTGCACTCAACTCACAGAGTTGA

              *     *  *               *        *  * *       *               
     490084 ACATTTCTATTGATAGAGCAGTTTTGAGACACTCTTCTTTTGGAATCTGCAAGTGGATATTTGGA
         66 ACCTTTCTTTTCATAGAGCAGTTTTGAAACACTCTTTTTGTAGAATCTGGAAGTGGATATTTGGA

            ** *                        *     *      
     490149 TAGATTTGAGGATTTCGTTGGAAACGG-GAT-T-AT-ATAT
        131 GCGCTTTGAGGATTTCGTTGGAAACGGAAATATCTTCATAT

                 *       *               *                 **  *   *         
     490186 AAAAAGTAGACAGCAGCATTCTCAGAAACTTCTTTGTGATGTTTGCATCCAGCTCTCAGAGTTGA
          1 AAAAACTAGACAGAAGCATTCTCAGAAACATCTTTGTGATGTTTGCACTCAACTCACAGAGTTGA

              *  * *          *  *        *        *   **        *  **       
     490251 ACATTCCCTTTCATAGAGTAGGTTTGAAACCCTCTTTTTATAGTGTCTGGAAGCGGGCATTTGGA
         66 ACCTTTCTTTTCATAGAGCAGTTTTGAAACACTCTTTTTGTAGAATCTGGAAGTGGATATTTGGA

                *  *    * * **  **  **          * *  *
     490316 GCGCATTCAGGCCTATGCTTAAAATAGGAAATATCTACCTAC
        131 GCGCTTTGAGG-ATTTCGTTGGAAACGGAAATATCTTCATAT

             *                    *    *  **          ** *        *    *     
     490358 AGAAACTAGACAGAAGCATTCTGAGAATCACGTTTGTGATGTGGGTACTCAACTAACAGTGTTGA
          1 AAAAACTAGACAGAAGCATTCTCAGAAACATCTTTGTGATGTTTGCACTCAACTCACAGAGTTGA

            *  *       *   *            *   *                *   *           
     490423 TCCATTCTTTTGATACAGCAGTTTTGAACCACACTTTTTGTAGAATCTGCAAGAGGATATTTGGA
         66 ACCTTTCTTTTCATAGAGCAGTTTTGAAACACTCTTTTTGTAGAATCTGGAAGTGGATATTTGGA

            **   *                     *   *      * *
     490488 TAGCTGTGAGGATTTCGTTGGAAACGGGAATGTCTTCAAAG
        131 GCGCTTTGAGGATTTCGTTGGAAACGGAAATATCTTCATAT

                *                         *   *            *    *            
     490529 AAAATCTAGACAGAAGCATTCTCAGAAACACCTTCGTGATGTTTGCAATCAAGTCACAGAGTTGA
          1 AAAAACTAGACAGAAGCATTCTCAGAAACATCTTTGTGATGTTTGCACTCAACTCACAGAGTTGA

                 * *             *  *           *      **            *       
     490594 ACCTTCCGTTTCATAGAGCAGGTTGGAAACACTCTTATTGTAGTGTCTGGAAGTGGACATTTGGA
         66 ACCTTTCTTTTCATAGAGCAGTTTTGAAACACTCTTTTTGTAGAATCTGGAAGTGGATATTTGGA

                   *   ** ** *  *   *            **  
     490659 GCGCTTTCAGGCCTATGGTGAAAAAGGAAATATCTTCCCAT
        131 GCGCTTTGAGGATTTCGTTGGAAACGGAAATATCTTCATAT

                  * * *      **      *   * *   *     ** ** *      *    *     
     490700 AAAAACGACATAGAAGCTATCTCAGGAACTTGTTTATGATGCATCTAATCAACTAACAGTGTTGA
          1 AAAAACTAGACAGAAGCATTCTCAGAAACATCTTTGTGATGTTTGCACTCAACTCACAGAGTTGA

                    ** * *                         * *       *               
     490765 ACCTTTCTACTGACAGAGCAG-TTTGAAACACTCTTTTTTTGGAATCTGCAAGTGGATATTTGGA
         66 ACCTTTCTTTTCATAGAGCAGTTTTGAAACACTCTTTTTGTAGAATCTGGAAGTGGATATTTGGA

            *                           *   *   *    
     490829 TCGCTTTGAGGATTTCGTTGGAAACGG-GAT-GC--AATAT
        131 GCGCTTTGAGGATTTCGTTGGAAACGGAAATATCTTCATAT

                **  *    *     *                 **  *   *  *    *   *     * 
     490866 AAAACGTACACAGCAGCATACTCAGAAA-ATACTTTGCCATATTTCCATTCAAGTCAGAGAGTGG
          1 AAAAACTAGACAGAAGCATTCTCAGAAACAT-CTTTGTGATGTTTGCACTCAACTCACAGAGTTG

               *  * **            *                  *  *             *      
     490930 AACATTCCCATTCATAGAGCAGGTTTGAAACACTCTTTTTGGAGTATCTGGAAGTGGACATTTGG
         65 AACCTTTCTTTTCATAGAGCAGTTTTGAAACACTCTTTTTGTAGAATCTGGAAGTGGATATTTGG

                    ** * * ** *  *   *            **  
     490995 AGCGCTTTCTGAACTATGGTGAAAAAGGAAATATCTTCCAAT
        130 AGCGCTTTGAGGATTTCGTTGGAAACGGAAATATCTTCATAT

            *     *               *      * *          *  **      **  *  *    
     491037 GAAAACAAGACAGAAGCATTCTGAGAAACTTATTTGTGATGTGTGTCCTCAACAAACGGACTTGA
          1 AAAAACTAGACAGAAGCATTCTCAGAAACATCTTTGTGATGTTTGCACTCAACTCACAGAGTTGA

                   *      **  * *  *  *             *   *    * * *           
     491102 ACCTTTCGTTTCATGCAGTACTTCTGGAACACTCTTTTTGAAGATTCTGCATGCGGATATTTGGA
         66 ACCTTTCTTTTCATAGAGCAGTTTTGAAACACTCTTTTTGTAGAATCTGGAAGTGGATATTTGGA

            **              *             **   *   * 
     491167 TAGCTTTGAGGATTTCTTTGGAAACGG---GCT-TACATGT
        131 GCGCTTTGAGGATTTCGTTGGAAACGGAAATATCTTCATAT

                 *       *               *        *   *    *    *       *    
     491204 AAAAATTAGACAGCAGCATTCTCAGAAACTTCTTTGTGGTGTCTGCATTCAAGTCACAGAATTGA
          1 AAAAACTAGACAGAAGCATTCTCAGAAACATCTTTGTGATGTTTGCACTCAACTCACAGAGTTGA

              * ** * **            *  * *      *       *             *       
     491269 ACTTCCCCTCACATAGAGCAGTTGTGCAGCACTCTATTTGTAGTATCTGGAAGTGGACATTTGGA
         66 ACCTTTCTTTTCATAGAGCAGTTTTGAAACACTCTTTTTGTAGAATCTGGAAGTGGATATTTGGA

             *         ** *    * *   *            **  
     491334 GGGCTTTGTAGCCTATC-TGGAAAAAGGAAATATCTTCCCAT
        131 GCGCTTTG-AGGATTTCGTTGGAAACGGAAATATCTTCATAT

            *  ** *   *     * *            *   *  *   * **        *  * * *   
     491375 GAATGCGAGATAGAAGTAATCTCAGAAACATGTTTATGCTGTATCTACTCAACTAACTGTGCTGA
          1 AAAAACTAGACAGAAGCATTCTCAGAAACATCTTTGTGATGTTTGCACTCAACTCACAGAGTTGA

              *     *  *               *        *  * *       *               
     491440 ACATTTCTATTGATAGAGCAGTTTTGAGACACTCTTCTTTTGGAATCTGCAAGTGGATATTTGGA
         66 ACCTTTCTTTTCATAGAGCAGTTTTGAAACACTCTTTTTGTAGAATCTGGAAGTGGATATTTGGA

            ** *                        *     *      
     491505 TAGATTTGAGGATTTCGTTGGAAACGG-GAT-T-AT-ATAT
        131 GCGCTTTGAGGATTTCGTTGGAAACGGAAATATCTTCATAT

            *    *       *               *                 **  *   *         
     491542 CAAAAGTAGACAGCAGCATTCTCAGAAACTTCTTTGTGATGTTTGCATCCAGCTCTCAGAGTTGA
          1 AAAAACTAGACAGAAGCATTCTCAGAAACATCTTTGTGATGTTTGCACTCAACTCACAGAGTTGA

              *  * *          *  *        *        *   **        *  **       
     491607 ACATTCCCTTTCATAGAGTAGGTTTGAAACCCTCTTTTTATAGTGTCTGGAAGCGGGCATTTGGA
         66 ACCTTTCTTTTCATAGAGCAGTTTTGAAACACTCTTTTTGTAGAATCTGGAAGTGGATATTTGGA

                   *    * * **  **  **          * *  *
     491672 GCGCTTTCAGGCCTATGCTTAAAATAGGAAATATCTACCTAC
        131 GCGCTTTGAGG-ATTTCGTTGGAAACGGAAATATCTTCATAT

             *                    *    *  **          ** *        *    *     
     491714 AGAAACTAGACAGAAGCATTCTGAGAATCACGTTTGTGATGTGGGTACTCAACTAACAGTGTTGA
          1 AAAAACTAGACAGAAGCATTCTCAGAAACATCTTTGTGATGTTTGCACTCAACTCACAGAGTTGA

            *  *       *   *            *   *                *   *           
     491779 TCCATTCTTTTGATACAGCAGTTTTGAACCACACTTTTTGTAGAATCTGCAAGAGGATATTTGGA
         66 ACCTTTCTTTTCATAGAGCAGTTTTGAAACACTCTTTTTGTAGAATCTGGAAGTGGATATTTGGA

            **   *                     *   *      * *
     491844 TAGCTGTGAGGATTTCGTTGGAAACGGGAATGTCTTCAAAG
        131 GCGCTTTGAGGATTTCGTTGGAAACGGAAATATCTTCATAT

                *                         *   *            *    *            
     491885 AAAATCTAGACAGAAGCATTCTCAGAAACACCTTCGTGATGTTTGCAATCAAGTCACAGAGTTGA
          1 AAAAACTAGACAGAAGCATTCTCAGAAACATCTTTGTGATGTTTGCACTCAACTCACAGAGTTGA

                 * *             *  *           *      *             *       
     491950 ACCTTCCGTTTCATAGAGCAGGTTGGAAACACTCTTATTGTAGTATCTGGAAGTGGACATTTGGA
         66 ACCTTTCTTTTCATAGAGCAGTTTTGAAACACTCTTTTTGTAGAATCTGGAAGTGGATATTTGGA

                   *   ** ** *  *   *            **  
     492015 GCGCTTTCAGGCCTATGGTGAAAAAGGAAATATCTTCCCAT
        131 GCGCTTTGAGGATTTCGTTGGAAACGGAAATATCTTCATAT

                  * * *      **      *   * *   *     ** ** *      *    *     
     492056 AAAAACGACATAGAAGCTATCTCAGGAACTTGTTTATGATGCATCTAATCAACTAACAGTGTTGA
          1 AAAAACTAGACAGAAGCATTCTCAGAAACATCTTTGTGATGTTTGCACTCAACTCACAGAGTTGA

                  * ** * *                         * *       *               
     492121 ACCTTTGTACTGACAGAGCAG-TTTGAAACACTCTTTTTTTGGAATCTGCAAGTGGATATTTGGA
         66 ACCTTTCTTTTCATAGAGCAGTTTTGAAACACTCTTTTTGTAGAATCTGGAAGTGGATATTTGGA

            *                           *   *   *    
     492185 TCGCTTTGAGGATTTCGTTGGAAACGG-GAT-GC--AATAT
        131 GCGCTTTGAGGATTTCGTTGGAAACGGAAATATCTTCATAT

                **  *    *     *                 **  *   *  *    *         * 
     492222 AAAACGTACACAGCAGCATACTCAGAAA-ATACTTTGCCATATTTCCATTCAAGTCACAGAGTGG
          1 AAAAACTAGACAGAAGCATTCTCAGAAACAT-CTTTGTGATGTTTGCACTCAACTCACAGAGTTG

               *  * **       *    *  *               *  *             *      
     492286 AACATTCCCATTCATAGCGCAGGTTGGAAACACTCTTTTTGGAGTATCTGGAAGTGGACATTTGG
         65 AACCTTTCTTTTCATAGAGCAGTTTTGAAACACTCTTTTTGTAGAATCTGGAAGTGGATATTTGG

                    ** * * ** *  *   *           * *  
     492351 AGCGCTTTCTGAACTATGGTGAAAAAGGAAATATCTTAAAAT
        130 AGCGCTTTGAGGATTTCGTTGGAAACGGAAATATCTTCATAT

            *     *               *      * *          *  **      **  *  *    
     492393 GAAAACAAGACAGAAGCATTCTGAGAAACTTATTTGTGATGTGTGTCCTCAACAAACGGACTTGA
          1 AAAAACTAGACAGAAGCATTCTCAGAAACATCTTTGTGATGTTTGCACTCAACTCACAGAGTTGA

                   *      **  * *  *  *             *   *    * * *           
     492458 ACCTTTCGTTTCATGCAGTACTTCTGGAACACTCTTTTTGAAGATTCTGCATGCGGATATTTGGA
         66 ACCTTTCTTTTCATAGAGCAGTTTTGAAACACTCTTTTTGTAGAATCTGGAAGTGGATATTTGGA

            **                            **   *   * 
     492523 TAGCTTTGAGGATTTCGTTGGAAACGG---GCT-TACATGT
        131 GCGCTTTGAGGATTTCGTTGGAAACGGAAATATCTTCATAT

                 *       *               *        *   *    *    *       *    
     492560 AAAAATTAGACAGCAGCATTCTCAGAAACTTCTTTGTGGTGTCTGCATTCAAGTCACAGAATTGA
          1 AAAAACTAGACAGAAGCATTCTCAGAAACATCTTTGTGATGTTTGCACTCAACTCACAGAGTTGA

              * ** * **            *  * *      *       *    *        *       
     492625 ACTTCCCCTCACATAGAGCAGTTGTGCAGCACTCTATTTGTAGTATCTCGAAGTGGACATTTGGA
         66 ACCTTTCTTTTCATAGAGCAGTTTTGAAACACTCTTTTTGTAGAATCTGGAAGTGGATATTTGGA

             *         ** *    * *   *            **  
     492690 GGGCTTTGTAGCCTATC-TGGAAAAAGGAAATATCTTCCCAT
        131 GCGCTTTG-AGGATTTCGTTGGAAACGGAAATATCTTCATAT

            *  ** *   *     * *            *   *  *   * **        *  * * *   
     492731 GAATGCGAGATAGAAGTAATCTCAGAAACATGTTTATGCTGTATCTACTCAACTAACTGTGCTGA
          1 AAAAACTAGACAGAAGCATTCTCAGAAACATCTTTGTGATGTTTGCACTCAACTCACAGAGTTGA

              *     *  *               *        *  * *       *               
     492796 ACATTTCTATTGATAGAGCAGTTTTGAGACACTCTTCTTTTGGAATCTGCAAGTGGATATTTGGA
         66 ACCTTTCTTTTCATAGAGCAGTTTTGAAACACTCTTTTTGTAGAATCTGGAAGTGGATATTTGGA

            ** *                        *     *      
     492861 TAGATTTGAGGATTTCGTTGGAAACGG-GAT-T-AT-ATAT
        131 GCGCTTTGAGGATTTCGTTGGAAACGGAAATATCTTCATAT

                 *       *               *                 **  *   *         
     492898 AAAAAGTAGACAGCAGCATTCTCAGAAACTTCTTTGTGATGTTTGCATCCAGCTCTCAGAGTTGA
          1 AAAAACTAGACAGAAGCATTCTCAGAAACATCTTTGTGATGTTTGCACTCAACTCACAGAGTTGA

              *  * *          *  *        *        *   **        *  **       
     492963 ACATTCCCTTTCATAGAGTAGGTTTGAAACCCTCTTTTTATAGTGTCTGGAAGCGGGCATTTGGA
         66 ACCTTTCTTTTCATAGAGCAGTTTTGAAACACTCTTTTTGTAGAATCTGGAAGTGGATATTTGGA

                   *    * * **  **  **          * *   
     493028 GCGCTTTCAGGCCTATGCTTAAAATAGGAAATATCTACCTAT
        131 GCGCTTTGAGG-ATTTCGTTGGAAACGGAAATATCTTCATAT

             *                    *    *  **          ** *        *    *     
     493070 AGAAACTAGACAGAAGCATTCTGAGAATCACGTTTGTGATGTGGGTACTCAACTAACAGTGTTGA
          1 AAAAACTAGACAGAAGCATTCTCAGAAACATCTTTGTGATGTTTGCACTCAACTCACAGAGTTGA

            *  *       *   *            *   *                *               
     493135 TCCATTCTTTTGATACAGCAGTTTTGAACCACACTTTTTGTAGAATCTGCAAGTGGATATTTGGA
         66 ACCTTTCTTTTCATAGAGCAGTTTTGAAACACTCTTTTTGTAGAATCTGGAAGTGGATATTTGGA

            **   *                     *   *        *
     493200 TAGCTGTGAGGATTTCGTTGGAAACGGGAATGTCTTCATAG
        131 GCGCTTTGAGGATTTCGTTGGAAACGGAAATATCTTCATAT

                **                     * * **         *  **   *   *          
     493241 AAAATTTAGACAGAAGCATTCTCAGAACCTTGATTGTGATGTGTGTTCTCCACTAACAGAGTTGA
          1 AAAAACTAGACAGAAGCATTCTCAGAAACATCTTTGTGATGTTTGCACTCAACTCACAGAGTTGA

                       * *   * *   *       *       *                         
     493306 ACCTTTCTTTTGACAGAACTGTTCTGAAACATTCTTTTTATAGAATCTGGAAGTGGATATTTGGA
         66 ACCTTTCTTTTCATAGAGCAGTTTTGAAACACTCTTTTTGTAGAATCTGGAAGTGGATATTTGGA

            **                         *          *  
     493371 AAGCTTTGAGGATTTCGTTGGAAACGGGAATATCTTCAAAT
        131 GCGCTTTGAGGATTTCGTTGGAAACGGAAATATCTTCATAT

            *   *    *            *           * *       *  *    *            
     493412 CAAATCTAGCCAGAAGCATTCTAAGAAACATCTTAGGGATGTTTACATTCAAGTCACAGAGTTGA
          1 AAAAACTAGACAGAAGCATTCTCAGAAACATCTTTGTGATGTTTGCACTCAACTCACAGAGTTGA

              *  * *     *       *         *    * *   **      *      *    *  
     493477 ACATTCCCTTTCACAGAGCAGGTTTGAAACAATCTTCTCGTACTATCTGGCAGTGGACATTTTGA
         66 ACCTTTCTTTTCATAGAGCAGTTTTGAAACACTCTTTTTGTAGAATCTGGAAGTGGATATTTGGA

              * *   *  ** ** *  *  **            ** *
     493542 GCTCCTTGGGGCCTATGCTGAAATAGGAAATATCTTCCGAC
        131 GCGCTTTGAGGATTTCGTTGGAAACGGAAATATCTTCATAT

                                 *     *  **          *           **    *    
     493583 AAAAACTAGACAGAAGCATTCGCAGAATCACGTTTGTGATGTGTGCACTCAACTGTCAGAATTGA
          1 AAAAACTAGACAGAAGCATTCTCAGAAACATCTTTGTGATGTTTGCACTCAACTCACAGAGTTGA

                 **   ** *      *                            * *            *
     493648 ACCTTGGTTTGGACAGAGCACTTTTGAAACACTCTTTTTGTAGAATCTGCAGGTGGATATTTGGC
         66 ACCTTTCTTTTCATAGAGCAGTTTTGAAACACTCTTTTTGTAGAATCTGGAAGTGGATATTTGGA

            **                         *   *      * *
     493713 TAGCTTTGAGGATTTCGTTGGAAACGGTAATGTCTTCAAAG
        131 GCGCTTTGAGGATTTCGTTGGAAACGGAAATATCTTCATAT

                *                         *   *            *    *            
     493754 AAAATCTAGACAGAAGCATTCTCAGAAACAACTTCGTGATGTTTGCAATCAAGTCACAGAGTTGA
          1 AAAAACTAGACAGAAGCATTCTCAGAAACATCTTTGTGATGTTTGCACTCAACTCACAGAGTTGA

                 * *             *  *                  *             *       
     493819 ACCTTCCGTTTCATAGAGCAGGTTGGAAACACTC-TTTTGTAGTATCTGGAAGTGGACATTTGGA
         66 ACCTTTCTTTTCATAGAGCAGTTTTGAAACACTCTTTTTGTAGAATCTGGAAGTGGATATTTGGA

             *         ** *    * *   *            **  
     493883 GGGCTTTGTAGCCTATC-TGGAAAAAGGAAATATCTTCCCAT
        131 GCGCTTTG-AGGATTTCGTTGGAAACGGAAATATCTTCATAT

            *  ** *   *     * *            *   *  *   * **        *  * * *   
     493924 GAATGCGAGATAGAAGTAATCTCAGAAACATGTTTATGCTGTATCTACTCAACTAACTGTGCTGA
          1 AAAAACTAGACAGAAGCATTCTCAGAAACATCTTTGTGATGTTTGCACTCAACTCACAGAGTTGA

              *     *  *               *        *  * *       *               
     493989 ACATTTCTATTGATAGAGCAGTTTTGAGACACTCTTCTTTTGGAATCTGCAAGTGGATATTTGGA
         66 ACCTTTCTTTTCATAGAGCAGTTTTGAAACACTCTTTTTGTAGAATCTGGAAGTGGATATTTGGA

            ** *                       **     *      
     494054 TAGATTTGAGGATTTCGTTGGAAAC-GCGAT-T-AT-ATAT
        131 GCGCTTTGAGGATTTCGTTGGAAACGGAAATATCTTCATAT

                 *       *               *                 **  *   *         
     494091 AAAAAGTAGACAGCAGCATTCTCAGAAACTTCTTTGTGATGTTTGCATCCAGCTCTCAGAGTTGA
          1 AAAAACTAGACAGAAGCATTCTCAGAAACATCTTTGTGATGTTTGCACTCAACTCACAGAGTTGA

            * *  * *          *  *        *        *   **        *  **       
     494156 GCATTCCCTTTCATAGAGTAGGTTTGAAACCCTCTTTTTATAGTGTCTGGAAGCGGGCATTTGGA
         66 ACCTTTCTTTTCATAGAGCAGTTTTGAAACACTCTTTTTGTAGAATCTGGAAGTGGATATTTGGA

                   *    * * **  **  **          * *  *
     494221 GCGCTTTCAGGCCTATGCTTAAAATAGGAAATATCTACCTAC
        131 GCGCTTTGAGG-ATTTCGTTGGAAACGGAAATATCTTCATAT

             *                    *    *  **          ** *        *    *     
     494263 AGAAACTAGACAGAAGCATTCTGAGAATCACGTTTGTGATGTGGGTACTCAACTAACAGTGTTGA
          1 AAAAACTAGACAGAAGCATTCTCAGAAACATCTTTGTGATGTTTGCACTCAACTCACAGAGTTGA

            *  *       *   *            *   *                *               
     494328 TCCATTCTTTTGATACAGCAGTTTTGAACCACACTTTTTGTAGAATCTGCAAGTGGATATTTGGA
         66 ACCTTTCTTTTCATAGAGCAGTTTTGAAACACTCTTTTTGTAGAATCTGGAAGTGGATATTTGGA

            **   *                     *   *      * *
     494393 TAGCTGTGAGGATTTCGTTGGAAACGGTAATGTCTTCAAAG
        131 GCGCTTTGAGGATTTCGTTGGAAACGGAAATATCTTCATAT

                *                         *   *            *    *            
     494434 AAAATCTAGACAGAAGCATTCTCAGAAACACCTTCGTGATGTTTGCAATCAAGTCACAGAGTTGA
          1 AAAAACTAGACAGAAGCATTCTCAGAAACATCTTTGTGATGTTTGCACTCAACTCACAGAGTTGA

                 * *    *        *  *           *      *             *       
     494499 ACCTTCCGTTTCGTAGAGCAGGTTGGAAACACTCTTATTGTAGTATCTGGAAGTGGACATTTGGA
         66 ACCTTTCTTTTCATAGAGCAGTTTTGAAACACTCTTTTTGTAGAATCTGGAAGTGGATATTTGGA

                   *   ** ** *  *   *            **  
     494564 GCGCTTTCAGGCCTATGGTGAAAAAGGAAATATCTTCCCAT
        131 GCGCTTTGAGGATTTCGTTGGAAACGGAAATATCTTCATAT

                  * * *      **      *   * *   *     ** ** *      *    *     
     494605 AAAAACGACATAGAAGCTATCTCAGGAACTTGTTTCTGATGCATCTAATCAACTAACAGTGTTGA
          1 AAAAACTAGACAGAAGCATTCTCAGAAACATCTTTGTGATGTTTGCACTCAACTCACAGAGTTGA

                  * ** * *       *                 * *       *               
     494670 ACCTTTGTACTGACAGAGCA-CTTTGAAACACTCTTTTTTTGGAATCTGCAAGTGGATATTTGGA
         66 ACCTTTCTTTTCATAGAGCAGTTTTGAAACACTCTTTTTGTAGAATCTGGAAGTGGATATTTGGA

            *                           *   *   *    
     494734 TCGCTTTGAGGATTTCGTTGGAAACGG-GAT-GC--AATAT
        131 GCGCTTTGAGGATTTCGTTGGAAACGGAAATATCTTCATAT

                **  *    *     *                 **  *   *  *    *         * 
     494771 AAAACGTACACAGCAGCATACTCAGAAA-ATACTTTGCCATATTTCCATTCAAGTCACAGAGTGG
          1 AAAAACTAGACAGAAGCATTCTCAGAAACAT-CTTTGTGATGTTTGCACTCAACTCACAGAGTTG

               *  * **            *  *               *  *             *      
     494835 AACATTCCCATTCATAGAGCAGGTTGGAAACACTCTTTTTGGAGTATCTGGAAGTGGACATTTGG
         65 AACCTTTCTTTTCATAGAGCAGTTTTGAAACACTCTTTTTGTAGAATCTGGAAGTGGATATTTGG

                    ** * * ** *  *   *            **  
     494900 AGCGCTTTCTGAACTATGGTGAAAAAGGAAATATCTTCCAAT
        130 AGCGCTTTGAGGATTTCGTTGGAAACGGAAATATCTTCATAT

            *     *               *      * *          *  **      **  *  *    
     494942 GAAAACAAGACAGAAGCATTCTGAGAAACTTATTTGTGATGTGTGTCCTCAACAAACGGACTTGA
          1 AAAAACTAGACAGAAGCATTCTCAGAAACATCTTTGTGATGTTTGCACTCAACTCACAGAGTTGA

                   *      **  * *  *  *             *   *    * * *           
     495007 ACCTTTCGTTTCATGCAGTACTTCTGGAACACTCTTTTTGAAGATTCTGCATGCGGATATTTGGA
         66 ACCTTTCTTTTCATAGAGCAGTTTTGAAACACTCTTTTTGTAGAATCTGGAAGTGGATATTTGGA

            **                            **   *   * 
     495072 TAGCTTTGAGGATTTCGTTGGAAACGG---GCT-TACATGT
        131 GCGCTTTGAGGATTTCGTTGGAAACGGAAATATCTTCATAT

                 *       *               *        *   *    *    *       *    
     495109 AAAAATTAGACAGCAGCATTCTCAGAAACTTCTTTGTGGTGTCTGCATTCAAGTCACAGAATTGA
          1 AAAAACTAGACAGAAGCATTCTCAGAAACATCTTTGTGATGTTTGCACTCAACTCACAGAGTTGA

              * ** * **            *  * *      *       *             *       
     495174 ACTTCCCCTCACATAGAGCAGTTGTGCAGCACTCTATTTGTAGTATCTGGAAGTGGACATTTGGA
         66 ACCTTTCTTTTCATAGAGCAGTTTTGAAACACTCTTTTTGTAGAATCTGGAAGTGGATATTTGGA

             *         ** *    * *   *            **  
     495239 GGGCTTTGTAGCCTATC-TGGAAAAAGGAAATATCTTCCCAT
        131 GCGCTTTG-AGGATTTCGTTGGAAACGGAAATATCTTCATAT

            *  ** *   *     * *            *   *  *   * **        *  * * *   
     495280 GAATGCGAGATAGAAGTAATCTCAGAAACATGTTTATGCTGTATCTACTCAACTAACTGTGCTGA
          1 AAAAACTAGACAGAAGCATTCTCAGAAACATCTTTGTGATGTTTGCACTCAACTCACAGAGTTGA

              *     *  *               *        *  * *       *               
     495345 ACATTTCTATTGATAGAGCAGTTTTGAGACACTCTTCTTTTGGAATCTGCAAGTGGATATTTGGA
         66 ACCTTTCTTTTCATAGAGCAGTTTTGAAACACTCTTTTTGTAGAATCTGGAAGTGGATATTTGGA

            ** *                        *     *      
     495410 TAGATTTGAGGATTTCGTTGGAAACGG-GAT-T-AT-ATAT
        131 GCGCTTTGAGGATTTCGTTGGAAACGGAAATATCTTCATAT

            *    *       *               *                 **  *   *         
     495447 CAAAAGTAGACAGCAGCATTCTCAGAAACTTCTTTGTGATGTTTGCATCCAGCTCTCAGAGTTGA
          1 AAAAACTAGACAGAAGCATTCTCAGAAACATCTTTGTGATGTTTGCACTCAACTCACAGAGTTGA

              *  * *          *  *        *        *   **        *  **       
     495512 ACATTCCCTTTCATAGAGTAGGTTTGAAACCCTCTTTTTATAGTGTCTGGAAGCGGGCATTTGGA
         66 ACCTTTCTTTTCATAGAGCAGTTTTGAAACACTCTTTTTGTAGAATCTGGAAGTGGATATTTGGA

                   *    * * **  **  **          * *  *
     495577 GCGCTTTCAGGCCTATGCTTAAAATAGGAAATATCTACCTAC
        131 GCGCTTTGAGG-ATTTCGTTGGAAACGGAAATATCTTCATAT

             *                    *    *  **          ** *        *    *     
     495619 AGAAACTAGACAGAAGCATTCTGAGAATCACGTTTGTGATGTGGGTACTCAACTAACAGTGTTGA
          1 AAAAACTAGACAGAAGCATTCTCAGAAACATCTTTGTGATGTTTGCACTCAACTCACAGAGTTGA

            *  *       *   *            *   *                *   *           
     495684 TCCATTCTTTTGATACAGCAGTTTTGAACCACACTTTTTGTAGAATCTGCAAGAGGATATTTGGA
         66 ACCTTTCTTTTCATAGAGCAGTTTTGAAACACTCTTTTTGTAGAATCTGGAAGTGGATATTTGGA

            **   *                     *   *      * *
     495749 TAGCTGTGAGGATTTCGTTGGAAACGGGAATGTCTTCAAAG
        131 GCGCTTTGAGGATTTCGTTGGAAACGGAAATATCTTCATAT

                *                         *   *            *    *            
     495790 AAAATCTAGACAGAAGCATTCTCAGAAACACCTTCGTGATGTTTGCAATCAAGTCACAGAGTTGA
          1 AAAAACTAGACAGAAGCATTCTCAGAAACATCTTTGTGATGTTTGCACTCAACTCACAGAGTTGA

                 * *             *  *           *      *             *       
     495855 ACCTTCCGTTTCATAGAGCAGGTTGGAAACACTCTTATTGTAGTATCTGGAAGTGGACATTTGGA
         66 ACCTTTCTTTTCATAGAGCAGTTTTGAAACACTCTTTTTGTAGAATCTGGAAGTGGATATTTGGA

                   *   ** ** *  *   *            **  
     495920 GCGCTTTCAGGCCTATGGTGAAAAAGGAAATATCTTCCCAT
        131 GCGCTTTGAGGATTTCGTTGGAAACGGAAATATCTTCATAT

                  * * **     **      *   * *   *     ** ** *      *    *     
     495961 AAAAACGACATCGAAGCTATCTCAGGAACTTGTTTATGATGCATCTAATCAACTAACAGTGTTGA
          1 AAAAACTAGACAGAAGCATTCTCAGAAACATCTTTGTGATGTTTGCACTCAACTCACAGAGTTGA

                  * ** * *                         * *       *               
     496026 ACCTTTGTACTGACAGAGCAG-TTTGAAACACTCTTTTTTTGGAATCTGCAAGTGGATATTTGGA
         66 ACCTTTCTTTTCATAGAGCAGTTTTGAAACACTCTTTTTGTAGAATCTGGAAGTGGATATTTGGA

            *                           *   *   *    
     496090 TCGCTTTGAGGATTTCGTTGGAAACGG-GAT-GC--AATAT
        131 GCGCTTTGAGGATTTCGTTGGAAACGGAAATATCTTCATAT

                **  *    *     *                 **  *   *  *    *         * 
     496127 AAAACGTACACAGCAGCATACTCAGAAA-ATACTTTGCCATATTTCCATTCAAGTCACAGAGTGG
          1 AAAAACTAGACAGAAGCATTCTCAGAAACAT-CTTTGTGATGTTTGCACTCAACTCACAGAGTTG

               *  * **            *                  *  *             *      
     496191 AACATTCCCATTCATAGAGCAGGTTTGAAACACTCTTTTTGGAGTATCTGGAAGTGGACATTTGG
         65 AACCTTTCTTTTCATAGAGCAGTTTTGAAACACTCTTTTTGTAGAATCTGGAAGTGGATATTTGG

                    ** * * ** *  *   *            **  
     496256 AGCGCTTTCTGAACTATGGTGAAAAAGGAAATATCTTCCAAT
        130 AGCGCTTTGAGGATTTCGTTGGAAACGGAAATATCTTCATAT

            *     *               *      * *          *  **      **  *  *    
     496298 GAAAACAAGACAGAAGCATTCTGAGAAACTTATTTGTGATGTGTGTCCTCAACAAACGGACTTGA
          1 AAAAACTAGACAGAAGCATTCTCAGAAACATCTTTGTGATGTTTGCACTCAACTCACAGAGTTGA

                   *      **  * *  *  *             *   *    * * *           
     496363 ACCTTTCGTTTCATGCAGTACTTCTGGAACACTCTTTTTGAAGATTCTGCATGCGGATATTTGGA
         66 ACCTTTCTTTTCATAGAGCAGTTTTGAAACACTCTTTTTGTAGAATCTGGAAGTGGATATTTGGA

            **                          **    *    * 
     496428 TAGCTTTGAGGATTTCGTTGGAAACGG-GCT-T-AT-ATGT
        131 GCGCTTTGAGGATTTCGTTGGAAACGGAAATATCTTCATAT

                 *       *               *        *   *    *    *       *    
     496465 AAAAATTAGACAGCAGCATTCTCAGAAACTTCTTTGTGGTGTCTGCATTCAAGTCACAGAATTGA
          1 AAAAACTAGACAGAAGCATTCTCAGAAACATCTTTGTGATGTTTGCACTCAACTCACAGAGTTGA

              * ** * **            *  * *      *       *             *       
     496530 ACTTCCCCTCACATAGAGCAGTTGTGCAGCACTCTATTTGTAGTATCTGGAAGTGGACATTTGGA
         66 ACCTTTCTTTTCATAGAGCAGTTTTGAAACACTCTTTTTGTAGAATCTGGAAGTGGATATTTGGA

             *         ** *    * *   *            **  
     496595 GGGCTTTGTAGCCTATC-TGGAAAAAGGAAATATCTTCCCAT
        131 GCGCTTTG-AGGATTTCGTTGGAAACGGAAATATCTTCATAT

            *  ** *   *     * *            *   *  *   * **        *  * * *   
     496636 GAATGCGAGATAGAAGTAATCTCAGAAACATGTTTATGCTGTATCTACTCAACTAACTGTGCTGA
          1 AAAAACTAGACAGAAGCATTCTCAGAAACATCTTTGTGATGTTTGCACTCAACTCACAGAGTTGA

              *     *  *               *        *  * *       *               
     496701 ACATTTCTATTGATAGAGCAGTTTTGAGACACTCTTCTTTTGGAATCTGCAAGTGGATATTTGGA
         66 ACCTTTCTTTTCATAGAGCAGTTTTGAAACACTCTTTTTGTAGAATCTGGAAGTGGATATTTGGA

            ** *                        *     *      
     496766 TAGATTTGAGGATTTCGTTGGAAACGG-GAT-T-AT-ATAT
        131 GCGCTTTGAGGATTTCGTTGGAAACGGAAATATCTTCATAT

            *    *       *               *                 **  *   *         
     496803 CAAAAGTAGACAGCAGCATTCTCAGAAACTTCTTTGTGATGTTTGCATCCAGCTCTCAGAGTTGA
          1 AAAAACTAGACAGAAGCATTCTCAGAAACATCTTTGTGATGTTTGCACTCAACTCACAGAGTTGA

              *  * *          *  *        *        *   **        *  **       
     496868 ACATTCCCTTTCATAGAGTAGGTTTGAAACCCTCTTTTTATAGTGTCTGGAAGCGGGCATTTGGA
         66 ACCTTTCTTTTCATAGAGCAGTTTTGAAACACTCTTTTTGTAGAATCTGGAAGTGGATATTTGGA

                   *    * * **  **  **          * *  *
     496933 GCGCTTTCAGGCCTATGCTTAAAATAGGAAATATCTACCTAC
        131 GCGCTTTGAGG-ATTTCGTTGGAAACGGAAATATCTTCATAT

             *                    *    *  **          ** *        *    *     
     496975 AGAAACTAGACAGAAGCATTCTGAGAATCACGTTTGTGATGTGGGTACTCAACTAACAGTGTTGA
          1 AAAAACTAGACAGAAGCATTCTCAGAAACATCTTTGTGATGTTTGCACTCAACTCACAGAGTTGA

            *  *       *   *            *   *                *   *           
     497040 TCCATTCTTTTGATACAGCAGTTTTGAACCACACTTTTTGTAGAATCTGCAAGAGGATATTTGGA
         66 ACCTTTCTTTTCATAGAGCAGTTTTGAAACACTCTTTTTGTAGAATCTGGAAGTGGATATTTGGA

            **   *                     *   *      * *
     497105 TAGCTGTGAGGATTTCGTTGGAAACGGGAATGTCTTCAAAG
        131 GCGCTTTGAGGATTTCGTTGGAAACGGAAATATCTTCATAT

                *                         *   *            *    *            
     497146 AAAATCTAGACAGAAGCATTCTCAGAAACACCTTCGTGATGTTTGCAATCAAGTCACAGAGTTGA
          1 AAAAACTAGACAGAAGCATTCTCAGAAACATCTTTGTGATGTTTGCACTCAACTCACAGAGTTGA

                 * *             *  *           *      *             *       
     497211 ACCTTCCGTTTCATAGAGCAGGTTGGAAACACTCTTATTGTAGTATCTGGAAGTGGACATTTGGA
         66 ACCTTTCTTTTCATAGAGCAGTTTTGAAACACTCTTTTTGTAGAATCTGGAAGTGGATATTTGGA

                   *   ** ** *  *   *            **  
     497276 GCGCTTTCAGGCCTATGGTGAAAAAGGAAATATCTTCCCAT
        131 GCGCTTTGAGGATTTCGTTGGAAACGGAAATATCTTCATAT

                  * * *      **      *   * *   *     ** ** *      *    *     
     497317 AAAAACGACATAGAAGCTATCTCAGGAACTTGTTTATGATGCATCTAATCAACTAACAGTGTTGA
          1 AAAAACTAGACAGAAGCATTCTCAGAAACATCTTTGTGATGTTTGCACTCAACTCACAGAGTTGA

                  * ** * *                         * *       *               
     497382 ACCTTTGTACTGACAGAGCAG-TTTGAAACACTCTTTTTTTGGAATCTGCAAGTGGATATTTGGA
         66 ACCTTTCTTTTCATAGAGCAGTTTTGAAACACTCTTTTTGTAGAATCTGGAAGTGGATATTTGGA

            *                           *   *   *    
     497446 TCGCTTTGAGGATTTCGTTGGAAACGG-GAT-GC--AATAT
        131 GCGCTTTGAGGATTTCGTTGGAAACGGAAATATCTTCATAT

                **  *    *     *                 **  *   *  *    *         * 
     497483 AAAACGTACACAGCAGCATACTCAGAAA-ATACTTTGCCATATTTCCATTCAAGTCACAGAGTGG
          1 AAAAACTAGACAGAAGCATTCTCAGAAACAT-CTTTGTGATGTTTGCACTCAACTCACAGAGTTG

               *  * **            *                  *  *             *      
     497547 AACATTCCCATTCATAGAGCAGGTTTGAAACACTCTTTTTGGAGTATCTGGAAGTGGACATTTGG
         65 AACCTTTCTTTTCATAGAGCAGTTTTGAAACACTCTTTTTGTAGAATCTGGAAGTGGATATTTGG

                    ** * * ** *  *   *            **  
     497612 AGCGCTTTCTGAACTATGGTGAAAAAGGAAATATCTTCCAAT
        130 AGCGCTTTGAGGATTTCGTTGGAAACGGAAATATCTTCATAT

            *     *               *      * *          *  **      **  *  *    
     497654 GAAAACAAGACAGAAGCATTCTGAGAAACTTATTTGTGATGTGTGTCCTCAACAAACGGACTTGA
          1 AAAAACTAGACAGAAGCATTCTCAGAAACATCTTTGTGATGTTTGCACTCAACTCACAGAGTTGA

                   *      **  * *  *  *             *   *    * * *           
     497719 ACCTTTCGTTTCATGCAGTACTTCTGGAACACTCTTTTTGAAGATTCTGCATGCGGATATTTGGA
         66 ACCTTTCTTTTCATAGAGCAGTTTTGAAACACTCTTTTTGTAGAATCTGGAAGTGGATATTTGGA

            **                            **   *   * 
     497784 TAGCTTTGAGGATTTCGTTGGAAACGG---GCT-TACATGT
        131 GCGCTTTGAGGATTTCGTTGGAAACGGAAATATCTTCATAT

                 *       *               *        *   *    *    *       *    
     497821 AAAAATTAGACAGCAGCATTCTCAGAAACTTCTTTGTGGTGTCTGCATTCAAGTCACAGAATTGA
          1 AAAAACTAGACAGAAGCATTCTCAGAAACATCTTTGTGATGTTTGCACTCAACTCACAGAGTTGA

              * ** * **            *  * *      *       *             *       
     497886 ACTTCCCCTCACATAGAGCAGTTGTGCAGCACTCTATTTGTAGTATCTGGAAGTGGACATTTGGA
         66 ACCTTTCTTTTCATAGAGCAGTTTTGAAACACTCTTTTTGTAGAATCTGGAAGTGGATATTTGGA

             *         ** *    * *   *            **  
     497951 GGGCTTTGTAGCCTATC-TGGAAAAAGGAAATATCTTCCCAT
        131 GCGCTTTG-AGGATTTCGTTGGAAACGGAAATATCTTCATAT

            *  ** *   *     * *            *   *  *   * **        *  * * *   
     497992 GAATGCGAGATAGAAGTAATCTCAGAAACATGTTTATGCTGTATCTACTCAACTAACTGTGCTGA
          1 AAAAACTAGACAGAAGCATTCTCAGAAACATCTTTGTGATGTTTGCACTCAACTCACAGAGTTGA

              *     *  *               *        *  * *       *               
     498057 ACATTTCTATTGATAGAGCAGTTTTGAGACACTCTTCTTTTGGAATCTGCAAGTGGATATTTGGA
         66 ACCTTTCTTTTCATAGAGCAGTTTTGAAACACTCTTTTTGTAGAATCTGGAAGTGGATATTTGGA

            ** *                        *     *      
     498122 TAGATTTGAGGATTTCGTTGGAAACGG-GAT-T-AT-ATAT
        131 GCGCTTTGAGGATTTCGTTGGAAACGGAAATATCTTCATAT

                 *       *               *                 **  *   *         
     498159 AAAAAGTAGACAGCAGCATTCTCAGAAACTTCTTTGTGATGTTTGCATCCAGCTCTCAGAGTTGA
          1 AAAAACTAGACAGAAGCATTCTCAGAAACATCTTTGTGATGTTTGCACTCAACTCACAGAGTTGA

              *  * *          *  *        *        *   **        *  **       
     498224 ACATTCCCTTTCATAGAGTAGGTTTGAAACCCTCTTTTTATAGTGTCTGGAAGCGGGCATTTGGA
         66 ACCTTTCTTTTCATAGAGCAGTTTTGAAACACTCTTTTTGTAGAATCTGGAAGTGGATATTTGGA

                   *    * * **  **  **          * *  *
     498289 GCGCTTTCAGGCCTATGCTTAAAATAGGAAATATCTACCTAC
        131 GCGCTTTGAGG-ATTTCGTTGGAAACGGAAATATCTTCATAT

             *                    *    *  **          ** *        *    *     
     498331 AGAAACTAGACAGAAGCATTCTGAGAATCACGTTTGTGATGTGGGTACTCAACTAACAGTGTTGA
          1 AAAAACTAGACAGAAGCATTCTCAGAAACATCTTTGTGATGTTTGCACTCAACTCACAGAGTTGA

            *  *       *   *            *   *                *   *           
     498396 TCCATTCTTTTGATACAGCAGTTTTGAACCACACTTTTTGTAGAATCTGCAAGAGGATATTTGGA
         66 ACCTTTCTTTTCATAGAGCAGTTTTGAAACACTCTTTTTGTAGAATCTGGAAGTGGATATTTGGA

            **   *                     *   *      * *
     498461 TAGCTGTGAGGATTTCGTTGGAAACGGGAATGTCTTCAAAG
        131 GCGCTTTGAGGATTTCGTTGGAAACGGAAATATCTTCATAT

                *                         *   *            *    *            
     498502 AAAATCTAGACAGAAGCATTCTCAGAAACACCTTCGTGATGTTTGCAATCAAGTCACAGAGTTGA
          1 AAAAACTAGACAGAAGCATTCTCAGAAACATCTTTGTGATGTTTGCACTCAACTCACAGAGTTGA

                 * *             *  *           *      *             *       
     498567 ACCTTCCGTTTCATAGAGCAGGTTGGAAACACTCTTATTGTAGTATCTGGAAGTGGACATTTGGA
         66 ACCTTTCTTTTCATAGAGCAGTTTTGAAACACTCTTTTTGTAGAATCTGGAAGTGGATATTTGGA

                   *   ** ** *  *   *            **  
     498632 GCGCTTTCAGGCCTATGGTGAAAAAGGAAATATCTTCCCAT
        131 GCGCTTTGAGGATTTCGTTGGAAACGGAAATATCTTCATAT

              *   * * *      **      *   * *   *     ** ** *      *    *     
     498673 AACAACGACATAGAAGCTATCTCAGGAACTTGTTTATGATGCATCTAATCAACTAACAGTGTTGA
          1 AAAAACTAGACAGAAGCATTCTCAGAAACATCTTTGTGATGTTTGCACTCAACTCACAGAGTTGA

                    ** * *                         * *       *               
     498738 ACCTTTCTACTGACAGAGCAG-TTTGAAACACTCTTTTTTTGGAATCTGCAAGTGGATATTTGGA
         66 ACCTTTCTTTTCATAGAGCAGTTTTGAAACACTCTTTTTGTAGAATCTGGAAGTGGATATTTGGA

            *                           *   *   *    
     498802 TCGCTTTGAGGATTTCGTTGGAAACGG-GAT-GC--AATAT
        131 GCGCTTTGAGGATTTCGTTGGAAACGGAAATATCTTCATAT

                **  *    *     *                 **  *   *  *    *   *     * 
     498839 AAAACGTACACAGCAGCATACTCAGAAA-ATACTTTGCCATATTTCCATTCAAGTCAGAGAGTGG
          1 AAAAACTAGACAGAAGCATTCTCAGAAACAT-CTTTGTGATGTTTGCACTCAACTCACAGAGTTG

               *  * **            *                  *  *             *      
     498903 AACATTCCCATTCATAGAGCAGGTTTGAAACACTCTTTTTGGAGTATCTGGAAGTGGACATTTGG
         65 AACCTTTCTTTTCATAGAGCAGTTTTGAAACACTCTTTTTGTAGAATCTGGAAGTGGATATTTGG

                    ** * * ** *  *   *            **  
     498968 AGCGCTTTCTGAACTATGGTGAAAAAGGAAATATCTTCCAAT
        130 AGCGCTTTGAGGATTTCGTTGGAAACGGAAATATCTTCATAT

            *     *               *      * *          *  **      **  *  *    
     499010 GAAAACAAGACAGAAGCATTCTGAGAAACTTATTTGTGATGTGTGTCCTCAACAAACGGACTTGA
          1 AAAAACTAGACAGAAGCATTCTCAGAAACATCTTTGTGATGTTTGCACTCAACTCACAGAGTTGA

                   *      **  * *  *  *             *   *    * * *           
     499075 ACCTTTCGTTTCATGCAGTACTTCTGGAACACTCTTTTTGAAGATTCTGCATGCGGATATTTGGA
         66 ACCTTTCTTTTCATAGAGCAGTTTTGAAACACTCTTTTTGTAGAATCTGGAAGTGGATATTTGGA

            **                            **   *   * 
     499140 TAGCTTTGAGGATTTCGTTGGAAACGG---GCT-TACATGT
        131 GCGCTTTGAGGATTTCGTTGGAAACGGAAATATCTTCATAT

                 *       *               *        *   *    *    *       *    
     499177 AAAAATTAGACAGCAGCATTCTCAGAAACTTCTTTGTGGTGTCTGCATTCAAGTCACAGAATTGA
          1 AAAAACTAGACAGAAGCATTCTCAGAAACATCTTTGTGATGTTTGCACTCAACTCACAGAGTTGA

              * ** * **            *  * *      *       *             *       
     499242 ACTTCCCCTCACATAGAGCAGTTGTGCAGCACTCTATTTGTAGTATCTGGAAGTGGACATTTGGA
         66 ACCTTTCTTTTCATAGAGCAGTTTTGAAACACTCTTTTTGTAGAATCTGGAAGTGGATATTTGGA

             *         ** *    * *   *            **  
     499307 GGGCTTTGTAGCCTATC-TGGAAAAAGGAAATATCTTCCCAT
        131 GCGCTTTG-AGGATTTCGTTGGAAACGGAAATATCTTCATAT

            *  ** *   *     * *            *   *  *   * **        *  * * *   
     499348 GAATGCGAGATAGAAGTAATCTCAGAAACATGTTTATGCTGTATCTACTCAACTAACTGTGCTGA
          1 AAAAACTAGACAGAAGCATTCTCAGAAACATCTTTGTGATGTTTGCACTCAACTCACAGAGTTGA

              *     *  *               *        *  * *       *               
     499413 ACATTTCTATTGATAGAGCAGTTTTGAGACACTCTTCTTTTGGAATCTGCAAGTGGATATTTGGA
         66 ACCTTTCTTTTCATAGAGCAGTTTTGAAACACTCTTTTTGTAGAATCTGGAAGTGGATATTTGGA

            ** *                        *     *      
     499478 TAGATTTGAGGATTTCGTTGGAAACGG-GAT-T-AT-ATAT
        131 GCGCTTTGAGGATTTCGTTGGAAACGGAAATATCTTCATAT

            *    **      *               *                 **  *   *         
     499515 CAAAAGCAGACAGCAGCATTCTCAGAAACTTCTTTGTGATGTTTGCATCCAGCTCTCAGAGTTGA
          1 AAAAACTAGACAGAAGCATTCTCAGAAACATCTTTGTGATGTTTGCACTCAACTCACAGAGTTGA

              *  * *          *  *        *        *   **        *  **       
     499580 ACATTCCCTTTCATAGAGTAGGTTTGAAACCCTCTTTTTATAGTGTCTGGAAGCGGGCATTTGGA
         66 ACCTTTCTTTTCATAGAGCAGTTTTGAAACACTCTTTTTGTAGAATCTGGAAGTGGATATTTGGA

                   *    * * **  **  **          * *  *
     499645 GCGCTTTCAGGCCTATGCTTAAAATAGGAAATATCTACCTAC
        131 GCGCTTTGAGG-ATTTCGTTGGAAACGGAAATATCTTCATAT

             *                    *    *  **          ** *        *    *     
     499687 AGAAACTAGACAGAAGCATTCTGAGAATCACGTTTGTGATGTGGGTACTCAACTAACAGTGTTGA
          1 AAAAACTAGACAGAAGCATTCTCAGAAACATCTTTGTGATGTTTGCACTCAACTCACAGAGTTGA

            *  *       *   *            *   *                *   *           
     499752 TCCATTCTTTTGATACAGCAGTTTTGAACCACACTTTTTGTAGAATCTGCAAGAGGATATTTGGA
         66 ACCTTTCTTTTCATAGAGCAGTTTTGAAACACTCTTTTTGTAGAATCTGGAAGTGGATATTTGGA

            **   *                     *   *      * *
     499817 TAGCTGTGAGGATTTCGTTGGAAACGGGAATGTCTTCAAAG
        131 GCGCTTTGAGGATTTCGTTGGAAACGGAAATATCTTCATAT

                *                         *   *            *    *            
     499858 AAAATCTAGACAGAAGCATTCTCAGAAACACCTTCGTGATGTTTGCAATCAAGTCACAGAGTTGA
          1 AAAAACTAGACAGAAGCATTCTCAGAAACATCTTTGTGATGTTTGCACTCAACTCACAGAGTTGA

                 * *             *  *           *      *             *       
     499923 ACCTTCCGTTTCATAGAGCAGGTTGGAAACACTCTTATTGTAGTATCTGGAAGTGGACATTTGGA
         66 ACCTTTCTTTTCATAGAGCAGTTTTGAAACACTCTTTTTGTAGAATCTGGAAGTGGATATTTGGA

                   *   ** ** *  *   *            **  
     499988 GCGCTTTCAGGCCTATGGTGAAAAAGGAAATATCTTCCCAT
        131 GCGCTTTGAGGATTTCGTTGGAAACGGAAATATCTTCATAT

                  * * *      **      *   * *   *     ** ** *      *    *     
     500029 AAAAACGACATAGAAGCTATCTCAGGAACTTGTTTATGATGCATCTAATCAACTAACAGTGTTGA
          1 AAAAACTAGACAGAAGCATTCTCAGAAACATCTTTGTGATGTTTGCACTCAACTCACAGAGTTGA

                  * ** * *                         * *       *               
     500094 ACCTTTGTACTGACAGAGCAG-TTTGAAACACTCTTTTTTTGGAATCTGCAAGTGGATATTTGGA
         66 ACCTTTCTTTTCATAGAGCAGTTTTGAAACACTCTTTTTGTAGAATCTGGAAGTGGATATTTGGA

            *                           *   *   *    
     500158 TCGCTTTGAGGATTTCGTTGGAAACGG-GAT-GC--AATAT
        131 GCGCTTTGAGGATTTCGTTGGAAACGGAAATATCTTCATAT

                **  *    *     *                 **  *   *  *    *         * 
     500195 AAAACGTACACAGCAGCATACTCAGAAA-ATACTTTGCCATATTTCCATTCAAGTCACAGAGTGG
          1 AAAAACTAGACAGAAGCATTCTCAGAAACAT-CTTTGTGATGTTTGCACTCAACTCACAGAGTTG

               *  * **            *          *       *  *             *      
     500259 AACATTCCCATTCATAGAGCAGGTTTGAAACACACTTTTTGGAGTATCTGGAAGTGGACATTTGG
         65 AACCTTTCTTTTCATAGAGCAGTTTTGAAACACTCTTTTTGTAGAATCTGGAAGTGGATATTTGG

                    ** * * ** *  *   *            **  
     500324 AGCGCTTTCTGAACTATGGTGAAAAAGGAAATATCTTCCAAT
        130 AGCGCTTTGAGGATTTCGTTGGAAACGGAAATATCTTCATAT

            *     *               *      * *          *  **      **  *  *    
     500366 GAAAACAAGACAGAAGCATTCTGAGAAACTTATTTGTGATGTGTGTCCTCAACAAACGGACTTGA
          1 AAAAACTAGACAGAAGCATTCTCAGAAACATCTTTGTGATGTTTGCACTCAACTCACAGAGTTGA

                   *      **  * *  *  *             *   *    * * *           
     500431 ACCTTTCGTTTCATGCAGTACTTCTGGAACACTCTTTTTGAAGATTCTGCATGCGGATATTTGGA
         66 ACCTTTCTTTTCATAGAGCAGTTTTGAAACACTCTTTTTGTAGAATCTGGAAGTGGATATTTGGA

            **                            **   *   * 
     500496 TAGCTTTGAGGATTTCGTTGGAAACGG---GCT-TACATGT
        131 GCGCTTTGAGGATTTCGTTGGAAACGGAAATATCTTCATAT

                 *       *               *        *   *    *    *       *    
     500533 AAAAATTAGACAGCAGCATTCTCAGAAACTTCTTTGTGGTGTCTGCATTCAAGTCACAGAATTGA
          1 AAAAACTAGACAGAAGCATTCTCAGAAACATCTTTGTGATGTTTGCACTCAACTCACAGAGTTGA

              * ** * **            *  * *      *       *    *        *       
     500598 ACTTCCCCTCACATAGAGCAGTTGTGCAGCACTCTATTTGTAGTATCTCGAAGTGGACATTTGGA
         66 ACCTTTCTTTTCATAGAGCAGTTTTGAAACACTCTTTTTGTAGAATCTGGAAGTGGATATTTGGA

             *         ** *    * *   *            **  
     500663 GGGCTTTGTAGCCTATC-TGGAAAAAGGAAATATCTTCCCAT
        131 GCGCTTTG-AGGATTTCGTTGGAAACGGAAATATCTTCATAT

            *  ** *   *     * *            *   *  *   * **        *  * * *   
     500704 GAATGCGAGATAGAAGTAATCTCAGAAACATGTTTATGCTGTATCTACTCAACTAACTGTGCTGA
          1 AAAAACTAGACAGAAGCATTCTCAGAAACATCTTTGTGATGTTTGCACTCAACTCACAGAGTTGA

              *     *  *               *        *  * *       *               
     500769 ACATTTCTATTGATAGAGCAGTTTTGAGACACTCTTCTTTTGGAATCTGCAAGTGGATATTTGGA
         66 ACCTTTCTTTTCATAGAGCAGTTTTGAAACACTCTTTTTGTAGAATCTGGAAGTGGATATTTGGA

            ** *                        *     *      
     500834 TAGATTTGAGGATTTCGTTGGAAACGG-GAT-T-AT-ATAT
        131 GCGCTTTGAGGATTTCGTTGGAAACGGAAATATCTTCATAT

                 *       *               *                 **  *   *         
     500871 AAAAAGTAGACAGCAGCATTCTCAGAAACTTCTTTGTGATGTTTGCATCCAGCTCTCAGAGTTGA
          1 AAAAACTAGACAGAAGCATTCTCAGAAACATCTTTGTGATGTTTGCACTCAACTCACAGAGTTGA

              *  * *          *  *        *        *   **        *  **       
     500936 ACATTCCCTTTCATAGAGTAGGTTTGAAACCCTCTTTTTATAGTGTCTGGAAGCGGGCATTTGGA
         66 ACCTTTCTTTTCATAGAGCAGTTTTGAAACACTCTTTTTGTAGAATCTGGAAGTGGATATTTGGA

                   *    * * **  **  **          * *   
     501001 GCGCTTTCAGGCCTATGCTTAAAATAGGAAATATCTACCTAT
        131 GCGCTTTGAGG-ATTTCGTTGGAAACGGAAATATCTTCATAT

             *                    *    *  **          ** *        *    *     
     501043 AGAAACTAGACAGAAGCATTCTGAGAATCACGTTTGTGATGTGGGTACTCAACTAACAGTGTTGA
          1 AAAAACTAGACAGAAGCATTCTCAGAAACATCTTTGTGATGTTTGCACTCAACTCACAGAGTTGA

            *  *       *   *            *   *                *               
     501108 TCCATTCTTTTGATACAGCAGTTTTGAACCACACTTTTTGTAGAATCTGCAAGTGGATATTTGGA
         66 ACCTTTCTTTTCATAGAGCAGTTTTGAAACACTCTTTTTGTAGAATCTGGAAGTGGATATTTGGA

            **   *                     *   *        *
     501173 TAGCTGTGAGGATTTCGTTGGAAACGGGAATGTCTTCATAG
        131 GCGCTTTGAGGATTTCGTTGGAAACGGAAATATCTTCATAT

                **                     * * **         *  **   *   *          
     501214 AAAATTTAGACAGAAGCATTCTCAGAACCTTGATTGTGATGTGTGTTCTCCACTAACAGAGTTGA
          1 AAAAACTAGACAGAAGCATTCTCAGAAACATCTTTGTGATGTTTGCACTCAACTCACAGAGTTGA

                       * *   * *   *       *       *                         
     501279 ACCTTTCTTTTGACAGAACTGTTCTGAAACATTCTTTTTATAGAATCTGGAAGTGGATATTTGGA
         66 ACCTTTCTTTTCATAGAGCAGTTTTGAAACACTCTTTTTGTAGAATCTGGAAGTGGATATTTGGA

            **                         *          *  
     501344 AAGCTTTGAGGATTTCGTTGGAAACGGGAATATCTTCAAAT
        131 GCGCTTTGAGGATTTCGTTGGAAACGGAAATATCTTCATAT

            *   *    *            *           * *       *  *    *            
     501385 CAAATCTAGCCAGAAGCATTCTAAGAAACATCTTAGGGATGTTTACATTCAAGTCACAGAGTTGA
          1 AAAAACTAGACAGAAGCATTCTCAGAAACATCTTTGTGATGTTTGCACTCAACTCACAGAGTTGA

              *  * *     *       *         *    * *   **      *      *    *  
     501450 ACATTCCCTTTCACAGAGCAGGTTTGAAACAATCTTCTCGTACTATCTGGCAGTGGACATTTTGA
         66 ACCTTTCTTTTCATAGAGCAGTTTTGAAACACTCTTTTTGTAGAATCTGGAAGTGGATATTTGGA

              * *   *  ** ** *  *   *            ** *
     501515 GCTCCTTGGGGCCTATGCTGAAAAAGGAAATATCTTCCGAC
        131 GCGCTTTGAGGATTTCGTTGGAAACGGAAATATCTTCATAT

                                 *     *  **          *           **    *    
     501556 AAAAACTAGACAGAAGCATTCGCAGAATCACGTTTGTGATGTGTGCACTCAACTGTCAGAATTGA
          1 AAAAACTAGACAGAAGCATTCTCAGAAACATCTTTGTGATGTTTGCACTCAACTCACAGAGTTGA

                 **   ** *      *                            * *            *
     501621 ACCTTGGTTTGGACAGAGCACTTTTGAAACACTCTTTTTGTAGAATCTGCAGGTGGATATTTGGC
         66 ACCTTTCTTTTCATAGAGCAGTTTTGAAACACTCTTTTTGTAGAATCTGGAAGTGGATATTTGGA

            **                         *   *      * *
     501686 TAGCTTTGAGGATTTCGTTGGAAACGGTAATGTCTTCAAAG
        131 GCGCTTTGAGGATTTCGTTGGAAACGGAAATATCTTCATAT

                *                         *   *            *    *            
     501727 AAAATCTAGACAGAAGCATTCTCAGAAACAACTTCGTGATGTTTGCAATCAAGTCACAGAGTTGA
          1 AAAAACTAGACAGAAGCATTCTCAGAAACATCTTTGTGATGTTTGCACTCAACTCACAGAGTTGA

                 * *             *  *                  *             *       
     501792 ACCTTCCGTTTCATAGAGCAGGTTGGAAACACTCTTTTTGTAGTATCTGGAAGTGGACATTTGGA
         66 ACCTTTCTTTTCATAGAGCAGTTTTGAAACACTCTTTTTGTAGAATCTGGAAGTGGATATTTGGA

             *         ** *    * *   *            **  
     501857 GGGCTTTGTAGCCTATC-TGGAAAAAGGAAATATCTTCCCAT
        131 GCGCTTTG-AGGATTTCGTTGGAAACGGAAATATCTTCATAT

            *  ** *   *     * *            *   *  *   * **        *  * * *   
     501898 GAATGCGAGATAGAAGTAATCTCAGAAACATGTTTATGCTGTATCTACTCAACTAACTGTGCTGA
          1 AAAAACTAGACAGAAGCATTCTCAGAAACATCTTTGTGATGTTTGCACTCAACTCACAGAGTTGA

              *     *  *               *        *  * *       *               
     501963 ACATTTCTATTGATAGAGCAGTTTTGAGACACTCTTCTTTTGGAATCTGCAAGTGGATATTTGGA
         66 ACCTTTCTTTTCATAGAGCAGTTTTGAAACACTCTTTTTGTAGAATCTGGAAGTGGATATTTGGA

            ** *                        *     *      
     502028 TAGATTTGAGGATTTCGTTGGAAACGG-GAT-T-AT-ATAT
        131 GCGCTTTGAGGATTTCGTTGGAAACGGAAATATCTTCATAT

                 *       *               *                 **  *   *         
     502065 AAAAAGTAGACAGCAGCATTCTCAGAAACTTCTTTGTGATGTTTGCATCCAGCTCTCAGAGTTGA
          1 AAAAACTAGACAGAAGCATTCTCAGAAACATCTTTGTGATGTTTGCACTCAACTCACAGAGTTGA

              *  * *          *  *        *        *   **        *  **       
     502130 ACATTCCCTTTCATAGAGTAGGTTTGAAACCCTCTTTTTATAGTGTCTGGAAGCGGGCATTTGGA
         66 ACCTTTCTTTTCATAGAGCAGTTTTGAAACACTCTTTTTGTAGAATCTGGAAGTGGATATTTGGA

                   *    * * **  **  **          * *  *
     502195 GCGCTTTCAGGCCTATGCTTAAAATAGGAAATATCTACCTAC
        131 GCGCTTTGAGG-ATTTCGTTGGAAACGGAAATATCTTCATAT

             *                    *    *  **          ** *        *    *     
     502237 AGAAACTAGACAGAAGCATTCTGAGAATCACGTTTGTGATGTGGGTACTCAACTAACAGTGTTGA
          1 AAAAACTAGACAGAAGCATTCTCAGAAACATCTTTGTGATGTTTGCACTCAACTCACAGAGTTGA

            *  *       *   *            *   *                *   *           
     502302 TCCATTCTTTTGATACAGCAGTTTTGAACCACACTTTTTGTAGAATCTGCAAGAGGATATTTGGA
         66 ACCTTTCTTTTCATAGAGCAGTTTTGAAACACTCTTTTTGTAGAATCTGGAAGTGGATATTTGGA

            **   *                     *   *      * *
     502367 TAGCTGTGAGGATTTCGTTGGAAACGGGAATGTCTTCAAAG
        131 GCGCTTTGAGGATTTCGTTGGAAACGGAAATATCTTCATAT

                *                         *   *            *    *            
     502408 AAAATCTAGACAGAAGCATTCTCAGAAACACCTTCGTGATGTTTGCAATCAAGTCACAGAGTTGA
          1 AAAAACTAGACAGAAGCATTCTCAGAAACATCTTTGTGATGTTTGCACTCAACTCACAGAGTTGA

                 * *             *  *           *      *             *       
     502473 ACCTTCCGTTTCATAGAGCAGGTTGGAAACACTCTTATTGTAGTATCTGGAAGTGGACATTTGGA
         66 ACCTTTCTTTTCATAGAGCAGTTTTGAAACACTCTTTTTGTAGAATCTGGAAGTGGATATTTGGA

                   *   ** ** *  *   *            **  
     502538 GCGCTTTCAGGCCTATGGTGAAAAAGGAAATATCTTCCCAT
        131 GCGCTTTGAGGATTTCGTTGGAAACGGAAATATCTTCATAT

                  * * *      **      *   * *   *     ** ** *      *    *     
     502579 AAAAACGACATAGAAGCTATCTCAGGAACTTGTTTATGATGCATCTAATCAACTAACAGTGTTGA
          1 AAAAACTAGACAGAAGCATTCTCAGAAACATCTTTGTGATGTTTGCACTCAACTCACAGAGTTGA

                  * ** * *                         * *       *               
     502644 ACCTTTGTACTGACAGAGCAG-TTTGAAACACTCTTTTTTTGGAATCTGCAAGTGGATATTTGGA
         66 ACCTTTCTTTTCATAGAGCAGTTTTGAAACACTCTTTTTGTAGAATCTGGAAGTGGATATTTGGA

            *                           *   *   *    
     502708 TCGCTTTGAGGATTTCGTTGGAAACGG-GAT-GC--AATAT
        131 GCGCTTTGAGGATTTCGTTGGAAACGGAAATATCTTCATAT

                **  *    *     *                 **  *   *  *    *         * 
     502745 AAAACATACACAGCAGCATACTCAGAAA-ATACTTTGCCATATTTCCATTCAAGTCACAGAGTGG
          1 AAAAACTAGACAGAAGCATTCTCAGAAACAT-CTTTGTGATGTTTGCACTCAACTCACAGAGTTG

               *  * **            *  *               *  *             *      
     502809 AACATTCCCATTCATAGAGCAGGTTGGAAACACTCTTTTTGGAGTATCTGGAAGTGGACATTTGG
         65 AACCTTTCTTTTCATAGAGCAGTTTTGAAACACTCTTTTTGTAGAATCTGGAAGTGGATATTTGG

                    ** * * ** *  *   *            **  
     502874 AGCGCTTTCTGAACTATGGTGAAAAAGGAAATATCTTCCAAT
        130 AGCGCTTTGAGGATTTCGTTGGAAACGGAAATATCTTCATAT

            *     *               *      * *          *  **      **  *  *    
     502916 GAAAACAAGACAGAAGCATTCTGAGAAACTTATTTGTGATGTGTGTCCTCAACAAACGGACTTGA
          1 AAAAACTAGACAGAAGCATTCTCAGAAACATCTTTGTGATGTTTGCACTCAACTCACAGAGTTGA

                   *      **  * *  *  *             *   *    * * *           
     502981 ACCTTTCGTTTCATGCAGTACTTCTGGAACACTCTTTTTGAAGATTCTGCATGCGGATATTTGGA
         66 ACCTTTCTTTTCATAGAGCAGTTTTGAAACACTCTTTTTGTAGAATCTGGAAGTGGATATTTGGA

            **                            **   *   * 
     503046 TAGCTTTGAGGATTTCGTTGGAAACGG---GCT-TACATGT
        131 GCGCTTTGAGGATTTCGTTGGAAACGGAAATATCTTCATAT

                 *       *               *        *   *    *    *       *    
     503083 AAAAATTAGACAGCAGCATTCTCAGAAACTTCTTTGTGGTGTCTGCATTCAAGTCACAGAATTGA
          1 AAAAACTAGACAGAAGCATTCTCAGAAACATCTTTGTGATGTTTGCACTCAACTCACAGAGTTGA

              * ** * **            *  * *      *       *             *       
     503148 ACTTCCCCTCACATAGAGCAGTTGTGCAGCACTCTATTTGTAGTATCTGGAAGTGGACATTTGGA
         66 ACCTTTCTTTTCATAGAGCAGTTTTGAAACACTCTTTTTGTAGAATCTGGAAGTGGATATTTGGA

             *         ** *    * *   *            **  
     503213 GGGCTTTGTAGCCTATC-TGGAAAAAGGAAATATCTTCCCAT
        131 GCGCTTTG-AGGATTTCGTTGGAAACGGAAATATCTTCATAT

            *  ** *   *     * *            *   *  *   * **        *  * * *   
     503254 GAATGCGAGATAGAAGTAATCTCAGAAACATGTTTATGCTGTATCTACTCAACTAACTGTGCTGA
          1 AAAAACTAGACAGAAGCATTCTCAGAAACATCTTTGTGATGTTTGCACTCAACTCACAGAGTTGA

              *     *  *               *        *  * *       *               
     503319 ACATTTCTATTGATAGAGCAGTTTTGAGACACTCTTCTTTTGGAATCTGCAAGTGGATATTTGGA
         66 ACCTTTCTTTTCATAGAGCAGTTTTGAAACACTCTTTTTGTAGAATCTGGAAGTGGATATTTGGA

            ** *                        *     *      
     503384 TAGATTTGAGGATTTCGTTGGAAACGG-GAT-T-AT-ATAT
        131 GCGCTTTGAGGATTTCGTTGGAAACGGAAATATCTTCATAT

                 *       *               *                 **  *   *         
     503421 AAAAAGTAGACAGCAGCATTCTCAGAAACTTCTTTGTGATGTTTGCATCCAGCTCTCAGAGTTGA
          1 AAAAACTAGACAGAAGCATTCTCAGAAACATCTTTGTGATGTTTGCACTCAACTCACAGAGTTGA

              *  * *          *  *        *        *   **        *  **       
     503486 ACATTCCCTTTCATAGAGTAGGTTTGAAACCCTCTTTTTATAGTGTCTGGAAGCGGGCATTTGGA
         66 ACCTTTCTTTTCATAGAGCAGTTTTGAAACACTCTTTTTGTAGAATCTGGAAGTGGATATTTGGA

                *  *    * * **  **  **          * *  *
     503551 GCGCATTCAGGCCTATGCTTAAAATAGGAAATATCTACCTAC
        131 GCGCTTTGAGG-ATTTCGTTGGAAACGGAAATATCTTCATAT

             *                    *    *  **          ** *        *    *     
     503593 AGAAACTAGACAGAAGCATTCTGAGAATCACGTTTGTGATGTGGGTACTCAACTAACAGTGTTGA
          1 AAAAACTAGACAGAAGCATTCTCAGAAACATCTTTGTGATGTTTGCACTCAACTCACAGAGTTGA

            *  *       *   *            *   *                *   *           
     503658 TCCATTCTTTTGATACAGCAGTTTTGAACCACACTTTTTGTAGAATCTGCAAGAGGATATTTGGA
         66 ACCTTTCTTTTCATAGAGCAGTTTTGAAACACTCTTTTTGTAGAATCTGGAAGTGGATATTTGGA

            **   *                     *   *      * *
     503723 TAGCTGTGAGGATTTCGTTGGAAACGGGAATGTCTTCAAAG
        131 GCGCTTTGAGGATTTCGTTGGAAACGGAAATATCTTCATAT

                *                         *   *            *    *            
     503764 AAAATCTAGACAGAAGCATTCTCAGAAACACCTTCGTGATGTTTGCAATCAAGTCACAGAGTTGA
          1 AAAAACTAGACAGAAGCATTCTCAGAAACATCTTTGTGATGTTTGCACTCAACTCACAGAGTTGA

                 * *             *  *           *      **            *       
     503829 ACCTTCCGTTTCATAGAGCAGGTTGGAAACACTCTTATTGTAGTGTCTGGAAGTGGACATTTGGA
         66 ACCTTTCTTTTCATAGAGCAGTTTTGAAACACTCTTTTTGTAGAATCTGGAAGTGGATATTTGGA

                   *   ** ** *  *   *            **  
     503894 GCGCTTTCAGGCCTATGGTGAAAAAGGAAATATCTTCCCAT
        131 GCGCTTTGAGGATTTCGTTGGAAACGGAAATATCTTCATAT

                  * * *      **      *   * *   *     ** ** *      *    *     
     503935 AAAAACGACATAGAAGCTATCTCAGGAACTTGTTTATGATGCATCTAATCAACTAACAGTGTTGA
          1 AAAAACTAGACAGAAGCATTCTCAGAAACATCTTTGTGATGTTTGCACTCAACTCACAGAGTTGA

                    ** * *                         * *       *               
     504000 ACCTTTCTACTGACAGAGCAG-TTTGAAACACTCTTTTTTTGGAATCTGCAAGTGGATATTTGGA
         66 ACCTTTCTTTTCATAGAGCAGTTTTGAAACACTCTTTTTGTAGAATCTGGAAGTGGATATTTGGA

            *                           *   *   *    
     504064 TCGCTTTGAGGATTTCGTTGGAAACGG-GAT-GC--AATAT
        131 GCGCTTTGAGGATTTCGTTGGAAACGGAAATATCTTCATAT

                **  *    *     *                 **  *   *  *    *   *     * 
     504101 AAAACGTACACAGCAGCATACTCAGAAA-ATACTTTGCCATATTTCCATTCAAGTCAGAGAGTGG
          1 AAAAACTAGACAGAAGCATTCTCAGAAACAT-CTTTGTGATGTTTGCACTCAACTCACAGAGTTG

               *  * **            *                  *  *             *      
     504165 AACATTCCCATTCATAGAGCAGGTTTGAAACACTCTTTTTGGAGTATCTGGAAGTGGACATTTGG
         65 AACCTTTCTTTTCATAGAGCAGTTTTGAAACACTCTTTTTGTAGAATCTGGAAGTGGATATTTGG

                    ** * * ** *  *   *            **  
     504230 AGCGCTTTCTGAACTATGGTGAAAAAGGAAATATCTTCCAAT
        130 AGCGCTTTGAGGATTTCGTTGGAAACGGAAATATCTTCATAT

            *     *               *      * *          *  **      **  *  *    
     504272 GAAAACAAGACAGAAGCATTCTGAGAAACTTATTTGTGATGTGTGTCCTCAACAAACGGACTTGA
          1 AAAAACTAGACAGAAGCATTCTCAGAAACATCTTTGTGATGTTTGCACTCAACTCACAGAGTTGA

                   *      **  * *  *  *             *   *    * * *           
     504337 ACCTTTCGTTTCATGCAGTACTTCTGGAACACTCTTTTTGAAGATTCTGCATGCGGATATTTGGA
         66 ACCTTTCTTTTCATAGAGCAGTTTTGAAACACTCTTTTTGTAGAATCTGGAAGTGGATATTTGGA

            **              *             **   *   * 
     504402 TAGCTTTGAGGATTTCTTTGGAAACGG---GCT-TACATGT
        131 GCGCTTTGAGGATTTCGTTGGAAACGGAAATATCTTCATAT

                 *       *               *        *   *    *    *       *    
     504439 AAAAATTAGACAGCAGCATTCTCAGAAACTTCTTTGTGGTGTCTGCATTCAAGTCACAGAATTGA
          1 AAAAACTAGACAGAAGCATTCTCAGAAACATCTTTGTGATGTTTGCACTCAACTCACAGAGTTGA

              * ** * **            *  * *      *       *             *       
     504504 ACTTCCCCTCACATAGAGCAGTTGTGCAGCACTCTATTTGTAGTATCTGGAAGTGGACATTTGGA
         66 ACCTTTCTTTTCATAGAGCAGTTTTGAAACACTCTTTTTGTAGAATCTGGAAGTGGATATTTGGA

             *         ** *    * *   *            **  
     504569 GGGCTTTGTAGCCTATC-TGGAAAAAGGAAATATCTTCCCAT
        131 GCGCTTTG-AGGATTTCGTTGGAAACGGAAATATCTTCATAT

            *  ** *   *     * *            *   *  *   * **        *  * * *   
     504610 GAATGCGAGATAGAAGTAATCTCAGAAACATGTTTATGCTGTATCTACTCAACTAACTGTGCTGA
          1 AAAAACTAGACAGAAGCATTCTCAGAAACATCTTTGTGATGTTTGCACTCAACTCACAGAGTTGA

              *     *  *               *        *  * *       *               
     504675 ACATTTCTATTGATAGAGCAGTTTTGAGACACTCTTCTTTTGGAATCTGCAAGTGGATATTTGGA
         66 ACCTTTCTTTTCATAGAGCAGTTTTGAAACACTCTTTTTGTAGAATCTGGAAGTGGATATTTGGA

            ** *                        *     *      
     504740 TAGATTTGAGGATTTCGTTGGAAACGG-GAT-T-AT-ATAT
        131 GCGCTTTGAGGATTTCGTTGGAAACGGAAATATCTTCATAT

            *    *       *               *                 **  *   *         
     504777 CAAAAGTAGACAGCAGCATTCTCAGAAACTTCTTTGTGATGTTTGCATCCAGCTCTCAGAGTTGA
          1 AAAAACTAGACAGAAGCATTCTCAGAAACATCTTTGTGATGTTTGCACTCAACTCACAGAGTTGA

              *  * *          *  *        *        *   **        *  **       
     504842 ACATTCCCTTTCATAGAGTAGGTTTGAAACCCTCTTTTTATAGTGTCTGGAAGCGGGCATTTGGA
         66 ACCTTTCTTTTCATAGAGCAGTTTTGAAACACTCTTTTTGTAGAATCTGGAAGTGGATATTTGGA

                   *    * * **  **  **          * *  *
     504907 GCGCTTTCAGGCCTATGCTTAAAATAGGAAATATCTACCTAC
        131 GCGCTTTGAGG-ATTTCGTTGGAAACGGAAATATCTTCATAT

             *                    *    *  **          ** *        *    *     
     504949 AGAAACTAGACAGAAGCATTCTGAGAATCACGTTTGTGATGTGGGTACTCAACTAACAGTGTTGA
          1 AAAAACTAGACAGAAGCATTCTCAGAAACATCTTTGTGATGTTTGCACTCAACTCACAGAGTTGA

            *  *       *   *            *   *                *   *           
     505014 TCCATTCTTTTGATACAGCAGTTTTGAACCACACTTTTTGTAGAATCTGCAAGAGGATATTTGGA
         66 ACCTTTCTTTTCATAGAGCAGTTTTGAAACACTCTTTTTGTAGAATCTGGAAGTGGATATTTGGA

            **   *                     *   *      * *
     505079 TAGCTGTGAGGATTTCGTTGGAAACGGGAATGTCTTCAAAG
        131 GCGCTTTGAGGATTTCGTTGGAAACGGAAATATCTTCATAT

                *                         *   *            *    *            
     505120 AAAATCTAGACAGAAGCATTCTCAGAAACACCTTCGTGATGTTTGCAATCAAGTCACAGAGTTGA
          1 AAAAACTAGACAGAAGCATTCTCAGAAACATCTTTGTGATGTTTGCACTCAACTCACAGAGTTGA

                 * *             *  *           *      *             *       
     505185 ACCTTCCGTTTCATAGAGCAGGTTGGAAACACTCTTATTGTAGTATCTGGAAGTGGACATTTGGA
         66 ACCTTTCTTTTCATAGAGCAGTTTTGAAACACTCTTTTTGTAGAATCTGGAAGTGGATATTTGGA

                   *   ** ** *  *   *            **  
     505250 GCGCTTTCAGGCCTATGGTGAAAAAGGAAATATCTTCCCAT
        131 GCGCTTTGAGGATTTCGTTGGAAACGGAAATATCTTCATAT

                  * * *      **      *   * *   *     ** ** *      *    *     
     505291 AAAAACGACATAGAAGCTATCTCAGGAACTTGTTTATGATGCATCTAATCAACTAACAGTGTTGA
          1 AAAAACTAGACAGAAGCATTCTCAGAAACATCTTTGTGATGTTTGCACTCAACTCACAGAGTTGA

                  * ** * *                         * *       *               
     505356 ACCTTTGTACTGACAGAGCAG-TTTGAAACACTCTTTTTTTGGAATCTGCAAGTGGATATTTGGA
         66 ACCTTTCTTTTCATAGAGCAGTTTTGAAACACTCTTTTTGTAGAATCTGGAAGTGGATATTTGGA

            *                           *   *   *    
     505420 TCGCTTTGAGGATTTCGTTGGAAACGG-GAT-GC--AATAT
        131 GCGCTTTGAGGATTTCGTTGGAAACGGAAATATCTTCATAT

                **  *    *     *                 **  *   *  *    *         * 
     505457 AAAACGTACACAGCAGCATACTCAGAAA-ATACTTTGCCATATTTCCATTCAAGTCACAGAGTGG
          1 AAAAACTAGACAGAAGCATTCTCAGAAACAT-CTTTGTGATGTTTGCACTCAACTCACAGAGTTG

               *  * **       *    *  *               *  *             *      
     505521 AACATTCCCATTCATAGCGCAGGTTGGAAACACTCTTTTTGGAGTATCTGGAAGTGGACATTTGG
         65 AACCTTTCTTTTCATAGAGCAGTTTTGAAACACTCTTTTTGTAGAATCTGGAAGTGGATATTTGG

                    ** * * ** *  *   *           * *  
     505586 AGCGCTTTCTGAACTATGGTGAAAAAGGAAATATCTTAAAAT
        130 AGCGCTTTGAGGATTTCGTTGGAAACGGAAATATCTTCATAT

            *     *               *      * *          *  **      **  *  *    
     505628 GAAAACAAGACAGAAGCATTCTGAGAAACTTATTTGTGATGTGTGTCCTCAACAAACGGACTTGA
          1 AAAAACTAGACAGAAGCATTCTCAGAAACATCTTTGTGATGTTTGCACTCAACTCACAGAGTTGA

                   *      **  * *  *  *             *   *    * * *           
     505693 ACCTTTCGTTTCATGCAGTACTTCTGGAACACTCTTTTTGAAGATTCTGCATGCGGATATTTGGA
         66 ACCTTTCTTTTCATAGAGCAGTTTTGAAACACTCTTTTTGTAGAATCTGGAAGTGGATATTTGGA

            **                            **   *   * 
     505758 TAGCTTTGAGGATTTCGTTGGAAACGG---GCT-TACATGT
        131 GCGCTTTGAGGATTTCGTTGGAAACGGAAATATCTTCATAT

                 *       *               *        *   *    *    *       *    
     505795 AAAAATTAGACAGCAGCATTCTCAGAAACTTCTTTGTGGTGTCTGCATTCAAGTCACAGAATTGA
          1 AAAAACTAGACAGAAGCATTCTCAGAAACATCTTTGTGATGTTTGCACTCAACTCACAGAGTTGA

              * ** * **            *  * *      *       *    *        *       
     505860 ACTTCCCCTCACATAGAGCAGTTGTGCAGCACTCTATTTGTAGTATCTCGAAGTGGACATTTGGA
         66 ACCTTTCTTTTCATAGAGCAGTTTTGAAACACTCTTTTTGTAGAATCTGGAAGTGGATATTTGGA

             *         ** *    * *   *            **  
     505925 GGGCTTTGTAGCCTATC-TGGAAAAAGGAAATATCTTCCCAT
        131 GCGCTTTG-AGGATTTCGTTGGAAACGGAAATATCTTCATAT

            *  ** *   *     * *            *   *  *   * **        *  * * *   
     505966 GAATGCGAGATAGAAGTAATCTCAGAAACATGTTTATGCTGTATCTACTCAACTAACTGTGCTGA
          1 AAAAACTAGACAGAAGCATTCTCAGAAACATCTTTGTGATGTTTGCACTCAACTCACAGAGTTGA

              *     *  *               *        *  * *       *               
     506031 ACATTTCTATTGATAGAGCAGTTTTGAGACACTCTTCTTTTGGAATCTGCAAGTGGATATTTGGA
         66 ACCTTTCTTTTCATAGAGCAGTTTTGAAACACTCTTTTTGTAGAATCTGGAAGTGGATATTTGGA

            ** *                        *     *      
     506096 TAGATTTGAGGATTTCGTTGGAAACGG-GAT-T-AT-ATAT
        131 GCGCTTTGAGGATTTCGTTGGAAACGGAAATATCTTCATAT

                 *       *               *                 **  *   *         
     506133 AAAAAGTAGACAGCAGCATTCTCAGAAACTTCTTTGTGATGTTTGCATCCAGCTCTCAGAGTTGA
          1 AAAAACTAGACAGAAGCATTCTCAGAAACATCTTTGTGATGTTTGCACTCAACTCACAGAGTTGA

              *  * *          *  *        *        *   **        *  **       
     506198 ACATTCCCTTTCATAGAGTAGGTTTGAAACCCTCTTTTTATAGTGTCTGGAAGCGGGCATTTGGA
         66 ACCTTTCTTTTCATAGAGCAGTTTTGAAACACTCTTTTTGTAGAATCTGGAAGTGGATATTTGGA

                   *    * * **  **  **          * *   
     506263 GCGCTTTCAGGCCTATGCTTAAAATAGGAAATATCTACCTAT
        131 GCGCTTTGAGG-ATTTCGTTGGAAACGGAAATATCTTCATAT

             *                    *    *  **          ** *        *    *     
     506305 AGAAACTAGACAGAAGCATTCTGAGAATCACGTTTGTGATGTGGGTACTCAACTAACAGTGTTGA
          1 AAAAACTAGACAGAAGCATTCTCAGAAACATCTTTGTGATGTTTGCACTCAACTCACAGAGTTGA

            *  *       *   *            *   *                *               
     506370 TCCATTCTTTTGATACAGCAGTTTTGAACCACACTTTTTGTAGAATCTGCAAGTGGATATTTGGA
         66 ACCTTTCTTTTCATAGAGCAGTTTTGAAACACTCTTTTTGTAGAATCTGGAAGTGGATATTTGGA

            **   *                     *   *        *
     506435 TAGCTGTGAGGATTTCGTTGGAAACGGGAATGTCTTCATAG
        131 GCGCTTTGAGGATTTCGTTGGAAACGGAAATATCTTCATAT

                **                     * * **         *  **   *   *          
     506476 AAAATTTAGACAGAAGCATTCTCAGAACCTTGATTGTGATGTGTGTTCTCCACTAACAGAGTTGA
          1 AAAAACTAGACAGAAGCATTCTCAGAAACATCTTTGTGATGTTTGCACTCAACTCACAGAGTTGA

                       * *   * *   *       *       *                         
     506541 ACCTTTCTTTTGACAGAACTGTTCTGAAACATTCTTTTTATAGAATCTGGAAGTGGATATTTGGA
         66 ACCTTTCTTTTCATAGAGCAGTTTTGAAACACTCTTTTTGTAGAATCTGGAAGTGGATATTTGGA

            **                         *          *  
     506606 AAGCTTTGAGGATTTCGTTGGAAACGGGAATATCTTCAAAT
        131 GCGCTTTGAGGATTTCGTTGGAAACGGAAATATCTTCATAT

            *   *    *            *           * *       *  *    *            
     506647 CAAATCTAGCCAGAAGCATTCTAAGAAACATCTTAGGGATGTTTACATTCAAGTCACAGAGTTGA
          1 AAAAACTAGACAGAAGCATTCTCAGAAACATCTTTGTGATGTTTGCACTCAACTCACAGAGTTGA

              *  * *     *       *         *    * *   **      *      *    *  
     506712 ACATTCCCTTTCACAGAGCAGGTTTGAAACAATCTTCTCGTACTATCTGGCAGTGGACATTTTGA
         66 ACCTTTCTTTTCATAGAGCAGTTTTGAAACACTCTTTTTGTAGAATCTGGAAGTGGATATTTGGA

              * *   *  ** ** *  *  **            ** *
     506777 GCTCCTTGGGGCCTATGCTGAAATAGGAAATATCTTCCGAC
        131 GCGCTTTGAGGATTTCGTTGGAAACGGAAATATCTTCATAT

                                 *     *  **          *           **    *    
     506818 AAAAACTAGACAGAAGCATTCGCAGAATCACGTTTGTGATGTGTGCACTCAACTGTCAGAATTGA
          1 AAAAACTAGACAGAAGCATTCTCAGAAACATCTTTGTGATGTTTGCACTCAACTCACAGAGTTGA

                 **   ** *      *                            * *            *
     506883 ACCTTGGTTTGGACAGAGCACTTTTGAAACACTCTTTTTGTAGAATCTGCAGGTGGATATTTGGC
         66 ACCTTTCTTTTCATAGAGCAGTTTTGAAACACTCTTTTTGTAGAATCTGGAAGTGGATATTTGGA

            **                         *   *      * *
     506948 TAGCTTTGAGGATTTCGTTGGAAACGGTAATGTCTTCAAAG
        131 GCGCTTTGAGGATTTCGTTGGAAACGGAAATATCTTCATAT

                *                         *   *            *    *            
     506989 AAAATCTAGACAGAAGCATTCTCAGAAACAACTTCGTGATGTTTGCAATCAAGTCACAGAGTTGA
          1 AAAAACTAGACAGAAGCATTCTCAGAAACATCTTTGTGATGTTTGCACTCAACTCACAGAGTTGA

                 * *             *  *                  *             *       
     507054 ACCTTCCGTTTCATAGAGCAGGTTGGAAACACTC-TTTTGTAGTATCTGGAAGTGGACATTTGGA
         66 ACCTTTCTTTTCATAGAGCAGTTTTGAAACACTCTTTTTGTAGAATCTGGAAGTGGATATTTGGA

             *         ** *    * *   *            **  
     507118 GGGCTTTGTAGCCTATC-TGGAAAAAGGAAATATCTTCCCAT
        131 GCGCTTTG-AGGATTTCGTTGGAAACGGAAATATCTTCATAT

            *  ** *   *     * *            *   *  *   * **        *  * * *   
     507159 GAATGCGAGATAGAAGTAATCTCAGAAACATGTTTATGCTGTATCTACTCAACTAACTGTGCTGA
          1 AAAAACTAGACAGAAGCATTCTCAGAAACATCTTTGTGATGTTTGCACTCAACTCACAGAGTTGA

              *     *  *               *        *  * *       *               
     507224 ACATTTCTATTGATAGAGCAGTTTTGAGACACTCTTCTTTTGGAATCTGCAAGTGGATATTTGGA
         66 ACCTTTCTTTTCATAGAGCAGTTTTGAAACACTCTTTTTGTAGAATCTGGAAGTGGATATTTGGA

            ** *                       **     *      
     507289 TAGATTTGAGGATTTCGTTGGAAAC-GCGAT-T-AT-ATAT
        131 GCGCTTTGAGGATTTCGTTGGAAACGGAAATATCTTCATAT

                 *       *               *                 **  *   *         
     507326 AAAAAGTAGACAGCAGCATTCTCAGAAACTTCTTTGTGATGTTTGCATCCAGCTCTCAGAGTTGA
          1 AAAAACTAGACAGAAGCATTCTCAGAAACATCTTTGTGATGTTTGCACTCAACTCACAGAGTTGA

            * *  * *          *  *        *        *   **        *  **       
     507391 GCATTCCCTTTCATAGAGTAGGTTTGAAACCCTCTTTTTATAGTGTCTGGAAGCGGGCATTTGGA
         66 ACCTTTCTTTTCATAGAGCAGTTTTGAAACACTCTTTTTGTAGAATCTGGAAGTGGATATTTGGA

                   *    * * **  **  **          * *  *
     507456 GCGCTTTCAGGCCTATGCTTAAAATAGGAAATATCTACCTAC
        131 GCGCTTTGAGG-ATTTCGTTGGAAACGGAAATATCTTCATAT

             *                    *    *  **          ** *        *    *     
     507498 AGAAACTAGACAGAAGCATTCTGAGAATCACGTTTGTGATGTGGGTACTCAACTAACAGTGTTGA
          1 AAAAACTAGACAGAAGCATTCTCAGAAACATCTTTGTGATGTTTGCACTCAACTCACAGAGTTGA

            *  *       *   *            *   *                *               
     507563 TCCATTCTTTTGATACAGCAGTTTTGAACCACACTTTTTGTAGAATCTGCAAGTGGATATTTGGA
         66 ACCTTTCTTTTCATAGAGCAGTTTTGAAACACTCTTTTTGTAGAATCTGGAAGTGGATATTTGGA

            **   *                     *   *      * *
     507628 TAGCTGTGAGGATTTCGTTGGAAACGGGAATGTCTTCAAAG
        131 GCGCTTTGAGGATTTCGTTGGAAACGGAAATATCTTCATAT

                *                         *   *            *    *            
     507669 AAAATCTAGACAGAAGCATTCTCAGAAACACCTTCGTGATGTTTGCAATCAAGTCACAGAGTTGA
          1 AAAAACTAGACAGAAGCATTCTCAGAAACATCTTTGTGATGTTTGCACTCAACTCACAGAGTTGA

                 * *    *        *  *           *      *             *       
     507734 ACCTTCCGTTTCGTAGAGCAGGTTGGAAACACTCTTATTGTAGTATCTGGAAGTGGACATTTGGA
         66 ACCTTTCTTTTCATAGAGCAGTTTTGAAACACTCTTTTTGTAGAATCTGGAAGTGGATATTTGGA

                   *   ** ** *  *   *            **  
     507799 GCGCTTTCAGGCCTATGGTGAAAAAGGAAATATCTTCCCAT
        131 GCGCTTTGAGGATTTCGTTGGAAACGGAAATATCTTCATAT

                  * * *      **      *   * *   *     ** ** *      *    *     
     507840 AAAAACGACATAGAAGCTATCTCAGGAACTTGTTTCTGATGCATCTAATCAACTAACAGTGTTGA
          1 AAAAACTAGACAGAAGCATTCTCAGAAACATCTTTGTGATGTTTGCACTCAACTCACAGAGTTGA

                  * ** * *       *                 * *       *               
     507905 ACCTTTGTACTGACAGAGCA-CTTTGAAACACTCTTTTTTTGGAATCTGCAAGTGGATATTTGGA
         66 ACCTTTCTTTTCATAGAGCAGTTTTGAAACACTCTTTTTGTAGAATCTGGAAGTGGATATTTGGA

            *                           *   *   *    
     507969 TCGCTTTGAGGATTTCGTTGGAAACGG-GAT-GC--AATAT
        131 GCGCTTTGAGGATTTCGTTGGAAACGGAAATATCTTCATAT

                **  *    *     *                 **  *   *  *    *         * 
     508006 AAAACGTACACAGCAGCATACTCAGAAA-ATACTTTGCCATATTTCCATTCAAGTCACAGAGTGG
          1 AAAAACTAGACAGAAGCATTCTCAGAAACAT-CTTTGTGATGTTTGCACTCAACTCACAGAGTTG

               *  * **            *  *               *  *             *      
     508070 AACATTCCCATTCATAGAGCAGGTTGGAAACACTCTTTTTGGAGTATCTGGAAGTGGACATTTGG
         65 AACCTTTCTTTTCATAGAGCAGTTTTGAAACACTCTTTTTGTAGAATCTGGAAGTGGATATTTGG

                    ** * * ** *  *   *            **  
     508135 AGCGCTTTCTGAACTATGGTGAAAAAGGAAATATCTTCCAAT
        130 AGCGCTTTGAGGATTTCGTTGGAAACGGAAATATCTTCATAT

            *     *               *      * *          *  **      **  *  *    
     508177 GAAAACAAGACAGAAGCATTCTGAGAAACTTATTTGTGATGTGTGTCCTCAACAAACGGACTTGA
          1 AAAAACTAGACAGAAGCATTCTCAGAAACATCTTTGTGATGTTTGCACTCAACTCACAGAGTTGA

                   *      **  * *  *  *             *   *    * * *           
     508242 ACCTTTCGTTTCATGCAGTACTTCTGGAACACTCTTTTTGAAGATTCTGCATGCGGATATTTGGA
         66 ACCTTTCTTTTCATAGAGCAGTTTTGAAACACTCTTTTTGTAGAATCTGGAAGTGGATATTTGGA

            **                            **   *   * 
     508307 TAGCTTTGAGGATTTCGTTGGAAACGG---GCT-TACATGT
        131 GCGCTTTGAGGATTTCGTTGGAAACGGAAATATCTTCATAT

                 *       *               *        *   *    *    *       *    
     508344 AAAAATTAGACAGCAGCATTCTCAGAAACTTCTTTGTGGTGTCTGCATTCAAGTCACAGAATTGA
          1 AAAAACTAGACAGAAGCATTCTCAGAAACATCTTTGTGATGTTTGCACTCAACTCACAGAGTTGA

              * ** * **            *  * *      *       *             *       
     508409 ACTTCCCCTCACATAGAGCAGTTGTGCAGCACTCTATTTGTAGTATCTGGAAGTGGACATTTGGA
         66 ACCTTTCTTTTCATAGAGCAGTTTTGAAACACTCTTTTTGTAGAATCTGGAAGTGGATATTTGGA

             *         ** *    * *   *            **  
     508474 GGGCTTTGTAGCCTATC-TGGAAAAAGGAAATATCTTCCCAT
        131 GCGCTTTG-AGGATTTCGTTGGAAACGGAAATATCTTCATAT

            *  ** *   *     * *            *   *  *   * **        *  * * *   
     508515 GAATGCGAGATAGAAGTAATCTCAGAAACATGTTTATGCTGTATCTACTCAACTAACTGTGCTGA
          1 AAAAACTAGACAGAAGCATTCTCAGAAACATCTTTGTGATGTTTGCACTCAACTCACAGAGTTGA

              *     *  *               *        *  * *       *               
     508580 ACATTTCTATTGATAGAGCAGTTTTGAGACACTCTTCTTTTGGAATCTGCAAGTGGATATTTGGA
         66 ACCTTTCTTTTCATAGAGCAGTTTTGAAACACTCTTTTTGTAGAATCTGGAAGTGGATATTTGGA

            ** *                        *     *      
     508645 TAGATTTGAGGATTTCGTTGGAAACGG-GAT-T-AT-ATAT
        131 GCGCTTTGAGGATTTCGTTGGAAACGGAAATATCTTCATAT

            *    *       *               *                 **  *   *         
     508682 CAAAAGTAGACAGCAGCATTCTCAGAAACTTCTTTGTGATGTTTGCATCCAGCTCTCAGAGTTGA
          1 AAAAACTAGACAGAAGCATTCTCAGAAACATCTTTGTGATGTTTGCACTCAACTCACAGAGTTGA

              *  * *          *  *        *        *   **        *  **       
     508747 ACATTCCCTTTCATAGAGTAGGTTTGAAACCCTCTTTTTATAGTGTCTGGAAGCGGGCATTTGGA
         66 ACCTTTCTTTTCATAGAGCAGTTTTGAAACACTCTTTTTGTAGAATCTGGAAGTGGATATTTGGA

                   *    * * **  **  **          * *  *
     508812 GCGCTTTCAGGCCTATGCTTAAAATAGGAAATATCTACCTAC
        131 GCGCTTTGAGG-ATTTCGTTGGAAACGGAAATATCTTCATAT

             *                    *    *  **          ** *        *    *     
     508854 AGAAACTAGACAGAAGCATTCTGAGAATCACGTTTGTGATGTGGGTACTCAACTAACAGTGTTGA
          1 AAAAACTAGACAGAAGCATTCTCAGAAACATCTTTGTGATGTTTGCACTCAACTCACAGAGTTGA

            *  *       *   *            *   *                *   *           
     508919 TCCATTCTTTTGATACAGCAGTTTTGAACCACACTTTTTGTAGAATCTGCAAGAGGATATTTGGA
         66 ACCTTTCTTTTCATAGAGCAGTTTTGAAACACTCTTTTTGTAGAATCTGGAAGTGGATATTTGGA

            **   *                     *   *      * *
     508984 TAGCTGTGAGGATTTCGTTGGAAACGGGAATGTCTTCAAAG
        131 GCGCTTTGAGGATTTCGTTGGAAACGGAAATATCTTCATAT

                *                         *   *            *    *            
     509025 AAAATCTAGACAGAAGCATTCTCAGAAACACCTTCGTGATGTTTGCAATCAAGTCACAGAGTTGA
          1 AAAAACTAGACAGAAGCATTCTCAGAAACATCTTTGTGATGTTTGCACTCAACTCACAGAGTTGA

                 * *             *  *           *      *             *       
     509090 ACCTTCCGTTTCATAGAGCAGGTTGGAAACACTCTTATTGTAGTATCTGGAAGTGGACATTTGGA
         66 ACCTTTCTTTTCATAGAGCAGTTTTGAAACACTCTTTTTGTAGAATCTGGAAGTGGATATTTGGA

                   *   ** ** *  *   *            **  
     509155 GCGCTTTCAGGCCTATGGTGAAAAAGGAAATATCTTCCCAT
        131 GCGCTTTGAGGATTTCGTTGGAAACGGAAATATCTTCATAT

                  * * **     **      *   * *   *     ** ** *      *    *     
     509196 AAAAACGACATCGAAGCTATCTCAGGAACTTGTTTATGATGCATCTAATCAACTAACAGTGTTGA
          1 AAAAACTAGACAGAAGCATTCTCAGAAACATCTTTGTGATGTTTGCACTCAACTCACAGAGTTGA

                  * ** * *                         * *       *               
     509261 ACCTTTGTACTGACAGAGCAG-TTTGAAACACTCTTTTTTTGGAATCTGCAAGTGGATATTTGGA
         66 ACCTTTCTTTTCATAGAGCAGTTTTGAAACACTCTTTTTGTAGAATCTGGAAGTGGATATTTGGA

            *                           *   *   *    
     509325 TCGCTTTGAGGATTTCGTTGGAAACGG-GAT-GC--AATAT
        131 GCGCTTTGAGGATTTCGTTGGAAACGGAAATATCTTCATAT

                **  *    *     *                 **  *   *  *    *         * 
     509362 AAAACGTACACAGCAGCATACTCAGAAA-ATACTTTGCCATATTTCCATTCAAGTCACAGAGTGG
          1 AAAAACTAGACAGAAGCATTCTCAGAAACAT-CTTTGTGATGTTTGCACTCAACTCACAGAGTTG

               *  * **            *                  *  *             *      
     509426 AACATTCCCATTCATAGAGCAGGTTTGAAACACTCTTTTTGGAGTATCTGGAAGTGGACATTTGG
         65 AACCTTTCTTTTCATAGAGCAGTTTTGAAACACTCTTTTTGTAGAATCTGGAAGTGGATATTTGG

                    ** * * ** *  *   *            **  
     509491 AGCGCTTTCTGAACTATGGTGAAAAAGGAAATATCTTCCAAT
        130 AGCGCTTTGAGGATTTCGTTGGAAACGGAAATATCTTCATAT

            *     *               *      * *          *  **      **  *  *    
     509533 GAAAACAAGACAGAAGCATTCTGAGAAACTTATTTGTGATGTGTGTCCTCAACAAACGGACTTGA
          1 AAAAACTAGACAGAAGCATTCTCAGAAACATCTTTGTGATGTTTGCACTCAACTCACAGAGTTGA

                   *      **  * *  *  *             *   *    * * *           
     509598 ACCTTTCGTTTCATGCAGTACTTCTGGAACACTCTTTTTGAAGATTCTGCATGCGGATATTTGGA
         66 ACCTTTCTTTTCATAGAGCAGTTTTGAAACACTCTTTTTGTAGAATCTGGAAGTGGATATTTGGA

            **                          **    *    * 
     509663 TAGCTTTGAGGATTTCGTTGGAAACGG-GCT-T-AT-ATGT
        131 GCGCTTTGAGGATTTCGTTGGAAACGGAAATATCTTCATAT

                 *       *               *        *   *    *    *       *    
     509700 AAAAATTAGACAGCAGCATTCTCAGAAACTTCTTTGTGGTGTCTGCATTCAAGTCACAGAATTGA
          1 AAAAACTAGACAGAAGCATTCTCAGAAACATCTTTGTGATGTTTGCACTCAACTCACAGAGTTGA

              * ** * **            *  * *      *       *             *       
     509765 ACTTCCCCTCACATAGAGCAGTTGTGCAGCACTCTATTTGTAGTATCTGGAAGTGGACATTTGGA
         66 ACCTTTCTTTTCATAGAGCAGTTTTGAAACACTCTTTTTGTAGAATCTGGAAGTGGATATTTGGA

             *         ** *    * *   *            **  
     509830 GGGCTTTGTAGCCTATC-TGGAAAAAGGAAATATCTTCCCAT
        131 GCGCTTTG-AGGATTTCGTTGGAAACGGAAATATCTTCATAT

            *  ** *   *     * *            *   *  *   * **        *  * * *   
     509871 GAATGCGAGATAGAAGTAATCTCAGAAACATGTTTATGCTGTATCTACTCAACTAACTGTGCTGA
          1 AAAAACTAGACAGAAGCATTCTCAGAAACATCTTTGTGATGTTTGCACTCAACTCACAGAGTTGA

              *     *  *               *        *  * *       *               
     509936 ACATTTCTATTGATAGAGCAGTTTTGAGACACTCTTCTTTTGGAATCTGCAAGTGGATATTTGGA
         66 ACCTTTCTTTTCATAGAGCAGTTTTGAAACACTCTTTTTGTAGAATCTGGAAGTGGATATTTGGA

            ** *                        *     *      
     510001 TAGATTTGAGGATTTCGTTGGAAACGG-GAT-T-AT-ATAT
        131 GCGCTTTGAGGATTTCGTTGGAAACGGAAATATCTTCATAT

            *    *       *               *                 **  *   *         
     510038 CAAAAGTAGACAGCAGCATTCTCAGAAACTTCTTTGTGATGTTTGCATCCAGCTCTCAGAGTTGA
          1 AAAAACTAGACAGAAGCATTCTCAGAAACATCTTTGTGATGTTTGCACTCAACTCACAGAGTTGA

              *  * *          *  *        *        *   **        *  **       
     510103 ACATTCCCTTTCATAGAGTAGGTTTGAAACCCTCTTTTTATAGTGTCTGGAAGCGGGCATTTGGA
         66 ACCTTTCTTTTCATAGAGCAGTTTTGAAACACTCTTTTTGTAGAATCTGGAAGTGGATATTTGGA

                   *    * * **  **  **          * *  *
     510168 GCGCTTTCAGGCCTATGCTTAAAATAGGAAATATCTACCTAC
        131 GCGCTTTGAGG-ATTTCGTTGGAAACGGAAATATCTTCATAT

             *                    *    *  **          ** *        *    *     
     510210 AGAAACTAGACAGAAGCATTCTGAGAATCACGTTTGTGATGTGGGTACTCAACTAACAGTGTTGA
          1 AAAAACTAGACAGAAGCATTCTCAGAAACATCTTTGTGATGTTTGCACTCAACTCACAGAGTTGA

            *  *       *   *            *   *                *   *           
     510275 TCCATTCTTTTGATACAGCAGTTTTGAACCACACTTTTTGTAGAATCTGCAAGAGGATATTTGGA
         66 ACCTTTCTTTTCATAGAGCAGTTTTGAAACACTCTTTTTGTAGAATCTGGAAGTGGATATTTGGA

            **   *                     *   *      * *
     510340 TAGCTGTGAGGATTTCGTTGGAAACGGGAATGTCTTCAAAG
        131 GCGCTTTGAGGATTTCGTTGGAAACGGAAATATCTTCATAT

                *                         *   *            *    *            
     510381 AAAATCTAGACAGAAGCATTCTCAGAAACACCTTCGTGATGTTTGCAATCAAGTCACAGAGTTGA
          1 AAAAACTAGACAGAAGCATTCTCAGAAACATCTTTGTGATGTTTGCACTCAACTCACAGAGTTGA

                 * *             *  *           *      *             *       
     510446 ACCTTCCGTTTCATAGAGCAGGTTGGAAACACTCTTATTGTAGTATCTGGAAGTGGACATTTGGA
         66 ACCTTTCTTTTCATAGAGCAGTTTTGAAACACTCTTTTTGTAGAATCTGGAAGTGGATATTTGGA

                   *   ** ** *  *   *            **  
     510511 GCGCTTTCAGGCCTATGGTGAAAAAGGAAATATCTTCCCAT
        131 GCGCTTTGAGGATTTCGTTGGAAACGGAAATATCTTCATAT

                  * * *      **      *   * *   *     ** ** *      *    *     
     510552 AAAAACGACATAGAAGCTATCTCAGGAACTTGTTTATGATGCATCTAATCAACTAACAGTGTTGA
          1 AAAAACTAGACAGAAGCATTCTCAGAAACATCTTTGTGATGTTTGCACTCAACTCACAGAGTTGA

                  * ** * *                         * *       *               
     510617 ACCTTTGTACTGACAGAGCAG-TTTGAAACACTCTTTTTTTGGAATCTGCAAGTGGATATTTGGA
         66 ACCTTTCTTTTCATAGAGCAGTTTTGAAACACTCTTTTTGTAGAATCTGGAAGTGGATATTTGGA

            *                           *   *   *    
     510681 TCGCTTTGAGGATTTCGTTGGAAACGG-GAT-GC--AATAT
        131 GCGCTTTGAGGATTTCGTTGGAAACGGAAATATCTTCATAT

                **  *    *     *                 **  *   *  *    *         * 
     510718 AAAACGTACACAGCAGCATACTCAGAAA-ATACTTTGCCATATTTCCATTCAAGTCACAGAGTGG
          1 AAAAACTAGACAGAAGCATTCTCAGAAACAT-CTTTGTGATGTTTGCACTCAACTCACAGAGTTG

               *  * **            *                  *  *             *      
     510782 AACATTCCCATTCATAGAGCAGGTTTGAAACACTCTTTTTGGAGTATCTGGAAGTGGACATTTGG
         65 AACCTTTCTTTTCATAGAGCAGTTTTGAAACACTCTTTTTGTAGAATCTGGAAGTGGATATTTGG

                    ** * * ** *  *   *            **  
     510847 AGCGCTTTCTGAACTATGGTGAAAAAGGAAATATCTTCCAAT
        130 AGCGCTTTGAGGATTTCGTTGGAAACGGAAATATCTTCATAT

            *     *               *      * *          *  **      **  *  *    
     510889 GAAAACAAGACAGAAGCATTCTGAGAAACTTATTTGTGATGTGTGTCCTCAACAAACGGACTTGA
          1 AAAAACTAGACAGAAGCATTCTCAGAAACATCTTTGTGATGTTTGCACTCAACTCACAGAGTTGA

                   *      **  * *  *  *             *   *    * * *           
     510954 ACCTTTCGTTTCATGCAGTACTTCTGGAACACTCTTTTTGAAGATTCTGCATGCGGATATTTGGA
         66 ACCTTTCTTTTCATAGAGCAGTTTTGAAACACTCTTTTTGTAGAATCTGGAAGTGGATATTTGGA

            **                            **   *   * 
     511019 TAGCTTTGAGGATTTCGTTGGAAACGG---GCT-TACATGT
        131 GCGCTTTGAGGATTTCGTTGGAAACGGAAATATCTTCATAT

                 *       *               *        *   *    *    *       *    
     511056 AAAAATTAGACAGCAGCATTCTCAGAAACTTCTTTGTGGTGTCTGCATTCAAGTCACAGAATTGA
          1 AAAAACTAGACAGAAGCATTCTCAGAAACATCTTTGTGATGTTTGCACTCAACTCACAGAGTTGA

              * ** * **            *  * *      *       *             *       
     511121 ACTTCCCCTCACATAGAGCAGTTGTGCAGCACTCTATTTGTAGTATCTGGAAGTGGACATTTGGA
         66 ACCTTTCTTTTCATAGAGCAGTTTTGAAACACTCTTTTTGTAGAATCTGGAAGTGGATATTTGGA

             *         ** *    * *   *            **  
     511186 GGGCTTTGTAGCCTATC-TGGAAAAAGGAAATATCTTCCCAT
        131 GCGCTTTG-AGGATTTCGTTGGAAACGGAAATATCTTCATAT

            *  ** *   *     * *            *   *  *   * **        *  * * *   
     511227 GAATGCGAGATAGAAGTAATCTCAGAAACATGTTTATGCTGTATCTACTCAACTAACTGTGCTGA
          1 AAAAACTAGACAGAAGCATTCTCAGAAACATCTTTGTGATGTTTGCACTCAACTCACAGAGTTGA

              *     *  *               *        *  * *       *               
     511292 ACATTTCTATTGATAGAGCAGTTTTGAGACACTCTTCTTTTGGAATCTGCAAGTGGATATTTGGA
         66 ACCTTTCTTTTCATAGAGCAGTTTTGAAACACTCTTTTTGTAGAATCTGGAAGTGGATATTTGGA

            ** *                        *     *      
     511357 TAGATTTGAGGATTTCGTTGGAAACGG-GAT-T-AT-ATAT
        131 GCGCTTTGAGGATTTCGTTGGAAACGGAAATATCTTCATAT

                 *       *               *                 **  *   *         
     511394 AAAAAGTAGACAGCAGCATTCTCAGAAACTTCTTTGTGATGTTTGCATCCAGCTCTCAGAGTTGA
          1 AAAAACTAGACAGAAGCATTCTCAGAAACATCTTTGTGATGTTTGCACTCAACTCACAGAGTTGA

              *  * *          *  *        *        *   **        *  **       
     511459 ACATTCCCTTTCATAGAGTAGGTTTGAAACCCTCTTTTTATAGTGTCTGGAAGCGGGCATTTGGA
         66 ACCTTTCTTTTCATAGAGCAGTTTTGAAACACTCTTTTTGTAGAATCTGGAAGTGGATATTTGGA

                   *    * * **  **  **          * *  *
     511524 GCGCTTTCAGGCCTATGCTTAAAATAGGAAATATCTACCTAC
        131 GCGCTTTGAGG-ATTTCGTTGGAAACGGAAATATCTTCATAT

             *                    *    *  **          ** *        *    *     
     511566 AGAAACTAGACAGAAGCATTCTGAGAATCACGTTTGTGATGTGGGTACTCAACTAACAGTGTTGA
          1 AAAAACTAGACAGAAGCATTCTCAGAAACATCTTTGTGATGTTTGCACTCAACTCACAGAGTTGA

            *  *       *   *            *   *                *   *           
     511631 TCCATTCTTTTGATACAGCAGTTTTGAACCACACTTTTTGTAGAATCTGCAAGAGGATATTTGGA
         66 ACCTTTCTTTTCATAGAGCAGTTTTGAAACACTCTTTTTGTAGAATCTGGAAGTGGATATTTGGA

            **   *                     *   *      * *
     511696 TAGCTGTGAGGATTTCGTTGGAAACGGGAATGTCTTCAAAG
        131 GCGCTTTGAGGATTTCGTTGGAAACGGAAATATCTTCATAT

                *                         *   *            *    *            
     511737 AAAATCTAGACAGAAGCATTCTCAGAAACACCTTCGTGATGTTTGCAATCAAGTCACAGAGTTGA
          1 AAAAACTAGACAGAAGCATTCTCAGAAACATCTTTGTGATGTTTGCACTCAACTCACAGAGTTGA

                 * *             *  *           *      *             *       
     511802 ACCTTCCGTTTCATAGAGCAGGTTGGAAACACTCTTATTGTAGTATCTGGAAGTGGACATTTGGA
         66 ACCTTTCTTTTCATAGAGCAGTTTTGAAACACTCTTTTTGTAGAATCTGGAAGTGGATATTTGGA

                   *   ** ** *  *   *            **  
     511867 GCGCTTTCAGGCCTATGGTGAAAAAGGAAATATCTTCCCAT
        131 GCGCTTTGAGGATTTCGTTGGAAACGGAAATATCTTCATAT

                  * * *      **      *   * *   *     ** ** *      *    *     
     511908 AAAAACGACATAGAAGCTATCTCAGGAACTTGTTTATGATGCATCTAATCAACTAACAGTGTTGA
          1 AAAAACTAGACAGAAGCATTCTCAGAAACATCTTTGTGATGTTTGCACTCAACTCACAGAGTTGA

                  * ** * *                         * *       *               
     511973 ACCTTTGTACTGACAGAGCAG-TTTGAAACACTCTTTTTTTGGAATCTGCAAGTGGATATTTGGA
         66 ACCTTTCTTTTCATAGAGCAGTTTTGAAACACTCTTTTTGTAGAATCTGGAAGTGGATATTTGGA

            *                           *   *   *    
     512037 TCGCTTTGAGGATTTCGTTGGAAACGG-GAT-GC--AATAT
        131 GCGCTTTGAGGATTTCGTTGGAAACGGAAATATCTTCATAT

                **  *    *     *                 **  *   *  *    *   *     * 
     512074 AAAACGTACACAGCAGCATACTCAGAAA-ATACTTTGCCATATTTCCATTCAAGTCAGAGAGTGG
          1 AAAAACTAGACAGAAGCATTCTCAGAAACAT-CTTTGTGATGTTTGCACTCAACTCACAGAGTTG

               *  * **            *                  *  *             *      
     512138 AACATTCCCATTCATAGAGCAGGTTTGAAACACTCTTTTTGGAGTATCTGGAAGTGGACATTTGG
         65 AACCTTTCTTTTCATAGAGCAGTTTTGAAACACTCTTTTTGTAGAATCTGGAAGTGGATATTTGG

                    ** * * ** *  *   *            **  
     512203 AGCGCTTTCTGAACTATGGTGAAAAAGGAAATATCTTCCAAT
        130 AGCGCTTTGAGGATTTCGTTGGAAACGGAAATATCTTCATAT

            *     *               *      * *          *  **      **  *  *    
     512245 GAAAACAAGACAGAAGCATTCTGAGAAACTTATTTGTGATGTGTGTCCTCAACAAACGGACTTGA
          1 AAAAACTAGACAGAAGCATTCTCAGAAACATCTTTGTGATGTTTGCACTCAACTCACAGAGTTGA

                   *      **  * *  *  *             *   *    * * *           
     512310 ACCTTTCGTTTCATGCAGTACTTCTGGAACACTCTTTTTGAAGATTCTGCATGCGGATATTTGGA
         66 ACCTTTCTTTTCATAGAGCAGTTTTGAAACACTCTTTTTGTAGAATCTGGAAGTGGATATTTGGA

            **                            **   *   * 
     512375 TAGCTTTGAGGATTTCGTTGGAAACGG---GCT-TACATGT
        131 GCGCTTTGAGGATTTCGTTGGAAACGGAAATATCTTCATAT

                 *       *               *        *   *    *    *       *    
     512412 AAAAATTAGACAGCAGCATTCTCAGAAACTTCTTTGTGGTGTCTGCATTCAAGTCACAGAATTGA
          1 AAAAACTAGACAGAAGCATTCTCAGAAACATCTTTGTGATGTTTGCACTCAACTCACAGAGTTGA

              * ** * **            *  * *      *       *             *       
     512477 ACTTCCCCTCACATAGAGCAGTTGTGCAGCACTCTATTTGTAGTATCTGGAAGTGGACATTTGGA
         66 ACCTTTCTTTTCATAGAGCAGTTTTGAAACACTCTTTTTGTAGAATCTGGAAGTGGATATTTGGA

             *         ** *    * *   *            **  
     512542 GGGCTTTGTAGCCTATC-TGGAAAAAGGAAATATCTTCCCAT
        131 GCGCTTTG-AGGATTTCGTTGGAAACGGAAATATCTTCATAT

            *  ** *   *     * *            *   *  *   * **        *  * * *   
     512583 GAATGCGAGATAGAAGTAATCTCAGAAACATGTTTATGCTGTATCTACTCAACTAACTGTGCTGA
          1 AAAAACTAGACAGAAGCATTCTCAGAAACATCTTTGTGATGTTTGCACTCAACTCACAGAGTTGA

              *     *  *               *        *  * *       *               
     512648 ACATTTCTATTGATAGAGCAGTTTTGAGACACTCTTCTTTTGGAATCTGCAAGTGGATATTTGGA
         66 ACCTTTCTTTTCATAGAGCAGTTTTGAAACACTCTTTTTGTAGAATCTGGAAGTGGATATTTGGA

            ** *                        *     *      
     512713 TAGATTTGAGGATTTCGTTGGAAACGG-GAT-T-AT-ATAT
        131 GCGCTTTGAGGATTTCGTTGGAAACGGAAATATCTTCATAT

            *    *       *               *                 **  *   *         
     512750 CAAAAGTAGACAGCAGCATTCTCAGAAACTTCTTTGTGATGTTTGCATCCAGCTCTCAGAGTTGA
          1 AAAAACTAGACAGAAGCATTCTCAGAAACATCTTTGTGATGTTTGCACTCAACTCACAGAGTTGA

              *  * *          *  *        *        *   **        *  **       
     512815 ACATTCCCTTTCATAGAGTAGGTTTGAAACCCTCTTTTTATAGTGTCTGGAAGCGGGCATTTGGA
         66 ACCTTTCTTTTCATAGAGCAGTTTTGAAACACTCTTTTTGTAGAATCTGGAAGTGGATATTTGGA

                   *    * * **  **  **          * *  *
     512880 GCGCTTTCAGGCCTATGCTTAAAATAGGAAATATCTACCTAC
        131 GCGCTTTGAGG-ATTTCGTTGGAAACGGAAATATCTTCATAT

             *                    *    *  **          ** *        *    *     
     512922 AGAAACTAGACAGAAGCATTCTGAGAATCACGTTTGTGATGTGGGTACTCAACTAACAGTGTTGA
          1 AAAAACTAGACAGAAGCATTCTCAGAAACATCTTTGTGATGTTTGCACTCAACTCACAGAGTTGA

            *  *       *   *            *   *                *   *           
     512987 TCCATTCTTTTGATACAGCAGTTTTGAACCACACTTTTTGTAGAATCTGCAAGAGGATATTTGGA
         66 ACCTTTCTTTTCATAGAGCAGTTTTGAAACACTCTTTTTGTAGAATCTGGAAGTGGATATTTGGA

            **   *                     *   *      * *
     513052 TAGCTGTGAGGATTTCGTTGGAAACGGGAATGTCTTCAAAG
        131 GCGCTTTGAGGATTTCGTTGGAAACGGAAATATCTTCATAT

                *                         *   *            *    *            
     513093 AAAATCTAGACAGAAGCATTCTCAGAAACACCTTCGTGATGTTTGCAATCAAGTCACAGAGTTGA
          1 AAAAACTAGACAGAAGCATTCTCAGAAACATCTTTGTGATGTTTGCACTCAACTCACAGAGTTGA

                 * *             *  *           *      *             *       
     513158 ACCTTCCGTTTCATAGAGCAGGTTGGAAACACTCTTATTGTAGTATCTGGAAGTGGACATTTGGA
         66 ACCTTTCTTTTCATAGAGCAGTTTTGAAACACTCTTTTTGTAGAATCTGGAAGTGGATATTTGGA

                   *   ** ** *  *   *            **  
     513223 GCGCTTTCAGGCCTATGGTGAAAAAGGAAATATCTTCCCAT
        131 GCGCTTTGAGGATTTCGTTGGAAACGGAAATATCTTCATAT

                  * * *      **      *   * *   *     ** ** *      *    *     
     513264 AAAAACGACATAGAAGCTATCTCAGGAACTTGTTTATGATGCATCTAATCAACTAACAGTGTTGA
          1 AAAAACTAGACAGAAGCATTCTCAGAAACATCTTTGTGATGTTTGCACTCAACTCACAGAGTTGA

                  * ** * *                         * *       *               
     513329 ACCTTTGTACTGACAGAGCAG-TTTGAAACACTCTTTTTTTGGAATCTGCAAGTGGATATTTGGA
         66 ACCTTTCTTTTCATAGAGCAGTTTTGAAACACTCTTTTTGTAGAATCTGGAAGTGGATATTTGGA

            *                           *   *   *    
     513393 TCGCTTTGAGGATTTCGTTGGAAACGG-GAT-GC--AATAT
        131 GCGCTTTGAGGATTTCGTTGGAAACGGAAATATCTTCATAT

                **  *    *     *                 **  *   *  *    *   *     * 
     513430 AAAACGTACACAGCAGCATACTCAGAAA-ATACTTTGCCATATTTCCATTCAAGTCAGAGAGTGG
          1 AAAAACTAGACAGAAGCATTCTCAGAAACAT-CTTTGTGATGTTTGCACTCAACTCACAGAGTTG

               *  * **            *                  *  *             *      
     513494 AACATTCCCATTCATAGAGCAGGTTTGAAACACTCTTTTTGGAGTATCTGGAAGTGGACATTTGG
         65 AACCTTTCTTTTCATAGAGCAGTTTTGAAACACTCTTTTTGTAGAATCTGGAAGTGGATATTTGG

                    ** * * ** *  *   *            **  
     513559 AGCGCTTTCTGAACTATGGTGAAAAAGGAAATATCTTCCAAT
        130 AGCGCTTTGAGGATTTCGTTGGAAACGGAAATATCTTCATAT

            *     *               *      * *          *  **      **  *  *    
     513601 GAAAACAAGACAGAAGCATTCTGAGAAACTTATTTGTGATGTGTGTCCTCAACAAACGGACTTGA
          1 AAAAACTAGACAGAAGCATTCTCAGAAACATCTTTGTGATGTTTGCACTCAACTCACAGAGTTGA

                   *      **  * *  *  *             *   *    * * *           
     513666 ACCTTTCGTTTCATGCAGTACTTCTGGAACACTCTTTTTGAAGATTCTGCATGCGGATATTTGGA
         66 ACCTTTCTTTTCATAGAGCAGTTTTGAAACACTCTTTTTGTAGAATCTGGAAGTGGATATTTGGA

            **              *             **   *   * 
     513731 TAGCTTTGAGGATTTCTTTGGAAACGG---GCT-TACATGT
        131 GCGCTTTGAGGATTTCGTTGGAAACGGAAATATCTTCATAT

                 *       *               *        *   *    *    *       *    
     513768 AAAAATTAGACAGCAGCATTCTCAGAAACTTCTTTGTGGTGTCTGCATTCAAGTCACAGAATTGA
          1 AAAAACTAGACAGAAGCATTCTCAGAAACATCTTTGTGATGTTTGCACTCAACTCACAGAGTTGA

              * ** * **            *  * *      *       *             *       
     513833 ACTTCCCCTCACATAGAGCAGTTGTGCAGCACTCTATTTGTAGTATCTGGAAGTGGACATTTGGA
         66 ACCTTTCTTTTCATAGAGCAGTTTTGAAACACTCTTTTTGTAGAATCTGGAAGTGGATATTTGGA

             *         ** *    * *   *            **  
     513898 GGGCTTTGTAGCCTATC-TGGAAAAAGGAAATATCTTCCCAT
        131 GCGCTTTG-AGGATTTCGTTGGAAACGGAAATATCTTCATAT

            *  ** *   *     * *            *   *  *   * **        *  * * *   
     513939 GAATGCGAGATAGAAGTAATCTCAGAAACATGTTTATGCTGTATCTACTCAACTAACTGTGCTGA
          1 AAAAACTAGACAGAAGCATTCTCAGAAACATCTTTGTGATGTTTGCACTCAACTCACAGAGTTGA

              *     *  *               *        *  * *       *               
     514004 ACATTTCTATTGATAGAGCAGTTTTGAGACACTCTTCTTTTGGAATCTGCAAGTGGATATTTGGA
         66 ACCTTTCTTTTCATAGAGCAGTTTTGAAACACTCTTTTTGTAGAATCTGGAAGTGGATATTTGGA

            ** *                        *     *      
     514069 TAGATTTGAGGATTTCGTTGGAAACGG-GAT-T-AT-ATAT
        131 GCGCTTTGAGGATTTCGTTGGAAACGGAAATATCTTCATAT

            *    *       *               *                 **  *   *         
     514106 CAAAAGTAGACAGCAGCATTCTCAGAAACTTCTTTGTGATGTTTGCATCCAGCTCTCAGAGTTGA
          1 AAAAACTAGACAGAAGCATTCTCAGAAACATCTTTGTGATGTTTGCACTCAACTCACAGAGTTGA

              *  * *          *  *        *        *   **        *  **       
     514171 ACATTCCCTTTCATAGAGTAGGTTTGAAACCCTCTTTTTATAGTGTCTGGAAGCGGGCATTTGGA
         66 ACCTTTCTTTTCATAGAGCAGTTTTGAAACACTCTTTTTGTAGAATCTGGAAGTGGATATTTGGA

                   *    * * **  **  **          * *  *
     514236 GCGCTTTCAGGCCTATGCTTAAAATAGGAAATATCTACCTAC
        131 GCGCTTTGAGG-ATTTCGTTGGAAACGGAAATATCTTCATAT

             *                    *    *  **          ** *        *    *     
     514278 AGAAACTAGACAGAAGCATTCTGAGAATCACGTTTGTGATGTGGGTACTCAACTAACAGTGTTGA
          1 AAAAACTAGACAGAAGCATTCTCAGAAACATCTTTGTGATGTTTGCACTCAACTCACAGAGTTGA

            *  *       *   *            *   *                *   *           
     514343 TCCATTCTTTTGATACAGCAGTTTTGAACCACACTTTTTGTAGAATCTGCAAGAGGATATTTGGA
         66 ACCTTTCTTTTCATAGAGCAGTTTTGAAACACTCTTTTTGTAGAATCTGGAAGTGGATATTTGGA

            **   *                     *   *      * *
     514408 TAGCTGTGAGGATTTCGTTGGAAACGGGAATGTCTTCAAAG
        131 GCGCTTTGAGGATTTCGTTGGAAACGGAAATATCTTCATAT

                *                         *   *            *    *            
     514449 AAAATCTAGACAGAAGCATTCTCAGAAACACCTTCGTGATGTTTGCAATCAAGTCACAGAGTTGA
          1 AAAAACTAGACAGAAGCATTCTCAGAAACATCTTTGTGATGTTTGCACTCAACTCACAGAGTTGA

                 * *             *  *           *      *             *       
     514514 ACCTTCCGTTTCATAGAGCAGGTTGGAAACACTCTTATTGTAGTATCTGGAAGTGGACATTTGGA
         66 ACCTTTCTTTTCATAGAGCAGTTTTGAAACACTCTTTTTGTAGAATCTGGAAGTGGATATTTGGA

                   *   ** ** *  *   *            **  
     514579 GCGCTTTCAGGCCTATGGTGAAAAAGGAAATATCTTCCCAT
        131 GCGCTTTGAGGATTTCGTTGGAAACGGAAATATCTTCATAT

                  * * *      **      *   * *   *     ** ** *      *    *     
     514620 AAAAACGACATAGAAGCTATCTCAGGAACTTGTTTATGATGCATCTAATCAACTAACAGTGTTGA
          1 AAAAACTAGACAGAAGCATTCTCAGAAACATCTTTGTGATGTTTGCACTCAACTCACAGAGTTGA

                  * ** * *                         * *       *               
     514685 ACCTTTGTACTGACAGAGCAG-TTTGAAACACTCTTTTTTTGGAATCTGCAAGTGGATATTTGGA
         66 ACCTTTCTTTTCATAGAGCAGTTTTGAAACACTCTTTTTGTAGAATCTGGAAGTGGATATTTGGA

            *                           *   *   *    
     514749 TCGCTTTGAGGATTTCGTTGGAAACGG-GAT-GC--AATAT
        131 GCGCTTTGAGGATTTCGTTGGAAACGGAAATATCTTCATAT

                **  *    *     *                 **  *   *  *    *         * 
     514786 AAAACGTACACAGCAGCATACTCAGAAA-ATACTTTGCCATATTTCCATTCAAGTCACAGAGTGG
          1 AAAAACTAGACAGAAGCATTCTCAGAAACAT-CTTTGTGATGTTTGCACTCAACTCACAGAGTTG

               *  * **            *                  *  *             *      
     514850 AACATTCCCATTCATAGAGCAGGTTTGAAACACTCTTTTTGGAGTATCTGGAAGTGGACATTTGG
         65 AACCTTTCTTTTCATAGAGCAGTTTTGAAACACTCTTTTTGTAGAATCTGGAAGTGGATATTTGG

                    ** * * ** *  *   *            **  
     514915 AGCGCTTTCTGAACTATGGTGAAAAAGGAAATATCTTCCAAT
        130 AGCGCTTTGAGGATTTCGTTGGAAACGGAAATATCTTCATAT

            *     *               *      * *          *  **      **  *  *    
     514957 GAAAACAAGACAGAAGCATTCTGAGAAACTTATTTGTGATGTGTGTCCTCAACAAACGGACTTGA
          1 AAAAACTAGACAGAAGCATTCTCAGAAACATCTTTGTGATGTTTGCACTCAACTCACAGAGTTGA

                   *      **  * *  *  *             *   *    * * *           
     515022 ACCTTTCGTTTCATGCAGTACTTCTGGAACACTCTTTTTGAAGATTCTGCATGCGGATATTTGGA
         66 ACCTTTCTTTTCATAGAGCAGTTTTGAAACACTCTTTTTGTAGAATCTGGAAGTGGATATTTGGA

            **                            **   *   * 
     515087 TAGCTTTGAGGATTTCGTTGGAAACGG---GCT-TACATGT
        131 GCGCTTTGAGGATTTCGTTGGAAACGGAAATATCTTCATAT

                 *       *               *        *   *    *    *       *    
     515124 AAAAATTAGACAGCAGCATTCTCAGAAACTTCTTTGTGGTGTCTGCATTCAAGTCACAGAATTGA
          1 AAAAACTAGACAGAAGCATTCTCAGAAACATCTTTGTGATGTTTGCACTCAACTCACAGAGTTGA

              * ** * **            *  * *      *       *             *       
     515189 ACTTCCCCTCACATAGAGCAGTTGTGCAGCACTCTATTTGTAGTATCTGGAAGTGGACATTTGGA
         66 ACCTTTCTTTTCATAGAGCAGTTTTGAAACACTCTTTTTGTAGAATCTGGAAGTGGATATTTGGA

             *         ****    * *   **           **  
     515254 GGGCTTTGTAGCCCATC-TGGAAAAAAGAAATATCTTCCCAT
        131 GCGCTTTG-AGGATTTCGTTGGAAACGGAAATATCTTCATAT

            *  ** *   *     * *            *   *  *   * **        *  * * *   
     515295 GAATGCGAGATAGAAGTAATCTCAGAAACATGTTTATGCTGTATCTACTCAACTAACTGTGCTGA
          1 AAAAACTAGACAGAAGCATTCTCAGAAACATCTTTGTGATGTTTGCACTCAACTCACAGAGTTGA

              *     *  *               *        *  * *       *               
     515360 ACATTTCTATTGATAGAGCAGTTTTGAGACACTCTTCTTTTGGAATCTGCAAGTGGATATTTGGA
         66 ACCTTTCTTTTCATAGAGCAGTTTTGAAACACTCTTTTTGTAGAATCTGGAAGTGGATATTTGGA

            ** *                        *     *      
     515425 TAGATTTGAGGATTTCGTTGGAAACGG-GAT-T-AT-ATAT
        131 GCGCTTTGAGGATTTCGTTGGAAACGGAAATATCTTCATAT

                 *       *               *                 **  *   *         
     515462 AAAAAGTAGACAGCAGCATTCTCAGAAACTTCTTTGTGATGTTTGCATCCAGCTCTCAGAGTTGA
          1 AAAAACTAGACAGAAGCATTCTCAGAAACATCTTTGTGATGTTTGCACTCAACTCACAGAGTTGA

              *  * *          *  *        *        *   **    *   *  **       
     515527 ACATTCCCTTTCATAGAGTAGGTTTGAAACCCTCTTTTTATAGTGTCTGCAAGCGGGCATTTGGA
         66 ACCTTTCTTTTCATAGAGCAGTTTTGAAACACTCTTTTTGTAGAATCTGGAAGTGGATATTTGGA

                   *    * * **  **  **    *     * *  *
     515592 GCGCTTTCAGGCCTATGCTTAAAATAGGAATTATCTACCTAC
        131 GCGCTTTGAGG-ATTTCGTTGGAAACGGAAATATCTTCATAT

             *                    *    *  **          ** *        *    *     
     515634 AGAAACTAGACAGAAGCATTCTGAGAATCACGTTTGTGATGTGGGTACTCAACTAACAGTGTTGA
          1 AAAAACTAGACAGAAGCATTCTCAGAAACATCTTTGTGATGTTTGCACTCAACTCACAGAGTTGA

            *  *       *   *            *   *      *         *   *           
     515699 TCCATTCTTTTGATACAGCAGTTTTGAACCACACTTTTTATAGAATCTGCAAGAGGATATTTGGA
         66 ACCTTTCTTTTCATAGAGCAGTTTTGAAACACTCTTTTTGTAGAATCTGGAAGTGGATATTTGGA

            **   *                     *   *      * *
     515764 TAGCTGTGAGGATTTCGTTGGAAACGGGAATGTCTTCAAAG
        131 GCGCTTTGAGGATTTCGTTGGAAACGGAAATATCTTCATAT

                *                         *   *            *    *            
     515805 AAAATCTAGACAGAAGCATTCTCAGAAACACCTTCGTGATGTTTGCAATCAAGTCACAGAGTTGA
          1 AAAAACTAGACAGAAGCATTCTCAGAAACATCTTTGTGATGTTTGCACTCAACTCACAGAGTTGA

                 * *             *  *           *      *             *       
     515870 ACCTTCCGTTTCATAGAGCAGGTTGGAAACACTCTTATTGTAGTATCTGGAAGTGGACATTTGGA
         66 ACCTTTCTTTTCATAGAGCAGTTTTGAAACACTCTTTTTGTAGAATCTGGAAGTGGATATTTGGA

                   *   ** ** *  *   *            **  
     515935 GCGCTTTCAGGCCTATGGTGAAAAAGGAAATATCTTCCCAT
        131 GCGCTTTGAGGATTTCGTTGGAAACGGAAATATCTTCATAT

                  * * *      **      *   * *   *     ** ** *      *    *     
     515976 AAAAACGACATAGAAGCTATCTCAGGAACTTGTTTATGATGCATCTAATCAACTAACAGTGTTGA
          1 AAAAACTAGACAGAAGCATTCTCAGAAACATCTTTGTGATGTTTGCACTCAACTCACAGAGTTGA

                  * ** * *                         * *       *               
     516041 ACCTTTGTACTGACAGAGCAG-TTTGAAACACTCTTTTTTTGGAATCTGCAAGTGGATATTTGGA
         66 ACCTTTCTTTTCATAGAGCAGTTTTGAAACACTCTTTTTGTAGAATCTGGAAGTGGATATTTGGA

            *                           *   *   *    
     516105 TCGCTTTGAGGATTTCGTTGGAAACGG-GAT-GC--AATAT
        131 GCGCTTTGAGGATTTCGTTGGAAACGGAAATATCTTCATAT

                **  *    *     *                 **  *   *  *    *         * 
     516142 AAAACGTACACAGCAGCATACTCAGAAA-ATACTTTGCCATATTTCCATTCAAGTCACAGAGTGG
          1 AAAAACTAGACAGAAGCATTCTCAGAAACAT-CTTTGTGATGTTTGCACTCAACTCACAGAGTTG

               *  * **            *                  *  *             *      
     516206 AACATTCCCATTCATAGAGCAGGTTTGAAACACTCTTTTTGGAGTATCTGGAAGTGGACATTTGG
         65 AACCTTTCTTTTCATAGAGCAGTTTTGAAACACTCTTTTTGTAGAATCTGGAAGTGGATATTTGG

                    ** * * ** *  *   *            **  
     516271 AGCGCTTTCTGAACTATGGTGAAAAAGGAAATATCTTCCAAT
        130 AGCGCTTTGAGGATTTCGTTGGAAACGGAAATATCTTCATAT

            *     *               *      * *          *  **      **  *  *    
     516313 GAAAACAAGACAGAAGCATTCTGAGAAACTTATTTGTGATGTGTGTCCTCAACAAACGGACTTGA
          1 AAAAACTAGACAGAAGCATTCTCAGAAACATCTTTGTGATGTTTGCACTCAACTCACAGAGTTGA

                   *      **  * *  *  *             *   *    * * *           
     516378 ACCTTTCGTTTCATGCAGTACTTCTGGAACACTCTTTTTGAAGATTCTGCATGCGGATATTTGGA
         66 ACCTTTCTTTTCATAGAGCAGTTTTGAAACACTCTTTTTGTAGAATCTGGAAGTGGATATTTGGA

            **                            **   *   * 
     516443 TAGCTTTGAGGATTTCGTTGGAAACGG---GCT-TACATGT
        131 GCGCTTTGAGGATTTCGTTGGAAACGGAAATATCTTCATAT

                 *       *               *        *   *    *    *       *    
     516480 AAAAATTAGACAGCAGCATTCTCAGAAACTTCTTTGTGGTGTCTGCATTCAAGTCACAGAATTGA
          1 AAAAACTAGACAGAAGCATTCTCAGAAACATCTTTGTGATGTTTGCACTCAACTCACAGAGTTGA

              * ** * **            *  * *      *       *    *        *       
     516545 ACTTCCCCTCACATAGAGCAGTTGTGCAGCACTCTATTTGTAGTATCTCGAAGTGGACATTTGGA
         66 ACCTTTCTTTTCATAGAGCAGTTTTGAAACACTCTTTTTGTAGAATCTGGAAGTGGATATTTGGA

             *         ** *    * *   *            **  
     516610 GGGCTTTGTAGCCTATC-TGGAAAAAGGAAATATCTTCCCAT
        131 GCGCTTTG-AGGATTTCGTTGGAAACGGAAATATCTTCATAT

            *  ** *   *     * *            *   *  *   * ***       *  * * *   
     516651 GAATGCGAGATAGAAGTAATCTCAGAAACATGTTTATGCTGTATCTTCTCAACTAACTGTGCTGA
          1 AAAAACTAGACAGAAGCATTCTCAGAAACATCTTTGTGATGTTTGCACTCAACTCACAGAGTTGA

              *     *  *               *        *  * *       *               
     516716 ACATTTCTATTGATAGAGCAGTTTTGAGACACTCTTCTTTTGGAATCTGCAAGTGGATATTTGGA
         66 ACCTTTCTTTTCATAGAGCAGTTTTGAAACACTCTTTTTGTAGAATCTGGAAGTGGATATTTGGA

            ** *                        *     *      
     516781 TAGATTTGAGGATTTCGTTGGAAACGG-GAT-T-AT-ATAT
        131 GCGCTTTGAGGATTTCGTTGGAAACGGAAATATCTTCATAT

                 *       *               *                 **  *   *         
     516818 AAAAAGTAGACAGCAGCATTCTCAGAAACTTCTTTGTGATGTTTGCATCCAGCTCTCAGAGTTGA
          1 AAAAACTAGACAGAAGCATTCTCAGAAACATCTTTGTGATGTTTGCACTCAACTCACAGAGTTGA

              *  * *          *  *        *        *   **        *  **       
     516883 ACATTCCCTTTCATAGAGTAGGTTTGAAACCCTCTTTTTATAGTGTCTGGAAGCGGGCATTTGGA
         66 ACCTTTCTTTTCATAGAGCAGTTTTGAAACACTCTTTTTGTAGAATCTGGAAGTGGATATTTGGA

                   *    * * **  **  **          * *  *
     516948 GCGCTTTCAGGCCTATGCTTAAAATAGGAAATATCTACCTAC
        131 GCGCTTTGAGG-ATTTCGTTGGAAACGGAAATATCTTCATAT

             *                    *    *  **          ** *        *    *     
     516990 AGAAACTAGACAGAAGCATTCTGAGAATCACGTTTGTGATGTGGGTACTCAACTAACAGTGTTGA
          1 AAAAACTAGACAGAAGCATTCTCAGAAACATCTTTGTGATGTTTGCACTCAACTCACAGAGTTGA

            *  *       *   *            *   *                *   *           
     517055 TCCATTCTTTTGATACAGCAGTTTTGAACCACACTTTTTGTAGAATCTGCAAGAGGATATTTGGA
         66 ACCTTTCTTTTCATAGAGCAGTTTTGAAACACTCTTTTTGTAGAATCTGGAAGTGGATATTTGGA

            **   *                     *   *      * *
     517120 TAGCTGTGAGGATTTCGTTGGAAACGGGAATGTCTTCAAAG
        131 GCGCTTTGAGGATTTCGTTGGAAACGGAAATATCTTCATAT

                *                         *   *            *    *            
     517161 AAAATCTAGACAGAAGCATTCTCAGAAACACCTTCGTGATGTTTGCAATCAAGTCACAGAGTTGA
          1 AAAAACTAGACAGAAGCATTCTCAGAAACATCTTTGTGATGTTTGCACTCAACTCACAGAGTTGA

                 * *             *  *           *      *             *       
     517226 ACCTTCCGTTTCATAGAGCAGGTTGGAAACACTCTTATTGTAGTATCTGGAAGTGGACATTTGGA
         66 ACCTTTCTTTTCATAGAGCAGTTTTGAAACACTCTTTTTGTAGAATCTGGAAGTGGATATTTGGA

                   *   ** ** *  *   *            **  
     517291 GCGCTTTCAGGCCTATGGTGAAAAAGGAAATATCTTCCCAT
        131 GCGCTTTGAGGATTTCGTTGGAAACGGAAATATCTTCATAT

                  * * *      **      *   * *   *     ** ** *      *    *     
     517332 AAAAACGACATAGAAGCTATCTCAGGAACTTGTTTATGATGCATCTAATCAACTAACAGTGTTGA
          1 AAAAACTAGACAGAAGCATTCTCAGAAACATCTTTGTGATGTTTGCACTCAACTCACAGAGTTGA

                  * ** * *                         * *       *               
     517397 ACCTTTGTACTGACAGAGCAG-TTTGAAACACTCTTTTTTTGGAATCTGCAAGTGGATATTTGGA
         66 ACCTTTCTTTTCATAGAGCAGTTTTGAAACACTCTTTTTGTAGAATCTGGAAGTGGATATTTGGA

            *                           *   *   *    
     517461 TCGCTTTGAGGATTTCGTTGGAAACGG-GAT-GC--AATAT
        131 GCGCTTTGAGGATTTCGTTGGAAACGGAAATATCTTCATAT

                **  *    *     *                 **  *   *  *    *         * 
     517498 AAAACGTACACAGCAGCATACTCAGAAA-ATACTTTGCCATATTTCCATTCAAGTCACAGAGTGG
          1 AAAAACTAGACAGAAGCATTCTCAGAAACAT-CTTTGTGATGTTTGCACTCAACTCACAGAGTTG

               *  * **            *                  *  *             *      
     517562 AACATTCCCATTCATAGAGCAGGTTTGAAACACTCTTTTTGGAGTATCTGGAAGTGGACATTTGG
         65 AACCTTTCTTTTCATAGAGCAGTTTTGAAACACTCTTTTTGTAGAATCTGGAAGTGGATATTTGG

                    ** * * ** *  *   *            **  
     517627 AGCGCTTTCTGAACTATGGTGAAAAAGGAAATATCTTCCAAT
        130 AGCGCTTTGAGGATTTCGTTGGAAACGGAAATATCTTCATAT

            *     *               *      * *          *  **      **  *  *    
     517669 GAAAACAAGACAGAAGCATTCTGAGAAACTTATTTGTGATGTGTGTCCTCAACAAACGGACTTGA
          1 AAAAACTAGACAGAAGCATTCTCAGAAACATCTTTGTGATGTTTGCACTCAACTCACAGAGTTGA

                   *      **  * *  *  *             *   *    * * *           
     517734 ACCTTTCGTTTCATGCAGTACTTCTGGAACACTCTTTTTGAAGATTCTGCATGCGGATATTTGGA
         66 ACCTTTCTTTTCATAGAGCAGTTTTGAAACACTCTTTTTGTAGAATCTGGAAGTGGATATTTGGA

            **                            **   *   * 
     517799 TAGCTTTGAGGATTTCGTTGGAAACGG---GCT-TACATGT
        131 GCGCTTTGAGGATTTCGTTGGAAACGGAAATATCTTCATAT

                 *       *               *        *   *    *    *       *    
     517836 AAAAATTAGACAGCAGCATTCTCAGAAACTTCTTTGTGGTGTCTGCATTCAAGTCACAGAATTGA
          1 AAAAACTAGACAGAAGCATTCTCAGAAACATCTTTGTGATGTTTGCACTCAACTCACAGAGTTGA

              * ** * **            *  * *      *       *             *       
     517901 ACTTCCCCTCACATAGAGCAGTTGTGCAGCACTCTATTTGTAGTATCTGGAAGTGGACATTTGGA
         66 ACCTTTCTTTTCATAGAGCAGTTTTGAAACACTCTTTTTGTAGAATCTGGAAGTGGATATTTGGA

             *         ** *    * *   *            **  
     517966 GGGCTTTGTAGCCTATC-TGGAAAAAGGAAATATCTTCCCAT
        131 GCGCTTTG-AGGATTTCGTTGGAAACGGAAATATCTTCATAT

            *  ** *   *     * *            *   *  *   * **        *  * * *   
     518007 GAATGCGAGATAGAAGTAATCTCAGAAACATGTTTATGCTGTATCTACTCAACTAACTGTGCTGA
          1 AAAAACTAGACAGAAGCATTCTCAGAAACATCTTTGTGATGTTTGCACTCAACTCACAGAGTTGA

              *     *  *               *        *  * *       *               
     518072 ACATTTCTATTTATAGAGCAGTTTTGAGACACTCTTCTTTTGGAATCTGCAAGTGGATATTTGGA
         66 ACCTTTCTTTTCATAGAGCAGTTTTGAAACACTCTTTTTGTAGAATCTGGAAGTGGATATTTGGA

            ** *                        *     *      
     518137 TAGATTTGAGGATTTCGTTGGAAACGG-GAT-T-AT-ATAT
        131 GCGCTTTGAGGATTTCGTTGGAAACGGAAATATCTTCATAT

            *    *       *               *                 **  *   *         
     518174 CAAAAGTAGACAGCAGCATTCTCAGAAACTTCTTTGTGATGTTTGCATCCAGCTCTCAGAGTTGA
          1 AAAAACTAGACAGAAGCATTCTCAGAAACATCTTTGTGATGTTTGCACTCAACTCACAGAGTTGA

              *  * *          *  *        *        *   **    *   *  **       
     518239 ACATTCCCTTTCATAGAGTAGGTTTGAAACCCTCTTTTTATAGTGTCTGCAAGCGGGCATTTGGA
         66 ACCTTTCTTTTCATAGAGCAGTTTTGAAACACTCTTTTTGTAGAATCTGGAAGTGGATATTTGGA

                   *    * * **  **  **          * *  *
     518304 GCGCTTTCAGGCCTATGCTTAAAATAGGAAATATCTACCTAC
        131 GCGCTTTGAGG-ATTTCGTTGGAAACGGAAATATCTTCATAT

             *                    *    *  **          ** *        *    *     
     518346 AGAAACTAGACAGAAGCATTCTGAGAATCACGTTTGTGATGTGGGTACTCAACTAACAGTGTTGA
          1 AAAAACTAGACAGAAGCATTCTCAGAAACATCTTTGTGATGTTTGCACTCAACTCACAGAGTTGA

            *  *       *   *            *   *                *  **           
     518411 TCCATTCTTTTGATACAGCAGTTTTGAACCACACTTTTTGTAGAATCTGCAACAGGATATTTGGA
         66 ACCTTTCTTTTCATAGAGCAGTTTTGAAACACTCTTTTTGTAGAATCTGGAAGTGGATATTTGGA

            **   *                     *   *      * *
     518476 TAGCTGTGAGGATTTCGTTGGAAACGGGAATGTCTTCAAAG
        131 GCGCTTTGAGGATTTCGTTGGAAACGGAAATATCTTCATAT

                *                         *   *            *    *            
     518517 AAAATCTAGACAGAAGCATTCTCAGAAACACCTTCGTGATGTTTGCAATCAAGTCACAGAGTTGA
          1 AAAAACTAGACAGAAGCATTCTCAGAAACATCTTTGTGATGTTTGCACTCAACTCACAGAGTTGA

                 * *             *  *           *      *             *       
     518582 ACCTTCCGTTTCATAGAGCAGGTTGGAAACACTCTTATTGTAGTATCTGGAAGTGGACATTTGGA
         66 ACCTTTCTTTTCATAGAGCAGTTTTGAAACACTCTTTTTGTAGAATCTGGAAGTGGATATTTGGA

                   *   ** ** *  *   *            **  
     518647 GCGCTTTCAGGCCTATGGTGAAAAAGGAAATATCTTCCCAT
        131 GCGCTTTGAGGATTTCGTTGGAAACGGAAATATCTTCATAT

                  * * *      **      *   * *   *     ** ** *      *    *     
     518688 AAAAACGACATAGAAGCTATCTCAGGAACTTGTTTATGATGCATCTAATCAACTAACAGTGTTGA
          1 AAAAACTAGACAGAAGCATTCTCAGAAACATCTTTGTGATGTTTGCACTCAACTCACAGAGTTGA

                  * ** * *                         * *       *               
     518753 ACCTTTGTACTGACAGAGCAG-TTTGAAACACTCTTTTTTTGGAATCTGCAAGTGGATATTTGGA
         66 ACCTTTCTTTTCATAGAGCAGTTTTGAAACACTCTTTTTGTAGAATCTGGAAGTGGATATTTGGA

            *                           *   *   *    
     518817 TCGCTTTGAGGATTTCGTTGGAAACGG-CAT-GC--AATAT
        131 GCGCTTTGAGGATTTCGTTGGAAACGGAAATATCTTCATAT

                **  *    *     *                 **  *   *  *    *         * 
     518854 AAAACGTACACAGCAGCATACTCAGAAA-ATACTTTGCCATATTTCCATTCAAGTCACAGAGTGG
          1 AAAAACTAGACAGAAGCATTCTCAGAAACAT-CTTTGTGATGTTTGCACTCAACTCACAGAGTTG

               *  * **            *                  *  *             *      
     518918 AACATTCCCATTCATAGAGCAGGTTTGAAACACTCTTTTTGGAGTATCTGGAAGTGGACATTTGG
         65 AACCTTTCTTTTCATAGAGCAGTTTTGAAACACTCTTTTTGTAGAATCTGGAAGTGGATATTTGG

                    ** * * ** *  *   *            **  
     518983 AGCGCTTTCTGAACTATGGTGAAAAAGGAAATATCTTCCAAT
        130 AGCGCTTTGAGGATTTCGTTGGAAACGGAAATATCTTCATAT

            *     *               *      * *          *  **      **  *  *    
     519025 GAAAACAAGACAGAAGCATTCTGAGAAACTTATTTGTGATGTGTGTCCTCAACAAACGGACTTGA
          1 AAAAACTAGACAGAAGCATTCTCAGAAACATCTTTGTGATGTTTGCACTCAACTCACAGAGTTGA

                   *      **  * *                    *   *    * * *          
     519090 ACCTTTCGTTTCATGCAGTACTTCTTG-AACACTCTTTTTGAAGATTCTGCATGCGGATATTTGG
         66 ACCTTTCTTTTCATAGAGCAGTT-TTGAAACACTCTTTTTGTAGAATCTGGAAGTGGATATTTGG

             **                            **   *   * 
     519154 ATAGCTTTGAGGATTTCGTTGGAAACGG---GCT-TACATGT
        130 AGCGCTTTGAGGATTTCGTTGGAAACGGAAATATCTTCATAT

                 *       *               *        *   *    *    *       *    
     519192 AAAAATTAGACAGCAGCATTCTCAGAAACTTCTTTGTGGTGTCTGCATTCAAGTCACAGAATTGA
          1 AAAAACTAGACAGAAGCATTCTCAGAAACATCTTTGTGATGTTTGCACTCAACTCACAGAGTTGA

              * ** * **            *  * *      *       *             *       
     519257 ACTTCCCCTCACATAGAGCAGTTGTGCAGCACTCTATTTGTAGTATCTGGAAGTGGACATTTGGA
         66 ACCTTTCTTTTCATAGAGCAGTTTTGAAACACTCTTTTTGTAGAATCTGGAAGTGGATATTTGGA

             *         ** *    * *   *            **  
     519322 GGGCTTTGTAGCCTATC-TGGAAAAAGGAAATATCTTCCCAT
        131 GCGCTTTG-AGGATTTCGTTGGAAACGGAAATATCTTCATAT

            *  ** *   *     * *            *   *  *   * **        *  * * *   
     519363 GAATGCGAGATAGAAGTAATCTCAGAAACATGTTTATGCTGTATCTACTCAACTAACTGTGCTGA
          1 AAAAACTAGACAGAAGCATTCTCAGAAACATCTTTGTGATGTTTGCACTCAACTCACAGAGTTGA

              *     *  *               *        *  * *       *               
     519428 ACATTTCTATTGATAGAGCAGTTTTGAGACACTCTTCTTTTGGAATCTGCAAGTGGATATTTGGA
         66 ACCTTTCTTTTCATAGAGCAGTTTTGAAACACTCTTTTTGTAGAATCTGGAAGTGGATATTTGGA

            ** *                        *     *      
     519493 TAGATTTGAGGATTTCGTTGGAAACGG-GAT-T-AT-ATAT
        131 GCGCTTTGAGGATTTCGTTGGAAACGGAAATATCTTCATAT

            *    *       *               *                 **  *   *         
     519530 CAAAAGTAGACAGCAGCATTCTCAGAAACTTCTTTGTGATGTTTGCATCCAGCTCTCAGAGTTGA
          1 AAAAACTAGACAGAAGCATTCTCAGAAACATCTTTGTGATGTTTGCACTCAACTCACAGAGTTGA

              *  * *          *  *        *        *   **    *   *  **       
     519595 ACATTCCCTTTCATAGAGTAGGTTTGAAACCCTCTTTTTATAGTGTCTGCAAGCGGGCATTTGGA
         66 ACCTTTCTTTTCATAGAGCAGTTTTGAAACACTCTTTTTGTAGAATCTGGAAGTGGATATTTGGA

                   *    * * **  **  **          * *  *
     519660 GCGCTTTCAGGCCTATGCTTAAAATAGGAAATATCTACCTAC
        131 GCGCTTTGAGG-ATTTCGTTGGAAACGGAAATATCTTCATAT

             *                    *    *  **          ** *        *    *     
     519702 AGAAACTAGACAGAAGCATTCTGAGAATCACGTTTGTGATGTGGGTACTCAACTAACAGTGTTGA
          1 AAAAACTAGACAGAAGCATTCTCAGAAACATCTTTGTGATGTTTGCACTCAACTCACAGAGTTGA

            *  *       *   *            *   *                *   *           
     519767 TCCATTCTTTTGATACAGCAGTTTTGAACCACACTTTTTGTAGAATCTGCAAGAGGATATTTGGA
         66 ACCTTTCTTTTCATAGAGCAGTTTTGAAACACTCTTTTTGTAGAATCTGGAAGTGGATATTTGGA

            **   *                     *   *      * *
     519832 TAGCTGTGAGGATTTCGTTGGAAACGGGAATGTCTTCAAAG
        131 GCGCTTTGAGGATTTCGTTGGAAACGGAAATATCTTCATAT

                *                         *   *            *    *            
     519873 AAAATCTAGACAGAAGCATTCTCAGAAACACCTTCGTGATGTTTGCAATCAAGTCACAGAGTTGA
          1 AAAAACTAGACAGAAGCATTCTCAGAAACATCTTTGTGATGTTTGCACTCAACTCACAGAGTTGA

                 * *             *  *           *      *             *       
     519938 ACCTTCCGTTTCATAGAGCAGGTTGGAAACACTCTTATTGTAGTATCTGGAAGTGGACATTTGGA
         66 ACCTTTCTTTTCATAGAGCAGTTTTGAAACACTCTTTTTGTAGAATCTGGAAGTGGATATTTGGA

                   *   *  ** *  *   *            **  
     520003 GCGCTTTCAGGCTTATGGTGAAAAAGGAAATATCTTCCCAT
        131 GCGCTTTGAGGATTTCGTTGGAAACGGAAATATCTTCATAT

                  * * *      **      *   * *   *     ** ** *      *    *     
     520044 AAAAACGACATAGAAGCTATCTCAGGAACTTGTTTATGATGCATCTAATCAACTAACAGTGTTGA
          1 AAAAACTAGACAGAAGCATTCTCAGAAACATCTTTGTGATGTTTGCACTCAACTCACAGAGTTGA

                  * ** * *                         * *       *               
     520109 ACCTTTGTACTGACAGAGCAG-TTTGAAACACTCTTTTTTTGGAATCTGCAAGTGGATATTTGGA
         66 ACCTTTCTTTTCATAGAGCAGTTTTGAAACACTCTTTTTGTAGAATCTGGAAGTGGATATTTGGA

            *                           *   *   *    
     520173 TCGCTTTGAGGATTTCGTTGGAAACGG-GAT-GC--AATAT
        131 GCGCTTTGAGGATTTCGTTGGAAACGGAAATATCTTCATAT

                **  *    *     *                 **  *   *  *    *         * 
     520210 AAAACGTACACAGCAGCATACTCAGAAA-ATACTTTGCCATATTTCCATTCAAGTCACAGAGTGG
          1 AAAAACTAGACAGAAGCATTCTCAGAAACAT-CTTTGTGATGTTTGCACTCAACTCACAGAGTTG

               *  * **            *                  *  *             *      
     520274 AACATTCCCATTCATAGAGCAGGTTTGAAACACTCTTTTTGGAGTATCTGGAAGTGGACATTTGG
         65 AACCTTTCTTTTCATAGAGCAGTTTTGAAACACTCTTTTTGTAGAATCTGGAAGTGGATATTTGG

                    ** * * ** *  *   *            **  
     520339 AGCGCTTTCTGAACTATGGTGAAAAAGGAAATATCTTCCAAT
        130 AGCGCTTTGAGGATTTCGTTGGAAACGGAAATATCTTCATAT

            *     *               *      * *          *  **      **  *  *    
     520381 GAAAACAAGACAGAAGCATTCTGAGAAACTTATTTGTGATGTGTGTCCTCAACAAACGGACTTGA
          1 AAAAACTAGACAGAAGCATTCTCAGAAACATCTTTGTGATGTTTGCACTCAACTCACAGAGTTGA

                  **      **  * *                    *   *    * * *          
     520446 ACCTTTTGTTTCATGCAGTACTTCTTG-AACACTCTTTTTGAAGATTCTGCATGCGGATATTTGG
         66 ACCTTTCTTTTCATAGAGCAGTT-TTGAAACACTCTTTTTGTAGAATCTGGAAGTGGATATTTGG

             **                            **   *   * 
     520510 ATAGCTTTGAGGATTTCGTTGGAAACGG---GCT-TACATGT
        130 AGCGCTTTGAGGATTTCGTTGGAAACGGAAATATCTTCATAT

                 *       *               *        *   *    *    *       *    
     520548 AAAAATTAGACAGCAGCATTCTCAGAAACTTCTTTGTGGTGTCTGCATTCAAGTCACAGAATTGA
          1 AAAAACTAGACAGAAGCATTCTCAGAAACATCTTTGTGATGTTTGCACTCAACTCACAGAGTTGA

              * ** * **            *  * *      *       *             *       
     520613 ACTTCCCCTCACATAGAGCAGTTGTGCAGCACTCTATTTGTAGTATCTGGAAGTGGACATTTGGA
         66 ACCTTTCTTTTCATAGAGCAGTTTTGAAACACTCTTTTTGTAGAATCTGGAAGTGGATATTTGGA

             *         ** *    * *   *            **  
     520678 GGGCTTTGTAGCCTATC-TGGAAAAAGGAAATATCTTCCCAT
        131 GCGCTTTG-AGGATTTCGTTGGAAACGGAAATATCTTCATAT

            *  ** *   *     * *            *   *  *   * **        *  * * *   
     520719 GAATGCGAGATAGAAGTAATCTCAGAAACATGTTTATGCTGTATCTACTCAACTAACTGTGCTGA
          1 AAAAACTAGACAGAAGCATTCTCAGAAACATCTTTGTGATGTTTGCACTCAACTCACAGAGTTGA

              *     *  *               *        *  * *       *               
     520784 ACATTTCTATTGATAGAGCAGTTTTGAGACACTCTTCTTTTGGAATCTGCAAGTGGATATTTGGA
         66 ACCTTTCTTTTCATAGAGCAGTTTTGAAACACTCTTTTTGTAGAATCTGGAAGTGGATATTTGGA

            ** *                        *     *      
     520849 TAGATTTGAGGATTTCGTTGGAAACGG-GAT-T-AT-ATAT
        131 GCGCTTTGAGGATTTCGTTGGAAACGGAAATATCTTCATAT

            *    *       *               *                 **  *   *         
     520886 CAAAAGTAGACAGCAGCATTCTCAGAAACTTCTTTGTGATGTTTGCATCCAGCTCTCAGAGTTGA
          1 AAAAACTAGACAGAAGCATTCTCAGAAACATCTTTGTGATGTTTGCACTCAACTCACAGAGTTGA

              *  * *          *  *        *        *   **    *   *  **       
     520951 ACATTCCCTTTCATAGAGTAGGTTTGAAACCCTCTTTTTATAGTGTCTGCAAGCGGGCATTTGGA
         66 ACCTTTCTTTTCATAGAGCAGTTTTGAAACACTCTTTTTGTAGAATCTGGAAGTGGATATTTGGA

                   *    * * **  **  **          ***  *
     521016 GCGCTTTCAGGCCTATGCTTAAAATAGGAAATATCTAACTAC
        131 GCGCTTTGAGG-ATTTCGTTGGAAACGGAAATATCTTCATAT

             *                    *    *  **          ** *        *    *     
     521058 AGAAACTAGACAGAAGCATTCTGAGAATCACGTTTGTGATGTGGGTACTCAACTAACAGTGTTGA
          1 AAAAACTAGACAGAAGCATTCTCAGAAACATCTTTGTGATGTTTGCACTCAACTCACAGAGTTGA

            *  *       *   *            *   *                *   *           
     521123 TCCATTCTTTTGATACAGCAGTTTTGAACCACACTTTTTGTAGAATCTGCAAGAGGATATTTGGA
         66 ACCTTTCTTTTCATAGAGCAGTTTTGAAACACTCTTTTTGTAGAATCTGGAAGTGGATATTTGGA

            **   *                     *   *      * *
     521188 TAGCTGTGAGGATTTCGTTGGAAACGGGAATGTCTTCAAAG
        131 GCGCTTTGAGGATTTCGTTGGAAACGGAAATATCTTCATAT

                *                         *   *            *    *            
     521229 AAAATCTAGACAGAAGCATTCTCAGAAACACCTTCGTGATGTTTGCAATCAAGTCACAGAGTTGA
          1 AAAAACTAGACAGAAGCATTCTCAGAAACATCTTTGTGATGTTTGCACTCAACTCACAGAGTTGA

                 * *             *  *           *      *             *       
     521294 ACCTTCCGTTTCATAGAGCAGGTTGGAAACACTCTTATTGTAGTATCTGGAAGTGGACATTTGGA
         66 ACCTTTCTTTTCATAGAGCAGTTTTGAAACACTCTTTTTGTAGAATCTGGAAGTGGATATTTGGA

                   *   ** ** *  *   *            **  
     521359 GCGCTTTCAGGCCTATGGTGAAAAAGGAAATATCTTCCCAT
        131 GCGCTTTGAGGATTTCGTTGGAAACGGAAATATCTTCATAT

                  * * *      **      *   * *   *     ** ** *      *    *     
     521400 AAAAACGACATAGAAGCTATCTCAGGAACTTGTTTATGATGCATCTAATCAACTAACAGTGTTGA
          1 AAAAACTAGACAGAAGCATTCTCAGAAACATCTTTGTGATGTTTGCACTCAACTCACAGAGTTGA

                  * ** * *                         * *       *               
     521465 ACCTTTGTACTGACAGAGCAG-TTTGAAACACTCTTTTTTTGGAATCTGCAAGTGGATATTTGGA
         66 ACCTTTCTTTTCATAGAGCAGTTTTGAAACACTCTTTTTGTAGAATCTGGAAGTGGATATTTGGA

            *                           *   *   *    
     521529 TCGCTTTGAGGATTTCGTTGGAAACGG-GAT-GC--AATAT
        131 GCGCTTTGAGGATTTCGTTGGAAACGGAAATATCTTCATAT

                **  *    *     *                 **  *   *  *    *         * 
     521566 AAAACGTACACAGCAGCATACTCAGAAA-ATACTTTGCCATATTTCCATTCAAGTCACAGAGTGG
          1 AAAAACTAGACAGAAGCATTCTCAGAAACAT-CTTTGTGATGTTTGCACTCAACTCACAGAGTTG

               *  * **            *                  *  *             *      
     521630 AACATTCCCATTCATAGAGCAGGTTTGAAACACTCTTTTTGGAGTATCTGGAAGTGGACATTTGG
         65 AACCTTTCTTTTCATAGAGCAGTTTTGAAACACTCTTTTTGTAGAATCTGGAAGTGGATATTTGG

                    ** * * ** *  *   *            **  
     521695 AGCGCTTTCTGAACTATGGTGAAAAAGGAAATATCTTCCAAT
        130 AGCGCTTTGAGGATTTCGTTGGAAACGGAAATATCTTCATAT

            *     *               *      * *          *  **      **  *  *    
     521737 GAAAACAAGACAGAAGCATTCTGAGAAACTTATTTGTGATGTGTGTCCTCAACAAACGGACTTGA
          1 AAAAACTAGACAGAAGCATTCTCAGAAACATCTTTGTGATGTTTGCACTCAACTCACAGAGTTGA

                   *      **  * *  *  *             *   *    * * *           
     521802 ACCTTTCGTTTCATGCAGTACTTCTGGAACACTCTTTTTGAAGATTCTGCATGCGGATATTTGGA
         66 ACCTTTCTTTTCATAGAGCAGTTTTGAAACACTCTTTTTGTAGAATCTGGAAGTGGATATTTGGA

            **                            **   *   * 
     521867 TAGCTTTGAGGATTTCGTTGGAAACGG---GCT-TACATGT
        131 GCGCTTTGAGGATTTCGTTGGAAACGGAAATATCTTCATAT

                 *       *               *        *   *    *    *       *    
     521904 AAAAATTAGACAGCAGCATTCTCAGAAACTTCTTTGTGGTGTCTGCATTCAAGTCACAGAATTGA
          1 AAAAACTAGACAGAAGCATTCTCAGAAACATCTTTGTGATGTTTGCACTCAACTCACAGAGTTGA

              * ** * **            *  * *      *       *             *       
     521969 ACTTCCCCTCACATAGAGCAGTTGTGCAGCACTCTATTTGTAGTATCTGGAAGTGGACATTTGGA
         66 ACCTTTCTTTTCATAGAGCAGTTTTGAAACACTCTTTTTGTAGAATCTGGAAGTGGATATTTGGA

             *         ** *    * *   *            **  
     522034 GGGCTTTGTAGCCTATC-TGGAAAAAGGAAATATCTTCCCAT
        131 GCGCTTTG-AGGATTTCGTTGGAAACGGAAATATCTTCATAT

            *  ** *   *     * *            *   *  *   * **        *  * * *   
     522075 GAATGCGAGATAGAAGTAATCTCAGAAACATGTTTATGCTGTATCTACTCAACTAACTGTGCTGA
          1 AAAAACTAGACAGAAGCATTCTCAGAAACATCTTTGTGATGTTTGCACTCAACTCACAGAGTTGA

              *     *  *               *        *  * *       *               
     522140 ACATTTCTATTGATAGAGCAGTTTTGAGACACTCTTCTTTTGGAATCTGCAAGTGGATATTTGGA
         66 ACCTTTCTTTTCATAGAGCAGTTTTGAAACACTCTTTTTGTAGAATCTGGAAGTGGATATTTGGA

             * *                        *     *      
     522205 GAGATTTGAGGATTTCGTTGGAAACGG-GAT-T-AT-ATAT
        131 GCGCTTTGAGGATTTCGTTGGAAACGGAAATATCTTCATAT

                 *       *               *                 **  *   *         
     522242 AAAAAGTAGACAGCAGCATTCTCAGAAACTTCTTTGTGATGTTTGCATCCAGCTCTCAGAGTTGA
          1 AAAAACTAGACAGAAGCATTCTCAGAAACATCTTTGTGATGTTTGCACTCAACTCACAGAGTTGA

              *  * *          *  *        *        *   **        *  **       
     522307 ACATTCCCTTTCATAGAGTAGGTTTGAAACCCTCTTTTTATAGTGTCTGGAAGCGGGCATTTGGA
         66 ACCTTTCTTTTCATAGAGCAGTTTTGAAACACTCTTTTTGTAGAATCTGGAAGTGGATATTTGGA

                   *    * * **  **  **          * *  *
     522372 GCGCTTTCAGGCCTATGCTTAAAATAGGAAATATCTACCTAC
        131 GCGCTTTGAGG-ATTTCGTTGGAAACGGAAATATCTTCATAT

             *                    *    *  **          ** *        *    *     
     522414 AGAAACTAGACAGAAGCATTCTGAGAATCACGTTTGTGATGTGGGTACTCAACTAACAGTGTTGA
          1 AAAAACTAGACAGAAGCATTCTCAGAAACATCTTTGTGATGTTTGCACTCAACTCACAGAGTTGA

            *  *       *   *            *   *                *               
     522479 TCCATTCTTTTGATACAGCAGTTTTGAACCACACTTTTTGTAGAATCTGCAAGTGGATATTTGGA
         66 ACCTTTCTTTTCATAGAGCAGTTTTGAAACACTCTTTTTGTAGAATCTGGAAGTGGATATTTGGA

            **   *                     *   *      * *
     522544 TAGCTGTGAGGATTTCGTTGGAAACGGGAATGTCTTCAAAG
        131 GCGCTTTGAGGATTTCGTTGGAAACGGAAATATCTTCATAT

                *                         *   *            *    *            
     522585 AAAATCTAGACAGAAGCATTCTCAGAAACACCTTCGTGATGTTTGCAATCAAGTCACAGAGTTGA
          1 AAAAACTAGACAGAAGCATTCTCAGAAACATCTTTGTGATGTTTGCACTCAACTCACAGAGTTGA

                 * *             *  *           *      *             *       
     522650 ACCTTCCGTTTCATAGAGCAGGTTGGAAACACTCTTATTGTAGTATCTGGAAGTGGACATTTGGA
         66 ACCTTTCTTTTCATAGAGCAGTTTTGAAACACTCTTTTTGTAGAATCTGGAAGTGGATATTTGGA

                   *   ** ** *  *   *            **  
     522715 GCGCTTTCAGGCCTATGGTGAAAAAGGAAATATCTTCCCAT
        131 GCGCTTTGAGGATTTCGTTGGAAACGGAAATATCTTCATAT

                  * * *      **      *   * *   *     ** ** *      *    *     
     522756 AAAAACGACATAGAAGCTATCTCAGGAACTTGTTTATGATGCATCTAATCAACTAACAGTGTTGA
          1 AAAAACTAGACAGAAGCATTCTCAGAAACATCTTTGTGATGTTTGCACTCAACTCACAGAGTTGA

                  * ** * *    *                    * *       *               
     522821 ACCTTTGTACTGACAGAGGAG-TTTGAAACACTCTTTTTTTGGAATCTGCAAGTGGATATTTGGA
         66 ACCTTTCTTTTCATAGAGCAGTTTTGAAACACTCTTTTTGTAGAATCTGGAAGTGGATATTTGGA

            *                           *   *   *    
     522885 TCGCTTTGAGGATTTCGTTGGAAACGG-GAT-GC--AATAT
        131 GCGCTTTGAGGATTTCGTTGGAAACGGAAATATCTTCATAT

                **  *    *     *                 **  *   *  *    *         * 
     522922 AAAACGTACACAGCAGCATACTCAGAAA-ATACTTTGCCATATTTCCATTCAAGTCACAGAGTGG
          1 AAAAACTAGACAGAAGCATTCTCAGAAACAT-CTTTGTGATGTTTGCACTCAACTCACAGAGTTG

               *  * **            *  *               *  *             *      
     522986 AACATTCCCATTCATAGAGCAGGTTGGAAACACTCTTTTTGGAGTATCTGGAAGTGGACATTTGG
         65 AACCTTTCTTTTCATAGAGCAGTTTTGAAACACTCTTTTTGTAGAATCTGGAAGTGGATATTTGG

                    ** * * ** *  *   *            **  
     523051 AGCGCTTTCTGAACTATGGTGAAAAAGGAAATATCTTCCAAT
        130 AGCGCTTTGAGGATTTCGTTGGAAACGGAAATATCTTCATAT

            *     *               *      * *          *  **      **  *  *    
     523093 GAAAACAAGACAGAAGCATTCTGAGAAACTTATTTGTGATGTGTGTCCTCAACAAACGGACTTGA
          1 AAAAACTAGACAGAAGCATTCTCAGAAACATCTTTGTGATGTTTGCACTCAACTCACAGAGTTGA

                   *      **  * *  *  *             *   *    * * *           
     523158 ACCTTTCGTTTCATGCAGTACTTCTGGAACACTCTTTTTGAAGATTCTGCATGCGGATATTTGGA
         66 ACCTTTCTTTTCATAGAGCAGTTTTGAAACACTCTTTTTGTAGAATCTGGAAGTGGATATTTGGA

            **                            **   *   * 
     523223 TAGCTTTGAGGATTTCGTTGGAAACGG---GCT-TACATGT
        131 GCGCTTTGAGGATTTCGTTGGAAACGGAAATATCTTCATAT

                 *       *               *        *   *    *    *       *    
     523260 AAAAATTAGACAGCAGCATTCTCAGAAACTTCTTTGTGGTGTCTGCATTCAAGTCACAGAATTGA
          1 AAAAACTAGACAGAAGCATTCTCAGAAACATCTTTGTGATGTTTGCACTCAACTCACAGAGTTGA

              * ** * **            *  * *      *       *             *       
     523325 ACTTCCCCTCACATAGAGCAGTTGTGCAGCACTCTATTTGTAGTATCTGGAAGTGGACATTTGGA
         66 ACCTTTCTTTTCATAGAGCAGTTTTGAAACACTCTTTTTGTAGAATCTGGAAGTGGATATTTGGA

             *         ** *    * *   *            **  
     523390 GGGCTTTGTAGCCTATC-TGGAAAAAGGAAATATCTTCCCAT
        131 GCGCTTTG-AGGATTTCGTTGGAAACGGAAATATCTTCATAT

            *  ** *   *     * *            *   *  *   * **        *  * * *   
     523431 GAATGCGAGATAGAAGTAATCTCAGAAACATGTTTATGCTGTATCTACTCAACTAACTGTGCTGA
          1 AAAAACTAGACAGAAGCATTCTCAGAAACATCTTTGTGATGTTTGCACTCAACTCACAGAGTTGA

              *     *  *               *        *  * *       *               
     523496 ACATTTCTATTGATAGAGCAGTTTTGAGACACTCTTCTTTTGGAATCTGCAAGTGGATATTTGGA
         66 ACCTTTCTTTTCATAGAGCAGTTTTGAAACACTCTTTTTGTAGAATCTGGAAGTGGATATTTGGA

            ** *                        *     *      
     523561 TAGATTTGAGGATTTCGTTGGAAACGG-GAT-T-AT-ATAT
        131 GCGCTTTGAGGATTTCGTTGGAAACGGAAATATCTTCATAT

            *    *       *               *                 **  *   *         
     523598 CAAAAGTAGACAGCAGCATTCTCAGAAACTTCTTTGTGATGTTTGCATCCAGCTCTCAGAGTTGA
          1 AAAAACTAGACAGAAGCATTCTCAGAAACATCTTTGTGATGTTTGCACTCAACTCACAGAGTTGA

              *  * *          *  *        *        *   **        *  **       
     523663 ACATTCCCTTTCATAGAGTAGGTTTGAAACCCTCTTTTTATAGTGTCTGGAAGCGGGCATTTGGA
         66 ACCTTTCTTTTCATAGAGCAGTTTTGAAACACTCTTTTTGTAGAATCTGGAAGTGGATATTTGGA

                   *    * * **  **  **          * *  *
     523728 GCGCTTTCAGGCCTATGCTTAAAATAGGAAATATCTACCTAC
        131 GCGCTTTGAGG-ATTTCGTTGGAAACGGAAATATCTTCATAT

             *                    *    *  **          ** *        *    *     
     523770 AGAAACTAGACAGAAGCATTCTGAGAATCACGTTTGTGATGTGGGTACTCAACTAACAGTGTTGA
          1 AAAAACTAGACAGAAGCATTCTCAGAAACATCTTTGTGATGTTTGCACTCAACTCACAGAGTTGA

            *  *       *   *            *   *                *   *           
     523835 TCCATTCTTTTGATACAGCAGTTTTGAACCACACTTTTTGTAGAATCTGCAAGAGGATATTTGGA
         66 ACCTTTCTTTTCATAGAGCAGTTTTGAAACACTCTTTTTGTAGAATCTGGAAGTGGATATTTGGA

            **   *                     *   *      * *
     523900 TAGCTGTGAGGATTTCGTTGGAAACGGGAATGTCTTCAAAG
        131 GCGCTTTGAGGATTTCGTTGGAAACGGAAATATCTTCATAT

                *                         *   *            *    *            
     523941 AAAATCTAGACAGAAGCATTCTCAGAAACACCTTCGTGATGTTTGCAATCAAGTCACAGAGTTGA
          1 AAAAACTAGACAGAAGCATTCTCAGAAACATCTTTGTGATGTTTGCACTCAACTCACAGAGTTGA

                 * *             *  *           *      *             *       
     524006 ACCTTCCGTTTCATAGAGCAGGTTGGAAACACTCTTATTGTAGTATCTGGAAGTGGACATTTGGA
         66 ACCTTTCTTTTCATAGAGCAGTTTTGAAACACTCTTTTTGTAGAATCTGGAAGTGGATATTTGGA

                   *   ** ** *  *   *            **  
     524071 GCGCTTTCAGGCCTATGGTGAAAAAGGAAATATCTTCCCAT
        131 GCGCTTTGAGGATTTCGTTGGAAACGGAAATATCTTCATAT

                  * * *    * **      *   * *   *     ** ** *      *    *     
     524112 AAAAACGACATAGAAACTATCTCAGGAACTTGTTTATGATGCATCTAATCAACTAACAGTGTTGA
          1 AAAAACTAGACAGAAGCATTCTCAGAAACATCTTTGTGATGTTTGCACTCAACTCACAGAGTTGA

                  * ** * *    *                    * *       *               
     524177 ACCTTTGTACTGACAGAGGAG-TTTGAAACACTCTTTTTTTGGAATCTGCAAGTGGATATTTGGA
         66 ACCTTTCTTTTCATAGAGCAGTTTTGAAACACTCTTTTTGTAGAATCTGGAAGTGGATATTTGGA

            *                           *   *   *    
     524241 TCGCTTTGAGGATTTCGTTGGAAACGG-GAT-GC--AATAT
        131 GCGCTTTGAGGATTTCGTTGGAAACGGAAATATCTTCATAT

                **  *    *     *                 **  *   *  *    *         * 
     524278 AAAACGTACACAGCAGCATACTCAGAAA-ATACTTTGCCATATTTCCATTCAAGTCACAGAGTGG
          1 AAAAACTAGACAGAAGCATTCTCAGAAACAT-CTTTGTGATGTTTGCACTCAACTCACAGAGTTG

               *  * **            *                  *  *             *      
     524342 AACATTCCCATTCATAGAGCAGGTTTGAAACACTCTTTTTGGAGTATCTGGAAGTGGACATTTGG
         65 AACCTTTCTTTTCATAGAGCAGTTTTGAAACACTCTTTTTGTAGAATCTGGAAGTGGATATTTGG

                    ** * * ** *  *   *            **  
     524407 AGCGCTTTCTGAACTATGGTGAAAAAGGAAATATCTTCCAAT
        130 AGCGCTTTGAGGATTTCGTTGGAAACGGAAATATCTTCATAT

            *     *               *      * *          *  **      **  *  *    
     524449 GAAAACAAGACAGAAGCATTCTGAGAAACTTATTTGTGATGTGTGTCCTCAACAAACGGACTTGA
          1 AAAAACTAGACAGAAGCATTCTCAGAAACATCTTTGTGATGTTTGCACTCAACTCACAGAGTTGA

                   *      **  * *  *  *             *   *    * * *           
     524514 ACCTTTCGTTTCATGCAGTACTTCTGGAACACTCTTTTTGAAGATTCTGCATGCGGATATTTGGA
         66 ACCTTTCTTTTCATAGAGCAGTTTTGAAACACTCTTTTTGTAGAATCTGGAAGTGGATATTTGGA

            **                            **   *   * 
     524579 TAGCTTTGAGGATTTCGTTGGAAACGG---GCT-TACATGT
        131 GCGCTTTGAGGATTTCGTTGGAAACGGAAATATCTTCATAT

                 *       *               *        *   *    *    *       *    
     524616 AAAAATTAGACAGCAGCATTCTCAGAAACTTCTTTGTGGTGTCTGCATTCAAGTCACAGAATTGA
          1 AAAAACTAGACAGAAGCATTCTCAGAAACATCTTTGTGATGTTTGCACTCAACTCACAGAGTTGA

              * ** * **            *  * *      *       *    *        *       
     524681 ACTTCCCCTCACATAGAGCAGTTGTGCAGCACTCTATTTGTAGTATCTCGAAGTGGACATTTGGA
         66 ACCTTTCTTTTCATAGAGCAGTTTTGAAACACTCTTTTTGTAGAATCTGGAAGTGGATATTTGGA

             *         ** *    * *   *            **  
     524746 GGGCTTTGTAGCCTATC-TGGAAAAAGGAAATATCTTCCCAT
        131 GCGCTTTG-AGGATTTCGTTGGAAACGGAAATATCTTCATAT

            *  ** *   *     * *            *   *  *   * ***       *  * * *   
     524787 GAATGCGAGATAGAAGTAATCTCAGAAACATGTTTATGCTGTATCTTCTCAACTAACTGTGCTGA
          1 AAAAACTAGACAGAAGCATTCTCAGAAACATCTTTGTGATGTTTGCACTCAACTCACAGAGTTGA

              *     *  *               *        *  * *       *               
     524852 ACATTTCTATTGATAGAGCAGTTTTGAGACACTCTTCTTTTGGAATCTGCAAGTGGATATTTGGA
         66 ACCTTTCTTTTCATAGAGCAGTTTTGAAACACTCTTTTTGTAGAATCTGGAAGTGGATATTTGGA

            ** *                        *     *      
     524917 TAGATTTGAGGATTTCGTTGGAAACGG-GAT-T-AT-ATAT
        131 GCGCTTTGAGGATTTCGTTGGAAACGGAAATATCTTCATAT

            *    *       *               *                 **  *   *         
     524954 CAAAAGTAGACAGCAGCATTCTCAGAAACTTCTTTGTGATGTTTGCATCCAGCTCTCAGAGTTGA
          1 AAAAACTAGACAGAAGCATTCTCAGAAACATCTTTGTGATGTTTGCACTCAACTCACAGAGTTGA

              *  * *          *  *        *        *   **        *  **       
     525019 ACATTCCCTTTCATAGAGTAGGTTTGAAACCCTCTTTTTATAGTGTCTGGAAGCGGGCATTTGGA
         66 ACCTTTCTTTTCATAGAGCAGTTTTGAAACACTCTTTTTGTAGAATCTGGAAGTGGATATTTGGA

                   *   ** ** *  *   *          * *   
     525084 GCGCTTTCAGGCCTATGCTGAAAAAGGAAATATCTACCTAT
        131 GCGCTTTGAGGATTTCGTTGGAAACGGAAATATCTTCATAT

             *                    *    *  **          ** *        *    *     
     525125 AGAAACTAGACAGAAGCATTCTGAGAATCACGTTTGTGATGTGGGTACTCAACTAACAGTGTTGA
          1 AAAAACTAGACAGAAGCATTCTCAGAAACATCTTTGTGATGTTTGCACTCAACTCACAGAGTTGA

            *  *       *   *            *   *                *               
     525190 TCCATTCTTTTGATACAGCAGTTTTGAACCACACTTTTTGTAGAATCTGCAAGTGGATATTTGGA
         66 ACCTTTCTTTTCATAGAGCAGTTTTGAAACACTCTTTTTGTAGAATCTGGAAGTGGATATTTGGA

            **   *                     *   *        *
     525255 TAGCTGTGAGGATTTCGTTGGAAACGGGAATGTCTTCATAG
        131 GCGCTTTGAGGATTTCGTTGGAAACGGAAATATCTTCATAT

                **                     * * **     *   *  **   *   *          
     525296 AAAATTTAGACAGAAGCATTCTCAGAACCTTGATTGTGGTGTGTGTTCTCCACTAACAGAGTTGA
          1 AAAAACTAGACAGAAGCATTCTCAGAAACATCTTTGTGATGTTTGCACTCAACTCACAGAGTTGA

                       * *   * *   *       *       *                         
     525361 ACCTTTCTTTTGACAGAACTGTTCTGAAACATTCTTTTTATAGAATCTGGAAGTGGATATTTGGA
         66 ACCTTTCTTTTCATAGAGCAGTTTTGAAACACTCTTTTTGTAGAATCTGGAAGTGGATATTTGGA

            **              *          *          *  
     525426 AAGCTTTGAGGATTTCATTGGAAACGGGAATATCTTCAAAT
        131 GCGCTTTGAGGATTTCGTTGGAAACGGAAATATCTTCATAT

                *    *            *           * *       *  *    *            
     525467 AAAATCTAGCCAGAAGCATTCTAAGAAACATCTTAGGGATGTTTACATTCAAGTCACAGAGTTGA
          1 AAAAACTAGACAGAAGCATTCTCAGAAACATCTTTGTGATGTTTGCACTCAACTCACAGAGTTGA

              *  * *     *       *         *    * *   **      *      *   **  
     525532 ACATTCCCTTTCACAGAGCAGATTTGAAACAATCTTCTCGTACTATCTGGCAGTGGACATTGTGA
         66 ACCTTTCTTTTCATAGAGCAGTTTTGAAACACTCTTTTTGTAGAATCTGGAAGTGGATATTTGGA

              * *   *  ** ** *  *   *            ** *
     525597 GCTCCTTGGGGCCTATGCTGAAAAAGGAAATATCTTCCGAC
        131 GCGCTTTGAGGATTTCGTTGGAAACGGAAATATCTTCATAT

                                 *     *  **          *           **    *    
     525638 AAAAACTAGACAGAAGCATTCGCAGAATCACGTTTGTGATGTGTGCACTCAACTGTCAGAATTGA
          1 AAAAACTAGACAGAAGCATTCTCAGAAACATCTTTGTGATGTTTGCACTCAACTCACAGAGTTGA

                 **   ** *      *                            * *            *
     525703 ACCTTGGTTTGGACAGAGCACTTTTGAAACACTCTTTTTGTAGAATCTGCAGGTGGATATTTGGC
         66 ACCTTTCTTTTCATAGAGCAGTTTTGAAACACTCTTTTTGTAGAATCTGGAAGTGGATATTTGGA

            **                         *   *      * *
     525768 TAGCTTTGAGGATTTCGTTGGAAACGGTAATGTCTTCAAAG
        131 GCGCTTTGAGGATTTCGTTGGAAACGGAAATATCTTCATAT

                *                         *   *            *    *            
     525809 AAAATCTAGACAGAAGCATTCTCAGAAACACCTTCGTGATGTTTGCAATCAAGTCACAGAGTTGA
          1 AAAAACTAGACAGAAGCATTCTCAGAAACATCTTTGTGATGTTTGCACTCAACTCACAGAGTTGA

                 * *             *  *                  *             *       
     525874 ACCTTCCGTTTCATAGAGCAGGTTGGAAACACTCTTTTTGTAGTATCTGGAAGTGGACATTTGGA
         66 ACCTTTCTTTTCATAGAGCAGTTTTGAAACACTCTTTTTGTAGAATCTGGAAGTGGATATTTGGA

             *         ** *    * *   *            **  
     525939 GGGCTTTGTAGCCTATC-TGGAAAAAGGAAATATCTTCCCAT
        131 GCGCTTTG-AGGATTTCGTTGGAAACGGAAATATCTTCATAT

            *  ** *   *     * *            *   *  *   * **        *  * * *   
     525980 GAATGCGAGATAGAAGTAATCTCAGAAACATGTTTATGCTGTATCTACTCAACTAACTGTGCTGA
          1 AAAAACTAGACAGAAGCATTCTCAGAAACATCTTTGTGATGTTTGCACTCAACTCACAGAGTTGA

              *     *  *               *        *  * *       *               
     526045 ACATTTCTATTGATAGAGCAGTTTTGAGACACTCTTCTTTTGGAATCTGCAAGTGGATATTTGGA
         66 ACCTTTCTTTTCATAGAGCAGTTTTGAAACACTCTTTTTGTAGAATCTGGAAGTGGATATTTGGA

            ** *                        *     *      
     526110 TAGATTTGAGGATTTCGTTGGAAACGG-GAT-T-AT-ATAT
        131 GCGCTTTGAGGATTTCGTTGGAAACGGAAATATCTTCATAT

                 *       *               *                 **  *   *         
     526147 AAAAAGTAGACAGCAGCATTCTCAGAAACTTCTTTGTGATGTTTGCATCCAGCTCTCAGAGTTGA
          1 AAAAACTAGACAGAAGCATTCTCAGAAACATCTTTGTGATGTTTGCACTCAACTCACAGAGTTGA

              *  * *          *  *        *        *   **        *  **       
     526212 ACATTCCCTTTCATAGAGTAGGTTTGAAACCCTCTTTTTATAGTGTCTGGAAGCGGGCATTTGGA
         66 ACCTTTCTTTTCATAGAGCAGTTTTGAAACACTCTTTTTGTAGAATCTGGAAGTGGATATTTGGA

                   *    * * **  **  **          * *  *
     526277 GCGCTTTCAGGCCTATGCTTAAAATAGGAAATATCTACCTAC
        131 GCGCTTTGAGG-ATTTCGTTGGAAACGGAAATATCTTCATAT

             *                    *    *  **          ** *        *    *     
     526319 AGAAACTAGACAGAAGCATTCTGAGAATCACGTTTGTGATGTGGGTACTCAACTAACAGTGTTGA
          1 AAAAACTAGACAGAAGCATTCTCAGAAACATCTTTGTGATGTTTGCACTCAACTCACAGAGTTGA

            *  *       *   *            *   *                *   *           
     526384 TCCATTCTTTTGATACAGCAGTTTTGAACCACACTTTTTGTAGAATCTGCAAGAGGATATTTGGA
         66 ACCTTTCTTTTCATAGAGCAGTTTTGAAACACTCTTTTTGTAGAATCTGGAAGTGGATATTTGGA

            **   *                     *   *      * *
     526449 TAGCTGTGAGGATTTCGTTGGAAACGGGAATGTCTTCAAAG
        131 GCGCTTTGAGGATTTCGTTGGAAACGGAAATATCTTCATAT

                *                         *   *            *    *            
     526490 AAAATCTAGACAGAAGCATTCTCAGAAACACCTTCGTGATGTTTGCAATCAAGTCACAGAGTTGA
          1 AAAAACTAGACAGAAGCATTCTCAGAAACATCTTTGTGATGTTTGCACTCAACTCACAGAGTTGA

                 * *             *  *           *      *             *       
     526555 ACCTTCCGTTTCATAGAGCAGGTTGGAAACACTCTTATTGTAGTATCTGGAAGTGGACATTTGGA
         66 ACCTTTCTTTTCATAGAGCAGTTTTGAAACACTCTTTTTGTAGAATCTGGAAGTGGATATTTGGA

                   *   ** ** *  *   *            **  
     526620 GCGCTTTCAGGCCTATGGTGAAAAAGGAAATATCTTCCCAT
        131 GCGCTTTGAGGATTTCGTTGGAAACGGAAATATCTTCATAT

                  * * *      **      *   * *   *     ** ** *      *    *     
     526661 AAAAACGACATAGAAGCTATCTCAGGAACTTGTTTATGATGCATCTAATCAACTAACAGTGTTGA
          1 AAAAACTAGACAGAAGCATTCTCAGAAACATCTTTGTGATGTTTGCACTCAACTCACAGAGTTGA

                  * ** * *                         * *       *               
     526726 ACCTTTGTACTGACAGAGCAG-TTTGAAACACTCTTTTTTTGGAATCTGCAAGTGGATATTTGGA
         66 ACCTTTCTTTTCATAGAGCAGTTTTGAAACACTCTTTTTGTAGAATCTGGAAGTGGATATTTGGA

            *                           *   *   *    
     526790 TCGCTTTGAGGATTTCGTTGGAAACGG-GAT-GC--AATAT
        131 GCGCTTTGAGGATTTCGTTGGAAACGGAAATATCTTCATAT

                **  *    *     *                 **  *   *  *    *   *     * 
     526827 AAAACGTACACAGCAGCATACTCAGAAA-ATACTTTGCCATATTTCCATTCAAGTCAGAGAGTGG
          1 AAAAACTAGACAGAAGCATTCTCAGAAACAT-CTTTGTGATGTTTGCACTCAACTCACAGAGTTG

               *  * **            *                  *  *             *      
     526891 AACATTCCCATTCATAGAGCAGGTTTGAAACACTCTTTTTGGAGTATCTGGAAGTGGACATTTGG
         65 AACCTTTCTTTTCATAGAGCAGTTTTGAAACACTCTTTTTGTAGAATCTGGAAGTGGATATTTGG

                    ** * * ** *  *   *            **  
     526956 AGCGCTTTCGGAACTATGGTGAAAAAGGAAATATCTTCCAAT
        130 AGCGCTTTGAGGATTTCGTTGGAAACGGAAATATCTTCATAT

            *     *               *      * *          *  **      **  *  *    
     526998 GAAAACAAGACAGAAGCATTCTGAGAAACTTATTTGTGATGTGTGTCCTCAACAAACGGACTTGA
          1 AAAAACTAGACAGAAGCATTCTCAGAAACATCTTTGTGATGTTTGCACTCAACTCACAGAGTTGA

                   *      **  * *  *  *             *   *    * * *           
     527063 ACCTTTCGTTTCATGCAGTACTTCTGGAACACTCTTTTTGAAGATTCTGCATGCGGATATTTGGA
         66 ACCTTTCTTTTCATAGAGCAGTTTTGAAACACTCTTTTTGTAGAATCTGGAAGTGGATATTTGGA

            **                            **   *   * 
     527128 TAGCTTTGAGGATTTCGTTGGAAACGG---GCT-TACATGT
        131 GCGCTTTGAGGATTTCGTTGGAAACGGAAATATCTTCATAT

                 *       *               *        *   *    *    *       *    
     527165 AAAAATTAGACAGCAGCATTCTCAGAAACTTCTTTGTGGTGTCTGCATTCAAGTCACAGAATTGA
          1 AAAAACTAGACAGAAGCATTCTCAGAAACATCTTTGTGATGTTTGCACTCAACTCACAGAGTTGA

              * ** * **            *  * *      *       *             *       
     527230 ACTTCCCCTCACATAGAGCAGTTGTGCAGCACTCTATTTGTAGTATCTGGAAGTGGACATTTGGA
         66 ACCTTTCTTTTCATAGAGCAGTTTTGAAACACTCTTTTTGTAGAATCTGGAAGTGGATATTTGGA

             *         ** *    * *   *            **  
     527295 GGGCTTTGTAGCCTATC-TGGAAAAAGGAAATATCTTCCCAT
        131 GCGCTTTG-AGGATTTCGTTGGAAACGGAAATATCTTCATAT

            *  ** *   *     * *            *   *  *   * **        *  * * *   
     527336 GAATGCGAGATAGAAGTAATCTCAGAAACATGTTTATGCTGTATCTACTCAACTAACTGTGCTGA
          1 AAAAACTAGACAGAAGCATTCTCAGAAACATCTTTGTGATGTTTGCACTCAACTCACAGAGTTGA

              *     *  *             * *        *  * *       *               
     527401 ACATTTCTATTGATAGAGCAGTTTTCAGACACTCTTCTTTTGGAATCTGCAAGTGGATATTTGGA
         66 ACCTTTCTTTTCATAGAGCAGTTTTGAAACACTCTTTTTGTAGAATCTGGAAGTGGATATTTGGA

            ** *                        *     *      
     527466 TAGATTTGAGGATTTCGTTGGAAACGG-GAT-T-AT-ATAT
        131 GCGCTTTGAGGATTTCGTTGGAAACGGAAATATCTTCATAT

                 *       *               *                 **  *   *         
     527503 AAAAAGTAGACAGCAGCATTCTCAGAAACTTCTTTGTGATGTTTGCATCCAGCTCTCAGAGTTGA
          1 AAAAACTAGACAGAAGCATTCTCAGAAACATCTTTGTGATGTTTGCACTCAACTCACAGAGTTGA

              *  * *          *  *        *        *   **        *  **       
     527568 ACATTCCCTTTCATAGAGTAGGTTTGAAACCCTCTTTTTATAGTGTCTGGAAGCGGGCATTTGGA
         66 ACCTTTCTTTTCATAGAGCAGTTTTGAAACACTCTTTTTGTAGAATCTGGAAGTGGATATTTGGA

                   *    * * **  **  **          * *  *
     527633 GCGCTTTCAGGCCTATGCTTAAAATAGGAAATATCTACCTAC
        131 GCGCTTTGAGG-ATTTCGTTGGAAACGGAAATATCTTCATAT

             **                   *    *  **          ** *        *    *     
     527675 AGGAACTAGACAGAAGCATTCTGAGAATCACGTTTGTGATGTGGGTACTCAACTAACAGTGTTGA
          1 AAAAACTAGACAGAAGCATTCTCAGAAACATCTTTGTGATGTTTGCACTCAACTCACAGAGTTGA

            *  *       *   *            *   *                *   *           
     527740 TCCATTCTTTTGATACAGCAGTTTTGAACCACACTTTTTGTAGAATCTGCAAGAGGATATTTGGA
         66 ACCTTTCTTTTCATAGAGCAGTTTTGAAACACTCTTTTTGTAGAATCTGGAAGTGGATATTTGGA

            **   *                     *   *      * *
     527805 TAGCTGTGAGGATTTCGTTGGAAACGGGAATGTCTTCAAAG
        131 GCGCTTTGAGGATTTCGTTGGAAACGGAAATATCTTCATAT

                *                         *   *            *    *            
     527846 AAAATCTAGACAGAAGCATTCTCAGAAACACCTTCGTGATGTTTGCAATCAAGTCACAGAGTTGA
          1 AAAAACTAGACAGAAGCATTCTCAGAAACATCTTTGTGATGTTTGCACTCAACTCACAGAGTTGA

                 * *             *  *           *      *             *       
     527911 ACCTTCCGTTTCATAGAGCAGGTTGGAAACACTCTTATTGTAGTATCTGGAAGTGGACATTTGGA
         66 ACCTTTCTTTTCATAGAGCAGTTTTGAAACACTCTTTTTGTAGAATCTGGAAGTGGATATTTGGA

                   *   ** ** *  *   *            **  
     527976 GCGCTTTCAGGCCTATGGTGAAAAAGGAAATATCTTCCCAT
        131 GCGCTTTGAGGATTTCGTTGGAAACGGAAATATCTTCATAT

                  * * *      **      *   * *   *     ** ** *      *    *     
     528017 AAAAACGACATAGAAGCTATCTCAGGAACTTGTTTATGATGCATCTAATCAACTAACAGTGTTGA
          1 AAAAACTAGACAGAAGCATTCTCAGAAACATCTTTGTGATGTTTGCACTCAACTCACAGAGTTGA

                  * ** * *                         * *       *               
     528082 ACCTTTGTACTGACAGAGCAG-TTTGAAACACTCTTTTTTTGGAATCTGCAAGTGGATATTTGGA
         66 ACCTTTCTTTTCATAGAGCAGTTTTGAAACACTCTTTTTGTAGAATCTGGAAGTGGATATTTGGA

            *                           *   *   *    
     528146 TCGCTTTGAGGATTTCGTTGGAAACGG-GAT-GC--AATAT
        131 GCGCTTTGAGGATTTCGTTGGAAACGGAAATATCTTCATAT

                **  *    *     *                 **  *   *  *    *         * 
     528183 AAAACGTACACAGCAGCATACTCAGAAA-ATACTTTGCCATATTTCCATTCAAGTCACAGAGTGG
          1 AAAAACTAGACAGAAGCATTCTCAGAAACAT-CTTTGTGATGTTTGCACTCAACTCACAGAGTTG

               *  * **            *                  *  *             *      
     528247 AACATTCCCATTCATAGAGCAGGTTTGAAACACTCTTTTTGGAGTATCTGGAAGTGGACATTTGG
         65 AACCTTTCTTTTCATAGAGCAGTTTTGAAACACTCTTTTTGTAGAATCTGGAAGTGGATATTTGG

                    ** * * ** *  *   *            **  
     528312 AGCGCTTTCTGAACTATGGTGAAAAAGGAAATATCTTCCAAT
        130 AGCGCTTTGAGGATTTCGTTGGAAACGGAAATATCTTCATAT

            *     *               *      * *          *  **      **  *  *    
     528354 GAAAACAAGACAGAAGCATTCTGAGAAACTTATTTGTGATGTGTGTCCTCAACAAACGGACTTGA
          1 AAAAACTAGACAGAAGCATTCTCAGAAACATCTTTGTGATGTTTGCACTCAACTCACAGAGTTGA

                   *      **  * *  *  *             *   *    * * *           
     528419 ACCTTTCGTTTCATGCAGTACTTCTGGAACACTCTTTTTGAAGATTCTGCATGCGGATATTTGGA
         66 ACCTTTCTTTTCATAGAGCAGTTTTGAAACACTCTTTTTGTAGAATCTGGAAGTGGATATTTGGA

            **                            **   *   * 
     528484 TAGCTTTGAGGATTTCGTTGGAAACGG---GCT-TACATGT
        131 GCGCTTTGAGGATTTCGTTGGAAACGGAAATATCTTCATAT

                 *       *               *        *   *    *    *       *    
     528521 AAAAATTAGACAGCAGCATTCTCAGAAACTTCTTTGTGGTGTCTGCATTCAAGTCACAGAATTGA
          1 AAAAACTAGACAGAAGCATTCTCAGAAACATCTTTGTGATGTTTGCACTCAACTCACAGAGTTGA

              * ** * **            *  * *      *       *             *       
     528586 ACTTCCCCTCACATAGAGCAGTTGTGCAGCACTCTATTTGTAGTATCTGGAAGTGGACATTTGGA
         66 ACCTTTCTTTTCATAGAGCAGTTTTGAAACACTCTTTTTGTAGAATCTGGAAGTGGATATTTGGA

             *         ** *    * *   *            **  
     528651 GGGCTTTGTAGCCTATC-TGGAAAAAGGAAATATCTTCCCAT
        131 GCGCTTTG-AGGATTTCGTTGGAAACGGAAATATCTTCATAT

            *  ** *   *     * *            *   *  *   * **        *  * * *   
     528692 GAATGCGAGATAGAAGTAATCTCAGAAACATGTTTATGCTGTATCTACTCAACTAACTGTGCTGA
          1 AAAAACTAGACAGAAGCATTCTCAGAAACATCTTTGTGATGTTTGCACTCAACTCACAGAGTTGA

              *     *  *               *        *  * *       *               
     528757 ACATTTCTATTGATAGAGCAGTTTTGAGACACTCTTCTTTTGGAATCTGCAAGTGGATATTTGGA
         66 ACCTTTCTTTTCATAGAGCAGTTTTGAAACACTCTTTTTGTAGAATCTGGAAGTGGATATTTGGA

            ** *                        *     *      
     528822 TAGATTTGAGGATTTCGTTGGAAACGG-GAT-T-AT-ATAT
        131 GCGCTTTGAGGATTTCGTTGGAAACGGAAATATCTTCATAT

            *    *       *               *                 **  *   *         
     528859 CAAAAGTAGACAGCAGCATTCTCAGAAACTTCTTTGTGATGTTTGCATCCAGCTCTCAGAGTTGA
          1 AAAAACTAGACAGAAGCATTCTCAGAAACATCTTTGTGATGTTTGCACTCAACTCACAGAGTTGA

              *  * *          *  *        *        *   **        *  **       
     528924 ACATTCCCTTTCATAGAGTAGGTTTGAAACCCTCTTTTTATAGTGTCTGGAAGCGGGCATTTGGA
         66 ACCTTTCTTTTCATAGAGCAGTTTTGAAACACTCTTTTTGTAGAATCTGGAAGTGGATATTTGGA

                   *    * * **  **  **          * *  *
     528989 GCGCTTTCAGGCCTATGCTTAAAATAGGAAATATCTACCTAC
        131 GCGCTTTGAGG-ATTTCGTTGGAAACGGAAATATCTTCATAT

             *                    *    *  **          ** *        *    *     
     529031 AGAAACTAGACAGAAGCATTCTGAGAATCACGTTTGTGATGTGGGTACTCAACTAACAGTGTTGA
          1 AAAAACTAGACAGAAGCATTCTCAGAAACATCTTTGTGATGTTTGCACTCAACTCACAGAGTTGA

            *  *       *   *            *   *                *   *           
     529096 TCCATTCTTTTGATACAGCAGTTTTGAACCACACTTTTTGTAGAATCTGCAAGAGGATATTTGGA
         66 ACCTTTCTTTTCATAGAGCAGTTTTGAAACACTCTTTTTGTAGAATCTGGAAGTGGATATTTGGA

            **   *                     *   *      * *
     529161 TAGCTGTGAGGATTTCGTTGGAAACGGGAATGTCTTCAAAG
        131 GCGCTTTGAGGATTTCGTTGGAAACGGAAATATCTTCATAT

                *                         *   *            *    *            
     529202 AAAATCTAGACAGAAGCATTCTCAGAAACACCTTCGTGATGTTTGCAATCAAGTCACAGAGTTGA
          1 AAAAACTAGACAGAAGCATTCTCAGAAACATCTTTGTGATGTTTGCACTCAACTCACAGAGTTGA

                 * *             *  *           *      *             *       
     529267 ACCTTCCGTTTCATAGAGCAGGTTGGAAACACTCTTATTGTAGTATCTGGAAGTGGACATTTGGA
         66 ACCTTTCTTTTCATAGAGCAGTTTTGAAACACTCTTTTTGTAGAATCTGGAAGTGGATATTTGGA

                   *   ** ** *  *   *            **  
     529332 GCGCTTTCAGGCCTATGGTGAAAAAGGAAATATCTTCCCAT
        131 GCGCTTTGAGGATTTCGTTGGAAACGGAAATATCTTCATAT

                  * * *      **      *   * *   *     ** ** *      *    *     
     529373 AAAAACGACATAGAAGCTATCTCAGGAACTTGTTTATGATGCATCTAATCAACTAACAGTGTTGA
          1 AAAAACTAGACAGAAGCATTCTCAGAAACATCTTTGTGATGTTTGCACTCAACTCACAGAGTTGA

                  * ** * *                         * *       *               
     529438 ACCTTTGTACTGACAGAGCAG-TTTGAAACACTCTTTTTTTGGAATCTGCAAGTGGATATTTGGA
         66 ACCTTTCTTTTCATAGAGCAGTTTTGAAACACTCTTTTTGTAGAATCTGGAAGTGGATATTTGGA

            *                           *   *   *    
     529502 TCGCTTTGAGGATTTCGTTGGAAACGG-GAT-GC--AATAT
        131 GCGCTTTGAGGATTTCGTTGGAAACGGAAATATCTTCATAT

                **  *    *     *                 **  *   *  *    *         * 
     529539 AAAACGTACACAGCAGCATACTCAGAAA-ATACTTTGCCATATTTCCATTCAAGTCACAGAGTGG
          1 AAAAACTAGACAGAAGCATTCTCAGAAACAT-CTTTGTGATGTTTGCACTCAACTCACAGAGTTG

               *  * **            *  *               *  *             *      
     529603 AACATTCCCATTCATAGAGCAGGTTGGAAACACTCTTTTTGGAGTATCTGGAAGTGGACATTTGG
         65 AACCTTTCTTTTCATAGAGCAGTTTTGAAACACTCTTTTTGTAGAATCTGGAAGTGGATATTTGG

                    ** * * ** *  *   *            **  
     529668 AGCGCTTTCTGAACTATGGTGAAAAAGGAAATATCTTCCAAT
        130 AGCGCTTTGAGGATTTCGTTGGAAACGGAAATATCTTCATAT

            *     *               *      * *          *  **      **  *  *    
     529710 GAAAACAAGACAGAAGCATTCTGAGAAACTTATTTGTGATGTCTGTCCTCAACAAACGGACTTGA
          1 AAAAACTAGACAGAAGCATTCTCAGAAACATCTTTGTGATGTTTGCACTCAACTCACAGAGTTGA

                   *      **  * *  *  *             *   *    * * *           
     529775 ACCTTTCGTTTCATGCAGTACTTCTGGAACACTCTTTTTGAAGATTCTGCATGCGGATATTTGGA
         66 ACCTTTCTTTTCATAGAGCAGTTTTGAAACACTCTTTTTGTAGAATCTGGAAGTGGATATTTGGA

            **                            **   *   * 
     529840 TAGCTTTGAGGATTTCGTTGGAAACGG---GCT-TACATGT
        131 GCGCTTTGAGGATTTCGTTGGAAACGGAAATATCTTCATAT

                 *       *               *        *   *    *    *       *    
     529877 AAAAATTAGACAGCAGCATTCTCAGAAACTTCTTTGTGGTGTCTGCATTCAAGTCACAGAATTGA
          1 AAAAACTAGACAGAAGCATTCTCAGAAACATCTTTGTGATGTTTGCACTCAACTCACAGAGTTGA

              * ** * **            *  * *      *       *    *        *       
     529942 ACTTCCCCTCACATAGAGCAGTTGTGCAGCACTCTATTTGTAGTATCTCGAAGTGGACATTTGGA
         66 ACCTTTCTTTTCATAGAGCAGTTTTGAAACACTCTTTTTGTAGAATCTGGAAGTGGATATTTGGA

             *         ** *    * *   *            **  
     530007 GGGCTTTGTAGCCTATC-TGGAAAAAGGAAATATCTTCCCAT
        131 GCGCTTTG-AGGATTTCGTTGGAAACGGAAATATCTTCATAT

            *  ** *   *     * *            *   *  *   * ***       *  * * *   
     530048 GAATGCGAGATAGAAGTAATCTCAGAAACATGTTTATGCTGTATCTTCTCAACTAACTGTGCTGA
          1 AAAAACTAGACAGAAGCATTCTCAGAAACATCTTTGTGATGTTTGCACTCAACTCACAGAGTTGA

              *     *  *               *        *  * *       *               
     530113 ACATTTCTATTGATAGAGCAGTTTTGAGACACTCTTCTTTTGGAATCTGCAAGTGGATATTTGGA
         66 ACCTTTCTTTTCATAGAGCAGTTTTGAAACACTCTTTTTGTAGAATCTGGAAGTGGATATTTGGA

            ** *                        *     *      
     530178 TAGATTTGAGGATTTCGTTGGAAACGG-GAT-T-AT-ATAT
        131 GCGCTTTGAGGATTTCGTTGGAAACGGAAATATCTTCATAT

                 *       *               *                 **  *   *         
     530215 AAAAAGTAGACAGCAGCATTCTCAGAAACTTCTTTGTGATGTTTGCATCCAGCTCTCAGAGTTGA
          1 AAAAACTAGACAGAAGCATTCTCAGAAACATCTTTGTGATGTTTGCACTCAACTCACAGAGTTGA

              *  * *          *  *        *        *   **        *  **       
     530280 ACATTCCCTTTCATAGAGTAGGTTTGAAACCCTCTTTTTATAGTGTCTGGAAGCGGGCATTTGGA
         66 ACCTTTCTTTTCATAGAGCAGTTTTGAAACACTCTTTTTGTAGAATCTGGAAGTGGATATTTGGA

                   *   ** ** *  *   *          * *   
     530345 GCGCTTTCAGGCCTATGCTGAAAAAGGAAATATCTACCTAT
        131 GCGCTTTGAGGATTTCGTTGGAAACGGAAATATCTTCATAT

             *                    *    *  **          ** *        *    *     
     530386 AGAAACTAGACAGAAGCATTCTGAGAATCACGTTTGTGATGTGGGTACTCAACTAACAGTGTTGA
          1 AAAAACTAGACAGAAGCATTCTCAGAAACATCTTTGTGATGTTTGCACTCAACTCACAGAGTTGA

            *  *       *   *            *   *                *               
     530451 TCCATTCTTTTGATACAGCAGTTTTGAACCACACTTTTTGTAGAATCTGCAAGTGGATATTTGGA
         66 ACCTTTCTTTTCATAGAGCAGTTTTGAAACACTCTTTTTGTAGAATCTGGAAGTGGATATTTGGA

            **   *                     *   *        *
     530516 TAGCTGTGAGGATTTCGTTGGAAACGGGAATGTCTTCATAG
        131 GCGCTTTGAGGATTTCGTTGGAAACGGAAATATCTTCATAT

                **                     * * **     *   *  **   *   *          
     530557 AAAATTTAGACAGAAGCATTCTCAGAACCTTGATTGTGGTGTGTGTTCTCCACTAACAGAGTTGA
          1 AAAAACTAGACAGAAGCATTCTCAGAAACATCTTTGTGATGTTTGCACTCAACTCACAGAGTTGA

                       * *   * *   *       *       *                         
     530622 ACCTTTCTTTTGACAGAACTGTTCTGAAACATTCTTTTTATAGAATCTGGAAGTGGATATTTGGA
         66 ACCTTTCTTTTCATAGAGCAGTTTTGAAACACTCTTTTTGTAGAATCTGGAAGTGGATATTTGGA

            **              *          *          *  
     530687 AAGCTTTGAGGATTTCATTGGAAACGGGAATATCTTCAAAT
        131 GCGCTTTGAGGATTTCGTTGGAAACGGAAATATCTTCATAT

                *    *            *           * *       *  *    *            
     530728 AAAATCTAGCCAGAAGCATTCTAAGAAACATCTTAGGGATGTTTACATTCAAGTCACAGAGTTGA
          1 AAAAACTAGACAGAAGCATTCTCAGAAACATCTTTGTGATGTTTGCACTCAACTCACAGAGTTGA

              *  * *     *       *         *    * *   **      *      *    *  
     530793 ACATTCCCTTTCACAGAGCAGGTTTGAAACAATCTTCTCGTACTATCTGGCAGTGGACATTTTGA
         66 ACCTTTCTTTTCATAGAGCAGTTTTGAAACACTCTTTTTGTAGAATCTGGAAGTGGATATTTGGA

              * *   *  ** ** *  *   *            ** *
     530858 GCTCCTTGGGGCCTATGCTGAAAAAGGAAATATCTTCCGAC
        131 GCGCTTTGAGGATTTCGTTGGAAACGGAAATATCTTCATAT

                                 *     *  **          *           **    *    
     530899 AAAAACTAGACAGAAGCATTCGCAGAATCACGTTTGTGATGTGTGCACTCAACTGTCAGAATTGA
          1 AAAAACTAGACAGAAGCATTCTCAGAAACATCTTTGTGATGTTTGCACTCAACTCACAGAGTTGA

                 **   ** *      *                            * *            *
     530964 ACCTTGGTTTGGACAGAGCACTTTTGAAACACTCTTTTTGTAGAATCTGCAGGTGGATATTTGGC
         66 ACCTTTCTTTTCATAGAGCAGTTTTGAAACACTCTTTTTGTAGAATCTGGAAGTGGATATTTGGA

            **                         *   *      * *
     531029 TAGCTTTGAGGATTTCGTTGGAAACGGTAATGTCTTCAAAG
        131 GCGCTTTGAGGATTTCGTTGGAAACGGAAATATCTTCATAT

                *                         *   *            *    *            
     531070 AAAATCTAGACAGAAGCATTCTCAGAAACACCTTCGTGATGTTTGCAATCAAGTCACAGAGTTGA
          1 AAAAACTAGACAGAAGCATTCTCAGAAACATCTTTGTGATGTTTGCACTCAACTCACAGAGTTGA

                 * *             *  *                  *             *       
     531135 ACCTTCCGTTTCATAGAGCAGGTTGGAAACACTCTTTTTGTAGTATCTGGAAGTGGACATTTGGA
         66 ACCTTTCTTTTCATAGAGCAGTTTTGAAACACTCTTTTTGTAGAATCTGGAAGTGGATATTTGGA

             *         ** *    * *   *            **  
     531200 GGGCTTTGTAGCCTATC-TGGAAAAAGGAAATATCTTCCCAT
        131 GCGCTTTG-AGGATTTCGTTGGAAACGGAAATATCTTCATAT

            *  ** *   *     * *           **   *  *   * **        *  * * *   
     531241 GAATGCGAGATAGAAGTAATCTCAGAAACACGTTTATGCTGTATCTACTCAACTAACTGTGCTGA
          1 AAAAACTAGACAGAAGCATTCTCAGAAACATCTTTGTGATGTTTGCACTCAACTCACAGAGTTGA

              *     *  *               *        *  * *       *               
     531306 ACATTTCTATTGATAGAGCAGTTTTGAGACACTCTTCTTTTGGAATCTGCAAGTGGATATTTGGA
         66 ACCTTTCTTTTCATAGAGCAGTTTTGAAACACTCTTTTTGTAGAATCTGGAAGTGGATATTTGGA

            ** *                        *     *      
     531371 TAGATTTGAGGATTTCGTTGGAAACGG-GAT-T-AT-ATAT
        131 GCGCTTTGAGGATTTCGTTGGAAACGGAAATATCTTCATAT

                 *       *               *                 **  *   *         
     531408 AAAAAGTAGACAGCAGCATTCTCAGAAACTTCTTTGTGATGTTTGCATCCAGCTCTCAGAGTTGA
          1 AAAAACTAGACAGAAGCATTCTCAGAAACATCTTTGTGATGTTTGCACTCAACTCACAGAGTTGA

              *  * *          *  *        *        *   **        *  **       
     531473 ACATTCCCTTTCATAGAGTAGGTTTGAAACCCTCTTTTTATAGTGTCTGGAAGCGGGCATTTGGA
         66 ACCTTTCTTTTCATAGAGCAGTTTTGAAACACTCTTTTTGTAGAATCTGGAAGTGGATATTTGGA

                   *    * * **  **  **          * *  *
     531538 GCGCTTTCAGGCCTATGCTTAAAATAGGAAATATCTACCTAC
        131 GCGCTTTGAGG-ATTTCGTTGGAAACGGAAATATCTTCATAT

             *                    *    *  **          ** *        *    *     
     531580 AGAAACTAGACAGAAGCATTCTGAGAATCACGTTTGTGATGTGGGTACTCAACTAACAGTGTTGA
          1 AAAAACTAGACAGAAGCATTCTCAGAAACATCTTTGTGATGTTTGCACTCAACTCACAGAGTTGA

            * **       *   *            *   *                *   *           
     531645 TCTATTCTTTTGATACAGCAGTTTTGAACCACACTTTTTGTAGAATCTGCAAGAGGATATTTGGA
         66 ACCTTTCTTTTCATAGAGCAGTTTTGAAACACTCTTTTTGTAGAATCTGGAAGTGGATATTTGGA

            **   *                     *   *      * *
     531710 TAGCTGTGAGGATTTCGTTGGAAACGGGAATGTCTTCAAAG
        131 GCGCTTTGAGGATTTCGTTGGAAACGGAAATATCTTCATAT

                *                       * *   *            *    *            
     531751 AAAATCTAGACAGAAGCATTCTCAGAAATACCTTCGTGATGTTTGCAATCAAGTCACAGAGTTGA
          1 AAAAACTAGACAGAAGCATTCTCAGAAACATCTTTGTGATGTTTGCACTCAACTCACAGAGTTGA

                 * *             *  *           *      *             *       
     531816 ACCTTCCGTTTCATAGAGCAGGTTGGAAACACTCTTATTGTAGTATCTGGAAGTGGACATTTGGA
         66 ACCTTTCTTTTCATAGAGCAGTTTTGAAACACTCTTTTTGTAGAATCTGGAAGTGGATATTTGGA

                   *   ** ** *  *   *            **  
     531881 GCGCTTTCAGGCCTATGGTGAAAAAGGAAATATCTTCCCAT
        131 GCGCTTTGAGGATTTCGTTGGAAACGGAAATATCTTCATAT

                  * * *      **      *   * *   *     ** ** *      *    *     
     531922 AAAAACGATATAGAAGCTATCTCAGGAACTTGTTTATGATGCATCTAATCAACTAACAGTGTTGA
          1 AAAAACTAGACAGAAGCATTCTCAGAAACATCTTTGTGATGTTTGCACTCAACTCACAGAGTTGA

              *   * ** * *                         * *       *               
     531987 ACTTTTGTACTGACAGAGCAG-TTTGAAACACTCTTTTTTTGGAATCTGCAAGTGGATATTTGGA
         66 ACCTTTCTTTTCATAGAGCAGTTTTGAAACACTCTTTTTGTAGAATCTGGAAGTGGATATTTGGA

            *                           *   *   *    
     532051 TCGCTTTGAGGATTTCGTTGGAAACGG-GAT-GC--AATAT
        131 GCGCTTTGAGGATTTCGTTGGAAACGGAAATATCTTCATAT

                **  *    *     *                 **  *   *  *    *         * 
     532088 AAAACGTACACAGCAGCATACTCAGAAA-ATACTTTGCCATATTTCCATTCAAGTCACAGAGTGG
          1 AAAAACTAGACAGAAGCATTCTCAGAAACAT-CTTTGTGATGTTTGCACTCAACTCACAGAGTTG

               *  * **            *                  *  *             *      
     532152 AACATTCCCATTCATAGAGCAGGTTTGAAACACTCTTTTTGGAGTATCTGGAAGTGGACATTTGG
         65 AACCTTTCTTTTCATAGAGCAGTTTTGAAACACTCTTTTTGTAGAATCTGGAAGTGGATATTTGG

                    ** * * ** *  *   *            **  
     532217 AGCGCTTTCTGAACTATGGTGAAAAAGGAAATATCTTCCAAT
        130 AGCGCTTTGAGGATTTCGTTGGAAACGGAAATATCTTCATAT

            *     *               *      * *          *  **      **  *  *    
     532259 GAAAACAAGACAGAAGCATTCTGAGAAACTTATTTGTGATGTGTGTCCTCAACAAACGGACTTGA
          1 AAAAACTAGACAGAAGCATTCTCAGAAACATCTTTGTGATGTTTGCACTCAACTCACAGAGTTGA

                   *      **  * *  *  *             *   *    * * *           
     532324 ACCTTTCGTTTCATGCAGTACTTCTGGAACACTCTTTTTGAAGATTCTGCATGCGGATATTTGGA
         66 ACCTTTCTTTTCATAGAGCAGTTTTGAAACACTCTTTTTGTAGAATCTGGAAGTGGATATTTGGA

            **                            **   *   * 
     532389 TAGCTTTGAGGATTTCGTTGGAAACGG---GCT-TACATGT
        131 GCGCTTTGAGGATTTCGTTGGAAACGGAAATATCTTCATAT

                 *       *               *        *   *    *    *       *    
     532426 AAAAATTAGACAGCAGCATTCTCAGAAACTTCTTTGTGGTGTCTGCATTCAAGTCACAGAATTGA
          1 AAAAACTAGACAGAAGCATTCTCAGAAACATCTTTGTGATGTTTGCACTCAACTCACAGAGTTGA

              * ** * **            *  * *      *       *             *       
     532491 ACTTCCCCTCACATAGAGCAGTTGTGCAGCACTCTATTTGTAGTATCTGGAAGTGGACATTTGGA
         66 ACCTTTCTTTTCATAGAGCAGTTTTGAAACACTCTTTTTGTAGAATCTGGAAGTGGATATTTGGA

             *         ** *    * *   *            **  
     532556 GGGCTTTGTAGCCTATC-TGGAAAAAGGAAATATCTTCCCAT
        131 GCGCTTTG-AGGATTTCGTTGGAAACGGAAATATCTTCATAT

            *  ** *   *     * *            *   *  *   * **        *  * * *   
     532597 GAATGCGAGATAGAAGTAATCTCAGAAACATGTTTATGCTGTATCTACTCAACTAACTGTGCTGA
          1 AAAAACTAGACAGAAGCATTCTCAGAAACATCTTTGTGATGTTTGCACTCAACTCACAGAGTTGA

              *     *  *               *        *  * *       *               
     532662 ACATTTCTATTGATAGAGCAGTTTTGAGACACTCTTCTTTTGGAATCTGCAAGTGGATATTTGGA
         66 ACCTTTCTTTTCATAGAGCAGTTTTGAAACACTCTTTTTGTAGAATCTGGAAGTGGATATTTGGA

            ** *                        *     *      
     532727 TAGATTTGAGGATTTCGTTGGAAACGG-GAT-T-AT-ATAT
        131 GCGCTTTGAGGATTTCGTTGGAAACGGAAATATCTTCATAT

                 *       *               *                 **  *   *         
     532764 AAAAAGTAGACAGCAGCATTCTCAGAAACTTCTTTGTGATGTTTGCATCCAGCTCTCAGAGTTGA
          1 AAAAACTAGACAGAAGCATTCTCAGAAACATCTTTGTGATGTTTGCACTCAACTCACAGAGTTGA

              *  * *          *  *        *        *   **    *   *  **       
     532829 ACATTCCCTTTCATAGAGTAGGTTTGAAACCCTCTTTTTATAGTGTCTGCAAGCGGGCATTTGGA
         66 ACCTTTCTTTTCATAGAGCAGTTTTGAAACACTCTTTTTGTAGAATCTGGAAGTGGATATTTGGA

                   *    * * **  **  **          * *  *
     532894 GCGCTTTCAGGCCTATGCTTAAAATAGGAAATATCTACCTAC
        131 GCGCTTTGAGG-ATTTCGTTGGAAACGGAAATATCTTCATAT

             *                    *    *  **          ** *        *    *     
     532936 AGAAACTAGACAGAAGCATTCTGAGAATCACGTTTGTGATGTGGGTACTCAACTAACAGTGTTGA
          1 AAAAACTAGACAGAAGCATTCTCAGAAACATCTTTGTGATGTTTGCACTCAACTCACAGAGTTGA

            *  *       *   *            *   *                *   *           
     533001 TCCATTCTTTTGATACAGCAGTTTTGAACCACACTTTTTGTAGAATCTGCAAGAGGATATTTGGA
         66 ACCTTTCTTTTCATAGAGCAGTTTTGAAACACTCTTTTTGTAGAATCTGGAAGTGGATATTTGGA

            **   *                     *   *      * *
     533066 TAGCTGTGAGGATTTCGTTGGAAACGGGAATGTCTTCAAAG
        131 GCGCTTTGAGGATTTCGTTGGAAACGGAAATATCTTCATAT

                *                         *   *            *    *            
     533107 AAAATCTAGACAGAAGCATTCTCAGAAACACCTTCGTGATGTTTGCAATCAAGTCACAGAGTTGA
          1 AAAAACTAGACAGAAGCATTCTCAGAAACATCTTTGTGATGTTTGCACTCAACTCACAGAGTTGA

                 * *             *  *           *      *             *       
     533172 ACCTTCCGTTTCATAGAGCAGGTTGGAAACACTCTTATTGTAGTATCTGGAAGTGGACATTTGGA
         66 ACCTTTCTTTTCATAGAGCAGTTTTGAAACACTCTTTTTGTAGAATCTGGAAGTGGATATTTGGA

                   *   ** ** *  *   *            **  
     533237 GCGCTTTCAGGCCTATGGTGAAAAAGGAAATATCTTCCCAT
        131 GCGCTTTGAGGATTTCGTTGGAAACGGAAATATCTTCATAT

                  * * *      **      *   * *   *     ** ** *      *    *     
     533278 AAAAACGACATAGAAGCTATCTCAGGAACTTGTTTATGATGCATCTAATCAACTAACAGTGTTGA
          1 AAAAACTAGACAGAAGCATTCTCAGAAACATCTTTGTGATGTTTGCACTCAACTCACAGAGTTGA

                  * ** * *                         * *       *               
     533343 ACCTTTGTACTGACAGAGCAG-TTTGAAACACTCTTTTTTTGGAATCTGCAAGTGGATATTTGGA
         66 ACCTTTCTTTTCATAGAGCAGTTTTGAAACACTCTTTTTGTAGAATCTGGAAGTGGATATTTGGA

            *                           *   *   *    
     533407 TCGCTTTGAGGATTTCGTTGGAAACGG-GAT-GC--AATAT
        131 GCGCTTTGAGGATTTCGTTGGAAACGGAAATATCTTCATAT

                **  *    *     *                 **  *   *  *    *         * 
     533444 AAAACGTACACAGCAGCATACTCAGAAA-ATACTTTGCCATATTTCCATTCAAGTCACAGAGTGG
          1 AAAAACTAGACAGAAGCATTCTCAGAAACAT-CTTTGTGATGTTTGCACTCAACTCACAGAGTTG

               *  * **            *                  *  *             *      
     533508 AACATTCCCATTCATAGAGCAGGTTTGAAACACTCTTTTTGGAGTATCTGGAAGTGGACATTTGG
         65 AACCTTTCTTTTCATAGAGCAGTTTTGAAACACTCTTTTTGTAGAATCTGGAAGTGGATATTTGG

                    ** * * ** *  *   *            **  
     533573 AGCGCTTTCTGAACTATGGTGAAAAAGGAAATATCTTCCAAT
        130 AGCGCTTTGAGGATTTCGTTGGAAACGGAAATATCTTCATAT

            *     *               *      * *          *  **      **  *  *    
     533615 GAAAACAAGACAGAAGCATTCTGAGAAACTTATTTGTGATGTGTGTCCTCAACAAACGGACTTGA
          1 AAAAACTAGACAGAAGCATTCTCAGAAACATCTTTGTGATGTTTGCACTCAACTCACAGAGTTGA

                   *      **  * *  *  *             *   *    * * *           
     533680 ACCTTTCGTTTCATGCAGTACTTCTGGAACACTCTTTTTGAAGATTCTGCATGCGGATATTTGGA
         66 ACCTTTCTTTTCATAGAGCAGTTTTGAAACACTCTTTTTGTAGAATCTGGAAGTGGATATTTGGA

            **                            **   *   * 
     533745 TAGCTTTGAGGATTTCGTTGGAAACGG---GCT-TACATGT
        131 GCGCTTTGAGGATTTCGTTGGAAACGGAAATATCTTCATAT

                 *       *               *        *   *    *    *       *    
     533782 AAAAATTAGACAGCAGCATTCTCAGAAACTTCTTTGTGGTGTCTGCATTCAAGTCACAGAATTGA
          1 AAAAACTAGACAGAAGCATTCTCAGAAACATCTTTGTGATGTTTGCACTCAACTCACAGAGTTGA

              * ** * **            *  * *      *       *             *       
     533847 ACTTCCCCTCACATAGAGCAGTTGTGCAGCACTCTATTTGTAGTATCTGGAAGTGGACATTTGGA
         66 ACCTTTCTTTTCATAGAGCAGTTTTGAAACACTCTTTTTGTAGAATCTGGAAGTGGATATTTGGA

             *         ** *    * *   *            **  
     533912 GGGCTTTGTAGCCTATC-TGGAAAAAGGAAATATCTTCCCAT
        131 GCGCTTTG-AGGATTTCGTTGGAAACGGAAATATCTTCATAT

            *  ** *   *     * *            *   *  *   * **        *  * * *   
     533953 GAATGCGAGATAGAAGTAATCTCAGAAACATGTTTATGCTGTATCTACTCAACTAACTGTGCTGA
          1 AAAAACTAGACAGAAGCATTCTCAGAAACATCTTTGTGATGTTTGCACTCAACTCACAGAGTTGA

              *     *  *               *        *  * *       *               
     534018 ACATTTCTATTTATAGAGCAGTTTTGAGACACTCTTCTTTTGGAATCTGCAAGTGGATATTTGGA
         66 ACCTTTCTTTTCATAGAGCAGTTTTGAAACACTCTTTTTGTAGAATCTGGAAGTGGATATTTGGA

            ** *                        *     *      
     534083 TAGATTTGAGGATTTCGTTGGAAACGG-GAT-T-AT-ATAT
        131 GCGCTTTGAGGATTTCGTTGGAAACGGAAATATCTTCATAT

            *    *       *               *                 **  *   *         
     534120 CAAAAGTAGACAGCAGCATTCTCAGAAACTTCTTTGTGATGTTTGCATCCAGCTCTCAGAGTTGA
          1 AAAAACTAGACAGAAGCATTCTCAGAAACATCTTTGTGATGTTTGCACTCAACTCACAGAGTTGA

              *  * *          *  *        *        *   **    *   *  **       
     534185 ACATTCCCTTTCATAGAGTAGGTTTGAAACCCTCTTTTTATAGTGTCTGCAAGCGGGCATTTGGA
         66 ACCTTTCTTTTCATAGAGCAGTTTTGAAACACTCTTTTTGTAGAATCTGGAAGTGGATATTTGGA

                   *    * * **  **  **          * *  *
     534250 GCGCTTTCAGGCCTATGCTTAAAATAGGAAATATCTACCTAC
        131 GCGCTTTGAGG-ATTTCGTTGGAAACGGAAATATCTTCATAT

             *                    *    *  **          ** *        *    *     
     534292 AGAAACTAGACAGAAGCATTCTGAGAATCACGTTTGTGATGTGGGTACTCAACTAACAGTGTTGA
          1 AAAAACTAGACAGAAGCATTCTCAGAAACATCTTTGTGATGTTTGCACTCAACTCACAGAGTTGA

            *          *   *            *   *                *   *           
     534357 TCCTTTCTTTTGATACAGCAGTTTTGAACCACACTTTTTGTAGAATCTGCAAGAGGATATTTGGA
         66 ACCTTTCTTTTCATAGAGCAGTTTTGAAACACTCTTTTTGTAGAATCTGGAAGTGGATATTTGGA

            **   *                     *   *      * *
     534422 TAGCTGTGAGGATTTCGTTGGAAACGGGAATGTCTTCAAAG
        131 GCGCTTTGAGGATTTCGTTGGAAACGGAAATATCTTCATAT

                *                         *   *            *    *            
     534463 AAAATCTAGACAGAAGCATTCTCAGAAACACCTTCGTGATGTTTGCAATCAAGTCACAGAGTTGA
          1 AAAAACTAGACAGAAGCATTCTCAGAAACATCTTTGTGATGTTTGCACTCAACTCACAGAGTTGA

                 * *             *  *           *      *             *       
     534528 ACCTTCCGTTTCATAGAGCAGGTTGGAAACACTCTTATTGTAGTATCTGGAAGTGGACATTTGGA
         66 ACCTTTCTTTTCATAGAGCAGTTTTGAAACACTCTTTTTGTAGAATCTGGAAGTGGATATTTGGA

                   *   ** ** *  *   *            **  
     534593 GCGCTTTCAGGCCTATGGTGAAAAAGGAAATATCTTCCCAT
        131 GCGCTTTGAGGATTTCGTTGGAAACGGAAATATCTTCATAT

                  * * *      **      *   * *   *     ** ** *      *    *     
     534634 AAAAACGACATAGAAGCTATCTCAGGAACTTGTTTATGATGCATCTAATCAACTAACAGTGTTGA
          1 AAAAACTAGACAGAAGCATTCTCAGAAACATCTTTGTGATGTTTGCACTCAACTCACAGAGTTGA

                  * ** * *                         * *       *               
     534699 ACCTTTGTACTGACAGAGCAG-TTTGAAACACTCTTTTTTTGGAATCTGCAAGTGGATATTTGGA
         66 ACCTTTCTTTTCATAGAGCAGTTTTGAAACACTCTTTTTGTAGAATCTGGAAGTGGATATTTGGA

            *                           *   *   *    
     534763 TCGCTTTGAGGATTTCGTTGGAAACGG-GAT-GC--AATAT
        131 GCGCTTTGAGGATTTCGTTGGAAACGGAAATATCTTCATAT

                **  *    *     *                 **  *   *  *    *         * 
     534800 AAAACGTACACAGCAGCATACTCAGAAA-ATACTTTGCCATATTTCCATTCAAGTCACAGAGTGG
          1 AAAAACTAGACAGAAGCATTCTCAGAAACAT-CTTTGTGATGTTTGCACTCAACTCACAGAGTTG

               *  * **            *                  *  *             *      
     534864 AACATTCCCATTCATAGAGCAGGTTTGAAACACTCTTTTTGGAGTATCTGGAAGTGGACATTTGG
         65 AACCTTTCTTTTCATAGAGCAGTTTTGAAACACTCTTTTTGTAGAATCTGGAAGTGGATATTTGG

                    ** * * ** *  *   *            **  
     534929 AGCGCTTTCTGAACTATGGTGAAAAAGGAAATATCTTCCAAT
        130 AGCGCTTTGAGGATTTCGTTGGAAACGGAAATATCTTCATAT

            *     *               *      * *          *  **      **  *  *    
     534971 GAAAACAAGACAGAAGCATTCTGAGAAACTTATTTGTGATGTGTGTCCTCAACAAACGGACTTGA
          1 AAAAACTAGACAGAAGCATTCTCAGAAACATCTTTGTGATGTTTGCACTCAACTCACAGAGTTGA

                   *      **  * *                    *   *    * * *          
     535036 ACCTTTCGTTTCATGCAGTACTTCTTG-AACACTCTTTTTGAAGATTCTGCATGCGGATATTTGG
         66 ACCTTTCTTTTCATAGAGCAGTT-TTGAAACACTCTTTTTGTAGAATCTGGAAGTGGATATTTGG

             **                   *        **   *   * 
     535100 ATAGCTTTGAGGATTTCGTTGGCAACGG---GCT-TACATGT
        130 AGCGCTTTGAGGATTTCGTTGGAAACGGAAATATCTTCATAT

                 *       *               *        *   *    *    *       *    
     535138 AAAAATTAGACAGCAGCATTCTCAGAAACTTCTTTGTGGTGTCTGCATTCAAGTCACAGAATTGA
          1 AAAAACTAGACAGAAGCATTCTCAGAAACATCTTTGTGATGTTTGCACTCAACTCACAGAGTTGA

              * ** * **            *  * *      *       *             *       
     535203 ACTTCCCCTCACATAGAGCAGTTGTGCAGCACTCTATTTGTAGTATCTGGAAGTGGACATTTGGA
         66 ACCTTTCTTTTCATAGAGCAGTTTTGAAACACTCTTTTTGTAGAATCTGGAAGTGGATATTTGGA

             *         ** *    * *   *            **  
     535268 GGGCTTTGTAGCCTATC-TGGAAAAAGGAAATATCTTCCCAT
        131 GCGCTTTG-AGGATTTCGTTGGAAACGGAAATATCTTCATAT

            *  ** *   *     * *            *   *  *   * **        *  * * *   
     535309 GAATGCGAGATAGAAGTAATCTCAGAAACATGTTTATGCTGTATCTACTCAACTAACTGTGCTGA
          1 AAAAACTAGACAGAAGCATTCTCAGAAACATCTTTGTGATGTTTGCACTCAACTCACAGAGTTGA

              *     *  *               *        *  * *       *               
     535374 ACATTTCTATTGATAGAGCAGTTTTGAGACACTCTTCTTTTGGAATCTGCAAGTGGATATTTGGA
         66 ACCTTTCTTTTCATAGAGCAGTTTTGAAACACTCTTTTTGTAGAATCTGGAAGTGGATATTTGGA

            ** *                        *     *      
     535439 TAGATTTGAGGATTTCGTTGGAAACGG-GAT-T-AT-ATAT
        131 GCGCTTTGAGGATTTCGTTGGAAACGGAAATATCTTCATAT

            *    *       *               *                 **  *   *         
     535476 CAAAAGTAGACAGCAGCATTCTCAGAAACTTCTTTGTGATGTTTGCATCCAGCTCTCAGAGTTGA
          1 AAAAACTAGACAGAAGCATTCTCAGAAACATCTTTGTGATGTTTGCACTCAACTCACAGAGTTGA

              *  * *          *  *        *        *   **    *   *  **       
     535541 ACATTCCCTTTCATAGAGTAGGTTTGAAACCCTCTTTTTATAGTGTCTGCAAGCGGGCATTTGGA
         66 ACCTTTCTTTTCATAGAGCAGTTTTGAAACACTCTTTTTGTAGAATCTGGAAGTGGATATTTGGA

                   *    * * **  **  **          * *  *
     535606 GCGCTTTCAGGCCTATGCTTAAAATAGGAAATATCTACCTAC
        131 GCGCTTTGAGG-ATTTCGTTGGAAACGGAAATATCTTCATAT

             *                    *    *  **          ** *        *    *     
     535648 AGAAACTAGACAGAAGCATTCTGAGAATCACGTTTGTGATGTGGGTACTCAACTAACAGTGTTGA
          1 AAAAACTAGACAGAAGCATTCTCAGAAACATCTTTGTGATGTTTGCACTCAACTCACAGAGTTGA

            *  *       *   *            *   *                *   *           
     535713 TCCATTCTTTTGATACAGCAGTTTTGAACCACACTTTTTGTAGAATCTGCAAGAGGATATTTGGA
         66 ACCTTTCTTTTCATAGAGCAGTTTTGAAACACTCTTTTTGTAGAATCTGGAAGTGGATATTTGGA

            **   *                     *   *      * *
     535778 TAGCTGTGAGGATTTCGTTGGAAACGGGAATGTCTTCAAAG
        131 GCGCTTTGAGGATTTCGTTGGAAACGGAAATATCTTCATAT

                *                         *   *            *    *            
     535819 AAAATCTAGACAGAAGCATTCTCAGAAACACCTTCGTGATGTTTGCAATCAAGTCACAGAGTTGA
          1 AAAAACTAGACAGAAGCATTCTCAGAAACATCTTTGTGATGTTTGCACTCAACTCACAGAGTTGA

                 * *             *  *           *      *             *       
     535884 ACCTTCCGTTTCATAGAGCAGGTTGGAAACACTCTTATTGTAGTATCTGGAAGTGGACATTTGGA
         66 ACCTTTCTTTTCATAGAGCAGTTTTGAAACACTCTTTTTGTAGAATCTGGAAGTGGATATTTGGA

                   *   ** ** *  *   *            **  
     535949 GCGCTTTCAGGCCTATGGTGAAAAAGGAAATATCTTCCCAT
        131 GCGCTTTGAGGATTTCGTTGGAAACGGAAATATCTTCATAT

                  * * *      **      *   * *   *     ** ** *      *    *     
     535990 AAAAACGACATAGAAGCTATCTCAGGAACTTGTTTATGATGCATCTAATCAACTAACAGTGTTGA
          1 AAAAACTAGACAGAAGCATTCTCAGAAACATCTTTGTGATGTTTGCACTCAACTCACAGAGTTGA

                  * ** * *                         * *       *               
     536055 ACCTTTGTACTGACAGAGCAG-TTTGAAACACTCTTTTTTTGGAATCTGCAAGTGGATATTTGGA
         66 ACCTTTCTTTTCATAGAGCAGTTTTGAAACACTCTTTTTGTAGAATCTGGAAGTGGATATTTGGA

            *                           *   *   *    
     536119 TCGCTTTGAGGATTTCGTTGGAAACGG-GAT-GC--AATAT
        131 GCGCTTTGAGGATTTCGTTGGAAACGGAAATATCTTCATAT

                **  *    *     *                 **  *   *  *    *   *     * 
     536156 AAAACGTACACAGCAGCATACTCAGAAA-ATACTTTGCCATATTTCCATTCAAGTCAGAGAGTGG
          1 AAAAACTAGACAGAAGCATTCTCAGAAACAT-CTTTGTGATGTTTGCACTCAACTCACAGAGTTG

               *  * **            *                  *  *             *      
     536220 AACATTCCCATTCATAGAGCAGGTTTGAAACACTCTTTTTGGAGTATCTGGAAGTGGACATTTGG
         65 AACCTTTCTTTTCATAGAGCAGTTTTGAAACACTCTTTTTGTAGAATCTGGAAGTGGATATTTGG

                    ** * * ** *  *   *            **  
     536285 AGCGCTTTCTGAACTATGGTGAAAAAGGAAATATCTTCCAAT
        130 AGCGCTTTGAGGATTTCGTTGGAAACGGAAATATCTTCATAT

            *     *               *      * *          *  **      **  *  *    
     536327 GAAAACAAGACAGAAGCATTCTGAGAAACTTATTTGTGATGTGTGTCCTCAACAAACGGACTTGA
          1 AAAAACTAGACAGAAGCATTCTCAGAAACATCTTTGTGATGTTTGCACTCAACTCACAGAGTTGA

                   *      **  * *  *  *             *   *    * * *           
     536392 ACCTTTCGTTTCATGCAGTACTTCTGGAACACTCTTTTTGAAGATTCTGCATGCGGATATTTGGA
         66 ACCTTTCTTTTCATAGAGCAGTTTTGAAACACTCTTTTTGTAGAATCTGGAAGTGGATATTTGGA

            **                            **   *   * 
     536457 TAGCTTTGAGGATTTCGTTGGAAACGG---GCT-TACATGT
        131 GCGCTTTGAGGATTTCGTTGGAAACGGAAATATCTTCATAT

                 *       *               *        *   *    *    *       *    
     536494 AAAAATTAGACAGCAGCATTCTCAGAAACTTCTTTGTGGTGTCTGCATTCAAGTCACAGAATTGA
          1 AAAAACTAGACAGAAGCATTCTCAGAAACATCTTTGTGATGTTTGCACTCAACTCACAGAGTTGA

              * ** * **            *  * *      *       *             *       
     536559 ACTTCCCCTCACATAGAGCAGTTGTGCAGCACTCTATTTGTAGTATCTGGAAGTGGACATTTGGA
         66 ACCTTTCTTTTCATAGAGCAGTTTTGAAACACTCTTTTTGTAGAATCTGGAAGTGGATATTTGGA

             *         ** *    * *   *            **  
     536624 GGGCTTTGTAGCCTATC-TGGAAAAAGGAAATATCTTCCCAT
        131 GCGCTTTG-AGGATTTCGTTGGAAACGGAAATATCTTCATAT

            *  ** *   *     * *            *   *  *   * **        *  * * *   
     536665 GAATGCGAGATAGAAGTAATCTCAGAAACATGTTTATGCTGTATCTACTCAACTAACTGTGCTGA
          1 AAAAACTAGACAGAAGCATTCTCAGAAACATCTTTGTGATGTTTGCACTCAACTCACAGAGTTGA

              *     *  *               *        *  * *       *               
     536730 ACATTTCTATTTATAGAGCAGTTTTGAGACACTCTTCTTTTGGAATCTGCAAGTGGATATTTGGA
         66 ACCTTTCTTTTCATAGAGCAGTTTTGAAACACTCTTTTTGTAGAATCTGGAAGTGGATATTTGGA

            ** *                        *     *      
     536795 TAGATTTGAGGATTTCGTTGGAAACGG-GAT-T-AT-ATAT
        131 GCGCTTTGAGGATTTCGTTGGAAACGGAAATATCTTCATAT

            *    *       *               *                 **  *   *         
     536832 CAAAAGTAGACAGCAGCATTCTCAGAAACTTCTTTGTGATGTTTGCATCCAGCTCTCAGAGTTGA
          1 AAAAACTAGACAGAAGCATTCTCAGAAACATCTTTGTGATGTTTGCACTCAACTCACAGAGTTGA

              *  * *          *  *        *        *   **        *  **       
     536897 ACATTCCCTTTCATAGAGTAGGTTTGAAACCCTCTTTTTATAGTGTCTGGAAGCGGGCATTTGGA
         66 ACCTTTCTTTTCATAGAGCAGTTTTGAAACACTCTTTTTGTAGAATCTGGAAGTGGATATTTGGA

                   *    * * **  **  **          * *  *
     536962 GCGCTTTCAGGCCTATGCTTAAAATAGGAAATATCTACCTAC
        131 GCGCTTTGAGG-ATTTCGTTGGAAACGGAAATATCTTCATAT

             *                    *    *  **          ** *        *    *     
     537004 AGAAACTAGACAGAAGCATTCTGAGAATCACGTTTGTGATGTGGGTACTCAACTAACAGTGTTGA
          1 AAAAACTAGACAGAAGCATTCTCAGAAACATCTTTGTGATGTTTGCACTCAACTCACAGAGTTGA

            *  *       *   *            *   *                *               
     537069 TCCATTCTTTTGATACAGCAGTTTTGAACCACACTTTTTGTAGAATCTGCAAGTGGATATTTGGA
         66 ACCTTTCTTTTCATAGAGCAGTTTTGAAACACTCTTTTTGTAGAATCTGGAAGTGGATATTTGGA

            **   *                     *   *      * *
     537134 TAGCTGTGAGGATTTCGTTGGAAACGGGAATGTCTTCAAAG
        131 GCGCTTTGAGGATTTCGTTGGAAACGGAAATATCTTCATAT

                *                         *   *            *    *            
     537175 AAAATCTAGACAGAAGCATTCTCAGAAACACCTTCGTGATGTTTGCAATCAAGTCACAGAGTTGA
          1 AAAAACTAGACAGAAGCATTCTCAGAAACATCTTTGTGATGTTTGCACTCAACTCACAGAGTTGA

                 * *             *  *           *      *             *       
     537240 ACCTTCCGTTTCATAGAGCAGGTTGGAAACACTCTTATTGTAGTATCTGGAAGTGGACATTTGGA
         66 ACCTTTCTTTTCATAGAGCAGTTTTGAAACACTCTTTTTGTAGAATCTGGAAGTGGATATTTGGA

                   *   ** ** *  *   *            **  
     537305 GCGCTTTCAGGCCTATGGTGAAAAAGGAAATATCTTCCCAT
        131 GCGCTTTGAGGATTTCGTTGGAAACGGAAATATCTTCATAT

                  * * *      **      *   * *   *     ** ** *      *    *     
     537346 AAAAACGACATAGAAGCTATCTCAGGAACTTGTTTATGATGCATCTAATCAACTAACAGTGTTGA
          1 AAAAACTAGACAGAAGCATTCTCAGAAACATCTTTGTGATGTTTGCACTCAACTCACAGAGTTGA

                  * ** * *    *                    * *       *               
     537411 ACCTTTGTACTGACAGAGGAG-TTTGAAACACTCTTTTTTTGGAATCTGCAAGTGGATATTTGGA
         66 ACCTTTCTTTTCATAGAGCAGTTTTGAAACACTCTTTTTGTAGAATCTGGAAGTGGATATTTGGA

            *                           *   *   *    
     537475 TCGCTTTGAGGATTTCGTTGGAAACGG-GAT-GC--AATAT
        131 GCGCTTTGAGGATTTCGTTGGAAACGGAAATATCTTCATAT

                **  *    *     *                 **  *   *  *    *   *     * 
     537512 AAAACGTACACAGCAGCATACTCAGAAA-ATACTTTGCCATATTTCCATTCAAGTCAGAGAGTGG
          1 AAAAACTAGACAGAAGCATTCTCAGAAACAT-CTTTGTGATGTTTGCACTCAACTCACAGAGTTG

               *  * **            *  *               *  *             *      
     537576 AACATTCCCATTCATAGAGCAGGTTGGAAACACTCTTTTTGGAGTATCTGGAAGTGGACATTTGG
         65 AACCTTTCTTTTCATAGAGCAGTTTTGAAACACTCTTTTTGTAGAATCTGGAAGTGGATATTTGG

                    ** * * ** *  *   *            **  
     537641 AGCGCTTTCTGAACTATGGTGAAAAAGGAAATATCTTCCAAT
        130 AGCGCTTTGAGGATTTCGTTGGAAACGGAAATATCTTCATAT

            *     *               *      * *          *  **      **  *  *    
     537683 GAAAACAAGACAGAAGCATTCTGAGAAACTTATTTGTGATGTGTGTCCTCAACAAACGGACTTGA
          1 AAAAACTAGACAGAAGCATTCTCAGAAACATCTTTGTGATGTTTGCACTCAACTCACAGAGTTGA

                   *      **  * *  *  *             *   *    * * *           
     537748 ACCTTTCGTTTCATGCAGTACTTCTGGAACACTCTTTTTGAAGATTCTGCATGCGGATATTTGGA
         66 ACCTTTCTTTTCATAGAGCAGTTTTGAAACACTCTTTTTGTAGAATCTGGAAGTGGATATTTGGA

            **                            **   *   * 
     537813 TAGCTTTGAGGATTTCGTTGGAAACGG---GCT-TACATGT
        131 GCGCTTTGAGGATTTCGTTGGAAACGGAAATATCTTCATAT

                 *       *               *        *   *    *    *       *    
     537850 AAAAATTAGACAGCAGCATTCTCAGAAACTTCTTTGTGGTGTCTGCATTCAAGTCACAGAATTGA
          1 AAAAACTAGACAGAAGCATTCTCAGAAACATCTTTGTGATGTTTGCACTCAACTCACAGAGTTGA

              * ** * **            *  * *      *       *             *       
     537915 ACTTCCCCTCACATAGAGCAGTTGTGCAGCACTCTATTTGTAGTATCTGGAAGTGGACATTTGGA
         66 ACCTTTCTTTTCATAGAGCAGTTTTGAAACACTCTTTTTGTAGAATCTGGAAGTGGATATTTGGA

             *         ** *    * *   *            **  
     537980 GGGCTTTGTAGCCTATC-TGGAAAAAGGAAATATCTTCCCAT
        131 GCGCTTTG-AGGATTTCGTTGGAAACGGAAATATCTTCATAT

            *  ** *   *     * *            *   *  *   * **        *  * * *   
     538021 GAATGCGAGATAGAAGTAATCTCAGAAACATGTTTATGCTGTATCTACTCAACTAACTGTGCTGA
          1 AAAAACTAGACAGAAGCATTCTCAGAAACATCTTTGTGATGTTTGCACTCAACTCACAGAGTTGA

              *     *  *               *        *  * *       *               
     538086 ACATTTCTATTGATAGAGCAGTTTTGAGACACTCTTCTTTTGGAATCTGCAAGTGGATATTTGGA
         66 ACCTTTCTTTTCATAGAGCAGTTTTGAAACACTCTTTTTGTAGAATCTGGAAGTGGATATTTGGA

            ** *                        *     *      
     538151 TAGATTTGAGGATTTCGTTGGAAACGG-GAT-T-AT-ATAT
        131 GCGCTTTGAGGATTTCGTTGGAAACGGAAATATCTTCATAT

            *    *       *               *                 **  *   *         
     538188 CAAAAGTAGACAGCAGCATTCTCAGAAACTTCTTTGTGATGTTTGCATCCAGCTCTCAGAGTTGA
          1 AAAAACTAGACAGAAGCATTCTCAGAAACATCTTTGTGATGTTTGCACTCAACTCACAGAGTTGA

              *  * *          *  *        *        *   **    *   *  **       
     538253 ACATTCCCTTTCATAGAGTAGGTTTGAAACCCTCTTTTTATAGTGTCTGCAAGCGGGCATTTGGA
         66 ACCTTTCTTTTCATAGAGCAGTTTTGAAACACTCTTTTTGTAGAATCTGGAAGTGGATATTTGGA

                   *    * * **  **  **          * *  *
     538318 GCGCTTTCAGGCCTATGCTTAAAATAGGAAATATCTACCTAC
        131 GCGCTTTGAGG-ATTTCGTTGGAAACGGAAATATCTTCATAT

             *                    *    *  **          ** *        *    *     
     538360 AGAAACTAGACAGAAGCATTCTGAGAATCACGTTTGTGATGTGGGTACTCAACTAACAGTGTTGA
          1 AAAAACTAGACAGAAGCATTCTCAGAAACATCTTTGTGATGTTTGCACTCAACTCACAGAGTTGA

            *  *       *   *            *   *                *               
     538425 TCCATTCTTTTGATACAGCAGTTTTGAACCACACTTTTTGTAGAATCTGCAAGTGGATATTTGGA
         66 ACCTTTCTTTTCATAGAGCAGTTTTGAAACACTCTTTTTGTAGAATCTGGAAGTGGATATTTGGA

            **   *                     *   *      * *
     538490 TAGCTGTGAGGATTTCGTTGGAAACGGGAATGTCTTCAAAG
        131 GCGCTTTGAGGATTTCGTTGGAAACGGAAATATCTTCATAT

                *                         *   *            *    *            
     538531 AAAATCTAGACAGAAGCATTCTCAGAAACACCTTCGTGATGTTTGCAATCAAGTCACAGAGTTGA
          1 AAAAACTAGACAGAAGCATTCTCAGAAACATCTTTGTGATGTTTGCACTCAACTCACAGAGTTGA

                 * *             *  *           *      *             *       
     538596 ACCTTCCGTTTCATAGAGCAGGTTGGAAACACTCTTATTGTAGTATCTGGAAGTGGACATTTGGA
         66 ACCTTTCTTTTCATAGAGCAGTTTTGAAACACTCTTTTTGTAGAATCTGGAAGTGGATATTTGGA

                   *   ** ** *  *   *            **  
     538661 GCGCTTTCAGGCCTATGGTGAAAAAGGAAATATCTTCCCAT
        131 GCGCTTTGAGGATTTCGTTGGAAACGGAAATATCTTCATAT

                  * * *      **      *   * *   *     ** ** *      *    *     
     538702 AAAAACGACATAGAAGCTATCTCAGGAACTTGTTTATGATGCATCTAATCAACTAACAGTGTTGA
          1 AAAAACTAGACAGAAGCATTCTCAGAAACATCTTTGTGATGTTTGCACTCAACTCACAGAGTTGA

                  * ** * *    *                    * *       *               
     538767 ACCTTTGTACTGACAGAGGAG-TTTGAAACACTCTTTTTTTGGAATCTGCAAGTGGATATTTGGA
         66 ACCTTTCTTTTCATAGAGCAGTTTTGAAACACTCTTTTTGTAGAATCTGGAAGTGGATATTTGGA

            *                       *   *   *   *    
     538831 TCGCTTTGAGGATTTCGTTGGAAAAGG-GAT-GC--AATAT
        131 GCGCTTTGAGGATTTCGTTGGAAACGGAAATATCTTCATAT

                **  *    *     *                 **  *   *  *    *   *     * 
     538868 AAAACGTACACAGCAGCATACTCAGAAA-ATACTTTGCCATATTTCCATTCAAGTCAGAGAGTGG
          1 AAAAACTAGACAGAAGCATTCTCAGAAACAT-CTTTGTGATGTTTGCACTCAACTCACAGAGTTG

               *  * **            *  *               *  *             *      
     538932 AACATTCCCATTCATAGAGCAGGTTGGAAACACTCTTTTTGGAGTATCTGGAAGTGGACATTTGG
         65 AACCTTTCTTTTCATAGAGCAGTTTTGAAACACTCTTTTTGTAGAATCTGGAAGTGGATATTTGG

                    ** * * ** *  *   *            **  
     538997 AGCGCTTTCTGAACTATGGTGAAAAAGGAAATATCTTCCAAT
        130 AGCGCTTTGAGGATTTCGTTGGAAACGGAAATATCTTCATAT

            *     *               *      * *          *  **      **  *  *    
     539039 GAAAACAAGACAGAAGCATTCTGAGAAACTTATTTGTGATGTGTGTCCTCAACAAACGGACTTGA
          1 AAAAACTAGACAGAAGCATTCTCAGAAACATCTTTGTGATGTTTGCACTCAACTCACAGAGTTGA

                   *      **  * *  *  *             *   *    * * *           
     539104 ACCTTTCGTTTCATGCAGTACTTCTGGAACACTCTTTTTGAAGATTCTGCATGCGGATATTTGGA
         66 ACCTTTCTTTTCATAGAGCAGTTTTGAAACACTCTTTTTGTAGAATCTGGAAGTGGATATTTGGA

            **                            **   *   * 
     539169 TAGCTTTGAGGATTTCGTTGGAAACGG---GCT-TACATGT
        131 GCGCTTTGAGGATTTCGTTGGAAACGGAAATATCTTCATAT

                 *       *               *        *   *    *    *       *    
     539206 AAAAATTAGACAGCAGCATTCTCAGAAACTTCTTTGTGGTGTCTGCATTCAAGTCACAGAATTGA
          1 AAAAACTAGACAGAAGCATTCTCAGAAACATCTTTGTGATGTTTGCACTCAACTCACAGAGTTGA

              * ** * **            *  * *      *       *             *       
     539271 ACTTCCCCTCACATAGAGCAGTTGTGCAGCACTCTATTTGTAGTATCTGGAAGTGGACATTTGGA
         66 ACCTTTCTTTTCATAGAGCAGTTTTGAAACACTCTTTTTGTAGAATCTGGAAGTGGATATTTGGA

             *         ** *    * *   *            **  
     539336 GGGCTTTGTAGCCTATC-TGGAAAAAGGAAATATCTTCCCAT
        131 GCGCTTTG-AGGATTTCGTTGGAAACGGAAATATCTTCATAT

            *  ** *   *     * *            *   *  *   * **        *  * * *   
     539377 GAATGCGAGATAGAAGTAATCTCAGAAACATGTTTATGCTGTATCTACTCAACTAACTGTGCTGA
          1 AAAAACTAGACAGAAGCATTCTCAGAAACATCTTTGTGATGTTTGCACTCAACTCACAGAGTTGA

              *     *  *               *        *  * *       *               
     539442 ACATTTCTATTGATAGAGCAGTTTTGAGACACTCTTCTTTTGGAATCTGCAAGTGGATATTTGGA
         66 ACCTTTCTTTTCATAGAGCAGTTTTGAAACACTCTTTTTGTAGAATCTGGAAGTGGATATTTGGA

            ** *                        *     *      
     539507 TAGATTTGAGGATTTCGTTGGAAACGG-GAT-T-AT-ATAT
        131 GCGCTTTGAGGATTTCGTTGGAAACGGAAATATCTTCATAT

                 *       *               *                 **  *   *         
     539544 AAAAAGTAGACAGCAGCATTCTCAGAAACTTCTTTGTGATGTTTGCATCCAGCTCTCAGAGTTGA
          1 AAAAACTAGACAGAAGCATTCTCAGAAACATCTTTGTGATGTTTGCACTCAACTCACAGAGTTGA

              *  * *          *  *        *        *   **    *   *  **       
     539609 ACATTCCCTTTCATAGAGTAGGTTTGAAACCCTCTTTTTATAGTGTCTGCAAGCGGGCATTTGGA
         66 ACCTTTCTTTTCATAGAGCAGTTTTGAAACACTCTTTTTGTAGAATCTGGAAGTGGATATTTGGA

                   *    * * **  **  **          * *  *
     539674 GCGCTTTCAGGCCTATGCTTAAAATAGGAAATATCTACCTAC
        131 GCGCTTTGAGG-ATTTCGTTGGAAACGGAAATATCTTCATAT

             *                    *    *  **          ** *        *    *     
     539716 AGAAACTAGACAGAAGCATTCTGAGAATCACGTTTGTGATGTGGGTACTCAACTAACAGTGTTGA
          1 AAAAACTAGACAGAAGCATTCTCAGAAACATCTTTGTGATGTTTGCACTCAACTCACAGAGTTGA

            *  *       *   *            *   *                *   *           
     539781 TCCATTCTTTTGATACAGCAGTTTTGAACCACACTTTTTGTAGAATCTGCAAGAGGATATTTGGA
         66 ACCTTTCTTTTCATAGAGCAGTTTTGAAACACTCTTTTTGTAGAATCTGGAAGTGGATATTTGGA

            **   *                     *   *      * *
     539846 TAGCTGTGAGGATTTCGTTGGAAACGGGAATGTCTTCAAAG
        131 GCGCTTTGAGGATTTCGTTGGAAACGGAAATATCTTCATAT

                *                         *   *            *    *            
     539887 AAAATCTAGACAGAAGCATTCTCAGAAACACCTTCGTGATGTTTGCAATCAAGTCACAGAGTTGA
          1 AAAAACTAGACAGAAGCATTCTCAGAAACATCTTTGTGATGTTTGCACTCAACTCACAGAGTTGA

                 * *             *  *           *      *             *       
     539952 ACCTTCCGTTTCATAGAGCAGGTTGGAAACACTCTTATTGTAGTATCTGGAAGTGGACATTTGGA
         66 ACCTTTCTTTTCATAGAGCAGTTTTGAAACACTCTTTTTGTAGAATCTGGAAGTGGATATTTGGA

                   *   ** ** *  *   *            **  
     540017 GCGCTTTCAGGCCTATGGTGAAAAAGGAAATATCTTCCCAT
        131 GCGCTTTGAGGATTTCGTTGGAAACGGAAATATCTTCATAT

                  * * *      **      *   * *   *     ** ** *      *    *     
     540058 AAAAACGACATAGAAGCTATCTCAGGAACTTGTTTATGATGCATCTAATCAACTAACAGTGTTGA
          1 AAAAACTAGACAGAAGCATTCTCAGAAACATCTTTGTGATGTTTGCACTCAACTCACAGAGTTGA

                  * ** * *                         * *       *               
     540123 ACCTTTGTACTGACAGAGCAG-TTTGAAACACTCTTTTTTTGGAATCTGCAAGTGGATATTTGGA
         66 ACCTTTCTTTTCATAGAGCAGTTTTGAAACACTCTTTTTGTAGAATCTGGAAGTGGATATTTGGA

            *                           *   *   *    
     540187 TCGCTTTGAGGATTTCGTTGGAAACGG-GAT-GC--AATAT
        131 GCGCTTTGAGGATTTCGTTGGAAACGGAAATATCTTCATAT

                **  *    *     *                 **  *   *  *    *         * 
     540224 AAAACGTACACAGCAGCATACTCAGAAA-ATACTTTGCCATATTTCCATTCAAGTCACAGAGTGG
          1 AAAAACTAGACAGAAGCATTCTCAGAAACAT-CTTTGTGATGTTTGCACTCAACTCACAGAGTTG

               *  * **            *                  *  *             *      
     540288 AACATTCCCATTCATAGAGCAGGTTTGAAACACTCTTTTTGGAGTATCTGGAAGTGGACATTTGG
         65 AACCTTTCTTTTCATAGAGCAGTTTTGAAACACTCTTTTTGTAGAATCTGGAAGTGGATATTTGG

                    ** * * ** *  *   *            **  
     540353 AGCGCTTTCTGAACTATGGTGAAAAAGGAAATATCTTCCAAT
        130 AGCGCTTTGAGGATTTCGTTGGAAACGGAAATATCTTCATAT

            *     *               *      * *          *  **      **  *  *    
     540395 GAAAACAAGACAGAAGCATTCTGAGAAACTTATTTGTGATGTGTGTCCTCAACAAACGGACTTGA
          1 AAAAACTAGACAGAAGCATTCTCAGAAACATCTTTGTGATGTTTGCACTCAACTCACAGAGTTGA

                   *      **  * *  *  *             *   *    * * *           
     540460 ACCTTTCGTTTCATGCAGTACTTCTGGAACACTCTTTTTGAAGATTCTGCATGCGGATATTTGGA
         66 ACCTTTCTTTTCATAGAGCAGTTTTGAAACACTCTTTTTGTAGAATCTGGAAGTGGATATTTGGA

            **                            **   *   * 
     540525 TAGCTTTGAGGATTTCGTTGGAAACGG---GCT-TACATGT
        131 GCGCTTTGAGGATTTCGTTGGAAACGGAAATATCTTCATAT

                 *       *               *        *   *    *    *       *    
     540562 AAAAATTAGACAGCAGCATTCTCAGAAACTTCTTTGTGGTGTCTGCATTCAAGTCACAGAATTGA
          1 AAAAACTAGACAGAAGCATTCTCAGAAACATCTTTGTGATGTTTGCACTCAACTCACAGAGTTGA

              * ** * **            *  * *      *       *    *        *       
     540627 ACTTCCCCTCACATAGAGCAGTTGTGCAGCACTCTATTTGTAGTATCTCGAAGTGGACATTTGGA
         66 ACCTTTCTTTTCATAGAGCAGTTTTGAAACACTCTTTTTGTAGAATCTGGAAGTGGATATTTGGA

             *         ** *    * *   *            **  
     540692 GGGCTTTGTAGCCTATC-TGGAAAAAGGAAATATCTTCCCAT
        131 GCGCTTTG-AGGATTTCGTTGGAAACGGAAATATCTTCATAT

            *  ** *   *     * *          * *   *  *   * ***       *  * * *   
     540733 GAATGCGAGATAGAAGTAATCTCAGAAACGTGTTTATGCTGTATCTTCTCAACTAACTGTGCTGA
          1 AAAAACTAGACAGAAGCATTCTCAGAAACATCTTTGTGATGTTTGCACTCAACTCACAGAGTTGA

              *     *  *               *        *  * *       *               
     540798 ACATTTCTATTGATAGAGCAGTTTTGAGACACTCTTCTTTTGGAATCTGCAAGTGGATATTTGGA
         66 ACCTTTCTTTTCATAGAGCAGTTTTGAAACACTCTTTTTGTAGAATCTGGAAGTGGATATTTGGA

            ** *                        *     *      
     540863 TAGATTTGAGGATTTCGTTGGAAACGG-GAT-T-AT-ATAT
        131 GCGCTTTGAGGATTTCGTTGGAAACGGAAATATCTTCATAT

            *    *       *               *                 **  *   *         
     540900 CAAAAGTAGACAGCAGCATTCTCAGAAACTTCTTTGTGATGTTTGCATCCAGCTCTCAGAGTTGA
          1 AAAAACTAGACAGAAGCATTCTCAGAAACATCTTTGTGATGTTTGCACTCAACTCACAGAGTTGA

              *  * *          *  *        *        *   **        *  **       
     540965 ACATTCCCTTTCATAGAGTAGGTTTGAAACCCTCTTTTTATAGTGTCTGGAAGCGGGCATTTGGA
         66 ACCTTTCTTTTCATAGAGCAGTTTTGAAACACTCTTTTTGTAGAATCTGGAAGTGGATATTTGGA

                   *   ** **    *   *          * *   
     541030 GCGCTTTCAGGCCTATGTTGAAAAAGGAAATATCTACCTAT
        131 GCGCTTTGAGGATTTCGTTGGAAACGGAAATATCTTCATAT

             *                    *    *  **          ** *        *    *     
     541071 AGAAACTAGACAGAAGCATTCTGAGAATCACGTTTGTGATGTGGGTACTCAACTAACAGTGTTGA
          1 AAAAACTAGACAGAAGCATTCTCAGAAACATCTTTGTGATGTTTGCACTCAACTCACAGAGTTGA

            *  *       *   *            *   *                *               
     541136 TCCATTCTTTTGATACAGCAGTTTTGAACCACACTTTTTGTAGAATCTGCAAGTGGATATTTGGA
         66 ACCTTTCTTTTCATAGAGCAGTTTTGAAACACTCTTTTTGTAGAATCTGGAAGTGGATATTTGGA

            **   *                     *   *        *
     541201 TAGCTGTGAGGATTTCGTTGGAAACGGGAATGTCTTCATAG
        131 GCGCTTTGAGGATTTCGTTGGAAACGGAAATATCTTCATAT

                **                     * * **     *   *  **   *   *          
     541242 AAAATTTAGACAGAAGCATTCTCAGAACCTTGATTGTGGTGTGTGTTCTCCACTAACAGAGTTGA
          1 AAAAACTAGACAGAAGCATTCTCAGAAACATCTTTGTGATGTTTGCACTCAACTCACAGAGTTGA

                       * *   * *   *       *       *                         
     541307 ACCTTTCTTTTGACAGAACTGTTCTGAAACATTCTTTTTATAGAATCTGGAAGTGGATATTTGGA
         66 ACCTTTCTTTTCATAGAGCAGTTTTGAAACACTCTTTTTGTAGAATCTGGAAGTGGATATTTGGA

            **              *          *          *  
     541372 AAGCTTTGAGGATTTCATTGGAAACGGGAATATCTTCAAAT
        131 GCGCTTTGAGGATTTCGTTGGAAACGGAAATATCTTCATAT

                *    *            *           * *       *  *    *            
     541413 AAAATCTAGCCAGAAGCATTCTAAGAAACATCTTAGGGATGTTTACATTCAAGTCACAGAGTTGA
          1 AAAAACTAGACAGAAGCATTCTCAGAAACATCTTTGTGATGTTTGCACTCAACTCACAGAGTTGA

              *  * *     *       *         *    * *   **      *      *   **  
     541478 ACATTCCCTTTCACAGAGCAGATTTGAAACAATCTTCTCGTACTATCTGGCAGTGGACATTGTGA
         66 ACCTTTCTTTTCATAGAGCAGTTTTGAAACACTCTTTTTGTAGAATCTGGAAGTGGATATTTGGA

              * *   *  ** ** *  *   *            ** *
     541543 GCTCCTTGGGGCCTATGCTGAAAAAGGAAATATCTTCCGAC
        131 GCGCTTTGAGGATTTCGTTGGAAACGGAAATATCTTCATAT

                                 *     *  **       *  *           **    *    
     541584 AAAAACTAGACAGAAGCATTCGCAGAATCACGTTTGTGAAGTGTGCACTCAACTGTCAGAATTGA
          1 AAAAACTAGACAGAAGCATTCTCAGAAACATCTTTGTGATGTTTGCACTCAACTCACAGAGTTGA

                 **   ** *      *                            * *            *
     541649 ACCTTGGTTTGGACAGAGCACTTTTGAAACACTCTTTTTGTAGAATCTGCAGGTGGATATTTGGC
         66 ACCTTTCTTTTCATAGAGCAGTTTTGAAACACTCTTTTTGTAGAATCTGGAAGTGGATATTTGGA

            **                         *   *      * *
     541714 TAGCTTTGAGGATTTCGTTGGAAACGGTAATGTCTTCAAAG
        131 GCGCTTTGAGGATTTCGTTGGAAACGGAAATATCTTCATAT

                *                         *   *            *    *            
     541755 AAAATCTAGACAGAAGCATTCTCAGAAACACCTTCGTGATGTTTGCAATCAAGTCACAGAGTTGA
          1 AAAAACTAGACAGAAGCATTCTCAGAAACATCTTTGTGATGTTTGCACTCAACTCACAGAGTTGA

                 * *             *  *                  *             *       
     541820 ACCTTCCGTTTCATAGAGCAGGTTGGAAACACTCTTTTTGTAGTATCTGGAAGTGGACATTTGGA
         66 ACCTTTCTTTTCATAGAGCAGTTTTGAAACACTCTTTTTGTAGAATCTGGAAGTGGATATTTGGA

             *         ** *    * *   *            **  
     541885 GGGCTTTGTAGCCTATC-TGGAAAAAGGAAATATCTTCCCAT
        131 GCGCTTTG-AGGATTTCGTTGGAAACGGAAATATCTTCATAT

            *  ** *   *     * *            *   *  *   * **        *  * * *   
     541926 GAATGCGAGATAGAAGTAATCTCAGAAACATGTTTATGCTGTATCTACTCAACTAACTGTGCTGA
          1 AAAAACTAGACAGAAGCATTCTCAGAAACATCTTTGTGATGTTTGCACTCAACTCACAGAGTTGA

              *     *  *               *        *  * *       *               
     541991 ACATTTCTATTGATAGAGCAGTTTTGAGACACTCTTCTTTTGGAATCTGCAAGTGGATATTTGGA
         66 ACCTTTCTTTTCATAGAGCAGTTTTGAAACACTCTTTTTGTAGAATCTGGAAGTGGATATTTGGA

            ** *                        *     *      
     542056 TAGATTTGAGGATTTCGTTGGAAACGG-GAT-T-AT-ATAT
        131 GCGCTTTGAGGATTTCGTTGGAAACGGAAATATCTTCATAT

                 *       *               *                 **  *   *         
     542093 AAAAAGTAGACAGCAGCATTCTCAGAAACTTCTTTGTGATGTTTGCATCCAGCTCTCAGAGTTGA
          1 AAAAACTAGACAGAAGCATTCTCAGAAACATCTTTGTGATGTTTGCACTCAACTCACAGAGTTGA

              *  * *          *  *        *        *   **        *  **       
     542158 ACATTCCCTTTCATAGAGTAGGTTTGAAACCCTCTTTTTATAGTGTCTGGAAGCGGGCATTTGGA
         66 ACCTTTCTTTTCATAGAGCAGTTTTGAAACACTCTTTTTGTAGAATCTGGAAGTGGATATTTGGA

                   *    * * **  **  **          * *  *
     542223 GCGCTTTCAGGCCTATGCTTAAAATAGGAAATATCTACCTAC
        131 GCGCTTTGAGG-ATTTCGTTGGAAACGGAAATATCTTCATAT

             *                    *    *  **          ** *        *    *     
     542265 AGAAACTAGACAGAAGCATTCTGAGAATCACGTTTGTGATGTGGGTACTCAACTAACAGTGTTGA
          1 AAAAACTAGACAGAAGCATTCTCAGAAACATCTTTGTGATGTTTGCACTCAACTCACAGAGTTGA

            *  *       *   *            *   *                *               
     542330 TCCATTCTTTTGATACAGCAGTTTTGAACCACACTTTTTGTAGAATCTGCAAGTGGATATTTGGA
         66 ACCTTTCTTTTCATAGAGCAGTTTTGAAACACTCTTTTTGTAGAATCTGGAAGTGGATATTTGGA

            **   *                     *   *      * *
     542395 TAGCTGTGAGGATTTCGTTGGAAACGGGAATGTCTTCAAAG
        131 GCGCTTTGAGGATTTCGTTGGAAACGGAAATATCTTCATAT

                *                         *   *            *    *            
     542436 AAAATCTAGACAGAAGCATTCTCAGAAACACCTTCGTGATGTTTGCAATCAAGTCACAGAGTTGA
          1 AAAAACTAGACAGAAGCATTCTCAGAAACATCTTTGTGATGTTTGCACTCAACTCACAGAGTTGA

                 * *             *  *           *      *             *       
     542501 ACCTTCCGTTTCATAGAGCAGGTTGGAAACACTCTTATTGTAGTATCTGGAAGTGGACATTTGGA
         66 ACCTTTCTTTTCATAGAGCAGTTTTGAAACACTCTTTTTGTAGAATCTGGAAGTGGATATTTGGA

                   *   ** ** *  *   *            **  
     542566 GCGCTTTCAGGCCTATGGTGAAAAAGGAAATATCTTCCCAT
        131 GCGCTTTGAGGATTTCGTTGGAAACGGAAATATCTTCATAT

                  * * *      **      *   * *   *     ** ** *      *    *     
     542607 AAAAACGACATAGAAGCTATCTCAGGAACTTGTTTATGATGCATCTAATCAACTAACAGTGTTGA
          1 AAAAACTAGACAGAAGCATTCTCAGAAACATCTTTGTGATGTTTGCACTCAACTCACAGAGTTGA

                  * ** * *                         * *       *               
     542672 ACCTTTGTACTGACAGAGCAG-TTTGAAACACTCTTTTTTTGGAATCTGCAAGTGGATATTTGGA
         66 ACCTTTCTTTTCATAGAGCAGTTTTGAAACACTCTTTTTGTAGAATCTGGAAGTGGATATTTGGA

            *                           *   *   *    
     542736 TCGCTTTGAGGATTTCGTTGGAAACGG-GAT-GC--AATAT
        131 GCGCTTTGAGGATTTCGTTGGAAACGGAAATATCTTCATAT

                **  *    *     *                 **  *   *  *    *   *     * 
     542773 AAAACGTACACAGCAGCATACTCAGAAA-ATACTTTGCCATATTTCCATTCAAGTCAGAGAGTGG
          1 AAAAACTAGACAGAAGCATTCTCAGAAACAT-CTTTGTGATGTTTGCACTCAACTCACAGAGTTG

               *  * **            *                  *  *             *      
     542837 AACATTCCCATTCATAGAGCAGGTTTGAAACACTCTTTTTGGAGTATCTGGAAGTGGACATTTGG
         65 AACCTTTCTTTTCATAGAGCAGTTTTGAAACACTCTTTTTGTAGAATCTGGAAGTGGATATTTGG

                    ** * * ** *  *   *            **  
     542902 AGCGCTTTCTGAACTATGGTGAAAAAGGAAATATCTTCCAAT
        130 AGCGCTTTGAGGATTTCGTTGGAAACGGAAATATCTTCATAT

            *     *               *      * *          *  **      **  *  *    
     542944 GAAAACAAGACAGAAGCATTCTGAGAAACTTATTTGTGATGTGTGTCCTCAACAAACGGACTTGA
          1 AAAAACTAGACAGAAGCATTCTCAGAAACATCTTTGTGATGTTTGCACTCAACTCACAGAGTTGA

                   *      **  * *  *  *             *   *    * * *           
     543009 ACCTTTCGTTTCATGCAGTACTTCTGGAACACTCTTTTTGAAGATTCTGCATGCGGATATTTGGA
         66 ACCTTTCTTTTCATAGAGCAGTTTTGAAACACTCTTTTTGTAGAATCTGGAAGTGGATATTTGGA

            **                            **   *   * 
     543074 TAGCTTTGAGGATTTCGTTGGAAACGG---GCT-TACATGT
        131 GCGCTTTGAGGATTTCGTTGGAAACGGAAATATCTTCATAT

                 *       *               *        *   *    *    *       *    
     543111 AAAAATTAGACAGCAGCATTCTCAGAAACTTCTTTGTGGTGTCTGCATTCAAGTCACAGAATTGA
          1 AAAAACTAGACAGAAGCATTCTCAGAAACATCTTTGTGATGTTTGCACTCAACTCACAGAGTTGA

              * ** * **            *  * *      *       *             *       
     543176 ACTTCCCCTCACATAGAGCAGTTGTGCAGCACTCTATTTGTAGTATCTGGAAGTGGACATTTGGA
         66 ACCTTTCTTTTCATAGAGCAGTTTTGAAACACTCTTTTTGTAGAATCTGGAAGTGGATATTTGGA

             *         ** *    * *   *            **  
     543241 GGGCTTTGTAGCCTATC-TGGAAAAAGGAAATATCTTCCCAT
        131 GCGCTTTG-AGGATTTCGTTGGAAACGGAAATATCTTCATAT

            *  ** *   *     * *            *   *  *   * **        *  * * *   
     543282 GAATGCGAGATAGAAGTAATCTCAGAAACATGTTTATGCTGTATCTACTCAACTAACTGTGCTGA
          1 AAAAACTAGACAGAAGCATTCTCAGAAACATCTTTGTGATGTTTGCACTCAACTCACAGAGTTGA

              *     *  *               *        *  * *       *               
     543347 ACATTTCTATTGATAGAGCAGTTTTGAGACACTCTTCTTTTGGAATCTGCAAGTGGATATTTGGA
         66 ACCTTTCTTTTCATAGAGCAGTTTTGAAACACTCTTTTTGTAGAATCTGGAAGTGGATATTTGGA

            ** *                        *     *      
     543412 TAGATTTGAGGATTTCGTTGGAAACGG-GAT-T-AT-ATAT
        131 GCGCTTTGAGGATTTCGTTGGAAACGGAAATATCTTCATAT

                 *       *               *                 **  *   *         
     543449 AAAAAGTAGACAGCAGCATTCTCAGAAACTTCTTTGTGATGTTTGCATCCAGCTCTCAGAGTTGA
          1 AAAAACTAGACAGAAGCATTCTCAGAAACATCTTTGTGATGTTTGCACTCAACTCACAGAGTTGA

              *  * *          *  *        *        *   **        *  **       
     543514 ACATTCCCTTTCATAGAGTAGGTTTGAAACCCTCTTTTTATAGTGTCTGGAAGCGGGCATTTGGA
         66 ACCTTTCTTTTCATAGAGCAGTTTTGAAACACTCTTTTTGTAGAATCTGGAAGTGGATATTTGGA

                   *    * * **  **  **          * *  *
     543579 GCGCTTTCAGGCCTATGCTTAAAATAGGAAATATCTACCTAC
        131 GCGCTTTGAGG-ATTTCGTTGGAAACGGAAATATCTTCATAT

             *                    *    *  **          ** *        *    *     
     543621 AGAAACTAGACAGAAGCATTCTGAGAATCACGTTTGTGATGTGGGTACTCAACTAACAGTGTTGA
          1 AAAAACTAGACAGAAGCATTCTCAGAAACATCTTTGTGATGTTTGCACTCAACTCACAGAGTTGA

            *  *       *   *            *   *                *   *           
     543686 TCCATTCTTTTGATACAGCAGTTTTGAACCACACTTTTTGTAGAATCTGCAAGAGGATATTTGGA
         66 ACCTTTCTTTTCATAGAGCAGTTTTGAAACACTCTTTTTGTAGAATCTGGAAGTGGATATTTGGA

            **   *                     *   *      * *
     543751 TAGCTGTGAGGATTTCGTTGGAAACGGGAATGTCTTCAAAG
        131 GCGCTTTGAGGATTTCGTTGGAAACGGAAATATCTTCATAT

                *                         *   *            *    *            
     543792 AAAATCTAGACAGAAGCATTCTCAGAAACACCTTCGTGATGTTTGCAATCAAGTCACAGAGTTGA
          1 AAAAACTAGACAGAAGCATTCTCAGAAACATCTTTGTGATGTTTGCACTCAACTCACAGAGTTGA

                 * *             *  *           *      *             *       
     543857 ACCTTCCGTTTCATAGAGCAGGTTGGAAACACTCTTATTGTAGTATCTGGAAGTGGACATTTGGA
         66 ACCTTTCTTTTCATAGAGCAGTTTTGAAACACTCTTTTTGTAGAATCTGGAAGTGGATATTTGGA

                   *   ** ** *  *   *            **  
     543922 GCGCTTTCAGGCCTATGGTGAAAAAGGAAATATCTTCCCAT
        131 GCGCTTTGAGGATTTCGTTGGAAACGGAAATATCTTCATAT

                  * * *      **      *   * *   *     ** ** *      *    *     
     543963 AAAAACGACATAGAAGCTATCTCAGGAACTTGTTTATGATGCATCTAATCAACTAACAGTGTTGA
          1 AAAAACTAGACAGAAGCATTCTCAGAAACATCTTTGTGATGTTTGCACTCAACTCACAGAGTTGA

                  * ** * *                         * *       *               
     544028 ACCTTTGTACTGACAGAGCAG-TTTGAAACACTCTTTTTTTGGAATCTGCAAGTGGATATTTGGA
         66 ACCTTTCTTTTCATAGAGCAGTTTTGAAACACTCTTTTTGTAGAATCTGGAAGTGGATATTTGGA

            *                           *   *   *    
     544092 TCGCTTTGAGGATTTCGTTGGAAACGG-GAT-GC--AATAT
        131 GCGCTTTGAGGATTTCGTTGGAAACGGAAATATCTTCATAT

                **  *    *     *                 **  *   *  *    *   *     * 
     544129 AAAACGTACACAGCAGCATACTCAGAAA-ATACTTTGCCATATTTCCATTCAAGTCAGAGAGTGG
          1 AAAAACTAGACAGAAGCATTCTCAGAAACAT-CTTTGTGATGTTTGCACTCAACTCACAGAGTTG

               *  * **            *                  *  *             *      
     544193 AACATTCCCATTCATAGAGCAGGTTTGAAACACTCTTTTTGGAGTATCTGGAAGTGGACATTTGG
         65 AACCTTTCTTTTCATAGAGCAGTTTTGAAACACTCTTTTTGTAGAATCTGGAAGTGGATATTTGG

                    ** * * ** *  *   *            **  
     544258 AGCGCTTTCTGAACTATGGTGAAAAAGGAAATATCTTCCAAT
        130 AGCGCTTTGAGGATTTCGTTGGAAACGGAAATATCTTCATAT

            *     *               *      * *          *  **      **  *  *    
     544300 GAAAACAAGACAGAAGCATTCTGAGAAACTTATTTGTGATGTGTGTCCTCAACAAACGGACTTGA
          1 AAAAACTAGACAGAAGCATTCTCAGAAACATCTTTGTGATGTTTGCACTCAACTCACAGAGTTGA

                   *      **  * *  *  *             *   *    * * *           
     544365 ACCTTTCGTTTCATGCAGTACTTCTGGAACACTCTTTTTGAAGATTCTGCATGCGGATATTTGGA
         66 ACCTTTCTTTTCATAGAGCAGTTTTGAAACACTCTTTTTGTAGAATCTGGAAGTGGATATTTGGA

            **                            **   *   * 
     544430 TAGCTTTGAGGATTTCGTTGGAAACGG---GCT-TACATGT
        131 GCGCTTTGAGGATTTCGTTGGAAACGGAAATATCTTCATAT

                 *       *               *        *   *    *    *       *    
     544467 AAAAATTAGACAGCAGCATTCTCAGAAACTTCTTTGTGGTGTCTGCATTCAAGTCACAGAATTGA
          1 AAAAACTAGACAGAAGCATTCTCAGAAACATCTTTGTGATGTTTGCACTCAACTCACAGAGTTGA

              * ** * **            *  * *      *       *             *       
     544532 ACTTCCCCTCACATAGAGCAGTTGTGCAGCACTCTATTTGTAGTATCTGGAAGTGGACATTTGGA
         66 ACCTTTCTTTTCATAGAGCAGTTTTGAAACACTCTTTTTGTAGAATCTGGAAGTGGATATTTGGA

             *         ** *    * *   *            **  
     544597 GGGCTTTGTAGCCTATC-TGGAAAAAGGAAATATCTTCCCAT
        131 GCGCTTTG-AGGATTTCGTTGGAAACGGAAATATCTTCATAT

            *  ** *   *     * *            *   *  *   * **        *  * * *   
     544638 GAATGCGAGATAGAAGTAATCTCAGAAACATGTTTATGCTGTATCTACTCAACTAACTGTGCTGA
          1 AAAAACTAGACAGAAGCATTCTCAGAAACATCTTTGTGATGTTTGCACTCAACTCACAGAGTTGA

              *     *  *             * *        *  * *       *               
     544703 ACATTTCTATTGATAGAGCAGTTTTCAGACACTCTTCTTTTGGAATCTGCAAGTGGATATTTGGA
         66 ACCTTTCTTTTCATAGAGCAGTTTTGAAACACTCTTTTTGTAGAATCTGGAAGTGGATATTTGGA

            ** *                        *     *      
     544768 TAGATTTGAGGATTTCGTTGGAAACGG-GAT-T-AT-ATAT
        131 GCGCTTTGAGGATTTCGTTGGAAACGGAAATATCTTCATAT

                 *       *               *                 **  *   *         
     544805 AAAAAGTAGACAGCAGCATTCTCAGAAACTTCTTTGTGATGTTTGCATCCAGCTCTCAGAGTTGA
          1 AAAAACTAGACAGAAGCATTCTCAGAAACATCTTTGTGATGTTTGCACTCAACTCACAGAGTTGA

              *  * *          *  *        *        *   **        *  **       
     544870 ACATTCCCTTTCATAGAGTAGGTTTGAAACCCTCTTTTTATAGTGTCTGGAAGCGGGCATTTGGA
         66 ACCTTTCTTTTCATAGAGCAGTTTTGAAACACTCTTTTTGTAGAATCTGGAAGTGGATATTTGGA

                   *    * * **  **  **          * *  *
     544935 GCGCTTTCAGGCCTATGCTTAAAATAGGAAATATCTACCTAC
        131 GCGCTTTGAGG-ATTTCGTTGGAAACGGAAATATCTTCATAT

             *                    *    *  **          ** *        *    *     
     544977 AGAAACTAGACAGAAGCATTCTGAGAATCACGTTTGTGATGTGGGTACTCAACTAACAGTGTTGA
          1 AAAAACTAGACAGAAGCATTCTCAGAAACATCTTTGTGATGTTTGCACTCAACTCACAGAGTTGA

            *  *       *   *            *   *                *   *           
     545042 TCCATTCTTTTGATACAGCAGTTTTGAACCACACTTTTTGTAGAATCTGCAAGAGGATATTTGGA
         66 ACCTTTCTTTTCATAGAGCAGTTTTGAAACACTCTTTTTGTAGAATCTGGAAGTGGATATTTGGA

            **   *                     *   *      * *
     545107 TAGCTGTGAGGATTTCGTTGGAAACGGGAATGTCTTCAAAG
        131 GCGCTTTGAGGATTTCGTTGGAAACGGAAATATCTTCATAT

                *                         *   *            *    *            
     545148 AAAATCTAGACAGAAGCATTCTCAGAAACACCTTCGTGATGTTTGCAATCAAGTCACAGAGTTGA
          1 AAAAACTAGACAGAAGCATTCTCAGAAACATCTTTGTGATGTTTGCACTCAACTCACAGAGTTGA

                 * **            *  *           *      *             *       
     545213 ACCTTCCGATTCATAGAGCAGGTTGGAAACACTCTTATTGTAGTATCTGGAAGTGGACATTTGGA
         66 ACCTTTCTTTTCATAGAGCAGTTTTGAAACACTCTTTTTGTAGAATCTGGAAGTGGATATTTGGA

                   *   ** ** *  *   *            **  
     545278 GCGCTTTCAGGCCTATGGTGAAAAAGGAAATATCTTCCCAT
        131 GCGCTTTGAGGATTTCGTTGGAAACGGAAATATCTTCATAT

                  * * *      **      *   * *   *     ** ** *      *    *     
     545319 AAAAACGACATAGAAGCTATCTCAGGAACTTGTTTATGATGCATCTAATCAACTAACAGTGTTGA
          1 AAAAACTAGACAGAAGCATTCTCAGAAACATCTTTGTGATGTTTGCACTCAACTCACAGAGTTGA

                  * ** * *                         * *       *               
     545384 ACCTTTGTACTGACAGAGCAG-TTTGAAACACTCTTTTTTTGGAATCTGCAAGTGGATATTTGGA
         66 ACCTTTCTTTTCATAGAGCAGTTTTGAAACACTCTTTTTGTAGAATCTGGAAGTGGATATTTGGA

            *                           *   *   *    
     545448 TCGCTTTGAGGATTTCGTTGGAAACGG-GAT-GC--AATAT
        131 GCGCTTTGAGGATTTCGTTGGAAACGGAAATATCTTCATAT

                **  *    *     *                 **  *   *  *    *         * 
     545485 AAAACGTACACAGCAGCATACTCAGAAA-ATACTTTGCCATATTTCCATTCAAGTCACAGAGTGG
          1 AAAAACTAGACAGAAGCATTCTCAGAAACAT-CTTTGTGATGTTTGCACTCAACTCACAGAGTTG

               *  * **            *  *               *  *             *      
     545549 AACATTCCCATTCATAGAGCAGGTTGGAAACACTCTTTTTGGAGTATCTGGAAGTGGACATTTGG
         65 AACCTTTCTTTTCATAGAGCAGTTTTGAAACACTCTTTTTGTAGAATCTGGAAGTGGATATTTGG

                    ** * * ** *  *   *            **  
     545614 AGCGCTTTCTGAACTATGGTGAAAAAGGAAATATCTTCCAAT
        130 AGCGCTTTGAGGATTTCGTTGGAAACGGAAATATCTTCATAT

            *     *               *      * *          *  **      **  *  *    
     545656 GAAAACAAGACAGAAGCATTCTGAGAAACTTATTTGTGATGTGTGTCCTCAACAAACGGACTTGA
          1 AAAAACTAGACAGAAGCATTCTCAGAAACATCTTTGTGATGTTTGCACTCAACTCACAGAGTTGA

                   *      **  * *  *  *             *   *    * * *           
     545721 ACCTTTCGTTTCATGCAGTACTTCTGGAACACTCTTTTTGAAGATTCTGCATGCGGATATTTGGA
         66 ACCTTTCTTTTCATAGAGCAGTTTTGAAACACTCTTTTTGTAGAATCTGGAAGTGGATATTTGGA

            **                            **   *   * 
     545786 TAGCTTTGAGGATTTCGTTGGAAACGG---GCT-TACATGT
        131 GCGCTTTGAGGATTTCGTTGGAAACGGAAATATCTTCATAT

                 *       *               *        *   *    *    *       *    
     545823 AAAAATTAGACAGCAGCATTCTCAGAAACTTCTTTGTGGTGTCTGCATTCAAGTCACAGAATTGA
          1 AAAAACTAGACAGAAGCATTCTCAGAAACATCTTTGTGATGTTTGCACTCAACTCACAGAGTTGA

              * ** * **            *  * *      *       *             *       
     545888 ACTTCCCCTCACATAGAGCAGTTGTGCAGCACTCTATTTGTAGTATCTGGAAGTGGACATTTGGA
         66 ACCTTTCTTTTCATAGAGCAGTTTTGAAACACTCTTTTTGTAGAATCTGGAAGTGGATATTTGGA

             *         ** *    * *   *            **  
     545953 GGGCTTTGTAGCCTATC-TGGAAAAAGGAAATATCTTCCCAT
        131 GCGCTTTG-AGGATTTCGTTGGAAACGGAAATATCTTCATAT

            *  ** *   *     * *            *   *  *   * **        *  * * *   
     545994 GAATGCGAGATAGAAGTAATCTCAGAAACATGTTTATGCTGTATCTACTCAACTAACTGTGCTGA
          1 AAAAACTAGACAGAAGCATTCTCAGAAACATCTTTGTGATGTTTGCACTCAACTCACAGAGTTGA

              *     *  *               *        *  * *       *               
     546059 ACATTTCTATTGATAGAGCAGTTTTGAGACACTCTTCTTTTGGAATCTGCAAGTGGATATTTGGA
         66 ACCTTTCTTTTCATAGAGCAGTTTTGAAACACTCTTTTTGTAGAATCTGGAAGTGGATATTTGGA

            ** *                        *     *      
     546124 TAGATTTGAGGATTTCGTTGGAAACGG-GAT-T-AT-ATAT
        131 GCGCTTTGAGGATTTCGTTGGAAACGGAAATATCTTCATAT

            *    *       *               *                 **  *   *         
     546161 CAAAAGTAGACAGCAGCATTCTCAGAAACTTCTTTGTGATGTTTGCATCCAGCTCTCAGAGTTGA
          1 AAAAACTAGACAGAAGCATTCTCAGAAACATCTTTGTGATGTTTGCACTCAACTCACAGAGTTGA

              *  * *          *  *        *        *   **        *  **       
     546226 ACATTCCCTTTCATAGAGTAGGTTTGAAACCCTCTTTTTATAGTGTCTGGAAGCGGGCATTTGGA
         66 ACCTTTCTTTTCATAGAGCAGTTTTGAAACACTCTTTTTGTAGAATCTGGAAGTGGATATTTGGA

                   *    * * **  **  **          * *  *
     546291 GCGCTTTCAGGCCTATGCTTAAAATAGGAAATATCTACCTAC
        131 GCGCTTTGAGG-ATTTCGTTGGAAACGGAAATATCTTCATAT

             *                    *    *  **          ** *        *    *     
     546333 AGAAACTAGACAGAAGCATTCTGAGAATCACGTTTGTGATGTGGGTACTCAACTAACAGTGTTGA
          1 AAAAACTAGACAGAAGCATTCTCAGAAACATCTTTGTGATGTTTGCACTCAACTCACAGAGTTGA

            *  *       *   *            *   *                *   *           
     546398 TCCATTCTTTTGATACAGCAGTTTTGAACCACACTTTTTGTAGAATCTGCAAGAGGATATTTGGA
         66 ACCTTTCTTTTCATAGAGCAGTTTTGAAACACTCTTTTTGTAGAATCTGGAAGTGGATATTTGGA

            **   *                     *   *      * *
     546463 TAGCTGTGAGGATTTCGTTGGAAACGGGAATGTCTTCAAAG
        131 GCGCTTTGAGGATTTCGTTGGAAACGGAAATATCTTCATAT

                *                         *   *            *    *            
     546504 AAAATCTAGACAGAAGCATTCTCAGAAACACCTTCGTGATGTTTGCAATCAAGTCACAGAGTTGA
          1 AAAAACTAGACAGAAGCATTCTCAGAAACATCTTTGTGATGTTTGCACTCAACTCACAGAGTTGA

                 * *             *  *           *      *             *       
     546569 ACCTTCCGTTTCATAGAGCAGGTTGGAAACACTCTTATTGTAGTATCTGGAAGTGGACATTTGGA
         66 ACCTTTCTTTTCATAGAGCAGTTTTGAAACACTCTTTTTGTAGAATCTGGAAGTGGATATTTGGA

                   *   ** ** *  *   *            **  
     546634 GCGCTTTCAGGCCTATGGTGAAAAAGGAAATATCTTCCCAT
        131 GCGCTTTGAGGATTTCGTTGGAAACGGAAATATCTTCATAT

                  * * *      **      *   * *   *     ** ** *      *    *     
     546675 AAAAACGACATAGAAGCTATCTCAGGAACTTGTTTATGATGCATCTAATCAACTAACAGTGTTGA
          1 AAAAACTAGACAGAAGCATTCTCAGAAACATCTTTGTGATGTTTGCACTCAACTCACAGAGTTGA

                  * ** * *                         * *       *               
     546740 ACCTTTGTACTGACAGAGCAG-TTTGAAACACTCTTTTTTTGGAATCTGCAAGTGGATATTTGGA
         66 ACCTTTCTTTTCATAGAGCAGTTTTGAAACACTCTTTTTGTAGAATCTGGAAGTGGATATTTGGA

            *                           *   *   *    
     546804 TCGCTTTGAGGATTTCGTTGGAAACGG-GAT-GC--AATAT
        131 GCGCTTTGAGGATTTCGTTGGAAACGGAAATATCTTCATAT
[truncated: 21,445,054 more chars]
