# Supplementary material for: Tigerfish designs oligonucleotide-based in situ hybridization probes targeting intervals of highly repetitive DNA at the scale of genomes
Source: Nat Commun. 2024 Feb 3;15:1027. doi: 10.1038/s41467-024-45385-x (PMC10838309; doi:10.1038/s41467-024-45385-x)
Supplement: Supplementary file 10 — Source Data [file 41467_2024_45385_MOESM10_ESM.gz › Tigerfish_full_data/Figure_S1_HOR_TRF_Analysis/TRF/trf_output/chr2_8_21_Y_hor.fa.s1.2.5.7.80.10.50.2000.1.txt.html]

chr2\_8\_21\_Y\_hor.fa.s1.2.5.7.80.10.50.2000.txt.html

```
Tandem Repeats Finder Program written by:

                 Gary Benson
      Program in Bioinformatics
          Boston University

Version 4.09

Sequence: chr2:92297386-94695067

Parameters: 2 5 7 80 10 50 2000

Pmatch=0.80,Pindel=0.10
tuple sizes 0,4,5,7
tuple distances 0, 29, 159, 2000

Length: 2397681
ACGTcount: A:0.29, C:0.17, G:0.21, T:0.32


Found at i:361 original size:171 final size:171

Alignment explanation  
  

    Indices: 41--1342  Score: 1191
    Period size: 171  Copynumber: 7.6  Consensus size: 171

         31 ATTTCTTAAG

               *  *      *                          *            *        *  
         41 AAACGGGAATATCCTCATATAAAATCTAGACAGAAGCATTATCAGAAACATCTGTGTGATGTTTG
          1 AAAAGGAAATATCTTCATATAAAATCTAGACAGAAGCATTCTCAGAAACATCTTTGTGATGTGTG

            * *                                         *      ** *          
        106 CATTCAAGTCACAGAGTTGAACATTCCTTTTCATAGAGCAGGTTCGAAACACTGATTTTGTAGTA
         66 TACTCAAGTCACAGAGTTGAACATTCCTTTTCATAGAGCAGGTTTGAAACATCGTTTTTGTAGTA

                      *                              
        171 TCTGCAACAGAACATTTGGATCGCTTTGTGGCATATGGTGA
        131 TCTGCAACAGGACATTTGGATCGCTTTGTGGCATATGGTGA

                            **      **              *        ***             
        212 AAAAGGAAATATCTTCCCATAAAAAATAGACAGAAGCATTATCAGAAACTAGTTTGTGATGTGTG
          1 AAAAGGAAATATCTTCATATAAAATCTAGACAGAAGCATTCTCAGAAACATCTTTGTGATGTGTG

                   *              *  *     *        **  * *                * 
        277 TACTCAACTCACAGAGTTGAACCTTTCTTTTGATAGAGCATTTTAGCAACATCGTTTTTGTAGAA
         66 TACTCAAGTCACAGAGTTGAACATTCCTTTTCATAGAGCAGGTTTGAAACATCGTTTTTGTAGTA

                   *    *        **     *     *   *  *
        342 TCTGCAAGAGGATATTTGGATACCTTTGAGG-ATTTCGTTGG
        131 TCTGCAACAGGACATTTGGATCGCTTTGTGGCATAT-GGTGA

               *  *                                              *        *  
        383 AAACGGGAATATCTTCATATAAAATCTAGACAGAAGCATTCTCAGAAACATCTGTGTGATGTTTG
          1 AAAAGGAAATATCTTCATATAAAATCTAGACAGAAGCATTCTCAGAAACATCTTTGTGATGTGTG

            * *       *                *                     *               
        448 AATTCAAGTCCCAGAGTTGAACATTCCCTTTCATAGAGCAGGTTTGAAATACTC-TTTTTGTAGT
         66 TACTCAAGTCACAGAGTTGAACATTCCTTTTCATAGAGCAGGTTTGAAACA-TCGTTTTTGTAGT

                 *    **   *  *         *  * *        
        512 ATCTGGAAC-TCACAATTTGATCGCTTTAT-TCTTATGGTGA
        130 ATCTGCAACAGGACATTTGGATCGCTTTGTGGCATATGGTGA

                        *   **      *   *                      **            
        552 AAAAGGAAATATTTTCCCATAAAAGCTACACAGAAGCATTCTCAGAAAC-TTGTTGTGATGTGTG
          1 AAAAGGAAATATCTTCATATAAAATCTAGACAGAAGCATTCTCAGAAACATCTTTGTGATGTGTG

               *   * *           *   *    **     *   *                      *
        616 TACACAACTAACAGAGTTGAAAATTTCTTTGGATAGACCAGTTTTGAAACACTC-TTTTTGTAGA
         66 TACTCAAGTCACAGAGTTGAACATTCCTTTTCATAGAGCAGGTTTGAAACA-TCGTTTTTGTAGT

                    *  * *        * *       ** *  **  *
        680 ATCTGCAAGAGCATATTTGGATAGATTTGTGGATTTTCTTTGG
        130 ATCTGCAACAGGACATTTGGATCGCTTTGTGGCATAT-GGTGA

               *  *                  **       *   *              *        *  
        723 AAACGGGAATATCTTCATATAAAATACAGACAGAGGCAGTCTCAGAAACATCTCTGTGATGTTTG
          1 AAAAGGAAATATCTTCATATAAAATCTAGACAGAAGCATTCTCAGAAACATCTTTGTGATGTGTG

            * *             *          *       *               ** *       *  
        788 CATTCAAGTCACAGAGGTGAACATTCCCTTTCATACAGCAGGTTTGAAACACTGATTTTGTATTA
         66 TACTCAAGTCACAGAGTTGAACATTCCTTTTCATAGAGCAGGTTTGAAACATCGTTTTTGTAGTA

                    *      * *  * **   *   **  *     
        853 TCTGCAACCGGACATGTTGAGCCATTTTTGGTCTACGGTGA
        131 TCTGCAACAGGACATTTGGATCGCTTTGTGGCATATGGTGA

                            ** *    *          *                *            
        894 AAAAGGAAATATCTTCCCAAAAAAACTAGACAGAACCATTCTCAGAAA-ATAGTTTGTGATGTGT
          1 AAAAGGAAATATCTTCATATAAAATCTAGACAGAAGCATTCTCAGAAACAT-CTTTGTGATGTGT

                *   * * *          *  *     *         **      *            * 
        958 GTACACAAATAAAAGAGTTGAACCTTTCTTTTGATAGAGCAGCATTGAAAGACTC-TTTTTGTTG
         65 GTACTCAAGTCACAGAGTTGAACATTCCTTTTCATAGAGCAGGTTTGAAACA-TCGTTTTTGTAG

            *        **   *        *      *     *  **  *
       1022 AATCTGCAAGTGGATATTTGGATAGCTTTGAGG-ATTTCATTGG
        129 TATCTGCAACAGGACATTTGGATCGCTTTGTGGCATAT-GGTGA

                               *                                 ** *     *  
       1065 AAAAGGAAATATCTTCATACAAAATCTAGACAGAAGCATTCTCAGAAACATCTCGGCGATGTTTG
          1 AAAAGGAAATATCTTCATATAAAATCTAGACAGAAGCATTCTCAGAAACATCTTTGTGATGTGTG

            * *               *        *                      * *   *        
       1130 CATTCAAGTCACAGAGTTAAACATTCCCTTTCA-AGAAGCAGGTTTGAAAAAACTGATTTTGTAG
         66 TACTCAAGTCACAGAGTTGAACATTCCTTTTCATAG-AGCAGGTTTGAAACATC-GTTTTTGTAG

             * *  *   *      *    * *** *     * *      
       1194 TTTGTGGAACTGGACATGTGGAGCCAGTGGTGGCCTGTGGTGA
        129 TATCTGCAACAGGACATTTGGATCGCTTTGTGGCATATGGTGA

                                     *   ** *    *            ***            
       1237 AAAAGGAAATATCTTCCAT-TAAAAACTACGCGGAAGGATTCTCAGAAACTAGTTTGTGATGTGT
          1 AAAAGGAAATATCTT-CATATAAAATCTAGACAGAAGCATTCTCAGAAACATCTTTGTGATGTGT

            *    *  * **  *        *  *     *         
       1301 TTACTGAACTACCAAAGTTGAACCTTTCTTTTGATAGAGCAG
         65 GTACTCAAGTCACAGAGTTGAACATTCCTTTTCATAGAGCAG

       1343 ATTCATGCTG


Statistics
Matches: 820,  Mismatches: 293, Indels: 35
        0.71            0.26        0.03

Matches are distributed among these distances:
 168   67  0.08
 169   58  0.07
 170   25  0.03
 171  480  0.59
 172  183  0.22
 173    7  0.01

ACGTcount: A:0.33, C:0.16, G:0.20, T:0.31


Consensus pattern (171 bp):   
AAAAGGAAATATCTTCATATAAAATCTAGACAGAAGCATTCTCAGAAACATCTTTGTGATGTGTG
TACTCAAGTCACAGAGTTGAACATTCCTTTTCATAGAGCAGGTTTGAAACATCGTTTTTGTAGTA
TCTGCAACAGGACATTTGGATCGCTTTGTGGCATATGGTGA

Found at i:2380 original size:171 final size:171

Alignment explanation  
  

    Indices: 1851--2397681  Score: 2246883
    Period size: 171  Copynumber: 14098.2  Consensus size: 171

       1841 AAAAAAAAAG

                     *         *           *     *                           
       1851 ATAGAGCAGATTTGAAACAATCTTTTTGTAGAATCTGCAAG-GGATATTTGGATAGCTTTGAGGA
          1 ATAGAGCAGGTTTGAAACACTCTTTTTGTAGTATCTGGAAGTGGATATTTGGATAGCTTTGAGGA

                *        *              *    *   *                    *      
       1915 TTTCATTGGAAACTGGAATATCTTCATACAAAATCTACACAGAAGCATTCTCAGAAACATCTTTG
         66 TTTCGTTGGAAACAGGAATATCTTCATATAAAAGCTAGACAGAAGCATTCTCAGAAACTTCTTTG

            *                   *  *                 
       1980 GGATGTTTGCATTCAAGTAATAGAGTTGAACATTCCCTTTC
        131 TGATGTTTGCATTCAAGTAACAGTGTTGAACATTCCCTTTC

                               *         * *                      ** * *     
       2021 ATAGAGCAGGTTTGAAACATTCTTTTTGTGGAATCTGGAAGTGGATATTTGGATCACATGGAGG-
          1 ATAGAGCAGGTTTGAAACACTCTTTTTGTAGTATCTGGAAGTGGATATTTGGATAGCTTTGAGGA

            ** **       **              *      **     *           **      ** 
       2085 CCTAATTGTGAACAAGGAGATATCTTCACATAAAAAGTAGACGGAAGCATTCTCCTAAACTTGCT
         66 TTTCGTTG-GAAACAGGA-ATATCTTCATATAAAAGCTAGACAGAAGCATTCTCAGAAACTTCTT

              **    *  * * *  *      *     * *  * *   *
       2150 TGGAATGTGTGTACTTAACTAACAGAGTTGAGCCTTTCTTTTG
        129 TGTGATGTTTGCATTCAAGTAACAGTGTTGAACATTCCCTTTC

                     *                 *   *     * *                *        
       2193 ATAGAGCAGTTTTGAAACACTCTTTTTCTAGAATCTGCATGTGGATATTTGGATAGATTTGAGGA
          1 ATAGAGCAGGTTTGAAACACTCTTTTTGTAGTATCTGGAAGTGGATATTTGGATAGCTTTGAGGA

                                         *   * *     *                *      
       2258 TTTCGTTGGAAACAGGAATATCTTCATATCAAATCAAGACACAAGCATTCTCAGAAACATCTTTG
         66 TTTCGTTGGAAACAGGAATATCTTCATATAAAAGCTAGACAGAAGCATTCTCAGAAACTTCTTTG

            *      *      *   * *  *                 
       2323 GGATGTTAGCATTCTAGTCAAAGAGTTGAACATTCCCTTTC
        131 TGATGTTTGCATTCAAGTAACAGTGTTGAACATTCCCTTTC

                               *       *         *       *    **  *        **
       2364 ATAGAGCAGGTTTGAAACATTCTTTTTATAGTATCTGCAAGTGGACATTTTCATCGCTTTGAGAC
          1 ATAGAGCAGGTTTGAAACACTCTTTTTGTAGTATCTGGAAGTGGATATTTGGATAGCTTTGAGGA

            * ** *      *             *       *          *            ***    
       2429 CTATGGT-GAAAAAGGAAATATCTTCGTATAAAAACTAGACAGAAACATTCTCAGAAATAACTTT
         66 TTTCGTTGGAAACAGG-AATATCTTCATATAAAAGCTAGACAGAAGCATTCTCAGAAACTTCTTT

                 * * ** *    *     **       *  * *   *
       2493 GTGATATGTTTACTCAACTAACACAGTTGAACCTTTCTTTTG
        130 GTGATGTTTGCATTCAAGTAACAGTGTTGAACATTCCCTTTC

                                 *         *     *                           
       2535 ATAGAGCA-GTTTGAAACACTTTTTTTGTAGAATCTGAAAGTGGATATTTGGATAGCTTTGAGGA
          1 ATAGAGCAGGTTTGAAACACTCTTTTTGTAGTATCTGGAAGTGGATATTTGGATAGCTTTGAGGA

                *    *  **  *    *           *                        *   *  
       2599 TTTCTTTGGGAAAGGGGATATGTTCATATAAAATCTAGACAGAAGCATTCTCAGAAACATCTCTG
         66 TTTCGTTGGAAACAGGAATATCTTCATATAAAAGCTAGACAGAAGCATTCTCAGAAACTTCTTTG

               * *            *        *  *          
       2664 TGAGGATTGCATTCAAGTCACAG-GTTAAATATTCCCTTTC
        131 TGATGTTTGCATTCAAGTAACAGTGTTGAACATTCCCTTTC

                         *     * ***          *     *    *       **      *   
       2704 ATAGAGCAGGTTTTAAACAATGAATTTGTAGTATGTGGAATTGGACATTTGGAGCGCTTTGTGG-
          1 ATAGAGCAGGTTTGAAACACTCTTTTTGTAGTATCTGGAAGTGGATATTTGGATAGCTTTGAGGA

            ** ** **    *             ** *    *           * *     *     * *  
       2768 CCTAATGTGAAAAAGGAAATATCTTCCCACAAAAACTAGACAGAAGTACTCTCATAAACTACCTT
         66 TTTCGTTGGAAACAGG-AATATCTTCATATAAAAGCTAGACAGAAGCATTCTCAGAAACTTCTTT

             *     *  ***    *      * *        * *   *
       2833 GCGATGTGTGTGCTCAACTAACAGAGCTGAACATTTCGTTTG
        130 GTGATGTTTGCATTCAAGTAACAGTGTTGAACATTCCCTTTC

                     *                     *     * *                         
       2875 ATAGAGCAGTTTTGAAACACTCTTTTTGTAGAATCTGCATGTGG--ATTTGGATAGCTTTGAGGA
          1 ATAGAGCAGGTTTGAAACACTCTTTTTGTAGTATCTGGAAGTGGATATTTGGATAGCTTTGAGGA

                    *   **         **        *                *       *  *   
       2938 TTTCGTTGCAAAGGGGAATATCTCTATATAAAATCTAGACAGAAGCATTCACAGAAACATCCTTG
         66 TTTCGTTGGAAACAGGAATATCTTCATATAAAAGCTAGACAGAAGCATTCTCAGAAACTTCTTTG

            * *    *          *    *  *        *     
       3003 GGGTGTTAGCATTCAAGTCACAGAGTGGAACATTCACTTTC
        131 TGATGTTTGCATTCAAGTAACAGTGTTGAACATTCCCTTTC

                                 *       * *             *    *   *         *
       3044 ATAGAGCAGGTTTGAAACACTATTTTTGTGGAATCTGGAAGTGGACATTTTGATCGCTTTGAGGC
          1 ATAGAGCAGGTTTGAAACACTCTTTTTGTAGTATCTGGAAGTGGATATTTGGATAGCTTTGAGGA

            * ** *  *   *             **      *     *     *         *  *     
       3109 CTATGGTGAAAAAAGGGAATATCTTCGCATAAAAACTAGAGAGAAGTATTCTCAGACACATCTTT
         66 TTTCGTTGGAAACA-GGAATATCTTCATATAAAAGCTAGACAGAAGCATTCTCAGAAACTTCTTT

             *      *      * * *    *     *           
       3174 GGGATGTTAGCATTCTAATGACAGAGTTGATCATTCCCTTTC
        130 GTGATGTTTGCATTCAAGTAACAGTGTTGAACATTCCCTTTC

                 *     *                                 *    *   **        *
       3216 ATAGAACAGGTATGAAACACTCTTTTTGTAGTATCTGGAAGTGGACATTTTGATCACTTTGAGGC
          1 ATAGAGCAGGTTTGAAACACTCTTTTTGTAGTATCTGGAAGTGGATATTTGGATAGCTTTGAGGA

            * ** *  *   **        *  **      *                   *      *    
       3281 CTATGGTGAAAAAGGGAATATCATCGCATAAAAACTAGACAGAAGCATTCTCATAAACTTGTTTG
         66 TTTCGTTGGAAACAGGAATATCTTCATATAAAAGCTAGACAGAAGCATTCTCAGAAACTTCTTTG

                  *  * *    **     * *        * *   *
       3346 TGATGTGTGAACTCAACAAACAGAGGTGAAC-TTTCTTTTG
        131 TGATGTTTGCATTCAAGTAACAGTGTTGAACATTCCCTTTC

                     *          *          *     * *          *        *     
       3386 ATAGAGCAGATTTGAAACACACTTTTTGTAGAATCTGCATGTGGATATTTAGATAGCTTCGAGGA
          1 ATAGAGCAGGTTTGAAACACTCTTTTTGTAGTATCTGGAAGTGGATATTTGGATAGCTTTGAGGA

                *  *     *    *          *   ***     *                *      
       3451 TTTCATTAGAAACGGGAAAATCTTCATATGAAATAGAGACACAAGCATTCTCAGAAACCTCTTTG
         66 TTTCGTTGGAAACAGGAATATCTTCATATAAAAGCTAGACAGAAGCATTCTCAGAAACTTCTTTG

            *      *      **  **   *     *  *        
       3516 GGATGTTAGCATTCGTGTCGCAGAGTTGATCACTCCCTTTC
        131 TGATGTTTGCATTCAAGTAACAGTGTTGAACATTCCCTTTC

                                         **              *    *   *          
       3557 ATAGAGCAGGTTTGAAACACTCTTTTTGTCATATCTGGAAGTGGACATTTTGATCGCTTTGAGGA
          1 ATAGAGCAGGTTTGAAACACTCTTTTTGTAGTATCTGGAAGTGGATATTTGGATAGCTTTGAGGA

            * ** *      *             ** *    *   *                     *   *
       3622 GTAAGGT-GAAAAAGGAAATATCTTCCCACAAAAACTACACAGAAGCATTCTCAGAAACTACTTA
         66 TTTCGTTGGAAACAGG-AATATCTTCATATAAAAGCTAGACAGAAGCATTCTCAGAAACTTCTTT

              *    * ** *    *    * *  *    *  * *   *
       3686 GTTATGTGTTTACTCAACTAACGGAGTAGAACCTTTCTTTTG
        130 GTGATGTTTGCATTCAAGTAACAGTGTTGAACATTCCCTTTC

                 *   *                 *   *     *            *              
       3728 ATAGACCAGTTTTGAAACACTCTTTTTATAGAATCTGCAAGTGGATATTTTGATAGCTTTGAGGA
          1 ATAGAGCAGGTTTGAAACACTCTTTTTGTAGTATCTGGAAGTGGATATTTGGATAGCTTTGAGGA

                *       **        *          *     * *                **     
       3793 TTTCCTTGGAAATGGGAATATCCTCATATAAAATCTAGATAAAAGCATTCTCAGAAACACCTTTG
         66 TTTCGTTGGAAACAGGAATATCTTCATATAAAAGCTAGACAGAAGCATTCTCAGAAACTTCTTTG

                  *      *    * *  *   **            
       3858 TGATGTCTGCATTGAAGTCAGAGAGTTCTACATTCCCTTTC
        131 TGATGTTTGCATTCAAGTAACAGTGTTGAACATTCCCTTTC

                 *   *           *                  *    *    *   *         *
       3899 ATAGAACAGCTTTGAAACACTATTTTTGTAGTATCTGGAAATGGACATTTAGATCGCTTTGAGGC
          1 ATAGAGCAGGTTTGAAACACTCTTTTTGTAGTATCTGGAAGTGGATATTTGGATAGCTTTGAGGA

            * ** *      *            * *      *      *      *  * **      *  *
       3964 CTATGGT-GAAAAAGGAAATATCTTTACATAAAAACTAGACGGAAGCAGTCACCCAAACTTATTG
         66 TTTCGTTGGAAACAGG-AATATCTTCATATAAAAGCTAGACAGAAGCATTCTCAGAAACTTCTTT

             **    *  * *    *    * **   *  *  *     *
       4028 GGAATGTGTGTACTCAACTAACCGATTTGGA-TTTTCCTTTG
        130 GTGATGTTTGCATTCAAGTAACAGTGTTGAACATTCCCTTTC

                     *                   * *     *                           
       4069 ATAGAGCAGTTTTGAAACACTCTTTTTGTGGAATCTGCAAGTGGATATTTGGATAGCTTTGAGGA
          1 ATAGAGCAGGTTTGAAACACTCTTTTTGTAGTATCTGGAAGTGGATATTTGGATAGCTTTGAGGA

              **         *      **   *       *    *                   *   *  
       4134 TTAGGTTGGAAACGGGAATAAGTTCTTATAAAACCTAGGCAGAAGCATTCTCAGAAACATCTCTG
         66 TTTCGTTGGAAACAGGAATATCTTCATATAAAAGCTAGACAGAAGCATTCTCAGAAACTTCTTTG

               * *            **   *                 
       4199 TGAGGATTGCATTCAAGTCCCAGAGTTGAACATTCCCTTTC
        131 TGATGTTTGCATTCAAGTAACAGTGTTGAACATTCCCTTTC

                     * *          **           *     * *  *       **      *  
       4240 ATAGAGCAGTTATGAAATCA-TGATTTTGTAGTATGTGGAACTAGACATTTGGAGTGCTTTGTGG
          1 ATAGAGCAGGTTTGAAA-CACTCTTTTTGTAGTATCTGGAAGTGGATATTTGGATAGCTTTGAGG

             ** *  **    *             **      *    *                   ***  
       4304 -CCTAGTGTGAAAAAGGAAATATCTTCCCATAAAAACTAGGCAGAAGCATTCTCAGAAACCAGTT
         65 ATTTCGTTGGAAACAGG-AATATCTTCATATAAAAGCTAGACAGAAGCATTCTCAGAAACTTCTT

                  * *  * **   *      *          * * * *
       4368 TGTGATATGTGTACACAACTAACAGGGTTGAACATTTCTTCTG
        129 TGTGATGTTTGCATTCAAGTAACAGTGTTGAACATTCCCTTTC

             *    * ***                    **    *      *                    
       4411 AGAGAGGACTCTTGAAACACTCTTTTTGTAGACTCTGCAAG-GGGTATTTGGATAGCTTTGAGGA
          1 ATAGAGCAGGTTTGAAACACTCTTTTTGTAGTATCTGGAAGTGGATATTTGGATAGCTTTGAGGA

                   *     **          *       *                        *      
       4475 TTTCGTTAGAAACGTGAATATCTTCGTATAAAATCTAGACAGAAGCATTCTCAGAAACATCTTTG
         66 TTTCGTTGGAAACAGGAATATCTTCATATAAAAGCTAGACAGAAGCATTCTCAGAAACTTCTTTG

            *   *             * ** *              *  
       4540 GGATTTTTGCATTCAAGTCAAGGAGTTGAACATTCCCTATC
        131 TGATGTTTGCATTCAAGTAACAGTGTTGAACATTCCCTTTC

              *           *  *           * * *     *     *    *   *  *      *
       4581 ATGGAGCAGGTTTGCAAAACTCTTTTTGTGGAAACTGGACGTGGACATTTAGATCGCATTGAGGC
          1 ATAGAGCAGGTTTGAAACACTCTTTTTGTAGTATCTGGAAGTGGATATTTGGATAGCTTTGAGGA

            * ** *  *   *            **      *     *             *  *  **    
       4646 CTGTGGTGAAAAAAGGAATATCTTCGAATAAAAACTAGAGAGAAGCATTCTCATAATCTAGTTTG
         66 TTTCGTTGGAAACAGGAATATCTTCATATAAAAGCTAGACAGAAGCATTCTCAGAAACTTCTTTG

              *   *  ***    *      * *     *  * *    
       4711 TGGTGTGTGTGCTCAACTAACAGAGCTGAACGTTTCTTTTC
        131 TGATGTTTGCATTCAAGTAACAGTGTTGAACATTCCCTTTC

               *     **                *   *    ****             *           
       4752 ATAAAGCAGTATTGAAACACTCTTTTTTTAGAATCTACCTGTGGATATTTGGAAAGCTTTGAGGA
          1 ATAGAGCAGGTTTGAAACACTCTTTTTGTAGTATCTGGAAGTGGATATTTGGATAGCTTTGAGGA

                         **          *       *     *                  *      
       4817 TTTCGTTGGAAACGCGAATATCTTCTTATAAAATCTAGATAGAAGCATTCTCAGAAACATCTTTG
         66 TTTCGTTGGAAACAGGAATATCTTCATATAAAAGCTAGACAGAAGCATTCTCAGAAACTTCTTTG

            * *    *          * *  *                 
       4882 GGGTGTTAGCATTCAAGTCAAAGAGTTGAACATTCCCTTTC
        131 TGATGTTTGCATTCAAGTAACAGTGTTGAACATTCCCTTTC

                                         * *          *  *    *   *         *
       4923 ATAGAGCAGGTTTGAAACACTCTTTTTGTGGAATCTGGAAGTAGACATTTTGATCGCTTTGAGGC
          1 ATAGAGCAGGTTTGAAACACTCTTTTTGTAGTATCTGGAAGTGGATATTTGGATAGCTTTGAGGA

            * ** *  *   **           **      *           *       *     ***   
       4988 CTGTGGTGAAAAAGGGAATATCTTCGCATAAAAACTAGACAGAAGTATTCTCATAAACTAGGTTG
         66 TTTCGTTGGAAACAGGAATATCTTCATATAAAAGCTAGACAGAAGCATTCTCAGAAACTTCTTTG

                  *  ***    *      *  *   **  * *   *
       5053 TGATGTGTGTGCTCAACTAACAGAGTCGAAACTTTCTTTTG
        131 TGATGTTTGCATTCAAGTAACAGTGTTGAACATTCCCTTTC

                     *                     *  *  * *             *   *       
       5094 ATAGAGCAGTTTTGAAACACTCTTTTTGTAGAATTTGCATGTGGATATTTGGACAGCATTGAGGA
          1 ATAGAGCAGGTTTGAAACACTCTTTTTGTAGTATCTGGAAGTGGATATTTGGATAGCTTTGAGGA

             *         * *    *    *     *   *** *   *                *      
       5159 TGTCGTTGGAATCGGGAAAATCTGCATATGAAATGGACACACAAGCATTCTCAGAAACCTCTTTG
         66 TTTCGTTGGAAACAGGAATATCTTCATATAAAAGCTAGACAGAAGCATTCTCAGAAACTTCTTTG

            *      *      *   **   *           *     
       5224 GGATGTTAGCATTCCAGTCGCAGAGTTGAACATTCTCTTTC
        131 TGATGTTTGCATTCAAGTAACAGTGTTGAACATTCCCTTTC

                                                         *    *   *         *
       5265 ATAGAGCAGGTTTGAAACACTCTTTTTGTA-TATCTGGAAGTGGACATTTTGATCGCTTTGAGGC
          1 ATAGAGCAGGTTTGAAACACTCTTTTTGTAGTATCTGGAAGTGGATATTTGGATAGCTTTGAGGA

            **** *      *             ** *    *   *       *             *    
       5329 GAAAGGT-GAAAAAGGAAATATCTTCCCACAAAAACTACACAGAAGTATTCTCAGAAACTACTTT
         66 TTTCGTTGGAAACAGG-AATATCTTCATATAAAAGCTAGACAGAAGCATTCTCAGAAACTTCTTT

                   * ** *    *      * *      *  ***   *
       5393 GTGATGTGTTTACTCAACTAACAGAGGTGAACCTTTTTTTTTG
        130 GTGATGTTTGCATTCAAGTAACAGTGTTGAA-CATTCCCTTTC

                 *  ***                *   *     *            *              
       5436 ATAGACCACTCTTGAAACACTCTTTTTATAGAATCTGCAAGTGGATATTTTGATAGCTTTGAGGA
          1 ATAGAGCAGGTTTGAAACACTCTTTTTGTAGTATCTGGAAGTGGATATTTGGATAGCTTTGAGGA

                * **     ***                 *     *                  **  *  
       5501 TTTCCTCAGAAACGATAATATCTTCATATAAAATCTAGAGAGAAGCATTCTCAGAAACAACTCTG
         66 TTTCGTTGGAAACAGGAATATCTTCATATAAAAGCTAGACAGAAGCATTCTCAGAAACTTCTTTG

                      *       * *  *    *            
       5566 TGATGTTTGCGTTCAAGTCAGAGAGTTGTACATTCCCTTTC
        131 TGATGTTTGCATTCAAGTAACAGTGTTGAACATTCCCTTTC

                           *                        *    *    *   **        *
       5607 ATAGAGCAGGTTTGAGACACTCTTTTTGTAGTATCTGGAAATGGACATTTAGATCCCTTTGAGGC
          1 ATAGAGCAGGTTTGAAACACTCTTTTTGTAGTATCTGGAAGTGGATATTTGGATAGCTTTGAGGA

              ** *  *   ** *         **      **     *      *    **      *    
       5672 TTATGGTGAAAAACGAAATATCTTCGCATAAAAAATAGACGGAAGCAGTCTCCAAAACTTGTTTG
         66 TTTCGTTGGAAACAGGAATATCTTCATATAAAAGCTAGACAGAAGCATTCTCAGAAACTTCTTTG

            **    *  * *    *      *      **  * *   *
       5737 GAATGTGTGTACTCAACTAACAGAGTTGAATCTTTCTTTTG
        131 TGATGTTTGCATTCAAGTAACAGTGTTGAACATTCCCTTTC

                     *                     *     *   *           *           
       5778 ATAGAGCAGTTTTGAAACACTCTTTTTGTAGAATCTGCAAGCGGATATTTTTGTATAGCTTTGAG
          1 ATAGAGCAGGTTTGAAACACTCTTTTTGTAGTATCTGGAAGTGGATA--TTTGGATAGCTTTGAG

                       *  **       *           **                   *   *   *
       5843 GATTTCGTTGGGAATGGGAATATGTTCATATAAAATATAGACAGAAGCATTCTCAGGAACATCTC
         64 GATTTCGTTGGAAACAGGAATATCTTCATATAAAAGCTAGACAGAAGCATTCTCAGAAACTTCTT

                 * *            **   *                 
       5908 TGTGAGGATTGCATTCAAGTCCCAGAGTTGAACATTCCCTTTC
        129 TGTGATGTTTGCATTCAAGTAACAGTGTTGAACATTCCCTTTC

                     * *          **           *     * *  *       **      *  
       5951 ATAGAGCAGTTATGAAATCA-TGATTTTGTAGTATGTGGAACTAGACATTTGGAGTGCTTTGTGG
          1 ATAGAGCAGGTTTGAAA-CACTCTTTTTGTAGTATCTGGAAGTGGATATTTGGATAGCTTTGAGG

             ** *  **    *             **      *    *                   ***  
       6015 -CCTAGTGTGAAAAAGGAAATATCTTCCCATAAAAACTAGGCAGAAGCATTCTCAGAAACCAGTT
         65 ATTTCGTTGGAAACAGG-AATATCTTCATATAAAAGCTAGACAGAAGCATTCTCAGAAACTTCTT

                  * *  * **   *      *          * * * *
       6079 TGTGATATGTGTACACAACTAACAGGGTTGAACATTTCTTCTG
        129 TGTGATGTTTGCATTCAAGTAACAGTGTTGAACATTCCCTTTC

             *    * ***                    **    *      *                    
       6122 AGAGAGGACTCTTGAAACACTCTTTTTGTAGACTCTGCAAG-GGGTATTTGGATAGCTTTGAGGA
          1 ATAGAGCAGGTTTGAAACACTCTTTTTGTAGTATCTGGAAGTGGATATTTGGATAGCTTTGAGGA

                   *     **          *       *                        *      
       6186 TTTCGTTAGAAACGTGAATATCTTCGTATAAAATCTAGACAGAAGCATTCTCAGAAACATCTTTG
         66 TTTCGTTGGAAACAGGAATATCTTCATATAAAAGCTAGACAGAAGCATTCTCAGAAACTTCTTTG

            *   *             * ** *              *  
       6251 GGATTTTTGCATTCAAGTCAAGGAGTTGAACATTCCCTATC
        131 TGATGTTTGCATTCAAGTAACAGTGTTGAACATTCCCTTTC

              *           *  *           * * *     *     *        *  *      *
       6292 ATGGAGCAGGTTTGCAAAACTCTTTTTGTGGAAACTGGACGTGGACATTTGGATCGCATTGAGGC
          1 ATAGAGCAGGTTTGAAACACTCTTTTTGTAGTATCTGGAAGTGGATATTTGGATAGCTTTGAGGA

            * *  *  *   *            **      *     *             *  *  **    
       6357 CTGCGGTGAAAAAAGGAATATCTTCGAATAAAAACTAGAGAGAAGCATTCTCATAATCTAGTTTG
         66 TTTCGTTGGAAACAGGAATATCTTCATATAAAAGCTAGACAGAAGCATTCTCAGAAACTTCTTTG

              *   *  ***    *      * *     *  * *    
       6422 TGGTGTGTGTGCTCAACTAACAGAGCTGAACGTTTCTTTTC
        131 TGATGTTTGCATTCAAGTAACAGTGTTGAACATTCCCTTTC

               *     **                *   *    ****             *  *        
       6463 ATAAAGCAGTATTGAAACACTCTTTTTTTAGAATCTACCTGTGGATA-TTGGAAAGTTTTGAGGA
          1 ATAGAGCAGGTTTGAAACACTCTTTTTGTAGTATCTGGAAGTGGATATTTGGATAGCTTTGAGGA

                         **          *       *     *                  *      
       6527 TTTCGTTGGAAACGCGAATATCTTCTTATAAAATCTAGATAGAAGCATTCTCAGAAACATCTTTG
         66 TTTCGTTGGAAACAGGAATATCTTCATATAAAAGCTAGACAGAAGCATTCTCAGAAACTTCTTTG

            * *    *          * *  *                 
       6592 GGGTGTTAGCATTCAAGTCAAAGAGTTGAACATTCCCTTTC
        131 TGATGTTTGCATTCAAGTAACAGTGTTGAACATTCCCTTTC

                                         * *          *  *    *   *         *
       6633 ATAGAGCAGGTTTGAAACACTCTTTTTGTGGAATCTGGAAGTAGACATTTTGATCGCTTTGAGGC
          1 ATAGAGCAGGTTTGAAACACTCTTTTTGTAGTATCTGGAAGTGGATATTTGGATAGCTTTGAGGA

            * ** *  *   **           **      *           *       *     ***   
       6698 CTGTGGTGAAAAAGGGAATATCTTCGCATAAAAACTAGACAGAAGTATTCTCATAAACTAGGTTG
         66 TTTCGTTGGAAACAGGAATATCTTCATATAAAAGCTAGACAGAAGCATTCTCAGAAACTTCTTTG

                  *  ***    *      *       *  * *   *
       6763 TGATGTGTGTGCTCAACTAACAGAGTTGAACCTTTCTTTTG
        131 TGATGTTTGCATTCAAGTAACAGTGTTGAACATTCCCTTTC

                     *           *         *  *  * *             *   *       
       6804 ATAGAGCAGTTTTGAAACACTTTTTTTGTAGAATTTGCATGTGGATATTTGGACAGCATTGAGGA
          1 ATAGAGCAGGTTTGAAACACTCTTTTTGTAGTATCTGGAAGTGGATATTTGGATAGCTTTGAGGA

             *         * *    *    *     *   *** *   *                *      
       6869 TGTCGTTGGAATCGGGAAAATCTGCATATGAAATGGACACACAAGCATTCTCAGAAACCTCTTTG
         66 TTTCGTTGGAAACAGGAATATCTTCATATAAAAGCTAGACAGAAGCATTCTCAGAAACTTCTTTG

            *      *      *   **   *           *     
       6934 GGATGTTAGCATTCCAGTCGCAGAGTTGAACATTCTCTTTC
        131 TGATGTTTGCATTCAAGTAACAGTGTTGAACATTCCCTTTC

                                                         *    *   *         *
       6975 ATAGAGCAGGTTTGAAACACTCTTTTTGTA-TATCTGGAAGTGGACATTTTGATCGCTTTGAGGC
          1 ATAGAGCAGGTTTGAAACACTCTTTTTGTAGTATCTGGAAGTGGATATTTGGATAGCTTTGAGGA

            **** *      *             ** *    *   *       *             *    
       7039 AAAAGGT-GAAAAAGGAAATATCTTCCCACAAAAACTACACAGAAGTATTCTCAGAAACTACTTT
         66 TTTCGTTGGAAACAGG-AATATCTTCATATAAAAGCTAGACAGAAGCATTCTCAGAAACTTCTTT

                   * ** *    *      * *     *  ***   *
       7103 GTGATGTGTTTACTCAACTAACAGAGGTGAACCTTTTTTTTG
        130 GTGATGTTTGCATTCAAGTAACAGTGTTGAACATTCCCTTTC

                 *  ***                *   *     *            *              
       7145 ATAGACCACTCTTGAAACACTCTTTTTATAGAATCTGCAAGTGGATATTTTGATAGCTTTGAGGA
          1 ATAGAGCAGGTTTGAAACACTCTTTTTGTAGTATCTGGAAGTGGATATTTGGATAGCTTTGAGGA

                * **     ***                 *     *                  **  *  
       7210 TTTCCTCAGAAACGATAATATCTTCATATAAAATCTAGAGAGAAGCATTCTCAGAAACAACTCTG
         66 TTTCGTTGGAAACAGGAATATCTTCATATAAAAGCTAGACAGAAGCATTCTCAGAAACTTCTTTG

                              * *  *    *            
       7275 TGATGTTTGCATTCAAGTCAGAGAGTTGTACATTCCCTTTC
        131 TGATGTTTGCATTCAAGTAACAGTGTTGAACATTCCCTTTC

                     *                              *    *    *   **        *
       7316 ATAGAGCAGCTTTGAAACACTCTTTTTGTAGTATCTGGAAATGGACATTTAGATCCCTTTGAGGC
          1 ATAGAGCAGGTTTGAAACACTCTTTTTGTAGTATCTGGAAGTGGATATTTGGATAGCTTTGAGGA

              ** *      *             ** *    **    **      *    **      *   
       7381 TTATGGT-GAAAAAGGAAATATCTTCGCAAAAAAAATAGATGGAAGCAGTCTCCAAAACTTGTTT
         66 TTTCGTTGGAAACAGG-AATATCTTCATATAAAAGCTAGACAGAAGCATTCTCAGAAACTTCTTT

             **    *  * *    *      *      **  * *   *
       7445 GGAATGTGTGTACTCAACTAACAGAGTTGAATCTTTCTTTTG
        130 GTGATGTTTGCATTCAAGTAACAGTGTTGAACATTCCCTTTC

                     *                     *     *               *           
       7487 ATAGAGCAGTTTTGAAACACTCTTTTTGTAGAATCTGCAAGTGGATATTTTTGTATAGCTTTGAG
          1 ATAGAGCAGGTTTGAAACACTCTTTTTGTAGTATCTGGAAGTGGATA--TTTGGATAGCTTTGAG

                       *  **       *           **                   *   *   *
       7552 GATTTCGTTGGGAATGGGAATATGTTCATATAAAATATAGACAGAAGCATTCTCAGGAACATCTC
         64 GATTTCGTTGGAAACAGGAATATCTTCATATAAAAGCTAGACAGAAGCATTCTCAGAAACTTCTT

                 * *            **   *                 
       7617 TGTGAGGATTGCATTCAAGTCCCAGAGTTGAACATTCCCTTTC
        129 TGTGATGTTTGCATTCAAGTAACAGTGTTGAACATTCCCTTTC

                     * *          **         * *     *    *       **       * 
       7660 ATAGAGCAGTTATGAAATCA-TGATTTTGTAGTGTGTGGAACTGGACATTTGGAGTGCTTTG-TG
          1 ATAGAGCAGGTTTGAAA-CACTCTTTTTGTAGTATCTGGAAGTGGATATTTGGATAGCTTTGAGG

              ****        *             **      ** * *                   *** 
       7723 -TCCTATTGTGAAAAAGGAAATATCTTCCCATAAAAAATTGGCAGAAGCATTCTCAGAAACCAGT
         65 ATTTCGTTG-GAAACAGG-AATATCTTCATATAAAAGCTAGACAGAAGCATTCTCAGAAACTTCT

                     *  * *    *      *       ** * *   *
       7787 TTGTGATGTGTGTACTCAACTAACAGGGTTGAACCCTTCTTTTG
        128 TTGTGATGTTTGCATTCAAGTAACAGTGTTGAACATTCCCTTTC

             *      *** *                  **    *                           
       7831 AGAGAGCACTCTAGAAACACTCTTTTTGTAGAGTCTGCAAGTGGATATTTGGATAGCTTTGAGGA
          1 ATAGAGCAGGTTTGAAACACTCTTTTTGTAGTATCTGGAAGTGGATATTTGGATAGCTTTGAGGA

                *        * *                 *   *     *              *      
       7896 TTTCCTTGGAAACGGCAATATCTTCATATAAAATCTAAACAGACG-ATTCTCAGAAACATCTTTG
         66 TTTCGTTGGAAACAGGAATATCTTCATATAAAAGCTAGACAGAAGCATTCTCAGAAACTTCTTTG

            *                 *    *                 
       7960 GGATGTTTGCATTCAAGTCACAGAGTTGAACATTCCCTTTC
        131 TGATGTTTGCATTCAAGTAACAGTGTTGAACATTCCCTTTC

              *         **      *        ***             *        *  *   *  *
       8001 ATGGAGCAGGTTGAAAACACCCTTTTTGTGCAATCTGGAAGTGGACATTTGGATCGCATTGGGGC
          1 ATAGAGCAGGTTTGAAACACTCTTTTTGTAGTATCTGGAAGTGGATATTTGGATAGCTTTGAGGA

            * ** *  *   **        *  **      *                   *   ****    
       8066 CTATGGTGAAAAAGGGAATATCCTCGAATAAAAACTAGACAGAAGCATTCTCATAAAGCAGTTTG
         66 TTTCGTTGGAAACAGGAATATCTTCATATAAAAGCTAGACAGAAGCATTCTCAGAAACTTCTTTG

                  *  ***    *      * *     *  * *    
       8131 TGATGTGTGTGCTCAACTAACAGAGCTGAACCTTTCTTTTC
        131 TGATGTTTGCATTCAAGTAACAGTGTTGAACATTCCCTTTC

                  *  *                   * *     * *             *  *        
       8172 ATAGAGTAGTTTTGAAACACTCTTTTTGTGGAATCTGCATGTGGATATTTGGAAAGTTTTGAGGA
          1 ATAGAGCAGGTTTGAAACACTCTTTTTGTAGTATCTGGAAGTGGATATTTGGATAGCTTTGAGGA

                *       **           *       *       *          *     *      
       8237 TTTCTTTGGAAAAGGGAATATCTTCCTATAAAATCTAGACAAAAGCATTCTCGGAAACATCTTTG
         66 TTTCGTTGGAAACAGGAATATCTTCATATAAAAGCTAGACAGAAGCATTCTCAGAAACTTCTTTG

            *   *  *  *       *    *      *      * * 
       8302 GGATATTAGCCTTCAAGTCACAGAGTTGAATATTCCCATAC
        131 TGATGTTTGCATTCAAGTAACAGTGTTGAACATTCCCTTTC

                                         * *             **       *         *
       8343 ATAGAGCAGGTTTGAAACACTCTTTTTGTTGCATCTGGAAGTGGACTTTTGGATCGCTTTGAGGC
          1 ATAGAGCAGGTTTGAAACACTCTTTTTGTAGTATCTGGAAGTGGATATTTGGATAGCTTTGAGGA

            * ** *  *   **           **      *   *       *       *     **    
       8408 GTGTGGTGAAAAAGGGAATATCTTCGCATAAAAACTATACAGAAGTATTCTCATAAACTAGTTTG
         66 TTTCGTTGGAAACAGGAATATCTTCATATAAAAGCTAGACAGAAGCATTCTCAGAAACTTCTTTG

                  *  ***    *      **  *      * *   *
       8473 TGATGTGTGTTCTCAACTAACAGATTTCAACATTTCTTTTG
        131 TGATGTTTGCATTCAAGTAACAGTGTTGAACATTCCCTTTC

                   * *                     *  *  * *   *       * * *         
       8514 ATAGAGCGGTTTTGAAACACTCTTTTTGTAGAATTTGCATGTGAATATTTGTACAACTTTGAGGA
          1 ATAGAGCAGGTTTGAAACACTCTTTTTGTAGTATCTGGAAGTGGATATTTGGATAGCTTTGAGGA

               *  *       *           *   *   * *                  *  **     
       8579 TTTTGTCGGAAACCTGGAA-ATCTTCCTATGAAATCGAGAC--AAGCATTCTCAGGAATCTCTTT
         66 TTTCGTTGGAAA-CAGGAATATCTTCATATAAAAGCTAGACAGAAGCATTCTCAGAAACTTCTTT

             *      *      *   *    *                 
       8641 GGGATGTTAGCATTCGAGTCACAGAGTTGAACATTCCCTTTC
        130 GTGATGTTTGCATTCAAGTAACAGTGTTGAACATTCCCTTTC

                                            *            *        *     *   *
       8683 ATAGAGCAGGTTTGAAACACTCTTTTTGTAGTGTCTGGAAGTGGACATTTGGATTGCTTTCAGGC
          1 ATAGAGCAGGTTTGAAACACTCTTTTTGTAGTATCTGGAAGTGGATATTTGGATAGCTTTGAGGA

              ** *      *             ** *    *   *                     *    
       8748 TTAAGGT-GAAAAAGGAAATATCTTCCCACAAAAACTACACAGAAGCATTCTCAGAAACTACTTT
         66 TTTCGTTGGAAACAGG-AATATCTTCATATAAAAGCTAGACAGAAGCATTCTCAGAAACTTCTTT

                   * ** *    *  *   *          * *   *
       8812 GTGATGTGTTTACTCAACT-TCAGAGTTGAACATTTCTTTTG
        130 GTGATGTTTGCATTCAAGTAACAGTGTTGAACATTCCCTTTC

                     *                     *     *   *           *           
       8853 ATAGAGCAGTTTTGAAACACTCTTTTTGTAGAATCTGCAAGAGGATATTTGGAGAGCTTTGAGGA
          1 ATAGAGCAGGTTTGAAACACTCTTTTTGTAGTATCTGGAAGTGGATATTTGGATAGCTTTGAGGA

                         *   *               *                      * ***    
       8918 TTTC-TTGGAAACGGGACTATCTTCATATAAAATCTAGACAGAAGCATTCTCAGAAGCAAATTTG
         66 TTTCGTTGGAAACAGGAATATCTTCATATAAAAGCTAGACAGAAGCATTCTCAGAAACTTCTTTG

                    * *  *    * *  *    *            
       8982 TGATGTTTACCTTGAAGTCAGAGAGTTGTACATTCCCTTTC
        131 TGATGTTTGCATTCAAGTAACAGTGTTGAACATTCCCTTTC

                       *                      *     *    *    *   * *     ***
       9023 ATAGAGCAGGTCTGAAACACTCTTTTTGTAGTATGTGGAAATGGACATTTAGATCGTTTTGACAC
          1 ATAGAGCAGGTTTGAAACACTCTTTTTGTAGTATCTGGAAGTGGATATTTGGATAGCTTTGAGGA

            * ** *      *              *      *      *      *    **      *   
       9088 CTATGGT-GAAAAAGGAAATATCTTCACATAAAAACTAGACGGAAGCAATCTCCAAAACTTGTTT
         66 TTTCGTTGGAAACAGG-AATATCTTCATATAAAAGCTAGACAGAAGCATTCTCAGAAACTTCTTT

             **    *  * *   **      *      **  ***   *
       9152 GGAATGTGTGTACTCAGCTAACAGAGTTGAATCTTTTTTTTG
        130 GTGATGTTTGCATTCAAGTAACAGTGTTGAACATTCCCTTTC

                     *                    **     *           *               
       9194 ATAGA-CAGTTTTGAAACACTCTTTTTGTAAAATCTGCAAGTGGATATTGGGATAGCTTTGAGGA
          1 ATAGAGCAGGTTTGAAACACTCTTTTTGTAGTATCTGGAAGTGGATATTTGGATAGCTTTGAGGA

                *       **       *        *  *           *   *        *   *  
       9258 TTTCCTTGGAAAGGGGAATATGTTCATATACAAACTAGACAGAAGAATTTTCAGAAACATCTCTG
         66 TTTCGTTGGAAACAGGAATATCTTCATATAAAAGCTAGACAGAAGCATTCTCAGAAACTTCTTTG

               * *            **   *                 
       9323 TGAGGATTGCATTCAAGTCCCAGAGTTGAACATTCCCTTTC
        131 TGATGTTTGCATTCAAGTAACAGTGTTGAACATTCCCTTTC

                       *     **  **           *     *   **              *  * 
       9364 ATAGAGCAGGTATGAAAACCTGATTTTGTAGTATGTGGAACTGGTCATTTGGAGTA-CTTCG-TG
          1 ATAGAGCAGGTTTGAAACACTCTTTTTGTAGTATCTGGAAGTGGATATTTGGA-TAGCTTTGAGG

             **  *    *   **          ***      *    **     *   *        ***  
       9427 ACCT-ATTGTCAAAAGGGAATATCTTGCCATAAAAACTAGGTAGAAGTATTTTCAGAAACAAGTT
         65 ATTTCGTTG-GAAACAGGAATATCTTCATATAAAAGCTAGACAGAAGCATTCTCAGAAACTTCTT

                    * ** *    *      **     **  * *   *
       9491 TGTGATGTGTATACTCAACTAACAGGTTTGAAACTTTCTTTTG
        129 TGTGATGTTTGCATTCAAGTAACAGTGTTGAACATTCCCTTTC

                    ***    *               **    *   *                       
       9534 ATAGAGCACTCTTGATACACTCTTTTTGTAGACTCTGCAAGGGGATATTTGGATAGCTTTGAGGA
          1 ATAGAGCAGGTTTGAAACACTCTTTTTGTAGTATCTGGAAGTGGATATTTGGATAGCTTTGAGGA

                *        *          *    *   *                        *      
       9599 TTTCTTTGGAAACGGGAATATCTTAATATGAAATCTAGACAGAAGCATTCTCAGAAACATCTTTG
         66 TTTCGTTGGAAACAGGAATATCTTCATATAAAAGCTAGACAGAAGCATTCTCAGAAACTTCTTTG

            *   *             * *  *                 
       9664 GGATATTTGCATTCAAGTCAGAGAGTTGAACATTCCCTTTC
        131 TGATGTTTGCATTCAAGTAACAGTGTTGAACATTCCCTTTC

              *            *             ***             *        *  *   *  *
       9705 ATGGAGCAGGTTTGAGACACTCTTTTTGTGCAATCTGGAAGTGGACATTTGGATCGCATTGGGGC
          1 ATAGAGCAGGTTTGAAACACTCTTTTTGTAGTATCTGGAAGTGGATATTTGGATAGCTTTGAGGA

            * ** *  *   ** *         **      *                   *     ***   
       9770 CTATGGTGAAAATGGAAATATCTTCGAATAAAAACTAGACAGAAGCATTCTCATAAACTAGCTTG
         66 TTTCGTTGGAAACAGGAATATCTTCATATAAAAGCTAGACAGAAGCATTCTCAGAAACTTCTTTG

                  *  ***    * *    * **    *  * *   *
       9835 TGATGTGTGGGCTCAACTGACAGAGCGGAACCTTTCTTTTG
        131 TGATGTTTGCATTCAAGTAACAGTGTTGAACATTCCCTTTC

             *      ***                   ***    *   *           *           
       9876 AGAGAGCACTGTTGAAACACTCTTTTTGTACACTCTGCAAGGGGATATTTGGACAGCTTTGAGGA
          1 ATAGAGCAGGTTTGAAACACTCTTTTTGTAGTATCTGGAAGTGGATATTTGGATAGCTTTGAGGA

                                             *  *           *         *      
       9941 TTTCGTTGGAAACAGG-ATATCTTCATATAAAATCTCGACAGAAGCATCCTCAGAAACATCTTTG
         66 TTTCGTTGGAAACAGGAATATCTTCATATAAAAGCTAGACAGAAGCATTCTCAGAAACTTCTTTG

            *          *      *    *                 
      10005 GGATGTTTGCAATCAAGTCACAGAGTTGAACATTCCCTTTC
        131 TGATGTTTGCATTCAAGTAACAGTGTTGAACATTCCCTTTC

              *                          * *             *        *         *
      10046 ATGGAGCAGGTTTGAAACACTCTTTTTGTGGAATCTGGAAGTGGACATTTGGATCGCTTTGAGGC
          1 ATAGAGCAGGTTTGAAACACTCTTTTTGTAGTATCTGGAAGTGGATATTTGGATAGCTTTGAGGA

            * *  *  *   **           **      *                   *     **    
      10111 CTGCGGTGAAAAAGGGAATATCTTCGAATAAAATCTAGACAGAAGCATTCTCATAAACTAGTTTG
         66 TTTCGTTGGAAACAGGAATATCTTCATATAAAAGCTAGACAGAAGCATTCTCAGAAACTTCTTTG

                              *      * *     *  * *    
      10176 TGATGTGTGTGC-TT-AACTAACAGAGCTGAACCTTTCTTTTC
        131 TGATGT-T-TGCATTCAAGTAACAGTGTTGAACATTCCCTTTC

                   * *                     *     * *             *           
      10217 ATAGAGCGGTTTTGAAACACTCTTTTTGTAGAATCTGCATGTGGATATTTGGAAAGCTTTGAGGA
          1 ATAGAGCAGGTTTGAAACACTCTTTTTGTAGTATCTGGAAGTGGATATTTGGATAGCTTTGAGGA

                *        *            **     *                        *      
      10282 TTTCTTTGGAAACGGGAATATCTTCACTTAAAATCTAGACAGAAGCATTCTCAGAAACGTCTTTG
         66 TTTCGTTGGAAACAGGAATATCTTCATATAAAAGCTAGACAGAAGCATTCTCAGAAACTTCTTTG

            * *    *          *   **       *    *    
      10347 GGGTGTTAGCATTCAAGTCACATAGTTGAACGTTCCTTTTC
        131 TGATGTTTGCATTCAAGTAACAGTGTTGAACATTCCCTTTC

                     *                   * *             *     *  *         *
      10388 ATAGAGCAGTTTTGAAACACTCTTTTTGTGGAATCTGGAAGTGGACATTTGCATCGCTTTGAGGC
          1 ATAGAGCAGGTTTGAAACACTCTTTTTGTAGTATCTGGAAGTGGATATTTGGATAGCTTTGAGGA

            * *  *     **   *    *   **      *           *       *     **    
      10453 CTGCGGT-GAAGAAGGTATATTTTCGCATAAAAACTAGACAGAAGTATTCTCATAAACTAGTTTG
         66 TTTCGTTGGAAACAGGAATATCTTCATATAAAAGCTAGACAGAAGCATTCTCAGAAACTTCTTTG

                  *  **     *  *   *       *  * *   *
      10517 TGATGTGTGTGTTCAACTACCAGAGTTGAACCTTTCTTTTG
        131 TGATGTTTGCATTCAAGTAACAGTGTTGAACATTCCCTTTC

                     *                         *  * *             *          
      10558 ATAGAGCAGTTTTGAAACACTCTTTTTGT-GTAATTTGCATGTGGATATTTGGACAGCTTTGAGG
          1 ATAGAGCAGGTTTGAAACACTCTTTTTGTAGT-ATCTGGAAGTGGATATTTGGATAGCTTTGAGG

                *         *    *          *   * *     *                *     
      10622 ATTTTGTTGGAAACGGGAAAATCTTCATATGAAATCGAGACACAAGCATTCTCAGAAACCTCTTT
         65 ATTTCGTTGGAAACAGGAATATCTTCATATAAAAGCTAGACAGAAGCATTCTCAGAAACTTCTTT

             *      *  *   *   *    *     *  *        
      10687 GGGATGTTAGCGTTCGAGTCACAGAGTTGATCACTCCCTTTC
        130 GTGATGTTTGCATTCAAGTAACAGTGTTGAACATTCCCTTTC

                  *  *      *      *      *              **   *   *         *
      10729 ATAGAGTAGCTTTGAAGCACTCTGTTTGTAATATCTGGAAGTGGACGTTTTGATCGCTTTGAGGC
          1 ATAGAGCAGGTTTGAAACACTCTTTTTGTAGTATCTGGAAGTGGATATTTGGATAGCTTTGAGGA

            * ** *      *           **** *    *   *                     * *  
      10794 GTAAGGT-GAAAAAGGAAATATCTGGCCACAAAAACTACACAGAAGCATTCTCAGAAACTACGTT
         66 TTTCGTTGGAAACAGG-AATATCTTCATATAAAAGCTAGACAGAAGCATTCTCAGAAACTTCTTT

                   * ** *    *      *       *  * *   *
      10858 GTGATGTGTTTACTCAACTAACAGAGTTGAACCTTTCTTTTG
        130 GTGATGTTTGCATTCAAGTAACAGTGTTGAACATTCCCTTTC

                     *                  *  *     * *                         
      10900 ATAGAGCAGTTTTGAAACACTCTTTTTGGAGAATCTGCAGGTGGATATTTGGATAGCTTTGAGGA
          1 ATAGAGCAGGTTTGAAACACTCTTTTTGTAGTATCTGGAAGTGGATATTTGGATAGCTTTGAGGA

                *       **                   *            *   *       **     
      10965 TTTCCTTGGAAAAGGGAATATCTTCATATAAAATCTAGACAGAAGCCTTCGCAGAAACAACTTTG
         66 TTTCGTTGGAAACAGGAATATCTTCATATAAAAGCTAGACAGAAGCATTCTCAGAAACTTCTTTG

                         *    * *  *    *            
      11030 TGATGTTTGCATTGAAGTCAGAGAGTTGTACATTCCCTTTC
        131 TGATGTTTGCATTCAAGTAACAGTGTTGAACATTCCCTTTC

                     *   *                         **    *    **  *         *
      11071 ATAGAGCAGCTTTCAAACACTCTTTTTGTAGTATCTGGAGATGGACATTTACATCGCTTTGAGGC
          1 ATAGAGCAGGTTTGAAACACTCTTTTTGTAGTATCTGGAAGTGGATATTTGGATAGCTTTGAGGA

            * ** *      *       * *   **      *      *      *    **      **  
      11136 CTATGGT-GAAATAGGAAATCTGTTCGCATAAAAACTAGACGGAAGCAGTCTCCAAAACTTGCTT
         66 TTTCGTTGGAAACAGG-AATATCTTCATATAAAAGCTAGACAGAAGCATTCTCAGAAACTTCTTT

             **    *  * *    *      *      ** ** *   *
      11200 GGAATGTGTGTACTCAACTAACAGAGTTGAATCTCTCTTTTG
        130 GTGATGTTTGCATTCAAGTAACAGTGTTGAACATTCCCTTTC

                     *       *             **   **  *                  *     
      11242 ATAGAGCAGTTTTGAAATACTCTTTTTGTAGAGTCTACAATTGGATATTTGGATAGCTTAGAGGA
          1 ATAGAGCAGGTTTGAAACACTCTTTTTGTAGTATCTGGAAGTGGATATTTGGATAGCTTTGAGGA

                         *       * *    *    *                       **   *  
      11307 TTTCGTTGGAAACGGGAATATGTCCATACAAAACCTAGACAGAAGCATTCTCAGAAAAATCTCTG
         66 TTTCGTTGGAAACAGGAATATCTTCATATAAAAGCTAGACAGAAGCATTCTCAGAAACTTCTTTG

               * *            **                     
      11372 TGAGGATTGCATTCAAGTCCCAGTGTTGAACATTCCCTTTC
        131 TGATGTTTGCATTCAAGTAACAGTGTTGAACATTCCCTTTC

               *       *         ***           *     *    *       **       * 
      11413 ATAAAGCAGGTGTG-AACACAAGATTTTGTAGTATATGGAACTGGACATTTGGAGTGCTTTG-TG
          1 ATAGAGCAGGTTTGAAACAC-TCTTTTTGTAGTATCTGGAAGTGGATATTTGGATAGCTTTGAGG

             **  *        **            **   *  *    *                   ****
      11476 ACCT-ATTGTGAAAAGGGAAATATCTTCCCATATAAACTAGGCAGAAGCATTCTCAGAAACCAGA
         65 ATTTCGTTG-GAAACAGG-AATATCTTCATATAAAAGCTAGACAGAAGCATTCTCAGAAACTTCT

                     *  * *    *      * *     *    *   *
      11540 TTGTGATGTGTGTACTCAACTAACAGGGGTGAACCTTCCTTTTG
        128 TTGTGATGTTTGCATTCAAGTAACAGTGTTGAACATTCCCTTTC

             *      ***                    **    *  **           *           
      11584 AGAGAGCACTCTTGAAACACTCTTTTTGTAGACTCTGCAATGGGATATTTGGACAGCTTTGAGGA
          1 ATAGAGCAGGTTTGAAACACTCTTTTTGTAGTATCTGGAAGTGGATATTTGGATAGCTTTGAGGA

                            *                *  *           *         *      
      11649 TTTCGTTGGAAAC-GGGATATCTTCATATAAAATCTCGACAGAAGCATCCTCAGAAACATCTTTG
         66 TTTCGTTGGAAACAGGAATATCTTCATATAAAAGCTAGACAGAAGCATTCTCAGAAACTTCTTTG

            *                 *    *              *  
      11713 GGATGTTTGCATTCAAGTCACAGAGTTGAACATTCCCTATC
        131 TGATGTTTGCATTCAAGTAACAGTGTTGAACATTCCCTTTC

              *                          * *             *        *  *      *
      11754 ATGGAGCAGGTTTGAAACACTCTTTTTGTGGAATCTGGAAGTGGACATTTGGATCGCATTGAGGC
          1 ATAGAGCAGGTTTGAAACACTCTTTTTGTAGTATCTGGAAGTGGATATTTGGATAGCTTTGAGGA

            * *  *  *   **           **      *   * *             *     **    
      11819 CTACGGTGAAAAAGGGAATATCTTCGAATAAAAACTATATAGAAGCATTCTCATAAACTAGTTTG
         66 TTTCGTTGGAAACAGGAATATCTTCATATAAAAGCTAGACAGAAGCATTCTCAGAAACTTCTTTG

                              *      * *     *  * *    
      11884 TGATGTGTGTGC-TT-AACTAACAGAGCTGAACCTTTCTTTTC
        131 TGATGT-T-TGCATTCAAGTAACAGTGTTGAACATTCCCTTTC

                   * *                     *     * *             *           
      11925 ATAGAGCGGTTTTGAAACACTCTTTTTGTAGAATCTGCATGTGGATATTTGGAAAGCTTTGAGGA
          1 ATAGAGCAGGTTTGAAACACTCTTTTTGTAGTATCTGGAAGTGGATATTTGGATAGCTTTGAGGA

             *           *            *      *                *       *      
      11990 TATCGTTGGAAACGGGAATATCTTCACATAAAATCTAGACAGAAGCATTCACAGAAACGTCTTTG
         66 TTTCGTTGGAAACAGGAATATCTTCATATAAAAGCTAGACAGAAGCATTCTCAGAAACTTCTTTG

            * *    *          *    *       *    *    
      12055 GGGTGTTAGCATTCAAGTCACAGAGTTGAACGTTCCTTTTC
        131 TGATGTTTGCATTCAAGTAACAGTGTTGAACATTCCCTTTC

                                         * *             **       *         *
      12096 ATAGAGCAGGTTTGAAACACTCTTTTTGTGGAATCTGGAAGTGGACCTTTGGATCGCTTTGAGGC
          1 ATAGAGCAGGTTTGAAACACTCTTTTTGTAGTATCTGGAAGTGGATATTTGGATAGCTTTGAGGA

            * *  *      *   *       ***      *       *  **       **    **    
      12161 CTGCGGT-GAAAAAGGTATATCTTTGCATAAAAACTAGACACAAATATTCTCATGAACTAGTTTG
         66 TTTCGTTGGAAACAGGAATATCTTCATATAAAAGCTAGACAGAAGCATTCTCAGAAACTTCTTTG

                  *  ***    *  *   *       *  * *   *
      12225 TGATGTGTGTGCTCAACTACCAGAGTTGAACCTTTCTTTTG
        131 TGATGTTTGCATTCAAGTAACAGTGTTGAACATTCCCTTTC

                     *   *                     *  * *             *          
      12266 ATAGAGCAGTTTTTAAACACTCTTTTTGT-GTAATTTGCATGTGGATATTTGGACAGCTTTGAGG
          1 ATAGAGCAGGTTTGAAACACTCTTTTTGTAGT-ATCTGGAAGTGGATATTTGGATAGCTTTGAGG

                          *    *                *     *                *     
      12330 ATTTCGTTGGAAACGGGAAAATCTTCATAT-----CGAGACACAAGCATTCTCAGAAACCTCTTT
         65 ATTTCGTTGGAAACAGGAATATCTTCATATAAAAGCTAGACAGAAGCATTCTCAGAAACTTCTTT

             *      *  *   *   *    *     *  *        
      12390 GGGATGTTAGCGTTCGAGTCACAGAGTTGATCACTCCCTTTC
        130 GTGATGTTTGCATTCAAGTAACAGTGTTGAACATTCCCTTTC

                  *  *      *      *                     **   *   *         *
      12432 ATAGAGTAGCTTTGAAGCACTCTGTTTGTAGTATCTGGAAGTGGACGTTTTGATCGCTTTGAGGC
          1 ATAGAGCAGGTTTGAAACACTCTTTTTGTAGTATCTGGAAGTGGATATTTGGATAGCTTTGAGGA

            * ** *      *            *** *    **  *                     * *  
      12497 GTAAGGT-GAAAAAGGAAATATCTTGCCACAAAAAATACACAGAAGCATTCTCAGAAACTACGTT
         66 TTTCGTTGGAAACAGG-AATATCTTCATATAAAAGCTAGACAGAAGCATTCTCAGAAACTTCTTT

                   * ** *    *      *       *  * *   *
      12561 GTGATGTGTTTACTCAACTAACAGAGTTGAACCTTTCGTTTG
        130 GTGATGTTTGCATTCAAGTAACAGTGTTGAACATTCCCTTTC

                     *          *       *        * *                         
      12603 ATAGAGCAGTTTTGAAACACACTTTTTGGAGTATCTGCAGGTGGATATTTGGATAGCTTTGAGGA
          1 ATAGAGCAGGTTTGAAACACTCTTTTTGTAGTATCTGGAAGTGGATATTTGGATAGCTTTGAGGA

                *       **                   *            *   *        *     
      12668 TTTCCTTGGAAAAGGGAATATCTTCATATAAAATCTAGACAGAAGCCTTCGCAGAAACTCCTTTG
         66 TTTCGTTGGAAACAGGAATATCTTCATATAAAAGCTAGACAGAAGCATTCTCAGAAACTTCTTTG

                         *    * *  *    *            
      12733 TGATGTTTGCATTGAAGTCAGAGAGTTGTACATTCCCTTTC
        131 TGATGTTTGCATTCAAGTAACAGTGTTGAACATTCCCTTTC

                 *   *    *                        **    *    *   *         *
      12774 ATAGAACAGCTTTGGAACACTCTTTTTGTAGTATCTGGAGATGGACATTTAGATCGCTTTGAGGC
          1 ATAGAGCAGGTTTGAAACACTCTTTTTGTAGTATCTGGAAGTGGATATTTGGATAGCTTTGAGGA

            * ** *      *             ** *    *      *  *   *    **      *  *
      12839 CTATGGT-GAAATAGGAAATATCTTCGCACAAAAACTAGACGGATGCAGTCTCCAAAACTTGTTG
         66 TTTCGTTGGAAACAGG-AATATCTTCATATAAAAGCTAGACAGAAGCATTCTCAGAAACTTCTTT

             **    *  * *    *      *      **  * *   *
      12903 GGAATGTGTGTACTCAACTAACAGAGTTGAATCTTTCTTTTG
        130 GTGATGTTTGCATTCAAGTAACAGTGTTGAACATTCCCTTTC

                     *            *        **    *                     *     
      12945 ATAGAGCAGTTTTGAAACACTCCTTTTGTAGGGTCTGCAAGTGGATATTTGGATAGCTTAGAGGA
          1 ATAGAGCAGGTTTGAAACACTCTTTTTGTAGTATCTGGAAGTGGATATTTGGATAGCTTTGAGGA

                                 * *    *    *                       **   *  
      13010 TTTCGTTGGAAAC-GGAATATGTCCATACAAAACCTAGACAGAAGCATTCTCAGAAAAATCTCTG
         66 TTTCGTTGGAAACAGGAATATCTTCATATAAAAGCTAGACAGAAGCATTCTCAGAAACTTCTTTG

               * *            **                     
      13074 TGAGGATTGCATTCAAGTCCCAGTGTTGAACATTCCCTTTC
        131 TGATGTTTGCATTCAAGTAACAGTGTTGAACATTCCCTTTC

               *       *         ***           *     *    *       **       * 
      13115 ATAAAGCAGGTGTG-AACACAAGATTTTGTAGTATATGGAACTGGACATTTGGAGTGCTTTG-TG
          1 ATAGAGCAGGTTTGAAACAC-TCTTTTTGTAGTATCTGGAAGTGGATATTTGGATAGCTTTGAGG

             **  *        *             **   *  *    *              *   **** 
      13178 ACCT-ATTGTGAAAAAGGAAATATCTTCCCATATAAACTAGGCAGAAGCATTCTCATAAAGCAGT
         65 ATTTCGTTG-GAAACAGG-AATATCTTCATATAAAAGCTAGACAGAAGCATTCTCAGAAACTTCT

                   * *  * *    *      *       *  * *   *
      13242 TTGTGATCTGTGTACTCAACTAACAGGGTTGAACCTTTCTTTTG
        128 TTGTGATGTTTGCATTCAAGTAACAGTGTTGAACATTCCCTTTC

             *      ***                    **    *  **           *           
      13286 AGAGAGCACTCTTGAAACACTCTTTTTGTAGACTCTGCAATGGGATATTTGGAAAGCTTTGAGGA
          1 ATAGAGCAGGTTTGAAACACTCTTTTTGTAGTATCTGGAAGTGGATATTTGGATAGCTTTGAGGA

                                             *  *           *         *      
      13351 TTTCGTTGGAAACAGG-ATATCTTCATATAAAATCTCGACAGAAGCATCCTCAGAAACATCTTTG
         66 TTTCGTTGGAAACAGGAATATCTTCATATAAAAGCTAGACAGAAGCATTCTCAGAAACTTCTTTG

            *                 *    *              *  
      13415 GGATGTTTGCATTCAAGTCACAGAGTTGAACATTCCCTATC
        131 TGATGTTTGCATTCAAGTAACAGTGTTGAACATTCCCTTTC

              *                          ***             *    *   *  *      *
      13456 ATGGAGCAGGTTTGAAACACTCTTTTTGTGAAATCTGGAAGTGGACATTTCGATCGCATTGAGGC
          1 ATAGAGCAGGTTTGAAACACTCTTTTTGTAGTATCTGGAAGTGGATATTTGGATAGCTTTGAGGA

              *  *  *   **           **      *                   *     **    
      13521 TTACGGTGAAAAAGGGAATATCTTCGAATAAAAACTAGACAGAAGCATTCTCATAAACTAGTTTG
         66 TTTCGTTGGAAACAGGAATATCTTCATATAAAAGCTAGACAGAAGCATTCTCAGAAACTTCTTTG

                              *      * *     *  * *    
      13586 TGATGTGTGTGC-TT-AACTAACAGAGCTGAACCTTTCTTTTC
        131 TGATGT-T-TGCATTCAAGTAACAGTGTTGAACATTCCCTTTC

                   * *                     *     * *          *  *           
      13627 ATAGAGCGGTTTTGAAACACTCTTTTTGTAGAATCTGCATGTGGATATTTAGAAAGCTTTGAGGA
          1 ATAGAGCAGGTTTGAAACACTCTTTTTGTAGTATCTGGAAGTGGATATTTGGATAGCTTTGAGGA

                         *    *       *      *                        *      
      13692 TTTCGTTGGAAACGGGAAAATCTTCACATAAAATCTAGACAGAAGCATTCTCAGAAACGTCTTTG
         66 TTTCGTTGGAAACAGGAATATCTTCATATAAAAGCTAGACAGAAGCATTCTCAGAAACTTCTTTG

            * *  * **         *    *       *    *    
      13757 GGGTGCTAACATTCAAGTCACAGAGTTGAACGTTCCTTTTC
        131 TGATGTTTGCATTCAAGTAACAGTGTTGAACATTCCCTTTC

                  *      *               * *             *        *         *
      13798 ATAGAGGAGGTTTCAAACACTCTTTTTGTGGAATCTGGAAGTGGACATTTGGATCGCTTTGAGGC
          1 ATAGAGCAGGTTTGAAACACTCTTTTTGTAGTATCTGGAAGTGGATATTTGGATAGCTTTGAGGA

            * ** *      *   *  *     **      *           *       **    **    
      13863 CTGTGGT-GAAAAAGGTATTTCTTCGCATAAAAACTAGACAGAAGTATTCTCATGAACTAGTTTG
         66 TTTCGTTGGAAACAGGAATATCTTCATATAAAAGCTAGACAGAAGCATTCTCAGAAACTTCTTTG

                  *  ***    *  *  **       *  * *   *
      13927 TGATGTGTGTGCTCAACTACCACAGTTGAACCTTTCTTTTG
        131 TGATGTTTGCATTCAAGTAACAGTGTTGAACATTCCCTTTC

                     *   *                     *  * *         *   *          
      13968 ATAGAGCAGTTTTTAAACACTCTTTTTGT-GTAATTTGCATGTGGATATTAGGACAGCTTTGAGG
          1 ATAGAGCAGGTTTGAAACACTCTTTTTGTAGT-ATCTGGAAGTGGATATTTGGATAGCTTTGAGG

                          *    *          *   * *     *                *     
      14032 ATTTCGTTGGAAACGGGAAAATCTTCATATGAAATCGAGACACAAGCATTCTCAGAAACCTCTTT
         65 ATTTCGTTGGAAACAGGAATATCTTCATATAAAAGCTAGACAGAAGCATTCTCAGAAACTTCTTT

             *      *  *   *   *    *     *  *        
      14097 GGGATGTTAGCGTTCGAGTCACAGAGTTGATCACTCCCTTTC
        130 GTGATGTTTGCATTCAAGTAACAGTGTTGAACATTCCCTTTC

                    *       *               *       *  * **   *   *         *
      14139 ATAGAGCAAGTTTGAAGCACTCTTTTTGTAGTTTCTGGAATTGAACGTTTTGATCGCTTTGAGGC
          1 ATAGAGCAGGTTTGAAACACTCTTTTTGTAGTATCTGGAAGTGGATATTTGGATAGCTTTGAGGA

            * ** *      *            * * *    **  *        *            * *  
      14204 GTAAGGT-GAAAAAGGAAATATCTTGACACAAAAAATACACAGAAGCCTTCTCAGAAACTACGTT
         66 TTTCGTTGGAAACAGG-AATATCTTCATATAAAAGCTAGACAGAAGCATTCTCAGAAACTTCTTT

                   * ** *    *      * *     *  * *   *
      14268 GTGATGTGTTTACTCAACTAACAGAGCTGAACCTTTCTTTTG
        130 GTGATGTTTGCATTCAAGTAACAGTGTTGAACATTCCCTTTC

                     *                  *  *      *                          
      14310 ATAGAGCAGTTTTGAAACACTCTTTTTGGAGAATC-GGCAGTTGGATATTTGGATAGCTTTGAGG
          1 ATAGAGCAGGTTTGAAACACTCTTTTTGTAGTATCTGGAAG-TGGATATTTGGATAGCTTTGAGG

                 *       **    *              *            *   *       **    
      14374 ATTTCCTTGGAAAAGGGAACATCTTCATATAAAATCTAGACAGAAGCCTTCGCAGAAACACCTTT
         65 ATTTCGTTGGAAACAGGAATATCTTCATATAAAAGCTAGACAGAAGCATTCTCAGAAACTTCTTT

                          *    * *  *    *            
      14439 GTGATGTTTGCATTGAAGTCAGAGAGTTGTACATTCCCTTTC
        130 GTGATGTTTGCATTCAAGTAACAGTGTTGAACATTCCCTTTC

                     *                             **    *    *   *         *
      14481 ATAGAGCAGCTTTGAAACACTCTTTTTGTAGTATCTGGAGATGGACATTTAGATCGCTTTGAGGC
          1 ATAGAGCAGGTTTGAAACACTCTTTTTGTAGTATCTGGAAGTGGATATTTGGATAGCTTTGAGGA

            * ** *      *             **      *      *      *    **       *  
      14546 CTATGGT-GAAATAGGAAATATCTTCGCATAAAAACTAGACGGAAGCACTCTCCAAAACTTCCTT
         66 TTTCGTTGGAAACAGG-AATATCTTCATATAAAAGCTAGACAGAAGCATTCTCAGAAACTTCTTT

             **    *  ***    *      **    ***  * *   *
      14610 GGAATGTGTGTTCTCAACTAACAGATTTGAGTCTTTCTTTTG
        130 GTGATGTTTGCATTCAAGTAACAGTGTTGAACATTCCCTTTC

                     *                     **    *    *                *     
      14652 ATAGAGCAGTTTTGAAACACTCTTTTTGTAGAGTCTGCAAGTCGATATTTGGATAGCTTAGAGGA
          1 ATAGAGCAGGTTTGAAACACTCTTTTTGTAGTATCTGGAAGTGGATATTTGGATAGCTTTGAGGA

                                 * *    *    *                       **   *  
      14717 TTTCGTTGGAAAC-GGAATATGTCCATAGAAAACCTAGACAGAAGCATTCTCAGAAAAATCTCTG
         66 TTTCGTTGGAAACAGGAATATCTTCATATAAAAGCTAGACAGAAGCATTCTCAGAAACTTCTTTG

               * *            **   *                 
      14781 TGAGGATTGCATTCAAGTCCCAGGGTTGAACATTCCCTTTC
        131 TGATGTTTGCATTCAAGTAACAGTGTTGAACATTCCCTTTC

               *    * **         ***           *     *    *       **       * 
      14822 ATAAAGCATGCGTG-AACACAAGATTTTGTAGTATATGGAACTGGACATTTGGAGTGCTTTG-TG
          1 ATAGAGCAGGTTTGAAACAC-TCTTTTTGTAGTATCTGGAAGTGGATATTTGGATAGCTTTGAGG

             **  *        *             **   *  *    *                  **** 
      14885 ACCT-ATTGTGAAAAAGGAAATATCTTCCCATATAAACTAGGCAGAAGCATTCTCAGAAAACAGT
         65 ATTTCGTTG-GAAACAGG-AATATCTTCATATAAAAGCTAGACAGAAGCATTCTCAGAAACTTCT

                     *  * *    *      *       *  * *   *
      14949 TTGTGATGTGTGTACTCAACTAACAGGGTTGAACCTTTCTTTTG
        128 TTGTGATGTTTGCATTCAAGTAACAGTGTTGAACATTCCCTTTC

             *      ***                    **    *   **       *  *        *  
      14993 AGAGAGCACTCTTGAAACACTC--TTTGTAGACTCTGCAAGGCGATATTTTGACAGCTTTGAAGA
          1 ATAGAGCAGGTTTGAAACACTCTTTTTGTAGTATCTGGAAGTGGATATTTGGATAGCTTTGAGGA

                            *                *  *           *         *      
      15056 TTTCGTTGGAAAC-GGGATATCTTCATATAAAATCTCGACAGAAGCATCCTCAGAAACATCTTTG
         66 TTTCGTTGGAAACAGGAATATCTTCATATAAAAGCTAGACAGAAGCATTCTCAGAAACTTCTTTG

            *                 *    *                 
      15120 GGATGTTTGCATTCAAGTCACAGAGTTGAACATTCCCTTTC
        131 TGATGTTTGCATTCAAGTAACAGTGTTGAACATTCCCTTTC

              *                          * *             * *      *  *      *
      15161 ATGGAGCAGGTTTGAAACACTCTTTTTGTGGAATCTGGAAGTGGACACTTGGATCGCATTGAGGC
          1 ATAGAGCAGGTTTGAAACACTCTTTTTGTAGTATCTGGAAGTGGATATTTGGATAGCTTTGAGGA

            * *  *  *   **           **      *   *               *     **    
      15226 CTACGGTGAAAAAGGGAATATCTTCGAATAAAAACTATACAGAAGCATTCTCATAAACTAGTTTG
         66 TTTCGTTGGAAACAGGAATATCTTCATATAAAAGCTAGACAGAAGCATTCTCAGAAACTTCTTTG

                              *        *     *  * *    
      15291 TGATGTGTGTGC-TT-AACTAACAGTGCTGAACCTTTCTTTTC
        131 TGATGT-T-TGCATTCAAGTAACAGTGTTGAACATTCCCTTTC

                   * *                     *     * *             *           
      15332 ATAGAGCGGTTTTGAAACACTCTTTTTGTAGAATCTGCATGTGGATATTTGGAAAGCTTTGAGGA
          1 ATAGAGCAGGTTTGAAACACTCTTTTTGTAGTATCTGGAAGTGGATATTTGGATAGCTTTGAGGA

                         *            *      *                        *      
      15397 TTTCGTTGGAAACGGGAATATCTTCACATAAAATCTAGACAGAAGCATTCTCAGAAACGTCTTTG
         66 TTTCGTTGGAAACAGGAATATCTTCATATAAAAGCTAGACAGAAGCATTCTCAGAAACTTCTTTG

            * *    *          *    *       *    *    
      15462 GGGTGTTAGCATTCAAGTCACAGAGTTGAACGTTCCTTTTC
        131 TGATGTTTGCATTCAAGTAACAGTGTTGAACATTCCCTTTC

                                         * *             *        *         *
      15503 ATAGAGCAGGTTTGAAACACTCTTTTTGTGGAATCTGGAAGTGGACATTTGGATCGCTTTGAGGC
          1 ATAGAGCAGGTTTGAAACACTCTTTTTGTAGTATCTGGAAGTGGATATTTGGATAGCTTTGAGGA

            * *  *      *   *        **      *           *       **    ** *  
      15568 CTGCGGT-GAAAAAGGTATATCTTCGCATAAAAACTAGACAGAAGTATTCTCATTAACTAGTCTG
         66 TTTCGTTGGAAACAGGAATATCTTCATATAAAAGCTAGACAGAAGCATTCTCAGAAACTTCTTTG

                  *  ***    *  *   *       *  * *   *
      15632 TGATGTGTGTGCTCAACTACCAGAGTTGAACCTTTCTTTTG
        131 TGATGTTTGCATTCAAGTAACAGTGTTGAACATTCCCTTTC

                     *       *         *   *  *  * *             *     *     
      15673 ATAGAGCAGTTTTGAAAAACTCTTTTTCTAGAATTTGCATGTGGATATTTGGACAGCTTAGAGGA
          1 ATAGAGCAGGTTTGAAACACTCTTTTTGTAGTATCTGGAAGTGGATATTTGGATAGCTTTGAGGA

                         *    *          *   * *     *                *      
      15738 TTTCGTTGGAAACGGGAAAATCTTCATATGAAATCGAGACACAAGCATTCTCAGAAACGTCTTTG
         66 TTTCGTTGGAAACAGGAATATCTTCATATAAAAGCTAGACAGAAGCATTCTCAGAAACTTCTTTG

            *      *  *   *  **    *     *  *        
      15803 GGATGTTAGCGTTCGAGGCACAGAGTTGATCACTCCCTTTC
        131 TGATGTTTGCATTCAAGTAACAGTGTTGAACATTCCCTTTC

                     *      *                            **   *   *         *
      15844 ATAGAGCAGCTTTGAAGCACTCTTTTTGTAGTATCTGGAAGTGGACGTTTTGATCGCTTTGAGGC
          1 ATAGAGCAGGTTTGAAACACTCTTTTTGTAGTATCTGGAAGTGGATATTTGGATAGCTTTGAGGA

            * ** *      *            *** *    *   *              *  *   * *  
      15909 GTAAGGT-GAAAAAGGAAATATCTTGCCACAAAAACTACACAGAAGCATTCTCGGACACTACGTT
         66 TTTCGTTGGAAACAGG-AATATCTTCATATAAAAGCTAGACAGAAGCATTCTCAGAAACTTCTTT

                   * ** *    *      *       *  *      
      15973 GTGATGTGTTTACTCAACTAACAGAGTTGAACCTTTCCTTTC
        130 GTGATGTTTGCATTCAAGTAACAGTGTTGAACATTCCCTTTC

                     *         *        *  *     * *                         
      16015 ATAGAGCAGTTTTGAAACAGTCTTTTTGGAGAATCTGCAGGTGGATATTTGGATAGCTTTGAGGA
          1 ATAGAGCAGGTTTGAAACACTCTTTTTGTAGTATCTGGAAGTGGATATTTGGATAGCTTTGAGGA

                *       **                   *                *       **     
      16080 TTTCCTTGGAAAAGGGAATATCTTCATATAAAATCTAGACAGAAGCATTCGCAGAAACACCTTTG
         66 TTTCGTTGGAAACAGGAATATCTTCATATAAAAGCTAGACAGAAGCATTCTCAGAAACTTCTTTG

            *            *    * *  *    *            
      16145 GGATGTTTGCATTGAAGTCAGAGAGTTGTACATTCCCTTTC
        131 TGATGTTTGCATTCAAGTAACAGTGTTGAACATTCCCTTTC

                     *                        *    **    *    *   *         *
      16186 ATAGAGCAGCTTTGAAACACTCTTTTTGTAGTATGTGGAGATGGACATTTAGATCGCTTTGAGGC
          1 ATAGAGCAGGTTTGAAACACTCTTTTTGTAGTATCTGGAAGTGGATATTTGGATAGCTTTGAGGA

            * ** *      *             **      *      *      *    **     *    
      16251 CTATGGT-GAAATAGGAAATATCTTCGCATAAAAACTAGACGGAAGCAGTCTCCAAAACTGC-TT
         66 TTTCGTTGGAAACAGG-AATATCTTCATATAAAAGCTAGACAGAAGCATTCTCAGAAACTTCTTT

             **    *  * *    *      *      **  * **  *
      16314 GGAATGTGTGTACTCAACTAACAGAGTTGAATCTTTCTGTTG
        130 GTGATGTTTGCATTCAAGTAACAGTGTTGAACATTCCCTTTC

                     *                     **    *                     *     
      16356 ATAGAGCAGTTTTGAAACACTCTTTTTGTAGAGTCTGCAAGTGGATATTTGGATAGCTTAGAGGA
          1 ATAGAGCAGGTTTGAAACACTCTTTTTGTAGTATCTGGAAGTGGATATTTGGATAGCTTTGAGGA

                         *       * *    *    *                       **   *  
      16421 TTTCGTTGGAAACGGGAATATGTCCATACAAAACCTAGACAGAAGCATTCTCAGAAAAATCTCTG
         66 TTTCGTTGGAAACAGGAATATCTTCATATAAAAGCTAGACAGAAGCATTCTCAGAAACTTCTTTG

               * *         *  **                     
      16486 TGAGGATTGCATTCATGTCCCAGTGTTGAACATTCCCTTTC
        131 TGATGTTTGCATTCAAGTAACAGTGTTGAACATTCCCTTTC

               *       *         ***           *     *    *       **  *    * 
      16527 ATAAAGCAGGTGTG-AACACAAGATTTTGTAGTATATGGAACTGGACATTTGGAGTGCCTTG-TG
          1 ATAGAGCAGGTTTGAAACAC-TCTTTTTGTAGTATCTGGAAGTGGATATTTGGATAGCTTTGAGG

             **  *        *             **   *  *    *                   ****
      16590 ACCT-ATTGTGAAAAAGGAAATATCTTCCCATATAAACTAGGCAGAAGCATTCTCAGAAACCAGA
         65 ATTTCGTTG-GAAACAGG-AATATCTTCATATAAAAGCTAGACAGAAGCATTCTCAGAAACTTCT

                     ** * *    *      *       *  * *   *
      16654 TTGTGATGTGAGTACTCAACTAACAGGGTTGAACCTTTCTTTTG
        128 TTGTGATGTTTGCATTCAAGTAACAGTGTTGAACATTCCCTTTC

             *      ***                    **    *  **           *           
      16698 AGAGAGCACTCTTGAAACACTCTTTTTGTAGACTCTGCAATGGGATATTTGGACAGCTTTGAGGA
          1 ATAGAGCAGGTTTGAAACACTCTTTTTGTAGTATCTGGAAGTGGATATTTGGATAGCTTTGAGGA

                            *                *  *           *         *      
      16763 TTTCGTTGGAAAC-GGGATATCTTCATATAAAATCTCGACAGAAGCATCCTCAGAAACATCTTTG
         66 TTTCGTTGGAAACAGGAATATCTTCATATAAAAGCTAGACAGAAGCATTCTCAGAAACTTCTTTG

            *                 *    *        *     *  
      16827 GGATGTTTGCATTCAAGTCACAGAGTTGAACACTCCCTATC
        131 TGATGTTTGCATTCAAGTAACAGTGTTGAACATTCCCTTTC

              *              *            **             *        *  *      *
      16868 ATGGAGCAGGTTTGAAAAACTCTTTTTGTAAAATCTGGAAGTGGACATTTGGATCGCATTGAGGC
          1 ATAGAGCAGGTTTGAAACACTCTTTTTGTAGTATCTGGAAGTGGATATTTGGATAGCTTTGAGGA

              *  *  *   **           **      *     *         *   *     **    
      16933 TTACGGTGAAAAAGGGAATATCTTCGAATAAAAACTAGATAGAAGCATTATCATAAACTAGTTTG
         66 TTTCGTTGGAAACAGGAATATCTTCATATAAAAGCTAGACAGAAGCATTCTCAGAAACTTCTTTG

                              *      * *     *  * *    
      16998 TGATGTGTGTGC-TT-AACTAACAGAGCTGAACCTTTCTTTTC
        131 TGATGT-T-TGCATTCAAGTAACAGTGTTGAACATTCCCTTTC

                   * *   *                 *     * *             *           
      17039 ATAGAGCGGTTTTAAAACACTCTTTTTGTAGAATCTGCATGTGGATATTTGGAAAGCTTTGAGGA
          1 ATAGAGCAGGTTTGAAACACTCTTTTTGTAGTATCTGGAAGTGGATATTTGGATAGCTTTGAGGA

                         *    *       *      *                     * **      
      17104 TTTCGTTGGAAACGGGAAAATCTTCACATAAAATCTAGACAGAAGCATTCTCAGAGAAGTCTTTG
         66 TTTCGTTGGAAACAGGAATATCTTCATATAAAAGCTAGACAGAAGCATTCTCAGAAACTTCTTTG

            * *  * **         *    *       *    *    
      17169 GGGTGCTAACATTCAAGTCACAGAGTTGAACGTTCCTTTTC
        131 TGATGTTTGCATTCAAGTAACAGTGTTGAACATTCCCTTTC

                  *      *               * *             *        *         *
      17210 ATAGAGGAGGTTTCAAACACTCTTTTTGTGGAATCTGGAAGTGGACATTTGGATCGCTTTGAGGC
          1 ATAGAGCAGGTTTGAAACACTCTTTTTGTAGTATCTGGAAGTGGATATTTGGATAGCTTTGAGGA

            * ** *      *   *  *     **      *           *       **    **    
      17275 CTGTGGT-GAAAAAGGTATTTCTTCGCATAAAAACTAGACAGAAGTATTCTCATGAACTAGTTTG
         66 TTTCGTTGGAAACAGGAATATCTTCATATAAAAGCTAGACAGAAGCATTCTCAGAAACTTCTTTG

                  *  ***    *  *  **       *  * *   *
      17339 TGATGTGTGTGCTCAACTACCACAGTTGAACCTTTCTTTTG
        131 TGATGTTTGCATTCAAGTAACAGTGTTGAACATTCCCTTTC

                     *   *                     *  * *             *          
      17380 ATAGAGCAGTTTTTAAACACTCTTTTTGT-GTAATTTGCATGTGGATATTTGGACAGCTTTGAGG
          1 ATAGAGCAGGTTTGAAACACTCTTTTTGTAGT-ATCTGGAAGTGGATATTTGGATAGCTTTGAGG

                          *    *          *   * *     *                *     
      17444 ATTTCGTTGGAAACGGGAAAATCTTCATATGAAATCGAGACACAAGCATTCTCAGAAACCTCTTT
         65 ATTTCGTTGGAAACAGGAATATCTTCATATAAAAGCTAGACAGAAGCATTCTCAGAAACTTCTTT

             *      *  *   *   *   **     *  *        
      17509 GGGATGTTAGCGTTCGAGTCACACAGTTGATCACTCCCTTTC
        130 GTGATGTTTGCATTCAAGTAACAGTGTTGAACATTCCCTTTC

                     *      *      *                     **   *   *         *
      17551 ATAGAGCAGCTTTGAAGCACTCTGTTTGTAGTATCTGGAAGTGGACGTTTTGATCGCTTTGAGGC
          1 ATAGAGCAGGTTTGAAACACTCTTTTTGTAGTATCTGGAAGTGGATATTTGGATAGCTTTGAGGA

            *  * *      *            *** *    *   *                     * *  
      17616 GTTAGAT-GAAAAAGGAAATATCTTGCCACAAAAACTACACAGAAGCATTCTCAGAAACTACGTT
         66 TTTCGTTGGAAACAGG-AATATCTTCATATAAAAGCTAGACAGAAGCATTCTCAGAAACTTCTTT

                   * ** *    *      *       *  * *   *
      17680 GTGATGTGTTTACTCAACTAACAGAGTTGAACCTTTCTTTTG
        130 GTGATGTTTGCATTCAAGTAACAGTGTTGAACATTCCCTTTC

                     *                  *  *     * *                         
      17722 ATAGAGCAGTTTTGAAACACTCTTTTTGGAGAATCTGCAGGTGGATATTTGGATAGCTTTGAGGA
          1 ATAGAGCAGGTTTGAAACACTCTTTTTGTAGTATCTGGAAGTGGATATTTGGATAGCTTTGAGGA

                *       **                   *            *   *       **     
      17787 TTTCCTTGGAAAAGGGAATATCTTCATATAAAATCTAGACAGAAGCCTTCGCAGAAACACCTTTG
         66 TTTCGTTGGAAACAGGAATATCTTCATATAAAAGCTAGACAGAAGCATTCTCAGAAACTTCTTTG

                         *    * *  *    *            
      17852 TGATGTTTGCATTGAAGTCAGAGAGTTGTACATTCCCTTTC
        131 TGATGTTTGCATTCAAGTAACAGTGTTGAACATTCCCTTTC

                     *   *                         **    *    **  *         *
      17893 ATAGAGCAGCTTTCAAACACTCTTTTTGTAGTATCTGGAGATGGACATTTACATCGCTTTGAGGC
          1 ATAGAGCAGGTTTGAAACACTCTTTTTGTAGTATCTGGAAGTGGATATTTGGATAGCTTTGAGGA

            * ** *      *       *     **      *      *      *    **      **  
      17958 CTATGGT-GAAATAGGAAATCTCTTCGCATAAAAACTAGACGGAAGCAGTCTCCAAAACTTGCTT
         66 TTTCGTTGGAAACAGG-AATATCTTCATATAAAAGCTAGACAGAAGCATTCTCAGAAACTTCTTT

             **    *  * *    *      *      **  * *   *
      18022 GGAATGTGTGTACTCAACTAACAGAGTTGAATCTTTCTTTTG
        130 GTGATGTTTGCATTCAAGTAACAGTGTTGAACATTCCCTTTC

                     *                     **   **                     *     
      18064 ATAGAGCAGTTTTGAAACACTCTTTTTGTAGAGTCTACAAGTGGATATTTGGATAGCTTAGAGGA
          1 ATAGAGCAGGTTTGAAACACTCTTTTTGTAGTATCTGGAAGTGGATATTTGGATAGCTTTGAGGA

                         *       * *    *    *                       **   *  
      18129 TTTCGTTGGAAACGGGAATATGTCCATACAAAACCTAGACAGAAGCATTCTCAGAAAAATCTCTG
         66 TTTCGTTGGAAACAGGAATATCTTCATATAAAAGCTAGACAGAAGCATTCTCAGAAACTTCTTTG

               * *  *         **                     
      18194 TGAGGATTACATTCAAGTGCCAGTGTTGAACATTCCCTTTC
        131 TGATGTTTGCATTCAAGTAACAGTGTTGAACATTCCCTTTC

               *       *         ***           *     *    *      ***      * *
      18235 ATAAAGCAGGTGTG-AACACAAGATTTTGTAGTATATGGAACTGGACATTTGGGGTGCTTTGTGA
          1 ATAGAGCAGGTTTGAAACAC-TCTTTTTGTAGTATCTGGAAGTGGATATTTGGATAGCTTTGAGG

            ** ** *      *             **   *  *    *                   ***  
      18299 CCTATGGT-GAAAAAGGAAATATCTTCCCATATAAACTAGGCAGAAGCATTCTCAGAAACCGGTT
         65 ATTTCGTTGGAAACAGG-AATATCTTCATATAAAAGCTAGACAGAAGCATTCTCAGAAACTTCTT

                    *  * *    *      *       * ** *   *
      18363 TGTGATGTGTGTACTCAACTAACAGGGTTGAACCTATCTTTTG
        129 TGTGATGTTTGCATTCAAGTAACAGTGTTGAACATTCCCTTTC

             *      ***                    **    *   *     ** *  *           
      18406 AGAGAGCACTCTTGAAACACTCTTTTTGTAGACTCTGCAAGGGGATACGTTGACAGCTTTGAGGA
          1 ATAGAGCAGGTTTGAAACACTCTTTTTGTAGTATCTGGAAGTGGATATTTGGATAGCTTTGAGGA

                         *            *    * *               *      * *      
      18471 TTTCGTTGGAAACTGGAATATCTTCACATAATATCTAGACAGAAGCATTTTCAGAACCGTCTTTG
         66 TTTCGTTGGAAACAGGAATATCTTCATATAAAAGCTAGACAGAAGCATTCTCAGAAACTTCTTTG

            * *   **          *    **      * *  *    
      18536 GGGTGTCAGCATTCAAGTCACAGATTTGAACGTACCTTTTC
        131 TGATGTTTGCATTCAAGTAACAGTGTTGAACATTCCCTTTC

                               *     *   ***             *  *     *  *      *
      18577 ATAGAGCAGGTTTGAAACATTCTTTCTGTGCAATCTGGAAGTGGACATGTGGATCGCATTGAGGC
          1 ATAGAGCAGGTTTGAAACACTCTTTTTGTAGTATCTGGAAGTGGATATTTGGATAGCTTTGAGGA

            * *  *      *   **       **      *           *       **    **    
      18642 CTGCGGT-GAAAAAGGTGTATCTTCGCATAAAAACTAGACAGAAGTATTCTCATGAACTAGTTTG
         66 TTTCGTTGGAAACAGGAATATCTTCATATAAAAGCTAGACAGAAGCATTCTCAGAAACTTCTTTG

                  *  ***    *  *   *       *  * *   *
      18706 TGATGTGTGTGCTCAACTACCAGAGTTGAACCTTTCTTTTG
        131 TGATGTTTGCATTCAAGTAACAGTGTTGAACATTCCCTTTC

               *     *   *               * *  *  * *             *           
      18747 ATACAGCAGTTTTTAAACACTCTTTTTGTGGAATTTGCATGTGGATATTTGGACAGCTTTGAGGA
          1 ATAGAGCAGGTTTGAAACACTCTTTTTGTAGTATCTGGAAGTGGATATTTGGATAGCTTTGAGGA

                         *               *   * *     *                *      
      18812 TTTCGTTGGAAACGGGAATATCTTCATATGAAATCGAGACACAAGCATTCTCAGAAACCTCTTTG
         66 TTTCGTTGGAAACAGGAATATCTTCATATAAAAGCTAGACAGAAGCATTCTCAGAAACTTCTTTG

            *      *  *   *   *    *     *  *        
      18877 GGATGTTAGCGTTCGAGTCACAGAGTTGATCACTCCCTTTC
        131 TGATGTTTGCATTCAAGTAACAGTGTTGAACATTCCCTTTC

                            *                   *        **   *   *         *
      18918 ATAGAGCAGGTTTGAAGCACTCTTTTTGTAGTATCTAGAAGTGGACGTTTTGATCGCTTTGAGGC
          1 ATAGAGCAGGTTTGAAACACTCTTTTTGTAGTATCTGGAAGTGGATATTTGGATAGCTTTGAGGA

            * ** *      *      *       *  *    *   *                   *  *  
      18983 GTAAGGT-GAAAAAGGAAAAATCTTGCCT-CAAAAACTACACAGAAGCATTCTCAGAAATTTATT
         66 TTTCGTTGGAAACAGG-AATATCTT-CATATAAAAGCTAGACAGAAGCATTCTCAGAAACTTCTT

                    **  **    *              *  * *   *
      19046 TGTGATGTGCGCCCTCAACTAACAGTGTTGAACCTTTCTTTTG
        129 TGTGATGTTTGCATTCAAGTAACAGTGTTGAACATTCCCTTTC

                     *                    **     *   ***         *           
      19089 ATAGAGCAGTTTTGAAACACTCTTTTTGTAAAATCTGCAAGAACATATTTGGACAGCTTTGAGGA
          1 ATAGAGCAGGTTTGAAACACTCTTTTTGTAGTATCTGGAAGTGGATATTTGGATAGCTTTGAGGA

                         *   * *            **                           *   
      19154 TTTCGTTGGAAACGGGATTGTCTTCATATAAACTCTAGACAGAAGCATTCTCAGAAACTTCATTG
         66 TTTCGTTGGAAACAGGAATATCTTCATATAAAAGCTAGACAGAAGCATTCTCAGAAACTTCTTTG

            *       **   *    **            *        
      19219 GGATGTTTCTATTGAAGTCGCAGTGTTGAACAGTCCCTTTC
        131 TGATGTTTGCATTCAAGTAACAGTGTTGAACATTCCCTTTC

              *                                          *     * **     *   *
      19260 ATGGAGCAGGTTTGAAACACTCTTTTTGTAGTATCTGGAAGTGGACATTTGTAGCGCTTTCAGGG
          1 ATAGAGCAGGTTTGAAACACTCTTTTTGTAGTATCTGGAAGTGGATATTTGGATAGCTTTGAGGA

            * **        *             **      *                  *      **   
      19325 CTATGTT-GAAAAAGGAAATATCTTCCCATAAAAACTAGACAGAAGCATTCTCTGAAACTAGTTT
         66 TTTCGTTGGAAACAGG-AATATCTTCATATAAAAGCTAGACAGAAGCATTCTCAGAAACTTCTTT

            *          ***    *      *      *   * *   *
      19389 CTGA-GTTGTGTCCTCAAATAACAGAGTTGAATATTTCTTTTG
        130 GTGATGTT-TGCATTCAAGTAACAGTGTTGAACATTCCCTTTC

             *   *   *   **              * *     *            * * *          
      19431 ACAGAACAGTTTTTTAACACTCTTTTTGTGGAATCTGCAAGTGGATATTTTGCTGGCTTTGAGGA
          1 ATAGAGCAGGTTTGAAACACTCTTTTTGTAGTATCTGGAAGTGGATATTTGGATAGCTTTGAGGA

                         *        *   *               *   *  * *      *     *
      19496 TTTCGTTGGAAACGGGAATA-CAT-ACA-AAAAGC-AGACAGCAGCGTTGTGAGAAACGTCTTTA
         66 TTTCGTTGGAAACAGGAATATCTTCATATAAAAGCTAGACAGAAGCATTCTCAGAAACTTCTTTG

                              *    *       *    * *  
      19557 TGATGTTTGCATTCAAGTCACAGAGTTGAACGTTCCGTATC
        131 TGATGTTTGCATTCAAGTAACAGTGTTGAACATTCCCTTTC

                        *       * *      **            ***       **  *  *   *
      19598 ATAGAGCAGGTTGGAAACACGCCTTTTGTCATATCTGGAAGTGTCCATTTGGAGTGCATTCAGGC
          1 ATAGAGCAGGTTTGAAACACTCTTTTTGTAGTATCTGGAAGTGGATATTTGGATAGCTTTGAGGA

              **        *        *    **   *  * *                        *   
      19663 TTGTGTT-GAAAAAGGAAATACCTTCCCATAGAAACCAGACAGAAGCATTCTCAGAAACTTATTT
         66 TTTCGTTGGAAACAGG-AATATCTTCATATAAAAGCTAGACAGAAGCATTCTCAGAAACTTCTTT

                   *  * *    *      **  *    *  **   *
      19727 GTGATGTGTGTACTCAACTAACAGAATTCAACAATCGTTTTG
        130 GTGATGTTTGCATTCAAGTAACAGTGTTGAACATTCCCTTTC

             **      *                   * *     *     *      * *   *        
      19769 AAGGAGCAGTTTTGAAACACTCTTTTTGTGGAATCTGCAAGTGCATATTTAGCTAGATTTGAGGA
          1 ATAGAGCAGGTTTGAAACACTCTTTTTGTAGTATCTGGAAGTGGATATTTGGATAGCTTTGAGGA

                         *   *    *               * * *                      
      19834 TTTCGTTGGAAACGGGATTA-CAT-ATA-AAAAGC-AGGCCGCAGCATTCTCAGAAACTTCTTTG
         66 TTTCGTTGGAAACAGGAATATCTTCATATAAAAGCTAGACAGAAGCATTCTCAGAAACTTCTTTG

                              *    *                 
      19895 TGATGTTTGCATTCAAGTCACAGAGTTGAACATTCCCTTTC
        131 TGATGTTTGCATTCAAGTAACAGTGTTGAACATTCCCTTTC

                      *      *             *       *     *       **     *   *
      19936 ATAGAGCAGGATTGAAAAACTCTTTTTGTAGAATCTGGATGTGGACATTTGGAGCGCTTTCAGGC
          1 ATAGAGCAGGTTTGAAACACTCTTTTTGTAGTATCTGGAAGTGGATATTTGGATAGCTTTGAGGA

            * ** *      *             *** *   *                          *   
      20001 CTATGGT-GAAAAAGGAAATATCTTCCCCTGAAAACTAGACAGAAGCATTCTCAGAAACTTATTT
         66 TTTCGTTGGAAACAGG-AATATCTTCATATAAAAGCTAGACAGAAGCATTCTCAGAAACTTCTTT

                   *   **    *     *        *  * *   *
      20065 GTGATGTGTGCCCTCAACTAACAATGTTGAACCTTTCTTTTG
        130 GTGATGTTTGCATTCAAGTAACAGTGTTGAACATTCCCTTTC

                     *            *      ***     *   *                       
      20107 ATAGAGCAGTTTTGAAACACT-ATTTTGTTAAATCTGCAAGAGGATATTTGGATAGCTTTGAGGA
          1 ATAGAGCAGGTTTGAAACACTCTTTTTGTAGTATCTGGAAGTGGATATTTGGATAGCTTTGAGGA

                         *   * *            **                       *       
      20171 TTTCGTTGGAAACGGGATTGTCTTCATATAAACTCTAGACAGAAGCATTCTCAGAAATTTCTTTG
         66 TTTCGTTGGAAACAGGAATATCTTCATATAAAAGCTAGACAGAAGCATTCTCAGAAACTTCTTTG

            *       **   *    *                     *
      20236 GGATGTTTCAATTGAAGTCACAGTGTTGAACATTCCCTTTG
        131 TGATGTTTGCATTCAAGTAACAGTGTTGAACATTCCCTTTC

            *                       *                    *       **   * *    
      20277 TTAGAGCAGGTTTGAAACACTCTTCTTGTAGTATCTGGAAGTGGACATTTGGAGCGCTCTCAGGA
          1 ATAGAGCAGGTTTGAAACACTCTTTTTGTAGTATCTGGAAGTGGATATTTGGATAGCTTTGAGGA

            * * **      *             **  *         *       * *          *   
      20342 CTACCGT-GAAAAAGGAAATATCTTCCAATGAAAGCTAGATAGAAGCAATGTCAGAAACTTTTTT
         66 TTTCGTTGGAAACAGG-AATATCTTCATATAAAAGCTAGACAGAAGCATTCTCAGAAACTTCTTT

            *                 **      *       *  * *   *
      20406 ATGATGTATCTGC--TCAGCTAACAGAGTTGAACCTTTCTTTTG
        130 GTGATGT-T-TGCATTCAAGTAACAGTGTTGAACATTCCCTTTC

             *       *      *            * *  *  *             **            
      20448 AGAGAGCAGCTTTGAAGCACTCTTTTTGTGGAATATGCAAGTGGATATTTGTCTAGCTTTGAGGA
          1 ATAGAGCAGGTTTGAAACACTCTTTTTGTAGTATCTGGAAGTGGATATTTGGATAGCTTTGAGGA

                         *   *    *                   *       *              
      20513 TTTCGTTGGAAACGGGATTA-CAT-ATA-AAAAGC-AGACAGCAGCATTCCCAGAAACTTCTTTG
         66 TTTCGTTGGAAACAGGAATATCTTCATATAAAAGCTAGACAGAAGCATTCTCAGAAACTTCTTTG

                              *    *                 
      20574 TGATGTTTGCATTCAAGTCACAGAGTTGAACATTCCCTTTC
        131 TGATGTTTGCATTCAAGTAACAGTGTTGAACATTCCCTTTC

                                                   *     *       **     *   *
      20615 ATAGAGCAGGTTTGAAACACTCTTTTTGTAGTATCTGGATGTGGACATTTGGAGCGCTTTCAGGC
          1 ATAGAGCAGGTTTGAAACACTCTTTTTGTAGTATCTGGAAGTGGATATTTGGATAGCTTTGAGGA

            * ** *      *             *** *   *                          *   
      20680 CTATGGT-GAAAAAGGAAATATCTTCCCCTGAAAACTAGACAGAAGCATTCTCAGAAACTTATTT
         66 TTTCGTTGGAAACAGG-AATATCTTCATATAAAAGCTAGACAGAAGCATTCTCAGAAACTTCTTT

                   **  **                      * *   *
      20744 -TGATGTGCGCCCTCAAGTAACAGTGTTGAACATTTCTTTTG
        130 GTGATGTTTGCATTCAAGTAACAGTGTTGAACATTCCCTTTC

                     *                     *     *                   * *     
      20785 ATAGAGCAGTTTTGAAACACTCTTTTTGTAGAATCTGCAAGTGGATATTTGGATAGCCTAGAGGA
          1 ATAGAGCAGGTTTGAAACACTCTTTTTGTAGTATCTGGAAGTGGATATTTGGATAGCTTTGAGGA

                         *       * *    *    *                       **   *  
      20850 TTTCGTTGGAAACGGGAATATGTCCATACAAAACCTAGACAGAAGCATTCTCAGAAAAATCTCTG
         66 TTTCGTTGGAAACAGGAATATCTTCATATAAAAGCTAGACAGAAGCATTCTCAGAAACTTCTTTG

               * *            **                     
      20915 TGAGGATTGCATTCAAGTCCCAGTGTTGAACATTCCCTTTC
        131 TGATGTTTGCATTCAAGTAACAGTGTTGAACATTCCCTTTC

               *       *         ***           *   * *    *       **       * 
      20956 ATAAAGCAGGTGTG-AACACAAGATTTTGTAGTATATGGTACTGGACATTTGGAGTGCTTTG-TG
          1 ATAGAGCAGGTTTGAAACAC-TCTTTTTGTAGTATCTGGAAGTGGATATTTGGATAGCTTTGAGG

             **  *        *             **   *  *    **                  *** 
      21019 ACCT-ATTGTGAAAAAGGAAATATCTTCCCATATAAACTAGGAAGAAGCATTCTCAGAAACCAGT
         65 ATTTCGTTG-GAAACAGG-AATATCTTCATATAAAAGCTAGACAGAAGCATTCTCAGAAACTTCT

                     ** * *    *      *       *  * *   *
      21083 TTGTGATGTGCGTACTCAACTAACAGGGTTGAACCTTTCTTTTG
        128 TTGTGATGTTTGCATTCAAGTAACAGTGTTGAACATTCCCTTTC

             *      * *      *             **    *   *           *           
      21127 AGAGAGCATGCTTGAAAAACTCTTTTTGTAGACTCTGCAAGGGGATATTTGGACAGCTTTGAGGA
          1 ATAGAGCAGGTTTGAAACACTCTTTTTGTAGTATCTGGAAGTGGATATTTGGATAGCTTTGAGGA

                            *                *  *           *         *  *   
      21192 TTTCGTTGGAAAC-GGGATATCTTCATATAAAATCTCGACAGAAGCATCCTCAGAAACATCCTTG
         66 TTTCGTTGGAAACAGGAATATCTTCATATAAAAGCTAGACAGAAGCATTCTCAGAAACTTCTTTG

            *           *     *    *       *         
      21256 GGATGTTTGCATGCAAGTCACAGAGTTGAACTTTCCCTTTC
        131 TGATGTTTGCATTCAAGTAACAGTGTTGAACATTCCCTTTC

              *                          * *                      *  *      *
      21297 ATGGAGCAGGTTTGAAACACTCTTTTTGTGGAATCTGGAAGTGGATATTTGGATCGCATTGAGGC
          1 ATAGAGCAGGTTTGAAACACTCTTTTTGTAGTATCTGGAAGTGGATATTTGGATAGCTTTGAGGA

            * *  *  *   **           **      *          *        *     *     
      21362 CTACGGTGAAAAAGGGAATATCTTCGAATAAAAACTAGACAGAAACATTCTCATAAACTACTTTG
         66 TTTCGTTGGAAACAGGAATATCTTCATATAAAAGCTAGACAGAAGCATTCTCAGAAACTTCTTTG

                  *  *** *  *      * *     *  * *    
      21427 TGATGTGTGTGGTTAACTAACAGAGCTGAACCTTTCTTTTC
        131 TGATGTTTGCATTCAAGTAACAGTGTTGAACATTCCCTTTC

                                           **     ***             *          
      21468 ATAGAGC-GGTTTGGAAACACTCTTTTTGTACAATCTGCCTGTGGATATTTGGAAAGCTTTGAGG
          1 ATAGAGCAGGTTT-GAAACACTCTTTTTGTAGTATCTGGAAGTGGATATTTGGATAGCTTTGAGG

                 *        *            *      *                        *     
      21532 ATTTCTTTGGAAACGGGAATATCTTCACATAAAATCTAGACAGAAGCATTCTCAGAAACGTCTTT
         65 ATTTCGTTGGAAACAGGAATATCTTCATATAAAAGCTAGACAGAAGCATTCTCAGAAACTTCTTT

             * *    *          *    *       *    *    
      21597 GGGGTGTTAGCATTCAAGTCACAGAGTTGAACGTTCCTTTTC
        130 GTGATGTTTGCATTCAAGTAACAGTGTTGAACATTCCCTTTC

                                         * *             *        *         *
      21639 ATAGAGCAGGTTTGAAACACTCTTTTTGTGGAATCTGGAAGTGGACATTTGGATCGCTTTGAGGC
          1 ATAGAGCAGGTTTGAAACACTCTTTTTGTAGTATCTGGAAGTGGATATTTGGATAGCTTTGAGGA

            * *   **    *   *   *    **                 **       **    ***   
      21704 CTGCG-GAGAAAAAGGTATACCTTCGCATAAAAGCTAGACAGAATTATTCTCACGAACTAGCTTG
         66 TTTCGTTGGAAACAGGAATATCTTCATATAAAAGCTAGACAGAAGCATTCTCAGAAACTTCTTTG

                 **  ***    *  *   *       *  * *   *
      21768 TGATGAGTGTGCTCAACTACCAGAGTTGAACCTTTCTTTTG
        131 TGATGTTTGCATTCAAGTAACAGTGTTGAACATTCCCTTTC

                     *                     *  *  * *    *        * *         
      21809 ATAGAGCAGTTTTGAAACACTCTTTTTGTAGAATTTGCATGTGGCTATTTGGACACCTTTGAGGA
          1 ATAGAGCAGGTTTGAAACACTCTTTTTGTAGTATCTGGAAGTGGATATTTGGATAGCTTTGAGGA

                         *    *          *   * *     *                *      
      21874 TTTCGTTGGAAACGGGAAAATCTTCATATGAAATCGAGACACAAGCATTCTCAGAAACCTCTTTG
         66 TTTCGTTGGAAACAGGAATATCTTCATATAAAAGCTAGACAGAAGCATTCTCAGAAACTTCTTTG

            *     **  *       *   **     * **        
      21939 GGATGTCAGCGTTCAAGTCACACAGTTGATCCCT-CCTTTC
        131 TGATGTTTGCATTCAAGTAACAGTGTTGAACATTCCCTTTC

                            *                            **   *   *         *
      21979 ATAGAGCAGGTTTGAAGCACTCTTTTTGTAGTATCTGGAAGTGGACGTTTTGATTGCTTTGAGGC
          1 ATAGAGCAGGTTTGAAACACTCTTTTTGTAGTATCTGGAAGTGGATATTTGGATAGCTTTGAGGA

            * ** *      *            *** *    *   *                     * *  
      22044 GTAAGGT-GAAAAAGGAAATATCTTGCCACAAAAACTACACAGAAGCATTCTCAGAAACTACGTT
         66 TTTCGTTGGAAACAGG-AATATCTTCATATAAAAGCTAGACAGAAGCATTCTCAGAAACTTCTTT

                   * ** *    *      *       *  * *   *
      22108 GTGATGTGTTTACTCAATTAACAGAGTTGAACCTTTCTTTTG
        130 GTGATGTTTGCATTCAAGTAACAGTGTTGAACATTCCCTTTC

                     *                  *  *  *  ***         *           *  *
      22150 ATAGAGCAGTTTTGAAACACTCTTTTTGGAGAATATGCCGGTGGATATTGGGATAGCTTTGTGGT
          1 ATAGAGCAGGTTTGAAACACTCTTTTTGTAGTATCTGGAAGTGGATATTTGGATAGCTTTGAGGA

                **      **                   *                *       **     
      22215 TTTCCGTGGAAAAGGGAATATCTTCATATAAAATCTAGACAGAAGCATTCGCAGAAACACCTTTG
         66 TTTCGTTGGAAACAGGAATATCTTCATATAAAAGCTAGACAGAAGCATTCTCAGAAACTTCTTTG

            *       *    *    * *  *   **          * 
      22280 CGATGTTTACATTGAAGTCAGAGAGTTCTACATTCCCTTAC
        131 TGATGTTTGCATTCAAGTAACAGTGTTGAACATTCCCTTTC

                     *                             **    *    *   *         *
      22321 ATAGAGCAGCTTTGAAACACTCTTTTTGTAGTATCTGGAGATGGACATTTAGATCGCTTTGAGGC
          1 ATAGAGCAGGTTTGAAACACTCTTTTTGTAGTATCTGGAAGTGGATATTTGGATAGCTTTGAGGA

            * ** *      *     *      **      *     **      *    **      **   
      22386 CTATGGT-GAAATAGGAAAATCTTCGCATAAAAACTAGATTGAAGCAGTCTCCAAAACTTGCTTG
         66 TTTCGTTGGAAACAGGAATATCTTCATATAAAAGCTAGACAGAAGCATTCTCAGAAACTTCTTTG

            **    *  * *    *      *      **  * *   *
      22450 GAATGTGTGTACTCAACTAACAGAGTTGAATCTTTCTTTTG
        131 TGATGTTTGCATTCAAGTAACAGTGTTGAACATTCCCTTTC

                     *                     **    *                     *     
      22491 ATAGAGCAGTTTTGAAACACTCTTTTTGTAGAGTCTGCAAGTGGATATTTGGATAGCTTAGAGGA
          1 ATAGAGCAGGTTTGAAACACTCTTTTTGTAGTATCTGGAAGTGGATATTTGGATAGCTTTGAGGA

               * *       *       * *    *    *                       **   *  
      22556 TTTTGCTGGAAACGGGAATATGTCCATACAAAACCTAGACAGAAGCATTCTCAGAAAAATCTCTG
         66 TTTCGTTGGAAACAGGAATATCTTCATATAAAAGCTAGACAGAAGCATTCTCAGAAACTTCTTTG

               * *            ** *                   
      22621 TGAGGATTGCATTCAAGTCCCGGTGTTGAACATTCCCTTTC
        131 TGATGTTTGCATTCAAGTAACAGTGTTGAACATTCCCTTTC

               *       *         ***           *          *       **       * 
      22662 ATAAAGCAGGTGTG-AACACAAGATTTTGTAGTATATGGAAGTGGACATTTGGAGTGCTTTG-TG
          1 ATAGAGCAGGTTTGAAACAC-TCTTTTTGTAGTATCTGGAAGTGGATATTTGGATAGCTTTGAGG

             **  *        *             **   *  *    *      *            *** 
      22725 ACCT-TTTGTGAAAAAGGAAATATCTTCCCATATAAACTAGGCAGAAGAATTCTCAGAAACCAGT
         65 ATTTCGTTG-GAAACAGG-AATATCTTCATATAAAAGCTAGACAGAAGCATTCTCAGAAACTTCT

                     *  * *    *      *       *  * *   *
      22789 TTGTGATGTGTGTACTCAACTAACAGGGTTGAACCTTTCTTTTG
        128 TTGTGATGTTTGCATTCAAGTAACAGTGTTGAACATTCCCTTTC

             *      ***                    *     *   *        * **           
      22833 AGAGAGCACTCTTGAAACACTCTTTTTGTAGAATCTGCAAGGGGATATTTTGTCAGCTTTGAGGA
          1 ATAGAGCAGGTTTGAAACACTCTTTTTGTAGTATCTGGAAGTGGATATTTGGATAGCTTTGAGGA

                            *                *  *           *         *      
      22898 TTTCGTTGGAAA-AGGGATATCTTCATATAAAATCTCGACAGAAGCATCCTCAGAAACATCTTTG
         66 TTTCGTTGGAAACAGGAATATCTTCATATAAAAGCTAGACAGAAGCATTCTCAGAAACTTCTTTG

            *                 *    *                 
      22962 GGATGTTTGCATTCAAGTCACAGAGTTGAACATTCCCTTTC
        131 TGATGTTTGCATTCAAGTAACAGTGTTGAACATTCCCTTTC

              *                          * *          *  *        *  *      *
      23003 ATGGAGCAGGTTTGAAACACTCTTTTTGTGGAATCTGGAAGTAGACATTTGGATCGCATTGAGGC
          1 ATAGAGCAGGTTTGAAACACTCTTTTTGTAGTATCTGGAAGTGGATATTTGGATAGCTTTGAGGA

            * *  *  *   **           **      *                   *     **    
      23068 CTACGGTGAAAAAGGGAATATCTTCGAATAAAAACTAGACAGAAGCATTCTCATAAACTAGTTTG
         66 TTTCGTTGGAAACAGGAATATCTTCATATAAAAGCTAGACAGAAGCATTCTCAGAAACTTCTTTG

                    *         *      * *     *  * *    
      23133 TGATCTGTGTGC-TT-AACTAACAGAGCTGAACCTTTCTTTTC
        131 TGA--TGTTTGCATTCAAGTAACAGTGTTGAACATTCCCTTTC

                   * *                     *     * *             *           
      23174 ATAGAGCGGTTTTGAAACACTCTTTTTGTAGAATCTGCATGTGGATATTTGGAAAGCTTTGAGGA
          1 ATAGAGCAGGTTTGAAACACTCTTTTTGTAGTATCTGGAAGTGGATATTTGGATAGCTTTGAGGA

                         * *          *      *                        *      
      23239 TTTCGTTGGAAACCGTAATATCTTCACATAAAATCTAGACAGAAGCATTCTCAGAAACGTCTTTG
         66 TTTCGTTGGAAACAGGAATATCTTCATATAAAAGCTAGACAGAAGCATTCTCAGAAACTTCTTTG

            * *    *          *    *       *    *    
      23304 GGGTGTTAGCATTCAAGTCACAGAGTTGAACGTTCCTTTTC
        131 TGATGTTTGCATTCAAGTAACAGTGTTGAACATTCCCTTTC

                                         * *             *        *         *
      23345 ATAGAGCAGGTTTGAAACACTCTTTTTGTGGAATCTGGAAGTGGACATTTGGATCGCTTTGAGGC
          1 ATAGAGCAGGTTTGAAACACTCTTTTTGTAGTATCTGGAAGTGGATATTTGGATAGCTTTGAGGA

            * *  *      *   *        **      *           *       **    **    
      23410 CTGCGGT-GAAAAAGGTATATCTTCGCATAAAAACTAGACAGAAGTATTCTCATGAACTAGTTTG
         66 TTTCGTTGGAAACAGGAATATCTTCATATAAAAGCTAGACAGAAGCATTCTCAGAAACTTCTTTG

                  *  ***    *  *   *       *  * *  **
      23474 TGATGTGTGTGCTCAACTACCAGAGTTGAACCTTTCTTTGG
        131 TGATGTTTGCATTCAAGTAACAGTGTTGAACATTCCCTTTC

                     *                     *  *  * *             *           
      23515 ATAGAGCAGTTTTGAAACACTCTTTTTGTAGAATTTGCATGTGGATATTTGGACAGCTTTGAGGA
          1 ATAGAGCAGGTTTGAAACACTCTTTTTGTAGTATCTGGAAGTGGATATTTGGATAGCTTTGAGGA

                 *       *    *          *   * *     *                     * 
      23580 TTTCGCTGGAAACGGGAAAATCTTCATATGAAATCGAGACACAAGCATTCTCAGAAACTTCTTAG
         66 TTTCGTTGGAAACAGGAATATCTTCATATAAAAGCTAGACAGAAGCATTCTCAGAAACTTCTTTG

            *      *  *   *   *   **     *  *        
      23645 GGATGTTAGCGTTCGAGTCACACAGTTGATCACTCCCTTTC
        131 TGATGTTTGCATTCAAGTAACAGTGTTGAACATTCCCTTTC

                            *                   *        **   *   *        **
      23686 ATAGAGCAGGTTTGAAGCACTCTTTTTGTAGTATCTTGAAGTGGACGTTTTGATCGCTTTGAGAC
          1 ATAGAGCAGGTTTGAAACACTCTTTTTGTAGTATCTGGAAGTGGATATTTGGATAGCTTTGAGGA

            * ** *      *            *** *    *   *                     * *  
      23751 ATAAGGT-GAAAAAGGAAATATCTTGCCACAAAAACTACACAGAAGCATTCTCAGAAACTACGTT
         66 TTTCGTTGGAAACAGG-AATATCTTCATATAAAAGCTAGACAGAAGCATTCTCAGAAACTTCTTT

                   * ** *    *      *       *  * *   *
      23815 GTGATGTGTTTACTCAACTAACAGAGTTGAACCTTTCTTTTG
        130 GTGATGTTTGCATTCAAGTAACAGTGTTGAACATTCCCTTTC

                     *                  *  *     * *                *        
      23857 ATAGAGCAGTTTTGAAACACTCTTTTTGGAGAATCTGCAGGTGGATATTTGGATAGATTTGAGGA
          1 ATAGAGCAGGTTTGAAACACTCTTTTTGTAGTATCTGGAAGTGGATATTTGGATAGCTTTGAGGA

                *       **                   *      *     *   *       **     
      23922 TTTCCTTGGAAAAGGGAATATCTTCATATAAAATCTAGACCGAAGCCTTCGCAGAAACACCTTTG
         66 TTTCGTTGGAAACAGGAATATCTTCATATAAAAGCTAGACAGAAGCATTCTCAGAAACTTCTTTG

                         *    * *  *    *         *  
      23987 TGATGTTTGCATTGAAGTCAGAGAGTTGTACATTCCCTGTC
        131 TGATGTTTGCATTCAAGTAACAGTGTTGAACATTCCCTTTC

                     *                             **  * * *  *   ** *      *
      24028 ATAGAGCAGCTTTGAAACACTCTTTTTGTAGTATCTGGAGATGCACAGTTAGATCACGTTGAGGC
          1 ATAGAGCAGGTTTGAAACACTCTTTTTGTAGTATCTGGAAGTGGATATTTGGATAGCTTTGAGGA

            * ** *      *             **      *      *      *    **      **  
      24093 CTATGGT-GAAATAGGAAATATCTTCGCATAAAAACTAGACGGAAGCAGTCTCCAAAACTTGCTT
         66 TTTCGTTGGAAACAGG-AATATCTTCATATAAAAGCTAGACAGAAGCATTCTCAGAAACTTCTTT

             **    *  * *    *      *      **  * *   *
      24157 GGAATGTGTGTACTCAACTAACAGAGTTGAATCTTTCTTTTG
        130 GTGATGTTTGCATTCAAGTAACAGTGTTGAACATTCCCTTTC

                     *                     **    *                     *     
      24199 ATAGAGCAGTTTTGAAACACTCTTTTTGTAGAGTCTGCAAGTGGATATTTGGATAGCTTAGAGGA
          1 ATAGAGCAGGTTTGAAACACTCTTTTTGTAGTATCTGGAAGTGGATATTTGGATAGCTTTGAGGA

                         *       * *    *    *                       **   *  
      24264 TTTCGTTGGAAACGGGAATATGTCCATAAAAAACCTAGACAGAAGCATTCTCAGAAAAATCTCTG
         66 TTTCGTTGGAAACAGGAATATCTTCATATAAAAGCTAGACAGAAGCATTCTCAGAAACTTCTTTG

               * *            **                     
      24329 TGAGGATTGCATTCAAGTCCCAGTGTTGAACATTCCCTTTC
        131 TGATGTTTGCATTCAAGTAACAGTGTTGAACATTCCCTTTC

              **       *         *** *         *          *       **       * 
      24370 ATGAAGCAGGTGTG-AACACAAGATGTTGTAGTATATGGAAGTGGACATTTGGAGTGCTTTG-TG
          1 ATAGAGCAGGTTTGAAACAC-TCTTTTTGTAGTATCTGGAAGTGGATATTTGGATAGCTTTGAGG

             **  *        *             **   *  *    *                   ****
      24433 ACCT-ATTGTGAAAAAGGAAATATCTTCCCATATAAACTAGGCAGAAGCATTCTCAGAAACCAGG
         65 ATTTCGTTG-GAAACAGG-AATATCTTCATATAAAAGCTAGACAGAAGCATTCTCAGAAACTTCT

                     *  * *    *      *       *  * *   *
      24497 TTGTGATGTGTGTACTCAACTAACAGGGTTGAACCTTTCTTTTG
        128 TTGTGATGTTTGCATTCAAGTAACAGTGTTGAACATTCCCTTTC

             *      * *      *             **    *  **           *           
      24541 AGAGAGCACGCTTGAAAAACTCTTTTTGTAGACTCTGCAATGGGATATTTGGACAGCTTTGAGGA
          1 ATAGAGCAGGTTTGAAACACTCTTTTTGTAGTATCTGGAAGTGGATATTTGGATAGCTTTGAGGA

                            *                *  *           *         *      
      24606 TTTCGTTGGAAAC-GGGATATCTTCATATAAAATCTCGACAGAAGCATCCTCAGAAACATCTTTG
         66 TTTCGTTGGAAACAGGAATATCTTCATATAAAAGCTAGACAGAAGCATTCTCAGAAACTTCTTTG

            *                 *    *                 
      24670 GGATGTTTGCATTCAAGTCACAGAGTTGAACATTCCCTTTC
        131 TGATGTTTGCATTCAAGTAACAGTGTTGAACATTCCCTTTC

              *                          * *         *   **     * *  *      *
      24711 ATGGAGCAGGTTTGAAACACTCTTTTTGTGGAATCTGGAAGGGGACTTTTGGGTCGCATTGAGGC
          1 ATAGAGCAGGTTTGAAACACTCTTTTTGTAGTATCTGGAAGTGGATATTTGGATAGCTTTGAGGA

            * *  *  *   **           **      *                   *     **    
      24776 CTACGGTGAAAAAGGGAATATCTTCGAATAAAAACTAGACAGAAGCATTCTCATAAACTAGTTTG
         66 TTTCGTTGGAAACAGGAATATCTTCATATAAAAGCTAGACAGAAGCATTCTCAGAAACTTCTTTG

                              *      * *     *  * *    
      24841 TGATGTGTGTGC-TT-AACTAACAGAGCTGAACCTTTCTTTTC
        131 TGATGT-T-TGCATTCAAGTAACAGTGTTGAACATTCCCTTTC

              *    * *                     *     *              * *   *      
      24882 ATTGAGCGGTTTTGAAACACTCTTTTTGTAGAATCTGCAAGTGGATATTTGGCTGGCTCTGAGGA
          1 ATAGAGCAGGTTTGAAACACTCTTTTTGTAGTATCTGGAAGTGGATATTTGGATAGCTTTGAGGA

                         *        *  *              * *           *          
      24947 TTTCGTTGGAAACGGGAATA-CAT-TTA-AAAAGC-AGACGGCAGCATTCTCAGCAACTTCTTTG
         66 TTTCGTTGGAAACAGGAATATCTTCATATAAAAGCTAGACAGAAGCATTCTCAGAAACTTCTTTG

                    *    *    **                *   *
      25008 TGATGTTTCCATTGAAGTCCCAGTGTTGAACATTCCATTTG
        131 TGATGTTTGCATTCAAGTAACAGTGTTGAACATTCCCTTTC

                                * *      ** *           ***       *** *  **  
      25049 ATAGAGCAGGTTTGAAACACGCCTTTTGTCATGTCTGGAAGCTGTCCATTTGGAGCACATTCCGG
          1 ATAGAGCAGGTTTGAAACACTCTTTTTGTAGTATCTGGAAG-TGGATATTTGGATAGCTTTGAGG

            *  **        *          *  **      *                  *       *  
      25114 CTTGTGTT-GAAAAAGGAAATATCCTCTCATAAAAACTAGACAGAAGCATTCTCTGAAACTTATT
         65 ATTTCGTTGGAAACAGG-AATATCTTCATATAAAAGCTAGACAGAAGCATTCTCAGAAACTTCTT

                    *  * *    *      **      **  **   *
      25178 TGTGATGTGTGTACTCAACTAACAGAATTGAACCATCGTTTTG
        129 TGTGATGTTTGCATTCAAGTAACAGTGTTGAACATTCCCTTTC

             *      **                 * * *     *    *        **    *       
      25221 AAAGAGCAATTTTGAAACACTCTTTTTCTGGAATCTGCAAGTCGATATTTGTCTAGCATTGAGGA
          1 ATAGAGCAGGTTTGAAACACTCTTTTTGTAGTATCTGGAAGTGGATATTTGGATAGCTTTGAGGA

                              *    *                  *       *              
      25286 TTTCGTTGGAAAC-GG--GAT-TACATATAAAAGC-AGACAGCAGCATTCCCAGAAACTTCTTTG
         66 TTTCGTTGGAAACAGGAATATCTTCATATAAAAGCTAGACAGAAGCATTCTCAGAAACTTCTTTG

                              *    *   *             
      25346 TGATGTTTGCATTCAAGTCACAGAGTTTAACATTCCCTTTC
        131 TGATGTTTGCATTCAAGTAACAGTGTTGAACATTCCCTTTC

                                                   *     *       **     *   *
      25387 ATAGAGCAGGTTTGAAACACTCTTTTTGTAGTATCTGGATGTGGACATTTGGAGCGCTTTCAGGC
          1 ATAGAGCAGGTTTGAAACACTCTTTTTGTAGTATCTGGAAGTGGATATTTGGATAGCTTTGAGGA

            * ** *      *             *** *   *                      *   *   
      25452 CTATGGT-GAAAAAGGAAATATCTTCCCCTGAAAACTAGACAGAAGCATTCTCAGAATCTTATTT
         66 TTTCGTTGGAAACAGG-AATATCTTCATATAAAAGCTAGACAGAAGCATTCTCAGAAACTTCTTT

                   **  ***   *              *  * *   *
      25516 GTGATGTGCGCCCCCAACTAACAGTGTTGAACCTTTCTTTTG
        130 GTGATGTTTGCATTCAAGTAACAGTGTTGAACATTCCCTTTC

                     *          *         **     *   **                      
      25558 ATAGAGCAGTTTTGAAACACACTTTTTGTAAAATCTGCAAGAAGATATTTGGATAGCTTTGAGGA
          1 ATAGAGCAGGTTTGAAACACTCTTTTTGTAGTATCTGGAAGTGGATATTTGGATAGCTTTGAGGA

                         *   * *            **            *              *   
      25623 TTTCGTTGGAAACGGGATTGTCTTCATATAAACTCTAGACAGAAGCTTTCTCAGAAACTTCATTG
         66 TTTCGTTGGAAACAGGAATATCTTCATATAAAAGCTAGACAGAAGCATTCTCAGAAACTTCTTTG

                    ** * *    *             *        
      25688 TGATGTTTCAACTGAAGTCACAGTGTTGAACAGTCCCTTTC
        131 TGATGTTTGCATTCAAGTAACAGTGTTGAACATTCCCTTTC

                                                         *       **   * *    
      25729 ATAGAGCAGGTTTGAAACACTCTTTTTGTAGTATCTGGAAGTGGACATTTGGAGCGCTCTCAGGA
          1 ATAGAGCAGGTTTGAAACACTCTTTTTGTAGTATCTGGAAGTGGATATTTGGATAGCTTTGAGGA

            * *   **    *             **            *       * *          *  *
      25794 CTAC-TGTGAAAAAGGAAATATCTTCCAATAAAAGCTAGATAGAAGCAATGTCAGAAA-TTTTTC
         66 TTTCGTTGGAAACAGG-AATATCTTCATATAAAAGCTAGACAGAAGCATTCTCAGAAACTTCTTT

            *      * ** *   **      *       *  * *   *
      25857 ATGATGTATCTACTCAGCTAACAGAGTTGAACCTTTCTTTTG
        130 GTGATGTTTGCATTCAAGTAACAGTGTTGAACATTCCCTTTC

             *   *   *   *              ** *  *  *           *  **     *     
      25899 AGAGACCAGTTTTAAAACACTCTTTTTGGGGAATATGCAAGTGGATATTAGGCCAGCTTGGAGGA
          1 ATAGAGCAGGTTTGAAACACTCTTTTTGTAGTATCTGGAAGTGGATATTTGGATAGCTTTGAGGA

                         *      * *                   *      *              *
      25964 TTTCGTTGGAAACGGGAAT-CCAT-ATA-AAAAGC-AGACAGCAGCATTGTCAGAAACTTCTTTC
         66 TTTCGTTGGAAACAGGAATATCTTCATATAAAAGCTAGACAGAAGCATTCTCAGAAACTTCTTTG

             *           *    **   *   *            *
      26025 TCATGTTTGCATTGAAGTCCCAGAGTTCAACATTCCCTTTA
        131 TGATGTTTGCATTCAAGTAACAGTGTTGAACATTCCCTTTC

                       *        * *      **        *    ***       **  *  **  
      26066 ATAGAGCAGGTCTGAAACACGCCTTTTGTCATATCTGGACGTTGTCCATTTGGAGCGCATTCCGG
          1 ATAGAGCAGGTTTGAAACACTCTTTTTGTAGTATCTGGAAG-TGGATATTTGGATAGCTTTGAGG

            *             *          *  **      *     *                    * 
      26131 CTTGTC-TT-GAAAAAGGAAATATCCTCCCATAAAAACTAGATAGAAGCATTCTCAGAAACTTAT
         65 ATT-TCGTTGGAAACAGG-AATATCTTCATATAAAAGCTAGACAGAAGCATTCTCAGAAACTTCT

                     *  * *    *      **      **  ** * *
      26194 TTGTGATGTGTGTACTCAACTAACAGAATTGAACCATCGTTGTG
        128 TTGTGATGTTTGCATTCAAGTAACAGTGTTGAACATTCCCTTTC

            **       *  *                * *     *             **            
      26238 GAAGAGCAGTTTGGAAACACTCTTTTTGTGGAATCTGCAAGTGGATATTTGTCTAGCTTTGAGGA
          1 ATAGAGCAGGTTTGAAACACTCTTTTTGTAGTATCTGGAAGTGGATATTTGGATAGCTTTGAGGA

                         *   *    *               * * *       *              
      26303 TTTCGTTGGAAACGGGATTA-CAT-ATA-AAAAGC-AGGCCGCAGCATTCCCAGAAACTTCTTTG
         66 TTTCGTTGGAAACAGGAATATCTTCATATAAAAGCTAGACAGAAGCATTCTCAGAAACTTCTTTG

               * *            *    *                 
      26364 TGACGGTTGCATTCAAGTCACAGAGTTGAACATTCCCTTTC
        131 TGATGTTTGCATTCAAGTAACAGTGTTGAACATTCCCTTTC

                  *                                *     *       **     *   *
      26405 ATAGAGAAGGTTTGAAACACTCTTTTTGTAGTATCTGGATGTGGACATTTGGAGCGCTTTCAGGC
          1 ATAGAGCAGGTTTGAAACACTCTTTTTGTAGTATCTGGAAGTGGATATTTGGATAGCTTTGAGGA

            * ** *      *             *** *   *                          *   
      26470 CTATGGT-GAAAAAGGAAATATCTTCCCCTGAAAACTAGACAGAAGCATTCTCAGAAACTTATTT
         66 TTTCGTTGGAAACAGG-AATATCTTCATATAAAAGCTAGACAGAAGCATTCTCAGAAACTTCTTT

                   **  **                   *  * *   *
      26534 GTGATGTGCGCCCTCAAGTAACAGTGTTGAACCTTTCTTTTG
        130 GTGATGTTTGCATTCAAGTAACAGTGTTGAACATTCCCTTTC

                     *                    **     *   *                       
      26576 ATAGAGCAGTTTTGAAACACTCTTTTTGTAAAATCTGCAAGAGGATATTTGGATAGCTTTGAGGA
          1 ATAGAGCAGGTTTGAAACACTCTTTTTGTAGTATCTGGAAGTGGATATTTGGATAGCTTTGAGGA

                         *   * *           ***                   *   *       
      26641 TTTCGTTGGAAACGGGATTGTCTTCATATAATCTCTAGACAGAAGCATTCTCATAAATTTCTTTG
         66 TTTCGTTGGAAACAGGAATATCTTCATATAAAAGCTAGACAGAAGCATTCTCAGAAACTTCTTTG

            *       **   *    *                   *  
      26706 GGATGTTTTAATTGAAGTCACAGTGTTGAACATTCCCTGTC
        131 TGATGTTTGCATTCAAGTAACAGTGTTGAACATTCCCTTTC

                                    *                    *       **   * *    
      26747 ATAGAGCAGGTTTGAAACACTCTTCTTGTAGTATCTGGAAGTGGACATTTGGAGCGCTCTCAGGA
          1 ATAGAGCAGGTTTGAAACACTCTTTTTGTAGTATCTGGAAGTGGATATTTGGATAGCTTTGAGGA

            * * **      *             **            *       * *          *  *
      26812 CTACAGT-GAAAAAGGAAATATCTTCCAATAAAAGCTAGATAGAAGCAATGTCAGAAACTTTTTC
         66 TTTCGTTGGAAACAGG-AATATCTTCATATAAAAGCTAGACAGAAGCATTCTCAGAAACTTCTTT

            *                 **      *       *  * *   *
      26876 ATGATGTATCTGC--TCAGCTAACAGAGTTGAACCTTTCTTTTG
        130 GTGATGT-T-TGCATTCAAGTAACAGTGTTGAACATTCCCTTTC

             *       *   *               * *     *             **         *  
      26918 AGAGAGCAGTTTTTAAACACTCTTTTTGTGGAATCTGCAAGTGGATATTTGTCTAGCTTTGATGA
          1 ATAGAGCAGGTTTGAAACACTCTTTTTGTAGTATCTGGAAGTGGATATTTGGATAGCTTTGAGGA

                         *   *    *                   *       *              
      26983 TTTCGTTGGAAACGGGATTA-CAT-ATA-AAAAGC-AGACAGCAGCATTCCCAGAAACTTCTTTG
         66 TTTCGTTGGAAACAGGAATATCTTCATATAAAAGCTAGACAGAAGCATTCTCAGAAACTTCTTTG

                              *    *                 
      27044 TGATGTTTGCATTCAAGTCACAGAGTTGAACATTCCCTTTC
        131 TGATGTTTGCATTCAAGTAACAGTGTTGAACATTCCCTTTC

                     *                             *     *       **     *   *
      27085 ATAGAGCAGCTTTGAAACACTCTTTTTGTAGTATCTGGATGTGGACATTTGGAGCGCTTTCAGGC
          1 ATAGAGCAGGTTTGAAACACTCTTTTTGTAGTATCTGGAAGTGGATATTTGGATAGCTTTGAGGA

            * ** *      *             *** *   *                          *   
      27150 CTATGGT-GAAAAAGGAAATATCTTCCCCTGAAAACTAGACAGAAGCATTCTCAGAAACTTATTT
         66 TTTCGTTGGAAACAGG-AATATCTTCATATAAAAGCTAGACAGAAGCATTCTCAGAAACTTCTTT

                   **  **    *              *  * *   *
      27214 GTGATGTGCGCCCTCAACTAACAGTGTTGAACCTTTCTTTTG
        130 GTGATGTTTGCATTCAAGTAACAGTGTTGAACATTCCCTTTC

                     *                     **    *                     *     
      27256 ATAGAGCAGTTTTGAAACACTCTTTTTGTAGAGTCTGCAAGTGGATATTTGGATAGCTTAGAGGA
          1 ATAGAGCAGGTTTGAAACACTCTTTTTGTAGTATCTGGAAGTGGATATTTGGATAGCTTTGAGGA

                         *       * *    *    *                       **   *  
      27321 TTTCGTTGGAAACGGGAATATGTCCATACAAAACCTAGACAGAAGCATTCTCAGAAAAATCTCTG
         66 TTTCGTTGGAAACAGGAATATCTTCATATAAAAGCTAGACAGAAGCATTCTCAGAAACTTCTTTG

               * *            **                     
      27386 TGAGGATTGCATTCAAGTCCCAGTGTTGAACATTCCCTTTC
        131 TGATGTTTGCATTCAAGTAACAGTGTTGAACATTCCCTTTC

               *       *         *** *     *   *     *    *       **       * 
      27427 ATAAAGCAGGTGTG-AACACAAGATGTTGTAATATATGGAACTGGACATTTGGAGTGCTTTG-TG
          1 ATAGAGCAGGTTTGAAACAC-TCTTTTTGTAGTATCTGGAAGTGGATATTTGGATAGCTTTGAGG

             **  *        *             **   *  *    *                   ****
      27490 ACCT-ATTGTGAAAAAGGAAATATCTTCCCATATAAACTAGGCAGAAGCATTCTCAGAAACCAGG
         65 ATTTCGTTG-GAAACAGG-AATATCTTCATATAAAAGCTAGACAGAAGCATTCTCAGAAACTTCT

                     *  * *    *      *       *  * *   *
      27554 TTGTGATGTGTGTACTCAACTAACAGGGTTGAACCTTTCTTTTG
        128 TTGTGATGTTTGCATTCAAGTAACAGTGTTGAACATTCCCTTTC

             *      * *      *             **    *   *           *           
      27598 AGAGAGCACGCTTGAAAAACTCTTTTTGTAGACTCTGCAAGGGGATATTTGGACAGCTTTGAGGA
          1 ATAGAGCAGGTTTGAAACACTCTTTTTGTAGTATCTGGAAGTGGATATTTGGATAGCTTTGAGGA

                            *                *  *                            
      27663 TTTCGTTGGAAA-AGGGATATCTTCATATAAAATCTCGACAGAAGCATTCTCAGAAACATCTTTC
         66 TTTCGTTGGAAACAGGAATATCTTCATATAAAAGCTAGACAGAAGCATTCTCAG-AA-A-C-TTC

                *           *     *    *       *         
      27727 TTTGGGATGTTTGCATGCAAGTCACAGAGTTGAACTTTCCCTTTC
        127 TTTGTGATGTTTGCATTCAAGTAACAGTGTTGAACATTCCCTTTC

              *                          * *          *  *        *  *      *
      27772 ATGGAGCAGGTTTGAAACACTCTTTTTGTGGAATCTGGAAGTAGACATTTGGATCGCATTGAGGC
          1 ATAGAGCAGGTTTGAAACACTCTTTTTGTAGTATCTGGAAGTGGATATTTGGATAGCTTTGAGGA

            * *  *  *   **           **      *      *            *     *     
      27837 CTACGGTGAAAAAGGGAATATCTTCGAATAAAAACTAGACGGAAGCATTCTCATAAACTACTTTG
         66 TTTCGTTGGAAACAGGAATATCTTCATATAAAAGCTAGACAGAAGCATTCTCAGAAACTTCTTTG

                  *   ** *  *      * *     *  * *    
      27902 TGATGTGTGCGGTTAACTAACAGAGCTGAACCTTTCTTTTC
        131 TGATGTTTGCATTCAAGTAACAGTGTTGAACATTCCCTTTC

                   * *                 *   *     ***             *           
      27943 ATAGAGCGGTTTTGAAACACTCTTTTTTTAGAATCTGCCTGTGGATATTTGGAAAGCTTTGAGGA
          1 ATAGAGCAGGTTTGAAACACTCTTTTTGTAGTATCTGGAAGTGGATATTTGGATAGCTTTGAGGA

                *        *            *      **                       *      
      28008 TTTCTTTGGAAACGGGAATATCTTCACATAAAATATAGACAGAAGCATTCTCAGAAACGTCTTTG
         66 TTTCGTTGGAAACAGGAATATCTTCATATAAAAGCTAGACAGAAGCATTCTCAGAAACTTCTTTG

            * *    *          *    *       **   *    
      28073 GGGTGTTAGCATTCAAGTCACAGAGTTGAACGCTCCTTTTC
        131 TGATGTTTGCATTCAAGTAACAGTGTTGAACATTCCCTTTC

                               *         * *             *        *         *
      28114 ATAGAGCAGGTTTGAAACATTCTTTTTGTGGAATCTGGAAGTGGACATTTGGATCGCTTTGAGGC
          1 ATAGAGCAGGTTTGAAACACTCTTTTTGTAGTATCTGGAAGTGGATATTTGGATAGCTTTGAGGA

            * *  *      *   *        **      *           *        *    **    
      28179 CTGCGGT-GAAAAAGGTATATCTTCGCATAAAAACTAGACAGAAGTATTCTCAGGAACTAGTTTG
         66 TTTCGTTGGAAACAGGAATATCTTCATATAAAAGCTAGACAGAAGCATTCTCAGAAACTTCTTTG

                  *  ***    *  *   *       *  * *   *
      28243 TGATGTGTGTGCTCAACTACCAGAGTTGAACCTTTCTTTTG
        131 TGATGTTTGCATTCAAGTAACAGTGTTGAACATTCCCTTTC

                     *                     *  *  * *    *   *    *           
      28284 ATAGAGCAGTTTTGAAACACTCTTTTTGTAGAATTTGCATGTGGCTATCTGGACAGCTTTGAGGA
          1 ATAGAGCAGGTTTGAAACACTCTTTTTGTAGTATCTGGAAGTGGATATTTGGATAGCTTTGAGGA

                         *    *          *   * *     *                *   *  
      28349 TTTCGTTGGAAACGGGAAAATCTTCATATGAAATCGAGACACAAGCATTCTCAGAAACCTCTCTG
         66 TTTCGTTGGAAACAGGAATATCTTCATATAAAAGCTAGACAGAAGCATTCTCAGAAACTTCTTTG

            *      *  *   *   *   **     *  *        
      28414 GGATGTTAGCGTTCGAGTCACACAGTTGATCACTCCCTTTC
        131 TGATGTTTGCATTCAAGTAACAGTGTTGAACATTCCCTTTC

                            *                            **   *   *         *
      28455 ATAGAGCAGGTTTGAAGCACTCTTTTTGTAGTATCTGGAAGTGGACGTTTTGATCGCTTTGAGGC
          1 ATAGAGCAGGTTTGAAACACTCTTTTTGTAGTATCTGGAAGTGGATATTTGGATAGCTTTGAGGA

            * ** *      *            *** *    *   *                     * *  
      28520 GTAAGGT-GAAAAAGGAAATATCTTGCCACAAAAACTACACAGAAGCATTCTCAGAAACTACGTT
         66 TTTCGTTGGAAACAGG-AATATCTTCATATAAAAGCTAGACAGAAGCATTCTCAGAAACTTCTTT

                   * ** *    *      *       *  * *   *
      28584 GTGATGTGTTTACTCAACTAACAGAGTTGAACCTTTCTTTTG
        130 GTGATGTTTGCATTCAAGTAACAGTGTTGAACATTCCCTTTC

                     *                  *  *     * *               *         
      28626 ATAGAGCAGTTTTGAAACACTCTTTTTGGAGAATCTGCAGGTGGATATTTGGATAACTTTGAGGA
          1 ATAGAGCAGGTTTGAAACACTCTTTTTGTAGTATCTGGAAGTGGATATTTGGATAGCTTTGAGGA

                *       **                   *      *     *   *       **     
      28691 TTTCCTTGGAAAAGGGAATATCTTCATATAAAATCTAGACCGAAGCCTTCGCAGAAACACCTTTG
         66 TTTCGTTGGAAACAGGAATATCTTCATATAAAAGCTAGACAGAAGCATTCTCAGAAACTTCTTTG

                         *    * *  *    *         *  
      28756 TGATGTTTGCATTGAAGTCAGAGAGTTGTACATTCCCTGTC
        131 TGATGTTTGCATTCAAGTAACAGTGTTGAACATTCCCTTTC

                     *                             **  * * *  *   ** *      *
      28797 ATAGAGCAGCTTTGAAACACTCTTTTTGTAGTATCTGGAGATGCACAGTTAGATCACGTTGAGGC
          1 ATAGAGCAGGTTTGAAACACTCTTTTTGTAGTATCTGGAAGTGGATATTTGGATAGCTTTGAGGA

            * ** *      *             **      *      *      *    **      **  
      28862 CTATGGT-GAAATAGGAAATATCTTCGCATAAAAACTAGACGGAAGCAGTCTCCAAAACTTGCTT
         66 TTTCGTTGGAAACAGG-AATATCTTCATATAAAAGCTAGACAGAAGCATTCTCAGAAACTTCTTT

             **    *  * *    *      *      **  * *   *
      28926 GGAATGTGTGTACTCAACTAACAGAGTTGAATCTTTCTTTTG
        130 GTGATGTTTGCATTCAAGTAACAGTGTTGAACATTCCCTTTC

                     *                     **    *                     *     
      28968 ATAGAGCAGTTTTGAAACACTCTTTTTGTAGAGTCTGCAAGTGGATATTTGGATAGCTTAGAGGA
          1 ATAGAGCAGGTTTGAAACACTCTTTTTGTAGTATCTGGAAGTGGATATTTGGATAGCTTTGAGGA

                         *       * *    *    *                       **   *  
      29033 TTTCGTTGGAAACGGGAATATGTCCATAAAAAACCTAGACAGAAGCATTCTCAGAAAAATCTCTG
         66 TTTCGTTGGAAACAGGAATATCTTCATATAAAAGCTAGACAGAAGCATTCTCAGAAACTTCTTTG

               * *            **                     
      29098 TGAGGATTGCATTCAAGTCCCAGTGTTGAACATTCCCTTTC
        131 TGATGTTTGCATTCAAGTAACAGTGTTGAACATTCCCTTTC

              **       *         ***           *          *       **      *  
      29139 ATGAAGCAGGTGTG-AACACAAGATTTTGTAGTATATGGAAGTGGACATTTGGAGTGCTTTGTGA
          1 ATAGAGCAGGTTTGAAACAC-TCTTTTTGTAGTATCTGGAAGTGGATATTTGGATAGCTTTGAG-

            *    *        *             **   *  *    *                  **** 
      29203 CATTTTG-T-GAAAAAGGAAGTATCTTCCCATATAAACTAGGCAGAAGCATTCTCAGAAAGCAGT
         64 GATTTCGTTGGAAACAGGAA-TATCTTCATATAAAAGCTAGACAGAAGCATTCTCAGAAACTTCT

                   * *  * **   *      *       *  * *   *
      29266 TTGTGATCTGTGTACACAACTAACAGGGTTGAACCTTTCTTTTG
        128 TTGTGATGTTTGCATTCAAGTAACAGTGTTGAACATTCCCTTTC

             *   *  ***                    **    *   *        *  *           
      29310 AGAGAACACTCTTGAAACACTCTTTTTGTAGACTCTGCAAGGGGATATTTTGACAGCTTTGAGGA
          1 ATAGAGCAGGTTTGAAACACTCTTTTTGTAGTATCTGGAAGTGGATATTTGGATAGCTTTGAGGA

                            *                *  *           *         *      
      29375 TTTCGTTGGAAAC-GGGATATCTTCATATAAAATCTCGACAGAAGCATCCTCAGAAACATCTTTG
         66 TTTCGTTGGAAACAGGAATATCTTCATATAAAAGCTAGACAGAAGCATTCTCAGAAACTTCTTTG

            *                 *    *                 
      29439 GGATGTTTGCATTCAAGTCACAGAGTTGAACATTCCCTTTC
        131 TGATGTTTGCATTCAAGTAACAGTGTTGAACATTCCCTTTC

              *                          * *         *   **     * *  *      *
      29480 ATGGAGCAGGTTTGAAACACTC-TTTTGTGGAATCTGGAAGGGGACTTTTGGGTCGCATTGAGGC
          1 ATAGAGCAGGTTTGAAACACTCTTTTTGTAGTATCTGGAAGTGGATATTTGGATAGCTTTGAGGA

            * *  *  *   **           **      *                   *     **    
      29544 CTACGGTGAAAAAGGGAATATCTTCGAATAAAAACTAGACAGAAGCATTCTCATAAACTAGTTTG
         66 TTTCGTTGGAAACAGGAATATCTTCATATAAAAGCTAGACAGAAGCATTCTCAGAAACTTCTTTG

                              *      * *     *  * *    
      29609 TGATGTGTGTGC-TT-AACTAACAGAGCTGAACCTTTCTTTTC
        131 TGATGT-T-TGCATTCAAGTAACAGTGTTGAACATTCCCTTTC

              *    * *                     *     *              * *   *      
      29650 ATTGAGCGGTTTTGAAACACTCTTTTTGTAGAATCTGCAAGTGGATATTTGGCTGGCTCTGAGGA
          1 ATAGAGCAGGTTTGAAACACTCTTTTTGTAGTATCTGGAAGTGGATATTTGGATAGCTTTGAGGA

                         *        *  *              * *    *                 
      29715 TTTCGTTGGAAACGGGAATA-CAT-TTA-AAAAGC-AGACGGCAGCAGTCTCAGAAACTTCTTTG
         66 TTTCGTTGGAAACAGGAATATCTTCATATAAAAGCTAGACAGAAGCATTCTCAGAAACTTCTTTG

                    *    *    **                *   *
      29776 TGATGTTTCCATTGAAGTCCCAGTGTTGAACATTCCATTTG
        131 TGATGTTTGCATTCAAGTAACAGTGTTGAACATTCCCTTTC

             *                  * *      ** *           ***       *** *  **  
      29817 AGAGAGCAGGTTTGAAACACGCCTTTTGTCATGTCTGGAAGCTGTCCATTTGGAGCACATTCCGG
          1 ATAGAGCAGGTTTGAAACACTCTTTTTGTAGTATCTGGAAG-TGGATATTTGGATAGCTTTGAGG

            *  **        *          *  **      *                  *       *  
      29882 CTTGTGTT-GAAAAAGGAAATATCCTCTCATAAAAACTAGACAGAAGCATTCTCTGAAACTTATT
         65 ATTTCGTTGGAAACAGG-AATATCTTCATATAAAAGCTAGACAGAAGCATTCTCAGAAACTTCTT

                    *  * *    *      **      **  **   *
      29946 TGTGATGTGTGTACTCAACTAACAGAATTGAACCATCGTTTTG
        129 TGTGATGTTTGCATTCAAGTAACAGTGTTGAACATTCCCTTTC

             *      **                 * * *     *    *        **    *       
      29989 AAAGAGCAATTTTGAAACACTCTTTTTCTGGAATCTGCAAGTCGATATTTGTCTAGCATTGAGGA
          1 ATAGAGCAGGTTTGAAACACTCTTTTTGTAGTATCTGGAAGTGGATATTTGGATAGCTTTGAGGA

                         *   *     **                 *       *       *      
      30054 TTTCGTTGGAAACGGGATTA-C-AAATA-AAAAGC-AGACAGCAGCATTCCCAGAAACATCTTTG
         66 TTTCGTTGGAAACAGGAATATCTTCATATAAAAGCTAGACAGAAGCATTCTCAGAAACTTCTTTG

            *                 *  * *  *              
      30115 CGATGTTTGCATTCAAGTCACTGAGTAGAACATTCCCTTTC
        131 TGATGTTTGCATTCAAGTAACAGTGTTGAACATTCCCTTTC

                                                   *     *       **     *   *
      30156 ATAGAGCAGGTTTGAAACACTCTTTTTGTAGTATCTGGATGTGGACATTTGGAGCGCTTTCAGGC
          1 ATAGAGCAGGTTTGAAACACTCTTTTTGTAGTATCTGGAAGTGGATATTTGGATAGCTTTGAGGA

            * ** *      *             *** *   *                      *   *   
      30221 CTATGGT-GAAAAAGGAAATATCTTCCCCTGAAAACTAGACAGAAGCATTCTCAGAATCTTATTT
         66 TTTCGTTGGAAACAGG-AATATCTTCATATAAAAGCTAGACAGAAGCATTCTCAGAAACTTCTTT

                   **  ***   *              *  * *   *
      30285 GTGATGTGCGCCCCCAACTAACAGTGTTGAACCTTTCTTTTG
        130 GTGATGTTTGCATTCAAGTAACAGTGTTGAACATTCCCTTTC

                     *          *         **     *   **                      
      30327 ATAGAGCAGTTTTGAAACACACTTTTTGTAAAATCTGCAAGAAGATATTTGGATAGCTTTGAGGA
          1 ATAGAGCAGGTTTGAAACACTCTTTTTGTAGTATCTGGAAGTGGATATTTGGATAGCTTTGAGGA

                         *   * *            **            *              *   
      30392 TTTCGTTGGAAACGGGATTGTCTTCATATAAACTCTAGACAGAAGCTTTCTCAGAAACTTCATTG
         66 TTTCGTTGGAAACAGGAATATCTTCATATAAAAGCTAGACAGAAGCATTCTCAGAAACTTCTTTG

                    ** * *    *             *        
      30457 TGATGTTTCAACTGAAGTCACAGTGTTGAACAGTCCCTTTC
        131 TGATGTTTGCATTCAAGTAACAGTGTTGAACATTCCCTTTC

                                                         *       **   * *    
      30498 ATAGAGCAGGTTTGAAACACTCTTTTTGTAGTATCTGGAAGTGGACATTTGGAGCGCTCTCAGGA
          1 ATAGAGCAGGTTTGAAACACTCTTTTTGTAGTATCTGGAAGTGGATATTTGGATAGCTTTGAGGA

            * *   **    *             **            *       * *          *  *
      30563 CTAC-TGTGAAAAAGGAAATATCTTCCAATAAAAGCTAGATAGAAGCAATGTCAGAAA-TTTTTC
         66 TTTCGTTGGAAACAGG-AATATCTTCATATAAAAGCTAGACAGAAGCATTCTCAGAAACTTCTTT

            *      * ** *   **      *       *  * *   *
      30626 ATGATGTATCTACTCAGCTAACAGAGTTGAACCTTTCTTTTG
        130 GTGATGTTTGCATTCAAGTAACAGTGTTGAACATTCCCTTTC

             *   *   *   *              ** *  *  *           *  **     *     
      30668 AGAGACCAGTTTTAAAACACTCTTTTTGGGGAATATGCAAGTGGATATTAGGCCAGCTTGGAGGA
          1 ATAGAGCAGGTTTGAAACACTCTTTTTGTAGTATCTGGAAGTGGATATTTGGATAGCTTTGAGGA

                         *      * *                   *      *               
      30733 TTTCGTTGGAAACGGGAAT-CCAT-ATA-AAAAGC-AGACAGCAGCATTGTCAGAAACTTCTTTG
         66 TTTCGTTGGAAACAGGAATATCTTCATATAAAAGCTAGACAGAAGCATTCTCAGAAACTTCTTTG

             *           *    **   *   *            *
      30794 TCATGTTTGCATTGAAGTCCCAGAGTTCAACATTCCCTTTA
        131 TGATGTTTGCATTCAAGTAACAGTGTTGAACATTCCCTTTC

                       *        * *      **        *    ***       **  *  **  
      30835 ATAGAGCAGGTCTGAAACACGCCTTTTGTCATATCTGGACGTTGTCCATTTGGAGCGCATTCCGG
          1 ATAGAGCAGGTTTGAAACACTCTTTTTGTAGTATCTGGAAG-TGGATATTTGGATAGCTTTGAGG

            *  **        *          *  **      *     *                    *  
      30900 CTTGTGTT-GAAAAAGGAAATATCCTCCCATAAAAACTAGATAGAAGCATTCTCAGAAACTTATT
         65 ATTTCGTTGGAAACAGG-AATATCTTCATATAAAAGCTAGACAGAAGCATTCTCAGAAACTTCTT

                    *  * *    *      **      **  ** * *
      30964 TGTGATGTGTGTACTCAACTAACAGAATTGAACCATCGTTGTG
        129 TGTGATGTTTGCATTCAAGTAACAGTGTTGAACATTCCCTTTC

            **       *  *                * *     *             **            
      31007 GAAGAGCAGTTTGGAAACACTCTTTTTGTGGAATCTGCAAGTGGATATTTGTCTAGCTTTGAGGA
          1 ATAGAGCAGGTTTGAAACACTCTTTTTGTAGTATCTGGAAGTGGATATTTGGATAGCTTTGAGGA

                         *   *    *               * * *       *              
      31072 TTTCGTTGGAAACGGGATTA-CAT-ATA-AAAAGC-AGGCCGCAGCATTCCCAGAAACTTCTTTG
         66 TTTCGTTGGAAACAGGAATATCTTCATATAAAAGCTAGACAGAAGCATTCTCAGAAACTTCTTTG

               * *            *    *                 
      31133 TGACGGTTGCATTCAAGTCACAGAGTTGAACATTCCCTTTC
        131 TGATGTTTGCATTCAAGTAACAGTGTTGAACATTCCCTTTC

                  *                                *     *       **     *   *
      31174 ATAGAGAAGGTTTGAAACACTCTTTTTGTAGTATCTGGATGTGGACATTTGGAGCGCTTTCAGGC
          1 ATAGAGCAGGTTTGAAACACTCTTTTTGTAGTATCTGGAAGTGGATATTTGGATAGCTTTGAGGA

            * ** *      *             *** *   *                          *   
      31239 CTATGGT-GAAAAAGGAAATATCTTCCCCTGAAAACTAGACAGAAGCATTCTCAGAAACTTATTT
         66 TTTCGTTGGAAACAGG-AATATCTTCATATAAAAGCTAGACAGAAGCATTCTCAGAAACTTCTTT

                   **  **                   * ** *   *
      31303 GTGATGTGCGCCCTCAAGTAACAGTGTTGAACCTGTCTTTTG
        130 GTGATGTTTGCATTCAAGTAACAGTGTTGAACATTCCCTTTC

                     *                    **     *   *                       
      31345 ATAGAGCAGTTTTGAAACACTCTTTTTGTAAAATCTGCAAGAGGATATTTGGATAGCTTTGAGGA
          1 ATAGAGCAGGTTTGAAACACTCTTTTTGTAGTATCTGGAAGTGGATATTTGGATAGCTTTGAGGA

                         *   * *            **                   *   *       
      31410 TTTCGTTGGAAACGGGATTGTCTTCATATAAACTCTAGACAGAAGCATTCTCATAAATTTCTTTG
         66 TTTCGTTGGAAACAGGAATATCTTCATATAAAAGCTAGACAGAAGCATTCTCAGAAACTTCTTTG

            *       **   *    *                   *  
      31475 GGATGTTTCAATTGAAGTCACAGTGTTGAACATTCCCTGTC
        131 TGATGTTTGCATTCAAGTAACAGTGTTGAACATTCCCTTTC

                                    *                    *       **   * *    
      31516 ATAGAGCAGGTTTGAAACACTCTTCTTGTAGTATCTGGAAGTGGACATTTGGAGCGCTCTCAGGA
          1 ATAGAGCAGGTTTGAAACACTCTTTTTGTAGTATCTGGAAGTGGATATTTGGATAGCTTTGAGGA

            * * **      *             **            *       * *          *  *
      31581 CTACAGT-GAAAAAGGAAATATCTTCCAATAAAAGCTAGATAGAAGCAATGTCAGAAACTTTTTC
         66 TTTCGTTGGAAACAGG-AATATCTTCATATAAAAGCTAGACAGAAGCATTCTCAGAAACTTCTTT

            *                 **      *       *  * *   *
      31645 ATGATGTATCTGC--TCAGCTAACAGAGTTGAACCTTTCTTTTG
        130 GTGATGT-T-TGCATTCAAGTAACAGTGTTGAACATTCCCTTTC

             *       *                   * *     *             **            
      31687 AGAGAGCAGTTTTGAAACACTCTTTTTGTGGAATCTGCAAGTGGATATTTGTCTAGCTTTGAGGA
          1 ATAGAGCAGGTTTGAAACACTCTTTTTGTAGTATCTGGAAGTGGATATTTGGATAGCTTTGAGGA

                         *   *    *                   *       *              
      31752 TTTCGTTGGAAACGGGATTA-CAT-ATA-AAAAGC-AGACAGCAGCATTCCCAGAAACTTCTTTG
         66 TTTCGTTGGAAACAGGAATATCTTCATATAAAAGCTAGACAGAAGCATTCTCAGAAACTTCTTTG

                              *    *                 
      31813 TGATGTTTGCATTCAAGTCACAGAGTTGAACATTCCCTTTC
        131 TGATGTTTGCATTCAAGTAACAGTGTTGAACATTCCCTTTC

               *     *                             *     *       **     *   *
      31854 ATACAGCAGCTTTGAAACACTCTTTTTGTAGTATCTGGATGTGGACATTTGGAGCGCTTTCAGGC
          1 ATAGAGCAGGTTTGAAACACTCTTTTTGTAGTATCTGGAAGTGGATATTTGGATAGCTTTGAGGA

            * ** *      *         *   *** *   *                          *   
      31919 CTATGGT-GAAAAAGGAAATATGTTCTCCTGAAAACTAGACAGAAGCATTCTCAGAAACTTATTT
         66 TTTCGTTGGAAACAGG-AATATCTTCATATAAAAGCTAGACAGAAGCATTCTCAGAAACTTCTTT

                   **  **    *              *  * *   *
      31983 GTGATGTGCGCCCTCAACTAACAGTGTTGAACCTTTCTTTTG
        130 GTGATGTTTGCATTCAAGTAACAGTGTTGAACATTCCCTTTC

                     *                     **    *                     *     
      32025 ATAGAGCAGTTTTGAAACACTCTTTTTGTAGAGTCTGCAAGTGGATATTTGGATAGCTTAGAGGA
          1 ATAGAGCAGGTTTGAAACACTCTTTTTGTAGTATCTGGAAGTGGATATTTGGATAGCTTTGAGGA

                         *       * *    *    *                       **   *  
      32090 TTTCGTTGGAAACGGGAATATGTCCATACAAAACCTAGACAGAAGCATTCTCAGAAAAATCTCTG
         66 TTTCGTTGGAAACAGGAATATCTTCATATAAAAGCTAGACAGAAGCATTCTCAGAAACTTCTTTG

               * *            **               *     
      32155 TGAGGATTGCATTCAAGTCCCAGTGTTGAACATTCTCTTTC
        131 TGATGTTTGCATTCAAGTAACAGTGTTGAACATTCCCTTTC

               *       *         *** *         *     *    **      **       * 
      32196 ATAAAGCAGGTGTG-AACACAGGATGTTGTAGTATATGGAACTGGAATTTTGGAGTGCTTTG-TG
          1 ATAGAGCAGGTTTGAAACAC-TCTTTTTGTAGTATCTGGAAGTGGATATTTGGATAGCTTTGAGG

             **  *        *             **   *  *    *                   ****
      32259 ACCT-ATTGTGAAAAAGGAAATATCTTCCCATATAAACTAGGCAGAAGCATTCTCAGAAACCAGG
         65 ATTTCGTTG-GAAACAGG-AATATCTTCATATAAAAGCTAGACAGAAGCATTCTCAGAAACTTCT

                     *  * *    *      *       *  * *   *
      32323 TTGTGATGTGTGTACTCAACTAACAGGGTTGAACCTTTCTTTTG
        128 TTGTGATGTTTGCATTCAAGTAACAGTGTTGAACATTCCCTTTC

             *      * *      *             **    *   *           *           
      32367 AGAGAGCACGCTTGAAAAACTCTTTTTGTAGACTCTGCAAGGGGATATTTGGACAGCTTTGAGGA
          1 ATAGAGCAGGTTTGAAACACTCTTTTTGTAGTATCTGGAAGTGGATATTTGGATAGCTTTGAGGA

                            *                *  *                            
      32432 TTTCGTTGGAAAC-GGGATATCTTCATATAAAATCTCGACAGAAGCATTCTCAGAAACATCTTTC
         66 TTTCGTTGGAAACAGGAATATCTTCATATAAAAGCTAGACAGAAGCATTCTCAG-AA-A-C-TTC

                *           *     *    *       *         
      32496 TTTGGGATGTTTGCATGCAAGTCACAGAGTTGAACTTTCCCTTTC
        127 TTTGTGATGTTTGCATTCAAGTAACAGTGTTGAACATTCCCTTTC

              *                          * *          *  *        *  *      *
      32541 ATGGAGCAGGTTTGAAACACTCTTTTTGTGGAATCTGGAAGTAGACATTTGGATCGCATTGAGGC
          1 ATAGAGCAGGTTTGAAACACTCTTTTTGTAGTATCTGGAAGTGGATATTTGGATAGCTTTGAGGA

            * *  *  *   **           **      *                   *     *     
      32606 CTACGGTGAAAAAGGGAATATCTTCGAATAAAAACTAGACAGAAGCATTCTCATAAACTACTTTG
         66 TTTCGTTGGAAACAGGAATATCTTCATATAAAAGCTAGACAGAAGCATTCTCAGAAACTTCTTTG

                  *   ** *  *      * *     *  * *    
      32671 TGATGTGTGCGGTTAACTAACAGAGCTGAACCTTTCTTTTC
        131 TGATGTTTGCATTCAAGTAACAGTGTTGAACATTCCCTTTC

                   * *                 *   *     ***             *           
      32712 ATAGAGCGGTTTTGAAACACTCTTTTTTTAGAATCTGCCTGTGGATATTTGGAAAGCTTTGAGGA
          1 ATAGAGCAGGTTTGAAACACTCTTTTTGTAGTATCTGGAAGTGGATATTTGGATAGCTTTGAGGA

                *        *            *      **                       *      
      32777 TTTCTTTGGAAACGGGAATATCTTCACATAAAATATAGACAGAAGCATTCTCAGAAACGTCTTTG
         66 TTTCGTTGGAAACAGGAATATCTTCATATAAAAGCTAGACAGAAGCATTCTCAGAAACTTCTTTG

            * *    *          *    *       **   *    
      32842 GGGTGTTAGCATTCAAGTCACAGAGTTGAACGCTCCTTTTC
        131 TGATGTTTGCATTCAAGTAACAGTGTTGAACATTCCCTTTC

                               *         * *             *        *         *
      32883 ATAGAGCAGGTTTGAAACATTCTTTTTGTGGAATCTGGAAGTGGACATTTGGATCGCTTTGAGGC
          1 ATAGAGCAGGTTTGAAACACTCTTTTTGTAGTATCTGGAAGTGGATATTTGGATAGCTTTGAGGA

            * *  *      *   *        **      *           *        *    **    
      32948 CTGCGGT-GAAAAAGGTATATCTTCGCATAAAAACTAGACAGAAGTATTCTCAGGAACTAGTTTG
         66 TTTCGTTGGAAACAGGAATATCTTCATATAAAAGCTAGACAGAAGCATTCTCAGAAACTTCTTTG

                  *  ***    *  *   *       *  * *   *
      33012 TGATGTGTGTGCTCAACTACCAGAGTTGAACCTTTCTTTTG
        131 TGATGTTTGCATTCAAGTAACAGTGTTGAACATTCCCTTTC

                     *                     *  *  * *    *   *    *           
      33053 ATAGAGCAGTTTTGAAACACTCTTTTTGTAGAATTTGCATGTGGCTATCTGGACAGCTTTGAGGA
          1 ATAGAGCAGGTTTGAAACACTCTTTTTGTAGTATCTGGAAGTGGATATTTGGATAGCTTTGAGGA

                         *    *          *   * *     *                *      
      33118 TTTCGTTGGAAACGGGAAAATCTTCATATGAAATCGAGACACAAGCATTCTCAGAAACCTCTTTG
         66 TTTCGTTGGAAACAGGAATATCTTCATATAAAAGCTAGACAGAAGCATTCTCAGAAACTTCTTTG

            *      *  *   *   *   **     *  *        
      33183 GGATGTTAGCCTTCGAGTCACACAGTTGATCACTCCCTTTC
        131 TGATGTTTGCATTCAAGTAACAGTGTTGAACATTCCCTTTC

             *              *                            **   *   *         *
      33224 ACAGAGCAGGTTTGAAGCACTCTTTTTGTAGTATCTGGAAGTGGACGTTTTGATCGCTTTGAGGC
          1 ATAGAGCAGGTTTGAAACACTCTTTTTGTAGTATCTGGAAGTGGATATTTGGATAGCTTTGAGGA

            * ** *      *            *** *    *   *                  *  * *  
      33289 ATAAGGT-GAAAAAGGAAATATCTTGCCACAAAAACTACACAGAAGCATTCTCAGAATCTACGTT
         66 TTTCGTTGGAAACAGG-AATATCTTCATATAAAAGCTAGACAGAAGCATTCTCAGAAACTTCTTT

                   * ** *    *      *       *  * *   *
      33353 GTGATGTGTTTACTCAACTAACAGAGTTGAACCTTTCTTTTG
        130 GTGATGTTTGCATTCAAGTAACAGTGTTGAACATTCCCTTTC

                     *         *        *  *     * *  *                      
      33395 ATAGAGCAGTTTTGAAACAGTCTTTTTGGAGAATCTGCAGGTAGATATTTGGATAGCTTTGAGGA
          1 ATAGAGCAGGTTTGAAACACTCTTTTTGTAGTATCTGGAAGTGGATATTTGGATAGCTTTGAGGA

                *       **                   *            *   *       **     
      33460 TTTCCTTGGAAAAGGGAATATCTTCATATAAAATCTAGACAGAAGCCTTCGCAGAAACACCTTTG
         66 TTTCGTTGGAAACAGGAATATCTTCATATAAAAGCTAGACAGAAGCATTCTCAGAAACTTCTTTG

            *            *    * *  *    *    *       
      33525 GGATGTTTGCATTGAAGTCAGAGAGTTGTACATCCCCTTTC
        131 TGATGTTTGCATTCAAGTAACAGTGTTGAACATTCCCTTTC

                     **                            **  * *    *   ** *      *
      33566 ATAGAGCAGCATTGAAACACTCTTTTTGTAGTATCTGGAGATGCACATTTAGATCACGTTGAGGC
          1 ATAGAGCAGGTTTGAAACACTCTTTTTGTAGTATCTGGAAGTGGATATTTGGATAGCTTTGAGGA

            * ** *      *             **      *     **      *    **      **  
      33631 CTATGGT-GAAATAGGAAATATCTTCGCATAAAAACTAGATGGAAGCAGTCTCCAAAACTTGCTT
         66 TTTCGTTGGAAACAGG-AATATCTTCATATAAAAGCTAGACAGAAGCATTCTCAGAAACTTCTTT

             **    *  * *    *      *      **  * *   *
      33695 GGAATGTGTGTACTCAACTAACAGAGTTGAATCTTTCTTTTG
        130 GTGATGTTTGCATTCAAGTAACAGTGTTGAACATTCCCTTTC

                     *                     **    *                     *     
      33737 ATAGAGCAGTTTTGAAACACTCTTTTTGTAGAGTCTGCAAGTGGATATTTGGATAGCTTAGAGGA
          1 ATAGAGCAGGTTTGAAACACTCTTTTTGTAGTATCTGGAAGTGGATATTTGGATAGCTTTGAGGA

                         *       * *    *    *                       **   *  
      33802 TTTCGTTGGAAACGGGAATATGTCCATACAAAACCTAGACAGAAGCATTCTCAGAAAAATCTCTG
         66 TTTCGTTGGAAACAGGAATATCTTCATATAAAAGCTAGACAGAAGCATTCTCAGAAACTTCTTTG

              ** *    *       ** *                   
      33867 TGGGGATTGCTTTCAAGTCCCGGTGTTGAACATTCCCTTTC
        131 TGATGTTTGCATTCAAGTAACAGTGTTGAACATTCCCTTTC

               *       *         ***           *          *       **      *  
      33908 ATAAAGCAGGTGTG-AACACAAGATTTTGTAGTATATGGAAGTGGACATTTGGAGTGCTTTGTGA
          1 ATAGAGCAGGTTTGAAACAC-TCTTTTTGTAGTATCTGGAAGTGGATATTTGGATAGCTTTGAG-

            *    *        *             **   *  *    *     **            ****
      33972 CATTTTG-T-GAAAAAGGAAATATCTTCCCATATAAACTAGGCAGAAAAATTCTCAGAAACCAGG
         64 GATTTCGTTGGAAACAGG-AATATCTTCATATAAAAGCTAGACAGAAGCATTCTCAGAAACTTCT

                   * *  * *    *      *       *  * *   *
      34035 TTGTGATCTGTGTACTCAACTAACAGGGTTGAACCTTTCTTTTG
        128 TTGTGATGTTTGCATTCAAGTAACAGTGTTGAACATTCCCTTTC

             *      ***              *     **    *   *        *  *           
      34079 AGAGAGCACTCTTGAAACACTCTTTCTGTAGACTCTGCAAGGGGATATTTTGACAGCTTTGAGGA
          1 ATAGAGCAGGTTTGAAACACTCTTTTTGTAGTATCTGGAAGTGGATATTTGGATAGCTTTGAGGA

                            *                *  *           *         *      
      34144 TTTCGTTGGAAAC-GGGATATCTTCATATAAAATCTCGACAGAAGCATCCTCAGAAACATCTTTG
         66 TTTCGTTGGAAACAGGAATATCTTCATATAAAAGCTAGACAGAAGCATTCTCAGAAACTTCTTTG

            *                 *    *                 
      34208 GGATGTTTGCATTCAAGTCACAGAGTTGAACATTCCCTTTC
        131 TGATGTTTGCATTCAAGTAACAGTGTTGAACATTCCCTTTC

              *                          * *         *   **     * *  *      *
      34249 ATGGAGCAGGTTTGAAACACTCTTTTTGTGGAATCTGGAAGGGGACCTTTGGGTCGCATTGAGGC
          1 ATAGAGCAGGTTTGAAACACTCTTTTTGTAGTATCTGGAAGTGGATATTTGGATAGCTTTGAGGA

            * *  *  *   **           **      *      *            *     **    
      34314 CTACGGTGAAAAAGGGAATATCTTCGAATAAAAACTAGACGGAAGCATTCTCATAAACTAGTTTG
         66 TTTCGTTGGAAACAGGAATATCTTCATATAAAAGCTAGACAGAAGCATTCTCAGAAACTTCTTTG

                  *  * * *  *      * *     *  * *    
      34379 TGATGTGTGTACTTAACTAACAGAGCTGAACCTTTCTTTTC
        131 TGATGTTTGCATTCAAGTAACAGTGTTGAACATTCCCTTTC

                   * *                 *   *     * *             *           
      34420 ATAGAGCGGTTTTGAAACACTCTTTTTATAGAATCTGCATGTGGATATTTGGAAAGCTTTGAGGA
          1 ATAGAGCAGGTTTGAAACACTCTTTTTGTAGTATCTGGAAGTGGATATTTGGATAGCTTTGAGGA

             *           *            *      **                       *      
      34485 TATCGTTGGAAACGGGAATATCTTCACATAAAATATAGACAGAAGCATTCTCAGAAACGTCTTTG
         66 TTTCGTTGGAAACAGGAATATCTTCATATAAAAGCTAGACAGAAGCATTCTCAGAAACTTCTTTG

            * *    *          *    *       *    *    
      34550 GGGTGTTAGCATTCAAGTCACAGAGTTGAACGTTCCTTTTC
        131 TGATGTTTGCATTCAAGTAACAGTGTTGAACATTCCCTTTC

                            * *          * *             *        *         *
      34591 ATAGAGCAGGTTTGAAGCCCTCTTTTTGTGGAATCTGGAAGTGGACATTTGGATCGCTTTGAGGC
          1 ATAGAGCAGGTTTGAAACACTCTTTTTGTAGTATCTGGAAGTGGATATTTGGATAGCTTTGAGGA

            * *  *      **  *         *      *  *        *       **    **   *
      34656 CTGCGGT-GAAAATGGTATATCTTCACATAAAAACTGGACAGAAGTATTCTCACGAACTAGTTTC
         66 TTTCGTTGGAAACAGGAATATCTTCATATAAAAGCTAGACAGAAGCATTCTCAGAAACTTCTTTG

                  *  ***    *      *       *  * *   *
      34720 TGATGTGTGTGCTCAACTAACAGAGTTGAACCTTTCTTTTG
        131 TGATGTTTGCATTCAAGTAACAGTGTTGAACATTCCCTTTC

             *       *   *              *  *     * *                    *    
      34761 ACAGAGCAGTTTTTAAACACTCTTTTTGGAGAATCTGCAGGTGGATATTTGGATAGCTTTTAGGA
          1 ATAGAGCAGGTTTGAAACACTCTTTTTGTAGTATCTGGAAGTGGATATTTGGATAGCTTTGAGGA

                *       **                   *            *   * *     **    *
      34826 TTTCCTTGGAAAAGGGAATATCTTCATATAAAATCTAGACAGAAGCCTTCGCGGAAACACCTTTT
         66 TTTCGTTGGAAACAGGAATATCTTCATATAAAAGCTAGACAGAAGCATTCTCAGAAACTTCTTTG

                         *    * *  **   *            
      34891 TGATGTTTGCATTGAAGTCAGAGAATTGTACATTCCCTTTC
        131 TGATGTTTGCATTCAAGTAACAGTGTTGAACATTCCCTTTC

                     *          *                  **    *    *   *         *
      34932 ATAGAGCAGCTTTGAAACACGCTTTTTGTAGTATCTGGAGATGGACATTTAGATCGCTTTGAGGG
          1 ATAGAGCAGGTTTGAAACACTCTTTTTGTAGTATCTGGAAGTGGATATTTGGATAGCTTTGAGGA

            * ** *      **            **    * *    * *      *    **      *   
      34997 CTATGGT-GAAATGGGAAATATCTTCGGATAACAACTAGTCGGAAGCAGTCTCCAAAACTTGTTT
         66 TTTCGTTGGAAACAGG-AATATCTTCATATAAAAGCTAGACAGAAGCATTCTCAGAAACTTCTTT

             **    *  * *    *      *      **  * *   *
      35061 GGAATGTGTGTACTCAACTAACAGAGTTGAATCTTTCTTTTG
        130 GTGATGTTTGCATTCAAGTAACAGTGTTGAACATTCCCTTTC

                     *                 *  ***    *                     *     
      35103 ATAGAGCAGTTTTGAAACACTCTTTTTTTACAGTCTGCAAGTGGATATTTGGATAGCTTAGAGGA
          1 ATAGAGCAGGTTTGAAACACTCTTTTTGTAGTATCTGGAAGTGGATATTTGGATAGCTTTGAGGA

                         *       * *    *    *                       **   *  
      35168 TTTCGTTGGAAACGGGAATATGTCCATACAAAACCTAGACAGAAGCATTCTCAGAAAAATCTCTG
         66 TTTCGTTGGAAACAGGAATATCTTCATATAAAAGCTAGACAGAAGCATTCTCAGAAACTTCTTTG

               * * *          **               *     
      35233 TGAGGATGGCATTCAAGTGCCAGTGTTGAACATTCTCTTTC
        131 TGATGTTTGCATTCAAGTAACAGTGTTGAACATTCCCTTTC

               *       *         ***           *     *    *      ***       * 
      35274 ATAAAGCAGGTGTG-AACACAAGATTTTGTAGTATATGGAACTGGACATTTGGGGTGCTTTG-TG
          1 ATAGAGCAGGTTTGAAACAC-TCTTTTTGTAGTATCTGGAAGTGGATATTTGGATAGCTTTGAGG

             **  *        *             **   *  *   **                   *** 
      35337 ACCT-ATTGTGAAAAAGGAAATATCTTCCCATATAAACTACGCAGAAGCATTCTCAGAAACCGGT
         65 ATTTCGTTG-GAAACAGG-AATATCTTCATATAAAAGCTAGACAGAAGCATTCTCAGAAACTTCT

                     *  * *    *      *       * ** *   *
      35401 TTGTGATGTGTGTACTCAACTAACAGGGTTGAACCTATCTTTTG
        128 TTGTGATGTTTGCATTCAAGTAACAGTGTTGAACATTCCCTTTC

             *      ***                           *   *        *  *          
      35445 AGAGAGCACTCTTGAAACACTCTTTTTGTA-TATTCTGCAAGGGGATATTTTGACAGCTTTGAGG
          1 ATAGAGCAGGTTTGAAACACTCTTTTTGTAGTA-TCTGGAAGTGGATATTTGGATAGCTTTGAGG

                    *     *            *      *               *      * *     
      35509 ATTTCGTTCGAAACTGGAATATCTTCACATAAAATCTAGACAGAAGCATTTTCAGAACCGTCTTT
         65 ATTTCGTTGGAAACAGGAATATCTTCATATAAAAGCTAGACAGAAGCATTCTCAGAAACTTCTTT

             * *   **          *   ***      *    *    
      35574 GGGGTGTCAGCATTCAAGTCACATATTTGAACGTTCCTTTTC
        130 GTGATGTTTGCATTCAAGTAACAGTGTTGAACATTCCCTTTC

                                     *   * *             *  *     *         *
      35616 ATAGAGCAGGTTTGAAACACTCTTTCTGTGGAATCTGGAAGTGGACATGTGGATCGCTTTGAGGC
          1 ATAGAGCAGGTTTGAAACACTCTTTTTGTAGTATCTGGAAGTGGATATTTGGATAGCTTTGAGGA

            * ** *      *   **       **      *           *       **    **    
      35681 CTGTGGT-GAAAAAGGTGTATCTTCGCATAAAAACTAGACAGAAGTATTCTCATGAACTAGTTTG
         66 TTTCGTTGGAAACAGGAATATCTTCATATAAAAGCTAGACAGAAGCATTCTCAGAAACTTCTTTG

                  *  ***    *  *   *       ** * *   *
      35745 TGATGTGTGTGCTCAACTACCAGAGTTGAACCCTTCTTTTG
        131 TGATGTTTGCATTCAAGTAACAGTGTTGAACATTCCCTTTC

                        ** *             * *  *  * *             *           
      35786 ATAGAGCA-GTTATATACACTCTTTTTGTGGAATTTGCATGTGGATATTTGGACAGCTTTGAGGA
          1 ATAGAGCAGGTTTGAAACACTCTTTTTGTAGTATCTGGAAGTGGATATTTGGATAGCTTTGAGGA

                         *    *  *       *   * *     *                *  *   
      35850 TTTCGTTGGAAACGGGAAAATTTTCATATGAAATCGAGACACAAGCATTCTCAGAAACCTCCTTG
         66 TTTCGTTGGAAACAGGAATATCTTCATATAAAAGCTAGACAGAAGCATTCTCAGAAACTTCTTTG

            *      *  *   *   *    *     *  *       *
      35915 GGATGTTAGCGTTCGAGTCACAGAGTTGATCACTCCCTTTA
        131 TGATGTTTGCATTCAAGTAACAGTGTTGAACATTCCCTTTC

                            *                            **   *  **         *
      35956 ATAGAGCAGGTTTGAAGCACTCTTTTTGTAGTATCTGGAAGTGGACGTTTTGAACGCTTTGAGGC
          1 ATAGAGCAGGTTTGAAACACTCTTTTTGTAGTATCTGGAAGTGGATATTTGGATAGCTTTGAGGA

            * ** *      *              *  *    *   *                      *  
      36021 GTAAGGT-GAAATAGGAAATATCTTGCCT-CAAAAACTACACAGAAGCATTCTCAGAAACTTATT
         66 TTTCGTTGGAAACAGG-AATATCTT-CATATAAAAGCTAGACAGAAGCATTCTCAGAAACTTCTT

                    **  **    *              *  * *   *
      36084 TGTGATGTGCGCCCTCAACTAACAGTGTTGAACCTTTCTTTTG
        129 TGTGATGTTTGCATTCAAGTAACAGTGTTGAACATTCCCTTTC

                     *                    **     *   **                      
      36127 ATAGAGCAGTTTTGAAACACTCTTTTTGTAAAATCTGCAAGAAGATATTTGGATAGCTTTGAGGA
          1 ATAGAGCAGGTTTGAAACACTCTTTTTGTAGTATCTGGAAGTGGATATTTGGATAGCTTTGAGGA

                         *   * *            **                           *   
      36192 TTTCGTTGGAAACGGGATTCTCTTCATATAAACTCTAGACAGAAGCATTCTCAGAAACTTCATTG
         66 TTTCGTTGGAAACAGGAATATCTTCATATAAAAGCTAGACAGAAGCATTCTCAGAAACTTCTTTG

            *       **   *    **            *        
      36257 GGATGTTTCTATTGAAGTCGCAGTGTTGAACAGTCCCTTTC
        131 TGATGTTTGCATTCAAGTAACAGTGTTGAACATTCCCTTTC

              *                                    *     *     * **     *   *
      36298 ATGGAGCAGGTTTGAAACACTCTTTTTGTAGTATCTGGACGTGGACATTTGTAGCGCTTTCAGGG
          1 ATAGAGCAGGTTTGAAACACTCTTTTTGTAGTATCTGGAAGTGGATATTTGGATAGCTTTGAGGA

            * ***       *             **      *                  *   *  **   
      36363 CTATATT-GAAAAAGGAAATATCTTCCCATAAAAACTAGACAGAAGCATTCTCTGAACCTAGTTT
         66 TTTCGTTGGAAACAGG-AATATCTTCATATAAAAGCTAGACAGAAGCATTCTCAGAAACTTCTTT

            *   ** *  ***    *      *          * *   *
      36427 CTGAGATGTGTCCTCAACTAACAGAGTTGAACATTTCTTTTG
        130 GTGATGTTTGCATTCAAGTAACAGTGTTGAACATTCCCTTTC

             *   *   *                   * *     *         *  * * *          
      36469 ACAGAACAGTTTTGAAACACTCTTTTTGTGGAATCTGCAAGTGGATACTTTGCTGGCTTTGAGGA
          1 ATAGAGCAGGTTTGAAACACTCTTTTTGTAGTATCTGGAAGTGGATATTTGGATAGCTTTGAGGA

                         *        *                   *   *  * *             
      36534 TTTCGTTGGAAACGGGAATA-CAT-ATA-AAAAGC-AGACAGCAGCGTTGTGAGAAACTTCTTTG
         66 TTTCGTTGGAAACAGGAATATCTTCATATAAAAGCTAGACAGAAGCATTCTCAGAAACTTCTTTG

                              *    *       *    * *  
      36595 TGATGTTTGCATTCAAGTCACAGAGTTGAACGTTCCGTATC
        131 TGATGTTTGCATTCAAGTAACAGTGTTGAACATTCCCTTTC

                        *      ** *      **            ***     * **  *  * ***
      36636 ATAGAGCAGGTTGGAAACATGCCTTTTGTCATATCTGGAAGTGTCCATTTGAAGCGCATTCAACC
          1 ATAGAGCAGGTTTGAAACACTCTTTTTGTAGTATCTGGAAGTGGATATTTGGATAGCTTTGAGGA

              **        *        **    *   *  * *                        *   
      36701 TTGTGTT-GAAAAAGGAAATACTTTCAAATAGAAACCAGACAGAAGCATTCTCAGAAACTTATTT
         66 TTTCGTTGGAAACAGG-AATATCTTCATATAAAAGCTAGACAGAAGCATTCTCAGAAACTTCTTT

                   *  * *    *      **  *    *  **   *
      36765 GTGATGTGTGTACTCAAATAACAGAATTCAACAATCGTTTTG
        130 GTGATGTTTGCATTCAAGTAACAGTGTTGAACATTCCCTTTC

             **      *                   * *     *     *      * *   *     *  
      36807 AAGGAGCAGTTTTGAAACACTCTTTTTGTGGAATCTGCAAGTGCATATTTAGCTAGATTTGACGA
          1 ATAGAGCAGGTTTGAAACACTCTTTTTGTAGTATCTGGAAGTGGATATTTGGATAGCTTTGAGGA

                         *   *    *                   *                *     
      36872 TTTCGTTGGAAACGGGATTA-CAT-ATA-AAAAGC-AGACAGCAGCATTCTCAGAAACTCCTTTG
         66 TTTCGTTGGAAACAGGAATATCTTCATATAAAAGCTAGACAGAAGCATTCTCAGAAACTTCTTTG

                              *    *                 
      36933 TGATGTTTGCATTCAAGTCACAGAGTTGAACATTCCCTTTC
        131 TGATGTTTGCATTCAAGTAACAGTGTTGAACATTCCCTTTC

                      *      *             *       *     *       **     *   *
      36974 ATAGAGCAGGATTGAAAAACTCTTTTTGTAGAATCTGGATGTGGACATTTGGAGTGCTTTCAGGC
          1 ATAGAGCAGGTTTGAAACACTCTTTTTGTAGTATCTGGAAGTGGATATTTGGATAGCTTTGAGGA

            * ** *      *             *** *                 *            **  
      37039 CTATGGT-GAAAAAGGAAATATCTTCCCCTGAAAGCTAGACAGAAGCACTCTCAGAAACTTAATT
         66 TTTCGTTGGAAACAGG-AATATCTTCATATAAAAGCTAGACAGAAGCATTCTCAGAAACTTCTTT

                   *   **    *              *  * *    
      37103 GTGATGTGTGCCCTCAACTAACAGTGTTGAACCTTTCTTTTC
        130 GTGATGTTTGCATTCAAGTAACAGTGTTGAACATTCCCTTTC

                     *           **      ***     *   *                       
      37145 ATAGAGCAGATTTGAAACAC-AATTTTGTTAAATCTGCAAGAGGATATTTGGATAGCTTTGAGGA
          1 ATAGAGCAGGTTTGAAACACTCTTTTTGTAGTATCTGGAAGTGGATATTTGGATAGCTTTGAGGA

                         *   * *            **                 *     *       
      37209 TTTCGTTGGAAACGGGATTGTCTTCATATAAACTCTAGACAGAAGCATTCTTAGAAATTTCTTTG
         66 TTTCGTTGGAAACAGGAATATCTTCATATAAAAGCTAGACAGAAGCATTCTCAGAAACTTCTTTG

            *       **   * *  *                     *
      37274 GGATGTTTCAATTGACGTCACAGTGTTGAACATTCCCTTTG
        131 TGATGTTTGCATTCAAGTAACAGTGTTGAACATTCCCTTTC

                                    *                    *       **   * *    
      37315 ATAGAGCAGGTTTGAAACACTCTTCTTGTAGTATCTGGAAGTGGACATTTGGAGCGCTCTCAGGA
          1 ATAGAGCAGGTTTGAAACACTCTTTTTGTAGTATCTGGAAGTGGATATTTGGATAGCTTTGAGGA

            * * **      *             **            *       * *          *  *
      37380 CTACAGT-GAAAAAGGAAATATCTTCCAATAAAAGCTAGATAGAAGCAATGTCAGAAACTTTTTC
         66 TTTCGTTGGAAACAGG-AATATCTTCATATAAAAGCTAGACAGAAGCATTCTCAGAAACTTCTTT

            *                 **      * *     *  * *   *
      37444 ATGATGTATCTGC--TCAGCTAACAGAGATGAACCTTTCTTTTG
        130 GTGATGT-T-TGCATTCAAGTAACAGTGTTGAACATTCCCTTTC

             *       *                   * *  *  *             **            
      37486 AGAGAGCAGCTTTGAAACACTCTTTTTGTGGAATATGCAAGTGGATATTTGTCTAGCTTTGAGGA
          1 ATAGAGCAGGTTTGAAACACTCTTTTTGTAGTATCTGGAAGTGGATATTTGGATAGCTTTGAGGA

                         *   *    *                   *       *              
      37551 TTTCGTTGGAAACGGGATTA-CAT-ATA-AAAAGC-AGACAGCAGCATTCCCAGAAACTTCTTTG
         66 TTTCGTTGGAAACAGGAATATCTTCATATAAAAGCTAGACAGAAGCATTCTCAGAAACTTCTTTG

                              *    *                 
      37612 TGATGTTTGCATTCAAGTCACAGAGTTGAACATTCCCTTTC
        131 TGATGTTTGCATTCAAGTAACAGTGTTGAACATTCCCTTTC

             *                            *        *     *       **     *   *
      37653 ACAGAGCAGGTTTGAAACACTCTTTTTGTAATATCTGGATGTGGACATTTGGAGCGCTTTCAGGC
          1 ATAGAGCAGGTTTGAAACACTCTTTTTGTAGTATCTGGAAGTGGATATTTGGATAGCTTTGAGGA

            * ** *      *             *** *   *                          *   
      37718 CTATGGT-GAAAAAGGAAATATCTTCCCCTGAAAACTAGACAGAAGCATTCTCAGAAACTTATTT
         66 TTTCGTTGGAAACAGG-AATATCTTCATATAAAAGCTAGACAGAAGCATTCTCAGAAACTTCTTT

                   *  * *    *              *  * *   *
      37782 GTGATGTGTGTAGTCAACTAACAGTGTTGAACCTTTCTTTTG
        130 GTGATGTTTGCATTCAAGTAACAGTGTTGAACATTCCCTTTC

            *        *                    **     *   *                       
      37824 GTAGAGCAGTTTTGAAACACTCTTTTTGTAAAATCTGCAAGAGGATATTTGGATAGCTTTGAGGA
          1 ATAGAGCAGGTTTGAAACACTCTTTTTGTAGTATCTGGAAGTGGATATTTGGATAGCTTTGAGGA

                         *   * *            **                       *      *
      37889 TTTCGTTGGAAACGGGATTGTCTTCATATAAACTCTAGACAGAAGCATTCTCAGAAATTTCTTTC
         66 TTTCGTTGGAAACAGGAATATCTTCATATAAAAGCTAGACAGAAGCATTCTCAGAAACTTCTTTG

            *       **   *    *                   *  
      37954 GGATGTTTCAATTGAAGTCACAGTGTTGAACATTCCCTGTC
        131 TGATGTTTGCATTCAAGTAACAGTGTTGAACATTCCCTTTC

                                                         *       **   * *    
      37995 ATAGAGCAGGTTTGAAACACTCTTTTTGTAGTATCTGGAAGTGGACATTTGGAGCGCTCTCAGGA
          1 ATAGAGCAGGTTTGAAACACTCTTTTTGTAGTATCTGGAAGTGGATATTTGGATAGCTTTGAGGA

            * * **      *             **            *       * *          *  *
      38060 CTACAGT-GAAAAAGGAAATATCTTCCAATAAAAGCTAGATAGAAGCAATGTCAGAAACTTTTTC
         66 TTTCGTTGGAAACAGG-AATATCTTCATATAAAAGCTAGACAGAAGCATTCTCAGAAACTTCTTT

            *      * ** *   **      *       *  * *   *
      38124 ATGATGTATCTACTCAGCTAACAGAGTTGAACCTTTCTTTTG
        130 GTGATGTTTGCATTCAAGTAACAGTGTTGAACATTCCCTTTC

             *       *                   * *     *             **            
      38166 AGAGAGCAGTTTTGAAACACTCTTTTTGTGGAATCTGCAAGTGGATATTTGTCTAGCTTTGAGGA
          1 ATAGAGCAGGTTTGAAACACTCTTTTTGTAGTATCTGGAAGTGGATATTTGGATAGCTTTGAGGA

                         *   *    *                   *       *              
      38231 TTTCGTTGGAAACGGGATTA-CAT-ATA-AAAAGC-AGACAGCAGCATTCCCAGAAACTTCTTTG
         66 TTTCGTTGGAAACAGGAATATCTTCATATAAAAGCTAGACAGAAGCATTCTCAGAAACTTCTTTG

                              *    *                 
      38292 TGATGTTTGCATTCAAGTCACAGAGTTGAACATTCCCTTTC
        131 TGATGTTTGCATTCAAGTAACAGTGTTGAACATTCCCTTTC

                                          *        *     *       **     *   *
      38333 ATAGAGCAGGTTTGAAACACTCTTTTTGTACTATCTGGATGTGGACATTTGGAGGGCTTTCAGGC
          1 ATAGAGCAGGTTTGAAACACTCTTTTTGTAGTATCTGGAAGTGGATATTTGGATAGCTTTGAGGA

            * ** *  *                 *** *   *                          *   
      38398 CTATGGTGCAAA-AGGAAATATCTTCCCCTGAAAACTAGACAGAAGCATTCTCAGAAACTTATTT
         66 TTTCGTTGGAAACAGG-AATATCTTCATATAAAAGCTAGACAGAAGCATTCTCAGAAACTTCTTT

                   **  **    *              *  * *    
      38462 GTGATGTGCGCCCTCAACTAACAGTGTTGAACCTTTCTTTTC
        130 GTGATGTTTGCATTCAAGTAACAGTGTTGAACATTCCCTTTC

                     *   *             *  **     *   *                       
      38504 ATAGAGCAGTTTTTAAACACTCTTTTTCTAAAATCTGCAAGAGGATATTTGGATAGCTTTGAGGA
          1 ATAGAGCAGGTTTGAAACACTCTTTTTGTAGTATCTGGAAGTGGATATTTGGATAGCTTTGAGGA

                         *   * *            **                           *   
      38569 TTTCGTTGGAAACGGGATTGTCTTCATATAAACTCTAGACAGAAGCATTCTCAGAAACTTCATTG
         66 TTTCGTTGGAAACAGGAATATCTTCATATAAAAGCTAGACAGAAGCATTCTCAGAAACTTCTTTG

            *       **   *    *             *        
      38634 GGATGTTTCAATTGAAGTCACAGTGTTGAACAGTCCCTTTC
        131 TGATGTTTGCATTCAAGTAACAGTGTTGAACATTCCCTTTC

                                *     *                  *    *  **   * *    
      38675 ATAGAGCAGGTTTGAAACACACTTTTCGTAGTATCTGGAAGTGGACATTTAGAGCGCTCTCAGGA
          1 ATAGAGCAGGTTTGAAACACTCTTTTTGTAGTATCTGGAAGTGGATATTTGGATAGCTTTGAGGA

            * *  *      *            *               *       * *          *  
      38740 CTACGGT-GAAAAAGGAAATATTCTCCA-ATAAAAGCTAGATAGAAGCAATGTCAGAAACTTTTT
         66 TTTCGTTGGAAACAGG-AATA-TCTTCATATAAAAGCTAGACAGAAGCATTCTCAGAAACTTCTT

            **      * ** *   **      *       *  * *   *
      38803 CATGATGTATCTACTCAGCTAACAGAGTTGAACCTTTCTTTTG
        129 TGTGATGTTTGCATTCAAGTAACAGTGTTGAACATTCCCTTTC

             *       *                   * *  *  *             **            
      38846 AGAGAGCAGTTTTGAAACACTCTTTTTGTGGAATATGCAAGTGGATATTTGTCTAGCTTTGAGGA
          1 ATAGAGCAGGTTTGAAACACTCTTTTTGTAGTATCTGGAAGTGGATATTTGGATAGCTTTGAGGA

                         *   *    *                 ***       *        **   *
      38911 TTTCGTTGGAAACGGGATTA-CAT-ATA-AAAAGC-AGACCCCAGCATTCACAGAAACTGTTTTT
         66 TTTCGTTGGAAACAGGAATATCTTCATATAAAAGCTAGACAGAAGCATTCTCAGAAACTTCTTTG

                              *    *            *    
      38972 TGATGTTTGCATTCAAGTCACAGAGTTGAACATTCCTTTTC
        131 TGATGTTTGCATTCAAGTAACAGTGTTGAACATTCCCTTTC

             *                                     *     *       **     *   *
      39013 ACAGAGCAGGTTTGAAACACTCTTTTTGTAGTATCTGGATGTGGACATTTGGAGCGCTTTCAGGC
          1 ATAGAGCAGGTTTGAAACACTCTTTTTGTAGTATCTGGAAGTGGATATTTGGATAGCTTTGAGGA

            * ** *      *             *** *   *              *           *   
      39078 CTATGGT-GAAAAAGGAAATATCTTCCCCTGAAAACTAGACAGAAGCATACTCAGAAACTTATTT
         66 TTTCGTTGGAAACAGG-AATATCTTCATATAAAAGCTAGACAGAAGCATTCTCAGAAACTTCTTT

                   **  **    *             **  * **  *
      39142 GTGATGTGCGCCCTCAACTAACAGTGTTGAAACTTTCTGTTG
        130 GTGATGTTTGCATTCAAGTAACAGTGTTGAACATTCCCTTTC

                     *                    **     *  ****                     
      39184 ATAGAGCAGTTTTGAAACACTCTTTTTGTAAAATCTGCAAAAATATATTTGGATAGCTTTGAGGA
          1 ATAGAGCAGGTTTGAAACACTCTTTTTGTAGTATCTGGAAGTGGATATTTGGATAGCTTTGAGGA

                         *   * *            **                           *   
      39249 TTTCGTTGGAAACGGGATTGTCTTCATATAAACTCTAGACAGAAGCATTCTCAGAAACTTCATTG
         66 TTTCGTTGGAAACAGGAATATCTTCATATAAAAGCTAGACAGAAGCATTCTCAGAAACTTCTTTG

            *       **   *    *             *        
      39314 GGATGTTTCAATTGAAGTCACAGTGTTGAACAGTCCCTTTC
        131 TGATGTTTGCATTCAAGTAACAGTGTTGAACATTCCCTTTC

                                                   *     *       **     *   *
      39355 ATAGAGCAGGTTTGAAACACTCTTTTTGTAGTATCTGGACGTGGACATTTGGAGCGCTTTCAGGC
          1 ATAGAGCAGGTTTGAAACACTCTTTTTGTAGTATCTGGAAGTGGATATTTGGATAGCTTTGAGGA

            * **      * *             **      * *  *             *     ***   
      39420 CTATGTT-GACAAAGGAAATATCTTCCCATAAAAACAAGGCAGAAGCATTCTCTGAAACCGGTTT
         66 TTTCGTTGGAAACAGG-AATATCTTCATATAAAAGCTAGACAGAAGCATTCTCAGAAACTTCTTT

            *   *  *  ***    *      *          * *   *
      39484 CTGAGGTGTGTCCTCAACTAACAGAGTTGAACATTTCTTTTG
        130 GTGATGTTTGCATTCAAGTAACAGTGTTGAACATTCCCTTTC

             *   *   *   *               * *     *     *        * *  *       
      39526 ACAGAACAGTTTTCAAACACTCTTTTTGTGGAATCTGCAAGTGAATATTTGGCTGGCCTTGAGGA
          1 ATAGAGCAGGTTTGAAACACTCTTTTTGTAGTATCTGGAAGTGGATATTTGGATAGCTTTGAGGA

                         *                            *      * *             
      39591 TTTCGTTGGAAACGGGAATA-CTT-ATA-AAAAGC-AGACAGCAGCATTGTGAGAAACTTCTTTG
         66 TTTCGTTGGAAACAGGAATATCTTCATATAAAAGCTAGACAGAAGCATTCTCAGAAACTTCTTTG

                              *    *   *  **   ** *  
      39652 TGATGTTTGCATTCAAGTCACAGAGTTCAAAGTTCAGTATC
        131 TGATGTTTGCATTCAAGTAACAGTGTTGAACATTCCCTTTC

            *           *       * *      **            ***       *** *  *   *
      39693 CTAGAGCAGGTTGGAAACACGCCTTTTGTCATATCTGGAAGTGTCCATTTGGAGCCCATTCAGGC
          1 ATAGAGCAGGTTTGAAACACTCTTTTTGTAGTATCTGGAAGTGGATATTTGGATAGCTTTGAGGA

              **        *             ** * *  * *                        *   
      39758 TTGTGTT-GAAAAAGGAAATATCTTCCCAAAGAAACCAGACAGAAGCATTCTCAGAAACTTATTT
         66 TTTCGTTGGAAACAGG-AATATCTTCATATAAAAGCTAGACAGAAGCATTCTCAGAAACTTCTTT

                   *  * *    *      **  *    *  **   *
      39822 GTGATGTGTGTACTCAACTAACAGAATTCAACAATCGTTTTG
        130 GTGATGTTTGCATTCAAGTAACAGTGTTGAACATTCCCTTTC

             **      *                   * *     *     *    * * *   *        
      39864 AAGGAGCAGTTTTGAAACACTCTTTTTGTGGAATCTGCAAGTGCATATGTAGCTAGATTTGAGGA
          1 ATAGAGCAGGTTTGAAACACTCTTTTTGTAGTATCTGGAAGTGGATATTTGGATAGCTTTGAGGA

                         *   *    *                   *       *              
      39929 TTTCGTTGGAAACGGGATTA-CAT-ATA-AAAAGC-AGACAGCAGCATTCCCAGAAACTTCTTTG
         66 TTTCGTTGGAAACAGGAATATCTTCATATAAAAGCTAGACAGAAGCATTCTCAGAAACTTCTTTG

                    *    *    *    *                 
      39990 TGATGTTTCCATTGAAGTCACAGAGTTGAACATTCCCTTTC
        131 TGATGTTTGCATTCAAGTAACAGTGTTGAACATTCCCTTTC

                                *                        *    *  **   * *    
      40031 ATAGAGCAGGTTTGAAACACAC-TTTTGTAGTATCTGGAAGTGGACATTTAGAGCGCTCTCAGGA
          1 ATAGAGCAGGTTTGAAACACTCTTTTTGTAGTATCTGGAAGTGGATATTTGGATAGCTTTGAGGA

            * *  *      *             **            *       * *          *  *
      40095 CTACGGT-GAAAAAGGAAATATCTTCCAATAAAAGCTAGATAGAAGCAATGTCAGAAACTTTTTC
         66 TTTCGTTGGAAACAGG-AATATCTTCATATAAAAGCTAGACAGAAGCATTCTCAGAAACTTCTTT

            *      * ** *   **      *      **  * *   *
      40159 ATGATGTATCTACTCAGCTAACAGAGTTGAATCTTTCTTTTG
        130 GTGATGTTTGCATTCAAGTAACAGTGTTGAACATTCCCTTTC

             *       *   *     *        ** *  *  *           ** **     *     
      40201 AGAGAGCAGTTTTAAAACAGTCTTTTTGGGGAATATGCAAGTGGATATTAAGCCAGCTTGGAGGA
          1 ATAGAGCAGGTTTGAAACACTCTTTTTGTAGTATCTGGAAGTGGATATTTGGATAGCTTTGAGGA

                         *      * *                   *              *       
      40266 TTTCGTTGGAAACGGGAAT-CCAT-ATA-AAAAGC-AGACAGCAGCATTCTCAGAAATTTCTTTG
         66 TTTCGTTGGAAACAGGAATATCTTCATATAAAAGCTAGACAGAAGCATTCTCAGAAACTTCTTTG

                         *    **                      
      40327 TGATGTTTGCATTGAAGTCCCAGATG-TGAACATTCCCTTTC
        131 TGATGTTTGCATTCAAGTAACAG-TGTTGAACATTCCCTTTC

               *                * *      **             ***       **  *  **  
      40368 ATAAAGCAGGTTTGAAACACGCCTTTTGTCATATCTGGAAGTTGTCCATTTGGAGGGCATTCCGG
          1 ATAGAGCAGGTTTGAAACACTCTTTTTGTAGTATCTGGAAG-TGGATATTTGGATAGCTTTGAGG

            *  **      * *          *  **      *     *                    *  
      40433 CTTGTGTT-GACAAAGGAAATATCCTCCCATAAAAACTAGATAGAA-CATTCTCAGAAACTTATT
         65 ATTTCGTTGGAAACAGG-AATATCTTCATATAAAAGCTAGACAGAAGCATTCTCAGAAACTTCTT

                    *  * *    *      **      **  **   *
      40496 TGTGATGTGTGTACTCAACTAACAGAATTGAACCATCGTTTTG
        129 TGTGATGTTTGCATTCAAGTAACAGTGTTGAACATTCCCTTTC

             *       *            *      * *     *             **            
      40539 AAAGAGCAGTTTTGAAACACTCCTTTTGTGGAATCTGCAAGTGGATATTTGTCTAGCTTTGAGGA
          1 ATAGAGCAGGTTTGAAACACTCTTTTTGTAGTATCTGGAAGTGGATATTTGGATAGCTTTGAGGA

                         *   *    *                   *       *              
      40604 TTTCGTTGGAAACGGGATTA-CAT-ATA-AAAAGC-AGACAGCAGCATTCCCAGAAACTTCTTTG
         66 TTTCGTTGGAAACAGGAATATCTTCATATAAAAGCTAGACAGAAGCATTCTCAGAAACTTCTTTG

                              *  * *  *       *      
      40665 TGATGTTTGCATTCAAGTCACTGAGTAGAACATTGCCTTTC
        131 TGATGTTTGCATTCAAGTAACAGTGTTGAACATTCCCTTTC

                                                   *     *       **     *   *
      40706 ATAGAGCAGGTTTGAAACACTCTTTTTGTAGTATCTGGATGTGGACATTTGGAGCGCTTTCAGGC
          1 ATAGAGCAGGTTTGAAACACTCTTTTTGTAGTATCTGGAAGTGGATATTTGGATAGCTTTGAGGA

            * ** *      *             *** *   *   *                      *   
      40771 CTATGGT-GAAAAAGGAAATATCTTCCCCTGAAAACTACACAGAAGCATTCTCAGAAACTTATTT
         66 TTTCGTTGGAAACAGG-AATATCTTCATATAAAAGCTAGACAGAAGCATTCTCAGAAACTTCTTT

                   **  **    *              *  * *   *
      40835 GTGATGTGCGCCCTCAACTAACAGTGTTGAACCTTTCTTTTG
        130 GTGATGTTTGCATTCAAGTAACAGTGTTGAACATTCCCTTTC

                     *          *         **     *   *                       
      40877 ATAGAGCAGTTTTGAAACACCCTTTTTGTAAAATCTGCAAGAGGATATTTGGATAGCTTTGAGGA
          1 ATAGAGCAGGTTTGAAACACTCTTTTTGTAGTATCTGGAAGTGGATATTTGGATAGCTTTGAGGA

                         *   * *            **                            *  
      40942 TTTCGTTGGAAACGGGATTGTCTTCATATAAACTCTAGACAGAAGCATTCTC-GAAAGCTTCATT
         66 TTTCGTTGGAAACAGGAATATCTTCATATAAAAGCTAGACAGAAGCATTCTCAGAAA-CTTCTTT

             *       **   *    *             *        
      41006 GGGATGTTTCAATTGAAGTCACAGTGTTGAACAGTCCCTTTC
        130 GTGATGTTTGCATTCAAGTAACAGTGTTGAACATTCCCTTTC

                                                   *     *       **     *   *
      41048 ATAGAGCAGGTTTGAAACACTCTTTTTGTAGTATCTGGATGTGGACATTTGGAGCGCTTTCAGGC
          1 ATAGAGCAGGTTTGAAACACTCTTTTTGTAGTATCTGGAAGTGGATATTTGGATAGCTTTGAGGA

            * ** *      *             *** *   *                          *   
      41113 CTATGGT-GAAAAAGGAAGTATCTTCCCCTGAAAACTAGACAGAAGCATTCTCAGAAACTTATTT
         66 TTTCGTTGGAAACAGGAA-TATCTTCATATAAAAGCTAGACAGAAGCATTCTCAGAAACTTCTTT

                   **  **    *              *  * *   *
      41177 GTGATGTGCGCCCTCAACTAACAGTGTTGAACCTTTCTTTTG
        130 GTGATGTTTGCATTCAAGTAACAGTGTTGAACATTCCCTTTC

                *    *      *        *    **     *   *                       
      41219 ATAGGGCAGTTTTGAAGCACTCTTTGTGTAAAATCTGCAAGAGGATATTTGGATAGCTTTGAGGA
          1 ATAGAGCAGGTTTGAAACACTCTTTTTGTAGTATCTGGAAGTGGATATTTGGATAGCTTTGAGGA

                         *   * *            **                    *      *   
      41284 TTTCGTTGGAAACGGGATTGTCTTCATATAAACTCTAGACAGAAGCATTCTCAGTAACTTCATTG
         66 TTTCGTTGGAAACAGGAATATCTTCATATAAAAGCTAGACAGAAGCATTCTCAGAAACTTCTTTG

            *       **   *    *             *        
      41349 GGATGTTTCAATTGAAGTCACAGTGTTGAACAGTCCCTTTC
        131 TGATGTTTGCATTCAAGTAACAGTGTTGAACATTCCCTTTC

                           *             *               *       **   * *    
      41390 ATAGAGCAGGTTTGAGACACTCTTTTTGTGGTATCTGGAAGTGGACATTTGGAGCGCTGTCAGGA
          1 ATAGAGCAGGTTTGAAACACTCTTTTTGTAGTATCTGGAAGTGGATATTTGGATAGCTTTGAGGA

            * *  *      *             **            *       * *       *  ** *
      41455 CTACGGT-GAAAAAGGAAATATCTTCCAATAAAAGCTAGATAGAAGCAATGTCAGAAAATTTGTC
         66 TTTCGTTGGAAACAGG-AATATCTTCATATAAAAGCTAGACAGAAGCATTCTCAGAAACTTCTTT

            *      * ** *   **      *       *  * *   *
      41519 ATGATGTATCTACTCAGCTAACAGAGTTGAACCTTTCTTTTG
        130 GTGATGTTTGCATTCAAGTAACAGTGTTGAACATTCCCTTTC

             *       *                   * *     *             **   *        
      41561 AGAGAGCAGTTTTGAAACACTCTTTTTGTGGAATCTGCAAGTGGATATTTGTCTAGATTTGAGGA
          1 ATAGAGCAGGTTTGAAACACTCTTTTTGTAGTATCTGGAAGTGGATATTTGGATAGCTTTGAGGA

                         *   *    *                   *       *              
      41626 TTTCGTTGGAAACGGGATTA-CAT-ATA-AAAAGC-AGACAGCAGCATTCCCAGAAACTTCTTTG
         66 TTTCGTTGGAAACAGGAATATCTTCATATAAAAGCTAGACAGAAGCATTCTCAGAAACTTCTTTG

                              *                      
      41687 TGATGTTTGCATTCAAGTCACAGTGTTGAACATTCCCTTTC
        131 TGATGTTTGCATTCAAGTAACAGTGTTGAACATTCCCTTTC

                                        *  ** * *  *     *              *   *
      41728 ATAGAGCAGGTTTGAAACACTCTTTTTGAAGAGTTTAGATGTGGACATTTGGAT-GCTTTCAGGC
          1 ATAGAGCAGGTTTGAAACACTCTTTTTGTAGTATCTGGAAGTGGATATTTGGATAGCTTTGAGGA

            * ** *      *             *** *   *                      *   *   
      41792 CTATGGT-GAAAAAGGAAATATCTTCCCCTGAAAACTAGACAGAAGCATTCTCAGAATCTTATTT
         66 TTTCGTTGGAAACAGG-AATATCTTCATATAAAAGCTAGACAGAAGCATTCTCAGAAACTTCTTT

                   **  **    *             **  * *   *
      41856 GTGATGTGCGCCCTCAACTAACAGTGTTGAAGCTTTCTTTTG
        130 GTGATGTTTGCATTCAAGTAACAGTGTTGAACATTCCCTTTC

                *    *      *        *    **     *   *                       
      41898 ATAGGGCAGTTTTGAAGCACTCTTTGTGTAAAATCTGCAAGAGGATATTTGGATAGCTTTGAGGA
          1 ATAGAGCAGGTTTGAAACACTCTTTTTGTAGTATCTGGAAGTGGATATTTGGATAGCTTTGAGGA

                         **  * *            **                      *    *   
      41963 TTTCGTTGGAAACGAGATTGTCTTCATATAAACTCTAGACAGAAGCATTCTCAGAAGCTTCATTG
         66 TTTCGTTGGAAACAGGAATATCTTCATATAAAAGCTAGACAGAAGCATTCTCAGAAACTTCTTTG

            *       **   *    *             *        
      42028 GGATGTTTCAATTGAAGTCACAGTGTTGAACAGTCCCTTTC
        131 TGATGTTTGCATTCAAGTAACAGTGTTGAACATTCCCTTTC

                                          *              *       **   * *    
      42069 ATAGAGCAGGTTTGAAACACTCTTTTTGTAATATCTGGAAGTGGACATTTGGAGCGCTCTCAGGA
          1 ATAGAGCAGGTTTGAAACACTCTTTTTGTAGTATCTGGAAGTGGATATTTGGATAGCTTTGAGGA

            * *  *      *  *          **            *       * *          *  *
      42134 CTGCGGT-GAAAAAGTAATTATCTTCCAATAAAAGCTAGATAGAAGCAATGTCAGAAACTTTTTC
         66 TTTCGTTGGAAACAGGAA-TATCTTCATATAAAAGCTAGACAGAAGCATTCTCAGAAACTTCTTT

            *      * ** *   **      *       *  * *   *
      42198 ATGATGTATCTACTCAGCTAACAGAGTTGAACCTTTCTTTTG
        130 GTGATGTTTGCATTCAAGTAACAGTGTTGAACATTCCCTTTC

             *       *                   * *     *          *  **            
      42240 AGAGAGCAGTTTTGAAACACTCTTTTTGTGGAATCTGCAAGTGGATATGTGTCTAGCTTTGAGGA
          1 ATAGAGCAGGTTTGAAACACTCTTTTTGTAGTATCTGGAAGTGGATATTTGGATAGCTTTGAGGA

                      *  *   *    *                *  *       *              
      42305 TTTCGTTGGATACGGGATTA-CAT-ATA-AAAAGC-AGAAAGCAGCATTCCCAGAAACTTCTTTG
         66 TTTCGTTGGAAACAGGAATATCTTCATATAAAAGCTAGACAGAAGCATTCTCAGAAACTTCTTTG

                     *        *    *                 
      42366 TGATGTTTGTATTCAAGTCACAGAGTTGAACATTCCCTTTC
        131 TGATGTTTGCATTCAAGTAACAGTGTTGAACATTCCCTTTC

                       *                           *     *       **     *   *
      42407 ATAGAGCAGGTATGAAACA--CTTTTTGTAGTATCTGGATGTGGACATTTGGAGCGCTTTCAGGC
          1 ATAGAGCAGGTTTGAAACACTCTTTTTGTAGTATCTGGAAGTGGATATTTGGATAGCTTTGAGGA

            * ** *      *             *** *   *                          * * 
      42470 CTATGGT-GAAAAAGGAAATATCTTCCCCTGAAAACTAGACAGAAGCATTCTCAGAAACTTATCT
         66 TTTCGTTGGAAACAGG-AATATCTTCATATAAAAGCTAGACAGAAGCATTCTCAGAAACTTCTTT

                   **  **    *              *  * *   *
      42534 GTGATGTGCGCCCTCAACTAACAGTGTTGAACCTTTCTTTTG
        130 GTGATGTTTGCATTCAAGTAACAGTGTTGAACATTCCCTTTC

                     *                    **     *   *                       
      42576 ATAGAGCAGTTTTGAAACACTCTTTTTGTAAAATCTGCAAGAGGATATTTGGATAGCTTTGAGGA
          1 ATAGAGCAGGTTTGAAACACTCTTTTTGTAGTATCTGGAAGTGGATATTTGGATAGCTTTGAGGA

                         *   * *            **                      *    *   
      42641 TTTCGTTGGAAACGGGATTGTCTTCATATAAACTCTAGACAGAAGCATTCTCAGAAGCTTCATTG
         66 TTTCGTTGGAAACAGGAATATCTTCATATAAAAGCTAGACAGAAGCATTCTCAGAAACTTCTTTG

            *       **   *    *             *        
      42706 GGATGTTTCAATTGAAGTCACAGTGTTGAACAGTCCCTTTC
        131 TGATGTTTGCATTCAAGTAACAGTGTTGAACATTCCCTTTC

                                                         *       **   * *    
      42747 ATAGAGCAGGTTTGAAACACTCTTTTTGTAGTATCTGGAAGTGGACATTTGGAGCGCTCTCAGGA
          1 ATAGAGCAGGTTTGAAACACTCTTTTTGTAGTATCTGGAAGTGGATATTTGGATAGCTTTGAGGA

            * *  *      *             **          * *  *    * *          *  *
      42812 CTACGGT-GAAAAAGGAAATATCTTCCAATAAAAGCTACATAGGAGCAATGTCAGAAACTTTTTC
         66 TTTCGTTGGAAACAGG-AATATCTTCATATAAAAGCTAGACAGAAGCATTCTCAGAAACTTCTTT

            *      * ** *   **      *       *  * *   *
      42876 ATGATGTATCTACTCAGCTAACAGAGTTGAACCTTTCTTTTG
        130 GTGATGTTTGCATTCAAGTAACAGTGTTGAACATTCCCTTTC

             *       *                   * *     *             **            
      42918 AGAGAGCAGTTTTGAAACACTCTTTTTGTGGAATCTGCAAGTGGATATTTGTCTAGCTTTGAGGA
          1 ATAGAGCAGGTTTGAAACACTCTTTTTGTAGTATCTGGAAGTGGATATTTGGATAGCTTTGAGGA

                         *   *    *               *   *       *            * 
      42983 TTTCGTTGGAAACGGGATTA-CAT-ATA-AAAAGC-AGTCAGCAGCATTCCCAGAAACTTCTTGG
         66 TTTCGTTGGAAACAGGAATATCTTCATATAAAAGCTAGACAGAAGCATTCTCAGAAACTTCTTTG

                              *    *                 
      43044 TGATGTTTGCATTCAAGTCACAGAGTTGAACATTCCCTTTC
        131 TGATGTTTGCATTCAAGTAACAGTGTTGAACATTCCCTTTC

                                                   *     *       **     * *  
      43085 ATAGAGCAGGTTTGAAACACTCTTTTTGTAGTATCTGGATGTGGACATTTGGAGCGCTTTCACGA
          1 ATAGAGCAGGTTTGAAACACTCTTTTTGTAGTATCTGGAAGTGGATATTTGGATAGCTTTGAGGA

            * ** *      *             *** *   *                          *   
      43150 CTATGGT-GAAAAAGGAAATATCTTCCCCTGAAAACTAGACAGAAGCATTCTCAGAAACTTATTT
         66 TTTCGTTGGAAACAGG-AATATCTTCATATAAAAGCTAGACAGAAGCATTCTCAGAAACTTCTTT

                   **  **    *              *  * *   *
      43214 GTGATGTGCGCCCTCAACTAACAGTGTTGAACCTTTCTTTTG
        130 GTGATGTTTGCATTCAAGTAACAGTGTTGAACATTCCCTTTC

                     *                    **     *   *                       
      43256 ATAGAGCAGTTTTGAAACACTCTTTTTGTAAAATCTGCAAGAGGATATTTGGATAGCTTTGAGGA
          1 ATAGAGCAGGTTTGAAACACTCTTTTTGTAGTATCTGGAAGTGGATATTTGGATAGCTTTGAGGA

                         *   * *            **                      *    *   
      43321 TTTCGTTGGAAACGGGATTGTCTTCATATAAACTCTAGACAGAAGCATTCTCAGAAGCTTCATTG
         66 TTTCGTTGGAAACAGGAATATCTTCATATAAAAGCTAGACAGAAGCATTCTCAGAAACTTCTTTG

            * *     **   *    *             *        
      43386 GGTTGTTTCAATTGAAGTCACAGTGTTGAACAGTCCCTTTC
        131 TGATGTTTGCATTCAAGTAACAGTGTTGAACATTCCCTTTC

                                                         *       **   * *    
      43427 ATAGAGCAGGTTTGAAACACTCTTTTTGTAGTATCTGGAAGTGGACATTTGGAGCGCTCTCAGGA
          1 ATAGAGCAGGTTTGAAACACTCTTTTTGTAGTATCTGGAAGTGGATATTTGGATAGCTTTGAGGA

            * *  *      *             **            *       * *          *  *
      43492 CTGCGGT-GAAAAAGGAAATATCTTCCAATAAAAGCTAGATAGAAGCAATGTCAGAAACTTTTTC
         66 TTTCGTTGGAAACAGG-AATATCTTCATATAAAAGCTAGACAGAAGCATTCTCAGAAACTTCTTT

            *   *  * ** *   **      *       *  * *   *
      43556 ATGACGTATCTACTCAGCTAACAGAGTTGAACCTTTCTTTTG
        130 GTGATGTTTGCATTCAAGTAACAGTGTTGAACATTCCCTTTC

             *       *                   * *     *             **            
      43598 AGAGAGCAGTTTTGAAACACTCTTTTTGTGGAATCTGCAAGTGGATATTTGTTTAGCTTTGAGGA
          1 ATAGAGCAGGTTTGAAACACTCTTTTTGTAGTATCTGGAAGTGGATATTTGGATAGCTTTGAGGA

                              *    *         * *      *       *   *          
      43663 TTTCGTTGGAAAC-GG--GAT-TACATATAAAAACCAGACAGCAGCATTCCCAGTAACTTCTTTG
         66 TTTCGTTGGAAACAGGAATATCTTCATATAAAAGCTAGACAGAAGCATTCTCAGAAACTTCTTTG

                              *    *                 
      43724 TGATGTTTGCATTCAAGTCACAGAGTTGAACATTCCCTTTC
        131 TGATGTTTGCATTCAAGTAACAGTGTTGAACATTCCCTTTC

                                 *                 *     *       **    **   *
      43765 ATAGAGCAGGTTTGAAACACTTTTTTTGTAGTATCTGGATGTGGACATTTGGAGCGCTTGCAGGC
          1 ATAGAGCAGGTTTGAAACACTCTTTTTGTAGTATCTGGAAGTGGATATTTGGATAGCTTTGAGGA

            * *  *      *             *** *   *     *        *       *   *   
      43830 CTACGGT-GAAAAAGGAAATATCTTCCCCTGAAAACTAGAGAGAAGCATACTCAGAATCTTATTT
         66 TTTCGTTGGAAACAGG-AATATCTTCATATAAAAGCTAGACAGAAGCATTCTCAGAAACTTCTTT

                   **  **    *          *   *  * *   *
      43894 GTGATGTGCGCCCTCAATTAACAGTGTTCAACCTTTCTTTTG
        130 GTGATGTTTGCATTCAAGTAACAGTGTTGAACATTCCCTTTC

                     *                    **     *   *                       
      43936 ATAGAGCAGTTTTGAAACACTCTTTTTGTAAAATCTGCAAGAGGATATTTGGATAGCTTTGAGGA
          1 ATAGAGCAGGTTTGAAACACTCTTTTTGTAGTATCTGGAAGTGGATATTTGGATAGCTTTGAGGA

                         *   * *            **                      *    *   
      44001 TTTCGTTGGAAACGGGATTGTCTTCATATAAACTCTAGACAGAAGCATTCTCAGAAGCTTCATTG
         66 TTTCGTTGGAAACAGGAATATCTTCATATAAAAGCTAGACAGAAGCATTCTCAGAAACTTCTTTG

            *       **   *    *             *        
      44066 GGATGTTTCAATTGAAGTCACAGTGTTGAACAGTCCCTTTC
        131 TGATGTTTGCATTCAAGTAACAGTGTTGAACATTCCCTTTC

                                                         *       *  * * *    
      44107 ATAGAGCAGGTTTGAAACACTCTTTTTGTAGTATCTGGAAGTGGACATTTGGAGAGTTCTCAGGA
          1 ATAGAGCAGGTTTGAAACACTCTTTTTGTAGTATCTGGAAGTGGATATTTGGATAGCTTTGAGGA

            * *  *      *             **          * *       * *      *   *  *
      44172 ATACGGT-GAAAAAGGAAATATCTTCCAATAAAAGCTACATAGAAGCAATGTCAGAACCTTTTTC
         66 TTTCGTTGGAAACAGG-AATATCTTCATATAAAAGCTAGACAGAAGCATTCTCAGAAACTTCTTT

            *      * ** *   **   *  *       *  * *   *
      44236 ATGATGTATCTACTCAGCTAATAGAGTTGAACCTTTCTTTTG
        130 GTGATGTTTGCATTCAAGTAACAGTGTTGAACATTCCCTTTC

             *       *                   * *     *             **            
      44278 AGAGAGCAGTTTTGAAACACTCTTTTTGTGGAATCTGCAAGTGGATATTTGTCTAGCTTTGAGGA
          1 ATAGAGCAGGTTTGAAACACTCTTTTTGTAGTATCTGGAAGTGGATATTTGGATAGCTTTGAGGA

                         *   *    *                   *       *              
      44343 TTTCGTTGGAAACGGGATTA-CAT-ATA-AAAAGC-AGACAGCAGCATTCCCAGAAACTTCTTTG
         66 TTTCGTTGGAAACAGGAATATCTTCATATAAAAGCTAGACAGAAGCATTCTCAGAAACTTCTTTG

                              *    *                 
      44404 TGATGTTTGCATTCAAGTCACAGAGTTGAACATTCCCTTTC
        131 TGATGTTTGCATTCAAGTAACAGTGTTGAACATTCCCTTTC

                     *          *                * *     *       **     *   *
      44445 ATAGAGCAGATTTGAAACACACTTTTTGTAGTATCTGTATGTGGACATTTGGAGCGCTTTCAGGC
          1 ATAGAGCAGGTTTGAAACACTCTTTTTGTAGTATCTGGAAGTGGATATTTGGATAGCTTTGAGGA

            * ** *      *             *** *   *                      *   *   
      44510 CTATGGT-GAAAAAGGAAATATCTTCCCCTGAAAACTAGACAGAAGCATTCTCAGAATCTTATTT
         66 TTTCGTTGGAAACAGG-AATATCTTCATATAAAAGCTAGACAGAAGCATTCTCAGAAACTTCTTT

                   **  **  * *       *     **  * *   *
      44574 GTGATGTGCGCCCTCCACTAACAGTCTTGAAGCTTTCTTTTG
        130 GTGATGTTTGCATTCAAGTAACAGTGTTGAACATTCCCTTTC

                     *                    **     *   *                       
      44616 ATAGAGCAGTTTTGAAACACTCTTTTTGTAAAATCTGCAAGAGGATATTTGGATAGCTTTGAGGA
          1 ATAGAGCAGGTTTGAAACACTCTTTTTGTAGTATCTGGAAGTGGATATTTGGATAGCTTTGAGGA

                         *   * *            **                   *  *    *   
      44681 TTTCGTTGGAAACGGGATTGTCTTCATATAAACCCTAGACAGAAGCATTCTCACAAGCTTCATTG
         66 TTTCGTTGGAAACAGGAATATCTTCATATAAAAGCTAGACAGAAGCATTCTCAGAAACTTCTTTG

            *       **   *    *             *        
      44746 GGATGTTTCAATTGAAGTCACAGTGTTGAACAGTCCCTTTC
        131 TGATGTTTGCATTCAAGTAACAGTGTTGAACATTCCCTTTC

                                                         *       **   * *    
      44787 ATAGAGCAGGTTTGAAACACTCTTTTTGTAGTATCTGGAAGTGGACATTTGGAGCGCTCTCAGGA
          1 ATAGAGCAGGTTTGAAACACTCTTTTTGTAGTATCTGGAAGTGGATATTTGGATAGCTTTGAGGA

            * *  *      *             **            *       * *          *  *
      44852 CTGCGGT-GAAAAAGGAATTATCTTCCAATAAAAGCTAGATAGAAGCAATGTCAGAAACTTTTTC
         66 TTTCGTTGGAAACAGGAA-TATCTTCATATAAAAGCTAGACAGAAGCATTCTCAGAAACTTCTTT

            *      * ** *   **      *       *  * *   *
      44916 ATGATGTATCTACTCAGCTAACAGAGTTGAACCTTTCTTTTG
        130 GTGATGTTTGCATTCAAGTAACAGTGTTGAACATTCCCTTTC

             *       *              *    * *     *             **            
      44958 AGAGAGCAGTTTTGAAACACTCTTCTTGTGGAATCTGCAAGTGGATATTTGTCTAGCTTTGAGGA
          1 ATAGAGCAGGTTTGAAACACTCTTTTTGTAGTATCTGGAAGTGGATATTTGGATAGCTTTGAGGA

                         *   *    *                   *       *              
      45023 TTTCGTTGGAAACGGGATTA-CAT-ATA-AAAAGC-AGACAGCAGCATTCCCAGAAACTTCTTTG
         66 TTTCGTTGGAAACAGGAATATCTTCATATAAAAGCTAGACAGAAGCATTCTCAGAAACTTCTTTG

                *             *    *                 
      45084 TGATATTTGCATTCAAGTCACAGAGTTGAACATTCCCTTTC
        131 TGATGTTTGCATTCAAGTAACAGTGTTGAACATTCCCTTTC

                                                   *     *       **   * *   *
      45125 ATAGAGCAGGTTTGAAACACTCTTTTTGTAGTATCTGGATGTGGACATTTGGAGCGCTGTCAGGC
          1 ATAGAGCAGGTTTGAAACACTCTTTTTGTAGTATCTGGAAGTGGATATTTGGATAGCTTTGAGGA

            * ** *      *             *** *   *         *            *   *   
      45190 CTATGGT-GAAAAAGGAAATATCTTCCCCTGAAAACTAGACAGAGGCATTCTCAGAATCTTATTT
         66 TTTCGTTGGAAACAGG-AATATCTTCATATAAAAGCTAGACAGAAGCATTCTCAGAAACTTCTTT

             *     **  **    *             **  * *   *
      45254 GCGATGTGCGCCCTCAACTAACAGTGTTGAAGCTTTCTTTTG
        130 GTGATGTTTGCATTCAAGTAACAGTGTTGAACATTCCCTTTC

            *        *   *                **     *   *                       
      45296 CTAGAGCAGTTTTTAAACACTCTTTTTGTAAAATCTGCAAGAGGATATTTGGATAGCTTTGAGGA
          1 ATAGAGCAGGTTTGAAACACTCTTTTTGTAGTATCTGGAAGTGGATATTTGGATAGCTTTGAGGA

                         *     *            **                      *    *   
      45361 TTTCGTTGGAAACGGGAATGTCTTCATATAAACTCTAGACAGAAGCATTCTCAGAAGCTTCATTG
         66 TTTCGTTGGAAACAGGAATATCTTCATATAAAAGCTAGACAGAAGCATTCTCAGAAACTTCTTTG

            * *     **   *    *             *        
      45426 GGTTGTTTCAATTGAAGTCACAGTGTTGAACAGTCCCTTTC
        131 TGATGTTTGCATTCAAGTAACAGTGTTGAACATTCCCTTTC

                               *                      *  *       ***  * *    
      45467 ATAGAGCAGGTTTGAAACAGTCTTTTTGTAGTATCTGGAAGTAGACATTTGGAGCACTCTCAGGA
          1 ATAGAGCAGGTTTGAAACACTCTTTTTGTAGTATCTGGAAGTGGATATTTGGATAGCTTTGAGGA

            * *  *      *             **            *       * *          *  *
      45532 CTACGGT-GAAAAAGGAATTATCTTCCAATAAAAGCTAGATAGAAGCAATGTCAGAAACTTTTTC
         66 TTTCGTTGGAAACAGGAA-TATCTTCATATAAAAGCTAGACAGAAGCATTCTCAGAAACTTCTTT

            *      * ** *   **      *       *  * *   *
      45596 ATGATGTATCTACTCAGCTAACAGAGTTGAACCTTTCTTTTG
        130 GTGATGTTTGCATTCAAGTAACAGTGTTGAACATTCCCTTTC

             *       *                   * *     *             **            
      45638 AGAGAGCAGTTTTGAAACACTCTTTTTGTGGAATCTGCAAGTGGATATTTGTCTAGCTTTGAGGA
          1 ATAGAGCAGGTTTGAAACACTCTTTTTGTAGTATCTGGAAGTGGATATTTGGATAGCTTTGAGGA

                         *   *    *                   *       *   *  *       
      45703 TTTCGTTGGAAACGGGATTA-CAT-ATA-AAAAGC-AGACAGCAGCATTCCCAGTAAATTCTTTG
         66 TTTCGTTGGAAACAGGAATATCTTCATATAAAAGCTAGACAGAAGCATTCTCAGAAACTTCTTTG

                              *    *                 
      45764 TGATGTTTGCATTCAAGTCACAGAGTTGAACATTCCCTTTC
        131 TGATGTTTGCATTCAAGTAACAGTGTTGAACATTCCCTTTC

                                 *                 *     *    *  **     *   *
      45805 ATAGAGCAGGTTTGAAACACTTTTTTTGTAGTATCTGGATGTGGACATTTAGAGCGCTTTCAGGC
          1 ATAGAGCAGGTTTGAAACACTCTTTTTGTAGTATCTGGAAGTGGATATTTGGATAGCTTTGAGGA

            * ** *      *             *** *   *                      *   *   
      45870 CTATGGT-GAAAAAGGAAATATCTTCCCCTGAAAACTAGACAGAAGCATTCTCAGAATCTTATTT
         66 TTTCGTTGGAAACAGG-AATATCTTCATATAAAAGCTAGACAGAAGCATTCTCAGAAACTTCTTT

                   **  **    *              *  * *   *
      45934 GTGATGTGCGCCCTCAACTAACAGTGTTGAACCTTTCTTTTG
        130 GTGATGTTTGCATTCAAGTAACAGTGTTGAACATTCCCTTTC

                     *                    **     *   *                       
      45976 ATAGAGCAGTTTTGAAACACTCTTTTTGTAAAATCTGCAAGAGGATATTTGGATAGCTTTGAGGA
          1 ATAGAGCAGGTTTGAAACACTCTTTTTGTAGTATCTGGAAGTGGATATTTGGATAGCTTTGAGGA

                         *   * *            **                      *    *   
      46041 TTTCGTTGGAAACGGGATTGTCTTCATATAAACTCTAGACAGAAGCATTCTCAGAAGCTTCATTG
         66 TTTCGTTGGAAACAGGAATATCTTCATATAAAAGCTAGACAGAAGCATTCTCAGAAACTTCTTTG

            *       **   *    *             *        
      46106 GGATGTTTCAATTGAAGTCACAGTGTTGAACAGTCCCTTTC
        131 TGATGTTTGCATTCAAGTAACAGTGTTGAACATTCCCTTTC

                                                         *       *  * * *    
      46147 ATAGAGCAGGTTTGAAACACTCTTTTTGTAGTATCTGGAAGTGGACATTTGGAGAGTTCTCAGGA
          1 ATAGAGCAGGTTTGAAACACTCTTTTTGTAGTATCTGGAAGTGGATATTTGGATAGCTTTGAGGA

            * *  *      *               *            *       * *      *   *  
      46212 CTACGGT-GAAAAAGGAAATATCTTCCAAAT-AAAGCTAGATAGAAGCAATGTCAGAATCTTTTT
         66 TTTCGTTGGAAACAGG-AATATCTT-CATATAAAAGCTAGACAGAAGCATTCTCAGAAACTTCTT

            **      * ** *   **      *       *  * *   *
      46275 CATGATGTATCTACTCAGCTAACAGAGTTGAACCTTTCTTTTG
        129 TGTGATGTTTGCATTCAAGTAACAGTGTTGAACATTCCCTTTC

             *       *                   * *     *             **            
      46318 AGAGAGCAGTTTTGAAACACTCTTTTTGTGGAATCTGCAAGTGGATATTTGTCTAGCTTTGAGGA
          1 ATAGAGCAGGTTTGAAACACTCTTTTTGTAGTATCTGGAAGTGGATATTTGGATAGCTTTGAGGA

                         *   *    *                   *       *     *        
      46383 TTTCGTTGGAAACGGGATTA-CAT-ATA-AAAAGC-AGACAGCAGCATTCCCAGAATCTTCTTTG
         66 TTTCGTTGGAAACAGGAATATCTTCATATAAAAGCTAGACAGAAGCATTCTCAGAAACTTCTTTG

                              *    *                 
      46444 TGATGTTTGCATTCAAGTCACAGAGTTGAACATTCCCTTTC
        131 TGATGTTTGCATTCAAGTAACAGTGTTGAACATTCCCTTTC

                     *       *                     *     *       **     *   *
      46485 ATAGAGCAGATTTGAAAAACTCTTTTTGTAGTATCTGGATGTGGACATTTGGAGCGCTTTCAGGC
          1 ATAGAGCAGGTTTGAAACACTCTTTTTGTAGTATCTGGAAGTGGATATTTGGATAGCTTTGAGGA

            * ** *      *             *** *   *                      *   *   
      46550 CTATGGT-GAAAAAGGAAATATCTTCTCCTGAAAACTAGACAGAAGCATTCTCAGAATCTTATTT
         66 TTTCGTTGGAAACAGG-AATATCTTCATATAAAAGCTAGACAGAAGCATTCTCAGAAACTTCTTT

                   **  **    *             **  * *   *
      46614 GTGATGTGCGCCCTCAACTAACAGTGTTGAAGCTTTCTTTTG
        130 GTGATGTTTGCATTCAAGTAACAGTGTTGAACATTCCCTTTC

                     *                    **     *   *                       
      46656 ATAGAGCAGTTTTGAAACACTCTTTTTGTAAAATCTGCAAGAGGATATTTGGATAGCTTTGAGGA
          1 ATAGAGCAGGTTTGAAACACTCTTTTTGTAGTATCTGGAAGTGGATATTTGGATAGCTTTGAGGA

                         *  ** *       *    **      *               *    *   
      46721 TTTCGTTGGAAACGGGTTTGTCTTCATTTAAACTCTAGACGGAAGCATTCTCAGAAGCTTCATTG
         66 TTTCGTTGGAAACAGGAATATCTTCATATAAAAGCTAGACAGAAGCATTCTCAGAAACTTCTTTG

            *   *   **   *    *             *        
      46786 GGATATTTCAATTGAAGTCACAGTGTTGAACAGTCCCTTTC
        131 TGATGTTTGCATTCAAGTAACAGTGTTGAACATTCCCTTTC

                       *                                 *       **   * *    
      46827 ATAGAGCAGGTGTGAAACACTCTTTTTGTAGTATCTGGAAGTGGACATTTGGAGCGCTCTTAGGA
          1 ATAGAGCAGGTTTGAAACACTCTTTTTGTAGTATCTGGAAGTGGATATTTGGATAGCTTTGAGGA

            * *  *      *             **            *       * *          *  *
      46892 CTACGGT-GAAAAAGGAAATATCTTCCAATAAAAGCTAGATAGAAGCAATGTCAGAAACTTTTTC
         66 TTTCGTTGGAAACAGG-AATATCTTCATATAAAAGCTAGACAGAAGCATTCTCAGAAACTTCTTT

            *      * ** *   **      *       *  * *   *
      46956 ATGATGTATCTACTCAGCTAACAGAGTTGAACCTTTCTTTTG
        130 GTGATGTTTGCATTCAAGTAACAGTGTTGAACATTCCCTTTC

             *       *                   * *     *             **     *      
      46998 AGAGAGCAGTTTTGAAACACTCTTTTTGTGGAATCTGCAAGTGGATATTTGTCTAGCTCTGAGGA
          1 ATAGAGCAGGTTTGAAACACTCTTTTTGTAGTATCTGGAAGTGGATATTTGGATAGCTTTGAGGA

                         *   *    *                   *       *  **          
      47063 TTTCGTTGGAAACGGGATTA-CAT-ATA-AAAAGC-AGACAGCAGCATTCCCAATAACTTCTTTG
         66 TTTCGTTGGAAACAGGAATATCTTCATATAAAAGCTAGACAGAAGCATTCTCAGAAACTTCTTTG

                              *    *                 
      47124 TGATGTTTGCATTCAAGTCACAGAGTTGAACATTCCCTTTC
        131 TGATGTTTGCATTCAAGTAACAGTGTTGAACATTCCCTTTC

                            *                      *     *       ** *   *   *
      47165 ATAGAGCAGGTTTGAATCACTCTTTTTGTAGTATCTGGATGTGGACATTTGGAGCGATTTCAGGC
          1 ATAGAGCAGGTTTGAAACACTCTTTTTGTAGTATCTGGAAGTGGATATTTGGATAGCTTTGAGGA

            * ** *      *             *** *   *                      *   *   
      47230 CTATGGT-GAAAAAGGAAATATCTTCCCCTGAAAACTAGACAGAAGCATTCTCAGAATCTTATTT
         66 TTTCGTTGGAAACAGG-AATATCTTCATATAAAAGCTAGACAGAAGCATTCTCAGAAACTTCTTT

             **    **  **    *             **  * *   *
      47294 GCAATGTGCGCCCTCAACTAACAGTGTTGAAGCTTTCTTTTG
        130 GTGATGTTTGCATTCAAGTAACAGTGTTGAACATTCCCTTTC

            *        *                    **     *   *                       
      47336 CTAGAGCAGTTTTGAAACACTCTTTTTGTAAAATCTGCAAGAGGATATTTGGATAGCTTTGAGGA
          1 ATAGAGCAGGTTTGAAACACTCTTTTTGTAGTATCTGGAAGTGGATATTTGGATAGCTTTGAGGA

                    *    *   * *            **          *           *    *   
      47401 TTTCGTTGAAAACGGGATTGTCTTCATATAAACTCTAGACAGAAACATTCTCAGAAGCTTCATTG
         66 TTTCGTTGGAAACAGGAATATCTTCATATAAAAGCTAGACAGAAGCATTCTCAGAAACTTCTTTG

            *       **   *    *             *        
      47466 GGATGTTTCAATTGAAGTCACAGTGTTGAACAGTCCCTTTC
        131 TGATGTTTGCATTCAAGTAACAGTGTTGAACATTCCCTTTC

                                                                 **   * *    
      47507 ATAGAGCAGGTTTGAAACACTCTTTTTGTAGTATCTGGAAGTGGATATTTGGAGCGCTCTCAGGA
          1 ATAGAGCAGGTTTGAAACACTCTTTTTGTAGTATCTGGAAGTGGATATTTGGATAGCTTTGAGGA

            * *  *  *                 **          * *       * *          *  *
      47572 CTACGGTGTAAA-AGGAAATATCTTCCAATAAAAGCTACATAGAAGCAATGTCAGAAACTTTTTC
         66 TTTCGTTGGAAACAGG-AATATCTTCATATAAAAGCTAGACAGAAGCATTCTCAGAAACTTCTTT

            *      * ** *   **      *       *  * *   *
      47636 ATGATGTATCTACTCAGCTAACAGAGTTGAACCTTTCTTTTG
        130 GTGATGTTTGCATTCAAGTAACAGTGTTGAACATTCCCTTTC

             *       *                   * *     *             **            
      47678 AGAGAGCAGTTTTGAAACACTCTTTTTGTGGAATCTGCAAGTGGATATTTGTCTAGCTTTGAGGA
          1 ATAGAGCAGGTTTGAAACACTCTTTTTGTAGTATCTGGAAGTGGATATTTGGATAGCTTTGAGGA

                         *   *    *               *   *       *              
      47743 TTTCGTTGGAAACGGGATTA-CAT-ATA-AAAAGC-AGTCAGCAGCATTCCCAGAAACTTCTTTG
         66 TTTCGTTGGAAACAGGAATATCTTCATATAAAAGCTAGACAGAAGCATTCTCAGAAACTTCTTTG

                              *    *                 
      47804 TGATGTTTGCATTCAAGTCACAGAGTTGAACATTCCCTTTC
        131 TGATGTTTGCATTCAAGTAACAGTGTTGAACATTCCCTTTC

                 *                        *        *     *       **     * * *
      47845 ATAGAACAGGTTTGAAACACTCTTTTTGTACTATCTGGATGTGGACATTTGGAGCGCTTTCACGC
          1 ATAGAGCAGGTTTGAAACACTCTTTTTGTAGTATCTGGAAGTGGATATTTGGATAGCTTTGAGGA

            * ** *      *             *** *   *                          *   
      47910 CTAAGGT-GAAAAAGGAAATATCTTCCCCTGAAAACTAGACAGAAGCATTCTCAGAAACTTATTT
         66 TTTCGTTGGAAACAGG-AATATCTTCATATAAAAGCTAGACAGAAGCATTCTCAGAAACTTCTTT

                   **  **    *             **  * *   *
      47974 GTGATGTGCGCCCTCAACTAACAGTGTTGAAGCTTTCTTTTG
        130 GTGATGTTTGCATTCAAGTAACAGTGTTGAACATTCCCTTTC

                     *                    **     *   **                      
      48016 ATAGAGCAGTTTTGAAACACTCTTTTTGTAAAATCTGAAAGACGATATTTGGATAGCTTTGAGGA
          1 ATAGAGCAGGTTTGAAACACTCTTTTTGTAGTATCTGGAAGTGGATATTTGGATAGCTTTGAGGA

                             * *            **          *           *    *   
      48081 TTTCGTTGGAAACAGGATTGTCTTCATATAAACTCTAGACAGAATCATTCTCAGAAGCTTCATTG
         66 TTTCGTTGGAAACAGGAATATCTTCATATAAAAGCTAGACAGAAGCATTCTCAGAAACTTCTTTG

            *       ** * *    *             *        
      48146 GGATGTTTCAAATGAAGTCACAGTGTTGAACAGTCCCTTTC
        131 TGATGTTTGCATTCAAGTAACAGTGTTGAACATTCCCTTTC

                                                         *       **   * *    
      48187 ATAGAGCAGGTTTGAAACACTCTTTTTGTAGTATCTGGAAGTGGACATTTGGAGCGCTCTCAGGA
          1 ATAGAGCAGGTTTGAAACACTCTTTTTGTAGTATCTGGAAGTGGATATTTGGATAGCTTTGAGGA

            * * **      *             **            *       * *          *  *
      48252 CTACCGT-GAAAAAGGAATTATCTTCCAATAAAAGCTAGATAGAAGCAATGTCAGAAACTTTTTC
         66 TTTCGTTGGAAACAGGAA-TATCTTCATATAAAAGCTAGACAGAAGCATTCTCAGAAACTTCTTT

            *      * ** *   **      *       *  * *   *
      48316 ATGATGTATCTACTCAGCTAACAGAGTTGAACCTTTCTTTTG
        130 GTGATGTTTGCATTCAAGTAACAGTGTTGAACATTCCCTTTC

             *       *                   * *     *             **            
      48358 AGAGAGCAGTTTTGAAACACTCTTTTTGTGGAATCTGCAAGTGGATATTTGTCTAGCTTTGAGGA
          1 ATAGAGCAGGTTTGAAACACTCTTTTTGTAGTATCTGGAAGTGGATATTTGGATAGCTTTGAGGA

                         *   *    *                   *       *   *          
      48423 TTTCGTTGGAAACGGGATTA-CAT-ATA-AAAAGC-AGACAGCAGCATTCCCAGTAACTTCTTTG
         66 TTTCGTTGGAAACAGGAATATCTTCATATAAAAGCTAGACAGAAGCATTCTCAGAAACTTCTTTG

                              *    *                 
      48484 TGATGTTTGCATTCAAGTCACAGAGTTGAACATTCCCTTTC
        131 TGATGTTTGCATTCAAGTAACAGTGTTGAACATTCCCTTTC

                                 *                 *     *       **  *  * * *
      48525 ATAGAGCAGGTTTGAAACACTTTTTTTGTAGTATCTGGATGTGGACATTTGGAGCGCATTCATGC
          1 ATAGAGCAGGTTTGAAACACTCTTTTTGTAGTATCTGGAAGTGGATATTTGGATAGCTTTGAGGA

            * ** *      *             *** *   *                      *   *   
      48590 CTATGGT-GAAAAAGGAAATATCTTCCCCTGAAAACTAGACAGAAGCATTCTCAGAATCTTATTT
         66 TTTCGTTGGAAACAGG-AATATCTTCATATAAAAGCTAGACAGAAGCATTCTCAGAAACTTCTTT

                   **  **    *             **  * *   *
      48654 GTGATGTGCGCCCTCAACTAACAGTGTTGAAGCTTTCTTTTG
        130 GTGATGTTTGCATTCAAGTAACAGTGTTGAACATTCCCTTTC

                     *                  * **     *   *                       
      48696 ATAGAGCAGTTTTGAAACACTCTTTTTGAAAAATCTGCAAGAGGATATTTGGATAGCTTTGAGGA
          1 ATAGAGCAGGTTTGAAACACTCTTTTTGTAGTATCTGGAAGTGGATATTTGGATAGCTTTGAGGA

                         *   * *            **                      *    *   
      48761 TTTCGTTGGAAACGGGATTGTCTTCATATAAACTCTAGACAGAAGCATTCTCAGAAGCTTCATTG
         66 TTTCGTTGGAAACAGGAATATCTTCATATAAAAGCTAGACAGAAGCATTCTCAGAAACTTCTTTG

            *       **   *    *             *        
      48826 GGATGTTTCAATTGAAGTCACAGTGTTGAACAGTCCCTTTC
        131 TGATGTTTGCATTCAAGTAACAGTGTTGAACATTCCCTTTC

                                                         *       **   * *    
      48867 ATAGAGCAGGTTTGAAACACTCTTTTTGTAGTATCTGGAAGTGGACATTTGGAGCGCTCTCAGGA
          1 ATAGAGCAGGTTTGAAACACTCTTTTTGTAGTATCTGGAAGTGGATATTTGGATAGCTTTGAGGA

            * *  *      *             **            *       * *          *  *
      48932 CTGCGGT-GAAAAAGGAATTATCTTCCAATAAAAGCTAGATAGAAGCAATGTCAGAAACTTTTTC
         66 TTTCGTTGGAAACAGGAA-TATCTTCATATAAAAGCTAGACAGAAGCATTCTCAGAAACTTCTTT

            *      * ** *   **      *       *  * *   *
      48996 ATGATGTATCTACTCAGTTAACAGAGTTGAACCTTTCTTTTG
        130 GTGATGTTTGCATTCAAGTAACAGTGTTGAACATTCCCTTTC

             *       *                   * *     *             **            
      49038 AGAGAGCAGTTTTGAAACACTCTTTTTGTGGAATCTGCAAGTGGATATTTGTCTAGCTTTGAGGA
          1 ATAGAGCAGGTTTGAAACACTCTTTTTGTAGTATCTGGAAGTGGATATTTGGATAGCTTTGAGGA

                         *   *    *               *   *       *              
      49103 TTTCGTTGGAAACGGGATTA-CAT-ATA-AAAAGC-AGTCAGCAGCATTCCCAGAAACTTCTTTG
         66 TTTCGTTGGAAACAGGAATATCTTCATATAAAAGCTAGACAGAAGCATTCTCAGAAACTTCTTTG

                              *    *                 
      49164 TGATGTTTGCATTCAAGTCACAGAGTTGAACATTCCCTTTC
        131 TGATGTTTGCATTCAAGTAACAGTGTTGAACATTCCCTTTC

                                                   *     *       **     *   *
      49205 ATAGAGCAGGTTTGAAACACTCTTTTTGTAGTATCTGGATGTGGACATTTGGAGCGCTTTCAGGC
          1 ATAGAGCAGGTTTGAAACACTCTTTTTGTAGTATCTGGAAGTGGATATTTGGATAGCTTTGAGGA

            * ** *      *             *** *   *                      *   *   
      49270 CTATGGT-GAAAAAGGAAATATCTTCCCCTGAAAACTAGACAGAAGCATTCTCAGAATCTTATTT
         66 TTTCGTTGGAAACAGG-AATATCTTCATATAAAAGCTAGACAGAAGCATTCTCAGAAACTTCTTT

            *      **  **    *              *  * *   *
      49334 CTGATGTGCGCCCTCAACTAACAGTGTTGAACCTTTCTTTTG
        130 GTGATGTTTGCATTCAAGTAACAGTGTTGAACATTCCCTTTC

                     *                    **     *   *                       
      49376 ATAGAGCAGTTTTGAAACACTCTTTTTGTAAAATCTGCAAGAGGATATTTGGATAGCTTTGAGGA
          1 ATAGAGCAGGTTTGAAACACTCTTTTTGTAGTATCTGGAAGTGGATATTTGGATAGCTTTGAGGA

                *        *   * *            **                      *    *   
      49441 TTTCTTTGGAAACGGGATTGTCTTCATATAAACTCTAGACAGAAGCATTCTCAGAAGCTTCATTG
         66 TTTCGTTGGAAACAGGAATATCTTCATATAAAAGCTAGACAGAAGCATTCTCAGAAACTTCTTTG

            *       **   *    *                      
      49506 GGATGTTTCAATTGAAGTCACAGTGTTGAACATTCCCTTTC
        131 TGATGTTTGCATTCAAGTAACAGTGTTGAACATTCCCTTTC

                               *                   *     *       **     *   *
      49547 ATAGAGCAGGTTTGAAACAATCTTTTTGTAGTATCTGGATGTGGACATTTGGAGCGCTTTCAGGC
          1 ATAGAGCAGGTTTGAAACACTCTTTTTGTAGTATCTGGAAGTGGATATTTGGATAGCTTTGAGGA

            * ** *      *             *** *   *                          *   
      49612 CTAAGGT-GAAAAAGGAAATATCTTCCCCTGAAAACTAGACAGAAGCATTCTCAGAAACTTATTT
         66 TTTCGTTGGAAACAGG-AATATCTTCATATAAAAGCTAGACAGAAGCATTCTCAGAAACTTCTTT

                   **  **    *             **  * *   *
      49676 GTGATGTGCGCCCTCAACTAACAGTGTTGAAGCTTTCTTTTG
        130 GTGATGTTTGCATTCAAGTAACAGTGTTGAACATTCCCTTTC

                     *        *          * *     *             **            
      49718 ATAGAGCAGTTTTGAAACTCTCTTTTTGTGGAATCTGCAAGTGGATATTTGTCTAGCTTTGAGGA
          1 ATAGAGCAGGTTTGAAACACTCTTTTTGTAGTATCTGGAAGTGGATATTTGGATAGCTTTGAGGA

                         *   *    *                   *       *     *   *    
      49783 TTTCGTTGGAAACGGGATTA-CAT-ATA-AAAAGC-AGACAGCAGCATTCCCAGAATCTTGTTTG
         66 TTTCGTTGGAAACAGGAATATCTTCATATAAAAGCTAGACAGAAGCATTCTCAGAAACTTCTTTG

                              *                      
      49844 TGATGTTTGCATTCAAGTCACAGTGTTGAACATTCCCTTTC
        131 TGATGTTTGCATTCAAGTAACAGTGTTGAACATTCCCTTTC

             *   *                     *   *       *     *       **     *   *
      49885 AGAGACCAGGTTTGAAACACTCTTTTTATAGAATCTGGATGTGGACATTTGGAGCGCTTTCAGGC
          1 ATAGAGCAGGTTTGAAACACTCTTTTTGTAGTATCTGGAAGTGGATATTTGGATAGCTTTGAGGA

            * ** *      *             *** *   *                      *   *   
      49950 CTATGGT-GAAAAAGGAAATATCTTCCCCTGAAAACTAGACAGAAGCATTCTCAGAATCTTATTT
         66 TTTCGTTGGAAACAGG-AATATCTTCATATAAAAGCTAGACAGAAGCATTCTCAGAAACTTCTTT

                   **  **    *        *    **  * *   *
      50014 GTGATGTGCGCCCTCAACTAACAGTGATGAAGCTTTCTTTTG
        130 GTGATGTTTGCATTCAAGTAACAGTGTTGAACATTCCCTTTC

                     *                    ***    *   *             *  *      
      50056 ATAGAGCAGTTTTGAAACACTCTTTTTGTAAATTCTGCAAGAGGATATTTGGATATCTCTGAGGA
          1 ATAGAGCAGGTTTGAAACACTCTTTTTGTAGTATCTGGAAGTGGATATTTGGATAGCTTTGAGGA

                         *   * *            **                      *    *   
      50121 TTTCGTTGGAAACGGGATTGTCTTCATATAAACTCTAGACAGAAGCATTCTCAGAAGCTTCATTG
         66 TTTCGTTGGAAACAGGAATATCTTCATATAAAAGCTAGACAGAAGCATTCTCAGAAACTTCTTTG

            *       **   *    *             *        
      50186 GGATGTTTCAATTGAAGTCACAGTGTTGAACAGTCCCTTTC
        131 TGATGTTTGCATTCAAGTAACAGTGTTGAACATTCCCTTTC

                                                         *       **   * *    
      50227 ATAGAGCAGGTTTGAAACACTCTTTTTGTAGTATCTGGAAGTGGACATTTGGAGCGCTCTCAGGA
          1 ATAGAGCAGGTTTGAAACACTCTTTTTGTAGTATCTGGAAGTGGATATTTGGATAGCTTTGAGGA

            * *  *      *             **            *       * *  *       *  *
      50292 CTACGGT-GAAAAAGGAAATATCTTCCAATAAAAGCTAGATAGAAGCAATGTCGGAAACTTTTTC
         66 TTTCGTTGGAAACAGG-AATATCTTCATATAAAAGCTAGACAGAAGCATTCTCAGAAACTTCTTT

            *      * ** *   **      *       *  *     *
      50356 ATGATGTATCTACTCAGCTAACAGAGTTGAACCTTTCCTTTG
        130 GTGATGTTTGCATTCAAGTAACAGTGTTGAACATTCCCTTTC

             *       *                   * *     *             **   *        
      50398 AGAGAGCAGTTTTGAAACACTCTTTTTGTGGAATCTGCAAGTGGATATTTGTCTAGATTTGAGGA
          1 ATAGAGCAGGTTTGAAACACTCTTTTTGTAGTATCTGGAAGTGGATATTTGGATAGCTTTGAGGA

                         *   *    *  *                *       *              
      50463 TTTCGTTGGAAACGGGATTA-CAT-GTA-AAAAGC-AGACAGCAGCATTCCCAGAAACTTCTTTG
         66 TTTCGTTGGAAACAGGAATATCTTCATATAAAAGCTAGACAGAAGCATTCTCAGAAACTTCTTTG

                              **   *                 
      50524 TGATGTTTGCATTCAAGTGTCAGAGTTGAACATTCCCTTTC
        131 TGATGTTTGCATTCAAGTAACAGTGTTGAACATTCCCTTTC

                                                   *     *       **     *   *
      50565 ATAGAGCAGGTTTGAAACACTCTTTTTGTAGTATCTGGATGTGGACATTTGGAGCGCTTTCAGGC
          1 ATAGAGCAGGTTTGAAACACTCTTTTTGTAGTATCTGGAAGTGGATATTTGGATAGCTTTGAGGA

            * ** *      *             *** *   *                      *   *   
      50630 CTATGGT-GAAAAAGGAAATATCTTCCCCTGAAAACTAGACAGAAGCATTCTCAGAATCTTATTT
         66 TTTCGTTGGAAACAGG-AATATCTTCATATAAAAGCTAGACAGAAGCATTCTCAGAAACTTCTTT

                   **  **    *      *      **  * *   *
      50694 GTGATGTGCGCCCTCAACTAACAGAGTTGAAGCTTTCTTTTG
        130 GTGATGTTTGCATTCAAGTAACAGTGTTGAACATTCCCTTTC

                     *                    **     *   *                       
      50736 ATAGAGCAGTTTTGAAACACTCTTTTTGTAAAATCTGCAAGAGGATATTTGGATAGCTTTGAGGA
          1 ATAGAGCAGGTTTGAAACACTCTTTTTGTAGTATCTGGAAGTGGATATTTGGATAGCTTTGAGGA

                         *   * *            **                      *    *   
      50801 TTTCGTTGGAAACGGGATTGTCTTCATATAAACTCTAGACAGAAGCATTCTCAGAAGCTTCATTG
         66 TTTCGTTGGAAACAGGAATATCTTCATATAAAAGCTAGACAGAAGCATTCTCAGAAACTTCTTTG

            *       **   *    *             *        
      50866 GGATGTTTCAATTGAAGTCACAGTGTTGAACAGTCCCTTTC
        131 TGATGTTTGCATTCAAGTAACAGTGTTGAACATTCCCTTTC

                                                         *       *  * * *    
      50907 ATAGAGCAGGTTTGAAACACTCTTTTTGTAGTATCTGGAAGTGGACATTTGGAGAGATCTCAGGA
          1 ATAGAGCAGGTTTGAAACACTCTTTTTGTAGTATCTGGAAGTGGATATTTGGATAGCTTTGAGGA

            * *  *    * *             **       *    *       * *          *  *
      50972 ATACGGT-GATAAAGGAAATATCTTCCAATAAAAGGTAGATAGAAGCAATGTCAGAAACTTTTTC
         66 TTTCGTTGGAAACAGG-AATATCTTCATATAAAAGCTAGACAGAAGCATTCTCAGAAACTTCTTT

            *      * ** *   **   *  *       *  * *   *
      51036 ATGATGTATCTACTCAGCTAAAAGAGTTGAACCTTTCTTTTG
        130 GTGATGTTTGCATTCAAGTAACAGTGTTGAACATTCCCTTTC

             *       *                   * *     *             **            
      51078 AGAGAGCAGTTTTGAAACACTCTTTTTGTGGAATCTGCAAGTGGATATTTGTCTAGCTTTGAGGA
          1 ATAGAGCAGGTTTGAAACACTCTTTTTGTAGTATCTGGAAGTGGATATTTGGATAGCTTTGAGGA

                         *   *    *                   *       *              
      51143 TTTCGTTGGAAACGGGATTA-CAT-ATA-AAAAGC-AGACAGCAGCATTCCCAGAAACTTCTTTG
         66 TTTCGTTGGAAACAGGAATATCTTCATATAAAAGCTAGACAGAAGCATTCTCAGAAACTTCTTTG

                              *    *                 
      51204 TGATGTTTGCATTCAAGTCACAGAGTTGAACATTCCCTTTC
        131 TGATGTTTGCATTCAAGTAACAGTGTTGAACATTCCCTTTC

                                                   *     *       **     *   *
      51245 ATAGAGCAGGTTTGAAACACTCTTTTTGTAGTATCTGGATGTGGACATTTGGAGCGCTTTCAGGC
          1 ATAGAGCAGGTTTGAAACACTCTTTTTGTAGTATCTGGAAGTGGATATTTGGATAGCTTTGAGGA

            * ** *      *             *** *   *                      *   *   
      51310 CTATGGT-GAAAAAGGAAATATCTTCCCCTGAAAACTAGACAGAAGCATTCTCAGAATCTTATTT
         66 TTTCGTTGGAAACAGG-AATATCTTCATATAAAAGCTAGACAGAAGCATTCTCAGAAACTTCTTT

                   **  **    *             **  * *   *
      51374 GTGATGTGCGCCCTCAACTAACAGTGTTGAAGCTTTCTTTTG
        130 GTGATGTTTGCATTCAAGTAACAGTGTTGAACATTCCCTTTC

                     *                    *      *   *                       
      51416 ATAGAGCAGTTTTGAAACACTCTTTTTGTAATATCTGCAAGAGGATATTTGGATAGCTTTGAGGA
          1 ATAGAGCAGGTTTGAAACACTCTTTTTGTAGTATCTGGAAGTGGATATTTGGATAGCTTTGAGGA

                *     *  *   * *            **                      *    *   
      51481 TTTCTTTGGAGACGGGATTGTCTTCATATAAACTCTAGACAGAAGCATTCTCAGAAGCTTCATTG
         66 TTTCGTTGGAAACAGGAATATCTTCATATAAAAGCTAGACAGAAGCATTCTCAGAAACTTCTTTG

            *       **   *    *             *        
      51546 GGATGTTTCAATTGAAGTCACAGTGTTGAACAGTCCCTTTC
        131 TGATGTTTGCATTCAAGTAACAGTGTTGAACATTCCCTTTC

                                                    *    *       **   * **   
      51587 ATAGAGCAGGTTTGAAACACTCTTTTTGTAGTATCTGGAATTGGACATTTGGAGCGCTCTCCGGA
          1 ATAGAGCAGGTTTGAAACACTCTTTTTGTAGTATCTGGAAGTGGATATTTGGATAGCTTTGAGGA

            * *  *      *             **          * *    *  * *          *  *
      51652 CTACGGT-GAAAAAGGAAATATCTTCCAATAAAAGCTACATAGAAACAATGTCAGAAACTTTTTC
         66 TTTCGTTGGAAACAGG-AATATCTTCATATAAAAGCTAGACAGAAGCATTCTCAGAAACTTCTTT

            *      * ** *   **      *       *  * *   *
      51716 ATGATGTATCTACTCAGCTAACAGAGTTGAACCTTTCTTTTG
        130 GTGATGTTTGCATTCAAGTAACAGTGTTGAACATTCCCTTTC

             *       *              *    * *     *             **            
      51758 AGAGAGCAGTTTTGAAACACTCTTCTTGTGGAATCTGCAAGTGGATATTTGTCTAGCTTTGAGGA
          1 ATAGAGCAGGTTTGAAACACTCTTTTTGTAGTATCTGGAAGTGGATATTTGGATAGCTTTGAGGA

                         *   *    *                   *       *              
      51823 TTTCGTTGGAAACGGGATTA-CAT-ATA-AAAAGC-AGACAGCAGCATTCCCAGAAACTTCTTTG
         66 TTTCGTTGGAAACAGGAATATCTTCATATAAAAGCTAGACAGAAGCATTCTCAGAAACTTCTTTG

                              *    *                 
      51884 TGATGTTTGCATTCAAGTCACAGAGTTGAACATTCCCTTTC
        131 TGATGTTTGCATTCAAGTAACAGTGTTGAACATTCCCTTTC

                                                   *     *       **     *   *
      51925 ATAGAGCAGGTTTGAAACACTCTTTTTGTAGTATCTGGATGTGGACATTTGGAGCGCTTTCAGGC
          1 ATAGAGCAGGTTTGAAACACTCTTTTTGTAGTATCTGGAAGTGGATATTTGGATAGCTTTGAGGA

            * ** *      *             *** *   *                      *   *   
      51990 CTATGGT-GAAAAAGGAAATATCTTCCCCTGAAAACTAGACAGAAGCATTCTCAGAATCTTATTT
         66 TTTCGTTGGAAACAGG-AATATCTTCATATAAAAGCTAGACAGAAGCATTCTCAGAAACTTCTTT

                   **  **    *             **  * *   *
      52054 GTGATGTGCGCCCTCAACTAACAGTGTTGAAGCTTTCTTTTG
        130 GTGATGTTTGCATTCAAGTAACAGTGTTGAACATTCCCTTTC

                     *                    ***    *   *                       
      52096 ATAGAGCAGTTTTGAAACACTCTTTTTGTAAACTCTGCAAGAGGATATTTGGATAGCTTTGAGGA
          1 ATAGAGCAGGTTTGAAACACTCTTTTTGTAGTATCTGGAAGTGGATATTTGGATAGCTTTGAGGA

                         *   * *            **                      *    *   
      52161 TTTCGTTGGAAACGGGATTGTCTTCATATAAACTCTAGACAGAAGCATTCTCAGAAGCTTCATTG
         66 TTTCGTTGGAAACAGGAATATCTTCATATAAAAGCTAGACAGAAGCATTCTCAGAAACTTCTTTG

            *       **   *    *             *        
      52226 GGATGTTTCTATTGAAGTCACAGTGTTGAACAGTCCCTTTC
        131 TGATGTTTGCATTCAAGTAACAGTGTTGAACATTCCCTTTC

                                                    *    *  *    *  * * *    
      52267 ATAGAGCAGGTTTGAAACACTCTTTTTGTAGTATCTGGAACTGGACATATGGAGAGATCTCAGGA
          1 ATAGAGCAGGTTTGAAACACTCTTTTTGTAGTATCTGGAAGTGGATATTTGGATAGCTTTGAGGA

            * **      * *             ** *          *       * *          *  *
      52332 ATATGTT-GATAAAGGAAATATCTTCCAACAAAAGCTAGATAGAAGCAATGTCAGAAACTTTTTC
         66 TTTCGTTGGAAACAGG-AATATCTTCATATAAAAGCTAGACAGAAGCATTCTCAGAAACTTCTTT

            *      * ** *   **      *       *  * *   *
      52396 ATGATGTATCTACTCAGCTAACAGAGTTGAACCTTTCTTTTG
        130 GTGATGTTTGCATTCAAGTAACAGTGTTGAACATTCCCTTTC

             *  *    *    *              * *     *             **            
      52438 AGAGTGCAGTTTTGGAACACTCTTTTTGTGGAATCTGCAAGTGGATATTTGTCTAGCTTTGAGGA
          1 ATAGAGCAGGTTTGAAACACTCTTTTTGTAGTATCTGGAAGTGGATATTTGGATAGCTTTGAGGA

                         *   *    *                   *       *      *       
      52503 TTTCGTTGGAAACGGGATTA-CAT-ATA-AAAAGC-AGACAGCAGCATTCCCAGAAATTTCTTTG
         66 TTTCGTTGGAAACAGGAATATCTTCATATAAAAGCTAGACAGAAGCATTCTCAGAAACTTCTTTG

                              *    *          *      
      52564 TGATGTTTGCATTCAAGTCACAGAGTTGAACATTGCCTTTC
        131 TGATGTTTGCATTCAAGTAACAGTGTTGAACATTCCCTTTC

                                                   *     *       **     *   *
      52605 ATAGAGCAGGTTTGAAACACTCTTTTTGTAGTATCTGGATGTGGACATTTGGAGCGCTTTCAGGC
          1 ATAGAGCAGGTTTGAAACACTCTTTTTGTAGTATCTGGAAGTGGATATTTGGATAGCTTTGAGGA

            * ** *      *             *** *   *                      *   *   
      52670 CTATGGT-GAAAAAGGAAATATCTTCCCCTGAAAACTAGACAGAAGCATTCTCAGAATCTTATTT
         66 TTTCGTTGGAAACAGG-AATATCTTCATATAAAAGCTAGACAGAAGCATTCTCAGAAACTTCTTT

                   **  **    *  *          **  * *   *
      52734 GTGATGTGCGCCCTCAACTATCAGTGTTGAAGCTTTCTTTTG
        130 GTGATGTTTGCATTCAAGTAACAGTGTTGAACATTCCCTTTC

                     *          *         **     *   *                       
      52776 ATAGAGCAGTTTTGAAACACACTTTTTGTAAAATCTGCAAGAGGATATTTGGATAGCTTTGAGGA
          1 ATAGAGCAGGTTTGAAACACTCTTTTTGTAGTATCTGGAAGTGGATATTTGGATAGCTTTGAGGA

                         *   * *            **                      *     *  
      52841 TTTCGTTGGAAACGGGATTGTCTTCATATAAACTCTAGACAGAAGCATTCTCAGAAGCTTC-ATG
         66 TTTCGTTGGAAACAGGAATATCTTCATATAAAAGCTAGACAGAAGCATTCTCAGAAACTTCTTTG

            *       **   *    *             *        
      52905 GGATGTTTCAATTGAAGTCACAGTGTTGAACAGTCCCTTTC
        131 TGATGTTTGCATTCAAGTAACAGTGTTGAACATTCCCTTTC

                   *                     *         *     *       *  * * *    
      52946 ATAGAGCTGGTTTGAAACACTCTTTTTGTTGTATCTGGATGTGGACATTTGGAGAGATCTCAGGA
          1 ATAGAGCAGGTTTGAAACACTCTTTTTGTAGTATCTGGAAGTGGATATTTGGATAGCTTTGAGGA

            * *  *    * *             **            *       * *          *  *
      53011 ATACGGT-GATAAAGGAAATATCTTCCAATAAAAGCTAGATAGAAGCAATGTCAGAAACTTTTTC
         66 TTTCGTTGGAAACAGG-AATATCTTCATATAAAAGCTAGACAGAAGCATTCTCAGAAACTTCTTT

            *      * ** *   **      *       *  * *   *
      53075 ATGATGTATCTACTCAGCTAACAGAGTTGAACCTTTCTTTTG
        130 GTGATGTTTGCATTCAAGTAACAGTGTTGAACATTCCCTTTC

             *       *    *              * *     *             **            
      53117 AGAGAGCAGTTTTGGAACACTCTTTTTGTGGAATCTGCAAGTGGATATTTGTCTAGCTTTGAGGA
          1 ATAGAGCAGGTTTGAAACACTCTTTTTGTAGTATCTGGAAGTGGATATTTGGATAGCTTTGAGGA

                         *   *    *                   *       *      *       
      53182 TTTCGTTGGAAACGGGATTA-CAT-ATA-AAAAGC-AGACAGCAGCATTCCCAGAAATTTCTTTG
         66 TTTCGTTGGAAACAGGAATATCTTCATATAAAAGCTAGACAGAAGCATTCTCAGAAACTTCTTTG

                              *    *                 
      53243 TGATGTTTGCATTCAAGTCACAGAGTTGAACATTCCCTTTC
        131 TGATGTTTGCATTCAAGTAACAGTGTTGAACATTCCCTTTC

                               *                    *    *       *  * * *    
      53284 ATAGAGCAGGTTTGAAACAATCTTTTTGTAGTATCTGGAATTGGACATTTGGAGAGATCTCAGGA
          1 ATAGAGCAGGTTTGAAACACTCTTTTTGTAGTATCTGGAAGTGGATATTTGGATAGCTTTGAGGA

            * *  *    * *             **            *       * *          *  *
      53349 ATACGGT-GATAAAGGAAATATCTTCCAATAAAAGCTAGATAGAAGCAATGTCAGAAACTTTTTC
         66 TTTCGTTGGAAACAGG-AATATCTTCATATAAAAGCTAGACAGAAGCATTCTCAGAAACTTCTTT

            *      * ** *   **      *      **  * *   *
      53413 ATGATGTATCTACTCAGCTAACAGAGTTGAAACTTTCTTTTG
        130 GTGATGTTTGCATTCAAGTAACAGTGTTGAACATTCCCTTTC

             *       *                   * *     *  *          **            
      53455 AGAGAGCAGTTTTGAAACACTCTTTTTGTGGAATCTGCAACTGGATATTTGTCTAGCTTTGAGGA
          1 ATAGAGCAGGTTTGAAACACTCTTTTTGTAGTATCTGGAAGTGGATATTTGGATAGCTTTGAGGA

                         *   *    *                   *       *      *       
      53520 TTTCGTTGGAAACGGGATTA-CAT-ATA-AAAAGC-AGACAGCAGCATTCCCAGAAATTTCTTTG
         66 TTTCGTTGGAAACAGGAATATCTTCATATAAAAGCTAGACAGAAGCATTCTCAGAAACTTCTTTG

                              *    *                 
      53581 TGATGTTTGCATTCAAGTCACAGAGTTGAACATTCCCTTTC
        131 TGATGTTTGCATTCAAGTAACAGTGTTGAACATTCCCTTTC

                                                   *     *       **     *  **
      53622 ATAGAGCAGGTTTGAAACACTCTTTTTGTAGTATCTGGATGTGGACATTTGGAGCGCTTTCAGAC
          1 ATAGAGCAGGTTTGAAACACTCTTTTTGTAGTATCTGGAAGTGGATATTTGGATAGCTTTGAGGA

            * ** *      *             *** *                          *   *   
      53687 CTATGGT-GAAAAAGGAAATATCTTCCCCTGAAAGCTAGACAGAAGCATTCTCAGAATCTTATTT
         66 TTTCGTTGGAAACAGG-AATATCTTCATATAAAAGCTAGACAGAAGCATTCTCAGAAACTTCTTT

                   **  **    *             **  * *   *
      53751 GTGATGTGCGCCCTCAACTAACAGTGTTGAAGCTTTCTTTTG
        130 GTGATGTTTGCATTCAAGTAACAGTGTTGAACATTCCCTTTC

                     *                    **      *  *                      *
      53793 ATAGAGCAGTTTTGAAACACTCTTTTTGTAAAATCT-GCAGAGGATATTTGGATAGCTTTGAGGC
          1 ATAGAGCAGGTTTGAAACACTCTTTTTGTAGTATCTGGAAGTGGATATTTGGATAGCTTTGAGGA

                         *   ***        *    *                      *    *   
      53857 TTTCGTTGGAAACGGGATGGTCTTCATACAAAATCTAGACAGAAGCATTCTCAGAAGCTTCATTG
         66 TTTCGTTGGAAACAGGAATATCTTCATATAAAAGCTAGACAGAAGCATTCTCAGAAACTTCTTTG

            *       **   *                  *        
      53922 GGATGTTTCAATTGAAGTAACAGTGTTGAACAGTCCCTTTC
        131 TGATGTTTGCATTCAAGTAACAGTGTTGAACATTCCCTTTC

                                                         **      *  * * *    
      53963 ATAGAGCAGGTTTGAAACACTCTTTTTGTAGTATCTGGAAGTGGACGTTTGGAGAGTTCTCAGGA
          1 ATAGAGCAGGTTTGAAACACTCTTTTTGTAGTATCTGGAAGTGGATATTTGGATAGCTTTGAGGA

            * *  *    * *             **            *       * *          *  *
      54028 ATACGGT-GATAAAGGAAATATCTTCCAATAAAAGCTAGATAGAAGCAATGTCAGAAACTTTTTC
         66 TTTCGTTGGAAACAGG-AATATCTTCATATAAAAGCTAGACAGAAGCATTCTCAGAAACTTCTTT

            *      * ** *   **      *       *  * *   *
      54092 ATGATGTATCTACTCAGCTAACAGAGTTGAACCTTTCTTTTG
        130 GTGATGTTTGCATTCAAGTAACAGTGTTGAACATTCCCTTTC

             *       *                 * * *     *   *        ***            
      54134 AGAGAGCAGTTTTGAAACACTCTTTTTCTGGAATCTGCAAGCGGATATTTTTCTAGCTTTGAGGA
          1 ATAGAGCAGGTTTGAAACACTCTTTTTGTAGTATCTGGAAGTGGATATTTGGATAGCTTTGAGGA

                         *   *    *                   *       *              
      54199 TTTCGTTGGAAACGGGATTA-CCT-ATA-AAAAGC-AGACAGCAGCATTCCCAGAAACTTCTTTG
         66 TTTCGTTGGAAACAGGAATATCTTCATATAAAAGCTAGACAGAAGCATTCTCAGAAACTTCTTTG

                              *    *          *      
      54260 TGATGTTTGCATTCAAGTCACAGAGTTGAACATTGCCTTTC
        131 TGATGTTTGCATTCAAGTAACAGTGTTGAACATTCCCTTTC

                                                   *     *       **     *   *
      54301 ATAGAGCAGGTTTGAAACACTCTTTTTGTAGTATCTGGATGTGGACATTTGGAGCGCTTTCAGGC
          1 ATAGAGCAGGTTTGAAACACTCTTTTTGTAGTATCTGGAAGTGGATATTTGGATAGCTTTGAGGA

            * ** *      *             *** *   *                      *   *   
      54366 CTATGGT-GAAAAAGGAAATATCTTCCCCTGAAAACTAGACAGAAGCATTCTCAGAATCTTATTT
         66 TTTCGTTGGAAACAGG-AATATCTTCATATAAAAGCTAGACAGAAGCATTCTCAGAAACTTCTTT

                   *   **    *  *          **  * *   *
      54430 GTGATGTGTGCCCTCAACTATCAGTGTTGAAGCTTTCTTTTG
        130 GTGATGTTTGCATTCAAGTAACAGTGTTGAACATTCCCTTTC

                     *          *         **     *   *                       
      54472 ATAGAGCAGTTTTGAAACACACTTTTTGTAAAATCTGCAAGAGGATATTTGGATAGCTTTGAGGA
          1 ATAGAGCAGGTTTGAAACACTCTTTTTGTAGTATCTGGAAGTGGATATTTGGATAGCTTTGAGGA

                         * * * *            **                      *    *   
      54537 TTTCGTTGGAAACGGTATTGTCTTCATATAAACTCTAGACAGAAGCATTCTCAGAAGCTTCATTG
         66 TTTCGTTGGAAACAGGAATATCTTCATATAAAAGCTAGACAGAAGCATTCTCAGAAACTTCTTTG

            *       **   *    *             *        
      54602 GGATGTTTCAATTGAAGTCACAGTGTTGAACAGTCCCTTTC
        131 TGATGTTTGCATTCAAGTAACAGTGTTGAACATTCCCTTTC

                                                         *  *    *  * * *    
      54643 ATAGAGCAGGTTTGAAACACTCTTTTTGTAGTATCTGGAAGTGGACATGTGGAGAGATCTCAGGA
          1 ATAGAGCAGGTTTGAAACACTCTTTTTGTAGTATCTGGAAGTGGATATTTGGATAGCTTTGAGGA

            * *  *    * *             **            *       * *          *  *
      54708 ATACGGT-GATAAAGGAAATATCTTCCAATAAAAGCTAGATAGAAGCAATGTCAGAAACTTTTTC
         66 TTTCGTTGGAAACAGG-AATATCTTCATATAAAAGCTAGACAGAAGCATTCTCAGAAACTTCTTT

            *      * ** *   **      *       *  * *   *
      54772 ATGATGTATCTACTCAGCTAACAGAGTTGAACCTTTCTTTTG
        130 GTGATGTTTGCATTCAAGTAACAGTGTTGAACATTCCCTTTC

             *       *                   * *     *   *        ***            
      54814 AGAGAGCAGTTTTGAAACACTCTTTTTGTGGAATCTGCAAGCGGATATTTTTCTAGCTTTGAGGA
          1 ATAGAGCAGGTTTGAAACACTCTTTTTGTAGTATCTGGAAGTGGATATTTGGATAGCTTTGAGGA

                         *   *    *                   *       *              
      54879 TTTCGTTGGAAACGGGATTA-CAT-ATA-AAAAGC-AGACAGCAGCATTCCCAGAAACTTCTTTG
         66 TTTCGTTGGAAACAGGAATATCTTCATATAAAAGCTAGACAGAAGCATTCTCAGAAACTTCTTTG

                              *    *                 
      54940 TGATGTTTGCATTCAAGTCACAGAGTTGAACATTCCCTTTC
        131 TGATGTTTGCATTCAAGTAACAGTGTTGAACATTCCCTTTC

                                                   *     *       **     *   *
      54981 ATAGAGCAGGTTTGAAACACTCTTTTTGTAGTATCTGGATGTGGACATTTGGAGCGCTTTCAGGC
          1 ATAGAGCAGGTTTGAAACACTCTTTTTGTAGTATCTGGAAGTGGATATTTGGATAGCTTTGAGGA

            * ** *      *             * * *   *                          *   
      55046 CTATGGT-GAAAAAGGAAATATCTTCCTCTGAAAACTAGACAGAAGCATTCTCAGAAACTTATTT
         66 TTTCGTTGGAAACAGG-AATATCTTCATATAAAAGCTAGACAGAAGCATTCTCAGAAACTTCTTT

                   **  **    *             **  * *    
      55110 GTGATGTGCGCCCTCAACTAACAGTGTTGAAGCTTTCTTTTC
        130 GTGATGTTTGCATTCAAGTAACAGTGTTGAACATTCCCTTTC

                     *       *           * *     *             **            
      55152 ATAGAGCAGTTTTGAAAAACTCTTTTTGTGGAATCTGCAAGTGGATATTTGTCTAGCTTTGAGGA
          1 ATAGAGCAGGTTTGAAACACTCTTTTTGTAGTATCTGGAAGTGGATATTTGGATAGCTTTGAGGA

                         **  *    *                   *       *              
      55217 TTTCGTTGGAAACGTGATTA-CAT-ATA-AAAAGC-AGACAGCAGCATTCCCAGAAACTTCTTTG
         66 TTTCGTTGGAAACAGGAATATCTTCATATAAAAGCTAGACAGAAGCATTCTCAGAAACTTCTTTG

                              *    *                 
      55278 TGATGTTTGCATTCAAGTCACAGAGTTGAACATTCCCTTTC
        131 TGATGTTTGCATTCAAGTAACAGTGTTGAACATTCCCTTTC

                                                   *     *       **     *   *
      55319 ATAGAGCAGGTTTGAAACACTCTTTTTGTAGTATCTGGATGTGGACATTTGGAGCGCTTTCAGGC
          1 ATAGAGCAGGTTTGAAACACTCTTTTTGTAGTATCTGGAAGTGGATATTTGGATAGCTTTGAGGA

            * ** *      *             *** *   *                      *   *   
      55384 CTGTGGT-GAAAAAGGAAATATCTTCCCCTGAAAACTAGACAGAAGCATTCTCAGAATCTTATTT
         66 TTTCGTTGGAAACAGG-AATATCTTCATATAAAAGCTAGACAGAAGCATTCTCAGAAACTTCTTT

                   **  **    *             **  * *   *
      55448 GTGATGTGCGCCCTCAACTAACAGTGTTGAAGCTTTCTTTTG
        130 GTGATGTTTGCATTCAAGTAACAGTGTTGAACATTCCCTTTC

                     *                 *  **     *   *                       
      55490 ATAGAGCAGTTTTGAAACACTCTTTTTCTAAAATCTGCAAGAGGATATTTGGATAGCTTTGAGGA
          1 ATAGAGCAGGTTTGAAACACTCTTTTTGTAGTATCTGGAAGTGGATATTTGGATAGCTTTGAGGA

                         *   * *            **                      *    *   
      55555 TTTCGTTGGAAACGGGATTGTCTTCATATAAACTCTAGACAGAAGCATTCTCAGAAGCTTCATTG
         66 TTTCGTTGGAAACAGGAATATCTTCATATAAAAGCTAGACAGAAGCATTCTCAGAAACTTCTTTG

            *       **   *    *             *        
      55620 GGATGTTTCAATTGAAGTCACAGTGTTGAACAGTCCCTTTC
        131 TGATGTTTGCATTCAAGTAACAGTGTTGAACATTCCCTTTC

                                                         *       *  * * *    
      55661 ATAGAGCAGGTTTGAAACACTCTTTTTGTAGTATCTGGAAGTGGACATTTGGAGAGATCTCAGGA
          1 ATAGAGCAGGTTTGAAACACTCTTTTTGTAGTATCTGGAAGTGGATATTTGGATAGCTTTGAGGA

            * *  *      *        *    **            *       * *          *  *
      55726 CTACGGT-GAAAAAGGAAATAGCTTCCAATAAAAGCTAGATAGAAGCAATGTCAGAAACTTTTTC
         66 TTTCGTTGGAAACAGG-AATATCTTCATATAAAAGCTAGACAGAAGCATTCTCAGAAACTTCTTT

            *      * ** *   **      *       *  * *   *
      55790 ATGATGTGTCTACTCAGCTAACAGAGTTGAACCTTTCTTTTG
        130 GTGATGTTTGCATTCAAGTAACAGTGTTGAACATTCCCTTTC

             *       *                   * *     *             **            
      55832 AGAGAGCAGTTTTGAAACACTCTTTTTGTGGAATCTGCAAGTGGATATTTGTCTAGCTTTGAGGA
          1 ATAGAGCAGGTTTGAAACACTCTTTTTGTAGTATCTGGAAGTGGATATTTGGATAGCTTTGAGGA

                         *   *    *                   *       *              
      55897 TTTCGTTGGAAACGGGATTA-CAT-ATA-AAAAGC-AGACAGCAGCATTCCCAGAAACTTCTTTG
         66 TTTCGTTGGAAACAGGAATATCTTCATATAAAAGCTAGACAGAAGCATTCTCAGAAACTTCTTTG

                              *    *                 
      55958 TGATGTTTGCATTCAAGTCACAGAGTTGAACATTCCCTTTC
        131 TGATGTTTGCATTCAAGTAACAGTGTTGAACATTCCCTTTC

                                                   *     *       **     *   *
      55999 ATAGAGCAGGTTTGAAACACTCTTTTTGTAGTATCTGGATGTGGACATTTGGAGCGCTTTCAGGC
          1 ATAGAGCAGGTTTGAAACACTCTTTTTGTAGTATCTGGAAGTGGATATTTGGATAGCTTTGAGGA

            * ** *      *             *** *   *                      *   *   
      56064 CTATGGT-GAAAAAGGAAATATCTTCCCCTGAAAACTAGACAGAAGCATTCTCAGAATCTTATTT
         66 TTTCGTTGGAAACAGG-AATATCTTCATATAAAAGCTAGACAGAAGCATTCTCAGAAACTTCTTT

                   **  **    *             **  * *   *
      56128 GTGATGTGCGCCCTCAATTAACAGTGTTGAAGCTTTCTTTTG
        130 GTGATGTTTGCATTCAAGTAACAGTGTTGAACATTCCCTTTC

                     *      *             **     *   *                       
      56170 ATAGAGCAGTTTTGAACCACTCTTTTTGTAAAATCTGCAAGAGGATATTTGGATAGCTTTGAGGA
          1 ATAGAGCAGGTTTGAAACACTCTTTTTGTAGTATCTGGAAGTGGATATTTGGATAGCTTTGAGGA

                         *   * *            **                      * *  *   
      56235 TTTCGTTGGAAACGGGATTGTCTTCATATAAACTCTAGACAGAAGCATTCTCAGAAGCCTCATTG
         66 TTTCGTTGGAAACAGGAATATCTTCATATAAAAGCTAGACAGAAGCATTCTCAGAAACTTCTTTG

            *       **   *    *             *        
      56300 GGATGTTTCAATTGAAGTCACAGTGTTGAACAGTCCCTTTC
        131 TGATGTTTGCATTCAAGTAACAGTGTTGAACATTCCCTTTC

                                                   *     *       **     *   *
      56341 ATAGAGCAGGTTTGAAACACTCTTTTTGTAGTATCTGGATGTGGACATTTGGAGCGCTTTCAGGC
          1 ATAGAGCAGGTTTGAAACACTCTTTTTGTAGTATCTGGAAGTGGATATTTGGATAGCTTTGAGGA

            * *  *      *             *** *   *                          *   
      56406 CTACGGT-GAAAAAGGAAATATCTTCCCCTGAAAACTAGACAGAAGCATTCTCAGAAACTTATTT
         66 TTTCGTTGGAAACAGG-AATATCTTCATATAAAAGCTAGACAGAAGCATTCTCAGAAACTTCTTT

                   **  **    *             **  * *   *
      56470 GTGATGTGCGCCCTCAACTAACAGTGTTGAAGCTTTCTTTTG
        130 GTGATGTTTGCATTCAAGTAACAGTGTTGAACATTCCCTTTC

                     *                   * *     *             **            
      56512 ATAGAGCAGTTTTGAAACACTCTTTTTGTGGAATCTGCAAGTGGATATTTGTCTAGCTTTGAGGA
          1 ATAGAGCAGGTTTGAAACACTCTTTTTGTAGTATCTGGAAGTGGATATTTGGATAGCTTTGAGGA

                         *   *    *                   *       *              
      56577 TTTCGTTGGAAACGGGATTA-CAT-ATA-AAAAGC-AGACAGCAGCATTCCCAGAAACTTCTTTG
         66 TTTCGTTGGAAACAGGAATATCTTCATATAAAAGCTAGACAGAAGCATTCTCAGAAACTTCTTTG

                 *            *    *   *             
      56638 TGATGCTTGCATTCAAGTCACAGAGTTAAACATTCCCTTTC
        131 TGATGTTTGCATTCAAGTAACAGTGTTGAACATTCCCTTTC

                                                   *     *       ***    *   *
      56679 ATAGAGCAGGTTTGAAACACTCTTTTTGTAGTATCTGGATGTGGACATTTGGAGCACTTTCAGGC
          1 ATAGAGCAGGTTTGAAACACTCTTTTTGTAGTATCTGGAAGTGGATATTTGGATAGCTTTGAGGA

            * ** *      *             *** *   *                      *   *   
      56744 CTATGGT-GAAAAAGGAAATATCTTCCCCTGAAAACTAGACAGAAGCATTCTCAGAATCTTATTT
         66 TTTCGTTGGAAACAGG-AATATCTTCATATAAAAGCTAGACAGAAGCATTCTCAGAAACTTCTTT

                   **  **    *             **  * *   *
      56808 GTGATGTGCGCCCTCAACTAACAGTGTTGAAGCTTTCTTTTG
        130 GTGATGTTTGCATTCAAGTAACAGTGTTGAACATTCCCTTTC

                     *                    **     *   *                       
      56850 ATAGAGCAGTTTTGAAACACTCTTTTTGTAAAATCTGCAAGAGGATATTTGGATAGCTTTGAGGA
          1 ATAGAGCAGGTTTGAAACACTCTTTTTGTAGTATCTGGAAGTGGATATTTGGATAGCTTTGAGGA

                         *   * *        *    *                *     **   *   
      56915 TTTCGTTGGAAACGGGATTGTCTTCATACAAAATCTAGACAGAAGCATTCGCAGAAGATTCATTG
         66 TTTCGTTGGAAACAGGAATATCTTCATATAAAAGCTAGACAGAAGCATTCTCAGAAACTTCTTTG

            **      **   *    *     *       *        
      56980 GAATGTTTCAATTGAAGTCACAGTTTTGAACAGTCCCTTTC
        131 TGATGTTTGCATTCAAGTAACAGTGTTGAACATTCCCTTTC

                                                         *       **   * *    
      57021 ATAGAGCAGGTTTGAAACACTCTTTTTGTAGTATCTGGAAGTGGACATTTGGAGCGCTCTCAGGA
          1 ATAGAGCAGGTTTGAAACACTCTTTTTGTAGTATCTGGAAGTGGATATTTGGATAGCTTTGAGGA

            * **  **    *             **          * *       * *          *  *
      57086 CTATG-GCGAAAAAGGAAATATCTTCCAATAAAAGCTACATAGAAGCAATGTCAGAAACTTTTTC
         66 TTTCGTTGGAAACAGG-AATATCTTCATATAAAAGCTAGACAGAAGCATTCTCAGAAACTTCTTT

            *      * ** *   **      *       *  * *   *
      57150 ATGATGTATCTACTCAGCTAACAGAGTTGAACCTTTCTTTTG
        130 GTGATGTTTGCATTCAAGTAACAGTGTTGAACATTCCCTTTC

             *       *                   * *     *             **            
      57192 AGAGAGCAGTTTTGAAACACTCTTTTTGTGGAATCTGCAAGTGGATATTTGTCTAGCTTTGAGGA
          1 ATAGAGCAGGTTTGAAACACTCTTTTTGTAGTATCTGGAAGTGGATATTTGGATAGCTTTGAGGA

                         *   *    *                   *       *   * *        
      57257 TTTCGTTGGAAACGGGATTA-CAT-ATA-AAAAGC-AGACAGCAGCATTCCCAGTATCTTCTTTG
         66 TTTCGTTGGAAACAGGAATATCTTCATATAAAAGCTAGACAGAAGCATTCTCAGAAACTTCTTTG

                    *         *    *                 
      57318 TGATGTTTCCATTCAAGTGACAGAGTTGAACATTCCCTTTC
        131 TGATGTTTGCATTCAAGTAACAGTGTTGAACATTCCCTTTC

                                                   *     *       ***    *   *
      57359 ATAGAGCAGGTTTGAAACACTCTTTTTGTAGTATCTGGATGTGGACATTTGGAGCACTTTCAGGC
          1 ATAGAGCAGGTTTGAAACACTCTTTTTGTAGTATCTGGAAGTGGATATTTGGATAGCTTTGAGGA

            * ** *      *             *** *   *                      *   *   
      57424 CTATGGT-GAAAAAGGAAATATCTTCCCCTGAAAACTAGACAGAAGCATTCTCAGAATCTTATTT
         66 TTTCGTTGGAAACAGG-AATATCTTCATATAAAAGCTAGACAGAAGCATTCTCAGAAACTTCTTT

                   **  **    *             **  * *   *
      57488 GTGATGTGCGCCCTCAACTAACAGTGTTGAAGCTTTCTTTTG
        130 GTGATGTTTGCATTCAAGTAACAGTGTTGAACATTCCCTTTC

                    **            *       **     *   *                       
      57530 ATAGAGCACTTTTGAAACACTCCTTTTGTAAAATCTGCAAGAGGATATTTGGATAGCTTTGAGGA
          1 ATAGAGCAGGTTTGAAACACTCTTTTTGTAGTATCTGGAAGTGGATATTTGGATAGCTTTGAGGA

                         *   * *            **                      *    *   
      57595 TTTCGTTGGAAACGGGATTGTCTTCATATAAACTCTAGACAGAAGCATTCTCAGAAGCTTCATTG
         66 TTTCGTTGGAAACAGGAATATCTTCATATAAAAGCTAGACAGAAGCATTCTCAGAAACTTCTTTG

            *       **   *    *             *        
      57660 GGATGTTTCAATTGAAGTCACAGTGTTGAACAGTCCCTTTC
        131 TGATGTTTGCATTCAAGTAACAGTGTTGAACATTCCCTTTC

                                                   *     *       **     *   *
      57701 ATAGAGCAGGTTTGAAACACTCTTTTTGTAGTATCTGGATGTGGACATTTGGAGCGCTTTCAGGC
          1 ATAGAGCAGGTTTGAAACACTCTTTTTGTAGTATCTGGAAGTGGATATTTGGATAGCTTTGAGGA

            * ** *      *             *** *   *                          *   
      57766 CTATGGT-GAAAAAGGAAATATCTTCCCCTGAAAACTAGACAGAAGCATTCTCAGAAACTTATTT
         66 TTTCGTTGGAAACAGG-AATATCTTCATATAAAAGCTAGACAGAAGCATTCTCAGAAACTTCTTT

                   **  **    *             **  * *   *
      57830 GTGATGTGCGCCCTCAACTAACAGTGTTGAAGCTTTCTTTTG
        130 GTGATGTTTGCATTCAAGTAACAGTGTTGAACATTCCCTTTC

                     *                   * *     *             **            
      57872 ATAGAGCAGTTTTGAAACACTCTTTTTGTGGAATCTGCAAGTGGATATTTGTCTAGCTTTGAGGA
          1 ATAGAGCAGGTTTGAAACACTCTTTTTGTAGTATCTGGAAGTGGATATTTGGATAGCTTTGAGGA

                         *   *    *                   *       *     *        
      57937 TTTCGTTGGAAACGGGATTA-CAT-ATA-AAAAGC-AGACAGCAGCATTCCCAGAATCTTCTTTG
         66 TTTCGTTGGAAACAGGAATATCTTCATATAAAAGCTAGACAGAAGCATTCTCAGAAACTTCTTTG

                              **   *            *    
      57998 TGATGTTTGCATTCAAGTCCCAGAGTTGAACATTCCGTTTC
        131 TGATGTTTGCATTCAAGTAACAGTGTTGAACATTCCCTTTC

              *                        *           *     *       **     *   *
      58039 ATGGAGCAGGTTTGAAACACTCTTTTTATAGTATCTGGATGTGGACATTTGGAGCGCTTTCAGGC
          1 ATAGAGCAGGTTTGAAACACTCTTTTTGTAGTATCTGGAAGTGGATATTTGGATAGCTTTGAGGA

            * ** *      *             *** *   * *                    *   *   
      58104 CTATGGT-GAAAAAGGAAATATCTTCTCCTGAAAACAAGACAGAAGCATTCTCAGAATCTTATTT
         66 TTTCGTTGGAAACAGG-AATATCTTCATATAAAAGCTAGACAGAAGCATTCTCAGAAACTTCTTT

                   **  **   **             **  * *   *
      58168 GTGATGTGCGCCCTCAGCTAACAGTGTTGAAGCTTTCTTTTG
        130 GTGATGTTTGCATTCAAGTAACAGTGTTGAACATTCCCTTTC

                     *                    **     *   *                       
      58210 ATAGAGCAGTTTTGAAACACTCTTTTTGTAAAATCTGCAAGAGGATATTTGGATAGCTTTGAGGA
          1 ATAGAGCAGGTTTGAAACACTCTTTTTGTAGTATCTGGAAGTGGATATTTGGATAGCTTTGAGGA

                         *   * *            **                      * *  *   
      58275 TTTCGTTGGAAACGGGATTGTCTTCATATAAACTCTAGACAGAAGCATTCTCAGAAGCCTCATTG
         66 TTTCGTTGGAAACAGGAATATCTTCATATAAAAGCTAGACAGAAGCATTCTCAGAAACTTCTTTG

            *       **   *    *           * *        
      58340 GGATGTTTCAATTGAAGTCACAGTGTTGAAAAGTCCCTTTC
        131 TGATGTTTGCATTCAAGTAACAGTGTTGAACATTCCCTTTC

                                                         *       **   * *    
      58381 ATAGAGCAGGTTTGAAACACTCTTTTTGTAGTATCTGGAAGTGGACATTTGGAGCGCTCTCAGGA
          1 ATAGAGCAGGTTTGAAACACTCTTTTTGTAGTATCTGGAAGTGGATATTTGGATAGCTTTGAGGA

            * *  *      *             **          * *       * *          *  *
      58446 CTACGGT-GAAAAAGGAAATATCTTCCAATAAAAGCTACATAGAAGCAATGTCAGAAACTTTTTC
         66 TTTCGTTGGAAACAGG-AATATCTTCATATAAAAGCTAGACAGAAGCATTCTCAGAAACTTCTTT

            *      * ** *   **      *      **  * *   *
      58510 ATGATGTATCTACTCAGCTAACAGAGTTGAAACTTTCTTTTT
        130 GTGATGTTTGCATTCAAGTAACAGTGTTGAACATTCCCTTTC

             *       *                   * *                   **            
      58552 AGAGAGCAGTTTTGAAACACTCTTTTTGTGGAATCTGGAAGTGGATATTTGTCTAGCTTTGAGGA
          1 ATAGAGCAGGTTTGAAACACTCTTTTTGTAGTATCTGGAAGTGGATATTTGGATAGCTTTGAGGA

                         *   *    *                   *       *   *          
      58617 TTTCGTTGGAAACGGGATTA-CAT-ATA-AAAAGC-AGACAGCAGCATTCCCAGTAACTTCTTTG
         66 TTTCGTTGGAAACAGGAATATCTTCATATAAAAGCTAGACAGAAGCATTCTCAGAAACTTCTTTG

                              *    *            * *  
      58678 TGATGTTTGCATTCAAGTCACAGAGTTGAACATTCCTTCTC
        131 TGATGTTTGCATTCAAGTAACAGTGTTGAACATTCCCTTTC

                                                   *     *       **     *   *
      58719 ATAGAGCAGGTTTGAAACACTCTTTTTGTAGTATCTGGATGTGGACATTTGGAGCGCTTTCAGGC
          1 ATAGAGCAGGTTTGAAACACTCTTTTTGTAGTATCTGGAAGTGGATATTTGGATAGCTTTGAGGA

            * ** *      *             *** *   *                      *   *   
      58784 CTATGGT-GAAAAAGGAAATATCTTCCCCTGAAAACTAGACAGAAGCATTCTCAGAATCTTATTT
         66 TTTCGTTGGAAACAGG-AATATCTTCATATAAAAGCTAGACAGAAGCATTCTCAGAAACTTCTTT

                   **  **    *             **  * *   *
      58848 GTGATGTGCGCCCTCAACTAACAGTGTTGAAGCTTTCTTTTG
        130 GTGATGTTTGCATTCAAGTAACAGTGTTGAACATTCCCTTTC

                     *                    **     *   *                       
      58890 ATAGAGCAGTTTTGAAACACTCTTTTTGTAAAATCTGCAAGAGGATATTTGGATAGCTTTGAGGA
          1 ATAGAGCAGGTTTGAAACACTCTTTTTGTAGTATCTGGAAGTGGATATTTGGATAGCTTTGAGGA

                         *   * *            **                *     * *  *  *
      58955 TTTCGTTGGAAACGGGATTGTCTTCATATAAACTCTAGACAGAAGCATTCACAGAAGCCTCATTC
         66 TTTCGTTGGAAACAGGAATATCTTCATATAAAAGCTAGACAGAAGCATTCTCAGAAACTTCTTTG

            *       **   *    *             *        
      59020 GGATGTTTCAATTGAAGTCACAGTGTTGAACAGTCCCTTTC
        131 TGATGTTTGCATTCAAGTAACAGTGTTGAACATTCCCTTTC

                                                   *     *       **     *   *
      59061 ATAGAGCAGGTTTGAAACACTCTTTTTGTAGTATCTGGATGTGGACATTTGGAGCGCTTTCAGGC
          1 ATAGAGCAGGTTTGAAACACTCTTTTTGTAGTATCTGGAAGTGGATATTTGGATAGCTTTGAGGA

            * ** *      *             *** *   *                      *   *   
      59126 CTATGGT-GAAAAAGGAAATATCTTCCCCTGAAAACTAGACAGAAGCATTCTCAGAATCTTATTT
         66 TTTCGTTGGAAACAGG-AATATCTTCATATAAAAGCTAGACAGAAGCATTCTCAGAAACTTCTTT

                   **  **    *             **  * *   *
      59190 GTGATGTGCGCCCTCAACTAACAGTGTTGAAGCTTTCTTTTG
        130 GTGATGTTTGCATTCAAGTAACAGTGTTGAACATTCCCTTTC

                  * **                    **     *   *                       
      59232 ATAGAGTACTTTTGAAACACTCTTTTTGTAAAATCTGCAAGAGGATATTTGGATAGCTTTGAGGA
          1 ATAGAGCAGGTTTGAAACACTCTTTTTGTAGTATCTGGAAGTGGATATTTGGATAGCTTTGAGGA

                         *   * *            **                      *    *   
      59297 TTTCGTTGGAAACGGGATTGTCTTCATATAAACTCTAGACAGAAGCATTCTCAGAAGCTTCATTG
         66 TTTCGTTGGAAACAGGAATATCTTCATATAAAAGCTAGACAGAAGCATTCTCAGAAACTTCTTTG

            *       **   *    *             *        
      59362 GGATGTTTCAATTGAAGTCACAGTGTTGAACAGTCCCTTTC
        131 TGATGTTTGCATTCAAGTAACAGTGTTGAACATTCCCTTTC

                                                   *     *       **     *   *
      59403 ATAGAGCAGGTTTGAAACACTCTTTTTGTAGTATCTGGATGTGGACATTTGGAGCGCTTTCAGGC
          1 ATAGAGCAGGTTTGAAACACTCTTTTTGTAGTATCTGGAAGTGGATATTTGGATAGCTTTGAGGA

            * ** *      *             *** *   *                          *   
      59468 CTATGGT-GAAAAAGGAAATATCTTCCCCTGAAAACTAGACAGAAGCATTCTCAGAAACTTATTT
         66 TTTCGTTGGAAACAGG-AATATCTTCATATAAAAGCTAGACAGAAGCATTCTCAGAAACTTCTTT

                   **  **    *             **  * *   *
      59532 GTGATGTGCGCCCTCAACTAACAGTGTTGAAGCTTTCTTTTG
        130 GTGATGTTTGCATTCAAGTAACAGTGTTGAACATTCCCTTTC

                     *                   * *     *             **            
      59574 ATAGAGCAGTTTTGAAACACTCTTTTTGTGGAATCTGCAAGTGGATATTTGTCTAGCTTTGAGGA
          1 ATAGAGCAGGTTTGAAACACTCTTTTTGTAGTATCTGGAAGTGGATATTTGGATAGCTTTGAGGA

                         *   *    *                   *       *     *        
      59639 TTTCGTTGGAAACGGGATTA-CAT-ATA-AAAAGC-AGACAGCAGCATTCCCAGAATCTTCTTTG
         66 TTTCGTTGGAAACAGGAATATCTTCATATAAAAGCTAGACAGAAGCATTCTCAGAAACTTCTTTG

                              **   *            *    
      59700 TGATGTTTGCATTCAAGTCCCAGAGTTGAACATTCCGTTTC
        131 TGATGTTTGCATTCAAGTAACAGTGTTGAACATTCCCTTTC

                                       *           *     *       **     *   *
      59741 ATAGAGCAGGTTTGAAACACTCTTTTTATAGTATCTGGATGTGGACATTTGGAGCGCTTTCAGGC
          1 ATAGAGCAGGTTTGAAACACTCTTTTTGTAGTATCTGGAAGTGGATATTTGGATAGCTTTGAGGA

            * ** *      *             *** *   * *                    *   *   
      59806 CTATGGT-GAAAAAGGAAATATCTTCTCCTGAAAACAAGACAGAAGCATTCTCAGAATCTTATTT
         66 TTTCGTTGGAAACAGG-AATATCTTCATATAAAAGCTAGACAGAAGCATTCTCAGAAACTTCTTT

                   **  **   **             **  * *   *
      59870 GTGATGTGCGCCCTCAGCTAACAGTGTTGAAGCTTTCTTTTG
        130 GTGATGTTTGCATTCAAGTAACAGTGTTGAACATTCCCTTTC

                     *                    **     *   *                       
      59912 ATAGAGCAGTTTTGAAACACTCTTTTTGTAAAATCTGCAAGAGGATATTTGGATAGCTTTGAGGA
          1 ATAGAGCAGGTTTGAAACACTCTTTTTGTAGTATCTGGAAGTGGATATTTGGATAGCTTTGAGGA

                         *   * *            **                      *    *   
      59977 TTTCGTTGGAAACGGGATTGTCTTCATATAAACTCTAGACAGAAGCATTCTCAGAAGCTTCATTG
         66 TTTCGTTGGAAACAGGAATATCTTCATATAAAAGCTAGACAGAAGCATTCTCAGAAACTTCTTTG

            *       **   *    *             *        
      60042 GGATGTTTCAATTGAAGTCACAGTGTTGAACAGTCCCTTTC
        131 TGATGTTTGCATTCAAGTAACAGTGTTGAACATTCCCTTTC

                                                         *       *  * * *    
      60083 ATAGAGCAGGTTTGAAACACTCTTTTTGTAGTATCTGGAAGTGGACATTTGGAGAGATCTCAGGA
          1 ATAGAGCAGGTTTGAAACACTCTTTTTGTAGTATCTGGAAGTGGATATTTGGATAGCTTTGAGGA

            * *  *      *        *    **            *       * *          *  *
      60148 CTACGGT-GAAAAAGGAAATAGCTTCCAATAAAAGCTAGATAGAAGCAATGTCAGAAACTTTTTC
         66 TTTCGTTGGAAACAGG-AATATCTTCATATAAAAGCTAGACAGAAGCATTCTCAGAAACTTCTTT

            *      * ** *   **      *       *  * *   *
      60212 ATGATGTGTCTACTCAGCTAACAGAGTTGAACCTTTCTTTTG
        130 GTGATGTTTGCATTCAAGTAACAGTGTTGAACATTCCCTTTC

             *       *                   * *     *             **            
      60254 AGAGAGCAGTTTTGAAACACTCTTTTTGTGGAATCTGCAAGTGGATATTTGTCTAGCTTTGAGGA
          1 ATAGAGCAGGTTTGAAACACTCTTTTTGTAGTATCTGGAAGTGGATATTTGGATAGCTTTGAGGA

                         *   *    *                   *       *              
      60319 TTTCGTTGGAAACGGGATTA-CAT-ATA-AAAAGC-AGACAGCAGCATTCCCAGAAACTTCTTTG
         66 TTTCGTTGGAAACAGGAATATCTTCATATAAAAGCTAGACAGAAGCATTCTCAGAAACTTCTTTG

                              *    *                 
      60380 TGATGTTTGCATTCAAGTCACAGAGTTGAACATTCCCTTTC
        131 TGATGTTTGCATTCAAGTAACAGTGTTGAACATTCCCTTTC

                                                   *     *       ***    *   *
      60421 ATAGAGCAGGTTTGAAACACTCTTTTTGTAGTATCTGGATGTGGACATTTGGAGCACTTTCAGGC
          1 ATAGAGCAGGTTTGAAACACTCTTTTTGTAGTATCTGGAAGTGGATATTTGGATAGCTTTGAGGA

            * ** *      *             *** *   *                      *   *   
      60486 CTATGGT-GAAAAAGGAAATATCTTCCCCTGAAAACTAGACAGAAGCATTCTCAGAATCTTATTT
         66 TTTCGTTGGAAACAGG-AATATCTTCATATAAAAGCTAGACAGAAGCATTCTCAGAAACTTCTTT

                   **  **    *             **  * *   *
      60550 GTGATGTGCGCCCTCAACTAACAGTGTTGAAGCTTTCTTTTG
        130 GTGATGTTTGCATTCAAGTAACAGTGTTGAACATTCCCTTTC

                     *                    **     *   *                       
      60592 ATAGAGCAGTTTTGAAACACTCTTTTTGTAAAATCTGCAAGAGGATATTTGGATAGCTTTGAGGA
          1 ATAGAGCAGGTTTGAAACACTCTTTTTGTAGTATCTGGAAGTGGATATTTGGATAGCTTTGAGGA

                         *   * *            **                *     **   *   
      60657 TTTCGTTGGAAACGGGATTGTCTTCATATAAACTCTAGACAGAAGCATTCGCAGAAGATTCATTG
         66 TTTCGTTGGAAACAGGAATATCTTCATATAAAAGCTAGACAGAAGCATTCTCAGAAACTTCTTTG

            **      **   *    *     *       *        
      60722 GAATGTTTCAATTGAAGTCACAGTTTTGAACAGTCCCTTTC
        131 TGATGTTTGCATTCAAGTAACAGTGTTGAACATTCCCTTTC

                                                         *       **   * *    
      60763 ATAGAGCAGGTTTGAAACACTCTTTTTGTAGTATCTGGAAGTGGACATTTGGAGCGCTCTCAGGA
          1 ATAGAGCAGGTTTGAAACACTCTTTTTGTAGTATCTGGAAGTGGATATTTGGATAGCTTTGAGGA

            * **  **    *             **          * *       * *          *  *
      60828 CTATG-GCGAAAAAGGAAATATCTTCCAATAAAAGCTACATAGAAGCAATGTCAGAAACTTTTTC
         66 TTTCGTTGGAAACAGG-AATATCTTCATATAAAAGCTAGACAGAAGCATTCTCAGAAACTTCTTT

            *      * ** *   **      *       *  * *   *
      60892 ATGATGTATCTACTCAGCTAACAGAGTTGAACCTTTCTTTTG
        130 GTGATGTTTGCATTCAAGTAACAGTGTTGAACATTCCCTTTC

             *       *                   * *     *             **            
      60934 AGAGAGCAGTTTTGAAACACTCTTTTTGTGGAATCTGCAAGTGGATATTTGTCTAGCTTTGAGGA
          1 ATAGAGCAGGTTTGAAACACTCTTTTTGTAGTATCTGGAAGTGGATATTTGGATAGCTTTGAGGA

                         *   *    *                   *       *   *          
      60999 TTTCGTTGGAAACGGGATTA-CAT-ATA-AAAAGC-AGACAGCAGCATTCCCAGTAACTTCTTTG
         66 TTTCGTTGGAAACAGGAATATCTTCATATAAAAGCTAGACAGAAGCATTCTCAGAAACTTCTTTG

                    *         *    *                 
      61060 TGATGTTTCCATTCAAGTGACAGAGTTGAACATTCCCTTTC
        131 TGATGTTTGCATTCAAGTAACAGTGTTGAACATTCCCTTTC

                                                   *     *       **     *   *
      61101 ATAGAGCAGGTTTGAAACACTCTTTTTGTAGTATCTGGATGTGGACATTTGGAGCGCTTTCAGGC
          1 ATAGAGCAGGTTTGAAACACTCTTTTTGTAGTATCTGGAAGTGGATATTTGGATAGCTTTGAGGA

            * ** *      *             *** *   *                      *   *   
      61166 CTATGGT-GAAAAAGGAAATATCTTCCCCTGAAAACTAGACAGAAGCATTCTCAGAATCTTATTT
         66 TTTCGTTGGAAACAGG-AATATCTTCATATAAAAGCTAGACAGAAGCATTCTCAGAAACTTCTTT

                   **  **    *             **  * *   *
      61230 GTGATGTGCGCCCTCAACTAACAGTGTTGAAGCTTTCTTTTG
        130 GTGATGTTTGCATTCAAGTAACAGTGTTGAACATTCCCTTTC

                  * **                    **     *   *                       
      61272 ATAGAGTACTTTTGAAACACTCTTTTTGTAAAATCTGCAAGAGGATATTTGGATAGCTTTGAGGA
          1 ATAGAGCAGGTTTGAAACACTCTTTTTGTAGTATCTGGAAGTGGATATTTGGATAGCTTTGAGGA

                         *   * *            **                      *    *   
      61337 TTTCGTTGGAAACGGGATTGTCTTCATATAAACTCTAGACAGAAGCATTCTCAGAAGCTTCATTG
         66 TTTCGTTGGAAACAGGAATATCTTCATATAAAAGCTAGACAGAAGCATTCTCAGAAACTTCTTTG

            *       **   *    *             *        
      61402 GGATGTTTCAATTGAAGTCACAGTGTTGAACAGTCCCTTTC
        131 TGATGTTTGCATTCAAGTAACAGTGTTGAACATTCCCTTTC

                                                   *     *       **     *   *
      61443 ATAGAGCAGGTTTGAAACACTCTTTTTGTAGTATCTGGATGTGGACATTTGGAGCGCTTTCAGGC
          1 ATAGAGCAGGTTTGAAACACTCTTTTTGTAGTATCTGGAAGTGGATATTTGGATAGCTTTGAGGA

            * *  *      *             *** *   *                          *   
      61508 CTACGGT-GAAAAAGGAAATATCTTCCCCTGAAAACTAGACAGAAGCATTCTCAGAAACTTATTT
         66 TTTCGTTGGAAACAGG-AATATCTTCATATAAAAGCTAGACAGAAGCATTCTCAGAAACTTCTTT

                   **  **    *             **  * *   *
      61572 GTGATGTGCGCCCTCAACTAACAGTGTTGAAGCTTTCTTTTG
        130 GTGATGTTTGCATTCAAGTAACAGTGTTGAACATTCCCTTTC

                     *                   * *     *             **            
      61614 ATAGAGCAGTTTTGAAACACTCTTTTTGTGGAATCTGCAAGTGGATATTTGTCTAGCTTTGAGGA
          1 ATAGAGCAGGTTTGAAACACTCTTTTTGTAGTATCTGGAAGTGGATATTTGGATAGCTTTGAGGA

                         *   *    *                   *       *     *        
      61679 TTTCGTTGGAAACGGGATTA-CAT-ATA-AAAAGC-AGACAGCAGCATTCCCAGAATCTTCTTTG
         66 TTTCGTTGGAAACAGGAATATCTTCATATAAAAGCTAGACAGAAGCATTCTCAGAAACTTCTTTG

                              **   *            *    
      61740 TGATGTTTGCATTCAAGTCCCAGAGTTGAACATTCCGTTTC
        131 TGATGTTTGCATTCAAGTAACAGTGTTGAACATTCCCTTTC

                                       *      *    *     *     * **     *   *
      61781 ATAGAGCAGGTTTGAAACACTCTTTTTATAGTATATGGATGTGGACATTTGCAGCGCTTTCAGGC
          1 ATAGAGCAGGTTTGAAACACTCTTTTTGTAGTATCTGGAAGTGGATATTTGGATAGCTTTGAGGA

            * ** *      *             *** *   *                      *   *   
      61846 CTATGGT-GAAAAAGGAAATATCTTCCCCTGAAAACTAGACAGAAGCATTCTCAGAATCTTATTT
         66 TTTCGTTGGAAACAGG-AATATCTTCATATAAAAGCTAGACAGAAGCATTCTCAGAAACTTCTTT

                   **  **   **             **  * *   *
      61910 GTGATGTGCGCCCTCAGCTAACAGTGTTGAAGCTTTCTTTTG
        130 GTGATGTTTGCATTCAAGTAACAGTGTTGAACATTCCCTTTC

                     *         *          **     *   *                       
      61952 ATAGAGCAGTTTTGAAACAGTCTTTTTGTAAAATCTGCAAGAGGATATTTGGATAGCTTTGAGGA
          1 ATAGAGCAGGTTTGAAACACTCTTTTTGTAGTATCTGGAAGTGGATATTTGGATAGCTTTGAGGA

                *        *   * *            ** *                    *    *   
      62017 TTTCATTGGAAACGGGATTTTCTTCATATAAACTCAAGACAGAAGCATTCTCAGAAGCTTCATTG
         66 TTTCGTTGGAAACAGGAATATCTTCATATAAAAGCTAGACAGAAGCATTCTCAGAAACTTCTTTG

            *    *  **   *    *           * *        
      62082 GGATGATTCAATTGAAGTCACAGTGTTGAAAAGTCCCTTTC
        131 TGATGTTTGCATTCAAGTAACAGTGTTGAACATTCCCTTTC

                                                         *       **   * *    
      62123 ATAGAGCAGGTTTGAAACACTCTTTTTGTAGTATCTGGAAGTGGACATTTGGAGCGCTCTCAGGA
          1 ATAGAGCAGGTTTGAAACACTCTTTTTGTAGTATCTGGAAGTGGATATTTGGATAGCTTTGAGGA

            * *  *      *             **          * *       * *          *  *
      62188 CTACGGT-GAAAAAGGAAATATCTTCCAATAAAAGCTACATAGAAGCAATGTCAGAAACTTTTTC
         66 TTTCGTTGGAAACAGG-AATATCTTCATATAAAAGCTAGACAGAAGCATTCTCAGAAACTTCTTT

            *      * ** *   **      *      **  * *   *
      62252 ATGATGTATCTACTCAGCTAACAGAGTTGAAACTTTCTTTTT
        130 GTGATGTTTGCATTCAAGTAACAGTGTTGAACATTCCCTTTC

             *       *                   * *           *       **            
      62294 AGAGAGCAGTTTTGAAACACTCTTTTTGTGGAATCTGGAAGTGCATATTTGTCTAGCTTTGAGGA
          1 ATAGAGCAGGTTTGAAACACTCTTTTTGTAGTATCTGGAAGTGGATATTTGGATAGCTTTGAGGA

                         *   *    *                   *       *   *          
      62359 TTTCGTTGGAAACGGGATTA-CAT-ATA-AAAAGC-AGACAGCAGCATTCCCAGTAACTTCTTTG
         66 TTTCGTTGGAAACAGGAATATCTTCATATAAAAGCTAGACAGAAGCATTCTCAGAAACTTCTTTG

                              *    *                 
      62420 TGATGTTTGCATTCAAGTCACAGAGTTGAACATTCCCTTTC
        131 TGATGTTTGCATTCAAGTAACAGTGTTGAACATTCCCTTTC

                                                   *     *       **     *  **
      62461 ATAGAGCAGGTTTGAAACACTCTTTTTGTAGTATCTGGATGTGGACATTTGGAGCGCTTTCAGCC
          1 ATAGAGCAGGTTTGAAACACTCTTTTTGTAGTATCTGGAAGTGGATATTTGGATAGCTTTGAGGA

            * ** *  *   **           *** *   *                      *   *    
      62526 CTATGGTGAAAAAGGGAATATCTTCCCCTGAAAACTAGACAGAAGCATTCTCAGAATCTTATTTG
         66 TTTCGTTGGAAACAGGAATATCTTCATATAAAAGCTAGACAGAAGCATTCTCAGAAACTTCTTTG

                  **  **    *             **  * *   *
      62591 TGATGTGCGCCCTCAACTAACAGTGTTGAAGCTTTCTTTTG
        131 TGATGTTTGCATTCAAGTAACAGTGTTGAACATTCCCTTTC

                     *                    **     *   *                       
      62632 ATAGAGCAGTTTTGAAACACTCTTTTTGTAAAATCTGCAAGAGGATATTTGGATAGCTTTGAGGA
          1 ATAGAGCAGGTTTGAAACACTCTTTTTGTAGTATCTGGAAGTGGATATTTGGATAGCTTTGAGGA

                        **   * *            **                      * *  *   
      62697 TTTCGTTGGAAATGGGATTGTCTTCATATAAACTCTAGACAGAAGCATTCTCAGAAGCCTCATTG
         66 TTTCGTTGGAAACAGGAATATCTTCATATAAAAGCTAGACAGAAGCATTCTCAGAAACTTCTTTG

            *       **   *    *             *        
      62762 GGATGTTTCAATTGAAGTCACAGTGTTGAACAGTCCCTTTC
        131 TGATGTTTGCATTCAAGTAACAGTGTTGAACATTCCCTTTC

                                                   *     *       **     *   *
      62803 ATAGAGCAGGTTTGAAACACTCTTTTTGTAGTATCTGGATGTGGACATTTGGAGCGCTTTCAGGC
          1 ATAGAGCAGGTTTGAAACACTCTTTTTGTAGTATCTGGAAGTGGATATTTGGATAGCTTTGAGGA

            * ** *      *             * * *   *                          *   
      62868 CTATGGT-GAAAAAGGAAATATCTTCCTCTGAAAACTAGACAGAAGCATTCTCAGAAACTTATTT
         66 TTTCGTTGGAAACAGG-AATATCTTCATATAAAAGCTAGACAGAAGCATTCTCAGAAACTTCTTT

                   **   *    *             **  * *   *
      62932 GTGATGTGCGCAATCAACTAACAGTGTTGAAGCTTTCTTTTG
        130 GTGATGTTTGCATTCAAGTAACAGTGTTGAACATTCCCTTTC

                     *                   * *                   **            
      62974 ATAGAGCAGTTTTGAAACACTCTTTTTGTGGAATCTGGAAGTGGATATTTGTCTAGCTTTGAGGA
          1 ATAGAGCAGGTTTGAAACACTCTTTTTGTAGTATCTGGAAGTGGATATTTGGATAGCTTTGAGGA

                         *   *    *                   *       *   *          
      63039 TTTCGTTGGAAACGGGATTA-CAT-ATA-AAAAGC-AGACAGCAGCATTCCCAGTAACTTCTTTG
         66 TTTCGTTGGAAACAGGAATATCTTCATATAAAAGCTAGACAGAAGCATTCTCAGAAACTTCTTTG

                              *    *                 
      63100 TGATGTTTGCATTCAAGTCACAGAGTTGAACATTCCCTTTC
        131 TGATGTTTGCATTCAAGTAACAGTGTTGAACATTCCCTTTC

                                                   *     *       **     *   *
      63141 ATAGAGCAGGTTTGAAACACTCTTTTTGTAGTATCTGGATGTGGACATTTGGAGCGCTTTCAGGC
          1 ATAGAGCAGGTTTGAAACACTCTTTTTGTAGTATCTGGAAGTGGATATTTGGATAGCTTTGAGGA

            * ** *      *             *** *   *                      *   *   
      63206 CTAAGGT-GAAAAAGGAAATATCTTCCCCTGAAAACTAGACAGAAGCATTCTCAGAATCTTATTT
         66 TTTCGTTGGAAACAGG-AATATCTTCATATAAAAGCTAGACAGAAGCATTCTCAGAAACTTCTTT

                   **  **    *             **  * *   *
      63270 GTGATGTGCGCCCTCAACTAACAGTGTTGAAACTTTCTTTTG
        130 GTGATGTTTGCATTCAAGTAACAGTGTTGAACATTCCCTTTC

                     *                    **     *   *        *              
      63312 ATAGAGCAGTTTTGAAACACTCTTTTTGTAAAATCTGCAAGAGGATATTTCGATAGCTTTGAGGA
          1 ATAGAGCAGGTTTGAAACACTCTTTTTGTAGTATCTGGAAGTGGATATTTGGATAGCTTTGAGGA

                         *   * *            **                      *    *   
      63377 TTTCGTTGGAAACGGGATTGTCTTCATATAAACTCTAGACAGAAGCATTCTCAGAAGCTTCATTG
         66 TTTCGTTGGAAACAGGAATATCTTCATATAAAAGCTAGACAGAAGCATTCTCAGAAACTTCTTTG

            *       **   *    *             *        
      63442 GGATGTTTCAATTGAAGTCACAGTGTTGAACAGTCCCTTTC
        131 TGATGTTTGCATTCAAGTAACAGTGTTGAACATTCCCTTTC

                                                   *     *       **     *   *
      63483 ATAGAGCAGGTTTGAAACACTCTTTTTGTAGTATCTGGATGTGGACATTTGGAGCGCTTTCAGGC
          1 ATAGAGCAGGTTTGAAACACTCTTTTTGTAGTATCTGGAAGTGGATATTTGGATAGCTTTGAGGA

            * *  *      *             *** *   *                          *   
      63548 CTACGGT-GAAAAAGGAAATATCTTCCCCTGAAAACTAGACAGAAGCATTCTCAGAAACTTATTT
         66 TTTCGTTGGAAACAGG-AATATCTTCATATAAAAGCTAGACAGAAGCATTCTCAGAAACTTCTTT

                   **  **    *             **  * *   *
      63612 GTGATGTGCGCCCTCAACTAACAGTGTTGAAGCTTTCTTTTG
        130 GTGATGTTTGCATTCAAGTAACAGTGTTGAACATTCCCTTTC

                     *                   * *     *             **            
      63654 ATAGAGCAGTTTTGAAACACTCTTTTTGTGGAATCTGCAAGTGGATATTTGTCTAGCTTTGAGGA
          1 ATAGAGCAGGTTTGAAACACTCTTTTTGTAGTATCTGGAAGTGGATATTTGGATAGCTTTGAGGA

                         *   *    *              *    *       *     *        
      63719 TTTCGTTGGAAACGGGATTA-CAT-ATA-AAAAGC-ACACAGCAGCATTCCCAGAATCTTCTTTG
         66 TTTCGTTGGAAACAGGAATATCTTCATATAAAAGCTAGACAGAAGCATTCTCAGAAACTTCTTTG

                              **   *                 
      63780 TGATGTTTGCATTCAAGTCCCAGAGTTGAACATTCCCTTTC
        131 TGATGTTTGCATTCAAGTAACAGTGTTGAACATTCCCTTTC

                                       *           *     *       **     *   *
      63821 ATAGAGCAGGTTTGAAACACTCTTTTTATAGTATCTGGATGTGGACATTTGGAGCGCTTTCAGGC
          1 ATAGAGCAGGTTTGAAACACTCTTTTTGTAGTATCTGGAAGTGGATATTTGGATAGCTTTGAGGA

            * ** *      *             *** *   *                      *   *   
      63886 CTATGGT-GAAAAAGGAAATATCTTCTCCTGAAAACTAGACAGAAGCATTCTCAGAATCTTATTT
         66 TTTCGTTGGAAACAGG-AATATCTTCATATAAAAGCTAGACAGAAGCATTCTCAGAAACTTCTTT

                   **  ***  **             **  * *   *
      63950 GTGATGTGCGCCCACAGCTAACAGTGTTGAAGCTTTCTTTTG
        130 GTGATGTTTGCATTCAAGTAACAGTGTTGAACATTCCCTTTC

                     *         *         ***     *   *                       
      63992 ATAGAGCAGTTTTGAAACAGTCTTTTTGTGAAATCTGCAAGAGGATATTTGGATAGCTTTGAGGA
          1 ATAGAGCAGGTTTGAAACACTCTTTTTGTAGTATCTGGAAGTGGATATTTGGATAGCTTTGAGGA

                *        *   * *            **                      *    *   
      64057 TTTCATTGGAAACGGGATTCTCTTCATATAAACTCTAGACAGAAGCATTCTCAGAAGCTTCATTG
         66 TTTCGTTGGAAACAGGAATATCTTCATATAAAAGCTAGACAGAAGCATTCTCAGAAACTTCTTTG

            *       **   *    *             *        
      64122 GGATGTTTCAATTGAAGTCACAGTGTTGAACAGTCCCTTTC
        131 TGATGTTTGCATTCAAGTAACAGTGTTGAACATTCCCTTTC

                                                         *       **   * *    
      64163 ATAGAGCAGGTTTGAAACACTCTTTTTGTAGTATCTGGAAGTGGACATTTGGAGTGCTCTCAGGA
          1 ATAGAGCAGGTTTGAAACACTCTTTTTGTAGTATCTGGAAGTGGATATTTGGATAGCTTTGAGGA

            * *  *      *             **          * *       * *          *  *
      64228 CTACGGT-GAAAAAGGAAATATCTTCCAATAAAAGCTACATAGAAGCAATGTCAGAAACTTTTTC
         66 TTTCGTTGGAAACAGG-AATATCTTCATATAAAAGCTAGACAGAAGCATTCTCAGAAACTTCTTT

            *      * ** *   **      *      **  ***   *
      64292 ATGATGTATCTACTCAGCTAACAGAGTTGAAACTTTATTTTG
        130 GTGATGTTTGCATTCAAGTAACAGTGTTGAACATTCCCTTTC

             *       *                   * *                   **            
      64334 AGAGAGCAGTTTTGAAACACTCTTTTTGTGGAATCTGGAAGTGGATATTTGTCTAGCTTTGAGGA
          1 ATAGAGCAGGTTTGAAACACTCTTTTTGTAGTATCTGGAAGTGGATATTTGGATAGCTTTGAGGA

                         *   *    *                   *       * * *          
      64399 TTTCGTTGGAAACGGGATTA-CAT-ATA-AAAAGC-AGACAGCAGCATTCCCCGTAACTTCTTTG
         66 TTTCGTTGGAAACAGGAATATCTTCATATAAAAGCTAGACAGAAGCATTCTCAGAAACTTCTTTG

                              *    *              *  
      64460 TGATGTTTGCATTCAAGTCACAGAGTTGAACATTCCCTCTC
        131 TGATGTTTGCATTCAAGTAACAGTGTTGAACATTCCCTTTC

                                                   *     *       **     *   *
      64501 ATAGAGCAGGTTTGAAACACTCTTTTTGTAGTATCTGGATGTGGACATTTGGAGCGCTTTCAGGC
          1 ATAGAGCAGGTTTGAAACACTCTTTTTGTAGTATCTGGAAGTGGATATTTGGATAGCTTTGAGGA

            * ** *      *             *** *   *                      *   *   
      64566 CTATGGT-GAAAAAGGAAATATCTTCCCCTGAAAACTAGACAGAAGCATTCTCAGAATCTTATTT
         66 TTTCGTTGGAAACAGG-AATATCTTCATATAAAAGCTAGACAGAAGCATTCTCAGAAACTTCTTT

                   **  **    *             **  * *   *
      64630 GTGATGTGCGCCCTCAACTAACAGTGTTGAAGCTTTCTTTTG
        130 GTGATGTTTGCATTCAAGTAACAGTGTTGAACATTCCCTTTC

                     *                    **     *   *                       
      64672 ATAGAGCAGTTTTGAAACACTCTTTTTGTAAAATCTGCAAGAGGATATTTGGATAGCTTTGAGGA
          1 ATAGAGCAGGTTTGAAACACTCTTTTTGTAGTATCTGGAAGTGGATATTTGGATAGCTTTGAGGA

                         *   * *            **                *     * *  *   
      64737 TTTCGTTGGAAACGGGATTGTCTTCATATAAACTCTAGACAGAAGCATTCACAGAAGCCTCATTG
         66 TTTCGTTGGAAACAGGAATATCTTCATATAAAAGCTAGACAGAAGCATTCTCAGAAACTTCTTTG

            *       **   *    *             *        
      64802 GGATGTTTCAATTGAAGTCACAGTGTTGAACAGTCCCTTTC
        131 TGATGTTTGCATTCAAGTAACAGTGTTGAACATTCCCTTTC

                                                   *     *       **     *   *
      64843 ATAGAGCAGGTTTGAAACACTCTTTTTGTAGTATCTGGATGTGGACATTTGGAGCGCTTTCAGGC
          1 ATAGAGCAGGTTTGAAACACTCTTTTTGTAGTATCTGGAAGTGGATATTTGGATAGCTTTGAGGA

            * ** *      *             * * *   *                          *   
      64908 CTATGGT-GAAAAAGGAAATATCTTCCTCTGAAAACTAGACAGAAGCATTCTCAGAAACTTATTT
         66 TTTCGTTGGAAACAGG-AATATCTTCATATAAAAGCTAGACAGAAGCATTCTCAGAAACTTCTTT

                   **  **    *             **  * *    
      64972 GTGATGTGCGCCCTCAACTAACAGTGTTGAAGCTTTCTTTTC
        130 GTGATGTTTGCATTCAAGTAACAGTGTTGAACATTCCCTTTC

                     *       *           * *     *             **            
      65014 ATAGAGCAGTTTTGAAAAACTCTTTTTGTGGAATCTGCAAGTGGATATTTGTCTAGCTTTGAGGA
          1 ATAGAGCAGGTTTGAAACACTCTTTTTGTAGTATCTGGAAGTGGATATTTGGATAGCTTTGAGGA

                         **  *    *                   *       *              
      65079 TTTCGTTGGAAACGTGATTA-CAT-ATA-AAAAGC-AGACAGCAGCATTCCCAGAAACTTCTTTG
         66 TTTCGTTGGAAACAGGAATATCTTCATATAAAAGCTAGACAGAAGCATTCTCAGAAACTTCTTTG

                              *    *                 
      65140 TGATGTTTGCATTCAAGTCACAGAGTTGAACATTCCCTTTC
        131 TGATGTTTGCATTCAAGTAACAGTGTTGAACATTCCCTTTC

                                                 * *     *       **     *   *
      65181 ATAGAGCAGGTTTGAAACACTCTTTTTGTAGTATCTGAATGTGGACATTTGGAGCGCTTTCAGGC
          1 ATAGAGCAGGTTTGAAACACTCTTTTTGTAGTATCTGGAAGTGGATATTTGGATAGCTTTGAGGA

            * ** *  *                 *** *   *                      *   *   
      65246 CTATGGTGTAAA-AGGAAATATCTTCCCCTGAAAACTAGACAGAAGCATTCTCAGAATCTTATTT
         66 TTTCGTTGGAAACAGG-AATATCTTCATATAAAAGCTAGACAGAAGCATTCTCAGAAACTTCTTT

                   **  **    *             **  * *   *
      65310 GTGATGTGCGCCCTCAACTAACAGTGTTGAAGCTTTCTTTTG
        130 GTGATGTTTGCATTCAAGTAACAGTGTTGAACATTCCCTTTC

                     *                 *  **     *   *                       
      65352 ATAGAGCAGTTTTGAAACACTCTTTTTCTAAAATCTGCAAGAGGATATTTGGATAGCTTTGAGGA
          1 ATAGAGCAGGTTTGAAACACTCTTTTTGTAGTATCTGGAAGTGGATATTTGGATAGCTTTGAGGA

                         *   * *           ***                      *    *   
      65417 TTTCGTTGGAAACGGGATTGTCTTCATATAACCTCTAGACAGAAGCATTCTCAGAAGCTTCATTG
         66 TTTCGTTGGAAACAGGAATATCTTCATATAAAAGCTAGACAGAAGCATTCTCAGAAACTTCTTTG

            *       **   *    *             *        
      65482 GGATGTTTCAATTGAAGTCACAGTGTTGAACAGTCCCTTTC
        131 TGATGTTTGCATTCAAGTAACAGTGTTGAACATTCCCTTTC

                                                         *       **     *   *
      65523 ATAGAGCAGGTTTGAAACACTCTTTTTGTAGTATCTGGAAGTGGACATTTGGAGCGCTTTCAGGC
          1 ATAGAGCAGGTTTGAAACACTCTTTTTGTAGTATCTGGAAGTGGATATTTGGATAGCTTTGAGGA

            * ** *      *             * * *   *                          *   
      65588 CTATGGT-GAAAAAGGAAATATCTTCCTCTGAAAACTAGACAGAAGCATTCTCAGAAACTTATTT
         66 TTTCGTTGGAAACAGG-AATATCTTCATATAAAAGCTAGACAGAAGCATTCTCAGAAACTTCTTT

                   **  **    *             **  * *   *
      65652 GTGATGTGCGCCCTCAACTAACAGTGTTGAAGCTTTCTTTTG
        130 GTGATGTTTGCATTCAAGTAACAGTGTTGAACATTCCCTTTC

                     *                 *  **     *   *         **            
      65694 ATAGAGCAGTTTTGAAACACTCTTTTTCTAAAATCTGCAAGAGGATATTTGTCTAGCTTTGAGGA
          1 ATAGAGCAGGTTTGAAACACTCTTTTTGTAGTATCTGGAAGTGGATATTTGGATAGCTTTGAGGA

                         *   *    *                   *       *     *        
      65759 TTTCGTTGGAAACGGGATTA-CAT-ATA-AAAAGC-AGACAGCAGCATTCCCAGAATCTTCTTTG
         66 TTTCGTTGGAAACAGGAATATCTTCATATAAAAGCTAGACAGAAGCATTCTCAGAAACTTCTTTG

                              **   *            *    
      65820 TGATGTTTGCATTCAAGTCCCAGAGTTGAACATTCCGTTTC
        131 TGATGTTTGCATTCAAGTAACAGTGTTGAACATTCCCTTTC

                                       *           *     *       **     *   *
      65861 ATAGAGCAGGTTTGAAACACTCTTTTTATAGTATCTGGATGTGGACATTTGGAGCGCTTTCAGGC
          1 ATAGAGCAGGTTTGAAACACTCTTTTTGTAGTATCTGGAAGTGGATATTTGGATAGCTTTGAGGA

            * ** *      *             *** *   * *                    *   *   
      65926 CTATGGT-GAAAAAGGAAATATCTTCTCCTGAAAACAAGACAGAAGCATTCTCAGAATCTTATTT
         66 TTTCGTTGGAAACAGG-AATATCTTCATATAAAAGCTAGACAGAAGCATTCTCAGAAACTTCTTT

                   **  **   **             **  * *   *
      65990 GTGATGTGCGCCCTCAGCTAACAGTGTTGAAGCTTTCTTTTG
        130 GTGATGTTTGCATTCAAGTAACAGTGTTGAACATTCCCTTTC

                     *         *          **     *   *                       
      66032 ATAGAGCAGTTTTGAAACAGTCTTTTTGTAAAATCTGCAAGAGGATATTTGGATAGCTTTGAGGA
          1 ATAGAGCAGGTTTGAAACACTCTTTTTGTAGTATCTGGAAGTGGATATTTGGATAGCTTTGAGGA

                *        *   * **           ** *                    *    *   
      66097 TTTCATTGGAAACGGGATTTGCTTCATATAAACTCAAGACAGAAGCATTCTCAGAAGCTTCATTG
         66 TTTCGTTGGAAACAGGAATATCTTCATATAAAAGCTAGACAGAAGCATTCTCAGAAACTTCTTTG

            *    *  **   *    *           * *        
      66162 GGATGATTCAATTGAAGTCACAGTGTTGAAAAGTCCCTTTC
        131 TGATGTTTGCATTCAAGTAACAGTGTTGAACATTCCCTTTC

                                                         *       **   * *    
      66203 ATAGAGCAGGTTTGAAACACTCTTTTTGTAGTATCTGGAAGTGGACATTTGGAGCGCTCTCAGGA
          1 ATAGAGCAGGTTTGAAACACTCTTTTTGTAGTATCTGGAAGTGGATATTTGGATAGCTTTGAGGA

            * *  *      *             **          * *       * *          *  *
      66268 CTACGGT-GAAAAAGGAAATATCTTCCAATAAAAGCTACATAGAAGCAATGTCAGAAACTTTTTC
         66 TTTCGTTGGAAACAGG-AATATCTTCATATAAAAGCTAGACAGAAGCATTCTCAGAAACTTCTTT

            *      * ** *   **      *      **  * *   *
      66332 ATGATGTATCTACTCAGCTAACAGAGTTGAAACTTTCTTTTT
        130 GTGATGTTTGCATTCAAGTAACAGTGTTGAACATTCCCTTTC

             *       *                   * *           *       **            
      66374 AGAGAGCAGTTTTGAAACACTCTTTTTGTGGAATCTGGAAGTGCATATTTGTCTAGCTTTGAGGA
          1 ATAGAGCAGGTTTGAAACACTCTTTTTGTAGTATCTGGAAGTGGATATTTGGATAGCTTTGAGGA

                         *   *    *                   *       *   *          
      66439 TTTCGTTGGAAACGGGATTA-CAT-ATA-AAAAGC-AGACAGCAGCATTCCCAGTAACTTCTTTG
         66 TTTCGTTGGAAACAGGAATATCTTCATATAAAAGCTAGACAGAAGCATTCTCAGAAACTTCTTTG

                              *    *                 
      66500 TGATGTTTGCATTCAAGTCACAGAGTTGAACATTCCCTTTC
        131 TGATGTTTGCATTCAAGTAACAGTGTTGAACATTCCCTTTC

                                                   *     *       **     *  **
      66541 ATAGAGCAGGTTTGAAACACTCTTTTTGTAGTATCTGGATGTGGACATTTGGAGCGCTTTCAGCC
          1 ATAGAGCAGGTTTGAAACACTCTTTTTGTAGTATCTGGAAGTGGATATTTGGATAGCTTTGAGGA

            * ** *  *   **           *** *   *                      *   *    
      66606 CTATGGTGAAAAAGGGAATATCTTCCCCTGAAAACTAGACAGAAGCATTCTCAGAATCTTATTTG
         66 TTTCGTTGGAAACAGGAATATCTTCATATAAAAGCTAGACAGAAGCATTCTCAGAAACTTCTTTG

                  **  **    *             **  * *   *
      66671 TGATGTGCGCCCTCAACTAACAGTGTTGAAGCTTTCTTTTG
        131 TGATGTTTGCATTCAAGTAACAGTGTTGAACATTCCCTTTC

                     *                    **     *   *                       
      66712 ATAGAGCAGTTTTGAAACACTCTTTTTGTAAAATCTGCAAGAGGATATTTGGATAGCTTTGAGGA
          1 ATAGAGCAGGTTTGAAACACTCTTTTTGTAGTATCTGGAAGTGGATATTTGGATAGCTTTGAGGA

                         *   * *            **                      * *  *   
      66777 TTTCGTTGGAAACGGGATTGTCTTCATATAAACTCTAGACAGAAGCATTCTCAGAAGCCTCATTG
         66 TTTCGTTGGAAACAGGAATATCTTCATATAAAAGCTAGACAGAAGCATTCTCAGAAACTTCTTTG

            *       **   *    *             *        
      66842 GGATGTTTCAATTGAAGTCACAGTGTTGAACAGTCCCTTTC
        131 TGATGTTTGCATTCAAGTAACAGTGTTGAACATTCCCTTTC

              *                                    *     *       **     *   *
      66883 ATGGAGCAGGTTTGAAACACTCTTTTTGTAGTATCTGGATGTGGACATTTGGAGCGCTTTCAGGC
          1 ATAGAGCAGGTTTGAAACACTCTTTTTGTAGTATCTGGAAGTGGATATTTGGATAGCTTTGAGGA

            * *  *      *             *** *   *                          *   
      66948 CTACGGT-GAAAAAGGAAATATCTTCCCCTGAAAACTAGACAGAAGCATTCTCAGAAACTTATTT
         66 TTTCGTTGGAAACAGG-AATATCTTCATATAAAAGCTAGACAGAAGCATTCTCAGAAACTTCTTT

                   **  **    *             **  * *   *
      67012 GTGATGTGCGCCCTCAACTAACAGTGTTGAAGCTTTCTTTTG
        130 GTGATGTTTGCATTCAAGTAACAGTGTTGAACATTCCCTTTC

                     *                   * *     *             **            
      67054 ATAGAGCAGTTTTGAAACACTCTTTTTGTGGAATCTGCAAGTGGATATTTGTCTAGCTTTGAGGA
          1 ATAGAGCAGGTTTGAAACACTCTTTTTGTAGTATCTGGAAGTGGATATTTGGATAGCTTTGAGGA

                         *   *    *                   *       *     *        
      67119 TTTCGTTGGAAACGGGATTA-CAT-ATA-AAAAGC-AGACAGCAGCATTCCCAGAATCTTCTTTG
         66 TTTCGTTGGAAACAGGAATATCTTCATATAAAAGCTAGACAGAAGCATTCTCAGAAACTTCTTTG

                              **   *                 
      67180 TGATGTTTGCATTCAAGTCCCAGAGTTGAACATTCCCTTTC
        131 TGATGTTTGCATTCAAGTAACAGTGTTGAACATTCCCTTTC

                                       *           *     *       **     *   *
      67221 ATAGAGCAGGTTTGAAACACTCTTTTTATAGTATCTGGATGTGGACATTTGGAGCGCTTTCAGGC
          1 ATAGAGCAGGTTTGAAACACTCTTTTTGTAGTATCTGGAAGTGGATATTTGGATAGCTTTGAGGA

            * ** *      *             *** *   *                      *   *   
      67286 CTATGGT-GAAAAAGGAAATATCTTCTCCTGAAAACTAGACAGAAGCATTCTCAGAATCTTATTT
         66 TTTCGTTGGAAACAGG-AATATCTTCATATAAAAGCTAGACAGAAGCATTCTCAGAAACTTCTTT

                   **  ***  **             **  * *   *
      67350 GTGATGTGCGCCCACAGCTAACAGTGTTGAAGCTTTCTTTTG
        130 GTGATGTTTGCATTCAAGTAACAGTGTTGAACATTCCCTTTC

                     *         *          **     *   *                       
      67392 ATAGAGCAGTTTTGAAACAGTCTTTTTGTAAAATCTGCAAGAGGATATTTGGATAGCTTTGAGGA
          1 ATAGAGCAGGTTTGAAACACTCTTTTTGTAGTATCTGGAAGTGGATATTTGGATAGCTTTGAGGA

                *        *   * *            **                      *    *   
      67457 TTTCATTGGAAACGGGATTCTCTTCATATAAACTCTAGACAGAAGCATTCTCAGAAGCTTCATTG
         66 TTTCGTTGGAAACAGGAATATCTTCATATAAAAGCTAGACAGAAGCATTCTCAGAAACTTCTTTG

            *       **   *    *             *        
      67522 GGATGTTTCAATTGAAGTCACAGTGTTGAACAGTCCCTTTC
        131 TGATGTTTGCATTCAAGTAACAGTGTTGAACATTCCCTTTC

                     *                                   *       **   * *    
      67563 ATAGAGCAGTTTTGAAACACTCTTTTTGTAGTATCTGGAAGTGGACATTTGGAGTGCTCTCAGGA
          1 ATAGAGCAGGTTTGAAACACTCTTTTTGTAGTATCTGGAAGTGGATATTTGGATAGCTTTGAGGA

            * *  *      *             **          * *       * *          *  *
      67628 CTACGGT-GAAAAAGGAAATATCTTCCAATAAAAGCTACATAGAAGCAATGTCAGAAACTTTTTC
         66 TTTCGTTGGAAACAGG-AATATCTTCATATAAAAGCTAGACAGAAGCATTCTCAGAAACTTCTTT

            *      * ** *   **      *      **  ***   *
      67692 ATGATGTATCTACTCAGCTAACAGAGTTGAAACTTTATTTTG
        130 GTGATGTTTGCATTCAAGTAACAGTGTTGAACATTCCCTTTC

             *       *           *       * *     *             **            
      67734 AGAGAGCAGTTTTGAAACACTATTTTTGTGGAATCTGCAAGTGGATATTTGTCTAGCTTTGAGGA
          1 ATAGAGCAGGTTTGAAACACTCTTTTTGTAGTATCTGGAAGTGGATATTTGGATAGCTTTGAGGA

                         *   *    *                   *       *   *          
      67799 TTTCGTTGGAAACGGGATTA-CAT-ATA-AAAAGC-AGACAGCAGCATTCCCAGTAACTTCTTTG
         66 TTTCGTTGGAAACAGGAATATCTTCATATAAAAGCTAGACAGAAGCATTCTCAGAAACTTCTTTG

                              *    *              *  
      67860 TGATGTTTGCATTCAAGTCACAGAGTTGAACATTCCCTCTC
        131 TGATGTTTGCATTCAAGTAACAGTGTTGAACATTCCCTTTC

                                                   *     *       **     *   *
      67901 ATAGAGCAGGTTTGAAACACTCTTTTTGTAGTATCTGGATGTGGACATTTGGAGCGCTTTCAGGC
          1 ATAGAGCAGGTTTGAAACACTCTTTTTGTAGTATCTGGAAGTGGATATTTGGATAGCTTTGAGGA

            * ** *      *             *** *   *                      *   *   
      67966 CTATGGT-GAAAAAGGAAATATCTTCCCCTGAAAACTAGACAGAAGCATTCTCAGAATCTTATTT
         66 TTTCGTTGGAAACAGG-AATATCTTCATATAAAAGCTAGACAGAAGCATTCTCAGAAACTTCTTT

                   **  **    *             **  * *   *
      68030 GTGATGTGCGCCCTCAACTAACAGTGTTGAAGCTTTCTTTTG
        130 GTGATGTTTGCATTCAAGTAACAGTGTTGAACATTCCCTTTC

                     *                    **     *   *                       
      68072 ATAGAGCAGTTTTGAAACACTCTTTTTGTAAAATCTGCAAGAGGATATTTGGATAGCTTTGAGGA
          1 ATAGAGCAGGTTTGAAACACTCTTTTTGTAGTATCTGGAAGTGGATATTTGGATAGCTTTGAGGA

                         *   * *            **    *           *     * *  *   
      68137 TTTCGTTGGAAACGGGATTGTCTTCATATAAACTCTAGGCAGAAGCATTCACAGAAGCCTCATTG
         66 TTTCGTTGGAAACAGGAATATCTTCATATAAAAGCTAGACAGAAGCATTCTCAGAAACTTCTTTG

            *       **   *    *             *        
      68202 GGATGTTTCAATTGAAGTCACAGTGTTGAACAGTCCCTTTC
        131 TGATGTTTGCATTCAAGTAACAGTGTTGAACATTCCCTTTC

                                                   *     *       **     *   *
      68243 ATAGAGCAGGTTTGAAACACTCTTTTTGTAGTATCTGGATGTGGACATTTGGAGCGCTTTCAGGC
          1 ATAGAGCAGGTTTGAAACACTCTTTTTGTAGTATCTGGAAGTGGATATTTGGATAGCTTTGAGGA

            * ** *      *             * * *   *                          *   
      68308 CTATGGT-GAAAAAGGAAATATCTTCCTCTGAAAACTAGACAGAAGCATTCTCAGAAACTTATTT
         66 TTTCGTTGGAAACAGG-AATATCTTCATATAAAAGCTAGACAGAAGCATTCTCAGAAACTTCTTT

                   **  **    *             **  * *    
      68372 GTGATGTGCGCCCTCAACTAACAGTGTTGAAGCTTTCTTTTC
        130 GTGATGTTTGCATTCAAGTAACAGTGTTGAACATTCCCTTTC

                     *       *           * *     *             **            
      68414 ATAGAGCAGTTTTGAAAAACTCTTTTTGTGGAATCTGCAAGTGGATATTTGTCTAGCTTTGAGGA
          1 ATAGAGCAGGTTTGAAACACTCTTTTTGTAGTATCTGGAAGTGGATATTTGGATAGCTTTGAGGA

                         **  *    *                   *       *              
      68479 TTTCGTTGGAAACGTGATTA-CAT-ATA-AAAAGC-AGACAGCAGCATTCCCAGAAACTTCTTTG
         66 TTTCGTTGGAAACAGGAATATCTTCATATAAAAGCTAGACAGAAGCATTCTCAGAAACTTCTTTG

              *               *    *                 
      68540 TGTTGTTTGCATTCAAGTCACAGAGTTGAACATTCCCTTTC
        131 TGATGTTTGCATTCAAGTAACAGTGTTGAACATTCCCTTTC

                               *                 * *     *       **     *   *
      68581 ATAGAGCAGGTTTGAAACATTCTTTTTGTAGTATCTGAATGTGGACATTTGGAGCGCTTTCAGGC
          1 ATAGAGCAGGTTTGAAACACTCTTTTTGTAGTATCTGGAAGTGGATATTTGGATAGCTTTGAGGA

            * ** *  *                 *** *   *                      *   *   
      68646 CTATGGTGTAAA-AGGAAATATCTTCCCCTGAAAACTAGACAGAAGCATTCTCAGAATCTTATTT
         66 TTTCGTTGGAAACAGG-AATATCTTCATATAAAAGCTAGACAGAAGCATTCTCAGAAACTTCTTT

                   **  **    *             **  * *   *
      68710 GTGATGTGCGCCCTCAACTAACAGTGTTGAAGCTTTCTTTTG
        130 GTGATGTTTGCATTCAAGTAACAGTGTTGAACATTCCCTTTC

                     *                 *  **     *   *                       
      68752 ATAGAGCAGTTTTGAAACACTCTTTTTCTAAAATCTGCAAGAGGATATTTGGATAGCTTTGAGGA
          1 ATAGAGCAGGTTTGAAACACTCTTTTTGTAGTATCTGGAAGTGGATATTTGGATAGCTTTGAGGA

                         *   ***           ***                      *    *   
      68817 TTTCGTTGGAAACGGGATGGTCTTCATATAACCTCTAGACAGAAGCATTCTCAGAAGCTTCATTG
         66 TTTCGTTGGAAACAGGAATATCTTCATATAAAAGCTAGACAGAAGCATTCTCAGAAACTTCTTTG

            *       **   *    *             *        
      68882 GGATGTTTCAATTGAAGTCACAGTGTTGAACAGTCCCTTTC
        131 TGATGTTTGCATTCAAGTAACAGTGTTGAACATTCCCTTTC

                                                         *       **     *   *
      68923 ATAGAGCAGGTTTGAAACACTCTTTTTGTAGTATCTGGAAGTGGACATTTGGAGCGCTTTCAGGC
          1 ATAGAGCAGGTTTGAAACACTCTTTTTGTAGTATCTGGAAGTGGATATTTGGATAGCTTTGAGGA

            * ** *      *             * * *   *                          *   
      68988 CTATGGT-GAAAAAGGAAATATCTTCCTCTGAAATCTAGACAGAAGCATTCTCAGAAACTTATTT
         66 TTTCGTTGGAAACAGG-AATATCTTCATATAAAAGCTAGACAGAAGCATTCTCAGAAACTTCTTT

                   **  **    *             **  * *   *
      69052 GTGATGTGCGCCCTCAACTAACAGTGTTGAAGCTTTCTTTTG
        130 GTGATGTTTGCATTCAAGTAACAGTGTTGAACATTCCCTTTC

                     *                 *  **     *   * *                     
      69094 ATAGAGCAGTTTTGAAACACTCTTTTTCTAAAATCTGCAAGAGAATATTTGGATAGCTTTGAGGA
          1 ATAGAGCAGGTTTGAAACACTCTTTTTGTAGTATCTGGAAGTGGATATTTGGATAGCTTTGAGGA

                         *   * *            **      *          *    *    *   
      69159 TTTCGTTGGAAACGGGATTGTCTTCATATAAACTCTAGACCGAAGCATTCTGAGAAGCTTCATTG
         66 TTTCGTTGGAAACAGGAATATCTTCATATAAAAGCTAGACAGAAGCATTCTCAGAAACTTCTTTG

            *       **   *    *             *        
      69224 GGATGTTTCAATTGAAGTCACAGTGTTGAACAGTCCCTTTC
        131 TGATGTTTGCATTCAAGTAACAGTGTTGAACATTCCCTTTC

                                                         *       *  * * *    
      69265 ATAGAGCAGGTTTGAAACACTCTTTTTGTAGTATCTGGAAGTGGACATTTGGAGAGATCTCAGGA
          1 ATAGAGCAGGTTTGAAACACTCTTTTTGTAGTATCTGGAAGTGGATATTTGGATAGCTTTGAGGA

            * *  *      *             **            *       * *          *  *
      69330 CTACGGT-GAAAAAGGAAATATCTTCCAATAAAAGCTAGATAGAAGCAATGTCAGAAACTTTTTC
         66 TTTCGTTGGAAACAGG-AATATCTTCATATAAAAGCTAGACAGAAGCATTCTCAGAAACTTCTTT

            *      * ** *   **      *       *  * *   *
      69394 ATGATGTGTCTACTCAGCTAACAGAGTTGAACCTTTCTTTTG
        130 GTGATGTTTGCATTCAAGTAACAGTGTTGAACATTCCCTTTC

             *       *                   * *     * *           **            
      69436 AGAGAGCAGTTTTGAAACACTCTTTTTGTGGAATCTGCATGTGGATATTTGTCTAGCTTTGAGGA
          1 ATAGAGCAGGTTTGAAACACTCTTTTTGTAGTATCTGGAAGTGGATATTTGGATAGCTTTGAGGA

                         *   *    *                   *       *     *        
      69501 TTTCGTTGGAAACGGGATTA-CAT-ATA-AAAAGC-AGACAGCAGCATTCCCAGAATCTTCTTTG
         66 TTTCGTTGGAAACAGGAATATCTTCATATAAAAGCTAGACAGAAGCATTCTCAGAAACTTCTTTG

                              *    *            *    
      69562 TGATGTTTGCATTCAAGTCACAGAGTTGAACATTCCTTTTC
        131 TGATGTTTGCATTCAAGTAACAGTGTTGAACATTCCCTTTC

                                                   *     *       **     *   *
      69603 ATAGAGCAGGTTTGAAACACTCTTTTTGTAGTATCTGGATGTGGACATTTGGAGCGCTTTCAGGC
          1 ATAGAGCAGGTTTGAAACACTCTTTTTGTAGTATCTGGAAGTGGATATTTGGATAGCTTTGAGGA

            * ** *      *             *** *   *                      *   *   
      69668 CTATGGT-GAAAAAGGAAATATCTTCCCCTGAAAACTAGACAGAAGCATTCTCAGAATCTTATTT
         66 TTTCGTTGGAAACAGG-AATATCTTCATATAAAAGCTAGACAGAAGCATTCTCAGAAACTTCTTT

                   **  **    *             **  * *   *
      69732 GTGATGTGCGCCCTCAACTAACAGTGTTGAAGCTTTCTTTTG
        130 GTGATGTTTGCATTCAAGTAACAGTGTTGAACATTCCCTTTC

                     *                    **     *   *                       
      69774 ATAGAGCAGTTTTGAAACACTCTTTTTGTAAAATCTGCAAGAGGATATTTGGATAGCTTTGAGGA
          1 ATAGAGCAGGTTTGAAACACTCTTTTTGTAGTATCTGGAAGTGGATATTTGGATAGCTTTGAGGA

                         *   * *            **          *           *    *   
      69839 TTTCGTTGGAAACGGGATTGTCTTCATATAAACTCTAGACAGAAACATTCTCAGAAGCTTCATTG
         66 TTTCGTTGGAAACAGGAATATCTTCATATAAAAGCTAGACAGAAGCATTCTCAGAAACTTCTTTG

            *       **   *    *             *        
      69904 GGATGTTTCAATTGAAGTCACAGTGTTGAACAGTCCCTTTC
        131 TGATGTTTGCATTCAAGTAACAGTGTTGAACATTCCCTTTC

                                                         *       *  * * *    
      69945 ATAGAGCAGGTTTGAAACACTCTTTTTGTAGTATCTGGAAGTGGACATTTGGAGAGATCTCAGGA
          1 ATAGAGCAGGTTTGAAACACTCTTTTTGTAGTATCTGGAAGTGGATATTTGGATAGCTTTGAGGA

            * *  *      *             **            *       * *          *  *
      70010 CTACGGT-GAAAAAGGAAATATCTTCCAATAAAAGCTAGATAGAAGCAATGTCAGAAACTTTTTC
         66 TTTCGTTGGAAACAGG-AATATCTTCATATAAAAGCTAGACAGAAGCATTCTCAGAAACTTCTTT

            *      * ** *   **      *          * *   *
      70074 ATGATGTATCTACTCAGCTAACAGAGTTGAACATTTCTTTTG
        130 GTGATGTTTGCATTCAAGTAACAGTGTTGAACATTCCCTTTC

             *       *            *      * *     *             **            
      70116 AGAGAGCAGTTTTGAAACACTCCTTTTGTGGAATCTGCAAGTGGATATTTGTCTAGCTTTGAGGA
          1 ATAGAGCAGGTTTGAAACACTCTTTTTGTAGTATCTGGAAGTGGATATTTGGATAGCTTTGAGGA

                            *                          *       *             
      70181 TTTCGTTGGAAAC-GGGAT-TC--CATATAAAAAGC-AGACAGCAGCATTCCCAGAAACTTCTTT
         66 TTTCGTTGGAAACAGGAATATCTTCATAT-AAAAGCTAGACAGAAGCATTCTCAGAAACTTCTTT

                               *    *                 
      70241 GTGATGTTTGCATTCAAGTCACAGAGTTGAACATTCCCTTTC
        130 GTGATGTTTGCATTCAAGTAACAGTGTTGAACATTCCCTTTC

                                                 * * *   *       **     *   *
      70283 ATAGAGCAGGTTTGAAACACTCTTTTTGTAGTATCTGTATGGGGACATTTGGAGCGCTTTCAGGC
          1 ATAGAGCAGGTTTGAAACACTCTTTTTGTAGTATCTGGAAGTGGATATTTGGATAGCTTTGAGGA

            * ** *      *             *** *   *                          *   
      70348 CTATGGT-GAAAAAGGAAATATCTTCCCCTGAAAACTAGACAGAAGCATTCTCAGAAACTTATTT
         66 TTTCGTTGGAAACAGG-AATATCTTCATATAAAAGCTAGACAGAAGCATTCTCAGAAACTTCTTT

                   **  **    *             **  * *   *
      70412 GTGATGTGCGCCCTCAACTAACAGTGTTGAAGCTTTCTTTTG
        130 GTGATGTTTGCATTCAAGTAACAGTGTTGAACATTCCCTTTC

                     *                    **  *  *   *       **              
      70454 ATAGAGCAGTTTTGAAACACTCTTTTTGTAAAATATGCAAGAGGATATTAAGATAGCTTTGAGGA
          1 ATAGAGCAGGTTTGAAACACTCTTTTTGTAGTATCTGGAAGTGGATATTTGGATAGCTTTGAGGA

                         *   * *            **                      *    *   
      70519 TTTCGTTGGAAACGGGATTGTCTTCATATAAACTCTAGACAGAAGCATTCTCAGAAGCTTCATTG
         66 TTTCGTTGGAAACAGGAATATCTTCATATAAAAGCTAGACAGAAGCATTCTCAGAAACTTCTTTG

            *       **   *    *             * *      
      70584 GGATGTTTCAATTGAAGTCACAGTGTTGAACAGTTCCTTTC
        131 TGATGTTTGCATTCAAGTAACAGTGTTGAACATTCCCTTTC

                    *                                    *       **   * **   
      70625 ATAGAGCAAGTTTGAAACACTCTTTTTGTAGTATCTGGAAGTGGACATTTGGAGCGCTCTCCGGA
          1 ATAGAGCAGGTTTGAAACACTCTTTTTGTAGTATCTGGAAGTGGATATTTGGATAGCTTTGAGGA

            * * **      *             **          * *       * *          *  *
      70690 CTACAGT-GAAAAAGGAAATATCTTCCAATAAAAGCTACATAGAAGCAATGTCAGAAACTTTTTC
         66 TTTCGTTGGAAACAGG-AATATCTTCATATAAAAGCTAGACAGAAGCATTCTCAGAAACTTCTTT

            *  *   * ** *   **      *       *  *     *
      70754 ATGTTGTATCTACTCAGCTAACAGAGTTGAACCTTTCCTTTG
        130 GTGATGTTTGCATTCAAGTAACAGTGTTGAACATTCCCTTTC

             **      *                   * *     *             **            
      70796 AGGGAGCAGTTTTGAAACACTCTTTTTGTGGAATCTGCAAGTGGATATTTGTCTAGCTTTGAGGA
          1 ATAGAGCAGGTTTGAAACACTCTTTTTGTAGTATCTGGAAGTGGATATTTGGATAGCTTTGAGGA

                         *   *    *                   *       *   *          
      70861 TTTCGTTGGAAACGGGATTA-CAT-ATA-AAAAGC-AGACAGCAGCATTCCCAGTAACTTCTTTG
         66 TTTCGTTGGAAACAGGAATATCTTCATATAAAAGCTAGACAGAAGCATTCTCAGAAACTTCTTTG

                              *    *                 
      70922 TGATGTTTGCATTCAAGTCACAGAGTTGAACATTCCCTTTC
        131 TGATGTTTGCATTCAAGTAACAGTGTTGAACATTCCCTTTC

                                                   *     *       **     *   *
      70963 ATAGAGCAGGTTTGAAACACTCTTTTTGTAGTATCTGGATGTGGACATTTGGAGCGCTTTCAGGC
          1 ATAGAGCAGGTTTGAAACACTCTTTTTGTAGTATCTGGAAGTGGATATTTGGATAGCTTTGAGGA

            * ** *      *             *** *   *                          *   
      71028 CTATGGT-GAAAAAGGAAATATCTTCCCCTGAAAACTAGACAGAAGCATTCTCAGAAACTTATTT
         66 TTTCGTTGGAAACAGG-AATATCTTCATATAAAAGCTAGACAGAAGCATTCTCAGAAACTTCTTT

                   **  **    *             **  * *   *
      71092 GTGATGTGCGCCCTCAACTAACAGTGTTGAAGCTTTCTTTTG
        130 GTGATGTTTGCATTCAAGTAACAGTGTTGAACATTCCCTTTC

                     *  *                * *     *             **   *        
      71134 ATAGAGCAGTTTGGAAACACTCTTTTTGTGGAATCTGCAAGTGGATATTTGTCTAGGTTTGAGGA
          1 ATAGAGCAGGTTTGAAACACTCTTTTTGTAGTATCTGGAAGTGGATATTTGGATAGCTTTGAGGA

             *           *   *    *                   *       *     *   *    
      71199 TATCGTTGGAAACGGGATTA-CAT-ATA-AAAAGC-AGACAGCAGCATTCCCAGAATCTTGTTTG
         66 TTTCGTTGGAAACAGGAATATCTTCATATAAAAGCTAGACAGAAGCATTCTCAGAAACTTCTTTG

                *             *    **                
      71260 TGATTTTTGCATTCAAGTCACAGACTTGAACATTCCCTTTC
        131 TGATGTTTGCATTCAAGTAACAGTGTTGAACATTCCCTTTC

             *     *                   *   *       *     *       **     *   *
      71301 AGAGAGCCGGTTTGAAACACTCTTTTTATAGAATCTGGATGTGGACATTTGGAGCGCTTTCAGGC
          1 ATAGAGCAGGTTTGAAACACTCTTTTTGTAGTATCTGGAAGTGGATATTTGGATAGCTTTGAGGA

            * ** *      *             *** *   *                      *   *   
      71366 CTATGGT-GAAAAAGGAAATATCTTCCCCTGAAAACTAGACAGAAGCATTCTCAGAATCTTATTT
         66 TTTCGTTGGAAACAGG-AATATCTTCATATAAAAGCTAGACAGAAGCATTCTCAGAAACTTCTTT

                   **  **    *             **  * *   *
      71430 GTGATGTGCGCCCTCAACTAACAGTGTTGAAGTTTTCTTTTG
        130 GTGATGTTTGCATTCAAGTAACAGTGTTGAACATTCCCTTTC

                     *                    **     *   *                       
      71472 ATAGAGCAGTTTTGAAACACTCTTTTTGTAAAATCTGCAAGAGGATATTTGGATAGCTTTGAGGA
          1 ATAGAGCAGGTTTGAAACACTCTTTTTGTAGTATCTGGAAGTGGATATTTGGATAGCTTTGAGGA

                *        *   * *            **                      *    *   
      71537 TTTCCTTGGAAACGGGATTGTCTTCATATAAACTCTAGACAGAAGCATTCTCAGAAGCTTCATTG
         66 TTTCGTTGGAAACAGGAATATCTTCATATAAAAGCTAGACAGAAGCATTCTCAGAAACTTCTTTG

            *       **   *    *             *        
      71602 GGATGTTTCAATTGAAGTCACAGTGTTGAACAGTCCCTTTC
        131 TGATGTTTGCATTCAAGTAACAGTGTTGAACATTCCCTTTC

                                                         *       *  * * *  * 
      71643 ATAGAGCAGGTTTGAAACACTCTTTTTGTAGTATCTGGAAGTGGACATTTGGAGAGATCTCAGTA
          1 ATAGAGCAGGTTTGAAACACTCTTTTTGTAGTATCTGGAAGTGGATATTTGGATAGCTTTGAGGA

            * *  *      *             **            *       * *          *  *
      71708 CTACGGT-GAAAAAGGAAATATCTTCCAATAAAAGCTAGATAGAAGCAATGTCAGAAACTTTTTC
         66 TTTCGTTGGAAACAGG-AATATCTTCATATAAAAGCTAGACAGAAGCATTCTCAGAAACTTCTTT

            *      * ** *   **      *       *  * *   *
      71772 ATGATGTATCTACTCAGCTAACAGAGTTGAACCTTTCTTTTG
        130 GTGATGTTTGCATTCAAGTAACAGTGTTGAACATTCCCTTTC

             *       *                   * *     *             **            
      71814 AGAGAGCAGTTTTGAAACACTCTTTTTGTGGAATCTGCAAGTGGATATTTGTCTAGCTTTGAGGA
          1 ATAGAGCAGGTTTGAAACACTCTTTTTGTAGTATCTGGAAGTGGATATTTGGATAGCTTTGAGGA

                *        *   *    *                   *       *   *          
      71879 TTTCCTTGGAAACGGGATTA-CAT-ATA-AAAAGC-AGACAGCAGCATTCCCAGTAACTTCTTTG
         66 TTTCGTTGGAAACAGGAATATCTTCATATAAAAGCTAGACAGAAGCATTCTCAGAAACTTCTTTG

                              *    **                
      71940 TGATGTTTGCATTCAAGTCACAGAATTGAACATTCCCTTTC
        131 TGATGTTTGCATTCAAGTAACAGTGTTGAACATTCCCTTTC

                                *                  *     *       ***    *   *
      71981 ATAGAGCAGGTTTGAAACACACTTTTTGTAGTATCTGGATGTGGACATTTGGAGCACTTTCAGGC
          1 ATAGAGCAGGTTTGAAACACTCTTTTTGTAGTATCTGGAAGTGGATATTTGGATAGCTTTGAGGA

            * ** *      *             *** *   *            *         *   *   
      72046 CTATGGT-GAAAAAGGAAATATCTTCCCCTGAAAACTAGACAGAAGCCTTCTCAGAATCTTATTT
         66 TTTCGTTGGAAACAGG-AATATCTTCATATAAAAGCTAGACAGAAGCATTCTCAGAAACTTCTTT

                   **  **    *             **  * *   *
      72110 GTGATGTGCGCCCTCAACTAACAGTGTTGAAGCTTTCTTTTG
        130 GTGATGTTTGCATTCAAGTAACAGTGTTGAACATTCCCTTTC

                     *   *       *       * *     *             **            
      72152 ATAGAGCAGTTTTCAAACACTGTTTTTGTGGAATCTGCAAGTGGATATTTGTCTAGCTTTGAGGA
          1 ATAGAGCAGGTTTGAAACACTCTTTTTGTAGTATCTGGAAGTGGATATTTGGATAGCTTTGAGGA

                         *   *    *                   *       *     *        
      72217 TTTCGTTGGAAACGGGATTA-CAT-ATA-AAAAGC-AGACAGCAGCATTCCCAGAATCTTCTTTG
         66 TTTCGTTGGAAACAGGAATATCTTCATATAAAAGCTAGACAGAAGCATTCTCAGAAACTTCTTTG

                              *    *                 
      72278 TGATGTTTGCATTCAAGTCACAGAGTTGAACATTCCCTTTC
        131 TGATGTTTGCATTCAAGTAACAGTGTTGAACATTCCCTTTC

                                                   *     *       **     *   *
      72319 ATAGAGCAGGTTTGAAACACTCTTTTTGTAGTATCTGGATGTGGACATTTGGAGCGCTTTCAGGC
          1 ATAGAGCAGGTTTGAAACACTCTTTTTGTAGTATCTGGAAGTGGATATTTGGATAGCTTTGAGGA

            * ** *      *             *****   *                      *   *   
      72384 CTATGGT-GAAAAAGGAAATATCTTCCCCCGAAAACTAGACAGAAGCATTCTCAGAATCTTATTT
         66 TTTCGTTGGAAACAGG-AATATCTTCATATAAAAGCTAGACAGAAGCATTCTCAGAAACTTCTTT

                   **  **    *             **  * *   *
      72448 GTGATGTGCGCCCTCAACTAACAGTGTTGAAGCTTTCTTTTG
        130 GTGATGTTTGCATTCAAGTAACAGTGTTGAACATTCCCTTTC

                     *                    **     *   *                       
      72490 ATAGAGCAGTTTTGAAACACTCTTTTTGTAAAATCTGCAAGAGGATATTTGGATAGCTTTGAGGA
          1 ATAGAGCAGGTTTGAAACACTCTTTTTGTAGTATCTGGAAGTGGATATTTGGATAGCTTTGAGGA

                         *   * *           ***                      * *  *   
      72555 TTTCGTTGGAAACGGGATTGTCTTCATATAACCTCTAGAC-GAAGCATTCTCAGAAGCCTCATTG
         66 TTTCGTTGGAAACAGGAATATCTTCATATAAAAGCTAGACAGAAGCATTCTCAGAAACTTCTTTG

            *       **   *    *             *        
      72619 GGATGTTTCAATTGAAGTCACAGTGTTGAACAGTCCCTTTC
        131 TGATGTTTGCATTCAAGTAACAGTGTTGAACATTCCCTTTC

                                                   *     *       **     *   *
      72660 ATAGAGCAGGTTTGAAACACTCTTTTTGTAGTATCTGGATGTGGACATTTGGAGCGCTTTCAGGC
          1 ATAGAGCAGGTTTGAAACACTCTTTTTGTAGTATCTGGAAGTGGATATTTGGATAGCTTTGAGGA

            * ** *      *             * * *   *                          *   
      72725 CTATGGT-GAAAAAGGAAATATCTTCCTCTGAAAACTAGACAGAAGCATTCTCAGAAACTTATTT
         66 TTTCGTTGGAAACAGG-AATATCTTCATATAAAAGCTAGACAGAAGCATTCTCAGAAACTTCTTT

                   **  **    *             **  * *   *
      72789 GTGATGTGCGCCCTCAACTAACAGTGTTGAAGCTTTCTTTTG
        130 GTGATGTTTGCATTCAAGTAACAGTGTTGAACATTCCCTTTC

                     *                   * *     *             **            
      72831 ATAGAGCAGTTTTGAAACACTCTTTTTGTGGAATCTGCAAGTGGATATTTGTCTAGCTTTGAGGA
          1 ATAGAGCAGGTTTGAAACACTCTTTTTGTAGTATCTGGAAGTGGATATTTGGATAGCTTTGAGGA

                         *   *    *                   *       *              
      72896 TTTCGTTGGAAACGGGATTA-CAT-ATA-AAAAGC-AGACAGCAGCATTCCCAGAAACTTCTTTG
         66 TTTCGTTGGAAACAGGAATATCTTCATATAAAAGCTAGACAGAAGCATTCTCAGAAACTTCTTTG

                              *    *                 
      72957 TGATGTTTGCATTCAAGTCACAGAGTTGAACATTCCCTTTC
        131 TGATGTTTGCATTCAAGTAACAGTGTTGAACATTCCCTTTC

                                                   *     *       **     *   *
      72998 ATAGAGCAGGTTTGAAACACTCTTTTTGTAGTATCTGGATGTGGACATTTGGAGCGCTTTCAGGC
          1 ATAGAGCAGGTTTGAAACACTCTTTTTGTAGTATCTGGAAGTGGATATTTGGATAGCTTTGAGGA

            * ** *      *             *** *   *                      *   *   
      73063 CTATGGT-GAAAAAGGAAATATCTTCCCCTGAAAACTAGACAGAAGCATTCTCAGAATCTTATTT
         66 TTTCGTTGGAAACAGG-AATATCTTCATATAAAAGCTAGACAGAAGCATTCTCAGAAACTTCTTT

                   **  **    *        *    **  * *   *
      73127 GTGATGTGCGCCCTCAACTAACAGTGCTGAAGCTTTCTTTTG
        130 GTGATGTTTGCATTCAAGTAACAGTGTTGAACATTCCCTTTC

                     *                    **     *   *                       
      73169 ATAGAGCAGTTTTGAAACACTCTTTTTGTAAAATCTGCAAGAGGATATTTGGATAGCTTTGAGGA
          1 ATAGAGCAGGTTTGAAACACTCTTTTTGTAGTATCTGGAAGTGGATATTTGGATAGCTTTGAGGA

                         *   * *            **                      *    *   
      73234 TTTCGTTGGAAACGGGATTGTCTTCATATAAACTCTAGACAGAAGCATTCTCAGAAGCTTCATTG
         66 TTTCGTTGGAAACAGGAATATCTTCATATAAAAGCTAGACAGAAGCATTCTCAGAAACTTCTTTG

            *       **   *    *             *        
      73299 GGATGTTTCAATTGAAGTCACAGTGTTGAACAGTCCCTTTC
        131 TGATGTTTGCATTCAAGTAACAGTGTTGAACATTCCCTTTC

                                                         *       *  * * *    
      73340 ATAGAGCAGGTTTGAAACACTCTTTTTGTAGTATCTGGAAGTGGACATTTGGAGAGATCTCAGGA
          1 ATAGAGCAGGTTTGAAACACTCTTTTTGTAGTATCTGGAAGTGGATATTTGGATAGCTTTGAGGA

            * *  *      *             **            *     * ***          *  *
      73405 CTACGGT-GAAAAAGGAAATATCTTCCAATAAAAGCTAGATAGAAGAAAAGTCAGAAACTTTTTC
         66 TTTCGTTGGAAACAGG-AATATCTTCATATAAAAGCTAGACAGAAGCATTCTCAGAAACTTCTTT

            *      * ** *   **      *       *  * *   *
      73469 ATGATGTATCTACTCAGCTAACAGAGTTGAACCTTTCTTTTG
        130 GTGATGTTTGCATTCAAGTAACAGTGTTGAACATTCCCTTTC

             *       *           *       * *     *             **   *        
      73511 AGAGAGCAGTTTTGAAACACTGTTTTTGTGGAATCTGCAAGTGGATATTTGTCTAGTTTTGAGGA
          1 ATAGAGCAGGTTTGAAACACTCTTTTTGTAGTATCTGGAAGTGGATATTTGGATAGCTTTGAGGA

                         *   *    *                   *       *              
      73576 TTTCGTTGGAAACGGGATTA-CAT-ATA-AAAAGC-AGACAGCAGCATTCCCAGAAACTTCTTTG
         66 TTTCGTTGGAAACAGGAATATCTTCATATAAAAGCTAGACAGAAGCATTCTCAGAAACTTCTTTG

                              *    *                 
      73637 TGATG-TTGCATTCAAGTCACAGAGTTGAACATTCCCTTTC
        131 TGATGTTTGCATTCAAGTAACAGTGTTGAACATTCCCTTTC

                        *                          *     *       **     *   *
      73677 ATAGAGCAGGTTGGAAACACTCTTTTTGTAGTATCTGGATGTGGACATTTGGAGCGCTTTCAGGC
          1 ATAGAGCAGGTTTGAAACACTCTTTTTGTAGTATCTGGAAGTGGATATTTGGATAGCTTTGAGGA

            * ** *      *             *** *   *                          *   
      73742 CTATGGT-GAAAAAGGAAATATCTTCCCCTGAAAACTAGACAGAAGCATTCTCAGAAACTTATTT
         66 TTTCGTTGGAAACAGG-AATATCTTCATATAAAAGCTAGACAGAAGCATTCTCAGAAACTTCTTT

                   **  ** *  *             **  * *   *
      73806 GTGATGTGCGCCCTGAACTAACAGTGTTGAAGCTTTCTTTTG
        130 GTGATGTTTGCATTCAAGTAACAGTGTTGAACATTCCCTTTC

              *      *                    **    **   *                       
      73848 ATTGAGCAGTTTTGAAACACTCTTTTTGTAAAATCTACAAGAGGATATTTGGATAGCTTTGAGGA
          1 ATAGAGCAGGTTTGAAACACTCTTTTTGTAGTATCTGGAAGTGGATATTTGGATAGCTTTGAGGA

                 *       *   * *            **                      *    *   
      73913 TTTCGCTGGAAACGGGATTGTCTTCATATAAACTCTAGACAGAAGCATTCTCAGAAGCTTCATTG
         66 TTTCGTTGGAAACAGGAATATCTTCATATAAAAGCTAGACAGAAGCATTCTCAGAAACTTCTTTG

            *       **   *    *             *        
      73978 GGATGTTTCAATTGAAGTCACAGTGTTGAACAGTCCCTTTC
        131 TGATGTTTGCATTCAAGTAACAGTGTTGAACATTCCCTTTC

                                                         *       *  * * *    
      74019 ATAGAGCAGGTTTGAAACACTCTTTTTGTAGTATCTGGAAGTGGACATTTGGAGAGATCTCAGGA
          1 ATAGAGCAGGTTTGAAACACTCTTTTTGTAGTATCTGGAAGTGGATATTTGGATAGCTTTGAGGA

            * ** *      *             **            *     * * *          *  *
      74084 CTATGGT-GAAAAAGGAAATATCTTCCAATAAAAGCTAGATAGAAGAAATGTCAGAAACTTTTTC
         66 TTTCGTTGGAAACAGG-AATATCTTCATATAAAAGCTAGACAGAAGCATTCTCAGAAACTTCTTT

            *      * ** *   **      **      *  * *   *
      74148 ATGATGTATCTACTCAGCTAACAGATTTGAACCTTTCTTTTG
        130 GTGATGTTTGCATTCAAGTAACAGTGTTGAACATTCCCTTTC

             *       *                   * *     *             **            
      74190 AGAGAGCAGTTTTGAAACACTCTTTTTGTGGAATCTGCAAGTGGATATTTGTCTAGCTTTGAGGA
          1 ATAGAGCAGGTTTGAAACACTCTTTTTGTAGTATCTGGAAGTGGATATTTGGATAGCTTTGAGGA

                         *   *    *                   *       *              
      74255 TTTCGTTGGAAACGGGATTA-CAT-ATA-AAAAGC-AGACAGCAGCATTCCCAGAAACTTCTTTG
         66 TTTCGTTGGAAACAGGAATATCTTCATATAAAAGCTAGACAGAAGCATTCTCAGAAACTTCTTTG

                    *       * *    *                 
      74316 TGATG-TTACATTCAACTCACAGAGTTGAACATTCCCTTTC
        131 TGATGTTTGCATTCAAGTAACAGTGTTGAACATTCCCTTTC

                                                   *     *       **     *   *
      74356 ATAGAGCAGGTTTGAAACACTCTTTTTGTAGTATCTGGATGTGGACATTTGGAGCGCTTTCAGGC
          1 ATAGAGCAGGTTTGAAACACTCTTTTTGTAGTATCTGGAAGTGGATATTTGGATAGCTTTGAGGA

            * ** *      *          *  *** *   *                      *   *   
      74421 CTATGGT-GAAAAAGGAAATATCCTCCCCTGAAAACTAGACAGAAGCATTCTCAGAATCTTATTT
         66 TTTCGTTGGAAACAGG-AATATCTTCATATAAAAGCTAGACAGAAGCATTCTCAGAAACTTCTTT

                   **  **    *             **  * *   *
      74485 GTGATGTGCGCCCTCAACTAACAGTGTTGAAGCTTTCTTTTG
        130 GTGATGTTTGCATTCAAGTAACAGTGTTGAACATTCCCTTTC

                     *                    **     *   *                       
      74527 ATAGAGCAGTTTTGAAACACTCTTTTTGTAAAATCTGCAAGAGGATATTTGGATAGCTTTGAGGA
          1 ATAGAGCAGGTTTGAAACACTCTTTTTGTAGTATCTGGAAGTGGATATTTGGATAGCTTTGAGGA

                         *   * *            **                      *    *   
      74592 TTTCGTTGGAAACGGGATTGTCTTCATATAAACTCTAGACAGAAGCATTCTCAGAAGCTTCATTG
         66 TTTCGTTGGAAACAGGAATATCTTCATATAAAAGCTAGACAGAAGCATTCTCAGAAACTTCTTTG

            *       **   *    *             *        
      74657 GGATGTTTCAATTGAAGTCACAGTGTTGAACAGTCCCTTTC
        131 TGATGTTTGCATTCAAGTAACAGTGTTGAACATTCCCTTTC

                                             *      *    *       **   * *    
      74698 ATAGAGCAGGTTTGAAACACTCTTTTTGTAGTACCTGGAAATGGACATTTGGAGCGCTCTCAGGA
          1 ATAGAGCAGGTTTGAAACACTCTTTTTGTAGTATCTGGAAGTGGATATTTGGATAGCTTTGAGGA

            * *  *      *             **          * *       * *          *  *
      74763 CTACGGT-GAAAAAGGAAATATCTTCCAATAAAAGCTACATAGAAGCAATGTCAGAAACTTTTTC
         66 TTTCGTTGGAAACAGG-AATATCTTCATATAAAAGCTAGACAGAAGCATTCTCAGAAACTTCTTT

            *      * ** *   **      *       *  * *   *
      74827 ATGATGTATCTACTCAGCTAACAGAGTTGAACCTTTCTTTTG
        130 GTGATGTTTGCATTCAAGTAACAGTGTTGAACATTCCCTTTC

             *       *                   * *     *             **            
      74869 AGAGAGCAGTTTTGAAACACTCTTTTTGTGGAATCTGCAAGTGGATATTTGTCTAGCTTTGAGGA
          1 ATAGAGCAGGTTTGAAACACTCTTTTTGTAGTATCTGGAAGTGGATATTTGGATAGCTTTGAGGA

                         *   *    *                   *       *   * *   *    
      74934 TTTCGTTGGAAACGGGATTA-CAT-ATA-AAAAGC-AGACAGCAGCATTCCCAGTATCTTGTTTG
         66 TTTCGTTGGAAACAGGAATATCTTCATATAAAAGCTAGACAGAAGCATTCTCAGAAACTTCTTTG

                    *         *    *                 
      74995 TGATGTTTCCATTCAAGTCACAGAGTTGAACATTCCCTTTC
        131 TGATGTTTGCATTCAAGTAACAGTGTTGAACATTCCCTTTC

                                                   *     *       **     *   *
      75036 ATAGAGCAGGTTTGAAACACTCTTTTTGTAGTATCTGGATGTGGACATTTGGAGCGCTTTCAGGC
          1 ATAGAGCAGGTTTGAAACACTCTTTTTGTAGTATCTGGAAGTGGATATTTGGATAGCTTTGAGGA

            * ** *      *             *** *   *           *              *   
      75101 CTATGGT-GAAAAAGGAAATATCTTCCCCTGAAAACTAGACAGAAGGATTCTCAGAAACTTATTT
         66 TTTCGTTGGAAACAGG-AATATCTTCATATAAAAGCTAGACAGAAGCATTCTCAGAAACTTCTTT

                   **  **    *             **  * *   *
      75165 GTGATGTGCGCCCTCAACTAACAGTGTTGAAGCTTTCTTTTG
        130 GTGATGTTTGCATTCAAGTAACAGTGTTGAACATTCCCTTTC

                     *                    **     *   *                       
      75207 ATAGAGCAGTTTTGAAACACTCTTTTTGTAAAATCTGCAAGAGGATATTTGGATAGCTTTGAGGA
          1 ATAGAGCAGGTTTGAAACACTCTTTTTGTAGTATCTGGAAGTGGATATTTGGATAGCTTTGAGGA

                         *   * *            **                      *    *   
      75272 TTTCGTTGGAAACGGGATTGTCTTCATATAAACTCTAGACAGAAGCATTCTCAGAAGCTTCATTG
         66 TTTCGTTGGAAACAGGAATATCTTCATATAAAAGCTAGACAGAAGCATTCTCAGAAACTTCTTTG

            *       **   *    *             *        
      75337 GGATGTTTCAATTGAAGTCACAGTGTTGAACAGTCCCTTTC
        131 TGATGTTTGCATTCAAGTAACAGTGTTGAACATTCCCTTTC

                                                   *     *       **     *   *
      75378 ATAGAGCAGGTTTGAAACACTCTTTTTGTAGTATCTGGATGTGGACATTTGGAGCGCTTTCAGGC
          1 ATAGAGCAGGTTTGAAACACTCTTTTTGTAGTATCTGGAAGTGGATATTTGGATAGCTTTGAGGA

            * ** *      *             *** *   *                          *   
      75443 CTATGGT-GAAAAAGGAAATATCTTCCCCTGAAAACTAGACAGAAGCATTCTCAGAAACTTATTT
         66 TTTCGTTGGAAACAGG-AATATCTTCATATAAAAGCTAGACAGAAGCATTCTCAGAAACTTCTTT

                   **  **    *             **  * *   *
      75507 GTGATGTGCGCCCTCAACTAACAGTGTTGAAGCTTTCTTTTG
        130 GTGATGTTTGCATTCAAGTAACAGTGTTGAACATTCCCTTTC

                     *                   * *     *             **       *    
      75549 ATAGAGCAGTTTTGAAACACTCTTTTTGTGGAATCTGCAAGTGGATATTTGTCTAGCTTTAAGGA
          1 ATAGAGCAGGTTTGAAACACTCTTTTTGTAGTATCTGGAAGTGGATATTTGGATAGCTTTGAGGA

                         *   *    *                   *       *     *        
      75614 TTTCGTTGGAAACGGGATTA-CAT-ATA-AAAAGC-AGACAGCAGCATTCCCAGAATCTTCTTTG
         66 TTTCGTTGGAAACAGGAATATCTTCATATAAAAGCTAGACAGAAGCATTCTCAGAAACTTCTTTG

                              **   *            *    
      75675 TGATGTTTGCATTCAAGTCCCAGAGTTGAACATTCCGTTTC
        131 TGATGTTTGCATTCAAGTAACAGTGTTGAACATTCCCTTTC

                                       *           *     *       **     *   *
      75716 ATAGAGCAGGTTTGAAACACTCTTTTTATAGTATCTGGATGTGGACATTTGGAGCGCTTTCAGGC
          1 ATAGAGCAGGTTTGAAACACTCTTTTTGTAGTATCTGGAAGTGGATATTTGGATAGCTTTGAGGA

            * ** *      *             *** *   * *                    *   *   
      75781 CTATGGT-GAAAAAGGAAATATCTTCTCCTGAAAACAAGACAGAAGCATTCTCAGAATCTTATTT
         66 TTTCGTTGGAAACAGG-AATATCTTCATATAAAAGCTAGACAGAAGCATTCTCAGAAACTTCTTT

                   **  **   **             **  * *   *
      75845 GTGATGTGCGCCCTCAGCTAACAGTGTTGAAGCTTTCTTTTG
        130 GTGATGTTTGCATTCAAGTAACAGTGTTGAACATTCCCTTTC

                     *         *          **     *   *                       
      75887 ATAGAGCAGTTTTGAAACAATCTTTTTGTAAAATCTGCAAGAGGATATTTGGATAGCTTTGAGGA
          1 ATAGAGCAGGTTTGAAACACTCTTTTTGTAGTATCTGGAAGTGGATATTTGGATAGCTTTGAGGA

                *        *   * *            ** *                    *    *   
      75952 TTTCATTGGAAACGGGATTTTCTTCATATAAACTCAAGACAGAAGCATTCTCAGAAGCTTCATTG
         66 TTTCGTTGGAAACAGGAATATCTTCATATAAAAGCTAGACAGAAGCATTCTCAGAAACTTCTTTG

            *       **   *    *             *        
      76017 GGATGTTTCAATTGAAGTCACAGTGTTGAACAGTCCCTTTC
        131 TGATGTTTGCATTCAAGTAACAGTGTTGAACATTCCCTTTC

                                                         *       **   *      
      76058 ATAGAGCAGGTTTGAAACACTCTTTTTGTAGTATCTGGAAGTGGACATTTGGAGCGCTCTGAGGA
          1 ATAGAGCAGGTTTGAAACACTCTTTTTGTAGTATCTGGAAGTGGATATTTGGATAGCTTTGAGGA

            * *  *      *             **          * *       * *          *  *
      76123 CTACGCT-GAAAAAGGAAATATCTTCCAATAAAAGCTACATAGAAGCAATGTCAGAAACTTTTTC
         66 TTTCGTTGGAAACAGG-AATATCTTCATATAAAAGCTAGACAGAAGCATTCTCAGAAACTTCTTT

            *      * ** *   **      *      **  * *   *
      76187 ATGATGTATCTACTCAGCTAACAGAGTTGAAACTTTCTTTTG
        130 GTGATGTTTGCATTCAAGTAACAGTGTTGAACATTCCCTTTC

             *       *                   * *                   **            
      76229 AGAGAGCAGTTTTGAAACACTCTTTTTGTGGAATCTGGAAGTGGATATTTGTCTAGCTTTGAGGA
          1 ATAGAGCAGGTTTGAAACACTCTTTTTGTAGTATCTGGAAGTGGATATTTGGATAGCTTTGAGGA

                         *   *    *                   *       *   *          
      76294 TTTCGTTGGAAACGGGATTA-CAT-ATA-AAAAGC-AGACAGCAGCATTCCCAGTAACTTCTTTG
         66 TTTCGTTGGAAACAGGAATATCTTCATATAAAAGCTAGACAGAAGCATTCTCAGAAACTTCTTTG

                              *    *                 
      76355 TGATGTTTGCATTCAAGTCACAGAGTTGAACATTCCCTTTC
        131 TGATGTTTGCATTCAAGTAACAGTGTTGAACATTCCCTTTC

                                                   *     *       **     *   *
      76396 ATAGAGCAGGTTTGAAACACTCTTTTTGTAGTATCTGGATGTGGACATTTGGAGCGCTTTCAGGC
          1 ATAGAGCAGGTTTGAAACACTCTTTTTGTAGTATCTGGAAGTGGATATTTGGATAGCTTTGAGGA

            * ** *      *             *** *   *                      *   *   
      76461 CTATGGT-GAAAAAGGAAATATCTTCCCCTGAAAACTAGACAGAAGCATTCTCAGAATCTTATTT
         66 TTTCGTTGGAAACAGG-AATATCTTCATATAAAAGCTAGACAGAAGCATTCTCAGAAACTTCTTT

                   **  **    *             **  * *   *
      76525 GTGATGTGCGCCCTCAACTAACAGTGTTGAAGCTTTCTTTTG
        130 GTGATGTTTGCATTCAAGTAACAGTGTTGAACATTCCCTTTC

                     *                    **     *   *                       
      76567 ATAGAGCAGTTTTGAAACACTCTTTTTGTAAAATCTGCAAGAGGATATTTGGATAGCTTTGAGGA
          1 ATAGAGCAGGTTTGAAACACTCTTTTTGTAGTATCTGGAAGTGGATATTTGGATAGCTTTGAGGA

                         *   * *            **                      * *  *   
      76632 TTTCGTTGGAAACGGGATTGTCTTCATATAAACTCTAGAC-GAAGCATTCTCAGAAGCCTCATTG
         66 TTTCGTTGGAAACAGGAATATCTTCATATAAAAGCTAGACAGAAGCATTCTCAGAAACTTCTTTG

            *       **   *    *             *        
      76696 GGATGTTTCAATTGAAGTCACAGTGTTGAACAGTCCCTTTC
        131 TGATGTTTGCATTCAAGTAACAGTGTTGAACATTCCCTTTC

                                                   *     *       **     *   *
      76737 ATAGAGCAGGTTTGAAACACTCTTTTTGTAGTATCTGGATGTGGACATTTGGAGCGCTTTCAGGC
          1 ATAGAGCAGGTTTGAAACACTCTTTTTGTAGTATCTGGAAGTGGATATTTGGATAGCTTTGAGGA

            * ** *      *             * * *   *                          *   
      76802 CTATGGT-GAAAAAGGAAATATCTTCCTCTGAAAACTAGACAGAAGCATTCTCAGAAACTTATTT
         66 TTTCGTTGGAAACAGG-AATATCTTCATATAAAAGCTAGACAGAAGCATTCTCAGAAACTTCTTT

                   **  **    *             **  * *   *
      76866 GTGATGTGCGCCCTCAACTAACAGTGTTGAAGCTTTCTTTTG
        130 GTGATGTTTGCATTCAAGTAACAGTGTTGAACATTCCCTTTC

                     *                   * *     *             **            
      76908 ATAGAGCAGTTTTGAAACACTCTTTTTGTGGAATCTGCAAGTGGATATTTGTCTAGCTTTGAGGA
          1 ATAGAGCAGGTTTGAAACACTCTTTTTGTAGTATCTGGAAGTGGATATTTGGATAGCTTTGAGGA

             *           *   *    *                   *       *              
      76973 TATCGTTGGAAACGGGATTA-CAT-ATA-AAAAGC-AGACAGCAGCATTCCCAGAAACTTCTTTG
         66 TTTCGTTGGAAACAGGAATATCTTCATATAAAAGCTAGACAGAAGCATTCTCAGAAACTTCTTTG

                              *    *                 
      77034 TGATGTTTGCATTCAAGTCACAGAGTTGAACATTCCCTTTC
        131 TGATGTTTGCATTCAAGTAACAGTGTTGAACATTCCCTTTC

                                                   *     *       **     *   *
      77075 ATAGAGCAGGTTTGAAACACTCTTTTTGTAGTATCTGGATGTGGACATTTGGAGCGCTTTCAGGC
          1 ATAGAGCAGGTTTGAAACACTCTTTTTGTAGTATCTGGAAGTGGATATTTGGATAGCTTTGAGGA

            * ** *      *             *** *   *                      *   *   
      77140 CTATGGT-GAAAAAGGAAATATCTTCCCCTGAAAACTAGACAGAAGCATTCTCAGAATCTTATTT
         66 TTTCGTTGGAAACAGG-AATATCTTCATATAAAAGCTAGACAGAAGCATTCTCAGAAACTTCTTT

                   **  **    *        *    **  * *   *
      77204 GTGATGTGCGCCCTCAACTAACAGTGCTGAAGCTTTCTTTTG
        130 GTGATGTTTGCATTCAAGTAACAGTGTTGAACATTCCCTTTC

                     *                    **     *   *                       
      77246 ATAGAGCAGTTTTGAAACACTCTTTTTGTAAAATCTGCAAGAGGATATTTGGATAGCTTTGAGGA
          1 ATAGAGCAGGTTTGAAACACTCTTTTTGTAGTATCTGGAAGTGGATATTTGGATAGCTTTGAGGA

                         *   * *            **                      *    *   
      77311 TTTCGTTGGAAACGGGATTGTCTTCATATAAACTCTAGACAGAAGCATTCTCAGAAGCTTCATTG
         66 TTTCGTTGGAAACAGGAATATCTTCATATAAAAGCTAGACAGAAGCATTCTCAGAAACTTCTTTG

            *       **   *    *             *        
      77376 GGATGTTTCAATTGAAGTCACAGTGTTGAACAGTCCCTTTC
        131 TGATGTTTGCATTCAAGTAACAGTGTTGAACATTCCCTTTC

                                                         *       *  * * *    
      77417 ATAGAGCAGGTTTGAAACACTCTTTTTGTAGTATCTGGAAGTGGACATTTGGAGAGATCTCAGGA
          1 ATAGAGCAGGTTTGAAACACTCTTTTTGTAGTATCTGGAAGTGGATATTTGGATAGCTTTGAGGA

            * *  *      *             **            *     * ***          *  *
      77482 CTACGGT-GAAAAAGGAAATATCTTCCAATAAAAGCTAGATAGAAGAAAAGTCAGAAACTTTTTC
         66 TTTCGTTGGAAACAGG-AATATCTTCATATAAAAGCTAGACAGAAGCATTCTCAGAAACTTCTTT

            *      * ** *   **      *       *  * *   *
      77546 ATGATGTATCTACTCAGCTAACAGAGTTGAACCTTTCTTTTG
        130 GTGATGTTTGCATTCAAGTAACAGTGTTGAACATTCCCTTTC

             *       *           *       * *     *             **            
      77588 AGAGAGCAGTTTTGAAACACTGTTTTTGTGGAATCTGCAAGTGGATATTTGTCTAGCTTTGAGGA
          1 ATAGAGCAGGTTTGAAACACTCTTTTTGTAGTATCTGGAAGTGGATATTTGGATAGCTTTGAGGA

                         *   *    *                   *       *              
      77653 TTTCGTTGGAAACGGGATTA-CAT-ATA-AAAAGC-AGACAGCAGCATTCCCAGAAACTTCTTTG
         66 TTTCGTTGGAAACAGGAATATCTTCATATAAAAGCTAGACAGAAGCATTCTCAGAAACTTCTTTG

                              *    *                 
      77714 TGATGTTTGCATTCAAGTCACAGAGTTGAACATTCCCTTTC
        131 TGATGTTTGCATTCAAGTAACAGTGTTGAACATTCCCTTTC

                                                   *     *       **     *   *
      77755 ATAGAGCAGGTTTGAAACACTCTTTTTGTAGTATCTGGATGTGGACATTTGGAGCGCTTTCAGGC
          1 ATAGAGCAGGTTTGAAACACTCTTTTTGTAGTATCTGGAAGTGGATATTTGGATAGCTTTGAGGA

            * ** *      *             *** *   *                      *   *   
      77820 CTATGGT-GAAAAAGGAAATATCTTCCCCTGAAAACTAGACAGAAGCATTCTCAGAATCTTATTT
         66 TTTCGTTGGAAACAGG-AATATCTTCATATAAAAGCTAGACAGAAGCATTCTCAGAAACTTCTTT

                   **  **    *             **  * *   *
      77884 GTGATGTCCGCCCTCAACTAACAGTGTTGAAGCTTTCTTTTG
        130 GTGATGTTTGCATTCAAGTAACAGTGTTGAACATTCCCTTTC

                     *                    **     *   *                       
      77926 ATAGAGCAGTTTTGAAACACTCTTTTTGTAAAATCTGCAAGAGGATATTTGGATAGCTTTGAGGA
          1 ATAGAGCAGGTTTGAAACACTCTTTTTGTAGTATCTGGAAGTGGATATTTGGATAGCTTTGAGGA

                         *   * *            **                      *    *   
      77991 TTTCGTTGGAAACGGGATTGTCTTCATATAAACTCTAGACAGAAGCATTCTCAGAAGCTTCATTG
         66 TTTCGTTGGAAACAGGAATATCTTCATATAAAAGCTAGACAGAAGCATTCTCAGAAACTTCTTTG

            *       **   *    *   *         *        
      78056 GGATGTTTCAATTGAAGTCACATTGTTGAACAGTCCCTTTC
        131 TGATGTTTGCATTCAAGTAACAGTGTTGAACATTCCCTTTC

                                           * *      *    *    *  **   * *    
      78097 ATAGAGCAGGTTTGAAACACTCTTTTTGTAGCACCTGGAAATGGACATTTTGAGCGCTCTCAGGA
          1 ATAGAGCAGGTTTGAAACACTCTTTTTGTAGTATCTGGAAGTGGATATTTGGATAGCTTTGAGGA

            * *  *      *             **          * *       * *          *  *
      78162 CTACGGT-GAAAAAGGAAATATCTTCCAATAAAAGCTACATAGAAGCAATGTCAGAAACTTTTTC
         66 TTTCGTTGGAAACAGG-AATATCTTCATATAAAAGCTAGACAGAAGCATTCTCAGAAACTTCTTT

            *      * ** *   **      *       *  * *   *
      78226 ATGATGTATCTACTCAGCTAACAGAGTTGAACCTTTCTTTTG
        130 GTGATGTTTGCATTCAAGTAACAGTGTTGAACATTCCCTTTC

             *       *       *           * *     *             **  *         
      78268 AGAGAGCAGTTTTGAAATACTCTTTTTGTGGAATCTGCAAGTGGATATTTGTCTACCTTTGAGGA
          1 ATAGAGCAGGTTTGAAACACTCTTTTTGTAGTATCTGGAAGTGGATATTTGGATAGCTTTGAGGA

                         *   *    *                   *       *              
      78333 TTTCGTTGGAAACGGGATTA-CAT-ATA-AAAAGC-AGACAGCAGCATTCCCAGAAACTTCTTTG
         66 TTTCGTTGGAAACAGGAATATCTTCATATAAAAGCTAGACAGAAGCATTCTCAGAAACTTCTTTG

                    *       * *    *      *          
      78394 TGATG-TTACATTCAACTCACAGAGTTGAATATTCCCTTTC
        131 TGATGTTTGCATTCAAGTAACAGTGTTGAACATTCCCTTTC

                                                   *     *       **     *   *
      78434 ATAGAGCAGGTTTGAAACACTCTTTTTGTAGTATCTGGATGTGGACATTTGGAGCGCTTTCAGGC
          1 ATAGAGCAGGTTTGAAACACTCTTTTTGTAGTATCTGGAAGTGGATATTTGGATAGCTTTGAGGA

            * ** *      *             *** *   *                      *   *   
      78499 CTATGGT-GAAAAAGGAAATATCTTCCCCTGAAAACTAGACAGAAGCATTCTCAGAATCTTATTT
         66 TTTCGTTGGAAACAGG-AATATCTTCATATAAAAGCTAGACAGAAGCATTCTCAGAAACTTCTTT

                   **  **    *             **  * *   *
      78563 GTGATGTGCGCCCTCAACTAACAGTGTTGAAGCTTTCTTTTG
        130 GTGATGTTTGCATTCAAGTAACAGTGTTGAACATTCCCTTTC

                 *   *                   * *     *             **            
      78605 ATAGACCAGTTTTGAAACACTCTTTTTGTGGAATCTGCAAGTGGATATTTGTCTAGCTTTGAGGA
          1 ATAGAGCAGGTTTGAAACACTCTTTTTGTAGTATCTGGAAGTGGATATTTGGATAGCTTTGAGGA

                         *   *    *                   *       *     *        
      78670 TTTCGTTGGAAACGGGATTA-CAT-ATA-AAAAGC-AGACAGCAGCATTCCCAGAATCTTCTTTG
         66 TTTCGTTGGAAACAGGAATATCTTCATATAAAAGCTAGACAGAAGCATTCTCAGAAACTTCTTTG

                            * **   *    *            
      78731 TGATGTTTGCATTCAACTCCCAGAGTTGGACATTCCCTTTC
        131 TGATGTTTGCATTCAAGTAACAGTGTTGAACATTCCCTTTC

                                                   *     *       **     *   *
      78772 ATAGAGCAGGTTTGAAACACTCTTTTTGTAGTATCTGGATGTGGACATTTGGAGCGCTTTCAGGC
          1 ATAGAGCAGGTTTGAAACACTCTTTTTGTAGTATCTGGAAGTGGATATTTGGATAGCTTTGAGGA

            * ** *      *             *** *   *    *                 *   *   
      78837 CTATGGT-GAAAAAGGAAATATCTTCTCCTGAAAACTAGGCAGAAGCATTCTCAGAATCTTATTT
         66 TTTCGTTGGAAACAGG-AATATCTTCATATAAAAGCTAGACAGAAGCATTCTCAGAAACTTCTTT

                   **   *   **             **  * *   *
      78901 GTGATGTGCGCACTCAGCTAACAGTGTTGAAGCTTTCTTTTG
        130 GTGATGTTTGCATTCAAGTAACAGTGTTGAACATTCCCTTTC

                     *                    **     *   *                       
      78943 ATAGAGCAGTTTTGAAACACTCTTTTTGTAAAATCTGCAAGAGGATATTTGGATAGCTTTGAGGA
          1 ATAGAGCAGGTTTGAAACACTCTTTTTGTAGTATCTGGAAGTGGATATTTGGATAGCTTTGAGGA

                         *   * *            **                      *    *   
      79008 TTTCGTTGGAAACGGGATTGTCTTCATATAAACTCTAGACAGAAGCATTCTCAGAAGCTTCATTG
         66 TTTCGTTGGAAACAGGAATATCTTCATATAAAAGCTAGACAGAAGCATTCTCAGAAACTTCTTTG

            *       **   *    *           * *        
      79073 GGATGTTTCAATTGAAGTCACAGTGTTGAAGAGTCCCTTTC
        131 TGATGTTTGCATTCAAGTAACAGTGTTGAACATTCCCTTTC

                                                         *       **   * *    
      79114 ATAGAGCAGGTTTGAAACACTCTTTTTGTAGTATCTGGAAGTGGACATTTGGAGCGCTCTCAGGA
          1 ATAGAGCAGGTTTGAAACACTCTTTTTGTAGTATCTGGAAGTGGATATTTGGATAGCTTTGAGGA

            * *  *      *             **            *       * *          *  *
      79179 CTGCGGT-GAAAAAGGAAGTATCTTCCAATAAAAGCTAGAGAGAAGCAATGTCAGAAACTTTTTC
         66 TTTCGTTGGAAACAGGAA-TATCTTCATATAAAAGCTAGACAGAAGCATTCTCAGAAACTTCTTT

            *      * ** *   **      *       *  * *   *
      79243 ATGATGTATCTACTCAGCTAACAGAGTTGAACCTTTCTTTTG
        130 GTGATGTTTGCATTCAAGTAACAGTGTTGAACATTCCCTTTC

             *       *            *      * *  *  *             **         *  
      79285 AGAGAGCAGTTTTGAAACACTCCTTTTGTGGAATATGCAAGTGGATATTTGTCTAGCTTTGAAGA
          1 ATAGAGCAGGTTTGAAACACTCTTTTTGTAGTATCTGGAAGTGGATATTTGGATAGCTTTGAGGA

                         *   *    *                   *       *              
      79350 TTTCGTTGGAAACGGGATTA-CAT-ATA-AAAAGC-AGACAGCAGCATTCCCAGAAACTTCTTTG
         66 TTTCGTTGGAAACAGGAATATCTTCATATAAAAGCTAGACAGAAGCATTCTCAGAAACTTCTTTG

                              *    *                 
      79411 TGATGTTTGCATTCAAGTCACAGAGTTGAACATTCCCTTTC
        131 TGATGTTTGCATTCAAGTAACAGTGTTGAACATTCCCTTTC

                                                   *     *       **     *   *
      79452 ATAGAGCAGGTTTGAAACACTCTTTTTGTAGTATCTGGATGTGGACATTTGGAGCGCTTTCAGGC
          1 ATAGAGCAGGTTTGAAACACTCTTTTTGTAGTATCTGGAAGTGGATATTTGGATAGCTTTGAGGA

            * ** *      *             *** *   *                      *   *   
      79517 CTATGGT-GAAAAAGGAAATATCTTCCCCTGAAAACTAGACAGAAGCATTCTCAGAATCTTATTT
         66 TTTCGTTGGAAACAGG-AATATCTTCATATAAAAGCTAGACAGAAGCATTCTCAGAAACTTCTTT

                   **  **    *         *   **  * *   *
      79581 GTGATGTGCGCCCTCAACTAACAGTGTAGAAGCTTTCTTTTG
        130 GTGATGTTTGCATTCAAGTAACAGTGTTGAACATTCCCTTTC

                     *                    **     *   *                       
      79623 ATAGAGCAGTTTTGAAACACTCTTTTTGTAAAATCTGCAAGAGGATATTTGGATAGCTTTGAGGA
          1 ATAGAGCAGGTTTGAAACACTCTTTTTGTAGTATCTGGAAGTGGATATTTGGATAGCTTTGAGGA

                         *   * *            **                      *    *   
      79688 TTTCGTTGGAAACGGGATTGTCTTCATATAAACTCTAGACAGAAGCATTCTCAGAAGCTTCATTG
         66 TTTCGTTGGAAACAGGAATATCTTCATATAAAAGCTAGACAGAAGCATTCTCAGAAACTTCTTTG

            *       **   *    *             *  *     
      79753 GGATGTTTCAATTGAAGTCACAGTGTTGAACAGTCACTTTC
        131 TGATGTTTGCATTCAAGTAACAGTGTTGAACATTCCCTTTC

                                                   *     *       **     *   *
      79794 ATAGAGCAGGTTTGAAACACTCTTTTTGTAGTATCTGGATGTGGACATTTGGAGCGCTTTCAGGC
          1 ATAGAGCAGGTTTGAAACACTCTTTTTGTAGTATCTGGAAGTGGATATTTGGATAGCTTTGAGGA

            * ** *      *             *** *   *                          *   
      79859 CTATGGT-GAAAAAGGAAATATCTTCCCCTGAAAACTAGACAGAAGCATTCTCAGAAACTTATTT
         66 TTTCGTTGGAAACAGG-AATATCTTCATATAAAAGCTAGACAGAAGCATTCTCAGAAACTTCTTT

                   **  **    *             **  * *   *
      79923 GTGATGTGCGCCCTCAACTAACAGTGTTGAAGTTTTCTTTTG
        130 GTGATGTTTGCATTCAAGTAACAGTGTTGAACATTCCCTTTC

                    **                   * *     * *           **            
      79965 ATAGAGCATTTTTGAAACACTCTTTTTGTGGAATCTGCATGTGGATATTTGTCTAGCTTTGAGGA
          1 ATAGAGCAGGTTTGAAACACTCTTTTTGTAGTATCTGGAAGTGGATATTTGGATAGCTTTGAGGA

                         *   *    *                   *       *     *        
      80030 TTTCGTTGGAAACGGGATTA-CAT-ATA-AAAAGC-AGACAGCAGCATTCCCAGAATCTTCTTTG
         66 TTTCGTTGGAAACAGGAATATCTTCATATAAAAGCTAGACAGAAGCATTCTCAGAAACTTCTTTG

                              *    *                 
      80091 TGATGTTTGCATTCAAGTCACAGAGTTGAACATTCCCTTTC
        131 TGATGTTTGCATTCAAGTAACAGTGTTGAACATTCCCTTTC

                                                   *     *       **     *   *
      80132 ATAGAGCAGGTTTGAAACACTCTTTTTGTAGTATCTGGATGTGGACATTTGGAGCGCTTTCAGGC
          1 ATAGAGCAGGTTTGAAACACTCTTTTTGTAGTATCTGGAAGTGGATATTTGGATAGCTTTGAGGA

            * ** *      *             *** *   *                          *   
      80197 CTATGGT-GAAAAAGGAAATATCTTCCCCTGAAAACTAGACAGAAGCATTCTCAGAAACTTATTT
         66 TTTCGTTGGAAACAGG-AATATCTTCATATAAAAGCTAGACAGAAGCATTCTCAGAAACTTCTTT

                   **  **    *             **  * *   *
      80261 GTGATGTGCGCCCTCAACTAACAGTGTTGAAGCTTTCTTTTG
        130 GTGATGTTTGCATTCAAGTAACAGTGTTGAACATTCCCTTTC

                     *                    **     *   *                       
      80303 ATAGAGCAGTTTTGAAACACTCTTTTTGTAAAATCTGCAAGAGGATATTTGGATAGCTTTGAGGA
          1 ATAGAGCAGGTTTGAAACACTCTTTTTGTAGTATCTGGAAGTGGATATTTGGATAGCTTTGAGGA

                         *   * *            **                      *    *   
      80368 TTTCGTTGGAAACGGGATTGTCTTCATATAAACTCTAGACAGAAGCATTCTCAGAAGCTTCATTG
         66 TTTCGTTGGAAACAGGAATATCTTCATATAAAAGCTAGACAGAAGCATTCTCAGAAACTTCTTTG

            *       **   *    *             * *      
      80433 GGATGTTTCAATTGAAGTCACAGTGTTGAACAGTTCCTTTC
        131 TGATGTTTGCATTCAAGTAACAGTGTTGAACATTCCCTTTC

                                                         *       **   * *    
      80474 ATAGAGCAGGTTTGAAACACTCTTTTTGTAGTATCTGGAAGTGGACATTTGGAGCGCTCTCAGGA
          1 ATAGAGCAGGTTTGAAACACTCTTTTTGTAGTATCTGGAAGTGGATATTTGGATAGCTTTGAGGA

            * *  *      *             **          * *       ***          *  *
      80539 CTACGGT-GAAAAAGGAAATATCTTCCAATAAAAGCTACATAGAAGCAAGGTCAGAAACTTTTTC
         66 TTTCGTTGGAAACAGG-AATATCTTCATATAAAAGCTAGACAGAAGCATTCTCAGAAACTTCTTT

            *      * ** *   **      *       *  *     *
      80603 ATGATGTATCTACTCAGCTAACAGAGTTGAACCTTTCCTTTG
        130 GTGATGTTTGCATTCAAGTAACAGTGTTGAACATTCCCTTTC

             **      *        *          * *     *             **            
      80645 AGGGAGCAGTTTTGAAACTCTCTTTTTGTGGAATCTGCAAGTGGATATTTGTCTAGCTTTGAGGA
          1 ATAGAGCAGGTTTGAAACACTCTTTTTGTAGTATCTGGAAGTGGATATTTGGATAGCTTTGAGGA

                         *   *    *                   *       *   *          
      80710 TTTCGTTGGAAACGGGATTA-CAT-ATA-AAAAGC-AGACAGCAGCATTCCCAGTAACTTCTTTG
         66 TTTCGTTGGAAACAGGAATATCTTCATATAAAAGCTAGACAGAAGCATTCTCAGAAACTTCTTTG

                              *    *                 
      80771 TGATGTTTGCATTCAAGTCACAGAGTTGAACATTCCCTTTC
        131 TGATGTTTGCATTCAAGTAACAGTGTTGAACATTCCCTTTC

                                                   *     *       **     *   *
      80812 ATAGAGCAGGTTTGAAACACTCTTTTTGTAGTATCTGGATGTGGACATTTGGAGCGCTTTCAGGC
          1 ATAGAGCAGGTTTGAAACACTCTTTTTGTAGTATCTGGAAGTGGATATTTGGATAGCTTTGAGGA

            * ** *      *             *** *   *                          *   
      80877 CTATGGT-GAAAAAGGAAATATCTTCCCCTGAAAACTAGACAGAAGCATTCTCAGAAACTTATTT
         66 TTTCGTTGGAAACAGG-AATATCTTCATATAAAAGCTAGACAGAAGCATTCTCAGAAACTTCTTT

                   **  **    *             **  * *   *
      80941 GTGATGTGCGCCCTCAACTAACAGTGTTGAAGCTTTCTTTTG
        130 GTGATGTTTGCATTCAAGTAACAGTGTTGAACATTCCCTTTC

                     *                    **     *   *                       
      80983 ATAGAGCAGTTTTGAAACACTCTTTTTGTAAAATCTGCAAGAGGATATTTGGATAGCTTTGAGGA
          1 ATAGAGCAGGTTTGAAACACTCTTTTTGTAGTATCTGGAAGTGGATATTTGGATAGCTTTGAGGA

                         *   * *            **                      *    *   
      81048 TTTCGTTGGAAACGGGATTGTCTTCATATAAACTCTAGACAGAAGCATTCTCAGAAGCTTCATTG
         66 TTTCGTTGGAAACAGGAATATCTTCATATAAAAGCTAGACAGAAGCATTCTCAGAAACTTCTTTG

            *       **   *    *             * *      
      81113 GGATGTTTCAATTGAAGTCACAGTGTTGAACAGTTCCTTTC
        131 TGATGTTTGCATTCAAGTAACAGTGTTGAACATTCCCTTTC

                                                         *       **   * *    
      81154 ATAGAGCAGGTTTGAAACACTCTTTTTGTAGTATCTGGAAGTGGACATTTGGAGCGCTCTCAGGA
          1 ATAGAGCAGGTTTGAAACACTCTTTTTGTAGTATCTGGAAGTGGATATTTGGATAGCTTTGAGGA

            * *  *      *             **            *       * *          *  *
      81219 CTACGGT-GAAAAAGGAAATATCTTCCAATAAAAGCTAGATAGAAGCAATGTCAGAAACTTTTTC
         66 TTTCGTTGGAAACAGG-AATATCTTCATATAAAAGCTAGACAGAAGCATTCTCAGAAACTTCTTT

            *      * ** *   **      *       *  * *   *
      81283 ATGATGTATCTACTCAGCTAACAGAGTTGAACCTTTCTTTTG
        130 GTGATGTTTGCATTCAAGTAACAGTGTTGAACATTCCCTTTC

             *     * *                   * *     *             **            
      81325 AGAGAGCCGTTTTGAAACACTCTTTTTGTGGAATCTGCAAGTGGATATTTGTCTAGCTTTGAGGA
          1 ATAGAGCAGGTTTGAAACACTCTTTTTGTAGTATCTGGAAGTGGATATTTGGATAGCTTTGAGGA

                         *   *    *      *            *       *     *        
      81390 TTTCGTTGGAAACGGGATTAT-AT-ATA-GAAAGC-AGACAGCAGCATTCCCAGAATCTTCTTTG
         66 TTTCGTTGGAAACAGGAATATCTTCATATAAAAGCTAGACAGAAGCATTCTCAGAAACTTCTTTG

                              *    *                 
      81451 TGATGTTTGCATTCAAGTCACAGAGTTGAACATTCCCTTTC
        131 TGATGTTTGCATTCAAGTAACAGTGTTGAACATTCCCTTTC

                                                   *     *       **     *   *
      81492 ATAGAGCAGGTTTGAAACACTCTTTTTGTAGTATCTGGATGTGGACATTTGGAGCGCTTTCAGGC
          1 ATAGAGCAGGTTTGAAACACTCTTTTTGTAGTATCTGGAAGTGGATATTTGGATAGCTTTGAGGA

            * ** *      *             *** *   *                      *   *   
      81557 CTATGGT-GAAAAAGGAAATATCTTCCCCTGAAAACTAGACAGAAGCATTCTCAGAATCTTATTT
         66 TTTCGTTGGAAACAGG-AATATCTTCATATAAAAGCTAGACAGAAGCATTCTCAGAAACTTCTTT

                   **  **    *             **  * *   *
      81621 GTGATGTGCGCCCTCAACTAACAGTGTTGAAGCTTTCTTTTG
        130 GTGATGTTTGCATTCAAGTAACAGTGTTGAACATTCCCTTTC

                     *                    **     *   *                       
      81663 ATAGAGCAGTTTTGAAACACTCTTTTTGTAAAATCTGCAAGAGGATATTTGGATAGCTTTGAGGA
          1 ATAGAGCAGGTTTGAAACACTCTTTTTGTAGTATCTGGAAGTGGATATTTGGATAGCTTTGAGGA

                *   *    *   * *            **                      *    *   
      81728 TTTCATTGCAAACGGGATTGTCTTCATATAAACTCTAGACAGAAGCATTCTCAGAAGCTTCATTG
         66 TTTCGTTGGAAACAGGAATATCTTCATATAAAAGCTAGACAGAAGCATTCTCAGAAACTTCTTTG

            *       **   *    *             *        
      81793 GGATGTTTCAATTGAAGTCACAGTGTTGAACAGTCCCTTTC
        131 TGATGTTTGCATTCAAGTAACAGTGTTGAACATTCCCTTTC

                                      *                  *       **   * *    
      81834 ATAGAGCAGGTTTGAAACACTCTTTTGGTAGTATCTGGAAGTGGACATTTGGAGCGCTCTCAGGA
          1 ATAGAGCAGGTTTGAAACACTCTTTTTGTAGTATCTGGAAGTGGATATTTGGATAGCTTTGAGGA

            * *  *      *             **            *       * *          *  *
      81899 CTGCGGT-GAAAAAGGAAATATCTTCCAATAAAAGCTAGATAGAAGCAATGTCAGAAACTTTTTC
         66 TTTCGTTGGAAACAGG-AATATCTTCATATAAAAGCTAGACAGAAGCATTCTCAGAAACTTCTTT

            *      * ** *   **      *       *  * *   *
      81963 ATGATGTATCTACTCAGCTAACAGAGTTGAACCTTTCTTTTG
        130 GTGATGTTTGCATTCAAGTAACAGTGTTGAACATTCCCTTTC

             *       *                   * *     *             **            
      82005 AGAGAGCAGTTTTGAAACACTCTTTTTGTGGAATCTGCAAGTGGATATTTGTCTAGCTTTGAGGA
          1 ATAGAGCAGGTTTGAAACACTCTTTTTGTAGTATCTGGAAGTGGATATTTGGATAGCTTTGAGGA

                         *   *    *                   *       *              
      82070 TTTCGTTGGAAACGGGATTA-CAT-ATA-AAAAGC-AGACAGCAGCATTCCCAGAAACTTCTTTG
         66 TTTCGTTGGAAACAGGAATATCTTCATATAAAAGCTAGACAGAAGCATTCTCAGAAACTTCTTTG

               *              *    *                 
      82131 TGACGTTTGCATTCAAGTCACAGAGTTGAACATTCCCTTTC
        131 TGATGTTTGCATTCAAGTAACAGTGTTGAACATTCCCTTTC

                                                 * *     *       **     *  **
      82172 ATAGAGCAGGTTTGAAACACTCTTTTTGTAGTATCTGTATGTGGACATTTGGAGCGCTTTCAGTC
          1 ATAGAGCAGGTTTGAAACACTCTTTTTGTAGTATCTGGAAGTGGATATTTGGATAGCTTTGAGGA

            * ** *      *             *** *   *                          *   
      82237 CTATGGT-GAAAAAGGAAATATCTTCCCCTGAAAACTAGACAGAAGCATTCTCAGAAACTTATTT
         66 TTTCGTTGGAAACAGG-AATATCTTCATATAAAAGCTAGACAGAAGCATTCTCAGAAACTTCTTT

                   **  **    *             **  * *   *
      82301 GTGATGTGCGCCCTCAACTAACAGTGTTGAAGCTTTCTTTTG
        130 GTGATGTTTGCATTCAAGTAACAGTGTTGAACATTCCCTTTC

                     *                    **     *   *                       
      82343 ATAGAGCAGTTTTGAAACACTCTTTTTGTAAAATCTGCAAGAGGATATTTGGATAGCTTTGAGGA
          1 ATAGAGCAGGTTTGAAACACTCTTTTTGTAGTATCTGGAAGTGGATATTTGGATAGCTTTGAGGA

                         *   * *            **                      *    *   
      82408 TTTCGTTGGAAACGGGACTGTCTTCATATAAACTCTAGACAGAAGCATTCTCAGAAGCTTCATTG
         66 TTTCGTTGGAAACAGGAATATCTTCATATAAAAGCTAGACAGAAGCATTCTCAGAAACTTCTTTG

            *       **   *    *             *        
      82473 GGATGTTTCAATTGAAGTCACAGTGTTGAACAGTCCCTTTC
        131 TGATGTTTGCATTCAAGTAACAGTGTTGAACATTCCCTTTC

                                      *                  *       **   * *    
      82514 ATAGAGCAGGTTTGAAACACTCTTTTGGTAGTATCTGGAAGTGGACATTTGGAGCGCTCTCAGGA
          1 ATAGAGCAGGTTTGAAACACTCTTTTTGTAGTATCTGGAAGTGGATATTTGGATAGCTTTGAGGA

            * *  *      *             **            *       * *          *  *
      82579 CTGCGGT-GAAAAAGGAAATATCTTCCAATAAAAGCTAGATAGAAGCAATGTCAGAAACTTTTTC
         66 TTTCGTTGGAAACAGG-AATATCTTCATATAAAAGCTAGACAGAAGCATTCTCAGAAACTTCTTT

            *      * ** *   **      *       *  * *   *
      82643 ATGATGTATCTACTCAGCTAACAGAGTTGAACCTTTCTTTTG
        130 GTGATGTTTGCATTCAAGTAACAGTGTTGAACATTCCCTTTC

             *       *                   * *     *             **            
      82685 AGAGAGCAGTTTTGAAACACTCTTTTTGTGGAATCTGCAAGTGGATATTTGTCTAGCTTTGAGGA
          1 ATAGAGCAGGTTTGAAACACTCTTTTTGTAGTATCTGGAAGTGGATATTTGGATAGCTTTGAGGA

                         *   *    *                   *       *   *          
      82750 TTTCGTTGGAAACGGGATTA-CAT-ATA-AAAAGC-AGACAGCAGCATTCCCAGTAACTTCTTTG
         66 TTTCGTTGGAAACAGGAATATCTTCATATAAAAGCTAGACAGAAGCATTCTCAGAAACTTCTTTG

                              *    *                 
      82811 TGATGTTTGCATTCAAGTCACAGAGTTGAACATTCCCTTTC
        131 TGATGTTTGCATTCAAGTAACAGTGTTGAACATTCCCTTTC

                               *                   *     *       **     *   *
      82852 ATAGAGCAGGTTTGAAACAGTCTTTTTGTAGTATCTGGATGTGGACATTTGGAGCGCTTTCAGGC
          1 ATAGAGCAGGTTTGAAACACTCTTTTTGTAGTATCTGGAAGTGGATATTTGGATAGCTTTGAGGA

            * ** *      *             *** *   *                      *   *   
      82917 CTATGGT-GAAAAAGGAAATATCTTCCCCTGAAAACTAGACAGAAGCATTCTCAGAATCTTATTT
         66 TTTCGTTGGAAACAGG-AATATCTTCATATAAAAGCTAGACAGAAGCATTCTCAGAAACTTCTTT

                   **  **    *             **  * *   *
      82981 GTGATGTGCGCCCTCAACTAACAGTGTTGAAGCTTTCTTTTG
        130 GTGATGTTTGCATTCAAGTAACAGTGTTGAACATTCCCTTTC

                     *                    **     *   *                       
      83023 ATAGAGCAGTTTTGAAACACTCTTTTTGTAAAATCTGCAAGAGGATATTTGGATAGCTTTGAGGA
          1 ATAGAGCAGGTTTGAAACACTCTTTTTGTAGTATCTGGAAGTGGATATTTGGATAGCTTTGAGGA

                         *   * *            **                      *    *   
      83088 TTTCGTTGGAAACGGGATTGTCTTCATATAAACTCTAGACAGAAGCATTCTCAGAAGCTTCATTG
         66 TTTCGTTGGAAACAGGAATATCTTCATATAAAAGCTAGACAGAAGCATTCTCAGAAACTTCTTTG

            *       **   *    *             *        
      83153 GGATGTTTCAATTGAAGTCACAGTGTTGAACAGTCCCTTTC
        131 TGATGTTTGCATTCAAGTAACAGTGTTGAACATTCCCTTTC

                                                         *       **   * *    
      83194 ATAGAGCAGGTTTGAAACACTCTTTTTGTAGTATCTGGAAGTGGACATTTGGAGCGCTCTCAGGA
          1 ATAGAGCAGGTTTGAAACACTCTTTTTGTAGTATCTGGAAGTGGATATTTGGATAGCTTTGAGGA

            * *  *      *             **          * *       * *          *  *
      83259 CTACGGT-GAAAAAGGAATTATCTTCCAATAAAAGCTACATAGAAGCAATGTCAGAAACTTTTTC
         66 TTTCGTTGGAAACAGGAA-TATCTTCATATAAAAGCTAGACAGAAGCATTCTCAGAAACTTCTTT

            *      * ** *   **      *       *  * *   *
      83323 ATGATGTATCTACTCAGCTAACAGAGTTGAACCTTTCTTTTG
        130 GTGATGTTTGCATTCAAGTAACAGTGTTGAACATTCCCTTTC

             *       *                   * *     *             **     *      
      83365 AGAGAGCAGTTTTGAAACACTCTTTTTGTGGAATCTGCAAGTGGATATTTGTCTAGCTGTGAGGA
          1 ATAGAGCAGGTTTGAAACACTCTTTTTGTAGTATCTGGAAGTGGATATTTGGATAGCTTTGAGGA

                         *   *    *                   *       *              
      83430 TTTCGTTGGAAACGGGATTA-CAT-ATA-AAAAGC-AGACAGCAGCATTCCCAGAAACTTCTTTG
         66 TTTCGTTGGAAACAGGAATATCTTCATATAAAAGCTAGACAGAAGCATTCTCAGAAACTTCTTTG

                              *    *                 
      83491 TGATGTTTGCATTCAAGTCACAGAGTTGAACATTCCCTTTC
        131 TGATGTTTGCATTCAAGTAACAGTGTTGAACATTCCCTTTC

                                                   *     *       *** *  *   *
      83532 ATAGAGCAGGTTTGAAACACTCTTTTTGTAGTATCTGGATGTGGACATTTGGAGCACGTTCAGGC
          1 ATAGAGCAGGTTTGAAACACTCTTTTTGTAGTATCTGGAAGTGGATATTTGGATAGCTTTGAGGA

            * ** *  *                 *** *   *                      *   *   
      83597 CTATGGTGTAAA-AGGAAATATCTTCCCCTGAAAACTAGACAGAAGCATTCTCAGAATCTTATTT
         66 TTTCGTTGGAAACAGG-AATATCTTCATATAAAAGCTAGACAGAAGCATTCTCAGAAACTTCTTT

                   **  **    *             **  * *   *
      83661 GTGATGTGCGCCCTCAACTAACAGTGTTGAAGCTTTCTTTTG
        130 GTGATGTTTGCATTCAAGTAACAGTGTTGAACATTCCCTTTC

                     *                    **     *   *              *        
      83703 ATAGAGCAGTTTTGAAACACTCTTTTTGTAAAATCTGCAAGAGGATATTTGGATAGATTTGAGGA
          1 ATAGAGCAGGTTTGAAACACTCTTTTTGTAGTATCTGGAAGTGGATATTTGGATAGCTTTGAGGA

                         *   * *            **                      *    *   
      83768 TTTCGTTGGAAACGGGATTGTCTTCATATAAACTCTAGACAGAAGCATTCTCAGAAGCTTCATTG
         66 TTTCGTTGGAAACAGGAATATCTTCATATAAAAGCTAGACAGAAGCATTCTCAGAAACTTCTTTG

            *       **   *    *             *        
      83833 GGATGTTTCAATTGAAGTCACAGTGTTGAACAGTCCCTTTC
        131 TGATGTTTGCATTCAAGTAACAGTGTTGAACATTCCCTTTC

                                                   *     *       **     *   *
      83874 ATAGAGCAGGTTTGAAACACTCTTTTTGTAGTATCTGGATGTGGACATTTGGAGCGCTTTCAGGC
          1 ATAGAGCAGGTTTGAAACACTCTTTTTGTAGTATCTGGAAGTGGATATTTGGATAGCTTTGAGGA

            * ** *      *             *** *   *                          *   
      83939 CTATGGT-GAAAAAGGAAATATCTTCCCCTGAAAACTAGACAGAAGCATTCTCAGAAACTTATTT
         66 TTTCGTTGGAAACAGG-AATATCTTCATATAAAAGCTAGACAGAAGCATTCTCAGAAACTTCTTT

                   **  **    *             **  * *   *
      84003 GTGATGTGCGCCCTCAACTAACAGTGTTGAAGCTTTCTTTTG
        130 GTGATGTTTGCATTCAAGTAACAGTGTTGAACATTCCCTTTC

                     *                   ***     *             **            
      84045 ATAGAGCAGTTTTGAAACACTCTTTTTGTGCAATCTGCAAGTGGATATTTGTCTAGCTTTGAGGA
          1 ATAGAGCAGGTTTGAAACACTCTTTTTGTAGTATCTGGAAGTGGATATTTGGATAGCTTTGAGGA

                         *   *    *         *         *       *     *        
      84110 TTTCGTTGGAAACGGGATTA-CAT-ATA-AAATGC-AGACAGCAGCATTCCCAGAATCTTCTTTG
         66 TTTCGTTGGAAACAGGAATATCTTCATATAAAAGCTAGACAGAAGCATTCTCAGAAACTTCTTTG

                              *    *                 
      84171 TGATGTTTGCATTCAAGTCACAGAGTTGAACATTCCCTTTC
        131 TGATGTTTGCATTCAAGTAACAGTGTTGAACATTCCCTTTC

                                       *        *  *     *       **     *   *
      84212 ATAGAGCAGGTTTGAAACACTCTTTTTATAGTATCTTGATGTGGACATTTGGAGCGCTTTCAGGC
          1 ATAGAGCAGGTTTGAAACACTCTTTTTGTAGTATCTGGAAGTGGATATTTGGATAGCTTTGAGGA

            **** *      *             *** *   *                      *   *   
      84277 CCATGGT-GAAAAAGGAAATATCTTCTCCTGAAAACTAGACAGAAGCATTCTCAGAATCTTATTT
         66 TTTCGTTGGAAACAGG-AATATCTTCATATAAAAGCTAGACAGAAGCATTCTCAGAAACTTCTTT

                   **  **    *             **  * *   *
      84341 GTGATGTGCGCCCTCAACTAACAGTGTTGAAGCTTTCTTTTG
        130 GTGATGTTTGCATTCAAGTAACAGTGTTGAACATTCCCTTTC

            *        *                    **     *   *                *      
      84383 TTAGAGCAGTTTTGAAACACTCTTTTTGTAAAATCTGCAAGAGGATATTTGGATAGCTATGAGGA
          1 ATAGAGCAGGTTTGAAACACTCTTTTTGTAGTATCTGGAAGTGGATATTTGGATAGCTTTGAGGA

                *        *   * *            **                      *    *   
      84448 TTTCATTGGAAACGGGATTGTCTTCATATAAACTCTAGACAGAAGCATTCTCAGAAGCTTCATTG
         66 TTTCGTTGGAAACAGGAATATCTTCATATAAAAGCTAGACAGAAGCATTCTCAGAAACTTCTTTG

            *       **   *    *             *        
      84513 GGATGTTTCAATTGAAGTCACAGTGTTGAACAGTCCCTTTC
        131 TGATGTTTGCATTCAAGTAACAGTGTTGAACATTCCCTTTC

                                                         *       **   * *    
      84554 ATAGAGCAGGTTTGAAACACTCTTTTTGTAGTATCTGGAAGTGGACATTTGGAGCGCTCTCAGGA
          1 ATAGAGCAGGTTTGAAACACTCTTTTTGTAGTATCTGGAAGTGGATATTTGGATAGCTTTGAGGA

            * *  *      *             **          * *       * *          *  *
      84619 CTACGGT-GAAAAAGGAAATATCTTCCAATAAAAGCTACATAGAAGCAATGTCAGAAACTTTTTC
         66 TTTCGTTGGAAACAGG-AATATCTTCATATAAAAGCTAGACAGAAGCATTCTCAGAAACTTCTTT

            *      * ** *   **      *       *  * *   *
      84683 ATGATGTATCTACTCAGCTAACAGAGTTGAACCTTTCTTTTG
        130 GTGATGTTTGCATTCAAGTAACAGTGTTGAACATTCCCTTTC

             *       *                   * *                   **            
      84725 AGAGAGCAGTTTTGAAACACTCTTTTTGTGGAATCTGGAAGTGGATATTTGTCTAGCTTTGAGGA
          1 ATAGAGCAGGTTTGAAACACTCTTTTTGTAGTATCTGGAAGTGGATATTTGGATAGCTTTGAGGA

                         *   *    *                   *       *   *          
      84790 TTTCGTTGGAAACGGGATTA-CAT-ATA-AAAAGC-AGACAGCAGCATTCCCAGTAACTTCTTTG
         66 TTTCGTTGGAAACAGGAATATCTTCATATAAAAGCTAGACAGAAGCATTCTCAGAAACTTCTTTG

                              *    *                 
      84851 TGATGTTTGCATTCAAGTCACAGAGTTGAACATTCCCTTTC
        131 TGATGTTTGCATTCAAGTAACAGTGTTGAACATTCCCTTTC

                                               *   *     *       **     *   *
      84892 ATAGAGCAGGTTTGAAACACTCTTTTTGTAGTATCAGGATGTGGACATTTGGAGCGCTTTCAGGC
          1 ATAGAGCAGGTTTGAAACACTCTTTTTGTAGTATCTGGAAGTGGATATTTGGATAGCTTTGAGGA

            * ** *      *             *** *   *                      *   *   
      84957 CTATGGT-GAAAAAGGAAATATCTTCCCCTGAAAACTAGACAGAAGCATTCTCAGAATCTTATTT
         66 TTTCGTTGGAAACAGG-AATATCTTCATATAAAAGCTAGACAGAAGCATTCTCAGAAACTTCTTT

                   **  **    *             **  * *   *
      85021 GTGATGTGCGCCCTCAACTAACAGTGTTGAAGCTTTCTTTTG
        130 GTGATGTTTGCATTCAAGTAACAGTGTTGAACATTCCCTTTC

                     *                    **     *   *                       
      85063 ATAGAGCAGTTTTGAAACACTCTTTTTGTAAAATCTGCAAGAGGATATTTGGATAGCTTTGAGGA
          1 ATAGAGCAGGTTTGAAACACTCTTTTTGTAGTATCTGGAAGTGGATATTTGGATAGCTTTGAGGA

                         *   * *            **                      *    *   
      85128 TTTCGTTGG-AACGGGATTGTCTTCATATAAACTCTAGACAGAAGCATTCTCAGAAGCTTCATTG
         66 TTTCGTTGGAAACAGGAATATCTTCATATAAAAGCTAGACAGAAGCATTCTCAGAAACTTCTTTG

            *       **   *    *             *        
      85192 GGATGTTTCAATTGAAGTCACAGTGTTGAACAGTCCCTTTC
        131 TGATGTTTGCATTCAAGTAACAGTGTTGAACATTCCCTTTC

                                                          *       *  * * *   
      85233 ATAGAGCAGGTTTAGAAACACTCTTTTTGTAGTATCTGGAAGTGGACATTTGGAGAGATCTCAGG
          1 ATAGAGCAGGTTT-GAAACACTCTTTTTGTAGTATCTGGAAGTGGATATTTGGATAGCTTTGAGG

             * *  *      *             **            *     * * *          *  
      85298 ACTACGGT-GAAAAAGGAAATATCTTCCAATAAAAGCTAGATAGAAGAAATGTCAGAAACTTTTT
         65 ATTTCGTTGGAAACAGG-AATATCTTCATATAAAAGCTAGACAGAAGCATTCTCAGAAACTTCTT

            **      * ** *   **      *       *  * *   *
      85362 CATGATGTATCTACTCAGCTAACAGAGTTGAACCTTTCTTTTG
        129 TGTGATGTTTGCATTCAAGTAACAGTGTTGAACATTCCCTTTC

             *       *                   * *  *  *             **            
      85405 AGAGAGCAGTTTTGAAACACTCTTTTTGTGGAATATGCAAGTGGATATTTGTCTAGCTTTGAGGA
          1 ATAGAGCAGGTTTGAAACACTCTTTTTGTAGTATCTGGAAGTGGATATTTGGATAGCTTTGAGGA

                         *   *    *                   *       *              
      85470 TTTCGTTGGAAACGGGATTA-CAT-ATA-AAAAGC-AGACAGCAGCATTCCCAGAAACTTCTTTG
         66 TTTCGTTGGAAACAGGAATATCTTCATATAAAAGCTAGACAGAAGCATTCTCAGAAACTTCTTTG

                              *    *                 
      85531 TGATGTTTGCATTCAAGTCACAGAGTTGAACATTCCCTTTC
        131 TGATGTTTGCATTCAAGTAACAGTGTTGAACATTCCCTTTC

                                                   *     *       ***    *   *
      85572 ATAGAGCAGGTTTGAAACACTCTTTTTGTAGTATCTGGATGTGGACATTTGGAGCACTTTCAGGC
          1 ATAGAGCAGGTTTGAAACACTCTTTTTGTAGTATCTGGAAGTGGATATTTGGATAGCTTTGAGGA

            * ** *      *             *****   *                      **  *   
      85637 CTATGGT-GAAAAAGGAAATATCTTCCCCCGAAAACTAGACAGAAGCATTCTCAGAATGTTATTT
         66 TTTCGTTGGAAACAGG-AATATCTTCATATAAAAGCTAGACAGAAGCATTCTCAGAAACTTCTTT

                   **  **    *             **  * *   *
      85701 GTGATGTGCGCCCTCAACTAACAGTGTTGAAGCTTTCTTTTG
        130 GTGATGTTTGCATTCAAGTAACAGTGTTGAACATTCCCTTTC

                     *                    **     *   *                       
      85743 ATAGAGCAGTTTTGAAACACTCTTTTTGTAAAATCTGCAAGAGGATATTTGGATAGCTTTGAGGA
          1 ATAGAGCAGGTTTGAAACACTCTTTTTGTAGTATCTGGAAGTGGATATTTGGATAGCTTTGAGGA

                         *   * *            **                      *    *   
      85808 TTTCGTTGGAAACGGGATTGTCTTCATATAAACTCTAGACAGAAGCATTCTCAGAAGCTTCATTG
         66 TTTCGTTGGAAACAGGAATATCTTCATATAAAAGCTAGACAGAAGCATTCTCAGAAACTTCTTTG

            *       **   *    *        *    *        
      85873 GGATGTTTCAATTGAAGTCACAGTGTTTAACAGTCCCTTTC
        131 TGATGTTTGCATTCAAGTAACAGTGTTGAACATTCCCTTTC

                                *                        *      ***   * *    
      85914 ATAGAGCAGGTTTGAAACACACTTTTTGTAGTATCTGGAAGTGGACATTTGGGGCGCTCTCAGGA
          1 ATAGAGCAGGTTTGAAACACTCTTTTTGTAGTATCTGGAAGTGGATATTTGGATAGCTTTGAGGA

            * *  *      *             **            *       * *          *  *
      85979 CTACGGT-GAAAAAGGAAATATCTTCCAATAAAAGCTAGATAGAAGCAATGTCAGAAACTTTTTC
         66 TTTCGTTGGAAACAGG-AATATCTTCATATAAAAGCTAGACAGAAGCATTCTCAGAAACTTCTTT

            *      * ** *   **      **      *  * *   *
      86043 ATGATGTATCTACTCAGCTAACAGATTTGAACCTTTCTTTTG
        130 GTGATGTTTGCATTCAAGTAACAGTGTTGAACATTCCCTTTC

             *       *   *               * *  *  *             **            
      86085 AGAGAGCAGTTTTAAAACACTCTTTTTGTGGAATATGCAAGTGGATATTTGTCTAGCTTTGAGGA
          1 ATAGAGCAGGTTTGAAACACTCTTTTTGTAGTATCTGGAAGTGGATATTTGGATAGCTTTGAGGA

                *            *    *                   *       *     *   *    
      86150 TTTCTTTGGAAACAGGATTA-CAT-ATA-AAAAGC-AGACAGCAGCATTCCCAGAATCTTGTTTG
         66 TTTCGTTGGAAACAGGAATATCTTCATATAAAAGCTAGACAGAAGCATTCTCAGAAACTTCTTTG

                     *        *    *    *            
      86211 TGATGTTTGTATTCAAGTCACAGGGTTGTACATTCCCTTTC
        131 TGATGTTTGCATTCAAGTAACAGTGTTGAACATTCCCTTTC

             *                         *   *       *     *       **     *   *
      86252 AGAGAGCAGGTTTGAAACACTCTTTTTATAGAATCTGGATGTGGACATTTGGAGCGCTTTCAGGC
          1 ATAGAGCAGGTTTGAAACACTCTTTTTGTAGTATCTGGAAGTGGATATTTGGATAGCTTTGAGGA

            * ** *      *             *** *   *                      *   *   
      86317 CTATGGT-GAAAAAGGAAATATCTTCTCCTGAAAACTAGACAGAAGCATTCTCAGAATCTTATTT
         66 TTTCGTTGGAAACAGG-AATATCTTCATATAAAAGCTAGACAGAAGCATTCTCAGAAACTTCTTT

                   **  **    *             **  * *   *
      86381 GTGATGTGCGCCCTCAACTAACAGTGTTGAAGCTTTCTTTTG
        130 GTGATGTTTGCATTCAAGTAACAGTGTTGAACATTCCCTTTC

                     *                    **     *   *                       
      86423 ATAGAGCAGTTTTGAAACACTCTTTTTGTAAAATCTGCAAGAGGATATTTGGATAGCTTTGAGGA
          1 ATAGAGCAGGTTTGAAACACTCTTTTTGTAGTATCTGGAAGTGGATATTTGGATAGCTTTGAGGA

                *        *   * *            **                      *    *   
      86488 TTTCATTGGAAACGGGATTGTCTTCATATAAACTCTAGACAGAAGCATTCTCAGAAGCTTCATTG
         66 TTTCGTTGGAAACAGGAATATCTTCATATAAAAGCTAGACAGAAGCATTCTCAGAAACTTCTTTG

            *       **   *    *             *        
      86553 GGATGTTTCAATTGAAGTCACAGTGTTGAACAGTCCCTTTC
        131 TGATGTTTGCATTCAAGTAACAGTGTTGAACATTCCCTTTC

                                                 *       *       **   * *    
      86594 ATAGAGCAGGTTTGAAACACTCTTTTTGTAGTATCTGCAAGTGGACATTTGGAGCGCTCTCAGGA
          1 ATAGAGCAGGTTTGAAACACTCTTTTTGTAGTATCTGGAAGTGGATATTTGGATAGCTTTGAGGA

            * *  *      *             **          * *       * *          *  *
      86659 CTACGGT-GAAAAAGGAAATATCTTCCAATAAAAGCTACATAGAAGCAATGTCAGAAACTTTTTC
         66 TTTCGTTGGAAACAGG-AATATCTTCATATAAAAGCTAGACAGAAGCATTCTCAGAAACTTCTTT

            *      * ** *   **      *       *  * *   *
      86723 ATGATGTATCTACTCAGCTAACAGAGTTGAACCTTTCTTTTG
        130 GTGATGTTTGCATTCAAGTAACAGTGTTGAACATTCCCTTTC

             *      **                   * *     *             **            
      86765 AGAGAGCACTTTTGAAACACTCTTTTTGTGGAATCTGCAAGTGGATATTTGTCTAGCTTTGAGGA
          1 ATAGAGCAGGTTTGAAACACTCTTTTTGTAGTATCTGGAAGTGGATATTTGGATAGCTTTGAGGA

                         *   *    *                   *       *   *          
      86830 TTTCGTTGGAAACGGGATTA-CAT-ATA-AAAAGC-AGACAGCAGCATTCCCAGTAACTTCTTTG
         66 TTTCGTTGGAAACAGGAATATCTTCATATAAAAGCTAGACAGAAGCATTCTCAGAAACTTCTTTG

                              *    *                 
      86891 TGATGTTTGCATTCAAGTCACAGAGTTGAACATTCCCTTTC
        131 TGATGTTTGCATTCAAGTAACAGTGTTGAACATTCCCTTTC

                                                   *     *       **     *   *
      86932 ATAGAGCAGGTTTGAAACACTCTTTTTGTAGTATCTGGATGTGGACATTTGGAGCGCTTTCAGGC
          1 ATAGAGCAGGTTTGAAACACTCTTTTTGTAGTATCTGGAAGTGGATATTTGGATAGCTTTGAGGA

            * ** *      *             *** *   *                      *   *   
      86997 CTATGGT-GAAAAAGGAAATATCTTCCCCTGAAAACTAGACAGAAGCATTCTCAGAATCTTATTT
         66 TTTCGTTGGAAACAGG-AATATCTTCATATAAAAGCTAGACAGAAGCATTCTCAGAAACTTCTTT

                   **  **    *             **  * *   *
      87061 GTGATGTGCGCCCTCAACTAACAGTGTTGAAGCTTTCTTTTG
        130 GTGATGTTTGCATTCAAGTAACAGTGTTGAACATTCCCTTTC

                     *             *      **     *   *                *      
      87103 ATAGAGCAGTTTTGAAACACTCTCTTTGTAAAATCTGCAAGAGGATATTTGGATAGCTGTGAGGA
          1 ATAGAGCAGGTTTGAAACACTCTTTTTGTAGTATCTGGAAGTGGATATTTGGATAGCTTTGAGGA

                         *   * *            **                      *    *   
      87168 TTTCGTTGGAAACGGGATTGTCTTCATATAAACTCTAGACAGAAGCATTCTCAGAAGCTTCATTG
         66 TTTCGTTGGAAACAGGAATATCTTCATATAAAAGCTAGACAGAAGCATTCTCAGAAACTTCTTTG

            *       **   *    *             *        
      87233 GGATGTTTCAATTGAAGTCACAGTGTTGAACAGTCCCTTTC
        131 TGATGTTTGCATTCAAGTAACAGTGTTGAACATTCCCTTTC

                                                   *     *       **     *   *
      87274 ATAGAGCAGGTTTGAAACACTCTTTTTGTAGTATCTGGATGTGGACATTTGGAGCGCTTTCAGGT
          1 ATAGAGCAGGTTTGAAACACTCTTTTTGTAGTATCTGGAAGTGGATATTTGGATAGCTTTGAGGA

            * ** *      *             *** *   *                          *   
      87339 CTATGGT-GAAAAAGGAAATATCTTCCCCTGAAAACTAGACAGAAGCATTCTCAGAAACTTATTT
         66 TTTCGTTGGAAACAGG-AATATCTTCATATAAAAGCTAGACAGAAGCATTCTCAGAAACTTCTTT

                   **  **    *             **  * *   *
      87403 GTGATGTGCGCCCTCAACTAACAGTGTTGAAGCTTTCTTTTG
        130 GTGATGTTTGCATTCAAGTAACAGTGTTGAACATTCCCTTTC

                     *                   * *     *             **            
      87445 ATAGAGCAGTTTTGAAACACTCTTTTTGTGGAATCTGCAAGTGGATATTTGTCTAGCTTTGAGGA
          1 ATAGAGCAGGTTTGAAACACTCTTTTTGTAGTATCTGGAAGTGGATATTTGGATAGCTTTGAGGA

                             *    *                   *       *   *          
      87510 TTTCGTTGGAAACAGGATTA-CAT-ATA-AAAAGC-AGACAGCAGCATTCCCAGTAACTTCTTTG
         66 TTTCGTTGGAAACAGGAATATCTTCATATAAAAGCTAGACAGAAGCATTCTCAGAAACTTCTTTG

                            * **   *                 
      87571 TGATGTTTGCATTCAACTCCCAGAGTTGAACATTCCCTTTC
        131 TGATGTTTGCATTCAAGTAACAGTGTTGAACATTCCCTTTC

                                                   *     *       **     *   *
      87612 ATAGAGCAGGTTTGAAACACTCTTTTTGTAGTATCTGGATGTGGAAATTTGGAGCGCTTTCAGGC
          1 ATAGAGCAGGTTTGAAACACTCTTTTTGTAGTATCTGGAAGTGGATATTTGGATAGCTTTGAGGA

            * ** *      *             *** *   *                      *   *   
      87677 CTATGGT-GAAAAAGGAAATATCTTCCCCTGAAAACTAGACAGAAGCATTCTCAGAATCTTATTT
         66 TTTCGTTGGAAACAGG-AATATCTTCATATAAAAGCTAGACAGAAGCATTCTCAGAAACTTCTTT

                   **  **    *             **  * *   *
      87741 GTGATGTGCGCCCTCAACTAACAGTGTTGAAGCTTTCTTTTG
        130 GTGATGTTTGCATTCAAGTAACAGTGTTGAACATTCCCTTTC

                     *                    **     *   *                *      
      87783 ATAGAGCAGTTTTGAAACACTCTTTTTGTAAAATCTGCAAGAGGATATTTGGATAGCTGTGAGGA
          1 ATAGAGCAGGTTTGAAACACTCTTTTTGTAGTATCTGGAAGTGGATATTTGGATAGCTTTGAGGA

                         *   * *            **        *       *     *        
      87848 TTTCGTTGGAAACGGGATTGTCTTCATATAAACTCTAGACAGCAGCATTCCCAGAATCTTCTTTG
         66 TTTCGTTGGAAACAGGAATATCTTCATATAAAAGCTAGACAGAAGCATTCTCAGAAACTTCTTTG

                            * **   * *               
      87913 TGATGTTTGCATTCAACTCCCAGAGATGAACATTCCCTTTC
        131 TGATGTTTGCATTCAAGTAACAGTGTTGAACATTCCCTTTC

                                                 *       *       **   * *    
      87954 ATAGAGCAGGTTTGAAACACTCTTTTTGTAGTATCTGCAAGTGGACATTTGGAGCGCTCTCAGGA
          1 ATAGAGCAGGTTTGAAACACTCTTTTTGTAGTATCTGGAAGTGGATATTTGGATAGCTTTGAGGA

            * *  *      *             **            *       * *          *  *
      88019 CTACGGT-GAAAAAGGAAATATCTTCCAATAAAAGCTAGATAGAAGCAATGTCAGAAACTTTTTC
         66 TTTCGTTGGAAACAGG-AATATCTTCATATAAAAGCTAGACAGAAGCATTCTCAGAAACTTCTTT

            *      * ** *   **      *       *  * *   *
      88083 ATGATGTATCTACTCAGCTAACAGAGTTGAACCTTTCTTTTG
        130 GTGATGTTTGCATTCAAGTAACAGTGTTGAACATTCCCTTTC

             *       *           *       * *     *             **            
      88125 AGAGAGCAGTTTTGAAACACTGTTTTTGTGGAATCTGCAAGTGGATATTTGTCTAGCTTTGAGGA
          1 ATAGAGCAGGTTTGAAACACTCTTTTTGTAGTATCTGGAAGTGGATATTTGGATAGCTTTGAGGA

                         *   *    *                   *       *              
      88190 TTTCGTTGGAAACGGGATTA-CAT-ATA-AAAAGC-AGACAGCAGCATTCCCAGAAACTTCTTTG
         66 TTTCGTTGGAAACAGGAATATCTTCATATAAAAGCTAGACAGAAGCATTCTCAGAAACTTCTTTG

               *              *    *                 
      88251 TGAAGTTTGCATTCAAGTCACAGAGTTGAACATTCCCTTTC
        131 TGATGTTTGCATTCAAGTAACAGTGTTGAACATTCCCTTTC

                                                 * *     *       **     *   *
      88292 ATAGAGCAGGTTTGAAACACTCTTTTTGTAGTATCTGTATGTGGACATTTGGAGCGCTTTCAGGC
          1 ATAGAGCAGGTTTGAAACACTCTTTTTGTAGTATCTGGAAGTGGATATTTGGATAGCTTTGAGGA

            * ** *      *             *** *   *                          *   
      88357 CTATGGT-GAAAAAGGAAATATCTTCCCCTGAAAACTAGACAGAAGCATTCTCAGAAACTTATTT
         66 TTTCGTTGGAAACAGG-AATATCTTCATATAAAAGCTAGACAGAAGCATTCTCAGAAACTTCTTT

                   **  **    *             **  * *   *
      88421 GTGATGTGCGCCCTCAACTAACAGTGTTGAAGCTTTCTTTTG
        130 GTGATGTTTGCATTCAAGTAACAGTGTTGAACATTCCCTTTC

                     *                    **     *   *                       
      88463 ATAGAGCAGTTTTGAAACACTCTTTTTGTAAAATCTGCAAGAGGATATTTGGATAGCTTTGAGGA
          1 ATAGAGCAGGTTTGAAACACTCTTTTTGTAGTATCTGGAAGTGGATATTTGGATAGCTTTGAGGA

                         *   * *       *    **                      *    *   
      88528 TTTCGTTGGAAACGGGATTGTCTTCATGTAAACTCTAGACAGAAGCATTCTCAGAAGCTTCATTG
         66 TTTCGTTGGAAACAGGAATATCTTCATATAAAAGCTAGACAGAAGCATTCTCAGAAACTTCTTTG

            *       **   *    *             *        
      88593 GGATGTTTCAATTGAAGTCACAGTGTTGAACAGTCCCTTTC
        131 TGATGTTTGCATTCAAGTAACAGTGTTGAACATTCCCTTTC

                                                         *       *  * * *    
      88634 ATAGAGCAGGTTTGAAACACTCTTTTTGTAGTATCTGGAAGTGGACATTTGGAGAGATCTCAGGA
          1 ATAGAGCAGGTTTGAAACACTCTTTTTGTAGTATCTGGAAGTGGATATTTGGATAGCTTTGAGGA

            * *  *    * *             **            *     * * *          *  *
      88699 CTACGGT-GACAAAGGAAATATCTTCCAATAAAAGCTAGATAGAAGAAATGTCAGAAACTTTTTC
         66 TTTCGTTGGAAACAGG-AATATCTTCATATAAAAGCTAGACAGAAGCATTCTCAGAAACTTCTTT

            *      * ** *   **      *       *  * *   *
      88763 ATGATGTATCTACTCAGCTAACAGAGTTGAACCTTTCTTTTG
        130 GTGATGTTTGCATTCAAGTAACAGTGTTGAACATTCCCTTTC

             *       *                   * *     *             **            
      88805 AGAGAGCAGTTTTGAAACACTCTTTTTGTGGAATCTGCAAGTGGATATTTGTCTAGCTTTGAGGA
          1 ATAGAGCAGGTTTGAAACACTCTTTTTGTAGTATCTGGAAGTGGATATTTGGATAGCTTTGAGGA

                         *   *    *                   *       *              
      88870 TTTCGTTGGAAACGGGATTA-CAT-ATA-AAAAGC-AGACAGCAGCATTCCCAGAAACTTCTTTG
         66 TTTCGTTGGAAACAGGAATATCTTCATATAAAAGCTAGACAGAAGCATTCTCAGAAACTTCTTTG

                              *    *       *         
      88931 TGATG-TTGCATTCAAGTCACAGAGTTGAACGTTCCCTTTC
        131 TGATGTTTGCATTCAAGTAACAGTGTTGAACATTCCCTTTC

                                                   *     *       **     *   *
      88971 ATAGAGCAGGTTTGAAACACTCTTTTTGTAGTATCTGGATGTGGACATTTGGAGCGCTTTCAGGC
          1 ATAGAGCAGGTTTGAAACACTCTTTTTGTAGTATCTGGAAGTGGATATTTGGATAGCTTTGAGGA

            * ** *      *             *** *   *                      *   *   
      89036 CTATGGT-GAAAAAGGAAATATCTTCCCCTGAAAACTAGACAGAAGCATTCTCAGAATCTTATTT
         66 TTTCGTTGGAAACAGG-AATATCTTCATATAAAAGCTAGACAGAAGCATTCTCAGAAACTTCTTT

                   **  **    *             **  * *   *
      89100 GTGATGTGCGCCCTCAACTAACAGTGTTGAAGCTTTCTTTTG
        130 GTGATGTTTGCATTCAAGTAACAGTGTTGAACATTCCCTTTC

                     *                    **     *   *                       
      89142 ATAGAGCAGTTTTGAAACACTCTTTTTGTAAAATCTGCAAGAGGATATTTGGATAGCTTTGAGGA
          1 ATAGAGCAGGTTTGAAACACTCTTTTTGTAGTATCTGGAAGTGGATATTTGGATAGCTTTGAGGA

                         *   * *            **                      *    *   
      89207 TTTCGTTGGAAACGGGATTGTCTTCATATAAACTCTAGACAGAAGCATTCTCAGAAGCTTCATTG
         66 TTTCGTTGGAAACAGGAATATCTTCATATAAAAGCTAGACAGAAGCATTCTCAGAAACTTCTTTG

            *       **   *    *             *        
      89272 GGATGTTTCAATTGAAGTCACAGTGTTGAACAGTCCCTTTC
        131 TGATGTTTGCATTCAAGTAACAGTGTTGAACATTCCCTTTC

                                                     *   *       **   * *    
      89313 ATAGAGCAGGTTTGAAACACTCTTTTTGTAGTATCTGGAAGAGGACATTTGGAGCGCTCTCAGGA
          1 ATAGAGCAGGTTTGAAACACTCTTTTTGTAGTATCTGGAAGTGGATATTTGGATAGCTTTGAGGA

            * *  *      *             **          * *       * *          *  *
      89378 CTACGGT-GAAAAAGGAAATATCTTCTAATAAAAGCTACATAGAAGCAATGTCAGAAACTTTTTC
         66 TTTCGTTGGAAACAGG-AATATCTTCATATAAAAGCTAGACAGAAGCATTCTCAGAAACTTCTTT

            *      * ** *   **      *       *  *     *
      89442 ATGATGTATCTACTCAGCTAACAGAGTTGAACCTTTCCTTTG
        130 GTGATGTTTGCATTCAAGTAACAGTGTTGAACATTCCCTTTC

             *       *                   * *     *             **            
      89484 AGAGAGCAGTTTTGAAACACTCTTTTTGTGGAATCTGCAAGTGGATATTTGTCTAGCTTTGAGGA
          1 ATAGAGCAGGTTTGAAACACTCTTTTTGTAGTATCTGGAAGTGGATATTTGGATAGCTTTGAGGA

                         *   *    *                   *       *     *        
      89549 TTTCGTTGGAAACGGGATTA-CAT-ATA-AAAAGC-AGACAGCAGCATTCCCAGAATCTTCTTTG
         66 TTTCGTTGGAAACAGGAATATCTTCATATAAAAGCTAGACAGAAGCATTCTCAGAAACTTCTTTG

                              *    *                 
      89610 TGATGTTTGCATTCAAGTCACAGAGTTGAACATTCCCTTTC
        131 TGATGTTTGCATTCAAGTAACAGTGTTGAACATTCCCTTTC

             *                         *   *       *     *       **     *   *
      89651 AGAGAGCAGGTTTGAAACACTCTTTTTATAGAATCTGGATGTGGACATTTGGAGCGCTTTCAGGC
          1 ATAGAGCAGGTTTGAAACACTCTTTTTGTAGTATCTGGAAGTGGATATTTGGATAGCTTTGAGGA

            * ** *      *             * * *   *                      *   *   
      89716 CTATGGT-GAAAAAGGAAATATCTTCCTCTGAAAACTAGACAGAAGCATTCTCAGAATCTTATTT
         66 TTTCGTTGGAAACAGG-AATATCTTCATATAAAAGCTAGACAGAAGCATTCTCAGAAACTTCTTT

                   **  **    *             **  * *   *
      89780 GTGATGTGCGCCCTCAACTAACAGTGTTGAAGCTTTCTTTTG
        130 GTGATGTTTGCATTCAAGTAACAGTGTTGAACATTCCCTTTC

                 *   *          *         **     *   *                       
      89822 ATAGATCAGTTTTGAAACACACTTTTTGTAAAATCTGCAAGAGGATATTTGGATAGCTTTGAGGA
          1 ATAGAGCAGGTTTGAAACACTCTTTTTGTAGTATCTGGAAGTGGATATTTGGATAGCTTTGAGGA

                *        *   * *        **  **                      *    *   
      89887 TTTCATTGGAAACGGGACTGTCTTCATAATAACTCTAGACAGAAGCATTCTCAGAAGCTTCATTG
         66 TTTCGTTGGAAACAGGAATATCTTCATATAAAAGCTAGACAGAAGCATTCTCAGAAACTTCTTTG

            *       **   *    *             *        
      89952 GGATGTTTCAATTGAAGTCACAGTGTTGAACAGTCCCTTTC
        131 TGATGTTTGCATTCAAGTAACAGTGTTGAACATTCCCTTTC

                                                         *       *  * * *    
      89993 ATAGAGCAGGTTTGAAACACTCTTTTTGTAGTATCTGGAAGTGGACATTTGGAGAGATCTCAGGA
          1 ATAGAGCAGGTTTGAAACACTCTTTTTGTAGTATCTGGAAGTGGATATTTGGATAGCTTTGAGGA

            * *  *      *             **            *       * *          *  *
      90058 CTACGGT-GAAAAAGGAAATATCTTCCAATAAAAGCTAGATAGAAGCAATGTCAGAAACTTTTTC
         66 TTTCGTTGGAAACAGG-AATATCTTCATATAAAAGCTAGACAGAAGCATTCTCAGAAACTTCTTT

            *      * ** *   **      *       *  * *   *
      90122 ATGATGTGTCTACTCAGCTAACAGAGTTGAACCTTTCTTTTG
        130 GTGATGTTTGCATTCAAGTAACAGTGTTGAACATTCCCTTTC

             *       *            *      * *     *             **            
      90164 AGAGAGCAGTTTTGAAACACTCATTTTGTGGAATCTGCAAGTGGATATTTGTCTAGCTTTGAGGA
          1 ATAGAGCAGGTTTGAAACACTCTTTTTGTAGTATCTGGAAGTGGATATTTGGATAGCTTTGAGGA

                         *   *    *                   *       *   *          
      90229 TTTCGTTGGAAACGGGATTA-CAT-ATA-AAAAGC-AGACAGCAGCATTCCCAGTAACTTCTTTG
         66 TTTCGTTGGAAACAGGAATATCTTCATATAAAAGCTAGACAGAAGCATTCTCAGAAACTTCTTTG

                              *    *                 
      90290 TGATGTTTGCATTCAAGTCACAGAGTTGAACATTCCCTTTC
        131 TGATGTTTGCATTCAAGTAACAGTGTTGAACATTCCCTTTC

                                                *  *     *       ** *   *   *
      90331 ATAGAGCAGGTTTGAAACACTCTTTTTGTAGTATCTCGATGTGGACATTTGGAGCGTTTTCAGGC
          1 ATAGAGCAGGTTTGAAACACTCTTTTTGTAGTATCTGGAAGTGGATATTTGGATAGCTTTGAGGA

            * ** *      *             *** *   *                      *   *   
      90396 CTATGGT-GAAAAAGGAAATATCTTCCCCTGAAAACTAGACAGAAGCATTCTCAGAATCTTATTT
         66 TTTCGTTGGAAACAGG-AATATCTTCATATAAAAGCTAGACAGAAGCATTCTCAGAAACTTCTTT

                   **  **    *             **  * *   *
      90460 GTGATGTGCGCCCTCAACTAACAGTGTTGAAGCTTTCTTTTG
        130 GTGATGTTTGCATTCAAGTAACAGTGTTGAACATTCCCTTTC

                     *                    **     *   *                       
      90502 ATAGAGCAGTTTTGAAACACTCTTTTTGTAAAATCTGCAAGAGGATATTTGGATAGCTTTGAGGA
          1 ATAGAGCAGGTTTGAAACACTCTTTTTGTAGTATCTGGAAGTGGATATTTGGATAGCTTTGAGGA

               **    *   *   * *            **                      *    *   
      90567 TTTAATTGGTAACGGGATTGTCTTCATATAAACTCTAGACAGAAGCATTCTCAGAAGCTTCATTG
         66 TTTCGTTGGAAACAGGAATATCTTCATATAAAAGCTAGACAGAAGCATTCTCAGAAACTTCTTTG

            *       **   *  * *             *        
      90632 GGATGTTTCAATTGAATTCACAGTGTTGAACAGTCCCTTTC
        131 TGATGTTTGCATTCAAGTAACAGTGTTGAACATTCCCTTTC

                                                *        *       **   * *    
      90673 ATAGAGCAGGTTTGAAACACTCTTTTTGTAGTATCTTGAAGTGGACATTTGGAGCGCTCTCAGGA
          1 ATAGAGCAGGTTTGAAACACTCTTTTTGTAGTATCTGGAAGTGGATATTTGGATAGCTTTGAGGA

            * *  *      *             **            **      * *          *  *
      90738 CTGCGGT-GAAAAAGGAAATATCTTCCAATAAAAGCTAGATGGAAGCAATGTCAGAAACTTTTTC
         66 TTTCGTTGGAAACAGG-AATATCTTCATATAAAAGCTAGACAGAAGCATTCTCAGAAACTTCTTT

            *      * ** *   **      *       *  * *   *
      90802 ATGATGTATCTACTCAGCTAACAGAGTTGAACCTTTCTTTTG
        130 GTGATGTTTGCATTCAAGTAACAGTGTTGAACATTCCCTTTC

             *       *                   * *     *         *   **            
      90844 AGAGAGCAGTTTTGAAACACTCTTTTTGTGGAATCTGCAAGTGGATACTTGTCTAGCTTTGAGGA
          1 ATAGAGCAGGTTTGAAACACTCTTTTTGTAGTATCTGGAAGTGGATATTTGGATAGCTTTGAGGA

                         *   *    *               *   *       **             
      90909 TTTCGTTGGAAACGGGATTA-CAT-ATA-AAAAGC-AGGCAGCAGCATTCCTAGAAACTTCTTTG
         66 TTTCGTTGGAAACAGGAATATCTTCATATAAAAGCTAGACAGAAGCATTCTCAGAAACTTCTTTG

                              *    *    *            
      90970 TGATGTTTGCATTCAAGTCACAGAGTTGCACATTCCCTTTC
        131 TGATGTTTGCATTCAAGTAACAGTGTTGAACATTCCCTTTC

                                                   *     *       **     *   *
      91011 ATAGAGCAGGTTTGAAACACTCTTTTTGTAGTATCTGGATGTGGACATTTGGAGCGCTTTCAGGC
          1 ATAGAGCAGGTTTGAAACACTCTTTTTGTAGTATCTGGAAGTGGATATTTGGATAGCTTTGAGGA

            * ** *      *             ***                                 *  
      91076 CTATGGT-GAAAAAGGAAATATCTTCCCCTGAAAAG-TAGACAGAAGCATTCTCAGAAACTTATT
         66 TTTCGTTGGAAACAGG-AATATCTTCATAT-AAAAGCTAGACAGAAGCATTCTCAGAAACTTCTT

                    **  **    *             **  * *   *
      91139 TGTGATGTGCGCCCTCAACTAACAGTGTTGAAGCTTTCTTTTG
        129 TGTGATGTTTGCATTCAAGTAACAGTGTTGAACATTCCCTTTC

                     *                    **     *   *                       
      91182 ATAGAGCAGTTTTGAAACACTCTTTTTGTAAAATCTGCAAGAGGATATTTGGATAGCTTTGAGGA
          1 ATAGAGCAGGTTTGAAACACTCTTTTTGTAGTATCTGGAAGTGGATATTTGGATAGCTTTGAGGA

                         *   * *            **                      *    *   
      91247 TTTCGTTGGAAACGGGATTGTCTTCATATAAACTCTAGACAGAAGCATTCTCAGAAGCTTCATTG
         66 TTTCGTTGGAAACAGGAATATCTTCATATAAAAGCTAGACAGAAGCATTCTCAGAAACTTCTTTG

            *       **   *    *             * *      
      91312 GGATGTTTCAATTGAAGTCACAGTGTTGAACAGTTCCTTTC
        131 TGATGTTTGCATTCAAGTAACAGTGTTGAACATTCCCTTTC

                                                         *       **  ** *    
      91353 ATAGAGCAGGTTTGAAACACTCTTTTTGTAGTATCTGGAAGTGGACATTTGGAGCGCCCTCAGGA
          1 ATAGAGCAGGTTTGAAACACTCTTTTTGTAGTATCTGGAAGTGGATATTTGGATAGCTTTGAGGA

            * *  *      *             **          * *       * *          *  *
      91418 CTACGGT-GAAAAAGGAAATATCTTCCAATAAAAGCTACATAGAAGCAATGTCAGAAACTTTTTC
         66 TTTCGTTGGAAACAGG-AATATCTTCATATAAAAGCTAGACAGAAGCATTCTCAGAAACTTCTTT

            *      * ** *   **      *       *        *
      91482 ATGATGTATCTACTCAGCTAACAGAGTTGAACCTT-CCTTTG
        130 GTGATGTTTGCATTCAAGTAACAGTGTTGAACATTCCCTTTC

             *       *                   * *     *             **            
      91523 AGAGAGCAGTTTTGAAACACTCTTTTTGTGGAATCTGCAAGTGGATATTTGTCTAGCTTTGAGGA
          1 ATAGAGCAGGTTTGAAACACTCTTTTTGTAGTATCTGGAAGTGGATATTTGGATAGCTTTGAGGA

                         *   *    *          *        *       *              
      91588 TTTCGTTGGAAACGGGATTA-CAT-ATA-AAAAAC-AGACAGCAGCATTCCCAGAAACTTCTTTG
         66 TTTCGTTGGAAACAGGAATATCTTCATATAAAAGCTAGACAGAAGCATTCTCAGAAACTTCTTTG

               *              *    *                 
      91649 TGAAGTTTGCATTCAAGTCACAGAGTTGAACATTCCCTTTC
        131 TGATGTTTGCATTCAAGTAACAGTGTTGAACATTCCCTTTC

                                 *               * *     *       **     *   *
      91690 ATAGAGCAGGTTTGAAACACTATTTTTGTAGTATCTGTATGTGGACATTTGGAGCGCTTTCAGGC
          1 ATAGAGCAGGTTTGAAACACTCTTTTTGTAGTATCTGGAAGTGGATATTTGGATAGCTTTGAGGA

            * ** *      *             *** *   *                      *   *   
      91755 CTATGGT-GAAAAAGGAAATATCTTCCCCTGAAAACTAGACAGAAGCATTCTCAGAATCTTATTT
         66 TTTCGTTGGAAACAGG-AATATCTTCATATAAAAGCTAGACAGAAGCATTCTCAGAAACTTCTTT

                   **  **    *             **  * *   *
      91819 GTGATGTGCGCCCTCAACTAACAGTGTTGAAGCTTTCTTTTG
        130 GTGATGTTTGCATTCAAGTAACAGTGTTGAACATTCCCTTTC

                     *                    **     *  **                       
      91861 ATAGAGCAGTTTTGAAACACTCTTTTTGTAAAATCTGCAACAGGATATTTGGATAGCTTTGAGGA
          1 ATAGAGCAGGTTTGAAACACTCTTTTTGTAGTATCTGGAAGTGGATATTTGGATAGCTTTGAGGA

                *        *   * *            **                      *    *  *
      91926 TTTCATTGGAAACGGGATTGTCTTCATATAAACTCTAGACAGAAGCATTCTCAGAAGCTTCATTC
         66 TTTCGTTGGAAACAGGAATATCTTCATATAAAAGCTAGACAGAAGCATTCTCAGAAACTTCTTTG

            *       **   *    *             *        
      91991 GGATGTTTCAATTGAAGTCACAGTGTTGAACAGTCCCTTTC
        131 TGATGTTTGCATTCAAGTAACAGTGTTGAACATTCCCTTTC

                                                         *       **   * *    
      92032 ATAGAGCAGGTTTGAAACACTCTTTTTGTAGTATCTGGAAGTGGACATTTGGAGCGCTCTCAGGA
          1 ATAGAGCAGGTTTGAAACACTCTTTTTGTAGTATCTGGAAGTGGATATTTGGATAGCTTTGAGGA

            * *  *      *             **            *       * *          *  *
      92097 CTGCGGT-GAAAAAGGAAATATCTTCCAATAAAAGCTAGATAGAAGCAATGTCAGAAACTTTTTC
         66 TTTCGTTGGAAACAGG-AATATCTTCATATAAAAGCTAGACAGAAGCATTCTCAGAAACTTCTTT

            *      * ** *            *       *  * *   *
      92161 ATGATGTATCTACT-AAGCTAACAGAGTTGAACCTTTCTTTTG
        130 GTGATGTTTGCATTCAAG-TAACAGTGTTGAACATTCCCTTTC

             *       *                   * *     *  *      *   **            
      92203 AGAGAGCAGTTTTGAAACACTCTTTTTGTGGAATCTGCAACTGGATACTTGTCTAGCTTTGAGGA
          1 ATAGAGCAGGTTTGAAACACTCTTTTTGTAGTATCTGGAAGTGGATATTTGGATAGCTTTGAGGA

                         *   *    *                   *       *              
      92268 TTTCGTTGGAAACGGGATTA-CAT-ATA-AAAAGC-AGACAGCAGCATTCCCAGAAACTTCTTTG
         66 TTTCGTTGGAAACAGGAATATCTTCATATAAAAGCTAGACAGAAGCATTCTCAGAAACTTCTTTG

                              *    *                 
      92329 TGATGTTTGCATTCAAGTCACAGAGTTGAACATTCCCTTTC
        131 TGATGTTTGCATTCAAGTAACAGTGTTGAACATTCCCTTTC

                                                   *     *       **     *   *
      92370 ATAGAGCAGGTTTGAAACACTCTTTTTGTAGTATCTGGATGTGGACATTTGGAGCGCTTTCAGGC
          1 ATAGAGCAGGTTTGAAACACTCTTTTTGTAGTATCTGGAAGTGGATATTTGGATAGCTTTGAGGA

            * ** *      *             *** *   *                          *   
      92435 CTATGGT-GAAAAAGGAAATATCTTCCCCTGAAAACTAGACAGAAGCATTCTCAGAAACTTATTT
         66 TTTCGTTGGAAACAGG-AATATCTTCATATAAAAGCTAGACAGAAGCATTCTCAGAAACTTCTTT

                   **  **    *             **  * *   *
      92499 GTGATGTGCGCCCTCAACTAACAGTGTTGAAGCTTTCTTTTG
        130 GTGATGTTTGCATTCAAGTAACAGTGTTGAACATTCCCTTTC

                     *                    **     *   *                       
      92541 ATAGAGCAGTTTTGAAACACTCTTTTTGTAAAATCTGCAAGAGGATATTTGGATAGCTTTGAGGA
          1 ATAGAGCAGGTTTGAAACACTCTTTTTGTAGTATCTGGAAGTGGATATTTGGATAGCTTTGAGGA

                         *   * *            **                      *    *   
      92606 TTTCGTTGGAAACGGGATTGTCTTCATATAAACTCTAGACAGAAGCATTCTCAGAAGCTTCATTG
         66 TTTCGTTGGAAACAGGAATATCTTCATATAAAAGCTAGACAGAAGCATTCTCAGAAACTTCTTTG

            *       **   *    *             *        
      92671 GGATGTTTCAATTGAAGTCACAGTGTTGAACACTCCCTTTC
        131 TGATGTTTGCATTCAAGTAACAGTGTTGAACATTCCCTTTC

                    *       *                            *       **   * *    
      92712 ATAGAGCACGTTTGAATCACTCTTTTTGTAGTATCTGGAAGTGGACATTTGGAGCGCTCTCAGGA
          1 ATAGAGCAGGTTTGAAACACTCTTTTTGTAGTATCTGGAAGTGGATATTTGGATAGCTTTGAGGA

            * *  *      *             **          * *       * *          *  *
      92777 CTACGGT-GAAAAAGGAAATATCTTCTAATAAAAGCTACATAGAAGCAATGTCAGAAACTTTTTC
         66 TTTCGTTGGAAACAGG-AATATCTTCATATAAAAGCTAGACAGAAGCATTCTCAGAAACTTCTTT

            *      * ** *   **     **       *  *     *
      92841 ATGATGTATCTACTCAGCTAACACAGTTGAACCTTTCCTTTG
        130 GTGATGTTTGCATTCAAGTAACAGTGTTGAACATTCCCTTTC

             *       *                   * *     *             **   *        
      92883 AGAGAGCAGTTTTGAAACACTCTTTTTGTGGAATCTGCAAGTGGATATTTGTCTAGATTTGAGGA
          1 ATAGAGCAGGTTTGAAACACTCTTTTTGTAGTATCTGGAAGTGGATATTTGGATAGCTTTGAGGA

                         *   *    *                   *       *     *   *    
      92948 TTTCGTTGGAAACGGGATTA-CAT-ATA-AAAAGC-AGACAGCAGCATTCCCAGAATCTTGTTTG
         66 TTTCGTTGGAAACAGGAATATCTTCATATAAAAGCTAGACAGAAGCATTCTCAGAAACTTCTTTG

                              *    *                 
      93009 TGATGTTTGCATTCAAGTCACAGAGTTGAACATTCCCTTTC
        131 TGATGTTTGCATTCAAGTAACAGTGTTGAACATTCCCTTTC

             *                         *   *       *     *       **     *   *
      93050 AGAGAGCAGGTTTGAAACACTCTTTTTATAGAATCTGGATGTGGACATTTGGAGCGCTTTCAGGC
          1 ATAGAGCAGGTTTGAAACACTCTTTTTGTAGTATCTGGAAGTGGATATTTGGATAGCTTTGAGGA

            * ** *      *             *** *   *                      *   *   
      93115 CTATGGT-GAAAAAGGAAATATCTTCTCCTGAAAACTAGACAGAAGCATTCTCAGAATCTTATTT
         66 TTTCGTTGGAAACAGG-AATATCTTCATATAAAAGCTAGACAGAAGCATTCTCAGAAACTTCTTT

                   **  **    *             **  * *   *
      93179 GTGATGTGCGCCCTCAACTAACAGTGTTGAAGCTTTCTTTTG
        130 GTGATGTTTGCATTCAAGTAACAGTGTTGAACATTCCCTTTC

                     *          *         **     *   *                       
      93221 ATAGAGCAGTTTTGAAACACACTTTTTGTAAAATCTGCAAGAGGATATTTGGATAGCTTTGAGGA
          1 ATAGAGCAGGTTTGAAACACTCTTTTTGTAGTATCTGGAAGTGGATATTTGGATAGCTTTGAGGA

                *        *   * *            **                      *    *   
      93286 TTTCATTGGAAACGGGACTGTCTTCATATAAACTCTAGACAGAAGCATTCTCAGAAGCTTCATTG
         66 TTTCGTTGGAAACAGGAATATCTTCATATAAAAGCTAGACAGAAGCATTCTCAGAAACTTCTTTG

            *       **   *    *             *        
      93351 GGATGTTTCAATTGAAGTCACAGTGTTGAACAGTCCCTTTC
        131 TGATGTTTGCATTCAAGTAACAGTGTTGAACATTCCCTTTC

                                                         *       *  * * *    
      93392 ATAGAGCAGGTTTGAAACACTCTTTTTGTAGTATCTGGAAGTGGACATTTGGAGAGATCTCAGGA
          1 ATAGAGCAGGTTTGAAACACTCTTTTTGTAGTATCTGGAAGTGGATATTTGGATAGCTTTGAGGA

            * *  *      *             **            *       * *          *  *
      93457 CTACGGT-GAAAAAGGAAATATCTTCCAATAAAAGCTAGATAGAAGCAATGTCAGAAACTTTTTC
         66 TTTCGTTGGAAACAGG-AATATCTTCATATAAAAGCTAGACAGAAGCATTCTCAGAAACTTCTTT

            *      * ** *   **      *       *  * *   *
      93521 ATGATGTATCTACTCAGCTAACAGAGTTGAACCTTTCTTTTG
        130 GTGATGTTTGCATTCAAGTAACAGTGTTGAACATTCCCTTTC

             *       *                   * *                   **            
      93563 AGAGAGCAGATTTGAAACACTCTTTTTGTGGAATCTGGAAGTGGATATTTGTCTAGCTTTGAGGA
          1 ATAGAGCAGGTTTGAAACACTCTTTTTGTAGTATCTGGAAGTGGATATTTGGATAGCTTTGAGGA

                         *   *    *                   *       *   *          
      93628 TTTCGTTGGAAACGGGATTA-CAT-ATA-AAAAGC-AGACAGCAGCATTCCCAGTAACTTCTTTG
         66 TTTCGTTGGAAACAGGAATATCTTCATATAAAAGCTAGACAGAAGCATTCTCAGAAACTTCTTTG

                            * *    *                 
      93689 TGATGTTTGCATTCAACTCACAGAGTTGAACATTCCCTTTC
        131 TGATGTTTGCATTCAAGTAACAGTGTTGAACATTCCCTTTC

                                                   *     *       **     *   *
      93730 ATAGAGCAGGTTTGAAACACTCTTTTTGTAGTATCTGGATGTGGACATTTGGAGCGCTTTCAGGC
          1 ATAGAGCAGGTTTGAAACACTCTTTTTGTAGTATCTGGAAGTGGATATTTGGATAGCTTTGAGGA

            * ** *      *             *** *   *                      *   *   
      93795 CTATGGT-GAAAAAGGAAATATCTTCCCCTGAAAACTAGACAGAAGCATTCTCAGAATCTTATTT
         66 TTTCGTTGGAAACAGG-AATATCTTCATATAAAAGCTAGACAGAAGCATTCTCAGAAACTTCTTT

                   **  **    *             **  * *   *
      93859 GTGATGTGCGCCCTCAACTAACAGTGTTGAAGCTTTCTTTTG
        130 GTGATGTTTGCATTCAAGTAACAGTGTTGAACATTCCCTTTC

                     *                    **     *   *                       
      93901 ATAGAGCAGTTTTGAAACACTCTTTTTGTAAAATCTGCAAGAGGATATTTGGATAGCTTTGAGGA
          1 ATAGAGCAGGTTTGAAACACTCTTTTTGTAGTATCTGGAAGTGGATATTTGGATAGCTTTGAGGA

                         *   * *            **                      *    *   
      93966 TTTCGTTGGAAACGGGATTGTCTTCATATAAACTCTAGACAGAAGCATTCTCAGAAGCTTCATTG
         66 TTTCGTTGGAAACAGGAATATCTTCATATAAAAGCTAGACAGAAGCATTCTCAGAAACTTCTTTG

            *       **   *    *             *        
      94031 GGATGTTTCAATTGAAGTCACAGTGTTGAACAGTCCCTTTC
        131 TGATGTTTGCATTCAAGTAACAGTGTTGAACATTCCCTTTC

                                                         *       **   * * *  
      94072 ATAGAGCAGGTTTGAAACACTCTTTTTGTAGTATCTGGAAGTGGACATTTGGAGCGCTCTCACGA
          1 ATAGAGCAGGTTTGAAACACTCTTTTTGTAGTATCTGGAAGTGGATATTTGGATAGCTTTGAGGA

            * *  *      *             **            *       * *          *  *
      94137 CTACGGT-GAAAAAGGAAGTATCTTCCAATAAAAGCTAGATAGAAGCAATGTCAGAAACTTTTTC
         66 TTTCGTTGGAAACAGGAA-TATCTTCATATAAAAGCTAGACAGAAGCATTCTCAGAAACTTCTTT

            *      * ** *   **      *       *  * *   *
      94201 ATGATGTATCTACTCAGCTAACAGAGTTGAACCTTTCTTTTG
        130 GTGATGTTTGCATTCAAGTAACAGTGTTGAACATTCCCTTTC

             *       *       *           * *                   **            
      94243 AGAGAGCAGTTTTGAAAAACTCTTTTTGTGGAATCTGGAAGTGGATATTTGTCTAGCTTTGAGGA
          1 ATAGAGCAGGTTTGAAACACTCTTTTTGTAGTATCTGGAAGTGGATATTTGGATAGCTTTGAGGA

                         *   *    *                   *       *   *          
      94308 TTTCGTTGGAAACGGGATTA-CAT-ATA-AAAAGC-AGACAGCAGCATTCCCAGTAACTTCTTTG
         66 TTTCGTTGGAAACAGGAATATCTTCATATAAAAGCTAGACAGAAGCATTCTCAGAAACTTCTTTG

                              *    *                 
      94369 TGATGTTTGCATTCAAGTCACAGAGTTGAACATTCCCTTTC
        131 TGATGTTTGCATTCAAGTAACAGTGTTGAACATTCCCTTTC

                                             *     *     *     * **     *   *
      94410 ATAGAGCAGGTTTGAAACACTCTTTTTGTAGTAACTGGATGTGGACATTTGCAGCGCTTTCAGGC
          1 ATAGAGCAGGTTTGAAACACTCTTTTTGTAGTATCTGGAAGTGGATATTTGGATAGCTTTGAGGA

            * ** *      *             *** *   *                          *   
      94475 CTAAGGT-GAAAAAGGAAATATCTTCCCCTGAAAACTAGACAGAAGCATTCTCAGAAACTTATTT
         66 TTTCGTTGGAAACAGG-AATATCTTCATATAAAAGCTAGACAGAAGCATTCTCAGAAACTTCTTT

                   **  **    *     *       **  * *   *
      94539 GTGATGTGCGCCCTCAACTAACAATGTTGAAGCTTTCTTTTG
        130 GTGATGTTTGCATTCAAGTAACAGTGTTGAACATTCCCTTTC

                     *                   * *     *             **            
      94581 ATAGAGCAGTTTTGAAACACTCTTTTTGTGGAATCTGCAAGTGGATATTTGTCTAGCTTTGAGGA
          1 ATAGAGCAGGTTTGAAACACTCTTTTTGTAGTATCTGGAAGTGGATATTTGGATAGCTTTGAGGA

                         *   *    *                   *       *     *        
      94646 TTTCGTTGGAAACGGGATTA-CAT-ATA-AAAAGC-AGACAGCAGCATTCCCAGAATCTTCTTTG
         66 TTTCGTTGGAAACAGGAATATCTTCATATAAAAGCTAGACAGAAGCATTCTCAGAAACTTCTTTG

                              *    *                 
      94707 TGATGTTTGCATTCAAGTCACAGAGTTGAACATTCCCTTTC
        131 TGATGTTTGCATTCAAGTAACAGTGTTGAACATTCCCTTTC

                                                   *     *       **     *   *
      94748 ATAGAGCAGGTTTGAAACACTCTTTTTGTAGTATCTGGATGTGGACATTTGGAGCGCTTTCAGGC
          1 ATAGAGCAGGTTTGAAACACTCTTTTTGTAGTATCTGGAAGTGGATATTTGGATAGCTTTGAGGA

            * ** *      *             *** *   *                      *   *   
      94813 CTATGGT-GAAAAAGGAAATATCTTCCCCTGAAAACTAGACAGAAGCATTCTCAGAATCTTATTT
         66 TTTCGTTGGAAACAGG-AATATCTTCATATAAAAGCTAGACAGAAGCATTCTCAGAAACTTCTTT

                   **  **    *             **  * *   *
      94877 GTGATGTGCGCCCTCAACTAACAGTGTTGAAGCTTTCTTTTG
        130 GTGATGTTTGCATTCAAGTAACAGTGTTGAACATTCCCTTTC

                     *                    **     *   *     *                 
      94919 ATAGAGCAGTTTTGAAACACTCTTTTTGTAAAATCTGCAAGAGGATACTTGGATAGCTTTGAGGA
          1 ATAGAGCAGGTTTGAAACACTCTTTTTGTAGTATCTGGAAGTGGATATTTGGATAGCTTTGAGGA

                             * *            **                      *    *   
      94984 TTTCGTTGGAAACAGGATTGTCTTCATATAAACTCTAGACAGAAGCATTCTCAGAAGCTTCATTG
         66 TTTCGTTGGAAACAGGAATATCTTCATATAAAAGCTAGACAGAAGCATTCTCAGAAACTTCTTTG

            *       **   *    *             *        
      95049 GGATGTTTCAATTGAAGTCACAGTGTTGAACAGTCCCTTTC
        131 TGATGTTTGCATTCAAGTAACAGTGTTGAACATTCCCTTTC

                                                         *       *  * * *    
      95090 ATAGAGCAGGTTTGAAACACTCTTTTTGTAGTATCTGGAAGTGGACATTTGGAGAGATCTCAGGA
          1 ATAGAGCAGGTTTGAAACACTCTTTTTGTAGTATCTGGAAGTGGATATTTGGATAGCTTTGAGGA

            * *  *    * *             **            *       * *          *  *
      95155 ATACGGT-GATAAAGGAAATATCTTCCAATAAAAGCTAGATAGAAGCAATGTCAGAAACTTTTTC
         66 TTTCGTTGGAAACAGG-AATATCTTCATATAAAAGCTAGACAGAAGCATTCTCAGAAACTTCTTT

            *      * ** *   **      *       *  * *   *
      95219 ATGATGTATCTACTCAGCTAACAGAGTTGAACCTTTCTTTTG
        130 GTGATGTTTGCATTCAAGTAACAGTGTTGAACATTCCCTTTC

             *   *   *                   * *     *             **            
      95261 AGAGAACAGTTTTGAAACACTCTTTTTGTGGAATCTGCAAGTGGATATTTGTCTAGCTTTGAGGA
          1 ATAGAGCAGGTTTGAAACACTCTTTTTGTAGTATCTGGAAGTGGATATTTGGATAGCTTTGAGGA

                         *   *    *                   *       *              
      95326 TTTCGTTGGAAACGGGATTA-CAT-ATA-AAAAGC-AGACAGCAGCATTCCCAGAAACTTCTTTG
         66 TTTCGTTGGAAACAGGAATATCTTCATATAAAAGCTAGACAGAAGCATTCTCAGAAACTTCTTTG

                              *  * *                 
      95387 TGATGTTTGCATTCAAGTCACCGAGTTGAACATTCCCTTTC
        131 TGATGTTTGCATTCAAGTAACAGTGTTGAACATTCCCTTTC

                                                   *     *    *  **     *  **
      95428 ATAGAGCAGGTTTGAAACACTCTTTTTGTAGTATCTGGATGTGGACATTTAGAGCGCTTTCAGTC
          1 ATAGAGCAGGTTTGAAACACTCTTTTTGTAGTATCTGGAAGTGGATATTTGGATAGCTTTGAGGA

            * ** *      *             *** *   *                      *   *   
      95493 CTATGGT-GAAAAAGGAAATATCTTCCCCTGAAAACTAGACAGAAGCATTCTCAGAATCTTATTT
         66 TTTCGTTGGAAACAGG-AATATCTTCATATAAAAGCTAGACAGAAGCATTCTCAGAAACTTCTTT

                   **  **    *             **  * *   *
      95557 GTGATGTGCGCCCTCAACTAACAGTGTTGAAGCTTTCTTTTG
        130 GTGATGTTTGCATTCAAGTAACAGTGTTGAACATTCCCTTTC

             *       *                    **     *   *                       
      95599 AAAGAGCAGTTTTGAAACACTCTTTTTGTAAAATCTGCAAGAGGATATTTGGATAGCTTTGAGGA
          1 ATAGAGCAGGTTTGAAACACTCTTTTTGTAGTATCTGGAAGTGGATATTTGGATAGCTTTGAGGA

                *        *   * *   *        **                      *    *   
      95664 TTTCATTGGAAACGGGATTGTCTCCATATAAACTCTAGACAGAAGCATTCTCAGAAGCTTCATTG
         66 TTTCGTTGGAAACAGGAATATCTTCATATAAAAGCTAGACAGAAGCATTCTCAGAAACTTCTTTG

            *       **   *    *             *        
      95729 GGATGTTTCAATTGAAGTCACAGTGTTGAACAGTCCCTTTC
        131 TGATGTTTGCATTCAAGTAACAGTGTTGAACATTCCCTTTC

                                                         *       **   * *    
      95770 ATAGAGCAGGTTTGAAACACTCTTTTTGTAGTATCTGGAAGTGGACATTTGGAGCGCTCTCAGGA
          1 ATAGAGCAGGTTTGAAACACTCTTTTTGTAGTATCTGGAAGTGGATATTTGGATAGCTTTGAGGA

            * *  *      *             **          * *       * *          *  *
      95835 CTACGGT-GAAAAAGGAAATATCTTCCAATAAAAGCTACATAGAAGCAATATCAGAAACTTTTTC
         66 TTTCGTTGGAAACAGG-AATATCTTCATATAAAAGCTAGACAGAAGCATTCTCAGAAACTTCTTT

            *      * ** *   **      *       *  * *   *
      95899 ATGATGTATCTACTCAGCTAACAGAGTTGAACCTTTCTTTTG
        130 GTGATGTTTGCATTCAAGTAACAGTGTTGAACATTCCCTTTC

             *       *  *                * *     *             **            
      95941 AGAGAGCAGTTTGGAAACACTCTTTTTGTGGAATCTGCAAGTGGATATTTGTCTAGCTTTGAGGA
          1 ATAGAGCAGGTTTGAAACACTCTTTTTGTAGTATCTGGAAGTGGATATTTGGATAGCTTTGAGGA

                         *   *    *      *            *       *              
      96006 TTTCGTTGGAAACGGGATTA-CAT-ATA-GAAAGC-AGACAGCAGCATTCCCAGAAACTTCTTTG
         66 TTTCGTTGGAAACAGGAATATCTTCATATAAAAGCTAGACAGAAGCATTCTCAGAAACTTCTTTG

                              *    *                 
      96067 TGATGTTTGCATTCAAGTCACAGAGTTGAACATTCCCTTTC
        131 TGATGTTTGCATTCAAGTAACAGTGTTGAACATTCCCTTTC

                                                   *     *       **     *   *
      96108 ATAGAGCAGGTTTGAAACACTCTTTTTGTAGTATCTGGATGTGGACATTTGGAGCGCTTTCAGGC
          1 ATAGAGCAGGTTTGAAACACTCTTTTTGTAGTATCTGGAAGTGGATATTTGGATAGCTTTGAGGA

            * ** *      *             *** *   *                      *   *   
      96173 CTATGGT-GAAAAAGGAAATATCTTCCCCTGAAAACTAGACAGAAGCATTCTCAGAATCTTATTT
         66 TTTCGTTGGAAACAGG-AATATCTTCATATAAAAGCTAGACAGAAGCATTCTCAGAAACTTCTTT

                   **  **    *             **  * *   *
      96237 GTGATGTGCGCCCTCAACTAACAGTGTTGAAGCTTTCTTTTG
        130 GTGATGTTTGCATTCAAGTAACAGTGTTGAACATTCCCTTTC

                     *                    **     *   *     *                 
      96279 ATAGAGCAGTTTTGAAACACTCTTTTTGTAAAATCTGCAAGAGGATACTTGGATAGCTTTGAGGA
          1 ATAGAGCAGGTTTGAAACACTCTTTTTGTAGTATCTGGAAGTGGATATTTGGATAGCTTTGAGGA

                         *   * *            **                      *    *   
      96344 TTTCGTTGGAAACGGGATTGTCTTCATATAAACTCTAGACAGAAGCATTCTCAGAAGCTTCATTG
         66 TTTCGTTGGAAACAGGAATATCTTCATATAAAAGCTAGACAGAAGCATTCTCAGAAACTTCTTTG

            *       **   *    *             *        
      96409 GGATGTTTCAATTGAAGTCACAGTGTTGAACAGTCCCTTTC
        131 TGATGTTTGCATTCAAGTAACAGTGTTGAACATTCCCTTTC

                                                    *    *       *  * * *    
      96450 ATAGAGCAGGTTTGAAACACTCTTTTTGTAGTATCTGGAAATGGACATTTGGAGAGATCTCAGGA
          1 ATAGAGCAGGTTTGAAACACTCTTTTTGTAGTATCTGGAAGTGGATATTTGGATAGCTTTGAGGA

            * *  *    * *             **            *       * *          *  *
      96515 ATACGGT-GATAAAGGAAATATCTTCCAATAAAAGCTAGATAGAAGCAATGTCAGAAACTTTTTC
         66 TTTCGTTGGAAACAGG-AATATCTTCATATAAAAGCTAGACAGAAGCATTCTCAGAAACTTCTTT

            *      * ** *   **      *       *  * *   *
      96579 ATGATGTATCTACTCAGCTAACAGAGTTGAACCTTTCTTTTG
        130 GTGATGTTTGCATTCAAGTAACAGTGTTGAACATTCCCTTTC

             *   *   *                   * *     *             **    *       
      96621 AGAGAACAGTTTTGAAACACTCTTTTTGTGGAATCTGCAAGTGGATATTTGTCTAGCATTGAGGA
          1 ATAGAGCAGGTTTGAAACACTCTTTTTGTAGTATCTGGAAGTGGATATTTGGATAGCTTTGAGGA

                         *   *    *                   *       *              
      96686 TTTCGTTGGAAACGGGATTA-CAT-ATA-AAAAGC-AGACAGCAGCATTCCCAGAAACTTCTTTG
         66 TTTCGTTGGAAACAGGAATATCTTCATATAAAAGCTAGACAGAAGCATTCTCAGAAACTTCTTTG

                              *  * *                 
      96747 TGATGTTTGCATTCAAGTCACCGAGTTGAACATTCCCTTTC
        131 TGATGTTTGCATTCAAGTAACAGTGTTGAACATTCCCTTTC

                                                   *     *       **     *   *
      96788 ATAGAGCAGGTTTGAAACACTCTTTTTGTAGTATCTGGATGTGGACATTTGGAGCGCTTTCAGGC
          1 ATAGAGCAGGTTTGAAACACTCTTTTTGTAGTATCTGGAAGTGGATATTTGGATAGCTTTGAGGA

            * ** *      *             *** *   *                      *   *   
      96853 CTATGGT-GAAAAAGGAAATATCTTCCCCTGAAAACTAGACAGAAGCATTCTCAGAATCTTATTT
         66 TTTCGTTGGAAACAGG-AATATCTTCATATAAAAGCTAGACAGAAGCATTCTCAGAAACTTCTTT

                   **  **    *             **  * *   *
      96917 GTGATGTGCGCCCTCAACTAACAGTGTTGAAGCTTTCTTTTG
        130 GTGATGTTTGCATTCAAGTAACAGTGTTGAACATTCCCTTTC

                     *                    **     *   *                       
      96959 ATAGAGCAGTTTTGAAACACTCTTTTTGTAAAATCTGCAAGAGGATATTTGGATAGCTTTGAGGA
          1 ATAGAGCAGGTTTGAAACACTCTTTTTGTAGTATCTGGAAGTGGATATTTGGATAGCTTTGAGGA

                *        *   * *            **                      *  * *   
      97024 TTTCATTGGAAACGGGATTGTCTTCATATAAACTCTAGACAGAAGCATTCTCAGAAGCTACATTG
         66 TTTCGTTGGAAACAGGAATATCTTCATATAAAAGCTAGACAGAAGCATTCTCAGAAACTTCTTTG

            *       **   *    *             *        
      97089 GGATGTTTCAATTGAAGTCACAGTGTTGAACAGTCCCTTTC
        131 TGATGTTTGCATTCAAGTAACAGTGTTGAACATTCCCTTTC

                                                   *     *       **     *   *
      97130 ATAGAGCAGGTTTGAAACACTCTTTTTGTAGTATCTGGATGTGGACATTTGGAGCGCTTTCAGGC
          1 ATAGAGCAGGTTTGAAACACTCTTTTTGTAGTATCTGGAAGTGGATATTTGGATAGCTTTGAGGA

            * ** *      *             *** *   *                          *   
      97195 CTATGGT-GAAAAAGGAAATATCTTCCCCTGAAAACTAGACAGAAGCATTCTCAGAAACTTATTT
         66 TTTCGTTGGAAACAGG-AATATCTTCATATAAAAGCTAGACAGAAGCATTCTCAGAAACTTCTTT

                   **  **    *             **  * *   *
      97259 GTGATGTGCGCCCTCAACTAACAGTGTTGAAGCTTTCTTTTG
        130 GTGATGTTTGCATTCAAGTAACAGTGTTGAACATTCCCTTTC

                     *                  ** *     *            ***            
      97301 ATAGAGCAGTTTTGAAACACTCTTTTTGGGGAATCTGCAAGTGGATATTTTTCTAGCTTTGAGGA
          1 ATAGAGCAGGTTTGAAACACTCTTTTTGTAGTATCTGGAAGTGGATATTTGGATAGCTTTGAGGA

                         *   *    *                   *       * *   *   *    
      97366 TTTCGTTGGAAACGGGATTA-CAT-ATA-AAAAGC-AGACAGCAGCATTCCCGGAATCTTGTTTG
         66 TTTCGTTGGAAACAGGAATATCTTCATATAAAAGCTAGACAGAAGCATTCTCAGAAACTTCTTTG

                              *    *                 
      97427 TGATGTTTGCATTCAAGTCACAGAGTTGAACATTCCCTTTC
        131 TGATGTTTGCATTCAAGTAACAGTGTTGAACATTCCCTTTC

             *                                     *     *     * **     *   *
      97468 AGAGAGCAGGTTTGAAACACTCTTTTTGTAGTATCTGGATGTGGACATTTGCAGCGCTTTCAGGC
          1 ATAGAGCAGGTTTGAAACACTCTTTTTGTAGTATCTGGAAGTGGATATTTGGATAGCTTTGAGGA

            * ** *      *             *** *   *                      *   *   
      97533 CTATGGT-GAAAAAGGAAATATCTTCTCCTGAAAACTAGACAGAAGCATTCTCAGAATCTTGTTT
         66 TTTCGTTGGAAACAGG-AATATCTTCATATAAAAGCTAGACAGAAGCATTCTCAGAAACTTCTTT

                   **  **    *             **  * *   *
      97597 GTGATGTGCGCCCTCAACTAACAGTGTTGAAGCTTTCTTTTG
        130 GTGATGTTTGCATTCAAGTAACAGTGTTGAACATTCCCTTTC

                     *                *   **     *   *        *              
      97639 ATAGAGCAGTTTTGAAACACTCTTTTCGTAAAATCTGCAAGAGGATATTTTGATAGCTTTGAGGA
          1 ATAGAGCAGGTTTGAAACACTCTTTTTGTAGTATCTGGAAGTGGATATTTGGATAGCTTTGAGGA

                   *     *   * *            **                      *    *   
      97704 TTTCGTTAGAAACGGGATTGTCTTCATATAAACTCTAGACAGAAGCATTCTCAGAAGCTTCATTG
         66 TTTCGTTGGAAACAGGAATATCTTCATATAAAAGCTAGACAGAAGCATTCTCAGAAACTTCTTTG

            *       **   *    *             *     *  
      97769 GGATGTTTCAATTGAAGTCACAGTGTTGAACAGTCCCTGTC
        131 TGATGTTTGCATTCAAGTAACAGTGTTGAACATTCCCTTTC

                                                         *       *  * * *    
      97810 ATAGAGCAGGTTTGAAACACTCTTTTTGTAGTATCTGGAAGTGGACATTTGGAGAGATCTCAGGA
          1 ATAGAGCAGGTTTGAAACACTCTTTTTGTAGTATCTGGAAGTGGATATTTGGATAGCTTTGAGGA

            * *  *    * *             **           **       * *          *  *
      97875 ATACGGT-GATAAAGGAAATATCTTCCAATAAAAGCTAGTTAGAAGCAATGTCAGAAACTTTTTC
         66 TTTCGTTGGAAACAGG-AATATCTTCATATAAAAGCTAGACAGAAGCATTCTCAGAAACTTCTTT

            *      * ** *   **      *       *  * *   *
      97939 ATGATGTATCTACTCAGCTAACAGAGTTGAACCTTTCTTTTG
        130 GTGATGTTTGCATTCAAGTAACAGTGTTGAACATTCCCTTTC

             *       *                                   *       **   * *    
      97981 AGAGAGCAGTTTTGAAACACTCTTTTTGTAGTATCTGGAAGTGGACATTTGGAACGCTCTCAGGA
          1 ATAGAGCAGGTTTGAAACACTCTTTTTGTAGTATCTGGAAGTGGATATTTGGATAGCTTTGAGGA

            * *  *      *             **            *       * **  *      *  *
      98046 CTGCGGT-GAAAAAGGAAATATCTTCCAATAAAAGCTAGATAGAAGCAATGCCACAAACTTTTTC
         66 TTTCGTTGGAAACAGG-AATATCTTCATATAAAAGCTAGACAGAAGCATTCTCAGAAACTTCTTT

            *    * * ** *   **      *    *  *  * *   *
      98110 ATGATATATCTACTCAGCTAACAGAGTTGGACCTTTCTTTTG
        130 GTGATGTTTGCATTCAAGTAACAGTGTTGAACATTCCCTTTC

             *       *                   * *     *             **            
      98152 AGAGAGCAGTTTTGAAACACTCTTTTTGTGGAATCTGCAAGTGGATATTTGTCTAGCTTTGAGGA
          1 ATAGAGCAGGTTTGAAACACTCTTTTTGTAGTATCTGGAAGTGGATATTTGGATAGCTTTGAGGA

                         *   *    *                   *       *              
      98217 TTTCGTTGGAAACGGGATTA-CAT-ATA-AAAAGC-AGACAGCAGCATTCCCAGAAACTTCTTTG
         66 TTTCGTTGGAAACAGGAATATCTTCATATAAAAGCTAGACAGAAGCATTCTCAGAAACTTCTTTG

                         *    *    *                 
      98278 TGATGTTTGCATTTAAGTCACAGAGTTGAACATTCCCTTTC
        131 TGATGTTTGCATTCAAGTAACAGTGTTGAACATTCCCTTTC

                                           *     * *   * *       **     *   *
      98319 ATAGAGCAGGTTTGAAACACTCTTTTTGTAGAATCTGTATGTGTACATTTGGAGCGCTTTCAGGC
          1 ATAGAGCAGGTTTGAAACACTCTTTTTGTAGTATCTGGAAGTGGATATTTGGATAGCTTTGAGGA

            * ** *      *             *** *   *                          *   
      98384 CTATGGT-GAAAAAGGAAATATCTTCCCCTGAAAACTAGACAGAAGCATTCTCAGAAACTTATTT
         66 TTTCGTTGGAAACAGG-AATATCTTCATATAAAAGCTAGACAGAAGCATTCTCAGAAACTTCTTT

                   **  **    *              *  * *   *
      98448 GTGATGTGCGCCCTCAACTAACAGTGTTGAACCTTTCTTTTG
        130 GTGATGTTTGCATTCAAGTAACAGTGTTGAACATTCCCTTTC

                   * *                    *      *   *                       
      98490 ATAGAGCCGTTTTGAAACACTCTTTTTGTAATATCTGCAAGAGGATATTTGGATAGCTTTGAGGA
          1 ATAGAGCAGGTTTGAAACACTCTTTTTGTAGTATCTGGAAGTGGATATTTGGATAGCTTTGAGGA

                             * *            **                      *    *   
      98555 TTTCGTTGGAAACAGGATTGTCTTCATATAAACTCTAGACAGAAGCATTCTCAGAAGCTTCATTG
         66 TTTCGTTGGAAACAGGAATATCTTCATATAAAAGCTAGACAGAAGCATTCTCAGAAACTTCTTTG

            *       **   *    *             *        
      98620 GGATGTTTCAATTGAAGTCACAGTGTTGAACAGTCCCTTTC
        131 TGATGTTTGCATTCAAGTAACAGTGTTGAACATTCCCTTTC

                                                         *       **   * *    
      98661 ATAGAGCAGGTTTGAAACACTCTTTTTGTAGTATCTGGAAGTGGACATTTGGAGCGCTCTCAGGA
          1 ATAGAGCAGGTTTGAAACACTCTTTTTGTAGTATCTGGAAGTGGATATTTGGATAGCTTTGAGGA

            * *  *      *             **     *    * *       * *          *  *
      98726 CTGCGGT-GAAAAAGGAAATATCTTCCAATAAAGGCTACATAGAAGCAATGTCAGAAACTTTTTC
         66 TTTCGTTGGAAACAGG-AATATCTTCATATAAAAGCTAGACAGAAGCATTCTCAGAAACTTCTTT

            *      * ** *   **      *       *        *
      98790 ATGATGTATCTACTCAGCTAACAGAGTTGAACCTT-CCTTTG
        130 GTGATGTTTGCATTCAAGTAACAGTGTTGAACATTCCCTTTC

             *       *                   * *     *             **            
      98831 AGAGAGCAGTTTTGAAACACTCTTTTTGTGGAATCTGCAAGTGGATATTTGTCTAGCTTTGAGGA
          1 ATAGAGCAGGTTTGAAACACTCTTTTTGTAGTATCTGGAAGTGGATATTTGGATAGCTTTGAGGA

                         *   *    *                   *       *              
      98896 TTTCGTTGGAAACGGGATTA-CAT-ATA-AAAAGC-AGACAGCAGCATTCCCAGAAACTTCTTTG
         66 TTTCGTTGGAAACAGGAATATCTTCATATAAAAGCTAGACAGAAGCATTCTCAGAAACTTCTTTG

                              *    *                 
      98957 TGATGTTTGCATTCAAGTCACAGAGTTGAACATTCCCTTTC
        131 TGATGTTTGCATTCAAGTAACAGTGTTGAACATTCCCTTTC

                      *                            *     *       **     *   *
      98998 ATAGAGCAGGATTGAAACACTCTTTTTGTAGTATCTGGATGTGGACATTTGGAGCGCTTTCAGGC
          1 ATAGAGCAGGTTTGAAACACTCTTTTTGTAGTATCTGGAAGTGGATATTTGGATAGCTTTGAGGA

            * ** *      *             *** *   *                          *   
      99063 CTAAGGT-GAAAAAGGAAATATCTTCCCCTGAAAACTAGACAGAAGCATTCTCAGAAACTTATTT
         66 TTTCGTTGGAAACAGG-AATATCTTCATATAAAAGCTAGACAGAAGCATTCTCAGAAACTTCTTT

                   **  **    *             **  * *   *
      99127 GTGATGTGCGCCCTCAACTAACAGTGTTGAAGCTTTCTTTTG
        130 GTGATGTTTGCATTCAAGTAACAGTGTTGAACATTCCCTTTC

                     *                   * *     *            ***            
      99169 ATAGAGCAGTTTTGAAACACTCTTTTTGTGGAATCTGCAAGTGGATATTTTTCTAGCTTTGAGGA
          1 ATAGAGCAGGTTTGAAACACTCTTTTTGTAGTATCTGGAAGTGGATATTTGGATAGCTTTGAGGA

                         *   *    *                   *       *     *   *    
      99234 TTTCGTTGGAAACGGGATTA-CAT-ATA-AAAAGC-AGACAGCAGCATTCCCAGAATCTTGTTTG
         66 TTTCGTTGGAAACAGGAATATCTTCATATAAAAGCTAGACAGAAGCATTCTCAGAAACTTCTTTG

                            * *    *                 
      99295 TGATGTTTGCATTCAACTCACAGAGTTGAACATTCCCTTTC
        131 TGATGTTTGCATTCAAGTAACAGTGTTGAACATTCCCTTTC

             *                         *           *     *       **     *   *
      99336 AGAGAGCAGGTTTGAAACACTCTTTTTATAGTATCTGGATGTGGACATTTGGAGCGCTTTCAGGC
          1 ATAGAGCAGGTTTGAAACACTCTTTTTGTAGTATCTGGAAGTGGATATTTGGATAGCTTTGAGGA

            * ** *      *             *** *   *                      *   *   
      99401 CTATGGT-GAAAAAGGAAATATCTTCTCCTGAAAACTAGACAGAAGCATTCTCAGAATCTTATTT
         66 TTTCGTTGGAAACAGG-AATATCTTCATATAAAAGCTAGACAGAAGCATTCTCAGAAACTTCTTT

                   *** **    *             **  * *   *
      99465 GTGATGTGCTCCCTCAACTAACAGTGTTGAAGCTTTCTTTTG
        130 GTGATGTTTGCATTCAAGTAACAGTGTTGAACATTCCCTTTC

                     *                *   **     *   *        *     *        
      99507 ATAGAGCAGTTTTGAAACACTCTTTTCGTAAAATCTGCAAGAGGATATTTTGATAGATTTGAGGA
          1 ATAGAGCAGGTTTGAAACACTCTTTTTGTAGTATCTGGAAGTGGATATTTGGATAGCTTTGAGGA

                         *   * *            **                      *    *   
      99572 TTTCGTTGGAAACGGGATTGTCTTCATATAAACTCTAGACAGAAGCATTCTCAGAAGCTTCATTG
         66 TTTCGTTGGAAACAGGAATATCTTCATATAAAAGCTAGACAGAAGCATTCTCAGAAACTTCTTTG

            *       **   *    *             *     *  
      99637 GGATGTTTCAATTGAAGTCACAGTGTTGAACAGTCCCTGTC
        131 TGATGTTTGCATTCAAGTAACAGTGTTGAACATTCCCTTTC

                                                    *    *       *  * * *    
      99678 ATAGAGCAGGTTTGAAACACTCTTTTTGTAGTATCTGGAACTGGACATTTGGAGAGATCTCAGGA
          1 ATAGAGCAGGTTTGAAACACTCTTTTTGTAGTATCTGGAAGTGGATATTTGGATAGCTTTGAGGA

            * *  *    * *             **            *       * *          *  *
      99743 ATACGGT-GATAAAGGAAATATCTTCCAATAAAAGCTAGATAGAAGCAATGTCAGAAACTTTTTC
         66 TTTCGTTGGAAACAGG-AATATCTTCATATAAAAGCTAGACAGAAGCATTCTCAGAAACTTCTTT

            *      * ** *   **      *       *  * *   *
      99807 ATGATGTATCTACTCAGCTAACAGAGTTGAACCTTTCTTTTG
        130 GTGATGTTTGCATTCAAGTAACAGTGTTGAACATTCCCTTTC

             *       *                                   *       **   * *    
      99849 AGAGAGCAGTTTTGAAACACTCTTTTTGTAGTATCTGGAAGTGGACATTTGGAGCGCTCTCAGGA
          1 ATAGAGCAGGTTTGAAACACTCTTTTTGTAGTATCTGGAAGTGGATATTTGGATAGCTTTGAGGA

            * *  *      *             **            *    *  * *          *  *
      99914 CTGCGGT-GAAAAAGGAAATATCTTCCAATAAAAGCTAGATAGAACCAATGTCAGAAACTTTTTC
         66 TTTCGTTGGAAACAGG-AATATCTTCATATAAAAGCTAGACAGAAGCATTCTCAGAAACTTCTTT

            *      * *  *   **      *       *  * *   *
      99978 ATGATGTATCCACTCAGCTAACAGAGTTGAACCTTTCTTTTG
        130 GTGATGTTTGCATTCAAGTAACAGTGTTGAACATTCCCTTTC

             *       *                   * *     *             **  **        
     100020 AGAGAGCAGTTTTGAAACACTCTTTTTGTGGAATCTGCAAGTGGATATTTGTCTACATTTGAGGA
          1 ATAGAGCAGGTTTGAAACACTCTTTTTGTAGTATCTGGAAGTGGATATTTGGATAGCTTTGAGGA

                         *   *    *                   *       *              
     100085 TTTCGTTGGAAACGGGATTA-CAT-ATA-AAAAGC-AGACAGCAGCATTCCCAGAAACTTCTTTG
         66 TTTCGTTGGAAACAGGAATATCTTCATATAAAAGCTAGACAGAAGCATTCTCAGAAACTTCTTTG

                              *    *                 
     100146 TGATGTTTGCATTCAAGTCACAGAGTTGAACATTCCCTTTC
        131 TGATGTTTGCATTCAAGTAACAGTGTTGAACATTCCCTTTC

                                           *     * *     *       **     *   *
     100187 ATAGAGCAGGTTTGAAACACTCTTTTTGTAGAATCTGTATGTGGACATTTGGAGCGCTTTCAGGC
          1 ATAGAGCAGGTTTGAAACACTCTTTTTGTAGTATCTGGAAGTGGATATTTGGATAGCTTTGAGGA

            * ** *      *             *** *   *                          *   
     100252 CTATGGT-GAAAAAGGAAATATCTTCCCCTGAAAACTAGACAGAAGCATTCTCAGAAACTTATTT
         66 TTTCGTTGGAAACAGG-AATATCTTCATATAAAAGCTAGACAGAAGCATTCTCAGAAACTTCTTT

                   **  **    *              *  * *   *
     100316 GTGATGTGCGCCCTCAACTAACAGTGTTGAACCTTTCTTTTG
        130 GTGATGTTTGCATTCAAGTAACAGTGTTGAACATTCCCTTTC

                     *                    *      *   *        *              
     100358 ATAGAGCAGTTTTGAAACACTCTTTTTGTAATATCTGCAAGAGGATATTTTGATAGCTTTGAGGA
          1 ATAGAGCAGGTTTGAAACACTCTTTTTGTAGTATCTGGAAGTGGATATTTGGATAGCTTTGAGGA

                         *   * *            **                      *    **  
     100423 TTTCGTTGGAAACGGGATTGTCTTCATATAAACTCTAGACAGAAGCATTCTCAGAAGCTTCAATG
         66 TTTCGTTGGAAACAGGAATATCTTCATATAAAAGCTAGACAGAAGCATTCTCAGAAACTTCTTTG

            *       **   *    *             * *      
     100488 GGATGTTTCAATTGAAGTCACAGTGTTGAACAGTTCCTTTC
        131 TGATGTTTGCATTCAAGTAACAGTGTTGAACATTCCCTTTC

                                                         *       **   * *    
     100529 ATAGAGCAGGTTTGAAACACTCTTTTTGTAGTATCTGGAAGTGGACATTTGGAGCGCTCTCAGGA
          1 ATAGAGCAGGTTTGAAACACTCTTTTTGTAGTATCTGGAAGTGGATATTTGGATAGCTTTGAGGA

            * ** *    * *             **            **      * *          *  *
     100594 CTAAGGT-GATAAAGGAAATATCTTCCAATAAAAGCTAGATTGAAGCAATGTCAGAAACTTTTTC
         66 TTTCGTTGGAAACAGG-AATATCTTCATATAAAAGCTAGACAGAAGCATTCTCAGAAACTTCTTT

            *   *  * ** *   **      *      **  *     *
     100658 ATGACGTATCTACTCAGCTAACAGAGTTGAAACTTTCCTTTG
        130 GTGATGTTTGCATTCAAGTAACAGTGTTGAACATTCCCTTTC

             *       *                   * *     *             **            
     100700 AGAGAGCAGTTTTGAAACACTCTTTTTGTGGAATCTGCAAGTGGATATTTGTCTAGCTTTGAGGA
          1 ATAGAGCAGGTTTGAAACACTCTTTTTGTAGTATCTGGAAGTGGATATTTGGATAGCTTTGAGGA

                         *   *    *                   *       *   *          
     100765 TTTCGTTGGAAACGGGATTA-CAT-ATA-AAAAGC-AGACAGCAGCATTCCCAGTAACTTCTTTG
         66 TTTCGTTGGAAACAGGAATATCTTCATATAAAAGCTAGACAGAAGCATTCTCAGAAACTTCTTTG

                              *    *                 
     100826 TGATGTTTGCATTCAAGTCACAGAGTTGAACATTCCCTTTC
        131 TGATGTTTGCATTCAAGTAACAGTGTTGAACATTCCCTTTC

                                                   *     *       **     *   *
     100867 ATAGAGCAGGTTTGAAACACTCTTTTTGTAGTATCTGGATGTGGACATTTGGAGCGCTTTCAGGC
          1 ATAGAGCAGGTTTGAAACACTCTTTTTGTAGTATCTGGAAGTGGATATTTGGATAGCTTTGAGGA

            * ** *      *             *** *   *                      *   *   
     100932 CTATGGT-GAAAAAGGAAATATCTTCTCCTGAAAACTAGACAGAAGCATTCTCAGAATCTTATTT
         66 TTTCGTTGGAAACAGG-AATATCTTCATATAAAAGCTAGACAGAAGCATTCTCAGAAACTTCTTT

                   **  **    *             **  * *   *
     100996 GTGATGTGCGCCCTCAACTAACAGTGTTGAAGCTTTCTTTTG
        130 GTGATGTTTGCATTCAAGTAACAGTGTTGAACATTCCCTTTC

                     *                *   **     *   *        *              
     101038 ATAGAGCAGTTTTGAAACACTCTTTTCGTAAAATCTGCAAGAGGATATTTTGATAGCTTTGAGGA
          1 ATAGAGCAGGTTTGAAACACTCTTTTTGTAGTATCTGGAAGTGGATATTTGGATAGCTTTGAGGA

                         **  * *            **   *                  *    *   
     101103 TTTCGTTGGAAACGAGATTGTCTTCATATAAACTCTACACAGAAGCATTCTCAGAAGCTTCATTG
         66 TTTCGTTGGAAACAGGAATATCTTCATATAAAAGCTAGACAGAAGCATTCTCAGAAACTTCTTTG

            *       **   *    *             *     *  
     101168 GGATGTTTCAATTGAAGTCACAGTGTTGAACAGTCCCTGTC
        131 TGATGTTTGCATTCAAGTAACAGTGTTGAACATTCCCTTTC

               *             *                           *       *  * * *    
     101209 ATACAGCAGGTTTGAAAAACTCTTTTTGTAGTATCTGGAAGTGGACATTTGGAGAGATCTCAGGA
          1 ATAGAGCAGGTTTGAAACACTCTTTTTGTAGTATCTGGAAGTGGATATTTGGATAGCTTTGAGGA

            * *  *    * *             **            *       * *          *  *
     101274 ATACGGT-GATAAAGGAAATATCTTCCAATAAAAGCTAGATAGAAGCAATGTCAGAAACTTTTTC
         66 TTTCGTTGGAAACAGG-AATATCTTCATATAAAAGCTAGACAGAAGCATTCTCAGAAACTTCTTT

            *      * *  *   **      *       *  * *   *
     101338 ATGATGTATCCACTCAGCTAACAGAGTTGAACCTTTCTTTTG
        130 GTGATGTTTGCATTCAAGTAACAGTGTTGAACATTCCCTTTC

             *       *                                   *       ** * * *    
     101380 AGAGAGCAGTTTTGAAACACTCTTTTTGTAGTATCTGGAAGTGGACATTTGGAGCGTTCTCAGGA
          1 ATAGAGCAGGTTTGAAACACTCTTTTTGTAGTATCTGGAAGTGGATATTTGGATAGCTTTGAGGA

            * *  *      *             **            *       * *    *     *  *
     101445 CTGCGGT-GAAAAAGGAAATATCTTCCAATAAAAGCTAGATAGAAGCAATGTCAGCAACTTTTTC
         66 TTTCGTTGGAAACAGG-AATATCTTCATATAAAAGCTAGACAGAAGCATTCTCAGAAACTTCTTT

            *      * ** *   **      *       *  * *   *
     101509 ATGATGTATCTACTCAGCTAACAGAGTTGAACCTTTCTTTTG
        130 GTGATGTTTGCATTCAAGTAACAGTGTTGAACATTCCCTTTC

             *       *                   * *     *             **            
     101551 AGAGAGCAGTTTTGAAACACTCTTTTTGTGGAATCTGCAAGTGGATATTTGTCTAGCTTTGAGGA
          1 ATAGAGCAGGTTTGAAACACTCTTTTTGTAGTATCTGGAAGTGGATATTTGGATAGCTTTGAGGA

                         *   *    *  *                *       *              
     101616 TTTCGTTGGAAACGGGATTA-CAT-TTA-AAAAGC-AGACAGCAGCATTCCCAG-AACTTCTTTG
         66 TTTCGTTGGAAACAGGAATATCTTCATATAAAAGCTAGACAGAAGCATTCTCAGAAACTTCTTTG

                              *    *                 
     101676 TGATGTTTGCATTCAAGTCACAGAGTTGAACATTCCCTTTC
        131 TGATGTTTGCATTCAAGTAACAGTGTTGAACATTCCCTTTC

                                                 * *     *       **     *   *
     101717 ATAGAGCAGGTTTGAAACACTCTTTTTGTAGTATCTGTATGTGGACATTTGGAGCGCTTTCAGGC
          1 ATAGAGCAGGTTTGAAACACTCTTTTTGTAGTATCTGGAAGTGGATATTTGGATAGCTTTGAGGA

            * ** *      *             *** *   *                          *   
     101782 CTATGGT-GAAAAAGGAAATATCTTCCCCTGAAAACTAGACAGAAGCATTCTCAGAAACTTATTT
         66 TTTCGTTGGAAACAGG-AATATCTTCATATAAAAGCTAGACAGAAGCATTCTCAGAAACTTCTTT

                   **  **    *              *  * *   *
     101846 GTGATGTGCGCCCTCAACTAACAGTGTTGAACCTTTCTTTTG
        130 GTGATGTTTGCATTCAAGTAACAGTGTTGAACATTCCCTTTC

                     *                    *      *   *                       
     101888 ATAGAGCAGTTTTGAAACACTCTTTTTGTAATATCTGCAAGAGGATATTTGGATAGCTTTGAGGA
          1 ATAGAGCAGGTTTGAAACACTCTTTTTGTAGTATCTGGAAGTGGATATTTGGATAGCTTTGAGGA

                         *   * *            **                      *    **  
     101953 TTTCGTTGGAAACGGGATTGTCTTCATATAAACTCTAGACAGAAGCATTCTCAGAAGCTTCAATG
         66 TTTCGTTGGAAACAGGAATATCTTCATATAAAAGCTAGACAGAAGCATTCTCAGAAACTTCTTTG

            *       **   *    *           * * *      
     102018 GGATGTTTCAATTGAAGTCACAGTGTTGAAGAGTTCCTTTC
        131 TGATGTTTGCATTCAAGTAACAGTGTTGAACATTCCCTTTC

                                                         *       **   * *    
     102059 ATAGAGCAGGTTTGAAACACTCTTTTTGTAGTATCTGGAAGTGGACATTTGGAGCGCTCTCAGGA
          1 ATAGAGCAGGTTTGAAACACTCTTTTTGTAGTATCTGGAAGTGGATATTTGGATAGCTTTGAGGA

            * ** *    * *             **            **      * *          *  *
     102124 CTAAGGT-GATAAAGGATATATCTTCCAATAAAAGCTAGATTGAAGCAATGTCAGAAACTTTTTC
         66 TTTCGTTGGAAACAGGA-ATATCTTCATATAAAAGCTAGACAGAAGCATTCTCAGAAACTTCTTT

            *   *  * ** *   **      *      **  *     *
     102188 ATGACGTATCTACTCAGCTAACAGAGTTGAAACTTTCCTTTG
        130 GTGATGTTTGCATTCAAGTAACAGTGTTGAACATTCCCTTTC

             *       *                   * *     *             **            
     102230 AGAGAGCAGTTTTGAAACACTCTTTTTGTGGAATCTGCAAGTGGATATTTGTCTAGCTTTGAGGA
          1 ATAGAGCAGGTTTGAAACACTCTTTTTGTAGTATCTGGAAGTGGATATTTGGATAGCTTTGAGGA

                         *   *    *                   *       *   *          
     102295 TTTCGTTGGAAACGGGATTA-CAT-ATA-AAAAGC-AGACAGCAGCATTCCCAGTAACTTCTTTG
         66 TTTCGTTGGAAACAGGAATATCTTCATATAAAAGCTAGACAGAAGCATTCTCAGAAACTTCTTTG

                              *    *                 
     102356 TGATGTTTGCATTCAAGTCACAGAGTTGAACATTCCCTTTC
        131 TGATGTTTGCATTCAAGTAACAGTGTTGAACATTCCCTTTC

                                                 * *     *       **     *  **
     102397 ATAGAGCAGGTTTGAAACACTCTTTTTGTAGTATCTGTATGTGGACATTTGGAGCGCTTTCAGTC
          1 ATAGAGCAGGTTTGAAACACTCTTTTTGTAGTATCTGGAAGTGGATATTTGGATAGCTTTGAGGA

            * ** *      *             *** *   *                          *   
     102462 CTATGGT-GAAAAAGGAAATATCTTCCCCTGAAAACTAGACAGAAGCATTCTCAGAAACTTATTT
         66 TTTCGTTGGAAACAGG-AATATCTTCATATAAAAGCTAGACAGAAGCATTCTCAGAAACTTCTTT

                   **  **    *              *  * *   *
     102526 GTGATGTGCGCCCTCAACTAACAGTGTTGAACCTTTCTTTTG
        130 GTGATGTTTGCATTCAAGTAACAGTGTTGAACATTCCCTTTC

                     *                    *      *   *              *        
     102568 ATAGAGCAGTTTTGAAACACTCTTTTTGTAATATCTGCAAGAGGATATTTGGATAGATTTGAGGA
          1 ATAGAGCAGGTTTGAAACACTCTTTTTGTAGTATCTGGAAGTGGATATTTGGATAGCTTTGAGGA

                *       **   * *            **                      *    *   
     102633 TTTCTTTGGAAAGGGGATTGTCTTCATATAAACTCTAGACAGAAGCATTCTCAGAAGCTTCATTG
         66 TTTCGTTGGAAACAGGAATATCTTCATATAAAAGCTAGACAGAAGCATTCTCAGAAACTTCTTTG

            *       **   *    *             * *      
     102698 GGATGTTTCAATTGAAGTCACAGTGTTGAACAGTTCCTTTC
        131 TGATGTTTGCATTCAAGTAACAGTGTTGAACATTCCCTTTC

                                                         *     * **   * *    
     102739 ATAGAGCAGGTTTGAAACACTCTTTTTGTAGTATCTGGAAGTGGACATTTGCAGCGCTCTCAGGA
          1 ATAGAGCAGGTTTGAAACACTCTTTTTGTAGTATCTGGAAGTGGATATTTGGATAGCTTTGAGGA

            * *  *    * *             **            *       * *          *  *
     102804 CTACGGT-GATAAAGGAAATATCTTCCAATAAAAGCTAGATAGAAGCAATGTCAGAAACTTTTTC
         66 TTTCGTTGGAAACAGG-AATATCTTCATATAAAAGCTAGACAGAAGCATTCTCAGAAACTTCTTT

            *      * ** *   **      *      **  *     *
     102868 ATGATGTATCTACTCAGCTAACAGAGTTGAAACTTTCCTTTG
        130 GTGATGTTTGCATTCAAGTAACAGTGTTGAACATTCCCTTTC

             *       *                   * *     *             **            
     102910 AGAGAGCAGTTTTGAAACACTCTTTTTGTGGAATCTGCAAGTGGATATTTGTCTAGCTTTGAGGA
          1 ATAGAGCAGGTTTGAAACACTCTTTTTGTAGTATCTGGAAGTGGATATTTGGATAGCTTTGAGGA

                         *   *     *                  *       *  **          
     102975 TTTCGTTGGAAACGGGATTA-C-ACATA-AAAAGC-AGACAGCAGCATTCCCATTAACTTCTTTG
         66 TTTCGTTGGAAACAGGAATATCTTCATATAAAAGCTAGACAGAAGCATTCTCAGAAACTTCTTTG

                              *    *                 
     103036 TGATGTTTGCATTCAAGTCACAGAGTTGAACATTCCCTTTC
        131 TGATGTTTGCATTCAAGTAACAGTGTTGAACATTCCCTTTC

                                                   *     *       **     *   *
     103077 ATAGAGCAGGTTTGAAACACTCTTTTTGTAGTATCTGGATGTGGACATTTGGAGCGCTTTCAGGC
          1 ATAGAGCAGGTTTGAAACACTCTTTTTGTAGTATCTGGAAGTGGATATTTGGATAGCTTTGAGGA

            * ** *      *             *** *   *                          *   
     103142 CTATGGT-GAAAAAGGAAATATCTTCCCCTGAAAACTAGACAGAAGCATTCTCAGAAACTTATTT
         66 TTTCGTTGGAAACAGG-AATATCTTCATATAAAAGCTAGACAGAAGCATTCTCAGAAACTTCTTT

                   **  **    *              *  * *   *
     103206 GTGATGTGCGCCCTCAACTAACAGTGTTGAACCTTTCTTTTG
        130 GTGATGTTTGCATTCAAGTAACAGTGTTGAACATTCCCTTTC

                     *                    *      *   *                       
     103248 ATAGAGCAGTTTTGAAACACTCTTTTTGTAATATCTGCAAGAGGATATTTGGATAGCTTTGAGGA
          1 ATAGAGCAGGTTTGAAACACTCTTTTTGTAGTATCTGGAAGTGGATATTTGGATAGCTTTGAGGA

                         *   * **           **                      *    *   
     103313 TTTCGTTGGAAACGGGATTGACTTCATATAAACTCTAGACAGAAGCATTCTCAGAAGCTTCATTG
         66 TTTCGTTGGAAACAGGAATATCTTCATATAAAAGCTAGACAGAAGCATTCTCAGAAACTTCTTTG

            *       **   *    *             *        
     103378 GGATGTTTCAATTGAAGTCACAGTGTTGAACAGTCCCTTTC
        131 TGATGTTTGCATTCAAGTAACAGTGTTGAACATTCCCTTTC

                                                         *       *  * * *    
     103419 ATAGAGCAGGTTTGAAACACTCTTTTTGTAGTATCTGGAAGTGGACATTTGGAGAGATCTCAGGA
          1 ATAGAGCAGGTTTGAAACACTCTTTTTGTAGTATCTGGAAGTGGATATTTGGATAGCTTTGAGGA

            * ** *    * *             **            *       * *       *  ** *
     103484 ATAAGGT-GACAAAGGAAATATCTTCCAATAAAAGCTAGATAGAAGCAATGTCAGAAAATTTCTC
         66 TTTCGTTGGAAACAGG-AATATCTTCATATAAAAGCTAGACAGAAGCATTCTCAGAAACTTCTTT

            *      * ** *   **      *       *  * *   *
     103548 ATGATGTATCTACTCAGCTAACAGAGTTGAACCTTTCTTTTG
        130 GTGATGTTTGCATTCAAGTAACAGTGTTGAACATTCCCTTTC

             *       *                   * *     *             **            
     103590 AGAGAGCAGTTTTGAAACACTCTTTTTGTGGAATCTGCAAGTGGATATTTGTCTAGCTTTGAGGA
          1 ATAGAGCAGGTTTGAAACACTCTTTTTGTAGTATCTGGAAGTGGATATTTGGATAGCTTTGAGGA

             *           *   *    *                   *       *   *          
     103655 TATCGTTGGAAACGGGATTA-CAT-ATA-AAAAGC-AGACAGCAGCATTCCCAGTAACTTCTTTG
         66 TTTCGTTGGAAACAGGAATATCTTCATATAAAAGCTAGACAGAAGCATTCTCAGAAACTTCTTTG

                              *    *                 
     103716 TGATGTTTGCATTCAAGTCACAGAGTTGAACATTCCCTTTC
        131 TGATGTTTGCATTCAAGTAACAGTGTTGAACATTCCCTTTC

                                                   *     *       **     *   *
     103757 ATAGAGCAGGTTTGAAACACTCTTTTTGTAGTATCTGGATGTGGACATTTGGAGCGCTTTCAGGC
          1 ATAGAGCAGGTTTGAAACACTCTTTTTGTAGTATCTGGAAGTGGATATTTGGATAGCTTTGAGGA

            * ** *      *             *** *   *                      *   *   
     103822 CTATGGT-GAAAAAGGAAATATCTTCCCCTGAAAACTAGACAGAAGCATTCTCAGAATCTTATTT
         66 TTTCGTTGGAAACAGG-AATATCTTCATATAAAAGCTAGACAGAAGCATTCTCAGAAACTTCTTT

                   **  **    *             **  * *   *
     103886 GTGATGTGCGCCCTCAACTAACAGTGTTGAAGCTTTCTTTTG
        130 GTGATGTTTGCATTCAAGTAACAGTGTTGAACATTCCCTTTC

                     *          *         **     *   *                       
     103928 ATAGAGCAGTTTTGAAACACCCTTTTTGTAAAATCTGCAAGAGGATATTTGGATAGCTTTGAGGA
          1 ATAGAGCAGGTTTGAAACACTCTTTTTGTAGTATCTGGAAGTGGATATTTGGATAGCTTTGAGGA

                             * *            **                      *    *   
     103993 TTTCGTTGGAAACAGGATTGTCTTCATATAAACTCTAGACAGAAGCATTCTCAGAAGCTTCATTG
         66 TTTCGTTGGAAACAGGAATATCTTCATATAAAAGCTAGACAGAAGCATTCTCAGAAACTTCTTTG

            *       **   *    *             *        
     104058 GGATGTTTCAATTGAAGTCACAGTGTTGAACAGTCCCTTTC
        131 TGATGTTTGCATTCAAGTAACAGTGTTGAACATTCCCTTTC

                                                         *       **   * *    
     104099 ATAGAGCAGGTTTGAAACACTCTTTTTGTAGTATCTGGAAGTGGACATTTGGAGCGCTCTCAGGA
          1 ATAGAGCAGGTTTGAAACACTCTTTTTGTAGTATCTGGAAGTGGATATTTGGATAGCTTTGAGGA

            * * **      *             **     *    * *       * *          *  *
     104164 CTACAGT-GAAAAAGGAAATATCTTCCAATAAAGGCTACATAGAAGCAATGTCAGAAACTTTTTC
         66 TTTCGTTGGAAACAGG-AATATCTTCATATAAAAGCTAGACAGAAGCATTCTCAGAAACTTCTTT

            *      * ** *   **      **      *  * *   *
     104228 ATGATGTATCTACTCAGCTAACAGATTTGAACCTTTCTTTTG
        130 GTGATGTTTGCATTCAAGTAACAGTGTTGAACATTCCCTTTC

             *       *                   * *     *             **            
     104270 AGAGAGCAGTTTTGAAACACTCTTTTTGTGGAATCTGCAAGTGGATATTTGTCTAGCTTTGAGGA
          1 ATAGAGCAGGTTTGAAACACTCTTTTTGTAGTATCTGGAAGTGGATATTTGGATAGCTTTGAGGA

                         *   *    *                   *       *              
     104335 TTTCGTTGGAAACGGGATTA-CAT-ATA-AAAAGC-AGACAGCAGCATTCCCAGAAACTTCTTTG
         66 TTTCGTTGGAAACAGGAATATCTTCATATAAAAGCTAGACAGAAGCATTCTCAGAAACTTCTTTG

                              *    *                 
     104396 TGATGTTTGCATTCAAGTCACAGAGTTGAACATTCCCTTTC
        131 TGATGTTTGCATTCAAGTAACAGTGTTGAACATTCCCTTTC

                                                 * *     *       **     *   *
     104437 ATAGAGCAGGTTTGAAACACTCTTTTTGTAGTATCTGTATGTGGACATTTGGAGCGCTTTCAGGC
          1 ATAGAGCAGGTTTGAAACACTCTTTTTGTAGTATCTGGAAGTGGATATTTGGATAGCTTTGAGGA

            * ** *      *             *** *   **                      *  *   
     104502 CTATGGT-GAAAAAGGAAATATCTTCCCCTGAAAATTAGACAGAAGCATTCTCAGAAATTTATTT
         66 TTTCGTTGGAAACAGG-AATATCTTCATATAAAAGCTAGACAGAAGCATTCTCAGAAACTTCTTT

                   **  **    *             **  * *   *
     104566 GTGATGTGCGCCCTCAACTAACAGTGTTGAAGCTTTCTTTTG
        130 GTGATGTTTGCATTCAAGTAACAGTGTTGAACATTCCCTTTC

                *    *                    **     *   *                       
     104608 ATAGCGCAGTTTTGAAACACTCTTTTTGTAAAATCTGCAAGAGGATATTTGGATAGCTTTGAGGA
          1 ATAGAGCAGGTTTGAAACACTCTTTTTGTAGTATCTGGAAGTGGATATTTGGATAGCTTTGAGGA

                         *   * *            **                      *    *   
     104673 TTTCGTTGGAAACGGGATTGTCTTCATATAAACTCTAGACAGAAGCATTCTCAGAAGCTTCATTG
         66 TTTCGTTGGAAACAGGAATATCTTCATATAAAAGCTAGACAGAAGCATTCTCAGAAACTTCTTTG

            *       **   *    *             *        
     104738 GGATGTTTCAATTGAAGTCACAGTGTTGAACAGTCCCTTTC
        131 TGATGTTTGCATTCAAGTAACAGTGTTGAACATTCCCTTTC

                                                         *       **   * *    
     104779 ATAGAGCAGGTTTGAAACACTCTTTTTGTAGTATCTGGAAGTGGACATTTGGAGCGCTCTCAGGA
          1 ATAGAGCAGGTTTGAAACACTCTTTTTGTAGTATCTGGAAGTGGATATTTGGATAGCTTTGAGGA

            * *  *      *             **            *       * *          *  *
     104844 CTGCGGT-GAAAAAGGAAATATCTTCCAATAAAAGCTAGATAGAAGCAATGTCAGAAACTTTTTC
         66 TTTCGTTGGAAACAGG-AATATCTTCATATAAAAGCTAGACAGAAGCATTCTCAGAAACTTCTTT

            *      * ** *   **      *       *        *
     104908 ATGATGTATCTACTCAGCTAACAGAGTTGAACCTT-CCTTTG
        130 GTGATGTTTGCATTCAAGTAACAGTGTTGAACATTCCCTTTC

             *       *                   ***     *             **            
     104949 AGAGAGCAGTTTTGAAACACTCTTTTTGTGAAATCTGCAAGTGGATATTTGTCTAGCTTTGAGGA
          1 ATAGAGCAGGTTTGAAACACTCTTTTTGTAGTATCTGGAAGTGGATATTTGGATAGCTTTGAGGA

                         *   *    *                   *  *    *   *          
     105014 TTTCGTTGGAAACGGGATTA-CAT-ATA-AAAAGC-AGACAGCAGTATTCCCAGTAACTTCTTTG
         66 TTTCGTTGGAAACAGGAATATCTTCATATAAAAGCTAGACAGAAGCATTCTCAGAAACTTCTTTG

                              *    *                 
     105075 TGATGTTTGCATTCAAGTCACAGAGTTGAACATTCCCTTTC
        131 TGATGTTTGCATTCAAGTAACAGTGTTGAACATTCCCTTTC

                                                   *     *       **     *   *
     105116 ATAGAGCAGGTTTGAAACACTCTTTTTGTAGTATCTGGATGTGGACATTTGGAGTGCTTTCAGGC
          1 ATAGAGCAGGTTTGAAACACTCTTTTTGTAGTATCTGGAAGTGGATATTTGGATAGCTTTGAGGA

            * ** *      *             *** *   *                      *   *   
     105181 CTATGGT-GAAAAAGGAAATATCTTCCCCTGAAAACTAGACAGAAGCATTCTCAGAATCTTATTT
         66 TTTCGTTGGAAACAGG-AATATCTTCATATAAAAGCTAGACAGAAGCATTCTCAGAAACTTCTTT

                   **  **    *             **  * *   *
     105245 GTGATGTGCGCCCTCAACTAACAGTGTTGAAGTTTTCTTTTG
        130 GTGATGTTTGCATTCAAGTAACAGTGTTGAACATTCCCTTTC

                     *                    **     *   * *                     
     105287 ATAGAGCAGTTTTGAAACACTCTTTTTGTAAAATCTGCAAGAGTATATTTGGATAGCTTTGAGGA
          1 ATAGAGCAGGTTTGAAACACTCTTTTTGTAGTATCTGGAAGTGGATATTTGGATAGCTTTGAGGA

                         *   * *            **                      * *  *   
     105352 TTTCGTTGGAAACGGGATTGTCTTCATATAAACTCTAGACAGAAGCATTCTCAGAAGCCTCATTG
         66 TTTCGTTGGAAACAGGAATATCTTCATATAAAAGCTAGACAGAAGCATTCTCAGAAACTTCTTTG

            *       **   *    *             *        
     105417 GGATGTTTCAATTGAAGTCACAGTGTTGAACAGTCCCTTTC
        131 TGATGTTTGCATTCAAGTAACAGTGTTGAACATTCCCTTTC

                                                   *     *       **     *   *
     105458 ATAGAGCAGGTTTGAAACACTCTTTTTGTAGTATCTGGATGTGGACATTTGGAGCGCTTTCAGGC
          1 ATAGAGCAGGTTTGAAACACTCTTTTTGTAGTATCTGGAAGTGGATATTTGGATAGCTTTGAGGA

            * ** *      *             *** *   *                      *   *   
     105523 CTATGGT-GAAAAAGGAAATATCTTCCCCTGAAAACTAGACAGAAGCATTCTCAGAATCTTATTT
         66 TTTCGTTGGAAACAGG-AATATCTTCATATAAAAGCTAGACAGAAGCATTCTCAGAAACTTCTTT

                   **  **    *             **  * *   *
     105587 GTGATGTGCGCCCTCAACTAACAGTGTTGAAGCTTTCTTTTG
        130 GTGATGTTTGCATTCAAGTAACAGTGTTGAACATTCCCTTTC

                     *                   * *     *             **            
     105629 ATAGAGCAGTTTTGAAACACTCTTTTTGTGGAATCTGCAAGTGGATATTTGTCTAGCTTTGAGGA
          1 ATAGAGCAGGTTTGAAACACTCTTTTTGTAGTATCTGGAAGTGGATATTTGGATAGCTTTGAGGA

                         *   *    *                   *       *              
     105694 TTTCGTTGGAAACGGGATTA-CAT-ATA-AAAAGC-AGACAGCAGCATTCCCAGAAACTTCTTTG
         66 TTTCGTTGGAAACAGGAATATCTTCATATAAAAGCTAGACAGAAGCATTCTCAGAAACTTCTTTG

                              *    *                 
     105755 TGATGTTTGCATTCAAGTCACAGAGTTGAACATTCCCTTTC
        131 TGATGTTTGCATTCAAGTAACAGTGTTGAACATTCCCTTTC

                                                   *     *       **     *   *
     105796 ATAGAGCAGGTTTGAAACACTCTTTTTGTAGTATCTGGATGTGGACATTTGGACCGCTTTCAGGC
          1 ATAGAGCAGGTTTGAAACACTCTTTTTGTAGTATCTGGAAGTGGATATTTGGATAGCTTTGAGGA

            * ** *      *             *** *   *                          *   
     105861 CTATGGT-GAAAAAGGAAATATCTTCCCCTGAAAACTAGACAGAAGCATTCTCAGAAACTTATTT
         66 TTTCGTTGGAAACAGG-AATATCTTCATATAAAAGCTAGACAGAAGCATTCTCAGAAACTTCTTT

                   **  **    *             **  * *   *
     105925 GTGATGTGCGCCCTCAACTAACAGTGTTGAAGCTTTCTTTTG
        130 GTGATGTTTGCATTCAAGTAACAGTGTTGAACATTCCCTTTC

                     *                    *      *   *                       
     105967 ATAGAGCAGTTTTGAAACACTCTTTTTGTAATATCTGCAAGAGGATATTTGGATAGCTTTGAGGA
          1 ATAGAGCAGGTTTGAAACACTCTTTTTGTAGTATCTGGAAGTGGATATTTGGATAGCTTTGAGGA

                         *   * *            **                      *    *   
     106032 TTTCGTTGGAAACGGGATTGTCTTCATATAAACTCTAGACAGAAGCATTCTCAGAAGCTTCATTG
         66 TTTCGTTGGAAACAGGAATATCTTCATATAAAAGCTAGACAGAAGCATTCTCAGAAACTTCTTTG

            *       **   *    *             *        
     106097 GGATGTTTCAATTGAAGTCACAGTGTTGAACAGTCCCTTTC
        131 TGATGTTTGCATTCAAGTAACAGTGTTGAACATTCCCTTTC

                                                         *       **   * *    
     106138 ATAGAGCAGGTTTGAAACACTCTTTTTGTAGTATCTGGAAGTGGACATTTGGAGCGCTCTCAGGA
          1 ATAGAGCAGGTTTGAAACACTCTTTTTGTAGTATCTGGAAGTGGATATTTGGATAGCTTTGAGGA

            * *  *      *             **            *       * *          *  *
     106203 CTGCGGT-GAAAAAGGAAATATCTTCCAATAAAAGCTAGATAGAAGCAATGTCAGAAACTTTTTC
         66 TTTCGTTGGAAACAGG-AATATCTTCATATAAAAGCTAGACAGAAGCATTCTCAGAAACTTCTTT

            *      * ** *   **      *       *        *
     106267 ATGATGTATCTACTCAGCTAACAGAGTTGAACCTT-CCTTTG
        130 GTGATGTTTGCATTCAAGTAACAGTGTTGAACATTCCCTTTC

             *       *                   * *     *             **            
     106308 AGAGAGCAGTTTTGAAACACTCTTTTTGTGGAATCTGCAAGTGGATATTTGTCTAGCTTTGAGGA
          1 ATAGAGCAGGTTTGAAACACTCTTTTTGTAGTATCTGGAAGTGGATATTTGGATAGCTTTGAGGA

                         *   *    *                   *       *              
     106373 TTTCGTTGGAAACGGGATTA-CAT-ATA-AAAAGC-AGACAGCAGCATTCCCAGAAACTTCTTTG
         66 TTTCGTTGGAAACAGGAATATCTTCATATAAAAGCTAGACAGAAGCATTCTCAGAAACTTCTTTG

                              *    *                 
     106434 TGATGTTTGCATTCAAGTCACAGAGTTGAACATTCCCTTTC
        131 TGATGTTTGCATTCAAGTAACAGTGTTGAACATTCCCTTTC

                      *                            *     *       **     *   *
     106475 ATAGAGCAGGATTGAAACACTCTTTTTGTAGTATCTGGATGTGGACATTTGGAGCGCTTTCAGGC
          1 ATAGAGCAGGTTTGAAACACTCTTTTTGTAGTATCTGGAAGTGGATATTTGGATAGCTTTGAGGA

            * ** *      *             *** *   *                          *   
     106540 CTAAGGT-GAAAAAGGAAATATCTTCCCCTGAAAACTAGACAGAAGCATTCTCAGAAACTTATTT
         66 TTTCGTTGGAAACAGG-AATATCTTCATATAAAAGCTAGACAGAAGCATTCTCAGAAACTTCTTT

                   **  **    *             **  * *   *
     106604 GTGATGTGCGCCCTCAACTAACAGTGTTGAAGCTTTCTTTTG
        130 GTGATGTTTGCATTCAAGTAACAGTGTTGAACATTCCCTTTC

                     *                   * *     *            ***            
     106646 ATAGAGCAGTTTTGAAACACTCTTTTTGTGGAATCTGCAAGTGGATATTTTTCTAGCTTTGAGGA
          1 ATAGAGCAGGTTTGAAACACTCTTTTTGTAGTATCTGGAAGTGGATATTTGGATAGCTTTGAGGA

                         *   *    *                   *       *     *   *    
     106711 TTTCGTTGGAAACGGGATTA-CAT-ATA-AAAAGC-AGACAGCAGCATTCCCAGAATCTTGTTTG
         66 TTTCGTTGGAAACAGGAATATCTTCATATAAAAGCTAGACAGAAGCATTCTCAGAAACTTCTTTG

                            * *    *                 
     106772 TGATGTTTGCATTCAACTCACAGAGTTGAACATTCCCTTTC
        131 TGATGTTTGCATTCAAGTAACAGTGTTGAACATTCCCTTTC

             *                         *           *     *       **     *   *
     106813 AGAGAGCAGGTTTGAAACACTCTTTTTATAGTATCTGGATGTGGACATTTGGAGCGCTTTCAGGC
          1 ATAGAGCAGGTTTGAAACACTCTTTTTGTAGTATCTGGAAGTGGATATTTGGATAGCTTTGAGGA

            * ** *      *             *** *   *                      *   *   
     106878 CTATGGT-GAAAAAGGAAATATCTTCTCCTGAAAACTAGACAGAAGCATTCTCAGAATCTTATTT
         66 TTTCGTTGGAAACAGG-AATATCTTCATATAAAAGCTAGACAGAAGCATTCTCAGAAACTTCTTT

                   *** **    *             **  * *   *
     106942 GTGATGTGCTCCCTCAACTAACAGTGTTGAAGCTTTCTTTTG
        130 GTGATGTTTGCATTCAAGTAACAGTGTTGAACATTCCCTTTC

                     *                *   **     *   *        *     *        
     106984 ATAGAGCAGTTTTGAAACACTCTTTTCGTAAAATCTGCAAGAGGATATTTTGATAGATTTGAGGA
          1 ATAGAGCAGGTTTGAAACACTCTTTTTGTAGTATCTGGAAGTGGATATTTGGATAGCTTTGAGGA

                         *   * *            **                      *    *   
     107049 TTTCGTTGGAAACGGGATTGTCTTCATATAAACTCTAGACAGAAGCATTCTCAGAAGCTTCATTG
         66 TTTCGTTGGAAACAGGAATATCTTCATATAAAAGCTAGACAGAAGCATTCTCAGAAACTTCTTTG

            *       **   *    *             *     *  
     107114 GGATGTTTCAATTGAAGTCACAGTGTTGAACAGTCCCTGTC
        131 TGATGTTTGCATTCAAGTAACAGTGTTGAACATTCCCTTTC

                                                    *    *       *  * * *    
     107155 ATAGAGCAGGTTTGAAACACTCTTTTTGTAGTATCTGGAACTGGACATTTGGAGAGATCTCAGGA
          1 ATAGAGCAGGTTTGAAACACTCTTTTTGTAGTATCTGGAAGTGGATATTTGGATAGCTTTGAGGA

            * *  *    * *             **            *       * *          *  *
     107220 ATACGGT-GATAAAGGAAATATCTTCCAATAAAAGCTAGATAGAAGCAATGTCAGAAACTTTTTC
         66 TTTCGTTGGAAACAGG-AATATCTTCATATAAAAGCTAGACAGAAGCATTCTCAGAAACTTCTTT

            *      * ** *   **      *       *  * *   *
     107284 ATGATGTATCTACTCAGCTAACAGAGTTGAACCTTTCTTTTG
        130 GTGATGTTTGCATTCAAGTAACAGTGTTGAACATTCCCTTTC

             *       *                                   *       **   * *    
     107326 AGAGAGCAGTTTTGAAACACTCTTTTTGTAGTATCTGGAAGTGGACATTTGGAGCGCTCTCAGGA
          1 ATAGAGCAGGTTTGAAACACTCTTTTTGTAGTATCTGGAAGTGGATATTTGGATAGCTTTGAGGA

            * *  *      *             **            *    *  * *          *  *
     107391 CTGCGGT-GAAAAAGGAAATATCTTCCAATAAAAGCTAGATAGAACCAATGTCAGAAACTTTTTC
         66 TTTCGTTGGAAACAGG-AATATCTTCATATAAAAGCTAGACAGAAGCATTCTCAGAAACTTCTTT

            *      * *  *   **      *       *  * *   *
     107455 ATGATGTATCCACTCAGCTAACAGAGTTGAACCTTTCTTTTG
        130 GTGATGTTTGCATTCAAGTAACAGTGTTGAACATTCCCTTTC

             *       *                   * *     *             **  **        
     107497 AGAGAGCAGTTTTGAAACACTCTTTTTGTGGAATCTGCAAGTGGATATTTGTCTACATTTGAGGA
          1 ATAGAGCAGGTTTGAAACACTCTTTTTGTAGTATCTGGAAGTGGATATTTGGATAGCTTTGAGGA

                         *   *    *                   *       *              
     107562 TTTCGTTGGAAACGGGATTA-CAT-ATA-AAAAGC-AGACAGCAGCATTCCCAGAAACTTCTTTG
         66 TTTCGTTGGAAACAGGAATATCTTCATATAAAAGCTAGACAGAAGCATTCTCAGAAACTTCTTTG

                              *    *                 
     107623 TGATGTTTGCATTCAAGTCACAGAGTTGAACATTCCCTTTC
        131 TGATGTTTGCATTCAAGTAACAGTGTTGAACATTCCCTTTC

                                           *     * *     *       **     *   *
     107664 ATAGAGCAGGTTTGAAACACTCTTTTTGTAGAATCTGTATGTGGACATTTGGAGCGCTTTCAGGC
          1 ATAGAGCAGGTTTGAAACACTCTTTTTGTAGTATCTGGAAGTGGATATTTGGATAGCTTTGAGGA

            * ** *      *             *** *   *                          *   
     107729 CTATGGT-GAAAAAGGAAATATCTTCCCCTGAAAACTAGACAGAAGCATTCTCAGAAACTTATTT
         66 TTTCGTTGGAAACAGG-AATATCTTCATATAAAAGCTAGACAGAAGCATTCTCAGAAACTTCTTT

                   **  **    *              *  * *   *
     107793 GTGATGTGCGCCCTCAACTAACAGTGTTGAACCTTTCTTTTG
        130 GTGATGTTTGCATTCAAGTAACAGTGTTGAACATTCCCTTTC

                     *                    *      *   *                       
     107835 ATAGAGCAGTTTTGAAACACTCTTTTTGTAATATCTGCAAGAGGATATTTGGATAGCTTTGAGGA
          1 ATAGAGCAGGTTTGAAACACTCTTTTTGTAGTATCTGGAAGTGGATATTTGGATAGCTTTGAGGA

                         *   * *            **                      *    **  
     107900 TTTCGTTGGAAACGGGATTGTCTTCATATAAACTCTAGACAGAAGCATTCTCAGAAGCTTCAATG
         66 TTTCGTTGGAAACAGGAATATCTTCATATAAAAGCTAGACAGAAGCATTCTCAGAAACTTCTTTG

            *       **   *    *             * *      
     107965 GGATGTTTCAATTGAAGTCACAGTGTTGAACAGTTCCTTTC
        131 TGATGTTTGCATTCAAGTAACAGTGTTGAACATTCCCTTTC

                                                         *       **   * *    
     108006 ATAGAGCAGGTTTGAAACACTCTTTTTGTAGTATCTGGAAGTGGACATTTGGAGCGCTCTCAGGA
          1 ATAGAGCAGGTTTGAAACACTCTTTTTGTAGTATCTGGAAGTGGATATTTGGATAGCTTTGAGGA

            * ** *    * *             **            **      * *          *  *
     108071 CTAAGGT-GATAAAGGAAATATCTTCCAATAAAAGCTAGATTGAAGCAATGTCAGAAACTTTTTC
         66 TTTCGTTGGAAACAGG-AATATCTTCATATAAAAGCTAGACAGAAGCATTCTCAGAAACTTCTTT

            *   *  * ** *   **      *      **  *     *
     108135 ATGACGTATCTACTCAGCTAACAGAGTTGAAACTTTCCTTTG
        130 GTGATGTTTGCATTCAAGTAACAGTGTTGAACATTCCCTTTC

             *       *                   * *     *             **            
     108177 AGAGAGCAGTTTTGAAACACTCTTTTTGTGGAATCTGCAAGTGGATATTTGTCTAGCTTTGAGGA
          1 ATAGAGCAGGTTTGAAACACTCTTTTTGTAGTATCTGGAAGTGGATATTTGGATAGCTTTGAGGA

                         *   *    *                   *       *   *          
     108242 TTTCGTTGGAAACGGGATTA-CAT-ATA-AAAAGC-AGACAGCAGCATTCCCAGTAACTTCTTTG
         66 TTTCGTTGGAAACAGGAATATCTTCATATAAAAGCTAGACAGAAGCATTCTCAGAAACTTCTTTG

                              *    *                 
     108303 TGATGTTTGCATTCAAGTCACAGAGTTGAACATTCCCTTTC
        131 TGATGTTTGCATTCAAGTAACAGTGTTGAACATTCCCTTTC

                                                   *     *       **     *   *
     108344 ATAGAGCAGGTTTGAAACACTCTTTTTGTAGTATCTGGATGTGGACATTTGGAGCGCTTTCAGGC
          1 ATAGAGCAGGTTTGAAACACTCTTTTTGTAGTATCTGGAAGTGGATATTTGGATAGCTTTGAGGA

            * ** *      *             *** *   *                      *   *   
     108409 CTATGGT-GAAAAAGGAAATATCTTCTCCTGAAAACTAGACAGAAGCATTCTCAGAATCTTATTT
         66 TTTCGTTGGAAACAGG-AATATCTTCATATAAAAGCTAGACAGAAGCATTCTCAGAAACTTCTTT

                   **  **    *             **  * *   *
     108473 GTGATGTGCGCCCTCAACTAACAGTGTTGAAGCTTTCTTTTG
        130 GTGATGTTTGCATTCAAGTAACAGTGTTGAACATTCCCTTTC

                     *                *   **     *   *        *              
     108515 ATAGAGCAGTTTTGAAACACTCTTTTCGTAAAATCTGCAAGAGGATATTTTGATAGCTTTGAGGA
          1 ATAGAGCAGGTTTGAAACACTCTTTTTGTAGTATCTGGAAGTGGATATTTGGATAGCTTTGAGGA

                         **  * *            **   *                  *    *   
     108580 TTTCGTTGGAAACGAGATTGTCTTCATATAAACTCTACACAGAAGCATTCTCAGAAGCTTCATTG
         66 TTTCGTTGGAAACAGGAATATCTTCATATAAAAGCTAGACAGAAGCATTCTCAGAAACTTCTTTG

            *       **   *    *             *     *  
     108645 GGATGTTTCAATTGAAGTCACAGTGTTGAACAGTCCCTGTC
        131 TGATGTTTGCATTCAAGTAACAGTGTTGAACATTCCCTTTC

               *             *                           *       *  * * *    
     108686 ATACAGCAGGTTTGAAAAACTCTTTTTGTAGTATCTGGAAGTGGACATTTGGAGAGATATCAGGA
          1 ATAGAGCAGGTTTGAAACACTCTTTTTGTAGTATCTGGAAGTGGATATTTGGATAGCTTTGAGGA

            * *  *    * *             **            *   *   * *          *  *
     108751 ATACGGT-GATAAAGGAAATATCTTCCAATAAAAGCTAGATAGAGGCAATGTCAGAAACTTTTTC
         66 TTTCGTTGGAAACAGG-AATATCTTCATATAAAAGCTAGACAGAAGCATTCTCAGAAACTTCTTT

            *      * ** *   **      *       *  * *   *
     108815 ATGATGTATCTACTCAGCTAACAGAGTTGAACCTTTCTTTTG
        130 GTGATGTTTGCATTCAAGTAACAGTGTTGAACATTCCCTTTC

             *       *                                   *       ** * * *    
     108857 AGAGAGCAGTTTTGAAACACTCTTTTTGTAGTATCTGGAAGTGGACATTTGGAGCGTTCTCAGGA
          1 ATAGAGCAGGTTTGAAACACTCTTTTTGTAGTATCTGGAAGTGGATATTTGGATAGCTTTGAGGA

            * *  *      *             **            *       * *    *     *  *
     108922 CTACGGT-GAAAAAGGAAATATCTTCCAATAAAAGCTAGATAGAAGCAATGTCAGCAACTTTTTC
         66 TTTCGTTGGAAACAGG-AATATCTTCATATAAAAGCTAGACAGAAGCATTCTCAGAAACTTCTTT

            *      * ** *   **      *       *  * *   *
     108986 ATGATGTATCTACTCAGCTAACAGAGTTGAACCTTTCTTTTG
        130 GTGATGTTTGCATTCAAGTAACAGTGTTGAACATTCCCTTTC

             *       *                   * *     *             **            
     109028 AGAGAGCAGTTTTGAAACACTCTTTTTGTGGAATCTGCAAGTGGATATTTGTCTAGCTTTGAGGA
          1 ATAGAGCAGGTTTGAAACACTCTTTTTGTAGTATCTGGAAGTGGATATTTGGATAGCTTTGAGGA

                         *   *    *  *                *       *              
     109093 TTTCGTTGGAAACGGGATTA-CAT-TTA-AAAAGC-AGACAGCAGCATTCCCAG-AACTTCTTTG
         66 TTTCGTTGGAAACAGGAATATCTTCATATAAAAGCTAGACAGAAGCATTCTCAGAAACTTCTTTG

                              *    *                 
     109153 TGATGTTTGCATTCAAGTCACAGAGTTGAACATTCCCTTTC
        131 TGATGTTTGCATTCAAGTAACAGTGTTGAACATTCCCTTTC

                                                 * *     *       **     *   *
     109194 ATAGAGCAGGTTTGAAACACTCTTTTTGTAGTATCTGTATGTGGACATTTGGAGCGCTTTCAGGC
          1 ATAGAGCAGGTTTGAAACACTCTTTTTGTAGTATCTGGAAGTGGATATTTGGATAGCTTTGAGGA

            * ** *      *             *** *   *                      *   *   
     109259 CTATGGT-GAAAAAGGAAATATCTTCCCCTGAAAACTAGACAGAAGCATTCTCAGAATCTTATTT
         66 TTTCGTTGGAAACAGG-AATATCTTCATATAAAAGCTAGACAGAAGCATTCTCAGAAACTTCTTT

                  ***  **    *              *  * *   *
     109323 GTGATGCGCGCCCTCAACTAACAGTGTTGAACCTTTCTTTTG
        130 GTGATGTTTGCATTCAAGTAACAGTGTTGAACATTCCCTTTC

                     *                    *      *   *                       
     109365 ATAGAGCAGTTTTGAAACACTCTTTTTGTAATATCTGCAAGAGGATATTTGGATAGCTTTGAGGA
          1 ATAGAGCAGGTTTGAAACACTCTTTTTGTAGTATCTGGAAGTGGATATTTGGATAGCTTTGAGGA

                         *   * *            **                      *    *   
     109430 TTTCGTTGGAAACGGGATTGTCTTCATATAAACTCTAGACAGAAGCATTCTCAGAAGCTTCATTG
         66 TTTCGTTGGAAACAGGAATATCTTCATATAAAAGCTAGACAGAAGCATTCTCAGAAACTTCTTTG

            *       **   *    *             *        
     109495 GGATGTTTCAATTGAAGTCACAGTGTTGAACAGTCCCTTTC
        131 TGATGTTTGCATTCAAGTAACAGTGTTGAACATTCCCTTTC

                                                         *       *  * * *    
     109536 ATAGAGCAGGTTTGAAACACTCTTTTTGTAGTATCTGGAAGTGGACATTTGGAGAGATCTCAGGA
          1 ATAGAGCAGGTTTGAAACACTCTTTTTGTAGTATCTGGAAGTGGATATTTGGATAGCTTTGAGGA

            * *  *    * *             **            *       * *          *  *
     109601 ATACGGT-GATAAAGGAAATATCTTCCAATAAAAGCTAGATAGAAGCAATGTCAGAAACTTTTTC
         66 TTTCGTTGGAAACAGG-AATATCTTCATATAAAAGCTAGACAGAAGCATTCTCAGAAACTTCTTT

            *      * ** *   **      *       *  * *   *
     109665 ATGATGTATCTACTCAGCTAACAGAGTTGAACCTTTCTTTTG
        130 GTGATGTTTGCATTCAAGTAACAGTGTTGAACATTCCCTTTC

            **       *                   * *     *             **            
     109707 CGAGAGCAGTTTTGAAACACTCTTTTTGTGGAATCTGCAAGTGGATATTTGTCTAGCTTTGAGGA
          1 ATAGAGCAGGTTTGAAACACTCTTTTTGTAGTATCTGGAAGTGGATATTTGGATAGCTTTGAGGA

                   *     *   *    *                   *       *              
     109772 TTTCGTTTGAAACGGGATTA-CAT-ATA-AAAAGC-AGACAGCAGCATTCCCAGAAACTTCTTTG
         66 TTTCGTTGGAAACAGGAATATCTTCATATAAAAGCTAGACAGAAGCATTCTCAGAAACTTCTTTG

                      *       *    *                 
     109833 TGATGTTTGCCTTCAAGTCACAGAGTTGAACATTCCCTTTC
        131 TGATGTTTGCATTCAAGTAACAGTGTTGAACATTCCCTTTC

                     *                           * *     *       **     *   *
     109874 ATAGAGCAGTTTTGAAACACTCTTTTTGTAGTATCTGTATGTGGACATTTGGAGCGCTTTCAGGC
          1 ATAGAGCAGGTTTGAAACACTCTTTTTGTAGTATCTGGAAGTGGATATTTGGATAGCTTTGAGGA

            * ** *      *             *** *   *                          *   
     109939 CTATGGT-GAAAAAGGAAATATCTTCCCCTGAAAACTAGACAGAAGCATTCTCAGAAACTTATTT
         66 TTTCGTTGGAAACAGG-AATATCTTCATATAAAAGCTAGACAGAAGCATTCTCAGAAACTTCTTT

              *    **  **    *              *  * *   *
     110003 GTAATGTGCGCCCTCAACTAACAGTGTTGAACTTTTCTTTTG
        130 GTGATGTTTGCATTCAAGTAACAGTGTTGAACATTCCCTTTC

                     *                    *      *   *                       
     110045 ATAGAGCAGTTTTGAAACACTCTTTTTGTAATATCTGCAAGAGGATATTTGGATAGCTTTGAGGA
          1 ATAGAGCAGGTTTGAAACACTCTTTTTGTAGTATCTGGAAGTGGATATTTGGATAGCTTTGAGGA

                         *   * *            **                      *    *   
     110110 TTTCGTTGGAAACGGGATTGTCTTCATATAAACTCTAGACAGAAGCATTCTCAGAAGCTTCATTG
         66 TTTCGTTGGAAACAGGAATATCTTCATATAAAAGCTAGACAGAAGCATTCTCAGAAACTTCTTTG

            *       **   *    *             *        
     110175 GGATGTTTCAATTGAAGTCACAGTGTTGAACAGTCCCTTTC
        131 TGATGTTTGCATTCAAGTAACAGTGTTGAACATTCCCTTTC

                                                   *     *       **     *   *
     110216 ATAGAGCAGGTTTGAAACACTCTTTTTGTAGTATCTGGATGTGGACATTTGGAGCGCTTTAAGGC
          1 ATAGAGCAGGTTTGAAACACTCTTTTTGTAGTATCTGGAAGTGGATATTTGGATAGCTTTGAGGA

            * ** *      *             *** *   *                          *   
     110281 CTATGGT-GAAAAAGGAAATATCTTCCCCTGAAAACTAGACAGAAGCATTCTCAGAAACTTATTT
         66 TTTCGTTGGAAACAGG-AATATCTTCATATAAAAGCTAGACAGAAGCATTCTCAGAAACTTCTTT

                   **  **   **             **  * *   *
     110345 GTGATGTGCGCCCTCAGCTAACAGTGTTGAAGCTTTCTTTTG
        130 GTGATGTTTGCATTCAAGTAACAGTGTTGAACATTCCCTTTC

                     *                   * *     *             **         ** 
     110387 ATAGAGCAGTTTTGAAACACTCTTTTTGTGGAATCTGCAAGTGGATATTTGTCTAGCTTTGATTA
          1 ATAGAGCAGGTTTGAAACACTCTTTTTGTAGTATCTGGAAGTGGATATTTGGATAGCTTTGAGGA

                         *   *     *                  *       *   *          
     110452 TTTCGTTGGAAACGGGATTA-C-ACATA-AAAAGC-AGACAGCAGCATTCCCAGTAACTTCTTTG
         66 TTTCGTTGGAAACAGGAATATCTTCATATAAAAGCTAGACAGAAGCATTCTCAGAAACTTCTTTG

                              *    *                 
     110513 TGATGTTTGCATTCAAGTCACAGAGTTGAACATTCCCTTTC
        131 TGATGTTTGCATTCAAGTAACAGTGTTGAACATTCCCTTTC

                                                   *     *       **     *   *
     110554 ATAGAGCAGGTTTGAAACACTCTTTTTGTAGTATCTGGATGTGGACATTTGGAGCGCTTTCAGGC
          1 ATAGAGCAGGTTTGAAACACTCTTTTTGTAGTATCTGGAAGTGGATATTTGGATAGCTTTGAGGA

            * ** *      *             *** *   *                          *   
     110619 CTATGGT-GAAAAAGGAAATATCTTCCCCTGAAAACTAGACAGAAGCATTCTCAGAAACTTATTT
         66 TTTCGTTGGAAACAGG-AATATCTTCATATAAAAGCTAGACAGAAGCATTCTCAGAAACTTCTTT

                   **  **    *             **  * *   *
     110683 GTGATGTGCGCCCTCAACTAACAGTGTTGAAGCTTTCTTTTG
        130 GTGATGTTTGCATTCAAGTAACAGTGTTGAACATTCCCTTTC

                     *                    *      *   *                       
     110725 ATAGAGCAGTTTTGAAACACTCTTTTTGTAATATCTGCAAGAGGATATTTGGATAGCTTTGAGGA
          1 ATAGAGCAGGTTTGAAACACTCTTTTTGTAGTATCTGGAAGTGGATATTTGGATAGCTTTGAGGA

                         *   * **           **                      *    *   
     110790 TTTCGTTGGAAACGGGATTGACTTCATATAAACTCTAGACAGAAGCATTCTCAGAAGCTTCATTG
         66 TTTCGTTGGAAACAGGAATATCTTCATATAAAAGCTAGACAGAAGCATTCTCAGAAACTTCTTTG

            *       **   *    *             *        
     110855 GGATGTTTCAATTGAAGTCACAGTGTTGAACAGTCCCTTTC
        131 TGATGTTTGCATTCAAGTAACAGTGTTGAACATTCCCTTTC

                                                         *       *  * * *    
     110896 ATAGAGCAGGTTTGAAACACTCTTTTTGTAGTATCTGGAAGTGGACATTTGGAGAGATCTCAGGA
          1 ATAGAGCAGGTTTGAAACACTCTTTTTGTAGTATCTGGAAGTGGATATTTGGATAGCTTTGAGGA

            * ** *    * *             **            *       * *       *  ** *
     110961 ATAAGGT-GACAAAGGAAATATCTTCCAATAAAAGCTAGATAGAAGCAATGTCAGAAAATTTCTC
         66 TTTCGTTGGAAACAGG-AATATCTTCATATAAAAGCTAGACAGAAGCATTCTCAGAAACTTCTTT

            *      * ** *   **      *       *  ***   *
     111025 ATGATGTATCTACTCAGCTAACAGAGTTGAACCTTTGTTTTG
        130 GTGATGTTTGCATTCAAGTAACAGTGTTGAACATTCCCTTTC

             *       *                   * *     *             **            
     111067 AGAGAGCAGTTTTGAAACACTCTTTTTGTGGAATCTGCAAGTGGATATTTGTCTAGCTTTGAGGA
          1 ATAGAGCAGGTTTGAAACACTCTTTTTGTAGTATCTGGAAGTGGATATTTGGATAGCTTTGAGGA

             *           *   *    *                   *       *   *          
     111132 TATCGTTGGAAACGGGATTA-CAT-ATA-AAAAGC-AGACAGCAGCATTCCCAGTAACTTCTTTG
         66 TTTCGTTGGAAACAGGAATATCTTCATATAAAAGCTAGACAGAAGCATTCTCAGAAACTTCTTTG

                              *    *                 
     111193 TGATGTTTGCATTCAAGTCACAGAGTTGAACATTCCCTTTC
        131 TGATGTTTGCATTCAAGTAACAGTGTTGAACATTCCCTTTC

                                                   *     *       **     *   *
     111234 ATAGAGCAGGTTTGAAACACTCTTTTTGTAGTATCTGGATGTGGACATTTGGAGCGCTTTCAGGC
          1 ATAGAGCAGGTTTGAAACACTCTTTTTGTAGTATCTGGAAGTGGATATTTGGATAGCTTTGAGGA

            * ** *      *             *** *   *                      *   *   
     111299 CTATGGT-GAAAAAGGAAATATCTTCCCCTGAAAACTAGACAGAAGCATTCTCAGAATCTTATTT
         66 TTTCGTTGGAAACAGG-AATATCTTCATATAAAAGCTAGACAGAAGCATTCTCAGAAACTTCTTT

                   **  **    *             **  * *   *
     111363 GTGATGTGCGCCCTCAACTAACAGTGTTGAAGCTTTCTTTTG
        130 GTGATGTTTGCATTCAAGTAACAGTGTTGAACATTCCCTTTC

                     *                    **     *   *                       
     111405 ATAGAGCAGTTTTGAAACACTCTTTTTGTAAAATCTGCAAGAGGATATTTGGATAGCTTTGAGGA
          1 ATAGAGCAGGTTTGAAACACTCTTTTTGTAGTATCTGGAAGTGGATATTTGGATAGCTTTGAGGA

                             * *            **                      *    *   
     111470 TTTCGTTGGAAACAGGATTGTCTTCATATAAACTCTAGACAGAAGCATTCTCAGAAGCTTCATTG
         66 TTTCGTTGGAAACAGGAATATCTTCATATAAAAGCTAGACAGAAGCATTCTCAGAAACTTCTTTG

            *       **   *    *             *        
     111535 GGATGTTTCAATTGAAGTCACAGTGTTGAACAGTCCCTTTC
        131 TGATGTTTGCATTCAAGTAACAGTGTTGAACATTCCCTTTC

                                                         *       **   * *    
     111576 ATAGAGCAGGTTTGAAACACTCTTTTTGTAGTATCTGGAAGTGGACATTTGGAGCGCTCTCAGGA
          1 ATAGAGCAGGTTTGAAACACTCTTTTTGTAGTATCTGGAAGTGGATATTTGGATAGCTTTGAGGA

            * *  *      *             **     *    * *       * *          *  *
     111641 CTACGGT-GAAAAAGGAAATATCTTCCAATAAAGGCTACATAGAAGCAATGTCAGAAACTTTTTC
         66 TTTCGTTGGAAACAGG-AATATCTTCATATAAAAGCTAGACAGAAGCATTCTCAGAAACTTCTTT

            *      * ** *   **      **      *  * *   *
     111705 ATGATGTATCTACTCAGCTAACAGATTTGAACCTTTCTTTTG
        130 GTGATGTTTGCATTCAAGTAACAGTGTTGAACATTCCCTTTC

             *       *                   * *     *             **            
     111747 AGAGAGCAGTTTTGAAACACTCTTTTTGTGGAATCTGCAAGTGGATATTTGTCTAGCTTTGAGGA
          1 ATAGAGCAGGTTTGAAACACTCTTTTTGTAGTATCTGGAAGTGGATATTTGGATAGCTTTGAGGA

                         *   *    *                   *       *              
     111812 TTTCGTTGGAAACGGGATTA-CAT-ATA-AAAAGC-AGACAGCAGCATTCCCAGAAACTTCTTTG
         66 TTTCGTTGGAAACAGGAATATCTTCATATAAAAGCTAGACAGAAGCATTCTCAGAAACTTCTTTG

                              *    *                 
     111873 TGATGTTTGCATTCAAGTCACAGAGTTGAACATTCCCTTTC
        131 TGATGTTTGCATTCAAGTAACAGTGTTGAACATTCCCTTTC

                                                 * *     *       **     *   *
     111914 ATAGAGCAGGTTTGAAACACTCTTTTTGTAGTATCTGTATGTGGACATTTGGAGCGCTTTCAGGC
          1 ATAGAGCAGGTTTGAAACACTCTTTTTGTAGTATCTGGAAGTGGATATTTGGATAGCTTTGAGGA

            * ** *      *             *** *   **                      *  *   
     111979 CTATGGT-GAAAAAGGAAATATCTTCCCCTGAAAATTAGACAGAAGCATTCTCAGAAATTTATTT
         66 TTTCGTTGGAAACAGG-AATATCTTCATATAAAAGCTAGACAGAAGCATTCTCAGAAACTTCTTT

                   **  **    *             **  * *   *
     112043 GTGATGTGCGCCCTCAACTAACAGTGTTGAAGCTTTCTTTTG
        130 GTGATGTTTGCATTCAAGTAACAGTGTTGAACATTCCCTTTC

                *    *                    **     *   *                       
     112085 ATAGCGCAGTTTTGAAACACTCTTTTTGTAAAATCTGCAAGAGGATATTTGGATAGCTTTGAGGA
          1 ATAGAGCAGGTTTGAAACACTCTTTTTGTAGTATCTGGAAGTGGATATTTGGATAGCTTTGAGGA

                         *   * *            **                      *    *   
     112150 TTTCGTTGGAAACGGGATTGTCTTCATATAAACTCTAGACAGAAGCATTCTCAGAAGCTTCATTG
         66 TTTCGTTGGAAACAGGAATATCTTCATATAAAAGCTAGACAGAAGCATTCTCAGAAACTTCTTTG

            *       **   *    *             *        
     112215 GGATGTTTCAATTGAAGTCACAGTGTTGAACAGTCCCTTTC
        131 TGATGTTTGCATTCAAGTAACAGTGTTGAACATTCCCTTTC

                                                   *     *       **     *   *
     112256 ATAGAGCAGGTTTGAAACACTCTTTTTGTAGTATCTGGATGTGGACATTTGGAGCGCTTTAAGGC
          1 ATAGAGCAGGTTTGAAACACTCTTTTTGTAGTATCTGGAAGTGGATATTTGGATAGCTTTGAGGA

            * ** *      *             *** *   *                          *   
     112321 CTATGGT-GAAAAAGGAAATATCTTCCCCTGAAAACTAGACAGAAGCATTCTCAGAAACTTATTT
         66 TTTCGTTGGAAACAGG-AATATCTTCATATAAAAGCTAGACAGAAGCATTCTCAGAAACTTCTTT

                   **  **   **             **  * *   *
     112385 GTGATGTGCGCCCTCAGCTAACAGTGTTGAAGCTTTCTTTTG
        130 GTGATGTTTGCATTCAAGTAACAGTGTTGAACATTCCCTTTC

                     *                   * *     *             **         ** 
     112427 ATAGAGCAGTTTTGAAACACTCTTTTTGTGGAATCTGCAAGTGGATATTTGTCTAGCTTTGATTA
          1 ATAGAGCAGGTTTGAAACACTCTTTTTGTAGTATCTGGAAGTGGATATTTGGATAGCTTTGAGGA

                         **  *     *                  *       *   *          
     112492 TTTCGTTGGAAACGAGATTA-C-ACATA-AAAAGC-AGACAGCAGCATTCCCAGTAACTTCTTTG
         66 TTTCGTTGGAAACAGGAATATCTTCATATAAAAGCTAGACAGAAGCATTCTCAGAAACTTCTTTG

                              *    *                 
     112553 TGATGTTTGCATTCAAGTCACAGAGTTGAACATTCCCTTTC
        131 TGATGTTTGCATTCAAGTAACAGTGTTGAACATTCCCTTTC

                                                   *     *       **     *   *
     112594 ATAGAGCAGGTTTGAAACACTCTTTTTGTAGTATCTGGATGTGGACATTTGGAGCGCTTTCAGGC
          1 ATAGAGCAGGTTTGAAACACTCTTTTTGTAGTATCTGGAAGTGGATATTTGGATAGCTTTGAGGA

            * ** *      *             *** *   *                          *   
     112659 CTATGGT-GAAAAAGGAAATATCTTCCCCTGAAAACTAGACAGAAGCATTCTCAGAAACTTATTT
         66 TTTCGTTGGAAACAGG-AATATCTTCATATAAAAGCTAGACAGAAGCATTCTCAGAAACTTCTTT

                   **  **    *              *  * *   *
     112723 GTGATGTGCGCCCTCAACTAACAGTGTTGAACCTTTCTTTTG
        130 GTGATGTTTGCATTCAAGTAACAGTGTTGAACATTCCCTTTC

                     *                    *      *   *                       
     112765 ATAGAGCAGTTTTGAAACACTCTTTTTGTAATATCTGCAAGAGGATATTTGGATAGCTTTGAGGA
          1 ATAGAGCAGGTTTGAAACACTCTTTTTGTAGTATCTGGAAGTGGATATTTGGATAGCTTTGAGGA

                         *   * **           **                      *    *   
     112830 TTTCGTTGGAAACGGGATTGACTTCATATAAACTCTAGACAGAAGCATTCTCAGAAGCTTCATTG
         66 TTTCGTTGGAAACAGGAATATCTTCATATAAAAGCTAGACAGAAGCATTCTCAGAAACTTCTTTG

            *       **   *    *             *        
     112895 GGATGTTTCAATTGAAGTCACAGTGTTGAACAGTCCCTTTC
        131 TGATGTTTGCATTCAAGTAACAGTGTTGAACATTCCCTTTC

                                                         *       *  * * *    
     112936 ATAGAGCAGGTTTGAAACACTCTTTTTGTAGTATCTGGAAGTGGACATTTGGAGAGATCTCAGGA
          1 ATAGAGCAGGTTTGAAACACTCTTTTTGTAGTATCTGGAAGTGGATATTTGGATAGCTTTGAGGA

            * ** *    * *             **            *       * *       *  ** *
     113001 ATAAGGT-GACAAAGGAAATATCTTCCAATAAAAGCTAGATAGAAGCAATGTCAGAAAATTTCTC
         66 TTTCGTTGGAAACAGG-AATATCTTCATATAAAAGCTAGACAGAAGCATTCTCAGAAACTTCTTT

            *      * ** *   **      *       *    *   *
     113065 ATGATGTATCTACTCAGCTAACAGAGTTGAACCTT-CTTTTG
        130 GTGATGTTTGCATTCAAGTAACAGTGTTGAACATTCCCTTTC

             *       *                   * *     *             **            
     113106 AGAGAGCAGTTTTGAAACACTCTTTTTGTGGAATCTGCAAGTGGATATTTGTCTAGCTTTGAGGA
          1 ATAGAGCAGGTTTGAAACACTCTTTTTGTAGTATCTGGAAGTGGATATTTGGATAGCTTTGAGGA

             *           *   *    *                   *       *              
     113171 TATCGTTGGAAACGGGATTA-CAT-ATA-AAAAGC-AGACAGCAGCATTCCCAGAAACTTCTTTG
         66 TTTCGTTGGAAACAGGAATATCTTCATATAAAAGCTAGACAGAAGCATTCTCAGAAACTTCTTTG

                              *    *                 
     113232 TGATGTTTGCATTCAAGTCACAGAGTTGAACATTCCCTTTC
        131 TGATGTTTGCATTCAAGTAACAGTGTTGAACATTCCCTTTC

                                                   *     *       **     *   *
     113273 ATAGAGCAGGTTTGAAACACTCTTTTTGTAGTATCTGGATGTGGACATTTGGAGCGCTTTCAGGC
          1 ATAGAGCAGGTTTGAAACACTCTTTTTGTAGTATCTGGAAGTGGATATTTGGATAGCTTTGAGGA

            * ** *      *             *** *   **                      *  *   
     113338 CTATGGT-GAAAAAGGAAATATCTTCCCCTGAAAATTAGACAGAAGCATTCTCAGAAATTTATTT
         66 TTTCGTTGGAAACAGG-AATATCTTCATATAAAAGCTAGACAGAAGCATTCTCAGAAACTTCTTT

                   **  **    *             **  * *   *
     113402 GTGATGTGCGCCCTCAACTAACAGTGTTGAAGCTTTCTTTTG
        130 GTGATGTTTGCATTCAAGTAACAGTGTTGAACATTCCCTTTC

                *    *                    **     *   *                       
     113444 ATAGCGCAGTTTTGAAACACTCTTTTTGTAAAATCTGCAAGAGGATATTTGGATAGCTTTGAGGA
          1 ATAGAGCAGGTTTGAAACACTCTTTTTGTAGTATCTGGAAGTGGATATTTGGATAGCTTTGAGGA

                         *   * *            **                      *    *   
     113509 TTTCGTTGGAAACGGGATTGTCTTCATATAAACTCTAGACAGAAGCATTCTCAGAAGCTTCATTG
         66 TTTCGTTGGAAACAGGAATATCTTCATATAAAAGCTAGACAGAAGCATTCTCAGAAACTTCTTTG

            *       **   *    *             *        
     113574 GGATGTTTCAATTGAAGTCACAGTGTTGAACAGTCCCTTTC
        131 TGATGTTTGCATTCAAGTAACAGTGTTGAACATTCCCTTTC

                                                         *       **   * *    
     113615 ATAGAGCAGGTTTGAAACACTCTTTTTGTAGTATCTGGAAGTGGACATTTGGAGCGCTCTCAGGA
          1 ATAGAGCAGGTTTGAAACACTCTTTTTGTAGTATCTGGAAGTGGATATTTGGATAGCTTTGAGGA

            * *  *      *             **            *       * *          *  *
     113680 CTGCGGT-GAAAAAGGAAATATCTTCCAATAAAAGCTAGATAGAAGCAATGTCAGAAACTTTTTC
         66 TTTCGTTGGAAACAGG-AATATCTTCATATAAAAGCTAGACAGAAGCATTCTCAGAAACTTCTTT

            *      * ** *   **      *  *    *        *
     113744 ATGATGTATCTACTCAGCTAACAGAGTCGAACCTT-CCTTTG
        130 GTGATGTTTGCATTCAAGTAACAGTGTTGAACATTCCCTTTC

             *       *                   ***     *             **            
     113785 AGAGAGCAGTTTTGAAACACTCTTTTTGTGAAATCTGCAAGTGGATATTTGTCTAGCTTTGAGGA
          1 ATAGAGCAGGTTTGAAACACTCTTTTTGTAGTATCTGGAAGTGGATATTTGGATAGCTTTGAGGA

                         *   *    *                   *  *    *   *          
     113850 TTTCGTTGGAAACGGGATTA-CAT-ATA-AAAAGC-AGACAGCAGTATTCCCAGTAACTTCTTTG
         66 TTTCGTTGGAAACAGGAATATCTTCATATAAAAGCTAGACAGAAGCATTCTCAGAAACTTCTTTG

                              *    *                 
     113911 TGATGTTTGCATTCAAGTCACAGAGTTGAACATTCCCTTTC
        131 TGATGTTTGCATTCAAGTAACAGTGTTGAACATTCCCTTTC

                                                   *     *       **     *   *
     113952 ATAGAGCAGGTTTGAAACACTCTTTTTGTAGTATCTGGATGTGGACATTTGGAGTGCTTTCAGGC
          1 ATAGAGCAGGTTTGAAACACTCTTTTTGTAGTATCTGGAAGTGGATATTTGGATAGCTTTGAGGA

            * ** *      *             *** *   *                      *   *   
     114017 CTATGGT-GAAAAAGGAAATATCTTCCCCTGAAAACTAGACAGAAGCATTCTCAGAATCTTATTT
         66 TTTCGTTGGAAACAGG-AATATCTTCATATAAAAGCTAGACAGAAGCATTCTCAGAAACTTCTTT

                   **  **    *             **  * *   *
     114081 GTGATGTGCGCCCTCAACTAACAGTGTTGAAGTTTTCTTTTG
        130 GTGATGTTTGCATTCAAGTAACAGTGTTGAACATTCCCTTTC

                     *                    **     *   * *                     
     114123 ATAGAGCAGTTTTGAAACACTCTTTTTGTAAAATCTGCAAGAGTATATTTGGATAGCTTTGAGGA
          1 ATAGAGCAGGTTTGAAACACTCTTTTTGTAGTATCTGGAAGTGGATATTTGGATAGCTTTGAGGA

                         *   * *            **                      * *  *   
     114188 TTTCGTTGGAAACGGGATTGTCTTCATATAAACTCTAGACAGAAGCATTCTCAGAAGCCTCATTG
         66 TTTCGTTGGAAACAGGAATATCTTCATATAAAAGCTAGACAGAAGCATTCTCAGAAACTTCTTTG

            *       **   *    *             *        
     114253 GGATGTTTCAATTGAAGTCACAGTGTTGAACAGTCCCTTTC
        131 TGATGTTTGCATTCAAGTAACAGTGTTGAACATTCCCTTTC

                                                   *     *       **     *   *
     114294 ATAGAGCAGGTTTGAAACACTCTTTTTGTAGTATCTGGATGTGGACATTTGGAGCGCTTTCAGGC
          1 ATAGAGCAGGTTTGAAACACTCTTTTTGTAGTATCTGGAAGTGGATATTTGGATAGCTTTGAGGA

            * ** *      *             *** *   *                          *   
     114359 CTATGGT-GAAAAAGGAAATATCTTCCCCTGAAAACTAGACAGAAGCATTCTCAGAAACTTATTT
         66 TTTCGTTGGAAACAGG-AATATCTTCATATAAAAGCTAGACAGAAGCATTCTCAGAAACTTCTTT

                   **  **    *             **  * *   *
     114423 GTGATGTGCGCCCTCAACTAACAGTGTTGAAGCTTTCTTTTG
        130 GTGATGTTTGCATTCAAGTAACAGTGTTGAACATTCCCTTTC

                     *                   * *     *             **            
     114465 ATAGAGCAGTTTTGAAACACTCTTTTTGTGGAATCTGCAAGTGGATATTTGTCTAGCTTTGAGGA
          1 ATAGAGCAGGTTTGAAACACTCTTTTTGTAGTATCTGGAAGTGGATATTTGGATAGCTTTGAGGA

                         *   *    *                   *       *              
     114530 TTTCGTTGGAAACGGGATTA-CAT-ATA-AAAAGC-AGACAGCAGCATTCCCAGAAACTTCTTTG
         66 TTTCGTTGGAAACAGGAATATCTTCATATAAAAGCTAGACAGAAGCATTCTCAGAAACTTCTTTG

                              *    *       *         
     114591 TGATGTTTGCATTCAAGTCACAGAGTTGAACTTTCCCTTTC
        131 TGATGTTTGCATTCAAGTAACAGTGTTGAACATTCCCTTTC

                                                 * *   * *       **     *   *
     114632 ATAGAGCAGGTTTGAAACACTCTTTTTGTAGTATCTGTATGTGCACATTTGGAGCGCTTTCAGGC
          1 ATAGAGCAGGTTTGAAACACTCTTTTTGTAGTATCTGGAAGTGGATATTTGGATAGCTTTGAGGA

            * ** *      *             *** *   *                      *   *   
     114697 CTATGGT-GAAAAAGGAAATATCTTCCCCTGAAAACTAGACAGAAGCATTCTCAGAATCTTATTT
         66 TTTCGTTGGAAACAGG-AATATCTTCATATAAAAGCTAGACAGAAGCATTCTCAGAAACTTCTTT

                   **  **    *             **  * *   *
     114761 GTGATGTGCGCCCTCAACTAACAGTGTTGAAGCTTTCTTTTG
        130 GTGATGTTTGCATTCAAGTAACAGTGTTGAACATTCCCTTTC

                     *                    **     *   *                       
     114803 ATAGAGCAGTTTTGAAACACTCTTTTTGTAAAATCTGCAAGAGGATATTTGGATAGCTTTGAGGA
          1 ATAGAGCAGGTTTGAAACACTCTTTTTGTAGTATCTGGAAGTGGATATTTGGATAGCTTTGAGGA

                *        *   * *            **                      *    *   
     114868 TTTCATTGGAAACGGGATTGTCTTCATATAAACTCTAGACAGAAGCATTCTCAGAAGCTTCATTG
         66 TTTCGTTGGAAACAGGAATATCTTCATATAAAAGCTAGACAGAAGCATTCTCAGAAACTTCTTTG

            *       **   *    *             *        
     114933 GGATGTTTCAATTGAAGTCACAGTGTTGAACAGTCCCTTTC
        131 TGATGTTTGCATTCAAGTAACAGTGTTGAACATTCCCTTTC

                                                         *       *  * * *    
     114974 ATAGAGCAGGTTTGAAACACTCTTTTTGTAGTATCTGGAAGTGGACATTTGGAGAGATCTCAGGA
          1 ATAGAGCAGGTTTGAAACACTCTTTTTGTAGTATCTGGAAGTGGATATTTGGATAGCTTTGAGGA

            * *  *      *             **            *       * *          *  *
     115039 CTACGGT-GAAAAAGGAAATATCTTCCAATAAAAGCTAGATAGAAGCAATGTCAGAAACTTTTTC
         66 TTTCGTTGGAAACAGG-AATATCTTCATATAAAAGCTAGACAGAAGCATTCTCAGAAACTTCTTT

            *      * ** *   **      *       *  * *   *
     115103 ATGATGTGTCTACTCAGCTAACAGAGTTGAACCTTTCTTTTG
        130 GTGATGTTTGCATTCAAGTAACAGTGTTGAACATTCCCTTTC

             *       *        *          * *     *             **   *        
     115145 AGAGAGCAGTTTTGAAACCCTCTTTTTGTGGAATCTGCAAGTGGATATTTGTCTAGTTTTGAGGA
          1 ATAGAGCAGGTTTGAAACACTCTTTTTGTAGTATCTGGAAGTGGATATTTGGATAGCTTTGAGGA

                             *    *                   *       *              
     115210 TTTCGTTGGAAAC-GGATTA-CAT-ATA-AAAAGC-AGACAGCAGCATTCCCAGAAACTTCTTTG
         66 TTTCGTTGGAAACAGGAATATCTTCATATAAAAGCTAGACAGAAGCATTCTCAGAAACTTCTTTG

                              *    *                 
     115270 TGATGTTTGCATTCAAGTCACAGAGTTGAACATTCCCTTTC
        131 TGATGTTTGCATTCAAGTAACAGTGTTGAACATTCCCTTTC

                                                   *     *       **     *   *
     115311 ATAGAGCAGGTTTGAAACACTCTTTTTGTAGTATCTGGATGTGGACATTTGGAGCGCTTTCAGGC
          1 ATAGAGCAGGTTTGAAACACTCTTTTTGTAGTATCTGGAAGTGGATATTTGGATAGCTTTGAGGA

            **** *  *                 *** *   *                      *   *   
     115376 CAATGGTGTAAA-AGGAAATATCTTCCCCTGAAAACTAGACAGAAGCATTCTCAGAATCTTATTT
         66 TTTCGTTGGAAACAGG-AATATCTTCATATAAAAGCTAGACAGAAGCATTCTCAGAAACTTCTTT

                   **  **    *             **  * *   *
     115440 GTGATGTGCGCCCTCAACTAACAGTGTTGAAGCTTTCTTTTG
        130 GTGATGTTTGCATTCAAGTAACAGTGTTGAACATTCCCTTTC

                     *                    *      *   *        *              
     115482 ATAGAGCAGTTTTGAAACACTCTTTTTGTAATATCTGCAAGAGGATATTTCGATAGCTTTGAGGA
          1 ATAGAGCAGGTTTGAAACACTCTTTTTGTAGTATCTGGAAGTGGATATTTGGATAGCTTTGAGGA

                         *   * *            **                      *    *   
     115547 TTTCGTTGGAAACGGGATTGTCTTCATATAAACTCTAGACAGAAGCATTCTCAGAACCTTCATTG
         66 TTTCGTTGGAAACAGGAATATCTTCATATAAAAGCTAGACAGAAGCATTCTCAGAAACTTCTTTG

            *       **   *    *             * *      
     115612 GGATGTTTCAATTGAAGTCACAGTGTTGAACAGTTCCTTTC
        131 TGATGTTTGCATTCAAGTAACAGTGTTGAACATTCCCTTTC

              *           *    *                         *       **   * *    
     115653 ATGGAGCAGGTTTGGAACAGTCTTTTTGTAGTATCTGGAAGTGGACATTTGGAGCGCTCTCAGGA
          1 ATAGAGCAGGTTTGAAACACTCTTTTTGTAGTATCTGGAAGTGGATATTTGGATAGCTTTGAGGA

            * *  *      *             **          * *       * *          *  *
     115718 ATACGGT-GAAAAAGGAAATATCTTCCAATAAAAGCTACATAGAAGCAATGTCAGAAACTTTTTC
         66 TTTCGTTGGAAACAGG-AATATCTTCATATAAAAGCTAGACAGAAGCATTCTCAGAAACTTCTTT

            *      * ** *   **      *       *  * *   *
     115782 ATGATGTATCTACTCAGCTAACAGAGTTGAACCTTTCTTTTG
        130 GTGATGTTTGCATTCAAGTAACAGTGTTGAACATTCCCTTTC

             *       *                   * *     *             **            
     115824 AGAGAGCAGTTTTGAAACACTCTTTTTGTGGAATCTGCAAGTGGATATTTGTCTAGCTTTGAGGA
          1 ATAGAGCAGGTTTGAAACACTCTTTTTGTAGTATCTGGAAGTGGATATTTGGATAGCTTTGAGGA

                         **  *    *                   *       *   *          
     115889 TTTCGTTGGAAACGTGATTA-CAT-ATA-AAAAGC-AGACAGCAGCATTCCCAGTAACTTCTTTG
         66 TTTCGTTGGAAACAGGAATATCTTCATATAAAAGCTAGACAGAAGCATTCTCAGAAACTTCTTTG

                              *    *                 
     115950 TGATGTTTGCATTCAAGTCACAGAGTTGAACATTCCCTTTC
        131 TGATGTTTGCATTCAAGTAACAGTGTTGAACATTCCCTTTC

                                                   *     *       **     *   *
     115991 ATAGAGCAGGTTTGAAACACTCTTTTTGTAGTATCTGGATGTGGAGATTTGGAGCGCTTTCAGGC
          1 ATAGAGCAGGTTTGAAACACTCTTTTTGTAGTATCTGGAAGTGGATATTTGGATAGCTTTGAGGA

            * ** *      *             *** *   *                          *   
     116056 CTATGGT-GAAAAAGGAAATATCTTCCCCTGAAAACTAGACAGAAGCATTCTCAGAAACTTATTT
         66 TTTCGTTGGAAACAGG-AATATCTTCATATAAAAGCTAGACAGAAGCATTCTCAGAAACTTCTTT

                *  **  **    *   *          *  * *    
     116120 GTGACGTGCGCCATCAACTAAGAGTGTTGAACCTTTCTTTTC
        130 GTGATGTTTGCATTCAAGTAACAGTGTTGAACATTCCCTTTC

                     *                    *      *   *                       
     116162 ATAGAGCAGTTTTGAAACACTCTTTTTGTAATATCTGCAAGAGGATATTTGGATAGCTTTGAGGA
          1 ATAGAGCAGGTTTGAAACACTCTTTTTGTAGTATCTGGAAGTGGATATTTGGATAGCTTTGAGGA

                         *   * *            **                      *    *   
     116227 TTTCGTTGGAAACGGGATTGTCTTCATATAAACTCTAGACAGAAGCATTCTCAGAAGCTTCATTG
         66 TTTCGTTGGAAACAGGAATATCTTCATATAAAAGCTAGACAGAAGCATTCTCAGAAACTTCTTTG

            *       **   *    *             *        
     116292 GGATGTTTCAATTGAAGTCACAGTGTTGAACAGTCCCTTTC
        131 TGATGTTTGCATTCAAGTAACAGTGTTGAACATTCCCTTTC

                                                         *       *  * * *    
     116333 ATAGAGCAGGTTTGAAACACTCTTTTTGTAGTATCTGGAAGTGGACATTTGGAGAGATCTCAGGA
          1 ATAGAGCAGGTTTGAAACACTCTTTTTGTAGTATCTGGAAGTGGATATTTGGATAGCTTTGAGGA

            * *  *    * *             **            *       * *          *  *
     116398 ATACGGT-GATAAAGGAAATATCTTCCAATAAAAGCTAGATAGAAGCAATGTCAGAAACTTTTTC
         66 TTTCGTTGGAAACAGG-AATATCTTCATATAAAAGCTAGACAGAAGCATTCTCAGAAACTTCTTT

            *      * ** *   **      *       *  *     *
     116462 ATGATGTATCTACTCAGCTAACAGAGTTGAACCTTTCCTTTG
        130 GTGATGTTTGCATTCAAGTAACAGTGTTGAACATTCCCTTTC

             *       *                   * *     *             **            
     116504 AGAGAGCAGTTTTGAAACACTCTTTTTGTGGAATCTGCAAGTGGATATTTGTCTAGCTTTGAGGA
          1 ATAGAGCAGGTTTGAAACACTCTTTTTGTAGTATCTGGAAGTGGATATTTGGATAGCTTTGAGGA

                         *   *    *                   *       *   *          
     116569 TTTCGTTGGAAACGGGATTA-CAT-ATA-AAAAGC-AGACAGCAGCATTCCCAGTAACTTCTTTG
         66 TTTCGTTGGAAACAGGAATATCTTCATATAAAAGCTAGACAGAAGCATTCTCAGAAACTTCTTTG

                              *    *                 
     116630 TGATGTTTGCATTCAAGTCACAGAGTTGAACATTCCCTTTC
        131 TGATGTTTGCATTCAAGTAACAGTGTTGAACATTCCCTTTC

              *   *                                *     *       **     *   *
     116671 ATGGAGGAGGTTTGAAACACTCTTTTTGTAGTATCTGGATGTGGACATTTGGAGCGCTTTCAGGC
          1 ATAGAGCAGGTTTGAAACACTCTTTTTGTAGTATCTGGAAGTGGATATTTGGATAGCTTTGAGGA

            * ** *      *             *** *   *                      *   *   
     116736 CTATGGT-GAAAAAGGAAATATCTTCCACTGAAAACTAGACAGAAGCATTCTCAGAATCTTATTT
         66 TTTCGTTGGAAACAGG-AATATCTTCATATAAAAGCTAGACAGAAGCATTCTCAGAAACTTCTTT

                   **  **    *             **  * *   *
     116800 GTGATGTGCGCCCTCAACTAACAGTGTTGAAGCTTTCTTTTG
        130 GTGATGTTTGCATTCAAGTAACAGTGTTGAACATTCCCTTTC

                     *                    **     *   *                       
     116842 ATAGAGCAGTTTTGAAACACTCTTTTTGTAAAATCTGCAAGAGGATATTTGGATAGCTTTGAGGA
          1 ATAGAGCAGGTTTGAAACACTCTTTTTGTAGTATCTGGAAGTGGATATTTGGATAGCTTTGAGGA

                         *   * *            **                      *    *   
     116907 TTTCGTTGGAAACGGGATTGTCTTCATATAAACTCTAGACAGAAGCATTCTCAGAAGCTTCATTG
         66 TTTCGTTGGAAACAGGAATATCTTCATATAAAAGCTAGACAGAAGCATTCTCAGAAACTTCTTTG

            *       **   *    *             *        
     116972 GGATGTTTCAATTGAAGTCACAGTGTTGAACAGTCCCTTTC
        131 TGATGTTTGCATTCAAGTAACAGTGTTGAACATTCCCTTTC

                                                         *       *  * * *    
     117013 ATAGAGCAGGTTTGAAACACTCTTTTTGTAGTATCTGGAAGTGGACATTTGGAGAGATCTCAGGA
          1 ATAGAGCAGGTTTGAAACACTCTTTTTGTAGTATCTGGAAGTGGATATTTGGATAGCTTTGAGGA

            * *  *    * *             **            *       * *          *  *
     117078 ATACGGT-GATAAAGGAAATATCTTCCAATAAAAGCTAGATAGAAGCAATGTCAGAAACTTTTTC
         66 TTTCGTTGGAAACAGG-AATATCTTCATATAAAAGCTAGACAGAAGCATTCTCAGAAACTTCTTT

            *      * ** *   **      *       *  * *   *
     117142 ATGATGTATCTACTCAGCTAACAGAGTTGAACCTTTCTTTTG
        130 GTGATGTTTGCATTCAAGTAACAGTGTTGAACATTCCCTTTC

            **       *                   * *     *             **            
     117184 CGAGAGCAGTTTTGAAACACTCTTTTTGTGGAATCTGCAAGTGGATATTTGTCTAGCTTTGAGGA
          1 ATAGAGCAGGTTTGAAACACTCTTTTTGTAGTATCTGGAAGTGGATATTTGGATAGCTTTGAGGA

                         *   *    *                   *       *              
     117249 TTTCGTTGGAAACGGGATTA-CAT-ATA-AAAAGC-AGACAGCAGCATTCCCAGAAACTTCTTTG
         66 TTTCGTTGGAAACAGGAATATCTTCATATAAAAGCTAGACAGAAGCATTCTCAGAAACTTCTTTG

                      *       *    *                 
     117310 TGATGTTTGCCTTCAAGTCACAGAGTTGAACATTCCCTTTC
        131 TGATGTTTGCATTCAAGTAACAGTGTTGAACATTCCCTTTC

                     *                           * *     *       **     *   *
     117351 ATAGAGCAGTTTTGAAACACTCTTTTTGTAGTATCTGTATGTGGACATTTGGAGCGCTTTCAGGC
          1 ATAGAGCAGGTTTGAAACACTCTTTTTGTAGTATCTGGAAGTGGATATTTGGATAGCTTTGAGGA

            * ** *      *             *** *   *                          *   
     117416 CTATGGT-GAAAAAGGAAATATCTTCCCCTGAAAACTAGACAGAAGCATTCTCAGAAACTTATTT
         66 TTTCGTTGGAAACAGG-AATATCTTCATATAAAAGCTAGACAGAAGCATTCTCAGAAACTTCTTT

              *    **  **    *              *  * *   *
     117480 GTAATGTGCGCCCTCAACTAACAGTGTTGAACTTTTCTTTTG
        130 GTGATGTTTGCATTCAAGTAACAGTGTTGAACATTCCCTTTC

                     *                    *      *   *                       
     117522 ATAGAGCAGTTTTGAAACACTCTTTTTGTAATATCTGCAAGAGGATATTTGGATAGCTTTGAGGA
          1 ATAGAGCAGGTTTGAAACACTCTTTTTGTAGTATCTGGAAGTGGATATTTGGATAGCTTTGAGGA

                         *   * *            **                      *    *   
     117587 TTTCGTTGGAAACGGGATTGTCTTCATATAAACTCTAGACAGAAGCATTCTCAGAAGCTTCATTG
         66 TTTCGTTGGAAACAGGAATATCTTCATATAAAAGCTAGACAGAAGCATTCTCAGAAACTTCTTTG

            *       **   *    *             * *      
     117652 GGATGTTTCAATTGAAGTCACAGTGTTGAACAGTTCCTTTC
        131 TGATGTTTGCATTCAAGTAACAGTGTTGAACATTCCCTTTC

                                                         *       **   * *    
     117693 ATAGAGCAGGTTTGAAACACTCTTTTTGTAGTATCTGGAAGTGGACATTTGGAGCGCTCTCAGGA
          1 ATAGAGCAGGTTTGAAACACTCTTTTTGTAGTATCTGGAAGTGGATATTTGGATAGCTTTGAGGA

            ***  *      *             **          * *       * * *        *  *
     117758 CGACGGT-GAAAAAGGAAATATCTTCCAATAAAAGCTACATAGAAGCAATGTGAGAAACTTTTTC
         66 TTTCGTTGGAAACAGG-AATATCTTCATATAAAAGCTAGACAGAAGCATTCTCAGAAACTTCTTT

            *   *  * ** *   **              *  * *   *
     117822 ATGACGTATCTACTCAGCTAACAGTGTTGAACCTTTCTTTTG
        130 GTGATGTTTGCATTCAAGTAACAGTGTTGAACATTCCCTTTC

                     *                   * *     *             **            
     117864 ATAGAGCAGTTTTGAAACACTCTTTTTGTGGAATCTGCAAGTGGATATTTGTCTAGCTTTGAGGA
          1 ATAGAGCAGGTTTGAAACACTCTTTTTGTAGTATCTGGAAGTGGATATTTGGATAGCTTTGAGGA

                         *   *    *                   *       *   *    *     
     117929 TTTCGTTGGAAACGGGATTA-CAT-ATA-AAAAGC-AGACAGCAGCATTCCCAGTAACTACTTTG
         66 TTTCGTTGGAAACAGGAATATCTTCATATAAAAGCTAGACAGAAGCATTCTCAGAAACTTCTTTG

                              *    *                 
     117990 TGATGTTTGCATTCAAGTCACAGAGTTGAACATTCCCTTTC
        131 TGATGTTTGCATTCAAGTAACAGTGTTGAACATTCCCTTTC

                                                   *     *       **     *   *
     118031 ATAGAGCAGGTTTGAAACACTCTTTTTGTAGTATCTGGATGTGGACATTTGGAGCGCTTTCAGGC
          1 ATAGAGCAGGTTTGAAACACTCTTTTTGTAGTATCTGGAAGTGGATATTTGGATAGCTTTGAGGA

              ** *      *             *** *   *                       *  *   
     118096 TTATGGT-GAAAAAGGAAATATCTTCCCCTGAAAACTAGACAGAAGCATTCTCAGAAAATTATTT
         66 TTTCGTTGGAAACAGG-AATATCTTCATATAAAAGCTAGACAGAAGCATTCTCAGAAACTTCTTT

                   **  **    *              *  * *   *
     118160 GTGATGTGCGCCCTCAACTAACAGTGTTGAACCTTTCTTTTG
        130 GTGATGTTTGCATTCAAGTAACAGTGTTGAACATTCCCTTTC

                     *                    *      *   *                       
     118202 ATAGAGCAGATTTGAAACACTCTTTTTGTAATATCTGCAAGAGGATATTTGGATAGCTTTGAGGA
          1 ATAGAGCAGGTTTGAAACACTCTTTTTGTAGTATCTGGAAGTGGATATTTGGATAGCTTTGAGGA

                         *   * *            **                      *    *   
     118267 TTTCGTTGGAAACGGGATTGTCTTCATATAAACTCTAGACAGAAGCATTCTCAGAAGCTTCATTG
         66 TTTCGTTGGAAACAGGAATATCTTCATATAAAAGCTAGACAGAAGCATTCTCAGAAACTTCTTTG

            *       **   *    *             *        
     118332 GGATGTTTCAATTGAAGTCACAGTGTTGAACAGTCCCTTTC
        131 TGATGTTTGCATTCAAGTAACAGTGTTGAACATTCCCTTTC

                                                         *       *  * **     
     118373 ATAGAGCAGGTTTGAAACACTCTTTTTGTAGTATCTGGAAGTGGACATTTGGAGAGATCCGAGGA
          1 ATAGAGCAGGTTTGAAACACTCTTTTTGTAGTATCTGGAAGTGGATATTTGGATAGCTTTGAGGA

            * *  *    * *             *             *       * *       *  *  *
     118438 ATACGGT-GACAAAGGAAATATCTTCCTATAAAAGCTAGATAGAAGCAATGTCAGAAAATTTTTC
         66 TTTCGTTGGAAACAGG-AATATCTTCATATAAAAGCTAGACAGAAGCATTCTCAGAAACTTCTTT

            *      * ** *   **      *       *  * *   *
     118502 ATGATGTATCTACTCAGCTAACAGAGTTGAACCTTTCTTTTG
        130 GTGATGTTTGCATTCAAGTAACAGTGTTGAACATTCCCTTTC

             *       *                   * *     *             **            
     118544 AGAGAGCAGTTTTGAAACACTCTTTTTGTGGAATCTGCAAGTGGATATTTGTCTAGCTTTGAGGA
          1 ATAGAGCAGGTTTGAAACACTCTTTTTGTAGTATCTGGAAGTGGATATTTGGATAGCTTTGAGGA

                         *   *    *                  **       *              
     118609 TTTCGTTGGAAACGGGATTA-CAT-ATA-AAAAGC-AGACAACAGCATTCCCAGAAACTTCTTTG
         66 TTTCGTTGGAAACAGGAATATCTTCATATAAAAGCTAGACAGAAGCATTCTCAGAAACTTCTTTG

               *              *    *                 
     118670 TGAAGTTTGCATTCAAGTCACAGAGTTGAACATTCCCTTTC
        131 TGATGTTTGCATTCAAGTAACAGTGTTGAACATTCCCTTTC

                                                   *     *       **  *  *   *
     118711 ATAGAGCAGGTTTGAAACACTCTTTTTGTAGTATCTGGATGTGGACATTTGGAGCGCGTTCAGGC
          1 ATAGAGCAGGTTTGAAACACTCTTTTTGTAGTATCTGGAAGTGGATATTTGGATAGCTTTGAGGA

            * ** *      *             *****   *                      *   *   
     118776 CTATGGT-GAAAAAGGCAATATCTTCCCCCGAAAACTAGACAGAAGCATTCTCAGAATCTTATTT
         66 TTTCGTTGGAAACAGG-AATATCTTCATATAAAAGCTAGACAGAAGCATTCTCAGAAACTTCTTT

                   **  **    *             *   * *   *
     118840 GTGATGTGCGCCCTCAACTAACAGTGTTGAAGATTTCTTTTG
        130 GTGATGTTTGCATTCAAGTAACAGTGTTGAACATTCCCTTTC

                     *                    **     *   *                       
     118882 ATAGAGCAGTTTTGAAACACTCTTTTTGTAAAATCTGCAAGAGGATATTTGGATAGCTTTGAGGA
          1 ATAGAGCAGGTTTGAAACACTCTTTTTGTAGTATCTGGAAGTGGATATTTGGATAGCTTTGAGGA

                *        *   * *            **                      *    *   
     118947 TTTCCTTGGAAACGGGATTGTCTTCATATAAACTCTAGACAGAAGCATTCTCAGAAGCTTCATTG
         66 TTTCGTTGGAAACAGGAATATCTTCATATAAAAGCTAGACAGAAGCATTCTCAGAAACTTCTTTG

            *       **   *    *             *        
     119012 GGATGTTTCAATTGAAGTCACAGTGTTGAACAGTCCCTTTC
        131 TGATGTTTGCATTCAAGTAACAGTGTTGAACATTCCCTTTC

                                                         *       *  * * *    
     119053 ATAGAGCAGGTTTGAAACACTCTTTTTGTAGTATCTGGAAGTGGACATTTGGAGAGATCTCAGGA
          1 ATAGAGCAGGTTTGAAACACTCTTTTTGTAGTATCTGGAAGTGGATATTTGGATAGCTTTGAGGA

            * *  *    * *             **            *       * *          *  *
     119118 ATACGGT-GATAAAGGAAATATCTTCCAATAAAAGCTAGATAGAAGCAATGTCAGAAACTTTTTC
         66 TTTCGTTGGAAACAGG-AATATCTTCATATAAAAGCTAGACAGAAGCATTCTCAGAAACTTCTTT

            *      * ** *   **      *       *  * *   *
     119182 ATGATGTATCTACTCAGCTAACAGAGTTGAACCTTTCTTTTG
        130 GTGATGTTTGCATTCAAGTAACAGTGTTGAACATTCCCTTTC

            **       *                   * **    *             **            
     119224 CGAGAGCAGTTTTGAAACACTCTTTTTGTGGACTCTGCAAGTGGATATTTGTCTAGCTTTGAGGA
          1 ATAGAGCAGGTTTGAAACACTCTTTTTGTAGTATCTGGAAGTGGATATTTGGATAGCTTTGAGGA

                         *   *    *                   *       *              
     119289 TTTCGTTGGAAACGGGATTA-CAT-ATA-AAAAGC-AGACAGCAGCATTCCCAGAAACTTCTTTG
         66 TTTCGTTGGAAACAGGAATATCTTCATATAAAAGCTAGACAGAAGCATTCTCAGAAACTTCTTTG

                      *       *    *                 
     119350 TGATGTTTGCCTTCAAGTCACAGAGTTGAACATTCCCTTTC
        131 TGATGTTTGCATTCAAGTAACAGTGTTGAACATTCCCTTTC

                     *                           * *     *       **     *   *
     119391 ATAGAGCAGTTTTGAAACACTCTTTTTGTAGTATCTGTATGTGGACATTTGGAGCGCTTTCAGGC
          1 ATAGAGCAGGTTTGAAACACTCTTTTTGTAGTATCTGGAAGTGGATATTTGGATAGCTTTGAGGA

            * ** *      *             *** *   *                          *   
     119456 CTATGGT-GAAAAAGGAAATATCTTCCCCTGAAAACTAGACAGAAGCATTCTCAGAAACTTATTT
         66 TTTCGTTGGAAACAGG-AATATCTTCATATAAAAGCTAGACAGAAGCATTCTCAGAAACTTCTTT

              *    **  **    *              *  * *   *
     119520 GTAATGTGCGCCCTCAACTAACAGTGTTGAACTTTTCTTTTG
        130 GTGATGTTTGCATTCAAGTAACAGTGTTGAACATTCCCTTTC

                     *                    *      *   *                       
     119562 ATAGAGCAGTTTTGAAACACTCTTTTTGTAATATCTGCAAGAGGATATTTGGATAGCTTTGAGGA
          1 ATAGAGCAGGTTTGAAACACTCTTTTTGTAGTATCTGGAAGTGGATATTTGGATAGCTTTGAGGA

                         *   * *            **                      *    *   
     119627 TTTCGTTGGAAACGGGATTGTCTTCATATAAACTCTAGACAGAAGCATTCTCAGAAGCTTCATTG
         66 TTTCGTTGGAAACAGGAATATCTTCATATAAAAGCTAGACAGAAGCATTCTCAGAAACTTCTTTG

            *       **   *    *             * *      
     119692 GGATGTTTCAATTGAAGTCACAGTGTTGAACAGTTCCTTTC
        131 TGATGTTTGCATTCAAGTAACAGTGTTGAACATTCCCTTTC

                                                         *       **   * *    
     119733 ATAGAGCAGGTTTGAAACACTCTTTTTGTAGTATCTGGAAGTGGACATTTGGAGCGCTCTCAGGA
          1 ATAGAGCAGGTTTGAAACACTCTTTTTGTAGTATCTGGAAGTGGATATTTGGATAGCTTTGAGGA

            ***  *      *             **          * *       * * *        *  *
     119798 CGACGGT-GAAAAAGGAAATATCTTCCAATAAAAGCTACATAGAAGCAATGTGAGAAACTTTTTC
         66 TTTCGTTGGAAACAGG-AATATCTTCATATAAAAGCTAGACAGAAGCATTCTCAGAAACTTCTTT

            *   *  * ** *   **              *  * *   *
     119862 ATGACGTATCTACTCAGCTAACAGTGTTGAACCTTTCTTTTG
        130 GTGATGTTTGCATTCAAGTAACAGTGTTGAACATTCCCTTTC

                     *                   * *     *             **            
     119904 ATAGAGCAGTTTTGAAACACTCTTTTTGTGGAATCTGCAAGTGGATATTTGTCTAGCTTTGAGGA
          1 ATAGAGCAGGTTTGAAACACTCTTTTTGTAGTATCTGGAAGTGGATATTTGGATAGCTTTGAGGA

                         *   *    *                   *       *   *    *     
     119969 TTTCGTTGGAAACGGGATTA-CAT-ATA-AAAAGC-AGACAGCAGCATTCCCAGTAACTACTTTG
         66 TTTCGTTGGAAACAGGAATATCTTCATATAAAAGCTAGACAGAAGCATTCTCAGAAACTTCTTTG

                              *    *                 
     120030 TGATGTTTGCATTCAAGTCACAGAGTTGAACATTCCCTTTC
        131 TGATGTTTGCATTCAAGTAACAGTGTTGAACATTCCCTTTC

                                                   *     *       **     *   *
     120071 ATAGAGCAGGTTTGAAACACTCTTTTTGTAGTATCTGGATGTGGACATTTGGAGCGCTTTCAGGC
          1 ATAGAGCAGGTTTGAAACACTCTTTTTGTAGTATCTGGAAGTGGATATTTGGATAGCTTTGAGGA

              ** *      *             *** *   *                       *  *   
     120136 TTATGGT-GAAAAAGGAAATATCTTCCCCTGAAAACTAGACAGAAGCATTCTCAGAAAATTATTT
         66 TTTCGTTGGAAACAGG-AATATCTTCATATAAAAGCTAGACAGAAGCATTCTCAGAAACTTCTTT

                   **  **    *              *  * *   *
     120200 GTGATGTGCGCCCTCAACTAACAGTGTTGAACCTTTCTTTTG
        130 GTGATGTTTGCATTCAAGTAACAGTGTTGAACATTCCCTTTC

                     *                    *      *   *                       
     120242 ATAGAGCAGATTTGAAACACTCTTTTTGTAATATCTGCAAGAGGATATTTGGATAGCTTTGAGGA
          1 ATAGAGCAGGTTTGAAACACTCTTTTTGTAGTATCTGGAAGTGGATATTTGGATAGCTTTGAGGA

                         *   * *            **                      *    *   
     120307 TTTCGTTGGAAACGGGATTGTCTTCATATAAACTCTAGACAGAAGCATTCTCAGAAGCTTCATTG
         66 TTTCGTTGGAAACAGGAATATCTTCATATAAAAGCTAGACAGAAGCATTCTCAGAAACTTCTTTG

            *       **   *    *             *        
     120372 GGATGTTTCAATTGAAGTCACAGTGTTGAACAGTCCCTTTC
        131 TGATGTTTGCATTCAAGTAACAGTGTTGAACATTCCCTTTC

                                                         *       *  * **     
     120413 ATAGAGCAGGTTTGAAACACTCTTTTTGTAGTATCTGGAAGTGGACATTTGGAGAGATCCGAGGA
          1 ATAGAGCAGGTTTGAAACACTCTTTTTGTAGTATCTGGAAGTGGATATTTGGATAGCTTTGAGGA

            * *  *    * *             *             *       * *       *  *  *
     120478 ATACGGT-GACAAAGGAAATATCTTCCTATAAAAGCTAGATAGAAGCAATGTCAGAAAATTTTTC
         66 TTTCGTTGGAAACAGG-AATATCTTCATATAAAAGCTAGACAGAAGCATTCTCAGAAACTTCTTT

            *      * ** *   **      *       *  * *   *
     120542 ATGATGTATCTACTCAGCTAACAGAGTTGAACCTTTCTTTTG
        130 GTGATGTTTGCATTCAAGTAACAGTGTTGAACATTCCCTTTC

             *       *                   * *     *             **            
     120584 AGAGAGCAGTTTTGAAACACTCTTTTTGTGGAATCTGCAAGTGGATATTTGTCTAGCTTTGAGGA
          1 ATAGAGCAGGTTTGAAACACTCTTTTTGTAGTATCTGGAAGTGGATATTTGGATAGCTTTGAGGA

                         *   *    *                  **       *              
     120649 TTTCGTTGGAAACGGGATTA-CAT-ATA-AAAAGC-AGACAACAGCATTCCCAGAAACTTCTTTG
         66 TTTCGTTGGAAACAGGAATATCTTCATATAAAAGCTAGACAGAAGCATTCTCAGAAACTTCTTTG

               *              *    *                 
     120710 TGAAGTTTGCATTCAAGTCACAGAGTTGAACATTCCCTTTC
        131 TGATGTTTGCATTCAAGTAACAGTGTTGAACATTCCCTTTC

                                                   *     *       **  *  *   *
     120751 ATAGAGCAGGTTTGAAACACTCTTTTTGTAGTATCTGGATGTGGACATTTGGAGCGCGTTCAGGC
          1 ATAGAGCAGGTTTGAAACACTCTTTTTGTAGTATCTGGAAGTGGATATTTGGATAGCTTTGAGGA

            * ** *      *             *****   *                      *   *   
     120816 CTATGGT-GAAAAAGGCAATATCTTCCCCCGAAAACTAGACAGAAGCATTCTCAGAATCTTATTT
         66 TTTCGTTGGAAACAGG-AATATCTTCATATAAAAGCTAGACAGAAGCATTCTCAGAAACTTCTTT

                   **  **    *             *   * *   *
     120880 GTGATGTGCGCCCTCAACTAACAGTGTTGAAGATTTCTTTTG
        130 GTGATGTTTGCATTCAAGTAACAGTGTTGAACATTCCCTTTC

                     *                    **     *   *                       
     120922 ATAGAGCAGTTTTGAAACACTCTTTTTGTAAAATCTGCAAGAGGATATTTGGATAGCTTTGAGGA
          1 ATAGAGCAGGTTTGAAACACTCTTTTTGTAGTATCTGGAAGTGGATATTTGGATAGCTTTGAGGA

                *        *   * *            **                      *    *   
     120987 TTTCCTTGGAAACGGGATTGTCTTCATATAAACTCTAGACAGAAGCATTCTCAGAAGCTTCATTG
         66 TTTCGTTGGAAACAGGAATATCTTCATATAAAAGCTAGACAGAAGCATTCTCAGAAACTTCTTTG

            *       **   *    *             *        
     121052 GGATGTTTCAATTGAAGTCACAGTGTTGAACAGTCCCTTTC
        131 TGATGTTTGCATTCAAGTAACAGTGTTGAACATTCCCTTTC

                                                   *     *       **     *   *
     121093 ATAGAGCAGGTTTGAAACACTCTTTTTGTAGTATCTGGATGTGGACATTTGGAGCGCTTTAAGGC
          1 ATAGAGCAGGTTTGAAACACTCTTTTTGTAGTATCTGGAAGTGGATATTTGGATAGCTTTGAGGA

            * ** *      *             *** *   *                          *   
     121158 CTATGGT-GAAAAAGGAAATATCTTCCCCTGAAAACTAGACAGAAGCATTCTCAGAAACTTATTT
         66 TTTCGTTGGAAACAGG-AATATCTTCATATAAAAGCTAGACAGAAGCATTCTCAGAAACTTCTTT

                   **  **   **             **  * *   *
     121222 GTGATGTGCGCCCTCAGCTAACAGTGTTGAAGCTTTCTTTTG
        130 GTGATGTTTGCATTCAAGTAACAGTGTTGAACATTCCCTTTC

                     *    *              * *     *             **            
     121264 ATAGAGCAGTTTTGGAACACTCTTTTTGTGGAATCTGCAAGTGGATATTTGTCTAGCTTTGAGGA
          1 ATAGAGCAGGTTTGAAACACTCTTTTTGTAGTATCTGGAAGTGGATATTTGGATAGCTTTGAGGA

                         *   *    *                   *       *     *        
     121329 TTTCGTTGGAAACGGGATTA-CAT-ATA-AAAAGC-AGACAGCAGCATTCCCAGAATCTTCTTTG
         66 TTTCGTTGGAAACAGGAATATCTTCATATAAAAGCTAGACAGAAGCATTCTCAGAAACTTCTTTG

                              *    *                 
     121390 TGATGTTTGCATTCAAGTCACAGAGTTGAACATTCCCTTTC
        131 TGATGTTTGCATTCAAGTAACAGTGTTGAACATTCCCTTTC

                                       *           * *   *       **     *   *
     121431 ATAGAGCAGGTTTGAAACACTCTTTTTATAGTATCTGGATGAGGACATTTGGAGCGCTTTCAGGC
          1 ATAGAGCAGGTTTGAAACACTCTTTTTGTAGTATCTGGAAGTGGATATTTGGATAGCTTTGAGGA

            * ** *      *             *** *   *                      *   *   
     121496 CTATGGT-GAAAAAGGAAATATCTTCCCCTGAAAACTAGACAGAAGCATTCTCAGAATCTTATTT
         66 TTTCGTTGGAAACAGG-AATATCTTCATATAAAAGCTAGACAGAAGCATTCTCAGAAACTTCTTT

                   **  **    *             **  * *   *
     121560 GTGATGTGCGCCCTCAACTAACAGTGTTGAAGCTTTCTTTTG
        130 GTGATGTTTGCATTCAAGTAACAGTGTTGAACATTCCCTTTC

                     *                    **     *   *                       
     121602 ATAGAGCAGTTTTGAAACACTCTTTTTGTAAAATCTGCAAGAGGATATTTGGATAGCTTTGAGGA
          1 ATAGAGCAGGTTTGAAACACTCTTTTTGTAGTATCTGGAAGTGGATATTTGGATAGCTTTGAGGA

                         *   * *            **                      *    *   
     121667 TTTCGTTGGAAACGGGATTGTCTTCATATAAACTCTAGACAGAAGCATTCTCAGAAGCTTCATTG
         66 TTTCGTTGGAAACAGGAATATCTTCATATAAAAGCTAGACAGAAGCATTCTCAGAAACTTCTTTG

            *       **   *    *             *        
     121732 GGATGTTTCAATTGAAGTCACAGTGTTGAACAGTCCCTTTC
        131 TGATGTTTGCATTCAAGTAACAGTGTTGAACATTCCCTTTC

                                                   *     *       **     *   *
     121773 ATAGAGCAGGTTTGAAACACTCTTTTTGTAGTATCTGGATGTGGACATTTGGAGCGCTTTCAGGC
          1 ATAGAGCAGGTTTGAAACACTCTTTTTGTAGTATCTGGAAGTGGATATTTGGATAGCTTTGAGGA

            * ** *      *             *** *   *                          *   
     121838 CTATGGT-GAAAAAGGAAATATCTTCCCCTGAAAACTAGACAGAAGCATTCTCAGAAACTTATTT
         66 TTTCGTTGGAAACAGG-AATATCTTCATATAAAAGCTAGACAGAAGCATTCTCAGAAACTTCTTT

                   **  **    *             **  * *   *
     121902 GTGATGTGCGCCCTCAACTAACAGTGTTGAAGCTTTCTTTTG
        130 GTGATGTTTGCATTCAAGTAACAGTGTTGAACATTCCCTTTC

                     *                   * *     *             **            
     121944 ATAGAGCAGTTTTGAAACACTCTTTTTGTGGAATCTGCAAGTGGATATTTGTCTAGCTTTGAGGA
          1 ATAGAGCAGGTTTGAAACACTCTTTTTGTAGTATCTGGAAGTGGATATTTGGATAGCTTTGAGGA

                         *   *    *                   *       *              
     122009 TTTCGTTGGAAACCGGATTA-CAT-ATA-AAAAGC-AGACAGCAGCATTCCCAGAAACTTCTTTG
         66 TTTCGTTGGAAACAGGAATATCTTCATATAAAAGCTAGACAGAAGCATTCTCAGAAACTTCTTTG

                              *    *                 
     122070 TGATGTTTGCATTCAAGTCACAGAGTTGAACATTCCCTTTC
        131 TGATGTTTGCATTCAAGTAACAGTGTTGAACATTCCCTTTC

                                                   *     *       **     *   *
     122111 ATAGAGCAGGTTTGAAACACTCTTTTTGTAGTATCTGGATGTGGACATTTGGAGCGCTTTCAGGC
          1 ATAGAGCAGGTTTGAAACACTCTTTTTGTAGTATCTGGAAGTGGATATTTGGATAGCTTTGAGGA

            * ** *      *             *** *   *                          *   
     122176 CTATGGT-GAAAAAGGCAATATCTTCCCCTGAAAACTAGACAGAAGCATTCTCAGAAACTTATTT
         66 TTTCGTTGGAAACAGG-AATATCTTCATATAAAAGCTAGACAGAAGCATTCTCAGAAACTTCTTT

                *  **  **    *              *  * *   *
     122240 GTGACGTGCGCCCTCAACTAACAGTGTTGAACCTTTCTTTTG
        130 GTGATGTTTGCATTCAAGTAACAGTGTTGAACATTCCCTTTC

                     *                    *      *   *                       
     122282 ATAGAGCAGTTTTGAAACACTCTTTTTGTAATATCTGCAAGAGGATATTTGGATAGCTTTGAGGA
          1 ATAGAGCAGGTTTGAAACACTCTTTTTGTAGTATCTGGAAGTGGATATTTGGATAGCTTTGAGGA

                         *   * *            **                      *    *   
     122347 TTTCGTTGGAAACGGGATTGTCTTCATATAAACTCTAGACAGAAGCATTCTCAGAAGCTTCATTG
         66 TTTCGTTGGAAACAGGAATATCTTCATATAAAAGCTAGACAGAAGCATTCTCAGAAACTTCTTTG

            *       **   *    *             *        
     122412 GGATGTTTCAATTGAAGTCACAGTGTTGAACAGTCCCTTTC
        131 TGATGTTTGCATTCAAGTAACAGTGTTGAACATTCCCTTTC

                     *                 *                 *       *  * * *    
     122453 ATAGAGCAGTTTTGAAACACTCTTTTTTTAGTATCTGGAAGTGGACATTTGGAGAGATCTCAGGA
          1 ATAGAGCAGGTTTGAAACACTCTTTTTGTAGTATCTGGAAGTGGATATTTGGATAGCTTTGAGGA

            * *  *    * *             ** *          *       * *          *  *
     122518 ATACGGT-GACAAAGGAAATATCTTCCAACAAAAGCTAGATAGAAGCAATGTCAGAAACTTTTTC
         66 TTTCGTTGGAAACAGG-AATATCTTCATATAAAAGCTAGACAGAAGCATTCTCAGAAACTTCTTT

            *      * ** *   **      *       *  ***   *
     122582 ATGATGTATCTACTCAGCTAACAGAGTTGAACCTTTATTTTG
        130 GTGATGTTTGCATTCAAGTAACAGTGTTGAACATTCCCTTTC

             *       *                   * *     *             **            
     122624 AGAGAGCAGTTTTGAAACACTCTTTTTGTGGAATCTGCAAGTGGATATTTGTCTAGCTTTGAGGA
          1 ATAGAGCAGGTTTGAAACACTCTTTTTGTAGTATCTGGAAGTGGATATTTGGATAGCTTTGAGGA

                         *   *    *                   *       *   *          
     122689 TTTCGTTGGAAACGGGATTA-CAT-ATA-AAAAGC-AGACAGCAGCATTCCCAGTAACTTCTTTG
         66 TTTCGTTGGAAACAGGAATATCTTCATATAAAAGCTAGACAGAAGCATTCTCAGAAACTTCTTTG

                              *    *                 
     122750 TGATGTTTGCATTCAAGTCACAGAGTTGAACATTCCCTTTC
        131 TGATGTTTGCATTCAAGTAACAGTGTTGAACATTCCCTTTC

                                                 * *     *       **     *   *
     122791 ATAGAGCAGGTTTGAAACACTCTTTTTGTAGTATCTGTATGTGGACATTTGGAGCGCTTTCAGGC
          1 ATAGAGCAGGTTTGAAACACTCTTTTTGTAGTATCTGGAAGTGGATATTTGGATAGCTTTGAGGA

            * ** *      *             *** *   *                      *   *   
     122856 CTATGGT-GAAAAAGGAAATATCTTCCCCTGAAAACTAGACAGAAGCATTCTCAGAATCTTATTT
         66 TTTCGTTGGAAACAGG-AATATCTTCATATAAAAGCTAGACAGAAGCATTCTCAGAAACTTCTTT

                   **  **    *             **  *     *
     122920 GTGATGTGCGCCCTCAACTAACAGTGTTGAAGCTTTCCTTTG
        130 GTGATGTTTGCATTCAAGTAACAGTGTTGAACATTCCCTTTC

                     *                    **     *   *                       
     122962 ATAGAGCAGTTTTGAAACACTCTTTTTGTAAAATCTGCAAGAGGATATTTGGATAGCTTTGAGGA
          1 ATAGAGCAGGTTTGAAACACTCTTTTTGTAGTATCTGGAAGTGGATATTTGGATAGCTTTGAGGA

                         *   * *            **                           *   
     123027 TTTCGTTGGAAACGGGATTGTCTTCATATAAACTCTAGACAGAAGCATTCTCAGAAACTTCATTG
         66 TTTCGTTGGAAACAGGAATATCTTCATATAAAAGCTAGACAGAAGCATTCTCAGAAACTTCTTTG

            *       **   *    *             *        
     123092 GGATGTTTCAATTGAAGTCACAGTGTTGAACAGTCCCTTTC
        131 TGATGTTTGCATTCAAGTAACAGTGTTGAACATTCCCTTTC

                                                         *       **   * *    
     123133 ATAGAGCAGGTTTGAAACACTCTTTTTGTAGTATCTGGAAGTGGACATTTGGAGCGCTCTCAGGA
          1 ATAGAGCAGGTTTGAAACACTCTTTTTGTAGTATCTGGAAGTGGATATTTGGATAGCTTTGAGGA

            * ** *      *             **     *    * *       * *          *  *
     123198 CTATGGT-GAAAAAGGAAATATCTTCCAATAAAGGCTACATAGAAGCAATGTCAGAAACTTTTTC
         66 TTTCGTTGGAAACAGG-AATATCTTCATATAAAAGCTAGACAGAAGCATTCTCAGAAACTTCTTT

            *      * ** *   **      *       *  * *   *
     123262 ATGATGTATCTACTCAGCTAACAGAGTTGAACCTTTCTTTTG
        130 GTGATGTTTGCATTCAAGTAACAGTGTTGAACATTCCCTTTC

             *       *                   * *     *             **            
     123304 AGAGAGCAGTTTTGAAACACTCTTTTTGTGGAATCTGCAAGTGGATATTTGTCTAGCTTTGAGGA
          1 ATAGAGCAGGTTTGAAACACTCTTTTTGTAGTATCTGGAAGTGGATATTTGGATAGCTTTGAGGA

                         *   *    *                   *       *              
     123369 TTTCGTTGGAAACGGGATTA-CAT-ATA-AAAAGC-AGACAGCAGCATTCCCAGAAACTTCTTTG
         66 TTTCGTTGGAAACAGGAATATCTTCATATAAAAGCTAGACAGAAGCATTCTCAGAAACTTCTTTG

                              *    *                 
     123430 TGATGTTTGCATTCAAGTCACAGAGTTGAACATTCCCTTTC
        131 TGATGTTTGCATTCAAGTAACAGTGTTGAACATTCCCTTTC

                                    *              *     *       **     *   *
     123471 ATAGAGCAGGTTTGAAACACTCTTCTTGTAGTATCTGGATGTGGACATTTGGAGCGCTTTCAGGC
          1 ATAGAGCAGGTTTGAAACACTCTTTTTGTAGTATCTGGAAGTGGATATTTGGATAGCTTTGAGGA

            * ** *      *             *** *   *                          *   
     123536 CTAAGGT-GAAAAAGGAAATATCTTCCCCTGAAAACTAGACAGAAGCATTCTCAGAAACTTATTT
         66 TTTCGTTGGAAACAGG-AATATCTTCATATAAAAGCTAGACAGAAGCATTCTCAGAAACTTCTTT

             *     **  **    *             **  * *   *
     123600 GCGATGTGCGCCCTCAACTAACAGTGTTGAAGCTTTCTTTTG
        130 GTGATGTTTGCATTCAAGTAACAGTGTTGAACATTCCCTTTC

                     *    *              * *     *             **            
     123642 ATAGAGCAGTTTTGTAACACTCTTTTTGTGGAATCTGCAAGTGGATATTTGTCTAGCTTTGAGGA
          1 ATAGAGCAGGTTTGAAACACTCTTTTTGTAGTATCTGGAAGTGGATATTTGGATAGCTTTGAGGA

                         *   *    *                   *       *     *        
     123707 TTTCGTTGGAAACGGGATTA-CAT-ATA-AAAAGC-AGACAGCAGCATTCCCAGAATCTTCTTTG
         66 TTTCGTTGGAAACAGGAATATCTTCATATAAAAGCTAGACAGAAGCATTCTCAGAAACTTCTTTG

                              *    *           *     
     123768 TGATGTTTGCATTCAAGTCACAGGGTTGAACATTCTCTTTC
        131 TGATGTTTGCATTCAAGTAACAGTGTTGAACATTCCCTTTC

                                   *               *     *       **     *    
     123809 ATAGAGCAGGTTTGAAACACTCTGTTTGTAGTATCTGGATGTGGACATTTGGAGCGCTTTCAGGC
          1 ATAGAGCAGGTTTGAAACACTCTTTTTGTAGTATCTGGAAGTGGATATTTGGATAGCTTTGAGG-

            * * *    *                 *** *   *                          *  
     123874 CTATGGTT-TAAA-AGGAAATATCTTCCCCTGAAAACTAGACAGAAGCATTCTCAGAAACTTATT
         65 ATTTCGTTGGAAACAGG-AATATCTTCATATAAAAGCTAGACAGAAGCATTCTCAGAAACTTCTT

                    **  **    *                    *   *
     123937 TGTGATGTGCGCCCTCAACTAACAGTGTTGAAGCATT-CTTTTG
        129 TGTGATGTTTGCATTCAAGTAACAGTGTTGAA-CATTCCCTTTC

                     *                   * *     *             **        *   
     123980 ATAGAGCAGTTTTGAAACACTCTTTTTGTGGAATCTGCAAGTGGATATTTGTCTAGCTTTGTGGA
          1 ATAGAGCAGGTTTGAAACACTCTTTTTGTAGTATCTGGAAGTGGATATTTGGATAGCTTTGAGGA

                        **   *    *                   *       *              
     124045 TTTCGTTGGAAAGGGGATTA-CAT-ATA-AAAAGC-AGACAGCAGCATTCCCAGAAACTTCTTTG
         66 TTTCGTTGGAAACAGGAATATCTTCATATAAAAGCTAGACAGAAGCATTCTCAGAAACTTCTTTG

               *              *    *                 
     124106 TGAAGTTTGCATTCAAGTCACAGAGTTGAACATTCCCTTTC
        131 TGATGTTTGCATTCAAGTAACAGTGTTGAACATTCCCTTTC

                                                   *     *       **     *   *
     124147 ATAGAGCAGGTTTGAAACACTCTTTTTGTAGTATCTGGATGTGGACATTTGGAGCGCTTTCAGGC
          1 ATAGAGCAGGTTTGAAACACTCTTTTTGTAGTATCTGGAAGTGGATATTTGGATAGCTTTGAGGA

            * ** *      *             *** *   *                          *   
     124212 CTATGGT-GAAAAAGGAAATATCTTCCCCTGAAAACTAGACAGAAGCATTCTCAGAAACTTATTT
         66 TTTCGTTGGAAACAGG-AATATCTTCATATAAAAGCTAGACAGAAGCATTCTCAGAAACTTCTTT

                   **  **    *             **  * *   *
     124276 GTGATGTGCGCCCTCAACTAACAGTGTTGAAGCTTTCTTTTG
        130 GTGATGTTTGCATTCAAGTAACAGTGTTGAACATTCCCTTTC

                     *                    *      *   *                       
     124318 ATAGAGCAGTTTTGAAACACTCTTTTTGTAATATCTGCAAGAGGATATTTGGATAGCTTTGAGGA
          1 ATAGAGCAGGTTTGAAACACTCTTTTTGTAGTATCTGGAAGTGGATATTTGGATAGCTTTGAGGA

                         *   * *            **      *               *    *   
     124383 TTTCGTTGGAAACGGGATTGTCTTCATATAAACTCTAGACTGAAGCATTCTCAGAAGCTTCATTG
         66 TTTCGTTGGAAACAGGAATATCTTCATATAAAAGCTAGACAGAAGCATTCTCAGAAACTTCTTTG

            *       **   *    *             *        
     124448 GGATGTTTCAATTGAAGTCACAGTGTTGAACAGTCCCTTTC
        131 TGATGTTTGCATTCAAGTAACAGTGTTGAACATTCCCTTTC

                                                         *       **   * *    
     124489 ATAGAGCAGGTTTGAAACACTCTTTTTGTAGTATCTGGAAGTGGACATTTGGAGCGCTCTCAGGA
          1 ATAGAGCAGGTTTGAAACACTCTTTTTGTAGTATCTGGAAGTGGATATTTGGATAGCTTTGAGGA

            * *  *    * *             **            *       ***          *  *
     124554 CTACGGT-GATAAAGGAAATATCTTCCAATAAAAGCTAGATAGAAGCAACGTCAGAAACTTTTTC
         66 TTTCGTTGGAAACAGG-AATATCTTCATATAAAAGCTAGACAGAAGCATTCTCAGAAACTTCTTT

            *      * ** *   **      *       *  * *   *
     124618 ATGATGTATCTACTCAGCTAACAGAGTTGAACCTTTCTTTTG
        130 GTGATGTTTGCATTCAAGTAACAGTGTTGAACATTCCCTTTC

             *       *                   * *     *   *         **            
     124660 AGAGAGCAGTTTTGAAACACTCTTTTTGTGGAATCTGCAAGAGGATATTTGTCTAGCTTTGAGGA
          1 ATAGAGCAGGTTTGAAACACTCTTTTTGTAGTATCTGGAAGTGGATATTTGGATAGCTTTGAGGA

                         *   *    *                   *       *     *        
     124725 TTTCGTTGGAAACGGGATTA-CAT-ATA-AAAAGC-AGACAGCAGCATTCCCAGAATCTTCTTTG
         66 TTTCGTTGGAAACAGGAATATCTTCATATAAAAGCTAGACAGAAGCATTCTCAGAAACTTCTTTG

                              *    *   *             
     124786 TGATGTTTGCATTCAAGTCACAGAGTTCAACATTCCCTTTC
        131 TGATGTTTGCATTCAAGTAACAGTGTTGAACATTCCCTTTC

                                                   *     *       **     *   *
     124827 ATAGAGCAGGTTTGAAACACTCTTTTTGTAGTATCTGGATGTGGACATTTGGAGCGCTTTCAGGC
          1 ATAGAGCAGGTTTGAAACACTCTTTTTGTAGTATCTGGAAGTGGATATTTGGATAGCTTTGAGGA

            * ** *  *   *             *** *   *                          *   
     124892 CTATGGT-AAAAAAGGAAATATCTTCCCCTGAAAACTAGACAGAAGCATTCTCAGAAACTTATTT
         66 TTTCGTTGGAAACAGG-AATATCTTCATATAAAAGCTAGACAGAAGCATTCTCAGAAACTTCTTT

                   **  **    *             **  * *   *
     124956 GTGATGTGCGCCCTCAACTAACAGTGTTGAAGCTTTCTTTTG
        130 GTGATGTTTGCATTCAAGTAACAGTGTTGAACATTCCCTTTC

                     *                   * *     *             **            
     124998 ATAGAGCAGTTTTGAAACACTCTTTTTGTGGAATCTGCAAGTGGATATTTGTCTAGCTTTGAGGA
          1 ATAGAGCAGGTTTGAAACACTCTTTTTGTAGTATCTGGAAGTGGATATTTGGATAGCTTTGAGGA

                    *    *   *    *                   *       *     *        
     125063 TTTCGTTGCAAACGGGATTA-CAT-ATA-AAAAGC-AGACAGCAGCATTCCCAGAATCTTCTTTG
         66 TTTCGTTGGAAACAGGAATATCTTCATATAAAAGCTAGACAGAAGCATTCTCAGAAACTTCTTTG

                              *    *                 
     125124 TGATGTTTGCATTCAAGTCACAGGGTTGAACATTCCCTTTC
        131 TGATGTTTGCATTCAAGTAACAGTGTTGAACATTCCCTTTC

                                  *    *           *     *       **     *   *
     125165 ATAGAGCAGGTTTGAAACACTCGTTTTATAGTATCTGGATGTGGACATTTGGAGCGCTTTCAGGC
          1 ATAGAGCAGGTTTGAAACACTCTTTTTGTAGTATCTGGAAGTGGATATTTGGATAGCTTTGAGGA

            * ** *      *             *** *   *                      *   *   
     125230 CTATGGT-GAAAAAGGAAATATCTTCTCCTGAAAACTAGACAGAAGCATTCTCAGAATCTTATTT
         66 TTTCGTTGGAAACAGG-AATATCTTCATATAAAAGCTAGACAGAAGCATTCTCAGAAACTTCTTT

                   **  ** *  *             **  * *   *
     125294 GTGATGTGCGCCCTAAACTAACAGTGTTGAAGCTTTCTTTTG
        130 GTGATGTTTGCATTCAAGTAACAGTGTTGAACATTCCCTTTC

                     *          *     *   **     *   *                       
     125336 ATAGAGCAGTTTTGAAACACACTTTTAGTAAAATCTGCAAGAGGATATTTGGATAGCTTTGAGGA
          1 ATAGAGCAGGTTTGAAACACTCTTTTTGTAGTATCTGGAAGTGGATATTTGGATAGCTTTGAGGA

                         *   * *            **                      *    *   
     125401 TTTCGTTGGAAACGGGATTGTCTTCATATAAACTCTAGACAGAAGCATTCTCAGAATCTTCATTG
         66 TTTCGTTGGAAACAGGAATATCTTCATATAAAAGCTAGACAGAAGCATTCTCAGAAACTTCTTTG

            *       **   *    *             *      **
     125466 GGATGTTTCAATTGAAGTCACAGTGTTGAACAGTCCCTTAA
        131 TGATGTTTGCATTCAAGTAACAGTGTTGAACATTCCCTTTC

                                                         *       **   * *    
     125507 ATAGAGCAGGTTTGAAACACTCTTTTTGTAGTATCTGGAAGTGGACATTTGGAGCGCTCTCAGGA
          1 ATAGAGCAGGTTTGAAACACTCTTTTTGTAGTATCTGGAAGTGGATATTTGGATAGCTTTGAGGA

            * *  *      *             **          * *       * *          *  *
     125572 CTGCGGT-GAAAAAGGAAATATCTTCCAATAAAAGCTACATAGAAGCAATGTCAGAAACTTTTTC
         66 TTTCGTTGGAAACAGG-AATATCTTCATATAAAAGCTAGACAGAAGCATTCTCAGAAACTTCTTT

            *      * ** *   **      *       *        *
     125636 ATGATGTATCTACTCAGCTAACAGAGTTGAACCTT-CCTTTG
        130 GTGATGTTTGCATTCAAGTAACAGTGTTGAACATTCCCTTTC

             *       *                   * *     *             **            
     125677 AGAGAGCAGTTTTGAAACACTCTTTTTGTGGAATCTGCAAGTGGATATTTGTCTAGCTTTGAGGA
          1 ATAGAGCAGGTTTGAAACACTCTTTTTGTAGTATCTGGAAGTGGATATTTGGATAGCTTTGAGGA

                         *   *    *                   *       *   *          
     125742 TTTCGTTGGAAACGGGATTA-CAT-ATA-AAAAGC-AGACAGCAGCATTCCCAGTAACTTCTTTG
         66 TTTCGTTGGAAACAGGAATATCTTCATATAAAAGCTAGACAGAAGCATTCTCAGAAACTTCTTTG

                              *    *                 
     125803 TGATGTTTGCATTCAAGTCACAGAGTTGAACATTCCCTTTC
        131 TGATGTTTGCATTCAAGTAACAGTGTTGAACATTCCCTTTC

                                                   *     *       **     *   *
     125844 ATAGAGCAGGTTTGAAACACTCTTTTTGTAGTATCTGGATGTGGACATTTGGAGCGCTTTCAGGC
          1 ATAGAGCAGGTTTGAAACACTCTTTTTGTAGTATCTGGAAGTGGATATTTGGATAGCTTTGAGGA

            * ** *      *             *** *   *                      *   *   
     125909 CTATGGT-GAAAAAGGTAATATCTTCCCCTGAAAACTAGACAGAAGCATTCTCAGAATCTTATTT
         66 TTTCGTTGGAAACAGG-AATATCTTCATATAAAAGCTAGACAGAAGCATTCTCAGAAACTTCTTT

                   **  **    *             **  * *   *
     125973 GTGATGTGCGCCCTCAACTAACAGTGTTGAAGCTTTCTTTTG
        130 GTGATGTTTGCATTCAAGTAACAGTGTTGAACATTCCCTTTC

                     *                    **     *   *                       
     126015 ATAGAGCAGTTTTGAAACACTCTTTTTGTAAAATCTGCAAGAGGATATTTGGATAGCTTTGAGGA
          1 ATAGAGCAGGTTTGAAACACTCTTTTTGTAGTATCTGGAAGTGGATATTTGGATAGCTTTGAGGA

                         *   * *            **                      * *  *   
     126080 TTTCGTTGGAAACGGGATTGTCTTCATATAAACTCTAGACAGAAGCATTCTCAGAAGCGTCATTG
         66 TTTCGTTGGAAACAGGAATATCTTCATATAAAAGCTAGACAGAAGCATTCTCAGAAACTTCTTTG

            *       **   *    *             *        
     126145 GGATGTTTCAATTGAAGTCACAGTGTTGAACAGTCCCTTTC
        131 TGATGTTTGCATTCAAGTAACAGTGTTGAACATTCCCTTTC

                                                   *     *       **     *    
     126186 ATAGAGCAGGTTTGAAACACTCTTTTTGTAGTATCTGGATGTGGACATTTGGAGCGCTTTCAGGC
          1 ATAGAGCAGGTTTGAAACACTCTTTTTGTAGTATCTGGAAGTGGATATTTGGATAGCTTTGAGG-

            * * *    *                 *** *   *                          *  
     126251 CTATGGTT-TAAA-AGGAAATATCTTCCCCTGAAAACTAGACAGAAGCATTCTCAGAAACTTATT
         65 ATTTCGTTGGAAACAGG-AATATCTTCATATAAAAGCTAGACAGAAGCATTCTCAGAAACTTCTT

                    **  **    *                    * * *
     126314 TGTGATGTGCGCCCTCAACTAACAGTGTTGAAGCATT-CTTCTG
        129 TGTGATGTTTGCATTCAAGTAACAGTGTTGAA-CATTCCCTTTC

                     *                   * *     *            ***            
     126357 ATAGAGCAGTTTTGAAACACTCTTTTTGTGGAATCTGCAAGTGGATATTTTTCTAGCTTTGAGGA
          1 ATAGAGCAGGTTTGAAACACTCTTTTTGTAGTATCTGGAAGTGGATATTTGGATAGCTTTGAGGA

                *  *     *   *    *                   *       *              
     126422 TTTCCTTAGAAACGGGATTA-CAT-ATA-AAAAGC-AGACAGCAGCATTCCCAGAAACTTCTTTG
         66 TTTCGTTGGAAACAGGAATATCTTCATATAAAAGCTAGACAGAAGCATTCTCAGAAACTTCTTTG

                              *    *   *             
     126483 TGATGTTTGCATTCAAGTCACAGAGTTAAACATTCCCTTTC
        131 TGATGTTTGCATTCAAGTAACAGTGTTGAACATTCCCTTTC

                                                   *     *       **     *   *
     126524 ATAGAGCAGGTTTGAAACACTCTTTTTGTAGTATCTGGATGTGGACATTTGGAGCGCTTTCAGGC
          1 ATAGAGCAGGTTTGAAACACTCTTTTTGTAGTATCTGGAAGTGGATATTTGGATAGCTTTGAGGA

            * ** *      *             *** *   *                      **  *   
     126589 CTATGGT-GAAAAAGGAAATATCTTCCCCTGAAAACTAGACAGAAGCATTCTCAGAATGTTATTT
         66 TTTCGTTGGAAACAGG-AATATCTTCATATAAAAGCTAGACAGAAGCATTCTCAGAAACTTCTTT

                   **  **    *             **  * *   *
     126653 GTGATGTGCGCCCTCAACTAACAGTGTTGAAGCTTTCTTTTG
        130 GTGATGTTTGCATTCAAGTAACAGTGTTGAACATTCCCTTTC

                     *                    **     *   *                       
     126695 ATAGAGCAGTTTTGAAACACTCTTTTTGTAAAATCTGCAAGAGGATATTTGGATAGCTTTGAGGA
          1 ATAGAGCAGGTTTGAAACACTCTTTTTGTAGTATCTGGAAGTGGATATTTGGATAGCTTTGAGGA

                         *   * *            **                      *    *   
     126760 TTTCGTTGGAAACGGGATTGTCTTCATATAAACTCTAGACAGAAGCATTCTCAGAAGCTTCATTG
         66 TTTCGTTGGAAACAGGAATATCTTCATATAAAAGCTAGACAGAAGCATTCTCAGAAACTTCTTTG

            *       **   *    *             *       *
     126825 GGATGTTTCAATTGAAGTCACAGTGTTGAACAGTCCCTTTA
        131 TGATGTTTGCATTCAAGTAACAGTGTTGAACATTCCCTTTC

                                                         *       **   * *    
     126866 ATAGAGCAGGTTTGAAACACTCTTTTTGTAGTATCTGGAAGTGGACATTTGGAGCGCTCTCAGGA
          1 ATAGAGCAGGTTTGAAACACTCTTTTTGTAGTATCTGGAAGTGGATATTTGGATAGCTTTGAGGA

            * *  *      *             **            *       * *          *  *
     126931 CTGCGGT-GAAAAAGGAAATATCTTCCAATAAAAGCTAGATAGAAGCAATGTCAGAAACTTTTTC
         66 TTTCGTTGGAAACAGG-AATATCTTCATATAAAAGCTAGACAGAAGCATTCTCAGAAACTTCTTT

            *      * ** *   **      *       *        *
     126995 ATGATGTATCTACTCAGCTAACAGAGTTGAACCTT-CCTTTG
        130 GTGATGTTTGCATTCAAGTAACAGTGTTGAACATTCCCTTTC

             *       *                   * *     *             **            
     127036 AGAGAGCAGTTTTGAAACACTCTTTTTGTGGAATCTGCAAGTGGATATTTGTCTAGCTTTGAGGA
          1 ATAGAGCAGGTTTGAAACACTCTTTTTGTAGTATCTGGAAGTGGATATTTGGATAGCTTTGAGGA

               *         *   *    *                   *   *   *              
     127101 TTTTGTTGGAAACGGGATTA-CAT-ATA-AAAAGC-AGACAGCAGCGTTCCCAGAAACTTCTTTG
         66 TTTCGTTGGAAACAGGAATATCTTCATATAAAAGCTAGACAGAAGCATTCTCAGAAACTTCTTTG

                              *    *                 
     127162 TGATGTTTGCATTCAAGTCACAGAGTTGAACATTCCCTTTC
        131 TGATGTTTGCATTCAAGTAACAGTGTTGAACATTCCCTTTC

                                                   *     *     * ** *   *   *
     127203 ATAGAGCAGGTTTGAAACACTCTTTTTGTAGTATCTGGATGTGGACATTTGCAGCGATTTCAGGC
          1 ATAGAGCAGGTTTGAAACACTCTTTTTGTAGTATCTGGAAGTGGATATTTGGATAGCTTTGAGGA

            * ** *      *             *** *   * *                        * * 
     127268 CTAAGGT-GAAAAAGGAAATATCTTCCCCTGAAAACGAGACAGAAGCATTCTCAGAAACTTATCT
         66 TTTCGTTGGAAACAGG-AATATCTTCATATAAAAGCTAGACAGAAGCATTCTCAGAAACTTCTTT

                   **  **    *              *  * *   *
     127332 GTGATGTGCGCCCTCAACTAACAGTGTTGAACCTTTCTTTTG
        130 GTGATGTTTGCATTCAAGTAACAGTGTTGAACATTCCCTTTC

                     *                    *      *   *                       
     127374 ATAGAGCAGTTTTGAAACACTCTTTTTGTAATATCTGCAAGAGGATATTTGGATAGCTTTGAGGA
          1 ATAGAGCAGGTTTGAAACACTCTTTTTGTAGTATCTGGAAGTGGATATTTGGATAGCTTTGAGGA

                         *   * *            **                      *    *   
     127439 TTTCGTTGGAAACGGGATTGTCTTCATATAAACTCTAGACAGAAGCATTCTCAGAAGCTTCATTG
         66 TTTCGTTGGAAACAGGAATATCTTCATATAAAAGCTAGACAGAAGCATTCTCAGAAACTTCTTTG

            *       **   *    *     *       * *      
     127504 GGATGTTTCAATTGAAGTCACAGTATTGAACAGTTCCTTTC
        131 TGATGTTTGCATTCAAGTAACAGTGTTGAACATTCCCTTTC

                 *                                     * *       **   * *    
     127545 ATAGAACAGGTTTGAAACACTCTTTTTGTAGTATCTGGAAGTGCACATTTGGAGCGCTCTCAGGA
          1 ATAGAGCAGGTTTGAAACACTCTTTTTGTAGTATCTGGAAGTGGATATTTGGATAGCTTTGAGGA

            * ** *      *             **          * *       * *          *  *
     127610 CTATGGT-GAAAAAGGAAATATCTTCCAATAAAAGCTACATAGAAGCAATGTCAGAAACTTTTTC
         66 TTTCGTTGGAAACAGG-AATATCTTCATATAAAAGCTAGACAGAAGCATTCTCAGAAACTTCTTT

            *      * ** *   **      *       *  *     *
     127674 ATGATGTATCTACTCAGCTAACAGAGTTGAACCTTTCCTTTG
        130 GTGATGTTTGCATTCAAGTAACAGTGTTGAACATTCCCTTTC

             *       *                   * *     *             **            
     127716 AGAGAGCAGTTTTGAAACACTCTTTTTGTGGAATCTGCAAGTGGATATTTGTCTAGCTTTGAGGA
          1 ATAGAGCAGGTTTGAAACACTCTTTTTGTAGTATCTGGAAGTGGATATTTGGATAGCTTTGAGGA

                         *   *    *                   *       *   *          
     127781 TTTCGTTGGAAACGGGATTA-CAT-ATA-AAAAGC-AGACAGCAGCATTCCCAGTAACTTCTTTG
         66 TTTCGTTGGAAACAGGAATATCTTCATATAAAAGCTAGACAGAAGCATTCTCAGAAACTTCTTTG

                              *    *                 
     127842 TGATGTTTGCATTCAAGTCACAGAGTTGAACATTCCCTTTC
        131 TGATGTTTGCATTCAAGTAACAGTGTTGAACATTCCCTTTC

                                                   *     *       **     *   *
     127883 ATAGAGCAGGTTTGAAACACTCTTTTTGTAGTATCTGGATGTGGACATTTGGAGCGCTTTCAGGC
          1 ATAGAGCAGGTTTGAAACACTCTTTTTGTAGTATCTGGAAGTGGATATTTGGATAGCTTTGAGGA

            * ** *      *            **** *   *                          *   
     127948 CTATGGT-GAAAAAGGAAATATCTTTCCCTGAAAACTAGACAGAAGCATTCTCAGAAACTTATTT
         66 TTTCGTTGGAAACAGG-AATATCTTCATATAAAAGCTAGACAGAAGCATTCTCAGAAACTTCTTT

                   **  **    *              *  * *   *
     128012 GTGATGTGCGCCCTCAACTAACAGTGTTGAACCTTTCTTTTG
        130 GTGATGTTTGCATTCAAGTAACAGTGTTGAACATTCCCTTTC

                     *                    *      *   *                       
     128054 ATAGAGCAGTTTTGAAACACTCTTTTTGTAATATCTGCAAGAGGATATTTGGATAGCTTTGAGGA
          1 ATAGAGCAGGTTTGAAACACTCTTTTTGTAGTATCTGGAAGTGGATATTTGGATAGCTTTGAGGA

                         *   *    *      *            *       *     *   *    
     128119 TTTCGTTGGAAACGGGATTA-CAT-ATA-CAAAGC-AGACAGCAGCATTCCCAGAATCTTGTTTG
         66 TTTCGTTGGAAACAGGAATATCTTCATATAAAAGCTAGACAGAAGCATTCTCAGAAACTTCTTTG

                              *    *                 
     128180 TGATGTTTGCATTCAAGTCACAGAGTTGAACATTCCCTTTC
        131 TGATGTTTGCATTCAAGTAACAGTGTTGAACATTCCCTTTC

             *                         *           *     *       **     *   *
     128221 AGAGAGCAGGTTTGAAACACTCTTTTTATAGTATCTGGATGTGGACATTTGGAGCGCTTTCAGGC
          1 ATAGAGCAGGTTTGAAACACTCTTTTTGTAGTATCTGGAAGTGGATATTTGGATAGCTTTGAGGA

            * ** *      *             ***     *                      *   *   
     128286 CTGTGGT-GAAAAAGGAAATATCTTCTCCTAAAAACTAGACAGAAGCATTCTCAGAATCTTATTT
         66 TTTCGTTGGAAACAGG-AATATCTTCATATAAAAGCTAGACAGAAGCATTCTCAGAAACTTCTTT

                   **  **    *             **  * *   *
     128350 GTGATGTGCGCCCTCAACTAACAGTGTTGAAGCTTTCTTTTG
        130 GTGATGTTTGCATTCAAGTAACAGTGTTGAACATTCCCTTTC

                     *                    **     *   *                       
     128392 ATAGAGCAGTTTTGAAACACTCTTTTTGTAAAATCTGCAAGAGGATATTTGGATAGCTTTGAGGA
          1 ATAGAGCAGGTTTGAAACACTCTTTTTGTAGTATCTGGAAGTGGATATTTGGATAGCTTTGAGGA

                         *   * *            **                      *        
     128457 TTTCGTTGGAAACGGGATTGTCTTCATATAAACTCTAGACAGAAGCATTCTCAGAAGCTTCTTTG
         66 TTTCGTTGGAAACAGGAATATCTTCATATAAAAGCTAGACAGAAGCATTCTCAGAAACTTCTTTG

            *             *    *             *        
     128522 GGATGTTT-CAGTTGAAGTCACAGTGTTGAACAGTCCCTTTC
        131 TGATGTTTGCA-TTCAAGTAACAGTGTTGAACATTCCCTTTC

                                                   *     *       ***  * *    
     128563 ATAGAGCAGGTTTGAAACACTCTTTTTGTAGTATCTGGATGTGGACATTTGGAGCTCTCTCAGGA
          1 ATAGAGCAGGTTTGAAACACTCTTTTTGTAGTATCTGGAAGTGGATATTTGGATAGCTTTGAGGA

              *  *      *             **          * *       * *          *  *
     128628 TTGCGGT-GAAAAAGGAAATATCTTCCAATAAAAGCTACATAGAAGCAATGTCAGAAACTTTTTC
         66 TTTCGTTGGAAACAGG-AATATCTTCATATAAAAGCTAGACAGAAGCATTCTCAGAAACTTCTTT

            *      * ** *   **      *       *        *
     128692 ATGATGTATCTACTCAGCTAACAGAGTTGAACCTT-CCTTTG
        130 GTGATGTTTGCATTCAAGTAACAGTGTTGAACATTCCCTTTC

             *       *                   * *     *             **            
     128733 AGAGAGCAGTTTTGAAACACTCTTTTTGTGGAATCTGCAAGTGGATATTTGTCTAGCTTTGAGGA
          1 ATAGAGCAGGTTTGAAACACTCTTTTTGTAGTATCTGGAAGTGGATATTTGGATAGCTTTGAGGA

                         *   *    *                   *       *              
     128798 TTTCGTTGGAAACGGGATTA-CAT-ATA-AAAAGC-AGACAGCAGCATTCCCAGAAACTTCTTTG
         66 TTTCGTTGGAAACAGGAATATCTTCATATAAAAGCTAGACAGAAGCATTCTCAGAAACTTCTTTG

                              *    *                 
     128859 TGATGTTTGCATTCAAGTCACAGAGTTGAACATTCCCTTTC
        131 TGATGTTTGCATTCAAGTAACAGTGTTGAACATTCCCTTTC

             *                         *           *     *       **         *
     128900 AGAGAGCAGGTTTGAAACACTCTTTTTATAGTATCTGGATGTGGACATTTGGAGCGCTTTGAGGC
          1 ATAGAGCAGGTTTGAAACACTCTTTTTGTAGTATCTGGAAGTGGATATTTGGATAGCTTTGAGGA

            * ** *      *             *** *   *                      *   *   
     128965 CTATGGT-GAAAAAGGAAATATCTTCTCCTGAAAACTAGACAGAAGCATTCTCAGAATCTTATTT
         66 TTTCGTTGGAAACAGG-AATATCTTCATATAAAAGCTAGACAGAAGCATTCTCAGAAACTTCTTT

              *    **  **    *             **  * *   *
     129029 GTAATGTGCGCCCTCAACTAACAGTGTTGAAGCTTTCTTTTG
        130 GTGATGTTTGCATTCAAGTAACAGTGTTGAACATTCCCTTTC

                     *                    **     *   *                       
     129071 ATAGAGCAGTTTTGAAACACTCTTTTTGTAAAATCTGAAAGAGGATATTTGGATAGCTTTGAGGA
          1 ATAGAGCAGGTTTGAAACACTCTTTTTGTAGTATCTGGAAGTGGATATTTGGATAGCTTTGAGGA

                         *   * *            **                      * *  *   
     129136 TTTCGTTGGAAACGGGATTGTCTTCATATAAACTCTAGACAGAAGCATTCTCAGAAGCGTCATTG
         66 TTTCGTTGGAAACAGGAATATCTTCATATAAAAGCTAGACAGAAGCATTCTCAGAAACTTCTTTG

            *       **   *    *             *        
     129201 GGATGTTTCAATTGAAGTCACAGTGTTGAACAGTCCCTTTC
        131 TGATGTTTGCATTCAAGTAACAGTGTTGAACATTCCCTTTC

                                                   *     *       **     *    
     129242 ATAGAGCAGGTTTGAAACACTCTTTTTGTAGTATCTGGATGTGGACATTTGGAGCGCTTTCAGGC
          1 ATAGAGCAGGTTTGAAACACTCTTTTTGTAGTATCTGGAAGTGGATATTTGGATAGCTTTGAGG-

            * * *    *                 *** *   *                          *  
     129307 CTATGGTT-TAAA-AGGAAATATCTTCCCCTGAAAACTAGACAGAAGCATTCTCAGAAACTTATT
         65 ATTTCGTTGGAAACAGG-AATATCTTCATATAAAAGCTAGACAGAAGCATTCTCAGAAACTTCTT

                    **  **    *                    *   *
     129370 TGTGATGTGCGCCCTCAACTAACAGTGTTGAACCATT-CTTTTG
        129 TGTGATGTTTGCATTCAAGTAACAGTGTTGAA-CATTCCCTTTC

                     *                   * *     *             **            
     129413 ATAGAGCAGTTTTGAAACACTCTTTTTGTGGAATCTGCAAGTGGATATTTGTCTAGCTTTGAGGA
          1 ATAGAGCAGGTTTGAAACACTCTTTTTGTAGTATCTGGAAGTGGATATTTGGATAGCTTTGAGGA

                         *   *    *                   *       *              
     129478 TTTCGTTGGAAACGGGATTA-CAT-ATA-AAAAGC-AGACAGCAGCATTCCCAGAAACTTCTTTG
         66 TTTCGTTGGAAACAGGAATATCTTCATATAAAAGCTAGACAGAAGCATTCTCAGAAACTTCTTTG

                              *    *                 
     129539 TGATGTTTGCATTCAAGTCACAGAGTTGAACATTCCCTTTC
        131 TGATGTTTGCATTCAAGTAACAGTGTTGAACATTCCCTTTC

                                                   *     *       **     *   *
     129580 ATAGAGCAGGTTTGAAACACTCTTTTTGTAGTATCTGGATGTGGACATTTGGAGCGCTTTCAGGC
          1 ATAGAGCAGGTTTGAAACACTCTTTTTGTAGTATCTGGAAGTGGATATTTGGATAGCTTTGAGGA

            * ** *      *             *** *   *                      *   *   
     129645 CTATGGT-GAAAAAGGAAATATCTTCCCCTGAAAACTAGACAGAAGCATTCTCAGAATCTTATTT
         66 TTTCGTTGGAAACAGG-AATATCTTCATATAAAAGCTAGACAGAAGCATTCTCAGAAACTTCTTT

                   **  **    *             **  * *   *
     129709 GTGATGTGCGCCCTCAACTAACAGTGTTGAAGCTTTCTTTTG
        130 GTGATGTTTGCATTCAAGTAACAGTGTTGAACATTCCCTTTC

                     *            *       **     *   *                       
     129751 ATAGAGCAGTTTTGAAACACTCCTTTTGTAAAATCTGCAAGAGGATATTTGGATAGCTTTGAGGA
          1 ATAGAGCAGGTTTGAAACACTCTTTTTGTAGTATCTGGAAGTGGATATTTGGATAGCTTTGAGGA

                         *   * *            **                      *    *   
     129816 TTTCGTTGGAAACGGGATTGTCTTCATATAAACTCTAGACAGAAGCATTCTCAGAAGCTTCATTG
         66 TTTCGTTGGAAACAGGAATATCTTCATATAAAAGCTAGACAGAAGCATTCTCAGAAACTTCTTTG

            *       **   *    *             *        
     129881 GGATGTTTCAATTGAAGTCACAGTGTTGAACAGTCCCTTTC
        131 TGATGTTTGCATTCAAGTAACAGTGTTGAACATTCCCTTTC

                                                         *       **   * *    
     129922 ATAGAGCAGGTTTGAAACACTCTTTTTGTAGTATCTGGAAGTGGACATTTGGAGCGCTCTCAGGA
          1 ATAGAGCAGGTTTGAAACACTCTTTTTGTAGTATCTGGAAGTGGATATTTGGATAGCTTTGAGGA

            * *  *      *             **            *       * *          *  *
     129987 CTGCGGT-GAAAAAGGAAATATCTTCCAATAAAAGCTAGATAGAAGCAATGTCAGAAACTTTTTC
         66 TTTCGTTGGAAACAGG-AATATCTTCATATAAAAGCTAGACAGAAGCATTCTCAGAAACTTCTTT

            *      * ** *   **      *       *  * *   *
     130051 ATGATGTATCTACTCAGCTAACAGAGTTGAACCTTTCTTTTG
        130 GTGATGTTTGCATTCAAGTAACAGTGTTGAACATTCCCTTTC

             *       *                   * *     *             **            
     130093 AGAGAGCAGTTTTGAAACACTCTTTTTGTGGAATCTGCAAGTGGATATTTGTCTAGCTTTGAGGA
          1 ATAGAGCAGGTTTGAAACACTCTTTTTGTAGTATCTGGAAGTGGATATTTGGATAGCTTTGAGGA

                         *   *    *                   *       *              
     130158 TTTCGTTGGAAACGGGATTA-CAT-ATA-AAAAGC-AGACAGCAGCATTCCCAGAAACTTCTTTG
         66 TTTCGTTGGAAACAGGAATATCTTCATATAAAAGCTAGACAGAAGCATTCTCAGAAACTTCTTTG

                              *    *                 
     130219 TGATGTTTGCATTCAAGTCACAGAGTTGAACATTCCCTTTC
        131 TGATGTTTGCATTCAAGTAACAGTGTTGAACATTCCCTTTC

                                                         *       **   * *    
     130260 ATAGAGCAGGTTTGAAACACTCTTTTTGTAGTATCTGGAAGTGGACATTTGGAGCGCTCTCAGGA
          1 ATAGAGCAGGTTTGAAACACTCTTTTTGTAGTATCTGGAAGTGGATATTTGGATAGCTTTGAGGA

            * *  *      *             **            *       * *          *  *
     130325 CTGCGGT-GAAAAAGGAAATATCTTCCAATAAAAGCTAGATAGAAGCAATGTCAGAAACTTTTTC
         66 TTTCGTTGGAAACAGG-AATATCTTCATATAAAAGCTAGACAGAAGCATTCTCAGAAACTTCTTT

            *      * ** *   **      *       *  * *   *
     130389 ATGATGTATCTACTCAGCTAACAGAGTTGAACCTTTCTTTTG
        130 GTGATGTTTGCATTCAAGTAACAGTGTTGAACATTCCCTTTC

             *       *                   * *     *             **            
     130431 AGAGAGCAGTTTTGAAACACTCTTTTTGTGGAATCTGCAAGTGGATATTTGTCTAGCTTTGAGGA
          1 ATAGAGCAGGTTTGAAACACTCTTTTTGTAGTATCTGGAAGTGGATATTTGGATAGCTTTGAGGA

                         *   *    *                   *       *   *          
     130496 TTTCGTTGGAAACGGGATTA-CAT-ATA-AAAAGC-AGACAGCAGCATTCCCAGTAACTTCTTTG
         66 TTTCGTTGGAAACAGGAATATCTTCATATAAAAGCTAGACAGAAGCATTCTCAGAAACTTCTTTG

                   *          *    *                 
     130557 TGATGTTCGCATTCAAGTCACAGAGTTGAACATTCCCTTTC
        131 TGATGTTTGCATTCAAGTAACAGTGTTGAACATTCCCTTTC

                                                   *     *       **     *   *
     130598 ATAGAGCAGGTTTGAAACACTCTTTTTGTAGTATCTGGATGTGGACATTTGGAGCGCTTTCAGGC
          1 ATAGAGCAGGTTTGAAACACTCTTTTTGTAGTATCTGGAAGTGGATATTTGGATAGCTTTGAGGA

            * ** *      *             *** *   *   *                  *   *   
     130663 CTATGGT-GAAAAAGGAAATATCTTCCCCTGAAAACTACACAGAAGCATTCTCAGAATCTTATTT
         66 TTTCGTTGGAAACAGG-AATATCTTCATATAAAAGCTAGACAGAAGCATTCTCAGAAACTTCTTT

                   **  **    *             **  * *    
     130727 GTGATGTGCGCCCTCAACTAACAGTGTTGAAGCTTTCTTTTC
        130 GTGATGTTTGCATTCAAGTAACAGTGTTGAACATTCCCTTTC

                     *                    **     *   *                       
     130769 ATAGAGCAGTTTTGAAACACTCTTTTTGTAAAATCTGCAAGAGGATATTTGGATAGCTTTGAGGA
          1 ATAGAGCAGGTTTGAAACACTCTTTTTGTAGTATCTGGAAGTGGATATTTGGATAGCTTTGAGGA

                         *   * *            **                      *    *   
     130834 TTTCGTTGGAAACGGGATTGTCTTCATATAAACTCTAGACAGAAGCATTCTCAGAAGCTTCATTG
         66 TTTCGTTGGAAACAGGAATATCTTCATATAAAAGCTAGACAGAAGCATTCTCAGAAACTTCTTTG

            *       **   *    *             *        
     130899 GGATGTTTCAATTGAAGTCACAGTGTTGAACAGTCCCTTTC
        131 TGATGTTTGCATTCAAGTAACAGTGTTGAACATTCCCTTTC

                                                   *     *       **     *    
     130940 ATAGAGCAGGTTTGAAACACTCTTTTTGTAGTATCTGGATGTGGACATTTGGAGCGCTTTCAGGC
          1 ATAGAGCAGGTTTGAAACACTCTTTTTGTAGTATCTGGAAGTGGATATTTGGATAGCTTTGAGG-

            * * *    *                 *** *   *     *                    *  
     131005 CTATGGTT-TAAA-AGGAAATATCTTCCCCTGAAAACTAGAGAGAAGCATTCTCAGAAACTTATT
         65 ATTTCGTTGGAAACAGG-AATATCTTCATATAAAAGCTAGACAGAAGCATTCTCAGAAACTTCTT

                    **  **    **            *      *   *
     131068 TGTGATGTGCGCCCTCAACAAACAGTGTTGTAGCATT-CTTTTG
        129 TGTGATGTTTGCATTCAAGTAACAGTGTTG-AACATTCCCTTTC

                     *                   * *     *             **   *   *    
     131111 ATAGAGCAGTTTTGAAACACTCTTTTTGTGGAATCTGCAAGTGGATATTTGTCTAGTTTTCAGGA
          1 ATAGAGCAGGTTTGAAACACTCTTTTTGTAGTATCTGGAAGTGGATATTTGGATAGCTTTGAGGA

                         *   *    *                *  *       *              
     131176 TTTCGTTGGAAACGGGATTA-CAT-ATA-AAAAGC-AGAAAGCAGCATTCCCAGAAACTTCTTTG
         66 TTTCGTTGGAAACAGGAATATCTTCATATAAAAGCTAGACAGAAGCATTCTCAGAAACTTCTTTG

                              *    *                 
     131237 TGATGTTTGCATTCAAGTCACAGAGTTGAACATTCCCTTTC
        131 TGATGTTTGCATTCAAGTAACAGTGTTGAACATTCCCTTTC

                                                   *     *       **     *   *
     131278 ATAGAGCAGGTTTGAAACACTCTTTTTGTAGTATCTGGATGTGGACATTTGGAGCGCTTTCAGGC
          1 ATAGAGCAGGTTTGAAACACTCTTTTTGTAGTATCTGGAAGTGGATATTTGGATAGCTTTGAGGA

            * ** *      *             *** *   *                          *   
     131343 CTATGGT-GAAAAAGGAAATATCTTCCCCTGAAAACTAGACAGAAGCATTCTCAGAAACTTATTT
         66 TTTCGTTGGAAACAGG-AATATCTTCATATAAAAGCTAGACAGAAGCATTCTCAGAAACTTCTTT

                   **  **    *              *  * *   *
     131407 GTGATGTGCGCCCTCAACTAACAGTGTTGAACCTTTCTTTTG
        130 GTGATGTTTGCATTCAAGTAACAGTGTTGAACATTCCCTTTC

                     *       *            *      *   *                       
     131449 ATAGAGCAGTTTTGAAAAACTCTTTTTGTAATATCTGCAAGAGGATATTTGGATAGCTTTGAGGA
          1 ATAGAGCAGGTTTGAAACACTCTTTTTGTAGTATCTGGAAGTGGATATTTGGATAGCTTTGAGGA

                         *   *    *                   *       *     *   *    
     131514 TTTCGTTGGAAACGGGATTA-CAT-ATA-AAAAGC-AGACAGCAGCATTCCCAGAATCTTGTTTG
         66 TTTCGTTGGAAACAGGAATATCTTCATATAAAAGCTAGACAGAAGCATTCTCAGAAACTTCTTTG

                              * *  *                 
     131575 TGATGTTTGCATTCAAGTCAGAGAGTTGAACATTCCCTTTC
        131 TGATGTTTGCATTCAAGTAACAGTGTTGAACATTCCCTTTC

                                       *           *     *       **     *   *
     131616 ATAGAGCAGGTTTGAAACACTCTTTTTATAGTATCTGGATGTGGACATTTGGAGCGCTTTCAGGC
          1 ATAGAGCAGGTTTGAAACACTCTTTTTGTAGTATCTGGAAGTGGATATTTGGATAGCTTTGAGGA

            * ** *      *             *** *   *                      *   *   
     131681 CTATGGT-GAAAAAGGATATATCTTCTCCTGAAAACTAGACAGAAGCATTCTCAGAATCTTATTT
         66 TTTCGTTGGAAACAGGA-ATATCTTCATATAAAAGCTAGACAGAAGCATTCTCAGAAACTTCTTT

                   **  **    *             **  * *   *
     131745 GTGATGTGCGCCCTCAACTAACAGTGTTGAAGCTTTCTTTTG
        130 GTGATGTTTGCATTCAAGTAACAGTGTTGAACATTCCCTTTC

                     *                *   **     *   *        *              
     131787 ATAGAGCAGTTTTGAAACACTCTTTTCGTAAAATCTGCAAGAGGATATTTTGATAGCTTTGAGGA
          1 ATAGAGCAGGTTTGAAACACTCTTTTTGTAGTATCTGGAAGTGGATATTTGGATAGCTTTGAGGA

                         *   * *            **                      *    *   
     131852 TTTCGTTGGAAACGGGATTGTCTTCATATAAACTCTAGACAGAAGCATTCTCAGAAGCTTCATTG
         66 TTTCGTTGGAAACAGGAATATCTTCATATAAAAGCTAGACAGAAGCATTCTCAGAAACTTCTTTG

            *       **   *    *             *        
     131917 GGATGTTTCAATTGAAGTCACAGTGTTGAACAGTCCCTTTC
        131 TGATGTTTGCATTCAAGTAACAGTGTTGAACATTCCCTTTC

                                                         *       *  * * *    
     131958 ATAGAGCAGGTTTGAAACACTCTTTTTGTAGTATCTGGAAGTGGACATTTGGAGAGATCTCAGGA
          1 ATAGAGCAGGTTTGAAACACTCTTTTTGTAGTATCTGGAAGTGGATATTTGGATAGCTTTGAGGA

            *    *    * *             **            *       * *          *  *
     132023 ATTCGGT-GATAAAGGAAATATCTTCCAATAAAAGCTAGATAGAAGCAATGTCAGAAACTTTTTC
         66 TTTCGTTGGAAACAGG-AATATCTTCATATAAAAGCTAGACAGAAGCATTCTCAGAAACTTCTTT

            *      * ** *   ** *    *       *  * *   *
     132087 ATGATGTATCTACTCAGCTGACAGAGTTGAACCTTTCTTTTG
        130 GTGATGTTTGCATTCAAGTAACAGTGTTGAACATTCCCTTTC

             *       *                   * *     *             **            
     132129 AGAGAGCAGTTTTGAAACACTCTTTTTGTGGAATCTGCAAGTGGATATTTGTCTAGCTTTGAGGA
          1 ATAGAGCAGGTTTGAAACACTCTTTTTGTAGTATCTGGAAGTGGATATTTGGATAGCTTTGAGGA

                         *   *    *                   *       *             *
     132194 TTTCGTTGGAAACGGGATTA-CAT-ATA-AAAAGC-AGACAGCAGCATTCCCAGAAACTTCTTTC
         66 TTTCGTTGGAAACAGGAATATCTTCATATAAAAGCTAGACAGAAGCATTCTCAGAAACTTCTTTG

                              *    *                 
     132255 TGATGTTTGCATTCAAGTCACAGAGTTGAACATTCCCTTTC
        131 TGATGTTTGCATTCAAGTAACAGTGTTGAACATTCCCTTTC

                                                   *     *       **     *   *
     132296 ATAGAGCAGGTTTGAAACACTCTTTTTGTAGTATCTGGATGTGGACATTTGGAGCGCTTTCAGGC
          1 ATAGAGCAGGTTTGAAACACTCTTTTTGTAGTATCTGGAAGTGGATATTTGGATAGCTTTGAGGA

            * ** *      *             *** *   *           *          *   *   
     132361 CTATGGT-GAAAAAGGAAATATCTTCCCCTGAAAACTAGACAGAAGAATTCTCAGAATCTTATTT
         66 TTTCGTTGGAAACAGG-AATATCTTCATATAAAAGCTAGACAGAAGCATTCTCAGAAACTTCTTT

                   **  **    *             **  * *   *
     132425 GTGATGTGCGCCCTCAACTAACAGTGTTGAAGCTTTCTTTTG
        130 GTGATGTTTGCATTCAAGTAACAGTGTTGAACATTCCCTTTC

                     *                    **     *   *               *       
     132467 ATAGAGCAGTTTTGAAACACTCTTTTTGTAAAATCTGCAAGAGGATATTTGGATAGCCTTGAGGA
          1 ATAGAGCAGGTTTGAAACACTCTTTTTGTAGTATCTGGAAGTGGATATTTGGATAGCTTTGAGGA

                         *   * *            **                      * *  *   
     132532 TTTCGTTGGAAACGGGATTGTCTTCATATAAACTCTAGACAGAAGCATTCTCAGAAGCGTCATTG
         66 TTTCGTTGGAAACAGGAATATCTTCATATAAAAGCTAGACAGAAGCATTCTCAGAAACTTCTTTG

            *       **   *    *             *        
     132597 GGATGTTTCAATTGAAGTCACAGTGTTGAACAGTCCCTTTC
        131 TGATGTTTGCATTCAAGTAACAGTGTTGAACATTCCCTTTC

                                                         *       **   * *    
     132638 ATAGAGCAGGTTTGAAACACTCTTTTTGTAGTATCTGGAAGTGGACATTTGGAGCGCTCTCAGGA
          1 ATAGAGCAGGTTTGAAACACTCTTTTTGTAGTATCTGGAAGTGGATATTTGGATAGCTTTGAGGA

            * *  *      *             **            *       * *          *  *
     132703 CTGCGGT-GAAAAAGGAAATATCTTCCAATAAAAGCTAGATAGAAGCAATGTCAGAAACTTTTTC
         66 TTTCGTTGGAAACAGG-AATATCTTCATATAAAAGCTAGACAGAAGCATTCTCAGAAACTTCTTT

            *      * ** *   **      *       *        *
     132767 ATGATGTATCTACTCAGCTAACAGAGTTGAACCTT-CCTTTG
        130 GTGATGTTTGCATTCAAGTAACAGTGTTGAACATTCCCTTTC

             *       *                   * *     *             **            
     132808 AGAGAGCAGTTTTGAAACACTCTTTTTGTGGAATCTGCAAGTGGATATTTGTCTAGCTTTGAGGA
          1 ATAGAGCAGGTTTGAAACACTCTTTTTGTAGTATCTGGAAGTGGATATTTGGATAGCTTTGAGGA

                         *   *    *                   *       *              
     132873 TTTCGTTGGAAACGGGATTA-CAT-ATA-AAAAGC-AGACAGCAGCATTCCCAGAAACTTCTTTG
         66 TTTCGTTGGAAACAGGAATATCTTCATATAAAAGCTAGACAGAAGCATTCTCAGAAACTTCTTTG

               *              *    *                 
     132934 TGAGGTTTGCATTCAAGTCACAGAGTTGAACATTCCCTTTC
        131 TGATGTTTGCATTCAAGTAACAGTGTTGAACATTCCCTTTC

                                                   *     *     * **     *   *
     132975 ATAGAGCAGGTTTGAAACACTCTTTTTGTAGTATCTGGATGTGGACATTTGCAGCGCTTTCAGGC
          1 ATAGAGCAGGTTTGAAACACTCTTTTTGTAGTATCTGGAAGTGGATATTTGGATAGCTTTGAGGA

            * ** *      *             *** *   *                          *   
     133040 CTAAGGT-GAAAAAGGAAATATCTTCCCCTGAAAACTAGACAGAAGCATTCTCAGAAACTTATTT
         66 TTTCGTTGGAAACAGG-AATATCTTCATATAAAAGCTAGACAGAAGCATTCTCAGAAACTTCTTT

                   **  **    *             **  * *   *
     133104 GTGATGTGCGCCCTCAACTAACAGTGTTGAAGCTTTCTTTTG
        130 GTGATGTTTGCATTCAAGTAACAGTGTTGAACATTCCCTTTC

                     *                    *      *   *       *               
     133146 ATAGAGCAGTTTTGAAACACTCTTTTTGTAATATCTGCAAGAGGATATTAGGATAGCTTTGAGGA
          1 ATAGAGCAGGTTTGAAACACTCTTTTTGTAGTATCTGGAAGTGGATATTTGGATAGCTTTGAGGA

                         *   * *            **                      *    *   
     133211 TTTCGTTGGAAACGGGATTGTCTTCATATAAACTCTAGACAGAAGCATTCTCAGAAGCTTCATTG
         66 TTTCGTTGGAAACAGGAATATCTTCATATAAAAGCTAGACAGAAGCATTCTCAGAAACTTCTTTG

            *       **   *    *             * *      
     133276 GGATGTTTCAATTGAAGTCACAGTGTTGAACAGTTCCTTTC
        131 TGATGTTTGCATTCAAGTAACAGTGTTGAACATTCCCTTTC

                 *                                       *       ***  * *    
     133317 ATAGAACAGGTTTGAAACACTCTTTTTGTAGTATCTGGAAGTGGACATTTGGAGCACTCTCAGGA
          1 ATAGAGCAGGTTTGAAACACTCTTTTTGTAGTATCTGGAAGTGGATATTTGGATAGCTTTGAGGA

            * ** *      *             **          * *       * *          *  *
     133382 CTATGGT-GAAAAAGGAAATATCTTCCAATAAAAGCTACATAGAAGCAATGTCAGAAACTTTTTC
         66 TTTCGTTGGAAACAGG-AATATCTTCATATAAAAGCTAGACAGAAGCATTCTCAGAAACTTCTTT

            *      * ** *   **      *       *  *     *
     133446 ATGATGTATCTACTCAGCTAACAGAGTTGAACCTTTCCTTTG
        130 GTGATGTTTGCATTCAAGTAACAGTGTTGAACATTCCCTTTC

             *       *                   * *     *             **            
     133488 AGAGAGCAGTTTTGAAACACTCTTTTTGTGGAATCTGCAAGTGGATATTTGTCTAGCTTTGAGGA
          1 ATAGAGCAGGTTTGAAACACTCTTTTTGTAGTATCTGGAAGTGGATATTTGGATAGCTTTGAGGA

                         *   *    *                   *       * * *          
     133553 TTTCGTTGGAAACGGGATTA-CAT-ATA-AAAAGC-AGACAGCAGCATTCCCTGTAACTTCTTTG
         66 TTTCGTTGGAAACAGGAATATCTTCATATAAAAGCTAGACAGAAGCATTCTCAGAAACTTCTTTG

                   *          *    *                 
     133614 TGATGTTCGCATTCAAGTCACAGAGTTGAACATTCCCTTTC
        131 TGATGTTTGCATTCAAGTAACAGTGTTGAACATTCCCTTTC

                    *                              *     *       **     *   *
     133655 ATAGAGCACGTTTGAAACACTCTTTTTGTAGTATCTGGATGTGGACATTTGGAGCGCTTTCAGGC
          1 ATAGAGCAGGTTTGAAACACTCTTTTTGTAGTATCTGGAAGTGGATATTTGGATAGCTTTGAGGA

            * ** *      *             *** *   *   *                  *   *   
     133720 CTATGGT-GAAAAAGGAAATATCTTCCCCTGAAAACTACACAGAAGCATTCTCAGAATCTTATTT
         66 TTTCGTTGGAAACAGG-AATATCTTCATATAAAAGCTAGACAGAAGCATTCTCAGAAACTTCTTT

                   **  **    *             **  * *   *
     133784 GTGATGTGCGCCCTCAACTAACAGTGTTGAAGCTTTCTTTTG
        130 GTGATGTTTGCATTCAAGTAACAGTGTTGAACATTCCCTTTC

                     *                    **     *   *                       
     133826 ATAGAGCAGTTTTGAAACACTCTTTTTGTAAAATCTGCAAGAGGATATTTGGATAGCTTTGAGGA
          1 ATAGAGCAGGTTTGAAACACTCTTTTTGTAGTATCTGGAAGTGGATATTTGGATAGCTTTGAGGA

                         *   * *            **                      *    *   
     133891 TTTCGTTGGAAACGGGATTGTCTTCATATAAACTCTAGACAGAAGCATTCTCAGAAGCTTCATTG
         66 TTTCGTTGGAAACAGGAATATCTTCATATAAAAGCTAGACAGAAGCATTCTCAGAAACTTCTTTG

            *       **   *    *             *        
     133956 GGATGTTTCAATTGAAGTCACAGTGTTGAACAGTCCCTTTC
        131 TGATGTTTGCATTCAAGTAACAGTGTTGAACATTCCCTTTC

                                                   *     *       ***    *    
     133997 ATAGAGCAGGTTTGAAACACTCTTTTTGTAGTATCTGGATGTGGACATTTGGAGCACTTTCAGGC
          1 ATAGAGCAGGTTTGAAACACTCTTTTTGTAGTATCTGGAAGTGGATATTTGGATAGCTTTGAGG-

            * * *    *                 *** *   *                          *  
     134062 CTATGGTT-TAAA-AGGAAATATCTTCCCCTGAAAACTAGACAGAAGCATTCTCAGAAACTTATT
         65 ATTTCGTTGGAAACAGG-AATATCTTCATATAAAAGCTAGACAGAAGCATTCTCAGAAACTTCTT

                    **  **    **            *      *   *
     134125 TGTGATGTGCGCCCTCAACAAACAGTGTTGTAGCATT-CTTTTG
        129 TGTGATGTTTGCATTCAAGTAACAGTGTTG-AACATTCCCTTTC

                     *          *        * *     *             **            
     134168 ATAGAGCAGTTTTGAAACACACTTTTTGTGGAATCTGCAAGTGGATATTTGTCTAGCTTTGAGGA
          1 ATAGAGCAGGTTTGAAACACTCTTTTTGTAGTATCTGGAAGTGGATATTTGGATAGCTTTGAGGA

                         *   *    *                *  *       *              
     134233 TTTCGTTGGAAACGGGATTA-CAT-ATA-AAAAGC-AGAAAGCAGCATTCCCAGAAACTTCTTTG
         66 TTTCGTTGGAAACAGGAATATCTTCATATAAAAGCTAGACAGAAGCATTCTCAGAAACTTCTTTG

                              *    *                 
     134294 TGATGTTTGCATTCAAGTCACAGAGTTGAACATTCCCTTTC
        131 TGATGTTTGCATTCAAGTAACAGTGTTGAACATTCCCTTTC

                                                   *     *     * **     *   *
     134335 ATAGAGCAGGTTTGAAACACTCTTTTTGTAGTATCTGGATGTGGACATTTGCAGCGCTTTCAGGC
          1 ATAGAGCAGGTTTGAAACACTCTTTTTGTAGTATCTGGAAGTGGATATTTGGATAGCTTTGAGGA

            * ** *      *             *** *   *                          *   
     134400 CTATGGT-GAAAAAGGAAATATCTTCCCCTGAAAACTAGACAGAAGCATTCTCAGAAACTTATTT
         66 TTTCGTTGGAAACAGG-AATATCTTCATATAAAAGCTAGACAGAAGCATTCTCAGAAACTTCTTT

                   **  **    *              *  * *   *
     134464 GTGATGTGCGCCCTCAACTAACAGTGTTGAACCTTTCTTTTG
        130 GTGATGTTTGCATTCAAGTAACAGTGTTGAACATTCCCTTTC

                     *                    *      *   *              *        
     134506 ATAGAGCAGTTTTGAAACACTCTTTTTGTAATATCTGCAAGAGGATATTTGGATAGATTTGAGGA
          1 ATAGAGCAGGTTTGAAACACTCTTTTTGTAGTATCTGGAAGTGGATATTTGGATAGCTTTGAGGA

                         *   *    *                   *       *     *   *    
     134571 TTTCGTTGGAAACGGGATTA-CAT-ATA-AAAAGC-AGACAGCAGCATTCCCAGAATCTTGTTTG
         66 TTTCGTTGGAAACAGGAATATCTTCATATAAAAGCTAGACAGAAGCATTCTCAGAAACTTCTTTG

                              *    *                 
     134632 TGATGTTTGCATTCAAGTCACAGAGTTGAACATTCCCTTTC
        131 TGATGTTTGCATTCAAGTAACAGTGTTGAACATTCCCTTTC

                                       *           *     *       **     *   *
     134673 ATAGAGCAGGTTTGAAACACTCTTTTTATAGTATCTGGATGTGGACATTTGGAGCGCTTTCAGGC
          1 ATAGAGCAGGTTTGAAACACTCTTTTTGTAGTATCTGGAAGTGGATATTTGGATAGCTTTGAGGA

            * ** *      *             *** *   *                      **  *   
     134738 CTATGGT-GAAAAAGGATATATCTTCTCCTGAAAACTAGACAGAAGCATTCTCAGAATTTTATTT
         66 TTTCGTTGGAAACAGGA-ATATCTTCATATAAAAGCTAGACAGAAGCATTCTCAGAAACTTCTTT

                   **  **    *             **  * *   *
     134802 GTGATGTGCGCCCTCAACTAACAGTGTTGAAGCTTTCTTTTG
        130 GTGATGTTTGCATTCAAGTAACAGTGTTGAACATTCCCTTTC

                     *                    **     *   *        *              
     134844 ATAGAGCAGTTTTGAAACACTCTTTTTGTAAAATCTGCAAGAGGATATTTTGATAGCTTTGAGGA
          1 ATAGAGCAGGTTTGAAACACTCTTTTTGTAGTATCTGGAAGTGGATATTTGGATAGCTTTGAGGA

                         *   * *            **                      *    *   
     134909 TTTCGTTGGAAACGGGATTGTCTTCATATAAACTCTAGACAGAAGCATTCTCAGAAGCTTCATTG
         66 TTTCGTTGGAAACAGGAATATCTTCATATAAAAGCTAGACAGAAGCATTCTCAGAAACTTCTTTG

            *       **   *    *             *        
     134974 GGATGTTTCAATTGAAGTCACAGTGTTGAACAGTCCCTTTC
        131 TGATGTTTGCATTCAAGTAACAGTGTTGAACATTCCCTTTC

                                                     *   *       *  * * *    
     135015 ATAGAGCAGGTTTGAAACACTCTTTTTGTAGTATCTGGAAGAGGACATTTGGAGAGATCTCAGGA
          1 ATAGAGCAGGTTTGAAACACTCTTTTTGTAGTATCTGGAAGTGGATATTTGGATAGCTTTGAGGA

            * *  *    * *             **            *       * *          *  *
     135080 ATACGGT-GATAAAGGAAATATCTTCCAATAAAAGCTAGATAGAAGCAATGTCAGAAACTTTTTC
         66 TTTCGTTGGAAACAGG-AATATCTTCATATAAAAGCTAGACAGAAGCATTCTCAGAAACTTCTTT

            *      * ** *   **      *       *  * *   *
     135144 ATGATGTATCTACTCAGCTAACAGAGTTGAACCTTTCTTTTG
        130 GTGATGTTTGCATTCAAGTAACAGTGTTGAACATTCCCTTTC

             *       *                   * *     *             **            
     135186 AGAGAGCAGTTTTGAAACACTCTTTTTGTGGAATCTGCAAGTGGATATTTGTCTAGCTTTGAGGA
          1 ATAGAGCAGGTTTGAAACACTCTTTTTGTAGTATCTGGAAGTGGATATTTGGATAGCTTTGAGGA

                         *   *    *                   *       *              
     135251 TTTCGTTGGAAACGGGATTA-CAT-ATA-AAAAGC-AGACAGCAGCATTCCCAGAAACTTCTTTG
         66 TTTCGTTGGAAACAGGAATATCTTCATATAAAAGCTAGACAGAAGCATTCTCAGAAACTTCTTTG

                              *    *                 
     135312 TGATGTTTGCATTCAAGTCACAGAGTTGAACATTCCCTTTC
        131 TGATGTTTGCATTCAAGTAACAGTGTTGAACATTCCCTTTC

                                       *           *     *       **     *   *
     135353 ATAGAGCAGGTTTGAAACACTCTTTTTATAGTATCTGGATGTGGACATTTGGAGCGCTTTCAGGC
          1 ATAGAGCAGGTTTGAAACACTCTTTTTGTAGTATCTGGAAGTGGATATTTGGATAGCTTTGAGGA

            * ** *      *             *** *   *                      *   *   
     135418 CTATGGT-GAAAAAGGAAATATCTTCCCCTGAAAACTAGACAGAAGCATTCTCAGAATCTTATTT
         66 TTTCGTTGGAAACAGG-AATATCTTCATATAAAAGCTAGACAGAAGCATTCTCAGAAACTTCTTT

                   **  **    *             **  * *   *
     135482 GTGATGTGCGCCCTCAACTAACAGTGTTGAAACTTTCTTTTG
        130 GTGATGTTTGCATTCAAGTAACAGTGTTGAACATTCCCTTTC

                     *                *   **     *   *        *              
     135524 ATAGAGCAGTTTTGAAACACTCTTTTCGTAAAATCTGCAAGAGGATATTTTGATAGCTTTGAGGA
          1 ATAGAGCAGGTTTGAAACACTCTTTTTGTAGTATCTGGAAGTGGATATTTGGATAGCTTTGAGGA

                         *   * *            **                      *    *   
     135589 TTTCGTTGGAAACGGGATTGTCTTCATATAAACTCTAGACAGAAGCATTCTCAGAAGCTTCATTG
         66 TTTCGTTGGAAACAGGAATATCTTCATATAAAAGCTAGACAGAAGCATTCTCAGAAACTTCTTTG

            *       **   *    *             *        
     135654 GGATGTTTCAATTGAAGTCACAGTGTTGAACAGTCCCTTTC
        131 TGATGTTTGCATTCAAGTAACAGTGTTGAACATTCCCTTTC

                         *                               *       *  * * *    
     135695 ATAGAGCAGGTTTAAAACACTCTTTTTGTAGTATCTGGAAGTGGACATTTGGAGAGATCTCAGGA
          1 ATAGAGCAGGTTTGAAACACTCTTTTTGTAGTATCTGGAAGTGGATATTTGGATAGCTTTGAGGA

            * *  *    * *             **            *       * *          *  *
     135760 ATACGGT-GATAAAGGAAATATCTTCCAATAAAAGCTAGATAGAAGCAATGTCAGAAACTTTTTC
         66 TTTCGTTGGAAACAGG-AATATCTTCATATAAAAGCTAGACAGAAGCATTCTCAGAAACTTCTTT

            *      * ** *   **      *       *  * *   *
     135824 ATGATGTATCTACTCAGCTAACAGAGTTGAACCTTTCTTTTG
        130 GTGATGTTTGCATTCAAGTAACAGTGTTGAACATTCCCTTTC

             *       *                   * *     *     *       **       *    
     135866 AGAGAGCAGTTTTGAAACACTCTTTTTGTGGAATCTGCAAGTGTATATTTGTCTAGCTTTTAGGA
          1 ATAGAGCAGGTTTGAAACACTCTTTTTGTAGTATCTGGAAGTGGATATTTGGATAGCTTTGAGGA

                         *   *    *                   *       *   *          
     135931 TTTCGTTGGAAACGGGATTA-CAT-ATA-AAAAGC-AGACAGCAGCATTCCCAGTAACTTCTTTG
         66 TTTCGTTGGAAACAGGAATATCTTCATATAAAAGCTAGACAGAAGCATTCTCAGAAACTTCTTTG

                              *    *                 
     135992 TGATGTTTGCATTCAAGTCACAGAGTTGAACATTCCCTTTC
        131 TGATGTTTGCATTCAAGTAACAGTGTTGAACATTCCCTTTC

                                                   *     *       **     *   *
     136033 ATAGAGCAGGTTTGAAACACTCTTTTTGTAGTATCTGGATGTGGACATTTGGAGCGCTTTCAGGC
          1 ATAGAGCAGGTTTGAAACACTCTTTTTGTAGTATCTGGAAGTGGATATTTGGATAGCTTTGAGGA

            * ** *      *             *** *   *     *     *          *   *   
     136098 CTATGGT-GAAAAAGGAAATATCTTCCCCTGAAAACTAGAGAGAAGAATTCTCAGAATCTTATTT
         66 TTTCGTTGGAAACAGG-AATATCTTCATATAAAAGCTAGACAGAAGCATTCTCAGAAACTTCTTT

              *    **  **    *             **  * *   *
     136162 GTAATGTGCGCCCTCAACTAACAGTGTTGAAGCTTTCTTTTG
        130 GTGATGTTTGCATTCAAGTAACAGTGTTGAACATTCCCTTTC

                     *                    **     *   *                       
     136204 ATAGAGCAGTTTTGAAACACTCTTTTTGTAAAATCTGAAAGAGGATATTTGGATAGCTTTGAGGA
          1 ATAGAGCAGGTTTGAAACACTCTTTTTGTAGTATCTGGAAGTGGATATTTGGATAGCTTTGAGGA

                         *   * *            **                      * *  *   
     136269 TTTCGTTGGAAACGGGATTGTCTTCATATAAACTCTAGACAGAAGCATTCTCAGAAGCGTCATTG
         66 TTTCGTTGGAAACAGGAATATCTTCATATAAAAGCTAGACAGAAGCATTCTCAGAAACTTCTTTG

            *       **   *    *             *        
     136334 GGATGTTTCAATTGAAGTCACAGTGTTGAACAGTCCCTTTC
        131 TGATGTTTGCATTCAAGTAACAGTGTTGAACATTCCCTTTC

                                                   *     *       **     *    
     136375 ATAGAGCAGGTTTGAAACACTCTTTTTGTAGTATCTGGATGTGGACATTTGGAGCGCTTTCAGGC
          1 ATAGAGCAGGTTTGAAACACTCTTTTTGTAGTATCTGGAAGTGGATATTTGGATAGCTTTGAGG-

            * * *    *                 *** *   *                          *  
     136440 CTATGGTT-TAAA-AGGAAATATCTTCCCCTGAAAACTAGACAGAAGCATTCTCAGAAACTTATT
         65 ATTTCGTTGGAAACAGG-AATATCTTCATATAAAAGCTAGACAGAAGCATTCTCAGAAACTTCTT

                    **  **    *                    *   *
     136503 TGTGATGTGCGCCCTCAACTAACAGTGTTGAAGCATT-CTTTTG
        129 TGTGATGTTTGCATTCAAGTAACAGTGTTGAA-CATTCCCTTTC

                     *                   * *     *             **            
     136546 ATAGAGCAGTTTTGAAACACTCTTTTTGTGGAATCTGCAAGTGGATATTTGTCTAGCTTTGAGGA
          1 ATAGAGCAGGTTTGAAACACTCTTTTTGTAGTATCTGGAAGTGGATATTTGGATAGCTTTGAGGA

                         *   *    *                   *       *              
     136611 TTTCGTTGGAAACGGGATTA-CAT-ATA-AAAAGC-AGACAGCAGCATTCCCAGAAACTTCTTTG
         66 TTTCGTTGGAAACAGGAATATCTTCATATAAAAGCTAGACAGAAGCATTCTCAGAAACTTCTTTG

                              *    *                 
     136672 TGATGTTTGCATTCAAGTCACAGAGTTGAACATTCCCTTTC
        131 TGATGTTTGCATTCAAGTAACAGTGTTGAACATTCCCTTTC

                                                   *     *     * **     *   *
     136713 ATAGAGCAGGTTTGAAACACTCTTTTTGTAGTATCTGGATGTGGACATTTGCAGCGCTTTCAGGC
          1 ATAGAGCAGGTTTGAAACACTCTTTTTGTAGTATCTGGAAGTGGATATTTGGATAGCTTTGAGGA

            * ** *      *             *** *   *                      *   *   
     136778 CTATGGT-GAAAAAGGAAATATCTTCCCCTGAAAACTAGACAGAAGCATTCTCAGAATCTTATTT
         66 TTTCGTTGGAAACAGG-AATATCTTCATATAAAAGCTAGACAGAAGCATTCTCAGAAACTTCTTT

                   **  **    *             **  * *   *
     136842 GTGATGTGCGCCCTCAACTAACAGTGTTGAAGCTTTCTTTTG
        130 GTGATGTTTGCATTCAAGTAACAGTGTTGAACATTCCCTTTC

                     *            *       **     *   *                       
     136884 ATAGAGCAGTTTTGAAACACTCCTTTTGTAAAATCTGCAAGAGGATATTTGGATAGCTTTGAGGA
          1 ATAGAGCAGGTTTGAAACACTCTTTTTGTAGTATCTGGAAGTGGATATTTGGATAGCTTTGAGGA

                         *   * *            **                      *    *   
     136949 TTTCGTTGGAAACGGGATTGTCTTCATATAAACTCTAGACAGAAGCATTCTCAGAAGCTTCATTG
         66 TTTCGTTGGAAACAGGAATATCTTCATATAAAAGCTAGACAGAAGCATTCTCAGAAACTTCTTTG

            *       **   *    *             *        
     137014 GGATGTTTCAATTGAAGTCACAGTGTTGAACAGTCCCTTTC
        131 TGATGTTTGCATTCAAGTAACAGTGTTGAACATTCCCTTTC

                                                         *       **   * *    
     137055 ATAGAGCAGGTTTGAAACACTCTTTTTGTAGTATCTGGAAGTGGACATTTGGAGCGCTCTCAGGA
          1 ATAGAGCAGGTTTGAAACACTCTTTTTGTAGTATCTGGAAGTGGATATTTGGATAGCTTTGAGGA

            * *  *      *             **            *       * *          *  *
     137120 CTGCGGT-GAAAAAGGAAATATCTTCCAATAAAAGCTAGATAGAAGCAATGTCAGAAACTTTTTC
         66 TTTCGTTGGAAACAGG-AATATCTTCATATAAAAGCTAGACAGAAGCATTCTCAGAAACTTCTTT

            *      * ** *   **      *       *  * *   *
     137184 ATGATGTATCTACTCAGCTAACAGAGTTGAACCTTTCTTTTG
        130 GTGATGTTTGCATTCAAGTAACAGTGTTGAACATTCCCTTTC

             *       *                   * *     *             **            
     137226 AGAGAGCAGTTTTGAAACACTCTTTTTGTGGAATCTGCAAGTGGATATTTGTCTAGCTTTGAGGA
          1 ATAGAGCAGGTTTGAAACACTCTTTTTGTAGTATCTGGAAGTGGATATTTGGATAGCTTTGAGGA

                         *   *    *                   *       *              
     137291 TTTCGTTGGAAACGGGATTA-CAT-ATA-AAAAGC-AGACAGCAGCATTCCCAGAAACTTCTTTG
         66 TTTCGTTGGAAACAGGAATATCTTCATATAAAAGCTAGACAGAAGCATTCTCAGAAACTTCTTTG

                              *   **                 
     137352 TGATGTTTGCATTCAAGTCACACAGTTGAACATTCCCTTTC
        131 TGATGTTTGCATTCAAGTAACAGTGTTGAACATTCCCTTTC

                                                         *       **   * *    
     137393 ATAGAGCAGGTTTGAAACACTCTTTTTGTAGTATCTGGAAGTGGACATTTGGAGCGCTCTCAGGA
          1 ATAGAGCAGGTTTGAAACACTCTTTTTGTAGTATCTGGAAGTGGATATTTGGATAGCTTTGAGGA

            * *  *      *             **            *       * *          *  *
     137458 CTGCGGT-GAAAAAGGAAATATCTTCCAATAAAAGCTAGATAGAAGCAATGTCAGAAACTTTTTC
         66 TTTCGTTGGAAACAGG-AATATCTTCATATAAAAGCTAGACAGAAGCATTCTCAGAAACTTCTTT

            *      * ** *   **      *       *  * *   *
     137522 ATGATGTATCTACTCAGCTAACAGAGTTGAACCTTTCTTTTG
        130 GTGATGTTTGCATTCAAGTAACAGTGTTGAACATTCCCTTTC

             *       *                   * *     *             **            
     137564 AGAGAGCAGTTTTGAAACACTCTTTTTGTGGAATCTGCAAGTGGATATTTGTCTAGCTTTGAGGA
          1 ATAGAGCAGGTTTGAAACACTCTTTTTGTAGTATCTGGAAGTGGATATTTGGATAGCTTTGAGGA

                         *   *    *                   *       *   *          
     137629 TTTCGTTGGAAACGGGATTA-CAT-ATA-AAAAGC-AGACAGCAGCATTCCCAGTAACTTCTTTG
         66 TTTCGTTGGAAACAGGAATATCTTCATATAAAAGCTAGACAGAAGCATTCTCAGAAACTTCTTTG

                   *          *    *                 
     137690 TGATGTTCGCATTCAAGTCACAGAGTTGAACATTCCCTTTC
        131 TGATGTTTGCATTCAAGTAACAGTGTTGAACATTCCCTTTC

                                                   *     *       **     *   *
     137731 ATAGAGCAGGTTTGAAACACTCTTTTTGTAGTATCTGGATGTGGACATTTGGAGCGCTTTCAGGC
          1 ATAGAGCAGGTTTGAAACACTCTTTTTGTAGTATCTGGAAGTGGATATTTGGATAGCTTTGAGGA

            * ** *      *             *** *   *   *                  *   *   
     137796 CTATGGT-GAAAAAGGAAATATCTTCCCCTGAAAACTACACAGAAGCATTCTCAGAATCTTATTT
         66 TTTCGTTGGAAACAGG-AATATCTTCATATAAAAGCTAGACAGAAGCATTCTCAGAAACTTCTTT

                   **  **    *             **  * *   *
     137860 GTGATGTGCGCCCTCAACTAACAGTGTTGAAGCTTTCTTTTG
        130 GTGATGTTTGCATTCAAGTAACAGTGTTGAACATTCCCTTTC

                     *                    **     *   *                       
     137902 ATAGAGCAGTTTTGAAACACTCTTTTTGTAAAATCTGCAAGAGGATATTTGGATAGCTTTGAGGA
          1 ATAGAGCAGGTTTGAAACACTCTTTTTGTAGTATCTGGAAGTGGATATTTGGATAGCTTTGAGGA

                         *   * *            **                      *    *   
     137967 TTTCGTTGGAAACGGGATTGTCTTCATATAAACTCTAGACAGAAGCATTCTCAGAAGCTTCATTG
         66 TTTCGTTGGAAACAGGAATATCTTCATATAAAAGCTAGACAGAAGCATTCTCAGAAACTTCTTTG

            *       **   *    *             *        
     138032 GGATGTTTCAATTGAAGTCACAGTGTTGAACAGTCCCTTTC
        131 TGATGTTTGCATTCAAGTAACAGTGTTGAACATTCCCTTTC

                                *                  *     *       **     *    
     138073 ATAGAGCAGGTTTGAAACACACTTTTTGTAGTATCTGGATGTGGACATTTGGAGCGCTTTCAGGC
          1 ATAGAGCAGGTTTGAAACACTCTTTTTGTAGTATCTGGAAGTGGATATTTGGATAGCTTTGAGG-

            * * *    *                 *** *   *        *                 *  
     138138 CTATGGTT-TAAA-AGGAAATATCTTCCCCTGAAAACTAGACAGGAGCATTCTCAGAAACTTATT
         65 ATTTCGTTGGAAACAGG-AATATCTTCATATAAAAGCTAGACAGAAGCATTCTCAGAAACTTCTT

                    **  **    **            *      *   *
     138201 TGTGATGTGCGCCCTCAACAAACAGTGTTGTAGCATT-CTTTTG
        129 TGTGATGTTTGCATTCAAGTAACAGTGTTG-AACATTCCCTTTC

                     *                   * *     *             **       *    
     138244 ATAGAGCAGTTTTGAAACACTCTTTTTGTGGAATCTGCAAGTGGATATTTGTCTAGCTTTCAGGA
          1 ATAGAGCAGGTTTGAAACACTCTTTTTGTAGTATCTGGAAGTGGATATTTGGATAGCTTTGAGGA

                         *   *    *                *  *       *              
     138309 TTTCGTTGGAAACGGGATTA-CAT-ATA-AAAAGC-AGAAAGCAGCATTCCCAGAAACTTCTTTG
         66 TTTCGTTGGAAACAGGAATATCTTCATATAAAAGCTAGACAGAAGCATTCTCAGAAACTTCTTTG

                              *    *                 
     138370 TGATGTTTGCATTCAAGTCACAGAGTTGAACATTCCCTTTC
        131 TGATGTTTGCATTCAAGTAACAGTGTTGAACATTCCCTTTC

                                                   *     *       **     *   *
     138411 ATAGAGCAGGTTTGAAACACTCTTTTTGTAGTATCTGGATGTGGACATTTGGAGCGCTTTCAGGC
          1 ATAGAGCAGGTTTGAAACACTCTTTTTGTAGTATCTGGAAGTGGATATTTGGATAGCTTTGAGGA

            * ** *      *             *** *   *                          *   
     138476 CTATGGT-GAAAAAGGAAATATCTTCCCCTGAAAACTAGACAGAAGCATTCTCAGAAACTTATTT
         66 TTTCGTTGGAAACAGG-AATATCTTCATATAAAAGCTAGACAGAAGCATTCTCAGAAACTTCTTT

                   **  **    *              *  * *   *
     138540 GTGATGTGCGCCCTCAACTAACAGTGTTGAACCTTTCTTTTG
        130 GTGATGTTTGCATTCAAGTAACAGTGTTGAACATTCCCTTTC

                     *       *            *      *   *                       
     138582 ATAGAGCAGTTTTGAAAAACTCTTTTTGTAATATCTGCAAGAGGATATTTGGATAGCTTTGAGGA
          1 ATAGAGCAGGTTTGAAACACTCTTTTTGTAGTATCTGGAAGTGGATATTTGGATAGCTTTGAGGA

                         *   *    *                   *       *     *   *    
     138647 TTTCGTTGGAAACGGGATTA-CAT-ATA-AAAAGC-AGACAGCAGCATTCCCAGAATCTTGTTTG
         66 TTTCGTTGGAAACAGGAATATCTTCATATAAAAGCTAGACAGAAGCATTCTCAGAAACTTCTTTG

                              *    *                 
     138708 TGATGTTTGCATTCAAGTCACAGAGTTGAACATTCCCTTTC
        131 TGATGTTTGCATTCAAGTAACAGTGTTGAACATTCCCTTTC

                                       *          ** *   *       **     *   *
     138749 ATAGAGCAGGTTTGAAACACTCTTTTTATAGTATCTGGGTGGGGACATTTGGAGCGCTTTCAGGC
          1 ATAGAGCAGGTTTGAAACACTCTTTTTGTAGTATCTGGAAGTGGATATTTGGATAGCTTTGAGGA

            * ** *      *             *** *   *                      *   *  *
     138814 CTATGGT-GAAAAAGGATATATCTTCTCCTGAAAACTAGACAGAAGCATTCTCAGAATCTTATTC
         66 TTTCGTTGGAAACAGGA-ATATCTTCATATAAAAGCTAGACAGAAGCATTCTCAGAAACTTCTTT

                   **  **    *             **  * *   *
     138878 GTGATGTGCGCCCTCAACTAACAGTGTTGAAGCTTTCTTTTG
        130 GTGATGTTTGCATTCAAGTAACAGTGTTGAACATTCCCTTTC

                     *                *   **     *   *        *              
     138920 ATAGAGCAGTTTTGAAACACTCTTTTCGTAAAATCTGCAAGAGGATATTTTGATAGCTTTGAGGA
          1 ATAGAGCAGGTTTGAAACACTCTTTTTGTAGTATCTGGAAGTGGATATTTGGATAGCTTTGAGGA

                         *   * *            **                      *    *   
     138985 TTTCGTTGGAAACGGGATTGTCTTCATATAAACTCTAGACAGAAGCATTCTCAGAAGCTTCATTG
         66 TTTCGTTGGAAACAGGAATATCTTCATATAAAAGCTAGACAGAAGCATTCTCAGAAACTTCTTTG

            *       **   *    *             *        
     139050 GGATGTTTCAATTGAAGTCACAGTGTTGAACAGTCCCTTTC
        131 TGATGTTTGCATTCAAGTAACAGTGTTGAACATTCCCTTTC

                                                         *       *  * * *    
     139091 ATAGAGCAGGTTTGAAACACTCTTTTTGTAGTATCTGGAAGTGGACATTTGGAGAGATCTCAGGA
          1 ATAGAGCAGGTTTGAAACACTCTTTTTGTAGTATCTGGAAGTGGATATTTGGATAGCTTTGAGGA

            *    *    * *             **            *       * *          *  *
     139156 ATTCGGT-GATAAAGGAAATATCTTCCAATAAAAGCTAGATAGAAGCAATGTCAGAAACTTTTTC
         66 TTTCGTTGGAAACAGG-AATATCTTCATATAAAAGCTAGACAGAAGCATTCTCAGAAACTTCTTT

            *      * ** *   **      *       *  * *   *
     139220 ATGATGTATCTACTCAGCTAACAGAGTTGAACCTTTCTTTTG
        130 GTGATGTTTGCATTCAAGTAACAGTGTTGAACATTCCCTTTC

             *       *                   * *     *             **            
     139262 AGAGAGCAGTTTTGAAACACTCTTTTTGTGGAATCTGCAAGTGGATATTTGTCTAGCTTTGAGGA
          1 ATAGAGCAGGTTTGAAACACTCTTTTTGTAGTATCTGGAAGTGGATATTTGGATAGCTTTGAGGA

                         *   *    *                   *       *              
     139327 TTTCGTTGGAAACGGGATTA-CAT-ATA-AAAAGC-AGACAGCAGCATTCCCAGAAACTTCTTTG
         66 TTTCGTTGGAAACAGGAATATCTTCATATAAAAGCTAGACAGAAGCATTCTCAGAAACTTCTTTG

                              *    *                 
     139388 TGATGTTTGCATTCAAGTCACAGAGTTGAACATTCCCTTTC
        131 TGATGTTTGCATTCAAGTAACAGTGTTGAACATTCCCTTTC

                                                   *     *       **     *   *
     139429 ATAGAGCAGGTTTGAAACACTCTTTTTGTAGTATCTGGATGTGGACATTTGGAGCGCTTTCAGGC
          1 ATAGAGCAGGTTTGAAACACTCTTTTTGTAGTATCTGGAAGTGGATATTTGGATAGCTTTGAGGA

            * ** *      *             *** *   *           *          *   *   
     139494 CTATGGT-GAAAAAGGAAATATCTTCCCCTGAAAACTAGACAGAAGAATTCTCAGAATCTTATTT
         66 TTTCGTTGGAAACAGG-AATATCTTCATATAAAAGCTAGACAGAAGCATTCTCAGAAACTTCTTT

                   **  **    *             **  * *   *
     139558 GTGATGTGCGCCCTCAACTAACAGTGTTGAAGCTTTCTTTTG
        130 GTGATGTTTGCATTCAAGTAACAGTGTTGAACATTCCCTTTC

                     *                    **     *   *               *       
     139600 ATAGAGCAGTTTTGAAACACTCTTTTTGTAAAATCTGCAAGAGGATATTTGGATAGCCTTGAGGA
          1 ATAGAGCAGGTTTGAAACACTCTTTTTGTAGTATCTGGAAGTGGATATTTGGATAGCTTTGAGGA

                         *   * *       *    **                      * *  *   
     139665 TTTCGTTGGAAACGGGATTGTCTTCATGTAAACTCTAGACAGAAGCATTCTCAGAAGCGTCATTG
         66 TTTCGTTGGAAACAGGAATATCTTCATATAAAAGCTAGACAGAAGCATTCTCAGAAACTTCTTTG

            *       **   *    *             *        
     139730 GGATGTTTCAATTGAAGTCACAGTGTTGAACAGTCCCTTTC
        131 TGATGTTTGCATTCAAGTAACAGTGTTGAACATTCCCTTTC

                                                   *     *       **     *    
     139771 ATAGAGCAGGTTTGAAACACTCTTTTTGTAGTATCTGGATGTGGACATTTGGAGCGCTTTCAGGC
          1 ATAGAGCAGGTTTGAAACACTCTTTTTGTAGTATCTGGAAGTGGATATTTGGATAGCTTTGAGG-

            * * *    *                 *** *   *                          *  
     139836 CTATGGTT-TAAA-AGGAAATATCTTCCCCTGAAAACTAGACAGAAGCATTCTCAGAAACTTATT
         65 ATTTCGTTGGAAACAGG-AATATCTTCATATAAAAGCTAGACAGAAGCATTCTCAGAAACTTCTT

                    **  **    *                    *   *
     139899 TGTGATGTGCGCCCTCAACTAACAGTGTTGAAGCATT-CTTTTG
        129 TGTGATGTTTGCATTCAAGTAACAGTGTTGAA-CATTCCCTTTC

                     *                   * *     *             **            
     139942 ATAGAGCAGTTTTGAAACACTCTTTTTGTGGAATCTGCAAGTGGATATTTGTCTAGCTTTGAGGA
          1 ATAGAGCAGGTTTGAAACACTCTTTTTGTAGTATCTGGAAGTGGATATTTGGATAGCTTTGAGGA

                         *   *    *                   *       *              
     140007 TTTCGTTGGAAACGGGATTA-CAT-ATA-AAAAGC-AGACAGCAGCATTCCCAGAAACTTCTTTG
         66 TTTCGTTGGAAACAGGAATATCTTCATATAAAAGCTAGACAGAAGCATTCTCAGAAACTTCTTTG

                            * *    *                 
     140068 TGATGTTTGCATTCAATTCACAGAGTTGAACATTCCCTTTC
        131 TGATGTTTGCATTCAAGTAACAGTGTTGAACATTCCCTTTC

                                                   *     *       **     *   *
     140109 ATAGAGCAGGTTTGAAACACTCTTTTTGTAGTATCTGGATGTGGACATTTGGAGCGCTTTCAGGC
          1 ATAGAGCAGGTTTGAAACACTCTTTTTGTAGTATCTGGAAGTGGATATTTGGATAGCTTTGAGGA

            * *  *      *             *** *   *                      *   *   
     140174 CTACGGT-GAAAAAGGAAATATCTTCCCCTGAAAACTAGACAGAAGCATTCTCAGAATCTTATTT
         66 TTTCGTTGGAAACAGG-AATATCTTCATATAAAAGCTAGACAGAAGCATTCTCAGAAACTTCTTT

                   **  **    *      *      **  * *   *
     140238 GTGATGTGCGCCCTCAACTAACAGGGTTGAAGCTTTCTTTTG
        130 GTGATGTTTGCATTCAAGTAACAGTGTTGAACATTCCCTTTC

                     *                    **     *   *                       
     140280 ATAGAGCAGTTTTGAAACACTCTTTTTGTAAAATCTGCAAGAGGATATTTGGATAGCTTTGAGGA
          1 ATAGAGCAGGTTTGAAACACTCTTTTTGTAGTATCTGGAAGTGGATATTTGGATAGCTTTGAGGA

                         *   * *            **                      *    *   
     140345 TTTCGTTGGAAACGGGATTGTCTTCATATAAACTCTAGACAGAAGCATTCTCAGAAGCTTCATTG
         66 TTTCGTTGGAAACAGGAATATCTTCATATAAAAGCTAGACAGAAGCATTCTCAGAAACTTCTTTG

            *       **   *    *             *        
     140410 GGATGTTTCAATTGAAGTCACAGTGTTGAACAGTCCCTTTC
        131 TGATGTTTGCATTCAAGTAACAGTGTTGAACATTCCCTTTC

                                                         *       **   * *    
     140451 ATAGAGCAGGTTTGAAACACTCTTTTTGTAGTATCTGGAAGTGGACATTTGGAGCGCTCTCAGGA
          1 ATAGAGCAGGTTTGAAACACTCTTTTTGTAGTATCTGGAAGTGGATATTTGGATAGCTTTGAGGA

            * *  *      *             **            **      * *          *  *
     140516 CTGCGGT-GAAAAAGGAAATATCTTCCAATAAAAGCTAGATTGAAGCAATGTCAGAAACTTTTTC
         66 TTTCGTTGGAAACAGG-AATATCTTCATATAAAAGCTAGACAGAAGCATTCTCAGAAACTTCTTT

            *      * ** *   **      *       *        *
     140580 ATGATGTATCTACTCAGCTAACAGAGTTGAACCTT-CCTTTG
        130 GTGATGTTTGCATTCAAGTAACAGTGTTGAACATTCCCTTTC

             *       *                   * *     *             **            
     140621 AGAGAGCAGTTTTGAAACACTCTTTTTGTGGAATCTGCAAGTGGATATTTGTCTAGCTTTGAGGA
          1 ATAGAGCAGGTTTGAAACACTCTTTTTGTAGTATCTGGAAGTGGATATTTGGATAGCTTTGAGGA

                         *   *    *                   *       * * *          
     140686 TTTCGTTGGAAACGGGATTA-CAT-ATA-AAAAGC-AGACAGCAGCATTCCCTGTAACTTCTTTG
         66 TTTCGTTGGAAACAGGAATATCTTCATATAAAAGCTAGACAGAAGCATTCTCAGAAACTTCTTTG

                   *          *    *                 
     140747 TGATGTTCGCATTCAAGTCACAGAGTTGAACATTCCCTTTC
        131 TGATGTTTGCATTCAAGTAACAGTGTTGAACATTCCCTTTC

                                                   *     *       **     *   *
     140788 ATAGAGCAGGTTTGAAACACTCTTTTTGTAGTATCTGGATGTGGACATTTGGAGCGCTTTCAGGC
          1 ATAGAGCAGGTTTGAAACACTCTTTTTGTAGTATCTGGAAGTGGATATTTGGATAGCTTTGAGGA

            * ** *      *             *** *   *   *                  *   *   
     140853 CTATGGT-GAAAAAGGAAATATCTTCCCCTGAAAACTACACAGAAGCATTCTCAGAATCTTATTT
         66 TTTCGTTGGAAACAGG-AATATCTTCATATAAAAGCTAGACAGAAGCATTCTCAGAAACTTCTTT

                   **  **    *             **  * *   *
     140917 GTGATGTGCGCCCTCAACTAACAGTGTTGAAGCTTTCTTTTG
        130 GTGATGTTTGCATTCAAGTAACAGTGTTGAACATTCCCTTTC

                     *                    **     *   *                       
     140959 ATAGAGCAGTTTTGAAACACTCTTTTTGTAAAATCTGCAAGAGGATATTTGGATAGCTTTGAGGA
          1 ATAGAGCAGGTTTGAAACACTCTTTTTGTAGTATCTGGAAGTGGATATTTGGATAGCTTTGAGGA

                         *   * *            **                      *    *   
     141024 TTTCGTTGGAAACGGGATTGTCTTCATATAAACTCTAGACAGAAGCATTCTCAGAAGCTTCATTG
         66 TTTCGTTGGAAACAGGAATATCTTCATATAAAAGCTAGACAGAAGCATTCTCAGAAACTTCTTTG

            *       **   *    *             *        
     141089 GGATGTTTCAATTGAAGTCACAGTGTTGAACAGTCCCTTTC
        131 TGATGTTTGCATTCAAGTAACAGTGTTGAACATTCCCTTTC

                                                   *     *       **     *    
     141130 ATAGAGCAGGTTTGAAACACTCTTTTTGTAGTATCTGGATGTGGACATTTGGAGCGCTTTCAGGC
          1 ATAGAGCAGGTTTGAAACACTCTTTTTGTAGTATCTGGAAGTGGATATTTGGATAGCTTTGAGG-

            * * *    *                 *** *   *                          *  
     141195 CTATGGTT-TAAA-AGGAAATATCTTCCCCTGAAAACTAGACAGAAGCATTCTCAGAAACTTATT
         65 ATTTCGTTGGAAACAGG-AATATCTTCATATAAAAGCTAGACAGAAGCATTCTCAGAAACTTCTT

                    *   **    **            *      *   *
     141258 TGTGATGTGTGCCCTCAACAAACAGTGTTGTAGCATT-CTTTTG
        129 TGTGATGTTTGCATTCAAGTAACAGTGTTG-AACATTCCCTTTC

                     *                   * *     *     *       **            
     141301 ATAGAGCAGTTTTGAAACACTCTTTTTGTGGAATCTGCAAGTGTATATTTGTCTAGCTTTGAGGA
          1 ATAGAGCAGGTTTGAAACACTCTTTTTGTAGTATCTGGAAGTGGATATTTGGATAGCTTTGAGGA

                         *   *    *                *  *       *              
     141366 TTTCGTTGGAAACGGGATTA-CAT-ATA-AAAAGC-AGAAAGCAGCATTCCCAGAAACTTCTTTG
         66 TTTCGTTGGAAACAGGAATATCTTCATATAAAAGCTAGACAGAAGCATTCTCAGAAACTTCTTTG

                              *    *                 
     141427 TGATGTTTGCATTCAAGTCACAGAGTTGAACATTCCCTTTC
        131 TGATGTTTGCATTCAAGTAACAGTGTTGAACATTCCCTTTC

                                                   *     *       **     *   *
     141468 ATAGAGCAGGTTTGAAACACTCTTTTTGTAGTATCTGGATGTGGACATTTGGAGCGCTTTCAGGC
          1 ATAGAGCAGGTTTGAAACACTCTTTTTGTAGTATCTGGAAGTGGATATTTGGATAGCTTTGAGGA

            * ** *      *             *** *   *                          *   
     141533 CTATGGT-GAAAAAGGAAATATCTTCCCCTGAAAACTAGACAGAAGCATTCTCAGAAACTTATTT
         66 TTTCGTTGGAAACAGG-AATATCTTCATATAAAAGCTAGACAGAAGCATTCTCAGAAACTTCTTT

                   **  **    *              *  * *   *
     141597 GTGATGTGCGCCCTCAACTAACAGTGTTGAACCTTTCTTTTG
        130 GTGATGTTTGCATTCAAGTAACAGTGTTGAACATTCCCTTTC

                     *                    *      *   *              *   *    
     141639 ATAGAGCAGTTTTGAAACACTCTTTTTGTAATATCTGCAAGAGGATATTTGGATAGATTTAAGGA
          1 ATAGAGCAGGTTTGAAACACTCTTTTTGTAGTATCTGGAAGTGGATATTTGGATAGCTTTGAGGA

                         *   *    *                   *       *              
     141704 TTTCGTTGGAAACGGGATTA-CAT-ATA-AAAAGC-AGACAGCAGCATTCCCAGAAACTTCTTTG
         66 TTTCGTTGGAAACAGGAATATCTTCATATAAAAGCTAGACAGAAGCATTCTCAGAAACTTCTTTG

                              *    *                 
     141765 TGATGTTTGCATTCAAGTCACAGAGTTGAACATTCCCTTTC
        131 TGATGTTTGCATTCAAGTAACAGTGTTGAACATTCCCTTTC

                                                   *     *       **     *   *
     141806 ATAGAGCAGGTTTGAAACACTCTTTTTGTAGTATCTGGATGTGGACATTTGGAGCGCTTTCAGGC
          1 ATAGAGCAGGTTTGAAACACTCTTTTTGTAGTATCTGGAAGTGGATATTTGGATAGCTTTGAGGA

            * ** *      *             *** *   *                      *   *   
     141871 CTATGGT-GAAAAAGGAAATATCTTCCCCTGAAAACTAGACAGAAGCATTCTCAGAATCTTATTT
         66 TTTCGTTGGAAACAGG-AATATCTTCATATAAAAGCTAGACAGAAGCATTCTCAGAAACTTCTTT

                   **  **    *             **  * *   *
     141935 GTGATGTGCGCCCTCAACTAACAGTGTTGAAGCTTTCTTTTG
        130 GTGATGTTTGCATTCAAGTAACAGTGTTGAACATTCCCTTTC

                     *                *   **     *   *        *              
     141977 ATAGAGCAGTTTTGAAACACTCTTTTCGTAAAATCTGCAAGAGGATATTTTGATAGCTTTGAGGA
          1 ATAGAGCAGGTTTGAAACACTCTTTTTGTAGTATCTGGAAGTGGATATTTGGATAGCTTTGAGGA

                         *   * *            **                      *    *   
     142042 TTTCGTTGGAAACGGGATTGTCTTCATATAAACTCTAGACAGAAGCATTCTCAGAAGCTTCATTG
         66 TTTCGTTGGAAACAGGAATATCTTCATATAAAAGCTAGACAGAAGCATTCTCAGAAACTTCTTTG

            *       **   *    *             *        
     142107 GGATGTTTCAATTGAAGTCACAGTGTTGAACAGTCCCTTTC
        131 TGATGTTTGCATTCAAGTAACAGTGTTGAACATTCCCTTTC

                         *                               *       ***  * *    
     142148 ATAGAGCAGGTTTAAAACACTCTTTTTGTAGTATCTGGAAGTGGACATTTGGAGCACTCTCAGGA
          1 ATAGAGCAGGTTTGAAACACTCTTTTTGTAGTATCTGGAAGTGGATATTTGGATAGCTTTGAGGA

            * ** *      *             **            *       * *          *  *
     142213 CTATGGT-GAAAAAGGAAATATCTTCCAATAAAAGCTAGATAGAAGCAATGTCAGAAACTTTTTC
         66 TTTCGTTGGAAACAGG-AATATCTTCATATAAAAGCTAGACAGAAGCATTCTCAGAAACTTCTTT

            *      * ** *   **      *       *  *     *
     142277 ATGATGTATCTACTCAGCTAACAGAGTTGAACCTTTCCTTTG
        130 GTGATGTTTGCATTCAAGTAACAGTGTTGAACATTCCCTTTC

             *       *                   * *     *             **            
     142319 AGAGAGCAGTTTTGAAACACTCTTTTTGTGGAATCTGCAAGTGGATATTTGTCTAGCTTTGAGGA
          1 ATAGAGCAGGTTTGAAACACTCTTTTTGTAGTATCTGGAAGTGGATATTTGGATAGCTTTGAGGA

                         *   *    *                   *       *              
     142384 TTTCGTTGGAAACGGGATTA-CAT-ATA-AAAAGC-AGACAGCAGCATTCCCAGAAACTTCTTTG
         66 TTTCGTTGGAAACAGGAATATCTTCATATAAAAGCTAGACAGAAGCATTCTCAGAAACTTCTTTG

                              *   **                 
     142445 TGATGTTTGCATTCAAGTCACACAGTTGAACATTCCCTTTC
        131 TGATGTTTGCATTCAAGTAACAGTGTTGAACATTCCCTTTC

                                                   *     *       **     *   *
     142486 ATAGAGCAGGTTTGAAACACTCTTTTTGTAGTATCTGGATGTGGACATTTGGAGCGCTTTCAGGC
          1 ATAGAGCAGGTTTGAAACACTCTTTTTGTAGTATCTGGAAGTGGATATTTGGATAGCTTTGAGGA

            * ** *      *             *** *   *                      *   *   
     142551 CTATGGT-GAAAAAGGAAATATCTTCCCCTGAAAACTAGACAGAAGCATTCTCAGAATCTTATTT
         66 TTTCGTTGGAAACAGG-AATATCTTCATATAAAAGCTAGACAGAAGCATTCTCAGAAACTTCTTT

                   **  **    *             **  * *   *
     142615 GTGATGTGCGCCCTCAACTAACAGTGTTGAAGCTTTCTTTTG
        130 GTGATGTTTGCATTCAAGTAACAGTGTTGAACATTCCCTTTC

                     *                    **     *   *                       
     142657 ATAGAGCAGTTTTGAAACACTCTTTTTGTAAAATCTGCAAGAGGATATTTGGATAGCTTTGAGGA
          1 ATAGAGCAGGTTTGAAACACTCTTTTTGTAGTATCTGGAAGTGGATATTTGGATAGCTTTGAGGA

                         *   * *            **                      *    *   
     142722 TTTCGTTGGAAACGGGATTGTCTTCATATAAACTCTAGACAGAAGCATTCTCAGAAGCTTCATTG
         66 TTTCGTTGGAAACAGGAATATCTTCATATAAAAGCTAGACAGAAGCATTCTCAGAAACTTCTTTG

            *       **   *    *             *        
     142787 GGATGTTTCAATTGAAGTCACAGTGTTGAACAGTCCCTTTC
        131 TGATGTTTGCATTCAAGTAACAGTGTTGAACATTCCCTTTC

                                                   *     *       **     *    
     142828 ATAGAGCAGGTTTGAAACACTCTTTTTGTAGTATCTGGATGTGGACATTTGGAGCGCTTTCAGGC
          1 ATAGAGCAGGTTTGAAACACTCTTTTTGTAGTATCTGGAAGTGGATATTTGGATAGCTTTGAGG-

            * * *    *                 *** *   *                          *  
     142893 CTATGGTT-TAAA-AGGAAATATCTTCCACTGAAAACTAGACAGAAGCATTCTCAGAAACTTATT
         65 ATTTCGTTGGAAACAGG-AATATCTTCATATAAAAGCTAGACAGAAGCATTCTCAGAAACTTCTT

                    **  **    **            *      *   *
     142956 TGTGATGTGCGCCCTCAACAAACAGTGTTGTAGCATT-CTTTTG
        129 TGTGATGTTTGCATTCAAGTAACAGTGTTG-AACATTCCCTTTC

                     *                   * *     *             **            
     142999 ATAGAGCAGTTTTGAAACACTCTTTTTGTGGAATCTGCAAGTGGATATTTGTCTAGCTTTGAGGA
          1 ATAGAGCAGGTTTGAAACACTCTTTTTGTAGTATCTGGAAGTGGATATTTGGATAGCTTTGAGGA

                         *   *    *                *  *       *     *        
     143064 TTTCGTTGGAAACGGGATTA-CAT-ATA-AAAAGC-AGAAAGCAGCATTCCCAGAATCTTCTTTG
         66 TTTCGTTGGAAACAGGAATATCTTCATATAAAAGCTAGACAGAAGCATTCTCAGAAACTTCTTTG

                              *    *                 
     143125 TGATGTTTGCATTCAAGTCACAGAGTTGAACATTCCCTTTC
        131 TGATGTTTGCATTCAAGTAACAGTGTTGAACATTCCCTTTC

                                                   *   * *     * **     *   *
     143166 ATAGAGCAGGTTTGAAACACTCTTTTTGTAGTATCTGGATGTGAACATTTGCAGCGCTTTCAGGC
          1 ATAGAGCAGGTTTGAAACACTCTTTTTGTAGTATCTGGAAGTGGATATTTGGATAGCTTTGAGGA

            * ** *      *             *** *   *                          *   
     143231 CTAAGGT-GAAAAAGGAAGTATCTTCCCCTGAAAACTAGACAGAAGCATTCTCAGAAACTTATTT
         66 TTTCGTTGGAAACAGGAA-TATCTTCATATAAAAGCTAGACAGAAGCATTCTCAGAAACTTCTTT

                   **  **    *             **  * *   *
     143295 GTGATGTGCGCCCTCAACTAACAGTGTTGAAGCTTTCTTTTG
        130 GTGATGTTTGCATTCAAGTAACAGTGTTGAACATTCCCTTTC

                     *                    **     *   *                       
     143337 ATAGAGCAGTTTTGAAACACTCTTTTTGTAAAATCTGCAAGAGGATATTTGGATAGCTTTGAGGA
          1 ATAGAGCAGGTTTGAAACACTCTTTTTGTAGTATCTGGAAGTGGATATTTGGATAGCTTTGAGGA

                         *   * *            **                      *    *   
     143402 TTTCGTTGGAAACGGGATTGTCTTCATATAAACTCTAGACAGAAGCATTCTCAGAAGCTTCATTG
         66 TTTCGTTGGAAACAGGAATATCTTCATATAAAAGCTAGACAGAAGCATTCTCAGAAACTTCTTTG

            *       **   *    *             *        
     143467 GGATGTTTCAATTGAAGTCACAGTGTTGAACAGTCCCTTTC
        131 TGATGTTTGCATTCAAGTAACAGTGTTGAACATTCCCTTTC

                               *                   *     *       **     *    
     143508 ATAGAGCAGGTTTGAAACATTCTTTTTGTAGTATCTGGATGTGGACATTTGGAGCGCTTTCAGGC
          1 ATAGAGCAGGTTTGAAACACTCTTTTTGTAGTATCTGGAAGTGGATATTTGGATAGCTTTGAGG-

            * * *    *                 *** *   *                          *  
     143573 CTATGGTT-TAAA-AGGAAATATCTTCCCCTGAAAACTAGACAGAAGCATTCTCAGAAACTTATT
         65 ATTTCGTTGGAAACAGG-AATATCTTCATATAAAAGCTAGACAGAAGCATTCTCAGAAACTTCTT

                    **  **    *                    *   *
     143636 TGTGATGTGCGCCCTCAACTAACAGTGTTGAAGCATT-CTTTTG
        129 TGTGATGTTTGCATTCAAGTAACAGTGTTGAA-CATTCCCTTTC

                     *                   * *     *             **            
     143679 ATAGAGCAGTTTTGAAACACTCTTTTTGTGGAATCTGCAAGTGGATATTTGTCTAGCTTTGAGGA
          1 ATAGAGCAGGTTTGAAACACTCTTTTTGTAGTATCTGGAAGTGGATATTTGGATAGCTTTGAGGA

                         *   *    *                   *       *              
     143744 TTTCGTTGGAAACGGGATTA-CAT-ATA-AAAAGC-AGACAGCAGCATTCCCAGAAACTTCTTTG
         66 TTTCGTTGGAAACAGGAATATCTTCATATAAAAGCTAGACAGAAGCATTCTCAGAAACTTCTTTG

                              *    *                 
     143805 TGATGTTTGCATTCAAGTCACAGAGTTGAACATTCCCTTTC
        131 TGATGTTTGCATTCAAGTAACAGTGTTGAACATTCCCTTTC

                                           *       *     *       **     *   *
     143846 ATAGAGCAGGTTTGAAACACTCTTTTTGTAGCATCTGGATGTGGACATTTGGAGCGCTTTCAGGC
          1 ATAGAGCAGGTTTGAAACACTCTTTTTGTAGTATCTGGAAGTGGATATTTGGATAGCTTTGAGGA

            * ** *      *             *** *   *           *          *   *   
     143911 CTATGGT-GAAAAAGGAAATATCTTCCCCTGAAAACTAGACAGAAGAATTCTCAGAATCTTATTT
         66 TTTCGTTGGAAACAGG-AATATCTTCATATAAAAGCTAGACAGAAGCATTCTCAGAAACTTCTTT

                   **  **    *             **  * *   *
     143975 GTGATGTGCGCCCTCAACTAACAGTGTTGAAGCTTTCTTTTG
        130 GTGATGTTTGCATTCAAGTAACAGTGTTGAACATTCCCTTTC

                     *                    *      *   *               *       
     144017 ATAGAGCAGTTTTGAAACACTCTTTTTGTAATATCTGCAAGAGGATATTTGGATAGCCTTGAGGA
          1 ATAGAGCAGGTTTGAAACACTCTTTTTGTAGTATCTGGAAGTGGATATTTGGATAGCTTTGAGGA

                         *   * *            **                      * *  *   
     144082 TTTCGTTGGAAACGGGATTGTCTTCATATAAACTCTAGACAGAAGCATTCTCAGAAGCGTCATTG
         66 TTTCGTTGGAAACAGGAATATCTTCATATAAAAGCTAGACAGAAGCATTCTCAGAAACTTCTTTG

            *       **   *    *             *        
     144147 GGATGTTTCAATTGAAGTCACAGTGTTGAACAGTCCCTTTC
        131 TGATGTTTGCATTCAAGTAACAGTGTTGAACATTCCCTTTC

                                                   *     *       **     *    
     144188 ATAGAGCAGGTTTGAAACACTCTTTTTGTAGTATCTGGATGTGGACATTTGGAGCGCTTTCAGGC
          1 ATAGAGCAGGTTTGAAACACTCTTTTTGTAGTATCTGGAAGTGGATATTTGGATAGCTTTGAGG-

            * * *    *                 *** *   *                          *  
     144253 CTATGGTT-TAAA-AGGAAATATCTTCCCCTGAAAACTAGACAGAAGCATTCTCAGAAACTTATT
         65 ATTTCGTTGGAAACAGG-AATATCTTCATATAAAAGCTAGACAGAAGCATTCTCAGAAACTTCTT

                    **  **    *                    *   *
     144316 TGTGATGTGCGCCCTCAACTAACAGTGTTGAAGCATT-CTTTTG
        129 TGTGATGTTTGCATTCAAGTAACAGTGTTGAA-CATTCCCTTTC

                     *                   * *     *             **            
     144359 ATAGAGCAGTTTTGAAACACTCTTTTTGTGGAATCTGCAAGTGGATATTTGTCTAGCTTTGAGGA
          1 ATAGAGCAGGTTTGAAACACTCTTTTTGTAGTATCTGGAAGTGGATATTTGGATAGCTTTGAGGA

                         *   *    *                   *       *              
     144424 TTTCGTTGGAAACGGGATTA-CAT-ATA-AAAAGC-AGACAGCAGCATTCCCAGAAACTTCTTTG
         66 TTTCGTTGGAAACAGGAATATCTTCATATAAAAGCTAGACAGAAGCATTCTCAGAAACTTCTTTG

                            * *    *                 
     144485 TGATGTTTGCATTCAATTCACAGAGTTGAACATTCCCTTTC
        131 TGATGTTTGCATTCAAGTAACAGTGTTGAACATTCCCTTTC

                                                   *     *       **     *   *
     144526 ATAGAGCAGGTTTGAAACACTCTTTTTGTAGTATCTGGATGTGGACATTTGGAGCGCTTTCAGGC
          1 ATAGAGCAGGTTTGAAACACTCTTTTTGTAGTATCTGGAAGTGGATATTTGGATAGCTTTGAGGA

            * ** *      *             *** *   *                      *   *   
     144591 CTATGGT-GAAAAAGGAAATATCTTCCCCTGAAAACTAGACAGAAGCATTCTCAGAATCTTATTT
         66 TTTCGTTGGAAACAGG-AATATCTTCATATAAAAGCTAGACAGAAGCATTCTCAGAAACTTCTTT

                   **  **    *             **  * *   *
     144655 GTGATGTGCGCCCTCAACTAACAGTGTTGAAGCTTTCTTTTG
        130 GTGATGTTTGCATTCAAGTAACAGTGTTGAACATTCCCTTTC

                     *                    **     *   *                       
     144697 ATAGAGCAGTTTTGAAACACTCTTTTTGTAAAATCTGCAAGAGGATATTTGGATAGCTTTGAGGA
          1 ATAGAGCAGGTTTGAAACACTCTTTTTGTAGTATCTGGAAGTGGATATTTGGATAGCTTTGAGGA

                         *   * *            **                      *    *   
     144762 TTTCGTTGGAAACGGGATTGTCTTCATATAAACTCTAGACAGAAGCATTCTCAGAAGCTTCATTG
         66 TTTCGTTGGAAACAGGAATATCTTCATATAAAAGCTAGACAGAAGCATTCTCAGAAACTTCTTTG

            *       **   *    *             *        
     144827 GGATGTTTCAATTGAAGTCACAGTGTTGAACAGTCCCTTTC
        131 TGATGTTTGCATTCAAGTAACAGTGTTGAACATTCCCTTTC

                                                         *       **   * *    
     144868 ATAGAGCAGGTTTGAAACACTCTTTTTGTAGTATCTGGAAGTGGACATTTGGAGCGCTCTCAGGA
          1 ATAGAGCAGGTTTGAAACACTCTTTTTGTAGTATCTGGAAGTGGATATTTGGATAGCTTTGAGGA

            * *  *      *             **            *       * *          *  *
     144933 CTGCGGT-GAAAAAGGAAATATCTTCCAATAAAAGCTAGATAGAAGCAATGTCAGAAACTTTTTC
         66 TTTCGTTGGAAACAGG-AATATCTTCATATAAAAGCTAGACAGAAGCATTCTCAGAAACTTCTTT

            *      * ** *   **      *       *        *
     144997 ATGATGTATCTACTCAGCTAACAGAGTTGAACCTT-CCTTTG
        130 GTGATGTTTGCATTCAAGTAACAGTGTTGAACATTCCCTTTC

             *       *                   * *     *             **            
     145038 AGAGAGCAGTTTTGAAACACTCTTTTTGTGGAATCTGCAAGTGGATATTTGTCTAGCTTTGAGGA
          1 ATAGAGCAGGTTTGAAACACTCTTTTTGTAGTATCTGGAAGTGGATATTTGGATAGCTTTGAGGA

                         *   *    *                   *       *              
     145103 TTTCGTTGGAAACCGGATTA-CAT-ATA-AAAAGC-AGACAGCAGCATTCCCAGAAACTTCTTTG
         66 TTTCGTTGGAAACAGGAATATCTTCATATAAAAGCTAGACAGAAGCATTCTCAGAAACTTCTTTG

               *              *    *                 
     145164 TGAGGTTTGCATTCAAGTCACAGAGTTGAACATTCCCTTTC
        131 TGATGTTTGCATTCAAGTAACAGTGTTGAACATTCCCTTTC

                                                   *     *     * **     *   *
     145205 ATAGAGCAGGTTTGAAACACTCTTTTTGTAGTATCTGGATGTGGACATTTGCAGCGCTTTCAGGC
          1 ATAGAGCAGGTTTGAAACACTCTTTTTGTAGTATCTGGAAGTGGATATTTGGATAGCTTTGAGGA

            * ** *      *             *** *   *                          *   
     145270 CTAAGGT-GAAAAAGGAAATATCTTCCCCTGAAAACTAGACAGAAGCATTCTCAGAAACTTATTT
         66 TTTCGTTGGAAACAGG-AATATCTTCATATAAAAGCTAGACAGAAGCATTCTCAGAAACTTCTTT

                   **  **    *             **  * *   *
     145334 GTGATGTGCGCCCTCAACTAACAGTGTTGAAGCTTTCTTTTG
        130 GTGATGTTTGCATTCAAGTAACAGTGTTGAACATTCCCTTTC

                     *                    *      *   *       *               
     145376 ATAGAGCAGTTTTGAAACACTCTTTTTGTAATATCTGCAAGAGGATATTAGGATAGCTTTGAGGA
          1 ATAGAGCAGGTTTGAAACACTCTTTTTGTAGTATCTGGAAGTGGATATTTGGATAGCTTTGAGGA

                         *   * *            **                      *    *   
     145441 TTTCGTTGGAAACGGGATTGTCTTCATATAAACTCTAGACAGAAGCATTCTCAGAAGCTTCATTG
         66 TTTCGTTGGAAACAGGAATATCTTCATATAAAAGCTAGACAGAAGCATTCTCAGAAACTTCTTTG

            *       **   *    *             * *      
     145506 GGATGTTTCAATTGAAGTCACAGTGTTGAACAGTTCCTTTC
        131 TGATGTTTGCATTCAAGTAACAGTGTTGAACATTCCCTTTC

                 *                                       *       ***  * *    
     145547 ATAGAACAGGTTTGAAACACTCTTTTTGTAGTATCTGGAAGTGGACATTTGGAGCACTCTCAGGA
          1 ATAGAGCAGGTTTGAAACACTCTTTTTGTAGTATCTGGAAGTGGATATTTGGATAGCTTTGAGGA

            * ** *      *             **          * *       * *          *  *
     145612 CTATGGT-GAAAAAGGAAATATCTTCCAATAAAAGCTACATAGAAGCAATGTCAGAAACTTTTTC
         66 TTTCGTTGGAAACAGG-AATATCTTCATATAAAAGCTAGACAGAAGCATTCTCAGAAACTTCTTT

            *      * ** *   **      *       *  *     *
     145676 ATGATGTATCTACTCAGCTAACAGAGTTGAACCTTTCCTTTG
        130 GTGATGTTTGCATTCAAGTAACAGTGTTGAACATTCCCTTTC

             *       *                   * *     *             **            
     145718 AGAGAGCAGTTTTGAAACACTCTTTTTGTGGAATCTGCAAGTGGATATTTGTCTAGCTTTGAGGA
          1 ATAGAGCAGGTTTGAAACACTCTTTTTGTAGTATCTGGAAGTGGATATTTGGATAGCTTTGAGGA

                         *   *    *                   *       *   *          
     145783 TTTCGTTGGAAACGGGATTA-CAT-ATA-AAAAGC-AGACAGCAGCATTCCCAGTAACTTCTTTG
         66 TTTCGTTGGAAACAGGAATATCTTCATATAAAAGCTAGACAGAAGCATTCTCAGAAACTTCTTTG

                   *          *    *                 
     145844 TGATGTTCGCATTCAAGTCACAGAGTTGAACATTCCCTTTC
        131 TGATGTTTGCATTCAAGTAACAGTGTTGAACATTCCCTTTC

                                                   *     *       **     *   *
     145885 ATAGAGCAGGTTTGAAACACTCTTTTTGTAGTATCTGGATGTGGACATTTGGAGCGCTTTCAGGC
          1 ATAGAGCAGGTTTGAAACACTCTTTTTGTAGTATCTGGAAGTGGATATTTGGATAGCTTTGAGGA

            * ** *      *             *** *   *   *                  *   *   
     145950 CTATGGT-GAAAAAGGAAATATCTTCCCCTGAAAACTACACAGAAGCATTCTCAGAATCTTATTT
         66 TTTCGTTGGAAACAGG-AATATCTTCATATAAAAGCTAGACAGAAGCATTCTCAGAAACTTCTTT

                   **  **    *             **  * *   *
     146014 GTGATGTGCGCCCTCAACTAACAGTGTTGAAGCTTTCTTTTG
        130 GTGATGTTTGCATTCAAGTAACAGTGTTGAACATTCCCTTTC

                     *                    **     *   *                       
     146056 ATAGAGCAGTTTTGAAACACTCTTTTTGTAAAATCTGCAAGAGGATATTTGGATAGCTTTGAGGA
          1 ATAGAGCAGGTTTGAAACACTCTTTTTGTAGTATCTGGAAGTGGATATTTGGATAGCTTTGAGGA

                         *   * *            **                      *    *   
     146121 TTTCGTTGGAAACGGGATTGTCTTCATATAAACTCTAGACAGAAGCATTCTCAGAAGCTTCATTG
         66 TTTCGTTGGAAACAGGAATATCTTCATATAAAAGCTAGACAGAAGCATTCTCAGAAACTTCTTTG

            *       **   *    *             *        
     146186 GGATGTTTCAATTGAAGTCACAGTGTTGAACAGTCCCTTTC
        131 TGATGTTTGCATTCAAGTAACAGTGTTGAACATTCCCTTTC

                                                   *     *       ***    *    
     146227 ATAGAGCAGGTTTGAAACACTCTTTTTGTAGTATCTGGATGTGGACATTTGGAGCTCTTTCAGGC
          1 ATAGAGCAGGTTTGAAACACTCTTTTTGTAGTATCTGGAAGTGGATATTTGGATAGCTTTGAGG-

            * * *    *                 *** *   *                          *  
     146292 CTATGGTT-TAAA-AGGAAATATCTTCCCCTGAAAACTAGACAGAAGCATTCTCAGAAACTTATT
         65 ATTTCGTTGGAAACAGG-AATATCTTCATATAAAAGCTAGACAGAAGCATTCTCAGAAACTTCTT

                    **  **    **            *      *   *
     146355 TGTGATGTGCGCCCTCAACAAACAGTGTTGTAGCATT-CTTTTG
        129 TGTGATGTTTGCATTCAAGTAACAGTGTTG-AACATTCCCTTTC

                     *                   * *     *             **       *    
     146398 ATAGAGCAGTTTTGAAACACTCTTTTTGTGGAATCTGCAAGTGGATATTTGTCTAGCTTTCAGGA
          1 ATAGAGCAGGTTTGAAACACTCTTTTTGTAGTATCTGGAAGTGGATATTTGGATAGCTTTGAGGA

                         *   *    *                *  *       *              
     146463 TTTCGTTGGAAACGGGATTA-CAT-ATA-AAAAGC-AGAAAGCAGCATTCCCAGAAACTTCTTTG
         66 TTTCGTTGGAAACAGGAATATCTTCATATAAAAGCTAGACAGAAGCATTCTCAGAAACTTCTTTG

                              *    *                 
     146524 TGATGTTTGCATTCAAGTCACAGAGTTGAACATTCCCTTTC
        131 TGATGTTTGCATTCAAGTAACAGTGTTGAACATTCCCTTTC

                                                   *     *       **     *   *
     146565 ATAGAGCAGGTTTGAAACACTCTTTTTGTAGTATCTGGATGTGGACATTTGGAGCGCTTTCAGGC
          1 ATAGAGCAGGTTTGAAACACTCTTTTTGTAGTATCTGGAAGTGGATATTTGGATAGCTTTGAGGA

            * ** *      *             *** *   *                          *   
     146630 CTATGGT-GAAAAAGGAAATATCTTCCCCTGAAAACTAGACAGAAGCATTCTCAGAAACTTATTT
         66 TTTCGTTGGAAACAGG-AATATCTTCATATAAAAGCTAGACAGAAGCATTCTCAGAAACTTCTTT

                   **  **    *              *  * *   *
     146694 GTGATGTGCGCCCTCAACTAACAGTGTTGAACCTTTCTTTTG
        130 GTGATGTTTGCATTCAAGTAACAGTGTTGAACATTCCCTTTC

                     *                    *      *   *              *        
     146736 ATAGAGCAGTTTTGAAACACTCTTTTTGTAATATCTGCAAGAGGATATTTGGATAGATTTGAGGA
          1 ATAGAGCAGGTTTGAAACACTCTTTTTGTAGTATCTGGAAGTGGATATTTGGATAGCTTTGAGGA

                         *   *    *                   *       *     *   *    
     146801 TTTCGTTGGAAACGGGATTA-CAT-ATA-AAAAGC-AGACAGCAGCATTCCCAGAATCTTGTTTG
         66 TTTCGTTGGAAACAGGAATATCTTCATATAAAAGCTAGACAGAAGCATTCTCAGAAACTTCTTTG

                              *    *                 
     146862 TGATGTTTGCATTCAAGTCACAGAGTTGAACATTCCCTTTC
        131 TGATGTTTGCATTCAAGTAACAGTGTTGAACATTCCCTTTC

                    *                  *           *     *       **     *   *
     146903 ATAGAGCATGTTTGAAACACTCTTTTTATAGTATCTGGATGTGGACATTTGGAGCGCTTTCAGGC
          1 ATAGAGCAGGTTTGAAACACTCTTTTTGTAGTATCTGGAAGTGGATATTTGGATAGCTTTGAGGA

            * ** *      *             *** *   *                      *   *   
     146968 CTATGGT-GAAAAAGGATATATCTTCTCCTGAAAACTAGACAGAAGCATTCTCAGAATCTTATTT
         66 TTTCGTTGGAAACAGGA-ATATCTTCATATAAAAGCTAGACAGAAGCATTCTCAGAAACTTCTTT

                   **  **    *             **  * *   *
     147032 GTGATGTGCGCCCTCAACTAACAGTGTTGAAGCTTTCTTTTG
        130 GTGATGTTTGCATTCAAGTAACAGTGTTGAACATTCCCTTTC

                     *                *   **     *   *        *              
     147074 ATAGAGCAGTTTTGAAACACTCTTTTCGTAAAATCTGCAAGAGGATATTTTGATAGCTTTGAGGA
          1 ATAGAGCAGGTTTGAAACACTCTTTTTGTAGTATCTGGAAGTGGATATTTGGATAGCTTTGAGGA

                         *   * *            **                      *    *   
     147139 TTTCGTTGGAAACGGGATTGTCTTCATATAAACTCTAGACAGAAGCATTCTCAGAAGCTTCATTG
         66 TTTCGTTGGAAACAGGAATATCTTCATATAAAAGCTAGACAGAAGCATTCTCAGAAACTTCTTTG

            *       **   *    *             *        
     147204 GGATGTTTCAATTGAAGTCACAGTGTTGAACAGTCCCTTTC
        131 TGATGTTTGCATTCAAGTAACAGTGTTGAACATTCCCTTTC

                                                         *       *  * * *    
     147245 ATAGAGCAGGTTTGAAACACTCTTTTTGTAGTATCTGGAAGTGGACATTTGGAGAGATCTCAGGA
          1 ATAGAGCAGGTTTGAAACACTCTTTTTGTAGTATCTGGAAGTGGATATTTGGATAGCTTTGAGGA

            * *  *    * *             **            *       * *          *  *
     147310 ATACGGT-GATAAAGGAAATATCTTCCAATAAAAGCTAGATAGAAGCAATGTCAGAAACTTTTTC
         66 TTTCGTTGGAAACAGG-AATATCTTCATATAAAAGCTAGACAGAAGCATTCTCAGAAACTTCTTT

            *      * ** *   **      *       *  * *   *
     147374 ATGATGTATCTACTCAGCTAACAGAGTTGAACCTTTCTTTTG
        130 GTGATGTTTGCATTCAAGTAACAGTGTTGAACATTCCCTTTC

             *       *                   * *     *             **            
     147416 AGAGAGCAGTTTTGAAACACTCTTTTTGTGGAATCTGCAAGTGGATATTTGTCTAGCTTTGAGGA
          1 ATAGAGCAGGTTTGAAACACTCTTTTTGTAGTATCTGGAAGTGGATATTTGGATAGCTTTGAGGA

                         *   *    *                   *       *              
     147481 TTTCGTTGGAAACGGGATTA-CAT-ATA-AAAAGC-AGACAGCAGCATTCCCAGAAACTTCTTTG
         66 TTTCGTTGGAAACAGGAATATCTTCATATAAAAGCTAGACAGAAGCATTCTCAGAAACTTCTTTG

                              *    *                 
     147542 TGATGTTTGCATTCAAGTCACAGAGTTGAACATTCCCTTTC
        131 TGATGTTTGCATTCAAGTAACAGTGTTGAACATTCCCTTTC

                                                   *     *       **     *   *
     147583 ATAGAGCAGGTTTGAAACACTCTTTTTGTAGTATCTGGATGTGGACATTTGGAGCGCTTTCAGGC
          1 ATAGAGCAGGTTTGAAACACTCTTTTTGTAGTATCTGGAAGTGGATATTTGGATAGCTTTGAGGA

            * ** *      *             *** *   *           *          *   *   
     147648 CTATGGT-GAAAAAGGAAATATCTTCCCCTGAAAACTAGACAGAAGAATTCTCAGAATCTTATTT
         66 TTTCGTTGGAAACAGG-AATATCTTCATATAAAAGCTAGACAGAAGCATTCTCAGAAACTTCTTT

                   **  **    *             **  * *   *
     147712 GTGATGTGCGCCCTCAACTAACAGTGTTGAAGCTTTCTTTTG
        130 GTGATGTTTGCATTCAAGTAACAGTGTTGAACATTCCCTTTC

                     *                    **     *   *         *     *       
     147754 ATAGAGCAGTTTTGAAACACTCTTTTTGTAAAATCTGCAAGAGGATATTTGTATAGCCTTGAGGA
          1 ATAGAGCAGGTTTGAAACACTCTTTTTGTAGTATCTGGAAGTGGATATTTGGATAGCTTTGAGGA

                         * * * *            **                      * *  *   
     147819 TTTCGTTGGAAACGGTATTGTCTTCATATAAACTCTAGACAGAAGCATTCTCAGAAGCGTCATTG
         66 TTTCGTTGGAAACAGGAATATCTTCATATAAAAGCTAGACAGAAGCATTCTCAGAAACTTCTTTG

            *       **   *    *           * *        
     147884 GGATGTTTCAATTGAAGTCACAGTGTTGAAGAGTCCCTTTC
        131 TGATGTTTGCATTCAAGTAACAGTGTTGAACATTCCCTTTC

                                                   *     *       **     *    
     147925 ATAGAGCAGGTTTGAAACACTCTTTTTGTAGTATCTGGATGTGGACATTTGGAGCGCTTTCAGGC
          1 ATAGAGCAGGTTTGAAACACTCTTTTTGTAGTATCTGGAAGTGGATATTTGGATAGCTTTGAGG-

            * * *    *                  * * *   *                          * 
     147990 CTATGGTT-TAAA-AGGAAATATCTTCCCTTTGAAAACTAGACAGAAGCATTCTCAGAAACTTAT
         65 ATTTCGTTGGAAACAGG-AATATCTT-CATATAAAAGCTAGACAGAAGCATTCTCAGAAACTTCT

                     **  **    *                    *   *
     148053 TTGTGATGTGCGCCCTCAACTAACAGTGTTGAAGCATT-CTTTTG
        128 TTGTGATGTTTGCATTCAAGTAACAGTGTTGAA-CATTCCCTTTC

                     *                   * *     *             **            
     148097 ATAGAGCAGTTTTGAAACACTCTTTTTGTGGAATCTGCAAGTGGATATTTGTCTAGCTTTGAGGA
          1 ATAGAGCAGGTTTGAAACACTCTTTTTGTAGTATCTGGAAGTGGATATTTGGATAGCTTTGAGGA

                         *   *    *                   *       *              
     148162 TTTCGTTGGAAACGGGATTA-CAT-ATA-AAAAGC-AGACAGCAGCATTCCCAGAAACTTCTTTG
         66 TTTCGTTGGAAACAGGAATATCTTCATATAAAAGCTAGACAGAAGCATTCTCAGAAACTTCTTTG

                              *    *   *             
     148223 TGATGTTTGCATTCAAGTCACAGAGTTCAACATTCCCTTTC
        131 TGATGTTTGCATTCAAGTAACAGTGTTGAACATTCCCTTTC

                                                   *     *       **     *   *
     148264 ATAGAGCAGGTTTGAAACACTCTTTTTGTAGTATCTGGATGTGGACATTTGGAGCGCTTTCAGGC
          1 ATAGAGCAGGTTTGAAACACTCTTTTTGTAGTATCTGGAAGTGGATATTTGGATAGCTTTGAGGA

            * ** *      *             *** *   *                      *   *   
     148329 CTATGGT-GAAAAAGGAAATATCTTCCCCTGAAAACTAGACAGAAGCATTCTCAGAATCTTATTT
         66 TTTCGTTGGAAACAGG-AATATCTTCATATAAAAGCTAGACAGAAGCATTCTCAGAAACTTCTTT

                   **  **    *             **  * *   *
     148393 GTGATGTGCGCCCTCAACTAACAGTGTTGAAGCTTTCTTTTG
        130 GTGATGTTTGCATTCAAGTAACAGTGTTGAACATTCCCTTTC

                     *          *         **     *   *                       
     148435 ATAGAGCAGTTTTGAAACACACTTTTTGTAAAATCTGCAAGAGGATATTTGGATAGCTTTGAGGA
          1 ATAGAGCAGGTTTGAAACACTCTTTTTGTAGTATCTGGAAGTGGATATTTGGATAGCTTTGAGGA

                         *   * *            **                      *    *   
     148500 TTTCGTTGGAAACGGGATTGTCTTCATATAAACTCTAGACAGAAGCATTCTCAGAAGCTTCATTG
         66 TTTCGTTGGAAACAGGAATATCTTCATATAAAAGCTAGACAGAAGCATTCTCAGAAACTTCTTTG

            *       **   *    *             *        
     148565 GGATGTTTCAATTGAAGTCACAGTGTTGAACAGTCCCTTTC
        131 TGATGTTTGCATTCAAGTAACAGTGTTGAACATTCCCTTTC

                                                         *       **   * *    
     148606 ATAGAGCAGGTTTGAAACACTCTTTTTGTAGTATCTGGAAGTGGACATTTGGAGCGCTCTCAGGA
          1 ATAGAGCAGGTTTGAAACACTCTTTTTGTAGTATCTGGAAGTGGATATTTGGATAGCTTTGAGGA

            * *  *      *             **            **      * *          *  *
     148671 CTGCGGT-GAAAAAGGAAATATCTTCCAATAAAAGCTAGATGGAAGCAATGTCAGAAACTTTTTC
         66 TTTCGTTGGAAACAGG-AATATCTTCATATAAAAGCTAGACAGAAGCATTCTCAGAAACTTCTTT

            *      * ** *   **      *       *        *
     148735 ATGATGTATCTACTCAGCTAACAGAGTTGAACCTT-CCTTTG
        130 GTGATGTTTGCATTCAAGTAACAGTGTTGAACATTCCCTTTC

             *       *                   * *     *             **            
     148776 AGAGAGCAGTTTTGAAACACTCTTTTTGTGGAATCTGCAAGTGGATATTTGTCTAGCTTTGAGGA
          1 ATAGAGCAGGTTTGAAACACTCTTTTTGTAGTATCTGGAAGTGGATATTTGGATAGCTTTGAGGA

                         *   *    *                   *       *              
     148841 TTTCGTTGGAAACGGGATTA-CAT-ATA-AAAAGC-AGACAGCAGCATTCCCAGAAACTTCTTTG
         66 TTTCGTTGGAAACAGGAATATCTTCATATAAAAGCTAGACAGAAGCATTCTCAGAAACTTCTTTG

               *              *    *                 
     148902 TGAGGTTTGCATTCAAGTCACAGAGTTGAACATTCCCTTTC
        131 TGATGTTTGCATTCAAGTAACAGTGTTGAACATTCCCTTTC

                                                   *     *       **     *   *
     148943 ATAGAGCAGGTTTGAAACACTCTTTTTGTAGTATCTGGATGTGGACATTTGGAGCGCTTTCAGGC
          1 ATAGAGCAGGTTTGAAACACTCTTTTTGTAGTATCTGGAAGTGGATATTTGGATAGCTTTGAGGA

            * ** *      *             *** *   *                          *   
     149008 CTATGGT-GAAAAAGGAAATATCTTCCCCTGAAAACTAGACAGAAGCATTCTCAGAAACTTATTT
         66 TTTCGTTGGAAACAGG-AATATCTTCATATAAAAGCTAGACAGAAGCATTCTCAGAAACTTCTTT

                   **  **    *             ** ** *   *
     149072 GTGATGTGCGCCCTCAACTAACAGTGTTGAAGCTCTCTTTTG
        130 GTGATGTTTGCATTCAAGTAACAGTGTTGAACATTCCCTTTC

                     *                    **     *   *                       
     149114 ATAGAGCAGTTTTGAAACACTCTTTTTGTAAAATCTGCAAGAGGATATTTGGATAGCTTTGAGGA
          1 ATAGAGCAGGTTTGAAACACTCTTTTTGTAGTATCTGGAAGTGGATATTTGGATAGCTTTGAGGA

                         *   * *            **                      *    *   
     149179 TTTCGTTGGAAACGGGATTGTCTTCATATAAACTCTAGACAGAAGCATTCTCAGAAGCTTCATTG
         66 TTTCGTTGGAAACAGGAATATCTTCATATAAAAGCTAGACAGAAGCATTCTCAGAAACTTCTTTG

            *       **   *    *             *        
     149244 GGATGTTTCAATTGAAGTCACAGTGTTGAACAGTCCCTTTC
        131 TGATGTTTGCATTCAAGTAACAGTGTTGAACATTCCCTTTC

                               *                   *     *       **     *    
     149285 ATAGAGCAGGTTTGAAACATTCTTTTTGTAGTATCTGGATGTGGACATTTGGAGCGCTTTCAGGC
          1 ATAGAGCAGGTTTGAAACACTCTTTTTGTAGTATCTGGAAGTGGATATTTGGATAGCTTTGAGG-

            * * *    *                 *** *   *                          *  
     149350 CTATGGTT-TAAA-AGGAAATATCTTCCCCTGAAAACTAGACAGAAGCATTCTCAGAAACTTATT
         65 ATTTCGTTGGAAACAGG-AATATCTTCATATAAAAGCTAGACAGAAGCATTCTCAGAAACTTCTT

                    **  **    *                    *   *
     149413 TGTGATGTGCGCCCTCAACTAACAGTGTTGAAGCATT-CTTTTG
        129 TGTGATGTTTGCATTCAAGTAACAGTGTTGAA-CATTCCCTTTC

                     *                   * *     *             **            
     149456 ATAGAGCAGTTTTGAAACACTCTTTTTGTGGAATCTGCAAGTGGATATTTGTCTAGCTTTGAGGA
          1 ATAGAGCAGGTTTGAAACACTCTTTTTGTAGTATCTGGAAGTGGATATTTGGATAGCTTTGAGGA

                         *   *    *                   *       *              
     149521 TTTCGTTGGAAACGGGATTA-CAT-ATA-AAAAGC-AGACAGCAGCATTCCCAGAAACTTCTTTG
         66 TTTCGTTGGAAACAGGAATATCTTCATATAAAAGCTAGACAGAAGCATTCTCAGAAACTTCTTTG

                              *  * *                 
     149582 TGATGTTTGCATTCAAGTCACTGAGTTGAACATTCCCTTTC
        131 TGATGTTTGCATTCAAGTAACAGTGTTGAACATTCCCTTTC

                                                   *     *       **     *   *
     149623 ATAGAGCAGGTTTGAAACACTCTTTTTGTAGTATCTGGATGTGGACATTTGGAGCGCTTTCAGGC
          1 ATAGAGCAGGTTTGAAACACTCTTTTTGTAGTATCTGGAAGTGGATATTTGGATAGCTTTGAGGA

            * ** *      *             *** *   *                      *   * * 
     149688 CTATGGT-GAAAAAGGATATATCTTCCCCTGAAAACTAGACAGAAGCATTCTCAGAATCTTATAT
         66 TTTCGTTGGAAACAGGA-ATATCTTCATATAAAAGCTAGACAGAAGCATTCTCAGAAACTTCTTT

                   **  **    *             **  * *   *
     149752 GTGATGTGCGCCCTCAACTAACAGTGTTGAAGGTTTCTTTTG
        130 GTGATGTTTGCATTCAAGTAACAGTGTTGAACATTCCCTTTC

                     *                    **     *   **                      
     149794 ATAGAGCAGTTTTGAAACACTCTTTTTGTAAAATCTGCAAGAAGATATTTGGATAGCTTTGAGGA
          1 ATAGAGCAGGTTTGAAACACTCTTTTTGTAGTATCTGGAAGTGGATATTTGGATAGCTTTGAGGA

                         *   * *            **                      *    *   
     149859 TTTCGTTGGAAACGGGATTGTCTTCATATAAACTCTAGACAGAAGCATTCTCAGAAGCTTCATTG
         66 TTTCGTTGGAAACAGGAATATCTTCATATAAAAGCTAGACAGAAGCATTCTCAGAAACTTCTTTG

            *       **   *    *             *        
     149924 GGATGTTTCAATTGAAGTCACAGTGTTGAACAGTCCCTTTC
        131 TGATGTTTGCATTCAAGTAACAGTGTTGAACATTCCCTTTC

                                                         *       **   * *    
     149965 ATAGAGCAGGTTTGAAACACTCTTTTTGTAGTATCTGGAAGTGGACATTTGGAGCGCTCTCAGGA
          1 ATAGAGCAGGTTTGAAACACTCTTTTTGTAGTATCTGGAAGTGGATATTTGGATAGCTTTGAGGA

            * *  *      *             **            *       * *          *  *
     150030 CTGCGGT-GAAAAAGGAAATATCTTCCAATAAAAGCTAGATAGAAGCAATGTCAGAAACTTTTTC
         66 TTTCGTTGGAAACAGG-AATATCTTCATATAAAAGCTAGACAGAAGCATTCTCAGAAACTTCTTT

            *      * ** *   **      *       *        *
     150094 ATGATGTATCTACTCAGCTAACAGAGTTGAACCTT-CCTTTG
        130 GTGATGTTTGCATTCAAGTAACAGTGTTGAACATTCCCTTTC

             *       *               *   * *     *             **            
     150135 AGAGAGCAGTTTTGAAACACTCTTTCTGTGGAATCTGCAAGTGGATATTTGTCTAGCTTTGAGGA
          1 ATAGAGCAGGTTTGAAACACTCTTTTTGTAGTATCTGGAAGTGGATATTTGGATAGCTTTGAGGA

                *        *   *    *                   *       *              
     150200 TTTCCTTGGAAACGGGATTA-CAT-ATA-AAAAGC-AGACAGCAGCATTCCCAGAAACTTCTTTG
         66 TTTCGTTGGAAACAGGAATATCTTCATATAAAAGCTAGACAGAAGCATTCTCAGAAACTTCTTTG

                              *    *                 
     150261 TGATGTTTGCATTCAAGTCACAGAGTTGAACATTCCCTTTC
        131 TGATGTTTGCATTCAAGTAACAGTGTTGAACATTCCCTTTC

                                             *     **    *     * **     *   *
     150302 ATAGAGCAGGTTTGAAACACTCTTTTTGTAGTACCTGGATTTGGACATTTGCAGCGCTTTCAGGC
          1 ATAGAGCAGGTTTGAAACACTCTTTTTGTAGTATCTGGAAGTGGATATTTGGATAGCTTTGAGGA

            * ** *      *             *** *   *                          *   
     150367 CTAAGGT-GAAAAAGGAAATATCTTCCCCTGAAAACTAGACAGAAGCATTCTCAGAAACTTATTT
         66 TTTCGTTGGAAACAGG-AATATCTTCATATAAAAGCTAGACAGAAGCATTCTCAGAAACTTCTTT

                   **  **    *             **  * *   *
     150431 GTGATGTGCGCCCTCAACTAACAGTGTTGAAGCTTTCTTTTG
        130 GTGATGTTTGCATTCAAGTAACAGTGTTGAACATTCCCTTTC

                     *                   * *     *   *         **            
     150473 ATAGAGCAGTTTTGAAACACTCTTTTTGTGGAATCTGCAAGAGGATATTTGTCTAGCTTTGAGGA
          1 ATAGAGCAGGTTTGAAACACTCTTTTTGTAGTATCTGGAAGTGGATATTTGGATAGCTTTGAGGA

                         *   *    *                   *       *     *   *    
     150538 TTTCGTTGGAAACGGGATTA-CAT-ATA-AAAAGC-AGACAGCAGCATTCCCAGAATCTTGTTTG
         66 TTTCGTTGGAAACAGGAATATCTTCATATAAAAGCTAGACAGAAGCATTCTCAGAAACTTCTTTG

                              *    *   *  *          
     150599 TGATGTTTGCATTCAAGTCACAGAGTTCAAAATTCCCTTTC
        131 TGATGTTTGCATTCAAGTAACAGTGTTGAACATTCCCTTTC

             *                         *  *        *     *       **     *   *
     150640 AGAGAGCAGGTTTGAAACACTCTTTTTATACTATCTGGATGTGGACATTTGGAGCGCTTTCAGGC
          1 ATAGAGCAGGTTTGAAACACTCTTTTTGTAGTATCTGGAAGTGGATATTTGGATAGCTTTGAGGA

            * ** *      *             *** *   *                      *   *   
     150705 CTATGGT-GAAAAAGGAAATATCTTCTCCTGAAAACTAGACAGAAGCATTCTCAGAATCTTATTT
         66 TTTCGTTGGAAACAGG-AATATCTTCATATAAAAGCTAGACAGAAGCATTCTCAGAAACTTCTTT

                   ** ***    *             **  * *   *
     150769 GTGATGTGCGTCCTCAACTAACAGTGTTGAAGCTTTCTTTTG
        130 GTGATGTTTGCATTCAAGTAACAGTGTTGAACATTCCCTTTC

                     *                    **     *   *        *              
     150811 ATAGAGCAGTTTTGAAACACTCTTTTTGTAAAATCTGCAAGAGGATATTTTGATAGCTTTGAGGA
          1 ATAGAGCAGGTTTGAAACACTCTTTTTGTAGTATCTGGAAGTGGATATTTGGATAGCTTTGAGGA

                         *   * *            **                      *    *   
     150876 TTTCGTTGGAAACGGGATTGTCTTCATATAAATTCTAGACAGAAGCATTCTCAGAAGCTTCATTG
         66 TTTCGTTGGAAACAGGAATATCTTCATATAAAAGCTAGACAGAAGCATTCTCAGAAACTTCTTTG

            *       **   *    *             *        
     150941 GGATGTTTCAATTGAAGTCACAGTGTTGAACAGTCCCTTTC
        131 TGATGTTTGCATTCAAGTAACAGTGTTGAACATTCCCTTTC

                                                         *       *  * * *    
     150982 ATAGAGCAGGTTTGAAACACTCTTTTTGTAGTATCTGGAAGTGGACATTTGGAGAGATCTCAGGA
          1 ATAGAGCAGGTTTGAAACACTCTTTTTGTAGTATCTGGAAGTGGATATTTGGATAGCTTTGAGGA

            * *  *  **  ** *         **            *       * *          *  **
     151047 ATACGGTGATAAGGGAAATATCTTCCAATAAAAGCTAGATAGAAGCAATGTCAGAAACTTTTTCA
         66 TTTCGTTGGAAACAGGAATATCTTCATATAAAAGCTAGACAGAAGCATTCTCAGAAACTTCTTTG

                  * ** *   **      *       *        *
     151112 TGATGTATCTACTCAGCTAACAGAGTTGAACCTT-CCTTTG
        131 TGATGTTTGCATTCAAGTAACAGTGTTGAACATTCCCTTTC

             *       *   *               * *     *             **            
     151152 AGAGAGCAGTTTTTAAACACTCTTTTTGTGGAATCTGCAAGTGGATATTTGTCTAGCTTTGAGGA
          1 ATAGAGCAGGTTTGAAACACTCTTTTTGTAGTATCTGGAAGTGGATATTTGGATAGCTTTGAGGA

                              *    *         *        *       *              
     151217 TTTCGTTGGAAAC-GG--GAT-TACATATAAAAAC-AGACAGCAGCATTCCCAGAAACTTCTTTG
         66 TTTCGTTGGAAACAGGAATATCTTCATATAAAAGCTAGACAGAAGCATTCTCAGAAACTTCTTTG

                              *    **                
     151277 TGATGTTTGCATTCAAGTCACAGACTTGAACATTCCCTTTC
        131 TGATGTTTGCATTCAAGTAACAGTGTTGAACATTCCCTTTC

                                                   *           * ** *   *   *
     151318 ATAGAGCAGGTTTGAAACACTCTTTTTGTAGTATCTGGATGTGGATATTTGCAGCGATTTCAGGC
          1 ATAGAGCAGGTTTGAAACACTCTTTTTGTAGTATCTGGAAGTGGATATTTGGATAGCTTTGAGGA

            * ** *      *             *** *   *                          *   
     151383 CTAAGGT-GAAAAAGGAAATATCTTCCCCTGAAAACTAGACAGAAGCATTCTCAGAAACTTATTT
         66 TTTCGTTGGAAACAGG-AATATCTTCATATAAAAGCTAGACAGAAGCATTCTCAGAAACTTCTTT

                   **  **    *              *  * *   *
     151447 GTGATGTGCGCCCTCAACTAACAGTGTTGAACCTTTCTTTTG
        130 GTGATGTTTGCATTCAAGTAACAGTGTTGAACATTCCCTTTC

                     *                    *      *   *                       
     151489 ATAGAGCAGTTTTGAAACACTCTTTTTGTAATATCTGCAAGAGGATATTTGGATAGCTTTGAGGA
          1 ATAGAGCAGGTTTGAAACACTCTTTTTGTAGTATCTGGAAGTGGATATTTGGATAGCTTTGAGGA

                         *   * *            **                      *    *   
     151554 TTTCGTTGGAAACGGGATTGTCTTCATATAAACTCTAGACAGAAGCATTCTCAGAAGCTTCATTG
         66 TTTCGTTGGAAACAGGAATATCTTCATATAAAAGCTAGACAGAAGCATTCTCAGAAACTTCTTTG

            *       **   *    *             * *      
     151619 GGATGTTTCAATTGAAGTCACAGTGTTGAACAGTTCCTTTC
        131 TGATGTTTGCATTCAAGTAACAGTGTTGAACATTCCCTTTC

                 *                                       *       **   * *    
     151660 ATAGAACAGGTTTGAAACACTCTTTTTGTAGTATCTGGAAGTGGACATTTGGAGCGCTCTCAGGA
          1 ATAGAGCAGGTTTGAAACACTCTTTTTGTAGTATCTGGAAGTGGATATTTGGATAGCTTTGAGGA

            * ** *      *             **          * *       * *          *  *
     151725 CTATGGT-GAAAAAGGAAATATCTTCCAATAAAAGCTACATAGAAGCAATGTCAGAAACTTTTTC
         66 TTTCGTTGGAAACAGG-AATATCTTCATATAAAAGCTAGACAGAAGCATTCTCAGAAACTTCTTT

            *      * ** *   **      *       *  *     *
     151789 ATGATGTATCTACTCAGCTAACAGAGTTGAACCTTTCCTTTG
        130 GTGATGTTTGCATTCAAGTAACAGTGTTGAACATTCCCTTTC

             *       *   *               * *     *             **            
     151831 AGAGAGCAGTTTTTAAACACTCTTTTTGTGGAATCTGCAAGTGGATATTTGTCTAGCTTTGAGGA
          1 ATAGAGCAGGTTTGAAACACTCTTTTTGTAGTATCTGGAAGTGGATATTTGGATAGCTTTGAGGA

                            *  *  *                   *       *   *          
     151896 TTTCGTTGGAAAC-GGGATTTCAT-ATA-AAAAGC-AGACAGCAGCATTCCCAGTAACTTCTTTG
         66 TTTCGTTGGAAACAGGAATATCTTCATATAAAAGCTAGACAGAAGCATTCTCAGAAACTTCTTTG

            *                 *    *                 
     151957 GGATGTTTGCATTCAAGTCACAGAGTTGAACATTCCCTTTC
        131 TGATGTTTGCATTCAAGTAACAGTGTTGAACATTCCCTTTC

                                                   *     *       **     *   *
     151998 ATAGAGCAGGTTTGAAACACTCTTTTTGTAGTATCTGGATGTGGACATTTGGAGCGCTTTCAGGC
          1 ATAGAGCAGGTTTGAAACACTCTTTTTGTAGTATCTGGAAGTGGATATTTGGATAGCTTTGAGGA

            * ** *      *             *** *   *                          *   
     152063 CTATGGT-GAAAAAGGAAATATCTTCCCCTGAAAACTAGACAGAAGCATTCTCAGAAACTTATTT
         66 TTTCGTTGGAAACAGG-AATATCTTCATATAAAAGCTAGACAGAAGCATTCTCAGAAACTTCTTT

                   **  **    *              *  * *   *
     152127 GTGATGTGCGCCCTCAACTAACAGTGTTGAACCTTTCTTTTG
        130 GTGATGTTTGCATTCAAGTAACAGTGTTGAACATTCCCTTTC

                     *                    *      *   *                       
     152169 ATAGAGCAGTTTTGAAACACTCTTTTTGTAATATCTGCAAGAGGATATTTGGATAGCTTTGAGGA
          1 ATAGAGCAGGTTTGAAACACTCTTTTTGTAGTATCTGGAAGTGGATATTTGGATAGCTTTGAGGA

                         *   *    *                   *       *     *   *    
     152234 TTTCGTTGGAAACGGGATTA-CAT-ATA-AAAAGC-AGACAGCAGCATTCCCAGAATCTTGTTTG
         66 TTTCGTTGGAAACAGGAATATCTTCATATAAAAGCTAGACAGAAGCATTCTCAGAAACTTCTTTG

                              *    *                 
     152295 TGATGTTTGCATTCAAGTCACAGAGTTGAACATTCCCTTTC
        131 TGATGTTTGCATTCAAGTAACAGTGTTGAACATTCCCTTTC

             *                         **          *     *       **     *   *
     152336 AGAGAGCAGGTTTGAAACACTCTTTTTAAAGTATCTGGATGTGGACATTTGGAGCGCTTTCAGGC
          1 ATAGAGCAGGTTTGAAACACTCTTTTTGTAGTATCTGGAAGTGGATATTTGGATAGCTTTGAGGA

            * ** *      *             *** *   *               *      *   *   
     152401 CTATGGT-GAAAAAGGAAATATCTTCTCCTGAAAACTAGACAGAAGCATTTTCAGAATCTTATTT
         66 TTTCGTTGGAAACAGG-AATATCTTCATATAAAAGCTAGACAGAAGCATTCTCAGAAACTTCTTT

            *      **  **    *             **  * *   *
     152465 TTGATGTGCGCCCTCAACTAACAGTGTTGAAGCTTTCTTTTG
        130 GTGATGTTTGCATTCAAGTAACAGTGTTGAACATTCCCTTTC

                     *                *   **     *   *        *              
     152507 ATAGAGCAGTTTTGAAACACTCTTTTCGTAAAATCTGCAAGAGGATATTTTGATAGCTTTGAGGA
          1 ATAGAGCAGGTTTGAAACACTCTTTTTGTAGTATCTGGAAGTGGATATTTGGATAGCTTTGAGGA

                         *   * *            **                      *    *   
     152572 TTTCGTTGGAAACGGGATTGTCTTCATATAAACTCTAGACAGAAGCATTCTCAGAAGCTTCATTG
         66 TTTCGTTGGAAACAGGAATATCTTCATATAAAAGCTAGACAGAAGCATTCTCAGAAACTTCTTTG

            *       **   *    *         *   *        
     152637 GGATGTTTCAATTGAAGTCACAGTGTTGGACAGTCCCTTTC
        131 TGATGTTTGCATTCAAGTAACAGTGTTGAACATTCCCTTTC

                                                         *    *  *  * * *    
     152678 ATAGAGCAGGTTTGAAACACTCTTTTTGTAGTATCTGGAAGTGGACATTTTGAGAGATCTCAGGA
          1 ATAGAGCAGGTTTGAAACACTCTTTTTGTAGTATCTGGAAGTGGATATTTGGATAGCTTTGAGGA

            * *  *    * *             **            *       * *          *  *
     152743 ATACGGT-GATAAAGGAAATATCTTCCAATAAAAGCTAGATAGAAGCAATGTCAGAAACTTTTTC
         66 TTTCGTTGGAAACAGG-AATATCTTCATATAAAAGCTAGACAGAAGCATTCTCAGAAACTTCTTT

            *      * ** *   **      *       *  * *   *
     152807 ATGATGTATCTACTCAGCTAACAGAGTTGAACCTTTCTTTTG
        130 GTGATGTTTGCATTCAAGTAACAGTGTTGAACATTCCCTTTC

             *       *                   * *     *             **            
     152849 AGAGAGCAGTTTTGAAACACTCTTTTTGTGGAATCTGCAAGTGGATATTTGTCTAGCTTTGAGGA
          1 ATAGAGCAGGTTTGAAACACTCTTTTTGTAGTATCTGGAAGTGGATATTTGGATAGCTTTGAGGA

                         *   *                        *       *   *          
     152914 TTTCGTTGGAAACGGGATTA-CTT-ATA-AAAAGC-AGACAGCAGCATTCCCAGTAACTTCTTTG
         66 TTTCGTTGGAAACAGGAATATCTTCATATAAAAGCTAGACAGAAGCATTCTCAGAAACTTCTTTG

                              *    *                 
     152975 TGATGTTTGCATTCAAGTCACAGAGTTGAACATTCCCTTTC
        131 TGATGTTTGCATTCAAGTAACAGTGTTGAACATTCCCTTTC

                                                   *     *       **     *   *
     153016 ATAGAGCAGGTTTGAAACACTCTTTTTGTAGTATCTGGATGTGGACATTTGGAGCGCTTTCAGGC
          1 ATAGAGCAGGTTTGAAACACTCTTTTTGTAGTATCTGGAAGTGGATATTTGGATAGCTTTGAGGA

            * ** *      *             *** *   *                      *   *   
     153081 CTATGGT-GAAAAAGGAAATATCTTCCCCTGAAAACTAGACAGAAGCATTCTCAGAATCTTATTT
         66 TTTCGTTGGAAACAGG-AATATCTTCATATAAAAGCTAGACAGAAGCATTCTCAGAAACTTCTTT

                   **  **    *             **  * *   *
     153145 GTGATGTGCGCCCTCAACTAACAGTGTTGAAGCTTTCTTTTG
        130 GTGATGTTTGCATTCAAGTAACAGTGTTGAACATTCCCTTTC

                     *                    **     *   *                       
     153187 ATAGAGCAGTTTTGAAACACTCTTTTTGTAAAATCTGCAAGAGGATATTTGGATAGCTTTGAGGA
          1 ATAGAGCAGGTTTGAAACACTCTTTTTGTAGTATCTGGAAGTGGATATTTGGATAGCTTTGAGGA

                         *   * *            **                      * *  *   
     153252 TTTCGTTGGAAACGGGATTGTCTTCATATAAACTCTAGACAGAAGCATTCTCAGAAGCGTCATTG
         66 TTTCGTTGGAAACAGGAATATCTTCATATAAAAGCTAGACAGAAGCATTCTCAGAAACTTCTTTG

            *       **   *    *             *        
     153317 GGATGTTTCAATTGAAGTCACAGTGTTGAACAGTCCCTTTC
        131 TGATGTTTGCATTCAAGTAACAGTGTTGAACATTCCCTTTC

                                                   *     *       **     *    
     153358 ATAGAGCAGGTTTGAAACACTCTTTTTGTAGTATCTGGATGTGGACATTTGGAGCGCTTTCAGGC
          1 ATAGAGCAGGTTTGAAACACTCTTTTTGTAGTATCTGGAAGTGGATATTTGGATAGCTTTGAGG-

            * * *    *                 *** *   *                          *  
     153423 CTATGGTT-TAAA-AGGAAATATCTTCCCCTGAAAACTAGACAGAAGCATTCTCAGAAACTTATT
         65 ATTTCGTTGGAAACAGG-AATATCTTCATATAAAAGCTAGACAGAAGCATTCTCAGAAACTTCTT

                    **  **    *                    *   *
     153486 TGTGATGTGCGCCCTCAACTAACAGTGTTGAAGCATT-CTTTTG
        129 TGTGATGTTTGCATTCAAGTAACAGTGTTGAA-CATTCCCTTTC

                   * *                   * *     *      *      **            
     153529 ATAGAGCCGTTTTGAAACACTCTTTTTGTGGAATCTGCAAGTGGTTATTTGTCTAGCTTTGAGGA
          1 ATAGAGCAGGTTTGAAACACTCTTTTTGTAGTATCTGGAAGTGGATATTTGGATAGCTTTGAGGA

                         *   *    *                   *       *              
     153594 TTTCGTTGGAAACGGGATTA-CAT-ATA-AAAAGC-AGACAGCAGCATTCCCAGAAACTTCTTTG
         66 TTTCGTTGGAAACAGGAATATCTTCATATAAAAGCTAGACAGAAGCATTCTCAGAAACTTCTTTG

                              *    *                 
     153655 TGATGTTTGCATTCAAGTCACAGAGTTGAACATTCCCTTTC
        131 TGATGTTTGCATTCAAGTAACAGTGTTGAACATTCCCTTTC

                                                   *     *       **     *   *
     153696 ATAGAGCAGGTTTGAAACACTCTTTTTGTAGTATCTGGATGTGGACATTTGGAGCGCTTTCAGGC
          1 ATAGAGCAGGTTTGAAACACTCTTTTTGTAGTATCTGGAAGTGGATATTTGGATAGCTTTGAGGA

            * ** *      *             *** *   *                      *   *   
     153761 CTATGGT-GAAAAAGGAAATATCTTCCCCTGAAAACTAGACAGAAGCATTCTCAGAATCTTATTT
         66 TTTCGTTGGAAACAGG-AATATCTTCATATAAAAGCTAGACAGAAGCATTCTCAGAAACTTCTTT

                   **  **    *             **  * *   *
     153825 GTGATGTGCGCCCTCAACTAACAGTGTTGAAGCTTTCTTTTG
        130 GTGATGTTTGCATTCAAGTAACAGTGTTGAACATTCCCTTTC

                     *                    **     *   *                       
     153867 ATAGAGCAGTTTTGAAACACTCTTTTTGTAAAATCTGCAAGAGGATATTTGGATAGCTTTGAGGA
          1 ATAGAGCAGGTTTGAAACACTCTTTTTGTAGTATCTGGAAGTGGATATTTGGATAGCTTTGAGGA

                         *   * *            **                      *    *   
     153932 TTTCGTTGGAAACGGGATTGTCTTCATATAAACTCTAGACAGAAGCATTCTCAGAAGCTTCATTG
         66 TTTCGTTGGAAACAGGAATATCTTCATATAAAAGCTAGACAGAAGCATTCTCAGAAACTTCTTTG

            *             *    *             *        
     153997 GGATGTTT-CACTTGAAGTCACAGTGTTGAACAGTCCCTTTC
        131 TGATGTTTGCA-TTCAAGTAACAGTGTTGAACATTCCCTTTC

                                                         *       **   * *    
     154038 ATAGAGCAGGTTTGAAACACTCTTTTTGTAGTATCTGGAAGTGGACATTTGGAGCGCTCTCAGGA
          1 ATAGAGCAGGTTTGAAACACTCTTTTTGTAGTATCTGGAAGTGGATATTTGGATAGCTTTGAGGA

            * *  *      *             **            *       * *          *  *
     154103 CTGCGGT-GAAAAAGGAAATATCTTCCAATAAAAGCTAGATAGAAGCAATGTCAGAAACTTTTTC
         66 TTTCGTTGGAAACAGG-AATATCTTCATATAAAAGCTAGACAGAAGCATTCTCAGAAACTTCTTT

            *      * ** *   **      *       *        *
     154167 ATGATGTATCTACTCAGCTAACAGAGTTGAACCTT-CCTTTG
        130 GTGATGTTTGCATTCAAGTAACAGTGTTGAACATTCCCTTTC

             *       *                   * *     *             **   *        
     154208 AGAGAGCAGTTTTGAAACACTCTTTTTGTGGAATCTGCAAGTGGATATTTGTCTAGATTTGAGGA
          1 ATAGAGCAGGTTTGAAACACTCTTTTTGTAGTATCTGGAAGTGGATATTTGGATAGCTTTGAGGA

                         *   *    *                   *       *              
     154273 TTTCGTTGGAAACGGGATTA-CAT-ATA-AAAAGC-AGACAGCAGCATTCCCAGAAACTTCTTTG
         66 TTTCGTTGGAAACAGGAATATCTTCATATAAAAGCTAGACAGAAGCATTCTCAGAAACTTCTTTG

                              *    *                 
     154334 TGATGTTTGCATTCAAGTCACAGAGTTGAACATTCCCTTTC
        131 TGATGTTTGCATTCAAGTAACAGTGTTGAACATTCCCTTTC

                                             *     **    *     * **     *   *
     154375 ATAGAGCAGGTTTGAAACACTCTTTTTGTAGTACCTGGATTTGGACATTTGCAGCGCTTTCAGGC
          1 ATAGAGCAGGTTTGAAACACTCTTTTTGTAGTATCTGGAAGTGGATATTTGGATAGCTTTGAGGA

            * ** *      *             *** *   *                          *   
     154440 CTAAGGT-GAAAAAGGAAATATCTTCCCCTGAAAACTAGACAGAAGCATTCTCAGAAACTTATTT
         66 TTTCGTTGGAAACAGG-AATATCTTCATATAAAAGCTAGACAGAAGCATTCTCAGAAACTTCTTT

                   **  **    *             **  * *   *
     154504 GTGATGTGCGCCCTCAACTAACAGTGTTGAAGCTTTCTTTTG
        130 GTGATGTTTGCATTCAAGTAACAGTGTTGAACATTCCCTTTC

                     *               *   * *     *   *         **            
     154546 ATAGAGCAGTTTTGAAACACTCTTTCTGTGGAATCTGCAAGAGGATATTTGTCTAGCTTTGAGGA
          1 ATAGAGCAGGTTTGAAACACTCTTTTTGTAGTATCTGGAAGTGGATATTTGGATAGCTTTGAGGA

                         *   *    *                   *       *     *   *    
     154611 TTTCGTTGGAAACGGGATTA-CAT-ATA-AAAAGC-AGACAGCAGCATTCCCAGAATCTTGTTTG
         66 TTTCGTTGGAAACAGGAATATCTTCATATAAAAGCTAGACAGAAGCATTCTCAGAAACTTCTTTG

                              *    *   *             
     154672 TGATGTTTGCATTCAAGTCACAGAGTTCAACATTCCCTTTC
        131 TGATGTTTGCATTCAAGTAACAGTGTTGAACATTCCCTTTC

             *        *                *  *        *     *       **     *   *
     154713 AGAGAGCAGGATTGAAACACTCTTTTTATACTATCTGGATGTGGACATTTGGAGCGCTTTCAGGC
          1 ATAGAGCAGGTTTGAAACACTCTTTTTGTAGTATCTGGAAGTGGATATTTGGATAGCTTTGAGGA

            * ** *      *             *** *   *                      *   *   
     154778 CTATGGT-GAAAAAGGAAATATCTTCTCCTGAAAACTAGACAGAAGCATTCTCAGAATCTTATTT
         66 TTTCGTTGGAAACAGG-AATATCTTCATATAAAAGCTAGACAGAAGCATTCTCAGAAACTTCTTT

                   ** ***    *             **  * *   *
     154842 GTGATGTGCGTCCTCAACTAACAGTGTTGAAGCTTTCTTTTG
        130 GTGATGTTTGCATTCAAGTAACAGTGTTGAACATTCCCTTTC

                     *          *         **     *   *        *              
     154884 ATAGAGCAGTTTTGAAACACACTTTTTGTAAAATCTGCAAGAGGATATTTTGATAGCTTTGAGGA
          1 ATAGAGCAGGTTTGAAACACTCTTTTTGTAGTATCTGGAAGTGGATATTTGGATAGCTTTGAGGA

                         *   * *            **                      *    *   
     154949 TTTCGTTGGAAACGGGATTGTCTTCATATAAATTCTAGACAGAAGCATTCTCAGAAGCTTCATTG
         66 TTTCGTTGGAAACAGGAATATCTTCATATAAAAGCTAGACAGAAGCATTCTCAGAAACTTCTTTG

            *       **   *    *             *        
     155014 GGATGTTTCAATTGAAGTCACAGTGTTGAACAGTCCCTTTC
        131 TGATGTTTGCATTCAAGTAACAGTGTTGAACATTCCCTTTC

                                                         *       *  * * *    
     155055 ATAGAGCAGGTTTGAAACACTCTTTTTGTAGTATCTGGAAGTGGACATTTGGAGAGATCTCAGGA
          1 ATAGAGCAGGTTTGAAACACTCTTTTTGTAGTATCTGGAAGTGGATATTTGGATAGCTTTGAGGA

            * *  *    * *             **            *       * *          *  *
     155120 ATACGGT-GATAAAGGAAATATCTTCCAATAAAAGCTAGATAGAAGCAATGTCAGAAACTTTTTC
         66 TTTCGTTGGAAACAGG-AATATCTTCATATAAAAGCTAGACAGAAGCATTCTCAGAAACTTCTTT

            *      * ** *   **      *       *        *
     155184 ATGATGTATCTACTCAGCTAACAGAGTTGAACCTT-CCTTTG
        130 GTGATGTTTGCATTCAAGTAACAGTGTTGAACATTCCCTTTC

             *       *                   * *     *             **            
     155225 AGAGAGCAGTTTTGAAACACTCTTTTTGTGGAATCTGCAAGTGGATATTTGTCTAGCTTTGAGGA
          1 ATAGAGCAGGTTTGAAACACTCTTTTTGTAGTATCTGGAAGTGGATATTTGGATAGCTTTGAGGA

                              *    *     *   *        *       *              
     155290 TTTCGTTGGAAAC-GG--GAT-TACATATGAAAAC-AGACAGCAGCATTCCCAGAAACTTCTTTG
         66 TTTCGTTGGAAACAGGAATATCTTCATATAAAAGCTAGACAGAAGCATTCTCAGAAACTTCTTTG

                              *    **                
     155350 TGATGTTTGCATTCAAGTCACAGACTTGAACATTCCCTTTC
        131 TGATGTTTGCATTCAAGTAACAGTGTTGAACATTCCCTTTC

                                                   *           * ** *   *   *
     155391 ATAGAGCAGGTTTGAAACACTCTTTTTGTAGTATCTGGATGTGGATATTTGCAGCGATTTCAGGC
          1 ATAGAGCAGGTTTGAAACACTCTTTTTGTAGTATCTGGAAGTGGATATTTGGATAGCTTTGAGGA

            * ** *      *             *** *   *                          *   
     155456 CTAAGGT-GAAAAAGGAAATATCTTCCCCTGAAAACTAGACAGAAGCATTCTCAGAAACTTATTT
         66 TTTCGTTGGAAACAGG-AATATCTTCATATAAAAGCTAGACAGAAGCATTCTCAGAAACTTCTTT

                   **  **    *              *  * *   *
     155520 GTGATGTGCGCCCTCAACTAACAGTGTTGAACCTTTCTTTTG
        130 GTGATGTTTGCATTCAAGTAACAGTGTTGAACATTCCCTTTC

                     *                    *      *   *                       
     155562 ATAGAGCAGTTTTGAAACACTCTTTTTGTAATATCTGCAAGAGGATATTTGGATAGCTTTGAGGA
          1 ATAGAGCAGGTTTGAAACACTCTTTTTGTAGTATCTGGAAGTGGATATTTGGATAGCTTTGAGGA

                         *   * *            **                      *  * *   
     155627 TTTCGTTGGAAACGGGATTGTCTTCATATAAACTCTAGACAGAAGCATTCTCAGAAGCTCCATTG
         66 TTTCGTTGGAAACAGGAATATCTTCATATAAAAGCTAGACAGAAGCATTCTCAGAAACTTCTTTG

            *       **   *    *             * *      
     155692 GGATGTTTCAATTGAAGTCACAGTGTTGAACAGTTCCTTTC
        131 TGATGTTTGCATTCAAGTAACAGTGTTGAACATTCCCTTTC

                 *                                       *       **   * *    
     155733 ATAGAACAGGTTTGAAACACTCTTTTTGTAGTATCTGGAAGTGGACATTTGGAGCGCTCTCAGGA
          1 ATAGAGCAGGTTTGAAACACTCTTTTTGTAGTATCTGGAAGTGGATATTTGGATAGCTTTGAGGA

            * ** *      *             **          * *       * *          *  *
     155798 CTATGGT-GAAAAAGGAAATATCTTCCAATAAAAGCTACATAGAAGCAATGTCAGAAACTTTTTC
         66 TTTCGTTGGAAACAGG-AATATCTTCATATAAAAGCTAGACAGAAGCATTCTCAGAAACTTCTTT

            *      * ** *   **      *       *  *     *
     155862 ATGATGTATCTACTCAGCTAACAGAGTTGAACCTTTCCTTTG
        130 GTGATGTTTGCATTCAAGTAACAGTGTTGAACATTCCCTTTC

             *       *   *               ***     *             **            
     155904 AGAGAGCAGTTTTTAAACACTCTTTTTGTGAAATCTGCAAGTGGATATTTGTCTAGCTTTGAGGA
          1 ATAGAGCAGGTTTGAAACACTCTTTTTGTAGTATCTGGAAGTGGATATTTGGATAGCTTTGAGGA

                         *   *    *                   *       *   *          
     155969 TTTCGTTGGAAACGGGATTA-CAT-ATA-AAAAGC-AGACAGCAGCATTCCCAGTAACTTCTTTG
         66 TTTCGTTGGAAACAGGAATATCTTCATATAAAAGCTAGACAGAAGCATTCTCAGAAACTTCTTTG

            *                 *    *                 
     156030 GGATGTTTGCATTCAAGTCACAGAGTTGAACATTCCCTTTC
        131 TGATGTTTGCATTCAAGTAACAGTGTTGAACATTCCCTTTC

                                                   *     *       **     *   *
     156071 ATAGAGCAGGTTTGAAACACTCTTTTTGTAGTATCTGGATGTGGACATTTGGAGCGCTTTCAGGC
          1 ATAGAGCAGGTTTGAAACACTCTTTTTGTAGTATCTGGAAGTGGATATTTGGATAGCTTTGAGGA

            * ** *      *             *** *   *                          *   
     156136 CTATGGT-GAAAAAGGAAATATCTTCCCCTGAAAACTAGACAGAAGCATTCTCAGAAACTTATTT
         66 TTTCGTTGGAAACAGG-AATATCTTCATATAAAAGCTAGACAGAAGCATTCTCAGAAACTTCTTT

                   **  **    *              *  * *   *
     156200 GTGATGTGCGCCCTCAACTAACAGTGTTGAACCTTTCTTTTG
        130 GTGATGTTTGCATTCAAGTAACAGTGTTGAACATTCCCTTTC

                     *                    *      *   *                       
     156242 ATAGAGCAGTTTTGAAACACTCTTTTTGTAATATCTGCAAGAGGATATTTGGATAGCTTTGAGGA
          1 ATAGAGCAGGTTTGAAACACTCTTTTTGTAGTATCTGGAAGTGGATATTTGGATAGCTTTGAGGA

                         *   *    *                   *       *     *   *    
     156307 TTTCGTTGGAAACGGGATTA-CAT-ATA-AAAAGC-AGACAGCAGCATTCCCAGAATCTTGTTTG
         66 TTTCGTTGGAAACAGGAATATCTTCATATAAAAGCTAGACAGAAGCATTCTCAGAAACTTCTTTG

                              *    *                 
     156368 TGATGTTTGCATTCAAGTCACAGAGTTGAACATTCCCTTTC
        131 TGATGTTTGCATTCAAGTAACAGTGTTGAACATTCCCTTTC

             *                         **          *     *       **     *   *
     156409 AGAGAGCAGGTTTGAAACACTCTTTTTAAAGTATCTGGATGTGGACATTTGGAGCGCTTTCAGGC
          1 ATAGAGCAGGTTTGAAACACTCTTTTTGTAGTATCTGGAAGTGGATATTTGGATAGCTTTGAGGA

            * ** *      *             *** *   *                      *   *   
     156474 CTATGGT-GAAAAAGGAAATATCTTCTCCTGAAAACTAGACAGAAGCATTCTCAGAATCTTATTT
         66 TTTCGTTGGAAACAGG-AATATCTTCATATAAAAGCTAGACAGAAGCATTCTCAGAAACTTCTTT

                   **  **    *             **  * *   *
     156538 GTGATGTGCGCCCTCAACTAACAGTGTTGAAGCTTTCTTTTG
        130 GTGATGTTTGCATTCAAGTAACAGTGTTGAACATTCCCTTTC

                     *                *   **     *   *        *              
     156580 ATAGAGCAGTTTTGAAACACTCTTTTCGTAAAATCTGCAAGAGGATATTTTGATAGCTTTGAGGA
          1 ATAGAGCAGGTTTGAAACACTCTTTTTGTAGTATCTGGAAGTGGATATTTGGATAGCTTTGAGGA

                         *   * *            **                      *  * *   
     156645 TTTCGTTGGAAACGGGATTGTCTTCATATAAACTCTAGACAGAAGCATTCTCAGAAGCTACATTG
         66 TTTCGTTGGAAACAGGAATATCTTCATATAAAAGCTAGACAGAAGCATTCTCAGAAACTTCTTTG

            *       **   *    *         *   *        
     156710 GGATGTTTCAATTGAAGTCACAGTGTTGGACAGTCCCTTTC
        131 TGATGTTTGCATTCAAGTAACAGTGTTGAACATTCCCTTTC

                                                         *    *  *  * * *    
     156751 ATAGAGCAGGTTTGAAACACTCTTTTTGTAGTATCTGGAAGTGGACATTTTGAGAGATCTCAGGA
          1 ATAGAGCAGGTTTGAAACACTCTTTTTGTAGTATCTGGAAGTGGATATTTGGATAGCTTTGAGGA

            * *  *    * *             **            *       * *          *  *
     156816 ATACGGT-GATAAAGGAAATATCTTCCAATAAAAGCTAGATAGAAGCAATGTCAGAAACTTTTTC
         66 TTTCGTTGGAAACAGG-AATATCTTCATATAAAAGCTAGACAGAAGCATTCTCAGAAACTTCTTT

            *      * ** *   **      *       *  * *   *
     156880 ATGATGTATCTACTCAGCTAACAGAGTTGAACCTTTCTTTTG
        130 GTGATGTTTGCATTCAAGTAACAGTGTTGAACATTCCCTTTC

             *       *                   * *     *             **            
     156922 AGAGAGCAGTTTTGAAACACTCTTTTTGTGGAATCTGCAAGTGGATATTTGTCTAGCTTTGAGGA
          1 ATAGAGCAGGTTTGAAACACTCTTTTTGTAGTATCTGGAAGTGGATATTTGGATAGCTTTGAGGA

                         *   *                        *       *   *          
     156987 TTTCGTTGGAAACGGGATTA-CTT-ATA-AAAAGC-AGACAGCAGCATTCCCAGTAACTTCTTTG
         66 TTTCGTTGGAAACAGGAATATCTTCATATAAAAGCTAGACAGAAGCATTCTCAGAAACTTCTTTG

                              *    *                 
     157048 TGATGTTTGCATTCAAGTCACAGAGTTGAACATTCCCTTTC
        131 TGATGTTTGCATTCAAGTAACAGTGTTGAACATTCCCTTTC

                                                   *     *       **     *   *
     157089 ATAGAGCAGGTTTGAAACACTCTTTTTGTAGTATCTGGATGTGGACATTTGGAGCGCTTTCAGGC
          1 ATAGAGCAGGTTTGAAACACTCTTTTTGTAGTATCTGGAAGTGGATATTTGGATAGCTTTGAGGA

            * ** *      *             *** *   *                          *   
     157154 CTATGGT-GAAAAAGGAAATATCTTCCCCTGAAAACTAGACAGAAGCATTCTCAGAAACTTATTT
         66 TTTCGTTGGAAACAGG-AATATCTTCATATAAAAGCTAGACAGAAGCATTCTCAGAAACTTCTTT

                   **  **    *                    *   *
     157218 GTGATGTGCGCCCTCAACTAACAGTGTTGAAGCATT-CTTTTG
        130 GTGATGTTTGCATTCAAGTAACAGTGTTGAA-CATTCCCTTTC

                   * *                   * *     *      *      **            
     157260 ATAGAGCCGTTTTGAAACACTCTTTTTGTGGAATCTGCAAGTGGTTATTTGTCTAGCTTTGAGGA
          1 ATAGAGCAGGTTTGAAACACTCTTTTTGTAGTATCTGGAAGTGGATATTTGGATAGCTTTGAGGA

                         *   *    *                   *       *              
     157325 TTTCGTTGGAAACGGGATTA-CAT-ATA-AAAAGC-AGACAGCAGCATTCCCAGAAACTTCTTTG
         66 TTTCGTTGGAAACAGGAATATCTTCATATAAAAGCTAGACAGAAGCATTCTCAGAAACTTCTTTG

                              *    *                 
     157386 TGATGTTTGCATTCAAGTCACAGAGTTGAACATTCCCTTTC
        131 TGATGTTTGCATTCAAGTAACAGTGTTGAACATTCCCTTTC

                                                   *     *       **     *   *
     157427 ATAGAGCAGGTTTGAAACACTCTTTTTGTAGTATCTGGATGTGGACATTTGGAGCGCTTTCAGGC
          1 ATAGAGCAGGTTTGAAACACTCTTTTTGTAGTATCTGGAAGTGGATATTTGGATAGCTTTGAGGA

            * ** *      *             *** *   *                      *   *   
     157492 CTATGGT-GAAAAAGGAAATATCTTCCCCTGAAAACTAGACAGAAGCATTCTCAGAATCTTATTT
         66 TTTCGTTGGAAACAGG-AATATCTTCATATAAAAGCTAGACAGAAGCATTCTCAGAAACTTCTTT

                   **  **    *             **  * *   *
     157556 GTGATGTGCGCCCTCAACTAACAGTGTTGAAGCTTTCTTTTG
        130 GTGATGTTTGCATTCAAGTAACAGTGTTGAACATTCCCTTTC

                     *                    **     *   *                       
     157598 ATAGAGCAGTTTTGAAACACTCTTTTTGTAAAATCTGCAAGAGGATATTTGGATAGCTTTGAGGA
          1 ATAGAGCAGGTTTGAAACACTCTTTTTGTAGTATCTGGAAGTGGATATTTGGATAGCTTTGAGGA

                         *   * *            **                      *    *   
     157663 TTTCGTTGGAAACGGGATTGTCTTCATATAAACTCTAGACAGAAGCATTCTCAGAAGCTTCATTG
         66 TTTCGTTGGAAACAGGAATATCTTCATATAAAAGCTAGACAGAAGCATTCTCAGAAACTTCTTTG

            *             *    *             *        
     157728 GGATGTTT-CACTTGAAGTCACAGTGTTGAACAGTCCCTTTC
        131 TGATGTTTGCA-TTCAAGTAACAGTGTTGAACATTCCCTTTC

                                                         *       **   * *    
     157769 ATAGAGCAGGTTTGAAACACTCTTTTTGTAGTATCTGGAAGTGGACATTTGGAGCGCTCTCAGGA
          1 ATAGAGCAGGTTTGAAACACTCTTTTTGTAGTATCTGGAAGTGGATATTTGGATAGCTTTGAGGA

            * *  *      *             **            *       * *          *  *
     157834 CTGCGGT-GAAAAAGGAAATATCTTCCAATAAAAGCTAGATAGAAGCAATGTCAGAAACTTTTTC
         66 TTTCGTTGGAAACAGG-AATATCTTCATATAAAAGCTAGACAGAAGCATTCTCAGAAACTTCTTT

            *      * ** *   **      *       *        *
     157898 ATGATGTATCTACTCAGCTAACAGAGTTGAACCTT-CCTTTG
        130 GTGATGTTTGCATTCAAGTAACAGTGTTGAACATTCCCTTTC

             *    *  *                   * *     *             **   *        
     157939 AGAGAGGAGTTTTGAAACACTCTTTTTGTGGAATCTGCAAGTGGATATTTGTCTAGATTTGAGGA
          1 ATAGAGCAGGTTTGAAACACTCTTTTTGTAGTATCTGGAAGTGGATATTTGGATAGCTTTGAGGA

                         *   *    *                   *       *              
     158004 TTTCGTTGGAAACGGGATTA-CAT-ATA-AAAAGC-AGACAGCAGCATTCCCAGAAACTTCTTTG
         66 TTTCGTTGGAAACAGGAATATCTTCATATAAAAGCTAGACAGAAGCATTCTCAGAAACTTCTTTG

               *              *   **                 
     158065 TGAGGTTTGCATTCAAGTCACATAGTTGAACATTCCCTTTC
        131 TGATGTTTGCATTCAAGTAACAGTGTTGAACATTCCCTTTC

                                                   *     *     * **     *   *
     158106 ATAGAGCAGGTTTGAAACACTCTTTTTGTAGTATCTGGATGTGGACATTTGCAGCGCTTTCAGGC
          1 ATAGAGCAGGTTTGAAACACTCTTTTTGTAGTATCTGGAAGTGGATATTTGGATAGCTTTGAGGA

            * ** *      *             *** *   *                          *   
     158171 CTAAGGT-GAAAAAGGAAATATCTTCCCCTGAAAACTAGACAGAAGCATTCTCAGAAACTTATTT
         66 TTTCGTTGGAAACAGG-AATATCTTCATATAAAAGCTAGACAGAAGCATTCTCAGAAACTTCTTT

                   **  **    *             **  * *   *
     158235 GTGATGTGCGCCCTCAACTAACAGTGTTGAAGCTTTCTTTTG
        130 GTGATGTTTGCATTCAAGTAACAGTGTTGAACATTCCCTTTC

                     *                    *      *   *       *               
     158277 ATAGAGCAGTTTTGAAACACTCTTTTTGTAATATCTGCAAGAGGATATTAGGATAGCTTTGAGGA
          1 ATAGAGCAGGTTTGAAACACTCTTTTTGTAGTATCTGGAAGTGGATATTTGGATAGCTTTGAGGA

                         *   * *            **                      *  * *   
     158342 TTTCGTTGGAAACGGGATTGTCTTCATATAAACTCTAGACAGAAGCATTCTCAGAAGCTCCATTG
         66 TTTCGTTGGAAACAGGAATATCTTCATATAAAAGCTAGACAGAAGCATTCTCAGAAACTTCTTTG

            *       **   **   *             *        
     158407 GGATGTTTCAATTGCAGTCACAGTGTTGAACAGTCCCTTTC
        131 TGATGTTTGCATTCAAGTAACAGTGTTGAACATTCCCTTTC

                                                         **      **   * *    
     158448 ATAGAGCAGGTTTGAAACACTCTTTTTGTAGTATCTGGAAGTGGACTTTTGGAGCGCTCTCAGGA
          1 ATAGAGCAGGTTTGAAACACTCTTTTTGTAGTATCTGGAAGTGGATATTTGGATAGCTTTGAGGA

            * *  *      *             **            *       * *        * *  *
     158513 CTGCGGT-GAAAAAGGAAATATCTTCCAATAAAAGCTAGATAGAAGCAATGTCAGAAACATTTTC
         66 TTTCGTTGGAAACAGG-AATATCTTCATATAAAAGCTAGACAGAAGCATTCTCAGAAACTTCTTT

            *      * ** *   **      *       *        *
     158577 ATGATGTATCTACTCAGCTAACAGAGTTGAACCTT-CCTTTG
        130 GTGATGTTTGCATTCAAGTAACAGTGTTGAACATTCCCTTTC

             *       *                   * *     *             **            
     158618 AGAGAGCAGTTTTGAAACACTCTTTTTGTGGAATCTGCAAGTGGATATTTGTCTAGCTTTGAGGA
          1 ATAGAGCAGGTTTGAAACACTCTTTTTGTAGTATCTGGAAGTGGATATTTGGATAGCTTTGAGGA

                         *   *    *                   *       *              
     158683 TTTCGTTGGAAACGGGATTA-CAT-ATA-AAAAGC-AGACAGCAGCATTCCCAGAAACTTCTTTG
         66 TTTCGTTGGAAACAGGAATATCTTCATATAAAAGCTAGACAGAAGCATTCTCAGAAACTTCTTTG

                              *    *                 
     158744 TGATGTTTGCATTCAAGTCACAGAGTTGAACATTCCCTTTC
        131 TGATGTTTGCATTCAAGTAACAGTGTTGAACATTCCCTTTC

                                                   *     *       ** *   *   *
     158785 ATAGAGCAGGTTTGAAACACTCTTTTTGTAGTATCTGGATGTGGACATTTGGAGCGTTTTCAGGC
          1 ATAGAGCAGGTTTGAAACACTCTTTTTGTAGTATCTGGAAGTGGATATTTGGATAGCTTTGAGGA

            * ** *      *             *** *   *                      *   *   
     158850 CTATGGT-GAAAAAGGAAATATCTTCCCCTGAAAACTAGACAGAAGCATTCTCAGAATCTTATTT
         66 TTTCGTTGGAAACAGG-AATATCTTCATATAAAAGCTAGACAGAAGCATTCTCAGAAACTTCTTT

                   **  **    *              *  * *   *
     158914 GTGATGTGCGCCCTCAACTAACAGTGTTGAACCTTTCTTTTG
        130 GTGATGTTTGCATTCAAGTAACAGTGTTGAACATTCCCTTTC

                     *                    **     *   *                       
     158956 ATAGAGCAGTTTTGAAACACTCTTTTTGTAAAATCTGCAAGAGGATATTTGGATAGCTTTGAGGA
          1 ATAGAGCAGGTTTGAAACACTCTTTTTGTAGTATCTGGAAGTGGATATTTGGATAGCTTTGAGGA

                         *   * *            **                      *    *   
     159021 TTTCGTTGGAAACGGGATTGTCTTCATATAAACTCTAGACAGAAGCATTCTCAGAAGCTTCATTG
         66 TTTCGTTGGAAACAGGAATATCTTCATATAAAAGCTAGACAGAAGCATTCTCAGAAACTTCTTTG

            *       **   *    *             *        
     159086 GGATGTTTCAATTGAAGTCACAGTGTTGAACAGTCCCTTTC
        131 TGATGTTTGCATTCAAGTAACAGTGTTGAACATTCCCTTTC

                                                      *  *       **   * *    
     159127 ATAGAGCAGGTTTGAAACACTCTTTTTGTAGTATCTGGAAGTTGACATTTGGAGCGCTCTCAGGA
          1 ATAGAGCAGGTTTGAAACACTCTTTTTGTAGTATCTGGAAGTGGATATTTGGATAGCTTTGAGGA

            * *  *      *         *   **            *       * *          *  *
     159192 CTACGGT-GAAAAAGGAAATATGTTCCAATAAAAGCTAGATAGAAGCAATGTCAGAAACTTTTTC
         66 TTTCGTTGGAAACAGG-AATATCTTCATATAAAAGCTAGACAGAAGCATTCTCAGAAACTTCTTT

            *      * ** *   **      *       *  * *   *
     159256 ATGATGTATCTACTCAGCTAACAGAGTTGAACCTTTCTTTTG
        130 GTGATGTTTGCATTCAAGTAACAGTGTTGAACATTCCCTTTC

             *       *                   * *                   **            
     159298 AGAGAGCAGTTTTGAAACACTCTTTTTGTGGAATCTGGAAGTGGATATTTGTCTAGCTTTGAGGA
          1 ATAGAGCAGGTTTGAAACACTCTTTTTGTAGTATCTGGAAGTGGATATTTGGATAGCTTTGAGGA

                         *   *    *                   *       *   *          
     159363 TTTCGTTGGAAACGGGATTA-CAT-ATA-AAAAGC-AGACAGCAGCATTCCCAGTAACTTCTTTG
         66 TTTCGTTGGAAACAGGAATATCTTCATATAAAAGCTAGACAGAAGCATTCTCAGAAACTTCTTTG

                              *    *                 
     159424 TGATGTTTGCATTCAAGTCACAGAGTTGAACATTCCCTTTC
        131 TGATGTTTGCATTCAAGTAACAGTGTTGAACATTCCCTTTC

                                                   *     *       **     *   *
     159465 ATAGAGCAGGTTTGAAACACTCTTTTTGTAGTATCTGGATGTGGACATTTGGAGCGCTTTCAGGC
          1 ATAGAGCAGGTTTGAAACACTCTTTTTGTAGTATCTGGAAGTGGATATTTGGATAGCTTTGAGGA

            * ** *      *             *** *   *                      *   *   
     159530 CTATGGT-GAAAAAGGAAATATCTTCCCCTGAAAACTAGACAGAAGCATTCTCAGAATCTTATTT
         66 TTTCGTTGGAAACAGG-AATATCTTCATATAAAAGCTAGACAGAAGCATTCTCAGAAACTTCTTT

                   **  **    *             **  * *   *
     159594 GTGATGTGCGCCCTCAACTAACAGTGTTGAAGCTTTCTTTTG
        130 GTGATGTTTGCATTCAAGTAACAGTGTTGAACATTCCCTTTC

                     *                    **     *   *                       
     159636 ATAGAGCAGTTTTGAAACACTCTTTTTGTAAAATCTGCAAGAGGATATTTGGATAGCTTTGAGGA
          1 ATAGAGCAGGTTTGAAACACTCTTTTTGTAGTATCTGGAAGTGGATATTTGGATAGCTTTGAGGA

                         *   * *            **                      *    *   
     159701 TTTCGTTGGAAACGGGATTGTCTTCATATAAACTCTAGACAGAAGCATTCTCAGAAGCTTCATTG
         66 TTTCGTTGGAAACAGGAATATCTTCATATAAAAGCTAGACAGAAGCATTCTCAGAAACTTCTTTG

            *       **   *    *             * *      
     159766 GGATGTTTCAATTGAAGTCACAGTGTTGAACAGTTCCTTTC
        131 TGATGTTTGCATTCAAGTAACAGTGTTGAACATTCCCTTTC

               * *                                     * *       **   * *    
     159807 ATATAACAGGTTTGAAACACTCTTTTTGTAGTATCTGGAAGTGTACATTTGGAGCGCTCTCAGGA
          1 ATAGAGCAGGTTTGAAACACTCTTTTTGTAGTATCTGGAAGTGGATATTTGGATAGCTTTGAGGA

            * ** *      *             **          * *       * *          *  *
     159872 CTATGCT-GAAAAAGGAAATATCTTCCAATAAAAGCTACATAGAAGCAATGTCAGAAACTTTTTC
         66 TTTCGTTGGAAACAGG-AATATCTTCATATAAAAGCTAGACAGAAGCATTCTCAGAAACTTCTTT

            *      * ** *   **      *       *  *     *
     159936 ATGATGTATCTACTCAGCTAACAGAGTTGAACCTTTCCTTTG
        130 GTGATGTTTGCATTCAAGTAACAGTGTTGAACATTCCCTTTC

             *       *                   * *     *             **            
     159978 AGAGAGCAGTTTTGAAACACTCTTTTTGTGGAATCTGCAAGTGGATATTTGTCTAGCTTTGAGGA
          1 ATAGAGCAGGTTTGAAACACTCTTTTTGTAGTATCTGGAAGTGGATATTTGGATAGCTTTGAGGA

                         *   *    *                   *     * *   *         *
     160043 TTTCGTTGGAAACGGGATTA-CAT-ATA-AAAAGC-AGACAGCAGCATGCCCAGTAACTTCTTTC
         66 TTTCGTTGGAAACAGGAATATCTTCATATAAAAGCTAGACAGAAGCATTCTCAGAAACTTCTTTG

                              *    *                 
     160104 TGATGTTTGCATTCAAGTCACAGAGTTGAACATTCCCTTTC
        131 TGATGTTTGCATTCAAGTAACAGTGTTGAACATTCCCTTTC

                                                   *     *       **     *   *
     160145 ATAGAGCAGGTTTGAAACACTCTTTTTGTAGTATCTGGATGTGGACATTTGGAGCGCTTTCAGGC
          1 ATAGAGCAGGTTTGAAACACTCTTTTTGTAGTATCTGGAAGTGGATATTTGGATAGCTTTGAGGA

            * ** *      *             *** *   *                      *   *   
     160210 CTATGGT-GAAAAAGGAAATATCTTCCCCTGAAAACTAGACAGAAGCATTCTCAGAATCTTATTT
         66 TTTCGTTGGAAACAGG-AATATCTTCATATAAAAGCTAGACAGAAGCATTCTCAGAAACTTCTTT

                   **  **    *             **  * *   *
     160274 GTGATGTGCGCCCTCAACTAACAGTGTTGAAGCTTTCTTTTG
        130 GTGATGTTTGCATTCAAGTAACAGTGTTGAACATTCCCTTTC

                     *                    **     *   *                       
     160316 ATAGAGCAGTTTTGAAACACTCTTTTTGTAAAATCTGCAAGAGGATATTTGGATAGCTTTGAGGA
          1 ATAGAGCAGGTTTGAAACACTCTTTTTGTAGTATCTGGAAGTGGATATTTGGATAGCTTTGAGGA

                         *   * *            **                      * *  *   
     160381 TTTCGTTGGAAACGGGATTGTCTTCATATAAACTCTAGACAGAAGCATTCTCAGAAGCGTCATTG
         66 TTTCGTTGGAAACAGGAATATCTTCATATAAAAGCTAGACAGAAGCATTCTCAGAAACTTCTTTG

            *       **   *    *             *        
     160446 GGATGTTTCAATTGAAGTCACAGTGTTGAACAGTCCCTTTC
        131 TGATGTTTGCATTCAAGTAACAGTGTTGAACATTCCCTTTC

                                                   *     *       **     *    
     160487 ATAGAGCAGGTTTGAAACACTCTTTTTGTAGTATCTGGATGTGGACATTTGGAGCGCTTTCAGGC
          1 ATAGAGCAGGTTTGAAACACTCTTTTTGTAGTATCTGGAAGTGGATATTTGGATAGCTTTGAGG-

            * * *    *                 *** *   *                          *  
     160552 CTATGGTT-TAAA-AGGAAATATCTTCCCCTGAAAACTAGACAGAAGCATTCTCAGAAACTTATT
         65 ATTTCGTTGGAAACAGG-AATATCTTCATATAAAAGCTAGACAGAAGCATTCTCAGAAACTTCTT

                    **  **    *                    *   *
     160615 TGTGATGTGCGCCCTCAACTAACAGTGTTGAAGCATT-CTTTTG
        129 TGTGATGTTTGCATTCAAGTAACAGTGTTGAA-CATTCCCTTTC

                     *                   * *     *             **            
     160658 ATAGAGCAGTTTTGAAACACTCTTTTTGTGGAATCTGCAAGTGGATATTTGTCTAGCTTTGAGGA
          1 ATAGAGCAGGTTTGAAACACTCTTTTTGTAGTATCTGGAAGTGGATATTTGGATAGCTTTGAGGA

                         *   *    *                   *       *              
     160723 TTTCGTTGGAAACGGGATTA-CAT-ATA-AAAAGC-AGACAGCAGCATTCCCAGAAACTTCTTTG
         66 TTTCGTTGGAAACAGGAATATCTTCATATAAAAGCTAGACAGAAGCATTCTCAGAAACTTCTTTG

             *                *    *                 
     160784 TTATGTTTGCATTCAAGTCACAGAGTTGAACATTCCCTTTC
        131 TGATGTTTGCATTCAAGTAACAGTGTTGAACATTCCCTTTC

                                                   *     *       **     *   *
     160825 ATAGAGCAGGTTTGAAACACTCTTTTTGTAGTATCTGGATGTGGACATTTGGAGCGCTTTCAGGC
          1 ATAGAGCAGGTTTGAAACACTCTTTTTGTAGTATCTGGAAGTGGATATTTGGATAGCTTTGAGGA

            * ** *      *             *** *   *       *                  *   
     160890 CTATGGT-GAAAAAGGAAATATCTTCCCCTGAAAACTAGACAAAAGCATTCTCAGAAACTTATTT
         66 TTTCGTTGGAAACAGG-AATATCTTCATATAAAAGCTAGACAGAAGCATTCTCAGAAACTTCTTT

                   **  **    *              *  * *   *
     160954 GTGATGTGCGCCCTCAACTAACAGTGTTGAACCTTTCTTTTG
        130 GTGATGTTTGCATTCAAGTAACAGTGTTGAACATTCCCTTTC

                     *                    *      *   *                       
     160996 ATAGAGCAGTTTTGAAACACTCTTTTTGTAATATCTGCAAGAGGATATTTGGATAGCTTTGAGGA
          1 ATAGAGCAGGTTTGAAACACTCTTTTTGTAGTATCTGGAAGTGGATATTTGGATAGCTTTGAGGA

                         *   * *            **                      *    *   
     161061 TTTCGTTGGAAACGGGATTGTCTTCATATAAACTCTAGACAGAAGCATTCTCAGAAGCTTCATTG
         66 TTTCGTTGGAAACAGGAATATCTTCATATAAAAGCTAGACAGAAGCATTCTCAGAAACTTCTTTG

            *       **   *    *             *        
     161126 GGATGTTTCAATTGAAGTCACAGTGTTGAACAGTCCCTTTC
        131 TGATGTTTGCATTCAAGTAACAGTGTTGAACATTCCCTTTC

                                                         *       * ** * *    
     161167 ATAGAGCAGGTTTGAAACACTCTTTTTGTAGTATCTGGAAGTGGACATTTGGAGAAATCTCAGGA
          1 ATAGAGCAGGTTTGAAACACTCTTTTTGTAGTATCTGGAAGTGGATATTTGGATAGCTTTGAGGA

            * *  *    * *             **            *       * *          *  *
     161232 ATACGGT-GATAAAGGAAATATCTTCCAATAAAAGCTAGATAGAAGCAATGTCAGAAACTTTTTC
         66 TTTCGTTGGAAACAGG-AATATCTTCATATAAAAGCTAGACAGAAGCATTCTCAGAAACTTCTTT

            *      * ** *   **      *       *  * *   *
     161296 ATGATGTATCTACTCAGCTAACAGAGTTGAACCTTTCTTTTG
        130 GTGATGTTTGCATTCAAGTAACAGTGTTGAACATTCCCTTTC

             *       *                   * *     *             **            
     161338 AGAGAGCAGTTTTGAAACACTCTTTTTGTGGAATCTGCAAGTGGATATTTGTCTAGCTTTGAGGA
          1 ATAGAGCAGGTTTGAAACACTCTTTTTGTAGTATCTGGAAGTGGATATTTGGATAGCTTTGAGGA

                         *   *    *                   *       *     *        
     161403 TTTCGTTGGAAACGGGATTA-CAT-ATA-AAAAGC-AGACAGCAGCATTCCCAGAATCTTCTTTG
         66 TTTCGTTGGAAACAGGAATATCTTCATATAAAAGCTAGACAGAAGCATTCTCAGAAACTTCTTTG

                *             *    *   *             
     161464 TGATATTTGCATTCAAGTCACAGAGTTCAACATTCCCTTTC
        131 TGATGTTTGCATTCAAGTAACAGTGTTGAACATTCCCTTTC

             *                         *           *     *       **     *   *
     161505 AGAGAGCAGGTTTGAAACACTCTTTTTATAGTATCTGGATGTGGACATTTGGAGCGCTTTCAGGC
          1 ATAGAGCAGGTTTGAAACACTCTTTTTGTAGTATCTGGAAGTGGATATTTGGATAGCTTTGAGGA

            * ** *      *             *** *   *                      *   *   
     161570 CTATGGT-GAAAAAGGAAATATCTTCTCCTGAAAACTAGACAGAAGCATTCTCAGAATCTTATTT
         66 TTTCGTTGGAAACAGG-AATATCTTCATATAAAAGCTAGACAGAAGCATTCTCAGAAACTTCTTT

                   ** ***    *             **  * *   *
     161634 GTGATGTGCGTCCTCAACTAACAGTGTTGAAGCTTTCTTTTG
        130 GTGATGTTTGCATTCAAGTAACAGTGTTGAACATTCCCTTTC

                     *                *   **     *   *        *              
     161676 ATAGAGCAGTTTTGAAACACTCTTTTCGTAAAATCTGCAAGAGGATATTTTGATAGCTTTGAGGA
          1 ATAGAGCAGGTTTGAAACACTCTTTTTGTAGTATCTGGAAGTGGATATTTGGATAGCTTTGAGGA

                         *   * *            **                      *    *   
     161741 TTTCGTTGGAAACGGGATTGTCTTCATATAAACTCTAGACAGAAGCATTCTCAGAAGCTTCATTG
         66 TTTCGTTGGAAACAGGAATATCTTCATATAAAAGCTAGACAGAAGCATTCTCAGAAACTTCTTTG

            *       **   *    **            *        
     161806 GGATGTTTCAATTGAAGTCCCAGTGTTGAACAGTCCCTTTC
        131 TGATGTTTGCATTCAAGTAACAGTGTTGAACATTCCCTTTC

                                                         *       *  * * *    
     161847 ATAGAGCAGGTTTGAAACACTCTTTTTGTAGTATCTGGAAGTGGACATTTGGAGAGATCTCAGGA
          1 ATAGAGCAGGTTTGAAACACTCTTTTTGTAGTATCTGGAAGTGGATATTTGGATAGCTTTGAGGA

            * *  *    * *             **            *       * *      *   *  *
     161912 ATACGGT-GATAAAGGAAATATCTTCCAATAAAAGCTAGATAGAAGCAATGTCAGAACCTTTTTC
         66 TTTCGTTGGAAACAGG-AATATCTTCATATAAAAGCTAGACAGAAGCATTCTCAGAAACTTCTTT

            *      * ** *   **      *       *  * *   *
     161976 ATGATGTATCTACTCAGCTAACAGAGTTGAACCTTTCTTTTG
        130 GTGATGTTTGCATTCAAGTAACAGTGTTGAACATTCCCTTTC

             *       *                   * *     *             **            
     162018 AGAGAGCAGTTTTGAAACACTCTTTTTGTGGAATCTGCAAGTGGATATTTGTCTAGCTTTGAGGA
          1 ATAGAGCAGGTTTGAAACACTCTTTTTGTAGTATCTGGAAGTGGATATTTGGATAGCTTTGAGGA

                  *      *   *    *                   *       *     *        
     162083 TTTCGTAGGAAACGGGATTA-CAT-ATA-AAAAGC-AGACAGCAGCATTCCCAGAATCTTCTTTG
         66 TTTCGTTGGAAACAGGAATATCTTCATATAAAAGCTAGACAGAAGCATTCTCAGAAACTTCTTTG

                              *    *                 
     162144 TGATGTTTGCATTCAAGTCACAGAGTTGAACATTCCCTTTC
        131 TGATGTTTGCATTCAAGTAACAGTGTTGAACATTCCCTTTC

                                                   *     *       **     *   *
     162185 ATAGAGCAGGTTTGAAACACTCTTTTTGTAGTATCTGGATGTGGACATTTGGAGCGCTTTCAGGC
          1 ATAGAGCAGGTTTGAAACACTCTTTTTGTAGTATCTGGAAGTGGATATTTGGATAGCTTTGAGGA

            * ** *      *             *** *   *                      *   *   
     162250 CTATGGT-GAAAAAGGAAATATCTTCTCCTGAAAACTAGACAGAAGCATTCTCAGAATCTTATTT
         66 TTTCGTTGGAAACAGG-AATATCTTCATATAAAAGCTAGACAGAAGCATTCTCAGAAACTTCTTT

                   **  **    *             **  * *   *
     162314 GTGATGTGCGCCCTCAACTAACAGTGTTGAAGCTTTCTTTTG
        130 GTGATGTTTGCATTCAAGTAACAGTGTTGAACATTCCCTTTC

                     *                    **     *   *                       
     162356 ATAGAGCAGTTTTGAAACACTCTTTTTGTAAAATCTGCAAGAGGATATTTGGATAGCTTTGAGGA
          1 ATAGAGCAGGTTTGAAACACTCTTTTTGTAGTATCTGGAAGTGGATATTTGGATAGCTTTGAGGA

                *        *   * *            **                      *    *   
     162421 TTTCATTGGAAACGGGATTGTCTTCATATAAACTCTAGACAGAAGCATTCTCAGAAGCTTCATTG
         66 TTTCGTTGGAAACAGGAATATCTTCATATAAAAGCTAGACAGAAGCATTCTCAGAAACTTCTTTG

            *             *    *             *        
     162486 GGATGTTT-CAGTTGAAGTCACAGTGTTGAACAGTCCCTTTC
        131 TGATGTTTGCA-TTCAAGTAACAGTGTTGAACATTCCCTTTC

                                                      *  *  *    **   * *    
     162527 ATAGAGCAGGTTTGAAACACTCTTTTTGTAGTATCTGGAAGTTGACATGTGGAGCGCTCTCAGGA
          1 ATAGAGCAGGTTTGAAACACTCTTTTTGTAGTATCTGGAAGTGGATATTTGGATAGCTTTGAGGA

            * *  *      *       *     **            *       * *          *  *
     162592 CTACGGT-GAAAAAGGAAATGTCTTCCAATAAAAGCTAGATAGAAGCAATGTCAGAAACTTTTTC
         66 TTTCGTTGGAAACAGG-AATATCTTCATATAAAAGCTAGACAGAAGCATTCTCAGAAACTTCTTT

            *      * ** *   **      *       *  * *   *
     162656 ATGATGTATCTACTCAGCTAACAGAGTTGAACCTTTCTTTTG
        130 GTGATGTTTGCATTCAAGTAACAGTGTTGAACATTCCCTTTC

             *       *                   * *                   **            
     162698 AGAGAGCAGTTTTGAAACACTCTTTTTGTGGAATCTGGAAGTGGATATTTGTCTAGCTTTGAGGA
          1 ATAGAGCAGGTTTGAAACACTCTTTTTGTAGTATCTGGAAGTGGATATTTGGATAGCTTTGAGGA

                         *   *    *                   *       *   *          
     162763 TTTCGTTGGAAACGGGATTA-CAT-ATA-AAAAGC-AGACAGCAGCATTCCCAGTAACTTCTTTG
         66 TTTCGTTGGAAACAGGAATATCTTCATATAAAAGCTAGACAGAAGCATTCTCAGAAACTTCTTTG

                              *    *  *              
     162824 TGATGTTTGCATTCAAGTCACAGAGTGGAACATTCCCTTTC
        131 TGATGTTTGCATTCAAGTAACAGTGTTGAACATTCCCTTTC

                                                   *     *       **     *   *
     162865 ATAGAGCAGGTTTGAAACACTCTTTTTGTAGTATCTGGATGTGGACATTTGGAGCGCTTTCAGGC
          1 ATAGAGCAGGTTTGAAACACTCTTTTTGTAGTATCTGGAAGTGGATATTTGGATAGCTTTGAGGA

            * ** *      *             *** *   *                      *   *   
     162930 CTATGGT-GAAAAAGGAAATATCTTCCCCTGAAAACTAGACAGAAGCATTCTCAGAATCTTATTT
         66 TTTCGTTGGAAACAGG-AATATCTTCATATAAAAGCTAGACAGAAGCATTCTCAGAAACTTCTTT

                   **  **    *             **  * *   *
     162994 GTGATGTGCGCCCTCAACTAACAGTGTTGAAGCTTTCTTTTG
        130 GTGATGTTTGCATTCAAGTAACAGTGTTGAACATTCCCTTTC

                     *                *   **  *  *   *                       
     163036 ATAGAGCAGTTTTGAAACACTCTTTTCGTAAAATGTGCAAGAGGATATTTGGATAGCTTTGAGGA
          1 ATAGAGCAGGTTTGAAACACTCTTTTTGTAGTATCTGGAAGTGGATATTTGGATAGCTTTGAGGA

                         *   * *            **                      *    *   
     163101 TTTCGTTGGAAACGGGATTGTCTTCATATAAACTCTAGACAGAAGCATTCTCAGAAGCTTCATTG
         66 TTTCGTTGGAAACAGGAATATCTTCATATAAAAGCTAGACAGAAGCATTCTCAGAAACTTCTTTG

            *       **   *    *             *        
     163166 GGATGTTTCAATTGAAGTCACAGTGTTGAACAGTCCCTTTC
        131 TGATGTTTGCATTCAAGTAACAGTGTTGAACATTCCCTTTC

                                                         *       **   * *    
     163207 ATAGAGCAGGTTTGAAACACTCTTTTTGTAGTATCTGGAAGTGGACATTTGGAGCGCTCTCAGGA
          1 ATAGAGCAGGTTTGAAACACTCTTTTTGTAGTATCTGGAAGTGGATATTTGGATAGCTTTGAGGA

            * *  *      *             **          * *       * *          *  *
     163272 CTGCGGT-GAAAAAGGAAATATCTTCCAATAAAAGCTACATAGAAGCAATGTCAGAAACTTTTTC
         66 TTTCGTTGGAAACAGG-AATATCTTCATATAAAAGCTAGACAGAAGCATTCTCAGAAACTTCTTT

            *      * ** *   **      *       *        *
     163336 ATGATGTATCTACTCAGCTAACAGAGTTGAACCTT-CCTTTG
        130 GTGATGTTTGCATTCAAGTAACAGTGTTGAACATTCCCTTTC

             *       *                   * *     *             **            
     163377 AGAGAGCAGTTTTGAAACACTCTTTTTGTGGAATCTGCAAGTGGATATTTGTCTAGCTTTGAGGA
          1 ATAGAGCAGGTTTGAAACACTCTTTTTGTAGTATCTGGAAGTGGATATTTGGATAGCTTTGAGGA

                         *   *    *                   *       *              
     163442 TTTCGTTGGAAACGGGATTA-CAT-ATA-AAAAGC-AGACAGCAGCATTCCCAGAAACTTCTTTG
         66 TTTCGTTGGAAACAGGAATATCTTCATATAAAAGCTAGACAGAAGCATTCTCAGAAACTTCTTTG

                              *    *                 
     163503 TGATGTTTGCATTCAAGTCACAGAGTTGAACATTCCCTTTC
        131 TGATGTTTGCATTCAAGTAACAGTGTTGAACATTCCCTTTC

                                                   *     *     * **     *   *
     163544 ATAGAGCAGGTTTGAAACACTCTTTTTGTAGTATCTGGATGTGGACATTTGCAGCGCTTTCAGGC
          1 ATAGAGCAGGTTTGAAACACTCTTTTTGTAGTATCTGGAAGTGGATATTTGGATAGCTTTGAGGA

            * ** *      *             *** *   *        *                 *   
     163609 CTAAGGT-GAAAAAGGAAATATCTTCCCCTGAAAACTAGACAGCAGCATTCTCAGAAACTTATTT
         66 TTTCGTTGGAAACAGG-AATATCTTCATATAAAAGCTAGACAGAAGCATTCTCAGAAACTTCTTT

                   **  **    *             **  * *   *
     163673 GTGATGTGCGCCCTCAACTAACAGTGTTGAAGCTTTCTTTTG
        130 GTGATGTTTGCATTCAAGTAACAGTGTTGAACATTCCCTTTC

                     *                   * *     *   *         **            
     163715 ATAGAGCAGTTTTGAAACACTCTTTTTGTGGAATCTGCAAGAGGATATTTGTCTAGCTTTGAGGA
          1 ATAGAGCAGGTTTGAAACACTCTTTTTGTAGTATCTGGAAGTGGATATTTGGATAGCTTTGAGGA

                         *   *    *                   *       *     *        
     163780 TTTCGTTGGAAACGGGATTA-CAT-ATA-AAAAGC-AGACAGCAGCATTCCCAGAATCTTCTTTG
         66 TTTCGTTGGAAACAGGAATATCTTCATATAAAAGCTAGACAGAAGCATTCTCAGAAACTTCTTTG

                *             *    *   *             
     163841 TGATATTTGCATTCAAGTCACAGAGTTCAACATTCCCTTTC
        131 TGATGTTTGCATTCAAGTAACAGTGTTGAACATTCCCTTTC

             *                         *           *     *       **     *   *
     163882 AGAGAGCAGGTTTGAAACACTCTTTTTATAGTATCTGGATGTGGACATTTGGAGCGCTTTCAGGC
          1 ATAGAGCAGGTTTGAAACACTCTTTTTGTAGTATCTGGAAGTGGATATTTGGATAGCTTTGAGGA

            * ** *      *             *** *   *                      *   *   
     163947 CTATGGT-GAAAAAGGAAATATCTTCTCCTGAAAACTAGACAGAAGCATTCTCAGAATCTTATTT
         66 TTTCGTTGGAAACAGG-AATATCTTCATATAAAAGCTAGACAGAAGCATTCTCAGAAACTTCTTT

                   ** ***    *             **  * *   *
     164011 GTGATGTGCGTCCTCAACTAACAGTGTTGAAGCTTTCTTTTG
        130 GTGATGTTTGCATTCAAGTAACAGTGTTGAACATTCCCTTTC

                     *                *   **     *   *        *              
     164053 ATAGAGCAGTTTTGAAACACTCTTTTCGTAAAATCTGCAAGAGGATATTTTGATAGCTTTGAGGA
          1 ATAGAGCAGGTTTGAAACACTCTTTTTGTAGTATCTGGAAGTGGATATTTGGATAGCTTTGAGGA

                         *   * *            **                      *    *   
     164118 TTTCGTTGGAAACGGGATTGTCTTCATATAAACTCTAGACAGAAGCATTCTCAGAAGCTTCATTG
         66 TTTCGTTGGAAACAGGAATATCTTCATATAAAAGCTAGACAGAAGCATTCTCAGAAACTTCTTTG

            *       **   *    **            *        
     164183 GGATGTTTCAATTGAAGTCCCAGTGTTGAACAGTCCCTTTC
        131 TGATGTTTGCATTCAAGTAACAGTGTTGAACATTCCCTTTC

                                                         *       *  * * *    
     164224 ATAGAGCAGGTTTGAAACACTCTTTTTGTAGTATCTGGAAGTGGACATTTGGAGAGATCTCAGGA
          1 ATAGAGCAGGTTTGAAACACTCTTTTTGTAGTATCTGGAAGTGGATATTTGGATAGCTTTGAGGA

            * *  *    * *             **            *       * *      *   *  *
     164289 ATACGGT-GATAAAGGAAATATCTTCCAATAAAAGCTAGATAGAAGCAATGTCAGAACCTTTTTC
         66 TTTCGTTGGAAACAGG-AATATCTTCATATAAAAGCTAGACAGAAGCATTCTCAGAAACTTCTTT

            *      * ** *   **      *       *  * *   *
     164353 ATGATGTATCTACTCAGCTAACAGAGTTGAACCTTTCTTTTG
        130 GTGATGTTTGCATTCAAGTAACAGTGTTGAACATTCCCTTTC

             *       *                   * *     *             **            
     164395 AGAGAGCAGTTTTGAAACACTCTTTTTGTGGAATCTGCAAGTGGATATTTGTCTAGCTTTGAGGA
          1 ATAGAGCAGGTTTGAAACACTCTTTTTGTAGTATCTGGAAGTGGATATTTGGATAGCTTTGAGGA

                  *      *   *    *                   *       *     *        
     164460 TTTCGTAGGAAACGGGATTA-CAT-ATA-AAAAGC-AGACAGCAGCATTCCCAGAATCTTCTTTG
         66 TTTCGTTGGAAACAGGAATATCTTCATATAAAAGCTAGACAGAAGCATTCTCAGAAACTTCTTTG

                              *    *                 
     164521 TGATGTTTGCATTCAAGTCACAGAGTTGAACATTCCCTTTC
        131 TGATGTTTGCATTCAAGTAACAGTGTTGAACATTCCCTTTC

                                                   *     *       **     *   *
     164562 ATAGAGCAGGTTTGAAACACTCTTTTTGTAGTATCTGGATGTGGACATTTGGAGCGCTTTCAGGC
          1 ATAGAGCAGGTTTGAAACACTCTTTTTGTAGTATCTGGAAGTGGATATTTGGATAGCTTTGAGGA

            * ** *      *             *** *   *                      *   *   
     164627 CTATGGT-GAAAAAGGAAATATCTTCTCCTGAAAACTAGACAGAAGCATTCTCAGAATCTTATTT
         66 TTTCGTTGGAAACAGG-AATATCTTCATATAAAAGCTAGACAGAAGCATTCTCAGAAACTTCTTT

                   **  **    *             **  * *   *
     164691 GTGATGTGCGCCCTCAACTAACAGTGTTGAAGCTTTCTTTTG
        130 GTGATGTTTGCATTCAAGTAACAGTGTTGAACATTCCCTTTC

                     *                    **     *   *                       
     164733 ATAGAGCAGTTTTGAAACACTCTTTTTGTAAAATCTGCAAGAGGATATTTGGATAGCTTTGAGGA
          1 ATAGAGCAGGTTTGAAACACTCTTTTTGTAGTATCTGGAAGTGGATATTTGGATAGCTTTGAGGA

                *        *   * *            **                      *    *   
     164798 TTTCATTGGAAACGGGATTGTCTTCATATAAACTCTAGACAGAAGCATTCTCAGAAGCTTCATTG
         66 TTTCGTTGGAAACAGGAATATCTTCATATAAAAGCTAGACAGAAGCATTCTCAGAAACTTCTTTG

            *             *    *             *        
     164863 GGATGTTT-CAGTTGAAGTCACAGTGTTGAACAGTCCCTTTC
        131 TGATGTTTGCA-TTCAAGTAACAGTGTTGAACATTCCCTTTC

                                                      *  *  *    **   * *    
     164904 ATAGAGCAGGTTTGAAACACTCTTTTTGTAGTATCTGGAAGTTGACATGTGGAGCGCTCTCAGGA
          1 ATAGAGCAGGTTTGAAACACTCTTTTTGTAGTATCTGGAAGTGGATATTTGGATAGCTTTGAGGA

            * *  *      *       *     **            *       * *          *  *
     164969 CTACGGT-GAAAAAGGAAATGTCTTCCAATAAAAGCTAGATAGAAGCAATGTCAGAAACTTTTTC
         66 TTTCGTTGGAAACAGG-AATATCTTCATATAAAAGCTAGACAGAAGCATTCTCAGAAACTTCTTT

            *      * ** *   **      *       *  * *   *
     165033 ATGATGTATCTACTCAGCTAACAGAGTTGAACCTTTCTTTTG
        130 GTGATGTTTGCATTCAAGTAACAGTGTTGAACATTCCCTTTC

             *       *                   * *                   **            
     165075 AGAGAGCAGTTTTGAAACACTCTTTTTGTGGAATCTGGAAGTGGATATTTGTCTAGCTTTGAGGA
          1 ATAGAGCAGGTTTGAAACACTCTTTTTGTAGTATCTGGAAGTGGATATTTGGATAGCTTTGAGGA

                         *   *    *                   *       *   *          
     165140 TTTCGTTGGAAACGGGATTA-CAT-ATA-AAAAGC-AGACAGCAGCATTCCCAGTAACTTCTTTG
         66 TTTCGTTGGAAACAGGAATATCTTCATATAAAAGCTAGACAGAAGCATTCTCAGAAACTTCTTTG

                              *    *  *              
     165201 TGATGTTTGCATTCAAGTCACAGAGTGGAACATTCCCTTTC
        131 TGATGTTTGCATTCAAGTAACAGTGTTGAACATTCCCTTTC

                                                   *     *       **     *   *
     165242 ATAGAGCAGGTTTGAAACACTCTTTTTGTAGTATCTGGATGTGGACATTTGGAGCGCTTTCAGGC
          1 ATAGAGCAGGTTTGAAACACTCTTTTTGTAGTATCTGGAAGTGGATATTTGGATAGCTTTGAGGA

            * ** *      *             *** *   *                      *   *   
     165307 CTATGGT-GAAAAAGGAAATATCTTCCCCTGAAAACTAGACAGAAGCATTCTCAGAATCTTATTT
         66 TTTCGTTGGAAACAGG-AATATCTTCATATAAAAGCTAGACAGAAGCATTCTCAGAAACTTCTTT

                   **  **    *             **  * *   *
     165371 GTGATGTGCGCCCTCAACTAACAGTGTTGAAGCTTTCTTTTG
        130 GTGATGTTTGCATTCAAGTAACAGTGTTGAACATTCCCTTTC

                     *                    **     *   *                       
     165413 ATAGAGCAGTTTTGAAACACTCTTTTTGTAAAATCTGCAAGAGGATATTTGGATAGCTTTGAGGA
          1 ATAGAGCAGGTTTGAAACACTCTTTTTGTAGTATCTGGAAGTGGATATTTGGATAGCTTTGAGGA

                         *   * *            **                      *    *   
     165478 TTTCGTTGGAAACGGGATTGTCTTCATATAAACTCTAGACAGAAGCATTCTCAGAAGCTTCATTG
         66 TTTCGTTGGAAACAGGAATATCTTCATATAAAAGCTAGACAGAAGCATTCTCAGAAACTTCTTTG

            *       **   *    *             *        
     165543 GGATGTTTCAATTGAAGTCACAGTGTTGAACAGTCCCTTTC
        131 TGATGTTTGCATTCAAGTAACAGTGTTGAACATTCCCTTTC

                                                         *       **   * *    
     165584 ATAGAGCAGGTTTGAAACACTCTTTTTGTAGTATCTGGAAGTGGACATTTGGAGCGCTCTCAGGA
          1 ATAGAGCAGGTTTGAAACACTCTTTTTGTAGTATCTGGAAGTGGATATTTGGATAGCTTTGAGGA

            * *  *      *             **          * *       * *          *  *
     165649 CTGCGGT-GAAAAAGGAAATATCTTCCAATAAAAGCTACATAGAAGCAATGTCAGAAACTTTTTC
         66 TTTCGTTGGAAACAGG-AATATCTTCATATAAAAGCTAGACAGAAGCATTCTCAGAAACTTCTTT

            *      * ** *   **      *       *        *
     165713 ATGATGTATCTACTCAGCTAACAGAGTTGAACCTT-CCTTTG
        130 GTGATGTTTGCATTCAAGTAACAGTGTTGAACATTCCCTTTC

             *       *                   * *     *             **            
     165754 AGAGAGCAGTTTTGAAACACTCTTTTTGTGGAATCTGCAAGTGGATATTTGTCTAGCTTTGAGGA
          1 ATAGAGCAGGTTTGAAACACTCTTTTTGTAGTATCTGGAAGTGGATATTTGGATAGCTTTGAGGA

                         *   *    *                   *       *              
     165819 TTTCGTTGGAAACGGGATTA-CAT-ATA-AAAAGC-AGACAGCAGCATTCCCAGAAACTTCTTTG
         66 TTTCGTTGGAAACAGGAATATCTTCATATAAAAGCTAGACAGAAGCATTCTCAGAAACTTCTTTG

                              *    **                
     165880 TGATGTTTGCATTCAAGTCACAGATTTGAACATTCCCTTTC
        131 TGATGTTTGCATTCAAGTAACAGTGTTGAACATTCCCTTTC

                                                   *     *       **     *   *
     165921 ATAGAGCAGGTTTGAAACACTCTTTTTGTAGTATCTGGATGTGGACATTTGGAGCGCTTTCAGGC
          1 ATAGAGCAGGTTTGAAACACTCTTTTTGTAGTATCTGGAAGTGGATATTTGGATAGCTTTGAGGA

            * ** *      *             *** *   *                      *   *   
     165986 CTATGGT-GAAAAAGGAAATATCTTCCCCTGAAAACTAGACAGAAGCATTCTCAGAATCTTATTT
         66 TTTCGTTGGAAACAGG-AATATCTTCATATAAAAGCTAGACAGAAGCATTCTCAGAAACTTCTTT

                   **  **    *             **  * *   *
     166050 GTGATGTGCGCCCTCAACTAACAGTGTTGAAGCTTTCTTTTG
        130 GTGATGTTTGCATTCAAGTAACAGTGTTGAACATTCCCTTTC

                     *                    **     *   *                       
     166092 ATAGAGCAGTTTTGAAACACTCTTTTTGTAAAATCTGCAAGAGGATATTTGGATAGCTTTGAGGA
          1 ATAGAGCAGGTTTGAAACACTCTTTTTGTAGTATCTGGAAGTGGATATTTGGATAGCTTTGAGGA

                         *   * *            **                      *    *   
     166157 TTTCGTTGGAAACGGGATTGTCTTCATATAAACTCTAGACAGAAGCATTCTCAGAAGCTTCATTG
         66 TTTCGTTGGAAACAGGAATATCTTCATATAAAAGCTAGACAGAAGCATTCTCAGAAACTTCTTTG

            *             *    *             *        
     166222 GGATGTTT-CAGTTGAAGTCACAGTGTTGAACAGTCCCTTTC
        131 TGATGTTTGCA-TTCAAGTAACAGTGTTGAACATTCCCTTTC

                               *                         *       **   * *    
     166263 ATAGAGCAGGTTTGAAACAGTCTTTTTGTAGTATCTGGAAGTGGACATTTGGAGCGCTCTCAGGA
          1 ATAGAGCAGGTTTGAAACACTCTTTTTGTAGTATCTGGAAGTGGATATTTGGATAGCTTTGAGGA

            * *  *      *             **            *       * *          *  *
     166328 CTGCGGT-GAAAAAGGAAATATCTTCCAATAAAAGCTAGATAGAAGCAATGTCAGAAACTTTTTC
         66 TTTCGTTGGAAACAGG-AATATCTTCATATAAAAGCTAGACAGAAGCATTCTCAGAAACTTCTTT

            *      * ** *   **      *       *        *
     166392 ATGATGTATCTACTCAGCTAACAGAGTTGAACCTT-CCTTTG
        130 GTGATGTTTGCATTCAAGTAACAGTGTTGAACATTCCCTTTC

             *       *                   * *     *             **            
     166433 AGAGAGCAGTTTTGAAACACTCTTTTTGTGGAATCTGCAAGTGGATATTTGTCTAGCTTTGAGGA
          1 ATAGAGCAGGTTTGAAACACTCTTTTTGTAGTATCTGGAAGTGGATATTTGGATAGCTTTGAGGA

                         *   *    *  *                *       *              
     166498 TTTCGTTGGAAACGGGATTA-CAT-GTA-AAAAGC-AGACAGCAGCATTCCCAGAAACTTCTTTG
         66 TTTCGTTGGAAACAGGAATATCTTCATATAAAAGCTAGACAGAAGCATTCTCAGAAACTTCTTTG

                              *    *                 
     166559 TGATGTTTGCATTCAAGTCACAGAGTTGAACATTCCCTTTC
        131 TGATGTTTGCATTCAAGTAACAGTGTTGAACATTCCCTTTC

                                                   *     *     * **     *   *
     166600 ATAGAGCAGGTTTGAAACACTCTTTTTGTAGTATCTGGATGTGGACATTTGCAGCGCTTTCAGGC
          1 ATAGAGCAGGTTTGAAACACTCTTTTTGTAGTATCTGGAAGTGGATATTTGGATAGCTTTGAGGA

            * ** *      *             *** *   *                          *   
     166665 CTAAGGT-GAAAAAGGAAATATCTTCCCCTGAAAACTAGACAGAAGCATTCTCAGAAACTTATTT
         66 TTTCGTTGGAAACAGG-AATATCTTCATATAAAAGCTAGACAGAAGCATTCTCAGAAACTTCTTT

                   **  **    *             **  * *   *
     166729 GTGATGTGCGCCCTCAACTAACAGTGTTGAAGCTTTCTTTTG
        130 GTGATGTTTGCATTCAAGTAACAGTGTTGAACATTCCCTTTC

                     *   *               * *                   **            
     166771 ATAGAGCAGTTTTTAAACACTCTTTTTGTGGAATCTGGAAGTGGATATTTGTCTAGCTTTGAGGA
          1 ATAGAGCAGGTTTGAAACACTCTTTTTGTAGTATCTGGAAGTGGATATTTGGATAGCTTTGAGGA

                         *   *    *                   *       *     *   *    
     166836 TTTCGTTGGAAACGGGATTA-CAT-ATA-AAAAGC-AGACAGCAGCATTCCCAGAATCTTGTTTG
         66 TTTCGTTGGAAACAGGAATATCTTCATATAAAAGCTAGACAGAAGCATTCTCAGAAACTTCTTTG

                              *    *                 
     166897 TGATGTTTGCATTCAAGTCACAGAGTTGAACATTCCCTTTC
        131 TGATGTTTGCATTCAAGTAACAGTGTTGAACATTCCCTTTC

             *      *                  *           *     *       **     *   *
     166938 AGAGAGCACGTTTGAAACACTCTTTTTATAGTATCTGGATGTGGACATTTGGAGCGCTTTCAGGC
          1 ATAGAGCAGGTTTGAAACACTCTTTTTGTAGTATCTGGAAGTGGATATTTGGATAGCTTTGAGGA

            * ** *      *             *** *   *                      *   *   
     167003 CTATGGT-GAAAAAGGAAATATCTTCTCCTGAAAACTAGACAGAAGCATTCTCAGAATCTTATTT
         66 TTTCGTTGGAAACAGG-AATATCTTCATATAAAAGCTAGACAGAAGCATTCTCAGAAACTTCTTT

                   **  **    *             **  * *   *
     167067 GTGATGTGCGCCCTCAACTAACAGTGTTGAAGCTTTCTTTTG
        130 GTGATGTTTGCATTCAAGTAACAGTGTTGAACATTCCCTTTC

                     *     *    *     *   **     *   *        *              
     167109 ATAGAGCAGTTTTGAGACACACTTTTCGTAAAATCTGCAAGAGGATATTTTGATAGCTTTGAGGA
          1 ATAGAGCAGGTTTGAAACACTCTTTTTGTAGTATCTGGAAGTGGATATTTGGATAGCTTTGAGGA

                         *   * *            **                      *    *   
     167174 TTTCGTTGGAAACGGGATTGTCTTCATATAAACTCTAGACAGAAGCATTCTCAGAAGCTTCATTG
         66 TTTCGTTGGAAACAGGAATATCTTCATATAAAAGCTAGACAGAAGCATTCTCAGAAACTTCTTTG

            *       **   *    *             *        
     167239 GGATGTTTCAATTGAAGTCACAGTGTTGAACAGTCCCTTTC
        131 TGATGTTTGCATTCAAGTAACAGTGTTGAACATTCCCTTTC

                                                         *       *  * * *    
     167280 ATAGAGCAGGTTTGAAACACTCTTTTTGTAGTATCTGGAAGTGGACATTTGGAGAGATCTCAGGA
          1 ATAGAGCAGGTTTGAAACACTCTTTTTGTAGTATCTGGAAGTGGATATTTGGATAGCTTTGAGGA

            * *  *    * *             **            *       * *          *  *
     167345 ATACGGT-GATAAAGGAAATATCTTCCAATAAAAGCTAGATAGAAGCAATGTCAGAAACTTTTTC
         66 TTTCGTTGGAAACAGG-AATATCTTCATATAAAAGCTAGACAGAAGCATTCTCAGAAACTTCTTT

            *  *   * ** *   **      *       *  * *   *
     167409 ATGTTGTATCTACTCAGCTAACAGAGTTGAACTTTTCTTTTG
        130 GTGATGTTTGCATTCAAGTAACAGTGTTGAACATTCCCTTTC

             *       *                   * *                   **            
     167451 AGAGAGCAGTTTTGAAACACTCTTTTTGTGGAATCTGGAAGTGGATATTTGTCTAGCTTTGAGGA
          1 ATAGAGCAGGTTTGAAACACTCTTTTTGTAGTATCTGGAAGTGGATATTTGGATAGCTTTGAGGA

                         *   *    *                   *       *              
     167516 TTTCGTTGGAAACGGGATTA-CAT-ATA-AAAAGC-AGACAGCAGCATTCCCAGAAACTTCTTTG
         66 TTTCGTTGGAAACAGGAATATCTTCATATAAAAGCTAGACAGAAGCATTCTCAGAAACTTCTTTG

                              *    *                 
     167577 TGATGTTTGCATTCAAGTCACAGAGTTGAACATTCCCTTTC
        131 TGATGTTTGCATTCAAGTAACAGTGTTGAACATTCCCTTTC

                                                   *     *       **     *   *
     167618 ATAGAGCAGGTTTGAAACACTCTTTTTGTAGTATCTGGATGTGGACATTTGGAACGCTTTCAGGC
          1 ATAGAGCAGGTTTGAAACACTCTTTTTGTAGTATCTGGAAGTGGATATTTGGATAGCTTTGAGGA

            * ** *      *             *** *   *                      *   *   
     167683 CTATGGT-GAAAAAGGAAATATCTTCTCCTGAAAACTAGACAGAAGCATTCTCAGAATCTTATTT
         66 TTTCGTTGGAAACAGG-AATATCTTCATATAAAAGCTAGACAGAAGCATTCTCAGAAACTTCTTT

                   **  **    *             **  * *   *
     167747 GTGATGTGCGCCCTCAACTAACAGTGTTGAAGCTTTCTTTTG
        130 GTGATGTTTGCATTCAAGTAACAGTGTTGAACATTCCCTTTC

                     *                    **     *   *                       
     167789 ATAGAGCAGTTTTGAAACACTCTTTTTGTAAAATCTGCAAGAGGATATTTGGATAGCTTTGAGGA
          1 ATAGAGCAGGTTTGAAACACTCTTTTTGTAGTATCTGGAAGTGGATATTTGGATAGCTTTGAGGA

                         *   * *            **                      *    *   
     167854 TTTCGTTGGAAACGGGATTGTCTTCATATAAACTCTAGACAGAAGCATTCTCAGAAGCTTCATTG
         66 TTTCGTTGGAAACAGGAATATCTTCATATAAAAGCTAGACAGAAGCATTCTCAGAAACTTCTTTG

            *             *    *             *  *     
     167919 GGATGTTT-CAGTTGAAGTCACAGTGTTGAACAGTCGCTTTC
        131 TGATGTTTGCA-TTCAAGTAACAGTGTTGAACATTCCCTTTC

                                                   *     *       **     *   *
     167960 ATAGAGCAGGTTTGAAACACTCTTTTTGTAGTATCTGGATGTGGACATTTGGAGCGCTTTCAGGC
          1 ATAGAGCAGGTTTGAAACACTCTTTTTGTAGTATCTGGAAGTGGATATTTGGATAGCTTTGAGGA

            * ** *      *             *** *   *                      *   *   
     168025 CTATGGT-GAAAAAGGAAATATCTTCCCCTGAAAACTAGACAGAAGCATTCTCAGAATCTTATTT
         66 TTTCGTTGGAAACAGG-AATATCTTCATATAAAAGCTAGACAGAAGCATTCTCAGAAACTTCTTT

                   **  **    *             **  * *   *
     168089 GTGATGTGCGCCCTCAACTAACAGTGTTGAAGCTTTCTTTTG
        130 GTGATGTTTGCATTCAAGTAACAGTGTTGAACATTCCCTTTC

                     *                    **     *   *                       
     168131 ATAGAGCAGTTTTGAAACACTCTTTTTGTAAAATCTGCAAGAGGATATTTGGATAGCTTTGAGGA
          1 ATAGAGCAGGTTTGAAACACTCTTTTTGTAGTATCTGGAAGTGGATATTTGGATAGCTTTGAGGA

                         *   * *            **                      * *  *   
     168196 TTTCGTTGGAAACGGGATTGTCTTCATATAAACTCTAGACAGAAGCATTCTCAGAAGCGTCATTG
         66 TTTCGTTGGAAACAGGAATATCTTCATATAAAAGCTAGACAGAAGCATTCTCAGAAACTTCTTTG

            *       **   *    *             *        
     168261 GGATGTTTCAATTGAAGTCACAGTGTTGAACAGTCCCTTTC
        131 TGATGTTTGCATTCAAGTAACAGTGTTGAACATTCCCTTTC

                                       *           *     *    *  **     *    
     168302 ATAGAGCAGGTTTGAAACACTCTTTTTATAGTATCTGGATGTGGACATTTTGAGCGCTTTCAGGC
          1 ATAGAGCAGGTTTGAAACACTCTTTTTGTAGTATCTGGAAGTGGATATTTGGATAGCTTTGAGG-

            * * *    *                 *** *   *                          *  
     168367 CTATGGTT-TAAA-AGGAAATATCTTCCCCTGAAAACTAGACAGAAGCATTCTCAGAAACTTATT
         65 ATTTCGTTGGAAACAGG-AATATCTTCATATAAAAGCTAGACAGAAGCATTCTCAGAAACTTCTT

                    **  **    *       *            *   *
     168430 TGTGATGTGCGCCCTCAACTAACAGTATTGAAGCATT-CTTTTG
        129 TGTGATGTTTGCATTCAAGTAACAGTGTTGAA-CATTCCCTTTC

                  *  *                   * *     *             **            
     168473 ATAGAGTAGTTTTGAAACACTCTTTTTGTGGAATCTGCAAGTGGATATTTGTCTAGCTTTGAGGA
          1 ATAGAGCAGGTTTGAAACACTCTTTTTGTAGTATCTGGAAGTGGATATTTGGATAGCTTTGAGGA

                         *   *    *                   *       *              
     168538 TTTCGTTGGAAACGGGATTA-CAT-ATA-AAAAGC-AGACAGCAGCATTCCCAGAAACTTCTTTG
         66 TTTCGTTGGAAACAGGAATATCTTCATATAAAAGCTAGACAGAAGCATTCTCAGAAACTTCTTTG

                              *    *  *              
     168599 TGATGTTTGCATTCAAGTCACAGAGTGGAACATTCCCTTTC
        131 TGATGTTTGCATTCAAGTAACAGTGTTGAACATTCCCTTTC

                                                   *     *       **     *   *
     168640 ATAGAGCAGGTTTGAAACACTCTTTTTGTAGTATCTGGATGTGGACATTTGGAGCGCTTTCAGGC
          1 ATAGAGCAGGTTTGAAACACTCTTTTTGTAGTATCTGGAAGTGGATATTTGGATAGCTTTGAGGA

            * ** *      *             *** *   *                      *   *   
     168705 CTATGGT-GAAAAAGGAAATATCTTCCCCTGAAAACTAGACAGAAGCATTCTCAGAATCTTATTT
         66 TTTCGTTGGAAACAGG-AATATCTTCATATAAAAGCTAGACAGAAGCATTCTCAGAAACTTCTTT

                   **  **    *             **  * *   *
     168769 GTGATGTGCGCCCTCAACTAACAGTGTTGAAGCTTTCTTTTG
        130 GTGATGTTTGCATTCAAGTAACAGTGTTGAACATTCCCTTTC

                     *                    **     *   *                       
     168811 ATAGAGCAGTTTTGAAACACTCTTTTTGTAAAATCTGCAAGAGGATATTTGGATAGCTTTGAGGA
          1 ATAGAGCAGGTTTGAAACACTCTTTTTGTAGTATCTGGAAGTGGATATTTGGATAGCTTTGAGGA

                         *   * *            **                      *    *   
     168876 TTTCGTTGGAAACGGGATTGTCTTCATATAAACTCTAGACAGAAGCATTCTCAGAAGCTTCATTG
         66 TTTCGTTGGAAACAGGAATATCTTCATATAAAAGCTAGACAGAAGCATTCTCAGAAACTTCTTTG

            *       **   *    *             *        
     168941 GGATGTTTCAATTGAAGTCACAGTGTTGAACAGTCCCTTTC
        131 TGATGTTTGCATTCAAGTAACAGTGTTGAACATTCCCTTTC

                                                         *       **   * *    
     168982 ATAGAGCAGGTTTGAAACACTCTTTTTGTAGTATCTGGAAGTGGACATTTGGAGCGCTCTCAGGA
          1 ATAGAGCAGGTTTGAAACACTCTTTTTGTAGTATCTGGAAGTGGATATTTGGATAGCTTTGAGGA

            * *  *      *             **          * *       * *          *  *
     169047 CTGCGGT-GAAAAAGGAAATATCTTCCAATAAAAGCTACATAGAAGCAATGTCAGAAACTTTTTC
         66 TTTCGTTGGAAACAGG-AATATCTTCATATAAAAGCTAGACAGAAGCATTCTCAGAAACTTCTTT

            *      * ** *   **      *       *        *
     169111 ATGATGTATCTACTCAGCTAACAGAGTTGAACCTT-CCTTTG
        130 GTGATGTTTGCATTCAAGTAACAGTGTTGAACATTCCCTTTC

             *       *                   * *     *             **            
     169152 AGAGAGCAGTTTTGAAACACTCTTTTTGTGGAATCTGCAAGTGGATATTTGTCTAGCTTTGAGGA
          1 ATAGAGCAGGTTTGAAACACTCTTTTTGTAGTATCTGGAAGTGGATATTTGGATAGCTTTGAGGA

                         *   *    *                   *       *              
     169217 TTTCGTTGGAAACGGGATTA-CAT-ATA-AAAAGC-AGACAGCAGCATTCCCAGAAACTTCTTTG
         66 TTTCGTTGGAAACAGGAATATCTTCATATAAAAGCTAGACAGAAGCATTCTCAGAAACTTCTTTG

                              *    *                 
     169278 TGATGTTTGCATTCAAGTCACAGAGTTGAACATTCCCTTTC
        131 TGATGTTTGCATTCAAGTAACAGTGTTGAACATTCCCTTTC

                                                   *     *       **     *   *
     169319 ATAGAGCAGGTTTGAAACACTCTTTTTGTAGTATCTGGATGTGGACATTTGGAGCGCTTTCAGGC
          1 ATAGAGCAGGTTTGAAACACTCTTTTTGTAGTATCTGGAAGTGGATATTTGGATAGCTTTGAGGA

            * ** *      *             *** *   *                      *   *   
     169384 CTATGGT-GAAAAAGGAAATATCTTCCCCTGAAAACTAGACAGAAGCATTCTCAGAATCTTATTT
         66 TTTCGTTGGAAACAGG-AATATCTTCATATAAAAGCTAGACAGAAGCATTCTCAGAAACTTCTTT

                   **  **    *             **  * *   *
     169448 GTGATGTGCGCCCTCAACTAACAGTGTTGAAGCTTTCTTTTG
        130 GTGATGTTTGCATTCAAGTAACAGTGTTGAACATTCCCTTTC

                     *                    **     *   *                       
     169490 ATAGAGCAGTTTTGAAACACTCTTTTTGTAAAATCTGCAAGCGGATATTTGGATAGCTTTGAGGA
          1 ATAGAGCAGGTTTGAAACACTCTTTTTGTAGTATCTGGAAGTGGATATTTGGATAGCTTTGAGGA

                         *   * * *          **                      *    *   
     169555 TTTCGTTGGAAACGGGATTGTATTCATATAAACTCTAGACAGAAGCATTCTCAGAAGCTTCATTG
         66 TTTCGTTGGAAACAGGAATATCTTCATATAAAAGCTAGACAGAAGCATTCTCAGAAACTTCTTTG

            *             *    *             *        
     169620 GGATGTTT-CAGTTGAAGTCACAGTGTTGAACAGTCCCTTTC
        131 TGATGTTTGCA-TTCAAGTAACAGTGTTGAACATTCCCTTTC

                               *                         *       **   * *    
     169661 ATAGAGCAGGTTTGAAACAGTCTTTTTGTAGTATCTGGAAGTGGACATTTGGAGCGCTCTCAGGA
          1 ATAGAGCAGGTTTGAAACACTCTTTTTGTAGTATCTGGAAGTGGATATTTGGATAGCTTTGAGGA

            * *  *      *             **            *       * *          *  *
     169726 CTGCGGT-GAAAAAGGAAATATCTTCCAATAAAAGCTAGATAGAAGCAATGTCAGAAACTTTTTC
         66 TTTCGTTGGAAACAGG-AATATCTTCATATAAAAGCTAGACAGAAGCATTCTCAGAAACTTCTTT

            *      * ** *   **      *       *        *
     169790 ATGATGTATCTACTCAGCTAACAGAGTTGAACCTT-CCTTTG
        130 GTGATGTTTGCATTCAAGTAACAGTGTTGAACATTCCCTTTC

             *       *                   * *     *             **            
     169831 AGAGAGCAGTTTTGAAACACTCTTTTTGTGGAATCTGCAAGTGGATATTTGTCTAGCTTTGAGGA
          1 ATAGAGCAGGTTTGAAACACTCTTTTTGTAGTATCTGGAAGTGGATATTTGGATAGCTTTGAGGA

                         *   *    *  *                *       *              
     169896 TTTCGTTGGAAACGGGATTA-CAT-GTA-AAAAGC-AGACAGCAGCATTCCCAGAAACTTCTTTG
         66 TTTCGTTGGAAACAGGAATATCTTCATATAAAAGCTAGACAGAAGCATTCTCAGAAACTTCTTTG

                              *    *                 
     169957 TGATGTTTGCATTCAAGTCACAGAGTTGAACATTCCCTTTC
        131 TGATGTTTGCATTCAAGTAACAGTGTTGAACATTCCCTTTC

                                                   *     *     * **     *   *
     169998 ATAGAGCAGGTTTGAAACACTCTTTTTGTAGTATCTGGATGTGGACATTTGCAGCGCTTTCAGGC
          1 ATAGAGCAGGTTTGAAACACTCTTTTTGTAGTATCTGGAAGTGGATATTTGGATAGCTTTGAGGA

            * ** *      *             *** *   *                          *   
     170063 CTAAGGT-GAAAAAGGAAATATCTTCCCCTGAAAACTAGACAGAAGCATTCTCAGAAACTTATTT
         66 TTTCGTTGGAAACAGG-AATATCTTCATATAAAAGCTAGACAGAAGCATTCTCAGAAACTTCTTT

                   **  **    *             **  * *   *
     170127 GTGATGTGCGCCCTCAACTAACAGTGTTGAAGCTTTCTTTTG
        130 GTGATGTTTGCATTCAAGTAACAGTGTTGAACATTCCCTTTC

                     *                   * *                   **            
     170169 ATAGAGCAGTTTTGAAACACTCTTTTTGTGGAATCTGGAAGTGGATATTTGTCTAGCTTTGAGGA
          1 ATAGAGCAGGTTTGAAACACTCTTTTTGTAGTATCTGGAAGTGGATATTTGGATAGCTTTGAGGA

                         *   *    *                   *       *     *   *    
     170234 TTTCGTTGGAAACGGGATTA-CAT-ATA-AAAAGC-AGACAGCAGCATTCCCAGAATCTTGTTTG
         66 TTTCGTTGGAAACAGGAATATCTTCATATAAAAGCTAGACAGAAGCATTCTCAGAAACTTCTTTG

                              *    *                 
     170295 TGATGTTTGCATTCAAGTCACAGAGTTGAACATTCCCTTTC
        131 TGATGTTTGCATTCAAGTAACAGTGTTGAACATTCCCTTTC

             *                         *           *     *       **     *   *
     170336 AGAGAGCAGGTTTGAAACACTCTTTTTATAGTATCTGGATGTGGACATTTGGAGCGCTTTCAGGC
          1 ATAGAGCAGGTTTGAAACACTCTTTTTGTAGTATCTGGAAGTGGATATTTGGATAGCTTTGAGGA

            * ** *      *             *** *   *                      *   *   
     170401 CTATGGT-GAAAAAGGAAATATCTTCTCCTGAAAACTAGACAGAAGCATTCTCAGAATCTTATTT
         66 TTTCGTTGGAAACAGG-AATATCTTCATATAAAAGCTAGACAGAAGCATTCTCAGAAACTTCTTT

                   **  **    *             **  * *   *
     170465 GTGATGTGCGCCCTCAACTAACAGTGTTGAAGCTTTCTTTTG
        130 GTGATGTTTGCATTCAAGTAACAGTGTTGAACATTCCCTTTC

                     *     *    *     *   **     *   *        *              
     170507 ATAGAGCAGTTTTGAGACACACTTTTCGTAAAATCTGCAAGAGGATATTTTGATAGCTTTGAGGA
          1 ATAGAGCAGGTTTGAAACACTCTTTTTGTAGTATCTGGAAGTGGATATTTGGATAGCTTTGAGGA

                         *   * *            **                      *    *   
     170572 TTTCGTTGGAAACGGGATTGTCTTCATATAAACTCTAGACAGAAGCATTCTCAGAAGCTTCATTG
         66 TTTCGTTGGAAACAGGAATATCTTCATATAAAAGCTAGACAGAAGCATTCTCAGAAACTTCTTTG

            *       **   *    *             *        
     170637 GGATGTTTCAATTGAAGTCACAGTGTTGAACAGTCCCTTTC
        131 TGATGTTTGCATTCAAGTAACAGTGTTGAACATTCCCTTTC

                                                         *       *  * * *    
     170678 ATAGAGCAGGTTTGAAACACTCTTTTTGTAGTATCTGGAAGTGGACATTTGGAGAGATCTCAGGA
          1 ATAGAGCAGGTTTGAAACACTCTTTTTGTAGTATCTGGAAGTGGATATTTGGATAGCTTTGAGGA

            * *  *    * *             **            *       * *          *  *
     170743 ATACGGT-GATAAAGGAAATATCTTCCAATAAAAGCTAGATAGAAGCAATGTCAGAAACTTTTTC
         66 TTTCGTTGGAAACAGG-AATATCTTCATATAAAAGCTAGACAGAAGCATTCTCAGAAACTTCTTT

            *  *   * ** *   **      *       *  * *   *
     170807 ATGTTGTATCTACTCAGCTAACAGAGTTGAACCTTTCTTTTG
        130 GTGATGTTTGCATTCAAGTAACAGTGTTGAACATTCCCTTTC

             *       *                   * *                   **            
     170849 AGAGAGCAGTTTTGAAACACTCTTTTTGTGGAATCTGGAAGTGGATATTTGTCTAGCTTTGAGGA
          1 ATAGAGCAGGTTTGAAACACTCTTTTTGTAGTATCTGGAAGTGGATATTTGGATAGCTTTGAGGA

                         *   *    *                   *       *              
     170914 TTTCGTTGGAAACGGGATTA-CAT-ATA-AAAAGC-AGACAGCAGCATTCCCAGAAACTTCTTTG
         66 TTTCGTTGGAAACAGGAATATCTTCATATAAAAGCTAGACAGAAGCATTCTCAGAAACTTCTTTG

                              *    *                 
     170975 TGATGTTTGCATTCAAGTCACAGAGTTGAACATTCCCTTTC
        131 TGATGTTTGCATTCAAGTAACAGTGTTGAACATTCCCTTTC

                                                   *     *       **     *   *
     171016 ATAGAGCAGGTTTGAAACACTCTTTTTGTAGTATCTGGATGTGGACATTTGGAGCGCTTTCAGGC
          1 ATAGAGCAGGTTTGAAACACTCTTTTTGTAGTATCTGGAAGTGGATATTTGGATAGCTTTGAGGA

            * ** *      *             *** *   *                      *   *   
     171081 CTATGGT-GAAAAAGGAAATATCTTCCCCTGAAAACTAGACAGAAGCATTCTCAGAATCTTATTT
         66 TTTCGTTGGAAACAGG-AATATCTTCATATAAAAGCTAGACAGAAGCATTCTCAGAAACTTCTTT

                   **  **    *             **  * *   *
     171145 GTGATGTGCGCCCTCAACTAACAGTGTTGAAGCTTTCTTTTG
        130 GTGATGTTTGCATTCAAGTAACAGTGTTGAACATTCCCTTTC

                     *        *           **     *   *              *        
     171187 ATAGAGCAGTTTTGAAACCCTCTTTTTGTAAAATCTGCAAGAGGATATTTGGATAGATTTGAGGA
          1 ATAGAGCAGGTTTGAAACACTCTTTTTGTAGTATCTGGAAGTGGATATTTGGATAGCTTTGAGGA

                         *   * *            **                      *    *   
     171252 TTTCGTTGGAAACGGGATTGTCTTCATATAAACTCTAGACAGAAGCATTCTCAGAAGCTTCATTG
         66 TTTCGTTGGAAACAGGAATATCTTCATATAAAAGCTAGACAGAAGCATTCTCAGAAACTTCTTTG

            *       **   *    *             *        
     171317 GGATGTTTCAATTGAAGTCACAGTGTTGAACAGTCCCTTTC
        131 TGATGTTTGCATTCAAGTAACAGTGTTGAACATTCCCTTTC

                                                         *       **   * *    
     171358 ATAGAGCAGGTTTGAAACACTCTTTTTGTAGTATCTGGAAGTGGACATTTGGAGCGCTCTCAGGA
          1 ATAGAGCAGGTTTGAAACACTCTTTTTGTAGTATCTGGAAGTGGATATTTGGATAGCTTTGAGGA

            * *  *      *             **            *       * *          *  *
     171423 CTGCGGT-GAAAAAGGAAATATCTTCCAATAAAAGCTAGATAGAAGCAATGTCAGAAACTTTTTC
         66 TTTCGTTGGAAACAGG-AATATCTTCATATAAAAGCTAGACAGAAGCATTCTCAGAAACTTCTTT

            *      * ** *   **      *          *     *
     171487 ATGATGTATCTACTCAGCTAACAGAGTTGAAC-TTTCCTTTG
        130 GTGATGTTTGCATTCAAGTAACAGTGTTGAACATTCCCTTTC

             *       *                   * *     *             **            
     171528 AGAGAGCAGTTTTGAAACACTCTTTTTGTGGAATCTGCAAGTGGATATTTGTCTAGCTTTGAGGA
          1 ATAGAGCAGGTTTGAAACACTCTTTTTGTAGTATCTGGAAGTGGATATTTGGATAGCTTTGAGGA

                *        *   *    *               *   *   *   *              
     171593 TTTCTTTGGAAACGGGATTA-CAT-ATA-AAAAGC-AGCCAGCAGCGTTCCCAGAAACTTCTTTG
         66 TTTCGTTGGAAACAGGAATATCTTCATATAAAAGCTAGACAGAAGCATTCTCAGAAACTTCTTTG

                             **    *                 
     171654 TGATGTTTGCATTCAAGCCACAGAGTTGAACATTCCCTTTC
        131 TGATGTTTGCATTCAAGTAACAGTGTTGAACATTCCCTTTC

                                                  **     *     * **     *   *
     171695 ATAGAGCAGGTTTGAAACACTCTTTTTGTAGTATCTGGTTGTGGACATTTGCAGCGCTTTCAGGC
          1 ATAGAGCAGGTTTGAAACACTCTTTTTGTAGTATCTGGAAGTGGATATTTGGATAGCTTTGAGGA

            * ** *      *             *** *   *      *  *                *   
     171760 CTAAGGT-GAAAAAGGAAATATCTTCCCCTGAAAACTAGACCGACGCATTCTCAGAAACTTATTT
         66 TTTCGTTGGAAACAGG-AATATCTTCATATAAAAGCTAGACAGAAGCATTCTCAGAAACTTCTTT

                   **  **    *             **  * *   *
     171824 GTGATGTGCGCCCTCAACTAACAGTGTTGAAGCTTTCTTTTG
        130 GTGATGTTTGCATTCAAGTAACAGTGTTGAACATTCCCTTTC

                     *                    *      *   *                       
     171866 ATAGAGCAGTTTTGAAACACTCTTTTTGTAATATCTGCAAGAGGATATTTGGATAGCTTTGAGGA
          1 ATAGAGCAGGTTTGAAACACTCTTTTTGTAGTATCTGGAAGTGGATATTTGGATAGCTTTGAGGA

                         *   * *            **                      *    *   
     171931 TTTCGTTGGAAACGGGATTGTCTTCATATAAACTCTAGACAGAAGCATTCTCAGAAGCTTCATTG
         66 TTTCGTTGGAAACAGGAATATCTTCATATAAAAGCTAGACAGAAGCATTCTCAGAAACTTCTTTG

            *       **   *    *             * *      
     171996 GGATGTTTCAATTGAAGTCACAGTGTTGAACAGTTCCTTTC
        131 TGATGTTTGCATTCAAGTAACAGTGTTGAACATTCCCTTTC

                 *                                       *       **   * *    
     172037 ATAGAACAGGTTTGAAACACTCTTTTTGTAGTATCTGGAAGTGGACATTTGGAGCGCTCTCAGGA
          1 ATAGAGCAGGTTTGAAACACTCTTTTTGTAGTATCTGGAAGTGGATATTTGGATAGCTTTGAGGA

            * ** *      *             **          * *       * *          *  *
     172102 CTATGGT-GAAAAAGGAAATATCTTCCAATAAAAGCTACATAGAAGCAATGTCAGAAACTTTTTC
         66 TTTCGTTGGAAACAGG-AATATCTTCATATAAAAGCTAGACAGAAGCATTCTCAGAAACTTCTTT

            *      * ** *   **      *       *  *     *
     172166 ATGATGTATCTACTCAGCTAACAGAGTTGAACCTTTCCTTTG
        130 GTGATGTTTGCATTCAAGTAACAGTGTTGAACATTCCCTTTC

             *       *                   * *     *             **            
     172208 AGAGAGCAGTTTTGAAACACTCTTTTTGTGGAATCTGCAAGTGGATATTTGTCTAGCTTTGAGGA
          1 ATAGAGCAGGTTTGAAACACTCTTTTTGTAGTATCTGGAAGTGGATATTTGGATAGCTTTGAGGA

                         *   *    *                   *       *   *          
     172273 TTTCGTTGGAAACGGGATTA-CAT-ATA-AAAAGC-AGACAGCAGCATTCCCAGTAACTTCTTTG
         66 TTTCGTTGGAAACAGGAATATCTTCATATAAAAGCTAGACAGAAGCATTCTCAGAAACTTCTTTG

                              *    *          *      
     172334 TGATGTTTGCATTCAAGTCACAGAGTTGAACATTGCCTTTC
        131 TGATGTTTGCATTCAAGTAACAGTGTTGAACATTCCCTTTC

                                                *  *     *     * **     *   *
     172375 ATAGAGCAGGTTTGAAACACTCTTTTTGTAGTATCTAGATGTGGACATTTGCAGCGCTTTCAGGC
          1 ATAGAGCAGGTTTGAAACACTCTTTTTGTAGTATCTGGAAGTGGATATTTGGATAGCTTTGAGGA

            * ** *      *             *** *   *                      *   *   
     172440 CTGTGGT-GAAAAAGGAAATATCTTCTCCTGAAAACTAGACAGAAGCATTCTCAGAATCTTATTT
         66 TTTCGTTGGAAACAGG-AATATCTTCATATAAAAGCTAGACAGAAGCATTCTCAGAAACTTCTTT

                   **  **    *             **  * *   *
     172504 GTGATGTGCGCCCTCAACTAACAGTGTTGAAGCTTTCTTTTG
        130 GTGATGTTTGCATTCAAGTAACAGTGTTGAACATTCCCTTTC

                     *                    **     *   *        *              
     172546 ATAGAGCAGTTTTGAAACACTCTTTTTGTAAAATCTGCAAGAGGATATTTCGATAGCTTTGAGGA
          1 ATAGAGCAGGTTTGAAACACTCTTTTTGTAGTATCTGGAAGTGGATATTTGGATAGCTTTGAGGA

                *        *   * *            **                      *    *   
     172611 TTTCATTGGAAACGGGATTGTCTTCATATAAACTCTAGACAGAAGCATTCTCAGAAGCTTCATTG
         66 TTTCGTTGGAAACAGGAATATCTTCATATAAAAGCTAGACAGAAGCATTCTCAGAAACTTCTTTG

            *       **   *    *             *        
     172676 GGATGTTTCAATTGAAGTCACAGTGTTGAACAGTCCCTTTC
        131 TGATGTTTGCATTCAAGTAACAGTGTTGAACATTCCCTTTC

                                                      *  *       **   * *    
     172717 ATAGAGCAGGTTTGAAACACTCTTTTTGTAGTATCTGGAAGTTGACATTTGGAGCGCTCTCAGGA
          1 ATAGAGCAGGTTTGAAACACTCTTTTTGTAGTATCTGGAAGTGGATATTTGGATAGCTTTGAGGA

            * *  *      *             **            *       * *          *  *
     172782 CTACGGT-GAAAAAGGAAATATCTTCCAATAAAAGCTAGATAGAAGCAATGTCAGAAACTTTTTC
         66 TTTCGTTGGAAACAGG-AATATCTTCATATAAAAGCTAGACAGAAGCATTCTCAGAAACTTCTTT

            *      * ** *   **      *       *  * *   *
     172846 ATGATGTATCTACTCAGCTAACAGAGTTGAACCTTTCTTTTG
        130 GTGATGTTTGCATTCAAGTAACAGTGTTGAACATTCCCTTTC

             *       *                   * *                   **            
     172888 AGAGAGCAGTTTTGAAACACTCTTTTTGTGGAATCTGGAAGTGGATATTTGTCTAGCTTTGAGGA
          1 ATAGAGCAGGTTTGAAACACTCTTTTTGTAGTATCTGGAAGTGGATATTTGGATAGCTTTGAGGA

                         *   *    *                   *       *   *          
     172953 TTTCGTTGGAAACGGGATTA-CAT-ATA-AAAAGC-AGACAGCAGCATTCCCAGTAACTTCTTTG
         66 TTTCGTTGGAAACAGGAATATCTTCATATAAAAGCTAGACAGAAGCATTCTCAGAAACTTCTTTG

                              *    *                 
     173014 TGATGTTTGCATTCAAGTCACAGAGTTGAACATTCCCTTTC
        131 TGATGTTTGCATTCAAGTAACAGTGTTGAACATTCCCTTTC

                                                   *     *       **     *   *
     173055 ATAGAGCAGGTTTGAAACACTCTTTTTGTAGTATCTGGATGTGGACATTTGGAGCGCTTTCAGGC
          1 ATAGAGCAGGTTTGAAACACTCTTTTTGTAGTATCTGGAAGTGGATATTTGGATAGCTTTGAGGA

            * ** *      *             *** *   *                          *   
     173120 CTATGGT-GAAAAAGGAAATATCTTCCCCTGAAAACTAGACAGAAGCATTCTCAGAAACTTATTT
         66 TTTCGTTGGAAACAGG-AATATCTTCATATAAAAGCTAGACAGAAGCATTCTCAGAAACTTCTTT

                   **  **    *             **  * *   *
     173184 GTGATGTGCGCCCTCAACTAACAGTGTTGAAGCTTTCTTTTG
        130 GTGATGTTTGCATTCAAGTAACAGTGTTGAACATTCCCTTTC

                     *                    *      *   *                       
     173226 ATAGAGCAGTTTTGAAACACTCTTTTTGTAATATCTGCAAGAGGATATTTGGATAGCTTTGAGGA
          1 ATAGAGCAGGTTTGAAACACTCTTTTTGTAGTATCTGGAAGTGGATATTTGGATAGCTTTGAGGA

                         *   * *            **                      *    *   
     173291 TTTCGTTGGAAACGGGATTGTCTTCATATAAACTCTAGACAGAAGCATTCTCAGAAGCTTCATTG
         66 TTTCGTTGGAAACAGGAATATCTTCATATAAAAGCTAGACAGAAGCATTCTCAGAAACTTCTTTG

            *       **   *    *             *        
     173356 GGATGTTTCAATTGAAGTCACAGTGTTGAACAGTCCCTTTC
        131 TGATGTTTGCATTCAAGTAACAGTGTTGAACATTCCCTTTC

                                                         *       **   * *    
     173397 ATAGAGCAGGTTTGAAACACTCTTTTTGTAGTATCTGGAAGTGGACATTTGGAGCGCTCTCAGGA
          1 ATAGAGCAGGTTTGAAACACTCTTTTTGTAGTATCTGGAAGTGGATATTTGGATAGCTTTGAGGA

            * *  *    * *             **            *       * *          *  *
     173462 CTACGGT-GATAAAGGAAATATCTTCCAATAAAAGCTAGATAGAAGCAATGTCAGAAACTTTTTC
         66 TTTCGTTGGAAACAGG-AATATCTTCATATAAAAGCTAGACAGAAGCATTCTCAGAAACTTCTTT

            *      * ** *   **      *       *  * *   *
     173526 ATGATGTATCTACTCAGCTAACAGAGTTGAACCTTTCTTTTG
        130 GTGATGTTTGCATTCAAGTAACAGTGTTGAACATTCCCTTTC

             *       *                   * *     *             **            
     173568 AGAGAGCAGTTTTGAAACACTCTTTTTGTGGAATCTGCAAGTGGATATTTGTCTAGCTTTGAGGA
          1 ATAGAGCAGGTTTGAAACACTCTTTTTGTAGTATCTGGAAGTGGATATTTGGATAGCTTTGAGGA

                         *   *    *                *  *       *     *        
     173633 TTTCGTTGGAAACGGGATTA-CAT-ATA-AAAAGC-AGATAGCAGCATTCCCAGAATCTTCTTTG
         66 TTTCGTTGGAAACAGGAATATCTTCATATAAAAGCTAGACAGAAGCATTCTCAGAAACTTCTTTG

                              *    *                 
     173694 TGATGTTTGCATTCAAGTCACAGAGTTGAACATTCCCTTTC
        131 TGATGTTTGCATTCAAGTAACAGTGTTGAACATTCCCTTTC

                                                   *     *       **     *   *
     173735 ATAGAGCAGGTTTGAAACACTCTTTTTGTAGTATCTGGATGTGGACATTTGGAGCGCTTTCAGGC
          1 ATAGAGCAGGTTTGAAACACTCTTTTTGTAGTATCTGGAAGTGGATATTTGGATAGCTTTGAGGA

            * ** *      *             *** *   *                      *   *   
     173800 CTATGGT-GAAAAAGGAAATATCTTCTCCTGAAAACTAGACAGAAGCATTCTCAGAATCTTATTT
         66 TTTCGTTGGAAACAGG-AATATCTTCATATAAAAGCTAGACAGAAGCATTCTCAGAAACTTCTTT

                   **  **    *             **  * *   *
     173864 GTGATGTGCGCCCTCAACTAACAGTGTTGAAGCTTTCTTTTG
        130 GTGATGTTTGCATTCAAGTAACAGTGTTGAACATTCCCTTTC

                     *                    **     *   *                       
     173906 ATAGAGCAGTTTTGAAACACTCTTTTTGTAAAATCTGCAAGAGGATATTTGGATAGCTTTGAGGA
          1 ATAGAGCAGGTTTGAAACACTCTTTTTGTAGTATCTGGAAGTGGATATTTGGATAGCTTTGAGGA

                *        *   * *            **                      *    *   
     173971 TTTCATTGGAAACGGGATTGTCTTCATATAAACTCTAGACAGAAGCATTCTCAGAAGCTTCATTG
         66 TTTCGTTGGAAACAGGAATATCTTCATATAAAAGCTAGACAGAAGCATTCTCAGAAACTTCTTTG

            *       **   *    *             *        
     174036 GGATGTTTCAATTGAAGTCACAGTGTTGAACAGTCCCTTTC
        131 TGATGTTTGCATTCAAGTAACAGTGTTGAACATTCCCTTTC

                               *                      *  *       **   * *    
     174077 ATAGAGCAGGTTTGAAACAGTCTTTTTGTAGTATCTGGAAGTTGACATTTGGAGCGCTCTCAGGA
          1 ATAGAGCAGGTTTGAAACACTCTTTTTGTAGTATCTGGAAGTGGATATTTGGATAGCTTTGAGGA

            * *  *      *             **            *       * *          *  *
     174142 CTACGGT-GAAAAAGGAAATATCTTCCAATAAAAGCTAGATAGAAGCAATGTCAGAAACTTTTTC
         66 TTTCGTTGGAAACAGG-AATATCTTCATATAAAAGCTAGACAGAAGCATTCTCAGAAACTTCTTT

            *      * ** *   **      *       *  * *   *
     174206 ATGATGTATCTACTCAGCTAACAGAGTTGAACCTTTCTTTTG
        130 GTGATGTTTGCATTCAAGTAACAGTGTTGAACATTCCCTTTC

             *       *                   * *                   **            
     174248 AGAGAGCAGTTTTGAAACACTCTTTTTGTGGAATCTGGAAGTGGATATTTGTCTAGCTTTGAGGA
          1 ATAGAGCAGGTTTGAAACACTCTTTTTGTAGTATCTGGAAGTGGATATTTGGATAGCTTTGAGGA

                         *   *    *                   *       *   *          
     174313 TTTCGTTGGAAACGGGATTA-CAT-ATA-AAAAGC-AGACAGCAGCATTCCCAGTAACTTCTTTG
         66 TTTCGTTGGAAACAGGAATATCTTCATATAAAAGCTAGACAGAAGCATTCTCAGAAACTTCTTTG

                              *    *                 
     174374 TGATGTTTGCATTCAAGTCACAGAGTTGAACATTCCCTTTC
        131 TGATGTTTGCATTCAAGTAACAGTGTTGAACATTCCCTTTC

                                                   *     *       **     *   *
     174415 ATAGAGCAGGTTTGAAACACTCTTTTTGTAGTATCTGGATGTGGACATTTGGAGCGCTTTCAGGC
          1 ATAGAGCAGGTTTGAAACACTCTTTTTGTAGTATCTGGAAGTGGATATTTGGATAGCTTTGAGGA

            * ** *      *             *** *   *                      *   *   
     174480 CTATGGT-GAAAAAGGAAATATCTTCCCCTGAAAACTAGACAGAAGCATTCTCAGAATCTTATTT
         66 TTTCGTTGGAAACAGG-AATATCTTCATATAAAAGCTAGACAGAAGCATTCTCAGAAACTTCTTT

                   **  **    *             **  * *   *
     174544 GTGATGTGCGCCCTCAACTAACAGTGTTGAAGCTTTCTTTTG
        130 GTGATGTTTGCATTCAAGTAACAGTGTTGAACATTCCCTTTC

                     *                    **     *   *                       
     174586 ATAGAGCAGTTTTGAAACACTCTTTTTGTAAAATCTGCAAGAGGATATTTGGATAGCTTTGAGGA
          1 ATAGAGCAGGTTTGAAACACTCTTTTTGTAGTATCTGGAAGTGGATATTTGGATAGCTTTGAGGA

                         *   * *            **                      *    *   
     174651 TTTCGTTGGAAACGGGATTGTCTTCATATAAACTCTAGACAGAAGCATTCTCAGAAGCTTCATTG
         66 TTTCGTTGGAAACAGGAATATCTTCATATAAAAGCTAGACAGAAGCATTCTCAGAAACTTCTTTG

            *       **   *    *             *        
     174716 GGATGTTTCAATTGAAGTCACAGTGTTGAACAGTCCCTTTC
        131 TGATGTTTGCATTCAAGTAACAGTGTTGAACATTCCCTTTC

                                                       * *       **   * *    
     174757 ATAGAGCAGGTTTGAAACACTCTTTTTGTAGTATCTGGAAGTGCACATTTGGAGCGCTCTCAGGA
          1 ATAGAGCAGGTTTGAAACACTCTTTTTGTAGTATCTGGAAGTGGATATTTGGATAGCTTTGAGGA

            * *  *      *             **            *       * *          *  *
     174822 CTGCGGT-GAAAAAGGAAATATCTTCCAATAAAAGCTAGATAGAAGCAATGTCAGAAACTTTTTC
         66 TTTCGTTGGAAACAGG-AATATCTTCATATAAAAGCTAGACAGAAGCATTCTCAGAAACTTCTTT

            *      * ** *   **      *       *        *
     174886 ATGATGTATCTACTCAGCTAACAGAGTTGAACCTT-CCTTTG
        130 GTGATGTTTGCATTCAAGTAACAGTGTTGAACATTCCCTTTC

             *       *                   * *     *             **            
     174927 AGAGAGCAGTTTTGAAACACTCTTTTTGTGGAATCTGCAAGTGGATATTTGTCTAGCTTTGAGGA
          1 ATAGAGCAGGTTTGAAACACTCTTTTTGTAGTATCTGGAAGTGGATATTTGGATAGCTTTGAGGA

                         *   *    *                   *       *              
     174992 TTTCGTTGGAAACGGGATTA-CAT-ATA-AAAAGC-AGACAGCAGCATTCCCAGAAACTTCTTTG
         66 TTTCGTTGGAAACAGGAATATCTTCATATAAAAGCTAGACAGAAGCATTCTCAGAAACTTCTTTG

                              *    *                 
     175053 TGATGTTTGCATTCAAGTCACAGAGTTGAACATTCCCTTTC
        131 TGATGTTTGCATTCAAGTAACAGTGTTGAACATTCCCTTTC

                                                   *     *       **     *   *
     175094 ATAGAGCAGGTTTGAAACACTCTTTTTGTAGTATCTGGATGTGGACATTTGGAGCGCTTTCAGGC
          1 ATAGAGCAGGTTTGAAACACTCTTTTTGTAGTATCTGGAAGTGGATATTTGGATAGCTTTGAGGA

            * ** *      *             *** *   *                      *   *   
     175159 CTATGGT-GAAAGAGGAAATATCTTCCCCTGAAAACTAGACAGAAGCATTCTCAGAATCTTATTT
         66 TTTCGTTGGAAACAGG-AATATCTTCATATAAAAGCTAGACAGAAGCATTCTCAGAAACTTCTTT

                   **  **    *             **  * *   *
     175223 GTGATGTGCGCCCTCAACTAACAGTGTTGAAGCTTTCTTTTG
        130 GTGATGTTTGCATTCAAGTAACAGTGTTGAACATTCCCTTTC

                     *                    **     *   *                       
     175265 ATAGAGCAGTTTTGAAACACTCTTTTTGTAAAATCTGCAAGAGGATATTTGGATAGCTTTGAGGA
          1 ATAGAGCAGGTTTGAAACACTCTTTTTGTAGTATCTGGAAGTGGATATTTGGATAGCTTTGAGGA

                         *   * *            **                      *    *   
     175330 TTTCGTTGGAAACGGGATTGTCTTCATATAAACTCTAGACAGAAGCATTCTCAGAAGCTTCATTG
         66 TTTCGTTGGAAACAGGAATATCTTCATATAAAAGCTAGACAGAAGCATTCTCAGAAACTTCTTTG

            *       **   *    *             *        
     175395 GGATGTTTCAATTGAAGTCACAGTGTTGAACAGTCCCTTTC
        131 TGATGTTTGCATTCAAGTAACAGTGTTGAACATTCCCTTTC

                                                         *       **   * *    
     175436 ATAGAGCAGGTTTGAAACACTCTTTTTGTAGTATCTGGAAGTGGACATTTGGAGCGCTCTCAGGA
          1 ATAGAGCAGGTTTGAAACACTCTTTTTGTAGTATCTGGAAGTGGATATTTGGATAGCTTTGAGGA

            * *  *      *             **          * *       * *          *  *
     175501 CTGCGGT-GAAAAAGGAAATATCTTCCAATAAAAGCTACATAGAAGCAATGTCAGAAACTTTTTC
         66 TTTCGTTGGAAACAGG-AATATCTTCATATAAAAGCTAGACAGAAGCATTCTCAGAAACTTCTTT

            *      * ** *   **      *       *        *
     175565 ATGATGTATCTACTCAGCTAACAGAGTTGAACCTT-CCTTTG
        130 GTGATGTTTGCATTCAAGTAACAGTGTTGAACATTCCCTTTC

             *       *                   * *     *             **            
     175606 AGAGAGCAGTTTTGAAACACTCTTTTTGTGGAATCTGCAAGTGGATATTTGTCTAGCTTTGAGGA
          1 ATAGAGCAGGTTTGAAACACTCTTTTTGTAGTATCTGGAAGTGGATATTTGGATAGCTTTGAGGA

                         *   *    *  *                *       *              
     175671 TTTCGTTGGAAACGGGATTA-CAT-GTA-AAAAGC-AGACAGCAGCATTCCCAGAAACTTCTTTG
         66 TTTCGTTGGAAACAGGAATATCTTCATATAAAAGCTAGACAGAAGCATTCTCAGAAACTTCTTTG

                              *    *                 
     175732 TGATGTTTGCATTCAAGTCACAGAGTTGAACATTCCCTTTC
        131 TGATGTTTGCATTCAAGTAACAGTGTTGAACATTCCCTTTC

                                                   *     *     * **     *   *
     175773 ATAGAGCAGGTTTGAAACACTCTTTTTGTAGTATCTGGATGTGGACATTTGCAGCGCTTTCAGGC
          1 ATAGAGCAGGTTTGAAACACTCTTTTTGTAGTATCTGGAAGTGGATATTTGGATAGCTTTGAGGA

            * ** *      *             *** *   *                          *   
     175838 CTAAGGT-GAAAAAGGAAATATCTTCCCCTGAAAACTAGACAGAAGCATTCTCAGAAACTTATTT
         66 TTTCGTTGGAAACAGG-AATATCTTCATATAAAAGCTAGACAGAAGCATTCTCAGAAACTTCTTT

                   **  **    *             **  * *   *
     175902 GTGATGTGCGCCCTCAACTAACAGTGTTGAAGCTTTCTTTTG
        130 GTGATGTTTGCATTCAAGTAACAGTGTTGAACATTCCCTTTC

                     *                   * *                  ***            
     175944 ATAGAGCAGTTTTGAAACACTCTTTTTGTGGAATCTGGAAGTGGATATTTTTCTAGCTTTGAGGA
          1 ATAGAGCAGGTTTGAAACACTCTTTTTGTAGTATCTGGAAGTGGATATTTGGATAGCTTTGAGGA

                         *   *    *                   *       *     *   *    
     176009 TTTCGTTGGAAACGGGATTA-CAT-ATA-AAAAGC-AGACAGCAGCATTCCCAGAATCTTGTTTG
         66 TTTCGTTGGAAACAGGAATATCTTCATATAAAAGCTAGACAGAAGCATTCTCAGAAACTTCTTTG

                              *    *                 
     176070 TGATGTTTGCATTCAAGTCACAGAGTTGAACATTCCCTTTC
        131 TGATGTTTGCATTCAAGTAACAGTGTTGAACATTCCCTTTC

             *                         *           *     *       **     *   *
     176111 AGAGAGCAGGTTTGAAACACTCTTTTTATAGTATCTGGATGTGGACATTTGGAGCGCTTTCAGGC
          1 ATAGAGCAGGTTTGAAACACTCTTTTTGTAGTATCTGGAAGTGGATATTTGGATAGCTTTGAGGA

            * ** *      *             *** *   *                      *   *   
     176176 GTATGGT-GAAAAAGGAAATATCTTCTCCTGAAAACTAGACAGAAGCATTCTCAGAATCTTATTT
         66 TTTCGTTGGAAACAGG-AATATCTTCATATAAAAGCTAGACAGAAGCATTCTCAGAAACTTCTTT

                   **  **    *             **  * *   *
     176240 GTGATGTGCGCCCTCAACTAACAGTGTTGAAGCTTTCTTTTG
        130 GTGATGTTTGCATTCAAGTAACAGTGTTGAACATTCCCTTTC

                     *     *    *     *   **     *   *        *              
     176282 ATAGAGCAGTTTTGAGACACACTTTTCGTAAAATCTGCAAGAGGATATTTTGATAGCTTTGAGGA
          1 ATAGAGCAGGTTTGAAACACTCTTTTTGTAGTATCTGGAAGTGGATATTTGGATAGCTTTGAGGA

                         *   * *            **                      *    *   
     176347 TTTCGTTGGAAACGGGATTGTCTTCATATAAACTCTAGACAGAAGCATTCTCAGAAGCTTCATTG
         66 TTTCGTTGGAAACAGGAATATCTTCATATAAAAGCTAGACAGAAGCATTCTCAGAAACTTCTTTG

            *       **   *    *      *      *        
     176412 GGATGTTTCAATTGAAGTCACAGTGGTGAACAGTCCCTTTC
        131 TGATGTTTGCATTCAAGTAACAGTGTTGAACATTCCCTTTC

                                                         *       **   * *    
     176453 ATAGAGCAGGTTTGAAACACTCTTTTTGTAGTATCTGGAAGTGGACATTTGGAGCGCTCTCAGGA
          1 ATAGAGCAGGTTTGAAACACTCTTTTTGTAGTATCTGGAAGTGGATATTTGGATAGCTTTGAGGA

            * *  *      *             **          * *       * *          *  *
     176518 CTGCGGT-GAAAAAGGAAATATCTTCCAATAAAAGCTACATAGAAGCAATGTCAGAAACTTTTTC
         66 TTTCGTTGGAAACAGG-AATATCTTCATATAAAAGCTAGACAGAAGCATTCTCAGAAACTTCTTT

            *      * ** *   **      *       *  *     *
     176582 ATGATGTATCTACTCAGCTAACAGAGTTGAACCTTTCCTTTG
        130 GTGATGTTTGCATTCAAGTAACAGTGTTGAACATTCCCTTTC

             *       *                   * *     *             **            
     176624 AGAGAGCAGTTTTGAAACACTCTTTTTGTGGAATCTGCAAGTGGATATTTGCCTAGCTTTGAGGA
          1 ATAGAGCAGGTTTGAAACACTCTTTTTGTAGTATCTGGAAGTGGATATTTGGATAGCTTTGAGGA

                         *   *    *                   *       *   *          
     176689 TTTCGTTGGAAACGGGATTA-CAT-ATA-AAAAGC-AGACAGCAGCATTCCCAGTAACTTCTTTG
         66 TTTCGTTGGAAACAGGAATATCTTCATATAAAAGCTAGACAGAAGCATTCTCAGAAACTTCTTTG

                              *    *          *      
     176750 TGATGTTTGCATTCAAGTCACAGAGTTGAACATTGCCTTTC
        131 TGATGTTTGCATTCAAGTAACAGTGTTGAACATTCCCTTTC

                                                   *     *    *  **     *   *
     176791 ATAGAGCAGGTTTGAAACACTCTTTTTGTAGTATCTGGATGTGGACATTTAGAGCGCTTTCAGGC
          1 ATAGAGCAGGTTTGAAACACTCTTTTTGTAGTATCTGGAAGTGGATATTTGGATAGCTTTGAGGA

            * ** *      *             *** *   *                      *   *   
     176856 CTGTGGT-GAAAAAGGAAATATCTTCTCCTGAAAACTAGACAGAAGCATTCTCAGAATCTTATTT
         66 TTTCGTTGGAAACAGG-AATATCTTCATATAAAAGCTAGACAGAAGCATTCTCAGAAACTTCTTT

                   **  **    *             **  * *   *
     176920 GTGATGTGCGCCCTCAACTAACAGTGTTGAAGCTTTCTTTTG
        130 GTGATGTTTGCATTCAAGTAACAGTGTTGAACATTCCCTTTC

                     *                    **     *   *        *              
     176962 ATAGAGCAGTTTTGAAACACTCTTTTTGTAAAATCTGCAAGAGGATATTTCGATAGCTTTGAGGA
          1 ATAGAGCAGGTTTGAAACACTCTTTTTGTAGTATCTGGAAGTGGATATTTGGATAGCTTTGAGGA

                *        *   * *            **                      *    *   
     177027 TTTCATTGGAAACGGGATTGTCTTCATATAAACTCTAGACAGAAGCATTCTCAGAAGCTTCATTG
         66 TTTCGTTGGAAACAGGAATATCTTCATATAAAAGCTAGACAGAAGCATTCTCAGAAACTTCTTTG

            *       **   *    *             *        
     177092 GGATGTTTCAATTGAAGTCACAGTGTTGAACAGTCCCTTTC
        131 TGATGTTTGCATTCAAGTAACAGTGTTGAACATTCCCTTTC

                                                      *  *       **   * *    
     177133 ATAGAGCAGGTTTGAAACACTCTTTTTGTAGTATCTGGAAGTTGACATTTGGAGCGCTCTCAGGA
          1 ATAGAGCAGGTTTGAAACACTCTTTTTGTAGTATCTGGAAGTGGATATTTGGATAGCTTTGAGGA

            * *  *      *             **            *       * *          *  *
     177198 CTACGGT-GAAAAAGGAAATATCTTCCAATAAAAGCTAGATAGAAGCAATGTCAGAAACTTTTTC
         66 TTTCGTTGGAAACAGG-AATATCTTCATATAAAAGCTAGACAGAAGCATTCTCAGAAACTTCTTT

            *      * ** *   **      *       *  * *    
     177262 ATGATGTATCTACTCAGTTAACAGAGTTGAACCTTTCTTTTC
        130 GTGATGTTTGCATTCAAGTAACAGTGTTGAACATTCCCTTTC

             *       *                   * *                   **            
     177304 AGAGAGCAGTTTTGAAACACTCTTTTTGTGGAATCTGGAAGTGGATATTTGTCTAGCTTTGAGGA
          1 ATAGAGCAGGTTTGAAACACTCTTTTTGTAGTATCTGGAAGTGGATATTTGGATAGCTTTGAGGA

                         *   *    *                   *       *   *          
     177369 TTTCGTTGGAAACGGGATTA-CAT-ATA-AAAAGC-AGACAGCAGCATTCCCAGTAACTTCTTTG
         66 TTTCGTTGGAAACAGGAATATCTTCATATAAAAGCTAGACAGAAGCATTCTCAGAAACTTCTTTG

                              *    *                 
     177430 TGATGTTTGCATTCAAGTCACAGAGTTGAACATTCCCTTTC
        131 TGATGTTTGCATTCAAGTAACAGTGTTGAACATTCCCTTTC

                                                   *     *       **     *   *
     177471 ATAGAGCAGGTTTGAAACACTCTTTTTGTAGTATCTGGATGTGGACATTTGGAGCGCTTTCAGGC
          1 ATAGAGCAGGTTTGAAACACTCTTTTTGTAGTATCTGGAAGTGGATATTTGGATAGCTTTGAGGA

            * ** *      *             *** *   *                      *   *   
     177536 CTATGGT-GAAAAAGGAAATATCTTCCCCTGAAAACTAGACAGAAGCATTCTCAGAATCTTATTT
         66 TTTCGTTGGAAACAGG-AATATCTTCATATAAAAGCTAGACAGAAGCATTCTCAGAAACTTCTTT

                   **  **    *             **  * *   *
     177600 GTGATGTGCGCCCTCAACTAACAGTGTTGAAGCTTTCTTTTG
        130 GTGATGTTTGCATTCAAGTAACAGTGTTGAACATTCCCTTTC

                     *                    **     *   *                       
     177642 ATAGAGCAGTTTTGAAACACTCTTTTTGTAAAATCTGCAAGAGGATATTTGGATAGCTTTGAGGA
          1 ATAGAGCAGGTTTGAAACACTCTTTTTGTAGTATCTGGAAGTGGATATTTGGATAGCTTTGAGGA

                         *   * *            **                      *    *   
     177707 TTTCGTTGGAAACGGGATTGTCTTCATATAAACTCTAGACAGAAGCATTCTCAGAAGCTTCATTG
         66 TTTCGTTGGAAACAGGAATATCTTCATATAAAAGCTAGACAGAAGCATTCTCAGAAACTTCTTTG

            *       **   *    *             *        
     177772 GGATGTTTCAATTGAAGTCACAGTGTTGAACAGTCCCTTTC
        131 TGATGTTTGCATTCAAGTAACAGTGTTGAACATTCCCTTTC

                                                         *       **   * *    
     177813 ATAGAGCAGGTTTGAAACACTCTTTTTGTAGTATCTGGAAGTGGACATTTGGAGCGCTCTCAGGA
          1 ATAGAGCAGGTTTGAAACACTCTTTTTGTAGTATCTGGAAGTGGATATTTGGATAGCTTTGAGGA

            * *  *      *             **          * *       * *          *  *
     177878 CTGCGGT-GAAAAAGGAAATATCTTCCAATAAAAGCTACATAGAAGCAATGTCAGAAACTTTTTC
         66 TTTCGTTGGAAACAGG-AATATCTTCATATAAAAGCTAGACAGAAGCATTCTCAGAAACTTCTTT

            *      * ** *   **      *       *        *
     177942 ATGATGTATCTACTCAGCTAACAGAGTTGAACCTT-CCTTTG
        130 GTGATGTTTGCATTCAAGTAACAGTGTTGAACATTCCCTTTC

             *       *                   * *     *             **            
     177983 AGAGAGCAGTTTTGAAACACTCTTTTTGTGGAATCTGCAAGTGGATATTTGTCTAGCTTTGAGGA
          1 ATAGAGCAGGTTTGAAACACTCTTTTTGTAGTATCTGGAAGTGGATATTTGGATAGCTTTGAGGA

                         *   *    *                   *       *              
     178048 TTTCGTTGGAAACGGGATTA-CAT-ATA-AAAAGC-AGACAGCAGCATTCCCAGAAACTTCTTTG
         66 TTTCGTTGGAAACAGGAATATCTTCATATAAAAGCTAGACAGAAGCATTCTCAGAAACTTCTTTG

                              *    *                 
     178109 TGATGTTTGCATTCAAGTCACAGAGTTGAACATTCCCTTTC
        131 TGATGTTTGCATTCAAGTAACAGTGTTGAACATTCCCTTTC

                                                   *     *       **     *   *
     178150 ATAGAGCAGGTTTGAAACACTCTTTTTGTAGTATCTGGATGTGGACATTTGGAGCGCTTTCAGGC
          1 ATAGAGCAGGTTTGAAACACTCTTTTTGTAGTATCTGGAAGTGGATATTTGGATAGCTTTGAGGA

            * ** *      *             *** *   *                      *   *   
     178215 CTATGGT-GAAAAAGGAAATATCTTCCCCTGAAAACTAGACAGAAGCATTCTCAGAATCTTATTT
         66 TTTCGTTGGAAACAGG-AATATCTTCATATAAAAGCTAGACAGAAGCATTCTCAGAAACTTCTTT

                   **  **    *             **  * *   *
     178279 GTGATGTGCGCCCTCAACTAACAGTGTTGAAGCTTTCTTTTG
        130 GTGATGTTTGCATTCAAGTAACAGTGTTGAACATTCCCTTTC

                     *                    **     *   *                       
     178321 ATAGAGCAGTTTTGAAACACTCTTTTTGTAAAATCTGCAAGAGGATATTTGGATAGCTTTGAGGA
          1 ATAGAGCAGGTTTGAAACACTCTTTTTGTAGTATCTGGAAGTGGATATTTGGATAGCTTTGAGGA

                         *   * *            **                      *    *   
     178386 TTTCGTTGGAAACGGGATTGTCTTCATATAAACTCTAGACAGAAGCATTCTCAGAAGCTTCATTG
         66 TTTCGTTGGAAACAGGAATATCTTCATATAAAAGCTAGACAGAAGCATTCTCAGAAACTTCTTTG

            *             *    *             *        
     178451 GGATGTTT-CAGTTGAAGTCACAGTGTTGAACAGTCCCTTTC
        131 TGATGTTTGCA-TTCAAGTAACAGTGTTGAACATTCCCTTTC

                     *                                   *       **   * *    
     178492 ATAGAGCAGCTTTGAAACACTCTTTTTGTAGTATCTGGAAGTGGACATTTGGAGCGCTCTCAGGA
          1 ATAGAGCAGGTTTGAAACACTCTTTTTGTAGTATCTGGAAGTGGATATTTGGATAGCTTTGAGGA

            * *  *      *             **          * *    *  * *          *  *
     178557 CTGCGGT-GAAAAAGGAAATATCTTCCAATAAAAGCTACATAGAAACAATGTCAGAAACTTTTTC
         66 TTTCGTTGGAAACAGG-AATATCTTCATATAAAAGCTAGACAGAAGCATTCTCAGAAACTTCTTT

            *      * ** *   **      *       *        *
     178621 ATGATGTATCTACTCAGCTAACAGAGTTGAACCTT-CCTTTG
        130 GTGATGTTTGCATTCAAGTAACAGTGTTGAACATTCCCTTTC

             *       *                   * *     *             **            
     178662 AGAGAGCAGTTTTGAAACACTCTTTTTGTGGAATCTGCAAGTGGATATTTGTCTAGCTTTGAGGA
          1 ATAGAGCAGGTTTGAAACACTCTTTTTGTAGTATCTGGAAGTGGATATTTGGATAGCTTTGAGGA

                         *   *    *                   *       *              
     178727 TTTCGTTGGAAACGGGATTA-CAT-ATA-AAAAGC-AGACAGCAGCATTCCCAGAAACTTCTTTG
         66 TTTCGTTGGAAACAGGAATATCTTCATATAAAAGCTAGACAGAAGCATTCTCAGAAACTTCTTTG

                              *    *                 
     178788 TGATGTTTGCATTCAAGTCACAGAGTTGAACATTCCCTTTC
        131 TGATGTTTGCATTCAAGTAACAGTGTTGAACATTCCCTTTC

                                                   *     *       **     *   *
     178829 ATAGAGCAGGTTTGAAACACTCTTTTTGTAGTATCTGGATGTGGACATTTGGAGCGCTTTCAGGC
          1 ATAGAGCAGGTTTGAAACACTCTTTTTGTAGTATCTGGAAGTGGATATTTGGATAGCTTTGAGGA

            * ** *      *             *** *   *                      *   *   
     178894 CTATGGT-GAAAAAGGAAATATCTTCCCCTGAAAACTAGACAGAAGCATTCTCAGAATCTTATTT
         66 TTTCGTTGGAAACAGG-AATATCTTCATATAAAAGCTAGACAGAAGCATTCTCAGAAACTTCTTT

                   **  **    *             **  * *   *
     178958 GTGATGTGCGCCCTCAACTAACAGTGTTGAAGCTTTCTTTTG
        130 GTGATGTTTGCATTCAAGTAACAGTGTTGAACATTCCCTTTC

                     *        *           **     *   *                       
     179000 ATAGAGCAGTTTTGAAACGCTCTTTTTGTAAAATCTGCAAGAGGATATTTGGATAGCTTTGAGGA
          1 ATAGAGCAGGTTTGAAACACTCTTTTTGTAGTATCTGGAAGTGGATATTTGGATAGCTTTGAGGA

                         *   * *            **            *         *    *   
     179065 TTTCGTTGGAAACGGGATTGTCTTCATATAAACTCTAGACAGAAGCTTTCTCAGAAGCTTCATTG
         66 TTTCGTTGGAAACAGGAATATCTTCATATAAAAGCTAGACAGAAGCATTCTCAGAAACTTCTTTG

            *       **   *    *             *        
     179130 GGATGTTTCAATTGAAGTCACAGTGTTGAACAGTCCCTTTC
        131 TGATGTTTGCATTCAAGTAACAGTGTTGAACATTCCCTTTC

                                                         *       **   * *    
     179171 ATAGAGCAGGTTTGAAACACTCTTTTTGTAGTATCTGGAAGTGGACATTTGGAGCGCTCTCAGGA
          1 ATAGAGCAGGTTTGAAACACTCTTTTTGTAGTATCTGGAAGTGGATATTTGGATAGCTTTGAGGA

            * *  *      *             **            *       * *          *  *
     179236 CTGCGGT-GAAAAAGGAAATATCTTCCAATAAAAGCTAGATAGAAGCAATGTCAGAAACTTTTTC
         66 TTTCGTTGGAAACAGG-AATATCTTCATATAAAAGCTAGACAGAAGCATTCTCAGAAACTTCTTT

            *      * ** *   **      *       *        *
     179300 ATGATGTATCTACTCAGCTAACAGAGTTGAACCTT-CCTTTG
        130 GTGATGTTTGCATTCAAGTAACAGTGTTGAACATTCCCTTTC

             *       *                   * *     *             **            
     179341 AGAGAGCAGTTTTGAAACACTCTTTTTGTGGAATCTGCAAGTGGATATTTGTCTAGCTTTGAGGA
          1 ATAGAGCAGGTTTGAAACACTCTTTTTGTAGTATCTGGAAGTGGATATTTGGATAGCTTTGAGGA

                *        *   *    *               *   *   *   *              
     179406 TTTCTTTGGAAACGGGATTA-CAT-ATA-AAAAGC-AGCCAGCAGCGTTCCCAGAAACTTCTTTG
         66 TTTCGTTGGAAACAGGAATATCTTCATATAAAAGCTAGACAGAAGCATTCTCAGAAACTTCTTTG

                             **    *                 
     179467 TGATGTTTGCATTCAAGCCACAGAGTTGAACATTCCCTTTC
        131 TGATGTTTGCATTCAAGTAACAGTGTTGAACATTCCCTTTC

                                                  **     *     * **     *   *
     179508 ATAGAGCAGGTTTGAAACACTCTTTTTGTAGTATCTGGTTGTGGACATTTGCAGCGCTTTCAGGC
          1 ATAGAGCAGGTTTGAAACACTCTTTTTGTAGTATCTGGAAGTGGATATTTGGATAGCTTTGAGGA

            * ** *      *             *** *   *         *                *   
     179573 CTAAGGT-GAAAAAGGAAATATCTTCCCCTGAAAACTAGACAGACGCATTCTCAGAAACTTATTT
         66 TTTCGTTGGAAACAGG-AATATCTTCATATAAAAGCTAGACAGAAGCATTCTCAGAAACTTCTTT

                   **  **    *             **  * *   *
     179637 GTGATGTGCGCCCTCAACTAACAGTGTTGAAGCTTTCTTTTG
        130 GTGATGTTTGCATTCAAGTAACAGTGTTGAACATTCCCTTTC

                     *                    *      *   *                       
     179679 ATAGAGCAGTTTTGAAACACTCTTTTTGTAATATCTGCAAGAGGATATTTGGATAGCTTTGAGGA
          1 ATAGAGCAGGTTTGAAACACTCTTTTTGTAGTATCTGGAAGTGGATATTTGGATAGCTTTGAGGA

                         *   * *            **                      *    *   
     179744 TTTCGTTGGAAACGGGATTGTCTTCATATAAACTCTAGACAGAAGCATTCTCAGAAGCTTCATTG
         66 TTTCGTTGGAAACAGGAATATCTTCATATAAAAGCTAGACAGAAGCATTCTCAGAAACTTCTTTG

            *       **   *    *             * *      
     179809 GGATGTTTCAATTGAAGTCACAGTGTTGAACAGTTCCTTTC
        131 TGATGTTTGCATTCAAGTAACAGTGTTGAACATTCCCTTTC

                 *                                       *       **   * *    
     179850 ATAGAACAGGTTTGAAACACTCTTTTTGTAGTATCTGGAAGTGGACATTTGGAGCGCTCTCAGGA
          1 ATAGAGCAGGTTTGAAACACTCTTTTTGTAGTATCTGGAAGTGGATATTTGGATAGCTTTGAGGA

            * ** *      *             **          * *       * **         *  *
     179915 CTATGGT-GAAAAAGGAAATATCTTCCAATAAAAGCTACATAGAAGCAATGACAGAAACTTTTTC
         66 TTTCGTTGGAAACAGG-AATATCTTCATATAAAAGCTAGACAGAAGCATTCTCAGAAACTTCTTT

            *      * ** *   **   *  *       *  *     *
     179979 ATGATGTATCTACTCAGCTAATAGAGTTGAACCTTTCCTTTG
        130 GTGATGTTTGCATTCAAGTAACAGTGTTGAACATTCCCTTTC

             *       *                   * *     *             **            
     180021 AGAGAGCAGTTTTGAAACACTCTTTTTGTGGAATCTGCAAGTGGATATTTGTCTAGCTTTGAGGA
          1 ATAGAGCAGGTTTGAAACACTCTTTTTGTAGTATCTGGAAGTGGATATTTGGATAGCTTTGAGGA

                         *   *    *        *          *       *   *          
     180086 TTTCGTTGGAAACGGGATTA-CAT-ATA-AACAGC-AGACAGCAGCATTCCCAGTAACTTCTTTG
         66 TTTCGTTGGAAACAGGAATATCTTCATATAAAAGCTAGACAGAAGCATTCTCAGAAACTTCTTTG

                              *    *          *      
     180147 TGATGTTTGCATTCAAGTCACAGAGTTGAACATTGCCTTTC
        131 TGATGTTTGCATTCAAGTAACAGTGTTGAACATTCCCTTTC

                                                   *     *       **     *   *
     180188 ATAGAGCAGGTTTGAAACACTCTTTTTGTAGTATCTGGATGTGGACATTTGGAGCGCTTTCAGGC
          1 ATAGAGCAGGTTTGAAACACTCTTTTTGTAGTATCTGGAAGTGGATATTTGGATAGCTTTGAGGA

            * ** *      *             *** *   *                      *   *   
     180253 CTGTGGT-GAAAAAGGAAATATCTTCTCCTGAAAACTAGACAGAAGCATTCTCAGAATCTTATTT
         66 TTTCGTTGGAAACAGG-AATATCTTCATATAAAAGCTAGACAGAAGCATTCTCAGAAACTTCTTT

                   **  **    *             **  * *   *
     180317 GTGATGTGCGCCCTCAACTAACAGTGTTGAAGCTTTCTTTTG
        130 GTGATGTTTGCATTCAAGTAACAGTGTTGAACATTCCCTTTC

                     *                    **     *   *        *              
     180359 ATAGAGCAGTTTTGAAACACTCTTTTTGTAAAATCTGCAAGAGGATATTTCGATAGCTTTGAGGA
          1 ATAGAGCAGGTTTGAAACACTCTTTTTGTAGTATCTGGAAGTGGATATTTGGATAGCTTTGAGGA

                *        *   * *            **                      *    *   
     180424 TTTCATTGGAAACGGGATTGTCTTCATATAAACTCTAGACAGAAGCATTCTCAGAAGCTTCATTG
         66 TTTCGTTGGAAACAGGAATATCTTCATATAAAAGCTAGACAGAAGCATTCTCAGAAACTTCTTTG

            *       **   *    *             *        
     180489 GGATGTTTCAATTGAAGTCACAGTGTTGAACAGTCCCTTTC
        131 TGATGTTTGCATTCAAGTAACAGTGTTGAACATTCCCTTTC

                                                      *  *       **   * *    
     180530 ATAGAGCAGGTTTGAAACACTCTTTTTGTAGTATCTGGAAGTTGACATTTGGAGCGCTCTCAGGA
          1 ATAGAGCAGGTTTGAAACACTCTTTTTGTAGTATCTGGAAGTGGATATTTGGATAGCTTTGAGGA

            * *  *      *             **            *       * *          *  *
     180595 CTACGGT-GAAAAAGGAAATATCTTCCAATAAAAGCTAGATAGAAGCAATGTCAGAAACTTTTTC
         66 TTTCGTTGGAAACAGG-AATATCTTCATATAAAAGCTAGACAGAAGCATTCTCAGAAACTTCTTT

            *      * ** *   **      *       *  * *   *
     180659 ATGATGTATCTACTCAGCTAACAGAGTTGAACCTTTCTTTTG
        130 GTGATGTTTGCATTCAAGTAACAGTGTTGAACATTCCCTTTC

             *       *                   * *                   **            
     180701 AGAGAGCAGTTTTGAAACACTCTTTTTGTGGAATCTGGAAGTGGATATTTGTCTAGCTTTGAGGA
          1 ATAGAGCAGGTTTGAAACACTCTTTTTGTAGTATCTGGAAGTGGATATTTGGATAGCTTTGAGGA

                         *   *    *                  **       *              
     180766 TTTCGTTGGAAACGGGATTA-CAT-ATA-AAAAGC-AGACAACAGCATTCCCAGAAACTTCTTTG
         66 TTTCGTTGGAAACAGGAATATCTTCATATAAAAGCTAGACAGAAGCATTCTCAGAAACTTCTTTG

                              *    *          *      
     180827 TGATGTTTGCATTCAAGTCACAGAGTTGAACATTTCCTTTC
        131 TGATGTTTGCATTCAAGTAACAGTGTTGAACATTCCCTTTC

                                                   *     *     * **     *   *
     180868 ATAGAGCAGGTTTGAAACACTCTTTTTGTAGTATCTGGATGTGGACATTTGCAGCGCTTTCAGGC
          1 ATAGAGCAGGTTTGAAACACTCTTTTTGTAGTATCTGGAAGTGGATATTTGGATAGCTTTGAGGA

            * ** *      *             *** *   *                          *   
     180933 CTAAGGT-GAAAAAGGAAATATCTTCCCCTGAAAACTAGACAGAAGCATTCTCAGAAACTTATTT
         66 TTTCGTTGGAAACAGG-AATATCTTCATATAAAAGCTAGACAGAAGCATTCTCAGAAACTTCTTT

                   **  **    *             **  * *   *
     180997 GTGATGTGCGCCCTCAACTAACAGTGTTGAAGCTTTCTTTTG
        130 GTGATGTTTGCATTCAAGTAACAGTGTTGAACATTCCCTTTC

                     *                   * *     *             **            
     181039 ATAGAGCAGTTTTGAAACACTCTTTTTGTGGAATCTGCAAGTGGATATTTGTCTAGCTTTGAGGA
          1 ATAGAGCAGGTTTGAAACACTCTTTTTGTAGTATCTGGAAGTGGATATTTGGATAGCTTTGAGGA

                         *   *    *                   *       *     *        
     181104 TTTCGTTGGAAACGGGATTA-CAT-ATA-AAAAGC-AGACAGCAGCATTCCCAGAATCTTCTTTG
         66 TTTCGTTGGAAACAGGAATATCTTCATATAAAAGCTAGACAGAAGCATTCTCAGAAACTTCTTTG

                              *    *   *             
     181165 TGATGTTTGCATTCAAGTCACAGAGTTCAACATTCCCTTTC
        131 TGATGTTTGCATTCAAGTAACAGTGTTGAACATTCCCTTTC

             *                         * *         *     *       **     *   *
     181206 AGAGAGCAGGTTTGAAACACTCTTTTTATCGTATCTGGATGTGGACATTTGGAGCGCTTTCAGGC
          1 ATAGAGCAGGTTTGAAACACTCTTTTTGTAGTATCTGGAAGTGGATATTTGGATAGCTTTGAGGA

            * ** *      *             *** *   *                      *   *   
     181271 CTATGGT-GAAAAAGGAAATATCTTCTCCTGAAAACTAGACAGAAGCATTCTCAGAATCTTATTT
         66 TTTCGTTGGAAACAGG-AATATCTTCATATAAAAGCTAGACAGAAGCATTCTCAGAAACTTCTTT

                   **  **    *             **  * *   *
     181335 GTGATGTGCGCCCTCAACTAACAGTGTTGAAGCTTTCTTTTG
        130 GTGATGTTTGCATTCAAGTAACAGTGTTGAACATTCCCTTTC

                     *                *   **     *   *        *              
     181377 ATAGAGCAGTTTTGAAACACTCTTTTCGTAAAATCTGCAAGAGGATATTTTGATAGCTTTGAGGA
          1 ATAGAGCAGGTTTGAAACACTCTTTTTGTAGTATCTGGAAGTGGATATTTGGATAGCTTTGAGGA

                         *   * *            **                      *    *   
     181442 TTTCGTTGGAAACGGGATTGTCTTCATATAAACTCTAGACAGAAGCATTCTCAGAAGCTTCATTG
         66 TTTCGTTGGAAACAGGAATATCTTCATATAAAAGCTAGACAGAAGCATTCTCAGAAACTTCTTTG

            *       **   *    *             *        
     181507 GGATGTTTCAATTGAAGTCACAGTGTTGAACAGTCCCTTTC
        131 TGATGTTTGCATTCAAGTAACAGTGTTGAACATTCCCTTTC

                                                         *       *  * * *    
     181548 ATAGAGCAGGTTTGAAACACTCTTTTTGTAGTATCTGGAAGTGGACATTTGGAGAGATCTCAGGA
          1 ATAGAGCAGGTTTGAAACACTCTTTTTGTAGTATCTGGAAGTGGATATTTGGATAGCTTTGAGGA

            * *  *    * *             **            *       * *          *  *
     181613 ATACGGT-GATAAAGGAAATATCTTCCAATAAAAGCTAGATAGAAGCAATGTCAGAAACTTTTTC
         66 TTTCGTTGGAAACAGG-AATATCTTCATATAAAAGCTAGACAGAAGCATTCTCAGAAACTTCTTT

            *      * ** *   **      *       *  * *   *
     181677 ATGATGTATCTACTCAGCTAACAGAGTTGAACCTTTCTTTTG
        130 GTGATGTTTGCATTCAAGTAACAGTGTTGAACATTCCCTTTC

             *       *                   * *     *             **            
     181719 AGAGAGCAGTTTTGAAACACTCTTTTTGTGGAATCTGCAAGTGGATATTTGTCTAGCTTTGAGGA
          1 ATAGAGCAGGTTTGAAACACTCTTTTTGTAGTATCTGGAAGTGGATATTTGGATAGCTTTGAGGA

                         *   *    *                   *       *              
     181784 TTTCGTTGGAAACGGGATTA-CAT-ATA-AAAAGC-AGACAGCAGCATTCCCAGAAACTTCTTTG
         66 TTTCGTTGGAAACAGGAATATCTTCATATAAAAGCTAGACAGAAGCATTCTCAGAAACTTCTTTG

                              *    *                 
     181845 TGATGTTTGCATTCAAGTCACAGAGTTGAACATTCCCTTTC
        131 TGATGTTTGCATTCAAGTAACAGTGTTGAACATTCCCTTTC

            *                                      *     *       **     *   *
     181886 CTAGAGCAGGTTTGAAACACTCTTTTTGTAGTATCTGGATGTGGACATTTGGAGCGCTTTCAGGC
          1 ATAGAGCAGGTTTGAAACACTCTTTTTGTAGTATCTGGAAGTGGATATTTGGATAGCTTTGAGGA

            * ** *      *             *** *   *                      *   *   
     181951 CTATGGT-GAAAAAGGAAATATCTTCCCCTGAAAACTAGACAGAAGCATTCTCAGAATCTTATTT
         66 TTTCGTTGGAAACAGG-AATATCTTCATATAAAAGCTAGACAGAAGCATTCTCAGAAACTTCTTT

                   **  **    *             **  * *   *
     182015 GTGATGTGCGCCCTCAACTAACAGTGTTGAAGCTTTCTTTTG
        130 GTGATGTTTGCATTCAAGTAACAGTGTTGAACATTCCCTTTC

                     *                    **     *   *                       
     182057 ATAGAGCAGTTTTGAAACACTCTTTTTGTAAAATCTGCAAGAGGATATTTGGATAGCTTTGAGGA
          1 ATAGAGCAGGTTTGAAACACTCTTTTTGTAGTATCTGGAAGTGGATATTTGGATAGCTTTGAGGA

                         *   * *            **                      *    *   
     182122 TTTCGTTGGAAACGGGATTGTCTTCATATAAACTCTAGACAGAAGCATTCTCAGAAGCTTCATTG
         66 TTTCGTTGGAAACAGGAATATCTTCATATAAAAGCTAGACAGAAGCATTCTCAGAAACTTCTTTG

            *       **   *    *             *        
     182187 GGATGTTTCAATTGAAGTCACAGTGTTGAACAGTCCCTTTC
        131 TGATGTTTGCATTCAAGTAACAGTGTTGAACATTCCCTTTC

                                                         *       **   * *    
     182228 ATAGAGCAGGTTTGAAACACTCTTTTTGTAGTATCTGGAAGTGGACATTTGGAGCGCTCTCAGGA
          1 ATAGAGCAGGTTTGAAACACTCTTTTTGTAGTATCTGGAAGTGGATATTTGGATAGCTTTGAGGA

            * *  *      *             **          * *       * *          *  *
     182293 CTGCGGT-GAAAAAGGAAATATCTTCCAATAAAAGCTACATAGAAGCAATGTCAGAAACTTTTTC
         66 TTTCGTTGGAAACAGG-AATATCTTCATATAAAAGCTAGACAGAAGCATTCTCAGAAACTTCTTT

            *      * ** *   **      *       *        *
     182357 ATGATGTATCTACTCAGCTAACAGAGTTGAACCTT-CCTTTG
        130 GTGATGTTTGCATTCAAGTAACAGTGTTGAACATTCCCTTTC

             *       *                   * *     *             **            
     182398 AGAGAGCAGTTTTGAAACACTCTTTTTGTGGAATCTGCAAGTGGATATTTGTCTAGCTTTGAGGA
          1 ATAGAGCAGGTTTGAAACACTCTTTTTGTAGTATCTGGAAGTGGATATTTGGATAGCTTTGAGGA

                         *   *    *               *   *   *   *              
     182463 TTTCGTTGGAAACGGGATTA-CAT-ATA-AAAAGC-AGCCAGCAGCGTTCCCAGAAACTTCTTTG
         66 TTTCGTTGGAAACAGGAATATCTTCATATAAAAGCTAGACAGAAGCATTCTCAGAAACTTCTTTG

                              *    *                 
     182524 TGATGTTTGCATTCAAGTCACAGAGTTGAACATTCCCTTTC
        131 TGATGTTTGCATTCAAGTAACAGTGTTGAACATTCCCTTTC

                                                  **     *     * **     *   *
     182565 ATAGAGCAGGTTTGAAACACTCTTTTTGTAGTATCTGGTTGTGGACATTTGCAGCGCTTTCAGGC
          1 ATAGAGCAGGTTTGAAACACTCTTTTTGTAGTATCTGGAAGTGGATATTTGGATAGCTTTGAGGA

            * ** *      *             *** *   *                       *  *   
     182630 CTAAGGT-GAAAAAGGAAATATCTTCCCCTGAAAACTAGACAGAAGCATTCTCAGAAAGTTATTT
         66 TTTCGTTGGAAACAGG-AATATCTTCATATAAAAGCTAGACAGAAGCATTCTCAGAAACTTCTTT

                   **  **    *             **  * *   *
     182694 GTGATGTGCGCCCTCAACTAACAGTGTTGAAGCTTTCTTTTG
        130 GTGATGTTTGCATTCAAGTAACAGTGTTGAACATTCCCTTTC

                     *                    *      *   *           *           
     182736 ATAGAGCAGTTTTGAAACACTCTTTTTGTAATATCTGCAAGAGGATATTTGGACAGCTTTGAGGA
          1 ATAGAGCAGGTTTGAAACACTCTTTTTGTAGTATCTGGAAGTGGATATTTGGATAGCTTTGAGGA

                         *   * *            **                      *    *   
     182801 TTTCGTTGGAAACGGGATTGTCTTCATATAAACTCTAGACAGAAGCATTCTCAGAAGCTTCATTG
         66 TTTCGTTGGAAACAGGAATATCTTCATATAAAAGCTAGACAGAAGCATTCTCAGAAACTTCTTTG

            *       **   *    *             * *      
     182866 GGATGTTTCAATTGAAGTCACAGTGTTGAACAGTTCCTTTC
        131 TGATGTTTGCATTCAAGTAACAGTGTTGAACATTCCCTTTC

                 *                                       *       **   * *    
     182907 ATAGAACAGGTTTGAAACACTCTTTTTGTAGTATCTGGAAGTGGACATTTGGAGCGCTCTCAGGA
          1 ATAGAGCAGGTTTGAAACACTCTTTTTGTAGTATCTGGAAGTGGATATTTGGATAGCTTTGAGGA

            * ** *      *             **          * *       * *          *  *
     182972 CTATGGT-GAAAAAGGAAATATCTTCCAATAAAAGCTACATAGAAGCAATGTCAGAAACTTTTTC
         66 TTTCGTTGGAAACAGG-AATATCTTCATATAAAAGCTAGACAGAAGCATTCTCAGAAACTTCTTT

            *      * ** *   **      *       *  *     *
     183036 ATGATGTATCTACTCAGCTAACAGAGTTGAACCTTTCCTTTG
        130 GTGATGTTTGCATTCAAGTAACAGTGTTGAACATTCCCTTTC

             *       *                   * *     *             **            
     183078 AGAGAGCAGTTTTGAAACACTCTTTTTGTGGAATCTGCAAGTGGATATTTGTCTAGCTTTGAGGA
          1 ATAGAGCAGGTTTGAAACACTCTTTTTGTAGTATCTGGAAGTGGATATTTGGATAGCTTTGAGGA

                         *   *    *                   *       *   *          
     183143 TTTCGTTGGAAACGGGATTA-CAT-ATA-AAAAGC-AGACAGCAGCATTCCCAGTAACTTCTTTG
         66 TTTCGTTGGAAACAGGAATATCTTCATATAAAAGCTAGACAGAAGCATTCTCAGAAACTTCTTTG

                             **    *                 
     183204 TGATGTTTGCATTCAAGACACAGAGTTGAACATTCCCTTTC
        131 TGATGTTTGCATTCAAGTAACAGTGTTGAACATTCCCTTTC

                                        *          *     *       **     *   *
     183245 ATAGAGCAGGTTTGAAACACTCTTTTTGAAGTATCTGGATGTGGACATTTGGAGCGCTTTCAGGC
          1 ATAGAGCAGGTTTGAAACACTCTTTTTGTAGTATCTGGAAGTGGATATTTGGATAGCTTTGAGGA

            * ** *      *             *** *   *                          *   
     183310 CTATGGT-GAAAAAGGAAATATCTTCCCCTGAAAACTAGACAGAAGCATTCTCAGAAACTTATTT
         66 TTTCGTTGGAAACAGG-AATATCTTCATATAAAAGCTAGACAGAAGCATTCTCAGAAACTTCTTT

                   **  **    *              *  * *   *
     183374 GTGATGTGCGCCCTCAACTAACAGTGTTGAACCTTTCTTTTG
        130 GTGATGTTTGCATTCAAGTAACAGTGTTGAACATTCCCTTTC

                 *   *                    *      *   *                       
     183416 ATAGAACAGTTTTGAAACACTCTTTTTGTAATATCTGCAAGAGGATATTTGGATAGCTTTGAGGA
          1 ATAGAGCAGGTTTGAAACACTCTTTTTGTAGTATCTGGAAGTGGATATTTGGATAGCTTTGAGGA

                         *   * *            **                      *    *   
     183481 TTTCGTTGGAAACGGGATTGTCTTCATATAAACTCTAGACAGAAGCATTCTCAGAAGCTTCATTG
         66 TTTCGTTGGAAACAGGAATATCTTCATATAAAAGCTAGACAGAAGCATTCTCAGAAACTTCTTTG

            *       **   *    *             *        
     183546 GGATGTTTCAATTGAAGTCACAGTGTTGAACAGTCCCTTTC
        131 TGATGTTTGCATTCAAGTAACAGTGTTGAACATTCCCTTTC

                                                         *       **   * *    
     183587 ATAGAGCAGGTTTGAAACACTCTTTTTGTAGTATCTGGAAGTGGACATTTGGAGCGCTCTCAGGA
          1 ATAGAGCAGGTTTGAAACACTCTTTTTGTAGTATCTGGAAGTGGATATTTGGATAGCTTTGAGGA

            * *  *    * *             **          * *       * *          *  *
     183652 CTACGGT-GATAAAGGAAATATCTTCCAATAAAAGCTACATAGAAGCAATGTCAGAAACTTTTTC
         66 TTTCGTTGGAAACAGG-AATATCTTCATATAAAAGCTAGACAGAAGCATTCTCAGAAACTTCTTT

            *      * ** *   **      *       *  * *   *
     183716 ATGATGTATCTACTCAGCTAACAGAGTTGAACCTTTCTTTTG
        130 GTGATGTTTGCATTCAAGTAACAGTGTTGAACATTCCCTTTC

             *       **                  * *     *             **       *    
     183758 AGAGAGCAGTATTGAAACACTCTTTTTGTGGAATCTGCAAGTGGATATTTGTCTAGCTTTCAGGA
          1 ATAGAGCAGGTTTGAAACACTCTTTTTGTAGTATCTGGAAGTGGATATTTGGATAGCTTTGAGGA

                             *    *                   *       *              
     183823 TTTCGTTGGAAACAGGATTA-CAT-ATA-AAAAGC-AGACAGCAGCATTCCCAGAAACTTCTTTG
         66 TTTCGTTGGAAACAGGAATATCTTCATATAAAAGCTAGACAGAAGCATTCTCAGAAACTTCTTTG

                              *    *                 
     183884 TGATGTTTGCATTCAAGTCACAGAGTTGAACATTCCCTTTC
        131 TGATGTTTGCATTCAAGTAACAGTGTTGAACATTCCCTTTC

                                                   *     *       **     *   *
     183925 ATAGAGCAGGTTTGAAACACTCTTTTTGTAGTATCTGGATGTGGACATTTGGAGCGCTTTCAGGC
          1 ATAGAGCAGGTTTGAAACACTCTTTTTGTAGTATCTGGAAGTGGATATTTGGATAGCTTTGAGGA

            * ** *      *             *** *   *                      **  *   
     183990 CTATGGT-GAAAAAGGAAATATCTTCCCCTGAAAACTAGACAGAAGCATTCTCAGAATTTTATTT
         66 TTTCGTTGGAAACAGG-AATATCTTCATATAAAAGCTAGACAGAAGCATTCTCAGAAACTTCTTT

                   **  **    *             **  * *   *
     184054 GTGATGTGCGCCCTCAACTAACAGTGTTGAAGCTTTCTTTTG
        130 GTGATGTTTGCATTCAAGTAACAGTGTTGAACATTCCCTTTC

               *     *                    **     **  *                       
     184096 ATACAGCAGTTTTGAAACACTCTTTTTGTAAAATCTGCTAGAGGATATTTGGATAGCTTTGAGGA
          1 ATAGAGCAGGTTTGAAACACTCTTTTTGTAGTATCTGGAAGTGGATATTTGGATAGCTTTGAGGA

                *        *   * *            **                     **    *   
     184161 TTTCTTTGGAAACGGGATTGTCTTCATATAAACTCTAGACAGAAGCATTCTCAGATGCTTCATTG
         66 TTTCGTTGGAAACAGGAATATCTTCATATAAAAGCTAGACAGAAGCATTCTCAGAAACTTCTTTG

            *       **   *    *             *        
     184226 GGATGTTTCAATTGAAGTCACAGTGTTGAACAGTCCCTTTC
        131 TGATGTTTGCATTCAAGTAACAGTGTTGAACATTCCCTTTC

                                                   *     *       **     *   *
     184267 ATAGAGCAGGTTTGAAACACTCTTTTTGTAGTATCTGGATGTGGACATTTGGAGCGCTTTCAGGC
          1 ATAGAGCAGGTTTGAAACACTCTTTTTGTAGTATCTGGAAGTGGATATTTGGATAGCTTTGAGGA

            * ** *      *             *** *   *                          *   
     184332 CTATGGT-GAAAAAGGAAATATCTTCCCCTGAAAACTAGACAGAAGCATTCTCAGAAACTTATTT
         66 TTTCGTTGGAAACAGG-AATATCTTCATATAAAAGCTAGACAGAAGCATTCTCAGAAACTTCTTT

                   *** **    *             **  * *   *
     184396 GTGATGTGCCCCCTCAACTAACAGTGTTGAAGCTTTCTTTTG
        130 GTGATGTTTGCATTCAAGTAACAGTGTTGAACATTCCCTTTC

                     *                   * *     *             **            
     184438 ATAGAGCAGTTTTGAAACACTCTTTTTGTGGAATCTGCAAGTGGATATTTGTCTAGCTTTGAGGA
          1 ATAGAGCAGGTTTGAAACACTCTTTTTGTAGTATCTGGAAGTGGATATTTGGATAGCTTTGAGGA

                         *   *    *                   *       *     *        
     184503 TTTCGTTGGAAACGGGATTA-CAT-ATA-AAAAGC-AGACAGCAGCATTCCCAGAATCTTCTTTG
         66 TTTCGTTGGAAACAGGAATATCTTCATATAAAAGCTAGACAGAAGCATTCTCAGAAACTTCTTTG

                              *    *                 
     184564 TGATGTTTGCATTCAAGTCACAGAGTTGAACATTCCCTTTC
        131 TGATGTTTGCATTCAAGTAACAGTGTTGAACATTCCCTTTC

                                                *  *     *       **     *   *
     184605 ATAGAGCAGGTTTGAAACACTCTTTTTGTAGTATCTCGATGTGGACATTTGGAGCGCTTTCAGGC
          1 ATAGAGCAGGTTTGAAACACTCTTTTTGTAGTATCTGGAAGTGGATATTTGGATAGCTTTGAGGA

            * ** *      *             *** *   *                      *   *   
     184670 CTATGGT-GAAAAAGGAAATATCTTCTCCTGAAAACTAGACAGAAGCATTCTCAGAATCTTATTT
         66 TTTCGTTGGAAACAGG-AATATCTTCATATAAAAGCTAGACAGAAGCATTCTCAGAAACTTCTTT

                   **  **    *             **  * *   *
     184734 GTGATGTGCGCCCTCAACTAACAGTGTTGAAGCTTTCTTTTG
        130 GTGATGTTTGCATTCAAGTAACAGTGTTGAACATTCCCTTTC

                     *                    **     *   *         *             
     184776 ATAGAGCAGTTTTGAAACACTCTTTTTGTAAAATCTGCAAGAGGATATTTGCATAGCTTTGAGGA
          1 ATAGAGCAGGTTTGAAACACTCTTTTTGTAGTATCTGGAAGTGGATATTTGGATAGCTTTGAGGA

                *        *   * *            **                      *    *   
     184841 TTTCATTGGAAACGGGATTGTCTTCATATAAACTCTAGACAGAAGCATTCTCAGAAGCTTCATTG
         66 TTTCGTTGGAAACAGGAATATCTTCATATAAAAGCTAGACAGAAGCATTCTCAGAAACTTCTTTG

            *       **   *    *             *        
     184906 GGATGTTTCAATTGAAGTCACAGTGTTGAACAGTCCCTTTC
        131 TGATGTTTGCATTCAAGTAACAGTGTTGAACATTCCCTTTC

                                                         *       **   * *    
     184947 ATAGAGCAGGTTTGAAACACTCTTTTTGTAGTATCTGGAAGTGGACATTTGGAGCGCTCTCAGGA
          1 ATAGAGCAGGTTTGAAACACTCTTTTTGTAGTATCTGGAAGTGGATATTTGGATAGCTTTGAGGA

            * *  *      *             **          * *       * *          *  *
     185012 CTACGGT-GAAAAAGGAAATATCTTCCAATAAAAGCTACATAGAAGCAATGTCAGAAACTTTTTC
         66 TTTCGTTGGAAACAGG-AATATCTTCATATAAAAGCTAGACAGAAGCATTCTCAGAAACTTCTTT

            *      * ** *   **      *       *  * *   *
     185076 ATGATGTATCTACTCAGCTAACAGAGTTGAACCTTTCTTTTG
        130 GTGATGTTTGCATTCAAGTAACAGTGTTGAACATTCCCTTTC

             *       *                   * *     *             **            
     185118 AGAGAGCAGTTTTGAAACACTCTTTTTGTGGAATCTGCAAGTGGATATTTGTCTAGCTTTGAGGA
          1 ATAGAGCAGGTTTGAAACACTCTTTTTGTAGTATCTGGAAGTGGATATTTGGATAGCTTTGAGGA

                             *    *                   *       *              
     185183 TTTCGTTGGAAACAGGATTA-CAT-ATA-AAAAGC-AGACAGCAGCATTCCCAGAAACTTCTTTG
         66 TTTCGTTGGAAACAGGAATATCTTCATATAAAAGCTAGACAGAAGCATTCTCAGAAACTTCTTTG

                              *    *                 
     185244 TGATGTTTGCATTCAAGTCACAGAGTTGAACATTCCCTTTC
        131 TGATGTTTGCATTCAAGTAACAGTGTTGAACATTCCCTTTC

                                                   *     *       **     *   *
     185285 ATAGAGCAGGTTTGAAACACTCTTTTTGTAGTATCTGGATGTGGACATTTGGAGCGCTTTCAGGC
          1 ATAGAGCAGGTTTGAAACACTCTTTTTGTAGTATCTGGAAGTGGATATTTGGATAGCTTTGAGGA

            * ** *      *             * * *   *                      **  *   
     185350 CTATGGT-GAAAAAGGAAATATCTTCCTCTGAAAACTAGACAGAAGCATTCTCAGAATTTTATTT
         66 TTTCGTTGGAAACAGG-AATATCTTCATATAAAAGCTAGACAGAAGCATTCTCAGAAACTTCTTT

                   **  **    *             **  * *   *
     185414 GTGATGTGCGCCCTCAACTAACAGTGTTGAAGCTTTCTTTTG
        130 GTGATGTTTGCATTCAAGTAACAGTGTTGAACATTCCCTTTC

                     *                    **     **  *                       
     185456 ATAGAGCAGTTTTGAAACACTCTTTTTGTAAAATCTGCTAGAGGATATTTGGATAGCTTTGAGGA
          1 ATAGAGCAGGTTTGAAACACTCTTTTTGTAGTATCTGGAAGTGGATATTTGGATAGCTTTGAGGA

                *        *   * *            **                     **    *   
     185521 TTTCTTTGGAAACGGGATTGTCTTCATATAAACTCTAGACAGAAGCATTCTCAGATGCTTCATTG
         66 TTTCGTTGGAAACAGGAATATCTTCATATAAAAGCTAGACAGAAGCATTCTCAGAAACTTCTTTG

            *       **   *    *             *    *   
     185586 GGATGTTTCAATTGAAGTCACAGTGTTGAACAGTCCCATTC
        131 TGATGTTTGCATTCAAGTAACAGTGTTGAACATTCCCTTTC

                                                   *     *       **     *   *
     185627 ATAGAGCAGGTTTGAAACACTCTTTTTGTAGTATCTGGATGTGGACATTTGGAGCGCTTTCAGGC
          1 ATAGAGCAGGTTTGAAACACTCTTTTTGTAGTATCTGGAAGTGGATATTTGGATAGCTTTGAGGA

            * ** *  *   *             *** *   *                          *   
     185692 CTATGGT-AAAAAAGGAAATATCTTCCCCTGAAAACTAGACAGAAGCATTCTCAGAAACTTATTT
         66 TTTCGTTGGAAACAGG-AATATCTTCATATAAAAGCTAGACAGAAGCATTCTCAGAAACTTCTTT

                   *** **    *             **  * *   *
     185756 GTGATGTGCCCCCTCAACTAACAGTGTTGAAGCTTTCTTTTG
        130 GTGATGTTTGCATTCAAGTAACAGTGTTGAACATTCCCTTTC

                     *  *                * *     *             **            
     185798 ATAGAGCAGTTTAGAAACACTCTTTTTGTGGAATCTGCAAGTGGATATTTGTCTAGCTTTGAGGA
          1 ATAGAGCAGGTTTGAAACACTCTTTTTGTAGTATCTGGAAGTGGATATTTGGATAGCTTTGAGGA

                         *   *    *                   *       *     *        
     185863 TTTCGTTGGAAACGGGATTA-CAT-ATA-AAAAGC-AGACAGCAGCATTCCCAGAATCTTCTTTG
         66 TTTCGTTGGAAACAGGAATATCTTCATATAAAAGCTAGACAGAAGCATTCTCAGAAACTTCTTTG

                              *    *                 
     185924 TGATGTTTGCATTCAAGTCACAGAGTTGAACATTCCCTTTC
        131 TGATGTTTGCATTCAAGTAACAGTGTTGAACATTCCCTTTC

                                                *  *     *       **     *   *
     185965 ATAGAGCAGGTTTGAAACACTCTTTTTGTAGTATCTCGATGTGGACATTTGGAGCGCTTTCAGGC
          1 ATAGAGCAGGTTTGAAACACTCTTTTTGTAGTATCTGGAAGTGGATATTTGGATAGCTTTGAGGA

            * ** *      *             *** *   *                      *   *   
     186030 CTATGGT-GAAAAAGGAAATATCTTCTCCTGAAAACTAGACAGAAGCATTCTCAGAATCTTATTT
         66 TTTCGTTGGAAACAGG-AATATCTTCATATAAAAGCTAGACAGAAGCATTCTCAGAAACTTCTTT

                   **  **    *             **  * *   *
     186094 GTGATGTGCGCCCTCAACTAACAGTGTTGAAGCTTTCTTTTG
        130 GTGATGTTTGCATTCAAGTAACAGTGTTGAACATTCCCTTTC

                     *                    **     *   *         *             
     186136 ATAGAGCAGATTTGAAACACTCTTTTTGTAAAATCTGCAAGAGGATATTTGCATAGCTTTGAGGA
          1 ATAGAGCAGGTTTGAAACACTCTTTTTGTAGTATCTGGAAGTGGATATTTGGATAGCTTTGAGGA

                *        *   * *      *     **                      *    *   
     186201 TTTCATTGGAAACGGGATTGTCTTCAAATAAACTCTAGACAGAAGCATTCTCAGAAGCTTCATTG
         66 TTTCGTTGGAAACAGGAATATCTTCATATAAAAGCTAGACAGAAGCATTCTCAGAAACTTCTTTG

            *       **   *    *             *        
     186266 GGATGTTTCAATTGAAGTCACAGTGTTGAACAGTCCCTTTC
        131 TGATGTTTGCATTCAAGTAACAGTGTTGAACATTCCCTTTC

                                                         *       **   * *    
     186307 ATAGAGCAGGTTTGAAACACTCTTTTTGTAGTATCTGGAAGTGGACATTTGGAGCGCTCTCAGGA
          1 ATAGAGCAGGTTTGAAACACTCTTTTTGTAGTATCTGGAAGTGGATATTTGGATAGCTTTGAGGA

            * *  *      *             **          * *       * *          *  *
     186372 CTACGGT-GAAAAAGGAAATATCTTCCAATAAAAGCTACATAGAAGCAATGTCAGAAACTTTTTC
         66 TTTCGTTGGAAACAGG-AATATCTTCATATAAAAGCTAGACAGAAGCATTCTCAGAAACTTCTTT

            *      * ** *   **      *       *  * *   *
     186436 ATGATGTATCTACTCAGCTAACAGAGTTGAACCTTTCTTTTG
        130 GTGATGTTTGCATTCAAGTAACAGTGTTGAACATTCCCTTTC

             *       *                   * *     *             **            
     186478 AGAGAGCAGTTTTGAAACACTCTTTTTGTGGAATCTGCAAGTGGATATTTGTCTAGCTTTGAGGA
          1 ATAGAGCAGGTTTGAAACACTCTTTTTGTAGTATCTGGAAGTGGATATTTGGATAGCTTTGAGGA

                             *    *                   *       *              
     186543 TTTCGTTGGAAACAGGATTA-CAT-ATA-AAAAGC-AGACAGCAGCATTCCCAGAAACTTCTTTG
         66 TTTCGTTGGAAACAGGAATATCTTCATATAAAAGCTAGACAGAAGCATTCTCAGAAACTTCTTTG

                    *         *    *                 
     186604 TGATGTTTACATTCAAGTCACAGAGTTGAACATTCCCTTTC
        131 TGATGTTTGCATTCAAGTAACAGTGTTGAACATTCCCTTTC

                                                   *     *       **     *   *
     186645 ATAGAGCAGGTTTGAAACACTCTTTTTGTAGTATCTGGATGTGGACATTTGGAGCGCTTTCAGGC
          1 ATAGAGCAGGTTTGAAACACTCTTTTTGTAGTATCTGGAAGTGGATATTTGGATAGCTTTGAGGA

            * ** *      *             *** *   *                      **  *   
     186710 CTATGGT-GAAAAAGGAAATATCTTCCCCTGAAAACTAGACAGAAGCATTCTCAGAATTTTATTT
         66 TTTCGTTGGAAACAGG-AATATCTTCATATAAAAGCTAGACAGAAGCATTCTCAGAAACTTCTTT

                   **  **    *             **  * *   *
     186774 GTGATGTGCGCCCTCAACTAACAGTGTTGAAGCTTTCTTTTG
        130 GTGATGTTTGCATTCAAGTAACAGTGTTGAACATTCCCTTTC

                     *                    **     **  *                       
     186816 ATAGAGCAGTTTTGAAACACTCTTTTTGTAAAATCTGCTAGAGGATATTTGGATAGCTTTGAGGA
          1 ATAGAGCAGGTTTGAAACACTCTTTTTGTAGTATCTGGAAGTGGATATTTGGATAGCTTTGAGGA

                *        *   * *            **                     **    *   
     186881 TTTCTTTGGAAACGGGATTGTCTTCATATAAACTCTAGACAGAAGCATTCTCAGATGCTTCATTG
         66 TTTCGTTGGAAACAGGAATATCTTCATATAAAAGCTAGACAGAAGCATTCTCAGAAACTTCTTTG

            *       **   *    *             *        
     186946 GGATGTTTCAATTGAAGTCACAGTGTTGAACAGTCCCTTTC
        131 TGATGTTTGCATTCAAGTAACAGTGTTGAACATTCCCTTTC

                                                   *     *       **     *   *
     186987 ATAGAGCAGGTTTGAAACACTCTTTTTGTAGTATCTGGATGTGGACATTTGGAGCGCTTTCAGGC
          1 ATAGAGCAGGTTTGAAACACTCTTTTTGTAGTATCTGGAAGTGGATATTTGGATAGCTTTGAGGA

            * ** *      *             *** *   *                          *   
     187052 CTATGGT-GAAAAAGGAAATATCTTCCCCTGAAAACTAGACAGAAGCATTCTCAGAAACTTATTT
         66 TTTCGTTGGAAACAGG-AATATCTTCATATAAAAGCTAGACAGAAGCATTCTCAGAAACTTCTTT

                   *** **    *             **  * *   *
     187116 GTGATGTGCCCCCTCAACTAACAGTGTTGAAGCTTTCTTTTG
        130 GTGATGTTTGCATTCAAGTAACAGTGTTGAACATTCCCTTTC

                     *                   * *     *             **            
     187158 ATAGAGCAGTTTTGAAACACTCTTTTTGTGGAATCTGCAAGTGGATATTTGTCTAGCTTTGAGGA
          1 ATAGAGCAGGTTTGAAACACTCTTTTTGTAGTATCTGGAAGTGGATATTTGGATAGCTTTGAGGA

                         *   *    *                   *       *     *        
     187223 TTTCGTTGGAAACGGGATTA-CAT-ATA-AAAAGC-AGACAGCAGCATTCCCAGAATCTTCTTTG
         66 TTTCGTTGGAAACAGGAATATCTTCATATAAAAGCTAGACAGAAGCATTCTCAGAAACTTCTTTG

                              *    *                 
     187284 TGATGTTTGCATTCAAGTCACAGAGTTGAACATTCCCTTTC
        131 TGATGTTTGCATTCAAGTAACAGTGTTGAACATTCCCTTTC

                                                *  *     *       **     *   *
     187325 ATAGAGCAGGTTTGAAACACTCTTTTTGTAGTATCTCGATGTGGACATTTGGAGCGCTTTCAGGC
          1 ATAGAGCAGGTTTGAAACACTCTTTTTGTAGTATCTGGAAGTGGATATTTGGATAGCTTTGAGGA

            * ** *      *             *** *   *                      *   *   
     187390 CTATGGT-GAAAAAGGAAATATCTTCTCCTGAAAACTAGACAGAAGCATTCTCAGAATCTTATTT
         66 TTTCGTTGGAAACAGG-AATATCTTCATATAAAAGCTAGACAGAAGCATTCTCAGAAACTTCTTT

                   **  **    *             **  * *   *
     187454 GTGATGTGCGCCCTCAACTAACAGTGTTGAAGCTTTCTTTTG
        130 GTGATGTTTGCATTCAAGTAACAGTGTTGAACATTCCCTTTC

                     *                    **     *   *         *             
     187496 ATAGAGCAGATTTGAAACACTCTTTTTGTAAAATCTGCAAGAGGATATTTGCATAGCTTTGAGGA
          1 ATAGAGCAGGTTTGAAACACTCTTTTTGTAGTATCTGGAAGTGGATATTTGGATAGCTTTGAGGA

                *        *   * *      *     **                      *    *   
     187561 TTTCATTGGAAACGGGATTGTCTTCAAATAAACTCTAGACAGAAGCATTCTCAGAAGCTTCATTG
         66 TTTCGTTGGAAACAGGAATATCTTCATATAAAAGCTAGACAGAAGCATTCTCAGAAACTTCTTTG

            *       **   *    *             *        
     187626 GGATGTTTCAATTGAAGTCACAGTGTTGAACAGTCCCTTTC
        131 TGATGTTTGCATTCAAGTAACAGTGTTGAACATTCCCTTTC

                                                         **      **   * *    
     187667 ATAGAGCAGGTTTGAAACACTCTTTTTGTAGTATCTGGAAGTGGACGTTTGGAGCGCTCTCAGGA
          1 ATAGAGCAGGTTTGAAACACTCTTTTTGTAGTATCTGGAAGTGGATATTTGGATAGCTTTGAGGA

            * *  *      *             **          * *       * *          *  *
     187732 CTACGGT-GAAAAAGGAAATATCTTCCAATAAAAGCTACATAGAAGCAATGTCAGAAACTTTTTC
         66 TTTCGTTGGAAACAGG-AATATCTTCATATAAAAGCTAGACAGAAGCATTCTCAGAAACTTCTTT

            *      * ** *   **      *       *  * *   *
     187796 ATGATGTATCTACTCAGCTAACAGAGTTGAACCTTTCTTTTG
        130 GTGATGTTTGCATTCAAGTAACAGTGTTGAACATTCCCTTTC

             *    *  *                   * *     *             **            
     187838 AGAGAGAAGTTTTGAAACACTCTTTTTGTGGAATCTGCAAGTGGATATTTGTCTAGCTTTGAGGA
          1 ATAGAGCAGGTTTGAAACACTCTTTTTGTAGTATCTGGAAGTGGATATTTGGATAGCTTTGAGGA

                         *   *    *               *   *   *   *              
     187903 TTTCGTTGGAAACGGGATTA-CAT-ATA-AAAAGC-AGCCAGCAGCGTTCCCAGAAACTTCTTTG
         66 TTTCGTTGGAAACAGGAATATCTTCATATAAAAGCTAGACAGAAGCATTCTCAGAAACTTCTTTG

                              *    *                 
     187964 TGATGTTTGCATTCAAGTCACAGAGTTGAACATTCCCTTTC
        131 TGATGTTTGCATTCAAGTAACAGTGTTGAACATTCCCTTTC

                                                  **     *     * **     *   *
     188005 ATAGAGCAGGTTTGAAACACTCTTTTTGTAGTATCTGGTTGTGGACATTTGCAGCGCTTTCAGGC
          1 ATAGAGCAGGTTTGAAACACTCTTTTTGTAGTATCTGGAAGTGGATATTTGGATAGCTTTGAGGA

            * ** *      *             *** *   *                          *   
     188070 CTAAGGT-GAAAAAGGAAATATCTTCCCCTGAAAACTAGACAGAAGCATTCTCAGAAACTTATTT
         66 TTTCGTTGGAAACAGG-AATATCTTCATATAAAAGCTAGACAGAAGCATTCTCAGAAACTTCTTT

                   **  **    *             **  * *   *
     188134 GTGATGTGCGCCCTCAACTAACAGTGTTGAAGCTTTCTTTTG
        130 GTGATGTTTGCATTCAAGTAACAGTGTTGAACATTCCCTTTC

                     *                    *      *   *           *           
     188176 ATAGAGCAGTTTTGAAACACTCTTTTTGTAATATCTGCAAGAGGATATTTGGACAGCTTTGAGGA
          1 ATAGAGCAGGTTTGAAACACTCTTTTTGTAGTATCTGGAAGTGGATATTTGGATAGCTTTGAGGA

                         *   * *            **                      *    *   
     188241 TTTCGTTGGAAACGGGATTGTCTTCATATAAACTCTAGACAGAAGCATTCTCAGAAGCTTCATTG
         66 TTTCGTTGGAAACAGGAATATCTTCATATAAAAGCTAGACAGAAGCATTCTCAGAAACTTCTTTG

            *       **   *    *             * *      
     188306 GGATGTTTCAATTGAAGTCACAGTGTTGAACAGTTCCTTTC
        131 TGATGTTTGCATTCAAGTAACAGTGTTGAACATTCCCTTTC

                 *                                       *       **   * *    
     188347 ATAGAACAGGTTTGAAACACTCTTTTTGTAGTATCTGGAAGTGGACATTTGGAGCGCTCTCAGGA
          1 ATAGAGCAGGTTTGAAACACTCTTTTTGTAGTATCTGGAAGTGGATATTTGGATAGCTTTGAGGA

            * ** *      *             **          * *       * *          *  *
     188412 CTATGGT-GAAAAAGGAAATATCTTCCAATAAAAGCTACATAGAAGCAATGTCAGAAACTTTTTC
         66 TTTCGTTGGAAACAGG-AATATCTTCATATAAAAGCTAGACAGAAGCATTCTCAGAAACTTCTTT

            *      * ** *   **      *       *  *     *
     188476 ATGATGTATCTACTCAGCTAACAGAGTTGAACCTTTCCTTTG
        130 GTGATGTTTGCATTCAAGTAACAGTGTTGAACATTCCCTTTC

             *       *                   * *     *             **            
     188518 AGAGAGCAGTTTTGAAACACTCTTTTTGTGGAATCTGCAAGTGGATATTTGTCTAGCTTTGAGGA
          1 ATAGAGCAGGTTTGAAACACTCTTTTTGTAGTATCTGGAAGTGGATATTTGGATAGCTTTGAGGA

               *         *   *    *                   *       *   *          
     188583 TTTGGTTGGAAACGGGATTA-CAT-ATA-AAAAGC-AGACAGCAGCATTCCCAGTAACTTCTTTG
         66 TTTCGTTGGAAACAGGAATATCTTCATATAAAAGCTAGACAGAAGCATTCTCAGAAACTTCTTTG

                             **    *                 
     188644 TGATGTTTGCATTCAAGACACAGAGTTGAACATTCCCTTTC
        131 TGATGTTTGCATTCAAGTAACAGTGTTGAACATTCCCTTTC

                                        *          *     *       **     *   *
     188685 ATAGAGCAGGTTTGAAACACTCTTTTTGAAGTATCTGGATGTGGACATTTGGAGCGCTTTCAGGC
          1 ATAGAGCAGGTTTGAAACACTCTTTTTGTAGTATCTGGAAGTGGATATTTGGATAGCTTTGAGGA

            * ** *      *             *** *   *                          *   
     188750 CTATGGT-GAAAAAGGAAATATCTTCCCCTGAAAACTAGACAGAAGCATTCTCAGAAACTTATTT
         66 TTTCGTTGGAAACAGG-AATATCTTCATATAAAAGCTAGACAGAAGCATTCTCAGAAACTTCTTT

                   **  **    *              *  * *   *
     188814 GTGATGTGCGCCCTCAACTAACAGTGTTGAACCTTTCTTTTG
        130 GTGATGTTTGCATTCAAGTAACAGTGTTGAACATTCCCTTTC

                 *   *                    *      *   *                       
     188856 ATAGAACAGTTTTGAAACACTCTTTTTGTAATATCTGCAAGAGGATATTTGGATAGCTTTGAGGA
          1 ATAGAGCAGGTTTGAAACACTCTTTTTGTAGTATCTGGAAGTGGATATTTGGATAGCTTTGAGGA

                         *   * *            **                      *    *   
     188921 TTTCGTTGGAAACGGGATTGTCTTCATATAAACTCTAGACAGAAGCATTCTCAGAAGCTTCATTG
         66 TTTCGTTGGAAACAGGAATATCTTCATATAAAAGCTAGACAGAAGCATTCTCAGAAACTTCTTTG

            *       **   *    *             *        
     188986 GGATGTTTCAATTGAAGTCACAGTGTTGAACAGTCCCTTTC
        131 TGATGTTTGCATTCAAGTAACAGTGTTGAACATTCCCTTTC

                                                         *       **   * *    
     189027 ATAGAGCAGGTTTGAAACACTCTTTTTGTAGTATCTGGAAGTGGACATTTGGAGCGCTCTCAGGA
          1 ATAGAGCAGGTTTGAAACACTCTTTTTGTAGTATCTGGAAGTGGATATTTGGATAGCTTTGAGGA

            * *  *    * *             **          * *       * *          *  *
     189092 CTACGGT-GATAAAGGAAATATCTTCCAATAAAAGCTACATAGAAGCAATGTCAGAAACTTTTTC
         66 TTTCGTTGGAAACAGG-AATATCTTCATATAAAAGCTAGACAGAAGCATTCTCAGAAACTTCTTT

            *      * ** *   **      *       *  * *   *
     189156 ATGATGTATCTACTCAGCTAACAGAGTTGAACCTTTCTTTTG
        130 GTGATGTTTGCATTCAAGTAACAGTGTTGAACATTCCCTTTC

             *       **                  * *     *             **       *    
     189198 AGAGAGCAGTATTGAAACACTCTTTTTGTGGAATCTGCAAGTGGATATTTGTCTAGCTTTCAGGA
          1 ATAGAGCAGGTTTGAAACACTCTTTTTGTAGTATCTGGAAGTGGATATTTGGATAGCTTTGAGGA

                             *    *                   *       *              
     189263 TTTCGTTGGAAACAGGATTA-CAT-ATA-AAAAGC-AGACAGCAGCATTCCCAGAAACTTCTTTG
         66 TTTCGTTGGAAACAGGAATATCTTCATATAAAAGCTAGACAGAAGCATTCTCAGAAACTTCTTTG

                              *    *                 
     189324 TGATGTTTGCATTCAAGTCACAGAGTTGAACATTCCCTTTC
        131 TGATGTTTGCATTCAAGTAACAGTGTTGAACATTCCCTTTC

                                                   *     *       **     *   *
     189365 ATAGAGCAGGTTTGAAACACTCTTTTTGTAGTATCTGGATGTGGACATTTGGAGCGCTTTCAGGC
          1 ATAGAGCAGGTTTGAAACACTCTTTTTGTAGTATCTGGAAGTGGATATTTGGATAGCTTTGAGGA

            * ** *      *             *** *   *                      **  *   
     189430 CTATGGT-GAAAAAGGAAATATCTTCCCCTGAAAACTAGACAGAAGCATTCTCAGAATTTTATTT
         66 TTTCGTTGGAAACAGG-AATATCTTCATATAAAAGCTAGACAGAAGCATTCTCAGAAACTTCTTT

                   **  **    *             **  * *   *
     189494 GTGATGTGCGCCCTCAACTAACAGTGTTGAAGCTTTCTTTTG
        130 GTGATGTTTGCATTCAAGTAACAGTGTTGAACATTCCCTTTC

               *     *                    **     **  *                       
     189536 ATACAGCAGTTTTGAAACACTCTTTTTGTAAAATCTGCTAGAGGATATTTGGATAGCTTTGAGGA
          1 ATAGAGCAGGTTTGAAACACTCTTTTTGTAGTATCTGGAAGTGGATATTTGGATAGCTTTGAGGA

                *        *   * *            **                     **    *   
     189601 TTTCTTTGGAAACGGGATTGTCTTCATATAAACTCTAGACAGAAGCATTCTCAGATGCTTCATTG
         66 TTTCGTTGGAAACAGGAATATCTTCATATAAAAGCTAGACAGAAGCATTCTCAGAAACTTCTTTG

            *       **   *    *             *        
     189666 GGATGTTTCAATTGAAGTCACAGTGTTGAACAGTCCCTTTC
        131 TGATGTTTGCATTCAAGTAACAGTGTTGAACATTCCCTTTC

                                                   *     *       **     *   *
     189707 ATAGAGCAGGTTTGAAACACTCTTTTTGTAGTATCTGGATGTGGACATTTGGAGCGCTTTCAGGC
          1 ATAGAGCAGGTTTGAAACACTCTTTTTGTAGTATCTGGAAGTGGATATTTGGATAGCTTTGAGGA

            * ** *      *             *** *   *                          *   
     189772 CTATGGT-GAAAAAGGAAATATCTTCCCCTGAAAACTAGACAGAAGCATTCTCAGAAACTTATTT
         66 TTTCGTTGGAAACAGG-AATATCTTCATATAAAAGCTAGACAGAAGCATTCTCAGAAACTTCTTT

                   *** **    *             **  * *   *
     189836 GTGATGTGCCCCCTCAACTAACAGTGTTGAAGCTTTCTTTTG
        130 GTGATGTTTGCATTCAAGTAACAGTGTTGAACATTCCCTTTC

                     *                   * *     *             **            
     189878 ATAGAGCAGTTTTGAAACACTCTTTTTGTGGAATCTGCAAGTGGATATTTGTCTAGCTTTGAGGA
          1 ATAGAGCAGGTTTGAAACACTCTTTTTGTAGTATCTGGAAGTGGATATTTGGATAGCTTTGAGGA

                         *   *    *                   *       *     *        
     189943 TTTCGTTGGAAACGGGATTA-CAT-ATA-AAAAGC-AGACAGCAGCATTCCCAGAATCTTCTTTG
         66 TTTCGTTGGAAACAGGAATATCTTCATATAAAAGCTAGACAGAAGCATTCTCAGAAACTTCTTTG

                              *    *                 
     190004 TGATGTTTGCATTCAAGTCACAGAGTTGAACATTCCCTTTC
        131 TGATGTTTGCATTCAAGTAACAGTGTTGAACATTCCCTTTC

                                                *  *     *       **     *   *
     190045 ATAGAGCAGGTTTGAAACACTCTTTTTGTAGTATCTCGATGTGGACATTTGGAGCGCTTTCAGGC
          1 ATAGAGCAGGTTTGAAACACTCTTTTTGTAGTATCTGGAAGTGGATATTTGGATAGCTTTGAGGA

            * ** *      *             *** *   *                      *   *   
     190110 CTATGGT-GAAAAAGGAAATATCTTCTCCTGAAAACTAGACAGAAGCATTCTCAGAATCTTATTT
         66 TTTCGTTGGAAACAGG-AATATCTTCATATAAAAGCTAGACAGAAGCATTCTCAGAAACTTCTTT

                   **  **    *             **  * *   *
     190174 GTGATGTGCGCCCTCAACTAACAGTGTTGAAGCTTTCTTTTG
        130 GTGATGTTTGCATTCAAGTAACAGTGTTGAACATTCCCTTTC

                     *                    **     *   *         *             
     190216 ATAGAGCAGTTTTGAAACACTCTTTTTGTAAAATCTGCAAGAGGATATTTGCATAGCTTTGAGGA
          1 ATAGAGCAGGTTTGAAACACTCTTTTTGTAGTATCTGGAAGTGGATATTTGGATAGCTTTGAGGA

                *        *   * *            **                      *    *   
     190281 TTTCATTGGAAACGGGATTGTCTTCATATAAACTCTAGACAGAAGCATTCTCAGAAGCTTCATTG
         66 TTTCGTTGGAAACAGGAATATCTTCATATAAAAGCTAGACAGAAGCATTCTCAGAAACTTCTTTG

            *       **   *    *             *        
     190346 GGATGTTTCAATTGAAGTCACAGTGTTGAACAGTCCCTTTC
        131 TGATGTTTGCATTCAAGTAACAGTGTTGAACATTCCCTTTC

                                                         *       **   * *    
     190387 ATAGAGCAGGTTTGAAACACTCTTTTTGTAGTATCTGGAAGTGGACATTTGGAGCGCTCTCAGGA
          1 ATAGAGCAGGTTTGAAACACTCTTTTTGTAGTATCTGGAAGTGGATATTTGGATAGCTTTGAGGA

            * *  *      *             **          * *       * *          *  *
     190452 CTACGGT-GAAAAAGGAAATATCTTCCAATAAAAGCTACATAGAAGCAATGTCAGAAACTTTTTC
         66 TTTCGTTGGAAACAGG-AATATCTTCATATAAAAGCTAGACAGAAGCATTCTCAGAAACTTCTTT

            *      * ** *   **      *       *  * *   *
     190516 ATGATGTATCTACTCAGCTAACAGAGTTGAACCTTTCTTTTG
        130 GTGATGTTTGCATTCAAGTAACAGTGTTGAACATTCCCTTTC

             *       *                   * *     *             **            
     190558 AGAGAGCAGTTTTGAAACACTCTTTTTGTGGAATCTGCAAGTGGATATTTGTCTAGCTTTGAGGA
          1 ATAGAGCAGGTTTGAAACACTCTTTTTGTAGTATCTGGAAGTGGATATTTGGATAGCTTTGAGGA

                             *    *                   *       *              
     190623 TTTCGTTGGAAACAGGATTA-CAT-ATA-AAAAGC-AGACAGCAGCATTCCCAGAAACTTCTTTG
         66 TTTCGTTGGAAACAGGAATATCTTCATATAAAAGCTAGACAGAAGCATTCTCAGAAACTTCTTTG

                              *    *                 
     190684 TGATGTTTGCATTCAAGTCACAGAGTTGAACATTCCCTTTC
        131 TGATGTTTGCATTCAAGTAACAGTGTTGAACATTCCCTTTC

                                                   *     *       **     *   *
     190725 ATAGAGCAGGTTTGAAACACTCTTTTTGTAGTATCTGGATGTGGACATTTGGAGCGCTTTCAGGC
          1 ATAGAGCAGGTTTGAAACACTCTTTTTGTAGTATCTGGAAGTGGATATTTGGATAGCTTTGAGGA

            * ** *      *             * * *   *                      **  *   
     190790 CTATGGT-GAAAAAGGAAATATCTTCCTCTGAAAACTAGACAGAAGCATTCTCAGAATTTTATTT
         66 TTTCGTTGGAAACAGG-AATATCTTCATATAAAAGCTAGACAGAAGCATTCTCAGAAACTTCTTT

                   **  **    *             **  * *   *
     190854 GTGATGTGCGCCCTCAACTAACAGTGTTGAAGCTTTCTTTTG
        130 GTGATGTTTGCATTCAAGTAACAGTGTTGAACATTCCCTTTC

                     *                    **     **  *                       
     190896 ATAGAGCAGTTTTGAAACACTCTTTTTGTAAAATCTGCTAGAGGATATTTGGATAGCTTTGAGGA
          1 ATAGAGCAGGTTTGAAACACTCTTTTTGTAGTATCTGGAAGTGGATATTTGGATAGCTTTGAGGA

                *        *   * *            **                     **    *   
     190961 TTTCTTTGGAAACGGGATTGTCTTCATATAAACTCTAGACAGAAGCATTCTCAGATGCTTCATTG
         66 TTTCGTTGGAAACAGGAATATCTTCATATAAAAGCTAGACAGAAGCATTCTCAGAAACTTCTTTG

            *       **   *    *             *    *   
     191026 GGATGTTTCAATTGAAGTCACAGTGTTGAACAGTCCCATTC
        131 TGATGTTTGCATTCAAGTAACAGTGTTGAACATTCCCTTTC

                                                   *     *       **     *   *
     191067 ATAGAGCAGGTTTGAAACACTCTTTTTGTAGTATCTGGATGTGGACATTTGGAGCGCTTTCAGGC
          1 ATAGAGCAGGTTTGAAACACTCTTTTTGTAGTATCTGGAAGTGGATATTTGGATAGCTTTGAGGA

            * ** *  *   *             *** *   *                          *   
     191132 CTATGGT-AAAAAAGGAAATATCTTCCCCTGAAAACTAGACAGAAGCATTCTCAGAAACTTATTT
         66 TTTCGTTGGAAACAGG-AATATCTTCATATAAAAGCTAGACAGAAGCATTCTCAGAAACTTCTTT

                   *** **    *             **  * *   *
     191196 GTGATGTGCCCCCTCAACTAACAGTGTTGAAGCTTTCTTTTG
        130 GTGATGTTTGCATTCAAGTAACAGTGTTGAACATTCCCTTTC

                     *  *                * *     *             **            
     191238 ATAGAGCAGTTTAGAAACACTCTTTTTGTGGAATCTGCAAGTGGATATTTGTCTAGCTTTGAGGA
          1 ATAGAGCAGGTTTGAAACACTCTTTTTGTAGTATCTGGAAGTGGATATTTGGATAGCTTTGAGGA

                         *   *    *                   *       *     *        
     191303 TTTCGTTGGAAACGGGATTA-CAT-ATA-AAAAGC-AGACAGCAGCATTCCCAGAATCTTCTTTG
         66 TTTCGTTGGAAACAGGAATATCTTCATATAAAAGCTAGACAGAAGCATTCTCAGAAACTTCTTTG

                              *    *                 
     191364 TGATGTTTGCATTCAAGTCACAGAGTTGAACATTCCCTTTC
        131 TGATGTTTGCATTCAAGTAACAGTGTTGAACATTCCCTTTC

                                                *  *     *       **     *   *
     191405 ATAGAGCAGGTTTGAAACACTCTTTTTGTAGTATCTCGATGTGGACATTTGGAGCGCTTTCAGGC
          1 ATAGAGCAGGTTTGAAACACTCTTTTTGTAGTATCTGGAAGTGGATATTTGGATAGCTTTGAGGA

            * ** *      *             *** *   *                      *   *   
     191470 CTATGGT-GAAAAAGGAAATATCTTCTCCTGAAAACTAGACAGAAGCATTCTCAGAATCTTATTT
         66 TTTCGTTGGAAACAGG-AATATCTTCATATAAAAGCTAGACAGAAGCATTCTCAGAAACTTCTTT

                   **  **    *             **  * *   *
     191534 GTGATGTGCGCCCTCAACTAACAGTGTTGAAGCTTTCTTTTG
        130 GTGATGTTTGCATTCAAGTAACAGTGTTGAACATTCCCTTTC

                     *                    **     *   *         *             
     191576 ATAGAGCAGATTTGAAACACTCTTTTTGTAAAATCTGCAAGAGGATATTTGCATAGCTTTGAGGA
          1 ATAGAGCAGGTTTGAAACACTCTTTTTGTAGTATCTGGAAGTGGATATTTGGATAGCTTTGAGGA

                *        *   * *      *     **                      *    *   
     191641 TTTCATTGGAAACGGGATTGTCTTCAAATAAACTCTAGACAGAAGCATTCTCAGAAGCTTCATTG
         66 TTTCGTTGGAAACAGGAATATCTTCATATAAAAGCTAGACAGAAGCATTCTCAGAAACTTCTTTG

            *       **   *    *             *        
     191706 GGATGTTTCAATTGAAGTCACAGTGTTGAACAGTCCCTTTC
        131 TGATGTTTGCATTCAAGTAACAGTGTTGAACATTCCCTTTC

                                                         *       **   * *    
     191747 ATAGAGCAGGTTTGAAACACTCTTTTTGTAGTATCTGGAAGTGGACATTTGGAGCGCTCTCAGGA
          1 ATAGAGCAGGTTTGAAACACTCTTTTTGTAGTATCTGGAAGTGGATATTTGGATAGCTTTGAGGA

            * *  *      *             **          * *       * *          *  *
     191812 CTACGGT-GAAAAAGGAAATATCTTCCAATAAAAGCTACATAGAAGCAATGTCAGAAACTTTTTC
         66 TTTCGTTGGAAACAGG-AATATCTTCATATAAAAGCTAGACAGAAGCATTCTCAGAAACTTCTTT

            *      * ** *   **      *       *  * *   *
     191876 ATGATGTATCTACTCAGCTAACAGAGTTGAACCTTTCTTTTG
        130 GTGATGTTTGCATTCAAGTAACAGTGTTGAACATTCCCTTTC

             *       *                   * *     *             **            
     191918 AGAGAGCAGTTTTGAAACACTCTTTTTGTGGAATCTGCAAGTGGATATTTGTCTAGCTTTGAGGA
          1 ATAGAGCAGGTTTGAAACACTCTTTTTGTAGTATCTGGAAGTGGATATTTGGATAGCTTTGAGGA

                             *    *                   *       *              
     191983 TTTCGTTGGAAACAGGATTA-CAT-ATA-AAAAGC-AGACAGCAGCATTCCCAGAAACTTCTTTG
         66 TTTCGTTGGAAACAGGAATATCTTCATATAAAAGCTAGACAGAAGCATTCTCAGAAACTTCTTTG

                              *    *                 
     192044 TGATGTTTGCATTCAAGTCACAGAGTTGAACATTCCCTTTC
        131 TGATGTTTGCATTCAAGTAACAGTGTTGAACATTCCCTTTC

                                                   *     *       **     *   *
     192085 ATAGAGCAGGTTTGAAACACTCTTTTTGTAGTATCTGGATGTGGACATTTGGAGCGCTTTCAGGC
          1 ATAGAGCAGGTTTGAAACACTCTTTTTGTAGTATCTGGAAGTGGATATTTGGATAGCTTTGAGGA

            * ** *      *             * * *   *                      **  *   
     192150 CTATGGT-GAAAAAGGAAATATCTTCCTCTGAAAACTAGACAGAAGCATTCTCAGAATTTTATTT
         66 TTTCGTTGGAAACAGG-AATATCTTCATATAAAAGCTAGACAGAAGCATTCTCAGAAACTTCTTT

                   **  **    *             **  * *   *
     192214 GTGATGTGCGCCCTCAACTAACAGTGTTGAAGCTTTCTTTTG
        130 GTGATGTTTGCATTCAAGTAACAGTGTTGAACATTCCCTTTC

                     *                    **  *  **  *                       
     192256 ATAGAGCAGTTTTGAAACACTCTTTTTGTAAAATATGCTAGAGGATATTTGGATAGCTTTGAGGA
          1 ATAGAGCAGGTTTGAAACACTCTTTTTGTAGTATCTGGAAGTGGATATTTGGATAGCTTTGAGGA

                *        *   * *            **                     **    *   
     192321 TTTCTTTGGAAACGGGATTGTCTTCATATAAACTCTAGACAGAAGCATTCTCAGATGCTTCATTG
         66 TTTCGTTGGAAACAGGAATATCTTCATATAAAAGCTAGACAGAAGCATTCTCAGAAACTTCTTTG

            *       **   *    *             *    *   
     192386 GGATGTTTCAATTGAAGTCACAGTGTTGAACAGTCCCATTC
        131 TGATGTTTGCATTCAAGTAACAGTGTTGAACATTCCCTTTC

                                                   *     *       **     *   *
     192427 ATAGAGCAGGTTTGAAACACTCTTTTTGTAGTATCTGGATGTGGACATTTGGAGCGCTTTCAGGC
          1 ATAGAGCAGGTTTGAAACACTCTTTTTGTAGTATCTGGAAGTGGATATTTGGATAGCTTTGAGGA

            * ** *  *   *             *** *   *                          *   
     192492 CTATGGT-AAAAAAGGAAATATCTTCCCCTGAAAACTAGACAGAAGCATTCTCAGAAACTTATTT
         66 TTTCGTTGGAAACAGG-AATATCTTCATATAAAAGCTAGACAGAAGCATTCTCAGAAACTTCTTT

                   *** **    *             **  * *   *
     192556 GTGATGTGCCCCCTCAACTAACAGTGTTGAAGCTTTCTTTTG
        130 GTGATGTTTGCATTCAAGTAACAGTGTTGAACATTCCCTTTC

                     *  *                * *     *             **            
     192598 ATAGAGCAGTTTAGAAACACTCTTTTTGTGGAATCTGCAAGTGGATATTTGTCTAGCTTTGAGGA
          1 ATAGAGCAGGTTTGAAACACTCTTTTTGTAGTATCTGGAAGTGGATATTTGGATAGCTTTGAGGA

                         *   *    *                   *       *     *        
     192663 TTTCGTTGGAAACGGGATTA-CAT-ATA-AAAAGC-AGACAGCAGCATTCCCAGAATCTTCTTTG
         66 TTTCGTTGGAAACAGGAATATCTTCATATAAAAGCTAGACAGAAGCATTCTCAGAAACTTCTTTG

                              *    *                 
     192724 TGATGTTTGCATTCAAGTCACAGAGTTGAACATTCCCTTTC
        131 TGATGTTTGCATTCAAGTAACAGTGTTGAACATTCCCTTTC

                                                *  *     *       **     *   *
     192765 ATAGAGCAGGTTTGAAACACTCTTTTTGTAGTATCTCGATGTGGACATTTGGAGCGCTTTCAGGC
          1 ATAGAGCAGGTTTGAAACACTCTTTTTGTAGTATCTGGAAGTGGATATTTGGATAGCTTTGAGGA

            * ** *      *             *** *   *                      *   *   
     192830 CTATGGT-GAAAAAGGAAATATCTTCTCCTGAAAACTAGACAGAAGCATTCTCAGAATCTTATTT
         66 TTTCGTTGGAAACAGG-AATATCTTCATATAAAAGCTAGACAGAAGCATTCTCAGAAACTTCTTT

                   **  **    *             **  * *   *
     192894 GTGATGTGCGCCCTCAACTAACAGTGTTGAAGCTTTCTTTTG
        130 GTGATGTTTGCATTCAAGTAACAGTGTTGAACATTCCCTTTC

                     *                    **     *   *         *             
     192936 ATAGAGCAGATTTGAAACACTCTTTTTGTAAAATCTGCAAGAGGATATTTGCATAGCTTTGAGGA
          1 ATAGAGCAGGTTTGAAACACTCTTTTTGTAGTATCTGGAAGTGGATATTTGGATAGCTTTGAGGA

                *        *   * *      *     **                      *    *   
     193001 TTTCATTGGAAACGGGATTGTCTTCAAATAAACTCTAGACAGAAGCATTCTCAGAAGCTTCATTG
         66 TTTCGTTGGAAACAGGAATATCTTCATATAAAAGCTAGACAGAAGCATTCTCAGAAACTTCTTTG

            *       **   *    *             *        
     193066 GGATGTTTCAATTGAAGTCACAGTGTTGAACAGTCCCTTTC
        131 TGATGTTTGCATTCAAGTAACAGTGTTGAACATTCCCTTTC

                                                         *       **   * *    
     193107 ATAGAGCAGGTTTGAAACACTCTTTTTGTAGTATCTGGAAGTGGACATTTGGAGCGCTCTCAGGA
          1 ATAGAGCAGGTTTGAAACACTCTTTTTGTAGTATCTGGAAGTGGATATTTGGATAGCTTTGAGGA

            * *  *      *             **          * *       * *          *  *
     193172 CTACGGT-GAAAAAGGAAATATCTTCCAATAAAAGCTACATAGAAGCAATGTCAGAAACTTTTTC
         66 TTTCGTTGGAAACAGG-AATATCTTCATATAAAAGCTAGACAGAAGCATTCTCAGAAACTTCTTT

            *      * ** *   **      *       *  * *   *
     193236 ATGATGTATCTACTCAGCTAACAGAGTTGAACCTTTCTTTTG
        130 GTGATGTTTGCATTCAAGTAACAGTGTTGAACATTCCCTTTC

             *       *                   * *     *             **            
     193278 AGAGAGCAGTTTTGAAACACTCTTTTTGTGGAATCTGCAAGTGGATATTTGTCTAGCTTTGAGGA
          1 ATAGAGCAGGTTTGAAACACTCTTTTTGTAGTATCTGGAAGTGGATATTTGGATAGCTTTGAGGA

                             *    *                   *       *              
     193343 TTTCGTTGGAAACAGGATTA-CAT-ATA-AAAAGC-AGACAGCAGCATTCCCAGAAACTTCTTTG
         66 TTTCGTTGGAAACAGGAATATCTTCATATAAAAGCTAGACAGAAGCATTCTCAGAAACTTCTTTG

                    *         *    *                 
     193404 TGATGTTTACATTCAAGTCACAGAGTTGAACATTCCCTTTC
        131 TGATGTTTGCATTCAAGTAACAGTGTTGAACATTCCCTTTC

                                                   *     *       **     *   *
     193445 ATAGAGCAGGTTTGAAACACTCTTTTTGTAGTATCTGGATGTGGACATTTGGAGCGCTTTCAGGC
          1 ATAGAGCAGGTTTGAAACACTCTTTTTGTAGTATCTGGAAGTGGATATTTGGATAGCTTTGAGGA

            * ** *      *             *** *   *                      **  *   
     193510 CTATGGT-GAAAAAGGAAATATCTTCCCCTGAAAACTAGACAGAAGCATTCTCAGAATTTTATTT
         66 TTTCGTTGGAAACAGG-AATATCTTCATATAAAAGCTAGACAGAAGCATTCTCAGAAACTTCTTT

                   **  **    *             **  * *   *
     193574 GTGATGTGCGCCCTCAACTAACAGTGTTGAAGCTTTCTTTTG
        130 GTGATGTTTGCATTCAAGTAACAGTGTTGAACATTCCCTTTC

                     *                    **     **  *                       
     193616 ATAGAGCAGTTTTGAAACACTCTTTTTGTAAAATCTGCTAGAGGATATTTGGATAGCTTTGAGGA
          1 ATAGAGCAGGTTTGAAACACTCTTTTTGTAGTATCTGGAAGTGGATATTTGGATAGCTTTGAGGA

                *        *   * *            **                     **    *   
     193681 TTTCTTTGGAAACGGGATTGTCTTCATATAAACTCTAGACAGAAGCATTCTCAGATGCTTCATTG
         66 TTTCGTTGGAAACAGGAATATCTTCATATAAAAGCTAGACAGAAGCATTCTCAGAAACTTCTTTG

            *       **   *    *             *        
     193746 GGATGTTTCAATTGAAGTCACAGTGTTGAACAGTCCCTTTC
        131 TGATGTTTGCATTCAAGTAACAGTGTTGAACATTCCCTTTC

                                                   *     *       **     *   *
     193787 ATAGAGCAGGTTTGAAACACTCTTTTTGTAGTATCTGGATGTGGACATTTGGAGCGCTTTCAGGC
          1 ATAGAGCAGGTTTGAAACACTCTTTTTGTAGTATCTGGAAGTGGATATTTGGATAGCTTTGAGGA

            * ** *      *             *** *   *                          *   
     193852 CTATGGT-GAAAAAGGAAATATCTTCCCCTGAAAACTAGACAGAAGCATTCTCAGAAACTTATTT
         66 TTTCGTTGGAAACAGG-AATATCTTCATATAAAAGCTAGACAGAAGCATTCTCAGAAACTTCTTT

                   *** **    *             **  * *   *
     193916 GTGATGTGCCCCCTCAACTAACAGTGTTGAAGCTTTCTTTTG
        130 GTGATGTTTGCATTCAAGTAACAGTGTTGAACATTCCCTTTC

                     *                   * *     *             **            
     193958 ATAGAGCAGTTTTGAAACACTCTTTTTGTGGAATCTGCAAGTGGATATTTGTCTAGCTTTGAGGA
          1 ATAGAGCAGGTTTGAAACACTCTTTTTGTAGTATCTGGAAGTGGATATTTGGATAGCTTTGAGGA

                         *   *    *                   *       **    *        
     194023 TTTCGTTGGAAACGGGATTA-CAT-ATA-AAAAGC-AGACAGCAGCATTCCGAGAATCTTCTTTG
         66 TTTCGTTGGAAACAGGAATATCTTCATATAAAAGCTAGACAGAAGCATTCTCAGAAACTTCTTTG

                              *    *                 
     194084 TGATGTTTGCATTCAAGTCACAGAGTTGAACATTCCCTTTC
        131 TGATGTTTGCATTCAAGTAACAGTGTTGAACATTCCCTTTC

                                                *  *     *       **     *   *
     194125 ATAGAGCAGGTTTGAAACACTCTTTTTGTAGTATCTCGATGTGGACATTTGGAGCGCTTTCAGGC
          1 ATAGAGCAGGTTTGAAACACTCTTTTTGTAGTATCTGGAAGTGGATATTTGGATAGCTTTGAGGA

            * ** *      *             *** *   *                      *   *   
     194190 CTATGGT-GAAAAAGGAAATATCTTCTCCTGAAAACTAGACAGAAGCATTCTCAGAATCTTATTT
         66 TTTCGTTGGAAACAGG-AATATCTTCATATAAAAGCTAGACAGAAGCATTCTCAGAAACTTCTTT

                   **  **    *             **  * *   *
     194254 GTGATGTGCGCCCTCAACTAACAGTGTTGAAGCTTTCTTTTG
        130 GTGATGTTTGCATTCAAGTAACAGTGTTGAACATTCCCTTTC

                     *                    **     *   *         *             
     194296 ATAGAGCAGATTTGAAACACTCTTTTTGTAAAATCTGCAAGAGGATATTTGCATAGCTTTGAGGA
          1 ATAGAGCAGGTTTGAAACACTCTTTTTGTAGTATCTGGAAGTGGATATTTGGATAGCTTTGAGGA

                *        *   * *      *     **                      *    *   
     194361 TTTCATTGGAAACGGGATTGTCTTCAAATAAACTCTAGACAGAAGCATTCTCAGAAGCTTCATTG
         66 TTTCGTTGGAAACAGGAATATCTTCATATAAAAGCTAGACAGAAGCATTCTCAGAAACTTCTTTG

            *       **   *    *             *        
     194426 GGATGTTTCAATTGAAGTCACAGTGTTGAACAGTCCCTTTC
        131 TGATGTTTGCATTCAAGTAACAGTGTTGAACATTCCCTTTC

                                                         **      **   * *    
     194467 ATAGAGCAGGTTTGAAACACTCTTTTTGTAGTATCTGGAAGTGGACGTTTGGAGCGCTCTCAGGA
          1 ATAGAGCAGGTTTGAAACACTCTTTTTGTAGTATCTGGAAGTGGATATTTGGATAGCTTTGAGGA

            * *  *      *             **          * *       * *          *  *
     194532 CTACGGT-GAAAAAGGAAATATCTTCCAATAAAAGCTACATAGAAGCAATGTCAGAAACTTTTTC
         66 TTTCGTTGGAAACAGG-AATATCTTCATATAAAAGCTAGACAGAAGCATTCTCAGAAACTTCTTT

            *      * ** *   **      *       *  * *   *
     194596 ATGATGTATCTACTCAGCTAACAGAGTTGAACCTTTCTTTTG
        130 GTGATGTTTGCATTCAAGTAACAGTGTTGAACATTCCCTTTC

             *    *  *                   * *     *             **            
     194638 AGAGAGAAGTTTTGAAACACTCTTTTTGTGGAATCTGCAAGTGGATATTTGTCTAGCTTTGAGGA
          1 ATAGAGCAGGTTTGAAACACTCTTTTTGTAGTATCTGGAAGTGGATATTTGGATAGCTTTGAGGA

                         *   *    *                   *       *              
     194703 TTTCGTTGGAAACGGGATTA-CAT-ATA-AAAAGC-AGACAGCAGCATTCCCAGAAACTTCTTTG
         66 TTTCGTTGGAAACAGGAATATCTTCATATAAAAGCTAGACAGAAGCATTCTCAGAAACTTCTTTG

                              *    *                 
     194764 TGATGTTTGCATTCAAGTCACAGAGTTGAACATTCCCTTTC
        131 TGATGTTTGCATTCAAGTAACAGTGTTGAACATTCCCTTTC

                                                *  *     *       **     *   *
     194805 ATAGAGCAGGTTTGAAACACTCTTTTTGTAGTATCTCGATGTGGACATTTGGAGCGCTTTCAGGC
          1 ATAGAGCAGGTTTGAAACACTCTTTTTGTAGTATCTGGAAGTGGATATTTGGATAGCTTTGAGGA

            * ** *      *             *** *   *           *          *   *   
     194870 CTATGGT-GAAAAAGGAAATATCTTCCCCTGAAAACTAGACAGAAGAATTCTCAGAATCTTATTT
         66 TTTCGTTGGAAACAGG-AATATCTTCATATAAAAGCTAGACAGAAGCATTCTCAGAAACTTCTTT

                   **  **    *             **  * *   *
     194934 GTGATGTGCGCCCTCAACTAACAGTGTTGAAGCTTTCTTTTG
        130 GTGATGTTTGCATTCAAGTAACAGTGTTGAACATTCCCTTTC

                     *                    **     *   *                       
     194976 ATAGAGCAGTTTTGAAACACTCTTTTTGTAAAATCTGCAAGAGGATATTTGGATAGCTTTGAGGA
          1 ATAGAGCAGGTTTGAAACACTCTTTTTGTAGTATCTGGAAGTGGATATTTGGATAGCTTTGAGGA

                         *   * *            **                      * *  *   
     195041 TTTCGTTGGAAACGGGATTGTCTTCATATAAACTCTAGACAGAAGCATTCTCAGAAGCGTCATTG
         66 TTTCGTTGGAAACAGGAATATCTTCATATAAAAGCTAGACAGAAGCATTCTCAGAAACTTCTTTG

            *       **   *    *             *        
     195106 GGATGTTTCAATTGAAGTCACAGTGTTGAACAGTCCCTTTC
        131 TGATGTTTGCATTCAAGTAACAGTGTTGAACATTCCCTTTC

                                                   *     *       **     *    
     195147 ATAGAGCAGGTTTGAAACACTCTTTTTGTAGTATCTGGATGTGGACATTTGGAGCGCTTTCAGGC
          1 ATAGAGCAGGTTTGAAACACTCTTTTTGTAGTATCTGGAAGTGGATATTTGGATAGCTTTGAGG-

            * * *    *                 *** *   * *                        *  
     195212 CTATGGTT-TAAA-AGGAAATATCTTCCCCTGAAAACCAGACAGAAGCATTCTCAGAAACTTATT
         65 ATTTCGTTGGAAACAGG-AATATCTTCATATAAAAGCTAGACAGAAGCATTCTCAGAAACTTCTT

                    **  **    *                    *   *
     195275 TGTGATGTGCGCCCTCAACTAACAGTGTTGAAGCATT-CTTTTG
        129 TGTGATGTTTGCATTCAAGTAACAGTGTTGAA-CATTCCCTTTC

                     **                  * *     *             **            
     195318 ATAGAGCAGTATTGAAACACTCTTTTTGTGGAATCTGCAAGTGGATATTTGTCTAGCTTTGAGGA
          1 ATAGAGCAGGTTTGAAACACTCTTTTTGTAGTATCTGGAAGTGGATATTTGGATAGCTTTGAGGA

                         *   *    *                   *       *              
     195383 TTTCGTTGGAAACGGGATTA-CAT-ATA-AAAAGC-AGACAGCAGCATTCCCAGAAACTTCTTTG
         66 TTTCGTTGGAAACAGGAATATCTTCATATAAAAGCTAGACAGAAGCATTCTCAGAAACTTCTTTG

                              *    *                 
     195444 TGATGTTTGCATTCAAGTCACAGAGTTGAACATTCCCTTTC
        131 TGATGTTTGCATTCAAGTAACAGTGTTGAACATTCCCTTTC

                                          *        *     *       **     *   *
     195485 ATAGAGCAGGTTTGAAACACTCTTTTTGTACTATCTGGATGTGGACATTTGGAGCGCTTTCAGGC
          1 ATAGAGCAGGTTTGAAACACTCTTTTTGTAGTATCTGGAAGTGGATATTTGGATAGCTTTGAGGA

            * ** *      *             *** *   *                      *   *   
     195550 CTATGGT-GAAAAAGGAAATATCTTCCCCTGAAAACTAGACAGAAGCATTCTCAGAATCTTATTT
         66 TTTCGTTGGAAACAGG-AATATCTTCATATAAAAGCTAGACAGAAGCATTCTCAGAAACTTCTTT

                   **  **    *             **  * *   *
     195614 GTGATGTGCGCCCTCAACTAACAGTGTTGAAGCTTTCTTTTG
        130 GTGATGTTTGCATTCAAGTAACAGTGTTGAACATTCCCTTTC

                     *   *   *            **     *   *                    *  
     195656 ATAGAGCAGTTTTCAAAGACTCTTTTTGTAAAATCTGCAAGAGGATATTTGGATAGCTTTGACGA
          1 ATAGAGCAGGTTTGAAACACTCTTTTTGTAGTATCTGGAAGTGGATATTTGGATAGCTTTGAGGA

                         *   * *            **                      *    *   
     195721 TTTCGTTGGAAACGGGATTGTCTTCATATAAACTCTAGACAGAAGCATTCTCAGAAGCTTCATTG
         66 TTTCGTTGGAAACAGGAATATCTTCATATAAAAGCTAGACAGAAGCATTCTCAGAAACTTCTTTG

            *       **   *    *             *        
     195786 GGATGTTTCAATTGAAGTCACAGTGTTGAACAGTCCCTTTC
        131 TGATGTTTGCATTCAAGTAACAGTGTTGAACATTCCCTTTC

                                                         *   *   *  * * *    
     195827 ATAGAGCAGGTTTGAAACACTCTTTTTGTAGTATCTGGAAGTGGACATTGGGAGAGATCTCAGGA
          1 ATAGAGCAGGTTTGAAACACTCTTTTTGTAGTATCTGGAAGTGGATATTTGGATAGCTTTGAGGA

            * *  *    * *             **            *       * *          *  *
     195892 ATACGGT-GATAAAGGAAATATCTTCCAATAAAAGCTAGATAGAAGCAATGTCAGAAACTTTTTC
         66 TTTCGTTGGAAACAGG-AATATCTTCATATAAAAGCTAGACAGAAGCATTCTCAGAAACTTCTTT

            *      * ** *   **      *       *  * *   *
     195956 ATGATGTATCTACTCAGCTAACAGAGTTGAACCTTTCTTTTG
        130 GTGATGTTTGCATTCAAGTAACAGTGTTGAACATTCCCTTTC

             *       *                   * *     *             **            
     195998 AGAGAGCAGTTTTGAAACACTCTTTTTGTGGAATCTGCAAGTGGATATTTGTCTAGCTTTGAGGA
          1 ATAGAGCAGGTTTGAAACACTCTTTTTGTAGTATCTGGAAGTGGATATTTGGATAGCTTTGAGGA

                             *    *                *  *       *              
     196063 TTTCGTTGGAAACAGGATTA-CAT-ATA-AAAAGC-AGATAGCAGCATTCCCAGAAACTTCTTTG
         66 TTTCGTTGGAAACAGGAATATCTTCATATAAAAGCTAGACAGAAGCATTCTCAGAAACTTCTTTG

                              *    * **              
     196124 TGATGTTTGCATTCAAGTCACAGAGAAGAACATTCCCTTTC
        131 TGATGTTTGCATTCAAGTAACAGTGTTGAACATTCCCTTTC

                                                   *     *       **     *   *
     196165 ATAGAGCAGGTTTGAAACACTCTTTTTGTAGTATCTGGATGTGGACATTTGGAGCGCTTTCAGGC
          1 ATAGAGCAGGTTTGAAACACTCTTTTTGTAGTATCTGGAAGTGGATATTTGGATAGCTTTGAGGA

            * ** *      *             *** *   *                      **  *   
     196230 CTATGGT-GAAAAAGGAAATATCTTCCCCTGAAAACTAGACAGAAGCATTCTCAGAATTTTATTT
         66 TTTCGTTGGAAACAGG-AATATCTTCATATAAAAGCTAGACAGAAGCATTCTCAGAAACTTCTTT

                   **  **    *             **  * *   *
     196294 GTGATGTGCGCCCTCAACTAACAGTGTTGAAGCTTTCTTTTG
        130 GTGATGTTTGCATTCAAGTAACAGTGTTGAACATTCCCTTTC

                     *                    **     **  *                       
     196336 ATAGAGCAGTTTTGAAACACTCTTTTTGTAAAATCTGCTAGAGGATATTTGGATAGCTTTGAGGA
          1 ATAGAGCAGGTTTGAAACACTCTTTTTGTAGTATCTGGAAGTGGATATTTGGATAGCTTTGAGGA

                *        *   * * *          **                     **    *   
     196401 TTTCTTTGGAAACGGGATTGTGTTCATATAAACTCTAGACAGAAGCATTCTCAGATGCTTCATTG
         66 TTTCGTTGGAAACAGGAATATCTTCATATAAAAGCTAGACAGAAGCATTCTCAGAAACTTCTTTG

            *       **   *    *             *        
     196466 GGATGTTTCAATTGAAGTCACAGTGTTGAACAGTCCCTTTC
        131 TGATGTTTGCATTCAAGTAACAGTGTTGAACATTCCCTTTC

                                                   *     *       **     *   *
     196507 ATAGAGCAGGTTTGAAACACTCTTTTTGTAGTATCTGGATGTGGACATTTGGAGCGCTTTCAGGC
          1 ATAGAGCAGGTTTGAAACACTCTTTTTGTAGTATCTGGAAGTGGATATTTGGATAGCTTTGAGGA

            * ** *      *             *** *   *                          *   
     196572 CTATGGT-GAAAAAGGAAATATCTTCCCCTGAAAACTAGACAGAAGCATTCTCAGAAACTTATTT
         66 TTTCGTTGGAAACAGG-AATATCTTCATATAAAAGCTAGACAGAAGCATTCTCAGAAACTTCTTT

                   *** **    *                    *   *
     196636 GTGATGTGCCCCCTCAACTAACAGTGTTGAAGCATT-CTTTTG
        130 GTGATGTTTGCATTCAAGTAACAGTGTTGAA-CATTCCCTTTC

                     *                   * *     *             **            
     196678 ATAGAGCAGTTTTGAAACACTCTTTTTGTGGAATCTGCAAGTGGATATTTGTCTAGCTTTGAGGA
          1 ATAGAGCAGGTTTGAAACACTCTTTTTGTAGTATCTGGAAGTGGATATTTGGATAGCTTTGAGGA

                         *   *    *                   *       *     *        
     196743 TTTCGTTGGAAACGGGATTA-CAT-ATA-AAAAGC-AGACAGCAGCATTCCCAGAATCTTCTTTG
         66 TTTCGTTGGAAACAGGAATATCTTCATATAAAAGCTAGACAGAAGCATTCTCAGAAACTTCTTTG

                              *    *                 
     196804 TGATGTTTGCATTCAAGTCACAGAGTTGAACATTCCCTTTC
        131 TGATGTTTGCATTCAAGTAACAGTGTTGAACATTCCCTTTC

                                                   *     *       **     *   *
     196845 ATAGAGCAGGTTTGAAACACTCTTTTTGTAGTATCTGGATGTGGACATTTGGAGCGCTTTCAGGC
          1 ATAGAGCAGGTTTGAAACACTCTTTTTGTAGTATCTGGAAGTGGATATTTGGATAGCTTTGAGGA

            * ** *      *             *** *   *                      *   *   
     196910 CTATGGT-GAAAAAGGAAATATCTTCTCCTGAAAACTAGACAGAAGCATTCTCAGAATCTTATTT
         66 TTTCGTTGGAAACAGG-AATATCTTCATATAAAAGCTAGACAGAAGCATTCTCAGAAACTTCTTT

                   *** **    *                    *   *
     196974 GTGATGTGCCCCCTCAACTAACAGTGTTGAAGCATT-CTTTTG
        130 GTGATGTTTGCATTCAAGTAACAGTGTTGAA-CATTCCCTTTC

                     *                   * *     *             **            
     197016 ATAGAGCAGTTTTGAAACACTCTTTTTGTGGAATCTGCAAGTGGATATTTGTCTAGCTTTGAGGA
          1 ATAGAGCAGGTTTGAAACACTCTTTTTGTAGTATCTGGAAGTGGATATTTGGATAGCTTTGAGGA

                         *   *    *                   *       *     *        
     197081 TTTCGTTGGAAACGGGATTA-CAT-ATA-AAAAGC-AGACAGCAGCATTCCCAGAATCTTCTTTG
         66 TTTCGTTGGAAACAGGAATATCTTCATATAAAAGCTAGACAGAAGCATTCTCAGAAACTTCTTTG

                              *    *                 
     197142 TGATGTTTGCATTCAAGTCACAGAGTTGAACATTCCCTTTC
        131 TGATGTTTGCATTCAAGTAACAGTGTTGAACATTCCCTTTC

                                                *  *     *       **     *   *
     197183 ATAGAGCAGGTTTGAAACACTCTTTTTGTAGTATCTCGATGTGGACATTTGGAGCGCTTTCAGGC
          1 ATAGAGCAGGTTTGAAACACTCTTTTTGTAGTATCTGGAAGTGGATATTTGGATAGCTTTGAGGA

            * ** *      *             *** *   *                      *   *   
     197248 CTATGGT-GAAAAAGGAAATATCTTCTCCTGAAAACTAGACAGAAGCATTCTCAGAATCTTATTT
         66 TTTCGTTGGAAACAGG-AATATCTTCATATAAAAGCTAGACAGAAGCATTCTCAGAAACTTCTTT

                   **  **    *             **  * *   *
     197312 GTGATGTGCGCCCTCAACTAACAGTGTTGAAGCTTTCTTTTG
        130 GTGATGTTTGCATTCAAGTAACAGTGTTGAACATTCCCTTTC

                     *                    **     *   *         *             
     197354 ATAGAGCAGATTTGAAACACTCTTTTTGTAAAATCTGCAAGAGGATATTTGCATAGCTTTGAGGA
          1 ATAGAGCAGGTTTGAAACACTCTTTTTGTAGTATCTGGAAGTGGATATTTGGATAGCTTTGAGGA

                *        *   * *      *     **                      *    *   
     197419 TTTCATTGGAAACGGGATTGTCTTCAAATAAACTCTAGACAGAAGCATTCTCAGAAGCTTCATTG
         66 TTTCGTTGGAAACAGGAATATCTTCATATAAAAGCTAGACAGAAGCATTCTCAGAAACTTCTTTG

            *       **   *    *             *        
     197484 GGATGTTTCAATTGAAGTCACAGTGTTGAACAGTCCCTTTC
        131 TGATGTTTGCATTCAAGTAACAGTGTTGAACATTCCCTTTC

                                                         *       *  * * *    
     197525 ATAGAGCAGGTTTGAAACACTCTTTTTGTAGTATCTGGAAGTGGACATTTGGAGAGATCTCAGGA
          1 ATAGAGCAGGTTTGAAACACTCTTTTTGTAGTATCTGGAAGTGGATATTTGGATAGCTTTGAGGA

            * *  *    * *             **            *       * *          *  *
     197590 ATACGGT-GATAAAGGAAATATCTTCCAATAAAAGCTAGATAGAAGCAATGTCAGAAACTTTTTC
         66 TTTCGTTGGAAACAGG-AATATCTTCATATAAAAGCTAGACAGAAGCATTCTCAGAAACTTCTTT

            *      * ** *   **      *       *  * *   *
     197654 ATGATGTATCTACTCAGCTAACAGAGTTGAACCTTTCTTTTG
        130 GTGATGTTTGCATTCAAGTAACAGTGTTGAACATTCCCTTTC

             *       *                   * *     *             **            
     197696 AGAGAGCAGTTTTGAAACACTCTTTTTGTGGAATCTGCAAGTGGATATTTGTCTAGCTTTGAGGA
          1 ATAGAGCAGGTTTGAAACACTCTTTTTGTAGTATCTGGAAGTGGATATTTGGATAGCTTTGAGGA

                             *    *                   *       *              
     197761 TTTCGTTGGAAACAGGATTA-CAT-ATA-AAAAGC-AGACAGCAGCATTCCCAGAAACTTCTTTG
         66 TTTCGTTGGAAACAGGAATATCTTCATATAAAAGCTAGACAGAAGCATTCTCAGAAACTTCTTTG

                              *    *                 
     197822 TGATGTTTGCATTCAAGTCACAGAGTTGAACATTCCCTTTC
        131 TGATGTTTGCATTCAAGTAACAGTGTTGAACATTCCCTTTC

                                                   *     *       **     *   *
     197863 ATAGAGCAGGTTTGAAACACTCTTTTTGTAGTATCTGGATGTGGACATTTGGAGCGCTTTCAGGC
          1 ATAGAGCAGGTTTGAAACACTCTTTTTGTAGTATCTGGAAGTGGATATTTGGATAGCTTTGAGGA

            * ** *      *             *** *   *                      **  *   
     197928 CTATGGT-GAAAAAGGAAATATCTTCCCCTGAAAACTAGACAGAAGCATTCTCAGAATTTTATTT
         66 TTTCGTTGGAAACAGG-AATATCTTCATATAAAAGCTAGACAGAAGCATTCTCAGAAACTTCTTT

                   **  **    *             **  * *   *
     197992 GTGATGTGCGCCCTCAACTAACAGTGTTGAAGCTTTCTTTTG
        130 GTGATGTTTGCATTCAAGTAACAGTGTTGAACATTCCCTTTC

                     * *                  **     **  *                       
     198034 ATAGAGCAGTTCTGAAACACTCTTTTTGTAAAATCTGCTAGAGGATATTTGGATAGCTTTGAGGA
          1 ATAGAGCAGGTTTGAAACACTCTTTTTGTAGTATCTGGAAGTGGATATTTGGATAGCTTTGAGGA

                *        *   * *            **                     **    *   
     198099 TTTCTTTGGAAACGGGATTGTCTTCATATAAACTCTAGACAGAAGCATTCTCAGATGCTTCATTG
         66 TTTCGTTGGAAACAGGAATATCTTCATATAAAAGCTAGACAGAAGCATTCTCAGAAACTTCTTTG

            *       **   *    *             *        
     198164 GGATGTTTCAATTGAAGTCACAGTGTTGAACAGTCCCTTTC
        131 TGATGTTTGCATTCAAGTAACAGTGTTGAACATTCCCTTTC

                                                   *     *       **     *   *
     198205 ATAGAGCAGGTTTGAAACACTCTTTTTGTAGTATCTGGATGTGGACATTTGGAACGCTTTCAGGC
          1 ATAGAGCAGGTTTGAAACACTCTTTTTGTAGTATCTGGAAGTGGATATTTGGATAGCTTTGAGGA

            * ** *      *             *** *   *                          *   
     198270 CTATGGT-GAAAAAGGAAATATCTTCCCCTGAAAACTAGACAGAAGCATTCTCAGAAACTTATTT
         66 TTTCGTTGGAAACAGG-AATATCTTCATATAAAAGCTAGACAGAAGCATTCTCAGAAACTTCTTT

                   *** **    *             **  * *   *
     198334 GTGATGTGCCCCCTCAACTAACAGTGTTGAAGCTTTCTTTTG
        130 GTGATGTTTGCATTCAAGTAACAGTGTTGAACATTCCCTTTC

                     *                   * *     *             **            
     198376 ATAGAGCAGTTTTGAAACACTCTTTTTGTGGAATCTGCAAGTGGATATTTGTCTAGCTTTGAGGA
          1 ATAGAGCAGGTTTGAAACACTCTTTTTGTAGTATCTGGAAGTGGATATTTGGATAGCTTTGAGGA

                         *   *    *                   *       *     *        
     198441 TTTCGTTGGAAACGGGATTA-CAT-ATA-AAAAGC-AGACAGCAGCATTCCCAGAATCTTCTTTG
         66 TTTCGTTGGAAACAGGAATATCTTCATATAAAAGCTAGACAGAAGCATTCTCAGAAACTTCTTTG

                              *    *                 
     198502 TGATGTTTGCATTCAAGTCACAGAGTTGAACATTCCCTTTC
        131 TGATGTTTGCATTCAAGTAACAGTGTTGAACATTCCCTTTC

                                                *  *     *       **     *   *
     198543 ATAGAGCAGGTTTGAAACACTCTTTTTGTAGTATCTCGATGTGGACATTTGGAGCGCTTTCAGGC
          1 ATAGAGCAGGTTTGAAACACTCTTTTTGTAGTATCTGGAAGTGGATATTTGGATAGCTTTGAGGA

            * ** *      *             *** *   *                      *   *   
     198608 CTATGGT-GAAAAAGGAAATATCTTCTCCTGAAAACTAGACAGAAGCATTCTCAGAATCTTATTT
         66 TTTCGTTGGAAACAGG-AATATCTTCATATAAAAGCTAGACAGAAGCATTCTCAGAAACTTCTTT

                   **  **    *             **  * *   *
     198672 GTGATGTGCGCCCTCAACTAACAGTGTTGAAGCTTTCTTTTG
        130 GTGATGTTTGCATTCAAGTAACAGTGTTGAACATTCCCTTTC

                     *                    **     *   *         *             
     198714 ATAGAGCAGATTTGAAACACTCTTTTTGTAAAATCTGCAAGAGGATATTTGCATAGCTTTGAGGA
          1 ATAGAGCAGGTTTGAAACACTCTTTTTGTAGTATCTGGAAGTGGATATTTGGATAGCTTTGAGGA

                *        *   * *      *     **                      *    *   
     198779 TTTCATTGGAAACGGGATTGTCTTCAAATAAACTCTAGACAGAAGCATTCTCAGAAGCTTCATTG
         66 TTTCGTTGGAAACAGGAATATCTTCATATAAAAGCTAGACAGAAGCATTCTCAGAAACTTCTTTG

            *       **   *    *             *        
     198844 GGATGTTTCAATTGAAGTCACAGTGTTGAACAGTCCCTTTC
        131 TGATGTTTGCATTCAAGTAACAGTGTTGAACATTCCCTTTC

                                                         *       **   * *    
     198885 ATAGAGCAGGTTTGAAACACTCTTTTTGTAGTATCTGGAAGTGGACATTTGGAGCGCTCTCAGGA
          1 ATAGAGCAGGTTTGAAACACTCTTTTTGTAGTATCTGGAAGTGGATATTTGGATAGCTTTGAGGA

            * *  *      *             **          * *       * *          *  *
     198950 CTACGGT-GAAAAAGGAAATATCTTCCAATAAAAGCTACATAGAAGCAATGTCAGAAACTTTTTC
         66 TTTCGTTGGAAACAGG-AATATCTTCATATAAAAGCTAGACAGAAGCATTCTCAGAAACTTCTTT

            *      * ** *   **      *       *  * *   *
     199014 ATGATGTATCTACTCAGCTAACAGAGTTGAACCTTTCTTTTG
        130 GTGATGTTTGCATTCAAGTAACAGTGTTGAACATTCCCTTTC

             *       *                   * *     *             **            
     199056 AGAGAGCAGTTTTGAAACACTCTTTTTGTGGAATCTGCAAGTGGATATTTGTCTAGCTTTGAGGA
          1 ATAGAGCAGGTTTGAAACACTCTTTTTGTAGTATCTGGAAGTGGATATTTGGATAGCTTTGAGGA

                         *   *    *                   *       *   *          
     199121 TTTCGTTGGAAACGGGATTA-CAT-ATA-AAAAGC-AGACAGCAGCATTCCCAGTAACTTCTTTG
         66 TTTCGTTGGAAACAGGAATATCTTCATATAAAAGCTAGACAGAAGCATTCTCAGAAACTTCTTTG

                              *    *                 
     199182 TGATGTTTGCATTCAAGTCACAGAGTTGAACATTCCCTTTC
        131 TGATGTTTGCATTCAAGTAACAGTGTTGAACATTCCCTTTC

                                                   *     *       **     *   *
     199223 ATAGAGCAGGTTTGAAACACTCTTTTTGTAGTATCTGGATGTGGACATTTGGAGCGCTTTCAGGC
          1 ATAGAGCAGGTTTGAAACACTCTTTTTGTAGTATCTGGAAGTGGATATTTGGATAGCTTTGAGGA

            * ** *      *             *** *   *           *          *   *   
     199288 CTATGGT-GAAAAAGGAAATATCTTCCCCTGAAAACTAGACAGAAGAATTCTCAGAATCTTATTT
         66 TTTCGTTGGAAACAGG-AATATCTTCATATAAAAGCTAGACAGAAGCATTCTCAGAAACTTCTTT

                   **  **    *             **  * *   *
     199352 GTGATGTGCGCCCTCAACTAACAGTGTTGAAGCTTTCTTTTG
        130 GTGATGTTTGCATTCAAGTAACAGTGTTGAACATTCCCTTTC

                     *                    **     *   *         *             
     199394 ATAGAGCAGTTTTGAAACACTCTTTTTGTAAAATCTGCAAGAGGATATTTGCATAGCTTTGAGGA
          1 ATAGAGCAGGTTTGAAACACTCTTTTTGTAGTATCTGGAAGTGGATATTTGGATAGCTTTGAGGA

                         *   * *            **                      * *  *   
     199459 TTTCGTTGGAAACGGGATTGTCTTCATATAAACTCTAGACAGAAGCATTCTCAGAAGCGTCATTG
         66 TTTCGTTGGAAACAGGAATATCTTCATATAAAAGCTAGACAGAAGCATTCTCAGAAACTTCTTTG

            *       **   *    *             *        
     199524 GGATGTTTCAATTGAAGTCACAGTGTTGAACAGTCCCTTTC
        131 TGATGTTTGCATTCAAGTAACAGTGTTGAACATTCCCTTTC

                                                   *     *       **     *    
     199565 ATAGAGCAGGTTTGAAACACTCTTTTTGTAGTATCTGGATGTGGACATTTGGAGCGCTTTCAGGC
          1 ATAGAGCAGGTTTGAAACACTCTTTTTGTAGTATCTGGAAGTGGATATTTGGATAGCTTTGAGG-

            * * *    *                 *** *   *                          *  
     199630 CTATGGTT-TAAA-AGGAAATATCTTCCCCTGAAAACTAGACAGAAGCATTCTCAGAAACTTATT
         65 ATTTCGTTGGAAACAGG-AATATCTTCATATAAAAGCTAGACAGAAGCATTCTCAGAAACTTCTT

                    **  **    *                    *   *
     199693 TGTGATGTGCGCCCTCAACTAACAGTGTTGAAGCATT-CTTTTG
        129 TGTGATGTTTGCATTCAAGTAACAGTGTTGAA-CATTCCCTTTC

                     **                  * *     *             **            
     199736 ATAGAGCAGTATTGAAACACTCTTTTTGTGGAATCTGCAAGTGGATATTTGTCTAGCTTTGAGGA
          1 ATAGAGCAGGTTTGAAACACTCTTTTTGTAGTATCTGGAAGTGGATATTTGGATAGCTTTGAGGA

                               ** *                   *       *              
     199801 TTTCGTTGGAAA-AGGAATTACAT-ATA-AAAAGC-AGACAGCAGCATTCCCAGAAACTTCTTTG
         66 TTTCGTTGGAAACAGGAATATCTTCATATAAAAGCTAGACAGAAGCATTCTCAGAAACTTCTTTG

                              *    *                 
     199862 TGATGTTTGCATTCAAGTCACAGGGTTGAACATTCCCTTTC
        131 TGATGTTTGCATTCAAGTAACAGTGTTGAACATTCCCTTTC

                                                   *     *       **     *   *
     199903 ATAGAGCAGGTTTGAAACACTCTTTTTGTAGTATCTGGATGTGGACATTTGGAGCGCTTTCAGGC
          1 ATAGAGCAGGTTTGAAACACTCTTTTTGTAGTATCTGGAAGTGGATATTTGGATAGCTTTGAGGA

            * ** *      *             *** *   *                          *   
     199968 CTATGGT-GAAAAAGGAAATATCTTCCCCTGAAAACTAGACAGAAGCATTCTCAGAAACTTATTT
         66 TTTCGTTGGAAACAGG-AATATCTTCATATAAAAGCTAGACAGAAGCATTCTCAGAAACTTCTTT

                   **  **    *             **  * *   *
     200032 GTGATGTGCGCCCTCAACTAACAGTGTTGAAGCTTTCTTTTG
        130 GTGATGTTTGCATTCAAGTAACAGTGTTGAACATTCCCTTTC

                     *   *                **     *   *                       
     200074 ATAGAGCAGTTTTCAAACACTCTTTTTGTAAAATCTGCAAGAGGATATTTGGATAGCTTTGAGGA
          1 ATAGAGCAGGTTTGAAACACTCTTTTTGTAGTATCTGGAAGTGGATATTTGGATAGCTTTGAGGA

                         *   * *            **                      *    *   
     200139 TTTCGTTGGAAACGGGATTGTCTTCATATAAACTCTAGACAGAAGCATTCTCAGAAGCTTCATTG
         66 TTTCGTTGGAAACAGGAATATCTTCATATAAAAGCTAGACAGAAGCATTCTCAGAAACTTCTTTG

            *       **   *    *             *        
     200204 GGATGTTTCAATTGAAGTCACAGTGTTGAACAGTCCCTTTC
        131 TGATGTTTGCATTCAAGTAACAGTGTTGAACATTCCCTTTC

                                                         *       *  * * *    
     200245 ATAGAGCAGGTTTGAAACACTCTTTTTGTAGTATCTGGAAGTGGACATTTGGAGAGATCTCAGGA
          1 ATAGAGCAGGTTTGAAACACTCTTTTTGTAGTATCTGGAAGTGGATATTTGGATAGCTTTGAGGA

            * *  *    * *             **            *       * *          *  *
     200310 ATACGGT-GATAAAGGAAATATCTTCCAATAAAAGCTAGATAGAAGCAATGTCAGAAACTTTTTC
         66 TTTCGTTGGAAACAGG-AATATCTTCATATAAAAGCTAGACAGAAGCATTCTCAGAAACTTCTTT

            *      * ** *   **      *       *  * *   *
     200374 ATGATGTATCTACTCAGCTAACAGAGTTGAACCTTTCTTTTG
        130 GTGATGTTTGCATTCAAGTAACAGTGTTGAACATTCCCTTTC

             *       *                   * *     *             **            
     200416 AGAGAGCAGTTTTGAAACACTCTTTTTGTGGAATCTGCAAGTGGATATTTGTCTAGCTTTGAGGA
          1 ATAGAGCAGGTTTGAAACACTCTTTTTGTAGTATCTGGAAGTGGATATTTGGATAGCTTTGAGGA

                             *    *                   *       *              
     200481 TTTCGTTGGAAACAGGATTA-CAT-ATA-AAAAGC-AGACAGCAGCATTCCCAGAAACTTCTTTG
         66 TTTCGTTGGAAACAGGAATATCTTCATATAAAAGCTAGACAGAAGCATTCTCAGAAACTTCTTTG

                              *    *                 
     200542 TGATGTTTGCATTCAAGTCACAGAGTTGAACATTCCCTTTC
        131 TGATGTTTGCATTCAAGTAACAGTGTTGAACATTCCCTTTC

                                                *  *     *       **     *   *
     200583 ATAGAGCAGGTTTGAAACACTCTTTTTGTAGTATCTCGATGTGGACATTTGGAGCGCTTTCAGGC
          1 ATAGAGCAGGTTTGAAACACTCTTTTTGTAGTATCTGGAAGTGGATATTTGGATAGCTTTGAGGA

            * ** *      *             *** *   *                      *   *   
     200648 CTATGGT-GAAAAAGGAAATATCTTCTCCTGAAAACTAGACAGAAGCATTCTCAGAACCTTATTT
         66 TTTCGTTGGAAACAGG-AATATCTTCATATAAAAGCTAGACAGAAGCATTCTCAGAAACTTCTTT

                   **  **    *             **  * *   *
     200712 GTGATGTGCGCCCTCAACTAACAGTGTTGAAGCTTTCTTTTG
        130 GTGATGTTTGCATTCAAGTAACAGTGTTGAACATTCCCTTTC

                     *                    **     *   *         *             
     200754 ATAGAGCAGTTTTGAAACACTCTTTTTGTAAAATCTGCAAGAGGATATTTGCATAGCTTTGAGGA
          1 ATAGAGCAGGTTTGAAACACTCTTTTTGTAGTATCTGGAAGTGGATATTTGGATAGCTTTGAGGA

                *        *   * *            **                      *    *   
     200819 TTTCATTGGAAACGGGATTGTCTTCATATAAACTCTAGACAGAAGCATTCTCAGAAGCTTCATTG
         66 TTTCGTTGGAAACAGGAATATCTTCATATAAAAGCTAGACAGAAGCATTCTCAGAAACTTCTTTG

            *       *    *    *             *        
     200884 GGATGTTTCCATTGAAGTCACAGTGTTGAACAGTCCCTTTC
        131 TGATGTTTGCATTCAAGTAACAGTGTTGAACATTCCCTTTC

                                                         *       **   * *    
     200925 ATAGAGCAGGTTTGAAACACTCTTTTTGTAGTATCTGGAAGTGGACATTTGGAGCGCTCTCAGGA
          1 ATAGAGCAGGTTTGAAACACTCTTTTTGTAGTATCTGGAAGTGGATATTTGGATAGCTTTGAGGA

            * *  *      *             **          * *       * *          *  *
     200990 CTACGGT-GAAAAAGGAAATATCTTCCAATAAAAGCTACATAGAAGCAATGTCAGAAACTTTTTC
         66 TTTCGTTGGAAACAGG-AATATCTTCATATAAAAGCTAGACAGAAGCATTCTCAGAAACTTCTTT

            *      * ** *   **      *       *  * *   *
     201054 ATGATGTATCTACTCAGCTAACAGAGTTGAACCTTTCTTTTG
        130 GTGATGTTTGCATTCAAGTAACAGTGTTGAACATTCCCTTTC

             *       *                   * *     *             **            
     201096 AGAGAGCAGTTTTGAAACACTCTTTTTGTGGAATCTGCAAGTGGATATTTGTCTAGCTTTGAGGA
          1 ATAGAGCAGGTTTGAAACACTCTTTTTGTAGTATCTGGAAGTGGATATTTGGATAGCTTTGAGGA

                        *    *    *                   *       *              
     201161 TTTCGTTGGAAATAGGATTA-CAT-ATA-AAAAGC-AGACAGCAGCATTCCCAGAAACTTCTTTG
         66 TTTCGTTGGAAACAGGAATATCTTCATATAAAAGCTAGACAGAAGCATTCTCAGAAACTTCTTTG

                              *    *                 
     201222 TGATGTTTGCATTCAAGTCACAGAGTTGAACATTCCCTTTC
        131 TGATGTTTGCATTCAAGTAACAGTGTTGAACATTCCCTTTC

                                                   *     *       **     *   *
     201263 ATAGAGCAGGTTTGAAACACTCTTTTTGTAGTATCTGGATGTGGACATTTGGAGCGCTTTCAGGC
          1 ATAGAGCAGGTTTGAAACACTCTTTTTGTAGTATCTGGAAGTGGATATTTGGATAGCTTTGAGGA

            * ** *      *             *** *   **                     **  *   
     201328 CTATGGT-GAAAAAGGAAATATCTTCCCCTGAAAATTAGACAGAAGCATTCTCAGAATTTTATTT
         66 TTTCGTTGGAAACAGG-AATATCTTCATATAAAAGCTAGACAGAAGCATTCTCAGAAACTTCTTT

                   **  **    *             **  * * * *
     201392 GTGATGTGCGCCCTCAACTAACAGTGTTGAAGCTTTCTTGTG
        130 GTGATGTTTGCATTCAAGTAACAGTGTTGAACATTCCCTTTC

                     *                    **     **  * *                     
     201434 ATAGAGCAGTTTTGAAACACTCTTTTTGTAAAATCTGCTAGAGAATATTTGGATAGCTTTGAGGA
          1 ATAGAGCAGGTTTGAAACACTCTTTTTGTAGTATCTGGAAGTGGATATTTGGATAGCTTTGAGGA

                *        *   * *            **                     **    *   
     201499 TTTCTTTGGAAACGGGATTTTCTTCATATAAACTCTAGACAGAAGCATTCTCAGATGCTTCATTG
         66 TTTCGTTGGAAACAGGAATATCTTCATATAAAAGCTAGACAGAAGCATTCTCAGAAACTTCTTTG

            *       **   *    *     *       *        
     201564 GGATGTTTCAATTGAAGTCACAGTATTGAACAGTCCCTTTC
        131 TGATGTTTGCATTCAAGTAACAGTGTTGAACATTCCCTTTC

            *  *                           *       *     *       **     * * *
     201605 TTACAGCAGGTTTGAAACACTCTTTTTGTAGAATCTGGATGTGGACATTTGGAGCGCTTTCATGC
          1 ATAGAGCAGGTTTGAAACACTCTTTTTGTAGTATCTGGAAGTGGATATTTGGATAGCTTTGAGGA

            * ** *      *             *** *   *                          *   
     201670 CTATGGT-GAAAAAGGAAATATCTTCCCCTGAAAACTAGACAGAAGCATTCTCAGAAACTTATTT
         66 TTTCGTTGGAAACAGG-AATATCTTCATATAAAAGCTAGACAGAAGCATTCTCAGAAACTTCTTT

                   *** **    *             **  * *   *
     201734 GTGATGTGCCCCCTCAACTAACAGTGTTGAAGCTTTCTTTTG
        130 GTGATGTTTGCATTCAAGTAACAGTGTTGAACATTCCCTTTC

                     *   *                **     *   *                       
     201776 ATAGAGCAGTTTTCAAACACTCTTTTTGTAAAATCTGCAAGAGGATATTTGGATAGCTTTGAGGA
          1 ATAGAGCAGGTTTGAAACACTCTTTTTGTAGTATCTGGAAGTGGATATTTGGATAGCTTTGAGGA

                         *   * *            **                      *    *   
     201841 TTTCGTTGGAAACGGGATTGTCTTCATATAAACTCTAGACAGAAGCATTCTCAGAAGCTTCATTG
         66 TTTCGTTGGAAACAGGAATATCTTCATATAAAAGCTAGACAGAAGCATTCTCAGAAACTTCTTTG

            *       **   *    *             *        
     201906 GGATGTTTCAATTGAAGTCACAGTGTTGAACAGTCCCTTTC
        131 TGATGTTTGCATTCAAGTAACAGTGTTGAACATTCCCTTTC

                                                         *       *  * * *    
     201947 ATAGAGCAGGTTTGAAACACTCTTTTTGTAGTATCTGGAAGTGGACATTTGGAGAGATCTCAGGA
          1 ATAGAGCAGGTTTGAAACACTCTTTTTGTAGTATCTGGAAGTGGATATTTGGATAGCTTTGAGGA

            * *  *    * *             **            *       * *          *  *
     202012 ATACGGT-GATAAAGGAAATATCTTCCAATAAAAGCTAGATAGAAGCAATGTCAGAAACTTTTTC
         66 TTTCGTTGGAAACAGG-AATATCTTCATATAAAAGCTAGACAGAAGCATTCTCAGAAACTTCTTT

            *      * ** *   **      *       *  * *   *
     202076 ATGATGTATCTACTCAGCTAACAGAGTTGAACCTTTCTTTTG
        130 GTGATGTTTGCATTCAAGTAACAGTGTTGAACATTCCCTTTC

             *       *                   * *     *             **            
     202118 AGAGAGCAGTTTTGAAACACTCTTTTTGTGGAATCTGCAAGTGGATATTTGTCTAGCTTTGAGGA
          1 ATAGAGCAGGTTTGAAACACTCTTTTTGTAGTATCTGGAAGTGGATATTTGGATAGCTTTGAGGA

                             *    *                   *       *              
     202183 TTTCGTTGGAAACAGGATTA-CAT-ATA-AAAAGC-AGACAGCAGCATTCCCAGAAACTTCTTTG
         66 TTTCGTTGGAAACAGGAATATCTTCATATAAAAGCTAGACAGAAGCATTCTCAGAAACTTCTTTG

                              *    *                 
     202244 TGATGTTTGCATTCAAGTCACAGAGTTGAACATTCCCTTTC
        131 TGATGTTTGCATTCAAGTAACAGTGTTGAACATTCCCTTTC

                                                   *     *       **     *   *
     202285 ATAGAGCAGGTTTGAAACACTCTTTTTGTAGTATCTGGATGTGGACATTTGGAGCGCTTTCAGGC
          1 ATAGAGCAGGTTTGAAACACTCTTTTTGTAGTATCTGGAAGTGGATATTTGGATAGCTTTGAGGA

            * ** *      *             *** *   *                 *        *   
     202350 CTATGGT-GAAAAAGGAAATATCTTCCCCTGAAAACTAGACAGAAGCATTCTAAGAAACTTATTT
         66 TTTCGTTGGAAACAGG-AATATCTTCATATAAAAGCTAGACAGAAGCATTCTCAGAAACTTCTTT

                   **  **    *             **  * *   *
     202414 GTGATGTGCGCCCTCAACTAACAGTGTTGAAGCTTTCTTTTG
        130 GTGATGTTTGCATTCAAGTAACAGTGTTGAACATTCCCTTTC

                     *                    **     *   *                   *   
     202456 ATAGAGCAGTTTTGAAACACTCTTTTTGTAAAATCTGCAAGAGGATATTTGGATAGCTTTGTGGA
          1 ATAGAGCAGGTTTGAAACACTCTTTTTGTAGTATCTGGAAGTGGATATTTGGATAGCTTTGAGGA

                *        *   * *            **                     **    *   
     202521 TTTCTTTGGAAACGGGATTGTCTTCATATAAACTCTAGACAGAAGCATTCTCAGATGCTTCATTG
         66 TTTCGTTGGAAACAGGAATATCTTCATATAAAAGCTAGACAGAAGCATTCTCAGAAACTTCTTTG

            *       **   *    *             *        
     202586 GGATGTTTCAATTGAAGTCACAGTGTTGAACAGTCCCTTTC
        131 TGATGTTTGCATTCAAGTAACAGTGTTGAACATTCCCTTTC

                                                   *     *       **     *   *
     202627 ATAGAGCAGGTTTGAAACACTCTTTTTGTAGTATCTGGATGTGGACATTTGGAGCGCTTTCAGGT
          1 ATAGAGCAGGTTTGAAACACTCTTTTTGTAGTATCTGGAAGTGGATATTTGGATAGCTTTGAGGA

            * ** *      *             *** *   *                          *   
     202692 CTATGGT-GAAAAAGGAAATATCTTCCCCTGAAAACTAGACAGAAGCATTCTCAGAAACTTATTT
         66 TTTCGTTGGAAACAGG-AATATCTTCATATAAAAGCTAGACAGAAGCATTCTCAGAAACTTCTTT

                   *** **    *             **  * *   *
     202756 GTGATGTGCCCCCTCAACTAACAGTGTTGAAGCTTTCTTTTG
        130 GTGATGTTTGCATTCAAGTAACAGTGTTGAACATTCCCTTTC

                     *                   * *     *             **            
     202798 ATAGAGCAGTTTTGAAACACTCTTTTTGTGGAATCTGCAAGTGGATATTTGTCTAGCTTTGAGGA
          1 ATAGAGCAGGTTTGAAACACTCTTTTTGTAGTATCTGGAAGTGGATATTTGGATAGCTTTGAGGA

                         *   *    *                   *       *     *        
     202863 TTTCGTTGGAAACGGGATTA-CAT-ATA-AAAAGC-AGACAGCAGCATTCCCAGAATCTTCTTTG
         66 TTTCGTTGGAAACAGGAATATCTTCATATAAAAGCTAGACAGAAGCATTCTCAGAAACTTCTTTG

                              *    *                 
     202924 TGATGTTTGCATTCAAGTCACAGAGTTGAACATTCCCTTTC
        131 TGATGTTTGCATTCAAGTAACAGTGTTGAACATTCCCTTTC

                                                *  *     *       **     *   *
     202965 ATAGAGCAGGTTTGAAACACTCTTTTTGTAGTATCTCGATGTGGACATTTGGAGCGCTTTCAGGC
          1 ATAGAGCAGGTTTGAAACACTCTTTTTGTAGTATCTGGAAGTGGATATTTGGATAGCTTTGAGGA

            * ** *      *             *** *   *                      *   *   
     203030 CTATGGT-GAAAAAGGAAATATCTTCTCCTGAAAACTAGACAGAAGCATTCTCAGAATCTTATTT
         66 TTTCGTTGGAAACAGG-AATATCTTCATATAAAAGCTAGACAGAAGCATTCTCAGAAACTTCTTT

                   **  **    *             **  * *   *
     203094 GTGATGTGCGCCCTCAACTAACAGTGTTGAAGCTTTCTTTTG
        130 GTGATGTTTGCATTCAAGTAACAGTGTTGAACATTCCCTTTC

                     *                    **     *   *         *             
     203136 ATAGAGCAGTTTTGAAACACTCTTTTTGTAAAATCTGCAAGAGGATATTTGCATAGCTTTGAGGA
          1 ATAGAGCAGGTTTGAAACACTCTTTTTGTAGTATCTGGAAGTGGATATTTGGATAGCTTTGAGGA

                *        *   * *            **                      *    *   
     203201 TTTCATTGGAAACGGGATTGTCTTCATATAAACTCTAGACAGAAGCATTCTCAGAAGCTTCATTG
         66 TTTCGTTGGAAACAGGAATATCTTCATATAAAAGCTAGACAGAAGCATTCTCAGAAACTTCTTTG

            *       **   *    *             *        
     203266 GGATGTTTCAATTGAAGTCACAGTGTTGAACAGTCCCTTTC
        131 TGATGTTTGCATTCAAGTAACAGTGTTGAACATTCCCTTTC

                                                         *       **   * *    
     203307 ATAGAGCAGGTTTGAAACACTCTTTTTGTAGTATCTGGAAGTGGACATTTGGAGCGCTCTCAGGA
          1 ATAGAGCAGGTTTGAAACACTCTTTTTGTAGTATCTGGAAGTGGATATTTGGATAGCTTTGAGGA

            * *  *      *             **          * *       * *          *  *
     203372 CTACGGT-GAAAAAGGAAATATCTTCCAATAAAAGCTACATAGAAGCAATGTCAGAAACTTTTTC
         66 TTTCGTTGGAAACAGG-AATATCTTCATATAAAAGCTAGACAGAAGCATTCTCAGAAACTTCTTT

            *      *  * *   **      *       *  * *   *
     203436 ATGATGTATGTACTCAGCTAACAGAGTTGAACCTTTCTTTTG
        130 GTGATGTTTGCATTCAAGTAACAGTGTTGAACATTCCCTTTC

             *       *                   * *     *             **          * 
     203478 AGAGAGCAGTTTTGAAACACTCTTTTTGTGGAATCTGCAAGTGGATATTTGTCTAGCTTTGAGAA
          1 ATAGAGCAGGTTTGAAACACTCTTTTTGTAGTATCTGGAAGTGGATATTTGGATAGCTTTGAGGA

                             *    *                   *       *              
     203543 TTTCGTTGGAAACAGGATTA-CAT-ATA-AAAAGC-AGACAGCAGCATTCCCAGAAACTTCTTTG
         66 TTTCGTTGGAAACAGGAATATCTTCATATAAAAGCTAGACAGAAGCATTCTCAGAAACTTCTTTG

                              *    *                 
     203604 TGATGTTTGCATTCAAGTCACAGAGTTGAACATTCCCTTTC
        131 TGATGTTTGCATTCAAGTAACAGTGTTGAACATTCCCTTTC

                                                   *     *       **     *   *
     203645 ATAGAGCAGGTTTGAAACACTCTTTTTGTAGTATCTGGATGTGGACATTTGGAGCGCTTTCAGGC
          1 ATAGAGCAGGTTTGAAACACTCTTTTTGTAGTATCTGGAAGTGGATATTTGGATAGCTTTGAGGA

            * ** *      *             *** *   *                      **  *   
     203710 CTATGGT-GAAAAAGGAAATATCTTCCCCTGAAAACTAGACAGAAGCATTCTCAGAATTTTATTT
         66 TTTCGTTGGAAACAGG-AATATCTTCATATAAAAGCTAGACAGAAGCATTCTCAGAAACTTCTTT

                   **  **    *             **  * *   *
     203774 GTGATGTGCGCCCTCAACTAACAGTGTTGAAGCTTTCTTTTG
        130 GTGATGTTTGCATTCAAGTAACAGTGTTGAACATTCCCTTTC

                     *                    **     **  *                       
     203816 ATAGAGCAGTTTTGAAACACTCTTTTTGTAAAATCTGCTAGAGGATATTTGGATAGCTTTGAGGA
          1 ATAGAGCAGGTTTGAAACACTCTTTTTGTAGTATCTGGAAGTGGATATTTGGATAGCTTTGAGGA

                         *   * *            **                     **    *   
     203881 TTTCGTTGGAAACGGGATTGTCTTCATATAAACTCTAGACAGAAGCATTCTCAGATGCTTCATTG
         66 TTTCGTTGGAAACAGGAATATCTTCATATAAAAGCTAGACAGAAGCATTCTCAGAAACTTCTTTG

            *       **   *    *             *        
     203946 GGATGTTTCAATTGAAGTCACAGTGTTGAACAGTCCCTTTC
        131 TGATGTTTGCATTCAAGTAACAGTGTTGAACATTCCCTTTC

                                                   *     *       **     *   *
     203987 ATAGAGCAGGTTTGAAACACTCTTTTTGTAGTATCTGGATGTGGACATTTGGAGCGCTTTCAGGC
          1 ATAGAGCAGGTTTGAAACACTCTTTTTGTAGTATCTGGAAGTGGATATTTGGATAGCTTTGAGGA

            * ** *      *             *** *   *                          *   
     204052 CTATGGT-GAAAAAGGAAATATCTTCCCCTGAAAACTAGACAGAAGCATTCTCAGAAACTTATTT
         66 TTTCGTTGGAAACAGG-AATATCTTCATATAAAAGCTAGACAGAAGCATTCTCAGAAACTTCTTT

            *      *** **    *             **  * *   *
     204116 CTGATGTGCCCCCTCAACTAACAGTGTTGAAGCTTTCTTTTG
        130 GTGATGTTTGCATTCAAGTAACAGTGTTGAACATTCCCTTTC

                     *                   * *     *             **            
     204158 ATAGAGCAGTTTTGAAACACTCTTTTTGTGGAATCTGCAAGTGGATATTTGTCTAGCTTTGAGGA
          1 ATAGAGCAGGTTTGAAACACTCTTTTTGTAGTATCTGGAAGTGGATATTTGGATAGCTTTGAGGA

                         *   *    *                   *       *     *        
     204223 TTTCGTTGGAAACGGGATTA-CAT-ATA-AAAAGC-AGACAGCAGCATTCCCAGAATCTTCTTTG
         66 TTTCGTTGGAAACAGGAATATCTTCATATAAAAGCTAGACAGAAGCATTCTCAGAAACTTCTTTG

                              *    *                 
     204284 TGATGTTTGCATTCAAGTCACAGAGTTGAACATTCCCTTTC
        131 TGATGTTTGCATTCAAGTAACAGTGTTGAACATTCCCTTTC

                                                *  *     *       **     *   *
     204325 ATAGAGCAGGTTTGAAACACTCTTTTTGTAGTATCTCGATGTGGACATTTGGAGCGCTTTCAGGC
          1 ATAGAGCAGGTTTGAAACACTCTTTTTGTAGTATCTGGAAGTGGATATTTGGATAGCTTTGAGGA

            * ** *      *             *** *   *                      *   *   
     204390 CTATGGT-GAAAAAGGAAATATCTTCTCCTGAAAACTAGACAGAAGCATTCTCAGAATCTTATTT
         66 TTTCGTTGGAAACAGG-AATATCTTCATATAAAAGCTAGACAGAAGCATTCTCAGAAACTTCTTT

                   **  **    *              *  * *    
     204454 GTGATGTGCGCTCTCAACTAACAGTGTTGAACCTTTCTTTTC
        130 GTGATGTTTGCATTCAAGTAACAGTGTTGAACATTCCCTTTC

                  *  *                    **     *   *         *             
     204496 ATAGAGTAGTTTTGAAACACTCTTTTTGTAAAATCTGCAAGAGGATATTTGCATAGCTTTGAGGA
          1 ATAGAGCAGGTTTGAAACACTCTTTTTGTAGTATCTGGAAGTGGATATTTGGATAGCTTTGAGGA

                *        *   * *            **                      *    *   
     204561 TTTCATTGGAAACGGGATTGTCTTCATATAAACTCTAGACAGAAGCATTCTCAGAAGCTTCATTG
         66 TTTCGTTGGAAACAGGAATATCTTCATATAAAAGCTAGACAGAAGCATTCTCAGAAACTTCTTTG

            *       **   *    *             *        
     204626 GGATGTTTCAATTGAAGTCACAGTGTTGAACAGTCCCTTTC
        131 TGATGTTTGCATTCAAGTAACAGTGTTGAACATTCCCTTTC

                                                         *       **   * *    
     204667 ATAGAGCAGGTTTGAAACACTCTTTTTGTAGTATCTGGAAGTGGACATTTGGAGCGCTCTCAGGA
          1 ATAGAGCAGGTTTGAAACACTCTTTTTGTAGTATCTGGAAGTGGATATTTGGATAGCTTTGAGGA

            * *  *      *             **          * *       * *          *  *
     204732 CTACGGT-GAAAAAGGAAATATCTTCCAATAAAAGCTACATAGAAGCAATGTCAGAAACTTTTTC
         66 TTTCGTTGGAAACAGG-AATATCTTCATATAAAAGCTAGACAGAAGCATTCTCAGAAACTTCTTT

            *      * ** *   **      *       *  * *   *
     204796 ATGATGTATCTACTCAGCTAACAGAGTTGAACCTTTCTTTTG
        130 GTGATGTTTGCATTCAAGTAACAGTGTTGAACATTCCCTTTC

             *       *                   * *     *             **            
     204838 AGAGAGCAGTTTTGAAACACTCTTTTTGTGGAATCTGCAAGTGGATATTTGTCTAGCTTTGAGGA
          1 ATAGAGCAGGTTTGAAACACTCTTTTTGTAGTATCTGGAAGTGGATATTTGGATAGCTTTGAGGA

                             *    *                   *       *              
     204903 TTTCGTTGGAAACAGGATTA-CAT-ATA-AAAAGC-AGACAGCAGCATTCCCAGAAACTTCTTTG
         66 TTTCGTTGGAAACAGGAATATCTTCATATAAAAGCTAGACAGAAGCATTCTCAGAAACTTCTTTG

                              *    *                 
     204964 TGATGTTTGCATTCAAGTCACAGAGTTGAACATTCCCTTTC
        131 TGATGTTTGCATTCAAGTAACAGTGTTGAACATTCCCTTTC

                                                   *     *       **     *   *
     205005 ATAGAGCAGGTTTGAAACACTCTTTTTGTAGTATCTGGATGTGGACATTTGGAGCGCTTTCAGGC
          1 ATAGAGCAGGTTTGAAACACTCTTTTTGTAGTATCTGGAAGTGGATATTTGGATAGCTTTGAGGA

            * ** *      *             *** *   *                      **  *   
     205070 CTATGGT-GAAAAAGGAAATATCTTCCCCTGAAAACTAGACAGAAGCATTCTCAGAATTTTATTT
         66 TTTCGTTGGAAACAGG-AATATCTTCATATAAAAGCTAGACAGAAGCATTCTCAGAAACTTCTTT

                   **  **    *             **  * *   *
     205134 GTGATGTGCGCCCTCAACTAACAGTGTTGAAGCTTTCTTTTG
        130 GTGATGTTTGCATTCAAGTAACAGTGTTGAACATTCCCTTTC

                     *                    **     **  *        *              
     205176 ATAGAGCAGTTTTGAAACACTCTTTTTGTAAAATCTGCTAGAGGATATTTCGATAGCTTTGAGGA
          1 ATAGAGCAGGTTTGAAACACTCTTTTTGTAGTATCTGGAAGTGGATATTTGGATAGCTTTGAGGA

                         *   * *            **                     **    *   
     205241 TTTCGTTGGAAACGGGATTGTCTTCATATAAACTCTAGACAGAAGCATTCTCAGATGCTTCATTG
         66 TTTCGTTGGAAACAGGAATATCTTCATATAAAAGCTAGACAGAAGCATTCTCAGAAACTTCTTTG

            *       **   *    *             *        
     205306 GGATGTTTCAATTGAAGTCACAGTGTTGAACAGTCCCTTTC
        131 TGATGTTTGCATTCAAGTAACAGTGTTGAACATTCCCTTTC

                                                   *     *       **     *   *
     205347 ATAGAGCAGGTTTGAAACACTCTTTTTGTAGTATCTGGATGTGGACATTTGGAGCGCTTTCAGGC
          1 ATAGAGCAGGTTTGAAACACTCTTTTTGTAGTATCTGGAAGTGGATATTTGGATAGCTTTGAGGA

            * ** *      *             *** *   *                          *   
     205412 CTATGGT-GAAAAAGGAAATATCTTCCCCTGAAAACTAGACAGAAGCATTCTCAGAAACTTATTT
         66 TTTCGTTGGAAACAGG-AATATCTTCATATAAAAGCTAGACAGAAGCATTCTCAGAAACTTCTTT

            *      *** **    *             **  * *   *
     205476 CTGATGTGCCCCCTCAACTAACAGTGTTGAAGCTTTCTTTTG
        130 GTGATGTTTGCATTCAAGTAACAGTGTTGAACATTCCCTTTC

                     *                   * *     *             **            
     205518 ATAGAGCAGTTTTGAAACACTCTTTTTGTGGAATCTGCAAGTGGATATTTGTCTAGCTTTGAGGA
          1 ATAGAGCAGGTTTGAAACACTCTTTTTGTAGTATCTGGAAGTGGATATTTGGATAGCTTTGAGGA

                         *   *    *                   *       *     *  *     
     205583 TTTCGTTGGAAACGGGATTA-CAT-ATA-AAAAGC-AGACAGCAGCATTCCCAGAATCTCCTTTG
         66 TTTCGTTGGAAACAGGAATATCTTCATATAAAAGCTAGACAGAAGCATTCTCAGAAACTTCTTTG

                              *    *                 
     205644 TGATGTTTGCATTCAAGTCACAGAGTTGAACATTCCCTTTC
        131 TGATGTTTGCATTCAAGTAACAGTGTTGAACATTCCCTTTC

                                                *  *     *       **     *   *
     205685 ATAGAGCAGGTTTGAAACACTCTTTTTGTAGTATCTCGATGTGGACATTTGGAGCGCTTTCAGGC
          1 ATAGAGCAGGTTTGAAACACTCTTTTTGTAGTATCTGGAAGTGGATATTTGGATAGCTTTGAGGA

            * ** *      *             *** *   *                      *   *   
     205750 CTATGGT-GAAAAAGGAAATATCTTCTCCTGAAAACTAGACAGAAGCATTCTCAGAATCTTATTT
         66 TTTCGTTGGAAACAGG-AATATCTTCATATAAAAGCTAGACAGAAGCATTCTCAGAAACTTCTTT

                   **  **    *             ** ** *   *
     205814 GTGATGTGCGCCCTCAACTAACAGTGTTGAAGCTATCTTTTG
        130 GTGATGTTTGCATTCAAGTAACAGTGTTGAACATTCCCTTTC

                     *                    **     *   *         *             
     205856 ATAGAGCAGTTTTGAAACACTCTTTTTGTAAAATCTGCAAGAGGATATTTGCATAGCTTTGAGGA
          1 ATAGAGCAGGTTTGAAACACTCTTTTTGTAGTATCTGGAAGTGGATATTTGGATAGCTTTGAGGA

                *        *   * *            **                      *    *   
     205921 TTTCATTGGAAACGGGATTGTCTTCATATAAACTCTAGACAGAAGCATTCTCAGAAGCTTCATTG
         66 TTTCGTTGGAAACAGGAATATCTTCATATAAAAGCTAGACAGAAGCATTCTCAGAAACTTCTTTG

            *       **   *    *             *        
     205986 GGATGTTTCAATTGAAGTCACAGTGTTGAACAGTCCCTTTC
        131 TGATGTTTGCATTCAAGTAACAGTGTTGAACATTCCCTTTC

                                                         *       **   * *    
     206027 ATAGAGCAGGTTTGAAACACTCTTTTTGTAGTATCTGGAAGTGGACATTTGGAGCGCTCTCAGGA
          1 ATAGAGCAGGTTTGAAACACTCTTTTTGTAGTATCTGGAAGTGGATATTTGGATAGCTTTGAGGA

            * *  *      *             **          * *       * *          *  *
     206092 CTACGGT-GAAAAAGGAAATATCTTCCAATAAAAGCTACATAGAAGCAATGTCAGAAACTTTTTC
         66 TTTCGTTGGAAACAGG-AATATCTTCATATAAAAGCTAGACAGAAGCATTCTCAGAAACTTCTTT

            *      * ** *   **      *       *  * *   *
     206156 ATGATGTATCTACTCAGCTAACAGAGTTGAACCTTTCTTTTG
        130 GTGATGTTTGCATTCAAGTAACAGTGTTGAACATTCCCTTTC

             *       *                   * *     *             **            
     206198 AGAGAGCAGTTTTGAAACACTCTTTTTGTGGAATCTGCAAGTGGATATTTGTCTAGCTTTGAGGA
          1 ATAGAGCAGGTTTGAAACACTCTTTTTGTAGTATCTGGAAGTGGATATTTGGATAGCTTTGAGGA

                             *    *                   *       *              
     206263 TTTCGTTGGAAACAGGATTA-CAT-ATA-AAAAGC-AGACAGCAGCATTCCCAGAAACTTCTTTG
         66 TTTCGTTGGAAACAGGAATATCTTCATATAAAAGCTAGACAGAAGCATTCTCAGAAACTTCTTTG

                              *    *                 
     206324 TGATGTTTGCATTCAAGTCACAGAGTTGAACATTCCCTTTC
        131 TGATGTTTGCATTCAAGTAACAGTGTTGAACATTCCCTTTC

                                                   *     *       **     *   *
     206365 ATAGAGCAGGTTTGAAACACTCTTTTTGTAGTATCTGGATGTGGACATTTGGAGCGCTTTCAGGC
          1 ATAGAGCAGGTTTGAAACACTCTTTTTGTAGTATCTGGAAGTGGATATTTGGATAGCTTTGAGGA

            * ** *      *             *** *   *                      **  *   
     206430 CTATGGT-GAAAAAGGAAATATCTTCCCCTGAAAACTAGACAGAAGCATTCTCAGAATTTTATTT
         66 TTTCGTTGGAAACAGG-AATATCTTCATATAAAAGCTAGACAGAAGCATTCTCAGAAACTTCTTT

                   **  **    *             **  * *   *
     206494 GTGATGTGCGCCCTCAACTAACAGTGTTGAAGCTTTCTTTTG
        130 GTGATGTTTGCATTCAAGTAACAGTGTTGAACATTCCCTTTC

                     *       *            **     **  *        *              
     206536 ATAGAGCAGTTTTGAAAAACTCTTTTTGTAAAATCTGCTAGAGGATATTTCGATAGCTTTGAGGA
          1 ATAGAGCAGGTTTGAAACACTCTTTTTGTAGTATCTGGAAGTGGATATTTGGATAGCTTTGAGGA

                         *   * *            **                     **    *   
     206601 TTTCGTTGGAAACGGGATTGTCTTCATATAAACTCTAGACAGAAGCATTCTCAGATGCTTCATTG
         66 TTTCGTTGGAAACAGGAATATCTTCATATAAAAGCTAGACAGAAGCATTCTCAGAAACTTCTTTG

            *       **   *    *             *        
     206666 GGATGTTTCAATTGAAGTCACAGTGTTGAACAGTCCCTTTC
        131 TGATGTTTGCATTCAAGTAACAGTGTTGAACATTCCCTTTC

                                                   *     *       **     *   *
     206707 ATAGAGCAGGTTTGAAACACTCTTTTTGTAGTATCTGGATGTGGACATTTGGAGCGCTTTCAGGC
          1 ATAGAGCAGGTTTGAAACACTCTTTTTGTAGTATCTGGAAGTGGATATTTGGATAGCTTTGAGGA

            * ** *      *             *** *   *                          *   
     206772 CTATGGT-GAAAAAGGAAATATCTTCCCCTGAAAACTAGACAGAAGCATTCTCAGAAACTTATTT
         66 TTTCGTTGGAAACAGG-AATATCTTCATATAAAAGCTAGACAGAAGCATTCTCAGAAACTTCTTT

            *      *** **    *             **  * *   *
     206836 CTGATGTGCCCCCTCAACTAACAGTGTTGAAGCTTTCTTTTG
        130 GTGATGTTTGCATTCAAGTAACAGTGTTGAACATTCCCTTTC

                     *                   * *     *             **            
     206878 ATAGAGCAGTTTTGAAACACTCTTTTTGTGGAATCTGCAAGTGGATATTTGTCTAGCTTTGAGGA
          1 ATAGAGCAGGTTTGAAACACTCTTTTTGTAGTATCTGGAAGTGGATATTTGGATAGCTTTGAGGA

                         *   *    *                   *       *     *  *     
     206943 TTTCGTTGGAAACGGGATTA-CAT-ATA-AAAAGC-AGACAGCAGCATTCCCAGAATCTCCTTTG
         66 TTTCGTTGGAAACAGGAATATCTTCATATAAAAGCTAGACAGAAGCATTCTCAGAAACTTCTTTG

                              *    *                 
     207004 TGATGTTTGCATTCAAGTCACAGAGTTGAACATTCCCTTTC
        131 TGATGTTTGCATTCAAGTAACAGTGTTGAACATTCCCTTTC

                                                *  *     *       **     *   *
     207045 ATAGAGCAGGTTTGAAACACTCTTTTTGTAGTATCTCGATGTGGACATTTGGAGCGCTTTCAGGC
          1 ATAGAGCAGGTTTGAAACACTCTTTTTGTAGTATCTGGAAGTGGATATTTGGATAGCTTTGAGGA

            * ** *      *             *** *   *                      *   *   
     207110 CTATGGT-GAAAAAGGAAATATCTTCTCCTGAAAACTAGACAGAAGCATTCTCAGAATCTTATTT
         66 TTTCGTTGGAAACAGG-AATATCTTCATATAAAAGCTAGACAGAAGCATTCTCAGAAACTTCTTT

                   **  **    *             ** ** *   *
     207174 GTGATGTGCGCCCTCAACTAACAGTGTTGAAGCTATCTTTTG
        130 GTGATGTTTGCATTCAAGTAACAGTGTTGAACATTCCCTTTC

                     *                    **     *   *         *             
     207216 ATAGAGCAGTTTTGAAACACTCTTTTTGTAAAATCTGCAAGAGGATATTTGCATAGCTTTGAGGA
          1 ATAGAGCAGGTTTGAAACACTCTTTTTGTAGTATCTGGAAGTGGATATTTGGATAGCTTTGAGGA

                *        *   * *            **                      *    *   
     207281 TTTCATTGGAAACGGGATTGTCTTCATATAAACTCTAGACAGAAGCATTCTCAGAAGCTTCATTG
         66 TTTCGTTGGAAACAGGAATATCTTCATATAAAAGCTAGACAGAAGCATTCTCAGAAACTTCTTTG

            *       **   *    *             *        
     207346 GGATGTTTCAATTGAAGTCACAGTGTTGAACAGTCCCTTTC
        131 TGATGTTTGCATTCAAGTAACAGTGTTGAACATTCCCTTTC

                                                         *       **   * *    
     207387 ATAGAGCAGGTTTGAAACACTCTTTTTGTAGTATCTGGAAGTGGACATTTGGAGCGCTCTCAGGA
          1 ATAGAGCAGGTTTGAAACACTCTTTTTGTAGTATCTGGAAGTGGATATTTGGATAGCTTTGAGGA

            * *  *      *             **          * *       * *          *  *
     207452 CTACGGT-GAAAAAGGAAATATCTTCCAATAAAAGCTACATAGAAGCAATGTCAGAAACTTTTTC
         66 TTTCGTTGGAAACAGG-AATATCTTCATATAAAAGCTAGACAGAAGCATTCTCAGAAACTTCTTT

            *      * ** *   **      *       *  * *   *
     207516 ATGATGTATCTACTCAGCTAACAGAGTTGAACCTTTCTTTTG
        130 GTGATGTTTGCATTCAAGTAACAGTGTTGAACATTCCCTTTC

             *       *                   * *     *             **            
     207558 AGAGAGCAGTTTTGAAACACTCTTTTTGTGGAATCTGCAAGTGGATATTTGTCTAGCTTTGAGGA
          1 ATAGAGCAGGTTTGAAACACTCTTTTTGTAGTATCTGGAAGTGGATATTTGGATAGCTTTGAGGA

                             *    *                   *       *              
     207623 TTTCGTTGGAAACAGGATTA-CAT-ATA-AAAAGC-AGACAGCAGCATTCCCAGAAACTTCTTTG
         66 TTTCGTTGGAAACAGGAATATCTTCATATAAAAGCTAGACAGAAGCATTCTCAGAAACTTCTTTG

                              *    *                 
     207684 TGATGTTTGCATTCAAGTCACAGAGTTGAACATTCCCTTTC
        131 TGATGTTTGCATTCAAGTAACAGTGTTGAACATTCCCTTTC

                                                   *     *       **     *   *
     207725 ATAGAGCAGGTTTGAAACACTCTTTTTGTAGTATCTGGATGTGGACATTTGGAGCGCTTTCAGGC
          1 ATAGAGCAGGTTTGAAACACTCTTTTTGTAGTATCTGGAAGTGGATATTTGGATAGCTTTGAGGA

            * ** *      *             *** *   *                      **  *   
     207790 CTATGGT-GAAAAAGGAAATATCTTCCCCTGAAAACTAGACAGAAGCATTCTCAGAATTTTATTT
         66 TTTCGTTGGAAACAGG-AATATCTTCATATAAAAGCTAGACAGAAGCATTCTCAGAAACTTCTTT

                   **  **    *             **  * *   *
     207854 GTGATGTGCGCCCTCAACTAACAGTGTTGAAGCTTTCTTTTG
        130 GTGATGTTTGCATTCAAGTAACAGTGTTGAACATTCCCTTTC

                                          **     **  *                       
     207896 ATAGAGCAGGTTTGAAACACTCTTTTTGTAAAATCTGCTAGAGGATATTTGGATAGCTTTGAGGA
          1 ATAGAGCAGGTTTGAAACACTCTTTTTGTAGTATCTGGAAGTGGATATTTGGATAGCTTTGAGGA

                *        *   * *            **                     **    *   
     207961 TTTCTTTGGAAACGGGATTGTCTTCATATAAACTCTAGACAGAAGCATTCTCAGATGCTTCATTG
         66 TTTCGTTGGAAACAGGAATATCTTCATATAAAAGCTAGACAGAAGCATTCTCAGAAACTTCTTTG

            *       **   *    *             *        
     208026 GGATGTTTCAATTGAAGTCACAGTGTTGAACAGTCCCTTTC
        131 TGATGTTTGCATTCAAGTAACAGTGTTGAACATTCCCTTTC

                                                   *     *       **     *   *
     208067 ATAGAGCAGGTTTGAAACACTCTTTTTGTAGTATCTGGATGTGGACATTTGGAGCGCTTTCAGGC
          1 ATAGAGCAGGTTTGAAACACTCTTTTTGTAGTATCTGGAAGTGGATATTTGGATAGCTTTGAGGA

            * ** *      *             *** *   *                          *   
     208132 CTATGGT-GAAAAAGGAAATATCTTCCCCTGAAAACTAGACAGAAGCATTCTCAGAAACTTATTT
         66 TTTCGTTGGAAACAGG-AATATCTTCATATAAAAGCTAGACAGAAGCATTCTCAGAAACTTCTTT

            *      *** **    *             **  * *   *
     208196 CTGATGTGCCCCCTCAACTAACAGTGTTGAAGCTTTCTTTTG
        130 GTGATGTTTGCATTCAAGTAACAGTGTTGAACATTCCCTTTC

                                         * *     *             **            
     208238 ATAGAGCAGGTTTGAAACACTCTTTTTGTGGAATCTGCAAGTGGATATTTGTCTAGCTTTGAGGA
          1 ATAGAGCAGGTTTGAAACACTCTTTTTGTAGTATCTGGAAGTGGATATTTGGATAGCTTTGAGGA

                         *   *    *                   *       *     *        
     208303 TTTCGTTGGAAACGGGATTA-CAT-ATA-AAAAGC-AGACAGCAGCATTCCCAGAATCTTCTTTG
         66 TTTCGTTGGAAACAGGAATATCTTCATATAAAAGCTAGACAGAAGCATTCTCAGAAACTTCTTTG

                              *    *                 
     208364 TGATGTTTGCATTCAAGTCACAGAGTTGAACATTCCCTTTC
        131 TGATGTTTGCATTCAAGTAACAGTGTTGAACATTCCCTTTC

                                                *  *     *       **     *   *
     208405 ATAGAGCAGGTTTGAAACACTCTTTTTGTAGTATCTCGATGTGGACATTTGGAGCGCTTTCAGGC
          1 ATAGAGCAGGTTTGAAACACTCTTTTTGTAGTATCTGGAAGTGGATATTTGGATAGCTTTGAGGA

            * ** *      *             *** *   *   *                  *   *   
     208470 CTATGGT-GAAAAAGGAATTATCTTCTCCTGAAAACTACACAGAAGCATTCTCAGAATCTTATTT
         66 TTTCGTTGGAAACAGGAA-TATCTTCATATAAAAGCTAGACAGAAGCATTCTCAGAAACTTCTTT

                   **  **    *             **  * *   *
     208534 GTGATGTGCGCCCTCAACTAACAGTGTTGAAGCTTTCTTTTG
        130 GTGATGTTTGCATTCAAGTAACAGTGTTGAACATTCCCTTTC

                     *         *          **     *   *         *             
     208576 ATAGAGCAGTTTTGAAACATTCTTTTTGTAAAATCTGCAAGAGGATATTTGCATAGCTTTGAGGA
          1 ATAGAGCAGGTTTGAAACACTCTTTTTGTAGTATCTGGAAGTGGATATTTGGATAGCTTTGAGGA

                *        *   * *            **                      *    *   
     208641 TTTCATTGGAAACGGGATTGTCTTCATATAAACTCTAGACAGAAGCATTCTCAGAAGCTTCATTG
         66 TTTCGTTGGAAACAGGAATATCTTCATATAAAAGCTAGACAGAAGCATTCTCAGAAACTTCTTTG

            *       **   *    *             *        
     208706 GGATGTTTCAATTGAAGTCACAGTGTTGAACAGTCCCTTTC
        131 TGATGTTTGCATTCAAGTAACAGTGTTGAACATTCCCTTTC

                                          *              *       **   * *    
     208747 ATAGAGCAGGTTTGAAACACTCTTTTTGTAATATCTGGAAGTGGACATTTGGAGCGCTCTCAGGA
          1 ATAGAGCAGGTTTGAAACACTCTTTTTGTAGTATCTGGAAGTGGATATTTGGATAGCTTTGAGGA

            * *  *      *             **          * *       * *          *  *
     208812 CTACGGT-GAAAAAGGAAATATCTTCCAATAAAAGCTACATAGAAGCAATGTCAGAAACTTTTTC
         66 TTTCGTTGGAAACAGG-AATATCTTCATATAAAAGCTAGACAGAAGCATTCTCAGAAACTTCTTT

            *      * ** *   **      *       *  * *   *
     208876 ATGATGTATCTACTCAGCTAACAGAGTTGAACCTTTCTTTTG
        130 GTGATGTTTGCATTCAAGTAACAGTGTTGAACATTCCCTTTC

             *       *                   * *     *             **            
     208918 AGAGAGCAGTTTTGAAACACTCTTTTTGTGGAATCTGCAAGTGGATATTTGTCTAGCTTTGAGGA
          1 ATAGAGCAGGTTTGAAACACTCTTTTTGTAGTATCTGGAAGTGGATATTTGGATAGCTTTGAGGA

                             *    *                   *       *              
     208983 TTTCGTTGGAAACAGGATTA-CAT-ATA-AAAAGC-AGACAGCAGCATTCCCAGAAACTTCTTTG
         66 TTTCGTTGGAAACAGGAATATCTTCATATAAAAGCTAGACAGAAGCATTCTCAGAAACTTCTTTG

                              *    *                 
     209044 TGATGTTTGCATTCAAGTCACAGAGTTGAACATTCCCTTTC
        131 TGATGTTTGCATTCAAGTAACAGTGTTGAACATTCCCTTTC

                                                   *     *       **     *   *
     209085 ATAGAGCAGGTTTGAAACACTCTTTTTGTAGTATCTGGATGTGGACATTTGGAGCGCTTTCAGGC
          1 ATAGAGCAGGTTTGAAACACTCTTTTTGTAGTATCTGGAAGTGGATATTTGGATAGCTTTGAGGA

            * ** *      *             *** *   *     *                **  *   
     209150 CTATGGT-GAAAAAGGAAATATCTTCCCCTGAAAACTAGAGAGAAGCATTCTCAGAATTTTATTT
         66 TTTCGTTGGAAACAGG-AATATCTTCATATAAAAGCTAGACAGAAGCATTCTCAGAAACTTCTTT

                   **  **    *             **  * *   *
     209214 GTGATGTGCGCCCTCAACTAACAGTGTTGAAGCTTTCTTTTG
        130 GTGATGTTTGCATTCAAGTAACAGTGTTGAACATTCCCTTTC

                     *                    **     **  *                       
     209256 ATAGAGCAGTTTTGAAACACTCTTTTTGTAAAATCTGCTAGAGGATATTTGGATAGCTTTGAGGA
          1 ATAGAGCAGGTTTGAAACACTCTTTTTGTAGTATCTGGAAGTGGATATTTGGATAGCTTTGAGGA

                *        *   * *            **          *          **    *   
     209321 TTTCTTTGGAAACGGGATTGTCTTCATATAAACTCTAGACAGAATCATTCTCAGATGCTTCATTG
         66 TTTCGTTGGAAACAGGAATATCTTCATATAAAAGCTAGACAGAAGCATTCTCAGAAACTTCTTTG

            *       **   *    *             *        
     209386 GGATGTTTCAATTGAAGTCACAGTGTTGAACAGTCCCTTTC
        131 TGATGTTTGCATTCAAGTAACAGTGTTGAACATTCCCTTTC

                                                   *     *       **     *   *
     209427 ATAGAGCAGGTTTGAAACACTCTTTTTGTAGTATCTGGATGTGGACATTTGGAGCGCTTTCAGGC
          1 ATAGAGCAGGTTTGAAACACTCTTTTTGTAGTATCTGGAAGTGGATATTTGGATAGCTTTGAGGA

            * ** *      *             *** *   *                          *   
     209492 CTATGGT-GAAAAAGGAAATATCTTCCCCTGAAAACTAGACAGAAGCATTCTCAGAAACTTATTT
         66 TTTCGTTGGAAACAGG-AATATCTTCATATAAAAGCTAGACAGAAGCATTCTCAGAAACTTCTTT

                   *** **    *             **  * *   *
     209556 GTGATGTGCCCCCTCAACTAACAGTGTTGAAGCTTTCTTTTG
        130 GTGATGTTTGCATTCAAGTAACAGTGTTGAACATTCCCTTTC

                     *                   * *     *             **            
     209598 ATAGAGCAGTTTTGAAACACTCTTTTTGTGGAATCTGCAAGTGGATATTTGTCTAGCTTTGAGGA
          1 ATAGAGCAGGTTTGAAACACTCTTTTTGTAGTATCTGGAAGTGGATATTTGGATAGCTTTGAGGA

                         *   *    *                   *       *     *        
     209663 TTTCGTTGGAAACGGGATTA-CAT-ATA-AAAAGC-AGACAGCAGCATTCCCAGAATCTTCTTTG
         66 TTTCGTTGGAAACAGGAATATCTTCATATAAAAGCTAGACAGAAGCATTCTCAGAAACTTCTTTG

                              *    *                 
     209724 TGATGTTTGCATTCAAGTCACAGAGTTGAACATTCCCTTTC
        131 TGATGTTTGCATTCAAGTAACAGTGTTGAACATTCCCTTTC

                                                *  *     *       **     *   *
     209765 ATAGAGCAGGTTTGAAACACTCTTTTTGTAGTATCTCGATGTGGACATTTGGAGCGCTTTCAGGC
          1 ATAGAGCAGGTTTGAAACACTCTTTTTGTAGTATCTGGAAGTGGATATTTGGATAGCTTTGAGGA

            * ** *      *             *** *   *                      *   *   
     209830 CTATGGT-GAAAAAGGAAATATCTTCTCCTGAAAACTAGACAGAAGCATTCTCAGAATCTTATTT
         66 TTTCGTTGGAAACAGG-AATATCTTCATATAAAAGCTAGACAGAAGCATTCTCAGAAACTTCTTT

                   **  **    *             **  * *   *
     209894 GTGATGTGCGCCCTCAACTAACAGTGTTGAAGCTTTCTTTTG
        130 GTGATGTTTGCATTCAAGTAACAGTGTTGAACATTCCCTTTC

                     *                    **     *   *                       
     209936 ATAGAGCAGTTTTGAAACACTCTTTTTGTAAAATCTGCAAGAGGATATTTGGATAGCTTTGAGGA
          1 ATAGAGCAGGTTTGAAACACTCTTTTTGTAGTATCTGGAAGTGGATATTTGGATAGCTTTGAGGA

                         *   * *            **                      *    *   
     210001 TTTCGTTGGAAACGGGATTGTCTTCATATAAACTCTAGACAGAAGCATTCTCAGAAGCTTCATTG
         66 TTTCGTTGGAAACAGGAATATCTTCATATAAAAGCTAGACAGAAGCATTCTCAGAAACTTCTTTG

            *       **   *    *             *        
     210066 GGATGTTTCAATTGAAGTCACAGTGTTGAACAGTCCCTTTC
        131 TGATGTTTGCATTCAAGTAACAGTGTTGAACATTCCCTTTC

            *                                            *       **   * *    
     210107 GTAGAGCAGGTTTGAAACACTCTTTTTGTAGTATCTGGAAGTGGACATTTGGAGCGCTCTCAGGA
          1 ATAGAGCAGGTTTGAAACACTCTTTTTGTAGTATCTGGAAGTGGATATTTGGATAGCTTTGAGGA

            * *  *      *             **         *  *       * *          * **
     210172 CTACGGT-GAAAAAGGAAGTATCTTCCAATAAAAGCTTGATAGAAGCAATGTCAGAAACTTTTAC
         66 TTTCGTTGGAAACAGGAA-TATCTTCATATAAAAGCTAGACAGAAGCATTCTCAGAAACTTCTTT

            *      * ** *   **      *       *  * *   *
     210236 ATGATGTATCTACTCAGCTAACAGAGTTGAACCTTTCTTTTG
        130 GTGATGTTTGCATTCAAGTAACAGTGTTGAACATTCCCTTTC

             *       *                   * *     *             **            
     210278 AGAGAGCAGTTTTGAAACACTCTTTTTGTGGAATCTGCAAGTGGATATTTGTCTAGCTTTGAGGA
          1 ATAGAGCAGGTTTGAAACACTCTTTTTGTAGTATCTGGAAGTGGATATTTGGATAGCTTTGAGGA

                         *   *    *                *  *       *              
     210343 TTTCGTTGGAAACGGGATTA-CAT-ATA-AAAAGC-AGAAAGCAGCATTCCCAGAAACTTCTTTG
         66 TTTCGTTGGAAACAGGAATATCTTCATATAAAAGCTAGACAGAAGCATTCTCAGAAACTTCTTTG

                              *    *      *          
     210404 TGATGTTTGCATTCAAGTCACAGAGTTGAAAATTCCCTTTC
        131 TGATGTTTGCATTCAAGTAACAGTGTTGAACATTCCCTTTC

                                                   *     *       **     *   *
     210445 ATAGAGCAGGTTTGAAACACTCTTTTTGTAGTATCTGGATGTGGACATTTGGAGCGCTTTCAGGC
          1 ATAGAGCAGGTTTGAAACACTCTTTTTGTAGTATCTGGAAGTGGATATTTGGATAGCTTTGAGGA

            * ** *      *             *** *   *                      **  *   
     210510 CTATGGT-GAAAAAGGAAATATCTTCCCCTGAAAACTAGACAGAAGCATTCTCAGAATTTTATTT
         66 TTTCGTTGGAAACAGG-AATATCTTCATATAAAAGCTAGACAGAAGCATTCTCAGAAACTTCTTT

            *      **  **  * *             **  * *   *
     210574 CTGATGTGCGCCCTCGACTAACAGTGTTGAAGCTTTCTTTTG
        130 GTGATGTTTGCATTCAAGTAACAGTGTTGAACATTCCCTTTC

                     *          *         **     *   *                       
     210616 ATAGAGCAGTTTTGAAACACACTTTTTGTAAAATCTGCAAGAGGATATTTGGATAGCTTTGAGGA
          1 ATAGAGCAGGTTTGAAACACTCTTTTTGTAGTATCTGGAAGTGGATATTTGGATAGCTTTGAGGA

                         *   * *            **                      *    *   
     210681 TTTCGTTGGAAACGGGATTGTCTTCATATAAACTCTAGACAGAAGCATTCTCAGAAGCTTCATTG
         66 TTTCGTTGGAAACAGGAATATCTTCATATAAAAGCTAGACAGAAGCATTCTCAGAAACTTCTTTG

            *       **   *    *             *        
     210746 GGATGTTTCAATTGAAGTCACAGTGTTGAACAGTCCCTTTC
        131 TGATGTTTGCATTCAAGTAACAGTGTTGAACATTCCCTTTC

                                                   *     *       **     *   *
     210787 ATAGAGCAGGTTTGAAACACTCTTTTTGTAGTATCTGGATGTGGACATTTGGAGCGCTTTCAGGC
          1 ATAGAGCAGGTTTGAAACACTCTTTTTGTAGTATCTGGAAGTGGATATTTGGATAGCTTTGAGGA

            * ** *      *             *** *   *                          *   
     210852 CTATGGT-GAAAAAGGAAATATCTTCCCCTGAAAACTAGACAGAAGCATTCTCAGAAACTTATTT
         66 TTTCGTTGGAAACAGG-AATATCTTCATATAAAAGCTAGACAGAAGCATTCTCAGAAACTTCTTT

                   *** **    *             **  * *   *
     210916 GTGATGTGCCCCCTCAACTAACAGTGTTGAAGCTTTCTTTTG
        130 GTGATGTTTGCATTCAAGTAACAGTGTTGAACATTCCCTTTC

                  *  *                   * *     *             **            
     210958 ATAGAGGAGTTTTGAAACACTCTTTTTGTGGAATCTGCAAGTGGATATTTGTCTAGCTTTGAGGA
          1 ATAGAGCAGGTTTGAAACACTCTTTTTGTAGTATCTGGAAGTGGATATTTGGATAGCTTTGAGGA

                              *    *                   *       *     *       
     211023 TTTCGTTGGAAAC-GG--GAT-TGCATATAAGAAGC-AGACAGCAGCATTCCCAGAATCTTCTTT
         66 TTTCGTTGGAAACAGGAATATCTTCATATAA-AAGCTAGACAGAAGCATTCTCAGAAACTTCTTT

                               *    *                 
     211083 GTGATGTTTGCATTCAAGTCACAGAGTTGAACATTCCCTTTC
        130 GTGATGTTTGCATTCAAGTAACAGTGTTGAACATTCCCTTTC

                                                *  *     *       **     *   *
     211125 ATAGAGCAGGTTTGAAACACTCTTTTTGTAGTATCTCGATGTGGACATTTGGAGCGCTTTCAGGC
          1 ATAGAGCAGGTTTGAAACACTCTTTTTGTAGTATCTGGAAGTGGATATTTGGATAGCTTTGAGGA

            * ** *      *             *** *   *                      *   *   
     211190 CTATGGT-GAAAAAGGAAATATCTTCTCCTGAAAACTAGACAGAAGCATTCTCAGAATCTTATTT
         66 TTTCGTTGGAAACAGG-AATATCTTCATATAAAAGCTAGACAGAAGCATTCTCAGAAACTTCTTT

                 * **  **    *             **  * *   *
     211254 GTGATATGCGCCCTCAACTAACAGTGTTGAAGCTTTCTTTTG
        130 GTGATGTTTGCATTCAAGTAACAGTGTTGAACATTCCCTTTC

                     *                    **     *   *         *             
     211296 ATAGAGCAGATTTGAAACACTCTTTTTGTAAAATCTGCAAGAGGATATTTGCATAGCTTTGAGGA
          1 ATAGAGCAGGTTTGAAACACTCTTTTTGTAGTATCTGGAAGTGGATATTTGGATAGCTTTGAGGA

                *        *   * *            **                      *    *   
     211361 TTTCATTGGAAACGGGATTGTCTTCATATAAACTCTAGACAGAAGCATTCTCAGAAGCTTCATTG
         66 TTTCGTTGGAAACAGGAATATCTTCATATAAAAGCTAGACAGAAGCATTCTCAGAAACTTCTTTG

            *       **   *    *           * *        
     211426 GGATGTTTCAATTGAAGTCACAGTGTTGAAGAGTCCCTTTC
        131 TGATGTTTGCATTCAAGTAACAGTGTTGAACATTCCCTTTC

                                                         *       **   * *    
     211467 ATAGAGCAGGTTTGAAACACTCTTTTTGTAGTATCTGGAAGTGGACATTTGGAGCGCTCTCAGGA
          1 ATAGAGCAGGTTTGAAACACTCTTTTTGTAGTATCTGGAAGTGGATATTTGGATAGCTTTGAGGA

            * *  *      *             **          * *       * *     *    *  *
     211532 CTACGGT-GAAAAAGGAAATATCTTCCAATAAAAGCTACATAGAAGCAATGTCAGATACTTTTTC
         66 TTTCGTTGGAAACAGG-AATATCTTCATATAAAAGCTAGACAGAAGCATTCTCAGAAACTTCTTT

            *      * ** *   **      *       *  * *   *
     211596 ATGATGTATCTACTCAGCTAACAGAGTTGAACCTTTCTTTTG
        130 GTGATGTTTGCATTCAAGTAACAGTGTTGAACATTCCCTTTC

             *       *               *   * *     *             **            
     211638 AGAGAGCAGTTTTGAAACACTCTTTCTGTGGAATCTGCAAGTGGATATTTGTCTAGCTTTGAGGA
          1 ATAGAGCAGGTTTGAAACACTCTTTTTGTAGTATCTGGAAGTGGATATTTGGATAGCTTTGAGGA

                              *    *         *   * *  *       *              
     211703 TTTCGTTGGAAAC-GG--GAT-TACATATAAAAAC-ACAAAGCAGCATTCCCAGAAACTTCTTTG
         66 TTTCGTTGGAAACAGGAATATCTTCATATAAAAGCTAGACAGAAGCATTCTCAGAAACTTCTTTG

                              *    *                 
     211763 TGATGTTTGCATTCAAGTCACAGAGTTGAACATTCCCTTTC
        131 TGATGTTTGCATTCAAGTAACAGTGTTGAACATTCCCTTTC

                                                   *     *       **     *   *
     211804 ATAGAGCAGGTTTGAAACACTCTTTTTGTAGTATCTGGATGTGGACATTTGGAGCGCTTTCAGGC
          1 ATAGAGCAGGTTTGAAACACTCTTTTTGTAGTATCTGGAAGTGGATATTTGGATAGCTTTGAGGA

            * ** *      *             *** *   *                      **  *   
     211869 CTATGGT-GAAAAAGGAAATATCTTCCCCTGAAAACTAGACAGAAGCATTCTCAGAATTTTATTT
         66 TTTCGTTGGAAACAGG-AATATCTTCATATAAAAGCTAGACAGAAGCATTCTCAGAAACTTCTTT

                   **  **    *       *     **  * *   *
     211933 GTGATGTGCGCCCTCAACTAACAGTATTGAAGCTTTCTTTTG
        130 GTGATGTTTGCATTCAAGTAACAGTGTTGAACATTCCCTTTC

                     *                    **     **  *                       
     211975 ATAGAGCAGTTTTGAAACACTCTTTTTGTAAAATCTGCTAGAGGATATTTGGATAGCTTTGAGGA
          1 ATAGAGCAGGTTTGAAACACTCTTTTTGTAGTATCTGGAAGTGGATATTTGGATAGCTTTGAGGA

                         *   * *            **                      *    *   
     212040 TTTCGTTGGAAACGGGATTGTCTTCATATAAACTCTAGACAGAAGCATTCTCAGAAGCTTCATTG
         66 TTTCGTTGGAAACAGGAATATCTTCATATAAAAGCTAGACAGAAGCATTCTCAGAAACTTCTTTG

            *       **   *    *             *        
     212105 GGATGTTTCAATTGAAGTCACAGTGTTGAACAGTCCCTTTC
        131 TGATGTTTGCATTCAAGTAACAGTGTTGAACATTCCCTTTC

                                  *                *     *       **     *   *
     212146 ATAGAGCAGGTTTGAAACACTCCTTTTGTAGTATCTGGATGTGGACATTTGGAGCGCTTTCAGGC
          1 ATAGAGCAGGTTTGAAACACTCTTTTTGTAGTATCTGGAAGTGGATATTTGGATAGCTTTGAGGA

            * ** *      *             *** *   *            *             *  *
     212211 CTATGGT-GAAAAAGGAAATATCTTCCCCTGAAAACTAGACAGAAGCCTTCTCAGAAACTTATTG
         66 TTTCGTTGGAAACAGG-AATATCTTCATATAAAAGCTAGACAGAAGCATTCTCAGAAACTTCTTT

                   **  **    *             **  * *   *
     212275 GTGATGTGCGCCCTCAACTAACAGTGTTGAAGCTTTCTTTTG
        130 GTGATGTTTGCATTCAAGTAACAGTGTTGAACATTCCCTTTC

                     *                   * *     *             **            
     212317 ATAGAGCAGTTTTGAAACACTCTTTTTGTGGAATCTGCAAGTGGATATTTGTCTAGCTTTGAGGA
          1 ATAGAGCAGGTTTGAAACACTCTTTTTGTAGTATCTGGAAGTGGATATTTGGATAGCTTTGAGGA

                         *   *    *                   *       *     *        
     212382 TTTCGTTGGAAACGGGATTA-CAT-ATA-AAAAGC-AGACAGCAGCATTCCCAGAATCTTCTTTG
         66 TTTCGTTGGAAACAGGAATATCTTCATATAAAAGCTAGACAGAAGCATTCTCAGAAACTTCTTTG

                              *    *                 
     212443 TGATGTTTGCATTCAAGTCACAGAGTTGAACATTCCCTTTC
        131 TGATGTTTGCATTCAAGTAACAGTGTTGAACATTCCCTTTC

                                       *           *     *       **     *   *
     212484 ATAGAGCAGGTTTGAAACACTCTTTTTATAGTATCTGGATGTGGACATTTGGAGCGCTTTCAGGC
          1 ATAGAGCAGGTTTGAAACACTCTTTTTGTAGTATCTGGAAGTGGATATTTGGATAGCTTTGAGGA

            * ** *      *             **  *   *                      **  * * 
     212549 CTATGGT-GAAAAAGGAAATATCTTCTCATGAAAACTAGACAGAAGCATTCTCAGAATTTTATCT
         66 TTTCGTTGGAAACAGG-AATATCTTCATATAAAAGCTAGACAGAAGCATTCTCAGAAACTTCTTT

                   **  **    *             **  * *   *
     212613 GTGATGTGCGCCCTCAACTAACAGTGTTGAAGCTTTCTTTTG
        130 GTGATGTTTGCATTCAAGTAACAGTGTTGAACATTCCCTTTC

                     *                    **     *   *        ***            
     212655 ATAGAGCAGTTTTGAAACACTCTTTTTGTAAAATCTGCAAGAGGATATTTTTCTAGCTTTGAGGA
          1 ATAGAGCAGGTTTGAAACACTCTTTTTGTAGTATCTGGAAGTGGATATTTGGATAGCTTTGAGGA

                         *   *    *            *   *  *       *              
     212720 TTTCGTTGGAAACGGGATTA-CAT-ATA-AAAAG-AAGAAAGCAGCATTCCCAGAAACTTCTTTG
         66 TTTCGTTGGAAACAGGAATATCTTCATATAAAAGCTAGACAGAAGCATTCTCAGAAACTTCTTTG

                           *  *    *                 
     212781 TGATGTTTGCATTCATGTCACAGAGTTGAACATTCCCTTTC
        131 TGATGTTTGCATTCAAGTAACAGTGTTGAACATTCCCTTTC

                                       *           *     *       **     *   *
     212822 ATAGAGCAGGTTTGAAACACTCTTTTTATAGTATCTGGATGTGGACATTTGGAGCGCTTTCAGGC
          1 ATAGAGCAGGTTTGAAACACTCTTTTTGTAGTATCTGGAAGTGGATATTTGGATAGCTTTGAGGA

            * ** *      *             *** *   *                      *   * * 
     212887 CTATGGT-GAAAAAGGAAATATCTTCTCCTGAAAACTAGACAGAAGCATTCTCAGAATCTTATCT
         66 TTTCGTTGGAAACAGG-AATATCTTCATATAAAAGCTAGACAGAAGCATTCTCAGAAACTTCTTT

                   *** **    *             **  * *   *
     212951 GTGATGTGCACCCTCAACTAACAGTGTTGAAGCTTTCTTTTG
        130 GTGATGTTTGCATTCAAGTAACAGTGTTGAACATTCCCTTTC

                     *                    **     *   *         *             
     212993 ATAGAGCAGTTTTGAAACACTCTTTTTGTAAAATCTGCAAGAGGATATTTGCATAGCTTTGAGGA
          1 ATAGAGCAGGTTTGAAACACTCTTTTTGTAGTATCTGGAAGTGGATATTTGGATAGCTTTGAGGA

                *        *   * *            **                      *    *   
     213058 TTTCATTGGAAACGGGATTGTCTTCATATAAACTCTAGACAGAAGCATTCTCAGAAGCTTCATTG
         66 TTTCGTTGGAAACAGGAATATCTTCATATAAAAGCTAGACAGAAGCATTCTCAGAAACTTCTTTG

            *       **   *    *             *        
     213123 GGATGTTTCAATTGAAGTCACAGTGTTGAACAGTCCCTTTC
        131 TGATGTTTGCATTCAAGTAACAGTGTTGAACATTCCCTTTC

                                                         *    *  *  * * *    
     213164 ATAGAGCAGGTTTGAAACACTCTTTTTGTAGTATCTGGAAGTGGACATTTTGAGAGATCTCAGGA
          1 ATAGAGCAGGTTTGAAACACTCTTTTTGTAGTATCTGGAAGTGGATATTTGGATAGCTTTGAGGA

            * *  *    * *             **            *       * *          *  *
     213229 ATACGGT-GATAAAGGAAATATCTTCCAATAAAAGCTAGATAGAAGCAATGTCAGAAACTTTTTC
         66 TTTCGTTGGAAACAGG-AATATCTTCATATAAAAGCTAGACAGAAGCATTCTCAGAAACTTCTTT

            *      * ** *   **      *       *  * *   *
     213293 ATGATGTATCTACTCAGCTAACAGAGTTGAACCTTTCTTTTG
        130 GTGATGTTTGCATTCAAGTAACAGTGTTGAACATTCCCTTTC

             *       *                   * *     *             **      *     
     213335 AGAGAGCAGTTTTGAAACACTCTTTTTGTGGAATCTGCAAGTGGATATTTGTCTAGCTTAGAGGA
          1 ATAGAGCAGGTTTGAAACACTCTTTTTGTAGTATCTGGAAGTGGATATTTGGATAGCTTTGAGGA

            *            *   *    *                   *       *              
     213400 ATTCGTTGGAAACGGGATTA-CAT-ATA-AAAAGC-AGACAGCAGCATTCCCAGAAACTTCTTTG
         66 TTTCGTTGGAAACAGGAATATCTTCATATAAAAGCTAGACAGAAGCATTCTCAGAAACTTCTTTG

                              *    *                 
     213461 TGATGTTTGCATTCAAGTCACAGAGTTGAACATTCCCTTTC
        131 TGATGTTTGCATTCAAGTAACAGTGTTGAACATTCCCTTTC

                                               *   *     *       **     *   *
     213502 ATAGAGCAGGTTTGAAACACTCTTTTTGTAGTATCGGGATGTGGACATTTGGAGCGCTTTCAGGC
          1 ATAGAGCAGGTTTGAAACACTCTTTTTGTAGTATCTGGAAGTGGATATTTGGATAGCTTTGAGGA

            * ** *      *             *** *   *                      **  *   
     213567 CTATGGT-GAAAAAGGAAATATCTTCCCCTGAAAACTAGACAGAAGCATTCTCAGAATTTTATTT
         66 TTTCGTTGGAAACAGG-AATATCTTCATATAAAAGCTAGACAGAAGCATTCTCAGAAACTTCTTT

                   **  **    *              *  * *   *
     213631 GTGATGTGCGCCCTCAACTAACAGTGTTGAACCTTTCTTTTG
        130 GTGATGTTTGCATTCAAGTAACAGTGTTGAACATTCCCTTTC

                     *         *          **     *   *                       
     213673 ATAGAGCAGTTTTGAAACAATCTTTTTGTAAAATCTGCAAGAGGATATTTGGATAGCTTTGAGGA
          1 ATAGAGCAGGTTTGAAACACTCTTTTTGTAGTATCTGGAAGTGGATATTTGGATAGCTTTGAGGA

                         *   * *    *       **                      *    *   
     213738 TTTCGTTGGAAACGGGATTGTCTTAATATAAACTCTAGACAGAAGCATTCTCAGAAGCTTCATTG
         66 TTTCGTTGGAAACAGGAATATCTTCATATAAAAGCTAGACAGAAGCATTCTCAGAAACTTCTTTG

            *       **   *    *             *        
     213803 GGATGTTTCAATTGAAGTCACAGTGTTGAACAGTCCCTTTC
        131 TGATGTTTGCATTCAAGTAACAGTGTTGAACATTCCCTTTC

                                                   *     *       **     *   *
     213844 ATAGAGCAGGTTTGAAACACTCTTTTTGTAGTATCTGGATGTGGACATTTGGAGCGCTTTCAGGC
          1 ATAGAGCAGGTTTGAAACACTCTTTTTGTAGTATCTGGAAGTGGATATTTGGATAGCTTTGAGGA

            * ***  **                 *** *   *                          *   
     213909 CTATTTTTTAAA-AGGAAATATCTTCCCCTGAAAACTAGACAGAAGCATTCTCAGAAACTTATTT
         66 TTTCGTTGGAAACAGG-AATATCTTCATATAAAAGCTAGACAGAAGCATTCTCAGAAACTTCTTT

                   **  **    *                    *   *
     213973 GTGATGTGCGCCCTCAACTAACAGTGTTGAAGCATT-CTTTTG
        130 GTGATGTTTGCATTCAAGTAACAGTGTTGAA-CATTCCCTTTC

                     *                   * *     *             **            
     214015 ATAGAGCAGTTTTGAAACACTCTTTTTGTGGAATCTGCAAGTGGATATTTGTCTAGCTTTGAGGA
          1 ATAGAGCAGGTTTGAAACACTCTTTTTGTAGTATCTGGAAGTGGATATTTGGATAGCTTTGAGGA

                         *   *    *                   *       *     *        
     214080 TTTCGTTGGAAACGGGATTA-CAT-ATA-AAAAGC-AGACAGCAGCATTCCCAGAATCTTCTTTG
         66 TTTCGTTGGAAACAGGAATATCTTCATATAAAAGCTAGACAGAAGCATTCTCAGAAACTTCTTTG

                              *    *                 
     214141 TGATGTTTGCATTCAAGTCACAGAGTTGAACATTCCCTTTC
        131 TGATGTTTGCATTCAAGTAACAGTGTTGAACATTCCCTTTC

                                                *  *     *       **     *   *
     214182 ATAGAGCAGGTTTGAAACACTCTTTTTGTAGTATCTCGATGTGGACATTTGGAGCGCTTTCAGGC
          1 ATAGAGCAGGTTTGAAACACTCTTTTTGTAGTATCTGGAAGTGGATATTTGGATAGCTTTGAGGA

            * ** *      *             *** *   *                      *   *   
     214247 CTATGGT-GAAAAAGGAAATATCTTCTCCTGAAAACTAGACAGAAGCATTCTCAGAATCTTATTT
         66 TTTCGTTGGAAACAGG-AATATCTTCATATAAAAGCTAGACAGAAGCATTCTCAGAAACTTCTTT

                   **  **    *             **  * * * *
     214311 GTGATGTGCGCCCTCAACTAACAGTGTTGAAGCTTTCTTCTG
        130 GTGATGTTTGCATTCAAGTAACAGTGTTGAACATTCCCTTTC

                     *                    **     *   *         *             
     214353 ATAGAGCAGTTTTGAAACACTCTTTTTGTAAAATCTGCAAGAGGATATTTGCATAGCTTTGAGGA
          1 ATAGAGCAGGTTTGAAACACTCTTTTTGTAGTATCTGGAAGTGGATATTTGGATAGCTTTGAGGA

                *        *   * *            **                      *    *   
     214418 TTTCATTGGAAACGGGATTGTCTTCATATAAACTCTAGACAGAAGCATTCTCAGAAGCTTCATTG
         66 TTTCGTTGGAAACAGGAATATCTTCATATAAAAGCTAGACAGAAGCATTCTCAGAAACTTCTTTG

            *       **   *    *             *        
     214483 GGATGTTTCAATTGAAGTCACAGTGTTGAACAGTCCCTTTC
        131 TGATGTTTGCATTCAAGTAACAGTGTTGAACATTCCCTTTC

                                                         *       **   * *    
     214524 ATAGAGCAGGTTTGAAACACTCTTTTTGTAGTATCTGGAAGTGGACATTTGGAGCGCTCTCAGGA
          1 ATAGAGCAGGTTTGAAACACTCTTTTTGTAGTATCTGGAAGTGGATATTTGGATAGCTTTGAGGA

            * *  *      *             **          * *       * *          *  *
     214589 CTACGGT-GAAAAAGGAAATATCTTCCAATAAAAGCTACATAGAAGCAATGTCAGAAACTTTTTC
         66 TTTCGTTGGAAACAGG-AATATCTTCATATAAAAGCTAGACAGAAGCATTCTCAGAAACTTCTTT

            *      * ** *   **      *       *  * *   *
     214653 ATGATGTATCTACTCAGCTAACAGAGTTGAACCTTTCTTTTG
        130 GTGATGTTTGCATTCAAGTAACAGTGTTGAACATTCCCTTTC

             *       *                            *  *          **           
     214695 AGAGAGCAGTTTTGAAACACTCTTTTTGT-GTAATCTGCAACTGGATATTTGTCTAGCTTTGAGG
          1 ATAGAGCAGGTTTGAAACACTCTTTTTGTAGT-ATCTGGAAGTGGATATTTGGATAGCTTTGAGG

             *            *   *    *                   *       *             
     214759 AATTCGTTGGAAACGGGATTA-CAT-ATA-AAAAGC-AGACAGCAGCATTCCCAGAAACTTCTTT
         65 ATTTCGTTGGAAACAGGAATATCTTCATATAAAAGCTAGACAGAAGCATTCTCAGAAACTTCTTT

                               *    *                 
     214820 GTGATGTTTGCATTCAAGTCACAGAGTTGAACATTCCCTTTC
        130 GTGATGTTTGCATTCAAGTAACAGTGTTGAACATTCCCTTTC

                                                   *     *       **     *   *
     214862 ATAGAGCAGGTTTGAAACACTCTTTTTGTAGTATCTGGATGTGGACATTTGGAGCGCTTTCAGGC
          1 ATAGAGCAGGTTTGAAACACTCTTTTTGTAGTATCTGGAAGTGGATATTTGGATAGCTTTGAGGA

            * ** *      *             *** *   *                      *   *   
     214927 CTATGGT-GAAAAAGGAAATATCTTCCCCTGAAAACTAGACAGAAGCATTCTCAGAATCTTATTT
         66 TTTCGTTGGAAACAGG-AATATCTTCATATAAAAGCTAGACAGAAGCATTCTCAGAAACTTCTTT

                   **  **    *             **  * *   *
     214991 GTGATGTGCGCCCTCAACTAACAGTGTTGAAGCTTTCTTTTG
        130 GTGATGTTTGCATTCAAGTAACAGTGTTGAACATTCCCTTTC

                     *   *    *           **     *   *                       
     215033 ATAGAGCAGTTTTCAAACTCTCTTTTTGTAAAATCTGCAAGAGGATATTTGGATAGCTTTGAGGA
          1 ATAGAGCAGGTTTGAAACACTCTTTTTGTAGTATCTGGAAGTGGATATTTGGATAGCTTTGAGGA

                *        *   * *            **                     **    *   
     215098 TTTCTTTGGAAACGGGATTGTCTTCATATAAACTCTAGACAGAAGCATTCTCAGATGCTTCATTG
         66 TTTCGTTGGAAACAGGAATATCTTCATATAAAAGCTAGACAGAAGCATTCTCAGAAACTTCTTTG

            *       **   *    *             *        
     215163 GGATGTTTCAATTGAAGTCACAGTGTTGAACAGTCCCTTTC
        131 TGATGTTTGCATTCAAGTAACAGTGTTGAACATTCCCTTTC

                                                   *     *       **     *   *
     215204 ATAGAGCAGGTTTGAAACACTCTTTTTGTAGTATCTGGATGTGGACATTTGGAGCGCTTTCAGGC
          1 ATAGAGCAGGTTTGAAACACTCTTTTTGTAGTATCTGGAAGTGGATATTTGGATAGCTTTGAGGA

            * ** *      *             *** *   *                          *   
     215269 CTATGGT-GAAAAAGGAAATATCTTCCCCTGAAAACTAGACAGAAGCATTCTCAGAAACTTATTT
         66 TTTCGTTGGAAACAGG-AATATCTTCATATAAAAGCTAGACAGAAGCATTCTCAGAAACTTCTTT

                   **  **    *             **  * *   *
     215333 GTGATGTGCGCCCTCAACTAACAGTGTTGAAGCTTTCTTTTG
        130 GTGATGTTTGCATTCAAGTAACAGTGTTGAACATTCCCTTTC

                     *                   * *     **            **            
     215375 ATAGAGCAGTTTTGAAACACTCTTTTTGTGGAATCTGCTAGTGGATATTTGTCTAGCTTTGAGGA
          1 ATAGAGCAGGTTTGAAACACTCTTTTTGTAGTATCTGGAAGTGGATATTTGGATAGCTTTGAGGA

                         *   *    *                   *       *   *          
     215440 TTTCGTTGGAAACGGGATTA-CAT-ATA-AAAAGC-AGACAGCAGCATTCCCAGTAACTTCTTTG
         66 TTTCGTTGGAAACAGGAATATCTTCATATAAAAGCTAGACAGAAGCATTCTCAGAAACTTCTTTG

                              *    *                 
     215501 TGATGTTTGCATTCAAGTCACAGAGTTGAACATTCCCTTTC
        131 TGATGTTTGCATTCAAGTAACAGTGTTGAACATTCCCTTTC

                                                   *     *       **     *   *
     215542 ATAGAGCAGGTTTGAAACACTCTTTTTGTAGTATCTGGATGTGGACATTTGGAGCGCTTTCAGGC
          1 ATAGAGCAGGTTTGAAACACTCTTTTTGTAGTATCTGGAAGTGGATATTTGGATAGCTTTGAGGA

            * ** *      *             *** *   *                      *   *   
     215607 CTATGGT-GAAAAAGGAAATATCTTCCCCTGAAAACTAGACAGAAGCATTCTCAGAATCTTATTT
         66 TTTCGTTGGAAACAGG-AATATCTTCATATAAAAGCTAGACAGAAGCATTCTCAGAAACTTCTTT

                   **  **    *             **  * *   *
     215671 GTGATGTGCGCCCTCAACTAACAGTGTTGAAGCTTTCTTTTG
        130 GTGATGTTTGCATTCAAGTAACAGTGTTGAACATTCCCTTTC

                     *                    **     *   *                       
     215713 ATAGAGCAGTTTTGAAACACTCTTTTTGTAAAATCTGCAAGAGGATATTTGGATAGCTTTGAGGA
          1 ATAGAGCAGGTTTGAAACACTCTTTTTGTAGTATCTGGAAGTGGATATTTGGATAGCTTTGAGGA

                    *    *   * *            **                      *    *   
     215778 TTTCGTTGAAAACGGGATTGTCTTCATATAAACTCTAGACAGAAGCATTCTCAGAAGCTTCATTG
         66 TTTCGTTGGAAACAGGAATATCTTCATATAAAAGCTAGACAGAAGCATTCTCAGAAACTTCTTTG

            *       **   *    *             *        
     215843 GGATGTTTCAATTGAAGTCACAGTGTTGAACAGTCCCTTTC
        131 TGATGTTTGCATTCAAGTAACAGTGTTGAACATTCCCTTTC

                                                       * *       **   * *    
     215884 ATAGAGCAGGTTTGAAACACTCTTTTTGTAGTATCTGGAAGTGAACATTTGGAGCGCTCTCAGGA
          1 ATAGAGCAGGTTTGAAACACTCTTTTTGTAGTATCTGGAAGTGGATATTTGGATAGCTTTGAGGA

            * *  *  *   *  * *       **         *  *       * *          *  **
     215949 CTACGGTGAAAAAAGAAGTATCTTCCAATAAAAGCTTGATAGAAGCAATGTCAGAAACTTTTTCA
         66 TTTCGTTGGAAACAGGAATATCTTCATATAAAAGCTAGACAGAAGCATTCTCAGAAACTTCTTTG

                  * ** *   **      *       *  * *   *
     216014 TGATGTATCTACTCAGCTAACAGAGTTGAACCTTTCTTTTG
        131 TGATGTTTGCATTCAAGTAACAGTGTTGAACATTCCCTTTC

             *       *                   * *     *             **            
     216055 AGAGAGCAGTTTTGAAACACTCTTTTTGTGGAATCTGCAAGTGGATATTTGACTAGCTTTGAGGA
          1 ATAGAGCAGGTTTGAAACACTCTTTTTGTAGTATCTGGAAGTGGATATTTGGATAGCTTTGAGGA

                         *   *    *                *  *       *              
     216120 TTTCGTTGGAAACGGGATTA-CAT-ATA-AAAAGC-AGAAAGCAGCATTCCCAGAAACTTCTTTG
         66 TTTCGTTGGAAACAGGAATATCTTCATATAAAAGCTAGACAGAAGCATTCTCAGAAACTTCTTTG

                              *    *                 
     216181 TGATGTTTGCATTCAAGTCACAGAGTTGAACATTCCCTTTC
        131 TGATGTTTGCATTCAAGTAACAGTGTTGAACATTCCCTTTC

                                               *   *     *       **     *   *
     216222 ATAGAGCAGGTTTGAAACACTCTTTTTGTAGTATCGGGATGTGGACATTTGGAGCGCTTTCAGGC
          1 ATAGAGCAGGTTTGAAACACTCTTTTTGTAGTATCTGGAAGTGGATATTTGGATAGCTTTGAGGA

            * ** *      *             *** *   *                      **  *   
     216287 CTATGGT-GAAAAAGGAAATATCTTCCCCTGAAAACTAGACAGAAGCATTCTCAGAATTTTATTT
         66 TTTCGTTGGAAACAGG-AATATCTTCATATAAAAGCTAGACAGAAGCATTCTCAGAAACTTCTTT

                   **  **    *              *  * *   *
     216351 GTGATGTGCGCCCTCAACTAACAGTGTTGAACCTTTCTTTTG
        130 GTGATGTTTGCATTCAAGTAACAGTGTTGAACATTCCCTTTC

                     *         *          **     *   *                       
     216393 ATAGAGCAGTTTTGAAACAATCTTTTTGTAAAATCTGCAAGAGGATATTTGGATAGCTTTGAGGA
          1 ATAGAGCAGGTTTGAAACACTCTTTTTGTAGTATCTGGAAGTGGATATTTGGATAGCTTTGAGGA

                         *   * *    *       **                      *    *   
     216458 TTTCGTTGGAAACGGGATTGTCTTAATATAAACTCTAGACAGAAGCATTCTCAGAAGCTTCATTG
         66 TTTCGTTGGAAACAGGAATATCTTCATATAAAAGCTAGACAGAAGCATTCTCAGAAACTTCTTTG

            *       **   *    *             *        
     216523 GGATGTTTCAATTGAAGTCACAGTGTTGAACAGTCCCTTTC
        131 TGATGTTTGCATTCAAGTAACAGTGTTGAACATTCCCTTTC

                                                   *     *       **     *   *
     216564 ATAGAGCAGGTTTGAAACACTCTTTTTGTAGTATCTGGATGTGGACATTTGGAGCGCTTTCAGGC
          1 ATAGAGCAGGTTTGAAACACTCTTTTTGTAGTATCTGGAAGTGGATATTTGGATAGCTTTGAGGA

            * ***  **                 *** *   *                          *   
     216629 CTATTTTTTAAA-AGGAAATATCTTCCCCTGAAAACTAGACAGAAGCATTCTCAGAAACTTATTT
         66 TTTCGTTGGAAACAGG-AATATCTTCATATAAAAGCTAGACAGAAGCATTCTCAGAAACTTCTTT

                   **  **    *                    *   *
     216693 GTGATGTGCGCCCTCAACTAACAGTGTTGAAGCATT-CTTTTG
        130 GTGATGTTTGCATTCAAGTAACAGTGTTGAA-CATTCCCTTTC

                     *                   * *     *             **            
     216735 ATAGAGCAGTTTTGAAACACTCTTTTTGTGGAATCTGCAAGTGGATATTTGTCTAGCTTTGAGGA
          1 ATAGAGCAGGTTTGAAACACTCTTTTTGTAGTATCTGGAAGTGGATATTTGGATAGCTTTGAGGA

                         *   *    *                   *       *              
     216800 TTTCGTTGGAAACGGGATTA-CAT-ATA-AAAAGC-AGACAGCAGCATTCCCAGAAACTTCTTTG
         66 TTTCGTTGGAAACAGGAATATCTTCATATAAAAGCTAGACAGAAGCATTCTCAGAAACTTCTTTG

                              *    *                *
     216861 TGATGTTTGCATTCAAGTCACAGAGTTGAACATTCCCTTTG
        131 TGATGTTTGCATTCAAGTAACAGTGTTGAACATTCCCTTTC

                                                 * *     *       **     *   *
     216902 ATAGAGCAGGTTTGAAACACTCTTTTTGTAGTATCTGTATGTGGACATTTGGAGCGCTTTCAGGC
          1 ATAGAGCAGGTTTGAAACACTCTTTTTGTAGTATCTGGAAGTGGATATTTGGATAGCTTTGAGGA

            * ** *      *             *** *   *          *           *   *   
     216967 CTATGGT-GAAAAAGGAAATATCTTCCCCTGAAAACTAGACAGAAACATTCTCAGAATCTTATTT
         66 TTTCGTTGGAAACAGG-AATATCTTCATATAAAAGCTAGACAGAAGCATTCTCAGAAACTTCTTT

                   **  **    *             **  * *   *
     217031 GTGATGTGCGCCCTCAACTAACAGTGTTGAAGCTTTCTTTTG
        130 GTGATGTTTGCATTCAAGTAACAGTGTTGAACATTCCCTTTC

                     *                    **     *   *                       
     217073 ATAGAGCAGTTTTGAAACACTCTTTTTGTAAAATCTGCAAGAGGATATTTGGATAGCTTTGAGGA
          1 ATAGAGCAGGTTTGAAACACTCTTTTTGTAGTATCTGGAAGTGGATATTTGGATAGCTTTGAGGA

                         *   * *            **                      *    *   
     217138 TTTCGTTGGAAACGGGATTGTCTTCATATAAACTCTAGACAGAAGCATTCTCAGAAGCTTCATTG
         66 TTTCGTTGGAAACAGGAATATCTTCATATAAAAGCTAGACAGAAGCATTCTCAGAAACTTCTTTG

            *       **   *    *             *        
     217203 GGATGTTTCAATTGAAGTCACAGTGTTGAACAGTCCCTTTC
        131 TGATGTTTGCATTCAAGTAACAGTGTTGAACATTCCCTTTC

                                                       * *       **   * *    
     217244 ATAGAGCAGGTTTGAAACACTCTTTTTGTAGTATCTGGAAGTGAACATTTGGAGCGCTCTCAGGA
          1 ATAGAGCAGGTTTGAAACACTCTTTTTGTAGTATCTGGAAGTGGATATTTGGATAGCTTTGAGGA

            * *  *      *             **          * *       * *          *  *
     217309 CTACGGT-GAAAAAGGAAATATCTTCCAATAAAAGCTACATAGAAGCAATGTCAGAAACTTTTTC
         66 TTTCGTTGGAAACAGG-AATATCTTCATATAAAAGCTAGACAGAAGCATTCTCAGAAACTTCTTT

            *      * ** *   **      *       *  * *   *
     217373 ATGATGTATCTACTCAGCTAACAGAGTTGAACCTTTCTTTTG
        130 GTGATGTTTGCATTCAAGTAACAGTGTTGAACATTCCCTTTC

             *       *                   * *    **             **            
     217415 AGAGAGCAGTTTTGAAACACTCTTTTTGTGGAATCTTCAAGTGGATATTTGTCTAGCTTTGAGGA
          1 ATAGAGCAGGTTTGAAACACTCTTTTTGTAGTATCTGGAAGTGGATATTTGGATAGCTTTGAGGA

                             *    *                   *       *              
     217480 TTTCGTTGGAAACAGGATTA-CAT-ATA-AAAAGC-AGACAGCAGCATTCCCAGAAACTTCTTTG
         66 TTTCGTTGGAAACAGGAATATCTTCATATAAAAGCTAGACAGAAGCATTCTCAGAAACTTCTTTG

                              *    *                 
     217541 TGATGTTTGCATTCAAGTCACAGAGTTGAACATTCCCTTTC
        131 TGATGTTTGCATTCAAGTAACAGTGTTGAACATTCCCTTTC

                                                   *     *       **     *   *
     217582 ATAGAGCAGGTTTGAAACACTCTTTTTGTAGTATCTGGATGTGGACATTTGGAGCGCTTTCAGGC
          1 ATAGAGCAGGTTTGAAACACTCTTTTTGTAGTATCTGGAAGTGGATATTTGGATAGCTTTGAGGA

            * ** *      *             *** *   *                      **  *   
     217647 CTATGGT-GAAAAAGGAAATATCTTCCCCTGAAAACTAGACAGAAGCATTCTCAGAATTTTATTT
         66 TTTCGTTGGAAACAGG-AATATCTTCATATAAAAGCTAGACAGAAGCATTCTCAGAAACTTCTTT

                   **  **    *             **  * *   *
     217711 GTGATGTGCGCCCTCAACTAACAGTGTTGAAGCTTTCTTTTG
        130 GTGATGTTTGCATTCAAGTAACAGTGTTGAACATTCCCTTTC

                     *                    **     **  *                       
     217753 ATAGAGCAGTTTTGAAACACTCTTTTTGTAAAATCTGCTAGAGGATATTTGGATAGCTTTGAGGA
          1 ATAGAGCAGGTTTGAAACACTCTTTTTGTAGTATCTGGAAGTGGATATTTGGATAGCTTTGAGGA

                *        *   * *            **                     **    *   
     217818 TTTCTTTGGAAACGGGATTGTCTTCATATAAACTCTAGACAGAAGCATTCTCAGATGCTTCATTG
         66 TTTCGTTGGAAACAGGAATATCTTCATATAAAAGCTAGACAGAAGCATTCTCAGAAACTTCTTTG

            *       **   *    *             *        
     217883 GGATGTTTCAATTGAAGTCACAGTGTTGAACAGTCCCTTTC
        131 TGATGTTTGCATTCAAGTAACAGTGTTGAACATTCCCTTTC

                                                   *     *       **     * * *
     217924 ATAGAGCAGGTTTGAAACACTCTTTTTGTAGTATCTGGATGTGGACATTTGGAGCGCTTTCATGC
          1 ATAGAGCAGGTTTGAAACACTCTTTTTGTAGTATCTGGAAGTGGATATTTGGATAGCTTTGAGGA

            * ** *      *             *** *   *                          *   
     217989 CTATGGT-GAAAAAGGAAATATCTTCCCCTGAAAACTAGACAGAAGCATTCTCAGAAACTTATTT
         66 TTTCGTTGGAAACAGG-AATATCTTCATATAAAAGCTAGACAGAAGCATTCTCAGAAACTTCTTT

            *      *** **    *             **  * *   *
     218053 CTGATGTGCCCCCTCAACTAACAGTGTTGAAGCTTTCTTTTG
        130 GTGATGTTTGCATTCAAGTAACAGTGTTGAACATTCCCTTTC

                     *                   * *     *             **            
     218095 ATAGAGCAGTTTTGAAACACTCTTTTTGTGGAATCTGCAAGTGGATATTTGTCTAGCTTTGAGGA
          1 ATAGAGCAGGTTTGAAACACTCTTTTTGTAGTATCTGGAAGTGGATATTTGGATAGCTTTGAGGA

                         *   *    *                   *       *     *        
     218160 TTTCGTTGGAAACGGGATTA-CAT-ATA-AAAAGC-AGACAGCAGCATTCCCAGAATCTTCTTTG
         66 TTTCGTTGGAAACAGGAATATCTTCATATAAAAGCTAGACAGAAGCATTCTCAGAAACTTCTTTG

                              *    *                 
     218221 TGATGTTTGCATTCAAGTCACAGAGTTGAACATTCCCTTTC
        131 TGATGTTTGCATTCAAGTAACAGTGTTGAACATTCCCTTTC

                                                *  *     *       **     *   *
     218262 ATAGAGCAGGTTTGAAACACTCTTTTTGTAGTATCTCGATGTGGACATTTGGAGCGCTTTCAGGC
          1 ATAGAGCAGGTTTGAAACACTCTTTTTGTAGTATCTGGAAGTGGATATTTGGATAGCTTTGAGGA

            * ** *      *             *** *   *                      *   *   
     218327 CTATGGT-GAAAAAGGAAATATCTTCTCCTGAAAACTAGACAGAAGCATTCTCAGAATCTTATTT
         66 TTTCGTTGGAAACAGG-AATATCTTCATATAAAAGCTAGACAGAAGCATTCTCAGAAACTTCTTT

                   **  **    *             **  * * * *
     218391 GTGATGTGCGCCCTCAACTAACAGTGTTGAAGCTTTCTTCTG
        130 GTGATGTTTGCATTCAAGTAACAGTGTTGAACATTCCCTTTC

                     *                    **     *   *         *             
     218433 ATAGAGCAGTTTTGAAACACTCTTTTTGTAAAATCTGCAAGAGGATATTTGCATAGCTTTGAGGA
          1 ATAGAGCAGGTTTGAAACACTCTTTTTGTAGTATCTGGAAGTGGATATTTGGATAGCTTTGAGGA

                *        *   * *            **                      *    *   
     218498 TTTCATTGGAAACGGGATTGTCTTCATATAAACTCTAGACAGAAGCATTCTCAGAAGCTTCATTG
         66 TTTCGTTGGAAACAGGAATATCTTCATATAAAAGCTAGACAGAAGCATTCTCAGAAACTTCTTTG

            *       **   *    *             *        
     218563 GGATGTTTCAATTGAAGTCACAGTGTTGAACAGTCCCTTTC
        131 TGATGTTTGCATTCAAGTAACAGTGTTGAACATTCCCTTTC

                                                         *       **   * *    
     218604 ATAGAGCAGGTTTGAAACACTCTTTTTGTAGTATCTGGAAGTGGACATTTGGAGCGCTCTCAGGA
          1 ATAGAGCAGGTTTGAAACACTCTTTTTGTAGTATCTGGAAGTGGATATTTGGATAGCTTTGAGGA

            * *  *      *             **          * *       * *          *  *
     218669 CTACGGT-GAAAAAGGAAATATCTTCCAATAAAAGCTACATAGAAGCAATGTCAGAAACTTTTTC
         66 TTTCGTTGGAAACAGG-AATATCTTCATATAAAAGCTAGACAGAAGCATTCTCAGAAACTTCTTT

            *      * ** *   **      *       *  * *   *
     218733 ATGATGTATCTACTCAGCTAACAGAGTTGAACCTTTCTTTTG
        130 GTGATGTTTGCATTCAAGTAACAGTGTTGAACATTCCCTTTC

             *       *                            *  *          **           
     218775 AGAGAGCAGTTTTGAAACACTCTTTTTGT-GTAATCTGCAACTGGATATTTGTCTAGCTTTGAGG
          1 ATAGAGCAGGTTTGAAACACTCTTTTTGTAGT-ATCTGGAAGTGGATATTTGGATAGCTTTGAGG

             *            *   *    *                   *       *             
     218839 AATTCGTTGGAAACGGGATTA-CAT-ATA-AAAAGC-AGACAGCAGCATTCCCAGAAACTTCTTT
         65 ATTTCGTTGGAAACAGGAATATCTTCATATAAAAGCTAGACAGAAGCATTCTCAGAAACTTCTTT

                               *    *                 
     218900 GTGATGTTTGCATTCAAGTCACAGAGTTGAACATTCCCTTTC
        130 GTGATGTTTGCATTCAAGTAACAGTGTTGAACATTCCCTTTC

                                                   *     *       **     *   *
     218942 ATAGAGCAGGTTTGAAACACTCTTTTTGTAGTATCTGGATGTGGACATTTGGAGCGCTTTCAGGC
          1 ATAGAGCAGGTTTGAAACACTCTTTTTGTAGTATCTGGAAGTGGATATTTGGATAGCTTTGAGGA

            * ** *      *             *** *   *                      *   *   
     219007 CTATGGT-GAAAAAGGAAATATCTTCCCCTGAAAACTAGACAGAAGCATTCTCAGAATCTTATTT
         66 TTTCGTTGGAAACAGG-AATATCTTCATATAAAAGCTAGACAGAAGCATTCTCAGAAACTTCTTT

                   **  **    *             **  * *   *
     219071 GTGATGTGCGCCCTCAACTAACAGTGTTGAAGCTTTCTTTTG
        130 GTGATGTTTGCATTCAAGTAACAGTGTTGAACATTCCCTTTC

                     *   *    *           **     *   *                       
     219113 ATAGAGCAGTTTTCAAACTCTCTTTTTGTAAAATCTGCAAGAGGATATTTGGATAGCTTTGAGGA
          1 ATAGAGCAGGTTTGAAACACTCTTTTTGTAGTATCTGGAAGTGGATATTTGGATAGCTTTGAGGA

                *        *   * *            **                     **    *   
     219178 TTTCTTTGGAAACGGGATTGTCTTCATATAAACTCTAGACAGAAGCATTCTCAGATGCTTCATTG
         66 TTTCGTTGGAAACAGGAATATCTTCATATAAAAGCTAGACAGAAGCATTCTCAGAAACTTCTTTG

            *       **   *    *             *        
     219243 GGATGTTTCAATTGAAGTCACAGTGTTGAACAGTCCCTTTC
        131 TGATGTTTGCATTCAAGTAACAGTGTTGAACATTCCCTTTC

                                                   *     *       **     *   *
     219284 ATAGAGCAGGTTTGAAACACTCTTTTTGTAGTATCTGGATGTGGACATTTGGAGCGCTTTCAGGC
          1 ATAGAGCAGGTTTGAAACACTCTTTTTGTAGTATCTGGAAGTGGATATTTGGATAGCTTTGAGGA

            * ** *      *             *** *   *                          *   
     219349 CTATGGT-GAAAAAGGAAATATCTTCCCCTGAAAACTAGACAGAAGCATTCTCAGAAACTTATTT
         66 TTTCGTTGGAAACAGG-AATATCTTCATATAAAAGCTAGACAGAAGCATTCTCAGAAACTTCTTT

                   **  **    *             **  * *   *
     219413 GTGATGTGCGCCCTCAACTAACAGTGTTGAAGCTTTCTTTTG
        130 GTGATGTTTGCATTCAAGTAACAGTGTTGAACATTCCCTTTC

                     *                   * *     **            **            
     219455 ATAGAGCAGTTTTGAAACACTCTTTTTGTGGAATCTGCTAGTGGATATTTGTCTAGCTTTGAGGA
          1 ATAGAGCAGGTTTGAAACACTCTTTTTGTAGTATCTGGAAGTGGATATTTGGATAGCTTTGAGGA

                         *   *    *                   *       *   *          
     219520 TTTCGTTGGAAACGGGATTA-CAT-ATA-AAAAGC-AGACAGCAGCATTCCCAGTAACTTCTTTG
         66 TTTCGTTGGAAACAGGAATATCTTCATATAAAAGCTAGACAGAAGCATTCTCAGAAACTTCTTTG

                              *    *                 
     219581 TGATGTTTGCATTCAAGTCACAGAGTTGAACATTCCCTTTC
        131 TGATGTTTGCATTCAAGTAACAGTGTTGAACATTCCCTTTC

                                                   *     *       **     *   *
     219622 ATAGAGCAGGTTTGAAACACTCTTTTTGTAGTATCTGGATGTGGACATTTGGAGCGCTTTCAGGC
          1 ATAGAGCAGGTTTGAAACACTCTTTTTGTAGTATCTGGAAGTGGATATTTGGATAGCTTTGAGGA

            * ** *      *             *** *   *                      *   *   
     219687 CTATGGT-GAAAAAGGAAATATCTTCCCCTGAAAACTAGACAGAAGCATTCTCAGAATCTTATTT
         66 TTTCGTTGGAAACAGG-AATATCTTCATATAAAAGCTAGACAGAAGCATTCTCAGAAACTTCTTT

                   **  **    *             **  * *   *
     219751 GTGATGTGCGCCCTCAACTAACAGTGTTGAAGCTTTCTTTTG
        130 GTGATGTTTGCATTCAAGTAACAGTGTTGAACATTCCCTTTC

                     *                    **     *   *                       
     219793 ATAGAGCAGTTTTGAAACACTCTTTTTGTAAAATCTGCAAGAGGATATTTGGATAGCTTTGAGGA
          1 ATAGAGCAGGTTTGAAACACTCTTTTTGTAGTATCTGGAAGTGGATATTTGGATAGCTTTGAGGA

                    *    *   * *            **                      *    *   
     219858 TTTCGTTGAAAACGGGATTGTCTTCATATAAACTCTAGACAGAAGCATTCTCAGAAGCTTCATTG
         66 TTTCGTTGGAAACAGGAATATCTTCATATAAAAGCTAGACAGAAGCATTCTCAGAAACTTCTTTG

            *       **   *    *             *        
     219923 GGATGTTTCAATTGAAGTCACAGTGTTGAACAGTCCCTTTC
        131 TGATGTTTGCATTCAAGTAACAGTGTTGAACATTCCCTTTC

                                                       * *       **   * *    
     219964 ATAGAGCAGGTTTGAAACACTCTTTTTGTAGTATCTGGAAGTGAACATTTGGAGCGCTCTCAGGA
          1 ATAGAGCAGGTTTGAAACACTCTTTTTGTAGTATCTGGAAGTGGATATTTGGATAGCTTTGAGGA

            * *  *  *   *  * *       **         *  *       * *          *  **
     220029 CTACGGTGAAAAAAGAAGTATCTTCCAATAAAAGCTTGATAGAAGCAATGTCAGAAACTTTTTCA
         66 TTTCGTTGGAAACAGGAATATCTTCATATAAAAGCTAGACAGAAGCATTCTCAGAAACTTCTTTG

                  * ** *   **      *       *  * *   *
     220094 TGATGTATCTACTCAGCTAACAGAGTTGAACCTTTCTTTTG
        131 TGATGTTTGCATTCAAGTAACAGTGTTGAACATTCCCTTTC

             *       *                   * *     *             **            
     220135 AGAGAGCAGTTTTGAAACACTCTTTTTGTGGAATCTGCAAGTGGATATTTGACTAGCTTTGAGGA
          1 ATAGAGCAGGTTTGAAACACTCTTTTTGTAGTATCTGGAAGTGGATATTTGGATAGCTTTGAGGA

                         *   *    *                *  *       *              
     220200 TTTCGTTGGAAACGGGATTA-CAT-ATA-AAAAGC-AGAAAGCAGCATTCCCAGAAACTTCTTTG
         66 TTTCGTTGGAAACAGGAATATCTTCATATAAAAGCTAGACAGAAGCATTCTCAGAAACTTCTTTG

                              *    *                 
     220261 TGATGTTTGCATTCAAGTCACAGAGTTGAACATTCCCTTTC
        131 TGATGTTTGCATTCAAGTAACAGTGTTGAACATTCCCTTTC

                                 *                 *     *       **     *   *
     220302 ATAGAGCAGGTTTGAAACACTGTTTTTGTAGTATCTGGATGTGGACATTTGGAGCGCTTTCAGGC
          1 ATAGAGCAGGTTTGAAACACTCTTTTTGTAGTATCTGGAAGTGGATATTTGGATAGCTTTGAGGA

            * ** *      *             *** *   *                      **  *   
     220367 CTATGGT-GAAAAAGGAAATATCTTCCCCTGAAAACTAGACAGAAGCATTCTCAGAATTTTATTT
         66 TTTCGTTGGAAACAGG-AATATCTTCATATAAAAGCTAGACAGAAGCATTCTCAGAAACTTCTTT

                   **  **  * *             **  * *   *
     220431 GTGATGTGCGCCCTCGACTAACAGTGTTGAAGCTTTCTTTTG
        130 GTGATGTTTGCATTCAAGTAACAGTGTTGAACATTCCCTTTC

                     *          *         **     *   *                       
     220473 ATAGAGCAGTTTTGAAACACACTTTTTGTAAAATCTGCAAGAGGATATTTGGATAGCTTTGAGGA
          1 ATAGAGCAGGTTTGAAACACTCTTTTTGTAGTATCTGGAAGTGGATATTTGGATAGCTTTGAGGA

                         *   * *     *      **          *           *    *   
     220538 TTTCGTTGGAAACGGGATTGTCTTCCTATAAACTCTAGACAGAATCATTCTCAGAAGCTTCATTG
         66 TTTCGTTGGAAACAGGAATATCTTCATATAAAAGCTAGACAGAAGCATTCTCAGAAACTTCTTTG

            *  *    **   *    *             *        
     220603 GGAAGTTTCAATTGAAGTCACAGTGTTGAACAGTCCCTTTC
        131 TGATGTTTGCATTCAAGTAACAGTGTTGAACATTCCCTTTC

                                  *                *     *       **     *   *
     220644 ATAGAGCAGGTTTGAAACACTCCTTTTGTAGTATCTGGATGTGGACATTTGGAGCGCTTTCAGGC
          1 ATAGAGCAGGTTTGAAACACTCTTTTTGTAGTATCTGGAAGTGGATATTTGGATAGCTTTGAGGA

            * ** *      *             *** *   *                      *   *   
     220709 CTATGGT-GAAAAAGGAAATATCTTCCCCTGAAAACTAGACAGAAGCATTCTCAGAATCTTATTT
         66 TTTCGTTGGAAACAGG-AATATCTTCATATAAAAGCTAGACAGAAGCATTCTCAGAAACTTCTTT

                   **  **    *             **  * *   *
     220773 GTGATGTGCGCCCTCAACTAACAGTGTTGAAGCTTTCTTTTG
        130 GTGATGTTTGCATTCAAGTAACAGTGTTGAACATTCCCTTTC

                     *                    **     *   *                       
     220815 ATAGAGCAGTTTTGAAACACTCTTTTTGTAAAATCTGCAAGAGGATATTTGGATAGCTTTGAGGA
          1 ATAGAGCAGGTTTGAAACACTCTTTTTGTAGTATCTGGAAGTGGATATTTGGATAGCTTTGAGGA

                         *   * *            **                      *    *   
     220880 TTTCGTTGGAAACGGGATTGTCTTCATATAAACTCTAGACAGAAGCATTCTCAGAAGCTTCATTG
         66 TTTCGTTGGAAACAGGAATATCTTCATATAAAAGCTAGACAGAAGCATTCTCAGAAACTTCTTTG

            *       **   *    *             *        
     220945 GGATGTTTCAATTGAAGTCACAGTGTTGAACAGTCCCTTTC
        131 TGATGTTTGCATTCAAGTAACAGTGTTGAACATTCCCTTTC

                                                         *       ***  * *    
     220986 ATAGAGCAGGTTTGAAACACTCTTTTTGTAGTATCTGGAAGTGGACATTTGGAGCACTCTCAGGA
          1 ATAGAGCAGGTTTGAAACACTCTTTTTGTAGTATCTGGAAGTGGATATTTGGATAGCTTTGAGGA

            * *  *      *              * *        * *       * *          *  *
     221051 CTGCGGT-GAAAAAGGAAATATCTTCAAACAAAAGCTACATAGAAGCAATGTCAGAAACTTTTTC
         66 TTTCGTTGGAAACAGG-AATATCTTCATATAAAAGCTAGACAGAAGCATTCTCAGAAACTTCTTT

            *      * ** *   **      *       *        *
     221115 ATGATGTATCTACTCAGCTAACAGAGTTGAACCTT-CCTTTG
        130 GTGATGTTTGCATTCAAGTAACAGTGTTGAACATTCCCTTTC

             *       *                   * *     *             **            
     221156 AGAGAGCAGTTTTGAAACACTCTTTTTGTGGAATCTGCAAGTGGATATTTGTCTAGCTTTGAGGA
          1 ATAGAGCAGGTTTGAAACACTCTTTTTGTAGTATCTGGAAGTGGATATTTGGATAGCTTTGAGGA

                         *   *    *                   *       *              
     221221 TTTCGTTGGAAACGGGATTA-CAT-ATA-AAAAGC-AGACAGCAGCATTCCCAGAAACTTCTTTG
         66 TTTCGTTGGAAACAGGAATATCTTCATATAAAAGCTAGACAGAAGCATTCTCAGAAACTTCTTTG

                              *    *                 
     221282 TGATGTTTGCATTCAAGTCACAGAGTTGAACATTCCCTTTC
        131 TGATGTTTGCATTCAAGTAACAGTGTTGAACATTCCCTTTC

                                                   *     *   *   **     *   *
     221323 ATAGAGCAGGTTTGAAACACTCTTTTTGTAGTATCTGGATGTGGACATTCGGAGCGCTTTCAGGC
          1 ATAGAGCAGGTTTGAAACACTCTTTTTGTAGTATCTGGAAGTGGATATTTGGATAGCTTTGAGGA

            * ** *      *             *** *   *                      *   *   
     221388 CTATGGT-GAAAAAGGAAATATCTTCCCCTGAAAACTAGACAGAAGCATTCTCAGAATCTTATTT
         66 TTTCGTTGGAAACAGG-AATATCTTCATATAAAAGCTAGACAGAAGCATTCTCAGAAACTTCTTT

                   **  **    *             **  * *   *
     221452 GTGATGTGCGCCCTCAACTAACAGTGTTGAAGCTTTCTTTTG
        130 GTGATGTTTGCATTCAAGTAACAGTGTTGAACATTCCCTTTC

                     *                    **     *   *                       
     221494 ATAGAGCAGTTTTGAAACACTCTTTTTGTAAAATCTGCAAGAGGATATTTGGATAGCTTTGAGGA
          1 ATAGAGCAGGTTTGAAACACTCTTTTTGTAGTATCTGGAAGTGGATATTTGGATAGCTTTGAGGA

                         *   * *            **                      *    *   
     221559 TTTCGTTGGAAACGGGATTGTCTTCATATAAACTCTAGACAGAAGCATTCTCAGAAGCTTCATTG
         66 TTTCGTTGGAAACAGGAATATCTTCATATAAAAGCTAGACAGAAGCATTCTCAGAAACTTCTTTG

            *             *    *             *        
     221624 GGATGTTT-CAGTTGAAGTCACAGTGTTGAACAGTCCCTTTC
        131 TGATGTTTGCA-TTCAAGTAACAGTGTTGAACATTCCCTTTC

                                                         *       **   * *    
     221665 ATAGAGCAGGTTTGAAACACTCTTTTTGTAGTATCTGGAAGTGGACATTTGGAGCGCTCTCAGGA
          1 ATAGAGCAGGTTTGAAACACTCTTTTTGTAGTATCTGGAAGTGGATATTTGGATAGCTTTGAGGA

            * *  *      *             **            *       * *          *  *
     221730 CTGCGGT-GAAAAAGGAAATATCTTCCAATAAAAGCTAGATAGAAGCAATGTCAGAAACTTTTTC
         66 TTTCGTTGGAAACAGG-AATATCTTCATATAAAAGCTAGACAGAAGCATTCTCAGAAACTTCTTT

            *      * ** *   **      *    *  *        *
     221794 ATGATGTATCTACTCAGCTAACAGAGTTGGACCTT-CCTTTG
        130 GTGATGTTTGCATTCAAGTAACAGTGTTGAACATTCCCTTTC

             *       *                   * *     *             **   *        
     221835 AGAGAGCAGTTTTGAAACACTCTTTTTGTGGAATCTGCAAGTGGATATTTGTCTAGATTTGAGGA
          1 ATAGAGCAGGTTTGAAACACTCTTTTTGTAGTATCTGGAAGTGGATATTTGGATAGCTTTGAGGA

                              *    *    *             *       *              
     221900 TTTCGTTGGAAAC-GG--GAT-TACATAAAAAAGC-AGACAGCAGCATTCCCAGAAACTTCTTTG
         66 TTTCGTTGGAAACAGGAATATCTTCATATAAAAGCTAGACAGAAGCATTCTCAGAAACTTCTTTG

                              *    **                
     221960 TGATGTTTGCATTCAAGTCACAGAATTGAACATTCCCTTTC
        131 TGATGTTTGCATTCAAGTAACAGTGTTGAACATTCCCTTTC

                                                   *     *     * **     *   *
     222001 ATAGAGCAGGTTTGAAACACTCTTTTTGTAGTATCTGGATGTGGACATTTGCAGCGCTTTCAGGC
          1 ATAGAGCAGGTTTGAAACACTCTTTTTGTAGTATCTGGAAGTGGATATTTGGATAGCTTTGAGGA

            * ** *      *             *** *   *                          *   
     222066 CTAAGGT-GAAAAAGGAAATATCTTCCCCTGAAAACTAGACAGAAGCATTCTCAGAAACTTATTT
         66 TTTCGTTGGAAACAGG-AATATCTTCATATAAAAGCTAGACAGAAGCATTCTCAGAAACTTCTTT

                   **  **    *             **  * *   *
     222130 GTGATGTGCGCCCTCAACTAACAGTGTTGAAGCTTTCTTTTG
        130 GTGATGTTTGCATTCAAGTAACAGTGTTGAACATTCCCTTTC

                     *                   * *     *             **            
     222172 ATAGAGCAGTTTTGAAACACTCTTTTTGTGGAATCTGCAAGTGGATATTTGTCTAGCTTTGAGGA
          1 ATAGAGCAGGTTTGAAACACTCTTTTTGTAGTATCTGGAAGTGGATATTTGGATAGCTTTGAGGA

                   *     *   *    *                   *       *     *   *    
     222237 TTTCGTTTGAAACGGGATTA-CAT-ATA-AAAAGC-AGACAGCAGCATTCCCAGAATCTTGTTTG
         66 TTTCGTTGGAAACAGGAATATCTTCATATAAAAGCTAGACAGAAGCATTCTCAGAAACTTCTTTG

               *              *    *                 
     222298 TGAAGTTTGCATTCAAGTCACAGAGTTGAACATTCCCTTTC
        131 TGATGTTTGCATTCAAGTAACAGTGTTGAACATTCCCTTTC

             *                         *           *     *       **     *   *
     222339 AGAGAGCAGGTTTGAAACACTCTTTTTATAGTATCTGGATGTGGACATTTGGAGCGCTTTCAGGC
          1 ATAGAGCAGGTTTGAAACACTCTTTTTGTAGTATCTGGAAGTGGATATTTGGATAGCTTTGAGGA

            * ** *      *             *** *   *                      *   *   
     222404 CTATGGT-GAAAAAGGAAATATCTTCTCCTGAAAACTAGACAGAAGCATTCTCAGAATCTTATTT
         66 TTTCGTTGGAAACAGG-AATATCTTCATATAAAAGCTAGACAGAAGCATTCTCAGAAACTTCTTT

                   **  **    *             **  * *   *
     222468 GTGATGTGCGCCCTCAACTAACAGTGTTGAAGCTTTCTTTTG
        130 GTGATGTTTGCATTCAAGTAACAGTGTTGAACATTCCCTTTC

                     *                *   **     *   *        *              
     222510 ATAGAGCAGTTTTGAAACACTCTTTTCGTAAAATCTGCAAGAGGATATTTTGATAGCTTTGAGGA
          1 ATAGAGCAGGTTTGAAACACTCTTTTTGTAGTATCTGGAAGTGGATATTTGGATAGCTTTGAGGA

                         *   * *            **               *      *    *   
     222575 TTTCGTTGGAAACGGGATTGTCTTCATATAAACTCTAGACAGAAGCATTATCAGAAGCTTCATTG
         66 TTTCGTTGGAAACAGGAATATCTTCATATAAAAGCTAGACAGAAGCATTCTCAGAAACTTCTTTG

            *       **   *    *             *        
     222640 GGATGTTTCAATTGAAGTCACAGTGTTGAACAGTCCCTTTC
        131 TGATGTTTGCATTCAAGTAACAGTGTTGAACATTCCCTTTC

                                                         *       **   * *    
     222681 ATAGAGCAGGTTTGAAACACTCTTTTTGTAGTATCTGGAAGTGGACATTTGGAGCGCTCTCAGGA
          1 ATAGAGCAGGTTTGAAACACTCTTTTTGTAGTATCTGGAAGTGGATATTTGGATAGCTTTGAGGA

            * *  *      *       *     **          * *       * *          *  *
     222746 CTGCGGT-GAAAAAGGAAATTTCTTCCAATAAAAGCTACATAGAAGCAATGTCAGAAACTTTTTC
         66 TTTCGTTGGAAACAGG-AATATCTTCATATAAAAGCTAGACAGAAGCATTCTCAGAAACTTCTTT

            *      * ** *   **      *       *        *
     222810 ATGATGTATCTACTCAGCTAACAGAGTTGAACCTT-CCTTTG
        130 GTGATGTTTGCATTCAAGTAACAGTGTTGAACATTCCCTTTC

             *       *                   * *     *             **            
     222851 AGAGAGCAGTTTTGAAACACTCTTTTTGTGGAATCTGCAAGTGGATATTTGTCTAGCTTTGAGGA
          1 ATAGAGCAGGTTTGAAACACTCTTTTTGTAGTATCTGGAAGTGGATATTTGGATAGCTTTGAGGA

                       * *   *    *                   *       *           *  
     222916 TTTCGTTGGAATCGGGATTA-CAT-ATA-AAAAGC-AGACAGCAGCATTCCCAGAAACTTCTCTG
         66 TTTCGTTGGAAACAGGAATATCTTCATATAAAAGCTAGACAGAAGCATTCTCAGAAACTTCTTTG

                              *    *                 
     222977 TGATGTTTGCATTCAAGTCACAGAGTTGAACATTCCCTTTC
        131 TGATGTTTGCATTCAAGTAACAGTGTTGAACATTCCCTTTC

                                                   *     *       **     *   *
     223018 ATAGAGCAGGTTTGAAACACTCTTTTTGTAGTATCTGGATGTGGACATTTGGAGCGCTTTCAGGC
          1 ATAGAGCAGGTTTGAAACACTCTTTTTGTAGTATCTGGAAGTGGATATTTGGATAGCTTTGAGGA

            * ** *      *             *** *   *                      *   *   
     223083 CTATGGT-GAAAAAGGAAATATCTTCCCCTGAAAACTAGACAGAAGCATTCTCAGAATCTTATTT
         66 TTTCGTTGGAAACAGG-AATATCTTCATATAAAAGCTAGACAGAAGCATTCTCAGAAACTTCTTT

                   **  **    *             **  * *   *
     223147 GTGATGTGCGCCCTCAACTAACAGTGTTGAAGCTTTCTTTTG
        130 GTGATGTTTGCATTCAAGTAACAGTGTTGAACATTCCCTTTC

                     *                    **     *   *                       
     223189 ATAGAGCAGTTTTGAAACACTCTTTTTGTAAAATCTGCAAGAGGATATTTGGATAGCTTTGAGGA
          1 ATAGAGCAGGTTTGAAACACTCTTTTTGTAGTATCTGGAAGTGGATATTTGGATAGCTTTGAGGA

                         *   * *            **                      *    *   
     223254 TTTCGTTGGAAACGGGATTGTCTTCATATAAACTCTAGACAGAAGCATTCTCAGAAGCTTCATTG
         66 TTTCGTTGGAAACAGGAATATCTTCATATAAAAGCTAGACAGAAGCATTCTCAGAAACTTCTTTG

            *             *    *             *        
     223319 GGATGTTT-CAGTTGAAGTCACAGTGTTGAACAGTCCCTTTC
        131 TGATGTTTGCA-TTCAAGTAACAGTGTTGAACATTCCCTTTC

                                                         *       **   * *    
     223360 ATAGAGCAGGTTTGAAACACTCTTTTTGTAGTATCTGGAAGTGGACATTTGGAGCGCTCTCAGGA
          1 ATAGAGCAGGTTTGAAACACTCTTTTTGTAGTATCTGGAAGTGGATATTTGGATAGCTTTGAGGA

            * *  *      *             **            *       * *          *  *
     223425 CTGCGGT-GAAAAAGGAAATATCTTCCAATAAAAGCTAGATAGAAGCAATGTCAGAAACTTTTTC
         66 TTTCGTTGGAAACAGG-AATATCTTCATATAAAAGCTAGACAGAAGCATTCTCAGAAACTTCTTT

            *      * ** *   **      *    *  *        *
     223489 ATGATGTATCTACTCAGCTAACAGAGTTGGACCTT-CCTTTG
        130 GTGATGTTTGCATTCAAGTAACAGTGTTGAACATTCCCTTTC

             *       *                   * *     *             **            
     223530 AGAGAGCAGTTTTGAAACACTCTTTTTGTGGAATCTGCAAGTGGATATTTGTCTAGCTTTGAGGA
          1 ATAGAGCAGGTTTGAAACACTCTTTTTGTAGTATCTGGAAGTGGATATTTGGATAGCTTTGAGGA

                         *   *    *                   *       *              
     223595 TTTCGTTGGAAACGGGATTA-CAT-ATA-AAAAGC-AGACAGCAGCATTCCCAGAAACTTCTTTG
         66 TTTCGTTGGAAACAGGAATATCTTCATATAAAAGCTAGACAGAAGCATTCTCAGAAACTTCTTTG

                              *    *                 
     223656 TGATGTTTGCATTCAAGTCACAGAGTTGAACATTCCCTTTC
        131 TGATGTTTGCATTCAAGTAACAGTGTTGAACATTCCCTTTC

                                                   *     *     * **     *   *
     223697 ATAGAGCAGGTTTGAAACACTCTTTTTGTAGTATCTGGATGTGGACATTTGCAGCGCTTTCAGGC
          1 ATAGAGCAGGTTTGAAACACTCTTTTTGTAGTATCTGGAAGTGGATATTTGGATAGCTTTGAGGA

            * ** *      *             *** *   *                          *   
     223762 CTAAGGT-GAAAAAGGAAATATCTTCCCCTGAAAACTAGACAGAAGCATTCTCAGAAACTTATTT
         66 TTTCGTTGGAAACAGG-AATATCTTCATATAAAAGCTAGACAGAAGCATTCTCAGAAACTTCTTT

                   **  **    *             **  * *   *
     223826 GTGATGTGCGCCCTCAACTAACAGTGTTGAAGCTTTCTTTTG
        130 GTGATGTTTGCATTCAAGTAACAGTGTTGAACATTCCCTTTC

                     *                   * *  *  *             **            
     223868 ATAGAGCAGTTTTGAAACACTCTTTTTGTGGAATTTGCAAGTGGATATTTGTCTAGCTTTGAGGA
          1 ATAGAGCAGGTTTGAAACACTCTTTTTGTAGTATCTGGAAGTGGATATTTGGATAGCTTTGAGGA

                         *   *    *                   *       *     *   *    
     223933 TTTCGTTGGAAACGGGATTA-CAT-ATA-AAAAGC-AGACAGCAGCATTCCCAGAATCTTGTTTG
         66 TTTCGTTGGAAACAGGAATATCTTCATATAAAAGCTAGACAGAAGCATTCTCAGAAACTTCTTTG

                              *    *                 
     223994 TGATGTTTGCATTCAAGTCACAGAGTTGAACATTCCCTTTC
        131 TGATGTTTGCATTCAAGTAACAGTGTTGAACATTCCCTTTC

             *                         *           *     *       **     *   *
     224035 AGAGAGCAGGTTTGAAACACTCTTTTTATAGTATCTGGATGTGGACATTTGGAGCGCTTTCAGGC
          1 ATAGAGCAGGTTTGAAACACTCTTTTTGTAGTATCTGGAAGTGGATATTTGGATAGCTTTGAGGA

            * ** *  *                 *** *   *                      *   *   
     224100 CTATGGTGTAAA-AGGAAATATCTTCTCCTGAAAACTAGACAGAAGCATTCTCAGAATCTTATTT
         66 TTTCGTTGGAAACAGG-AATATCTTCATATAAAAGCTAGACAGAAGCATTCTCAGAAACTTCTTT

                   **  **    *             **  * *   *
     224164 GTGATGTGCGCCCTCAACTAACAGTGTTGAAGCTTTCTTTTG
        130 GTGATGTTTGCATTCAAGTAACAGTGTTGAACATTCCCTTTC

                     *                **  **     *   *        *              
     224206 ATAGAGCAGTTTTGAAACACTCTTTTCCTAAAATCTGCAAGAGGATATTTTGATAGCTTTGAGGA
          1 ATAGAGCAGGTTTGAAACACTCTTTTTGTAGTATCTGGAAGTGGATATTTGGATAGCTTTGAGGA

                         *   * *            **                      *    *   
     224271 TTTCGTTGGAAACGGGATTGTCTTCATATAAACTCTAGACAGAAGCATTCTCAGAAGCTTCATTG
         66 TTTCGTTGGAAACAGGAATATCTTCATATAAAAGCTAGACAGAAGCATTCTCAGAAACTTCTTTG

            *       **   *    *             *        
     224336 GGATGTTTCAATTGAAGTCACAGTGTTGAACAGTCCCTTTC
        131 TGATGTTTGCATTCAAGTAACAGTGTTGAACATTCCCTTTC

                                                         *       **   * *    
     224377 ATAGAGCAGGTTTGAAACACTCTTTTTGTAGTATCTGGAAGTGGACATTTGGAGCGCTCTCAGGA
          1 ATAGAGCAGGTTTGAAACACTCTTTTTGTAGTATCTGGAAGTGGATATTTGGATAGCTTTGAGGA

            * *  *      *             **            *       * *          *  *
     224442 CTGCGGT-GAAAAAGGAAATATCTTCCAATAAAAGCTAGATAGAAGCAATGTCAGAAACTTTTTC
         66 TTTCGTTGGAAACAGG-AATATCTTCATATAAAAGCTAGACAGAAGCATTCTCAGAAACTTCTTT

            *      * ** *   **      *       *        *
     224506 ATGATGTATCTACTCAGCTAACAGAGTTGAACCTT-CCTTTG
        130 GTGATGTTTGCATTCAAGTAACAGTGTTGAACATTCCCTTTC

             *       *                   * *     *             **            
     224547 AGAGAGCAGTTTTGAAACACTCTTTTTGTGGAATCTGCAAGTGGATATTTGTCTAGCTTTGAGGA
          1 ATAGAGCAGGTTTGAAACACTCTTTTTGTAGTATCTGGAAGTGGATATTTGGATAGCTTTGAGGA

                         *   *    *                   *       *              
     224612 TTTCGTTGGAAACGGGATTA-CAT-ATA-AAAAGC-AGACAGCAGCATTCCCAGAAACTTCTTTG
         66 TTTCGTTGGAAACAGGAATATCTTCATATAAAAGCTAGACAGAAGCATTCTCAGAAACTTCTTTG

                              *    *                 
     224673 TGATGTTTGCATTCAAGTCACAGAGTTGAACATTCCCTTTC
        131 TGATGTTTGCATTCAAGTAACAGTGTTGAACATTCCCTTTC

                                                   *     *     * **     *   *
     224714 ATAGAGCAGGTTTGAAACACTCTTTTTGTAGTATCTGGATGTGGACATTTGCAGCGCTTTCAGGC
          1 ATAGAGCAGGTTTGAAACACTCTTTTTGTAGTATCTGGAAGTGGATATTTGGATAGCTTTGAGGA

            * ** *      *             *** *   *                          *   
     224779 CTATGGT-GAAAAAGGAAATATCTTCCCCTGAAAACTAGACAGAAGCATTCTCAGAAACTTATTT
         66 TTTCGTTGGAAACAGG-AATATCTTCATATAAAAGCTAGACAGAAGCATTCTCAGAAACTTCTTT

                   **  **    *               *    *   *
     224843 GTGATGTGCGCCCTCAACTAACAGTGTTGAAGCCTT-CTTTTG
        130 GTGATGTTTGCATTCAAGTAACAGTGTTGAA-CATTCCCTTTC

                     *                   * *     *             **            
     224885 ATAGAGCAGTTTTGAAACACTCTTTTTGTGGAATCTGCAAGTGGATATTTGTCTAGCTTTGAGGA
          1 ATAGAGCAGGTTTGAAACACTCTTTTTGTAGTATCTGGAAGTGGATATTTGGATAGCTTTGAGGA

                         *   *    *                   *       *     *   *    
     224950 TTTCGTTGGAAACGGGATTA-CAT-ATA-AAAAGC-AGACAGCAGCATTCCCAGAATCTTGTTTG
         66 TTTCGTTGGAAACAGGAATATCTTCATATAAAAGCTAGACAGAAGCATTCTCAGAAACTTCTTTG

                              *    *                 
     225011 TGATGTTTGCATTCAAGTCACAGAGTTGAACATTCCCTTTC
        131 TGATGTTTGCATTCAAGTAACAGTGTTGAACATTCCCTTTC

             *                         *           *     *       **     *   *
     225052 AGAGAGCAGGTTTGAAACACTCTTTTTATAGTATCTGGATGTGGACATTTGGAGCGCTTTCAGGC
          1 ATAGAGCAGGTTTGAAACACTCTTTTTGTAGTATCTGGAAGTGGATATTTGGATAGCTTTGAGGA

            * ** *      *             *** *   *                      *   * * 
     225117 CTATGGT-GAAAAAGGAAATATCTTCTCCTGAAAACTAGACAGAAGCATTCTCAGAATCTTATCT
         66 TTTCGTTGGAAACAGG-AATATCTTCATATAAAAGCTAGACAGAAGCATTCTCAGAAACTTCTTT

                   **  **    *             **  * *   *
     225181 GTGATGTGCGCCCTCAACTAACAGTGTTGAAGCTTTCTTTTG
        130 GTGATGTTTGCATTCAAGTAACAGTGTTGAACATTCCCTTTC

                     *                *   **     *   *        *              
     225223 ATAGAGCAGTTTTGAAACACTCTTTTCGTAAAATCTGCAAGAGGATATTTTGATAGCTTTGAGGA
          1 ATAGAGCAGGTTTGAAACACTCTTTTTGTAGTATCTGGAAGTGGATATTTGGATAGCTTTGAGGA

                         *   * *            **                      *    **  
     225288 TTTCGTTGGAAACGGGATTGTCTTCATATAAACTCTAGACAGAAGCATTCTCAGAAGCTTCAGTG
         66 TTTCGTTGGAAACAGGAATATCTTCATATAAAAGCTAGACAGAAGCATTCTCAGAAACTTCTTTG

            *       **   *    *             *        
     225353 GGATGTTTCAATTGAAGTCACAGTGTTGAACAGTCCCTTTC
        131 TGATGTTTGCATTCAAGTAACAGTGTTGAACATTCCCTTTC

                                 *                       *       *  * * *    
     225394 ATAGAGCAGGTTTGAAACACTGTTTTTGTAGTATCTGGAAGTGGACATTTGGAGAGATCTCAGGA
          1 ATAGAGCAGGTTTGAAACACTCTTTTTGTAGTATCTGGAAGTGGATATTTGGATAGCTTTGAGGA

            * *  *    * *             **            *       * *          *  *
     225459 ATACGAT-GATAAAGGAAATATCTTCCAATAAAAGCTAGATAGAAGCAATGTCAGAAACTTTTTC
         66 TTTCGTTGGAAACAGG-AATATCTTCATATAAAAGCTAGACAGAAGCATTCTCAGAAACTTCTTT

            *      * ** *   **      *       *  * *   *
     225523 ATGATGTATCTACTCAGCTAACAGAGTTGAACCTTTCTTTTG
        130 GTGATGTTTGCATTCAAGTAACAGTGTTGAACATTCCCTTTC

             *       *                   * *                   **            
     225565 AGAGAGCAGTTTTGAAACACTCTTTTTGTGGAATCTGGAAGTGGATATTTGTCTAGCTTTGAGGA
          1 ATAGAGCAGGTTTGAAACACTCTTTTTGTAGTATCTGGAAGTGGATATTTGGATAGCTTTGAGGA

                         *   *    *                   *       *   *          
     225630 TTTCGTTGGAAACGGGATTA-CAT-ATA-AAAAGC-AGACAGCAGCATTCCCAGTAACTTCTTTG
         66 TTTCGTTGGAAACAGGAATATCTTCATATAAAAGCTAGACAGAAGCATTCTCAGAAACTTCTTTG

                              *    *                 
     225691 TGATGTTTGCATTCAAGTCACAGAGTTGAACATTCCCTTTC
        131 TGATGTTTGCATTCAAGTAACAGTGTTGAACATTCCCTTTC

             *                                     *     *       **     *   *
     225732 AGAGAGCAGGTTTGAAACACTCTTTTTGTAGTATCTGGATGTGGACATTTGGAGCGCTTTCAGGC
          1 ATAGAGCAGGTTTGAAACACTCTTTTTGTAGTATCTGGAAGTGGATATTTGGATAGCTTTGAGGA

            * ** *      *             *** *   *                      *   *   
     225797 CTATGGT-GAAAAAGGAAATATCTTCCCCTGAAAACTAGACAGAAGCATTCTCAGAATCTTATTT
         66 TTTCGTTGGAAACAGG-AATATCTTCATATAAAAGCTAGACAGAAGCATTCTCAGAAACTTCTTT

                   **  **    *             **  * *   *
     225861 GTGATGTGCGCCCTCAACTAACAGTGTTGAAGCTTTCTTTTG
        130 GTGATGTTTGCATTCAAGTAACAGTGTTGAACATTCCCTTTC

                     *                    **     *   *                       
     225903 ATAGAGCAGTTTTGAAACACTCTTTTTGTAAAATCTGCAAGAGGATATTTGGATAGCTTTGAGGA
          1 ATAGAGCAGGTTTGAAACACTCTTTTTGTAGTATCTGGAAGTGGATATTTGGATAGCTTTGAGGA

                         *   * *            **                      *    *   
     225968 TTTCGTTGGAAACGGGATTGTCTTCATATAAACTCTAGACAGAAGCATTCTCAGAAGCTTCATTG
         66 TTTCGTTGGAAACAGGAATATCTTCATATAAAAGCTAGACAGAAGCATTCTCAGAAACTTCTTTG

            *             *    *             *        
     226033 GGATGTTT-CAGTTGAAGTCACAGTGTTGAACAGTCCCTTTC
        131 TGATGTTTGCA-TTCAAGTAACAGTGTTGAACATTCCCTTTC

                                                         *       **   * *    
     226074 ATAGAGCAGGTTTGAAACACTCTTTTTGTAGTATCTGGAAGTGGACATTTGGAGCGCTCTCAGGA
          1 ATAGAGCAGGTTTGAAACACTCTTTTTGTAGTATCTGGAAGTGGATATTTGGATAGCTTTGAGGA

            * *  *      *             **          * *       * *          *  *
     226139 CTGCGGT-GAAAAAGGAAATATCTTCCAATAAAAGCTACATAGAAGCAATGTCAGAAACTTTTTC
         66 TTTCGTTGGAAACAGG-AATATCTTCATATAAAAGCTAGACAGAAGCATTCTCAGAAACTTCTTT

            *      * ** *   **      *    *  *        *
     226203 ATGATGTATCTACTCAGCTAACAGAGTTGGACCTT-CCTTTG
        130 GTGATGTTTGCATTCAAGTAACAGTGTTGAACATTCCCTTTC

             *       *                   * *     *             **            
     226244 AGAGAGCAGTTTTGAAACACTCTTTTTGTGGAATCTGCAAGTGGATATTTGTCTAGCTTTGAGGA
          1 ATAGAGCAGGTTTGAAACACTCTTTTTGTAGTATCTGGAAGTGGATATTTGGATAGCTTTGAGGA

                         *   *    *                   *       *      *       
     226309 TTTCGTTGGAAACCGGATTA-CAT-ATA-AAAAGC-AGACAGCAGCATTCCCAGAAATTTCTTTG
         66 TTTCGTTGGAAACAGGAATATCTTCATATAAAAGCTAGACAGAAGCATTCTCAGAAACTTCTTTG

                              *    *                 
     226370 TGATGTTTGCATTCAAGTCACAGAGTTGAACATTCCCTTTC
        131 TGATGTTTGCATTCAAGTAACAGTGTTGAACATTCCCTTTC

                                                   *     *     * **     *   *
     226411 ATAGAGCAGGTTTGAAACACTCTTTTTGTAGTATCTGGATGTGGACATTTGCAGCGCTTTCAGGC
          1 ATAGAGCAGGTTTGAAACACTCTTTTTGTAGTATCTGGAAGTGGATATTTGGATAGCTTTGAGGA

            * ** *      *             *** *   *                          *   
     226476 CTAAGGT-GAAAAAGGAAATATCTTCCCCTGAAAACTAGACAGAAGCATTCTCAGAAACTTATTT
         66 TTTCGTTGGAAACAGG-AATATCTTCATATAAAAGCTAGACAGAAGCATTCTCAGAAACTTCTTT

                   **  **    *             **  * *   *
     226540 GTGATGTGCGCCCTCAACTAACAGTGTTGAAGCTTTCTTTTG
        130 GTGATGTTTGCATTCAAGTAACAGTGTTGAACATTCCCTTTC

                     *                   * *     *             **            
     226582 ATAGAGCAGTTTTGAAACACTCTTTTTGTGGAATCTGCAAGTGGATATTTGTCTAGCTTTGAGGA
          1 ATAGAGCAGGTTTGAAACACTCTTTTTGTAGTATCTGGAAGTGGATATTTGGATAGCTTTGAGGA

                         *   *    *                   *       *     *   *    
     226647 TTTCGTTGGAAACGGGATTA-CAT-ATA-AAAAGC-AGACAGCAGCATTCCCAGAATCTTGTTTG
         66 TTTCGTTGGAAACAGGAATATCTTCATATAAAAGCTAGACAGAAGCATTCTCAGAAACTTCTTTG

                              *    *                 
     226708 TGATGTTTGCATTCAAGTCACAGAGTTGAACATTCCCTTTC
        131 TGATGTTTGCATTCAAGTAACAGTGTTGAACATTCCCTTTC

             *                         *           *     *       **     *   *
     226749 AGAGAGCAGGTTTGAAACACTCTTTTTATAGTATCTGGATGTGGACATTTGGAGCGCTTTCAGGC
          1 ATAGAGCAGGTTTGAAACACTCTTTTTGTAGTATCTGGAAGTGGATATTTGGATAGCTTTGAGGA

            * ** *      *             *** *   *                      *   *   
     226814 CTATGGT-GAAAAAGGAAATATCTTCTCCTGAAAACTAGACAGAAGCATTCTCAGAATCTTATTT
         66 TTTCGTTGGAAACAGG-AATATCTTCATATAAAAGCTAGACAGAAGCATTCTCAGAAACTTCTTT

                   *** **    *             **  * *   *
     226878 GTGATGTGCCCCCTCAACTAACAGTGTTGAAGCTTTCTTTTG
        130 GTGATGTTTGCATTCAAGTAACAGTGTTGAACATTCCCTTTC

                     *                *   **     *   *        *              
     226920 ATAGAGCAGTTTTGAAACACTCTTTTCGTAAAATCTGCAAGAGGATATTTTGATAGCTTTGAGGA
          1 ATAGAGCAGGTTTGAAACACTCTTTTTGTAGTATCTGGAAGTGGATATTTGGATAGCTTTGAGGA

                         *   * *            **                      *    *   
     226985 TTTCGTTGGAAACGGGATTGTCTTCATATAAACTCTAGACAGAAGCATTCTCAGAAGCTTCATTG
         66 TTTCGTTGGAAACAGGAATATCTTCATATAAAAGCTAGACAGAAGCATTCTCAGAAACTTCTTTG

            *       **   *    *             *       *
     227050 GGATGTTTCAATTGAAGTCACAGTGTTGAACAGTCCCTTTA
        131 TGATGTTTGCATTCAAGTAACAGTGTTGAACATTCCCTTTC

                                                         *       **   * *    
     227091 ATAGAGCAGGTTTGAAACACTCTTTTTGTAGTATCTGGAAGTGGACATTTGGAGCGCTCTCAGGA
          1 ATAGAGCAGGTTTGAAACACTCTTTTTGTAGTATCTGGAAGTGGATATTTGGATAGCTTTGAGGA

            * *  *      *             **            *       * *          *  *
     227156 CTACGGT-GAAAAAGGAAATATCTTCCAATAAAAGCTAGATAGAAGCAATGTCAGAAACTTTTTC
         66 TTTCGTTGGAAACAGG-AATATCTTCATATAAAAGCTAGACAGAAGCATTCTCAGAAACTTCTTT

            *      * ** *   **      *       *        *
     227220 ATGATGTATCTACTCAGCTAACAGAGTTGAACCTT-CCTTTG
        130 GTGATGTTTGCATTCAAGTAACAGTGTTGAACATTCCCTTTC

             *       *                   * *     *             **            
     227261 AGAGAGCAGTTTTGAAACACTCTTTTTGTGGAATCTGCAAGTGGATATTTGTCTAGCTTTGAGGA
          1 ATAGAGCAGGTTTGAAACACTCTTTTTGTAGTATCTGGAAGTGGATATTTGGATAGCTTTGAGGA

                         *   *    *                   *       *              
     227326 TTTCGTTGGAAACGGGATTA-CAT-ATA-AAAAGC-AGACAGCAGCATTCCCAGAAACTTCTTTG
         66 TTTCGTTGGAAACAGGAATATCTTCATATAAAAGCTAGACAGAAGCATTCTCAGAAACTTCTTTG

                              *    *                 
     227387 TGATGTTTGCATTCAAGTCACAGAGTTGAACATTCCCTTTC
        131 TGATGTTTGCATTCAAGTAACAGTGTTGAACATTCCCTTTC

                                                   *     *     * **     *   *
     227428 ATAGAGCAGGTTTGAAACACTCTTTTTGTAGTATCTGGATGTGGACATTTGCAGCGCTTTCAGGC
          1 ATAGAGCAGGTTTGAAACACTCTTTTTGTAGTATCTGGAAGTGGATATTTGGATAGCTTTGAGGA

            * ** *      *             *** *   *                          *   
     227493 CTATGGT-GAAAAAGGAAATATCTTCCCCTGAAAACTAGACAGAAGCATTCTCAGAAACTTATTT
         66 TTTCGTTGGAAACAGG-AATATCTTCATATAAAAGCTAGACAGAAGCATTCTCAGAAACTTCTTT

                   **  **    *               *    *   *
     227557 GTGATGTGCGCCCTCAACTAACAGTGTTGAAGCCTT-CTTTTG
        130 GTGATGTTTGCATTCAAGTAACAGTGTTGAA-CATTCCCTTTC

                     *                   * *     *             **            
     227599 ATAGAGCAGTTTTGAAACACTCTTTTTGTGGAATCTGCAAGTGGATATTTGTCTAGCTTTGAGGA
          1 ATAGAGCAGGTTTGAAACACTCTTTTTGTAGTATCTGGAAGTGGATATTTGGATAGCTTTGAGGA

                         *   *    *                   *       *     *   *    
     227664 TTTCGTTGGAAACGGGATTA-CAT-ATA-AAAAGC-AGACAGCAGCATTCCCAGAATCTTGTTTG
         66 TTTCGTTGGAAACAGGAATATCTTCATATAAAAGCTAGACAGAAGCATTCTCAGAAACTTCTTTG

                              *    *                 
     227725 TGATGTTTGCATTCAAGTCACAGAGTTGAACATTCCCTTTC
        131 TGATGTTTGCATTCAAGTAACAGTGTTGAACATTCCCTTTC

             *                         *           *     *       **     *   *
     227766 AGAGAGCAGGTTTGAAACACTCTTTTTATAGTATCTGGATGTGGACATTTGGAGCGCTTTCAGGC
          1 ATAGAGCAGGTTTGAAACACTCTTTTTGTAGTATCTGGAAGTGGATATTTGGATAGCTTTGAGGA

            * ** *      *             *** *   *       *              *   * * 
     227831 CTATGGT-GAAAAAGGAAATATCTTCTCCTGAAAACTAGACACAAGCATTCTCAGAATCTTATCT
         66 TTTCGTTGGAAACAGG-AATATCTTCATATAAAAGCTAGACAGAAGCATTCTCAGAAACTTCTTT

                   **  **    *             **  * *   *
     227895 GTGATGTGCGCCCTCAACTAACAGTGTTGAAGCTTTCTTTTG
        130 GTGATGTTTGCATTCAAGTAACAGTGTTGAACATTCCCTTTC

                     *                *   **     *   *        *              
     227937 ATAGAGCAGTTTTGAAACACTCTTTTCGTAAAATCTGCAAGAGGATATTTTGATAGCTTTGAGGA
          1 ATAGAGCAGGTTTGAAACACTCTTTTTGTAGTATCTGGAAGTGGATATTTGGATAGCTTTGAGGA

                         *   * *            **                      *    **  
     228002 TTTCGTTGGAAACGGGATTGTCTTCATATAAACTCTAGACAGAAGCATTCTCAGAAGCTTCAGTG
         66 TTTCGTTGGAAACAGGAATATCTTCATATAAAAGCTAGACAGAAGCATTCTCAGAAACTTCTTTG

            *       **   *    *             *        
     228067 GGATGTTTCAATTGAAGTCACAGTGTTGAACAGTCCCTTTC
        131 TGATGTTTGCATTCAAGTAACAGTGTTGAACATTCCCTTTC

                                 *                       *       *  * * *    
     228108 ATAGAGCAGGTTTGAAACACTGTTTTTGTAGTATCTGGAAGTGGACATTTGGAGAGATCTCAGGA
          1 ATAGAGCAGGTTTGAAACACTCTTTTTGTAGTATCTGGAAGTGGATATTTGGATAGCTTTGAGGA

            * *  *    * *             **            *       * *          *  *
     228173 ATACGGT-GATAAAGGAAATATCTTCCAATAAAAGCTAGATAGAAGCAATGTCAGAAACTTTTTC
         66 TTTCGTTGGAAACAGG-AATATCTTCATATAAAAGCTAGACAGAAGCATTCTCAGAAACTTCTTT

            *      * ** *   **      *       *  * *   *
     228237 ATGATGTATCTACTCAGCTAACAGAGTTGAACCTTTCTTTTG
        130 GTGATGTTTGCATTCAAGTAACAGTGTTGAACATTCCCTTTC

             *       *                   * *                   **            
     228279 AGAGAGCAGTTTTGAAACACTCTTTTTGTGGAATCTGGAAGTGGATATTTGTCTAGCTTTGAGGA
          1 ATAGAGCAGGTTTGAAACACTCTTTTTGTAGTATCTGGAAGTGGATATTTGGATAGCTTTGAGGA

                         *   *    *                   *       *   *          
     228344 TTTCGTTGGAAACGGGATTA-CAT-ATA-AAAAGC-AGACAGCAGCATTCCCAGTAACTTCTTTG
         66 TTTCGTTGGAAACAGGAATATCTTCATATAAAAGCTAGACAGAAGCATTCTCAGAAACTTCTTTG

                              *    *                 
     228405 TGATGTTTGCATTCAAGTCACAGAGTTGAACATTCCCTTTC
        131 TGATGTTTGCATTCAAGTAACAGTGTTGAACATTCCCTTTC

             *                                     *     *       **     *   *
     228446 AGAGAGCAGGTTTGAAACACTCTTTTTGTAGTATCTGGATGTGGACATTTGGAGCGCTTTCAGGC
          1 ATAGAGCAGGTTTGAAACACTCTTTTTGTAGTATCTGGAAGTGGATATTTGGATAGCTTTGAGGA

            * ** *      *             *** *   *                      *   *   
     228511 CTATGGT-GAAAAAGGAAATATCTTCCCCTGAAAACTAGACAGAAGCATTCTCAGAATCTTATTT
         66 TTTCGTTGGAAACAGG-AATATCTTCATATAAAAGCTAGACAGAAGCATTCTCAGAAACTTCTTT

                   **  **    *             **  * *   *
     228575 GTGATGTGCGCCCTCAACTAACAGTGTTGAAGCTTTCTTTTG
        130 GTGATGTTTGCATTCAAGTAACAGTGTTGAACATTCCCTTTC

                     *                    **     *   *                       
     228617 ATAGAGCAGTTTTGAAACACTCTTTTTGTAAAATCTGCAAGAGGATATTTGGATAGCTTTGAGGA
          1 ATAGAGCAGGTTTGAAACACTCTTTTTGTAGTATCTGGAAGTGGATATTTGGATAGCTTTGAGGA

                         *   * *            **                      *    *   
     228682 TTTCGTTGGAAACGGGATTGTCTTCATATAAACTCTAGACAGAAGCATTCTCAGAAGCTTCATTG
         66 TTTCGTTGGAAACAGGAATATCTTCATATAAAAGCTAGACAGAAGCATTCTCAGAAACTTCTTTG

            *             *    *                      
     228747 GGATGTTT-CAGTTGAAGTCACAGTGTTGAACATTCCCTTTC
        131 TGATGTTTGCA-TTCAAGTAACAGTGTTGAACATTCCCTTTC

                                                   *     *       **     *   *
     228788 ATAGAGCAGGTTTGAAACACTCTTTTTGTAGTATCTGGATGTGGACATTTGGAGCGCTTTCAGGC
          1 ATAGAGCAGGTTTGAAACACTCTTTTTGTAGTATCTGGAAGTGGATATTTGGATAGCTTTGAGGA

            * ** *      *      *      *** *   *                      *   *   
     228853 CTATGGT-GAAAAAGGAAACATCTTCTCCTGAAAACTAGACAGAAGCATTCTCAGAATCTTATTT
         66 TTTCGTTGGAAACAGG-AATATCTTCATATAAAAGCTAGACAGAAGCATTCTCAGAAACTTCTTT

                   **  **    *             **  * *   *
     228917 GTGATGTGCGCCCTCAACTAACAGTGTTGAAGCTTTCTTTTG
        130 GTGATGTTTGCATTCAAGTAACAGTGTTGAACATTCCCTTTC

                     *                *   **     *   *        *              
     228959 ATAGAGCAGTTTTGAAACACTCTTTTCGTAAAATCTGCAAGAGGATATTTTGATAGCTTTGAGGA
          1 ATAGAGCAGGTTTGAAACACTCTTTTTGTAGTATCTGGAAGTGGATATTTGGATAGCTTTGAGGA

                         *   * *            **                      *    **  
     229024 TTTCGTTGGAAACGGGATTGTCTTCATATAAACTCTAGACAGAAGCATTCTCAGAAGCTTCAGTG
         66 TTTCGTTGGAAACAGGAATATCTTCATATAAAAGCTAGACAGAAGCATTCTCAGAAACTTCTTTG

            *       **   *    *             *        
     229089 GGATGTTTCAATTGAAGTCACAGTGTTGAACAGTCCCTTTC
        131 TGATGTTTGCATTCAAGTAACAGTGTTGAACATTCCCTTTC

                                                         *       *  * * *    
     229130 ATAGAGCAGGTTTGAAACACTCTTTTTGTAGTATCTGGAAGTGGACATTTGGAGAGATCTCAGGA
          1 ATAGAGCAGGTTTGAAACACTCTTTTTGTAGTATCTGGAAGTGGATATTTGGATAGCTTTGAGGA

            * *  *    * *             **            *       * *          *  *
     229195 ATACGGT-GATAAAGGAAATATCTTCCAATAAAAGCTAGATAGAAGCAATGTCAGAAACTTTTTC
         66 TTTCGTTGGAAACAGG-AATATCTTCATATAAAAGCTAGACAGAAGCATTCTCAGAAACTTCTTT

            *      * ** *   **      *       *  * *   *
     229259 ATGATGTATCTACTCAGCTAACAGAGTTGAACCTTTCTTTTG
        130 GTGATGTTTGCATTCAAGTAACAGTGTTGAACATTCCCTTTC

             *       *                   * *     *             **            
     229301 AGAGAGCAGTTTTGAAACACTCTTTTTGTGGAATCTGCAAGTGGATATTTGTCTAGCTTTGAGGA
          1 ATAGAGCAGGTTTGAAACACTCTTTTTGTAGTATCTGGAAGTGGATATTTGGATAGCTTTGAGGA

                         *   *    *                   *       *   *     *    
     229366 TTTCGTTGGAAACGGGATTA-CAT-ATA-AAAAGC-AGACAGCAGCATTCCCAGTAACTTGTTTG
         66 TTTCGTTGGAAACAGGAATATCTTCATATAAAAGCTAGACAGAAGCATTCTCAGAAACTTCTTTG

                              *    *                 
     229427 TGATGTTTGCATTCAAGTCACAGAGTTGAACATTCCCTTTC
        131 TGATGTTTGCATTCAAGTAACAGTGTTGAACATTCCCTTTC

                  *                                *     *       **     *   *
     229468 ATAGAGAAGGTTTGAAACACTCTTTTTGTAGTATCTGGATGTGGACATTTGGAGCGCTTTCAGGC
          1 ATAGAGCAGGTTTGAAACACTCTTTTTGTAGTATCTGGAAGTGGATATTTGGATAGCTTTGAGGA

            * ** *      *             *** *   *                      *   *   
     229533 CTATGGT-GAAAAAGGAAATATCTTCCCCTGAAAACTAGACAGAAGCATTCTCAGAATCTTATTT
         66 TTTCGTTGGAAACAGG-AATATCTTCATATAAAAGCTAGACAGAAGCATTCTCAGAAACTTCTTT

                   **  **    *             **  * *   *
     229597 GTGATGTGCGCCCTCAACTAACAGTGTTGAAGCTTTCTTTTG
        130 GTGATGTTTGCATTCAAGTAACAGTGTTGAACATTCCCTTTC

                     *                    **     *   *                       
     229639 ATAGAGCAGTTTTGAAACACTCTTTTTGTAAAATCTGCAAGAGGATATTTGGATAGCTTTGAGGA
          1 ATAGAGCAGGTTTGAAACACTCTTTTTGTAGTATCTGGAAGTGGATATTTGGATAGCTTTGAGGA

                         *   * *            **                      *    *   
     229704 TTTCGTTGGAAACGGGATTGTCTTCATATAAACTCTAGACAGAAGCATTCTCAGAAGCTTCATTG
         66 TTTCGTTGGAAACAGGAATATCTTCATATAAAAGCTAGACAGAAGCATTCTCAGAAACTTCTTTG

            *       **   *    *             *        
     229769 GGATGTTTCAATTGAAGTCACAGTGTTGAACAGTCCCTTTC
        131 TGATGTTTGCATTCAAGTAACAGTGTTGAACATTCCCTTTC

                                                         *       **   * *    
     229810 ATAGAGCAGGTTTGAAACACTCTTTTTGTAGTATCTGGAAGTGGACATTTGGAGCGCTCTCAGGA
          1 ATAGAGCAGGTTTGAAACACTCTTTTTGTAGTATCTGGAAGTGGATATTTGGATAGCTTTGAGGA

            * ** *      *             **          * *       * *          *  *
     229875 CTGTGGT-GAAAAAGGAAATATCTTCCAATAAAAGCTACATAGAAGCAATGTCAGAAACTTTTTC
         66 TTTCGTTGGAAACAGG-AATATCTTCATATAAAAGCTAGACAGAAGCATTCTCAGAAACTTCTTT

            *      * ** *   **      *       *        *
     229939 ATGATGTATCTACTCAGCTAACAGAGTTGAACCTT-CCTTTG
        130 GTGATGTTTGCATTCAAGTAACAGTGTTGAACATTCCCTTTC

             *       *                   * *     *            ***            
     229980 AGAGAGCAGTTTTGAAACACTCTTTTTGTGGGATCTGCAAGTGGATATTTTTCTAGCTTTGAGGA
          1 ATAGAGCAGGTTTGAAACACTCTTTTTGTAGTATCTGGAAGTGGATATTTGGATAGCTTTGAGGA

                         *   *    *                   *       *              
     230045 TTTCGTTGGAAACGGGATTA-CAT-ATA-AAAAGC-AGACAGCAGCATTCCCAGAAACTTCTTTG
         66 TTTCGTTGGAAACAGGAATATCTTCATATAAAAGCTAGACAGAAGCATTCTCAGAAACTTCTTTG

                              *    *                 
     230106 TGATGTTTGCATTCAAGTCACAGAGTTGAACATTCCCTTTC
        131 TGATGTTTGCATTCAAGTAACAGTGTTGAACATTCCCTTTC

                                                   *     *       **     *   *
     230147 ATAGAGCAGGTTTGAAACACTCTTTTTGTAGTATCTGGATGTGGACATTTGGAGCGCTTTCAGGC
          1 ATAGAGCAGGTTTGAAACACTCTTTTTGTAGTATCTGGAAGTGGATATTTGGATAGCTTTGAGGA

            * ** *      *             *** *   *                      *   *   
     230212 CTATGGT-GAAAAAGGAAATATCTTCCCCTGAAAACTAGACAGAAGCATTCTCAGAATCTTATTT
         66 TTTCGTTGGAAACAGG-AATATCTTCATATAAAAGCTAGACAGAAGCATTCTCAGAAACTTCTTT

                   **  **    *             **  * *   *
     230276 GTGATGTGCGCCCTCAACTAACAGTGTTGAAGCTTTCTTTTG
        130 GTGATGTTTGCATTCAAGTAACAGTGTTGAACATTCCCTTTC

                     *                    **     *   *                       
     230318 ATAGAGCAGTTTTGAAACACTCTTTTTGTAAAATCTGCAAGAGGATATTTGGATAGCTTTGAGGA
          1 ATAGAGCAGGTTTGAAACACTCTTTTTGTAGTATCTGGAAGTGGATATTTGGATAGCTTTGAGGA

                         *   * *            **                      *    *   
     230383 TTTCGTTGGAAACGGGATTGTCTTCATATAAACTCTAGACAGAAGCATTCTCAGAAGCTTCATTG
         66 TTTCGTTGGAAACAGGAATATCTTCATATAAAAGCTAGACAGAAGCATTCTCAGAAACTTCTTTG

            *             *    *             *        
     230448 GGATGTTT-CAGTTGAAGTCACAGTGTTGAACAGTCCCTTTC
        131 TGATGTTTGCA-TTCAAGTAACAGTGTTGAACATTCCCTTTC

                                                         *       **   * *    
     230489 ATAGAGCAGGTTTGAAACACTCTTTTTGTAGTATCTGGAAGTGGACATTTGGAGCGCTCTCAGGA
          1 ATAGAGCAGGTTTGAAACACTCTTTTTGTAGTATCTGGAAGTGGATATTTGGATAGCTTTGAGGA

            * *  *      *             **            *       * *          *  *
     230554 CTGCGGT-GAAAAAGGAAATATCTTCCAATAAAAGCTAGATAGAAGCAATGTCAGAAACTTTTTC
         66 TTTCGTTGGAAACAGG-AATATCTTCATATAAAAGCTAGACAGAAGCATTCTCAGAAACTTCTTT

            *      * ** *   **      *       *        *
     230618 ATGATGTATCTACTCAGCTAACAGAGTTGAACCTT-CCTTTG
        130 GTGATGTTTGCATTCAAGTAACAGTGTTGAACATTCCCTTTC

             *       *                   * *     *             **            
     230659 AGAGAGCAGTTTTGAAACACTCTTTTTGTGGAATCTGCAAGTGGATATTTGTCTAGCTTTGAGGA
          1 ATAGAGCAGGTTTGAAACACTCTTTTTGTAGTATCTGGAAGTGGATATTTGGATAGCTTTGAGGA

                         *   *    *                   *       *     *   *    
     230724 TTTCGTTGGAAACGGGATTA-CAT-ATA-AAAAGC-AGACAGCAGCATTCCCAGAATCTTGTTTG
         66 TTTCGTTGGAAACAGGAATATCTTCATATAAAAGCTAGACAGAAGCATTCTCAGAAACTTCTTTG

                              *    *                 
     230785 TGATGTTTGCATTCAAGTCACAGAGTTGAACATTCCCTTTC
        131 TGATGTTTGCATTCAAGTAACAGTGTTGAACATTCCCTTTC

             *                         *           *     *       **     *   *
     230826 AGAGAGCAGGTTTGAAACACTCTTTTTATAGTATCTGGATGTGGACATTTGGAGCGCTTTCAGGC
          1 ATAGAGCAGGTTTGAAACACTCTTTTTGTAGTATCTGGAAGTGGATATTTGGATAGCTTTGAGGA

            * ** *      *             *** *   *                      *   *   
     230891 CTATGGT-GAAAAAGGAAATATCTTCTCCTGAAAACTAGACAGAAGCATTCTCAGAATCTTATTT
         66 TTTCGTTGGAAACAGG-AATATCTTCATATAAAAGCTAGACAGAAGCATTCTCAGAAACTTCTTT

                   **  **    *             **  * *   *
     230955 GTGATGTGCGCCCTCAACTAACAGTGTTGAAGCTTTCTTTTG
        130 GTGATGTTTGCATTCAAGTAACAGTGTTGAACATTCCCTTTC

                     *                *   **     *   *        *              
     230997 ATAGAGCAGTTTTGAAACACTCTTTTCGTAAAATCTGCAAGAGGATATTTTGATAGCTTTGAGGA
          1 ATAGAGCAGGTTTGAAACACTCTTTTTGTAGTATCTGGAAGTGGATATTTGGATAGCTTTGAGGA

                         *   * *            **                      *    *   
     231062 TTTCGTTGGAAACGGGATTGTCTTCATATAAACTCTAGACAGAAGCATTCTCAGAAGCTTCATTG
         66 TTTCGTTGGAAACAGGAATATCTTCATATAAAAGCTAGACAGAAGCATTCTCAGAAACTTCTTTG

            *       **   *    *             *        
     231127 GGATGTTTCAATTGAAGTCACAGTGTTGAACAGTCCCTTTC
        131 TGATGTTTGCATTCAAGTAACAGTGTTGAACATTCCCTTTC

                                                    *    * *     **   * **   
     231168 ATAGAGCAGGTTTGAAACACTCTTTTTGTAGTATCTGGAATTGGACAATTGGAGCGCTCTCCGGA
          1 ATAGAGCAGGTTTGAAACACTCTTTTTGTAGTATCTGGAAGTGGATATTTGGATAGCTTTGAGGA

            * *  *    * *             **            *       * *          *  *
     231233 CTACGGT-GATAAAGGAAATATCTTCCAATAAAAGCTAGATAGAAGCAATGTCAGAAACTTTTTC
         66 TTTCGTTGGAAACAGG-AATATCTTCATATAAAAGCTAGACAGAAGCATTCTCAGAAACTTCTTT

            *      * ** *   **      *       *  * *   *
     231297 ATGATGTATCTACTCAGCTAACAGAGTTGAACCTTTCTTTTG
        130 GTGATGTTTGCATTCAAGTAACAGTGTTGAACATTCCCTTTC

             *       *                   * *     *             **            
     231339 AGAGAGCAGTTTTGAAACACTCTTTTTGTGGAATCTGCAAGTGGATATTTGTTTAGCTTTGAGGA
          1 ATAGAGCAGGTTTGAAACACTCTTTTTGTAGTATCTGGAAGTGGATATTTGGATAGCTTTGAGGA

                         *   *    *                   *       *              
     231404 TTTCGTTGGAAACGGGATTA-CAT-ATA-AAAAGC-AGACAGCAGCATTCCCAGAAACTTCTTTG
         66 TTTCGTTGGAAACAGGAATATCTTCATATAAAAGCTAGACAGAAGCATTCTCAGAAACTTCTTTG

            *                 *    *                 
     231465 AGATGTTTGCATTCAAGTCACAGAGTTGAACATTCCCTTTC
        131 TGATGTTTGCATTCAAGTAACAGTGTTGAACATTCCCTTTC

                                                   *     *       **     *   *
     231506 ATAGAGCAGGTTTGAAACACTCTTTTTGTAGTATCTGGATGTGGACATTTGGAGCGCTTTCAGGC
          1 ATAGAGCAGGTTTGAAACACTCTTTTTGTAGTATCTGGAAGTGGATATTTGGATAGCTTTGAGGA

            * ** *      *             *** *   *                      *   *   
     231571 CTATGGT-GAAAAAGGAAATATCTTCCCCTGAAAACTAGACAGAAGCATTCTCAGAATCTTATTT
         66 TTTCGTTGGAAACAGG-AATATCTTCATATAAAAGCTAGACAGAAGCATTCTCAGAAACTTCTTT

                   **  **    *             **  * *   *
     231635 GTGATGTGCGCCCTCAACTAACAGTGTTGAAGCTTTCTTTTG
        130 GTGATGTTTGCATTCAAGTAACAGTGTTGAACATTCCCTTTC

                  *  *                    **  *  *   *                       
     231677 ATAGAGGAGTTTTGAAACACTCTTTTTGTAAAATATGCAAGAGGATATTTGGATAGCTTTGAGGA
          1 ATAGAGCAGGTTTGAAACACTCTTTTTGTAGTATCTGGAAGTGGATATTTGGATAGCTTTGAGGA

                         *   * *            **                      *    *   
     231742 TTTCGTTGGAAACGGGATTGTCTTCATATAAACTCTAGACAGAAGCATTCTCAGAAGCTTCATTG
         66 TTTCGTTGGAAACAGGAATATCTTCATATAAAAGCTAGACAGAAGCATTCTCAGAAACTTCTTTG

            *             *    *             *        
     231807 GGATGTTT-CAGTTGAAGTCACAGTGTTGAACAGTCCCTTTC
        131 TGATGTTTGCA-TTCAAGTAACAGTGTTGAACATTCCCTTTC

                                                         *       **   * *    
     231848 ATAGAGCAGGTTTGAAACACTCTTTTTGTAGTATCTGGAAGTGGACATTTGGAGCGCTCTCAGGA
          1 ATAGAGCAGGTTTGAAACACTCTTTTTGTAGTATCTGGAAGTGGATATTTGGATAGCTTTGAGGA

            * *  *      *             **            *       * *          *  *
     231913 CTGCGGT-GAAAAAGGAAATATCTTCCAATAAAAGCTAGATAGAAGCAATGTCAGAAACTTTTTC
         66 TTTCGTTGGAAACAGG-AATATCTTCATATAAAAGCTAGACAGAAGCATTCTCAGAAACTTCTTT

            *      * ** *   **      *       *        *
     231977 ATGATGTATCTACTCAGCTAACAGAGTTGAACCTT-CCTTTG
        130 GTGATGTTTGCATTCAAGTAACAGTGTTGAACATTCCCTTTC

             *       *                   * *     *             **            
     232018 AGAGAGCAGTTTTGAAACACTCTTTTTGTGGAATCTGCAAGTGGATATTTGTCTAGCTTTGAGGA
          1 ATAGAGCAGGTTTGAAACACTCTTTTTGTAGTATCTGGAAGTGGATATTTGGATAGCTTTGAGGA

                         *   *    *                   *       *              
     232083 TTTCGTTGGAAACGGGATTA-CAT-ATA-AAAAGC-AGACAGCAGCATTCCCAGAAACTTCTTTG
         66 TTTCGTTGGAAACAGGAATATCTTCATATAAAAGCTAGACAGAAGCATTCTCAGAAACTTCTTTG

                              *    *                 
     232144 TGATGTTTGCATTCAAGTCACAGAGTTGAACATTCCCTTTC
        131 TGATGTTTGCATTCAAGTAACAGTGTTGAACATTCCCTTTC

                                                   *     *     * **     *   *
     232185 ATAGAGCAGGTTTGAAACACTCTTTTTGTAGTATCTGGATGTGGACATTTGCAGCGCTTTCAGGC
          1 ATAGAGCAGGTTTGAAACACTCTTTTTGTAGTATCTGGAAGTGGATATTTGGATAGCTTTGAGGA

            * ** *      *             *** *   *                          *   
     232250 CTAAGGT-GAAAAAGGAAATATCTTCCCCTGAAAACTAGACAGAAGCATTCTCAGAAACTTATTT
         66 TTTCGTTGGAAACAGG-AATATCTTCATATAAAAGCTAGACAGAAGCATTCTCAGAAACTTCTTT

                   **  **    *             **  * *   *
     232314 GTGATGTGCGCCCTCAACTAACAGTGTTGAAGCTTTCTTTTG
        130 GTGATGTTTGCATTCAAGTAACAGTGTTGAACATTCCCTTTC

                     *                   * *     *             **            
     232356 ATAGAGCAGTTTTGAAACACTCTTTTTGTGGAATCTGCAAGTGGATATTTGTCTAGCTTTGAGGA
          1 ATAGAGCAGGTTTGAAACACTCTTTTTGTAGTATCTGGAAGTGGATATTTGGATAGCTTTGAGGA

                         *   *    *                   *       *     *   *    
     232421 TTTCGTTGGAAACGGGATTA-CAT-ATA-AAAAGC-AGACAGCAGCATTCCCAGAATCTTGTTTG
         66 TTTCGTTGGAAACAGGAATATCTTCATATAAAAGCTAGACAGAAGCATTCTCAGAAACTTCTTTG

                              *    *                 
     232482 TGATGTTTGCATTCAAGTCACAGAGTTGAACATTCCCTTTC
        131 TGATGTTTGCATTCAAGTAACAGTGTTGAACATTCCCTTTC

             *                         *           *             **     *   *
     232523 AGAGAGCAGGTTTGAAACACTCTTTTTATAGTATCTGGATGTGGATATTTGGAGCGCTTTCAGGC
          1 ATAGAGCAGGTTTGAAACACTCTTTTTGTAGTATCTGGAAGTGGATATTTGGATAGCTTTGAGGA

            * ** *      *             *** *   *                      *   *   
     232588 CTATGGT-GAAAAAGGAAATATCTTCTCCTGAAAACTAGACAGAAGCATTCTCAGAATCTTATTT
         66 TTTCGTTGGAAACAGG-AATATCTTCATATAAAAGCTAGACAGAAGCATTCTCAGAAACTTCTTT

                   **  **    *             **  * *   *
     232652 GTGATGTGCGCCCTCAACTAACAGTGTTGAAGCTTTCTTTTG
        130 GTGATGTTTGCATTCAAGTAACAGTGTTGAACATTCCCTTTC

                     *                *   **     *   *        *              
     232694 ATAGAGCAGTTTTGAAACACTCTTTTCGTAAAATCTGCAAGAGGATATTTTGATAGCTTTGAGGA
          1 ATAGAGCAGGTTTGAAACACTCTTTTTGTAGTATCTGGAAGTGGATATTTGGATAGCTTTGAGGA

                         *   * *            **                      *    *   
     232759 TTTCGTTGGAAACGGGATTGTCTTCATATAAACTCTAGACAGAAGCATTCTCAGAAGCTTCATTG
         66 TTTCGTTGGAAACAGGAATATCTTCATATAAAAGCTAGACAGAAGCATTCTCAGAAACTTCTTTG

            *             *    *             *        
     232824 GGATGTTT-CAGTTGAAGTCACAGTGTTGAACAGTCCCTTTC
        131 TGATGTTTGCA-TTCAAGTAACAGTGTTGAACATTCCCTTTC

                                                         *       *  * * *    
     232865 ATAGAGCAGGTTTGAAACACTCTTTTTGTAGTATCTGGAAGTGGACATTTGGAGAGATCTCAGGA
          1 ATAGAGCAGGTTTGAAACACTCTTTTTGTAGTATCTGGAAGTGGATATTTGGATAGCTTTGAGGA

            * *  *    * *             **            *       * *          *  *
     232930 ATACGGT-GATAAAGGAAATATCTTCCAATAAAAGCTAGATAGAAGCAATGTCAGAAACTTTTTC
         66 TTTCGTTGGAAACAGG-AATATCTTCATATAAAAGCTAGACAGAAGCATTCTCAGAAACTTCTTT

            *      * ** *   **      *       *  * *   *
     232994 ATGATGTATCTACTCAGCTAACAGAGTTGAACCTTTCTTTTG
        130 GTGATGTTTGCATTCAAGTAACAGTGTTGAACATTCCCTTTC

             *    *  *                   * *     *             **            
     233036 AGAGAGGAGTTTTGAAACACTCTTTTTGTGGAATCTGCAAGTGGATATTTGTCTAGCTTTGAGGA
          1 ATAGAGCAGGTTTGAAACACTCTTTTTGTAGTATCTGGAAGTGGATATTTGGATAGCTTTGAGGA

                                   *                   *       *             
     233101 TTTCGTTGGAAAC-GG--TAT-TACATATAAAAAGC-AGACAGCAGCATTCCCAGAAACTTCTTT
         66 TTTCGTTGGAAACAGGAATATCTTCATAT-AAAAGCTAGACAGAAGCATTCTCAGAAACTTCTTT

                               *    *                 
     233161 GTGATGTTTGCATTCAAGTCACAGAGTTGAACATTCCCTTTC
        130 GTGATGTTTGCATTCAAGTAACAGTGTTGAACATTCCCTTTC

                                                   *     *       **     *   *
     233203 ATAGAGCAGGTTTGAAACACTCTTTTTGTAGTATCTGGATGTGGACATTTGGAGCGCTTTCAGGC
          1 ATAGAGCAGGTTTGAAACACTCTTTTTGTAGTATCTGGAAGTGGATATTTGGATAGCTTTGAGGA

            * ** *      *             *** *   *                      *   *   
     233268 CTATGGT-GAAAAAGGAAATATCTTCCCCTGAAAACTAGACAGAAGCATTCTCAGAATCTTATTT
         66 TTTCGTTGGAAACAGG-AATATCTTCATATAAAAGCTAGACAGAAGCATTCTCAGAAACTTCTTT

                   **  **    *             **  * *   *
     233332 GTGATGTGCGCCCTCAACTAACAGTGTTGAAGCTTTCTTTTG
        130 GTGATGTTTGCATTCAAGTAACAGTGTTGAACATTCCCTTTC

                     *                *   **     *   *        *              
     233374 ATAGAGCAGTTTTGAAACACTCTTTTCGTAAAATCTGCAAGAGGATATTTTGATAGCTTTGAGGA
          1 ATAGAGCAGGTTTGAAACACTCTTTTTGTAGTATCTGGAAGTGGATATTTGGATAGCTTTGAGGA

                         *   * *            **                      *    *   
     233439 TTTCGTTGGAAACGGGATTGTCTTCATATAAACTCTAGACAGAAGCATTCTCAGAAGCTTCATTG
         66 TTTCGTTGGAAACAGGAATATCTTCATATAAAAGCTAGACAGAAGCATTCTCAGAAACTTCTTTG

            *             *    *             *        
     233504 GGATGTTT-CAGTTGAAGTCACAGTGTTGAACAGTCCCTTTC
        131 TGATGTTTGCA-TTCAAGTAACAGTGTTGAACATTCCCTTTC

                                                         *       **   * *    
     233545 ATAGAGCAGGTTTGAAACACTCTTTTTGTAGTATCTGGAAGTGGACATTTGGAGCGCTCTCAGGA
          1 ATAGAGCAGGTTTGAAACACTCTTTTTGTAGTATCTGGAAGTGGATATTTGGATAGCTTTGAGGA

            * *  *      *             **          * *       * *          *  *
     233610 CTGCGGT-GAAAAAGGAAATATCTTCCAATAAAAGCTACATAGAAGCAATGTCAGAAACTTTTTC
         66 TTTCGTTGGAAACAGG-AATATCTTCATATAAAAGCTAGACAGAAGCATTCTCAGAAACTTCTTT

            *      * ** *   **      *    *  *        *
     233674 ATGATGTATCTACTCAGCTAACAGAGTTGGACCTT-CCTTTG
        130 GTGATGTTTGCATTCAAGTAACAGTGTTGAACATTCCCTTTC

             *       *                   * *     *             **            
     233715 AGAGAGCAGTTTTGAAACACTCTTTTTGTGGAATCTGCAAGTGGATATTTGTCTAGCTTTGAGGA
          1 ATAGAGCAGGTTTGAAACACTCTTTTTGTAGTATCTGGAAGTGGATATTTGGATAGCTTTGAGGA

                         *   *    *                   *       *              
     233780 TTTCGTTGGAAACGGGATTA-CAT-ATA-AAAAGC-AGACAGCAGCATTCCCAGAAACTTCTTTG
         66 TTTCGTTGGAAACAGGAATATCTTCATATAAAAGCTAGACAGAAGCATTCTCAGAAACTTCTTTG

                              *    *                 
     233841 TGATGTTTGCATTCAAGTCACAGAGTTGAACATTCCCTTTC
        131 TGATGTTTGCATTCAAGTAACAGTGTTGAACATTCCCTTTC

                                                   *     *       **     *   *
     233882 ATAGAGCAGGTTTGAAACACTCTTTTTGTAGTATCTGGATGTGGACATTTGGAGCGCTTTCAGGC
          1 ATAGAGCAGGTTTGAAACACTCTTTTTGTAGTATCTGGAAGTGGATATTTGGATAGCTTTGAGGA

            * ** *      *             *** *   *                      *   *   
     233947 CTATGGT-GAAAAAGGAAATATCTTCCCCTGAAAACTAGACAGAAGCATTCTCAGAATCTTATTT
         66 TTTCGTTGGAAACAGG-AATATCTTCATATAAAAGCTAGACAGAAGCATTCTCAGAAACTTCTTT

                   **  **    *             **  * *   *
     234011 GTGATGTGCGCCCTCAACTAACAGTGTTGAAGCTTTCTTTTG
        130 GTGATGTTTGCATTCAAGTAACAGTGTTGAACATTCCCTTTC

                     *                    **     *   *                       
     234053 ATAGAGCAGTTTTGAAACACTCTTTTTGTAAAATCTGCAAGAGGATATTTGGATAGCTTTGAGGA
          1 ATAGAGCAGGTTTGAAACACTCTTTTTGTAGTATCTGGAAGTGGATATTTGGATAGCTTTGAGGA

                         *   * *            **                      *    *   
     234118 TTTCGTTGGAAACGGGATTGTCTTCATATAAACTCTAGACAGAAGCATTCTCAGAAGCTTCATTG
         66 TTTCGTTGGAAACAGGAATATCTTCATATAAAAGCTAGACAGAAGCATTCTCAGAAACTTCTTTG

            *             *    *             *        
     234183 GGATGTTT-CAGTTGAAGTCACAGTGTTGAACAGTCCCTTTC
        131 TGATGTTTGCA-TTCAAGTAACAGTGTTGAACATTCCCTTTC

                                                         *       **   * *    
     234224 ATAGAGCAGGTTTGAAACACTCTTTTTGTAGTATCTGGAAGTGGACATTTGGAGCGCTCTCAGGA
          1 ATAGAGCAGGTTTGAAACACTCTTTTTGTAGTATCTGGAAGTGGATATTTGGATAGCTTTGAGGA

            * *  *      *             **            *       * *          *  *
     234289 CTGCGGT-GAAAAAGGAAATATCTTCCAATAAAAGCTAGATAGAAGCAATGTCAGAAACTTTTTC
         66 TTTCGTTGGAAACAGG-AATATCTTCATATAAAAGCTAGACAGAAGCATTCTCAGAAACTTCTTT

            *      * ** *   **      *       *        *
     234353 ATGATGTATCTACTCAGCTAACAGAGTTGAACCTT-CCTTTG
        130 GTGATGTTTGCATTCAAGTAACAGTGTTGAACATTCCCTTTC

             *       *                   * *     *             **            
     234394 AGAGAGCAGTTTTGAAACACTCTTTTTGTGGAATCTGCAAGTGGATATTTGTCTAGCTTTGAGGA
          1 ATAGAGCAGGTTTGAAACACTCTTTTTGTAGTATCTGGAAGTGGATATTTGGATAGCTTTGAGGA

                         *   *    *                   *       *     *   *    
     234459 TTTCGTTGGAAACGGGATTA-CAT-ATA-AAAAGC-AGACAGCAGCATTCCCAGAATCTTGTTTG
         66 TTTCGTTGGAAACAGGAATATCTTCATATAAAAGCTAGACAGAAGCATTCTCAGAAACTTCTTTG

                              *    *                 
     234520 TGATGTTTGCATTCAAGTCACAGAGTTGAACATTCCCTTTC
        131 TGATGTTTGCATTCAAGTAACAGTGTTGAACATTCCCTTTC

             *                         *           *     *       **     *   *
     234561 AGAGAGCAGGTTTGAAACACTCTTTTTATAGTATCTGGATGTGGACATTTGGAGCGCTTTCAGGC
          1 ATAGAGCAGGTTTGAAACACTCTTTTTGTAGTATCTGGAAGTGGATATTTGGATAGCTTTGAGGA

            * ** *      *             *** *   *                      *   *   
     234626 CTATGGT-GAAAAAGGAAATATCTTCTCCTGAAAACTAGACAGAAGCATTCTCAGAATCTTATTT
         66 TTTCGTTGGAAACAGG-AATATCTTCATATAAAAGCTAGACAGAAGCATTCTCAGAAACTTCTTT

                   **  **    *             **  * *   *
     234690 GTGATGTGCGCCCTCAACTAACAGTGTTGAAGCTTTCTTTTG
        130 GTGATGTTTGCATTCAAGTAACAGTGTTGAACATTCCCTTTC

                     *                *   **     *   *        *              
     234732 ATAGAGCAGTTTTGAAACACTCTTTTCGTAAAATCTGCAAGAGGATATTTTGATAGCTTTGAGGA
          1 ATAGAGCAGGTTTGAAACACTCTTTTTGTAGTATCTGGAAGTGGATATTTGGATAGCTTTGAGGA

                         *   * *            **                      *    **  
     234797 TTTCGTTGGAAACGGGATTGTCTTCATATAAACTCTAGACAGAAGCATTCTCAGAAGCTTCAGTG
         66 TTTCGTTGGAAACAGGAATATCTTCATATAAAAGCTAGACAGAAGCATTCTCAGAAACTTCTTTG

            *       **   *    *             *        
     234862 GGATGTTTCAATTGAAGTCACAGTGTTGAACAGTCCCTTTC
        131 TGATGTTTGCATTCAAGTAACAGTGTTGAACATTCCCTTTC

                                                         *       *  * * *    
     234903 ATAGAGCAGGTTTGAAACACTCTTTTTGTAGTATCTGGAAGTGGACATTTGGAGAGATCTCAGGA
          1 ATAGAGCAGGTTTGAAACACTCTTTTTGTAGTATCTGGAAGTGGATATTTGGATAGCTTTGAGGA

            * *  *    * *             **            *       * *          *  *
     234968 ATACGGT-GATAAAGGAAATATCTTCCAATAAAAGCTAGATAGAAGCAATGTCAGAAACTTTTTC
         66 TTTCGTTGGAAACAGG-AATATCTTCATATAAAAGCTAGACAGAAGCATTCTCAGAAACTTCTTT

            *      * ** *   **      *       *  * *   *
     235032 ATGATGTATCTACTCAGCTAACAGAGTTGAACCTTTCTTTTG
        130 GTGATGTTTGCATTCAAGTAACAGTGTTGAACATTCCCTTTC

             *       *                   * *     *             **            
     235074 AGAGAGCAGTTTTGAAACACTCTTTTTGTGGAATCTGCAAGTGGATATTTGTCTAGCTTTGAGGA
          1 ATAGAGCAGGTTTGAAACACTCTTTTTGTAGTATCTGGAAGTGGATATTTGGATAGCTTTGAGGA

                         *   *    *                   *       *   *          
     235139 TTTCGTTGGAAACGGGATTA-CAT-ATA-AAAAGC-AGACAGCAGCATTCCCAGTAACTTCTTTG
         66 TTTCGTTGGAAACAGGAATATCTTCATATAAAAGCTAGACAGAAGCATTCTCAGAAACTTCTTTG

                              *    *                 
     235200 TGATGTTTGCATTCAAGTCACAGAGTTGAACATTCCCTTTC
        131 TGATGTTTGCATTCAAGTAACAGTGTTGAACATTCCCTTTC

                                                   *     *     * **     *   *
     235241 ATAGAGCAGGTTTGAAACACTCTTTTTGTAGTATCTGGATGTGGACATTTGCAGCGCTTTCAGGC
          1 ATAGAGCAGGTTTGAAACACTCTTTTTGTAGTATCTGGAAGTGGATATTTGGATAGCTTTGAGGA

            * ** *      *             *** *   * *                        *   
     235306 CTAAGGT-GAAAAAGGAAATATCTTCCCCTGAAAACCAGACAGAAGCATTCTCAGAAACTTATTT
         66 TTTCGTTGGAAACAGG-AATATCTTCATATAAAAGCTAGACAGAAGCATTCTCAGAAACTTCTTT

                   **  **    *             **  * *   *
     235370 GTGATGTGCGCCCTCAACTAACAGTGTTGAAGCTTTCTTTTG
        130 GTGATGTTTGCATTCAAGTAACAGTGTTGAACATTCCCTTTC

                     *                   * *     *             **            
     235412 ATAGAGCAGCTTTGAAACACTCTTTTTGTGGAATCTGCAAGTGGATATTTGTCTAGCTTTGAGGA
          1 ATAGAGCAGGTTTGAAACACTCTTTTTGTAGTATCTGGAAGTGGATATTTGGATAGCTTTGAGGA

                         *   *    *                   *       *     *   *    
     235477 TTTCGTTGGAAACGGGATTA-CAT-ATA-AAAAGC-AGACAGCAGCATTCCCAGAATCTTGTTTG
         66 TTTCGTTGGAAACAGGAATATCTTCATATAAAAGCTAGACAGAAGCATTCTCAGAAACTTCTTTG

                              *    *                 
     235538 TGATGTTTGCATTCAAGTCACAGAGTTGAACATTCCCTTTC
        131 TGATGTTTGCATTCAAGTAACAGTGTTGAACATTCCCTTTC

             *                         *           *     *       **     *   *
     235579 AGAGAGCAGGTTTGAAACACTCTTTTTATAGTATCTGGATGTGGACATTTGGAGCGCTTTCAGGC
          1 ATAGAGCAGGTTTGAAACACTCTTTTTGTAGTATCTGGAAGTGGATATTTGGATAGCTTTGAGGA

            * ** *      *             *** *   *                      *   *   
     235644 CTATGGT-GAAAAAGGAAATATCTTCTCCTGAAAACTAGACAGAAGCATTCTCAGAATCTTATTT
         66 TTTCGTTGGAAACAGG-AATATCTTCATATAAAAGCTAGACAGAAGCATTCTCAGAAACTTCTTT

                   **  **    *             **  * *   *
     235708 GTGATGTGCGCCCTCAACTAACAGTGTTGAAGCTTTCTTTTG
        130 GTGATGTTTGCATTCAAGTAACAGTGTTGAACATTCCCTTTC

                     *                *   **     *   *        *              
     235750 ATAGAGCAGTTTTGAAACACTCTTTTCGTAAAATCTGCAAGAGGATATTTTGATAGCTTTGAGGA
          1 ATAGAGCAGGTTTGAAACACTCTTTTTGTAGTATCTGGAAGTGGATATTTGGATAGCTTTGAGGA

                         *   * *            **                      *    *   
     235815 TTTCGTTGGAAACGGGATTGTCTTCATATAAACTCTAGACAGAAGCATTCTCAGAAGCTTCATTG
         66 TTTCGTTGGAAACAGGAATATCTTCATATAAAAGCTAGACAGAAGCATTCTCAGAAACTTCTTTG

            *             *    *             *        
     235880 GGATGTTT-CAGTTGAAGTCACAGTGTTGAACAGTCCCTTTC
        131 TGATGTTTGCA-TTCAAGTAACAGTGTTGAACATTCCCTTTC

                                                         *       **   * *    
     235921 ATAGAGCAGGTTTGAAACACTCTTTTTGTAGTATCTGGAAGTGGACATTTGGAGCGCTCTCAGGA
          1 ATAGAGCAGGTTTGAAACACTCTTTTTGTAGTATCTGGAAGTGGATATTTGGATAGCTTTGAGGA

            * *  *      *             **          * *       * *          *  *
     235986 CTGCGGT-GAAAAAGGAAATATCTTCCAATAAAAGCTACATAGAAGCAATGTCAGAAACTTTTTC
         66 TTTCGTTGGAAACAGG-AATATCTTCATATAAAAGCTAGACAGAAGCATTCTCAGAAACTTCTTT

            *      * ** *   **      *    *  *        *
     236050 ATGATGTATCTACTCAGCTAACAGAGTTGGACCTT-CCTTTG
        130 GTGATGTTTGCATTCAAGTAACAGTGTTGAACATTCCCTTTC

             *       *                   * *     *             **            
     236091 AGAGAGCAGTTTTGAAACACTCTTTTTGTGGAATCTGCAAGTGGATATTTGTCTAGCTTTGAGGA
          1 ATAGAGCAGGTTTGAAACACTCTTTTTGTAGTATCTGGAAGTGGATATTTGGATAGCTTTGAGGA

                         *   *    *                   *       *              
     236156 TTTCGTTGGAAACGGGATTA-CAT-ATA-AAAAGC-AGACAGCAGCATTCCCAGAAACTTCTTTG
         66 TTTCGTTGGAAACAGGAATATCTTCATATAAAAGCTAGACAGAAGCATTCTCAGAAACTTCTTTG

                              *    *                 
     236217 TGATGTTTGCATTCAAGTCACAGAGTTGAACATTCCCTTTC
        131 TGATGTTTGCATTCAAGTAACAGTGTTGAACATTCCCTTTC

                                                   *     *       **     *   *
     236258 ATAGAGCAGGTTTGAAACACTCTTTTTGTAGTATCTGGATGTGGACATTTGGAGCGCTTTCAGGC
          1 ATAGAGCAGGTTTGAAACACTCTTTTTGTAGTATCTGGAAGTGGATATTTGGATAGCTTTGAGGA

            * ** *      *             *** *   *                          *   
     236323 CTATGGT-GAAAAAGGAAATATCTTCCCCTGAAAACTAGACAGAAGCATTCTCAGAAACTTATTT
         66 TTTCGTTGGAAACAGG-AATATCTTCATATAAAAGCTAGACAGAAGCATTCTCAGAAACTTCTTT

                   *** **    *             **  * *   *
     236387 GTGATGTGCCCCCTCAACTAACAGTGTTGAAGCTTTCTTTTG
        130 GTGATGTTTGCATTCAAGTAACAGTGTTGAACATTCCCTTTC

                     *                   * *     *             **            
     236429 ATAGAGCAGTTTTGAAACACTCTTTTTGTGGAATCTGCAAGTGGATATTTGTCTAGCTTTGAGGA
          1 ATAGAGCAGGTTTGAAACACTCTTTTTGTAGTATCTGGAAGTGGATATTTGGATAGCTTTGAGGA

                         *   *    *                   *       *     *   *    
     236494 TTTCGTTGGAAACGGGATTA-CAT-ATA-AAAAGC-AGACAGCAGCATTCCCAGAATCTTGTTTG
         66 TTTCGTTGGAAACAGGAATATCTTCATATAAAAGCTAGACAGAAGCATTCTCAGAAACTTCTTTG

                              *    *                 
     236555 TGATGTTTGCATTCAAGTCACAGAGTTGAACATTCCCTTTC
        131 TGATGTTTGCATTCAAGTAACAGTGTTGAACATTCCCTTTC

             *                         *           *     *       **     *   *
     236596 AGAGAGCAGGTTTGAAACACTCTTTTTATAGTATCTGGATGTGGACATTTGGAGCGCTTTCAGGC
          1 ATAGAGCAGGTTTGAAACACTCTTTTTGTAGTATCTGGAAGTGGATATTTGGATAGCTTTGAGGA

            * ** *      *             *** *   *                      *   *   
     236661 CTATGGT-GAAAAAGGAAATATCTTCTCCTGAAAACTAGACAGAAGCATTCTCAGAATCTTATTT
         66 TTTCGTTGGAAACAGG-AATATCTTCATATAAAAGCTAGACAGAAGCATTCTCAGAAACTTCTTT

                   **  **    *             **  * *   *
     236725 GTGATGTGCGCCCTCAACTAACAGTGTTGAAGCTTTCTTTTG
        130 GTGATGTTTGCATTCAAGTAACAGTGTTGAACATTCCCTTTC

                     *             *  *   **     *   *        *   *          
     236767 ATAGAGCAGTTTTGAAACACTCTGTTCGTAAAATCTGCAAGAGGATATTTTGATTGCTTTGAGGA
          1 ATAGAGCAGGTTTGAAACACTCTTTTTGTAGTATCTGGAAGTGGATATTTGGATAGCTTTGAGGA

                      *  *   * *            **                      *    *   
     236832 TTTCGTTGGATACGGGATTGTCTTCATATAAACTCTAGACAGAAGCATTCTCAGAAGCTTCATTG
         66 TTTCGTTGGAAACAGGAATATCTTCATATAAAAGCTAGACAGAAGCATTCTCAGAAACTTCTTTG

            *       **   *    *             *        
     236897 GGATGTTTCAATTGAAGTCACAGTGTTGAACAGTCCCTTTC
        131 TGATGTTTGCATTCAAGTAACAGTGTTGAACATTCCCTTTC

                                                         *       *  * * *    
     236938 ATAGAGCAGGTTTGAAACACTCTTTTTGTAGTATCTGGAAGTGGACATTTGGAGAGATCTCAGGA
          1 ATAGAGCAGGTTTGAAACACTCTTTTTGTAGTATCTGGAAGTGGATATTTGGATAGCTTTGAGGA

            * *  *    * *             **            *       * *          *  *
     237003 ATACGGT-GATAAAGGAAATATCTTCCAATAAAAGCTAGATAGAAGCAATGTCAGAAACTTTTTC
         66 TTTCGTTGGAAACAGG-AATATCTTCATATAAAAGCTAGACAGAAGCATTCTCAGAAACTTCTTT

            *      * ** *   **      *       *  * *   *
     237067 ATGATGTATCTACTCAGCTAACAGAGTTGAACCTTTCTTTTG
        130 GTGATGTTTGCATTCAAGTAACAGTGTTGAACATTCCCTTTC

             *       *                   * *     *             **            
     237109 AGAGAGCAGTTTTGAAACACTCTTTTTGTGGAATCTGCAAGTGGATATTTGTCTAGCTTTGAGGA
          1 ATAGAGCAGGTTTGAAACACTCTTTTTGTAGTATCTGGAAGTGGATATTTGGATAGCTTTGAGGA

                         *   *    *                   *       *   *          
     237174 TTTCGTTGGAAACGGGATTA-CAT-ATA-AAAAGC-AGACAGCAGCATTCCCAGTAACTTCTTTG
         66 TTTCGTTGGAAACAGGAATATCTTCATATAAAAGCTAGACAGAAGCATTCTCAGAAACTTCTTTG

                              *    *                 
     237235 TGATGTTTGCATTCAAGTCACAGAGTTGAACATTCCCTTTC
        131 TGATGTTTGCATTCAAGTAACAGTGTTGAACATTCCCTTTC

                                                   *     *       **     *   *
     237276 ATAGAGCAGGTTTGAAACACTCTTTTTGTAGTATCTGGATGTGGACATTTGGAGCGCTTTCAGGC
          1 ATAGAGCAGGTTTGAAACACTCTTTTTGTAGTATCTGGAAGTGGATATTTGGATAGCTTTGAGGA

            * ** *      *             *** *   *                      *   *   
     237341 CTATGGT-GAAAAAGGAAATATCTTCCCCTGAAAACTAGACAGAAGCATTCTCAGAATCTTATTT
         66 TTTCGTTGGAAACAGG-AATATCTTCATATAAAAGCTAGACAGAAGCATTCTCAGAAACTTCTTT

                   **  **    *             **  * *   *
     237405 GTGATGTGCGCCCTCAACTAACAGTGTTGAAGCTTTCTTTTG
        130 GTGATGTTTGCATTCAAGTAACAGTGTTGAACATTCCCTTTC

                     *                    **     *   *                       
     237447 ATAGAGCAGTTTTGAAACACTCTTTTTGTAAAATCTGCAAGAGGATATTTGGATAGCTTTGAGGA
          1 ATAGAGCAGGTTTGAAACACTCTTTTTGTAGTATCTGGAAGTGGATATTTGGATAGCTTTGAGGA

                         *   * *            **                      *    *   
     237512 TTTCGTTGGAAACGGGATTGTCTTCATATAAACTCTAGACAGAAGCATTCTCAGAAGCTTCATTG
         66 TTTCGTTGGAAACAGGAATATCTTCATATAAAAGCTAGACAGAAGCATTCTCAGAAACTTCTTTG

            *       ***  *    *             *        
     237577 GGATGTTTTAGTTGAAGTCACAGTGTTGAACAGTCCCTTTC
        131 TGATGTTTGCATTCAAGTAACAGTGTTGAACATTCCCTTTC

                                                         *       **   * *    
     237618 ATAGAGCAGGTTTGAAACACTCTTTTTGTAGTATCTGGAAGTGGACATTTGGAGCGCTCTCAGGA
          1 ATAGAGCAGGTTTGAAACACTCTTTTTGTAGTATCTGGAAGTGGATATTTGGATAGCTTTGAGGA

            * *  *      *             **            *       * *          *  *
     237683 CTGCGGT-GAAAAAGGAAATATCTTCCAATAAAAGCTAGATAGAAGCAATGTCAGAAACTTTTTC
         66 TTTCGTTGGAAACAGG-AATATCTTCATATAAAAGCTAGACAGAAGCATTCTCAGAAACTTCTTT

            *      * ** *   **      *       *        *
     237747 ATGATGTATCTACTCAGCTAACAGAGTTGAACCTT-CCTTTG
        130 GTGATGTTTGCATTCAAGTAACAGTGTTGAACATTCCCTTTC

             *       *                   * *     *             **            
     237788 AGAGAGCAGTTTTGAAACACTCTTTTTGTGGAATCTGCAAGTGGATATTTGTCTAGCTTTGAGGA
          1 ATAGAGCAGGTTTGAAACACTCTTTTTGTAGTATCTGGAAGTGGATATTTGGATAGCTTTGAGGA

                         *   *    *                   *       *              
     237853 TTTCGTTGGAAACGGGATTA-CAT-ATA-AAAAGC-AGACAGCAGCATTCCCAGAAACTTCTTTG
         66 TTTCGTTGGAAACAGGAATATCTTCATATAAAAGCTAGACAGAAGCATTCTCAGAAACTTCTTTG

                              *    *                 
     237914 TGATGTTTGCATTCAAGTCACAGAGTTGAACATTCCCTTTC
        131 TGATGTTTGCATTCAAGTAACAGTGTTGAACATTCCCTTTC

                             *            *        *     *     * **     *   *
     237955 ATAGAGCAGGTTTGAAAAACTCTTTTTGTAATATCTGGATGTGGACATTTGCAGCGCTTTCAGGC
          1 ATAGAGCAGGTTTGAAACACTCTTTTTGTAGTATCTGGAAGTGGATATTTGGATAGCTTTGAGGA

            * ** *      *             *** *   *                          *   
     238020 CTAAGGT-GAAAAAGGAAATATCTTCCCCTGAAAACTAGACAGAAGCATTCTCAGAAACTTATTT
         66 TTTCGTTGGAAACAGG-AATATCTTCATATAAAAGCTAGACAGAAGCATTCTCAGAAACTTCTTT

                   **  **    *             **  * *   *
     238084 GTGATGTGCGCCCTCAACTAACAGTGTTGAAGCTTTCTTTTG
        130 GTGATGTTTGCATTCAAGTAACAGTGTTGAACATTCCCTTTC

                     *                   * *     *             **            
     238126 ATAGAGCAGTTTTGAAACACTCTTTTTGTGGAATCTGCAAGTGGATATTTGTCTAGCTTTGAGGA
          1 ATAGAGCAGGTTTGAAACACTCTTTTTGTAGTATCTGGAAGTGGATATTTGGATAGCTTTGAGGA

                         *   *    *                   *       **    *   *    
     238191 TTTCGTTGGAAACGGGATTA-CAT-ATA-AAAAGC-AGACAGCAGCATTCCTAGAATCTTGTTTG
         66 TTTCGTTGGAAACAGGAATATCTTCATATAAAAGCTAGACAGAAGCATTCTCAGAAACTTCTTTG

                            **     *                 
     238252 TGATGTTTGCATTCAAAAAACAGAGTTGAACATTCCCTTTC
        131 TGATGTTTGCATTCAAGTAACAGTGTTGAACATTCCCTTTC

             *                         *           *     *       **     *   *
     238293 AGAGAGCAGGTTTGAAACACTCTTTTTATAGTATCTGGATGTGGACATTTGGAGCGCTTTCAGGC
          1 ATAGAGCAGGTTTGAAACACTCTTTTTGTAGTATCTGGAAGTGGATATTTGGATAGCTTTGAGGA

            * ** *      *             *** *   *                      *   *   
     238358 CTATGGT-GAAAAAGGAAATATCTTCTCCTGAAAACTAGACAGAAGCATTCTCAGAATCTTATTT
         66 TTTCGTTGGAAACAGG-AATATCTTCATATAAAAGCTAGACAGAAGCATTCTCAGAAACTTCTTT

                   **  **    *             **  * *   *
     238422 GTGATGTGGGCCCTCAACTAACAGTGTTGAAGCTTTCTTTTG
        130 GTGATGTTTGCATTCAAGTAACAGTGTTGAACATTCCCTTTC

                     *                *   **     *   *        *              
     238464 ATAGAGCAGTTTTGAAACACTCTTTTCGTAAAATCTGCAAGAGGATATTTTGATAGCTTTGAGGA
          1 ATAGAGCAGGTTTGAAACACTCTTTTTGTAGTATCTGGAAGTGGATATTTGGATAGCTTTGAGGA

                         *   * *            **                      *    **  
     238529 TTTCGTTGGAAACGGGATTGTCTTCATATAAACTCTAGACAGAAGCATTCTCAGAAGCTTCAGTG
         66 TTTCGTTGGAAACAGGAATATCTTCATATAAAAGCTAGACAGAAGCATTCTCAGAAACTTCTTTG

            *       **   * *  *             *        
     238594 GGATGTTTCAATTGATGTCACAGTGTTGAACAGTCCCTTTC
        131 TGATGTTTGCATTCAAGTAACAGTGTTGAACATTCCCTTTC

                                                         *       *  * * *    
     238635 ATAGAGCAGGTTTGAAACACTCTTTTTGTAGTATCTGGAAGTGGACATTTGGAGAGATCTCAGGA
          1 ATAGAGCAGGTTTGAAACACTCTTTTTGTAGTATCTGGAAGTGGATATTTGGATAGCTTTGAGGA

            * *  *    * *             **            *       * *          *  *
     238700 ATACGGT-GATAAAGGAAATATCTTCCAATAAAAGCTAGATAGAAGCAATGTCAGAAACTTTTTC
         66 TTTCGTTGGAAACAGG-AATATCTTCATATAAAAGCTAGACAGAAGCATTCTCAGAAACTTCTTT

            *      * ** *   **      *       *  * *   *
     238764 ATGATGTATCTACTCAGCTAACAGAGTTGAACCTTTCTTTTG
        130 GTGATGTTTGCATTCAAGTAACAGTGTTGAACATTCCCTTTC

             *       *                   * *     *             **            
     238806 AGAGAGCAGTTTTGAAACACTCTTTTTGTGGAATCTGCAAGTGGATATTTGTCTAGCTTTGAGGA
          1 ATAGAGCAGGTTTGAAACACTCTTTTTGTAGTATCTGGAAGTGGATATTTGGATAGCTTTGAGGA

                         *   *    *                   *       *              
     238871 TTTCGTTGGAAACGGGATTA-CAT-ATA-AAAAGC-AGACAGCAGCATTCCCAGAAACTTCTTTG
         66 TTTCGTTGGAAACAGGAATATCTTCATATAAAAGCTAGACAGAAGCATTCTCAGAAACTTCTTTG

                              *    *                 
     238932 TGATGTTTGCATTCAAGTCACAGAGTTGAACATTCCCTTTC
        131 TGATGTTTGCATTCAAGTAACAGTGTTGAACATTCCCTTTC

                                                 * *     *       **     *   *
     238973 ATAGAGCAGGTTTGAAACACTCTTTTTGTAGTATCTGTATGTGGACATTTGGAGCGCTTTCAGGC
          1 ATAGAGCAGGTTTGAAACACTCTTTTTGTAGTATCTGGAAGTGGATATTTGGATAGCTTTGAGGA

              ** *      *             *** *   *                          *   
     239038 TTATGGT-GAAAAAGGAAATATCTTCCCCTGAAAACTAGACAGAAGCATTCTCAGAAACTTATTT
         66 TTTCGTTGGAAACAGG-AATATCTTCATATAAAAGCTAGACAGAAGCATTCTCAGAAACTTCTTT

                   **  **    *              *  * *   *
     239102 GTGATGTGCGCCCTCAACTAACAGTGTTGAACCTTTCTTTTG
        130 GTGATGTTTGCATTCAAGTAACAGTGTTGAACATTCCCTTTC

                     *                    *      *   *                       
     239144 ATAGAGCAGTTTTGAAACACTCTTTTTGTAATATCTGCAAGAGGATATTTGGATAGCTTTGAGGA
          1 ATAGAGCAGGTTTGAAACACTCTTTTTGTAGTATCTGGAAGTGGATATTTGGATAGCTTTGAGGA

             *           *   * *            **                      *        
     239209 TATCGTTGGAAACGGGATTGTCTTCATATAAACTCTAGACAGAAGCATTCTCAGAAGCTTCATTT
         66 TTTCGTTGGAAACAGGAATATCTTCATATAAAAGCTAGACAGAAGCATTCTCAGAAACTTC-TTT

                     **   *    *             *        
     239274 G-GATGTTTCAATTGAAGTCACAGTGTTGAACAGTCCCTTTC
        130 GTGATGTTTGCATTCAAGTAACAGTGTTGAACATTCCCTTTC

                                                         *       **   * *    
     239315 ATAGAGCAGGTTTGAAACACTCTTTTTGTAGTATCTGGAAGTGGACATTTGGAGCGCTCTCAGGA
          1 ATAGAGCAGGTTTGAAACACTCTTTTTGTAGTATCTGGAAGTGGATATTTGGATAGCTTTGAGGA

            * *  *      *      *      **          * *       * *          *  *
     239380 CTGCGGT-GAAAAAGGAAAAATCTTCCAATAAAAGCTACATAGAAGCAATGTCAGAAACTTTTTC
         66 TTTCGTTGGAAACAGG-AATATCTTCATATAAAAGCTAGACAGAAGCATTCTCAGAAACTTCTTT

            *      * ** *   **      *                *
     239444 ATGATGTATCTACTCAGCTAACAGAGTTGAACATT-CCTTTG
        130 GTGATGTTTGCATTCAAGTAACAGTGTTGAACATTCCCTTTC

             *       *                   * *     *             **            
     239485 AGAGAGCAGTTTTGAAACACTCTTTTTGTGGAATCTGCAAGTGGATATTTGTCTAGCTTTGAGGA
          1 ATAGAGCAGGTTTGAAACACTCTTTTTGTAGTATCTGGAAGTGGATATTTGGATAGCTTTGAGGA

                         *   *    *                   *       *              
     239550 TTTCGTTGGAAACGGGATTA-CAT-ATA-AAAAGC-AGACAGCAGCATTCCCAGAAACTTCTTTG
         66 TTTCGTTGGAAACAGGAATATCTTCATATAAAAGCTAGACAGAAGCATTCTCAGAAACTTCTTTG

                              *    *                 
     239611 TGATGTTTGCATTCAAGTCACAGAGTTGAACATTCCCTTTC
        131 TGATGTTTGCATTCAAGTAACAGTGTTGAACATTCCCTTTC

                                                   *     *       **     *   *
     239652 ATAGAGCAGGTTTGAAACACTCTTTTTGTAGTATCTGGATGTGGACATTTGGAGCGCTTTCAGGC
          1 ATAGAGCAGGTTTGAAACACTCTTTTTGTAGTATCTGGAAGTGGATATTTGGATAGCTTTGAGGA

            * ** *      *             *** *   *                      *   *   
     239717 CTATGGT-GAAAAAGGAAATATCTTCCCCTGAAAACTAGACAGAAGCATTCTCAGAATCTTATTT
         66 TTTCGTTGGAAACAGG-AATATCTTCATATAAAAGCTAGACAGAAGCATTCTCAGAAACTTCTTT

                   **  **    *             **  * *   *
     239781 GTGATGTGCGCCCTCAACTAACAGTGTTGAAGCTTTCTTTTG
        130 GTGATGTTTGCATTCAAGTAACAGTGTTGAACATTCCCTTTC

                     *                    **     *   *     *  *              
     239823 ATAGAGCAGTTTTGAAACACTCTTTTTGTAAAATCTGCAAGAGGATAATTTGATAGCTTTGAGGA
          1 ATAGAGCAGGTTTGAAACACTCTTTTTGTAGTATCTGGAAGTGGATATTTGGATAGCTTTGAGGA

            *            *   * *            **                      *    *   
     239888 ATTCGTTGGAAACGGGATTGTCTTCATATAAACTCTAGACAGAAGCATTCTCAGAAGCTTCATTG
         66 TTTCGTTGGAAACAGGAATATCTTCATATAAAAGCTAGACAGAAGCATTCTCAGAAACTTCTTTG

            *             *    *             *        
     239953 GGATGTTT-CAGTTGAAGTCACAGTGTTGAACAGTCCCTTTC
        131 TGATGTTTGCA-TTCAAGTAACAGTGTTGAACATTCCCTTTC

                                                         *       **   * *    
     239994 ATAGAGCAGGTTTGAAACACTCTTTTTGTAGTATCTGGAAGTGGACATTTGGAGCGCTCTCAGGA
          1 ATAGAGCAGGTTTGAAACACTCTTTTTGTAGTATCTGGAAGTGGATATTTGGATAGCTTTGAGGA

            * *  *      *             **            *       * *          *  *
     240059 CTGCGGT-GAAAAAGGAAATATCTTCCAATAAAAGCTAGATAGAAGCAATGTCAGAAACTTTTTC
         66 TTTCGTTGGAAACAGG-AATATCTTCATATAAAAGCTAGACAGAAGCATTCTCAGAAACTTCTTT

            *      * ** *   **      *       *        *
     240123 ATGATGTATCTACTCAGCTAACAGAGTTGAACCTT-CCTTTG
        130 GTGATGTTTGCATTCAAGTAACAGTGTTGAACATTCCCTTTC

             *       *            *      * *     *             **            
     240164 AGAGAGCAGTTTTGAAACACTCCTTTTGTGGAATCTGCAAGTGGATATTTGTCTAGCTTTGAGGA
          1 ATAGAGCAGGTTTGAAACACTCTTTTTGTAGTATCTGGAAGTGGATATTTGGATAGCTTTGAGGA

                         *   *    *                   *       *              
     240229 TTTCGTTGGAAACGGGATTA-CAT-ATA-AAAAGC-AGACAGCAGCATTCCCAGAAACTTCTTTG
         66 TTTCGTTGGAAACAGGAATATCTTCATATAAAAGCTAGACAGAAGCATTCTCAGAAACTTCTTTG

                              *    *                 
     240290 TGATGTTTGCATTCAAGTCACAGAGTTGAACATTCCCTTTC
        131 TGATGTTTGCATTCAAGTAACAGTGTTGAACATTCCCTTTC

                                **                 *     *     * **     *   *
     240331 ATAGAGCAGGTTTGAAACACATTTTTTGTAGTATCTGGATGTGGACATTTGCAGCGCTTTCAGGC
          1 ATAGAGCAGGTTTGAAACACTCTTTTTGTAGTATCTGGAAGTGGATATTTGGATAGCTTTGAGGA

            * ** *      *             *** *   *                          *   
     240396 CTAAGGT-GAAAAAGGAAATATCTTCCCCTGAAAACTAGACAGAAGCATTCTCAGAAACTTATTT
         66 TTTCGTTGGAAACAGG-AATATCTTCATATAAAAGCTAGACAGAAGCATTCTCAGAAACTTCTTT

                   **  **    *             **  * *   *
     240460 GTGATGTGCGCCCTCAACTAACAGTGTTGAAGCTTTCTTTTG
        130 GTGATGTTTGCATTCAAGTAACAGTGTTGAACATTCCCTTTC

                     *                   * *     *             **            
     240502 ATAGAGCAGTTTTGAAACACTCTTTTTGTGGAATCTGCAAGTGGATATTTGTCTAGCTTTGAGGA
          1 ATAGAGCAGGTTTGAAACACTCTTTTTGTAGTATCTGGAAGTGGATATTTGGATAGCTTTGAGGA

                              *    *          **      *       *     *   *    
     240567 TTTCGTTGGAAAC-GG--GAT-TACATATAAAAGGCAGACAGCAGCATTCCCAGAATCTTGTTTG
         66 TTTCGTTGGAAACAGGAATATCTTCATATAAAAGCTAGACAGAAGCATTCTCAGAAACTTCTTTG

                              *    *   *             
     240628 TGATGTTTGCATTCAAGTCACAGAGTTCAACATTCCCTTTC
        131 TGATGTTTGCATTCAAGTAACAGTGTTGAACATTCCCTTTC

             *                         *           *     *       **     *   *
     240669 AGAGAGCAGGTTTGAAACACTCTTTTTATAGTATCTGGATGTGGACATTTGGAGCGCTTTCAGGC
          1 ATAGAGCAGGTTTGAAACACTCTTTTTGTAGTATCTGGAAGTGGATATTTGGATAGCTTTGAGGA

              ** *      *             *** *   *                      *   *   
     240734 TTATGGT-GAAAAAGGAAATATCTTCTCCTGAAAACTAGACAGAAGCATTCTCAGAATCTTATTT
         66 TTTCGTTGGAAACAGG-AATATCTTCATATAAAAGCTAGACAGAAGCATTCTCAGAAACTTCTTT

                   ** ***    *             **  * *   *
     240798 GTGATGTGCGTCCTCAACTAACAGTGTTGAAGCTTTCTTTTG
        130 GTGATGTTTGCATTCAAGTAACAGTGTTGAACATTCCCTTTC

                     *                *   **     *   *        *              
     240840 ATAGAGCAGTTTTGAAACACTCTTTTCGTAAAATCTGCAAGAGGATATTTTGATAGCTTTGAGGA
          1 ATAGAGCAGGTTTGAAACACTCTTTTTGTAGTATCTGGAAGTGGATATTTGGATAGCTTTGAGGA

                         *   * *            **                      *    *   
     240905 TTTCGTTGGAAACGGGATTGTCTTCATATAAACTCTAGACAGAAGCATTCTCAGAAGCTTCATTG
         66 TTTCGTTGGAAACAGGAATATCTTCATATAAAAGCTAGACAGAAGCATTCTCAGAAACTTCTTTG

            *       **   *    *             *        
     240970 GGATGTTTCAATTGAAGTCACAGTGTTGAACAGTCCCTTTC
        131 TGATGTTTGCATTCAAGTAACAGTGTTGAACATTCCCTTTC

                                                         *       *  * * *    
     241011 ATAGAGCAGGTTTGAAACACTCTTTTTGTAGTATCTGGAAGTGGACATTTGGAGAGATCTCAGGA
          1 ATAGAGCAGGTTTGAAACACTCTTTTTGTAGTATCTGGAAGTGGATATTTGGATAGCTTTGAGGA

            * *  *    * *             **            *       * *          *  *
     241076 ATACGGT-GATAAAGGAAATATCTTCCAATAAAAGCTAGATAGAAGCAATGTCAGAAACTTTTTC
         66 TTTCGTTGGAAACAGG-AATATCTTCATATAAAAGCTAGACAGAAGCATTCTCAGAAACTTCTTT

            *      * ** *   **      *       *  * *   *
     241140 ATGATGTATCTACTCAGCTAACAGAGTTGAACCTTTCTTTTG
        130 GTGATGTTTGCATTCAAGTAACAGTGTTGAACATTCCCTTTC

             *       *        *          * *     *             **            
     241182 AGAGAGCAGTTTTGAAACTCTCTTTTTGTGGAATCTGCAAGTGGATATTTGTCTAGCTTTGAGGA
          1 ATAGAGCAGGTTTGAAACACTCTTTTTGTAGTATCTGGAAGTGGATATTTGGATAGCTTTGAGGA

                         *   *    *  *                *       *        *     
     241247 TTTCGTTGGAAACGGGATTA-CAT-GTA-AAAAGC-AGACAGCAGCATTCCCAGAAACTACTTTG
         66 TTTCGTTGGAAACAGGAATATCTTCATATAAAAGCTAGACAGAAGCATTCTCAGAAACTTCTTTG

                              *    *                 
     241308 TGATGTTTGCATTCAAGTCACAGAGTTGAACATTCCCTTTC
        131 TGATGTTTGCATTCAAGTAACAGTGTTGAACATTCCCTTTC

                                                 * *     *       ***    *   *
     241349 ATAGAGCAGGTTTGAAACACTCTTTTTGTAGTATCTGTATGTGGACATTTGGAGCACTTTCAGGC
          1 ATAGAGCAGGTTTGAAACACTCTTTTTGTAGTATCTGGAAGTGGATATTTGGATAGCTTTGAGGA

            * ** *      *             *** *   *                          *   
     241414 CTATGGT-GAAAAAGGAAATATCTTCCCCTGAAAACTAGACAGAAGCATTCTCAGAAACTTATTT
         66 TTTCGTTGGAAACAGG-AATATCTTCATATAAAAGCTAGACAGAAGCATTCTCAGAAACTTCTTT

                   **  **    *              *  * *   *
     241478 GTGATGTGCGCCCTCAACTAACAGTGTTGAACCTTTCTTTTG
        130 GTGATGTTTGCATTCAAGTAACAGTGTTGAACATTCCCTTTC

                     *                    *      *   *                       
     241520 ATAGAGCAGTTTTGAAACACTCTTTTTGTAATATCTGCAAGAGGATATTTGGATAGCTTTGAGGA
          1 ATAGAGCAGGTTTGAAACACTCTTTTTGTAGTATCTGGAAGTGGATATTTGGATAGCTTTGAGGA

                         *   * *            **                      *    *   
     241585 TTTCGTTGGAAACGGGATTGTCTTCATATAAACTCTAGACAGAAGCATTCTCAGAAGCTTCATTG
         66 TTTCGTTGGAAACAGGAATATCTTCATATAAAAGCTAGACAGAAGCATTCTCAGAAACTTCTTTG

            *       **   *  * *            ** *      
     241650 GGATGTTTCAATTGAACTCACAGTGTTGAACTGTTCCTTTC
        131 TGATGTTTGCATTCAAGTAACAGTGTTGAACATTCCCTTTC

                 *  *                                    *       **   * *    
     241691 ATAGAACAAGTTTGAAACACTCTTTTTGTAGTATCTGGAAGTGGACATTTGGAGCGCTCTCAGGA
          1 ATAGAGCAGGTTTGAAACACTCTTTTTGTAGTATCTGGAAGTGGATATTTGGATAGCTTTGAGGA

            * *  *  *   ** *         **          * *       * *          *  **
     241756 CTACGGTGAAAATGGAAATATCTTCCAATAAAAGCTACATAGAAGCAATGTCAGAAACTTTTTCA
         66 TTTCGTTGGAAACAGGAATATCTTCATATAAAAGCTAGACAGAAGCATTCTCAGAAACTTCTTTG

                  * ** *   **      *       *  *     *
     241821 TGATGTATCTACTCAGCTAACAGAGTTGAACCTTTCCTTTG
        131 TGATGTTTGCATTCAAGTAACAGTGTTGAACATTCCCTTTC

             *       *                   * *     *             **            
     241862 AGAGAGCAGTTTTGAAACACTCTTTTTGTGGAATCTGCAAGTGGATATTTGTCTAGCTTTGAGGA
          1 ATAGAGCAGGTTTGAAACACTCTTTTTGTAGTATCTGGAAGTGGATATTTGGATAGCTTTGAGGA

                         *   *    *                   *       *   *          
     241927 TTTCGTTGGAAACGGGATTA-CAT-ATA-AAAAGC-AGACAGCAGCATTCCCAGTAACTTCTTTG
         66 TTTCGTTGGAAACAGGAATATCTTCATATAAAAGCTAGACAGAAGCATTCTCAGAAACTTCTTTG

                              *    *            *    
     241988 TGATGTTTGCATTCAAGTCACAGAGTTGAACATTCCGTTTC
        131 TGATGTTTGCATTCAAGTAACAGTGTTGAACATTCCCTTTC

                                                   *     *       **          
     242029 ATAGAGCAGGTTTGAAACACTCTTTTTGTAGTATCTGGATGTGGACATTTGGAGCG-TTTGCAGG
          1 ATAGAGCAGGTTTGAAACACTCTTTTTGTAGTATCTGGAAGTGGATATTTGGATAGCTTTG-AGG

             * * *    *                 *** *   *                          * 
     242093 CCTATGGTT-TAAA-AGGAAATATCTTCCCCTGAAAACTAGACAGAAGCATTCTCAGAAACTTAT
         65 -ATTTCGTTGGAAACAGG-AATATCTTCATATAAAAGCTAGACAGAAGCATTCTCAGAAACTTCT

                     **  **    *       *            *   *
     242156 TTGTGATGTGCGCCCTCAACTAACAGTTTTGAAGCATT-CTTTTG
        128 TTGTGATGTTTGCATTCAAGTAACAGTGTTGAA-CATTCCCTTTC

                     *                   * *     *             **       *    
     242200 ATAGAGCAGTTTTGAAACACTCTTTTTGTGGAATCTGCAAGTGGATATTTGTCTAGCTTTCAGGA
          1 ATAGAGCAGGTTTGAAACACTCTTTTTGTAGTATCTGGAAGTGGATATTTGGATAGCTTTGAGGA

                         *   *    *                   *       *     *   *    
     242265 TTTCGTTGGAAACGGGATTA-CAT-ATA-AAAAGC-AGACAGCAGCATTCCCAGAATCTTGTTTG
         66 TTTCGTTGGAAACAGGAATATCTTCATATAAAAGCTAGACAGAAGCATTCTCAGAAACTTCTTTG

                              *    *                 
     242326 TGATG-TTGCATTCAAGTCACAGAGTTGAACATTCCCTTTC
        131 TGATGTTTGCATTCAAGTAACAGTGTTGAACATTCCCTTTC

                                       *           *     *       **     *   *
     242366 ATAGAGCAGGTTTGAAACACTCTTTTTATAGTATCTGGATGTGGACATTTGGAGCGCTTTCAGGC
          1 ATAGAGCAGGTTTGAAACACTCTTTTTGTAGTATCTGGAAGTGGATATTTGGATAGCTTTGAGGA

            * ** *      *             *** *   *   *                  *   *   
     242431 CTGTGGT-GAAAAAGGAAATATCTTCTCCTGAAAACTACACAGAAGCATTCTCAGAATCTTATTT
         66 TTTCGTTGGAAACAGG-AATATCTTCATATAAAAGCTAGACAGAAGCATTCTCAGAAACTTCTTT

                   **  **    *              *  * *   *
     242495 GTGATGTGCGCCCTCAACTAACAGTGTTGAACCTTTCTTTTG
        130 GTGATGTTTGCATTCAAGTAACAGTGTTGAACATTCCCTTTC

                     *                    *      *   *                       
     242537 ATAGAGCAGTTTTGAAACACTC-TTTTGTAATATCTGCAAGAGGATATTTGGATAGCTTTGAGGA
          1 ATAGAGCAGGTTTGAAACACTCTTTTTGTAGTATCTGGAAGTGGATATTTGGATAGCTTTGAGGA

                         *   *    *                   *       *     *   **   
     242601 TTTCGTTGGAAACGGGATTA-CAT-ATA-AAAAGC-AGACAGCAGCATTCCCAGAATCTTGATTG
         66 TTTCGTTGGAAACAGGAATATCTTCATATAAAAGCTAGACAGAAGCATTCTCAGAAACTTCTTTG

                              *    *                 
     242662 TGATGTTTGCATTCAAGTCACAGAGTTGAACATTCCCTTTC
        131 TGATGTTTGCATTCAAGTAACAGTGTTGAACATTCCCTTTC

             *                         *           *     *       **     *   *
     242703 AGAGAGCAGGTTTGAAACACTCTTTTTATAGTATCTGGATGTGGACATTTGGAGCGCTTTCAGGC
          1 ATAGAGCAGGTTTGAAACACTCTTTTTGTAGTATCTGGAAGTGGATATTTGGATAGCTTTGAGGA

            * ** *      *             *** *   *                      *   *   
     242768 CTATGGT-GAAAAAGGAAATATCTTCTCCTGAAAACTAGACAGAAGCATTCTCAGAATCTTATTT
         66 TTTCGTTGGAAACAGG-AATATCTTCATATAAAAGCTAGACAGAAGCATTCTCAGAAACTTCTTT

                   **  **    *             **  * *   *
     242832 GTGATGTGCGCCCTCAACTAACAGTGTTGAAGCTTTCTTTTG
        130 GTGATGTTTGCATTCAAGTAACAGTGTTGAACATTCCCTTTC

                     *                *   **     *   *        *              
     242874 ATAGAGCAGTTTTGAAACACTCTTTTCGTAAAATCTGCAAGAGGATATTTTGATAGCTTTGAGGA
          1 ATAGAGCAGGTTTGAAACACTCTTTTTGTAGTATCTGGAAGTGGATATTTGGATAGCTTTGAGGA

                         *   * *            **                      *    *   
     242939 TTTCGTTGGAAACGGGATTGTCTTCATATAAACTCTAGACAGAAGCATTCTCAGAAGCTTCATTG
         66 TTTCGTTGGAAACAGGAATATCTTCATATAAAAGCTAGACAGAAGCATTCTCAGAAACTTCTTTG

            *       **   *    *             *        
     243004 GGATGTTTCAATTGAAGTCACAGTGTTGAACACTCCCTTTC
        131 TGATGTTTGCATTCAAGTAACAGTGTTGAACATTCCCTTTC

                                                         *       *  * * *    
     243045 ATAGAGCAGGTTTGAAACACTCTTTTTGTAGTATCTGGAAGTGGACATTTGGAGAGATCTCAGGA
          1 ATAGAGCAGGTTTGAAACACTCTTTTTGTAGTATCTGGAAGTGGATATTTGGATAGCTTTGAGGA

            * *  *    * *             **            *       * *          *  *
     243110 ATACGGT-GATAAAGGAAATATCTTCCAATAAAAGCTAGATAGAAGCAATGTCAGAAACTTTTTC
         66 TTTCGTTGGAAACAGG-AATATCTTCATATAAAAGCTAGACAGAAGCATTCTCAGAAACTTCTTT

            *      * ** *   **  *   *       *  * *   *
     243174 ATGATGTATCTACTCAGCTACCAGAGTTGAACCTTTCTTTTG
        130 GTGATGTTTGCATTCAAGTAACAGTGTTGAACATTCCCTTTC

             *       *                   * *     *             **            
     243216 AGAGAGCAGTTTTGAAACACTCTTTTTGTGGGATCTGCAAGTGGATATTTGTCTAGCTTTGAGGA
          1 ATAGAGCAGGTTTGAAACACTCTTTTTGTAGTATCTGGAAGTGGATATTTGGATAGCTTTGAGGA

                         *   *    *                   *       *              
     243281 TTTCGTTGGAAACGGGATTA-CAT-ATA-AAAAGC-AGACAGCAGCATTCCCAGAAACTTCTTTG
         66 TTTCGTTGGAAACAGGAATATCTTCATATAAAAGCTAGACAGAAGCATTCTCAGAAACTTCTTTG

               *              *    *                 
     243342 TGAAGTTTGCATTCAAGTCACAGAGTTGAACATTCCCTTTC
        131 TGATGTTTGCATTCAAGTAACAGTGTTGAACATTCCCTTTC

                                                   *     *       **     *   *
     243383 ATAGAGCAGGTTTGAAACACTCTTTTTGTAGTATCTGGATGTGGACATTTGGAGCGCTTTCAGGC
          1 ATAGAGCAGGTTTGAAACACTCTTTTTGTAGTATCTGGAAGTGGATATTTGGATAGCTTTGAGGA

            * ** *      *             *** *   *                          *   
     243448 CTATGGT-GAAAAAGGAAATATCTTCCCCTGAAAACTAGACAGAAGCATTCTCAGAAACTTATTT
         66 TTTCGTTGGAAACAGG-AATATCTTCATATAAAAGCTAGACAGAAGCATTCTCAGAAACTTCTTT

                   **  **    *             **  * *   *
     243512 GTGATGTGCGCCCTCAACTAACAGTGTTGAAGCTTTCTTTTG
        130 GTGATGTTTGCATTCAAGTAACAGTGTTGAACATTCCCTTTC

                     *                    *      *   *                       
     243554 ATAGAGCAGTTTTGAAACACTCTTTTTGTAATATCTGCAAGAGGATATTTGGATAGCTTTGAGGA
          1 ATAGAGCAGGTTTGAAACACTCTTTTTGTAGTATCTGGAAGTGGATATTTGGATAGCTTTGAGGA

                         *   * *            **                      *    *   
     243619 TTTCGTTGGAAACGGGATTGTCTTCATATAAACTCTAGACAGAAGCATTCTCAGAAGCTTCATTG
         66 TTTCGTTGGAAACAGGAATATCTTCATATAAAAGCTAGACAGAAGCATTCTCAGAAACTTCTTTG

            *       **   *    *             * *      
     243684 GGATGTTTCAATTGAAGTCACAGTGTTGAACAGTTCCTTTC
        131 TGATGTTTGCATTCAAGTAACAGTGTTGAACATTCCCTTTC

                 *                                       *       **   * *    
     243725 ATAGAACAGGTTTGAAACACTCTTTTTGTAGTATCTGGAAGTGGACATTTGGAGCGCTCTCAGGA
          1 ATAGAGCAGGTTTGAAACACTCTTTTTGTAGTATCTGGAAGTGGATATTTGGATAGCTTTGAGGA

            * ** *      *             **          * *       * *          *  *
     243790 CTATGGT-GAAAAAGGAAATATCTTCCAATAAAAGCTACATAGAAGCAATGTCAGAAACTTTTTC
         66 TTTCGTTGGAAACAGG-AATATCTTCATATAAAAGCTAGACAGAAGCATTCTCAGAAACTTCTTT

            *      * ** *   **      *       *  *     *
     243854 ATGATGTATCTACTCAGCTAACAGAGTTGAACCTTTCCTTTG
        130 GTGATGTTTGCATTCAAGTAACAGTGTTGAACATTCCCTTTC

             *       *                   * *     *             **            
     243896 AGAGAGCAGTTTTGAAACACTCTTTTTGTGGAATCTGCAAGTGGATATTTGTCTAGCTTTGAGGA
          1 ATAGAGCAGGTTTGAAACACTCTTTTTGTAGTATCTGGAAGTGGATATTTGGATAGCTTTGAGGA

                         *   *    *                   *       *     *   **   
     243961 TTTCGTTGGAAACGGGATTA-CAT-ATA-AAAAGC-AGACAGCAGCATTCCCAGAATCTTGATTG
         66 TTTCGTTGGAAACAGGAATATCTTCATATAAAAGCTAGACAGAAGCATTCTCAGAAACTTCTTTG

                              *    *   *             
     244022 TGATGTTTGCATTCAAGTCACAGAGTTCAACATTCCCTTTC
        131 TGATGTTTGCATTCAAGTAACAGTGTTGAACATTCCCTTTC

             *                         *           *     *       **     *   *
     244063 AGAGAGCAGGTTTGAAACACTCTTTTTATAGTATCTGGATGTGGACATTTGGAGCGCTTTCAGGC
          1 ATAGAGCAGGTTTGAAACACTCTTTTTGTAGTATCTGGAAGTGGATATTTGGATAGCTTTGAGGA

            * ** *      *             *** *   *                      *   *   
     244128 CTATGGT-GAAAAAGGAAATATCTTCTCCTGAAAACTAGACAGAAGCATTCTCAGAATCTTATTT
         66 TTTCGTTGGAAACAGG-AATATCTTCATATAAAAGCTAGACAGAAGCATTCTCAGAAACTTCTTT

                   **  **    *             **  * *   *
     244192 GTGATGTGCGCCCTCAACTAACAGTGTTGAAGCTTTCTTTTG
        130 GTGATGTTTGCATTCAAGTAACAGTGTTGAACATTCCCTTTC

                     *                *   **     *   *        *              
     244234 ATAGAGCAGTTTTGAAACACTCTTTTCGTAAAATCTGCAAGAGGATATTTTGATAGCTTTGAGGA
          1 ATAGAGCAGGTTTGAAACACTCTTTTTGTAGTATCTGGAAGTGGATATTTGGATAGCTTTGAGGA

                         *   * *            **                      *    *   
     244299 TTTCGTTGGAAACGGGATTGTCTTCATATAAACTCTAGACAGAAGCATTCTCAGAAGCTTCATTG
         66 TTTCGTTGGAAACAGGAATATCTTCATATAAAAGCTAGACAGAAGCATTCTCAGAAACTTCTTTG

            *       **   *    *             *        
     244364 GGATGTTTCAATTGAAGTCACAGTGTTGAACAGTCCCTTTC
        131 TGATGTTTGCATTCAAGTAACAGTGTTGAACATTCCCTTTC

                                                         *       *  * * *    
     244405 ATAGAGCAGGTTTGAAACACTCTTTTTGTAGTATCTGGAAGTGGACATTTGGAGAGATCTCAGGA
          1 ATAGAGCAGGTTTGAAACACTCTTTTTGTAGTATCTGGAAGTGGATATTTGGATAGCTTTGAGGA

            * *  *    * *             **            *       * *          *  *
     244470 ATACGGT-GATAAAGGAAATATCTTCCAATAAAAGCTAGATAGAAGCAATGTCAGAAACTTTTTC
         66 TTTCGTTGGAAACAGG-AATATCTTCATATAAAAGCTAGACAGAAGCATTCTCAGAAACTTCTTT

            *      * ** *   **      *       *  * *   *
     244534 ATGATGTATCTACTCAGCTAACAGAGTTGAACCTTTCTTTTG
        130 GTGATGTTTGCATTCAAGTAACAGTGTTGAACATTCCCTTTC

             *       *                   * *    **             **            
     244576 AGAGAGCAGTTTTGAAACACTCTTTTTGTGGAATCTACAAGTGGATATTTGTCTAGCTTTGAGGA
          1 ATAGAGCAGGTTTGAAACACTCTTTTTGTAGTATCTGGAAGTGGATATTTGGATAGCTTTGAGGA

                         *   *    *                   *       *              
     244641 TTTCGTTGGAAACGGGATTA-CAT-ATA-AAAAGC-AGACAGCAGCATTCCCAGAAACTTCTTTG
         66 TTTCGTTGGAAACAGGAATATCTTCATATAAAAGCTAGACAGAAGCATTCTCAGAAACTTCTTTG

                              *    *                 
     244702 TGATGTTTGCATTCAAGTCACAGAGTTGAACATTCCCTTTC
        131 TGATGTTTGCATTCAAGTAACAGTGTTGAACATTCCCTTTC

                                                   *     *       **         *
     244743 ATAGAGCAGGTTTGAAACACTCTTTTTGTAGTATCTGGATGTGGACATTTGGAGCGCTTTGAGGC
          1 ATAGAGCAGGTTTGAAACACTCTTTTTGTAGTATCTGGAAGTGGATATTTGGATAGCTTTGAGGA

            * ** *      *             *** *   *                          *   
     244808 CTATGGT-GAAAAAGGAAATATCTTCCCCTGAAAACTAGACAGAAGCATTCTCAGAAACTTATTT
         66 TTTCGTTGGAAACAGG-AATATCTTCATATAAAAGCTAGACAGAAGCATTCTCAGAAACTTCTTT

                   **  **    *             **  * *   *
     244872 GTGATGTGCGCCCTCAACTAACAGTGTTGAAGCTTTCTTTTG
        130 GTGATGTTTGCATTCAAGTAACAGTGTTGAACATTCCCTTTC

                     *                    *      *   *                       
     244914 ATAGAGCAGTTTTGAAACACTCTTTTTGTAATATCTGCAAGAGGATATTTGGATAGCTTTGAGGA
          1 ATAGAGCAGGTTTGAAACACTCTTTTTGTAGTATCTGGAAGTGGATATTTGGATAGCTTTGAGGA

                         *   * *            **                      *    *   
     244979 TTTCGTTGGAAACGGGATTGTCTTCATATAAACTCTAGACAGAAGCATTCTCAGAAGCTTCATTG
         66 TTTCGTTGGAAACAGGAATATCTTCATATAAAAGCTAGACAGAAGCATTCTCAGAAACTTCTTTG

            *       **   *    *             * *      
     245044 GGATGTTTCAATTGAAGTCACAGTGTTGAACAGTTCCTTTC
        131 TGATGTTTGCATTCAAGTAACAGTGTTGAACATTCCCTTTC

                 *                                       *       **   * *    
     245085 ATAGAACAGGTTTGAAACACTCTTTTTGTAGTATCTGGAAGTGGACATTTGGAGCGCTCTCAGGA
          1 ATAGAGCAGGTTTGAAACACTCTTTTTGTAGTATCTGGAAGTGGATATTTGGATAGCTTTGAGGA

            * ** *      *             **          * *       * *          *  *
     245150 CTATGGT-GAAAAAGGAAATATCTTCCAATAAAAGCTACATAGAAGCAATGTCAGAAACTTTTTC
         66 TTTCGTTGGAAACAGG-AATATCTTCATATAAAAGCTAGACAGAAGCATTCTCAGAAACTTCTTT

            *      * ** *   **      *       *  *     *
     245214 ATGATGTATCTACTCAGCTAACAGAGTTGAACCTTTCCTTTG
        130 GTGATGTTTGCATTCAAGTAACAGTGTTGAACATTCCCTTTC

             *       *                   * *     *             **            
     245256 AGAGAGCAGTTTTGAAACACTCTTTTTGTGGAATCTGCAAGTGGATATTTGTCTAGCTTTGAGGA
          1 ATAGAGCAGGTTTGAAACACTCTTTTTGTAGTATCTGGAAGTGGATATTTGGATAGCTTTGAGGA

                         *   *    *                   *       *     *   **   
     245321 TTTCGTTGGAAACGGGATTA-CAT-ATA-AAAAGC-AGACAGCAGCATTCCCAGAATCTTGATTG
         66 TTTCGTTGGAAACAGGAATATCTTCATATAAAAGCTAGACAGAAGCATTCTCAGAAACTTCTTTG

                              *    *   *             
     245382 TGATGTTTGCATTCAAGTCACAGAGTTCAACATTCCCTTTC
        131 TGATGTTTGCATTCAAGTAACAGTGTTGAACATTCCCTTTC

             *                         *           *     *       **     *   *
     245423 AGAGAGCAGGTTTGAAACACTCTTTTTATAGTATCTGGATGTGGACATTTGGAGCGCTTTCAGGC
          1 ATAGAGCAGGTTTGAAACACTCTTTTTGTAGTATCTGGAAGTGGATATTTGGATAGCTTTGAGGA

            * ** *      *             *** *   *                      *   *   
     245488 CTATGGT-GAAAAAGGAAATATCTTCTCCTGAAAACTAGACAGAAGCATTCTCAGAATCTTATTT
         66 TTTCGTTGGAAACAGG-AATATCTTCATATAAAAGCTAGACAGAAGCATTCTCAGAAACTTCTTT

                   **  **    *             **  * *   *
     245552 GTGATGTGCGCCCTCAACTAACAGTGTTGAAGCTTTCTTTTG
        130 GTGATGTTTGCATTCAAGTAACAGTGTTGAACATTCCCTTTC

                     *                *   **     *   *        *              
     245594 ATAGAGCAGTTTTGAAACACTCTTTTCGTAAAATCTGCAAGAGGATATTTTGATAGCTTTGAGGA
          1 ATAGAGCAGGTTTGAAACACTCTTTTTGTAGTATCTGGAAGTGGATATTTGGATAGCTTTGAGGA

                         *   * *          * **                      *    *   
     245659 TTTCGTTGGAAACGGGATTGTCTTCATATATACTCTAGACAGAAGCATTCTCAGAAGCTTCATTG
         66 TTTCGTTGGAAACAGGAATATCTTCATATAAAAGCTAGACAGAAGCATTCTCAGAAACTTCTTTG

            *       **   *    *             *        
     245724 GGATGTTTCAATTGAAGTCACAGTGTTGAACAGTCCCTTTC
        131 TGATGTTTGCATTCAAGTAACAGTGTTGAACATTCCCTTTC

                                                         *       *  * * *    
     245765 ATAGAGCAGGTTTGAAACACTCTTTTTGTAGTATCTGGAAGTGGACATTTGGAGAGATCTCAGGA
          1 ATAGAGCAGGTTTGAAACACTCTTTTTGTAGTATCTGGAAGTGGATATTTGGATAGCTTTGAGGA

            * *  *    * *             **            *       * *          *  *
     245830 ATACGGT-GATAAAGGAAATATCTTCCAATAAAAGCTAGATAGAAGCAATGTCAGAAACTTTTTC
         66 TTTCGTTGGAAACAGG-AATATCTTCATATAAAAGCTAGACAGAAGCATTCTCAGAAACTTCTTT

            *      * ** *   **      *       *  * *   *
     245894 ATGATGTATCTACTCAGCTAACAGAGTTGAACCTTTCTTTTG
        130 GTGATGTTTGCATTCAAGTAACAGTGTTGAACATTCCCTTTC

             *       *                   * *     *             **            
     245936 AGAGAGCAGTTTTGAAACACTCTTTTTGTGGAATCTGCAAGTGGATATTTGTCTAGCTTTGAGGA
          1 ATAGAGCAGGTTTGAAACACTCTTTTTGTAGTATCTGGAAGTGGATATTTGGATAGCTTTGAGGA

                         *   *    *                   *       *              
     246001 TTTCGTTGGAAACGGGATTA-CAT-ATA-AAAAGC-AGACAGCAGCATTCCCAGAAACTTCTTTG
         66 TTTCGTTGGAAACAGGAATATCTTCATATAAAAGCTAGACAGAAGCATTCTCAGAAACTTCTTTG

                              *    *                 
     246062 TGATGTTTGCATTCAAGTCACAGAGTTGAACATTCCCTTTC
        131 TGATGTTTGCATTCAAGTAACAGTGTTGAACATTCCCTTTC

                                                   *     *       **     *   *
     246103 ATAGAGCAGGTTTGAAACACTCTTTTTGTAGTATCTGGATGTGGACATTTGGAGCGCTTTCAGGC
          1 ATAGAGCAGGTTTGAAACACTCTTTTTGTAGTATCTGGAAGTGGATATTTGGATAGCTTTGAGGA

            * ** *      *             *** *   *                          *   
     246168 CTATGGT-GAAAAAGGAAATATCTTCCCCTGAAAACTAGACAGAAGCATTCTCAGAAACTTATTT
         66 TTTCGTTGGAAACAGG-AATATCTTCATATAAAAGCTAGACAGAAGCATTCTCAGAAACTTCTTT

                   **  **    *             **  * *   *
     246232 GTGATGTGCGCCCTCAACTAACAGTGTTGAAGCTTTCTTTTG
        130 GTGATGTTTGCATTCAAGTAACAGTGTTGAACATTCCCTTTC

                     *                    *      *   *                    *  
     246274 ATAGAGCAGTTTTGAAACACTCTTTTTGTAATATCTGCAAGAGGATATTTGGATAGCTTTGATGA
          1 ATAGAGCAGGTTTGAAACACTCTTTTTGTAGTATCTGGAAGTGGATATTTGGATAGCTTTGAGGA

                         *   * *            **                      *    *   
     246339 TTTCGTTGGAAACGGGATTGTCTTCATATAAACTCTAGACAGAAGCATTCTCAGAAGCTTCATTG
         66 TTTCGTTGGAAACAGGAATATCTTCATATAAAAGCTAGACAGAAGCATTCTCAGAAACTTCTTTG

            *    *  **   * *  *             * *      
     246404 GGATGCTTCAATTGATGTCACAGTGTTGAACAGTTCCTTTC
        131 TGATGTTTGCATTCAAGTAACAGTGTTGAACATTCCCTTTC

                 *                                       *       **   * **   
     246445 ATAGAACAGGTTTGAAACACTCTTTTTGTAGTATCTGGAAGTGGACATTTGGAGCGCTCTCTGGA
          1 ATAGAGCAGGTTTGAAACACTCTTTTTGTAGTATCTGGAAGTGGATATTTGGATAGCTTTGAGGA

            * ** *      *             **          * *       * *          *  *
     246510 CTATGGT-GAAAAAGGAAATATCTTCCAATAAAAGCTACATAGAAGCAATGTCAGAAACTTTTTC
         66 TTTCGTTGGAAACAGG-AATATCTTCATATAAAAGCTAGACAGAAGCATTCTCAGAAACTTCTTT

            *      * ** *   **      *       *  *     *
     246574 ATGATGTATCTACTCAGCTAACAGAGTTGAACCTTTCCTTTG
        130 GTGATGTTTGCATTCAAGTAACAGTGTTGAACATTCCCTTTC

             *       *                   * *     *             **            
     246616 AGAGAGCAGTTTTGAAACACTCTTTTTGTGGAATCTGCAAGTGGATATTTGTCTAGCTTTGAGGA
          1 ATAGAGCAGGTTTGAAACACTCTTTTTGTAGTATCTGGAAGTGGATATTTGGATAGCTTTGAGGA

                         *   *    *                   *       *   *          
     246681 TTTCGTTGGAAACGGGATTA-CAT-ATA-AAAAGC-AGACAGCAGCATTCCCAGTAACTTCTTTG
         66 TTTCGTTGGAAACAGGAATATCTTCATATAAAAGCTAGACAGAAGCATTCTCAGAAACTTCTTTG

                              *    *                 
     246742 TGATGTTTGCATTCAAGTCACAGAGTTGAACATTCCCTTTC
        131 TGATGTTTGCATTCAAGTAACAGTGTTGAACATTCCCTTTC

                                                   *     *       **     *   *
     246783 ATAGAGCAGGTTTGAAACACTCTTTTTGTAGTATCTGGATGTGGACATTTGGAGCGCTTTCAGGC
          1 ATAGAGCAGGTTTGAAACACTCTTTTTGTAGTATCTGGAAGTGGATATTTGGATAGCTTTGAGGA

            * ** *      *             *** *   *           *          *   *   
     246848 CTATGGT-GAAAAAGGAAATATCTTCCCCTGAAAACTAGACAGAAGAATTCTCAGAATCTTATTT
         66 TTTCGTTGGAAACAGG-AATATCTTCATATAAAAGCTAGACAGAAGCATTCTCAGAAACTTCTTT

                   **  **    *             **  * *   *
     246912 GTGATGTGCGCCCTCAACTAACAGTGTTGAAGCTTTCTTTTG
        130 GTGATGTTTGCATTCAAGTAACAGTGTTGAACATTCCCTTTC

                     *                   ***     *   *                       
     246954 ATAGAGCAGTTTTGAAACACTCTTTTTGTTAAATCTGCAAGAGGATATTTGGATAGCTTTGAGGA
          1 ATAGAGCAGGTTTGAAACACTCTTTTTGTAGTATCTGGAAGTGGATATTTGGATAGCTTTGAGGA

                         *   * *            **                      * *  *   
     247019 TTTCGTTGGAAACGGGATTGTCTTCATATAAACTCTAGACAGAAGCATTCTCAGAAGCGTCATTG
         66 TTTCGTTGGAAACAGGAATATCTTCATATAAAAGCTAGACAGAAGCATTCTCAGAAACTTCTTTG

            *       **   *    *             *        
     247084 GGATGTTTCAATTGAAGTCACAGTGTTGAACAGTCCCTTTC
        131 TGATGTTTGCATTCAAGTAACAGTGTTGAACATTCCCTTTC

                                                   *     *       **     *    
     247125 ATAGAGCAGGTTTGAAACACTCTTTTTGTAGTATCTGGATGTGGACATTTGGAGCGCTTTCAGGC
          1 ATAGAGCAGGTTTGAAACACTCTTTTTGTAGTATCTGGAAGTGGATATTTGGATAGCTTTGAGG-

            * * *    *                 *** *   *                          *  
     247190 CTATGGTT-TAAA-AGGAAATATCTTCCCCTGAAAACTAGACAGAAGCATTCTCAGAAACTTATT
         65 ATTTCGTTGGAAACAGG-AATATCTTCATATAAAAGCTAGACAGAAGCATTCTCAGAAACTTCTT

                    **  **    *                    *   *
     247253 TGTGATGTGCGCCCTCAACTAACAGTGTTGAAGCATT-CTTTTG
        129 TGTGATGTTTGCATTCAAGTAACAGTGTTGAA-CATTCCCTTTC

                     *                   * *     *             **            
     247296 ATAGAGCAGTTTTGAAACACTCTTTTTGTGGAATCTGCAAGTGGATATTTGTCTAGCTTTGAGGA
          1 ATAGAGCAGGTTTGAAACACTCTTTTTGTAGTATCTGGAAGTGGATATTTGGATAGCTTTGAGGA

                         *   *    *                   *       *              
     247361 TTTCGTTGGAAACGGGATTA-CAT-ATA-AAAAGC-AGACAGCAGCATTCCCAGAAACTTCTTTG
         66 TTTCGTTGGAAACAGGAATATCTTCATATAAAAGCTAGACAGAAGCATTCTCAGAAACTTCTTTG

                              *    *                 
     247422 TGATGTTTGCATTCAAGTCACAGAGTTGAACATTCCCTTTC
        131 TGATGTTTGCATTCAAGTAACAGTGTTGAACATTCCCTTTC

                                                   *     **      **     *   *
     247463 ATAGAGCAGGTTTGAAACACTCTTTTTGTAGTATCTGGATGTGGACTTTTGGAGCGCTTTCAGGC
          1 ATAGAGCAGGTTTGAAACACTCTTTTTGTAGTATCTGGAAGTGGATATTTGGATAGCTTTGAGGA

            * ** *      *             ***     *                      *   *   
     247528 CTATGGT-GAAAAAGGAAATATCTTCCCCTAAAAACTAGACAGAAGCATTCTCAGAATCTTATTT
         66 TTTCGTTGGAAACAGG-AATATCTTCATATAAAAGCTAGACAGAAGCATTCTCAGAAACTTCTTT

                   **  **    *             **  * *   *
     247592 GTGATGTGCGCCCTCAACTAACAGTGTTGAAGCTTTCTTTTG
        130 GTGATGTTTGCATTCAAGTAACAGTGTTGAACATTCCCTTTC

                     *                    **     *   *                     * 
     247634 ATAGAGCAGTTTTGAAACACTCTTTTTGTAAAATCTGCAAGAGGATATTTGGATAGCTTTGAGAA
          1 ATAGAGCAGGTTTGAAACACTCTTTTTGTAGTATCTGGAAGTGGATATTTGGATAGCTTTGAGGA

                         *   * *            **                      *    *   
     247699 TTTCGTTGGAAACGGGATTGTCTTCATATAAACTCTAGACAGAAGCATTCTCAGAAGCTTCATTG
         66 TTTCGTTGGAAACAGGAATATCTTCATATAAAAGCTAGACAGAAGCATTCTCAGAAACTTCTTTG

            *       **   *    *             *        
     247764 GGATGTTTCAATTGAAGTCACAGTGTTGAACAGTCCCTTTC
        131 TGATGTTTGCATTCAAGTAACAGTGTTGAACATTCCCTTTC

                                                         *       **   * *    
     247805 ATAGAGCAGGTTTGAAACACTCTTTTTGTAGTATCTGGAAGTGGACATTTGGAGCGCTCTCAGGA
          1 ATAGAGCAGGTTTGAAACACTCTTTTTGTAGTATCTGGAAGTGGATATTTGGATAGCTTTGAGGA

            * *  *      *             **            *       * *          *  *
     247870 CTGCGGT-GAAAAAGGAAATATCTTCCAATAAAAGCTAGATAGAAGCAATGTCAGAAACTTTTTC
         66 TTTCGTTGGAAACAGG-AATATCTTCATATAAAAGCTAGACAGAAGCATTCTCAGAAACTTCTTT

            *      * ** *   **      *       *        *
     247934 ATGATGTATCTACTCAGCTAACAGAGTTGAACCTT-CCTTTG
        130 GTGATGTTTGCATTCAAGTAACAGTGTTGAACATTCCCTTTC

             *       *            *      * *     *             **            
     247975 AGAGAGCAGTTTTGAAACACTCGTTTTGTGGAATCTGCAAGTGGATATTTGTCTAGCTTTGAGGA
          1 ATAGAGCAGGTTTGAAACACTCTTTTTGTAGTATCTGGAAGTGGATATTTGGATAGCTTTGAGGA

                         *   *     **                 *       *              
     248040 TTTCGTTGGAAACGGGATTA-C-AAATA-AAAAGC-AGACAGCAGCATTCCCAGAAACTTCTTTG
         66 TTTCGTTGGAAACAGGAATATCTTCATATAAAAGCTAGACAGAAGCATTCTCAGAAACTTCTTTG

                              *    *                 
     248101 TGATGTTTGCATTCAAGTCACAGAGTTGAACATTCCCTTTC
        131 TGATGTTTGCATTCAAGTAACAGTGTTGAACATTCCCTTTC

                                           *       *     *       **         *
     248142 ATAGAGCAGGTTTGAAACACTCTTTTTGTAGCATCTGGATGTGGACATTTGGAGCGCTTTGAGGC
          1 ATAGAGCAGGTTTGAAACACTCTTTTTGTAGTATCTGGAAGTGGATATTTGGATAGCTTTGAGGA

            * ** *      *             *** *   *                          *   
     248207 CTATGGT-GAAAAAGGAAATATCTTCCCCTGAAAACTAGACAGAAGCATTCTCAGAAACTTATTT
         66 TTTCGTTGGAAACAGG-AATATCTTCATATAAAAGCTAGACAGAAGCATTCTCAGAAACTTCTTT

                   **  **    *              *  * *   *
     248271 GTGATGTGCGCCCTCAACTAACAGTGTTGAACCTTTCTTTTG
        130 GTGATGTTTGCATTCAAGTAACAGTGTTGAACATTCCCTTTC

                     *                    *      *  **                       
     248313 ATAGAGCAGTTTTGAAACACTCTTTTTGTAATATCTGCAATAGGATATTTGGATAGCTTTGAGGA
          1 ATAGAGCAGGTTTGAAACACTCTTTTTGTAGTATCTGGAAGTGGATATTTGGATAGCTTTGAGGA

                         *   *    *                   *       *     *   **   
     248378 TTTCGTTGGAAACGGGATTA-CAT-ATA-AAAAGC-AGACAGCAGCATTCCCAGAATCTTGATTG
         66 TTTCGTTGGAAACAGGAATATCTTCATATAAAAGCTAGACAGAAGCATTCTCAGAAACTTCTTTG

                           *  *    *                 
     248439 TGATGTTTGCATTCATGTCACAGAGTTGAACATTCCCTTTC
        131 TGATGTTTGCATTCAAGTAACAGTGTTGAACATTCCCTTTC

             *                         *           *     *       **     *   *
     248480 AGAGAGCAGGTTTGAAACACTCTTTTTATAGTATCTGGATGTGGACATTTGGAGCGCTTTCAGGC
          1 ATAGAGCAGGTTTGAAACACTCTTTTTGTAGTATCTGGAAGTGGATATTTGGATAGCTTTGAGGA

            * ** *      *             *** *   *                      *   *   
     248545 CTATGGT-GAAAAAGGAAATATCTTCTCCTGAAAACTAGACAGAAGCATTCTCAGAATCTTATTT
         66 TTTCGTTGGAAACAGG-AATATCTTCATATAAAAGCTAGACAGAAGCATTCTCAGAAACTTCTTT

                   **  **    *             **  * *   *
     248609 GTGATGTGCGCCCTCAACTAACAGTGTTGAAGCTTTCTTTTG
        130 GTGATGTTTGCATTCAAGTAACAGTGTTGAACATTCCCTTTC

                     *                *   **     *   *        *              
     248651 ATAGAGCAGTTTTGAAACACTCTTTTCGTAAAATCTGCAAGAGGATATTTTGATAGCTTTGAGGA
          1 ATAGAGCAGGTTTGAAACACTCTTTTTGTAGTATCTGGAAGTGGATATTTGGATAGCTTTGAGGA

                         *   * *            **                      *    *   
     248716 TTTCGTTGGAAACGGGATTGTCTTCATATAAACTCTAGACAGAAGCATTCTCAGAAGCTTCATTG
         66 TTTCGTTGGAAACAGGAATATCTTCATATAAAAGCTAGACAGAAGCATTCTCAGAAACTTCTTTG

            *       **   *    *             *        
     248781 GGATGTTTCAATTGAAGTCACAGTGTTGAACAGTCCCTTTC
        131 TGATGTTTGCATTCAAGTAACAGTGTTGAACATTCCCTTTC

                                                         *       *  * * *    
     248822 ATAGAGCAGGTTTGAAACACTCTTTTTGTAGTATCTGGAAGTGGACATTTGGAGAGATCTCAGGA
          1 ATAGAGCAGGTTTGAAACACTCTTTTTGTAGTATCTGGAAGTGGATATTTGGATAGCTTTGAGGA

            * *  *    * *             **            *       * *          *  *
     248887 ATACGGT-GATAAAGGAAATATCTTCCAATAAAAGCTAGATAGAAGCAATGTCAGAAACTTTTTC
         66 TTTCGTTGGAAACAGG-AATATCTTCATATAAAAGCTAGACAGAAGCATTCTCAGAAACTTCTTT

            *      * ** *   **      *       *  * *   *
     248951 ATGATGTATCTACTCAGCTAACAGAGTTGAACCTTTCTTTTG
        130 GTGATGTTTGCATTCAAGTAACAGTGTTGAACATTCCCTTTC

             *       *                   * *     *             **            
     248993 AGAGAGCAGTTTTGAAACACTCTTTTTGTGGAATCTGCAAGTGGATATTTGTCTAGCTTTGAGGA
          1 ATAGAGCAGGTTTGAAACACTCTTTTTGTAGTATCTGGAAGTGGATATTTGGATAGCTTTGAGGA

                         *   *    *                   *       *              
     249058 TTTCGTTGGAAACGGGATTA-CAT-ATA-AAAAGC-AGACAGCAGCATTCCCAGAAACTTCTTTG
         66 TTTCGTTGGAAACAGGAATATCTTCATATAAAAGCTAGACAGAAGCATTCTCAGAAACTTCTTTG

                              *    *                 
     249119 TGATGTTTGCATTCAAGTCACAGAGTTGAACATTCCCTTTC
        131 TGATGTTTGCATTCAAGTAACAGTGTTGAACATTCCCTTTC

                                                   *     *       **     *   *
     249160 ATAGAGCAGGTTTGAAACACTCTTTTTGTAGTATCTGGATGTGGACATTTGGAGCGCTTTCAGGC
          1 ATAGAGCAGGTTTGAAACACTCTTTTTGTAGTATCTGGAAGTGGATATTTGGATAGCTTTGAGGA

            * ** *      *             *** *   *                          *   
     249225 CTATGGT-GAAAAAGGAAATATCTTCCCCTGAAAACTAGACAGAAGCATTCTCAGAAACTTATTT
         66 TTTCGTTGGAAACAGG-AATATCTTCATATAAAAGCTAGACAGAAGCATTCTCAGAAACTTCTTT

                   **  **    *             **  * *   *
     249289 GTGATGTGCGCCCTCAACTAACAGTGTTGAAGCTTTCTTTTG
        130 GTGATGTTTGCATTCAAGTAACAGTGTTGAACATTCCCTTTC

                     *                    *      *   *                      *
     249331 ATAGAGCAGTTTTGAAACACTCTTTTTGTAATATCTGCAAGAGGATATTTGGATAGCTTTGAGGT
          1 ATAGAGCAGGTTTGAAACACTCTTTTTGTAGTATCTGGAAGTGGATATTTGGATAGCTTTGAGGA

                         *   * *             *                      *    *   
     249396 TTTCGTTGGAAACGGGATTGTCTTCATATAAAATCTAGACAGAAGCATTCTCAGAAGCTTCATTG
         66 TTTCGTTGGAAACAGGAATATCTTCATATAAAAGCTAGACAGAAGCATTCTCAGAAACTTCTTTG

            *       **   *    *             * *      
     249461 GGATGTTTCAATTGAAGTCACAGTGTTGAACAGTTCCTTTC
        131 TGATGTTTGCATTCAAGTAACAGTGTTGAACATTCCCTTTC

                 *                                       *       **   * *  * 
     249502 ATAGAACAGGTTTGAAACACTCTTTTTGTAGTATCTGGAAGTGGACATTTGGAGCGCTCTCAGTA
          1 ATAGAGCAGGTTTGAAACACTCTTTTTGTAGTATCTGGAAGTGGATATTTGGATAGCTTTGAGGA

            * ** *      *             *** *   *                          *   
     249567 CTATGGT-GAAAAAGGAAATATCTTCCCCTGAAAACTAGACAGAAGCATTCTCAGAAACTTATTT
         66 TTTCGTTGGAAACAGG-AATATCTTCATATAAAAGCTAGACAGAAGCATTCTCAGAAACTTCTTT

                   **  **    *                    *   *
     249631 GTGATGTGCGCCCTCAACTAACAGTGTTGAAGCATT-CTTTTG
        130 GTGATGTTTGCATTCAAGTAACAGTGTTGAA-CATTCCCTTTC

                     *                   * *     *             **            
     249673 ATAGAGCAGTTTTGAAACACTCTTTTTGTGGAATCTGCAAGTGGATATTTGTCTAGCTTTGAGGA
          1 ATAGAGCAGGTTTGAAACACTCTTTTTGTAGTATCTGGAAGTGGATATTTGGATAGCTTTGAGGA

                         *   *    *                   *       *              
     249738 TTTCGTTGGAAACGGGATTA-CAT-ATA-AAAAGC-AGACAGCAGCATTCCCAGAAACTTCTTTG
         66 TTTCGTTGGAAACAGGAATATCTTCATATAAAAGCTAGACAGAAGCATTCTCAGAAACTTCTTTG

                 *            *    *                 
     249799 TGATGCTTGCATTCAAGTCACAGAGTTGAACATTCCCTTTC
        131 TGATGTTTGCATTCAAGTAACAGTGTTGAACATTCCCTTTC

                                                   *     *       **     *   *
     249840 ATAGAGCAGGTTTGAAACACTCTTTTTGTAGTATCTGGATGTGGACATTTGGAGCGCTTTCAGGC
          1 ATAGAGCAGGTTTGAAACACTCTTTTTGTAGTATCTGGAAGTGGATATTTGGATAGCTTTGAGGA

            * ** *      *             *** *   *                          *   
     249905 CTATGGT-GAAAAAGGAAATATCTTCCCCTGAAAACTAGACAGAAGCATTCTCAGAAACTTATTT
         66 TTTCGTTGGAAACAGG-AATATCTTCATATAAAAGCTAGACAGAAGCATTCTCAGAAACTTCTTT

                   **  **    *              *  * *   *
     249969 GTGATGTGCGCCCTCAACTAACAGTGTTGAACCTTTCTTTTG
        130 GTGATGTTTGCATTCAAGTAACAGTGTTGAACATTCCCTTTC

                     *                    *      *   *                       
     250011 ATAGAGCAGTTTTGAAACACTCTTTTTGTAATATCTGCAAGAGGATATTTGGATAGCTTTGAGGA
          1 ATAGAGCAGGTTTGAAACACTCTTTTTGTAGTATCTGGAAGTGGATATTTGGATAGCTTTGAGGA

                         *   * *            **                      *    *   
     250076 TTTCGTTGGAAACGGGATTGTCTTCATATAAACTCTAGACAGAAGCATTCTCAGAAGCTTCATTG
         66 TTTCGTTGGAAACAGGAATATCTTCATATAAAAGCTAGACAGAAGCATTCTCAGAAACTTCTTTG

            *       **   *    *             *        
     250141 GGATGTTTCAATTGAAGTCACAGTGTTGAACAGTCCCTTTC
        131 TGATGTTTGCATTCAAGTAACAGTGTTGAACATTCCCTTTC

                                                         *       *  * * *    
     250182 ATAGAGCAGGTTTGAAACACTCTTTTTGTAGTATCTGGAAGTGGACATTTGGAGAGATCTCAGGA
          1 ATAGAGCAGGTTTGAAACACTCTTTTTGTAGTATCTGGAAGTGGATATTTGGATAGCTTTGAGGA

            * *  *    * *             **            *       * *          *  *
     250247 ATACGGT-GATAAAGGAAATATCTTCCAATAAAAGCTAGATAGAAGCAATGTCAGAAACTTTTTC
         66 TTTCGTTGGAAACAGG-AATATCTTCATATAAAAGCTAGACAGAAGCATTCTCAGAAACTTCTTT

            *      * ** *   **      *       *  * *   *
     250311 ATGATGTATCTACTCAGCTAACAGAGTTGAACCTTTCTTTTG
        130 GTGATGTTTGCATTCAAGTAACAGTGTTGAACATTCCCTTTC

             *       *                   * *     *             **            
     250353 AGAGAGCAGTTTTGAAACACTCTTTTTGTGGAATCTGCAAGTGGATATTTGTCTAGCTTTGAGGA
          1 ATAGAGCAGGTTTGAAACACTCTTTTTGTAGTATCTGGAAGTGGATATTTGGATAGCTTTGAGGA

                         *   *    *                   *       *              
     250418 TTTCGTTGGAAACGGGATTA-CAT-ATA-AAAAGC-AGACAGCAGCATTCCCAGAAACTTCTTTG
         66 TTTCGTTGGAAACAGGAATATCTTCATATAAAAGCTAGACAGAAGCATTCTCAGAAACTTCTTTG

                              *    *                 
     250479 TGATGTTTGCATTCAAGTCACAGAGTTGAACATTCCCTTTC
        131 TGATGTTTGCATTCAAGTAACAGTGTTGAACATTCCCTTTC

                                                 * *     *       **     *   *
     250520 ATAGAGCAGGTTTGAAACACTCTTTTTGTAGTATCTGTATGTGGACATTTGGAGCGCTTTCAGGC
          1 ATAGAGCAGGTTTGAAACACTCTTTTTGTAGTATCTGGAAGTGGATATTTGGATAGCTTTGAGGA

              ** *      *             *** *   *                        * *   
     250585 TTATGGT-GAAAAAGGAAATATCTTCCCCTGAAAACTAGACAGAAGCATTCTCAGAAACCTATTT
         66 TTTCGTTGGAAACAGG-AATATCTTCATATAAAAGCTAGACAGAAGCATTCTCAGAAACTTCTTT

                   **  **    *          *   *  * *   *
     250649 GTGATGTGCGCCCTCAACTAACAGTGTTTAACCTTTCTTTTG
        130 GTGATGTTTGCATTCAAGTAACAGTGTTGAACATTCCCTTTC

                     *                    *      *   **                      
     250691 ATAGAGCAGTTTTGAAACACTCTTTTTGTAATATCTGCAAGAAGATATTTGGATAGCTTTGAGGA
          1 ATAGAGCAGGTTTGAAACACTCTTTTTGTAGTATCTGGAAGTGGATATTTGGATAGCTTTGAGGA

                         *   * *            **                      *    *   
     250756 TTTCGTTGGAAACGGGATTGTCTTCATATAAACTCTAGACAGAAGCATTCTCAGAAGCTTCATTG
         66 TTTCGTTGGAAACAGGAATATCTTCATATAAAAGCTAGACAGAAGCATTCTCAGAAACTTCTTTG

            *       **   *    *             * *      
     250821 GGATGTTTCAATTGAAGTCACAGTGTTGAACAGTTCCTTTC
        131 TGATGTTTGCATTCAAGTAACAGTGTTGAACATTCCCTTTC

                 *                                       *       **   * *    
     250862 ATAGAACAGGTTTGAAACACTCTTTTTGTAGTATCTGGAAGTGGACATTTGGAGCGCTCTCAGGA
          1 ATAGAGCAGGTTTGAAACACTCTTTTTGTAGTATCTGGAAGTGGATATTTGGATAGCTTTGAGGA

            * *  *      *             **          * *       * *       *  *  *
     250927 CTACGGT-GAAAAAGGAAATATCTTCCAATAAAAGCTACATAGAAGCAATGTCAGAAAGTTTTTC
         66 TTTCGTTGGAAACAGG-AATATCTTCATATAAAAGCTAGACAGAAGCATTCTCAGAAACTTCTTT

            *      * ** *   **      *       *  *   * *
     250991 ATGATGTATCTACTCAGCTAACAGAGTTGAACCTTTCCTATG
        130 GTGATGTTTGCATTCAAGTAACAGTGTTGAACATTCCCTTTC

             *       *                   * *     *             **            
     251033 AGAGAGCAGTTTTGAAACACTCTTTTTGTGGAATCTGCAAGTGGATATTTGTCTAGCTTTGAGGA
          1 ATAGAGCAGGTTTGAAACACTCTTTTTGTAGTATCTGGAAGTGGATATTTGGATAGCTTTGAGGA

                         *   *    *                   *       *   *          
     251098 TTTCGTTGGAAACGGGATTA-CAT-ATA-AAAAGC-AGACAGCAGCATTCCCAGTAACTTCTTTG
         66 TTTCGTTGGAAACAGGAATATCTTCATATAAAAGCTAGACAGAAGCATTCTCAGAAACTTCTTTG

             *                ***  *                 
     251159 TTATGTTTGCATTCAAGTCTGAGAGTTGAACATTCCCTTTC
        131 TGATGTTTGCATTCAAGTAACAGTGTTGAACATTCCCTTTC

                     *                  *          *     *       **     *   *
     251200 ATAGAGCAGTTTTGAAACACTCTTTTTGAAGTATCTGGATGTGGACATTTGGAGCGCTTTCAGGC
          1 ATAGAGCAGGTTTGAAACACTCTTTTTGTAGTATCTGGAAGTGGATATTTGGATAGCTTTGAGGA

            * ** *      *             *** *   *                          *   
     251265 CTATGGT-GAAAAAGGAAATATCTTCCCCTGAAAACTAGACAGAAGCATTCTCAGAAACTTATTT
         66 TTTCGTTGGAAACAGG-AATATCTTCATATAAAAGCTAGACAGAAGCATTCTCAGAAACTTCTTT

                   **  **    *              *  * *   *
     251329 GTGATGTGCGCCCTCAACTAACAGTGTTGAACCTTTCTTTTG
        130 GTGATGTTTGCATTCAAGTAACAGTGTTGAACATTCCCTTTC

                     *                    *   *  *   *                       
     251371 ATAGAGCAGTTTTGAAACACTCTTTTTGTAATATTTGCAAGAGGATATTTGGATAGCTTTGAGGA
          1 ATAGAGCAGGTTTGAAACACTCTTTTTGTAGTATCTGGAAGTGGATATTTGGATAGCTTTGAGGA

                         *   * *            **                      *    *   
     251436 TTTCGTTGGAAACGGGATTGTCTTCATATAAACTCTAGACAGAAGCATTCTCAGAAGCTTCATTG
         66 TTTCGTTGGAAACAGGAATATCTTCATATAAAAGCTAGACAGAAGCATTCTCAGAAACTTCTTTG

            *       **   *    *             *        
     251501 GGATGTTTCAATTGAAGTCACAGTGTTGAACAGTCCCTTTC
        131 TGATGTTTGCATTCAAGTAACAGTGTTGAACATTCCCTTTC

                                                         *       **   * *    
     251542 ATAGAGCAGGTTTGAAACACTCTTTTTGTAGTATCTGGAAGTGGACATTTGGAGCGCTCTCAGGA
          1 ATAGAGCAGGTTTGAAACACTCTTTTTGTAGTATCTGGAAGTGGATATTTGGATAGCTTTGAGGA

            * *  *    * *             **            *     * * *          *  *
     251607 CTACGGT-GATAAAGGAAATATCTTCCAATAAAAGCTAGATAGAAGGAATGTCAGAAACTTTTTC
         66 TTTCGTTGGAAACAGG-AATATCTTCATATAAAAGCTAGACAGAAGCATTCTCAGAAACTTCTTT

            * *    * ** *   **   *  *       *  * *   *
     251671 ATAATGTATCTACTCAGCTAAGAGAGTTGAACCTTTCTTTTG
        130 GTGATGTTTGCATTCAAGTAACAGTGTTGAACATTCCCTTTC

             *       *                   * *     *             **            
     251713 AGAGAGCAGTTTTGAAACACTCTTTTTGTGGAATCTGCAAGTGGATATTTGTCTAGCTTTGAGGA
          1 ATAGAGCAGGTTTGAAACACTCTTTTTGTAGTATCTGGAAGTGGATATTTGGATAGCTTTGAGGA

                         *   *    *                   *       *     *        
     251778 TTTCGTTGGAAACGGGATTA-CAT-ATA-AAAAGC-AGACAGCAGCATTCCCAGAATCTTCTTTG
         66 TTTCGTTGGAAACAGGAATATCTTCATATAAAAGCTAGACAGAAGCATTCTCAGAAACTTCTTTG

                              * *  *                 
     251839 TGATGTTTGCATTCAAGTCAGAGAGTTGAACATTCCCTTTC
        131 TGATGTTTGCATTCAAGTAACAGTGTTGAACATTCCCTTTC

            *                                      *     *       **     *   *
     251880 TTAGAGCAGGTTTGAAACACTCTTTTTGTAGTATCTGGATGTGGACATTTGGAGCGCTTTCAGGC
          1 ATAGAGCAGGTTTGAAACACTCTTTTTGTAGTATCTGGAAGTGGATATTTGGATAGCTTTGAGGA

            * ** *      *             *** *   *                      *   *   
     251945 CTATGGT-GAAAAAGGAAATATCTTCCCCTGAAAACTAGACAGAAGCATTCTCAGAATCTTATTT
         66 TTTCGTTGGAAACAGG-AATATCTTCATATAAAAGCTAGACAGAAGCATTCTCAGAAACTTCTTT

                   **  **    *             **  * *   *
     252009 GTGATGTGCGCCCTCAACTAACAGTGTTGAAGCTTTCTTTTG
        130 GTGATGTTTGCATTCAAGTAACAGTGTTGAACATTCCCTTTC

                     *                    **     *   *                       
     252051 ATAGAGCAGTTTTGAAACACTCTTTTTGTAAAATCTGCAAGAGGATATTTGGATAGCTTTGAGGA
          1 ATAGAGCAGGTTTGAAACACTCTTTTTGTAGTATCTGGAAGTGGATATTTGGATAGCTTTGAGGA

                         *   * *            **                      *    *   
     252116 TTTCGTTGGAAACGGGATTGTCTTCATATAAACTCTAGACAGAAGCATTCTCAGAAGCTTCATTG
         66 TTTCGTTGGAAACAGGAATATCTTCATATAAAAGCTAGACAGAAGCATTCTCAGAAACTTCTTTG

            *       **   *    *             *        
     252181 GGATGTTTCAATTGAAGTCACAGTGTTGAACAATCCCTTTC
        131 TGATGTTTGCATTCAAGTAACAGTGTTGAACATTCCCTTTC

                  *                    *                 *       **   * *    
     252222 ATAGAGTAGGTTTGAAACACTCTTTTTTTAGTATCTGGAAGTGGACATTTGGAGCGCTCTCAGGA
          1 ATAGAGCAGGTTTGAAACACTCTTTTTGTAGTATCTGGAAGTGGATATTTGGATAGCTTTGAGGA

            * *  *      *             **            *       * *              
     252287 CTGCGGT-GAAAAAGGAAATATCTTCCAATAAAAGCTAGATAGAAGCAATGTCAGAAACTT-TTT
         66 TTTCGTTGGAAACAGG-AATATCTTCATATAAAAGCTAGACAGAAGCATTCTCAGAAACTTCTTT

                    * ** *   **      *       **   *   *
     252350 GATGATGTATCTACTCAGCTAACAGAGTTGAACCCT-CATTTG
        130 G-TGATGTTTGCATTCAAGTAACAGTGTTGAACATTCCCTTTC

             *       *            *      * *     *             **            
     252392 AGAGAGCAGTTTTGAAACACTCGTTTTGTGGAATCTGCAAGTGGATATTTGTCTAGCTTTGAGGA
          1 ATAGAGCAGGTTTGAAACACTCTTTTTGTAGTATCTGGAAGTGGATATTTGGATAGCTTTGAGGA

                         **  *    *                  **       *              
     252457 TTTCGTTGGAAACGTGATTA-CAT-ATA-AAAAGC-AGACAACAGCATTCCCAGAAACTTCTTTG
         66 TTTCGTTGGAAACAGGAATATCTTCATATAAAAGCTAGACAGAAGCATTCTCAGAAACTTCTTTG

                              *    *                 
     252518 TGATGTTTGCATTCAAGTCACAGAGTTGAACATTCCCTTTC
        131 TGATGTTTGCATTCAAGTAACAGTGTTGAACATTCCCTTTC

                                                   *     *     * **     *   *
     252559 ATAGAGCAGGTTTGAAACACTCTTTTTGTAGTATCTGGATGTGGACATTTGCAGCGCTTTCAGGC
          1 ATAGAGCAGGTTTGAAACACTCTTTTTGTAGTATCTGGAAGTGGATATTTGGATAGCTTTGAGGA

            * ** *      *             *** *   *                          *   
     252624 CTAAGGT-GAAAAAGGAAATATCTTCCCCTGAAAACTAGACAGAAGCATTCTCAGAAACTTTTTT
         66 TTTCGTTGGAAACAGG-AATATCTTCATATAAAAGCTAGACAGAAGCATTCTCAGAAACTTCTTT

                   **  **    *             **  * *   *
     252688 GTGATGTGCGCCCTCAACTAACAGTGTTGAAGCTTTCTTTTG
        130 GTGATGTTTGCATTCAAGTAACAGTGTTGAACATTCCCTTTC

                     *                    *    * *   *                       
     252730 ATAGAGCAGTTTTGAAACACTCTTTTTGTAATATCCGCAAGAGGATATTTGGATAGCTTTGAGGA
          1 ATAGAGCAGGTTTGAAACACTCTTTTTGTAGTATCTGGAAGTGGATATTTGGATAGCTTTGAGGA

                         *   * *          * **                      *    *   
     252795 TTTCGTTGGAAACGGGATTGTCTTCATATATACTCTAGACAGAAGCATTCTCAGAAGCTTCATTG
         66 TTTCGTTGGAAACAGGAATATCTTCATATAAAAGCTAGACAGAAGCATTCTCAGAAACTTCTTTG

            *       **   *    *             *        
     252860 GGATGTTTCAATTGAAGTCACAGTGTTGAACAGTCCCTTTC
        131 TGATGTTTGCATTCAAGTAACAGTGTTGAACATTCCCTTTC

                                                         *       *  * * *    
     252901 ATAGAGCAGGTTTGAAACACTCTTTTTGTAGTATCTGGAAGTGGACATTTGGAGAGATCTCAGGA
          1 ATAGAGCAGGTTTGAAACACTCTTTTTGTAGTATCTGGAAGTGGATATTTGGATAGCTTTGAGGA

            * *  *    * *             **            *       * *          *  *
     252966 ATACGGT-GATAAAGGAAATATCTTCCAATAAAAGCTAGATAGAAGCAATGTCAGAAACTTTTTC
         66 TTTCGTTGGAAACAGG-AATATCTTCATATAAAAGCTAGACAGAAGCATTCTCAGAAACTTCTTT

            *                 **      *       *  * *   *
     253030 ATGATGTATCTGC--TCAGCTAACAGAGTTGAACCTTTCTTTTG
        130 GTGATGT-T-TGCATTCAAGTAACAGTGTTGAACATTCCCTTTC

             *       *         *         * *     *             **            
     253072 AGAGAGCAGTTTTGAAACAATCTTTTTGTGGAATCTGCAAGTGGATATTTGTCTAGCTTTGAGGA
          1 ATAGAGCAGGTTTGAAACACTCTTTTTGTAGTATCTGGAAGTGGATATTTGGATAGCTTTGAGGA

                         *   *    *                   *       *              
     253137 TTTCGTTGGAAACGGGATTA-CAT-ATA-AAAAGC-AGACAGCAGCATTCCCAGAAACTTCTTTG
         66 TTTCGTTGGAAACAGGAATATCTTCATATAAAAGCTAGACAGAAGCATTCTCAGAAACTTCTTTG

                              *    *                 
     253198 TGATGTTTGCATTCAAGTCACAGAGTTGAACATTCCCTTTC
        131 TGATGTTTGCATTCAAGTAACAGTGTTGAACATTCCCTTTC

                                                   *     *       **     *   *
     253239 ATAGAGCAGGTTTGAAACACTCTTTTTGTAGTATCTGGATGTGGACATTTGGAGCGCTTTCAGGC
          1 ATAGAGCAGGTTTGAAACACTCTTTTTGTAGTATCTGGAAGTGGATATTTGGATAGCTTTGAGGA

            * ** *      *             *** *   *                          *   
     253304 CTATGGT-GAAAAAGGAAATATCTTCCCCTGAAAACTAGACAGAAGCATTCTCAGAAACTTATTT
         66 TTTCGTTGGAAACAGG-AATATCTTCATATAAAAGCTAGACAGAAGCATTCTCAGAAACTTCTTT

                   **  **    *             **  * *   *
     253368 GTGATGTGCGCCCTCAACTAACAGTGTTGAAGCTTTCTTTTG
        130 GTGATGTTTGCATTCAAGTAACAGTGTTGAACATTCCCTTTC

               *     *                    *      *   *                       
     253410 ATACAGCAGTTTTGAAACACTCTTTTTGTAATATCTGCAAGAGGATATTTGGATAGCTTTGAGGA
          1 ATAGAGCAGGTTTGAAACACTCTTTTTGTAGTATCTGGAAGTGGATATTTGGATAGCTTTGAGGA

                         *   * *            **                      *    *   
     253475 TTTCGTTGGAAACGGGATTGTCTTCATATAAACTCTAGACAGAAGCATTCTCAGAAGCTTCATTG
         66 TTTCGTTGGAAACAGGAATATCTTCATATAAAAGCTAGACAGAAGCATTCTCAGAAACTTCTTTG

            *       **   *    *             * *      
     253540 GGATGTTTCAATTGAAGTCACAGTGTTGAACAGTTCCTTTC
        131 TGATGTTTGCATTCAAGTAACAGTGTTGAACATTCCCTTTC

                 *                                       *       **   * *    
     253581 ATAGAACAGGTTTGAAACACTCTTTTTGTAGTATCTGGAAGTGGACATTTGGAGCGCTCTCAGGA
          1 ATAGAGCAGGTTTGAAACACTCTTTTTGTAGTATCTGGAAGTGGATATTTGGATAGCTTTGAGGA

            * ** *      *             **  *       * *       * *          *  *
     253646 CTATGGT-GAAAAAGGAAATATCTTCCAATGAAAGCTACATAGAAGCAATGTCAGAAACTTTTTC
         66 TTTCGTTGGAAACAGG-AATATCTTCATATAAAAGCTAGACAGAAGCATTCTCAGAAACTTCTTT

            *      * ** *   **      *       *  *     *
     253710 ATGATGTATCTACTCAGCTAACAGAGTTGAACCTTTCCTTTG
        130 GTGATGTTTGCATTCAAGTAACAGTGTTGAACATTCCCTTTC

             *       *                   * *     *             **            
     253752 AGAGAGCAGTTTTGAAACACTCTTTTTGTGGCATCTGCAAGTGGATATTTGTCTAGCTTTGAGGA
          1 ATAGAGCAGGTTTGAAACACTCTTTTTGTAGTATCTGGAAGTGGATATTTGGATAGCTTTGAGGA

                         *   *    *                   *       *   *          
     253817 TTTCGTTGGAAACGGGATTA-CAT-ATA-AAAAGC-AGACAGCAGCATTCCCAGTAACTTCTTTG
         66 TTTCGTTGGAAACAGGAATATCTTCATATAAAAGCTAGACAGAAGCATTCTCAGAAACTTCTTTG

                              *    *                 
     253878 TGATGTTTGCATTCAAGTCACAGAGTTGAACATTCCCTTTC
        131 TGATGTTTGCATTCAAGTAACAGTGTTGAACATTCCCTTTC

                                                   *     *       **     *   *
     253919 ATAGAGCAGGTTTGAAACACTCTTTTTGTAGTATCTGGATGTGGACATTTGGAGCGCTTTCAGGC
          1 ATAGAGCAGGTTTGAAACACTCTTTTTGTAGTATCTGGAAGTGGATATTTGGATAGCTTTGAGGA

            * ** *      *             *** *   *           *          *   *   
     253984 CTATGGT-GAAAAAGGAAATATCTTCCCCTGAAAACTAGACAGAAGAATTCTCAGAATCTTATTT
         66 TTTCGTTGGAAACAGG-AATATCTTCATATAAAAGCTAGACAGAAGCATTCTCAGAAACTTCTTT

                   **  **    *             **  * *   *
     254048 GTGATGTGCGCCCTCAACTAACAGTGTTGAAGCTTTCTTTTG
        130 GTGATGTTTGCATTCAAGTAACAGTGTTGAACATTCCCTTTC

                     *                   ***     *   *                       
     254090 ATAGAGCAGTTTTGAAACACTCTTTTTGTTAAATCTGCAAGAGGATATTTGGATAGCTTTGAGGA
          1 ATAGAGCAGGTTTGAAACACTCTTTTTGTAGTATCTGGAAGTGGATATTTGGATAGCTTTGAGGA

                         *   * *            **                      * *  *   
     254155 TTTCGTTGGAAACGGGATTGTCTTCATATAAACTCTAGACAGAAGCATTCTCAGAAGCGTCATTG
         66 TTTCGTTGGAAACAGGAATATCTTCATATAAAAGCTAGACAGAAGCATTCTCAGAAACTTCTTTG

            *       **   *    *             *        
     254220 GGATGTTTCAATTGAAGTCACAGTGTTGAACAGTCCCTTTC
        131 TGATGTTTGCATTCAAGTAACAGTGTTGAACATTCCCTTTC

                                                   *     *       **     *    
     254261 ATAGAGCAGGTTTGAAACACTCTTTTTGTAGTATCTGGATGTGGACATTTGGAGCGCTTTCAGGC
          1 ATAGAGCAGGTTTGAAACACTCTTTTTGTAGTATCTGGAAGTGGATATTTGGATAGCTTTGAGG-

            * * *    *                 *** *   *                          *  
     254326 CTATGGTT-TAAA-AGGATATATCTTCCCCTGAAAACTAGACAGAAGCATTCTCAGAAACTTATT
         65 ATTTCGTTGGAAACAGGA-ATATCTTCATATAAAAGCTAGACAGAAGCATTCTCAGAAACTTCTT

                    **  **    *                    *   *
     254389 TGTGATGTGCGCCCTCAACTAACAGTGTTGAAGCATT-CTTTTG
        129 TGTGATGTTTGCATTCAAGTAACAGTGTTGAA-CATTCCCTTTC

                     *                   * *     *             **            
     254432 ATAGAGCAGTTTTGAAACACTCTTTTTGTGGAATCTGCAAGTGGATATTTGTCTAGCTTTGAGGA
          1 ATAGAGCAGGTTTGAAACACTCTTTTTGTAGTATCTGGAAGTGGATATTTGGATAGCTTTGAGGA

                         *   *    *                   *       *   *          
     254497 TTTCGTTGGAAACGGGATTA-CAT-ATA-AAAAGC-AGACAGCAGCATTCCCAGGAACTTCTTTG
         66 TTTCGTTGGAAACAGGAATATCTTCATATAAAAGCTAGACAGAAGCATTCTCAGAAACTTCTTTG

                              *    *                 
     254558 TGATGTTTGCATTCAAGTCACAGAGTTGAACATTCCCTTTC
        131 TGATGTTTGCATTCAAGTAACAGTGTTGAACATTCCCTTTC

                                                   *     **      **     *   *
     254599 ATAGAGCAGGTTTGAAACACTCTTTTTGTAGTATCTGGATGTGGACCTTTGGAGCGCTTTCAGGC
          1 ATAGAGCAGGTTTGAAACACTCTTTTTGTAGTATCTGGAAGTGGATATTTGGATAGCTTTGAGGA

            * ** *      *             *** *   *                      *   *   
     254664 CTATGGT-GAAAAAGGAAATATCTTCCCCTGAAAACTAGACAGAAGCATTCTCAGAATCTTATTT
         66 TTTCGTTGGAAACAGG-AATATCTTCATATAAAAGCTAGACAGAAGCATTCTCAGAAACTTCTTT

                   **  **    *             **  * *   *
     254728 GTGATGTGCGCCCTCAACTAACAGTGTTGAAGCTTTCTTTTG
        130 GTGATGTTTGCATTCAAGTAACAGTGTTGAACATTCCCTTTC

                     *                    **     *   *                     * 
     254770 ATAGAGCAGTTTTGAAACACTCTTTTTGTAAAATCTGCAAGAGGATATTTGGATAGCTTTGAGCA
          1 ATAGAGCAGGTTTGAAACACTCTTTTTGTAGTATCTGGAAGTGGATATTTGGATAGCTTTGAGGA

                         *   * *            **                      *    *   
     254835 TTTCGTTGGAAACGGGATTGTCTTCATATAAACTCTAGACAGAAGCATTCTCAGAAGCTTCATTG
         66 TTTCGTTGGAAACAGGAATATCTTCATATAAAAGCTAGACAGAAGCATTCTCAGAAACTTCTTTG

            *       **   *    *             *        
     254900 GGATGTTTCAATTGAAGTCACAGTGTTGAACAGTCCCTTTC
        131 TGATGTTTGCATTCAAGTAACAGTGTTGAACATTCCCTTTC

                                                         *       **   * *    
     254941 ATAGAGCAGGTTTGAAACACTCTTTTTGTAGTATCTGGAAGTGGACATTTGGAGCGCTCTCAGGA
          1 ATAGAGCAGGTTTGAAACACTCTTTTTGTAGTATCTGGAAGTGGATATTTGGATAGCTTTGAGGA

            * *  *      *             **          * *       * *          *  *
     255006 CTACGGT-GAAAAAGGAAATATCTTCCAATAAAAGCTACATAGAAGCAATGTCAGAAACTTTTTC
         66 TTTCGTTGGAAACAGG-AATATCTTCATATAAAAGCTAGACAGAAGCATTCTCAGAAACTTCTTT

            *      * ** *   **      *       *        *
     255070 ATGATGTATCTACTCAGCTAACAGAGTTGAACCTT-CCTTTG
        130 GTGATGTTTGCATTCAAGTAACAGTGTTGAACATTCCCTTTC

             *       *            *      ***     *             **            
     255111 AGAGAGCAGTTTTGAAACACTCGTTTTGTGCAATCTGCAAGTGGATATTTGTCTAGCTTTGAGGA
          1 ATAGAGCAGGTTTGAAACACTCTTTTTGTAGTATCTGGAAGTGGATATTTGGATAGCTTTGAGGA

                         *   *    *                   *       *              
     255176 TTTCGTTGGAAACGGGATTA-CAT-ATA-AAAAGC-AGACAGCAGCATTCCCAGAAACTTCTTTG
         66 TTTCGTTGGAAACAGGAATATCTTCATATAAAAGCTAGACAGAAGCATTCTCAGAAACTTCTTTG

                              *    *                 
     255237 TGATGTTTGCATTCAAGTCACAGAGTTGAACATTCCCTTTC
        131 TGATGTTTGCATTCAAGTAACAGTGTTGAACATTCCCTTTC

                                                   *     *     * **     *   *
     255278 ATAGAGCAGGTTTGAAACACTCTTTTTGTAGTATCTGGATGTGGACATTTGCAGCGCTTTCAGGC
          1 ATAGAGCAGGTTTGAAACACTCTTTTTGTAGTATCTGGAAGTGGATATTTGGATAGCTTTGAGGA

            * ** *      *             *** *   *                          *   
     255343 CTATGGT-GAAAAAGGAAATATCTTCCCCTGAAAACTAGACAGAAGCATTCTCAGAAACTTATTT
         66 TTTCGTTGGAAACAGG-AATATCTTCATATAAAAGCTAGACAGAAGCATTCTCAGAAACTTCTTT

                   **  **    *             **  * *   *
     255407 GTGATGTGCGCCCTCAACTAACAGTGTTGAAGCTTTCTTTTG
        130 GTGATGTTTGCATTCAAGTAACAGTGTTGAACATTCCCTTTC

                     *                    *      *   *                       
     255449 ATAGAGCAGTTTTGAAACACTCTTTTTGTAATATCTGCAAGAGGATATTTGGATAGCTTTGAGGA
          1 ATAGAGCAGGTTTGAAACACTCTTTTTGTAGTATCTGGAAGTGGATATTTGGATAGCTTTGAGGA

                         *   * *    *       **                      *    *   
     255514 TTTCGTTGGAAACGGGATTGTCTTAATATAAACTCTAGACAGAAGCATTCTCAGAAGCTTCATTG
         66 TTTCGTTGGAAACAGGAATATCTTCATATAAAAGCTAGACAGAAGCATTCTCAGAAACTTCTTTG

            *       **   *    *             * *      
     255579 GGATGTTTCAATTGAAGTCACAGTGTTGAACAGTTCCTTTC
        131 TGATGTTTGCATTCAAGTAACAGTGTTGAACATTCCCTTTC

                 *                                       *       **   * *    
     255620 ATAGAACAGGTTTGAAACACTCTTTTTGTAGTATCTGGAAGTGGACATTTGGAGCGCTCTCAGGA
          1 ATAGAGCAGGTTTGAAACACTCTTTTTGTAGTATCTGGAAGTGGATATTTGGATAGCTTTGAGGA

            * ** *      *             **  *       * *       * *          *  *
     255685 CTATGAT-GAAAAAGGAAATATCTTCCAATGAAAGCTACATAGAAGCAATGTCAGAAACTTTTTC
         66 TTTCGTTGGAAACAGG-AATATCTTCATATAAAAGCTAGACAGAAGCATTCTCAGAAACTTCTTT

            *      * ** *   **      *       *  *     *
     255749 ATGATGTATCTACTCAGCTAACAGAGTTGAACCTTTCCTTTG
        130 GTGATGTTTGCATTCAAGTAACAGTGTTGAACATTCCCTTTC

             *       *                   * *     *             **            
     255791 AGAGAGCAGTTTTGAAACACTCTTTTTGTGGAATCTGCAAGTGGATATTTGTCTAGCTTTGAGGA
          1 ATAGAGCAGGTTTGAAACACTCTTTTTGTAGTATCTGGAAGTGGATATTTGGATAGCTTTGAGGA

                         *   *    *                   *       *   *          
     255856 TTTCGTTGGAAACGGGATTA-CAT-ATA-AAAAGC-AGACAGCAGCATTCCCAGTAACTTCTTTG
         66 TTTCGTTGGAAACAGGAATATCTTCATATAAAAGCTAGACAGAAGCATTCTCAGAAACTTCTTTG

                              *    *                 
     255917 TGATGTTTGCATTCAAGTCACAGAGTTGAACATTCCCTTTC
        131 TGATGTTTGCATTCAAGTAACAGTGTTGAACATTCCCTTTC

                                                   *     *       **     *   *
     255958 ATAGAGCAGGTTTGAAACACTCTTTTTGTAGTATCTGGATGTGGACATTTGGAGCGCTTTCAGGC
          1 ATAGAGCAGGTTTGAAACACTCTTTTTGTAGTATCTGGAAGTGGATATTTGGATAGCTTTGAGGA

            * ** *      *             *** *   *           *          *   *   
     256023 CTATGGT-GAAAAAGGAAATATCTTCCCCTGAAAACTAGACAGAAGAATTCTCAGAATCTTATTT
         66 TTTCGTTGGAAACAGG-AATATCTTCATATAAAAGCTAGACAGAAGCATTCTCAGAAACTTCTTT

                   **  **    *             **  * *   *
     256087 GTGATGTGCGCCCTCAACTAACAGTGTTGAAGCTTTCTTTTG
        130 GTGATGTTTGCATTCAAGTAACAGTGTTGAACATTCCCTTTC

                     *                   ***     *   *                       
     256129 ATAGAGCAGTTTTGAAACACTCTTTTTGTTAAATCTGCAAGAGGATATTTGGATAGCTTTGAGGA
          1 ATAGAGCAGGTTTGAAACACTCTTTTTGTAGTATCTGGAAGTGGATATTTGGATAGCTTTGAGGA

                         *   * *            **                      * *  *   
     256194 TTTCGTTGGAAACGGGATTGTCTTCATATAAACTCTAGACAGAAGCATTCTCAGAAGCGTCATTG
         66 TTTCGTTGGAAACAGGAATATCTTCATATAAAAGCTAGACAGAAGCATTCTCAGAAACTTCTTTG

            *       **   *    *             *        
     256259 GGATGTTTCAATTGAAGTCACAGTGTTGAACAGTCCCTTTC
        131 TGATGTTTGCATTCAAGTAACAGTGTTGAACATTCCCTTTC

                                                   *     *       **     *    
     256300 ATAGAGCAGGTTTGAAACACTCTTTTTGTAGTATCTGGATGTGGACATTTGGAGCGCTTTCAGGC
          1 ATAGAGCAGGTTTGAAACACTCTTTTTGTAGTATCTGGAAGTGGATATTTGGATAGCTTTGAGG-

            * * *    *                 *** *   *                          *  
     256365 CTATGGTT-TAAA-AGGAAATATCTTCCCCTGAAAACTAGACAGAAGCATTCTCAGAAACTTATT
         65 ATTTCGTTGGAAACAGG-AATATCTTCATATAAAAGCTAGACAGAAGCATTCTCAGAAACTTCTT

                    **  **    *          *         *   *
     256428 TGTGATGTGCGCCCTCAACTAACAGTGTTAAAGCATT-CTTTTG
        129 TGTGATGTTTGCATTCAAGTAACAGTGTTGAA-CATTCCCTTTC

                     *                   * *     *             **            
     256471 ATAGAGCAGTTTTGAAACACTCTTTTTGTGGAATCTGCAAGTGGATATTTGTCTAGCTTTGAGGA
          1 ATAGAGCAGGTTTGAAACACTCTTTTTGTAGTATCTGGAAGTGGATATTTGGATAGCTTTGAGGA

                         *   *    *                   *       *              
     256536 TTTCGTTGGAAACGGGATTA-CAT-ATA-AAAAGC-AGACAGCAGCATTCCCAGAAACTTCTTTG
         66 TTTCGTTGGAAACAGGAATATCTTCATATAAAAGCTAGACAGAAGCATTCTCAGAAACTTCTTTG

                              *    *                 
     256597 TGATGTTTGCATTCAAGTCACAGAGTTGAACATTCCCTTTC
        131 TGATGTTTGCATTCAAGTAACAGTGTTGAACATTCCCTTTC

                                                   *     **      **     *   *
     256638 ATAGAGCAGGTTTGAAACACTCTTTTTGTAGTATCTGGATGTGGACCTTTGGAGCGCTTTCAGGC
          1 ATAGAGCAGGTTTGAAACACTCTTTTTGTAGTATCTGGAAGTGGATATTTGGATAGCTTTGAGGA

            * ** *      *             *** *   *                      *   *   
     256703 CTATGGT-GAAAAAGGAAATATCTTCCCCTGAAAACTAGACAGAAGCATTCTCAGAATCTTATTT
         66 TTTCGTTGGAAACAGG-AATATCTTCATATAAAAGCTAGACAGAAGCATTCTCAGAAACTTCTTT

                   **  **    *             **  * *   *
     256767 GTGATGTGCGCCCTCAACTAACAGTGTTGAAGCTTTCTTTTG
        130 GTGATGTTTGCATTCAAGTAACAGTGTTGAACATTCCCTTTC

                     *                    **     *   *                     * 
     256809 ATAGAGCAGTTTTGAAACACTCTTTTTGTAAAATCTGCAAGAGGATATTTGGATAGCTTTGAGAA
          1 ATAGAGCAGGTTTGAAACACTCTTTTTGTAGTATCTGGAAGTGGATATTTGGATAGCTTTGAGGA

                         *   * *            **                      *    *   
     256874 TTTCGTTGGAAACGGGATTGTCTTCATATAAACTCTAGACAGAAGCATTCTCAGAAGCTTCATTG
         66 TTTCGTTGGAAACAGGAATATCTTCATATAAAAGCTAGACAGAAGCATTCTCAGAAACTTCTTTG

            *       **   *    *             *        
     256939 GGATGTTTCAATTGAAGTCACAGTGTTGAACAGT-CCTTTC
        131 TGATGTTTGCATTCAAGTAACAGTGTTGAACATTCCCTTTC

                                                         *       **   * *    
     256979 ATAGAGCAGGTTTGAAACACTCTTTTTGTAGTATCTGGAAGTGGACATTTGGAGCGCTCTCAGGA
          1 ATAGAGCAGGTTTGAAACACTCTTTTTGTAGTATCTGGAAGTGGATATTTGGATAGCTTTGAGGA

            * *  *      *             **          * *       * *          *  *
     257044 CTGCGGT-GAAAAAGGAAATATCTTCCAATAAAAGCTACATAGAAGCAATGTCAGAAACTTTTTC
         66 TTTCGTTGGAAACAGG-AATATCTTCATATAAAAGCTAGACAGAAGCATTCTCAGAAACTTCTTT

            *      * ** *   **      *       *        *
     257108 ATGATGTATCTACTCAGCTAACAGGGTTGAACCTT-CCTTTG
        130 GTGATGTTTGCATTCAAGTAACAGTGTTGAACATTCCCTTTC

             *       *            *      * *     *             **            
     257149 AGAGAGCAGTTTTGAAACACTCGTTTTGTGGAATCTGCAAGTGGATATTTGTCTAGCTTTGAGGA
          1 ATAGAGCAGGTTTGAAACACTCTTTTTGTAGTATCTGGAAGTGGATATTTGGATAGCTTTGAGGA

                         *   *    *                   *       *              
     257214 TTTCGTTGGAAACGGGATTA-CAT-ATA-AAAAGC-AGACAGCAGCATTCCCAGAAACTTCTTTG
         66 TTTCGTTGGAAACAGGAATATCTTCATATAAAAGCTAGACAGAAGCATTCTCAGAAACTTCTTTG

                              *    *                 
     257275 TGATGTTTGCATTCAAGTCACAGAGTTGAACATTCCCTTTC
        131 TGATGTTTGCATTCAAGTAACAGTGTTGAACATTCCCTTTC

                                                   *     *     * **     *   *
     257316 ATAGAGCAGGTTTGAAACACTCTTTTTGTAGTATCTGGATGTGGACATTTGCAGCGCTTTCAGGC
          1 ATAGAGCAGGTTTGAAACACTCTTTTTGTAGTATCTGGAAGTGGATATTTGGATAGCTTTGAGGA

            * ** *      *             *** *   *                          *   
     257381 CTATGGT-GAAAAAGGAAATATCTTCCCCTGAAAACTAGACAGAAGCATTCTCAGAAACTTATTT
         66 TTTCGTTGGAAACAGG-AATATCTTCATATAAAAGCTAGACAGAAGCATTCTCAGAAACTTCTTT

                   **  **    *       *     **  * *   *
     257445 GTGATGTGCGCCCTCAACTAACAGTTTTGAAGCTTTCTTTTG
        130 GTGATGTTTGCATTCAAGTAACAGTGTTGAACATTCCCTTTC

                     *                   * *     *             **            
     257487 ATAGAGCAGTTTTGAAACACTCTTTTTGTGGAATCTGCAAGTGGATATTTGTCTAGCTTTGAGGA
          1 ATAGAGCAGGTTTGAAACACTCTTTTTGTAGTATCTGGAAGTGGATATTTGGATAGCTTTGAGGA

                *        *   *    *                   *       *              
     257552 TTTCATTGGAAACGGGATTA-CAT-ATA-AAAAGC-AGACAGCAGCATTCCCAGAAACTTCTTTG
         66 TTTCGTTGGAAACAGGAATATCTTCATATAAAAGCTAGACAGAAGCATTCTCAGAAACTTCTTTG

                   *          *   **                 
     257613 TGATGTTCGCATTCAAGTCACACAGTTGAACATTCCCTTTC
        131 TGATGTTTGCATTCAAGTAACAGTGTTGAACATTCCCTTTC

                                                   *     *       **     *   *
     257654 ATAGAGCAGGTTTGAAACACTCTTTTTGTAGTATCTGGATGTGGACATTTGGAGCGCTTTCAGGC
          1 ATAGAGCAGGTTTGAAACACTCTTTTTGTAGTATCTGGAAGTGGATATTTGGATAGCTTTGAGGA

            * ** *      *             *** *   *                          *   
     257719 CTATGGT-GAAAAAGGATATATCTTCCCCTGAAAACTAGACAGAAGCATTCTCAGAAACTTATTT
         66 TTTCGTTGGAAACAGGA-ATATCTTCATATAAAAGCTAGACAGAAGCATTCTCAGAAACTTCTTT

                   **  **    *              *  * *    
     257783 GTGATGTGCGCCCTCAACTAACAGTGTTGAACCTTTCTTTTC
        130 GTGATGTTTGCATTCAAGTAACAGTGTTGAACATTCCCTTTC

                     *                    *      *   *                       
     257825 ATAGAGCAGTTTTGAAACACTCTTTTTGTAATATCTGCAAGAGGATATTTGGATAGCTTTGAGGA
          1 ATAGAGCAGGTTTGAAACACTCTTTTTGTAGTATCTGGAAGTGGATATTTGGATAGCTTTGAGGA

                         *   * *            **                      *    *   
     257890 TTTCGTTGGAAACGGGATTGTCTTCATATAAACTCTAGACAGAAGCATTCTCAGAAGCTTCATTG
         66 TTTCGTTGGAAACAGGAATATCTTCATATAAAAGCTAGACAGAAGCATTCTCAGAAACTTCTTTG

            *       **   *    *        *    *        
     257955 GGATGTTTCAATTGAAGTCACAGTGTTTAACAGTCCCTTTC
        131 TGATGTTTGCATTCAAGTAACAGTGTTGAACATTCCCTTTC

                                                         *       *  * * *    
     257996 ATAGAGCAGGTTTGAAACACTCTTTTTGTAGTATCTGGAAGTGGACATTTGGAGAGATCTCAGGA
          1 ATAGAGCAGGTTTGAAACACTCTTTTTGTAGTATCTGGAAGTGGATATTTGGATAGCTTTGAGGA

            * *  *    * *             **            *       * *          *  *
     258061 ATACGGT-GATAAAGGAAATATCTTCCAATAAAAGCTAGATAGAAGCAATGTCAGAAACTTTTTC
         66 TTTCGTTGGAAACAGG-AATATCTTCATATAAAAGCTAGACAGAAGCATTCTCAGAAACTTCTTT

            *      * ** *   **      *       *  * *     *
     258125 ATGATGTATCTACTCAGCTAACAGAGTTGAACCTTTCTTTTGAG
        130 GTGATGTTTGCATTCAAGTAACAGTGTTGAACATTCCCTTT--C

             *       *                   * *     *             **            
     258169 AGAGAGCAGTTTTGAAACACTCTTTTTGTGGAATCTGCAAGTGGATATTTGTCTAGCTTTGAGGA
          1 ATAGAGCAGGTTTGAAACACTCTTTTTGTAGTATCTGGAAGTGGATATTTGGATAGCTTTGAGGA

                         *   *    *              *    *       *              
     258234 TTTCGTTGGAAACGGGATTA-CAT-ATA-AAAAGC-ACACAGCAGCATTCCCAGAAACTTCTTTG
         66 TTTCGTTGGAAACAGGAATATCTTCATATAAAAGCTAGACAGAAGCATTCTCAGAAACTTCTTTG

               *              *    *                 
     258295 TGAAGTTTGCATTCAAGTCACAGAGTTGAACATTCCCTTTC
        131 TGATGTTTGCATTCAAGTAACAGTGTTGAACATTCCCTTTC

                                            *    * *     *       **     *   *
     258336 ATAGAGCAGGTTTGAAACACTCTTTTTGTAGTGTCTGTATGTGGACATTTGGAGCGCTTTCAGGC
          1 ATAGAGCAGGTTTGAAACACTCTTTTTGTAGTATCTGGAAGTGGATATTTGGATAGCTTTGAGGA

            * ** *     **             *** *   *                      *   *   
     258401 CTATGGT-GAATAAGGAAATATCTTCCCCTGAAAACTAGACAGAAGCATTCTCAGAATCTTATTT
         66 TTTCGTTGGAAACAGG-AATATCTTCATATAAAAGCTAGACAGAAGCATTCTCAGAAACTTCTTT

                   **  **    *             **  * *   *
     258465 GTGATGTGCGCCCTCAACTAACAGTGTTGAAGCTTTCTTTTG
        130 GTGATGTTTGCATTCAAGTAACAGTGTTGAACATTCCCTTTC

                     *                    **     *   *                       
     258507 ATAGAGCAGTTTTGAAACACTCTTTTTGTAAAATCTGCAAGAGGATATTTGGATAGCTTTGAGGA
          1 ATAGAGCAGGTTTGAAACACTCTTTTTGTAGTATCTGGAAGTGGATATTTGGATAGCTTTGAGGA

                         *   * *            **                      *    *   
     258572 TTTCGTTGGAAACGGGATTGTCTTCATATAAACTCTAGACAGAAGCATTCTCAGAAGCTTCATTG
         66 TTTCGTTGGAAACAGGAATATCTTCATATAAAAGCTAGACAGAAGCATTCTCAGAAACTTCTTTG

            *       **   *    *             * *      
     258637 GGATGTTTCAATTGAAGTCACAGTGTTGAACAGTTCCTTTC
        131 TGATGTTTGCATTCAAGTAACAGTGTTGAACATTCCCTTTC

                 *                                       *       **   * *    
     258678 ATAGAACAGGTTTGAAACACTCTTTTTGTAGTATCTGGAAGTGGACATTTGGAGCGCTCTCAGGA
          1 ATAGAGCAGGTTTGAAACACTCTTTTTGTAGTATCTGGAAGTGGATATTTGGATAGCTTTGAGGA

            * *  *      *             **          * *       * *          *  *
     258743 CTACGGT-GAAAAAGGAAATATCTTCCAATAAAAGCTACATAGAAGCAATGTCAGAAACTTTTTC
         66 TTTCGTTGGAAACAGG-AATATCTTCATATAAAAGCTAGACAGAAGCATTCTCAGAAACTTCTTT

            *      * ** *   **      *       *  *     *
     258807 ATGATGTATCTACTCAGCTAACAGAGTTGAACCTTTCCTTTG
        130 GTGATGTTTGCATTCAAGTAACAGTGTTGAACATTCCCTTTC

             *       *                   * *     *             **            
     258849 AGAGAGCAGTTTTGAAACACTCTTTTTGTTGAATCTGCAAGTGGATATTTGTCTAGCTTTGAGGA
          1 ATAGAGCAGGTTTGAAACACTCTTTTTGTAGTATCTGGAAGTGGATATTTGGATAGCTTTGAGGA

                *        *   *    *                   *       *   *          
     258914 TTTCATTGGAAACGGGATTA-CAT-ATA-AAAAGC-AGACAGCAGCATTCCCAGTAACTTCTTTG
         66 TTTCGTTGGAAACAGGAATATCTTCATATAAAAGCTAGACAGAAGCATTCTCAGAAACTTCTTTG

                              *    *                 
     258975 TGATGTTTGCATTCAAGTCACAGAGTTGAACATTCCCTTTC
        131 TGATGTTTGCATTCAAGTAACAGTGTTGAACATTCCCTTTC

                                                   *     *       **     *   *
     259016 ATAGAGCAGGTTTGAAACACTCTTTTTGTAGTATCTGGATGTGGACATTTGGAGCGCTTTCAGGC
          1 ATAGAGCAGGTTTGAAACACTCTTTTTGTAGTATCTGGAAGTGGATATTTGGATAGCTTTGAGGA

            * ** *      *             * * *   *                          *   
     259081 CTATGGT-GAAAAAGGAAATATCTTCCTCTGAAAACTAGACAGAAGCATTCTCAGAAACTTATTT
         66 TTTCGTTGGAAACAGG-AATATCTTCATATAAAAGCTAGACAGAAGCATTCTCAGAAACTTCTTT

                   **  **    *              *  * *   *
     259145 GTGATGTGCGCCCTCAATTAACAGTGTTGAACCTTTCTTTTG
        130 GTGATGTTTGCATTCAAGTAACAGTGTTGAACATTCCCTTTC

                     *                    *      *   *                       
     259187 ATAGAGCAGTTTTGAAACACTCTTTTTGTAATATCTGCAAGAGGATATTTGGATAGCTTTGAGGA
          1 ATAGAGCAGGTTTGAAACACTCTTTTTGTAGTATCTGGAAGTGGATATTTGGATAGCTTTGAGGA

                         *   * *            **                      *    *   
     259252 TTTCGTTGGAAACGGGATTGTCTTCATATAAACTCTAGACAGAAGCATTCTCAGAACCTTCATTG
         66 TTTCGTTGGAAACAGGAATATCTTCATATAAAAGCTAGACAGAAGCATTCTCAGAAACTTCTTTG

            *       **   *    *             *        
     259317 GGATGTTTCAATTGAAGTCACAGTGTTGAACAGTCCCTTTC
        131 TGATGTTTGCATTCAAGTAACAGTGTTGAACATTCCCTTTC

                                                         *       **   * *    
     259358 ATAGAGCAGGTTTGAAACACTCTTTTTGTAGTATCTGGAAGTGGACATTTGGAGCGCTCTCAGGA
          1 ATAGAGCAGGTTTGAAACACTCTTTTTGTAGTATCTGGAAGTGGATATTTGGATAGCTTTGAGGA

            * *  *    * *             **            *       * *          *  *
     259423 CTCCGGT-GATAAAGGAAATATCTTCCAATAAAAGCTAGATAGAAGCAATGTCAGAAACTTTTTC
         66 TTTCGTTGGAAACAGG-AATATCTTCATATAAAAGCTAGACAGAAGCATTCTCAGAAACTTCTTT

            *      * ** *   **      *       *  * *   *
     259487 ATGATGTATCTACTCAGCTAACAGAGTTGAACCTTTCTTTTG
        130 GTGATGTTTGCATTCAAGTAACAGTGTTGAACATTCCCTTTC

             *       *                   * *     *             **            
     259529 AGAGAGCAGTTTTGAAACACTCTTTTTGTGGAATCTGCAAGTGGATATTTGTCTAGCTTTGAGGA
          1 ATAGAGCAGGTTTGAAACACTCTTTTTGTAGTATCTGGAAGTGGATATTTGGATAGCTTTGAGGA

                         *   *    *                   *       *     *        
     259594 TTTCGTTGGAAACGGGATTA-CAT-ATA-AAAAGC-AGACAGCAGCATTCCCAGAATCTTCTTTG
         66 TTTCGTTGGAAACAGGAATATCTTCATATAAAAGCTAGACAGAAGCATTCTCAGAAACTTCTTTG

                    *         *    *                 
     259655 TGATGTTTCCATTCAAGTCACAGAGTTGAACATTCCCTTTC
        131 TGATGTTTGCATTCAAGTAACAGTGTTGAACATTCCCTTTC

                                                   *     *       **     *   *
     259696 ATAGAGCAGGTTTGAAACACTCTTTTTGTAGTATCTGGATGTGGACATTTGGAGCGCTTTCAGGC
          1 ATAGAGCAGGTTTGAAACACTCTTTTTGTAGTATCTGGAAGTGGATATTTGGATAGCTTTGAGGA

            * ** *      *             *** *   *                      *   *   
     259761 CTATGGT-GAAAAAGGAAATATCTTCCCCTGAAAACTAGACAGAAGCATTCTCAGAATCTTATTT
         66 TTTCGTTGGAAACAGG-AATATCTTCATATAAAAGCTAGACAGAAGCATTCTCAGAAACTTCTTT

                   **  **    *             **  * *   *
     259825 GTGATGTGCGCCCTCAACTAACAGTGTTGAAGCTTTCTTTTG
        130 GTGATGTTTGCATTCAAGTAACAGTGTTGAACATTCCCTTTC

                     *                    **     *   *                       
     259867 ATAGAGCAGTTTTGAAACACTCTTTTTGTAAAATCTGCAAGAGGATATTTGGATAGCTTTGAGGA
          1 ATAGAGCAGGTTTGAAACACTCTTTTTGTAGTATCTGGAAGTGGATATTTGGATAGCTTTGAGGA

                         *   * *            **                      *    *   
     259932 TTTCGTTGGAAACGGGATTGTCTTCATATAAACTCTAGACAGAAGCATTCTCAGAAGCTTCATTG
         66 TTTCGTTGGAAACAGGAATATCTTCATATAAAAGCTAGACAGAAGCATTCTCAGAAACTTCTTTG

            *       **   *    *             *        
     259997 GGATGTTTCAATTGAAGTCACAGTGTTGAACAGTCCCTTTC
        131 TGATGTTTGCATTCAAGTAACAGTGTTGAACATTCCCTTTC

                                                         *       **   * *    
     260038 ATAGAGCAGGTTTGAAACACTCTTTTTGTAGTATCTGGAAGTGGACATTTGGAGCGCTCTCAGGA
          1 ATAGAGCAGGTTTGAAACACTCTTTTTGTAGTATCTGGAAGTGGATATTTGGATAGCTTTGAGGA

            * ** *      *             **            *       * *          *  *
     260103 CTGGGGT-GAAAAAGGAAATATCTTCCAATAAAAGCTAGATAGAAGCAATGTCAGAAACTTTTTC
         66 TTTCGTTGGAAACAGG-AATATCTTCATATAAAAGCTAGACAGAAGCATTCTCAGAAACTTCTTT

            *      * ** *   **      *       *        *
     260167 ATGATGTATCTACTCAGCTAACAGAGTTGAACCTT-CCTTTG
        130 GTGATGTTTGCATTCAAGTAACAGTGTTGAACATTCCCTTTC

             *       *            *      * *     *             **            
     260208 AGAGAGCAGTTTTGAAACACTCGTTTTGTGGAATCTGCAAGTGGATATTTGTCTAGCTTTGAGGA
          1 ATAGAGCAGGTTTGAAACACTCTTTTTGTAGTATCTGGAAGTGGATATTTGGATAGCTTTGAGGA

                         *   *    *                   *       *              
     260273 TTTCGTTGGAAACGGGATTA-CAT-ATA-AAAAGC-AGACAGCAGCATTCCCAGAAACTTCTTTG
         66 TTTCGTTGGAAACAGGAATATCTTCATATAAAAGCTAGACAGAAGCATTCTCAGAAACTTCTTTG

                              *    *                 
     260334 TGATGTTTGCATTCAAGTCACAGAGTTGAACATTCCCTTTC
        131 TGATGTTTGCATTCAAGTAACAGTGTTGAACATTCCCTTTC

                                                   *     *     * **     *   *
     260375 ATAGAGCAGGTTTGAAACACTCTTTTTGTAGTATCTGGATGTGGACATTTGCAGCGCTTTCAGGC
          1 ATAGAGCAGGTTTGAAACACTCTTTTTGTAGTATCTGGAAGTGGATATTTGGATAGCTTTGAGGA

            * ** *      *             *** *   *                          *   
     260440 CTAAGGT-GAAAAAGGAAATATCTTCCCCTGAAAACTAGACAGAAGCATTCTCAGAAACTTATTT
         66 TTTCGTTGGAAACAGG-AATATCTTCATATAAAAGCTAGACAGAAGCATTCTCAGAAACTTCTTT

                   **  **    *             **  * *   *
     260504 GTGATGTGCGCCCTCAACTAACAGTGTTGAAGCTTTCTTTTG
        130 GTGATGTTTGCATTCAAGTAACAGTGTTGAACATTCCCTTTC

                     *                    *      *   *                       
     260546 ATAGAGCAGTTTTGAAACACTCTTTTTGTAATATCTGCAAGAGGATATTTGGATAGCTTTGAGGA
          1 ATAGAGCAGGTTTGAAACACTCTTTTTGTAGTATCTGGAAGTGGATATTTGGATAGCTTTGAGGA

                         *   * *            **                      *    *   
     260611 TTTCGTTGGAAACGGGATTGTCTTCATATAAACTCTAGACAGAAGCATTCTCAGAAGCTTCATTG
         66 TTTCGTTGGAAACAGGAATATCTTCATATAAAAGCTAGACAGAAGCATTCTCAGAAACTTCTTTG

            *       **   *    *             * *      
     260676 GGATGTTTCAATTGAAGTCACAGTGTTGAACAGTTCCTTTC
        131 TGATGTTTGCATTCAAGTAACAGTGTTGAACATTCCCTTTC

                 *                                       *       **   * *    
     260717 ATAGAACAGGTTTGAAACACTCTTTTTGTAGTATCTGGAAGTGGACATTTGGAGCGCTCTCAGGA
          1 ATAGAGCAGGTTTGAAACACTCTTTTTGTAGTATCTGGAAGTGGATATTTGGATAGCTTTGAGGA

            * *  *      *             **          * *       * *          *  *
     260782 CTACGGT-GAAAAAGGAAATATCTTCCAATAAAAGCTACATAGAAGCAATGTCAGAAACTTTTTC
         66 TTTCGTTGGAAACAGG-AATATCTTCATATAAAAGCTAGACAGAAGCATTCTCAGAAACTTCTTT

            *      * ** *   **      *       *  *     *
     260846 ATGATGTATCTACTCAGCTAACAGAGTTGAACCTTTCCTTTG
        130 GTGATGTTTGCATTCAAGTAACAGTGTTGAACATTCCCTTTC

             *       *                   * *     *             **            
     260888 AGAGAGCAGTTTTGAAACACTCTTTTTGTTGAATCTGCAAGTGGATATTTGTCTAGCTTTGAGGA
          1 ATAGAGCAGGTTTGAAACACTCTTTTTGTAGTATCTGGAAGTGGATATTTGGATAGCTTTGAGGA

                         *   *    *                   *       *   *          
     260953 TTTCGTTGGAAACGGGATTA-CAT-ATA-AAAAGC-AGACAGCAGCATTCCCAGTAACTTCTTTG
         66 TTTCGTTGGAAACAGGAATATCTTCATATAAAAGCTAGACAGAAGCATTCTCAGAAACTTCTTTG

                              *    *                 
     261014 TGATGTTTGCATTCAAGTCACAGAGTTGAACATTCCCTTTC
        131 TGATGTTTGCATTCAAGTAACAGTGTTGAACATTCCCTTTC

                                *                  *     *       **     *   *
     261055 ATAGAGCAGGTTTGAAACACACTTTTTGTAGTATCTGGATGTGGACATTTGGAGCGCTTTCAGGC
          1 ATAGAGCAGGTTTGAAACACTCTTTTTGTAGTATCTGGAAGTGGATATTTGGATAGCTTTGAGGA

            * ** *      *             *** *   *                      *   *   
     261120 CTATGGT-GAAAAAGGAAATATCTTCCCCTGAAAACTAGACAGAAGCATTCTCAGAATCTTATTT
         66 TTTCGTTGGAAACAGG-AATATCTTCATATAAAAGCTAGACAGAAGCATTCTCAGAAACTTCTTT

                   **  **    *             **  * *   *
     261184 GTGATGTGCGCCCTCAACTAACAGTGTTGAAGCTTTCTTTTG
        130 GTGATGTTTGCATTCAAGTAACAGTGTTGAACATTCCCTTTC

                     *                    **     *   *                       
     261226 ATAGAGCAGTTTTGAAACACTCTTTTTGTAAAATCTGCAAGAGGATATTTGGATAGCTTTGAGGA
          1 ATAGAGCAGGTTTGAAACACTCTTTTTGTAGTATCTGGAAGTGGATATTTGGATAGCTTTGAGGA

                         *   * *            **                      *    *   
     261291 TTTCGTTGGAAACGGGATTGTCTTCATATAAACTCTAGACAGAAGCATTCTCAGAAGCTTCATTG
         66 TTTCGTTGGAAACAGGAATATCTTCATATAAAAGCTAGACAGAAGCATTCTCAGAAACTTCTTTG

            *       **   *    *             *        
     261356 GGATGTTTCAATTGAAGTCACAGTGTTGAACAGTCCCTTTC
        131 TGATGTTTGCATTCAAGTAACAGTGTTGAACATTCCCTTTC

                                                         *       **   * *    
     261397 ATAGAGCAGGTTTGAAACACTCTTTTTGTAGTATCTGGAAGTGGACATTTGGAGCGCTCTCAGGA
          1 ATAGAGCAGGTTTGAAACACTCTTTTTGTAGTATCTGGAAGTGGATATTTGGATAGCTTTGAGGA

            * *  *      *             **            *       * *          *  *
     261462 CTGCGGT-GAAAAAGGAAATATCTTCCAATAAAAGCTAGATAGAAGCAATGTCAGAAACTTTTTC
         66 TTTCGTTGGAAACAGG-AATATCTTCATATAAAAGCTAGACAGAAGCATTCTCAGAAACTTCTTT

            *      * ** *   **      *       *        *
     261526 ATGATGTATCTACTCAGCTAACAGAGTTGAACCTT-CCTTTG
        130 GTGATGTTTGCATTCAAGTAACAGTGTTGAACATTCCCTTTC

             *       *            *      * *     *             **            
     261567 AGAGAGCAGTTTTGAAACACTCGTTTTGTGGAATCTGCAAGTGGATATTTGTCTAGCTTTGAGGA
          1 ATAGAGCAGGTTTGAAACACTCTTTTTGTAGTATCTGGAAGTGGATATTTGGATAGCTTTGAGGA

                *        *   *    *                   *       *              
     261632 TTTCCTTGGAAACGGGATTA-CAT-ATA-AAAAGC-AGACAGCAGCATTCCCAGAAACTTCTTTG
         66 TTTCGTTGGAAACAGGAATATCTTCATATAAAAGCTAGACAGAAGCATTCTCAGAAACTTCTTTG

                              *    *                 
     261693 TGATGTTTGCATTCAAGTCACAGAGTTGAACATTCCCTTTC
        131 TGATGTTTGCATTCAAGTAACAGTGTTGAACATTCCCTTTC

                                                   *     *     * **     *   *
     261734 ATAGAGCAGGTTTGAAACACTCTTTTTGTAGTATCTGGATGTGGACATTTGCAGCGCTTTCAGGC
          1 ATAGAGCAGGTTTGAAACACTCTTTTTGTAGTATCTGGAAGTGGATATTTGGATAGCTTTGAGGA

            * ** *      *             *** *   *                          *   
     261799 CTAAGGT-GAAAAAGGAAATATCTTCCCCTGAAAACTAGACAGAAGCATTCTCAGAAACTTATTT
         66 TTTCGTTGGAAACAGG-AATATCTTCATATAAAAGCTAGACAGAAGCATTCTCAGAAACTTCTTT

                   **  **    *             **  * *   *
     261863 GTGATGTGCGCCCTCAACTAACAGTGTTGAAGCTTTCTTTTG
        130 GTGATGTTTGCATTCAAGTAACAGTGTTGAACATTCCCTTTC

                     *                    *      *   *                       
     261905 ATAGAGCAGTTTTGAAACACTCTTTTTGTAATATCTGCAAGAGGATATTTGGATAGCTTTGAGGA
          1 ATAGAGCAGGTTTGAAACACTCTTTTTGTAGTATCTGGAAGTGGATATTTGGATAGCTTTGAGGA

                         *   * *            **                      *    *   
     261970 TTTCGTTGGAAACGGGATTGTCTTCATATAAACTCTAGACAGAAGCATTCTCAGAAGCTTCATTG
         66 TTTCGTTGGAAACAGGAATATCTTCATATAAAAGCTAGACAGAAGCATTCTCAGAAACTTCTTTG

            *       **   *    *             * *      
     262035 GGATGTTTCAATTGAAGTCACAGTGTTGAACAGTTCCTTTC
        131 TGATGTTTGCATTCAAGTAACAGTGTTGAACATTCCCTTTC

                 *                                       *       **   ***    
     262076 ATAGAACAGGTTTGAAACACTCTTTTTGTAGTATCTGGAAGTGGACATTTGGAGCGCTCCCAGGA
          1 ATAGAGCAGGTTTGAAACACTCTTTTTGTAGTATCTGGAAGTGGATATTTGGATAGCTTTGAGGA

            * ** *      *             **          * *       * *          *  *
     262141 CTATGGT-GAAAAAGGAAATATCTTCCAATAAAAGCTACATAGAAGCAATGTCAGAAACTTTTTC
         66 TTTCGTTGGAAACAGG-AATATCTTCATATAAAAGCTAGACAGAAGCATTCTCAGAAACTTCTTT

            *      * ** *   ** *    *      **  *     *
     262205 ATGATGTATCTACTCAGCTTACAGAGTTGAAACTTTCCTTTG
        130 GTGATGTTTGCATTCAAGTAACAGTGTTGAACATTCCCTTTC

             *       *                   * *     *             **            
     262247 AGAGAGCAGTTTTGAAACACTCTTTTTGTGGAATCTGCAAGTGGATATTTGTCTAGCTTTGAGGA
          1 ATAGAGCAGGTTTGAAACACTCTTTTTGTAGTATCTGGAAGTGGATATTTGGATAGCTTTGAGGA

                         *   *    *                   *       *   *          
     262312 TTTCGTTGGAAACGGGATTA-CAT-ATA-AAAAGC-AGACAGCAGCATTCCCAGTAACTTCTTTG
         66 TTTCGTTGGAAACAGGAATATCTTCATATAAAAGCTAGACAGAAGCATTCTCAGAAACTTCTTTG

                              *    *                 
     262373 TGATGTTTGCATTCAAGTCACAGAGTTGAACATTCCCTTTC
        131 TGATGTTTGCATTCAAGTAACAGTGTTGAACATTCCCTTTC

                             *    *                *     *       **     *   *
     262414 ATAGAGCAGGTTTGAAAAACTCCTTTTGTAGTATCTGGATGTGGACATTTGGAGCGCTTTCAGGC
          1 ATAGAGCAGGTTTGAAACACTCTTTTTGTAGTATCTGGAAGTGGATATTTGGATAGCTTTGAGGA

            * ** *      *             *** * * *                          *   
     262479 CTATGGT-GAAAAAGGAAATATCTTCCCCTGAGAACTAGACAGAAGCATTCTCAGAAACTTATTT
         66 TTTCGTTGGAAACAGG-AATATCTTCATATAAAAGCTAGACAGAAGCATTCTCAGAAACTTCTTT

                   **  **    *              *  * *   *
     262543 GTGATGTGCGCCCTCAACTAACAGTGTTGAACCTTTCTTTTG
        130 GTGATGTTTGCATTCAAGTAACAGTGTTGAACATTCCCTTTC

                     *                    *      *   *                       
     262585 ATAGAGCAGTTTTGAAACACTCTTTTTGTAATATCTGCAAGAGGATATTTGGATAGCTTTGAGGA
          1 ATAGAGCAGGTTTGAAACACTCTTTTTGTAGTATCTGGAAGTGGATATTTGGATAGCTTTGAGGA

                         *   *    *                   *       *     *   *    
     262650 TTTCGTTGGAAACGGGATTA-CAT-ATA-AAAAGC-AGACAGCAGCATTCCCAGAATCTTGTTTG
         66 TTTCGTTGGAAACAGGAATATCTTCATATAAAAGCTAGACAGAAGCATTCTCAGAAACTTCTTTG

                    *         *    *                 
     262711 TGATGTTTCCATTCAAGTCACAGAGTTGAACATTCCCTTTC
        131 TGATGTTTGCATTCAAGTAACAGTGTTGAACATTCCCTTTC

             *                         *           *     *       **     *   *
     262752 AGAGAGCAGGTTTGAAACACTCTTTTTATAGTATCTGGATGTGGACATTTGGAGCGCTTTCAGGC
          1 ATAGAGCAGGTTTGAAACACTCTTTTTGTAGTATCTGGAAGTGGATATTTGGATAGCTTTGAGGA

            * ** *      *             *** *   *                      *   *   
     262817 CTATGGT-GAAAAAGGAAATATCTTCTCCTGAAAACTAGACAGAAGCATTCTCAGAATCTTATTT
         66 TTTCGTTGGAAACAGG-AATATCTTCATATAAAAGCTAGACAGAAGCATTCTCAGAAACTTCTTT

                   *** **    *             **  * *   *
     262881 GTGATGTGCCCCCTCAACTAACAGTGTTGAAGCTTTCTTTTG
        130 GTGATGTTTGCATTCAAGTAACAGTGTTGAACATTCCCTTTC

                     *                *   **     *   *        *              
     262923 ATAGAGCAGTTTTGAAACACTCTTTTCGTAAAATCTGCAAGAGGATATTTTGATAGCTTTGAGGA
          1 ATAGAGCAGGTTTGAAACACTCTTTTTGTAGTATCTGGAAGTGGATATTTGGATAGCTTTGAGGA

                *        *   * *            **                      *    *   
     262988 TTTCATTGGAAACGGGATTGTCTTCATATAAACTCTAGACAGAAGCATTCTCAGAAGCTTCATTG
         66 TTTCGTTGGAAACAGGAATATCTTCATATAAAAGCTAGACAGAAGCATTCTCAGAAACTTCTTTG

            *       **   *    *             *        
     263053 GGATGTTTCAATTGAAGTCACAGTGTTGAACAGTCCCTTTC
        131 TGATGTTTGCATTCAAGTAACAGTGTTGAACATTCCCTTTC

                                                         *       *  * * *    
     263094 ATAGAGCAGGTTTGAAACACTCTTTTTGTAGTATCTGGAAGTGGACATTTGGAGAGATCTCAGGA
          1 ATAGAGCAGGTTTGAAACACTCTTTTTGTAGTATCTGGAAGTGGATATTTGGATAGCTTTGAGGA

            * *  *    * *             **            *       * *          *  *
     263159 ATACGGT-GATAAAGGAAATATCTTCCAATAAAAGCTAGATAGAAGCAATGTCAGAAACTTTTTC
         66 TTTCGTTGGAAACAGG-AATATCTTCATATAAAAGCTAGACAGAAGCATTCTCAGAAACTTCTTT

            *      * ** *   **      *       *  * *   *
     263223 ATGATGTATCTACTCAGCTAACAGAGTTGAACCTTTCTTTTG
        130 GTGATGTTTGCATTCAAGTAACAGTGTTGAACATTCCCTTTC

             *       *                   * *     *             **            
     263265 AGAGAGCAGTTTTGAAACACTCTTTTTGTGGAATCTGCAAGTGGATATTTGTCTAGCTTTGAGGA
          1 ATAGAGCAGGTTTGAAACACTCTTTTTGTAGTATCTGGAAGTGGATATTTGGATAGCTTTGAGGA

                         *   *    *                   *       *              
     263330 TTTCGTTGGAAACGGGATTA-CAT-ATA-AAAAGC-AGACAGCAGCATTCCCAGAAACTTCTTTG
         66 TTTCGTTGGAAACAGGAATATCTTCATATAAAAGCTAGACAGAAGCATTCTCAGAAACTTCTTTG

                              *    *                 
     263391 TGATGTTTGCATTCAAGTCACAGAGTTGAACATTCCCTTTC
        131 TGATGTTTGCATTCAAGTAACAGTGTTGAACATTCCCTTTC

                                        *          *     *       **     *   *
     263432 ATAGAGCAGGTTTGAAACACTCTTTTTGAAGTATCTGGATGTGGACATTTGGAGCGCTTTCAGGC
          1 ATAGAGCAGGTTTGAAACACTCTTTTTGTAGTATCTGGAAGTGGATATTTGGATAGCTTTGAGGA

              ** *      *             *** *   *           *              *   
     263497 TTATGGT-GAAAAAGGAAATATCTTCCCCTGAAAACTAGACAGAAGAATTCTCAGAAACTTGTTT
         66 TTTCGTTGGAAACAGG-AATATCTTCATATAAAAGCTAGACAGAAGCATTCTCAGAAACTTCTTT

                   **  **    *             **  * *   *
     263561 GTGATGTGCGCCCTCAACTAACAGTGTTGAAGCTTTCTTTTG
        130 GTGATGTTTGCATTCAAGTAACAGTGTTGAACATTCCCTTTC

                     *                    **     *   *                       
     263603 ATAGAGCAGTTTTGAAACACTCTTTTTGTAAAATCTGCAAGAGGATATTTGGATAGCTTTGAGGA
          1 ATAGAGCAGGTTTGAAACACTCTTTTTGTAGTATCTGGAAGTGGATATTTGGATAGCTTTGAGGA

                         *   * *            **                      * *  *   
     263668 TTTCGTTGGAAACGGGATTGTCTTCATATAAACTCTAGACAGAAGCATTCTCAGAAGCGTCATTG
         66 TTTCGTTGGAAACAGGAATATCTTCATATAAAAGCTAGACAGAAGCATTCTCAGAAACTTCTTTG

            *       **   *    *             *        
     263733 GGATGTTTCAATTGAAGTCACAGTGTTGAACAGTCCCTTTC
        131 TGATGTTTGCATTCAAGTAACAGTGTTGAACATTCCCTTTC

                                                   *     *  *    **     *    
     263774 ATAGAGCAGGTTTGAAACACTCTTTTTGTAGTATCTGGATGTGGACATGTGGAGCGCTTTCAGGC
          1 ATAGAGCAGGTTTGAAACACTCTTTTTGTAGTATCTGGAAGTGGATATTTGGATAGCTTTGAGG-

            * * *    *                 *** *   *                          *  
     263839 CTATGGTT-TAAA-AGGAAATATCTTCCCCTGAAAACTAGACAGAAGCATTCTCAGAAACTTATT
         65 ATTTCGTTGGAAACAGG-AATATCTTCATATAAAAGCTAGACAGAAGCATTCTCAGAAACTTCTT

                    **  **    *     *              *   *
     263902 TGTGATGTGCGCCCTCAACTAACACTGTTGAAGCATT-CTTTTG
        129 TGTGATGTTTGCATTCAAGTAACAGTGTTGAA-CATTCCCTTTC

              *      *                   * *     *             **            
     263945 ATTGAGCAGTTTTGAAACACTCTTTTTGTGGAATCTGCAAGTGGATATTTGTCTAGCTTTGAGGA
          1 ATAGAGCAGGTTTGAAACACTCTTTTTGTAGTATCTGGAAGTGGATATTTGGATAGCTTTGAGGA

                 *       *   *    *                   *       *              
     264010 TTTCGCTGGAAACGGGATTA-CAT-ATA-AAAAGC-AGACAGCAGCATTCCCAGAAACTTCTTTG
         66 TTTCGTTGGAAACAGGAATATCTTCATATAAAAGCTAGACAGAAGCATTCTCAGAAACTTCTTTG

                              *    *                 
     264071 TGATGTTTGCATTCAAGTCACAGAGTTGAACATTCCCTTTC
        131 TGATGTTTGCATTCAAGTAACAGTGTTGAACATTCCCTTTC

                                                   *     *       ** *   *   *
     264112 ATAGAGCAGGTTTGAAACACTCTTTTTGTAGTATCTGGATGTGGACATTTGGAGCGATTTCAGGC
          1 ATAGAGCAGGTTTGAAACACTCTTTTTGTAGTATCTGGAAGTGGATATTTGGATAGCTTTGAGGA

            * ** *      *             *** *   *                    * *   *   
     264177 CTATGGT-GAAAAAGGAAATATCTTCCCCTGAAAACTAGACAGAAGCATTCTCAGGATCTTATTT
         66 TTTCGTTGGAAACAGG-AATATCTTCATATAAAAGCTAGACAGAAGCATTCTCAGAAACTTCTTT

                   **  **    *             **  * **  *
     264241 GTGATGTGCGCCCTCAACTAACAGTGTTGAAGCTTTCTATTG
        130 GTGATGTTTGCATTCAAGTAACAGTGTTGAACATTCCCTTTC

                     *                    **     **  *                       
     264283 ATAGAGCAGTTTTGAAACACTCTTTTTGTAAAATCTGCTAGAGGATATTTGGATAGCTTTGAGGA
          1 ATAGAGCAGGTTTGAAACACTCTTTTTGTAGTATCTGGAAGTGGATATTTGGATAGCTTTGAGGA

                *        *   * *            **                     **    *   
     264348 TTTCTTTGGAAACGGGATTGTCTTCATATAAACTCTAGACAGAAGCATTCTCAGATGCTTCATTG
         66 TTTCGTTGGAAACAGGAATATCTTCATATAAAAGCTAGACAGAAGCATTCTCAGAAACTTCTTTG

            *       **   *    *             *        
     264413 GGATGTTTCAATTGAAGTCACAGTGTTGAACAGTCCCTTTC
        131 TGATGTTTGCATTCAAGTAACAGTGTTGAACATTCCCTTTC

                                                   *     *       **   * *    
     264454 ATAGAGCAGGTTTGAAACACTCTTTTTGTAGTATCTGGATGTGGACATTTGGAGCGCTCTCAGGA
          1 ATAGAGCAGGTTTGAAACACTCTTTTTGTAGTATCTGGAAGTGGATATTTGGATAGCTTTGAGGA

            * *  *      *             **     *    * *       * *         **  *
     264519 CTACGGT-GAAAAAGGAAATATCTTCCAATAAAGGCTACATAGAAGCAATGTCAGAAACTATTTC
         66 TTTCGTTGGAAACAGG-AATATCTTCATATAAAAGCTAGACAGAAGCATTCTCAGAAACTTCTTT

            *      * ** *   **      *       *  * *   *
     264583 ATGATGTATCTACTCAGCTAACAGAGTTGAACCTTTCTTTTG
        130 GTGATGTTTGCATTCAAGTAACAGTGTTGAACATTCCCTTTC

             *       *                   * *                   **       *    
     264625 AGAGAGCAGTTTTGAAACACTCTTTTTGTGGAATCTGGAAGTGGATATTTGTCTAGCTTTTAGGA
          1 ATAGAGCAGGTTTGAAACACTCTTTTTGTAGTATCTGGAAGTGGATATTTGGATAGCTTTGAGGA

                         *   *    *                   *  *    *   *          
     264690 TTTCGTTGGAAACGGGATTA-CAT-ATA-AAAAGC-AGACAGCAGGATTCCCAGTAACTTCTTTG
         66 TTTCGTTGGAAACAGGAATATCTTCATATAAAAGCTAGACAGAAGCATTCTCAGAAACTTCTTTG

                              *    *                 
     264751 TGATGTTTGCATTCAAGTCACAGAGTTGAACATTCCCTTTC
        131 TGATGTTTGCATTCAAGTAACAGTGTTGAACATTCCCTTTC

                                                 * *     *       **     *   *
     264792 ATAGAGCAGGTTTGAAACACTCTTTTTGTAGTATCTGTATGTGGACATTTGGAGCGCTTTCAGGC
          1 ATAGAGCAGGTTTGAAACACTCTTTTTGTAGTATCTGGAAGTGGATATTTGGATAGCTTTGAGGA

            * ** *      *             *** *   *                      *   *   
     264857 CTATGGT-GAAAAAGGAAATATCTTCCCCTGAAAACTAGACAGAAGCATTCTCAGAATCTTATTT
         66 TTTCGTTGGAAACAGG-AATATCTTCATATAAAAGCTAGACAGAAGCATTCTCAGAAACTTCTTT

                  ***  **    *             **  * *   *
     264921 GTGATGAGCGCCCTCAACTAACAGTGTTGAAGCTTTCTTTTG
        130 GTGATGTTTGCATTCAAGTAACAGTGTTGAACATTCCCTTTC

                     *                    **     *   *                       
     264963 ATAGAGCAGTTTTGAAACACTCTTTTTGTAAAATCTGCAAGAGGATATTTGGATAGCTTTGAGGA
          1 ATAGAGCAGGTTTGAAACACTCTTTTTGTAGTATCTGGAAGTGGATATTTGGATAGCTTTGAGGA

                         *   * *            **                      *    *   
     265028 TTTCGTTGGAAACGGGATTGTCTTCATATAAACTCTAGACAGAAGCATTCTCAGAAGCTTCATTG
         66 TTTCGTTGGAAACAGGAATATCTTCATATAAAAGCTAGACAGAAGCATTCTCAGAAACTTCTTTG

            *       **   *    *             *        
     265093 GGATGTTTCAATTGAAGTCACAGTGTTGAACAGTCCCTTTC
        131 TGATGTTTGCATTCAAGTAACAGTGTTGAACATTCCCTTTC

                                                   *     *       **     *   *
     265134 ATAGAGCAGGTTTGAAACACTCTTTTTGTAGTATCTGGATGTGGACATTTGGAGCGCTTTCAGGC
          1 ATAGAGCAGGTTTGAAACACTCTTTTTGTAGTATCTGGAAGTGGATATTTGGATAGCTTTGAGGA

            * ** *      *             *** *   *                          *   
     265199 CTATGGT-GAAAAAGGAAATATCTTCCCCTGAAAACTAGACAGAAGCATTCTCAGAAACTTATTT
         66 TTTCGTTGGAAACAGG-AATATCTTCATATAAAAGCTAGACAGAAGCATTCTCAGAAACTTCTTT

                   **  **    *             **  * *   *
     265263 GTGATGTGCGCCCTCAACTAACAGTGTTGAAGCTTTCTTTTG
        130 GTGATGTTTGCATTCAAGTAACAGTGTTGAACATTCCCTTTC

                     *                   * *     *             **            
     265305 ATAGAGCAGTTTTGAAACACTCTTTTTGTGGAATCTGCAAGTGGATATTTGTCTAGCTTTGAGGA
          1 ATAGAGCAGGTTTGAAACACTCTTTTTGTAGTATCTGGAAGTGGATATTTGGATAGCTTTGAGGA

                *        *   *    *                   *       *     *        
     265370 TTTCCTTGGAAACGGGATTA-CAT-ATA-AAAAGC-AGACAGCAGCATTCCCAGAATCTTCTTTG
         66 TTTCGTTGGAAACAGGAATATCTTCATATAAAAGCTAGACAGAAGCATTCTCAGAAACTTCTTTG

                              *    *                 
     265431 TGATGTTTGCATTCAAGTCACAGAGTTGAACATTCCCTTTC
        131 TGATGTTTGCATTCAAGTAACAGTGTTGAACATTCCCTTTC

                                                   *     *       **     *   *
     265472 ATAGAGCAGGTTTGAAACACTCTTTTTGTAGTATCTGGATGTGGACATTTGGAGCGCTTTCAGGC
          1 ATAGAGCAGGTTTGAAACACTCTTTTTGTAGTATCTGGAAGTGGATATTTGGATAGCTTTGAGGA

            * ** *      *             *** *   *                      *   *   
     265537 CTATGGT-GAAAAAGGAAATATCTTCTCCTGAAAACTAGACAGAAGCATTCTCAGAATCTTATTT
         66 TTTCGTTGGAAACAGG-AATATCTTCATATAAAAGCTAGACAGAAGCATTCTCAGAAACTTCTTT

                   **  **    *             **  * *   *
     265601 GTGATGTGCGCCCTCAACTAACAGTGTTGAAGCTTTCTTTTG
        130 GTGATGTTTGCATTCAAGTAACAGTGTTGAACATTCCCTTTC

                     *                    **     *   *                       
     265643 ATAGAGCAGTTTTGAAACACTCTTTTTGTAAAATCTGCAAGAGGATATTTGGATAGCTTTGAGGA
          1 ATAGAGCAGGTTTGAAACACTCTTTTTGTAGTATCTGGAAGTGGATATTTGGATAGCTTTGAGGA

                *        *   * *            **           *          *    *   
     265708 TTTCATTGGAAACGGGATTGTCTTCATATAAACTCTAGACAGAAGTATTCTCAGAAGCTTCATTG
         66 TTTCGTTGGAAACAGGAATATCTTCATATAAAAGCTAGACAGAAGCATTCTCAGAAACTTCTTTG

            *       **   *    *             *        
     265773 GGATGTTTCAATTGAAGTCACAGTGTTGAACAGTCCCTTTC
        131 TGATGTTTGCATTCAAGTAACAGTGTTGAACATTCCCTTTC

                                                         *       **   * *    
     265814 ATAGAGCAGGTTTGAAACACTCTTTTTGTAGTATCTGGAAGTGGACATTTGGAGCGCTCTCAGGA
          1 ATAGAGCAGGTTTGAAACACTCTTTTTGTAGTATCTGGAAGTGGATATTTGGATAGCTTTGAGGA

            * *  *      *             **          * *       * *          *  *
     265879 CTACGGT-GAAAAAGGAAATATCTTCCAATAAAAGCTACATAGAAGCAATGTCAGAAACTTTTTC
         66 TTTCGTTGGAAACAGG-AATATCTTCATATAAAAGCTAGACAGAAGCATTCTCAGAAACTTCTTT

            *      * ** *   **      *       *  * *   *
     265943 ATGATGTATCTACTCAGCTAACAGAGTTGAACCTTTCTTTTG
        130 GTGATGTTTGCATTCAAGTAACAGTGTTGAACATTCCCTTTC

             *       *                   * *                   **            
     265985 AGAGAGCAGTTTTGAAACACTCTTTTTGTGGAATCTGGAAGTGGATATTTGTCTAGCTTTGAGGA
          1 ATAGAGCAGGTTTGAAACACTCTTTTTGTAGTATCTGGAAGTGGATATTTGGATAGCTTTGAGGA

                         *   *    *                   *       *   *          
     266050 TTTCGTTGGAAACGGGATTA-CAT-ATA-AAAAGC-AGACAGCAGCATTCCCAGTAACTTCTTTG
         66 TTTCGTTGGAAACAGGAATATCTTCATATAAAAGCTAGACAGAAGCATTCTCAGAAACTTCTTTG

                              *    *                 
     266111 TGATGTTTGCATTCAAGTCACAGAGTTGAACATTCCCTTTC
        131 TGATGTTTGCATTCAAGTAACAGTGTTGAACATTCCCTTTC

                                                   *     *       **     *   *
     266152 ATAGAGCAGGTTTGAAACACTCTTTTTGTAGTATCTGGATGTGGACATTTGGAGCGCTTTCAGGC
          1 ATAGAGCAGGTTTGAAACACTCTTTTTGTAGTATCTGGAAGTGGATATTTGGATAGCTTTGAGGA

            * ** *      *             *** *   *                      *   *   
     266217 CTATGGT-GAAAAAGGAAATATCTTCCCCTGAAAACTAGACAGAAGCATTCTCAGAATCTTATTT
         66 TTTCGTTGGAAACAGG-AATATCTTCATATAAAAGCTAGACAGAAGCATTCTCAGAAACTTCTTT

                   **  **    *             **  * *   *
     266281 GTGATGTGCGCCCTCAACTAACAGTGTTGAAGCTTTCTTTTG
        130 GTGATGTTTGCATTCAAGTAACAGTGTTGAACATTCCCTTTC

                     *                    **     *   *                       
     266323 ATAGAGCAGTTTTGAAACACTCTTTTTGTAAAATCTGCAAGAGGATATTTGGATAGCTTTGAGGA
          1 ATAGAGCAGGTTTGAAACACTCTTTTTGTAGTATCTGGAAGTGGATATTTGGATAGCTTTGAGGA

                *        *   * *            **                      *    *   
     266388 TTTCTTTGGAAACGGGATTGTCTTCATATAAACTCTAGACAGAAGCATTCTCAGAAGCTTCATTG
         66 TTTCGTTGGAAACAGGAATATCTTCATATAAAAGCTAGACAGAAGCATTCTCAGAAACTTCTTTG

            *       **   *    *             *        
     266453 GGATGTTTCAATTGAAGTCACAGTGTTGAACAGTCCCTTTC
        131 TGATGTTTGCATTCAAGTAACAGTGTTGAACATTCCCTTTC

                                                   *     *       **     *   *
     266494 ATAGAGCAGGTTTGAAACACTCTTTTTGTAGTATCTGGATGTGGACATTTGGAGCGCTTTCAGGC
          1 ATAGAGCAGGTTTGAAACACTCTTTTTGTAGTATCTGGAAGTGGATATTTGGATAGCTTTGAGGA

            * ** *      *             *** *   *                          *   
     266559 CTATGGT-GAAAAAGGAAATATCTTCCCCTGAAAACTAGACAGAAGCATTCTCAGAAACTTATTT
         66 TTTCGTTGGAAACAGG-AATATCTTCATATAAAAGCTAGACAGAAGCATTCTCAGAAACTTCTTT

                   **  **    *             **  * *   *
     266623 GTGATGTGCGCCCTCAACTAACAGTGTTGAAGCTTTCTTTTG
        130 GTGATGTTTGCATTCAAGTAACAGTGTTGAACATTCCCTTTC

                  *  *   *               * *     *    *   *   ***            
     266665 ATAGAGTAGTTTTTAAACACTCTTTTTGTGGAATCTGCAAGTTGATGTTTTTCTAGCTTTGAGGA
          1 ATAGAGCAGGTTTGAAACACTCTTTTTGTAGTATCTGGAAGTGGATATTTGGATAGCTTTGAGGA

                         *   *    *                   *       *     *   *    
     266730 TTTCGTTGGAAACGGGATTA-CAT-ATA-AAAAGC-AGACAGCAGCATTCCCAGAATCTTGTTTG
         66 TTTCGTTGGAAACAGGAATATCTTCATATAAAAGCTAGACAGAAGCATTCTCAGAAACTTCTTTG

                              *    *                 
     266791 TGATGTTTGCATTCAAGTCACAGAGTTGAACATTCCCTTTC
        131 TGATGTTTGCATTCAAGTAACAGTGTTGAACATTCCCTTTC

             *                         *           *     *       **     *   *
     266832 AGAGAGCAGGTTTGAAACACTCTTTTTATAGTATCTGGATGTGGACATTTGGAGCGCTTTCAGGC
          1 ATAGAGCAGGTTTGAAACACTCTTTTTGTAGTATCTGGAAGTGGATATTTGGATAGCTTTGAGGA

            * ** *  *   *  *         *** *   *                      *   *   *
     266897 CTATGGTGAAAAAAGAAATATCTTCCCCTGAAAACTAGACAGAAGCATTCTCAGAATCTTATTTC
         66 TTTCGTTGGAAACAGGAATATCTTCATATAAAAGCTAGACAGAAGCATTCTCAGAAACTTCTTTG

                  **  **    *             **  * *   *
     266962 TGATGTGCGCCCTCAACTAACAGTGTTGAAGCTTTCTTTTG
        131 TGATGTTTGCATTCAAGTAACAGTGTTGAACATTCCCTTTC

                     *                *   **     *   *        *              
     267003 ATAGAGCAGTTTTGAAACACTCTTTTCGTAAAATCTGCAAGAGGATATTTTGATAGCTTTGAGGA
          1 ATAGAGCAGGTTTGAAACACTCTTTTTGTAGTATCTGGAAGTGGATATTTGGATAGCTTTGAGGA

                         *   * *            **                      *    *   
     267068 TTTCGTTGGAAACGGGATTGTCTTCATATAAACTCTAGACAGAAGCATTCTCAGAAGCTTCATTG
         66 TTTCGTTGGAAACAGGAATATCTTCATATAAAAGCTAGACAGAAGCATTCTCAGAAACTTCTTTG

            *       **   *    *    *        *        
     267133 GGATGTTTCAATTGAAGTCACAGGGTTGAACAGTCCCTTTC
        131 TGATGTTTGCATTCAAGTAACAGTGTTGAACATTCCCTTTC

                                                         *       *  * * *    
     267174 ATAGAGCAGGTTTGAAACACTCTTTTTGTAGTATCTGGAAGTGGACATTTGGAGAGATCTCAGGA
          1 ATAGAGCAGGTTTGAAACACTCTTTTTGTAGTATCTGGAAGTGGATATTTGGATAGCTTTGAGGA

            * *  *    * *             **            *       * *          *  *
     267239 ATACGGT-GATAAAGGAAATATCTTCCAATAAAAGCTAGATAGAAGCAATGTCAGAAACTTTTTC
         66 TTTCGTTGGAAACAGG-AATATCTTCATATAAAAGCTAGACAGAAGCATTCTCAGAAACTTCTTT

            *      * ** *   **      *       *  * *   *
     267303 ATGATGTATCTACTCAGCTAACAGAGTTGAACCTTTCTTTTG
        130 GTGATGTTTGCATTCAAGTAACAGTGTTGAACATTCCCTTTC

             *       *                   * *     *             **            
     267345 AGAGAGCAGTTTTGAAACACTCTTTTTGTGGAATCTGCAAGTGGATATTTGTCTAGCTTTGAGGA
          1 ATAGAGCAGGTTTGAAACACTCTTTTTGTAGTATCTGGAAGTGGATATTTGGATAGCTTTGAGGA

                             *    *                   *       *              
     267410 TTTCGTTGGAAACAGGATTA-CAT-ATA-AAAAGC-AGACAGCAGCATTCCCAGAAACTTCTTTG
         66 TTTCGTTGGAAACAGGAATATCTTCATATAAAAGCTAGACAGAAGCATTCTCAGAAACTTCTTTG

                              *    *                 
     267471 TGATGTTTGCATTCAAGTCACAGAGTTGAACATTCCCTTTC
        131 TGATGTTTGCATTCAAGTAACAGTGTTGAACATTCCCTTTC

                               *                   *     *       **     *   *
     267512 ATAGAGCAGGTTTGAAACAGTCTTTTTGTAGTATCTGGATGTGGACATTTGGAGCGCTTTCAGGC
          1 ATAGAGCAGGTTTGAAACACTCTTTTTGTAGTATCTGGAAGTGGATATTTGGATAGCTTTGAGGA

            * ** *      *             *** *   * *                    **  *   
     267577 CTATGGT-GAAAAAGGAAATATCTTCCCCTGAAAACCAGACAGAAGCATTCTCAGAATTTTATTT
         66 TTTCGTTGGAAACAGG-AATATCTTCATATAAAAGCTAGACAGAAGCATTCTCAGAAACTTCTTT

              *    **  **    *             **  * *   *
     267641 GTCATGTGCGCCCTCAACTAACAGTGTTGAAGCTTTCTTTTG
        130 GTGATGTTTGCATTCAAGTAACAGTGTTGAACATTCCCTTTC

                     *                    **     **  *                       
     267683 ATAGAGCAGTTTTGAAACACTCTTTTTGTAAAATCTGCTAGAGGATATTTGGATAGCTTTGAGGA
          1 ATAGAGCAGGTTTGAAACACTCTTTTTGTAGTATCTGGAAGTGGATATTTGGATAGCTTTGAGGA

                *        *   * *            **                     **    *   
     267748 TTTCTTTGGAAACGGGATTGTCTTCATATAAACTCTAGACAGAAGCATTCTCAGATGCTTCATTG
         66 TTTCGTTGGAAACAGGAATATCTTCATATAAAAGCTAGACAGAAGCATTCTCAGAAACTTCTTTG

            *       **   *    *             *        
     267813 GGATGTTTCAATTGAAGTCACAGTGTTGAACAGTCCCTTTC
        131 TGATGTTTGCATTCAAGTAACAGTGTTGAACATTCCCTTTC

                                                   *     *       **     *   *
     267854 ATAGAGCAGGTTTGAAACACTCTTTTTGTAGTATCTGGATGTGGACATTTGGAGCGCTTTCAGGC
          1 ATAGAGCAGGTTTGAAACACTCTTTTTGTAGTATCTGGAAGTGGATATTTGGATAGCTTTGAGGA

            * ** *      *             *** *   *                          *   
     267919 CTATGGT-GAAAAAGGAAATATCTTCCCCTGAAAACTAGACAGAAGCATTCTCAGAAACTTATTT
         66 TTTCGTTGGAAACAGG-AATATCTTCATATAAAAGCTAGACAGAAGCATTCTCAGAAACTTCTTT

                   **  **    *       *     **  * *   *
     267983 GTGATGTGCGCCCTCAACTAACAGTTTTGAAGCTTTCTTTTG
        130 GTGATGTTTGCATTCAAGTAACAGTGTTGAACATTCCCTTTC

                     *                   * *     *             **            
     268025 ATAGAGCAGTTTTGAAACACTCTTTTTGTGGAATCTGCAAGTGGATATTTGTCTAGCTTTGAGGA
          1 ATAGAGCAGGTTTGAAACACTCTTTTTGTAGTATCTGGAAGTGGATATTTGGATAGCTTTGAGGA

                *        *   *    *                   *       *              
     268090 TTTCATTGGAAACGGGATTA-CAT-ATA-AAAAGC-AGACAGCAGCATTCCCAGAAACTTCTTTG
         66 TTTCGTTGGAAACAGGAATATCTTCATATAAAAGCTAGACAGAAGCATTCTCAGAAACTTCTTTG

                   *          *   **                 
     268151 TGATGTTCGCATTCAAGTCACACAGTTGAACATTCCCTTTC
        131 TGATGTTTGCATTCAAGTAACAGTGTTGAACATTCCCTTTC

                                                   *     *       **     *   *
     268192 ATAGAGCAGGTTTGAAACACTCTTTTTGTAGTATCTGGATGTGGACATTTGGAGCGCTTTCAGGC
          1 ATAGAGCAGGTTTGAAACACTCTTTTTGTAGTATCTGGAAGTGGATATTTGGATAGCTTTGAGGA

            * ** *      *             *** *   *                          *   
     268257 CTATGGT-GAAAAAGGATATATCTTCCCCTGAAAACTAGACAGAAGCATTCTCAGAAACTTATTT
         66 TTTCGTTGGAAACAGGA-ATATCTTCATATAAAAGCTAGACAGAAGCATTCTCAGAAACTTCTTT

                   **  **    *              *  * *    
     268321 GTGATGTGCGCCCTCAACTAACAGTGTTGAACCTTTCTTTTC
        130 GTGATGTTTGCATTCAAGTAACAGTGTTGAACATTCCCTTTC

                     *                    *      *   *                       
     268363 ATAGAGCAGTTTTGAAACACTCTTTTTGTAATATCTGCAAGAGGATATTTGGATAGCTTTGAGGA
          1 ATAGAGCAGGTTTGAAACACTCTTTTTGTAGTATCTGGAAGTGGATATTTGGATAGCTTTGAGGA

                         *   * *            **     *                *    *   
     268428 TTTCGTTGGAAACGGGATTGTCTTCATATAAACTCTAGATAGAAGCATTCTCAGAAGCTTCATTG
         66 TTTCGTTGGAAACAGGAATATCTTCATATAAAAGCTAGACAGAAGCATTCTCAGAAACTTCTTTG

            *       **   *    *             *        
     268493 GGATGTTTCAATTGAAGTCACAGTGTTGAACAGTCCCTTTC
        131 TGATGTTTGCATTCAAGTAACAGTGTTGAACATTCCCTTTC

                                                         *       *  * * *    
     268534 ATAGAGCAGGTTTGAAACACTCTTTTTGTAGTATCTGGAAGTGGACATTTGGAGAGATCTCAGGA
          1 ATAGAGCAGGTTTGAAACACTCTTTTTGTAGTATCTGGAAGTGGATATTTGGATAGCTTTGAGGA

            * *  *      *             **            *       * *          *  *
     268599 ATACGGT-GAAAAAGGAAATATCTTCCAATAAAAGCTAGATAGAAGCAATGTCAGAAACTTTTTC
         66 TTTCGTTGGAAACAGG-AATATCTTCATATAAAAGCTAGACAGAAGCATTCTCAGAAACTTCTTT

            *      * ** *   **      *       *  * *     *
     268663 ATGATGTATCTACTCAGCTAACAGAGTTGAACCTTTCTTTTGAG
        130 GTGATGTTTGCATTCAAGTAACAGTGTTGAACATTCCCTTT--C

             *       *                   * *     *             **            
     268707 AGAGAGCAGTTTTGAAACACTCTTTTTGTGGAATCTGCAAGTGGATATTTGTCTAGCTTTGAGGA
          1 ATAGAGCAGGTTTGAAACACTCTTTTTGTAGTATCTGGAAGTGGATATTTGGATAGCTTTGAGGA

                         *   *    *              *    *       *              
     268772 TTTCGTTGGAAACGGGATTA-CAT-ATA-AAAAGC-ACACAGCAGCATTCCCAGAAACTTCTTTG
         66 TTTCGTTGGAAACAGGAATATCTTCATATAAAAGCTAGACAGAAGCATTCTCAGAAACTTCTTTG

               *              *    *                 
     268833 TGAAGTTTGCATTCAAGTCACAGAGTTGAACATTCCCTTTC
        131 TGATGTTTGCATTCAAGTAACAGTGTTGAACATTCCCTTTC

                                                 * *     *       **     *   *
     268874 ATAGAGCAGGTTTGAAACACTCTTTTTGTAGTATCTGTATGTGGACATTTGGAGCGCTTTCAGGC
          1 ATAGAGCAGGTTTGAAACACTCTTTTTGTAGTATCTGGAAGTGGATATTTGGATAGCTTTGAGGA

            * ** *      *             *** *   *                      *   *   
     268939 CTATGGT-GAAAAAGGAAATATCTTCCCCTGAAAACTAGACAGAAGCATTCTCAGAATCTTATTT
         66 TTTCGTTGGAAACAGG-AATATCTTCATATAAAAGCTAGACAGAAGCATTCTCAGAAACTTCTTT

                   **  **    *             **  * *   *
     269003 GTGATGTGCGCCCTCAACTAACAGTGTTGAAGCTTTCTTTTG
        130 GTGATGTTTGCATTCAAGTAACAGTGTTGAACATTCCCTTTC

                     *                    **     *   *                       
     269045 ATAGAGCAGTTTTGAAACACTCTTTTTGTAAAATCTGCAAGAGGATATTTGGATAGCTTTGAGGA
          1 ATAGAGCAGGTTTGAAACACTCTTTTTGTAGTATCTGGAAGTGGATATTTGGATAGCTTTGAGGA

                         *   * *            **                      *    *   
     269110 TTTCGTTGGAAACGGGATTGTCTTCATATAAACTCTAGACAGAAGCATTCTCAGAAGCTTCATTG
         66 TTTCGTTGGAAACAGGAATATCTTCATATAAAAGCTAGACAGAAGCATTCTCAGAAACTTCTTTG

            *       **   *    *    *                 
     269175 GGATGTTTCAATTGAAGTCACAGAGTTGAACATTCCCTTTC
        131 TGATGTTTGCATTCAAGTAACAGTGTTGAACATTCCCTTTC

                                                   *     *     * **     *   *
     269216 ATAGAGCAGGTTTGAAACACTCTTTTTGTAGTATCTGGATGTGGACATTTGCAGCGCTTTCAGGC
          1 ATAGAGCAGGTTTGAAACACTCTTTTTGTAGTATCTGGAAGTGGATATTTGGATAGCTTTGAGGA

            * ** *      *             *** *   *                 *    *    *  
     269281 CTAAGGT-GAAAAAGGAAATATCTTCCCCTGAAAACTAGACAGAAGCATTCTAAGAAGCTTCATT
         66 TTTCGTTGGAAACAGG-AATATCTTCATATAAAAGCTAGACAGAAGCATTCTCAGAAACTTCTTT

             *       **   *    *             *        
     269345 GGGATGTTTCAATTGAAGTCACAGTGTTGAACAGTCCCTTTC
        130 GTGATGTTTGCATTCAAGTAACAGTGTTGAACATTCCCTTTC

                                                         *       *  * * *    
     269387 ATAGAGCAGGTTTGAAACACTCTTTTTGTAGTATCTGGAAGTGGACATTTGGAGAGATCTCAGGA
          1 ATAGAGCAGGTTTGAAACACTCTTTTTGTAGTATCTGGAAGTGGATATTTGGATAGCTTTGAGGA

            * *  *    * *             **            *       * *          *  *
     269452 ATACGGT-GATAAAGGAAATATCTTCCAATAAAAGCTAGATAGAAGCAATGTCAGAAACTTTTTC
         66 TTTCGTTGGAAACAGG-AATATCTTCATATAAAAGCTAGACAGAAGCATTCTCAGAAACTTCTTT

            *      * ** *   **      *       *  * *   *
     269516 ATGATGTATCTACTCAGCTAACAGAGTTGAACCTTTCTTTTG
        130 GTGATGTTTGCATTCAAGTAACAGTGTTGAACATTCCCTTTC

             *       *                   * *     *             **            
     269558 AGAGAGCAGTTTTGAAACACTCTTTTTGTGGAATCTGCAAGTGGATATTTGTCTAGCTTTGAGGA
          1 ATAGAGCAGGTTTGAAACACTCTTTTTGTAGTATCTGGAAGTGGATATTTGGATAGCTTTGAGGA

                         *   *    *                   *       *              
     269623 TTTCGTTGGAAACGGGATTA-CAT-ATA-AAAAGC-AGACAGCAGCATTCCCAGAAACTTCTTTG
         66 TTTCGTTGGAAACAGGAATATCTTCATATAAAAGCTAGACAGAAGCATTCTCAGAAACTTCTTTG

                              *    *                 
     269684 TGATGTTTGCATTCAAGTCACAGAGTTGAACATTCCCTTTC
        131 TGATGTTTGCATTCAAGTAACAGTGTTGAACATTCCCTTTC

                           *                       *     *       **     *   *
     269725 ATAGAGCAGGTTTGAGACACTCTTTTTGTAGTATCTGGATGTGGACATTTGGAGCGCTTTCAGGC
          1 ATAGAGCAGGTTTGAAACACTCTTTTTGTAGTATCTGGAAGTGGATATTTGGATAGCTTTGAGGA

            * ** *      *             *** *   *                          *   
     269790 CTATGGT-GAAAAAGGAAATATCTTCCCCTGAAAACTAGACAGAAGCATTCTCAGAAACTTATTT
         66 TTTCGTTGGAAACAGG-AATATCTTCATATAAAAGCTAGACAGAAGCATTCTCAGAAACTTCTTT

                   **  **    *             **  * *   *
     269854 GTGATGTGCGCCCTCAACTAACAGTGTTGAAGCTTTCTTTTG
        130 GTGATGTTTGCATTCAAGTAACAGTGTTGAACATTCCCTTTC

                     *                    *      *   *                       
     269896 ATAGAGCAGTTTTGAAACACTCTTTTTGTAATATCTGCAAGAGGATATTTGGATAGCTTTGAGGA
          1 ATAGAGCAGGTTTGAAACACTCTTTTTGTAGTATCTGGAAGTGGATATTTGGATAGCTTTGAGGA

                         *   * *            **                      *    *   
     269961 TTTCGTTGGAAACGGGATTGTCTTCATATAAACTCTAGACAGAAGCATTCTCAGAAGCTTCATTG
         66 TTTCGTTGGAAACAGGAATATCTTCATATAAAAGCTAGACAGAAGCATTCTCAGAAACTTCTTTG

            * *     **   *    *             * *      
     270026 GGTTGTTTCAATTGAAGTCACAGTGTTGAACAGTTCCTTTC
        131 TGATGTTTGCATTCAAGTAACAGTGTTGAACATTCCCTTTC

                 *                                       *       **   * *    
     270067 ATAGAACAGGTTTGAAACACTCTTTTTGTAGTATCTGGAAGTGGACATTTGGAGGGCTCTCAGGA
          1 ATAGAGCAGGTTTGAAACACTCTTTTTGTAGTATCTGGAAGTGGATATTTGGATAGCTTTGAGGA

            * ** *      *             **          * *       * *          *  *
     270132 CTATGGT-GAAAAAGGAAATATCTTCCAATAAAAGCTACATAGAAGCAATGTCAGAAACTTTTTC
         66 TTTCGTTGGAAACAGG-AATATCTTCATATAAAAGCTAGACAGAAGCATTCTCAGAAACTTCTTT

            *      * ** *   **      * *     *  *     *
     270196 ATGATGTATCTACTCAGCTAACAGAGGTGAACCTTTCCTTTG
        130 GTGATGTTTGCATTCAAGTAACAGTGTTGAACATTCCCTTTC

             *       *                   * *     *             **            
     270238 AGAGAGCAGTTTTGAAACACTCTTTTTGTGGAATCTGCAAGTGGATATTTGTCTAGCTTTGAGGA
          1 ATAGAGCAGGTTTGAAACACTCTTTTTGTAGTATCTGGAAGTGGATATTTGGATAGCTTTGAGGA

                         *   *    *                   *       *   *          
     270303 TTTCGTTGGAAACGGGATTA-CAT-ATA-AAAAGC-AGACAGCAGCATTCCCAGTAACTTCTTTG
         66 TTTCGTTGGAAACAGGAATATCTTCATATAAAAGCTAGACAGAAGCATTCTCAGAAACTTCTTTG

                              ***  *                 
     270364 TGATGTTTGCATTCAAGTCTGAGAGTTGAACATTCCCTTTC
        131 TGATGTTTGCATTCAAGTAACAGTGTTGAACATTCCCTTTC

                                        *          *     *       **     *   *
     270405 ATAGAGCAGGTTTGAAACACTCTTTTTGAAGTATCTGGATGTGGACATTTGGAGCGCTTTCAGGC
          1 ATAGAGCAGGTTTGAAACACTCTTTTTGTAGTATCTGGAAGTGGATATTTGGATAGCTTTGAGGA

            * ** *      *             *** *   *                          *   
     270470 CTATGGT-GAAAAAGGAAATATCTTCCCCTGAAAACTAGACAGAAGCATTCTCAGAAACTTATTT
         66 TTTCGTTGGAAACAGG-AATATCTTCATATAAAAGCTAGACAGAAGCATTCTCAGAAACTTCTTT

                   **  **    *              *  * *   *
     270534 GTGATGTGCGCCCTCAACTAACAGTGTTGAACCTTTCTTTTG
        130 GTGATGTTTGCATTCAAGTAACAGTGTTGAACATTCCCTTTC

                     *                    *      *   *                       
     270576 ATAGAGCAGTTTTGAAACACTCTTTTTGTAATATCTGCAAGAGGATATTTGGATAGCTTTGAGGA
          1 ATAGAGCAGGTTTGAAACACTCTTTTTGTAGTATCTGGAAGTGGATATTTGGATAGCTTTGAGGA

                         *   * *            **                      *    *   
     270641 TTTCGTTGGAAACGGGATTGTCTTCATATAAACTCTAGACAGAAGCATTCTCAGAAGCTTCATTG
         66 TTTCGTTGGAAACAGGAATATCTTCATATAAAAGCTAGACAGAAGCATTCTCAGAAACTTCTTTG

            *       **   *    *             *        
     270706 GGATGTTTCAATTGAAGTCACAGTGTTGAACAGTCCCTTTC
        131 TGATGTTTGCATTCAAGTAACAGTGTTGAACATTCCCTTTC

                                                         *       **   * *    
     270747 ATAGAGCAGGTTTGAAACACTCTTTTTGTAGTATCTGGAAGTGGACATTTGGAGCGCTCTCAGGA
          1 ATAGAGCAGGTTTGAAACACTCTTTTTGTAGTATCTGGAAGTGGATATTTGGATAGCTTTGAGGA

            * *  *    * *             **            *       * *          *  *
     270812 CTACGGT-GATAAAGGAAATATCTTCCAATAAAAGCTAGATAGAAGCAATGTCAGAAACTTTTTC
         66 TTTCGTTGGAAACAGG-AATATCTTCATATAAAAGCTAGACAGAAGCATTCTCAGAAACTTCTTT

            * *    * ** *   **      *       *  * *   *
     270876 ATAATGTATCTACTCAGCTAACAGAGTTGAACCTTTCTTTTG
        130 GTGATGTTTGCATTCAAGTAACAGTGTTGAACATTCCCTTTC

             *       *                   * *     *             **   *        
     270918 AGAGAGCAGTTTTGAAACACTCTTTTTGTGGAATCTGCAAGTGGATATTTGTCTAGATTTGAGGA
          1 ATAGAGCAGGTTTGAAACACTCTTTTTGTAGTATCTGGAAGTGGATATTTGGATAGCTTTGAGGA

                         *   *    *                   *       *     *        
     270983 TTTCGTTGGAAACGGGATTA-CAT-ATA-AAAAGC-AGACAGCAGCATTCCCAGAATCTTCTTTG
         66 TTTCGTTGGAAACAGGAATATCTTCATATAAAAGCTAGACAGAAGCATTCTCAGAAACTTCTTTG

                              * *  *                 
     271044 TGATGTTTGCATTCAAGTCAGAGAGTTGAACATTCCCTTTC
        131 TGATGTTTGCATTCAAGTAACAGTGTTGAACATTCCCTTTC

                                                   *     *     * **     *   *
     271085 ATAGAGCAGGTTTGAAACACTCTTTTTGTAGTATCTGGATGTGGACATTTGCAGCGCTTTCAGGC
          1 ATAGAGCAGGTTTGAAACACTCTTTTTGTAGTATCTGGAAGTGGATATTTGGATAGCTTTGAGGA

            * ** *      *             *** *   *                          *   
     271150 CTAAGGT-GAAAAAGGAAATATCTTCCCCTGAAAACTAGACAGAAGCATTCTCAGAAACTTATTT
         66 TTTCGTTGGAAACAGG-AATATCTTCATATAAAAGCTAGACAGAAGCATTCTCAGAAACTTCTTT

                   **  **    *             **  * *   *
     271214 GTGATGTGCGCCCTCAACTAACAGTGTTGAAGCTTTCTTTTG
        130 GTGATGTTTGCATTCAAGTAACAGTGTTGAACATTCCCTTTC

                     *                   * *     *             **            
     271256 ATAGAGCAGTTTTGAAACACTCTTTTTGTGGAATCTGCAAGTGGATATTTGTCTAGCTTTGAGGA
          1 ATAGAGCAGGTTTGAAACACTCTTTTTGTAGTATCTGGAAGTGGATATTTGGATAGCTTTGAGGA

                         *   *    *                   *       *     *   *  * 
     271321 TTTCGTTGGAAACGGGATTA-CAT-ATA-AAAAGC-AGACAGCAGCATTCCCAGAATCTTGTTCG
         66 TTTCGTTGGAAACAGGAATATCTTCATATAAAAGCTAGACAGAAGCATTCTCAGAAACTTCTTTG

                              *    *                 
     271382 TGATGTTTGCATTCAAGTCACAGAGTTGAACATTCCCTTTC
        131 TGATGTTTGCATTCAAGTAACAGTGTTGAACATTCCCTTTC

             *                         *           *     *       **     *   *
     271423 AGAGAGCAGGTTTGAAACACTCTTTTTATAGTATCTGGATGTGGACATTTGGAGCGCTTTCAGGC
          1 ATAGAGCAGGTTTGAAACACTCTTTTTGTAGTATCTGGAAGTGGATATTTGGATAGCTTTGAGGA

            * ** *      *             *** *   *                      *   *   
     271488 CTATGGT-GAAAAAGGAAATATCTTCTCCTGAAAACTAGACAGAAGCATTCTCAGAATCTTATTT
         66 TTTCGTTGGAAACAGG-AATATCTTCATATAAAAGCTAGACAGAAGCATTCTCAGAAACTTCTTT

                   *** **    *             **  * *   *
     271552 GTGATGTGCACCCTCAACTAACAGTGTTGAAGCTTTCTTTTG
        130 GTGATGTTTGCATTCAAGTAACAGTGTTGAACATTCCCTTTC

                     *                *   **     *   *                       
     271594 ATAGAGCAGTTTTGAAACACTCTTTTCGTAAAATCTGCAAGAGGATATTTGGATAGCTTTGAGGA
          1 ATAGAGCAGGTTTGAAACACTCTTTTTGTAGTATCTGGAAGTGGATATTTGGATAGCTTTGAGGA

                         *   * *            **                      *    *   
     271659 TTTCGTTGGAAACGGGATTGTCTTCATATAAACTCTAGACAGAAGCATTCTCAGAAGCTTCATTG
         66 TTTCGTTGGAAACAGGAATATCTTCATATAAAAGCTAGACAGAAGCATTCTCAGAAACTTCTTTG

            *       **   *    *             * *      
     271724 GGATGTTTCAATTGAAGTCACAGTGTTGAACAGTTCCTTTC
        131 TGATGTTTGCATTCAAGTAACAGTGTTGAACATTCCCTTTC

                 *                                       *       *  * * *    
     271765 ATAGAACAGGTTTGAAACACTCTTTTTGTAGTATCTGGAAGTGGACATTTGGAGAGATCTCAGGA
          1 ATAGAGCAGGTTTGAAACACTCTTTTTGTAGTATCTGGAAGTGGATATTTGGATAGCTTTGAGGA

            * * **    * *             **          * *       * *          *  *
     271830 ATACAGT-GATAAAGGAAATATCTTCCAATAAAAGCTACATAGAAGCAATGTCAGAAACTTTTTC
         66 TTTCGTTGGAAACAGG-AATATCTTCATATAAAAGCTAGACAGAAGCATTCTCAGAAACTTCTTT

            *      * ** *   **      *       *  * *   *
     271894 ATGATGTATCTACTCAGCTAACAGAGTTGAACCTTTCTTTTG
        130 GTGATGTTTGCATTCAAGTAACAGTGTTGAACATTCCCTTTC

             *       *                   * *     *             **            
     271936 AGAGAGCAGTTTTGAAACACTCTTTTTGTGGAATCTGCAAGTGGATATTTGTCTAGCTTTGAGGA
          1 ATAGAGCAGGTTTGAAACACTCTTTTTGTAGTATCTGGAAGTGGATATTTGGATAGCTTTGAGGA

                         *  **    *                   *       *              
     272001 TTTCGTTGGAAACGGGTTTA-CAT-ATA-AAAAGC-AGACAGCAGCATTCCCAGAAACTTCTTTG
         66 TTTCGTTGGAAACAGGAATATCTTCATATAAAAGCTAGACAGAAGCATTCTCAGAAACTTCTTTG

               *              *    *                 
     272062 TGAAGTTTGCATTCAAGTCACAGAGTTGAACATTCCCTTTC
        131 TGATGTTTGCATTCAAGTAACAGTGTTGAACATTCCCTTTC

                                                 * *     *       **     *    
     272103 ATAGAGCAGGTTTGAAACACTCTTTTTGTAGTATCTGTATGTGGACATTTGGAGCGCTTTCAGGA
          1 ATAGAGCAGGTTTGAAACACTCTTTTTGTAGTATCTGGAAGTGGATATTTGGATAGCTTTGAGGA

            * ** *      *             *** *   *                      *   *   
     272168 CTATGGT-GAAAAAGGAAATATCTTCCCCTGAAAACTAGACAGAAGCATTCTCAGAATCTTATTT
         66 TTTCGTTGGAAACAGG-AATATCTTCATATAAAAGCTAGACAGAAGCATTCTCAGAAACTTCTTT

                   **  **    *             **  * *   *
     272232 GTGATGTGCGCCCTCAACTAACAGTGTTGAAGCTTTCTTTTG
        130 GTGATGTTTGCATTCAAGTAACAGTGTTGAACATTCCCTTTC

                  *  *                    **     *   *                       
     272274 ATAGAGGAGTTTTGAAACACTCTTTTTGTAAAATCTGCAAGAGGATATTTGGATAGCTTTGAGGA
          1 ATAGAGCAGGTTTGAAACACTCTTTTTGTAGTATCTGGAAGTGGATATTTGGATAGCTTTGAGGA

                         *   * *            **    *                 *    *   
     272339 TTTCGTTGGAAACGGGATTGTCTTCATATAAACTCTAGGCAGAAGCATTCTCAGAAGCTTCATTG
         66 TTTCGTTGGAAACAGGAATATCTTCATATAAAAGCTAGACAGAAGCATTCTCAGAAACTTCTTTG

            *       **   *    *             *        
     272404 GGATGTTTCAATTGAAGTCACAGTGTTGAACAGTCCCTTTC
        131 TGATGTTTGCATTCAAGTAACAGTGTTGAACATTCCCTTTC

                 *                                 *     *       **     *    
     272445 ATAGAACAGGTTTGAAACACTCTTTTTGTAGTATCTGGATGTGGACATTTGGAGCGCTTTCAGGC
          1 ATAGAGCAGGTTTGAAACACTCTTTTTGTAGTATCTGGAAGTGGATATTTGGATAGCTTTGAGG-

            * * *    *                 *** *   *                          *  
     272510 CTATGGTT-TAAA-AGGAAATATCTTCCCCTGAAAACTAGACAGAAGCATTCTCAGAAACTTATT
         65 ATTTCGTTGGAAACAGG-AATATCTTCATATAAAAGCTAGACAGAAGCATTCTCAGAAACTTCTT

                    **  **    *                    *   *
     272573 TGTGATGTGCGCCCTCAACTAACAGTGTTGAAGCATT-CTTTTG
        129 TGTGATGTTTGCATTCAAGTAACAGTGTTGAA-CATTCCCTTTC

                     *                 * * *     *             **            
     272616 ATAGAGCAGTTTTGAAACACTCTTTTTCTGGAATCTGCAAGTGGATATTTGTCTAGCTTTGAGGA
          1 ATAGAGCAGGTTTGAAACACTCTTTTTGTAGTATCTGGAAGTGGATATTTGGATAGCTTTGAGGA

                         *   *    *                   *       *              
     272681 TTTCGTTGGAAACGGGATTA-CAT-ATA-AAAAGC-AGACAGCAGCATTCCCAGAAACTTCTTTG
         66 TTTCGTTGGAAACAGGAATATCTTCATATAAAAGCTAGACAGAAGCATTCTCAGAAACTTCTTTG

                              *    *                 
     272742 TGATGTTTGCATTCAAGTCACAGAGTTGAACATTCCCTTTC
        131 TGATGTTTGCATTCAAGTAACAGTGTTGAACATTCCCTTTC

                                                   *     *       **     *   *
     272783 ATAGAGCAGGTTTGAAACACTCTTTTTGTAGTATCTGGATGTGGACATTTGGAGCGCTTTCAGGC
          1 ATAGAGCAGGTTTGAAACACTCTTTTTGTAGTATCTGGAAGTGGATATTTGGATAGCTTTGAGGA

            * ** *      *             *** *   *                      *   *   
     272848 CTATGGT-GAAAAAGGAAATATCTTCCCCTGAAAACTAGACAGAAGCATTCTCAGAATCTTATTT
         66 TTTCGTTGGAAACAGG-AATATCTTCATATAAAAGCTAGACAGAAGCATTCTCAGAAACTTCTTT

                   **  **    *             **  * *   *
     272912 GTGATGTGCGCCCTCAACTAACAGTGTTGAAGCTTTCTTTTG
        130 GTGATGTTTGCATTCAAGTAACAGTGTTGAACATTCCCTTTC

                     *                    **     *   *                       
     272954 ATAGAGCAGTTTTGAAACACTCTTTTTGTAAAATCTGCAAGAGGATATTTGGATAGCTTTGAGGA
          1 ATAGAGCAGGTTTGAAACACTCTTTTTGTAGTATCTGGAAGTGGATATTTGGATAGCTTTGAGGA

                         *   * *            **                      *    *   
     273019 TTTCGTTGGAAACGGGATTGTCTTCATATAAACTCTAGACAGAAGCATTCTCAGAAGCTTCATTG
         66 TTTCGTTGGAAACAGGAATATCTTCATATAAAAGCTAGACAGAAGCATTCTCAGAAACTTCTTTG

            *       **   *    *                      
     273084 GGATGTTTCAATTGAAGTCACAGTGTTGAACATTCCCTTTC
        131 TGATGTTTGCATTCAAGTAACAGTGTTGAACATTCCCTTTC

                                                         *       *  * * *    
     273125 ATAGAGCAGGTTTGAAACACTCTTTTTGTAGTATCTGGAAGTGGACATTTGGAGAGATCTCAGGA
          1 ATAGAGCAGGTTTGAAACACTCTTTTTGTAGTATCTGGAAGTGGATATTTGGATAGCTTTGAGGA

            * *  *    * *             **            *     * * *          *  *
     273190 ATACGGT-GATAAAGGAAATATCTTCCAATAAAAGCTAGATAGAAGAAATGTCAGAAACTTTTTC
         66 TTTCGTTGGAAACAGG-AATATCTTCATATAAAAGCTAGACAGAAGCATTCTCAGAAACTTCTTT

            *      * ** *   **      *       *  * *   *
     273254 ATGATGTATCTACTCAGCTAACAGAGTTGAACCTTTCTTTTG
        130 GTGATGTTTGCATTCAAGTAACAGTGTTGAACATTCCCTTTC

             *       *                   * *     *             **            
     273296 AGAGAGCAGTTTTGAAACACTCTTTTTGTGGAATCTGCAAGTGGATATTTGTCTAGCTTTGAGGA
          1 ATAGAGCAGGTTTGAAACACTCTTTTTGTAGTATCTGGAAGTGGATATTTGGATAGCTTTGAGGA

                             *    *                   *       *              
     273361 TTTCGTTGGAAACAGGATTA-CAT-ATA-AAAAGC-AGACAGCAGCATTCCCAGAAACTTCTTTG
         66 TTTCGTTGGAAACAGGAATATCTTCATATAAAAGCTAGACAGAAGCATTCTCAGAAACTTCTTTG

                              *    *          *      
     273422 TGATGTTTGCATTCAAGTCACAGAGTTGAACATTGCCTTTC
        131 TGATGTTTGCATTCAAGTAACAGTGTTGAACATTCCCTTTC

                               *                   *     *       **         *
     273463 ATAGAGCAGGTTTGAAACAGTCTTTTTGTAGTATCTGGATGTGGACATTTGGAGCGCTTTGAGGC
          1 ATAGAGCAGGTTTGAAACACTCTTTTTGTAGTATCTGGAAGTGGATATTTGGATAGCTTTGAGGA

            * ** *      *             *** *   *                      **  *   
     273528 CTATGGT-GAAAAAGGAAATATCTTCCCCTGAAAACTAGACAGAAGCATTCTCAGAATTTTATTT
         66 TTTCGTTGGAAACAGG-AATATCTTCATATAAAAGCTAGACAGAAGCATTCTCAGAAACTTCTTT

                   **  **    *             **  * **  *
     273592 GTGATGTGCGCCCTCAACTAACAGTGTTGAAGCTTTCTATTG
        130 GTGATGTTTGCATTCAAGTAACAGTGTTGAACATTCCCTTTC

                     *            *       **     **  *                       
     273634 ATAGAGCAGTTTTGAAACACTCCTTTTGTAAAATCTGCTAGAGGATATTTGGATAGCTTTGAGGA
          1 ATAGAGCAGGTTTGAAACACTCTTTTTGTAGTATCTGGAAGTGGATATTTGGATAGCTTTGAGGA

                *        *   * *            **                     **    *   
     273699 TTTCTTTGGAAACGGGATTGTCTTCATATAAACTCTAGACAGAAGCATTCTCAGATGCTTCATTG
         66 TTTCGTTGGAAACAGGAATATCTTCATATAAAAGCTAGACAGAAGCATTCTCAGAAACTTCTTTG

            *       **   *    *             *        
     273764 GGATGTTTCAATTGAAGTCACAGTGTTGAACAGTCCCTTTC
        131 TGATGTTTGCATTCAAGTAACAGTGTTGAACATTCCCTTTC

                                                   *     *       **   * *    
     273805 ATAGAGCAGGTTTGAAACACTCTTTTTGTAGTATCTGGATGTGGACATTTGGAGCGCTCTCAGGA
          1 ATAGAGCAGGTTTGAAACACTCTTTTTGTAGTATCTGGAAGTGGATATTTGGATAGCTTTGAGGA

            * *  *      *             **          * *       * *         **  *
     273870 CTACGGT-GAAAAAGGAAATATCTTCCAATAAAAGCTACATAGAAGCAATGTCAGAAACTATTTC
         66 TTTCGTTGGAAACAGG-AATATCTTCATATAAAAGCTAGACAGAAGCATTCTCAGAAACTTCTTT

            *      * ** *   **      *       *  * *   *
     273934 ATGATGTATCTACTCAGCTAACAGAGTTGAACCTTTCTTTTG
        130 GTGATGTTTGCATTCAAGTAACAGTGTTGAACATTCCCTTTC

             *       *                   * *                   **            
     273976 AGAGAGCAGTTTTGAAACACTCTTTTTGTGGAATCTGGAAGTGGATATTTGTCTAGCTTTGAGGA
          1 ATAGAGCAGGTTTGAAACACTCTTTTTGTAGTATCTGGAAGTGGATATTTGGATAGCTTTGAGGA

                         *   *    *                   *       *     *   *    
     274041 TTTCGTTGGAAACGGGATTA-CAT-ATA-AAAAGC-AGACAGCAGCATTCCCAGAATCTTGTTTG
         66 TTTCGTTGGAAACAGGAATATCTTCATATAAAAGCTAGACAGAAGCATTCTCAGAAACTTCTTTG

                              *    *                 
     274102 TGATGTTTGCATTCAAGTCACAGAGTTGAACATTCCCTTTC
        131 TGATGTTTGCATTCAAGTAACAGTGTTGAACATTCCCTTTC

             *                         *           *     *       **     *   *
     274143 AGAGAGCAGGTTTGAAACACTCTTTTTATAGTATCTGGATGTGGACATTTGGAGCGCTTTCAGGC
          1 ATAGAGCAGGTTTGAAACACTCTTTTTGTAGTATCTGGAAGTGGATATTTGGATAGCTTTGAGGA

            * ** *      *             *** *   *                 *    *   *   
     274208 CTATGGT-GAAAAAGGAAATATCTTCCCCTGAAAACTAGACAGAAGCATTCTTAGAATCTTATTT
         66 TTTCGTTGGAAACAGG-AATATCTTCATATAAAAGCTAGACAGAAGCATTCTCAGAAACTTCTTT

            *      **  **    *             **  * *   *
     274272 CTGATGTGCGCCCTCAACTAACAGTGTTGAAGCTTTCTTTTG
        130 GTGATGTTTGCATTCAAGTAACAGTGTTGAACATTCCCTTTC

                     *                *   **     *   *        *              
     274314 ATAGAGCAGTTTTGAAACACTCTTTTCGTAAAATCTGCAAGAGGATATTTTGATAGCTTTGAGGA
          1 ATAGAGCAGGTTTGAAACACTCTTTTTGTAGTATCTGGAAGTGGATATTTGGATAGCTTTGAGGA

                         *   * *            **                      *    *   
     274379 TTTCGTTGGAAACGGGATTGTCTTCATATAAACTCTAGACAGAAGCATTCTCAGAAGCTTCATTG
         66 TTTCGTTGGAAACAGGAATATCTTCATATAAAAGCTAGACAGAAGCATTCTCAGAAACTTCTTTG

            *       **   *    *    *        *        
     274444 GGATGTTTCAATTGAAGTCACAGGGTTGAACAGTCCCTTTC
        131 TGATGTTTGCATTCAAGTAACAGTGTTGAACATTCCCTTTC

                                                         *       *  * * *    
     274485 ATAGAGCAGGTTTGAAACACTCTTTTTGTAGTATCTGGAAGTGGACATTTGGAGAGATGTCAGGA
          1 ATAGAGCAGGTTTGAAACACTCTTTTTGTAGTATCTGGAAGTGGATATTTGGATAGCTTTGAGGA

            * *  *    * *             **            *       * *          *  *
     274550 ATACGGT-GATAAAGGAAATATCTTCCAATAAAAGCTAGATAGAAGCAATGTCAGAAACTTTTTC
         66 TTTCGTTGGAAACAGG-AATATCTTCATATAAAAGCTAGACAGAAGCATTCTCAGAAACTTCTTT

            *      * ** *   **      *       *  * *   *
     274614 ATGATGTATCTACTCAGCTAACAGAGTTGAACCTTTCTTTTG
        130 GTGATGTTTGCATTCAAGTAACAGTGTTGAACATTCCCTTTC

             *       *                   * *     *             **            
     274656 AGAGAGCAGTTTTGAAACACTCTTTTTGTGGAATCTGCAAGTGGATATTTGTCTAGCTTTGAGGA
          1 ATAGAGCAGGTTTGAAACACTCTTTTTGTAGTATCTGGAAGTGGATATTTGGATAGCTTTGAGGA

                             *    *                   *       *              
     274721 TTTCGTTGGAAACAGGATTA-CAT-ATA-AAAAGC-AGACAGCAGCATTCCCAGAAACTTCTTTG
         66 TTTCGTTGGAAACAGGAATATCTTCATATAAAAGCTAGACAGAAGCATTCTCAGAAACTTCTTTG

                              *    *                 
     274782 TGATGTTTGCATTCAAGTCACAGAGTTGAACATTCCCTTTC
        131 TGATGTTTGCATTCAAGTAACAGTGTTGAACATTCCCTTTC

                               *                   *     *       **     *   *
     274823 ATAGAGCAGGTTTGAAACAGTCTTTTTGTAGTATCTGGATGTGGACATTTGGAGCGCTTTCAGGC
          1 ATAGAGCAGGTTTGAAACACTCTTTTTGTAGTATCTGGAAGTGGATATTTGGATAGCTTTGAGGA

            * ** *      *             *** *   *                      **  *   
     274888 CTATGGT-GAAAAAGGAAATATCTTCCACTGAAAACTAGACAGAAGCATTCTCAGAATTTTATTT
         66 TTTCGTTGGAAACAGG-AATATCTTCATATAAAAGCTAGACAGAAGCATTCTCAGAAACTTCTTT

              *    **  **    *             **  * *   *
     274952 GTCATGTGCGCCCTCAACTAACAGTGTTGAAGCTTTCTTTTG
        130 GTGATGTTTGCATTCAAGTAACAGTGTTGAACATTCCCTTTC

                     *                    **     **  *                       
     274994 ATAGAGCAGTTTTGAAACACTCTTTTTGTAAAATCTGCTAGAGGATATTTGGATAGCTTTGAGGA
          1 ATAGAGCAGGTTTGAAACACTCTTTTTGTAGTATCTGGAAGTGGATATTTGGATAGCTTTGAGGA

                *        *   * *            **                     **    *   
     275059 TTTCTTTGGAAACGGGATTGTCTTCATATAAACTCTAGACAGAAGCATTCTCAGATGCTTCATTG
         66 TTTCGTTGGAAACAGGAATATCTTCATATAAAAGCTAGACAGAAGCATTCTCAGAAACTTCTTTG

            *       **   *    *             *        
     275124 GGATGTTTCAATTGAAGTCACAGTGTTGAACAGTCCCTTTC
        131 TGATGTTTGCATTCAAGTAACAGTGTTGAACATTCCCTTTC

                                                   *     *       **     *   *
     275165 ATAGAGCAGGTTTGAAACACTCTTTTTGTAGTATCTGGATGTGGACATTTGGAGCGCTTTCAGGC
          1 ATAGAGCAGGTTTGAAACACTCTTTTTGTAGTATCTGGAAGTGGATATTTGGATAGCTTTGAGGA

            * ** *      *             *** *   *                          *   
     275230 CTATGGT-GAAAAAGGAAATATCTTCCCCTGAAAACTAGACAGAAGCATTCTCAGAAACTTATTT
         66 TTTCGTTGGAAACAGG-AATATCTTCATATAAAAGCTAGACAGAAGCATTCTCAGAAACTTCTTT

                   **  **    *       *     **  * *   *
     275294 GTGATGTGCGCCCTCAACTAACAGTTTTGAAGCTTTCTTTTG
        130 GTGATGTTTGCATTCAAGTAACAGTGTTGAACATTCCCTTTC

                     *                   * *     *             **            
     275336 ATAGAGCAGTTTTGAAACACTCTTTTTGTGGAATCTGCAAGTGGATATTTGTCTAGCTTTGAGGA
          1 ATAGAGCAGGTTTGAAACACTCTTTTTGTAGTATCTGGAAGTGGATATTTGGATAGCTTTGAGGA

                *        *   *    *                   *       *              
     275401 TTTCATTGGAAACGGGATTA-CAT-ATA-AAAAGC-AGACAGCAGCATTCCCAGAAACTTCTTTG
         66 TTTCGTTGGAAACAGGAATATCTTCATATAAAAGCTAGACAGAAGCATTCTCAGAAACTTCTTTG

                   *          *   **                 
     275462 TGATGTTCGCATTCAAGTCACACAGTTGAACATTCCCTTTC
        131 TGATGTTTGCATTCAAGTAACAGTGTTGAACATTCCCTTTC

                                                   *     *       **     *   *
     275503 ATAGAGCAGGTTTGAAACACTCTTTTTGTAGTATCTGGATGTGGACATTTGGAGCGCTTTCAGGC
          1 ATAGAGCAGGTTTGAAACACTCTTTTTGTAGTATCTGGAAGTGGATATTTGGATAGCTTTGAGGA

            * ** *      *             *** *   *                          *   
     275568 CTATGGT-GAAAAAGGATATATCTTCCCCTGAAAACTAGACAGAAGCATTCTCAGAAACTTATTT
         66 TTTCGTTGGAAACAGGA-ATATCTTCATATAAAAGCTAGACAGAAGCATTCTCAGAAACTTCTTT

                   **  **    *              *  * *   *
     275632 GTGATGTGCGCCCTCAACTAACAGTGTTGAACCTTTCTTTTG
        130 GTGATGTTTGCATTCAAGTAACAGTGTTGAACATTCCCTTTC

                     *                    *      *   *                       
     275674 ATAGAGCAGTTTTGAAACACTCTTTTTGTAATATCTGCAAGAGGATATTTGGATAGCTTTGAGGA
          1 ATAGAGCAGGTTTGAAACACTCTTTTTGTAGTATCTGGAAGTGGATATTTGGATAGCTTTGAGGA

                         *   * *            **                      *    *   
     275739 TTTCGTTGGAAACGGGATTGTCTTCATATAAACTCTAGACAGAAGCATTCTCAGAAGCTTCATTG
         66 TTTCGTTGGAAACAGGAATATCTTCATATAAAAGCTAGACAGAAGCATTCTCAGAAACTTCTTTG

            *       **   *    *             *        
     275804 GGATGTTTCAATTGAAGTCACAGTGTTGAACAGTCCCTTTC
        131 TGATGTTTGCATTCAAGTAACAGTGTTGAACATTCCCTTTC

                                                         *       *  * * *    
     275845 ATAGAGCAGGTTTGAAACACTCTTTTTGTAGTATCTGGAAGTGGACATTTGGAGAGATCTCAGGA
          1 ATAGAGCAGGTTTGAAACACTCTTTTTGTAGTATCTGGAAGTGGATATTTGGATAGCTTTGAGGA

            * *  *      *             **            *       * *          *  *
     275910 ATACGGT-GAAAAAGGAAATATCTTCCAATAAAAGCTAGATAGAAGCAATGTCAGAAACTTTTTC
         66 TTTCGTTGGAAACAGG-AATATCTTCATATAAAAGCTAGACAGAAGCATTCTCAGAAACTTCTTT

            *      * ** *   **      *       *  * *     *
     275974 ATGATGTATCTACTCAGCTAACAGAGTTGAACCTTTCTTTTGAG
        130 GTGATGTTTGCATTCAAGTAACAGTGTTGAACATTCCCTTT--C

             *       *                   * *     *             **            
     276018 AGAGAGCAGTTTTGAAACACTCTTTTTGTGGAATCTGCAAGTGGATATTTGTCTAGCTTTGAGGA
          1 ATAGAGCAGGTTTGAAACACTCTTTTTGTAGTATCTGGAAGTGGATATTTGGATAGCTTTGAGGA

                         *   *    *              *    *       *              
     276083 TTTCGTTGGAAACGGGATTA-CAT-ATA-AAAAGC-ACACAGCAGCATTCCCAGAAACTTCTTTG
         66 TTTCGTTGGAAACAGGAATATCTTCATATAAAAGCTAGACAGAAGCATTCTCAGAAACTTCTTTG

               *              *    *                 
     276144 TGAAGTTTGCATTCAAGTCACAGAGTTGAACATTCCCTTTC
        131 TGATGTTTGCATTCAAGTAACAGTGTTGAACATTCCCTTTC

                                *                * *     *       **     *   *
     276185 ATAGAGCAGGTTTGAAACACACTTTTTGTAGTATCTGTATGTGGACATTTGGAGCGCTTTCAGGC
          1 ATAGAGCAGGTTTGAAACACTCTTTTTGTAGTATCTGGAAGTGGATATTTGGATAGCTTTGAGGA

            * ** *      *             *** *   *                      *   *   
     276250 CTATGGT-GAAAAAGGAAATATCTTCCCCTGAAAACTAGACAGAAGCATTCTCAGAATCTTATTT
         66 TTTCGTTGGAAACAGG-AATATCTTCATATAAAAGCTAGACAGAAGCATTCTCAGAAACTTCTTT

                   **  **    *             **  * *   *
     276314 GTGATGTGCGCCCTCAACTAACAGTGTTGAAGCTTTCTTTTG
        130 GTGATGTTTGCATTCAAGTAACAGTGTTGAACATTCCCTTTC

                     *                    **     *   *                       
     276356 ATAGAGCAGTTTTGAAACACTCTTTTTGTAAAATCTGCAAGAGGATATTTGGATAGCTTTGAGGA
          1 ATAGAGCAGGTTTGAAACACTCTTTTTGTAGTATCTGGAAGTGGATATTTGGATAGCTTTGAGGA

                         *   * *   *        **                      *    *   
     276421 TTTCGTTGGAAACGGGATTGTCTACATATAAACTCTAGACAGAAGCATTCTCAGAAGCTTCATTG
         66 TTTCGTTGGAAACAGGAATATCTTCATATAAAAGCTAGACAGAAGCATTCTCAGAAACTTCTTTG

            *       **   *    *             *        
     276486 GGATGTTTCAATTGAAGTCACAGTGTTGAACAGTCCCTTTC
        131 TGATGTTTGCATTCAAGTAACAGTGTTGAACATTCCCTTTC

                                                         *       **   * *    
     276527 ATAGAGCAGGTTTGAAACACTCTTTTTGTAGTATCTGGAAGTGGACATTTGGAGCGCTCTCAGGA
          1 ATAGAGCAGGTTTGAAACACTCTTTTTGTAGTATCTGGAAGTGGATATTTGGATAGCTTTGAGGA

            * *  *      *             **            *       * *          *  *
     276592 CTGCGGT-GAAAAAGGAAATATCTTCCAATAAAAGCTAGATAGAAGCAATGTCAGAAACTTTTTC
         66 TTTCGTTGGAAACAGG-AATATCTTCATATAAAAGCTAGACAGAAGCATTCTCAGAAACTTCTTT

            *      * ** *   **      *       *        *
     276656 ATGATGTATCTACTCAGCTAACAGAGTTGAACCTT-CCTTTG
        130 GTGATGTTTGCATTCAAGTAACAGTGTTGAACATTCCCTTTC

             *       *                   * *     *             **            
     276697 AGAGAGCAGTTTTGAAACACTCTTTTTGTGGAATCTGCAAGTGGATATTTGTCTAGCTTTGAGGA
          1 ATAGAGCAGGTTTGAAACACTCTTTTTGTAGTATCTGGAAGTGGATATTTGGATAGCTTTGAGGA

                         *   *    *   *               *       *     *        
     276762 TTTCGTTGGAAACGGGATTA-CAT-AGA-AAAAGC-AGACAGCAGCATTCCCAGAATCTTCTTTG
         66 TTTCGTTGGAAACAGGAATATCTTCATATAAAAGCTAGACAGAAGCATTCTCAGAAACTTCTTTG

                              *    *                 
     276823 TGATGTTTGCATTCAAGTCACAGAGTTGAACATTCCCTTTC
        131 TGATGTTTGCATTCAAGTAACAGTGTTGAACATTCCCTTTC

                                                   *     *     * **     *   *
     276864 ATAGAGCAGGTTTGAAACACTCTTTTTGTAGTATCTGGATGTGGACATTTGCAGCGCTTTCAGGC
          1 ATAGAGCAGGTTTGAAACACTCTTTTTGTAGTATCTGGAAGTGGATATTTGGATAGCTTTGAGGA

            * ** *      *             *** *   *                          *   
     276929 CTAAGGT-GAAAAAGGAAATATCTTCCCCTGAAAACTAGACAGAAGCATTCTCAGAAACTTATTT
         66 TTTCGTTGGAAACAGG-AATATCTTCATATAAAAGCTAGACAGAAGCATTCTCAGAAACTTCTTT

                   **  **    *             **  * *   *
     276993 GTGATGTGCGCCCTC-ACTAACAGTGTTGAAGCTTTCTTTTG
        130 GTGATGTTTGCATTCAAGTAACAGTGTTGAACATTCCCTTTC

                     *                   * *     *             **            
     277034 ATAGAGCAGTTTTGAAACACTCTTTTTGTGGAATCTGCAAGTGGATATTTGTCTAGCTTTGAGGA
          1 ATAGAGCAGGTTTGAAACACTCTTTTTGTAGTATCTGGAAGTGGATATTTGGATAGCTTTGAGGA

                         *   *    *                   *       *     *   *  * 
     277099 TTTCGTTGGAAACGGGATTA-CAT-ATA-AAAAGC-AGACAGCAGCATTCCCAGAATCTTGTTCG
         66 TTTCGTTGGAAACAGGAATATCTTCATATAAAAGCTAGACAGAAGCATTCTCAGAAACTTCTTTG

                              *    *                 
     277160 TGATGTTTGCATTCAAGTCACAGAGTTGAACATTCCCTTTC
        131 TGATGTTTGCATTCAAGTAACAGTGTTGAACATTCCCTTTC

             *                         *           *     * *     **     *   *
     277201 AGAGAGCAGGTTTGAAACACTCTTTTTATAGTATCTGGATGTGGACACTTGGAGCGCTTTCAGGC
          1 ATAGAGCAGGTTTGAAACACTCTTTTTGTAGTATCTGGAAGTGGATATTTGGATAGCTTTGAGGA

            * ** *      *             *** *   *                      *   *   
     277266 CTATGGT-GAAAAAGGAAATATCTTCTCCTGAAAACTAGACAGAAGCATTCTCAGAATCTTATTT
         66 TTTCGTTGGAAACAGG-AATATCTTCATATAAAAGCTAGACAGAAGCATTCTCAGAAACTTCTTT

                   *** **    *             **  * *   *
     277330 GTGATGTGCACCCTCAACTAACAGTGTTGAAGCTTTCTTTTG
        130 GTGATGTTTGCATTCAAGTAACAGTGTTGAACATTCCCTTTC

                     *                *   **     *   *                       
     277372 ATAGAGCAGTTTTGAAACACTCTTTTCGTAAAATCTGCAAGAGGATATTTGGATAGCTTTGAGGA
          1 ATAGAGCAGGTTTGAAACACTCTTTTTGTAGTATCTGGAAGTGGATATTTGGATAGCTTTGAGGA

                         *   * *  *         **                      *    *   
     277437 TTTCGTTGGAAACGGGATTGTCGTCATATAAACTCTAGACAGAAGCATTCTCAGAAGCTTCATTG
         66 TTTCGTTGGAAACAGGAATATCTTCATATAAAAGCTAGACAGAAGCATTCTCAGAAACTTCTTTG

            *       **   *    *             *  *     
     277502 GGATGTTTCAATTGAAGTCACAGTGTTGAACAGTCTCTTTC
        131 TGATGTTTGCATTCAAGTAACAGTGTTGAACATTCCCTTTC

                                                         *       *  * * *    
     277543 ATAGAGCAGGTTTGAAACACTCTTTTTGTAGTATCTGGAAGTGGACATTTGGAGAGATCTCAGGA
          1 ATAGAGCAGGTTTGAAACACTCTTTTTGTAGTATCTGGAAGTGGATATTTGGATAGCTTTGAGGA

            * *  *    * *             **          * *       * *          *  *
     277608 ATACGGT-GATAAAGGAAATATCTTCCAATAAAAGCTACATAGAAGCAATGTCAGAAACTTTTTC
         66 TTTCGTTGGAAACAGG-AATATCTTCATATAAAAGCTAGACAGAAGCATTCTCAGAAACTTCTTT

            *      * ** *   **      *       *  * *   *
     277672 ATGATGTATCTACTCAGCTAACAGAGTTGAACCTTTCTTTTG
        130 GTGATGTTTGCATTCAAGTAACAGTGTTGAACATTCCCTTTC

             *       *                   * *     *             **            
     277714 AGAGAGCAGTTTTGAAACACTCTTTTTGTGGAATCTGCAAGTGGATATTTGTCTAGCTTTGAGGA
          1 ATAGAGCAGGTTTGAAACACTCTTTTTGTAGTATCTGGAAGTGGATATTTGGATAGCTTTGAGGA

                         *  **    *                   *       *      *       
     277779 TTTCGTTGGAAACGGGTTTA-CAT-ATA-AAAAGC-AGACAGCAGCATTCCCAGAAAGTTCTTTG
         66 TTTCGTTGGAAACAGGAATATCTTCATATAAAAGCTAGACAGAAGCATTCTCAGAAACTTCTTTG

               **             *    *                 
     277840 TGAATTTTGCATTCAAGTCACAGAGTTGAACATTCCCTTTC
        131 TGATGTTTGCATTCAAGTAACAGTGTTGAACATTCCCTTTC

                                                   *     *       **     *   *
     277881 ATAGAGCAGGTTTGAAACACTCTTTTTGTAGTATCTGGATGTGGACATTTGGAGCGCTTTCAGGC
          1 ATAGAGCAGGTTTGAAACACTCTTTTTGTAGTATCTGGAAGTGGATATTTGGATAGCTTTGAGGA

            * ** *      *             *** *   *                      *   *   
     277946 CTATGGT-GAAAAAGGAAATATCTTCCCCTGAAAACTAGACAGAAGCATTCTCAGAATCTTATTT
         66 TTTCGTTGGAAACAGG-AATATCTTCATATAAAAGCTAGACAGAAGCATTCTCAGAAACTTCTTT

                   **  **    *             **  * *   *
     278010 GTGATGTGCGCCCTCAACTAACAGTGTTGAAGCTTTCTTTTG
        130 GTGATGTTTGCATTCAAGTAACAGTGTTGAACATTCCCTTTC

                     *                    **     *   *           *  *        
     278052 ATAGAGCAGTTTTGAAACACTCTTTTTGTAAAATCTGCAAGAGGATATTTGGAGAGTTTTGAGGA
          1 ATAGAGCAGGTTTGAAACACTCTTTTTGTAGTATCTGGAAGTGGATATTTGGATAGCTTTGAGGA

                *        *   * *            **                      *    *   
     278117 TTTCTTTGGAAACGGGATTGTCTTCATATAAACTCTAGACAGAAGCATTCTCAGAAGCTTCATTG
         66 TTTCGTTGGAAACAGGAATATCTTCATATAAAAGCTAGACAGAAGCATTCTCAGAAACTTCTTTG

            *       **   *    *             *        
     278182 GGATGTTTCAATTGAAGTCACAGTGTTGAACAGTCCCTTTC
        131 TGATGTTTGCATTCAAGTAACAGTGTTGAACATTCCCTTTC

                                *                  **    *       **     *   *
     278223 ATAGAGCAGGTTTGAAACACCCTTTTTGTAGTATCTGGATATGGACATTTGGAGCGCTTTCAGGC
          1 ATAGAGCAGGTTTGAAACACTCTTTTTGTAGTATCTGGAAGTGGATATTTGGATAGCTTTGAGGA

            * ** *      *             *** *   *                          *   
     278288 CTATGGT-GAAAAAGGAAATATCTTCCCCTGAAAACTAGACAGAAGCATTCTCAGAAACTTATTT
         66 TTTCGTTGGAAACAGG-AATATCTTCATATAAAAGCTAGACAGAAGCATTCTCAGAAACTTCTTT

                   **  **    *             **  * *   *
     278352 GTGATGTGCGCCCTCAACTAACAGTGTTGAAGCTTTCTTTTG
        130 GTGATGTTTGCATTCAAGTAACAGTGTTGAACATTCCCTTTC

                     *                   * *     *        *    **            
     278394 ATAGAGCAGCTTTGAAACACTCTTTTTGTGGAATCTGCAAGTGGATGTTTGTCTAGCTTTGAGGA
          1 ATAGAGCAGGTTTGAAACACTCTTTTTGTAGTATCTGGAAGTGGATATTTGGATAGCTTTGAGGA

                         *   *    *                   *       *     *   *    
     278459 TTTCGTTGGAAACGGGATTA-CAT-ATA-AAAAGC-AGACAGCAGCATTCCCAGAATCTTGTTTG
         66 TTTCGTTGGAAACAGGAATATCTTCATATAAAAGCTAGACAGAAGCATTCTCAGAAACTTCTTTG

                              *    *                 
     278520 TGATGTTTGCATTCAAGTCACAGAGTTGAACATTCCCTTTC
        131 TGATGTTTGCATTCAAGTAACAGTGTTGAACATTCCCTTTC

             *    *                    *           *     *       **     *   *
     278561 AGAGAGAAGGTTTGAAACACTCTTTTTATAGTATCTGGATGTGGACATTTGGAGCGCTTTCAGGC
          1 ATAGAGCAGGTTTGAAACACTCTTTTTGTAGTATCTGGAAGTGGATATTTGGATAGCTTTGAGGA

            * ** *      *             *** *   *       *   *          *   *   
     278626 CTATGGT-GAAAAAGGAAATATCTTCTCCTGAAAACTAGACACAAGAATTCTCAGAATCTTATTT
         66 TTTCGTTGGAAACAGG-AATATCTTCATATAAAAGCTAGACAGAAGCATTCTCAGAAACTTCTTT

                   **  **    *             **  * *   *
     278690 GTGATGTGCGCCCTCAACTAACAGTGTTGAAGCTTTCTTTTG
        130 GTGATGTTTGCATTCAAGTAACAGTGTTGAACATTCCCTTTC

                     *                *   **     *   *        *              
     278732 ATAGAGCAGTTTTGAAACACTCTTTTCGTAAAATCTGCAAGAGGATATTTTGATAGCTTTGAGGA
          1 ATAGAGCAGGTTTGAAACACTCTTTTTGTAGTATCTGGAAGTGGATATTTGGATAGCTTTGAGGA

                         *   * *            **                      *    *   
     278797 TTTCGTTGGAAACGGGATTGTCTTCATATAAACTCTAGACAGAAGCATTCTCAGAAGCTTCATTG
         66 TTTCGTTGGAAACAGGAATATCTTCATATAAAAGCTAGACAGAAGCATTCTCAGAAACTTCTTTG

            *       **   *    *             *        
     278862 GGATGTTTCAATTGAAGTCACAGTGTTGAACAGTCCCTTTC
        131 TGATGTTTGCATTCAAGTAACAGTGTTGAACATTCCCTTTC

                                                         *       *  * * *    
     278903 ATAGAGCAGGTTTGAAACACTCTTTTTGTAGTATCTGGAAGTGGACATTTGGAGAGATCTCAGGA
          1 ATAGAGCAGGTTTGAAACACTCTTTTTGTAGTATCTGGAAGTGGATATTTGGATAGCTTTGAGGA

            * *  *      *             **            *       * *          *  *
     278968 ATACGGT-GAAAAAGGAAATATCTTCCAATAAAAGCTAGATAGAAGCAATGTCAGAAACTTTTTC
         66 TTTCGTTGGAAACAGG-AATATCTTCATATAAAAGCTAGACAGAAGCATTCTCAGAAACTTCTTT

            *      * ** *   **      *       *  * *   *
     279032 ATGATGTATCTACTCAGCTAACAGAGTTGAACCTTTCTTTTG
        130 GTGATGTTTGCATTCAAGTAACAGTGTTGAACATTCCCTTTC

             *       *                   * *     *             **            
     279074 AGAGAGCAGTTTTGAAACACTCTTTTTGTGGAATCTGCAAGTGGATATTTGTCTAGCTTTGAGGA
          1 ATAGAGCAGGTTTGAAACACTCTTTTTGTAGTATCTGGAAGTGGATATTTGGATAGCTTTGAGGA

                         *        *                   *       *              
     279139 TTTCGTTGGAAACGGGAATA-CAT-ATA-AAAAGC-AGACAGCAGCATTCCCAGAAACTTCTTTG
         66 TTTCGTTGGAAACAGGAATATCTTCATATAAAAGCTAGACAGAAGCATTCTCAGAAACTTCTTTG

                              *    *                 
     279200 TGATGTTTGCATTCAAGTCACAGAGTTGAACATTCCCTTTC
        131 TGATGTTTGCATTCAAGTAACAGTGTTGAACATTCCCTTTC

                    *                  *           *   * *       **     *   *
     279241 ATAGAGCACGTTTGAAACACTCTTTTTATAGTATCTGGATGTGCACATTTGGAGCGCTTTCAGGC
          1 ATAGAGCAGGTTTGAAACACTCTTTTTGTAGTATCTGGAAGTGGATATTTGGATAGCTTTGAGGA

            * ** *  *   ** *         *** *   *                      *   *    
     279306 CTATGGTGAAAAGGGAAATATCTTCTCCTGAAAACTAGACAGAAGCATTCTCAGAATCTTATTTG
         66 TTTCGTTGGAAACAGGAATATCTTCATATAAAAGCTAGACAGAAGCATTCTCAGAAACTTCTTTG

                  **  **    *             **  * *    
     279371 TGATGTGCGCCCTCAACTAACAGTGTTGAAGCTTTCTTTTC
        131 TGATGTTTGCATTCAAGTAACAGTGTTGAACATTCCCTTTC

                     *                    **     *   *                 *     
     279412 ATAGAGCAGTTTTGAAACACTCTTTTTGTAAAATCTGCAAGAGGATATTTGGATAGCTTGGAGGA
          1 ATAGAGCAGGTTTGAAACACTCTTTTTGTAGTATCTGGAAGTGGATATTTGGATAGCTTTGAGGA

                         *   * *            **                      *    *   
     279477 TTTCGTTGGAAACGGGATTGTCTTCATATAAACTCTAGACAGAAGCATTCTCAGAAGCTTCATTG
         66 TTTCGTTGGAAACAGGAATATCTTCATATAAAAGCTAGACAGAAGCATTCTCAGAAACTTCTTTG

            *       **   *    *             *        
     279542 GGATGTTTCAATTGAAGTCACAGTGTTGAACAGTCCCTTTC
        131 TGATGTTTGCATTCAAGTAACAGTGTTGAACATTCCCTTTC

                                                   *     *       **     *    
     279583 ATAGAGCAGGTTTGAAACACTCTTTTTGTAGTATCTGGATGTGGACATTTGGAGCGCTTTCAGGT
          1 ATAGAGCAGGTTTGAAACACTCTTTTTGTAGTATCTGGAAGTGGATATTTGGATAGCTTTGAGG-

            * * *    *                 *** *   *                          *  
     279648 CTATGGTT-TAAA-AGGAAATATCTTCCCCTGAAAACTAGACAGAAGCATTCTCAGAAACTTATT
         65 ATTTCGTTGGAAACAGG-AATATCTTCATATAAAAGCTAGACAGAAGCATTCTCAGAAACTTCTT

                    **  **    *                    *   *
     279711 TGTGATGTGCGCCCTCAACTAACAGTGTTGAAGCATT-CTTTTG
        129 TGTGATGTTTGCATTCAAGTAACAGTGTTGAA-CATTCCCTTTC

                     *                 * * *     *             **            
     279754 ATAGAGCAGTTTTGAAACACTCTTTTTCTGGAATCTGCAAGTGGATATTTGTCTAGCTTTGAGGA
          1 ATAGAGCAGGTTTGAAACACTCTTTTTGTAGTATCTGGAAGTGGATATTTGGATAGCTTTGAGGA

                         *   *    *                   *       *              
     279819 TTTCGTTGGAAACGGGATTA-CAT-ATA-AAAAGC-AGACAGCAGCATTCCCAGAAACTTCTTTG
         66 TTTCGTTGGAAACAGGAATATCTTCATATAAAAGCTAGACAGAAGCATTCTCAGAAACTTCTTTG

                              *    *                 
     279880 TGATGTTTGCATTCAAGTCACAGAGTTGAACATTCCCTTTC
        131 TGATGTTTGCATTCAAGTAACAGTGTTGAACATTCCCTTTC

                                                   *     *     * **     *   *
     279921 ATAGAGCAGGTTTGAAACACTCTTTTTGTAGTATCTGGATGTGGACATTTGCAGGGCTTTCAGGC
          1 ATAGAGCAGGTTTGAAACACTCTTTTTGTAGTATCTGGAAGTGGATATTTGGATAGCTTTGAGGA

            * ** *      *             *** *   *                          *   
     279986 CTAAGGT-GAAAAAGGAAATATCTTCCCCTGAAAACTAGACAGAAGCATTCTCAGAAACTTATTT
         66 TTTCGTTGGAAACAGG-AATATCTTCATATAAAAGCTAGACAGAAGCATTCTCAGAAACTTCTTT

                   **  **    *             **  * *   *
     280050 GTGATGTGCGCCCTCAACTAACAGTGTTGAAGCTTTCTTTTG
        130 GTGATGTTTGCATTCAAGTAACAGTGTTGAACATTCCCTTTC

                     *                   * *     *             **            
     280092 ATAGAGCAGTTTTGAAACACTCTTTTTGTGGAATCTGCAAGTGGATATTTGTCTAGCTTTGAGGA
          1 ATAGAGCAGGTTTGAAACACTCTTTTTGTAGTATCTGGAAGTGGATATTTGGATAGCTTTGAGGA

                         *   *    *                *  *       *     *   *    
     280157 TTTCGTTGGAAACGGGATTA-CAT-ATA-AAAAGC-AGAAAGCAGCATTCCCAGAATCTTGTTTG
         66 TTTCGTTGGAAACAGGAATATCTTCATATAAAAGCTAGACAGAAGCATTCTCAGAAACTTCTTTG

                              *    *                 
     280218 TGATGTTTGCATTCAAGTCACAGAGTTGAACATTCCCTTTC
        131 TGATGTTTGCATTCAAGTAACAGTGTTGAACATTCCCTTTC

             *                         *           *   * *       **   * *   *
     280259 AGAGAGCAGGTTTGAAACACTCTTTTTATAGTATCTGGATGTGTACATTTGGAGCGCTGTCAGGC
          1 ATAGAGCAGGTTTGAAACACTCTTTTTGTAGTATCTGGAAGTGGATATTTGGATAGCTTTGAGGA

            * ** *      *             *** *   *                      *   *   
     280324 CTATGGT-GAAAAAGGAAATATCTTCCCCTGAAAACTAGACAGAAGCATTCTCAGAATCTTATTT
         66 TTTCGTTGGAAACAGG-AATATCTTCATATAAAAGCTAGACAGAAGCATTCTCAGAAACTTCTTT

                   **  **    *             **  * *   *
     280388 GTGATGTGCGCCCTCAACTAACAGTGTTGAAGCTTTCTTTTG
        130 GTGATGTTTGCATTCAAGTAACAGTGTTGAACATTCCCTTTC

                     *                *   *      *   *        *              
     280430 ATAGAGCAGTTTTGAAACACTCTTTTCGTAATATCTGCAAGAGGATATTTTGATAGCTTTGAGGA
          1 ATAGAGCAGGTTTGAAACACTCTTTTTGTAGTATCTGGAAGTGGATATTTGGATAGCTTTGAGGA

                         *   * *            **                      *    *   
     280495 TTTCGTTGGAAACGGGATTGTCTTCATATAAACTCTAGACAGAAGCATTCTCAGAAGCTTCATTG
         66 TTTCGTTGGAAACAGGAATATCTTCATATAAAAGCTAGACAGAAGCATTCTCAGAAACTTCTTTG

            *       **   *    *             *        
     280560 GGATGTTTCAATTGAAGTCACAGTGTTGAACAGTCCCTTTC
        131 TGATGTTTGCATTCAAGTAACAGTGTTGAACATTCCCTTTC

                                                         *       *  * * *    
     280601 ATAGAGCAGGTTTGAAACACTCTTTTTGTAGTATCTGGAAGTGGACATTTGGAGAGATCTCAGGA
          1 ATAGAGCAGGTTTGAAACACTCTTTTTGTAGTATCTGGAAGTGGATATTTGGATAGCTTTGAGGA

            * *  *    * *             **       *    *       * *          *  *
     280666 ATACGGT-GATAAAGGAAATATCTTCCAATAAAAGATAGATAGAAGCAATATCAGAAACTTTTTC
         66 TTTCGTTGGAAACAGG-AATATCTTCATATAAAAGCTAGACAGAAGCATTCTCAGAAACTTCTTT

            *      *                  *       *  * *   *
     280730 ATGATGTAT-C-TTCACAGCTAACAGAGTTGAACCTTTCTTTTG
        130 GTGATGTTTGCATTCA-AG-TAACAGTGTTGAACATTCCCTTTC

             *       *                   * *     *             **            
     280772 AGAGAGCAGTTTTGAAACACTCTTTTTGTGGAATCTGCAAGTGGATATTTGTCTAGCTTTGAGGA
          1 ATAGAGCAGGTTTGAAACACTCTTTTTGTAGTATCTGGAAGTGGATATTTGGATAGCTTTGAGGA

                         *   *    *                   *       *              
     280837 TTTCGTTGGAAACGGGATTA-CAT-ATA-AAAAGC-AGACAGCAGCATTCCCAGAAACTTCTTTG
         66 TTTCGTTGGAAACAGGAATATCTTCATATAAAAGCTAGACAGAAGCATTCTCAGAAACTTCTTTG

                              *    *                 
     280898 TGATGTTTGCATTCAAGTCACAGAGTTGAACATTCCCTTTC
        131 TGATGTTTGCATTCAAGTAACAGTGTTGAACATTCCCTTTC

                                                   *     *     * **     *   *
     280939 ATAGAGCAGGTTTGAAACACTC-TTTTGTAGTATCTGGATGTGGACATTTGCAGCGCTTTCAGGC
          1 ATAGAGCAGGTTTGAAACACTCTTTTTGTAGTATCTGGAAGTGGATATTTGGATAGCTTTGAGGA

            * ** *      *             *** *   *                          *   
     281003 CTATGGT-GAAAAAGGAAATATCTTCCCCTGAAAACTAGACAGAAGCATTCTCAGAAACTTATTT
         66 TTTCGTTGGAAACAGG-AATATCTTCATATAAAAGCTAGACAGAAGCATTCTCAGAAACTTCTTT

                   **  **    *              *  * *   *
     281067 GTGATGTGCGCCCTCAACTAACAGTGTTGAACCTTTCTTTTG
        130 GTGATGTTTGCATTCAAGTAACAGTGTTGAACATTCCCTTTC

                     *                    *      *   *                       
     281109 ATAGAGCAGTTTTGAAACACTCTTTTTGTAATATCTGCAAGAGGATATTTGGATAGCTTTGAGGA
          1 ATAGAGCAGGTTTGAAACACTCTTTTTGTAGTATCTGGAAGTGGATATTTGGATAGCTTTGAGGA

                *        *   * *            **                      *    *   
     281174 TTTCCTTGGAAACGGGATTGTCTTCATATAAACTCTAGACAGAAGCATTCTCAGAAGCTTCATTG
         66 TTTCGTTGGAAACAGGAATATCTTCATATAAAAGCTAGACAGAAGCATTCTCAGAAACTTCTTTG

            *       **   *    *             * *      
     281239 GGATGTTTCAATTGAAGTCACAGTGTTGAACAGTTCCTTTC
        131 TGATGTTTGCATTCAAGTAACAGTGTTGAACATTCCCTTTC

                 *                                       *       **   * *    
     281280 ATAGAACAGGTTTGAAACACTCTTTTTGTAGTATCTGGAAGTGGACATTTGGAGCGCTCTCAGGA
          1 ATAGAGCAGGTTTGAAACACTCTTTTTGTAGTATCTGGAAGTGGATATTTGGATAGCTTTGAGGA

            * ** *      *             **          * *       * *          *  *
     281345 CTATGGT-GAAAAAGGAAATATCTTCCAATAAAAGCTACATAGAAGCAATGTCAGAAACTTTTTC
         66 TTTCGTTGGAAACAGG-AATATCTTCATATAAAAGCTAGACAGAAGCATTCTCAGAAACTTCTTT

            *      * ** *   **      *       *  *     *
     281409 ATGATGTATCTACTCAGCTAACAGAGTTGAACCTTTCCTTTG
        130 GTGATGTTTGCATTCAAGTAACAGTGTTGAACATTCCCTTTC

             *       *                 * * *     *             **            
     281451 AGAGAGCAGTTTTGAAACACTCTTTTTCTGGAATCTGCAAGTGGATATTTGTCTAGCTTTGAGGA
          1 ATAGAGCAGGTTTGAAACACTCTTTTTGTAGTATCTGGAAGTGGATATTTGGATAGCTTTGAGGA

                         *   *    *                   *       *   *          
     281516 TTTCGTTGGAAACGGGATTA-CAT-ATA-AAAAGC-AGACAGCAGCATTCCCAGTAACTTCTTTG
         66 TTTCGTTGGAAACAGGAATATCTTCATATAAAAGCTAGACAGAAGCATTCTCAGAAACTTCTTTG

                              *    *                 
     281577 TGATGTTTGCATTCAAGTCACAGAGTTGAACATTCCCTTTC
        131 TGATGTTTGCATTCAAGTAACAGTGTTGAACATTCCCTTTC

                                                 * *     *       **     *   *
     281618 ATAGAGCAGGTTTGAAACACTCTTTTTGTAGTATCTGTATGTGGACATTTGGAGCGCTTTCAGGC
          1 ATAGAGCAGGTTTGAAACACTCTTTTTGTAGTATCTGGAAGTGGATATTTGGATAGCTTTGAGGA

            * ** *      *             *** *   *                          *   
     281683 CTATGGT-GAAAAAGGAAATATCTTCCCCTGAAAACTAGACAGAAGCATTCTCAGAAACTTATTT
         66 TTTCGTTGGAAACAGG-AATATCTTCATATAAAAGCTAGACAGAAGCATTCTCAGAAACTTCTTT

                   **  **    *              *  * *   *
     281747 GTGATGTGCGCCCTCAACTAACAGTGTTGAACCTTTCTTTTG
        130 GTGATGTTTGCATTCAAGTAACAGTGTTGAACATTCCCTTTC

                     *                    **     *   *                       
     281789 ATAGAGCAGTTTTGAAACACTCTTTTTGTAAAATCTGCAAGAGGATATTTGGATAGCTTTGAGGA
          1 ATAGAGCAGGTTTGAAACACTCTTTTTGTAGTATCTGGAAGTGGATATTTGGATAGCTTTGAGGA

                         *   * *            **                      *    *   
     281854 TTTCGTTGGAAACGGGATTGTCTTCATATAAACTCTAGACAGAAGCATTCTCAGAAGCTTCATTG
         66 TTTCGTTGGAAACAGGAATATCTTCATATAAAAGCTAGACAGAAGCATTCTCAGAAACTTCTTTG

            *       **   * *  *             * *      
     281919 GGATGTTTCAATTGAGGTCACAGTGTTGAACAGTTCCTTTC
        131 TGATGTTTGCATTCAAGTAACAGTGTTGAACATTCCCTTTC

                 *                                       *       **   * *    
     281960 ATAGAACAGGTTTGAAACACTCTTTTTGTAGTATCTGGAAGTGGACATTTGGAGCGCTCTCAGGA
          1 ATAGAGCAGGTTTGAAACACTCTTTTTGTAGTATCTGGAAGTGGATATTTGGATAGCTTTGAGGA

            * *  *  *   ** *                      * *       * *          *  *
     282025 CTACGGTGAAAATGGAAATATCTTTCA-ATAAAAGCTACATAGAAGCAATGTCAGAAACTTTTTC
         66 TTTCGTTGGAAACAGGAATATC-TTCATATAAAAGCTAGACAGAAGCATTCTCAGAAACTTCTTT

            *      * ** *   **      *       *  *     *
     282089 ATGATGTATCTACTCAGCTAACAGAGTTGAACCTTTCCTTTG
        130 GTGATGTTTGCATTCAAGTAACAGTGTTGAACATTCCCTTTC

             *       *          *      * * *     *             **            
     282131 AGAGAGCAGTTTTGAAACACACTTTTTCTGGAATCTGCAAGTGGATATTTGTCTAGCTTTGAGGA
          1 ATAGAGCAGGTTTGAAACACTCTTTTTGTAGTATCTGGAAGTGGATATTTGGATAGCTTTGAGGA

                         *   *    *                   *       *     *        
     282196 TTTCGTTGGAAACGGGATTA-CAT-ATA-AAAAGC-AGACAGCAGCATTCCCAGAATCTTCTTTG
         66 TTTCGTTGGAAACAGGAATATCTTCATATAAAAGCTAGACAGAAGCATTCTCAGAAACTTCTTTG

                              *    *                 
     282257 TGATGTTTGCATTCAAGTCACAGAGTTGAACATTCCCTTTC
        131 TGATGTTTGCATTCAAGTAACAGTGTTGAACATTCCCTTTC

                                                *  *     *       **     *   *
     282298 ATAGAGCAGGTTTGAAACACTCTTTTTGTAGTATCTTGATGTGGACATTTGGAGCGCTTTCAGGC
          1 ATAGAGCAGGTTTGAAACACTCTTTTTGTAGTATCTGGAAGTGGATATTTGGATAGCTTTGAGGA

            * ** *      *             *** *   *                      *   *   
     282363 CTATGGT-GAAAAAGGAAATATCTTCTCCTGAAAACTAGACAGAAGCATTCTCAGAATCTTATTT
         66 TTTCGTTGGAAACAGG-AATATCTTCATATAAAAGCTAGACAGAAGCATTCTCAGAAACTTCTTT

                   **  **    *                 * *   *
     282427 GTGATGTGCGCCCTCAACTAACAGTGTTGAAC-TTTCTTTTG
        130 GTGATGTTTGCATTCAAGTAACAGTGTTGAACATTCCCTTTC

                     *             *      **     *   *                       
     282468 ATAGAGCAGTTTTGAAACACTC-ATTTGTAAAATCTGCAAGAGGATATTTGGATAGCTTTGAGGA
          1 ATAGAGCAGGTTTGAAACACTCTTTTTGTAGTATCTGGAAGTGGATATTTGGATAGCTTTGAGGA

                *        *   * *            **                      *    *   
     282532 TTTCATTGGAAACGGGATTCTCTTCATATAAACTCTAGACAGAAGCATTCTCAGAAGCTTCATTG
         66 TTTCGTTGGAAACAGGAATATCTTCATATAAAAGCTAGACAGAAGCATTCTCAGAAACTTCTTTG

            *       **   *    *             *        
     282597 GGATGTTTCAATTGAAGTCACAGTGTTGAACAGTCCCTTTC
        131 TGATGTTTGCATTCAAGTAACAGTGTTGAACATTCCCTTTC

                                                      *  *       **   * *   *
     282638 ATAGAGCAGGTTTGAAACACTCTTTTTGTAGTATCTGGAAGTTGACATTTGGAGCGCTCTCAGGG
          1 ATAGAGCAGGTTTGAAACACTCTTTTTGTAGTATCTGGAAGTGGATATTTGGATAGCTTTGAGGA

            * *  *      *             **            *       * *          *  *
     282703 CTACGGT-GAAAAAGGAAATATCTTCCAATAAAAGCTAGATAGAAGCAATGTCAGAAACTTTTTC
         66 TTTCGTTGGAAACAGG-AATATCTTCATATAAAAGCTAGACAGAAGCATTCTCAGAAACTTCTTT

            *      * ** *   **      *   *   *  * *   *
     282767 ATGATGTATCTACTCAGCTAACAGAGTTAAACCTTTCTTTTG
        130 GTGATGTTTGCATTCAAGTAACAGTGTTGAACATTCCCTTTC

             *       *       *           * *     *             **            
     282809 AGAGAGCAGTTTTGAAATACTCTTTTTGTGGAATCTGCAAGTGGATATTTGTCTAGCTTTGAGGA
          1 ATAGAGCAGGTTTGAAACACTCTTTTTGTAGTATCTGGAAGTGGATATTTGGATAGCTTTGAGGA

                         *   *    *                   *       *   *          
     282874 TTTCGTTGGAAACGGGATTA-CAT-ATA-AAAAGC-AGACAGCAGCATTCCCAGTAACTTCTTTG
         66 TTTCGTTGGAAACAGGAATATCTTCATATAAAAGCTAGACAGAAGCATTCTCAGAAACTTCTTTG

                              *    *                 
     282935 TGATGTTTGCATTCAAGTCACAGAGTTGAACATTCCCTTTC
        131 TGATGTTTGCATTCAAGTAACAGTGTTGAACATTCCCTTTC

                                                   *     *       **     *   *
     282976 ATAGAGCAGGTTTGAAACACTCTTTTTGTAGTATCTGGATGTGGACATTTGGAGCGCTTTCAGGC
          1 ATAGAGCAGGTTTGAAACACTCTTTTTGTAGTATCTGGAAGTGGATATTTGGATAGCTTTGAGGA

            * ** *      *             *** *   *                      *   *   
     283041 CTATGGT-GAAAAAGGAAATATCTTCCCCTGAAAACTAGACAGAAGCATTCTCAGAATCTTATTT
         66 TTTCGTTGGAAACAGG-AATATCTTCATATAAAAGCTAGACAGAAGCATTCTCAGAAACTTCTTT

                   **  **    *             **  * *   *
     283105 GTGATGTGCGCCCTCAACTAACAGTGTTGAAGCTTTCTTTTG
        130 GTGATGTTTGCATTCAAGTAACAGTGTTGAACATTCCCTTTC

                     *                    **     *   *                       
     283147 ATAGAGCAGTTTTGAAACACTCTTTTTGTAAAATCTGCAAGAGGATATTTGGATAGCTTTGAGGA
          1 ATAGAGCAGGTTTGAAACACTCTTTTTGTAGTATCTGGAAGTGGATATTTGGATAGCTTTGAGGA

                         *   * *        *   **                      *    *   
     283212 TTTCGTTGGAAACGGGATTGTCTTCATAAAAACTCTAGACAGAAGCATTCTCAGAAGCTTCATTG
         66 TTTCGTTGGAAACAGGAATATCTTCATATAAAAGCTAGACAGAAGCATTCTCAGAAACTTCTTTG

            *             *    *             *        
     283277 GGATGTTT-CACTTGAAGTCACAGTGTTGAACAGTCCCTTTC
        131 TGATGTTTGCA-TTCAAGTAACAGTGTTGAACATTCCCTTTC

                           *                             *       *  * * *    
     283318 ATAGAGCAGGTTTGAGACACTCTTTTTGTAGTATCTGGAAGTGGACATTTGGAGAGATCTCAGGA
          1 ATAGAGCAGGTTTGAAACACTCTTTTTGTAGTATCTGGAAGTGGATATTTGGATAGCTTTGAGGA

            * *  *      *             **            *       * *          *  *
     283383 ATACGGT-GAAAAAGGAAATATCTTCCAATAAAAGCTAGATAGAAGCAATGTCAGAAACTTTTTC
         66 TTTCGTTGGAAACAGG-AATATCTTCATATAAAAGCTAGACAGAAGCATTCTCAGAAACTTCTTT

            *      * ** *   **   *  *       *  * *   *
     283447 ATGATGTATCTACTCAGCTAAGAGAGTTGAACCTTTCTTTTG
        130 GTGATGTTTGCATTCAAGTAACAGTGTTGAACATTCCCTTTC

             *       *                   * *     *             **            
     283489 AGAGAGCAGTTTTGAAACACTCTTTTTGTGGAATCTGCAAGTGGATATTTGTCTAGCTTTGAGGA
          1 ATAGAGCAGGTTTGAAACACTCTTTTTGTAGTATCTGGAAGTGGATATTTGGATAGCTTTGAGGA

                         *        *                   *       *              
     283554 TTTCGTTGGAAACGGGAATA-CAT-ATA-AAAAGC-AGACAGCAGCATTCCCAGAAACTTCTTTG
         66 TTTCGTTGGAAACAGGAATATCTTCATATAAAAGCTAGACAGAAGCATTCTCAGAAACTTCTTTG

                              *    *                 
     283615 TGATGTTTGCATTCAAGTCACAGAGTTGAACATTCCCTTTC
        131 TGATGTTTGCATTCAAGTAACAGTGTTGAACATTCCCTTTC

                                                   *     *       **     *   *
     283656 ATAGAGCAGGTTTGAAACACTCTTTTTGTAGTATCTGGATGTGGACATTTGGAGCGCTTTCAGGC
          1 ATAGAGCAGGTTTGAAACACTCTTTTTGTAGTATCTGGAAGTGGATATTTGGATAGCTTTGAGGA

            * ** *      *             *** *   *                      *   *   
     283721 CTATGGT-GAAAAAGGAAATATCTTCCCCTGAAAACTAGACAGAAGCATTCTCAGAATCTTATTT
         66 TTTCGTTGGAAACAGG-AATATCTTCATATAAAAGCTAGACAGAAGCATTCTCAGAAACTTCTTT

                   **  **    *             **  * *   *
     283785 GTGATGTGCGCCCTCAACTAACAGTGTTGAAGCTTTCTTTTG
        130 GTGATGTTTGCATTCAAGTAACAGTGTTGAACATTCCCTTTC

                     *                    **     *   *           *  *        
     283827 ATAGAGCAGTTTTGAAACACTCTTTTTGTAAAATCTGCAAGAGGATATTTGGAGAGTTTTGAGGA
          1 ATAGAGCAGGTTTGAAACACTCTTTTTGTAGTATCTGGAAGTGGATATTTGGATAGCTTTGAGGA

                *        *   * *         *  **                      *    *   
     283892 TTTCTTTGGAAACGGGATTGTCTTCATATTAACTCTAGACAGAAGCATTCTCAGAAGCTTCATTG
         66 TTTCGTTGGAAACAGGAATATCTTCATATAAAAGCTAGACAGAAGCATTCTCAGAAACTTCTTTG

            *       **   *    *             *        
     283957 GGATGTTTCAATTGAAGTCACAGTGTTGAACAGTCCCTTTC
        131 TGATGTTTGCATTCAAGTAACAGTGTTGAACATTCCCTTTC

                                *                  **    *       **     *   *
     283998 ATAGAGCAGGTTTGAAACACCCTTTTTGTAGTATCTGGATATGGACATTTGGAGCGCTTTCAGGC
          1 ATAGAGCAGGTTTGAAACACTCTTTTTGTAGTATCTGGAAGTGGATATTTGGATAGCTTTGAGGA

            * ** *      *             *** * * *                          *   
     284063 CTATGGT-GAAAAAGGAAATATCTTCCCCTGAGAACTAGACAGAAGCATTCTCAGAAACTTATTT
         66 TTTCGTTGGAAACAGG-AATATCTTCATATAAAAGCTAGACAGAAGCATTCTCAGAAACTTCTTT

                   **  **    *             **  * *   *
     284127 GTGATGTGCGCCCTCAACTAACAGTGTTGAAGCTTTCTTTTG
        130 GTGATGTTTGCATTCAAGTAACAGTGTTGAACATTCCCTTTC

                     *                   * *     *        *    **            
     284169 ATAGAGCAGCTTTGAAACACTCTTTTTGTGGAATCTGCAAGTGGATGTTTGTCTAGCTTTGAGGA
          1 ATAGAGCAGGTTTGAAACACTCTTTTTGTAGTATCTGGAAGTGGATATTTGGATAGCTTTGAGGA

                         *   *    *                   *       *     *   *    
     284234 TTTCGTTGGAAACGGGATTA-CAT-ATA-AAAAGC-AGACAGCAGCATTCCCAGAATCTTGTTTG
         66 TTTCGTTGGAAACAGGAATATCTTCATATAAAAGCTAGACAGAAGCATTCTCAGAAACTTCTTTG

                              *    *                 
     284295 TGATGTTTGCATTCAAGTCACAGAGTTGAACATTCCCTTTC
        131 TGATGTTTGCATTCAAGTAACAGTGTTGAACATTCCCTTTC

             *    *                    *           *     *       **     *   *
     284336 AGAGAGAAGGTTTGAAACACTCTTTTTATAGTATCTGGATGTGGACATTTGGAGCGCTTTCAGGC
          1 ATAGAGCAGGTTTGAAACACTCTTTTTGTAGTATCTGGAAGTGGATATTTGGATAGCTTTGAGGA

            * ** *      *             *** *   *       *   *          *   *   
     284401 CTATGGT-GAAAAAGGAAATATCTTCTCCTGAAAACTAGACACAAGAATTCTCAGAATCTTATTT
         66 TTTCGTTGGAAACAGG-AATATCTTCATATAAAAGCTAGACAGAAGCATTCTCAGAAACTTCTTT

                   **  **    *             **  * *   *
     284465 GTGATGTGCGCCCTCAACTAACAGTGTTGAAGCTTTCTTTTG
        130 GTGATGTTTGCATTCAAGTAACAGTGTTGAACATTCCCTTTC

                     *                *   **     *   *        *              
     284507 ATAGAGCAGTTTTGAAACACTCTTTTCGTAAAATCTGCAAGAGGATATTTTGATAGCTTTGAGGA
          1 ATAGAGCAGGTTTGAAACACTCTTTTTGTAGTATCTGGAAGTGGATATTTGGATAGCTTTGAGGA

                         *   * *            **                      *    *   
     284572 TTTCGTTGGAAACGGGATTGTCTTCATATAAACTCTAGACAGAAGCATTCTCAGAAGCTTCATTG
         66 TTTCGTTGGAAACAGGAATATCTTCATATAAAAGCTAGACAGAAGCATTCTCAGAAACTTCTTTG

            *       **   *    *             *        
     284637 GGATGTTTCAATTGAAGTCACAGTGTTGAACAGTCCCTTTC
        131 TGATGTTTGCATTCAAGTAACAGTGTTGAACATTCCCTTTC

                                                         *       *  * * *    
     284678 ATAGAGCAGGTTTGAAACACTCTTTTTGTAGTATCTGGAAGTGGACATTTGGAGAGATCTCAGGA
          1 ATAGAGCAGGTTTGAAACACTCTTTTTGTAGTATCTGGAAGTGGATATTTGGATAGCTTTGAGGA

            * *  *    * *             **            *       * *          *  *
     284743 ATACGGT-GATAAAGGAAATATCTTCCAATAAAAGCTAGATAGAAGCAATGTCAGAAACTTTTTC
         66 TTTCGTTGGAAACAGG-AATATCTTCATATAAAAGCTAGACAGAAGCATTCTCAGAAACTTCTTT

            *      * ** *   **      *       *  * *   *
     284807 ATGATGTATCTACTCAGCTAACAGAGTTGAACCTTTCTTTTG
        130 GTGATGTTTGCATTCAAGTAACAGTGTTGAACATTCCCTTTC

             *       *                   * *     *             **            
     284849 AGAGAGCAGTTTTGAAACACTCTTTTTGTGGAATCTGCAAGTGGATATTTGTCTAGCTTTGAGGA
          1 ATAGAGCAGGTTTGAAACACTCTTTTTGTAGTATCTGGAAGTGGATATTTGGATAGCTTTGAGGA

                         *   *    *                   *       *   *          
     284914 TTTCGTTGGAAACGGGATTA-CAT-ATA-AAAAGC-AGACAGCAGCATTCCCAGTAACTTCTTTG
         66 TTTCGTTGGAAACAGGAATATCTTCATATAAAAGCTAGACAGAAGCATTCTCAGAAACTTCTTTG

                              *    *                 
     284975 TGATGTTTGCATTCAAGTCACAGAGTTGAACATTCCCTTTC
        131 TGATGTTTGCATTCAAGTAACAGTGTTGAACATTCCCTTTC

                                                 * *     *       **     *   *
     285016 ATAGAGCAGGTTTGAAACACTCTTTTTGTAGTATCTGTATGTGGACATTTGGAGCGCTTTCAGGC
          1 ATAGAGCAGGTTTGAAACACTCTTTTTGTAGTATCTGGAAGTGGATATTTGGATAGCTTTGAGGA

            * ** *      *             *** *   *           *              *   
     285081 CTATGGT-GAAAAAGGAAATATCTTCCCCTGAAAACTAGACAGAAGAATTCTCAGAAACTTGTTT
         66 TTTCGTTGGAAACAGG-AATATCTTCATATAAAAGCTAGACAGAAGCATTCTCAGAAACTTCTTT

                   **  **    *             **  * *   *
     285145 GTGATGTGCGCCCTCAACTAACAGTGTTGAAGCTTTCTTTTG
        130 GTGATGTTTGCATTCAAGTAACAGTGTTGAACATTCCCTTTC

                     *                    **     *   *                       
     285187 ATAGAGCAGTTTTGAAACACTCTTTTTGTAAAATCTGCAAGAGGATATTTGGATAGCTTTGAGGA
          1 ATAGAGCAGGTTTGAAACACTCTTTTTGTAGTATCTGGAAGTGGATATTTGGATAGCTTTGAGGA

                         *   * *            **                      * *  *   
     285252 TTTCGTTGGAAACGGGATTGTCTTCATATAAACTCTAGACAGAAGCATTCTCAGAAGCGTCATTG
         66 TTTCGTTGGAAACAGGAATATCTTCATATAAAAGCTAGACAGAAGCATTCTCAGAAACTTCTTTG

            *       **   *    *             *        
     285317 GGATGTTTCAATTGAAGTCACAGTGTTGAACAGTCCCTTTC
        131 TGATGTTTGCATTCAAGTAACAGTGTTGAACATTCCCTTTC

                                                   *     *       **     *    
     285358 ATAGAGCAGGTTTGAAACACTCTTTTTGTAGTATCTGGATGTGGACATTTGGAGCGCTTTCAGGC
          1 ATAGAGCAGGTTTGAAACACTCTTTTTGTAGTATCTGGAAGTGGATATTTGGATAGCTTTGAGG-

            * * *    *                 *** *   *                          *  
     285423 CTATGGTT-TAAA-AGGAAATATCTTCCCCTGAAAACTAGACAGAAGCATTCTCAGAAACTTATT
         65 ATTTCGTTGGAAACAGG-AATATCTTCATATAAAAGCTAGACAGAAGCATTCTCAGAAACTTCTT

                    **  **    *                    *   *
     285486 TGTGATGTGCGCCCTCAACTAACAGTGTTGAAGCATT-CTTTTG
        129 TGTGATGTTTGCATTCAAGTAACAGTGTTGAA-CATTCCCTTTC

              *      *                   * *     *             **            
     285529 ATTGAGCAGTTTTGAAACACTCTTTTTGTGGAATCTGCAAGTGGATATTTGTCTAGCTTTGAGGA
          1 ATAGAGCAGGTTTGAAACACTCTTTTTGTAGTATCTGGAAGTGGATATTTGGATAGCTTTGAGGA

                 *       *   *    *                   *       *              
     285594 TTTCGCTGGAAACGGGATTA-CAT-ATA-AAAAGC-AGACAGCAGCATTCCCAGAAACTTCTTTG
         66 TTTCGTTGGAAACAGGAATATCTTCATATAAAAGCTAGACAGAAGCATTCTCAGAAACTTCTTTG

                              *    *                 
     285655 TGATGTTTGCATTCAAGTCACAGAGTTGAACATTCCCTTTC
        131 TGATGTTTGCATTCAAGTAACAGTGTTGAACATTCCCTTTC

                                                   *     *       *  * * *    
     285696 ATAGAGCAGGTTTGAAACACTCTTTTTGTAGTATCTGGATGTGGACATTTGGAGAGATCTCAGGA
          1 ATAGAGCAGGTTTGAAACACTCTTTTTGTAGTATCTGGAAGTGGATATTTGGATAGCTTTGAGGA

            * *  *      *             **            *       * *          *  *
     285761 ATACGGT-GAAAAAGGAAATATCTTCCAATAAAAGCTAGATAGAAGCAATGTCAGAAACTTTTTC
         66 TTTCGTTGGAAACAGG-AATATCTTCATATAAAAGCTAGACAGAAGCATTCTCAGAAACTTCTTT

            *      * ** *   **      *       *  * *   *
     285825 ATGATGTATCTACTCAGCTAACAGAGTTGAACCTTTCTTTTG
        130 GTGATGTTTGCATTCAAGTAACAGTGTTGAACATTCCCTTTC

             *       *                   * *     *             **            
     285867 AGAGAGCAGTTTTGAAACACTCTTTTTGTGGAATCTGCAAGTGGATATTTGTCTAGCTTTGAGGA
          1 ATAGAGCAGGTTTGAAACACTCTTTTTGTAGTATCTGGAAGTGGATATTTGGATAGCTTTGAGGA

                         *        *                   *       *              
     285932 TTTCGTTGGAAACGGGAATA-CAT-ATA-AAAAGC-AGACAGCAGCATTCCCAGAAACTTCTTTG
         66 TTTCGTTGGAAACAGGAATATCTTCATATAAAAGCTAGACAGAAGCATTCTCAGAAACTTCTTTG

                              *    *                 
     285993 TGATGTTTGCATTCAAGTCACAGAGTTGAACATTCCCTTTC
        131 TGATGTTTGCATTCAAGTAACAGTGTTGAACATTCCCTTTC

                    *                  *           *   * *       **     *   *
     286034 ATAGAGCACGTTTGAAACACTCTTTTTATAGTATCTGGATGTGCACATTTGGAGCGCTTTCAGGC
          1 ATAGAGCAGGTTTGAAACACTCTTTTTGTAGTATCTGGAAGTGGATATTTGGATAGCTTTGAGGA

            * ** *  *   ** *         *** *   *                      *   *    
     286099 CTATGGTGAAAAGGGAAATATCTTCTCCTGAAAACTAGACAGAAGCATTCTCAGAATCTTATTTG
         66 TTTCGTTGGAAACAGGAATATCTTCATATAAAAGCTAGACAGAAGCATTCTCAGAAACTTCTTTG

                  **  **    *             **  * *    
     286164 TGATGTGCGCCCTCAACTAACAGTGTTGAAGCTTTCTTTTC
        131 TGATGTTTGCATTCAAGTAACAGTGTTGAACATTCCCTTTC

                     *                    **     *   *                 *     
     286205 ATAGAGCAGTTTTGAAACACTCTTTTTGTAAAATCTGCAAGAGGATATTTGGATAGCTTGGAGGA
          1 ATAGAGCAGGTTTGAAACACTCTTTTTGTAGTATCTGGAAGTGGATATTTGGATAGCTTTGAGGA

                         *   * *            **                      *    *   
     286270 TTTCGTTGGAAACGGGATTGTCTTCATATAAACTCTAGACAGAAGCATTCTCAGAAGCTTCATTG
         66 TTTCGTTGGAAACAGGAATATCTTCATATAAAAGCTAGACAGAAGCATTCTCAGAAACTTCTTTG

            *       **   *    *             *        
     286335 GGATGTTTCAATTGAAGTCACAGTGTTGAACAGTCCCTTTC
        131 TGATGTTTGCATTCAAGTAACAGTGTTGAACATTCCCTTTC

                                                   *     *       **     *    
     286376 ATAGAGCAGGTTTGAAACACTCTTTTTGTAGTATCTGGATGTGGACATTTGGAGCGCTTTCAGGT
          1 ATAGAGCAGGTTTGAAACACTCTTTTTGTAGTATCTGGAAGTGGATATTTGGATAGCTTTGAGG-

            * * *    *                 *** *   *                          *  
     286441 CTATGGTT-TAAA-AGGAAATATCTTCCCCTGAAAACTAGACAGAAGCATTCTCAGAAACTTATT
         65 ATTTCGTTGGAAACAGG-AATATCTTCATATAAAAGCTAGACAGAAGCATTCTCAGAAACTTCTT

                    **  **    *                    *   *
     286504 TGTGATGTGCGCCCTCAACTAACAGTGTTGAAGCATT-CTTTTG
        129 TGTGATGTTTGCATTCAAGTAACAGTGTTGAA-CATTCCCTTTC

                     *                 * * *     *             **            
     286547 ATAGAGCAGTTTTGAAACACTCTTTTTCTGGAATCTGCAAGTGGATATTTGTCTAGCTTTGAGGA
          1 ATAGAGCAGGTTTGAAACACTCTTTTTGTAGTATCTGGAAGTGGATATTTGGATAGCTTTGAGGA

                         *   *    *                   *       *              
     286612 TTTCGTTGGAAACGGGATTA-CAT-ATA-AAAAGC-AGACAGCAGCATTCCCAGAAACTTCTTTG
         66 TTTCGTTGGAAACAGGAATATCTTCATATAAAAGCTAGACAGAAGCATTCTCAGAAACTTCTTTG

                              *    *                 
     286673 TGATGTTTGCATTCAAGTCACAGAGTTGAACATTCCCTTTC
        131 TGATGTTTGCATTCAAGTAACAGTGTTGAACATTCCCTTTC

                                                   *     *     * **     *   *
     286714 ATAGAGCAGGTTTGAAACACTCTTTTTGTAGTATCTGGATGTGGACATTTGCAGGGCTTTCAGGC
          1 ATAGAGCAGGTTTGAAACACTCTTTTTGTAGTATCTGGAAGTGGATATTTGGATAGCTTTGAGGA

            * ** *      *             *** *   *                          *   
     286779 CTAAGGT-GAAAAAGGAAATATCTTCCCCTGAAAACTAGACAGAAGCATTCTCAGAAACTTATTT
         66 TTTCGTTGGAAACAGG-AATATCTTCATATAAAAGCTAGACAGAAGCATTCTCAGAAACTTCTTT

                   **  **    *             **  * *   *
     286843 GTGATGTGCGCCCTCAACTAACAGTGTTGAAGCTTTCTTTTG
        130 GTGATGTTTGCATTCAAGTAACAGTGTTGAACATTCCCTTTC

                                         * *     *             **            
     286885 ATAGAGCA-GTTTGAAACACTCTTTTTGTGGAATCTGCAAGTGGATATTTGTCTAGCTTTGAGGA
          1 ATAGAGCAGGTTTGAAACACTCTTTTTGTAGTATCTGGAAGTGGATATTTGGATAGCTTTGAGGA

                         *   *    *                *  *       *     *   *    
     286949 TTTCGTTGGAAACGGGATTA-CAT-ATA-AAAAGC-AGAAAGCAGCATTCCCAGAATCTTGTTTG
         66 TTTCGTTGGAAACAGGAATATCTTCATATAAAAGCTAGACAGAAGCATTCTCAGAAACTTCTTTG

                              *    *                 
     287010 TGATGTTTGCATTCAAGTCACAGAGTTGAACATTCCCTTTC
        131 TGATGTTTGCATTCAAGTAACAGTGTTGAACATTCCCTTTC

             *                         *           *   * *       **   * *   *
     287051 AGAGAGCAGGTTTGAAACACTCTTTTTATAGTATCTGGATGTGTACATTTGGAGCGCTGTCAGGC
          1 ATAGAGCAGGTTTGAAACACTCTTTTTGTAGTATCTGGAAGTGGATATTTGGATAGCTTTGAGGA

            * ** *      *             *** *   *                      *   *   
     287116 CTATGGT-GAAAAAGGAAATATCTTCTCCTGAAAACTAGACAGAAGCATTCTCAGAATCTTATTT
         66 TTTCGTTGGAAACAGG-AATATCTTCATATAAAAGCTAGACAGAAGCATTCTCAGAAACTTCTTT

                *  **  **    *             **  * *   *
     287180 GTGAGGTGCGCCCTCAACTAACAGTGTTGAAGCTTTCTTTTG
        130 GTGATGTTTGCATTCAAGTAACAGTGTTGAACATTCCCTTTC

                     *                *   *      *   *        *              
     287222 ATAGAGCAGTTTTGAAACACTCTTTTCGTAATATCTGCAAGAGGATATTTTGATAGCTTTGAGGA
          1 ATAGAGCAGGTTTGAAACACTCTTTTTGTAGTATCTGGAAGTGGATATTTGGATAGCTTTGAGGA

                         *   * *            **                      *    *   
     287287 TTTCGTTGGAAACGGGATTGTCTTCATATAAACTCTAGACAGAAGCATTCTCAGAAGCTTCATTG
         66 TTTCGTTGGAAACAGGAATATCTTCATATAAAAGCTAGACAGAAGCATTCTCAGAAACTTCTTTG

            *       **   *    *             *        
     287352 GGATGTTTCAATTGAAGTCACAGTGTTGAACAGTCCCTTTC
        131 TGATGTTTGCATTCAAGTAACAGTGTTGAACATTCCCTTTC

                                                         *       *  * * *    
     287393 ATAGAGCAGGTTTGAAACACTCTTTTTGTAGTATCTGGAAGTGGACATTTGGAGAGATCTCAGGA
          1 ATAGAGCAGGTTTGAAACACTCTTTTTGTAGTATCTGGAAGTGGATATTTGGATAGCTTTGAGGA

            * *  *    * *             **       *    *       * *          *  *
     287458 ATACGGT-GATAAAGGAAATATCTTCCAATAAAAGATAGATAGAAGCAATATCAGAAACTTTTTC
         66 TTTCGTTGGAAACAGG-AATATCTTCATATAAAAGCTAGACAGAAGCATTCTCAGAAACTTCTTT

            *      *                  *       *  * *   *
     287522 ATGATGTAT-C-TTCACAGCTAACAGAGTTGAACCTTTCTTTTG
        130 GTGATGTTTGCATTCA-AG-TAACAGTGTTGAACATTCCCTTTC

             *       *                   * *     *             **            
     287564 AGAGAGCAGTTTTGAAACACTCTTTTTGTGGAATCTGCAAGTGGATATTTGTCTAGCTTTGAGGA
          1 ATAGAGCAGGTTTGAAACACTCTTTTTGTAGTATCTGGAAGTGGATATTTGGATAGCTTTGAGGA

                             *    *                   *       *              
     287629 TTTCGTTGGAAACAGGATTA-CAT-ATA-AAAAGC-AGACAGCAGCATTCCCAGAAACTTCTTTG
         66 TTTCGTTGGAAACAGGAATATCTTCATATAAAAGCTAGACAGAAGCATTCTCAGAAACTTCTTTG

                              *    *                 
     287690 TGATGTTTGCATTCAAGTCACAGAGTTGAACATTCCCTTTC
        131 TGATGTTTGCATTCAAGTAACAGTGTTGAACATTCCCTTTC

                                                   *     *     * **     *   *
     287731 ATAGAGCAGGTTTGAAACACTC-TTTTGTAGTATCTGGATGTGGACATTTGCAGCGCTTTCAGGC
          1 ATAGAGCAGGTTTGAAACACTCTTTTTGTAGTATCTGGAAGTGGATATTTGGATAGCTTTGAGGA

            * ** *      *             *** *   *                          *   
     287795 CTATGGT-GAAAAAGGAAATATCTTCCCCTGAAAACTAGACAGAAGCATTCTCAGAAACTTATTT
         66 TTTCGTTGGAAACAGG-AATATCTTCATATAAAAGCTAGACAGAAGCATTCTCAGAAACTTCTTT

                   **  **    *              *  * *   *
     287859 GTGATGTGCGCCCTCAACTAACAGTGTTGAACCTTTCTTTTG
        130 GTGATGTTTGCATTCAAGTAACAGTGTTGAACATTCCCTTTC

                     *                    *      *   *                       
     287901 ATAGAGCAGTTTTGAAACACTCTTTTTGTAATATCTGCAAGAGGATATTTGGATAGCTTTGAGGA
          1 ATAGAGCAGGTTTGAAACACTCTTTTTGTAGTATCTGGAAGTGGATATTTGGATAGCTTTGAGGA

                *        *   * *            **                      *    *   
     287966 TTTCCTTGGAAACGGGATTGTCTTCATATAAACTCTAGACAGAAGCATTCTCAGAAGCTTCATTG
         66 TTTCGTTGGAAACAGGAATATCTTCATATAAAAGCTAGACAGAAGCATTCTCAGAAACTTCTTTG

            *       **   *    *             * *      
     288031 GGATGTTTCAATTGAAGTCACAGTGTTGAACAGTTCCTTTC
        131 TGATGTTTGCATTCAAGTAACAGTGTTGAACATTCCCTTTC

                 *                                       *       **   * *    
     288072 ATAGAACAGGTTTGAAACACTCTTTTTGTAGTATCTGGAAGTGGACATTTGGAGCGCTCTCAGGA
          1 ATAGAGCAGGTTTGAAACACTCTTTTTGTAGTATCTGGAAGTGGATATTTGGATAGCTTTGAGGA

            * ** *      *             **          * *       * *          *  *
     288137 CTATGGT-GAAAAAGGAAATATCTTCCAATAAAAGCTACATAGAAGCAATGTCAGAAACTTTTTC
         66 TTTCGTTGGAAACAGG-AATATCTTCATATAAAAGCTAGACAGAAGCATTCTCAGAAACTTCTTT

            *      * ** *   **      *       *  *     *
     288201 ATGATGTATCTACTCAGCTAACAGAGTTGAACCTTTCCTTTG
        130 GTGATGTTTGCATTCAAGTAACAGTGTTGAACATTCCCTTTC

             *       *                 * * *     *             **            
     288243 AGAGAGCAGTTTTGAAACACTCTTTTTCTGGAATCTGCAAGTGGATATTTGTCTAGCTTTGAGGA
          1 ATAGAGCAGGTTTGAAACACTCTTTTTGTAGTATCTGGAAGTGGATATTTGGATAGCTTTGAGGA

                         *   *    *                   *       *   *          
     288308 TTTCGTTGGAAACGGGATTA-CAT-ATA-AAAAGC-AGACAGCAGCATTCCCAGTAACTTCTTTG
         66 TTTCGTTGGAAACAGGAATATCTTCATATAAAAGCTAGACAGAAGCATTCTCAGAAACTTCTTTG

                              *    *                 
     288369 TGATGTTTGCATTCAAGTCACAGAGTTGAACATTCCCTTTC
        131 TGATGTTTGCATTCAAGTAACAGTGTTGAACATTCCCTTTC

                                                 * *     *       **     *   *
     288410 ATAGAGCAGGTTTGAAACACTCTTTTTGTAGTATCTGTATGTGGACATTTGGAGCGCTTTCAGGC
          1 ATAGAGCAGGTTTGAAACACTCTTTTTGTAGTATCTGGAAGTGGATATTTGGATAGCTTTGAGGA

            * ** *      *             *** *   *                          *   
     288475 CTATGGT-GAAAAAGGAAATATCTTCCCCTGAAAACTAGACAGAAGCATTCTCAGAAACTTATTT
         66 TTTCGTTGGAAACAGG-AATATCTTCATATAAAAGCTAGACAGAAGCATTCTCAGAAACTTCTTT

                   **  **    *              *  * *   *
     288539 GTGATGTGCGCCCTCAACTAACAGTGTTGAACCTTTCTTTTG
        130 GTGATGTTTGCATTCAAGTAACAGTGTTGAACATTCCCTTTC

                     *                    **     *   *                       
     288581 ATAGAGCAGTTTTGAAACACTCTTTTTGTAAAATCTGCAAGAGGATATTTGGATAGCTTTGAGGA
          1 ATAGAGCAGGTTTGAAACACTCTTTTTGTAGTATCTGGAAGTGGATATTTGGATAGCTTTGAGGA

                         *   * *            **                      *    *   
     288646 TTTCGTTGGAAACGGGATTGTCTTCATATAAACTCTAGACAGAAGCATTCTCAGAAGCTTCATTG
         66 TTTCGTTGGAAACAGGAATATCTTCATATAAAAGCTAGACAGAAGCATTCTCAGAAACTTCTTTG

            *       **   * *  *             * *      
     288711 GGATGTTTCAATTGAGGTCACAGTGTTGAACAGTTCCTTTC
        131 TGATGTTTGCATTCAAGTAACAGTGTTGAACATTCCCTTTC

                 *                                       *       **   * *    
     288752 ATAGAACAGGTTTGAAACACTCTTTTTGTAGTATCTGGAAGTGGACATTTGGAGCGCTCTCAGGA
          1 ATAGAGCAGGTTTGAAACACTCTTTTTGTAGTATCTGGAAGTGGATATTTGGATAGCTTTGAGGA

            * *  *  *   ** *                      * *       * *          *  *
     288817 CTACGGTGAAAATGGAAATATCTTTCA-ATAAAAGCTACATAGAAGCAATGTCAGAAACTTTTTC
         66 TTTCGTTGGAAACAGGAATATC-TTCATATAAAAGCTAGACAGAAGCATTCTCAGAAACTTCTTT

            *      * ** *   **      *       *  *     *
     288881 ATGATGTATCTACTCAGCTAACAGAGTTGAACCTTTCCTTTG
        130 GTGATGTTTGCATTCAAGTAACAGTGTTGAACATTCCCTTTC

             *       *                 * * *     *             **            
     288923 AGAGAGCAGTTTTGAAACACTCTTTTTCTGGAATCTGCAAGTGGATATTTGTCTAGCTTTGAGGA
          1 ATAGAGCAGGTTTGAAACACTCTTTTTGTAGTATCTGGAAGTGGATATTTGGATAGCTTTGAGGA

                         *   *    *                   *       *     *        
     288988 TTTCGTTGGAAACGGGATTA-CAT-ATA-AAAAGC-AGACAGCAGCATTCCCAGAATCTTCTTTG
         66 TTTCGTTGGAAACAGGAATATCTTCATATAAAAGCTAGACAGAAGCATTCTCAGAAACTTCTTTG

                              *    *                 
     289049 TGATGTTTGCATTCAAGTCACAGAGTTGAACATTCCCTTTC
        131 TGATGTTTGCATTCAAGTAACAGTGTTGAACATTCCCTTTC

                                                *  *     *       **     *   *
     289090 ATAGAGCAGGTTTGAAACACTCTTTTTGTAGTATCTTGATGTGGACATTTGGAGCGCTTTCAGGC
          1 ATAGAGCAGGTTTGAAACACTCTTTTTGTAGTATCTGGAAGTGGATATTTGGATAGCTTTGAGGA

            * ** *      *             *** *   *                      *   *   
     289155 CTATGGT-GAAAAAGGAAATATCTTCTCCTGAAAACTAGACAGAAGCATTCTCAGAATCTTATTT
         66 TTTCGTTGGAAACAGG-AATATCTTCATATAAAAGCTAGACAGAAGCATTCTCAGAAACTTCTTT

                   **  **    *                 * *   *
     289219 GTGATGTGCGCCCTCAACTAACAGTGTTGAAC-TTTCTTTTG
        130 GTGATGTTTGCATTCAAGTAACAGTGTTGAACATTCCCTTTC

                     *             *      **     *   *                       
     289260 ATAGAGCAGTTTTGAAACACTC-ATTTGTAAAATCTGCAAGAGGATATTTGGATAGCTTTGAGGA
          1 ATAGAGCAGGTTTGAAACACTCTTTTTGTAGTATCTGGAAGTGGATATTTGGATAGCTTTGAGGA

                *        *   * *            **   *                  *    *   
     289324 TTTCATTGGAAACGGGATTCTCTTCATATAAACTCTACACAGAAGCATTCTCAGAAGCTTCATTG
         66 TTTCGTTGGAAACAGGAATATCTTCATATAAAAGCTAGACAGAAGCATTCTCAGAAACTTCTTTG

            *       **   *    *             *        
     289389 GGATGTTTCAATTGAAGTCACAGTGTTGAACAGTCCCTTTC
        131 TGATGTTTGCATTCAAGTAACAGTGTTGAACATTCCCTTTC

                                                      *  *       **   * *   *
     289430 ATAGAGCAGGTTTGAAACACTCTTTTTGTAGTATCTGGAAGTTGACATTTGGAGCGCTCTCAGGG
          1 ATAGAGCAGGTTTGAAACACTCTTTTTGTAGTATCTGGAAGTGGATATTTGGATAGCTTTGAGGA

            * *  *      *             **            *       * *          *  *
     289495 CTACGGT-GAAAAAGGAAATATCTTCCAATAAAAGCTAGATAGAAGCAATGTCAGAAACTTTTTC
         66 TTTCGTTGGAAACAGG-AATATCTTCATATAAAAGCTAGACAGAAGCATTCTCAGAAACTTCTTT

            *      * ** *   **      *   *   *  * *   *
     289559 ATGATGTATCTACTCAGCTAACAGAGTTAAACCTTTCTTTTG
        130 GTGATGTTTGCATTCAAGTAACAGTGTTGAACATTCCCTTTC

             *       *                   * *     *             **            
     289601 AGAGAGCAGTTTTGAAACACTCTTTTTGTGGAATCTGCAAGTGGATATTTGTCTAGCTTTGAGGA
          1 ATAGAGCAGGTTTGAAACACTCTTTTTGTAGTATCTGGAAGTGGATATTTGGATAGCTTTGAGGA

                         *        *                   *       *              
     289666 TTTCGTTGGAAACGGGAATA-CAT-ATA-AAAAGC-AGACAGCAGCATTCCCAGAAACTTCTTTG
         66 TTTCGTTGGAAACAGGAATATCTTCATATAAAAGCTAGACAGAAGCATTCTCAGAAACTTCTTTG

                              *    *                 
     289727 TGATGTTTGCATTCAAGTCACAGAGTTGAACATTCCCTTTC
        131 TGATGTTTGCATTCAAGTAACAGTGTTGAACATTCCCTTTC

                                                   *     *       **     *   *
     289768 ATAGAGCAGGTTTGAAACACTCTTTTTGTAGTATCTGGATGTGGACATTTGGAGCGCTTTCAGGC
          1 ATAGAGCAGGTTTGAAACACTCTTTTTGTAGTATCTGGAAGTGGATATTTGGATAGCTTTGAGGA

            * ** *      *             *** *   *                      *   *   
     289833 CTATGGT-GAAAAAGGAAATATCTTCCCCTGAAAACTAGACAGAAGCATTCTCAGAATCTTATTT
         66 TTTCGTTGGAAACAGG-AATATCTTCATATAAAAGCTAGACAGAAGCATTCTCAGAAACTTCTTT

                   **  **    *             **  * *   *
     289897 GTGATGTGCGCCCTCAACTAACAGTGTTGAAGCTTTCTTTTG
        130 GTGATGTTTGCATTCAAGTAACAGTGTTGAACATTCCCTTTC

                     *                    **     *   *                       
     289939 ATAGAGCAGTTTTGAAACACTCTTTTTGTAAAATCTGCAAGAGGATATTTGGATAGCTTTGAGGA
          1 ATAGAGCAGGTTTGAAACACTCTTTTTGTAGTATCTGGAAGTGGATATTTGGATAGCTTTGAGGA

                         *   * *            **                      *    *   
     290004 TTTCGTTGGAAACGGGATTGTCTTCATATAAACTCTAGACAGAAGCATTCTCAGAAGCTTCATTG
         66 TTTCGTTGGAAACAGGAATATCTTCATATAAAAGCTAGACAGAAGCATTCTCAGAAACTTCTTTG

            *       **   *    *             *        
     290069 GGATGTTTCAATTGAAGTCACAGTGTTGAACAGTCCCTTTC
        131 TGATGTTTGCATTCAAGTAACAGTGTTGAACATTCCCTTTC

                                                   *     *       **     *    
     290110 ATAGAGCAGGTTTGAAACACTCTTTTTGTAGTATCTGGATGTGGACATTTGGAGCGCTTTCAGGC
          1 ATAGAGCAGGTTTGAAACACTCTTTTTGTAGTATCTGGAAGTGGATATTTGGATAGCTTTGAGG-

            * * *    *                 *** *   *                          *  
     290175 CTATGGTT-TAAA-AGGAAATATCTTCCCCTGAAAACTAGACAGAAGCATTCTCAGAAACTTATT
         65 ATTTCGTTGGAAACAGG-AATATCTTCATATAAAAGCTAGACAGAAGCATTCTCAGAAACTTCTT

                    **  **    *                    *   *
     290238 TGTGATGTGCGCCCTCAACTAACAGTGTTGAAGCATT-CTTTTG
        129 TGTGATGTTTGCATTCAAGTAACAGTGTTGAA-CATTCCCTTTC

                     *                   * *     *             **            
     290281 ATAGAGCAGTTTTGAAACACTCTTTTTGTGGAATCTGCAAGTGGATATTTGTCTAGCTTTGAGGA
          1 ATAGAGCAGGTTTGAAACACTCTTTTTGTAGTATCTGGAAGTGGATATTTGGATAGCTTTGAGGA

                         *   *    *                   *       *              
     290346 TTTCGTTGGAAACGGGATTA-CAT-ATA-AAAAGC-AGACAGCAGCATTCCCAGAAACTTCTTTG
         66 TTTCGTTGGAAACAGGAATATCTTCATATAAAAGCTAGACAGAAGCATTCTCAGAAACTTCTTTG

                              *    *                 
     290407 TGATGTTTGCATTCAAGTCACAGAGTTGAACATTCCCTTTC
        131 TGATGTTTGCATTCAAGTAACAGTGTTGAACATTCCCTTTC

                                                   *     *       **     *   *
     290448 ATAGAGCAGGTTTGAAACACTCTTTTTGTAGTATCTGGATGTGGACATTTGGAGCGCTTTCAGGC
          1 ATAGAGCAGGTTTGAAACACTCTTTTTGTAGTATCTGGAAGTGGATATTTGGATAGCTTTGAGGA

            * ** *      *             *** *   *                          *   
     290513 CTATGGT-GAAAAAGGAAATATCTTCCCCTGAAAACTAGACAGAAGCATTCTCAGAAACTTATTT
         66 TTTCGTTGGAAACAGG-AATATCTTCATATAAAAGCTAGACAGAAGCATTCTCAGAAACTTCTTT

                   *   **    *             **  * *   *
     290577 GTGATGTGTGCCCTCAACTAACAGTGTTGAAGCTTTCTTTTG
        130 GTGATGTTTGCATTCAAGTAACAGTGTTGAACATTCCCTTTC

                     *                    **     *   *              *        
     290619 ATAGAGCAGTTTTGAAACACTCTTTTTGTAAAATCTGCAAGAGGATATTTGGATAGATTTGAGGA
          1 ATAGAGCAGGTTTGAAACACTCTTTTTGTAGTATCTGGAAGTGGATATTTGGATAGCTTTGAGGA

                         *   * *            **                      *    *   
     290684 TTTCGTTGGAAACGGGATTGTCTTCATATAAACTCTAGACAGAAGCATTCTCAGAAGCTTCATTG
         66 TTTCGTTGGAAACAGGAATATCTTCATATAAAAGCTAGACAGAAGCATTCTCAGAAACTTCTTTG

            *       **   *    *           * *        
     290749 GGATGTTTCAATTGAAGTCACAGTGTTGAAAAGTCCCTTTC
        131 TGATGTTTGCATTCAAGTAACAGTGTTGAACATTCCCTTTC

                                                         *       **   * *    
     290790 ATAGAGCAGGTTTGAAACACTCTTTTTGTAGTATCTGGAAGTGGACATTTGGAGCGCTCTCAGGA
          1 ATAGAGCAGGTTTGAAACACTCTTTTTGTAGTATCTGGAAGTGGATATTTGGATAGCTTTGAGGA

            * *  *      *         *   **            *       * *          *  *
     290855 CTGCGGT-GAAAAAGGAAATATATTCCAATAAAAGCTAGATAGAAGCAATGTCAGAAACTTTTTC
         66 TTTCGTTGGAAACAGG-AATATCTTCATATAAAAGCTAGACAGAAGCATTCTCAGAAACTTCTTT

            *      * ** *   **      *       *    *   *
     290919 ATGATGTATCTACTCAGCTAACAGAGTTGAACCTT-CATTTG
        130 GTGATGTTTGCATTCAAGTAACAGTGTTGAACATTCCCTTTC

             *       *            *      * *     *             **            
     290960 AGAGAGCAGTTTTGAAACACTCGTTTTGTGGAATCTGCAAGTGGATATTTGTCTAGCTTTGAGGA
          1 ATAGAGCAGGTTTGAAACACTCTTTTTGTAGTATCTGGAAGTGGATATTTGGATAGCTTTGAGGA

                         *   *    *                   *       *              
     291025 TTTCGTTGGAAACGGGATTA-CAT-ATA-AAAAGC-AGACAGCAGCATTCCCAGAAACTTCTTTG
         66 TTTCGTTGGAAACAGGAATATCTTCATATAAAAGCTAGACAGAAGCATTCTCAGAAACTTCTTTG

                              *    *                 
     291086 TGATGTTTGCATTCAAGTCACAGAGTTGAACATTCCCTTTC
        131 TGATGTTTGCATTCAAGTAACAGTGTTGAACATTCCCTTTC

                                                   *     *     * **     *   *
     291127 ATAGAGCAGGTTTGAAACACTCTTTTTGTAGTATCTGGATGTGGACATTTGCAGCGCTTTCAGGC
          1 ATAGAGCAGGTTTGAAACACTCTTTTTGTAGTATCTGGAAGTGGATATTTGGATAGCTTTGAGGA

            * ** *      *             *** *   *                          *   
     291192 CTAAGGT-GAAAAAGGAAATATCTTCCCCTGAAAACTAGACAGAAGCATTCTCAGAAACTTATTT
         66 TTTCGTTGGAAACAGG-AATATCTTCATATAAAAGCTAGACAGAAGCATTCTCAGAAACTTCTTT

                   **  **    *             **  * *   *
     291256 GTGATGTGCGCCCTCAACTAACAGTGTTGAAGCTTTCTTTTG
        130 GTGATGTTTGCATTCAAGTAACAGTGTTGAACATTCCCTTTC

                     *                    *      *   *                       
     291298 ATAGAGCAGTTTTGAAACACTCTTTTTGTAATATCTGCAAGAGGATATTTGGATAGCTTTGAGGA
          1 ATAGAGCAGGTTTGAAACACTCTTTTTGTAGTATCTGGAAGTGGATATTTGGATAGCTTTGAGGA

                         *   * *            **                      *    *   
     291363 TTTCGTTGGAAACGGGATTGTCTTCATATAAACTCTAGACAGAAGCATTCTCAGAAGCTTCATTG
         66 TTTCGTTGGAAACAGGAATATCTTCATATAAAAGCTAGACAGAAGCATTCTCAGAAACTTCTTTG

            *       **   *    *             *  *     
     291428 GGATGTTTCAATTGAAGTCACAGTGTTGAACAGTCTCTTTC
        131 TGATGTTTGCATTCAAGTAACAGTGTTGAACATTCCCTTTC

                 *                                       *       **   * *    
     291469 ATAGAACAGGTTTGAAACACTCTTTTTGTAGTATCTGGAAGTGGACATTTGGAGCGCTCTCAGGA
          1 ATAGAGCAGGTTTGAAACACTCTTTTTGTAGTATCTGGAAGTGGATATTTGGATAGCTTTGAGGA

            * ** *      *             **          * *       * *          *  *
     291534 CTATGGT-GAAAAAGGAAATATCTTCCAATAAAAGCTACATAGAAGCAATGTCAGAAACTTTTTC
         66 TTTCGTTGGAAACAGG-AATATCTTCATATAAAAGCTAGACAGAAGCATTCTCAGAAACTTCTTT

            *      * ** *   **      *       *  *     *
     291598 ATGATGTATCTACTCAGCTAACAGAGTTGAACCTTTCCTTTG
        130 GTGATGTTTGCATTCAAGTAACAGTGTTGAACATTCCCTTTC

             *       *                   * *     *             **            
     291640 AGAGAGCAGTTTTGAAACACTCTTTTTGTGGAATCTGCAAGTGGATATTTGTCTAGCTTTGAGGA
          1 ATAGAGCAGGTTTGAAACACTCTTTTTGTAGTATCTGGAAGTGGATATTTGGATAGCTTTGAGGA

            *            *   *    *                   *       *   *          
     291705 CTTCGTTGGAAACGGGATTAT-AT-ATA-AAAAGC-AGACAGCAGCATTCCCAGTAACTTCTTTG
         66 TTTCGTTGGAAACAGGAATATCTTCATATAAAAGCTAGACAGAAGCATTCTCAGAAACTTCTTTG

                              *    *                 
     291766 TGATGTTTGCATTCAAGTCACAGAGTTGAACATTCCCTTTC
        131 TGATGTTTGCATTCAAGTAACAGTGTTGAACATTCCCTTTC

                                                   *     *       **     *   *
     291807 ATAGAGCAGGTTTGAAACACTCTTTTTGTAGTATCTGGATGTGGACATTTGGAGCGCTTTCAGGC
          1 ATAGAGCAGGTTTGAAACACTCTTTTTGTAGTATCTGGAAGTGGATATTTGGATAGCTTTGAGGA

            * ** *      *             *** *   *                          *   
     291872 CTATGGT-GAAAAAGGAAATATCTTCCCCTGAAAACTAGACAGAAGCATTCTCAGAAACTTATTT
         66 TTTCGTTGGAAACAGG-AATATCTTCATATAAAAGCTAGACAGAAGCATTCTCAGAAACTTCTTT

                   **  **    *              *  * *   *
     291936 GTGATGTGCGCCCTCAACTAACAGTGTTGAACCTTTCTTTTG
        130 GTGATGTTTGCATTCAAGTAACAGTGTTGAACATTCCCTTTC

                     *                    *      *   *                       
     291978 ATAGAGCAGTTTTGAAACACTCTTTTTGTAATATCTGCAAGAGGATATTTGGATAGCTTTGAGGA
          1 ATAGAGCAGGTTTGAAACACTCTTTTTGTAGTATCTGGAAGTGGATATTTGGATAGCTTTGAGGA

                         *   *    *                   *       *     *   *    
     292043 TTTCGTTGGAAACGGGATTA-CAT-ATA-AAAAGC-AGACAGCAGCATTCCCAGAATCTTGTTTG
         66 TTTCGTTGGAAACAGGAATATCTTCATATAAAAGCTAGACAGAAGCATTCTCAGAAACTTCTTTG

                              *    *                 
     292104 TGATGTTTGCATTCAAGTCACAGAGTTGAACATTCCCTTTC
        131 TGATGTTTGCATTCAAGTAACAGTGTTGAACATTCCCTTTC

             *                         *           *     *       **     *   *
     292145 AGAGAGCAGGTTTGAAACACTCTTTTTATAGTATCTGGATGTGGACATTTGGAGCGCTTTCAGGC
          1 ATAGAGCAGGTTTGAAACACTCTTTTTGTAGTATCTGGAAGTGGATATTTGGATAGCTTTGAGGA

            * ** *      *                  *   *                      *   *  
     292210 CTATGGT-GAAAAAGGAAATATCTTC-TACTGAAAACTAGACAGAAGCATTCTCAGAATCTTATT
         66 TTTCGTTGGAAACAGG-AATATCTTCATA-TAAAAGCTAGACAGAAGCATTCTCAGAAACTTCTT

                    **  **    *             **  * *   *
     292273 TGTGATGTGCGCCCTCAACTAACAGTGTTGAAGCTTTCTTTTG
        129 TGTGATGTTTGCATTCAAGTAACAGTGTTGAACATTCCCTTTC

                     *                *   **     *   *        *              
     292316 ATAGAGCAGTTTTGAAACACTCTTTTCGTAAAATCTGCAAGAGGATATTTTGATAGCTTTGAGGA
          1 ATAGAGCAGGTTTGAAACACTCTTTTTGTAGTATCTGGAAGTGGATATTTGGATAGCTTTGAGGA

                *       **   * *            **                      *    *   
     292381 TTTCATTGGAAATGGGATTGTCTTCATATAAACTCTAGACAGAAGCATTCTCAGAAGCTTCATTG
         66 TTTCGTTGGAAACAGGAATATCTTCATATAAAAGCTAGACAGAAGCATTCTCAGAAACTTCTTTG

            *       **   *    *             *        
     292446 GGATGTTTCAATTGAAGTCACAGTGTTGAACAGTCCCTTTC
        131 TGATGTTTGCATTCAAGTAACAGTGTTGAACATTCCCTTTC

               *                                         *       *  * * *    
     292487 ATACAGCAGGTTTGAAACACTCTTTTTGTAGTATCTGGAAGTGGACATTTGGAGAGATCTCAGGA
          1 ATAGAGCAGGTTTGAAACACTCTTTTTGTAGTATCTGGAAGTGGATATTTGGATAGCTTTGAGGA

            * *  *    * *             **            *       * *          *  *
     292552 ATACGGT-GATAAAGGAAATATCTTCCAATAAAAGCTAGATAGAAGCAATGTCAGAAACTTTTTC
         66 TTTCGTTGGAAACAGG-AATATCTTCATATAAAAGCTAGACAGAAGCATTCTCAGAAACTTCTTT

            *      * ** *   **      *       *  * *   *
     292616 ATGATGTATCTACTCAGCTAACAGAGTTGAACCTTTCTTTTG
        130 GTGATGTTTGCATTCAAGTAACAGTGTTGAACATTCCCTTTC

             *       *                   * *     *             **            
     292658 AGAGAGCAGTTTTGAAACACTCTTTTTGTGGAATCTGCAAGTGGATATTTGTCTAGCTTTGAGGA
          1 ATAGAGCAGGTTTGAAACACTCTTTTTGTAGTATCTGGAAGTGGATATTTGGATAGCTTTGAGGA

                         *   *    *                   *       *   *          
     292723 TTTCGTTGGAAACGGGATTA-CAT-ATA-AAAAGC-AGACAGCAGCATTCCCAGTAACTTCTTTG
         66 TTTCGTTGGAAACAGGAATATCTTCATATAAAAGCTAGACAGAAGCATTCTCAGAAACTTCTTTG

                              *    *                 
     292784 TGATGTTTGCATTCAAGTCACAGAGTTGAACATTCCCTTTC
        131 TGATGTTTGCATTCAAGTAACAGTGTTGAACATTCCCTTTC

                                                   *     *    *  **         *
     292825 ATAGAGCAGGTTTGAAACACTCTTTTTGTAGTATCTGGATGTGGACATTTTGAGCGCTTTGAGGC
          1 ATAGAGCAGGTTTGAAACACTCTTTTTGTAGTATCTGGAAGTGGATATTTGGATAGCTTTGAGGA

            * ** *      *             *** *   *           *          *   *   
     292890 CTATGGT-GAAAAAGGAAATATCTTCCCCTGAAAACTAGACAGAAGAATTCTCAGAATCTTATTT
         66 TTTCGTTGGAAACAGG-AATATCTTCATATAAAAGCTAGACAGAAGCATTCTCAGAAACTTCTTT

                   **  **    *             **  * *   *
     292954 GTGATGTGCGCCCTCAACTAACAGTGTTGAAGCTTTCTTTTG
        130 GTGATGTTTGCATTCAAGTAACAGTGTTGAACATTCCCTTTC

                     *                    **     *   *                       
     292996 ATAGAGCAGTTTTGAAACACTCTTTTTGTAAAATCTGCAAGAGGATATTTGGATAGCTTTGAGGA
          1 ATAGAGCAGGTTTGAAACACTCTTTTTGTAGTATCTGGAAGTGGATATTTGGATAGCTTTGAGGA

                         *   * *            **                      * *  *   
     293061 TTTCGTTGGAAACGGGATTGTCTTCATATAAACTCTAGACAGAAGCATTCTCAGAAGCGTCATTG
         66 TTTCGTTGGAAACAGGAATATCTTCATATAAAAGCTAGACAGAAGCATTCTCAGAAACTTCTTTG

            *       **   *    *             *        
     293126 GGATGTTTCAATTGAAGTCACAGTGTTGAACAGTCCCTTTC
        131 TGATGTTTGCATTCAAGTAACAGTGTTGAACATTCCCTTTC

                                                   *     *       **     *    
     293167 ATAGAGCAGGTTTGAAACACTCTTTTTGTAGTATCTGGATGTGGACATTTGGAGCGCTTTCAGGC
          1 ATAGAGCAGGTTTGAAACACTCTTTTTGTAGTATCTGGAAGTGGATATTTGGATAGCTTTGAGG-

            * * *    * *               *** *   *                          *  
     293232 CTATGGTT-TACA-AGGAAATATCTTCCCCTGAAAACTAGACAGAAGCATTCTCAGAAACTTATT
         65 ATTTCGTTGGAAACAGG-AATATCTTCATATAAAAGCTAGACAGAAGCATTCTCAGAAACTTCTT

                    **  **    *                    *   *
     293295 TGTGATGTGCGCCCTCAACTAACAGTGTTGAAGCATT-CTTTTG
        129 TGTGATGTTTGCATTCAAGTAACAGTGTTGAA-CATTCCCTTTC

                     *                   * *     *             **            
     293338 ATAGAGCAGTTTTGAAACACTCTTTTTGTGGAATCTGCAAGTGGATATTTGTCTAGCTTTGAGGA
          1 ATAGAGCAGGTTTGAAACACTCTTTTTGTAGTATCTGGAAGTGGATATTTGGATAGCTTTGAGGA

                         *   *    *                   *       *              
     293403 TTTCGTTGGAAACGGGATTA-CAT-ATA-AAAAGC-AGACAGCAGCATTCCCAGAAACTTCTTTG
         66 TTTCGTTGGAAACAGGAATATCTTCATATAAAAGCTAGACAGAAGCATTCTCAGAAACTTCTTTG

                              *    *                 
     293464 TGATGTTTGCATTCAAGTCACAGAGTTGAACATTCCCTTTC
        131 TGATGTTTGCATTCAAGTAACAGTGTTGAACATTCCCTTTC

                                                   *           * **     *   *
     293505 ATAGAGCAGGTTTGAAACACTCTTTTTGTAGTATCTGGATGTGGATATTTGCAGCGCTTTCAGGC
          1 ATAGAGCAGGTTTGAAACACTCTTTTTGTAGTATCTGGAAGTGGATATTTGGATAGCTTTGAGGA

            * ** *      *             *** *   *                          *   
     293570 CTAAGGT-GAAAAAGGAAATATCTTCCCCTGAAAACTAGACAGAAGCATTCTCAGAAACTTATTT
         66 TTTCGTTGGAAACAGG-AATATCTTCATATAAAAGCTAGACAGAAGCATTCTCAGAAACTTCTTT

                   **  **    *             **  * *   *
     293634 GTGATGTGCGCCCTCAACTAACAGTGTTGAAGCTTTCTTTTG
        130 GTGATGTTTGCATTCAAGTAACAGTGTTGAACATTCCCTTTC

                     *                    *      *   *              *        
     293676 ATAGAGCAGTTTTGAAACACTCTTTTTGTAATATCTGCAAGAGGATATTTGGATAGGTTTGAGGA
          1 ATAGAGCAGGTTTGAAACACTCTTTTTGTAGTATCTGGAAGTGGATATTTGGATAGCTTTGAGGA

                *        *   * *            **                      *    *   
     293741 TTTCCTTGGAAACGGGATTGTCTTCATATAAACTCTAGACAGAAGCATTCTCAGAAGCTTCATTG
         66 TTTCGTTGGAAACAGGAATATCTTCATATAAAAGCTAGACAGAAGCATTCTCAGAAACTTCTTTG

            *       **   *    *             * *      
     293806 GGATGTTTCAATTGAAGTCACAGTGTTGAACAGTTCCTTTC
        131 TGATGTTTGCATTCAAGTAACAGTGTTGAACATTCCCTTTC

                 *                                       *       **   * *    
     293847 ATAGAACAGGTTTGAAACACTCTTTTTGTAGTATCTGGAAGTGGACATTTGGAGCGCTCTCAGGA
          1 ATAGAGCAGGTTTGAAACACTCTTTTTGTAGTATCTGGAAGTGGATATTTGGATAGCTTTGAGGA

            * *  *      *             **          * *       * *          *  *
     293912 CTACGGT-GAAAAAGGAAATATCTTCCAATAAAAGCTACATAGAAGCAATGTCAGAAACTTTTTC
         66 TTTCGTTGGAAACAGG-AATATCTTCATATAAAAGCTAGACAGAAGCATTCTCAGAAACTTCTTT

            *      * ** *   **      *       *  *     *
     293976 ATGATGTATCTACTCAGCTAACAGAGTTGAACCTTTCCTTTG
        130 GTGATGTTTGCATTCAAGTAACAGTGTTGAACATTCCCTTTC

             *       *                   * *     *             **            
     294018 AGAGAGCAGTTTTGAAACACTCTTTTTGTTGAATCTGCAAGTGGATATTTGTCTAGCTTTGAGGA
          1 ATAGAGCAGGTTTGAAACACTCTTTTTGTAGTATCTGGAAGTGGATATTTGGATAGCTTTGAGGA

                         *   *    *                   *       *   *          
     294083 TTTCGTTGGAAACGGGATTA-CAT-ATA-AAAAGC-AGACAGCAGCATTCCCAGTAACTTCTTTG
         66 TTTCGTTGGAAACAGGAATATCTTCATATAAAAGCTAGACAGAAGCATTCTCAGAAACTTCTTTG

                              *    *                 
     294144 TGATGTTTGCATTCAAGTCACAGAGTTGAACATTCCCTTTC
        131 TGATGTTTGCATTCAAGTAACAGTGTTGAACATTCCCTTTC

                                                   *     *  *    **     *   *
     294185 ATAGAGCAGGTTTGAAACACTCTTTTTGTAGTATCTGGATGTGGACATATGGAGCGCTTTCAGGC
          1 ATAGAGCAGGTTTGAAACACTCTTTTTGTAGTATCTGGAAGTGGATATTTGGATAGCTTTGAGGA

            * ** *      *             *** *   *                          *   
     294250 CTATGGT-GAAAAAGGAAATATCTTCCCCTGAAAACTAGACAGAAGCATTCTCAGAAACTTATTT
         66 TTTCGTTGGAAACAGG-AATATCTTCATATAAAAGCTAGACAGAAGCATTCTCAGAAACTTCTTT

                   **  **    *              *  * *   *
     294314 GTGATGTGCGCCCTCAATTAACAGTGTTGAACCTTTCTTTTG
        130 GTGATGTTTGCATTCAAGTAACAGTGTTGAACATTCCCTTTC

                     *                    *      *   *                       
     294356 ATAGAGCAGTTTTGAAACACTCTTTTTGTAATATCTGCAAGAGGATATTTGGATAGCTTTGAGGA
          1 ATAGAGCAGGTTTGAAACACTCTTTTTGTAGTATCTGGAAGTGGATATTTGGATAGCTTTGAGGA

                         *   *    *                   *       *     *   *    
     294421 TTTCGTTGGAAACGGGATTA-CAT-ATA-AAAAGC-AGACAGCAGCATTCCCAGAATCTTGTTTG
         66 TTTCGTTGGAAACAGGAATATCTTCATATAAAAGCTAGACAGAAGCATTCTCAGAAACTTCTTTG

                    *              *                 
     294482 TGATGTTTCCATTCAAGTAACAGAGTTGAACATTCCCTTTC
        131 TGATGTTTGCATTCAAGTAACAGTGTTGAACATTCCCTTTC

             *                         *           *     *       **     *   *
     294523 AGAGAGCAGGTTTGAAACACTCTTTTTATAGTATCTGGATGTGGACATTTGGAGCGCTTTCAGGC
          1 ATAGAGCAGGTTTGAAACACTCTTTTTGTAGTATCTGGAAGTGGATATTTGGATAGCTTTGAGGA

            * ** *      *             *** *   *                      *   *   
     294588 CTATGGT-GAAAAAGGAAATATCTTCTCCTGAAAACTAGACAGAAGCATTCTCAGAATCTTATTT
         66 TTTCGTTGGAAACAGG-AATATCTTCATATAAAAGCTAGACAGAAGCATTCTCAGAAACTTCTTT

                   *** **    *             **  * *   *
     294652 GTGATGTGCCCCCTCAACTAACAGTGTTGAAGCTTTCTTTTG
        130 GTGATGTTTGCATTCAAGTAACAGTGTTGAACATTCCCTTTC

                     *                *   **     *   *        *     *        
     294694 ATAGAGCAGTTTTGAAACACTCTTTTCGTAAAATCTGCAAGAGGATATTTTGATAGATTTGAGGA
          1 ATAGAGCAGGTTTGAAACACTCTTTTTGTAGTATCTGGAAGTGGATATTTGGATAGCTTTGAGGA

                         *   * *            **                      *    *   
     294759 TTTCGTTGGAAACGGGATTGTCTTCATATAAACTCTAGACAGAAGCATTCTCAGAAGCTTCATTG
         66 TTTCGTTGGAAACAGGAATATCTTCATATAAAAGCTAGACAGAAGCATTCTCAGAAACTTCTTTG

            *       **   *    *             *        
     294824 GGATGTTTCAATTGAAGTCACAGTGTTGAACAGTCCCTTTC
        131 TGATGTTTGCATTCAAGTAACAGTGTTGAACATTCCCTTTC

                                                         *       *  * * *    
     294865 ATAGAGCAGGTTTGAAACACTCTTTTTGTAGTATCTGGAAGTGGACATTTGGAGAGATCTCAGGA
          1 ATAGAGCAGGTTTGAAACACTCTTTTTGTAGTATCTGGAAGTGGATATTTGGATAGCTTTGAGGA

            * *  *    * *             **            *       * *          *  *
     294930 ATACGGT-GATAAAGGAAATATCTTCCAATAAAAGCTAGATAGAAGCAATGTCAGAAACTTTTTC
         66 TTTCGTTGGAAACAGG-AATATCTTCATATAAAAGCTAGACAGAAGCATTCTCAGAAACTTCTTT

            *      * ** *   **      *       *  * *   *
     294994 ATGATGTATCTACTCAGCTAACAGAGTTGAACCTTTCTTTTG
        130 GTGATGTTTGCATTCAAGTAACAGTGTTGAACATTCCCTTTC

             *       *                   * *     *             **            
     295036 AGAGAGCAGTTTTGAAACACTCTTTTTGTGGAATCTGCAAGTGGATATTTGTCTAGCTTTGAGGA
          1 ATAGAGCAGGTTTGAAACACTCTTTTTGTAGTATCTGGAAGTGGATATTTGGATAGCTTTGAGGA

                         *   *    *                   *       *              
     295101 TTTCGTTGGAAACGGGATTA-CAT-ATA-AAAAGC-AGACAGCAGCATTCCCAGAAACTTCTTTG
         66 TTTCGTTGGAAACAGGAATATCTTCATATAAAAGCTAGACAGAAGCATTCTCAGAAACTTCTTTG

                              * *  *                 
     295162 TGATGTTTGCATTCAAGTCAGAGAGTTGAACATTCCCTTTC
        131 TGATGTTTGCATTCAAGTAACAGTGTTGAACATTCCCTTTC

                           *                       *     *       **     *   *
     295203 ATAGAGCAGGTTTGAGACACTCTTTTTGTAGTATCTGGATGTGGACATTTGGAGCGCTTTCAGGC
          1 ATAGAGCAGGTTTGAAACACTCTTTTTGTAGTATCTGGAAGTGGATATTTGGATAGCTTTGAGGA

            * ** *      *             *** *   *                          *   
     295268 CTATGGT-GAAAAAGGAAATATCTTCCCCTGAAAACTAGACAGAAGCATTCTCAGAAACTTATTT
         66 TTTCGTTGGAAACAGG-AATATCTTCATATAAAAGCTAGACAGAAGCATTCTCAGAAACTTCTTT

                   **  **    *             **  * *   *
     295332 GTGATGTGCGCCCTCAACTAACAGTGTTGAAGCTTTCTTTTG
        130 GTGATGTTTGCATTCAAGTAACAGTGTTGAACATTCCCTTTC

                     *                    *      *   *                       
     295374 ATAGAGCAGTTTTGAAACACTCTTTTTGTAATATCTGCAAGAGGATATTTGGATAGCTTTGAGGA
          1 ATAGAGCAGGTTTGAAACACTCTTTTTGTAGTATCTGGAAGTGGATATTTGGATAGCTTTGAGGA

                         *   * *       *    **                      *    *   
     295439 TTTCGTTGGAAACGGGATTGTCTTCATGTAAACTCTAGACAGAAGCATTCTCAGAAGCTTCATTG
         66 TTTCGTTGGAAACAGGAATATCTTCATATAAAAGCTAGACAGAAGCATTCTCAGAAACTTCTTTG

            * *     **   *    *             * *      
     295504 GGTTGTTTCAATTGAAGTCACAGTGTTGAACAGTTCCTTTC
        131 TGATGTTTGCATTCAAGTAACAGTGTTGAACATTCCCTTTC

                 *                                       *       **   * *    
     295545 ATAGAACAGGTTTGAAACACTCTTTTTGTAGTATCTGGAAGTGGACATTTGGAGGGCTCTCAGGA
          1 ATAGAGCAGGTTTGAAACACTCTTTTTGTAGTATCTGGAAGTGGATATTTGGATAGCTTTGAGGA

            * ** *      *             **          * *       * *          *  *
     295610 CTATGGT-GAAAAAGGAAATATCTTCCAATAAAAGCTACATAGAAGCAATGTCAGAAACTTTTTC
         66 TTTCGTTGGAAACAGG-AATATCTTCATATAAAAGCTAGACAGAAGCATTCTCAGAAACTTCTTT

            *      * ** *   **      * *     *  *     *
     295674 ATGATGTATCTACTCAGCTAACAGAGGTGAACCTTTCCTTTG
        130 GTGATGTTTGCATTCAAGTAACAGTGTTGAACATTCCCTTTC

             *       *                   * *     *             **            
     295716 AGAGAGCAGTTTTGAAACACTCTTTTTGTGGAATCTGCAAGTGGATATTTGTCTAGCTTTGAGGA
          1 ATAGAGCAGGTTTGAAACACTCTTTTTGTAGTATCTGGAAGTGGATATTTGGATAGCTTTGAGGA

                         *   *    *                   *       *   *          
     295781 TTTCGTTGGAAACGGGATTA-CAT-ATA-AAAAGC-AGACAGCAGCATTCCCAGTAACTTCTTTG
         66 TTTCGTTGGAAACAGGAATATCTTCATATAAAAGCTAGACAGAAGCATTCTCAGAAACTTCTTTG

                              *    *                 
     295842 TGATGTTTGCATTCAAGTCACAGAGTTGAACATTCCCTTTC
        131 TGATGTTTGCATTCAAGTAACAGTGTTGAACATTCCCTTTC

                              *       *            *     *       **     *   *
     295883 ATAGAGCAGGTTTGAAACCCTCTTTTGGTAGTATCTGGATGTGGACATTTGGAGCGCTTTCAGGC
          1 ATAGAGCAGGTTTGAAACACTCTTTTTGTAGTATCTGGAAGTGGATATTTGGATAGCTTTGAGGA

            * ** *      *             *** *   *                      *   *   
     295948 CTATGGT-GAAAAAGGAAATATCTTCCCCTGAAAACTAGACAGAAGCATTCTCAGAATCTTATTT
         66 TTTCGTTGGAAACAGG-AATATCTTCATATAAAAGCTAGACAGAAGCATTCTCAGAAACTTCTTT

                   **  **    *             **  * *   *
     296012 GTGATGTGCGCCCTCAACTAACAGTGTTGAAGCTTTCTTTTG
        130 GTGATGTTTGCATTCAAGTAACAGTGTTGAACATTCCCTTTC

                     *                    **     *   *                       
     296054 ATAGAGCAGTTTTGAAACACTCTTTTTGTAAAATCTGCAAGAGGATATTTGGATAGCTTTGAGGA
          1 ATAGAGCAGGTTTGAAACACTCTTTTTGTAGTATCTGGAAGTGGATATTTGGATAGCTTTGAGGA

                         *   * *            **                      *    *   
     296119 TTTCGTTGGAAACGGGATTGTCTTCATATAAACTCTAGACAGAAGCATTCTCAGAAGCTTCATTG
         66 TTTCGTTGGAAACAGGAATATCTTCATATAAAAGCTAGACAGAAGCATTCTCAGAAACTTCTTTG

            *       **   *    *             *        
     296184 GGATGTTTCAATTGAAGTCACAGTGTTGAACAGTCCCTTTC
        131 TGATGTTTGCATTCAAGTAACAGTGTTGAACATTCCCTTTC

                                                   *     *       **     *    
     296225 ATAGAGCAGGTTTGAAACACTCTTTTTGTAGTATCTGGATGTGGACATTTGGAGCGCTTTCAGGC
          1 ATAGAGCAGGTTTGAAACACTCTTTTTGTAGTATCTGGAAGTGGATATTTGGATAGCTTTGAGG-

            * * *    *                 *** *   *                          *  
     296290 CTATGGTT-TAAA-AGGAAATATCTTCCCCTGAAAACTAGACAGAAGCATTCTCAGAAACTTATT
         65 ATTTCGTTGGAAACAGG-AATATCTTCATATAAAAGCTAGACAGAAGCATTCTCAGAAACTTCTT

                    **  **    *                    *   *
     296353 TGTGATGTGCGCCCTCAACTAACAGTGTTGAAGCATT-CTTTTG
        129 TGTGATGTTTGCATTCAAGTAACAGTGTTGAA-CATTCCCTTTC

                     *                   * *     *             **            
     296396 ATAGAGCAGTTTTGAAACACTCTTTTTGTGGAATCTGCAAGTGGATATTTGTCTAGCTTTGAGGA
          1 ATAGAGCAGGTTTGAAACACTCTTTTTGTAGTATCTGGAAGTGGATATTTGGATAGCTTTGAGGA

                         *   *    *                   *       *              
     296461 TTTCGTTGGAAACGGGATTA-CAT-ATA-AAAAGC-AGACAGCAGCATTCCCAGAAACTTCTTTG
         66 TTTCGTTGGAAACAGGAATATCTTCATATAAAAGCTAGACAGAAGCATTCTCAGAAACTTCTTTG

                 *            *    *                 
     296522 TGATGCTTGCATTCAAGTCACAGAGTTGAACATTCCCTTTC
        131 TGATGTTTGCATTCAAGTAACAGTGTTGAACATTCCCTTTC

                                                   *     *       **     *   *
     296563 ATAGAGCAGGTTTGAAACACTCTTTTTGTAGTATCTGGATGTGGACATTTGGAGCGCTTTCAGGC
          1 ATAGAGCAGGTTTGAAACACTCTTTTTGTAGTATCTGGAAGTGGATATTTGGATAGCTTTGAGGA

            * ** *      *             *** *   *                          *   
     296628 CTATGGT-GAAAAAGGAAATATCTTCCCCTGAAAACTAGACAGAAGCATTCTCAGAAACTTATTT
         66 TTTCGTTGGAAACAGG-AATATCTTCATATAAAAGCTAGACAGAAGCATTCTCAGAAACTTCTTT

                   **  **    *              *  * *   *
     296692 GTGATGTGCGCCCTCAACTAACAGTGTTGAACCTTTCTTTTG
        130 GTGATGTTTGCATTCAAGTAACAGTGTTGAACATTCCCTTTC

                     *        *           *      *   *                       
     296734 ATAGAGCAGTTTTGAAACGCTCTTTTTGTAATATCTGCAAGAGGATATTTGGATAGCTTTGAGGA
          1 ATAGAGCAGGTTTGAAACACTCTTTTTGTAGTATCTGGAAGTGGATATTTGGATAGCTTTGAGGA

                         *   * *             *                      *    *   
     296799 TTTCGTTGGAAACGGGATTGTCTTCATATAAAATCTAGACAGAAGCATTCTCAGAAGCTTCATTG
         66 TTTCGTTGGAAACAGGAATATCTTCATATAAAAGCTAGACAGAAGCATTCTCAGAAACTTCTTTG

            *       **   *    *             *        
     296864 GGATGTTTCAATTGAAGTCACAGTGTTGAACAGTCCCTTTC
        131 TGATGTTTGCATTCAAGTAACAGTGTTGAACATTCCCTTTC

                                                         *       *  * * *    
     296905 ATAGAGCAGGTTTGAAACACTCTTTTTGTAGTATCTGGAAGTGGACATTTGGAGAGATCTCAGGA
          1 ATAGAGCAGGTTTGAAACACTCTTTTTGTAGTATCTGGAAGTGGATATTTGGATAGCTTTGAGGA

            * *  *    * *             **            *       * *          *  *
     296970 ATACGGT-GATAAAGGAAATATCTTCCAATAAAAGCTAGATAGAAGCAATGTCAGAAACTTTTTC
         66 TTTCGTTGGAAACAGG-AATATCTTCATATAAAAGCTAGACAGAAGCATTCTCAGAAACTTCTTT

            *      * ** *   **      *       *  * *   *
     297034 ATGATGTATCTACTCAGCTAACAGAGTTGAACCTTTCTTTTG
        130 GTGATGTTTGCATTCAAGTAACAGTGTTGAACATTCCCTTTC

             *       *                   * *     *             **            
     297076 AGAGAGCAGTTTTGAAACACTCTTTTTGTGGAATCTGCAAGTGGATATTTGTCTAGCTTTGAGGA
          1 ATAGAGCAGGTTTGAAACACTCTTTTTGTAGTATCTGGAAGTGGATATTTGGATAGCTTTGAGGA

                   *     *   *    *                   *       *              
     297141 TTTCGTTTGAAACGGGATTA-CAT-ATA-AAAAGC-AGACAGCAGCATTCCCAGAAACTTCTTTG
         66 TTTCGTTGGAAACAGGAATATCTTCATATAAAAGCTAGACAGAAGCATTCTCAGAAACTTCTTTG

                              *    **                
     297202 TGATGTTTGCATTCAAGTCACAGATTTGAACATTCCCTTTC
        131 TGATGTTTGCATTCAAGTAACAGTGTTGAACATTCCCTTTC

                                *                * *     *       ** *   *   *
     297243 ATAGAGCAGGTTTGAAACACACTTTTTGTAGTATCTGTATGTGGACATTTGGAGCGGTTTCAGGC
          1 ATAGAGCAGGTTTGAAACACTCTTTTTGTAGTATCTGGAAGTGGATATTTGGATAGCTTTGAGGA

            * ** *      *             *** *   *                        * *   
     297308 CTATGGT-GAAAAAGGAAATATCTTCCCCTGAAAACTAGACAGAAGCATTCTCAGAAACCTATTT
         66 TTTCGTTGGAAACAGG-AATATCTTCATATAAAAGCTAGACAGAAGCATTCTCAGAAACTTCTTT

                   **  **    *          *   *  * *   *
     297372 GTGATGTGCGCCCTCAACTAACAGTGTTTAACCTTTCTTTTG
        130 GTGATGTTTGCATTCAAGTAACAGTGTTGAACATTCCCTTTC

                     *                    *      *   *                       
     297414 ATAGAGCAGTTTTGAAACACTCTTTTTGTAATATCTGCAAGAGGATATTTGGATAGCTTTGAGGA
          1 ATAGAGCAGGTTTGAAACACTCTTTTTGTAGTATCTGGAAGTGGATATTTGGATAGCTTTGAGGA

                     *   *   * *            **                      *    *   
     297479 TTTCGTTGGCAACGGGATTGTCTTCATATAAACTCTAGACAGAAGCATTCTCAGAAGCTTCATTG
         66 TTTCGTTGGAAACAGGAATATCTTCATATAAAAGCTAGACAGAAGCATTCTCAGAAACTTCTTTG

            *       **   *    *   *         * *      
     297544 GGATGTTTCAATTGAAGTCACACTGTTGAACAGTTCCTTTC
        131 TGATGTTTGCATTCAAGTAACAGTGTTGAACATTCCCTTTC

                 *                                       *       **   * *    
     297585 ATAGAACAGGTTTGAAACACTCTTTTTGTAGTATCTGGAAGTGGACATTTGGAGCGCTCTCAGGA
          1 ATAGAGCAGGTTTGAAACACTCTTTTTGTAGTATCTGGAAGTGGATATTTGGATAGCTTTGAGGA

            * *  *      *             **          * *       * *          *  *
     297650 CTACGGT-GAAAAAGGAAATATCTTCCAATAAAAGCTACATAGAAGCAATGTCAGAAACTTTTTC
         66 TTTCGTTGGAAACAGG-AATATCTTCATATAAAAGCTAGACAGAAGCATTCTCAGAAACTTCTTT

            *      * ** *   **      *       *  *     *
     297714 ATGATGTATCTACTCAGCTAACAGAGTTGAACCTTTCCTTTG
        130 GTGATGTTTGCATTCAAGTAACAGTGTTGAACATTCCCTTTC

             *       *                   * *     *             **            
     297756 AGAGAGCAGTTTTGAAACACTCTTTTTGTGGAATCTGCAAGTGGATATTTGTCTAGCTTTGAGGA
          1 ATAGAGCAGGTTTGAAACACTCTTTTTGTAGTATCTGGAAGTGGATATTTGGATAGCTTTGAGGA

                         *   *    *                   *       *   *          
     297821 TTTCGTTGGAAACGGGATTA-CAT-ATA-AAAAGC-AGACAGCAGCATTCCCAGTAACTTCTTTG
         66 TTTCGTTGGAAACAGGAATATCTTCATATAAAAGCTAGACAGAAGCATTCTCAGAAACTTCTTTG

                              * *  *                 
     297882 TGATGTTTGCATTCAAGTCAGAGAGTTGAACATTCCCTTTC
        131 TGATGTTTGCATTCAAGTAACAGTGTTGAACATTCCCTTTC

                                        *    *     *     *       **     *   *
     297923 ATAGAGCAGGTTTGAAACACTCTTTTTGAAGTACCTGGATGTGGACATTTGGAGCGCTTTCAGGC
          1 ATAGAGCAGGTTTGAAACACTCTTTTTGTAGTATCTGGAAGTGGATATTTGGATAGCTTTGAGGA

            * ** *      *             *** *   *                          *   
     297988 CTATGGT-GAAAAAGGAAATATCTTCCCCTGAAAACTAGACAGAAGCATTCTCAGAAACTTATTT
         66 TTTCGTTGGAAACAGG-AATATCTTCATATAAAAGCTAGACAGAAGCATTCTCAGAAACTTCTTT

                   *** **    *              *  * *   *
     298052 GTGATGTGCACCCTCAACTAACAGTGTTGAACCTTTCTTTTG
        130 GTGATGTTTGCATTCAAGTAACAGTGTTGAACATTCCCTTTC

                     *                    *      *   *                       
     298094 ATAGAGCAGTTTTGAAACACTCTTTTTGTAATATCTGCAAGAGGATATTTGGATAGCTTTGAGGA
          1 ATAGAGCAGGTTTGAAACACTCTTTTTGTAGTATCTGGAAGTGGATATTTGGATAGCTTTGAGGA

                         *   * *            **                      *    *   
     298159 TTTCGTTGGAAACGGGATTGTCTTCATATAAACTCTAGACAGAAGCATTCTCAGAAGCTTCATTG
         66 TTTCGTTGGAAACAGGAATATCTTCATATAAAAGCTAGACAGAAGCATTCTCAGAAACTTCTTTG

            *  *    **   *    *             *        
     298224 GGAGGTTTCAATTGAAGTCACAGTGTTGAACAGTCCCTTTC
        131 TGATGTTTGCATTCAAGTAACAGTGTTGAACATTCCCTTTC

                                                         *       **   * *    
     298265 ATAGAGCAGGTTTGAAACACTCTTTTTGTAGTATCTGGAAGTGGACATTTGGAGCGCTCTCAGGA
          1 ATAGAGCAGGTTTGAAACACTCTTTTTGTAGTATCTGGAAGTGGATATTTGGATAGCTTTGAGGA

            * *  *    * *             **      *     *       * *          *  *
     298330 CTACGGT-GATAAAGGAAATATCTTCCAATAAAAACTAGATAGAAGCAATGTCAGAAACTTTTTC
         66 TTTCGTTGGAAACAGG-AATATCTTCATATAAAAGCTAGACAGAAGCATTCTCAGAAACTTCTTT

            *      * ** *   **      *       *  * *   *
     298394 ATGATGTATCTACTCAGCTAACAGAGTTGAACCTTTCTTTTG
        130 GTGATGTTTGCATTCAAGTAACAGTGTTGAACATTCCCTTTC

             *       *                   * *     *             **   *        
     298436 AGAGAGCAGTTTTGAAACACTCTTTTTGTGGAATCTGCAAGTGGATATTTGTCTAGATTTGAGGA
          1 ATAGAGCAGGTTTGAAACACTCTTTTTGTAGTATCTGGAAGTGGATATTTGGATAGCTTTGAGGA

                         *  **    *                   *       *     *       *
     298501 TTTCGTTGGAAACGGGTTTA-CAT-ATA-AAAAGC-AGACAGCAGCATTCCCAGAATCTTCTTTC
         66 TTTCGTTGGAAACAGGAATATCTTCATATAAAAGCTAGACAGAAGCATTCTCAGAAACTTCTTTG

                              *    *                 
     298562 TGATGTTTGCATTCAAGTCACAGAGTTGAACATTCCCTTTC
        131 TGATGTTTGCATTCAAGTAACAGTGTTGAACATTCCCTTTC

                                                   *     *       **     *   *
     298603 ATAGAGCAGGTTTGAAACACTCTTTTTGTAGTATCTGGATGTGGACATTTGGAGCGCTTTCAGGC
          1 ATAGAGCAGGTTTGAAACACTCTTTTTGTAGTATCTGGAAGTGGATATTTGGATAGCTTTGAGGA

            * ** *      *             *** * * *                          *   
     298668 CTATGGT-GAAAAAGGAAATATCTTCCCCTGAGAACTAGACAGAAGCATTCTCAGAAACTTATTT
         66 TTTCGTTGGAAACAGG-AATATCTTCATATAAAAGCTAGACAGAAGCATTCTCAGAAACTTCTTT

                   **  **    *   *          *  * *   *
     298732 GTGATGTGCGCCCTCAACTAAAAGTGTTGAACCTTTCTTTTG
        130 GTGATGTTTGCATTCAAGTAACAGTGTTGAACATTCCCTTTC

                     *                    *      *   *         *             
     298774 ATAGAGCAGTTTTGAAACACTCTTTTTGTAATATCTGCAAGAGGATATTTGTATAGCTTTGAGGA
          1 ATAGAGCAGGTTTGAAACACTCTTTTTGTAGTATCTGGAAGTGGATATTTGGATAGCTTTGAGGA

                         *   *    *                   *       *     *   *    
     298839 TTTCGTTGGAAACGGGATTA-CAT-ATA-AAAAGC-AGACAGCAGCATTCCCAGAATCTTGTTTG
         66 TTTCGTTGGAAACAGGAATATCTTCATATAAAAGCTAGACAGAAGCATTCTCAGAAACTTCTTTG

                    *         *    *                 
     298900 TGATGTTTCCATTCAAGTCACAGAGTTGAACATTCCCTTTC
        131 TGATGTTTGCATTCAAGTAACAGTGTTGAACATTCCCTTTC

             *                         *           *     *       **     *   *
     298941 AGAGAGCAGGTTTGAAACACTCTTTTTATAGTATCTGGATGTGGACATTTGGAGCGCTTTCAGGC
          1 ATAGAGCAGGTTTGAAACACTCTTTTTGTAGTATCTGGAAGTGGATATTTGGATAGCTTTGAGGA

            * ** *      *             *** *   *                      *   *   
     299006 CTATGGT-GAAAAAGGAAATATCTTCTCCTGAAAACTAGACAGAAGCATTCTCAGAATCTTATTT
         66 TTTCGTTGGAAACAGG-AATATCTTCATATAAAAGCTAGACAGAAGCATTCTCAGAAACTTCTTT

                   **  **    *             **  * *   *
     299070 GTGATGTGCGCCCTCAACTAACAGTGTTGAAGCTTTCTTTTG
        130 GTGATGTTTGCATTCAAGTAACAGTGTTGAACATTCCCTTTC

                     *                *   **     *   *        *              
     299112 ATAGAGCAGTTTTGAAACACTCTTTTCGTAAAATCTGCAAGAGGATATTTTGATAGCTTTGAGGA
          1 ATAGAGCAGGTTTGAAACACTCTTTTTGTAGTATCTGGAAGTGGATATTTGGATAGCTTTGAGGA

                         *   * *            **                      *    *   
     299177 TTTCGTTGGAAACGGGATTGTCTTCATATAAACTCTAGACAGAAGCATTCTCAGAAGCTTCATTG
         66 TTTCGTTGGAAACAGGAATATCTTCATATAAAAGCTAGACAGAAGCATTCTCAGAAACTTCTTTG

            *       **   *    *             *        
     299242 GGATGTTTCAATTGAAGTCACAGTGTTGAACAGTCCCTTTC
        131 TGATGTTTGCATTCAAGTAACAGTGTTGAACATTCCCTTTC

                                                         *       **   * *    
     299283 ATAGAGCAGGTTTGAAACACTCTTTTTGTAGTATCTGGAAGTGGACATTTGGAGCGCTCTCAGGA
          1 ATAGAGCAGGTTTGAAACACTCTTTTTGTAGTATCTGGAAGTGGATATTTGGATAGCTTTGAGGA

            * *  *    * *             **            *       * *          *  *
     299348 CTCCGGT-GATAAAGGAAATATCTTCCAATAAAAGCTAGATAGAAGCAATGTCAGAAACTTTTTC
         66 TTTCGTTGGAAACAGG-AATATCTTCATATAAAAGCTAGACAGAAGCATTCTCAGAAACTTCTTT

            *      * ** *   **      *       *  * *   *
     299412 ATGATGTATCTACTCAGCTAACAGAGTTGAACCTTTCTTTTG
        130 GTGATGTTTGCATTCAAGTAACAGTGTTGAACATTCCCTTTC

             *       *                   * *     *             **            
     299454 AGAGAGCAGTTTTGAAACACTCTTTTTGTGGAATCTGCAAGTGGATATTTGTCTAGCTTTGAGGA
          1 ATAGAGCAGGTTTGAAACACTCTTTTTGTAGTATCTGGAAGTGGATATTTGGATAGCTTTGAGGA

                         *   *    *                   *       *              
     299519 TTTCGTTGGAAACGGGATTA-CAT-ATA-AAAAGC-AGACAGCAGCATTCCCAGAAACTTCTTTG
         66 TTTCGTTGGAAACAGGAATATCTTCATATAAAAGCTAGACAGAAGCATTCTCAGAAACTTCTTTG

                       *      *    *        *        
     299580 TGATGTTTGCAATCAAGTCACAGAGTTGAACACTCCCTTTC
        131 TGATGTTTGCATTCAAGTAACAGTGTTGAACATTCCCTTTC

                                                   *     *     * **  *  *   *
     299621 ATAGAGCAGGTTTGAAACACTCTTTTTGTAGTATCTGGATGTGGACATTTGCAGCGCATTCAGGC
          1 ATAGAGCAGGTTTGAAACACTCTTTTTGTAGTATCTGGAAGTGGATATTTGGATAGCTTTGAGGA

            * ** *      *             *** *   *                          *   
     299686 CTAAGGT-GAAAAAGGAAATATCTTCCCCTGAAAACTAGACAGAAGCATTCTCAGAAACTTATTT
         66 TTTCGTTGGAAACAGG-AATATCTTCATATAAAAGCTAGACAGAAGCATTCTCAGAAACTTCTTT

                   **  **    *             **  * *   *
     299750 GTGATGTGCGCCCTCAACTAACAGTGTTGAAGCTTTCTTTTG
        130 GTGATGTTTGCATTCAAGTAACAGTGTTGAACATTCCCTTTC

                     *                    *      *   *                       
     299792 ATAGAGCAGTTTTGAAACACTCTTTTTGTAATATCTGCAAGAGGATATTTGGATAGCTTTGAGGA
          1 ATAGAGCAGGTTTGAAACACTCTTTTTGTAGTATCTGGAAGTGGATATTTGGATAGCTTTGAGGA

                         *   * *            **                      *    *   
     299857 TTTCGTTGGAAACGGGATTGTCTTCATATAAACTCTAGACAGAAGCATTCTCAGAAGCTTCATTG
         66 TTTCGTTGGAAACAGGAATATCTTCATATAAAAGCTAGACAGAAGCATTCTCAGAAACTTCTTTG

            *       **   *    *             * *      
     299922 GGATGTTTCAATTGAAGTCACAGTGTTGAACAGTTCCTTTC
        131 TGATGTTTGCATTCAAGTAACAGTGTTGAACATTCCCTTTC

                 *                                               **   * *    
     299963 ATAGAACAGGTTTGAAACACTCTTTTTGTAGTATCTGGAAGTGGATATTTGGAGCGCTCTCAGGA
          1 ATAGAGCAGGTTTGAAACACTCTTTTTGTAGTATCTGGAAGTGGATATTTGGATAGCTTTGAGGA

            * ** *      *             **          * *       * *          *  *
     300028 CTATGGT-GAAAAAGGAAATATCTTCCAATAAAAGCTACATAGAAGCAATGTCAGAAACTTTTTC
         66 TTTCGTTGGAAACAGG-AATATCTTCATATAAAAGCTAGACAGAAGCATTCTCAGAAACTTCTTT

            *      * ** *   **     **       *  *     *
     300092 ATGATGTATCTACTCAGCTAACACAGTTGAACCTTTCCTTTG
        130 GTGATGTTTGCATTCAAGTAACAGTGTTGAACATTCCCTTTC

             *       *                   * *     *             **            
     300134 AGAGAGCAGTTTTGAAACACTCTTTTTGTGGAATCTGCAAGTGGATATTTGTCTAGCTTTGAGGA
          1 ATAGAGCAGGTTTGAAACACTCTTTTTGTAGTATCTGGAAGTGGATATTTGGATAGCTTTGAGGA

                         *   *    *                   *       *   *          
     300199 TTTCGTTGGAAACGGGATTA-CAT-ATA-AAAAGC-AGACAGCAGCATTCCCAGTAACTTCTTTG
         66 TTTCGTTGGAAACAGGAATATCTTCATATAAAAGCTAGACAGAAGCATTCTCAGAAACTTCTTTG

                              *    *                 
     300260 TGATGTTTGCATTCAAGTCACAGAGTTGAACATTCCCTTTC
        131 TGATGTTTGCATTCAAGTAACAGTGTTGAACATTCCCTTTC

                                                   *     *       **     *   *
     300301 ATAGAGCAGGTTTGAAACACTCTTTTTGTAGTATCTGGATGTGGACATTTGGAGCGCTTTCAGGC
          1 ATAGAGCAGGTTTGAAACACTCTTTTTGTAGTATCTGGAAGTGGATATTTGGATAGCTTTGAGGA

            * ** *      *             *** *   *                      *   *   
     300366 CTATGGT-GAAAAAGGAAATATCTTCCCCTGAAAACTAGACAGAAGCATTCTCAGAATCTTATTT
         66 TTTCGTTGGAAACAGG-AATATCTTCATATAAAAGCTAGACAGAAGCATTCTCAGAAACTTCTTT

                   **  **    *             **  * *   *
     300430 GTGATGTGCGCCCTCAACTAACAGTGTTGAAGCTTTCTTTTG
        130 GTGATGTTTGCATTCAAGTAACAGTGTTGAACATTCCCTTTC

                     *                    **     *   *                       
     300472 ATAGAGCAGTTTTGAAACACTCTTTTTGTAAAATCTGCAAGAGGATATTTGGATAGCTTTGAGGA
          1 ATAGAGCAGGTTTGAAACACTCTTTTTGTAGTATCTGGAAGTGGATATTTGGATAGCTTTGAGGA

                         *   * *            **                      *    *   
     300537 TTTCGTTGGAAACGGGATTGTCTTCATATAAACTCTAGACAGAAGCATTCTCAGAAGCTTCATTG
         66 TTTCGTTGGAAACAGGAATATCTTCATATAAAAGCTAGACAGAAGCATTCTCAGAAACTTCTTTG

            *       **   *    *             *        
     300602 GGATGTTTCAATTGAAGTCACAGTGTTGAACAGTCCCTTTC
        131 TGATGTTTGCATTCAAGTAACAGTGTTGAACATTCCCTTTC

                                                   *     *       **     *    
     300643 ATAGAGCAGGTTTGAAACACTCTTTTTGTAGTATCTGGATGTGGACATTTGGAGCGCTTTCAGGC
          1 ATAGAGCAGGTTTGAAACACTCTTTTTGTAGTATCTGGAAGTGGATATTTGGATAGCTTTGAGG-

            * * *    *                 *** *   *                          *  
     300708 CTATGGTT-TAAA-AGGAAATATCTTCCCCTGAAAACTAGACAGAAGCATTCTCAGAAACTTATT
         65 ATTTCGTTGGAAACAGG-AATATCTTCATATAAAAGCTAGACAGAAGCATTCTCAGAAACTTCTT

                    **  **    *                    *   *
     300771 TGTGATGTGCGCCCTCAACTAACAGTGTTGAAGCATT-CTTTTG
        129 TGTGATGTTTGCATTCAAGTAACAGTGTTGAA-CATTCCCTTTC

                     *                   * *     *             **            
     300814 ATAGAGCAGTTTTGAAACACTCTTTTTGTGGAATCTGCAAGTGGATATTTGTCTAGCTTTGAGGA
          1 ATAGAGCAGGTTTGAAACACTCTTTTTGTAGTATCTGGAAGTGGATATTTGGATAGCTTTGAGGA

                         *   *    *                   *       *              
     300879 TTTCGTTGGAAACGGGATTA-CAT-ATA-AAAAGC-AGACAGCAGCATTCCCAGAAACTTCTTTG
         66 TTTCGTTGGAAACAGGAATATCTTCATATAAAAGCTAGACAGAAGCATTCTCAGAAACTTCTTTG

                              *    *                 
     300940 TGATGTTTGCATTCAAGTCACAGAGTTGAACATTCCCTTTC
        131 TGATGTTTGCATTCAAGTAACAGTGTTGAACATTCCCTTTC

                                                   *     *       **     *   *
     300981 ATAGAGCAGGTTTGAAACACTCTTTTTGTAGTATCTGGATGTGGACATTTGGAGCGCTTTCAGGC
          1 ATAGAGCAGGTTTGAAACACTCTTTTTGTAGTATCTGGAAGTGGATATTTGGATAGCTTTGAGGA

            * ** *      *             *** *   *                          *   
     301046 CTATGGT-GAAAAAGGAAATATCTTCCCCTGAAAACTAGACAGAAGCATTCTCAGAAACTTATTT
         66 TTTCGTTGGAAACAGG-AATATCTTCATATAAAAGCTAGACAGAAGCATTCTCAGAAACTTCTTT

                   **  **    *             **  * *   *
     301110 GTGATGTGCGCCCTCAACTAACAGTGTTGAAGCTTTCTTTTG
        130 GTGATGTTTGCATTCAAGTAACAGTGTTGAACATTCCCTTTC

                     *                    *      *   *                       
     301152 ATAGAGCAGTTTTGAAACACTCTTTTTGTAATATCTGCAAGAGGATATTTGGATAGCTTTGAGGA
          1 ATAGAGCAGGTTTGAAACACTCTTTTTGTAGTATCTGGAAGTGGATATTTGGATAGCTTTGAGGA

                         *   * *            **          *           *    *   
     301217 TTTCGTTGGAAACGGGATTGTCTTCATATAAACTCTAGACAGAACCATTCTCAGAAGCTTCATTG
         66 TTTCGTTGGAAACAGGAATATCTTCATATAAAAGCTAGACAGAAGCATTCTCAGAAACTTCTTTG

            *       **   *    *             *        
     301282 GGATGTTTCAATTGAAGTCACAGTGTTGAACAGTCCCTTTC
        131 TGATGTTTGCATTCAAGTAACAGTGTTGAACATTCCCTTTC

                                                         *       *  * * *    
     301323 ATAGAGCAGGTTTGAAACACTCTTTTTGTAGTATCTGGAAGTGGACATTTGGAGAGATCTCAGGA
          1 ATAGAGCAGGTTTGAAACACTCTTTTTGTAGTATCTGGAAGTGGATATTTGGATAGCTTTGAGGA

            * * **      *             **            *       * *          *  *
     301388 CTACAGT-GAAAAAGGAAATATCTTCCAATAAAAGCTAGATAGAAGCAATGTCAGAAACTTTTTC
         66 TTTCGTTGGAAACAGG-AATATCTTCATATAAAAGCTAGACAGAAGCATTCTCAGAAACTTCTTT

            *      * ** *   **      *       *  * *   *
     301452 ATGATGTATCTACTCAGCTAACAGAGTTGAACCTTTCTTTTG
        130 GTGATGTTTGCATTCAAGTAACAGTGTTGAACATTCCCTTTC

             *       *                   * *     *             **            
     301494 AGAGAGCAGTTTTGAAACACTCTTTTTGTGGAATCTGCAAGTGGATATTTGTCTAGCTTTGAGGA
          1 ATAGAGCAGGTTTGAAACACTCTTTTTGTAGTATCTGGAAGTGGATATTTGGATAGCTTTGAGGA

                *        *   *    *                   *       *              
     301559 TTTCCTTGGAAACGGGATTA-CAT-ATA-AAAAGC-AGACAGCAGCATTCCCAGAAACTTCTTTG
         66 TTTCGTTGGAAACAGGAATATCTTCATATAAAAGCTAGACAGAAGCATTCTCAGAAACTTCTTTG

                              *    *                 
     301620 TGATGTTTGCATTCAAGTCACAGAGTTGAACATTCCCTTTC
        131 TGATGTTTGCATTCAAGTAACAGTGTTGAACATTCCCTTTC

                                                   *     *       **     *   *
     301661 ATAGAGCAGGTTTGAAACACTCTTTTTGTAGTATCTGGATGTGGACATTTGGAGCGCTTTCAGGC
          1 ATAGAGCAGGTTTGAAACACTCTTTTTGTAGTATCTGGAAGTGGATATTTGGATAGCTTTGAGGA

            * ** *      *             *** *   *                          *   
     301726 CTATGGT-GAAAAAGGAAATATCTTCCCCTGAAAACTAGACAGAAGCATTCTCAGAAACTTATTT
         66 TTTCGTTGGAAACAGG-AATATCTTCATATAAAAGCTAGACAGAAGCATTCTCAGAAACTTCTTT

                   **  **    *              *  * *   *
     301790 GTGATGTGCGCCCTCAACTAACAGTGTTGAACCTTTCTTTTG
        130 GTGATGTTTGCATTCAAGTAACAGTGTTGAACATTCCCTTTC

                     *                    *      *   *     *                 
     301832 ATAGAGCAGTTTTGAAACACTCTTTTTGTAATATCTGCAAGAGGATACTTGGATAGCTTTGAGGA
          1 ATAGAGCAGGTTTGAAACACTCTTTTTGTAGTATCTGGAAGTGGATATTTGGATAGCTTTGAGGA

                         *   * *            **     *                *    *   
     301897 TTTCGTTGGAAACGGGATTGTCTTCATATAAACTCTAGATAGAAGCATTCTCAGAAGCTTCATTG
         66 TTTCGTTGGAAACAGGAATATCTTCATATAAAAGCTAGACAGAAGCATTCTCAGAAACTTCTTTG

            *       **   *    *             *        
     301962 GGATGTTTCAATTGAAGTCACAGTGTTGAACAGTCCCTTTC
        131 TGATGTTTGCATTCAAGTAACAGTGTTGAACATTCCCTTTC

                                                         *       **   * *    
     302003 ATAGAGCAGGTTTGAAACACTCTTTTTGTAGTATCTGGAAGTGGACATTTGGAGCGCTCTCAGGA
          1 ATAGAGCAGGTTTGAAACACTCTTTTTGTAGTATCTGGAAGTGGATATTTGGATAGCTTTGAGGA

            * *  *    * *             **            *       * *          *  *
     302068 CTACGGT-GATAAAGGAAATATCTTCCAATAAAAGCTAGATAGAAGCAATGTCAGAAACTTTTTC
         66 TTTCGTTGGAAACAGG-AATATCTTCATATAAAAGCTAGACAGAAGCATTCTCAGAAACTTCTTT

            *      **** *   **      *       *  * *   *
     302132 ATGATGTACCTACTCAGCTAACAGAGTTGAACCTTTCTTTTG
        130 GTGATGTTTGCATTCAAGTAACAGTGTTGAACATTCCCTTTC

             *       *                   * *     *             **   *        
     302174 AGAGAGCAGTTTTGAAACACTCTTTTTGTGGAATCTGCAAGTGGATATTTGTCTAGTTTTGAGGA
          1 ATAGAGCAGGTTTGAAACACTCTTTTTGTAGTATCTGGAAGTGGATATTTGGATAGCTTTGAGGA

                         *   *    *                   *      * *             
     302239 TTTCGTTGGAAACGGGATTA-CAT-ATA-AAAAGC-AGACAGCAGCATTGTGAGAAACTTCTTTG
         66 TTTCGTTGGAAACAGGAATATCTTCATATAAAAGCTAGACAGAAGCATTCTCAGAAACTTCTTTG

                              *    *                 
     302300 TGATGTTTGCATTCAAGTCACAGAGTTGAACATTCCCTTTC
        131 TGATGTTTGCATTCAAGTAACAGTGTTGAACATTCCCTTTC

                                                         *       **   * *    
     302341 ATAGAGCAGGTTTGAAACACTCTTTTTGTAGTATCTGGAAGTGGACATTTGGAGCGCTCTCAGGA
          1 ATAGAGCAGGTTTGAAACACTCTTTTTGTAGTATCTGGAAGTGGATATTTGGATAGCTTTGAGGA

            * *  *      *             **        *   *       * *          *  *
     302406 CTGCGGT-GAAAAAGGAAATATCTTCCAATAAAAGCAAGATAGAAGCAATGTCAGAAACTTTTTC
         66 TTTCGTTGGAAACAGG-AATATCTTCATATAAAAGCTAGACAGAAGCATTCTCAGAAACTTCTTT

            *      * ** *   **      *       *        *
     302470 ATGATGTATCTACTCAGCTAACAGAGTTGAACCTT-CCTTTG
        130 GTGATGTTTGCATTCAAGTAACAGTGTTGAACATTCCCTTTC

             *       *                   * *     *             **            
     302511 AGAGAGCAGTTTTGAAACACTCTTTTTGTGGAATCTGCAAGTGGATATTTGTCTAGCTTTGAGGA
          1 ATAGAGCAGGTTTGAAACACTCTTTTTGTAGTATCTGGAAGTGGATATTTGGATAGCTTTGAGGA

                         *   *    *                   *       *              
     302576 TTTCGTTGGAAACGGGATTA-CAT-ATA-AAAAGC-AGACAGCAGCATTCCCAGAAACTTCTTTG
         66 TTTCGTTGGAAACAGGAATATCTTCATATAAAAGCTAGACAGAAGCATTCTCAGAAACTTCTTTG

                              *    *                 
     302637 TGATGTTTGCATTCAAGTCACAGAGTTGAACATTCCCTTTC
        131 TGATGTTTGCATTCAAGTAACAGTGTTGAACATTCCCTTTC

               *                                   *             ** *   *   *
     302678 ATATAGCAGGTTTGAAACACTCTTTTTGTAGTATCTGGATGTGGATATTTGGAGCGATTTCAGGC
          1 ATAGAGCAGGTTTGAAACACTCTTTTTGTAGTATCTGGAAGTGGATATTTGGATAGCTTTGAGGA

            * ** *      *             *** *   *                      *   *   
     302743 CTATGGT-GAAAAAGGAAATATCTTCCCCTGAAAACTAGACAGAAGCATTCTCAGAATCTTATTT
         66 TTTCGTTGGAAACAGG-AATATCTTCATATAAAAGCTAGACAGAAGCATTCTCAGAAACTTCTTT

                   **  **    *             **  * *   *
     302807 GTGATGTGCGCCCTCAACTAACAGTGTTGAAGCTTTCTTTTG
        130 GTGATGTTTGCATTCAAGTAACAGTGTTGAACATTCCCTTTC

                     *         *          **     *   *                       
     302849 ATAGAGCAGTTTTGAAACATTCTTTTTGTAAAATCTGCAAGAGGATATTTGGATAGCTTTGAGGA
          1 ATAGAGCAGGTTTGAAACACTCTTTTTGTAGTATCTGGAAGTGGATATTTGGATAGCTTTGAGGA

                         *   * *            **                      *    *   
     302914 TTTCGTTGGAAACGGGATTGTCTTCATATAAACTCTAGACAGAAGCATTCTCAGAAGCTTCATTG
         66 TTTCGTTGGAAACAGGAATATCTTCATATAAAAGCTAGACAGAAGCATTCTCAGAAACTTCTTTG

            *             *    *                      
     302979 GGATGTTT-CAGTTGAAGTCACAGTGTTGAACATTCCCTTTC
        131 TGATGTTTGCA-TTCAAGTAACAGTGTTGAACATTCCCTTTC

                                                         *       **   * *    
     303020 ATAGAGCAGGTTTGAAACACTCTTTTTGTAGTATCTGGAAGTGGACATTTGGAGCGCTCTCAGGA
          1 ATAGAGCAGGTTTGAAACACTCTTTTTGTAGTATCTGGAAGTGGATATTTGGATAGCTTTGAGGA

            * *  *      *             **            *       * *          *  *
     303085 CTGCGGT-GAAAAAGGAAATATCTTCCAATAAAAGCTAGATAGAAGCAATGTCAGAAACTTTTTC
         66 TTTCGTTGGAAACAGG-AATATCTTCATATAAAAGCTAGACAGAAGCATTCTCAGAAACTTCTTT

            *      * ** *   **      *       *        *
     303149 ATGATGTATCTACTCAGCTAACAGAGTTGAACCTT-CCTTTG
        130 GTGATGTTTGCATTCAAGTAACAGTGTTGAACATTCCCTTTC

             *       *                   * *     *             **            
     303190 AGAGAGCAGTTTTGAAACACTCTTTTTGTGGAATCTGCAAGTGGATATTTGTCTAGCTTTGAGGA
          1 ATAGAGCAGGTTTGAAACACTCTTTTTGTAGTATCTGGAAGTGGATATTTGGATAGCTTTGAGGA

                         *   *    *                   *       *     *   *    
     303255 TTTCGTTGGAAACGGGATTA-CAT-ATA-AAAAGC-AGACAGCAGCATTCCCAGAATCTTGTTTG
         66 TTTCGTTGGAAACAGGAATATCTTCATATAAAAGCTAGACAGAAGCATTCTCAGAAACTTCTTTG

                              *    *                 
     303316 TGATGTTTGCATTCAAGTCACAGAGTTGAACATTCCCTTTC
        131 TGATGTTTGCATTCAAGTAACAGTGTTGAACATTCCCTTTC

             *                         *           *     *       **     *   *
     303357 AGAGAGCAGGTTTGAAACACTCTTTTTATAGTATCTGGATGTGGACATTTGGAGCGCTTTCAGGC
          1 ATAGAGCAGGTTTGAAACACTCTTTTTGTAGTATCTGGAAGTGGATATTTGGATAGCTTTGAGGA

            * ** *      *             *** *   *                      *   *   
     303422 CTATGGT-GAAAAAGGAAATATCTTCTCCTGAAAACTAGACAGAAGCATTCTCAGAATCTTATTT
         66 TTTCGTTGGAAACAGG-AATATCTTCATATAAAAGCTAGACAGAAGCATTCTCAGAAACTTCTTT

                   **  **    *             **  * *   *
     303486 GTGATGTGCGCCCTCAACTAACAGTGTTGAAGCTTTCTTTTG
        130 GTGATGTTTGCATTCAAGTAACAGTGTTGAACATTCCCTTTC

                     *          *     *   **     *   *        *    *         
     303528 ATAGAGCAGTTTTGAAACACACTTTTCGTAAAATCTGCAAGAGGATATTTTGATAACTTTGAGGA
          1 ATAGAGCAGGTTTGAAACACTCTTTTTGTAGTATCTGGAAGTGGATATTTGGATAGCTTTGAGGA

                         *   * *      *     **                      *    *   
     303593 TTTCGTTGGAAACGGGATTGTCTTCACATAAACTCTAGACAGAAGCATTCTCAGAAGCTTCATTG
         66 TTTCGTTGGAAACAGGAATATCTTCATATAAAAGCTAGACAGAAGCATTCTCAGAAACTTCTTTG

            *       **   *    *             *        
     303658 GGATGTTTCAATTGAAGTCACAGTGTTGAACAGTCCCTTTC
        131 TGATGTTTGCATTCAAGTAACAGTGTTGAACATTCCCTTTC

                                                         *       *  * * *    
     303699 ATAGAGCAGGTTTGAAACACTCTTTTTGTAGTATCTGGAAGTGGACATTTGGAGAGATCTCAGGA
          1 ATAGAGCAGGTTTGAAACACTCTTTTTGTAGTATCTGGAAGTGGATATTTGGATAGCTTTGAGGA

            * *  *    * *             **            *       * *          *  *
     303764 ATACGGT-GATAAAGGAAATATCTTCCAATAAAAGCTAGATAGAAGCAATGTCAGAAACTTTTTC
         66 TTTCGTTGGAAACAGG-AATATCTTCATATAAAAGCTAGACAGAAGCATTCTCAGAAACTTCTTT

            *      * ** *   **      *       *  * *   *
     303828 ATGATGTATCTACTCAGCTAACAGAGTTGAACCTTTCTTTTG
        130 GTGATGTTTGCATTCAAGTAACAGTGTTGAACATTCCCTTTC

             *       *                   * *     *             **            
     303870 AGAGAGCAGTTTTGAAACACTCTTTTTGTGGAATCTGCAAGTGGATATTTGTCTAGCTTTGAGGA
          1 ATAGAGCAGGTTTGAAACACTCTTTTTGTAGTATCTGGAAGTGGATATTTGGATAGCTTTGAGGA

                         *   *    *                   *       *   *          
     303935 TTTCGTTGGAAACGGGATTA-CAT-ATA-AAAAGC-AGACAGCAGCATTCCCAGTAACTTCTTTG
         66 TTTCGTTGGAAACAGGAATATCTTCATATAAAAGCTAGACAGAAGCATTCTCAGAAACTTCTTTG

                              *    *                 
     303996 TGATGTTTGCATTCAAGTCACAGAGTTGAACATTCCCTTTC
        131 TGATGTTTGCATTCAAGTAACAGTGTTGAACATTCCCTTTC

                                                   *     *       **     *   *
     304037 ATAGAGCAGGTTTGAAACACTCTTTTTGTAGTATCTGGATGTGGACATTTGGAGCGCTTTCAGGC
          1 ATAGAGCAGGTTTGAAACACTCTTTTTGTAGTATCTGGAAGTGGATATTTGGATAGCTTTGAGGA

            * ** *      *             *** *   *           *          *   *   
     304102 CTATGGT-GAAAAAGGAAATATCTTCCCCTGAAAACTAG-CAGAAGAATTCTCAGAATCTTATTT
         66 TTTCGTTGGAAACAGG-AATATCTTCATATAAAAGCTAGACAGAAGCATTCTCAGAAACTTCTTT

                   **  **    *             **  * *   *
     304165 GTGATGTGCGCCCTCAACTAACAGTGTTGAAGCTTTCTTTTG
        130 GTGATGTTTGCATTCAAGTAACAGTGTTGAACATTCCCTTTC

                     *                    **     *   *                       
     304207 ATAGAGCAGTTTTGAAACACTCTTTTTGTAAAATCTGCAAGAGGATATTTGGATAGCTTTGAGGA
          1 ATAGAGCAGGTTTGAAACACTCTTTTTGTAGTATCTGGAAGTGGATATTTGGATAGCTTTGAGGA

                         *   * *            **                      *    *   
     304272 TTTCGTTGGAAACGGGATTGTCTTCATATAAACTCTAGACAGAAGCATTCTCAGAAGCTTCATTG
         66 TTTCGTTGGAAACAGGAATATCTTCATATAAAAGCTAGACAGAAGCATTCTCAGAAACTTCTTTG

            *       **   *    *             *        
     304337 GGATGTTTCAATTGAAGTCACAGTGTTGAACAGTCCCTTTC
        131 TGATGTTTGCATTCAAGTAACAGTGTTGAACATTCCCTTTC

                                                         *       *  * * *    
     304378 ATAGAGCAGGTTTGAAACACTCTTTTTGTAGTATCTGGAAGTGGACATTTGGAGAGATCTCAGGA
          1 ATAGAGCAGGTTTGAAACACTCTTTTTGTAGTATCTGGAAGTGGATATTTGGATAGCTTTGAGGA

            * *  *    * *             **            *       * *          *  *
     304443 ATACGGT-GATAAAGGAAATATCTTCCAATAAAAGCTAGATAGAAGCAATGTCAGAAACTTTTTC
         66 TTTCGTTGGAAACAGG-AATATCTTCATATAAAAGCTAGACAGAAGCATTCTCAGAAACTTCTTT

            *      * ** *   **     **       *  * *   *
     304507 ATGATGTATCTACTCAGCTAACATAGTTGAACCTTTCTTTTG
        130 GTGATGTTTGCATTCAAGTAACAGTGTTGAACATTCCCTTTC

             *       *                   * *     *             **            
     304549 AGAGAGCAGTTTTGAAACACTCTTTTTGTGGAATCTGCAAGTGGATATTTGTCTAGCTTTGAGGA
          1 ATAGAGCAGGTTTGAAACACTCTTTTTGTAGTATCTGGAAGTGGATATTTGGATAGCTTTGAGGA

                         *   *    *                   *       *   *          
     304614 TTTCGTTGGAAACGGGATTA-CAT-ATA-AAAAGC-AGACAGCAGCATTCCCAGTAACTTCTTTG
         66 TTTCGTTGGAAACAGGAATATCTTCATATAAAAGCTAGACAGAAGCATTCTCAGAAACTTCTTTG

                              *    *                 
     304675 TGATGTTTGCATTCAAGTCACAGAGTTGAACATTCCCTTTC
        131 TGATGTTTGCATTCAAGTAACAGTGTTGAACATTCCCTTTC

                                                   *     *       **     *   *
     304716 ATAGAGCAGGTTTGAAACACTCTTTTTGTAGTATCTGGATGTGGACATTTGGAGCGCTTTCAGGC
          1 ATAGAGCAGGTTTGAAACACTCTTTTTGTAGTATCTGGAAGTGGATATTTGGATAGCTTTGAGGA

            * ** *      *             *** *   *                      *   *   
     304781 CTATGGT-GAAAAAGGAAATATCTTCCCCTGAAAACTAGACAGAAGCATTCTCAGAATCTTATTT
         66 TTTCGTTGGAAACAGG-AATATCTTCATATAAAAGCTAGACAGAAGCATTCTCAGAAACTTCTTT

                   **  **    *             **  * *   *
     304845 GTGATGTGCGCCCTCAACTAACAGTGTTGAAGCTTTCTTTTG
        130 GTGATGTTTGCATTCAAGTAACAGTGTTGAACATTCCCTTTC

                     *                    **     *   *                       
     304887 ATAGAGCAGTTTTGAAACACTCTTTTTGTAAAATCTGCAAGAGGATATTTGGATAGCTTTGAGGA
          1 ATAGAGCAGGTTTGAAACACTCTTTTTGTAGTATCTGGAAGTGGATATTTGGATAGCTTTGAGGA

                         *   * *            **           *          * *  *   
     304952 TTTCGTTGGAAACGGGATTGTCTTCATATAAACTCTAGACAGAAGAATTCTCAGAAGCGTCATTG
         66 TTTCGTTGGAAACAGGAATATCTTCATATAAAAGCTAGACAGAAGCATTCTCAGAAACTTCTTTG

            *       **   *    *             *        
     305017 GGATGTTTCAATTGAAGTCACAGTGTTGAACAGTCCCTTTC
        131 TGATGTTTGCATTCAAGTAACAGTGTTGAACATTCCCTTTC

                                                   *     *       **     *    
     305058 ATAGAGCAGGTTTGAAACACTCTTTTTGTAGTATCTGGATGTGGACATTTGGAGCGCTTTCAGGC
          1 ATAGAGCAGGTTTGAAACACTCTTTTTGTAGTATCTGGAAGTGGATATTTGGATAGCTTTGAGG-

            * * *    *                 *** *   *                          *  
     305123 CTATGGTT-TAAA-AGGAAATATCTTCCCCTGAAAACTAGACAGAAGCATTCTCAGAAACTTATT
         65 ATTTCGTTGGAAACAGG-AATATCTTCATATAAAAGCTAGACAGAAGCATTCTCAGAAACTTCTT

                    **  **    *                    *   *
     305186 TGTGATGTGCGCCCTCAACTAACAGTGTTGAAGCATT-CTTTTG
        129 TGTGATGTTTGCATTCAAGTAACAGTGTTGAA-CATTCCCTTTC

                     *                   * *     *             **            
     305229 ATAGAGCAGTTTTGAAACACTCTTTTTGTGGAATCTGCAAGTGGATATTTGTCTAGCTTTGAGGA
          1 ATAGAGCAGGTTTGAAACACTCTTTTTGTAGTATCTGGAAGTGGATATTTGGATAGCTTTGAGGA

                         *   *    *                   *       *              
     305294 TTTCGTTGGAAACGGGATTA-CAT-ATA-AAAAGC-AGACAGCAGCATTCCCAGAAACTTCTTTG
         66 TTTCGTTGGAAACAGGAATATCTTCATATAAAAGCTAGACAGAAGCATTCTCAGAAACTTCTTTG

                              *    *                 
     305355 TGATGTTTGCATTCAAGTCACAGAGTTGAACATTCCCTTTC
        131 TGATGTTTGCATTCAAGTAACAGTGTTGAACATTCCCTTTC

                                                   *     **      **     *   *
     305396 ATAGAGCAGGTTTGAAACACTCTTTTTGTAGTATCTGGATGTGGACCTTTGGAGCGCTTTCAGGC
          1 ATAGAGCAGGTTTGAAACACTCTTTTTGTAGTATCTGGAAGTGGATATTTGGATAGCTTTGAGGA

            * ** *      *             *** *   *                     **   *   
     305461 CTATGGT-GAAAAAGGAAATATCTTCCCCTGAAAACTAGACAGAAGCATTCTCAGAGTCTTATTT
         66 TTTCGTTGGAAACAGG-AATATCTTCATATAAAAGCTAGACAGAAGCATTCTCAGAAACTTCTTT

                   **  **    **            **  * *   *
     305525 GTGATGTGCGCCCTCAACGAACAGTGTTGAAGCTTTCTTTTG
        130 GTGATGTTTGCATTCAAGTAACAGTGTTGAACATTCCCTTTC

                     *                    *      *   *                       
     305567 ATAGAGCAGTTTTGAAACACTCTTTTTGTAATATCTGCAAGAGGATATTTGGATAGCTTTGAGGA
          1 ATAGAGCAGGTTTGAAACACTCTTTTTGTAGTATCTGGAAGTGGATATTTGGATAGCTTTGAGGA

                **       *   * *            **                      *    *   
     305632 TTTCTCTGGAAACGGGATTGTCTTCATATAAACTCTAGACAGAAGCATTCTCAGAAGCTTCATTG
         66 TTTCGTTGGAAACAGGAATATCTTCATATAAAAGCTAGACAGAAGCATTCTCAGAAACTTCTTTG

            *       **   *    *             *        
     305697 GGATGTTTCAATTGAAGTCACAGTGTTGAACAGTCCCTTTC
        131 TGATGTTTGCATTCAAGTAACAGTGTTGAACATTCCCTTTC

                                                         *       **   * *    
     305738 ATAGAGCAGGTTTGAAACACTCTTTTTGTAGTATCTGGAAGTGGACATTTGGAGCGCTCTCAGGA
          1 ATAGAGCAGGTTTGAAACACTCTTTTTGTAGTATCTGGAAGTGGATATTTGGATAGCTTTGAGGA

            * *  *      *             **            *       * *          *  *
     305803 CTGCGGT-GAAAAAGGAAATATCTTCCAATAAAAGCTAGATAGAAGCAATGTCAGAAACTTTTTC
         66 TTTCGTTGGAAACAGG-AATATCTTCATATAAAAGCTAGACAGAAGCATTCTCAGAAACTTCTTT

            *      * ** *   **      *       *        *
     305867 ATGATGTATCTACTCAGCTAACAGAGTTGAACCTT-CCTTTG
        130 GTGATGTTTGCATTCAAGTAACAGTGTTGAACATTCCCTTTC

             *       *                   * *     *             **            
     305908 AGAGAGCAGTTTTGAAACACTCTTTTTGTGGAATCTGCAAGTGGATATTTGTCTAGCTTTGAGGA
          1 ATAGAGCAGGTTTGAAACACTCTTTTTGTAGTATCTGGAAGTGGATATTTGGATAGCTTTGAGGA

                         *   *    *                   *       *              
     305973 TTTCGTTGGAAACGGGATTA-CAT-ATA-AAAAGC-AGACAGCAGCATTCCCAGAAACTTCTTTG
         66 TTTCGTTGGAAACAGGAATATCTTCATATAAAAGCTAGACAGAAGCATTCTCAGAAACTTCTTTG

                              *    *                 
     306034 TGATGTTTGCATTCAAGTCACAGAGTTGAACATTCCCTTTC
        131 TGATGTTTGCATTCAAGTAACAGTGTTGAACATTCCCTTTC

                                                   *     *     * **     *   *
     306075 ATAGAGCAGGTTTGAAACACTCTTTTTGTAGTATCTGGATGTGGACATTTGCAGCGCTTTCAGGC
          1 ATAGAGCAGGTTTGAAACACTCTTTTTGTAGTATCTGGAAGTGGATATTTGGATAGCTTTGAGGA

            * ** *      *             *** *   **                         *   
     306140 CTAAGGT-GAAAAAGGAAATATCTTCCCCTGAAAATTAGACAGAAGCATTCTCAGAAACTTATTT
         66 TTTCGTTGGAAACAGG-AATATCTTCATATAAAAGCTAGACAGAAGCATTCTCAGAAACTTCTTT

                   **  **    *             **  * *   *
     306204 GTGATGTGCGCCCTCAACTAACAGTGTTGAAGCTTTCTTTTG
        130 GTGATGTTTGCATTCAAGTAACAGTGTTGAACATTCCCTTTC

                     *   *                *      *   *                  *    
     306246 ATAGAGCAGTTTTCAAACACTCTTTTTGTAATATCTGCAAGAGGATATTTGGATAGCTTTCAGGA
          1 ATAGAGCAGGTTTGAAACACTCTTTTTGTAGTATCTGGAAGTGGATATTTGGATAGCTTTGAGGA

                         *   * *            **                      *    *   
     306311 TTTCGTTGGAAACGGGATTGTCTTCATATAAACTCTAGACAGAAGCATTCTCAGAAGCTTCATTG
         66 TTTCGTTGGAAACAGGAATATCTTCATATAAAAGCTAGACAGAAGCATTCTCAGAAACTTCTTTG

            *       **   *    *             * *      
     306376 GGATGTTTCAATTGAAGTCACAGTGTTGAACAGTTCCTTTC
        131 TGATGTTTGCATTCAAGTAACAGTGTTGAACATTCCCTTTC

                 *                                       *       **   * *    
     306417 ATAGAACAGGTTTGAAACACTCTTTTTGTAGTATCTGGAAGTGGACATTTGGAGCGCTCTCAGGA
          1 ATAGAGCAGGTTTGAAACACTCTTTTTGTAGTATCTGGAAGTGGATATTTGGATAGCTTTGAGGA

            * ** *      *             **          * *      ** *          *  *
     306482 CTATGGT-GAAAAAGGAAATATCTTCCAATAAAAGCTACATAGAAGCTATGTCAGAAACTTTTTC
         66 TTTCGTTGGAAACAGG-AATATCTTCATATAAAAGCTAGACAGAAGCATTCTCAGAAACTTCTTT

            *      * ** *   **      *       *  *     *
     306546 ATGATGTATCTACTCAGCTAACAGAGTTGAACCTTTCCTTTG
        130 GTGATGTTTGCATTCAAGTAACAGTGTTGAACATTCCCTTTC

             *       *                   * *     *             **            
     306588 AGAGAGCAGTTTTGAAACACTCTTTTTGTGGAATCTGCAAGTGGATATTTGTCTAGCTTTGAGGA
          1 ATAGAGCAGGTTTGAAACACTCTTTTTGTAGTATCTGGAAGTGGATATTTGGATAGCTTTGAGGA

                         *   *    *                   *       *   *          
     306653 TTTCGTTGGAAACGGGATTA-CAT-ATA-AAAAGC-AGACAGCAGCATTCCCAGTAACTTCTTTG
         66 TTTCGTTGGAAACAGGAATATCTTCATATAAAAGCTAGACAGAAGCATTCTCAGAAACTTCTTTG

                              *    *                 
     306714 TGATGTTTGCATTCAAGTCACAGAGTTGAACATTCCCTTTC
        131 TGATGTTTGCATTCAAGTAACAGTGTTGAACATTCCCTTTC

                                                   *     *       **     *   *
     306755 ATAGAGCAGGTTTGAAACACTCTTTTTGTAGTATCTGGATGTGGACATTTGGAGCGCTTTCAGGC
          1 ATAGAGCAGGTTTGAAACACTCTTTTTGTAGTATCTGGAAGTGGATATTTGGATAGCTTTGAGGA

            * ** *      *             *** *   *                      *   *   
     306820 CTATGGT-GAAAAAGGAAATATCTTCCCCTGAAAACTAGACAGAAGCATTCTCAGAATCTTATTT
         66 TTTCGTTGGAAACAGG-AATATCTTCATATAAAAGCTAGACAGAAGCATTCTCAGAAACTTCTTT

                   **  **    *             **  * *   *
     306884 GTGATGTGCGCCCTCAACTAACAGTGTTGAAGCTTTCTTTTG
        130 GTGATGTTTGCATTCAAGTAACAGTGTTGAACATTCCCTTTC

                     *                    **     *   *                       
     306926 ATAGAGCAGTTTTGAAACACTCTTTTTGTAAAATCTGCAAGAGGATATTTGGATAGCTTTGAGGA
          1 ATAGAGCAGGTTTGAAACACTCTTTTTGTAGTATCTGGAAGTGGATATTTGGATAGCTTTGAGGA

                         *   * *            **           *          *    *   
     306991 TTTCGTTGGAAACGGGATTGTCTTCATATAAACTCTAGACAGAAGAATTCTCAGAAGCTTCATTG
         66 TTTCGTTGGAAACAGGAATATCTTCATATAAAAGCTAGACAGAAGCATTCTCAGAAACTTCTTTG

            *       **   *    *             *        
     307056 GGATGTTTCAATTGAAGTCACAGTGTTGAACAGTCCCTTTC
        131 TGATGTTTGCATTCAAGTAACAGTGTTGAACATTCCCTTTC

                                                   *     *       **     *    
     307097 ATAGAGCAGGTTTGAAACACTCTTTTTGTAGTATCTGGATGTGGACATTTGGAGCGCTTTCAGGC
          1 ATAGAGCAGGTTTGAAACACTCTTTTTGTAGTATCTGGAAGTGGATATTTGGATAGCTTTGAGG-

            * * *    *                 *** *   *                          *  
     307162 CTATGGTT-TAAA-AGGAAATATCTTCCCCTGAAAACTAGACAGAAGCATTCTCAGAAACTTATT
         65 ATTTCGTTGGAAACAGG-AATATCTTCATATAAAAGCTAGACAGAAGCATTCTCAGAAACTTCTT

                    **  **    *                        *
     307225 TGTGATGTGCGCCCTCAACTAACAGTGTTGAAGCATT-CCTTTG
        129 TGTGATGTTTGCATTCAAGTAACAGTGTTGAA-CATTCCCTTTC

                     *                   * *     *             **            
     307268 ATAGAGCAGTTTTGAAACACTCTTTTTGTGGAATCTGCAAGTGGATATTTGTCTAGCTTTGAGGA
          1 ATAGAGCAGGTTTGAAACACTCTTTTTGTAGTATCTGGAAGTGGATATTTGGATAGCTTTGAGGA

                      *  *   *    *                   *       *              
     307333 TTTCGTTGGAGACGGGATTA-CAT-ATA-AAAAGC-AGACAGCAGCATTCCCAGAAACTTCTTTG
         66 TTTCGTTGGAAACAGGAATATCTTCATATAAAAGCTAGACAGAAGCATTCTCAGAAACTTCTTTG

                              *    *                 
     307394 TGATGTTTGCATTCAAGTCACAGAGTTGAACATTCCCTTTC
        131 TGATGTTTGCATTCAAGTAACAGTGTTGAACATTCCCTTTC

                                                   *     *     * **     *   *
     307435 ATAGAGCAGGTTTGAAACACTCTTTTTGTAGTATCTGGATGTGGACATTTGCAGCGCTTTCAGGC
          1 ATAGAGCAGGTTTGAAACACTCTTTTTGTAGTATCTGGAAGTGGATATTTGGATAGCTTTGAGGA

            * ** *      *             *** *   *                          *   
     307500 CTAAGGT-GAAAAAGGAAATATCTTCCCCTGAAAACTAGACAGAAGCATTCTCAGAAACTTATTT
         66 TTTCGTTGGAAACAGG-AATATCTTCATATAAAAGCTAGACAGAAGCATTCTCAGAAACTTCTTT

                   **  **    *             **  * *   *
     307564 GTGATGTGCGCCCTCAACTAACAGTGTTGAAGCTTTCTTTTG
        130 GTGATGTTTGCATTCAAGTAACAGTGTTGAACATTCCCTTTC

                     *                    *      *   *                  *    
     307606 ATAGAGCAGTTTTGAAACACTCTTTTTGTAATATCTGCAAGAGGATATTTGGATAGCTTTCAGGA
          1 ATAGAGCAGGTTTGAAACACTCTTTTTGTAGTATCTGGAAGTGGATATTTGGATAGCTTTGAGGA

                         *   * *            **                      *    *   
     307671 TTTCGTTGGAAACGGGATTGTCTTCATATAAACTCTAGACAGAAGCATTCTCAGAAGCTTCATTG
         66 TTTCGTTGGAAACAGGAATATCTTCATATAAAAGCTAGACAGAAGCATTCTCAGAAACTTCTTTG

            *       **   *    *               *      
     307736 GGATGTTTCAATTGAAGTCACAGTGTTGAACATTTCCTTTC
        131 TGATGTTTGCATTCAAGTAACAGTGTTGAACATTCCCTTTC

                 *                                       *       **   * *    
     307777 ATAGAACAGGTTTGAAACACTCTTTTTGTAGTATCTGGAAGTGGACATTTGGAGCGCTCTCAGGA
          1 ATAGAGCAGGTTTGAAACACTCTTTTTGTAGTATCTGGAAGTGGATATTTGGATAGCTTTGAGGA

            * ** *      *             **          * *       * *          *  *
     307842 CTATGGT-GAAAAAGGAAATATCTTCCAATAAAAGCTACATAGAAGCAATGTCAGAAACTTTTTC
         66 TTTCGTTGGAAACAGG-AATATCTTCATATAAAAGCTAGACAGAAGCATTCTCAGAAACTTCTTT

            *      * ** *   **     **       *  *     *
     307906 ATGATGTATCTACTCAGCTAACACAGTTGAACCTTTCCTTTG
        130 GTGATGTTTGCATTCAAGTAACAGTGTTGAACATTCCCTTTC

             *       *                   * *     *             **            
     307948 AGAGAGCAGTTTTGAAACACTCTTTTTGTGGAATCTGCAAGTGGATATTTGTCTAGCTTTGAGGA
          1 ATAGAGCAGGTTTGAAACACTCTTTTTGTAGTATCTGGAAGTGGATATTTGGATAGCTTTGAGGA

                         *   *    *                   *       *   *          
     308013 TTTCGTTGGAAACGGGATTA-CAT-ATA-AAAAGC-AGACAGCAGCATTCCCAGTAACTTCTTTG
         66 TTTCGTTGGAAACAGGAATATCTTCATATAAAAGCTAGACAGAAGCATTCTCAGAAACTTCTTTG

                              *    *                 
     308074 TGATGTTTGCATTCAAGTCACAGAGTTGAACATTCCCTTTC
        131 TGATGTTTGCATTCAAGTAACAGTGTTGAACATTCCCTTTC

                                                   *     *       **     *   *
     308115 ATAGAGCAGGTTTGAAACACTCTTTTTGTAGTATCTGGATGTGGACATTTGGAGCGCTTTCAGGC
          1 ATAGAGCAGGTTTGAAACACTCTTTTTGTAGTATCTGGAAGTGGATATTTGGATAGCTTTGAGGA

            * ** *      *             *** *   *                      *   *   
     308180 CTATGGT-GAAAAAGGAAATATCTTCCCCTGAAAACTAGACAGAAGCATTCTCAGAATCTTATTT
         66 TTTCGTTGGAAACAGG-AATATCTTCATATAAAAGCTAGACAGAAGCATTCTCAGAAACTTCTTT

                   **  **    *             **  * *   *
     308244 GTGATGTGCGCCCTCAACTAACAGTGTTGAAGCTTTCTTTTG
        130 GTGATGTTTGCATTCAAGTAACAGTGTTGAACATTCCCTTTC

                     *          *         **     *   *                       
     308286 ATAGAGCAGTTTTGAAACACACTTTTTGTAAAATCTGCAAGAGGATATTTGGATAGCTTTGAGGA
          1 ATAGAGCAGGTTTGAAACACTCTTTTTGTAGTATCTGGAAGTGGATATTTGGATAGCTTTGAGGA

                         *   * *            **                      *    *   
     308351 TTTCGTTGGAAACGGGATTGTCTTCATATAAACTCTAGACAGAAGCATTCTCAGAAGCTTCATTG
         66 TTTCGTTGGAAACAGGAATATCTTCATATAAAAGCTAGACAGAAGCATTCTCAGAAACTTCTTTG

            *       **   *    *             *        
     308416 GGATGTTTCAATTGAAGTCACAGTGTTGAACAGTCCCTTTC
        131 TGATGTTTGCATTCAAGTAACAGTGTTGAACATTCCCTTTC

                                                   *     *       **     *    
     308457 ATAGAGCAGGTTTGAAACACTCTTTTTGTAGTATCTGGATGTGGACATTTGGAGCGCTTTCAGGC
          1 ATAGAGCAGGTTTGAAACACTCTTTTTGTAGTATCTGGAAGTGGATATTTGGATAGCTTTGAGG-

            * * *    *                 *** *   *                          *  
     308522 CTATGGTT-TAAA-AGGAAATATCTTCCCCTGAAAACTAGACAGAAGCATTCTCAGAAACTTATT
         65 ATTTCGTTGGAAACAGG-AATATCTTCATATAAAAGCTAGACAGAAGCATTCTCAGAAACTTCTT

                *   **  **    *                    *   *
     308585 TGTGCTGTGCGCCCTCAACTAACAGTGTTGAAGCATT-CTTTTG
        129 TGTGATGTTTGCATTCAAGTAACAGTGTTGAA-CATTCCCTTTC

                     *        *          * *     *  *          **            
     308628 ATAGAGCAGTTTTGAAACCCTCTTTTTGTGGAATCTGCAATTGGATATTTGTCTAGCTTTGAGGA
          1 ATAGAGCAGGTTTGAAACACTCTTTTTGTAGTATCTGGAAGTGGATATTTGGATAGCTTTGAGGA

                         *   *    *                   *       *              
     308693 TTTCGTTGGAAACGGGATTA-CAT-ATA-AAAAGC-AGACAGCAGCATTCCCAGAAACTTCTTTG
         66 TTTCGTTGGAAACAGGAATATCTTCATATAAAAGCTAGACAGAAGCATTCTCAGAAACTTCTTTG

                              *    *                 
     308754 TGATGTTTGCATTCAAGTCACAGAGTTGAACATTCCCTTTC
        131 TGATGTTTGCATTCAAGTAACAGTGTTGAACATTCCCTTTC

                                                   *     *       **     *   *
     308795 ATAGAGCAGGTTTGAAACACTCTTTTTGTAGTATCTGGATGTGGACATTTGGAGCGCTTTCAGGC
          1 ATAGAGCAGGTTTGAAACACTCTTTTTGTAGTATCTGGAAGTGGATATTTGGATAGCTTTGAGGA

            * ** *      *             *** *   *                          *   
     308860 CTATGGT-GAAAAAGGAAATATCTTCCCCTGAAAACTAGACAGAAGCATTCTCAGAAACTTATTT
         66 TTTCGTTGGAAACAGG-AATATCTTCATATAAAAGCTAGACAGAAGCATTCTCAGAAACTTCTTT

                   **  **    *             **  * *   *
     308924 GTGATGTGCGCCCTCAACTAACAGTGTTGAAGCTTTCTTTTG
        130 GTGATGTTTGCATTCAAGTAACAGTGTTGAACATTCCCTTTC

                     *                    *      *   *                       
     308966 ATAGAGCAGTTTTGAAACACTCTTTTTGTAATATCTGCAAGAGGATATTTGGATAGCTTTGAGGA
          1 ATAGAGCAGGTTTGAAACACTCTTTTTGTAGTATCTGGAAGTGGATATTTGGATAGCTTTGAGGA

                         *   * *            **                      *    *   
     309031 TTTCGTTGGAAACGGGATTGTCTTCATATAAACTCTAGACAGAAGCATTCTCAGAAGCTTCATTG
         66 TTTCGTTGGAAACAGGAATATCTTCATATAAAAGCTAGACAGAAGCATTCTCAGAAACTTCTTTG

            *       **   *    *             *        
     309096 GGATGTTTCAATTGAAGTCACAGTGTTGAACAGTCCCTTTC
        131 TGATGTTTGCATTCAAGTAACAGTGTTGAACATTCCCTTTC

                                                         *       *  * * *    
     309137 ATAGAGCAGGTTTGAAACACTCTTTTTGTAGTATCTGGAAGTGGACATTTGGAGAGATCTCAGGA
          1 ATAGAGCAGGTTTGAAACACTCTTTTTGTAGTATCTGGAAGTGGATATTTGGATAGCTTTGAGGA

            * *  *    * *             **            *       * *          *  *
     309202 ATACGGT-GATAAAGGAAATATCTTCCAATAAAAGCTAGATAGAAGCAATGTCAGAAACTTTTTC
         66 TTTCGTTGGAAACAGG-AATATCTTCATATAAAAGCTAGACAGAAGCATTCTCAGAAACTTCTTT

            *      * ** *   **      *       *  * *   *
     309266 ATGATGTATCTACTCAGCTAACAGAGTTGAACCTTTCTTTTG
        130 GTGATGTTTGCATTCAAGTAACAGTGTTGAACATTCCCTTTC

             *       *                   * *     *             **            
     309308 AGAGAGCAGTTTTGAAACACTCTTTTTGTGGAATCTGCAAGTGGATATTTGTCTAGCTTTGAGGA
          1 ATAGAGCAGGTTTGAAACACTCTTTTTGTAGTATCTGGAAGTGGATATTTGGATAGCTTTGAGGA

                         *   *    *                   *       *              
     309373 TTTCGTTGGAAACGGGATTA-CAT-ATA-AAAAGC-AGACAGCAGCATTCCCAGAAACTTCTTTG
         66 TTTCGTTGGAAACAGGAATATCTTCATATAAAAGCTAGACAGAAGCATTCTCAGAAACTTCTTTG

                              *    *                 
     309434 TGATGTTTGCATTCAAGTCACAGAGTTGAACATTCCCTTTC
        131 TGATGTTTGCATTCAAGTAACAGTGTTGAACATTCCCTTTC

               *                                   *     *       **     *   *
     309475 ATATAGCAGGTTTGAAACACTCTTTTTGTAGTATCTGGATGTGGACATTTGGAGCGCTTTCAGGC
          1 ATAGAGCAGGTTTGAAACACTCTTTTTGTAGTATCTGGAAGTGGATATTTGGATAGCTTTGAGGA

            * ** *      *             *** *   *                      *   *   
     309540 CTATGGT-GAAAAAGGAAATATCTTCCCCTGAAAACTAGACAGAAGCATTCTCAGAATCTTATTT
         66 TTTCGTTGGAAACAGG-AATATCTTCATATAAAAGCTAGACAGAAGCATTCTCAGAAACTTCTTT

                   **  **    *             **  * *   *
     309604 GTGATGTGCGCCCTCAACTAACAGTGTTGAAGCTTTCTTTTG
        130 GTGATGTTTGCATTCAAGTAACAGTGTTGAACATTCCCTTTC

                     *                    **     *   *                       
     309646 ATAGAGCAGTTTTGAAACACTCTTTTTGTAAAATCTGCAAGAGGATATTTGGATAGCTTTGAGGA
          1 ATAGAGCAGGTTTGAAACACTCTTTTTGTAGTATCTGGAAGTGGATATTTGGATAGCTTTGAGGA

                         *   * *            **                      *    *   
     309711 TTTCGTTGGAAACGGGATTGTCTTCATATAAACTCTAGACAGAAGCATTCTCAGAAGCTTCATTG
         66 TTTCGTTGGAAACAGGAATATCTTCATATAAAAGCTAGACAGAAGCATTCTCAGAAACTTCTTTG

            *             *    *                      
     309776 GGATGTTT-CAGTTGAAGTCACAGTGTTGAACATTCCCTTTC
        131 TGATGTTTGCA-TTCAAGTAACAGTGTTGAACATTCCCTTTC

                                                         *       **   * *    
     309817 ATAGAGCAGGTTTGAAACACTCTTTTTGTAGTATCTGGAAGTGGACATTTGGAGCGCTCTCAGGA
          1 ATAGAGCAGGTTTGAAACACTCTTTTTGTAGTATCTGGAAGTGGATATTTGGATAGCTTTGAGGA

            * *  *      *             **            *       * *          *  *
     309882 CTGCGGT-GAAAAAGGAAATATCTTCCAATAAAAGCTAGATAGAAGCAATGTCAGAAACTTTTTC
         66 TTTCGTTGGAAACAGG-AATATCTTCATATAAAAGCTAGACAGAAGCATTCTCAGAAACTTCTTT

            *      * ** *   **      *       *        *
     309946 ATGATGTATCTACTCAGCTAACAGAGTTGAACCTT-CCTTTG
        130 GTGATGTTTGCATTCAAGTAACAGTGTTGAACATTCCCTTTC

             *       *                   * *     *             **            
     309987 AGAGAGCAGTTTTGAAACACTCTTTTTGTGGAATCTGCAAGTGGATATTTGTCTAGCTTTGAGGA
          1 ATAGAGCAGGTTTGAAACACTCTTTTTGTAGTATCTGGAAGTGGATATTTGGATAGCTTTGAGGA

                         *   *    *                   *       *     *   *    
     310052 TTTCGTTGGAAACGGGATTA-CAT-ATA-AAAAGC-AGACAGCAGCATTCCCAGAATCTTGTTTG
         66 TTTCGTTGGAAACAGGAATATCTTCATATAAAAGCTAGACAGAAGCATTCTCAGAAACTTCTTTG

                              *    *                 
     310113 TGATGTTTGCATTCAAGTCACAGAGTTGAACATTCCCTTTC
        131 TGATGTTTGCATTCAAGTAACAGTGTTGAACATTCCCTTTC

             *                         *           *     *       **     *   *
     310154 AGAGAGCAGGTTTGAAACACTCTTTTTATAGTATCTGGATGTGGACATTTGGAGCGCTTTCAGGC
          1 ATAGAGCAGGTTTGAAACACTCTTTTTGTAGTATCTGGAAGTGGATATTTGGATAGCTTTGAGGA

            * ** *      *             *** *   *                      *   *   
     310219 CTATGGT-GAAAAAGGAAATATCTTCTCCTGAAAACTAGACAGAAGCATTCTCAGAATCTTATTT
         66 TTTCGTTGGAAACAGG-AATATCTTCATATAAAAGCTAGACAGAAGCATTCTCAGAAACTTCTTT

                   **  **    *             **  * *   *
     310283 GTGATGTGCGCCCTCAACTAACAGTGTTGAAGCTTTCTTTTG
        130 GTGATGTTTGCATTCAAGTAACAGTGTTGAACATTCCCTTTC

                     *          *     *   **     *   *        *              
     310325 ATAGAGCAGTTTTGAAACACACTTTTCGTAAAATCTGCAAGAGGATATTTTGATAGCTTTGAGGA
          1 ATAGAGCAGGTTTGAAACACTCTTTTTGTAGTATCTGGAAGTGGATATTTGGATAGCTTTGAGGA

                         *   * *            **                      *    *   
     310390 TTTCGTTGGAAACGGGATTGTCTTCATATAAACTCTAGACAGAAGCATTCTCAGAAGCTTCATTG
         66 TTTCGTTGGAAACAGGAATATCTTCATATAAAAGCTAGACAGAAGCATTCTCAGAAACTTCTTTG

            *       **   *    *             *        
     310455 GGATGTTTCAATTGAAGTCACAGTGTTGAACAGTCCCTTTC
        131 TGATGTTTGCATTCAAGTAACAGTGTTGAACATTCCCTTTC

                                                         *       *  * * *    
     310496 ATAGAGCAGGTTTGAAACACTCTTTTTGTAGTATCTGGAAGTGGACATTTGGAGAGATCTCAGGA
          1 ATAGAGCAGGTTTGAAACACTCTTTTTGTAGTATCTGGAAGTGGATATTTGGATAGCTTTGAGGA

            * *  *    * *             **            *       * *          *  *
     310561 ATACGGT-GATAAAGGAAATATCTTCCAATAAAAGCTAGATAGAAGCAATGTCAGAAACTTTTTC
         66 TTTCGTTGGAAACAGG-AATATCTTCATATAAAAGCTAGACAGAAGCATTCTCAGAAACTTCTTT

            *      * ** *   **      *       *  * *   *
     310625 ATGATGTATCTACTCAGCTAACAGAGTTGAACCTTTCTTTTG
        130 GTGATGTTTGCATTCAAGTAACAGTGTTGAACATTCCCTTTC

             *       *                   * *     *             **            
     310667 AGAGAGCAGTTTTGAAACACTCTTTTTGTGGAATCTGCAAGTGGATATTTGTCTAGCTTTGAGGA
          1 ATAGAGCAGGTTTGAAACACTCTTTTTGTAGTATCTGGAAGTGGATATTTGGATAGCTTTGAGGA

                         *   *    *                   *       *   *          
     310732 TTTCGTTGGAAACGGGATTA-CAT-ATA-AAAAGC-AGACAGCAGCATTCCCAGTAACTTCTTTG
         66 TTTCGTTGGAAACAGGAATATCTTCATATAAAAGCTAGACAGAAGCATTCTCAGAAACTTCTTTG

                              *    *                 
     310793 TGATGTTTGCATTCAAGTCACAGAGTTGAACATTCCCTTTC
        131 TGATGTTTGCATTCAAGTAACAGTGTTGAACATTCCCTTTC

               *                                   *     *       **     *   *
     310834 ATACAGCAGGTTTGAAACACTCTTTTTGTAGTATCTGGATGTGGACATTTGGAGCGCTTTCAGGC
          1 ATAGAGCAGGTTTGAAACACTCTTTTTGTAGTATCTGGAAGTGGATATTTGGATAGCTTTGAGGA

            * ** *      *             *** *   *                      *   *   
     310899 CTATGGT-GAAAAAGGAAATATCTTCTCCTGAAAACTAGACAGAAGCATTCTCAGAATCTTATTT
         66 TTTCGTTGGAAACAGG-AATATCTTCATATAAAAGCTAGACAGAAGCATTCTCAGAAACTTCTTT

                   **  **    *             **  * *   *
     310963 GTGATGTGCGCCCTCAACTAACAGTGTTGAAGCTTTCTTTTG
        130 GTGATGTTTGCATTCAAGTAACAGTGTTGAACATTCCCTTTC

                     *                    *      *   *                       
     311005 ATAGAGCAGTTTTGAAACACTCTTTTTGTAATATCTGCAAGAGGATATTTGGATAGCTTTGAGGA
          1 ATAGAGCAGGTTTGAAACACTCTTTTTGTAGTATCTGGAAGTGGATATTTGGATAGCTTTGAGGA

                         *   * *            **                      *    *   
     311070 TTTCGTTGGAAACGGGATTGTCTTCATATAAACTCTAGACAGAAGCATTCTCAGAAGCTTCATTG
         66 TTTCGTTGGAAACAGGAATATCTTCATATAAAAGCTAGACAGAAGCATTCTCAGAAACTTCTTTG

            *       **   *    *             *        
     311135 GGATGTTTCAATTGAAGTCACAGTGTTGAACAGTCCCTTTC
        131 TGATGTTTGCATTCAAGTAACAGTGTTGAACATTCCCTTTC

                                                         *       *  * * *    
     311176 ATAGAGCAGGTTTGAAACACTCTTTTTGTAGTATCTGGAAGTGGACATTTGGAGAGATCTCAGGA
          1 ATAGAGCAGGTTTGAAACACTCTTTTTGTAGTATCTGGAAGTGGATATTTGGATAGCTTTGAGGA

            * *  *    * *             **            *       * *          *  *
     311241 ATACGGT-GATAAAGGAAATATCTTCCAATAAAAGCTAGATAGAAGCAATGTCAGAAACTTTTTC
         66 TTTCGTTGGAAACAGG-AATATCTTCATATAAAAGCTAGACAGAAGCATTCTCAGAAACTTCTTT

            *      * ** *   **      *       *  * *   *
     311305 ATGATGTATCTACTCAGCTAACAGAGTTGAACCTTTCTTTTG
        130 GTGATGTTTGCATTCAAGTAACAGTGTTGAACATTCCCTTTC

            ** *     *                   * *     *             **            
     311347 TGATAGCAGTTTTGAAACACTCTTTTTGTGGAATCTGCAAGTGGATATTTGTCTAGCTTTGAGGA
          1 ATAGAGCAGGTTTGAAACACTCTTTTTGTAGTATCTGGAAGTGGATATTTGGATAGCTTTGAGGA

                         *   *    *                   *       *   *          
     311412 TTTCGTTGGAAACGGGATTA-CAT-ATA-AAAAGC-AGACAGCAGCATTCCCAGTAACTTCTTTG
         66 TTTCGTTGGAAACAGGAATATCTTCATATAAAAGCTAGACAGAAGCATTCTCAGAAACTTCTTTG

                              *    *                 
     311473 TGATGTTTGCATTCAAGTCACAGAGTTGAACATTCCCTTTC
        131 TGATGTTTGCATTCAAGTAACAGTGTTGAACATTCCCTTTC

               *                          *        *     *       **     *   *
     311514 ATACAGCAGGTTTGAAACACTCTTTTTGTACTATCTGGATGTGGACATTTGGAGCGCTTTCAGGC
          1 ATAGAGCAGGTTTGAAACACTCTTTTTGTAGTATCTGGAAGTGGATATTTGGATAGCTTTGAGGA

            * ** *      *             *** *   *           *          *   *   
     311579 CTATGGT-GAAAAAGGAAATATCTTCCCCTGAAAACTAGACAGAAGAATTCTCAGAATCTTATTT
         66 TTTCGTTGGAAACAGG-AATATCTTCATATAAAAGCTAGACAGAAGCATTCTCAGAAACTTCTTT

                   **  **    *       *     **  * *   *
     311643 GTGATGTGCGCCCTCAACTAACAGTCTTGAAGCTTTCTTTTG
        130 GTGATGTTTGCATTCAAGTAACAGTGTTGAACATTCCCTTTC

                     *                    **     *   *                       
     311685 ATAGAGCAGTTTTGAAACACTCTTTTTGTAAAATCTGCAAGAGGATATTTGGATAGCTTTGAGGA
          1 ATAGAGCAGGTTTGAAACACTCTTTTTGTAGTATCTGGAAGTGGATATTTGGATAGCTTTGAGGA

                         *   * *            **                      **   *   
     311750 TTTCGTTGGAAACGGGATTGTCTTCATATAAACTCTAGACAGAAGCATTCTCAGAAGTTTCATTG
         66 TTTCGTTGGAAACAGGAATATCTTCATATAAAAGCTAGACAGAAGCATTCTCAGAAACTTCTTTG

            *       **   *    *             *        
     311815 GGATGTTTCAATTGAAGTCACAGTGTTGAACAGTCCCTTTC
        131 TGATGTTTGCATTCAAGTAACAGTGTTGAACATTCCCTTTC

                                **                       *       *  * * *    
     311856 ATAGAGCAGGTTTGAAACACAATTTTTGTAGTATCTGGAAGTGGACATTTGGAGAGATCTCAGGA
          1 ATAGAGCAGGTTTGAAACACTCTTTTTGTAGTATCTGGAAGTGGATATTTGGATAGCTTTGAGGA

            * *  *    * *             **     *    * *       * *          *  *
     311921 ATACGGT-GATAAAGGAAATATCTTCCAATAAACGCTACATAGAAGCAATGTCAGAAACTTTTTC
         66 TTTCGTTGGAAACAGG-AATATCTTCATATAAAAGCTAGACAGAAGCATTCTCAGAAACTTCTTT

            *      * ** *   **      *       *  * *   *
     311985 ATGATGTATCTACTCAGCTAACAGAGTTGAACCTTTCTTTTG
        130 GTGATGTTTGCATTCAAGTAACAGTGTTGAACATTCCCTTTC

             *       *                   * *     *             **            
     312027 AGAGAGCAGTTTTGAAACACTCTTTTTGTGGAATCTGCAAGTGGATATTTGTCTAGCTTTGAGGA
          1 ATAGAGCAGGTTTGAAACACTCTTTTTGTAGTATCTGGAAGTGGATATTTGGATAGCTTTGAGGA

                         *   *    *                   *       *              
     312092 TTTCGTTGGAAACGGGATTA-CAT-ATA-AAAAGC-AGACAGCAGCATTCCCAGAAACTTCTTTG
         66 TTTCGTTGGAAACAGGAATATCTTCATATAAAAGCTAGACAGAAGCATTCTCAGAAACTTCTTTG

                              *    *                 
     312153 TGATGTTTGCATTCAAGTCACAGAGTTGAACATTCCCTTTC
        131 TGATGTTTGCATTCAAGTAACAGTGTTGAACATTCCCTTTC

                                                   *     *     * **     *   *
     312194 ATAGAGCAGGTTTGAAACACTCTTTTTGTAGTATCTGGATGTGGACATTTGCAGCGCTTTCAGGC
          1 ATAGAGCAGGTTTGAAACACTCTTTTTGTAGTATCTGGAAGTGGATATTTGGATAGCTTTGAGGA

            * ** *      *             *** *   *                          *   
     312259 CTAAGGT-GAAAAAGGAAATATCTTCCCCTGAAAACTAGACAGAAGCATTCTCAGAAACTTATTT
         66 TTTCGTTGGAAACAGG-AATATCTTCATATAAAAGCTAGACAGAAGCATTCTCAGAAACTTCTTT

                   **  **    *             **  * *   *
     312323 GTGATGTGCGCCCTCAACTAACAGTGTTGAAGCTTTCTTTTG
        130 GTGATGTTTGCATTCAAGTAACAGTGTTGAACATTCCCTTTC

                     *                    *      *   *                       
     312365 ATAGAGCAGTTTTGAAACACTCTTTTTGTAATATCTGCAAGAGGATATTTGGATAGCTTTGAGGA
          1 ATAGAGCAGGTTTGAAACACTCTTTTTGTAGTATCTGGAAGTGGATATTTGGATAGCTTTGAGGA

                         *   * *            **                      *    *   
     312430 TTTCGTTGGAAACGGGATTGTCTTCATATAAACTCTAGACAGAAGCATTCTCAGAAGCTTCATTG
         66 TTTCGTTGGAAACAGGAATATCTTCATATAAAAGCTAGACAGAAGCATTCTCAGAAACTTCTTTG

            *       **   *    *     *       * *      
     312495 GGATGTTTCAATTGAAGTCACAGTATTGAACAGTTCCTTTC
        131 TGATGTTTGCATTCAAGTAACAGTGTTGAACATTCCCTTTC

                                                         *       *  * * *    
     312536 ATAGAGCAGGTTTGAAACACTCTTTTTGTAGTATCTGGAAGTGGACATTTGGAGAGATCTCAGGA
          1 ATAGAGCAGGTTTGAAACACTCTTTTTGTAGTATCTGGAAGTGGATATTTGGATAGCTTTGAGGA

            * *  *    * *             **            *       * *          *  *
     312601 ATACGGT-GATAAAGGAAATATCTTCCAATAAAAGCTAGATAGAAGCAATGTCAGAAACTTTTTC
         66 TTTCGTTGGAAACAGG-AATATCTTCATATAAAAGCTAGACAGAAGCATTCTCAGAAACTTCTTT

            *      * ** *   **      *       *  * *   *
     312665 ATGATGTATCTACTCAGCTAACAGAGTTGAACCTTTCTTTTG
        130 GTGATGTTTGCATTCAAGTAACAGTGTTGAACATTCCCTTTC

             *       *                   * *     *             **            
     312707 AGAGAGCAGTTTTGAAACACTCTTTTTGTGGAATCTGCAAGTGGATATTTGTCTAGCTTTGAGGA
          1 ATAGAGCAGGTTTGAAACACTCTTTTTGTAGTATCTGGAAGTGGATATTTGGATAGCTTTGAGGA

                         *   *    *                   *       *   *          
     312772 TTTCGTTGGAAACGGGATTA-CAT-ATA-AAAAGC-AGACAGCAGCATTCCCAGTAACTTCTTTG
         66 TTTCGTTGGAAACAGGAATATCTTCATATAAAAGCTAGACAGAAGCATTCTCAGAAACTTCTTTG

                              *    *                 
     312833 TGATGTTTGCATTCAAGTCACAGAGTTGAACATTCCCTTTC
        131 TGATGTTTGCATTCAAGTAACAGTGTTGAACATTCCCTTTC

               *                                   *     *       **     *   *
     312874 ATACAGCAGGTTTGAAACACTCTTTTTGTAGTATCTGGATGTGGACATTTGGAGCGCTTTCAGGC
          1 ATAGAGCAGGTTTGAAACACTCTTTTTGTAGTATCTGGAAGTGGATATTTGGATAGCTTTGAGGA

            * ** *      *             *** *   *           *          *   *   
     312939 CTATGGT-GAAAAAGGAAATATCTTCCCCTGAAAACTAGACAGAAGAATTCTCAGAATCTTATTT
         66 TTTCGTTGGAAACAGG-AATATCTTCATATAAAAGCTAGACAGAAGCATTCTCAGAAACTTCTTT

                   **  **    *       *     **  * *   *
     313003 GTGATGTGCGCCCTCAACTAACAGTCTTGAAGCTTTCTTTTG
        130 GTGATGTTTGCATTCAAGTAACAGTGTTGAACATTCCCTTTC

                     *                    **     *   *         **            
     313045 ATAGAGCAGTTTTGAAACACTCTTTTTGTAAAATCTGCAAGAGGATATTTGTCTAGCTTTGAGGA
          1 ATAGAGCAGGTTTGAAACACTCTTTTTGTAGTATCTGGAAGTGGATATTTGGATAGCTTTGAGGA

                         *   *    *                   *       *              
     313110 TTTCGTTGGAAACGGGATTA-CAT-ATA-AAAAGC-AGACAGCAGCATTCCCAGAAACTTCTTTG
         66 TTTCGTTGGAAACAGGAATATCTTCATATAAAAGCTAGACAGAAGCATTCTCAGAAACTTCTTTG

                              *    *                 
     313171 TGATGTTTGCATTCAAGTCACAGAGTTGAACATTCCCTTTC
        131 TGATGTTTGCATTCAAGTAACAGTGTTGAACATTCCCTTTC

                                                   *     *     * **     *   *
     313212 ATAGAGCAGGTTTGAAACACTCTTTTTGTAGTATCTGGATGTGGACATTTGCAGCGCTTTCAGGC
          1 ATAGAGCAGGTTTGAAACACTCTTTTTGTAGTATCTGGAAGTGGATATTTGGATAGCTTTGAGGA

            * ** *      *             *** *   *                          *   
     313277 CTAAGGT-GAAAAAGGAAATATCTTCCCCTGAAAACTAGACAGAAGCATTCTCAGAAACTTATTT
         66 TTTCGTTGGAAACAGG-AATATCTTCATATAAAAGCTAGACAGAAGCATTCTCAGAAACTTCTTT

                   **  **    *             **  * *   *
     313341 GTGATGTGCGCCCTCAACTAACAGTGTTGAAGCTTTCTTTTG
        130 GTGATGTTTGCATTCAAGTAACAGTGTTGAACATTCCCTTTC

                     *                    *      *   *                  *    
     313383 ATAGAGCAGTTTTGAAACACTCTTTTTGTAATATCTGCAAGAGGATATTTGGATAGCTTTCAGGA
          1 ATAGAGCAGGTTTGAAACACTCTTTTTGTAGTATCTGGAAGTGGATATTTGGATAGCTTTGAGGA

                         *   * *            **                      *    *   
     313448 TTTCGTTGGAAACGGGATTGTCTTCATATAAACTCTAGACAGAAGCATTCTCAGAAGCTTCATTG
         66 TTTCGTTGGAAACAGGAATATCTTCATATAAAAGCTAGACAGAAGCATTCTCAGAAACTTCTTTG

            *       **   *    *               *      
     313513 GGATGTTTCAATTGAAGTCACAGTGTTGAACATTTCCTTTC
        131 TGATGTTTGCATTCAAGTAACAGTGTTGAACATTCCCTTTC

                 *                                       *       **   * *    
     313554 ATAGAACAGGTTTGAAACACTCTTTTTGTAGTATCTGGAAGTGGACATTTGGAGCGCTCTCAGGA
          1 ATAGAGCAGGTTTGAAACACTCTTTTTGTAGTATCTGGAAGTGGATATTTGGATAGCTTTGAGGA

            * ** *      *             **          * *       * *          *  *
     313619 CTATGGT-GAAAAAGGAAATATCTTCCAATAAAAGCTACATAGAAGCAATGTCAGAAACTTTTTC
         66 TTTCGTTGGAAACAGG-AATATCTTCATATAAAAGCTAGACAGAAGCATTCTCAGAAACTTCTTT

            *      * ** *   **      *       *  *     *
     313683 ATGATGTATCTACTCAGCTAACAGAGTTGAACCTTTCCTTTG
        130 GTGATGTTTGCATTCAAGTAACAGTGTTGAACATTCCCTTTC

             *       *                   * *     *             **            
     313725 AGAGAGCAGTTTTGAAACACTCTTTTTGTGGAATCTGCAAGTGGATATTTGTCTAGCTTTGAGGA
          1 ATAGAGCAGGTTTGAAACACTCTTTTTGTAGTATCTGGAAGTGGATATTTGGATAGCTTTGAGGA

                         *   *    *                   *       *   *          
     313790 TTTCGTTGGAAACGGGATTA-CAT-ATA-AAAAGC-AGACAGCAGCATTCCCAGTAACTTCTTTG
         66 TTTCGTTGGAAACAGGAATATCTTCATATAAAAGCTAGACAGAAGCATTCTCAGAAACTTCTTTG

                              *    *                 
     313851 TGATGTTTGCATTCAAGTCACAGAGTTGAACATTCCCTTTC
        131 TGATGTTTGCATTCAAGTAACAGTGTTGAACATTCCCTTTC

                                                   *     *       **     *   *
     313892 ATAGAGCAGGTTTGAAACACTCTTTTTGTAGTATCTGGATGTGGACATTTGGAGCGCTTTCAGGC
          1 ATAGAGCAGGTTTGAAACACTCTTTTTGTAGTATCTGGAAGTGGATATTTGGATAGCTTTGAGGA

            * ** *      *             *** *   *                      *   *   
     313957 CTATGGT-GAAAAAGGAAATATCTTCCCCTGAAAACTAGACAGAAGCATTCTCAGAATCTTATTT
         66 TTTCGTTGGAAACAGG-AATATCTTCATATAAAAGCTAGACAGAAGCATTCTCAGAAACTTCTTT

                   **  **    *             **  * *   *
     314021 GTGATGTGCGCCCTCAACTAACAGTGTTGAAGCTTTCTTTTG
        130 GTGATGTTTGCATTCAAGTAACAGTGTTGAACATTCCCTTTC

                     *          *         **     *   *                       
     314063 ATAGAGCAGTTTTGAAACACACTTTTTGTAAAATCTGCAAGAGGATATTTGGATAGCTTTGAGGA
          1 ATAGAGCAGGTTTGAAACACTCTTTTTGTAGTATCTGGAAGTGGATATTTGGATAGCTTTGAGGA

                         *   * *            **                      *    *   
     314128 TTTCGTTGGAAACGGGATTGTCTTCATATAAACTCTAGACAGAAGCATTCTCAGAAGCTTCATTG
         66 TTTCGTTGGAAACAGGAATATCTTCATATAAAAGCTAGACAGAAGCATTCTCAGAAACTTCTTTG

            *       **   *    *             *        
     314193 GGATGTTTCAATTGAAGTCACAGTGTTGAACAGTCCCTTTC
        131 TGATGTTTGCATTCAAGTAACAGTGTTGAACATTCCCTTTC

                                                   *     *       **     *    
     314234 ATAGAGCAGGTTTGAAACACTCTTTTTGTAGTATCTGGATGTGGACATTTGGAGCGCTTTCAGGC
          1 ATAGAGCAGGTTTGAAACACTCTTTTTGTAGTATCTGGAAGTGGATATTTGGATAGCTTTGAGG-

            * * *    *                 *** *   *                          *  
     314299 CTATGGTT-TAAA-AGGAAATATCTTCCCCTGAAAACTAGACAGAAGCATTCTCAGAAACTTATT
         65 ATTTCGTTGGAAACAGG-AATATCTTCATATAAAAGCTAGACAGAAGCATTCTCAGAAACTTCTT

                    **  **    *                    *   *
     314362 TGTGATGTGCGCCCTCAACTAACAGTGTTGAAGCATT-CTTTTG
        129 TGTGATGTTTGCATTCAAGTAACAGTGTTGAA-CATTCCCTTTC

                     *                   * *     *             **            
     314405 ATAGAGCAGTTTTGAAACACTCTTTTTGTGGAATCTGCAAGTGGATATTTGTCTAGCTTTGAGGA
          1 ATAGAGCAGGTTTGAAACACTCTTTTTGTAGTATCTGGAAGTGGATATTTGGATAGCTTTGAGGA

                         *   *    *                   *       *              
     314470 TTTCGTTGGAAACGGGATTA-CAT-ATA-AAAAGC-AGACAGCAGCATTCCCAGAAACTTCTTTG
         66 TTTCGTTGGAAACAGGAATATCTTCATATAAAAGCTAGACAGAAGCATTCTCAGAAACTTCTTTG

                              *    *                 
     314531 TGATGTTTGCATTCAAGTCACAGAGTTGAACATTCCCTTTC
        131 TGATGTTTGCATTCAAGTAACAGTGTTGAACATTCCCTTTC

                                                   *     *       **     *   *
     314572 ATAGAGCAGGTTTGAAACACTCTTTTTGTAGTATCTGGATGTGGACATTTGGAGCGCTTTCAGGC
          1 ATAGAGCAGGTTTGAAACACTCTTTTTGTAGTATCTGGAAGTGGATATTTGGATAGCTTTGAGGA

            * ** *      *             *** *   *                          *   
     314637 CTATGGT-GAAAAAGGAAATATCTTCCCCTGAAAACTAGACAGAAGCATTCTCAGAAACTTATTT
         66 TTTCGTTGGAAACAGG-AATATCTTCATATAAAAGCTAGACAGAAGCATTCTCAGAAACTTCTTT

                   **  **    *              *  * *   *
     314701 GTGATGTGCGCCCTCAACTAACAGTGTTGAACCTTTCTTTTG
        130 GTGATGTTTGCATTCAAGTAACAGTGTTGAACATTCCCTTTC

                     *                    *      *   *                       
     314743 ATAGAGCAGTTTTGAAACACTCTTTTTGTAATATCTGCAAGAGGATATTTGGATAGCTTTGAGGA
          1 ATAGAGCAGGTTTGAAACACTCTTTTTGTAGTATCTGGAAGTGGATATTTGGATAGCTTTGAGGA

                         *   * *            **                      *    *   
     314808 TTTCGTTGGAAACGGGATTGTCTTCATATAAACTCTAGACAGAAGCATTCTCAGAAGCTTCATTG
         66 TTTCGTTGGAAACAGGAATATCTTCATATAAAAGCTAGACAGAAGCATTCTCAGAAACTTCTTTG

            *       **   *    *             *        
     314873 GGATGTTTCAATTGAAGTCACAGTGTTGAACAGTCCCTTTC
        131 TGATGTTTGCATTCAAGTAACAGTGTTGAACATTCCCTTTC

                 *                                       *       **   * *    
     314914 ATAGAACAGGTTTGAAACACTCTTTTTGTAGTATCTGGAAGTGGACATTTGGAGCGCTCTCAGGA
          1 ATAGAGCAGGTTTGAAACACTCTTTTTGTAGTATCTGGAAGTGGATATTTGGATAGCTTTGAGGA

            * *  *    * *             **      *   * *       * *          *  *
     314979 CTACGGT-GATAAAGGAAATATCTTCCAATAAAACCTACATAGAAGCAATGTCAGAAACTTTTTC
         66 TTTCGTTGGAAACAGG-AATATCTTCATATAAAAGCTAGACAGAAGCATTCTCAGAAACTTCTTT

            *      * ** *   **      *       *  * *   *
     315043 ATGATGTATCTACTCAGCTAACAGAGTTGAACCTTTCTTTTG
        130 GTGATGTTTGCATTCAAGTAACAGTGTTGAACATTCCCTTTC

             *       *          *        * *     *             **            
     315085 AGAGAGCAGTTTTGAAACACACTTTTTGTGGAATCTGCAAGTGGATATTTGTCTAGCTTTGAGGA
          1 ATAGAGCAGGTTTGAAACACTCTTTTTGTAGTATCTGGAAGTGGATATTTGGATAGCTTTGAGGA

                         *   *    *                   *       *              
     315150 TTTCGTTGGAAACGGGATTA-CAT-ATA-AAAAGC-AGACAGCAGCATTCCCAGAAACTTCTTTG
         66 TTTCGTTGGAAACAGGAATATCTTCATATAAAAGCTAGACAGAAGCATTCTCAGAAACTTCTTTG

                              *    *                 
     315211 TGATGTTTGCATTCAAGTCACAGAGTTGAACATTCCCTTTC
        131 TGATGTTTGCATTCAAGTAACAGTGTTGAACATTCCCTTTC

                                                   *     *       **     *   *
     315252 ATAGAGCAGGTTTGAAACACTCTTTTTGTAGTATCTGGATGTGGACATTTGGAGCGCTTTCAGGC
          1 ATAGAGCAGGTTTGAAACACTCTTTTTGTAGTATCTGGAAGTGGATATTTGGATAGCTTTGAGGA

            * ** *      *             *** *   *                      *   *   
     315317 CTATGGT-GAAAAAGGAAATATCTTCCCCTGAAAACTAGACAGAAGCATTCTCAGAATCTTATTT
         66 TTTCGTTGGAAACAGG-AATATCTTCATATAAAAGCTAGACAGAAGCATTCTCAGAAACTTCTTT

                   **  **    *                    *   *
     315381 GTGATGTGCGCCCTCAACTAACAGTGTTGAAGCATT-CTTTTG
        130 GTGATGTTTGCATTCAAGTAACAGTGTTGAA-CATTCCCTTTC

                     *                    **     *   *                       
     315423 ATAGAGCAGTTTTGAAACACTCTTTTTGTAAAATCTGCAAGAGGATATTTGGATAGCTTTGAGGA
          1 ATAGAGCAGGTTTGAAACACTCTTTTTGTAGTATCTGGAAGTGGATATTTGGATAGCTTTGAGGA

                         *   * *            **                           *   
     315488 TTTCGTTGGAAACGGGATTGTCTTCATATAAACTCTAGACAGAAGCATTCTCAGAAACTTCATTG
         66 TTTCGTTGGAAACAGGAATATCTTCATATAAAAGCTAGACAGAAGCATTCTCAGAAACTTCTTTG

            *       **   *    *             *        
     315553 GGATGTTTCAATTGAAGTCACAGTGTTGAACAGTCCCTTTC
        131 TGATGTTTGCATTCAAGTAACAGTGTTGAACATTCCCTTTC

                                                         *       **   * *    
     315594 ATAGAGCAGGTTTGAAACACTCTTTTTGTAGTATCTGGAAGTGGACATTTGGAGCGCTCTCAGGA
          1 ATAGAGCAGGTTTGAAACACTCTTTTTGTAGTATCTGGAAGTGGATATTTGGATAGCTTTGAGGA

            * *  *      *             **    *       *       * *          *  *
     315659 CTGCGGT-GAAAAAGGAAATATCTTCCAATAAGAGCTAGATAGAAGCAATGTCAGAAACTTTTTC
         66 TTTCGTTGGAAACAGG-AATATCTTCATATAAAAGCTAGACAGAAGCATTCTCAGAAACTTCTTT

            *      * ** *   **      *       *    *   *
     315723 ATGATGTATCTACTCAGCTAACAGAGTTGAACCTT-CATTTG
        130 GTGATGTTTGCATTCAAGTAACAGTGTTGAACATTCCCTTTC

             *       *            *      * *     *             **            
     315764 AGAGAGCAGTTTTGAAACACTCGTTTTGTGGAATCTGCAAGTGGATATTTGTCTAGCTTTGAGGA
          1 ATAGAGCAGGTTTGAAACACTCTTTTTGTAGTATCTGGAAGTGGATATTTGGATAGCTTTGAGGA

                         *   *    *                   *       *              
     315829 TTTCGTTGGAAACGGGATTA-CAT-ATA-AAAAGC-AGACAGCAGCATTCCCAGAAACTTCTTTG
         66 TTTCGTTGGAAACAGGAATATCTTCATATAAAAGCTAGACAGAAGCATTCTCAGAAACTTCTTTG

                              *    *                 
     315890 TGATGTTTGCATTCAAGTCACAGAGTTGAACATTCCCTTTC
        131 TGATGTTTGCATTCAAGTAACAGTGTTGAACATTCCCTTTC

                                                   *     *    *  **         *
     315931 ATAGAGCAGGTTTGAAACACTCTTTTTGTAGTATCTGGATGTGGACATTTTGAGCGCTTTGAGGC
          1 ATAGAGCAGGTTTGAAACACTCTTTTTGTAGTATCTGGAAGTGGATATTTGGATAGCTTTGAGGA

            * *  *      *       *     *** *   *           *          *   *   
     315996 CTACGGT-GAAAAAGGAAATCTCTTCCCCTGAAAACTAGACAGAAGAATTCTCAGAATCTTATTT
         66 TTTCGTTGGAAACAGG-AATATCTTCATATAAAAGCTAGACAGAAGCATTCTCAGAAACTTCTTT

                   *   **    *             **  * *   *
     316060 GTGATGTGTGCCCTCAACTAACAGTGTTGAAGCTTTCTTTTG
        130 GTGATGTTTGCATTCAAGTAACAGTGTTGAACATTCCCTTTC

                     *                   ***     *   *             *         
     316102 ATAGAGCAGTTTTGAAACACTCTTTTTGTTAAATCTGCAAGAGGATATTTGGATACCTTTGAGGA
          1 ATAGAGCAGGTTTGAAACACTCTTTTTGTAGTATCTGGAAGTGGATATTTGGATAGCTTTGAGGA

                         *   * *            **                      *    *   
     316167 TTTCGTTGGAAACGGGATTGTCTTCATATAAACTCTAGACAGAAGCATTCTCAGAAGCTTCATTG
         66 TTTCGTTGGAAACAGGAATATCTTCATATAAAAGCTAGACAGAAGCATTCTCAGAAACTTCTTTG

            *       **   *    *             *        
     316232 GGATGTTTCAATTGAAGTCACAGTGTTGAACAGTCCCTTTC
        131 TGATGTTTGCATTCAAGTAACAGTGTTGAACATTCCCTTTC

                                                   *     *     * **     *   *
     316273 ATAGAGCAGGTTTGAAACACTCTTTTTGTAGTATCTGGATGTGGACATTTGCAGCGCTTTCAGGC
          1 ATAGAGCAGGTTTGAAACACTCTTTTTGTAGTATCTGGAAGTGGATATTTGGATAGCTTTGAGGA

            * ** *      *             *** *   *                          *   
     316338 CTAAGGT-GAAAAAGGAAATATCTTCCCCTGAAAACTAGACAGAAGCATTCTCAGAAACTTATTT
         66 TTTCGTTGGAAACAGG-AATATCTTCATATAAAAGCTAGACAGAAGCATTCTCAGAAACTTCTTT

                   **  **    *             ** ** *   *
     316402 GTGATGTGCGCCCTCAACTAACAGTGTTGAAGCTGTCTTTTG
        130 GTGATGTTTGCATTCAAGTAACAGTGTTGAACATTCCCTTTC

                     *                    *      *   *                       
     316444 ATAGAGCAGTTTTGAAACACTCTTTTTGTAATATCTGCAAGAGGATATTTGGATAGCTTTGAGGA
          1 ATAGAGCAGGTTTGAAACACTCTTTTTGTAGTATCTGGAAGTGGATATTTGGATAGCTTTGAGGA

                         *   * *            **                      *    *   
     316509 TTTCGTTGGAAACGGGATTGTCTTCATATAAACTCTAGACAGAAGCATTCTCAGAAGCTTCATTG
         66 TTTCGTTGGAAACAGGAATATCTTCATATAAAAGCTAGACAGAAGCATTCTCAGAAACTTCTTTG

            *       **   *    *             * *      
     316574 GGATGTTTCAATTGAAGTCACAGTGTTGAACAGTTCCTTTC
        131 TGATGTTTGCATTCAAGTAACAGTGTTGAACATTCCCTTTC

                 *                                       *       **   * *    
     316615 ATAGAACAGGTTTGAAACACTCTTTTTGTAGTATCTGGAAGTGGACATTTGGAGCGCTCTCAGGA
          1 ATAGAGCAGGTTTGAAACACTCTTTTTGTAGTATCTGGAAGTGGATATTTGGATAGCTTTGAGGA

            * ** *      *             **          * *       * *          *  *
     316680 CTATGGT-GAAAAAGGAAATATCTTCCAATAAAAGCTACATAGAAGCAATGTCAGAAACTTTTTC
         66 TTTCGTTGGAAACAGG-AATATCTTCATATAAAAGCTAGACAGAAGCATTCTCAGAAACTTCTTT

            *      * ** *   **      *       *  *     *
     316744 ATGATGTATCTACTCAGCTAACAGAGTTGAACCTTTCCTTTG
        130 GTGATGTTTGCATTCAAGTAACAGTGTTGAACATTCCCTTTC

             *       *                   * *     *             **            
     316786 AGAGAGCAGTTTTGAAACACTCTTTTTGTGGAATCTGCAAGTGGATATTTGCTTAGCTTTGAGGA
          1 ATAGAGCAGGTTTGAAACACTCTTTTTGTAGTATCTGGAAGTGGATATTTGGATAGCTTTGAGGA

                        **   *    *                   *       *   *          
     316851 TTTCGTTGGAAATGGGATTA-CAT-ATA-AAAAGC-AGACAGCAGCATTCCCAGTAACTTCTTTG
         66 TTTCGTTGGAAACAGGAATATCTTCATATAAAAGCTAGACAGAAGCATTCTCAGAAACTTCTTTG

                              *    *                 
     316912 TGATGTTTGCATTCAAGTCACAGAGTTGAACATTCCCTTTC
        131 TGATGTTTGCATTCAAGTAACAGTGTTGAACATTCCCTTTC

                                                   *     *       **     *   *
     316953 ATAGAGCAGGTTTGAAACACTCTTTTTGTAGTATCTGGATGTGGACATTTGGAGCGCTTTCAGGC
          1 ATAGAGCAGGTTTGAAACACTCTTTTTGTAGTATCTGGAAGTGGATATTTGGATAGCTTTGAGGA

            * ** *      *             *** *   *                      *   *   
     317018 CTATGGT-GAAAAAGGAAATATCTTCCCCTGAAAACTAGACAGAAGCATTCTCAGAATCTTATTT
         66 TTTCGTTGGAAACAGG-AATATCTTCATATAAAAGCTAGACAGAAGCATTCTCAGAAACTTCTTT

                   **  **    *             **  * *   *
     317082 GTGATGTGCGCCCTCAACTAACAGTGTTGAAGCTTTCTTTTG
        130 GTGATGTTTGCATTCAAGTAACAGTGTTGAACATTCCCTTTC

                     *                    **     *   *                       
     317124 ATAGAGCAGTTTTGAAACACTCTTTTTGTAAAATCTGCAAGAGGATATTTGGATAGCTTTGAGGA
          1 ATAGAGCAGGTTTGAAACACTCTTTTTGTAGTATCTGGAAGTGGATATTTGGATAGCTTTGAGGA

                         *   * *            **                      *    *   
     317189 TTTCGTTGGAAACGGGATTGTCTTCATATAAACTCTAGACAGAAGCATTCTCAGAAGCTTCATTG
         66 TTTCGTTGGAAACAGGAATATCTTCATATAAAAGCTAGACAGAAGCATTCTCAGAAACTTCTTTG

            *       **   *    *             *        
     317254 GGATGTTTCAATTGAAGTCACAGTGTTGAACAGTCCCTTTC
        131 TGATGTTTGCATTCAAGTAACAGTGTTGAACATTCCCTTTC

                                                   *     *       **     *    
     317295 ATAGAGCAGGTTTGAAACACTCTTTTTGTAGTATCTGGATGTGGACATTTGGAGCGCTTTCAGGC
          1 ATAGAGCAGGTTTGAAACACTCTTTTTGTAGTATCTGGAAGTGGATATTTGGATAGCTTTGAGG-

            * * *    *                 *** *   *                          *  
     317360 CTATGGTT-TAAA-AGGAAATATCTTCCCCTGAAAACTAGACAGAAGCATTCTCAGAAACTTATT
         65 ATTTCGTTGGAAACAGG-AATATCTTCATATAAAAGCTAGACAGAAGCATTCTCAGAAACTTCTT

                    **  **    *                    *   *
     317423 TGTGATGTGCGCCCTCAACTAACAGTGTTGAAGCATT-CTTTTG
        129 TGTGATGTTTGCATTCAAGTAACAGTGTTGAA-CATTCCCTTTC

                     *                   * *     *             **            
     317466 ATAGAGCAGTTTTGAAACACTCTTTTTGTGGAATCTGCAAGTGGATATTTGTCTAGCTTTGAGGA
          1 ATAGAGCAGGTTTGAAACACTCTTTTTGTAGTATCTGGAAGTGGATATTTGGATAGCTTTGAGGA

                         *   *    *                   *       *              
     317531 TTTCGTTGGAAACGGGATTA-CAT-ATA-AAAAGC-AGACAGCAGCATTCCCAGAAACTTCTTTG
         66 TTTCGTTGGAAACAGGAATATCTTCATATAAAAGCTAGACAGAAGCATTCTCAGAAACTTCTTTG

                              *    *                 
     317592 TGATGTTTGCATTCAAGTCACAGAGTTGAACATTCCCTTTC
        131 TGATGTTTGCATTCAAGTAACAGTGTTGAACATTCCCTTTC

                                                   *     *       **     *   *
     317633 ATAGAGCAGGTTTGAAACACTCTTTTTGTAGTATCTGGATGTGGACATTTGGAGCGCTTTCAGGC
          1 ATAGAGCAGGTTTGAAACACTCTTTTTGTAGTATCTGGAAGTGGATATTTGGATAGCTTTGAGGA

            * ** *      *             *** *   *                          *   
     317698 CTATGGT-GAAAAAGGAAATATCTTCCCCTGAAAACTAGACAGAAGCATTCTCAGAAACTTATTT
         66 TTTCGTTGGAAACAGG-AATATCTTCATATAAAAGCTAGACAGAAGCATTCTCAGAAACTTCTTT

                   **  **   **             **  * *   *
     317762 GTGATGTGCGCCCTCAGCTAACAGTGTTGAAGCTTTCTTTTG
        130 GTGATGTTTGCATTCAAGTAACAGTGTTGAACATTCCCTTTC

                     *                    *      *   *                       
     317804 ATAGAGCAGTTTTGAAACACTCTTTTTGTAATATCTGCAAGAGGATATTTGGATAGCTTTGAGGA
          1 ATAGAGCAGGTTTGAAACACTCTTTTTGTAGTATCTGGAAGTGGATATTTGGATAGCTTTGAGGA

                         *   * *            **                      *    *   
     317869 TTTCGTTGGAAACGGGATTGTCTTCATATAAACTCTAGACAGAAGCATTCTCAGAAGCTTCATTG
         66 TTTCGTTGGAAACAGGAATATCTTCATATAAAAGCTAGACAGAAGCATTCTCAGAAACTTCTTTG

            *       **   *    *             *        
     317934 GGATGTTTCAATTGAAGTCACAGTGTTGAACAGTCCCTTTC
        131 TGATGTTTGCATTCAAGTAACAGTGTTGAACATTCCCTTTC

                                                         *       *  * * *    
     317975 ATAGAGCAGGTTTGAAACACTCTTTTTGTAGTATCTGGAAGTGGACATTTGGAGAGATCTCAGGA
          1 ATAGAGCAGGTTTGAAACACTCTTTTTGTAGTATCTGGAAGTGGATATTTGGATAGCTTTGAGGA

            * *  *      *             **            *       * *          *  *
     318040 CTACGGT-GAAAAAGGAAATATCTTCCAATAAAAGCTAGATAGAAGCAATGTCAGAAACTTTTTC
         66 TTTCGTTGGAAACAGG-AATATCTTCATATAAAAGCTAGACAGAAGCATTCTCAGAAACTTCTTT

            *      * ** *   **      *       *  * *   *
     318104 ATGATGTATCTACTCAGCTAACAGAGTTGAACCTTTCTTTTG
        130 GTGATGTTTGCATTCAAGTAACAGTGTTGAACATTCCCTTTC

             *       *                   * *     *             **            
     318146 AGAGAGCAGTTTTGAAACACTCTTTTTGTGGAATCTGCAAGTGGATATTTGTCTAGCTTTGAGGA
          1 ATAGAGCAGGTTTGAAACACTCTTTTTGTAGTATCTGGAAGTGGATATTTGGATAGCTTTGAGGA

                         *   *    *                   *       *              
     318211 TTTCGTTGGAAACGGGATTA-CAT-ATA-AAAAGC-AGACAGCAGCATTCCCAGAAACTTCTTTG
         66 TTTCGTTGGAAACAGGAATATCTTCATATAAAAGCTAGACAGAAGCATTCTCAGAAACTTCTTTG

                              *    *                 
     318272 TGATGTTTGCATTCAAGTCACAGAGTTGAACATTCCCTTTC
        131 TGATGTTTGCATTCAAGTAACAGTGTTGAACATTCCCTTTC

                                                   *     *    *  **     *   *
     318313 ATAGAGCAGGTTTGAAACACTCTTTTTGTAGTATCTGGATGTGGACATTTAGAGCGCTTTCAGGC
          1 ATAGAGCAGGTTTGAAACACTCTTTTTGTAGTATCTGGAAGTGGATATTTGGATAGCTTTGAGGA

            * ** *      *             *** *   *                          *   
     318378 CTATGGT-GAAAAAGGAAATATCTTCCCCTGAAAACTAGACAGAAGCATTCTCAGAAACTTATTT
         66 TTTCGTTGGAAACAGG-AATATCTTCATATAAAAGCTAGACAGAAGCATTCTCAGAAACTTCTTT

                   *** **    *              *  * *   *
     318442 GTGATGTGCTCCCTCAACTAACAGTGTTGAACCTTTCTTTTG
        130 GTGATGTTTGCATTCAAGTAACAGTGTTGAACATTCCCTTTC

                     *                    *      *   *                       
     318484 ATAGAGCAGTTTTGAAACACTCTTTTTGTAATATCTGCAAGAGGATATTTGGATAGCTTTGAGGA
          1 ATAGAGCAGGTTTGAAACACTCTTTTTGTAGTATCTGGAAGTGGATATTTGGATAGCTTTGAGGA

                         *   * *            **                      *    *   
     318549 TTTCGTTGGAAACGGGATTGTCTTCATATAAACTCTAGACAGAAGCATTCTCAGAAGCTTCATTG
         66 TTTCGTTGGAAACAGGAATATCTTCATATAAAAGCTAGACAGAAGCATTCTCAGAAACTTCTTTG

            *       **   *    *         *   *        
     318614 GGATGTTTCAATTGAAGTCACAGTGTTGTACAGTCCCTTTC
        131 TGATGTTTGCATTCAAGTAACAGTGTTGAACATTCCCTTTC

                                                         *       **   * *    
     318655 ATAGAGCAGGTTTGAAACACTCTTTTTGTAGTATCTGGAAGTGGACATTTGGAGCGCTCTCAGGA
          1 ATAGAGCAGGTTTGAAACACTCTTTTTGTAGTATCTGGAAGTGGATATTTGGATAGCTTTGAGGA

            * *  *    * *             **            *       * *          *  *
     318720 CTACGGT-GATAAAGGAAATATCTTCCAATAAAAGCTAGATAGAAGCAATGTCAGAAACTTTTTC
         66 TTTCGTTGGAAACAGG-AATATCTTCATATAAAAGCTAGACAGAAGCATTCTCAGAAACTTCTTT

            *      * ** *   **      *       *  * *   *
     318784 ATGATGTATCTACTCAGCTAACAGAGTTGAACCTTTCTTTTG
        130 GTGATGTTTGCATTCAAGTAACAGTGTTGAACATTCCCTTTC

             *       *                   * *     *             **            
     318826 AGAGAGCAGTTTTGAAACACTCTTTTTGTGGAATCTGCAAGTGGATATTTGTCTAGCTTTGAGGA
          1 ATAGAGCAGGTTTGAAACACTCTTTTTGTAGTATCTGGAAGTGGATATTTGGATAGCTTTGAGGA

                         *   *    *                   *       *              
     318891 TTTCGTTGGAAACGGGATTA-CAT-ATA-AAAAGC-AGACAGCAGCATTCCCAGAAACTTCTTTG
         66 TTTCGTTGGAAACAGGAATATCTTCATATAAAAGCTAGACAGAAGCATTCTCAGAAACTTCTTTG

                              *    *                 
     318952 TGATGTTTGCATTCAAGTCACAGAGTTGAACATTCCCTTTC
        131 TGATGTTTGCATTCAAGTAACAGTGTTGAACATTCCCTTTC

                                                   *     *       **     *   *
     318993 ATAGAGCAGGTTTGAAACACTCTTTTTGTAGTATCTGGATGTGGACATTTGGAGCGCTTTCAGGC
          1 ATAGAGCAGGTTTGAAACACTCTTTTTGTAGTATCTGGAAGTGGATATTTGGATAGCTTTGAGGA

            * ** *      *             *** *   *                          *   
     319058 CTATGGT-GAAAAAGGAAATATCTTCCCCTGAAAACTAGACAGAAGCATTCTCAGAAACTTATTT
         66 TTTCGTTGGAAACAGG-AATATCTTCATATAAAAGCTAGACAGAAGCATTCTCAGAAACTTCTTT

                   **  **   **             **  * *   *
     319122 GTGATGTGCGCCCTCAGCTAACAGTGTTGAAGCTTTCTTTTG
        130 GTGATGTTTGCATTCAAGTAACAGTGTTGAACATTCCCTTTC

                     *                    *      *   *                       
     319164 ATAGAGCAGTTTTGAAACACTCTTTTTGTAATATCTGCAAGAGGATATTTGGATAGCTTTGAGGA
          1 ATAGAGCAGGTTTGAAACACTCTTTTTGTAGTATCTGGAAGTGGATATTTGGATAGCTTTGAGGA

                         *   * *            **                      *    *   
     319229 TTTCGTTGGAAACGGGATTGTCTTCATATAAACTCTAGACAGAAGCATTCTCAGAAGCTTCATTG
         66 TTTCGTTGGAAACAGGAATATCTTCATATAAAAGCTAGACAGAAGCATTCTCAGAAACTTCTTTG

            *       **   *    *             *        
     319294 GGATGTTTCAATTGAAGTCACAGTGTTGAACAGTCCCTTTC
        131 TGATGTTTGCATTCAAGTAACAGTGTTGAACATTCCCTTTC

                                                         *       *  * * *    
     319335 ATAGAGCAGGTTTGAAACACTCTTTTTGTAGTATCTGGAAGTGGACATTTGGAGAGATCTCAGGA
          1 ATAGAGCAGGTTTGAAACACTCTTTTTGTAGTATCTGGAAGTGGATATTTGGATAGCTTTGAGGA

            * *  *      *             **            *       * *          *  *
     319400 CTACGGT-GAAAAAGGAAATATCTTCCAATAAAAGCTAGATAGAAGCAATGTCAGAAACTTTTTC
         66 TTTCGTTGGAAACAGG-AATATCTTCATATAAAAGCTAGACAGAAGCATTCTCAGAAACTTCTTT

            *      * ** *   **      *       *  * *   *
     319464 ATGATGTATCTACTCAGCTAACAGAGTTGAACCTTTCTTTTG
        130 GTGATGTTTGCATTCAAGTAACAGTGTTGAACATTCCCTTTC

             *       *                   * *     *             **            
     319506 AGAGAGCAGTTTTGAAACACTCTTTTTGTGGAATCTGCAAGTGGATATTTGTCTAGCTTTGAGGA
          1 ATAGAGCAGGTTTGAAACACTCTTTTTGTAGTATCTGGAAGTGGATATTTGGATAGCTTTGAGGA

                         *   *    *                   *       *              
     319571 TTTCGTTGGAAACGGGATTA-CAT-ATA-AAAAGC-AGACAGCAGCATTCCCAGAAACTTCTTTG
         66 TTTCGTTGGAAACAGGAATATCTTCATATAAAAGCTAGACAGAAGCATTCTCAGAAACTTCTTTG

                              *    *                 
     319632 TGATGTTTGCATTCAAGTCACAGAGTTGAACATTCCCTTTC
        131 TGATGTTTGCATTCAAGTAACAGTGTTGAACATTCCCTTTC

                                                   * *   *    *  **     *   *
     319673 ATAGAGCAGGTTTGAAACACTCTTTTTGTAGTATCTGGATGGGGACATTTAGAGCGCTTTCAGGC
          1 ATAGAGCAGGTTTGAAACACTCTTTTTGTAGTATCTGGAAGTGGATATTTGGATAGCTTTGAGGA

            * ** *      *             *** *   *                          *   
     319738 CTATGGT-GAAAAAGGAAATATCTTCCCCTGAAAACTAGACAGAAGCATTCTCAGAAACTTATTT
         66 TTTCGTTGGAAACAGG-AATATCTTCATATAAAAGCTAGACAGAAGCATTCTCAGAAACTTCTTT

                   *** **    *              *  * *   *
     319802 GTGATGTGCTCCCTCAACTAACAGTGTTGAACCTTTCTTTTG
        130 GTGATGTTTGCATTCAAGTAACAGTGTTGAACATTCCCTTTC

                     *                    *      *   *    *        *         
     319844 ATAGAGCAGTTTTGAAACACTCTTTTTGTAATATCTGCAAGAGGATGTTTGGATACCTTTGAGGA
          1 ATAGAGCAGGTTTGAAACACTCTTTTTGTAGTATCTGGAAGTGGATATTTGGATAGCTTTGAGGA

                         *   * *            **                      *    *   
     319909 TTTCGTTGGAAACGGGATTGTCTTCATATAAACTCTAGACAGAAGCATTCTCAGAAGCTTCATTG
         66 TTTCGTTGGAAACAGGAATATCTTCATATAAAAGCTAGACAGAAGCATTCTCAGAAACTTCTTTG

            *       **   *    *         *   *        
     319974 GGATGTTTCAATTGAAGTCACAGTGTTGTACAGTCCCTTTC
        131 TGATGTTTGCATTCAAGTAACAGTGTTGAACATTCCCTTTC

                                                         *       **   * *    
     320015 ATAGAGCAGGTTTGAAACACTCTTTTTGTAGTATCTGGAAGTGGACATTTGGAGCGCTCTCAGGA
          1 ATAGAGCAGGTTTGAAACACTCTTTTTGTAGTATCTGGAAGTGGATATTTGGATAGCTTTGAGGA

            * *  *    * *             **            *       * *          *  *
     320080 CTACGGT-GATAAAGGAAATATCTTCCAATAAAAGCTAGATAGAAGCAATGTCAGAAACTTTTTC
         66 TTTCGTTGGAAACAGG-AATATCTTCATATAAAAGCTAGACAGAAGCATTCTCAGAAACTTCTTT

            *      * ** *   **      *       *  * *   *
     320144 ATGATGTATCTACTCAGCTAACAGAGTTGAACCTTTCTTTTG
        130 GTGATGTTTGCATTCAAGTAACAGTGTTGAACATTCCCTTTC

             *       *                   * *     *             **            
     320186 AGAGAGCAGTTTTGAAACACTCTTTTTGTGGAATCTGCAAGTGGATATTTGTCTAGCTTTGAGGA
          1 ATAGAGCAGGTTTGAAACACTCTTTTTGTAGTATCTGGAAGTGGATATTTGGATAGCTTTGAGGA

                         *   *    *                   *       *              
     320251 TTTCGTTGGAAACGGGATTA-CAT-ATA-AAAAGC-AGACAGCAGCATTCCCAGAAACTTCTTTG
         66 TTTCGTTGGAAACAGGAATATCTTCATATAAAAGCTAGACAGAAGCATTCTCAGAAACTTCTTTG

                              *    *                 
     320312 TGATGTTTGCATTCAAGTCACAGAGTTGAACATTCCCTTTC
        131 TGATGTTTGCATTCAAGTAACAGTGTTGAACATTCCCTTTC

                                                   *     *       **     *   *
     320353 ATAGAGCAGGTTTGAAACACTCTTTTTGTAGTATCTGGATGTGGACATTTGGAGCGCTTTCAGGC
          1 ATAGAGCAGGTTTGAAACACTCTTTTTGTAGTATCTGGAAGTGGATATTTGGATAGCTTTGAGGA

            * ** *      *             *** *   *                      *   *   
     320418 CTATGGT-GAAAAAGGAAATATCTTCCCCTGAAAACTAGACAGAAGCATTCTCAGAATCTTATTT
         66 TTTCGTTGGAAACAGG-AATATCTTCATATAAAAGCTAGACAGAAGCATTCTCAGAAACTTCTTT

                   **  **    *             **  * *   *
     320482 GTGATGTGCGCCCTCAACTAACAGTGTTGAAGCTTTCTTTTG
        130 GTGATGTTTGCATTCAAGTAACAGTGTTGAACATTCCCTTTC

                     *                    *      *   *                       
     320524 ATAGAGCAGTTTTGAAACACTCTTTTTGTAATATCTGCAAGAGGATATTTGGATAGCTTTGAGGA
          1 ATAGAGCAGGTTTGAAACACTCTTTTTGTAGTATCTGGAAGTGGATATTTGGATAGCTTTGAGGA

                         *   * *            **                      *    *   
     320589 TTTCGTTGGAAACGGGATTGTCTTCATATAAACTCTAGACAGAAGCATTCTCAGAAGCTTCATTG
         66 TTTCGTTGGAAACAGGAATATCTTCATATAAAAGCTAGACAGAAGCATTCTCAGAAACTTCTTTG

            *       **   *    *             *        
     320654 GGATGTTTCAATTGAAGTCACAGTGTTGAACAGTCCCTTTC
        131 TGATGTTTGCATTCAAGTAACAGTGTTGAACATTCCCTTTC

                                                         *       **   * *  * 
     320695 ATAGAGCAGGTTTGAAACACTCTTTTTGTAGTATCTGGAAGTGGACATTTGGAGCGCTCTCAGCA
          1 ATAGAGCAGGTTTGAAACACTCTTTTTGTAGTATCTGGAAGTGGATATTTGGATAGCTTTGAGGA

            * *  *      *             **          * *       * *          *  *
     320760 CTGCGGT-GAAAAAGGAAATATCTTCCAATAAAAGCTACATAGAAGCAATGTCAGAAACTTTTTC
         66 TTTCGTTGGAAACAGG-AATATCTTCATATAAAAGCTAGACAGAAGCATTCTCAGAAACTTCTTT

            *      * ** *   **      *       *        *
     320824 ATGATGTATCTACTCAGCTAACAGAGTTGAACCTT-CCTTTG
        130 GTGATGTTTGCATTCAAGTAACAGTGTTGAACATTCCCTTTC

             *       *                   * *     *             **            
     320865 AGAGAGCAGTTTTGAAACACTCTTTTTGTGGAATCTGCAAGTGGATATTTGTCTAGCTTTGAGGA
          1 ATAGAGCAGGTTTGAAACACTCTTTTTGTAGTATCTGGAAGTGGATATTTGGATAGCTTTGAGGA

                         *   *    *                   *       *              
     320930 TTTCGTTGGAAACGGGATTA-CAT-ATA-AAAAGC-AGACAGCAGCATTCCCAGAAACTTCTTTG
         66 TTTCGTTGGAAACAGGAATATCTTCATATAAAAGCTAGACAGAAGCATTCTCAGAAACTTCTTTG

                              *    *                 
     320991 TGATGTTTGCATTCAAGTCACAGAGTTGAACATTCCCTTTC
        131 TGATGTTTGCATTCAAGTAACAGTGTTGAACATTCCCTTTC

             *                                     *     *     * **     *   *
     321032 AGAGAGCAGGTTTGAAACACTCTTTTTGTAGTATCTGGATGTGGACATTTGCAGCGCTTTCAGGC
          1 ATAGAGCAGGTTTGAAACACTCTTTTTGTAGTATCTGGAAGTGGATATTTGGATAGCTTTGAGGA

            * ** *      *       *     *** *   *                          *   
     321097 CTATGGT-GAAAAAGGAAATCTCTTCCCCTGAAAACTAGACAGAAGCATTCTCAGAAACTTATTT
         66 TTTCGTTGGAAACAGG-AATATCTTCATATAAAAGCTAGACAGAAGCATTCTCAGAAACTTCTTT

                   **  **    *             **  * *   *
     321161 GTGATGTGCGCCCTCAACTAACAGTGTTGAAGCTTTCTTTTG
        130 GTGATGTTTGCATTCAAGTAACAGTGTTGAACATTCCCTTTC

                     *                    *      *   *                       
     321203 ATAGAGCAGTTTTGAAACACTCTTTTTGTAATATCTGCAAGAGGATATTTGGATAGCTTTGAGGA
          1 ATAGAGCAGGTTTGAAACACTCTTTTTGTAGTATCTGGAAGTGGATATTTGGATAGCTTTGAGGA

                         *   * *            **                      *    *   
     321268 TTTCGTTGGAAACGGGATTGTCTTCATATAAACTCTAGACAGAAGCATTCTCAGAAGCTTCATTG
         66 TTTCGTTGGAAACAGGAATATCTTCATATAAAAGCTAGACAGAAGCATTCTCAGAAACTTCTTTG

            *       **   *    *               *      
     321333 GGATGTTTCAATTGAAGTCACAGTGTTGAACATTTCCTTTC
        131 TGATGTTTGCATTCAAGTAACAGTGTTGAACATTCCCTTTC

                 *                                       *       **   * *    
     321374 ATAGAACAGGTTTGAAACACTCTTTTTGTAGTATCTGGAAGTGGACATTTGGAGCGCTCTCAGGA
          1 ATAGAGCAGGTTTGAAACACTCTTTTTGTAGTATCTGGAAGTGGATATTTGGATAGCTTTGAGGA

            * ** *      *             **          * *       * *          *  *
     321439 CTATGGT-GAAAAAGGAAATATCTTCCAATAAAAGCTACATAGAAGCAATGTCAGAAACTTTTTC
         66 TTTCGTTGGAAACAGG-AATATCTTCATATAAAAGCTAGACAGAAGCATTCTCAGAAACTTCTTT

            *      * ** *   **      *       *  *     *
     321503 ATGATGTATCTACTCAGCTAACAGAGTTGAACCTTTCCTTTG
        130 GTGATGTTTGCATTCAAGTAACAGTGTTGAACATTCCCTTTC

             *       *                   * *     *             **            
     321545 AGAGAGCAGTTTTGAAACACTCTTTTTGTGGAATCTGCAAGTGGATATTTGTCTAGCTTTGAGGA
          1 ATAGAGCAGGTTTGAAACACTCTTTTTGTAGTATCTGGAAGTGGATATTTGGATAGCTTTGAGGA

                         *   *    *                   *       *   *          
     321610 TTTCGTTGGAAACGGGATTA-CAT-ATA-AAAAGC-AGACAGCAGCATTCCCAGTAACTTCTTTG
         66 TTTCGTTGGAAACAGGAATATCTTCATATAAAAGCTAGACAGAAGCATTCTCAGAAACTTCTTTG

                              *    *                 
     321671 TGATGTTTGCATTCAAGTCACAGAGTTGAACATTCCCTTTC
        131 TGATGTTTGCATTCAAGTAACAGTGTTGAACATTCCCTTTC

                                                   *     *       **     *   *
     321712 ATAGAGCAGGTTTGAAACACTCTTTTTGTAGTATCTGGATGTGGACATTTGGAGCGCTTTCAGGC
          1 ATAGAGCAGGTTTGAAACACTCTTTTTGTAGTATCTGGAAGTGGATATTTGGATAGCTTTGAGGA

            * ** *      *             *** *   *                      *   *   
     321777 CTATGGT-GAAAAAGGAAATATCTTCCCCTGAAAACTAGACAGAAGCATTCTCAGAATCTTATTT
         66 TTTCGTTGGAAACAGG-AATATCTTCATATAAAAGCTAGACAGAAGCATTCTCAGAAACTTCTTT

                   **  **    *                    *   *
     321841 GTGATGTGCGCCCTCAACTAACAGTGTTGAAGCATT-CTTTTG
        130 GTGATGTTTGCATTCAAGTAACAGTGTTGAA-CATTCCCTTTC

                     *                    **     *   *                       
     321883 ATAGAGCAGTTTTGAAACACTCTTTTTGTAAAATCTGCAAGAGGATATTTGGATAGCTTTGAGGA
          1 ATAGAGCAGGTTTGAAACACTCTTTTTGTAGTATCTGGAAGTGGATATTTGGATAGCTTTGAGGA

                         *   * *            **                           *   
     321948 TTTCGTTGGAAACGGGATTGTCTTCATATAAACTCTAGACAGAAGCATTCTCAGAAACTTCATTG
         66 TTTCGTTGGAAACAGGAATATCTTCATATAAAAGCTAGACAGAAGCATTCTCAGAAACTTCTTTG

            *       **   *    *             *        
     322013 GGATGTTTCAATTGAAGTCACAGTGTTGAACAGTCCCTTTC
        131 TGATGTTTGCATTCAAGTAACAGTGTTGAACATTCCCTTTC

                                                         *       **   * *    
     322054 ATAGAGCAGGTTTGAAACACTCTTTTTGTAGTATCTGGAAGTGGACATTTGGAGCGCTCTCAGGA
          1 ATAGAGCAGGTTTGAAACACTCTTTTTGTAGTATCTGGAAGTGGATATTTGGATAGCTTTGAGGA

            * *  *      *             **            *       * *          *  *
     322119 CTGCGGT-GAAAAAGGAAATATCTTCCAATAAAAGCTAGATAGAAGCAATGTCAGAAACTTTTTC
         66 TTTCGTTGGAAACAGG-AATATCTTCATATAAAAGCTAGACAGAAGCATTCTCAGAAACTTCTTT

            *      * ** *   **      *       *    *   *
     322183 ATGATGTATCTACTCAGCTAACAGAGTTGAACCTT-CATTTG
        130 GTGATGTTTGCATTCAAGTAACAGTGTTGAACATTCCCTTTC

             *       *            *      * *     *             **            
     322224 AGAGAGCAGTTTTGAAACACTCGTTTTGTGGAATCTGCAAGTGGATATTTGTCTAGCTTTGAGGA
          1 ATAGAGCAGGTTTGAAACACTCTTTTTGTAGTATCTGGAAGTGGATATTTGGATAGCTTTGAGGA

                         *   *    *                   *       *              
     322289 TTTCGTTGGAAACGGGATTA-CAT-ATA-AAAAGC-AGACAGCAGCATTCCCAGAAACTTCTTTG
         66 TTTCGTTGGAAACAGGAATATCTTCATATAAAAGCTAGACAGAAGCATTCTCAGAAACTTCTTTG

                              *    *                 
     322350 TGATGTTTGCATTCAAGTCACAGAGTTGAACATTCCCTTTC
        131 TGATGTTTGCATTCAAGTAACAGTGTTGAACATTCCCTTTC

                                                   *     *     * **     *   *
     322391 ATAGAGCAGGTTTGAAACACTCTTTTTGTAGTATCTGGATGTGGACATTTGCAGCGCTTTCAGGC
          1 ATAGAGCAGGTTTGAAACACTCTTTTTGTAGTATCTGGAAGTGGATATTTGGATAGCTTTGAGGA

            * ** *      *             *** *   *                          *   
     322456 CTAAGGT-GAAAAAGGAAATATCTTCCCCTGAAAACTAGACAGAAGCATTCTCAGAAACTTATTT
         66 TTTCGTTGGAAACAGG-AATATCTTCATATAAAAGCTAGACAGAAGCATTCTCAGAAACTTCTTT

                   *   **    *              *  * *   *
     322520 GTGATGTGTGCCCTCAACTAACAGTGTTGAACCTTTCTTTTG
        130 GTGATGTTTGCATTCAAGTAACAGTGTTGAACATTCCCTTTC

                     *                    *      *   *                       
     322562 ATAGAGCAGTTTTGAAACACTCTTTTTGTAATATCTGCAAGAGGATATTTGGATAGCTTTGAGGA
          1 ATAGAGCAGGTTTGAAACACTCTTTTTGTAGTATCTGGAAGTGGATATTTGGATAGCTTTGAGGA

                         *   * *            **                      *    *   
     322627 TTTCGTTGGAAACGGGATTGTCTTCATATAAACTCTAGACAGAAGCATTCTCAGAAGCTTCATTG
         66 TTTCGTTGGAAACAGGAATATCTTCATATAAAAGCTAGACAGAAGCATTCTCAGAAACTTCTTTG

            *       **   *    *     *       * *      
     322692 GGATGTTTCAATTGAAGTCACAGTATTGAACAGTTCCTTTC
        131 TGATGTTTGCATTCAAGTAACAGTGTTGAACATTCCCTTTC

                 *                                       *       **   ***    
     322733 ATAGAACAGGTTTGAAACACTCTTTTTGTAGTATCTGGAAGTGGACATTTGGAGCGCTCCCAGGA
          1 ATAGAGCAGGTTTGAAACACTCTTTTTGTAGTATCTGGAAGTGGATATTTGGATAGCTTTGAGGA

            * ** *      *             **          * *       * *          *  *
     322798 CTATGGT-GAAAAAGGAAATATCTTCCAATAAAAGCTACATAGAAGCAATGTCAGAAACTTTTTC
         66 TTTCGTTGGAAACAGG-AATATCTTCATATAAAAGCTAGACAGAAGCATTCTCAGAAACTTCTTT

            *      * ** *   **      *       *  *     *
     322862 ATGATGTATCTACTCAGCTAACAGAGTTGAACCTTTCCTTTG
        130 GTGATGTTTGCATTCAAGTAACAGTGTTGAACATTCCCTTTC

             *       *                   * *     *             **            
     322904 AGAGAGCAGTTTTGAAACACTCTTTTTGTGGAATCTGCAAGTGGATATTTGTCTAGCTTTGAGGA
          1 ATAGAGCAGGTTTGAAACACTCTTTTTGTAGTATCTGGAAGTGGATATTTGGATAGCTTTGAGGA

                         *   *    *                   *       *   *          
     322969 TTTCGTTGGAAACGGGATTA-CAT-ATA-AAAAGC-AGACAGCAGCATTCCCAGTAACTTCTTTG
         66 TTTCGTTGGAAACAGGAATATCTTCATATAAAAGCTAGACAGAAGCATTCTCAGAAACTTCTTTG

                              *    *                 
     323030 TGATGTTTGCATTCAAGTCACAGAGTTGAACATTCCCTTTC
        131 TGATGTTTGCATTCAAGTAACAGTGTTGAACATTCCCTTTC

                                                   *     *       **     *   *
     323071 ATAGAGCAGGTTTGAAACACTCTTTTTGTAGTATCTGGATGTGGACATTTGGAGCGCTTTCAGGC
          1 ATAGAGCAGGTTTGAAACACTCTTTTTGTAGTATCTGGAAGTGGATATTTGGATAGCTTTGAGGA

            * ** *      *             *** *   *                          *   
     323136 CTATGGT-GAAAAAGGAAATATCTTCCCCTGAAAACTAGACAGAAGCATTCTCAGAAACTTATTT
         66 TTTCGTTGGAAACAGG-AATATCTTCATATAAAAGCTAGACAGAAGCATTCTCAGAAACTTCTTT

                   **  **    *              *  * *   *
     323200 GTGATGTGCGCCCTCAACTAACAGTGTTGAACCTTTCTTTTG
        130 GTGATGTTTGCATTCAAGTAACAGTGTTGAACATTCCCTTTC

                     *                    *      *   * *            *        
     323242 ATAGAGCAGTTTTGAAACACTCTTTTTGTAATATCTGCAAGAGTATATTTGGATAGTTTTGAGGA
          1 ATAGAGCAGGTTTGAAACACTCTTTTTGTAGTATCTGGAAGTGGATATTTGGATAGCTTTGAGGA

                 *            *    *         *        *       *     *   *    
     323307 TTTCGCTGGAAAC-GG--GAT-TACATATAAAAAC-AGACAGCAGCATTCCCAGAATCTTGTTTG
         66 TTTCGTTGGAAACAGGAATATCTTCATATAAAAGCTAGACAGAAGCATTCTCAGAAACTTCTTTG

                    *         *    *                 
     323367 TGATGTTTCCATTCAAGTCACAGAGTTGAACATTCCCTTTC
        131 TGATGTTTGCATTCAAGTAACAGTGTTGAACATTCCCTTTC

             *                         *           *     *       **     *   *
     323408 ACAGAGCAGGTTTGAAACACTCTTTTTATAGTATCTGGATGTGGACATTTGGAGCGCTTTCAGGC
          1 ATAGAGCAGGTTTGAAACACTCTTTTTGTAGTATCTGGAAGTGGATATTTGGATAGCTTTGAGGA

            * ** *      *             *** *   *                      *   *   
     323473 CTATGGT-GAAAAAGGAAATATCTTCTCCTGAAAACTAGACAGAAGCATTCTCAGAATCTTATTT
         66 TTTCGTTGGAAACAGG-AATATCTTCATATAAAAGCTAGACAGAAGCATTCTCAGAAACTTCTTT

                   **  **    *             **  * *   *
     323537 GTGATGTGCGCCCTCAACTAACAGTGTTGAAGCTTTCTTTTG
        130 GTGATGTTTGCATTCAAGTAACAGTGTTGAACATTCCCTTTC

                     *                *   **     *   *        *              
     323579 ATAGAGCAGTTTTGAAACACTCTTTTCGTAAAATCTGCAAGAGGATATTTTGATAGCTTTGAGGA
          1 ATAGAGCAGGTTTGAAACACTCTTTTTGTAGTATCTGGAAGTGGATATTTGGATAGCTTTGAGGA

                         *   * *            **                      *    *   
     323644 TTTCGTTGGAAACGGGATTGTCTTCATATAAACTCTAGACAGAAGCATTCTCAGAAGCTTCATTG
         66 TTTCGTTGGAAACAGGAATATCTTCATATAAAAGCTAGACAGAAGCATTCTCAGAAACTTCTTTG

            *       **   *    *             *        
     323709 GGATGTTTCAATTGAAGTCACAGTGTTGAACAGTCCCTTTC
        131 TGATGTTTGCATTCAAGTAACAGTGTTGAACATTCCCTTTC

                                                         *       **   * *    
     323750 ATAGAGCAGGTTTGAAACACTCTTTTTGTAGTATCTGGAAGTGGACATTTGGAGCGCTCTCAGGA
          1 ATAGAGCAGGTTTGAAACACTCTTTTTGTAGTATCTGGAAGTGGATATTTGGATAGCTTTGAGGA

            * *  *      *             **            *       * *          *  *
     323815 CTGCGGT-GAAAAAGGAAATATCTTCCAATAAAAGCTAGATAGAAGCAATGTCAGAAACTTTTTC
         66 TTTCGTTGGAAACAGG-AATATCTTCATATAAAAGCTAGACAGAAGCATTCTCAGAAACTTCTTT

            *      * ** *   **      *       *        *
     323879 ATGATGTATCTACTCAGCTAACAGAGTTGAACCTT-CCTTTG
        130 GTGATGTTTGCATTCAAGTAACAGTGTTGAACATTCCCTTTC

             *       *                   * *     *             **            
     323920 AGAGAGCAGTTTTGAAACACTCTTTTTGTGGAATCTGCAAGTGGATATTTGTCTAGCTTTGAGGA
          1 ATAGAGCAGGTTTGAAACACTCTTTTTGTAGTATCTGGAAGTGGATATTTGGATAGCTTTGAGGA

                         **  *    *                   *       *              
     323985 TTTCGTTGGAAACGTGATTA-CAT-ATA-AAAAGC-AGACAGCAGCATTCCCAGAAACTTCTTTG
         66 TTTCGTTGGAAACAGGAATATCTTCATATAAAAGCTAGACAGAAGCATTCTCAGAAACTTCTTTG

                              *    **                
     324046 TGATGTTTGCATTCAAGTCACAGATTTGAACATTCCCTTTC
        131 TGATGTTTGCATTCAAGTAACAGTGTTGAACATTCCCTTTC

                                                   *     *     * **     *   *
     324087 ATAGAGCAGGTTTGAAACACTCTTTTTGTAGTATCTGGATGTGGACATTTGCAGCGCTTTCAGGC
          1 ATAGAGCAGGTTTGAAACACTCTTTTTGTAGTATCTGGAAGTGGATATTTGGATAGCTTTGAGGA

            * ** *      *             *** *   *   *                      *   
     324152 CTAAGGT-GAAAAAGGAAATATCTTCCCCTGAAAACTACACAGAAGCATTCTCAGAAACTTATTT
         66 TTTCGTTGGAAACAGG-AATATCTTCATATAAAAGCTAGACAGAAGCATTCTCAGAAACTTCTTT

                   **  **    *             **  * *   *
     324216 GTGATGTGCGCCCTCAACTAACAGTGTTGAAGCTTTCTTTTG
        130 GTGATGTTTGCATTCAAGTAACAGTGTTGAACATTCCCTTTC

                     *                    *      *   *                       
     324258 ATAGAGCAGTTTTGAAACACTCTTTTTGTAATATCTGCAAGAGGATATTTGGATAGCTTTGAGGA
          1 ATAGAGCAGGTTTGAAACACTCTTTTTGTAGTATCTGGAAGTGGATATTTGGATAGCTTTGAGGA

                         *   * *            **                      *    *   
     324323 TTTCGTTGGAAACGGGATTGTCTTCATATAAACTCTAGACAGAAGCATTCTCAGAAGCTTCATTG
         66 TTTCGTTGGAAACAGGAATATCTTCATATAAAAGCTAGACAGAAGCATTCTCAGAAACTTCTTTG

            *       **   *    *             * *      
     324388 GGATGTTTCAATTGAAGTCACAGTGTTGAACAGTTCCTTTC
        131 TGATGTTTGCATTCAAGTAACAGTGTTGAACATTCCCTTTC

                 *                                       *       **   * *    
     324429 ATAGAACAGGTTTGAAACACTCTTTTTGTAGTATCTGGAAGTGGACATTTGGAGCGCTCTCAGGA
          1 ATAGAGCAGGTTTGAAACACTCTTTTTGTAGTATCTGGAAGTGGATATTTGGATAGCTTTGAGGA

            * ** *      *             **          * *      ** *          *  *
     324494 CTATGGT-GAAAAAGGAAATATCTTCCAATAAAAGCTACATAGAAGCTATGTCAGAAACTTTTTC
         66 TTTCGTTGGAAACAGG-AATATCTTCATATAAAAGCTAGACAGAAGCATTCTCAGAAACTTCTTT

            *      * ** *   **      *       *  *     *
     324558 ATGATGTATCTACTCAGCTAACAGAGTTGAACCTTTCCTTTG
        130 GTGATGTTTGCATTCAAGTAACAGTGTTGAACATTCCCTTTC

             *       *                   * *     *             **            
     324600 AGAGAGCAGTTTTGAAACACTCTTTTTGTGGAATCTGCAAGTGGATATTTGTCTAGCTTTGAGGA
          1 ATAGAGCAGGTTTGAAACACTCTTTTTGTAGTATCTGGAAGTGGATATTTGGATAGCTTTGAGGA

                         *   *    *                   *       *   *          
     324665 TTTCGTTGGAAACGGGATTA-CAT-ATA-AAAAGC-AGACAGCAGCATTCCCAGTAACTTCTTTG
         66 TTTCGTTGGAAACAGGAATATCTTCATATAAAAGCTAGACAGAAGCATTCTCAGAAACTTCTTTG

                              *    *                 
     324726 TGATGTTTGCATTCAAGTCACAGAGTTGAACATTCCCTTTC
        131 TGATGTTTGCATTCAAGTAACAGTGTTGAACATTCCCTTTC

                                                ** *     *       **     *   *
     324767 ATAGAGCAGGTTTGAAACACTCTTTTTGTAGTATCTCTATGTGGACATTTGGAGCGCTTTCAGGC
          1 ATAGAGCAGGTTTGAAACACTCTTTTTGTAGTATCTGGAAGTGGATATTTGGATAGCTTTGAGGA

            * ** *      *             *** *   *                          *   
     324832 CTATGGT-GAAAAAGGAAATATCTTCCCCTGAAAACTAGACAGAAGCATTCTCAGAAACTTATTT
         66 TTTCGTTGGAAACAGG-AATATCTTCATATAAAAGCTAGACAGAAGCATTCTCAGAAACTTCTTT

                   **  **    *              *  * *   *
     324896 GTGATGTGCGCCCTCAACTAACAGTGTTGAACCTTTCTTTTG
        130 GTGATGTTTGCATTCAAGTAACAGTGTTGAACATTCCCTTTC

                     *                    *      *   *                       
     324938 ATAGAGCAGTTTTGAAACACTCTTTTTGTAATATCTGCAAGAGGATATTTGGATAGCTTTGAGGA
          1 ATAGAGCAGGTTTGAAACACTCTTTTTGTAGTATCTGGAAGTGGATATTTGGATAGCTTTGAGGA

                         *   *    *                   *       *     *   *    
     325003 TTTCGTTGGAAACGGGATTA-CAT-ATA-AAAAGC-AGACAGCAGCATTCCCAGAATCTTGTTTG
         66 TTTCGTTGGAAACAGGAATATCTTCATATAAAAGCTAGACAGAAGCATTCTCAGAAACTTCTTTG

                                   *                 
     325064 TGATGTTTGCATTCAAGT--CAGAGTTGAACATTCCCTTTC
        131 TGATGTTTGCATTCAAGTAACAGTGTTGAACATTCCCTTTC

             *                         *           *     *       **     *   *
     325103 AGAGAGCAGGTTTGAAACACTCTTTTTATAGTATCTGGATGTGGACATTTGGAGCGCTTTCAGGC
          1 ATAGAGCAGGTTTGAAACACTCTTTTTGTAGTATCTGGAAGTGGATATTTGGATAGCTTTGAGGA

            * ** *      *             *** *   *                      *   *   
     325168 CTATGGT-GAAAAAGGAAATATCTTCTCCTGAAAACTAGACAGAAGCATTCTCAGAATCTTATTT
         66 TTTCGTTGGAAACAGG-AATATCTTCATATAAAAGCTAGACAGAAGCATTCTCAGAAACTTCTTT

                   **  **    *             **  * *   *
     325232 GTGATGTGCGCCCTCAACTAACAGTGTTGAAGCTTTCTTTTG
        130 GTGATGTTTGCATTCAAGTAACAGTGTTGAACATTCCCTTTC

                     *                *   **     *   *        *              
     325274 ATAGAGCAGTTTTGAAACACTCTTTTCGTAAAATCTGCAAGAGGATATTTTGATAGCTTTGAGGA
          1 ATAGAGCAGGTTTGAAACACTCTTTTTGTAGTATCTGGAAGTGGATATTTGGATAGCTTTGAGGA

                         *   * *            **                      *    *   
     325339 TTTCGTTGGAAACGGGATTGTCTTCATATAAACTCTAGACAGAAGCATTCTCAGAAGCTTCATTG
         66 TTTCGTTGGAAACAGGAATATCTTCATATAAAAGCTAGACAGAAGCATTCTCAGAAACTTCTTTG

            *       **   *    *             *        
     325404 GGATGTTTCAATTGAAGTCACAGTGTTGAACAGTCCCTTTC
        131 TGATGTTTGCATTCAAGTAACAGTGTTGAACATTCCCTTTC

                                                         *       **   * *    
     325445 ATAGAGCAGGTTTGAAACACTCTTTTTGTAGTATCTGGAAGTGGACATTTGGAGCGCTCTCAGGA
          1 ATAGAGCAGGTTTGAAACACTCTTTTTGTAGTATCTGGAAGTGGATATTTGGATAGCTTTGAGGA

            * *  *      *             **            *       * *          *  *
     325510 CTGCGGT-GAAAAAGGAAATATCTTCCAATAAAAGCTAGATAGAAGCAATGTCAGAAACTTTTTC
         66 TTTCGTTGGAAACAGG-AATATCTTCATATAAAAGCTAGACAGAAGCATTCTCAGAAACTTCTTT

            *      * ** *   **      *       *        *
     325574 ATGATGTATCTACTCAGCTAACAGAGTTGAACCTT-CCTTTG
        130 GTGATGTTTGCATTCAAGTAACAGTGTTGAACATTCCCTTTC

             *       *                   * *     *             **            
     325615 AGAGAGCAGTTTTGAAACACTCTTTTTGTGGAATCTGCAAGTGGATATTTGTCTAGCTTTGAGGA
          1 ATAGAGCAGGTTTGAAACACTCTTTTTGTAGTATCTGGAAGTGGATATTTGGATAGCTTTGAGGA

                         *   *    *                   *       *              
     325680 TTTCGTTGGAAACGGGATTA-CAT-ATA-AAAAGC-AGACAGCAGCATTCCCAGAAACTTCTTTG
         66 TTTCGTTGGAAACAGGAATATCTTCATATAAAAGCTAGACAGAAGCATTCTCAGAAACTTCTTTG

                              *    **                
     325741 TGATGTTTGCATTCAAGTCACAGATTTGAACATTCCCTTTC
        131 TGATGTTTGCATTCAAGTAACAGTGTTGAACATTCCCTTTC

                                                   *     *     * **     *   *
     325782 ATAGAGCAGGTTTGAAACACTCTTTTTGTAGTATCTGGATGTGGACATTTGCAGCGCTTTCAGGC
          1 ATAGAGCAGGTTTGAAACACTCTTTTTGTAGTATCTGGAAGTGGATATTTGGATAGCTTTGAGGA

            * ** *      *             *** *   *                          *   
     325847 CTAAGGT-GAAAAAGGAAATATCTTCCCCTGAAAACTAGACAGAAGCATTCTCAGAAACTTATTT
         66 TTTCGTTGGAAACAGG-AATATCTTCATATAAAAGCTAGACAGAAGCATTCTCAGAAACTTCTTT

                   **  **    *             **  * *   *
     325911 GTGATGTGCGCCCTCAACTAACAGTGTTGAAGCTTTCTTTTG
        130 GTGATGTTTGCATTCAAGTAACAGTGTTGAACATTCCCTTTC

                     *                    *      *   *                       
     325953 ATAGAGCAGTTTTGAAACACTCTTTTTGTAATATCTGCAAGAGGATATTTGGATAGCTTTGAGGA
          1 ATAGAGCAGGTTTGAAACACTCTTTTTGTAGTATCTGGAAGTGGATATTTGGATAGCTTTGAGGA

                         *   * *            **                      *    *   
     326018 TTTCGTTGGAAACGGGATTGTCTTCATATAAACTCTAGACAGAAGCATTCTCAGAAGCTTCATTG
         66 TTTCGTTGGAAACAGGAATATCTTCATATAAAAGCTAGACAGAAGCATTCTCAGAAACTTCTTTG

            *       **   *    *             *        
     326083 GGATGTTTCAATTGAAGTCACAGTGTTGAACAGTCCCTTTC
        131 TGATGTTTGCATTCAAGTAACAGTGTTGAACATTCCCTTTC

                                                         *       **   * *    
     326124 ATAGAGCAGGTTTGAAACACTCTTTTTGTAGTATCTGGAAGTGGACATTTGGAGCGCTCTCAGGA
          1 ATAGAGCAGGTTTGAAACACTCTTTTTGTAGTATCTGGAAGTGGATATTTGGATAGCTTTGAGGA

            * ** *      *             **          * *       * *          *  *
     326189 CTGTGGT-GAAAAAGGAAATATCTTCCAATAAAAGCTACATAGAAGCAATGTCAGAAACTTTTTC
         66 TTTCGTTGGAAACAGG-AATATCTTCATATAAAAGCTAGACAGAAGCATTCTCAGAAACTTCTTT

            *      * ** *   **      *       *  *     *
     326253 ATGATGTATCTACTCAGCTAACAGAGTTGAACCTTTCCTTTG
        130 GTGATGTTTGCATTCAAGTAACAGTGTTGAACATTCCCTTTC

             *       *                   * *     *             **            
     326295 AGAGAGCAGTTTTGAAACACTCTTTTTGTGGAATCTGCAAGTGGATATTTGTCTAGCTTTGAGGA
          1 ATAGAGCAGGTTTGAAACACTCTTTTTGTAGTATCTGGAAGTGGATATTTGGATAGCTTTGAGGA

                         *   *    *                   *       *              
     326360 TTTCGTTGGAAACGGGATTA-CAT-ATA-AAAAGC-AGACAGCAGCATTCCCAGAAACTTCTTTG
         66 TTTCGTTGGAAACAGGAATATCTTCATATAAAAGCTAGACAGAAGCATTCTCAGAAACTTCTTTG

                              *    *                 
     326421 TGATGTTTGCATTCAAGTCACAGAGTTGAACATTCCCTTTC
        131 TGATGTTTGCATTCAAGTAACAGTGTTGAACATTCCCTTTC

                                                   *     *       **     *   *
     326462 ATAGAGCAGGTTTGAAACACTCTTTTTGTAGTATCTGGATGTGGACATTTGGAGCGCTTTCAGGC
          1 ATAGAGCAGGTTTGAAACACTCTTTTTGTAGTATCTGGAAGTGGATATTTGGATAGCTTTGAGGA

            * ** *      *             *** *   *                      *   *   
     326527 CTATGGT-GAAAAAGGAAATATCTTCTCCTGAAAACTAGACAGAAGCATTCTCAGAATCTTATTT
         66 TTTCGTTGGAAACAGG-AATATCTTCATATAAAAGCTAGACAGAAGCATTCTCAGAAACTTCTTT

                   **  **    *             **  * *   *
     326591 GTGATGTGCGCCCTCAACTAACAGTGTTGAAGCTTTCTTTTG
        130 GTGATGTTTGCATTCAAGTAACAGTGTTGAACATTCCCTTTC

                     *                    *      *   *                       
     326633 ATAGAGCAGTTTTGAAACACTCTTTTTGTAATATCTGCAAGAGGATATTTGGATAGCTTTGAGGA
          1 ATAGAGCAGGTTTGAAACACTCTTTTTGTAGTATCTGGAAGTGGATATTTGGATAGCTTTGAGGA

                         *   * *            **                      *    *   
     326698 TTTCGTTGGAAACGGGATTGTCTTCATATAAACTCTAGACAGAAGCATTCTCAGAAGCTTCATTG
         66 TTTCGTTGGAAACAGGAATATCTTCATATAAAAGCTAGACAGAAGCATTCTCAGAAACTTCTTTG

            *       **   *    *             *        
     326763 GGATGTTTCAATTGAAGTCACAGTGTTGAACAGTCCCTTTC
        131 TGATGTTTGCATTCAAGTAACAGTGTTGAACATTCCCTTTC

                        *                                *       *  * ***    
     326804 ATAGAGCAGGTTGGAAACACTCTTTTTGTAGTATCTGGAAGTGGACATTTGGAGAGATCGCAGGA
          1 ATAGAGCAGGTTTGAAACACTCTTTTTGTAGTATCTGGAAGTGGATATTTGGATAGCTTTGAGGA

            * *  *    * *             **            *       * *          *  *
     326869 ATACGGT-GATAAAGGAAATATCTTCCAATAAAAGCTAGATAGAAGCAATGTCAGAAACTTTTTC
         66 TTTCGTTGGAAACAGG-AATATCTTCATATAAAAGCTAGACAGAAGCATTCTCAGAAACTTCTTT

            *      * ** *   **      *       *  * * * *
     326933 ATGATGTATCTACTCAGCTAACAGAGTTGAACCTTTCTTCTG
        130 GTGATGTTTGCATTCAAGTAACAGTGTTGAACATTCCCTTTC

             *       *                   * *     *             **            
     326975 AGAGAGCAGTTTTGAAACACTCTTTTTGTGGAATCTGCAAGTGGATATTTGTCTAGCTTTGAGGA
          1 ATAGAGCAGGTTTGAAACACTCTTTTTGTAGTATCTGGAAGTGGATATTTGGATAGCTTTGAGGA

                         *   *    *                   *       *   *          
     327040 TTTCGTTGGAAACGGGATTA-CAT-ATA-AAAAGC-AGACAGCAGCATTCCCAGTAACTTCTTTG
         66 TTTCGTTGGAAACAGGAATATCTTCATATAAAAGCTAGACAGAAGCATTCTCAGAAACTTCTTTG

                              *    *                 
     327101 TGATGTTTGCATTCAAGTCACAGAGTTGAACATTCCCTTTC
        131 TGATGTTTGCATTCAAGTAACAGTGTTGAACATTCCCTTTC

               *                                   *     *       **     *   *
     327142 ATACAGCAGGTTTGAAACACTCTTTTTGTAGTATCTGGATGTGGACATTTGGAGCGCTTTCAGGC
          1 ATAGAGCAGGTTTGAAACACTCTTTTTGTAGTATCTGGAAGTGGATATTTGGATAGCTTTGAGGA

            * ** *      *             *** *   *           *          *   *   
     327207 CTATGGT-GAAAAAGGAAATATCTTCCCCTGAAAACTAGACAGAAGAATTCTCAGAATCTTATTT
         66 TTTCGTTGGAAACAGG-AATATCTTCATATAAAAGCTAGACAGAAGCATTCTCAGAAACTTCTTT

                   **  **    *       *     **  * *   *
     327271 GTGATGTGCGCCCTCAACTAACAGTCTTGAAGCTTTCTTTTG
        130 GTGATGTTTGCATTCAAGTAACAGTGTTGAACATTCCCTTTC

                     *         *          **     *   *                       
     327313 ATAGAGCAGTTTTGAAACAATCTTTTTGTAAAATCTGCAAGAGGATATTTGGATAGCTTTGAGGA
          1 ATAGAGCAGGTTTGAAACACTCTTTTTGTAGTATCTGGAAGTGGATATTTGGATAGCTTTGAGGA

                         *   * *            **                      *    *   
     327378 TTTCGTTGGAAACGGGATTGTCTTCATATAAACTCTAGACAGAAGCATTCTCAGAAGCTTCATTG
         66 TTTCGTTGGAAACAGGAATATCTTCATATAAAAGCTAGACAGAAGCATTCTCAGAAACTTCTTTG

            *       **   *    *             *        
     327443 GGATGTTTCAATTGAAGTCACAGTGTTGAACAGTCCCTTTC
        131 TGATGTTTGCATTCAAGTAACAGTGTTGAACATTCCCTTTC

                                                         *       *  * * *    
     327484 ATAGAGCAGGTTTGAAACACTCTTTTTGTAGTATCTGGAAGTGGACATTTGGAGAGATCTCAGGA
          1 ATAGAGCAGGTTTGAAACACTCTTTTTGTAGTATCTGGAAGTGGATATTTGGATAGCTTTGAGGA

            * *  *    * *             **     *    * *       * *          *  *
     327549 ATACGGT-GATAAAGGAAATATCTTCCAATAAACGCTACATAGAAGCAATGTCAGAAACTTTTTC
         66 TTTCGTTGGAAACAGG-AATATCTTCATATAAAAGCTAGACAGAAGCATTCTCAGAAACTTCTTT

            *      * ** *   **      *       *  * *   *
     327613 ATGATGTATCTACTCAGCTAACAGAGTTGAACCTTTCTTTTG
        130 GTGATGTTTGCATTCAAGTAACAGTGTTGAACATTCCCTTTC

             *       *                   * *     *             **            
     327655 AGAGAGCAGTTTTGAAACACTCTTTTTGTGGAATCTGCAAGTGGATATTTGTCTAGCTTTGAGGA
          1 ATAGAGCAGGTTTGAAACACTCTTTTTGTAGTATCTGGAAGTGGATATTTGGATAGCTTTGAGGA

                         *   *    *                   *       *      *       
     327720 TTTCGTTGGAAACGGGATTA-CAT-ATA-AAAAGC-AGACAGCAGCATTCCCAGAAATTTCTTTG
         66 TTTCGTTGGAAACAGGAATATCTTCATATAAAAGCTAGACAGAAGCATTCTCAGAAACTTCTTTG

                              *    *                 
     327781 TGATGTTTGCATTCAAGTCACAGAGTTGAACATTCCCTTTC
        131 TGATGTTTGCATTCAAGTAACAGTGTTGAACATTCCCTTTC

               *                                   *     *       **     *   *
     327822 ATACAGCAGGTTTGAAACACTCTTTTTGTAGTATCTGGATGTGGACATTTGGAGCGCTTTCAGGC
          1 ATAGAGCAGGTTTGAAACACTCTTTTTGTAGTATCTGGAAGTGGATATTTGGATAGCTTTGAGGA

            * ** *      *             *** *   *           *     *    *   *   
     327887 CTATGGT-GAAAAAGGAAATATCTTCCCCTGAAAACTAGACAGAAGAATTCTTAGAATCTTATTT
         66 TTTCGTTGGAAACAGG-AATATCTTCATATAAAAGCTAGACAGAAGCATTCTCAGAAACTTCTTT

                   **  **    *             **  * *   *
     327951 GTGATGTGCGCCCTCAACTAACAGTGTTGAAGCTTTCTTTTG
        130 GTGATGTTTGCATTCAAGTAACAGTGTTGAACATTCCCTTTC

                 *   *                    **     *   *                  *    
     327993 ATAGAACAGTTTTGAAACACTCTTTTTGTAAAATCTGCAAGAGGATATTTGGATAGCTTTAAGGA
          1 ATAGAGCAGGTTTGAAACACTCTTTTTGTAGTATCTGGAAGTGGATATTTGGATAGCTTTGAGGA

                         *   * *            **                      * *  *   
     328058 TTTCGTTGGAAACGGGATTGTCTTCATATAAACTCTAGACAGAAGCATTCTCAGAAGCGTCATTG
         66 TTTCGTTGGAAACAGGAATATCTTCATATAAAAGCTAGACAGAAGCATTCTCAGAAACTTCTTTG

            *       **   *    *             *        
     328123 GGATGTTTCAATTGAAGTCACAGTGTTGAACAGTCCCTTTC
        131 TGATGTTTGCATTCAAGTAACAGTGTTGAACATTCCCTTTC

                                                   *     *       **     *  **
     328164 ATAGAGCAGGTTTGAAACACTCTTTTTGTAGTATCTGGATGTGGACATTTGGAGCGCTTTAAGCC
          1 ATAGAGCAGGTTTGAAACACTCTTTTTGTAGTATCTGGAAGTGGATATTTGGATAGCTTTGAGGA

             * *    *                 *** *   *                          *   
     328229 TATGGTT-TAAA-AGGAAATATCTTCCCCTGAAAACTAGACAGAAGCATTCTCAGAAACTTATTT
         66 TTTCGTTGGAAACAGG-AATATCTTCATATAAAAGCTAGACAGAAGCATTCTCAGAAACTTCTTT

                   **  **    *                    *   *
     328292 GTGATGTGCGCCCTCAACTAACAGTGTTGAAGCATT-CTTTTG
        130 GTGATGTTTGCATTCAAGTAACAGTGTTGAA-CATTCCCTTTC

                     *                   * *     *             **            
     328334 ATAGAGCAGTTTTGAAACACTCTTTTTGTGGAATCTGCAAGTGGATATTTGTCTAGCTTTGAGGA
          1 ATAGAGCAGGTTTGAAACACTCTTTTTGTAGTATCTGGAAGTGGATATTTGGATAGCTTTGAGGA

                         *   *    *                   *       *              
     328399 TTTCGTTGGAAACGGGATTA-CAT-ATA-AAAAGC-AGACAGCAGCATTCCCAGAAACTTCTTTG
         66 TTTCGTTGGAAACAGGAATATCTTCATATAAAAGCTAGACAGAAGCATTCTCAGAAACTTCTTTG

                              *    *                 
     328460 TGATGTTTGCATTCAAGTCACAGAGTTGAACATTCCCTTTC
        131 TGATGTTTGCATTCAAGTAACAGTGTTGAACATTCCCTTTC

                                                   *     **      **     *   *
     328501 ATAGAGCAGGTTTGAAACACTCTTTTTGTAGTATCTGGATGTGGACCTTTGGAGCGCTTTCAGGC
          1 ATAGAGCAGGTTTGAAACACTCTTTTTGTAGTATCTGGAAGTGGATATTTGGATAGCTTTGAGGA

            * ** *      *             *** *   *                      *   *   
     328566 CTATGGT-GAAAAAGGAAATATCTTCCCCTGAAAACTAGACAGAAGCATTCTCAGAATCTTATTT
         66 TTTCGTTGGAAACAGG-AATATCTTCATATAAAAGCTAGACAGAAGCATTCTCAGAAACTTCTTT

                   **  **    **            **  * *   *
     328630 GTGATGTGCGCCCTCAACGAACAGTGTTGAAGCTTTCTTTTG
        130 GTGATGTTTGCATTCAAGTAACAGTGTTGAACATTCCCTTTC

                     *                    *      *   *                       
     328672 ATAGAGCAGTTTTGAAACACTCTTTTTGTAATATCTGCAAGAGGATATTTGGATAGCTTTGAGGA
          1 ATAGAGCAGGTTTGAAACACTCTTTTTGTAGTATCTGGAAGTGGATATTTGGATAGCTTTGAGGA

                         *   * *            **                      *    *   
     328737 TTTCGTTGGAAACGGGATTGTCTTCATATAAACTCTAGACAGAAGCATTCTCAGAAGCTTCATTG
         66 TTTCGTTGGAAACAGGAATATCTTCATATAAAAGCTAGACAGAAGCATTCTCAGAAACTTCTTTG

            *       **   *    *             *        
     328802 GGATGTTTCAATTGAAGTCACAGTGTTGAACAGTCCCTTTC
        131 TGATGTTTGCATTCAAGTAACAGTGTTGAACATTCCCTTTC

                                                         *       **   * *  * 
     328843 ATAGAGCAGGTTTGAAACACTCTTTTTGTAGTATCTGGAAGTGGACATTTGGAGCGCTCTCAGTA
          1 ATAGAGCAGGTTTGAAACACTCTTTTTGTAGTATCTGGAAGTGGATATTTGGATAGCTTTGAGGA

            * *  *      *             **            *       * *          *  *
     328908 CTGCGGT-GAAAAAGGAAATATCTTCCAATAAAAGCTAGATAGAAGCAATGTCAGAAACTTTTTC
         66 TTTCGTTGGAAACAGG-AATATCTTCATATAAAAGCTAGACAGAAGCATTCTCAGAAACTTCTTT

            *      * ** *   **      *       *        *
     328972 ATGATGTATCTACTCAGCTAACAGAGTTGAACCTT-CCTTTG
        130 GTGATGTTTGCATTCAAGTAACAGTGTTGAACATTCCCTTTC

             *       *                   * *     *             **            
     329013 AGAGAGCAGTTTTGAAACACTCTTTTTGTGGAATCTGCAAGTGGATATTTGTCTAGCTTTGAGGA
          1 ATAGAGCAGGTTTGAAACACTCTTTTTGTAGTATCTGGAAGTGGATATTTGGATAGCTTTGAGGA

                         *   *    *                   *       *              
     329078 TTTCGTTGGAAACGGGATTA-CAT-ATA-AAAAGC-AGACAGCAGCATTCCCAGAAACTTCTTTG
         66 TTTCGTTGGAAACAGGAATATCTTCATATAAAAGCTAGACAGAAGCATTCTCAGAAACTTCTTTG

                              *    *                 
     329139 TGATGTTTGCATTCAAGTCACAGAGTTGAACATTCCCTTTC
        131 TGATGTTTGCATTCAAGTAACAGTGTTGAACATTCCCTTTC

                           *                       *     *     * **     *   *
     329180 ATAGAGCAGGTTTGATACACTCTTTTTGTAGTATCTGGATGTGGACATTTGCAGCGCTTTCAGGC
          1 ATAGAGCAGGTTTGAAACACTCTTTTTGTAGTATCTGGAAGTGGATATTTGGATAGCTTTGAGGA

            * ** *      *             *** *   *                          *   
     329245 CTAAGGT-GAAAAAGGAAATATCTTCCCCTGAAAACTAGACAGAAGCATTCTCAGAAACTTATTT
         66 TTTCGTTGGAAACAGG-AATATCTTCATATAAAAGCTAGACAGAAGCATTCTCAGAAACTTCTTT

                   **  **    *             **  * *   *
     329309 GTGATGTGCGCCCTCAACTAACAGTGTTGAAGCTTTCTTTTG
        130 GTGATGTTTGCATTCAAGTAACAGTGTTGAACATTCCCTTTC

                     *                    *      *   *                       
     329351 ATAGAGCAGTTTTGAAACACTCTTTTTGTAATATCTGCAAGAGGATATTTGGATAGCTTTGAGGA
          1 ATAGAGCAGGTTTGAAACACTCTTTTTGTAGTATCTGGAAGTGGATATTTGGATAGCTTTGAGGA

                         *   * *            **                      *    *   
     329416 TTTCGTTGGAAACGGGATTGTCTTCATATAAACTCTAGACAGAAGCATTCTCAGAAGCTTCATTG
         66 TTTCGTTGGAAACAGGAATATCTTCATATAAAAGCTAGACAGAAGCATTCTCAGAAACTTCTTTG

            * *     **   *    *             * *      
     329481 GGTTGTTTCAATTGAAGTCACAGTGTTGAACAGTTCCTTTC
        131 TGATGTTTGCATTCAAGTAACAGTGTTGAACATTCCCTTTC

                 *                                       *       **   * *    
     329522 ATAGAACAGGTTTGAAACACTCTTTTTGTAGTATCTGGAAGTGGACATTTGGAGGGCTCTCAGGA
          1 ATAGAGCAGGTTTGAAACACTCTTTTTGTAGTATCTGGAAGTGGATATTTGGATAGCTTTGAGGA

            * ** *      *             **          ***       * *          *  *
     329587 CTATGGT-GAAAAAGGAAATATCTTCCAATAAAAGCTACTTAGAAGCAATGTCAGAAACTTTTTC
         66 TTTCGTTGGAAACAGG-AATATCTTCATATAAAAGCTAGACAGAAGCATTCTCAGAAACTTCTTT

            *      * ** *   **      * *     *  *     *
     329651 ATGATGTATCTACTCAGCTAACAGAGGTGAACCTTTCCTTTG
        130 GTGATGTTTGCATTCAAGTAACAGTGTTGAACATTCCCTTTC

             *       *                   * *     *             **            
     329693 AGAGAGCAGTTTTGAAACACTCTTTTTGTGGAATCTGCAAGTGGATATTTGTCTAGCTTTGAGGA
          1 ATAGAGCAGGTTTGAAACACTCTTTTTGTAGTATCTGGAAGTGGATATTTGGATAGCTTTGAGGA

                         *   *    *                   *       *   *          
     329758 TTTCGTTGGAAACGGGATTA-CAT-ATA-AAAAGC-AGACAGCAGCATTCCCAGTAACTTCTTTG
         66 TTTCGTTGGAAACAGGAATATCTTCATATAAAAGCTAGACAGAAGCATTCTCAGAAACTTCTTTG

                              *    *                 
     329819 TGATGTTTGCATTCAAGTCACAGAGTTGAACATTCCCTTTC
        131 TGATGTTTGCATTCAAGTAACAGTGTTGAACATTCCCTTTC

                                                   *     *       **     *   *
     329860 ATAGAGCAGGTTTGAAACACTCTTTTTGTAGTATCTGGATGTGGACATTTGGAGCGCTTTCAGGC
          1 ATAGAGCAGGTTTGAAACACTCTTTTTGTAGTATCTGGAAGTGGATATTTGGATAGCTTTGAGGA

            * ** *      *             *** *   *   *                  *   *   
     329925 CTATGGT-GAAAAAGGAAATATCTTCCCCTGAAAACTACACAGAAGCATTCTCAGAATCTTATTT
         66 TTTCGTTGGAAACAGG-AATATCTTCATATAAAAGCTAGACAGAAGCATTCTCAGAAACTTCTTT

                   **  **    *             **  * *   *
     329989 GTGATGTGCGCCCTCAACTAACAGTGTTGAAGCTTTCTTTTG
        130 GTGATGTTTGCATTCAAGTAACAGTGTTGAACATTCCCTTTC

                     *                    **     *   *                       
     330031 ATAGAGCAGTTTTGAAACACTCTTTTTGTAAAATCTGCAAGAGGATATTTGGATAGCTTTGAGGA
          1 ATAGAGCAGGTTTGAAACACTCTTTTTGTAGTATCTGGAAGTGGATATTTGGATAGCTTTGAGGA

                         *   * *            **                      *    *   
     330096 TTTCGTTGGAAACGGGATTGTCTTCATATAAACTCTAGACAGAAGCATTCTCAGAAGCTTCATTG
         66 TTTCGTTGGAAACAGGAATATCTTCATATAAAAGCTAGACAGAAGCATTCTCAGAAACTTCTTTG

            *       **   * *  *             *        
     330161 GGATGTTTCAATTGATGTCACAGTGTTGAACAGTCCCTTTC
        131 TGATGTTTGCATTCAAGTAACAGTGTTGAACATTCCCTTTC

                                                   *     *       **     *    
     330202 ATAGAGCAGGTTTGAAACACTCTTTTTGTAGTATCTGGATGTGGACATTTGGAGCGCTTTCAGGC
          1 ATAGAGCAGGTTTGAAACACTCTTTTTGTAGTATCTGGAAGTGGATATTTGGATAGCTTTGAGG-

            * * *    *                 *** *   *                          *  
     330267 CTATGGTT-TAAA-AGGAAATATCTTCCCCTGAAAACTAGACAGAAGCATTCTCAGAAACTTATT
         65 ATTTCGTTGGAAACAGG-AATATCTTCATATAAAAGCTAGACAGAAGCATTCTCAGAAACTTCTT

                    **  **    *             *      *   *
     330330 TGTGATGTGCGCCCTCAACTAACAGTGTTGTAGCATT-CTTTTG
        129 TGTGATGTTTGCATTCAAGTAACAGTGTTG-AACATTCCCTTTC

                     *                   * *     *             **            
     330373 ATAGAGCAGTTTTGAAACACTCTTTTTGTGGAATCTGCAAGTGGATATTTGTCTAGCTTTGAGGA
          1 ATAGAGCAGGTTTGAAACACTCTTTTTGTAGTATCTGGAAGTGGATATTTGGATAGCTTTGAGGA

                         *   *    *                   *       *              
     330438 TTTCGTTGGAAACGGGATTA-CAT-ATA-AAAAGC-AGACAGCAGCATTCCCAGAAACTTCTTTG
         66 TTTCGTTGGAAACAGGAATATCTTCATATAAAAGCTAGACAGAAGCATTCTCAGAAACTTCTTTG

                 *            *    *                 
     330499 TGATGCTTGCATTCAAGTCACAGAGTTGAACATTCCCTTTC
        131 TGATGTTTGCATTCAAGTAACAGTGTTGAACATTCCCTTTC

                                                   *     *       **     *    
     330540 ATAGAGCAGGTTTGAAACACTCTTTTTGTAGTATCTGGATGTGGACATTTGGAGCGCTTTCAGGC
          1 ATAGAGCAGGTTTGAAACACTCTTTTTGTAGTATCTGGAAGTGGATATTTGGATAGCTTTGAGG-

            * * *        *             *** *   *                          *  
     330605 CTATAG-T-GAAAAAGGAAATATCTTCCCCTGAAAACTAGACAGAAGCATTCTCAGAAACTTATT
         65 ATTTCGTTGGAAACAGG-AATATCTTCATATAAAAGCTAGACAGAAGCATTCTCAGAAACTTCTT

                    **  **    *             **  * *   *
     330668 TGTGATGTGCGCCCTCAACTAACAGTGTTGAAACTTTCTTTTG
        129 TGTGATGTTTGCATTCAAGTAACAGTGTTGAACATTCCCTTTC

                     *                    *      *   *                       
     330711 ATAGAGCAGTTTTGAAACACTCTTTTTGTAATATCTGCAAGAGGATATTTGGATAGCTTTGAGGA
          1 ATAGAGCAGGTTTGAAACACTCTTTTTGTAGTATCTGGAAGTGGATATTTGGATAGCTTTGAGGA

                         *   * *             *                      *    *   
     330776 TTTCGTTGGAAACGGGATTGTCTTCATATAAAATCTAGACAGAAGCATTCTCAGAAGCTTCATTG
         66 TTTCGTTGGAAACAGGAATATCTTCATATAAAAGCTAGACAGAAGCATTCTCAGAAACTTCTTTG

            *       **   *    *             *        
     330841 GGATGTTTCAATTGAAGTCACAGTGTTGAACAGTCCCTTTC
        131 TGATGTTTGCATTCAAGTAACAGTGTTGAACATTCCCTTTC

                                                         *       *  * * *    
     330882 ATAGAGCAGGTTTGAAACACTCTTTTTGTAGTATCTGGAAGTGGACATTTGGAGAGATCTCAGGA
          1 ATAGAGCAGGTTTGAAACACTCTTTTTGTAGTATCTGGAAGTGGATATTTGGATAGCTTTGAGGA

            * *  *    * *             **            *       * *          *  *
     330947 ATACGGT-GATAAAGGAAATATCTTCCAATAAAAGCTAGATAGAAGCAATGTCAGAAACTTTTTC
         66 TTTCGTTGGAAACAGG-AATATCTTCATATAAAAGCTAGACAGAAGCATTCTCAGAAACTTCTTT

            *      * ** *   **      *       *  * *   *
     331011 ATGATGTATCTACTCAGCTAACAGAGTTGAACCTTTCTTTTG
        130 GTGATGTTTGCATTCAAGTAACAGTGTTGAACATTCCCTTTC

             *       *                   * *     *             **            
     331053 AGAGAGCAGTTTTGAAACACTCTTTTTGTGGAATCTGCAAGTGGATATTTGTCTAGCTTTGAGGA
          1 ATAGAGCAGGTTTGAAACACTCTTTTTGTAGTATCTGGAAGTGGATATTTGGATAGCTTTGAGGA

                         *   *    *                   *       * *            
     331118 TTTCGTTGGAAACGGGATTA-CAT-ATA-AAAAGC-AGACAGCAGCATTCCCCGAAACTTCTTTG
         66 TTTCGTTGGAAACAGGAATATCTTCATATAAAAGCTAGACAGAAGCATTCTCAGAAACTTCTTTG

              *               *    **                
     331179 TGTTGTTTGCATTCAAGTCACAGATTTGAACATTCCCTTTC
        131 TGATGTTTGCATTCAAGTAACAGTGTTGAACATTCCCTTTC

                                                   *     *    *  **         *
     331220 ATAGAGCAGGTTTGAAACACTCTTTTTGTAGTATCTGGATGTGGACATTTTGAGCGCTTTGAGGC
          1 ATAGAGCAGGTTTGAAACACTCTTTTTGTAGTATCTGGAAGTGGATATTTGGATAGCTTTGAGGA

            * ** *      *       *     *** *   *           *          *   *   
     331285 CTATGGT-GAAAAAGGAAATCTCTTCCCCTGAAAACTAGACAGAAGAATTCTCAGAATCTTATTT
         66 TTTCGTTGGAAACAGG-AATATCTTCATATAAAAGCTAGACAGAAGCATTCTCAGAAACTTCTTT

                   **  **    *             **  * *   *
     331349 GTGATGTGCGCCCTCAACTAACAGTGTTGAAGCTTTCTTTTG
        130 GTGATGTTTGCATTCAAGTAACAGTGTTGAACATTCCCTTTC

                     *                   ***     *   *                       
     331391 ATAGAGCAGTTTTGAAACACTCTTTTTGTTAAATCTGCAAGAGGATATTTGGATAGCTTTGAGGA
          1 ATAGAGCAGGTTTGAAACACTCTTTTTGTAGTATCTGGAAGTGGATATTTGGATAGCTTTGAGGA

                         *   * *            **                      * *  *   
     331456 TTTCGTTGGAAACGGGATTGTCTTCATATAAACTCTAGACAGAAGCATTCTCAGAAGCGTCATTG
         66 TTTCGTTGGAAACAGGAATATCTTCATATAAAAGCTAGACAGAAGCATTCTCAGAAACTTCTTTG

            *       **   *    *             *        
     331521 GGATGTTTCAATTGAAGTCACAGTGTTGAACAGTCCCTTTC
        131 TGATGTTTGCATTCAAGTAACAGTGTTGAACATTCCCTTTC

                                                   *     *       **     *    
     331562 ATAGAGCAGGTTTGAAACACTCTTTTTGTAGTATCTGGATGTGGACATTTGGAGCGCTTTCAGGC
          1 ATAGAGCAGGTTTGAAACACTCTTTTTGTAGTATCTGGAAGTGGATATTTGGATAGCTTTGAGG-

            * * *    *                 *** *   *                          *  
     331627 CTATGGTT-TAAA-AGGAAATATCTTCCCCTGAAAACTAGACAGAAGCATTCTCAGAAACTTATT
         65 ATTTCGTTGGAAACAGG-AATATCTTCATATAAAAGCTAGACAGAAGCATTCTCAGAAACTTCTT

                    **  **    *                    *   *
     331690 TGTGATGTGCGCCCTCAACTAACAGTGTTGAAGCATT-CTTTTG
        129 TGTGATGTTTGCATTCAAGTAACAGTGTTGAA-CATTCCCTTTC

                     *                   * *     *             **            
     331733 ATAGAGCAGTTTTGAAACACTCTTTTTGTGGAATCTGCAAGTGGATATTTGTCTAGCTTTGAGGA
          1 ATAGAGCAGGTTTGAAACACTCTTTTTGTAGTATCTGGAAGTGGATATTTGGATAGCTTTGAGGA

                         *   *    *                   *       *              
     331798 TTTCGTTGGAAACGGGATTA-CAT-ATA-AAAAGC-AGACAGCAGCATTCCCAGAAACTTCTTTG
         66 TTTCGTTGGAAACAGGAATATCTTCATATAAAAGCTAGACAGAAGCATTCTCAGAAACTTCTTTG

                              *    *                 
     331859 TGATGTTTGCATTCAAGTCACAGAGTTGAACATTCCCTTTC
        131 TGATGTTTGCATTCAAGTAACAGTGTTGAACATTCCCTTTC

                                                   *     *       **     *   *
     331900 ATAGAGCAGGTTTGAAACACTCTTTTTGTAGTATCTGGATGTGGACATTTGGAGCGCTTTCAGGC
          1 ATAGAGCAGGTTTGAAACACTCTTTTTGTAGTATCTGGAAGTGGATATTTGGATAGCTTTGAGGA

            * ** *      *             *** *   *                      *   *   
     331965 CTATGGT-GAAAAAGGAAATATCTTCCCCTGAAAACTAGACAGAAGCATTCTCAGAATCTTATTT
         66 TTTCGTTGGAAACAGG-AATATCTTCATATAAAAGCTAGACAGAAGCATTCTCAGAAACTTCTTT

                   **  **    *             **  * *   *
     332029 GTGATGTGCGCCCTCAACTAACAGTGTTGAAGCTTTCTTTTG
        130 GTGATGTTTGCATTCAAGTAACAGTGTTGAACATTCCCTTTC

                     *          *         **     *  **                       
     332071 ATAGAGCAGTTTTGAAACACCCTTTTTGTAAAATCTGCAACAGGATATTTGGATAGCTTTGAGGA
          1 ATAGAGCAGGTTTGAAACACTCTTTTTGTAGTATCTGGAAGTGGATATTTGGATAGCTTTGAGGA

                         *   * *            **                      *    *   
     332136 TTTCGTTGGAAACGGGATTGTCTTCATATAAACTCTAGACAGAAGCATTCTCAGAAGCTTCATTG
         66 TTTCGTTGGAAACAGGAATATCTTCATATAAAAGCTAGACAGAAGCATTCTCAGAAACTTCTTTG

            *       **   *    *             *        
     332201 GGATGTTTCAATTGAAGTCACAGTGTTGAACAGTCCCTTTC
        131 TGATGTTTGCATTCAAGTAACAGTGTTGAACATTCCCTTTC

                                                         *       **  ** **   
     332242 ATAGAGCAGGTTTGAAACACTCTTTTTGTAGTATCTGGAAGTGGACATTTGGAGCGCACTCGGGA
          1 ATAGAGCAGGTTTGAAACACTCTTTTTGTAGTATCTGGAAGTGGATATTTGGATAGCTTTGAGGA

            * *  *      *             **            *       * *          *  *
     332307 CTGCGGT-GAAAAAGGAAATATCTTCCAATAAAAGCTAGATAGAAGCAATGTCAGAAACTTTTTC
         66 TTTCGTTGGAAACAGG-AATATCTTCATATAAAAGCTAGACAGAAGCATTCTCAGAAACTTCTTT

            *      * ** *   **      *       *    *   *
     332371 ATGATGTATCTACTCAGCTAACAGAGTTGAACCTT-CATTTG
        130 GTGATGTTTGCATTCAAGTAACAGTGTTGAACATTCCCTTTC

             *       *            *      * *     *             **            
     332412 AGAGAGCAGTTTTGAAACACTCGTTTTGTGGAATCTGCAAGTGGATATTTGTCTAGCTTTGAGGA
          1 ATAGAGCAGGTTTGAAACACTCTTTTTGTAGTATCTGGAAGTGGATATTTGGATAGCTTTGAGGA

               *         *   *    *                   *       *              
     332477 TTTTGTTGGAAACGGGATTA-CAT-ATA-AAAAGC-AGACAGCAGCATTCCCAGAAACTTCTTTG
         66 TTTCGTTGGAAACAGGAATATCTTCATATAAAAGCTAGACAGAAGCATTCTCAGAAACTTCTTTG

                              *    *                 
     332538 TGATGTTTGCATTCAAGTCACAGAGTTGAACATTCCCTTTC
        131 TGATGTTTGCATTCAAGTAACAGTGTTGAACATTCCCTTTC

                                                   *     *     * **     *   *
     332579 ATAGAGCAGGTTTGAAACACTCTTTTTGTAGTATCTGGATGTGGACATTTGCAGCGCTTTCAGGC
          1 ATAGAGCAGGTTTGAAACACTCTTTTTGTAGTATCTGGAAGTGGATATTTGGATAGCTTTGAGGA

            * ** *      *             *** *   *                          *   
     332644 CTAAGGT-GAAAAAGGAAATATCTTCCCCTGAAAACTAGACAGAAGCATTCTCAGAAACTTATTT
         66 TTTCGTTGGAAACAGG-AATATCTTCATATAAAAGCTAGACAGAAGCATTCTCAGAAACTTCTTT

                   **  **    *             **  * *   *
     332708 GTGATGTGCGCCCTCAACTAACAGTGTTGAAGCTTTCTTTTG
        130 GTGATGTTTGCATTCAAGTAACAGTGTTGAACATTCCCTTTC

                     *                    *      *   *                       
     332750 ATAGAGCAGTTTTGAAACACTCTTTTTGTAATATCTGCAAGAGGATATTTGGATAGCTTTGAGGA
          1 ATAGAGCAGGTTTGAAACACTCTTTTTGTAGTATCTGGAAGTGGATATTTGGATAGCTTTGAGGA

                         *   * *            **                      *    *   
     332815 TTTCGTTGGAAACGGGATTGTCTTCATATAAACTCTAGACAGAAGCATTCTCAGAAGCTTCATTG
         66 TTTCGTTGGAAACAGGAATATCTTCATATAAAAGCTAGACAGAAGCATTCTCAGAAACTTCTTTG

            *       **   *    *             *        
     332880 GGATGTTTCAATTGAAGTCACAGTGTTGAACAGTCCCTTTC
        131 TGATGTTTGCATTCAAGTAACAGTGTTGAACATTCCCTTTC

                 *                                       *       **   * *    
     332921 ATAGAACAGGTTTGAAACACTCTTTTTGTAGTATCTGGAAGTGGACATTTGGAGCGCTCTCAGGA
          1 ATAGAGCAGGTTTGAAACACTCTTTTTGTAGTATCTGGAAGTGGATATTTGGATAGCTTTGAGGA

            * ** *      *             **          * *       * *          *  *
     332986 CTATGGT-GAAAAAGGAAATATCTTCCAATAAAAGCTACATAGAAGCAATGTCAGAAACTTTTTC
         66 TTTCGTTGGAAACAGG-AATATCTTCATATAAAAGCTAGACAGAAGCATTCTCAGAAACTTCTTT

            *      * ** *   **      *       *  *     *
     333050 ATGATGTATCTACTCAGCTAACAGAGTTGAACCTTTCCTTTG
        130 GTGATGTTTGCATTCAAGTAACAGTGTTGAACATTCCCTTTC

             *       *                   * *     *             **            
     333092 AGAGAGCAGTTTTGAAACACTCTTTTTGTGGAATCTGCAAGTGGATATTTGTCTAGCTTTGAGGA
          1 ATAGAGCAGGTTTGAAACACTCTTTTTGTAGTATCTGGAAGTGGATATTTGGATAGCTTTGAGGA

                         *   *    *                   *       *   *          
     333157 TTTCGTTGGAAACGGGATTA-CAT-ATA-AAAAGC-AGACAGCAGCATTCCCAGTAACTTCTTTG
         66 TTTCGTTGGAAACAGGAATATCTTCATATAAAAGCTAGACAGAAGCATTCTCAGAAACTTCTTTG

                              *    *                 
     333218 TGATGTTTGCATTCAAGTCACAGAGTTGAACATTCCCTTTC
        131 TGATGTTTGCATTCAAGTAACAGTGTTGAACATTCCCTTTC

                                                   *     *       **     *   *
     333259 ATAGAGCAGGTTTGAAACACTCTTTTTGTAGTATCTGGATGTGGACATTTGGAGCGCTTTCAGGC
          1 ATAGAGCAGGTTTGAAACACTCTTTTTGTAGTATCTGGAAGTGGATATTTGGATAGCTTTGAGGA

            * ** *      *             *** *   *            *         *   *   
     333324 CTATGGT-GAAAAAGGAAATATCTTCTCCTGAAAACTAGACAGAAGCTTTCTCAGAATCTTATTT
         66 TTTCGTTGGAAACAGG-AATATCTTCATATAAAAGCTAGACAGAAGCATTCTCAGAAACTTCTTT

                   **  **    *             **  * *   *
     333388 GTGATGTGCGCCCTCAACTAACAGTGTTGAAGCTTTCTTTTG
        130 GTGATGTTTGCATTCAAGTAACAGTGTTGAACATTCCCTTTC

                     *                    *      *   *                       
     333430 ATAGAGCAGTTTTGAAACACTCTTTTTGTAATATCTGCAAGAGGATATTTGGATAGCTTTGAGGA
          1 ATAGAGCAGGTTTGAAACACTCTTTTTGTAGTATCTGGAAGTGGATATTTGGATAGCTTTGAGGA

                         *   * *            **                      *    *   
     333495 TTTCGTTGGAAACGGGATTGTCTTCATATAAACTCTAGACAGAAGCATTCTCAGAAGCTTCATTG
         66 TTTCGTTGGAAACAGGAATATCTTCATATAAAAGCTAGACAGAAGCATTCTCAGAAACTTCTTTG

            *       **   *    *             *        
     333560 AGATGTTTCAATTGAAGTCACAGTGTTGAACAGTCCCTTTC
        131 TGATGTTTGCATTCAAGTAACAGTGTTGAACATTCCCTTTC

                                                         *       *  * * *    
     333601 ATAGAGCAGGTTTGAAACACTCTTTTTGTAGTATCTGGAAGTGGACATTTGGAGAGATCTCAGGA
          1 ATAGAGCAGGTTTGAAACACTCTTTTTGTAGTATCTGGAAGTGGATATTTGGATAGCTTTGAGGA

            * *  *    * *             **            *       * *          *  *
     333666 ATACGGT-GATAAAGGAAATATCTTCCAATAAAAGCTAGATAGAAGCAATGTCAGAAACTTTTTC
         66 TTTCGTTGGAAACAGG-AATATCTTCATATAAAAGCTAGACAGAAGCATTCTCAGAAACTTCTTT

            *      * ** *   **      *       *  * *   *
     333730 ATGATGTATCTACTCAGCTAACAGAGTTGAACCTTTCTTTTG
        130 GTGATGTTTGCATTCAAGTAACAGTGTTGAACATTCCCTTTC

             *       *                   * *     *             **            
     333772 AGAGAGCAGTTTTGAAACACTCTTTTTGTGGAATCTGCAAGTGGATATTTGTCTAGCTTTGAGGA
          1 ATAGAGCAGGTTTGAAACACTCTTTTTGTAGTATCTGGAAGTGGATATTTGGATAGCTTTGAGGA

                         *   *    *                   *       *   *          
     333837 TTTCGTTGGAAACGGGATTA-CAT-ATA-AAAAGC-AGACAGCAGCATTCCCAGTAACTTCTTTG
         66 TTTCGTTGGAAACAGGAATATCTTCATATAAAAGCTAGACAGAAGCATTCTCAGAAACTTCTTTG

                              *    *                 
     333898 TGATGTTTGCATTCAAGTCACAGAGTTGAACATTCCCTTTC
        131 TGATGTTTGCATTCAAGTAACAGTGTTGAACATTCCCTTTC

               *                                   *     *       **     *   *
     333939 ATACAGCAGGTTTGAAACACTCTTTTTGTAGTATCTGGATGTGGACATTTGGAGCGCTTTCAGGC
          1 ATAGAGCAGGTTTGAAACACTCTTTTTGTAGTATCTGGAAGTGGATATTTGGATAGCTTTGAGGA

            * ** *      *             *** *   *           *     *    *   *   
     334004 CTATGGT-GAAAAAGGAAATATCTTCCCCTGAAAACTAGACAGAAGAATTCTTAGAATCTTATTT
         66 TTTCGTTGGAAACAGG-AATATCTTCATATAAAAGCTAGACAGAAGCATTCTCAGAAACTTCTTT

                   **  **    *             **  * *   *
     334068 GTGATGTGCGCCCTCAACTAACAGTGTTGAAGCTTTCTTTTG
        130 GTGATGTTTGCATTCAAGTAACAGTGTTGAACATTCCCTTTC

                     *                    **     *   *                       
     334110 ATAGAGCAGTTTTGAAACACTCTTTTTGTAAAATCTGCAAGAGGATATTTGGATAGCTTTGAGGA
          1 ATAGAGCAGGTTTGAAACACTCTTTTTGTAGTATCTGGAAGTGGATATTTGGATAGCTTTGAGGA

                         *   * *            **                      * *  *   
     334175 TTTCGTTGGAAACGGGATTGTCTTCATATAAACTCTAGACAGAAGCATTCTCAGAAGCGTCATTG
         66 TTTCGTTGGAAACAGGAATATCTTCATATAAAAGCTAGACAGAAGCATTCTCAGAAACTTCTTTG

            *       **   *    *             *        
     334240 GGATGTTTCAATTGAAGTCACAGTGTTGAACAGTCCCTTTC
        131 TGATGTTTGCATTCAAGTAACAGTGTTGAACATTCCCTTTC

                 *                                       *       **   * *    
     334281 ATAGAACAGGTTTGAAACACTCTTTTTGTAGTATCTGGAAGTGGACATTTGGAGCGCTCTCAGGA
          1 ATAGAGCAGGTTTGAAACACTCTTTTTGTAGTATCTGGAAGTGGATATTTGGATAGCTTTGAGGA

            * ** *      *             **          * *       * *          *  *
     334346 CTATGGT-GAAAAAGGAAATATCTTCCAATAAAAGCTACATAGAAGCAATGTCAGAAACTTTTTC
         66 TTTCGTTGGAAACAGG-AATATCTTCATATAAAAGCTAGACAGAAGCATTCTCAGAAACTTCTTT

            *      * ** *   **      *       *  *     *
     334410 ATGATGTATCTACTCAGCTAACAGAGTTGAACCTTTCCTTTG
        130 GTGATGTTTGCATTCAAGTAACAGTGTTGAACATTCCCTTTC

             *       *                   * *     *             **            
     334452 AGAGAGCAGTTTTGAAACACTCTTTTTGTGGAATCTGCAAGTGGATATTTGTCTAGCTTTGAGGA
          1 ATAGAGCAGGTTTGAAACACTCTTTTTGTAGTATCTGGAAGTGGATATTTGGATAGCTTTGAGGA

                         *   *    *                   *       *   *          
     334517 TTTCGTTGGAAACGGGATTA-CAT-ATA-AAAAGC-AGACAGCAGCATTCCCAGTAACTTCTTTG
         66 TTTCGTTGGAAACAGGAATATCTTCATATAAAAGCTAGACAGAAGCATTCTCAGAAACTTCTTTG

                              *    *                 
     334578 TGATGTTTGCATTCAAGTCACAGAGTTGAACATTCCCTTTC
        131 TGATGTTTGCATTCAAGTAACAGTGTTGAACATTCCCTTTC

                                                   *     *    *  **     *   *
     334619 ATAGAGCAGGTTTGAAACACTCTTTTTGTAGTATCTGGATGTGGACATTTAGAGCGCTTTCAGGC
          1 ATAGAGCAGGTTTGAAACACTCTTTTTGTAGTATCTGGAAGTGGATATTTGGATAGCTTTGAGGA

            * ** *      *             *** *   *            *         *   *   
     334684 CTATGGT-GAAAAAGGAAATATCTTCTCCTGAAAACTAGACAGAAGCTTTCTCAGAATCTTATTT
         66 TTTCGTTGGAAACAGG-AATATCTTCATATAAAAGCTAGACAGAAGCATTCTCAGAAACTTCTTT

                   **  **    *             **  * *   *
     334748 GTGATGTGCGCCCTCAACTAACAGTGTTGAAGCTTTCTTTTG
        130 GTGATGTTTGCATTCAAGTAACAGTGTTGAACATTCCCTTTC

                     *                    *      *   *                       
     334790 ATAGAGCAGTTTTGAAACACTCTTTTTGTAATATCTGCAAGAGGATATTTGGATAGCTTTGAGGA
          1 ATAGAGCAGGTTTGAAACACTCTTTTTGTAGTATCTGGAAGTGGATATTTGGATAGCTTTGAGGA

                         *   * *            **                      *    *   
     334855 TTTCGTTGGAAACGGGATTGTCTTCATATAAACTCTAGACAGAAGCATTCTCAGAAGCTTCATTG
         66 TTTCGTTGGAAACAGGAATATCTTCATATAAAAGCTAGACAGAAGCATTCTCAGAAACTTCTTTG

            *       **   *    *             *        
     334920 GGATGTTTCAATTGAAGTCACAGTGTTGAACAGTCCCTTTC
        131 TGATGTTTGCATTCAAGTAACAGTGTTGAACATTCCCTTTC

                                                         *       *  * * *    
     334961 ATAGAGCAGGTTTGAAACACTCTTTTTGTAGTATCTGGAAGTGGACATTTGGAGAGATCTCAGGA
          1 ATAGAGCAGGTTTGAAACACTCTTTTTGTAGTATCTGGAAGTGGATATTTGGATAGCTTTGAGGA

            * *  *    * *             **            *       * *          *  *
     335026 ATACGGT-GATAAAGGAAATATCTTCCAATAAAAGCTAGATAGAAGCAATGTCAGAAACTTTTTC
         66 TTTCGTTGGAAACAGG-AATATCTTCATATAAAAGCTAGACAGAAGCATTCTCAGAAACTTCTTT

            *      * ** *   **      *       *  * *   *
     335090 ATGATGTATCTACTCAGCTAACAGAGTTGAACCTTTCTTTTG
        130 GTGATGTTTGCATTCAAGTAACAGTGTTGAACATTCCCTTTC

             *       *                   * *     *             **            
     335132 AGAGAGCAGTTTTGAAACACTCTTTTTGTGGAATCTGCAAGTGGATATTTGTCTAGCTTTGAGGA
          1 ATAGAGCAGGTTTGAAACACTCTTTTTGTAGTATCTGGAAGTGGATATTTGGATAGCTTTGAGGA

                         *   *    *                   *       *   *          
     335197 TTTCGTTGGAAACGGGATTA-CAT-ATA-AAAAGC-AGACAGCAGCATTCCCAGTAACTTCTTTG
         66 TTTCGTTGGAAACAGGAATATCTTCATATAAAAGCTAGACAGAAGCATTCTCAGAAACTTCTTTG

                              *    *                 
     335258 TGATGTTTGCATTCAAGTCACAGAGTTGAACATTCCCTTTC
        131 TGATGTTTGCATTCAAGTAACAGTGTTGAACATTCCCTTTC

               *                                   *     *       **     *   *
     335299 ATACAGCAGGTTTGAAACACTCTTTTTGTAGTATCTGGATGTGGACATTTGGAGCGCTTTCAGGC
          1 ATAGAGCAGGTTTGAAACACTCTTTTTGTAGTATCTGGAAGTGGATATTTGGATAGCTTTGAGGA

            * ** *      *             *** *   *           *     *    *   *   
     335364 CTATGGT-GAAAAAGGAAATATCTTCCCCTGAAAACTAGACAGAAGAATTCTTAGAATCTTATTT
         66 TTTCGTTGGAAACAGG-AATATCTTCATATAAAAGCTAGACAGAAGCATTCTCAGAAACTTCTTT

                   **  **    *             **  * *   *
     335428 GTGATGTGCGCCCTCAACTAACAGTGTTGAAGCTTTCTTTTG
        130 GTGATGTTTGCATTCAAGTAACAGTGTTGAACATTCCCTTTC

                     *                    **     *   *                       
     335470 ATAGAGCAGTTTTGAAACACTCTTTTTGTAAAATCTGCAAGAGGATATTTGGATAGCTTTGAGGA
          1 ATAGAGCAGGTTTGAAACACTCTTTTTGTAGTATCTGGAAGTGGATATTTGGATAGCTTTGAGGA

                         *   * *            **                      * *  *   
     335535 TTTCGTTGGAAACGGGATTGTCTTCATATAAACTCTAGACAGAAGCATTCTCAGAAGCGTCATTG
         66 TTTCGTTGGAAACAGGAATATCTTCATATAAAAGCTAGACAGAAGCATTCTCAGAAACTTCTTTG

            *       **   *    *             *        
     335600 GGATGTTTCAATTGAAGTCACAGTGTTGAACAGTCCCTTTC
        131 TGATGTTTGCATTCAAGTAACAGTGTTGAACATTCCCTTTC

                                                   *     *       **     *  **
     335641 ATAGAGCAGGTTTGAAACACTCTTTTTGTAGTATCTGGATGTGGACATTTGGAGCGCTTTAAGCC
          1 ATAGAGCAGGTTTGAAACACTCTTTTTGTAGTATCTGGAAGTGGATATTTGGATAGCTTTGAGGA

             * *    *                 *** *   *                          *   
     335706 TATGGTT-TAAA-AGGAAATATCTTCCCCTGAAAACTAGACAGAAGCATTCTCAGAAACTTATTT
         66 TTTCGTTGGAAACAGG-AATATCTTCATATAAAAGCTAGACAGAAGCATTCTCAGAAACTTCTTT

                   **  **    *                    *   *
     335769 GTGATGTGCGCCCTCAACTAACAGTGTTGAAGCATT-CTTTTG
        130 GTGATGTTTGCATTCAAGTAACAGTGTTGAA-CATTCCCTTTC

                     *                   * *     *             **            
     335811 ATAGAGCAGTTTTGAAACACTCTTTTTGTGGAATCTGCAAGTGGATATTTGTCTAGCTTTGAGGA
          1 ATAGAGCAGGTTTGAAACACTCTTTTTGTAGTATCTGGAAGTGGATATTTGGATAGCTTTGAGGA

                         *   *    *                   *       *              
     335876 TTTCGTTGGAAACGGGATTA-CAT-ATA-AAAAGC-AGACAGCAGCATTCCCAGAAACTTCTTTG
         66 TTTCGTTGGAAACAGGAATATCTTCATATAAAAGCTAGACAGAAGCATTCTCAGAAACTTCTTTG

                              *    *                 
     335937 TGATGTTTGCATTCAAGTCACAGAGTTGAACATTCCCTTTC
        131 TGATGTTTGCATTCAAGTAACAGTGTTGAACATTCCCTTTC

                                                   *     *       **     *   *
     335978 ATAGAGCAGGTTTGAAACACTCTTTTTGTAGTATCTGGATGTGGACATTTGGAGCGCTTTCAGGC
          1 ATAGAGCAGGTTTGAAACACTCTTTTTGTAGTATCTGGAAGTGGATATTTGGATAGCTTTGAGGA

            * ** *      *             *** *   *                      *   *   
     336043 CTATGGT-GAAAAAGGAAATATCTTCCCCTGAAAACTAGACAGAAGCATTCTCAGAATCTTATTT
         66 TTTCGTTGGAAACAGG-AATATCTTCATATAAAAGCTAGACAGAAGCATTCTCAGAAACTTCTTT

                   **  **    **            **  * *   *
     336107 GTGATGTGCGCCCTCAACGAACAGTGTTGAAGCTTTCTTTTG
        130 GTGATGTTTGCATTCAAGTAACAGTGTTGAACATTCCCTTTC

             *       *                    *      *   *                       
     336149 AAAGAGCAGTTTTGAAACACTCTTTTTGTAATATCTGCAAGAGGATATTTGGATAGCTTTGAGGA
          1 ATAGAGCAGGTTTGAAACACTCTTTTTGTAGTATCTGGAAGTGGATATTTGGATAGCTTTGAGGA

                         *   * *            **                      *    *   
     336214 TTTCGTTGGAAACGGGATTGTCTTCATATAAACTCTAGACAGAAGCATTCTCAGAAGCTTCATTG
         66 TTTCGTTGGAAACAGGAATATCTTCATATAAAAGCTAGACAGAAGCATTCTCAGAAACTTCTTTG

            *       **   *    *             *        
     336279 GGATGTTTCAATTGAAGTCACAGTGTTGAACAGTCCCTTTC
        131 TGATGTTTGCATTCAAGTAACAGTGTTGAACATTCCCTTTC

                                                         *       **   * *  * 
     336320 ATAGAGCAGGTTTGAAACACTCTTTTTGTAGTATCTGGAAGTGGACATTTGGAGCGCTCTCAGTA
          1 ATAGAGCAGGTTTGAAACACTCTTTTTGTAGTATCTGGAAGTGGATATTTGGATAGCTTTGAGGA

            * *  *      *             **            *       * *          *  *
     336385 CTGCGGT-GAAAAAGGAAATATCTTCCAATAAAAGCTAGATAGAAGCAATGTCAGAAACTTTTTC
         66 TTTCGTTGGAAACAGG-AATATCTTCATATAAAAGCTAGACAGAAGCATTCTCAGAAACTTCTTT

            *      * ** *   **      *       *        *
     336449 ATGATGTATCTACTCAGCTAACAGAGTTGAACCTT-CCTTTG
        130 GTGATGTTTGCATTCAAGTAACAGTGTTGAACATTCCCTTTC

             *       *                   * *     *             **            
     336490 AGAGAGCAGTTTTGAAACACTCTTTTTGTGGAATCTGCAAGTGGATATTTGTCTAGCTTTGAGGA
          1 ATAGAGCAGGTTTGAAACACTCTTTTTGTAGTATCTGGAAGTGGATATTTGGATAGCTTTGAGGA

                         *   *    *                   *       *              
     336555 TTTCGTTGGAAACGGGATTA-CAT-ATA-AAAAGC-AGACAGCAGCATTCCCAGAAACTTCTTTG
         66 TTTCGTTGGAAACAGGAATATCTTCATATAAAAGCTAGACAGAAGCATTCTCAGAAACTTCTTTG

                              *    *                 
     336616 TGATGTTTGCATTCAAGTCACAGAGTTGAACATTCCCTTTC
        131 TGATGTTTGCATTCAAGTAACAGTGTTGAACATTCCCTTTC

                                                   *     *     * **     *   *
     336657 ATAGAGCAGGTTTGAAACACTCTTTTTGTAGTATCTGGATGTGGACATTTGCAGCGCTTTCAGGC
          1 ATAGAGCAGGTTTGAAACACTCTTTTTGTAGTATCTGGAAGTGGATATTTGGATAGCTTTGAGGA

            * ** *      *             *** *   *                      *   *   
     336722 CTAAGGT-GAAAAAGGAAATATCTTCCCCTGAAAACTAGACAGAAGCATTCTCAGAATCTTATTT
         66 TTTCGTTGGAAACAGG-AATATCTTCATATAAAAGCTAGACAGAAGCATTCTCAGAAACTTCTTT

                   **  **    *             **  * *   *
     336786 GTGATGTGCGCCCTCAACTAACAGTGTTGAAGCTTTCTTTTG
        130 GTGATGTTTGCATTCAAGTAACAGTGTTGAACATTCCCTTTC

                     *                    *      *   *                       
     336828 ATAGAGCAGTTTTGAAACACTCTTTTTGTAATATCTGCAAGAGGATATTTGGATAGCTTTGAGGA
          1 ATAGAGCAGGTTTGAAACACTCTTTTTGTAGTATCTGGAAGTGGATATTTGGATAGCTTTGAGGA

                         *   * *            **                      *    *   
     336893 TTTCGTTGGAAACGGGATTGTCTTCATATAAACTCTAGACAGAAGCATTCTCAGAAGCTTCATTG
         66 TTTCGTTGGAAACAGGAATATCTTCATATAAAAGCTAGACAGAAGCATTCTCAGAAACTTCTTTG

            *       **   *    *             *        
     336958 GGATGTTTCAATTGAAGTCACAGTGTTGAACAGTCCCTTTC
        131 TGATGTTTGCATTCAAGTAACAGTGTTGAACATTCCCTTTC

                 *                                       *       **   * *    
     336999 ATAGAACAGGTTTGAAACACTCTTTTTGTAGTATCTGGAAGTGGACATTTGGAGCGCTCTCAGGA
          1 ATAGAGCAGGTTTGAAACACTCTTTTTGTAGTATCTGGAAGTGGATATTTGGATAGCTTTGAGGA

            * ** *      *             **          * *       * *          *  *
     337064 CTATGGT-GAAAAAGGAAATATCTTCCAATAAAAGCTACATAGAAGCAATGTCAGAAACTTTTTC
         66 TTTCGTTGGAAACAGG-AATATCTTCATATAAAAGCTAGACAGAAGCATTCTCAGAAACTTCTTT

            *      * ** *   **      *       *  *     *
     337128 ATGATGTATCTACTCAGCTAACAGAGTTGAACCTTTCCTTTG
        130 GTGATGTTTGCATTCAAGTAACAGTGTTGAACATTCCCTTTC

             *       *                   * *     *             **            
     337170 AGAGAGCAGTTTTGAAACACTCTTTTTGTGGAATCTGCAAGTGGATATTTGTCTAGCTTTGAGGA
          1 ATAGAGCAGGTTTGAAACACTCTTTTTGTAGTATCTGGAAGTGGATATTTGGATAGCTTTGAGGA

                         *   *    *                   *       *   *          
     337235 TTTCGTTGGAAACGGGATTA-CAT-ATA-AAAAGC-AGACAGCAGCATTCCCAGTAACTTCTTTG
         66 TTTCGTTGGAAACAGGAATATCTTCATATAAAAGCTAGACAGAAGCATTCTCAGAAACTTCTTTG

                              *    *                 
     337296 TGATGTTTGCATTCAAGTCACAGAGTTGAACATTCCCTTTC
        131 TGATGTTTGCATTCAAGTAACAGTGTTGAACATTCCCTTTC

                                                   *     *       **     *   *
     337337 ATAGAGCAGGTTTGAAACACTCTTTTTGTAGTATCTGGATGTGGACATTTGGAGCGCTTTCAGGC
          1 ATAGAGCAGGTTTGAAACACTCTTTTTGTAGTATCTGGAAGTGGATATTTGGATAGCTTTGAGGA

            * ** *      *             *** *   *                      *   *   
     337402 CTATGGT-GAAAAAGGAAATATCTTCTCCTGAAAACTAGACAGAAGCATTCTCAGAATCTTATTT
         66 TTTCGTTGGAAACAGG-AATATCTTCATATAAAAGCTAGACAGAAGCATTCTCAGAAACTTCTTT

                   **  **    *             **  * *   *
     337466 GTGATGTGCGCCCTCAACTAACAGTGTTGAAGCTTTCTTTTG
        130 GTGATGTTTGCATTCAAGTAACAGTGTTGAACATTCCCTTTC

                     *                    **     *   *                       
     337508 ATAGAGCAGTTTTGAAACACTCTTTTTGTAAAATCTGCAAGAGGATATTTGGATAGCTTTGAGGA
          1 ATAGAGCAGGTTTGAAACACTCTTTTTGTAGTATCTGGAAGTGGATATTTGGATAGCTTTGAGGA

                         *   * *            **                      * *  *   
     337573 TTTCGTTGGAAACGGGATTGTCTTCATATAAACTCTAGACAGAAGCATTCTCAGAAGCGTCATTG
         66 TTTCGTTGGAAACAGGAATATCTTCATATAAAAGCTAGACAGAAGCATTCTCAGAAACTTCTTTG

            *       **   *    *             *        
     337638 GGATGTTTCAATTGAAGTCACAGTGTTGAACAGTCCCTTTC
        131 TGATGTTTGCATTCAAGTAACAGTGTTGAACATTCCCTTTC

                 *                                       *       **   * *    
     337679 ATAGAACAGGTTTGAAACACTCTTTTTGTAGTATCTGGAAGTGGACATTTGGAGCGCTCTCAGGA
          1 ATAGAGCAGGTTTGAAACACTCTTTTTGTAGTATCTGGAAGTGGATATTTGGATAGCTTTGAGGA

            * ** *      *             **          * *       * *          *  *
     337744 CTATGGT-GAAAAAGGAAATATCTTCCAATAAAAGCTACATAGAAGCAATGTCAGAAACTTTTTC
         66 TTTCGTTGGAAACAGG-AATATCTTCATATAAAAGCTAGACAGAAGCATTCTCAGAAACTTCTTT

            *      * ** *   **      *       *  *     *
     337808 ATGATGTATCTACTCAGCTAACAGAGTTGAACCTTTCCTTTG
        130 GTGATGTTTGCATTCAAGTAACAGTGTTGAACATTCCCTTTC

             *       *                   * *     *             **            
     337850 AGAGAGCAGTTTTGAAACACTCTTTTTGTGGAATCTGCAAGTGGATATTTGTCTAGCTTTGAGGA
          1 ATAGAGCAGGTTTGAAACACTCTTTTTGTAGTATCTGGAAGTGGATATTTGGATAGCTTTGAGGA

                         *   *    *                   *       *   *          
     337915 TTTCGTTGGAAACGGGATTA-CAT-ATA-AAAAGC-AGACAGCAGCATTCCCAGTAACTTCTTTG
         66 TTTCGTTGGAAACAGGAATATCTTCATATAAAAGCTAGACAGAAGCATTCTCAGAAACTTCTTTG

                              *    *                 
     337976 TGATGTTTGCATTCAAGTCACAGAGTTGAACATTCCCTTTC
        131 TGATGTTTGCATTCAAGTAACAGTGTTGAACATTCCCTTTC

                                                   *     *       **     *   *
     338017 ATAGAGCAGGTTTGAAACACTCTTTTTGTAGTATCTGGATGTGGACATTTGGAGCGCTTTCAGGC
          1 ATAGAGCAGGTTTGAAACACTCTTTTTGTAGTATCTGGAAGTGGATATTTGGATAGCTTTGAGGA

            * ** *      *             *** *   *                      *   *   
     338082 CTATGGT-GAAAAAGGAAATATCTTCTCCTGAAAACTAGACAGAAGCATTCTCAGAATCTTATTT
         66 TTTCGTTGGAAACAGG-AATATCTTCATATAAAAGCTAGACAGAAGCATTCTCAGAAACTTCTTT

                   **  **    *             **  * *   *
     338146 GTGATGTGCGCCCTCAACTAACAGTGTTGAAGCTTTCTTTTG
        130 GTGATGTTTGCATTCAAGTAACAGTGTTGAACATTCCCTTTC

                     *                    *      *   *                       
     338188 ATAGAGCAGTTTTGAAACACTCTTTTTGTAATATCTGCAAGAGGATATTTGGATAGCTTTGAGGA
          1 ATAGAGCAGGTTTGAAACACTCTTTTTGTAGTATCTGGAAGTGGATATTTGGATAGCTTTGAGGA

                         *   * *            **                      *    *   
     338253 TTTCGTTGGAAACGGGATTGTCTTCATATAAACTCTAGACAGAAGCATTCTCAGAAGCTTCATTG
         66 TTTCGTTGGAAACAGGAATATCTTCATATAAAAGCTAGACAGAAGCATTCTCAGAAACTTCTTTG

            *       **   *    *             *        
     338318 GGATGTTTCAATTGAAGTCACAGTGTTGAACAGTCCCTTTC
        131 TGATGTTTGCATTCAAGTAACAGTGTTGAACATTCCCTTTC

                                                         *       *  * * *    
     338359 ATAGAGCAGGTTTGAAACACTCTTTTTGTAGTATCTGGAAGTGGACATTTGGAGAGATCTCAGGA
          1 ATAGAGCAGGTTTGAAACACTCTTTTTGTAGTATCTGGAAGTGGATATTTGGATAGCTTTGAGGA

            * *  *    * *             **            *       * *          *  *
     338424 ATACGGT-GATAAAGGAAATATCTTCCAATAAAAGCTAGATAGAAGCAATGTCAGAAACTTTTTC
         66 TTTCGTTGGAAACAGG-AATATCTTCATATAAAAGCTAGACAGAAGCATTCTCAGAAACTTCTTT

            *      * ** *   **      *       *  * *   *
     338488 ATGATGTATCTACTCAGCTAACAGAGTTGAACCTTTCTTTTG
        130 GTGATGTTTGCATTCAAGTAACAGTGTTGAACATTCCCTTTC

             *       *                   * *     *             **            
     338530 AGAGAGCAGTTTTGAAACACTCTTTTTGTGGAATCTGCAAGTGGATATTTGTCTAGCTTTGAGGA
          1 ATAGAGCAGGTTTGAAACACTCTTTTTGTAGTATCTGGAAGTGGATATTTGGATAGCTTTGAGGA

                         *   *    *                   *       *   *          
     338595 TTTCGTTGGAAACGGGATTA-CAT-ATA-AAAAGC-AGACAGCAGCATTCCCAGTAACTTCTTTG
         66 TTTCGTTGGAAACAGGAATATCTTCATATAAAAGCTAGACAGAAGCATTCTCAGAAACTTCTTTG

                              *    *                 
     338656 TGATGTTTGCATTCAAGTCACAGAGTTGAACATTCCCTTTC
        131 TGATGTTTGCATTCAAGTAACAGTGTTGAACATTCCCTTTC

               *                                   *     *       **     *   *
     338697 ATACAGCAGGTTTGAAACACTCTTTTTGTAGTATCTGGATGTGGACATTTGGAGCGCTTTCAGGC
          1 ATAGAGCAGGTTTGAAACACTCTTTTTGTAGTATCTGGAAGTGGATATTTGGATAGCTTTGAGGA

            * ** *      *             *** *   *           *          *   *   
     338762 CTATGGT-GAAAAAGGAAATATCTTCCCCTGAAAACTAGACAGAAGAATTCTCAGAATCTTATTT
         66 TTTCGTTGGAAACAGG-AATATCTTCATATAAAAGCTAGACAGAAGCATTCTCAGAAACTTCTTT

                   **  **    *       *     **  * *   *
     338826 GTGATGTGCGCCCTCAACTAACAGTCTTGAAGCTTTCTTTTG
        130 GTGATGTTTGCATTCAAGTAACAGTGTTGAACATTCCCTTTC

                     *                    *      *   *                       
     338868 ATAGAGCAGTTTTGAAACACTCTTTTTGTAATATCTGCAAGAGGATATTTGGATAGCTTTGAGGA
          1 ATAGAGCAGGTTTGAAACACTCTTTTTGTAGTATCTGGAAGTGGATATTTGGATAGCTTTGAGGA

                         *   * *            **                      *    *   
     338933 TTTCGTTGGAAACGGGATTGTCTTCATATAAACTCTAGACAGAAGCATTCTCAGAAGCTTCATTG
         66 TTTCGTTGGAAACAGGAATATCTTCATATAAAAGCTAGACAGAAGCATTCTCAGAAACTTCTTTG

            *       **   *    *             *        
     338998 GGATGTTTCAATTGAAGTCACAGTGTTGAACAGTCCCTTTC
        131 TGATGTTTGCATTCAAGTAACAGTGTTGAACATTCCCTTTC

                                                         *       *  * * *    
     339039 ATAGAGCAGGTTTGAAACACTCTTTTTGTAGTATCTGGAAGTGGACATTTGGAGAGATCTCAGGA
          1 ATAGAGCAGGTTTGAAACACTCTTTTTGTAGTATCTGGAAGTGGATATTTGGATAGCTTTGAGGA

            * *  *    * *             **            *       * *          *  *
     339104 ATACGGT-GATAAAGGAAATATCTTCCAATAAAAGCTAGATAGAAGCAATGTCAGAAACTTTTTC
         66 TTTCGTTGGAAACAGG-AATATCTTCATATAAAAGCTAGACAGAAGCATTCTCAGAAACTTCTTT

            *      * ** *   **      *       *  * *   *
     339168 ATGATGTATCTACTCAGCTAACAGAGTTGAACCTTTCTTTTG
        130 GTGATGTTTGCATTCAAGTAACAGTGTTGAACATTCCCTTTC

             *       *                   * *     *             **            
     339210 AGAGAGCAGTTTTGAAACACTCTTTTTGTGGAATCTGCAAGTGGATATTTGTCTAGCTTTGAGGA
          1 ATAGAGCAGGTTTGAAACACTCTTTTTGTAGTATCTGGAAGTGGATATTTGGATAGCTTTGAGGA

                         *   *    *                   *       *   *          
     339275 TTTCGTTGGAAACGGGATTA-CAT-ATA-AAAAGC-AGACAGCAGCATTCCCAGTAACTTCTTTG
         66 TTTCGTTGGAAACAGGAATATCTTCATATAAAAGCTAGACAGAAGCATTCTCAGAAACTTCTTTG

                              *    *                 
     339336 TGATGTTTGCATTCAAGTCACAGAGTTGAACATTCCCTTTC
        131 TGATGTTTGCATTCAAGTAACAGTGTTGAACATTCCCTTTC

               *                                   *     *       **     *   *
     339377 ATACAGCAGGTTTGAAACACTCTTTTTGTAGTATCTGGATGTGGACATTTGGAGCGCTTTCAGGC
          1 ATAGAGCAGGTTTGAAACACTCTTTTTGTAGTATCTGGAAGTGGATATTTGGATAGCTTTGAGGA

            * ** *      *             *** *   *           *          *   *   
     339442 CTATGGT-GAAAAAGGAAATATCTTCCCCTGAAAACTAGACAGAAGAATTCTCAGAATCTTATTT
         66 TTTCGTTGGAAACAGG-AATATCTTCATATAAAAGCTAGACAGAAGCATTCTCAGAAACTTCTTT

                   **  **    *       *     **  * *   *
     339506 GTGATGTGCGCCCTCAACTAACAGTCTTGAAGCTTTCTTTTG
        130 GTGATGTTTGCATTCAAGTAACAGTGTTGAACATTCCCTTTC

                     *                    **     *   *                       
     339548 ATAGAGCAGTTTTGAAACACTCTTTTTGTAAAATCTGCAAGAGGATATTTGGATAGCTTTGAGGA
          1 ATAGAGCAGGTTTGAAACACTCTTTTTGTAGTATCTGGAAGTGGATATTTGGATAGCTTTGAGGA

                         *   * *            **                      *    *   
     339613 TTTCGTTGGAAACGGGATTGTCTTCATATAAACTCTAGACAGAAGCATTCTCAGAAGCTTCATTG
         66 TTTCGTTGGAAACAGGAATATCTTCATATAAAAGCTAGACAGAAGCATTCTCAGAAACTTCTTTG

            *       **   *    *             *        
     339678 GGATGTTTCAATTGAAGTCACAGTGTTGAACAGTCCCTTTC
        131 TGATGTTTGCATTCAAGTAACAGTGTTGAACATTCCCTTTC

                                                         *       *  * * *    
     339719 ATAGAGCAGGTTTGAAACACTCTTTTTGTAGTATCTGGAAGTGGACATTTGGAGAGATCTCAGGA
          1 ATAGAGCAGGTTTGAAACACTCTTTTTGTAGTATCTGGAAGTGGATATTTGGATAGCTTTGAGGA

            * *  *    * *             **     *    * *       * *          *  *
     339784 ATACGGT-GATAAAGGAAATATCTTCCAATAAACGCTACATAGAAGCAATGTCAGAAACTTTTTC
         66 TTTCGTTGGAAACAGG-AATATCTTCATATAAAAGCTAGACAGAAGCATTCTCAGAAACTTCTTT

            *      * ** *   **      *       *  * *   *
     339848 ATGATGTATCTACTCAGCTAACAGAGTTGAACCTTTCTTTTG
        130 GTGATGTTTGCATTCAAGTAACAGTGTTGAACATTCCCTTTC

             *       *                   * *     *             **            
     339890 AGAGAGCAGTTTTGAAACACTCTTTTTGTGGAATCTGCAAGTGGATATTTGTCTAGCTTTGAGGA
          1 ATAGAGCAGGTTTGAAACACTCTTTTTGTAGTATCTGGAAGTGGATATTTGGATAGCTTTGAGGA

                         *   *    *                   *       *   *          
     339955 TTTCGTTGGAAACGGGATTA-CAT-ATA-AAAAGC-AGACAGCAGCATTCCCAGTAACTTCTTTG
         66 TTTCGTTGGAAACAGGAATATCTTCATATAAAAGCTAGACAGAAGCATTCTCAGAAACTTCTTTG

                              *    *                 
     340016 TGATGTTTGCATTCAAGTCACAGAGTTGAACATTCCCTTTC
        131 TGATGTTTGCATTCAAGTAACAGTGTTGAACATTCCCTTTC

               *                                   *     *       **     *   *
     340057 ATACAGCAGGTTTGAAACACTCTTTTTGTAGTATCTGGATGTGGACATTTGGAGCGCTTTCAGGC
          1 ATAGAGCAGGTTTGAAACACTCTTTTTGTAGTATCTGGAAGTGGATATTTGGATAGCTTTGAGGA

            * ** *      *             *** *   *           *     *    *   *   
     340122 CTATGGT-GAAAAAGGAAATATCTTCCCCTGAAAACTAGACAGAAGAATTCTTAGAATCTTATTT
         66 TTTCGTTGGAAACAGG-AATATCTTCATATAAAAGCTAGACAGAAGCATTCTCAGAAACTTCTTT

                   **  **    *             **  * *   *
     340186 GTGATGTGCGCCCTCAACTAACAGTGTTGAAGCTTTCTTTTG
        130 GTGATGTTTGCATTCAAGTAACAGTGTTGAACATTCCCTTTC

                     *                    **     *   *                       
     340228 ATAGAGCAGTTTTGAAACACTCTTTTTGTAAAATCTGCAAGAGGATATTTGGATAGCTTTGAGGA
          1 ATAGAGCAGGTTTGAAACACTCTTTTTGTAGTATCTGGAAGTGGATATTTGGATAGCTTTGAGGA

                         *   * *            **                      * *  *   
     340293 TTTCGTTGGAAACGGGATTGTCTTCATATAAACTCTAGACAGAAGCATTCTCAGAAGCGTCATTG
         66 TTTCGTTGGAAACAGGAATATCTTCATATAAAAGCTAGACAGAAGCATTCTCAGAAACTTCTTTG

            *       **   *    *             *        
     340358 GGATGTTTCAATTGAAGTCACAGTGTTGAACAGTCCCTTTC
        131 TGATGTTTGCATTCAAGTAACAGTGTTGAACATTCCCTTTC

                                                   *     *       **     *  **
     340399 ATAGAGCAGGTTTGAAACACTCTTTTTGTAGTATCTGGATGTGGACATTTGGAGCGCTTTAAGCC
          1 ATAGAGCAGGTTTGAAACACTCTTTTTGTAGTATCTGGAAGTGGATATTTGGATAGCTTTGAGGA

             * *    *                 *** *   *                          *   
     340464 TATGGTT-TAAA-AGGAAATATCTTCCCCTGAAAACTAGACAGAAGCATTCTCAGAAACTTATTT
         66 TTTCGTTGGAAACAGG-AATATCTTCATATAAAAGCTAGACAGAAGCATTCTCAGAAACTTCTTT

                   **  **    *                    *   *
     340527 GTGATGTGCGCCCTCAACTAACAGTGTTGAAGCATT-CTTTTG
        130 GTGATGTTTGCATTCAAGTAACAGTGTTGAA-CATTCCCTTTC

                     *                   * *     *             **            
     340569 ATAGAGCAGTTTTGAAACACTCTTTTTGTGGAATCTGCAAGTGGATATTTGTCTAGCTTTGAGGA
          1 ATAGAGCAGGTTTGAAACACTCTTTTTGTAGTATCTGGAAGTGGATATTTGGATAGCTTTGAGGA

                         *   *    *                   *       *              
     340634 TTTCGTTGGAAACGGGATTA-CAT-ATA-AAAAGC-AGACAGCAGCATTCCCAGAAACTTCTTTG
         66 TTTCGTTGGAAACAGGAATATCTTCATATAAAAGCTAGACAGAAGCATTCTCAGAAACTTCTTTG

                              *    *                 
     340695 TGATGTTTGCATTCAAGTCACAGAGTTGAACATTCCCTTTC
        131 TGATGTTTGCATTCAAGTAACAGTGTTGAACATTCCCTTTC

                                                   *     **      **     *   *
     340736 ATAGAGCAGGTTTGAAACACTCTTTTTGTAGTATCTGGATGTGGACCTTTGGAGCGCTTTCAGGC
          1 ATAGAGCAGGTTTGAAACACTCTTTTTGTAGTATCTGGAAGTGGATATTTGGATAGCTTTGAGGA

            * ** *      *             *** *   *                      *   *   
     340801 CTATGGT-GAAAAAGGAAATATCTTCCCCTGAAAACTAGACAGAAGCATTCTCAGAATCTTATTT
         66 TTTCGTTGGAAACAGG-AATATCTTCATATAAAAGCTAGACAGAAGCATTCTCAGAAACTTCTTT

                   **  **    **            **  * *   *
     340865 GTGATGTGCGCCCTCAACGAACAGTGTTGAAGCTTTCTTTTG
        130 GTGATGTTTGCATTCAAGTAACAGTGTTGAACATTCCCTTTC

             *       *                    *      *   *                       
     340907 AAAGAGCAGTTTTGAAACACTCTTTTTGTAATATCTGCAAGAGGATATTTGGATAGCTTTGAGGA
          1 ATAGAGCAGGTTTGAAACACTCTTTTTGTAGTATCTGGAAGTGGATATTTGGATAGCTTTGAGGA

                         *   * *            **                      *    *   
     340972 TTTCGTTGGAAACGGGATTGTCTTCATATAAACTCTAGACAGAAGCATTCTCAGAAGCTTCATTG
         66 TTTCGTTGGAAACAGGAATATCTTCATATAAAAGCTAGACAGAAGCATTCTCAGAAACTTCTTTG

            *       **   *    *             *        
     341037 GGATGTTTCAATTGAAGTCACAGTGTTGAACAGTCCCTTTC
        131 TGATGTTTGCATTCAAGTAACAGTGTTGAACATTCCCTTTC

                                                         *       **   * *  * 
     341078 ATAGAGCAGGTTTGAAACACTCTTTTTGTAGTATCTGGAAGTGGACATTTGGAGCGCTCTCAGTA
          1 ATAGAGCAGGTTTGAAACACTCTTTTTGTAGTATCTGGAAGTGGATATTTGGATAGCTTTGAGGA

            * *  *      *             **            *       * *          *  *
     341143 CTGCGGT-GAAAAAGGAAATATCTTCCAATAAAAGCTAGATAGAAGCAATGTCAGAAACTTTTTC
         66 TTTCGTTGGAAACAGG-AATATCTTCATATAAAAGCTAGACAGAAGCATTCTCAGAAACTTCTTT

            *      * ** *   **      *       *        *
     341207 ATGATGTATCTACTCAGCTAACAGAGTTGAACCTT-CCTTTG
        130 GTGATGTTTGCATTCAAGTAACAGTGTTGAACATTCCCTTTC

             *       *                   * *     *             **            
     341248 AGAGAGCAGTTTTGAAACACTCTTTTTGTGGAATCTGCAAGTGGATATTTGTCTAGCTTTGAGGA
          1 ATAGAGCAGGTTTGAAACACTCTTTTTGTAGTATCTGGAAGTGGATATTTGGATAGCTTTGAGGA

                         *   *    *                   *       *              
     341313 TTTCGTTGGAAACGGGATTA-CAT-ATA-AAAAGC-AGACAGCAGCATTCCCAGAAACTTCTTTG
         66 TTTCGTTGGAAACAGGAATATCTTCATATAAAAGCTAGACAGAAGCATTCTCAGAAACTTCTTTG

                              *    *                 
     341374 TGATGTTTGCATTCAAGTCACAGAGTTGAACATTCCCTTTC
        131 TGATGTTTGCATTCAAGTAACAGTGTTGAACATTCCCTTTC

                                                   *     **    * **     *   *
     341415 ATAGAGCAGGTTTGAAACACTCTTTTTGTAGTATCTGGATGTGGACTTTTGCAGCGCTTTCAGGC
          1 ATAGAGCAGGTTTGAAACACTCTTTTTGTAGTATCTGGAAGTGGATATTTGGATAGCTTTGAGGA

            * ** *      *             *** *   *                          *   
     341480 CTAAGGT-GAAAAAGGAAATATCTTCCCCTGAAAACTAGACAGAAGCATTCTCAGAAACTTATTT
         66 TTTCGTTGGAAACAGG-AATATCTTCATATAAAAGCTAGACAGAAGCATTCTCAGAAACTTCTTT

                   **  **    *             **  * *   *
     341544 GTGATGTGCGCCCTCAACTAACAGTGTTGAAGCTTTCTTTTG
        130 GTGATGTTTGCATTCAAGTAACAGTGTTGAACATTCCCTTTC

                     *                    *      *   *                       
     341586 ATAGAGCAGTTTTGAAACACTCTTTTTGTAATATCTGCAAGAGGATATTTGGATAGCTTTGAGGA
          1 ATAGAGCAGGTTTGAAACACTCTTTTTGTAGTATCTGGAAGTGGATATTTGGATAGCTTTGAGGA

                         *   * *            **                      *    *   
     341651 TTTCGTTGGAAACGGGATTGTCTTCATATAAACTCTAGACAGAAGCATTCTCAGAAGCTTCATTG
         66 TTTCGTTGGAAACAGGAATATCTTCATATAAAAGCTAGACAGAAGCATTCTCAGAAACTTCTTTG

            *       **   *    *             *        
     341716 GGATGTTTCAATTGAAGTCACAGTGTTGAACAGTCCCTTTC
        131 TGATGTTTGCATTCAAGTAACAGTGTTGAACATTCCCTTTC

                 *                                       *       **   * *    
     341757 ATAGAACAGGTTTGAAACACTCTTTTTGTAGTATCTGGAAGTGGACATTTGGAGCGCTCTCAGGA
          1 ATAGAGCAGGTTTGAAACACTCTTTTTGTAGTATCTGGAAGTGGATATTTGGATAGCTTTGAGGA

            * ** *      *             **          * *       * *          *  *
     341822 CTATGGT-GAAAAAGGAAATATCTTCCAATAAAAGCTACATAGAAGCAATGTCAGAAACTTTTTC
         66 TTTCGTTGGAAACAGG-AATATCTTCATATAAAAGCTAGACAGAAGCATTCTCAGAAACTTCTTT

            *      * ** *   **      *       *  *     *
     341886 ATGATGTATCTACTCAGCTAACAGAGTTGAACCTTTCCTTTG
        130 GTGATGTTTGCATTCAAGTAACAGTGTTGAACATTCCCTTTC

             *       *                   * *     *             **            
     341928 AGAGAGCAGTTTTGAAACACTCTTTTTGTGGAATCTGCAAGTGGATATTTGTCTAGCTTTGAGGA
          1 ATAGAGCAGGTTTGAAACACTCTTTTTGTAGTATCTGGAAGTGGATATTTGGATAGCTTTGAGGA

                         *   *    *                   *       *   *          
     341993 TTTCGTTGGAAACGGGATTA-CAT-ATA-AAAAGC-AGACAGCAGCATTCCCAGTAACTTCTTTG
         66 TTTCGTTGGAAACAGGAATATCTTCATATAAAAGCTAGACAGAAGCATTCTCAGAAACTTCTTTG

                              * *  *                 
     342054 TGATGTTTGCATTCAAGTCAGAGAGTTGAACATTCCCTTTC
        131 TGATGTTTGCATTCAAGTAACAGTGTTGAACATTCCCTTTC

                                                   *     *       **     *   *
     342095 ATAGAGCAGGTTTGAAACACTCTTTTTGTAGTATCTGGATGTGGACATTTGGAGCGCTTTCAGGC
          1 ATAGAGCAGGTTTGAAACACTCTTTTTGTAGTATCTGGAAGTGGATATTTGGATAGCTTTGAGGA

            * ** *      *             *** *   *                      *   *   
     342160 CTATGGT-GAAAAAGGAAATATCTTCTCCTGAAAACTAGACAGAAGCATTCTCAGAATCTTATTT
         66 TTTCGTTGGAAACAGG-AATATCTTCATATAAAAGCTAGACAGAAGCATTCTCAGAAACTTCTTT

                   **  **    *             **  * *   *
     342224 GTGATGTGCGCCCTCAACTAACAGTGTTGAAGCTTTCTTTTG
        130 GTGATGTTTGCATTCAAGTAACAGTGTTGAACATTCCCTTTC

                     *                    *      *   *                       
     342266 ATAGAGCAGTTTTGAAACACTCTTTTTGTAATATCTGCAAGAGGATATTTGGATAGCTTTGAGGA
          1 ATAGAGCAGGTTTGAAACACTCTTTTTGTAGTATCTGGAAGTGGATATTTGGATAGCTTTGAGGA

                         *   * *            **                      *    *   
     342331 TTTCGTTGGAAACGGGATTGTCTTCATATAAACTCTAGACAGAAGCATTCTCAGAAGCTTCATTG
         66 TTTCGTTGGAAACAGGAATATCTTCATATAAAAGCTAGACAGAAGCATTCTCAGAAACTTCTTTG

            *       **   *    *             *        
     342396 GGATGTTTCAATTGAAGTCACAGTGTTGAACAGTCCCTTTC
        131 TGATGTTTGCATTCAAGTAACAGTGTTGAACATTCCCTTTC

                                                         *       *  * * *    
     342437 ATAGAGCAGGTTTGAAACACTCTTTTTGTAGTATCTGGAAGTGGACATTTGGAGAGATCTCAGGA
          1 ATAGAGCAGGTTTGAAACACTCTTTTTGTAGTATCTGGAAGTGGATATTTGGATAGCTTTGAGGA

            * *  *    * *             **            *       * *          *  *
     342502 ATACGGT-GATAAAGGAAATATCTTCCAATAAAAGCTAGATAGAAGCAATGTCAGAAACTTTTTC
         66 TTTCGTTGGAAACAGG-AATATCTTCATATAAAAGCTAGACAGAAGCATTCTCAGAAACTTCTTT

            *      * ** *   **      *          * *   *
     342566 ATGATGTATCTACTCAGCTAACAGAGTTGAACATTTCTTTTG
        130 GTGATGTTTGCATTCAAGTAACAGTGTTGAACATTCCCTTTC

             *       *                   * *     *             **            
     342608 AGAGAGCAGTTTTGAAACACTCTTTTTGTGGAATCTGCAAGTGGATATTTGTCTAGCTTTGAGGA
          1 ATAGAGCAGGTTTGAAACACTCTTTTTGTAGTATCTGGAAGTGGATATTTGGATAGCTTTGAGGA

               *         *   *    *                   *       *   *          
     342673 TTTAGTTGGAAACGGGATTA-CAT-ATA-AAAAGC-AGACAGCAGCATTCCCAGTAACTTCTTTG
         66 TTTCGTTGGAAACAGGAATATCTTCATATAAAAGCTAGACAGAAGCATTCTCAGAAACTTCTTTG

                              *    *                 
     342734 TGATGTTTGCATTCAAGTCACAGAGTTGAACATTCCCTTTC
        131 TGATGTTTGCATTCAAGTAACAGTGTTGAACATTCCCTTTC

               *                                   *     *       **     *   *
     342775 ATACAGCAGGTTTGAAACACTCTTTTTGTAGTATCTGGATGTGGACATTTGGAGCGCTTTCAGGC
          1 ATAGAGCAGGTTTGAAACACTCTTTTTGTAGTATCTGGAAGTGGATATTTGGATAGCTTTGAGGA

            * ** *      *             *** *   *           *          *   *   
     342840 CTATGGT-GAAAAAGGAAATATCTTCCCCTGAAAACTAGACAGAAGAATTCTCAGAATCTTATTT
         66 TTTCGTTGGAAACAGG-AATATCTTCATATAAAAGCTAGACAGAAGCATTCTCAGAAACTTCTTT

                   **  **    *       *     **  * *   *
     342904 GTGATGTGCGCCCTCAACTAACAGTCTTGAAGCTTTCTTTTG
        130 GTGATGTTTGCATTCAAGTAACAGTGTTGAACATTCCCTTTC

                     *                    **     *   *                       
     342946 ATAGAGCAGTTTTGAAACACTCTTTTTGTAAAATCTGCAAGAGGATATTTGGATAGCTTTGAGGA
          1 ATAGAGCAGGTTTGAAACACTCTTTTTGTAGTATCTGGAAGTGGATATTTGGATAGCTTTGAGGA

                         *   * *            **                      *    *   
     343011 TTTCGTTGGAAACGGGATTGTCTTCATATAAACTCTAGACAGAAGCATTCTCAGAAGCTTCATTG
         66 TTTCGTTGGAAACAGGAATATCTTCATATAAAAGCTAGACAGAAGCATTCTCAGAAACTTCTTTG

            *       **   *    *             *        
     343076 GGATGTTTCAATTGAAGTCACAGTGTTGAACAGTCCCTTTC
        131 TGATGTTTGCATTCAAGTAACAGTGTTGAACATTCCCTTTC

                                                         *       *  * * *    
     343117 ATAGAGCAGGTTTGAAACACTCTTTTTGTAGTATCTGGAAGTGGACATTTGGAGAGATCTCAGGA
          1 ATAGAGCAGGTTTGAAACACTCTTTTTGTAGTATCTGGAAGTGGATATTTGGATAGCTTTGAGGA

            * *  *    * *             **     *    * *       * *          *  *
     343182 ATACGGT-GATAAAGGAAATATCTTCCAATAAACGCTACATAGAAGCAATGTCAGAAACTTTTTC
         66 TTTCGTTGGAAACAGG-AATATCTTCATATAAAAGCTAGACAGAAGCATTCTCAGAAACTTCTTT

            *      * ** *   **      *       *        *
     343246 ATGATGTATCTACTCAGCTAACAGAGTTGAACCTT-CCTTTG
        130 GTGATGTTTGCATTCAAGTAACAGTGTTGAACATTCCCTTTC

             *       *                   * *     *             **            
     343287 AGAGAGCAGTTTTGAAACACTCTTTTTGTGGAATCTGCAAGTGGATATTTGTCTAGCTTTGAGGA
          1 ATAGAGCAGGTTTGAAACACTCTTTTTGTAGTATCTGGAAGTGGATATTTGGATAGCTTTGAGGA

                         *   *    *                   *       *              
     343352 TTTCGTTGGAAACGGGATTA-CAT-ATA-AAAAGC-AGACAGCAGCATTCCCAGAAACTTCTTTG
         66 TTTCGTTGGAAACAGGAATATCTTCATATAAAAGCTAGACAGAAGCATTCTCAGAAACTTCTTTG

                              *    *                 
     343413 TGATGTTTGCATTCAAGTCACAGAGTTGAACATTCCCTTTC
        131 TGATGTTTGCATTCAAGTAACAGTGTTGAACATTCCCTTTC

                                                   *     *     * **     *   *
     343454 ATAGAGCAGGTTTGAAACACTCTTTTTGTAGTATCTGGATGTGGACATTTGCAGCGCTTTCAGGC
          1 ATAGAGCAGGTTTGAAACACTCTTTTTGTAGTATCTGGAAGTGGATATTTGGATAGCTTTGAGGA

            * ** *      *             *** *   *                          *   
     343519 CTAAGGT-GAAAAAGGAAATATCTTCCCCTGAAAACTAGACAGAAGCATTCTCAGAAACTTATTT
         66 TTTCGTTGGAAACAGG-AATATCTTCATATAAAAGCTAGACAGAAGCATTCTCAGAAACTTCTTT

                   **  **    *             **  * *   *
     343583 GTGATGTGCGCCCTCAACTAACAGTGTTGAAGCTTTCTTTTG
        130 GTGATGTTTGCATTCAAGTAACAGTGTTGAACATTCCCTTTC

                     *                    *      *   *                       
     343625 ATAGAGCAGTTTTGAAACACTCTTTTTGTAATATCTGCAAGAGGATATTTGGATAGCTTTGAGGA
          1 ATAGAGCAGGTTTGAAACACTCTTTTTGTAGTATCTGGAAGTGGATATTTGGATAGCTTTGAGGA

                         *   * *            **                      *    *   
     343690 TTTCGTTGGAAACGGGATTGTCTTCATATAAACTCTAGACAGAAGCATTCTCAGAAGCTTCATTG
         66 TTTCGTTGGAAACAGGAATATCTTCATATAAAAGCTAGACAGAAGCATTCTCAGAAACTTCTTTG

            *       **   *    *             *        
     343755 GGATGTTTCAATTGAAGTCACAGTGTTGAACAGTCCCTTTC
        131 TGATGTTTGCATTCAAGTAACAGTGTTGAACATTCCCTTTC

                 *                                       *       **   * *    
     343796 ATAGAACAGGTTTGAAACACTCTTTTTGTAGTATCTGGAAGTGGACATTTGGAGCGCTCTCAGGA
          1 ATAGAGCAGGTTTGAAACACTCTTTTTGTAGTATCTGGAAGTGGATATTTGGATAGCTTTGAGGA

            * ** *      *             **          * *       * *          *  *
     343861 CTATGGT-GAAAAAGGAAATATCTTCCAATAAAAGCTACATAGAAGCAATGTCAGAAACTTTTTC
         66 TTTCGTTGGAAACAGG-AATATCTTCATATAAAAGCTAGACAGAAGCATTCTCAGAAACTTCTTT

            *      * ** *   **      *       *  *     *
     343925 ATGATGTATCTACTCAGCTAACAGAGTTGAACCTTTCCTTTG
        130 GTGATGTTTGCATTCAAGTAACAGTGTTGAACATTCCCTTTC

             *       *                   * *     *             **   *        
     343967 AGAGAGCAGTTTTGAAACACTCTTTTTGTGGAATCTGCAAGTGGATATTTGTCTAGATTTGAGGA
          1 ATAGAGCAGGTTTGAAACACTCTTTTTGTAGTATCTGGAAGTGGATATTTGGATAGCTTTGAGGA

                         *   *    *                   *       *   *          
     344032 TTTCGTTGGAAACGGGATTA-CAT-ATA-AAAAGC-AGACAGCAGCATTCCCAGTAACTTCTTTG
         66 TTTCGTTGGAAACAGGAATATCTTCATATAAAAGCTAGACAGAAGCATTCTCAGAAACTTCTTTG

                              *    *                 
     344093 TGATGTTTGCATTCAAGTCACAGAGTTGAACATTCCCTTTC
        131 TGATGTTTGCATTCAAGTAACAGTGTTGAACATTCCCTTTC

                                                   *     *       **     *   *
     344134 ATAGAGCAGGTTTGAAACACTCTTTTTGTAGTATCTGGATGTGGACATTTGGAGCGCTTTCAGGC
          1 ATAGAGCAGGTTTGAAACACTCTTTTTGTAGTATCTGGAAGTGGATATTTGGATAGCTTTGAGGA

            * ** *      *             *** *   *           *          *   *   
     344199 CTATGGT-GAAAAAGGAAATATCTTCCCCTGAAAACTAGACAGAAGAATTCTCAGAATCTTATTT
         66 TTTCGTTGGAAACAGG-AATATCTTCATATAAAAGCTAGACAGAAGCATTCTCAGAAACTTCTTT

                   **  **    *       *     **  * *   *
     344263 GTGATGTGCGCCCTCAACTAACAGTCTTGAAGCTTTCTTTTG
        130 GTGATGTTTGCATTCAAGTAACAGTGTTGAACATTCCCTTTC

                     *                *   **     *   *                       
     344305 ATAGAGCAGTTTTGAAACACTCTTTTCGTAAAATCTGCAAGAGGATATTTGGATAGCTTTGAGGA
          1 ATAGAGCAGGTTTGAAACACTCTTTTTGTAGTATCTGGAAGTGGATATTTGGATAGCTTTGAGGA

                         *   * *            **                      *    *   
     344370 TTTCGTTGGAAACGGGATTGTCTTCATATAAACTCTAGACAGAAGCATTCTCAGAAGCTTCATTG
         66 TTTCGTTGGAAACAGGAATATCTTCATATAAAAGCTAGACAGAAGCATTCTCAGAAACTTCTTTG

            *       **   *    *             *        
     344435 GGATGTTTCAATTGAAGTCACAGTGTTGAACAGTCCCTTTC
        131 TGATGTTTGCATTCAAGTAACAGTGTTGAACATTCCCTTTC

                                                         *       *  * * *    
     344476 ATAGAGCAGGTTTGAAACACTCTTTTTGTAGTATCTGGAAGTGGACATTTGGAGAGATCTCAGGA
          1 ATAGAGCAGGTTTGAAACACTCTTTTTGTAGTATCTGGAAGTGGATATTTGGATAGCTTTGAGGA

            * *  *    * *             **     *    * *       * *          *  *
     344541 ATACGGT-GATAAAGGAAATATCTTCCAATAAACGCTACATAGAAGCAATGTCAGAAACTTTTTC
         66 TTTCGTTGGAAACAGG-AATATCTTCATATAAAAGCTAGACAGAAGCATTCTCAGAAACTTCTTT

            *      * ** *   **      *       *  * *   *
     344605 ATGATGTATCTACTCAGCTAACAGAGTTGAACCTTTCTTTTG
        130 GTGATGTTTGCATTCAAGTAACAGTGTTGAACATTCCCTTTC

             *       *                   * *     *             **            
     344647 AGAGAGCAGTTTTGAAACACTCTTTTTGTGGAATCTGCAAGTGGATATTTGTCTAGCTTTGAGGA
          1 ATAGAGCAGGTTTGAAACACTCTTTTTGTAGTATCTGGAAGTGGATATTTGGATAGCTTTGAGGA

                         *   *    *                   *       *   *          
     344712 TTTCGTTGGAAACGGGATTA-CAT-ATA-AAAAGC-AGACAGCAGCATTCCCAGTAACTTCTTTG
         66 TTTCGTTGGAAACAGGAATATCTTCATATAAAAGCTAGACAGAAGCATTCTCAGAAACTTCTTTG

                              *    *                 
     344773 TGATGTTTGCATTCAAGTCACAGAGTTGAACATTCCCTTTC
        131 TGATGTTTGCATTCAAGTAACAGTGTTGAACATTCCCTTTC

               *                                   *     *       **     *   *
     344814 ATACAGCAGGTTTGAAACACTCTTTTTGTAGTATCTGGATGTGGACATTTGGAGCGCTTTCAGGC
          1 ATAGAGCAGGTTTGAAACACTCTTTTTGTAGTATCTGGAAGTGGATATTTGGATAGCTTTGAGGA

            * ** *      *             *** *   *           *     *    *   *   
     344879 CTATGGT-GAAAAAGGAAATATCTTCCCCTGAAAACTAGACAGAAGAATTCTTAGAATCTTATTT
         66 TTTCGTTGGAAACAGG-AATATCTTCATATAAAAGCTAGACAGAAGCATTCTCAGAAACTTCTTT

                   **  **    *             **  * *   *
     344943 GTGATGTGCGCCCTCAACTAACAGTGTTGAAGCTTTCTTTTG
        130 GTGATGTTTGCATTCAAGTAACAGTGTTGAACATTCCCTTTC

                     *                    **     *   *                       
     344985 ATAGAGCAGTTTTGAAACACTCTTTTTGTAAAATCTGCAAGAGGATATTTGGATAGCTTTGAGGA
          1 ATAGAGCAGGTTTGAAACACTCTTTTTGTAGTATCTGGAAGTGGATATTTGGATAGCTTTGAGGA

                         *   * *            **                      * *  *   
     345050 TTTCGTTGGAAACGGGATTGTCTTCATATAAACTCTAGACAGAAGCATTCTCAGAAGCGTCATTG
         66 TTTCGTTGGAAACAGGAATATCTTCATATAAAAGCTAGACAGAAGCATTCTCAGAAACTTCTTTG

            *       **   *    *             *        
     345115 GGATGTTTCAATTGAAGTCACAGTGTTGAACAGTCCCTTTC
        131 TGATGTTTGCATTCAAGTAACAGTGTTGAACATTCCCTTTC

                                                   *     *       **     *  **
     345156 ATAGAGCAGGTTTGAAACACTCTTTTTGTAGTATCTGGATGTGGACATTTGGAGCGCTTTAAGCC
          1 ATAGAGCAGGTTTGAAACACTCTTTTTGTAGTATCTGGAAGTGGATATTTGGATAGCTTTGAGGA

             * *    *                 *** *   *                          *   
     345221 TATGGTT-TAAA-AGGAAATATCTTCCCCTGAAAACTAGACAGAAGCATTCTCAGAAACTTATTT
         66 TTTCGTTGGAAACAGG-AATATCTTCATATAAAAGCTAGACAGAAGCATTCTCAGAAACTTCTTT

                   **  **    *                    *   *
     345284 GTGATGTGCGCCCTCAACTAACAGTGTTGAAGCATT-CTTTTG
        130 GTGATGTTTGCATTCAAGTAACAGTGTTGAA-CATTCCCTTTC

                     *                   * *     *             **            
     345326 ATAGAGCAGTTTTGAAACACTCTTTTTGTGGAATCTGCAAGTGGATATTTGTCTAGCTTTGAGGA
          1 ATAGAGCAGGTTTGAAACACTCTTTTTGTAGTATCTGGAAGTGGATATTTGGATAGCTTTGAGGA

                         *   *    *                   *       *              
     345391 TTTCGTTGGAAACGGGATTA-CAT-ATA-AAAAGC-AGACAGCAGCATTCCCAGAAACTTCTTTG
         66 TTTCGTTGGAAACAGGAATATCTTCATATAAAAGCTAGACAGAAGCATTCTCAGAAACTTCTTTG

                              *    *                 
     345452 TGATGTTTGCATTCAAGTCACAGAGTTGAACATTCCCTTTC
        131 TGATGTTTGCATTCAAGTAACAGTGTTGAACATTCCCTTTC

                                                   *     *     * **     *   *
     345493 ATAGAGCAGGTTTGAAACACTCTTTTTGTAGTATCTGGATGTGGACATTTGCAGCGCTTTCAGGC
          1 ATAGAGCAGGTTTGAAACACTCTTTTTGTAGTATCTGGAAGTGGATATTTGGATAGCTTTGAGGA

            * ** *      *             *** *   *                          *   
     345558 CTAAGGT-GAAAAAGGAAATATCTTCCCCTGAAAACTAGACAGAAGCATTCTCAGAAACTTATTT
         66 TTTCGTTGGAAACAGG-AATATCTTCATATAAAAGCTAGACAGAAGCATTCTCAGAAACTTCTTT

                   **  **    *             **  * *   *
     345622 GTGATGTGCGCCCTCAACTAACAGTGTTGAAGCTTTCTTTTG
        130 GTGATGTTTGCATTCAAGTAACAGTGTTGAACATTCCCTTTC

                     *                    *      *   *                       
     345664 ATAGAGCAGTTTTGAAACACTCTTTTTGTAATATCTGCAAGAGGATATTTGGATAGCTTTGAGGA
          1 ATAGAGCAGGTTTGAAACACTCTTTTTGTAGTATCTGGAAGTGGATATTTGGATAGCTTTGAGGA

                         *   * *            **                      *    *   
     345729 TTTCGTTGGAAACGGGATTGTCTTCATATAAACTCTAGACAGAAGCATTCTCAGAAGCTTCATTG
         66 TTTCGTTGGAAACAGGAATATCTTCATATAAAAGCTAGACAGAAGCATTCTCAGAAACTTCTTTG

            *       **   *    *             * *      
     345794 GGATGTTTCAATTGAAGTCACAGTGTTGAACAGTTCCTTTC
        131 TGATGTTTGCATTCAAGTAACAGTGTTGAACATTCCCTTTC

                 *                                       *       **   * *    
     345835 ATAGAACAGGTTTGAAACACTCTTTTTGTAGTATCTGGAAGTGGACATTTGGAGCGCTCTCAGGA
          1 ATAGAGCAGGTTTGAAACACTCTTTTTGTAGTATCTGGAAGTGGATATTTGGATAGCTTTGAGGA

            * ** *      *             *           * *      ** *          *  *
     345900 CTATGGT-GAAAAAGGAAATATCTTCCTATAAAAGCTACATAGAAGCTATGTCAGAAACTTTTTC
         66 TTTCGTTGGAAACAGG-AATATCTTCATATAAAAGCTAGACAGAAGCATTCTCAGAAACTTCTTT

            *      * ** *   **      *       *  *     *
     345964 ATGATGTATCTACTCAGCTAACAGAGTTGAACCTTTCCTTTG
        130 GTGATGTTTGCATTCAAGTAACAGTGTTGAACATTCCCTTTC

             *       *                   * *     *             **            
     346006 AGAGAGCAGTTTTGAAACACTCTTTTTGTGGAATCTGCAAGTGGATATTTGTCTAGCTTTGAGGA
          1 ATAGAGCAGGTTTGAAACACTCTTTTTGTAGTATCTGGAAGTGGATATTTGGATAGCTTTGAGGA

                         *   *    *                   *       *   *          
     346071 TTTCGTTGGAAACGGGATTA-CAT-ATA-AAAAGC-AGACAGCAGCATTCCCAGTAACTTCTTTG
         66 TTTCGTTGGAAACAGGAATATCTTCATATAAAAGCTAGACAGAAGCATTCTCAGAAACTTCTTTG

                              *    *                 
     346132 TGATGTTTGCATTCAAGTCACAGAGTTGAACATTCCCTTTC
        131 TGATGTTTGCATTCAAGTAACAGTGTTGAACATTCCCTTTC

                                                ** *     *       **     *   *
     346173 ATAGAGCAGGTTTGAAACACTCTTTTTGTAGTATCTCTATGTGGACATTTGGAGCGCTTTCAGGC
          1 ATAGAGCAGGTTTGAAACACTCTTTTTGTAGTATCTGGAAGTGGATATTTGGATAGCTTTGAGGA

            * ** *      *             *** *   *                          *   
     346238 CTATGGT-GAAAAAGGAAATATCTTCCCCTGAAAACTAGACAGAAGCATTCTCAGAAACTTATTT
         66 TTTCGTTGGAAACAGG-AATATCTTCATATAAAAGCTAGACAGAAGCATTCTCAGAAACTTCTTT

                   **  **    *              *  * *   *
     346302 GTGATGTGCGCCCTCAACTAACAGTGTTGAACCTTTCTTTTG
        130 GTGATGTTTGCATTCAAGTAACAGTGTTGAACATTCCCTTTC

                     *                    *      *   *                       
     346344 ATAGAGCAGTTTTGAAACACTCTTTTTGTAATATCTGCAAGAGGATATTTGGATAGCTTTGAGGA
          1 ATAGAGCAGGTTTGAAACACTCTTTTTGTAGTATCTGGAAGTGGATATTTGGATAGCTTTGAGGA

                         *   *    *                   *       *     *   *    
     346409 TTTCGTTGGAAACGGGATTA-CAT-ATA-AAAAGC-AGACAGCAGCATTCCCAGAATCTTGTTTG
         66 TTTCGTTGGAAACAGGAATATCTTCATATAAAAGCTAGACAGAAGCATTCTCAGAAACTTCTTTG

                                   *                 
     346470 TGATGTTTGCATTCAAGT--CAGAGTTGAACATTCCCTTTC
        131 TGATGTTTGCATTCAAGTAACAGTGTTGAACATTCCCTTTC

             *                         *           *     *       **     *   *
     346509 AGAGAGCAGGTTTGAAACACTCTTTTTATAGTATCTGGATGTGGACATTTGGAGCGCTTTCAGGC
          1 ATAGAGCAGGTTTGAAACACTCTTTTTGTAGTATCTGGAAGTGGATATTTGGATAGCTTTGAGGA

            * ** *      *             *** *   *                      *   *   
     346574 CTATGGT-GAAAAAGGAAATATCTTCTCCTGAAAACTAGACAGAAGCATTCTCAGAATCTTATTT
         66 TTTCGTTGGAAACAGG-AATATCTTCATATAAAAGCTAGACAGAAGCATTCTCAGAAACTTCTTT

                   **  **    *             **  * *   *
     346638 GTGATGTGCGCCCTCAACTAACAGTGTTGAAGCTTTCTTTTG
        130 GTGATGTTTGCATTCAAGTAACAGTGTTGAACATTCCCTTTC

                     *                *   **     *   *        *              
     346680 ATAGAGCAGTTTTGAAACACTCTTTTCGTAAAATCTGCAAGAGGATATTTTGATAGCTTTGAGGA
          1 ATAGAGCAGGTTTGAAACACTCTTTTTGTAGTATCTGGAAGTGGATATTTGGATAGCTTTGAGGA

                         *   * *            **                      *    *   
     346745 TTTCGTTGGAAACGGGATTGTCTTCATATAAACTCTAGACAGAAGCATTCTCAGAAGCTTCATTG
         66 TTTCGTTGGAAACAGGAATATCTTCATATAAAAGCTAGACAGAAGCATTCTCAGAAACTTCTTTG

            *       **   *    *             *        
     346810 GGATGTTTCAATTGAAGTCACAGTGTTGAACAGTCCCTTTC
        131 TGATGTTTGCATTCAAGTAACAGTGTTGAACATTCCCTTTC

                                                         *       *  * * *    
     346851 ATAGAGCAGGTTTGAAACACTCTTTTTGTAGTATCTGGAAGTGGACATTTGGAGAGATCTCAGGA
          1 ATAGAGCAGGTTTGAAACACTCTTTTTGTAGTATCTGGAAGTGGATATTTGGATAGCTTTGAGGA

            * *  *    * *             **            *       * *          *  *
     346916 ATACGGT-GATAAAGGAAATATCTTCCAATAAAAGCTAGATAGAAGCAATGTCAGAAACTTTTTC
         66 TTTCGTTGGAAACAGG-AATATCTTCATATAAAAGCTAGACAGAAGCATTCTCAGAAACTTCTTT

            *      * ** *   **              *  * *   *
     346980 ATGATGTATCTACTCAGCTAACAGTGTTGAACCTTTCTTTTG
        130 GTGATGTTTGCATTCAAGTAACAGTGTTGAACATTCCCTTTC

             *       *                   * *     *             **            
     347022 AGAGAGCAGTTTTGAAACACTCTTTTTGTGGAATCTGCAAGTGGATATTTGTCTAGCTTTGAGGA
          1 ATAGAGCAGGTTTGAAACACTCTTTTTGTAGTATCTGGAAGTGGATATTTGGATAGCTTTGAGGA

                         *   *    *                  **  *    *   *          
     347087 TTTCGTTGGAAACGGGATTA-CAT-ATA-AAAAGC-AGACACCAGAATTCCCAGTAACTTCTTTG
         66 TTTCGTTGGAAACAGGAATATCTTCATATAAAAGCTAGACAGAAGCATTCTCAGAAACTTCTTTG

                              *    *                 
     347148 TGATGTTTGCATTCAAGTCACAGAGTTGAACATTCCCTTTC
        131 TGATGTTTGCATTCAAGTAACAGTGTTGAACATTCCCTTTC

                         *                         *     *    *  **         *
     347189 ATAGAGCAGGTTTAAAACACTCTTTTTGTAGTATCTGGATGTGGACATTTTGAGCGCTTTGAGGC
          1 ATAGAGCAGGTTTGAAACACTCTTTTTGTAGTATCTGGAAGTGGATATTTGGATAGCTTTGAGGA

            * ** *      *       *     *** *   *           *          *   *   
     347254 CTATGGT-GAAAAAGGAAATCTCTTCCCCTGAAAACTAGACAGAAGAATTCTCAGAATCTTATTT
         66 TTTCGTTGGAAACAGG-AATATCTTCATATAAAAGCTAGACAGAAGCATTCTCAGAAACTTCTTT

                   **  **    *             **  * *   *
     347318 GTGATGTGCGCCCTCAACTAACAGTGTTGAAGCTTTCTTTTG
        130 GTGATGTTTGCATTCAAGTAACAGTGTTGAACATTCCCTTTC

                     *                   ***     *   *                       
     347360 ATAGAGCAGTTTTGAAACACTCTTTTTGTTAAATCTGCAAGAGGATATTTGGATAGCTTTGAGGA
          1 ATAGAGCAGGTTTGAAACACTCTTTTTGTAGTATCTGGAAGTGGATATTTGGATAGCTTTGAGGA

                         *   * *            **                      *    *   
     347425 TTTCGTTGGAAACGGGATTGTCTTCATATAAACTCTAGACAGAAGCATTCTCAGAACCTTCATTG
         66 TTTCGTTGGAAACAGGAATATCTTCATATAAAAGCTAGACAGAAGCATTCTCAGAAACTTCTTTG

            *       **   *    *             * *      
     347490 GGATGTTTCAATTGAAGTCACAGTGTTGAACAGTTCCTTTC
        131 TGATGTTTGCATTCAAGTAACAGTGTTGAACATTCCCTTTC

                 *                                       *       **   * *    
     347531 ATAGAACAGGTTTGAAACACTCTTTTTGTAGTATCTGGAAGTGGACATTTGGAGCGCTCTCAGGA
          1 ATAGAGCAGGTTTGAAACACTCTTTTTGTAGTATCTGGAAGTGGATATTTGGATAGCTTTGAGGA

            * ** *      *             **          * *       * *          *  *
     347596 CTATGGT-GAAAAAGGAAATATCTTCCAATAAAAGCTACATAGAAGCAATGTCAGAAACTTTTTC
         66 TTTCGTTGGAAACAGG-AATATCTTCATATAAAAGCTAGACAGAAGCATTCTCAGAAACTTCTTT

            *      * ** *   **      *       *  *     *
     347660 ATGATGTATCTACTCAGCTAACAGAGTTGAACCTTTCCTTTG
        130 GTGATGTTTGCATTCAAGTAACAGTGTTGAACATTCCCTTTC

             *       *                   * *     *             **            
     347702 AGAGAGCAGTTTTGAAACACTCTTTTTGTGGAATCTGCAAGTGGATATTTGCTTAGCTTTGAGGA
          1 ATAGAGCAGGTTTGAAACACTCTTTTTGTAGTATCTGGAAGTGGATATTTGGATAGCTTTGAGGA

                         *   *    *                   *       *   *          
     347767 TTTCGTTGGAAACGGGATTA-CAT-ATA-AAAAGC-AGACAGCAGCATTCCCAGTAACTTCTTTG
         66 TTTCGTTGGAAACAGGAATATCTTCATATAAAAGCTAGACAGAAGCATTCTCAGAAACTTCTTTG

                              *    *                 
     347828 TGATGTTTGCATTCAAGTCACAGAGTTGAACATTCCCTTTC
        131 TGATGTTTGCATTCAAGTAACAGTGTTGAACATTCCCTTTC

                                                   *     *       **     *   *
     347869 ATAGAGCAGGTTTGAAACACTCTTTTTGTAGTATCTGGATGTGGACATTTGGAGCGCTTTCAGGC
          1 ATAGAGCAGGTTTGAAACACTCTTTTTGTAGTATCTGGAAGTGGATATTTGGATAGCTTTGAGGA

            * ** *      *             *** *   *                      *   *   
     347934 CTATGGT-GAAAAAGGAAATATCTTCTCCTGAAAACTAGACAGAAGCATTCTCAGAATCTTATTT
         66 TTTCGTTGGAAACAGG-AATATCTTCATATAAAAGCTAGACAGAAGCATTCTCAGAAACTTCTTT

                   **  **    *       *     **  * *   *
     347998 GTGATGTGCGCCCTCAACTAACAGTCTTGAAGCTTTCTTTTG
        130 GTGATGTTTGCATTCAAGTAACAGTGTTGAACATTCCCTTTC

                     *                    **     *   *                       
     348040 ATAGAGCAGTTTTGAAACACTCTTTTTGTAAAATCTGCAAGAGGATATTTGGATAGCTTTGAGGA
          1 ATAGAGCAGGTTTGAAACACTCTTTTTGTAGTATCTGGAAGTGGATATTTGGATAGCTTTGAGGA

                         *   * *            **                      *    *   
     348105 TTTCGTTGGAAACGGGATTGTCTTCATATAAACTCTAGACAGAAGCATTCTCAGAAGCTTCATTG
         66 TTTCGTTGGAAACAGGAATATCTTCATATAAAAGCTAGACAGAAGCATTCTCAGAAACTTCTTTG

            *       **   *    *             *        
     348170 GGATGTTTCAATTGAAGTCACAGTGTTGAACAGTCCCTTTC
        131 TGATGTTTGCATTCAAGTAACAGTGTTGAACATTCCCTTTC

                                                         *       *  * * *    
     348211 ATAGAGCAGGTTTGAAACACTCTTTTTGTAGTATCTGGAAGTGGACATTTGGAGAGATCTCAGGA
          1 ATAGAGCAGGTTTGAAACACTCTTTTTGTAGTATCTGGAAGTGGATATTTGGATAGCTTTGAGGA

            * *  *    * *             **     *    * *       * *          *  *
     348276 ATACGGT-GATAAAGGAAATATCTTCCAATAAACGCTACATAGAAGCAATGTCAGAAACTTTTTC
         66 TTTCGTTGGAAACAGG-AATATCTTCATATAAAAGCTAGACAGAAGCATTCTCAGAAACTTCTTT

            *      * ** *   **      *       *  * *   *
     348340 ATGATGTATCTACTCAGCTAACAGAGTTGAACCTTTCTTTTG
        130 GTGATGTTTGCATTCAAGTAACAGTGTTGAACATTCCCTTTC

             *       *                   * *     *             **            
     348382 AGAGAGCAGTTTTGAAACACTCTTTTTGTGGAATCTGCAAGTGGATATTTGTCTAGCTTTGAGGA
          1 ATAGAGCAGGTTTGAAACACTCTTTTTGTAGTATCTGGAAGTGGATATTTGGATAGCTTTGAGGA

                         *   *    *                   *       *   *          
     348447 TTTCGTTGGAAACGGGATTA-CAT-ATA-AAAAGC-AGACAGCAGCATTCCCAGTAACTTCTTTG
         66 TTTCGTTGGAAACAGGAATATCTTCATATAAAAGCTAGACAGAAGCATTCTCAGAAACTTCTTTG

                              *    *                 
     348508 TGATGTTTGCATTCAAGTCACAGAGTTGAACATTCCCTTTC
        131 TGATGTTTGCATTCAAGTAACAGTGTTGAACATTCCCTTTC

               *                                   *     *       **     *   *
     348549 ATACAGCAGGTTTGAAACACTCTTTTTGTAGTATCTGGATGTGGACATTTGGAGCGCTTTCAGGC
          1 ATAGAGCAGGTTTGAAACACTCTTTTTGTAGTATCTGGAAGTGGATATTTGGATAGCTTTGAGGA

            * ** *      *             *** *   *           *     *    *   *   
     348614 CTATGGT-GAAAAAGGAAATATCTTCCCCTGAAAACTAGACAGAAGAATTCTTAGAATCTTATTT
         66 TTTCGTTGGAAACAGG-AATATCTTCATATAAAAGCTAGACAGAAGCATTCTCAGAAACTTCTTT

                   **  **    *             **  * *   *
     348678 GTGATGTGCGCCCTCAACTAACAGTGTTGAAGCTTTCTTTTG
        130 GTGATGTTTGCATTCAAGTAACAGTGTTGAACATTCCCTTTC

                     *          *         **     *   *                       
     348720 ATAGAGCAGTTTTGAAACACACTTTTTGTAAAATCTGCAAGAGGATATTTGGATAGCTTTGAGGA
          1 ATAGAGCAGGTTTGAAACACTCTTTTTGTAGTATCTGGAAGTGGATATTTGGATAGCTTTGAGGA

                         *   * *            **                      * *  *   
     348785 TTTCGTTGGAAACGGGATTGTCTTCATATAAACTCTAGACAGAAGCATTCTCAGAAGCGTCATTG
         66 TTTCGTTGGAAACAGGAATATCTTCATATAAAAGCTAGACAGAAGCATTCTCAGAAACTTCTTTG

            *       **   *    *             *        
     348850 GGATGTTTCAATTGAAGTCACAGTGTTGAACAGTCCCTTTC
        131 TGATGTTTGCATTCAAGTAACAGTGTTGAACATTCCCTTTC

                                                   *     *       **     *  **
     348891 ATAGAGCAGGTTTGAAACACTCTTTTTGTAGTATCTGGATGTGGACATTTGGAGCGCTTTAAGCC
          1 ATAGAGCAGGTTTGAAACACTCTTTTTGTAGTATCTGGAAGTGGATATTTGGATAGCTTTGAGGA

             * *    *                 *** *   *                          *   
     348956 TATGGTT-TAAA-AGGAAATATCTTCCCCTGAAAACTAGACAGAAGCATTCTCAGAAACTTATTT
         66 TTTCGTTGGAAACAGG-AATATCTTCATATAAAAGCTAGACAGAAGCATTCTCAGAAACTTCTTT

                   **  **    *                    *   *
     349019 GTGATGTGCGCCCTCAACTAACAGTGTTGAAGCATT-CTTTTG
        130 GTGATGTTTGCATTCAAGTAACAGTGTTGAA-CATTCCCTTTC

                     *                   * *     *             **            
     349061 ATAGAGCAGTTTTGAAACACTCTTTTTGTGGAATCTGCAAGTGGATATTTGTCTAGCTTTGAGGA
          1 ATAGAGCAGGTTTGAAACACTCTTTTTGTAGTATCTGGAAGTGGATATTTGGATAGCTTTGAGGA

                         *   *    *                   *       *              
     349126 TTTCGTTGGAAACGGGATTA-CAT-ATA-AAAAGC-AGACAGCAGCATTCCCAGAAACTTCTTTG
         66 TTTCGTTGGAAACAGGAATATCTTCATATAAAAGCTAGACAGAAGCATTCTCAGAAACTTCTTTG

                              *    *                 
     349187 TGATGTTTGCATTCAAGTCACAGAGTTGAACATTCCCTTTC
        131 TGATGTTTGCATTCAAGTAACAGTGTTGAACATTCCCTTTC

                                                   *     **      **     *   *
     349228 ATAGAGCAGGTTTGAAACACTCTTTTTGTAGTATCTGGATGTGGACCTTTGGAGCGCTTTCAGGC
          1 ATAGAGCAGGTTTGAAACACTCTTTTTGTAGTATCTGGAAGTGGATATTTGGATAGCTTTGAGGA

            * ** *      *             *** *   *                      *   *   
     349293 CTATGGT-GAAAAAGGAAATATCTTCCCCTGAAAACTAGACAGAAGCATTCTCAGAATCTTATTT
         66 TTTCGTTGGAAACAGG-AATATCTTCATATAAAAGCTAGACAGAAGCATTCTCAGAAACTTCTTT

                   **  **    *             **  * *   *
     349357 GTGATGTGCGCCCTCAACTAACAGTGTTGAAGCTTTCTTTTG
        130 GTGATGTTTGCATTCAAGTAACAGTGTTGAACATTCCCTTTC

                     *                    *      *   *                    ** 
     349399 ATAGAGCAGTTTTGAAACACTCTTTTTGTAATATCTGCAAGAGGATATTTGGATAGCTTTGAATA
          1 ATAGAGCAGGTTTGAAACACTCTTTTTGTAGTATCTGGAAGTGGATATTTGGATAGCTTTGAGGA

                         *   * *            **                      *    *   
     349464 TTTCGTTGGAAACGGGATTGTCTTCATATAAACTCTAGACAGAAGCATTCTCAGAAGCTTCATTG
         66 TTTCGTTGGAAACAGGAATATCTTCATATAAAAGCTAGACAGAAGCATTCTCAGAAACTTCTTTG

            *       **   *    *             *        
     349529 GGATGTTTCAATTGAAGTCACAGTGTTGAACAGTCCCTTTC
        131 TGATGTTTGCATTCAAGTAACAGTGTTGAACATTCCCTTTC

                                                         *       **   * *  * 
     349570 ATAGAGCAGGTTTGAAACACTCTTTTTGTAGTATCTGGAAGTGGACATTTGGAGCGCTCTCAGTA
          1 ATAGAGCAGGTTTGAAACACTCTTTTTGTAGTATCTGGAAGTGGATATTTGGATAGCTTTGAGGA

            * *  *      *             **            *       * *          *  *
     349635 CTGCGGT-GAAAAAGGAAATATCTTCCAATAAAAGCTAGATAGAAGCAATGTCAGAAACTTTTTC
         66 TTTCGTTGGAAACAGG-AATATCTTCATATAAAAGCTAGACAGAAGCATTCTCAGAAACTTCTTT

            *      * ** *   **      *       *        *
     349699 ATGATGTATCTACTCAGCTAACAGAGTTGAACCTT-CCTTTG
        130 GTGATGTTTGCATTCAAGTAACAGTGTTGAACATTCCCTTTC

             *       *                   * *     *             **            
     349740 AGAGAGCAGTTTTGAAACACTCTTTTTGTGGAATCTGCAAGTGGATATTTGTCTAGCTTTGAGGA
          1 ATAGAGCAGGTTTGAAACACTCTTTTTGTAGTATCTGGAAGTGGATATTTGGATAGCTTTGAGGA

                         *   *    *                   *       *              
     349805 TTTCGTTGGAAACGGGATTA-CAT-ATA-AAAAGC-AGACAGCAGCATTCCCAGAAACTTCTTTG
         66 TTTCGTTGGAAACAGGAATATCTTCATATAAAAGCTAGACAGAAGCATTCTCAGAAACTTCTTTG

                              *    *                 
     349866 TGATGTTTGCATTCAAGTCACAGAGTTGAACATTCCCTTTC
        131 TGATGTTTGCATTCAAGTAACAGTGTTGAACATTCCCTTTC

                                                   *     *     * **     *   *
     349907 ATAGAGCAGGTTTGAAACACTCTTTTTGTAGTATCTGGATGTGGACATTTGCAGCGCTTTCAGGC
          1 ATAGAGCAGGTTTGAAACACTCTTTTTGTAGTATCTGGAAGTGGATATTTGGATAGCTTTGAGGA

            * ** *      *             *** *   *                          *   
     349972 CTAAGGT-GAAAAAGGAAATATCTTCCCCTGAAAACTAGACAGAAGCATTCTCAGAAACTTATTT
         66 TTTCGTTGGAAACAGG-AATATCTTCATATAAAAGCTAGACAGAAGCATTCTCAGAAACTTCTTT

                   **  **    *             **  * *   *
     350036 GTGATGTGCGCCCTCAACTAACAGTGTTGAAGCTTTCTTTTG
        130 GTGATGTTTGCATTCAAGTAACAGTGTTGAACATTCCCTTTC

                     *                    *      *   *                       
     350078 ATAGAGCAGTTTTGAAACACTCTTTTTGTAATATCTGCAAGAGGATATTTGGATAGCTTTGAGGA
          1 ATAGAGCAGGTTTGAAACACTCTTTTTGTAGTATCTGGAAGTGGATATTTGGATAGCTTTGAGGA

                         *   * *            **                               
     350143 TTTCGTTGGAAACGGGATTGTCTTCATATAAACTCTAGACAGAAGCATTCTCAGAAACTTCTTTG
         66 TTTCGTTGGAAACAGGAATATCTTCATATAAAAGCTAGACAGAAGCATTCTCAGAAACTTCTTTG

            *       **   *    *             *        
     350208 GGATGTTTCAATTGAAGTCACAGTGTTGAACAGTCCCTTTC
        131 TGATGTTTGCATTCAAGTAACAGTGTTGAACATTCCCTTTC

                 *                                       *       **   * *    
     350249 ATAGAACAGGTTTGAAACACTCTTTTTGTAGTATCTGGAAGTGGACATTTGGAGCGCTCTCAGGA
          1 ATAGAGCAGGTTTGAAACACTCTTTTTGTAGTATCTGGAAGTGGATATTTGGATAGCTTTGAGGA

            * ** *      *             **          * *       * *          *  *
     350314 CTATGGT-GAAAAAGGAAATATCTTCCAATAAAAGCTACATAGAAGCAATGTCAGAAACTTTTTC
         66 TTTCGTTGGAAACAGG-AATATCTTCATATAAAAGCTAGACAGAAGCATTCTCAGAAACTTCTTT

            *      * ** *   **      *       *  *     *
     350378 ATGATGTATCTACTCAGCTAACAGAGTTGAACCTTTCCTTTG
        130 GTGATGTTTGCATTCAAGTAACAGTGTTGAACATTCCCTTTC

             *       *                   * *     *             **            
     350420 AGAGAGCAGTTTTGAAACACTCTTTTTGTGGAATCTGCAAGTGGATATTTGTCTAGCTTTGAGGA
          1 ATAGAGCAGGTTTGAAACACTCTTTTTGTAGTATCTGGAAGTGGATATTTGGATAGCTTTGAGGA

                         *   *    *                   *       *   *          
     350485 TTTCGTTGGAAACGGGATTA-CAT-ATA-AAAAGC-AGACAGCAGCATTCCCAGTAACTTCTTTG
         66 TTTCGTTGGAAACAGGAATATCTTCATATAAAAGCTAGACAGAAGCATTCTCAGAAACTTCTTTG

                              *    *                 
     350546 TGATGTTTGCATTCAAGTCACAGAGTTGAACATTCCCTTTC
        131 TGATGTTTGCATTCAAGTAACAGTGTTGAACATTCCCTTTC

                                                   *     *       **     *   *
     350587 ATAGAGCAGGTTTGAAACACTCTTTTTGTAGTATCTGGATGTGGACATTTGGAACGCTTTCAGGC
          1 ATAGAGCAGGTTTGAAACACTCTTTTTGTAGTATCTGGAAGTGGATATTTGGATAGCTTTGAGGA

            * ** *      *             *** *   *                      *   *   
     350652 CTATGGT-GAAAAAGGAAATATCTTCTCCTGAAAACTAGACAGAAGCATTCTCAGAATCTTATTT
         66 TTTCGTTGGAAACAGG-AATATCTTCATATAAAAGCTAGACAGAAGCATTCTCAGAAACTTCTTT

                   **  **    *             **  * *   *
     350716 GTGATGTGCGCCCTCAACTAACAGTGTTGAAGCTTTCTTTTG
        130 GTGATGTTTGCATTCAAGTAACAGTGTTGAACATTCCCTTTC

                     *                    *      *   *                       
     350758 ATAGAGCAGTTTTGAAACACTCTTTTTGTAATATCTGCAAGAGGATATTTGGATAGCTTTGAGGA
          1 ATAGAGCAGGTTTGAAACACTCTTTTTGTAGTATCTGGAAGTGGATATTTGGATAGCTTTGAGGA

                         *   * *            **                      *    *   
     350823 TTTCGTTGGAAACGGGATTGTCTTCATATAAACTCTAGACAGAAGCATTCTCAGAAGCTTCATTG
         66 TTTCGTTGGAAACAGGAATATCTTCATATAAAAGCTAGACAGAAGCATTCTCAGAAACTTCTTTG

            *       **   *    *             *        
     350888 GGATGTTTCAATTGAAGTCACAGTGTTGAACAGTCCCTTTC
        131 TGATGTTTGCATTCAAGTAACAGTGTTGAACATTCCCTTTC

                                                         *       *  * * *    
     350929 ATAGAGCAGGTTTGAAACACTCTTTTTGTAGTATCTGGAAGTGGACATTTGGAGAGATCTCAGGA
          1 ATAGAGCAGGTTTGAAACACTCTTTTTGTAGTATCTGGAAGTGGATATTTGGATAGCTTTGAGGA

            * *  *    * *             **            *       * *          *  *
     350994 ATACGGT-GATAAAGGAAATATCTTCCAATAAAAGCTAGATAGAAGCAATGTCAGAAACTTTTTC
         66 TTTCGTTGGAAACAGG-AATATCTTCATATAAAAGCTAGACAGAAGCATTCTCAGAAACTTCTTT

            *      * ** *   **      *       *  * *   *
     351058 ATGATGTATCTACTCAGCTAACAGAGTTGAACCTTTCTTTTG
        130 GTGATGTTTGCATTCAAGTAACAGTGTTGAACATTCCCTTTC

             *       *                   * *     *             **            
     351100 AGAGAGCAGTTTTGAAACACTCTTTTTGTGGAATCTGCAAGTGGATATTTGTCTAGCTTTGAGGA
          1 ATAGAGCAGGTTTGAAACACTCTTTTTGTAGTATCTGGAAGTGGATATTTGGATAGCTTTGAGGA

                         *   *    *                   *       *     *        
     351165 TTTCGTTGGAAACGGGATTA-CAT-ATA-AAAAGC-AGACAGCAGCATTCCCAGAATCTTCTTTG
         66 TTTCGTTGGAAACAGGAATATCTTCATATAAAAGCTAGACAGAAGCATTCTCAGAAACTTCTTTG

                              *    *                 
     351226 TGATGTTTGCATTCAAGTCACAGAGTTGAACATTCCCTTTC
        131 TGATGTTTGCATTCAAGTAACAGTGTTGAACATTCCCTTTC

               *                                   *     *       **     *   *
     351267 ATACAGCAGGTTTGAAACACTCTTTTTGTAGTATCTGGATGTGGACATTTGGAGCGCTTTCAGGC
          1 ATAGAGCAGGTTTGAAACACTCTTTTTGTAGTATCTGGAAGTGGATATTTGGATAGCTTTGAGGA

            * ** *      *             *** *   *           *          *   *   
     351332 CTATGGT-GAAAAAGGAAATATCTTCCCCTGAAAACTAGACAGAAGAATTCTCAGAATCTTATTT
         66 TTTCGTTGGAAACAGG-AATATCTTCATATAAAAGCTAGACAGAAGCATTCTCAGAAACTTCTTT

                   **  **    *       *     **  * *   *
     351396 GTGATGTGCGCCCTCAACTAACAGTCTTGAAGCTTTCTTTTG
        130 GTGATGTTTGCATTCAAGTAACAGTGTTGAACATTCCCTTTC

                     *                    **     *   *                       
     351438 ATAGAGCAGTTTTGAAACACTCTTTTTGTAAAATCTGCAAGAGGATATTTGGATAGCTTTGAGGA
          1 ATAGAGCAGGTTTGAAACACTCTTTTTGTAGTATCTGGAAGTGGATATTTGGATAGCTTTGAGGA

                         *   * *            **                      *    *   
     351503 TTTCGTTGGAAACGGGATTGTCTTCATATAAACTCTAGACAGAAGCATTCTCAGAAGCTTCATTG
         66 TTTCGTTGGAAACAGGAATATCTTCATATAAAAGCTAGACAGAAGCATTCTCAGAAACTTCTTTG

            *       **   *    *             *        
     351568 GGATGTTTCAATTGAAGTCACAGTGTTGAACAGTCCCTTTC
        131 TGATGTTTGCATTCAAGTAACAGTGTTGAACATTCCCTTTC

                                                         *       *  * * *    
     351609 ATAGAGCAGGTTTGAAACACTCTTTTTGTAGTATCTGGAAGTGGACATTTGGAGAGATCTCAGGA
          1 ATAGAGCAGGTTTGAAACACTCTTTTTGTAGTATCTGGAAGTGGATATTTGGATAGCTTTGAGGA

            * *  *    * *             **     *    * *       * *          *  *
     351674 ATACGGT-GATAAAGGAAATATCTTCCAATAAACGCTACATAGAAGCAATGTCAGAAACTTTTTC
         66 TTTCGTTGGAAACAGG-AATATCTTCATATAAAAGCTAGACAGAAGCATTCTCAGAAACTTCTTT

            *      * ** *   **      *       *  * *   *
     351738 ATGATGTATATACTCAGCTAACAGAGTTGAACCTTTCTTTTG
        130 GTGATGTTTGCATTCAAGTAACAGTGTTGAACATTCCCTTTC

             *       *                   * *     *             **            
     351780 AGAGAGCAGTTTTGAAACACTCTTTTTGTGGAATCTGCAAGTGGATATTTGTCTAGCTTTGAGGA
          1 ATAGAGCAGGTTTGAAACACTCTTTTTGTAGTATCTGGAAGTGGATATTTGGATAGCTTTGAGGA

                         *   *    *                   *       *   *          
     351845 TTTCGTTGGAAACGGGATTA-CAT-ATA-AAAAGC-AGACAGCAGCATTCCCAGTAACTTCTTTG
         66 TTTCGTTGGAAACAGGAATATCTTCATATAAAAGCTAGACAGAAGCATTCTCAGAAACTTCTTTG

                              *    *                 
     351906 TGATGTTTGCATTCAAGTCACAGAGTTGAACATTCCCTTTC
        131 TGATGTTTGCATTCAAGTAACAGTGTTGAACATTCCCTTTC

               *                                   *     *       **     *   *
     351947 ATACAGCAGGTTTGAAACACTCTTTTTGTAGTATCTGGATGTGGACATTTGGAGCGCTTTCAGGC
          1 ATAGAGCAGGTTTGAAACACTCTTTTTGTAGTATCTGGAAGTGGATATTTGGATAGCTTTGAGGA

            * ** *      *             *** *   *           *     *    *   *   
     352012 CTATGGT-GAAAAAGGAAATATCTTCCCCTGAAAACTAGACAGAAGAATTCTTAGAATCTTATTT
         66 TTTCGTTGGAAACAGG-AATATCTTCATATAAAAGCTAGACAGAAGCATTCTCAGAAACTTCTTT

                   **  **    *             **  * *   *
     352076 GTGATGTGCGCCCTCAACTAACAGTGTTGAAGCTTTCTTTTG
        130 GTGATGTTTGCATTCAAGTAACAGTGTTGAACATTCCCTTTC

                     *                    **     *   *                       
     352118 ATAGAGCAGTTTTGAAACACTCTTTTTGTAAAATCTGCAAGAGGATATTTGGATAGCTTTGAGGA
          1 ATAGAGCAGGTTTGAAACACTCTTTTTGTAGTATCTGGAAGTGGATATTTGGATAGCTTTGAGGA

                         *   * *            **                      * *  *   
     352183 TTTCGTTGGAAACGGGATTGTCTTCATATAAACTCTAGACAGAAGCATTCTCAGAAGCGTCATTG
         66 TTTCGTTGGAAACAGGAATATCTTCATATAAAAGCTAGACAGAAGCATTCTCAGAAACTTCTTTG

            *       **   *    *             *        
     352248 GGATGTTTCAATTGAAGTCACAGTGTTGAACAGTCCCTTTC
        131 TGATGTTTGCATTCAAGTAACAGTGTTGAACATTCCCTTTC

                                                   *     *       **     *  **
     352289 ATAGAGCAGGTTTGAAACACTCTTTTTGTAGTATCTGGATGTGGACATTTGGAGCGCTTTAAGCC
          1 ATAGAGCAGGTTTGAAACACTCTTTTTGTAGTATCTGGAAGTGGATATTTGGATAGCTTTGAGGA

             * *    *                 *** *   *                          *   
     352354 TATGGTT-TAAA-AGGAAATATCTTCCCCTGAAAACTAGACAGAAGCATTCTCAGAAACTTATTT
         66 TTTCGTTGGAAACAGG-AATATCTTCATATAAAAGCTAGACAGAAGCATTCTCAGAAACTTCTTT

                   **  **    *                    *   *
     352417 GTGATGTGCGCCCTCAACTAACAGTGTTGAAGCATT-CTTTTG
        130 GTGATGTTTGCATTCAAGTAACAGTGTTGAA-CATTCCCTTTC

                     *                   * *     *             **            
     352459 ATAGAGCAGTTTTGAAACACTCTTTTTGTGGAATCTGCAAGTGGATATTTGTCTAGCTTTGAGGA
          1 ATAGAGCAGGTTTGAAACACTCTTTTTGTAGTATCTGGAAGTGGATATTTGGATAGCTTTGAGGA

                         *   *    *                   *       *              
     352524 TTTCGTTGGAAACGGGATTA-CAT-ATA-AAAAGC-AGACAGCAGCATTCCCAGAAACTTCTTTG
         66 TTTCGTTGGAAACAGGAATATCTTCATATAAAAGCTAGACAGAAGCATTCTCAGAAACTTCTTTG

                              *    *                 
     352585 TGATGTTTGCATTCAAGTCACAGAGTTGAACATTCCCTTTC
        131 TGATGTTTGCATTCAAGTAACAGTGTTGAACATTCCCTTTC

                                                   *     **      **     *   *
     352626 ATAGAGCAGGTTTGAAACACTCTTTTTGTAGTATCTGGATGTGGACCTTTGGAGCGCTTTCAGGC
          1 ATAGAGCAGGTTTGAAACACTCTTTTTGTAGTATCTGGAAGTGGATATTTGGATAGCTTTGAGGA

            * ** *      *             *** *   *                      *   *   
     352691 CTATGGT-GAAAAAGGAAATATCTTCCCCTGAAAACTAGACAGAAGCATTCTCAGAATCTTATTT
         66 TTTCGTTGGAAACAGG-AATATCTTCATATAAAAGCTAGACAGAAGCATTCTCAGAAACTTCTTT

                   **  **    *             **  * *   *
     352755 GTGATGTGCGCCCTCAACTAACAGTGTTGAAGCTTTCTTTTG
        130 GTGATGTTTGCATTCAAGTAACAGTGTTGAACATTCCCTTTC

                     *                    *      *   *                    ** 
     352797 ATAGAGCAGTTTTGAAACACTCTTTTTGTAATATCTGCAAGAGGATATTTGGATAGCTTTGAATA
          1 ATAGAGCAGGTTTGAAACACTCTTTTTGTAGTATCTGGAAGTGGATATTTGGATAGCTTTGAGGA

                         *   * *            **                      *    *   
     352862 TTTCGTTGGAAACGGGATTGTCTTCATATAAACTCTAGACAGAAGCATTCTCAGAAGCTTCATTG
         66 TTTCGTTGGAAACAGGAATATCTTCATATAAAAGCTAGACAGAAGCATTCTCAGAAACTTCTTTG

            *       **   *    *             *        
     352927 GGATGTTTCAATTGAAGTCACAGTGTTGAACAGTCCCTTTC
        131 TGATGTTTGCATTCAAGTAACAGTGTTGAACATTCCCTTTC

                        *                                *       **   * *  * 
     352968 ATAGAGCAGGTTCGAAACACTCTTTTTGTAGTATCTGGAAGTGGACATTTGGAGCGCTCTCAGTA
          1 ATAGAGCAGGTTTGAAACACTCTTTTTGTAGTATCTGGAAGTGGATATTTGGATAGCTTTGAGGA

            * *  *      *             **            *       * *          *  *
     353033 CTGCGGT-GAAAAAGGAAATATCTTCCAATAAAAGCTAGATAGAAGCAATGTCAGAAACTTTTTC
         66 TTTCGTTGGAAACAGG-AATATCTTCATATAAAAGCTAGACAGAAGCATTCTCAGAAACTTCTTT

            *      * ** *   **      *       *        *
     353097 ATGATGTATCTACTCAGCTAACAGAGTTGAACCTT-CCTTTG
        130 GTGATGTTTGCATTCAAGTAACAGTGTTGAACATTCCCTTTC

             *       *                   * *     *             **            
     353138 AGAGAGCAGTTTTGAAACACTCTTTTTGTGGAATCTGCAAGTGGATATTTGTCTAGCTTTGAGGA
          1 ATAGAGCAGGTTTGAAACACTCTTTTTGTAGTATCTGGAAGTGGATATTTGGATAGCTTTGAGGA

                         *   *    *                   *       *              
     353203 TTTCGTTGGAAACGGGATTA-CAT-ATA-AAAAGC-AGACAGCAGCATTCCCAGAAACTTCTTTG
         66 TTTCGTTGGAAACAGGAATATCTTCATATAAAAGCTAGACAGAAGCATTCTCAGAAACTTCTTTG

                              *    *                 
     353264 TGATGTTTGCATTCAAGTCACAGAGTTGAACATTCCCTTTC
        131 TGATGTTTGCATTCAAGTAACAGTGTTGAACATTCCCTTTC

                                                   *     *     * **     *   *
     353305 ATAGAGCAGGTTTGAAACACTCTTTTTGTAGTATCTGGATGTGGACATTTGCAGCGCTTTCAGGC
          1 ATAGAGCAGGTTTGAAACACTCTTTTTGTAGTATCTGGAAGTGGATATTTGGATAGCTTTGAGGA

            * ** *      *             *** *   *                          *   
     353370 CTAAGGT-GAAAAAGGAAATATCTTCCCCTGAAAACTAGACAGAAGCATTCTCAGAAACTTATTT
         66 TTTCGTTGGAAACAGG-AATATCTTCATATAAAAGCTAGACAGAAGCATTCTCAGAAACTTCTTT

                   **  **    *             **  * *   *
     353434 GTGATGTGCGCCCTCAACTAACAGTGTTGAAGCTTTCTTTTG
        130 GTGATGTTTGCATTCAAGTAACAGTGTTGAACATTCCCTTTC

                     *                    *      *   *                       
     353476 ATAGAGCAGTTTTGAAACACTCTTTTTGTAATATCTGCAAGAGGATATTTGGATAGCTTTGAGGA
          1 ATAGAGCAGGTTTGAAACACTCTTTTTGTAGTATCTGGAAGTGGATATTTGGATAGCTTTGAGGA

                         *   * *            **                           *   
     353541 TTTCGTTGGAAACGGGATTGTCTTCATATAAACTCTAGACAGAAGCATTCTCAGAAACTTCATTG
         66 TTTCGTTGGAAACAGGAATATCTTCATATAAAAGCTAGACAGAAGCATTCTCAGAAACTTCTTTG

            *       **   *    *             *        
     353606 GGATGTTTCAATTGAAGTCACAGTGTTGAACAGTCCCTTTC
        131 TGATGTTTGCATTCAAGTAACAGTGTTGAACATTCCCTTTC

                 *                                       *       **   * *    
     353647 ATAGAACAGGTTTGAAACACTCTTTTTGTAGTATCTGGAAGTGGACATTTGGAGCGCTCTCAGGA
          1 ATAGAGCAGGTTTGAAACACTCTTTTTGTAGTATCTGGAAGTGGATATTTGGATAGCTTTGAGGA

            * ** *      *             **          * *       * *          *  *
     353712 CTATGGT-GAAAAAGGAAATATCTTCCAATAAAAGCTACATAGAAGCAATGTCAGAAACTTTTTC
         66 TTTCGTTGGAAACAGG-AATATCTTCATATAAAAGCTAGACAGAAGCATTCTCAGAAACTTCTTT

            *      * ** *   **      *       *  *     *
     353776 ATGATGTATCTACTCAGCTAACAGAGTTGAACCTTTCCTTTG
        130 GTGATGTTTGCATTCAAGTAACAGTGTTGAACATTCCCTTTC

             *       *                   * *     *             **            
     353818 AGAGAGCAGTTTTGAAACACTCTTTTTGTGGAATCTGCAAGTGGATATTTGTCTAGCTTTGAGGA
          1 ATAGAGCAGGTTTGAAACACTCTTTTTGTAGTATCTGGAAGTGGATATTTGGATAGCTTTGAGGA

                         *   *    *                   *       *   *          
     353883 TTTCGTTGGAAACGGGATTA-CAT-ATA-AAAAGC-AGACAGCAGCATTCCCAGTAACTTCTTTG
         66 TTTCGTTGGAAACAGGAATATCTTCATATAAAAGCTAGACAGAAGCATTCTCAGAAACTTCTTTG

                              *    *                 
     353944 TGATGTTTGCATTCAAGTCACAGAGTTGAACATTCCCTTTC
        131 TGATGTTTGCATTCAAGTAACAGTGTTGAACATTCCCTTTC

                                                   *     *       **     *   *
     353985 ATAGAGCAGGTTTGAAACACTCTTTTTGTAGTATCTGGATGTGGACATTTGGAACGCTTTCAGGC
          1 ATAGAGCAGGTTTGAAACACTCTTTTTGTAGTATCTGGAAGTGGATATTTGGATAGCTTTGAGGA

            * ** *      *             *** *   *                      *   *   
     354050 CTATGGT-GAAAAAGGAAATATCTTCTCCTGAAAACTAGACAGAAGCATTCTCAGAATCTTATTT
         66 TTTCGTTGGAAACAGG-AATATCTTCATATAAAAGCTAGACAGAAGCATTCTCAGAAACTTCTTT

                   **  **    *             **  * *   *
     354114 GTGATGTGCGCCCTCAACTAACAGTGTTGAAGCTTTCTTTTG
        130 GTGATGTTTGCATTCAAGTAACAGTGTTGAACATTCCCTTTC

                     *                    *      *   *                       
     354156 ATAGAGCAGTTTTGAAACACTCTTTTTGTAATATCTGCAAGAGGATATTTGGATAGCTTTGAGGA
          1 ATAGAGCAGGTTTGAAACACTCTTTTTGTAGTATCTGGAAGTGGATATTTGGATAGCTTTGAGGA

                         *   * *            **                      *    *   
     354221 TTTCGTTGGAAACGGGATTGTCTTCATATAAACTCTAGACAGAAGCATTCTCAGAAGCTTCATTG
         66 TTTCGTTGGAAACAGGAATATCTTCATATAAAAGCTAGACAGAAGCATTCTCAGAAACTTCTTTG

            *       **   *    *             *        
     354286 GGATGTTTCAATTGAAGTCACAGTGTTGAACAGTCCCTTTC
        131 TGATGTTTGCATTCAAGTAACAGTGTTGAACATTCCCTTTC

                                                         *       *  * * *    
     354327 ATAGAGCAGGTTTGAAACACTCTTTTTGTAGTATCTGGAAGTGGACATTTGGAGAGATCTCAGGA
          1 ATAGAGCAGGTTTGAAACACTCTTTTTGTAGTATCTGGAAGTGGATATTTGGATAGCTTTGAGGA

            * *  *    * *             **            *       * *          *  *
     354392 ATACGGT-GATAAAGGAAATATCTTCCAATAAAAGCTAGATAGAAGCAATGTCAGAAACTTTTTC
         66 TTTCGTTGGAAACAGG-AATATCTTCATATAAAAGCTAGACAGAAGCATTCTCAGAAACTTCTTT

            *      * ** *   **      *       *  * *   *
     354456 ATGATGTATCTACTCAGCTAACAGAGTTGAACCTTTCTTTTG
        130 GTGATGTTTGCATTCAAGTAACAGTGTTGAACATTCCCTTTC

             *       *                   * *     *             **            
     354498 AGAGAGCAGTTTTGAAACACTCTTTTTGTGGAATCTGCAAGTGGATATTTGTCTAGCTTTGAGGA
          1 ATAGAGCAGGTTTGAAACACTCTTTTTGTAGTATCTGGAAGTGGATATTTGGATAGCTTTGAGGA

                         *   *    *                   *       *   *          
     354563 TTTCGTTGGAAACGGGATTA-CAT-ATA-AAAAGC-AGACAGCAGCATTCCCAGTAACTTCTTTG
         66 TTTCGTTGGAAACAGGAATATCTTCATATAAAAGCTAGACAGAAGCATTCTCAGAAACTTCTTTG

                              *    *                 
     354624 TGATGTTTGCATTCAAGTCACAGAGTTGAACATTCCCTTTC
        131 TGATGTTTGCATTCAAGTAACAGTGTTGAACATTCCCTTTC

               *                                   *     *       **     *   *
     354665 ATACAGCAGGTTTGAAACACTCTTTTTGTAGTATCTGGATGTGGACATTTGGAGCGCTTTCAGGC
          1 ATAGAGCAGGTTTGAAACACTCTTTTTGTAGTATCTGGAAGTGGATATTTGGATAGCTTTGAGGA

            * ** *      *             *** *   *           *          *   *   
     354730 CTATGGT-GAAAAAGGAAATATCTTCCCCTGAAAACTAGACAGAAGAATTCTCAGAATCTTATTT
         66 TTTCGTTGGAAACAGG-AATATCTTCATATAAAAGCTAGACAGAAGCATTCTCAGAAACTTCTTT

                   **  **    *       *     **  * *   *
     354794 GTGATGTGCGCCCTCAACTAACAGTCTTGAAGCTTTCTTTTG
        130 GTGATGTTTGCATTCAAGTAACAGTGTTGAACATTCCCTTTC

                     *                    **     *   *                       
     354836 ATAGAGCAGTTTTGAAACACTCTTTTTGTAAAATCTGCAAGAGGATATTTGGATAGCTTTGAGGA
          1 ATAGAGCAGGTTTGAAACACTCTTTTTGTAGTATCTGGAAGTGGATATTTGGATAGCTTTGAGGA

                         *   * *            **                      *    *   
     354901 TTTCGTTGGAAACGGGATTGTCTTCATATAAACTCTAGACAGAAGCATTCTCAGAAGCTTCATTG
         66 TTTCGTTGGAAACAGGAATATCTTCATATAAAAGCTAGACAGAAGCATTCTCAGAAACTTCTTTG

            *       **   *    *             *        
     354966 GGATGTTTCAATTGAAGTCACAGTGTTGAACAGTCCCTTTC
        131 TGATGTTTGCATTCAAGTAACAGTGTTGAACATTCCCTTTC

                                                         *       *  * * *    
     355007 ATAGAGCAGGTTTGAAACACTCTTTTTGTAGTATCTGGAAGTGGACATTTGGAGAGATCTCAGGA
          1 ATAGAGCAGGTTTGAAACACTCTTTTTGTAGTATCTGGAAGTGGATATTTGGATAGCTTTGAGGA

            * *  *    * *             **     *    * *       * *          *  *
     355072 ATACGGT-GATAAAGGAAATATCTTCCAATAAACGCTACATAGAAGCAATGTCAGAAACTTTTTC
         66 TTTCGTTGGAAACAGG-AATATCTTCATATAAAAGCTAGACAGAAGCATTCTCAGAAACTTCTTT

            *      * ** *   **      *       *  *     *
     355136 ATGATGTATCTACTCAGCTAACAGAGTTGAACCTTTCCTTTG
        130 GTGATGTTTGCATTCAAGTAACAGTGTTGAACATTCCCTTTC

             *       *                   * *     *             **            
     355178 AGAGAGCAGTTTTGAAACACTCTTTTTGTGGAATCTGCAAGTGGATATTTGTCTAGCTTTGAGGA
          1 ATAGAGCAGGTTTGAAACACTCTTTTTGTAGTATCTGGAAGTGGATATTTGGATAGCTTTGAGGA

                         *   *    *                   *       *   *          
     355243 TTTCGTTGGAAACGGGATTA-CAT-ATA-AAAAGC-AGACAGCAGCATTCCCAGTAACTTCTTTG
         66 TTTCGTTGGAAACAGGAATATCTTCATATAAAAGCTAGACAGAAGCATTCTCAGAAACTTCTTTG

                              *    *                 
     355304 TGATGTTTGCATTCAAGTCACAGAGTTGAACATTCCCTTTC
        131 TGATGTTTGCATTCAAGTAACAGTGTTGAACATTCCCTTTC

               *                                   *     *       **     *   *
     355345 ATACAGCAGGTTTGAAACACTCTTTTTGTAGTATCTGGATGTGGACATTTGGAGCGCTTTCAGGC
          1 ATAGAGCAGGTTTGAAACACTCTTTTTGTAGTATCTGGAAGTGGATATTTGGATAGCTTTGAGGA

            * ** *  *   ****         *** *   *           *     *    *   *    
     355410 CTATGGTGAAAAACCAAATATCTTCCCCTGAAAACTAGACAGAAGAATTCTTAGAATCTTATTTG
         66 TTTCGTTGGAAACAGGAATATCTTCATATAAAAGCTAGACAGAAGCATTCTCAGAAACTTCTTTG

                  **  **    *             **  * *   *
     355475 TGATGTGCGCCCTCAACTAACAGTGTTGAAGCTTTCTTTTG
        131 TGATGTTTGCATTCAAGTAACAGTGTTGAACATTCCCTTTC

                     *         *          **     *   *                       
     355516 ATAGAGCAGTTTTGAAACAATCTTTTTGTAAAATCTGCAAGAGGATATTTGGATAGCTTTGAGGA
          1 ATAGAGCAGGTTTGAAACACTCTTTTTGTAGTATCTGGAAGTGGATATTTGGATAGCTTTGAGGA

                         *   * *             *                      * *  *   
     355581 TTTCGTTGGAAACGGGATTGTCTTCATATAAAATCTAGACAGAAGCATTCTCAGAAGCGTCATTG
         66 TTTCGTTGGAAACAGGAATATCTTCATATAAAAGCTAGACAGAAGCATTCTCAGAAACTTCTTTG

            *       **   *    *             *        
     355646 GGATGTTTCAATTGAAGTCACAGTGTTGAACAGTCCCTTTC
        131 TGATGTTTGCATTCAAGTAACAGTGTTGAACATTCCCTTTC

                                                   *     *       **     *  **
     355687 ATAGAGCAGGTTTGAAACACTCTTTTTGTAGTATCTGGATGTGGACATTTGGAGCGCTTTAAGCC
          1 ATAGAGCAGGTTTGAAACACTCTTTTTGTAGTATCTGGAAGTGGATATTTGGATAGCTTTGAGGA

             * *    *                 *** *   *                          *   
     355752 TATGGTT-TAAA-AGGAAATATCTTCCCCTGAAAACTAGACAGAAGCATTCTCAGAAACTTATTT
         66 TTTCGTTGGAAACAGG-AATATCTTCATATAAAAGCTAGACAGAAGCATTCTCAGAAACTTCTTT

                   **  **    *                    *   *
     355815 GTGATGTGCGCCCTCAACTAACAGTGTTGAAGCATT-CTTTTG
        130 GTGATGTTTGCATTCAAGTAACAGTGTTGAA-CATTCCCTTTC

                     *                   * *     *             **            
     355857 ATAGAGCAGTTTTGAAACACTCTTTTTGTGGAATCTGCAAGTGGATATTTGTCTAGCTTTGAGGA
          1 ATAGAGCAGGTTTGAAACACTCTTTTTGTAGTATCTGGAAGTGGATATTTGGATAGCTTTGAGGA

                         *   *    *                   *       *              
     355922 TTTCGTTGGAAACGGGATTA-CAT-ATA-AAAAGC-AGACAGCAGCATTCCCAGAAACTTCTTTG
         66 TTTCGTTGGAAACAGGAATATCTTCATATAAAAGCTAGACAGAAGCATTCTCAGAAACTTCTTTG

                              *    *                 
     355983 TGATGTTTGCATTCAAGTCACAGAGTTGAACATTCCCTTTC
        131 TGATGTTTGCATTCAAGTAACAGTGTTGAACATTCCCTTTC

                                                   *     **      **     *   *
     356024 ATAGAGCAGGTTTGAAACACTCTTTTTGTAGTATCTGGATGTGGACCTTTGGAGCGCTTTCAGGC
          1 ATAGAGCAGGTTTGAAACACTCTTTTTGTAGTATCTGGAAGTGGATATTTGGATAGCTTTGAGGA

            * ** *      *             *** *   *                      *   *   
     356089 CTATGGT-GAAAAAGGAAATATCTTCCCCTGAAAACTAGACAGAAGCATTCTCAGAATCTTATTT
         66 TTTCGTTGGAAACAGG-AATATCTTCATATAAAAGCTAGACAGAAGCATTCTCAGAAACTTCTTT

                   **  **    **            **  * *   *
     356153 GTGATGTGCGCCCTCAACGAACAGTGTTGAAGCTTTCTTTTG
        130 GTGATGTTTGCATTCAAGTAACAGTGTTGAACATTCCCTTTC

                     *                    *      *   *                       
     356195 ATAGAGCAGTTTTGAAACACTCTTTTTGTAATATCTGCAAGAGGATATTTGGATAGCTTTGAGGA
          1 ATAGAGCAGGTTTGAAACACTCTTTTTGTAGTATCTGGAAGTGGATATTTGGATAGCTTTGAGGA

                         * * * *            **                      *    *   
     356260 TTTCGTTGGAAACGGTATTGTCTTCATATAAACTCTAGACAGAAGCATTCTCAGAAGCTTCATTG
         66 TTTCGTTGGAAACAGGAATATCTTCATATAAAAGCTAGACAGAAGCATTCTCAGAAACTTCTTTG

            *       **   *    *             *        
     356325 GGATGTTTCAATTGAAGTCACAGTGTTGAACAGTCCCTTTC
        131 TGATGTTTGCATTCAAGTAACAGTGTTGAACATTCCCTTTC

                                                         *       ** * * *  * 
     356366 ATAGAGCAGGTTTGAAACACTCTTTTTGTAGTATCTGGAAGTGGACATTTGGAGCGTTCTCAGTA
          1 ATAGAGCAGGTTTGAAACACTCTTTTTGTAGTATCTGGAAGTGGATATTTGGATAGCTTTGAGGA

            * *  *      *             **            *       * *       *  *  *
     356431 CTGCGGT-GAAAAAGGAAATATCTTCCAATAAAAGCTAGATAGAAGCAATGTCAGAAAATTTTTC
         66 TTTCGTTGGAAACAGG-AATATCTTCATATAAAAGCTAGACAGAAGCATTCTCAGAAACTTCTTT

            *      * ** *   **      *       *        *
     356495 ATGATGTATCTACTCAGCTAACAGAGTTGAACCTT-CCTTTG
        130 GTGATGTTTGCATTCAAGTAACAGTGTTGAACATTCCCTTTC

             *       *                   * *     *             **            
     356536 AGAGAGCAGTTTTGAAACACTCTTTTTGTGGAATCTGCAAGTGGATATTTGTCTAGCTTTGAGGA
          1 ATAGAGCAGGTTTGAAACACTCTTTTTGTAGTATCTGGAAGTGGATATTTGGATAGCTTTGAGGA

                         *   *    *                   *       *              
     356601 TTTCGTTGGAAACGGGATTA-CAT-ATA-AAAAGC-AGACAGCAGCATTCCCAGAAACTTCTTTG
         66 TTTCGTTGGAAACAGGAATATCTTCATATAAAAGCTAGACAGAAGCATTCTCAGAAACTTCTTTG

                              *    *                 
     356662 TGATGTTTGCATTCAAGTCACAGAGTTGAACATTCCCTTTC
        131 TGATGTTTGCATTCAAGTAACAGTGTTGAACATTCCCTTTC

                                                   *     *     * **     *   *
     356703 ATAGAGCAGGTTTGAAACACTC-TTTTGTAGTATCTGGATGTGGACATTTGCAGCGCTTTCAGGC
          1 ATAGAGCAGGTTTGAAACACTCTTTTTGTAGTATCTGGAAGTGGATATTTGGATAGCTTTGAGGA

            * ** *      *             *** *   *                          *   
     356767 CTAAGGT-GAAAAAGGAAATATCTTCCCCTGAAAACTAGACAGAAGCATTCTCAGAAACTTATTT
         66 TTTCGTTGGAAACAGG-AATATCTTCATATAAAAGCTAGACAGAAGCATTCTCAGAAACTTCTTT

                   **  **    *             **  * *   *
     356831 GTGATGTGCGCCCTCAACTAACAGTGTTGAAGCTTTCTTTTG
        130 GTGATGTTTGCATTCAAGTAACAGTGTTGAACATTCCCTTTC

                     *                    *      *   *                       
     356873 ATAGAGCAGTTTTGAAACACTCTTTTTGTAATATCTGCAAGAGGATATTTGGATAGCTTTGAGGA
          1 ATAGAGCAGGTTTGAAACACTCTTTTTGTAGTATCTGGAAGTGGATATTTGGATAGCTTTGAGGA

              *          *   * *            **                           *   
     356938 TTCCGTTGGAAACGGGATTGTCTTCATATAAACTCTAGACAGAAGCATTCTCAGAAACTTCATTG
         66 TTTCGTTGGAAACAGGAATATCTTCATATAAAAGCTAGACAGAAGCATTCTCAGAAACTTCTTTG

            *       **   *    *             *        
     357003 GGATGTTTCAATTGAAGTCACAGTGTTGAACAGTCCCTTTC
        131 TGATGTTTGCATTCAAGTAACAGTGTTGAACATTCCCTTTC

                 *                                       *       **   * *    
     357044 ATAGAACAGGTTTGAAACACTCTTTTTGTAGTATCTGGAAGTGGACATTTGGAGCGCTCTCAGGA
          1 ATAGAGCAGGTTTGAAACACTCTTTTTGTAGTATCTGGAAGTGGATATTTGGATAGCTTTGAGGA

            * ** *      *             **          * *       * *          *  *
     357109 CTATGGT-GAAAAAGGAAATATCTTCCAATAAAAGCTACATAGAAGCAATGTCAGAAACTTTTTC
         66 TTTCGTTGGAAACAGG-AATATCTTCATATAAAAGCTAGACAGAAGCATTCTCAGAAACTTCTTT

            *      * ** *   **      *       *  *     *
     357173 ATGATGTATCTACTCAGCTAACAGAGTTGAACCTTTCCTTTG
        130 GTGATGTTTGCATTCAAGTAACAGTGTTGAACATTCCCTTTC

             *       *                   * *     *             **            
     357215 AGAGAGCAGTTTTGAAACACTCTTTTTGTGGAATCTGCAAGTGGATATTTGTCTAGCTTTGAGGA
          1 ATAGAGCAGGTTTGAAACACTCTTTTTGTAGTATCTGGAAGTGGATATTTGGATAGCTTTGAGGA

                         *   *    *                   *       *   *          
     357280 TTTCGTTGGAAACGGGATTA-CAT-ATA-AAAAGC-AGACAGCAGCATTCCCAGTAACTTCTTTG
         66 TTTCGTTGGAAACAGGAATATCTTCATATAAAAGCTAGACAGAAGCATTCTCAGAAACTTCTTTG

                              *    *                 
     357341 TGATGTTTGCATTCAAGTCACAGAGTTGAACATTCCCTTTC
        131 TGATGTTTGCATTCAAGTAACAGTGTTGAACATTCCCTTTC

                                                   *     *       **     *   *
     357382 ATAGAGCAGGTTTGAAACACTCTTTTTGTAGTATCTGGATGTGGACATTTGGAGCGCTTTCAGGC
          1 ATAGAGCAGGTTTGAAACACTCTTTTTGTAGTATCTGGAAGTGGATATTTGGATAGCTTTGAGGA

            * ** *      *             *** *   *                      *   *   
     357447 CTATGGT-GAAAAAGGAAATATCTTCTCCTGAAAACTAGACAGAAGCATTCTCAGAATCTTATTT
         66 TTTCGTTGGAAACAGG-AATATCTTCATATAAAAGCTAGACAGAAGCATTCTCAGAAACTTCTTT

                   **  **    *             **  * *   *
     357511 GTGATGTGCGCCCTCAACTAACAGTGTTGAAGCTTTCTTTTG
        130 GTGATGTTTGCATTCAAGTAACAGTGTTGAACATTCCCTTTC

                     *                    *      *   *                       
     357553 ATAGAGCAGTTTTGAAACACTCTTTTTGTAATATCTGCAAGAGGATATTTGGATAGCTTTGAGGA
          1 ATAGAGCAGGTTTGAAACACTCTTTTTGTAGTATCTGGAAGTGGATATTTGGATAGCTTTGAGGA

                         *   * *            **                      *    *   
     357618 TTTCGTTGGAAACGGGATTGTCTTCATATAAACTCTAGACAGAAGCATTCTCAGAAGCTTCATTG
         66 TTTCGTTGGAAACAGGAATATCTTCATATAAAAGCTAGACAGAAGCATTCTCAGAAACTTCTTTG

            *       **   *    *             *        
     357683 GGATGTTTCAATTGAAGTCACAGTGTTGAACAGTCCCTTTC
        131 TGATGTTTGCATTCAAGTAACAGTGTTGAACATTCCCTTTC

               *                                         *       *  * * *    
     357724 ATATAGCAGGTTTGAAACACTCTTTTTGTAGTATCTGGAAGTGGACATTTGGAGAGATCTCAGGA
          1 ATAGAGCAGGTTTGAAACACTCTTTTTGTAGTATCTGGAAGTGGATATTTGGATAGCTTTGAGGA

            * *  *    * *             **            *       * *          *  *
     357789 ATACGGT-GATAAAGGAAATATCTTCCAATAAAAGCTAGATAGAAGCAATGTCAGAAACTTTTTC
         66 TTTCGTTGGAAACAGG-AATATCTTCATATAAAAGCTAGACAGAAGCATTCTCAGAAACTTCTTT

            *      * ** *   **      *       *  * *   *
     357853 ATGATGTATCTACTCAGCTAACAGAGTTGAACCTTTCTTTTG
        130 GTGATGTTTGCATTCAAGTAACAGTGTTGAACATTCCCTTTC

             *       *                   * *     *             **            
     357895 AGAGAGCAGTTTTGAAACACTCTTTTTGTGGAATCTGCAAGTGGATATTTGTCTAGCTTTGAGGA
          1 ATAGAGCAGGTTTGAAACACTCTTTTTGTAGTATCTGGAAGTGGATATTTGGATAGCTTTGAGGA

                         *   *    *                   *       *   *          
     357960 TTTCGTTGGAAACGGGATTA-CAT-ATA-AAAAGC-AGACAGCAGCATTCCCAGTAACTTCTTTG
         66 TTTCGTTGGAAACAGGAATATCTTCATATAAAAGCTAGACAGAAGCATTCTCAGAAACTTCTTTG

                              *    *                 
     358021 TGATGTTTGCATTCAAGTCACAGAGTTGAACATTCCCTTTC
        131 TGATGTTTGCATTCAAGTAACAGTGTTGAACATTCCCTTTC

               *                                   *     *       **     *   *
     358062 ATACAGCAGGTTTGAAACACTCTTTTTGTAGTATCTGGATGTGGACATTTGGAGCGCTTTCAGGC
          1 ATAGAGCAGGTTTGAAACACTCTTTTTGTAGTATCTGGAAGTGGATATTTGGATAGCTTTGAGGA

            * ** *      *             *** *   *           *          *   *   
     358127 CTATGGT-GAAAAAGGAAATATCTTCCCCTGAAAACTAGACAGAAGAATTCTCAGAATCTTATTT
         66 TTTCGTTGGAAACAGG-AATATCTTCATATAAAAGCTAGACAGAAGCATTCTCAGAAACTTCTTT

                   **  **    *       *     **  * *   *
     358191 GTGATGTGCGCCCTCAACTAACAGTCTTGAAGCTTTCTTTTG
        130 GTGATGTTTGCATTCAAGTAACAGTGTTGAACATTCCCTTTC

                     *                    **     *   *    *                  
     358233 ATAGAGCAGTTTTGAAACACTCTTTTTGTAAAATCTGCAAGAGGATGTTTGGATAGCTTTGAGGA
          1 ATAGAGCAGGTTTGAAACACTCTTTTTGTAGTATCTGGAAGTGGATATTTGGATAGCTTTGAGGA

               *         *   * *            **                      *    *   
     358298 TTTTGTTGGAAACGGGATTGTCTTCATATAAACTCTAGACAGAAGCATTCTCAGAAGCTTCATTG
         66 TTTCGTTGGAAACAGGAATATCTTCATATAAAAGCTAGACAGAAGCATTCTCAGAAACTTCTTTG

            *       **   *    *             *        
     358363 GGATGTTTCAATTGAAGTCACAGTGTTGAACAGTCCCTTTC
        131 TGATGTTTGCATTCAAGTAACAGTGTTGAACATTCCCTTTC

                                                         *       *  * * *    
     358404 ATAGAGCAGGTTTGAAACACTCTTTTTGTAGTATCTGGAAGTGGACATTTGGAGAGATCTCAGGA
          1 ATAGAGCAGGTTTGAAACACTCTTTTTGTAGTATCTGGAAGTGGATATTTGGATAGCTTTGAGGA

            * *  *    * *             **     *    * *       * *          *  *
     358469 ATACGGT-GATAAAGGAAATATCTTCCAATAAACGCTACATAGAAGCAATGTCAGAAACTTTTTC
         66 TTTCGTTGGAAACAGG-AATATCTTCATATAAAAGCTAGACAGAAGCATTCTCAGAAACTTCTTT

            *      * ** *   **      *       *  * *   *
     358533 ATGATGTATATACTCAGCTAACAGAGTTGAACCTTTCTTTTG
        130 GTGATGTTTGCATTCAAGTAACAGTGTTGAACATTCCCTTTC

             *       *                   * *     *             **            
     358575 AGAGAGCAGTTTTGAAACACTCTTTTTGTGGAATCTGCAAGTGGATATTTGTCTAGCTTTGAGGA
          1 ATAGAGCAGGTTTGAAACACTCTTTTTGTAGTATCTGGAAGTGGATATTTGGATAGCTTTGAGGA

                         *   *    *                   *       *   *          
     358640 TTTCGTTGGAAACGGGATTA-CAT-ATA-AAAAGC-AGACAGCAGCATTCCCAGTAACTTCTTTG
         66 TTTCGTTGGAAACAGGAATATCTTCATATAAAAGCTAGACAGAAGCATTCTCAGAAACTTCTTTG

                              *    *                 
     358701 TGATGTTTGCATTCAAGTCACAGAGTTGAACATTCCCTTTC
        131 TGATGTTTGCATTCAAGTAACAGTGTTGAACATTCCCTTTC

               *                                   *     *       **     *   *
     358742 ATACAGCAGGTTTGAAACACTCTTTTTGTAGTATCTGGATGTGGACATTTGGAGCGCTTTCAGGC
          1 ATAGAGCAGGTTTGAAACACTCTTTTTGTAGTATCTGGAAGTGGATATTTGGATAGCTTTGAGGA

            * ** *      *             *** *   *           *     *    *   *   
     358807 CTATGGT-GAAAAAGGAAATATCTTCCCCTGAAAACTAGACAGAAGAATTCTTAGAATCTTATTT
         66 TTTCGTTGGAAACAGG-AATATCTTCATATAAAAGCTAGACAGAAGCATTCTCAGAAACTTCTTT

                   **  **    *             **  * *   *
     358871 GTGATGTGCGCCCTCAACTAACAGTGTTGAAGCTTTCTTTTG
        130 GTGATGTTTGCATTCAAGTAACAGTGTTGAACATTCCCTTTC

                     *                    **     *   *                       
     358913 ATAGAGCAGTTTTGAAACACTCTTTTTGTAAAATCTGCAAGAGGATATTTGGATAGCTTTGAGGA
          1 ATAGAGCAGGTTTGAAACACTCTTTTTGTAGTATCTGGAAGTGGATATTTGGATAGCTTTGAGGA

                         *   * *            **                      * *  *   
     358978 TTTCGTTGGAAACGGGATTGTCTTCATATAAACTCTAGACAGAAGCATTCTCAGAAGCGTCATTG
         66 TTTCGTTGGAAACAGGAATATCTTCATATAAAAGCTAGACAGAAGCATTCTCAGAAACTTCTTTG

            *       **   *    *             *        
     359043 GGATGTTTCAATTGAAGTCACAGTGTTGAACAGTCCCTTTC
        131 TGATGTTTGCATTCAAGTAACAGTGTTGAACATTCCCTTTC

                                                   *     *       **     *  **
     359084 ATAGAGCAGGTTTGAAACACTCTTTTTGTAGTATCTGGATGTGGACATTTGGAGCGCTTTAAGCC
          1 ATAGAGCAGGTTTGAAACACTCTTTTTGTAGTATCTGGAAGTGGATATTTGGATAGCTTTGAGGA

             * *    *                 *** *   *                          *   
     359149 TATGGTT-TAAA-AGGAAATATCTTCCCCTGAAAACTAGACAGAAGCATTCTCAGAAACTTATTT
         66 TTTCGTTGGAAACAGG-AATATCTTCATATAAAAGCTAGACAGAAGCATTCTCAGAAACTTCTTT

                   **  **    *                    *   *
     359212 GTGATGTGCGCCCTCAACTAACAGTGTTGAAGCATT-CTTTTG
        130 GTGATGTTTGCATTCAAGTAACAGTGTTGAA-CATTCCCTTTC

                     *                    *      *   *                       
     359254 ATAGAGCAGTTTTGAAACACTCTTTTTGTAATATCTGCAAGAGGATATTTGGATAGCTTTGAGGA
          1 ATAGAGCAGGTTTGAAACACTCTTTTTGTAGTATCTGGAAGTGGATATTTGGATAGCTTTGAGGA

                         *   *    *                   *       *     *   *    
     359319 TTTCGTTGGAAACGGGATTA-CAT-ATA-AAAAGC-AGACAGCAGCATTCCCAGAATCTTGTTTG
         66 TTTCGTTGGAAACAGGAATATCTTCATATAAAAGCTAGACAGAAGCATTCTCAGAAACTTCTTTG

                                   *                 
     359380 TGATGTTTGCATTCAAGT--CAGAGTTGAACATTCCCTTTC
        131 TGATGTTTGCATTCAAGTAACAGTGTTGAACATTCCCTTTC

             *                         *           *     *       **     *   *
     359419 AGAGAGCAGGTTTGAAACACTCTTTTTATAGTATCTGGATGTGGACATTTGGAGCGCTTTCAGGC
          1 ATAGAGCAGGTTTGAAACACTCTTTTTGTAGTATCTGGAAGTGGATATTTGGATAGCTTTGAGGA

            * ** *      *             *** *   *                      *   *   
     359484 CTATGGT-GAAAAAGGAAATATCTTCTCCTGAAAACTAGACAGAAGCATTCTCAGAATCTTATTT
         66 TTTCGTTGGAAACAGG-AATATCTTCATATAAAAGCTAGACAGAAGCATTCTCAGAAACTTCTTT

                   **  **    *             **  * *   *
     359548 GTGATGTGCGCCCTCAACTAACAGTGTTGAAGCTTTCTTTTG
        130 GTGATGTTTGCATTCAAGTAACAGTGTTGAACATTCCCTTTC

                     *                *   **     *   *        *              
     359590 ATAGAGCAGTTTTGAAACACTCTTTTCGTAAAATCTGCAAGAGGATATTTTGATAGCTTTGAGGA
          1 ATAGAGCAGGTTTGAAACACTCTTTTTGTAGTATCTGGAAGTGGATATTTGGATAGCTTTGAGGA

                         *   * *            **                      *    *   
     359655 TTTCGTTGGAAACGGGATTGTCTTCATATAAACTCTAGACAGAAGCATTCTCAGAAGCTTCATTG
         66 TTTCGTTGGAAACAGGAATATCTTCATATAAAAGCTAGACAGAAGCATTCTCAGAAACTTCTTTG

            *       **   *    *             * *      
     359720 GGATGTTTCAATTGAAGTCACAGTGTTGAACAGTTCCTTTC
        131 TGATGTTTGCATTCAAGTAACAGTGTTGAACATTCCCTTTC

                 *                                       *       **   * *    
     359761 ATAGAACAGGTTTGAAACACTCTTTTTGTAGTATCTGGAAGTGGACATTTGGAGCGCTCTCAGGA
          1 ATAGAGCAGGTTTGAAACACTCTTTTTGTAGTATCTGGAAGTGGATATTTGGATAGCTTTGAGGA

            * ** *      *             *           * *      ** *          *  *
     359826 CTATGGT-GAAAAAGGAAATATCTTCCTATAAAAGCTACATAGAAGCTATGTCAGAAACTTTTTC
         66 TTTCGTTGGAAACAGG-AATATCTTCATATAAAAGCTAGACAGAAGCATTCTCAGAAACTTCTTT

            *      * ** *   **      *       *  *     *
     359890 ATGATGTATCTACTCAGCTAACAGAGTTGAACCTTTCCTTTG
        130 GTGATGTTTGCATTCAAGTAACAGTGTTGAACATTCCCTTTC

             *       *                   * *    **             **            
     359932 AGAGAGCAGTTTTGAAACACTCTTTTTGTGGAATCTACAAGTGGATATTTGTCTAGCTTTGAGGA
          1 ATAGAGCAGGTTTGAAACACTCTTTTTGTAGTATCTGGAAGTGGATATTTGGATAGCTTTGAGGA

                         *   *    *                   *       *   *          
     359997 TTTCGTTGGAAACGGGATTA-CAT-ATA-AAAAGC-AGACAGCAGCATTCCCAGTAACTTCTTTG
         66 TTTCGTTGGAAACAGGAATATCTTCATATAAAAGCTAGACAGAAGCATTCTCAGAAACTTCTTTG

                              *    *                 
     360058 TGATGTTTGCATTCAAGTCACAGAGTTGAACATTCCCTTTC
        131 TGATGTTTGCATTCAAGTAACAGTGTTGAACATTCCCTTTC

                                                       * *       *  * * *    
     360099 ATAGAGCAGGTTTGAAACACTCTTTTTGTAGTATCTGGAAGTGCACATTTGGAGAGATCTCAGGA
          1 ATAGAGCAGGTTTGAAACACTCTTTTTGTAGTATCTGGAAGTGGATATTTGGATAGCTTTGAGGA

            * *  *    * *             **            *       * *          *  *
     360164 ATACGGT-GATAAAGGAAATATCTTCCAATAAAAGCTAGATAGAAGCAATGTCAGAAACTTTTTC
         66 TTTCGTTGGAAACAGG-AATATCTTCATATAAAAGCTAGACAGAAGCATTCTCAGAAACTTCTTT

            *      * ** *   **              *  * *   *
     360228 ATGATGTATCTACTCAGCTAACAGTGTTGAACCTTTCTTTTG
        130 GTGATGTTTGCATTCAAGTAACAGTGTTGAACATTCCCTTTC

             *       *                   * *     *             **            
     360270 AGAGAGCAGTTTTGAAACACTCTTTTTGTGGAATCTGCAAGTGGATATTTGTCTAGCTTTGAGGA
          1 ATAGAGCAGGTTTGAAACACTCTTTTTGTAGTATCTGGAAGTGGATATTTGGATAGCTTTGAGGA

                         *   *    *                   *  *    *   *          
     360335 TTTCGTTGGAAACGGGATTA-CAT-ATA-AAAAGC-AGACAGCAGAATTCCCAGTAACTTCTTTG
         66 TTTCGTTGGAAACAGGAATATCTTCATATAAAAGCTAGACAGAAGCATTCTCAGAAACTTCTTTG

                              *    *                 
     360396 TGATGTTTGCATTCAAGTCACAGAGTTGAACATTCCCTTTC
        131 TGATGTTTGCATTCAAGTAACAGTGTTGAACATTCCCTTTC

                                                   *     *    *  **         *
     360437 ATAGAGCAGGTTTGAAACACTCTTTTTGTAGTATCTGGATGTGGACATTTTGAGCGCTTTGAGGC
          1 ATAGAGCAGGTTTGAAACACTCTTTTTGTAGTATCTGGAAGTGGATATTTGGATAGCTTTGAGGA

            * ** *      *       *     *** *   *           *          *   *   
     360502 CTATGGT-GAAAAAGGAAATCTCTTCCCCTGAAAACTAGACAGAAGAATTCTCAGAATCTTATTT
         66 TTTCGTTGGAAACAGG-AATATCTTCATATAAAAGCTAGACAGAAGCATTCTCAGAAACTTCTTT

                   **  **    *              *  * *   *
     360566 GTGATGTGCGCCCTCAACTAACAGTGTTGAACCTTTCTTTTG
        130 GTGATGTTTGCATTCAAGTAACAGTGTTGAACATTCCCTTTC

                     *          *        ***     *   *                       
     360608 ATAGAGCAGTTTTGAAACACCCTTTTTGTTAAATCTGCAAGAGGATATTTGGATAGCTTTGAGGA
          1 ATAGAGCAGGTTTGAAACACTCTTTTTGTAGTATCTGGAAGTGGATATTTGGATAGCTTTGAGGA

                         *   * *            **                      *    *   
     360673 TTTCGTTGGAAACGGGATTGTCTTCATATAAACTCTAGACAGAAGCATTCTCAGAAGCTTCATTG
         66 TTTCGTTGGAAACAGGAATATCTTCATATAAAAGCTAGACAGAAGCATTCTCAGAAACTTCTTTG

            **      **   *    *             *        
     360738 GCATGTTTCAATTGAAGTCACAGTGTTGAACAGTCCCTTTC
        131 TGATGTTTGCATTCAAGTAACAGTGTTGAACATTCCCTTTC

                                                   *     *     * **     *   *
     360779 ATAGAGCAGGTTTGAAACACTCTTTTTGTAGTATCTGGATGTGGACATTTGCAGCGCTTTCAGGC
          1 ATAGAGCAGGTTTGAAACACTCTTTTTGTAGTATCTGGAAGTGGATATTTGGATAGCTTTGAGGA

            * ** *      *             *** *   *                          *   
     360844 CTAAGGT-GAAAAAGGAAATATCTTCCCCTGAAAACTAGACAGAAGCATTCTCAGAAACTTATTT
         66 TTTCGTTGGAAACAGG-AATATCTTCATATAAAAGCTAGACAGAAGCATTCTCAGAAACTTCTTT

                   **  **    *             **  * *   *
     360908 GTGATGTGCGCCCTCAACTAACAGTGTTGAAGCTTTCTTTTG
        130 GTGATGTTTGCATTCAAGTAACAGTGTTGAACATTCCCTTTC

                     *                    *      *   *                  *    
     360950 ATAGAGCAGTTTTGAAACACTCTTTTTGTAATATCTGCAAGAGGATATTTGGATAGCTTTCAGGA
          1 ATAGAGCAGGTTTGAAACACTCTTTTTGTAGTATCTGGAAGTGGATATTTGGATAGCTTTGAGGA

                         *   * *            **                      *    *   
     361015 TTTCGTTGGAAACGGGATTGTCTTCATATAAACTCTAGACAGAAGCATTCTCAGAAGCTTCATTG
         66 TTTCGTTGGAAACAGGAATATCTTCATATAAAAGCTAGACAGAAGCATTCTCAGAAACTTCTTTG

            *       **   *    *             * *      
     361080 GGATGTTTCAATTGAAGTCACAGTGTTGAACAGTTCCTTTC
        131 TGATGTTTGCATTCAAGTAACAGTGTTGAACATTCCCTTTC

                 *                                       *       **   * *    
     361121 ATAGAACAGGTTTGAAACACTCTTTTTGTAGTATCTGGAAGTGGACATTTGGAGCGCTCTCAGGA
          1 ATAGAGCAGGTTTGAAACACTCTTTTTGTAGTATCTGGAAGTGGATATTTGGATAGCTTTGAGGA

            * ** *      *             *           * *      ** *          *  *
     361186 CTATGGT-GAAAAAGGAAATATCTTCCTATAAAAGCTACATAGAAGCTATGTCAGAAACTTTTTC
         66 TTTCGTTGGAAACAGG-AATATCTTCATATAAAAGCTAGACAGAAGCATTCTCAGAAACTTCTTT

            *      * ** *   **      *       *  *     *
     361250 ATGATGTATCTACTCAGCTAACAGAGTTGAACCTTTCCTTTG
        130 GTGATGTTTGCATTCAAGTAACAGTGTTGAACATTCCCTTTC

             *       *                   * *    **             **            
     361292 AGAGAGCAGTTTTGAAACACTCTTTTTGTGGAATCTACAAGTGGATATTTGTCTAGCTTTGAGGA
          1 ATAGAGCAGGTTTGAAACACTCTTTTTGTAGTATCTGGAAGTGGATATTTGGATAGCTTTGAGGA

                         *   *    *                   *       *   *          
     361357 TTTCGTTGGAAACGGGATTA-CAT-ATA-AAAAGC-AGACAGCAGCATTCCCAGTAACTTCTTTG
         66 TTTCGTTGGAAACAGGAATATCTTCATATAAAAGCTAGACAGAAGCATTCTCAGAAACTTCTTTG

                              *    *                 
     361418 TGATGTTTGCATTCAAGTCACAGAGTTGAACATTCCCTTTC
        131 TGATGTTTGCATTCAAGTAACAGTGTTGAACATTCCCTTTC

                                                ** *     *       **     *   *
     361459 ATAGAGCAGGTTTGAAACACTCTTTTTGTAGTATCTCTATGTGGACATTTGGAGCGCTTTCAGGC
          1 ATAGAGCAGGTTTGAAACACTCTTTTTGTAGTATCTGGAAGTGGATATTTGGATAGCTTTGAGGA

            * ** *      *             *** *   *                          *   
     361524 CTATGGT-GAAAAAGGAAATATCTTCCCCTGAAAACTAGACAGAAGCATTCTCAGAAACTTATTT
         66 TTTCGTTGGAAACAGG-AATATCTTCATATAAAAGCTAGACAGAAGCATTCTCAGAAACTTCTTT

                   **  **    *              *  * *   *
     361588 GTGATGTGCGCCCTCAACTAACAGTGTTGAACCTTTCTTTTG
        130 GTGATGTTTGCATTCAAGTAACAGTGTTGAACATTCCCTTTC

                     *                    *      *   *                       
     361630 ATAGAGCAGTTTTGAAACACTCTTTTTGTAATATCTGCAAGAGGATATTTGGATAGCTTTGAGGA
          1 ATAGAGCAGGTTTGAAACACTCTTTTTGTAGTATCTGGAAGTGGATATTTGGATAGCTTTGAGGA

                         *   *    *                   *       *     *   *    
     361695 TTTCGTTGGAAACGGGATTA-CAT-ATA-AAAAGC-AGACAGCAGCATTCCCAGAATCTTGTTTG
         66 TTTCGTTGGAAACAGGAATATCTTCATATAAAAGCTAGACAGAAGCATTCTCAGAAACTTCTTTG

                                   *                 
     361756 TGATGTTTGCATTCAAGT--CAGAGTTGAACATTCCCTTTC
        131 TGATGTTTGCATTCAAGTAACAGTGTTGAACATTCCCTTTC

             *                         *           *     *       **     *   *
     361795 AGAGAGCAGGTTTGAAACACTCTTTTTATAGTATCTGGATGTGGACATTTGGAGCGCTTTCAGGC
          1 ATAGAGCAGGTTTGAAACACTCTTTTTGTAGTATCTGGAAGTGGATATTTGGATAGCTTTGAGGA

            * ** *      *             *** *   *                      *   *   
     361860 CTATGGT-GAAAAAGGAAATATCTTCTCCTGAAAACTAGACAGAAGCATTCTCAGAATCTTATTT
         66 TTTCGTTGGAAACAGG-AATATCTTCATATAAAAGCTAGACAGAAGCATTCTCAGAAACTTCTTT

                   **  **    *             **  * *   *
     361924 GTGATGTGCGCCCTCAACTAACAGTGTTGAAGCTTTCTTTTG
        130 GTGATGTTTGCATTCAAGTAACAGTGTTGAACATTCCCTTTC

                     *                *   **     *   *        *              
     361966 ATAGAGCAGTTTTGAAACACTCTTTTCGTAAAATCTGCAAGAGGATATTTTGATAGCTTTGAGGA
          1 ATAGAGCAGGTTTGAAACACTCTTTTTGTAGTATCTGGAAGTGGATATTTGGATAGCTTTGAGGA

                         *   * *            **                      *    *   
     362031 TTTCGTTGGAAACGGGATTGTCTTCATATAAACTCTAGACAGAAGCATTCTCAGAAGCTTCATTG
         66 TTTCGTTGGAAACAGGAATATCTTCATATAAAAGCTAGACAGAAGCATTCTCAGAAACTTCTTTG

            *       **   *    *             *        
     362096 GGATGTTTCAATTGAAGTCACAGTGTTGAACAGTCCCTTTC
        131 TGATGTTTGCATTCAAGTAACAGTGTTGAACATTCCCTTTC

                                                       * *       *  * * *    
     362137 ATAGAGCAGGTTTGAAACACTCTTTTTGTAGTATCTGGAAGTGCACATTTGGAGAGATCTCAGGA
          1 ATAGAGCAGGTTTGAAACACTCTTTTTGTAGTATCTGGAAGTGGATATTTGGATAGCTTTGAGGA

            * *  *    * *             **            *       * *          *  *
     362202 ATACGGT-GATAAAGGAAATATCTTCCAATAAAAGCTAGATAGAAGCAATGTCAGAAACTTTTTC
         66 TTTCGTTGGAAACAGG-AATATCTTCATATAAAAGCTAGACAGAAGCATTCTCAGAAACTTCTTT

            *      * ** *   **              *  * *   *
     362266 ATGATGTATCTACTCAGCTAACAGTGTTGAACCTTTCTTTTG
        130 GTGATGTTTGCATTCAAGTAACAGTGTTGAACATTCCCTTTC

             *       *                   * *     *             **            
     362308 AGAGAGCAGTTTTGAAACACTCTTTTTGTGGAATCTGCAAGTGGATATTTGTCTAGCTTTGAGGA
          1 ATAGAGCAGGTTTGAAACACTCTTTTTGTAGTATCTGGAAGTGGATATTTGGATAGCTTTGAGGA

                         *   *    *                   *  *    *   *          
     362373 TTTCGTTGGAAACGGGATTA-CAT-ATA-AAAAGC-AGACAGCAGAATTCCCAGTAACTTCTTTG
         66 TTTCGTTGGAAACAGGAATATCTTCATATAAAAGCTAGACAGAAGCATTCTCAGAAACTTCTTTG

                              *    *                 
     362434 TGATGTTTGCATTCAAGTCACAGAGTTGAACATTCCCTTTC
        131 TGATGTTTGCATTCAAGTAACAGTGTTGAACATTCCCTTTC

                                                   *     *    *  **         *
     362475 ATAGAGCAGGTTTGAAACACTCTTTTTGTAGTATCTGGATGTGGACATTTTGAGCGCTTTGAGGC
          1 ATAGAGCAGGTTTGAAACACTCTTTTTGTAGTATCTGGAAGTGGATATTTGGATAGCTTTGAGGA

            * ** *      *       *     *** *   *           *          *   *   
     362540 CTATGGT-GAAAAAGGAAATCTCTTCCCCTGAAAACTAGACAGAAGAATTCTCAGAATCTTATTT
         66 TTTCGTTGGAAACAGG-AATATCTTCATATAAAAGCTAGACAGAAGCATTCTCAGAAACTTCTTT

                   **  **    *              *  * *   *
     362604 GTGATGTGCGCCCTCAACTAACAGTGTTGAACCTTTCTTTTG
        130 GTGATGTTTGCATTCAAGTAACAGTGTTGAACATTCCCTTTC

                     *          *        ***     *   *                       
     362646 ATAGAGCAGTTTTGAAACACCCTTTTTGTTAAATCTGCAAGAGGATATTTGGATAGCTTTGAGGA
          1 ATAGAGCAGGTTTGAAACACTCTTTTTGTAGTATCTGGAAGTGGATATTTGGATAGCTTTGAGGA

                         *   * *            **                      *    *   
     362711 TTTCGTTGGAAACGGGATTGTCTTCATATAAACTCTAGACAGAAGCATTCTCAGAAGCTTCATTG
         66 TTTCGTTGGAAACAGGAATATCTTCATATAAAAGCTAGACAGAAGCATTCTCAGAAACTTCTTTG

            **      **   *    *             *        
     362776 GCATGTTTCAATTGAAGTCACAGTGTTGAACAGTCCCTTTC
        131 TGATGTTTGCATTCAAGTAACAGTGTTGAACATTCCCTTTC

                                                   *     *     * **     *   *
     362817 ATAGAGCAGGTTTGAAACACTCTTTTTGTAGTATCTGGATGTGGACATTTGCAGCGCTTTCAGGC
          1 ATAGAGCAGGTTTGAAACACTCTTTTTGTAGTATCTGGAAGTGGATATTTGGATAGCTTTGAGGA

            * ** *      *             *** *   *                          *   
     362882 CTAAGGT-GAAAAAGGAAATATCTTCCCCTGAAAACTAGACAGAAGCATTCTCAGAAACTTATTT
         66 TTTCGTTGGAAACAGG-AATATCTTCATATAAAAGCTAGACAGAAGCATTCTCAGAAACTTCTTT

                   **  **    *             **  * *   *
     362946 GTGATGTGCGCCCTCAACTAACAGTGTTGAAGCTTTCTTTTG
        130 GTGATGTTTGCATTCAAGTAACAGTGTTGAACATTCCCTTTC

                     *                    *      *   *                       
     362988 ATAGAGCAGTTTTGAAACACTCTTTTTGTAATATCTGCAAGAGGATATTTGGATAGCTTTGAGGA
          1 ATAGAGCAGGTTTGAAACACTCTTTTTGTAGTATCTGGAAGTGGATATTTGGATAGCTTTGAGGA

                *        *   * *            **                      *    *   
     363053 TTTCATTGGAAACGGGATTGTCTTCATATAAACTCTAGACAGAAGCATTCTCAGAAGCTTCATTG
         66 TTTCGTTGGAAACAGGAATATCTTCATATAAAAGCTAGACAGAAGCATTCTCAGAAACTTCTTTG

            *       **   *    *             * *      
     363118 GGATGTTTCAATTGAAGTCACAGTGTTGAACAGTTCCTTTC
        131 TGATGTTTGCATTCAAGTAACAGTGTTGAACATTCCCTTTC

                 *                                       *       **   * *    
     363159 ATAGAACAGGTTTGAAACACTCTTTTTGTAGTATCTGGAAGTGGACATTTGGAGCGCTCTCAGGA
          1 ATAGAGCAGGTTTGAAACACTCTTTTTGTAGTATCTGGAAGTGGATATTTGGATAGCTTTGAGGA

            * ** *      *             **          * *       * *          *  *
     363224 CTATGGT-GAAAAAGGAAATATCTTCCAATAAAAGCTACATAGAAGCAATGTCAGAAACTTTTTC
         66 TTTCGTTGGAAACAGG-AATATCTTCATATAAAAGCTAGACAGAAGCATTCTCAGAAACTTCTTT

            *      * ** *   **      *       *  *     *
     363288 ATGATGTATCTACTCAGCTAACAGAGTTGAACCTTTCCTTTG
        130 GTGATGTTTGCATTCAAGTAACAGTGTTGAACATTCCCTTTC

             *       *                   * *     *             **            
     363330 AGAGAGCAGTTTTGAAACACTCTTTTTGTGGAATCTGCAAGTGGATATTTGCTTAGCTTTGAGGA
          1 ATAGAGCAGGTTTGAAACACTCTTTTTGTAGTATCTGGAAGTGGATATTTGGATAGCTTTGAGGA

                         *   *    *                   *       *   *          
     363395 TTTCGTTGGAAACGGGATTA-CAT-ATA-AAAAGC-AGACAGCAGCATTCCCAGTAACTTCTTTG
         66 TTTCGTTGGAAACAGGAATATCTTCATATAAAAGCTAGACAGAAGCATTCTCAGAAACTTCTTTG

                              *    *                 
     363456 TGATGTTTGCATTCAAGTCACAGAGTTGAACATTCCCTTTC
        131 TGATGTTTGCATTCAAGTAACAGTGTTGAACATTCCCTTTC

                                                   *     *       **     *   *
     363497 ATAGAGCAGGTTTGAAACACTCTTTTTGTAGTATCTGGATGTGGACATTTGGAGCGCTTTCAGGC
          1 ATAGAGCAGGTTTGAAACACTCTTTTTGTAGTATCTGGAAGTGGATATTTGGATAGCTTTGAGGA

            * ** *      *             *** *   *                      *   *   
     363562 CTATGGT-GAAAAAGGAAATATCTTCCCCTGAAAACTAGACAGAAGCATTCTCAGAATCTTATTT
         66 TTTCGTTGGAAACAGG-AATATCTTCATATAAAAGCTAGACAGAAGCATTCTCAGAAACTTCTTT

                   **  **    *             **  * *   *
     363626 GTGATGTGCGCCCTCAACTAACAGTGTTGAAGCTTTCTTTTG
        130 GTGATGTTTGCATTCAAGTAACAGTGTTGAACATTCCCTTTC

                     *                    **     *   *                       
     363668 ATAGAGCAGTTTTGAAACACTCTTTTTGTAAAATCTGCAAGAGGATATTTGGATAGCTTTGAGGA
          1 ATAGAGCAGGTTTGAAACACTCTTTTTGTAGTATCTGGAAGTGGATATTTGGATAGCTTTGAGGA

                         *   * *            **                      *    *   
     363733 TTTCGTTGGAAACGGGATTGTCTTCATATAAACTCTAGACAGAAGCATTCTCAGAAGCTTCATTG
         66 TTTCGTTGGAAACAGGAATATCTTCATATAAAAGCTAGACAGAAGCATTCTCAGAAACTTCTTTG

            *       **   *    *             *        
     363798 GGATGTTTCAATTGAAGTCACAGTGTTGAACAGTCCCTTTC
        131 TGATGTTTGCATTCAAGTAACAGTGTTGAACATTCCCTTTC

                                                   *     *       **     *    
     363839 ATAGAGCAGGTTTGAAACACTCTTTTTGTAGTATCTGGATGTGGACATTTGGAGCGCTTTCAGGC
          1 ATAGAGCAGGTTTGAAACACTCTTTTTGTAGTATCTGGAAGTGGATATTTGGATAGCTTTGAGG-

            * * *    *                 *** *   *                          *  
     363904 CTATGGTT-TAAA-AGGAAATATCTTCCCCTGAAAACTAGACAGAAGCATTCTCAGAAACTTATT
         65 ATTTCGTTGGAAACAGG-AATATCTTCATATAAAAGCTAGACAGAAGCATTCTCAGAAACTTCTT

                    **  **    *                    *   *
     363967 TGTGATGTGCGCCCTCAACTAACAGTGTTGAAGCATT-CTTTTG
        129 TGTGATGTTTGCATTCAAGTAACAGTGTTGAA-CATTCCCTTTC

                     *                   * *     *             **            
     364010 ATAGAGCAGTTTTGAAACACTCTTTTTGTGGAATCTGCAAGTGGATATTTGTCTAGCTTTGAGGA
          1 ATAGAGCAGGTTTGAAACACTCTTTTTGTAGTATCTGGAAGTGGATATTTGGATAGCTTTGAGGA

                         *   *    *                   *       *              
     364075 TTTCGTTGGAAACGGGATTA-CAT-ATA-AAAAGC-AGACAGCAGCATTCCCAGAAACTTCTTTG
         66 TTTCGTTGGAAACAGGAATATCTTCATATAAAAGCTAGACAGAAGCATTCTCAGAAACTTCTTTG

                              *    *                 
     364136 TGATGTTTGCATTCAAGTCACAGAGTTGAACATTCCCTTTC
        131 TGATGTTTGCATTCAAGTAACAGTGTTGAACATTCCCTTTC

                                                   *     *       **     *   *
     364177 ATAGAGCAGGTTTGAAACACTCTTTTTGTAGTATCTGGATGTGGACATTTGGAGCGCTTTCAGGC
          1 ATAGAGCAGGTTTGAAACACTCTTTTTGTAGTATCTGGAAGTGGATATTTGGATAGCTTTGAGGA

            * ** *      *             *** *   *                          *   
     364242 CTATGGT-GAAAAAGGAAATATCTTCCCCTGAAAACTAGACAGAAGCATTCTCAGAAACTTATTT
         66 TTTCGTTGGAAACAGG-AATATCTTCATATAAAAGCTAGACAGAAGCATTCTCAGAAACTTCTTT

                   **  **    *             **  * *   *
     364306 GTGATGTGCGCCCTCAACTAACAGTGTTGAAGCTTTCTTTTG
        130 GTGATGTTTGCATTCAAGTAACAGTGTTGAACATTCCCTTTC

                     *                    *      *   *                       
     364348 ATAGAGCAGTTTTGAAACACTCTTTTTGTAATATCTGCAAGAGGATATTTGGATAGCTTTGAGGA
          1 ATAGAGCAGGTTTGAAACACTCTTTTTGTAGTATCTGGAAGTGGATATTTGGATAGCTTTGAGGA

                         *   * *            **                      *    *   
     364413 TTTCGTTGGAAACGGGATTGTCTTCATATAAACTCTAGACAGAAGCATTCTCAGAAGCTTCATTG
         66 TTTCGTTGGAAACAGGAATATCTTCATATAAAAGCTAGACAGAAGCATTCTCAGAAACTTCTTTG

            *       **   *    *             **       
     364478 GGATGTTTCAATTGAAGTCACAGTGTTGAACAGACCCTTTC
        131 TGATGTTTGCATTCAAGTAACAGTGTTGAACATTCCCTTTC

                                                         *       **   * *    
     364519 ATAGAGCAGGTTTGAAACACTCTTTTTGTAGTATCTGGAAGTGGACATTTGGAGCGCTCTCAGGA
          1 ATAGAGCAGGTTTGAAACACTCTTTTTGTAGTATCTGGAAGTGGATATTTGGATAGCTTTGAGGA

            * *  *      *             **          * *       * *          *  *
     364584 CTGCGGT-GAAAAAGGAAATATCTTCCAATAAAAGCTACATAGAAGCAATGTCAGAAACTTTTTC
         66 TTTCGTTGGAAACAGG-AATATCTTCATATAAAAGCTAGACAGAAGCATTCTCAGAAACTTCTTT

            *      * ** *   **      *       *        *
     364648 ATGATGTATCTACTCAGCTAACAGAGTTGAACCTT-CCTTTG
        130 GTGATGTTTGCATTCAAGTAACAGTGTTGAACATTCCCTTTC

             *       *                   * *     *             **            
     364689 AGAGAGCAGTTTTGAAACACTCTTTTTGTGGAATCTGCAAGTGGATATTTGTCTAGCTTTGAGGA
          1 ATAGAGCAGGTTTGAAACACTCTTTTTGTAGTATCTGGAAGTGGATATTTGGATAGCTTTGAGGA

                         *   *    *                   *       *  *           
     364754 TTTCGTTGGAAACGGGATTA-CAT-ATA-AAAAGC-AGACAGCAGCATTCCCACAAACTTCTTTG
         66 TTTCGTTGGAAACAGGAATATCTTCATATAAAAGCTAGACAGAAGCATTCTCAGAAACTTCTTTG

                              *    *                 
     364815 TGATGTTTGCATTCAAGTCACAGAGTTGAACATTCCCTTTC
        131 TGATGTTTGCATTCAAGTAACAGTGTTGAACATTCCCTTTC

                                                   *     *     * ***    *   *
     364856 ATAGAGCAGGTTTGAAACACTCTTTTTGTAGTATCTGGATGTGGACATTTGCAGCACTTTCAGGC
          1 ATAGAGCAGGTTTGAAACACTCTTTTTGTAGTATCTGGAAGTGGATATTTGGATAGCTTTGAGGA

            * ** *      *             *** *   *                          *   
     364921 CTAAGGT-GAAAAAGGAAATATCTTCCCCTGAAAACTAGACAGAAGCATTCTCAGAAACTTATTT
         66 TTTCGTTGGAAACAGG-AATATCTTCATATAAAAGCTAGACAGAAGCATTCTCAGAAACTTCTTT

                 * **  **    *             **  * *   *
     364985 GTGATATGCGCCCTCAACTAACAGTGTTGAAGCTTTCTTTTG
        130 GTGATGTTTGCATTCAAGTAACAGTGTTGAACATTCCCTTTC

                     *                    *      *   *        *              
     365027 ATAGAGCAGTTTTGAAACACTCTTTTTGTAATATCTGTAAGAGGATATTTTGATAGCTTTGAGGA
          1 ATAGAGCAGGTTTGAAACACTCTTTTTGTAGTATCTGGAAGTGGATATTTGGATAGCTTTGAGGA

                         *   * *            **                      *    *   
     365092 TTTCGTTGGAAACGGGATTGTCTTCATATAAACTCTAGACAGAAGCATTCTCAGAAGCTTCATTG
         66 TTTCGTTGGAAACAGGAATATCTTCATATAAAAGCTAGACAGAAGCATTCTCAGAAACTTCTTTG

            *       **   *    *               *      
     365157 GGATGTTTCAATTGAAGTCACAGTGTTGAACATTTCCTTTC
        131 TGATGTTTGCATTCAAGTAACAGTGTTGAACATTCCCTTTC

                 *                                       *       **   * *    
     365198 ATAGAACAGGTTTGAAACACTCTTTTTGTAGTATCTGGAAGTGGACATTTGGAGCGCTCTCAGGA
          1 ATAGAGCAGGTTTGAAACACTCTTTTTGTAGTATCTGGAAGTGGATATTTGGATAGCTTTGAGGA

            * ** *      *             *** *   *                      *   *   
     365263 CTATGGT-GAAAAAGGAAATATCTTCCCCTGAAAACTAGACAGAAGCATTCTCAGAATCTTATTT
         66 TTTCGTTGGAAACAGG-AATATCTTCATATAAAAGCTAGACAGAAGCATTCTCAGAAACTTCTTT

                   **  **    *                    *   *
     365327 GTGATGTGCGCCCTCAACTAACAGTGTTGAAGCATT-CTTTTG
        130 GTGATGTTTGCATTCAAGTAACAGTGTTGAA-CATTCCCTTTC

                     *                    **     *   *                       
     365369 ATAGAGCAGTTTTGAAACACTCTTTTTGTAAAATCTGCAAGAGGATATTTGGATAGCTTTGAGGA
          1 ATAGAGCAGGTTTGAAACACTCTTTTTGTAGTATCTGGAAGTGGATATTTGGATAGCTTTGAGGA

                         *   * *            **                      *    *   
     365434 TTTCGTTGGAAACGGGATTGTCTTCATATAAACTCTAGACAGAAGCATTCTCAGAAGCTTCATTG
         66 TTTCGTTGGAAACAGGAATATCTTCATATAAAAGCTAGACAGAAGCATTCTCAGAAACTTCTTTG

            *       **   *    *             *        
     365499 GGATGTTTCAATTGAAGTCACAGTGTTGAACAGTCCCTTTC
        131 TGATGTTTGCATTCAAGTAACAGTGTTGAACATTCCCTTTC

                                                         *       **   * *    
     365540 ATAGAGCAGGTTTGAAACACTCTTTTTGTAGTATCTGGAAGTGGACATTTGGAGCGCTCTCAGGA
          1 ATAGAGCAGGTTTGAAACACTCTTTTTGTAGTATCTGGAAGTGGATATTTGGATAGCTTTGAGGA

            * *  *      *             **            *       * *          *  *
     365605 CTGCGGT-GAAAAAGGAAATATCTTCCAATAAAAGCTAGATAGAAGCAATGTCAGAAACTTTTTC
         66 TTTCGTTGGAAACAGG-AATATCTTCATATAAAAGCTAGACAGAAGCATTCTCAGAAACTTCTTT

            *      * ** *   **      *       *    *   *
     365669 ATGATGTATCTACTCAGCTAACAGAGTTGAACCTT-CATTTG
        130 GTGATGTTTGCATTCAAGTAACAGTGTTGAACATTCCCTTTC

             *       *            *      * *     *             **            
     365710 AGAGAGCAGTTTTGAAACACTCGTTTTGTGGAATCTGCAAGTGGATATTTGTCTAGCTTTGAGGA
          1 ATAGAGCAGGTTTGAAACACTCTTTTTGTAGTATCTGGAAGTGGATATTTGGATAGCTTTGAGGA

                         *   *    *          *        *       *              
     365775 TTTCGTTGGAAACGGGATTA-CAT-ATA-AAAATC-AGACAGCAGCATTCCCAGAAACTTCTTTG
         66 TTTCGTTGGAAACAGGAATATCTTCATATAAAAGCTAGACAGAAGCATTCTCAGAAACTTCTTTG

                              *    *                 
     365836 TGATGTTTGCATTCAAGTCACAGAGTTGAACATTCCCTTTC
        131 TGATGTTTGCATTCAAGTAACAGTGTTGAACATTCCCTTTC

                                                   *     *     * **     *   *
     365877 ATAGAGCAGGTTTGAAACACTCTTTTTGTAGTATCTGGATGTGGACATTTGCAGCGCTTTCAGGC
          1 ATAGAGCAGGTTTGAAACACTCTTTTTGTAGTATCTGGAAGTGGATATTTGGATAGCTTTGAGGA

            * ** *      *             *** *   *                          *   
     365942 CTAAGGT-GAAAAAGGAAATATCTTCCCCTGAAAACTAGACAGAAGCATTCTCAGAAACTTATTT
         66 TTTCGTTGGAAACAGG-AATATCTTCATATAAAAGCTAGACAGAAGCATTCTCAGAAACTTCTTT

                   **  **    *              *  * **  *
     366006 GTGATGTGCGCCCTCAACTAACAGTGTTGAACCTTTCTGTTG
        130 GTGATGTTTGCATTCAAGTAACAGTGTTGAACATTCCCTTTC

                     *          *         *      *   *                       
     366048 ATAGAGCAGTTTTGAAACACACTTTTTGTAATATCTGCAAGAGGATATTTGGATAG-TTTGAGGA
          1 ATAGAGCAGGTTTGAAACACTCTTTTTGTAGTATCTGGAAGTGGATATTTGGATAGCTTTGAGGA

                         *   * *            **                      *    *   
     366112 TTTCGTTGGAAACGGGATTGTCTTCATATAAACTCTAGACAGAAGCATTCTCAGAAGCTTCATTG
         66 TTTCGTTGGAAACAGGAATATCTTCATATAAAAGCTAGACAGAAGCATTCTCAGAAACTTCTTTG

            *       **   *    *             * *      
     366177 GGATGTTTCAATTGAAGTCACAGTGTTGAACAGTTCCTTTC
        131 TGATGTTTGCATTCAAGTAACAGTGTTGAACATTCCCTTTC

                 *       *                               *       **   ***    
     366218 ATAGAACAGGTTTTAAACACTCTTTTTGTAGTATCTGGAAGTGGACATTTGGAGCGCTCCCAGGA
          1 ATAGAGCAGGTTTGAAACACTCTTTTTGTAGTATCTGGAAGTGGATATTTGGATAGCTTTGAGGA

            * ** *      *             **          * *       * *          *  *
     366283 CTATGGT-GAAAAAGGAAATATCTTCCAATAAAAGCTACATAGAAGCAATGTCAGAAACTTTTTC
         66 TTTCGTTGGAAACAGG-AATATCTTCATATAAAAGCTAGACAGAAGCATTCTCAGAAACTTCTTT

            *      * ** *   **      *       *  **    *
     366347 ATGATGTATCTACTCAGCTAACAGAGTTGAACCTTTACTTTG
        130 GTGATGTTTGCATTCAAGTAACAGTGTTGAACATTCCCTTTC

             *       *                   * *     *             **            
     366389 AGAGAGCAGTTTTGAAACACTCTTTTTGTGGAATCTGCAAGTGGATATTTGTCTAGCTTTGAGGA
          1 ATAGAGCAGGTTTGAAACACTCTTTTTGTAGTATCTGGAAGTGGATATTTGGATAGCTTTGAGGA

                         *   *    *                   *       *   *          
     366454 TTTCGTTGGAAACGGGATTA-CAT-ATA-AAAAGC-AGACAGCAGCATTCCCAGTAACTTCTTTG
         66 TTTCGTTGGAAACAGGAATATCTTCATATAAAAGCTAGACAGAAGCATTCTCAGAAACTTCTTTG

                              *    *                 
     366515 TGATGTTTGCATTCAAGTCACAGAGTTGAACATTCCCTTTC
        131 TGATGTTTGCATTCAAGTAACAGTGTTGAACATTCCCTTTC

                                                   *     *       **     *   *
     366556 ATAGAGCAGGTTTGAAACACTCTTTTTGTAGTATCTGGATGTGGACATTTGGAGCGCTTTCAGGC
          1 ATAGAGCAGGTTTGAAACACTCTTTTTGTAGTATCTGGAAGTGGATATTTGGATAGCTTTGAGGA

            * ** *      *             *** *   *                          *   
     366621 CTATGGT-GAAAAAGGAAATATCTTCCCCTGAAAACTAGACAGAAGCATTCTCAGAAACTTATTT
         66 TTTCGTTGGAAACAGG-AATATCTTCATATAAAAGCTAGACAGAAGCATTCTCAGAAACTTCTTT

                   **  **    *              *  * *   *
     366685 GTGATGTGCGCCCTCAACTAACAGTGTTGAACCTTTCTTTTG
        130 GTGATGTTTGCATTCAAGTAACAGTGTTGAACATTCCCTTTC

                     *                    *      *   *                       
     366727 ATAGAGCAGTTTTGAAACACTCTTTTTGTAATATCTGCAAGAGGATATTTGGATAGCTTTGAGGA
          1 ATAGAGCAGGTTTGAAACACTCTTTTTGTAGTATCTGGAAGTGGATATTTGGATAGCTTTGAGGA

                              *    *         *        *       *     *   *    
     366792 TTTCGTTGGAAAC-GG--GAT-TACATATAAAAAC-AGACAGCAGCATTCCCAGAATCTTGTTTG
         66 TTTCGTTGGAAACAGGAATATCTTCATATAAAAGCTAGACAGAAGCATTCTCAGAAACTTCTTTG

                                   *                 
     366852 TGATGTTTGCATTCAAGT--CAGAGTTGAACATTCCCTTTC
        131 TGATGTTTGCATTCAAGTAACAGTGTTGAACATTCCCTTTC

             *                         *           *     *       **     *   *
     366891 AGAGAGCAGGTTTGAAACACTCTTTTTATAGTATCTGGATGTGGACATTTGGAGCGCTTTCAGGC
          1 ATAGAGCAGGTTTGAAACACTCTTTTTGTAGTATCTGGAAGTGGATATTTGGATAGCTTTGAGGA

            * ** *      *             *** *   *                      *   *   
     366956 CTATGGT-GAAAAAGGAAATATCTTCTCCTGAAAACTAGACAGAAGCATTCTCAGAATCTTATTT
         66 TTTCGTTGGAAACAGG-AATATCTTCATATAAAAGCTAGACAGAAGCATTCTCAGAAACTTCTTT

                   **  **    *             **  * *   *
     367020 GTGATGTGCGCCCTCAACTAACAGTGTTGAAGCTTTCTTTTG
        130 GTGATGTTTGCATTCAAGTAACAGTGTTGAACATTCCCTTTC

                     *                *   **     *   *        *              
     367062 ATAGAGCAGTTTTGAAACACTCTTTTCGTAAAATCTGCAAGAGGATATTTTGATAGCTTTGAGGA
          1 ATAGAGCAGGTTTGAAACACTCTTTTTGTAGTATCTGGAAGTGGATATTTGGATAGCTTTGAGGA

                         *   * *            **                      *    *   
     367127 TTTCGTTGGAAACGGGATTGTCTTCATATAAACTCTAGACAGAAGCATTCTCAGAAGCTTCATTG
         66 TTTCGTTGGAAACAGGAATATCTTCATATAAAAGCTAGACAGAAGCATTCTCAGAAACTTCTTTG

            *       **   *    *  *          *        
     367192 GGATGTTTCAATTGAAGTCACTGTGTTGAACAGTCCCTTTC
        131 TGATGTTTGCATTCAAGTAACAGTGTTGAACATTCCCTTTC

                                                         *       **   * *    
     367233 ATAGAGCAGGTTTGAAACACTCTTTTTGTAGTATCTGGAAGTGGACATTTGGAGCGCTCTCAGGA
          1 ATAGAGCAGGTTTGAAACACTCTTTTTGTAGTATCTGGAAGTGGATATTTGGATAGCTTTGAGGA

            * *  *    * *             **            *   *   * *          *  *
     367298 CTACGGT-GATAAAGGAAATATCTTCCAATAAAAGCTAGATAGATGCAATGTCAGAAACTTTTTC
         66 TTTCGTTGGAAACAGG-AATATCTTCATATAAAAGCTAGACAGAAGCATTCTCAGAAACTTCTTT

            *      * ** *   **      *       *  *     *
     367362 ATGATGTATCTACTCAGCTAACAGAGTTGAACCTTTCCTTTG
        130 GTGATGTTTGCATTCAAGTAACAGTGTTGAACATTCCCTTTC

             *       *                   * *     *             **            
     367404 AGAGAGCAGTTTTGAAACACTCTTTTTGTTGAATCTGCAAGTGGATATTTGTCTAGCTTTGAGGA
          1 ATAGAGCAGGTTTGAAACACTCTTTTTGTAGTATCTGGAAGTGGATATTTGGATAGCTTTGAGGA

                         *   *    *              *    *       *   *          
     367469 TTTCGTTGGAAACGGGATTA-CAT-ATA-AAAAGC-ATACAGCAGCATTCCCAGTAACTTCTTTG
         66 TTTCGTTGGAAACAGGAATATCTTCATATAAAAGCTAGACAGAAGCATTCTCAGAAACTTCTTTG

                              *    *                 
     367530 TGATGTTTGCATTCAAGTCACAGAGTTGAACATTCCCTTTC
        131 TGATGTTTGCATTCAAGTAACAGTGTTGAACATTCCCTTTC

                                                   *     *       **     *   *
     367571 ATAGAGCAGGTTTGAAACACTCTTTTTGTAGTATCTGGATGTGGACATTTGGAGCGCTTTCAGGC
          1 ATAGAGCAGGTTTGAAACACTCTTTTTGTAGTATCTGGAAGTGGATATTTGGATAGCTTTGAGGA

            * ** *      *             *** *   *                          *   
     367636 CTATGGT-GAAAAAGGAAATATCTTCCCCTGAAAACTAGACAGAAGCATTCTCAGAAACTTATTT
         66 TTTCGTTGGAAACAGG-AATATCTTCATATAAAAGCTAGACAGAAGCATTCTCAGAAACTTCTTT

              *    **  **    *              *  * *   *
     367700 GTTATGTGCGCCCTCAATTAACAGTGTTGAACCTTTCTTTTG
        130 GTGATGTTTGCATTCAAGTAACAGTGTTGAACATTCCCTTTC

                     *                    *      *   *                       
     367742 ATAGAGCAGTTTTGAAACACTCTTTTTGTAATATCTGCAAGAGGATATTTGGATAGCTTTGAGGA
          1 ATAGAGCAGGTTTGAAACACTCTTTTTGTAGTATCTGGAAGTGGATATTTGGATAGCTTTGAGGA

                         *   *    *                   *       *     *   *    
     367807 TTTCGTTGGAAACGGGATTA-CAT-ATA-AAAAGC-AGACAGCAGCATTCCCAGAATCTTGTTTG
         66 TTTCGTTGGAAACAGGAATATCTTCATATAAAAGCTAGACAGAAGCATTCTCAGAAACTTCTTTG

                              *    *                 
     367868 TGATGTTTGCATTCAAGTCACAGAGTTGAACATTCCCTTTC
        131 TGATGTTTGCATTCAAGTAACAGTGTTGAACATTCCCTTTC

             *                         *           *     *       **     *   *
     367909 AGAGAGCAGGTTTGAAACACTCTTTTTATAGTATCTGGATGTGGACATTTGGAGCGCTTTCAGGC
          1 ATAGAGCAGGTTTGAAACACTCTTTTTGTAGTATCTGGAAGTGGATATTTGGATAGCTTTGAGGA

            * ** *      *             *** *   *                      *   *   
     367974 CTATGGT-GAAAAAGGAAATATCTTCTCCTGAAAACTAGACAGAAGCATTCTCAGAATCTTATTT
         66 TTTCGTTGGAAACAGG-AATATCTTCATATAAAAGCTAGACAGAAGCATTCTCAGAAACTTCTTT

                   **  **    *             **  * *   *
     368038 GTGATGTGCGCCCTCAACTAACAGTGTTGAAGCTTTCTTTTG
        130 GTGATGTTTGCATTCAAGTAACAGTGTTGAACATTCCCTTTC

                     *                    **     *   *                       
     368080 ATAGAGCAGTTTTGAAACACTCTTTTTGTAAAATCTGCAAGAGGATATTTGGATAGCTTTGAGGA
          1 ATAGAGCAGGTTTGAAACACTCTTTTTGTAGTATCTGGAAGTGGATATTTGGATAGCTTTGAGGA

                         *   * *            **                      *    *   
     368145 TTTCGTTGGAAACGGGATTGTCTTCATATAAACTCTAGACAGAAGCATTCTCAGAATCTTCATTG
         66 TTTCGTTGGAAACAGGAATATCTTCATATAAAAGCTAGACAGAAGCATTCTCAGAAACTTCTTTG

            *       **   *    *             *        
     368210 GGATGTTTCAATTGAAGTCACAGTGTTGAACAGTCCCTTTC
        131 TGATGTTTGCATTCAAGTAACAGTGTTGAACATTCCCTTTC

                                                         *       *  * * *    
     368251 ATAGAGCAGGTTTGAAACACTCTTTTTGTAGTATCTGGAAGTGGACATTTGGAGAGATCTCAGGA
          1 ATAGAGCAGGTTTGAAACACTCTTTTTGTAGTATCTGGAAGTGGATATTTGGATAGCTTTGAGGA

            * *  *    * *             **            *       * *          *  *
     368316 ATACGGT-GATAAAGGAAATATCTTCCAATAAAAGCTAGATAGAAGCAATGTCAGAAACTTTTTC
         66 TTTCGTTGGAAACAGG-AATATCTTCATATAAAAGCTAGACAGAAGCATTCTCAGAAACTTCTTT

            *      * ** *   **      *       *  * *   *
     368380 ATGATGTATCTACTCAGCTAACAGAGTTGAACCTTTCTTTTG
        130 GTGATGTTTGCATTCAAGTAACAGTGTTGAACATTCCCTTTC

             *       *                   * *  *  *             **            
     368422 AGAGAGCAGTTTTGAAACACTCTTTTTGTGGAATATGCAAGTGGATATTTGTCTAGCTTTGAGGA
          1 ATAGAGCAGGTTTGAAACACTCTTTTTGTAGTATCTGGAAGTGGATATTTGGATAGCTTTGAGGA

                         *   *    *                   *       *              
     368487 TTTCGTTGGAAACGGGATTA-CAT-ATA-AAAAGC-AGACAGCAGCATTCCCAGAAACTTCTTTG
         66 TTTCGTTGGAAACAGGAATATCTTCATATAAAAGCTAGACAGAAGCATTCTCAGAAACTTCTTTG

                              *    *                 
     368548 TGATGTTTGCATTCAAGTTACAGAGTTGAACATTCCCTTTC
        131 TGATGTTTGCATTCAAGTAACAGTGTTGAACATTCCCTTTC

                                                ** *     *       **     *   *
     368589 ATAGAGCAGGTTTGAAACACTCTTTTTGTAGTATCTCTATGTGGACATTTGGAGCGCTTTCAGGC
          1 ATAGAGCAGGTTTGAAACACTCTTTTTGTAGTATCTGGAAGTGGATATTTGGATAGCTTTGAGGA

            * ** *      *             *** *   *           *          *   *   
     368654 CTATGGT-GAAAAAGGAAATATCTTCCCCTGAAAACTAGACAGAAGAATTCTCAGAATCTTATTT
         66 TTTCGTTGGAAACAGG-AATATCTTCATATAAAAGCTAGACAGAAGCATTCTCAGAAACTTCTTT

                   **  **    *             **  * *   *
     368718 GTGATGTGCGCCCTCAACTAACAGTGTTGAAGCTTTCTTTTG
        130 GTGATGTTTGCATTCAAGTAACAGTGTTGAACATTCCCTTTC

                     *          *         **     *   *                       
     368760 ATAGAGCAGTTTTGAAACACCCTTTTTGTAAAATCTGCAAGAGGATATTTGGATAGCTTTGAGGA
          1 ATAGAGCAGGTTTGAAACACTCTTTTTGTAGTATCTGGAAGTGGATATTTGGATAGCTTTGAGGA

                         * * * *            **                      * *  *   
     368825 TTTCGTTGGAAACGGTATTGTCTTCATATAAACTCTAGACAGAAGCATTCTCAGAAGCGTCATTG
         66 TTTCGTTGGAAACAGGAATATCTTCATATAAAAGCTAGACAGAAGCATTCTCAGAAACTTCTTTG

            *       **   *    *             *        
     368890 GGATGTTTCAATTGAAGTCACAGTGTTGAACAGTCCCTTTC
        131 TGATGTTTGCATTCAAGTAACAGTGTTGAACATTCCCTTTC

                                                   *     *       **     *    
     368931 ATAGAGCAGGTTTGAAACACTCTTTTTGTAGTATCTGGATGTGGACATTTGGAGCGCTTTCAGGC
          1 ATAGAGCAGGTTTGAAACACTCTTTTTGTAGTATCTGGAAGTGGATATTTGGATAGCTTTGAGG-

            * * *    *                 *** *   *                          *  
     368996 CTATGGTT-TAAA-AGGAAATATCTTCCCCTGAAAACTAGACAGAAGCATTCTCAGAAACTTATT
         65 ATTTCGTTGGAAACAGG-AATATCTTCATATAAAAGCTAGACAGAAGCATTCTCAGAAACTTCTT

                    **  **    *                    *   *
     369059 TGTGATGTGCGCCCTCAACTAACAGTGTTGAAGCATT-CTTTTG
        129 TGTGATGTTTGCATTCAAGTAACAGTGTTGAA-CATTCCCTTTC

                     *                   * *     *             **            
     369102 ATAGAGCAGTTTTGAAACACTCTTTTTGTGGAATCTGCAAGTGGATATTTGTCTAGCTTTGAGGA
          1 ATAGAGCAGGTTTGAAACACTCTTTTTGTAGTATCTGGAAGTGGATATTTGGATAGCTTTGAGGA

                    **   *   *    *                   *       *              
     369167 TTTCGTTGTTAACGGGATTA-CAT-ATA-AAAAGC-AGACAGCAGCATTCCCAGAAACTTCTTTG
         66 TTTCGTTGGAAACAGGAATATCTTCATATAAAAGCTAGACAGAAGCATTCTCAGAAACTTCTTTG

                         *    *    *                 
     369228 TGATGTTTGCATTAAAGTCACAGAGTTGAACATTCCCTTTC
        131 TGATGTTTGCATTCAAGTAACAGTGTTGAACATTCCCTTTC

                                                   *     *       **     *   *
     369269 ATAGAGCAGGTTTGAAACACTCTTTTTGTAGTATCTGGATGTGGACATTTGGAGCGCTTTCAGGC
          1 ATAGAGCAGGTTTGAAACACTCTTTTTGTAGTATCTGGAAGTGGATATTTGGATAGCTTTGAGGA

            * ** *      *             *** *   *                      *   *   
     369334 CTATGGT-GAAAAAGGAAATATCTTCCCCTGAAAACTAGACAGAAGCATTCTCAGAATCTTATTT
         66 TTTCGTTGGAAACAGG-AATATCTTCATATAAAAGCTAGACAGAAGCATTCTCAGAAACTTCTTT

                   **  **    *             **  * *   *
     369398 GTGATGTGCGCCCTCAACTAACAGTGTTGAAGCTTTCTTTTG
        130 GTGATGTTTGCATTCAAGTAACAGTGTTGAACATTCCCTTTC

                     *                    **     *   *                       
     369440 ATAGAGCAGTTTTGAAACACTCTTTTTGTAAAATCTGCAAGAGGATATTTGGATAGCTTTGAGGA
          1 ATAGAGCAGGTTTGAAACACTCTTTTTGTAGTATCTGGAAGTGGATATTTGGATAGCTTTGAGGA

                         *   * *            **                      *    *   
     369505 TTTCGTTGGAAACGGGATTGTCTTCATATAAACTCTAGACAGAAGCATTCTCAGAAGCTTCATTG
         66 TTTCGTTGGAAACAGGAATATCTTCATATAAAAGCTAGACAGAAGCATTCTCAGAAACTTCTTTG

            *       **   *    *             *        
     369570 GGATGTTTCAATTGAAGTCACAGTGTTGAACAGTCCCTTTC
        131 TGATGTTTGCATTCAAGTAACAGTGTTGAACATTCCCTTTC

                                                         *       **   * *    
     369611 ATAGAGCAGGTTTGAAACACTCTTTTTGTAGTATCTGGAAGTGGACATTTGGAGCGCTCTCAGGA
          1 ATAGAGCAGGTTTGAAACACTCTTTTTGTAGTATCTGGAAGTGGATATTTGGATAGCTTTGAGGA

            * *  *      *             **            *       * *          *  *
     369676 CTGCGGT-GAAAAAGGAAATATCTTCCAATAAAAGCTAGATAGAAGCAATGTCAGAAACTTTTTC
         66 TTTCGTTGGAAACAGG-AATATCTTCATATAAAAGCTAGACAGAAGCATTCTCAGAAACTTCTTT

            *      * ** *   **      *       *        *
     369740 ATGATGTATCTACTCAGCTAACAGAGTTGAACCTT-CCTTTG
        130 GTGATGTTTGCATTCAAGTAACAGTGTTGAACATTCCCTTTC

             *       *   *               * *     *             **            
     369781 AGAGAGCAGTTTTTAAACACTCTTTTTGTGGAATCTGCAAGTGGATATTTGTCTAGCTTTGAGGA
          1 ATAGAGCAGGTTTGAAACACTCTTTTTGTAGTATCTGGAAGTGGATATTTGGATAGCTTTGAGGA

                         *   *    *                   *       *              
     369846 TTTCGTTGGAAACGGGATTA-CAT-ATA-AAAAGC-AGACAGCAGCATTCCCAGAAACTTCTTTG
         66 TTTCGTTGGAAACAGGAATATCTTCATATAAAAGCTAGACAGAAGCATTCTCAGAAACTTCTTTG

                              *    *                 
     369907 TGATGTTTGCATTCAAGTCACAGAGTTGAACATTCCCTTTC
        131 TGATGTTTGCATTCAAGTAACAGTGTTGAACATTCCCTTTC

                                                   *     *     * **     *   *
     369948 ATAGAGCAGGTTTGAAACACTCTTTTTGTAGTATCTGGATGTGGACATTTGCAGCGCTTTCAGGC
          1 ATAGAGCAGGTTTGAAACACTCTTTTTGTAGTATCTGGAAGTGGATATTTGGATAGCTTTGAGGA

            * ** *      *             *** *   *                          *   
     370013 CTAAGGT-GAAAAAGGAAATATCTTCCCCTGAAAACTAGACAGAAGCATTCTCAGAAACTTATTT
         66 TTTCGTTGGAAACAGG-AATATCTTCATATAAAAGCTAGACAGAAGCATTCTCAGAAACTTCTTT

                   **  **    *              *  * *   *
     370077 GTGATGTGCGCCCTCAACTAACAGTGTTGAACCTTTCTTTTG
        130 GTGATGTTTGCATTCAAGTAACAGTGTTGAACATTCCCTTTC

                     *                    *      *   *                       
     370119 ATAGAGCAGTTTTGAAACACTCTTTTTGTAATATCTGCAAGAGGATATTTGGATAGCTTTGAGGA
          1 ATAGAGCAGGTTTGAAACACTCTTTTTGTAGTATCTGGAAGTGGATATTTGGATAGCTTTGAGGA

                         *   * *            **                      *    *   
     370184 TTTCGTTGGAAACGGGATTGTCTTCATATAAACTCTAGACAGAAGCATTCTCAGAAGCTTCATTG
         66 TTTCGTTGGAAACAGGAATATCTTCATATAAAAGCTAGACAGAAGCATTCTCAGAAACTTCTTTG

            *       **   *    *               *      
     370249 GGATGTTTCAATTGAAGTCACAGTGTTGAACATTTCCTTTC
        131 TGATGTTTGCATTCAAGTAACAGTGTTGAACATTCCCTTTC

                 *                                       *       **   * *    
     370290 ATAGAACAGGTTTGAAACACTCTTTTTGTAGTATCTGGAAGTGGACATTTGGAGCGCTCTCAGGA
          1 ATAGAGCAGGTTTGAAACACTCTTTTTGTAGTATCTGGAAGTGGATATTTGGATAGCTTTGAGGA

            * ** *      *             **          * *       * *          *  *
     370355 CTATGGT-GAAAAAGGAAATATCTTCCAATAAAAGCTACATAGAAGCAATGTCAGAAACTTTTTC
         66 TTTCGTTGGAAACAGG-AATATCTTCATATAAAAGCTAGACAGAAGCATTCTCAGAAACTTCTTT

            *      * ** *   **      *       *  *     *
     370419 ATGATGTATCTACTCAGCTAACAGAGTTGAACCTTTCCTTTG
        130 GTGATGTTTGCATTCAAGTAACAGTGTTGAACATTCCCTTTC

             *       *                   * *     *             **            
     370461 AGAGAGCAGTTTTGAAACACTCTTTTTGTGGAATCTGCAAGTGGATATTTGTCTAGCTTTGAGGA
          1 ATAGAGCAGGTTTGAAACACTCTTTTTGTAGTATCTGGAAGTGGATATTTGGATAGCTTTGAGGA

                         *   *    *                   *       *   *          
     370526 TTTCGTTGGAAACGGGATTA-CAT-ATA-AAAAGC-AGACAGCAGCATTCCCAGTAACTTCTTTG
         66 TTTCGTTGGAAACAGGAATATCTTCATATAAAAGCTAGACAGAAGCATTCTCAGAAACTTCTTTG

                              *    *                 
     370587 TGATGTTTGCATTCAAGTCACAGAGTTGAACATTCCCTTTC
        131 TGATGTTTGCATTCAAGTAACAGTGTTGAACATTCCCTTTC

                                                   *     *       **     *   *
     370628 ATAGAGCAGGTTTGAAACACTCTTTTTGTAGTATCTGGATGTGGACATTTGGAGCGCTTTCAGGC
          1 ATAGAGCAGGTTTGAAACACTCTTTTTGTAGTATCTGGAAGTGGATATTTGGATAGCTTTGAGGA

            * *  *      *             *** *   *                      *   *   
     370693 CTACGGT-GAAAAAGGAAATATCTTCCCCTGAAAACTAGACAGAAGCATTCTCAGAATCTTATTT
         66 TTTCGTTGGAAACAGG-AATATCTTCATATAAAAGCTAGACAGAAGCATTCTCAGAAACTTCTTT

                   **  **    *             **  * *   *
     370757 GTGATGTGCGCCCTCAACTAACAGTGTTGAAGCTTTCTTTTG
        130 GTGATGTTTGCATTCAAGTAACAGTGTTGAACATTCCCTTTC

                     *                    **     *   *                       
     370799 ATAGAGCAGTTTTGAAACACTCTTTTTGTAAAATCTGCAAGAGGATATTTGGATAGCTTTGAGGA
          1 ATAGAGCAGGTTTGAAACACTCTTTTTGTAGTATCTGGAAGTGGATATTTGGATAGCTTTGAGGA

                         *   * *            **                      *    *   
     370864 TTTCGTTGGAAACGGGATTGTCTTCATATAAACTCTAGACAGAAGCATTCTCAGAAGCTTCATTG
         66 TTTCGTTGGAAACAGGAATATCTTCATATAAAAGCTAGACAGAAGCATTCTCAGAAACTTCTTTG

            *       **   *    *   *         * *      
     370929 GGATGTTTCAATTGAAGTCACACTGTTGAACAGTTCCTTTC
        131 TGATGTTTGCATTCAAGTAACAGTGTTGAACATTCCCTTTC

                 *                                       *       **   * *    
     370970 ATAGAACAGGTTTGAAACACTCTTTTTGTAGTATCTGGAAGTGGACATTTGGAGCGCTCTCAGGA
          1 ATAGAGCAGGTTTGAAACACTCTTTTTGTAGTATCTGGAAGTGGATATTTGGATAGCTTTGAGGA

            * *  *      *             **          * *       * *          *  *
     371035 CTACGGT-GAAAAAGGAAATATCTTCCAATAAAAGCTACATAGAAGCAATGTCAGAAACTTTTTC
         66 TTTCGTTGGAAACAGG-AATATCTTCATATAAAAGCTAGACAGAAGCATTCTCAGAAACTTCTTT

            *      * ** *   **      *       *  *     *
     371099 ATGATGTATCTACTCAGCTAACAGAGTTGAACCTTTCCTTTG
        130 GTGATGTTTGCATTCAAGTAACAGTGTTGAACATTCCCTTTC

             *       *                   * *     *             **            
     371141 AGAGAGCAGTTTTGAAACACTCTTTTTGTGGAATCTGCAAGTGGATATTTGTCTAGCTTTGAGGA
          1 ATAGAGCAGGTTTGAAACACTCTTTTTGTAGTATCTGGAAGTGGATATTTGGATAGCTTTGAGGA

                         *   *    *                   *       *   *          
     371206 TTTCGTTGGAAACGGGATTA-CAT-ATA-AAAAGC-AGACAGCAGCATTCCCAGTAACTTCTTTG
         66 TTTCGTTGGAAACAGGAATATCTTCATATAAAAGCTAGACAGAAGCATTCTCAGAAACTTCTTTG

                              * *  *                 
     371267 TGATGTTTGCATTCAAGTCAGAGAGTTGAACATTCCCTTTC
        131 TGATGTTTGCATTCAAGTAACAGTGTTGAACATTCCCTTTC

                                                   *     *       **     *   *
     371308 ATAGAGCAGGTTTGAAACACTCTTTTTGTAGTATCTGGATGTGGACATTTGGAGCGCTTTCAGGC
          1 ATAGAGCAGGTTTGAAACACTCTTTTTGTAGTATCTGGAAGTGGATATTTGGATAGCTTTGAGGA

            * ** *      *             *** *   *                          *   
     371373 CTATGGT-GAAAAAGGAAATATCTTCCCCTGAAAACTAGACAGAAGCATTCTCAGAAACTTATTT
         66 TTTCGTTGGAAACAGG-AATATCTTCATATAAAAGCTAGACAGAAGCATTCTCAGAAACTTCTTT

                   **  **    *              *  * *   *
     371437 GTGATGTGCGCCCTCAACTAACAGTGTTGAACCTTTCTTTTG
        130 GTGATGTTTGCATTCAAGTAACAGTGTTGAACATTCCCTTTC

                     *                    **     *   *                       
     371479 ATAGAGCAGTTTTGAAACACTCTTTTTGTAAAATCTGCAAGAGGATATTTGGATAGCTTTGAGGA
          1 ATAGAGCAGGTTTGAAACACTCTTTTTGTAGTATCTGGAAGTGGATATTTGGATAGCTTTGAGGA

                         *   * *            **                      *    *   
     371544 TTTCGTTGGAAACGGGATTGTCTTCATATAAACTCTAGACAGAAGCATTCTCAGAAGCTTCATTG
         66 TTTCGTTGGAAACAGGAATATCTTCATATAAAAGCTAGACAGAAGCATTCTCAGAAACTTCTTTG

            *  *    **   *    *             *        
     371609 GGAGGTTTCAATTGAAGTCACAGTGTTGAACAGTCCCTTTC
        131 TGATGTTTGCATTCAAGTAACAGTGTTGAACATTCCCTTTC

                                                         *       **   * *    
     371650 ATAGAGCAGGTTTGAAACACTCTTTTTGTAGTATCTGGAAGTGGACATTTGGAGCGCTCTCAGGA
          1 ATAGAGCAGGTTTGAAACACTCTTTTTGTAGTATCTGGAAGTGGATATTTGGATAGCTTTGAGGA

            * *  *    * *             **            *       * *          *  *
     371715 CTACGGT-GATAAAGGAAATATCTTCCAATAAAAGCTAGATAGAAGCAATGTCAGAAACTTTTTC
         66 TTTCGTTGGAAACAGG-AATATCTTCATATAAAAGCTAGACAGAAGCATTCTCAGAAACTTCTTT

            *      * ** *   **      *       *  *     *
     371779 ATGATGTATCTACTCAGCTAACAGAGTTGAACCTTTCCTTTG
        130 GTGATGTTTGCATTCAAGTAACAGTGTTGAACATTCCCTTTC

             *       *                   * *     *             **            
     371821 AGAGAGCAGTTTTGAAACACTCTTTTTGTGGAATCTGCAAGTGGATATTTGTCTAGCTTTGAGGA
          1 ATAGAGCAGGTTTGAAACACTCTTTTTGTAGTATCTGGAAGTGGATATTTGGATAGCTTTGAGGA

                    *    *  **    *                   *       *     *        
     371886 TTTCGTTGCAAACGGGTTTA-CAT-ATA-AAAAGC-AGACAGCAGCATTCCCAGAATCTTCTTTG
         66 TTTCGTTGGAAACAGGAATATCTTCATATAAAAGCTAGACAGAAGCATTCTCAGAAACTTCTTTG

                              *    *                 
     371947 TGATGTTTGCATTCAAGTCACAGAGTTGAACATTCCCTTTC
        131 TGATGTTTGCATTCAAGTAACAGTGTTGAACATTCCCTTTC

                                                   *     *       **     *   *
     371988 ATAGAGCAGGTTTGAAACACTCTTTTTGTAGTATCTGGATGTGGACATTTGGAGCGCTTTCAGGC
          1 ATAGAGCAGGTTTGAAACACTCTTTTTGTAGTATCTGGAAGTGGATATTTGGATAGCTTTGAGGA

            * ** *      *             *** *   *                      *   *   
     372053 CTATGGT-GAAAAAGGAAATATCTTCCCCTGAAAACTAGACAGAAGCATTCTCAGAATCTTATTT
         66 TTTCGTTGGAAACAGG-AATATCTTCATATAAAAGCTAGACAGAAGCATTCTCAGAAACTTCTTT

                   **  **    *             **  * *   *
     372117 GTGATGTGCGCCCTCAACTAACAGTGTTGAAGCTTTCTTTTG
        130 GTGATGTTTGCATTCAAGTAACAGTGTTGAACATTCCCTTTC

                     *                    **     *   *                       
     372159 ATAGAGCAGTTTTGAAACACTCTTTTTGTAAAATCTGCAAGAGGATATTTGGATAGCTTTGAGGA
          1 ATAGAGCAGGTTTGAAACACTCTTTTTGTAGTATCTGGAAGTGGATATTTGGATAGCTTTGAGGA

                         *   * *            **                      * *  *   
     372224 TTTCGTTGGAAACGGGATTGTCTTCATATAAACTCTAGACAGAAGCATTCTCAGAAGCGTCATTG
         66 TTTCGTTGGAAACAGGAATATCTTCATATAAAAGCTAGACAGAAGCATTCTCAGAAACTTCTTTG

            *       **   *    *             *        
     372289 GGATGTTTCAATTGAAGTCACAGTGTTGAACAGTCCCTTTC
        131 TGATGTTTGCATTCAAGTAACAGTGTTGAACATTCCCTTTC

                                                   *     *       **     *    
     372330 ATAGAGCAGGTTTGAAACACTCTTTTTGTAGTATCTGGATGTGGACATTTGGAGCGCTTTCAGGC
          1 ATAGAGCAGGTTTGAAACACTCTTTTTGTAGTATCTGGAAGTGGATATTTGGATAGCTTTGAGG-

            * * *    *                 *** *   *                          *  
     372395 CTATGGTT-TAAA-AGGAAATATCTTCCCCTGAAAACTAGACAGAAGCATTCTCAGAAACTTATT
         65 ATTTCGTTGGAAACAGG-AATATCTTCATATAAAAGCTAGACAGAAGCATTCTCAGAAACTTCTT

                    **  **    *                    *   *
     372458 TGTGATGTGCGCCCTCAACTAACAGTGTTGAAGCATT-CTTTTG
        129 TGTGATGTTTGCATTCAAGTAACAGTGTTGAA-CATTCCCTTTC

                 *   *                   * *     *             **            
     372501 ATAGAACAGTTTTGAAACACTCTTTTTGTGGAATCTGCAAGTGGATATTTGTCTAGCTTTGAGGA
          1 ATAGAGCAGGTTTGAAACACTCTTTTTGTAGTATCTGGAAGTGGATATTTGGATAGCTTTGAGGA

                         *   *    *                   *       *              
     372566 TTTCGTTGGAAACGGGATTA-CAT-ATA-AAAAGC-AGACAGCAGCATTCGCAGAAACTTCTTTG
         66 TTTCGTTGGAAACAGGAATATCTTCATATAAAAGCTAGACAGAAGCATTCTCAGAAACTTCTTTG

                              *    *                 
     372627 TGATGTTTGCATTCAAGTCACAGAGTTGAACATTCCCTTTC
        131 TGATGTTTGCATTCAAGTAACAGTGTTGAACATTCCCTTTC

                                                   *     *       **     *   *
     372668 ATAGAGCAGGTTTGAAACACTCTTTTTGTAGTATCTGGATGTGGACATTTGGAGCGCTTTCAGGC
          1 ATAGAGCAGGTTTGAAACACTCTTTTTGTAGTATCTGGAAGTGGATATTTGGATAGCTTTGAGGA

            * ** *      *             *** *   *                          *   
     372733 CTATGGT-GAAAAAGGAAATATCTTCCCCTGAAAACTAGACAGAAGCATTCTCAGAAACTTATTT
         66 TTTCGTTGGAAACAGG-AATATCTTCATATAAAAGCTAGACAGAAGCATTCTCAGAAACTTCTTT

                   **  **    *             **  * *   *
     372797 GTGATGTGCGCCCTCAACTAACAGTGTTGAAGCTTTCTTTTG
        130 GTGATGTTTGCATTCAAGTAACAGTGTTGAACATTCCCTTTC

                     *                    *      *   *                       
     372839 ATAGAGCAGTTTTGAAACACTCTTTTTGTAATATCTGCAAGAGGATATTTGGATAGCTTTGAGGA
          1 ATAGAGCAGGTTTGAAACACTCTTTTTGTAGTATCTGGAAGTGGATATTTGGATAGCTTTGAGGA

                         *   * *            **                      *    *   
     372904 TTTCGTTGGAAACGGGATTGTCTTCATATAAACTCTAGACAGAAGCATTCTCAGAAGCTTCATTG
         66 TTTCGTTGGAAACAGGAATATCTTCATATAAAAGCTAGACAGAAGCATTCTCAGAAACTTCTTTG

            *       **   *    *             *        
     372969 GGATGTTTCAATTGAAGTCACAGTGTTGAACAGTCCCTTTC
        131 TGATGTTTGCATTCAAGTAACAGTGTTGAACATTCCCTTTC

                                                   *     *       **     *    
     373010 ATAGAGCAGGTTTGAAACACTCTTTTTGTAGTATCTGGATGTGGACATTTGGAGCGCTTTCAGGC
          1 ATAGAGCAGGTTTGAAACACTCTTTTTGTAGTATCTGGAAGTGGATATTTGGATAGCTTTGAGG-

            * * *    *                 *** *   *                          *  
     373075 CTATGGTT-TAAA-AGGAAATATCTTCCCCTGAAAACTAGACAGAAGCATTCTCAGAAACTTATT
         65 ATTTCGTTGGAAACAGG-AATATCTTCATATAAAAGCTAGACAGAAGCATTCTCAGAAACTTCTT

                    **  **    *                    *   *
     373138 TGTGATGTGCGCCCTCAACTAACAGTGTTGAAGCATT-CTTTTG
        129 TGTGATGTTTGCATTCAAGTAACAGTGTTGAA-CATTCCCTTTC

                     *                   * *     *             **            
     373181 ATAGAGCAGTTTTGAAACACTCTTTTTGTGGAATCTGCAAGTGGATATTTGTCTAGCTTTGAGGA
          1 ATAGAGCAGGTTTGAAACACTCTTTTTGTAGTATCTGGAAGTGGATATTTGGATAGCTTTGAGGA

                         *   *    *                   *       *   *   *      
     373246 TTTCGTTGGAAACGGGATTA-CAT-ATA-AAAAGC-AGACAGCAGCATTCCCAGGAACATCTTTG
         66 TTTCGTTGGAAACAGGAATATCTTCATATAAAAGCTAGACAGAAGCATTCTCAGAAACTTCTTTG

                              *    *                 
     373307 TGATGTTTGCATTCAAGTCACAGAGTTGAACATTCCCTTTC
        131 TGATGTTTGCATTCAAGTAACAGTGTTGAACATTCCCTTTC

                                                   *     *       **     *   *
     373348 ATAGAGCAGGTTTGAAACACTCTTTTTGTAGTATCTGGATGTGGACATTTGGAGCGCTTTCAGGC
          1 ATAGAGCAGGTTTGAAACACTCTTTTTGTAGTATCTGGAAGTGGATATTTGGATAGCTTTGAGGA

            * ** *      *             *** *   *                          *   
     373413 CTATGGT-GAAAAAGGAAATATCTTCCCCTGAAAACTAGACAGAAGCATTCTCAGAAACTTATTT
         66 TTTCGTTGGAAACAGG-AATATCTTCATATAAAAGCTAGACAGAAGCATTCTCAGAAACTTCTTT

                   **  **    *              *  * *   *
     373477 GTGATGTGCGCCCTCAACTAACAGTGTTGAACCTTTCTTTTG
        130 GTGATGTTTGCATTCAAGTAACAGTGTTGAACATTCCCTTTC

                     *                    *      *   *                       
     373519 ATAGAGCAGTTTTGAAACACTCTTTTTGTAATATCTGCAAGAGGATATTTGGATAGCTTTGAGGA
          1 ATAGAGCAGGTTTGAAACACTCTTTTTGTAGTATCTGGAAGTGGATATTTGGATAGCTTTGAGGA

                         *   * *            **                      *    *   
     373584 TTTCGTTGGAAACGGGATTGTCTTCATATAAACTCTAGACAGAAGCATTCTCAGAAGCTTCATTG
         66 TTTCGTTGGAAACAGGAATATCTTCATATAAAAGCTAGACAGAAGCATTCTCAGAAACTTCTTTG

            *       **   *    *             *        
     373649 GGATGTTTCAATTGAAGTCACAGTGTTGAACAGTCCCTTTC
        131 TGATGTTTGCATTCAAGTAACAGTGTTGAACATTCCCTTTC

                                                         *       *  * * *    
     373690 ATAGAGCAGGTTTGAAACACTCTTTTTGTAGTATCTGGAAGTGGACATTTGGAGAGATCTCAGGA
          1 ATAGAGCAGGTTTGAAACACTCTTTTTGTAGTATCTGGAAGTGGATATTTGGATAGCTTTGAGGA

            * *  *    * *             **            *       * *          *  *
     373755 ATACGGT-GATAAAGGAAATATCTTCCAATAAAAGCTAGATAGAAGCAATGTCAGAAACTTTTTC
         66 TTTCGTTGGAAACAGG-AATATCTTCATATAAAAGCTAGACAGAAGCATTCTCAGAAACTTCTTT

            *      * ** *   **      *       *  * *   *
     373819 ATGATGTATCTACTCAGCTAACAGAGTTGAACCTTTCTTTTG
        130 GTGATGTTTGCATTCAAGTAACAGTGTTGAACATTCCCTTTC

             *       *                   * *     *             **            
     373861 AGAGAGCAGTTTTGAAACACTCTTTTTGTGGAATCTGCAAGTGGATATTTGTCTAGCTTTGAGGA
          1 ATAGAGCAGGTTTGAAACACTCTTTTTGTAGTATCTGGAAGTGGATATTTGGATAGCTTTGAGGA

                         *   *    *                   *       *              
     373926 TTTCGTTGGAAACGGGATTA-CAT-ATA-AAAAGC-AGACAGCAGCATTCCCAGAAACTTCTTTG
         66 TTTCGTTGGAAACAGGAATATCTTCATATAAAAGCTAGACAGAAGCATTCTCAGAAACTTCTTTG

                              *    *                 
     373987 TGATGTTTGCATTCAAGTCACAGAGTTGAACATTCCCTTTC
        131 TGATGTTTGCATTCAAGTAACAGTGTTGAACATTCCCTTTC

                                                   *     *       **     *   *
     374028 ATAGAGCAGGTTTGAAACACTCTTTTTGTAGTATCTGGATGTGGACATTTGGAGCGCTTTCAGGC
          1 ATAGAGCAGGTTTGAAACACTCTTTTTGTAGTATCTGGAAGTGGATATTTGGATAGCTTTGAGGA

            * ** *      *             *** * * *                          *   
     374093 CTATGGT-GAAAAAGGAAATATCTTCCCCTGAGAACTAGACAGAAGCATTCTCAGAAACTTATTT
         66 TTTCGTTGGAAACAGG-AATATCTTCATATAAAAGCTAGACAGAAGCATTCTCAGAAACTTCTTT

                   **  **    *              *  * *   *
     374157 GTGATGTGCGCCCTCAACTAACAGTGTTGAACCTTTCTTTTG
        130 GTGATGTTTGCATTCAAGTAACAGTGTTGAACATTCCCTTTC

                     *                    *      *   *                       
     374199 ATAGAGCAGTTTTGAAACACTCTTTTTGTAATATCTGCAAGAGGATATTTGGATAGCTTTGAGGA
          1 ATAGAGCAGGTTTGAAACACTCTTTTTGTAGTATCTGGAAGTGGATATTTGGATAGCTTTGAGGA

                         *   * *            **                      *    *   
     374264 TTTCGTTGGAAACGGGATTGTCTTCATATAAACTCTAGACAGAAGCATTCTCAGAAGCTTCATTG
         66 TTTCGTTGGAAACAGGAATATCTTCATATAAAAGCTAGACAGAAGCATTCTCAGAAACTTCTTTG

            *       **   *    *             *        
     374329 GGATGTTTCAATTGAAGTCACAGTGTTGAACAGTCCCTTTC
        131 TGATGTTTGCATTCAAGTAACAGTGTTGAACATTCCCTTTC

                                                   *     *       **     *    
     374370 ATAGAGCAGGTTTGAAACACTCTTTTTGTAGTATCTGGATGTGGACATTTGGAGCGCTTTCAGGC
          1 ATAGAGCAGGTTTGAAACACTCTTTTTGTAGTATCTGGAAGTGGATATTTGGATAGCTTTGAGG-

            * * *    *                 *** *   *                          *  
     374435 CTATGGTT-TAAA-AGGAAATATCTTCCCCTGAAAACTAGACAGAAGCATTCTCAGAAACTTATT
         65 ATTTCGTTGGAAACAGG-AATATCTTCATATAAAAGCTAGACAGAAGCATTCTCAGAAACTTCTT

                    **  **    *                        *
     374498 TGTGATGTGCGCCCTCAACTAACAGTGTTGAAGCATT-CCTTTG
        129 TGTGATGTTTGCATTCAAGTAACAGTGTTGAA-CATTCCCTTTC

                     *                   * *     *             **  *         
     374541 ATAGAGCAGTTTTGAAACACTCTTTTTGTGGAATCTGCAAGTGGATATTTGTCTATCTTTGAGGA
          1 ATAGAGCAGGTTTGAAACACTCTTTTTGTAGTATCTGGAAGTGGATATTTGGATAGCTTTGAGGA

                         *   *    *                   *       *              
     374606 TTTCGTTGGAAACGGGATTA-CAT-ATA-AAAAGC-AGACAGCAGCATTCCCAGAAACTTCTTTG
         66 TTTCGTTGGAAACAGGAATATCTTCATATAAAAGCTAGACAGAAGCATTCTCAGAAACTTCTTTG

                              *    *                 
     374667 TGATGTTTGCATTCAAGTCACAGAGTTGAACATTCCCTTTC
        131 TGATGTTTGCATTCAAGTAACAGTGTTGAACATTCCCTTTC

                                                   *     *       **     *   *
     374708 ATAGAGCAGGTTTGAAACACTCTTTTTGTAGTATCTGGATGTGGACATTTGGAGCGCTTTCAGGC
          1 ATAGAGCAGGTTTGAAACACTCTTTTTGTAGTATCTGGAAGTGGATATTTGGATAGCTTTGAGGA

            * ** *      *             *** *   *                          *   
     374773 CTATGGT-GAAAAAGGAAATATCTTCCCCTGAAAACTAGACAGAAGCATTCTCAGAAACTTATTT
         66 TTTCGTTGGAAACAGG-AATATCTTCATATAAAAGCTAGACAGAAGCATTCTCAGAAACTTCTTT

                   **  **    *              *  * *   *
     374837 GTGATGTGCGCCCTCAACTAACAGTGTTGAACCTTTCTTTTG
        130 GTGATGTTTGCATTCAAGTAACAGTGTTGAACATTCCCTTTC

                     *                    *      *   *                       
     374879 ATAGAGCAGTTTTGAAACACTCTTTTTGTAATATCTGCAAGAGGATATTTGGATAGCTTTGAGGA
          1 ATAGAGCAGGTTTGAAACACTCTTTTTGTAGTATCTGGAAGTGGATATTTGGATAGCTTTGAGGA

                         *   * *            **                      *    *   
     374944 TTTCGTTGGAAACGGGATTGTCTTCATATAAACTCTAGACAGAAGCATTCTCAGAAGCTTCATTG
         66 TTTCGTTGGAAACAGGAATATCTTCATATAAAAGCTAGACAGAAGCATTCTCAGAAACTTCTTTG

            *       **   *    *                       
     375009 GGATGTTTCAATTGAAGTCACAGTGTTGAACAGTTCCC-TTC
        131 TGATGTTTGCATTCAAGTAACAGTGTTGAACA-TTCCCTTTC

                 *                                       *       **   * *    
     375050 ATAGAACAGGTTTGAAACACTCTTTTTGTAGTATCTGGAAGTGGACATTTGGAGCGCTCTCAGGA
          1 ATAGAGCAGGTTTGAAACACTCTTTTTGTAGTATCTGGAAGTGGATATTTGGATAGCTTTGAGGA

            * ** *      *             **          * *       * *          *  *
     375115 CTATGGT-GAAAAAGGAAATATCTTCCAATAAAAGCTACATAGAAGCAATGTCAGAAACTTTTTC
         66 TTTCGTTGGAAACAGG-AATATCTTCATATAAAAGCTAGACAGAAGCATTCTCAGAAACTTCTTT

            *      * ** *   **      *       *  *     *
     375179 ATGATGTATCTACTCAGCTAACAGAGTTGAACCTTTCCTTTG
        130 GTGATGTTTGCATTCAAGTAACAGTGTTGAACATTCCCTTTC

             *       *                   * *     *             **            
     375221 AGAGAGCAGTTTTGAAACACTCTTTTTGTGGAATCTGCAAGTGGATATTTGCTTAGCTTTGAGGA
          1 ATAGAGCAGGTTTGAAACACTCTTTTTGTAGTATCTGGAAGTGGATATTTGGATAGCTTTGAGGA

                         *   *    *                   *       *   *          
     375286 TTTCGTTGGAAACGGGATTA-CAT-ATA-AAAAGC-AGACAGCAGCATTCCCAGTAACTTCTTTG
         66 TTTCGTTGGAAACAGGAATATCTTCATATAAAAGCTAGACAGAAGCATTCTCAGAAACTTCTTTG

                              *    *         *       
     375347 TGATGTTTGCATTCAAGTCACAGAGTTGAACATGCCCTTTC
        131 TGATGTTTGCATTCAAGTAACAGTGTTGAACATTCCCTTTC

                                                   *     *       **     *   *
     375388 ATAGAGCAGGTTTGAAACACTCTTTTTGTAGTATCTGGATGTGGACATTTGGAGCGCTTTCAGGC
          1 ATAGAGCAGGTTTGAAACACTCTTTTTGTAGTATCTGGAAGTGGATATTTGGATAGCTTTGAGGA

            * ** *      *             *** *   *                      *   *   
     375453 CTATGGT-GAAAAAGGAAATATCTTCCCCTGAAAACTAGACAGAAGCATTCTCAGAATCTTATTT
         66 TTTCGTTGGAAACAGG-AATATCTTCATATAAAAGCTAGACAGAAGCATTCTCAGAAACTTCTTT

                   **  **    *             **  * *   *
     375517 GTGATGTGCGCCCTCAACTAACAGTGTTGAAGCTTTCTTTTG
        130 GTGATGTTTGCATTCAAGTAACAGTGTTGAACATTCCCTTTC

                     *                    **     *   *                       
     375559 ATAGAGCAGTTTTGAAACACTCTTTTTGTAAAATCTGCAAGAGGATATTTGGATAGCTTTGAGGA
          1 ATAGAGCAGGTTTGAAACACTCTTTTTGTAGTATCTGGAAGTGGATATTTGGATAGCTTTGAGGA

                         *   * *            **                      *    *   
     375624 TTTCGTTGGAAACGGGATTGTCTTCATATAAACTCTAGACAGAAGCATTCTCAGAAGCTTCATTG
         66 TTTCGTTGGAAACAGGAATATCTTCATATAAAAGCTAGACAGAAGCATTCTCAGAAACTTCTTTG

            *       **   *    *             *        
     375689 GGATGTTTCAATTGAAGTCACAGTGTTGAACAGTCCCTTTC
        131 TGATGTTTGCATTCAAGTAACAGTGTTGAACATTCCCTTTC

                                                   *     *       **     *    
     375730 ATAGAGCAGGTTTGAAACACTCTTTTTGTAGTATCTGGATGTGGACATTTGGAGCGCTTTCAGGC
          1 ATAGAGCAGGTTTGAAACACTCTTTTTGTAGTATCTGGAAGTGGATATTTGGATAGCTTTGAGG-

            * * *    *                 *** *   *                          *  
     375795 CTATGGTT-TAAA-AGGAAATATCTTCCCCTGAAAACTAGACAGAAGCATTCTCAGAAACTTATT
         65 ATTTCGTTGGAAACAGG-AATATCTTCATATAAAAGCTAGACAGAAGCATTCTCAGAAACTTCTT

                    **  **    *                    *   *
     375858 TGTGATGTGCGCCCTCAACTAACAGTGTTGAAGCATT-CTTTTG
        129 TGTGATGTTTGCATTCAAGTAACAGTGTTGAA-CATTCCCTTTC

                     *                   * *     *             **            
     375901 ATAGAGCAGTTTTGAAACACTCTTTTTGTGGAATCTGCAAGTGGATATTTGTCTAGCTTTGAGGA
          1 ATAGAGCAGGTTTGAAACACTCTTTTTGTAGTATCTGGAAGTGGATATTTGGATAGCTTTGAGGA

                         *   *    *                   *       *              
     375966 TTTCGTTGGAAACGGGATTA-CAT-ATA-AAAAGC-AGACAGCAGCATTCCCAGAAACTTCTTTG
         66 TTTCGTTGGAAACAGGAATATCTTCATATAAAAGCTAGACAGAAGCATTCTCAGAAACTTCTTTG

                              *    *                 
     376027 TGATGTTTGCATTCAAGTCACAGAGTTGAACATTCCCTTTC
        131 TGATGTTTGCATTCAAGTAACAGTGTTGAACATTCCCTTTC

                                                   *     *       **     *   *
     376068 ATAGAGCAGGTTTGAAACACTCTTTTTGTAGTATCTGGATGTGGACATTTGGAGCGCTTTCAGGC
          1 ATAGAGCAGGTTTGAAACACTCTTTTTGTAGTATCTGGAAGTGGATATTTGGATAGCTTTGAGGA

            * ** *      *             *** *   *                          *  *
     376133 CTATGGT-GAAAAAGGAAATATCTTCCCCTGAAAACTAGACAGAAGCATTCTCAGAAACTTATTC
         66 TTTCGTTGGAAACAGG-AATATCTTCATATAAAAGCTAGACAGAAGCATTCTCAGAAACTTCTTT

                   **  **    *             **  * *   *
     376197 GTGATGTGCGCCCTCAACTAACAGTGTTGAAGCTTTCTTTTG
        130 GTGATGTTTGCATTCAAGTAACAGTGTTGAACATTCCCTTTC

                     *                    *      *   *                       
     376239 ATAGAGCAGTTTTGAAACACTCTTTTTGTAATATCTGCAAGAGGATATTTGGATAGCTTTGAGGA
          1 ATAGAGCAGGTTTGAAACACTCTTTTTGTAGTATCTGGAAGTGGATATTTGGATAGCTTTGAGGA

                         *   * *            **                      *    *   
     376304 TTTCGTTGGAAACGGGATTGTCTTCATATAAACTCTAGACAGAAGCATTCTCAGAAGCTTCATTG
         66 TTTCGTTGGAAACAGGAATATCTTCATATAAAAGCTAGACAGAAGCATTCTCAGAAACTTCTTTG

            *       **   *    *             *        
     376369 GGATGTTTCAATTGAAGTCACAGTGTTGAACAGTCCCTTTC
        131 TGATGTTTGCATTCAAGTAACAGTGTTGAACATTCCCTTTC

                                                   *     *       **     *    
     376410 ATAGAGCAGGTTTGAAACACTCTTTTTGTAGTATCTGGATGTGGACATTTGGAGCGCTTTCAGGC
          1 ATAGAGCAGGTTTGAAACACTCTTTTTGTAGTATCTGGAAGTGGATATTTGGATAGCTTTGAGG-

            * * *    *                 ***                                 * 
     376475 CTATGGTT-TAAA-AGGAAATATCTTCCCCTGAAAAG-TAGACAGAAGCATTCTCAGAAACTTAT
         65 ATTTCGTTGGAAACAGG-AATATCTTCATAT-AAAAGCTAGACAGAAGCATTCTCAGAAACTTCT

                     *   **    *                    *   *
     376537 TTGTGATGTGTGCCCTCAACTAACAGTGTTGAAGCATT-CTTTTG
        128 TTGTGATGTTTGCATTCAAGTAACAGTGTTGAA-CATTCCCTTTC

                     *                   * *     *             **            
     376581 ATAGAGCAGTTTTGAAACACTCTTTTTGTGGAATCTGCAAGTGGATATTTGTCTAGCTTTGAGGA
          1 ATAGAGCAGGTTTGAAACACTCTTTTTGTAGTATCTGGAAGTGGATATTTGGATAGCTTTGAGGA

                         *   *    *                   *       *              
     376646 TTTCGTTGGAAACGGGATTA-CAT-ATA-AAAAGC-AGACAGCAGCATTCCCAGAAACTTCTTTG
         66 TTTCGTTGGAAACAGGAATATCTTCATATAAAAGCTAGACAGAAGCATTCTCAGAAACTTCTTTG

                              *    *                 
     376707 TGATGTTTGCATTCAAGTCACAGAGTTGAACATTCCCTTTC
        131 TGATGTTTGCATTCAAGTAACAGTGTTGAACATTCCCTTTC

                                                   *     *       **     *   *
     376748 ATAGAGCAGGTTTGAAACACTCTTTTTGTAGTATCTGGATGTGGACATTTGGAGCGCTTTCAGGC
          1 ATAGAGCAGGTTTGAAACACTCTTTTTGTAGTATCTGGAAGTGGATATTTGGATAGCTTTGAGGA

            * ** *      *             *** *   *                          *   
     376813 CTATGGT-GAAAAAGGAAATATCTTCCCCTGAAAACTAGACAGAAGCATTCTCAGAAACTTATTT
         66 TTTCGTTGGAAACAGG-AATATCTTCATATAAAAGCTAGACAGAAGCATTCTCAGAAACTTCTTT

              *    **  **                   *  * *   *
     376877 GTAATGTGCGCCCTCAAGTAACAGTGTTGAACCTTTCTTTTG
        130 GTGATGTTTGCATTCAAGTAACAGTGTTGAACATTCCCTTTC

                     *                    *      *   *                       
     376919 ATAGAGCAGTTTTGAAACACTCTTTTTGTAATATCTGCAAGAGGATATTTGGATAGCTTTGAGGA
          1 ATAGAGCAGGTTTGAAACACTCTTTTTGTAGTATCTGGAAGTGGATATTTGGATAGCTTTGAGGA

                         *   * *             *                      *    *   
     376984 TTTCGTTGGAAACGGGATTGTCTTCATATAAAATCTAGACAGAAGCATTCTCAGAAGCTTCATTG
         66 TTTCGTTGGAAACAGGAATATCTTCATATAAAAGCTAGACAGAAGCATTCTCAGAAACTTCTTTG

            *       **   *    *             *        
     377049 GGATGTTTCAATTGAAGTCACAGTGTTGAACAGTCCCTTTC
        131 TGATGTTTGCATTCAAGTAACAGTGTTGAACATTCCCTTTC

                                                         *       *  * * *  * 
     377090 ATAGAGCAGGTTTGAAACACTCTTTTTGTAGTATCTGGAAGTGGACATTTGGAGAGATCTCAGCA
          1 ATAGAGCAGGTTTGAAACACTCTTTTTGTAGTATCTGGAAGTGGATATTTGGATAGCTTTGAGGA

            * *  *    * *             **            *       * *          *  *
     377155 ATACGGT-GATAAAGGAAATATCTTCCAATAAAAGCTAGATAGAAGCAATGTCAGAAACTTTTTC
         66 TTTCGTTGGAAACAGG-AATATCTTCATATAAAAGCTAGACAGAAGCATTCTCAGAAACTTCTTT

            *      * ** *   **      *       *  * *   *
     377219 ATGATGTATCTACTCAGCTAACAGAGTTGAACCTTTCTTTTG
        130 GTGATGTTTGCATTCAAGTAACAGTGTTGAACATTCCCTTTC

             *       *                   * *     *             **            
     377261 AGAGAGCAGTTTTGAAACACTCTTTTTGTGGAATCTGCAAGTGGATATTTGTCTAGCTTTGAGGA
          1 ATAGAGCAGGTTTGAAACACTCTTTTTGTAGTATCTGGAAGTGGATATTTGGATAGCTTTGAGGA

                         *   *    *                   *       *              
     377326 TTTCGTTGGAAACGGGATTA-CAT-ATA-AAAAGC-AGACAGCAGCATTCCCAGAAACTTCTTTG
         66 TTTCGTTGGAAACAGGAATATCTTCATATAAAAGCTAGACAGAAGCATTCTCAGAAACTTCTTTG

                              *    *                 
     377387 TGATGTTTGCATTCAAGTTACAGAGTTGAACATTCCCTTTC
        131 TGATGTTTGCATTCAAGTAACAGTGTTGAACATTCCCTTTC

                                                ** *     *       **     *   *
     377428 ATAGAGCAGGTTTGAAACACTCTTTTTGTAGTATCTCTATGTGGACATTTGGAGCGCTTTCAGGC
          1 ATAGAGCAGGTTTGAAACACTCTTTTTGTAGTATCTGGAAGTGGATATTTGGATAGCTTTGAGGA

            * ** *      *             *** *   *           *          *   *   
     377493 CTATGGT-GAAAAAGGAAATATCTTCCCCTGAAAACTAGACAGAAGAATTCTCAGAATCTTATTT
         66 TTTCGTTGGAAACAGG-AATATCTTCATATAAAAGCTAGACAGAAGCATTCTCAGAAACTTCTTT

                   **  **    *             **  * *   *
     377557 GTGATGTGCGCCCTCAACTAACAGTGTTGAAGCTTTCTTTTG
        130 GTGATGTTTGCATTCAAGTAACAGTGTTGAACATTCCCTTTC

                     *                    **     *   *                       
     377599 ATAGAGCAGTTTTGAAACACTCTTTTTGTAAAATCTGCAAGAGGATATTTGGATAGCTTTGAGGA
          1 ATAGAGCAGGTTTGAAACACTCTTTTTGTAGTATCTGGAAGTGGATATTTGGATAGCTTTGAGGA

                         * * * *            **                      * *  *   
     377664 TTTCGTTGGAAACGGTATTGTCTTCATATAAACTCTAGACAGAAGCATTCTCAGAAGCGTCATTG
         66 TTTCGTTGGAAACAGGAATATCTTCATATAAAAGCTAGACAGAAGCATTCTCAGAAACTTCTTTG

            *       **   *    *             *        
     377729 GGATGTTTCAATTGAAGTCACAGTGTTGAACAGTCCCTTTC
        131 TGATGTTTGCATTCAAGTAACAGTGTTGAACATTCCCTTTC

                                                   *     *       **     *    
     377770 ATAGAGCAGGTTTGAAACACTCTTTTTGTAGTATCTGGATGTGGACATTTGGAGCGCTTTCAGGC
          1 ATAGAGCAGGTTTGAAACACTCTTTTTGTAGTATCTGGAAGTGGATATTTGGATAGCTTTGAGG-

            * * *    *                 *** *   *                          *  
     377835 CTATGGTT-TAAA-AGGAAATATCTTCCCCTGAAAACTAGACAGAAGCATTCTCAGAAACTTATT
         65 ATTTCGTTGGAAACAGG-AATATCTTCATATAAAAGCTAGACAGAAGCATTCTCAGAAACTTCTT

                    **  **    *                    *   *
     377898 TGTGATGTGCGCCCTCAACTAACAGTGTTGAAGCATT-CTTTTG
        129 TGTGATGTTTGCATTCAAGTAACAGTGTTGAA-CATTCCCTTTC

                     *                   * *     *             **            
     377941 ATAGAGCAGTTTTGAAACACTCTTTTTGTGGAATCTGCAAGTGGATATTTGTCTAGCTTTGAGGA
          1 ATAGAGCAGGTTTGAAACACTCTTTTTGTAGTATCTGGAAGTGGATATTTGGATAGCTTTGAGGA

                    **   *   *    *                   *       *              
     378006 TTTCGTTGTTAACGGGATTA-CAT-ATA-AAAAGC-AGACAGCAGCATTCCCAGAAACTTCTTTG
         66 TTTCGTTGGAAACAGGAATATCTTCATATAAAAGCTAGACAGAAGCATTCTCAGAAACTTCTTTG

                         *    *    *                 
     378067 TGATGTTTGCATTAAAGTCACAGAGTTGAACATTCCCTTTC
        131 TGATGTTTGCATTCAAGTAACAGTGTTGAACATTCCCTTTC

                                                   *     *       **     *   *
     378108 ATAGAGCAGGTTTGAAACACTCTTTTTGTAGTATCTGGATGTGGACATTTGGAGCGCTTTCAGGC
          1 ATAGAGCAGGTTTGAAACACTCTTTTTGTAGTATCTGGAAGTGGATATTTGGATAGCTTTGAGGA

            * ** *      *             *** *   *                      *   *   
     378173 CTATGGT-GAAAAAGGAAATATCTTCCCCTGAAAACTAGACAGAAGCATTCTCAGAATCTTATTT
         66 TTTCGTTGGAAACAGG-AATATCTTCATATAAAAGCTAGACAGAAGCATTCTCAGAAACTTCTTT

                   **  **    *             **  * *   *
     378237 GTGATGTGCGCCCTCAACTAACAGTGTTGAAGCTTTCTTTTG
        130 GTGATGTTTGCATTCAAGTAACAGTGTTGAACATTCCCTTTC

                     *                    **     *   *                       
     378279 ATAGAGCAGTTTTGAAACACTCTTTTTGTAAAATCTGCAAGAGGATATTTGGATAGCTTTGAGGA
          1 ATAGAGCAGGTTTGAAACACTCTTTTTGTAGTATCTGGAAGTGGATATTTGGATAGCTTTGAGGA

                         *   * *            **                      *    *   
     378344 TTTCGTTGGAAACGGGATTGTCTTCATATAAACTCTAGACAGAAGCATTCTCAGAAGCTTCATTG
         66 TTTCGTTGGAAACAGGAATATCTTCATATAAAAGCTAGACAGAAGCATTCTCAGAAACTTCTTTG

            *       **   *    *             *        
     378409 GGATGTTTCAATTGAAGTCACAGTGTTGAACAGTCCCTTTC
        131 TGATGTTTGCATTCAAGTAACAGTGTTGAACATTCCCTTTC

                                                         *       **   * *    
     378450 ATAGAGCAGGTTTGAAACACTCTTTTTGTAGTATCTGGAAGTGGACATTTGGAGCGCTCTCAGGA
          1 ATAGAGCAGGTTTGAAACACTCTTTTTGTAGTATCTGGAAGTGGATATTTGGATAGCTTTGAGGA

            * *  *      *             **            *       * *          *  *
     378515 CTGCGGT-GAAAAAGGAAATATCTTCCAATAAAAGCTAGATAGAAGCAATGTCAGAAACTTTTTC
         66 TTTCGTTGGAAACAGG-AATATCTTCATATAAAAGCTAGACAGAAGCATTCTCAGAAACTTCTTT

            *      * ** *   **      *       *        *
     378579 ATGATGTATCTACTCAGCTAACAGAGTTGAACCTT-CCTTTG
        130 GTGATGTTTGCATTCAAGTAACAGTGTTGAACATTCCCTTTC

             *       *   *               * *     *             **            
     378620 AGAGAGCAGTTTTTAAACACTCTTTTTGTGGAATCTGCAAGTGGATATTTGTCTAGCTTTGAGGA
          1 ATAGAGCAGGTTTGAAACACTCTTTTTGTAGTATCTGGAAGTGGATATTTGGATAGCTTTGAGGA

                         *   *    *                   *       *              
     378685 TTTCGTTGGAAACGGGATTA-CAT-ATA-AAAAGC-AGACAGCAGCATTCCCAGAAACTTCTTTG
         66 TTTCGTTGGAAACAGGAATATCTTCATATAAAAGCTAGACAGAAGCATTCTCAGAAACTTCTTTG

                              *    *                 
     378746 TGATGTTTGCATTCAAGTCACAGAGTTGAACATTCCCTTTC
        131 TGATGTTTGCATTCAAGTAACAGTGTTGAACATTCCCTTTC

                                                   *     *     * **     *   *
     378787 ATAGAGCAGGTTTGAAACACTCTTTTTGTAGTATCTGGATGTGGACATTTGCAGCGCTTTCAGGC
          1 ATAGAGCAGGTTTGAAACACTCTTTTTGTAGTATCTGGAAGTGGATATTTGGATAGCTTTGAGGA

            * ** *      *             *** *   *                          *   
     378852 CTAAGGT-GAAAAAGGAAATATCTTCCCCTGAAAACTAGACAGAAGCATTCTCAGAAACTTATTT
         66 TTTCGTTGGAAACAGG-AATATCTTCATATAAAAGCTAGACAGAAGCATTCTCAGAAACTTCTTT

                   **  **    *              *  * *   *
     378916 GTGATGTGCGCCCTCAACTAACAGTGTTGAACCTTTCTTTTG
        130 GTGATGTTTGCATTCAAGTAACAGTGTTGAACATTCCCTTTC

                     *                    *      *   *                       
     378958 ATAGAGCAGTTTTGAAACACTCTTTTTGTAATATCTGCAAGAGGATATTTGGATAGCTTTGAGGA
          1 ATAGAGCAGGTTTGAAACACTCTTTTTGTAGTATCTGGAAGTGGATATTTGGATAGCTTTGAGGA

                         *   * *            **                      *    *   
     379023 TTTCGTTGGAAACGGGATTGTCTTCATATAAACTCTAGACAGAAGCATTCTCAGAAGCTTCATTG
         66 TTTCGTTGGAAACAGGAATATCTTCATATAAAAGCTAGACAGAAGCATTCTCAGAAACTTCTTTG

            *       **   *    *               *      
     379088 GGATGTTTCAATTGAAGTCACAGTGTTGAACATTTCCTTTC
        131 TGATGTTTGCATTCAAGTAACAGTGTTGAACATTCCCTTTC

                 *                                       *       **   * *    
     379129 ATAGAACAGGTTTGAAACACTCTTTTTGTAGTATCTGGAAGTGGACATTTGGAGCGCTCTCAGGA
          1 ATAGAGCAGGTTTGAAACACTCTTTTTGTAGTATCTGGAAGTGGATATTTGGATAGCTTTGAGGA

            * ** *      *             **          * *       * *          *  *
     379194 CTATGGT-GAAAAAGGAAATATCTTCCAATAAAAGCTACATAGAAGCAATGTCAGAAACTTTTTC
         66 TTTCGTTGGAAACAGG-AATATCTTCATATAAAAGCTAGACAGAAGCATTCTCAGAAACTTCTTT

            *      * ** *   **      *       *  *     *
     379258 ATGATGTATCTACTCAGCTAACAGAGTTGAACCTTTCCTTTG
        130 GTGATGTTTGCATTCAAGTAACAGTGTTGAACATTCCCTTTC

             *       *                   * *     *             **            
     379300 AGAGAGCAGTTTTGAAACACTCTTTTTGTGGAATCTGCAAGTGGATATTTGTCTAGCTTTGAGGA
          1 ATAGAGCAGGTTTGAAACACTCTTTTTGTAGTATCTGGAAGTGGATATTTGGATAGCTTTGAGGA

                         *   *    *                   *       *   *          
     379365 TTTCGTTGGAAACGGGATTA-CAT-ATA-AAAAGC-AGACAGCAGCATTCCCAGTAACTTCTTTG
         66 TTTCGTTGGAAACAGGAATATCTTCATATAAAAGCTAGACAGAAGCATTCTCAGAAACTTCTTTG

                              *    *                 
     379426 TGATGTTTGCATTCAAGTCACAGAGTTGAACATTCCCTTTC
        131 TGATGTTTGCATTCAAGTAACAGTGTTGAACATTCCCTTTC

                                                         *       *  * * *    
     379467 ATAGAGCAGGTTTGAAACACTCTTTTTGTAGTATCTGGAAGTGGACATTTGGAGAGATCTCAGGA
          1 ATAGAGCAGGTTTGAAACACTCTTTTTGTAGTATCTGGAAGTGGATATTTGGATAGCTTTGAGGA

            * *  *    * *             **            *       * *          *  *
     379532 ATACGGT-GATAAAGGAAATATCTTCCAATAAAAGCTAGATAGAAGCAATATCAGAAACTTTTTC
         66 TTTCGTTGGAAACAGG-AATATCTTCATATAAAAGCTAGACAGAAGCATTCTCAGAAACTTCTTT

            *      * ** *   **      *       *  * *   *
     379596 ATGATGTATCTACTCAGCTAACAGAGTTGAACCTTTCTTTTG
        130 GTGATGTTTGCATTCAAGTAACAGTGTTGAACATTCCCTTTC

             *       *                   * *     *             **            
     379638 AGAGAGCAGTTTTGAAACACTCTTTTTGTGGAATCTGCAAGTGGATATTTGTCTAGCTTTGAGGA
          1 ATAGAGCAGGTTTGAAACACTCTTTTTGTAGTATCTGGAAGTGGATATTTGGATAGCTTTGAGGA

                         *   *    *                   *       *              
     379703 TTTCGTTGGAAACGGGATTA-CAT-ATA-AAAAGC-AGACAGCAGCATTCCCAGAAACTTCTTTG
         66 TTTCGTTGGAAACAGGAATATCTTCATATAAAAGCTAGACAGAAGCATTCTCAGAAACTTCTTTG

                              *    **                
     379764 TGATGTTTGCATTCAAGTCACAGATTTGAACATTCCCTTTC
        131 TGATGTTTGCATTCAAGTAACAGTGTTGAACATTCCCTTTC

                                *                * *     *       **     *   *
     379805 ATAGAGCAGGTTTGAAACACACTTTTTGTAGTATCTGTATGTGGACATTTGGAGCGCTTTCAGGC
          1 ATAGAGCAGGTTTGAAACACTCTTTTTGTAGTATCTGGAAGTGGATATTTGGATAGCTTTGAGGA

            * ** *      *             *** *   *                        * *   
     379870 CTATGGT-GAAAAAGGAAATATCTTCCCCTGAAAACTAGACAGAAGCATTCTCAGAAACCTATTT
         66 TTTCGTTGGAAACAGG-AATATCTTCATATAAAAGCTAGACAGAAGCATTCTCAGAAACTTCTTT

                   **  **    *          *   *  * *   *
     379934 GTGATGTGCGCCCTCAACTAACAGTGTTTAACCTTTCTTTTG
        130 GTGATGTTTGCATTCAAGTAACAGTGTTGAACATTCCCTTTC

                     *                    *      *   *              *        
     379976 ATAGAGCAGTTTTGAAACACTCTTTTTGTAATATCTGCAAGAGGATATTTGGATAGATTTGAGGA
          1 ATAGAGCAGGTTTGAAACACTCTTTTTGTAGTATCTGGAAGTGGATATTTGGATAGCTTTGAGGA

                         *   * *            **                      *    *   
     380041 TTTCGTTGGAAACGGGATTGTCTTCATATAAACTCTAGACAGAAGCATTCTCAGAAGCTTCATTG
         66 TTTCGTTGGAAACAGGAATATCTTCATATAAAAGCTAGACAGAAGCATTCTCAGAAACTTCTTTG

            *       **   *    *   *         * *      
     380106 GGATGTTTCAATTGAAGTCACACTGTTGAACAGTTCCTTTC
        131 TGATGTTTGCATTCAAGTAACAGTGTTGAACATTCCCTTTC

                 *                                       *       **   * *    
     380147 ATAGAACAGGTTTGAAACACTCTTTTTGTAGTATCTGGAAGTGGACATTTGGAGCGCTCTCAGGA
          1 ATAGAGCAGGTTTGAAACACTCTTTTTGTAGTATCTGGAAGTGGATATTTGGATAGCTTTGAGGA

            * *  *      *             **          * *       * *          *  *
     380212 CTACGGT-GAAAAAGGAAATATCTTCCAATAAAAGCTACATAGAAGCAATGTCAGAAACTTTTTC
         66 TTTCGTTGGAAACAGG-AATATCTTCATATAAAAGCTAGACAGAAGCATTCTCAGAAACTTCTTT

            *      * ** *   **      *       *  *     *
     380276 ATGATGTATCTACTCAGCTAACAGAGTTGAACCTTTCCTTTG
        130 GTGATGTTTGCATTCAAGTAACAGTGTTGAACATTCCCTTTC

             *       *                   * *     *             **            
     380318 AGAGAGCAGTTTTGAAACACTCTTTTTGTGGAATCTGCAAGTGGATATTTGTCTAGCTTTGAGGA
          1 ATAGAGCAGGTTTGAAACACTCTTTTTGTAGTATCTGGAAGTGGATATTTGGATAGCTTTGAGGA

                         *   *    *                   *       *   *          
     380383 TTTCGTTGGAAACGGGATTA-CAT-ATA-AAAAGC-AGACAGCAGCATTCCCAGTAACTTCTTTG
         66 TTTCGTTGGAAACAGGAATATCTTCATATAAAAGCTAGACAGAAGCATTCTCAGAAACTTCTTTG

                              * *  *                 
     380444 TGATGTTTGCATTCAAGTCAGAGAGTTGAACATTCCCTTTC
        131 TGATGTTTGCATTCAAGTAACAGTGTTGAACATTCCCTTTC

                                                   *     *       **     *   *
     380485 ATAGAGCAGGTTTGAAACACTCTTTTTGTAGTATCTGGATGTGGACATTTGGAGCGCTTTCAGGC
          1 ATAGAGCAGGTTTGAAACACTCTTTTTGTAGTATCTGGAAGTGGATATTTGGATAGCTTTGAGGA

            * ** *      *             *** *   *                          *   
     380550 CTATGGT-GAAAAAGGAAATATCTTCCCCTGAAAACTAGACAGAAGCATTCTCAGAAACTTATTT
         66 TTTCGTTGGAAACAGG-AATATCTTCATATAAAAGCTAGACAGAAGCATTCTCAGAAACTTCTTT

                   **  **    *              *  * *   *
     380614 GTGATGTGCGCCCTCAACTAACAGTGTTGAACCTTTCTTTTG
        130 GTGATGTTTGCATTCAAGTAACAGTGTTGAACATTCCCTTTC

                     *                    **     *   *                       
     380656 ATAGAGCAGTTTTGAAACACTCTTTTTGTAAAATCTGCAAGAGGATATTTGGATAGCTTTGAGGA
          1 ATAGAGCAGGTTTGAAACACTCTTTTTGTAGTATCTGGAAGTGGATATTTGGATAGCTTTGAGGA

                         *   * *            **                      *    *   
     380721 TTTCGTTGGAAACGGGATTGTCTTCATATAAACTCTAGACAGAAGCATTCTCAGAAGCTTCATTG
         66 TTTCGTTGGAAACAGGAATATCTTCATATAAAAGCTAGACAGAAGCATTCTCAGAAACTTCTTTG

            *  *    **   *    *             *        
     380786 GGAGGTTTCAATTGAAGTCACAGTGTTGAACAGTCCCTTTC
        131 TGATGTTTGCATTCAAGTAACAGTGTTGAACATTCCCTTTC

                                                         *       **   * *    
     380827 ATAGAGCAGGTTTGAAACACTCTTTTTGTAGTATCTGGAAGTGGACATTTGGAGCGCTCTCAGGA
          1 ATAGAGCAGGTTTGAAACACTCTTTTTGTAGTATCTGGAAGTGGATATTTGGATAGCTTTGAGGA

            * *  *    * *             **            *       * *          *  *
     380892 CTACGGT-GATAAAGGAAATATCTTCCAATAAAAGCTAGATAGAAGCAATGTCAGAAACTTTTTC
         66 TTTCGTTGGAAACAGG-AATATCTTCATATAAAAGCTAGACAGAAGCATTCTCAGAAACTTCTTT

            *      * ** *   **      *       *  *     *
     380956 ATGATGTATCTACTCAGCTAACAGAGTTGAACCTTTCCTTTG
        130 GTGATGTTTGCATTCAAGTAACAGTGTTGAACATTCCCTTTC

             *       *                   * *     *             **            
     380998 AGAGAGCAGTTTTGAAACACTCTTTTTGTGGAATCTGCAAGTGGATATTTGTCTAGCTTTGAGGA
          1 ATAGAGCAGGTTTGAAACACTCTTTTTGTAGTATCTGGAAGTGGATATTTGGATAGCTTTGAGGA

                         *  **    *                   *       *     *        
     381063 TTTCGTTGGAAACGGGTTTA-CAT-ATA-AAAAGC-AGACAGCAGCATTCCCAGAATCTTCTTTG
         66 TTTCGTTGGAAACAGGAATATCTTCATATAAAAGCTAGACAGAAGCATTCTCAGAAACTTCTTTG

                              *    *                 
     381124 TGATGTTTGCATTCAAGTCACAGAGTTGAACATTCCCTTTC
        131 TGATGTTTGCATTCAAGTAACAGTGTTGAACATTCCCTTTC

              *                                    *     *       **     *   *
     381165 ATTGAGCAGGTTTGAAACACTCTTTTTGTAGTATCTGGATGTGGACATTTGGAGCGCTTTCAGGC
          1 ATAGAGCAGGTTTGAAACACTCTTTTTGTAGTATCTGGAAGTGGATATTTGGATAGCTTTGAGGA

            * ** *      *             *** *   *                      *   *   
     381230 CTATGGT-GAAAAAGGAAATATCTTCCCCTGAAAACTAGACAGAAGCATTCTCAGAATCTTATTT
         66 TTTCGTTGGAAACAGG-AATATCTTCATATAAAAGCTAGACAGAAGCATTCTCAGAAACTTCTTT

                   **  **    *             **  * *   *
     381294 GTGATGTGCGCCCTCAACTAACAGTGTTGAAGCTTTCTTTTG
        130 GTGATGTTTGCATTCAAGTAACAGTGTTGAACATTCCCTTTC

                     *                    **     *   *                       
     381336 ATAGAGCAGTTTTGAAACACTCTTTTTGTAAAATCTGCAAGAGGATATTTGGATAGCTTTGAGGA
          1 ATAGAGCAGGTTTGAAACACTCTTTTTGTAGTATCTGGAAGTGGATATTTGGATAGCTTTGAGGA

                         *   * *            **                      * *  *   
     381401 TTTCGTTGGAAACGGGATTGTCTTCATATAAACTCTAGACAGAAGCATTCTCAGAAGCGTCATTG
         66 TTTCGTTGGAAACAGGAATATCTTCATATAAAAGCTAGACAGAAGCATTCTCAGAAACTTCTTTG

            *       **   *    *             *        
     381466 GGATGTTTCAATTGAAGTCACAGTGTTGAACAGTCCCTTTC
        131 TGATGTTTGCATTCAAGTAACAGTGTTGAACATTCCCTTTC

                                                   *     *       **     *    
     381507 ATAGAGCAGGTTTGAAACACTCTTTTTGTAGTATCTGGATGTGGACATTTGGAGCGCTTTCAGGC
          1 ATAGAGCAGGTTTGAAACACTCTTTTTGTAGTATCTGGAAGTGGATATTTGGATAGCTTTGAGG-

            * * *    *                 *** *   *                          *  
     381572 CTATGGTT-TAAA-AGGAAATATCTTCCCCTGAAAACTAGACAGAAGCATTCTCAGAAACTTATT
         65 ATTTCGTTGGAAACAGG-AATATCTTCATATAAAAGCTAGACAGAAGCATTCTCAGAAACTTCTT

                    **  **    *                    *   *
     381635 TGTGATGTGCGCCCTCAACTAACAGTGTTGAAGCATT-CTTTTG
        129 TGTGATGTTTGCATTCAAGTAACAGTGTTGAA-CATTCCCTTTC

                     *                   * *     *             **            
     381678 ATAGAGCAGTTTTGAAACACTCTTTTTGTGGAATCTGCAAGTGGATATTTGTCTAGCTTTGAGGA
          1 ATAGAGCAGGTTTGAAACACTCTTTTTGTAGTATCTGGAAGTGGATATTTGGATAGCTTTGAGGA

                         *   *    *                   *       *              
     381743 TTTCGTTGGAAACGGGATTA-CAT-ATA-AAAAGC-AGACAGCAGCATTCCCAGAAACTTCTTTG
         66 TTTCGTTGGAAACAGGAATATCTTCATATAAAAGCTAGACAGAAGCATTCTCAGAAACTTCTTTG

                              *    *                 
     381804 TGATGTTTGCATTCAAGTCACAGAGTTGAACATTCCCTTTC
        131 TGATGTTTGCATTCAAGTAACAGTGTTGAACATTCCCTTTC

                                                   *     *       **     *   *
     381845 ATAGAGCAGGTTTGAAACACTCTTTTTGTAGTATCTGGATGTGGACATTTGGAGCGCTTTCAGGC
          1 ATAGAGCAGGTTTGAAACACTCTTTTTGTAGTATCTGGAAGTGGATATTTGGATAGCTTTGAGGA

            * ** *      *             *** *   *                          *   
     381910 CTATGGT-GAAAAAGGAAATATCTTCCCCTGAAAACTAGACAGAAGCATTCTCAGAAACTTATTT
         66 TTTCGTTGGAAACAGG-AATATCTTCATATAAAAGCTAGACAGAAGCATTCTCAGAAACTTCTTT

                   **  **    *             **  * *   *
     381974 GTGATGTGCGCCCTCAACTAACAGTGTTGAAGCTTTCTTTTG
        130 GTGATGTTTGCATTCAAGTAACAGTGTTGAACATTCCCTTTC

                     *                    *      *   *                       
     382016 ATAGAGCAGTTTTGAAACACTCTTTTTGTAATATCTGCAAGAGGATATTTGGATAGCTTTGAGGA
          1 ATAGAGCAGGTTTGAAACACTCTTTTTGTAGTATCTGGAAGTGGATATTTGGATAGCTTTGAGGA

                         *   * *            **                      *    *   
     382081 TTTCGTTGGAAACGGGATTGTCTTCATATAAACTCTAGACAGAAGCATTCTCAGAAGCTTCATTG
         66 TTTCGTTGGAAACAGGAATATCTTCATATAAAAGCTAGACAGAAGCATTCTCAGAAACTTCTTTG

            *       **   *    *             *        
     382146 GGATGTTTCAATTGAAGTCACAGTGTTGAACAGTCCCTTTC
        131 TGATGTTTGCATTCAAGTAACAGTGTTGAACATTCCCTTTC

                                                   *     *       **     *    
     382187 ATAGAGCAGGTTTGAAACACTCTTTTTGTAGTATCTGGATGTGGACATTTGGAGCGCTTTCAGGC
          1 ATAGAGCAGGTTTGAAACACTCTTTTTGTAGTATCTGGAAGTGGATATTTGGATAGCTTTGAGG-

            * * *    *                 *** *   *                          *  
     382252 CTATGGTT-TAAA-AGGAAATATCTTCCCCTGAAAACTAGACAGAAGCATTCTCAGAAACTTATT
         65 ATTTCGTTGGAAACAGG-AATATCTTCATATAAAAGCTAGACAGAAGCATTCTCAGAAACTTCTT

                    **  **    *                    *   *
     382315 TGTGATGTGCGCCCTCAACTAACAGTGTTGAAGCATT-CTTTTG
        129 TGTGATGTTTGCATTCAAGTAACAGTGTTGAA-CATTCCCTTTC

                     *                   * *     *             **            
     382358 ATAGAGCAGTTTTGAAACACTCTTTTTGTGGAATCTGCAAGTGGATATTTGTCTAGCTTTGAGGA
          1 ATAGAGCAGGTTTGAAACACTCTTTTTGTAGTATCTGGAAGTGGATATTTGGATAGCTTTGAGGA

                         *   *    *                   *       *   *   *      
     382423 TTTCGTTGGAAACGGGATTA-CAT-ATA-AAAAGC-AGACAGCAGCATTCCCAGGAACATCTTTG
         66 TTTCGTTGGAAACAGGAATATCTTCATATAAAAGCTAGACAGAAGCATTCTCAGAAACTTCTTTG

                              *    *                 
     382484 TGATGTTTGCATTCAAGTCACAGAGTTGAACATTCCCTTTC
        131 TGATGTTTGCATTCAAGTAACAGTGTTGAACATTCCCTTTC

                                                   *     *       **     *   *
     382525 ATAGAGCAGGTTTGAAACACTCTTTTTGTAGTATCTGGATGTGGACATTTGGAGCGCTTTCAGGC
          1 ATAGAGCAGGTTTGAAACACTCTTTTTGTAGTATCTGGAAGTGGATATTTGGATAGCTTTGAGGA

            * ** *      *             *** *   *                          *   
     382590 CTATGGT-GAAAAAGGAAATATCTTCCCCTGAAAACTAGACAGAAGCATTCTCAGAAACTTATTT
         66 TTTCGTTGGAAACAGG-AATATCTTCATATAAAAGCTAGACAGAAGCATTCTCAGAAACTTCTTT

                   **  **    *              *  * *   *
     382654 GTGATGTGCGCCCTCAACTAACAGTGTTGAACCTTTCTTTTG
        130 GTGATGTTTGCATTCAAGTAACAGTGTTGAACATTCCCTTTC

                     *                    *      *   *                       
     382696 ATAGAGCAGTTTTGAAACACTCTTTTTGTAATATCTGCAAGAGGATATTTGGATAGCTTTGAGGA
          1 ATAGAGCAGGTTTGAAACACTCTTTTTGTAGTATCTGGAAGTGGATATTTGGATAGCTTTGAGGA

                         *   * *            **                      *    *   
     382761 TTTCGTTGGAAACGGGATTGTCTTCATATAAACTCTAGACAGAAGCATTCTCAGAAGCTTCATTG
         66 TTTCGTTGGAAACAGGAATATCTTCATATAAAAGCTAGACAGAAGCATTCTCAGAAACTTCTTTG

            *       **   *    *             *        
     382826 GGATGTTTCAATTGAAGTCACAGTGTTGAACAGTCCCTTTC
        131 TGATGTTTGCATTCAAGTAACAGTGTTGAACATTCCCTTTC

                                                         *       *  * * *    
     382867 ATAGAGCAGGTTTGAAACACTCTTTTTGTAGTATCTGGAAGTGGACATTTGGAGAGATCTCAGGA
          1 ATAGAGCAGGTTTGAAACACTCTTTTTGTAGTATCTGGAAGTGGATATTTGGATAGCTTTGAGGA

            * *  *    * *             **            *       * *          *  *
     382932 ATACGGT-GATAAAGGAAATATCTTCCAATAAAAGCTAGATAGAAGCAATGTCAGAAACTTTTTC
         66 TTTCGTTGGAAACAGG-AATATCTTCATATAAAAGCTAGACAGAAGCATTCTCAGAAACTTCTTT

            *      * ** *   **      *       *  * *   *
     382996 ATGATGTATCTACTCAGCTAACAGAGTTGAACCTTTCTTTTG
        130 GTGATGTTTGCATTCAAGTAACAGTGTTGAACATTCCCTTTC

             *       *                   * *     *             **            
     383038 AGAGAGCAGTTTTGAAACACTCTTTTTGTGGAATCTGCAAGTGGATATTTGTCTAGCTTTGAGGA
          1 ATAGAGCAGGTTTGAAACACTCTTTTTGTAGTATCTGGAAGTGGATATTTGGATAGCTTTGAGGA

                         *   *    *                   *       *              
     383103 TTTCGTTGGAAACGGGATTA-CAT-ATA-AAAAGC-AGACAGCAGCATTCCCAGAAACTTCTTTG
         66 TTTCGTTGGAAACAGGAATATCTTCATATAAAAGCTAGACAGAAGCATTCTCAGAAACTTCTTTG

                              *    *                 
     383164 TGATGTTTGCATTCAAGTCACAGAGTTGAACATTCCCTTTC
        131 TGATGTTTGCATTCAAGTAACAGTGTTGAACATTCCCTTTC

                                                   *     *       **     *   *
     383205 ATAGAGCAGGTTTGAAACACTCTTTTTGTAGTATCTGGATGTGGACATTTGGAGCGCTTTCAGGC
          1 ATAGAGCAGGTTTGAAACACTCTTTTTGTAGTATCTGGAAGTGGATATTTGGATAGCTTTGAGGA

            * ** *      *             *** * * *                          *   
     383270 CTATGGT-GAAAAAGGAAATATCTTCCCCTGAGAACTAGACAGAAGCATTCTCAGAAACTTATTT
         66 TTTCGTTGGAAACAGG-AATATCTTCATATAAAAGCTAGACAGAAGCATTCTCAGAAACTTCTTT

                   **  **    *              *  * *   *
     383334 GTGATGTGCGCCCTCAACTAACAGTGTTGAACCTTTCTTTTG
        130 GTGATGTTTGCATTCAAGTAACAGTGTTGAACATTCCCTTTC

                     *                    *      *   *                       
     383376 ATAGAGCAGTTTTGAAACACTCTTTTTGTAATATCTGCAAGAGGATATTTGGATAGCTTTGAGGA
          1 ATAGAGCAGGTTTGAAACACTCTTTTTGTAGTATCTGGAAGTGGATATTTGGATAGCTTTGAGGA

                         *   * *            **                      *    *   
     383441 TTTCGTTGGAAACGGGATTGTCTTCATATAAACTCTAGACAGAAGCATTCTCAGAAGCTTCATTG
         66 TTTCGTTGGAAACAGGAATATCTTCATATAAAAGCTAGACAGAAGCATTCTCAGAAACTTCTTTG

            *       **   *    *             *        
     383506 GGATGTTTCAATTGAAGTCACAGTGTTGAACAGTCCCTTTC
        131 TGATGTTTGCATTCAAGTAACAGTGTTGAACATTCCCTTTC

                                                   *     *       **     *    
     383547 ATAGAGCAGGTTTGAAACACTCTTTTTGTAGTATCTGGATGTGGACATTTGGAGCGCTTTCAGGC
          1 ATAGAGCAGGTTTGAAACACTCTTTTTGTAGTATCTGGAAGTGGATATTTGGATAGCTTTGAGG-

            * * *    *                 *** *   *                          *  
     383612 CTATGGTT-TAAA-AGGAAATATCTTCCCCTGAAAACTAGACAGAAGCATTCTCAGAAACTTATT
         65 ATTTCGTTGGAAACAGG-AATATCTTCATATAAAAGCTAGACAGAAGCATTCTCAGAAACTTCTT

                    **  **    *                        *
     383675 TGTGATGTGCGCCCTCAACTAACAGTGTTGAAGCATT-CCTTTG
        129 TGTGATGTTTGCATTCAAGTAACAGTGTTGAA-CATTCCCTTTC

                     *                   * *     *             **  *         
     383718 ATAGAGCAGTTTTGAAACACTCTTTTTGTGGAATCTGCAAGTGGATATTTGTCTATCTTTGAGGA
          1 ATAGAGCAGGTTTGAAACACTCTTTTTGTAGTATCTGGAAGTGGATATTTGGATAGCTTTGAGGA

                         *   *    *                   *       *            * 
     383783 TTTCGTTGGAAACGGGATTA-CAT-ATA-AAAAGC-AGACAGCAGCATTCCCAGAAACTTCTTAG
         66 TTTCGTTGGAAACAGGAATATCTTCATATAAAAGCTAGACAGAAGCATTCTCAGAAACTTCTTTG

                              *    *                 
     383844 TGATGTTTGCATTCAAGTCACAGAGTTGAACATTCCCTTTC
        131 TGATGTTTGCATTCAAGTAACAGTGTTGAACATTCCCTTTC

                                                   *     *       **     *   *
     383885 ATAGAGCAGGTTTGAAACACTCTTTTTGTAGTATCTGGATGTGGACATTTGGAGCGCTTTCAGGC
          1 ATAGAGCAGGTTTGAAACACTCTTTTTGTAGTATCTGGAAGTGGATATTTGGATAGCTTTGAGGA

            * ** *      *             *** *   *                          *   
     383950 CTATGGT-GAAAAAGGAAATATCTTCCCCTGAAAACTAGACAGAAGCATTCTCAGAAACTTATTT
         66 TTTCGTTGGAAACAGG-AATATCTTCATATAAAAGCTAGACAGAAGCATTCTCAGAAACTTCTTT

                   **  **    *              *  * *   *
     384014 GTGATGTGCGCCCTCAACTAACAGTGTTGAACCTTTCTTTTG
        130 GTGATGTTTGCATTCAAGTAACAGTGTTGAACATTCCCTTTC

                     *                    *      *   *                       
     384056 ATAGAGCAGTTTTGAAACACTCTTTTTGTAATATCTGCAAGAGGATATTTGGATAGCTTTGAGGA
          1 ATAGAGCAGGTTTGAAACACTCTTTTTGTAGTATCTGGAAGTGGATATTTGGATAGCTTTGAGGA

                         *   * *            **                      *    *   
     384121 TTTCGTTGGAAACGGGATTGTCTTCATATAAACTCTAGACAGAAGCATTCTCAGAAGCTTCATTG
         66 TTTCGTTGGAAACAGGAATATCTTCATATAAAAGCTAGACAGAAGCATTCTCAGAAACTTCTTTG

            *       **   *    *             * *      
     384186 GGATGTTTCAATTGAAGTCACAGTGTTGAACAGTTCCTTTC
        131 TGATGTTTGCATTCAAGTAACAGTGTTGAACATTCCCTTTC

                 *                                       *       **   * *    
     384227 ATAGAACAGGTTTGAAACACTCTTTTTGTAGTATCTGGAAGTGGACATTTGGAGCGCTCTCAGGA
          1 ATAGAGCAGGTTTGAAACACTCTTTTTGTAGTATCTGGAAGTGGATATTTGGATAGCTTTGAGGA

            * ** *      *             **          * *       * *          *  *
     384292 CTATGGT-GAAAAAGGAAATATCTTCCAATAAAAGCTACATAGAAGCAATGTCAGAAACTTTTTC
         66 TTTCGTTGGAAACAGG-AATATCTTCATATAAAAGCTAGACAGAAGCATTCTCAGAAACTTCTTT

            *      * ** *   **      *       *  *     *
     384356 ATGATGTATCTACTCAGCTAACAGAGTTGAACCTTTCCTTTG
        130 GTGATGTTTGCATTCAAGTAACAGTGTTGAACATTCCCTTTC

             *       *                   * *     *             **            
     384398 AGAGAGCAGTTTTGAAACACTCTTTTTGTGGAATCTGCAAGTGGATATTTGCTTAGCTTTGAGGA
          1 ATAGAGCAGGTTTGAAACACTCTTTTTGTAGTATCTGGAAGTGGATATTTGGATAGCTTTGAGGA

                         *   *    *                   *       *              
     384463 TTTCGTTGGAAACGGGATTA-CAT-ATA-AAAAGC-AGACAGCAGCATTCCCAGAAACTTCTTTG
         66 TTTCGTTGGAAACAGGAATATCTTCATATAAAAGCTAGACAGAAGCATTCTCAGAAACTTCTTTG

                              *    *                 
     384524 TGATGTTTGCATTCAAGTCACAGAGTTGAACATTCCCTTTC
        131 TGATGTTTGCATTCAAGTAACAGTGTTGAACATTCCCTTTC

                                                   *     *       **     *   *
     384565 ATAGAGCAGGTTTGAAACACTCTTTTTGTAGTATCTGGATGTGGACATTTGGAGCGCTTTCAGGC
          1 ATAGAGCAGGTTTGAAACACTCTTTTTGTAGTATCTGGAAGTGGATATTTGGATAGCTTTGAGGA

            * ** *      *             *** *   *                          *   
     384630 CTATGGT-GAAAAAGGAAATATCTTCCCCTGAAAACTAGACAGAAGCATTCTCAGAAACTTATTT
         66 TTTCGTTGGAAACAGG-AATATCTTCATATAAAAGCTAGACAGAAGCATTCTCAGAAACTTCTTT

                   **  **    *             **  * *   *
     384694 GTGATGTGCGCCCTCAACTAACAGTGTTGAAGCTTTCTTTTG
        130 GTGATGTTTGCATTCAAGTAACAGTGTTGAACATTCCCTTTC

                     *                    *      *   *                       
     384736 ATAGAGCAGTTTTGAAACACTCTTTTTGTAATATCTGCAAGAGGATATTTGGATAGCTTTGAGGA
          1 ATAGAGCAGGTTTGAAACACTCTTTTTGTAGTATCTGGAAGTGGATATTTGGATAGCTTTGAGGA

                         *   * *            **                      *    *   
     384801 TTTCGTTGGAAACGGGATTGTCTTCATATAAACTCTAGACAGAAGCATTCTCAGAAGCTTCATTG
         66 TTTCGTTGGAAACAGGAATATCTTCATATAAAAGCTAGACAGAAGCATTCTCAGAAACTTCTTTG

            *       **   *    *             *        
     384866 GGATGTTTCAATTGAAGTCACAGTGTTGAACAGTCCCTTTC
        131 TGATGTTTGCATTCAAGTAACAGTGTTGAACATTCCCTTTC

                                                   *     *       **     *    
     384907 ATAGAGCAGGTTTGAAACACTCTTTTTGTAGTATCTGGATGTGGACATTTGGAGCGCTTTCAGGC
          1 ATAGAGCAGGTTTGAAACACTCTTTTTGTAGTATCTGGAAGTGGATATTTGGATAGCTTTGAGG-

            * * *    *                 *** *   *                          *  
     384972 CTATGGTT-TAAA-AGGAAATATCTTCCCCTGAAAACTAGACAGAAGCATTCTCAGAAACTTATT
         65 ATTTCGTTGGAAACAGG-AATATCTTCATATAAAAGCTAGACAGAAGCATTCTCAGAAACTTCTT

                    *   **    *                    *   *
     385035 TGTGATGTGTGCCCTCAACTAACAGTGTTGAAGCATT-CTTTTG
        129 TGTGATGTTTGCATTCAAGTAACAGTGTTGAA-CATTCCCTTTC

                     *                   * *     *             **            
     385078 ATAGAGCAGTTTTGAAACACTCTTTTTGTGGAATCTGCAAGTGGATATTTGTCTAGCTTTGAGGA
          1 ATAGAGCAGGTTTGAAACACTCTTTTTGTAGTATCTGGAAGTGGATATTTGGATAGCTTTGAGGA

                         *   *    *                   *       *              
     385143 TTTCGTTGGAAACGGGATTA-CAT-ATA-AAAAGC-AGACAGCAGCATTCCCAGAAACTTCTTTG
         66 TTTCGTTGGAAACAGGAATATCTTCATATAAAAGCTAGACAGAAGCATTCTCAGAAACTTCTTTG

                              *    *                 
     385204 TGATGTTTGCATTCAAGTCACAGAGTTGAACATTCCCTTTC
        131 TGATGTTTGCATTCAAGTAACAGTGTTGAACATTCCCTTTC

                                                   *     *       **     *   *
     385245 ATAGAGCAGGTTTGAAACACTCTTTTTGTAGTATCTGGATGTGGACATTTGGAGCGCTTTAAGGC
          1 ATAGAGCAGGTTTGAAACACTCTTTTTGTAGTATCTGGAAGTGGATATTTGGATAGCTTTGAGGA

            * ** *      *             *** *   *                          *   
     385310 CTATGGT-GAAAAAGGAAATATCTTCCCCTGAAAACTAGACAGAAGCATTCTCAGAAACTTATTT
         66 TTTCGTTGGAAACAGG-AATATCTTCATATAAAAGCTAGACAGAAGCATTCTCAGAAACTTCTTT

                   **  **                   *  * *   *
     385374 GTGATGTGCGCCCTCAAGTAACAGTGTTGAACCTTTCTTTTG
        130 GTGATGTTTGCATTCAAGTAACAGTGTTGAACATTCCCTTTC

                     *                    *      *   *                       
     385416 ATAGAGCAGTTTTGAAACACTCTTTTTGTAATATCTGCAAGAGGATATTTGGATAGCTTTGAGGA
          1 ATAGAGCAGGTTTGAAACACTCTTTTTGTAGTATCTGGAAGTGGATATTTGGATAGCTTTGAGGA

                         *   * *             *                      *    *   
     385481 TTTCGTTGGAAACGGGATTGTCTTCATATAAAATCTAGACAGAAGCATTCTCAGAAGCTTCATTG
         66 TTTCGTTGGAAACAGGAATATCTTCATATAAAAGCTAGACAGAAGCATTCTCAGAAACTTCTTTG

            *       **   *    *             *        
     385546 GGATGTTTCAATTGAAGTCACAGTGTTGAACAGTCCCTTTC
        131 TGATGTTTGCATTCAAGTAACAGTGTTGAACATTCCCTTTC

                                                         *       *  * * *  * 
     385587 ATAGAGCAGGTTTGAAACACTCTTTTTGTAGTATCTGGAAGTGGACATTTGGAGAGATCTCAGCA
          1 ATAGAGCAGGTTTGAAACACTCTTTTTGTAGTATCTGGAAGTGGATATTTGGATAGCTTTGAGGA

            * *  *    * *             **            *       * *          *  *
     385652 ATACGGT-GATAAAGGAAATATCTTCCAATAAAAGCTAGATAGAAGCAATGTCAGAAACTTTTTC
         66 TTTCGTTGGAAACAGG-AATATCTTCATATAAAAGCTAGACAGAAGCATTCTCAGAAACTTCTTT

            *      * ** *   **      *       *  * *   *
     385716 ATGATGTATCTACTCAGCTAACAGAGTTGAACCTTTCTTTTG
        130 GTGATGTTTGCATTCAAGTAACAGTGTTGAACATTCCCTTTC

             *       *                   * *     *             **            
     385758 AGAGAGCAGTTTTGAAACACTCTTTTTGTGGAATCTGCAAGTGGATATTTGTCTAGCTTTGAGGA
          1 ATAGAGCAGGTTTGAAACACTCTTTTTGTAGTATCTGGAAGTGGATATTTGGATAGCTTTGAGGA

                         *   *    *                   *       *              
     385823 TTTCGTTGGAAACGGGATTA-CAT-ATA-AAAAGC-AGACAGCAGCATTCCCAGAAACTTCTTTG
         66 TTTCGTTGGAAACAGGAATATCTTCATATAAAAGCTAGACAGAAGCATTCTCAGAAACTTCTTTG

                              *    **                
     385884 TGATGTTTGCATTCAAGTCACAGATTTGAACATTCCCTTTC
        131 TGATGTTTGCATTCAAGTAACAGTGTTGAACATTCCCTTTC

                                *                * *     *       **     *   *
     385925 ATAGAGCAGGTTTGAAACACACTTTTTGTAGTATCTGTATGTGGACATTTGGAGCGCTTTCAGGC
          1 ATAGAGCAGGTTTGAAACACTCTTTTTGTAGTATCTGGAAGTGGATATTTGGATAGCTTTGAGGA

            * ** *      *             *** *   *                        * *   
     385990 CTATGGT-GAAAAAGGAAATATCTTCCCCTGAAAACTAGACAGAAGCATTCTCAGAAACCTATTT
         66 TTTCGTTGGAAACAGG-AATATCTTCATATAAAAGCTAGACAGAAGCATTCTCAGAAACTTCTTT

                  ***  **    *          *   *  * *   *
     386054 GTGATGGGCGCCCTCAACTAACAGTGTTTAACCTTTCTTTTG
        130 GTGATGTTTGCATTCAAGTAACAGTGTTGAACATTCCCTTTC

                     *                    *      *   *                       
     386096 ATAGAGCAGTTTTGAAACACTCTTTTTGTAATATCTGCAAGAGGATATTTGGATAGCTTTGAGGA
          1 ATAGAGCAGGTTTGAAACACTCTTTTTGTAGTATCTGGAAGTGGATATTTGGATAGCTTTGAGGA

                         *   * *            **                      *    *   
     386161 TTTCGTTGGAAACGGGATTGTCTTCATATAAACTCTAGACAGAAGCATTCTCAGAAGCTTCATTG
         66 TTTCGTTGGAAACAGGAATATCTTCATATAAAAGCTAGACAGAAGCATTCTCAGAAACTTCTTTG

            *       **   *    *   *         * *      
     386226 GGATGTTTCAATTGAAGTCACACTGTTGAACAGTTCCTTTC
        131 TGATGTTTGCATTCAAGTAACAGTGTTGAACATTCCCTTTC

                 *                                       *       **   * *    
     386267 ATAGAACAGGTTTGAAACACTCTTTTTGTAGTATCTGGAAGTGGACATTTGGAGCGCTCTCAGGA
          1 ATAGAGCAGGTTTGAAACACTCTTTTTGTAGTATCTGGAAGTGGATATTTGGATAGCTTTGAGGA

            * *  *      *             **          * *       * *          *  *
     386332 CTACGGT-GAAAAAGGAAATATCTTCCAATAAAAGCTACATAGAAGCAATGTCAGAAACTTTTTC
         66 TTTCGTTGGAAACAGG-AATATCTTCATATAAAAGCTAGACAGAAGCATTCTCAGAAACTTCTTT

            *      * ** *   **      *       *  *     *
     386396 ATGATGTATCTACTCAGCTAACAGAGTTGAACCTTTCCTTTG
        130 GTGATGTTTGCATTCAAGTAACAGTGTTGAACATTCCCTTTC

             *       *                   * *     *             **            
     386438 AGAGAGCAGTTTTGAAACACTCTTTTTGTGGAATCTGCAAGTGGATATTTGTCTAGCTTTGAGGA
          1 ATAGAGCAGGTTTGAAACACTCTTTTTGTAGTATCTGGAAGTGGATATTTGGATAGCTTTGAGGA

                         *   *    *                   *       *   *         *
     386503 TTTCGTTGGAAACGGGATTA-CAT-ATA-AAAAGC-AGACAGCAGCATTCCCAGTAACTTCTTTA
         66 TTTCGTTGGAAACAGGAATATCTTCATATAAAAGCTAGACAGAAGCATTCTCAGAAACTTCTTTG

                              * *  *                 
     386564 TGATGTTTGCATTCAAGTCAGAGAGTTGAACATTCCCTTTC
        131 TGATGTTTGCATTCAAGTAACAGTGTTGAACATTCCCTTTC

                                        *          *     *       **     *   *
     386605 ATAGAGCAGGTTTGAAACACTCTTTTTGAAGTATCTGGATGTGGACATTTGGAGCGCTTTCAGGC
          1 ATAGAGCAGGTTTGAAACACTCTTTTTGTAGTATCTGGAAGTGGATATTTGGATAGCTTTGAGGA

            * ** *      *             *** *   *                          *   
     386670 CTATGGT-GAAAAAGGAAATATCTTCCCCTGAAAACTAGACAGAAGCATTCTCAGAAACTTATTT
         66 TTTCGTTGGAAACAGG-AATATCTTCATATAAAAGCTAGACAGAAGCATTCTCAGAAACTTCTTT

                   **  **    *              *  * *   *
     386734 GTGATGTGCGCCCTCAACTAACAGTGTTGAACCTTTCTTTTG
        130 GTGATGTTTGCATTCAAGTAACAGTGTTGAACATTCCCTTTC

                     *                    **     *   *                       
     386776 ATAGAGCAGTTTTGAAACACTCTTTTTGTAAAATCTGCAAGAGGATATTTGGATAGCTTTGAGGA
          1 ATAGAGCAGGTTTGAAACACTCTTTTTGTAGTATCTGGAAGTGGATATTTGGATAGCTTTGAGGA

                         *   * *            **                      *    *   
     386841 TTTCGTTGGAAACGGGATTGTCTTCATATAAACTCTAGACAGAAGCATTCTCAGAAGCTTCATTG
         66 TTTCGTTGGAAACAGGAATATCTTCATATAAAAGCTAGACAGAAGCATTCTCAGAAACTTCTTTG

            *  *    **   *    *             *        
     386906 GGAGGTTTCAATTGAAGTCACAGTGTTGAACAGTCCCTTTC
        131 TGATGTTTGCATTCAAGTAACAGTGTTGAACATTCCCTTTC

                                                         *       **   * *    
     386947 ATAGAGCAGGTTTGAAACACTCTTTTTGTAGTATCTGGAAGTGGACATTTGGAGCGCTCTCAGGA
          1 ATAGAGCAGGTTTGAAACACTCTTTTTGTAGTATCTGGAAGTGGATATTTGGATAGCTTTGAGGA

            * *  *    * *             **            *       * *          *  *
     387012 CTACGGT-GATAAAGGAAATATCTTCCAATAAAAGCTAGATAGAAGCAATGTCAGAAACTTTTTC
         66 TTTCGTTGGAAACAGG-AATATCTTCATATAAAAGCTAGACAGAAGCATTCTCAGAAACTTCTTT

            *      * ** *   **      *       *  * *   *
     387076 ATGATGTATCTACTCAGCTAACAGAGTTGAACCTTTCTTTTG
        130 GTGATGTTTGCATTCAAGTAACAGTGTTGAACATTCCCTTTC

             *       *                  ** *     *             **            
     387118 AGAGAGCAGTTTTGAAACACTCTTTTTGGGGAATCTGCAAGTGGATATTTGTCTAGCTTTGAGGA
          1 ATAGAGCAGGTTTGAAACACTCTTTTTGTAGTATCTGGAAGTGGATATTTGGATAGCTTTGAGGA

                         *  **    *                   *       *     *        
     387183 TTTCGTTGGAAACGGGTTTA-CAT-ATA-AAAAGC-AGACAGCAGCATTCCCAGAATCTTCTTTG
         66 TTTCGTTGGAAACAGGAATATCTTCATATAAAAGCTAGACAGAAGCATTCTCAGAAACTTCTTTG

                              *    *                 
     387244 TGATGTTTGCATTCAAGTCACAGAGTTGAACATTCCCTTTC
        131 TGATGTTTGCATTCAAGTAACAGTGTTGAACATTCCCTTTC

                                                   *     *       **     *   *
     387285 ATAGAGCAGGTTTGAAACACTCTTTTTGTAGTATCTGGATGTGGACATTTGGAGCGCTTTCAGGC
          1 ATAGAGCAGGTTTGAAACACTCTTTTTGTAGTATCTGGAAGTGGATATTTGGATAGCTTTGAGGA

            * ** *      *             *** *   *                      *   *   
     387350 CTATGGT-GAAAAAGGAAATATCTTCCCCTGAAAACTAGACAGAAGCATTCTCAGAATCTTATTT
         66 TTTCGTTGGAAACAGG-AATATCTTCATATAAAAGCTAGACAGAAGCATTCTCAGAAACTTCTTT

                   **  **    *             **  * *   *
     387414 GTGATGTGCGCCCTCAACTAACAGTGTTGAAGCTTTCTTTTG
        130 GTGATGTTTGCATTCAAGTAACAGTGTTGAACATTCCCTTTC

                     *                    **     *   *                       
     387456 ATAGAGCAGTTTTGAAACACTCTTTTTGTAAAATCTGCAAGAGGATATTTGGATAGCTTTGAGGA
          1 ATAGAGCAGGTTTGAAACACTCTTTTTGTAGTATCTGGAAGTGGATATTTGGATAGCTTTGAGGA

                         *   * *            **                      * *  *   
     387521 TTTCGTTGGAAACGGGATTGTCTTCATATAAACTCTAGACAGAAGCATTCTCAGAAGCGTCATTG
         66 TTTCGTTGGAAACAGGAATATCTTCATATAAAAGCTAGACAGAAGCATTCTCAGAAACTTCTTTG

            *       **   *    *             *        
     387586 GGATGTTTCAATTGAAGTCACAGTGTTGAACAGTCCCTTTC
        131 TGATGTTTGCATTCAAGTAACAGTGTTGAACATTCCCTTTC

                                                   *     *       **     *    
     387627 ATAGAGCAGGTTTGAAACACTCTTTTTGTAGTATCTGGATGTGGACATTTGGAGCGCTTTCAGGC
          1 ATAGAGCAGGTTTGAAACACTCTTTTTGTAGTATCTGGAAGTGGATATTTGGATAGCTTTGAGG-

            * * *    *                 *** *   *                          *  
     387692 CTATGGTT-TAAA-AGGAAATATCTTCCCCTGAAAACTAGACAGAAGCATTCTCAGAAACTTATT
         65 ATTTCGTTGGAAACAGG-AATATCTTCATATAAAAGCTAGACAGAAGCATTCTCAGAAACTTCTT

                    **  **    *                    *   *
     387755 TGTGATGTGCGCCCTCAACTAACAGTGTTGAAGCATT-CTTTTG
        129 TGTGATGTTTGCATTCAAGTAACAGTGTTGAA-CATTCCCTTTC

                     *                   * *     *             **            
     387798 ATAGAGCAGTTTTGAAACACTCTTTTTGTGGAATCTGCAAGTGGATATTTGTCTAGCTTTGAGGA
          1 ATAGAGCAGGTTTGAAACACTCTTTTTGTAGTATCTGGAAGTGGATATTTGGATAGCTTTGAGGA

                    **   *   *    *                   *       *              
     387863 TTTCGTTGTTAACGGGATTA-CAT-ATA-AAAAGC-AGACAGCAGCATTCCCAGAAACTTCTTTG
         66 TTTCGTTGGAAACAGGAATATCTTCATATAAAAGCTAGACAGAAGCATTCTCAGAAACTTCTTTG

                              *    *                 
     387924 TGATGTTTGCATTCAAGTCACAGAGTTGAACATTCCCTTTC
        131 TGATGTTTGCATTCAAGTAACAGTGTTGAACATTCCCTTTC

                                                 * *     *       **     *   *
     387965 ATAGAGCAGGTTTGAAACACTCTTTTTGTAGTATCTGTATGTGGACATTTGGAGCGCTTTCAGGC
          1 ATAGAGCAGGTTTGAAACACTCTTTTTGTAGTATCTGGAAGTGGATATTTGGATAGCTTTGAGGA

            * ** *      *             *** *   *                      *   *   
     388030 CTATGGT-GAAAAAGGAAATATCTTCCCCTGAAAACTAGACAGAAGCATTCTCAGAATCTTATTT
         66 TTTCGTTGGAAACAGG-AATATCTTCATATAAAAGCTAGACAGAAGCATTCTCAGAAACTTCTTT

                 * **  **    *             **  * *   *
     388094 GTGATTTGCGCCCTCAACTAACAGTGTTGAAGCTTTCTTTTG
        130 GTGATGTTTGCATTCAAGTAACAGTGTTGAACATTCCCTTTC

                     *                    **     *   *                       
     388136 ATAGAGCAGTTTTGAAACACTCTTTTTGTAAAATCTGCAAGAGGATATTTGGATAGCTTTGAGGA
          1 ATAGAGCAGGTTTGAAACACTCTTTTTGTAGTATCTGGAAGTGGATATTTGGATAGCTTTGAGGA

                         *   * *            **                      *    *   
     388201 TTTCGTTGGAAACGGGATTGTCTTCATATAAACTCTAGACAGAAGCATTCTCAGAAGCTTCATTG
         66 TTTCGTTGGAAACAGGAATATCTTCATATAAAAGCTAGACAGAAGCATTCTCAGAAACTTCTTTG

            *       **   *    *             *        
     388266 GGATGTTTCAATTGAAGTCACAGTGTTGAACAGTCCCTTTC
        131 TGATGTTTGCATTCAAGTAACAGTGTTGAACATTCCCTTTC

                                                         *       **   * *    
     388307 ATAGAGCAGGTTTGAAACACTCTTTTTGTAGTATCTGGAAGTGGACATTTGGAGCGCTCTCAGGA
          1 ATAGAGCAGGTTTGAAACACTCTTTTTGTAGTATCTGGAAGTGGATATTTGGATAGCTTTGAGGA

            * ** *      *             **          * *       * *          *  *
     388372 CTATGGT-GAAAAAGGAAATATCTTCCAATAAAAGCTACATAGAAGCAATGTCAGAAACTTTTTC
         66 TTTCGTTGGAAACAGG-AATATCTTCATATAAAAGCTAGACAGAAGCATTCTCAGAAACTTCTTT

            *      * ** *   **      *       *  *     *
     388436 ATGATGTATCTACTCAGCTAACAGAGTTGAACCTTTCCTTTG
        130 GTGATGTTTGCATTCAAGTAACAGTGTTGAACATTCCCTTTC

             *       *                   * *     *             **            
     388478 AGAGAGCAGTTTTGAAACACTCTTTTTGTGGAATCTGCAAGTGGATATTTGCTTAGCTTTGAGGA
          1 ATAGAGCAGGTTTGAAACACTCTTTTTGTAGTATCTGGAAGTGGATATTTGGATAGCTTTGAGGA

                         *   *    *                   *       *   *          
     388543 TTTCGTTGGAAACGGGATTA-CAT-ATA-AAAAGC-AGACAGCAGCATTCCCAGTAACTTCTTTG
         66 TTTCGTTGGAAACAGGAATATCTTCATATAAAAGCTAGACAGAAGCATTCTCAGAAACTTCTTTG

                              *    *         *       
     388604 TGATGTTTGCATTCAAGTCACAGAGTTGAACATGCCCTTTC
        131 TGATGTTTGCATTCAAGTAACAGTGTTGAACATTCCCTTTC

                                                   *     *       **     *   *
     388645 ATAGAGCAGGTTTGAAACACTCTTTTTGTAGTATCTGGATGTGGACATTTGGAGCGCTTTCAGGC
          1 ATAGAGCAGGTTTGAAACACTCTTTTTGTAGTATCTGGAAGTGGATATTTGGATAGCTTTGAGGA

            * ** *      *             *** *   *                      *   *   
     388710 CTATGGT-GAAAAAGGAAATATCTTCCCCTGAAAACTAGACAGAAGCATTCTCAGAATCTTATTT
         66 TTTCGTTGGAAACAGG-AATATCTTCATATAAAAGCTAGACAGAAGCATTCTCAGAAACTTCTTT

                   **  **    *             **  * *   *
     388774 GTGATGTGCGCCCTCAACTAACAGTGTTGAAGCTTTCTTTTG
        130 GTGATGTTTGCATTCAAGTAACAGTGTTGAACATTCCCTTTC

                     *                    **     *   *                       
     388816 ATAGAGCAGTTTTGAAACACTCTTTTTGTAAAATCTGCAAGAGGATATTTGGATAGCTTTGAGGA
          1 ATAGAGCAGGTTTGAAACACTCTTTTTGTAGTATCTGGAAGTGGATATTTGGATAGCTTTGAGGA

                         *   * *            **                      *    *   
     388881 TTTCGTTGGAAACGGGATTGTCTTCATATAAACTCTAGACAGAAGCATTCTCAGAAGCTTCATTG
         66 TTTCGTTGGAAACAGGAATATCTTCATATAAAAGCTAGACAGAAGCATTCTCAGAAACTTCTTTG

            *       **   *    *             *        
     388946 GGATGTTTCAATTGAAGTCACAGTGTTGAACAGTCCCTTTC
        131 TGATGTTTGCATTCAAGTAACAGTGTTGAACATTCCCTTTC

                                                   *     *       **     *    
     388987 ATAGAGCAGGTTTGAAACACTCTTTTTGTAGTATCTGGATGTGGACATTTGGAGCGCTTTCAGGC
          1 ATAGAGCAGGTTTGAAACACTCTTTTTGTAGTATCTGGAAGTGGATATTTGGATAGCTTTGAGG-

            * * *    *                 *** *   *                          *  
     389052 CTATGGTT-TAAA-AGGAAATATCTTCCCCTGAAAACTAGACAGAAGCATTCTCAGAAACTTATT
         65 ATTTCGTTGGAAACAGG-AATATCTTCATATAAAAGCTAGACAGAAGCATTCTCAGAAACTTCTT

                    **  **    *                    *   *
     389115 TGTGATGTGCGCCCTCAACTAACAGTGTTGAAGCATT-CTTTTG
        129 TGTGATGTTTGCATTCAAGTAACAGTGTTGAA-CATTCCCTTTC

                     *                   * *     *             **            
     389158 ATAGAGCAGTTTTGAAACACTCTTTTTGTGGAATCTGCAAGTGGATATTTGTCTAGCTTTGAGGA
          1 ATAGAGCAGGTTTGAAACACTCTTTTTGTAGTATCTGGAAGTGGATATTTGGATAGCTTTGAGGA

                         *   *    *                   *       *              
     389223 TTTCGTTGGAAACGGGATTA-CAT-ATA-AAAAGC-AGACAGCAGCATTCCCAGAAACTTCTTTG
         66 TTTCGTTGGAAACAGGAATATCTTCATATAAAAGCTAGACAGAAGCATTCTCAGAAACTTCTTTG

                              *    *                 
     389284 TGATGTTTGCATTCAAGTCACAGAGTTGAACATTCCCTTTC
        131 TGATGTTTGCATTCAAGTAACAGTGTTGAACATTCCCTTTC

                                                   *     *       **     *   *
     389325 ATAGAGCAGGTTTGAAACACTCTTTTTGTAGTATCTGGATGTGGACATTTGGAGCGCTTTCAGGC
          1 ATAGAGCAGGTTTGAAACACTCTTTTTGTAGTATCTGGAAGTGGATATTTGGATAGCTTTGAGGA

            * ** *      *             *** *   *                          *   
     389390 CTATGGT-GAAAAAGGAAATATCTTCCCCTGAAAACTAGACAGAAGCATTCTCAGAAACTTATTT
         66 TTTCGTTGGAAACAGG-AATATCTTCATATAAAAGCTAGACAGAAGCATTCTCAGAAACTTCTTT

                   **  **    *             **  * *   *
     389454 GTGATGTGCGCCCTCAACTAACAGTGTTGAAGCTTTCTTTTG
        130 GTGATGTTTGCATTCAAGTAACAGTGTTGAACATTCCCTTTC

                     *                    *      *   *                       
     389496 ATAGAGCAGTTTTGAAACACTCTTTTTGTAATATCTGCAAGAGGATATTTGGATAGCTTTGAGGA
          1 ATAGAGCAGGTTTGAAACACTCTTTTTGTAGTATCTGGAAGTGGATATTTGGATAGCTTTGAGGA

                         *   * *            **                      *    *   
     389561 TTTCGTTGGAAACGGGATTGTCTTCATATAAACTCTAGACAGAAGCATTCTCAGAAGCTTCATTG
         66 TTTCGTTGGAAACAGGAATATCTTCATATAAAAGCTAGACAGAAGCATTCTCAGAAACTTCTTTG

            *       **   *    *             *        
     389626 GGATGTTTCAATTGAAGTCACAGTGTTGAACAGTCCCTTTC
        131 TGATGTTTGCATTCAAGTAACAGTGTTGAACATTCCCTTTC

                                                   *     *       **     *    
     389667 ATAGAGCAGGTTTGAAACACTCTTTTTGTAGTATCTGGATGTGGACATTTGGAGCGCTTTCAGGC
          1 ATAGAGCAGGTTTGAAACACTCTTTTTGTAGTATCTGGAAGTGGATATTTGGATAGCTTTGAGG-

            * * *    *                 *** *   *                          *  
     389732 CTATGGTT-TAAA-AGGAAATATCTTCCCCTGAAAACTAGACAGAAGCATTCTCAGAAACTTATT
         65 ATTTCGTTGGAAACAGG-AATATCTTCATATAAAAGCTAGACAGAAGCATTCTCAGAAACTTCTT

                    **  **    *                    *   *
     389795 TGTGATGTGCGCCCTCAACTAACAGTGTTGAAGCATT-CTTTTG
        129 TGTGATGTTTGCATTCAAGTAACAGTGTTGAA-CATTCCCTTTC

                     *                   * *     *             **            
     389838 ATAGAGCAGTTTTGAAACACTCTTTTTGTGGAATCTGCAAGTGGATATTTGTCTAGCTTTGAGGA
          1 ATAGAGCAGGTTTGAAACACTCTTTTTGTAGTATCTGGAAGTGGATATTTGGATAGCTTTGAGGA

                         *   *    *                   *       *              
     389903 TTTCGTTGGAAACGGGATTA-CAT-ATA-AAAAGC-AGACAGCAGCATTCCCAGAAACTTCTTTG
         66 TTTCGTTGGAAACAGGAATATCTTCATATAAAAGCTAGACAGAAGCATTCTCAGAAACTTCTTTG

                              *    *                 
     389964 TGATGTTTGCATTCAAGTCACAGAGTTGAACATTCCCTTTC
        131 TGATGTTTGCATTCAAGTAACAGTGTTGAACATTCCCTTTC

                                                   *     *       **     *   *
     390005 ATAGAGCAGGTTTGAAACACTCTTTTTGTAGTATCTGGATGTGGACATTTGGAGCGCTTTCAGGC
          1 ATAGAGCAGGTTTGAAACACTCTTTTTGTAGTATCTGGAAGTGGATATTTGGATAGCTTTGAGGA

            * ** *      *             *** *   *                          *   
     390070 CTATGGT-GAAAAAGGAAATATCTTCCCCTGAAAACTAGACAGAAGCATTCTCAGAAACTTATTT
         66 TTTCGTTGGAAACAGG-AATATCTTCATATAAAAGCTAGACAGAAGCATTCTCAGAAACTTCTTT

                   **  **                   *  * *   *
     390134 GTGATGTGCGCCCTCAAGTAACAGTGTTGAACCTTTCTTTTG
        130 GTGATGTTTGCATTCAAGTAACAGTGTTGAACATTCCCTTTC

                     *                    *      *   *                       
     390176 ATAGAGCAGTTTTGAAACACTCTTTTTGTAATATCTGCAAGAGGATATTTGGATAGCTTTGAGGA
          1 ATAGAGCAGGTTTGAAACACTCTTTTTGTAGTATCTGGAAGTGGATATTTGGATAGCTTTGAGGA

                         *   * *             *                      *    *   
     390241 TTTCGTTGGAAACGGGATTGTCTTCATATAAAATCTAGACAGAAGCATTCTCAGAAGCTTCATTG
         66 TTTCGTTGGAAACAGGAATATCTTCATATAAAAGCTAGACAGAAGCATTCTCAGAAACTTCTTTG

            *       **   *    *             *        
     390306 GGATGTTTCAATTGAAGTCACAGTGTTGAACAGTCCCTTTC
        131 TGATGTTTGCATTCAAGTAACAGTGTTGAACATTCCCTTTC

                                                         *       *  * * *  * 
     390347 ATAGAGCAGGTTTGAAACACTCTTTTTGTAGTATCTGGAAGTGGACATTTGGAGAGATCTCAGCA
          1 ATAGAGCAGGTTTGAAACACTCTTTTTGTAGTATCTGGAAGTGGATATTTGGATAGCTTTGAGGA

            * *  *    * *             **            *       * *          *  *
     390412 ATACGGT-GATAAAGGAAATATCTTCCAATAAAAGCTAGATAGAAGCAATGTCAGAAACTTTTTC
         66 TTTCGTTGGAAACAGG-AATATCTTCATATAAAAGCTAGACAGAAGCATTCTCAGAAACTTCTTT

            *      * ** *   **      *       *  * *   *
     390476 ATGATGTATCTACTCAGCTAACAGAGTTGAACCTTTCTTTTG
        130 GTGATGTTTGCATTCAAGTAACAGTGTTGAACATTCCCTTTC

             *       *                   * *     *             **            
     390518 AGAGAGCAGTTTTGAAACACTCTTTTTGTGGAATCTGCAAGTGGATATTTGTCTAGCTTTGAGGA
          1 ATAGAGCAGGTTTGAAACACTCTTTTTGTAGTATCTGGAAGTGGATATTTGGATAGCTTTGAGGA

                         *   *    *                   *       *              
     390583 TTTCGTTGGAAACGGGATTA-CAT-ATA-AAAAGC-AGACAGCAGCATTCCCAGAAACTTCTTTG
         66 TTTCGTTGGAAACAGGAATATCTTCATATAAAAGCTAGACAGAAGCATTCTCAGAAACTTCTTTG

                              *    **                
     390644 TGATGTTTGCATTCAAGTCACAGATTTGAACATTCCCTTTC
        131 TGATGTTTGCATTCAAGTAACAGTGTTGAACATTCCCTTTC

                                *                * *     *       **     *   *
     390685 ATAGAGCAGGTTTGAAACACACTTTTTGTAGTATCTGTATGTGGACATTTGGAGCGCTTTCAGGC
          1 ATAGAGCAGGTTTGAAACACTCTTTTTGTAGTATCTGGAAGTGGATATTTGGATAGCTTTGAGGA

            * ** *      *             *** *   *                        * *   
     390750 CTATGGT-GAAAAAGGAAATATCTTCCCCTGAAAACTAGACAGAAGCATTCTCAGAAACCTATTT
         66 TTTCGTTGGAAACAGG-AATATCTTCATATAAAAGCTAGACAGAAGCATTCTCAGAAACTTCTTT

                   **  **   **          *   *  * *   *
     390814 GTGATGTGCGCCCTCAGCTAACAGTGTTTAACCTTTCTTTTG
        130 GTGATGTTTGCATTCAAGTAACAGTGTTGAACATTCCCTTTC

                     *                    *      *   *                       
     390856 ATAGAGCAGTTTTGAAACACTCTTTTTGTAATATCTGCAAGAGGATATTTGGATAGCTTTGAGGA
          1 ATAGAGCAGGTTTGAAACACTCTTTTTGTAGTATCTGGAAGTGGATATTTGGATAGCTTTGAGGA

                         *   * *            **                      *    *   
     390921 TTTCGTTGGAAACGGGATTGTCTTCATATAAACTCTAGACAGAAGCATTCTCAGAAGCTTCATTG
         66 TTTCGTTGGAAACAGGAATATCTTCATATAAAAGCTAGACAGAAGCATTCTCAGAAACTTCTTTG

            *       **   *    *   *         * *      
     390986 GGATGTTTCAATTGAAGTCACACTGTTGAACAGTTCCTTTC
        131 TGATGTTTGCATTCAAGTAACAGTGTTGAACATTCCCTTTC

                                                         *       **   * *    
     391027 ATAGAGCAGGTTTGAAACACTCTTTTTGTAGTATCTGGAAGTGGACATTTGGAGCGCTCTCAGGA
          1 ATAGAGCAGGTTTGAAACACTCTTTTTGTAGTATCTGGAAGTGGATATTTGGATAGCTTTGAGGA

            * *  *      *             **          * *       * *          *  *
     391092 CTACGGT-GAAAAAGGAAATATCTTCCAATAAAAGCTACATAGAAGCAATGTCAGAAACTTTTTC
         66 TTTCGTTGGAAACAGG-AATATCTTCATATAAAAGCTAGACAGAAGCATTCTCAGAAACTTCTTT

            *      * ** *   **      *       *  *     *
     391156 ATGATGTATCTACTCAGCTAACAGAGTTGAACCTTTCCTTTG
        130 GTGATGTTTGCATTCAAGTAACAGTGTTGAACATTCCCTTTC

             *       *                   * *     *             **            
     391198 AGAGAGCAGTTTTGAAACACTCTTTTTGTGGAATCTGCAAGTGGATATTTGTCTAGCTTTGAGGA
          1 ATAGAGCAGGTTTGAAACACTCTTTTTGTAGTATCTGGAAGTGGATATTTGGATAGCTTTGAGGA

                         *   *    *                   *       *   *          
     391263 TTTCGTTGGAAACGGGATTA-CAT-ATA-AAAAGC-AGACAGCAGCATTCCCAGTAACTTCTTTG
         66 TTTCGTTGGAAACAGGAATATCTTCATATAAAAGCTAGACAGAAGCATTCTCAGAAACTTCTTTG

                              *    *                 
     391324 TGATGTTTGCATTCAAGTCACAGAGTTGAACATTCCCTTTC
        131 TGATGTTTGCATTCAAGTAACAGTGTTGAACATTCCCTTTC

                                        *          *     *       **     *   *
     391365 ATAGAGCAGGTTTGAAACACTCTTTTTGAAGTATCTGGATGTGGACATTTGGAGCGCTTTCAGGC
          1 ATAGAGCAGGTTTGAAACACTCTTTTTGTAGTATCTGGAAGTGGATATTTGGATAGCTTTGAGGA

            * ** *      *             *** *   *                          *   
     391430 CTATGGT-GAAAAAGGAAATATCTTCCCCTGAAAACTAGACAGAAGCATTCTCAGAAACTTATTT
         66 TTTCGTTGGAAACAGG-AATATCTTCATATAAAAGCTAGACAGAAGCATTCTCAGAAACTTCTTT

                   **  **    *              *  * *   *
     391494 GTGATGTGCGCCCTCAACTAACAGTGTTGAACCTTTCTTTTG
        130 GTGATGTTTGCATTCAAGTAACAGTGTTGAACATTCCCTTTC

                     *                    **     *   *                       
     391536 ATAGAGCAGTTTTGAAACACTCTTTTTGTAAAATCTGCAAGAGGATATTTGGATAGCTTTGAGGA
          1 ATAGAGCAGGTTTGAAACACTCTTTTTGTAGTATCTGGAAGTGGATATTTGGATAGCTTTGAGGA

                         *   * *            **                      *    *   
     391601 TTTCGTTGGAAACGGGATTGTCTTCATATAAACTCTAGACAGAAGCATTCTCAGAAGCTTCATTG
         66 TTTCGTTGGAAACAGGAATATCTTCATATAAAAGCTAGACAGAAGCATTCTCAGAAACTTCTTTG

            *  *    **   *    *             *        
     391666 GGAGGTTTCAATTGAAGTCACAGTGTTGAACAGTCCCTTTC
        131 TGATGTTTGCATTCAAGTAACAGTGTTGAACATTCCCTTTC

                                                         *       **   * *    
     391707 ATAGAGCAGGTTTGAAACACTCTTTTTGTAGTATCTGGAAGTGGACATTTGGAGCGCTCTCAGGA
          1 ATAGAGCAGGTTTGAAACACTCTTTTTGTAGTATCTGGAAGTGGATATTTGGATAGCTTTGAGGA

            * *  *    * *             **            *       * *          *  *
     391772 CTACGGT-GATAAAGGAAATATCTTCCAATAAAAGCTAGATAGAAGCAATGTCAGAAACTTTTTC
         66 TTTCGTTGGAAACAGG-AATATCTTCATATAAAAGCTAGACAGAAGCATTCTCAGAAACTTCTTT

            *      * ** *   **      *       *  * *   *
     391836 ATGATGTATCTACTCAGCTAACAGAGTTGAACCTTTCTTTTG
        130 GTGATGTTTGCATTCAAGTAACAGTGTTGAACATTCCCTTTC

             *       *                   * *     *             **            
     391878 AGAGAGCAGTTTTGAAACACTCTTTTTGTGGAATCTGCAAGTGGATATTTGTCTAGCTTTGAGGA
          1 ATAGAGCAGGTTTGAAACACTCTTTTTGTAGTATCTGGAAGTGGATATTTGGATAGCTTTGAGGA

                         *  **    *                   *       *     *        
     391943 TTTCGTTGGAAACGGGTTTA-CAT-ATA-AAAAGC-AGACAGCAGCATTCCCAGAATCTTCTTTG
         66 TTTCGTTGGAAACAGGAATATCTTCATATAAAAGCTAGACAGAAGCATTCTCAGAAACTTCTTTG

                              *    *                 
     392004 TGATGTTTGCATTCAAGTCACAGAGTTGAACATTCCCTTTC
        131 TGATGTTTGCATTCAAGTAACAGTGTTGAACATTCCCTTTC

                                                   *     *       **     *   *
     392045 ATAGAGCAGGTTTGAAACACTCTTTTTGTAGTATCTGGATGTGGACATTTGGAGCGCTTTCAGGC
          1 ATAGAGCAGGTTTGAAACACTCTTTTTGTAGTATCTGGAAGTGGATATTTGGATAGCTTTGAGGA

            * ** *      *             *** *   *                      *   *   
     392110 CTATGGT-GAAAAAGGAAATATCTTCCCCTGAAAACTAGACAGAAGCATTCTCAGAATCTTATTT
         66 TTTCGTTGGAAACAGG-AATATCTTCATATAAAAGCTAGACAGAAGCATTCTCAGAAACTTCTTT

                   **  **    *             **  * *   *
     392174 GTGATGTGCGCCCTCAACTAACAGTGTTGAAGCTTTCTTTTG
        130 GTGATGTTTGCATTCAAGTAACAGTGTTGAACATTCCCTTTC

                     *                   ***     *   *              *        
     392216 ATAGAGCAGTTTTGAAACACTCTTTTTGTGAAATCTGCAAGAGGATATTTGGATAGATTTGAGGA
          1 ATAGAGCAGGTTTGAAACACTCTTTTTGTAGTATCTGGAAGTGGATATTTGGATAGCTTTGAGGA

                         * * * *            **                      *    *   
     392281 TTTCGTTGGAAACGGTATTGTCTTCATATAAACTCTAGACAGAAGCATTCTCAGAAGCTTCATTG
         66 TTTCGTTGGAAACAGGAATATCTTCATATAAAAGCTAGACAGAAGCATTCTCAGAAACTTCTTTG

            *       **   *    *             *        
     392346 GGATGTTTCAATTGAAGTCACAGTGTTGAACAGTCCCTTTC
        131 TGATGTTTGCATTCAAGTAACAGTGTTGAACATTCCCTTTC

                                                         *       **   * *    
     392387 ATAGAGCAGGTTTGAAACACTCTTTTTGTAGTATCTGGAAGTGGACATTTGGAGCGCTCTCAGGA
          1 ATAGAGCAGGTTTGAAACACTCTTTTTGTAGTATCTGGAAGTGGATATTTGGATAGCTTTGAGGA

            * * **      *             **            *       * *          *  *
     392452 CTGCAGT-GAAAAAGGAAATATCTTCCAATAAAAGCTAGATAGAAGCAATGTCAGAAACTTTTTC
         66 TTTCGTTGGAAACAGG-AATATCTTCATATAAAAGCTAGACAGAAGCATTCTCAGAAACTTCTTT

            *      * ** *   **      *       *        *
     392516 ATGATGTATCTACTCAGCTAACAGAGTTGAACCTT-CCTTTG
        130 GTGATGTTTGCATTCAAGTAACAGTGTTGAACATTCCCTTTC

            **       *                   * *     *             **            
     392557 CGAGAGCAGTTTTGAAACACTCTTTTTGTGGAATCTGCAAGTGGATATTTGTCTAGCTTTGAGGA
          1 ATAGAGCAGGTTTGAAACACTCTTTTTGTAGTATCTGGAAGTGGATATTTGGATAGCTTTGAGGA

                         *   *    *                   *       *              
     392622 TTTCGTTGGAAACGGGATTA-CAT-ATA-AAAAGC-AGACAGCAGCATTCCCAGAAACTTCTTTG
         66 TTTCGTTGGAAACAGGAATATCTTCATATAAAAGCTAGACAGAAGCATTCTCAGAAACTTCTTTG

                              *    *        *        
     392683 TGATGTTTGCATTCAAGTCACAGAGTTGAACACTCCCTTTC
        131 TGATGTTTGCATTCAAGTAACAGTGTTGAACATTCCCTTTC

                                                   *     *     * **     *   *
     392724 ATAGAGCAGGTTTGAAACACTCTTTTTGTAGTATCTGGATGTGGACATTTGCAGCGCTTTCAGGC
          1 ATAGAGCAGGTTTGAAACACTCTTTTTGTAGTATCTGGAAGTGGATATTTGGATAGCTTTGAGGA

            * ** *      *             *** *   *                          *   
     392789 CTAAGGT-GAAAAAGGAAATATCTTCCCCTGAAAACTAGACAGAAGCATTCTCAGAAACTTATTT
         66 TTTCGTTGGAAACAGG-AATATCTTCATATAAAAGCTAGACAGAAGCATTCTCAGAAACTTCTTT

                   **  **    *             **  * *   *
     392853 GTGATGTGCGCCCTCAACTAACAGTGTTGAAGCTTTCTTTTG
        130 GTGATGTTTGCATTCAAGTAACAGTGTTGAACATTCCCTTTC

                     *                    *      *   *                       
     392895 ATAGAGCAGTTTTGAAACACTCTTTTTGTAATATCTGCAAGAGGATATTTGGATAGCTTTGAGGA
          1 ATAGAGCAGGTTTGAAACACTCTTTTTGTAGTATCTGGAAGTGGATATTTGGATAGCTTTGAGGA

                         *   * *            **                     **    *   
     392960 TTTCGTTGGAAACGGGATTGTCTTCATATAAACTCTAGACAGAAGCATTCTCAGAGGCTTCATTG
         66 TTTCGTTGGAAACAGGAATATCTTCATATAAAAGCTAGACAGAAGCATTCTCAGAAACTTCTTTG

            *       **   *    *             * *      
     393025 GGATGTTTCAATTGAAGTCACAGTGTTGAACAGTTCCTTTC
        131 TGATGTTTGCATTCAAGTAACAGTGTTGAACATTCCCTTTC

                 *                             *         *       **   * *    
     393066 ATAGAACAGGTTTGAAACACTCTTTTTGTAGTATCGGGAAGTGGACATTTGGAGCGCTCTCAGGA
          1 ATAGAGCAGGTTTGAAACACTCTTTTTGTAGTATCTGGAAGTGGATATTTGGATAGCTTTGAGGA

            * ** *      *             **          * *       * *          *  *
     393131 CTATGGT-GAAAAAGGAAATATCTTCCAATAAAAGCTACATAGAAGCAATGTCAGAAACTTTTTC
         66 TTTCGTTGGAAACAGG-AATATCTTCATATAAAAGCTAGACAGAAGCATTCTCAGAAACTTCTTT

            *      * ** *   **      *       *  *     *
     393195 ATGATGTATCTACTCAGCTAACAGAGTTGAACCTTTCCTTTG
        130 GTGATGTTTGCATTCAAGTAACAGTGTTGAACATTCCCTTTC

             *       *                   * *     *             **            
     393237 AGAGAGCAGTTTTGAAACACTCTTTTTGTGGAATCTGCAAGTGGATATTTGCTTAGCTTTGAGGA
          1 ATAGAGCAGGTTTGAAACACTCTTTTTGTAGTATCTGGAAGTGGATATTTGGATAGCTTTGAGGA

                         *   *    *                   *       *   *          
     393302 TTTCGTTGGAAACGGGATTA-CAT-ATA-AAAAGC-AGACAGCAGCATTCCCAGTAACTTCTTTG
         66 TTTCGTTGGAAACAGGAATATCTTCATATAAAAGCTAGACAGAAGCATTCTCAGAAACTTCTTTG

                              *    *         *       
     393363 TGATGTTTGCATTCAAGTCACAGAGTTGAACATGCCCTTTC
        131 TGATGTTTGCATTCAAGTAACAGTGTTGAACATTCCCTTTC

                                                   *     *       **     *    
     393404 ATAGAGCAGGTTTGAAACACTCTTTTTGTAGTATCTGGATGTGGACATTTGGAGCGCTTTCAGGC
          1 ATAGAGCAGGTTTGAAACACTCTTTTTGTAGTATCTGGAAGTGGATATTTGGATAGCTTTGAGG-

            * *          *             *** *   *                      *   *  
     393469 CTATCG-T-GAAAAAGGAAATATCTTCTCCTGAAAACTAGACAGAAGCATTCTCAGAATCTTATT
         65 ATTTCGTTGGAAACAGG-AATATCTTCATATAAAAGCTAGACAGAAGCATTCTCAGAAACTTCTT

                    **  **    *             **  * *   *
     393532 TGTGATGTGCGCCCTCAACTAACAGTGTTGAAGCTTTCTTTTG
        129 TGTGATGTTTGCATTCAAGTAACAGTGTTGAACATTCCCTTTC

                     *         *          **     *   *                       
     393575 ATAGAGCAGTTTTGAAACAGTCTTTTTGTAAAATCTGCAAGAGGATATTTGGATAGCTTTGAGGA
          1 ATAGAGCAGGTTTGAAACACTCTTTTTGTAGTATCTGGAAGTGGATATTTGGATAGCTTTGAGGA

                         *   * *            **                      *    *   
     393640 TTTCGTTGGAAACGGGATTGTCTTCATATAAACTCTAGACAGAAGCATTCTCAGAAGCTTCATTG
         66 TTTCGTTGGAAACAGGAATATCTTCATATAAAAGCTAGACAGAAGCATTCTCAGAAACTTCTTTG

            *       **   *    *             *        
     393705 GGATGTTTCAATTGAAGTCACAGTGTTGAACAGTCCCTTTC
        131 TGATGTTTGCATTCAAGTAACAGTGTTGAACATTCCCTTTC

                                                   *     *    *  **     *    
     393746 ATAGAGCAGGTTTGAAACACTCTTTTTGTAGTATCTGGATGTGGACATTTAGAGCGCTTTCAGGC
          1 ATAGAGCAGGTTTGAAACACTCTTTTTGTAGTATCTGGAAGTGGATATTTGGATAGCTTTGAGG-

            * * *    *                 *** *   *                          *  
     393811 CTATGGTT-TAAA-AGGAAATATCTTCCCCTGAAAACTAGACAGAAGCATTCTCAGAAACTTATT
         65 ATTTCGTTGGAAACAGG-AATATCTTCATATAAAAGCTAGACAGAAGCATTCTCAGAAACTTCTT

                    **  **    *                    *   *
     393874 TGTGATGTGCGCCCTCAACTAACAGTGTTGAAGCATT-CTTTTG
        129 TGTGATGTTTGCATTCAAGTAACAGTGTTGAA-CATTCCCTTTC

                     *                   * *     *           * **  *         
     393917 ATAGAGCAGTTTTGAAACACTCTTTTTGTGGAATCTGCAAGTGGATATTGGTCTATCTTTGAGGA
          1 ATAGAGCAGGTTTGAAACACTCTTTTTGTAGTATCTGGAAGTGGATATTTGGATAGCTTTGAGGA

                             *    *                   *       *              
     393982 TTTCGTTGGAAACAGGATTA-CAT-ATA-AAAAGC-AGACAGCAGCATTCCCAGAAACTTCTTTG
         66 TTTCGTTGGAAACAGGAATATCTTCATATAAAAGCTAGACAGAAGCATTCTCAGAAACTTCTTTG

                              *    *                 
     394043 TGATGTTTGCATTCAAGTCACAGAGTTGAACATTCCCTTTC
        131 TGATGTTTGCATTCAAGTAACAGTGTTGAACATTCCCTTTC

                                                   *     *       **     *   *
     394084 ATAGAGCAGGTTTGAAACACTCTTTTTGTAGTATCTGGATGTGGACATTTGGAGCGCTTTCAGGC
          1 ATAGAGCAGGTTTGAAACACTCTTTTTGTAGTATCTGGAAGTGGATATTTGGATAGCTTTGAGGA

            * ** *      *             *** *   *                          *   
     394149 CTATGGT-GAAAAAGGAAATATCTTCCCCTGAAAACTAGACAGAAGCATTCTCAGAAACTTATTT
         66 TTTCGTTGGAAACAGG-AATATCTTCATATAAAAGCTAGACAGAAGCATTCTCAGAAACTTCTTT

                   **  **    *             **  * *   *
     394213 GTGATGTGCGCCCTCAACTAACAGTGTTGAAGCTTTCTTTTG
        130 GTGATGTTTGCATTCAAGTAACAGTGTTGAACATTCCCTTTC

             *       *                    *      *   *                       
     394255 ACAGAGCAGTTTTGAAACACTCTTTTTGTAATATCTGCAAGAGGATATTTGGATAGCTTTGAGGA
          1 ATAGAGCAGGTTTGAAACACTCTTTTTGTAGTATCTGGAAGTGGATATTTGGATAGCTTTGAGGA

                         *   * *            **                      *    *   
     394320 TTTCGTTGGAAACGGGATTGTCTTCATATAAACTCTAGACAGAAGCATTCTCAGAAGCTTCATTG
         66 TTTCGTTGGAAACAGGAATATCTTCATATAAAAGCTAGACAGAAGCATTCTCAGAAACTTCTTTG

            *       **   *    *             *        
     394385 GGATGTTTCAATTGAAGTCACAGTGTTGAACAGTCCCTTTC
        131 TGATGTTTGCATTCAAGTAACAGTGTTGAACATTCCCTTTC

                                                *  *     *       **     *    
     394426 ATAGAGCAGGTTTGAAACACTCTTTTTGTAGTATCTAGATGTGGACATTTGGAGCGCTTTCAGGC
          1 ATAGAGCAGGTTTGAAACACTCTTTTTGTAGTATCTGGAAGTGGATATTTGGATAGCTTTGAGG-

            * * *    *                 *** *   *                          *  
     394491 CTATGGTT-TAAA-AGGAAATATCTTCCCCTGAAAACTAGACAGAAGCATTCTCAGAAACTTATT
         65 ATTTCGTTGGAAACAGG-AATATCTTCATATAAAAGCTAGACAGAAGCATTCTCAGAAACTTCTT

                    **  **    *                    *   *
     394554 TGTGATGTGCGCCCTCAACTAACAGTGTTGAAGCATT-CTTTTG
        129 TGTGATGTTTGCATTCAAGTAACAGTGTTGAA-CATTCCCTTTC

                     *                   * *     *             **            
     394597 ATAGAGCAGTTTTGAAACACTCTTTTTGTGGAATCTGCAAGTGGATATTTGTCTAGCTTTGAGGA
          1 ATAGAGCAGGTTTGAAACACTCTTTTTGTAGTATCTGGAAGTGGATATTTGGATAGCTTTGAGGA

                         *   *    *                   *       *   *   *      
     394662 TTTCGTTGGAAACGGGATTA-CAT-ATA-AAAAGC-AGACAGCAGCATTCCCAGGAACATCTTTG
         66 TTTCGTTGGAAACAGGAATATCTTCATATAAAAGCTAGACAGAAGCATTCTCAGAAACTTCTTTG

                              *    *                 
     394723 TGATGTTTGCATTCAAGTCACAGAGTTGAACATTCCCTTTC
        131 TGATGTTTGCATTCAAGTAACAGTGTTGAACATTCCCTTTC

                                                   *     *       **     *   *
     394764 ATAGAGCAGGTTTGAAACACTCTTTTTGTAGTATCTGGATGTGGACATTTGGAGCGCTTTCAGGC
          1 ATAGAGCAGGTTTGAAACACTCTTTTTGTAGTATCTGGAAGTGGATATTTGGATAGCTTTGAGGA

            * ** *      *             *** *   *                          *   
     394829 CTATGGT-GAAAAAGGAAATATCTTCCCCTGAAAACTAGACAGAAGCATTCTCAGAAACTTATTT
         66 TTTCGTTGGAAACAGG-AATATCTTCATATAAAAGCTAGACAGAAGCATTCTCAGAAACTTCTTT

                   **  **    *              *  * *   *
     394893 GTGATGTGCGCCCTCAACTAACAGTGTTGAACCTTTCTTTTG
        130 GTGATGTTTGCATTCAAGTAACAGTGTTGAACATTCCCTTTC

                     *                    *      **  *                       
     394935 ATAGAGCAGTTTTGAAACACTCTTTTTGTAATATCTGCTAGAGGATATTTGGATAGCTTTGAGGA
          1 ATAGAGCAGGTTTGAAACACTCTTTTTGTAGTATCTGGAAGTGGATATTTGGATAGCTTTGAGGA

                         *   * *            **                      *    *   
     395000 TTTCGTTGGAAACGGGATTGTCTTCATATAAACTCTAGACAGAAGCATTCTCAGAAGCTTCATTG
         66 TTTCGTTGGAAACAGGAATATCTTCATATAAAAGCTAGACAGAAGCATTCTCAGAAACTTCTTTG

            *       **   *    *             *        
     395065 GGATGTTTCAATTGAAGTCACAGTGTTGAACAGTCCCTTTC
        131 TGATGTTTGCATTCAAGTAACAGTGTTGAACATTCCCTTTC

                                                         *       *  * * *    
     395106 ATAGAGCAGGTTTGAAACACTCTTTTTGTAGTATCTGGAAGTGGACATTTGGAGAGATCTCAGGA
          1 ATAGAGCAGGTTTGAAACACTCTTTTTGTAGTATCTGGAAGTGGATATTTGGATAGCTTTGAGGA

            * *  *    * *             **            *      ** *          *  *
     395171 ATACGGT-GATAAAGGAAATATCTTCCAATAAAAGCTAGATAGAAGCTATGTCAGAAACTTTTTC
         66 TTTCGTTGGAAACAGG-AATATCTTCATATAAAAGCTAGACAGAAGCATTCTCAGAAACTTCTTT

            *      * ** *   **      *       *  * *   *
     395235 ATGATGTATCTACTCAGCTAACAGAGTTGAACCTTTCTTTTG
        130 GTGATGTTTGCATTCAAGTAACAGTGTTGAACATTCCCTTTC

             *       *                   * *     *     *       **            
     395277 AGAGAGCAGTTTTGAAACACTCTTTTTGTGGAATCTGCAAGTGCATATTTGTCTAGCTTTGAGGA
          1 ATAGAGCAGGTTTGAAACACTCTTTTTGTAGTATCTGGAAGTGGATATTTGGATAGCTTTGAGGA

                         *   *    *                   *       *              
     395342 TTTCGTTGGAAACGGGATTA-CAT-ATA-AAAAGC-AGACAGCAGCATTCCCAGAAACTTCTTTG
         66 TTTCGTTGGAAACAGGAATATCTTCATATAAAAGCTAGACAGAAGCATTCTCAGAAACTTCTTTG

                              *    *                 
     395403 TGATGTTTGCATTCAAGTCACAGAGTTGAACATTCCCTTTC
        131 TGATGTTTGCATTCAAGTAACAGTGTTGAACATTCCCTTTC

                                                   *     *  *    **     *   *
     395444 ATAGAGCAGGTTTGAAACACTCTTTTTGTAGTATCTGGATGTGGACATATGGAGCGCTTTCAGGC
          1 ATAGAGCAGGTTTGAAACACTCTTTTTGTAGTATCTGGAAGTGGATATTTGGATAGCTTTGAGGA

            * ** *      *             *** * * *                 *    *   *   
     395509 CTATGGT-GAAAAAGGAAATATCTTCCCCTGAGAACTAGACAGAAGCATTCTGAGAATCTTATTT
         66 TTTCGTTGGAAACAGG-AATATCTTCATATAAAAGCTAGACAGAAGCATTCTCAGAAACTTCTTT

                   **  **    *             **  * *   *
     395573 GTGATGTGCGCCCTCAACTAACAGTGTTGAAGCTTTCTTTTG
        130 GTGATGTTTGCATTCAAGTAACAGTGTTGAACATTCCCTTTC

                     *                    **     *   *                       
     395615 ATAGAGCAGTTTTGAAACACTCTTTTTGTAAAATCTGCAAGAGGATATTTGGATAGCTTTGAGGA
          1 ATAGAGCAGGTTTGAAACACTCTTTTTGTAGTATCTGGAAGTGGATATTTGGATAGCTTTGAGGA

                         *   * *            **                      *    *   
     395680 TTTCGTTGGAAACGGGATTGTCTTCATATAAACTCTAGACAGAAGCATTCTCAGAAGCTTCATTG
         66 TTTCGTTGGAAACAGGAATATCTTCATATAAAAGCTAGACAGAAGCATTCTCAGAAACTTCTTTG

            *       **   *    *             *        
     395745 GGATGTTTCAATTGAAGTCACAGTGTTGAACAGTCCCTTTC
        131 TGATGTTTGCATTCAAGTAACAGTGTTGAACATTCCCTTTC

                                                         *       **   * *    
     395786 ATAGAGCAGGTTTGAAACACTCTTTTTGTAGTATCTGGAAGTGGACATTTGGAGCGCTCTCAGGA
          1 ATAGAGCAGGTTTGAAACACTCTTTTTGTAGTATCTGGAAGTGGATATTTGGATAGCTTTGAGGA

            * *  *      *             ** *          *       * *          *  *
     395851 CTGCGGT-GAAAAAGGAAATATCTTCCAACAAAAGCTAGATAGAAGCAATGTCAGAAACTTTTTC
         66 TTTCGTTGGAAACAGG-AATATCTTCATATAAAAGCTAGACAGAAGCATTCTCAGAAACTTCTTT

            *      * ** *   **      *       *        *
     395915 ATGATGTATCTACTCAGCTAACAGAGTTGAACCTT-CCTTTG
        130 GTGATGTTTGCATTCAAGTAACAGTGTTGAACATTCCCTTTC

             *       *                   * *     *             **            
     395956 AGAGAGCAGTTTTGAAACACTCTTTTTGTGGAATCTGCAAGTGGATATTTGTCTAGCTTTGAGGA
          1 ATAGAGCAGGTTTGAAACACTCTTTTTGTAGTATCTGGAAGTGGATATTTGGATAGCTTTGAGGA

                         *   *    *                   *       *              
     396021 TTTCGTTGGAAACGGGATTA-CAT-ATA-AAAAGC-AGACAGCAGCATTCCCAGAAACTTCTTTG
         66 TTTCGTTGGAAACAGGAATATCTTCATATAAAAGCTAGACAGAAGCATTCTCAGAAACTTCTTTG

                              *    *                 
     396082 TGATGTTTGCATTCAAGTCACAGAGTTGAACATTCCCTTTC
        131 TGATGTTTGCATTCAAGTAACAGTGTTGAACATTCCCTTTC

                                                   *     *     * **     *   *
     396123 ATAGAGCAGGTTTGAAACACTCTTTTTGTAGTATCTGGATGTGGACATTTGCAGCGCTTTCAGGC
          1 ATAGAGCAGGTTTGAAACACTCTTTTTGTAGTATCTGGAAGTGGATATTTGGATAGCTTTGAGGA

            * ** *      *             *** *   *                          *   
     396188 CTATGGT-GAAAAAGGAAATATCTTCCCCTGAAAACTAGACAGAAGCATTCTCAGAAACTTATTT
         66 TTTCGTTGGAAACAGG-AATATCTTCATATAAAAGCTAGACAGAAGCATTCTCAGAAACTTCTTT

                   **  **    *             **  * *   *
     396252 GTGATGTGCGCCCTCAACTAACAGTGTTGAAGCTTTCTTTTG
        130 GTGATGTTTGCATTCAAGTAACAGTGTTGAACATTCCCTTTC

               *     *                    *      *   *                  *    
     396294 ATAAAGCAGTTTTGAAACACTCTTTTTGTAATATCTGCAAGAGGATATTTGGATAGCTTTCAGGA
          1 ATAGAGCAGGTTTGAAACACTCTTTTTGTAGTATCTGGAAGTGGATATTTGGATAGCTTTGAGGA

                         *   * *            **                      *    *   
     396359 TTTCGTTGGAAACGGGATTGTCTTCATATAAACTCTAGACAGAAGCATTCTCAGAAGCTTCATTG
         66 TTTCGTTGGAAACAGGAATATCTTCATATAAAAGCTAGACAGAAGCATTCTCAGAAACTTCTTTG

            *       **   *    * *           *        
     396424 GGATGTTTCAATTGAAGTCAAAGTGTTGAACAGTCCCTTTC
        131 TGATGTTTGCATTCAAGTAACAGTGTTGAACATTCCCTTTC

            *                                   *        *       *  * * *    
     396465 GTAGAGCAGGTTTGAAACACTCTTTTTGTAGTATCTCGAAGTGGACATTTGGAGAGATCTCAGGA
          1 ATAGAGCAGGTTTGAAACACTCTTTTTGTAGTATCTGGAAGTGGATATTTGGATAGCTTTGAGGA

            * *  *    * *             **            *       * *          *  *
     396530 ATACGGT-GATAAAGGAAATATCTTCCAATAAAAGCTAGATAGAAGCAATGTCAGAAACTTTTTC
         66 TTTCGTTGGAAACAGG-AATATCTTCATATAAAAGCTAGACAGAAGCATTCTCAGAAACTTCTTT

            *      * ** *   **      *       *  *     *
     396594 ATGATGTATCTACTCAGCTAACAGAGTTGAACCTTTCCTTTG
        130 GTGATGTTTGCATTCAAGTAACAGTGTTGAACATTCCCTTTC

             *       *                   * *     *             **            
     396636 AGAGAGCAGTTTTGAAACACTCTTTTTGTGGAATCTGCAAGTGGATATTTGCTTAGCTTTGAGGA
          1 ATAGAGCAGGTTTGAAACACTCTTTTTGTAGTATCTGGAAGTGGATATTTGGATAGCTTTGAGGA

                         *   *    *                   *       *   *          
     396701 TTTCGTTGGAAACGGGATTA-CAT-ATA-AAAAGC-AGACAGCAGCATTCCCAGTAACTTCTTTG
         66 TTTCGTTGGAAACAGGAATATCTTCATATAAAAGCTAGACAGAAGCATTCTCAGAAACTTCTTTG

                              *    *                 
     396762 TGATGTTTGCATTCAAGTCACAGAGTTGAACATTCCCTTTC
        131 TGATGTTTGCATTCAAGTAACAGTGTTGAACATTCCCTTTC

                                                   *     *       **     *   *
     396803 ATAGAGCAGGTTTGAAACACTCTTTTTGTAGTATCTGGATGTGGACATTTGGAGCGCTTTCAGGC
          1 ATAGAGCAGGTTTGAAACACTCTTTTTGTAGTATCTGGAAGTGGATATTTGGATAGCTTTGAGGA

            * ** *      *             *** *   *                      *   *   
     396868 CTATGGT-GAAAAAGGAAATATCTTCCCCTGAAAACTAGACAGAAGCATTCTCAGAATCTTATTT
         66 TTTCGTTGGAAACAGG-AATATCTTCATATAAAAGCTAGACAGAAGCATTCTCAGAAACTTCTTT

                   **  **    *             **  * *   *
     396932 GTGATGTGCGCCCTCAACTAACAGTGTTGAAGCTTTCTTTTG
        130 GTGATGTTTGCATTCAAGTAACAGTGTTGAACATTCCCTTTC

                     *                    **     *   *                       
     396974 ATAGAGCAGTTTTGAAACACTCTTTTTGTAAAATCTGCAAGAGGATATTTGGATAGCTTTGAGGA
          1 ATAGAGCAGGTTTGAAACACTCTTTTTGTAGTATCTGGAAGTGGATATTTGGATAGCTTTGAGGA

                         *   * *            **                      *    *   
     397039 TTTCGTTGGAAACGGGATTGTCTTCATATAAACTCTAGACAGAAGCATTCTCAGAAGCTTCATTG
         66 TTTCGTTGGAAACAGGAATATCTTCATATAAAAGCTAGACAGAAGCATTCTCAGAAACTTCTTTG

            *       **   *    *             *        
     397104 GGATGTTTCAATTGAAGTCACAGTGTTGAACAGTCCCTTTC
        131 TGATGTTTGCATTCAAGTAACAGTGTTGAACATTCCCTTTC

                                                   *     *       **     *    
     397145 ATAGAGCAGGTTTGAAACACTCTTTTTGTAGTATCTGGATGTGGACATTTGGAGCGCTTTCAGGC
          1 ATAGAGCAGGTTTGAAACACTCTTTTTGTAGTATCTGGAAGTGGATATTTGGATAGCTTTGAGG-

            * * *    *                 *** *   *                          *  
     397210 CTATGGTT-TAAA-AGGAAATATCTTCCCCTGAAAACTAGACAGAAGCATTCTCAGAAACTTATT
         65 ATTTCGTTGGAAACAGG-AATATCTTCATATAAAAGCTAGACAGAAGCATTCTCAGAAACTTCTT

                    **  **    *                    *   *
     397273 TGTGATGTGCGCCCTCAACTAACAGTGTTGAAGCATT-CTTTTG
        129 TGTGATGTTTGCATTCAAGTAACAGTGTTGAA-CATTCCCTTTC

                     *                   * *     *             **            
     397316 ATAGAGCAGTTTTGAAACACTCTTTTTGTGGAATCTGCAAGTGGATATTTGTCTAGCTTTGAGGA
          1 ATAGAGCAGGTTTGAAACACTCTTTTTGTAGTATCTGGAAGTGGATATTTGGATAGCTTTGAGGA

                         *   *    *                   *       *              
     397381 TTTCGTTGGAAACGGGATTA-CAT-ATA-AAAAGC-AGACAGCAGCATTCCCAGAAACTTCTTTG
         66 TTTCGTTGGAAACAGGAATATCTTCATATAAAAGCTAGACAGAAGCATTCTCAGAAACTTCTTTG

                              *    *            *    
     397442 TGATGTTTGCATTCAAGTCACAGAGTTGAACATTCCGTTTC
        131 TGATGTTTGCATTCAAGTAACAGTGTTGAACATTCCCTTTC

                                                   *     *       **     *   *
     397483 ATAGAGCAGGTTTGAAACACTCTTTTTGTAGTATCTGGATGTGGACATTTGGAGCGCTTTCAGGC
          1 ATAGAGCAGGTTTGAAACACTCTTTTTGTAGTATCTGGAAGTGGATATTTGGATAGCTTTGAGGA

            * ** *      *             *** *   *                          *   
     397548 CTATGGT-GAAAAAGGAAATATCTTCCCCTGAAAACTAGACAGAAGCATTCTCAGAAACTTATTT
         66 TTTCGTTGGAAACAGG-AATATCTTCATATAAAAGCTAGACAGAAGCATTCTCAGAAACTTCTTT

                   **  **    *              *  * *   *
     397612 GTGATGTGCGCCCTCAACTAACAGTGTTGAACCTTTCTTTTG
        130 GTGATGTTTGCATTCAAGTAACAGTGTTGAACATTCCCTTTC

                     *                    *      *   *                       
     397654 ATAGAGCAGTTTTGAAACACTCTTTTTGTAATATCTGCAAGAGGATATTTGGATAGCTTTGAGGA
          1 ATAGAGCAGGTTTGAAACACTCTTTTTGTAGTATCTGGAAGTGGATATTTGGATAGCTTTGAGGA

                         *   * *            **                      *    *   
     397719 TTTCGTTGGAAACGGGATTGTCTTCATATAAACTCTAGACAGAAGCATTCTCAGAAGCTTCATTG
         66 TTTCGTTGGAAACAGGAATATCTTCATATAAAAGCTAGACAGAAGCATTCTCAGAAACTTCTTTG

            *       **   *    *             *        
     397784 GGATGTTTCAATTGAAGTCACAGTGTTGAACAGTCCCTTTC
        131 TGATGTTTGCATTCAAGTAACAGTGTTGAACATTCCCTTTC

                                                   *     *       **     *    
     397825 ATAGAGCAGGTTTGAAACACTCTTTTTGTAGTATCTGGATGTGGACATTTGGAGCGCTTTCAGGC
          1 ATAGAGCAGGTTTGAAACACTCTTTTTGTAGTATCTGGAAGTGGATATTTGGATAGCTTTGAGG-

            * * *    *                 *** *   *                          *  
     397890 CTATGGTT-TAAA-AGGAAATATCTTCCCCTGAAAACTAGACAGAAGCATTCTCAGAAACTTATT
         65 ATTTCGTTGGAAACAGG-AATATCTTCATATAAAAGCTAGACAGAAGCATTCTCAGAAACTTCTT

                    **  **    *                    *   *
     397953 TGTGATGTGCGCCCTCAACTAACAGTGTTGAAGCATT-CTTTTG
        129 TGTGATGTTTGCATTCAAGTAACAGTGTTGAA-CATTCCCTTTC

                     *                   * *     *             **            
     397996 ATAGAGCAGTTTTGAAACACTCTTTTTGTGGAATCTGCAAGTGGATATTTGTCTAGCTTTGAGGA
          1 ATAGAGCAGGTTTGAAACACTCTTTTTGTAGTATCTGGAAGTGGATATTTGGATAGCTTTGAGGA

                         *   *    *                   *       *              
     398061 TTTCGTTGGAAACGGGATTA-CAT-ATA-AAAAGC-AGACAGCAGCATTCCCAGAAACTTCTTTG
         66 TTTCGTTGGAAACAGGAATATCTTCATATAAAAGCTAGACAGAAGCATTCTCAGAAACTTCTTTG

                              *    *                 
     398122 TGATGTTTGCATTCAAGTCACAGAGTTGAACATTCCCTTTC
        131 TGATGTTTGCATTCAAGTAACAGTGTTGAACATTCCCTTTC

                                                   *     *       **     *   *
     398163 ATAGAGCAGGTTTGAAACACTCTTTTTGTAGTATCTGGATGTGGACATTTGGAGCGCTTTCAGGC
          1 ATAGAGCAGGTTTGAAACACTCTTTTTGTAGTATCTGGAAGTGGATATTTGGATAGCTTTGAGGA

            * ** *      *             *** *   *                          *   
     398228 CTATGGT-GAAAAAGGAAATATCTTCCCCTGAAAACTAGACAGAAGCATTCTCAGAAACTTATTT
         66 TTTCGTTGGAAACAGG-AATATCTTCATATAAAAGCTAGACAGAAGCATTCTCAGAAACTTCTTT

                   **  **    *             **  * *   *
     398292 GTGATGTGCGCCCTCAACTAACAGTGTTGAAGCTTTCTTTTG
        130 GTGATGTTTGCATTCAAGTAACAGTGTTGAACATTCCCTTTC

                     *                    **     *   *                       
     398334 ATAGAGCAGTTTTGAAACACTCTTTTTGTAAAATCTGCAAGAGGATATTTGGATAGCTTTGAGGA
          1 ATAGAGCAGGTTTGAAACACTCTTTTTGTAGTATCTGGAAGTGGATATTTGGATAGCTTTGAGGA

                         *   * *            **                      *    *   
     398399 TTTCGTTGGAAACGGGATTGTCTTCATATAAACTCTAGACAGAAGCATTCTCAGAAGCTTCATTG
         66 TTTCGTTGGAAACAGGAATATCTTCATATAAAAGCTAGACAGAAGCATTCTCAGAAACTTCTTTG

            *       **   *    *             *        
     398464 GGATGTTTCAATTGAAGTCACAGTGTTGAACAGTCCCTTTC
        131 TGATGTTTGCATTCAAGTAACAGTGTTGAACATTCCCTTTC

                                                   *     *       **     *    
     398505 ATAGAGCAGGTTTGAAACACTCTTTTTGTAGTATCTGGATGTGGACATTTGGAGCGCTTTCAGGC
          1 ATAGAGCAGGTTTGAAACACTCTTTTTGTAGTATCTGGAAGTGGATATTTGGATAGCTTTGAGG-

            * * *    *                 *** *   *                          *  
     398570 CTATGGTT-TAAA-AGGAAATATCTTCCCCTGAAAACTAGACAGAAGCATTCTCAGAAACTTATT
         65 ATTTCGTTGGAAACAGG-AATATCTTCATATAAAAGCTAGACAGAAGCATTCTCAGAAACTTCTT

                    **  **    *                    *   *
     398633 TGTGATGTGCGCCCTCAACTAACAGTGTTGAAGCATT-CTTTTG
        129 TGTGATGTTTGCATTCAAGTAACAGTGTTGAA-CATTCCCTTTC

                     *                   * *     *             **            
     398676 ATAGAGCAGTTTTGAAACACTCTTTTTGTGGAATCTGCAAGTGGATATTTGTCTAGCTTTGAGGA
          1 ATAGAGCAGGTTTGAAACACTCTTTTTGTAGTATCTGGAAGTGGATATTTGGATAGCTTTGAGGA

                         *   *    *                   *       *      *       
     398741 TTTCGTTGGAAACGGGATTA-CAT-ATA-AAAAGC-AGACAGCAGCATTCCCAGAAATTTCTTTG
         66 TTTCGTTGGAAACAGGAATATCTTCATATAAAAGCTAGACAGAAGCATTCTCAGAAACTTCTTTG

                              *    *            *    
     398802 TGATGTTTGCATTCAAGTCACAGAGTTGAACATTCCGTTTC
        131 TGATGTTTGCATTCAAGTAACAGTGTTGAACATTCCCTTTC

                                                   *     *       **     *   *
     398843 ATAGAGCAGGTTTGAAACACTCTTTTTGTAGTATCTGGATGTGGACATTTGGAGCGCTTTCAGGC
          1 ATAGAGCAGGTTTGAAACACTCTTTTTGTAGTATCTGGAAGTGGATATTTGGATAGCTTTGAGGA

            * ** *      *             *** *   *                          *   
     398908 CTATGGT-GAAAAAGGAAATATCTTCCCCTGAAAACTAGACAGAAGCATTCTCAGAAACTTATTT
         66 TTTCGTTGGAAACAGG-AATATCTTCATATAAAAGCTAGACAGAAGCATTCTCAGAAACTTCTTT

                   **  **    *              *  * *   *
     398972 GTGATGTGCGCCCTCAACTAACAGTGTTGAACCTTTCTTTTG
        130 GTGATGTTTGCATTCAAGTAACAGTGTTGAACATTCCCTTTC

                     *                    *      *   *                       
     399014 ATAGAGCAGTTTTGAAACACTCTTTTTGTAATATCTGCAAGAGGATATTTGGATAGCTTTGAGGA
          1 ATAGAGCAGGTTTGAAACACTCTTTTTGTAGTATCTGGAAGTGGATATTTGGATAGCTTTGAGGA

                         *   * *            **                      *    *   
     399079 TTTCGTTGGAAACGGGATTGTCTTCATATAAACTCTAGACAGAAGCATTCTCAGAAGCTTCATTG
         66 TTTCGTTGGAAACAGGAATATCTTCATATAAAAGCTAGACAGAAGCATTCTCAGAAACTTCTTTG

            *       **   *    *             *        
     399144 GGATGTTTCAATTGAAGTCACAGTGTTGAACAGTCCCTTTC
        131 TGATGTTTGCATTCAAGTAACAGTGTTGAACATTCCCTTTC

                                                   *     *       **     *    
     399185 ATAGAGCAGGTTTGAAACACTCTTTTTGTAGTATCTGGATGTGGACATTTGGAGCGCTTTCAGGC
          1 ATAGAGCAGGTTTGAAACACTCTTTTTGTAGTATCTGGAAGTGGATATTTGGATAGCTTTGAGG-

            * * *    *                 *** *   *                          *  
     399250 CTATGGTT-TAAA-AGGAAATATCTTCCCCTGAAAACTAGACAGAAGCATTCTCAGAAACTTATT
         65 ATTTCGTTGGAAACAGG-AATATCTTCATATAAAAGCTAGACAGAAGCATTCTCAGAAACTTCTT

                    **  **    *                    *   *
     399313 TGTGATGTGCGCCCTCAACTAACAGTGTTGAAGCATT-CTTTTG
        129 TGTGATGTTTGCATTCAAGTAACAGTGTTGAA-CATTCCCTTTC

                     *                   * *     *             **            
     399356 ATAGAGCAGTTTTGAAACACTCTTTTTGTGGAATCTGCAAGTGGATATTTGTCTAGCTTTGAGGA
          1 ATAGAGCAGGTTTGAAACACTCTTTTTGTAGTATCTGGAAGTGGATATTTGGATAGCTTTGAGGA

                         *   *    *      *            *       *              
     399421 TTTCGTTGGAAACGGGATTA-CAT-ATA-GAAAGC-AGACAGCAGCATTCCCAGAAACTTCTTTG
         66 TTTCGTTGGAAACAGGAATATCTTCATATAAAAGCTAGACAGAAGCATTCTCAGAAACTTCTTTG

                              *    *                 
     399482 TGATGTTTGCATTCAAGTCACAGAGTTGAACATTCCCTTTC
        131 TGATGTTTGCATTCAAGTAACAGTGTTGAACATTCCCTTTC

                                                   *     *       **     *   *
     399523 ATAGAGCAGGTTTGAAACACTCTTTTTGTAGTATCTGGATGTGGACATTTGGAGCGCTTTCAGGC
          1 ATAGAGCAGGTTTGAAACACTCTTTTTGTAGTATCTGGAAGTGGATATTTGGATAGCTTTGAGGA

            * ** *      *             *** *   *                          *   
     399588 CTATGGT-GAAAAAGGAAATATCTTCCCCTGAAAACTAGACAGAAGCATTCTCAGAAACTTATTT
         66 TTTCGTTGGAAACAGG-AATATCTTCATATAAAAGCTAGACAGAAGCATTCTCAGAAACTTCTTT

                   **  **    *              *  * *   *
     399652 GTGATGTGCGCCCTCAACTAACAGTGTTGAACCTTTCTTTTG
        130 GTGATGTTTGCATTCAAGTAACAGTGTTGAACATTCCCTTTC

                     *                    *      *   *                       
     399694 ATAGAGCAGTTTTGAAACACTCTTTTTGTAATATCTGCAAGAGGATATTTGGATAGCTTTGAGGA
          1 ATAGAGCAGGTTTGAAACACTCTTTTTGTAGTATCTGGAAGTGGATATTTGGATAGCTTTGAGGA

                         *   * *            **                      *    *   
     399759 TTTCGTTGGAAACGGGATTGTCTTCATATAAACTCTAGACAGAAGCATTCTCAGAAGCTTCATTG
         66 TTTCGTTGGAAACAGGAATATCTTCATATAAAAGCTAGACAGAAGCATTCTCAGAAACTTCTTTG

            *       **   *    *             *        
     399824 GGATGTTTCAATTGAAGTCACAGTGTTGAACAGTCCCTTTC
        131 TGATGTTTGCATTCAAGTAACAGTGTTGAACATTCCCTTTC

                                                         *       *  * * *    
     399865 ATAGAGCAGGTTTGAAACACTCTTTTTGTAGTATCTGGAAGTGGACATTTGGAGAGATCTCAGGA
          1 ATAGAGCAGGTTTGAAACACTCTTTTTGTAGTATCTGGAAGTGGATATTTGGATAGCTTTGAGGA

            * *  *    * *             **            *       * *          *  *
     399930 ATACGGT-GATAAAGGAAATATCTTCCAATAAAAGCTAGATAGAAGCAATGTCAGAAACTTTTTC
         66 TTTCGTTGGAAACAGG-AATATCTTCATATAAAAGCTAGACAGAAGCATTCTCAGAAACTTCTTT

            *      * ** *   **      *       *  * *   *
     399994 ATGATGTATCTACTCAGCTAACAGAGTTGAACCTTTCTTTTG
        130 GTGATGTTTGCATTCAAGTAACAGTGTTGAACATTCCCTTTC

             *       *                   * *     *             **            
     400036 AGAGAGCAGTTTTGAAACACTCTTTTTGTGGAATCTGCAAGTGGATATTTGTCTAGCTTTGAGGA
          1 ATAGAGCAGGTTTGAAACACTCTTTTTGTAGTATCTGGAAGTGGATATTTGGATAGCTTTGAGGA

                         *   *    *                   *       *              
     400101 TTTCGTTGGAAACGGGATTA-CAT-ATA-AAAAGC-AGACAGCAGCATTCCCAGAAACTTCTTTG
         66 TTTCGTTGGAAACAGGAATATCTTCATATAAAAGCTAGACAGAAGCATTCTCAGAAACTTCTTTG

                              *    *                 
     400162 TGATGTTTGCATTCAAGTCACAGAGTTGAACATTCCCTTTC
        131 TGATGTTTGCATTCAAGTAACAGTGTTGAACATTCCCTTTC

                                                 * *     *       **     *   *
     400203 ATAGAGCAGGTTTGAAACACTCTTTTTGTAGTATCTGTATGTGGACATTTGGAGCGCTTTCAGGC
          1 ATAGAGCAGGTTTGAAACACTCTTTTTGTAGTATCTGGAAGTGGATATTTGGATAGCTTTGAGGA

            * ** *      *             *** *   *                          *   
     400268 CTATGGT-GAAAAAGGAAATATCTTCCCCTGAAAACTAGACAGAAGCATTCTCAGAAACTTATTT
         66 TTTCGTTGGAAACAGG-AATATCTTCATATAAAAGCTAGACAGAAGCATTCTCAGAAACTTCTTT

                   *** **    *              *  * *   *
     400332 GTGATGTGCTCCCTCAACTAACAGTGTTGAACCTTTCTTTTG
        130 GTGATGTTTGCATTCAAGTAACAGTGTTGAACATTCCCTTTC

                     *                    *      *   *                       
     400374 ATAGAGCAGTTTTGAAACACTCTTTTTGTAATATCTGCAAGAGGATATTTGGATAGCTTTGAGGA
          1 ATAGAGCAGGTTTGAAACACTCTTTTTGTAGTATCTGGAAGTGGATATTTGGATAGCTTTGAGGA

                         *   * *            **                      *    *   
     400439 TTTCGTTGGAAACGGGATTGTCTTCATATAAACTCTAGACAGAAGCATTCTCAGAAGCTTCATTG
         66 TTTCGTTGGAAACAGGAATATCTTCATATAAAAGCTAGACAGAAGCATTCTCAGAAACTTCTTTG

            *       **   *    *             * *      
     400504 GGATGTTTCAATTGAAGTCACAGTGTTGAACAGTTCCTTTC
        131 TGATGTTTGCATTCAAGTAACAGTGTTGAACATTCCCTTTC

                 *                                       *       **   * *    
     400545 ATAGAACAGGTTTGAAACACTCTTTTTGTAGTATCTGGAAGTGGACATTTGGAGCGCTCTCAGGA
          1 ATAGAGCAGGTTTGAAACACTCTTTTTGTAGTATCTGGAAGTGGATATTTGGATAGCTTTGAGGA

            * *  *      *             **          * *       * *          *  *
     400610 CTACGGT-GAAAAAGGAAATATCTTCCAATAAAAGCTACATAGAAGCAATGTCAGAAACTTTTTC
         66 TTTCGTTGGAAACAGG-AATATCTTCATATAAAAGCTAGACAGAAGCATTCTCAGAAACTTCTTT

            *      * ** *   **      *       *  *     *
     400674 ATGATGTATCTACTCAGCTAACAGAGTTGAACCTTTCCTTTG
        130 GTGATGTTTGCATTCAAGTAACAGTGTTGAACATTCCCTTTC

             *       *                   * *     *             **            
     400716 AGAGAGCAGTTTTGAAACACTCTTTTTGTGGAATCTGCAAGTGGATATTTGTCTAGCTTTGAGGA
          1 ATAGAGCAGGTTTGAAACACTCTTTTTGTAGTATCTGGAAGTGGATATTTGGATAGCTTTGAGGA

                         *   *    *                   *       *   *          
     400781 TTTCGTTGGAAACGGGATTA-CAT-ATA-AAAAGC-AGACAGCAGCATTCCCAGTAACTTCTTTG
         66 TTTCGTTGGAAACAGGAATATCTTCATATAAAAGCTAGACAGAAGCATTCTCAGAAACTTCTTTG

                              *    *                 
     400842 TGATGTTTGCATTCAAGTCACAGAGTTGAACATTCCCTTTC
        131 TGATGTTTGCATTCAAGTAACAGTGTTGAACATTCCCTTTC

                                        *          *     *       **     *   *
     400883 ATAGAGCAGGTTTGAAACACTCTTTTTGAAGTATCTGGATGTGGACATTTGGAGCGCTTTCAGGC
          1 ATAGAGCAGGTTTGAAACACTCTTTTTGTAGTATCTGGAAGTGGATATTTGGATAGCTTTGAGGA

            * ** *      *             *** *   *                          *   
     400948 CTATGGT-GAAAAAGGAAATATCTTCCCCTGAAAACTAGACAGAAGCATTCTCAGAAACTTATTT
         66 TTTCGTTGGAAACAGG-AATATCTTCATATAAAAGCTAGACAGAAGCATTCTCAGAAACTTCTTT

                   **  **    *              *  * *   *
     401012 GTGATGTGCGCCCTCAACTAACAGTGTTGAACCTTTCTTTTG
        130 GTGATGTTTGCATTCAAGTAACAGTGTTGAACATTCCCTTTC

                     *                    *      *   *                       
     401054 ATAGAGCAGTTTTGAAACACTCTTTTTGTAATATCTGCAAGAGGATATTTGGATAGCTTTGAGGA
          1 ATAGAGCAGGTTTGAAACACTCTTTTTGTAGTATCTGGAAGTGGATATTTGGATAGCTTTGAGGA

                         *   * *            **                      *    *   
     401119 TTTCGTTGGAAACGGGATTGTCTTCATATAAACTCTAGACAGAAGCATTCTCAGAAGCTTCATTG
         66 TTTCGTTGGAAACAGGAATATCTTCATATAAAAGCTAGACAGAAGCATTCTCAGAAACTTCTTTG

            *       **   *    *             *        
     401184 GGATGTTTCAATTGAAGTCACAGTGTTGAACAGTCCCTTTC
        131 TGATGTTTGCATTCAAGTAACAGTGTTGAACATTCCCTTTC

                                                   *     *       **     *    
     401225 ATAGAGCAGGTTTGAAACACTCTTTTTGTAGTATCTGGATGTGGACATTTGGAGCGCTTTCAGGC
          1 ATAGAGCAGGTTTGAAACACTCTTTTTGTAGTATCTGGAAGTGGATATTTGGATAGCTTTGAGG-

            * * *    *                 *** *   *                          *  
     401290 CTATGGTT-TAAA-AGGAAATATCTTCCCCTGAAAACTAGACAGAAGCATTCTCAGAAACTTATT
         65 ATTTCGTTGGAAACAGG-AATATCTTCATATAAAAGCTAGACAGAAGCATTCTCAGAAACTTCTT

                    **  **    *                    *   *
     401353 TGTGATGTGCGCCCTCAACTAACAGTGTTGAAGCATT-CTTTTG
        129 TGTGATGTTTGCATTCAAGTAACAGTGTTGAA-CATTCCCTTTC

                     *                   * *     *             **            
     401396 ATAGAGCAGTTTTGAAACACTCTTTTTGTGGAATCTGCAAGTGGATATTTGTCTAGCTTTGAGGA
          1 ATAGAGCAGGTTTGAAACACTCTTTTTGTAGTATCTGGAAGTGGATATTTGGATAGCTTTGAGGA

                         *   *    *                   *       *              
     401461 TTTCGTTGGAAACGGGATTA-CAT-ATA-AAAAGC-AGACAGCAGCATTCCCAGAAACTTCTTTG
         66 TTTCGTTGGAAACAGGAATATCTTCATATAAAAGCTAGACAGAAGCATTCTCAGAAACTTCTTTG

                              *    *                 
     401522 TGATGTTTGCATTCAAGTCACAGAGTTGAACATTCCCTTTC
        131 TGATGTTTGCATTCAAGTAACAGTGTTGAACATTCCCTTTC

                                                   *     *       **     *   *
     401563 ATAGAGCAGGTTTGAAACACTCTTTTTGTAGTATCTGGATGTGGACATTTGGAGCGCTTTCAGGC
          1 ATAGAGCAGGTTTGAAACACTCTTTTTGTAGTATCTGGAAGTGGATATTTGGATAGCTTTGAGGA

            * ** *      *             *** *   *                          *   
     401628 CTATGGT-GAAAAAGGAAATATCTTCCCCTGAAAACTAGACAGAAGCATTCTCAGAAACTTATTT
         66 TTTCGTTGGAAACAGG-AATATCTTCATATAAAAGCTAGACAGAAGCATTCTCAGAAACTTCTTT

                   **  **    *             **  * *   *
     401692 GTGATGTGCGCCCTCAACTAACAGTGTTGAAGCTTTCTTTTG
        130 GTGATGTTTGCATTCAAGTAACAGTGTTGAACATTCCCTTTC

                     *                    *      *   *                  *    
     401734 ATAGAGCAGTTTTGAAACACTCTTTTTGTAATATCTGCAAGAGGATATTTGGATAGCTTTCAGGA
          1 ATAGAGCAGGTTTGAAACACTCTTTTTGTAGTATCTGGAAGTGGATATTTGGATAGCTTTGAGGA

                         *   * *            **          *           *    *   
     401799 TTTCGTTGGAAACGGGATTGTCTTCATATAAACTCTAGACAGAACCATTCTCAGAAGCTTCATTG
         66 TTTCGTTGGAAACAGGAATATCTTCATATAAAAGCTAGACAGAAGCATTCTCAGAAACTTCTTTG

            *       **   *    *             *        
     401864 GGATGTTTCAATTGAAGTCACAGTGTTGAACAGTCCCTTTC
        131 TGATGTTTGCATTCAAGTAACAGTGTTGAACATTCCCTTTC

                                                         *       *  * * *    
     401905 ATAGAGCAGGTTTGAAACACTCTTTTTGTAGTATCTGGAAGTGGACATTTGGAGAGATCTCAGGA
          1 ATAGAGCAGGTTTGAAACACTCTTTTTGTAGTATCTGGAAGTGGATATTTGGATAGCTTTGAGGA

            * *  *      *             **            *       * *          *  *
     401970 CTACGGT-GAAAAAGGAAATATCTTCCAATAAAAGCTAGATAGAAGCAATGTCAGAAACTTTTTC
         66 TTTCGTTGGAAACAGG-AATATCTTCATATAAAAGCTAGACAGAAGCATTCTCAGAAACTTCTTT

            *      * ** *   **      *       *  * *   *
     402034 ATGATGTATCTACTCAGCTAACAGAGTTGAACCTTTCTTTTG
        130 GTGATGTTTGCATTCAAGTAACAGTGTTGAACATTCCCTTTC

             *       *                   * *     *             **            
     402076 AGAGAGCAGTTTTGAAACACTCTTTTTGTGGAATCTGCAAGTGGATATTTGTCTAGCTTTGAGGA
          1 ATAGAGCAGGTTTGAAACACTCTTTTTGTAGTATCTGGAAGTGGATATTTGGATAGCTTTGAGGA

                         *   *    *                   *       *              
     402141 TTTCGTTGGAAACGGGATTA-CAT-ATA-AAAAGC-AGACAGCAGCATTCCCAGAAACTTCTTTG
         66 TTTCGTTGGAAACAGGAATATCTTCATATAAAAGCTAGACAGAAGCATTCTCAGAAACTTCTTTG

                              *    *                 
     402202 TGATGTTTGCATTCAAGTCACAGAGTTGAACATTCCCTTTC
        131 TGATGTTTGCATTCAAGTAACAGTGTTGAACATTCCCTTTC

                                                   *     *       **     *   *
     402243 ATAGAGCAGGTTTGAAACACTCTTTTTGTAGTATCTGGATGTGGACATTTGGAGCGCTTTCAGGC
          1 ATAGAGCAGGTTTGAAACACTCTTTTTGTAGTATCTGGAAGTGGATATTTGGATAGCTTTGAGGA

            * ** *      *             *** *   *                          *   
     402308 CTATGGT-GAAAAAGGAAATATCTTCCCCTGAAAACTAGACAGAAGCATTCTCAGAAACTTATTT
         66 TTTCGTTGGAAACAGG-AATATCTTCATATAAAAGCTAGACAGAAGCATTCTCAGAAACTTCTTT

                   **  **    *              *  * *   *
     402372 GTGATGTGCGCCCTCAACTAACAGTGTTGAACCTTTCTTTTG
        130 GTGATGTTTGCATTCAAGTAACAGTGTTGAACATTCCCTTTC

                     *                    *      *   *                       
     402414 ATAGAGCAGTTTTGAAACACTCTTTTTGTAATATCTGCAAGAGGATATTTGGATAGCTTTGAGGA
          1 ATAGAGCAGGTTTGAAACACTCTTTTTGTAGTATCTGGAAGTGGATATTTGGATAGCTTTGAGGA

                         *   * *            **                      *    *   
     402479 TTTCGTTGGAAACGGGATTGTCTTCATATAAACTCTAGACAGAAGCATTCTCAGAAGCTTCATTG
         66 TTTCGTTGGAAACAGGAATATCTTCATATAAAAGCTAGACAGAAGCATTCTCAGAAACTTCTTTG

            *       **   *    *             *        
     402544 GGATGTTTCAATTGAAGTCACAGTGTTGAACAGTCCCTTTC
        131 TGATGTTTGCATTCAAGTAACAGTGTTGAACATTCCCTTTC

                                                   *     *       **     *    
     402585 ATAGAGCAGGTTTGAAACACTCTTTTTGTAGTATCTGGATGTGGACATTTGGAGCGCTTTCAGGC
          1 ATAGAGCAGGTTTGAAACACTCTTTTTGTAGTATCTGGAAGTGGATATTTGGATAGCTTTGAGG-

            * * *    *                 *** *   *                          *  
     402650 CTATGGTT-TAAA-AGGAAATATCTTCCCCTGAAAACTAGACAGAAGCATTCTCAGAAACTTATT
         65 ATTTCGTTGGAAACAGG-AATATCTTCATATAAAAGCTAGACAGAAGCATTCTCAGAAACTTCTT

                    **  **    *                    *   *
     402713 TGTGATGTGCGCCCTCAACTAACAGTGTTGAAGCATT-CTTTTG
        129 TGTGATGTTTGCATTCAAGTAACAGTGTTGAA-CATTCCCTTTC

                     *                   * *     *             **            
     402756 ATAGAGCAGTTTTGAAACACTCTTTTTGTGGAATCTGCAAGTGGATATTTGTCTAGCTTTGAGGA
          1 ATAGAGCAGGTTTGAAACACTCTTTTTGTAGTATCTGGAAGTGGATATTTGGATAGCTTTGAGGA

                         *   *    *      *            *       *              
     402821 TTTCGTTGGAAACGGGATTA-CAT-ATA-GAAAGC-AGACAGCAGCATTCCCAGAAACTTCTTTG
         66 TTTCGTTGGAAACAGGAATATCTTCATATAAAAGCTAGACAGAAGCATTCTCAGAAACTTCTTTG

                              *    *                 
     402882 TGATGTTTGCATTCAAGTCACAGAGTTGAACATTCCCTTTC
        131 TGATGTTTGCATTCAAGTAACAGTGTTGAACATTCCCTTTC

                                                   *     *       **     *   *
     402923 ATAGAGCAGGTTTGAAACACTCTTTTTGTAGTATCTGGATGTGGACATTTGGAGCGCTTTCAGGC
          1 ATAGAGCAGGTTTGAAACACTCTTTTTGTAGTATCTGGAAGTGGATATTTGGATAGCTTTGAGGA

            * ** *      *             *** *   *                          *   
     402988 CTATGGT-GAAAAAGGAAATATCTTCCCCTGAAAACTAGACAGAAGCATTCTCAGAAACTTATTT
         66 TTTCGTTGGAAACAGG-AATATCTTCATATAAAAGCTAGACAGAAGCATTCTCAGAAACTTCTTT

                   **  **    *              *  * *   *
     403052 GTGATGTGCGCCCTCAACTAACAGTGTTGAACCTTTCTTTTG
        130 GTGATGTTTGCATTCAAGTAACAGTGTTGAACATTCCCTTTC

                     *                    *      *   *                       
     403094 ATAGAGCAGTTTTGAAACACTCTTTTTGTAATATCTGCAAGAGGATATTTGGATAGCTTTGAGGA
          1 ATAGAGCAGGTTTGAAACACTCTTTTTGTAGTATCTGGAAGTGGATATTTGGATAGCTTTGAGGA

                         *   * *            **                      *    *   
     403159 TTTCGTTGGAAACGGGATTGTCTTCATATAAACTCTAGACAGAAGCATTCTCAGAAGCTTCATTG
         66 TTTCGTTGGAAACAGGAATATCTTCATATAAAAGCTAGACAGAAGCATTCTCAGAAACTTCTTTG

            *       **   *    *             *        
     403224 GGATGTTTCAATTGAAGTCACAGTGTTGAACAGTCCCTTTC
        131 TGATGTTTGCATTCAAGTAACAGTGTTGAACATTCCCTTTC

                                                         *       *  * * *    
     403265 ATAGAGCAGGTTTGAAACACTCTTTTTGTAGTATCTGGAAGTGGACATTTGGAGAGATCTCAGGA
          1 ATAGAGCAGGTTTGAAACACTCTTTTTGTAGTATCTGGAAGTGGATATTTGGATAGCTTTGAGGA

            * *  *    * *             **            *       * *          *  *
     403330 ATACGGT-GATAAAGGAAATATCTTCCAATAAAAGCTAGATAGAAGCAATGTCAGAAACTTTTTC
         66 TTTCGTTGGAAACAGG-AATATCTTCATATAAAAGCTAGACAGAAGCATTCTCAGAAACTTCTTT

            *      * ** *   **      *       *  * *   *
     403394 ATGATGTATCTACTCAGCTAACAGAGTTGAACCTTTCTTTTG
        130 GTGATGTTTGCATTCAAGTAACAGTGTTGAACATTCCCTTTC

             *       *                   * *     *             **           *
     403436 AGAGAGCAGTTTTGAAACACTCTTTTTGTGGAATCTGCAAGTGGATATTTGTCTAGCTTTGAGGC
          1 ATAGAGCAGGTTTGAAACACTCTTTTTGTAGTATCTGGAAGTGGATATTTGGATAGCTTTGAGGA

                         *   *    *                   *       *              
     403501 TTTCGTTGGAAACGGGATTA-CAT-ATA-AAAAGC-AGACAGCAGCATTCCCAGAAACTTCTTTG
         66 TTTCGTTGGAAACAGGAATATCTTCATATAAAAGCTAGACAGAAGCATTCTCAGAAACTTCTTTG

                              *    *                 
     403562 TGATGTTTGCATTCAAGTCACAGAGTTGAACATTCCCTTTC
        131 TGATGTTTGCATTCAAGTAACAGTGTTGAACATTCCCTTTC

                                                 * *     *       **     *   *
     403603 ATAGAGCAGGTTTGAAACACTCTTTTTGTAGTATCTGTATGTGGACATTTGGAGCGCTTTCAGGC
          1 ATAGAGCAGGTTTGAAACACTCTTTTTGTAGTATCTGGAAGTGGATATTTGGATAGCTTTGAGGA

            * ** *      *             *** *   *                          *   
     403668 CTATGGT-GAAAAAGGAAATATCTTCCCCTGAAAACTAGACAGAAGCATTCTCAGAAACTTATTT
         66 TTTCGTTGGAAACAGG-AATATCTTCATATAAAAGCTAGACAGAAGCATTCTCAGAAACTTCTTT

                   *** **    *              *  * *   *
     403732 GTGATGTGCTCCCTCAACTAACAGTGTTGAACCTTTCTTTTG
        130 GTGATGTTTGCATTCAAGTAACAGTGTTGAACATTCCCTTTC

                     *                    *      *   *                       
     403774 ATAGAGCAGTTTTGAAACACTCTTTTTGTAATATCTGCAAGAGGATATTTGGATAGCTTTGAGGA
          1 ATAGAGCAGGTTTGAAACACTCTTTTTGTAGTATCTGGAAGTGGATATTTGGATAGCTTTGAGGA

                         *   * *            **                      *    *   
     403839 TTTCGTTGGAAACGGGATTGTCTTCATATAAACTCTAGACAGAAGCATTCTCAGAAGCTTCATTG
         66 TTTCGTTGGAAACAGGAATATCTTCATATAAAAGCTAGACAGAAGCATTCTCAGAAACTTCTTTG

            *       **   *    *             * *      
     403904 GGATGTTTCAATTGAAGTCACAGTGTTGAACAGTTCCTTTC
        131 TGATGTTTGCATTCAAGTAACAGTGTTGAACATTCCCTTTC

                 *         *                             *       **   * *    
     403945 ATAGAACAGGTTTGAGACACTCTTTTTGTAGTATCTGGAAGTGGACATTTGGAGCGCTCTCAGGA
          1 ATAGAGCAGGTTTGAAACACTCTTTTTGTAGTATCTGGAAGTGGATATTTGGATAGCTTTGAGGA

            * *  *      *             **          * *       * *          *  *
     404010 CTACGGT-GAAAAAGGAAATATCTTCCAATAAAAGCTACATAGAAGCAATGTCAGAAACTTTTTC
         66 TTTCGTTGGAAACAGG-AATATCTTCATATAAAAGCTAGACAGAAGCATTCTCAGAAACTTCTTT

            *      * ** *   **      *       *  *     *
     404074 ATGATGTATCTACTCAGCTAACAGAGTTGAACCTTTCCTTTG
        130 GTGATGTTTGCATTCAAGTAACAGTGTTGAACATTCCCTTTC

             *       *                   * *     *             **            
     404116 AGAGAGCAGTTTTGAAACACTCTTTTTGTGGAATCTGCAAGTGGATATTTGTCTAGCTTTGAGGA
          1 ATAGAGCAGGTTTGAAACACTCTTTTTGTAGTATCTGGAAGTGGATATTTGGATAGCTTTGAGGA

                         *   *    *                   *       *   *          
     404181 TTTCGTTGGAAACGGGATTA-CAT-ATA-AAAAGC-AGACAGCAGCATTCCCAGTAACTTCTTTG
         66 TTTCGTTGGAAACAGGAATATCTTCATATAAAAGCTAGACAGAAGCATTCTCAGAAACTTCTTTG

                              *    *                 
     404242 TGATGTTTGCATTCAAGTCACAGAGTTGAACATTCCCTTTC
        131 TGATGTTTGCATTCAAGTAACAGTGTTGAACATTCCCTTTC

                                        *          *     *       **     *   *
     404283 ATAGAGCAGGTTTGAAACACTCTTTTTGAAGTATCTGGATGTGGACATTTGGAGCGCTTTCAGGC
          1 ATAGAGCAGGTTTGAAACACTCTTTTTGTAGTATCTGGAAGTGGATATTTGGATAGCTTTGAGGA

            * ** *      *             *** *   *                          *   
     404348 CTATGGT-GAAAAAGGAAATATCTTCCCCTGAAAACTAGACAGAAGCATTCTCAGAAACTTATTT
         66 TTTCGTTGGAAACAGG-AATATCTTCATATAAAAGCTAGACAGAAGCATTCTCAGAAACTTCTTT

                   **  **    *              *  * *   *
     404412 GTGATGTGCGCCCTCAACTAACAGTGTTGAACCTTTCTTTTG
        130 GTGATGTTTGCATTCAAGTAACAGTGTTGAACATTCCCTTTC

                     *                    *      *   *                       
     404454 ATAGAGCAGTTTTGAAACACTCTTTTTGTAATATCTGCAAGAGGATATTTGGATAGCTTTGAGGA
          1 ATAGAGCAGGTTTGAAACACTCTTTTTGTAGTATCTGGAAGTGGATATTTGGATAGCTTTGAGGA

                         *   * *            **                      *    *   
     404519 TTTCGTTGGAAACGGGATTGTCTTCATATAAACTCTAGACAGAAGCATTCTCAGAAGCTTCATTG
         66 TTTCGTTGGAAACAGGAATATCTTCATATAAAAGCTAGACAGAAGCATTCTCAGAAACTTCTTTG

            *       **   *    *             *        
     404584 GGATGTTTCAATTGAAGTCACAGTGTTGAACAGTCCCTTTC
        131 TGATGTTTGCATTCAAGTAACAGTGTTGAACATTCCCTTTC

                                                   *     *       **     *    
     404625 ATAGAGCAGGTTTGAAACACTCTTTTTGTAGTATCTGGATGTGGACATTTGGAGCGCTTTCAGGC
          1 ATAGAGCAGGTTTGAAACACTCTTTTTGTAGTATCTGGAAGTGGATATTTGGATAGCTTTGAGG-

            * * *    *                 *** *   *                          *  
     404690 CTATGGTT-TAAA-AGGAAATATCTTCCCCTGAAAACTAGACAGAAGCATTCTCAGAAACTTATT
         65 ATTTCGTTGGAAACAGG-AATATCTTCATATAAAAGCTAGACAGAAGCATTCTCAGAAACTTCTT

                    **  **    *                    *   *
     404753 TGTGATGTGCGCCCTCAACTAACAGTGTTGAAGCATT-CTTTTG
        129 TGTGATGTTTGCATTCAAGTAACAGTGTTGAA-CATTCCCTTTC

                     *                   * *     *             **            
     404796 ATAGAGCAGTTTTGAAACACTCTTTTTGTGGAATCTGCAAGTGGATATTTGTCTAGCTTTGAGGA
          1 ATAGAGCAGGTTTGAAACACTCTTTTTGTAGTATCTGGAAGTGGATATTTGGATAGCTTTGAGGA

                         *   *    *                   *       *              
     404861 TTTCGTTGGAAACGGGATTA-CAT-ATA-AAAAGC-AGACAGCAGCATTCCCAGAAACTTCTTTG
         66 TTTCGTTGGAAACAGGAATATCTTCATATAAAAGCTAGACAGAAGCATTCTCAGAAACTTCTTTG

                              *    *                 
     404922 TGATGTTTGCATTCAAGTCACAGAGTTGAACATTCCCTTTC
        131 TGATGTTTGCATTCAAGTAACAGTGTTGAACATTCCCTTTC

                                                   *     *       **     *   *
     404963 ATAGAGCAGGTTTGAAACACTCTTTTTGTAGTATCTGGATGTGGACATTTGGAGCGCTTTCAGGC
          1 ATAGAGCAGGTTTGAAACACTCTTTTTGTAGTATCTGGAAGTGGATATTTGGATAGCTTTGAGGA

            * ** *      *             *** *   *                          *   
     405028 CTATGGT-GAAAAAGGAAATATCTTCCCCTGAAAACTAGACAGAAGCATTCTCAGAAACTTATTT
         66 TTTCGTTGGAAACAGG-AATATCTTCATATAAAAGCTAGACAGAAGCATTCTCAGAAACTTCTTT

                   **  **    *             **  * *   *
     405092 GTGATGTGCGCCCTCAACTAACAGTGTTGAAGCTTTCTTTTG
        130 GTGATGTTTGCATTCAAGTAACAGTGTTGAACATTCCCTTTC

                     *                    *      *   *                  *    
     405134 ATAGAGCAGTTTTGAAACACTCTTTTTGTAATATCTGCAAGAGGATATTTGGATAGCTTTCAGGA
          1 ATAGAGCAGGTTTGAAACACTCTTTTTGTAGTATCTGGAAGTGGATATTTGGATAGCTTTGAGGA

                         *   * *            **          *           *    *   
     405199 TTTCGTTGGAAACGGGATTGTCTTCATATAAACTCTAGACAGAACCATTCTCAGAAGCTTCATTG
         66 TTTCGTTGGAAACAGGAATATCTTCATATAAAAGCTAGACAGAAGCATTCTCAGAAACTTCTTTG

            *       **   *    *             *        
     405264 GGATGTTTCAATTGAAGTCACAGTGTTGAACAGTCCCTTTC
        131 TGATGTTTGCATTCAAGTAACAGTGTTGAACATTCCCTTTC

                                                         *       *  * * *    
     405305 ATAGAGCAGGTTTGAAACACTCTTTTTGTAGTATCTGGAAGTGGACATTTGGAGAGATCTCAGGA
          1 ATAGAGCAGGTTTGAAACACTCTTTTTGTAGTATCTGGAAGTGGATATTTGGATAGCTTTGAGGA

            * *  *      *             **            *       * *          *  *
     405370 CTACGGT-GAAAAAGGAAATATCTTCCAATAAAAGCTAGATAGAAGCAATGTCAGAAACTTTTTC
         66 TTTCGTTGGAAACAGG-AATATCTTCATATAAAAGCTAGACAGAAGCATTCTCAGAAACTTCTTT

            *      * ** *   **      *       *  * *   *
     405434 ATGATGTATCTACTCAGCTAACAGAGTTGAACCTTTCTTTTG
        130 GTGATGTTTGCATTCAAGTAACAGTGTTGAACATTCCCTTTC

             *       *                   * *     *             **            
     405476 AGAGAGCAGTTTTGAAACACTCTTTTTGTGGAATCTGCAAGTGGATATTTGTCTAGCTTTGAGGA
          1 ATAGAGCAGGTTTGAAACACTCTTTTTGTAGTATCTGGAAGTGGATATTTGGATAGCTTTGAGGA

                         *   *    *                   *       *              
     405541 TTTCGTTGGAAACGGGATTA-CAT-ATA-AAAAGC-AGACAGCAGCATTCCCAGAAACTTCTTTG
         66 TTTCGTTGGAAACAGGAATATCTTCATATAAAAGCTAGACAGAAGCATTCTCAGAAACTTCTTTG

                              *    *                 
     405602 TGATGTTTGCATTCAAGTCACAGAGTTGAACATTCCCTTTC
        131 TGATGTTTGCATTCAAGTAACAGTGTTGAACATTCCCTTTC

                                                   *     *       **     *   *
     405643 ATAGAGCAGGTTTGAAACACTCTTTTTGTAGTATCTGGATGTGGACATTTGGAGCGCTTTCAGGC
          1 ATAGAGCAGGTTTGAAACACTCTTTTTGTAGTATCTGGAAGTGGATATTTGGATAGCTTTGAGGA

            * ** *      *             *** *   *                          *   
     405708 CTATGGT-GAAAAAGGAAATATCTTCCCCTGAAAACTAGACAGAAGCATTCTCAGAAACTTATTT
         66 TTTCGTTGGAAACAGG-AATATCTTCATATAAAAGCTAGACAGAAGCATTCTCAGAAACTTCTTT

                   **  **    *              *  * *   *
     405772 GTGATGTGCGCCCTCAACTAACAGTGTTGAACCTTTCTTTTG
        130 GTGATGTTTGCATTCAAGTAACAGTGTTGAACATTCCCTTTC

                     *                    *      *   *     *                 
     405814 ATAGAGCAGTTTTGAAACACTCTTTTTGTAATATCTGCAAGAGGATACTTGGATAGCTTTGAGGA
          1 ATAGAGCAGGTTTGAAACACTCTTTTTGTAGTATCTGGAAGTGGATATTTGGATAGCTTTGAGGA

             *           *   * *            **                      *    *   
     405879 TATCGTTGGAAACGGGATTGTCTTCATATAAACTCTAGACAGAAGCATTCTCAGAAGCTTCATTG
         66 TTTCGTTGGAAACAGGAATATCTTCATATAAAAGCTAGACAGAAGCATTCTCAGAAACTTCTTTG

            *       **   *                  *        
     405944 GGATGTTTCAATTGAAGTAACAGTGTTGAACAGTCCCTTTC
        131 TGATGTTTGCATTCAAGTAACAGTGTTGAACATTCCCTTTC

                                                         *       **   * *    
     405985 ATAGAGCAGGTTTGAAACACTCTTTTTGTAGTATCTGGAAGTGGACATTTGGAGCGCTCTCAGGA
          1 ATAGAGCAGGTTTGAAACACTCTTTTTGTAGTATCTGGAAGTGGATATTTGGATAGCTTTGAGGA

            * *  *      *             ** *          *       * *       *  *  *
     406050 CTGCGGT-GAAAAAGGAAATATCTTCCAACAAAAGCTAGATAGAAGCAATGTCAGAAAATTTTTC
         66 TTTCGTTGGAAACAGG-AATATCTTCATATAAAAGCTAGACAGAAGCATTCTCAGAAACTTCTTT

            *      * ** *   **      *       *        *
     406114 ATGATGTATCTACTCAGCTAACAGAGTTGAACCTT-CCTTTG
        130 GTGATGTTTGCATTCAAGTAACAGTGTTGAACATTCCCTTTC

             *       *                   * *     *             **            
     406155 AGAGAGCAGTTTTGAAACACTCTTTTTGTGGAATCTGCAAGTGGATATTTGTCTAGCTTTGAGGA
          1 ATAGAGCAGGTTTGAAACACTCTTTTTGTAGTATCTGGAAGTGGATATTTGGATAGCTTTGAGGA

                         *   *    *                   *       *              
     406220 TTTCGTTGGAAACGGGATTA-CAT-ATA-AAAAGC-AGACAGCAGCATTCCCAGAAACTTCTTTG
         66 TTTCGTTGGAAACAGGAATATCTTCATATAAAAGCTAGACAGAAGCATTCTCAGAAACTTCTTTG

                              *    *                 
     406281 TGATGTTTGCATTCAAGTCACAGAGTTGAACATTCCCTTTC
        131 TGATGTTTGCATTCAAGTAACAGTGTTGAACATTCCCTTTC

                                                   *     *     * **     *   *
     406322 ATAGAGCAGGTTTGAAACACTCTTTTTGTAGTATCTGGATGTGGACATTTGCAGCGCTTTCAGGC
          1 ATAGAGCAGGTTTGAAACACTCTTTTTGTAGTATCTGGAAGTGGATATTTGGATAGCTTTGAGGA

            * ** *      *             *** *   *                          *   
     406387 CTATGGT-GAAAAAGGAAATATCTTCCCCTGAAAACTAGACAGAAGCATTCTCAGAAACTTATTT
         66 TTTCGTTGGAAACAGG-AATATCTTCATATAAAAGCTAGACAGAAGCATTCTCAGAAACTTCTTT

                   **  **    *             **  * *    
     406451 GTGATGTGCGCCCTCAACTAACAGTGTTGAAGCTTTCTTTTC
        130 GTGATGTTTGCATTCAAGTAACAGTGTTGAACATTCCCTTTC

                     *                    *      *   *                  *    
     406493 ATAGAGCAGTTTTGAAACACTCTTTTTGTAATATCTGCAAGAGGATATTTGGATAGCTTTCAGGA
          1 ATAGAGCAGGTTTGAAACACTCTTTTTGTAGTATCTGGAAGTGGATATTTGGATAGCTTTGAGGA

                         *   * *            **                      *    *   
     406558 TTTCGTTGGAAACGGGATTGTCTTCATATAAACTCTAGACAGAAGCATTCTCAGAAGCTTCATTG
         66 TTTCGTTGGAAACAGGAATATCTTCATATAAAAGCTAGACAGAAGCATTCTCAGAAACTTCTTTG

            *       **   *    *             *        
     406623 GGATGTTTCAATTGAAGTCACAGTGTTGAACAGTCCCTTTC
        131 TGATGTTTGCATTCAAGTAACAGTGTTGAACATTCCCTTTC

                                                   *     *       **     *    
     406664 ATAGAGCAGGTTTGAAACACTCTTTTTGTAGTATCTGGATGTGGACATTTGGAGCGCTTTCAGGC
          1 ATAGAGCAGGTTTGAAACACTCTTTTTGTAGTATCTGGAAGTGGATATTTGGATAGCTTTGAGG-

            * * *    *                 *** *   *                          *  
     406729 CTATGGTT-TAAA-AGGAAATATCTTCCCCTGAAAACTAGACAGAAGCATTCTCAGAAACTTATT
         65 ATTTCGTTGGAAACAGG-AATATCTTCATATAAAAGCTAGACAGAAGCATTCTCAGAAACTTCTT

                    **  **    *                    *   *
     406792 TGTGATGTGCGCCCTCAACTAACAGTGTTGAAGCATT-CTTTTG
        129 TGTGATGTTTGCATTCAAGTAACAGTGTTGAA-CATTCCCTTTC

                     *                   * *     *             **            
     406835 ATAGAGCAGTTTTGAAACACTCTTTTTGTGGAATCTGCAAGTGGATATTTGTCTAGCTTTGAGGA
          1 ATAGAGCAGGTTTGAAACACTCTTTTTGTAGTATCTGGAAGTGGATATTTGGATAGCTTTGAGGA

                         *   *    *                   *       *              
     406900 TTTCGTTGGAAACGGGATTA-CAT-ATA-AAAAGC-AGACAGCAGCATTCCCAGAAACTTCTTTG
         66 TTTCGTTGGAAACAGGAATATCTTCATATAAAAGCTAGACAGAAGCATTCTCAGAAACTTCTTTG

                              *    *                 
     406961 TGATGTTTGCATTCAAGTCACAGAGTTGAACATTCCCTTTC
        131 TGATGTTTGCATTCAAGTAACAGTGTTGAACATTCCCTTTC

                                                   *     *     * **     *   *
     407002 ATAGAGCAGGTTTGAAACACTCTTTTTGTAGTATCTGGATGTGGACATTTGCAGCGCTTTCAGGC
          1 ATAGAGCAGGTTTGAAACACTCTTTTTGTAGTATCTGGAAGTGGATATTTGGATAGCTTTGAGGA

            * ** *      *             *** *   *                          *   
     407067 CTAAGGT-GAAAAAGGAAATATCTTCCCCTGAAAACTAGACAGAAGCATTCTCAGAAACTTATTT
         66 TTTCGTTGGAAACAGG-AATATCTTCATATAAAAGCTAGACAGAAGCATTCTCAGAAACTTCTTT

                   **  **    *             **  * *   *
     407131 GTGATGTGCGCCCTCAACTAACAGTGTTGAAGCTTTCTTTTG
        130 GTGATGTTTGCATTCAAGTAACAGTGTTGAACATTCCCTTTC

                     *                    *      *   *     *                 
     407173 ATAGAGCAGTTTTGAAACACTCTTTTTGTAATATCTGCAAGAGGATACTTGGATAGCTTTGAGGA
          1 ATAGAGCAGGTTTGAAACACTCTTTTTGTAGTATCTGGAAGTGGATATTTGGATAGCTTTGAGGA

                         *   * *            **                      *    *   
     407238 TTTCGTTGGAAACGGGATTGTCTTCATATAAACTCTAGACAGAAGCATTCTCAGAAGCTTCATTG
         66 TTTCGTTGGAAACAGGAATATCTTCATATAAAAGCTAGACAGAAGCATTCTCAGAAACTTCTTTG

            *       **   *   **             *        
     407303 GGATGTTTCAATTGAAGACACAGTGTTGAACAGTCCCTTTC
        131 TGATGTTTGCATTCAAGTAACAGTGTTGAACATTCCCTTTC

                                                         *       **     *    
     407344 ATAGAGCAGGTTTGAAACACTCTTTTTGTAGTATCTGGAAGTGGACATTTGGAGCGCTTTCAGGC
          1 ATAGAGCAGGTTTGAAACACTCTTTTTGTAGTATCTGGAAGTGGATATTTGGATAGCTTTGAGG-

            * * *    *                 *** *   *                          *  
     407409 CTATGGTT-TAAA-AGGAAATATCTTCCCCTGAAAACTAGACAGAAGCATTCTCAGAAACTTATT
         65 ATTTCGTTGGAAACAGG-AATATCTTCATATAAAAGCTAGACAGAAGCATTCTCAGAAACTTCTT

                    **  **    *          *  **  * *   *
     407472 TGTGATGTGCGCCCTCAACTAACAGTGTTAAAGCTTTCTTTTG
        129 TGTGATGTTTGCATTCAAGTAACAGTGTTGAACATTCCCTTTC

                     *                   * *     *        *    ***           
     407515 ATAGAGCAGTTTTGAAACACTCTTTTTGTGGAATCTGCAAGTGGATGTTTGTCCAGCTTTGAGGA
          1 ATAGAGCAGGTTTGAAACACTCTTTTTGTAGTATCTGGAAGTGGATATTTGGATAGCTTTGAGGA

                         *   *    *          *        *       *     *   *    
     407580 TTTCGTTGGAAACGGGATTA-CAT-ATA-AAAAAC-AGACAGCAGCATTCCCAGAATCTTGTTTG
         66 TTTCGTTGGAAACAGGAATATCTTCATATAAAAGCTAGACAGAAGCATTCTCAGAAACTTCTTTG

                              *    *                 
     407641 TGATGTTTGCATTCAAGTCACAGAGTTGAACATTCCCTTTC
        131 TGATGTTTGCATTCAAGTAACAGTGTTGAACATTCCCTTTC

             *                         *           *   * *       **     *   *
     407682 AGAGAGCAGGTTTGAAACACTCTTTTTATAGTATCTGGATGTGAACATTTGGAGCGCTTTCAGGC
          1 ATAGAGCAGGTTTGAAACACTCTTTTTGTAGTATCTGGAAGTGGATATTTGGATAGCTTTGAGGA

            * ** *      *             *** *   *                      *   *   
     407747 CTATGGT-GAAAAAGGAAATATCTTCTCCTGAAAACTAGACAGAAGCATTCTCAGAATCTTATTT
         66 TTTCGTTGGAAACAGG-AATATCTTCATATAAAAGCTAGACAGAAGCATTCTCAGAAACTTCTTT

                   **  **    *     *       **  * *   *
     407811 GTGATGTGCGCCCTCAACTAACAATGTTGAAGCTTTCTTTTG
        130 GTGATGTTTGCATTCAAGTAACAGTGTTGAACATTCCCTTTC

                     *    *           *   **     *   *                       
     407853 ATAGAGCAGTTTTGCAACACTCTTTTCGTAAAATCTGCAAGAGGATATTTGGATAGCTTTGAGGA
          1 ATAGAGCAGGTTTGAAACACTCTTTTTGTAGTATCTGGAAGTGGATATTTGGATAGCTTTGAGGA

                         *   * *            **       *              *    *   
     407918 TTTCGTTGGAAACGGGATTGTCTTCATATAAACTCTAGACAAAAGCATTCTCAGAAGCTTCATTG
         66 TTTCGTTGGAAACAGGAATATCTTCATATAAAAGCTAGACAGAAGCATTCTCAGAAACTTCTTTG

            *       **   *    *             * *      
     407983 GGATGTTTCAATTGAAGTCACAGTGTTGAACAGTTCCTTTC
        131 TGATGTTTGCATTCAAGTAACAGTGTTGAACATTCCCTTTC

                 *                                       *       **   * *    
     408024 ATAGAACAGGTTTGAAACACTCTTTTTGTAGTATCTGGAAGTGGACATTTGGAGCGCTCTCAGGA
          1 ATAGAGCAGGTTTGAAACACTCTTTTTGTAGTATCTGGAAGTGGATATTTGGATAGCTTTGAGGA

            * ** *      *             **          * *       * *          *  *
     408089 CTATGGT-GAAAAAGGAAATATCTTCCAATAAAAGCTACATAGAAGCAATGTCAGAAACTTTTTC
         66 TTTCGTTGGAAACAGG-AATATCTTCATATAAAAGCTAGACAGAAGCATTCTCAGAAACTTCTTT

            *      * ** *   **      *       *  *     *
     408153 ATGATGTATCTACTCAGCTAACAGAGTTGAACCTTTCCTTTG
        130 GTGATGTTTGCATTCAAGTAACAGTGTTGAACATTCCCTTTC

             *       *                   * *     *             **            
     408195 AGAGAGCAGTTTTGAAACACTCTTTTTGTGGAATCTGCAAGTGGATATTTGTCTAGCTTTGAGGA
          1 ATAGAGCAGGTTTGAAACACTCTTTTTGTAGTATCTGGAAGTGGATATTTGGATAGCTTTGAGGA

                         *   *    *                   *       *   *          
     408260 TTTCGTTGGAAACGGGATTA-CAT-ATA-AAAAGC-AGACAGCAGCATTCCCAGTAACTTCTTTG
         66 TTTCGTTGGAAACAGGAATATCTTCATATAAAAGCTAGACAGAAGCATTCTCAGAAACTTCTTTG

                    *         *    *                 
     408321 TGATGTTTTCATTCAAGTCACAGAGTTGAACATTCCCTTTC
        131 TGATGTTTGCATTCAAGTAACAGTGTTGAACATTCCCTTTC

                                        *          *     *       **     *   *
     408362 ATAGAGCAGGTTTGAAACACTCTTTTTGAAGTATCTGGATGTGGACATTTGGAGCGCTTTCAGGC
          1 ATAGAGCAGGTTTGAAACACTCTTTTTGTAGTATCTGGAAGTGGATATTTGGATAGCTTTGAGGA

            * ** *      *             *** *   *                          *   
     408427 CTATGGT-GAAAAAGGAAATATCTTCCCCTGAAAACTAGACAGAAGCATTCTCAGAAACTTATTT
         66 TTTCGTTGGAAACAGG-AATATCTTCATATAAAAGCTAGACAGAAGCATTCTCAGAAACTTCTTT

                   **  **    *              *  * *   *
     408491 GTGATGTGCGCCCTCAACTAACAGTGTTGAACCTTTCTTTTG
        130 GTGATGTTTGCATTCAAGTAACAGTGTTGAACATTCCCTTTC

                     *                    *      *   *                       
     408533 ATAGAGCAGTTTTGAAACACTCTTTTTGTAATATCTGCAAGAGGATATTTGGATAGCTTTGAGGA
          1 ATAGAGCAGGTTTGAAACACTCTTTTTGTAGTATCTGGAAGTGGATATTTGGATAGCTTTGAGGA

                         *   * *            **                  *   *    *   
     408598 TTTCGTTGGAAACGGGATTGTCTTCATATAAACTCTAGACAGAAGCATTCTCCGAAGCTTCATTG
         66 TTTCGTTGGAAACAGGAATATCTTCATATAAAAGCTAGACAGAAGCATTCTCAGAAACTTCTTTG

            *       **   *    *             *        
     408663 GGATGTTTCAATTGAAGTCACAGTGTTGAACAGTCCCTTTC
        131 TGATGTTTGCATTCAAGTAACAGTGTTGAACATTCCCTTTC

                                                   *     *       **     *   *
     408704 ATAGAGCAGGTTTGAAACACTCTTTTTGTAGTATCTGGATGTGGACATTTGGAGCGCTTTCAGGC
          1 ATAGAGCAGGTTTGAAACACTCTTTTTGTAGTATCTGGAAGTGGATATTTGGATAGCTTTGAGGA

            * ** *      *             *** *   *                          *   
     408769 CTATGGT-GAAAAAGGAAATATCTTCCCCTGAAAACTAGACAGAAGCATTCTCAGAAACTTATTT
         66 TTTCGTTGGAAACAGG-AATATCTTCATATAAAAGCTAGACAGAAGCATTCTCAGAAACTTCTTT

                   **  **    *             **  * *   *
     408833 GTGATGTGCGCCCTCAACTAACAGTGTTGAAGCTTTCTTTTG
        130 GTGATGTTTGCATTCAAGTAACAGTGTTGAACATTCCCTTTC

                     *                    **     *   *            *          
     408875 ATAGAGCAGTTTTGAAACACTCTTTTTGTAAAATCTGCAAGAGGATATTTGGATGGCTTTGAGGA
          1 ATAGAGCAGGTTTGAAACACTCTTTTTGTAGTATCTGGAAGTGGATATTTGGATAGCTTTGAGGA

                *        *   * *            **                      *    *   
     408940 TTTCTTTGGAAACGGGATTGTCTTCATATAAACTCTAGACAGAAGCATTCTCAGAAGCTTCATTG
         66 TTTCGTTGGAAACAGGAATATCTTCATATAAAAGCTAGACAGAAGCATTCTCAGAAACTTCTTTG

            *       **   *    *             * *      
     409005 GGATGTTTCAATTGAAGTCACAGTGTTGAACAGTTCCTTTC
        131 TGATGTTTGCATTCAAGTAACAGTGTTGAACATTCCCTTTC

                 *                                       *       **   * *    
     409046 ATAGAACAGGTTTGAAACACTCTTTTTGTAGTATCTGGAAGTGGACATTTGGAGCGCTCTCAGGA
          1 ATAGAGCAGGTTTGAAACACTCTTTTTGTAGTATCTGGAAGTGGATATTTGGATAGCTTTGAGGA

            * ** *      *             **          * *       * *          *  *
     409111 CTATGGT-GAAAAAGGAAATATCTTCCAATAAAAGCTACATAGAAGCAATGTCAGAAACTTTTTC
         66 TTTCGTTGGAAACAGG-AATATCTTCATATAAAAGCTAGACAGAAGCATTCTCAGAAACTTCTTT

            *      * ** *   **      *       *  *     *
     409175 ATGATGTATCTACTCAGCTAACAGAGTTGAACCTTTCCTTTG
        130 GTGATGTTTGCATTCAAGTAACAGTGTTGAACATTCCCTTTC

             *       *                   * *     *        *    **            
     409217 AGAGAGCAGTTTTGAAACACTCTTTTTGTGGAATCTGCAAGTGGATGTTTGTCTAGCTTTGAGGA
          1 ATAGAGCAGGTTTGAAACACTCTTTTTGTAGTATCTGGAAGTGGATATTTGGATAGCTTTGAGGA

                         *  **    *                   *       *     *   *    
     409282 TTTCGTTGGAAACGGGTTTA-CAT-ATA-AAAAGC-AGACAGCAGCATTCCCAGAATCTTGTTTG
         66 TTTCGTTGGAAACAGGAATATCTTCATATAAAAGCTAGACAGAAGCATTCTCAGAAACTTCTTTG

                              *    *                 
     409343 TGATGTTTGCATTCAAGTCACAGAGTTGAACATTCCCTTTC
        131 TGATGTTTGCATTCAAGTAACAGTGTTGAACATTCCCTTTC

                                       *           *     *       **     *   *
     409384 ATAGAGCAGGTTTGAAACACTCTTTTTATAGTATCTGGATGTGGACATTTGGAGCGCTTTCAGGC
          1 ATAGAGCAGGTTTGAAACACTCTTTTTGTAGTATCTGGAAGTGGATATTTGGATAGCTTTGAGGA

            * ** *      *             *** *   *                      *   *   
     409449 CTATGGT-GAAAAAGGAAATATCTTCTCCTGAAAACTAGACAGAAGCATTCTCAGAATCTTATTT
         66 TTTCGTTGGAAACAGG-AATATCTTCATATAAAAGCTAGACAGAAGCATTCTCAGAAACTTCTTT

             *     **  **    *             **  * *   *
     409513 GCGATGTGCGCCCTCAACTAACAGTGTTGAAGCTTTCTTTTG
        130 GTGATGTTTGCATTCAAGTAACAGTGTTGAACATTCCCTTTC

                     *                *   **     *   *        *              
     409555 ATAGAGCAGTTTTGAAACACTCTTTTCGTAAAATCTGCAAGAGGATATTTTGATAGCTTTGAGGA
          1 ATAGAGCAGGTTTGAAACACTCTTTTTGTAGTATCTGGAAGTGGATATTTGGATAGCTTTGAGGA

             *           *   * *            **                  *   *    *   
     409620 TATCGTTGGAAACGGGATTGTCTTCATATAAACTCTAGACAGAAGCATTCTCTGAAGCTTCATTG
         66 TTTCGTTGGAAACAGGAATATCTTCATATAAAAGCTAGACAGAAGCATTCTCAGAAACTTCTTTG

            *       **   *    *             *        
     409685 GGATGTTTCAATTGAAGTCACAGTGTTGAACAGTCCCTTTC
        131 TGATGTTTGCATTCAAGTAACAGTGTTGAACATTCCCTTTC

                                                         *       *  * * * *  
     409726 ATAGAGCAGGTTTGAAACACTCTTTTTGTAGTATCTGGAAGTGGACATTTGGAGAGATCTCATGA
          1 ATAGAGCAGGTTTGAAACACTCTTTTTGTAGTATCTGGAAGTGGATATTTGGATAGCTTTGAGGA

            * *  *    * *             **            *       * *          *  *
     409791 ATACGGT-GATAAAGGAAATATCTTCCAATAAAAGCTAGATAGAAGCAATGTCAGAAACTTTTTC
         66 TTTCGTTGGAAACAGG-AATATCTTCATATAAAAGCTAGACAGAAGCATTCTCAGAAACTTCTTT

            *      * ** *   **      *       *  * *   *
     409855 ATGATGTATCTACTCAGCTAACAGAGTTGAACCTTTCTTTTG
        130 GTGATGTTTGCATTCAAGTAACAGTGTTGAACATTCCCTTTC

             *       *                   * *     *             **            
     409897 AGAGAGCAGTTTTGAAACACTCTTTTTGTGGAATCTGCAAGTGGATATTTGTCTAGCTTTGAGGA
          1 ATAGAGCAGGTTTGAAACACTCTTTTTGTAGTATCTGGAAGTGGATATTTGGATAGCTTTGAGGA

                         *   *    *                   *       *   *          
     409962 TTTCGTTGGAAACGGGATTA-CAT-ATA-AAAAGC-AGACAGCAGCATTCCCAGTAACTTCTTTG
         66 TTTCGTTGGAAACAGGAATATCTTCATATAAAAGCTAGACAGAAGCATTCTCAGAAACTTCTTTG

                              *    *        *        
     410023 TGATGTTTGCATTCAAGTCACAGAGTTGAACACTCCCTTTC
        131 TGATGTTTGCATTCAAGTAACAGTGTTGAACATTCCCTTTC

                                                   *     *     * **     *   *
     410064 ATAGAGCAGGTTTGAAACACTCTTTTTGTAGTATCTGGATGTGGACATTTGCAGCGCTTTCAGGC
          1 ATAGAGCAGGTTTGAAACACTCTTTTTGTAGTATCTGGAAGTGGATATTTGGATAGCTTTGAGGA

            * ** *      *             *** *   *                          *   
     410129 CTAAGGT-GAAAAAGGAAATATCTTCCCCTGAAAACTAGACAGAAGCATTCTCAGAAACTTATTT
         66 TTTCGTTGGAAACAGG-AATATCTTCATATAAAAGCTAGACAGAAGCATTCTCAGAAACTTCTTT

                   **  **    *             **  * *   *
     410193 GTGATGTGCGCCCTCAACTAACAGTGTTGAAGCTTTCTTTTG
        130 GTGATGTTTGCATTCAAGTAACAGTGTTGAACATTCCCTTTC

                *  * *                    *      *   *                       
     410235 ATAGTGCCGTTTTGAAACACTCTTTTTGTAATATCTGCAAGAGGATATTTGGATAGCTTTGAGGA
          1 ATAGAGCAGGTTTGAAACACTCTTTTTGTAGTATCTGGAAGTGGATATTTGGATAGCTTTGAGGA

                         *   * *            **                      *    * * 
     410300 TTTCGTTGGAAACGGGATTGTCTTCATATAAACTCTAGACAGAAGCATTCTCAGAAGCTTCATCG
         66 TTTCGTTGGAAACAGGAATATCTTCATATAAAAGCTAGACAGAAGCATTCTCAGAAACTTCTTTG

            *       **   *    *             * *      
     410365 GGATGTTTCAATTGAAGTCACAGTGTTGAACAGTTCCTTTC
        131 TGATGTTTGCATTCAAGTAACAGTGTTGAACATTCCCTTTC

                 *                                       *       **   * *    
     410406 ATAGAACAGGTTTGAAACACTCTTTTTGTAGTATCTGGAAGTGGACATTTGGAGCGCTCTCAGGA
          1 ATAGAGCAGGTTTGAAACACTCTTTTTGTAGTATCTGGAAGTGGATATTTGGATAGCTTTGAGGA

            * ** *      *             **          * *       * *          *  *
     410471 CTATGGT-GAAAAAGGAAATATCTTCCAATAAAAGCTACATAGAAGCAATGTCAGAAACTTTTTC
         66 TTTCGTTGGAAACAGG-AATATCTTCATATAAAAGCTAGACAGAAGCATTCTCAGAAACTTCTTT

            *      * ** *   **      *       *  *     *
     410535 ATGATGTATCTACTCAGCTAACAGAGTTGAACCTTTCCTTTG
        130 GTGATGTTTGCATTCAAGTAACAGTGTTGAACATTCCCTTTC

             *       *                   * *     *             **            
     410577 AGAGAGCAGTTTTGAAACACTCTTTTTGTGGAATCTGCAAGTGGATATTTGCTTAGCTTTGAGGA
          1 ATAGAGCAGGTTTGAAACACTCTTTTTGTAGTATCTGGAAGTGGATATTTGGATAGCTTTGAGGA

                         *   *    *                   *       *   *          
     410642 TTTCGTTGGAAACGGGATTA-CAT-ATA-AAAAGC-AGACAGCAGCATTCCCAGTAACTTCTTTG
         66 TTTCGTTGGAAACAGGAATATCTTCATATAAAAGCTAGACAGAAGCATTCTCAGAAACTTCTTTG

                              *    *                 
     410703 TGATGTTTGCATTCAAGTCACAGAGTTGAACATTCCCTTTC
        131 TGATGTTTGCATTCAAGTAACAGTGTTGAACATTCCCTTTC

                                                   *     *       **     *   *
     410744 ATAGAGCAGGTTTGAAACACTCTTTTTGTAGTATCTGGATGTGGACATTTGGAGCGCTTTCAGGC
          1 ATAGAGCAGGTTTGAAACACTCTTTTTGTAGTATCTGGAAGTGGATATTTGGATAGCTTTGAGGA

            * ** *      *             *** *   *                      *   *   
     410809 CTATGGT-GAAAAAGGAAATATCTTCCCCTGAAAACTAGACAGAAGCATTCTCAGAATCTTATTT
         66 TTTCGTTGGAAACAGG-AATATCTTCATATAAAAGCTAGACAGAAGCATTCTCAGAAACTTCTTT

                   **  **    *             **  * *   *
     410873 GTGATGTGCGCCCTCAACTAACAGTGTTGAAGCTTTCTTTTG
        130 GTGATGTTTGCATTCAAGTAACAGTGTTGAACATTCCCTTTC

                     *                    **     *   *               *       
     410915 ATAGAGCAGTTTTGAAACACTCTTTTTGTAAAATCTGCAAGAGGATATTTGGATAGCATTGAGGA
          1 ATAGAGCAGGTTTGAAACACTCTTTTTGTAGTATCTGGAAGTGGATATTTGGATAGCTTTGAGGA

                         *   * *            **                      *    *   
     410980 TTTCGTTGGAAACGGGATTGTCTTCATATAAACTCTAGACAGAAGCATTCTCAGAAGCTTCATTG
         66 TTTCGTTGGAAACAGGAATATCTTCATATAAAAGCTAGACAGAAGCATTCTCAGAAACTTCTTTG

            *       **   *    *             *        
     411045 GGATGTTTCAATTGAAGTCACAGTGTTGAACAGTCCCTTTC
        131 TGATGTTTGCATTCAAGTAACAGTGTTGAACATTCCCTTTC

                                                   *     *       **     *    
     411086 ATAGAGCAGGTTTGAAACACTCTTTTTGTAGTATCTGGATGTGGACATTTGGAGCGCTTTCAGGC
          1 ATAGAGCAGGTTTGAAACACTCTTTTTGTAGTATCTGGAAGTGGATATTTGGATAGCTTTGAGG-

            * * *    *                 *** *   *                          *  
     411151 CTATGGTT-TAAA-AGGAAATATCTTCCCCTGAAAACTAGACAGAAGCATTCTCAGAAACTTATT
         65 ATTTCGTTGGAAACAGG-AATATCTTCATATAAAAGCTAGACAGAAGCATTCTCAGAAACTTCTT

                    **  **    *                    *   *
     411214 TGTGATGTGCGCCCTCAACTAACAGTGTTGAAGCATT-CTTTTG
        129 TGTGATGTTTGCATTCAAGTAACAGTGTTGAA-CATTCCCTTTC

                     *                   * *  *  *             **            
     411257 ATAGAGCAGTTTTGAAACACTCTTTTTGTGGAATGTGCAAGTGGATATTTGTCTAGCTTTGAGGA
          1 ATAGAGCAGGTTTGAAACACTCTTTTTGTAGTATCTGGAAGTGGATATTTGGATAGCTTTGAGGA

                         *   *    *                   *       *              
     411322 TTTCGTTGGAAACGGGATTA-CAT-ATA-AAAAGC-AGACAGCAGCATTCCCAGAAACTTCTTTG
         66 TTTCGTTGGAAACAGGAATATCTTCATATAAAAGCTAGACAGAAGCATTCTCAGAAACTTCTTTG

                              *    *                 
     411383 TGATGTTTGCATTCAAGTCACAGAGTTGAACATTCCCTTTC
        131 TGATGTTTGCATTCAAGTAACAGTGTTGAACATTCCCTTTC

                                                   *     *       **     *   *
     411424 ATAGAGCAGGTTTGAAACACTCTTTTTGTAGTATCTGGATGTGGACATTTGGAGCGCTTTCAGGC
          1 ATAGAGCAGGTTTGAAACACTCTTTTTGTAGTATCTGGAAGTGGATATTTGGATAGCTTTGAGGA

            * ** *      *             *** *   *                          *   
     411489 CTATGGT-GAAAAAGGAAATATCTTCCCCTGAAAACTAGACAGAAGCATTCTCAGAAACTTATTT
         66 TTTCGTTGGAAACAGG-AATATCTTCATATAAAAGCTAGACAGAAGCATTCTCAGAAACTTCTTT

                   **  **    *              *  * *   *
     411553 GTGATGTGCGCCCTCAACTAACAGTGTTGAACCTTTCTTTTG
        130 GTGATGTTTGCATTCAAGTAACAGTGTTGAACATTCCCTTTC

                     *                    *      *   *                       
     411595 ATAGAGCAGTTTTGAAACACTCTTTTTGTAATATCTGCAAGAGGATATTTGGATAGCTTTGAGGA
          1 ATAGAGCAGGTTTGAAACACTCTTTTTGTAGTATCTGGAAGTGGATATTTGGATAGCTTTGAGGA

                         *   * *            **                      *    *   
     411660 TTTCGTTGGAAACGGGATTGTCTTCATATAAACTCTAGACAGAAGCATTCTCAGAAGCTTCATTG
         66 TTTCGTTGGAAACAGGAATATCTTCATATAAAAGCTAGACAGAAGCATTCTCAGAAACTTCTTTG

            *       **   *    *             *        
     411725 GGATGTTTCAATTGAAGTCACAGTGTTGAACAGTCCCTTTC
        131 TGATGTTTGCATTCAAGTAACAGTGTTGAACATTCCCTTTC

                                                   *     *       **     *   *
     411766 ATAGAGCAGGTTTGAAACACTCTTTTTGTAGTATCTGGATGTGGACATTTGGAGCGCTTTCAGGC
          1 ATAGAGCAGGTTTGAAACACTCTTTTTGTAGTATCTGGAAGTGGATATTTGGATAGCTTTGAGGA

            * ** *      *             *** *   *                          *   
     411831 CTATGGT-GAAAAAGGAAATATCTTCCCCTGAAAACTAGACAGAAGCATTCTCAGAAACTTTTTT
         66 TTTCGTTGGAAACAGG-AATATCTTCATATAAAAGCTAGACAGAAGCATTCTCAGAAACTTCTTT

                   **  **    *              *  * *   *
     411895 GTGATGTGCGCCCTCAACTAACAGTGTTGAACCTTTCTTTTG
        130 GTGATGTTTGCATTCAAGTAACAGTGTTGAACATTCCCTTTC

                 *   *                    *      *   *                       
     411937 ATAGACCAGTTTTGAAACACTCTTTTTGTAATATCTGCAAGAGGATATTTGGATAGCTTTGAGGA
          1 ATAGAGCAGGTTTGAAACACTCTTTTTGTAGTATCTGGAAGTGGATATTTGGATAGCTTTGAGGA

                         *   * *            **                      *    *   
     412002 TTTCGTTGGAAACGGGATTGTCTTCATATAAACTCTAGACAGAAGCATTCTCAGAAGCTTCATTG
         66 TTTCGTTGGAAACAGGAATATCTTCATATAAAAGCTAGACAGAAGCATTCTCAGAAACTTCTTTG

            *       **   *    *             *        
     412067 GGATGTTTCAATTGAAGTCACAGTGTTGAACAGTCCCTTTC
        131 TGATGTTTGCATTCAAGTAACAGTGTTGAACATTCCCTTTC

                                                         *    *  ** *** *    
     412108 ATAGAGCAGGTTTGAAACACTCTTTTTGTAGTATCTGGAAGTGGACATTTTGAGCGAGCTCAGGA
          1 ATAGAGCAGGTTTGAAACACTCTTTTTGTAGTATCTGGAAGTGGATATTTGGATAGCTTTGAGGA

            * *  *    * *             **            *       * *          *  *
     412173 CTACGGT-GATAAAGGAAATATCTTCCAATAAAAGCTAGATAGAAGCAATGTCAGAAACTTTTTC
         66 TTTCGTTGGAAACAGG-AATATCTTCATATAAAAGCTAGACAGAAGCATTCTCAGAAACTTCTTT

            *      * ** *   **      *       *  * *   *
     412237 ATGATGTATCTACTCAGCTAACAGAGTTGAACCTTTCTTTTG
        130 GTGATGTTTGCATTCAAGTAACAGTGTTGAACATTCCCTTTC

             *    *  *                   * *     *            ***            
     412279 AAAGAGAAGTTTTGAAACACTCTTTTTGTGGAATCTGCAAGTGGATATTTCTCTAGCTTTGAGGA
          1 ATAGAGCAGGTTTGAAACACTCTTTTTGTAGTATCTGGAAGTGGATATTTGGATAGCTTTGAGGA

                         *  **    *   *               *       *     *        
     412344 TTTCGTTGGAAACGGGTTTA-CAT-ACA-AAAAGC-AGACAGCAGCATTCCCAGAATCTTCTTTG
         66 TTTCGTTGGAAACAGGAATATCTTCATATAAAAGCTAGACAGAAGCATTCTCAGAAACTTCTTTG

                              *    *                 
     412405 TGATGTTTGCATTCAAGTCACAGAGTTGAACATTCCCTTTC
        131 TGATGTTTGCATTCAAGTAACAGTGTTGAACATTCCCTTTC

                                                   *     *       **     *   *
     412446 ATAGAGCAGGTTTGAAACACTCTTTTTGTAGTATCTGGATGTGGACATTTGGAGCGCTTTCAGGC
          1 ATAGAGCAGGTTTGAAACACTCTTTTTGTAGTATCTGGAAGTGGATATTTGGATAGCTTTGAGGA

            * ** *      *             *** *   *                      * * *   
     412511 CTATGGT-GAAAAAGGAAATATCTTCCCCTGAAAACTAGACAGAAGCATTCTCAGAATCATATTT
         66 TTTCGTTGGAAACAGG-AATATCTTCATATAAAAGCTAGACAGAAGCATTCTCAGAAACTTCTTT

                   **  **    *             **  * *   *
     412575 GTGATGTGCGCCCTCAACTAACAGTGTTGAAGCTTTCTTTTG
        130 GTGATGTTTGCATTCAAGTAACAGTGTTGAACATTCCCTTTC

                     *                    **     *   *                       
     412617 ATAGAGCAGTTTTGAAACACTCTTTTTGTAAAATCTGCAAGAGGATATTTGGATAGCTTTGAGGA
          1 ATAGAGCAGGTTTGAAACACTCTTTTTGTAGTATCTGGAAGTGGATATTTGGATAGCTTTGAGGA

                         * * * *            **                      *    *   
     412682 TTTCGTTGGAAACGGTATTGTCTTCATATAAATTCTAGACAGAAGCATTCTCAGAAGCTTCATTG
         66 TTTCGTTGGAAACAGGAATATCTTCATATAAAAGCTAGACAGAAGCATTCTCAGAAACTTCTTTG

            *       **   *    *             *        
     412747 GGATGTTTCAATTGAAGTCACAGTGTTGAACAGTCCCTTTC
        131 TGATGTTTGCATTCAAGTAACAGTGTTGAACATTCCCTTTC

                                                 * *     *       **     *    
     412788 ATAGAGCAGGTTTGAAACACTCTTTTTGTAGTATCTGTATGTGGACATTTGGAGCGCTTTCAGGC
          1 ATAGAGCAGGTTTGAAACACTCTTTTTGTAGTATCTGGAAGTGGATATTTGGATAGCTTTGAGG-

            * * *    *                 *** *   *                          *  
     412853 CTATGGTT-TAAA-AGGAAATATCTTCCCCTGAAAACTAGACAGAAGCATTCTCAGAAACTTATT
         65 ATTTCGTTGGAAACAGG-AATATCTTCATATAAAAGCTAGACAGAAGCATTCTCAGAAACTTCTT

                    **  **    *                    *   *
     412916 TGTGATGTGCGCCCTCAACTAACAGTGTTGAAGCATT-CTTTTG
        129 TGTGATGTTTGCATTCAAGTAACAGTGTTGAA-CATTCCCTTTC

                                         * *     *             **            
     412959 ATAGAGCA-GTTTGAAACACTCTTTTTGTGGAATCTGCAAGTGGATATTTGTCTAGCTTTGAGGA
          1 ATAGAGCAGGTTTGAAACACTCTTTTTGTAGTATCTGGAAGTGGATATTTGGATAGCTTTGAGGA

                         *   *    *      *            *       *              
     413023 TTTCGTTGGAAACGGGATTA-CAT-ATA-GAAAGC-AGACAGCAGCATTCCCAGAAACTTCTTTG
         66 TTTCGTTGGAAACAGGAATATCTTCATATAAAAGCTAGACAGAAGCATTCTCAGAAACTTCTTTG

                              *    *                 
     413084 TGATGTTTGCATTCAAGTCACAGAGTTGAACATTCCCTTTC
        131 TGATGTTTGCATTCAAGTAACAGTGTTGAACATTCCCTTTC

                                                   *     *       **     *   *
     413125 ATAGAGCAGGTTTGAAACACTCTTTTTGTAGTATCTGGATGTGGACATTTGGAGCGCTTTCAGGC
          1 ATAGAGCAGGTTTGAAACACTCTTTTTGTAGTATCTGGAAGTGGATATTTGGATAGCTTTGAGGA

            * ** *      *             *** *   *                          *   
     413190 CTATGGT-GAAAAAGGAAATATCTTCCCCTGAAAACTAGACAGAAGCATTCTCAGAAACTTATTT
         66 TTTCGTTGGAAACAGG-AATATCTTCATATAAAAGCTAGACAGAAGCATTCTCAGAAACTTCTTT

                   **  **    *             **  * *   *
     413254 GTGATGTGCGCCCTCAAATAACAGTGTTGAAGCTTTCTTTTG
        130 GTGATGTTTGCATTCAAGTAACAGTGTTGAACATTCCCTTTC

                     *                    *      *   *                       
     413296 ATAGAGCAGTTTTGAAACACTCTTTTTGTAATATCTGCAAGAGGATATTTGGATAGCTTTGAGGA
          1 ATAGAGCAGGTTTGAAACACTCTTTTTGTAGTATCTGGAAGTGGATATTTGGATAGCTTTGAGGA

                         *   * *            **                      *    *   
     413361 TTTCGTTGGAAACGGGATTGTCTTCATATAAACTCTAGACAGAAGCATTCTCAGAAGCTTCATTG
         66 TTTCGTTGGAAACAGGAATATCTTCATATAAAAGCTAGACAGAAGCATTCTCAGAAACTTCTTTG

            *       **   *    *             *        
     413426 GGATGTTTCAATTGAAGTCACAGTGTTGAACAGTCCCTTTC
        131 TGATGTTTGCATTCAAGTAACAGTGTTGAACATTCCCTTTC

                                                         *       *  * * *    
     413467 ATAGAGCAGGTTTGAAACACTCTTTTTGTAGTATCTGGAAGTGGACATTTGGAGAGATCTCAGGA
          1 ATAGAGCAGGTTTGAAACACTCTTTTTGTAGTATCTGGAAGTGGATATTTGGATAGCTTTGAGGA

            * * **      *             **            *       * *          *  *
     413532 CTACAGT-GAAAAAGGAAATATCTTCCAATAAAAGCTAGATAGAAGCAATGTCAGAAACTTTTTC
         66 TTTCGTTGGAAACAGG-AATATCTTCATATAAAAGCTAGACAGAAGCATTCTCAGAAACTTCTTT

            *      * ** *   **      *       *  * *   *
     413596 ATGATGTATCTACTCAGCTAACAGGGTTGAACCTTTCTTTTG
        130 GTGATGTTTGCATTCAAGTAACAGTGTTGAACATTCCCTTTC

             *       *                   * *     *             **            
     413638 AGAGAGCAGTTTTGAAACACTCTTTTTGTGGAATCTGCAAGTGGATATTTGTCTAGCTTTGAGGA
          1 ATAGAGCAGGTTTGAAACACTCTTTTTGTAGTATCTGGAAGTGGATATTTGGATAGCTTTGAGGA

                         *   *    *                   *       *              
     413703 TTTCGTTGGAAACGGGATTA-CAT-ATA-AAAAGC-AGACAGCAGCATTCCCAGAAACTTCTTTG
         66 TTTCGTTGGAAACAGGAATATCTTCATATAAAAGCTAGACAGAAGCATTCTCAGAAACTTCTTTG

                              *    *                 
     413764 TGATGTTTGCATTCAAGTCACAGAGTTGAACATTCCCTTTC
        131 TGATGTTTGCATTCAAGTAACAGTGTTGAACATTCCCTTTC

                                                   *     *       **     *   *
     413805 ATAGAGCAGGTTTGAAACACTCTTTTTGTAGTATCTGGATGTGGACATTTGGAGCGCTTTCAGGC
          1 ATAGAGCAGGTTTGAAACACTCTTTTTGTAGTATCTGGAAGTGGATATTTGGATAGCTTTGAGGA

            * ** *      *             *** *   *                          *   
     413870 CTATGGT-GAAAAAGGAAATATCTTCCCCTGAAAACTAGACAGAAGCATTCTCAGAAACTTATTT
         66 TTTCGTTGGAAACAGG-AATATCTTCATATAAAAGCTAGACAGAAGCATTCTCAGAAACTTCTTT

                   **  **    *              *  * *   *
     413934 GTGATGTGCGCCCTCAACTAACAGTGTTGAACCTTTCTTTTG
        130 GTGATGTTTGCATTCAAGTAACAGTGTTGAACATTCCCTTTC

                     *                    *      *   *                       
     413976 ATAGAGCAGTTTTGAAACACTCTTTTTGTAATATCTGCAAGAGGATATTTGGATAGCTTTGAGGA
          1 ATAGAGCAGGTTTGAAACACTCTTTTTGTAGTATCTGGAAGTGGATATTTGGATAGCTTTGAGGA

                         * * * *       *    **          *           *    *   
     414041 TTTCGTTGGAAACGGTATTGTCTTCATGTAAACTCTAGACAGAAACATTCTCAGAAGCTTCATTG
         66 TTTCGTTGGAAACAGGAATATCTTCATATAAAAGCTAGACAGAAGCATTCTCAGAAACTTCTTTG

            *       **   *    *             * *      
     414106 GGATGTTTCAATTGAAGTCACAGTGTTGAACAGTTCCTTTC
        131 TGATGTTTGCATTCAAGTAACAGTGTTGAACATTCCCTTTC

                 *       *                               *       **   * *    
     414147 ATAGAACAGGTTTCAAACACTCTTTTTGTAGTATCTGGAAGTGGACATTTGGAGCGCTCTCAGGA
          1 ATAGAGCAGGTTTGAAACACTCTTTTTGTAGTATCTGGAAGTGGATATTTGGATAGCTTTGAGGA

            * *  *      *             **          * *       * *          *  *
     414212 CTACGGT-GAAAAAGGAAATATCTTCCAATAAAAGCTACATAGAAGCAATGTCAGAAACTTTTTC
         66 TTTCGTTGGAAACAGG-AATATCTTCATATAAAAGCTAGACAGAAGCATTCTCAGAAACTTCTTT

            *      * ** *   **      *      **  *     *
     414276 ATGATGTATCTACTCAGCTAACAGAGTTGAAACTTTCCTTTG
        130 GTGATGTTTGCATTCAAGTAACAGTGTTGAACATTCCCTTTC

             *       *                   * *     *             **            
     414318 AGAGAGCAGTTTTGAAACACTCTTTTTGTGGAATCTGCAAGTGGATATTTGTCTAGCTTTGAGGA
          1 ATAGAGCAGGTTTGAAACACTCTTTTTGTAGTATCTGGAAGTGGATATTTGGATAGCTTTGAGGA

                         *   *    *                   *       *   *     *   *
     414383 TTTCGTTGGAAACGGGATTA-CAT-ATA-AAAAGC-AGACAGCAGCATTCCCAGTAACTTTTTTC
         66 TTTCGTTGGAAACAGGAATATCTTCATATAAAAGCTAGACAGAAGCATTCTCAGAAACTTCTTTG

                              *    *       *         
     414444 TGATGTTTGCATTCAAGTCACAGAGTTGAACGTTCCCTTTC
        131 TGATGTTTGCATTCAAGTAACAGTGTTGAACATTCCCTTTC

                                        *          *     *       **     *   *
     414485 ATAGAGCAGGTTTGAAACACTCTTTTTGAAGTATCTGGATGTGGACATTTGGAGCGCTTTCAGGC
          1 ATAGAGCAGGTTTGAAACACTCTTTTTGTAGTATCTGGAAGTGGATATTTGGATAGCTTTGAGGA

            * ** *      *             *** *   *                          *   
     414550 CTATGGT-GAAAAAGGAAATATCTTCCCCTGAAAACTAGACAGAAGCATTCTCAGAAACTTATTT
         66 TTTCGTTGGAAACAGG-AATATCTTCATATAAAAGCTAGACAGAAGCATTCTCAGAAACTTCTTT

                   **  **    *              *  * *   *
     414614 GTGATGTGCGCCCTCAACTAACAGTGTTGAACCTTTCTTTTG
        130 GTGATGTTTGCATTCAAGTAACAGTGTTGAACATTCCCTTTC

                     *                    *      *   *                       
     414656 ATAGAGCAGTTTTGAAACACTCTTTTTGTAATATCTGCAAGAGGATATTTGGATAGCTTTGAGGA
          1 ATAGAGCAGGTTTGAAACACTCTTTTTGTAGTATCTGGAAGTGGATATTTGGATAGCTTTGAGGA

                         *   * *            **                      *    *   
     414721 TTTCGTTGGAAACGGGATTGTCTTCATATAAACTCTAGACAGAAGCATTCTCAGAAGCTTCATTG
         66 TTTCGTTGGAAACAGGAATATCTTCATATAAAAGCTAGACAGAAGCATTCTCAGAAACTTCTTTG

            *       **   *    *             * *      
     414786 GGATGTTTCAATTGAAGTCACAGTGTTGAACAGTTCCTTTC
        131 TGATGTTTGCATTCAAGTAACAGTGTTGAACATTCCCTTTC

                 *                                       *       **   * *    
     414827 ATAGAACAGGTTTGAAACACTCTTTTTGTAGTATCTGGAAGTGGACATTTGGAGGGCTCTCAGGA
          1 ATAGAGCAGGTTTGAAACACTCTTTTTGTAGTATCTGGAAGTGGATATTTGGATAGCTTTGAGGA

            * ** *  *   ****         **          * *       * *          *  **
     414892 CTATGGTGAAAAATTAAATATCTTCCAATAAAAGCTACATAGAAGCAATGTCAGAAACTTTTTCA
         66 TTTCGTTGGAAACAGGAATATCTTCATATAAAAGCTAGACAGAAGCATTCTCAGAAACTTCTTTG

                  * ** *   **      * *     *  *     *
     414957 TGATGTATCTACTCAGCTAACAGAGGTGAACCTTTCCTTTG
        131 TGATGTTTGCATTCAAGTAACAGTGTTGAACATTCCCTTTC

             *       *                   * *     *             **            
     414998 AGAGAGCAGTTTTGAAACACTCTTTTTGTGGAATCTGCAAGTGGATATTTGTCTAGCTTTGAGGA
          1 ATAGAGCAGGTTTGAAACACTCTTTTTGTAGTATCTGGAAGTGGATATTTGGATAGCTTTGAGGA

                         *   *    *                   *       *   *          
     415063 TTTCGTTGGAAACGGGATTA-CAT-ATA-AAAAGC-AGACAGCAGCATTCCCAGTAACTTCTTTG
         66 TTTCGTTGGAAACAGGAATATCTTCATATAAAAGCTAGACAGAAGCATTCTCAGAAACTTCTTTG

                              *    *                 
     415124 TGATGTTTGCATTCAAGTCACAGAGTTGAACATTCCCTTTC
        131 TGATGTTTGCATTCAAGTAACAGTGTTGAACATTCCCTTTC

                                                   *     *       **     *   *
     415165 ATAGAGCAGGTTTGAAACACTCTTTTTGTAGTATCTGGATGTGGACATTTGGAGCGCTTTCAGGC
          1 ATAGAGCAGGTTTGAAACACTCTTTTTGTAGTATCTGGAAGTGGATATTTGGATAGCTTTGAGGA

            * ** *      *             *** *   *           *          *   *   
     415230 CTATGGT-GAAAAAGGAAATATCTTCCCCTGAAAACTAGACAGAAGAATTCTCAGAATCTTATTT
         66 TTTCGTTGGAAACAGG-AATATCTTCATATAAAAGCTAGACAGAAGCATTCTCAGAAACTTCTTT

                   **  **    *             **  * *   *
     415294 GTGATGTGCGCCCTCAACTAACAGTGTTGAAGCTTTCTTTTG
        130 GTGATGTTTGCATTCAAGTAACAGTGTTGAACATTCCCTTTC

                     *                    **     *   *                       
     415336 ATAGAGCAGTTTTGAAACACTCTTTTTGTAAAATCTGCAAGAGGATATTTGGATAGCTTTGAGGA
          1 ATAGAGCAGGTTTGAAACACTCTTTTTGTAGTATCTGGAAGTGGATATTTGGATAGCTTTGAGGA

                         *   * *            **   **                 * *  *   
     415401 TTTCGTTGGAAACGGGATTGTCTTCATATAAACTCTACGCAGAAGCATTCTCAGAAGCGTCATTG
         66 TTTCGTTGGAAACAGGAATATCTTCATATAAAAGCTAGACAGAAGCATTCTCAGAAACTTCTTTG

            *   *   **   *    *             *        
     415466 GGATATTTCAATTGAAGTCACAGTGTTGAACAGTCCCTTTC
        131 TGATGTTTGCATTCAAGTAACAGTGTTGAACATTCCCTTTC

                                                   *     *       **     *    
     415507 ATAGAGCAGGTTTGAAACACTCTTTTTGTAGTATCTGGATGTGGACATTTGGAGCGCTTTCAGGC
          1 ATAGAGCAGGTTTGAAACACTCTTTTTGTAGTATCTGGAAGTGGATATTTGGATAGCTTTGAGG-

            * * *     *  *             *** *   *                          *  
     415572 CTATGGTT--TAATAGGAAATATCTTCCCCTGAAAACTAGACAGAAGCATTCTCAGAAACTTATT
         65 ATTTCGTTGGAAACAGG-AATATCTTCATATAAAAGCTAGACAGAAGCATTCTCAGAAACTTCTT

                    **  **    *                    *   *
     415635 TGTGATGTGCGCCCTCAACTAACAGTGTTGAAGCATT-CTTTTG
        129 TGTGATGTTTGCATTCAAGTAACAGTGTTGAA-CATTCCCTTTC

                     *                   * *     *        *    **            
     415678 ATAGAGCAGTTTTGAAACACTCTTTTTGTGGAATCTGCAAGTGGATGTTTGTCTAGCTTTGAGGA
          1 ATAGAGCAGGTTTGAAACACTCTTTTTGTAGTATCTGGAAGTGGATATTTGGATAGCTTTGAGGA

                         *   *    *                   *       *              
     415743 TTTCGTTGGAAACGGGATTA-CAT-ATA-AAAAGC-AGACAGCAGCATTCCCAGAAACTTCTTTG
         66 TTTCGTTGGAAACAGGAATATCTTCATATAAAAGCTAGACAGAAGCATTCTCAGAAACTTCTTTG

             *                *    *                 
     415804 TAATGTTTGCATTCAAGTCACAGAGTTGAACATTCCCTTTC
        131 TGATGTTTGCATTCAAGTAACAGTGTTGAACATTCCCTTTC

                                                   *     *       ***    *   *
     415845 ATAGAGCAGGTTTGAAACACTCTTTTTGTAGTATCTGGATGTGGACATTTGGAGCCCTTTCAGGC
          1 ATAGAGCAGGTTTGAAACACTCTTTTTGTAGTATCTGGAAGTGGATATTTGGATAGCTTTGAGGA

            * ** *      *             *** *   *                      *   *   
     415910 CTATGGT-GAAAAAGGAAATATCTTCCCCTGAAAACTAGACAGAAGCATTCTCAGAATCTTATTT
         66 TTTCGTTGGAAACAGG-AATATCTTCATATAAAAGCTAGACAGAAGCATTCTCAGAAACTTCTTT

                   **  **    *             **  * *   *
     415974 GTGATGTGCGCCCTCAACTAACAGTGTTGAAGCTTTCTTTTG
        130 GTGATGTTTGCATTCAAGTAACAGTGTTGAACATTCCCTTTC

                     *                    **     *   *                       
     416016 ATAGAGCAGTTTTGAAACACTCTTTTTGTAAAATCTGCAAGAGGATATTTGGATAGCTTTGAGGA
          1 ATAGAGCAGGTTTGAAACACTCTTTTTGTAGTATCTGGAAGTGGATATTTGGATAGCTTTGAGGA

                         *   * *            **                      *    *   
     416081 TTTCGTTGGAAACGGGATTGTCTTCATATAAACTCTAGACAGAAGCATTCTCAGAAGCTTCATTG
         66 TTTCGTTGGAAACAGGAATATCTTCATATAAAAGCTAGACAGAAGCATTCTCAGAAACTTCTTTG

            *       **   *    *             *        
     416146 GGATGTTTCAATTGAAGTCACAGTGTTGAACAGTCCCTTTC
        131 TGATGTTTGCATTCAAGTAACAGTGTTGAACATTCCCTTTC

                                                         *          * * *    
     416187 ATAGAGCAGGTTTGAAACACTCTTTTTGTAGTATCTGGAAGTGGACATTTGGATAGATCTCAGGA
          1 ATAGAGCAGGTTTGAAACACTCTTTTTGTAGTATCTGGAAGTGGATATTTGGATAGCTTTGAGGA

            * *  *    * *             **            *       * *          *  *
     416252 ATACGGT-GATAAAGGAAATATCTTCCAATAAAAGCTAGATAGAAGCAATGTCAGAAACTTTTTC
         66 TTTCGTTGGAAACAGG-AATATCTTCATATAAAAGCTAGACAGAAGCATTCTCAGAAACTTCTTT

            *      * ** *   **      *       *  * *   *
     416316 ATGATGTATCTACTCAGCTAACAGAGTTGAACCTTTCTTTTG
        130 GTGATGTTTGCATTCAAGTAACAGTGTTGAACATTCCCTTTC

             *       *                   * *     *             **            
     416358 AGAGAGCAGTTTTGAAACACTCTTTTTGTGGAATCTGCAAGTGGATATTTGTCTAGCTTTGAGGA
          1 ATAGAGCAGGTTTGAAACACTCTTTTTGTAGTATCTGGAAGTGGATATTTGGATAGCTTTGAGGA

                         *   *    *                   *       *   *          
     416423 TTTCGTTGGAAACGGGATTA-CAT-ATA-AAAAGC-AGACAGCAGCATTCCCAGTAACTTCTTTG
         66 TTTCGTTGGAAACAGGAATATCTTCATATAAAAGCTAGACAGAAGCATTCTCAGAAACTTCTTTG

                              *    *                 
     416484 TGATGTTTGCATTCAAGTCACAGAGTTGAACATTCCCTTTC
        131 TGATGTTTGCATTCAAGTAACAGTGTTGAACATTCCCTTTC

                                                   *     *       **     *   *
     416525 ATAGAGCAGGTTTGAAACACTCTTTTTGTAGTATCTGGATGTGGACATTTGGAGCGCTTTCAGGC
          1 ATAGAGCAGGTTTGAAACACTCTTTTTGTAGTATCTGGAAGTGGATATTTGGATAGCTTTGAGGA

            ****  *     *             *** *   *     *     *          *   *   
     416590 CAATG-GGGAAAAAGGAAATATCTTCCCCTGAAAACTAGAGAGAAGAATTCTCAGAATCTTATTT
         66 TTTCGTTGGAAACAGG-AATATCTTCATATAAAAGCTAGACAGAAGCATTCTCAGAAACTTCTTT

                   **  **    *             **  * *   *
     416654 GTGATGTGCGCCCTCAACTAACAGTGTTGAAGCTTTCTTTTG
        130 GTGATGTTTGCATTCAAGTAACAGTGTTGAACATTCCCTTTC

                     *                    **     *   *                       
     416696 ATAGAGCAGTTTTGAAACACTCTTTTTGTAAAATCTGCAAGAGGATATTTGGATAGCTTTGAGGA
          1 ATAGAGCAGGTTTGAAACACTCTTTTTGTAGTATCTGGAAGTGGATATTTGGATAGCTTTGAGGA

                         *   * *        *   **      *               * *  *   
     416761 TTTCGTTGGAAACGGGATTGTCTTCATACAAATTCTAGACCGAAGCATTCTCAGAAGCGTCATTG
         66 TTTCGTTGGAAACAGGAATATCTTCATATAAAAGCTAGACAGAAGCATTCTCAGAAACTTCTTTG

            *       **   *    *             *        
     416826 GGATGTTTCAATTGAAGTCACAGTGTTGAACAGTCCCTTTC
        131 TGATGTTTGCATTCAAGTAACAGTGTTGAACATTCCCTTTC

                                                   *     *       **     *    
     416867 ATAGAGCAGGTTTGAAACACTCTTTTTGTAGTATCTGGATGTGGACATTTGGAGCGCTTTCAGGC
          1 ATAGAGCAGGTTTGAAACACTCTTTTTGTAGTATCTGGAAGTGGATATTTGGATAGCTTTGAGG-

            * * *    *             *   *** *   *                          *  
     416932 CTATGGTT-TAAA-AGGAAATATTTTCCCCTGAAAACTAGACAGAAGCATTCTCAGAAACTTATT
         65 ATTTCGTTGGAAACAGG-AATATCTTCATATAAAAGCTAGACAGAAGCATTCTCAGAAACTTCTT

                    **  **    *                   **   *
     416995 TGTGATGTGCGCCCTCAACTAACAGTGTTGAAGCATT-GTTTTG
        129 TGTGATGTTTGCATTCAAGTAACAGTGTTGAA-CATTCCCTTTC

                     *                   * *                   **          * 
     417038 ATAGAGCAGTTTTGAAACACTCTTTTTGTGGAATCTGGAAGTGGATATTTGTCTAGCTTTGAGAA
          1 ATAGAGCAGGTTTGAAACACTCTTTTTGTAGTATCTGGAAGTGGATATTTGGATAGCTTTGAGGA

                   *     *   *    *                   *       *         *    
     417103 TTTCGTTTGAAACGGGATTA-CAT-ATA-AAAAGC-AGACAGCAGCATTCCCAGAAACTTGTTTG
         66 TTTCGTTGGAAACAGGAATATCTTCATATAAAAGCTAGACAGAAGCATTCTCAGAAACTTCTTTG

                              *    *                 
     417164 TGATGTTTGCATTCAAGTCACAGAGTTGAACATTCCCTTTC
        131 TGATGTTTGCATTCAAGTAACAGTGTTGAACATTCCCTTTC

             *                         *           *     *       **     *   *
     417205 AGAGAGCAGGTTTGAAACACTCTTTTTATAGTATCTGGATGTGGACATTTGGAGCGCTTTCAGGC
          1 ATAGAGCAGGTTTGAAACACTCTTTTTGTAGTATCTGGAAGTGGATATTTGGATAGCTTTGAGGA

            * ** *  *   ** *         *** *   *          *               *    
     417270 CTATGGTGAAAAATGAAATATCTTCTCCTGAAAACTAGACAGAATCATTCTCAGAAACTTATTTG
         66 TTTCGTTGGAAACAGGAATATCTTCATATAAAAGCTAGACAGAAGCATTCTCAGAAACTTCTTTG

                  **  **    *              *  * *   *
     417335 TGATGTGCGCCCTCAACTAACAGTGTTGAACCTTTCTTTTG
        131 TGATGTTTGCATTCAAGTAACAGTGTTGAACATTCCCTTTC

                     *                    *   *  *  **                  *    
     417376 ATAGAGCAGTTTTGAAACACTCTTTTTGTAATATTTGCAAAAGGATATTTGGATAGCTTTAAGGA
          1 ATAGAGCAGGTTTGAAACACTCTTTTTGTAGTATCTGGAAGTGGATATTTGGATAGCTTTGAGGA

                         *   * *            **                      *    *   
     417441 TTTCGTTGGAAACGGGATTGTCTTCATATAAACTCTAGACAGAAGCATTCTCAGAAGCTTCATTG
         66 TTTCGTTGGAAACAGGAATATCTTCATATAAAAGCTAGACAGAAGCATTCTCAGAAACTTCTTTG

            *       **   *    *             *        
     417506 GGATGTTTCAATTGAAGTCACAGTGTTGAACAGTCCCTTTC
        131 TGATGTTTGCATTCAAGTAACAGTGTTGAACATTCCCTTTC

                                                   *     *    *  **          
     417547 ATAGAGCAGGTTTGAAACACTCTTTTTGTAGTATCTGGATGTGGACATTTAGAGCG-TTTGCAGG
          1 ATAGAGCAGGTTTGAAACACTCTTTTTGTAGTATCTGGAAGTGGATATTTGGATAGCTTTG-AGG

             * * *    *                 *** *   *                          * 
     417611 CCTATGGTT-TAAA-AGGAAATATCTTCCCCTGAAAACTAGACAGAAGCATTCTCAGAAACTTAT
         65 -ATTTCGTTGGAAACAGG-AATATCTTCATATAAAAGCTAGACAGAAGCATTCTCAGAAACTTCT

                     **  **    *                    *   *
     417674 TTGTGATGTGCGCCCTCAACTAACAGTGTTGAAGCATT-CTTTTG
        128 TTGTGATGTTTGCATTCAAGTAACAGTGTTGAA-CATTCCCTTTC

                     *       *           * *     *             **            
     417718 ATAGAGCAGTTTTGAAAAACTCTTTTTGTGGAATCTGCAAGTGGATATTTGTCTAGCTTTGAGGA
          1 ATAGAGCAGGTTTGAAACACTCTTTTTGTAGTATCTGGAAGTGGATATTTGGATAGCTTTGAGGA

                         *   *    *                   *       *              
     417783 TTTCGTTGGAAACGGGATTA-CAT-ATA-AAAAGC-AGACAGCAGCATTCCCAGAAACTTCTTTG
         66 TTTCGTTGGAAACAGGAATATCTTCATATAAAAGCTAGACAGAAGCATTCTCAGAAACTTCTTTG

                              *    *                 
     417844 TGATGTTTGCATTCAAGTCACAGAGTTGAACATTCCCTTTC
        131 TGATGTTTGCATTCAAGTAACAGTGTTGAACATTCCCTTTC

                                                   *     **      **     *   *
     417885 ATAGAGCAGGTTTGAAACACTCTTTTTGTAGTATCTGGATGTGGACCTTTGGAGCGCTTTCAGGC
          1 ATAGAGCAGGTTTGAAACACTCTTTTTGTAGTATCTGGAAGTGGATATTTGGATAGCTTTGAGGA

            * ** *      *             *** *   *                      *   *   
     417950 CTATGGT-GAAAAAGGAAATATCTTCCCCTGAAAACTAGACAGAAGCATTCTCAGAATCTTATTT
         66 TTTCGTTGGAAACAGG-AATATCTTCATATAAAAGCTAGACAGAAGCATTCTCAGAAACTTCTTT

                   **  **    *             **  * *   *
     418014 GTGATGTGCGCCCTCAACTAACAGTGTTGAAGCTTTCTTTTG
        130 GTGATGTTTGCATTCAAGTAACAGTGTTGAACATTCCCTTTC

                     *                    **     *   *                       
     418056 ATAGAGCAGTTTTGAAACACTCTTTTTGTAAAATCTGCAAGAGGATATTTGGATAGCTTTGAGGA
          1 ATAGAGCAGGTTTGAAACACTCTTTTTGTAGTATCTGGAAGTGGATATTTGGATAGCTTTGAGGA

                         *   * *            **                      *    *   
     418121 TTTCGTTGGAAACGGGATTGTCTTCATATAAACTCTAGACAGAAGCATTCTCAGAAGCTTCATTG
         66 TTTCGTTGGAAACAGGAATATCTTCATATAAAAGCTAGACAGAAGCATTCTCAGAAACTTCTTTG

            *       **   *    *             *        
     418186 GGATGTTTCAATTGAAGTCACAGTGTTGAACAGTCCCTTTC
        131 TGATGTTTGCATTCAAGTAACAGTGTTGAACATTCCCTTTC

                                                         *       **   * *    
     418227 ATAGAGCAGGTTTGAAACACTCTTTTTGTAGTATCTGGAAGTGGACATTTGGAGCGCTCTCAGGA
          1 ATAGAGCAGGTTTGAAACACTCTTTTTGTAGTATCTGGAAGTGGATATTTGGATAGCTTTGAGGA

            * *  *      *             **            *       * *          *  *
     418292 CTGCGGT-GAAAAAGGAAATATCTTCCAATAAAAGCTAGATAGAAGCAATGTCAGAAACTTTTTC
         66 TTTCGTTGGAAACAGG-AATATCTTCATATAAAAGCTAGACAGAAGCATTCTCAGAAACTTCTTT

            *      * ** *   **      *       *        *
     418356 ATGATGTATCTACTCAGCTAACAGAGTTGAACCTT-CCTTTG
        130 GTGATGTTTGCATTCAAGTAACAGTGTTGAACATTCCCTTTC

             *       *                   * *     *             **            
     418397 AGAGAGCAGTTTTGAAACACTCTTTTTGTGGAATCTGCAAGTGGATATTTGTCTAGCTTTGAGGA
          1 ATAGAGCAGGTTTGAAACACTCTTTTTGTAGTATCTGGAAGTGGATATTTGGATAGCTTTGAGGA

                         *   *    *                   *       *              
     418462 TTTCGTTGGAAACGGGATTA-CAT-ATA-AAAAGC-AGACAGCAGCATTCCCAGAAACTTCTTTG
         66 TTTCGTTGGAAACAGGAATATCTTCATATAAAAGCTAGACAGAAGCATTCTCAGAAACTTCTTTG

                              *    *                 
     418523 TGATGTTTGCATTCAAGTCACAGAGTTGAACATTCCCTTTC
        131 TGATGTTTGCATTCAAGTAACAGTGTTGAACATTCCCTTTC

                                 *                 *     *     * **     *   *
     418564 ATAGAGCAGGTTTGAAACACTGTTTTTGTAGTATCTGGATGTGGACATTTGCAGCGCTTTCAGGC
          1 ATAGAGCAGGTTTGAAACACTCTTTTTGTAGTATCTGGAAGTGGATATTTGGATAGCTTTGAGGA

            * ** *      *             *** *   *                          *   
     418629 CTAAGGT-GAAAAAGGAAATATCTTCCCCTGAAAACTAGACAGAAGCATTCTCAGAAACTTATTT
         66 TTTCGTTGGAAACAGG-AATATCTTCATATAAAAGCTAGACAGAAGCATTCTCAGAAACTTCTTT

                   **  **    *             **  * *   *
     418693 GTGATGTGCGCCCTCAACTAACAGTGTTGAAGCTTTCTTTTG
        130 GTGATGTTTGCATTCAAGTAACAGTGTTGAACATTCCCTTTC

                     *                   * *     *             **            
     418735 ATAGAGCAGTTTTGAAACACTCTTTTTGTGGAATCTGCAAGTGGATATTTGTCTAGCTTTGAGGA
          1 ATAGAGCAGGTTTGAAACACTCTTTTTGTAGTATCTGGAAGTGGATATTTGGATAGCTTTGAGGA

                         *   *    *                   *       *     *   *    
     418800 TTTCGTTGGAAACGGGATTA-CAT-ATA-AAAAGC-AGACAGCAGCATTCCCAGAATCTTGTTTG
         66 TTTCGTTGGAAACAGGAATATCTTCATATAAAAGCTAGACAGAAGCATTCTCAGAAACTTCTTTG

                              *    *                 
     418861 TGATGTTTGCATTCAAGTCACAGAGTTGAACATTCCCTTTC
        131 TGATGTTTGCATTCAAGTAACAGTGTTGAACATTCCCTTTC

             *                                           *    *  *  * * *    
     418902 AGAGAGCAGGTTTGAAACACTCTTTTTGTAGTATCTGGAAGTGGACATTTTGAGAGATCTCAGGA
          1 ATAGAGCAGGTTTGAAACACTCTTTTTGTAGTATCTGGAAGTGGATATTTGGATAGCTTTGAGGA

            * *  *    * *             **            *       * *          *  *
     418967 ATACGGT-GATAAAGGAAATATCTTCCAATAAAAGCTAGATAGAAGCAATGTCAGAAACTTTTTC
         66 TTTCGTTGGAAACAGG-AATATCTTCATATAAAAGCTAGACAGAAGCATTCTCAGAAACTTCTTT

            *      * ** *   **      *       *  * *   *
     419031 ATGATGTATCTACTCAGCTAACAGAGTTGAACCTTTCTTTTG
        130 GTGATGTTTGCATTCAAGTAACAGTGTTGAACATTCCCTTTC

             *       *                   * *     *             **            
     419073 AGAGAGCAGTTTTGAAACACTCTTTTTGTGGAATCTGCAAGTGGATATTTGTCTAGCTTTGAGGA
          1 ATAGAGCAGGTTTGAAACACTCTTTTTGTAGTATCTGGAAGTGGATATTTGGATAGCTTTGAGGA

                         *   *    *                   *       *              
     419138 TTTCGTTGGAAACGGGATTA-CAT-ATA-AAAAGC-AGACAGCAGCATTCCCAGAAACTTCTTTG
         66 TTTCGTTGGAAACAGGAATATCTTCATATAAAAGCTAGACAGAAGCATTCTCAGAAACTTCTTTG

                              *    *                 
     419199 TGATGTTTGCATTCAAGTCACAGAGTTGAACATTCCCTTTC
        131 TGATGTTTGCATTCAAGTAACAGTGTTGAACATTCCCTTTC

                                                   *     *       **     *   *
     419240 ATAGAGCAGGTTTGAAACACTCTTTTTGTAGTATCTGGATGTGGACATTTGGAGCGCTTTCAGGC
          1 ATAGAGCAGGTTTGAAACACTCTTTTTGTAGTATCTGGAAGTGGATATTTGGATAGCTTTGAGGA

            * ** *      *             *** *   *                          *   
     419305 CTATGGT-GAAAAAGGAAATATCTTCCCCTGAAAACTAGACAGAAGCATTCTCAGAAACTTATTT
         66 TTTCGTTGGAAACAGG-AATATCTTCATATAAAAGCTAGACAGAAGCATTCTCAGAAACTTCTTT

             *     **  **    *              *   *    *
     419369 GCGATGTGCGCCCTCAACTAACAGTGTTGAACCTT-TCTTTT
        130 GTGATGTTTGCATTCAAGTAACAGTGTTGAACATTCCCTTTC

               *     *                    *      *   *                       
     419410 ATACAGCAGTTTTGAAACACTCTTTTTGTAATATCTGCAAGAGGATATTTGGATAGCTTTGAGGA
          1 ATAGAGCAGGTTTGAAACACTCTTTTTGTAGTATCTGGAAGTGGATATTTGGATAGCTTTGAGGA

                         *   * *       *    **          *           *    *   
     419475 TTTCGTTGGAAACGGGATTGTCTTCATGTAAACTCTAGACAGAAACATTCTCAGAAGCTTCATTG
         66 TTTCGTTGGAAACAGGAATATCTTCATATAAAAGCTAGACAGAAGCATTCTCAGAAACTTCTTTG

            *       **   *    *             * *      
     419540 GGATGTTTCAATTGAAGTCACAGTGTTGAACAGTTCCTTTC
        131 TGATGTTTGCATTCAAGTAACAGTGTTGAACATTCCCTTTC

                 *                                       *       **   * *    
     419581 ATAGAACAGGTTTGAAACACTCTTTTTGTAGTATCTGGAAGTGGACATTTGGAGCGCTCTCAGGA
          1 ATAGAGCAGGTTTGAAACACTCTTTTTGTAGTATCTGGAAGTGGATATTTGGATAGCTTTGAGGA

            * *  *      *             **          * *       * *          *  *
     419646 CTACGGT-GAAAAAGGAAATATCTTCCAATAAAAGCTACATAGAAGCAATGTCAGAAACTTTTTC
         66 TTTCGTTGGAAACAGG-AATATCTTCATATAAAAGCTAGACAGAAGCATTCTCAGAAACTTCTTT

            *      * ** *   **      *       *  *     *
     419710 ATGATGTATCTACTCAGCTAACAGAGTTGAACCTTTCCTTTG
        130 GTGATGTTTGCATTCAAGTAACAGTGTTGAACATTCCCTTTC

             *       *                   * *     *             **            
     419752 AGAGAGCAGTTTTGAAACACTCTTTTTGTGGAATCTGCAAGTGGATATTTGTCTAGCTTTGAGGA
          1 ATAGAGCAGGTTTGAAACACTCTTTTTGTAGTATCTGGAAGTGGATATTTGGATAGCTTTGAGGA

                         *   *    *                   *       *   *         *
     419817 TTTCGTTGGAAACGGGATTA-CAT-ATA-AAAAGC-AGACAGCAGCATTCCCAGTAACTTCTTTC
         66 TTTCGTTGGAAACAGGAATATCTTCATATAAAAGCTAGACAGAAGCATTCTCAGAAACTTCTTTG

                              *    *      *          
     419878 TGATGTTTGCATTCAAGTCACAGAGTTGAAAATTCCCTTTC
        131 TGATGTTTGCATTCAAGTAACAGTGTTGAACATTCCCTTTC

                                                   *     *     * **     *   *
     419919 ATAGAGCAGGTTTGAAACACTCTTTTTGTAGTATCTGGATGTGGACATTTGCAGCGCTTTCAGGC
          1 ATAGAGCAGGTTTGAAACACTCTTTTTGTAGTATCTGGAAGTGGATATTTGGATAGCTTTGAGGA

            * **        *             *** *   *                          *   
     419984 CTAAGTT-GAAAAAGGAAATATCTTCCCCTGAAAACTAGACAGAAGCATTCTCAGAAACTTATTT
         66 TTTCGTTGGAAACAGG-AATATCTTCATATAAAAGCTAGACAGAAGCATTCTCAGAAACTTCTTT

                   **  **    *             **  * *   *
     420048 GTGATGTGCGCCCTCAACTAACAGTGTTGAAGCTTTCTTTTG
        130 GTGATGTTTGCATTCAAGTAACAGTGTTGAACATTCCCTTTC

                     *                   * *     *             **          * 
     420090 ATAGAGCAGTTTTGAAACACTCTTTTTGTGGAATCTGCAAGTGGATATTTGTCTAGCTTTGAGAA
          1 ATAGAGCAGGTTTGAAACACTCTTTTTGTAGTATCTGGAAGTGGATATTTGGATAGCTTTGAGGA

                         *   *    *                   *       *     *   *    
     420155 TTTCGTTGGAAACGGGATTA-CAT-ATA-AAAAGC-AGACAGCAGCATTCCCAGAATCTTGTTTG
         66 TTTCGTTGGAAACAGGAATATCTTCATATAAAAGCTAGACAGAAGCATTCTCAGAAACTTCTTTG

                              *    *                 
     420216 TGATGTTTGCATTCAAGTCACAGAGTTGAACATTCCCTTTC
        131 TGATGTTTGCATTCAAGTAACAGTGTTGAACATTCCCTTTC

             *                         *           *     *       **     *   *
     420257 AGAGAGCAGGTTTGAAACACTCTTTTTATAGTATCTGGATGTGGACATTTGGAGCGCTTTCAGGC
          1 ATAGAGCAGGTTTGAAACACTCTTTTTGTAGTATCTGGAAGTGGATATTTGGATAGCTTTGAGGA

            * ** *      *             *** *   *                      *   *   
     420322 CTATGGT-GAAAAAGGAAATATCTTCTCCTGAAAACTAGACAGAAGCATTCTCAGAATCTTATTT
         66 TTTCGTTGGAAACAGG-AATATCTTCATATAAAAGCTAGACAGAAGCATTCTCAGAAACTTCTTT

                   **  **    *             **  * *   *
     420386 GTGATGTGCGCCCTCAACTAACAGTGTTGAAGCTTTCTTTTG
        130 GTGATGTTTGCATTCAAGTAACAGTGTTGAACATTCCCTTTC

                     *                    *      *   *                       
     420428 ATAGAGCAGTTTTGAAACACTCTTTTTGTAATATCTGCAAGAGGATATTTGGATAGCTTTGAGGA
          1 ATAGAGCAGGTTTGAAACACTCTTTTTGTAGTATCTGGAAGTGGATATTTGGATAGCTTTGAGGA

                         *   * *            **                      *    *   
     420493 TTTCGTTGGAAACGGGATTGTCTTCATATAAACTCTAGACAGAAGCATTCTCAGAAGCTTCATTG
         66 TTTCGTTGGAAACAGGAATATCTTCATATAAAAGCTAGACAGAAGCATTCTCAGAAACTTCTTTG

            *       **   *    *             * *      
     420558 GGATGTTTCAATTGAAGTCACAGTGTTGAACAGTTCCTTTC
        131 TGATGTTTGCATTCAAGTAACAGTGTTGAACATTCCCTTTC

                 *                                       *       **   * *    
     420599 ATAGAACAGGTTTGAAACACTCTTTTTGTAGTATCTGGAAGTGGACATTTGGAGCGCTCTCAGGA
          1 ATAGAGCAGGTTTGAAACACTCTTTTTGTAGTATCTGGAAGTGGATATTTGGATAGCTTTGAGGA

            * ** *      *             **          * *       * *          *  *
     420664 CTATGGT-GAAAAAGGAAATATCTTCCAATAAAAGCTACATAGAAGCAATGTCAGAAACTTTTTC
         66 TTTCGTTGGAAACAGG-AATATCTTCATATAAAAGCTAGACAGAAGCATTCTCAGAAACTTCTTT

            *      * ** *   **      *       *  *     *
     420728 ATGATGTATCTACTCAGCTAACAGAGTTGAACCTTTCCTTTG
        130 GTGATGTTTGCATTCAAGTAACAGTGTTGAACATTCCCTTTC

             *       *                   * *     *             **            
     420770 AGAGAGCAGTTTTGAAACACTCTTTTTGTGGAATCTGCAAGTGGATATTTGTCTAGCTTTGAGGA
          1 ATAGAGCAGGTTTGAAACACTCTTTTTGTAGTATCTGGAAGTGGATATTTGGATAGCTTTGAGGA

                         *   *    *                 * *       *   *          
     420835 TTTCGTTGGAAACGGGATTA-CAT-ATA-AAAAGC-AGACGGCAGCATTCCCAGTAACTTCTTTG
         66 TTTCGTTGGAAACAGGAATATCTTCATATAAAAGCTAGACAGAAGCATTCTCAGAAACTTCTTTG

                    *         *    *                 
     420896 TGATGTTTTCATTCAAGTCACAGAGTTGAACATTCCCTTTC
        131 TGATGTTTGCATTCAAGTAACAGTGTTGAACATTCCCTTTC

                                                   *     *       **     *  **
     420937 ATAGAGCAGGTTTGAAACACTCTTTTTGTAGTATCTGGATGTGGACATTTGGAGCGCTTTCAGAC
          1 ATAGAGCAGGTTTGAAACACTCTTTTTGTAGTATCTGGAAGTGGATATTTGGATAGCTTTGAGGA

            * ** *      *             *** *   *                          *   
     421002 CTATGGT-GAAAAAGGAAATATCTTCCCCTGAAAACTAGACAGAAGCATTCTCAGAAACTTATTT
         66 TTTCGTTGGAAACAGG-AATATCTTCATATAAAAGCTAGACAGAAGCATTCTCAGAAACTTCTTT

                   **  **    *              *  * *   *
     421066 GTGATGTGCGCCCTCAACTAACAGTGTTGAACCTTTCTTTTG
        130 GTGATGTTTGCATTCAAGTAACAGTGTTGAACATTCCCTTTC

                     *                    *      *   *                       
     421108 ATAGAGCAGTTTTGAAACACTCTTTTTGTAATATCTGCAAGAGGATATTTGGATAGCTTTGAGGA
          1 ATAGAGCAGGTTTGAAACACTCTTTTTGTAGTATCTGGAAGTGGATATTTGGATAGCTTTGAGGA

                                *            **                      *    *  
     421173 TTTCGTTGGAAAC-GGAATTGTCTTCATATAAACTCTAGACAGAAGCATTCTCAGAAGCTTCATT
         66 TTTCGTTGGAAACAGGAA-TATCTTCATATAAAAGCTAGACAGAAGCATTCTCAGAAACTTCTTT

             *       **   *    *             *        
     421237 GGGATGTTTCAATTGAAGTCACAGTGTTGAACAGTCCCTTTC
        130 GTGATGTTTGCATTCAAGTAACAGTGTTGAACATTCCCTTTC

                                                         *       *  * * *    
     421279 ATAGAGCAGGTTTGAAACACTCTTTTTGTAGTATCTGGAAGTGGACATTTGGAGAGATCTCAGGA
          1 ATAGAGCAGGTTTGAAACACTCTTTTTGTAGTATCTGGAAGTGGATATTTGGATAGCTTTGAGGA

            * *  *    * *             **            *       * *          *  *
     421344 ATACGGT-GATAAAGGAAATATCTTCCAATAAAAGCTAGATAGAAGCAATGTCAGAAACTTTTTC
         66 TTTCGTTGGAAACAGG-AATATCTTCATATAAAAGCTAGACAGAAGCATTCTCAGAAACTTCTTT

            *      * ** *   **      *       *  * *   *
     421408 ATGATGTATCTACTCAGCTAACAGAGTTGAACCTTTCTTTTG
        130 GTGATGTTTGCATTCAAGTAACAGTGTTGAACATTCCCTTTC

             *  *    *                   * *     *             **            
     421450 AGAGTGCAGTTTTGAAACACTCTTTTTGTGGAATCTGCAAGTGGATATTTGTCTAGCTTTGAGGA
          1 ATAGAGCAGGTTTGAAACACTCTTTTTGTAGTATCTGGAAGTGGATATTTGGATAGCTTTGAGGA

                         *   *    *                   *       *      *       
     421515 TTTCGTTGGAAACGGGATTA-CAT-ATA-AAAAGC-AGACAGCAGCATTCCCAGAAATTTCTTTG
         66 TTTCGTTGGAAACAGGAATATCTTCATATAAAAGCTAGACAGAAGCATTCTCAGAAACTTCTTTG

               *              *    *         *       
     421576 TGAAGTTTGCATTCAAGTCACAGAGTTGAACATGCCCTTTC
        131 TGATGTTTGCATTCAAGTAACAGTGTTGAACATTCCCTTTC

                                                 * *     *       **     *   *
     421617 ATAGAGCAGGTTTGAAACACTCTTTTTGTAGTATCTGTATGTGGACATTTGGAGCGCTTTCAGGC
          1 ATAGAGCAGGTTTGAAACACTCTTTTTGTAGTATCTGGAAGTGGATATTTGGATAGCTTTGAGGA

            * ** *      *             *** *   *                          *   
     421682 CTATGGT-GAAAAAGGAAATATCTTCCCCTGAAAACTAGACAGAAGCATTCTCAGAAACTTATTT
         66 TTTCGTTGGAAACAGG-AATATCTTCATATAAAAGCTAGACAGAAGCATTCTCAGAAACTTCTTT

                   **  **    *              *  * *   *
     421746 GTGATGTGCGCCCTCAACTAACAGTGTTGAACCTTTCTTTTG
        130 GTGATGTTTGCATTCAAGTAACAGTGTTGAACATTCCCTTTC

                     *                    *   *  *  **                  *    
     421788 ATAGAGCAGTTTTGAAACACTCTTTTTGTAATATTTGCAAAAGGATATTTGGATAGCTTTAAGGA
          1 ATAGAGCAGGTTTGAAACACTCTTTTTGTAGTATCTGGAAGTGGATATTTGGATAGCTTTGAGGA

                         *   * *            **                      *    *   
     421853 TTTCGTTGGAAACGGGATTGTCTTCATATAAACTCTAGACAGAAGCATTCTCAGAAGCTTCATTG
         66 TTTCGTTGGAAACAGGAATATCTTCATATAAAAGCTAGACAGAAGCATTCTCAGAAACTTCTTTG

            *       **   *    *             *        
     421918 GGATGTTTCAATTGAAGTCACAGTGTTGAACAGTCCCTTTC
        131 TGATGTTTGCATTCAAGTAACAGTGTTGAACATTCCCTTTC

                                                         *       **   * *    
     421959 ATAGAGCAGGTTTGAAACACTCTTTTTGTAGTATCTGGAAGTGGACATTTGGAGCGCTCTCAGGA
          1 ATAGAGCAGGTTTGAAACACTCTTTTTGTAGTATCTGGAAGTGGATATTTGGATAGCTTTGAGGA

            * *  *      *             **            *       * *          *  *
     422024 CTGCGGT-GAAAAAGGAAATATCTTCCAATAAAAGCTAGATAGAAGCAATGTCAGAAACTTTTTC
         66 TTTCGTTGGAAACAGG-AATATCTTCATATAAAAGCTAGACAGAAGCATTCTCAGAAACTTCTTT

            *      * ** *   **      *       *        *
     422088 ATGATGTATCTACTCAGCTAACAGAGTTGAACCTT-CCTTTG
        130 GTGATGTTTGCATTCAAGTAACAGTGTTGAACATTCCCTTTC

             *       *                   * *     *             **            
     422129 AGAGAGCAGTTTTGAAACACTCTTTTTGTGGAATCTGCAAGTGGATATTTGTCTAGCTTTGAGGA
          1 ATAGAGCAGGTTTGAAACACTCTTTTTGTAGTATCTGGAAGTGGATATTTGGATAGCTTTGAGGA

                         *   *    *                   *       *              
     422194 TTTCGTTGGAAACGGGATTA-CAT-ATA-AAAAGC-AGACAGCAGCATTCCCAGAAACTTCTTTG
         66 TTTCGTTGGAAACAGGAATATCTTCATATAAAAGCTAGACAGAAGCATTCTCAGAAACTTCTTTG

                              *    *                 
     422255 TGATGTTTGCATTCAAGTCACAGAGTTGAACATTCCCTTTC
        131 TGATGTTTGCATTCAAGTAACAGTGTTGAACATTCCCTTTC

                                 *                 *     *     * **     *   *
     422296 ATAGAGCAGGTTTGAAACACTGTTTTTGTAGTATCTGGATGTGGACATTTGCAGCGCTTTCAGGC
          1 ATAGAGCAGGTTTGAAACACTCTTTTTGTAGTATCTGGAAGTGGATATTTGGATAGCTTTGAGGA

            * ** *      *             *** *   *                          *   
     422361 CTAAGGT-GAAAAAGGAAATATCTTCCCCTGAAAACTAGACAGAAGCATTCTCAGAAACTTATTT
         66 TTTCGTTGGAAACAGG-AATATCTTCATATAAAAGCTAGACAGAAGCATTCTCAGAAACTTCTTT

                   **  **    *             **  * *   *
     422425 GTGATGTGCGCCCTCAACTAACAGTGTTGAAGCTTTCTTTTG
        130 GTGATGTTTGCATTCAAGTAACAGTGTTGAACATTCCCTTTC

                     *                   * *     *             **            
     422467 ATAGAGCAGTTTTGAAACACTCTTTTTGTGGAATCTGCAAGTGGATATTTGTCTAGCTTTGAGGA
          1 ATAGAGCAGGTTTGAAACACTCTTTTTGTAGTATCTGGAAGTGGATATTTGGATAGCTTTGAGGA

                *  *     *   *    *                   *       *     *   *    
     422532 TTTCTTTTGAAACGGGATTA-CAT-ATA-AAAAGC-AGACAGCAGCATTCCCAGAATCTTGTTTG
         66 TTTCGTTGGAAACAGGAATATCTTCATATAAAAGCTAGACAGAAGCATTCTCAGAAACTTCTTTG

                              *    *                 
     422593 TGATGTTTGCATTCAAGTCACAGAGTTGAACATTCCCTTTC
        131 TGATGTTTGCATTCAAGTAACAGTGTTGAACATTCCCTTTC

             *                                           *    *  *  * * *    
     422634 AGAGAGCAGGTTTGAAACACTCTTTTTGTAGTATCTGGAAGTGGACATTTTGAGAGATCTCAGGA
          1 ATAGAGCAGGTTTGAAACACTCTTTTTGTAGTATCTGGAAGTGGATATTTGGATAGCTTTGAGGA

            * *  *    * *             **            *       * *          *  *
     422699 ATACGGT-GATAAAGGAAATATCTTCCAATAAAAGCTAGATAGAAGCAATGTCAGAAACTTTTTC
         66 TTTCGTTGGAAACAGG-AATATCTTCATATAAAAGCTAGACAGAAGCATTCTCAGAAACTTCTTT

            *      * ** *   **      *       *  * *   *
     422763 ATGATGTATCTACTCAGCTAACAGAGTTGAACCTTTCTTTTG
        130 GTGATGTTTGCATTCAAGTAACAGTGTTGAACATTCCCTTTC

             *       *                   * *     *             **            
     422805 AGAGAGCAGTTTTGAAACACTCTTTTTGTGGAATCTGCAAGTGGATATTTGTCTAGCTTTGAGGA
          1 ATAGAGCAGGTTTGAAACACTCTTTTTGTAGTATCTGGAAGTGGATATTTGGATAGCTTTGAGGA

                         *   *    *                   *       *              
     422870 TTTCGTTGGAAACGGGATTA-CAT-ATA-AAAAGC-AGACAGCAGCATTCCCAGAAACTTCTTTG
         66 TTTCGTTGGAAACAGGAATATCTTCATATAAAAGCTAGACAGAAGCATTCTCAGAAACTTCTTTG

                              *    *                 
     422931 TGATGTTTGCATTCAAGTCACAGAGTTGAACATTCCCTTTC
        131 TGATGTTTGCATTCAAGTAACAGTGTTGAACATTCCCTTTC

                                                   *     *       **     *   *
     422972 ATAGAGCAGGTTTGAAACACTCTTTTTGTAGTATCTGGATGTGGACATTTGGAGCGCTTTCAGGC
          1 ATAGAGCAGGTTTGAAACACTCTTTTTGTAGTATCTGGAAGTGGATATTTGGATAGCTTTGAGGA

            * ** *      *             *** *   *                        * *   
     423037 CTATGGT-GAAAAAGGAAATATCTTCCCCTGAAAACTAGACAGAAGCATTCTCAGAAACATATTT
         66 TTTCGTTGGAAACAGG-AATATCTTCATATAAAAGCTAGACAGAAGCATTCTCAGAAACTTCTTT

             *     **  **    *              *  * *   *
     423101 GCGATGTGCGCCCTCAACTAACAGTGTTGAACCTTTCTTTTG
        130 GTGATGTTTGCATTCAAGTAACAGTGTTGAACATTCCCTTTC

                     *                    *      *   *                       
     423143 ATAGAGCAGTTTTGAAACACTCTTTTTGTAATATCTGCAAGAGGATATTTGGATAGCTTTGAGGA
          1 ATAGAGCAGGTTTGAAACACTCTTTTTGTAGTATCTGGAAGTGGATATTTGGATAGCTTTGAGGA

                         *   * *       *    **          *           *    *   
     423208 TTTCGTTGGAAACGGGATTGTCTTCATGTAAACTCTAGACAGAAACATTCTCAGAAGCTTCATTG
         66 TTTCGTTGGAAACAGGAATATCTTCATATAAAAGCTAGACAGAAGCATTCTCAGAAACTTCTTTG

            *       **   *    *             * *      
     423273 GGATGTTTCAATTGAAGTCACAGTGTTGAACAGTTCCTTTC
        131 TGATGTTTGCATTCAAGTAACAGTGTTGAACATTCCCTTTC

                 *                                       *       **   * *    
     423314 ATAGAACAGGTTTGAAACACTCTTTTTGTAGTATCTGGAAGTGGACATTTGGAGCGCTCTCAGGA
          1 ATAGAGCAGGTTTGAAACACTCTTTTTGTAGTATCTGGAAGTGGATATTTGGATAGCTTTGAGGA

            * *  *      *             **          * *       * *          *  *
     423379 CTACGGT-GAAAAAGGAAATATCTTCCAATAAAAGCTACATAGAAGCAATGTCAGAAACTTTTTC
         66 TTTCGTTGGAAACAGG-AATATCTTCATATAAAAGCTAGACAGAAGCATTCTCAGAAACTTCTTT

            *      * ** *   **      *       *  *     *
     423443 ATGATGTATCTACTCAGCTAACAGAGTTGAACCTTTCCTTTG
        130 GTGATGTTTGCATTCAAGTAACAGTGTTGAACATTCCCTTTC

             *       *                   * *     *             **    *       
     423485 AGAGAGCAGTTTTGAAACACTCTTTTTGTGGAATCTGCAAGTGGATATTTGTCTAGCGTTGAGGA
          1 ATAGAGCAGGTTTGAAACACTCTTTTTGTAGTATCTGGAAGTGGATATTTGGATAGCTTTGAGGA

                         *   *    *                   *       *   *         *
     423550 TTTCGTTGGAAACGGGATTA-CAT-ATA-AAAAGC-AGACAGCAGCATTCCCAGTAACTTCTTTC
         66 TTTCGTTGGAAACAGGAATATCTTCATATAAAAGCTAGACAGAAGCATTCTCAGAAACTTCTTTG

                              *    *       *         
     423611 TGATGTTTGCATTCAAGTCACAGAGTTGAACGTTCCCTTTC
        131 TGATGTTTGCATTCAAGTAACAGTGTTGAACATTCCCTTTC

                                        *          *     *       **     *   *
     423652 ATAGAGCAGGTTTGAAACACTCTTTTTGAAGTATCTGGATGTGGACATTTGGAGCGCTTTCAGGC
          1 ATAGAGCAGGTTTGAAACACTCTTTTTGTAGTATCTGGAAGTGGATATTTGGATAGCTTTGAGGA

            * ** *      *             *** *   *                          *   
     423717 CTATGGT-GAAAAAGGAAATATCTTCCCCTGAAAACTAGACAGAAGCATTCTCAGAAACTTATTT
         66 TTTCGTTGGAAACAGG-AATATCTTCATATAAAAGCTAGACAGAAGCATTCTCAGAAACTTCTTT

                   **  **    *              *  * *   *
     423781 GTGATGTGCGCCCTCAACTAACAGTGTTGAACCTTTCTTTTG
        130 GTGATGTTTGCATTCAAGTAACAGTGTTGAACATTCCCTTTC

                     *                    *      *   *                       
     423823 ATAGAGCAGTTTTGAAACACTCTTTTTGTAATATCTGCAAGAGGATATTTGGATAGCTTTGAGGA
          1 ATAGAGCAGGTTTGAAACACTCTTTTTGTAGTATCTGGAAGTGGATATTTGGATAGCTTTGAGGA

                         *   * *            **                      *    *   
     423888 TTTCGTTGGAAACGGGATTGTCTTCATATAAACTCTAGACAGAAGCATTCTCAGAAGCTTCATTG
         66 TTTCGTTGGAAACAGGAATATCTTCATATAAAAGCTAGACAGAAGCATTCTCAGAAACTTCTTTG

            *       **   *    *             *        
     423953 GGATGTTTCAATTGAAGTCACAGTGTTGAACAGTCCCTTTC
        131 TGATGTTTGCATTCAAGTAACAGTGTTGAACATTCCCTTTC

                                                         *       **   * *    
     423994 ATAGAGCAGGTTTGAAACACTCTTTTTGTAGTATCTGGAAGTGGACATTTGGAGCGCTCTCAGGA
          1 ATAGAGCAGGTTTGAAACACTCTTTTTGTAGTATCTGGAAGTGGATATTTGGATAGCTTTGAGGA

            * *  *    * *             **            *       * *          *  *
     424059 CTCCGGT-GATAAAGGAAATATCTTCCAATAAAAGCTAGATAGAAGCAATGTCAGAAACTTTTTC
         66 TTTCGTTGGAAACAGG-AATATCTTCATATAAAAGCTAGACAGAAGCATTCTCAGAAACTTCTTT

            *      * ** *   **      *       *  * *   *
     424123 ATGATGTATCTACTCAGCTAACAGAGTTGAACCTTTCTTTTG
        130 GTGATGTTTGCATTCAAGTAACAGTGTTGAACATTCCCTTTC

             *       *                   * *  *                **  **        
     424165 AGAGAGCAGTTTTGAAACACTCTTTTTGTGGAATATGGAAGTGGATATTTGTCTAAATTTGAGGA
          1 ATAGAGCAGGTTTGAAACACTCTTTTTGTAGTATCTGGAAGTGGATATTTGGATAGCTTTGAGGA

                         *   *    *                   *       *     *        
     424230 TTTCGTTGGAAACGGGATTA-CAT-ATA-AAAAGC-AGACAGCAGCATTCCCAGAATCTTCTTTG
         66 TTTCGTTGGAAACAGGAATATCTTCATATAAAAGCTAGACAGAAGCATTCTCAGAAACTTCTTTG

                              *    *                 
     424291 TGATGTTTGCATTCAAGTCACAGAGTTGAACATTCCCTTTC
        131 TGATGTTTGCATTCAAGTAACAGTGTTGAACATTCCCTTTC

                                                   *     *       **     *   *
     424332 ATAGAGCAGGTTTGAAACACTCTTTTTGTAGTATCTGGATGTGGACATTTGGAGCGCTTTCAGGC
          1 ATAGAGCAGGTTTGAAACACTCTTTTTGTAGTATCTGGAAGTGGATATTTGGATAGCTTTGAGGA

            * ** *      *             *** *   *                      *   *   
     424397 CTATGGT-GAAAAAGGAAATATCTTCCCCTGAAAACTAGACAGAAGCATTCTCAGAATCTTATTT
         66 TTTCGTTGGAAACAGG-AATATCTTCATATAAAAGCTAGACAGAAGCATTCTCAGAAACTTCTTT

                   **  **    *             **  * *   *
     424461 GTGATGTGCGCCCTCAACTAACAGTGTTGAAGCTTTCTTTTG
        130 GTGATGTTTGCATTCAAGTAACAGTGTTGAACATTCCCTTTC

                     *                    **     *   *                       
     424503 ATAGAGCAGTTTTGAAACACTCTTTTTGTAAAATCTGCAAGAGGATATTTGGATAGCTTTGAGGA
          1 ATAGAGCAGGTTTGAAACACTCTTTTTGTAGTATCTGGAAGTGGATATTTGGATAGCTTTGAGGA

                         *   * *            **                      *    *   
     424568 TTTCGTTGGAAACGGGATTGTCTTCATATAAACTCTAGACAGAAGCATTCTCAGAAGCTTCATTG
         66 TTTCGTTGGAAACAGGAATATCTTCATATAAAAGCTAGACAGAAGCATTCTCAGAAACTTCTTTG

            *       **   *    *             *        
     424633 GGATGTTTCAATTGAAGTCACAGTGTTGAACAGTCCCTTTC
        131 TGATGTTTGCATTCAAGTAACAGTGTTGAACATTCCCTTTC

                                                         *       *  * * *    
     424674 ATAGAGCAGGTTTGAAACACTCTTTTTGTAGTATCTGGAAGTGGACATTTGGAGAGATCTCAGGA
          1 ATAGAGCAGGTTTGAAACACTCTTTTTGTAGTATCTGGAAGTGGATATTTGGATAGCTTTGAGGA

            * *  *    * *             **            *       * *          *  *
     424739 ATACGGT-GATAAAGGAAATATCTTCCAATAAAAGCTAGATAGAAGCAATGTCAGAAACTTTTTC
         66 TTTCGTTGGAAACAGG-AATATCTTCATATAAAAGCTAGACAGAAGCATTCTCAGAAACTTCTTT

            *      * ** *   **      *       *  * *   *
     424803 ATGATGTATCTACTCAGCTAACAGAGTTGAACCTTTCTTTTG
        130 GTGATGTTTGCATTCAAGTAACAGTGTTGAACATTCCCTTTC

             *       *                   * *     *             **            
     424845 AGAGAGCAGTTTTGAAACACTCTTTTTGTGGAATCTGCAAGTGGATATTTGTCTAGCTTTGAGGA
          1 ATAGAGCAGGTTTGAAACACTCTTTTTGTAGTATCTGGAAGTGGATATTTGGATAGCTTTGAGGA

                         *   *    *                   **      *              
     424910 TTTCGTTGGAAACGGGATTA-CAT-ATA-AAAAGC-AGACAGCGGCATTCCCAGAAACTTCTTTG
         66 TTTCGTTGGAAACAGGAATATCTTCATATAAAAGCTAGACAGAAGCATTCTCAGAAACTTCTTTG

                              *    *                 
     424971 TGATGTTTGCATTCAAGTCACAGAGTTGAACATTCCCTTTC
        131 TGATGTTTGCATTCAAGTAACAGTGTTGAACATTCCCTTTC

               *                                   *     *     * **     *   *
     425012 ATACAGCAGGTTTGAAACACTCTTTTTGTAGTATCTGGATGTGGACATTTGCAGCGCTTTCAGGC
          1 ATAGAGCAGGTTTGAAACACTCTTTTTGTAGTATCTGGAAGTGGATATTTGGATAGCTTTGAGGA

            * ** *      *             *** *   *                          *   
     425077 CTAAGGT-GAAAAAGGAAATATCTTCCCCTGAAAACTAGACAGAAGCATTCTCAGAAACTTATTT
         66 TTTCGTTGGAAACAGG-AATATCTTCATATAAAAGCTAGACAGAAGCATTCTCAGAAACTTCTTT

            *      **  **    *             **  * *   *
     425141 CTGATGTGCGCCCTCAACTAACAGTGTTGAAGCTTTCTTTTG
        130 GTGATGTTTGCATTCAAGTAACAGTGTTGAACATTCCCTTTC

                     *                   * *     *             **          * 
     425183 ATAGAGCAGTTTTGAAACACTCTTTTTGTGGAATCTGCAAGTGGATATTTGTCTAGCTTTGAGAA
          1 ATAGAGCAGGTTTGAAACACTCTTTTTGTAGTATCTGGAAGTGGATATTTGGATAGCTTTGAGGA

                         *   *    *                   *       *     *   *  * 
     425248 TTTCGTTGGAAACGGGATTA-CAT-ATA-AAAAGC-AGACAGCAGCATTCCCAGAATCTTGTTGG
         66 TTTCGTTGGAAACAGGAATATCTTCATATAAAAGCTAGACAGAAGCATTCTCAGAAACTTCTTTG

                              *    *          *      
     425309 TGATGTTTGCATTCAAGTCACAGAGTTGAACATTTCCTTTC
        131 TGATGTTTGCATTCAAGTAACAGTGTTGAACATTCCCTTTC

             *                         *           *     *       **     *   *
     425350 AGAGAGCAGGTTTGAAACACTCTTTTTATAGTATCTGGATGTGGACATTTGGAGCGCTTTCAGGC
          1 ATAGAGCAGGTTTGAAACACTCTTTTTGTAGTATCTGGAAGTGGATATTTGGATAGCTTTGAGGA

            * ** *      *             *** *   *                      *   *   
     425415 CTATGGT-GAAAAAGGAAATATCTTCTCCTGAAAACTAGACAGAAGCATTCTCAGAATCTTATTT
         66 TTTCGTTGGAAACAGG-AATATCTTCATATAAAAGCTAGACAGAAGCATTCTCAGAAACTTCTTT

                   **  **    *             **  * *   *
     425479 GTGATGTGCGCCCTCAACTAACAGTGTTGAAGCTTTCTTTTG
        130 GTGATGTTTGCATTCAAGTAACAGTGTTGAACATTCCCTTTC

                     *                    *      *   *                       
     425521 ATAGAGCAGTTTTGAAACACTCTTTTTGTAATATCTGCAAGAGGATATTTGGATAGCTTTGAGGA
          1 ATAGAGCAGGTTTGAAACACTCTTTTTGTAGTATCTGGAAGTGGATATTTGGATAGCTTTGAGGA

                         *   * *            **                      *    *   
     425586 TTTCGTTGGAAACGGGATTGTCTTCATATAAACTCTAGACAGAAGCATTCTCAGAAGCTTCATTG
         66 TTTCGTTGGAAACAGGAATATCTTCATATAAAAGCTAGACAGAAGCATTCTCAGAAACTTCTTTG

            *       **   *    *   *         * *      
     425651 GGATGTTTCAATTGAAGTCACACTGTTGAACAGTTCCTTTC
        131 TGATGTTTGCATTCAAGTAACAGTGTTGAACATTCCCTTTC

                 *                                       *       **   * *    
     425692 ATAGAACAGGTTTGAAACACTCTTTTTGTAGTATCTGGAAGTGGACATTTGGAGCGCTCTCAGGA
          1 ATAGAGCAGGTTTGAAACACTCTTTTTGTAGTATCTGGAAGTGGATATTTGGATAGCTTTGAGGA

            * ** *      *        *    **          * *       * *          *  *
     425757 CTATGGT-GAAAAAGGATATAACTTCCAATAAAAGCTACATAGAAGCAATGTCAGAAACTTTTTC
         66 TTTCGTTGGAAACAGGA-ATATCTTCATATAAAAGCTAGACAGAAGCATTCTCAGAAACTTCTTT

            *      * ** *   **      *       *  *     *
     425821 ATGATGTATCTACTCAGCTAACAGAGTTGAACCTTTCCTTTG
        130 GTGATGTTTGCATTCAAGTAACAGTGTTGAACATTCCCTTTC

             *       *                   * *     *             **            
     425863 AGAGAGCAGTTTTGAAACACTCTTTTTGTGGAATCTGCAAGTGGATATTTGTCTAGCTTTGAGGA
          1 ATAGAGCAGGTTTGAAACACTCTTTTTGTAGTATCTGGAAGTGGATATTTGGATAGCTTTGAGGA

                         *   *    *                   *       *   *          
     425928 TTTCGTTGGAAACGGGATTA-CAT-ATA-AAAAGC-AGACAGCAGCATTCCCAGTAACTTCTTTG
         66 TTTCGTTGGAAACAGGAATATCTTCATATAAAAGCTAGACAGAAGCATTCTCAGAAACTTCTTTG

                    *         *    *                 
     425989 TGATGTTTTCATTCAAGTCACAGAGTTGAACATTCCCTTTC
        131 TGATGTTTGCATTCAAGTAACAGTGTTGAACATTCCCTTTC

                                                   *     *       **     *  **
     426030 ATAGAGCAGGTTTGAAACACTCTTTTTGTAGTATCTGGATGTGGACATTTGGAGCGCTTTCAGAC
          1 ATAGAGCAGGTTTGAAACACTCTTTTTGTAGTATCTGGAAGTGGATATTTGGATAGCTTTGAGGA

            * ** *      *             *** *   *                          *   
     426095 CTATGGT-GAAAAAGGAAATATCTTCCCCTGAAAACTAGACAGAAGCATTCTCAGAAACTTATTT
         66 TTTCGTTGGAAACAGG-AATATCTTCATATAAAAGCTAGACAGAAGCATTCTCAGAAACTTCTTT

                   **  **    *              *  * *   *
     426159 GTGATGTGCGCCCTCAACTAACAGTGTTGAACCTTTCTTTTG
        130 GTGATGTTTGCATTCAAGTAACAGTGTTGAACATTCCCTTTC

                     *                    *      *   *                       
     426201 ATAGAGCAGTTTTGAAACACTCTTTTTGTAATATCTGCAAGAGGATATTTGGATAGCTTTGAGGA
          1 ATAGAGCAGGTTTGAAACACTCTTTTTGTAGTATCTGGAAGTGGATATTTGGATAGCTTTGAGGA

                                *            **                      *    *  
     426266 TTTCGTTGGAAAC-GGAATTGTCTTCATATAAACTCTAGACAGAAGCATTCTCAGAAGCTTCATT
         66 TTTCGTTGGAAACAGGAA-TATCTTCATATAAAAGCTAGACAGAAGCATTCTCAGAAACTTCTTT

             *       **   *    *             *        
     426330 GGGATGTTTCAATTGAAGTCACAGTGTTGAACAGTCCCTTTC
        130 GTGATGTTTGCATTCAAGTAACAGTGTTGAACATTCCCTTTC

                                                         *       *  * * *    
     426372 ATAGAGCAGGTTTGAAACACTCTTTTTGTAGTATCTGGAAGTGGACATTTGGAGAGATCTCAGGA
          1 ATAGAGCAGGTTTGAAACACTCTTTTTGTAGTATCTGGAAGTGGATATTTGGATAGCTTTGAGGA

            * *  *    * *             **            *    *  * *          *  *
     426437 ATACGGT-GATAAAGGAAATATCTTCCAATAAAAGCTAGATAGAAACAATGTCAGAAACTTTTTC
         66 TTTCGTTGGAAACAGG-AATATCTTCATATAAAAGCTAGACAGAAGCATTCTCAGAAACTTCTTT

            *      * ** *   **      *       *  * *   *
     426501 ATGATGTATCTACTCAGCTAACAGAGTTGAACCTTTCTTTTG
        130 GTGATGTTTGCATTCAAGTAACAGTGTTGAACATTCCCTTTC

             *       *                   * *     *             **            
     426543 AGAGAGCAGTTTTGAAACACTCTTTTTGTGGAATCTGCAAGTGGATATTTGTCTAGCTTTGAGGA
          1 ATAGAGCAGGTTTGAAACACTCTTTTTGTAGTATCTGGAAGTGGATATTTGGATAGCTTTGAGGA

                         *   *    *                   *       *      *       
     426608 TTTCGTTGGAAACGGGATTA-CAT-ATA-AAAAGC-AGACAGCAGCATTCCCAGAAATTTCTTTG
         66 TTTCGTTGGAAACAGGAATATCTTCATATAAAAGCTAGACAGAAGCATTCTCAGAAACTTCTTTG

               *              *    *         *       
     426669 TGAAGTTTGCATTCAAGTCACAGAGTTGAACATGCCCTTTC
        131 TGATGTTTGCATTCAAGTAACAGTGTTGAACATTCCCTTTC

                               *                 * *     *       **     *   *
     426710 ATAGAGCAGGTTTGAAACATTCTTTTTGTAGTATCTGTATGTGGACATTTGGAGCGCTTTCAGGC
          1 ATAGAGCAGGTTTGAAACACTCTTTTTGTAGTATCTGGAAGTGGATATTTGGATAGCTTTGAGGA

            * ** *      *             *** *   *                      *   *   
     426775 CTATGGT-GAAAAAGGAAATATCTTCCCCTGAAAACTAGACAGAAGCATTCTCAGAATCTTATTT
         66 TTTCGTTGGAAACAGG-AATATCTTCATATAAAAGCTAGACAGAAGCATTCTCAGAAACTTCTTT

                   **  **    *             **  * *   *
     426839 GTGATGTGCGCCCTCAACTAACAGTGTTGAAGCTTTCTTTTG
        130 GTGATGTTTGCATTCAAGTAACAGTGTTGAACATTCCCTTTC

                     *                    **     *   *                       
     426881 ATAGAGCAGTTTTGAAACACTCTTTTTGTAAAATCTGCAAGAGGATATTTGGATAGCTTTGAGGA
          1 ATAGAGCAGGTTTGAAACACTCTTTTTGTAGTATCTGGAAGTGGATATTTGGATAGCTTTGAGGA

                *        *   * *            **                      *    *   
     426946 TTTCTTTGGAAACGGGATTGTCTTCATATAAACTCTAGACAGAAGCATTCTCAGAAGCTTCATTG
         66 TTTCGTTGGAAACAGGAATATCTTCATATAAAAGCTAGACAGAAGCATTCTCAGAAACTTCTTTG

            *       **   *    *             *        
     427011 GGATGTTTCAATTGAAGTCACAGTGTTGAACAGTCCCTTTC
        131 TGATGTTTGCATTCAAGTAACAGTGTTGAACATTCCCTTTC

                                                   *     *       **     *   *
     427052 ATAGAGCAGGTTTGAAACACTCTTTTTGTAGTATCTGGATGTGGACATTTGGAGCGCTTTCAGGC
          1 ATAGAGCAGGTTTGAAACACTCTTTTTGTAGTATCTGGAAGTGGATATTTGGATAGCTTTGAGGA

            * ** *      *             *** *   *                      *   *   
     427117 CTATGGT-GAAAAAGGAAATATCTTCCCCTGAAAACTAGACAGAAGCATTCTCAGAATCTTATTT
         66 TTTCGTTGGAAACAGG-AATATCTTCATATAAAAGCTAGACAGAAGCATTCTCAGAAACTTCTTT

            *      **  **    *             **  * *   *
     427181 CTGATGTGCGCCCTCAACTAACAGTGTTGAAGCTTTCTTTTG
        130 GTGATGTTTGCATTCAAGTAACAGTGTTGAACATTCCCTTTC

                     *                    **     *   *                       
     427223 ATAGAGCAGTTTTGAAACACTCTTTTTGTAAAATCTGCAAGAGGATATTTGGATAGCTTTGAGGA
          1 ATAGAGCAGGTTTGAAACACTCTTTTTGTAGTATCTGGAAGTGGATATTTGGATAGCTTTGAGGA

                         *   * *            **     *                *    *   
     427288 TTTCGTTGGAAACGGGATTGTCTTCATATAAACTCTAGAAAGAAGCATTCTCAGAAGCTTCATTG
         66 TTTCGTTGGAAACAGGAATATCTTCATATAAAAGCTAGACAGAAGCATTCTCAGAAACTTCTTTG

            *             *    *                      
     427353 GGATGTTT-CAGTTGAAGTCACAGTGTTGAACATTCCCTTTC
        131 TGATGTTTGCA-TTCAAGTAACAGTGTTGAACATTCCCTTTC

                             *                           *       **   * *    
     427394 ATAGAGCAGGTTTGAAATACTCTTTTTGTAGTATCTGGAAGTGGACATTTGGAGCGCTGTCAGGA
          1 ATAGAGCAGGTTTGAAACACTCTTTTTGTAGTATCTGGAAGTGGATATTTGGATAGCTTTGAGGA

            * *  *      *             **            *       * *          *  *
     427459 CTGCGGT-GAAAAAGGAAATATCTTCCAATAAAAGCTAGATAGAAGCAATGTCAGAAACTTTTTC
         66 TTTCGTTGGAAACAGG-AATATCTTCATATAAAAGCTAGACAGAAGCATTCTCAGAAACTTCTTT

            *      * ** *   **      **      *        *
     427523 ATGATGTATCTACTCAGCTAACAGATTTGAACCTT-CCTTTG
        130 GTGATGTTTGCATTCAAGTAACAGTGTTGAACATTCCCTTTC

             *       *                   * *     *             **            
     427564 AGAGAGCAGTTTTGAAACACTCTTTTTGTGGAATCTGCAAGTGGATATTTGTCTAGCTTTGAGGA
          1 ATAGAGCAGGTTTGAAACACTCTTTTTGTAGTATCTGGAAGTGGATATTTGGATAGCTTTGAGGA

                        **   *    *     *             *       *              
     427629 TTTCGTTGGAAATGGGATTA-CAT-A-AAAAAAGC-AGACAGCAGCATTCCCAGAAACTTCTTTG
         66 TTTCGTTGGAAACAGGAATATCTTCATATAAAAGCTAGACAGAAGCATTCTCAGAAACTTCTTTG

                              *    *          *      
     427690 TGATGTTTGCATTCAAGTCACAGAGTTGAACATTTCCTTTC
        131 TGATGTTTGCATTCAAGTAACAGTGTTGAACATTCCCTTTC

                                                   *     *    ** **     *   *
     427731 ATAGAGCAGGTTTGAAACACTCTTTTTGTAGTATCTGGATGTGGACATTTTCAGCGCTTTCAGGC
          1 ATAGAGCAGGTTTGAAACACTCTTTTTGTAGTATCTGGAAGTGGATATTTGGATAGCTTTGAGGA

            * ** *      *             *** *   *                          *   
     427796 CTAAGGT-GAAAAAGGAAATATCTTCCCCTGAAAACTAGACAGAAGCATTCTCAGAAACTTATTT
         66 TTTCGTTGGAAACAGG-AATATCTTCATATAAAAGCTAGACAGAAGCATTCTCAGAAACTTCTTT

                   **  **    *             **  * *   *
     427860 GTGATGTGCGCCCTCAACTAACAGTGTTGAAGCTTTCTTTTG
        130 GTGATGTTTGCATTCAAGTAACAGTGTTGAACATTCCCTTTC

                     *                   * *     * *          ***            
     427902 ATAGAGCAGTTTTGAAACACTCTTTTTGTGGAATCTGCATGTGGATATTTTTCTAGCTTTGAGGA
          1 ATAGAGCAGGTTTGAAACACTCTTTTTGTAGTATCTGGAAGTGGATATTTGGATAGCTTTGAGGA

                         *   *    *                   *       *     *   *    
     427967 TTTCGTTGGAAACGGGATTA-CAT-ATA-AAAAGC-AGACAGCAGCATTCCCAGAATCTTGTTTG
         66 TTTCGTTGGAAACAGGAATATCTTCATATAAAAGCTAGACAGAAGCATTCTCAGAAACTTCTTTG

                              *    *                 
     428028 TGATGTTTGCATTCAAGTCACAGAGTTGAACATTCCCTTTC
        131 TGATGTTTGCATTCAAGTAACAGTGTTGAACATTCCCTTTC

             *                         *           *     *       **     *   *
     428069 AGAGAGCAGGTTTGAAACACTCTTTTTATAGTATCTGGATGTGGACATTTGGAGCGCTTTCAGGC
          1 ATAGAGCAGGTTTGAAACACTCTTTTTGTAGTATCTGGAAGTGGATATTTGGATAGCTTTGAGGA

            * ** *      *             *** *   *                      *   *   
     428134 CTATGGT-GAAAAAGGAAATATCTTCTCCTGAAAACTAGACAGAAGCATTCTCAGAATCTTATTT
         66 TTTCGTTGGAAACAGG-AATATCTTCATATAAAAGCTAGACAGAAGCATTCTCAGAAACTTCTTT

                   **  **    *  *          **  * *   *
     428198 GTGATGTGCGCCCTCAACTAGCAGTGTTGAAACTTTCTTTTG
        130 GTGATGTTTGCATTCAAGTAACAGTGTTGAACATTCCCTTTC

                     *                    **     *   *                       
     428240 ATAGAGCAGTTTTGAAACACTCTTTTTGTAAAATCTGCAAGAGGATATTTGGATAGCTTTGAGGA
          1 ATAGAGCAGGTTTGAAACACTCTTTTTGTAGTATCTGGAAGTGGATATTTGGATAGCTTTGAGGA

                         *   * *             *                   *  *    *   
     428305 TTTCGTTGGAAACGGGATTGTCTTCATATAAAATCTAGACAGAAGCATTCTCAAAAGCTTCATTG
         66 TTTCGTTGGAAACAGGAATATCTTCATATAAAAGCTAGACAGAAGCATTCTCAGAAACTTCTTTG

            *       ** * *    *             *        
     428370 GGATGTTTCAACTGAAGTCACAGTGTTGAACAGTCCCTTTC
        131 TGATGTTTGCATTCAAGTAACAGTGTTGAACATTCCCTTTC

                                      *                  *       **   * *    
     428411 ATAGAGCAGGTTTGAAACACTCTTTTGGTAGTATCTGGAAGTGGACATTTGGAGCGCTCTTAGGA
          1 ATAGAGCAGGTTTGAAACACTCTTTTTGTAGTATCTGGAAGTGGATATTTGGATAGCTTTGAGGA

            * *  *      *             **          * *       * *          *  *
     428476 CTACGGT-GAAAAAGGAAATATCTTCCAATAAAAGCTACATAGAAGCAATGTCAGAAACTTTTTC
         66 TTTCGTTGGAAACAGG-AATATCTTCATATAAAAGCTAGACAGAAGCATTCTCAGAAACTTCTTT

            *      * ** *   **      *       *  * *   *
     428540 ATGATGTATCTACTCAGCTAACAGAGTTGAACCTTTCTTTTG
        130 GTGATGTTTGCATTCAAGTAACAGTGTTGAACATTCCCTTTC

             *       *                   * *     *             **            
     428582 AGAGAGCAGTTTTGAAACACTCTTTTTGTGGAATCTGCAAGTGGATATTTGTCTAGCTTTGAGGA
          1 ATAGAGCAGGTTTGAAACACTCTTTTTGTAGTATCTGGAAGTGGATATTTGGATAGCTTTGAGGA

                         *        *                   *       *              
     428647 TTTCGTTGGAAACGGGAATA-CAT-ATA-AAAAGC-AGACAGCAGCATTCCCAGAAACTTCTTTG
         66 TTTCGTTGGAAACAGGAATATCTTCATATAAAAGCTAGACAGAAGCATTCTCAGAAACTTCTTTG

                              *    *                 
     428708 TGATGTTTGCATTCAAGTCACAGAGTTGAACATTCCCTTTC
        131 TGATGTTTGCATTCAAGTAACAGTGTTGAACATTCCCTTTC

                                                 * *     *       **     *   *
     428749 ATAGAGCAGGTTTGAAACACTCTTTTTGTAGTATCTGTATGTGGACATTTGGAGCGCTTTCAGGC
          1 ATAGAGCAGGTTTGAAACACTCTTTTTGTAGTATCTGGAAGTGGATATTTGGATAGCTTTGAGGA

            * ** *      *             *** *   *                      *   *   
     428814 CTATGGT-GAAAAAGGAAATATCTTCCCCTGAAAACTAGACAGAAGCATTCTCAGAATCTTATTT
         66 TTTCGTTGGAAACAGG-AATATCTTCATATAAAAGCTAGACAGAAGCATTCTCAGAAACTTCTTT

                   ** ***    *             **  * *   *
     428878 GTGATGTGCGACCTCAACTAACAGTGTTGAAGCTTTCTTTTG
        130 GTGATGTTTGCATTCAAGTAACAGTGTTGAACATTCCCTTTC

                     *                    **     *   *                       
     428920 ATAGAGCAGTTTTGAAACACTCTTTTTGTAAAATCTGCAAGAGGATATTTGGATAGCTTTGAGGA
          1 ATAGAGCAGGTTTGAAACACTCTTTTTGTAGTATCTGGAAGTGGATATTTGGATAGCTTTGAGGA

                         *   * *            **                      *    *   
     428985 TTTCGTTGGAAACGGGATTGTCTTCATATAAACTCTAGACAGAAGCATTCTCAGAAGCTTCATTG
         66 TTTCGTTGGAAACAGGAATATCTTCATATAAAAGCTAGACAGAAGCATTCTCAGAAACTTCTTTG

            *       **   *    *             *        
     429050 GGATGTTTCAATTGAAGTCACAGTGTTGAACAGTCCCTTTC
        131 TGATGTTTGCATTCAAGTAACAGTGTTGAACATTCCCTTTC

                                                         *       *  * * *    
     429091 ATAGAGCAGGTTTGAAACACTCTTTTTGTAGTATCTGGAAGTGGACATTTGGAGAGATCTCAGGA
          1 ATAGAGCAGGTTTGAAACACTCTTTTTGTAGTATCTGGAAGTGGATATTTGGATAGCTTTGAGGA

            * *  *      *             **            *       * *          *  *
     429156 ATACGGT-GAAAAAGGAAATATCTTCCAATAAAAGCTAGATAGAAGCAATGTCAGAAACTTTTTC
         66 TTTCGTTGGAAACAGG-AATATCTTCATATAAAAGCTAGACAGAAGCATTCTCAGAAACTTCTTT

            *      * ** *   **      *       *  * *   *
     429220 ATGATGTATCTACTCAGCTAACAGAGTTGAACCTTTCTTTTG
        130 GTGATGTTTGCATTCAAGTAACAGTGTTGAACATTCCCTTTC

             *       *                   * *     *             **            
     429262 AGAGAGCAGTTTTGAAACACTCTTTTTGTGGAATCTGCAAGTGGATATTTGTCTAGCTTTGAGGA
          1 ATAGAGCAGGTTTGAAACACTCTTTTTGTAGTATCTGGAAGTGGATATTTGGATAGCTTTGAGGA

                *        *        *               *   *       *     *        
     429327 TTTCTTTGGAAACGGGAATA-CAT-ATA-AAAAGC-AGTCAGCAGCATTCCCAGAATCTTCTTTG
         66 TTTCGTTGGAAACAGGAATATCTTCATATAAAAGCTAGACAGAAGCATTCTCAGAAACTTCTTTG

                              *    *                 
     429388 TGATGTTTGCATTCAAGTCACAGAGTTGAACATTCCCTTTC
        131 TGATGTTTGCATTCAAGTAACAGTGTTGAACATTCCCTTTC

                                                 * *     *       **     *   *
     429429 ATAGAGCAGGTTTGAAACACTCTTTTTGTAGTATCTGTATGTGGACATTTGGAGCGCTTTCAGGC
          1 ATAGAGCAGGTTTGAAACACTCTTTTTGTAGTATCTGGAAGTGGATATTTGGATAGCTTTGAGGA

            * ** *      *             *** *   *                      *   *   
     429494 CTATGGT-GAAAAAGGAAATATCTTCCCCTGAAAACTAGACAGAAGCATTCTCAGAATCTTATTT
         66 TTTCGTTGGAAACAGG-AATATCTTCATATAAAAGCTAGACAGAAGCATTCTCAGAAACTTCTTT

                   **  **    *             **  * *   *
     429558 GTGATGTGCGCCCTCAACTAACAGTGTTGAAGCTTTCTTTTG
        130 GTGATGTTTGCATTCAAGTAACAGTGTTGAACATTCCCTTTC

                     *                    **     *   *                       
     429600 ATAGAGCAGTTTTGAAACACTCTTTTTGTAAAATCTGCAAGAGGATATTTGGATAGCTTTGAGGA
          1 ATAGAGCAGGTTTGAAACACTCTTTTTGTAGTATCTGGAAGTGGATATTTGGATAGCTTTGAGGA

                         *   * *            **                      *    *   
     429665 TTTCGTTGGAAACGGGATTGTCTTCATATAAACTCTAGACAGAAGCATTCTCAGAAGCTTCATTG
         66 TTTCGTTGGAAACAGGAATATCTTCATATAAAAGCTAGACAGAAGCATTCTCAGAAACTTCTTTG

            *       **   *    *             *        
     429730 GGATGTTTCAATTGAAGTCACAGTGTTGAACAGTCCCTTTC
        131 TGATGTTTGCATTCAAGTAACAGTGTTGAACATTCCCTTTC

                                                   *     *       **     *    
     429771 ATAGAGCAGGTTTGAAACACTCTTTTTGTAGTATCTGGATGTGGACATTTGGAGCGCTTTCAGGC
          1 ATAGAGCAGGTTTGAAACACTCTTTTTGTAGTATCTGGAAGTGGATATTTGGATAGCTTTGAGG-

            * * *    *                 *** *   *                          *  
     429836 CTATGGTT-TAAA-AGGAAATATCTTCCCCTGAAAACTAGACAGAAGCATTCTCAGAAACTTATT
         65 ATTTCGTTGGAAACAGG-AATATCTTCATATAAAAGCTAGACAGAAGCATTCTCAGAAACTTCTT

                    **  **    *                    *   *
     429899 TGTGATGTGCGCCCTCAACTAACAGTGTTGAAGCATT-CTTTTG
        129 TGTGATGTTTGCATTCAAGTAACAGTGTTGAA-CATTCCCTTTC

                     *                   * *     *             **            
     429942 ATAGAGCAGTTTTGAAACACTCTTTTTGTGGAATCTGCAAGTGGATATTTGTCTAGCTTTGAGGA
          1 ATAGAGCAGGTTTGAAACACTCTTTTTGTAGTATCTGGAAGTGGATATTTGGATAGCTTTGAGGA

                *        *   *    *                   *       *              
     430007 TTTCTTTGGAAACGGGATTA-CAT-ATA-AAAAGC-AGACAGCAGCATTCCCAGAAACTTCTTTG
         66 TTTCGTTGGAAACAGGAATATCTTCATATAAAAGCTAGACAGAAGCATTCTCAGAAACTTCTTTG

                              *    *                 
     430068 TGATGTTTGCATTCAAGTCACAGAGTTGAACATTCCCTTTC
        131 TGATGTTTGCATTCAAGTAACAGTGTTGAACATTCCCTTTC

             *                       *             *     *       **     *   *
     430109 AGAGAGCAGGTTTGAAACACTCTTTATGTAGTATCTGGATGTGGACATTTGGAGCGCTTTCAGGC
          1 ATAGAGCAGGTTTGAAACACTCTTTTTGTAGTATCTGGAAGTGGATATTTGGATAGCTTTGAGGA

            * ** *      *             *** *   *                      *   *   
     430174 CTATGGT-GAAAAAGGAAATATCTTCCCCTGAAAACTAGACAGAAGCATTCTCAGAATCTTATTT
         66 TTTCGTTGGAAACAGG-AATATCTTCATATAAAAGCTAGACAGAAGCATTCTCAGAAACTTCTTT

                   **  **    *             **  * *   *
     430238 GTGATGTGCGCCCTCAACTAACAGTGTTGAAGCTTTCTTTTG
        130 GTGATGTTTGCATTCAAGTAACAGTGTTGAACATTCCCTTTC

                     *                    **     *   *                       
     430280 ATAGAGCAGTTTTGAAACACTCTTTTTGTAAAATCTGCAAGAGGATATTTGGATAGCTTTGAGGA
          1 ATAGAGCAGGTTTGAAACACTCTTTTTGTAGTATCTGGAAGTGGATATTTGGATAGCTTTGAGGA

                         *   * *            **          *           *    *   
     430345 TTTCGTTGGAAACGGGATTGTCTTCATATAAACTCTAGACAGAATCATTCTCAGAAGCTTCATTG
         66 TTTCGTTGGAAACAGGAATATCTTCATATAAAAGCTAGACAGAAGCATTCTCAGAAACTTCTTTG

            *       **   *    *             *        
     430410 GGATGTTTCAATTGAAGTCACAGTGTTGAACAGTCCCTTTC
        131 TGATGTTTGCATTCAAGTAACAGTGTTGAACATTCCCTTTC

                                                         *       **   * *    
     430451 ATAGAGCAGGTTTGAAACACTC--TTTGTAGTATCTGGAAGTGGACATTTGGAGCGCTCTCAGGA
          1 ATAGAGCAGGTTTGAAACACTCTTTTTGTAGTATCTGGAAGTGGATATTTGGATAGCTTTGAGGA

            * *  *      *             **            *       * *          *  *
     430514 CTACGGT-GAAAAAGGAAGTATCTTCCAATAAAAGCTAGATAGAAGCAATGTCAGAAACTTTTTC
         66 TTTCGTTGGAAACAGGAA-TATCTTCATATAAAAGCTAGACAGAAGCATTCTCAGAAACTTCTTT

            *      * ** *   **      *       *  * *   *
     430578 ATGATGTATCTACTCAGCTAACAGAGTTGAACCTTTCTTTTG
        130 GTGATGTTTGCATTCAAGTAACAGTGTTGAACATTCCCTTTC

             *       *                   * *     *            ***        *   
     430620 AGAGAGCAGTTTTGAAACACTCTTTTTGTGGAATCTGCAAGTGGATATTTCTCTAGCTTTGTGGA
          1 ATAGAGCAGGTTTGAAACACTCTTTTTGTAGTATCTGGAAGTGGATATTTGGATAGCTTTGAGGA

                         *   *    *                   *       *     *        
     430685 TTTCGTTGGAAACGGGATTA-CAT-ATA-AAAAGC-AGACAGCAGCATTCCCAGAATCTTCTTTG
         66 TTTCGTTGGAAACAGGAATATCTTCATATAAAAGCTAGACAGAAGCATTCTCAGAAACTTCTTTG

                              *    *                 
     430746 TGATGTTTGCATTCAAGTCACAGAGTTGAACATTCCCTTTC
        131 TGATGTTTGCATTCAAGTAACAGTGTTGAACATTCCCTTTC

                                                   *     *       **     *   *
     430787 ATAGAGCAGGTTTGAAACACTCTTTTTGTAGTATCTGGATGTGGACATTTGGAGCGCTTTCAGGC
          1 ATAGAGCAGGTTTGAAACACTCTTTTTGTAGTATCTGGAAGTGGATATTTGGATAGCTTTGAGGA

            * ** *      *             *** *   *                      *       
     430852 CTATGGT-GAAAAAGGAAATATCTTCCCCTGAAAACTAGACAGAAGCATTCTCAGAATCTTCTTT
         66 TTTCGTTGGAAACAGG-AATATCTTCATATAAAAGCTAGACAGAAGCATTCTCAGAAACTTCTTT

                   **  **    *              *  * *   *
     430916 GTGATGTGCGCCCTCAACTAACAGTGTTGAACCTTTCTTTTG
        130 GTGATGTTTGCATTCAAGTAACAGTGTTGAACATTCCCTTTC

                     *                    *      *   *                       
     430958 ATAGAGCAGTTTTGAAACACTCTTTTTGTAATATCTGCAAGAGGATATTTGGATAGCTTTGAGGA
          1 ATAGAGCAGGTTTGAAACACTCTTTTTGTAGTATCTGGAAGTGGATATTTGGATAGCTTTGAGGA

                         *   * *   *        **             *        *    *   
     431023 TTTCGTTGGAAACGGGATTGTCTGCATATAAACTCTAGACAGAAGCAGTCTCAGAAGCTTCATTG
         66 TTTCGTTGGAAACAGGAATATCTTCATATAAAAGCTAGACAGAAGCATTCTCAGAAACTTCTTTG

            *       **   *    *             *        
     431088 GGATGTTTCAATTGAAGTCACAGTGTTGAACAGTCCCTTTC
        131 TGATGTTTGCATTCAAGTAACAGTGTTGAACATTCCCTTTC

                                                         *       *  * * *    
     431129 ATAGAGCAGGTTTGAAACACTCTTTTTGTAGTATCTGGAAGTGGACATTTGGAGAGATCTCAGGA
          1 ATAGAGCAGGTTTGAAACACTCTTTTTGTAGTATCTGGAAGTGGATATTTGGATAGCTTTGAGGA

            * *  *      *             **            *       * *          *  *
     431194 ATACGGT-GAAAAAGGAAATATCTTCCAATAAAAGCTAGATAGAAGCAATGTCAGAAACTTTTTC
         66 TTTCGTTGGAAACAGG-AATATCTTCATATAAAAGCTAGACAGAAGCATTCTCAGAAACTTCTTT

            *      * ** *   **      *       *  * *   *
     431258 ATGATGTATCTACTCAGCTAACAGAGTTGAACCTTTCTTTTG
        130 GTGATGTTTGCATTCAAGTAACAGTGTTGAACATTCCCTTTC

             *       *                   * *     *           * **            
     431300 AGAGAGCAGTTTTGAAACACTCTTTTTGTGGAATCTGCAAGTGGATATTGGTCTAGCTTTGAGGA
          1 ATAGAGCAGGTTTGAAACACTCTTTTTGTAGTATCTGGAAGTGGATATTTGGATAGCTTTGAGGA

                *        *        *                   *       *     *        
     431365 TTTCTTTGGAAACGGGAATA-CAT-ATA-AAAAGC-AGACAGCAGCATTCCCAGAATCTTCTTTG
         66 TTTCGTTGGAAACAGGAATATCTTCATATAAAAGCTAGACAGAAGCATTCTCAGAAACTTCTTTG

                              *    *                 
     431426 TGATGTTTGCATTCAAGTCACAGAGTTGAACATTCCCTTTC
        131 TGATGTTTGCATTCAAGTAACAGTGTTGAACATTCCCTTTC

                                                 * *     *       **     *   *
     431467 ATAGAGCAGGTTTGAAACACTCTTTTTGTAGTATCTGTATGTGGACATTTGGAGCGCTTTCAGGC
          1 ATAGAGCAGGTTTGAAACACTCTTTTTGTAGTATCTGGAAGTGGATATTTGGATAGCTTTGAGGA

            * ** *      *             *** *   *                      *   *   
     431532 CTATGGT-GAAAAAGGAAATATCTTCCCCTGAAAACTAGACAGAAGCATTCTCAGAATCTTATTT
         66 TTTCGTTGGAAACAGG-AATATCTTCATATAAAAGCTAGACAGAAGCATTCTCAGAAACTTCTTT

                   **  **    *             **  * *   *
     431596 GTGATGTGCGCCCTCAACTAACAGTGTTGAAGCTTTCTTTTG
        130 GTGATGTTTGCATTCAAGTAACAGTGTTGAACATTCCCTTTC

                     *                    **     *   *                       
     431638 ATAGAGCAGTTTTGAAACACTCTTTTTGTAAAATCTGCAAGAGGATATTTGGATAGCTTTGAGGA
          1 ATAGAGCAGGTTTGAAACACTCTTTTTGTAGTATCTGGAAGTGGATATTTGGATAGCTTTGAGGA

                         *   * *            **          *           *    *   
     431703 TTTCGTTGGAAACGGGATTGTCTTCATATAAACTCTAGACAGAATCATTCTCAGAAGCTTCATTG
         66 TTTCGTTGGAAACAGGAATATCTTCATATAAAAGCTAGACAGAAGCATTCTCAGAAACTTCTTTG

            *       **   *    *             *        
     431768 GGATGTTTCAATTGAAGTCACAGTGTTGAACAGTCCCTTTC
        131 TGATGTTTGCATTCAAGTAACAGTGTTGAACATTCCCTTTC

                                                         *       **   * *    
     431809 ATAGAGCAGGTTTGAAACACTC--TTTGTAGTATCTGGAAGTGGACATTTGGAGCGCTCTCAGGA
          1 ATAGAGCAGGTTTGAAACACTCTTTTTGTAGTATCTGGAAGTGGATATTTGGATAGCTTTGAGGA

            * *  *      *             **            *       * *          *  *
     431872 CTACGGT-GAAAAAGGAAGTATCTTCCAATAAAAGCTAGATAGAAGCAATGTCAGAAACTTTTTC
         66 TTTCGTTGGAAACAGGAA-TATCTTCATATAAAAGCTAGACAGAAGCATTCTCAGAAACTTCTTT

            *      * ** *   **      *       *  * *   *
     431936 ATGATGTATCTACTCAGCTAACAGAGTTGAACCTTTCTTTTG
        130 GTGATGTTTGCATTCAAGTAACAGTGTTGAACATTCCCTTTC

             *       *                   * *     *             **            
     431978 AGAGAGCAGTTTTGAAACACTCTTTTTGTGGAATCTGCAAGTGGATATTTGTCTAGCTTTGAGGA
          1 ATAGAGCAGGTTTGAAACACTCTTTTTGTAGTATCTGGAAGTGGATATTTGGATAGCTTTGAGGA

              *          *   *    *                   *       *              
     432043 TTACGTTGGAAACGGGATTA-CAT-ATA-AAAAGC-AGACAGCAGCATTCCCAGAAACTTCTTTG
         66 TTTCGTTGGAAACAGGAATATCTTCATATAAAAGCTAGACAGAAGCATTCTCAGAAACTTCTTTG

               *   *          *   **                 
     432104 TGAAGTTAGCATTCAAGTCACAAAGTTGAACATTCCCTTTC
        131 TGATGTTTGCATTCAAGTAACAGTGTTGAACATTCCCTTTC

                                          *              *       **     *   *
     432145 ATAGAGCAGGTTTGAAACACTCTTTTTGTACTATCTGGAAGTGGACATTTGGAGCGCTTTCAGGC
          1 ATAGAGCAGGTTTGAAACACTCTTTTTGTAGTATCTGGAAGTGGATATTTGGATAGCTTTGAGGA

            * ** *      *             *** *   *                      *   *   
     432210 CTATGGT-GAAAAAGGAAATATCTTCCCCTGAAAACTAGACAGAAGCATTCTCAGAATCTTATTT
         66 TTTCGTTGGAAACAGG-AATATCTTCATATAAAAGCTAGACAGAAGCATTCTCAGAAACTTCTTT

            *      **  **    *              *  * *   *
     432274 TTGATGTGCGCCCTCAACTAACAGTGTTGAACCTTTCTTTTG
        130 GTGATGTTTGCATTCAAGTAACAGTGTTGAACATTCCCTTTC

                     *                    *      *   *                       
     432316 ATAGAGCAGTTTTGAAACACTCTTTTTGTAATATCTGCAAGAGGATATTTGGATAGCTTTGAGGA
          1 ATAGAGCAGGTTTGAAACACTCTTTTTGTAGTATCTGGAAGTGGATATTTGGATAGCTTTGAGGA

                         *   * *            **                      *    *   
     432381 TTTCGTTGGAAACGGGATTGTCTTCATATAAACTCTAGACAGAAGCATTCTCAGAAGCTTCATTG
         66 TTTCGTTGGAAACAGGAATATCTTCATATAAAAGCTAGACAGAAGCATTCTCAGAAACTTCTTTG

            *       **   *    *             *        
     432446 GGATGTTTCAATTGAAGTCACAGTGTTGAACAGTCCCTTTC
        131 TGATGTTTGCATTCAAGTAACAGTGTTGAACATTCCCTTTC

                                                         *       *  * * *    
     432487 ATAGAGCAGGTTTGAAACACTCTTTTTGTAGTATCTGGAAGTGGACATTTGGAGAGATCTCAGGA
          1 ATAGAGCAGGTTTGAAACACTCTTTTTGTAGTATCTGGAAGTGGATATTTGGATAGCTTTGAGGA

            * *  *    * *             **            *       * *          *  *
     432552 ATACGGT-GATAAAGGAAATATCTTCCAATAAAAGCTAGATAGAAGCAATGTCAGAAACTTTTTC
         66 TTTCGTTGGAAACAGG-AATATCTTCATATAAAAGCTAGACAGAAGCATTCTCAGAAACTTCTTT

            *      * ** *   **      *       *  * *   *
     432616 ATGATGTATCTACTCAGCTAACAGAGTTGAACCTTTCTTTTG
        130 GTGATGTTTGCATTCAAGTAACAGTGTTGAACATTCCCTTTC

             *       *                   * *     *             **            
     432658 AGAGAGCAGTTTTGAAACACTCTTTTTGTGGAATCTGCAAGTGGATATTTGTCTAGCTTTGAGGA
          1 ATAGAGCAGGTTTGAAACACTCTTTTTGTAGTATCTGGAAGTGGATATTTGGATAGCTTTGAGGA

                         *   *    *                   *       *              
     432723 TTTCGTTGGAAACGGGATTA-CAT-ATA-AAAAGC-AGACAGCAGCATTCCCAGAAACTTCTTTG
         66 TTTCGTTGGAAACAGGAATATCTTCATATAAAAGCTAGACAGAAGCATTCTCAGAAACTTCTTTG

               *   *          *    *                 
     432784 TGAAGTTAGCATTCAAGTCACAGAGTTGAACATTCCCTTTC
        131 TGATGTTTGCATTCAAGTAACAGTGTTGAACATTCCCTTTC

                                                   *     *       **     *   *
     432825 ATAGAGCAGGTTTGAAACACTCTTTTTGTAGTATCTGGATGTGGACATTTGGAGCGCTTTCAGGC
          1 ATAGAGCAGGTTTGAAACACTCTTTTTGTAGTATCTGGAAGTGGATATTTGGATAGCTTTGAGGA

            * ** *      *             *** *   *                          *   
     432890 CTATGGT-GAAAAAGGAAATATCTTCCCCTGAAAACTAGACAGAAGCATTCTCAGAAACTTATTT
         66 TTTCGTTGGAAACAGG-AATATCTTCATATAAAAGCTAGACAGAAGCATTCTCAGAAACTTCTTT

                  ***  **    *             **  * *   *
     432954 GTGATGCGCGCCCTCAACTAACAGTGTTGAAGCTTTCTTTTG
        130 GTGATGTTTGCATTCAAGTAACAGTGTTGAACATTCCCTTTC

                     *                   * *     *             **            
     432996 ATAGAGCAGTTTTGAAACACTCTTTTTGTGGAATCTGCAAGTGGATATTTGTCTAGCTTTGAGGA
          1 ATAGAGCAGGTTTGAAACACTCTTTTTGTAGTATCTGGAAGTGGATATTTGGATAGCTTTGAGGA

                         *   *    *                   *       *     *        
     433061 TTTCGTTGGAAACGGGATTA-CAT-ATA-AAAAGC-AGACAGCAGCATTCCCAGAATCTTCTTTG
         66 TTTCGTTGGAAACAGGAATATCTTCATATAAAAGCTAGACAGAAGCATTCTCAGAAACTTCTTTG

                              *    *                 
     433122 TGATGTTTGCATTCAAGTCACAGAGTTGAACATTCCCTTTC
        131 TGATGTTTGCATTCAAGTAACAGTGTTGAACATTCCCTTTC

                                       *           * *   *       **     *   *
     433163 ATAGAGCAGGTTTGAAACACTCTTTTTATAGTATCTGGATGAGGACATTTGGAGCGCTTTCAGGC
          1 ATAGAGCAGGTTTGAAACACTCTTTTTGTAGTATCTGGAAGTGGATATTTGGATAGCTTTGAGGA

            * ** *  *   ** *         *** *   *                      *   *    
     433228 CTATGGTGAAAAGGGAAATATCTTCTCCTGAAAACTAGACAGAAGCATTCTCAGAATCTTATTTG
         66 TTTCGTTGGAAACAGGAATATCTTCATATAAAAGCTAGACAGAAGCATTCTCAGAAACTTCTTTG

                  **  **    *             **  * *   *
     433293 TGATGTGCGCCCTCAACTAACAGTGTTGAAGCTTTCTTTTG
        131 TGATGTTTGCATTCAAGTAACAGTGTTGAACATTCCCTTTC

                     *                    **     *   *                       
     433334 ATAGAGCAGTTTTGAAACACTCTTTTTGTAAAATCTGCAAGAGGATATTTGGATAGCTTTGAGGA
          1 ATAGAGCAGGTTTGAAACACTCTTTTTGTAGTATCTGGAAGTGGATATTTGGATAGCTTTGAGGA

                *        *   * *            **                      *    *   
     433399 TTTCATTGGAAACGGGATTGTCTTCATATAAACTCTAGACAGAAGCATTCTCAGAAGCTTCATTG
         66 TTTCGTTGGAAACAGGAATATCTTCATATAAAAGCTAGACAGAAGCATTCTCAGAAACTTCTTTG

            *       **   *    *             *        
     433464 GGATGTTTCAATTGAAGTCACAGTGTTGAACAGTCCCTTTC
        131 TGATGTTTGCATTCAAGTAACAGTGTTGAACATTCCCTTTC

                                                         *       *  * * *    
     433505 ATAGAGCAGGTTTGAAACACTCTTTTTGTAGTATCTGGAAGTGGACATTTGGAGAGATCTCAGGA
          1 ATAGAGCAGGTTTGAAACACTCTTTTTGTAGTATCTGGAAGTGGATATTTGGATAGCTTTGAGGA

            * *  *    * *             **            *       * *          *  *
     433570 ATACGGT-GATAAAGGAAATATCTTCCAATAAAAGCTAGATAGAAGCAATGTCAGAAACTTTTTC
         66 TTTCGTTGGAAACAGG-AATATCTTCATATAAAAGCTAGACAGAAGCATTCTCAGAAACTTCTTT

            *      * ** *   **      *       *  * *   *
     433634 ATGATGTATCTACTCAGCTAACAGAGTTGAACCTTTCTTTTG
        130 GTGATGTTTGCATTCAAGTAACAGTGTTGAACATTCCCTTTC

             *       *                   * *     *             **            
     433676 AGAGAGCAGTTTTGAAACACTCTTTTTGTGGAATCTGCAAGTGGATATTTGTCTAGCTTTGAGGA
          1 ATAGAGCAGGTTTGAAACACTCTTTTTGTAGTATCTGGAAGTGGATATTTGGATAGCTTTGAGGA

                   *     *   *    *                   *       *              
     433741 TTTCGTTCGAAACGGGATTA-CAT-ATA-AAAAGC-AGACAGCAGCATTCCCAGAAACTTCTTTG
         66 TTTCGTTGGAAACAGGAATATCTTCATATAAAAGCTAGACAGAAGCATTCTCAGAAACTTCTTTG

               *              *    *          *      
     433802 TGAAGTTTGCATTCAAGTCACAGAGTTGAACATTGCCTTTC
        131 TGATGTTTGCATTCAAGTAACAGTGTTGAACATTCCCTTTC

                                                   *     *       **     *   *
     433843 ATAGAGCAGGTTTGAAACACTCTTTTTGTAGTATCTGGATGTGGACATTTGGAGCGCTTTCAGGC
          1 ATAGAGCAGGTTTGAAACACTCTTTTTGTAGTATCTGGAAGTGGATATTTGGATAGCTTTGAGGA

            * ** *      *             *** *   *                      *   *   
     433908 CTATGGT-GAAAAAGGAAATATCTTCCCCTGAAAACTAGACAGAAGCATTCTCAGAATCTTATTT
         66 TTTCGTTGGAAACAGG-AATATCTTCATATAAAAGCTAGACAGAAGCATTCTCAGAAACTTCTTT

                   **  **    *             **  * *   *
     433972 GTGATGTGCGCCCTCAACTAACAGTGTTGAAGCTTTCTTTTG
        130 GTGATGTTTGCATTCAAGTAACAGTGTTGAACATTCCCTTTC

                     *                    **     *   *                       
     434014 ATAGAGCAGTTTTGAAACACTCTTTTTGTAAAATCTGCAAGAGGATATTTGGATAGCTTTGAGGA
          1 ATAGAGCAGGTTTGAAACACTCTTTTTGTAGTATCTGGAAGTGGATATTTGGATAGCTTTGAGGA

               *         *   * *            **                      *    *   
     434079 TTTTGTTGGAAACGGGATTGTCTTCATATAAACTCTAGACAGAAGCATTCTCAGAAGCTTCATTG
         66 TTTCGTTGGAAACAGGAATATCTTCATATAAAAGCTAGACAGAAGCATTCTCAGAAACTTCTTTG

            *       **   *    *             *        
     434144 GGATGTTTCAATTGAAGTCACAGTGTTGAACAGTCCCTTTC
        131 TGATGTTTGCATTCAAGTAACAGTGTTGAACATTCCCTTTC

                                                         *       **   * *    
     434185 ATAGAGCAGGTTTGAAACACTC--TTTGTAGTATCTGGAAGTGGACATTTGGAGCGCTCTCAGGA
          1 ATAGAGCAGGTTTGAAACACTCTTTTTGTAGTATCTGGAAGTGGATATTTGGATAGCTTTGAGGA

            * *  *      *             **            *       * *          *  *
     434248 CTACGGT-GAAAAAGGAAGTATCTTCTAATAAAAGCTAGATAGAAGCAATGTCAGAAACTTTTTC
         66 TTTCGTTGGAAACAGGAA-TATCTTCATATAAAAGCTAGACAGAAGCATTCTCAGAAACTTCTTT

            *      * ** *   **      *       *  * *   *
     434312 ATGATGTATCTACTCAGCTAACAGAGTTGAACCTTTCTTTTG
        130 GTGATGTTTGCATTCAAGTAACAGTGTTGAACATTCCCTTTC

             *       *                   * *     *             **            
     434354 AGAGAGCAGTTTTGAAACACTCTTTTTGTGGAATCTGCAAGTGGATATTTGTCTAGCTTTGAGGA
          1 ATAGAGCAGGTTTGAAACACTCTTTTTGTAGTATCTGGAAGTGGATATTTGGATAGCTTTGAGGA

                         *   *    *                   *       *              
     434419 TTTCGTTGGAAACGGGATTA-CAT-ATA-AAAAGC-AGACAGCAGCATTCCCAGAAACTTCTTTG
         66 TTTCGTTGGAAACAGGAATATCTTCATATAAAAGCTAGACAGAAGCATTCTCAGAAACTTCTTTG

                              *   **       *         
     434480 TGATGTTTGCATTCAAGTCACACAGTTGAACTTTCCCTTTC
        131 TGATGTTTGCATTCAAGTAACAGTGTTGAACATTCCCTTTC

                                                   *     *       **     *   *
     434521 ATAGAGCAGGTTTGAAACACTCTTTTTGTAGTATCTGGATGTGGACATTTGGAGCGCTTTCAGGC
          1 ATAGAGCAGGTTTGAAACACTCTTTTTGTAGTATCTGGAAGTGGATATTTGGATAGCTTTGAGGA

              ** *      *   *         *** *   *             *        **  *   
     434586 TTATGGT-GAAAAAGTAAATATCTTCCCCTGAAAACTAGACAGAAGCACTCTCAGAATTTTATTT
         66 TTTCGTTGGAAACAG-GAATATCTTCATATAAAAGCTAGACAGAAGCATTCTCAGAAACTTCTTT

                   **  **    *             **  * *   *
     434650 GTGATGTGCGCCCTCAACTAACAGTGTTGAAGCTTTCTTTTG
        130 GTGATGTTTGCATTCAAGTAACAGTGTTGAACATTCCCTTTC

                     *                    **     *   *                       
     434692 ATAGAGCAGTTTTGAAACACTCTTTTTGTAAAATCTGCAAGAGGATATTTGGATAGCTTTGAGGA
          1 ATAGAGCAGGTTTGAAACACTCTTTTTGTAGTATCTGGAAGTGGATATTTGGATAGCTTTGAGGA

                *   *    *   * *            **                      *    *   
     434757 TTTCTTTGAAAACTGGATTGTCTTCATATAAACTCTAGACAGAAGCATTCTCAGAAGCTTCATTG
         66 TTTCGTTGGAAACAGGAATATCTTCATATAAAAGCTAGACAGAAGCATTCTCAGAAACTTCTTTG

            *       **   *    *             *        
     434822 GGATGTTTCAATTGAAGTCACAGTGTTGAACAGTCCCTTTC
        131 TGATGTTTGCATTCAAGTAACAGTGTTGAACATTCCCTTTC

                                                   *     *       **     *   *
     434863 ATAGAGCAGGTTTGAAACACTCTTTTTGTAGTATCTGGATGTGGACATTTGGAGCGCTTTCAGGC
          1 ATAGAGCAGGTTTGAAACACTCTTTTTGTAGTATCTGGAAGTGGATATTTGGATAGCTTTGAGGA

            * ** *      *             *** *   *                          *   
     434928 CTATGGT-GAAAAAGGAAATATCTTCCCCTGAAAACTAGACAGAAGCATTCTCAGAAACTTATTT
         66 TTTCGTTGGAAACAGG-AATATCTTCATATAAAAGCTAGACAGAAGCATTCTCAGAAACTTCTTT

                   **  **    *             **  * *   *
     434992 GTGATGTGCGCCCTCAACTAACAGTGTTGAAGCTTTCTTTTG
        130 GTGATGTTTGCATTCAAGTAACAGTGTTGAACATTCCCTTTC

                     *                   * *     *             **            
     435034 ATAGAGCAGTTTTGAAACACTCTTTTTGTGGAATCTGCAAGTGGATATTTGTCTAGCTTTGAGGA
          1 ATAGAGCAGGTTTGAAACACTCTTTTTGTAGTATCTGGAAGTGGATATTTGGATAGCTTTGAGGA

                         *   *    *                   *       *     *        
     435099 TTTCGTTGGAAACGGGATTA-CAT-ATA-AAAAGC-AGACAGCAGCATTCCCAGAATCTTCTTTG
         66 TTTCGTTGGAAACAGGAATATCTTCATATAAAAGCTAGACAGAAGCATTCTCAGAAACTTCTTTG

            *                 *    *                 
     435160 GGATGTTTGCATTCAAGTCACAGAGTTGAACATTCCCTTTC
        131 TGATGTTTGCATTCAAGTAACAGTGTTGAACATTCCCTTTC

                                       *           *     *       **     *   *
     435201 ATAGAGCAGGTTTGAAACACTCTTTTTATAGTATCTGGATGTGGACATTTGGAGCGCTTTCAGGC
          1 ATAGAGCAGGTTTGAAACACTCTTTTTGTAGTATCTGGAAGTGGATATTTGGATAGCTTTGAGGA

            * ** *  *   ** *         *** *   *                      *   *    
     435266 CTATGGTGAAAAGGGAAATATCTTCTCCTGAAAACTAGACAGAAGCATTCTCAGAATCTTATTTG
         66 TTTCGTTGGAAACAGGAATATCTTCATATAAAAGCTAGACAGAAGCATTCTCAGAAACTTCTTTG

                  **  **    *              *  * *   *
     435331 TGATGTGCGCCCTCAACTAACAGTGTTGAACCTTTCTTTTG
        131 TGATGTTTGCATTCAAGTAACAGTGTTGAACATTCCCTTTC

                     *                    **     *   *                       
     435372 ATAGAGCAGTTTTGAAACACTCTTTTTGTAAAATCTGCAAGAGGATATTTGGATAGCTTTGAGGA
          1 ATAGAGCAGGTTTGAAACACTCTTTTTGTAGTATCTGGAAGTGGATATTTGGATAGCTTTGAGGA

                *        *   * *            **                      *    *   
     435437 TTTCATTGGAAACGGGATTGTCTTCATATAAACTCTAGACAGAAGCATTCTCAGAAGCTTCATTG
         66 TTTCGTTGGAAACAGGAATATCTTCATATAAAAGCTAGACAGAAGCATTCTCAGAAACTTCTTTG

            *       **   *    *         *   *        
     435502 GGATGTTTCAATTGAAGTCACAGTGTTGGACAGTCCCTTTC
        131 TGATGTTTGCATTCAAGTAACAGTGTTGAACATTCCCTTTC

                                                         *       ** * * *    
     435543 ATAGAGCAGGTTTGAAACACTCTTTTTGTAGTATCTGGAAGTGGACATTTGGAGCGATCTCAGGA
          1 ATAGAGCAGGTTTGAAACACTCTTTTTGTAGTATCTGGAAGTGGATATTTGGATAGCTTTGAGGA

            * *  *      *             **          * *       * *          *  *
     435608 CTACGGT-GAAAAAGGAAATATCTTCCAATAAAAGCTACATAGAAGCAATGTCAGAAACTTTTTC
         66 TTTCGTTGGAAACAGG-AATATCTTCATATAAAAGCTAGACAGAAGCATTCTCAGAAACTTCTTT

            *      * ** *   **      *       *  * *   *
     435672 ATGATGTATCTACTCAGCTAACAGAGTTGAACCTTTCTTTTG
        130 GTGATGTTTGCATTCAAGTAACAGTGTTGAACATTCCCTTTC

             *       *                   * *     *             **            
     435714 AGAGAGCAGTTTTGAAACACTCTTTTTGTGGAATCTGCAAGTGGATATTTGTCTAGCTTTGAGGA
          1 ATAGAGCAGGTTTGAAACACTCTTTTTGTAGTATCTGGAAGTGGATATTTGGATAGCTTTGAGGA

                         *   *    *                   *       *   *          
     435779 TTTCGTTGGAAACGGGATTA-CAT-ATA-AAAAGC-AGACAGCAGCATTCCCAGTAACTTCTTTG
         66 TTTCGTTGGAAACAGGAATATCTTCATATAAAAGCTAGACAGAAGCATTCTCAGAAACTTCTTTG

                              *    *                 
     435840 TGATGTTTGCATTCAAGTCACAGAGTTGAACATTCCCTTTC
        131 TGATGTTTGCATTCAAGTAACAGTGTTGAACATTCCCTTTC

                                                   *     *       **     *   *
     435881 ATAGAGCAGGTTTGAAACACTCTTTTTGTAGTATCTGGATGTGGACATTTGGAGCGCTTTCAGGC
          1 ATAGAGCAGGTTTGAAACACTCTTTTTGTAGTATCTGGAAGTGGATATTTGGATAGCTTTGAGGA

            * ** *      *             *** *   *                      *   *   
     435946 CTATGGT-GAAAAAGGAAATATCTTCCCCTGAAAACTAGACAGAAGCATTCTCAGAATCTTATTT
         66 TTTCGTTGGAAACAGG-AATATCTTCATATAAAAGCTAGACAGAAGCATTCTCAGAAACTTCTTT

                 * **  **    *           * **  * *   *
     436010 GTGATATGCGCTCTCAACTAACAGTGTTGCAGCTTTCTTTTG
        130 GTGATGTTTGCATTCAAGTAACAGTGTTGAACATTCCCTTTC

                     *                    **     *   *                       
     436052 ATAGAGCAGTTTTGAAACACTCTTTTTGTAAAATCTGCAAGAGGATATTTGGATAGCTTTGAGGA
          1 ATAGAGCAGGTTTGAAACACTCTTTTTGTAGTATCTGGAAGTGGATATTTGGATAGCTTTGAGGA

                         *   * *            **                      *    *   
     436117 TTTCGTTGGAAACGGGATTGTCTTCATATAAACTCTAGACAGAAGCATTCTCAGAAGCTTCATTG
         66 TTTCGTTGGAAACAGGAATATCTTCATATAAAAGCTAGACAGAAGCATTCTCAGAAACTTCTTTG

            *       **   *    *             *        
     436182 GGATGTTTCAATTGAAGTCACAGTGTTGAACAGTCCCTTTC
        131 TGATGTTTGCATTCAAGTAACAGTGTTGAACATTCCCTTTC

                                                         *       *  * * *    
     436223 ATAGAGCAGGTTTGAAACACTCTTTTTGTAGTATCTGGAAGTGGACATTTGGAGAGATCTCAGGA
          1 ATAGAGCAGGTTTGAAACACTCTTTTTGTAGTATCTGGAAGTGGATATTTGGATAGCTTTGAGGA

            * *  *    * *             **            *       * *          *  *
     436288 ATACGGT-GATAAAGGAAATATCTTCCAATAAAAGCTAGATAGAAGCAATGTCAGAAACTTTTTC
         66 TTTCGTTGGAAACAGG-AATATCTTCATATAAAAGCTAGACAGAAGCATTCTCAGAAACTTCTTT

            *      * ** *   **      *       *  * *   *
     436352 ATGATGTATCTACTCAGCTAACAGAGTTGAACCTTTCTTTTG
        130 GTGATGTTTGCATTCAAGTAACAGTGTTGAACATTCCCTTTC

             *       *                   * *     *             **            
     436394 AGAGAGCAGTTTTGAAACACTCTTTTTGTGGAATCTGCAAGTGGATATTTGTCTAGCTTTGAGGA
          1 ATAGAGCAGGTTTGAAACACTCTTTTTGTAGTATCTGGAAGTGGATATTTGGATAGCTTTGAGGA

                         *   *     *               *  *       *  *           
     436459 TTTCGTTGGAAACGGGATTA-C-ACATA-AAAAGC-AGAGAGCAGCATTCCCACAAACTTCTTTG
         66 TTTCGTTGGAAACAGGAATATCTTCATATAAAAGCTAGACAGAAGCATTCTCAGAAACTTCTTTG

            *                 *    *                 
     436520 CGATGTTTGCATTCAAGTCACAGAGTTGAACATTCCCTTTC
        131 TGATGTTTGCATTCAAGTAACAGTGTTGAACATTCCCTTTC

                  *                       *        *     *       **     * * *
     436561 ATAGAGGAGGTTTGAAACACTCTTTTTGTACTATCTGGATGTGGACATTTGGAGCGCTTTCAAGC
          1 ATAGAGCAGGTTTGAAACACTCTTTTTGTAGTATCTGGAAGTGGATATTTGGATAGCTTTGAGGA

            * ** *      *             *** *   *                          *   
     436626 CTAAGGT-GAAAAAGGAAATATCTTCCCCTGAAAACTAGACAGAAGCATTCTCAGAAACTTATTT
         66 TTTCGTTGGAAACAGG-AATATCTTCATATAAAAGCTAGACAGAAGCATTCTCAGAAACTTCTTT

                   **  **    *     *        *  * **  *
     436690 GTGATGTGCGCCCTCAACTAACACTGTTGAACCTTTCTCTTG
        130 GTGATGTTTGCATTCAAGTAACAGTGTTGAACATTCCCTTTC

                     *                    *      *   *                       
     436732 ATAGAGCAGTTTTGAAACACTCTTTTTGTAATATCTGCAAGAGGATATTTGGATAGCTTTGAGGA
          1 ATAGAGCAGGTTTGAAACACTCTTTTTGTAGTATCTGGAAGTGGATATTTGGATAGCTTTGAGGA

                         *   * *            **                      *    *   
     436797 TTTCGTTGGAAACGGGATTGTCTTCATATAAACTCTAGACAGAAGCATTCTCAGAAGCTTCATTG
         66 TTTCGTTGGAAACAGGAATATCTTCATATAAAAGCTAGACAGAAGCATTCTCAGAAACTTCTTTG

            *       **   *    *             *        
     436862 GGATGTTTCAATTGAAGTCACAGTGTTGAACAGTCCCTTTC
        131 TGATGTTTGCATTCAAGTAACAGTGTTGAACATTCCCTTTC

                                                         *       *  * * *    
     436903 ATAGAGCAGGTTTGAAACACTCTTTTTGTAGTATCTGGAAGTGGACATTTGGAGAGATCTCAGGA
          1 ATAGAGCAGGTTTGAAACACTCTTTTTGTAGTATCTGGAAGTGGATATTTGGATAGCTTTGAGGA

            * *  *    * *             **            *       * *          *  *
     436968 ATACGGT-GATAAAGGAAATATCTTCCAATAAAAGCTAGATAGAAGCAATGTCAGAAACTTTTTC
         66 TTTCGTTGGAAACAGG-AATATCTTCATATAAAAGCTAGACAGAAGCATTCTCAGAAACTTCTTT

            *      * ** *   **      *       *  * *   *
     437032 ATGATGTATCTACTCAGCTAACAGAGTTGAACCTTTCTTTTG
        130 GTGATGTTTGCATTCAAGTAACAGTGTTGAACATTCCCTTTC

             *       *                   * *     *             **            
     437074 AGAGAGCAGTTTTGAAACACTCTTTTTGTGGAATCTGCAAGTGGATATTTGTCTAGCTTTGAGGA
          1 ATAGAGCAGGTTTGAAACACTCTTTTTGTAGTATCTGGAAGTGGATATTTGGATAGCTTTGAGGA

                         *   *    *                   *       *     *        
     437139 TTTCGTTGGAAACGGGATTA-CAT-ATA-AAAAGC-AGACAGCAGCATTCCCAGAATCTTCTTTG
         66 TTTCGTTGGAAACAGGAATATCTTCATATAAAAGCTAGACAGAAGCATTCTCAGAAACTTCTTTG

                              *    *                 
     437200 TGATGTTTGCATTCAAGTCACAGAGTTGAACATTCCCTTTC
        131 TGATGTTTGCATTCAAGTAACAGTGTTGAACATTCCCTTTC

                                       *           *     *       **     *   *
     437241 ATAGAGCAGGTTTGAAACACTCTTTTTATAGTATCTGGATGTGGACATTTGGAGCGCTTTCAGGC
          1 ATAGAGCAGGTTTGAAACACTCTTTTTGTAGTATCTGGAAGTGGATATTTGGATAGCTTTGAGGA

            * ** *      *             *** *   *                      *   *   
     437306 CTATGGT-GAAAAAGGAAATATCTTCCCCTGAAAACTAGACAGAAGCATTCTCAGAATCTTATTT
         66 TTTCGTTGGAAACAGG-AATATCTTCATATAAAAGCTAGACAGAAGCATTCTCAGAAACTTCTTT

                   **  **    *              *  * *   *
     437370 GTGATGTGCGCCCTCAACTAACAGTGTTGAACCTTTCTTTTG
        130 GTGATGTTTGCATTCAAGTAACAGTGTTGAACATTCCCTTTC

                     *                    *      *   *         *             
     437412 ATAGAGCAGTTTTGAAACACTCTTTTTGTAATATCTGCAAGAGGATATTTGCATAGCTTTGAGGA
          1 ATAGAGCAGGTTTGAAACACTCTTTTTGTAGTATCTGGAAGTGGATATTTGGATAGCTTTGAGGA

                         *   * *            **                      *    *   
     437477 TTTCGTTGGAAACGGGATTGTCTTCATATAAACTCTAGACAGAAGCATTCTCAGAAGCTTCATTG
         66 TTTCGTTGGAAACAGGAATATCTTCATATAAAAGCTAGACAGAAGCATTCTCAGAAACTTCTTTG

            *       **   *    *             *        
     437542 GGATGTTTCAATTGAAGTCACAGTGTTGAACAGTCCCTTTC
        131 TGATGTTTGCATTCAAGTAACAGTGTTGAACATTCCCTTTC

                                                         *       *  * * *    
     437583 ATAGAGCAGGTTTGAAACACTCTTTTTGTAGTATCTGGAAGTGGACATTTGGAGAGATCTTAGGA
          1 ATAGAGCAGGTTTGAAACACTCTTTTTGTAGTATCTGGAAGTGGATATTTGGATAGCTTTGAGGA

            * ** *    * *             **            *       * *          *  *
     437648 ATAAGGT-GATAAAGGAAATATCTTCCAATAAAAGCTAGATAGAAGCAATGTCAGAAACTTTTTC
         66 TTTCGTTGGAAACAGG-AATATCTTCATATAAAAGCTAGACAGAAGCATTCTCAGAAACTTCTTT

            *      * ** *   **      *       *  * *   *
     437712 ATGATGTATCTACTCAGCTAACAGAGTTGAACCTTTCTTTTG
        130 GTGATGTTTGCATTCAAGTAACAGTGTTGAACATTCCCTTTC

             *       *           *       * *     *             **    *       
     437754 AGAGAGCAGTTTTGAAACACTATTTTTGTGGAATCTGCAAGTGGATATTTGTCTAGCATTGAGGA
          1 ATAGAGCAGGTTTGAAACACTCTTTTTGTAGTATCTGGAAGTGGATATTTGGATAGCTTTGAGGA

                         *   *    *                   *       *              
     437819 TTTCGTTGGAAACGGGATTA-CAT-ATA-AAAAGC-AGACAGCAGCATTCCCAGAAACTTCTTTG
         66 TTTCGTTGGAAACAGGAATATCTTCATATAAAAGCTAGACAGAAGCATTCTCAGAAACTTCTTTG

               *   *          *    *                 
     437880 TGAAGTTAGCATTCAAGTCACAGAGTTGAACATTCCCTTTC
        131 TGATGTTTGCATTCAAGTAACAGTGTTGAACATTCCCTTTC

                                                   *     *       **     *   *
     437921 ATAGAGCAGGTTTGAAACACTCTTTTTGTAGTATCTGGATGTGGACATTTGGAGCGCTTTCAGGC
          1 ATAGAGCAGGTTTGAAACACTCTTTTTGTAGTATCTGGAAGTGGATATTTGGATAGCTTTGAGGA

            * ** *      *             *** *   *                      *   *   
     437986 CTATGGT-GAAAAAGGAAATATCTTCCCCTGAAAACTAGACAGAAGCATTCTCAGAATCTTATTT
         66 TTTCGTTGGAAACAGG-AATATCTTCATATAAAAGCTAGACAGAAGCATTCTCAGAAACTTCTTT

                   **  **    *             **  * *   *
     438050 GTGATGTGCGCCCTCAACTAACAGTGTTGAAGCTTTCTTTTG
        130 GTGATGTTTGCATTCAAGTAACAGTGTTGAACATTCCCTTTC

                *    *                    **     *   *                       
     438092 ATAGGGCAGTTTTGAAACACTCTTTTTGTAAAATCTGCAAGAGGATATTTGGATAGCTTTGAGGA
          1 ATAGAGCAGGTTTGAAACACTCTTTTTGTAGTATCTGGAAGTGGATATTTGGATAGCTTTGAGGA

                *        *   * *            **                      *    *   
     438157 TTTCTTTGGAAACTGGATTGTCTTCATATAAACTCTAGACAGAAGCATTCTCAGAAGCTTCATTG
         66 TTTCGTTGGAAACAGGAATATCTTCATATAAAAGCTAGACAGAAGCATTCTCAGAAACTTCTTTG

            *       **   *    *             *        
     438222 GGATGTTTCAATTGAAGTCACAGTGTTGAACAGTCCCTTTC
        131 TGATGTTTGCATTCAAGTAACAGTGTTGAACATTCCCTTTC

                                       *           *     *       **     *   *
     438263 ATAGAGCAGGTTTGAAACACTCTTTTTTTAGTATCTGGATGTGGACATTTGGAGCGCTTTCAGGC
          1 ATAGAGCAGGTTTGAAACACTCTTTTTGTAGTATCTGGAAGTGGATATTTGGATAGCTTTGAGGA

            * ** *      *             *** *   *             *        **  *   
     438328 CTATGGT-GAAAAAGGAAATATCTTCCCCTGAAAACTAGACAGAAGCACTCTCAGAATTTTATTT
         66 TTTCGTTGGAAACAGG-AATATCTTCATATAAAAGCTAGACAGAAGCATTCTCAGAAACTTCTTT

                   **  **    *             **  * *   *
     438392 GTGATGTGCGCCCTCAACTAACAGTGTTGAAGCTTTCTTTTG
        130 GTGATGTTTGCATTCAAGTAACAGTGTTGAACATTCCCTTTC

                     *           *        **     *   *                       
     438434 ATAGAGCAGTTTTGAAACACTGTTTTTGTAAAATCTGAAAGAGGATATTTGGATAGCTTTGAGGA
          1 ATAGAGCAGGTTTGAAACACTCTTTTTGTAGTATCTGGAAGTGGATATTTGGATAGCTTTGAGGA

                *        *   * *            **                      *    *   
     438499 TTTCTTTGGAAACTGGATTGTCTTCATATAAACTCTAGACAGAAGCATTCTCAGAAGCTTCATTG
         66 TTTCGTTGGAAACAGGAATATCTTCATATAAAAGCTAGACAGAAGCATTCTCAGAAACTTCTTTG

            *       **   *    *             *        
     438564 GGATGTTTCAATTGAAGTCACAGTGTTGAACAGTCCCTTTC
        131 TGATGTTTGCATTCAAGTAACAGTGTTGAACATTCCCTTTC

                                                   *     *       **     *    
     438605 ATAGAGCAGGTTTGAAACACTCTTTTTGTAGTATCTGGATGTGGACATTTGGAGCGCTTTCAGGC
          1 ATAGAGCAGGTTTGAAACACTCTTTTTGTAGTATCTGGAAGTGGATATTTGGATAGCTTTGAGG-

              *   *      *             *** *   *                          *  
     438670 ATAT-GGT-GAAAAAGGAAATATCTTCCCCTGAAAACTAGACAGAAGCATTCTCAGAAACTTATT
         65 ATTTCGTTGGAAACAGG-AATATCTTCATATAAAAGCTAGACAGAAGCATTCTCAGAAACTTCTT

                    **  **    *             **  * *   *
     438733 TGTGATGTGCGCCCTCAACTAACAGTGTTGAAGCTTTCTTTTG
        129 TGTGATGTTTGCATTCAAGTAACAGTGTTGAACATTCCCTTTC

                     *                   * *     *             **            
     438776 ATAGAGCAGTTTTGAAACACTCTTTTTGTGGAATCTGCAAGTGGATATTTGTCTAGCTTTGAGGA
          1 ATAGAGCAGGTTTGAAACACTCTTTTTGTAGTATCTGGAAGTGGATATTTGGATAGCTTTGAGGA

                         *        *                   *       *     *        
     438841 TTTCGTTGGAAACGGGAATA-CAT-ATA-AAAAGC-AGACAGCAGCATTCCCAGAATCTTCTTTG
         66 TTTCGTTGGAAACAGGAATATCTTCATATAAAAGCTAGACAGAAGCATTCTCAGAAACTTCTTTG

                              *    *                 
     438902 TGATGTTTGCATTCAAGTCACAGAGTTGAACATTCCCTTTC
        131 TGATGTTTGCATTCAAGTAACAGTGTTGAACATTCCCTTTC

                      *                *           *     *       **     *   *
     438943 ATAGAGCAGGATTGAAACACTCTTTTTATAGTATCTGGATGTGGACATTTGGAGCGCTTTCAGGC
          1 ATAGAGCAGGTTTGAAACACTCTTTTTGTAGTATCTGGAAGTGGATATTTGGATAGCTTTGAGGA

            * ** *  *   ** *         *** *   *                      *   *    
     439008 CTATGGTGAAAAGGGAAATATCTTCTCCTGAAAACTAGACAGAAGCATTCTCAGAATCTTATTTG
         66 TTTCGTTGGAAACAGGAATATCTTCATATAAAAGCTAGACAGAAGCATTCTCAGAAACTTCTTTG

                  **  **    *             **  * *   *
     439073 TGATGTGCGCCCTCAACTAACAGTGTTGAAGCTTTCTTTTG
        131 TGATGTTTGCATTCAAGTAACAGTGTTGAACATTCCCTTTC

             *       *                           *      *      **            
     439114 ACAGAGCAGTTTTGAAACACTC--TTT-T--TATCTGCAAGTGGTTATTTGTCTAGCTTTGAGGA
          1 ATAGAGCAGGTTTGAAACACTCTTTTTGTAGTATCTGGAAGTGGATATTTGGATAGCTTTGAGGA

                         *   *    *                   *       *     *   *    
     439174 TTTCGTTGGAAACGGGATTA-CAT-ATA-AAAAGC-AGACAGCAGCATTCCCAGAATCTTGTTTG
         66 TTTCGTTGGAAACAGGAATATCTTCATATAAAAGCTAGACAGAAGCATTCTCAGAAACTTCTTTG

                              *    *                 
     439235 TGATGTTTGCATTCAAGTCACAGAGTTGAACATTCCCTTTC
        131 TGATGTTTGCATTCAAGTAACAGTGTTGAACATTCCCTTTC

             *                         *           *     *       **     *   *
     439276 AGAGAGCAGGTTTGAAACACTCTTTTTATAGTATCTGGATGTGGACATTTGGAGCGCTTTCAGGC
          1 ATAGAGCAGGTTTGAAACACTCTTTTTGTAGTATCTGGAAGTGGATATTTGGATAGCTTTGAGGA

            * ** *      *             *** *   *                      *   *   
     439341 CTATGGT-GAAAAAGGAAATATCTTCTCCTGAAAACTAGACAGAAGCATTCTCAGAATCTTATTT
         66 TTTCGTTGGAAACAGG-AATATCTTCATATAAAAGCTAGACAGAAGCATTCTCAGAAACTTCTTT

                   **  **    *             **  * *   *
     439405 GTGATGTGCGCCCTCAACTAACAGTGTTGAAGCTTTCTTTTG
        130 GTGATGTTTGCATTCAAGTAACAGTGTTGAACATTCCCTTTC

                     *                    **     *   *                       
     439447 ATAGAGCAGTTTTGAAACACTCTTTTTGTAAAATCTGCAAGAGGATATTTGGATAGCTTTGAGGA
          1 ATAGAGCAGGTTTGAAACACTCTTTTTGTAGTATCTGGAAGTGGATATTTGGATAGCTTTGAGGA

                         *   * *            **                      *    *   
     439512 TTTCGTTGGAAACGGGATTGTCTTCATATAAACTCTAGACAGAAGCATTCTCAGAAGCTTCATTG
         66 TTTCGTTGGAAACAGGAATATCTTCATATAAAAGCTAGACAGAAGCATTCTCAGAAACTTCTTTG

            *       **   *    *             *        
     439577 GGATGTTTCAATTGAAGTCACAGTGTTGAACAGTCCCTTTC
        131 TGATGTTTGCATTCAAGTAACAGTGTTGAACATTCCCTTTC

                                                         *       *  * * *    
     439618 ATAGAGCAGGTTTGAAACACTCTTTTTGTAGTATCTGGAAGTGGACATTTGGAGAGATCTCAGGA
          1 ATAGAGCAGGTTTGAAACACTCTTTTTGTAGTATCTGGAAGTGGATATTTGGATAGCTTTGAGGA

            * *  *    * *             **            *       * *          *  *
     439683 ATACGGT-GATAAAGGAAATATCTTCCAATAAAAGCTAGATAGAAGCAATGTCAGAAACTTTTTC
         66 TTTCGTTGGAAACAGG-AATATCTTCATATAAAAGCTAGACAGAAGCATTCTCAGAAACTTCTTT

            *      * ** *   **      *       *  * *   *
     439747 ATGATGTATCTACTCAGCTAACAGAGTTGAACCTTTCTTTTG
        130 GTGATGTTTGCATTCAAGTAACAGTGTTGAACATTCCCTTTC

             *       *                  ** *     *             **            
     439789 AGAGAGCAGTTTTGAAACACTCTTTTTGGGGAATCTGCAAGTGGATATTTGTCTAGCTTTGAGGA
          1 ATAGAGCAGGTTTGAAACACTCTTTTTGTAGTATCTGGAAGTGGATATTTGGATAGCTTTGAGGA

                         *   *     *               *  *       *  *           
     439854 TTTCGTTGGAAACGGGATTA-C-ACATA-AAAAGC-AGAGAGCAGCATTCCCACAAACTTCTTTG
         66 TTTCGTTGGAAACAGGAATATCTTCATATAAAAGCTAGACAGAAGCATTCTCAGAAACTTCTTTG

            *                 *    *                 
     439915 CGATGTTTGCATTCAAGTCACAGAGTTGAACATTCCCTTTC
        131 TGATGTTTGCATTCAAGTAACAGTGTTGAACATTCCCTTTC

                                          *        *     *   *   **     * * *
     439956 ATAGAGCAGGTTTGAAACACTCTTTTTGTACTATCTGGATGTGGACATTGGGAGCGCTTTCAAGC
          1 ATAGAGCAGGTTTGAAACACTCTTTTTGTAGTATCTGGAAGTGGATATTTGGATAGCTTTGAGGA

            * ** *      *             *** *   *                          *   
     440021 CTATGGT-GAAAAAGGAAATATCTTCCCCTGAAAACTAGACAGAAGCATTCTCAGAAACTTATTT
         66 TTTCGTTGGAAACAGG-AATATCTTCATATAAAAGCTAGACAGAAGCATTCTCAGAAACTTCTTT

                   **  **    *     *        *  * **  *
     440085 GTGATGTGCGCCCTCAACTAACACTGTTGAACCTTTCTCTTG
        130 GTGATGTTTGCATTCAAGTAACAGTGTTGAACATTCCCTTTC

                     *                    *      *   *                       
     440127 ATAGAGCAGTTTTGAAACACTCTTTTTGTAATATCTGCAAGAGGATATTTGGATAGCTTTGAGGA
          1 ATAGAGCAGGTTTGAAACACTCTTTTTGTAGTATCTGGAAGTGGATATTTGGATAGCTTTGAGGA

                         *   * *            **                      *    *   
     440192 TTTCGTTGGAAACGGGATTGTCTTCATATAAACTCTAGACAGAAGCATTCTCAGAAGCTTCATTG
         66 TTTCGTTGGAAACAGGAATATCTTCATATAAAAGCTAGACAGAAGCATTCTCAGAAACTTCTTTG

            *       **   *    *             *        
     440257 GGATGTTTCAATTGAAGTCACAGTGTTGAACAGTCCCTTTC
        131 TGATGTTTGCATTCAAGTAACAGTGTTGAACATTCCCTTTC

                                                         *       *  * * *    
     440298 ATAGAGCAGGTTTGAAACACTCTTTTTGTAGTATCTGGAAGTGGACATTTGGAGAGATCTCAGGA
          1 ATAGAGCAGGTTTGAAACACTCTTTTTGTAGTATCTGGAAGTGGATATTTGGATAGCTTTGAGGA

            * *  *    * *             **            *       * *          *  *
     440363 ATACGGT-GATAAAGGAAATATCTTCCAATAAAAGCTAGATAGAAGCAATGTCAGAAACTTTTTC
         66 TTTCGTTGGAAACAGG-AATATCTTCATATAAAAGCTAGACAGAAGCATTCTCAGAAACTTCTTT

            *      * ** *   **      *       *  * *   *
     440427 ATGATGTATCTACTCAGCTAACAGAGTTGAACCTTTCTTTTG
        130 GTGATGTTTGCATTCAAGTAACAGTGTTGAACATTCCCTTTC

             *       *                   * *     *             **            
     440469 AGAGAGCAGTTTTGAAACACTCTTTTTGTGGAATCTGCAAGTGGATATTTGTCTAGCTTTGAGGA
          1 ATAGAGCAGGTTTGAAACACTCTTTTTGTAGTATCTGGAAGTGGATATTTGGATAGCTTTGAGGA

                         *   *    *                   *       *     *        
     440534 TTTCGTTGGAAACGGGATTA-CAT-ATA-AAAAGC-AGACAGCAGCATTCCCAGAATCTTCTTTG
         66 TTTCGTTGGAAACAGGAATATCTTCATATAAAAGCTAGACAGAAGCATTCTCAGAAACTTCTTTG

                              *    *                 
     440595 TGATGTTTGCATTCAAGTCACAGAGTTGAACATTCCCTTTC
        131 TGATGTTTGCATTCAAGTAACAGTGTTGAACATTCCCTTTC

                                       *           *     *       **     *   *
     440636 ATAGAGCAGGTTTGAAACACTCTTTTTATAGTATCTGGATGTGGACATTTGGAGCGCTTTCAGGC
          1 ATAGAGCAGGTTTGAAACACTCTTTTTGTAGTATCTGGAAGTGGATATTTGGATAGCTTTGAGGA

            * ** *      *             *** *   *                      *   *   
     440701 CTATGGT-GAAAAAGGAAATATCTTCCCCTGAAAACTAGACAGAAGCATTCTCAGAATCTTATTT
         66 TTTCGTTGGAAACAGG-AATATCTTCATATAAAAGCTAGACAGAAGCATTCTCAGAAACTTCTTT

                   **  **    *   *          *  * *   *
     440765 GTGATGTGCGCCCTCAACTAAGAGTGTTGAACCTTTCTTTTG
        130 GTGATGTTTGCATTCAAGTAACAGTGTTGAACATTCCCTTTC

                     *                    *      *   *         *             
     440807 ATAGAGCAGTTTTGAAACACTCTTTTTGTAATATCTGCAAGAGGATATTTGCATAGCTTTGAGGA
          1 ATAGAGCAGGTTTGAAACACTCTTTTTGTAGTATCTGGAAGTGGATATTTGGATAGCTTTGAGGA

                         *   * *            **                      *    *   
     440872 TTTCGTTGGAAACGGGATTGTCTTCATATAAACTCTAGACAGAAGCATTCTCAGAAGCTTCATTG
         66 TTTCGTTGGAAACAGGAATATCTTCATATAAAAGCTAGACAGAAGCATTCTCAGAAACTTCTTTG

            *       **   *    *             *        
     440937 GGATGTTTCAATTGAAGTCACAGTGTTGAACAGTCCCTTTC
        131 TGATGTTTGCATTCAAGTAACAGTGTTGAACATTCCCTTTC

                                                         *       *  * * *    
     440978 ATAGAGCAGGTTTGAAACACTCTTTTTGTAGTATCTGGAAGTGGACATTTGGAGAGATCTCAGGA
          1 ATAGAGCAGGTTTGAAACACTCTTTTTGTAGTATCTGGAAGTGGATATTTGGATAGCTTTGAGGA

            * *  *    * *             **            *       * *          *  *
     441043 ATACGGT-GATAAAGGAAATATCTTCCAATAAAAGCTAGATAGAAGCAATGTCAGAAACTTTTTC
         66 TTTCGTTGGAAACAGG-AATATCTTCATATAAAAGCTAGACAGAAGCATTCTCAGAAACTTCTTT

            *      * ** *   **      *       *  * *   *
     441107 ATGATGTATCTACTCAGCTAACAGAGTTGAACCTTTCTTTTG
        130 GTGATGTTTGCATTCAAGTAACAGTGTTGAACATTCCCTTTC

             *       *                   * *     *             **    *       
     441149 AGAGAGCAGTTTTGAAACACTCTTTTTGTGGAATCTGCAAGTGGATATTTGTCTAGCATTGAGGA
          1 ATAGAGCAGGTTTGAAACACTCTTTTTGTAGTATCTGGAAGTGGATATTTGGATAGCTTTGAGGA

                         *   *    *                   *       *              
     441214 TTTCGTTGGAAACGGGATTA-CAT-ATA-AAAAGC-AGACAGCAGCATTCCCAGAAACTTCTTTG
         66 TTTCGTTGGAAACAGGAATATCTTCATATAAAAGCTAGACAGAAGCATTCTCAGAAACTTCTTTG

               *   *          *    *                 
     441275 TGAAGTTAGCATTCAAGTCACAGAGTTGAACATTCCCTTTC
        131 TGATGTTTGCATTCAAGTAACAGTGTTGAACATTCCCTTTC

                                                   *     *       **     *   *
     441316 ATAGAGCAGGTTTGAAACACTCTTTTTGTAGTATCTGGATGTGGACATTTGGAGCGCTTTCAGGC
          1 ATAGAGCAGGTTTGAAACACTCTTTTTGTAGTATCTGGAAGTGGATATTTGGATAGCTTTGAGGA

            * ** *      *             *** *   *                      *   *   
     441381 CTATGGT-GAAAAAGGAAATATCTTCCCCTGAAAACTAGACAGAAGCATTCTCAGAATCTTATTT
         66 TTTCGTTGGAAACAGG-AATATCTTCATATAAAAGCTAGACAGAAGCATTCTCAGAAACTTCTTT

                   **  **    *             **  * *   *
     441445 GTGATGTGCGCCCTCAACTAACAGTGTTGAAGCTTTCTTTTG
        130 GTGATGTTTGCATTCAAGTAACAGTGTTGAACATTCCCTTTC

                     *                    **     *   *                       
     441487 ATAGAGCAGTTTTGAAACACTCTTTTTGTAAAATCTGCAAGAGGATATTTGGATAGCTTTGAGGA
          1 ATAGAGCAGGTTTGAAACACTCTTTTTGTAGTATCTGGAAGTGGATATTTGGATAGCTTTGAGGA

                *        *   * *            **                      *    *   
     441552 TTTCTTTGGAAACTGGATTGTCTTCATATAAACTCTAGACAGAAGCATTCTCAGAAGCTTCATTG
         66 TTTCGTTGGAAACAGGAATATCTTCATATAAAAGCTAGACAGAAGCATTCTCAGAAACTTCTTTG

            *       **   *    *             *        
     441617 GGATGTTTCAATTGAAGTCACAGTGTTGAACAGTCCCTTTC
        131 TGATGTTTGCATTCAAGTAACAGTGTTGAACATTCCCTTTC

                                       *           *     *       **     *   *
     441658 ATAGAGCAGGTTTGAAACACTCTTTTTTTAGTATCTGGATGTGGACATTTGGAGCGCTTTCAGGC
          1 ATAGAGCAGGTTTGAAACACTCTTTTTGTAGTATCTGGAAGTGGATATTTGGATAGCTTTGAGGA

            * ** *      *             *** *   *             *        **  *   
     441723 CTATGGT-GAAAAAGGAAATATCTTCCCCTGAAAACTAGACAGAAGCACTCTCAGAATTTTATTT
         66 TTTCGTTGGAAACAGG-AATATCTTCATATAAAAGCTAGACAGAAGCATTCTCAGAAACTTCTTT

                   **  **    *             **  * *   *
     441787 GTGATGTGCGCCCTCAACTAACAGTGTTGAAGCTTTCTTTTG
        130 GTGATGTTTGCATTCAAGTAACAGTGTTGAACATTCCCTTTC

                     *                    **     *   *                       
     441829 ATAGAGCAGTTTTGAAACACTCTTTTTGTAAAATCTGAAAGAGGATATTTGGATAGCTTTGAGGA
          1 ATAGAGCAGGTTTGAAACACTCTTTTTGTAGTATCTGGAAGTGGATATTTGGATAGCTTTGAGGA

                *        *   * *            **                      *    *   
     441894 TTTCTTTGGAAACTGGATTGTCTTCATATAAACTCTAGACAGAAGCATTCTCAGAAGCTTCATTG
         66 TTTCGTTGGAAACAGGAATATCTTCATATAAAAGCTAGACAGAAGCATTCTCAGAAACTTCTTTG

            *       **   *    *             *        
     441959 GGATGTTTCAATTGAAGTCACAGTGTTGAACAGTCCCTTTC
        131 TGATGTTTGCATTCAAGTAACAGTGTTGAACATTCCCTTTC

                                                   *     *       **     *    
     442000 ATAGAGCAGGTTTGAAACACTCTTTTTGTAGTATCTGGATGTGGACATTTGGAGCGCTTTCAGGC
          1 ATAGAGCAGGTTTGAAACACTCTTTTTGTAGTATCTGGAAGTGGATATTTGGATAGCTTTGAGG-

              *   *      *             *** *   *                          *  
     442065 ATAT-GGT-GAAAAAGGAAATATCTTCCCCTGAAAACTAGACAGAAGCATTCTCAGAAACTTATT
         65 ATTTCGTTGGAAACAGG-AATATCTTCATATAAAAGCTAGACAGAAGCATTCTCAGAAACTTCTT

                    **  **    *             **  * *   *
     442128 TGTGATGTGCGCCCTCAACTAACAGTGTTGAAGCTTTCTTTTG
        129 TGTGATGTTTGCATTCAAGTAACAGTGTTGAACATTCCCTTTC

                     *                   * *     *             **            
     442171 ATAGAGCAGTTTTGAAACACTCTTTTTGTGGAATCTGCAAGTGGATATTTGTCTAGCTTTGAGGA
          1 ATAGAGCAGGTTTGAAACACTCTTTTTGTAGTATCTGGAAGTGGATATTTGGATAGCTTTGAGGA

                         *        *                   *       *     *        
     442236 TTTCGTTGGAAACGGGAATA-CAT-ATA-AAAAGC-AGACAGCAGCATTCCCAGAATCTTCTTTG
         66 TTTCGTTGGAAACAGGAATATCTTCATATAAAAGCTAGACAGAAGCATTCTCAGAAACTTCTTTG

                              *    *                 
     442297 TGATGTTTGCATTCAAGTCACAGAGTTGAACATTCCCTTTC
        131 TGATGTTTGCATTCAAGTAACAGTGTTGAACATTCCCTTTC

                      *                *           *     *       **     *   *
     442338 ATAGAGCAGGATTGAAACACTCTTTTTATAGTATCTGGATGTGGACATTTGGAGCGCTTTCAGGC
          1 ATAGAGCAGGTTTGAAACACTCTTTTTGTAGTATCTGGAAGTGGATATTTGGATAGCTTTGAGGA

            * ** *  *   ** *         *** *   *                      *   *    
     442403 CTATGGTGAAAAGGGAAATATCTTCTCCTGAAAACTAGACAGAAGCATTCTCAGAATCTTATTTG
         66 TTTCGTTGGAAACAGGAATATCTTCATATAAAAGCTAGACAGAAGCATTCTCAGAAACTTCTTTG

                  **  **    *             **  * *   *
     442468 TGATGTGCGCCCTCAACTAACAGTGTTGAAGCTTTCTTTTG
        131 TGATGTTTGCATTCAAGTAACAGTGTTGAACATTCCCTTTC

             *       *                           *             **            
     442509 ACAGAGCAGTTTTGAAACACTC--TTT-T--TATCTGCAAGTGGATATTTGTCTAGCTTTGAGGA
          1 ATAGAGCAGGTTTGAAACACTCTTTTTGTAGTATCTGGAAGTGGATATTTGGATAGCTTTGAGGA

                         *   *    *                   *       *     *   *    
     442569 TTTCGTTGGAAACGGGATTA-CAT-ATA-AAAAGC-AGACAGCAGCATTCCCAGAATCTTGTTTG
         66 TTTCGTTGGAAACAGGAATATCTTCATATAAAAGCTAGACAGAAGCATTCTCAGAAACTTCTTTG

                              *    *                 
     442630 TGATGTTTGCATTCAAGTCACAGAGTTGAACATTCCCTTTC
        131 TGATGTTTGCATTCAAGTAACAGTGTTGAACATTCCCTTTC

             *                         *  *        *     *    *  **     *   *
     442671 AGAGAGCAGGTTTGAAACACTCTTTTTATATTATCTGGATGTGGACATTTTGAGCGCTTTCAGGC
          1 ATAGAGCAGGTTTGAAACACTCTTTTTGTAGTATCTGGAAGTGGATATTTGGATAGCTTTGAGGA

            * ** *      *             *** *   *                      *   *   
     442736 CTATGGT-GAAAAAGGAAATATCTTCTCCTGAAAACTAGACAGAAGCATTCTCAGAATCTTATTT
         66 TTTCGTTGGAAACAGG-AATATCTTCATATAAAAGCTAGACAGAAGCATTCTCAGAAACTTCTTT

                   **  **    *             **  * *   *
     442800 GTGATGTGCGCCCTCAACTAACAGTGTTGAAGCTTTCTTTTG
        130 GTGATGTTTGCATTCAAGTAACAGTGTTGAACATTCCCTTTC

                     *                    **  *  *   *                     * 
     442842 ATAGAGCAGTTTTGAAACACTCTTTTTGTAAAATTTGCAAGAGGATATTTGGATAGCTTTGAGAA
          1 ATAGAGCAGGTTTGAAACACTCTTTTTGTAGTATCTGGAAGTGGATATTTGGATAGCTTTGAGGA

                *        *   * *            **                      *    *   
     442907 TTTCATTGGAAACGGGATTGTCTTCATATAAACTCTAGACAGAAGCATTCTCAGAAGCTTCATTG
         66 TTTCGTTGGAAACAGGAATATCTTCATATAAAAGCTAGACAGAAGCATTCTCAGAAACTTCTTTG

            *       **   *    *             *        
     442972 GGATGTTTCAATTGAAGTCACAGTGTTGAACAGTCCCTTTC
        131 TGATGTTTGCATTCAAGTAACAGTGTTGAACATTCCCTTTC

                                                         *       **   * *    
     443013 ATAGAGCAGGTTTGAAACACTCTTTTTGTAGTATCTGGAAGTGGACATTTGGAGCGCTCTCAGGA
          1 ATAGAGCAGGTTTGAAACACTCTTTTTGTAGTATCTGGAAGTGGATATTTGGATAGCTTTGAGGA

            * *  *      *             **          * *       * *          *  *
     443078 CTACGGT-GAAAAAGGAAATATCTTCCAATAAAAGCTACATAGAAGCAATGTCAGAAACTTTTTC
         66 TTTCGTTGGAAACAGG-AATATCTTCATATAAAAGCTAGACAGAAGCATTCTCAGAAACTTCTTT

            *      * ** *   **      *       *  * *   *
     443142 ATGATGTATCTACTCAGCTAACAGAGTTGAACCTTTCTTTTG
        130 GTGATGTTTGCATTCAAGTAACAGTGTTGAACATTCCCTTTC

             *       *                   * *                   **            
     443184 AGAGAGCAGTTTTGAAACACTCTTTTTGTGGAATCTGGAAGTGGATATTTGTCTAGCTTTGAGGA
          1 ATAGAGCAGGTTTGAAACACTCTTTTTGTAGTATCTGGAAGTGGATATTTGGATAGCTTTGAGGA

                         *   *    *                   *       *   *          
     443249 TTTCGTTGGAAACGGGATTA-CAT-ATA-AAAAGC-AGACAGCAGCATTCCCAGTAACTTCTTTG
         66 TTTCGTTGGAAACAGGAATATCTTCATATAAAAGCTAGACAGAAGCATTCTCAGAAACTTCTTTG

                              *   **                 
     443310 TGATGTTTGCATTCAAGTCACAAAGTTGAACATTCCCTTTC
        131 TGATGTTTGCATTCAAGTAACAGTGTTGAACATTCCCTTTC

                                                   *     *       ***    *   *
     443351 ATAGAGCAGGTTTGAAACACTCTTTTTGTAGTATCTGGATGTGGACATTTGGAGCACTTTCAGGC
          1 ATAGAGCAGGTTTGAAACACTCTTTTTGTAGTATCTGGAAGTGGATATTTGGATAGCTTTGAGGA

            * ** *      *             *** *   *                      *   *   
     443416 CTATGGT-GAAAAAGGAAATATCTTCCCCTGAAAACTAGACAGAAGCATTCTCAGAATCTTATTT
         66 TTTCGTTGGAAACAGG-AATATCTTCATATAAAAGCTAGACAGAAGCATTCTCAGAAACTTCTTT

                   **  **    *              *  * *   *
     443480 GTGATGTGCGCCCTCAACTAACAGTGTTGAACCTTTCTTTTG
        130 GTGATGTTTGCATTCAAGTAACAGTGTTGAACATTCCCTTTC

                     *                    **     *   *                    *  
     443522 ATAGAGCAGTTTTGAAACACTCTTTTTGTAAAATCTGCAAGAGGATATTTGGATAGCTTTGAAGA
          1 ATAGAGCAGGTTTGAAACACTCTTTTTGTAGTATCTGGAAGTGGATATTTGGATAGCTTTGAGGA

                         *   * *            **                      *    *   
     443587 TTTCGTTGGAAACGGGATTGTCTTCATATAAACTCTAGACAGAAGCATTCTCAGAAGCTTCATTG
         66 TTTCGTTGGAAACAGGAATATCTTCATATAAAAGCTAGACAGAAGCATTCTCAGAAACTTCTTTG

            *       **   *    *             *        
     443652 GGATGTTTCAATTGAAGTCACAGTGTTGAACAGTCCCTTTC
        131 TGATGTTTGCATTCAAGTAACAGTGTTGAACATTCCCTTTC

                  *                  *                   *       ** * * *    
     443693 ATAGAGGAGGTTTGAAACACTCTTTATGTAGTATCTGGAAGTGGACATTTGGAGTGATCTCAGGA
          1 ATAGAGCAGGTTTGAAACACTCTTTTTGTAGTATCTGGAAGTGGATATTTGGATAGCTTTGAGGA

            * *  *    * *             **            *       * *          *  *
     443758 ATACGGT-GATAAAGGAAATATCTTCCAATAAAAGCTAGATAGAAGCAATGTCAGAAACTTTTTC
         66 TTTCGTTGGAAACAGG-AATATCTTCATATAAAAGCTAGACAGAAGCATTCTCAGAAACTTCTTT

            *      * ** *   **      *       *  * *   *
     443822 ATGATGTATCTACTCAGCTAACAGAGTTGAACCTTTCTTTTG
        130 GTGATGTTTGCATTCAAGTAACAGTGTTGAACATTCCCTTTC

             *       *                   * * *   *            ***            
     443864 AGAGAGCAGTTTTGAAACACTCTTTTTGTGGAAACTGCAAGTGGATATTTCTCTAGCTTTGAGGA
          1 ATAGAGCAGGTTTGAAACACTCTTTTTGTAGTATCTGGAAGTGGATATTTGGATAGCTTTGAGGA

                         *   *    *                   *       *              
     443929 TTTCGTTGGAAACGGGATTA-CAT-ATA-AAAAGC-AGACAGCAGCATTCCCAGAAACTTCTTTG
         66 TTTCGTTGGAAACAGGAATATCTTCATATAAAAGCTAGACAGAAGCATTCTCAGAAACTTCTTTG

                              *    *                 
     443990 TGATGTTTGCATTCAAGTCACAGAGTTGAACATTCCCTTTC
        131 TGATGTTTGCATTCAAGTAACAGTGTTGAACATTCCCTTTC

                                                   *     *       **     *   *
     444031 ATAGAGCAGGTTTGAAACACTCTTTTTGTAGTATCTGGATGTGGACATTTGGAGCGCTTTCAGGC
          1 ATAGAGCAGGTTTGAAACACTCTTTTTGTAGTATCTGGAAGTGGATATTTGGATAGCTTTGAGGA

            * ** *      *             *** *   *                          *   
     444096 CTATGGT-GAAAAAGGAAATATCTTCCCCTGAAAACTAGACAGAAGCATTCTCAGAAACTTATTT
         66 TTTCGTTGGAAACAGG-AATATCTTCATATAAAAGCTAGACAGAAGCATTCTCAGAAACTTCTTT

                   **  **    *              *  * *   *
     444160 GTGATGTGCGCCCTCAACTAACAGTGTTGAACCTTTCTTTTG
        130 GTGATGTTTGCATTCAAGTAACAGTGTTGAACATTCCCTTTC

                     *                  * *      *   *                       
     444202 ATAGAGCAGTTTTGAAACACTCTTTTTGCAATATCTGCAAGAGGATATTTGGATAGCTTTGAGGA
          1 ATAGAGCAGGTTTGAAACACTCTTTTTGTAGTATCTGGAAGTGGATATTTGGATAGCTTTGAGGA

                         *   * *            **                      *    *   
     444267 TTTCGTTGGAAACGGGATTGTCTTCATATAAACTCTAGACAGAAGCATTCTCAGAAGCTTCATTG
         66 TTTCGTTGGAAACAGGAATATCTTCATATAAAAGCTAGACAGAAGCATTCTCAGAAACTTCTTTG

            *       **   *    *             * *      
     444332 GGATGTTTCAATTGAAGTCACAGTGTTGAACAGTTCCTTTC
        131 TGATGTTTGCATTCAAGTAACAGTGTTGAACATTCCCTTTC

                 *                                     * *       ***  * *    
     444373 ATAGAACAGGTTTGAAACACTCTTTTTGTAGTATCTGGAAGTGAACATTTGGAGCACTCTCAGGA
          1 ATAGAGCAGGTTTGAAACACTCTTTTTGTAGTATCTGGAAGTGGATATTTGGATAGCTTTGAGGA

            * *  *      *             **            *       * *          *  *
     444438 CTGCGGT-GAAAAAGGAAATATCTTCCAATAAAAGCTAGATAGAAGCAATGTCAGAAACTTTTTC
         66 TTTCGTTGGAAACAGG-AATATCTTCATATAAAAGCTAGACAGAAGCATTCTCAGAAACTTCTTT

            *      * ** *   **      *       *        *
     444502 ATGATGTATCTACTCAGCTAACAGAGTTGAACCTT-CCTTTG
        130 GTGATGTTTGCATTCAAGTAACAGTGTTGAACATTCCCTTTC

             *       *                   * *     *             **            
     444543 AGAGAGCAGTTTTGAAACACTCTTTTTGTGGAATCTGCAAGTGGATATTTGCCTAGCTTTGAGGA
          1 ATAGAGCAGGTTTGAAACACTCTTTTTGTAGTATCTGGAAGTGGATATTTGGATAGCTTTGAGGA

                 *       *   *    *                   *       *              
     444608 TTTCGCTGGAAACGGGATTA-CAT-ATA-AAAAGC-AGACAGCAGCATTCCCAGAAACTTCTTTG
         66 TTTCGTTGGAAACAGGAATATCTTCATATAAAAGCTAGACAGAAGCATTCTCAGAAACTTCTTTG

                              *    *                 
     444669 TGATGTTTGCATTCAAGTCACAGAGTTGAACATTCCCTTTC
        131 TGATGTTTGCATTCAAGTAACAGTGTTGAACATTCCCTTTC

                                                 * *     *     * **     *   *
     444710 ATAGAGCAGGTTTGAAACACTCTTTTTGTAGTATCTGTATGTGGACATTTGCAGCGCTTTCAGGC
          1 ATAGAGCAGGTTTGAAACACTCTTTTTGTAGTATCTGGAAGTGGATATTTGGATAGCTTTGAGGA

            * ** *      *             *** *   *                          *   
     444775 CTAAGGT-GAAAAAGGAAATATCTTCCCCTGAAAACTAGACAGAAGCATTCTCAGAAACTTATTT
         66 TTTCGTTGGAAACAGG-AATATCTTCATATAAAAGCTAGACAGAAGCATTCTCAGAAACTTCTTT

            *      **  **    *             **  * *   *
     444839 TTGATGTGCGCCCTCAACTAACAGTGTTGAAGCTTTCTTTTG
        130 GTGATGTTTGCATTCAAGTAACAGTGTTGAACATTCCCTTTC

             *       *                           *             **            
     444881 ACAGAGCAGTTTTGAAACACTC--TTT-T--TATCTGCAAGTGGATATTTGTCTAGCTTTGAGGA
          1 ATAGAGCAGGTTTGAAACACTCTTTTTGTAGTATCTGGAAGTGGATATTTGGATAGCTTTGAGGA

                         *   *    *                   *       *     *   *    
     444941 TTTCGTTGGAAACGGGATTA-CAT-ATA-AAAAGC-AGACAGCAGCATTCCCAGAATCTTGTTTG
         66 TTTCGTTGGAAACAGGAATATCTTCATATAAAAGCTAGACAGAAGCATTCTCAGAAACTTCTTTG

                              *    *                 
     445002 TGATGTTTGCATTCAAGTCACAGAGTTGAACATTCCCTTTC
        131 TGATGTTTGCATTCAAGTAACAGTGTTGAACATTCCCTTTC

             *                         *           *     *       **     *   *
     445043 AGAGAGCAGGTTTGAAACACTCTTTTTATAGTATCTGGATGTGGACATTTGGAGCGCTTTCAGGC
          1 ATAGAGCAGGTTTGAAACACTCTTTTTGTAGTATCTGGAAGTGGATATTTGGATAGCTTTGAGGA

            * ** *      *             *** *   *                      *   *   
     445108 CTATGGT-GAAAAAGGAAATATCTTCTCCTGAAAACTAGACAGAAGCATTCTCAGAATCTTATTT
         66 TTTCGTTGGAAACAGG-AATATCTTCATATAAAAGCTAGACAGAAGCATTCTCAGAAACTTCTTT

                   **  **    *             **  * *   *
     445172 GTGATGTGCGCCCTCAACTAACAGTGTTGAAGCTTTCTTTTG
        130 GTGATGTTTGCATTCAAGTAACAGTGTTGAACATTCCCTTTC

                     *                    **     *   *                     * 
     445214 ATAGAGCAGTTTTGAAACACTCTTTTTGTAAAATCTGCAAGAGGATATTTGGATAGCTTTGAGAA
          1 ATAGAGCAGGTTTGAAACACTCTTTTTGTAGTATCTGGAAGTGGATATTTGGATAGCTTTGAGGA

                *        *   * *            **                      *    *   
     445279 TTTCATTGGAAACGGGATTGTCTTCATATAAACTCTAGACAGAAGCATTCTCAGAAGCTTCATTG
         66 TTTCGTTGGAAACAGGAATATCTTCATATAAAAGCTAGACAGAAGCATTCTCAGAAACTTCTTTG

            *       **   *    *             *        
     445344 GGATGTTTCAATTGAAGTCACAGTGTTGAACAGTCCCTTTC
        131 TGATGTTTGCATTCAAGTAACAGTGTTGAACATTCCCTTTC

                                                         *       **   * *    
     445385 ATAGAGCAGGTTTGAAACACTCTTTTTGTAGTATCTGGAAGTGGACATTTGGAGCGCTCTCAGGA
          1 ATAGAGCAGGTTTGAAACACTCTTTTTGTAGTATCTGGAAGTGGATATTTGGATAGCTTTGAGGA

            * *  *  *   ** *         **          * *       * *          *  **
     445450 CTACGGTGAAAAACGAAATATCTTCCAATAAAAGCTACATAGAAGCAATGTCAGAAACTTTTTCA
         66 TTTCGTTGGAAACAGGAATATCTTCATATAAAAGCTAGACAGAAGCATTCTCAGAAACTTCTTTG

                  * ** *   **      *       *  * *   *
     445515 TGATGTATCTACTCAGCTAACAGAGTTGAACCTTTCTTTTG
        131 TGATGTTTGCATTCAAGTAACAGTGTTGAACATTCCCTTTC

             *       *                   * *                   **            
     445556 AGAGAGCAGTTTTGAAACACTCTTTTTGTGGAATCTGGAAGTGGATATTTGTCTAGCTTTGAGGA
          1 ATAGAGCAGGTTTGAAACACTCTTTTTGTAGTATCTGGAAGTGGATATTTGGATAGCTTTGAGGA

                         *   *    *                   *       *   *          
     445621 TTTCGTTGGAAACGGGATTA-CAT-ATA-AAAAGC-AGACAGCAGCATTCCCAGTAACTTCTTTG
         66 TTTCGTTGGAAACAGGAATATCTTCATATAAAAGCTAGACAGAAGCATTCTCAGAAACTTCTTTG

                              *   **                 
     445682 TGATGTTTGCATTCAAGTCACAAAGTTGAACATTCCCTTTC
        131 TGATGTTTGCATTCAAGTAACAGTGTTGAACATTCCCTTTC

                                                   *     *       ***    *   *
     445723 ATAGAGCAGGTTTGAAACACTCTTTTTGTAGTATCTGGATGTGGACATTTGGAGCACTTTCAGGC
          1 ATAGAGCAGGTTTGAAACACTCTTTTTGTAGTATCTGGAAGTGGATATTTGGATAGCTTTGAGGA

            * ** *                    *** *   *                      *   *   
     445788 CTATGGTGGAAA-AGGAAATATCTTCCCCTGAAAACTAGACAGAAGCATTCTCAGAATCTTATTT
         66 TTTCGTTGGAAACAGG-AATATCTTCATATAAAAGCTAGACAGAAGCATTCTCAGAAACTTCTTT

                   **  **    *              *  * *   *
     445852 GTGATGTGCGCCCTCAACTAACAGTGTTGAACCTTTCTTTTG
        130 GTGATGTTTGCATTCAAGTAACAGTGTTGAACATTCCCTTTC

                     *                    **    **   *                    *  
     445894 ATAGAGCAGTTTTGAAACACTCTTTTTGTAAAATCTACAAGAGGATATTTGGATAGCTTTGAAGA
          1 ATAGAGCAGGTTTGAAACACTCTTTTTGTAGTATCTGGAAGTGGATATTTGGATAGCTTTGAGGA

                         *   * *            **                      *    *   
     445959 TTTCGTTGGAAACGGGATTGTCTTCATATAAACTCTAGACAGAAGCATTCTCAGAAGCTTCATTG
         66 TTTCGTTGGAAACAGGAATATCTTCATATAAAAGCTAGACAGAAGCATTCTCAGAAACTTCTTTG

            *       **   *    *             *        
     446024 GGATGTTTCAATTGAAGTCACAGTGTTGAACAGTCCCTTTC
        131 TGATGTTTGCATTCAAGTAACAGTGTTGAACATTCCCTTTC

                  *    *                                 *       ***  * *    
     446065 ATAGAGGAGGTGTGAAACACTCTTTTTGTAGTATCTGGAAGTGGACATTTGGAGCACTCTCAGGA
          1 ATAGAGCAGGTTTGAAACACTCTTTTTGTAGTATCTGGAAGTGGATATTTGGATAGCTTTGAGGA

            * * **      *             **            *       * *          *  *
     446130 CTGCAGT-GAAAAAGGAAATATCTTCCAATAAAAGCTAGATAGAAGCAATGTCAGAAACTTTTTC
         66 TTTCGTTGGAAACAGG-AATATCTTCATATAAAAGCTAGACAGAAGCATTCTCAGAAACTTCTTT

            *      * ** *   **      *       *        *
     446194 ATGATGTATCTACTCAGCTAACAGAGTTGAACCTT-CCTTTG
        130 GTGATGTTTGCATTCAAGTAACAGTGTTGAACATTCCCTTTC

             *       *                   * *     *             **            
     446235 AGAGAGCAGTTTTGAAACACTCTTTTTGTGGAATCTGCAAGTGGATATTTGTCTAGCTTTGAGGA
          1 ATAGAGCAGGTTTGAAACACTCTTTTTGTAGTATCTGGAAGTGGATATTTGGATAGCTTTGAGGA

                 *       *   *    *                   *       *  *           
     446300 TTTCGCTGGAAACGGGATTA-CAT-ATA-AAAAGC-AGACAGCAGCATTCCCAAAAACTTCTTTG
         66 TTTCGTTGGAAACAGGAATATCTTCATATAAAAGCTAGACAGAAGCATTCTCAGAAACTTCTTTG

                              *    *                 
     446361 TGATGTTTGCATTCAAGTCACAGAGTTGAACATTCCCTTTC
        131 TGATGTTTGCATTCAAGTAACAGTGTTGAACATTCCCTTTC

                                                 * *     *     * **     *   *
     446402 ATAGAGCAGGTTTGAAACACTCTTTTTGTAGTATCTGTATGTGGACATTTGCAGCGCTTTCAGGC
          1 ATAGAGCAGGTTTGAAACACTCTTTTTGTAGTATCTGGAAGTGGATATTTGGATAGCTTTGAGGA

            * ** *      *            **** *   *                          *   
     446467 CTAAGGT-GAAAAAGGAAATATCTTTCCCTGAAAACTAGACAGAAGCATTCTCAGAAACTTATTT
         66 TTTCGTTGGAAACAGG-AATATCTTCATATAAAAGCTAGACAGAAGCATTCTCAGAAACTTCTTT

                   **  **    *             **  * *   *
     446531 GTGATGTGCGCCCTCAACTAACAGTGTTGAAGCTTTCTTTTG
        130 GTGATGTTTGCATTCAAGTAACAGTGTTGAACATTCCCTTTC

             *       *         *                 *             **            
     446573 ACAGAGCAGTTTTGAAACAATC--TTT-T--TATCTGCAAGTGGATATTTGTCTAGCTTTGAGGA
          1 ATAGAGCAGGTTTGAAACACTCTTTTTGTAGTATCTGGAAGTGGATATTTGGATAGCTTTGAGGA

                         *   *    *                   *       *     *   *    
     446633 TTTCGTTGGAAACGGGATTA-CAT-ATA-AAAAGC-AGACAGCAGCATTCCCAGAATCTTGTTTG
         66 TTTCGTTGGAAACAGGAATATCTTCATATAAAAGCTAGACAGAAGCATTCTCAGAAACTTCTTTG

                              *    *                 
     446694 TGATGTTTGCATTCAAGTCACAGAGTTGAACATTCCCTTTC
        131 TGATGTTTGCATTCAAGTAACAGTGTTGAACATTCCCTTTC

             *                         *           *     *       **     *   *
     446735 AGAGAGCAGGTTTGAAACACTCTTTTTATAGTATCTGGATGTGGACATTTGGAGTGCTTTCAGGC
          1 ATAGAGCAGGTTTGAAACACTCTTTTTGTAGTATCTGGAAGTGGATATTTGGATAGCTTTGAGGA

            * ** *      *             *** *   *               *      *   *   
     446800 CTATGGT-GAAAAAGGAAATATCTTCTCCTGAAAACTAGACAGAAGCATTTTCAGAATCTTATTT
         66 TTTCGTTGGAAACAGG-AATATCTTCATATAAAAGCTAGACAGAAGCATTCTCAGAAACTTCTTT

                   **  **    *             **  * *   *
     446864 GTGATGTGCGCCCTCAACTAACAGTGTTGAAGCTTTCTTTTG
        130 GTGATGTTTGCATTCAAGTAACAGTGTTGAACATTCCCTTTC

                     *                *   **     *   *        *              
     446906 ATAGAGCAGTTTTGAAACACTCTTTTCGTAAAATCTGCAAGAGGATATTTTGATAGCTTTGAGGA
          1 ATAGAGCAGGTTTGAAACACTCTTTTTGTAGTATCTGGAAGTGGATATTTGGATAGCTTTGAGGA

                         *   * *            **                      *    *   
     446971 TTTCGTTGGAAACGGGATTGTCTTCATATAAACTCTAGACAGAAGCATTCTCAGAAGCTTCATTG
         66 TTTCGTTGGAAACAGGAATATCTTCATATAAAAGCTAGACAGAAGCATTCTCAGAAACTTCTTTG

            *       **   *    *             *        
     447036 GGATGTTTCAATTGAAGTCACAGTGTTGAACAGTCCCTTTC
        131 TGATGTTTGCATTCAAGTAACAGTGTTGAACATTCCCTTTC

                                                         *       *  * * *    
     447077 ATAGAGCAGGTTTGAAACACTCTTTTTGTAGTATCTGGAAGTGGACATTTGGAGAGATCTCAGGA
          1 ATAGAGCAGGTTTGAAACACTCTTTTTGTAGTATCTGGAAGTGGATATTTGGATAGCTTTGAGGA

            * *  *    * *             ***         * *       * *          *  *
     447142 ATACGGT-GATAAAGGAAATATCTTCCAGTAAAAGCTAAATAGAAGCAATGTCAGAAACTTTTTC
         66 TTTCGTTGGAAACAGG-AATATCTTCATATAAAAGCTAGACAGAAGCATTCTCAGAAACTTCTTT

            *      * ** *   **      *       *  *     *
     447206 ATGATGTATCTACTCAGCTAACAGAGTTGAACCTTTCCTTTG
        130 GTGATGTTTGCATTCAAGTAACAGTGTTGAACATTCCCTTTC

             *       *                   * *     *             **            
     447248 AGAGAGCAGTTTTGAAACACTCTTTTTGTGGAATCTGCAAGTGGATATTTGTCTAGCTTTGAGGA
          1 ATAGAGCAGGTTTGAAACACTCTTTTTGTAGTATCTGGAAGTGGATATTTGGATAGCTTTGAGGA

                         *   *    *                   *       *   *          
     447313 TTTCGTTGGAAACGGGATTA-CAT-ATA-AAAAGC-AGACAGCAGCATTCCCAGTAACTTCTTTG
         66 TTTCGTTGGAAACAGGAATATCTTCATATAAAAGCTAGACAGAAGCATTCTCAGAAACTTCTTTG

                              *    *                 
     447374 TGATGTTTGCATTCAAGTCACAGAGTTGAACATTCCCTTTC
        131 TGATGTTTGCATTCAAGTAACAGTGTTGAACATTCCCTTTC

                  *                                *     *       **     *    
     447415 ATAGAGGAGGTTTGAAACACTCTTTTTGTAGTATCTGGATGTGGACATTTGGAGCGCTTTCAGGC
          1 ATAGAGCAGGTTTGAAACACTCTTTTTGTAGTATCTGGAAGTGGATATTTGGATAGCTTTGAGG-

            * * *    *                 *** *   *                          *  
     447480 CTATGGTT-TAAA-AGGAAATATCTTCCCCTGAAAACTAGACAGAAGCATTCTCAGAAACTTATT
         65 ATTTCGTTGGAAACAGG-AATATCTTCATATAAAAGCTAGACAGAAGCATTCTCAGAAACTTCTT

                    **  **    *                    *   *
     447543 TGTGATGTGCGCCCTCAACTAACAGTGTTGAAGCATT-CTTTTG
        129 TGTGATGTTTGCATTCAAGTAACAGTGTTGAA-CATTCCCTTTC

                     *                   * *     *             **            
     447586 ATAGAGCAGTTTTGAAACACTCTTTTTGTGGAATCTGCAAGTGGATATTTGTCTAGCTTTGAGGA
          1 ATAGAGCAGGTTTGAAACACTCTTTTTGTAGTATCTGGAAGTGGATATTTGGATAGCTTTGAGGA

                            *  *  *                   *       *    *         
     447651 TTTCGTTGGAAAC-GGGATTTCAT-ATA-AAAAGC-AGACAGCAGCATTCGCAGAGACTTCTTTG
         66 TTTCGTTGGAAACAGGAATATCTTCATATAAAAGCTAGACAGAAGCATTCTCAGAAACTTCTTTG

                              *    *                 
     447712 TGATGTTTGCATTCAAGTCACAGAGTTGAACATTCCCTTTC
        131 TGATGTTTGCATTCAAGTAACAGTGTTGAACATTCCCTTTC

                                 *                 *     *       **     *   *
     447753 ATAGAGCAGGTTTGAAACACTGTTTTTGTAGTATCTGGATGTGGACATTTGGAGCGCTTTCAGGC
          1 ATAGAGCAGGTTTGAAACACTCTTTTTGTAGTATCTGGAAGTGGATATTTGGATAGCTTTGAGGA

            * ** *      *             *** *   *                          *   
     447818 CTATGGT-GAAAAAGGAAATATCTTCCCCTGAAAACTAGACAGAAGCATTCTCAGAAACTTATTT
         66 TTTCGTTGGAAACAGG-AATATCTTCATATAAAAGCTAGACAGAAGCATTCTCAGAAACTTCTTT

                   **  **    **            **  * *   *
     447882 GTGATGTGCGCCCTCAACGAACAGTGTTGAAGCTTTCTTTTG
        130 GTGATGTTTGCATTCAAGTAACAGTGTTGAACATTCCCTTTC

                     *                    *      *   *                       
     447924 ATAGAGCAGTTTTGAAACACTCTTTTTGTAATATCTGCAAGAGGATATTTGGATAGCTTTGAGGA
          1 ATAGAGCAGGTTTGAAACACTCTTTTTGTAGTATCTGGAAGTGGATATTTGGATAGCTTTGAGGA

                         *   * *            **                      *    *   
     447989 TTTCGTTGGAAACGGGATTGTCTTCATATAAACTCTAGACAGAAGCATTCTCAGAAGCTTCATTG
         66 TTTCGTTGGAAACAGGAATATCTTCATATAAAAGCTAGACAGAAGCATTCTCAGAAACTTCTTTG

            *       **   *    *             * *      
     448054 GGATGTTTCAATTGAAGTCACAGTGTTGAACAGTTCCTTTC
        131 TGATGTTTGCATTCAAGTAACAGTGTTGAACATTCCCTTTC

                       *                                 *       *  * * *    
     448095 ATAGAGCAGGTATGAAACACTCTTTTTGTAGTATCTGGAAGTGGACATTTGGAGAGATCTCAGGA
          1 ATAGAGCAGGTTTGAAACACTCTTTTTGTAGTATCTGGAAGTGGATATTTGGATAGCTTTGAGGA

            * *  *    * *             **            *       * *          *  *
     448160 ATACGGT-GATAAAGGAAATATCTTCCAATAAAAGCTAGATAGAAGCAATGTCAGAAACTTTTTC
         66 TTTCGTTGGAAACAGG-AATATCTTCATATAAAAGCTAGACAGAAGCATTCTCAGAAACTTCTTT

            *      * ** *   **      *       *  * *   *
     448224 ATGATGTATCTACTCAGCTAACAGAGTTGAACCTTTCTTTTG
        130 GTGATGTTTGCATTCAAGTAACAGTGTTGAACATTCCCTTTC

             *       *                   * *    **             **            
     448266 AGAGAGCAGTTTTGAAACACTCTTTTTGTGGAATCTACAAGTGGATATTTGTCTAGCTTTGAGGA
          1 ATAGAGCAGGTTTGAAACACTCTTTTTGTAGTATCTGGAAGTGGATATTTGGATAGCTTTGAGGA

                         *   *    *                   *       *              
     448331 TTTCGTTGGAAACGGGATTA-CAT-ATA-AAAAGC-AGACAGCAGCATTCCCAGAAACTTCTTTG
         66 TTTCGTTGGAAACAGGAATATCTTCATATAAAAGCTAGACAGAAGCATTCTCAGAAACTTCTTTG

                              *    *                 
     448392 TGATGTTTGCATTCAAGTCACAGAGTTGAACATTCCCTTTC
        131 TGATGTTTGCATTCAAGTAACAGTGTTGAACATTCCCTTTC

                                                   *     **      **     *   *
     448433 ATAGAGCAGGTTTGAAACACTCTTTTTGTAGTATCTGGATGTGGACCTTTGGAGCGCTTTCAGGC
          1 ATAGAGCAGGTTTGAAACACTCTTTTTGTAGTATCTGGAAGTGGATATTTGGATAGCTTTGAGGA

            * ** *      *             *** *   *                      *   *   
     448498 CTATGGT-GAAAAAGGAAATATCTTCCCCTGAAAACTAGACAGAAGCATTCTCAGAATCTTATTT
         66 TTTCGTTGGAAACAGG-AATATCTTCATATAAAAGCTAGACAGAAGCATTCTCAGAAACTTCTTT

                   **  **    **            **  * *   *
     448562 GTGATGTGCGCCCTCAACGAACAGTGTTGAAGCTTTCTTTTG
        130 GTGATGTTTGCATTCAAGTAACAGTGTTGAACATTCCCTTTC

                     *                    *      *   *                       
     448604 ATAGAGCAGTTTTGAAACACTCTTTTTGTAATATCTGCAAGAGGATATTTGGATAGCTTTGAGGA
          1 ATAGAGCAGGTTTGAAACACTCTTTTTGTAGTATCTGGAAGTGGATATTTGGATAGCTTTGAGGA

                         *   * *            **                      *    *   
     448669 TTTCGTTGGAAACGGGATTGTCTTCATATAAACTCTAGACAGAAGCATTCTCAGAAGCTTCATTG
         66 TTTCGTTGGAAACAGGAATATCTTCATATAAAAGCTAGACAGAAGCATTCTCAGAAACTTCTTTG

            *       **   *    *             *        
     448734 GGATGTTTCAATTGAAGTCACAGTGTTGAACAGTCCCTTTC
        131 TGATGTTTGCATTCAAGTAACAGTGTTGAACATTCCCTTTC

                                                         *       **   * *    
     448775 ATAGAGCAGGTTTGAAACACTCTTTTTGTAGTATCTGGAAGTGGACATTTGGAGCGCTCTCAGGA
          1 ATAGAGCAGGTTTGAAACACTCTTTTTGTAGTATCTGGAAGTGGATATTTGGATAGCTTTGAGGA

            * *  *      *             **            *       * *          *  *
     448840 CTGCGGT-GAAAAAGGAACTATCTTCCAATAAAAGCTAGATAGAAGCAATGTCAGAAACTTTTTC
         66 TTTCGTTGGAAACAGGAA-TATCTTCATATAAAAGCTAGACAGAAGCATTCTCAGAAACTTCTTT

            *      * ** *   **      *       *        *
     448904 ATGATGTATCTACTCAGCTAACAGAGTTGAACCTT-CCTTTG
        130 GTGATGTTTGCATTCAAGTAACAGTGTTGAACATTCCCTTTC

             *       *                   * *     *            ***            
     448945 AGAGAGCAGTTTTGAAACACTCTTTTTGTGGAATCTGCAAGTGGATATTTTTCTAGCTTTGAGGA
          1 ATAGAGCAGGTTTGAAACACTCTTTTTGTAGTATCTGGAAGTGGATATTTGGATAGCTTTGAGGA

                         *   *    *                   *       *   *          
     449010 TTTCGTTGGAAACGGGATTA-CAT-ATA-AAAAGC-AGACAGCAGCATTCCCAGTAACTTCTTTG
         66 TTTCGTTGGAAACAGGAATATCTTCATATAAAAGCTAGACAGAAGCATTCTCAGAAACTTCTTTG

                              *    *                 
     449071 TGATGTTTGCATTCAAGTCACAGAGTTGAACATTCCCTTTC
        131 TGATGTTTGCATTCAAGTAACAGTGTTGAACATTCCCTTTC

                                                   *     *       **     *   *
     449112 ATAGAGCAGGTTTGAAACACTCTTTTTGTAGTATCTGGATGTGGACATTTGGAGCGCTTTCAGGC
          1 ATAGAGCAGGTTTGAAACACTCTTTTTGTAGTATCTGGAAGTGGATATTTGGATAGCTTTGAGGA

            * ** *      *             *** *   *                      *   *   
     449177 CTATGGT-GAAAAAGGAAATATCTTCTCCTGAAAACTAGACAGAAGCATTCTCAGAATCTTATTT
         66 TTTCGTTGGAAACAGG-AATATCTTCATATAAAAGCTAGACAGAAGCATTCTCAGAAACTTCTTT

                   **  **    *             **  * *   *
     449241 GTGATGTGCGCCCTCAACTAACAGTGTTGAAGCTTTCTTTTG
        130 GTGATGTTTGCATTCAAGTAACAGTGTTGAACATTCCCTTTC

                     *                *   **     *   *                       
     449283 ATAGAGCAGTTTTGAAACACTCTTTTCGTAAAATCTGTAAGAGGATATTTGGATAGCTTTGAGGA
          1 ATAGAGCAGGTTTGAAACACTCTTTTTGTAGTATCTGGAAGTGGATATTTGGATAGCTTTGAGGA

                         *   * *            **       *              *    *   
     449348 TTTCGTTGGAAACGGGATTGTCTTCATATAAACTCTAGACAAAAGCATTCTCAGAAGCTTCATTG
         66 TTTCGTTGGAAACAGGAATATCTTCATATAAAAGCTAGACAGAAGCATTCTCAGAAACTTCTTTG

            *       **   *    *             * *      
     449413 GGATGTTTCAATTGAAGTCACAGTGTTGAACAGTTCCTTTC
        131 TGATGTTTGCATTCAAGTAACAGTGTTGAACATTCCCTTTC

                 *                                       *       **   ***    
     449454 ATAGAACAGGTTTGAAACACTCTTTTTGTAGTATCTGGAAGTGGACATTTGGAGCGCTCACAGGA
          1 ATAGAGCAGGTTTGAAACACTCTTTTTGTAGTATCTGGAAGTGGATATTTGGATAGCTTTGAGGA

            * *  *      *             **            *       * *        * *  *
     449519 CTGCGGT-GAAAAAGGAAATATCTTCCAATAAAAGCTAGATAGAAGCAATGTCAGAAACATTTTC
         66 TTTCGTTGGAAACAGG-AATATCTTCATATAAAAGCTAGACAGAAGCATTCTCAGAAACTTCTTT

            *      * ** *   **      *       *        *
     449583 ATGATGTATCTACTCAGCTAACAGAGTTGAACCTT-CCTTTG
        130 GTGATGTTTGCATTCAAGTAACAGTGTTGAACATTCCCTTTC

             *       *                   * *     *             **            
     449624 AGAGAGCAGTTTTGAAACACTCTTTTTGTGGAATCTGCAAGTGGATATTTGTCTAGCTTTGAGGA
          1 ATAGAGCAGGTTTGAAACACTCTTTTTGTAGTATCTGGAAGTGGATATTTGGATAGCTTTGAGGA

                         *   *    *               *   *   *   *              
     449689 TTTCGTTGGAAACGGGATTA-CAT-ATA-AAAAGC-AGCCAGCAGCGTTCCCAGAAACTTCTTTG
         66 TTTCGTTGGAAACAGGAATATCTTCATATAAAAGCTAGACAGAAGCATTCTCAGAAACTTCTTTG

                              *    *                 
     449750 TGATGTTTGCATTCAAGTCACAGAGTTGAACATTCCCTTTC
        131 TGATGTTTGCATTCAAGTAACAGTGTTGAACATTCCCTTTC

                                                  **     *     * **     *   *
     449791 ATAGAGCAGGTTTGAAACACTCTTTTTGTAGTATCTGGTTGTGGACATTTGCAGCGCTTTCAGGC
          1 ATAGAGCAGGTTTGAAACACTCTTTTTGTAGTATCTGGAAGTGGATATTTGGATAGCTTTGAGGA

            * ** *      *             *** *   *                          *   
     449856 CTAAGGT-GAAAAAGGAAATATCTTCCCCTGAAAACTAGACAGAAGCATTCTCAGAAACTTATTT
         66 TTTCGTTGGAAACAGG-AATATCTTCATATAAAAGCTAGACAGAAGCATTCTCAGAAACTTCTTT

                   **  **    *             **  * *   *
     449920 GTGATGTGCGCCCTCAACTAACAGTGTTGAAGCTTTCTTTTG
        130 GTGATGTTTGCATTCAAGTAACAGTGTTGAACATTCCCTTTC

                     *        *           *      * * *                       
     449962 ATAGAGCAGTTTTGAAACGCTCTTTTTGTAATATCTGCAGGAGGATATTTGGATAGCTTTGAGGA
          1 ATAGAGCAGGTTTGAAACACTCTTTTTGTAGTATCTGGAAGTGGATATTTGGATAGCTTTGAGGA

                         *   * *            **                      *    *   
     450027 TTTCGTTGGAAACGGGATTGTCTTCATATAAACTCTAGACAGAAGCATTCTCAGAAGCTTCATTG
         66 TTTCGTTGGAAACAGGAATATCTTCATATAAAAGCTAGACAGAAGCATTCTCAGAAACTTCTTTG

            *       **   *    *             *        
     450092 GGATGTTTCAATTGAAGTCACAGTGTTGAACAGTCCCTTTC
        131 TGATGTTTGCATTCAAGTAACAGTGTTGAACATTCCCTTTC

                                                         *       *  * * *    
     450133 ATAGAGCAGGTTTGAAACACTCTTTTTGTAGTATCTGGAAGTGGACATTTGGAGAGATCTCAGGA
          1 ATAGAGCAGGTTTGAAACACTCTTTTTGTAGTATCTGGAAGTGGATATTTGGATAGCTTTGAGGA

            * *  *    * *             **            *       * *          *  *
     450198 ATACGGT-GATAAAGGAAATATCTTCCAATAAAAGCTAGATAGAAGCAATGTCAGAAACTTTTTC
         66 TTTCGTTGGAAACAGG-AATATCTTCATATAAAAGCTAGACAGAAGCATTCTCAGAAACTTCTTT

            *      * ** *   **      *       *  * *   *
     450262 ATGATGTATCTACTCAGCTAACAGAGTTGAACCTTTCTTTTG
        130 GTGATGTTTGCATTCAAGTAACAGTGTTGAACATTCCCTTTC

             *       *                   * *     *             **            
     450304 AGAGAGCAGTTTTGAAACACTCTTTTTGTGGAATCTGCAAGTGGATATTTGTCTAGCTTTGAGGA
          1 ATAGAGCAGGTTTGAAACACTCTTTTTGTAGTATCTGGAAGTGGATATTTGGATAGCTTTGAGGA

                         *   *    *                   *       *  *           
     450369 TTTCGTTGGAAACGGGATTA-CAT-ATA-AAAAGC-AGACAGCAGCATTCCCACAAACTTCTTTG
         66 TTTCGTTGGAAACAGGAATATCTTCATATAAAAGCTAGACAGAAGCATTCTCAGAAACTTCTTTG

            *                 *    *                 
     450430 CGATGTTTGCATTCAAGTCACAGAGTTGAACATTCCCTTTC
        131 TGATGTTTGCATTCAAGTAACAGTGTTGAACATTCCCTTTC

                                                   *     *       **     *   *
     450471 ATAGAGCAGGTTTGAAACACTCTTTTTGTAGTATCTGGATGTGGACATTTGGAGCGCTTTCAGGC
          1 ATAGAGCAGGTTTGAAACACTCTTTTTGTAGTATCTGGAAGTGGATATTTGGATAGCTTTGAGGA

            * ** *      *             *** *   *      *                   *   
     450536 CTATGGT-GAAAAAGGAAATATCTTCCCCTGAAAACTAGACTGAAGCATTCTCAGAAACTTATTT
         66 TTTCGTTGGAAACAGG-AATATCTTCATATAAAAGCTAGACAGAAGCATTCTCAGAAACTTCTTT

                   **  **    *              *  * *   *
     450600 GTGATGTGCGCCCTCAACTAACAGTGTTGAACCTTTCTTTTG
        130 GTGATGTTTGCATTCAAGTAACAGTGTTGAACATTCCCTTTC

                     *                    **     *   *                       
     450642 ATAGAGCAGTTTTGAAACACTCTTTTTGTAAAATCTGCAAGAGGATATTTGGATAGCTTTGAGGA
          1 ATAGAGCAGGTTTGAAACACTCTTTTTGTAGTATCTGGAAGTGGATATTTGGATAGCTTTGAGGA

                         *   * *            **               *      *    *   
     450707 TTTCGTTGGAAACGGGATTGTCTTCATATAAACTCTAGACAGAAGCATTATCAGAAGCTTCATTG
         66 TTTCGTTGGAAACAGGAATATCTTCATATAAAAGCTAGACAGAAGCATTCTCAGAAACTTCTTTG

            *       **   *    *             *        
     450772 GGATGTTTCAATTGAAGTCACAGTGTTGAACAGTCCCTTTC
        131 TGATGTTTGCATTCAAGTAACAGTGTTGAACATTCCCTTTC

                                                         *    *  *  * * *    
     450813 ATAGAGCAGGTTTGAAACACTCTTTTTGTAGTATCTGGAAGTGGACATTTTGAGAGATCTCAGGA
          1 ATAGAGCAGGTTTGAAACACTCTTTTTGTAGTATCTGGAAGTGGATATTTGGATAGCTTTGAGGA

            * *  *    * *             **            *       * *          *  *
     450878 ATACGGT-GATAAAGGAAATATCTTCCAATAAAAGCTAGATAGAAGCAATGTCAGAAACTTTTTC
         66 TTTCGTTGGAAACAGG-AATATCTTCATATAAAAGCTAGACAGAAGCATTCTCAGAAACTTCTTT

            *      * ** *   **     **       *  * *    
     450942 ATGATGTATCTACTCAGCTAACACAGTTGAACCTTTCTTTTC
        130 GTGATGTTTGCATTCAAGTAACAGTGTTGAACATTCCCTTTC

             *       *                   * *     *             **            
     450984 AGAGAGCAGTTTTGAAACACTCTTTTTGTGGAATCTGCAAGTGGATATTTGTCTAGCTTTGAGGA
          1 ATAGAGCAGGTTTGAAACACTCTTTTTGTAGTATCTGGAAGTGGATATTTGGATAGCTTTGAGGA

                         *   *    *                   *       *            * 
     451049 TTTCGTTGGAAACGGGATTA-CAT-ATA-AAAAGC-AGACAGCAGCATTCCCAGAAACTTCTTAG
         66 TTTCGTTGGAAACAGGAATATCTTCATATAAAAGCTAGACAGAAGCATTCTCAGAAACTTCTTTG

               *              *    *                 
     451110 TGAAGTTTGCATTCAAGTCACAGAGTTGAACATTCCCTTTC
        131 TGATGTTTGCATTCAAGTAACAGTGTTGAACATTCCCTTTC

                                                 * *     *       **     *   *
     451151 ATAGAGCAGGTTTGAAACACTCTTTTTGTAGTATCTGTATGTGGACATTTGGAGCGCTTTCAGGC
          1 ATAGAGCAGGTTTGAAACACTCTTTTTGTAGTATCTGGAAGTGGATATTTGGATAGCTTTGAGGA

            * ** *      *             *** *   *                      *   *   
     451216 CTATGGT-GAAAAAGGAAATATCTTCCCCTGAAAACTAGACAGAAGCATTCTCAGAATCTTATTT
         66 TTTCGTTGGAAACAGG-AATATCTTCATATAAAAGCTAGACAGAAGCATTCTCAGAAACTTCTTT

                   **  **    *     *       **  * *   *
     451280 GTGATGTGCGCCCTCAACTAACAATGTTGAAGCTTTCTTTTG
        130 GTGATGTTTGCATTCAAGTAACAGTGTTGAACATTCCCTTTC

                     *                   * *     * *          ***            
     451322 ATAGAGCAGTTTTGAAACACTCTTTTTGTGGAATCTGCATGTGGATATTTTTCTAGCTTTGAGGA
          1 ATAGAGCAGGTTTGAAACACTCTTTTTGTAGTATCTGGAAGTGGATATTTGGATAGCTTTGAGGA

                         *   *    *                   *       *     *   *    
     451387 TTTCGTTGGAAACGGGATTA-CAT-ATA-AAAAGC-AGACAGCAGCATTCCCAGAATCTTGTTTG
         66 TTTCGTTGGAAACAGGAATATCTTCATATAAAAGCTAGACAGAAGCATTCTCAGAAACTTCTTTG

                              *    *                 
     451448 TGATGTTTGCATTCAAGTCACAGAGTTGAACATTCCCTTTC
        131 TGATGTTTGCATTCAAGTAACAGTGTTGAACATTCCCTTTC

             *                         *           *     *       **     *   *
     451489 AGAGAGCAGGTTTGAAACACTCTTTTTATAGTATCTGGATGTGGACATTTGGAGCGCTTTCAGGC
          1 ATAGAGCAGGTTTGAAACACTCTTTTTGTAGTATCTGGAAGTGGATATTTGGATAGCTTTGAGGA

            * ** *      *             *** *   *                      *   *   
     451554 CTATGGT-GAAAAAGGAAATATCTTCTCCTGAAAACTAGACAGAAGCATTCTCAGAATCTTATTT
         66 TTTCGTTGGAAACAGG-AATATCTTCATATAAAAGCTAGACAGAAGCATTCTCAGAAACTTCTTT

                   **  **    *  *          **  * *   *
     451618 GTGATGTGCGCCCTCAACTAGCAGTGTTGAAACTTTCTTTTG
        130 GTGATGTTTGCATTCAAGTAACAGTGTTGAACATTCCCTTTC

                     *          *         **     *   *                       
     451660 ATAGAGCAGTTTTGAAACACCCTTTTTGTAAAATCTGCAAGAGGATATTTGGATAGCTTTGAGGA
          1 ATAGAGCAGGTTTGAAACACTCTTTTTGTAGTATCTGGAAGTGGATATTTGGATAGCTTTGAGGA

                         *   * *             *                   *  *    *   
     451725 TTTCGTTGGAAACGGGATTGTCTTCATATAAAATCTAGACAGAAGCATTCTCAAAAGCTTCATTG
         66 TTTCGTTGGAAACAGGAATATCTTCATATAAAAGCTAGACAGAAGCATTCTCAGAAACTTCTTTG

            *       ** * *    *             *        
     451790 GGATGTTTCAACTGAAGTCACAGTGTTGAACAGTCCCTTTC
        131 TGATGTTTGCATTCAAGTAACAGTGTTGAACATTCCCTTTC

                                      *                  *       **   * *    
     451831 ATAGAGCAGGTTTGAAACACTCTTTTGGTAGTATCTGGAAGTGGACATTTGGAGCGCTCTCAGGA
          1 ATAGAGCAGGTTTGAAACACTCTTTTTGTAGTATCTGGAAGTGGATATTTGGATAGCTTTGAGGA

            * *  *      *             **            *       * *          *  *
     451896 CTGCGGT-GAAAAAGGAATTATCTTCCAATAAAAGCTAGATAGAAGCAATGTCAGAAACTTTTTC
         66 TTTCGTTGGAAACAGGAA-TATCTTCATATAAAAGCTAGACAGAAGCATTCTCAGAAACTTCTTT

            *      * ** *   **      *       *        *
     451960 ATGATGTATCTACTCAGCTAACAGAGTTGAACCTT-CCTTTG
        130 GTGATGTTTGCATTCAAGTAACAGTGTTGAACATTCCCTTTC

             *       *                   * *     *             **            
     452001 AGAGAGCAGTTTTGAAACACTCTTTTTGTGGAATCTGCAAGTGGATATTTGTCTAGCTTTGAGGA
          1 ATAGAGCAGGTTTGAAACACTCTTTTTGTAGTATCTGGAAGTGGATATTTGGATAGCTTTGAGGA

                         **  *    *                   **      *              
     452066 TTTCGTTGGAAACGAGATTA-CAT-ATA-AAAAGC-AGACAGCGGCATTCCCAGAAACTTCTTTG
         66 TTTCGTTGGAAACAGGAATATCTTCATATAAAAGCTAGACAGAAGCATTCTCAGAAACTTCTTTG

                              *    *                 
     452127 TGATGTTTGCATTCAAGTCACAGAGTTGAACATTCCCTTTC
        131 TGATGTTTGCATTCAAGTAACAGTGTTGAACATTCCCTTTC

                                          *        *     *       **     *   *
     452168 ATAGAGCAGGTTTGAAACACTCTTTTTGTACTATCTGGATGTGGACATTTGGAGCGCTTTCAGGC
          1 ATAGAGCAGGTTTGAAACACTCTTTTTGTAGTATCTGGAAGTGGATATTTGGATAGCTTTGAGGA

            * ** *      *             *** *   *                          *   
     452233 CTATGGT-GAAAAAGGAAATATCTTCCCCTGAAAACTAGACAGAAGCATTCTCAGAAACTTATTT
         66 TTTCGTTGGAAACAGG-AATATCTTCATATAAAAGCTAGACAGAAGCATTCTCAGAAACTTCTTT

                   **  **    *              *  * *   *
     452297 GTGATGTGCGCCCTCAACTAACAGTGTTGAACCTTTCTTTTG
        130 GTGATGTTTGCATTCAAGTAACAGTGTTGAACATTCCCTTTC

                     *                    *      *   *                       
     452339 ATAGAGCAGTTTTGAAACACTCTTTTTGTAATATCTGCAAGAGGATATTTGGATAGCTTTGAGGA
          1 ATAGAGCAGGTTTGAAACACTCTTTTTGTAGTATCTGGAAGTGGATATTTGGATAGCTTTGAGGA

                         *   * *            **                      *    *   
     452404 TTTCGTTGGAAACGGGATTGTCTTCATATAAACTCTAGACAGAAGCATTCTCAGAAGCTTCATTG
         66 TTTCGTTGGAAACAGGAATATCTTCATATAAAAGCTAGACAGAAGCATTCTCAGAAACTTCTTTG

            *       **   *    *             *        
     452469 GGATGTTTCAATTGAAGTCACAGTGTTGAACAGTCCCTTTC
        131 TGATGTTTGCATTCAAGTAACAGTGTTGAACATTCCCTTTC

                                                         *       *  * * *    
     452510 ATAGAGCAGGTTTGAAACACTCTTTTTGTAGTATCTGGAAGTGGACATTTGGAGAGATCTCAGGA
          1 ATAGAGCAGGTTTGAAACACTCTTTTTGTAGTATCTGGAAGTGGATATTTGGATAGCTTTGAGGA

            * *  *    * *             **            *       * *          *  *
     452575 ATACGGT-GATAAAGGAAATATCTTCCAATAAAAGCTAGATAGAAGCAATGTCAGAAACTTTTTC
         66 TTTCGTTGGAAACAGG-AATATCTTCATATAAAAGCTAGACAGAAGCATTCTCAGAAACTTCTTT

            *      * ** *   **      *       *  * *   *
     452639 ATGATGTATCTACTCAGCTAACAGAGTTGAACCTTTCTTTTG
        130 GTGATGTTTGCATTCAAGTAACAGTGTTGAACATTCCCTTTC

             *       *                   * *     *            ***            
     452681 AGAGAGCAGTTTTGAAACACTCTTTTTGTGGAATCTGCAAGTGGATATTTTTCTAGCTTTGAGGA
          1 ATAGAGCAGGTTTGAAACACTCTTTTTGTAGTATCTGGAAGTGGATATTTGGATAGCTTTGAGGA

                         *   *    *                   *       *              
     452746 TTTCGTTGGAAACGGGATTA-CAT-ATA-AAAAGC-AGACAGCAGCATTCCCAGAAACTTCTTTG
         66 TTTCGTTGGAAACAGGAATATCTTCATATAAAAGCTAGACAGAAGCATTCTCAGAAACTTCTTTG

               *              *                      
     452807 TGAAGTTTGCATTCAAGTCACAGTGTTGAACATTCCCTTTC
        131 TGATGTTTGCATTCAAGTAACAGTGTTGAACATTCCCTTTC

                                                 * *     *       **     *   *
     452848 ATAGAGCAGGTTTGAAACACTCTTTTTGTAGTATCTGTATGTGGACATTTGGAGCGCTTTCAGGC
          1 ATAGAGCAGGTTTGAAACACTCTTTTTGTAGTATCTGGAAGTGGATATTTGGATAGCTTTGAGGA

            * ** *      *             *** *   *            *         *   *   
     452913 CTATGGT-GAAAAAGGAAATATCTTCCCCTGAAAACTAGACAGAAGCGTTCTCAGAATCTTATTT
         66 TTTCGTTGGAAACAGG-AATATCTTCATATAAAAGCTAGACAGAAGCATTCTCAGAAACTTCTTT

                   **  **    *             **  * *    
     452977 GTGATGTGCGCCCTCAACTAACAGTGTTGAAGCTTTCTTTTC
        130 GTGATGTTTGCATTCAAGTAACAGTGTTGAACATTCCCTTTC

                     *                    **    **   *                       
     453019 ATAGAGCAGTTTTGAAACACTCTTTTTGTAAAATCTCCAAGAGGATATTTGGATAGCTTTGAGGA
          1 ATAGAGCAGGTTTGAAACACTCTTTTTGTAGTATCTGGAAGTGGATATTTGGATAGCTTTGAGGA

                *        *   * *            **                      *    *   
     453084 TTTCTTTGGAAACGGGATTGTCTTCATATAAACTCTAGACAGAAGCATTCTCAGAAGCTTCATTG
         66 TTTCGTTGGAAACAGGAATATCTTCATATAAAAGCTAGACAGAAGCATTCTCAGAAACTTCTTTG

            *       **   *    *             *        
     453149 GGATGTTTCAATTAAAGTCACAGTGTTGAACAGTCCCTTTC
        131 TGATGTTTGCATTCAAGTAACAGTGTTGAACATTCCCTTTC

                                                   *     *       **     *   *
     453190 ATAGAGCAGGTTTGAAACACTCTTTTTGTAGTATCTGGATGTGGACATTTGGAGCGCTTTCAGGC
          1 ATAGAGCAGGTTTGAAACACTCTTTTTGTAGTATCTGGAAGTGGATATTTGGATAGCTTTGAGGA

            * ** *      *             *** *   *                          *   
     453255 CTATGGT-GAAAAAGGAAATATCTTCCCCTGAAAACTAGACAGAAGCATTCTCAGAAACTTATTT
         66 TTTCGTTGGAAACAGG-AATATCTTCATATAAAAGCTAGACAGAAGCATTCTCAGAAACTTCTTT

                   **  **    *             **  * *   *
     453319 GTGATGTGCGCCCTCAACTAACAGTGTTGAAGCTTTCTTTTG
        130 GTGATGTTTGCATTCAAGTAACAGTGTTGAACATTCCCTTTC

                     *                   * *     *        *    **            
     453361 ATAGAGCAGTTTTGAAACACTCTTTTTGTGGAATCTGCAAGTGGATGTTTGTCTAGCTTTGAGGA
          1 ATAGAGCAGGTTTGAAACACTCTTTTTGTAGTATCTGGAAGTGGATATTTGGATAGCTTTGAGGA

                         *   *    *                   *       *     *   *    
     453426 TTTCGTTGGAAACGGGATTA-CAT-ATA-AAAAGC-AGACAGCAGCATTCCCAGAATCTTGTTTG
         66 TTTCGTTGGAAACAGGAATATCTTCATATAAAAGCTAGACAGAAGCATTCTCAGAAACTTCTTTG

                              *    *                 
     453487 TGATGTTTGCATTCAAGTCACAGAGTTGAACATTCCCTTTC
        131 TGATGTTTGCATTCAAGTAACAGTGTTGAACATTCCCTTTC

             *                         *           *     *       **     *   *
     453528 AGAGAGCAGGTTTGAAACACTCTTTTTATAGTATCTGGATGTGGACATTTGGAGCGCTTTCAGGC
          1 ATAGAGCAGGTTTGAAACACTCTTTTTGTAGTATCTGGAAGTGGATATTTGGATAGCTTTGAGGA

            * ** *      *             *** *   *                      *   *   
     453593 CTATGGT-GAAAAAGGAAATATCTTCTCCTGAAAACTAGACAGAAGCATTCTCAGAATCTTATTT
         66 TTTCGTTGGAAACAGG-AATATCTTCATATAAAAGCTAGACAGAAGCATTCTCAGAAACTTCTTT

                   **  **    *             **  * *   *
     453657 GTGATGTGCGCCCTCAACTAACAGTGTTGAAGCTTTCTTTTG
        130 GTGATGTTTGCATTCAAGTAACAGTGTTGAACATTCCCTTTC

                     *                *   **     *   *                       
     453699 ATAGAGCAGTTTTGAAACACTCTTTTCGTAAAATCTGCAAGAGGATATTTGGATAGCTTTGAGGA
          1 ATAGAGCAGGTTTGAAACACTCTTTTTGTAGTATCTGGAAGTGGATATTTGGATAGCTTTGAGGA

                         *   * *            **                      *    *   
     453764 TTTCGTTGGAAACGGGATTGTCTTCATATAAACTCTAGACAGAAGCATTCTCAGAAGCTTCATTG
         66 TTTCGTTGGAAACAGGAATATCTTCATATAAAAGCTAGACAGAAGCATTCTCAGAAACTTCTTTG

            *       **   *    *             *        
     453829 GGATGTTTCAATTGAAGTCACAGTGTTGAACAGTCCCTTTC
        131 TGATGTTTGCATTCAAGTAACAGTGTTGAACATTCCCTTTC

                                                         *       *  * * *    
     453870 ATAGAGCAGGTTTGAAACACTCTTTTTGTAGTATCTGGAAGTGGACATTTGGAGAGATCTCAGGA
          1 ATAGAGCAGGTTTGAAACACTCTTTTTGTAGTATCTGGAAGTGGATATTTGGATAGCTTTGAGGA

            * *  *    * *             **            *       * *          *  *
     453935 ATACGGT-GATAAAGGAAATATCTTCCAATAAAAGCTAGATAGAAGCAATGTCAGAAACTTTTTC
         66 TTTCGTTGGAAACAGG-AATATCTTCATATAAAAGCTAGACAGAAGCATTCTCAGAAACTTCTTT

            *      * ** *   **      *       *  * *   *
     453999 ATGATGTATCTACTCAGCTAACAGAGTTGAACCTTTCTTTTG
        130 GTGATGTTTGCATTCAAGTAACAGTGTTGAACATTCCCTTTC

             *       *                   * *     *             **            
     454041 AGAGAGCAGTTTTGAAACACTCTTTTTGTGGAATCTGCAAGTGGATATTTGTCTAGCTTTGAGGA
          1 ATAGAGCAGGTTTGAAACACTCTTTTTGTAGTATCTGGAAGTGGATATTTGGATAGCTTTGAGGA

                         *   *    *                   *       *   *          
     454106 TTTCGTTGGAAACGGGATTA-CAT-ATA-AAAAGC-AGACAGCAGCATTCCCAGTAACTTCTTTG
         66 TTTCGTTGGAAACAGGAATATCTTCATATAAAAGCTAGACAGAAGCATTCTCAGAAACTTCTTTG

                              *    *                 
     454167 TGATGTTTGCATTCAAGTCACAGAGTTGAACATTCCCTTTC
        131 TGATGTTTGCATTCAAGTAACAGTGTTGAACATTCCCTTTC

                                                 * ***   *     * **     *   *
     454208 ATAGAGCAGGTTTGAAACACTCTTTTTGTAGTATCTGTATACGGACATTTGCAGCGCTTTCAGGC
          1 ATAGAGCAGGTTTGAAACACTCTTTTTGTAGTATCTGGAAGTGGATATTTGGATAGCTTTGAGGA

            * ** *      *             *** *   *                          *   
     454273 CTAAGGT-GAAAAAGGAAATATCTTCCCCTGAAAACTAGACAGAAGCATTCTCAGAAACTTATTT
         66 TTTCGTTGGAAACAGG-AATATCTTCATATAAAAGCTAGACAGAAGCATTCTCAGAAACTTCTTT

                   **  ** *  **            **  * *   *
     454337 GTGATGTGCGCCCTTAACCAACAGTGTTGAAGCTTTCTTTTG
        130 GTGATGTTTGCATTCAAGTAACAGTGTTGAACATTCCCTTTC

             *       *                   * *     *             **            
     454379 ACAGAGCAGTTTTGAAACACTCTTTTTGTGGAATCTGCAAGTGGATATTTGTCTAGCTTTGAGGA
          1 ATAGAGCAGGTTTGAAACACTCTTTTTGTAGTATCTGGAAGTGGATATTTGGATAGCTTTGAGGA

                         *   *    *                   *       *     *   *    
     454444 TTTCGTTGGAAACGGGATTA-CAT-ATA-AAAAGC-AGACAGCAGCATTCCCAGAATCTTGTTTG
         66 TTTCGTTGGAAACAGGAATATCTTCATATAAAAGCTAGACAGAAGCATTCTCAGAAACTTCTTTG

                              *    *                 
     454505 TGATGTTTGCATTCAAGTCACAGAGTTGAACATTCCCTTTC
        131 TGATGTTTGCATTCAAGTAACAGTGTTGAACATTCCCTTTC

             *                         *           *     *       **     *   *
     454546 AGAGAGCAGGTTTGAAACACTCTTTTTATAGTATCTGGATGTGGACATTTGGAGCGCTTTCAGGC
          1 ATAGAGCAGGTTTGAAACACTCTTTTTGTAGTATCTGGAAGTGGATATTTGGATAGCTTTGAGGA

            * ** *      *             *** *   *                      *   *   
     454611 CTATGGT-GAAAAAGGAAATATCTTCTCCTGAAAACTAGACAGAAGCATTCTCAGAATCTTATTT
         66 TTTCGTTGGAAACAGG-AATATCTTCATATAAAAGCTAGACAGAAGCATTCTCAGAAACTTCTTT

                  ***  **    *             **  * *   *
     454675 GTGATGAGCGCCCTCAACTAACAGTGTTGAAGCTTTCTTTTG
        130 GTGATGTTTGCATTCAAGTAACAGTGTTGAACATTCCCTTTC

                     *                *   **     *   *        *         *    
     454717 ATAGAGCAGTTTTGAAACACTCTTTTCGTAAAATCTGCAAGAGGATATTTTGATAGCTTTCAGGA
          1 ATAGAGCAGGTTTGAAACACTCTTTTTGTAGTATCTGGAAGTGGATATTTGGATAGCTTTGAGGA

                         *   * *             *                 *    *    *   
     454782 TTTCGTTGGAAACGGGATTGTCTTCATATAAAATCTAGACAGAAGCATTCTGAGAAGCTTCATTG
         66 TTTCGTTGGAAACAGGAATATCTTCATATAAAAGCTAGACAGAAGCATTCTCAGAAACTTCTTTG

            *       **   *    *             *        
     454847 GGATGTTTCAATTGAAGTCACAGTGTTGAACAGTCCCTTTC
        131 TGATGTTTGCATTCAAGTAACAGTGTTGAACATTCCCTTTC

                                                         *       *  * * *    
     454888 ATAGAGCAGGTTTGAAACACTCTTTTTGTAGTATCTGGAAGTGGACATTTGGAGAGATCTCAGGA
          1 ATAGAGCAGGTTTGAAACACTCTTTTTGTAGTATCTGGAAGTGGATATTTGGATAGCTTTGAGGA

            * *  *    * *             **          * *       * *          *  *
     454953 ATACGGT-GATAAAGGAAATATCTTCCAATAAAAGCTACATAGAAGCAATGTCAGAAACTTTTTC
         66 TTTCGTTGGAAACAGG-AATATCTTCATATAAAAGCTAGACAGAAGCATTCTCAGAAACTTCTTT

            *      * ** *   **      *       *  *     *
     455017 ATGATGTATCTACTCAGCTAACAGAGTTGAACCTTTCCTTTG
        130 GTGATGTTTGCATTCAAGTAACAGTGTTGAACATTCCCTTTC

             *       *                   * *     *             **            
     455059 AGAGAGCAGTTTTGAAACACTCTTTTTGTGGAATCTGCAAGTGGATATTTGTCTAGCTTTGAGGA
          1 ATAGAGCAGGTTTGAAACACTCTTTTTGTAGTATCTGGAAGTGGATATTTGGATAGCTTTGAGGA

                         *   *              **                      *    *   
     455124 TTTCGTTGGAAACGGGATTATCTTCATATAAACTCTAGACAGAAGCATTCTCAGAAGCTTCATTG
         66 TTTCGTTGGAAACAGGAATATCTTCATATAAAAGCTAGACAGAAGCATTCTCAGAAACTTCTTTG

            *       **   *    *             *        
     455189 GGATGTTTCAATTGAAGTCACAGTGTTGAACAGTCCCTTTC
        131 TGATGTTTGCATTCAAGTAACAGTGTTGAACATTCCCTTTC

                                                   *     *       **     *    
     455230 ATAGAGCAGGTTTGAAACACTCTTTTTGTAGTATCTGGATGTGGACATTTGGAGCGCTTTCAGGC
          1 ATAGAGCAGGTTTGAAACACTCTTTTTGTAGTATCTGGAAGTGGATATTTGGATAGCTTTGAGG-

            * * *    *                 *** *   *                          *  
     455295 CTATGGTT-TAAA-AGGAAATATCTTCCCCTGAAAACTAGACAGAAGCATTCTCAGAAACTTATT
         65 ATTTCGTTGGAAACAGG-AATATCTTCATATAAAAGCTAGACAGAAGCATTCTCAGAAACTTCTT

                    **  **    *                    *   *
     455358 TGTGATGTGCGCCCTCAACTAACAGTGTTGAAGCATT-CTTTTG
        129 TGTGATGTTTGCATTCAAGTAACAGTGTTGAA-CATTCCCTTTC

                     *            *      * *     *             **            
     455401 ATAGAGCAGTTTTGAAACACTCGTTTTGTGGAATCTGCAAGTGGATATTTGTCTAGCTTTGAGGA
          1 ATAGAGCAGGTTTGAAACACTCTTTTTGTAGTATCTGGAAGTGGATATTTGGATAGCTTTGAGGA

                         *   *    *                   *       *              
     455466 TTTCGTTGGAAACGGGATTA-CAT-ATA-AAAAGC-AGACAGCAGCATTCCCAGAAACTTCTTTG
         66 TTTCGTTGGAAACAGGAATATCTTCATATAAAAGCTAGACAGAAGCATTCTCAGAAACTTCTTTG

                              *    *                 
     455527 TGATGTTTGCATTCAAGTCACAGAGTTGAACATTCCCTTTC
        131 TGATGTTTGCATTCAAGTAACAGTGTTGAACATTCCCTTTC

                                                   *     *     * **     *   *
     455568 ATAGAGCAGGTTTGAAACACTCTTTTTGTAGTATCTGGATGTGGACATTTGCAGCGCTTTCAGGC
          1 ATAGAGCAGGTTTGAAACACTCTTTTTGTAGTATCTGGAAGTGGATATTTGGATAGCTTTGAGGA

            * ** *      *             *** *   *                          *   
     455633 CTAAGGT-GAAAAAGGAAATATCTTCCCCTGAAAACTAGACAGAAGCATTCTCAGAAACTTATTT
         66 TTTCGTTGGAAACAGG-AATATCTTCATATAAAAGCTAGACAGAAGCATTCTCAGAAACTTCTTT

                   **  **    *             **  * *   *
     455697 GTGATGTGCGCCCTCAACTAACAGTGTTGAAGCTTTCTTTTG
        130 GTGATGTTTGCATTCAAGTAACAGTGTTGAACATTCCCTTTC

                     *                    *      *   *                       
     455739 ATAGAGCAGTTTTGAAACACTCTTTTTGTAATATCTGCAAGAGGATATTTGGATAGCTTTGAGGA
          1 ATAGAGCAGGTTTGAAACACTCTTTTTGTAGTATCTGGAAGTGGATATTTGGATAGCTTTGAGGA

                         *   * *            **                      *    *   
     455804 TTTCGTTGGAAACGGGATTGTCTTCATATAAACTCTAGACAGAAGCATTCTCAGAAGCTTCATTG
         66 TTTCGTTGGAAACAGGAATATCTTCATATAAAAGCTAGACAGAAGCATTCTCAGAAACTTCTTTG

            *       **   *    *             * *      
     455869 GGATGTTTCAATTGAAGTCACAGTGTTGAACAGTTCCTTTC
        131 TGATGTTTGCATTCAAGTAACAGTGTTGAACATTCCCTTTC

                 *                                       *       **   * *    
     455910 ATAGAACAGGTTTGAAACACTCTTTTTGTAGTATCTGGAAGTGGACATTTGGAGCGCTCTCAGGA
          1 ATAGAGCAGGTTTGAAACACTCTTTTTGTAGTATCTGGAAGTGGATATTTGGATAGCTTTGAGGA

            * ** *      *             **          * *       * *          *  *
     455975 CTATGGT-GAAAAAGGAAATATCTTCCAATAAAAGCTACATAGAAGCAATGTCAGAAACTTTTTC
         66 TTTCGTTGGAAACAGG-AATATCTTCATATAAAAGCTAGACAGAAGCATTCTCAGAAACTTCTTT

            *      * ** *   **      *       *  * *   *
     456039 ATGATGTATCTACTCAGCTAACAGAGTTGAACCTTTCTTTTG
        130 GTGATGTTTGCATTCAAGTAACAGTGTTGAACATTCCCTTTC

                     *                    *      *   *                       
     456081 ATAGAGCAGTTTTGAAACACTCTTTTTGTAATATCTGCAAGAGGATATTTGGATAGCTTTGAGGA
          1 ATAGAGCAGGTTTGAAACACTCTTTTTGTAGTATCTGGAAGTGGATATTTGGATAGCTTTGAGGA

                         *   *    *                   *       *     *   *    
     456146 TTTCGTTGGAAACGGGATTA-CAT-ATA-AAAAGC-AGACAGCAGCATTCCCAGAATCTTGTTTG
         66 TTTCGTTGGAAACAGGAATATCTTCATATAAAAGCTAGACAGAAGCATTCTCAGAAACTTCTTTG

            *                 *    *                 
     456207 CGATGTTTGCATTCAAGTCACAGAGTTGAACATTCCCTTTC
        131 TGATGTTTGCATTCAAGTAACAGTGTTGAACATTCCCTTTC

             *                         *           *     *       **     *   *
     456248 AGAGAGCAGGTTTGAAACACTCTTTTTATAGTATCTGGATGTGGACATTTGGAGCGCTTTCAGGC
          1 ATAGAGCAGGTTTGAAACACTCTTTTTGTAGTATCTGGAAGTGGATATTTGGATAGCTTTGAGGA

            * ** *      *             *** **  *                      *   *   
     456313 CTATGGT-GAAAAAGGAAATATCTTCTCCTGTAAACTAGACAGAAGCATTCTCAGAATCTTATTT
         66 TTTCGTTGGAAACAGG-AATATCTTCATATAAAAGCTAGACAGAAGCATTCTCAGAAACTTCTTT

                 ****  **    *             **  * *   *
     456377 GTGATAAGCGCCCTCAACTAACAGTGTTGAAGCTTTCTTTTG
        130 GTGATGTTTGCATTCAAGTAACAGTGTTGAACATTCCCTTTC

                     *                *   **     *   *                       
     456419 ATAGAGCAGTTTTGAAACACTCTTTTCGTAAAATCTGCAAGAGGATATTTGGATAGCTTTGAGGA
          1 ATAGAGCAGGTTTGAAACACTCTTTTTGTAGTATCTGGAAGTGGATATTTGGATAGCTTTGAGGA

                         *   * *            **                      *    *   
     456484 TTTCGTTGGAAACGGGATTGTCTTCATATAAACTCTAGACAGAAGCATTCTCAGAAGCTTCATTG
         66 TTTCGTTGGAAACAGGAATATCTTCATATAAAAGCTAGACAGAAGCATTCTCAGAAACTTCTTTG

            *       **   *    *             *        
     456549 GGATGTTTCAATTGAAGTCACAGTGTTGAACAGTCCCTTTC
        131 TGATGTTTGCATTCAAGTAACAGTGTTGAACATTCCCTTTC

                                                         *       *  * * *    
     456590 ATAGAGCAGGTTTGAAACACTCTTTTTGTAGTATCTGGAAGTGGACATTTGGAGAGTTCTCAGGA
          1 ATAGAGCAGGTTTGAAACACTCTTTTTGTAGTATCTGGAAGTGGATATTTGGATAGCTTTGAGGA

            * *  *    * *             **            *       * *          *  *
     456655 ATACGGT-GATAAAGGAAATATCTTCCAATAAAAGCTAGATAGAAGCAATGTCAGAAACTTTTTC
         66 TTTCGTTGGAAACAGG-AATATCTTCATATAAAAGCTAGACAGAAGCATTCTCAGAAACTTCTTT

            *      * ** *   **      *       *  * *   *
     456719 ATGATGTATCTACTCAGCTAACAGAGTTGAACCTTTCTTTTG
        130 GTGATGTTTGCATTCAAGTAACAGTGTTGAACATTCCCTTTC

             *       *                   * *     *             **            
     456761 AGAGAGCAGTTTTGAAACACTCTTTTTGTGGAATCTGCAAGTGGATATTTGTCTAGCTTTGAGGA
          1 ATAGAGCAGGTTTGAAACACTCTTTTTGTAGTATCTGGAAGTGGATATTTGGATAGCTTTGAGGA

                         *   *    *                   *       *   *          
     456826 TTTCGTTGGAAACGGGATTA-CAT-ATA-AAAAGC-AGACAGCAGCATTCCCAGTAACTTCTTTG
         66 TTTCGTTGGAAACAGGAATATCTTCATATAAAAGCTAGACAGAAGCATTCTCAGAAACTTCTTTG

                              *    *                 
     456887 TGATGTTTGCATTCAAGTCACAGAGTTGAACATTCCCTTTC
        131 TGATGTTTGCATTCAAGTAACAGTGTTGAACATTCCCTTTC

                                                   *     *     * **     *   *
     456928 ATAGAGCAGGTTTGAAACACTCTTTTTGTAGTATCTGGATGTGGACATTTGCAGCGCTTTCAGGC
          1 ATAGAGCAGGTTTGAAACACTCTTTTTGTAGTATCTGGAAGTGGATATTTGGATAGCTTTGAGGA

            * *  *      *             *** *   *                          *   
     456993 CTACGGT-GAAAAAGGAAATATCTTCCCCTGAAAACTAGACAGAAGCATTCTCAGAAACTTATTT
         66 TTTCGTTGGAAACAGG-AATATCTTCATATAAAAGCTAGACAGAAGCATTCTCAGAAACTTCTTT

             *    ***  **    *             **  * *   *
     457057 GCGATGGGCGCCCTCAACTAACAGTGTTGAAGCTTTCTTTTG
        130 GTGATGTTTGCATTCAAGTAACAGTGTTGAACATTCCCTTTC

                     *                *   **     *   *                       
     457099 ATAGAGCAGTTTTGAAACACTCTTTTCGTAAAATCTGCAAGAGGATATTTGGATAGCTTTGAGGA
          1 ATAGAGCAGGTTTGAAACACTCTTTTTGTAGTATCTGGAAGTGGATATTTGGATAGCTTTGAGGA

                         *   * *            **                      *    *   
     457164 TTTCGTTGGAAACGGGATTGTCTTCATATAAACTCTAGACAGAAGCATTCTCAGAAGCTTCATTG
         66 TTTCGTTGGAAACAGGAATATCTTCATATAAAAGCTAGACAGAAGCATTCTCAGAAACTTCTTTG

            *       **   *    *             *        
     457229 GGATGTTTCAATTGAAGTCACAGTGTTGAACAGTCCCTTTC
        131 TGATGTTTGCATTCAAGTAACAGTGTTGAACATTCCCTTTC

                                                         *    *  *  * * *    
     457270 ATAGAGCAGGTTTGAAACACTCTTTTTGTAGTATCTGGAAGTGGACATTTTGAGAGATCTCAGGA
          1 ATAGAGCAGGTTTGAAACACTCTTTTTGTAGTATCTGGAAGTGGATATTTGGATAGCTTTGAGGA

            * *  *    * *             **            *       * *          *  *
     457335 ATACGGT-GATAAAGGTAATATCTTCCAATAAAAGCTAGATAGAAGCAATGTCAGAAACTTTTTC
         66 TTTCGTTGGAAACAGG-AATATCTTCATATAAAAGCTAGACAGAAGCATTCTCAGAAACTTCTTT

            *      * ** *   **      *       *  *     *
     457399 ATGATGTATCTACTCAGCTAACAGAGTTGAACCTTTCCTTTG
        130 GTGATGTTTGCATTCAAGTAACAGTGTTGAACATTCCCTTTC

             *       *                   * *     *             **            
     457441 AGAGAGCAGTTTTGAAACACTCTTTTTGTGGAATCTGCAAGTGGATATTTGTCTAGCTTTGAGGA
          1 ATAGAGCAGGTTTGAAACACTCTTTTTGTAGTATCTGGAAGTGGATATTTGGATAGCTTTGAGGA

                         *   *    *                   *       *   *          
     457506 TTTCGTTGGAAACGGGATTA-CAT-ATA-AAAAGC-AGACAGCAGCATTCCCAGTAACTTCTTTG
         66 TTTCGTTGGAAACAGGAATATCTTCATATAAAAGCTAGACAGAAGCATTCTCAGAAACTTCTTTG

                              *    *      *          
     457567 TGATGTTTGCATTCAAGTCACAGAGTTGAATATTCCCTTTC
        131 TGATGTTTGCATTCAAGTAACAGTGTTGAACATTCCCTTTC

                                                   *     *       **     *   *
     457608 ATAGAGCAGGTTTGAAACACTCTTTTTGTAGTATCTGGATGTGGACATTTGGAGCGCTTTCAGGC
          1 ATAGAGCAGGTTTGAAACACTCTTTTTGTAGTATCTGGAAGTGGATATTTGGATAGCTTTGAGGA

            * ** *      *             *** *   *                          *   
     457673 CTATGGT-GAAAAAGGAAATATCTTCCCCTGAAAACTAGACAGAAGCATTCTCAGAAACTTATTT
         66 TTTCGTTGGAAACAGG-AATATCTTCATATAAAAGCTAGACAGAAGCATTCTCAGAAACTTCTTT

                   **  **    *     *        *  * *   *
     457737 GTGATGTGCGCCCTCAACTAACACTGTTGAACCTTTCTTTTG
        130 GTGATGTTTGCATTCAAGTAACAGTGTTGAACATTCCCTTTC

                     *                    *      *   *                       
     457779 ATAGAGCAGTTTTGAAACACTCTTTTTGTAATATCTGCAAGAGGATATTTGGATAGCTTTGAGGA
          1 ATAGAGCAGGTTTGAAACACTCTTTTTGTAGTATCTGGAAGTGGATATTTGGATAGCTTTGAGGA

                         *   *    *                   *  *    *              
     457844 TTTCGTTGGAAACGGGATTA-CAT-ATA-AAAAGC-AGACAGCAGGATTCCCAGAAACTTCTTTG
         66 TTTCGTTGGAAACAGGAATATCTTCATATAAAAGCTAGACAGAAGCATTCTCAGAAACTTCTTTG

                              *    *                 
     457905 TGATGTTTGCATTCAAGTCACAGAGTTGAACATTCCCTTTC
        131 TGATGTTTGCATTCAAGTAACAGTGTTGAACATTCCCTTTC

                                                   *     *     * **     *   *
     457946 ATAGAGCAGGTTTGAAACACTCTTTTTGTAGTATCTGGATGTGGACATTTGCAGCGCTTTCAGGC
          1 ATAGAGCAGGTTTGAAACACTCTTTTTGTAGTATCTGGAAGTGGATATTTGGATAGCTTTGAGGA

            * ** *      *             *** *   *                          *   
     458011 CTAAGGT-GAAAAAGGAAATATCTTCCCCTGAAAACTAGACAGAAGCATTCTCAGAAACTTATTT
         66 TTTCGTTGGAAACAGG-AATATCTTCATATAAAAGCTAGACAGAAGCATTCTCAGAAACTTCTTT

                   **  **    *    *         *  * *   *
     458075 GTGATGTGCGCCCTCAACTAACGGTGTTGAACCTTTCTTTTG
        130 GTGATGTTTGCATTCAAGTAACAGTGTTGAACATTCCCTTTC

                     *                    **     *   *                       
     458117 ATAGAGCAGTTTTGAAACACTCTTTTTGTAAAATCTGCAAGAGGATATTTGGATAGCTTTGAGGA
          1 ATAGAGCAGGTTTGAAACACTCTTTTTGTAGTATCTGGAAGTGGATATTTGGATAGCTTTGAGGA

                         *   * *            **                      *    *   
     458182 TTTCGTTGGAAACGGGATTGTCTTCATATAAACTCTAGACAGAAGCATTCTCAGAAGCTTCATTG
         66 TTTCGTTGGAAACAGGAATATCTTCATATAAAAGCTAGACAGAAGCATTCTCAGAAACTTCTTTG

            *       **   *    *             *        
     458247 GGATGTTTCAATTGAAGTCACAGTGTTGAACAGTCCCTTTC
        131 TGATGTTTGCATTCAAGTAACAGTGTTGAACATTCCCTTTC

                                                   *     *       **     *    
     458288 ATAGAGCAGGTTTGAAACACTCTTTTTGTAGTATCTGGATGTGGACATTTGGAGCGCTTTCAGGC
          1 ATAGAGCAGGTTTGAAACACTCTTTTTGTAGTATCTGGAAGTGGATATTTGGATAGCTTTGAGG-

            * * *    *                 *** *   *                          *  
     458353 CTATGGTT-TAAA-AGGAAATATCTTCCCCTGAAAACTAGACAGAAGCATTCTCAGAAACTTATT
         65 ATTTCGTTGGAAACAGG-AATATCTTCATATAAAAGCTAGACAGAAGCATTCTCAGAAACTTCTT

                    **  **    *                    *   *
     458416 TGTGATGTGCGCCCTCAACTAACAGTGTTGAAGCATT-CTTTTG
        129 TGTGATGTTTGCATTCAAGTAACAGTGTTGAA-CATTCCCTTTC

                   * *                   * *     *             **            
     458459 ATAGAGCTGTTTTGAAACACTCTTTTTGTGGAATCTGCAAGTGGATATTTGTCTAGCTTTGAGGA
          1 ATAGAGCAGGTTTGAAACACTCTTTTTGTAGTATCTGGAAGTGGATATTTGGATAGCTTTGAGGA

                         *   *   *                    *       *     *   *    
     458524 TTTCGTTGGAAACGGGATTA-ATT-ATA-AAAAGC-AGACAGCAGCATTCCCAGAATCTTGTTTG
         66 TTTCGTTGGAAACAGGAATATCTTCATATAAAAGCTAGACAGAAGCATTCTCAGAAACTTCTTTG

                              *    *                 
     458585 TGATGTTTGCATTCAAGTCACAGAGTTGAACATTCCCTTTC
        131 TGATGTTTGCATTCAAGTAACAGTGTTGAACATTCCCTTTC

             *                         *           *     *       **     *   *
     458626 AGAGAGCAGGTTTGAAACACTCTTTTTATAGTATCTGGATGTGGACATTTGGAGCGCTTTCAGGC
          1 ATAGAGCAGGTTTGAAACACTCTTTTTGTAGTATCTGGAAGTGGATATTTGGATAGCTTTGAGGA

            * ** *      *             *** *   *                      *   *   
     458691 CTATGAT-GAAAAAGGAAATATCTTCTCCTGAAAACTAGACAGAAGCATTCTCAGAATCTTATTT
         66 TTTCGTTGGAAACAGG-AATATCTTCATATAAAAGCTAGACAGAAGCATTCTCAGAAACTTCTTT

                   **  **    *             **  * *   *
     458755 GTGATGTGCGCCCTCAACTAACAGTGTTGAAGCTTTCTTTTG
        130 GTGATGTTTGCATTCAAGTAACAGTGTTGAACATTCCCTTTC

                     *                *   **     *   *                       
     458797 ATAGAGCAGTTTTGAAACACTCTTTTCGTAAAATCTGCAAGAGGATATTTGGATAGCTTTGAGGA
          1 ATAGAGCAGGTTTGAAACACTCTTTTTGTAGTATCTGGAAGTGGATATTTGGATAGCTTTGAGGA

                         *   * *            **                      *    *   
     458862 TTTCGTTGGAAACGGGATTGTCTTCATATAAACTCTAGACAGAAGCATTCTCAGAAGCTTCATTG
         66 TTTCGTTGGAAACAGGAATATCTTCATATAAAAGCTAGACAGAAGCATTCTCAGAAACTTCTTTG

            *       **   *    *             *        
     458927 GGATGTTTCAATTGAAGTCACAGTGTTGAACAGTCCCTTTC
        131 TGATGTTTGCATTCAAGTAACAGTGTTGAACATTCCCTTTC

                                                         *       *  * * *    
     458968 ATAGAGCAGGTTTGAAACACTCTTTTTGTAGTATCTGGAAGTGGACATTTGGAGAGATCTCAGGA
          1 ATAGAGCAGGTTTGAAACACTCTTTTTGTAGTATCTGGAAGTGGATATTTGGATAGCTTTGAGGA

            * *  *    * *             **            *       * *          *  *
     459033 ATACGGT-GATAAAGGTAATATCTTCCAATAAAAGCTAGATAGAAGCAATGTCAGAAACTTTTTC
         66 TTTCGTTGGAAACAGG-AATATCTTCATATAAAAGCTAGACAGAAGCATTCTCAGAAACTTCTTT

            *      * ** *   **      *       *  * *   *
     459097 ATGATGTATCTACTCAGCTAACAGAGTTGAACCTTTCTTTTG
        130 GTGATGTTTGCATTCAAGTAACAGTGTTGAACATTCCCTTTC

             *       *                   * *     *             **            
     459139 AGAGAGCAGTTTTGAAACACTCTTTTTGTGGAATCTGCAAGTGGATATTTGTCTAGCTTTGAGGA
          1 ATAGAGCAGGTTTGAAACACTCTTTTTGTAGTATCTGGAAGTGGATATTTGGATAGCTTTGAGGA

                         *   *    *                   *       *   *          
     459204 TTTCGTTGGAAACGGGATTA-CAT-ATA-AAAAGC-AGACAGCAGCATTCCCAGTAACTTCTTTG
         66 TTTCGTTGGAAACAGGAATATCTTCATATAAAAGCTAGACAGAAGCATTCTCAGAAACTTCTTTG

                              *    *                 
     459265 TGATGTTTGCATTCAAGTCACAGAGTTGAACATTCCCTTTC
        131 TGATGTTTGCATTCAAGTAACAGTGTTGAACATTCCCTTTC

                             *   *                 *     *       **     *   *
     459306 ATAGAGCAGGTTTGAAATACTTTTTTTGTAGTATCTGGATGTGGACATTTGGAGCGCTTTCAGGC
          1 ATAGAGCAGGTTTGAAACACTCTTTTTGTAGTATCTGGAAGTGGATATTTGGATAGCTTTGAGGA

            * ** *      *             **          * *       * *          *  *
     459371 CTATGGT-GAAAAAGGAAATATCTTCCAATAAAAGCTACATAGAAGCAATGTCAGAAACTTTTTC
         66 TTTCGTTGGAAACAGG-AATATCTTCATATAAAAGCTAGACAGAAGCATTCTCAGAAACTTCTTT

            *      * ** *   **      *       *  * *   *
     459435 ATGATGTATCTACTCAGCTAACAGAGTTGAACCTTTCTTTTG
        130 GTGATGTTTGCATTCAAGTAACAGTGTTGAACATTCCCTTTC

             *       *                    **     *   *                       
     459477 AGAGAGCAGTTTTGAAACACTCTTTTTGTAAAATCTGCAAGAGGATATTTGGATAGCTTTGAGGA
          1 ATAGAGCAGGTTTGAAACACTCTTTTTGTAGTATCTGGAAGTGGATATTTGGATAGCTTTGAGGA

                         *   * *            **                      * *  *   
     459542 TTTCGTTGGAAACGGGATTGTCTTCATATAAACTCTAGACAGAAGCATTCTCAGAAGCGTCATTG
         66 TTTCGTTGGAAACAGGAATATCTTCATATAAAAGCTAGACAGAAGCATTCTCAGAAACTTCTTTG

            *       **   *    *             *        
     459607 GGATGTTTCAATTGAAGTCACAGTGTTGAACAGTCCCTTTC
        131 TGATGTTTGCATTCAAGTAACAGTGTTGAACATTCCCTTTC

                                                   *     *       **     *   *
     459648 ATAGAGCAGGTTTGAAACACTCTTTTTGTAGTATCTGGATGTGGACATTTGGAGCGCTTTCAGGC
          1 ATAGAGCAGGTTTGAAACACTCTTTTTGTAGTATCTGGAAGTGGATATTTGGATAGCTTTGAGGA

            * ** *      *             *** *   *                          *   
     459713 CTATGGT-GAAAAAGGAAATATCTTCCCCTGAAAACTAGACAGAAGCATTCTCAGAAACTTATTT
         66 TTTCGTTGGAAACAGG-AATATCTTCATATAAAAGCTAGACAGAAGCATTCTCAGAAACTTCTTT

                   **  *     *                    *   *
     459777 GTGATGTGCGCCTTCAACTAACAGTGTTGAAGCATT-CTTTTG
        130 GTGATGTTTGCATTCAAGTAACAGTGTTGAA-CATTCCCTTTC

                     *                   * *     *             **            
     459819 ATAGAGCAGTTTTGAAACACTCTTTTTGTGGAATCTGCAAGTGGATATTTGTCTAGCTTTGAGGA
          1 ATAGAGCAGGTTTGAAACACTCTTTTTGTAGTATCTGGAAGTGGATATTTGGATAGCTTTGAGGA

                         *   *    *                   *       *              
     459884 TTTCGTTGGAAACGGGATTA-CAT-ATA-AAAAGC-AGACAGCAGCATTCCCAGAAACTTCTTTG
         66 TTTCGTTGGAAACAGGAATATCTTCATATAAAAGCTAGACAGAAGCATTCTCAGAAACTTCTTTG

                              *    *                 
     459945 TGATGTTTGCATTCAAGTCACAGAGTTGAACATTCCCTTTC
        131 TGATGTTTGCATTCAAGTAACAGTGTTGAACATTCCCTTTC

             *                                     *     *       **     *  **
     459986 AGAGAGCAGGTTTGAAACACTCTTTTTGTAGTATCTGGATGTGGACATTTGGAGCGCTTTCAGCC
          1 ATAGAGCAGGTTTGAAACACTCTTTTTGTAGTATCTGGAAGTGGATATTTGGATAGCTTTGAGGA

            * ** *      *             *** *   *                      *   *   
     460051 CTATGGT-GAAAAAGGAAATATCTTCCCCTGAAAACTAGACAGAAGCATTCTCAGAATCTTATTT
         66 TTTCGTTGGAAACAGG-AATATCTTCATATAAAAGCTAGACAGAAGCATTCTCAGAAACTTCTTT

                   **  **    *             **  * *   *
     460115 GTGATGTGCGCCCTCAACTAACAGTGTTGAAGCTTTCTTTTG
        130 GTGATGTTTGCATTCAAGTAACAGTGTTGAACATTCCCTTTC

                     *                    **     *   *                       
     460157 ATAGAGCAGTTTTGAAACACTCTTTTTGTAAAATCTGCAAGAGGATATTTGGATAGCTTTGAGGA
          1 ATAGAGCAGGTTTGAAACACTCTTTTTGTAGTATCTGGAAGTGGATATTTGGATAGCTTTGAGGA

                         *   * *     *      **                      *    *   
     460222 TTTCGTTGGAAACGGGATTGTCTTCCTATAAACTCTAGACAGAAGCATTCTCAGAAGCTTCATTG
         66 TTTCGTTGGAAACAGGAATATCTTCATATAAAAGCTAGACAGAAGCATTCTCAGAAACTTCTTTG

            *       **   *    *             *        
     460287 GGATGTTTCAATTGAAGTCACAGTGTTGAACAGTCCCTTTC
        131 TGATGTTTGCATTCAAGTAACAGTGTTGAACATTCCCTTTC

                                                         *       **   * *    
     460328 ATAGAGCAGGTTTGAAACACTCTTTTTGTAGTATCTGGAAGTGGACATTTGGAGCGCTCTCAGGA
          1 ATAGAGCAGGTTTGAAACACTCTTTTTGTAGTATCTGGAAGTGGATATTTGGATAGCTTTGAGGA

            * *  *      *         *   **            *       * *          *  *
     460393 CTGCGGT-GAAAAAGGAACTATTTTCCAATAAAAGCTAGATAGAAGCAATGTCAGAAACTTTTTC
         66 TTTCGTTGGAAACAGGAA-TATCTTCATATAAAAGCTAGACAGAAGCATTCTCAGAAACTTCTTT

            *      * ** *   **      *       *        *
     460457 ATGATGTATCTACTCAGCTAACAGAGTTGAACCTT-CCTTTG
        130 GTGATGTTTGCATTCAAGTAACAGTGTTGAACATTCCCTTTC

             *       *                   * *     *             **            
     460498 AGAGAGCAGTTTTGAAACACTCTTTTTGTGGAATCTGCAAGTGGATATTTGTCTAGCTTTGAGGA
          1 ATAGAGCAGGTTTGAAACACTCTTTTTGTAGTATCTGGAAGTGGATATTTGGATAGCTTTGAGGA

                         *   *    *                   *       *              
     460563 TTTCGTTGGAAACGGGATTA-CAT-ATA-AAAAGC-AGACAGCAGCATTCCCAGAAACTTCTTTG
         66 TTTCGTTGGAAACAGGAATATCTTCATATAAAAGCTAGACAGAAGCATTCTCAGAAACTTCTTTG

                              *   **                 
     460624 TGATGTTTGCATTCAAGTCACATAGTTGAACATTCCCTTTC
        131 TGATGTTTGCATTCAAGTAACAGTGTTGAACATTCCCTTTC

                                                   *     *     * **     *   *
     460665 ATAGAGCAGGTTTGAAACACTCTTTTTGTAGTATCTGGATGTGGACATTTGCAGCGCTTTCAGGC
          1 ATAGAGCAGGTTTGAAACACTCTTTTTGTAGTATCTGGAAGTGGATATTTGGATAGCTTTGAGGA

            * ** *      *             *** *   *                          *   
     460730 CTAAGGT-GAAAAAGGAAATATCTTCCCCTGAAAACTAGACAGAAGCATTCTCAGAAACTTATTT
         66 TTTCGTTGGAAACAGG-AATATCTTCATATAAAAGCTAGACAGAAGCATTCTCAGAAACTTCTTT

                   **  **    *             **  * *   *
     460794 GTGATGTGCGCCCTCAACTAACAGTGTTGAAGCTTTCTTTTG
        130 GTGATGTTTGCATTCAAGTAACAGTGTTGAACATTCCCTTTC

                     *                    *      *   *                       
     460836 ATAGAGCAGTTTTGAAACACTCTTTTTGTAATATCTGCAAGAGGATATTTGGATAGCTTTGAGGA
          1 ATAGAGCAGGTTTGAAACACTCTTTTTGTAGTATCTGGAAGTGGATATTTGGATAGCTTTGAGGA

                         *   * *            **                      *    *   
     460901 TTTCGTTGGAAACGGGATTGTCTTCATATAAACTCTAGACAGAAGCATTCTCAGAAGCTTCATTG
         66 TTTCGTTGGAAACAGGAATATCTTCATATAAAAGCTAGACAGAAGCATTCTCAGAAACTTCTTTG

            *       **   *    *             * *      
     460966 GGATGTTTCAATTGAAGTCACAGTGTTGAACAGTTCCTTTC
        131 TGATGTTTGCATTCAAGTAACAGTGTTGAACATTCCCTTTC

                 *                                       *       **   * *    
     461007 ATAGAACAGGTTTGAAACACTCTTTTTGTAGTATCTGGAAGTGGACATTTGGAGCGCTCTCAGGA
          1 ATAGAGCAGGTTTGAAACACTCTTTTTGTAGTATCTGGAAGTGGATATTTGGATAGCTTTGAGGA

            * *  *  *   ** *         **          * *       * *          *  **
     461072 CTACGGTGAAAATGGAAATATCTTCCAATAAAAGCTACATAGAAGCAATGTCAGAAACTTTTTCA
         66 TTTCGTTGGAAACAGGAATATCTTCATATAAAAGCTAGACAGAAGCATTCTCAGAAACTTCTTTG

                  * ** *   **      *       *  *     *
     461137 TGATGTATCTACTCAGCTAACAGAGTTGAACCTTTCCTTTG
        131 TGATGTTTGCATTCAAGTAACAGTGTTGAACATTCCCTTTC

             *       *                   * *     *             **            
     461178 AGAGAGCAGTTTTGAAACACTCTTTTTGTGGAATCTGCAAGTGGATATTTGTCTAGCTTTGAGGA
          1 ATAGAGCAGGTTTGAAACACTCTTTTTGTAGTATCTGGAAGTGGATATTTGGATAGCTTTGAGGA

                         *   *    *                   *       *     *        
     461243 TTTCGTTGGAAACGGGATTA-CAT-ATA-AAAAGC-AGACAGCAGCATTCCCAGAATCTTCTTTG
         66 TTTCGTTGGAAACAGGAATATCTTCATATAAAAGCTAGACAGAAGCATTCTCAGAAACTTCTTTG

                              *    *                 
     461304 TGATGTTTGCATTCAAGTCACAGAGTTGAACATTCCCTTTC
        131 TGATGTTTGCATTCAAGTAACAGTGTTGAACATTCCCTTTC

                                                   *     *       **     *   *
     461345 ATAGAGCAGGTTTGAAACACTCTTTTTGTAGTATCTGGATGTGGACATTTGGAGCGCTTTCAGGC
          1 ATAGAGCAGGTTTGAAACACTCTTTTTGTAGTATCTGGAAGTGGATATTTGGATAGCTTTGAGGA

            * ** *      *             *** *   *                      *   *   
     461410 CTATGGT-GAAAAAGGAAATATCTTCCCCTGAAAACTAGACAGAAGCATTCTCAGAATCTTATTT
         66 TTTCGTTGGAAACAGG-AATATCTTCATATAAAAGCTAGACAGAAGCATTCTCAGAAACTTCTTT

                   **  **    *             **  * *   *
     461474 GTGATGTGCGCCCTCAACTAACAGTGTTGAAGCTTTCTTTTG
        130 GTGATGTTTGCATTCAAGTAACAGTGTTGAACATTCCCTTTC

                     *        *           **     *   *                       
     461516 ATAGAGCAGTTTTGAAACTCTCTTTTTGTAAAATCTGCAAGAGGATATTTGGATAGCTTTGAGGA
          1 ATAGAGCAGGTTTGAAACACTCTTTTTGTAGTATCTGGAAGTGGATATTTGGATAGCTTTGAGGA

                         *   * *            **                      *    *   
     461581 TTTCGTTGGAAACGGGATTGTCTTCATATAAACTCTAGACAGAAGCATTCTCAGAAGCTTCATTG
         66 TTTCGTTGGAAACAGGAATATCTTCATATAAAAGCTAGACAGAAGCATTCTCAGAAACTTCTTTG

            *       **   *    *             *        
     461646 GGATGTTTCAATTGAAGTCACAGTGTTGAACAGTCCCTTTC
        131 TGATGTTTGCATTCAAGTAACAGTGTTGAACATTCCCTTTC

                                                         *       **   * *    
     461687 ATAGAGCAGGTTTGAAACACTCTTTTTGTAGTATCTGGAAGTGGACATTTGGAACGCTCTCAGGA
          1 ATAGAGCAGGTTTGAAACACTCTTTTTGTAGTATCTGGAAGTGGATATTTGGATAGCTTTGAGGA

            * *  *      *             **            *       * *          *  *
     461752 CTGCGGT-GAAAAAGGAAATATCTTCCAATAAAAGCTAGATAGAAGCAATGTCAGAAACTTTTTC
         66 TTTCGTTGGAAACAGG-AATATCTTCATATAAAAGCTAGACAGAAGCATTCTCAGAAACTTCTTT

            *      * ** *   **      *       *  *     *
     461816 ATGATGTATCTACTCAGCTAACAGAGTTGAACCTTTCCTTTG
        130 GTGATGTTTGCATTCAAGTAACAGTGTTGAACATTCCCTTTC

             *       *            *      * *     *             **  *         
     461858 AGAGAGCAGTTTTGAAACACTCGTTTTGTGGAATCTGCAAGTGGATATTTGTCTACCTTTGAGGA
          1 ATAGAGCAGGTTTGAAACACTCTTTTTGTAGTATCTGGAAGTGGATATTTGGATAGCTTTGAGGA

                         *   *    *                   *       *              
     461923 TTTCGTTGGAAACGGGATTA-CAT-ATA-AAAAGC-AGACAGCAGCATTCCCAGAAACTTCTTTG
         66 TTTCGTTGGAAACAGGAATATCTTCATATAAAAGCTAGACAGAAGCATTCTCAGAAACTTCTTTG

                              *    *                 
     461984 TGATGTTTGCATTCAAGTCACAGAGTTGAACATTCCCTTTC
        131 TGATGTTTGCATTCAAGTAACAGTGTTGAACATTCCCTTTC

                                                   *     *     * **     *   *
     462025 ATAGAGCAGGTTTGAAACACTCTTTTTGTAGTATCTGGATGTGGACATTTGCAGCGCTTTCAGGC
          1 ATAGAGCAGGTTTGAAACACTCTTTTTGTAGTATCTGGAAGTGGATATTTGGATAGCTTTGAGGA

            * ** *      *             *** *   *                          *   
     462090 CTAAGGT-GAAAAAGGAAATATCTTCCCCTGAAAACTAGACAGAAGCATTCTCAGAAACTTATTT
         66 TTTCGTTGGAAACAGG-AATATCTTCATATAAAAGCTAGACAGAAGCATTCTCAGAAACTTCTTT

                   **  **    *             **  * *   *
     462154 GTGATGTGCGCCCTCAACTAACAGTGTTGAAGCTTTCTTTTG
        130 GTGATGTTTGCATTCAAGTAACAGTGTTGAACATTCCCTTTC

                     *                    *      *   *                       
     462196 ATAGAGCAGTTTTGAAACACTCTTTTTGTAATATCTGCAAGAGGATATTTGGATAGCTTTGAGGA
          1 ATAGAGCAGGTTTGAAACACTCTTTTTGTAGTATCTGGAAGTGGATATTTGGATAGCTTTGAGGA

                         *   * *            **                      *    *   
     462261 TTTCGTTGGAAACGGGATTGTCTTCATATAAACTCTAGACAGAAGCATTCTCAGAAGCTTCATTG
         66 TTTCGTTGGAAACAGGAATATCTTCATATAAAAGCTAGACAGAAGCATTCTCAGAAACTTCTTTG

            *       **   *    *             *        
     462326 GGATGTTTCAATTGAAGTCACAGTGTTGAACAGTCCCTTTC
        131 TGATGTTTGCATTCAAGTAACAGTGTTGAACATTCCCTTTC

                                                         *       **   * *    
     462367 ATAGAGCAGGTTTGAAACACTCTTTTTGTAGTATCTGGAAGTGGACATTTGGAACGCTCTCAGGA
          1 ATAGAGCAGGTTTGAAACACTCTTTTTGTAGTATCTGGAAGTGGATATTTGGATAGCTTTGAGGA

            * *  *      *             **            *       * *          *  *
     462432 CTGCGGT-GAAAAAGGAAATATCTTCCAATAAAAGCTAGATAGAAGCAATGTCAGAAACTTTTTC
         66 TTTCGTTGGAAACAGG-AATATCTTCATATAAAAGCTAGACAGAAGCATTCTCAGAAACTTCTTT

            *      * ** *   **      *       *  * *   *
     462496 ATGATGTATCTACTCAGCTAACAGAGTTGAACCTTTCTTTTG
        130 GTGATGTTTGCATTCAAGTAACAGTGTTGAACATTCCCTTTC

                     *                   * *     *             **            
     462538 ATAGAGCAGTTTTGAAACACTCTTTTTGTGGAATCTGCAAGTGGATATTTGTCTAGCTTTGAGGA
          1 ATAGAGCAGGTTTGAAACACTCTTTTTGTAGTATCTGGAAGTGGATATTTGGATAGCTTTGAGGA

                         *   *    *                   *       *   *          
     462603 TTTCGTTGGAAACGGGATTA-CAT-ATA-AAAAGC-AGACAGCAGCATTCCCAGTAACTTCTTTG
         66 TTTCGTTGGAAACAGGAATATCTTCATATAAAAGCTAGACAGAAGCATTCTCAGAAACTTCTTTG

                              *    *                 
     462664 TGATGTTTGCATTCAAGTCACAGAGTTGAACATTCCCTTTC
        131 TGATGTTTGCATTCAAGTAACAGTGTTGAACATTCCCTTTC

                                 *                 *     *       **     *   *
     462705 ATAGAGCAGGTTTGAAACACTTTTTTTGTAGTATCTGGATGTGGACATTTGGAGCGCTTTCAGGC
          1 ATAGAGCAGGTTTGAAACACTCTTTTTGTAGTATCTGGAAGTGGATATTTGGATAGCTTTGAGGA

            * ** *      *             **          * * *     * *          *  *
     462770 CTATGGT-GAAAAAGGAAATATCTTCCAATAAAAGCTACATAAAAGCAATGTCAGAAACTTTTTC
         66 TTTCGTTGGAAACAGG-AATATCTTCATATAAAAGCTAGACAGAAGCATTCTCAGAAACTTCTTT

            *      * ** *   **      *       *  * *   *
     462834 ATGATGTATCTACTCAGCTAACAGAGTTGAACCTTTCTTTTG
        130 GTGATGTTTGCATTCAAGTAACAGTGTTGAACATTCCCTTTC

             *       *                   * *                   **            
     462876 AGAGAGCAGTTTTGAAACACTCTTTTTGTGGAATCTGGAAGTGGATATTTGTCTAGCTTTGAGGA
          1 ATAGAGCAGGTTTGAAACACTCTTTTTGTAGTATCTGGAAGTGGATATTTGGATAGCTTTGAGGA

                         *   *    *                   *       *   *          
     462941 TTTCGTTGGAAACGGGATTA-CAT-ATA-AAAAGC-AGACAGCAGCATTCCCAGTAACTTCTTTG
         66 TTTCGTTGGAAACAGGAATATCTTCATATAAAAGCTAGACAGAAGCATTCTCAGAAACTTCTTTG

                              *    *                 
     463002 TGATGTTTGCATTCAAGTCACAGAGTTGAACATTCCCTTTC
        131 TGATGTTTGCATTCAAGTAACAGTGTTGAACATTCCCTTTC

                                                   *     *       **     *    
     463043 ATAGAGCAGGTTTGAAACACTCTTTTTGTAGTATCTGGATGTGGACATTTGGAGCGCTTTCAGG-
          1 ATAGAGCAGGTTTGAAACACTCTTTTTGTAGTATCTGGAAGTGGATATTTGGATAGCTTTGAGGA

            **  *        *             *** *   *           *          *   *  
     463107 CCT-ATTGTGAAAAAGGAAATATCTTCCCCTGAAAACTAGACAGAAGAATTCTCAGAATCTTATT
         66 TTTCGTTG-GAAACAGG-AATATCTTCATATAAAAGCTAGACAGAAGCATTCTCAGAAACTTCTT

                    **  **    *             **  * *   *
     463171 TGTGATGTGCGCCCTCAACTAACAGTGTTGAAGCTTTCTTTTG
        129 TGTGATGTTTGCATTCAAGTAACAGTGTTGAACATTCCCTTTC

                     *                    **     *   *                       
     463214 ATAGAGCAGTTTTGAAACACTCTTTTTGTAAAATCTGCAAGAGGATATTTGGATAGCTTTGAGGA
          1 ATAGAGCAGGTTTGAAACACTCTTTTTGTAGTATCTGGAAGTGGATATTTGGATAGCTTTGAGGA

                         *   * *            **   *                  * *  *   
     463279 TTTCGTTGGAAACGGGATTGTCTTCATATAAACTCTACACAGAAGCATTCTCAGAAGCGTCATTG
         66 TTTCGTTGGAAACAGGAATATCTTCATATAAAAGCTAGACAGAAGCATTCTCAGAAACTTCTTTG

            *       **   *    *             *        
     463344 GGATGTTTCAATTGAAGTCACAGTGTTGAACAGTCCCTTTC
        131 TGATGTTTGCATTCAAGTAACAGTGTTGAACATTCCCTTTC

                                                   *     *       **     *    
     463385 ATAGAGCAGGTTTGAAACACTCTTTTTGTAGTATCTGGATGTGGACATTTGGAGCGCTTTCAGGC
          1 ATAGAGCAGGTTTGAAACACTCTTTTTGTAGTATCTGGAAGTGGATATTTGGATAGCTTTGAGG-

            * * *    *                 *** *   *                          *  
     463450 CTATGGTT-TAAA-AGGAAATATCTTCCCCTGAAAACTAGACAGAAGCATTCTCAGAAACTTATT
         65 ATTTCGTTGGAAACAGG-AATATCTTCATATAAAAGCTAGACAGAAGCATTCTCAGAAACTTCTT

                    **  *     *                    *   *
     463513 TGTGATGTGCGCCTTCAACTAACAGTGTTGAAGCATT-CTTTTG
        129 TGTGATGTTTGCATTCAAGTAACAGTGTTGAA-CATTCCCTTTC

                     *                   * *     *             **            
     463556 ATAGAGCAGTTTTGAAACACTCTTTTTGTGGAATCTGCAAGTGGATATTTGTCTAGCTTTGAGGA
          1 ATAGAGCAGGTTTGAAACACTCTTTTTGTAGTATCTGGAAGTGGATATTTGGATAGCTTTGAGGA

                         *   *    *                   *       *              
     463621 TTTCGTTGGAAACGGGATTA-CAT-ATA-AAAAGC-AGACAGCAGCATTCCCAGAAACTTCTTTG
         66 TTTCGTTGGAAACAGGAATATCTTCATATAAAAGCTAGACAGAAGCATTCTCAGAAACTTCTTTG

                              *    *                 
     463682 TGATGTTTGCATTCAAGTCACAGAGTTGAACATTCCCTTTC
        131 TGATGTTTGCATTCAAGTAACAGTGTTGAACATTCCCTTTC

             *                                     *     *       **     *   *
     463723 AGAGAGCAGGTTTGAAACACTCTTTTTGTAGTATCTGGATGTGGACATTTGGAGCGCTTTCAGGC
          1 ATAGAGCAGGTTTGAAACACTCTTTTTGTAGTATCTGGAAGTGGATATTTGGATAGCTTTGAGGA

            * ** *      *             *** *   *                      *   *   
     463788 CTATGGT-GAAAAAGGAAATATCTTCCCCTGAAAACTAGACAGAAGCATTCTCAGAATCTTATTT
         66 TTTCGTTGGAAACAGG-AATATCTTCATATAAAAGCTAGACAGAAGCATTCTCAGAAACTTCTTT

                   **  **    *             **  * *   *
     463852 GTGATGTGCGCCCTCAACTAACAGTGTTGAAGCTTTCTTTTG
        130 GTGATGTTTGCATTCAAGTAACAGTGTTGAACATTCCCTTTC

                     *                    **     *   *                       
     463894 ATAGAGCAGTTTTGAAACACTCTTTTTGTAAAATCTGCAAGAGGATATTTGGATAGCTTTGAGGA
          1 ATAGAGCAGGTTTGAAACACTCTTTTTGTAGTATCTGGAAGTGGATATTTGGATAGCTTTGAGGA

                         *   * *            **                      *    *   
     463959 TTTCGTTGGAAACGGGATTGTCTTCATATAAACTCTAGACAGAAGCATTCTCAGAAGCTTCATTG
         66 TTTCGTTGGAAACAGGAATATCTTCATATAAAAGCTAGACAGAAGCATTCTCAGAAACTTCTTTG

            *       **   *    *             *        
     464024 GGATGTTTCAATTGAAGTCACAGTGTTGAACAGTCCCTTTC
        131 TGATGTTTGCATTCAAGTAACAGTGTTGAACATTCCCTTTC

                                                         *       **   * *    
     464065 ATAGAGCAGGTTTGAAACACTCTTTTTGTAGTATCTGGAAGTGGACATTTGGAGCGCTCTCAGGA
          1 ATAGAGCAGGTTTGAAACACTCTTTTTGTAGTATCTGGAAGTGGATATTTGGATAGCTTTGAGGA

            * *  *      *             **            *       * *          *  *
     464130 CTGCGGT-GAAAAAGGAAATATCTTCCAATAAAAGCTAGATAGAAGCAATGTCAGAAACTTTTTC
         66 TTTCGTTGGAAACAGG-AATATCTTCATATAAAAGCTAGACAGAAGCATTCTCAGAAACTTCTTT

            *      * ** *   **      *       *        *
     464194 ATGATGTATCTACTCAGCTAACAGAGTTGAACCTT-CCTTTG
        130 GTGATGTTTGCATTCAAGTAACAGTGTTGAACATTCCCTTTC

             *       *                   * *     *             **            
     464235 AGAGAGCAGTTTTGAAACACTCTTTTTGTGGAATCTGCAAGTGGATATTTGTCTAGCTTTGAGGA
          1 ATAGAGCAGGTTTGAAACACTCTTTTTGTAGTATCTGGAAGTGGATATTTGGATAGCTTTGAGGA

                         *   * *            **                      *    *   
     464300 TTTCGTTGGAAACGGGATTGTCTTCATATAAACTCTAGACAGAAGCATTCTCAGAAGCTTCATTG
         66 TTTCGTTGGAAACAGGAATATCTTCATATAAAAGCTAGACAGAAGCATTCTCAGAAACTTCTTTG

            *             *    *             * *      
     464365 GGATGTTT-CATTTGAAGTCACAGTGTTGAACAGTTCCTTTC
        131 TGATGTTTGCA-TTCAAGTAACAGTGTTGAACATTCCCTTTC

                 *                                       *       **   * *    
     464406 ATAGAACAGGTTTGAAACACTCTTTTTGTAGTATCTGGAAGTGGACATTTGGAGCGCTCTCAGGA
          1 ATAGAGCAGGTTTGAAACACTCTTTTTGTAGTATCTGGAAGTGGATATTTGGATAGCTTTGAGGA

            * ** *      *             **          * *       * *          *  *
     464471 CTATGGT-GAAAAAGGAAATATCTTCCAATAAAAGCTACATAGAAGCAATGTCAGAAACTTTTTC
         66 TTTCGTTGGAAACAGG-AATATCTTCATATAAAAGCTAGACAGAAGCATTCTCAGAAACTTCTTT

            *      * ** *   **      *       *  *     *
     464535 ATGATGTATCTACTCAGCTAACAGAGTTGAACCTTTCCTTTG
        130 GTGATGTTTGCATTCAAGTAACAGTGTTGAACATTCCCTTTC

             *       *                   * *     * *           **            
     464577 AGAGAGCAGTTTTGAAACACTCTTTTTGTGGAATCTGCAGGTGGATATTTGTCTAGCTTTGAGGA
          1 ATAGAGCAGGTTTGAAACACTCTTTTTGTAGTATCTGGAAGTGGATATTTGGATAGCTTTGAGGA

                         *   *    *                   *       *   *          
     464642 TTTCGTTGGAAACGGGATTA-CAT-ATA-AAAAGC-AGACAGCAGCATTCCCAGTAACTTCTTTG
         66 TTTCGTTGGAAACAGGAATATCTTCATATAAAAGCTAGACAGAAGCATTCTCAGAAACTTCTTTG

                              *    *          *      
     464703 TGATGTTTGCATTCAAGTCACAGAGTTGAACATTGCCTTTC
        131 TGATGTTTGCATTCAAGTAACAGTGTTGAACATTCCCTTTC

                         *                         *     *       **     *   *
     464744 ATAGAGCAGGTTTCAAACACTCTTTTTGTAGTATCTGGATGTGGACATTTGGAGCGCTTTCAGGC
          1 ATAGAGCAGGTTTGAAACACTCTTTTTGTAGTATCTGGAAGTGGATATTTGGATAGCTTTGAGGA

            * ** *      *             *** *   *                      *   *   
     464809 CTATGGT-GAAAAAGGAAATATCTTCCCCTGAAAACTAGACAGAAGCATTCTCAGAATCTTATTT
         66 TTTCGTTGGAAACAGG-AATATCTTCATATAAAAGCTAGACAGAAGCATTCTCAGAAACTTCTTT

                   **  **    *             **  * *   *
     464873 GTGATGTGCGCCCTCAACTAACAGTGTTGAAGCTTTCTTTTG
        130 GTGATGTTTGCATTCAAGTAACAGTGTTGAACATTCCCTTTC

                     *                    **     *   *                       
     464915 ATAGAGCAGTTTTGAAACACTCTTTTTGTAAAATCTGCAAGAGGATATTTGGATAGCTTTGAGGA
          1 ATAGAGCAGGTTTGAAACACTCTTTTTGTAGTATCTGGAAGTGGATATTTGGATAGCTTTGAGGA

                         *   * *            **                      *    *   
     464980 TTTCGTTGGAAACGGGATTGTCTTCATATAAACTCTAGACAGAAGCATTCTCAGAAGCTTCATTG
         66 TTTCGTTGGAAACAGGAATATCTTCATATAAAAGCTAGACAGAAGCATTCTCAGAAACTTCTTTG

            *       **   *    *             *        
     465045 GGATGTTTCAATTGAAGTCACAGTGTTGAACAGTCCCTTTC
        131 TGATGTTTGCATTCAAGTAACAGTGTTGAACATTCCCTTTC

                         *                         *     *       **     *    
     465086 ATAGAGCAGGTTTCAAACACTCTTTTTGTAGTATCTGGATGTGGACATTTGGAGCGCTTTCAGGC
          1 ATAGAGCAGGTTTGAAACACTCTTTTTGTAGTATCTGGAAGTGGATATTTGGATAGCTTTGAGG-

            * * *    *                 *** *   *                          *  
     465151 CTATGGTT-TAAA-AGGAAATATCTTCCCCTGAAAACTAGACAGAAGCATTCTCAGAAACTTATT
         65 ATTTCGTTGGAAACAGG-AATATCTTCATATAAAAGCTAGACAGAAGCATTCTCAGAAACTTCTT

                    **  **    *                    *   *
     465214 TGTGATGTGCGCCCTCAACTAACAGTGTTGAAGCATT-CTTTTG
        129 TGTGATGTTTGCATTCAAGTAACAGTGTTGAA-CATTCCCTTTC

                     *                   * *     *             **            
     465257 ATAGAGCAGTTTTGAAACACTCTTTTTGTGGAATCTGCAAGTGGATATTTGTCTAGCTTTGAGGA
          1 ATAGAGCAGGTTTGAAACACTCTTTTTGTAGTATCTGGAAGTGGATATTTGGATAGCTTTGAGGA

                         *   *    *                   *       *              
     465322 TTTCGTTGGAAACGGGATTA-CAT-ATA-AAAAGC-AGACAGCAGCATTCCCAGAAACTTCTTTG
         66 TTTCGTTGGAAACAGGAATATCTTCATATAAAAGCTAGACAGAAGCATTCTCAGAAACTTCTTTG

                              *    *                 
     465383 TGATGTTTGCATTCAAGTCACAGAGTTGAACATTCCCTTTC
        131 TGATGTTTGCATTCAAGTAACAGTGTTGAACATTCCCTTTC

                                                  **     *       **     *   *
     465424 ATAGAGCAGGTTTGAAACACTCTTTTTGTAGTATCTGGTTGTGGACATTTGGAGCGCTTTCAGGC
          1 ATAGAGCAGGTTTGAAACACTCTTTTTGTAGTATCTGGAAGTGGATATTTGGATAGCTTTGAGGA

            * ** *      *             *** *   *                          *   
     465489 CTATGGT-GAAAAAGGAAATATCTTCCCCTGAAAACTAGACAGAAGCATTCTCAGAAACTTATTT
         66 TTTCGTTGGAAACAGG-AATATCTTCATATAAAAGCTAGACAGAAGCATTCTCAGAAACTTCTTT

                   **  **    *             **  * *   *
     465553 GTGATGTGCGCCCTCAACTAACAGTGTTGAAGCTTTCTTTTG
        130 GTGATGTTTGCATTCAAGTAACAGTGTTGAACATTCCCTTTC

                     *                    *      *   *                       
     465595 ATAGAGCAGTTTTGAAACACTCTTTTTGTAATATCTGCAAGAGGATATTTGGATAGCTTTGAGGA
          1 ATAGAGCAGGTTTGAAACACTCTTTTTGTAGTATCTGGAAGTGGATATTTGGATAGCTTTGAGGA

                         *   * *            **                      *    *   
     465660 TTTCGTTGGAAACGGGATTGTCTTCATATAAACTCTAGACAGAAGCATTCTCAGAAGCTTCATTG
         66 TTTCGTTGGAAACAGGAATATCTTCATATAAAAGCTAGACAGAAGCATTCTCAGAAACTTCTTTG

            *       **   *    *             *        
     465725 GGATGTTTCAATTGAAGTCACAGTGTTGAACAGTCCCTTTC
        131 TGATGTTTGCATTCAAGTAACAGTGTTGAACATTCCCTTTC

                                                         *       *  * * *    
     465766 ATAGAGCAGGTTTGAAACACTCTTTTTGTAGTATCTGGAAGTGGACATTTGGAGAGATCTCAGGA
          1 ATAGAGCAGGTTTGAAACACTCTTTTTGTAGTATCTGGAAGTGGATATTTGGATAGCTTTGAGGA

            ***  *    * *             **          * *       * *          *   
     465831 AAACGGT-GATAAAGGAAATATCTTCCAATAAAAGCTACATAGAAGCAATGTCAGAAACTTTTTT
         66 TTTCGTTGGAAACAGG-AATATCTTCATATAAAAGCTAGACAGAAGCATTCTCAGAAACTTCTTT

            *      * ** *   **      *       *  *     *
     465895 ATGATGTATCTACTCAGCTAACAGAGTTGAACCTTTCCTTTG
        130 GTGATGTTTGCATTCAAGTAACAGTGTTGAACATTCCCTTTC

             *       *                   * *     *     *       **   *        
     465937 AGAGAGCAGTTTTGAAACACTCTTTTTGTGGAATCTGCAAGTGTATATTTGTCTAGATTTGAGGA
          1 ATAGAGCAGGTTTGAAACACTCTTTTTGTAGTATCTGGAAGTGGATATTTGGATAGCTTTGAGGA

                         *   *    *                   *       *   *          
     466002 TTTCGTTGGAAACGGGATTA-CAT-ATA-AAAAGC-AGACAGCAGCATTCCCAGTAACTTCTTTG
         66 TTTCGTTGGAAACAGGAATATCTTCATATAAAAGCTAGACAGAAGCATTCTCAGAAACTTCTTTG

               *              *    *                 
     466063 TGAGGTTTGCATTCAAGTCACAGAGTTGAACATTCCCTTTC
        131 TGATGTTTGCATTCAAGTAACAGTGTTGAACATTCCCTTTC

                                                   *     *       **     *   *
     466104 ATAGAGCAGGTTTGAAACACTCTTTTTGTAGTATCTGGATGTGGACATTTGGAGCGCTTTCAGGC
          1 ATAGAGCAGGTTTGAAACACTCTTTTTGTAGTATCTGGAAGTGGATATTTGGATAGCTTTGAGGA

            * ** *      *             **          * *       * *          *  *
     466169 CTATGGT-GAAAAAGGAAATATCTTCCAATAAAAGCTACATAGAAGCAATGTCAGAAACTTTTTC
         66 TTTCGTTGGAAACAGG-AATATCTTCATATAAAAGCTAGACAGAAGCATTCTCAGAAACTTCTTT

            *      * ** *   **      *       *  * *   *
     466233 ATGATGTATCTACTCAGCTAACAGAGTTGAACCTTTCTTTTG
        130 GTGATGTTTGCATTCAAGTAACAGTGTTGAACATTCCCTTTC

             *       *                   * *                   **            
     466275 AGAGAGCAGTTTTGAAACACTCTTTTTGTGGAATCTGGAAGTGGATATTTGTCTAGCTTTGAGGA
          1 ATAGAGCAGGTTTGAAACACTCTTTTTGTAGTATCTGGAAGTGGATATTTGGATAGCTTTGAGGA

                         *   *    *                   *       *   *          
     466340 TTTCGTTGGAAACGGGATTA-CAT-ATA-AAAAGC-AGACAGCAGCATTCCCAGTAACTTCTTTG
         66 TTTCGTTGGAAACAGGAATATCTTCATATAAAAGCTAGACAGAAGCATTCTCAGAAACTTCTTTG

                              *    *                 
     466401 TGATGTTTGCATTCAAGTCACAGAGTTGAACATTCCCTTTC
        131 TGATGTTTGCATTCAAGTAACAGTGTTGAACATTCCCTTTC

                                                   *     *       **     *   *
     466442 ATAGAGCAGGTTTGAAACACTCTTTTTGTAGTATCTGGATGTGGACATTTGGAGCGCTTTCAGGC
          1 ATAGAGCAGGTTTGAAACACTCTTTTTGTAGTATCTGGAAGTGGATATTTGGATAGCTTTGAGGA

            * ** *      *             *** *   *           *         **   *   
     466507 CTATGGT-GAAAAAGGAAATATCTTCCCCTGAAAACTAGACAGAAGAATTCTCAGAGTCTTATTT
         66 TTTCGTTGGAAACAGG-AATATCTTCATATAAAAGCTAGACAGAAGCATTCTCAGAAACTTCTTT

                   **  **    *             **  * *   *
     466571 GTGATGTGCGCCCTCAACTAACAGTGTTGAAGCTTTCTTTTG
        130 GTGATGTTTGCATTCAAGTAACAGTGTTGAACATTCCCTTTC

                     *   *                **     *   *                       
     466613 ATAGAGCAGTTTTTAAACACTCTTTTTGTAAAATCTGCAAGAGGATATTTGGATAGCTTTGAGGA
          1 ATAGAGCAGGTTTGAAACACTCTTTTTGTAGTATCTGGAAGTGGATATTTGGATAGCTTTGAGGA

                         *   * *            **                      * *  *   
     466678 TTTCGTTGGAAACGGGATTGTCTTCATATAAACTCTAGACAGAAGCATTCTCAGAAGCGTCATTG
         66 TTTCGTTGGAAACAGGAATATCTTCATATAAAAGCTAGACAGAAGCATTCTCAGAAACTTCTTTG

            *       **   *    *             *        
     466743 GGATGTTTCAATTGAAGTCACAGTGTTGAACAGTCCCTTTC
        131 TGATGTTTGCATTCAAGTAACAGTGTTGAACATTCCCTTTC

                                                   *     *       **     *    
     466784 ATAGAGCAGGTTTGAAACACTCTTTTTGTAGTATCTGGATGTGGACATTTGGAGCGCTTTCAGGC
          1 ATAGAGCAGGTTTGAAACACTCTTTTTGTAGTATCTGGAAGTGGATATTTGGATAGCTTTGAGG-

            * * *    *                 *** *   *                          *  
     466849 CTATGGTT-TAAA-AGGAAATATCTTCCCCTGAAAACTAGACAGAAGCATTCTCAGAAACTTATT
         65 ATTTCGTTGGAAACAGG-AATATCTTCATATAAAAGCTAGACAGAAGCATTCTCAGAAACTTCTT

                    **  **    *                    *   *
     466912 TGTGATGTGCGCCCTCAACTAACAGTGTTGAAGCATT-CTTTTG
        129 TGTGATGTTTGCATTCAAGTAACAGTGTTGAA-CATTCCCTTTC

                     *                   * *     *             **            
     466955 ATAGAGCAGTTTTGAAACACTCTTTTTGTGGAATCTGCAAGTGGATATTTGTCTAGCTTTGAGGA
          1 ATAGAGCAGGTTTGAAACACTCTTTTTGTAGTATCTGGAAGTGGATATTTGGATAGCTTTGAGGA

                         *   *    *                   * *     *   *          
     467020 TTTCGTTGGAAACGGGATTA-CAT-ATA-AAAAGC-AGACAGCAACATTCCCAGTAACTTCTTTG
         66 TTTCGTTGGAAACAGGAATATCTTCATATAAAAGCTAGACAGAAGCATTCTCAGAAACTTCTTTG

                              *    *                 
     467081 TGATGTTTGCATTCAAGTCACAGAGTTGAACATTCCCTTTC
        131 TGATGTTTGCATTCAAGTAACAGTGTTGAACATTCCCTTTC

                                        *         **     *       **     *   *
     467122 ATAGAGCAGGTTTGAAACACTCTTTTTGAAGTATCTGGTTGTGGACATTTGGAGCGCTTTCAGGC
          1 ATAGAGCAGGTTTGAAACACTCTTTTTGTAGTATCTGGAAGTGGATATTTGGATAGCTTTGAGGA

            * ** *      *             *** *   *                          *   
     467187 CTATGGT-GAAAAAGGAAATATCTTCCCCTGAAAACTAGACAGAAGCATTCTCAGAAACTTATTT
         66 TTTCGTTGGAAACAGG-AATATCTTCATATAAAAGCTAGACAGAAGCATTCTCAGAAACTTCTTT

                   **  **    *              *  * *   *
     467251 GTGATGTGCGCCCTCAACTAACAGTGTTGAACCTTTCTTTTG
        130 GTGATGTTTGCATTCAAGTAACAGTGTTGAACATTCCCTTTC

                     *                    *      *   *     *  *     *        
     467293 ATAGAGCAGTTTTGAAACACTCTTTTTGTAATATCTGCAAGAGGATAATTTGATAGATTTGAGGA
          1 ATAGAGCAGGTTTGAAACACTCTTTTTGTAGTATCTGGAAGTGGATATTTGGATAGCTTTGAGGA

                         *   * *            **                      *    *   
     467358 TTTCGTTGGAAACGGGATTGTCTTCATATAAACTCTAGACAGAAGCATTCTCAGAAGCTTCATTG
         66 TTTCGTTGGAAACAGGAATATCTTCATATAAAAGCTAGACAGAAGCATTCTCAGAAACTTCTTTG

            *       **   *    *             *        
     467423 GGATGTTTCAATTGAAGTCACAGTGTTGAACAGTCCCTTTC
        131 TGATGTTTGCATTCAAGTAACAGTGTTGAACATTCCCTTTC

                                                   **    *       **   * *    
     467464 ATAGAGCAGGTTTGAAACACTCTTTTTGTAGTATCTGGAGATGGACATTTGGAGCGCTCTCAGGA
          1 ATAGAGCAGGTTTGAAACACTCTTTTTGTAGTATCTGGAAGTGGATATTTGGATAGCTTTGAGGA

            * *  *    * *             **            *       * *          *  *
     467529 CTACGGT-GATAAAGGAAATATCTTCCAATAAAAGCTAGATAGAAGCAATGTCAGAAACTTTTTC
         66 TTTCGTTGGAAACAGG-AATATCTTCATATAAAAGCTAGACAGAAGCATTCTCAGAAACTTCTTT

            *      * ** *   **      *       *  * *   *
     467593 ATGATGTATCTACTCAGCTAACAGAGTTGAACCTTTCTTTTG
        130 GTGATGTTTGCATTCAAGTAACAGTGTTGAACATTCCCTTTC

             *       *                   * *     *             **            
     467635 AGAGAGCAGTTTTGAAACACTCTTTTTGTGGAATCTGCAAGTGGATATTTGTCTAGCTTTGAGGA
          1 ATAGAGCAGGTTTGAAACACTCTTTTTGTAGTATCTGGAAGTGGATATTTGGATAGCTTTGAGGA

                         *   *    *                   *       *     *        
     467700 TTTCGTTGGAAACGGGATTA-CAT-ATA-AAAAGC-AGACAGCAGCATTCCCAGAATCTTCTTTG
         66 TTTCGTTGGAAACAGGAATATCTTCATATAAAAGCTAGACAGAAGCATTCTCAGAAACTTCTTTG

                              *    *                 
     467761 TGATGTTTGCATTCAAGTCACAGAGTTGAACATTCCCTTTC
        131 TGATGTTTGCATTCAAGTAACAGTGTTGAACATTCCCTTTC

                                           *       *     *       **     *   *
     467802 ATAGAGCAGGTTTGAAACACTCTTTTTGTAGCATCTGGATGTGGACATTTGGAGCGCTTTCAGGC
          1 ATAGAGCAGGTTTGAAACACTCTTTTTGTAGTATCTGGAAGTGGATATTTGGATAGCTTTGAGGA

            * ** *      *             *** *   *                      *   *   
     467867 CTATGGT-GAAAAAGGAAATATCTTCCCCTGAAAACTAGACAGAAGCATTCTCAGAATCTTATTT
         66 TTTCGTTGGAAACAGG-AATATCTTCATATAAAAGCTAGACAGAAGCATTCTCAGAAACTTCTTT

                   **  **    *             **  * *   *
     467931 GTGATGTGCGCCCTCAACTAACAGTGTTGAAGCTTTCTTTTG
        130 GTGATGTTTGCATTCAAGTAACAGTGTTGAACATTCCCTTTC

                     *                    **     *   *                       
     467973 ATAGAGCAGTTTTGAAACACTCTTTTTGTAAAATCTGCAAGAGGATATTTGGATAGCTTTGAGGA
          1 ATAGAGCAGGTTTGAAACACTCTTTTTGTAGTATCTGGAAGTGGATATTTGGATAGCTTTGAGGA

                         **  * *            **                      *    *   
     468038 TTTCGTTGGAAACGTGATTGTCTTCATATAAACTCTAGACAGAAGCATTCTCAGAAGCTTCATTG
         66 TTTCGTTGGAAACAGGAATATCTTCATATAAAAGCTAGACAGAAGCATTCTCAGAAACTTCTTTG

            *       **   *    *             *        
     468103 GGATGTTTCAATTGAAGTCACAGTGTTGAACAGTCCCTTTC
        131 TGATGTTTGCATTCAAGTAACAGTGTTGAACATTCCCTTTC

                                                         *       **   * *    
     468144 ATAGAGCAGGTTTGAAACACTCTTTTTGTAGTATCTGGAAGTGGACATTTGGAGCGCTCTCAGGA
          1 ATAGAGCAGGTTTGAAACACTCTTTTTGTAGTATCTGGAAGTGGATATTTGGATAGCTTTGAGGA

            * *  *      *             **            *       * *          *  *
     468209 CTGCGGT-GAAAAAGGAAATATCTTCCAATAAAAGCTAGATAGAAGCAATGTCAGAAACTTTTTC
         66 TTTCGTTGGAAACAGG-AATATCTTCATATAAAAGCTAGACAGAAGCATTCTCAGAAACTTCTTT

            *      * ** *   **      *       *    *   *
     468273 ATGATGTATCTACTCAGCTAACAGAGTTGAACCTT-CATTTG
        130 GTGATGTTTGCATTCAAGTAACAGTGTTGAACATTCCCTTTC

             *       *            *      * *     *             **  **        
     468314 AGAGAGCAGTTTTGAAACACTCGTTTTGTGGAATCTGCAAGTGGATATTTGTCTACATTTGAGGA
          1 ATAGAGCAGGTTTGAAACACTCTTTTTGTAGTATCTGGAAGTGGATATTTGGATAGCTTTGAGGA

                         *   *    *                   *       *     *        
     468379 TTTCGTTGGAAACGGGATTA-CAT-ATA-AAAAGC-AGACAGCAGCATTCCCAGAATCTTCTTTG
         66 TTTCGTTGGAAACAGGAATATCTTCATATAAAAGCTAGACAGAAGCATTCTCAGAAACTTCTTTG

                              *    *                 
     468440 TGATGTTTGCATTCAAGTCACAGAGTTGAACATTCCCTTTC
        131 TGATGTTTGCATTCAAGTAACAGTGTTGAACATTCCCTTTC

                                                   *     *    *  *  * * *    
     468481 ATAGAGCAGGTTTGAAACACTCTTTTTGTAGTATCTGGATGTGGACATTTTGAGAGATCTCAGGA
          1 ATAGAGCAGGTTTGAAACACTCTTTTTGTAGTATCTGGAAGTGGATATTTGGATAGCTTTGAGGA

            * *  *    * *             *** *   *                          *   
     468546 ATACGGT-GATAAAGGAAATATCTTCCCCTGAAAACTAGACAGAAGCATTCTCAGAAACTTATTT
         66 TTTCGTTGGAAACAGG-AATATCTTCATATAAAAGCTAGACAGAAGCATTCTCAGAAACTTCTTT

                   **  **    *             **  * *   *
     468610 GTGATGTGCGCCCTCAACTAACAGTGTTGAAGCTTTCTTTTG
        130 GTGATGTTTGCATTCAAGTAACAGTGTTGAACATTCCCTTTC

                     *                    *      *   *                       
     468652 ATAGAGCAGTTTTGAAACACTCTTTTTGTAATATCTGCAAGAGGATATTTGGATAGCTTTGAGGA
          1 ATAGAGCAGGTTTGAAACACTCTTTTTGTAGTATCTGGAAGTGGATATTTGGATAGCTTTGAGGA

                         *  ** *            **                      *    *   
     468717 TTTCGTTGGAAACGGGTTTGTCTTCATATAAACTCTAGACAGAAGCATTCTCAGAAGCTTCATTG
         66 TTTCGTTGGAAACAGGAATATCTTCATATAAAAGCTAGACAGAAGCATTCTCAGAAACTTCTTTG

            *       **   *    *             * *      
     468782 GGATGTTTCAATTGAAGTCACAGTGTTGAACAGTTCCTTTC
        131 TGATGTTTGCATTCAAGTAACAGTGTTGAACATTCCCTTTC

                 *                                       *       **   * *    
     468823 ATAGAACAGGTTTGAAACACTCTTTTTGTAGTATCTGGAAGTGGACATTTGGAGCGCTCTCAGGA
          1 ATAGAGCAGGTTTGAAACACTCTTTTTGTAGTATCTGGAAGTGGATATTTGGATAGCTTTGAGGA

            * ** *      *             **          * *       * *          *  *
     468888 CTATGGT-GAAAAAGGAAATATCTTCCAATAAAAGCTACATAGAAGCAATGTCAGAAACTTTTTC
         66 TTTCGTTGGAAACAGG-AATATCTTCATATAAAAGCTAGACAGAAGCATTCTCAGAAACTTCTTT

            *      * ** *   **      *       *  *     *
     468952 ATGATGTATCTACTCAGCTAACAGAGTTGAACCTTTCCTTTG
        130 GTGATGTTTGCATTCAAGTAACAGTGTTGAACATTCCCTTTC

             *       *                   * *     *             **            
     468994 AGAGAGCAGTTTTGAAACACTCTTTTTGTGGAATCTGCAAGTGGATATTTGTCTAGCTTTGAGGA
          1 ATAGAGCAGGTTTGAAACACTCTTTTTGTAGTATCTGGAAGTGGATATTTGGATAGCTTTGAGGA

                         *   *    *                   *       *   *          
     469059 TTTCGTTGGAAACGGGATTA-CAT-ATA-AAAAGC-AGACAGCAGCATTCCCAGTAACTTCTTTG
         66 TTTCGTTGGAAACAGGAATATCTTCATATAAAAGCTAGACAGAAGCATTCTCAGAAACTTCTTTG

                              *    *      *          
     469120 TGATGTTTGCATTCAAGTCACAGAGTTGAATATTCCCTTTC
        131 TGATGTTTGCATTCAAGTAACAGTGTTGAACATTCCCTTTC

                                                   *     *       **     *   *
     469161 ATAGAGCAGGTTTGAAACACTCTTTTTGTAGTATCTGGATGTGGACATTTGGAGCGCTTTCAGGC
          1 ATAGAGCAGGTTTGAAACACTCTTTTTGTAGTATCTGGAAGTGGATATTTGGATAGCTTTGAGGA

            * ** *      *             *** *   *         *                *   
     469226 CTATGGT-GAAAAAGGAAATATCTTCCCCTGAAAACTAGACAGAGGCATTCTCAGAAACTTATTT
         66 TTTCGTTGGAAACAGG-AATATCTTCATATAAAAGCTAGACAGAAGCATTCTCAGAAACTTCTTT

                   **  **    *                    *   *
     469290 GTGATGTGCGCCCTCAACTAACAGTGTTGAAGCATT-CTTTTG
        130 GTGATGTTTGCATTCAAGTAACAGTGTTGAA-CATTCCCTTTC

                     *                    *      *   *                       
     469332 ATAGAGCAGTTTTGAAACACTCTTTTTGTAATATCTGCAAGAGGATATTTGGATAGCTTTGAGGA
          1 ATAGAGCAGGTTTGAAACACTCTTTTTGTAGTATCTGGAAGTGGATATTTGGATAGCTTTGAGGA

                         *   *    *                   *       *              
     469397 TTTCGTTGGAAACTGGATTA-CAT-ATA-AAAAGC-AGACAGCAGCATTCCCAGAAACTTCTTTG
         66 TTTCGTTGGAAACAGGAATATCTTCATATAAAAGCTAGACAGAAGCATTCTCAGAAACTTCTTTG

                              *    *                 
     469458 TGATGTTTGCATTCAAGTCACAGAGTTGAACATTCCCTTTC
        131 TGATGTTTGCATTCAAGTAACAGTGTTGAACATTCCCTTTC

             *                         *           *     *       **     *  **
     469499 AGAGAGCAGGTTTGAAACACTCTTTTTCTAGTATCTGGATGTGGACATTTGGAGCGCTTTCAGCC
          1 ATAGAGCAGGTTTGAAACACTCTTTTTGTAGTATCTGGAAGTGGATATTTGGATAGCTTTGAGGA

            * ** *      *             *** *   *                      *   *   
     469564 CTATGGT-GAAAAAGGAAATATCTTCCCCTGAAAACTAGACAGAAGCATTCTCAGAATCTTATTT
         66 TTTCGTTGGAAACAGG-AATATCTTCATATAAAAGCTAGACAGAAGCATTCTCAGAAACTTCTTT

                   ** ***    *             **  * *   *
     469628 GTGATGTGCGTCCTCAACTAACAGTGTTGAAGCTTTCTTTTG
        130 GTGATGTTTGCATTCAAGTAACAGTGTTGAACATTCCCTTTC

                     *                    **     *   * *                     
     469670 ATAGAGCAGTTTTGAAACACTCTTTTTGTAAAATCTGCAAGAGAATATTTGGATAGCTTTGAGGA
          1 ATAGAGCAGGTTTGAAACACTCTTTTTGTAGTATCTGGAAGTGGATATTTGGATAGCTTTGAGGA

                         *   * *            **                      *    *   
     469735 TTTCGTTGGAAACGGGATTGTCTTCATATAAACTCTAGACAGAAGCATTCTCAGAAGCTTCATTG
         66 TTTCGTTGGAAACAGGAATATCTTCATATAAAAGCTAGACAGAAGCATTCTCAGAAACTTCTTTG

            *       **   *    *             *        
     469800 GGATGTTTCAATTGAAGTCACAGTGTTGAACAGTCCCTTTC
        131 TGATGTTTGCATTCAAGTAACAGTGTTGAACATTCCCTTTC

                         *                               *       **     *    
     469841 ATAGAGCAGGTTTCAAACACTCTTTTTGTAGTATCTGGAAGTGGACATTTGGAGCGCTTTCAGGC
          1 ATAGAGCAGGTTTGAAACACTCTTTTTGTAGTATCTGGAAGTGGATATTTGGATAGCTTTGAGG-

            * * *    *                 *** *   *                          * *
     469906 CTATGGTT-TAAA-AGGAAATATCTTCCCCTGAAAACTAGACAGAAGCATTCTCAGAAACTTATA
         65 ATTTCGTTGGAAACAGG-AATATCTTCATATAAAAGCTAGACAGAAGCATTCTCAGAAACTTCTT

                    **  **    *                    *   *
     469969 TGTGATGTGCGCCCTCAACTAACAGTGTTGAAGCATT-CTTTTG
        129 TGTGATGTTTGCATTCAAGTAACAGTGTTGAA-CATTCCCTTTC

                     *                   * *     *             **            
     470012 ATAGAGCAGTTTTGAAACACTCTTTTTGTGGAATCTGCAAGTGGATATTTGTCTAGCTTTGAGGA
          1 ATAGAGCAGGTTTGAAACACTCTTTTTGTAGTATCTGGAAGTGGATATTTGGATAGCTTTGAGGA

                         *   *    *     *             *       *              
     470077 TTTCGTTGGAAACGGGATTA-CAT-AT-GAAAAGC-AGACAGCAGCATTCCCAGAAACTTCTTTG
         66 TTTCGTTGGAAACAGGAATATCTTCATATAAAAGCTAGACAGAAGCATTCTCAGAAACTTCTTTG

                            * *    *                 
     470138 TGATGTTTGCATTCAACTCACAGAGTTGAACATTCCCTTTC
        131 TGATGTTTGCATTCAAGTAACAGTGTTGAACATTCCCTTTC

                                                   *     *       **     *   *
     470179 ATAGAGCAGGTTTGAAACACTCTTTTTGTAGTATCTGGATGTGGACATTTGGAGCGCTTTCAGGC
          1 ATAGAGCAGGTTTGAAACACTCTTTTTGTAGTATCTGGAAGTGGATATTTGGATAGCTTTGAGGA

            * ** *      *             *** *   *                          *   
     470244 CTATGGT-GAAAAAGGAAATATCTTCCCCTGAAAACTAGACAGAAGCATTCTCAGAAACTTATTT
         66 TTTCGTTGGAAACAGG-AATATCTTCATATAAAAGCTAGACAGAAGCATTCTCAGAAACTTCTTT

                   **  **    *             **  * *   *
     470308 GTGATGTGCGCCCTCAACTAACAGTGTTGAAGCTTTCTTTTG
        130 GTGATGTTTGCATTCAAGTAACAGTGTTGAACATTCCCTTTC

                     *                    *      *   *                       
     470350 ATAGAGCAGTTTTGAAACACTCTTTTTGTAATATCTGCAAGAGGATATTTGGATAGCTTTGAGGA
          1 ATAGAGCAGGTTTGAAACACTCTTTTTGTAGTATCTGGAAGTGGATATTTGGATAGCTTTGAGGA

                         *   *   *                    *       *     *   *    
     470415 TTTCGTTGGAAACGGGATTA-ATT-ATA-AAAAGC-AGACAGCAGCATTCCCAGAATCTTGTTTG
         66 TTTCGTTGGAAACAGGAATATCTTCATATAAAAGCTAGACAGAAGCATTCTCAGAAACTTCTTTG

                              *    *                 
     470476 TGATGTTTGCATTCAAGTGACAGAGTTGAACATTCCCTTTC
        131 TGATGTTTGCATTCAAGTAACAGTGTTGAACATTCCCTTTC

             *    *                    *           *     *       **     *   *
     470517 AGAGAGGAGGTTTGAAACACTCTTTTTATAGTATCTGGATGTGGACATTTGGACCGCTTTCAGGC
          1 ATAGAGCAGGTTTGAAACACTCTTTTTGTAGTATCTGGAAGTGGATATTTGGATAGCTTTGAGGA

            * ** *      *             *** *   *                      *   *   
     470582 CTATGGT-GAAAAAGGAAATATCTTCTCCTGAAAACTAGACAGAAGCATTCTCAGAATCTTATTT
         66 TTTCGTTGGAAACAGG-AATATCTTCATATAAAAGCTAGACAGAAGCATTCTCAGAAACTTCTTT

                   **  **    *             **  * *   *
     470646 GTGATGTGCGCCCTCAACTAACAGTGTTGAAGCTTTCTTTTG
        130 GTGATGTTTGCATTCAAGTAACAGTGTTGAACATTCCCTTTC

                     *                *   **     *   *        *              
     470688 ATAGAGCAGTTTTGAAACACTCTTTTCGTAAAATCTGCAAGAGGATATTTTGATAGCTTTGAGGA
          1 ATAGAGCAGGTTTGAAACACTCTTTTTGTAGTATCTGGAAGTGGATATTTGGATAGCTTTGAGGA

                         *   * *            **                      *    *   
     470753 TTTCGTTGGAAACGGGATTGTCTTCATATAAACTCTAGACAGAAGCATTCTCAGAAGCTTCATTG
         66 TTTCGTTGGAAACAGGAATATCTTCATATAAAAGCTAGACAGAAGCATTCTCAGAAACTTCTTTG

            *       **   *    *             *        
     470818 GGATGTTTCAATTGAAGTCACAGTGTTGAACAGTCCCTTTC
        131 TGATGTTTGCATTCAAGTAACAGTGTTGAACATTCCCTTTC

                                                         *       *  * * *    
     470859 ATAGAGCAGGTTTGAAACACTCTTTTTGTAGTATCTGGAAGTGGACATTTGGAGAGATCTCAGGA
          1 ATAGAGCAGGTTTGAAACACTCTTTTTGTAGTATCTGGAAGTGGATATTTGGATAGCTTTGAGGA

            * *  *    * *             **            *       * *          *  *
     470924 ATACGGT-GATAAAGGAAATATCTTCCAATAAAAGCTAGATAGAAGCAATGTCAGAAACTTTTTC
         66 TTTCGTTGGAAACAGG-AATATCTTCATATAAAAGCTAGACAGAAGCATTCTCAGAAACTTCTTT

            *      * ** *   **      *       *  * *   *
     470988 ATGATGTATCTACTCAGCTAACAGAGTTGAACCTTTCTTTTG
        130 GTGATGTTTGCATTCAAGTAACAGTGTTGAACATTCCCTTTC

             *       *                   * *                   **            
     471030 AGAGAGCAGTTTTGAAACACTCTTTTTGTGGAATCTGGAAGTGGATATTTGTCTAGCTTTGAGGA
          1 ATAGAGCAGGTTTGAAACACTCTTTTTGTAGTATCTGGAAGTGGATATTTGGATAGCTTTGAGGA

                         *   *    *                   *       *   *          
     471095 TTTCGTTGGAAACGGGATTA-CAT-ATA-AAAAGC-AGACAGCAGCATTCCCAGTAACTTCTTTG
         66 TTTCGTTGGAAACAGGAATATCTTCATATAAAAGCTAGACAGAAGCATTCTCAGAAACTTCTTTG

               *              *    *                 
     471156 TGACGTTTGCATTCAAGTCACAGAGTTGAACATTCCCTTTC
        131 TGATGTTTGCATTCAAGTAACAGTGTTGAACATTCCCTTTC

                                                   *     *    *  **     *   *
     471197 ATAGAGCAGGTTTGAAACACTCTTTTTGTAGTATCTGGATGTGGACATTTTGAGCGCTTTCAGGC
          1 ATAGAGCAGGTTTGAAACACTCTTTTTGTAGTATCTGGAAGTGGATATTTGGATAGCTTTGAGGA

            * ** *      *             *** *   *           *          *   *   
     471262 CTATGGT-GAAAAAGGAAATATCTTCCCCTGAAAACTAGACAGAAGAATTCTCAGAATCTTATTT
         66 TTTCGTTGGAAACAGG-AATATCTTCATATAAAAGCTAGACAGAAGCATTCTCAGAAACTTCTTT

                   **  **    *             **  * *   *
     471326 GTGATGTGCGCCATCAACTAACAGTGTTGAAGCTTTCTTTTG
        130 GTGATGTTTGCATTCAAGTAACAGTGTTGAACATTCCCTTTC

                     *                    **     *   *                       
     471368 ATAGAGCAGTTTTGAAACACTCTTTTTGTAAAATCTGCAAGAGGATATTTGGATAGCTTTGAGGA
          1 ATAGAGCAGGTTTGAAACACTCTTTTTGTAGTATCTGGAAGTGGATATTTGGATAGCTTTGAGGA

                      *  *   * *            **                      * *  *   
     471433 TTTCGTTGGAGACGGGATTGTCTTCATATAAACTCTAGACAGAAGCATTCTCAGAAGCGTCATTG
         66 TTTCGTTGGAAACAGGAATATCTTCATATAAAAGCTAGACAGAAGCATTCTCAGAAACTTCTTTG

            *       **   *    *                      
     471498 GGATGTTTCAATTGAAGTCACAGTGTTGAACATTCCCTTTC
        131 TGATGTTTGCATTCAAGTAACAGTGTTGAACATTCCCTTTC

                                                   *     *       **     *    
     471539 ATAGAGCAGGTTTGAAACACTCTTTTTGTAGTATCTGGATGTGGACATTTGGAGCGCTTTCAGGC
          1 ATAGAGCAGGTTTGAAACACTCTTTTTGTAGTATCTGGAAGTGGATATTTGGATAGCTTTGAGG-

            * * *    *                 *** *   *                          *  
     471604 CTATGGTT-TAAA-AGGAAATATCTTCCCCTGAAAACTAGACAGAAGCATTCTCAGAAACTTATT
         65 ATTTCGTTGGAAACAGG-AATATCTTCATATAAAAGCTAGACAGAAGCATTCTCAGAAACTTCTT

                    **  **    *                    *   *
     471667 TGTGATGTGCGCCCTCAACTAACAGTGTTGAAGCATT-CTTTTG
        129 TGTGATGTTTGCATTCAAGTAACAGTGTTGAA-CATTCCCTTTC

               *     *                   * *     *             **            
     471710 ATACAGCAGTTTTGAAACACTCTTTTTGTGGAATCTGCAAGTGGATATTTGTCTAGCTTTGAGGA
          1 ATAGAGCAGGTTTGAAACACTCTTTTTGTAGTATCTGGAAGTGGATATTTGGATAGCTTTGAGGA

                         *   *    *                   *       *   *          
     471775 TTTCGTTGGAAACGGGATTA-CAT-ATA-AAAAGC-AGACAGCAGCATTCCCAGTAACTTCTTTG
         66 TTTCGTTGGAAACAGGAATATCTTCATATAAAAGCTAGACAGAAGCATTCTCAGAAACTTCTTTG

                              * *  *                 
     471836 TGATGTTTGCATTCAAGTCAGAGAGTTGAACATTCCCTTTC
        131 TGATGTTTGCATTCAAGTAACAGTGTTGAACATTCCCTTTC

                                        *         **     *       **     *   *
     471877 ATAGAGCAGGTTTGAAACACTCTTTTTGAAGTATCTGGTTGTGGACATTTGGAGCGCTTTCAGGC
          1 ATAGAGCAGGTTTGAAACACTCTTTTTGTAGTATCTGGAAGTGGATATTTGGATAGCTTTGAGGA

            * ** *      *             *** *   *                          *   
     471942 CTATGGT-GAAAAAGGAAATATCTTCCCCTGAAAACTAGACAGAAGCATTCTCAGAAACTTATTT
         66 TTTCGTTGGAAACAGG-AATATCTTCATATAAAAGCTAGACAGAAGCATTCTCAGAAACTTCTTT

                   **  **    *              *  * *   *
     472006 GTGATGTGCGCCCTCAACTAACAGTGTTGAACCTTTCTTTTG
        130 GTGATGTTTGCATTCAAGTAACAGTGTTGAACATTCCCTTTC

                     *                    *      *   *                       
     472048 ATAGAGCAGTTTTGAAACACTCTTTTTGTAATATCTGCAAGAGGATATTTGGATAGCTTTGAGGA
          1 ATAGAGCAGGTTTGAAACACTCTTTTTGTAGTATCTGGAAGTGGATATTTGGATAGCTTTGAGGA

                         *   * *            **                      *    *   
     472113 TTTCGTTGGAAACGGGATTGTCTTCATATAAACTCTAGACAGAAGCATTCTCAGAAGCTTCATTG
         66 TTTCGTTGGAAACAGGAATATCTTCATATAAAAGCTAGACAGAAGCATTCTCAGAAACTTCTTTG

            *       **   *    *             *        
     472178 GGATGTTTCAATTGAAGTCACAGTGTTGAACAGTCCCTTTC
        131 TGATGTTTGCATTCAAGTAACAGTGTTGAACATTCCCTTTC

                                                         *       **   * *    
     472219 ATAGAGCAGGTTTGAAACACTCTTTTTGTAGTATCTGGAAGTGGACATTTGGAGCGCTCTCAGGA
          1 ATAGAGCAGGTTTGAAACACTCTTTTTGTAGTATCTGGAAGTGGATATTTGGATAGCTTTGAGGA

            * *  *      *             **            *       * *          *  *
     472284 CTGCGGT-GAAAAAGGAAATATCTTCCAATAAAAGCTAGATAGAAGCAATGTCAGAAACTTTTTC
         66 TTTCGTTGGAAACAGG-AATATCTTCATATAAAAGCTAGACAGAAGCATTCTCAGAAACTTCTTT

            *      * ** *   **      *       *    *   *
     472348 ATGATGTATCTACTCAGCTAACAGAGTTGAACCTT-CATTTG
        130 GTGATGTTTGCATTCAAGTAACAGTGTTGAACATTCCCTTTC

             *       *            *      * *     *             **            
     472389 AGAGAGCAGTTTTGAAACACTCGTTTTGTGGAATCTGCAAGTGGATATTTGTCTAGCTTTGAGGA
          1 ATAGAGCAGGTTTGAAACACTCTTTTTGTAGTATCTGGAAGTGGATATTTGGATAGCTTTGAGGA

                         *   *    *                   *       *   *          
     472454 TTTCGTTGGAAACGGGATTA-CAT-ATA-AAAAGC-AGACAGCAGCATTCCCAGTAACTTCTTTG
         66 TTTCGTTGGAAACAGGAATATCTTCATATAAAAGCTAGACAGAAGCATTCTCAGAAACTTCTTTG

                              *    *                 
     472515 TGATGTTTGCATTCAAGTCACAGAGTTGAACATTCCCTTTC
        131 TGATGTTTGCATTCAAGTAACAGTGTTGAACATTCCCTTTC

                                         *         *   * *     * **     *   *
     472556 ATAGAGCAGGTTTGAAACACTCTTTTTGTTGTATCTGGATGTGTACATTTGCAGCGCTTTCAGGC
          1 ATAGAGCAGGTTTGAAACACTCTTTTTGTAGTATCTGGAAGTGGATATTTGGATAGCTTTGAGGA

            * ** *      *             *** *   *                          *   
     472621 CTAAGGT-GAAAAAGGAAATATCTTCCCCTGAAAACTAGACAGAAGCATTCTCAGAAACTTATTT
         66 TTTCGTTGGAAACAGG-AATATCTTCATATAAAAGCTAGACAGAAGCATTCTCAGAAACTTCTTT

                   **  **    *             **  * *   *
     472685 GTGATGTGCGCCCTCAACTAACAGTGTTGAAGCTTTCTTTTG
        130 GTGATGTTTGCATTCAAGTAACAGTGTTGAACATTCCCTTTC

                     *                    *      *   *                       
     472727 ATAGAGCAGTTTTGAAACACTCTTTTTGTAATATCTGCAAGAGGATATTTGGATAGCTTTGAGGA
          1 ATAGAGCAGGTTTGAAACACTCTTTTTGTAGTATCTGGAAGTGGATATTTGGATAGCTTTGAGGA

                         *  ** *            **                      *    *   
     472792 TTTCGTTGGAAACGGGTTTGTCTTCATATAAACTCTAGACAGAAGCATTCTCAGAAGCTTCATTG
         66 TTTCGTTGGAAACAGGAATATCTTCATATAAAAGCTAGACAGAAGCATTCTCAGAAACTTCTTTG

            *       **   *    *             * *      
     472857 GGATGTTTCAATTGAAGTCACAGTGTTGAACAGTTCCTTTC
        131 TGATGTTTGCATTCAAGTAACAGTGTTGAACATTCCCTTTC

                 *                                       *       **   * *    
     472898 ATAGAACAGGTTTGAAACACTCTTTTTGTAGTATCTGGAAGTGGACATTTGGAGCGCTCTCAGGA
          1 ATAGAGCAGGTTTGAAACACTCTTTTTGTAGTATCTGGAAGTGGATATTTGGATAGCTTTGAGGA

            * ** *      *             **          * *       * *          *  *
     472963 CTATGGT-GAAAAAGGAAATATCTTCCAATAAAAGCTACATAGAAGCAATGTCAGAAACTTTTTC
         66 TTTCGTTGGAAACAGG-AATATCTTCATATAAAAGCTAGACAGAAGCATTCTCAGAAACTTCTTT

            *      * ** *   **      *       *  *     *
     473027 ATGATGTATCTACTCAGCTAACAGAGTTGAACCTTTCCTTTG
        130 GTGATGTTTGCATTCAAGTAACAGTGTTGAACATTCCCTTTC

             *       *                   * *     *             **            
     473069 AGAGAGCAGTTTTGAAACACTCTTTTTGTGGAATCTGCAAGTGGATATTTGTCTAGCTTTGAGGA
          1 ATAGAGCAGGTTTGAAACACTCTTTTTGTAGTATCTGGAAGTGGATATTTGGATAGCTTTGAGGA

                         *   *    *                   *    *  *   *          
     473134 TTTCGTTGGAAACGGGATTA-CAT-ATA-AAAAGC-AGACAGCAGCACTCCCAGTAACTTCTTTG
         66 TTTCGTTGGAAACAGGAATATCTTCATATAAAAGCTAGACAGAAGCATTCTCAGAAACTTCTTTG

                              *    *      *          
     473195 TGATGTTTGCATTCAAGTCACAGAGTTGAATATTCCCTTTC
        131 TGATGTTTGCATTCAAGTAACAGTGTTGAACATTCCCTTTC

                                                   *     *       **     *   *
     473236 ATAGAGCAGGTTTGAAACACTCTTTTTGTAGTATCTGGATGTGGACATTTGGAGCGCTTTCAGGC
          1 ATAGAGCAGGTTTGAAACACTCTTTTTGTAGTATCTGGAAGTGGATATTTGGATAGCTTTGAGGA

            * ** *      *             *** *   *                          *   
     473301 CTATGGT-GAAAAAGGAAATATCTTCCCCTGAAAACTAGACAGAAGCATTCTCAGAAACTTATTT
         66 TTTCGTTGGAAACAGG-AATATCTTCATATAAAAGCTAGACAGAAGCATTCTCAGAAACTTCTTT

                   **  **    *              *  * *   *
     473365 GTGATGTGCGCCCTCAACTAACAGTGTTGAACCTTTCTTTTG
        130 GTGATGTTTGCATTCAAGTAACAGTGTTGAACATTCCCTTTC

                     *                    *      *   *                       
     473407 ATAGAGCAGTTTTGAAACACTCTTTTTGTAATATCTGCAAGAGGATATTTGGATAGCTTTGAGGA
          1 ATAGAGCAGGTTTGAAACACTCTTTTTGTAGTATCTGGAAGTGGATATTTGGATAGCTTTGAGGA

                         *   *    *                   *       *   *          
     473472 TTTCGTTGGAAACGGGATTA-CAT-ATA-AAAAGC-AGACAGCAGCATTCCCAGTAACTTCTTTG
         66 TTTCGTTGGAAACAGGAATATCTTCATATAAAAGCTAGACAGAAGCATTCTCAGAAACTTCTTTG

               *              *    *                 
     473533 TGACGTTTGCATTCAAGTCACAGAGTTGAACATTCCCTTTC
        131 TGATGTTTGCATTCAAGTAACAGTGTTGAACATTCCCTTTC

                                                   *     *       **     *   *
     473574 ATAGAGCAGGTTTGAAACACTCTTTTTGTAGTATCTGGATGTGGACATTTGGAGCGCTTTCAGGC
          1 ATAGAGCAGGTTTGAAACACTCTTTTTGTAGTATCTGGAAGTGGATATTTGGATAGCTTTGAGGA

            * ** *      *           * *** *   *           *          *   *   
     473639 CTATGGT-GAAAAAGGAAATATCTACCCCTGAAAACTAGACAGAAGAATTCTCAGAATCTTATTT
         66 TTTCGTTGGAAACAGG-AATATCTTCATATAAAAGCTAGACAGAAGCATTCTCAGAAACTTCTTT

                   **  **    *             **  * *   *
     473703 GTGATGTGCGCCATCAACTAACAGTGTTGAAGCTTTCTTTTG
        130 GTGATGTTTGCATTCAAGTAACAGTGTTGAACATTCCCTTTC

                     *                    **     *   *                       
     473745 ATAGAGCAGTTTTGAAACACTCTTTTTGTAAAATCTGCAAGAGGATATTTGGATAGCTTTGAGGA
          1 ATAGAGCAGGTTTGAAACACTCTTTTTGTAGTATCTGGAAGTGGATATTTGGATAGCTTTGAGGA

                         *   * *            **                      * *  **  
     473810 TTTCGTTGGAAACGGGATTGTCTTCATATAAACTCTAGACAGAAGCATTCTCAGAAGCGTCACTG
         66 TTTCGTTGGAAACAGGAATATCTTCATATAAAAGCTAGACAGAAGCATTCTCAGAAACTTCTTTG

            *       **   *    *                      
     473875 GGATGTTTCAATTGAAGTCACAGTGTTGAACATTCCCTTTC
        131 TGATGTTTGCATTCAAGTAACAGTGTTGAACATTCCCTTTC

                                                   *     *       **     *    
     473916 ATAGAGCAGGTTTGAAACACTCTTTTTGTAGTATCTGGATGTGGACATTTGGAGCGCTTTCAGGC
          1 ATAGAGCAGGTTTGAAACACTCTTTTTGTAGTATCTGGAAGTGGATATTTGGATAGCTTTGAGG-

            * * *    *                 *** *   *                          *  
     473981 CTATGGTT-TAAA-AGGAAATATCTTCCCCTGAAAACTAGACAGAAGCATTCTCAGAAACTTATT
         65 ATTTCGTTGGAAACAGG-AATATCTTCATATAAAAGCTAGACAGAAGCATTCTCAGAAACTTCTT

                    **  **    *                    *   *
     474044 TGTGATGTGCGCCCTCAACTAACAGTGTTGAAGCATT-CTTTTG
        129 TGTGATGTTTGCATTCAAGTAACAGTGTTGAA-CATTCCCTTTC

                     *                   * *     *             **            
     474087 ATAGAGCAGTTTTGAAACACTCTTTTTGTGGAATCTGCAAGTGGATATTTGTCTAGCTTTGAGGA
          1 ATAGAGCAGGTTTGAAACACTCTTTTTGTAGTATCTGGAAGTGGATATTTGGATAGCTTTGAGGA

                         *   *    *                   *       *   *          
     474152 TTTCGTTGGAAACGGGATTA-CAT-ATA-AAAAGC-AGACAGCAGCATTCCCAGTAACTTCTTTG
         66 TTTCGTTGGAAACAGGAATATCTTCATATAAAAGCTAGACAGAAGCATTCTCAGAAACTTCTTTG

                              * *  *                 
     474213 TGATGTTTGCATTCAAGTCAGAGAGTTGAACATTCCCTTTC
        131 TGATGTTTGCATTCAAGTAACAGTGTTGAACATTCCCTTTC

                                        *         **     *       **     *   *
     474254 ATAGAGCAGGTTTGAAACACTCTTTTTGAAGTATCTGGTTGTGGACATTTGGAGCGCTTTCAGGC
          1 ATAGAGCAGGTTTGAAACACTCTTTTTGTAGTATCTGGAAGTGGATATTTGGATAGCTTTGAGGA

            * ** *      *             *** *   *                          *   
     474319 CTATGGT-GAAAAAGGAAATATCTTCCCCTGAAAACTAGACAGAAGCATTCTCAGAAACTTATTT
         66 TTTCGTTGGAAACAGG-AATATCTTCATATAAAAGCTAGACAGAAGCATTCTCAGAAACTTCTTT

                   **  **    *              *  * *   *
     474383 GTGATGTGCGCCCTCAACTAACAGTGTTGAACCTTTCTTTTG
        130 GTGATGTTTGCATTCAAGTAACAGTGTTGAACATTCCCTTTC

                     *                    *      *   *                       
     474425 ATAGAGCAGTTTTGAAACACTCTTTTTGTAATATCTGCAAGAGGATATTTGGATAGCTTTGAGGA
          1 ATAGAGCAGGTTTGAAACACTCTTTTTGTAGTATCTGGAAGTGGATATTTGGATAGCTTTGAGGA

                         *   * *            **                      *    *   
     474490 TTTCGTTGGAAACGGGATTGTCTTCATATAAACTCTAGACAGAAGCATTCTCAGAAGCTTCATTG
         66 TTTCGTTGGAAACAGGAATATCTTCATATAAAAGCTAGACAGAAGCATTCTCAGAAACTTCTTTG

            *       **   *    *             *        
     474555 GGATGTTTCAATTGAAGTCACAGTGTTGAACAGTCCCTTTC
        131 TGATGTTTGCATTCAAGTAACAGTGTTGAACATTCCCTTTC

                                                         *       **   * *    
     474596 ATAGAGCAGGTTTGAAACACTCTTTTTGTAGTATCTGGAAGTGGACATTTGGAGCGCTCTCAGGA
          1 ATAGAGCAGGTTTGAAACACTCTTTTTGTAGTATCTGGAAGTGGATATTTGGATAGCTTTGAGGA

            * *  *    * *             **            *       * *          *  *
     474661 CTACGAT-GATAAAGGAAATATCTTCCAATAAAAGCTAGATAGAAGCAATGTCAGAAACTTTTTC
         66 TTTCGTTGGAAACAGG-AATATCTTCATATAAAAGCTAGACAGAAGCATTCTCAGAAACTTCTTT

            *      * ** *   **      *          * *   *
     474725 ATGATGTATCTACTCAGCTAACAGAGTTGAACATTTCTTTTG
        130 GTGATGTTTGCATTCAAGTAACAGTGTTGAACATTCCCTTTC

             *       *                   * *     *             **            
     474767 AGAGAGCAGTTTTGAAACACTCTTTTTGTGGAATCTGCAAGTGGATATTTGTCTAGCTTTGAGGA
          1 ATAGAGCAGGTTTGAAACACTCTTTTTGTAGTATCTGGAAGTGGATATTTGGATAGCTTTGAGGA

                         *   *    *                   *       *     *        
     474832 TTTCGTTGGAAACGGGATTA-CAT-ATA-AAAAGC-AGACAGCAGCATTCCCAGAATCTTCTTTG
         66 TTTCGTTGGAAACAGGAATATCTTCATATAAAAGCTAGACAGAAGCATTCTCAGAAACTTCTTTG

                              *    *                 
     474893 TGATGTTTGCATTCAAGTCACAGAGTTGAACATTCCCTTTC
        131 TGATGTTTGCATTCAAGTAACAGTGTTGAACATTCCCTTTC

                                                   *     *       **     *   *
     474934 ATAGAGCAGGTTTGAAACACTCTTTTTGTAGTATCTGGATGTGGACATTTGGAGCGCTTTCAGGC
          1 ATAGAGCAGGTTTGAAACACTCTTTTTGTAGTATCTGGAAGTGGATATTTGGATAGCTTTGAGGA

            * ** *      *             *** *   *                      *   *   
     474999 CTATGGT-GAAAAAGGAAATATCTTCCCCTGAAAACTAGACAGAAGCATTCTCAGAATCTTATTT
         66 TTTCGTTGGAAACAGG-AATATCTTCATATAAAAGCTAGACAGAAGCATTCTCAGAAACTTCTTT

                   **  **    *             **  * *   *
     475063 GTGATGTGCGCCCTCAACTAACAGTGTTGAAGCTTTCTTTTG
        130 GTGATGTTTGCATTCAAGTAACAGTGTTGAACATTCCCTTTC

                     *                    **     *   *                       
     475105 ATAGAGCAGTTTTGAAACACTCTTTTTGTAAAATCTGCAAGAGGATATTTGGATAGCTTTGAGGA
          1 ATAGAGCAGGTTTGAAACACTCTTTTTGTAGTATCTGGAAGTGGATATTTGGATAGCTTTGAGGA

                         *   * *            **                      *    *   
     475170 TTTCGTTGGAAACGGGATTGTCTTCATATAAACTCTAGACAGAAGCATTCTCAGAAGCTTCATTG
         66 TTTCGTTGGAAACAGGAATATCTTCATATAAAAGCTAGACAGAAGCATTCTCAGAAACTTCTTTG

            *       **   *    *             *        
     475235 GGATGTTTCAATTGAAGTCACAGTGTTGAACAGTCCCTTTC
        131 TGATGTTTGCATTCAAGTAACAGTGTTGAACATTCCCTTTC

                                                         *       **   * *    
     475276 ATAGAGCAGGTTTGAAACACTCTTTTTGTAGTATCTGGAAGTGGACATTTGGAGCGCTCTCAGGA
          1 ATAGAGCAGGTTTGAAACACTCTTTTTGTAGTATCTGGAAGTGGATATTTGGATAGCTTTGAGGA

            * *  *      *             **            *       * *          *  *
     475341 CTGCGGT-GAAAAAGGAAATATCTTCCAATAAAAGCTAGATAGAAGCAATGTCAGAAACTTTTTC
         66 TTTCGTTGGAAACAGG-AATATCTTCATATAAAAGCTAGACAGAAGCATTCTCAGAAACTTCTTT

            *      * ** *   **      *       *    *   *
     475405 ATGATGTATCTACTCAGCTAACAGAGTTGAACCTT-CATTTG
        130 GTGATGTTTGCATTCAAGTAACAGTGTTGAACATTCCCTTTC

             *       *            *      * *     *             **            
     475446 AGAGAGCAGTTTTGAAACACTCGTTTTGTGGAATCTGCAAGTGGATATTTGTCTAGCTTTGAGGA
          1 ATAGAGCAGGTTTGAAACACTCTTTTTGTAGTATCTGGAAGTGGATATTTGGATAGCTTTGAGGA

                         *   *    *                   *       *              
     475511 TTTCGTTGGAAACGGGATTA-CAT-ATA-AAAAGC-AGACAGCAGCATTCCCAGAAACTTCTTTG
         66 TTTCGTTGGAAACAGGAATATCTTCATATAAAAGCTAGACAGAAGCATTCTCAGAAACTTCTTTG

                              *    *                 
     475572 TGATGTTTGCATTCAAGTCACAGAGTTGAACATTCCCTTTC
        131 TGATGTTTGCATTCAAGTAACAGTGTTGAACATTCCCTTTC

                                                   *   * *     * **     *   *
     475613 ATAGAGCAGGTTTGAAACACTCTTTTTGTAGTATCTGGATGTGTACATTTGCAGCGCTTTCAGGC
          1 ATAGAGCAGGTTTGAAACACTCTTTTTGTAGTATCTGGAAGTGGATATTTGGATAGCTTTGAGGA

            * ** *      *             *** *   *                          *   
     475678 CTAAGGT-GAAAAAGGAAATATCTTCCCCTGAAAACTAGACAGAAGCATTCTCAGAAACTTATTT
         66 TTTCGTTGGAAACAGG-AATATCTTCATATAAAAGCTAGACAGAAGCATTCTCAGAAACTTCTTT

                   **  **    *             **  * *   *
     475742 GTGATGTGCGCCCTCAACTAACAGTGTTGAAGCTTTCTTTTG
        130 GTGATGTTTGCATTCAAGTAACAGTGTTGAACATTCCCTTTC

                     *        *           *      *   *                       
     475784 ATAGAGCAGTTTTGAAACGCTCTTTTTGTAATATCTGCAAGAGGATATTTGGATAGCTTTGAGGA
          1 ATAGAGCAGGTTTGAAACACTCTTTTTGTAGTATCTGGAAGTGGATATTTGGATAGCTTTGAGGA

                         *  ** *            **                      *    *   
     475849 TTTCGTTGGAAACGGGTTTGTCTTCATATAAACTCTAGACAGAAGCATTCTCAGAAGCTTCATTG
         66 TTTCGTTGGAAACAGGAATATCTTCATATAAAAGCTAGACAGAAGCATTCTCAGAAACTTCTTTG

            *       **   *    *             * *      
     475914 GGATGTTTCAATTGAAGTCACAGTGTTGAACAGTTCCTTTC
        131 TGATGTTTGCATTCAAGTAACAGTGTTGAACATTCCCTTTC

                 *                                       *       **   * *    
     475955 ATAGAACAGGTTTGAAACACTCTTTTTGTAGTATCTGGAAGTGGACATTTGGAGCGCTCTCAGGA
          1 ATAGAGCAGGTTTGAAACACTCTTTTTGTAGTATCTGGAAGTGGATATTTGGATAGCTTTGAGGA

            * ** *      *             **          * *       * *          *  *
     476020 CTATGGT-GAAAAAGGAAATATCTTCCAATAAAAGCTACATAGAAGCAATGTCAGAAACTTTTTC
         66 TTTCGTTGGAAACAGG-AATATCTTCATATAAAAGCTAGACAGAAGCATTCTCAGAAACTTCTTT

            *      * ** *   **      *       *  *     *
     476084 ATGATGTATCTACTCAGCTAACAGAGTTGAACCTTTCCTTTG
        130 GTGATGTTTGCATTCAAGTAACAGTGTTGAACATTCCCTTTC

             *       *                   * *     *             **            
     476126 AGAGAGCAGTTTTGAAACACTCTTTTTGTGGAATCTGCAAGTGGATATTTGTCTAGCTTTGAGGA
          1 ATAGAGCAGGTTTGAAACACTCTTTTTGTAGTATCTGGAAGTGGATATTTGGATAGCTTTGAGGA

                         *   *    *                   *       *   *          
     476191 TTTCGTTGGAAACGGGATTA-CAT-ATA-AAAAGC-AGACAGCAGCATTCCCAGTAACTTCTTTG
         66 TTTCGTTGGAAACAGGAATATCTTCATATAAAAGCTAGACAGAAGCATTCTCAGAAACTTCTTTG

                              *    *      *          
     476252 TGATGTTTGCATTCAAGTCACAGAGTTGAATATTCCCTTTC
        131 TGATGTTTGCATTCAAGTAACAGTGTTGAACATTCCCTTTC

                                                   *     *       **     *   *
     476293 ATAGAGCAGGTTTGAAACACTCTTTTTGTAGTATCTGGATGTGGACATTTGGAGCGCTTTCAGGC
          1 ATAGAGCAGGTTTGAAACACTCTTTTTGTAGTATCTGGAAGTGGATATTTGGATAGCTTTGAGGA

            * ** *      *             *** *   *                          *   
     476358 CTATGGT-GAAAAAGGAAATATCTTCCCCTGAAAACTAGACAGAAGCATTCTCAGAAACTTATTT
         66 TTTCGTTGGAAACAGG-AATATCTTCATATAAAAGCTAGACAGAAGCATTCTCAGAAACTTCTTT

                   **  **    *              *  * *   *
     476422 GTGATGTGCGCCCTCAACTAACAGTGTTGAACCTTTCTTTTG
        130 GTGATGTTTGCATTCAAGTAACAGTGTTGAACATTCCCTTTC

                     *                    *      *   *                       
     476464 ATAGAGCAGTTTTGAAACACTCTTTTTGTAATATCTGCAAGAGGATATTTGGATAGCTTTGAGGA
          1 ATAGAGCAGGTTTGAAACACTCTTTTTGTAGTATCTGGAAGTGGATATTTGGATAGCTTTGAGGA

                         *   *    *                   *       *              
     476529 TTTCGTTGGAAACGGGATTA-CAT-ATA-AAAAGC-AGACAGCAGCATTCCCAGAAACTTCTTTG
         66 TTTCGTTGGAAACAGGAATATCTTCATATAAAAGCTAGACAGAAGCATTCTCAGAAACTTCTTTG

                              *    *                 
     476590 TGATGTTTGCATTCAAGTCACAGAGTTGAACATTCCCTTTC
        131 TGATGTTTGCATTCAAGTAACAGTGTTGAACATTCCCTTTC

                                                   *     *     * **     *   *
     476631 ATAGAGCAGGTTTGAAACACTCTTTTTGTAGTATCTGGATGTGGACATTTGCAGCGCTTTCAGGC
          1 ATAGAGCAGGTTTGAAACACTCTTTTTGTAGTATCTGGAAGTGGATATTTGGATAGCTTTGAGGA

            * ** *      *             *** *   *                          *   
     476696 CTAAGGT-GAAAAAGGAAATATCTTCCCCTGAAAACTAGACAGAAGCATTCTCAGAAACTTATTT
         66 TTTCGTTGGAAACAGG-AATATCTTCATATAAAAGCTAGACAGAAGCATTCTCAGAAACTTCTTT

                   **  **    *              *  * *   *
     476760 GTGATGTGCGCCCTCAACTAACAGTGTTGAACCTTTCTTTTG
        130 GTGATGTTTGCATTCAAGTAACAGTGTTGAACATTCCCTTTC

                     *                    *      *   *                       
     476802 ATAGAGCAGTTTTGAAACACTCTTTTTGTAATATCTGCAAGAGGATATTTGGATAGCTTTGAGGA
          1 ATAGAGCAGGTTTGAAACACTCTTTTTGTAGTATCTGGAAGTGGATATTTGGATAGCTTTGAGGA

                         *   * *            **                      *    *   
     476867 TTTCGTTGGAAACGGGATTGTCTTCATATAAACTCTAGACAGAAGCATTCTCAGAAGCTTCATTG
         66 TTTCGTTGGAAACAGGAATATCTTCATATAAAAGCTAGACAGAAGCATTCTCAGAAACTTCTTTG

            *       **   *    *             *        
     476932 GGATGTTTCAATTGAAGTCACAGTGTTGAACAGTCCCTTTC
        131 TGATGTTTGCATTCAAGTAACAGTGTTGAACATTCCCTTTC

                                                   *     *       **     *    
     476973 ATAGAGCAGGTTTGAAACACTCTTTTTGTAGTATCTGGATGTGGACATTTGGAGCGCTTTCAGGC
          1 ATAGAGCAGGTTTGAAACACTCTTTTTGTAGTATCTGGAAGTGGATATTTGGATAGCTTTGAGG-

            * * *    *                 *** *   *                          *  
     477038 CTATGGTT-TAAA-AGGAAATATCTTCCCCTGAAAACTAGACAGAAGCATTCTCAGAAACTTATT
         65 ATTTCGTTGGAAACAGG-AATATCTTCATATAAAAGCTAGACAGAAGCATTCTCAGAAACTTCTT

                    **  **    *                    *   *
     477101 TGTGATGTGCGCCCTCAACTAACAGTGTTGAAGCATT-CTTTTG
        129 TGTGATGTTTGCATTCAAGTAACAGTGTTGAA-CATTCCCTTTC

                     *                   * *     *             **            
     477144 ATAGAGCAGTTTTGAAACACTCTTTTTGTGGAATCTGCAAGTGGATATTTGTCTAGCTTTGAGGA
          1 ATAGAGCAGGTTTGAAACACTCTTTTTGTAGTATCTGGAAGTGGATATTTGGATAGCTTTGAGGA

                         *   *   *                    *       *     *   *    
     477209 TTTCGTTGGAAACCGGATTA-ATT-ATA-AAAAGC-AGACAGCAGCATTCCCAGAATCTTGTTTG
         66 TTTCGTTGGAAACAGGAATATCTTCATATAAAAGCTAGACAGAAGCATTCTCAGAAACTTCTTTG

                              *    *                 
     477270 TGATGTTTGCATTCAAGTCACAGAGTTGAACATTCCCTTTC
        131 TGATGTTTGCATTCAAGTAACAGTGTTGAACATTCCCTTTC

             *                         *           *     *       **     *   *
     477311 AGAGAGCAGGTTTGAAACACTCTTTTTATAGTATCTGGATGTGGACATTTGGAGCGCTTTCAGGC
          1 ATAGAGCAGGTTTGAAACACTCTTTTTGTAGTATCTGGAAGTGGATATTTGGATAGCTTTGAGGA

            * ** *      *             *** *   *                      *   *   
     477376 CTATGGT-GAAAAAGGAAATATCTTCTCCTGAAAACTAGACAGAAGCATTCTCAGAATCTTATTT
         66 TTTCGTTGGAAACAGG-AATATCTTCATATAAAAGCTAGACAGAAGCATTCTCAGAAACTTCTTT

                   **  **    *             **  * *   *
     477440 GTGATGTGCGCCCTCAACTAACAGTGTTGAAGCTTTCTTTTG
        130 GTGATGTTTGCATTCAAGTAACAGTGTTGAACATTCCCTTTC

                     *                *   **     *   *        *              
     477482 ATAGAGCAGTTTTGAAACACTCTTTTCGTAAAATCTGCAAGAGGATATTTTGATAGCTTTGAGGA
          1 ATAGAGCAGGTTTGAAACACTCTTTTTGTAGTATCTGGAAGTGGATATTTGGATAGCTTTGAGGA

                         *   * *            **                      *    *   
     477547 TTTCGTTGGAAACGGGATTGTCTTCATATAAACTCTAGACAGAAGCATTCTCAGAAGCTTCATTG
         66 TTTCGTTGGAAACAGGAATATCTTCATATAAAAGCTAGACAGAAGCATTCTCAGAAACTTCTTTG

            *       **   *    *             *        
     477612 GGATGTTTCAATTGAAGTCACAGTGTTGAACAGTCCCTTTC
        131 TGATGTTTGCATTCAAGTAACAGTGTTGAACATTCCCTTTC

                                                         *       *  * * *    
     477653 ATAGAGCAGGTTTGAAACACTCTTTTTGTAGTATCTGGAAGTGGACATTTGGAGAGATCTCAGGA
          1 ATAGAGCAGGTTTGAAACACTCTTTTTGTAGTATCTGGAAGTGGATATTTGGATAGCTTTGAGGA

            * *  *    * *             **          * *       * *          *  *
     477718 ATACGGT-GATAAAGGAAATATCTTCCAATAAAAGCTACATAGAAGCAATGTCAGAAACTTTTTC
         66 TTTCGTTGGAAACAGG-AATATCTTCATATAAAAGCTAGACAGAAGCATTCTCAGAAACTTCTTT

            *      * ** *   **      *       *  * *   *
     477782 ATGATGTATCTACTCAGCTAACAGAGTTGAACCTTTCTTTTG
        130 GTGATGTTTGCATTCAAGTAACAGTGTTGAACATTCCCTTTC

             *       *                *   **     *   *                       
     477824 AGAGAGCAGTTTTGAAACACTCTTTTCGTAAAATCTGCAAGAGGATATTTGGATAGCTTTGAGGA
          1 ATAGAGCAGGTTTGAAACACTCTTTTTGTAGTATCTGGAAGTGGATATTTGGATAGCTTTGAGGA

                         *   * *            **                      *    *   
     477889 TTTCGTTGGAAACGGGATTGTCTTCATATAAACTCTAGACAGAAGCATTCTCAGAAGCTTCATTG
         66 TTTCGTTGGAAACAGGAATATCTTCATATAAAAGCTAGACAGAAGCATTCTCAGAAACTTCTTTG

            *       **   *    *             *        
     477954 GGATGTTTCAATTGAAGTCACAGTGTTGAACAGTCCCTTTC
        131 TGATGTTTGCATTCAAGTAACAGTGTTGAACATTCCCTTTC

                                                   *     *       **     *    
     477995 ATAGAGCAGGTTTGAAACACTCTTTTTGTAGTATCTGGATGTGGACATTTGGAGCGCTTTCAGGC
          1 ATAGAGCAGGTTTGAAACACTCTTTTTGTAGTATCTGGAAGTGGATATTTGGATAGCTTTGAGG-

            * * *    *                 *** *   *                          *  
     478060 CTATGGTT-TAAA-AGGAAATATCTTCCCCTGAAAACTAGACAGAAGCATTCTCAGAAACTTATT
         65 ATTTCGTTGGAAACAGG-AATATCTTCATATAAAAGCTAGACAGAAGCATTCTCAGAAACTTCTT

                    **  *     *                    *   *
     478123 TGTGATGTGCGCCTTCAACTAACAGTGTTGAAGCATT-CTTTTG
        129 TGTGATGTTTGCATTCAAGTAACAGTGTTGAA-CATTCCCTTTC

                     *                   * *     *             **            
     478166 ATAGAGCAGTTTTGAAACACTCTTTTTGTGGAATCTGCAAGTGGATATTTGTCTAGCTTTGAGGA
          1 ATAGAGCAGGTTTGAAACACTCTTTTTGTAGTATCTGGAAGTGGATATTTGGATAGCTTTGAGGA

                         *   *    *     *             *       *              
     478231 TTTCGTTGGAAACGGGATTA-CAT-AT-GAAAAGC-AGACAGCAGCATTCCCAGAAACTTCTTTG
         66 TTTCGTTGGAAACAGGAATATCTTCATATAAAAGCTAGACAGAAGCATTCTCAGAAACTTCTTTG

                              *    *                 
     478292 TGATGTTTGCATTCAAGTCACAGAGTTGAACATTCCCTTTC
        131 TGATGTTTGCATTCAAGTAACAGTGTTGAACATTCCCTTTC

             *                                     *     *       **     *  **
     478333 AGAGAGCAGGTTTGAAACACTCTTTTTGTAGTATCTGGATGTGGACATTTGGAGCGCTTTCAGCC
          1 ATAGAGCAGGTTTGAAACACTCTTTTTGTAGTATCTGGAAGTGGATATTTGGATAGCTTTGAGGA

            * ** *      *             *** *   *                      *   *   
     478398 CTATGGT-GAAAAAGGAAATATCTTCCCCTGAAAACTAGACAGAAGCATTCTCAGAATCTTATTT
         66 TTTCGTTGGAAACAGG-AATATCTTCATATAAAAGCTAGACAGAAGCATTCTCAGAAACTTCTTT

                   **  **    *             **  * *   *
     478462 GTGATGTGCGCCCTCAACTAACAGTGTTGAAGCTTTCTTTTG
        130 GTGATGTTTGCATTCAAGTAACAGTGTTGAACATTCCCTTTC

                     *                    *      *   *                       
     478504 ATAGAGCAGTTTTGAAACACTCTTTTTGTAATATCTGCAAGAGGATATTTGGATAGCTTTGAGGA
          1 ATAGAGCAGGTTTGAAACACTCTTTTTGTAGTATCTGGAAGTGGATATTTGGATAGCTTTGAGGA

                         *   * *            **    *                 *    *   
     478569 TTTCGTTGGAAACGGGATTGTCTTCATATAAACTCTAGGCAGAAGCATTCTCAGAAGCTTCATTG
         66 TTTCGTTGGAAACAGGAATATCTTCATATAAAAGCTAGACAGAAGCATTCTCAGAAACTTCTTTG

            *       **   *    *             * *      
     478634 GGATGTTTCAATTGAAGTCACAGTGTTGAACAGTTCCTTTC
        131 TGATGTTTGCATTCAAGTAACAGTGTTGAACATTCCCTTTC

                 *                                       *       **   * *    
     478675 ATAGAACAGGTTTGAAACACTCTTTTTGTAGTATCTGGAAGTGGACATTTGGAGCGCTCTCAGGA
          1 ATAGAGCAGGTTTGAAACACTCTTTTTGTAGTATCTGGAAGTGGATATTTGGATAGCTTTGAGGA

            * *  *  *   ** *         **          * *       * *          *  **
     478740 CTACGGTGAAAATGGAAATATCTTCCAATAAAAGCTACATAGAAGCAATGTCAGAAACTTTTTCA
         66 TTTCGTTGGAAACAGGAATATCTTCATATAAAAGCTAGACAGAAGCATTCTCAGAAACTTCTTTG

                  * ** *   **      *       *  *     *
     478805 TGATGTATCTACTCAGCTAACAGAGTTGAACCTTTCCTTTG
        131 TGATGTTTGCATTCAAGTAACAGTGTTGAACATTCCCTTTC

             *       *                   * *     *             **            
     478846 AGAGAGCAGTTTTGAAACACTCTTTTTGTGGAATCTGCAAGTGGATATTTGTCTAGCTTTGAGGA
          1 ATAGAGCAGGTTTGAAACACTCTTTTTGTAGTATCTGGAAGTGGATATTTGGATAGCTTTGAGGA

                         *   *    *                   *       *     *        
     478911 TTTCGTTGGAAACGGGATTA-CAT-ATA-AAAAGC-AGACAGCAGCATTCCCAGAATCTTCTTTG
         66 TTTCGTTGGAAACAGGAATATCTTCATATAAAAGCTAGACAGAAGCATTCTCAGAAACTTCTTTG

                              *    *                 
     478972 TGATGTTTGCATTCAAGTCACAGAGTTGAACATTCCCTTTC
        131 TGATGTTTGCATTCAAGTAACAGTGTTGAACATTCCCTTTC

                                                   *     *       **     *   *
     479013 ATAGAGCAGGTTTGAAACACTCTTTTTGTAGTATCTGGATGTGGACATTTGGAGCGCTTTCAGGC
          1 ATAGAGCAGGTTTGAAACACTCTTTTTGTAGTATCTGGAAGTGGATATTTGGATAGCTTTGAGGA

            * ** *      *             *** *   *                      *   *   
     479078 CTATGGT-GAAAAAGGAAATATCTTCCCCTGAAAACTAGACAGAAGCATTCTCAGAATCTTATTT
         66 TTTCGTTGGAAACAGG-AATATCTTCATATAAAAGCTAGACAGAAGCATTCTCAGAAACTTCTTT

                   **  **    *             **  * *   *
     479142 GTGATGTGCGCCCTCAACTAACAGTGTTGAAGCTTTCTTTTG
        130 GTGATGTTTGCATTCAAGTAACAGTGTTGAACATTCCCTTTC

                     *                    **    **   *                       
     479184 ATAGAGCAGTTTTGAAACACTCTTTTTGTAAAATCTTCAAGAGGATATTTGGATAGCTTTGAGGA
          1 ATAGAGCAGGTTTGAAACACTCTTTTTGTAGTATCTGGAAGTGGATATTTGGATAGCTTTGAGGA

                         *   * *            **                      *    *   
     479249 TTTCGTTGGAAACGGGATTGTCTTCATATAAACTCTAGACAGAAGCATTCTCAGAAGCTTCATTG
         66 TTTCGTTGGAAACAGGAATATCTTCATATAAAAGCTAGACAGAAGCATTCTCAGAAACTTCTTTG

            *       **   *    *             *        
     479314 GGATGTTTCAATTGAAGTCACAGTGTTGAACAGTCCCTTTC
        131 TGATGTTTGCATTCAAGTAACAGTGTTGAACATTCCCTTTC

                                                         *       **   * *    
     479355 ATAGAGCAGGTTTGAAACACTCTTTTTGTAGTATCTGGAAGTGGACATTTGGAACGCTCTCAGGA
          1 ATAGAGCAGGTTTGAAACACTCTTTTTGTAGTATCTGGAAGTGGATATTTGGATAGCTTTGAGGA

            * *  *      *             **            *       * *          *  *
     479420 CTGCGGT-GAAAAAGGAAATATCTTCCAATAAAAGCTAGATAGAAGCAATGTCAGAAACTTTTTC
         66 TTTCGTTGGAAACAGG-AATATCTTCATATAAAAGCTAGACAGAAGCATTCTCAGAAACTTCTTT

            *      * ** *   **      *       *  * *   *
     479484 ATGATGTATCTACTCAGCTAACAGAGTTGAACCTTTCTTTTG
        130 GTGATGTTTGCATTCAAGTAACAGTGTTGAACATTCCCTTTC

             *       *            *      * *     *             **  *         
     479526 AGAGAGCAGTTTTGAAACACTCGTTTTGTGGAATCTGCAAGTGGATATTTGTCTACCTTTGAGGA
          1 ATAGAGCAGGTTTGAAACACTCTTTTTGTAGTATCTGGAAGTGGATATTTGGATAGCTTTGAGGA

                         *   *    *                   *       *              
     479591 TTTCGTTGGAAACGGGATTA-CAT-ATA-AAAAGC-AGACAGCAGCATTCCCAGAAACTTCTTTG
         66 TTTCGTTGGAAACAGGAATATCTTCATATAAAAGCTAGACAGAAGCATTCTCAGAAACTTCTTTG

                              *    *                 
     479652 TGATGTTTGCATTCAAGTCACAGAGTTGAACATTCCCTTTC
        131 TGATGTTTGCATTCAAGTAACAGTGTTGAACATTCCCTTTC

                                                   *     *     * **     *   *
     479693 ATAGAGCAGGTTTGAAACACTCTTTTTGTAGTATCTGGATGTGGACATTTGCAGCGCTTTCAGGC
          1 ATAGAGCAGGTTTGAAACACTCTTTTTGTAGTATCTGGAAGTGGATATTTGGATAGCTTTGAGGA

            * ** *      *             *** *   *                          *   
     479758 CTAAGGT-GAAAAAGGAAATATCTTCCCCTGAAAACTAGACAGAAGCATTCTCAGAAACTTATTT
         66 TTTCGTTGGAAACAGG-AATATCTTCATATAAAAGCTAGACAGAAGCATTCTCAGAAACTTCTTT

                   **  **    *             **  * *   *
     479822 GTGATGTGCGCCCTCAACTAACAGTGTTGAAGCTTTCTTTTG
        130 GTGATGTTTGCATTCAAGTAACAGTGTTGAACATTCCCTTTC

                     *                    *      *   *                       
     479864 ATAGAGCAGTTTTGAAACACTCTTTTTGTAATATCTGCAAGAGGATATTTGGATAGCTTTGAGGA
          1 ATAGAGCAGGTTTGAAACACTCTTTTTGTAGTATCTGGAAGTGGATATTTGGATAGCTTTGAGGA

                         *   * *            **                      *    *   
     479929 TTTCGTTGGAAACGGGATTGTCTTCATATAAACTCTAGACAGAAGCATTCTCAGAAGCTTCATTG
         66 TTTCGTTGGAAACAGGAATATCTTCATATAAAAGCTAGACAGAAGCATTCTCAGAAACTTCTTTG

            *       **   *    *             *        
     479994 GGATGTTTCAATTGAAGTCACAGTGTTGAACAGTCCCTTTC
        131 TGATGTTTGCATTCAAGTAACAGTGTTGAACATTCCCTTTC

                                                   *     *       **   * *    
     480035 ATAGAGCAGGTTTGAAACACTCTTTTTGTAGTATCTGGATGTGGACATTTGGAACGCTCTCAGGA
          1 ATAGAGCAGGTTTGAAACACTCTTTTTGTAGTATCTGGAAGTGGATATTTGGATAGCTTTGAGGA

            * *  *      *             **            *       * *          *  *
     480100 CTGCGGT-GAAAAAGGAAATATCTTCCAATAAAAGCTAGATAGAAGCAATGTCAGAAACTTTTTC
         66 TTTCGTTGGAAACAGG-AATATCTTCATATAAAAGCTAGACAGAAGCATTCTCAGAAACTTCTTT

            *  *   * ** *   **      *       *  * *   *
     480164 ATGCTGTATCTACTCAGCTAACAGAGTTGAACCTTTCTTTTG
        130 GTGATGTTTGCATTCAAGTAACAGTGTTGAACATTCCCTTTC

             *       *              *    * *     *             **            
     480206 AGAGAGCAGTTTTGAAACACTCTTCTTGTGGAATCTGCAAGTGGATATTTGTCTAGCTTTGAGGA
          1 ATAGAGCAGGTTTGAAACACTCTTTTTGTAGTATCTGGAAGTGGATATTTGGATAGCTTTGAGGA

                         *   *    *                   *       *   *          
     480271 TTTCGTTGGAAACGGGATTA-CAT-ATA-AAAAGC-AGACAGCAGCATTCCCAGTAACTTCTTTG
         66 TTTCGTTGGAAACAGGAATATCTTCATATAAAAGCTAGACAGAAGCATTCTCAGAAACTTCTTTG

                              *    *                 
     480332 TGATGTTTGCATTCAAGTCACAGAGTTGAACATTCCCTTTC
        131 TGATGTTTGCATTCAAGTAACAGTGTTGAACATTCCCTTTC

                       *                           *     *       **     *   *
     480373 ATAGAGCAGGTATGAAACACTCTTTTTGTAGTATCTGGATGTGGACATTTGGAGCGCTTTCAGGC
          1 ATAGAGCAGGTTTGAAACACTCTTTTTGTAGTATCTGGAAGTGGATATTTGGATAGCTTTGAGGA

            * ** *      *             **          * * *     * *          *  *
     480438 CTATGGT-GAAAAAGGAAATATCTTCCAATAAAAGCTACATAAAAGCAATGTCAGAAACTTTTTC
         66 TTTCGTTGGAAACAGG-AATATCTTCATATAAAAGCTAGACAGAAGCATTCTCAGAAACTTCTTT

            *      * ** *   **      *       *  * *   *
     480502 ATGATGTATCTACTCAGCTAACAGAGTTGAACCTTTCTTTTG
        130 GTGATGTTTGCATTCAAGTAACAGTGTTGAACATTCCCTTTC

             *       *                   * *                   **            
     480544 AGAGAGCAGTTTTGAAACACTCTTTTTGTGGAATCTGGAAGTGGATATTTGTCTAGCTTTGAGGA
          1 ATAGAGCAGGTTTGAAACACTCTTTTTGTAGTATCTGGAAGTGGATATTTGGATAGCTTTGAGGA

                         *   *    *                   *       *   *          
     480609 TTTCGTTGGAAACGGGATTA-CAT-ATA-AAAAGC-AGACAGCAGCATTCCCAGTAACTTCTTTG
         66 TTTCGTTGGAAACAGGAATATCTTCATATAAAAGCTAGACAGAAGCATTCTCAGAAACTTCTTTG

                              *    *                 
     480670 TGATGTTTGCATTCAAGTCACAGAGTTGAACATTCCCTTTC
        131 TGATGTTTGCATTCAAGTAACAGTGTTGAACATTCCCTTTC

                                                   *     *       **     *    
     480711 ATAGAGCAGGTTTGAAACACTCTTTTTGTAGTATCTGGATGTGGACATTTGGAGCGCTTTCAGG-
          1 ATAGAGCAGGTTTGAAACACTCTTTTTGTAGTATCTGGAAGTGGATATTTGGATAGCTTTGAGGA

            **  *        *             *** *   *           *          *   *  
     480775 CCT-ATTGTGAAAAAGGAAATATCTTCCCCTGAAAACTAGACAGAAGAATTCTCAGAATCTTATT
         66 TTTCGTTG-GAAACAGG-AATATCTTCATATAAAAGCTAGACAGAAGCATTCTCAGAAACTTCTT

                    **  **    *             **  * *   *
     480839 TGTGATGTGCGCCCTCAACTAACAGTGTTGAAGCTTTCTTTTG
        129 TGTGATGTTTGCATTCAAGTAACAGTGTTGAACATTCCCTTTC

                     *                    **     *   *                       
     480882 ATAGAGCAGTTTTGAAACACTCTTTTTGTAAAATCTGCAAGAGGATATTTGGATAGCTTTGAGGA
          1 ATAGAGCAGGTTTGAAACACTCTTTTTGTAGTATCTGGAAGTGGATATTTGGATAGCTTTGAGGA

                         *   * *            **   *                  * *  *   
     480947 TTTCGTTGGAAACGGGATTGTCTTCATATAAACTCTACACAGAAGCATTCTCAGAAGCGTCATTG
         66 TTTCGTTGGAAACAGGAATATCTTCATATAAAAGCTAGACAGAAGCATTCTCAGAAACTTCTTTG

            *       **   *    *             *        
     481012 GGATGTTTCAATTGAAGTCACAGTGTTGAACAGTCCCTTTC
        131 TGATGTTTGCATTCAAGTAACAGTGTTGAACATTCCCTTTC

                                                   *     *       **     *    
     481053 ATAGAGCAGGTTTGAAACACTCTTTTTGTAGTATCTGGATGTGGACATTTGGAGCGCTTTCAGGC
          1 ATAGAGCAGGTTTGAAACACTCTTTTTGTAGTATCTGGAAGTGGATATTTGGATAGCTTTGAGG-

            * * *    *                 *** *   *                          *  
     481118 CTATGGTT-TAAA-AGGAAATATCTTCCCCTGAAAACTAGACAGAAGCATTCTCAGAAACTTATT
         65 ATTTCGTTGGAAACAGG-AATATCTTCATATAAAAGCTAGACAGAAGCATTCTCAGAAACTTCTT

                    **  *     *                    *   *
     481181 TGTGATGTGCGCCTTCAACTAACAGTGTTGAAGCATT-CTTTTG
        129 TGTGATGTTTGCATTCAAGTAACAGTGTTGAA-CATTCCCTTTC

                     *                   * *     *             **            
     481224 ATAGAGCAGTTTTGAAACACTCTTTTTGTGGAATCTGCAAGTGGATATTTGTCTAGCTTTGAGGA
          1 ATAGAGCAGGTTTGAAACACTCTTTTTGTAGTATCTGGAAGTGGATATTTGGATAGCTTTGAGGA

                         *   *    *                   *       *              
     481289 TTTCGTTGGAAACGGGATTA-CAT-ATA-AAAAGC-AGACAGCAGCATTCCCAGAAACTTCTTTG
         66 TTTCGTTGGAAACAGGAATATCTTCATATAAAAGCTAGACAGAAGCATTCTCAGAAACTTCTTTG

                              *    *                 
     481350 TGATGTTTGCATTCAAGTCACAGAGTTGAACATTCCCTTTC
        131 TGATGTTTGCATTCAAGTAACAGTGTTGAACATTCCCTTTC

             *                                     *     *       **     *   *
     481391 AGAGAGCAGGTTTGAAACACTCTTTTTGTAGTATCTGGATGTGGACATTTGGAGCGCTTTCAGGC
          1 ATAGAGCAGGTTTGAAACACTCTTTTTGTAGTATCTGGAAGTGGATATTTGGATAGCTTTGAGGA

            * ** *      *             *** *   *                      *   *   
     481456 CTATGGT-GAAAAAGGAAATATCTTCCCCTGAAAACTAGACAGAAGCATTCTCAGAATCTTATTT
         66 TTTCGTTGGAAACAGG-AATATCTTCATATAAAAGCTAGACAGAAGCATTCTCAGAAACTTCTTT

                   **  **    *             **  * *   *
     481520 GTGATGTGCGCCCTCAACTAACAGTGTTGAAGCTTTCTTTTG
        130 GTGATGTTTGCATTCAAGTAACAGTGTTGAACATTCCCTTTC

                     *                    **     *   *                       
     481562 ATAGAGCAGTTTTGAAACACTCTTTTTGTAAAATCTGCAAGAGGATATTTGGATAGCTTTGAGGA
          1 ATAGAGCAGGTTTGAAACACTCTTTTTGTAGTATCTGGAAGTGGATATTTGGATAGCTTTGAGGA

                         *   * *            **                      *    *   
     481627 TTTCGTTGGAAACGGGATTGTCTTCATATAAACTCTAGACAGAAGCATTCTCAGAAGCTTCATTG
         66 TTTCGTTGGAAACAGGAATATCTTCATATAAAAGCTAGACAGAAGCATTCTCAGAAACTTCTTTG

            *       ** * *    *             *  *     
     481692 GGATGTTTCAAATGAAGTCACAGTGTTGAACAGTCTCTTTC
        131 TGATGTTTGCATTCAAGTAACAGTGTTGAACATTCCCTTTC

                                                         *       **   * *    
     481733 ATAGAGCAGGTTTGAAACACTCTTTTTGTAGTATCTGGAAGTGGACATTTGGAGCGCTCTCAGGA
          1 ATAGAGCAGGTTTGAAACACTCTTTTTGTAGTATCTGGAAGTGGATATTTGGATAGCTTTGAGGA

            * *  *      *             **            *       * *          *  *
     481798 CTGCGGT-GAAAAAGGAACTATCTTCCAATAAAAGCTAGATAGAAGCAATGTCAGAAACTTTTTC
         66 TTTCGTTGGAAACAGGAA-TATCTTCATATAAAAGCTAGACAGAAGCATTCTCAGAAACTTCTTT

            *      * ** *   **      *       *        *
     481862 ATGATGTATCTACTCAGCTAACAGAGTTGAACCTT-CCTTTG
        130 GTGATGTTTGCATTCAAGTAACAGTGTTGAACATTCCCTTTC

             *       *                   * *     *             **            
     481903 AGAGAGCAGTTTTGAAACACTCTTTTTGTGGAATCTGCAAGTGGATATTTGTCTAGCTTTGAGGA
          1 ATAGAGCAGGTTTGAAACACTCTTTTTGTAGTATCTGGAAGTGGATATTTGGATAGCTTTGAGGA

                         *   * *            **                      *    *   
     481968 TTTCGTTGGAAACGGGATTGTCTTCATATAAACTCTAGACAGAAGCATTCTCAGAAGCTTCATTG
         66 TTTCGTTGGAAACAGGAATATCTTCATATAAAAGCTAGACAGAAGCATTCTCAGAAACTTCTTTG

            *       **   *    *             * *      
     482033 GGATGTTTCAATTGAAGTCACAGTGTTGAACAGTTCCTTTC
        131 TGATGTTTGCATTCAAGTAACAGTGTTGAACATTCCCTTTC

                 *                                       *       **   ***    
     482074 ATAGAACAGGTTTGAAACACTCTTTTTGTAGTATCTGGAAGTGGACATTTGGAGCGCTCCCAGGA
          1 ATAGAGCAGGTTTGAAACACTCTTTTTGTAGTATCTGGAAGTGGATATTTGGATAGCTTTGAGGA

            * *  *      *             **          * *       * *          *  *
     482139 CTACGGT-GAAAAAGGAAATATCTTCCAATAAAAGCTACATAGAAGCAATGTCAGAAACTTTTTC
         66 TTTCGTTGGAAACAGG-AATATCTTCATATAAAAGCTAGACAGAAGCATTCTCAGAAACTTCTTT

            *      * ** *   **      *       *        *
     482203 ATGATGTATCTACTCAGCTAACAGAGTTGAACCTT-CCTTTG
        130 GTGATGTTTGCATTCAAGTAACAGTGTTGAACATTCCCTTTC

             *       *                   * *     *             **            
     482244 AGAGAGCAGTTTTGAAACACTCTTTTTGTGGAATCTGCAAGTGGATATTTGTCTAGCTTTGAGGA
          1 ATAGAGCAGGTTTGAAACACTCTTTTTGTAGTATCTGGAAGTGGATATTTGGATAGCTTTGAGGA

                         *   *    *                   *       *              
     482309 TTTCGTTGGAAACGGGATTA-CAT-ATA-AAAAGC-AGACAGCAGCATTCCCAGAAACTTCTTTG
         66 TTTCGTTGGAAACAGGAATATCTTCATATAAAAGCTAGACAGAAGCATTCTCAGAAACTTCTTTG

                              *    *                 
     482370 TGATGTTTGCATTCAAGTCACAGAGTTGAACATTCCCTTTC
        131 TGATGTTTGCATTCAAGTAACAGTGTTGAACATTCCCTTTC

                                                   *     *     * **     *   *
     482411 ATAGAGCAGGTTTGAAACACTCTTTTTGTAGTATCTGGATGTGGACATTTGCAGCGCTTTCAGGC
          1 ATAGAGCAGGTTTGAAACACTCTTTTTGTAGTATCTGGAAGTGGATATTTGGATAGCTTTGAGGA

            * ** *      *             *** *   *                          *   
     482476 CTAAGGT-GAAAAAGGAAATATCTTCCCCTGAAAACTAGACAGAAGCATTCTCAGAAACTTATTT
         66 TTTCGTTGGAAACAGG-AATATCTTCATATAAAAGCTAGACAGAAGCATTCTCAGAAACTTCTTT

                   **  **    *             **  * *   *
     482540 GTGATGTGCGCCCTCAACTAACAGTGTTGAAGCTTTCTTTTG
        130 GTGATGTTTGCATTCAAGTAACAGTGTTGAACATTCCCTTTC

                     *                    *      *   *                       
     482582 ATAGAGCAGTTTTGAAACACTCTTTTTGTAATATCTGCAAGAGGATATTTGGATAGCTTTGAGGA
          1 ATAGAGCAGGTTTGAAACACTCTTTTTGTAGTATCTGGAAGTGGATATTTGGATAGCTTTGAGGA

                         *   * *            **                      *    *   
     482647 TTTCGTTGGAAACGGGATTGTCTTCATATAAACTCTAGACAGAAGCATTCTCAGAAGCTTCATTG
         66 TTTCGTTGGAAACAGGAATATCTTCATATAAAAGCTAGACAGAAGCATTCTCAGAAACTTCTTTG

            *       **   *    *             * *      
     482712 GGATGTTTCAATTGAAGTCACAGTGTTGAACAGTTCCTTTC
        131 TGATGTTTGCATTCAAGTAACAGTGTTGAACATTCCCTTTC

                 *                                       *       **   * *    
     482753 ATAGAACAGGTTTGAAACACTCTTTTTGTAGTATCTGGAAGTGGACATTTGGAGCGCTCTCAGGA
          1 ATAGAGCAGGTTTGAAACACTCTTTTTGTAGTATCTGGAAGTGGATATTTGGATAGCTTTGAGGA

            * *  *  *   ** *         **          * *       * *          *  **
     482818 CTACGGTGAAAATGGAAATATCTTCCAATAAAAGCTACATAGAAGCAATGTCAGAAACTTTTTCA
         66 TTTCGTTGGAAACAGGAATATCTTCATATAAAAGCTAGACAGAAGCATTCTCAGAAACTTCTTTG

                  * ** *   **      *       *  *     *
     482883 TGATGTATCTACTCAGCTAACAGAGTTGAACCTTTCCTTTG
        131 TGATGTTTGCATTCAAGTAACAGTGTTGAACATTCCCTTTC

             *       *                   * *     *             **            
     482924 AGAGAGCAGTTTTGAAACACTCTTTTTGTGGAATCTGCAAGTGGATATTTGTCTAGCTTTGAGGA
          1 ATAGAGCAGGTTTGAAACACTCTTTTTGTAGTATCTGGAAGTGGATATTTGGATAGCTTTGAGGA

                         *   *    *                   *       *     *        
     482989 TTTCGTTGGAAACGGGATTA-CAT-ATA-AAAAGC-AGACAGCAGCATTCCCAGAATCTTCTTTG
         66 TTTCGTTGGAAACAGGAATATCTTCATATAAAAGCTAGACAGAAGCATTCTCAGAAACTTCTTTG

                              *    *                 
     483050 TGATGTTTGCATTCAAGTCACAGAGTTGAACATTCCCTTTC
        131 TGATGTTTGCATTCAAGTAACAGTGTTGAACATTCCCTTTC

                                                   *     *       **     *   *
     483091 ATAGAGCAGGTTTGAAACACTCTTTTTGTAGTATCTGGATGTGGACATTTGGAGCGCTTTCAGGC
          1 ATAGAGCAGGTTTGAAACACTCTTTTTGTAGTATCTGGAAGTGGATATTTGGATAGCTTTGAGGA

            * ** *      *             *** *   *      *               *   *   
     483156 CTATGGT-GAAAAAGGAAATATCTTCCCCTGAAAACTAGACGGAAGCATTCTCAGAATCTTATTT
         66 TTTCGTTGGAAACAGG-AATATCTTCATATAAAAGCTAGACAGAAGCATTCTCAGAAACTTCTTT

                   **  **    *             **  * *   *
     483220 GTGATGTGCGCCCTCAACTAACAGTGTTGAAGCTTTCTTTTG
        130 GTGATGTTTGCATTCAAGTAACAGTGTTGAACATTCCCTTTC

                     *                    **     *   *                       
     483262 ATAGAGCAGTTTTGAAACACTCTTTTTGTAAAATCTGCAAGAGGATATTTGGATAGCTTTGAGGA
          1 ATAGAGCAGGTTTGAAACACTCTTTTTGTAGTATCTGGAAGTGGATATTTGGATAGCTTTGAGGA

                         *   * *            **                      *    *   
     483327 TTTCGTTGGAAACGGGATTGTCTTCATATAAACTCTAGACAGAAGCATTCTCAGAAGCTTCATTG
         66 TTTCGTTGGAAACAGGAATATCTTCATATAAAAGCTAGACAGAAGCATTCTCAGAAACTTCTTTG

            *       **   *    *             *        
     483392 GGATGTTTCAATTGAAGTCACAGTGTTGAACAGTCCCTTTC
        131 TGATGTTTGCATTCAAGTAACAGTGTTGAACATTCCCTTTC

                                                         *       **   * *    
     483433 ATAGAGCAGGTTTGAAACACTCTTTTTGTAGTATCTGGAAGTGGACATTTGGAACGCTCTCAGGA
          1 ATAGAGCAGGTTTGAAACACTCTTTTTGTAGTATCTGGAAGTGGATATTTGGATAGCTTTGAGGA

            * *  *      *             **            *       * *          *  *
     483498 CTGCGGT-GAAAAAGGAAATATCTTCCAATAAAAGCTAGATAGAAGCAATGTCAGAAACTTTTTC
         66 TTTCGTTGGAAACAGG-AATATCTTCATATAAAAGCTAGACAGAAGCATTCTCAGAAACTTCTTT

            *      * ** *   **      *       *  * *   *
     483562 ATGATGTATCTACTCAGCTAACAGAGTTGAACCTTTCTTTTG
        130 GTGATGTTTGCATTCAAGTAACAGTGTTGAACATTCCCTTTC

             *       *                   * *     *             **   *        
     483604 AGAGAGCAGTTTTGAAACACTCTTTTTGTGGAATCTGCAAGTGGATATTTGTCTAGATTTGAGGA
          1 ATAGAGCAGGTTTGAAACACTCTTTTTGTAGTATCTGGAAGTGGATATTTGGATAGCTTTGAGGA

                         *   *    *                   *       *     *        
     483669 TTTCGTTGGAAACGGGATTA-CAT-ATA-AAAAGC-AGACAGCAGCATTCCCAGAATCTTCTTTG
         66 TTTCGTTGGAAACAGGAATATCTTCATATAAAAGCTAGACAGAAGCATTCTCAGAAACTTCTTTG

                              *    *                 
     483730 TGATGTTTGCATTCAAGTCACAGAGTTGAACATTCCCTTTC
        131 TGATGTTTGCATTCAAGTAACAGTGTTGAACATTCCCTTTC

                                 *                 *     *       **     *   *
     483771 ATAGAGCAGGTTTGAAACACTTTTTTTGTAGTATCTGGATGTGGACATTTGGAGCGCTTTCAGGC
          1 ATAGAGCAGGTTTGAAACACTCTTTTTGTAGTATCTGGAAGTGGATATTTGGATAGCTTTGAGGA

            * ** *      *             **          * * *     * * *        *  *
     483836 CTATGGT-GAAAAAGGAAATATCTTCCAATAAAAGCTACATAAAAGCAATGTGAGAAACTTTTTC
         66 TTTCGTTGGAAACAGG-AATATCTTCATATAAAAGCTAGACAGAAGCATTCTCAGAAACTTCTTT

            *      * ** *   **      **      *  * *   *
     483900 ATGATGTATCTACTCAGCTAACAGGCTTGAACCTTTCTTTTG
        130 GTGATGTTTGCATTCAAGTAACAGTGTTGAACATTCCCTTTC

             *       *                   * *                   **   *        
     483942 AGAGAGCAGTTTTGAAACACTCTTTTTGTGGAATCTGGAAGTGGATATTTGTCTAGTTTTGAGGA
          1 ATAGAGCAGGTTTGAAACACTCTTTTTGTAGTATCTGGAAGTGGATATTTGGATAGCTTTGAGGA

                         *   *    *                   *       *   *          
     484007 TTTCGTTGGAAACGGGATTA-CAT-ATA-AAAAGC-AGACAGCAGCATTCCCAGTAACTTCTTTG
         66 TTTCGTTGGAAACAGGAATATCTTCATATAAAAGCTAGACAGAAGCATTCTCAGAAACTTCTTTG

                              *    *                 
     484068 TGATGTTTGCATTCAAGTCACAGAGTTGAACATTCCCTTTC
        131 TGATGTTTGCATTCAAGTAACAGTGTTGAACATTCCCTTTC

                                                   *     *       **     *   *
     484109 ATAGAGCAGGTTTGAAACACTCTTTTTGTAGTATCTGGATGTGGACATTTGGAGCGCTTTCAGGC
          1 ATAGAGCAGGTTTGAAACACTCTTTTTGTAGTATCTGGAAGTGGATATTTGGATAGCTTTGAGGA

            * ** *      *             *** *   *                      *   *   
     484174 CTATGGT-GAAAAAGGAAATATCTTCCCCTGAAAACTAGACAGAAGCATTCTCAGAATCTTATTT
         66 TTTCGTTGGAAACAGG-AATATCTTCATATAAAAGCTAGACAGAAGCATTCTCAGAAACTTCTTT

                   *** **    *             **  * *   *
     484238 GTGATGTGCACCCTCAACTAACAGTGTTGAAGCTTTCTTTTG
        130 GTGATGTTTGCATTCAAGTAACAGTGTTGAACATTCCCTTTC

                     *                    **     *   *                       
     484280 ATAGAGCAGTTTTGAAACACTCTTTTTGTAAAATCTGCAAGAGGATATTTGGATAGCTTTGAGGA
          1 ATAGAGCAGGTTTGAAACACTCTTTTTGTAGTATCTGGAAGTGGATATTTGGATAGCTTTGAGGA

                         *   * *            **                      *    *   
     484345 TTTCGTTGGAAACGGGATTGTCTTCATATAAACTCTAGACAGAAGCATTCTCAGAAGCTTCATTG
         66 TTTCGTTGGAAACAGGAATATCTTCATATAAAAGCTAGACAGAAGCATTCTCAGAAACTTCTTTG

            *       **   *    *             *        
     484410 GGATGTTTCAATTGAAGTCACAGTGTTGAACAGTCCCTTTC
        131 TGATGTTTGCATTCAAGTAACAGTGTTGAACATTCCCTTTC

                                                         *       **   * *    
     484451 ATAGAGCAGGTTTGAAACACTCTTTTTGTAGTATCTGGAAGTGGACATTTGGAGCGCTCTCAGGA
          1 ATAGAGCAGGTTTGAAACACTCTTTTTGTAGTATCTGGAAGTGGATATTTGGATAGCTTTGAGGA

            * *  *      *             **            *       * *          *  *
     484516 CTGCGGT-GAAAAAGGAAATATCTTCCAATAAAAGCTAGATAGAAGCAATGTCAGAAACTTTTTC
         66 TTTCGTTGGAAACAGG-AATATCTTCATATAAAAGCTAGACAGAAGCATTCTCAGAAACTTCTTT

            *      * ** *   **      *       *    *   *
     484580 ATGATGTATCTACTCAGCTAACAGAGTTGAACCTT-CATTTG
        130 GTGATGTTTGCATTCAAGTAACAGTGTTGAACATTCCCTTTC

             *       *            *      * *     *             **            
     484621 AGAGAGCAGTTTTGAAACACTCGTTTTGTGGAATCTGCAAGTGGATATTTGTCTAGCTTTGAGGA
          1 ATAGAGCAGGTTTGAAACACTCTTTTTGTAGTATCTGGAAGTGGATATTTGGATAGCTTTGAGGA

                         *   *    *                   *       *              
     484686 TTTCGTTGGAAACGGGATTA-CAT-ATA-AAAAGC-AGACAGCAGCATTCCCAGAAACTTCTTTG
         66 TTTCGTTGGAAACAGGAATATCTTCATATAAAAGCTAGACAGAAGCATTCTCAGAAACTTCTTTG

                              *    *                 
     484747 TGATGTTTGCATTCAAGTCACAGAGTTGAACATTCCCTTTC
        131 TGATGTTTGCATTCAAGTAACAGTGTTGAACATTCCCTTTC

                                                   *   * *     * **     *   *
     484788 ATAGAGCAGGTTTGAAACACTCTTTTTGTAGTATCTGGATGTGTACATTTGCAGCGCTTTCAGGC
          1 ATAGAGCAGGTTTGAAACACTCTTTTTGTAGTATCTGGAAGTGGATATTTGGATAGCTTTGAGGA

            * ** *      *             *** *   *                          *   
     484853 CTAAGGT-GAAAAAGGAAATATCTTCCCCTGAAAACTAGACAGAAGCATTCTCAGAAACTTATTT
         66 TTTCGTTGGAAACAGG-AATATCTTCATATAAAAGCTAGACAGAAGCATTCTCAGAAACTTCTTT

                   **  **    *             **  * *   *
     484917 GTGATGTGCGCCCTCAACTAACAGTGTTGAAGCTTTCTTTTG
        130 GTGATGTTTGCATTCAAGTAACAGTGTTGAACATTCCCTTTC

                     *                    *      *   *                       
     484959 ATAGAGCAGTTTTGAAACACTCTTTTTGTAATATCTGCAAGAGGATATTTGGATAGCTTTGAGGA
          1 ATAGAGCAGGTTTGAAACACTCTTTTTGTAGTATCTGGAAGTGGATATTTGGATAGCTTTGAGGA

                         *  ** *            **                      *    *   
     485024 TTTCGTTGGAAACGGGTTTGTCTTCATATAAACTCTAGACAGAAGCATTCTCAGAAGCTTCATTG
         66 TTTCGTTGGAAACAGGAATATCTTCATATAAAAGCTAGACAGAAGCATTCTCAGAAACTTCTTTG

            *       **   *    *             * *      
     485089 GGATGTTTCAATTGAAGTCACAGTGTTGAACAGTTCCTTTC
        131 TGATGTTTGCATTCAAGTAACAGTGTTGAACATTCCCTTTC

                 *                                       *       **   * *    
     485130 ATAGAACAGGTTTGAAACACTCTTTTTGTAGTATCTGGAAGTGGACATTTGGAGCGCTCTCAGGA
          1 ATAGAGCAGGTTTGAAACACTCTTTTTGTAGTATCTGGAAGTGGATATTTGGATAGCTTTGAGGA

            * ** *      *             **          * *       * *          *  *
     485195 CTATGGT-GAAAAAGGAAATATCTTCCAATAAAAGCTACATAGAAGCAATGTCAGAAACTTTTTC
         66 TTTCGTTGGAAACAGG-AATATCTTCATATAAAAGCTAGACAGAAGCATTCTCAGAAACTTCTTT

            *      * ** *   **      *       *  *     *
     485259 ATGATGTATCTACTCAGCTAACAGAGTTGAACCTTTCCTTTG
        130 GTGATGTTTGCATTCAAGTAACAGTGTTGAACATTCCCTTTC

             *       *                   * *     *             **            
     485301 AGAGAGCAGTTTTGAAACACTCTTTTTGTGGAATCTGCAAGTGGATATTTGTCTAGCTTTGAGGA
          1 ATAGAGCAGGTTTGAAACACTCTTTTTGTAGTATCTGGAAGTGGATATTTGGATAGCTTTGAGGA

                         *   *    *                   *       *   *          
     485366 TTTCGTTGGAAACGGGATTA-CAT-ATA-AAAAGC-AGACAGCAGCATTCCCAGTAACTTCTTTG
         66 TTTCGTTGGAAACAGGAATATCTTCATATAAAAGCTAGACAGAAGCATTCTCAGAAACTTCTTTG

                              *    * *    *          
     485427 TGATGTTTGCATTCAAGTCACAGAGATGAATATTCCCTTTC
        131 TGATGTTTGCATTCAAGTAACAGTGTTGAACATTCCCTTTC

                                                   *     *       **     *   *
     485468 ATAGAGCAGGTTTGAAACACTCTTTTTGTAGTATCTGGATGTGGACATTTGGAGCGCTTTCAGGC
          1 ATAGAGCAGGTTTGAAACACTCTTTTTGTAGTATCTGGAAGTGGATATTTGGATAGCTTTGAGGA

            * ** *      *             *** *   *                          *   
     485533 CTATGGT-GAAAAAGGAAATATCTTCCCCTGAAAACTAGACAGAAGCATTCTCAGAAACTTATTT
         66 TTTCGTTGGAAACAGG-AATATCTTCATATAAAAGCTAGACAGAAGCATTCTCAGAAACTTCTTT

                   **  **    *              *  * *   *
     485597 GTGATGTGCGCCCTCAACTAACAGTGTTGAACCTTTCTTTTG
        130 GTGATGTTTGCATTCAAGTAACAGTGTTGAACATTCCCTTTC

                     *                    **     *   *                       
     485639 ATAGAGCAGTTTTGAAACACTCTTTTTGTAAAATCTGCAAGAGGATATTTGGATAGCTTTGAGGA
          1 ATAGAGCAGGTTTGAAACACTCTTTTTGTAGTATCTGGAAGTGGATATTTGGATAGCTTTGAGGA

                         *   * *            **                      *    *   
     485704 TTTCGTTGGAAACGGGATTGTCTTCATATAAACTCTAGACAGAAGCATTCTCAGAAGCTTCATTG
         66 TTTCGTTGGAAACAGGAATATCTTCATATAAAAGCTAGACAGAAGCATTCTCAGAAACTTCTTTG

            *       **   *    *             *        
     485769 GGATGTTTCAATTGAAGTCACAGTGTTGAACAGTCCCTTTC
        131 TGATGTTTGCATTCAAGTAACAGTGTTGAACATTCCCTTTC

                                                   *     *       **     *    
     485810 ATAGAGCAGGTTTGAAACACTCTTTTTGTAGTATCTGGATGTGGACATTTGGAGCGCTTTCAGGC
          1 ATAGAGCAGGTTTGAAACACTCTTTTTGTAGTATCTGGAAGTGGATATTTGGATAGCTTTGAGG-

            * * *    *                 *** *   *                          *  
     485875 CTATGGTT-TAAA-AGGAAATATCTTCCCCTGAAAACTAGACAGAAGCATTCTCAGAAACTTATT
         65 ATTTCGTTGGAAACAGG-AATATCTTCATATAAAAGCTAGACAGAAGCATTCTCAGAAACTTCTT

                    **  **    *                    *   *
     485938 TGTGATGTGCGCCCTCAACTAACAGTGTTGAAGCATT-CTTTTG
        129 TGTGATGTTTGCATTCAAGTAACAGTGTTGAA-CATTCCCTTTC

                     *                   * *     *             **            
     485981 ATAGAGCAGTTTTGAAACACTCTTTTTGTGGAATCTGCAAGTGGATATTTGTCTAGCTTTGAGGA
          1 ATAGAGCAGGTTTGAAACACTCTTTTTGTAGTATCTGGAAGTGGATATTTGGATAGCTTTGAGGA

                         *   *   *                    *       *     *   *    
     486046 TTTCGTTGGAAACGGGATTA-ATT-ATA-AAAAGC-AGACAGCAGCATTCCCAGAATCTTGTTTG
         66 TTTCGTTGGAAACAGGAATATCTTCATATAAAAGCTAGACAGAAGCATTCTCAGAAACTTCTTTG

                              *    *                 
     486107 TGATGTTTGCATTCAAGTCACAGAGTTGAACATTCCCTTTC
        131 TGATGTTTGCATTCAAGTAACAGTGTTGAACATTCCCTTTC

             *                         *           *     *       **     *   *
     486148 AGAGAGCAGGTTTGAAACACTCTTTTTATAGTATCTGGATGTGGACATTTGGAGCGCTTTCAGGC
          1 ATAGAGCAGGTTTGAAACACTCTTTTTGTAGTATCTGGAAGTGGATATTTGGATAGCTTTGAGGA

            * ** *      *             *** *   *                      *   *   
     486213 CTATGAT-GAAAAAGGAAATATCTTCTCCTGAAAACTAGACAGAAGCATTCTCAGAATCTTATTT
         66 TTTCGTTGGAAACAGG-AATATCTTCATATAAAAGCTAGACAGAAGCATTCTCAGAAACTTCTTT

                   **  **    *             **  * *   *
     486277 GTGATGTGCGCCCTCAACTAACAGTGTTGAAGCTTTCTTTTG
        130 GTGATGTTTGCATTCAAGTAACAGTGTTGAACATTCCCTTTC

                     *                *   **     *   *                       
     486319 ATAGAGCAGTTTTGAAACACTCTTTTCGTAAAATCTGCAAGAGGATATTTGGATAGCTTTGAGGA
          1 ATAGAGCAGGTTTGAAACACTCTTTTTGTAGTATCTGGAAGTGGATATTTGGATAGCTTTGAGGA

                         *   * *            **                      *    *   
     486384 TTTCGTTGGAAACGGGATTGTCTTCATATAAACTCTAGACAGAAGCATTCTCAGAAGCTTCATTG
         66 TTTCGTTGGAAACAGGAATATCTTCATATAAAAGCTAGACAGAAGCATTCTCAGAAACTTCTTTG

            *       **   *    *             *        
     486449 GGATGTTTCAATTGAAGTCACAGTGTTGAACAGTCCCTTTC
        131 TGATGTTTGCATTCAAGTAACAGTGTTGAACATTCCCTTTC

                                                         *       *  * * *    
     486490 ATAGAGCAGGTTTGAAACACTCTTTTTGTAGTATCTGGAAGTGGACATTTGGAGAGATCTCAGGA
          1 ATAGAGCAGGTTTGAAACACTCTTTTTGTAGTATCTGGAAGTGGATATTTGGATAGCTTTGAGGA

            * *  *    * *             **            *       ***          *  *
     486555 ATACGGT-GATAAAGGTAATATCTTCCAATAAAAGCTAGATAGAAGCAAAGTCAGAAACTTTTTC
         66 TTTCGTTGGAAACAGG-AATATCTTCATATAAAAGCTAGACAGAAGCATTCTCAGAAACTTCTTT

            *      * ** *   **      *       *  * *   *
     486619 ATGATGTATCTACTCAGCTAACAGAGTTGAACCTTTCTTTTG
        130 GTGATGTTTGCATTCAAGTAACAGTGTTGAACATTCCCTTTC

             *       *                   * *     *             **            
     486661 AGAGAGCAGTTTTGAAACACTCTTTTTGTGGAATCTGCAAGTGGATATTTGTCTAGCTTTGAGGA
          1 ATAGAGCAGGTTTGAAACACTCTTTTTGTAGTATCTGGAAGTGGATATTTGGATAGCTTTGAGGA

                         *   *    *                   *       *   *          
     486726 TTTCGTTGGAAACGGGATTA-CAT-ATA-AAAAGC-AGACAGCAGCATTCCCAGTAACTTCTTTG
         66 TTTCGTTGGAAACAGGAATATCTTCATATAAAAGCTAGACAGAAGCATTCTCAGAAACTTCTTTG

                              *    *                 
     486787 TGATGTTTGCATTCAAGTCACAGAGTTGAACATTCCCTTTC
        131 TGATGTTTGCATTCAAGTAACAGTGTTGAACATTCCCTTTC

                                 *                 *     *       **     *   *
     486828 ATAGAGCAGGTTTGAAACACTTTTTTTGTAGTATCTGGATGTGGACATTTGGAGCGCTTTCAGGC
          1 ATAGAGCAGGTTTGAAACACTCTTTTTGTAGTATCTGGAAGTGGATATTTGGATAGCTTTGAGGA

            * ** *      *             **            *       * *          *  *
     486893 CTATGGT-GAAAAAGGAAATATCTTCCAATAAAAGCTAGATAGAAGCAATGTCAGAAACTTTTTC
         66 TTTCGTTGGAAACAGG-AATATCTTCATATAAAAGCTAGACAGAAGCATTCTCAGAAACTTCTTT

            *      * ** *   **      *       *  * *   *
     486957 ATGATGTATCTACTCAGCTAACAGAGTTGAACCTTTCTTTTG
        130 GTGATGTTTGCATTCAAGTAACAGTGTTGAACATTCCCTTTC

             *       *                    **     *   *                       
     486999 AGAGAGCAGTTTTGAAACACTCTTTTTGTAAAATCTGCAAGAGGATATTTGGATAGCTTTGAGGA
          1 ATAGAGCAGGTTTGAAACACTCTTTTTGTAGTATCTGGAAGTGGATATTTGGATAGCTTTGAGGA

                         *   * *            **                      * *  *   
     487064 TTTCGTTGGAAACGGGATTGTCTTCATATAAACTCTAGACAGAAGCATTCTCAGAAGCGTCATTG
         66 TTTCGTTGGAAACAGGAATATCTTCATATAAAAGCTAGACAGAAGCATTCTCAGAAACTTCTTTG

            *       **   *    *             *        
     487129 GGATGTTTCAATTGAAGTCACAGTGTTGAACAGTCCCTTTC
        131 TGATGTTTGCATTCAAGTAACAGTGTTGAACATTCCCTTTC

                                                   *     *       **     *    
     487170 ATAGAGCAGGTTTGAAACACTCTTTTTGTAGTATCTGGATGTGGACATTTGGAGCGCTTTCAGGC
          1 ATAGAGCAGGTTTGAAACACTCTTTTTGTAGTATCTGGAAGTGGATATTTGGATAGCTTTGAGG-

            * * *    *                 *** *   *                          *  
     487235 CTATGGTT-TAAA-AGGAAATATCTTCCCCTGAAAACTAGACAGAAGCATTCTCAGAAACTTATT
         65 ATTTCGTTGGAAACAGG-AATATCTTCATATAAAAGCTAGACAGAAGCATTCTCAGAAACTTCTT

                    **  *     *                    *   *
     487298 TGTGATGTGCGCCTTCAACTAACAGTGTTGAAGCATT-CTTTTG
        129 TGTGATGTTTGCATTCAAGTAACAGTGTTGAA-CATTCCCTTTC

                     *                   * *     *             **            
     487341 ATAGAGCAGTTTTGAAACACTCTTTTTGTGGAATCTGCAAGTGGATATTTGTCTAGCTTTGAGGA
          1 ATAGAGCAGGTTTGAAACACTCTTTTTGTAGTATCTGGAAGTGGATATTTGGATAGCTTTGAGGA

                         *   *    *                   *       *              
     487406 TTTCGTTGGAAACGGGATTA-CAT-ATA-AAAAGC-AGACAGCAGCATTCCCAGAAACTTCTTTG
         66 TTTCGTTGGAAACAGGAATATCTTCATATAAAAGCTAGACAGAAGCATTCTCAGAAACTTCTTTG

                              *    *                 
     487467 TGATGTTTGCATTCAAGTCACAGAGTTGAACATTCCCTTTC
        131 TGATGTTTGCATTCAAGTAACAGTGTTGAACATTCCCTTTC

             *                                     *     *       **     *  **
     487508 AGAGAGCAGGTTTGAAACACTCTTTTTGTAGTATCTGGATGTGGACATTTGGAGCGCTTTCAGCC
          1 ATAGAGCAGGTTTGAAACACTCTTTTTGTAGTATCTGGAAGTGGATATTTGGATAGCTTTGAGGA

            * ** *      *             *** *   *                      *   *   
     487573 CTATGGT-GAAAAAGGAAATATCTTCCCCTGAAAACTAGACAGAAGCATTCTCAGAATCTTATTT
         66 TTTCGTTGGAAACAGG-AATATCTTCATATAAAAGCTAGACAGAAGCATTCTCAGAAACTTCTTT

                   **  **    *             **  * *   *
     487637 GTGATGTGCGCCCTCAACTAACAGTGTTGAAGCTTTCTTTTG
        130 GTGATGTTTGCATTCAAGTAACAGTGTTGAACATTCCCTTTC

                     *                    **     *   *                       
     487679 ATAGAGCAGTTTTGAAACACTCTTTTTGTAAAATCTGCAAGAGGATATTTGGATAGCTTTGAGGA
          1 ATAGAGCAGGTTTGAAACACTCTTTTTGTAGTATCTGGAAGTGGATATTTGGATAGCTTTGAGGA

                         *   * *            **                      *    *   
     487744 TTTCGTTGGAAACGGGATTGTCTTCATATAAACTCTAGACAGAAGCATTCTCAGAAGCTTCATTG
         66 TTTCGTTGGAAACAGGAATATCTTCATATAAAAGCTAGACAGAAGCATTCTCAGAAACTTCTTTG

            *       **   *    *             *        
     487809 GGATGTTTCAATTGAAGTCACAGTGTTGAACAGTCCCTTTC
        131 TGATGTTTGCATTCAAGTAACAGTGTTGAACATTCCCTTTC

                                                         *       **   * *    
     487850 ATAGAGCAGGTTTGAAACACTCTTTTTGTAGTATCTGGAAGTGGACATTTGGAGCGCTCTCAGGA
          1 ATAGAGCAGGTTTGAAACACTCTTTTTGTAGTATCTGGAAGTGGATATTTGGATAGCTTTGAGGA

            * *  *      *             **            *       * *          *  *
     487915 CTGCGGT-GAAAAAGGAACTATCTTCCAATAAAAGCTAGATAGAAGCAATGTCAGAAACTTTTTC
         66 TTTCGTTGGAAACAGGAA-TATCTTCATATAAAAGCTAGACAGAAGCATTCTCAGAAACTTCTTT

            *      * ** *   **      *       *  *     *
     487979 ATGATGTATCTACTCAGCTAACAGAGTTGAACCTTTCCTTTG
        130 GTGATGTTTGCATTCAAGTAACAGTGTTGAACATTCCCTTTC

             *       *                   * *     *             **            
     488021 AGAGAGCAGTTTTGAAACACTCTTTTTGTGGAATCTGCAAGTGGATATTTGTCTAGCTTTGAGGA
          1 ATAGAGCAGGTTTGAAACACTCTTTTTGTAGTATCTGGAAGTGGATATTTGGATAGCTTTGAGGA

                         *   *    *                   *       *   *          
     488086 TTTCGTTGGAAACGGGATTA-CAT-ATA-AAAAGC-AGACAGCAGCATTCCCAGTAACTTCTTTG
         66 TTTCGTTGGAAACAGGAATATCTTCATATAAAAGCTAGACAGAAGCATTCTCAGAAACTTCTTTG

                              *    *                 
     488147 TGATGTTTGCATTCAAGTCACAGAGTTGAACATTCCCTTTC
        131 TGATGTTTGCATTCAAGTAACAGTGTTGAACATTCCCTTTC

                                                   *     *       **     *   *
     488188 ATAGAGCAGGTTTGAAACACTCTTTTTGTAGTATCTGGATGTGGACATTTGGAGCGCTTTCAGGC
          1 ATAGAGCAGGTTTGAAACACTCTTTTTGTAGTATCTGGAAGTGGATATTTGGATAGCTTTGAGGA

            * ** *      *             *** *   *                          *   
     488253 CTATGGT-GAAAAAGGAAATATCTTCCCCTGAAAACTAGACAGAAGCATTCTCAGAAACTTATTT
         66 TTTCGTTGGAAACAGG-AATATCTTCATATAAAAGCTAGACAGAAGCATTCTCAGAAACTTCTTT

                   **  **    *              *  * *   *
     488317 GTGATGTGCGCCCTCAACTAACAGTGTTGAACCTTTCTTTTG
        130 GTGATGTTTGCATTCAAGTAACAGTGTTGAACATTCCCTTTC

                     *                    *      *   *                       
     488359 ATAGAGCAGTTTTGAAACACTCTTTTTGTAATATCTGCAAGAGGATATTTGGATAGCTTTGAGGA
          1 ATAGAGCAGGTTTGAAACACTCTTTTTGTAGTATCTGGAAGTGGATATTTGGATAGCTTTGAGGA

                         *   *    *                   *       *     *   *    
     488424 TTTCGTTGGAAACGGGATTA-CAT-ATA-AAAAGC-AGACAGCAGCATTCCCAGAATCTTGTTTG
         66 TTTCGTTGGAAACAGGAATATCTTCATATAAAAGCTAGACAGAAGCATTCTCAGAAACTTCTTTG

                              *    *                 
     488485 TGATGTTTGCATTCAAGTCACAGAGTTGAACATTCCCTTTC
        131 TGATGTTTGCATTCAAGTAACAGTGTTGAACATTCCCTTTC

             *                         *    *      *     *       **     *   *
     488526 AGAGAGCAGGTTTGAAACACTCTTTTTATAGTGTCTGGATGTGGACATTTGGAGCGCTTTCAGGC
          1 ATAGAGCAGGTTTGAAACACTCTTTTTGTAGTATCTGGAAGTGGATATTTGGATAGCTTTGAGGA

            * ** *      *             *** **  *                          *   
     488591 CTATGGT-GAAAAAGGAAATATCTTCTCCTGTAAACTAGACAGAAGCATTCTCAGAAACTTATTT
         66 TTTCGTTGGAAACAGG-AATATCTTCATATAAAAGCTAGACAGAAGCATTCTCAGAAACTTCTTT

                   **  **    *             **  * *   *
     488655 GTGATGTGCGCCCTCAACTAACAGTGTTGAAGCTTTCTTTTG
        130 GTGATGTTTGCATTCAAGTAACAGTGTTGAACATTCCCTTTC

                     *                    **     *   *                       
     488697 ATAGAGCAGTTTTGAAACACTCTTTTTGTAAAATCTGCAAGAGGATATTTGGATAGCTTTGAGGA
          1 ATAGAGCAGGTTTGAAACACTCTTTTTGTAGTATCTGGAAGTGGATATTTGGATAGCTTTGAGGA

                         *   * *            **                      *    *   
     488762 TTTCGTTGGAAACGGGATTGTCTTCATATAAACTCTAGACAGAAGCATTCTCAGAAGCTTCATTG
         66 TTTCGTTGGAAACAGGAATATCTTCATATAAAAGCTAGACAGAAGCATTCTCAGAAACTTCTTTG

            *       **   *    *             *        
     488827 GGATGTTTCAATTGAAGTCACAGTGTTGAACAGTCCCTTTC
        131 TGATGTTTGCATTCAAGTAACAGTGTTGAACATTCCCTTTC

                                                         *       **   * *    
     488868 ATAGAGCAGGTTTGAAACACTCTTTTTGTAGTATCTGGAAGTGGACATTTGGAACGCTCTCAGGA
          1 ATAGAGCAGGTTTGAAACACTCTTTTTGTAGTATCTGGAAGTGGATATTTGGATAGCTTTGAGGA

            * *  *      *             **          * *       * *          *  *
     488933 CTGCGGT-GAAAAAGGAAATATCTTCCAATAAAAGCTACATAGAAGCAATGTCAGAAACTTTTTC
         66 TTTCGTTGGAAACAGG-AATATCTTCATATAAAAGCTAGACAGAAGCATTCTCAGAAACTTCTTT

            *      * ** *   **      *       *  * *   *
     488997 ATGATGTATCTACTCAGCTAACAGAGTTGAACCTTTCTTTTG
        130 GTGATGTTTGCATTCAAGTAACAGTGTTGAACATTCCCTTTC

             *       *                   * *     *           * **            
     489039 AGAGAGCAGTTTTGAAACACTCTTTTTGTGGAATCTGCAAGTGGATATTAGTCTAGCTTTGAGGA
          1 ATAGAGCAGGTTTGAAACACTCTTTTTGTAGTATCTGGAAGTGGATATTTGGATAGCTTTGAGGA

                         *   *    *                   *       *   *          
     489104 TTTCGTTGGAAACGGGATTA-CAT-ATA-AAAAGC-AGACAGCAGCATTCCCAGTAACTTCTTTG
         66 TTTCGTTGGAAACAGGAATATCTTCATATAAAAGCTAGACAGAAGCATTCTCAGAAACTTCTTTG

                              *    *                 
     489165 TGATGTTTGCATTCAAGTCACAGAGTTGAACATTCCCTTTC
        131 TGATGTTTGCATTCAAGTAACAGTGTTGAACATTCCCTTTC

                                 *  *              *             **     *   *
     489206 ATAGAGCAGGTTTGAAACACTTTTCTTGTAGTATCTGGATGTGGATATTTGGAGCGCTTTCAGGC
          1 ATAGAGCAGGTTTGAAACACTCTTTTTGTAGTATCTGGAAGTGGATATTTGGATAGCTTTGAGGA

            * ** *      *             **          * *       * *          *  *
     489271 CTATGGT-GAAAAAGGAAATATCTTCCAATAAAAGCTACATAGAAGCAATGTCAGAAACTTTTTC
         66 TTTCGTTGGAAACAGG-AATATCTTCATATAAAAGCTAGACAGAAGCATTCTCAGAAACTTCTTT

            *      * ** *   **      *       *  * *   *
     489335 ATGATGTATCTACTCAGCTAACAGAGTTGAACCTTTCTTTTG
        130 GTGATGTTTGCATTCAAGTAACAGTGTTGAACATTCCCTTTC

             *       *                            *             **           
     489377 AGAGAGCAGTTTTGAAACACTCTTTTTGT-GTAATCTGAAAGTGGATATTTGTCTAGCTTTGAGG
          1 ATAGAGCAGGTTTGAAACACTCTTTTTGTAGT-ATCTGGAAGTGGATATTTGGATAGCTTTGAGG

                          *   *    *                   *       *   *         
     489441 ATTTCGTTGGAAACGGGATTA-CAT-ATA-AAAAGC-AGACAGCAGCATTCCCAGTAACTTCTTT
         65 ATTTCGTTGGAAACAGGAATATCTTCATATAAAAGCTAGACAGAAGCATTCTCAGAAACTTCTTT

                               *    *                 
     489502 GTGATGTTTGCATTCAAGTCACAGAGTTGAACATTCCCTTTC
        130 GTGATGTTTGCATTCAAGTAACAGTGTTGAACATTCCCTTTC

                                                   *     *       **     *    
     489544 ATAGAGCAGGTTTGAAACACTCTTTTTGTAGTATCTGGATGTGGACATTTGGAGCGCTTTCAGG-
          1 ATAGAGCAGGTTTGAAACACTCTTTTTGTAGTATCTGGAAGTGGATATTTGGATAGCTTTGAGGA

            **  *        *             *** *   *           *          *   *  
     489608 CCT-ATTGTGAAAAAGGAAATATCTTCCCCTGAAAACTAGACAGAAGAATTCTCAGAATCTTATT
         66 TTTCGTTG-GAAACAGG-AATATCTTCATATAAAAGCTAGACAGAAGCATTCTCAGAAACTTCTT

                    **  **    *             **  * *   *
     489672 TGTGATGTGCGCCCTCAACTAACAGTGTTGAAGCTTTCTTTTG
        129 TGTGATGTTTGCATTCAAGTAACAGTGTTGAACATTCCCTTTC

             *       *                    **     *   *                       
     489715 AGAGAGCAGTTTTGAAACACTCTTTTTGTAAAATCTGCAAGAGGATATTTGGATAGCTTTGAGGA
          1 ATAGAGCAGGTTTGAAACACTCTTTTTGTAGTATCTGGAAGTGGATATTTGGATAGCTTTGAGGA

                         *   * *        *   **   *                  * *  *   
     489780 TTTCGTTGGAAACGGGATTGTCTTCATAGAAACTCTACACAGAAGCATTCTCAGAAGCGTCATTG
         66 TTTCGTTGGAAACAGGAATATCTTCATATAAAAGCTAGACAGAAGCATTCTCAGAAACTTCTTTG

            *       **   *    *             *        
     489845 GGATGTTTCAATTGAAGTCACAGTGTTGAACAGTCCCTTTC
        131 TGATGTTTGCATTCAAGTAACAGTGTTGAACATTCCCTTTC

                                                         *       **   * *    
     489886 ATAGAGCAGGTTTGAAACACTCTTTTTGTAGTATCTGGAAGTGGACATTTGGAACGCTCTCAGGA
          1 ATAGAGCAGGTTTGAAACACTCTTTTTGTAGTATCTGGAAGTGGATATTTGGATAGCTTTGAGGA

            * *  *      *             **            *       * *          *  *
     489951 CTGCGGT-GAAAAAGGAAATATCTTCCAATAAAAGCTAGATAGAAGCAATGTCAGAAACTTTTTC
         66 TTTCGTTGGAAACAGG-AATATCTTCATATAAAAGCTAGACAGAAGCATTCTCAGAAACTTCTTT

            *      * ** *   **      *       *  * *   *
     490015 ATGATGTATCTACTCAGCTAACAGAGTTGAACCTTTCTTTTG
        130 GTGATGTTTGCATTCAAGTAACAGTGTTGAACATTCCCTTTC

             *       *            *      * *     *             **  *         
     490057 AGAGAGCAGTTTTGAAACACTCGTTTTGTGGAATCTGCAAGTGGATATTTGTCTACCTTTGAGGA
          1 ATAGAGCAGGTTTGAAACACTCTTTTTGTAGTATCTGGAAGTGGATATTTGGATAGCTTTGAGGA

                         *   *    *                   *       *              
     490122 TTTCGTTGGAAACGGGATTA-CAT-ATA-AAAAGC-AGACAGCAGCATTCCCAGAAACTTCTTTG
         66 TTTCGTTGGAAACAGGAATATCTTCATATAAAAGCTAGACAGAAGCATTCTCAGAAACTTCTTTG

                              *    *                 
     490183 TGATGTTTGCATTCAAGTCACAGAGTTGAACATTCCCTTTC
        131 TGATGTTTGCATTCAAGTAACAGTGTTGAACATTCCCTTTC

                                                   *     *     * **     *   *
     490224 ATAGAGCAGGTTTGAAACACTCTTTTTGTAGTATCTGGATGTGGACATTTGCAGCGCTTTCAGGC
          1 ATAGAGCAGGTTTGAAACACTCTTTTTGTAGTATCTGGAAGTGGATATTTGGATAGCTTTGAGGA

            * ** *      *             *** *   *                          *   
     490289 CTAAGGT-GAAAAAGGAAATATCTTCCCCTGAAAACTAGACAGAAGCATTCTCAGAAACTTATTT
         66 TTTCGTTGGAAACAGG-AATATCTTCATATAAAAGCTAGACAGAAGCATTCTCAGAAACTTCTTT

                   **  **    *             **  * *   *
     490353 GTGATGTGCGCCCTCAACTAACAGTGTTGAAGCTTTCTTTTG
        130 GTGATGTTTGCATTCAAGTAACAGTGTTGAACATTCCCTTTC

                     *                    *      *   *                       
     490395 ATAGAGCAGTTTTGAAACACTCTTTTTGTAATATCTGCAAGAGGATATTTGGATAGCTTTGAGGA
          1 ATAGAGCAGGTTTGAAACACTCTTTTTGTAGTATCTGGAAGTGGATATTTGGATAGCTTTGAGGA

                         *   * *            **                      *    *   
     490460 TTTCGTTGGAAACGGGATTGTCTTCATATAAACTCTAGACAGAAGCATTCTCAGAAGCTTCATTG
         66 TTTCGTTGGAAACAGGAATATCTTCATATAAAAGCTAGACAGAAGCATTCTCAGAAACTTCTTTG

            *       **   *    *             *        
     490525 GGATGTTTCAATTGAAGTCACAGTGTTGAACAGTCCCTTTC
        131 TGATGTTTGCATTCAAGTAACAGTGTTGAACATTCCCTTTC

                                                         *       **   * *    
     490566 ATAGAGCAGGTTTGAAACACTCTTTTTGTAGTATCTGGAAGTGGACATTTGGAACGCTCTCAGGA
          1 ATAGAGCAGGTTTGAAACACTCTTTTTGTAGTATCTGGAAGTGGATATTTGGATAGCTTTGAGGA

            * *  *      *             **            *       * *          *  *
     490631 CTGCGGT-GAAAAAGGAAATATCTTCCAATAAAAGCTAGATAGAAGCAATGTCAGAAACTTTTTC
         66 TTTCGTTGGAAACAGG-AATATCTTCATATAAAAGCTAGACAGAAGCATTCTCAGAAACTTCTTT

            *      * ** *   **      *       *  * *   *
     490695 ATGATGTATCTACTCAGCTAACAGAGTTGAACCTTTCTTTTG
        130 GTGATGTTTGCATTCAAGTAACAGTGTTGAACATTCCCTTTC

             *       *                   * *     *             **            
     490737 AGAGAGCAGTTTTGAAACACTCTTTTTGTGGAATCTGCAAGTGGATATTTGTCTAGCTTTGAGGA
          1 ATAGAGCAGGTTTGAAACACTCTTTTTGTAGTATCTGGAAGTGGATATTTGGATAGCTTTGAGGA

                         *   *    *                   *       *   *          
     490802 TTTCGTTGGAAACGGGATTA-CAT-ATA-AAAAGC-AGACAGCAGCATTCCCAGTAACTTCTTTG
         66 TTTCGTTGGAAACAGGAATATCTTCATATAAAAGCTAGACAGAAGCATTCTCAGAAACTTCTTTG

                              *    *                 
     490863 TGATGTTTGCATTCAAGTCACAGAGTTGAACATTCCCTTTC
        131 TGATGTTTGCATTCAAGTAACAGTGTTGAACATTCCCTTTC

                                 *                 *     *       **     *   *
     490904 ATAGAGCAGGTTTGAAACACTTTTTTTGTAGTATCTGGATGTGGACATTTGGAGCGCTTTCAGGC
          1 ATAGAGCAGGTTTGAAACACTCTTTTTGTAGTATCTGGAAGTGGATATTTGGATAGCTTTGAGGA

            * ** *      *             **          * * *     * *          *  *
     490969 CTATGGT-GAAAAAGGAAATATCTTCCAATAAAAGCTACATAAAAGCAATGTCAGAAACTTTTTC
         66 TTTCGTTGGAAACAGG-AATATCTTCATATAAAAGCTAGACAGAAGCATTCTCAGAAACTTCTTT

            *      * ** *   **      *       *  *     *
     491033 ATGATGTATCTACTCAGCTAACAGAGTTGAACCTTTCCTTTG
        130 GTGATGTTTGCATTCAAGTAACAGTGTTGAACATTCCCTTTC

             *       *                   * *     * *         * **            
     491075 AGAGAGCAGTTTTGAAACACTCTTTTTGTGGAATCTGCAGGTGGATATTGGTCTAGCTTTGAGGA
          1 ATAGAGCAGGTTTGAAACACTCTTTTTGTAGTATCTGGAAGTGGATATTTGGATAGCTTTGAGGA

                         *   *    *                   *       *   *          
     491140 TTTCGTTGGAAACGGGATTA-CAT-ATA-AAAAGC-AGACAGCAGCATTCCCAGTAACTTCTTTG
         66 TTTCGTTGGAAACAGGAATATCTTCATATAAAAGCTAGACAGAAGCATTCTCAGAAACTTCTTTG

                              *    *          *      
     491201 TGATGTTTGCATTCAAGTCACAGAGTTGAACATTGCCTTTC
        131 TGATGTTTGCATTCAAGTAACAGTGTTGAACATTCCCTTTC

                         *                         *     *       **     *   *
     491242 ATAGAGCAGGTTTCAAACACTCTTTTTGTAGTATCTGGATGTGGACATTTGGAGCGCTTTCAGGC
          1 ATAGAGCAGGTTTGAAACACTCTTTTTGTAGTATCTGGAAGTGGATATTTGGATAGCTTTGAGGA

            * ** *      *             *** *   *                      *   *   
     491307 CTATGGT-GAAAAAGGAAATATCTTCCCCTGAAAACTAGACAGAAGCATTCTCAGAATCTTATTT
         66 TTTCGTTGGAAACAGG-AATATCTTCATATAAAAGCTAGACAGAAGCATTCTCAGAAACTTCTTT

                   **  **    *             **  * *   *
     491371 GTGATGTGCGCCCTCAACTAACAGTGTTGAAGCTTTCTTTTG
        130 GTGATGTTTGCATTCAAGTAACAGTGTTGAACATTCCCTTTC

                     *                    **     *   *                       
     491413 ATAGAGCAGTTTTGAAACACTCTTTTTGTAAAATCTGCAAGAGGATATTTGGATAGCTTTGAGGA
          1 ATAGAGCAGGTTTGAAACACTCTTTTTGTAGTATCTGGAAGTGGATATTTGGATAGCTTTGAGGA

                         *   * *            **                      *    *   
     491478 TTTCGTTGGAAACGGGATTGTCTTCATATAAACTCTAGACAGAAGCATTCTCAGAAGCTTCATTG
         66 TTTCGTTGGAAACAGGAATATCTTCATATAAAAGCTAGACAGAAGCATTCTCAGAAACTTCTTTG

            *       **   *    *             *        
     491543 GGATGTTTCAATTGAAGTCACAGTGTTGAACAGTCCCTTTC
        131 TGATGTTTGCATTCAAGTAACAGTGTTGAACATTCCCTTTC

                         *                         *     *       **     *    
     491584 ATAGAGCAGGTTTCAAACACTCTTTTTGTAGTATCTGGATGTGGACATTTGGAGCGCTTTCAGGC
          1 ATAGAGCAGGTTTGAAACACTCTTTTTGTAGTATCTGGAAGTGGATATTTGGATAGCTTTGAGG-

            * * *    *                **** *   * *                        *  
     491649 CTATGGTT-TAAA-AGGAAATATCTTACCCTGAAAACCAGACAGAAGCATTCTCAGAAACTTATT
         65 ATTTCGTTGGAAACAGG-AATATCTTCATATAAAAGCTAGACAGAAGCATTCTCAGAAACTTCTT

                    **  **    *                    *   *
     491712 TGTGATGTGCGCCCTCAACTAACAGTGTTGAAGCATT-CTTTTG
        129 TGTGATGTTTGCATTCAAGTAACAGTGTTGAA-CATTCCCTTTC

                     *                   * *     *             **            
     491755 ATAGAGCAGTTTTGAAACACTCTTTTTGTGGAATCTGCAAGTGGATATTTGTCTAGCTTTGAGGA
          1 ATAGAGCAGGTTTGAAACACTCTTTTTGTAGTATCTGGAAGTGGATATTTGGATAGCTTTGAGGA

                         *   *    *     *             *       *              
     491820 TTTCGTTGGAAACGGGATTA-CAT-AT-GAAAAGC-AGACAGCAGCATTCCCAGAAACTTCTTTG
         66 TTTCGTTGGAAACAGGAATATCTTCATATAAAAGCTAGACAGAAGCATTCTCAGAAACTTCTTTG

                            * *    *                 
     491881 TGATGTTTGCATTCAACTCACAGAGTTGAACATTCCCTTTC
        131 TGATGTTTGCATTCAAGTAACAGTGTTGAACATTCCCTTTC

                                                   *     *       **     *   *
     491922 ATAGAGCAGGTTTGAAACACTCTTTTTGTAGTATCTGGATGTGGACATTTGGAGCGCTTTCAGGC
          1 ATAGAGCAGGTTTGAAACACTCTTTTTGTAGTATCTGGAAGTGGATATTTGGATAGCTTTGAGGA

            * ** *      *             *** *   *                          *   
     491987 CTATGGT-GAAAAAGGAAATATCTTCCCCTGAAAACTAGACAGAAGCATTCTCAGAAACTTATTT
         66 TTTCGTTGGAAACAGG-AATATCTTCATATAAAAGCTAGACAGAAGCATTCTCAGAAACTTCTTT

                   **  **    *             **  * *   *
     492051 GTGATGTGCGCCCTCAACTAACAGTGTTGAAGCTTTCTTTTG
        130 GTGATGTTTGCATTCAAGTAACAGTGTTGAACATTCCCTTTC

                     *                    *      *   *                       
     492093 ATAGAGCAGTTTTGAAACACTCTTTTTGTAATATCTGCAAGAGGATATTTGGATAGCTTTGAGGA
          1 ATAGAGCAGGTTTGAAACACTCTTTTTGTAGTATCTGGAAGTGGATATTTGGATAGCTTTGAGGA

                         *   *   *                    *       *     * * *    
     492158 TTTCGTTGGAAACGGGATTA-ATT-ATA-AAAAGC-AGACAGCAGCATTCCCAGAATCATGTTTG
         66 TTTCGTTGGAAACAGGAATATCTTCATATAAAAGCTAGACAGAAGCATTCTCAGAAACTTCTTTG

                              *    *                 
     492219 TGATGTTTGCATTCAAGTGACAGAGTTGAACATTCCCTTTC
        131 TGATGTTTGCATTCAAGTAACAGTGTTGAACATTCCCTTTC

             *                         *           *     *       **     *   *
     492260 AGAGAGCAGGTTTGAAACACTCTTTTTATAGTATCTGGATGTGGACATTTGGAGCGCTTTCAGGC
          1 ATAGAGCAGGTTTGAAACACTCTTTTTGTAGTATCTGGAAGTGGATATTTGGATAGCTTTGAGGA

            * ** *      *             *** *   *                      *   *   
     492325 CTATGGT-GAAAAAGGAAATATCTTCTCCTGAAAACTAGACAGAAGCATTCTCAGAATCTTATTT
         66 TTTCGTTGGAAACAGG-AATATCTTCATATAAAAGCTAGACAGAAGCATTCTCAGAAACTTCTTT

                   **  **    *             **  * *   *
     492389 GTGATGTGCGCCCTCAACTAACAGTGTTGAAGCTTTCTTTTG
        130 GTGATGTTTGCATTCAAGTAACAGTGTTGAACATTCCCTTTC

                     *                *   **     *   *        *              
     492431 ATAGAGCAGTTTTGAAACACTCTTTTCGTAAAATCTGCAAGAGGATATTTTGATAGCTTTGAGGA
          1 ATAGAGCAGGTTTGAAACACTCTTTTTGTAGTATCTGGAAGTGGATATTTGGATAGCTTTGAGGA

                         *   * *            **                      *    *   
     492496 TTTCGTTGGAAACGGGATTGTCTTCATATAAACTCTAGACAGAAGCATTCTCAGAAGCTTCATTG
         66 TTTCGTTGGAAACAGGAATATCTTCATATAAAAGCTAGACAGAAGCATTCTCAGAAACTTCTTTG

            *       **   *    *             *        
     492561 GGATGTTTCAATTGAAGTCACAGTGTTGAACAGTCCCTTTC
        131 TGATGTTTGCATTCAAGTAACAGTGTTGAACATTCCCTTTC

                                                         *       *  * * *    
     492602 ATAGAGCAGGTTTGAAACACTCTTTTTGTAGTATCTGGAAGTGGACATTTGGAGAGATCTCAGGA
          1 ATAGAGCAGGTTTGAAACACTCTTTTTGTAGTATCTGGAAGTGGATATTTGGATAGCTTTGAGGA

            * *  *    * *             **            *       * *          *  *
     492667 ATACGGT-GATAAAGGAAATATCTTCCAATAAAAGCTAGATAGAAGCAATGTCAGAAACTTTTTC
         66 TTTCGTTGGAAACAGG-AATATCTTCATATAAAAGCTAGACAGAAGCATTCTCAGAAACTTCTTT

            *      * ** *   **      *       *  * *   *
     492731 ATGATGTATCTACTCACCTAACAGAGTTGAACCTTTCTTTTG
        130 GTGATGTTTGCATTCAAGTAACAGTGTTGAACATTCCCTTTC

             *       *                   * *     *   *         **            
     492773 AGAGAGCAGTTTTGAAACACTCTTTTTGTGGAATCTGCAAGAGGATATTTGTCTAGCTTTGAGGA
          1 ATAGAGCAGGTTTGAAACACTCTTTTTGTAGTATCTGGAAGTGGATATTTGGATAGCTTTGAGGA

                         *   *    *                   *       *   *          
     492838 TTTCGTTGGAAACGGGATTA-CAT-ATA-AAAAGC-AGACAGCAGCATTCCCAGTAACTTCTTTG
         66 TTTCGTTGGAAACAGGAATATCTTCATATAAAAGCTAGACAGAAGCATTCTCAGAAACTTCTTTG

               *              *    *                 
     492899 TGAGGTTTGCATTCAAGTGACAGAGTTGAACATTCCCTTTC
        131 TGATGTTTGCATTCAAGTAACAGTGTTGAACATTCCCTTTC

                                                   *     *       **     *   *
     492940 ATAGAGCAGGTTTGAAACACTCTTTTTGTAGTATCTGGATGTGGACATTTGGAGCGCTTTCAGGC
          1 ATAGAGCAGGTTTGAAACACTCTTTTTGTAGTATCTGGAAGTGGATATTTGGATAGCTTTGAGGA

            * ** *      *             **          * *       * *          *  *
     493005 CTATGGT-GAAAAAGGAAATATCTTCCAATAAAAGCTACATAGAAGCAATGTCAGAAACTTTTTC
         66 TTTCGTTGGAAACAGG-AATATCTTCATATAAAAGCTAGACAGAAGCATTCTCAGAAACTTCTTT

            *      * ** *   **      *       *  * *   *
     493069 ATGATGTATCTACTCAGCTAACAGAGTTGAACCTTTCTTTTG
        130 GTGATGTTTGCATTCAAGTAACAGTGTTGAACATTCCCTTTC

             *       *                   * *                   **            
     493111 AGAGAGCAGTTTTGAAACACTCTTTTTGTGGAATCTGGAAGTGGATATTTGTCTAGCTTTGAGGA
          1 ATAGAGCAGGTTTGAAACACTCTTTTTGTAGTATCTGGAAGTGGATATTTGGATAGCTTTGAGGA

                         *   *    *                   *       *   *          
     493176 TTTCGTTGGAAACGGGATTA-CAT-ATA-AAAAGC-AGACAGCAGCATTCCCAGTAACTTCTTTG
         66 TTTCGTTGGAAACAGGAATATCTTCATATAAAAGCTAGACAGAAGCATTCTCAGAAACTTCTTTG

               *              *    *                 
     493237 TGACGTTTGCATTCAAGTCACAGAGTTGAACATTCCCTTTC
        131 TGATGTTTGCATTCAAGTAACAGTGTTGAACATTCCCTTTC

                                                   *     *       **     *   *
     493278 ATAGAGCAGGTTTGAAACACTCTTTTTGTAGTATCTGGATGTGGACATTTGGAGCGCTTTCAGGC
          1 ATAGAGCAGGTTTGAAACACTCTTTTTGTAGTATCTGGAAGTGGATATTTGGATAGCTTTGAGGA

            * ** *      *             *** *   *           *          *   *   
     493343 CTATGGT-GAAAAAGGAAATATCTTCCCCTGAAAACTAGACAGAAGAATTCTCAGAATCTTATTT
         66 TTTCGTTGGAAACAGG-AATATCTTCATATAAAAGCTAGACAGAAGCATTCTCAGAAACTTCTTT

                   **  **    *              *  * *   *
     493407 GTGATGTGCGCCATCAACTAACAGTGTTGAACCTTTCTTTTG
        130 GTGATGTTTGCATTCAAGTAACAGTGTTGAACATTCCCTTTC

                     *                    **     *   *                       
     493449 ATAGAGCAGTTTTGAAACACTCTTTTTGTAAAATCTGCAAGAGGATATTTGGATAGCTTTGAGGA
          1 ATAGAGCAGGTTTGAAACACTCTTTTTGTAGTATCTGGAAGTGGATATTTGGATAGCTTTGAGGA

                         *   * *            **                      * *  *   
     493514 TTTCGTTGGAAACGGGATTGTCTTCATATAAACTCTAGACAGAAGCATTCTCAGAAGCGTCATTG
         66 TTTCGTTGGAAACAGGAATATCTTCATATAAAAGCTAGACAGAAGCATTCTCAGAAACTTCTTTG

            *       **   *    *                      
     493579 GGATGTTTCAATTGAAGTCACAGTGTTGAACATTCCCTTTC
        131 TGATGTTTGCATTCAAGTAACAGTGTTGAACATTCCCTTTC

                                                   *     *       **     *    
     493620 ATAGAGCAGGTTTGAAACACTCTTTTTGTAGTATCTGGATGTGGACATTTGGAGCGCTTTCAGGC
          1 ATAGAGCAGGTTTGAAACACTCTTTTTGTAGTATCTGGAAGTGGATATTTGGATAGCTTTGAGG-

            * * *    *                 *** *   *                          *  
     493685 CTATGGTT-TAAA-AGGAAATATCTTCCCCTGAAAACTAGACAGAAGCATTCTCAGAAACTTATT
         65 ATTTCGTTGGAAACAGG-AATATCTTCATATAAAAGCTAGACAGAAGCATTCTCAGAAACTTCTT

                    **  **    *                    *   *
     493748 TGTGATGTGCGCCCTCAACTAACAGTGTTGAAGCATT-CTTTTG
        129 TGTGATGTTTGCATTCAAGTAACAGTGTTGAA-CATTCCCTTTC

                     *                   * *     *             **            
     493791 ATAGAGCAGTTTTGAAACACTCTTTTTGTGGAATCTGCAAGTGGATATTTGTCTAGCTTTGAGGA
          1 ATAGAGCAGGTTTGAAACACTCTTTTTGTAGTATCTGGAAGTGGATATTTGGATAGCTTTGAGGA

                         *   *    *                   *       *   *          
     493856 TTTCGTTGGAAACGGGATTA-CAT-ATA-AAAAGC-AGACAGCAGCATTCCCAGTAACTTCTTTG
         66 TTTCGTTGGAAACAGGAATATCTTCATATAAAAGCTAGACAGAAGCATTCTCAGAAACTTCTTTG

                              *    *                 
     493917 TGATGTTTGCATTCAAGTCACAGAGTTGAACATTCCCTTTC
        131 TGATGTTTGCATTCAAGTAACAGTGTTGAACATTCCCTTTC

                    *                   *         **     *       **     *   *
     493958 ATAGAGCATGTTTGAAACACTCTTTTTGAAGTATCTGGTTGTGGACATTTGGAGCGCTTTCAGGC
          1 ATAGAGCAGGTTTGAAACACTCTTTTTGTAGTATCTGGAAGTGGATATTTGGATAGCTTTGAGGA

            * ** *      *             *** *   *                          *   
     494023 CTATGGT-GAAAAAGGAAATATCTTCCCCTGAAAACTAGACAGAAGCATTCTCAGAAACTTATTT
         66 TTTCGTTGGAAACAGG-AATATCTTCATATAAAAGCTAGACAGAAGCATTCTCAGAAACTTCTTT

                   **  **    *              *  * *   *
     494087 GTGATGTGCGCCCTCAACTAACAGTGTTGAACCTTTCTTTTG
        130 GTGATGTTTGCATTCAAGTAACAGTGTTGAACATTCCCTTTC

                     *                    *      *   *                       
     494129 ATAGAGCAGTTTTGAAACACTCTTTTTGTAATATCTGCAAGAGGATATTTGGATAGCTTTGAGGA
          1 ATAGAGCAGGTTTGAAACACTCTTTTTGTAGTATCTGGAAGTGGATATTTGGATAGCTTTGAGGA

                         *   * *            **                      *    *   
     494194 TTTCGTTGGAAACGGGATTGTCTTCATATAAACTCTAGACAGAAGCATTCTCAGAAGCTTCATTG
         66 TTTCGTTGGAAACAGGAATATCTTCATATAAAAGCTAGACAGAAGCATTCTCAGAAACTTCTTTG

            *       **   *    *             *        
     494259 GGATGTTTCAATTGAAGTCACAGTGTTGAACAGTCCCTTTC
        131 TGATGTTTGCATTCAAGTAACAGTGTTGAACATTCCCTTTC

                                                         *       **   * *    
     494300 ATAGAGCAGGTTTGAAACACTCTTTTTGTAGTATCTGGAAGTGGACATTTGGAGCGCTCTCAGGA
          1 ATAGAGCAGGTTTGAAACACTCTTTTTGTAGTATCTGGAAGTGGATATTTGGATAGCTTTGAGGA

            * *  *    * *             **            *       * *          *  *
     494365 CTACGAT-GATAAAGGAAATATCTTCCAATAAAAGCTAGATAGAAGCAATGTCAGAAACTTTTTC
         66 TTTCGTTGGAAACAGG-AATATCTTCATATAAAAGCTAGACAGAAGCATTCTCAGAAACTTCTTT

            *      * ** *   **      *       *  * *   *
     494429 ATGATGTATCTACTCAGCTAACAGAGTTGAACCTTTCTTTTG
        130 GTGATGTTTGCATTCAAGTAACAGTGTTGAACATTCCCTTTC

             *       *                   * *     *             **            
     494471 AGAGAGCAGTTTTGAAACACTCTTTTTGTGGAATCTGCAAGTGGATATTTGTCTAGCTTTGAGGA
          1 ATAGAGCAGGTTTGAAACACTCTTTTTGTAGTATCTGGAAGTGGATATTTGGATAGCTTTGAGGA

                         *   *    *                   *       *     *        
     494536 TTTCGTTGGAAACGGGATTA-CAT-ATA-AAAAGC-AGACAGCAGCATTCCCAGAATCTTCTTTG
         66 TTTCGTTGGAAACAGGAATATCTTCATATAAAAGCTAGACAGAAGCATTCTCAGAAACTTCTTTG

                              *    *                 
     494597 TGATGTTTGCATTCAAGTCACAGAGTTGAACATTCCCTTTC
        131 TGATGTTTGCATTCAAGTAACAGTGTTGAACATTCCCTTTC

                                                   *     *       **     *   *
     494638 ATAGAGCAGGTTTGAAACACTCTTTTTGTAGTATCTGGATGTGGACATTTGGAGCGCTTTCAGGC
          1 ATAGAGCAGGTTTGAAACACTCTTTTTGTAGTATCTGGAAGTGGATATTTGGATAGCTTTGAGGA

            * ** *      *             *** *   *                      *   *   
     494703 CTATGGT-GAAAAAGGAAATATCTTCCCCTGAAAACTAGACAGAAGCATTCTCAGAATCTTATTT
         66 TTTCGTTGGAAACAGG-AATATCTTCATATAAAAGCTAGACAGAAGCATTCTCAGAAACTTCTTT

                   **  **    *             **  * *   *
     494767 GTGATGTGCGCCCTCAACTAACAGTGTTGAAGCTTTCTTTTG
        130 GTGATGTTTGCATTCAAGTAACAGTGTTGAACATTCCCTTTC

                     *                  * **     *   *                       
     494809 ATAGAGCAGTTTTGAAACACTCTTTTTGCAAAATCTGCAAGAGGATATTTGGATAGCTTTGAGGA
          1 ATAGAGCAGGTTTGAAACACTCTTTTTGTAGTATCTGGAAGTGGATATTTGGATAGCTTTGAGGA

                         *   * *            **                      *    *   
     494874 TTTCGTTGGAAACGGGATTGTCTTCATATAAACTCTAGACAGAAGCATTCTCAGAAGCTTCATTG
         66 TTTCGTTGGAAACAGGAATATCTTCATATAAAAGCTAGACAGAAGCATTCTCAGAAACTTCTTTG

            *       **   *    *             *        
     494939 GGATGTTTCAATTGAAGTCACAGTGTTGAACAGTCCCTTTC
        131 TGATGTTTGCATTCAAGTAACAGTGTTGAACATTCCCTTTC

                                                         *       **   * *    
     494980 ATAGAGCAGGTTTGAAACACTCTTTTTGTAGTATCTGGAAGTGGACATTTGGAGCGCTCTCAGGA
          1 ATAGAGCAGGTTTGAAACACTCTTTTTGTAGTATCTGGAAGTGGATATTTGGATAGCTTTGAGGA

            * *  *      *             **            *       * *          *  *
     495045 CTGCGGT-GAAAAAGGAAATATCTTCCAATAAAAGCTAGATAGAAGCAATGTCAGAAACTTTTTC
         66 TTTCGTTGGAAACAGG-AATATCTTCATATAAAAGCTAGACAGAAGCATTCTCAGAAACTTCTTT

            *      * ** *   **      *       *    *   *
     495109 ATGATGTATCTACTCAGCTAACAGAGTTGAACCTT-CATTTG
        130 GTGATGTTTGCATTCAAGTAACAGTGTTGAACATTCCCTTTC

             *       *            *      * *     *             **            
     495150 AGAGAGCAGTTTTGAAACACTCGTTTTGTGGAATCTGCAAGTGGATATTTGTCTAGCTTTGAGGA
          1 ATAGAGCAGGTTTGAAACACTCTTTTTGTAGTATCTGGAAGTGGATATTTGGATAGCTTTGAGGA

                         *   *    *                   *       *              
     495215 TTTCGTTGGAAACGGGATTA-CAT-ATA-AAAAGC-AGACAGCAGCATTCCCAGAAACTTCTTTG
         66 TTTCGTTGGAAACAGGAATATCTTCATATAAAAGCTAGACAGAAGCATTCTCAGAAACTTCTTTG

                              *    *                 
     495276 TGATGTTTGCATTCAAGTCACAGAGTTGAACATTCCCTTTC
        131 TGATGTTTGCATTCAAGTAACAGTGTTGAACATTCCCTTTC

                                                   *   * *     * **     *   *
     495317 ATAGAGCAGGTTTGAAACACTCTTTTTGTAGTATCTGGATGTGTACATTTGCAGCGCTTTCAGGC
          1 ATAGAGCAGGTTTGAAACACTCTTTTTGTAGTATCTGGAAGTGGATATTTGGATAGCTTTGAGGA

            * ** *      *             *** *   *                          *   
     495382 CTAAGGT-GAAAAAGGAAATATCTTCCCCTGAAAACTAGACAGAAGCATTCTCAGAAACTTATTT
         66 TTTCGTTGGAAACAGG-AATATCTTCATATAAAAGCTAGACAGAAGCATTCTCAGAAACTTCTTT

                   **  **    *     *       **  * *   *
     495446 GTGATGTGCGCCCTCAACTAACACTGTTGAAGCTTTCTTTTG
        130 GTGATGTTTGCATTCAAGTAACAGTGTTGAACATTCCCTTTC

                     * *                  *      *   *                       
     495488 ATAGAGCAGTTATGAAACACTCTTTTTGTAATATCTGCAAGAGGATATTTGGATAGCTTTGAGGA
          1 ATAGAGCAGGTTTGAAACACTCTTTTTGTAGTATCTGGAAGTGGATATTTGGATAGCTTTGAGGA

                         *  ** *            **     *                *    *   
     495553 TTTCGTTGGAAACGGGTTTGTCTTCATATAAACTCTAGAGAGAAGCATTCTCAGAAGCTTCATTG
         66 TTTCGTTGGAAACAGGAATATCTTCATATAAAAGCTAGACAGAAGCATTCTCAGAAACTTCTTTG

            *       **   *    *             * *      
     495618 GGATGTTTCAATTGAAGTCACAGTGTTGAACAGTTCCTTTC
        131 TGATGTTTGCATTCAAGTAACAGTGTTGAACATTCCCTTTC

                 *                     *                 *       **   * *    
     495659 ATAGAACAGGTTTGAAACACTCTTTTTATAGTATCTGGAAGTGGACATTTGGAGCGCTCTCAGGA
          1 ATAGAGCAGGTTTGAAACACTCTTTTTGTAGTATCTGGAAGTGGATATTTGGATAGCTTTGAGGA

            * ** *      *             **          * *       * *          *  *
     495724 CTATGGT-GAAAAAGGAAATATCTTCCAATAAAAGCTACATAGAAGCAATGTCAGAAACTTTTTC
         66 TTTCGTTGGAAACAGG-AATATCTTCATATAAAAGCTAGACAGAAGCATTCTCAGAAACTTCTTT

            *      * ** *   **      *       *  *     *
     495788 ATGATGTATCTACTCAGCTAACAGAGTTGAACCTTTCCTTTG
        130 GTGATGTTTGCATTCAAGTAACAGTGTTGAACATTCCCTTTC

             *       *                   * *     *             **            
     495830 AGAGAGCAGTTTTGAAACACTCTTTTTGTGGAATCTGCAAGTGGATATTTGTCTAGCTTTGAGGA
          1 ATAGAGCAGGTTTGAAACACTCTTTTTGTAGTATCTGGAAGTGGATATTTGGATAGCTTTGAGGA

                         *   *    *                   *       *   *          
     495895 TTTCGTTGGAAACGGGATTA-CAT-ATA-AAAAGC-AGACAGCAGCATTCCCAGTAACTTCTTTG
         66 TTTCGTTGGAAACAGGAATATCTTCATATAAAAGCTAGACAGAAGCATTCTCAGAAACTTCTTTG

                              *    *      *          
     495956 TGATGTTTGCATTCAAGTCACAGAGTTGAATATTCCCTTTC
        131 TGATGTTTGCATTCAAGTAACAGTGTTGAACATTCCCTTTC

                                                   *     *       **     *   *
     495997 ATAGAGCAGGTTTGAAACACTCTTTTTGTAGTATCTGGATGTGGACATTTGGAGCGCTTTCAGGC
          1 ATAGAGCAGGTTTGAAACACTCTTTTTGTAGTATCTGGAAGTGGATATTTGGATAGCTTTGAGGA

            * ** *      *             *** *   *                          *   
     496062 CTATGGT-GAAAAAGGAAATATCTTCCCCTGAAAACTAGACAGAAGCATTCTCAGAAACTTATTT
         66 TTTCGTTGGAAACAGG-AATATCTTCATATAAAAGCTAGACAGAAGCATTCTCAGAAACTTCTTT

                   **  **    *              *  * *   *
     496126 GTGATGTGCGCCCTCAACTAACAGTGTTGAACCTTTCTTTTG
        130 GTGATGTTTGCATTCAAGTAACAGTGTTGAACATTCCCTTTC

                     *                    *      *   *                       
     496168 ATAGAGCAGTTTTGAAACACTCTTTTTGTAATATCTGCAAGAGGATATTTGGATAGCTTTGAGGA
          1 ATAGAGCAGGTTTGAAACACTCTTTTTGTAGTATCTGGAAGTGGATATTTGGATAGCTTTGAGGA

                         *   *    *                   *       *   *          
     496233 TTTCGTTGGAAACGGGATTA-CAT-ATA-AAAAGC-AGACAGCAGCATTCCCAGTAACTTCTTTG
         66 TTTCGTTGGAAACAGGAATATCTTCATATAAAAGCTAGACAGAAGCATTCTCAGAAACTTCTTTG

               *              *    *                 
     496294 TGACGTTTGCATTCAAGTCACAGAGTTGAACATTCCCTTTC
        131 TGATGTTTGCATTCAAGTAACAGTGTTGAACATTCCCTTTC

            *           *                          *     *       **     **  *
     496335 GTAGAGCAGGTTGGAAACACTCTTTTTGTAGTATCTGGATGTGGACATTTGGAGCGCTTTCTGGC
          1 ATAGAGCAGGTTTGAAACACTCTTTTTGTAGTATCTGGAAGTGGATATTTGGATAGCTTTGAGGA

            *  * *      *             *** *   *                          *   
     496400 CTTTGGT-GAAAAAGGAAATATCTTCCCCTGAAAACTAGACAGAAGCATTCTCAGAAACTTATTT
         66 TTTCGTTGGAAACAGG-AATATCTTCATATAAAAGCTAGACAGAAGCATTCTCAGAAACTTCTTT

                   **  **    *              *  * *   *
     496464 GTGATGTGCGCCCTCAACTAACAGTGTTGAACCTTTCTTTTG
        130 GTGATGTTTGCATTCAAGTAACAGTGTTGAACATTCCCTTTC

                     *                    *      *   *                       
     496506 ATAGAGCAGTTTTGAAACACTCTTTTTGTAATATCTGCAAGAGGATATTTGGATAGCTTTGAGGA
          1 ATAGAGCAGGTTTGAAACACTCTTTTTGTAGTATCTGGAAGTGGATATTTGGATAGCTTTGAGGA

                         *   * *            **                      *    *   
     496571 TTTCGTTGGAAACGGGATTGTCTTCATATAAACTCTAGACAGAAGCATTCTCAGAAGCTTCATTG
         66 TTTCGTTGGAAACAGGAATATCTTCATATAAAAGCTAGACAGAAGCATTCTCAGAAACTTCTTTG

            *       **   *    *             *  *     
     496636 GGATGTTTCAATTGAAGTCACAGTGTTGAACAGTCGCTTTC
        131 TGATGTTTGCATTCAAGTAACAGTGTTGAACATTCCCTTTC

                                                         *       **   * *    
     496677 ATAGAGCAGGTTTGAAACACTCTTTTTGTAGTATCTGGAAGTGGACATTTGGAGCGCTCTCAGGA
          1 ATAGAGCAGGTTTGAAACACTCTTTTTGTAGTATCTGGAAGTGGATATTTGGATAGCTTTGAGGA

            * *  *    * *             **            *       * *          *  *
     496742 CTACGAT-GATAAAGGAAATATCTTCCAATAAAAGCTAGATAGAAGCAATGTCAGAAACTTTTTC
         66 TTTCGTTGGAAACAGG-AATATCTTCATATAAAAGCTAGACAGAAGCATTCTCAGAAACTTCTTT

            *      * ** *   **      *       *  * *   *
     496806 ATGATGTATCTACTCAGCTAACAGAGTTGAACCTTTCTTTTG
        130 GTGATGTTTGCATTCAAGTAACAGTGTTGAACATTCCCTTTC

             *       *                   * *     *             **            
     496848 AGAGAGCAGTTTTGAAACACTCTTTTTGTGGAATCTGCAAGTGGATATTTGTCTAGCTTTGAGGA
          1 ATAGAGCAGGTTTGAAACACTCTTTTTGTAGTATCTGGAAGTGGATATTTGGATAGCTTTGAGGA

                         *   *    *                   *       *     *        
     496913 TTTCGTTGGAAACGGGATTA-CAT-ATA-AAAAGC-AGACAGCAGCATTCCCAGAATCTTCTTTG
         66 TTTCGTTGGAAACAGGAATATCTTCATATAAAAGCTAGACAGAAGCATTCTCAGAAACTTCTTTG

                              *    *                 
     496974 TGATGTTTGCATTCAAGTCACAGAGTTGAACATTCCCTTTC
        131 TGATGTTTGCATTCAAGTAACAGTGTTGAACATTCCCTTTC

                                                   *     *       **     *   *
     497015 ATAGAGCAGGTTTGAAACACTCTTTTTGTAGTATCTGGATGTGGACATTTGGAGCGCTTTCAGGC
          1 ATAGAGCAGGTTTGAAACACTCTTTTTGTAGTATCTGGAAGTGGATATTTGGATAGCTTTGAGGA

            * ** *      *             *** *   *                      *   *   
     497080 CTATGGT-GAAAAAGGAAATATCTTCCCCTGAAAACTAGACAGAAGCATTCTCAGAATCTTATTT
         66 TTTCGTTGGAAACAGG-AATATCTTCATATAAAAGCTAGACAGAAGCATTCTCAGAAACTTCTTT

                   **  **    *             **  * *   *
     497144 GTGATGTGCGCCCTCAACTAACAGTGTTGAAGCTTTCTTTTG
        130 GTGATGTTTGCATTCAAGTAACAGTGTTGAACATTCCCTTTC

                     *                    **     *   *                       
     497186 ATAGAGCAGTTTTGAAACACTCTTTTTGTAAAATCTGCAAGAGGATATTTGGATAGCTTTGAGGA
          1 ATAGAGCAGGTTTGAAACACTCTTTTTGTAGTATCTGGAAGTGGATATTTGGATAGCTTTGAGGA

                         *   * *            **                      *    *   
     497251 TTTCGTTGGAAACGGGATTGTCTTCATATAAACTCTAGACAGAAGCATTCTCAGAAGCTTCATTG
         66 TTTCGTTGGAAACAGGAATATCTTCATATAAAAGCTAGACAGAAGCATTCTCAGAAACTTCTTTG

            *       **   *    * *           *        
     497316 GGATGTTTCAATTGAAGTCAGAGTGTTGAACAGTCCCTTTC
        131 TGATGTTTGCATTCAAGTAACAGTGTTGAACATTCCCTTTC

                                                         *       **   * *    
     497357 ATAGAGCAGGTTTGAAACACTCTTTTTGTAGTATCTGGAAGTGGACATTTGGAGCGCTCTCAGGA
          1 ATAGAGCAGGTTTGAAACACTCTTTTTGTAGTATCTGGAAGTGGATATTTGGATAGCTTTGAGGA

            * *  *      *             **            *       * *          *  *
     497422 CTGCGGT-GAAAAAGGAAATATCTTCCAATAAAAGCTAGATAGAAGCAATGTCAGAAACTTTTTC
         66 TTTCGTTGGAAACAGG-AATATCTTCATATAAAAGCTAGACAGAAGCATTCTCAGAAACTTCTTT

            *      * ** *   **      *       *    *   *
     497486 ATGATGTATCTACTCAGCTAACAGAGTTGAACCTT-CATTTG
        130 GTGATGTTTGCATTCAAGTAACAGTGTTGAACATTCCCTTTC

             *       *            *      * *     *             **            
     497527 AGAGAGCAGTTTTGAAACACTCGTTTTGTGGAATCTGCAAGTGGATATTTGTCTAGCTTTGAGGA
          1 ATAGAGCAGGTTTGAAACACTCTTTTTGTAGTATCTGGAAGTGGATATTTGGATAGCTTTGAGGA

                         *   *    *                   *       *              
     497592 TTTCGTTGGAAACGGGATTA-CAT-ATA-AAAAGC-AGACAGCAGCATTCCCAGAAACTTCTTTG
         66 TTTCGTTGGAAACAGGAATATCTTCATATAAAAGCTAGACAGAAGCATTCTCAGAAACTTCTTTG

                              *    *                 
     497653 TGATGTTTGCATTCAAGTCACAGAGTTGAACATTCCCTTTC
        131 TGATGTTTGCATTCAAGTAACAGTGTTGAACATTCCCTTTC

                                                   *   * *     * ** *   *   *
     497694 ATAGAGCAGGTTTGAAACACTCTTTTTGTAGTATCTGGATGTGTACATTTGCAGCGTTTTCAGGC
          1 ATAGAGCAGGTTTGAAACACTCTTTTTGTAGTATCTGGAAGTGGATATTTGGATAGCTTTGAGGA

            * ** *      *             *** *   *                          *   
     497759 CTAAGGT-GAAAAAGGAAATATCTTCCCCTGAAAACTAGACAGAAGCATTCTCAGAAACTTATTT
         66 TTTCGTTGGAAACAGG-AATATCTTCATATAAAAGCTAGACAGAAGCATTCTCAGAAACTTCTTT

                   **  **    *             **  * *   *
     497823 GTGATGTGCGCCCTCAACTAACAGTGTTGAAGCTTTCTTTTG
        130 GTGATGTTTGCATTCAAGTAACAGTGTTGAACATTCCCTTTC

                     *                    *      *   *                       
     497865 ATAGAGCAGTTTTGAAACACTCTTTTTGTAATATCTGCAAGAGGATATTTGGATAGCTTTGAGGA
          1 ATAGAGCAGGTTTGAAACACTCTTTTTGTAGTATCTGGAAGTGGATATTTGGATAGCTTTGAGGA

                         *  ** *            **                      *    *   
     497930 TTTCGTTGGAAACGGGTTTGTCTTCATATAAACTCTAGACAGAAGCATTCTCAGAAGCTTCATTG
         66 TTTCGTTGGAAACAGGAATATCTTCATATAAAAGCTAGACAGAAGCATTCTCAGAAACTTCTTTG

            *       **   *    *             * *      
     497995 GGATGTTTCAATTGAAGTCACAGTGTTGAACAGTTCCTTTC
        131 TGATGTTTGCATTCAAGTAACAGTGTTGAACATTCCCTTTC

                 *                                       *       **   * *    
     498036 ATAGAACAGGTTTGAAACACTCTTTTTGTAGTATCTGGAAGTGGACATTTGGAGCGCTCTCAGGA
          1 ATAGAGCAGGTTTGAAACACTCTTTTTGTAGTATCTGGAAGTGGATATTTGGATAGCTTTGAGGA

            * ** *      *             **          * *       * *          *  *
     498101 CTATGGT-GAAAAAGGAAATATCTTCCAATAAAAGCTACATAGAAGCAATGTCAGAAACTTTTTC
         66 TTTCGTTGGAAACAGG-AATATCTTCATATAAAAGCTAGACAGAAGCATTCTCAGAAACTTCTTT

            *      * ** *   **      *       *  *     *
     498165 ATGATGTATCTACTCAGCTAACAGAGTTGAACCTTTCCTTTG
        130 GTGATGTTTGCATTCAAGTAACAGTGTTGAACATTCCCTTTC

             *       *                   * *     *             **            
     498207 AGAGAGCAGTTTTGAAACACTCTTTTTGTGGAATCTGCAAGTGGATATTTGTCTAGCTTTGAGGA
          1 ATAGAGCAGGTTTGAAACACTCTTTTTGTAGTATCTGGAAGTGGATATTTGGATAGCTTTGAGGA

                         *   *    *                   *       **  *          
     498272 TTTCGTTGGAAACGGGATTA-CAT-ATA-AAAAGC-AGACAGCAGCATTCCGAGTAACTTCTTTG
         66 TTTCGTTGGAAACAGGAATATCTTCATATAAAAGCTAGACAGAAGCATTCTCAGAAACTTCTTTG

                              *    *      *          
     498333 TGATGTTTGCATTCAAGTCACAGAGTTGAATATTCCCTTTC
        131 TGATGTTTGCATTCAAGTAACAGTGTTGAACATTCCCTTTC

                         *                         *     *       **     *   *
     498374 ATAGAGCAGGTTTCAAACACTCTTTTTGTAGTATCTGGATGTGGACATTTGGAGCGCTTTCAGGC
          1 ATAGAGCAGGTTTGAAACACTCTTTTTGTAGTATCTGGAAGTGGATATTTGGATAGCTTTGAGGA

            * ** *      *             *** *   *                          *   
     498439 CTATGGT-GAAAAAGGAAATATCTTCCCCTGAAAACTAGACAGAAGCATTCTCAGAAACTTATTT
         66 TTTCGTTGGAAACAGG-AATATCTTCATATAAAAGCTAGACAGAAGCATTCTCAGAAACTTCTTT

                   **  **    *              *  * *   *
     498503 GTGATGTGCGCCCTCAACTAACAGTGTTGAACCTTTCTTTTG
        130 GTGATGTTTGCATTCAAGTAACAGTGTTGAACATTCCCTTTC

                     *                    *      *   *                  *    
     498545 ATAGAGCAGTTTTGAAACACTCTTTTTGTAATATCTGCAAGAGGATATTTGGATAGCTTTCAGGA
          1 ATAGAGCAGGTTTGAAACACTCTTTTTGTAGTATCTGGAAGTGGATATTTGGATAGCTTTGAGGA

                               ** *                   *       *              
     498610 TTTCGTTGGAAAC-GGAATTACAT-ATA-AAAAGC-AGACAGCAGCATTCCCAGAAACTTCTTTG
         66 TTTCGTTGGAAACAGGAATATCTTCATATAAAAGCTAGACAGAAGCATTCTCAGAAACTTCTTTG

                              *    *                 
     498671 TGATGTTTGCATTCAAGTCACAGAGTTGAACATTCCCTTTC
        131 TGATGTTTGCATTCAAGTAACAGTGTTGAACATTCCCTTTC

             *                                     *     *       **     *   *
     498712 AGAGAGCAGGTTTGAAACACTCTTTTTGTAGTATCTGGATGTGGACATTTGGAGCGCTTTCAGGC
          1 ATAGAGCAGGTTTGAAACACTCTTTTTGTAGTATCTGGAAGTGGATATTTGGATAGCTTTGAGGA

            * ** *      *             *** *   *                      *   *   
     498777 CTATGGT-GAAAAAGGAAATATCTTCCCCTGAAAACTAGACAGAAGCATTCTCAGAATCTTATTT
         66 TTTCGTTGGAAACAGG-AATATCTTCATATAAAAGCTAGACAGAAGCATTCTCAGAAACTTCTTT

                   **  **    *             **  * *   *
     498841 GTGATGTGCGCCCTCAACTAACAGTGTTGAAGCTTTCTTTTG
        130 GTGATGTTTGCATTCAAGTAACAGTGTTGAACATTCCCTTTC

                     *                    **     *   *                       
     498883 ATAGAGCAGTTTTGAAACACTCTTTTTGTAAAATCTGCAAGAGGATATTTGGATAGCTTTGAGGA
          1 ATAGAGCAGGTTTGAAACACTCTTTTTGTAGTATCTGGAAGTGGATATTTGGATAGCTTTGAGGA

                       * *   * *            **          **          *    *   
     498948 TTTCGTTGGAATCGGGATTGTCTTCATATAAACTCTAGACAGAATAATTCTCAGAAGCTTCATTG
         66 TTTCGTTGGAAACAGGAATATCTTCATATAAAAGCTAGACAGAAGCATTCTCAGAAACTTCTTTG

            *       **   *    *             * *      
     499013 GGATGTTTCAATTGAAGTCACAGTGTTGAACAGTTCCTTTC
        131 TGATGTTTGCATTCAAGTAACAGTGTTGAACATTCCCTTTC

                 *                                       *       **   * *    
     499054 ATAGAACAGGTTTGAAACACTCTTTTTGTAGTATCTGGAAGTGGACATTTGGAGCGCTCTCAGGA
          1 ATAGAGCAGGTTTGAAACACTCTTTTTGTAGTATCTGGAAGTGGATATTTGGATAGCTTTGAGGA

            * *  *  *   ** *         **          * *       * *          *  * 
     499119 CTACGGTGAAAATGGAAATATCTTCCAATAAAAGCTACATAGAAGCAATGTCAGAAACTTTTTCG
         66 TTTCGTTGGAAACAGGAATATCTTCATATAAAAGCTAGACAGAAGCATTCTCAGAAACTTCTTTG

                  * ** *   **      *       *  *     *
     499184 TGATGTATCTACTCAGCTAACAGAGTTGAACCTTTCCTTTG
        131 TGATGTTTGCATTCAAGTAACAGTGTTGAACATTCCCTTTC

             *       *                   * *     *             **            
     499225 AGAGAGCAGTTTTGAAACACTCTTTTTGTGGAATCTGCAAGTGGATATTTGTCTAGCTTTGAGGA
          1 ATAGAGCAGGTTTGAAACACTCTTTTTGTAGTATCTGGAAGTGGATATTTGGATAGCTTTGAGGA

                         *   *    *                   *       *     *        
     499290 TTTCGTTGGAAACGGGATTA-CAT-ATA-AAAAGC-AGACAGCAGCATTCCCAGAATCTTCTTTG
         66 TTTCGTTGGAAACAGGAATATCTTCATATAAAAGCTAGACAGAAGCATTCTCAGAAACTTCTTTG

                              *    *                 
     499351 TGATGTTTGCATTCAAGTCACAGAGTTGAACATTCCCTTTC
        131 TGATGTTTGCATTCAAGTAACAGTGTTGAACATTCCCTTTC

                                                   *     *       **     *   *
     499392 ATAGAGCAGGTTTGAAACACTCTTTTTGTAGTATCTGGATGTGGACATTTGGAGCGCTTTCAGGC
          1 ATAGAGCAGGTTTGAAACACTCTTTTTGTAGTATCTGGAAGTGGATATTTGGATAGCTTTGAGGA

            * ** *      *             *** *   *                      *   *   
     499457 CTATGGT-GAAAAAGGAAATATCTTCCCCTGAAAACTAGACAGAAGCATTCTCAGAATCTTATTT
         66 TTTCGTTGGAAACAGG-AATATCTTCATATAAAAGCTAGACAGAAGCATTCTCAGAAACTTCTTT

                   **  **    *             **  * *   *
     499521 GTGATGTGCGCCCTCAACTAACAGTGTTGAAGCTTTCTTTTG
        130 GTGATGTTTGCATTCAAGTAACAGTGTTGAACATTCCCTTTC

                     *                    **     *   *                       
     499563 ATAGAGCAGTTTTGAAACACTCTTTTTGTAAAATCTGCAAGAGGATATTTGGATAGCTTTGAGGA
          1 ATAGAGCAGGTTTGAAACACTCTTTTTGTAGTATCTGGAAGTGGATATTTGGATAGCTTTGAGGA

                         *   * *            **                      *    *   
     499628 TTTCGTTGGAAACGGGATTGTCTTCATATAAACTCTAGACAGAAGCATTCTCAGAAGCTTCATTG
         66 TTTCGTTGGAAACAGGAATATCTTCATATAAAAGCTAGACAGAAGCATTCTCAGAAACTTCTTTG

            *       **   *    *             *        
     499693 GGATGTTTCAATTGAAGTCACAGTGTTGAACAGTCCCTTTC
        131 TGATGTTTGCATTCAAGTAACAGTGTTGAACATTCCCTTTC

                                                         *       **   * *    
     499734 ATAGAGCAGGTTTGAAACACTCTTTTTGTAGTATCTGGAAGTGGACATTTGGAACGCTCTCAGGA
          1 ATAGAGCAGGTTTGAAACACTCTTTTTGTAGTATCTGGAAGTGGATATTTGGATAGCTTTGAGGA

            * *  *      *             **            *       * *          *  *
     499799 CTGCGGT-GAAAAAGGAAATATCTTCCAATAAAAGCTAGATAGAAGCAATGTCAGAAACTTTTTC
         66 TTTCGTTGGAAACAGG-AATATCTTCATATAAAAGCTAGACAGAAGCATTCTCAGAAACTTCTTT

            *      * ** *   **      *       *  * *   *
     499863 ATGATGTATCTACTCAGCTAACAGAGTTGAACCTTTCTTTTG
        130 GTGATGTTTGCATTCAAGTAACAGTGTTGAACATTCCCTTTC

             *       *            *      * *     *         *   **            
     499905 AGAGAGCAGTTTTGAAACACTCGTTTTGTGGAATCTGCAAGTGGATAATTGTCTAGCTTTGAGGA
          1 ATAGAGCAGGTTTGAAACACTCTTTTTGTAGTATCTGGAAGTGGATATTTGGATAGCTTTGAGGA

                         *   *    *                   *       *              
     499970 TTTCGTTGGAAACGGGATTA-CAT-ATA-AAAAGC-AGACAGCAGCATTCCCAGAAACTTCTTTG
         66 TTTCGTTGGAAACAGGAATATCTTCATATAAAAGCTAGACAGAAGCATTCTCAGAAACTTCTTTG

                              *    *                 
     500031 TGATGTTTGCATTCAAGTCACAGAGTTGAACATTCCCTTTC
        131 TGATGTTTGCATTCAAGTAACAGTGTTGAACATTCCCTTTC

                                                   *     *     * **     *   *
     500072 ATAGAGCAGGTTTGAAACACTCTTTTTGTAGTATCTGGATGTGGACATTTGCAGCGCTTTCAGGC
          1 ATAGAGCAGGTTTGAAACACTCTTTTTGTAGTATCTGGAAGTGGATATTTGGATAGCTTTGAGGA

            * ** *      *             *** *   *                          *   
     500137 CTAAGGT-GAAAAAGGAAATATCTTCCCCTGAAAACTAGACAGAAGCATTCTCAGAAACTTATTT
         66 TTTCGTTGGAAACAGG-AATATCTTCATATAAAAGCTAGACAGAAGCATTCTCAGAAACTTCTTT

                   **  **    *             **  * *   *
     500201 GTGATGTGCGCCCTCAACTAACAGTGTTGAAGCTTTCTTTTG
        130 GTGATGTTTGCATTCAAGTAACAGTGTTGAACATTCCCTTTC

                     *                    *      *   **    *                 
     500243 ATAGAGCAGTTTTGAAACACTCTTTTTGTAATATCTGCAAGACGATAATTGGATAGCTTTGAGGA
          1 ATAGAGCAGGTTTGAAACACTCTTTTTGTAGTATCTGGAAGTGGATATTTGGATAGCTTTGAGGA

                         *   * *            **                      *    *   
     500308 TTTCGTTGGAAACGGGATTGTCTTCATATAAACTCTAGACAGAAGCATTCTCAGAAGCTTCATTG
         66 TTTCGTTGGAAACAGGAATATCTTCATATAAAAGCTAGACAGAAGCATTCTCAGAAACTTCTTTG

            *       **   *    *             *        
     500373 GGATGTTTCAATTGAAGTCACAGTGTTGAACAGTCCCTTTC
        131 TGATGTTTGCATTCAAGTAACAGTGTTGAACATTCCCTTTC

                                                         *       **   * *    
     500414 ATAGAGCAGGTTTGAAACACTCTTTTTGTAGTATCTGGAAGTGGACATTTGGAACGCTCTCAGGA
          1 ATAGAGCAGGTTTGAAACACTCTTTTTGTAGTATCTGGAAGTGGATATTTGGATAGCTTTGAGGA

            * *  *      *             **            *       * *          *  *
     500479 CTGCGGT-GAAAAAGGAAATATCTTCCAATAAAAGCTAGATAGAAGCAATGTCAGAAACTTTTTC
         66 TTTCGTTGGAAACAGG-AATATCTTCATATAAAAGCTAGACAGAAGCATTCTCAGAAACTTCTTT

            *      * ** *   **      *       *  * *   *
     500543 ATGATGTATCTACTCAGCTAACAGAGTTGAACCTTTCTTTTG
        130 GTGATGTTTGCATTCAAGTAACAGTGTTGAACATTCCCTTTC

             *       *                   * *     *             **            
     500585 AGAGAGCAGTTTTGAAACACTCTTTTTGTGGAATCTGCAAGTGGATATTTGTCTAGCTTTGAGGA
          1 ATAGAGCAGGTTTGAAACACTCTTTTTGTAGTATCTGGAAGTGGATATTTGGATAGCTTTGAGGA

                         *   *    *                   *       *   *          
     500650 TTTCGTTGGAAACGGGATTA-CAT-ATA-AAAAGC-AGACAGCAGCATTCCCAGTAACTTCTTTG
         66 TTTCGTTGGAAACAGGAATATCTTCATATAAAAGCTAGACAGAAGCATTCTCAGAAACTTCTTTG

                              *    *                 
     500711 TGATGTTTGCATTCAAGTCACAGAGTTGAACATTCCCTTTC
        131 TGATGTTTGCATTCAAGTAACAGTGTTGAACATTCCCTTTC

                                 *                 *     *       **     *   *
     500752 ATAGAGCAGGTTTGAAACACTTTTTTTGTAGTATCTGGATGTGGACATTTGGAGCGCTTTCAGGC
          1 ATAGAGCAGGTTTGAAACACTCTTTTTGTAGTATCTGGAAGTGGATATTTGGATAGCTTTGAGGA

            * ** *      *             **          * * *     * *          *  *
     500817 CTATGGT-GAAAAAGGAAATATCTTCCAATAAAAGCTACATAAAAGCAATGTCAGAAACTTTTTC
         66 TTTCGTTGGAAACAGG-AATATCTTCATATAAAAGCTAGACAGAAGCATTCTCAGAAACTTCTTT

            *      * ** *   **      *       *  * *   *
     500881 ATGATGTATCTACTCAGCTAACAGAGTTGAACCTTTCTTTTG
        130 GTGATGTTTGCATTCAAGTAACAGTGTTGAACATTCCCTTTC

             *       *                   * *                   **            
     500923 AGAGAGCAGTTTTGAAACACTCTTTTTGTGGAATCTGGAAGTGGATATTTGTCTAGCTTTGAGGA
          1 ATAGAGCAGGTTTGAAACACTCTTTTTGTAGTATCTGGAAGTGGATATTTGGATAGCTTTGAGGA

                         *   *    *                   *       *   *          
     500988 TTTCGTTGGAAACGGGATTA-CAT-ATA-AAAAGC-AGACAGCAGCATTCCCAGTAACTTCTTTG
         66 TTTCGTTGGAAACAGGAATATCTTCATATAAAAGCTAGACAGAAGCATTCTCAGAAACTTCTTTG

                              *    *                 
     501049 TGATGTTTGCATTCAAGTCACAGAGTTGAACATTCCCTTTC
        131 TGATGTTTGCATTCAAGTAACAGTGTTGAACATTCCCTTTC

                                       *           *     *       **     *    
     501090 ATAGAGCAGGTTTGAAACACTCTTTTTTTAGTATCTGGATGTGGACATTTGGAGCGCTTTCAGG-
          1 ATAGAGCAGGTTTGAAACACTCTTTTTGTAGTATCTGGAAGTGGATATTTGGATAGCTTTGAGGA

            **  *        *             *** *   *           *          *   *  
     501154 CCT-ATTGTGAAAAAGGAAATATCTTCCCCTGAAAACTAGACAGAAGAATTCTCAGAATCTTATT
         66 TTTCGTTG-GAAACAGG-AATATCTTCATATAAAAGCTAGACAGAAGCATTCTCAGAAACTTCTT

                    **  **    *             **  * *   *
     501218 TGTGATGTGCGCCCTCAACTAACAGTGTTGAAGCTTTCTTTTG
        129 TGTGATGTTTGCATTCAAGTAACAGTGTTGAACATTCCCTTTC

                     *                 *  **     *   *                       
     501261 ATAGAGCAGTTTTGAAACACTCTTTTTATAAAATCTGCAAGAGGATATTTGGATAGCTTTGAGGA
          1 ATAGAGCAGGTTTGAAACACTCTTTTTGTAGTATCTGGAAGTGGATATTTGGATAGCTTTGAGGA

                         *   * *            **   *                  * *  *   
     501326 TTTCGTTGGAAACGGGATTGTCTTCATATAAACTCTACACAGAAGCATTCTCAGAAGCGTCATTG
         66 TTTCGTTGGAAACAGGAATATCTTCATATAAAAGCTAGACAGAAGCATTCTCAGAAACTTCTTTG

            *       **   *    *             *        
     501391 GGATGTTTCAATTGAAGTCACAGTGTTGAACAGTCCCTTTC
        131 TGATGTTTGCATTCAAGTAACAGTGTTGAACATTCCCTTTC

                                                   *     *       **     *    
     501432 ATAGAGCAGGTTTGAAACACTCTTTTTGTAGTATCTGGATGTGGACATTTGGAGCGCTTTCAGGC
          1 ATAGAGCAGGTTTGAAACACTCTTTTTGTAGTATCTGGAAGTGGATATTTGGATAGCTTTGAGG-

            * * *    *                 *** *   *                          *  
     501497 CTATGGTT-TAAA-AGGAAATATCTTCCCCTGAAAACTAGACAGAAGCATTCTCAGAAACTTATT
         65 ATTTCGTTGGAAACAGG-AATATCTTCATATAAAAGCTAGACAGAAGCATTCTCAGAAACTTCTT

                    **  *     *                    *   *
     501560 TGTGATGTGCGCCTTCAACTAACAGTGTTGAAGCATT-CTTTTG
        129 TGTGATGTTTGCATTCAAGTAACAGTGTTGAA-CATTCCCTTTC

                     *                   * *     *             **            
     501603 ATAGAGCAGTTTTGAAACACTCTTTTTGTGGAATCTGCAAGTGGATATTTGTCTAGCTTTGAGGA
          1 ATAGAGCAGGTTTGAAACACTCTTTTTGTAGTATCTGGAAGTGGATATTTGGATAGCTTTGAGGA

                         *   *    *                   *       *              
     501668 TTTCGTTGGAAACGGGATTA-CAT-ATA-AAAAGC-AGACAGCAGCATTCCCAGAAACTTCTTTG
         66 TTTCGTTGGAAACAGGAATATCTTCATATAAAAGCTAGACAGAAGCATTCTCAGAAACTTCTTTG

                              *    *                 
     501729 TGATGTTTGCATTCAAGTCACAGAGTTGAACATTCCCTTTC
        131 TGATGTTTGCATTCAAGTAACAGTGTTGAACATTCCCTTTC

             *                                     *     *       **     *  **
     501770 AGAGAGCAGGTTTGAAACACTCTTTTTGTAGTATCTGGATGTGGACATTTGGAGCGCTTTCAGCC
          1 ATAGAGCAGGTTTGAAACACTCTTTTTGTAGTATCTGGAAGTGGATATTTGGATAGCTTTGAGGA

            * ** *      *             *** *   *                      *   *   
     501835 CTATGGT-GAAAAAGGAAATATCTTCCCCTGAAAACTAGACAGAAGCATTCTCAGAATCTTATTT
         66 TTTCGTTGGAAACAGG-AATATCTTCATATAAAAGCTAGACAGAAGCATTCTCAGAAACTTCTTT

                   **  **    *             **  * *   *
     501899 GTGATGTGCGCCCTCAACTAACAGTGTTGAAGCTTTCTTTTG
        130 GTGATGTTTGCATTCAAGTAACAGTGTTGAACATTCCCTTTC

                     *                    **     *   *                       
     501941 ATAGAGCAGTTTTGAAACACTCTTTTTGTAAAATCTGCAAGAGGATATTTGGATAGCTTTGAGGA
          1 ATAGAGCAGGTTTGAAACACTCTTTTTGTAGTATCTGGAAGTGGATATTTGGATAGCTTTGAGGA

                         *   * *            **                      *    *   
     502006 TTTCGTTGGAAACGGGATTGTCTTCATATAAACTCTAGACAGAAGCATTCTCAGAAGCTTCATTG
         66 TTTCGTTGGAAACAGGAATATCTTCATATAAAAGCTAGACAGAAGCATTCTCAGAAACTTCTTTG

            *       **   *    *             *        
     502071 GGATGTTTCAATTGAAGTCACAGTGTTGAACAGTCCCTTTC
        131 TGATGTTTGCATTCAAGTAACAGTGTTGAACATTCCCTTTC

                                                         *       **   * *    
     502112 ATAGAGCAGGTTTGAAACACTCTTTTTGTAGTATCTGGAAGTGGACATTTGGAGCGCTCTCAGGA
          1 ATAGAGCAGGTTTGAAACACTCTTTTTGTAGTATCTGGAAGTGGATATTTGGATAGCTTTGAGGA

            * *  *      *             **            *       * *          *  *
     502177 CTGCGGT-GAAAAAGGAACTATCTTCCAATAAAAGCTAGATAGAAGCAATGTCAGAAACTTTTTC
         66 TTTCGTTGGAAACAGGAA-TATCTTCATATAAAAGCTAGACAGAAGCATTCTCAGAAACTTCTTT

            *      * ** *   **      *       *  *     *
     502241 ATGATGTATCTACTCAGCTAACAGAGTTGAACCTTTCCTTTG
        130 GTGATGTTTGCATTCAAGTAACAGTGTTGAACATTCCCTTTC

             *       *                   * *     *             **            
     502283 AGAGAGCAGTTTTGAAACACTCTTTTTGTGGAATCTGCAAGTGGATATTTGTCTAGCTTTGAGGA
          1 ATAGAGCAGGTTTGAAACACTCTTTTTGTAGTATCTGGAAGTGGATATTTGGATAGCTTTGAGGA

                         *   *    *                   *       *     *   *    
     502348 TTTCGTTGGAAACGGGATTA-CAT-ATA-AAAAGC-AGACAGCAGCATTCCCAGAATCTTGTTTG
         66 TTTCGTTGGAAACAGGAATATCTTCATATAAAAGCTAGACAGAAGCATTCTCAGAAACTTCTTTG

                              *    *                 
     502409 TGATGTTTGCATTCAAGTCACAGAGTTGAACATTCCCTTTC
        131 TGATGTTTGCATTCAAGTAACAGTGTTGAACATTCCCTTTC

             *                         *    *      *     *       **     *   *
     502450 AGAGAGCAGGTTTGAAACACTCTTTTTATAGTGTCTGGATGTGGACATTTGGAGCGCTTTCAGGC
          1 ATAGAGCAGGTTTGAAACACTCTTTTTGTAGTATCTGGAAGTGGATATTTGGATAGCTTTGAGGA

            * ** *      *             *** **  *                          *   
     502515 CTATGGT-GAAAAAGGAAATATCTTCTCCTGTAAACTAGACAGAAGCATTCTCAGAAACTTATTT
         66 TTTCGTTGGAAACAGG-AATATCTTCATATAAAAGCTAGACAGAAGCATTCTCAGAAACTTCTTT

                   **  **    *             **  * *   *
     502579 GTGATGTGCGCCCTCAACTAACAGTGTTGAAGCTTTCTTTTG
        130 GTGATGTTTGCATTCAAGTAACAGTGTTGAACATTCCCTTTC

                     *                    **     *   *                       
     502621 ATAGAGCAGTTTTGAAACACTCTTTTTGTAAAATCTGCAAGAGGATATTTGGATAGCTTTGAGGA
          1 ATAGAGCAGGTTTGAAACACTCTTTTTGTAGTATCTGGAAGTGGATATTTGGATAGCTTTGAGGA

                         *   * *            **                      *    *   
     502686 TTTCGTTGGAAACGGGATTGTCTTCATATAAACTCTAGACAGAAGCATTCTCAGAAGCTTCATTG
         66 TTTCGTTGGAAACAGGAATATCTTCATATAAAAGCTAGACAGAAGCATTCTCAGAAACTTCTTTG

            *       **   *    *             *        
     502751 GGATGTTTCAATTGAAGTCACAGTGTTGAACAGTCCCTTTC
        131 TGATGTTTGCATTCAAGTAACAGTGTTGAACATTCCCTTTC

                                                         *       **   * *    
     502792 ATAGAGCAGGTTTGAAACACTCTTTTTGTAGTATCTGGAAGTGGACATTTGGAACGCTCTCAGGA
          1 ATAGAGCAGGTTTGAAACACTCTTTTTGTAGTATCTGGAAGTGGATATTTGGATAGCTTTGAGGA

            * *  *      *             **            *       * *          *  *
     502857 CTGCGGT-GAAAAAGGAAATATCTTCCAATAAAAGCTAGATAGAAGCAATGTCAGAAACTTTTTC
         66 TTTCGTTGGAAACAGG-AATATCTTCATATAAAAGCTAGACAGAAGCATTCTCAGAAACTTCTTT

            *      * ** *   **      *       *  * *   *
     502921 ATGATGTATCTACTCAGCTAACAGAGTTGAACCTTTCTTTTG
        130 GTGATGTTTGCATTCAAGTAACAGTGTTGAACATTCCCTTTC

             *       *                   * *     *           * **            
     502963 AGAGAGCAGTTTTGAAACACTCTTTTTGTGGAATCTGCAAGTGGATATTAGTCTAGCTTTGAGGA
          1 ATAGAGCAGGTTTGAAACACTCTTTTTGTAGTATCTGGAAGTGGATATTTGGATAGCTTTGAGGA

                         *   *    *                   *       *   *          
     503028 TTTCGTTGGAAACGGGATTA-CAT-ATA-AAAAGC-AGACAGCAGCATTCCCAGTAACTTCTTTG
         66 TTTCGTTGGAAACAGGAATATCTTCATATAAAAGCTAGACAGAAGCATTCTCAGAAACTTCTTTG

                              *    *                 
     503089 TGATGTTTGCATTCAAGTCACAGAGTTGAACATTCCCTTTC
        131 TGATGTTTGCATTCAAGTAACAGTGTTGAACATTCCCTTTC

                                 *                 *             **     *   *
     503130 ATAGAGCAGGTTTGAAACACTTTTTTTGTAGTATCTGGATGTGGATATTTGGAGCGCTTTCAGGC
          1 ATAGAGCAGGTTTGAAACACTCTTTTTGTAGTATCTGGAAGTGGATATTTGGATAGCTTTGAGGA

            * ** *      *             **          * *       * *          *  *
     503195 CTATGGT-GAAAAAGGAAATATCTTCCAATAAAAGCTACATAGAAGCAATGTCAGAAACTTTTTC
         66 TTTCGTTGGAAACAGG-AATATCTTCATATAAAAGCTAGACAGAAGCATTCTCAGAAACTTCTTT

            *      * ** *   **      *       *  * *   *
     503259 ATGATGTATCTACTCAGCTAACAGAGTTGAACCTTTCTTTTG
        130 GTGATGTTTGCATTCAAGTAACAGTGTTGAACATTCCCTTTC

             *       *                            *             **           
     503301 AGAGAGCAGTTTTGAAACACTCTTTTTGT-GTAATCTGAAAGTGGATATTTGTCTAGCTTTGAGG
          1 ATAGAGCAGGTTTGAAACACTCTTTTTGTAGT-ATCTGGAAGTGGATATTTGGATAGCTTTGAGG

                          *   *    *                   *       *   *         
     503365 ATTTCGTTGGAAACGGGATTA-CAT-ATA-AAAAGC-AGACAGCAGCATTCCCAGTAACTTCTTT
         65 ATTTCGTTGGAAACAGGAATATCTTCATATAAAAGCTAGACAGAAGCATTCTCAGAAACTTCTTT

                *              *    *                 
     503426 GTGAAGTTTGCATTCAAGTCACAGAGTTGAACATTCCCTTTC
        130 GTGATGTTTGCATTCAAGTAACAGTGTTGAACATTCCCTTTC

                                                   *     *       **     *    
     503468 ATAGAGCAGGTTTGAAACACTCTTTTTGTAGTATCTGGATGTGGACATTTGGAGCGCTTTCAGG-
          1 ATAGAGCAGGTTTGAAACACTCTTTTTGTAGTATCTGGAAGTGGATATTTGGATAGCTTTGAGGA

            **  *        *             *** *   *           *          *   *  
     503532 CCT-ATTGTGAAAAAGGAAATATCTTCCCCTGAAAACTAGACAGAAGAATTCTCAGAATCTTATT
         66 TTTCGTTG-GAAACAGG-AATATCTTCATATAAAAGCTAGACAGAAGCATTCTCAGAAACTTCTT

                    **  **    *             **  * *   *
     503596 TGTGATGTGCGCCCTCAACTAACAGTGTTGAAGCTTTCTTTTG
        129 TGTGATGTTTGCATTCAAGTAACAGTGTTGAACATTCCCTTTC

                     *                    **     *   *                       
     503639 ATAGAGCAGTTTTGAAACACTCTTTTTGTAAAATCTGCAAGAGGATATTTGGATAGCTTTGAGGA
          1 ATAGAGCAGGTTTGAAACACTCTTTTTGTAGTATCTGGAAGTGGATATTTGGATAGCTTTGAGGA

                         *   * *            **   *                  * *  *   
     503704 TTTCGTTGGAAACGGGATTGTCTTCATATAAACTCTACACAGAAGCATTCTCAGAAGCGTCATTG
         66 TTTCGTTGGAAACAGGAATATCTTCATATAAAAGCTAGACAGAAGCATTCTCAGAAACTTCTTTG

            *       **   *    *             *        
     503769 GGATGTTTCAATTGAAGTCACAGTGTTGAACAGTCCCTTTC
        131 TGATGTTTGCATTCAAGTAACAGTGTTGAACATTCCCTTTC

                                                   *     *       **     *    
     503810 ATAGAGCAGGTTTGAAACACTCTTTTTGTAGTATCTGGATGTGGACATTTGGAGCGCTTTCAGGC
          1 ATAGAGCAGGTTTGAAACACTCTTTTTGTAGTATCTGGAAGTGGATATTTGGATAGCTTTGAGG-

            * * *    *                 *** *   *                          *  
     503875 CTATGGTT-TAAA-AGGAAATATCTTCCCCTGAAAACTAGACAGAAGCATTCTCAGAAACTTATT
         65 ATTTCGTTGGAAACAGG-AATATCTTCATATAAAAGCTAGACAGAAGCATTCTCAGAAACTTCTT

                    **  *     *                    *   *
     503938 TGTGATGTGCGCCTTCAACTAACAGTGTTGAAGCATT-CTTTTG
        129 TGTGATGTTTGCATTCAAGTAACAGTGTTGAA-CATTCCCTTTC

                     *                   * *     *             **            
     503981 ATAGAGCAGTTTTGAAACACTCTTTTTGTGGAATCTGCAAGTGGATATTTGTCTAGCTTTGAGGA
          1 ATAGAGCAGGTTTGAAACACTCTTTTTGTAGTATCTGGAAGTGGATATTTGGATAGCTTTGAGGA

                         *   *    *                   *       *              
     504046 TTTCGTTGGAAACGGGATTA-CAT-ATA-AAAAGC-AGACAGCAGCATTCCCAGAAACTTCTTTG
         66 TTTCGTTGGAAACAGGAATATCTTCATATAAAAGCTAGACAGAAGCATTCTCAGAAACTTCTTTG

                              *    *                 
     504107 TGATGTTTGCATTCAAGTCACAGAGTTGAACATTCCCTTTC
        131 TGATGTTTGCATTCAAGTAACAGTGTTGAACATTCCCTTTC

             *                                     *     *       **     *   *
     504148 AGAGAGCAGGTTTGAAACACTCTTTTTGTAGTATCTGGATGTGGACATTTGGAGCGCTTTCAGGC
          1 ATAGAGCAGGTTTGAAACACTCTTTTTGTAGTATCTGGAAGTGGATATTTGGATAGCTTTGAGGA

            * ** *      *             *** *   *                      *   *   
     504213 CTATGGT-GAAAAAGGAAATATCTTCCCCTGAAAACTAGACAGAAGCATTCTCAGAATCTTATTT
         66 TTTCGTTGGAAACAGG-AATATCTTCATATAAAAGCTAGACAGAAGCATTCTCAGAAACTTCTTT

                   **  **    *             **  * *   *
     504277 GTGATGTGCGCCCTCAACTAACAGTGTTGAAGCTTTCTTTTG
        130 GTGATGTTTGCATTCAAGTAACAGTGTTGAACATTCCCTTTC

                     *                    **     *   *                       
     504319 ATAGAGCAGTTTTGAAACACTCTTTTTGTAAAATCTGCAAGAGGATATTTGGATAGCTTTGAGGA
          1 ATAGAGCAGGTTTGAAACACTCTTTTTGTAGTATCTGGAAGTGGATATTTGGATAGCTTTGAGGA

                         *   * *            **                      *    *   
     504384 TTTCGTTGGAAACGGGATTGTCTTCATATAAACTCTAGACAGAAGCATTCTCAGAAGCTTCATTG
         66 TTTCGTTGGAAACAGGAATATCTTCATATAAAAGCTAGACAGAAGCATTCTCAGAAACTTCTTTG

            *       **   *    *             *        
     504449 GGATGTTTCAATTGAAGTCACAGTGTTGAACAGTCCCTTTC
        131 TGATGTTTGCATTCAAGTAACAGTGTTGAACATTCCCTTTC

                                *                        *       **   * *    
     504490 ATAGAGCAGGTTTGAAACACACTTTTTGTAGTATCTGGAAGTGGACATTTGGAGCGCTCTCAGGA
          1 ATAGAGCAGGTTTGAAACACTCTTTTTGTAGTATCTGGAAGTGGATATTTGGATAGCTTTGAGGA

            * *  *      *             **            *       * *          *  *
     504555 CTACGGT-GAAAAAGGAACTATCTTCCAATAAAAGCTAGATAGAAGCAATGTCAGAAACTTTTTC
         66 TTTCGTTGGAAACAGGAA-TATCTTCATATAAAAGCTAGACAGAAGCATTCTCAGAAACTTCTTT

            *      * ** *   **      *       *        *
     504619 ATGATGTATCTACTCAGCTAACAGAGTTGAACCTT-CCTTTG
        130 GTGATGTTTGCATTCAAGTAACAGTGTTGAACATTCCCTTTC

             *       *                   * *     *             **            
     504660 AGAGAGCAGTTTTGAAACACTCTTTTTGTGGAATCTGCAAGTGGATATTTGTCTAGCTTTGAGGA
          1 ATAGAGCAGGTTTGAAACACTCTTTTTGTAGTATCTGGAAGTGGATATTTGGATAGCTTTGAGGA

                         *   *    *                   *       *              
     504725 TTTCGTTGGAAACGGGATTA-CAT-ATA-AAAAGC-AGACAGCAGCATTCCCAGAAACTTCTTTG
         66 TTTCGTTGGAAACAGGAATATCTTCATATAAAAGCTAGACAGAAGCATTCTCAGAAACTTCTTTG

                              *    *                 
     504786 TGATGTTTGCATTCAAGTCACAGAGTTGAACATTCCCTTTC
        131 TGATGTTTGCATTCAAGTAACAGTGTTGAACATTCCCTTTC

                                                   *     *     * **     *   *
     504827 ATAGAGCAGGTTTGAAACACTCTTTTTGTAGTATCTGGATGTGGACATTTGCAGCGCTTTCAGGC
          1 ATAGAGCAGGTTTGAAACACTCTTTTTGTAGTATCTGGAAGTGGATATTTGGATAGCTTTGAGGA

            * ** *      *             *** *   *                          *   
     504892 CTAAGGT-GAAAAAGGAAATATCTTCCCCTGAAAACTAGACAGAAGCATTCTCAGAAACTTATTT
         66 TTTCGTTGGAAACAGG-AATATCTTCATATAAAAGCTAGACAGAAGCATTCTCAGAAACTTCTTT

                   **  **    *     *       **  * *   *
     504956 GTGATGTGCGCCCTCAACTAACAATGTTGAAGCTTTCTTTTG
        130 GTGATGTTTGCATTCAAGTAACAGTGTTGAACATTCCCTTTC

                     *                    *      *   *                       
     504998 ATAGAGCAGTTTTGAAACACTCTTTTTGTAATATCTGCAAGAGGATATTTGGATAGCTTTGAGGA
          1 ATAGAGCAGGTTTGAAACACTCTTTTTGTAGTATCTGGAAGTGGATATTTGGATAGCTTTGAGGA

                         *   * *            **                      *    *   
     505063 TTTCGTTGGAAACGGGATTGTCTTCATATAAACTCTAGACAGAAGCATTCTCAGAAGCTTCATTG
         66 TTTCGTTGGAAACAGGAATATCTTCATATAAAAGCTAGACAGAAGCATTCTCAGAAACTTCTTTG

            *       **   *    *             * *      
     505128 GGATGTTTCAATTGAAGTCACAGTGTTGAACAGTTCCTTTC
        131 TGATGTTTGCATTCAAGTAACAGTGTTGAACATTCCCTTTC

                 *                                       *       **   * *    
     505169 ATAGAACAGGTTTGAAACACTCTTTTTGTAGTATCTGGAAGTGGACATTTGGAGCGCTCTCAGGA
          1 ATAGAGCAGGTTTGAAACACTCTTTTTGTAGTATCTGGAAGTGGATATTTGGATAGCTTTGAGGA

            * *  *  *   ** *         **          * *       * *          *  **
     505234 CTACGGTGAAAATGGAAATATCTTCCAATAAAAGCTACATAGAAGCAATGTCAGAAACTTTTTCA
         66 TTTCGTTGGAAACAGGAATATCTTCATATAAAAGCTAGACAGAAGCATTCTCAGAAACTTCTTTG

                  * ** *   **      *       *  *     *
     505299 TGATGTATCTACTCAGCTAACAGAGTTGAACCTTTCCTTTG
        131 TGATGTTTGCATTCAAGTAACAGTGTTGAACATTCCCTTTC

             *       *                   * *     *             **            
     505340 AGAGAGCAGTTTTGAAACACTCTTTTTGTGGAATCTGCAAGTGGATATTTGTCTAGCTTTGAGGA
          1 ATAGAGCAGGTTTGAAACACTCTTTTTGTAGTATCTGGAAGTGGATATTTGGATAGCTTTGAGGA

                         *   *    *                   *       *     *        
     505405 TTTCGTTGGAAACGGGATTA-CAT-ATA-AAAAGC-AGACAGCAGCATTCCCAGAATCTTCTTTG
         66 TTTCGTTGGAAACAGGAATATCTTCATATAAAAGCTAGACAGAAGCATTCTCAGAAACTTCTTTG

                              *    *                 
     505466 TGATGTTTGCATTCAAGTCACAGAGTTGAACATTCCCTTTC
        131 TGATGTTTGCATTCAAGTAACAGTGTTGAACATTCCCTTTC

                                                   *     *       **     *   *
     505507 ATAGAGCAGGTTTGAAACACTCTTTTTGTAGTATCTGGATGTGGACATTTGGAGCGCTTTCAGGC
          1 ATAGAGCAGGTTTGAAACACTCTTTTTGTAGTATCTGGAAGTGGATATTTGGATAGCTTTGAGGA

            * ** *      *             *** *   *                      *   *   
     505572 CTATGGT-GAAAAAGGAAATATCTTCCCCTGAAAACTAGACAGAAGCATTCTCAGAATCTTATTT
         66 TTTCGTTGGAAACAGG-AATATCTTCATATAAAAGCTAGACAGAAGCATTCTCAGAAACTTCTTT

                   **  **    *             **  * *   *
     505636 GTGATGTGCGCCCTCAACTAACAGTGTTGAAGCTTTCTTTTG
        130 GTGATGTTTGCATTCAAGTAACAGTGTTGAACATTCCCTTTC

                     *                    **     *   *                       
     505678 ATAGAGCAGTTTTGAAACACTCTTTTTGTAAAATCTGCAAGAGGATATTTGGATAGCTTTGAGGA
          1 ATAGAGCAGGTTTGAAACACTCTTTTTGTAGTATCTGGAAGTGGATATTTGGATAGCTTTGAGGA

                         *   * *            **                      *    *   
     505743 TTTCGTTGGAAACGGGATTGTCTTCATATAAACTCTAGACAGAAGCATTCTCAGAAGCTTCATTG
         66 TTTCGTTGGAAACAGGAATATCTTCATATAAAAGCTAGACAGAAGCATTCTCAGAAACTTCTTTG

            *       **   *    *             *        
     505808 GGATGTTTCAATTGAAGTCACAGTGTTGAACAGTCCCTTTC
        131 TGATGTTTGCATTCAAGTAACAGTGTTGAACATTCCCTTTC

                                                         *       **   * *    
     505849 ATAGAGCAGGTTTGAAACACTCTTTTTGTAGTATCTGGAAGTGGACATTTGGAACGCTCTCAGGA
          1 ATAGAGCAGGTTTGAAACACTCTTTTTGTAGTATCTGGAAGTGGATATTTGGATAGCTTTGAGGA

            * *  *      *             **            *       * *          *  *
     505914 CTGCGGT-GAAAAAGGAAATATCTTCCAATAAAAGCTAGATAGAAGCAATGTCAGAAACTTTTTC
         66 TTTCGTTGGAAACAGG-AATATCTTCATATAAAAGCTAGACAGAAGCATTCTCAGAAACTTCTTT

            *      * ** *   **      *       *  * *   *
     505978 ATGATGTATCTACTCAGCTAACAGAGTTGAACCTTTCTTTTG
        130 GTGATGTTTGCATTCAAGTAACAGTGTTGAACATTCCCTTTC

             *       *                   * *     *             **            
     506020 AGAGAGCAGTTTTGAAACACTCTTTTTGTGGAATCTGCAAGTGGATATTTGTCTAGCTTTGAGGA
          1 ATAGAGCAGGTTTGAAACACTCTTTTTGTAGTATCTGGAAGTGGATATTTGGATAGCTTTGAGGA

                         *   *    *                   *       *   *          
     506085 TTTCGTTGGAAACGGGATTA-CAT-ATA-AAAAGC-AGACAGCAGCATTCCCAGTAACTTCTTTG
         66 TTTCGTTGGAAACAGGAATATCTTCATATAAAAGCTAGACAGAAGCATTCTCAGAAACTTCTTTG

                              *    *                 
     506146 TGATGTTTGCATTCAAGTCACAGAGTTGAACATTCCCTTTC
        131 TGATGTTTGCATTCAAGTAACAGTGTTGAACATTCCCTTTC

                                 *                 *     *       **     *   *
     506187 ATAGAGCAGGTTTGAAACACTTTTTTTGTAGTATCTGGATGTGGACATTTGGAGCGCTTTCAGGC
          1 ATAGAGCAGGTTTGAAACACTCTTTTTGTAGTATCTGGAAGTGGATATTTGGATAGCTTTGAGGA

            * ** *      *             **          * * *     * *          *  *
     506252 CTATGGT-GAAAAAGGAAATATCTTCCAATAAAAGCTACATAAAAGCAATGTCAGAAACTTTTTC
         66 TTTCGTTGGAAACAGG-AATATCTTCATATAAAAGCTAGACAGAAGCATTCTCAGAAACTTCTTT

            *      * ** *   **      *       *  * *   *
     506316 ATGATGTATCTACTCAGCTAACAGAGTTGAACCTTTCTTTTG
        130 GTGATGTTTGCATTCAAGTAACAGTGTTGAACATTCCCTTTC

             *       *                   * *                   **            
     506358 AGAGAGCAGTTTTGAAACACTCTTTTTGTGGAATCTGGAAGTGGATATTTGTCTAGCTTTGAGGA
          1 ATAGAGCAGGTTTGAAACACTCTTTTTGTAGTATCTGGAAGTGGATATTTGGATAGCTTTGAGGA

                         *   *    *                   *       *   *          
     506423 TTTCGTTGGAAACGGGATTA-CAT-ATA-AAAAGC-AGACAGCAGCATTCCCAGTAACTTCTTTG
         66 TTTCGTTGGAAACAGGAATATCTTCATATAAAAGCTAGACAGAAGCATTCTCAGAAACTTCTTTG

                              *    *      *          
     506484 TGATGTTTGCATTCAAGTCACAGAGTTGAATATTCCCTTTC
        131 TGATGTTTGCATTCAAGTAACAGTGTTGAACATTCCCTTTC

                                                   *     *       **     *    
     506525 ATAGAGCAGGTTTGAAACACTCTTTTTGTAGTATCTGGATGTGGACATTTGGAGCGCTTTCAGG-
          1 ATAGAGCAGGTTTGAAACACTCTTTTTGTAGTATCTGGAAGTGGATATTTGGATAGCTTTGAGGA

            **  *        *             *** *   *           *          *   *  
     506589 CCT-ATTGTGAAAAAGGAAATATCTTCCCCTGAAAACTAGACAGAAGAATTCTCAGAATCTTATT
         66 TTTCGTTG-GAAACAGG-AATATCTTCATATAAAAGCTAGACAGAAGCATTCTCAGAAACTTCTT

                    **  **    *             **  * *   *
     506653 TGTGATGTGCGCCCTCAACTAACAGTGTTGAAGCTTTCTTTTG
        129 TGTGATGTTTGCATTCAAGTAACAGTGTTGAACATTCCCTTTC

                     *                    **     *   *                       
     506696 ATAGAGCAGTTTTGAAACACTCTTTTTGTAAAATCTGCAAGAGGATATTTGGATAGCTTTGAGGA
          1 ATAGAGCAGGTTTGAAACACTCTTTTTGTAGTATCTGGAAGTGGATATTTGGATAGCTTTGAGGA

                         *   * * *          **   *                  * *  *   
     506761 TTTCGTTGGAAACGGGATTGTTTTCATATAAACTCTACACAGAAGCATTCTCAGAAGCGTCATTG
         66 TTTCGTTGGAAACAGGAATATCTTCATATAAAAGCTAGACAGAAGCATTCTCAGAAACTTCTTTG

            *       **   *    *             *        
     506826 GGATGTTTCAATTGAAGTCACAGTGTTGAACAGTCCCTTTC
        131 TGATGTTTGCATTCAAGTAACAGTGTTGAACATTCCCTTTC

                                                   *     *       **     *   *
     506867 ATAGAGCAGGTTTGAAACACTCTTTTTGTAGTATCTGGATGTGGACATTTGGAGCGCTTTCAGGC
          1 ATAGAGCAGGTTTGAAACACTCTTTTTGTAGTATCTGGAAGTGGATATTTGGATAGCTTTGAGGA

            * ** *      *             *** *   *                      *   *   
     506932 CTATGGT-GAAAAAGGAAATATCTTCCCCTGAAAACTAGACAGAAGCATTCTCAGAATCTTATTT
         66 TTTCGTTGGAAACAGG-AATATCTTCATATAAAAGCTAGACAGAAGCATTCTCAGAAACTTCTTT

                   **  **    *             **  * *   *
     506996 GTGATGTGCGCCCTCAACTAACAGTGTTGAAGCTTTCTTTTG
        130 GTGATGTTTGCATTCAAGTAACAGTGTTGAACATTCCCTTTC

                     *                    **     *   *                       
     507038 ATAGAGCAGTTTTGAAACACTCTTTTTGTAAAATCTGCAAGAGGATATTTGGATAGCTTTGAGGA
          1 ATAGAGCAGGTTTGAAACACTCTTTTTGTAGTATCTGGAAGTGGATATTTGGATAGCTTTGAGGA

                         *   * *            **                      *    *   
     507103 TTTCGTTGGAAACGGGATTGTCTTCATATAAACTCTAGACAGAAGCATTCTCAGAAGCTTCATTG
         66 TTTCGTTGGAAACAGGAATATCTTCATATAAAAGCTAGACAGAAGCATTCTCAGAAACTTCTTTG

            *       **   *    *             *        
     507168 GGATGTTTCAATTGAAGTCACAGTGTTGAACAGTCCCTTTC
        131 TGATGTTTGCATTCAAGTAACAGTGTTGAACATTCCCTTTC

                                                         *       **   * *    
     507209 ATAGAGCAGGTTTGAAACACTCTTTTTGTAGTATCTGGAAGTGGACATTTGGAGCGCTCTCAGGA
          1 ATAGAGCAGGTTTGAAACACTCTTTTTGTAGTATCTGGAAGTGGATATTTGGATAGCTTTGAGGA

            * *  *      *             **            *       * *          *  *
     507274 CTGCGGT-GAAAAAGGAACTATCTTCCAATAAAAGCTAGATAGAAGCAATGTCAGAAACTTTTTC
         66 TTTCGTTGGAAACAGGAA-TATCTTCATATAAAAGCTAGACAGAAGCATTCTCAGAAACTTCTTT

            *      * ** *   **      *       *        *
     507338 ATGATGTATCTACTCAGCTAACAGAGTTGAACCTT-CCTTTG
        130 GTGATGTTTGCATTCAAGTAACAGTGTTGAACATTCCCTTTC

             *       *                   * *     *             **            
     507379 AGAGAGCAGTTTTGAAACACTCTTTTTGTGGAATCTGCAAGTGGATATTTGTCTAGCTTTGAGGA
          1 ATAGAGCAGGTTTGAAACACTCTTTTTGTAGTATCTGGAAGTGGATATTTGGATAGCTTTGAGGA

                         *   * *            **                      *    *   
     507444 TTTCGTTGGAAACGGGATTGTCTTCATATAAACTCTAGACAGAAGCATTCTCAGAAGCTTCATTG
         66 TTTCGTTGGAAACAGGAATATCTTCATATAAAAGCTAGACAGAAGCATTCTCAGAAACTTCTTTG

            *       **   *    *             * *      
     507509 GGATGTTTCAATTGAAGTCACAGTGTTGAACAGTTCCTTTC
        131 TGATGTTTGCATTCAAGTAACAGTGTTGAACATTCCCTTTC

                 *                                       *       **   * *    
     507550 ATAGAACAGGTTTGAAACACTCTTTTTGTAGTATCTGGAAGTGGACATTTGGAGCGCTCTCAGGA
          1 ATAGAGCAGGTTTGAAACACTCTTTTTGTAGTATCTGGAAGTGGATATTTGGATAGCTTTGAGGA

            * *  *      *             **          * *       * *          *  *
     507615 CTACGGT-GAAAAAGGAAATATCTTCCAATAAAAGCTACATAGAAGCAATGTCAGAAACTTTTTC
         66 TTTCGTTGGAAACAGG-AATATCTTCATATAAAAGCTAGACAGAAGCATTCTCAGAAACTTCTTT

            * *    * ** *   **      *       *  *     *
     507679 ATCATGTATCTACTCAGCTAACAGAGTTGAACCTTTCCTTTG
        130 GTGATGTTTGCATTCAAGTAACAGTGTTGAACATTCCCTTTC

             *       *                   * *     *             **            
     507721 AGAGAGCAGTTTTGAAACACTCTTTTTGTGGAATCTGCAAGTGGATATTTGTCTAGCTTTGAGGA
          1 ATAGAGCAGGTTTGAAACACTCTTTTTGTAGTATCTGGAAGTGGATATTTGGATAGCTTTGAGGA

                         *   *    *                   *       *     *        
     507786 TTTCGTTGGAAACGGGATTA-CAT-ATA-AAAAGC-AGACAGCAGCATTCCCAGAATCTTCTTTG
         66 TTTCGTTGGAAACAGGAATATCTTCATATAAAAGCTAGACAGAAGCATTCTCAGAAACTTCTTTG

                              *    *                 
     507847 TGATGTTTGCATTCAAGTCACAGAGTTGAACATTCCCTTTC
        131 TGATGTTTGCATTCAAGTAACAGTGTTGAACATTCCCTTTC

                                                   *     *       **     *   *
     507888 ATAGAGCAGGTTTGAAACACTCTTTTTGTAGTATCTGGATGTGGACATTTGGAGCGCTTTCAGGC
          1 ATAGAGCAGGTTTGAAACACTCTTTTTGTAGTATCTGGAAGTGGATATTTGGATAGCTTTGAGGA

            * ** *      *             *** *   *                      *   *   
     507953 CTATGGT-GAAAAAGGAAATATCTTCCCCTGAAAACTAGACAGAAGCATTCTCAGAATCTTATTT
         66 TTTCGTTGGAAACAGG-AATATCTTCATATAAAAGCTAGACAGAAGCATTCTCAGAAACTTCTTT

                   **  **    *             **  * *   *
     508017 GTGATGTGCGCCCTCAACTAACAGTGTTGAAGCTTTCTTTTG
        130 GTGATGTTTGCATTCAAGTAACAGTGTTGAACATTCCCTTTC

                     *                    **     *   *                       
     508059 ATAGAGCAGTTTTGAAACACTCTTTTTGTAAAATCTGCAAGAGGATATTTGGATAGCTTTGAGGA
          1 ATAGAGCAGGTTTGAAACACTCTTTTTGTAGTATCTGGAAGTGGATATTTGGATAGCTTTGAGGA

                         *   * *            **                      *    *   
     508124 TTTCGTTGGAAACGGGATTGTCTTCATATAAACTCTAGACAGAAGCATTCTCAGAAGCTTCATTG
         66 TTTCGTTGGAAACAGGAATATCTTCATATAAAAGCTAGACAGAAGCATTCTCAGAAACTTCTTTG

            *       **   *    *             *        
     508189 GGATGTTTCAATTGAAGTCACAGTGTTGAACAGTCCCTTTC
        131 TGATGTTTGCATTCAAGTAACAGTGTTGAACATTCCCTTTC

                                                         *       **   * *    
     508230 ATAGAGCAGGTTTGAAACACTCTTTTTGTAGTATCTGGAAGTGGACATTTGGAACGCTCTCAGGA
          1 ATAGAGCAGGTTTGAAACACTCTTTTTGTAGTATCTGGAAGTGGATATTTGGATAGCTTTGAGGA

            * *  *      *             **            *       * *          *  *
     508295 CTGCGGT-GAAAAAGGAAATATCTTCCAATAAAAGCTAGATAGAAGCAATGTCAGAAACTTTTTC
         66 TTTCGTTGGAAACAGG-AATATCTTCATATAAAAGCTAGACAGAAGCATTCTCAGAAACTTCTTT

            *      * ** *   **      *       *  * *   *
     508359 ATGATGTATCTACTCAGCTAACAGAGTTGAACCTTTCTTTTG
        130 GTGATGTTTGCATTCAAGTAACAGTGTTGAACATTCCCTTTC

             *       *                    **     *   *                       
     508401 AGAGAGCAGTTTTGAAACACTCTTTTTGTAAAATCTGCAAGAGGATATTTGGATAGCTTTGAGGA
          1 ATAGAGCAGGTTTGAAACACTCTTTTTGTAGTATCTGGAAGTGGATATTTGGATAGCTTTGAGGA

                  *      *   * *            **                      * *  *   
     508466 TTTCGTAGGAAACGGGATTGTCTTCATATAAACTCTAGACAGAAGCATTCTCAGAAGCGTCATTG
         66 TTTCGTTGGAAACAGGAATATCTTCATATAAAAGCTAGACAGAAGCATTCTCAGAAACTTCTTTG

            *       **   *    *             *        
     508531 GGATGTTTCAATTGAAGTCACAGTGTTGAACAGTCCCTTTC
        131 TGATGTTTGCATTCAAGTAACAGTGTTGAACATTCCCTTTC

                                                   *     *       **     *    
     508572 ATAGAGCAGGTTTGAAACACTCTTTTTGTAGTATCTGGATGTGGACATTTGGAGCGCTTTCAGGC
          1 ATAGAGCAGGTTTGAAACACTCTTTTTGTAGTATCTGGAAGTGGATATTTGGATAGCTTTGAGG-

            * * *    *                 *** *   *                          *  
     508637 CTATGGTT-TAAA-AGGAAATATCTTCCCCTGAAAACTAGACAGAAGCATTCTCAGAAACTTATT
         65 ATTTCGTTGGAAACAGG-AATATCTTCATATAAAAGCTAGACAGAAGCATTCTCAGAAACTTCTT

                    **  **    *             **  * *   *
     508700 TGTGATGTGCGCCCTCAACTAACAGTGTTGAAGCTTTCTTTTG
        129 TGTGATGTTTGCATTCAAGTAACAGTGTTGAACATTCCCTTTC

                     *                    **     *   *                       
     508743 ATAGAGCAGTTTTGAAACACTCTTTTTGTAAAATCTGCAAGAGGATATTTGGATAGCTTTGAGGA
          1 ATAGAGCAGGTTTGAAACACTCTTTTTGTAGTATCTGGAAGTGGATATTTGGATAGCTTTGAGGA

                         *   * *            **                      *    *   
     508808 TTTCGTTGGAAACGGGATTGTCTTCATATAAACTCTAGACAGAAGCATTCTCAGAAGCTTCATTG
         66 TTTCGTTGGAAACAGGAATATCTTCATATAAAAGCTAGACAGAAGCATTCTCAGAAACTTCTTTG

            *       **   *    *             *        
     508873 GGATGTTTCAATTGAAGTCACAGTGTTGAACAGTCCCTTTC
        131 TGATGTTTGCATTCAAGTAACAGTGTTGAACATTCCCTTTC

                                                         *       **   * *    
     508914 ATAGAGCAGGTTTGAAACACTCTTTTTGTAGTATCTGGAAGTGGACATTTGGAGCGCTCTCAGGA
          1 ATAGAGCAGGTTTGAAACACTCTTTTTGTAGTATCTGGAAGTGGATATTTGGATAGCTTTGAGGA

            * *  *      *             **            *       * *          *  *
     508979 CTGCGGT-GAAAAAGGAACTATCTTCCAATAAAAGCTAGATAGAAGCAATGTCAGAAACTTTTTC
         66 TTTCGTTGGAAACAGGAA-TATCTTCATATAAAAGCTAGACAGAAGCATTCTCAGAAACTTCTTT

            *      * ** *   **      *       *        *
     509043 ATGATGTATCTACTCAGCTAACAGAGTTGAACCTT-CCTTTG
        130 GTGATGTTTGCATTCAAGTAACAGTGTTGAACATTCCCTTTC

             *       *                   * *     *             **            
     509084 AGAGAGCAGTTTTGAAACACTCTTTTTGTGGAATCTGCAAGTGGATATTTGTCTAGCTTTGAGGA
          1 ATAGAGCAGGTTTGAAACACTCTTTTTGTAGTATCTGGAAGTGGATATTTGGATAGCTTTGAGGA

                         *   *    *                   *       *              
     509149 TTTCGTTGGAAACGGGATTA-CAT-ATA-AAAAGC-AGACAGCAGCATTCCCAGAAACTTCTTTG
         66 TTTCGTTGGAAACAGGAATATCTTCATATAAAAGCTAGACAGAAGCATTCTCAGAAACTTCTTTG

                *             *    *                 
     509210 TGATATTTGCATTCAAGTCACAGAGTTGAACATTCCCTTTC
        131 TGATGTTTGCATTCAAGTAACAGTGTTGAACATTCCCTTTC

                                                   *     *     * **     *   *
     509251 ATAGAGCAGGTTTGAAACACTCTTTTTGTAGTATCTGGATGTGGACATTTGCAGCGCTTTCAGGC
          1 ATAGAGCAGGTTTGAAACACTCTTTTTGTAGTATCTGGAAGTGGATATTTGGATAGCTTTGAGGA

            * ** *      *             *** *   *                          *   
     509316 CTAAGGT-GAAAAAGGAAATATCTTCCCCTGAAAACTAGACAGAAGCATTCTCAGAAACTTATTT
         66 TTTCGTTGGAAACAGG-AATATCTTCATATAAAAGCTAGACAGAAGCATTCTCAGAAACTTCTTT

                   **  **    *             **  * *   *
     509380 GTGATGTGCGCCCTCAACTAACAGTGTTGAAGCTTTCTTTTG
        130 GTGATGTTTGCATTCAAGTAACAGTGTTGAACATTCCCTTTC

                     *                    *      *   *                       
     509422 ATAGAGCAGTTTTGAAACACTCTTTTTGTAATATCTGCAAGAGGATATTTGGATAGCTTTGAGGA
          1 ATAGAGCAGGTTTGAAACACTCTTTTTGTAGTATCTGGAAGTGGATATTTGGATAGCTTTGAGGA

                         *   * *            **                      *    *   
     509487 TTTCGTTGGAAACGGGATTGTCTTCATATAAACTCTAGACAGAAGCATTCTCAGAAGCTTCATTG
         66 TTTCGTTGGAAACAGGAATATCTTCATATAAAAGCTAGACAGAAGCATTCTCAGAAACTTCTTTG

            *       **   *    *             * *      
     509552 GGATGTTTCAATTGAAGTCACAGTGTTGAACAGTTCCTTTC
        131 TGATGTTTGCATTCAAGTAACAGTGTTGAACATTCCCTTTC

                 *                                       *       **   * *    
     509593 ATAGAACAGGTTTGAAACACTCTTTTTGTAGTATCTGGAAGTGGACATTTGGAGCGCTCTCAGGA
          1 ATAGAGCAGGTTTGAAACACTCTTTTTGTAGTATCTGGAAGTGGATATTTGGATAGCTTTGAGGA

            * *  *  *   ** *                      * *       * *          *  *
     509658 CTACGGTGAAAATGGAAATATCTTACA-ATAAAAGCTACATAGAAGCAATGTCAGAAACTTTTTC
         66 TTTCGTTGGAAACAGGAATATCTT-CATATAAAAGCTAGACAGAAGCATTCTCAGAAACTTCTTT

            *      * ** *   **      *       *  *     *
     509722 ATGATGTATCTACTCAGCTAACAGAGTTGAACCTTTCCTTTG
        130 GTGATGTTTGCATTCAAGTAACAGTGTTGAACATTCCCTTTC

             *       *                   * *     *             **            
     509764 AGAGAGCAGTTTTGAAACACTCTTTTTGTGGAATCTGCAAGTGGATATTTGTCTAGCTTTGAGGA
          1 ATAGAGCAGGTTTGAAACACTCTTTTTGTAGTATCTGGAAGTGGATATTTGGATAGCTTTGAGGA

                         *   *    *                   *       *     *        
     509829 TTTCGTTGGAAACGGGATTA-CAT-ATA-AAAAGC-AGACAGCAGCATTCCCAGAATCTTCTTTG
         66 TTTCGTTGGAAACAGGAATATCTTCATATAAAAGCTAGACAGAAGCATTCTCAGAAACTTCTTTG

                              *    *                 
     509890 TGATGTTTGCATTCAAGTCACAGAGTTGAACATTCCCTTTC
        131 TGATGTTTGCATTCAAGTAACAGTGTTGAACATTCCCTTTC

                         *                         *     *       **     *   *
     509931 ATAGAGCAGGTTTCAAACACTCTTTTTGTAGTATCTGGATGTGGACATTTGGAACGCTTTCAGGC
          1 ATAGAGCAGGTTTGAAACACTCTTTTTGTAGTATCTGGAAGTGGATATTTGGATAGCTTTGAGGA

            * ** *      *             *** *   *                      *   *   
     509996 CTATGGT-GAAAAAGGAAATATCTTCCCCTGAAAACTAGACAGAAGCATTCTCAGAATCTTATTT
         66 TTTCGTTGGAAACAGG-AATATCTTCATATAAAAGCTAGACAGAAGCATTCTCAGAAACTTCTTT

                   **  **    *             **  * *   *
     510060 GTGATGTGCGCCCTCAACTAACAGTGTTGAAGCTTTCTTTTG
        130 GTGATGTTTGCATTCAAGTAACAGTGTTGAACATTCCCTTTC

                     *                    **     *   *                       
     510102 ATAGAGCAGTTTTGAAACACTCTTTTTGTAAAATCTGCAAGAGGATATTTGGATAGCTTTGAGGA
          1 ATAGAGCAGGTTTGAAACACTCTTTTTGTAGTATCTGGAAGTGGATATTTGGATAGCTTTGAGGA

                         *   * *            **                      *    *   
     510167 TTTCGTTGGAAACGGGATTGTCTTCATATAAACTCTAGACAGAAGCATTCTCAGAAGCTTCATTG
         66 TTTCGTTGGAAACAGGAATATCTTCATATAAAAGCTAGACAGAAGCATTCTCAGAAACTTCTTTG

            *       **   *    *             *        
     510232 GGATGTTTCAATTGAAGTCACAGTGTTGAACAGTCCCTTTC
        131 TGATGTTTGCATTCAAGTAACAGTGTTGAACATTCCCTTTC

                                                         *       **   * *    
     510273 ATAGAGCAGGTTTGAAACACTCTTTTTGTAGTATCTGGAAGTGGACATTTGGAACGCTCTCAGGA
          1 ATAGAGCAGGTTTGAAACACTCTTTTTGTAGTATCTGGAAGTGGATATTTGGATAGCTTTGAGGA

            * *  *      *             **            *       * *          *  *
     510338 CTGCGGT-GAAAAAGGAAATATCTTCCAATAAAAGCTAGATAGAAGCAATGTCAGAAACTTTTTC
         66 TTTCGTTGGAAACAGG-AATATCTTCATATAAAAGCTAGACAGAAGCATTCTCAGAAACTTCTTT

            *      * ** *   **      *       *  * *   *
     510402 ATGATGTATCTACTCAGCTAACAGAGTTGAACCTTTCTTTTG
        130 GTGATGTTTGCATTCAAGTAACAGTGTTGAACATTCCCTTTC

             *       *            *      * *     *             **  *         
     510444 AGAGAGCAGTTTTGAAACACTCGTTTTGTGGAATCTGCAAGTGGATATTTGTCTACCTTTGAGGA
          1 ATAGAGCAGGTTTGAAACACTCTTTTTGTAGTATCTGGAAGTGGATATTTGGATAGCTTTGAGGA

                         *   *    *                   *       *              
     510509 TTTCGTTGGAAACGGGATTA-CAT-ATA-AAAAGC-AGACAGCAGCATTCCCAGAAACTTCTTTG
         66 TTTCGTTGGAAACAGGAATATCTTCATATAAAAGCTAGACAGAAGCATTCTCAGAAACTTCTTTG

                              *    *                 
     510570 TGATGTTTGCATTCAAGTCACAGAGTTGAACATTCCCTTTC
        131 TGATGTTTGCATTCAAGTAACAGTGTTGAACATTCCCTTTC

                                                   *     *     * **     *   *
     510611 ATAGAGCAGGTTTGAAACACTCTTTTTGTAGTATCTGGATGTGGACATTTGCAGCGCTTTCAGGC
          1 ATAGAGCAGGTTTGAAACACTCTTTTTGTAGTATCTGGAAGTGGATATTTGGATAGCTTTGAGGA

            * ** *      *             *** *   *                          *   
     510676 CTAAGGT-GAAAAAGGAAATATCTTCCCCTGAAAACTAGACAGAAGCATTCTCAGAAACTTATTT
         66 TTTCGTTGGAAACAGG-AATATCTTCATATAAAAGCTAGACAGAAGCATTCTCAGAAACTTCTTT

                   **  *     *                    *   *
     510740 GTGATGTGCGCCTTCAACTAACAGTGTTGAAGCATT-CTTTTG
        130 GTGATGTTTGCATTCAAGTAACAGTGTTGAA-CATTCCCTTTC

                     *                   * *     *             **            
     510782 ATAGAGCAGTTTTGAAACACTCTTTTTGTGGAATCTGCAAGTGGATATTTGTCTAGCTTTGAGGA
          1 ATAGAGCAGGTTTGAAACACTCTTTTTGTAGTATCTGGAAGTGGATATTTGGATAGCTTTGAGGA

                         *   *    *                   *       *              
     510847 TTTCGTTGGAAACGGGATTA-CAT-ATA-AAAAGC-AGACAGCAGCATTCCCAGAAACTTCTTTG
         66 TTTCGTTGGAAACAGGAATATCTTCATATAAAAGCTAGACAGAAGCATTCTCAGAAACTTCTTTG

                              *    *                 
     510908 TGATGTTTGCATTCAAGTCACAGAGTTGAACATTCCCTTTC
        131 TGATGTTTGCATTCAAGTAACAGTGTTGAACATTCCCTTTC

             *                                     *     *       **     *   *
     510949 AGAGAGCAGGTTTGAAACACTCTTTTTGTAGTATCTGGATGTGGACATTTGGAGCGCTTTCAGGC
          1 ATAGAGCAGGTTTGAAACACTCTTTTTGTAGTATCTGGAAGTGGATATTTGGATAGCTTTGAGGA

            * ** *      *             *** *   *                      *   *   
     511014 CTATGGT-GAAAAAGGAAATATCTTCCCCTGAAAACTAGACAGAAGCATTCTCAGAATCTTATTT
         66 TTTCGTTGGAAACAGG-AATATCTTCATATAAAAGCTAGACAGAAGCATTCTCAGAAACTTCTTT

                   **  **    *             **  * *   *
     511078 GTGATGTGCGCCCTCAACTAACAGTGTTGAAGCTTTCTTTTG
        130 GTGATGTTTGCATTCAAGTAACAGTGTTGAACATTCCCTTTC

                     *                    **     *   *                       
     511120 ATAGAGCAGTTTTGAAACACTCTTTTTGTAAAATCTGCAAGAGGATATTTGGATAGCTTTGAGGA
          1 ATAGAGCAGGTTTGAAACACTCTTTTTGTAGTATCTGGAAGTGGATATTTGGATAGCTTTGAGGA

                         *   * *            **                      *    *   
     511185 TTTCGTTGGAAACGGGATTGTCTTCATATAAACTCTAGACAGAAGCATTCTCAGAAGCTTCATTG
         66 TTTCGTTGGAAACAGGAATATCTTCATATAAAAGCTAGACAGAAGCATTCTCAGAAACTTCTTTG

            *       **   *    *             *        
     511250 GGATGTTTCAATTGAAGTCACAGTGTTGAACAGTCCCTTTC
        131 TGATGTTTGCATTCAAGTAACAGTGTTGAACATTCCCTTTC

                                *                        *       **   * *    
     511291 ATAGAGCAGGTTTGAAACACACTTTTTGTAGTATCTGGAAGTGGACATTTGGAGCGCTCTCAGGA
          1 ATAGAGCAGGTTTGAAACACTCTTTTTGTAGTATCTGGAAGTGGATATTTGGATAGCTTTGAGGA

            * *  *      *             **            *       * *          *  *
     511356 CTACGGT-GAAAAAGGAACTATCTTCCAATAAAAGCTAGATAGAAGCAATGTCAGAAACTTTTTC
         66 TTTCGTTGGAAACAGGAA-TATCTTCATATAAAAGCTAGACAGAAGCATTCTCAGAAACTTCTTT

            *      * ** *   **      *       *        *
     511420 ATGATGTATCTACTCAGCTAACAGAGTTGAACCTT-CCTTTG
        130 GTGATGTTTGCATTCAAGTAACAGTGTTGAACATTCCCTTTC

             *       *                   * *     *             **            
     511461 AGAGAGCAGTTTTGAAACACTCTTTTTGTGGAATCTGCAAGTGGATATTTGTCTAGCTTTGAGGA
          1 ATAGAGCAGGTTTGAAACACTCTTTTTGTAGTATCTGGAAGTGGATATTTGGATAGCTTTGAGGA

                         *   *    *                   *       *              
     511526 TTTCGTTGGAAACGGGATTA-CAT-ATA-AAAAGC-AGACAGCAGCATTCCCAGAAACTTCTTTG
         66 TTTCGTTGGAAACAGGAATATCTTCATATAAAAGCTAGACAGAAGCATTCTCAGAAACTTCTTTG

                              *    *                 
     511587 TGATGTTTGCATTCAAGTCACAGAGTTGAACATTCCCTTTC
        131 TGATGTTTGCATTCAAGTAACAGTGTTGAACATTCCCTTTC

                                                   *     *     * **     *   *
     511628 ATAGAGCAGGTTTGAAACACTCTTTTTGTAGTATCTGGATGTGGACATTTGCAGCGCTTTCAGGC
          1 ATAGAGCAGGTTTGAAACACTCTTTTTGTAGTATCTGGAAGTGGATATTTGGATAGCTTTGAGGA

            * ** *      *             *** *   *                          *   
     511693 CTAAGGT-GAAAAAGGAAATATCTTCCCCTGAAAACTAGACAGAAGCATTCTCAGAAACTTATTT
         66 TTTCGTTGGAAACAGG-AATATCTTCATATAAAAGCTAGACAGAAGCATTCTCAGAAACTTCTTT

                   **  **    *     *       **  * *   *
     511757 GTGATGTGCGCCCTCAACTAACAATGTTGAAGCTTTCTTTTG
        130 GTGATGTTTGCATTCAAGTAACAGTGTTGAACATTCCCTTTC

                     *                    *      *   *                       
     511799 ATAGAGCAGTTTTGAAACACTCTTTTTGTAATATCTGCAAGAGGATATTTGGATAGCTTTGAGGA
          1 ATAGAGCAGGTTTGAAACACTCTTTTTGTAGTATCTGGAAGTGGATATTTGGATAGCTTTGAGGA

                         *   * *            **                      *    *   
     511864 TTTCGTTGGAAACGGGATTGTCTTCATATAAACTCTAGACAGAAGCATTCTCAGAAGCTTCATTG
         66 TTTCGTTGGAAACAGGAATATCTTCATATAAAAGCTAGACAGAAGCATTCTCAGAAACTTCTTTG

            *       **   *    *             * *      
     511929 GGATGTTTCAATTGAAGTCACAGTGTTGAACAGTTCCTTTC
        131 TGATGTTTGCATTCAAGTAACAGTGTTGAACATTCCCTTTC

                 *                                       *       **   * *    
     511970 ATAGAACAGGTTTGAAACACTCTTTTTGTAGTATCTGGAAGTGGACATTTGGAGCGCTCTCAGGA
          1 ATAGAGCAGGTTTGAAACACTCTTTTTGTAGTATCTGGAAGTGGATATTTGGATAGCTTTGAGGA

            * *  *  *   ** *         **          * *       * *          *  **
     512035 CTACGGTGAAAATGGAAATATCTTCCAATAAAAGCTACATAGAAGCAATGTCAGAAACTTTTTCA
         66 TTTCGTTGGAAACAGGAATATCTTCATATAAAAGCTAGACAGAAGCATTCTCAGAAACTTCTTTG

                  * ** *   **      *       *  *     *
     512100 TGATGTATCTACTCAGCTAACAGAGTTGAACCTTTCCTTTG
        131 TGATGTTTGCATTCAAGTAACAGTGTTGAACATTCCCTTTC

             *       *                   * *     *             **            
     512141 AGAGAGCAGTTTTGAAACACTCTTTTTGTGGAATCTGCAAGTGGATATTTGTCTAGCTTTGAGGA
          1 ATAGAGCAGGTTTGAAACACTCTTTTTGTAGTATCTGGAAGTGGATATTTGGATAGCTTTGAGGA

                         *   *    *                   *       *     *        
     512206 TTTCGTTGGAAACGGGATTA-CAT-ATA-AAAAGC-AGACAGCAGCATTCCCAGAATCTTCTTTG
         66 TTTCGTTGGAAACAGGAATATCTTCATATAAAAGCTAGACAGAAGCATTCTCAGAAACTTCTTTG

                              *    *                 
     512267 TGATGTTTGCATTCAAGTCACAGAGTTGAACATTCCCTTTC
        131 TGATGTTTGCATTCAAGTAACAGTGTTGAACATTCCCTTTC

                                                   *     *       **     *   *
     512308 ATAGAGCAGGTTTGAAACACTCTTTTTGTAGTATCTGGATGTGGACATTTGGAGCGCTTTCAGGC
          1 ATAGAGCAGGTTTGAAACACTCTTTTTGTAGTATCTGGAAGTGGATATTTGGATAGCTTTGAGGA

            * ** *      *             *** *   *                      *   *   
     512373 CTATGGT-GAAAAAGGAAATATCTTCCCCTGAAAACTAGACAGAAGCATTCTCAGAATCTTATTT
         66 TTTCGTTGGAAACAGG-AATATCTTCATATAAAAGCTAGACAGAAGCATTCTCAGAAACTTCTTT

                   **  **    *             **  * *   *
     512437 GTGATGTGCGCCCTCAACTAACAGTGTTGAAGCTTTCTTTTG
        130 GTGATGTTTGCATTCAAGTAACAGTGTTGAACATTCCCTTTC

                     *                    **     *   *                       
     512479 ATAGAGCAGTTTTGAAACACTCTTTTTGTAAAATCTGCAAGAGGATATTTGGATAGCTTTGAGGA
          1 ATAGAGCAGGTTTGAAACACTCTTTTTGTAGTATCTGGAAGTGGATATTTGGATAGCTTTGAGGA

                         *   * *            **                      *    *   
     512544 TTTCGTTGGAAACGGGATTGTCTTCATATAAACTCTAGACAGAAGCATTCTCAGAAGCTTCATTG
         66 TTTCGTTGGAAACAGGAATATCTTCATATAAAAGCTAGACAGAAGCATTCTCAGAAACTTCTTTG

            *       **   *    *             *        
     512609 GGATGTTTCAATTGAAGTCACAGTGTTGAACAGTCCCTTTC
        131 TGATGTTTGCATTCAAGTAACAGTGTTGAACATTCCCTTTC

                                                         *       **   * *    
     512650 ATAGAGCAGGTTTGAAACACTCTTTTTGTAGTATCTGGAAGTGGACATTTGGAACGCTCTCAGGA
          1 ATAGAGCAGGTTTGAAACACTCTTTTTGTAGTATCTGGAAGTGGATATTTGGATAGCTTTGAGGA

            * *  *      *             **            *       * *          *  *
     512715 CTGCGGT-GAAAAAGGAAATATCTTCCAATAAAAGCTAGATAGAAGCAATGTCAGAAACTTTTTC
         66 TTTCGTTGGAAACAGG-AATATCTTCATATAAAAGCTAGACAGAAGCATTCTCAGAAACTTCTTT

            *      * ** *   **      *       *  * *   *
     512779 ATGATGTATCTACTCAGCTAACAGAGTTGAACCTTTCTTTTG
        130 GTGATGTTTGCATTCAAGTAACAGTGTTGAACATTCCCTTTC

             *       *                   * *     *             **            
     512821 AGAGAGCAGTTTTGAAACACTCTTTTTGTGGAATCTGCAAGTGGATATTTGTCTAGCTTTGAGGA
          1 ATAGAGCAGGTTTGAAACACTCTTTTTGTAGTATCTGGAAGTGGATATTTGGATAGCTTTGAGGA

                         *   *    *                   *       *   *          
     512886 TTTCGTTGGAAACGGGATTA-CAT-ATA-AAAAGC-AGACAGCAGCATTCCCAGTAACTTCTTTG
         66 TTTCGTTGGAAACAGGAATATCTTCATATAAAAGCTAGACAGAAGCATTCTCAGAAACTTCTTTG

                              *    *                 
     512947 TGATGTTTGCATTCAAGTCACAGAGTTGAACATTCCCTTTC
        131 TGATGTTTGCATTCAAGTAACAGTGTTGAACATTCCCTTTC

                                 *                 *     *       **     *   *
     512988 ATAGAGCAGGTTTGAAACACTTTTTTTGTAGTATCTGGATGTGGACATTTGGAGCGCTTTCAGGC
          1 ATAGAGCAGGTTTGAAACACTCTTTTTGTAGTATCTGGAAGTGGATATTTGGATAGCTTTGAGGA

            * ** *      *             **          * * *     * *          *  *
     513053 CTATGGT-GAAAAAGGAAATATCTTCCAATAAAAGCTACATAAAAGCAATGTCAGAAACTTTTTC
         66 TTTCGTTGGAAACAGG-AATATCTTCATATAAAAGCTAGACAGAAGCATTCTCAGAAACTTCTTT

            *      * ** *   **      *       *  * *   *
     513117 ATGATGTATCTACTCAGCTAACAGAGTTGAACCTTTCTTTTG
        130 GTGATGTTTGCATTCAAGTAACAGTGTTGAACATTCCCTTTC

             *       *                   * *                   **            
     513159 AGAGAGCAGTTTTGAAACACTCTTTTTGTGGAATCTGGAAGTGGATATTTGTCTAGCTTTGAGGA
          1 ATAGAGCAGGTTTGAAACACTCTTTTTGTAGTATCTGGAAGTGGATATTTGGATAGCTTTGAGGA

                         *   *    *                   *       *   *          
     513224 TTTCGTTGGAAACGGGATTA-CAT-ATA-AAAAGC-AGACAGCAGCATTCCCAGTAACTTCTTTG
         66 TTTCGTTGGAAACAGGAATATCTTCATATAAAAGCTAGACAGAAGCATTCTCAGAAACTTCTTTG

                              *    *                 
     513285 TGATGTTTGCATTCAAGTCACAGAGTTGAACATTCCCTTTC
        131 TGATGTTTGCATTCAAGTAACAGTGTTGAACATTCCCTTTC

                                                   *     *       **     *    
     513326 ATAGAGCAGGTTTGAAACACTCTTTTTGTAGTATCTGGATGTGGACATTTGGAGCGCTTTCAGG-
          1 ATAGAGCAGGTTTGAAACACTCTTTTTGTAGTATCTGGAAGTGGATATTTGGATAGCTTTGAGGA

            **  *        *             *** *   *           *          *   *  
     513390 CCT-ATTGTGAAAAAGGAAATATCTTCCCCTGAAAACTAGACAGAAGAATTCTCAGAATCTTATT
         66 TTTCGTTG-GAAACAGG-AATATCTTCATATAAAAGCTAGACAGAAGCATTCTCAGAAACTTCTT

                    **  **    *             **  * *   *
     513454 TGTGATGTGCGCCCTCAACTAACAGTGTTGAAGCTTTCTTTTG
        129 TGTGATGTTTGCATTCAAGTAACAGTGTTGAACATTCCCTTTC

                     *                    **     *   *                       
     513497 ATAGAGCAGTTTTGAAACACTCTTTTTGTAAAATCTGCAAGAGGATATTTGGATAGCTTTGAGGA
          1 ATAGAGCAGGTTTGAAACACTCTTTTTGTAGTATCTGGAAGTGGATATTTGGATAGCTTTGAGGA

                         *   * * *          **   *                  * *  *   
     513562 TTTCGTTGGAAACGGGATTGTTTTCATATAAACTCTACACAGAAGCATTCTCAGAAGCGTCATTG
         66 TTTCGTTGGAAACAGGAATATCTTCATATAAAAGCTAGACAGAAGCATTCTCAGAAACTTCTTTG

            *       **   *    *             *        
     513627 GGATGTTTCAATTGAAGTCACAGTGTTGAACAGTCCCTTTC
        131 TGATGTTTGCATTCAAGTAACAGTGTTGAACATTCCCTTTC

                                                   *     *       **     *   *
     513668 ATAGAGCAGGTTTGAAACACTCTTTTTGTAGTATCTGGATGTGGACATTTGGAGCGCTTTCAGGC
          1 ATAGAGCAGGTTTGAAACACTCTTTTTGTAGTATCTGGAAGTGGATATTTGGATAGCTTTGAGGA

            * ** *      *             *** *   *                      *   *   
     513733 CTATGGT-GAAAAAGGAAATATCTTCCCCTGAAAACTAGACAGAAGCATTCTCAGAATCTTATTT
         66 TTTCGTTGGAAACAGG-AATATCTTCATATAAAAGCTAGACAGAAGCATTCTCAGAAACTTCTTT

                   **  **    *             **  * *   *
     513797 GTGATGTGCGCCCTCAACTAACAGTGTTGAAGCTTTCTTTTG
        130 GTGATGTTTGCATTCAAGTAACAGTGTTGAACATTCCCTTTC

                     *                    **     *   *                       
     513839 ATAGAGCAGTTTTGAAACACTCTTTTTGTAAAATCTGCAAGAGGATATTTGGATAGCTTTGAGGA
          1 ATAGAGCAGGTTTGAAACACTCTTTTTGTAGTATCTGGAAGTGGATATTTGGATAGCTTTGAGGA

                         *   * *            **                      *    *   
     513904 TTTCGTTGGAAACGGGATTGTCTTCATATAAACTCTAGACAGAAGCATTCTCAGAAGCTTCATTG
         66 TTTCGTTGGAAACAGGAATATCTTCATATAAAAGCTAGACAGAAGCATTCTCAGAAACTTCTTTG

            *       **   *    *             *        
     513969 GGATGTTTCAATTGAAGTCACAGTGTTGAACAGTCCCTTTC
        131 TGATGTTTGCATTCAAGTAACAGTGTTGAACATTCCCTTTC

                                                         *       **   * *    
     514010 ATAGAGCAGGTTTGAAACACTCTTTTTGTAGTATCTGGAAGTGGACATTTGGAGCGCTCTCAGGA
          1 ATAGAGCAGGTTTGAAACACTCTTTTTGTAGTATCTGGAAGTGGATATTTGGATAGCTTTGAGGA

            * *  *      *             **            *       * *          *  *
     514075 CTGCGGT-GAAAAAGGAACTATCTTCCAATAAAAGCTAGATAGAAGCAATGTCAGAAACTTTTTC
         66 TTTCGTTGGAAACAGGAA-TATCTTCATATAAAAGCTAGACAGAAGCATTCTCAGAAACTTCTTT

            *      * ** *   **      *       *        *
     514139 ATGATGTATCTACTCAGCTAACAGAGTTGAACCTT-CCTTTG
        130 GTGATGTTTGCATTCAAGTAACAGTGTTGAACATTCCCTTTC

             *       *                   * *     *             **            
     514180 AGAGAGCAGTTTTGAAACACTCTTTTTGTGGAATCTGCAAGTGGATATTTGTCTAGCTTTGAGGA
          1 ATAGAGCAGGTTTGAAACACTCTTTTTGTAGTATCTGGAAGTGGATATTTGGATAGCTTTGAGGA

                         *   * *            **                      *    *   
     514245 TTTCGTTGGAAACGGGATTGTCTTCATATAAACTCTAGACAGAAGCATTCTCAGAAGCTTCATTG
         66 TTTCGTTGGAAACAGGAATATCTTCATATAAAAGCTAGACAGAAGCATTCTCAGAAACTTCTTTG

            *       **   *    *             * *      
     514310 GGATGTTTCAATTGAAGTCACAGTGTTGAACAGTTCCTTTC
        131 TGATGTTTGCATTCAAGTAACAGTGTTGAACATTCCCTTTC

                 *                                       *       **   * *    
     514351 ATAGAACAGGTTTGAAACACTCTTTTTGTAGTATCTGGAAGTGGACATTTGGAGCGCTCTCAGGA
          1 ATAGAGCAGGTTTGAAACACTCTTTTTGTAGTATCTGGAAGTGGATATTTGGATAGCTTTGAGGA

            * *  *      *             **          * *       * *          *  *
     514416 CTACGGT-GAAAAAGGAAATATCTTCCAATAAAAGCTACATAGAAGCAATGTCAGAAACTTTTTC
         66 TTTCGTTGGAAACAGG-AATATCTTCATATAAAAGCTAGACAGAAGCATTCTCAGAAACTTCTTT

            * *    * ** *   **      *       *  *     *
     514480 ATCATGTATCTACTCAGCTAACAGAGTTGAACCTTTCCTTTG
        130 GTGATGTTTGCATTCAAGTAACAGTGTTGAACATTCCCTTTC

             *       *                   * *     *             **            
     514522 AGAGAGCAGTTTTGAAACACTCTTTTTGTGGAATCTGCAAGTGGATATTTGTCTAGCTTTGAGGA
          1 ATAGAGCAGGTTTGAAACACTCTTTTTGTAGTATCTGGAAGTGGATATTTGGATAGCTTTGAGGA

                         *   *    *                   *       *     *        
     514587 TTTCGTTGGAAACGGGATTA-CAT-ATA-AAAAGC-AGACAGCAGCATTCCCAGAATCTTCTTTG
         66 TTTCGTTGGAAACAGGAATATCTTCATATAAAAGCTAGACAGAAGCATTCTCAGAAACTTCTTTG

                              *    *                 
     514648 TGATGTTTGCATTCAAGTCACAGAGTTGAACATTCCCTTTC
        131 TGATGTTTGCATTCAAGTAACAGTGTTGAACATTCCCTTTC

                                                   *     *       **     *   *
     514689 ATAGAGCAGGTTTGAAACACTCTTTTTGTAGTATCTGGATGTGGACATTTGGAGCGCTTTCAGGC
          1 ATAGAGCAGGTTTGAAACACTCTTTTTGTAGTATCTGGAAGTGGATATTTGGATAGCTTTGAGGA

            * ** *      *             *** *   *                      *   *   
     514754 CTATGGT-GAAAAAGGAAATATCTTCCCCTGAAAACTAGACAGAAGCATTCTCAGAATCTTATTT
         66 TTTCGTTGGAAACAGG-AATATCTTCATATAAAAGCTAGACAGAAGCATTCTCAGAAACTTCTTT

                   **  **    *             **  * *   *
     514818 GTGATGTGCGCCCTCAACTAACAGTGTTGAAGCTTTCTTTTG
        130 GTGATGTTTGCATTCAAGTAACAGTGTTGAACATTCCCTTTC

                     *                    **     *   *                       
     514860 ATAGAGCAGTTTTGAAACACTCTTTTTGTAAAATCTGCAAGAGGATATTTGGATAGCTTTGAGGA
          1 ATAGAGCAGGTTTGAAACACTCTTTTTGTAGTATCTGGAAGTGGATATTTGGATAGCTTTGAGGA

                         *   * *            **                      *    *   
     514925 TTTCGTTGGAAACGGGATTGTCTTCATATAAACTCTAGACAGAAGCATTCTCAGAAGCTTCATTG
         66 TTTCGTTGGAAACAGGAATATCTTCATATAAAAGCTAGACAGAAGCATTCTCAGAAACTTCTTTG

            *       **   *    *             *        
     514990 GGATGTTTCAATTGAAGTCACAGTGTTGAACAGTCCCTTTC
        131 TGATGTTTGCATTCAAGTAACAGTGTTGAACATTCCCTTTC

                                                         *       **   * *    
     515031 ATAGAGCAGGTTTGAAACACTCTTTTTGTAGTATCTGGAAGTGGACATTTGGAACGCTCTCAGGA
          1 ATAGAGCAGGTTTGAAACACTCTTTTTGTAGTATCTGGAAGTGGATATTTGGATAGCTTTGAGGA

            * *  *      *             **            *       * *          *  *
     515096 CTGCGGT-GAAAAAGGAAATATCTTCCAATAAAAGCTAGATAGAAGCAATGTCAGAAACTTTTTC
         66 TTTCGTTGGAAACAGG-AATATCTTCATATAAAAGCTAGACAGAAGCATTCTCAGAAACTTCTTT

            *      * ** *   **      *       *  * *   *
     515160 ATGATGTATCTACTCAGCTAACAGAGTTGAACCTTTCTTTTG
        130 GTGATGTTTGCATTCAAGTAACAGTGTTGAACATTCCCTTTC

             *       *                    **     *   *                       
     515202 AGAGAGCAGTTTTGAAACACTCTTTTTGTAAAATCTGCAAGAGGATATTTGGATAGCTTTGAGGA
          1 ATAGAGCAGGTTTGAAACACTCTTTTTGTAGTATCTGGAAGTGGATATTTGGATAGCTTTGAGGA

                  *      *   * *            **                      * *  *   
     515267 TTTCGTAGGAAACGGGATTGTCTTCATATAAACTCTAGACAGAAGCATTCTCAGAAGCGTCATTG
         66 TTTCGTTGGAAACAGGAATATCTTCATATAAAAGCTAGACAGAAGCATTCTCAGAAACTTCTTTG

            *       **   *    *             *        
     515332 GGATGTTTCAATTGAAGTCACAGTGTTGAACAGTCCCTTTC
        131 TGATGTTTGCATTCAAGTAACAGTGTTGAACATTCCCTTTC

                                                   *     *       **     *    
     515373 ATAGAGCAGGTTTGAAACACTCTTTTTGTAGTATCTGGATGTGGACATTTGGAGCGCTTTCAGGC
          1 ATAGAGCAGGTTTGAAACACTCTTTTTGTAGTATCTGGAAGTGGATATTTGGATAGCTTTGAGG-

            * * *    *                 *** *   *                          *  
     515438 CTATGGTT-TAAA-AGGAAATATCTTCCCCTGAAAACTAGACAGAAGCATTCTCAGAAACTTATT
         65 ATTTCGTTGGAAACAGG-AATATCTTCATATAAAAGCTAGACAGAAGCATTCTCAGAAACTTCTT

                    **  **    *             **  * *   *
     515501 TGTGATGTGCGCCCTCAACTAACAGTGTTGAAGCTTTCTTTTG
        129 TGTGATGTTTGCATTCAAGTAACAGTGTTGAACATTCCCTTTC

                     *                    **     *   *                       
     515544 ATAGAGCAGTTTTGAAACACTCTTTTTGTAAAATCTGCAAGAGGATATTTGGATAGCTTTGAGGA
          1 ATAGAGCAGGTTTGAAACACTCTTTTTGTAGTATCTGGAAGTGGATATTTGGATAGCTTTGAGGA

                         *   * *            **                      *    *   
     515609 TTTCGTTGGAAACGGGATTGTCTTCATATAAACTCTAGACAGAAGCATTCTCAGAAGCTTCATTG
         66 TTTCGTTGGAAACAGGAATATCTTCATATAAAAGCTAGACAGAAGCATTCTCAGAAACTTCTTTG

            *       **   *    *             *        
     515674 GGATGTTTCAATTGAAGTCACAGTGTTGAACAGTCCCTTTC
        131 TGATGTTTGCATTCAAGTAACAGTGTTGAACATTCCCTTTC

                                                         *       **   * *    
     515715 ATAGAGCAGGTTTGAAACACTCTTTTTGTAGTATCTGGAAGTGGACATTTGGAGCGCTCTCAGGA
          1 ATAGAGCAGGTTTGAAACACTCTTTTTGTAGTATCTGGAAGTGGATATTTGGATAGCTTTGAGGA

            * *  *      *             **            *       * *          *  *
     515780 CTGCGGT-GAAAAAGGAACTATCTTCCAATAAAAGCTAGATAGAAGCAATGTCAGAAACTTTTTC
         66 TTTCGTTGGAAACAGGAA-TATCTTCATATAAAAGCTAGACAGAAGCATTCTCAGAAACTTCTTT

            *      * ** *   **      *       *        *
     515844 ATGATGTATCTACTCAGCTAACAGAGTTGAACCTT-CCTTTG
        130 GTGATGTTTGCATTCAAGTAACAGTGTTGAACATTCCCTTTC

             *       *                   * *     *             **            
     515885 AGAGAGCAGTTTTGAAACACTCTTTTTGTGGAATCTGCAAGTGGATATTTGTCTAGCTTTGAGGA
          1 ATAGAGCAGGTTTGAAACACTCTTTTTGTAGTATCTGGAAGTGGATATTTGGATAGCTTTGAGGA

                         *   *    *                   *       *              
     515950 TTTCGTTGGAAACGGGATTA-CAT-ATA-AAAAGC-AGACAGCAGCATTCCCAGAAACTTCTTTG
         66 TTTCGTTGGAAACAGGAATATCTTCATATAAAAGCTAGACAGAAGCATTCTCAGAAACTTCTTTG

                              *    *                 
     516011 TGATGTTTGCATTCAAGTCACAGAGTTGAACATTCCCTTTC
        131 TGATGTTTGCATTCAAGTAACAGTGTTGAACATTCCCTTTC

                                                   *     *     * **     *   *
     516052 ATAGAGCAGGTTTGAAACACTCTTTTTGTAGTATCTGGATGTGGACATTTGCAGCGCTTTCAGGC
          1 ATAGAGCAGGTTTGAAACACTCTTTTTGTAGTATCTGGAAGTGGATATTTGGATAGCTTTGAGGA

            * ** *      *             *** *   *                          *   
     516117 CTAAGGT-GAAAAAGGAAATATCTTCCCCTGAAAACTAGACAGAAGCATTCTCAGAAACTTATTT
         66 TTTCGTTGGAAACAGG-AATATCTTCATATAAAAGCTAGACAGAAGCATTCTCAGAAACTTCTTT

                   **  **    *             **  * *   *
     516181 GTGATGTGCGCCCTCAACTAACAGTGTTGAAGCTTTCTTTTG
        130 GTGATGTTTGCATTCAAGTAACAGTGTTGAACATTCCCTTTC

                     *                    *      *   *                       
     516223 ATAGAGCAGTTTTGAAACACTCTTTTTGTAATATCTGCAAGAGGATATTTGGATAGCTTTGAGGA
          1 ATAGAGCAGGTTTGAAACACTCTTTTTGTAGTATCTGGAAGTGGATATTTGGATAGCTTTGAGGA

                         *   * *            **                      *    *   
     516288 TTTCGTTGGAAACGGGATTGTCTTCATATAAACTCTAGACAGAAGCATTCTCAGAAGCTTCATTG
         66 TTTCGTTGGAAACAGGAATATCTTCATATAAAAGCTAGACAGAAGCATTCTCAGAAACTTCTTTG

            *       **   *    *             * *      
     516353 GGATGTTTCAATTGAAGTCACAGTGTTGAACAGTTCCTTTC
        131 TGATGTTTGCATTCAAGTAACAGTGTTGAACATTCCCTTTC

                 *                                       *       **   * *    
     516394 ATAGAACAGGTTTGAAACACTCTTTTTGTAGTATCTGGAAGTGGACATTTGGAGCGCTCTCAGGA
          1 ATAGAGCAGGTTTGAAACACTCTTTTTGTAGTATCTGGAAGTGGATATTTGGATAGCTTTGAGGA

            * *  *  *   ** *                      * *       * *          *  *
     516459 CTACGGTGAAAATGGAAATATCTTACA-ATAAAAGCTACATAGAAGCAATGTCAGAAACTTTTTC
         66 TTTCGTTGGAAACAGGAATATCTT-CATATAAAAGCTAGACAGAAGCATTCTCAGAAACTTCTTT

            *      * ** *   **      *       *  *     *
     516523 ATGATGTATCTACTCAGCTAACAGAGTTGAACCTTTCCTTTG
        130 GTGATGTTTGCATTCAAGTAACAGTGTTGAACATTCCCTTTC

             *       *                   * *     *             **            
     516565 AGAGAGCAGTTTTGAAACACTCTTTTTGTGGAATCTGCAAGTGGATATTTGTCTAGCTTTGAGGA
          1 ATAGAGCAGGTTTGAAACACTCTTTTTGTAGTATCTGGAAGTGGATATTTGGATAGCTTTGAGGA

                         *   *    *                   *       *     *        
     516630 TTTCGTTGGAAACGGGATTA-CAT-ATA-AAAAGC-AGACAGCAGCATTCCCAGAATCTTCTTTG
         66 TTTCGTTGGAAACAGGAATATCTTCATATAAAAGCTAGACAGAAGCATTCTCAGAAACTTCTTTG

                              *    *                 
     516691 TGATGTTTGCATTCAAGTCACAGAGTTGAACATTCCCTTTC
        131 TGATGTTTGCATTCAAGTAACAGTGTTGAACATTCCCTTTC

                         *                         *     *       **     *   *
     516732 ATAGAGCAGGTTTCAAACACTCTTTTTGTAGTATCTGGATGTGGACATTTGGAACGCTTTCAGGC
          1 ATAGAGCAGGTTTGAAACACTCTTTTTGTAGTATCTGGAAGTGGATATTTGGATAGCTTTGAGGA

            * ** *      *             *** *   *                      *   *   
     516797 CTATGGT-GAAAAAGGAAATATCTTCCCCTGAAAACTAGACAGAAGCATTCTCAGAATCTTATTT
         66 TTTCGTTGGAAACAGG-AATATCTTCATATAAAAGCTAGACAGAAGCATTCTCAGAAACTTCTTT

                   **  **    *             **  * *   *
     516861 GTGATGTGCGCCCTCAACTAACAGTGTTGAAGCTTTCTTTTG
        130 GTGATGTTTGCATTCAAGTAACAGTGTTGAACATTCCCTTTC

                     *                    **     *   *                       
     516903 ATAGAGCAGTTTTGAAACACTCTTTTTGTAAAATCTGCAAGAGGATATTTGGATAGCTTTGAGGA
          1 ATAGAGCAGGTTTGAAACACTCTTTTTGTAGTATCTGGAAGTGGATATTTGGATAGCTTTGAGGA

                         *   * *            **                      *    *   
     516968 TTTCGTTGGAAACGGGATTGTCTTCATATAAACTCTAGACAGAAGCATTCTCAGAAGCTTCATTG
         66 TTTCGTTGGAAACAGGAATATCTTCATATAAAAGCTAGACAGAAGCATTCTCAGAAACTTCTTTG

            *       **   *    *             *        
     517033 GGATGTTTCAATTGAAGTCACAGTGTTGAACAGTCCCTTTC
        131 TGATGTTTGCATTCAAGTAACAGTGTTGAACATTCCCTTTC

                                                         *       **   * *    
     517074 ATAGAGCAGGTTTGAAACACTCTTTTTGTAGTATCTGGAAGTGGACATTTGGAACGCTCTCAGGA
          1 ATAGAGCAGGTTTGAAACACTCTTTTTGTAGTATCTGGAAGTGGATATTTGGATAGCTTTGAGGA

            * *  *      *             **            *       * *          *  *
     517139 CTGCGGT-GAAAAAGGAAATATCTTCCAATAAAAGCTAGATAGAAGCAATGTCAGAAACTTTTTC
         66 TTTCGTTGGAAACAGG-AATATCTTCATATAAAAGCTAGACAGAAGCATTCTCAGAAACTTCTTT

            *      * ** *   **      *       *  * *   *
     517203 ATGATGTATCTACTCAGCTAACAGAGTTGAACCTTTCTTTTG
        130 GTGATGTTTGCATTCAAGTAACAGTGTTGAACATTCCCTTTC

             *       *            *      * *     *             **  *         
     517245 AGAGAGCAGTTTTGAAACACTCGTTTTGTGGAATCTGCAAGTGGATATTTGTCTACCTTTGAGGA
          1 ATAGAGCAGGTTTGAAACACTCTTTTTGTAGTATCTGGAAGTGGATATTTGGATAGCTTTGAGGA

                         *   *    *                   *       *              
     517310 TTTCGTTGGAAACGGGATTA-CAT-ATA-AAAAGC-AGACAGCAGCATTCCCAGAAACTTCTTTG
         66 TTTCGTTGGAAACAGGAATATCTTCATATAAAAGCTAGACAGAAGCATTCTCAGAAACTTCTTTG

                              *    *                 
     517371 TGATGTTTGCATTCAAGTCACAGAGTTGAACATTCCCTTTC
        131 TGATGTTTGCATTCAAGTAACAGTGTTGAACATTCCCTTTC

                                                   *     *     * **     *   *
     517412 ATAGAGCAGGTTTGAAACACTCTTTTTGTAGTATCTGGATGTGGACATTTGCAGCGCTTTCAGGC
          1 ATAGAGCAGGTTTGAAACACTCTTTTTGTAGTATCTGGAAGTGGATATTTGGATAGCTTTGAGGA

            * ** *      *             *** *   *                          *   
     517477 CTAAGGT-GAAAAAGGAAATATCTTCCCCTGAAAACTAGACAGAAGCATTCTCAGAAACTTATTT
         66 TTTCGTTGGAAACAGG-AATATCTTCATATAAAAGCTAGACAGAAGCATTCTCAGAAACTTCTTT

                   **  **    *             **  * *   *
     517541 GTGATGTGCGCCCTCAACTAACAGTGTTGAAGCTTTCTTTTG
        130 GTGATGTTTGCATTCAAGTAACAGTGTTGAACATTCCCTTTC

                     * *                  *      *   *                       
     517583 ATAGAGCAGATATGAAACACTCTTTTTGTAATATCTGCAAGAGGATATTTGGATAGCTTTGAGGA
          1 ATAGAGCAGGTTTGAAACACTCTTTTTGTAGTATCTGGAAGTGGATATTTGGATAGCTTTGAGGA

                         *   * *            **                      *    *   
     517648 TTTCGTTGGAAACGGGATTGTCTTCATATAAACTCTAGACAGAAGCATTCTCAGAAGCTTCATTG
         66 TTTCGTTGGAAACAGGAATATCTTCATATAAAAGCTAGACAGAAGCATTCTCAGAAACTTCTTTG

            *       **   *    *             *        
     517713 GGATGTTTCAATTGAAGTCACAGTGTTGAACAGTCCCTTTC
        131 TGATGTTTGCATTCAAGTAACAGTGTTGAACATTCCCTTTC

                                                         *       **   * *    
     517754 ATAGAGCAGGTTTGAAACACTCTTTTTGTAGTATCTGGAAGTGGACATTTGGAACGCTCTCAGGA
          1 ATAGAGCAGGTTTGAAACACTCTTTTTGTAGTATCTGGAAGTGGATATTTGGATAGCTTTGAGGA

            * *  *      *             **            *       * *          *  *
     517819 CTGCGGT-GAAAAAGGAAATATCTTCCAATAAAAGCTAGATAGAAGCAATGTCAGAAACTTTTTC
         66 TTTCGTTGGAAACAGG-AATATCTTCATATAAAAGCTAGACAGAAGCATTCTCAGAAACTTCTTT

            *      * ** *   **      *       *  * *   *
     517883 ATGATGTATCTACTCAGCTAACAGAGTTGAACCTTTCTTTTG
        130 GTGATGTTTGCATTCAAGTAACAGTGTTGAACATTCCCTTTC

             *       *    *              * *     *             **   *        
     517925 AGAGAGCAGTTTTGTAACACTCTTTTTGTGGAATCTGCAAGTGGATATTTGTCTAGATTTGAGGA
          1 ATAGAGCAGGTTTGAAACACTCTTTTTGTAGTATCTGGAAGTGGATATTTGGATAGCTTTGAGGA

                         *   *    *                   *       *   *          
     517990 TTTCGTTGGAAACGGGATTA-CAT-ATA-AAAAGC-AGACAGCAGCATTCCCAGTAACTTCTTTG
         66 TTTCGTTGGAAACAGGAATATCTTCATATAAAAGCTAGACAGAAGCATTCTCAGAAACTTCTTTG

                              *    *                 
     518051 TGATGTTTGCATTCAAGTCACAGAGTTGAACATTCCCTTTC
        131 TGATGTTTGCATTCAAGTAACAGTGTTGAACATTCCCTTTC

                                 *                 *     *       **     *   *
     518092 ATAGAGCAGGTTTGAAACACTTTTTTTGTAGTATCTGGATGTGGACATTTGGAGCGCTTTCAGGC
          1 ATAGAGCAGGTTTGAAACACTCTTTTTGTAGTATCTGGAAGTGGATATTTGGATAGCTTTGAGGA

            * ** *      *             **          * * *     * *          *  *
     518157 CTATGGT-GAAAAAGGAAATATCTTCCAATAAAAGCTACATAAAAGCAATGTCAGAAACTTTTTC
         66 TTTCGTTGGAAACAGG-AATATCTTCATATAAAAGCTAGACAGAAGCATTCTCAGAAACTTCTTT

            *      * ** *   **      *       *  * *   *
     518221 ATGATGTATCTACTCAGCTAACAGAGTTGAACCTTTCTTTTG
        130 GTGATGTTTGCATTCAAGTAACAGTGTTGAACATTCCCTTTC

             *       *                   * *                   **   *        
     518263 AGAGAGCAGTTTTGAAACACTCTTTTTGTGGAATCTGGAAGTGGATATTTGTCTAGTTTTGAGGA
          1 ATAGAGCAGGTTTGAAACACTCTTTTTGTAGTATCTGGAAGTGGATATTTGGATAGCTTTGAGGA

               *         *   *    *                   *       *   * *    *   
     518328 TTTTGTTGGAAACGGGATTA-CAT-ATA-AAAAGC-AGACAGCAGCATTCCCAGTATCTTCCTTG
         66 TTTCGTTGGAAACAGGAATATCTTCATATAAAAGCTAGACAGAAGCATTCTCAGAAACTTCTTTG

                              *    *                 
     518389 TGATGTTTGCATTCAAGTCACAGAGTTGAACATTCCCTTTC
        131 TGATGTTTGCATTCAAGTAACAGTGTTGAACATTCCCTTTC

                                                   *     *       **     *    
     518430 ATAGAGCAGGTTTGAAACACTCTTTTTGTAGTATCTGGATGTGGACATTTGGAGCGCTTTCAGG-
          1 ATAGAGCAGGTTTGAAACACTCTTTTTGTAGTATCTGGAAGTGGATATTTGGATAGCTTTGAGGA

            **  *        *             *** *   *           *          *   *  
     518494 CCT-ATTGTGAAAAAGGAAATATCTTCCCCTGAAAACTAGACAGAAGAATTCTCAGAATCTTATT
         66 TTTCGTTG-GAAACAGG-AATATCTTCATATAAAAGCTAGACAGAAGCATTCTCAGAAACTTCTT

                    **  **    *             **  * *   *
     518558 TGTGATGTGCGCCCTCAACTAACAGTGTTGAAGCTTTCTTTTG
        129 TGTGATGTTTGCATTCAAGTAACAGTGTTGAACATTCCCTTTC

                     *                    **     *   *                       
     518601 ATAGAGCAGTTTTGAAACACTCTTTTTGTAAAATCTGCAAGAGGATATTTGGATAGCTTTGAGGA
          1 ATAGAGCAGGTTTGAAACACTCTTTTTGTAGTATCTGGAAGTGGATATTTGGATAGCTTTGAGGA

                         *   * *            **   *        *         * *  *   
     518666 TTTCGTTGGAAACGGGATTGTCTTCATATAAACTCTACACAGAAGCTTTCTCAGAAGCGTCATTG
         66 TTTCGTTGGAAACAGGAATATCTTCATATAAAAGCTAGACAGAAGCATTCTCAGAAACTTCTTTG

            *       **   *    *             *        
     518731 GGATGTTTCAATTGAAGTCACAGTGTTGAACAGTCCCTTTC
        131 TGATGTTTGCATTCAAGTAACAGTGTTGAACATTCCCTTTC

                                       *           *     *       **     *    
     518772 ATAGAGCAGGTTTGAAACACTCTTTTTCTAGTATCTGGATGTGGACATTTGGAGCGCTTTCAGGC
          1 ATAGAGCAGGTTTGAAACACTCTTTTTGTAGTATCTGGAAGTGGATATTTGGATAGCTTTGAGG-

            * * *    *                 *** *   *                          *  
     518837 CTATGGTT-TAAA-AGGAAATATCTTCCCCTGAAAACTAGACAGAAGCATTCTCAGAAACTTATT
         65 ATTTCGTTGGAAACAGG-AATATCTTCATATAAAAGCTAGACAGAAGCATTCTCAGAAACTTCTT

                    **  *     *                    *   *
     518900 TGTGATGTGCGCCTTCAACTAACAGTGTTGAAGCATT-CTTTTG
        129 TGTGATGTTTGCATTCAAGTAACAGTGTTGAA-CATTCCCTTTC

                     *                   * *     *             **            
     518943 ATAGAGCAGTTTTGAAACACTCTTTTTGTGGAATCTGCAAGTGGATATTTGTCTAGCTTTGAGGA
          1 ATAGAGCAGGTTTGAAACACTCTTTTTGTAGTATCTGGAAGTGGATATTTGGATAGCTTTGAGGA

                         *   *    *                   *       *              
     519008 TTTCGTTGGAAACGGGATTA-CAT-ATA-AAAAGC-AGACAGCAGCATTCCCAGAAACTTCTTTG
         66 TTTCGTTGGAAACAGGAATATCTTCATATAAAAGCTAGACAGAAGCATTCTCAGAAACTTCTTTG

                              *    *                 
     519069 TGATGTTTGCATTCAAGTCACAGAGTTGAACATTCCCTTTC
        131 TGATGTTTGCATTCAAGTAACAGTGTTGAACATTCCCTTTC

             *    *                    *           *     *       **     *   *
     519110 AGAGAGTAGGTTTGAAACACTCTTTTTATAGTATCTGGATGTGGACATTTGGAGCGCTTTCAGGC
          1 ATAGAGCAGGTTTGAAACACTCTTTTTGTAGTATCTGGAAGTGGATATTTGGATAGCTTTGAGGA

            * ** *      *             *** *   *                      *   *   
     519175 CTATGGT-GAAAAAGGAAATATCTTCTCCTGAAAACTAGACAGAAGCATTCTCAGAATCTTATTT
         66 TTTCGTTGGAAACAGG-AATATCTTCATATAAAAGCTAGACAGAAGCATTCTCAGAAACTTCTTT

                   **  **    *             **  * *   *
     519239 GTGATGTGCGCCCTCAACTAACAGTGTTGAAGCTTTCTTTTG
        130 GTGATGTTTGCATTCAAGTAACAGTGTTGAACATTCCCTTTC

                     *                    **     *   *        *              
     519281 ATAGAGCAGTTTTGAAACACTCTTTTTGTAAAATCTGCAAGAGGATATTTTGATAGCTTTGAGGA
          1 ATAGAGCAGGTTTGAAACACTCTTTTTGTAGTATCTGGAAGTGGATATTTGGATAGCTTTGAGGA

                         *   * *            **                      *    *   
     519346 TTTCGTTGGAAACGGGATTGTCTTCATATAAACTCTAGACAGAAGCATTCTCAGAAGCTTCATTG
         66 TTTCGTTGGAAACAGGAATATCTTCATATAAAAGCTAGACAGAAGCATTCTCAGAAACTTCTTTG

            *       **   *    *             *        
     519411 GGATGTTTCAATTGAAGTCACAGTGTTGAACAGTCCCTTTC
        131 TGATGTTTGCATTCAAGTAACAGTGTTGAACATTCCCTTTC

                                                         *       *  * * *    
     519452 ATAGAGCAGGTTTGAAACACTCTTTTTGTAGTATCTGGAAGTGGACATTTGGAGAGATCTCAGGA
          1 ATAGAGCAGGTTTGAAACACTCTTTTTGTAGTATCTGGAAGTGGATATTTGGATAGCTTTGAGGA

            * *  *    * *             **            *       * *          *  *
     519517 ATACGGT-GATAAAGGAAATATCTTCCAATAAAAGCTAGATAGAAGCAATGTCAGAAACTTTTTC
         66 TTTCGTTGGAAACAGG-AATATCTTCATATAAAAGCTAGACAGAAGCATTCTCAGAAACTTCTTT

            *      * ** *   **      *       *  * *   *
     519581 ATGATGTATCTACTCAGCTAACAGAGTTGAACCTTTCTTTTG
        130 GTGATGTTTGCATTCAAGTAACAGTGTTGAACATTCCCTTTC

             *       *                   * *     *             **            
     519623 AGAGAGCAGTTTTGAAACACTCTTTTTGTGGAATCTGCAAGTGGATATTTGTCTAGCTTTGAGGA
          1 ATAGAGCAGGTTTGAAACACTCTTTTTGTAGTATCTGGAAGTGGATATTTGGATAGCTTTGAGGA

                         *   *    *                   *       *   *          
     519688 TTTCGTTGGAAACGGGATTA-CAT-ATA-AAAAGC-AGACAGCAGCATTCCCAGTAACTTCTTTG
         66 TTTCGTTGGAAACAGGAATATCTTCATATAAAAGCTAGACAGAAGCATTCTCAGAAACTTCTTTG

               *              *    *                 
     519749 TGAGGTTTGCATTCAAGTGACAGAGTTGAACATTCCCTTTC
        131 TGATGTTTGCATTCAAGTAACAGTGTTGAACATTCCCTTTC

                                                   *     *       **     *   *
     519790 ATAGAGCAGGTTTGAAACACTCTTTTTGTAGTATCTGGATGTGGACATTTGGAGCGCTTTCAGGC
          1 ATAGAGCAGGTTTGAAACACTCTTTTTGTAGTATCTGGAAGTGGATATTTGGATAGCTTTGAGGA

            * ** *      *             **          * *       * *          *  *
     519855 CTATGGT-GAAAAAGGAAATATCTTCGAATAAAAGCTACATAGAAGCAATGTCAGAAACTTTTTC
         66 TTTCGTTGGAAACAGG-AATATCTTCATATAAAAGCTAGACAGAAGCATTCTCAGAAACTTCTTT

            *      * ** *   **      *       *  * *   *
     519919 ATGATGTATCTACTCAGCTAACAGAGTTGAACCTTTCTTTTG
        130 GTGATGTTTGCATTCAAGTAACAGTGTTGAACATTCCCTTTC

                     *                   * *                   **   *        
     519961 ATAGAGCAGTTTTGAAACACTCTTTTTGTGGAATCTGGAAGTGGATATTTGTCTAGATTTGAGGA
          1 ATAGAGCAGGTTTGAAACACTCTTTTTGTAGTATCTGGAAGTGGATATTTGGATAGCTTTGAGGA

                         *   *    *                   *       *   *          
     520026 TTTCGTTGGAAACGGGATTA-CAT-ATA-AAAAGC-AGACAGCAGCATTCCCAGTAACTTCTTTG
         66 TTTCGTTGGAAACAGGAATATCTTCATATAAAAGCTAGACAGAAGCATTCTCAGAAACTTCTTTG

               *              *    *                 
     520087 TGACGTTTGCATTCAAGTCACAGAGTTGAACATTCCCTTTC
        131 TGATGTTTGCATTCAAGTAACAGTGTTGAACATTCCCTTTC

                                                   *     *       **     *   *
     520128 ATAGAGCAGGTTTGAAACACTCTTTTTGTAGTATCTGGATGTGGACATTTGGAGCGCTTTCAGGC
          1 ATAGAGCAGGTTTGAAACACTCTTTTTGTAGTATCTGGAAGTGGATATTTGGATAGCTTTGAGGA

            * ** *      *             *** *   *           *          *   *   
     520193 CTATGGT-GAAAAAGGAAATATCTTCCCCTGAAAACTAGACAGAAGAATTCTCAGAATCTTATTT
         66 TTTCGTTGGAAACAGG-AATATCTTCATATAAAAGCTAGACAGAAGCATTCTCAGAAACTTCTTT

                   **  **    *             **  * *   *
     520257 GTGATGTGCGCCATCAACTAACAGTGTTGAAGCTTTCTTTTG
        130 GTGATGTTTGCATTCAAGTAACAGTGTTGAACATTCCCTTTC

                     *                    **     *   **                      
     520299 ATAGAGCAGTTTTGAAACACTCTTTTTGTAAAATCTGCAAGACGATATTTGGATAGCTTTGAGGA
          1 ATAGAGCAGGTTTGAAACACTCTTTTTGTAGTATCTGGAAGTGGATATTTGGATAGCTTTGAGGA

                         *   * *            **        *             * *  *   
     520364 TTTCGTTGGAAACGGGATTGTCTTCATATAAACTCTAGACAGCAGCATTCTCAGAAGCGTCATTG
         66 TTTCGTTGGAAACAGGAATATCTTCATATAAAAGCTAGACAGAAGCATTCTCAGAAACTTCTTTG

            *       **   *    *                      
     520429 GGATGTTTCAATTGAAGTCACAGTGTTGAACATTCCCTTTC
        131 TGATGTTTGCATTCAAGTAACAGTGTTGAACATTCCCTTTC

                                                   *     *       **     *    
     520470 ATAGAGCAGGTTTGAAACACTCTTTTTGTAGTATCTGGATGTGGACATTTGGAGCGCTTTCAGGC
          1 ATAGAGCAGGTTTGAAACACTCTTTTTGTAGTATCTGGAAGTGGATATTTGGATAGCTTTGAGG-

            * * *    *                 *** *   *                          *  
     520535 CTATGGTT-TAAA-AGGAAATATCTTCCCCTGAAAACTAGACAGAAGCATTCTCAGAAACTTATT
         65 ATTTCGTTGGAAACAGG-AATATCTTCATATAAAAGCTAGACAGAAGCATTCTCAGAAACTTCTT

                    **  **    *                    *  **
     520598 TGTGATGTGCGCCCTCAACTAACAGTGTTGAAGCATT-CTTTGG
        129 TGTGATGTTTGCATTCAAGTAACAGTGTTGAA-CATTCCCTTTC

                     *                   * *     *             **            
     520641 ATAGAGCAGTTTTGAAACACTCTTTTTGTGGAATCTGCAAGTGGATATTTGTCTAGCTTTGAGGA
          1 ATAGAGCAGGTTTGAAACACTCTTTTTGTAGTATCTGGAAGTGGATATTTGGATAGCTTTGAGGA

                         *   *    *                   *       *              
     520706 TTTCGTTGGAAACGGGATTA-CAT-ATA-AAAAGC-AGACAGCAGCATTCCCAGAAACTTCTTTG
         66 TTTCGTTGGAAACAGGAATATCTTCATATAAAAGCTAGACAGAAGCATTCTCAGAAACTTCTTTG

                              *    *                 
     520767 TGATGTTTGCATTCAAGTCACAGAGTTGAACATTCCCTTTC
        131 TGATGTTTGCATTCAAGTAACAGTGTTGAACATTCCCTTTC

                                                   *     *     * **     *   *
     520808 ATAGAGCAGGTTTGAAACACTCTTTTTGTAGTATCTGGATGTGGACATTTGCAGCGCTTTCAGGC
          1 ATAGAGCAGGTTTGAAACACTCTTTTTGTAGTATCTGGAAGTGGATATTTGGATAGCTTTGAGGA

            * ** *      *             *** *   *                          *   
     520873 CTAAGGT-GAAAAAGGAAATATCTTCCCCTGAAAACTAGACAGAAGCATTCTCAGAAACTTATTT
         66 TTTCGTTGGAAACAGG-AATATCTTCATATAAAAGCTAGACAGAAGCATTCTCAGAAACTTCTTT

                   **  **    *             **  * * * *
     520937 GTGATGTGCGCCCTCAACTAACAGTGTTGAAGCTTTCTTCTG
        130 GTGATGTTTGCATTCAAGTAACAGTGTTGAACATTCCCTTTC

                     *                    *      *   **                      
     520979 ATAGAGCAGTTTTGAAACACTCTTTTTGTAATATCTGCAAGACGATATTTGGATAGCTTTGAGGA
          1 ATAGAGCAGGTTTGAAACACTCTTTTTGTAGTATCTGGAAGTGGATATTTGGATAGCTTTGAGGA

                         *   * *            **                      *    *   
     521044 TTTCGTTGGAAACGGGATTGTCTTCATATAAACTCTAGACAGAAGCATTCTCAGAAGCTTCATTG
         66 TTTCGTTGGAAACAGGAATATCTTCATATAAAAGCTAGACAGAAGCATTCTCAGAAACTTCTTTG

            *       **   *    *             *        
     521109 GGATGTTTCAATTGAAGTCACAGTGTTGAACAGTCCCTTTC
        131 TGATGTTTGCATTCAAGTAACAGTGTTGAACATTCCCTTTC

                                                         *       **   * *    
     521150 ATAGAGCAGGTTTGAAACACTCTTTTTGTAGTATCTGGAAGTGGACATTTGGAACGCTCTCAGGA
          1 ATAGAGCAGGTTTGAAACACTCTTTTTGTAGTATCTGGAAGTGGATATTTGGATAGCTTTGAGGA

            * *  *      *             **            *       * *          *  *
     521215 CTGCGGT-GAAAAAGGAAATATCTTCCAATAAAAGCTAGATAGAAGCAATGTCAGAAACTTTTTC
         66 TTTCGTTGGAAACAGG-AATATCTTCATATAAAAGCTAGACAGAAGCATTCTCAGAAACTTCTTT

            *      * ** *   **      *       *  * *   *
     521279 ATGATGTATCTACTCAGCTAACAGAGTTGAACCTTTCTTTTG
        130 GTGATGTTTGCATTCAAGTAACAGTGTTGAACATTCCCTTTC

             *       *                   * *     *             **            
     521321 AGAGAGCAGTTTTGAAACACTCTTTTTGTGGAATCTGCAAGTGGATATTTGTCTAGCTTTGAGGA
          1 ATAGAGCAGGTTTGAAACACTCTTTTTGTAGTATCTGGAAGTGGATATTTGGATAGCTTTGAGGA

                         *   *    *                   *       *   *          
     521386 TTTCGTTGGAAACGGGATTA-CAT-ATA-AAAAGC-AGACAGCAGCATTCCCAGTAACTTCTTTG
         66 TTTCGTTGGAAACAGGAATATCTTCATATAAAAGCTAGACAGAAGCATTCTCAGAAACTTCTTTG

                              *    *                 
     521447 TGATGTTTGCATTCAAGTCACAGAGTTGAACATTCCCTTTC
        131 TGATGTTTGCATTCAAGTAACAGTGTTGAACATTCCCTTTC

                                 *                 *     *       **     *   *
     521488 ATAGAGCAGGTTTGAAACACTTTTTTTGTAGTATCTGGATGTGGACATTTGGAGCGCTTTCAGGC
          1 ATAGAGCAGGTTTGAAACACTCTTTTTGTAGTATCTGGAAGTGGATATTTGGATAGCTTTGAGGA

            * ** *      *             **          * * *     * *          *  *
     521553 CTATGGT-GAAAAAGGAAATATCTTCCAATAAAAGCTACATAAAAGCAATGTCAGAAACTTTTTC
         66 TTTCGTTGGAAACAGG-AATATCTTCATATAAAAGCTAGACAGAAGCATTCTCAGAAACTTCTTT

            *      * ** *   **      *       *  * *   *
     521617 ATGATGTATCTACTCAGCTAACAGAGTTGAACCTTTCTTTTG
        130 GTGATGTTTGCATTCAAGTAACAGTGTTGAACATTCCCTTTC

             *       *                   * *                   **            
     521659 AGAGAGCAGTTTTGAAACACTCTTTTTGTGGAATCTGGAAGTGGATATTTGTCTAGCTTTGAGGA
          1 ATAGAGCAGGTTTGAAACACTCTTTTTGTAGTATCTGGAAGTGGATATTTGGATAGCTTTGAGGA

                         *   *    *                   *       *   *          
     521724 TTTCGTTGGAAACGGGATTA-CAT-ATA-AAAAGC-AGACAGCAGCATTCCCAGTAACTTCTTTG
         66 TTTCGTTGGAAACAGGAATATCTTCATATAAAAGCTAGACAGAAGCATTCTCAGAAACTTCTTTG

                              *    *                 
     521785 TGATGTTTGCATTCAAGTCACAGAGTTGAACATTCCCTTTC
        131 TGATGTTTGCATTCAAGTAACAGTGTTGAACATTCCCTTTC

                                       *           *     *       **     *    
     521826 ATAGAGCAGGTTTGAAACACTCTTTTTTTAGTATCTGGATGTGGACATTTGGAGCGCTTTCAGG-
          1 ATAGAGCAGGTTTGAAACACTCTTTTTGTAGTATCTGGAAGTGGATATTTGGATAGCTTTGAGGA

            **  *        *             *** *   *           *          *   *  
     521890 CCT-ATTGTGAAAAAGGAAATATCTTCCCCTGAAAACTAGACAGAAGAATTCTCAGAATCTTATT
         66 TTTCGTTG-GAAACAGG-AATATCTTCATATAAAAGCTAGACAGAAGCATTCTCAGAAACTTCTT

                    **  **    *             **  * *   *
     521954 TGTGATGTGCGCCCTCAACTAACAGTGTTGAAGCTTTCTTTTG
        129 TGTGATGTTTGCATTCAAGTAACAGTGTTGAACATTCCCTTTC

                     *                 *  **     *   *                       
     521997 ATAGAGCAGTTTTGAAACACTCTTTTTATAAAATCTGCAAGAGGATATTTGGATAGCTTTGAGGA
          1 ATAGAGCAGGTTTGAAACACTCTTTTTGTAGTATCTGGAAGTGGATATTTGGATAGCTTTGAGGA

                         *   * *            **   *                  * *  *   
     522062 TTTCGTTGGAAACGGGATTGTCTTCATATAAACTCTACACAGAAGCATTCTCAGAAGCGTCATTG
         66 TTTCGTTGGAAACAGGAATATCTTCATATAAAAGCTAGACAGAAGCATTCTCAGAAACTTCTTTG

            *       **   *    *             *        
     522127 GGATGTTTCAATTGAAGTCACAGTGTTGAACAGTCCCTTTC
        131 TGATGTTTGCATTCAAGTAACAGTGTTGAACATTCCCTTTC

                                                         *       **   * *    
     522168 ATAGAGCAGGTTTGAAACACTCTTTTTGTAGTATCTGGAAGTGGACATTTGGAGCGCTCTCAGGA
          1 ATAGAGCAGGTTTGAAACACTCTTTTTGTAGTATCTGGAAGTGGATATTTGGATAGCTTTGAGGA

            * *  *      *             **            *       * *          *  *
     522233 CTGCGGT-GAAAAAGGAACTATCTTCCAATAAAAGCTAGATAGAAGCAATGTCAGAAACTTTTTC
         66 TTTCGTTGGAAACAGGAA-TATCTTCATATAAAAGCTAGACAGAAGCATTCTCAGAAACTTCTTT

            *      * ** *   **      *       *        *
     522297 ATGATGTATCTACTCAGCTAACAGAGTTGAACCTT-CCTTTG
        130 GTGATGTTTGCATTCAAGTAACAGTGTTGAACATTCCCTTTC

             *       *                   * *     *             **            
     522338 AGAGAGCAGTTTTGAAACACTCTTTTTGTGGAATCTGCAAGTGGATATTTGTCTAGCTTTGAGGA
          1 ATAGAGCAGGTTTGAAACACTCTTTTTGTAGTATCTGGAAGTGGATATTTGGATAGCTTTGAGGA

                         *   * *            **                      *    *   
     522403 TTTCGTTGGAAACGGGATTGTCTTCATATAAACTCTAGACAGAAGCATTCTCAGAAGCTTCATTG
         66 TTTCGTTGGAAACAGGAATATCTTCATATAAAAGCTAGACAGAAGCATTCTCAGAAACTTCTTTG

            *       **   *    *             * *      
     522468 GGATGTTTCAATTGAAGTCACAGTGTTGAACAGTTCCTTTC
        131 TGATGTTTGCATTCAAGTAACAGTGTTGAACATTCCCTTTC

                 *                                       *       **   * *    
     522509 ATAGAACAGGTTTGAAACACTCTTTTTGTAGTATCTGGAAGTGGACATTTGGAGCGCTCTCAGGA
          1 ATAGAGCAGGTTTGAAACACTCTTTTTGTAGTATCTGGAAGTGGATATTTGGATAGCTTTGAGGA

            * *  *      *             **          * *       * *          *  *
     522574 CTACGGT-GAAAAAGGAAATATCTTCCAATAAAAGCTACATAGAAGCAATGTCAGAAACTTTTTC
         66 TTTCGTTGGAAACAGG-AATATCTTCATATAAAAGCTAGACAGAAGCATTCTCAGAAACTTCTTT

            *      * ** *   **      *       *  *     *
     522638 ATGATGTATCTACTCAGCTAACAGAGTTGAACCTTTCCTTTG
        130 GTGATGTTTGCATTCAAGTAACAGTGTTGAACATTCCCTTTC

             *       *          *        * *     *             **            
     522680 AGAGAGCAGTTTTGAAACACGCTTTTTGTGGAATCTGCAAGTGGATATTTGTCTAGCTTTGAGGA
          1 ATAGAGCAGGTTTGAAACACTCTTTTTGTAGTATCTGGAAGTGGATATTTGGATAGCTTTGAGGA

                         *   *    *                   *       *     *        
     522745 TTTCGTTGGAAACGGGATTA-CAT-ATA-AAAAGC-AGACAGCAGCATTCCCAGAATCTTCTTTG
         66 TTTCGTTGGAAACAGGAATATCTTCATATAAAAGCTAGACAGAAGCATTCTCAGAAACTTCTTTG

                              *    *                 
     522806 TGATGTTTGCATTCAAGTCACAGAGTTGAACATTCCCTTTC
        131 TGATGTTTGCATTCAAGTAACAGTGTTGAACATTCCCTTTC

                                                   *     *       **     *   *
     522847 ATAGAGCAGGTTTGAAACACTCTTTTTGTAGTATCTGGATGTGGACATTTGGAGCGCTTTCAGGC
          1 ATAGAGCAGGTTTGAAACACTCTTTTTGTAGTATCTGGAAGTGGATATTTGGATAGCTTTGAGGA

            * ** *      *             *** *   *                      *   *   
     522912 CTATGGT-GAAAAAGGAAATATCTTCCCCTGAAAACTAGACAGAAGCATTCTCAGAATCTTATTT
         66 TTTCGTTGGAAACAGG-AATATCTTCATATAAAAGCTAGACAGAAGCATTCTCAGAAACTTCTTT

                   **  **    *             **  * *   *
     522976 GTGATGTGCGCCCTCAACTAACAGTGTTGAAGCTTTCTTTTG
        130 GTGATGTTTGCATTCAAGTAACAGTGTTGAACATTCCCTTTC

                     *                    **     *   *                       
     523018 ATAGAGCAGTTTTGAAACACTCTTTTTGTAAAATCTGCAAGAGGATATTTGGATAGCTTTGAGGA
          1 ATAGAGCAGGTTTGAAACACTCTTTTTGTAGTATCTGGAAGTGGATATTTGGATAGCTTTGAGGA

                         *   * *            **                      *    *   
     523083 TTTCGTTGGAAACGGGATTGTCTTCATATAAACTCTAGACAGAAGCATTCTCAGAAGCTTCATTG
         66 TTTCGTTGGAAACAGGAATATCTTCATATAAAAGCTAGACAGAAGCATTCTCAGAAACTTCTTTG

            *       **   *    *             *        
     523148 GGATGTTTCAATTGAAGTCACAGTGTTGAACAGTCCCTTTC
        131 TGATGTTTGCATTCAAGTAACAGTGTTGAACATTCCCTTTC

                                                         *       **   * **   
     523189 ATAGAGCAGGTTTGAAACACTCTTTTTGTAGTATCTGGAAGTGGACATTTGGAACGCTCTCCGGA
          1 ATAGAGCAGGTTTGAAACACTCTTTTTGTAGTATCTGGAAGTGGATATTTGGATAGCTTTGAGGA

            * *  *      *             **          * *       * *          *  *
     523254 CTGCGGT-GAAAAAGGAAATATCTTCCAATAAAAGCTACATAGAAGCAATGTCAGAAACTTTTTC
         66 TTTCGTTGGAAACAGG-AATATCTTCATATAAAAGCTAGACAGAAGCATTCTCAGAAACTTCTTT

            *      * ** *   **      *       *  * *   *
     523318 ATGATGTATCTACTCAGCTAACAGAGTTGAACCTTTCTTTTG
        130 GTGATGTTTGCATTCAAGTAACAGTGTTGAACATTCCCTTTC

             *       *                    **     *   *                       
     523360 AGAGAGCAGTTTTGAAACACTCTTTTTGTAAAATCTGCAAGAGGATATTTGGATAGCTTTGAGGA
          1 ATAGAGCAGGTTTGAAACACTCTTTTTGTAGTATCTGGAAGTGGATATTTGGATAGCTTTGAGGA

                         *   * *            **                      *    *   
     523425 TTTCGTTGGAAACGGGATTGTCTTCATATAAACTCTAGACAGAAGCATTCTCAGAAGCTTCATTG
         66 TTTCGTTGGAAACAGGAATATCTTCATATAAAAGCTAGACAGAAGCATTCTCAGAAACTTCTTTG

            *       **   *    *             *        
     523490 GGATGTTTCAATTGAAGTCACAGTGTTGAACAGTCCCTTTC
        131 TGATGTTTGCATTCAAGTAACAGTGTTGAACATTCCCTTTC

                                                   *     *       **     *    
     523531 ATAGAGCAGGTTTGAAACACTCTTTTTGTAGTATCTGGATGTGGACATTTGGAGCGCTTTCAGGC
          1 ATAGAGCAGGTTTGAAACACTCTTTTTGTAGTATCTGGAAGTGGATATTTGGATAGCTTTGAGG-

            * * *    *                 *** *   *                          *  
     523596 CTATGGTT-TAAA-AGGAAATATCTTCCCCTGAAAACTAGACAGAAGCATTCTCAGAAACTTATT
         65 ATTTCGTTGGAAACAGG-AATATCTTCATATAAAAGCTAGACAGAAGCATTCTCAGAAACTTCTT

                    **  *     *                    *   *
     523659 TGTGATGTGCGCCTTCAACTAACAGTGTTGAAGCATT-CTTTTG
        129 TGTGATGTTTGCATTCAAGTAACAGTGTTGAA-CATTCCCTTTC

                     *                   * *     *             **            
     523702 ATAGAGCAGTTTTGAAACACTCTTTTTGTGGAATCTGCAAGTGGATATTTGTCTAGCTTTGAGGA
          1 ATAGAGCAGGTTTGAAACACTCTTTTTGTAGTATCTGGAAGTGGATATTTGGATAGCTTTGAGGA

                         *   *    *                   *       *              
     523767 TTTCGTTGGAAACGGGATTA-CAT-ATA-AAAAGC-AGACAGCAGCATTCCCAGAAACTTCTTTG
         66 TTTCGTTGGAAACAGGAATATCTTCATATAAAAGCTAGACAGAAGCATTCTCAGAAACTTCTTTG

                              *    *                 
     523828 TGATGTTTGCATTCAAGTCACAGAGTTGAACATTCCCTTTC
        131 TGATGTTTGCATTCAAGTAACAGTGTTGAACATTCCCTTTC

             *                                     *     *       **     *  **
     523869 AGAGAGCAGGTTTGAAACACTCTTTTTGTAGTATCTGGATGTGGACATTTGGAGCGCTTTCAGCC
          1 ATAGAGCAGGTTTGAAACACTCTTTTTGTAGTATCTGGAAGTGGATATTTGGATAGCTTTGAGGA

            * ** *      *             *** *   *                      *   *   
     523934 CTATGGT-GAAAAAGGAAATATCTTCCCCTGAAAACTAGACAGAAGCATTCTCAGAATCTTATTT
         66 TTTCGTTGGAAACAGG-AATATCTTCATATAAAAGCTAGACAGAAGCATTCTCAGAAACTTCTTT

                   **  **    *             **  * *   *
     523998 GTGATGTGCGCCCTCAACTAACAGTGTTGAAGCTTTCTTTTG
        130 GTGATGTTTGCATTCAAGTAACAGTGTTGAACATTCCCTTTC

                     *                    **     *   *                       
     524040 ATAGAGCAGTTTTGAAACACTCTTTTTGTAAAATCTGCAAGAGGATATTTGGATAGCTTTGAGGA
          1 ATAGAGCAGGTTTGAAACACTCTTTTTGTAGTATCTGGAAGTGGATATTTGGATAGCTTTGAGGA

                         *   * *            **                      *    *   
     524105 TTTCGTTGGAAACGGGATTGTCTTCATATAAACTCTAGACAGAAGCATTCTCAGAAGCTTCATTG
         66 TTTCGTTGGAAACAGGAATATCTTCATATAAAAGCTAGACAGAAGCATTCTCAGAAACTTCTTTG

            *       **   *    *             *        
     524170 GGATGTTTCAATTGAAGTCACAGTGTTGAACAGTCCCTTTC
        131 TGATGTTTGCATTCAAGTAACAGTGTTGAACATTCCCTTTC

                                                         *       **   * *    
     524211 ATAGAGCAGGTTTGAAACACTCTTTTTGTAGTATCTGGAAGTGGACATTTGGAGCGCTCTCAGGA
          1 ATAGAGCAGGTTTGAAACACTCTTTTTGTAGTATCTGGAAGTGGATATTTGGATAGCTTTGAGGA

            * *  *      *             **            *       * *          *  *
     524276 CTGCGGT-GAAAAAGGAACTATCTTCCAATAAAAGCTAGATAGAAGCAATGTCAGAAACTTTTTC
         66 TTTCGTTGGAAACAGGAA-TATCTTCATATAAAAGCTAGACAGAAGCATTCTCAGAAACTTCTTT

            *      * ** *   **      *       *  *     *
     524340 ATGATGTATCTACTCAGCTAACAGAGTTGAACCTTTCCTTTG
        130 GTGATGTTTGCATTCAAGTAACAGTGTTGAACATTCCCTTTC

             *       *                   * *     *             **            
     524382 AGAGAGCAGTTTTGAAACACTCTTTTTGTGGAATCTGCAAGTGGATATTTGTCTAGCTTTGAGGA
          1 ATAGAGCAGGTTTGAAACACTCTTTTTGTAGTATCTGGAAGTGGATATTTGGATAGCTTTGAGGA

                         *   *    *                   *       *     *   *    
     524447 TTTCGTTGGAAACGGGATTA-CAT-ATA-AAAAGC-AGACAGCAGCATTCCCAGAATCTTGTTTG
         66 TTTCGTTGGAAACAGGAATATCTTCATATAAAAGCTAGACAGAAGCATTCTCAGAAACTTCTTTG

                              *    *                 
     524508 TGATGTTTGCATTCAAGTCACAGAGTTGAACATTCCCTTTC
        131 TGATGTTTGCATTCAAGTAACAGTGTTGAACATTCCCTTTC

             *                         *    *      *     *       **     *   *
     524549 AGAGAGCAGGTTTGAAACACTCTTTTTATAGTGTCTGGATGTGGACATTTGGAGCGCTTTCAGGC
          1 ATAGAGCAGGTTTGAAACACTCTTTTTGTAGTATCTGGAAGTGGATATTTGGATAGCTTTGAGGA

            * ** *      *             *** **  *                          *   
     524614 CTATGGT-GAAAAAGGAAATATCTTCTCCTGTAAACTAGACAGAAGCATTCTCAGAAACTTATTT
         66 TTTCGTTGGAAACAGG-AATATCTTCATATAAAAGCTAGACAGAAGCATTCTCAGAAACTTCTTT

                   **  **    *             **  * *   *
     524678 GTGATGTGCGCCCTCAACTAACAGTGTTGAAGCTTTCTTTTG
        130 GTGATGTTTGCATTCAAGTAACAGTGTTGAACATTCCCTTTC

                     *                    **     *   *                       
     524720 ATAGAGCAGTTTTGAAACACTCTTTTTGTAAAATCTGCAAGAGGATATTTGGATAGCTTTGAGGA
          1 ATAGAGCAGGTTTGAAACACTCTTTTTGTAGTATCTGGAAGTGGATATTTGGATAGCTTTGAGGA

                         *   * *            **                      *    *   
     524785 TTTCGTTGGAAACGGGATTGTCTTCATATAAACTCTAGACAGAAGCATTCTCAGAAGCTTCATTG
         66 TTTCGTTGGAAACAGGAATATCTTCATATAAAAGCTAGACAGAAGCATTCTCAGAAACTTCTTTG

            *       **   *    *             *        
     524850 GGATGTTTCAATTGAAGTCACAGTGTTGAACAGTCCCTTTC
        131 TGATGTTTGCATTCAAGTAACAGTGTTGAACATTCCCTTTC

                                                         *       **   * *    
     524891 ATAGAGCAGGTTTGAAACACTCTTTTTGTAGTATCTGGAAGTGGACATTTGGAACGCTCTCAGGA
          1 ATAGAGCAGGTTTGAAACACTCTTTTTGTAGTATCTGGAAGTGGATATTTGGATAGCTTTGAGGA

            * *  *      *             **            *       * *          *  *
     524956 CTGCGGT-GAAAAAGGAAATATCTTCCAATAAAAGCTAGATAGAAGCAATGTCAGAAACTTTTTC
         66 TTTCGTTGGAAACAGG-AATATCTTCATATAAAAGCTAGACAGAAGCATTCTCAGAAACTTCTTT

            *      * ** *   **      *       *  * *   *
     525020 ATGATGTATCTACTCAGCTAACAGAGTTGAACCTTTCTTTTG
        130 GTGATGTTTGCATTCAAGTAACAGTGTTGAACATTCCCTTTC

             *       *                            *             **           
     525062 AGAGAGCAGTTTTGAAACACTCTTTTTGT-GTAATCTGAAAGTGGATATTTGTCTAGCTTTGAGG
          1 ATAGAGCAGGTTTGAAACACTCTTTTTGTAGT-ATCTGGAAGTGGATATTTGGATAGCTTTGAGG

                          *   *    *                   *       *   *         
     525126 ATTTCGTTGGAAACGGGATTA-CAT-ATA-AAAAGC-AGACAGCAGCATTCCCAGTAACTTCTTT
         65 ATTTCGTTGGAAACAGGAATATCTTCATATAAAAGCTAGACAGAAGCATTCTCAGAAACTTCTTT

                *              *    *                 
     525187 GTGAAGTTTGCATTCAAGTCACAGAGTTGAACATTCCCTTTC
        130 GTGATGTTTGCATTCAAGTAACAGTGTTGAACATTCCCTTTC

                                                   *     *       **     *    
     525229 ATAGAGCAGGTTTGAAACACTCTTTTTGTAGTATCTGGATGTGGACATTTGGAGCGCTTTCAGG-
          1 ATAGAGCAGGTTTGAAACACTCTTTTTGTAGTATCTGGAAGTGGATATTTGGATAGCTTTGAGGA

            **  *        *             *** *   *           *          *   *  
     525293 CCT-ATTGTGAAAAAGGAAATATCTTCCCCTGAAAACTAGACAGAAGAATTCTCAGAATCTTATT
         66 TTTCGTTG-GAAACAGG-AATATCTTCATATAAAAGCTAGACAGAAGCATTCTCAGAAACTTCTT

                    **  **    *             **  * *   *
     525357 TGTGATGTGCGCCCTCAACTAACAGTGTTGAAGCTTTCTTTTG
        129 TGTGATGTTTGCATTCAAGTAACAGTGTTGAACATTCCCTTTC

                     *                    **     *   *                       
     525400 ATAGAGCAGTTTTGAAACACTCTTTTTGTAAAATCTGCAAGAGGATATTTGGATAGCTTTGAGGA
          1 ATAGAGCAGGTTTGAAACACTCTTTTTGTAGTATCTGGAAGTGGATATTTGGATAGCTTTGAGGA

                         *   * *            **   *                  * *  *   
     525465 TTTCGTTGGAAACGGGATTGTCTTCATATAAACTCTACACAGAAGCATTCTCAGAAGCGTCATTG
         66 TTTCGTTGGAAACAGGAATATCTTCATATAAAAGCTAGACAGAAGCATTCTCAGAAACTTCTTTG

            *       **   *    *             *        
     525530 GGATGTTTCAATTGAAGTCACAGTGTTGAACAGTCCCTTTC
        131 TGATGTTTGCATTCAAGTAACAGTGTTGAACATTCCCTTTC

                                                   *     *       **     *    
     525571 ATAGAGCAGGTTTGAAACACTCTTTTTGTAGTATCTGGATGTGGACATTTGGAGCGCTTTCAGGC
          1 ATAGAGCAGGTTTGAAACACTCTTTTTGTAGTATCTGGAAGTGGATATTTGGATAGCTTTGAGG-

            * * *    *                 *** *   *                          *  
     525636 CTATGGTT-TAAA-AGGAAATATCTTCCCCTGAAAACTAGACAGAAGCATTCTCAGAAACTTATT
         65 ATTTCGTTGGAAACAGG-AATATCTTCATATAAAAGCTAGACAGAAGCATTCTCAGAAACTTCTT

                    **  *     *                    *   *
     525699 TGTGATGTGCGCCTTCAACTAACAGTGTTGAAGCATT-CTTTTG
        129 TGTGATGTTTGCATTCAAGTAACAGTGTTGAA-CATTCCCTTTC

                     *                   * *     *             **            
     525742 ATAGAGCAGTTTTGAAACACTCTTTTTGTGGAATCTGCAAGTGGATATTTGTCTAGCTTTGAGGA
          1 ATAGAGCAGGTTTGAAACACTCTTTTTGTAGTATCTGGAAGTGGATATTTGGATAGCTTTGAGGA

                         *   *    *                   *       *              
     525807 TTTCGTTGGAAACGGGATTA-CAT-ATA-AAAAGC-AGACAGCAGCATTCCCAGAAACTTCTTTG
         66 TTTCGTTGGAAACAGGAATATCTTCATATAAAAGCTAGACAGAAGCATTCTCAGAAACTTCTTTG

                              *    *                 
     525868 TGATGTTTGCATTCAAGTCACAGAGTTGAACATTCCCTTTC
        131 TGATGTTTGCATTCAAGTAACAGTGTTGAACATTCCCTTTC

             *                                     *     *       **     *   *
     525909 AGAGAGCAGGTTTGAAACACTCTTTTTGTAGTATCTGGATGTGGACATTTGGAGCGCTTTCAGGC
          1 ATAGAGCAGGTTTGAAACACTCTTTTTGTAGTATCTGGAAGTGGATATTTGGATAGCTTTGAGGA

            * ** *      *             *** *   *                      *   *   
     525974 CTATGGT-GAAAAAGGAAATATCTTCCCCTGAAAACTAGACAGAAGCATTCTCAGAATCTTATTT
         66 TTTCGTTGGAAACAGG-AATATCTTCATATAAAAGCTAGACAGAAGCATTCTCAGAAACTTCTTT

                   **  **    *             **  * *   *
     526038 GTGATGTGCGCCCTCAACTAACAGTGTTGAAGCTTTCTTTTG
        130 GTGATGTTTGCATTCAAGTAACAGTGTTGAACATTCCCTTTC

                     *                    **     *   *                       
     526080 ATAGAGCAGTTTTGAAACACTCTTTTTGTAAAATCTGCAAGAGGATATTTGGATAGCTTTGAGGA
          1 ATAGAGCAGGTTTGAAACACTCTTTTTGTAGTATCTGGAAGTGGATATTTGGATAGCTTTGAGGA

                         *   * *            **                      *    *   
     526145 TTTCGTTGGAAACGGGATTGTCTTCATATAAACTCTAGACAGAAGCATTCTCAGAAGCTTCATTG
         66 TTTCGTTGGAAACAGGAATATCTTCATATAAAAGCTAGACAGAAGCATTCTCAGAAACTTCTTTG

            *       **   *    *             *        
     526210 GGATGTTTCAATTGAAGTCACAGTGTTGAACAGTCCCTTTC
        131 TGATGTTTGCATTCAAGTAACAGTGTTGAACATTCCCTTTC

                                *                        *       **   * *    
     526251 ATAGAGCAGGTTTGAAACACACTTTTTGTAGTATCTGGAAGTGGACATTTGGAGCGCTCTCAGGA
          1 ATAGAGCAGGTTTGAAACACTCTTTTTGTAGTATCTGGAAGTGGATATTTGGATAGCTTTGAGGA

            * *  *      *             **            *       * *          *  *
     526316 CTACGGT-GAAAAAGGAACTATCTTCCAATAAAAGCTAGATAGAAGCAATGTCAGAAACTTTTTC
         66 TTTCGTTGGAAACAGGAA-TATCTTCATATAAAAGCTAGACAGAAGCATTCTCAGAAACTTCTTT

            *      * ** *   **      *       *        *
     526380 ATGATGTATCTACTCAGCTAACAGAGTTGAACCTT-CCTTTG
        130 GTGATGTTTGCATTCAAGTAACAGTGTTGAACATTCCCTTTC

             *       *                   * *     *             **            
     526421 AGAGAGCAGTTTTGAAACACTCTTTTTGTGGAATCTGCAAGTGGATATTTGTCTAGCTTTGAGGA
          1 ATAGAGCAGGTTTGAAACACTCTTTTTGTAGTATCTGGAAGTGGATATTTGGATAGCTTTGAGGA

                         *   *    *                   *       *              
     526486 TTTCGTTGGAAACGGGATTA-CAT-ATA-AAAAGC-AGACAGCAGCATTCCCAGAAACTTCTTTG
         66 TTTCGTTGGAAACAGGAATATCTTCATATAAAAGCTAGACAGAAGCATTCTCAGAAACTTCTTTG

                              *    *                 
     526547 TGATGTTTGCATTCAAGTCACAGAGTTGAACATTCCCTTTC
        131 TGATGTTTGCATTCAAGTAACAGTGTTGAACATTCCCTTTC

                                                   *     *     * **     *   *
     526588 ATAGAGCAGGTTTGAAACACTCTTTTTGTAGTATCTGGATGTGGACATTTGCAGCGCTTTCAGGC
          1 ATAGAGCAGGTTTGAAACACTCTTTTTGTAGTATCTGGAAGTGGATATTTGGATAGCTTTGAGGA

            * ** *      *             *** *   *                          *   
     526653 CTAAGGT-GAAAAAGGAAATATCTTCCCCTGAAAACTAGACAGAAGCATTCTCAGAAACTTATTT
         66 TTTCGTTGGAAACAGG-AATATCTTCATATAAAAGCTAGACAGAAGCATTCTCAGAAACTTCTTT

                   **  **    *     *       **  * *   *
     526717 GTGATGTGCGCCCTCAACTAACAATGTTGAAGCTTTCTTTTG
        130 GTGATGTTTGCATTCAAGTAACAGTGTTGAACATTCCCTTTC

                     *                    *      *   *                       
     526759 ATAGAGCAGTTTTGAAACACTCTTTTTGTAATATCTGCAAGAGGATATTTGGATAGCTTTGAGGA
          1 ATAGAGCAGGTTTGAAACACTCTTTTTGTAGTATCTGGAAGTGGATATTTGGATAGCTTTGAGGA

                         *   * *            **                      *    *   
     526824 TTTCGTTGGAAACGGGATTGTCTTCATATAAACTCTAGACAGAAGCATTCTCAGAAGCTTCATTG
         66 TTTCGTTGGAAACAGGAATATCTTCATATAAAAGCTAGACAGAAGCATTCTCAGAAACTTCTTTG

            *       **   *    *             * *      
     526889 GGATGTTTCAATTGAAGTCACAGTGTTGAACAGTTCCTTTC
        131 TGATGTTTGCATTCAAGTAACAGTGTTGAACATTCCCTTTC

                 *                                       *       **   * *    
     526930 ATAGAACAGGTTTGAAACACTCTTTTTGTAGTATCTGGAAGTGGACATTTGGAGCGCTCTCAGGA
          1 ATAGAGCAGGTTTGAAACACTCTTTTTGTAGTATCTGGAAGTGGATATTTGGATAGCTTTGAGGA

            * *  *  *   ** *         **          * *       * *          *  **
     526995 CTACGGTGAAAATGGAAATATCTTCCAATAAAAGCTACATAGAAGCAATGTCAGAAACTTTTTCA
         66 TTTCGTTGGAAACAGGAATATCTTCATATAAAAGCTAGACAGAAGCATTCTCAGAAACTTCTTTG

                  * ** *   **      *       *  *     *
     527060 TGATGTATCTACTCAGCTAACAGAGTTGAACCTTTCCTTTT
        131 TGATGTTTGCATTCAAGTAACAGTGTTGAACATTCCCTTTC

             *       *                   * *     *             **            
     527101 AGAGAGCAGTTTTGAAACACTCTTTTTGTGGAATCTGCAAGTGGATATTTGTCTAGCTTTGAGGA
          1 ATAGAGCAGGTTTGAAACACTCTTTTTGTAGTATCTGGAAGTGGATATTTGGATAGCTTTGAGGA

                         *   *    *                   *       *     *        
     527166 TTTCGTTGGAAACGGGATTA-CAT-ATA-AAAAGC-AGACAGCAGCATTCCCAGAATCTTCTTTG
         66 TTTCGTTGGAAACAGGAATATCTTCATATAAAAGCTAGACAGAAGCATTCTCAGAAACTTCTTTG

                              *    *                 
     527227 TGATGTTTGCATTCAAGTCACAGAGTTGAACATTCCCTTTC
        131 TGATGTTTGCATTCAAGTAACAGTGTTGAACATTCCCTTTC

                         *                         *     *       **     *   *
     527268 ATAGAGCAGGTTTCAAACACTCTTTTTGTAGTATCTGGATGTGGACATTTGGAGCGCTTTCAGGC
          1 ATAGAGCAGGTTTGAAACACTCTTTTTGTAGTATCTGGAAGTGGATATTTGGATAGCTTTGAGGA

            * ** *      *             *** *   *                      *   *   
     527333 CTATGGT-GAAAAAGGAAATATCTTCCCCTGAAAACTAGACAGAAGCATTCTCAGAATCTTATTT
         66 TTTCGTTGGAAACAGG-AATATCTTCATATAAAAGCTAGACAGAAGCATTCTCAGAAACTTCTTT

                   **  **    *             **  * *   *
     527397 GTGATGTGCGCCCTCAACTAACAGTGTTGAAGCTTTCTTTTG
        130 GTGATGTTTGCATTCAAGTAACAGTGTTGAACATTCCCTTTC

                     *                    **     *   *                       
     527439 ATAGAGCAGTTTTGAAACACTCTTTTTGTAAAATCTGCAAGAGGATATTTGGATAGCTTTGAGGA
          1 ATAGAGCAGGTTTGAAACACTCTTTTTGTAGTATCTGGAAGTGGATATTTGGATAGCTTTGAGGA

                         *   * *            **                      *    *   
     527504 TTTCGTTGGAAACGGGATTGTCTTCATATAAACTCTAGACAGAAGCATTCTCAGAAGCTTCATTG
         66 TTTCGTTGGAAACAGGAATATCTTCATATAAAAGCTAGACAGAAGCATTCTCAGAAACTTCTTTG

            *       **   *    *             *        
     527569 GGATGTTTCAATTGAAGTCACAGTGTTGAACAGTCCCTTTC
        131 TGATGTTTGCATTCAAGTAACAGTGTTGAACATTCCCTTTC

                                                         *       **   * *    
     527610 ATAGAGCAGGTTTGAAACACTCTTTTTGTAGTATCTGGAAGTGGACATTTGGAACGCTCTCAGGA
          1 ATAGAGCAGGTTTGAAACACTCTTTTTGTAGTATCTGGAAGTGGATATTTGGATAGCTTTGAGGA

            * *  *      *             **            *       * *          *  *
     527675 CTGCGGT-GAAAAAGGAAATATCTTCCAATAAAAGCTAGATAGAAGCAATGTCAGAAACTTTTTC
         66 TTTCGTTGGAAACAGG-AATATCTTCATATAAAAGCTAGACAGAAGCATTCTCAGAAACTTCTTT

            *      * ** *   **      *       *  * *   *
     527739 ATGATGTATCTACTCAGCTAACAGAGTTGAACCTTTCTTTTG
        130 GTGATGTTTGCATTCAAGTAACAGTGTTGAACATTCCCTTTC

             *       *                   * *     *             **            
     527781 AGAGAGCAGTTTTGAAACACTCTTTTTGTGGAATCTGCAAGTGGATATTTGTCTAGCTTTGAGGA
          1 ATAGAGCAGGTTTGAAACACTCTTTTTGTAGTATCTGGAAGTGGATATTTGGATAGCTTTGAGGA

                         *   *    *                   *       *   *          
     527846 TTTCGTTGGAAACGGGATTA-CAT-ATA-AAAAGC-AGACAGCAGCATTCCCAGTAACTTCTTTG
         66 TTTCGTTGGAAACAGGAATATCTTCATATAAAAGCTAGACAGAAGCATTCTCAGAAACTTCTTTG

                              *    *                 
     527907 TGATGTTTGCATTCAAGTCACAGAGTTGAACATTCCCTTTC
        131 TGATGTTTGCATTCAAGTAACAGTGTTGAACATTCCCTTTC

                                 *                 *     *       **     *   *
     527948 ATAGAGCAGGTTTGAAACACTTTTTTTGTAGTATCTGGATGTGGACATTTGGAGCGCTTTCAGGC
          1 ATAGAGCAGGTTTGAAACACTCTTTTTGTAGTATCTGGAAGTGGATATTTGGATAGCTTTGAGGA

            * ** *      *             **          * * *     * *          *  *
     528013 CTATGGT-GAAAAAGGAAATATCTTCCAATAAAAGCTACATAAAAGCAATGTCAGAAACTTTTTC
         66 TTTCGTTGGAAACAGG-AATATCTTCATATAAAAGCTAGACAGAAGCATTCTCAGAAACTTCTTT

            *      * ** *   **      *       *  * *   *
     528077 ATGATGTATCTACTCAGCTAACAGAGTTGAACCTTTCTTTTG
        130 GTGATGTTTGCATTCAAGTAACAGTGTTGAACATTCCCTTTC

             *       *                   * *                   **            
     528119 AGAGAGCAGTTTTGAAACACTCTTTTTGTGGAATCTGGAAGTGGATATTTGTCTAGCTTTGAGGA
          1 ATAGAGCAGGTTTGAAACACTCTTTTTGTAGTATCTGGAAGTGGATATTTGGATAGCTTTGAGGA

                         *   *    *                   *       *   *          
     528184 TTTCGTTGGAAACGGGATTA-CAT-ATA-AAAAGC-AGACAGCAGCATTCCCAGTAACTTCTTTG
         66 TTTCGTTGGAAACAGGAATATCTTCATATAAAAGCTAGACAGAAGCATTCTCAGAAACTTCTTTG

                              *    *                 
     528245 TGATGTTTGCATTCAAGTCACAGAGTTGAACATTCCCTTTC
        131 TGATGTTTGCATTCAAGTAACAGTGTTGAACATTCCCTTTC

                                                   *     *       **     *    
     528286 ATAGAGCAGGTTTGAAACACTCTTTTTGTAGTATCTGGATGTGGACATTTGGAGCGCTTTCAGG-
          1 ATAGAGCAGGTTTGAAACACTCTTTTTGTAGTATCTGGAAGTGGATATTTGGATAGCTTTGAGGA

            **  *        *             *** *   *           *          *   *  
     528350 CCT-ATTGTGAAAAAGGAAATATCTTCCCCTGAAAACTAGACAGAAGAATTCTCAGAATCTTATT
         66 TTTCGTTG-GAAACAGG-AATATCTTCATATAAAAGCTAGACAGAAGCATTCTCAGAAACTTCTT

                    **  **    *             **  * *   *
     528414 TGTGATGTGCGCCCTCAACTAACAGTGTTGAAGCTTTCTTTTG
        129 TGTGATGTTTGCATTCAAGTAACAGTGTTGAACATTCCCTTTC

                     *                    **     *   *                       
     528457 ATAGAGCAGTTTTGAAACACTCTTTTTGTAAAATCTGCAAGAGGATATTTGGATAGCTTTGAGGA
          1 ATAGAGCAGGTTTGAAACACTCTTTTTGTAGTATCTGGAAGTGGATATTTGGATAGCTTTGAGGA

                         *   * * *          **   *                  * *  *   
     528522 TTTCGTTGGAAACGGGATTGTTTTCATATAAACTCTACACAGAAGCATTCTCAGAAGCGTCATTG
         66 TTTCGTTGGAAACAGGAATATCTTCATATAAAAGCTAGACAGAAGCATTCTCAGAAACTTCTTTG

            *       **   *    *             *        
     528587 GGATGTTTCAATTGAAGTCACAGTGTTGAACAGTCCCTTTC
        131 TGATGTTTGCATTCAAGTAACAGTGTTGAACATTCCCTTTC

                                                   *     *       **     *   *
     528628 ATAGAGCAGGTTTGAAACACTCTTTTTGTAGTATCTGGATGTGGACATTTGGAGCGCTTTCAGGC
          1 ATAGAGCAGGTTTGAAACACTCTTTTTGTAGTATCTGGAAGTGGATATTTGGATAGCTTTGAGGA

            * ** *      *             *** *   *                      *   *   
     528693 CTATGGT-GAAAAAGGAAATATCTTCCCCTGAAAACTAGACAGAAGCATTCTCAGAATCTTATTT
         66 TTTCGTTGGAAACAGG-AATATCTTCATATAAAAGCTAGACAGAAGCATTCTCAGAAACTTCTTT

                   **  **    *             **  * *   *
     528757 GTGATGTGCGCCCTCAACTAACAGTGTTGAAGCTTTCTTTTG
        130 GTGATGTTTGCATTCAAGTAACAGTGTTGAACATTCCCTTTC

                     *                    **     *   *                       
     528799 ATAGAGCAGTTTTGAAACACTCTTTTTGTAAAATCTGCAAGAGGATATTTGGATAGCTTTGAGGA
          1 ATAGAGCAGGTTTGAAACACTCTTTTTGTAGTATCTGGAAGTGGATATTTGGATAGCTTTGAGGA

                         *   * *            **                      *    *   
     528864 TTTCGTTGGAAACGGGATTGTCTTCATATAAACTCTAGACAGAAGCATTCTCAGAAGCTTCATTG
         66 TTTCGTTGGAAACAGGAATATCTTCATATAAAAGCTAGACAGAAGCATTCTCAGAAACTTCTTTG

            *       **   *    *             *        
     528929 GGATGTTTCAATTGAAGTCACAGTGTTGAACAGTCCCTTTC
        131 TGATGTTTGCATTCAAGTAACAGTGTTGAACATTCCCTTTC

                                                         *       **   * *    
     528970 ATAGAGCAGGTTTGAAACACTCTTTTTGTAGTATCTGGAAGTGGACATTTGGAGCGCTCTCAGGA
          1 ATAGAGCAGGTTTGAAACACTCTTTTTGTAGTATCTGGAAGTGGATATTTGGATAGCTTTGAGGA

            * *  *      *             **            *       * *          *  *
     529035 CTGCGGT-GAAAAAGGAACTATCTTCCAATAAAAGCTAGATAGAAGCAATGTCAGAAACTTTTTC
         66 TTTCGTTGGAAACAGGAA-TATCTTCATATAAAAGCTAGACAGAAGCATTCTCAGAAACTTCTTT

            *      * ** *   **      *       *        *
     529099 ATGATGTATCTACTCAGCTAACAGAGTTGAACCTT-CCTTTG
        130 GTGATGTTTGCATTCAAGTAACAGTGTTGAACATTCCCTTTC

             *       *                   * *     *             **            
     529140 AGAGAGCAGTTTTGAAACACTCTTTTTGTGGAATCTGCAAGTGGATATTTGTCTAGCTTTGAGGA
          1 ATAGAGCAGGTTTGAAACACTCTTTTTGTAGTATCTGGAAGTGGATATTTGGATAGCTTTGAGGA

                         *   * *            **                      *    *   
     529205 TTTCGTTGGAAACGGGATTGTCTTCATATAAACTCTAGACAGAAGCATTCTCAGAAGCTTCATTG
         66 TTTCGTTGGAAACAGGAATATCTTCATATAAAAGCTAGACAGAAGCATTCTCAGAAACTTCTTTG

            *       **   *    *             * *      
     529270 GGATGTTTCAATTGAAGTCACAGTGTTGAACAGTTCCTTTC
        131 TGATGTTTGCATTCAAGTAACAGTGTTGAACATTCCCTTTC

                 *                                       *       **   * *    
     529311 ATAGAACAGGTTTGAAACACTCTTTTTGTAGTATCTGGAAGTGGACATTTGGAGCGCTCTCAGGA
          1 ATAGAGCAGGTTTGAAACACTCTTTTTGTAGTATCTGGAAGTGGATATTTGGATAGCTTTGAGGA

            * *  *      *             **          * *       * *          *  *
     529376 CTACGGT-GAAAAAGGAAATATCTTCCAATAAAAGCTACATAGAAGCAATGTCAGAAACTTTTTC
         66 TTTCGTTGGAAACAGG-AATATCTTCATATAAAAGCTAGACAGAAGCATTCTCAGAAACTTCTTT

            * *    * ** *   **      *       *  *     *
     529440 ATCATGTATCTACTCAGCTAACAGAGTTGAACCTTTCCTTTG
        130 GTGATGTTTGCATTCAAGTAACAGTGTTGAACATTCCCTTTC

             *       *                   * *     *             **            
     529482 AGAGAGCAGTTTTGAAACACTCTTTTTGTGGAATCTGCAAGTGGATATTTGTCTAGCTTTGAGGA
          1 ATAGAGCAGGTTTGAAACACTCTTTTTGTAGTATCTGGAAGTGGATATTTGGATAGCTTTGAGGA

                         *   *    *                   *       *     *        
     529547 TTTCGTTGGAAACGGGATTA-CAT-ATA-AAAAGC-AGACAGCAGCATTCCCAGAATCTTCTTTG
         66 TTTCGTTGGAAACAGGAATATCTTCATATAAAAGCTAGACAGAAGCATTCTCAGAAACTTCTTTG

                              *    *                 
     529608 TGATGTTTGCATTCAAGTCACAGAGTTGAACATTCCCTTTC
        131 TGATGTTTGCATTCAAGTAACAGTGTTGAACATTCCCTTTC

                                                   *     *       **     *   *
     529649 ATAGAGCAGGTTTGAAACACTCTTTTTGTAGTATCTGGATGTGGACATTTGGAGCGCTTTCAGGC
          1 ATAGAGCAGGTTTGAAACACTCTTTTTGTAGTATCTGGAAGTGGATATTTGGATAGCTTTGAGGA

            * ** *      *             *** *   *                      *   *   
     529714 CTATGGT-GAAAAAGGAAATATCTTCCCCTGAAAACTAGACAGAAGCATTCTCAGAATCTTATTT
         66 TTTCGTTGGAAACAGG-AATATCTTCATATAAAAGCTAGACAGAAGCATTCTCAGAAACTTCTTT

                   **  **    *             **  * *   *
     529778 GTGATGTGCGCCCTCAACTAACAGTGTTGAAGCTTTCTTTTG
        130 GTGATGTTTGCATTCAAGTAACAGTGTTGAACATTCCCTTTC

                     *                    **     *   *                       
     529820 ATAGAGCAGTTTTGAAACACTCTTTTTGTAAAATCTGCAAGAGGATATTTGGATAGCTTTGAGGA
          1 ATAGAGCAGGTTTGAAACACTCTTTTTGTAGTATCTGGAAGTGGATATTTGGATAGCTTTGAGGA

                         *   * *            **                      *    *   
     529885 TTTCGTTGGAAACGGGATTGTCTTCATATAAACTCTAGACAGAAGCATTCTCAGAAGCTTCATTG
         66 TTTCGTTGGAAACAGGAATATCTTCATATAAAAGCTAGACAGAAGCATTCTCAGAAACTTCTTTG

            *       **   *    *             *        
     529950 GGATGTTTCAATTGAAGTCACAGTGTTGAACAGTCCCTTTC
        131 TGATGTTTGCATTCAAGTAACAGTGTTGAACATTCCCTTTC

                                                         *       **   * *    
     529991 ATAGAGCAGGTTTGAAACACTCTTTTTGTAGTATCTGGAAGTGGACATTTGGAACGCTCTCAGGA
          1 ATAGAGCAGGTTTGAAACACTCTTTTTGTAGTATCTGGAAGTGGATATTTGGATAGCTTTGAGGA

            * *  *      *             **            *       * *          *  *
     530056 CTGCGGT-GAAAAAGGAAATATCTTCCAATAAAAGCTAGATAGAAGCAATGTCAGAAACTTTTTC
         66 TTTCGTTGGAAACAGG-AATATCTTCATATAAAAGCTAGACAGAAGCATTCTCAGAAACTTCTTT

            *      * ** *   **      *       *  * *   *
     530120 ATGATGTATCTACTCAGCTAACAGAGTTGAACCTTTCTTTTG
        130 GTGATGTTTGCATTCAAGTAACAGTGTTGAACATTCCCTTTC

             *       *                    **     *   *                       
     530162 AGAGAGCAGTTTTGAAACACTCTTTTTGTAAAATCTGCAAGAGGATATTTGGATAGCTTTGAGGA
          1 ATAGAGCAGGTTTGAAACACTCTTTTTGTAGTATCTGGAAGTGGATATTTGGATAGCTTTGAGGA

                  *      *   * *            **                      * *  *   
     530227 TTTCGTAGGAAACGGGATTGTCTTCATATAAACTCTAGACAGAAGCATTCTCAGAAGCGTCATTG
         66 TTTCGTTGGAAACAGGAATATCTTCATATAAAAGCTAGACAGAAGCATTCTCAGAAACTTCTTTG

            *       **   *    *             *        
     530292 GGATGTTTCAATTGAAGTCACAGTGTTGAACAGTCCCTTTC
        131 TGATGTTTGCATTCAAGTAACAGTGTTGAACATTCCCTTTC

                                                   *     *       **     *    
     530333 ATAGAGCAGGTTTGAAACACTCTTTTTGTAGTATCTGGATGTGGACATTTGGAGCGCTTTCAGGC
          1 ATAGAGCAGGTTTGAAACACTCTTTTTGTAGTATCTGGAAGTGGATATTTGGATAGCTTTGAGG-

            * * *    *                 *** *   *                          *  
     530398 CTATGGTT-TAAA-AGGAAATATCTTCCCCTGAAAACTAGACAGAAGCATTCTCAGAAACTTATT
         65 ATTTCGTTGGAAACAGG-AATATCTTCATATAAAAGCTAGACAGAAGCATTCTCAGAAACTTCTT

                    **  **    *             **  * *   *
     530461 TGTGATGTGCGCCCTCAACTAACAGTGTTGAAGCTTTCTTTTG
        129 TGTGATGTTTGCATTCAAGTAACAGTGTTGAACATTCCCTTTC

                     *                    **     *   *                       
     530504 ATAGAGCAGTTTTGAAACACTCTTTTTGTAAAATCTGCAAGAGGATATTTGGATAGCTTTGAGGA
          1 ATAGAGCAGGTTTGAAACACTCTTTTTGTAGTATCTGGAAGTGGATATTTGGATAGCTTTGAGGA

                         *   * *            **                      *    *   
     530569 TTTCGTTGGAAACGGGATTGTCTTCATATAAACTCTAGACAGAAGCATTCTCAGAAGCTTCATTG
         66 TTTCGTTGGAAACAGGAATATCTTCATATAAAAGCTAGACAGAAGCATTCTCAGAAACTTCTTTG

            *       **   *    *             *        
     530634 GGATGTTTCAATTGAAGTCACAGTGTTGAACAGTCCCTTTC
        131 TGATGTTTGCATTCAAGTAACAGTGTTGAACATTCCCTTTC

                                                         *       **   * *    
     530675 ATAGAGCAGGTTTGAAACACTCTTTTTGTAGTATCTGGAAGTGGACATTTGGAGCGCTCTCAGGA
          1 ATAGAGCAGGTTTGAAACACTCTTTTTGTAGTATCTGGAAGTGGATATTTGGATAGCTTTGAGGA

            * *  *      *             **            *       * *          *  *
     530740 CTGCGGT-GAAAAAGGAACTATCTTCCAATAAAAGCTAGATAGAAGCAATGTCAGAAACTTTTTC
         66 TTTCGTTGGAAACAGGAA-TATCTTCATATAAAAGCTAGACAGAAGCATTCTCAGAAACTTCTTT

            *      * ** *   **      *       *        *
     530804 ATGATGTATCTACTCAGCTAACAGAGTTGAACCTT-CCTTTG
        130 GTGATGTTTGCATTCAAGTAACAGTGTTGAACATTCCCTTTC

             *       *                   * *     *             **            
     530845 AGAGAGCAGTTTTGAAACACTCTTTTTGTGGAATCTGCAAGTGGATATTTGTCTAGCTTTGAGGA
          1 ATAGAGCAGGTTTGAAACACTCTTTTTGTAGTATCTGGAAGTGGATATTTGGATAGCTTTGAGGA

                         *   *    *                   *       *              
     530910 TTTCGTTGGAAACGGGATTA-CAT-ATA-AAAAGC-AGACAGCAGCATTCCCAGAAACTTCTTTG
         66 TTTCGTTGGAAACAGGAATATCTTCATATAAAAGCTAGACAGAAGCATTCTCAGAAACTTCTTTG

                              *    *                 
     530971 TGATGTTTGCATTCAAGTCACAGAGTTGAACATTCCCTTTC
        131 TGATGTTTGCATTCAAGTAACAGTGTTGAACATTCCCTTTC

                                                   *     *     * **     *   *
     531012 ATAGAGCAGGTTTGAAACACTCTTTTTGTAGTATCTGGATGTGGACATTTGCAGCGCTTTCAGGC
          1 ATAGAGCAGGTTTGAAACACTCTTTTTGTAGTATCTGGAAGTGGATATTTGGATAGCTTTGAGGA

            * ** *      *             *** *   *                          *   
     531077 CTAAGGT-GAAAAAGGAAATATCTTCCCCTGAAAACTAGACAGAAGCATTCTCAGAAACTTATTT
         66 TTTCGTTGGAAACAGG-AATATCTTCATATAAAAGCTAGACAGAAGCATTCTCAGAAACTTCTTT

                   **  **    *             **  * *   *
     531141 GTGATGTGCGCCCTCAACTAACAGTGTTGAAGCTTTCTTTTG
        130 GTGATGTTTGCATTCAAGTAACAGTGTTGAACATTCCCTTTC

                     *                    *      *   *                       
     531183 ATAGAGCAGTTTTGAAACACTCTTTTTGTAATATCTGCAAGAGGATATTTGGATAGCTTTGAGGA
          1 ATAGAGCAGGTTTGAAACACTCTTTTTGTAGTATCTGGAAGTGGATATTTGGATAGCTTTGAGGA

                         *   * *            **                      *    *   
     531248 TTTCGTTGGAAACGGGATTGTCTTCATATAAACTCTAGACAGAAGCATTCTCAGAAGCTTCATTG
         66 TTTCGTTGGAAACAGGAATATCTTCATATAAAAGCTAGACAGAAGCATTCTCAGAAACTTCTTTG

            *       **   *    *             * *      
     531313 GGATGTTTCAATTGAAGTCACAGTGTTGAACAGTTCCTTTC
        131 TGATGTTTGCATTCAAGTAACAGTGTTGAACATTCCCTTTC

                 *                                       *       **   * *    
     531354 ATAGAACAGGTTTGAAACACTCTTTTTGTAGTATCTGGAAGTGGACATTTGGAGCGCTCTCAGGA
          1 ATAGAGCAGGTTTGAAACACTCTTTTTGTAGTATCTGGAAGTGGATATTTGGATAGCTTTGAGGA

            * *  *  *   ** *                      * *       * *          *  *
     531419 CTACGGTGAAAATGGAAATATCTTACA-ATAAAAGCTACATAGAAGCAATGTCAGAAACTTTTTC
         66 TTTCGTTGGAAACAGGAATATCTT-CATATAAAAGCTAGACAGAAGCATTCTCAGAAACTTCTTT

            *      * ** *   **      *       *  *     *
     531483 ATGATGTATCTACTCAGCTAACAGAGTTGAACCTTTCCTTTG
        130 GTGATGTTTGCATTCAAGTAACAGTGTTGAACATTCCCTTTC

             *       *                   * *     *             **            
     531525 AGAGAGCAGTTTTGAAACACTCTTTTTGTGGAATCTGCAAGTGGATATTTGTCTAGCTTTGAGGA
          1 ATAGAGCAGGTTTGAAACACTCTTTTTGTAGTATCTGGAAGTGGATATTTGGATAGCTTTGAGGA

                         *   *    *                   *       *     *        
     531590 TTTCGTTGGAAACGGGATTA-CAT-ATA-AAAAGC-AGACAGCAGCATTCCCAGAATCTTCTTTG
         66 TTTCGTTGGAAACAGGAATATCTTCATATAAAAGCTAGACAGAAGCATTCTCAGAAACTTCTTTG

                              *    *                 
     531651 TGATGTTTGCATTCAAGTCACAGAGTTGAACATTCCCTTTC
        131 TGATGTTTGCATTCAAGTAACAGTGTTGAACATTCCCTTTC

                         *                         *     *       **     *   *
     531692 ATAGAGCAGGTTTCAAACACTCTTTTTGTAGTATCTGGATGTGGACATTTGGAGCGCTTTCAGGC
          1 ATAGAGCAGGTTTGAAACACTCTTTTTGTAGTATCTGGAAGTGGATATTTGGATAGCTTTGAGGA

            * ** *      *             *** *   *                      *   *   
     531757 CTATGGT-GAAAAAGGAAATATCTTCCCCTGAAAACTAGACAGAAGCATTCTCAGAATCTTATTT
         66 TTTCGTTGGAAACAGG-AATATCTTCATATAAAAGCTAGACAGAAGCATTCTCAGAAACTTCTTT

                   **  **    *             **  * *   *
     531821 GTGATGTGCGCCCTCAACTAACAGTGTTGAAGCTTTCTTTTG
        130 GTGATGTTTGCATTCAAGTAACAGTGTTGAACATTCCCTTTC

                     *                    **     *   *                       
     531863 ATAGAGCAGTTTTGAAACACTCTTTTTGTAAAATCTGCAAGAGGATATTTGGATAGCTTTGAGGA
          1 ATAGAGCAGGTTTGAAACACTCTTTTTGTAGTATCTGGAAGTGGATATTTGGATAGCTTTGAGGA

                         *   * *            **                      *    *   
     531928 TTTCGTTGGAAACGGGATTGTCTTCATATAAACTCTAGACAGAAGCATTCTCAGAAGCTTCATTG
         66 TTTCGTTGGAAACAGGAATATCTTCATATAAAAGCTAGACAGAAGCATTCTCAGAAACTTCTTTG

            *       **   *    *             *        
     531993 GGATGTTTCAATTGAAGTCACAGTGTTGAACAGTCCCTTTC
        131 TGATGTTTGCATTCAAGTAACAGTGTTGAACATTCCCTTTC

                                                         *       **   * *    
     532034 ATAGAGCAGGTTTGAAACACTCTTTTTGTAGTATCTGGAAGTGGACATTTGGAACGCTCTCAGGA
          1 ATAGAGCAGGTTTGAAACACTCTTTTTGTAGTATCTGGAAGTGGATATTTGGATAGCTTTGAGGA

            * *  *      *             **            *       * *          *  *
     532099 CTGCGGT-GAAAAAGGAAATATCTTCCAATAAAAGCTAGATAGAAGCAATGTCAGAAACTTTTTC
         66 TTTCGTTGGAAACAGG-AATATCTTCATATAAAAGCTAGACAGAAGCATTCTCAGAAACTTCTTT

            *      * ** *   **      *       *  * *   *
     532163 ATGATGTATCTACTCAGCTAACAGAGTTGAACCTTTCTTTTG
        130 GTGATGTTTGCATTCAAGTAACAGTGTTGAACATTCCCTTTC

             *       *            *      * *     *             **  *         
     532205 AGAGAGCAGTTTTGAAACACTCGTTTTGTGGAATCTGCAAGTGGATATTTGTCTACCTTTGAGGA
          1 ATAGAGCAGGTTTGAAACACTCTTTTTGTAGTATCTGGAAGTGGATATTTGGATAGCTTTGAGGA

                         *   *    *                   *       *              
     532270 TTTCGTTGGAAACGGGATTA-CAT-ATA-AAAAGC-AGACAGCAGCATTCCCAGAAACTTCTTTG
         66 TTTCGTTGGAAACAGGAATATCTTCATATAAAAGCTAGACAGAAGCATTCTCAGAAACTTCTTTG

                              *    *                 
     532331 TGATGTTTGCATTCAAGTCACAGAGTTGAACATTCCCTTTC
        131 TGATGTTTGCATTCAAGTAACAGTGTTGAACATTCCCTTTC

                                                   *     *     * **     *   *
     532372 ATAGAGCAGGTTTGAAACACTCTTTTTGTAGTATCTGGATGTGGACATTTGCAGCGCTTTCAGGC
          1 ATAGAGCAGGTTTGAAACACTCTTTTTGTAGTATCTGGAAGTGGATATTTGGATAGCTTTGAGGA

            * ** *      *             *** *   *                          *   
     532437 CTAAGGT-GAAAAAGGAAATATCTTCCCCTGAAAACTAGACAGAAGCATTCTCAGAAACTTATTT
         66 TTTCGTTGGAAACAGG-AATATCTTCATATAAAAGCTAGACAGAAGCATTCTCAGAAACTTCTTT

                   **  **    *             **  * *   *
     532501 GTGATGTGCGCCCTCAACTAACAGTGTTGAAGCTTTCTTTTG
        130 GTGATGTTTGCATTCAAGTAACAGTGTTGAACATTCCCTTTC

                     *                    *      *   *                       
     532543 ATAGAGCAGTTTTGAAACACTCTTTTTGTAATATCTGCAAGAGGATATTTGGATAGCTTTGAGGA
          1 ATAGAGCAGGTTTGAAACACTCTTTTTGTAGTATCTGGAAGTGGATATTTGGATAGCTTTGAGGA

                         *   * *            **                      *    *   
     532608 TTTCGTTGGAAACGGGATTGTCTTCATATAAACTCTAGACAGAAGCATTCTCAGAAGCTTCATTG
         66 TTTCGTTGGAAACAGGAATATCTTCATATAAAAGCTAGACAGAAGCATTCTCAGAAACTTCTTTG

            *       **   *    *             *        
     532673 GGATGTTTCAATTGAAGTCACAGTGTTGAACAGTCCCTTTC
        131 TGATGTTTGCATTCAAGTAACAGTGTTGAACATTCCCTTTC

                                                         *       **   * *    
     532714 ATAGAGCAGGTTTGAAACACTCTTTTTGTAGTATCTGGAAGTGGACATTTGGAACGCTCTCAGGA
          1 ATAGAGCAGGTTTGAAACACTCTTTTTGTAGTATCTGGAAGTGGATATTTGGATAGCTTTGAGGA

            * *  *      *             **            *       * *          *  *
     532779 CTGCGGT-GAAAAAGGAAATATCTTCCAATAAAAGCTAGATAGAAGCAATGTCAGAAACTTTTTC
         66 TTTCGTTGGAAACAGG-AATATCTTCATATAAAAGCTAGACAGAAGCATTCTCAGAAACTTCTTT

            *      * ** *   **      *       *  * *   *
     532843 ATGATGTATCTACTCAGCTAACAGAGTTGAACCTTTCTTTTG
        130 GTGATGTTTGCATTCAAGTAACAGTGTTGAACATTCCCTTTC

             *       *    *              * *     *             **            
     532885 AGAGAGCAGTTTTGTAACACTCTTTTTGTGGAATCTGCAAGTGGATATTTGTCTAGCTTTGAGGA
          1 ATAGAGCAGGTTTGAAACACTCTTTTTGTAGTATCTGGAAGTGGATATTTGGATAGCTTTGAGGA

                         *   * *            **                      *    *   
     532950 TTTCGTTGGAAACGGGATTGTCTTCATATAAACTCTAGACAGAAGCATTCTCAGAAGCTTCATTG
         66 TTTCGTTGGAAACAGGAATATCTTCATATAAAAGCTAGACAGAAGCATTCTCAGAAACTTCTTTG

            *       **   *    *             * *      
     533015 GGATGTTTCAATTGAAGTCACAGTGTTGAACAGTTCCTTTC
        131 TGATGTTTGCATTCAAGTAACAGTGTTGAACATTCCCTTTC

                 *                                       *       **   * *    
     533056 ATAGAACAGGTTTGAAACACTCTTTTTGTAGTATCTGGAAGTGGACATTTGGAGCGCTCTCAGGA
          1 ATAGAGCAGGTTTGAAACACTCTTTTTGTAGTATCTGGAAGTGGATATTTGGATAGCTTTGAGGA

            * *  *      *             **          * *       * *          *  *
     533121 CTACGGT-GAAAAAGGAAATATCTTCCAATAAAAGCTACATAGAAGCAATGTCAGAAACTTTTTC
         66 TTTCGTTGGAAACAGG-AATATCTTCATATAAAAGCTAGACAGAAGCATTCTCAGAAACTTCTTT

            * *    * ** *   **      *       *  *     *
     533185 ATCATGTATCTACTCAGCTAACAGAGTTGAACCTTTCCTTTG
        130 GTGATGTTTGCATTCAAGTAACAGTGTTGAACATTCCCTTTC

             *       *                   * *     *             **            
     533227 AGAGAGCAGTTTTGAAACACTCTTTTTGTGGAATCTGCAAGTGGATATTTGTCTAGCTTTGAGGA
          1 ATAGAGCAGGTTTGAAACACTCTTTTTGTAGTATCTGGAAGTGGATATTTGGATAGCTTTGAGGA

                         *   *    *                   *       *     *        
     533292 TTTCGTTGGAAACGGGATTA-CAT-ATA-AAAAGC-AGACAGCAGCATTCCCAGAATCTTCTTTG
         66 TTTCGTTGGAAACAGGAATATCTTCATATAAAAGCTAGACAGAAGCATTCTCAGAAACTTCTTTG

                              *    *                 
     533353 TGATGTTTGCATTCAAGTCACAGAGTTGAACATTCCCTTTC
        131 TGATGTTTGCATTCAAGTAACAGTGTTGAACATTCCCTTTC

                                                   *     *       **     *   *
     533394 ATAGAGCAGGTTTGAAACACTCTTTTTGTAGTATCTGGATGTGGACATTTGGAGCGCTTTCAGGC
          1 ATAGAGCAGGTTTGAAACACTCTTTTTGTAGTATCTGGAAGTGGATATTTGGATAGCTTTGAGGA

            * ** *      *             *** *   *                      *   *   
     533459 CTATGGT-GAAAAAGGAAATATCTTCCCCTGAAAACTAGACAGAAGCATTCTCAGAATCTTATTT
         66 TTTCGTTGGAAACAGG-AATATCTTCATATAAAAGCTAGACAGAAGCATTCTCAGAAACTTCTTT

                   **  **    *             **  * *   *
     533523 GTGATGTGCGCCCTCAACTAACAGTGTTGAAGCTTTCTTTTG
        130 GTGATGTTTGCATTCAAGTAACAGTGTTGAACATTCCCTTTC

                     *                    **     *   *                       
     533565 ATAGAGCAGTTTTGAAACACTCTTTTTGTAAAATCTGCAAGAGGATATTTGGATAGCTTTGAGGA
          1 ATAGAGCAGGTTTGAAACACTCTTTTTGTAGTATCTGGAAGTGGATATTTGGATAGCTTTGAGGA

                         *   * *            **                      *    *   
     533630 TTTCGTTGGAAACGGGATTGTCTTCATATAAACTCTAGACAGAAGCATTCTCAGAAGCTTCATTG
         66 TTTCGTTGGAAACAGGAATATCTTCATATAAAAGCTAGACAGAAGCATTCTCAGAAACTTCTTTG

            *       **   *    *             *        
     533695 GGATGTTTCAATTGAAGTCACAGTGTTGAACAGTCCCTTTC
        131 TGATGTTTGCATTCAAGTAACAGTGTTGAACATTCCCTTTC

                                                         *       **   * *    
     533736 ATAGAGCAGGTTTGAAACACTCTTTTTGTAGTATCTGGAAGTGGACATTTGGAACGCTCTCAGGA
          1 ATAGAGCAGGTTTGAAACACTCTTTTTGTAGTATCTGGAAGTGGATATTTGGATAGCTTTGAGGA

            * *  *      *             **            *       * *          *  *
     533801 CTGCGGT-GAAAAAGGAAATATCTTCCAATAAAAGCTAGATAGAAGCAATGTCAGAAACTTTTTC
         66 TTTCGTTGGAAACAGG-AATATCTTCATATAAAAGCTAGACAGAAGCATTCTCAGAAACTTCTTT

            *      * ** *   **      *       *  * *   *
     533865 ATGATGTATCTACTCAGCTAACAGAGTTGAACCTTTCTTTTG
        130 GTGATGTTTGCATTCAAGTAACAGTGTTGAACATTCCCTTTC

             *       *                    **     *   *                       
     533907 AGAGAGCAGTTTTGAAACACTCTTTTTGTAAAATCTGCAAGAGGATATTTGGATAGCTTTGAGGA
          1 ATAGAGCAGGTTTGAAACACTCTTTTTGTAGTATCTGGAAGTGGATATTTGGATAGCTTTGAGGA

                  *      *   * *            **                      * *  *   
     533972 TTTCGTAGGAAACGGGATTGTCTTCATATAAACTCTAGACAGAAGCATTCTCAGAAGCGTCATTG
         66 TTTCGTTGGAAACAGGAATATCTTCATATAAAAGCTAGACAGAAGCATTCTCAGAAACTTCTTTG

            *       **   *    *             *        
     534037 GGATGTTTCAATTGAAGTCACAGTGTTGAACAGTCCCTTTC
        131 TGATGTTTGCATTCAAGTAACAGTGTTGAACATTCCCTTTC

                                                   *     *       **     *    
     534078 ATAGAGCAGGTTTGAAACACTCTTTTTGTAGTATCTGGATGTGGACATTTGGAGCGCTTTCAGGC
          1 ATAGAGCAGGTTTGAAACACTCTTTTTGTAGTATCTGGAAGTGGATATTTGGATAGCTTTGAGG-

            * * *    *                 *** *   *                          *  
     534143 CTATGGTT-TAAA-AGGAAATATCTTCCCCTGAAAACTAGACAGAAGCATTCTCAGAAACTTATT
         65 ATTTCGTTGGAAACAGG-AATATCTTCATATAAAAGCTAGACAGAAGCATTCTCAGAAACTTCTT

                    **  **    *             **  * *   *
     534206 TGTGATGTGCGCCCTCAACTAACAGTGTTGAAGCTTTCTTTTG
        129 TGTGATGTTTGCATTCAAGTAACAGTGTTGAACATTCCCTTTC

                     *                    **     *   *                       
     534249 ATAGAGCAGTTTTGAAACACTCTTTTTGTAAAATCTGCAAGAGGATATTTGGATAGCTTTGAGGA
          1 ATAGAGCAGGTTTGAAACACTCTTTTTGTAGTATCTGGAAGTGGATATTTGGATAGCTTTGAGGA

                         *   * *            **                      *    *   
     534314 TTTCGTTGGAAACGGGATTGTCTTCATATAAACTCTAGACAGAAGCATTCTCAGAAGCTTCATTG
         66 TTTCGTTGGAAACAGGAATATCTTCATATAAAAGCTAGACAGAAGCATTCTCAGAAACTTCTTTG

            *       **   *    *             *        
     534379 GGATGTTTCAATTGAAGTCACAGTGTTGAACAGTCCCTTTC
        131 TGATGTTTGCATTCAAGTAACAGTGTTGAACATTCCCTTTC

                                                         *       **   * *    
     534420 ATAGAGCAGGTTTGAAACACTCTTTTTGTAGTATCTGGAAGTGGACATTTGGAGCGCTCTCAGGA
          1 ATAGAGCAGGTTTGAAACACTCTTTTTGTAGTATCTGGAAGTGGATATTTGGATAGCTTTGAGGA

            * *  *      *             **            *       * *          *  *
     534485 CTGCGGT-GAAAAAGGAACTATCTTCCAATAAAAGCTAGATAGAAGCAATGTCAGAAACTTTTTC
         66 TTTCGTTGGAAACAGGAA-TATCTTCATATAAAAGCTAGACAGAAGCATTCTCAGAAACTTCTTT

            *      * ** *   **      *       *        *
     534549 ATGATGTATCTACTCAGCTAACAGAGTTGAACCTT-CCTTTG
        130 GTGATGTTTGCATTCAAGTAACAGTGTTGAACATTCCCTTTC

             *       *                   * *     *             **            
     534590 AGAGAGCAGTTTTGAAACACTCTTTTTGTGGAATCTGCAAGTGGATATTTGTCTAGCTTTGAGGA
          1 ATAGAGCAGGTTTGAAACACTCTTTTTGTAGTATCTGGAAGTGGATATTTGGATAGCTTTGAGGA

                         *   *    *                   *       *              
     534655 TTTCGTTGGAAACGGGATTA-CAT-ATA-AAAAGC-AGACAGCAGCATTCCCAGAAACTTCTTTG
         66 TTTCGTTGGAAACAGGAATATCTTCATATAAAAGCTAGACAGAAGCATTCTCAGAAACTTCTTTG

                              *    *                 
     534716 TGATGTTTGCATTCAAGTCACAGAGTTGAACATTCCCTTTC
        131 TGATGTTTGCATTCAAGTAACAGTGTTGAACATTCCCTTTC

                                                   *     *     * **     *   *
     534757 ATAGAGCAGGTTTGAAACACTCTTTTTGTAGTATCTGGATGTGGACATTTGCAGCGCTTTCAGGC
          1 ATAGAGCAGGTTTGAAACACTCTTTTTGTAGTATCTGGAAGTGGATATTTGGATAGCTTTGAGGA

            * ** *      *             *** *   *                          *   
     534822 CTAAGGT-GAAAAAGGAAATATCTTCCCCTGAAAACTAGACAGAAGCATTCTCAGAAACTTATTT
         66 TTTCGTTGGAAACAGG-AATATCTTCATATAAAAGCTAGACAGAAGCATTCTCAGAAACTTCTTT

                   **  **    *             **  * *   *
     534886 GTGATGTGCGCCCTCAACTAACAGTGTTGAAGCTTTCTTTTG
        130 GTGATGTTTGCATTCAAGTAACAGTGTTGAACATTCCCTTTC

                     *                    *      *   *                       
     534928 ATAGAGCAGTTTTGAAACACTCTTTTTGTAATATCTGCAAGAGGATATTTGGATAGCTTTGAGGA
          1 ATAGAGCAGGTTTGAAACACTCTTTTTGTAGTATCTGGAAGTGGATATTTGGATAGCTTTGAGGA

                         *   * *            **                      *    *   
     534993 TTTCGTTGGAAACGGGATTGTCTTCATATAAACTCTAGACAGAAGCATTCTCAGAAGCTTCATTG
         66 TTTCGTTGGAAACAGGAATATCTTCATATAAAAGCTAGACAGAAGCATTCTCAGAAACTTCTTTG

            *       **   *    *             * *      
     535058 GGATGTTTCAATTGAAGTCACAGTGTTGAACAGTTCCTTTC
        131 TGATGTTTGCATTCAAGTAACAGTGTTGAACATTCCCTTTC

                 *                                       *       **   * *    
     535099 ATAGAACAGGTTTGAAACACTCTTTTTGTAGTATCTGGAAGTGGACATTTGGAGCGCTCTCAGGA
          1 ATAGAGCAGGTTTGAAACACTCTTTTTGTAGTATCTGGAAGTGGATATTTGGATAGCTTTGAGGA

            * *  *  *   ** *                      * *       * *          *  *
     535164 CTACGGTGAAAATGGAAATATCTTACA-ATAAAAGCTACATAGAAGCAATGTCAGAAACTTTTTC
         66 TTTCGTTGGAAACAGGAATATCTT-CATATAAAAGCTAGACAGAAGCATTCTCAGAAACTTCTTT

            *      * ** *   **      *       *  *     *
     535228 ATGATGTATCTACTCAGCTAACAGAGTTGAACCTTTCCTTTG
        130 GTGATGTTTGCATTCAAGTAACAGTGTTGAACATTCCCTTTC

             *       *                   * *     *             **            
     535270 AGAGAGCAGTTTTGAAACACTCTTTTTGTGGAATCTGCAAGTGGATATTTGTCTAGCTTTGAGGA
          1 ATAGAGCAGGTTTGAAACACTCTTTTTGTAGTATCTGGAAGTGGATATTTGGATAGCTTTGAGGA

                         *   *    *                   *       *     *        
     535335 TTTCGTTGGAAACGGGATTA-CAT-ATA-AAAAGC-AGACAGCAGCATTCCCAGAATCTTCTTTG
         66 TTTCGTTGGAAACAGGAATATCTTCATATAAAAGCTAGACAGAAGCATTCTCAGAAACTTCTTTG

                              *    *                 
     535396 TGATGTTTGCATTCAAGTCACAGAGTTGAACATTCCCTTTC
        131 TGATGTTTGCATTCAAGTAACAGTGTTGAACATTCCCTTTC

                         *                         *     *       **     *   *
     535437 ATAGAGCAGGTTTCAAACACTCTTTTTGTAGTATCTGGATGTGGACATTTGGAGCGCTTTCAGGC
          1 ATAGAGCAGGTTTGAAACACTCTTTTTGTAGTATCTGGAAGTGGATATTTGGATAGCTTTGAGGA

            * ** *      *             *** *   *                      *   *   
     535502 CTATGGT-GAAAAAGGAAATATCTTCCCCTGAAAACTAGACAGAAGCATTCTCAGAATCTTATTT
         66 TTTCGTTGGAAACAGG-AATATCTTCATATAAAAGCTAGACAGAAGCATTCTCAGAAACTTCTTT
[truncated: 15,759,602 more chars]
