# Supplementary material for: Tigerfish designs oligonucleotide-based in situ hybridization probes targeting intervals of highly repetitive DNA at the scale of genomes
Source: Nat Commun. 2024 Feb 3;15:1027. doi: 10.1038/s41467-024-45385-x (PMC10838309; doi:10.1038/s41467-024-45385-x)
Supplement: Supplementary file 10 — Source Data [file 41467_2024_45385_MOESM10_ESM.gz › Tigerfish_full_data/Figure_S1_HOR_TRF_Analysis/TRF/trf_output/chr2_8_21_Y_hor.fa.2.5.7.80.10.50.2000.summary.html]

Output Summary

```
Tandem Repeats Finder Program written by:
Gary Benson
Program in Bioinformatics
Boston University
Version 4.09

Please cite:
G. Benson,
"Tandem repeats finder: a program to analyze DNA sequences"
Nucleic Acid Research(1999)
Vol. 27, No. 2, pp. 573-580.


Multiple Sequence Summary

Only sequences containing repeats are shown!

Click on sequence description to view repeat table.

|  |  |  |
| --- | --- | --- |
| Sequence Index | Sequence Description | Number of Repeats |
| 1 | chr2:92297386-94695067 | 9 |
| 2 | chr8:44214155-46350589 | 24 |
| 3 | chr21:10962853-11311467 | 4 |
| 4 | chrY:10565750-10883085 | 3 |
```
