# Supplementary material for: Tigerfish designs oligonucleotide-based in situ hybridization probes targeting intervals of highly repetitive DNA at the scale of genomes
Source: Nat Commun. 2024 Feb 3;15:1027. doi: 10.1038/s41467-024-45385-x (PMC10838309; doi:10.1038/s41467-024-45385-x)
Supplement: Supplementary file 10 — Source Data [file 41467_2024_45385_MOESM10_ESM.gz › Tigerfish_full_data/Figure_S1_HOR_TRF_Analysis/TRF/trf_output/chr2_8_21_Y_hor.fa.s4.2.5.7.80.10.50.2000.1.html]

chr2\_8\_21\_Y\_hor.fa.s4.2.5.7.80.10.50.2000.1.html  

```
Tandem Repeats Finder Program written by:
```

```
Gary Benson  
Program in Bioinformatics  
Boston University  
Version 4.09  

Please cite:
G. Benson,
"Tandem repeats finder: a program to analyze DNA sequences"
Nucleic Acid Research(1999)
Vol. 27, No. 2, pp. 573-580.

Sequence: chrY:10565750-10883085
Parameters: 2 5 7 80 10 50 2000
Length:  317335
```

```
Tables:   1   

This is table  1  of  1  ( 3 repeats found )
```

```
Click on indices to view alignment
```

Table Explanation  
  

|  |  |  |  |  |  |  |  |  |  |  |  |
| --- | --- | --- | --- | --- | --- | --- | --- | --- | --- | --- | --- |
| Indices | Period Size | Copy Number | Consensus Size | Percent Matches | Percent Indels | Score | A | C | G | T | Entropy (0-2) |
| 1--316722 | 171 | 1861.4 | 171 | 79 | 1 | 378844 | 33 | 20 | 16 | 29 | 1.95 |
| 3115--3163 | 26 | 1.9 | 25 | 79 | 4 | 61 | 42 | 14 | 16 | 26 | 1.86 |
| 313133--313181 | 26 | 1.9 | 25 | 79 | 4 | 61 | 42 | 14 | 16 | 26 | 1.86 |

```
Tables:   1
```

The End!
