# Supplementary material for: Tigerfish designs oligonucleotide-based in situ hybridization probes targeting intervals of highly repetitive DNA at the scale of genomes
Source: Nat Commun. 2024 Feb 3;15:1027. doi: 10.1038/s41467-024-45385-x (PMC10838309; doi:10.1038/s41467-024-45385-x)
Supplement: Supplementary file 10 — Source Data [file 41467_2024_45385_MOESM10_ESM.gz › Tigerfish_full_data/Figure_S1_HOR_TRF_Analysis/TRF/trf_output/chr2_8_21_Y_hor.fa.s2.2.5.7.80.10.50.2000.1.html]

chr2\_8\_21\_Y\_hor.fa.s2.2.5.7.80.10.50.2000.1.html  

```
Tandem Repeats Finder Program written by:
```

```
Gary Benson  
Program in Bioinformatics  
Boston University  
Version 4.09  

Please cite:
G. Benson,
"Tandem repeats finder: a program to analyze DNA sequences"
Nucleic Acid Research(1999)
Vol. 27, No. 2, pp. 573-580.

Sequence: chr8:44214155-46350589
Parameters: 2 5 7 80 10 50 2000
Length:  2136434
```

```
Tables:   1   

This is table  1  of  1  ( 24 repeats found )
```

```
Click on indices to view alignment
```

Table Explanation  
  

|  |  |  |  |  |  |  |  |  |  |  |  |
| --- | --- | --- | --- | --- | --- | --- | --- | --- | --- | --- | --- |
| Indices | Period Size | Copy Number | Consensus Size | Percent Matches | Percent Indels | Score | A | C | G | T | Entropy (0-2) |
| 949--9753 | 171 | 51.7 | 171 | 74 | 1 | 8047 | 30 | 16 | 19 | 33 | 1.94 |
| 17206--17504 | 149 | 2.0 | 149 | 82 | 0 | 416 | 30 | 16 | 17 | 35 | 1.92 |
| 20225--21039 | 169 | 5.0 | 153 | 71 | 12 | 509 | 31 | 16 | 19 | 31 | 1.95 |
| 20378--22357 | 511 | 3.9 | 511 | 79 | 2 | 2260 | 31 | 16 | 19 | 32 | 1.94 |
| 24649--24686 | 12 | 3.2 | 12 | 84 | 0 | 55 | 86 | 7 | 0 | 5 | 0.69 |
| 24652--24687 | 8 | 4.5 | 8 | 85 | 0 | 51 | 86 | 11 | 0 | 2 | 0.68 |
| 24682--2125761 | 171 | 12362.8 | 171 | 68 | 1 | 1651403 | 29 | 17 | 21 | 31 | 1.96 |
| 24682--2130766 | 1193 | 1770.3 | 1193 | 78 | 1 | 1591768 | 29 | 17 | 21 | 31 | 1.96 |
| 61324--61533 | 113 | 1.9 | 113 | 92 | 0 | 371 | 33 | 10 | 25 | 29 | 1.90 |
| 63265--63318 | 27 | 2.0 | 28 | 77 | 3 | 66 | 25 | 14 | 14 | 44 | 1.84 |
| 82289--82342 | 27 | 2.0 | 28 | 81 | 3 | 73 | 25 | 14 | 12 | 46 | 1.81 |
| 86067--86276 | 113 | 1.9 | 113 | 92 | 0 | 371 | 33 | 10 | 25 | 29 | 1.90 |
| 87882--88087 | 113 | 1.8 | 113 | 89 | 0 | 342 | 33 | 11 | 25 | 30 | 1.91 |
| 24682--2130766 | 1356 | 1549.0 | 1359 | 78 | 1 | 1589898 | 29 | 17 | 21 | 31 | 1.96 |
| 242390--242438 | 1 | 49.0 | 1 | 100 | 0 | 98 | 0 | 0 | 0 | 100 | 0.00 |
| 246799--246852 | 1 | 54.0 | 1 | 100 | 0 | 108 | 0 | 0 | 0 | 100 | 0.00 |
| 1907445--1907686 | 122 | 2.0 | 122 | 85 | 0 | 365 | 29 | 20 | 20 | 30 | 1.97 |
| 2117422--2117450 | 1 | 29.0 | 1 | 100 | 0 | 58 | 100 | 0 | 0 | 0 | 0.00 |
| 2117446--2130766 | 341 | 39.0 | 341 | 76 | 3 | 13840 | 31 | 16 | 19 | 33 | 1.94 |
| 2125616--2125914 | 149 | 2.0 | 149 | 82 | 0 | 409 | 30 | 16 | 16 | 36 | 1.91 |
| 2125765--2130766 | 171 | 29.1 | 171 | 72 | 5 | 4063 | 31 | 16 | 19 | 33 | 1.94 |
| 2128788--2130767 | 511 | 3.9 | 513 | 79 | 2 | 2298 | 31 | 16 | 19 | 32 | 1.94 |
| Indices | Period Size | Copy Number | Consensus Size | Percent Matches | Percent Indels | Score | A | C | G | T | Entropy (0-2) |
| 2133091--2136433 | 171 | 19.6 | 170 | 77 | 1 | 3735 | 31 | 16 | 18 | 33 | 1.93 |
| 2133087--2136433 | 1021 | 3.3 | 1018 | 81 | 1 | 4254 | 31 | 16 | 18 | 33 | 1.93 |

```
Tables:   1
```

The End!
